# Neglected Tropical Diseases Elimination in the Philippines: Challenges and Gaps

**DOI:** 10.3390/tropicalmed11040106

**Published:** 2026-04-17

**Authors:** Josephine Abrazaldo, Patrick de Vera, Sheila Grace Martin, John Leo Dayrit, Daryl Christian Mejos, Ferdinand Mortel

**Affiliations:** College of Medical Technology, Manila Central University, Caloocan 1400, Metro Manila, Philippines; jabrazaldo@faculty.mcu.edu.ph (J.A.); pdevera@faculty.mcu.edu.ph (P.d.V.); sgmartin@faculty.mcu.edu.ph (S.G.M.); jcdayrit@faculty.mcu.edu.ph (J.L.D.); famortel@mcu.edu.ph (F.M.)

**Keywords:** neglected tropical disease (NTD), elimination, surveillance, challenges, gaps, low-middle income countries (LMICs)

## Abstract

Neglected tropical diseases (NTDs) such as soil-transmitted helminthiasis, lymphatic filariasis, schistosomiasis, leprosy, rabies, and food-borne trematodiasis are endemic in the Philippines. Despite global and national elimination efforts, these six NTDs remain a persistent burden to the poor, those living in Geographically Isolated and Disadvantaged Areas (GIDAs), and other vulnerable groups. This narrative review synthesized data from Field Health Services Information System (FHSIS) reports of the Philippine Department of Health (DOH) from 2020 to 2024, the available literature from electronic databases, and DOH and WHO reports focusing on the challenges, barriers, and gaps in NTD control and elimination in the country. Core challenges include complex epidemiological landscapes, lapses in disease surveillance, infrastructure, and fragmented health care systems. Gaps include access to diagnostics, insufficient funding and human resource training, and scarcity of local studies focusing on endemic NTDs. With these challenges and gaps, this review highlights the need for a real-time feedback loop system in surveillance strategy, community-based interventions, full integration of NTDs in primary health care, and collaboration between government, NGOs and private entities. Addressing these challenges and gaps is key to shifting from control to elimination.

## 1. Introduction

Neglected tropical diseases (NTDs) are a diverse group of diseases affecting mostly low-income countries (LICs) and low-middle income countries (LMICs). It is estimated that NTDs affect more than one billion people globally [1]. Fifteen NTDs are endemic in twenty-nine countries in the Western Pacific Region where the Philippines is geographically located [2].

The Philippines is considered an LMIC according to the World Bank Classification. The country carries a high burden of NTDs. Factors such as poverty, poor living conditions, limited access to safe water, sanitation, and hygiene (WASH), and limited access to health services all equally contribute to increased risk of NTDs [3]. NTDs such as schistosomiasis, soil-transmitted helminthiasis (STH), lymphatic filariasis (LF), leprosy, and rabies remain endemic in geographically isolated and disadvantaged areas (GIDAs) in the Philippines. These diseases remain a persistent public health challenge despite decades of implementation of control and elimination efforts [4]. These challenges are shared among neighboring LMICs, where progress towards elimination varies.

The WHO Asia Subregion Report presents the current status of key NTDs endemic in the region. Leprosy, LF, and rabies are in the elimination phase as a public health problem, whereas STH, schistosomiasis, and food-borne trematodiasis (FBT) are all in the control phase [5]. Malaysia, Thailand, and Vietnam have achieved WHO validation of LF elimination as a public health problem and are maintaining high preventive chemotherapy (PC) coverage for STH. In the Philippines, local transmission of LF was identified in 46 provinces. Currently, LF has been eliminated as a public health problem in 44 provinces that were previously endemic for the disease. On the other hand, high STH burden continues to persist in GIDAs. Thailand and Vietnam continue to have high incidence of FBTs. Indonesia remains endemic for LF [6].

The continued presence of these NTDs poses a significant barrier to achieving the Sustainable Development Goals (SDGs) SDG1 (No Poverty) and SDG3 (Good Health and Well-Being). Persistence of NTDs continues to contribute to morbidity and loss of labor productivity, further exacerbating poverty and stalling economic growth, particularly in an LMIC such as the Philippines [7].

Recognizing the impacts of NTDs, the WHO launched the NTD Roadmap 2021–2030. The roadmap calls for integrated, country-led strategies to eliminate at least one NTD in 100 countries by 2030, and aims to reduce the number of people requiring treatment for NTDs by 90% [8]. Aligned with this global strategy, the Philippine Department of Health (DOH) has implemented disease-specific elimination programs and intensified mass drug administration (MDA) campaigns [Supplementary File S1].

While the Philippines aligned its national strategies with the WHO NTD Roadmap 2021–2030, it is not without gaps and challenges. This paper delves into the current state of NTDs in the Philippines, identifies programmatic gaps and key challenges, and proposes the development of a real-time surveillance loop for NTDs.

## 2. Methods

This narrative review synthesized data presented in the Philippine DOH Field Health Services and Information System (FHSIS) reports from 2020 to 2024. Reported annual new cases of endemic NTDs and MDA coverage were retrieved from the reports and presented in this review. Relevant data, policies, and contextual information were also retrieved from articles and studies from electronic databases such as Google Scholar and Science Direct, and reports from the WHO and the Philippine DOH. Keywords used to identify relevant references include “Philippines,” “neglected tropical diseases,” “NTDs,” “lymphatic filariasis,” “schistosomiasis,” “soil-transmitted helminthiasis,” “rabies,” “leprosy,” “food-borned trematodiasis,” “surveillance,” “elimination,” “control,” “challenges,” “gaps.” Appropriate Boolean operators are used with the selected keywords to search the targeted literature to review.

The selection of references was based on the relevance of the literature with the review objective. Non-relevant and non-English articles were excluded. More recent publications and literature were preferred; however, relevant older references were still included due to scarcity of literature in the local setting. The collected literature was analyzed narratively to provide a comprehensive overview on the current status of endemic NTDs in the Philippines, challenges and gaps in control and elimination efforts, and barriers in the implementation of the Multi-Disease Elimination Plan (MDEP) 2024–2030.

## 3. Prevalence and Burden of Key NTDs in the Philippines

**Figure 1:**
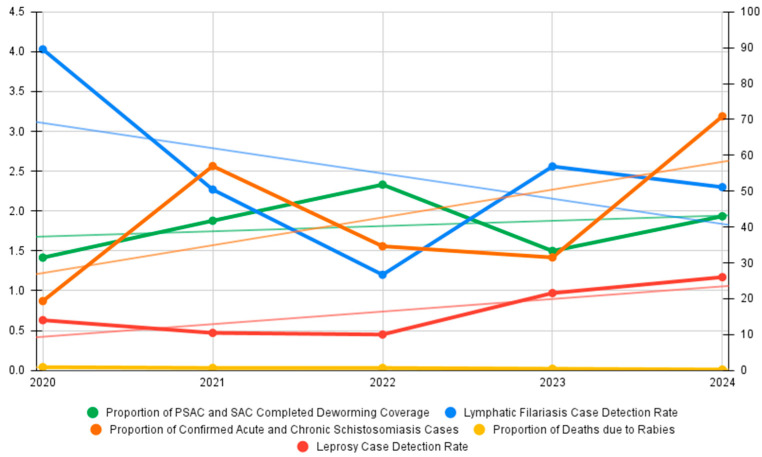
Trends in indicators of endemic NTDs in the Philippines from 2020 to 2024. Indicators of STH and schistosomiasis are measured against the right horizontal axis; while indicators for LF, rabies, and leprosy are measured against the left horizontal axis. Source: DOH FHSIS 2020–2024 [Supplementary Files S2–S6].

Figure 1 shows the trends in epidemiologic indicators of endemic NTDs in the Philippines from 2020 to 2024. Compared with other NTDs, surveillance reporting for STH in the Philippines is based on the coverage of preventive chemotherapy (PC) mass administration rather than annual reported cases of infection. The WHO Roadmap identifies pre-school-age children (PSAC) and school-age children as the primary target of regular PC to control STH. Over the period of 2020 to 2024, the coverage of completed PC courses among PSAC and SAC nationwide ranges between 31.45% and 51.81%. Despite the increasing trend, coverage is way below the 75% benchmark recommended by the WHO [9].

An initial decline in LF case detection rate is observed between 2020 (4.03%) and 2022 (1.2%). The trajectory of the trend, however, forms a “U-shape” as the case detection rebounds towards 2023. The overall trend shows a downward direction, but the case detection rate is still above the <1% national target.

The proportion of confirmed acute and chronic schistosomiasis cases shows an upward direction. The proportion of confirmed cases fluctuated greatly within the five-year period, starting at 19.33% in 2020 and ending at 70.87% in 2024. Schistosomiasis in the Philippines is uniquely difficult to address because of its zoonotic nature. Unlike leprosy that has purely human-to-human transmission, *Schistosoma japonicum* may infect animals such as water buffaloes (carabaos), dogs, and pigs. Infected animals serve as reservoirs and allow persistence of local transmission. Effective schistosomiasis elimination measures require a combination of MDA, management of human cases, and control of animal reservoirs to reduce transmission [10,11,12]. Farming and fishing remain the occupations with the highest risk based on the confirmed cases in the 2024 FHSIS report.

Rabies remains a significant public health concern. The proportion of deaths due to rabies shows a steady decline from 2020 (0.04%) to 2024 (0.01%). Continuous effort in case reporting and control of both animal and human rabies is necessary to keep the country on track to achieve a rabies-free Philippines by 2030.

Data for leprosy reveals a complex trajectory influenced heavily by the normalization of health services post-pandemic. Reported cases saw a significant artificial decline during the height of COVID-19 [13]. This may be due to the suspension of active case-finding activities and the repurposing of health workers. In 2023 and 2024, the Philippines saw an increase in case detection rate. This spike does not necessarily indicate an outbreak but rather reflects a catch-up in surveillance and clearance of backlogged undiagnosed cases. Leprosy continues to affect individuals of all ages; however, a large proportion of new cases reported occur among children under 15 years old. Multibacillary leprosy remains the most common form of leprosy, with lepromatous type being the most frequent clinical presentation [14].

### 3.1. Soil Transmitted Helminthiasis

STH is caused by infection of parasitic worms primarily transmitted by ingestion of embryonated eggs from contaminated soil and larval skin penetration in areas with poor sanitation. The main species that infect humans are giant intestinal roundworm (*Ascaris lumbricoides*), human whipworm (*Trichuris trichura*), and human hookworms (*Necator americanus* and *Ancylostoma duodenale*) [15]. *T. trichiura* and hookworms damage their host tissue and cause blood loss, whereas *A. lumbricoides* impairs host nutrition status via competition for nutrients and impaired absorption in heavy infections.

The WHO Preventive Chemotherapy and Transmission Control (PCT) Databank presents that the number of PSAC and SAC that require PC for STH in the Philippines is 37,310,807 as of 2024 [16]. The prevalence of *A. lumbricoides* (20%) and *Trichuris trichiura* (29.3%) is higher in Visayas Islands than in other parts of the country. Hookworms (1.3%) prevalence is highest in Mindanao [17]. Among the occupationally exposed population, *T. trichiura* (59.3%) has the highest prevalence, followed by *A. lumbricoides* (4.24%) and human hookworms (4.24%) [18].

Table 1 shows the annual national coverage of STH preventive chemotherapy (SPC) for PSAC and SAC based on the eligible population from 2020 to 2024. Annual coverage is inconsistent with the highest national coverage recorded in 2022 (51.81%) and the lowest in 2020 (31.21%). The PSAC and SAC SPC coverage varied significantly between regions within the five-year period. It is notable that some regions are far behind the target coverage. The Bangsamoro Administrative Region in Muslim Mindanao (BARMM), for instance, recorded the lowest coverage in 2020 and 2021 but has shown improvement since then. Region VII also recorded consistently low coverage. On the top-end, Region IX has shown the highest consistency in SPC coverage, particularly among SAC. An explicit gap in coverage between regions is present. The implementation of NTD control and elimination programs in the Philippines are led by local government units [19]. This decentralized system leads to variability in program implementation and differences in social mobilization activities. Moreover, the implementation of these programs is highly dependent on the availability of resources on the community level. Distribution of PC may be limited by challenges such as delayed supply distribution of anthelminthic drugs and lack of human resources [20]. Low community participation due to misconceptions on deworming and inconsistent participation of private schools in MDA also interfere in achieving high national SPC coverage among PSAC and SAC [19,21].

### 3.2. Lymphatic Filariasis

LF is an NTD transmitted to humans through mosquito vectors. There are three species of filarial worms endemic to the Philippines: *Wuchereria bancrofti*, which is responsible for 90% of the cases, *Brugia malayi,* and *Brugia timori* [22]. In 2015, eight Philippine endemic provinces were declared free of infection; three more provinces were declared LF-free in 2017. As of 2024, the DOH has reported that 44 out of the 46 provinces where local transmission was identified have been declared free of infection [23]. The two provinces that remain endemic for LF are Sultan Kudarat and Zamboanga del Norte [Supplementary File S2].

Table 2 shows that the burden of LF is shifting geographically across the archipelago. Persistent high-burden regions such as Region IV-B reported the highest number of cases twice (2020 and 2022). Emerging hotspots in Mindanao were identified. In 2023 and 2024, the highest burden shifted to Region XI and Region XII with 149 and 286 cases respectively. Region XII remains one of the two regions where LF is still endemic. Apart from regions with already zero cases, regions that are likely approaching elimination as a public health problem status are Region XIII and BARMM. These regions have managed to keep confirmed cases in the single digits or very low double digits. BARMM recording one case in 2023 and Region VI recording two cases in 2024 is a significant milestone, though it requires rigorous verification to ensure it is not a result of under-reporting.

### 3.3. Schistosomiasis

Schistosomiasis in Asian countries such as the People’s Republic of China, Indonesia, and the Philippines is primarily caused by *S. japonicum* [24]. Schistosomiasis is widespread across 28 provinces in the Philippines, affecting primarily those in the Visayas and Mindanao regions. The highest burden is in the Eastern Visayan islands, Leyte and Samar [25]. In Mindanao, almost all provinces are affected except for Misamis Oriental, Sarangani, and the Sulu Archipelago [21]. Endemicity foci were also identified in the Cagayan Valley located in the Northern Luzon area. Across the endemic provinces, it is estimated that 12.4 million Filipinos are at risk of contracting the infection and 2.7 million are directly exposed to the parasite [26]. Risk and exposure are primarily attributed to the agricultural nature of the common livelihood of Filipinos in endemic provinces.

The Philippines has seen a decreasing trend in prevalence of schistosomiasis since the establishment of endemicity in the country. A national prevalence of 20.0% was reported in 1940 when the first survey was conducted. Since then, prevalence has decreased to approximately 4.68% and 4.0% in 2017 [27] and 2019 [28], respectively. A mortality rate of 1.78% of the positive cases for schistosomiasis is recorded annually. Children, male, and those working as farmers (74.1% prevalence), fishermen, and tuber gatherers (60% prevalence) are the most vulnerable among the exposed population in endemic provinces [29]. Risk among children is associated with lack of proper hygiene, and play habits exposing them to infested water [30]. Distribution patterns of burden among different occupations show that livelihoods that primarily rely on activities requiring contact with potentially contaminated waters, such as farming and fishing in fresh bodies of water, have higher prevalence rates [10].

Spatial distribution of schistosomiasis endemicity in the Philippines is concentrated in areas stricken by rainfall throughout the year. Year-round precipitation supports reproduction and maintenance of habitats of the primary snail intermediate host of *S. japonicum* in the country [31]. Water dynamics is known to influence the vertical and horizontal distribution of snail populations. Previous studies have shown an inverse relationship between water levels and flooding time with snail population density [32]. Increased water levels and flooding time support the conduciveness of the environment for snail reproduction. Flooding and water overflows promote redistribution of cercaria and snail intermediate hosts in the environment. Increasing water flow velocity brought about by flooding also increases the overall transmission risk by promoting contact of schistosome cercaria with its appropriate intermediate host [33,34]. As a country located in the Western Pacific Region, the Philippines periodically experiences typhoons that bring heavy rainfall volume, particularly to those provinces facing the Pacific Ocean. Schistosomiasis-ridden provinces are mostly located in climatic region types II, III, and IV, which experience no dry season, a short dry season (1 to 3 months only), and evenly distributed rainfall throughout the year respectively [35].

Table 3 shows the number of acute and chronic schistosomiasis cases confirmed by stool examination and rectal biopsy in the Philippines from 2020 to 2024. Unlike other NTDs, schistosomiasis is heavily tied to specific environmental topographies, particularly freshwater basins and agricultural wetlands. Cases remain concentrated in traditional endemic regions. The burden is heavily skewed toward the southern and eastern parts of the country. In 2020, 2021, and 2024, Region X had the highest burden with 333, 467, and 218 cases respectively. Region XIII reported 205 cases in 2022, the highest among the endemic regions in that year.

A sharp increase in confirmed cases was reported in 2024, with the highest number coming from Leyte and Samar provinces in Region VIII. Many of the provinces experience year-round rainfall, which influences environmental conditions associated with schistosomiasis epidemiology [24,36]. The snail vector *Oncomelania hupensi quadrasi* is able to survive human manipulation of its pristine habitation in forest swamps and floodplains. Eventually, the snails settled in rice fields, irrigation canals, ponds, streams and even ditches [21].

### 3.4. Rabies

Rabies remains a critical public health issue in the Philippines, where dog-mediated infections are endemic. Despite efforts to control and prevent the disease, the Philippines reports one of the highest incidence rates of human rabies in Asia [37]. Rabies spreads to people and animals via exposure to saliva from infected animals. The virus is typically transmitted through bites, scratches, or direct contact with mucosa (e.g., eyes, mouth, or open wounds). Once clinical symptoms appear, rabies is virtually 100% fatal [38]. Ninety-eight percent of animal rabies cases are associated with dogs while the remaining two percent are attributed to cats and other domesticated animals such as carabao, cattle, pigs and goats [39]. A study in the Philippines shows that the incidence of canine rabies is highest among puppies, with 38.0% of cases occurring among dogs under 3 months old and 24.0% in those between 3 and 11 months old [40].

The Anti-Rabies Act of 2007 (RA 9482) was enacted to address the country’s growing concerns about rabies as a significant public health problem [41]. The National Rabies Prevention and Control Program Strategic Plan 2020–2025 targets to eliminate human rabies in the Philippines and to declare the country human rabies-free by 2030 [42].

Table 4 shows the specific regions that consistently carry the highest burden of rabies-related deaths. Region IVA was the primary hotspot early in the decade, accounting for 80 deaths in 2020 alone. Region III reported the highest burden from 2022 to 2024, reporting 54 deaths annually for two consecutive years and sharing the highest count in 2024 with Region XII. This indicates a northward to southward spread of high-fatality zones and an emerging concern in Mindanao. Although the total number of reported rabies-related deaths are increasing in the presented data, it is notable that the trend in the number of deaths in relation to the number of reported animal bite cases is decreasing from 0.04% in 2020 to 0.02% in 2024.

Certain regions have maintained exceptionally low mortality rates throughout the five-year period. The Cordillera Administrative Region (CAR) shows the lowest burden in four of the five years, never exceeding two deaths annually. Other low-burden regions including BARMM, Region VII, and Caraga have also recorded minimum deaths (one each) in specific years (2021 and 2023).

### 3.5. Leprosy

Leprosy is a chronic infectious disease caused mainly by *Mycobacterium leprae* [43]. Another etiologic agent of the disease is *Mycobacterium lepromatosis,* which was discovered in two patients who died of diffuse lepromatous leprosy. The novel *Mycobacterium* DNA was purified from two separate cases: one from a heavily infected, freshly frozen liver autopsy, and one from a paraffin-embedded skin tissue [44].

Elimination of leprosy as a public health problem was achieved in the WHO Western Pacific Region in the late 1980s; however, new cases continued to be reported in the Region [45]. The WHO identified leprosy as an eliminated global public health problem in the year 2000. Elimination as a public health problem is defined as the overall reduction in prevalence to less than one case per 10,000 people [46,47]. From 1985 to 2011, the recorded number of cases worldwide fell from 5.4 million to approximately 219,000. The prevalence rate in terms of 10,000 people dropped from approximately 21.1 in 1985 to 0.37 in 2011, excluding Europe [47]. Approximately 180,000 people globally were infected with leprosy in 2016, with most cases concentrated in Africa and Asia [48]. The Philippines has the highest number of leprosy cases in the Western Pacific Region. In 2014, the country recorded 1,665 cases, representing 38% of the regional total [49]. While the proportion of the population affected is low, leprosy persists in vulnerable populations. The WHO Western Pacific Regional Office classifies the Philippines as an outlier, being the country with the highest incidence of leprosy among the countries in the region [46].

Table 5 shows the regional hotspots of leprosy from 2020 to 2024. Region III reported the highest number of confirmed cases (1416) in 2020. A shift in distribution of confirmed cases towards the central and southern part of the country is noted from 2021 to 2024. Region VIII reported the highest number of cases in 2021 and 2023. Region X had the highest number of cases in 2022. There were 754 reported cases in Region IX, the highest in 2024. The lowest number of confirmed cases were recorded consistently in BARMM in 2020 and 2021. The National Capital Region (NCR) had the lowest number of reported cases from 2022 to 2024.

### 3.6. Food-Borne Trematodiasis

FBTs refer to a group of parasitic infections caused by various species of flukes transmitted through ingestion of food contaminated with metacercaria. This group includes flukes that establish infection in the liver, lungs, and the intestines. FBTs typically encompass fascioliasis, clonorchiasis, fasciolopsiasis, paragonimiasis and heterophyidiasis.

The Philippines is recognized as a major endemic hub of FBTs. *Paragonimus westermani* (and the proposed *Paragonimus philippinensis*) is reported in at least 12 provinces with estimated prevalence ranging from 0.7% to 52%. This prevalence is inextricably linked to traditional culinary practices, specifically the consumption of “*kinagang*,” a delicacy featuring mountain crabs briefly boiled in coconut milk. The potential of *Isolapotamon* spp. as an intermediate host of *P. westermani* in the Philippines aside from *Sundathelphusa philippina* has also been reported [50,51].

A landmark study among populations on Mindanao identified the presence of *Haplorchis taichui* [52]. A prevalence of 36% was identified via stool examination. These findings suggest that intestinal trematodiasis may be far more endemic than previously realized, particularly in regions where traditional raw fish dishes are common.

*Fasciola* spp. is endemic in the Northern Samar province, with a reported prevalence rate of 95.33% in 2015 [53]. *Heterophyes heterophyes*, on the other hand, is endemic in Davao del Norte, Southern Mindanao with a reported overall prevalence rate of 2.36% [54]. Currently there are no available national data or surveillance specific to FBTs.

### 3.7. Emerging and Re-Emerging Health Problems Due to NTDs

#### Yaws

Yaws is a chronic infectious disease caused by *Treponema pallidum* subsp. *pertenue*. The disease affects the bone, skin, and cartilage, resulting in destruction and deformation if left untreated. The infection is transmitted through contact with skin lesions. Poverty, poor socio-economic conditions, and lack of personal hygiene contribute and facilitate the spread of yaws.

Yaws was once widespread in the Philippines. The WHO conducted mass treatment campaigns in the 1950s to 1960s. It was thought to have been eliminated during the 1950s and surveillance was discontinued without formal verification. In 2021, the WHO included yaws in the list of NTDs. Yaws is endemic to the Philippines and reporting of cases is currently a challenge [55]. In 2017, cases of yaws were reported from Linguasan Marsh area in Central Mindanao. These are the first confirmed cases since the cessation of official reports in 1973 [56]. Table 6 summarizes the historical epidemiologic data of yaws in the Philippines.

## 4. Current NTD Elimination Strategies

The WHO NTD Roadmap 2021–2030 serves as a guiding framework for the control and elimination of NTDs globally. The roadmap emphasizes measurable health outcomes, strengthened country capacities, and shifts in organizational structures, and defined roles and responsibilities across sectors and levels of governance. It adopts an action-oriented approach to facilitate planning, generate political momentum, and enhance resource mobilization to support sustainable interventions against NTDs [57].

In line with the WHO roadmap, the Philippine DOH launched the MDEP in 2024. This strategic framework aims to eliminate key NTDs as a public health problem, along with other priority diseases for elimination by the year 2030. The strategic pillars of MDEP include surveillance and information systems, access to laboratory services, service delivery, safe and quality medicines, vaccines and technology, human resource and capacity building, environmental and social determinants of health, stewardship and finance, and research [Supplementary File S1]. Table 7 shows the desired outcomes of the MDEP strategic pillars, and the progress and achievements in 2024–2025.

**Figure 2:**
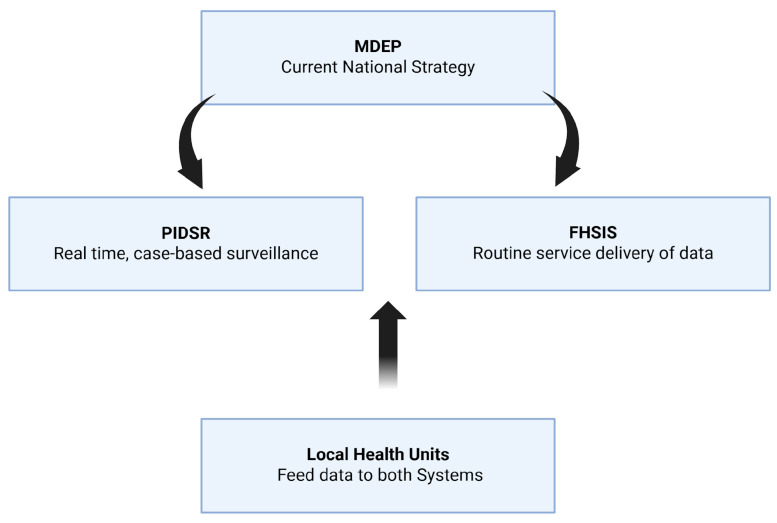
Integration of FHSIS and PIDSR in MDEP strategy (Created in BioRender. de Vera, P. (2026) https://BioRender.com/avy1rx2, accessed on 7 March 2026).

Surveillance serves as the backbone of effective control and elimination of NTDs. The Philippine Integrated Disease Surveillance and Response (PIDSR) and FHSIS predate MDEP. Figure 2 shows the aim of MDEP to integrate PIDSR and FHSIS in multi-disease surveillance and elimination to promote data-driven decision-making. This will result in data flow harmonization that can be used for planning, targeting, and evaluating elimination efforts.

Rapid disease detection mitigates transmission by placing timely control actions. To support the goal of MDEP, the DOH created the Office for Health Laboratories. The role of the Office for Health Laboratories is to provide overall directions, policies, and programs for the creation of the Philippine Health Laboratory System (PHLS), the leader of the overall system to deliver quality clinical and public health laboratory services. Vaccination, vector control, and MDA are included in the service delivery strategic pillar of the MDEP. Clinical Practice Guidelines and Operational Guidelines were created to provide a framework for human and animal vaccination as a measure to interrupt the chain of rabies transmission; however, insufficient budget allocations for rabies vaccine procurement are a persistent challenge. Measures such as habitat modification, biological control, and application of molluscicides are being implemented to control freshwater snail vectors. Currently, MDA is being implemented for LF and schistosomiasis. The Philippines has adopted post-exposure prophylaxis (PEP) for leprosy using single-dose rifampicin (SDR) to reduce incidence within endemic communities as endorsed by WHO.

## 5. Challenges and Gaps

### 5.1. Challenges

The Philippine DOH maintains strong partnership and collaboration with other government agencies and institutions to resolve the nation’s public health challenges. Despite the collective commitment, the country is yet to overcome established difficulties in controlling and eliminating endemic NTDs as a public health problem. The Republic Act No. 11223 or the Philippine Universal Health Care (UHC) Act enacted in 2019 aims to address entrenched health system challenges in achieving equitable access to quality health care [58]. Currently, the Philippine Health Insurance Corporation (PhilHealth) packages can only partially cover cases of schistosomiasis, LF, and STH. The management of cases of NTDs is mostly reliant on external donors and fundings such as with the United States Agency for International Development for LF [59], and Japan International Cooperation Agency/Korea International Cooperation Agency in improving health outcomes in BARMM [60]. There has been significant progress in operationalizing the One Health approach in efforts to eliminate endemic NTDs as a public health problem; however, total control and elimination of NTDs in the archipelago are constrained by its geography, environmental barriers, and persistent logistical and access barriers. Moving supplies from centralized stores to remote primary health care facilities is hindered by flooded roads, rough seas, or unpredictable weather conditions [61]. In recent years, flooding has increased due to heavier rainfall during the monsoon and wet seasons. Floods mobilize pathogens through sewage overflows, fecal contamination, and runoff, and also drive zoonotic transmission by bringing animal pathogens into closer contact with humans [62]. These continuously affect island provinces, remote agricultural zones, and even urban settlements, making routine surveillance and preventive services difficult to sustain.

The ability to identify endemic hotspots in real-time and the availability of integrated dashboards to survey and monitor NTDs are still in progress and limited to only a few diseases. The RabDash DC Project, a data analytics dashboard funded by the Department of Science and Technology-Philippine Council for Health Research and Development (DOST-PCHRD) and implemented by the University of the Philippines Mindanao in collaboration with the Davao City Veterinarian’s Office and the Philippine Genome Center Mindanao, is utilized to monitor rabies in the Davao Region only [63]. The Geographic Information System (GIS) has become an integral tool in surveillance by enabling spatial analysis, risk mapping, and predictive modeling that can be utilized for vector-borne diseases transmitted by *Aedes* or *Anopheles* mosquitos [64]. The absence of geospatial mapping platforms hampers evidence-based decision-making, targeted resource allocation, and timely outbreak response, particularly in high-risk and underserved communities. Weak surveillance leads to low case detection and late outbreak responses in cases of rabies [65].

Socioeconomic determinants play a central role in maintaining NTD transmission. Poverty and its associated characteristics are among the main factors which put individuals and families at risk. Poor and marginalized populations of indigenous people (IP) communities who settle in GIDAs, and those who reside in areas with peace and order conflicts are hardly reached by primary health care services. Although Animal Bite Treatment Centers that provide rabies PEP are established in every province across the Philippines, the burden of animal bite patients falls disproportionately among clinics according to their accessibility and the socioeconomic situation of the region [65]. Significant disparities in access to WASH and quality health education persist, particularly among the poorest households in the poorest areas [27,66,67,68].

The challenges identified in mainstreaming NTDs into the Philippine health care system are primarily related to inadequate organization and management at the point of service delivery [69]. Progress in reduction and elimination of cases is inhibited by limited governance mechanisms within inter-agency committees, fragmented risk assessment and surveillance, untapped opportunities for joint investigation and response, insufficient resources for capacity-building, and absence of comprehensive risk communication and community engagement initiatives [70]. Reporting mechanisms vary by disease and locality. For instance, traditional testing such as the Kato-Katz technique for STH has been shown to have limited sensitivity, particularly in low-intensity infections. This may result in potentially underestimated prevalence [24,71]. Disparities in service delivery remain pronounced in marginalized communities, primarily due to inefficiencies in local government units (LGUs) and uneven resource distribution [72]. This further restricts long-term investments in sanitation, vector control, and food safety measures, undermining disease control efforts.

Shortage and uneven distribution of health care workers (HCWs), coupled with high patient-to-provider ratios exacerbated by the “brain drain” phenomenon, limits the delivery of specialized services such as laboratory diagnostics, disability management for leprosy and LF, and emergency care for rabies exposures [73,74]. In most settings, frontline health workers are overburdened and lack disease-specific training. Further delays in this crucial process greatly affect case management and disease detection. This was evident during the COVID-19 pandemic (2020–2023). Frontline HCWs were central to the pandemic response and, given their exposure, soon emerged as one of the more affected groups [75,76].

The pandemic significantly disrupted implementation of NTD programs across the country. Diversion of resources, delayed MDA campaigns (e.g., for schistosomiasis), reduced animal vaccination coverage (e.g., dog rabies), and limited community-based surveillance and health education activities resulted in delayed diagnosis, reduced treatment coverage, and weakened preventive services, with residual effects persisting during the transition to the “new normal” post-pandemic [77,78,79].

Social and informational barriers continue to undermine NTD control efforts. Stigma and discrimination towards lepers in the community both discourage health-seeking behaviors and contribute to delayed diagnosis and long-term disability [80]. Cultural beliefs, misinformation, and rumors can also hinder the effective transmission of knowledge [81]. Many remote areas have poor to no access to the internet, resulting in reduced community participation in preventive programs and loss of data during uploading [82].

### 5.2. Gaps

Achieving reduced or zero new infections of priority diseases by 2030 through the Philippine MDEP will require continuous coordinated effort from head offices, organizations, and key institutions. Apart from the multiple challenges we are facing, the nation must fill in the gaps to achieve the target elimination of these endemic NTDs. Contingency planning for public health emergencies remains inadequate. This makes LMICs, like the Philippines, with weak health systems, particularly vulnerable as demonstrated during the COVID-19 pandemic and during natural disasters as in the case of typhoon Haiyan in 2013 [83,84].

Another gap is the lack of integrated, real-time surveillance dashboards capable of consolidating data across different diseases (e.g., monitoring system for non-canine rabies, FBTs, yaws). Despite considerable resources and coordination invested within a limited timeframe, fragmented data systems and lack of visibility of supply chain performance leads to substandard performance and inefficiencies in on-time delivery of medicine to in-country central medical stores. Sometimes, delivery targets may lag as much as 40%. This fails to meet the 80% WHO target, which requires all shipments to be delivered at least one month before the planned MDA date [85].

Many NTD programs lack adaptive mechanisms to maintain essential services due to the absence of clear policies for MDA and complexities in setting accurate targets for immunization initiatives (e.g., limited access to animal vaccination, PEP for leprosy, post-exposure prophylaxis for rabies). This calls for strengthened coordination across the human and animal disease surveillance and information system, and case investigation for diseases (e.g., schistosomiasis, rabies). Furthermore, institutionalization of the Expanded National Practice Guidelines has yet to achieve widespread circulation. Drug prices serve as a key barrier to health care access in the Philippines, where out-of-pocket payments remain the main form of health spending [86]. The Drug Price Reference Index (DPRI) stipulates lower prices compared to prevalent local market rates, undermining cost-effectiveness [86].

Persistent gaps also exist in investment for research, diagnostics, and innovation, particularly for locally appropriate tools. Project NOAH (Nationwide Operational Assessment of Hazards), the Philippines’ leading disaster science and early warning program, was effectively discontinued around early 2017 due to budget issues and internal conflicts [87]. Policy weaknesses and lack of funding were consistently raised as barriers to NTD management [88]. Recent advancements in schistosomiasis diagnostics have resulted in the development of assays that have superior diagnostic performance. The cost of adopting these novel techniques, however, remains a major limitation for developing economies and underfunded control and elimination programs like in the Philippines [89]. These constraints reduce the ability to adapt global strategies to local contexts and delay the adoption of innovative, cost-effective approaches tailored to Philippine settings. Many global health development aid projects provide funding for short-term cycles (usually 3–5 years), and once these periods end, sustaining the momentum can be difficult [90].

Table 8 summarizes challenges and gaps on the implementation of the eight strategic pillars of MDEP. The implementation of the MDEP in the Philippines is constrained by multiple challenges and gaps across surveillance, service delivery, logistics, financing, and research. Addressing these challenges and gaps demands strong leadership and political will. Well-coordinated and multisectoral approaches are key to integrating financing reform, data-driven decision- and policy-making, and equity-focused program designs to achieve long-term NTD control and elimination.

**Figure 3:**
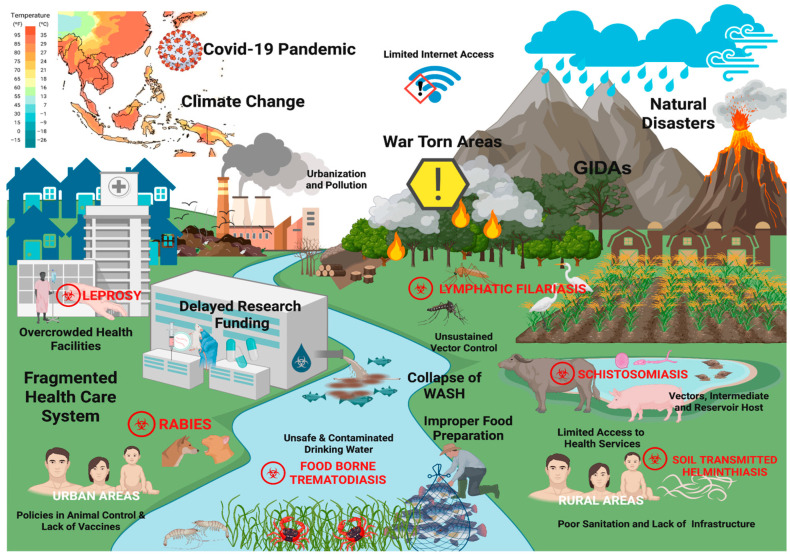
Challenges and gaps in the control and elimination of NTDs. (Created in BioRender. de Vera, P. (2026) https://BioRender.com/74hc4gd, accessed on 7 March 2026).

Figure 3 shows the persistent challenges that limited the Philippines’ progress toward the elimination of endemic NTDs in the past decade. The country’s geographic location and vulnerability to climate change-related disasters such as typhoons, flooding, and landslides have damaged infrastructure, contaminated water sources, and facilitated vector proliferation. As an agriculture-dependent country, rural populations remain highly exposed to parasitic infections due to environmental contact and the natural presence of intermediate and reservoir hosts. Fragmented health care systems, limited internet access in remote areas, and socioeconomic disadvantages restrict access to health services, surveillance, and public health information. These constraints were further compounded by delays in research funding, armed conflicts, the COVID-19 pandemic, and disruptions in prophylaxis availability, MDA, vector control, and animal health policies, leading to outbreaks, resurgence, and reinfection among vulnerable populations.

## 6. Recommendations

Table 9 shows a summary of the challenges and gaps identified in the Philippine MDEP and the corresponding recommendations. Initiation of elimination efforts is heavily rooted in data provided by intensive and robust surveillance systems. Implementation of surveillance–response systems that focus on rapid detection of existing, new or reintroduced cases, identification of areas of low transmission, understanding trends in disease incidence and prevalence, and detection of possible drug resistance is integral as countries approach elimination of NTDs as a public health problem [91].

**Figure 4:**
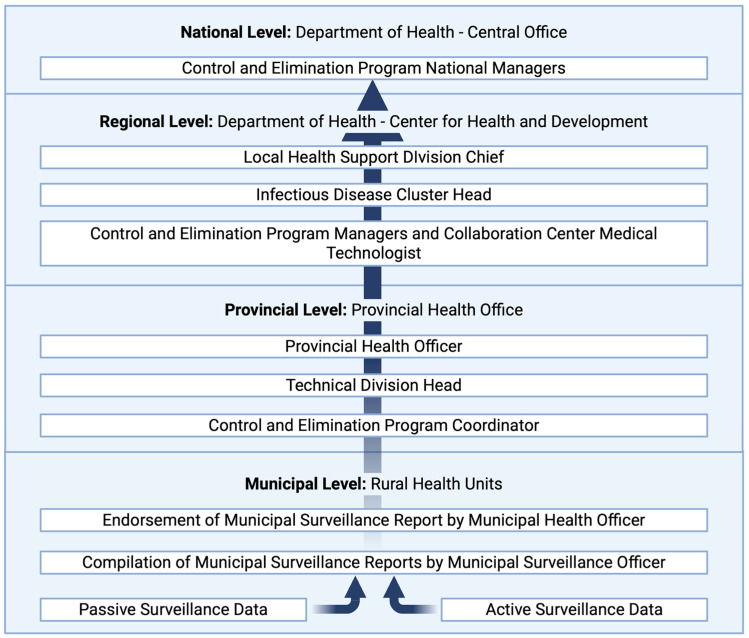
Current flow of surveillance data in the Philippines from the municipal level to the national level [93]. (Created in BioRender. de Vera, P. (2026) https://BioRender.com/0vs022c, accessed on 7 March 2026).

Robust case detection and disease monitoring, and health system capacity-building are integral in NTD elimination, as seen in multiple countries that have eliminated at least one NTD [92]. Delays in data reporting serve as a major barrier in surveillance and elimination efforts as they may lead to lags in response from pertinent organizations to address emerging outbreaks. Figure 4 shows the current flow of surveillance data in the Philippines from rural health centers to the DOH central office [93]. At the national level, the data are utilized for decision making—for the respective programs, provision of additional support, resource allocation, and subsequent actions. The process of recording, reporting, and transmitting surveillance data in the Philippines is still done through a paper-based approach, with data transmission from rural health units taking one week to six months to reach the next pertinent agency [93]. Real-time reporting is necessary to identify outbreaks and improve response planning and implementation [94]. This underscores the significance of real-time transmission of surveillance data, particularly in areas of low transmission, to initiate appropriate and timely responses for managing emerging cases and preventing resurgence. Moreover, surveillance data validates the effectiveness of control measures.

**Figure 5:**
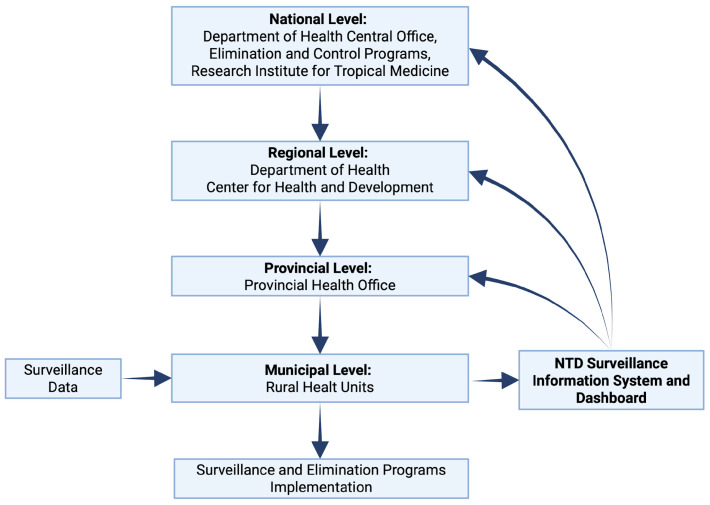
Proposed real-time feedback loop system. (Created in BioRender. de Vera, P. (2026) https://BioRender.com/7m7a97n, accessed on 7 March 2026).

To improve the efficiency of surveillance data flow and allow faster reflex measures towards emerging outbreaks, we propose a real-time feedback loop system as shown in Figure 5. In this feedback loop system, surveillance data such as number of suspected and confirmed cases, demographic and geographic distribution of cases, MDA coverage and compliance, vector control implementation and mapping, and treatment coverage are collected and compiled in rural health units (RHUs). The core of the proposed real-time feedback loop system is the implementation of an integrated digital NTD surveillance information system and dashboard. This would allow local government units at the municipal level to encode and update surveillance data to a national database on a real-time basis, thus opening opportunities for the continuous monitoring of cases. The use of geospatial mapping and live data may give way to identification of areas with increasing prevalence rates. It also aims to standardize surveillance data and case reporting across local government units in endemic areas. Elimination and control program committees and the DOH offices at different levels will have access to real-time data for faster decision-making and timely response such as immediate mobilization of resources and appropriation to the affected barangays or municipalities. Rapid response feedback loops may be automatically triggered for immediate intervention when the system detects low drug compliance or a sudden spike in cases.

Consistent data updates from the NTD surveillance information system and dashboard will allow the DOH central office to monitor cases across the endemic provinces and, subsequently, respond by reevaluating and revising existing modules, the establishment of partnerships with local and international agencies, the development of an elimination roadmap, the reshaping of technical guidelines based on the current needs of the endemic agencies, policy support, and resource mobilization and allocation. Regional policy adaptation and direction of technical assistance, capacity-building, and coordination of logistics from regional DOH-CHD, as well as logistics management and resources mobilization, technical assistance and capacity-building, coordination with municipal health offices, and outbreak investigation from provincial health are delivered promptly to identified transmission hotspots and high-risk populations. Implementation of surveillance and elimination programs is ultimately implemented at the municipal level. Timely and adequate support and resources to the granular level are integral to the success of elimination efforts.

The elimination of NTDs requires coordination and collaborative efforts between stakeholders, involving the national and local government, non-government organizations, health human resources, public health researchers, and community members. A lack of human resources and capacity, and acceptance from community members remain major challenges in the elimination of NTDs in the Philippines [19,20,95,96,97]. These factors continuously cripple the implementation of MDA, hindering the achievement of effective coverage and detection of cases. Furthermore, the participation of private institutions in elimination efforts is inconsistent, thus hampering progress [19,21]. Continuous implementation of capacity-building programs among the local health workforce is just as critical as adoption of intensive and robust models of surveillance–response systems. Aside from the local health workforce, capacity-building programs may also cover the community members to establish ownership of their health through self-care coupled with provision of kits for disease prevention and management. Exploring the factors that impact the compliance of community members towards currently existing surveillance and elimination programs is key to bridge gaps in community participation. Furthermore, measures to push community sensitization and mobilization, inter- and intrasectoral linkages, and partnerships are crucial to foster sustainable efforts towards NTD elimination in the country.

## 7. Conclusions

The Philippines, being an LMIC, consistently faces challenges in achieving the NTD elimination targets set by the WHO. NTD control and elimination efforts are in place; however, several key issues need to be addressed to advance progress toward national and global targets. The impact of the complex epidemiological landscape of NTDs such as LF and schistosomiasis is best reflected by the fluctuating epidemiologic indicators reported from 2020 to 2024. Rabies remains a persistent hazard and the human rabies-free Philippines by 2030 is still far from actualized. The Philippines remains an outlier in the Western Pacific Region, reporting more than 1000 new cases of leprosy annually. This paper also identified important programmatic gaps that hinder the implementation of the Philippine MDEP 2024–2030. The Philippines’ archipelagic terrain presents logistical challenges, particularly in GIDAs, where inadequate infrastructure and weak referral systems lead to diagnostic and treatment delays. Moreover, the analysis identified systemic concerns, including convoluted procurement methods, insufficient financing for important vaccines, particularly rabies, and a shortage of specialist laboratory professionals. Social barriers such as the propagation of disinformation in rural locations continue to limit community participation in preventive programs.

With these challenges and gaps in the elimination of NTDs, this review recommends establishing a real-time surveillance system for NTDs. Local governments might encode and update surveillance data in real time by combining existing technologies into a responsive, digital-based interface. This framework would provide the Department of Health (DOH) with the data it needs to revise technical standards in response to current demands.

Overall, the study argues that, while the Philippine MDEP 2024–2030 offers a strategic roadmap, its effectiveness is hindered by a mismatch between ambitious policy and logistics. To transition from “control” to “elimination,” the Philippines must bridge the gap between its archipelagic challenges and health care delivery through digital innovation, specifically, real-time surveillance, as well as an updated emphasis on addressing the social stigmas that conceal many illnesses.

## Figures and Tables

**Table 1:** Reported PSAC and SAC SPC coverage in the Philippines per region from 2020 to 2024.

Year	Reported National SPC Coverage (PSAC and SAC)	Highest PSAC SPC Coverage per Region	Lowest PSAC SPC Coverage per Region	Highest SAC SPC Coverage per Region	Lowest SAC SPC Coverage per Region
2020	6,300,438 (31.45%)	Region XIII (91.63%)	BARMM (0.05%)	Region XIII (64.48%)	BARMM (0.03%)
2021	8,379,341 (40.74%)	Region X (85.36%)	BARMM (0.09%)	Region IX (92.66%)	BARMM (0.08%)
2022	10,418,100 (51.81%)	Region III (89.74%)	BARMM (23.73%)	Region IX (83.89%)	Region VII (17.53%)
2023	6,890,693 (33.31%)	Region XI (81.19%)	BARMM (28.12%)	Region IX (72.35%)	Region II (8.35%)
2024	8,950,385 (42.99%)	Region IV-B (90.23%)	Region VII (31.16%)	Region IX (96.65%)	Region VII (8.32%)

Source: DOH FHSIS 2020–2024 [Supplementary Files S2–S6].

**Table 2:** Confirmed number of LF cases per region from 2020 to 2024.

Year	Number of Cases	Highest Number of Confirmed Cases per Region	Lowest Number of Confirmed Cases per Region
2020	372	Region IV-B (142)	Region XIII (39)
2021	245	Region VIII (126)	Region XIII (2)
2022	138	Region IV-B (121)	Region XI (2)
2023	289	Region XI (149)	BARMM (1)
2024	352	Region XII (286)	Region VI (2)

Source: DOH FHSIS 2020–2024 [Supplementary Files S2–S6].

**Table 3:** Number of confirmed cases of schistosomiasis per region from 2020 to 2024.

Year	Number of Confirmed Acute and Chronic Cases	Highest Number of Confirmed Cases per Region	Lowest Number of Confirmed Cases per Region
2020	673	Region X (333)	Region V (4)
2021	636	Region X (467)	Region V (5)
2022	583	Region XIII (205)	Region V (13)
2023	478	Region X (218)	Regions IV-B (1)
2024	2781	Region VIII (1009)	Region IVA (3)

Source: DOH FHSIS 2020–2024 [Supplementary Files S2–S6].

**Table 4:** Reported number of deaths due to rabies per region from 2020 to 2024.

Year	Number of Deaths	Highest Number of Deaths per Region	Lowest Number of Deaths per Region
2020	239	Region IVA (80)	CAR (1)
2021	230	Region IVA (34)	CAR (1), Region VII (1), and BARMM (1)
2022	284	Region III (54)	CAR (2)
2023	305	Region III (54)	Region XIII (1)
2024	299	Region III (39) and Region XII (39)	CAR (2)

Source: DOH FHSIS 2020–2024 [Supplementary Files S2–S6].

**Table 5:** Number of confirmed leprosy cases per region from 2020 to 2024.

Year	Number of Cases	Highest Number of Confirmed Cases per Region	Lowest Number of Confirmed Cases per Region
2020	5402	Region III (1416)	BARMM (38)
2021	929	Region VIII (241)	BARMM (6)
2022	2210	Region X (422)	NCR (7)
2023	1998	Region VIII (303)	NCR (9)
2024	2582	Region IX (754)	NCR (13)

Source: DOH FHSIS 2020–2024 [Supplementary Files S2–S6].

**Table 6:** Historical and epidemiologic data for yaws [56].

Year	Prevalence Rate	Location
1951	9.6%	Leyte, Samar and Southern Philippine provinces
1960	0.4%	Not mentioned
1961	13.5/100,000 (3864 cases)	Not mentioned
1973	1.1/100,000 (424 cases)	Last official report
2000	11.7% (82 cases)	BARMM, Liguasan Marsh area
2009	25.5% (Suspected yaws cases)	Pregnant women undergoing antenatal post and natal care (MSF) Mindanao
2017	14 suspected and confirmed cases	Mindanao
2020	54.3% 19 cases	Aeta Village, Luzon, Quezon, and Dumagat of Rizal

**Table 7:** Summary of the achievements for each MDEP strategic pillar.

**Strategic Pillar**	**Desired Outcome**	**Achievement 2024–2025**
Surveillance and Information System	Responsive, participatory, and localized surveillance systems for data needs to enable quick action at a local level	Inclusion of all thirteen diseases in the list of notifiable diseases which include some NTDs Training of public health workers to incorporate surveillance data in public health decision-making Inclusion of animal and environmental data for priority diseases in surveillance systems Joint DOH-DA-DILG administrative order and operation manual on integrated health information systems disseminated to all 17 centers for health development
Access to Laboratory Services	Diseases for elimination are incorporated in the public health laboratory network through the implementation of the Philippine Health Laboratory System	Construction of sub-national laboratories initiated (target 3 years construction) Sustainability plan for vaccine-preventable diseases (VPDs) referral laboratories developed including human resource, training, and budget plans
Service Delivery	Vaccination: A 95% immunization coverage for vaccine-preventable diseases and zero outbreaks/incidence for vaccine-preventable diseases Vector Control: Integrated vector management in high-risk population MDA/PC: A 50% reduction in new cases from baseline	Vaccination: Incremental increase in immunization coverage by 2% per year for vaccine-preventable diseases starting in 2024 and ending at a 14% increase by 2030 Vector control: At least 80% of malaria, LF and schistosomiasis high-burden endemic areas have updated vector maps MDA/PC: Rifampicin (for leprosy), azithromycin (for yaws) included in the Philippine National Formulary
Safe and Quality Medicines, Vaccines and Technology	Standard of care determined based on the recommended actions, and interventions	Mapping of MDEP 13 diseases which include some of the NTDs in terms of current available standard of care
Human Resource and Capacity Building	Medical and allied health graduates equipped with diseases for elimination prevention, control, and management competencies	Meeting with Commission of Higher Education (CHED) department and different Colleges and University Administrations to enhance curriculum on diseases for elimination
Environmental and Social Determinants of Health	A 50% improvement in social behavior towards diseases for elimination of the 13 priority diseases	Two studies on social behavior towards diseases for elimination
Stewardship and Finance	Multi-disease elimination guided by and overseen by functional groups with members of various key sectors across both public and private organizations, including societies	Establishment of scientific technical advisory group and technical working group
Research	80% increase in the utilization of high-quality local research as evidence for policy, guidelines or standard of care development	Approval of research agenda for diseases for elimination and compendium of research on diseases for elimination from the academe and other research institution

**Table 8:** Current challenges and gaps for elimination of NTDs according to the Philippine MDEP 2024–2030.

**MDEP Strategic Pillars**	**Challenges**	**Gaps**
Surveillance and Information System	Late and under reporting of cases. Post-validation of surveillance not in place. Diseases for elimination are captured by different information systems in place. Insufficient manpower for IS management. Inadequate utilization of IS at local level. Inadequate data sharing mechanisms among stakeholders.	Data requirements for diseases for elimination not in the present surveillance and information system (e.g., yaws). Strengthen coordination across the human and animal disease surveillance and information system, case investigation (e.g., SCH, rabies). Monitoring system for non-canine rabies not in place. Other NTDs are not yet on the health agenda.
Access to Laboratory Services	Limited capacity for timely confirmatory testing. Sustainability of the sub-national laboratories for VPDs. Insufficient animal laboratories for confirmatory testing (e.g., rabies). Diseases for elimination need quality assessment and quality improvement systems.	No specifics for STHs and food-borne trematodiasis.
Service delivery	Vaccination: Inadequate dissemination of information to the intended audience, leading to hesitancy. Accessibility issues stemming from geographical distances to sites and schedule conflicts. Prevailing concern pertains to an inadequate budget allocation for dog rabies PrEP and PEP vaccines (LGUs, Public ABTC/RHUs). Vector Control: Failure to achieve targeted utilization and insufficient supervisory mechanisms. Erosion of support from LGUs. Scarcity of communication volunteers available for implementation of related interventions. MDA and PCT: Lack of established guidelines for pre-transmission surveys (e.g., Filariasis). Limited only to LF and SCH. Diagnostics and Human Resource: Disruptions in the supply of test kits at primary health care facilities. Limited availability of qualified laboratory staff (medical technologists), leading to non-specialized personnel performing laboratory tests. Restricted access to laboratory services within GIDAs.	Vaccination: Complexities in setting accurate targets for immunization initiatives. MDA and PCT: Absence of clear policies for mass drug administration. Implementation of LPEP is not fully realized. Diagnostics and Human Resource: Integration of services provided by the private sector has not been fully realized within the program’s IS.
Safe and Quality Medicines, Vaccines and Technology Environmental and Social Determinants of health	The procurement system is convoluted and adherence to timelines. Delays in process and transportation logistics and bottlenecks in delivery and distribution pipelines. Insufficient warehouse infrastructures and capacity in all levels (poor storage, near expiring/expired, not utilized in some cases). International procurement avenues are limited in their scope.	Institutionalization of the Expanded National Practice Guidelines has yet to achieve widespread circulation. Drug Price Reference Index (DPRI) stipulates lower prices compared to prevalent local market rates, undermining cost-effectiveness.
Human Resource and Capacity Building	Existing allied medical programs do not encompass all diseases targeted for eradication/elimination. A scarcity of subject matter experts equipped to revise curriculum content Non-availability and outdated training modules tailored for specific disease elimination (rabies, leprosy).	Deficiency in subject matter experts for the development of comprehensive training manuals. Limited dissemination of clinical pathways (i.e., leprosy).
Stewardship and Finance	Scheduling conflicts. Unexplored territory (some vulnerable regions and endemic hotspots) and finding common ground. Limited coverage in PhilHealth benefit package.	Overlapping functions of oversight bodies. Establishing permanent representation. Hesitations on the level of commitment and decision-making processes. Insufficient funding for other diseases. Lack of mechanisms for fund pooling.
Research	Fragmented processes in initiating research studies and ecosystem.	Insufficient local research studies focusing on zoonotic diseases. Inadequate high-quality research activities relevant to diseases for elimination aligned with the research agenda.

**Table 9:** Corresponding recommendations for MDEP challenges and gaps.

**Challenges and Gaps**	**Recommendations**
Late reporting of cases Diseases for elimination are captured by different information systems in place Inadequate data sharing mechanisms among stakeholders	Adoption and implementation of a digital-based NTD surveillance information system and dashboard for real-time feedback loop system
Limited capacity for confirmatory testing Insufficient animal laboratories for confirmatory testing	Infrastructure building—internet provision, decentralization of confirmatory laboratories, establishment of more animal testing laboratories, WASH facilities
Non-availability and outdated training modules tailored for specific disease elimination	Update training modules tailored for specific disease elimination
Limited availability of qualified laboratory staff leading to non-specialized personnel performing laboratory tests Existing allied medical programs do not encompass all diseases targeted for eradication/elimination Scarcity of subject matter experts equipped to revise curriculum content Insufficient manpower for information management	Continuous capacity-building of health workforce, and other human resources involved in elimination and control programs from national to municipal level
Inadequate dissemination of information to intended audience leading to vaccine hesitancy	Community sensitization and mobilization towards participatory surveillance, and full integration of NTD elimination in primary health care
Inadequate budget allocation for dog rabies PrEP and PEP vaccines Limited coverage in PhilHealth benefit package	Sustainable financing, and resources allocation and mobilization
Under reporting of NTD cases due to low sensitivity of currently available screening methods	Research on feasible more sensitive and specific diagnostic methodologies
Other NTDs not yet on the health agenda	Incorporation of other endemic NTDs in the national health agenda and elimination plan
Lack of research activities relevant to diseases for elimination aligned with research agenda Insufficient local research studies focusing on zoonotic disease	Partnership and capacity-building with researchers, and funding allocation for research activities

## Data Availability

No new data was created in this study. Data sharing is not applicable to this article.

## Supplementary Materials

The following supporting information can be downloaded at: https://www.mdpi.com/article/10.3390/tropicalmed11040106/s1, Supplementary File S1: MDEP 2023-2030, Supplementary File S2: FHSIS 2020, Supplementary File S3: FHSIS 2021, Supplementary File S4: FHSIS 2022, Supplementary File S5: FHSIS 2023, Supplementary File S6: FHSIS 2024.

## Acronyms

ABTC	Animal Bite Treatment Center
CHD	Centers for Health Development
DOH	Department of Health
FHSIS	Field Health Services Information System
GIDAs	Geographically Isolated and Disadvantaged Areas
LF	Lymphatic Filariasis
LGU	Local Government Unit
LPEP	Leprosy Post-Exposure Prophylaxis
MDA	Mass Drug Administration
MDEP	Multi-Disease Elimination Plan
NGO	Non-Government Organization
NTD	Neglected Tropical Disease
PCT	Preventive Chemotherapy
PEP	Post-Exposure Prophylaxis
PHO	Provincial Health Officer
PIDSR	Philippine Integrated Disease Surveillance and Response
PSAC	Pre-School-Age Children
PrEP	Pre-Exposure Prophylaxis
SAC	School-Age Children
SCH	Schistosomiasis
STH	Soil-Transmitted Helminths
VPD	Vaccine-Preventable Diseases
WASH	Water, Sanitation, and Hygiene
WHO	World Health Organization

## References

[1] World Health Organization Global Report on Neglected Tropical Diseases 2024. https://www.who.int/teams/control-of-neglected-tropical-diseases/global-report-on-neglected-tropical-diseases-2024.

[2] World Health Organization Controlling and Eliminating Neglected Tropical Diseases. https://www.who.int/westernpacific/activities/controlling-and-eliminating-neglected-tropical-diseases.

[3] World Health Organization Global Report on Neglected Tropical Diseases 2025. https://www.who.int/publications/i/item/9789240114043.

[4] Lansang M.P.M. RITM probes into infectious and neglected tropical diseases in PH; highlights vaccination in SEA and employee satisfaction among HCWs. *Research Institute for Tropical Medicine*, 18 June 2024. https://ritm.gov.ph/ritm-probes-into-infectious-and-neglected-tropical-diseases-in-ph/.

[5] World Health Organization Programme Managers Meeting on Neglected Tropical Diseases in Asia Subregion. https://www.who.int/publications/i/item/RS-2018-GE-13-PHL.

[6] World Health Organization Strengthening Multi-Disease Elimination and Cross-Border Collaboration: Indonesia and Timor-Leste’s Collaborative Approach in Eliminating Malaria and Lymphatic Filariasis. https://www.who.int/southeastasia/news/detail/10-03-2025-strengthening-multi-disease-elimination-and-cross-border-collaboration--indonesia-and-timor-leste-s-collaborative-approach-in-eliminating-malaria-and-lymphatic-filariasis.

[7] United Nations Development Programme Sustainable Development Goals. https://www.undp.org/philippines/sustainable-development-goals.

[8] World Health Organization A Road Map for Neglected Tropical Diseases 2021–2030. https://www.who.int/publications/i/item/9789240010352.

[9] Mationg M.L.S., Tallo V.L., Williams G.M., Gordon C.A., Clements A.C.A., McManus D.P., Gray D.J. (2021). The Control of Soil-Transmitted Helminthiases in the Philippines: The Story Continues. Infect. Dis. Poverty.

[10] Gordon C.A., Acosta L.P., Gray D.J., Olveda R.M., Jarilla B., Gobert G.N., Ross A.G., McManus D.P. (2012). High Prevalence of *Schistosoma japonicum* Infection in Carabao from Samar Province, the Philippines: Implications for Transmission and Control. PLoS Negl. Trop. Dis..

[11] Wu H.-W., Qin Y.-F., Chu K., Meng R., Liu Y., McGarvey S.T., Olveda R., Acosta L., Ji M.-J., Fernandez T. (2010). High Prevalence of *Schistosoma japonicum* Infection in Water Buffaloes in the Philippines Assessed by Real-Time Polymerase Chain Reaction. Am. J. Trop. Med. Hyg..

[12] Tenorio J.C.B., Molina E.C. (2020). *Schistosoma japonicum* Infections in Cattle and Water Buffaloes of Farming Communities of Koronadal City, Philippines. Int. J. One Health.

[13] Peña K.D. World Leprosy Day: Seeking an end to age-old disease even as COVID attacks. *Inquirer*, 31 January 2022. https://newsinfo.inquirer.net/1547895/world-leprosy-day-seeking-an-end-to-age-old-disease-even-as-covid-attacks.

[14] Tankeh A.C., Mendoza A.M.B., Ramilo V.C. (2025). Demographic, Clinical, and Microbiological Profile and Compliance to Treatment of Newly Diagnosed Patients with Hansen’s Disease at a Tertiary Hospital and Sanitarium in Metro Manila: A 10-Year Review. Asian J. Public Health Pract..

[15] World Health Organization Soil-Transmitted Helminth Infections. https://www.who.int/news-room/fact-sheets/detail/soil-transmitted-helminth-infections.

[16] World Health Organization Soil-Transmitted Helminthiasis. https://www.who.int/data/gho/data/themes/topics/soil-transmitted-helminthiases.

[17] Tsheten T., Alene K.A., Restrepo A.C., Kelly M., Lau C., Clements A.C.A., Gray D.J., Daga C., Mapalo V.J., Espino F.E. (2024). Risk Mapping and Socio-Ecological Drivers of Soil-Transmitted Helminth Infections in the Philippines: A Spatial Modelling Study. Lancet Reg. Health-West. Pac..

[18] Coronado A.B., Galindon G.S., Ibabao D.D.A., Jose N.C.B.S., Dagamac N.H.A., Recopuerto-Medina L.M. (2025). Prevalence and Risk Assessment of Soil-Transmitted Helminths Among the Rice and Vegetable Farmers of Panay, Capiz, Philippines: A Cross-Sectional Study. Acta Parasitol..

[19] Delos Trinos J.P.C.R., Wulandari L.P.L., Clarke N., Belizario V., Kaldor J., Nery S.V. (2021). Prevalence of Soil-Transmitted Helminth Infections, Schistosomiasis, and Lymphatic Filariasis Before and After Preventive Chemotherapy Initiation in the Philippines: A Systematic Review and Meta-Analysis. PLoS Negl. Trop. Dis..

[20] Ladia M.A.J., Belizario V.Y., Lacuna J.M., Durano L.P., Alonte A.I. (2022). Schistosomiasis and Soil-Transmitted Helminthiasis Morbidity Control in Selected Communities in Eastern Visayas, Philippines: Post Haiyan. Acta Med. Philipp..

[21] Leonardo L., Hernandez L., Magturo T.C., Palasi W., Rubite J.M., De Cadiz A., Moendeg K., Fornillos R.J., Tabios I.K., Mistica M. (2020). Current Status of Neglected Tropical Diseases (NTDs) in the Philippines. Acta Trop..

[22] World Health Organization Lymphatic Filariasis. https://www.who.int/news-room/fact-sheets/detail/lymphatic-filariasis.

[23] Montemayor M.T. DOH: 44 of 46 identified provinces in PH already filariasis-free. *Philippine News Agency*, 14 July 2023. https://www.pna.gov.ph/articles/1205662.

[24] Tabilin E.J., Gray D.J., Jiz M.A., Mationg M.L., Inobaya M., Avenido-Cervantes E., Sato M., Sato M.O., Sako Y., Mu Y. (2025). Schistosomiasis in the Philippines: A Comprehensive Review of Epidemiology and Current Control. Trop. Med. Infect. Dis..

[25] Yao X., Chen Y., Chen K., Lin L., Zhong J., Shan C., Liu M., Chen X., Zhang Y., Li H. (2025). Prevention and Control of Schistosomiasis in the Philippines from a Health Education Perspective. Front. Public Health.

[26] Abeleda A.I., Lucero-Prisno D.E., Ogaya J.B. (2024). Addressing Schistosomiasis in the Philippines: Need for a Coordinated Intersectoral Effort. Asian Pac. J. Trop. Med..

[27] Tangcalagan D., Daga C., Tan A., Reyes R., Macalinao M.L., Mationg M.L., Alday P., Galit S., Luchavez J., Erce E. (2022). The 2013–2015 Nationwide Prevalence Survey of Soil-Transmitted Helminths (STH) and Schistosomiasis among School-Age Children in Public Schools in the Philippines. Pediatr. Infect. Dis. Soc. Philipp. J..

[28] Belizario V.Y., De Cadiz A.E., Navarro R.C., Flores M.J.C., Molina V.B., Dalisay S.N.M., Medina J.R.C., Lumangaya C.R. (2022). The Status of Schistosomiasis Japonica Control in the Philippines: The Need for an Integrated Approach to Address a Multidimensional Problem. Int. J. One Health.

[29] Chan J.M.P., Chan H., Flores M.C. (2021). Status of schistosomiasis in the Philippines: Prevalence, control and innovative methods for detection and elimination. DLSU Research Congress.

[30] Magalhães R.J.S., Salamat M.S., Leonardo L., Gray D.J., Carabin H., Halton K., McManus D.P., Williams G.M., Rivera P., Saniel O. (2014). Geographical Distribution of Human *Schistosoma japonicum* Infection in the Philippines: Tools to Support Disease Control and Further Elimination. Int. J. Parasitol..

[31] Leonardo L., Rivera P., Saniel O., Solon J.A., Chigusa Y., Villacorte E., Chua J.C., Moendeg K., Manalo D., Crisostomo B. (2015). New Endemic Foci of Schistosomiasis Infections in the Philippines. Acta Trop..

[32] Gong Y., Zhang S., Lin D., Cai Y., Lv S., Zheng M., Hu B., Lei X., Xu N., Wang J. (2025). Decoding the Impact of Environmental Shifts on Snail Density Dynamics in the Yangtze River Basin: A 26-Year Study. Parasites Vectors.

[33] Liu M.-M., Feng Y., Yang K. (2021). Impact of Micro-Environmental Factors on Survival, Reproduction and Distribution of Oncomelania Hupensis Snails. Infect. Dis. Poverty.

[34] Adekiya T.A., Aruleba R.T., Oyinloye B.E., Okosun K.O., Kappo A.P. (2019). The Effect of Climate Change and the Snail-Schistosome Cycle in Transmission and Bio-Control of Schistosomiasis in Sub-Saharan Africa. Int. J. Environ. Res. Public Health.

[35] Philippine Atmospheric, Geophysical and Astronomical Services Administration Climate of the Philippines. https://www.pagasa.dost.gov.ph/information/climate-philippines.

[36] Hong J., Agustin W., Yoon S., Park J.-S. (2022). Changes of Extreme Precipitation in the Philippines, Projected from the CMIP6 Multi-Model Ensemble. Weather Clim. Extrem..

[37] Smith C., Ortal A., Girasol M., Suzuki S., Coughlan C. (2024). Rabies in the Philippines: A Call to Action. Lancet Reg. Health-West. Pac..

[38] World Health Organization Rabies. https://www.who.int/news-room/fact-sheets/detail/rabies.

[39] Philippine Council for Health Research and Development Rabies-Free Philippines, Still Possible. https://www.pchrd.dost.gov.ph/news_and_updates/rabies-free-philippines-still-possible/.

[40] Mananggit M.R., Kimitsuki K., Saito N., Garcia A.M.G., Lacanilao P.M.T., Ongtangco J.T., Velasco C.R., Rosario M.V.D., Lagayan M.G.O., Yamada K. (2021). Background and Descriptive Features of Rabies-Suspected Animals in Central Luzon, Philippines. Trop. Med. Health.

[41] San Jose R., Magsino P.J., Bundalian R. (2019). Pet Owners’ Awareness on RA 9482 (Anti-Rabies Act of 2007) in Magalang, Pampanga Philippines. Heliyon.

[42] World Organization for Animal Health National Rabies Prevention and Control Program Stategic Plan 2020–2025. https://rr-asia.woah.org/app/uploads/2020/03/final-mtp-rabies_philippines.pdf.

[43] World Health Organization Leprosy. https://www.who.int/news-room/fact-sheets/detail/leprosy.

[44] Han X.Y., Seo Y.-H., Sizer K.C., Schoberle T., May G.S., Spencer J.S., Li W., Nair R.G. (2008). A New *Mycobacterium* Species Causing Diffuse Lepromatous Leprosy. Am. J. Clin. Pathol..

[45] Rahevar K., Morishita F., Oh K.H., Islam T. (2021). Epidemiological Review of Leprosy in WHO’s Western Pacific Region: 1991–2019. West. Pac. Surveill. Response.

[46] National Nutrition Council Leprosy Remains a Hidden, Persistent Problem in the Philippines. https://nnc.gov.ph/visayas-region/leprosy-remains-a-hidden-persistent-problem-in-the-philippines/.

[47] World Health Organization (2012). Global leprosy situation, 2012. Wkly. Epidemiol. Rec..

[48] Ramos A.J.O. Leprosy in the Philippines: History, Disease, and Challenges. *The Filipino Doctor, n.d.*. https://thefilipinodoctor.com/article/leprosy-in-the-philippines-history-disease-and-challenges.

[49] World Health Organization World Leprosy Day 2016: Tackling Stigma and Discrimination in the Philippines. https://www.who.int/philippines/news/feature-stories/detail/world-leprosy-day-2016-tackling-stigma-and-discrimination-in-the-philippines.

[50] Ricarte D.R., Cambronero J.M., Lorico C.H., Santos H.J., Arce N.S., De Cadiz A.E. (2025). First Report of *Isolapotamon* sp. as a Potential Intermediate Host of *Paragonimus westermani* in Davao Oriental, Philippines. Parasitologia.

[51] Paller V.G., Samudio J.A., Patagnan K.L., Santamaria L., Tolentino A.K., Ligalig C., Posa G.A., Amongo J.M. (2021). *Paragonimus westermani* Infection of Freshwater Crab *Sundathelphusa philippina* and Melaniid Snails in Cadacan River in Irosin, Sorsogon, Philippines. J. Parasit. Dis..

[52] Belizario V.Y., de Leon W.U., Bersabe M.J.J., Baird J.K., Bangs M.J. (2004). A Focus of Human Infection by *Haplorchis taichui* (Trematoda: Heterophyidae) in the Southern Philippines. J. Parasitol..

[53] Gordon C.A., Acosta L.P., Gobert G.N., Jiz M., Olveda R.M., Ross A.G., Gray D.J., Williams G.M., Harn D., Li Y. (2015). High Prevalence of *Schistosoma japonicum* and *Fasciola gigantica* in Bovines from Northern Samar, the Philippines. PLoS Negl. Trop. Dis..

[54] Moendeg K.J., Leonardo L.R., Isorena T.G., Hilotina F.C.A.S., Pates I.S., Cacayorin N.O. (2021). Prevalence and Spatial Distribution of Heterophyidiasis in Southern Philippines. Acta Trop..

[55] World Health Organization Yaws. https://www.who.int/news-room/fact-sheets/detail/yaws.

[56] Dofitas B.L., Kalim S.P., Toledo C.B., Richardus J.H. (2020). Yaws in the Philippines: First Reported Cases since the 1970s. Infect. Dis. Poverty.

[57] World Health Organization A Sustainability Framework for Action Against Neglected Tropical Diseases 2021–2030. Ending the Neglect to Attain the Sustainable Development Goals. https://www.who.int/publications/i/item/9789240019027.

[58] Co P.A., Vîlcu I., De Guzman D., Banzon E. (2024). Staying the Course: Reflections on the Progress and Challenges of the UHC Law in the Philippines. Health Syst. Reform.

[59] U.S. Embassy Manila Visiting USAID Official Highlights U.S.-Philippine Partnership in Health. https://ph.usembassy.gov/visiting-usaid-official-highlights-u-s-philippine-partnership-in-health/.

[60] Japan International Cooperation Agency Japan, Republic of Korea, United States Announce Partnership to Improve Health Outcomes in BARMM. https://www.jica.go.jp/english/overseas/philippine/information/press/2024/1552374_53492.html.

[61] Mupunga I., Dimech W., Izumi K., Rahevar K., Sanikullah K., Kelley J.F., Morishita F., Tran H., Yadav R.P. (2025). A Qualitative Evaluation of Access to Essential Laboratory Services for Communicable Diseases at the Primary Health Care Level in the Western Pacific Region. Trop. Med. Health.

[62] Tan B., De Vera P., Abrazaldo J., Ng C. (2025). Flood-Associated Disease Outbreaks and Transmission in Southeast Asia. Front. Microbiol..

[63] Philippine Council for Health Research and Development RabDash DC: Rabies Data Analytics Dashboard. https://www.pchrd.dost.gov.ph/heartnovation/rabdash-dc-rabies-data-analytics-dashboard/.

[64] Prakash Nayak P., Pai B.J., Govindan S. (2025). Leveraging Geographic Information System for Dengue Surveillance: A Scoping Review. Trop. Med. Health.

[65] Rysava K., Espineda J., Silo E.A.V., Carino S., Aringo A.M., Bernales R.P., Adonay F.F., Tildesley M.J., Hampson K. (2022). One Health Surveillance for Rabies: A Case Study of Integrated Bite Case Management in Albay Province, Philippines. Front. Trop. Dis..

[66] Uleb V.G., Talamayan J.T., Casas L.D., Villasenor J.M., Bacatan E. (2024). The Last-Mile Challenge: Water, Sanitation, and Hygiene (WASH) in the Philippines.

[67] Aagaard-Hansen J., Chaignat C.L. (2010). Neglected tropical diseases: Equity and social determinants. Equity, Social Determinants and Public Health Programmes.

[68] De Souza E.A., da Silva-Nunes M., dos Santos Malafronte R., Muniz P.T., Cardoso M.A., Ferreira M.U. (2007). Prevalence and Spatial Distribution of Intestinal Parasitic Infections in a Rural Amazonian Settlement, Acre State, Brazil. Cad. Saúde Pública.

[69] Hailu A., Gebre T., Seife F., Enbiale W., Tamiru A., Yohanes T., Yohannes A., Kitu M., Merdekios B., Kebede B. (2025). Challenges and Strategies for Mainstreaming Neglected Tropical Diseases Campaign Interventions in Ethiopia. Am. J. Trop. Med. Hyg..

[70] Dayapera L.Z.A., Sy J.C.Y., Valenzuela S., Eala S.J.L., Del Rosario C.M.I.P., Buensuceso K.N.C., Dy A.S., Morales D.A., Gibson A.G., Apostol G.L.C. (2024). One Health in the Philippines: A Review and Situational Analysis. One Health.

[71] Olveda R.M., Tallo V., Olveda D.U., Inobaya M.T., Chau T.N., Ross A.G. (2016). National Survey Data for Zoonotic Schistosomiasis in the Philippines Grossly Underestimates the True Burden of Disease within Endemic Zones: Implications for Future Control. Int. J. Infect. Dis..

[72] Wenlong Z., Tien N.H., Sibghatullah A., Asih D., Soelton M., Ramli Y. (2022). Impact of Energy Efficiency, Technology Innovation, Institutional Quality, and Trade Openness on Greenhouse Gas Emissions in Ten Asian Economies. Environ. Sci. Pollut. Res..

[73] Tan-Lim C.S.C., Javelosa M.A.U., Sanchez J.T., Dans L.F., Rey M.P., Elepano A.G., De Mesa R.Y.H., Dans A.L. (2024). Impact of Primary Care System Interventions on Healthcare Worker Satisfaction and Intention to Stay in the Philippines: A Follow-up Study. BMJ Open Qual..

[74] Ateneo de Manila University Ateneo study highlights workforce hurdles to Universal Health Care in the Philippines. *Ateneo de Manila University*, 22 May 2025. https://www.ateneo.edu/news/2025/05/22/ateneo-study-highlights-workforce-hurdles-universal-health-care-philippines.

[75] Stensland S.Ø., Bondjers K., Zwart J.-A., Rosseland L.A., Atar D., Christensen J.O., Matre D., Glad K.A., Wentzel-Larsen T., Wøien H. (2025). Development and Psychometric Validation of the Frontline Health Workers’ Occupational Risk and Characteristics in Emergencies Index (FORCE-Index)—The Covid Hospital Cohort Study. Public Health Pract..

[76] Hughes M.M., Groenewold M.R., Lessem S.E., Xu K., Ussery E.N., Wiegand R.E., Qin X., Do T., Thomas D., Tsai S. (2020). Update: Characteristics of Health Care Personnel with COVID-19—United States, February 12–July 16, 2020. MMWR Morb. Mortal. Wkly. Rep..

[77] Butala C.B., Cave R.N.R., Fyfe J., Coleman P.G., Yang G.-J., Welburn S.C. (2024). Impact of COVID-19 on the Neglected Tropical Diseases: A Scoping Review. Infect. Dis. Poverty.

[78] Evangelio F.K., Evangelio S.A., Macanan J.R., Lachica Z.P., Lagare A.P., Eng M.N., Sepulveda M.C., Baja E.S., Mata M.A. (2025). Effect of COVID-19, Vaccination Ratio, and Human Population on the Reported Canine Rabies Cases in Davao City, Philippines: A Panel Regression Analysis. Acta Med. Philipp..

[79] Nadal D., Beeching S., Cleaveland S., Cronin K., Hampson K., Steenson R., Abela-Ridder B. (2022). Rabies and the Pandemic: Lessons for One Health. Trans. R. Soc. Trop. Med. Hyg..

[80] Lufianti A., Mahanani S., Idris D.N.T. (2022). Stigma and Self-Concept of Leprosy Patients. Open Access Maced. J. Med. Sci..

[81] Pudasainee M., Paneru D.P., Acharya B., Sharma P., Khanal A., Rana B., Adhikari S., Adhikari C. (2025). Acceptability of Mass Drug Administration for Lymphatic Filariasis in Baglung Municipality of Nepal: A Mixed-Method Study. PLoS Glob. Public Health.

[82] Fornace K.M., Surendra H., Abidin T.R., Reyes R., Macalinao M.L.M., Stresman G., Luchavez J., Ahmad R.A., Supargiyono S., Espino F. (2018). Use of Mobile Technology-Based Participatory Mapping Approaches to Geolocate Health Facility Attendees for Disease Surveillance in Low Resource Settings. Int. J. Health Geogr..

[83] Amit A.M., Pepito V.C., Dayrit M. (2021). Early Response to COVID-19 in the Philippines. West. Pac. Surveill. Response.

[84] McPherson M., Counahan M., Hall J.L. (2015). Responding to Typhoon Haiyan in the Philippines. West. Pac. Surveill. Response.

[85] Kasparis E., Huang Y., Lin W., Vasilakis C. (2021). Improving Timeliness in the Neglected Tropical Diseases Preventive Chemotherapy Donation Supply Chain through Information Sharing: A Retrospective Empirical Analysis. PLoS Negl. Trop. Dis..

[86] Chua J.M.T., Capili J.T., Rabanal M.A.S., Mallabo L.A.C., Asuncion J.E.L. (2025). Towards a Drug Distributor Price Reference Index: A 2023 Survey of Pricing Discrepancies for Region 02 Medicines Procured from NCR and Local Distributors, Philippines. J. Appl. Pharm. Sci..

[87] Dimacali T.J. Gov’t to shut down Project NOAH. *GMA News Online*, 29 January 2017. https://www.gmanetwork.com/news/scitech/science/597540/gov-t-to-shut-down-project-noah/story/.

[88] Pfavayi L.T., Osakunor D.N.M., Chiombola C.E., Mutapi F. (2025). Identifying Gaps in the Care and Management of NTD Morbidity, Disability and Disfigurement in Africa. PLoS Negl. Trop. Dis..

[89] Tenorio J.C.B., Molina E.C. (2021). *Schistosoma japonicum* in the Philippines: Its Epidemiology, Diagnostics, Control, and Elimination. J. Agric. Res. Dev. Ext. Technol..

[90] Musuka H., Mano O., Iradukunda P.G., Pierre G., Munyonho F.T., Moyo E., Dzinamarira T. (2025). Global Health Development Aid Initiatives and the Quality of Medical Laboratory Services in Sub-Saharan Africa: A Narrative Review. Glob. Health J..

[91] Zhou X.-N., Bergquist R., Tanner M. (2013). Elimination of Tropical Disease Through Surveillance and Response. Infect. Dis. Poverty.

[92] Hietanen H., Pfavayi L.T., Mutapi F. (2025). Unlocking the Blueprint to Eliminating Neglected Tropical Diseases: A Review of Efforts in 50 Countries That Have Eliminated at Least 1 NTD. PLoS Negl. Trop. Dis..

[93] Belizario V.Y., Ampo S.A.M., Henderson K., de los Santos T., Gerth-Guyette E., de Guzman L.M., Lumangaya C., Siao T., Chua R.S.C.S. (2024). Surveillance of Soil-Transmitted Helminthiasis and Schistosomiasis in the Philippines: Review of Current Policies, Guidelines, and Practices. Southeast Asian J. Trop. Med. Public Health.

[94] Swedberg C., Miranda M.E.G., Bautista C., Anderson D., Basa-Tulio M., Chng N.R., Cruz V.D.D., Kundegorski M., Maestro J., Manalo D. (2023). Using Integrated Bite Case Management to Estimate the Burden of Rabies and Evaluate Surveillance in Oriental Mindoro, Philippines. One Health Implement. Res..

[95] Ortu G., Williams O. (2017). Neglected Tropical Diseases: Exploring Long Term Practical Approaches to Achieve Sustainable Disease Elimination and Beyond. Infect. Dis. Poverty.

[96] Belizario V.Y., Delos Trinos J.P.C.R., Lentejas N., Alonte A.J., Cuayzon A.N., Isiderio M.E., Delgado R., Tejero M., Molina V.B. (2021). Use of Geographic Information System as a Tool for Schistosomiasis Surveillance in an Endemic Municipality in Eastern Samar, the Philippines. Geospat. Health.

[97] Yuson M., Bautista C.T., Rees E.M., Bogaardt C., Cruz V.D.D., Durrant R., Formstone A., Manalo D.L., Manzanilla D.R., Kundergorski M. (2024). Combining Genomics and Epidemiology to Investigate a Zoonotic Outbreak of Rabies in Romblon Province, Philippines. Nat. Commun..

