# Supplementary material for: Neglected Tropical Diseases Elimination in the Philippines: Challenges and Gaps
Source: Trop Med Infect Dis. 2026 Apr 17;11(4):106. doi: 10.3390/tropicalmed11040106 (PMC13120366; doi:10.3390/tropicalmed11040106)
Supplement: Supplementary file 1 [file tropicalmed-11-00106-s001.zip › Supplementary File S1. MDEP Philippines 2023-2030.pdf]

PHILIPPINE MULTI-DISEASE ELIMINATION PLAN 2024-2030

Republic of the Philippines
Department of Health

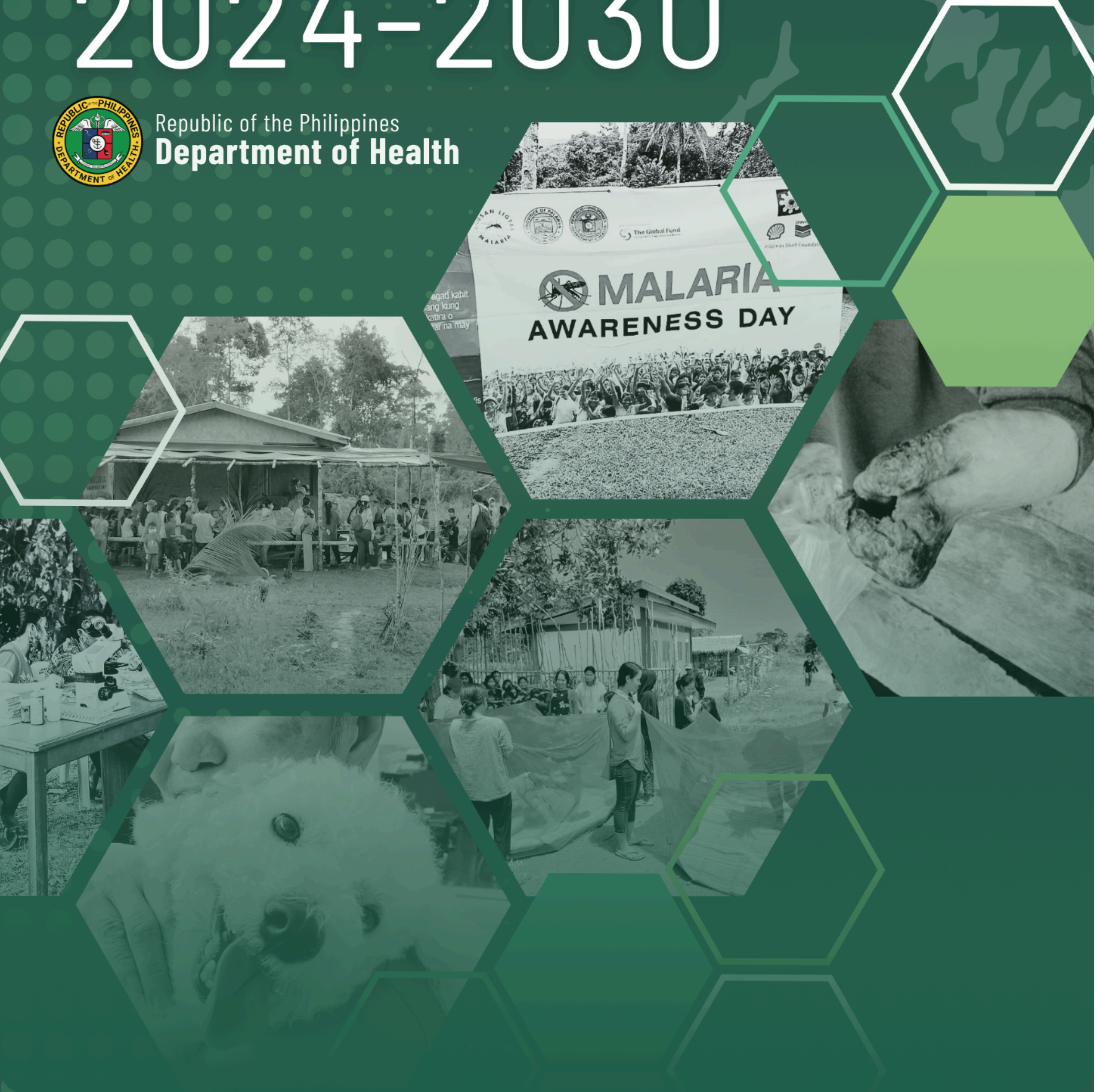

Philippine Multi-Disease Elimination Plan 2024-2030

© Republic of the Philippines – Department of Health 2023

Suggested citation. Philippine Multi-Disease Elimination Plan 2024-2030. Philippines: Department of Health. 2023

Table of Contents

ACKNOWLEDGEMENTS

ACRONYMS 4

ABOUT THE DOCUMENT 8

INTRODUCTION 9

VISION 16

GOALS 16

STRATEGIES AND MILESTONES 17

1. Surveillance and Information Systems 17

Component 1.1 A comprehensive, multi-disease surveillance and integrated programmatic HIS that can obtain data needed to estimate the burden of disease and determine risk factors of the diseases for eradication/elimination 18

Component 1.2 Engagement of all stakeholders, especially the private sector, in mandatory reporting of notifiable diseases and events 19

Component 1.3 Strengthen compliance of all disease reporting units (DRU) in reporting notifiable diseases to the national surveillance systems 20

2. Access to Laboratory Services 22

Component 2.1 Strengthening the public health laboratory network through the implementation of the Philippine Health Laboratory System 22

Component 2.2 Ensure quality assurance mechanisms are in place for laboratory services 24

3. Service Delivery 26

Component 3.1 Vaccination services 27

Component 3.2 Vector control 29

Component 3.3 Mass drug administration (MDA) / preventive chemotherapy (PCT) 31

Component 3.4 Intensified Case Management 32

Component 3.5 Ensure access to screening and diagnostic tests 33

Component 3.6 Health Care Provider Network 34

Component 3.7 Continuous Quality Improvement and Supportive Supervision 35

4. Safe and Quality Medicines, Vaccines, and Technology 36

Component 4.1: Facilitate the process of standard setting and Clinical Practice Guidelines development 36

Component 4.2 Ensure that regulatory and legal requirements on commodities are facilitated and enforced 37
Component 4.3 Ensure agile and responsive processes on the procurement of commodities 38
Component 4.4 Prepositioning and uninterrupted supply of high-quality medicines and other commodities at the facility through supply chain management 39
5. Human Resource and Capacity Building 41
Component 5.1 Align medical and allied health curriculum with the continuum of care of diseases for elimination 41
Component 5.2 Capability building and supportive supervision for primary care providers and facility-based health workers for effective delivery of services 42
6. Environment and Social Determinants of Health 44
Component 6.1 Improve health promotion activities directed towards diseases for elimination through social behavior change communication and demand generation activities 45
Component 6.2 Strengthen multisectoral collaboration and coordination at all levels to address common environmental and social determinants of health 46
7. Stewardship and Finance 48
Component 7.1 Stewardship through a multi-sectoral oversight committee 48
Component 7.2 Develop evidence-based policies 49
Component 7.3 Secure sufficient government and non-government financial resources in support of elimination strategies 50
8. Research 52
Component 8.1 Systematized identification of research needs 52
Component 8.2 Strengthening of research that supports new innovations supporting diseases for elimination and eradication 52
Component 8.3 Propose, recommend, and conduct quality and representative surveys, studies, and serosurveys based on international standard methodologies 53
MONITORING AND EVALUATION 55
BUDGET 57
REFERENCES 64

ACKNOWLEDGEMENTS

The Department of Health acknowledges all who contributed to the development of the Philippine Multi-Disease Elimination Plan including the Disease Prevention and Control Bureau leadership through Dir. Razel Nikka M. Hao, Dir. Jose Gerard B. Belimac, Dr. Raffy A. Deray and Dr. Kim Patrick S. Tejano. Special thanks goes to USAID's Act to End Neglected Tropical Diseases - East program implemented by RTI International, and the Global Fund through the Pilipinas Shell Foundation, Inc. and APMargin that all helped to facilitate the consultation workshops and provided technical assistance to the DOH in the development of this document. Gratitude is also extended to the following technical working group members: Dr. Maria Rosario Sylvia Uy, Ms. Sheen Angelou Juangco, Ms. Faye Yorainne Ebana, Ms. Camille Baladjay, Dr. Allan Fabella, Ms. Mary Joy Morin, Mr. Roland Sardan, Dr. Clarito Cairo, Jr., Mr. Vincent Sumergido, Mr. Gerald John Paz, Dr. Mara Jean Almazora-Millar, Dr. Ann Ysabel Andres, Dr. Janis Asuncion Bunoan-Macazo, Ms. Zenaida Recidoro, Ms. Dulce Elfa, Mr. Ken Borling, Dr. Diana Jean Vasquez, Ms. Princess Mhyco Esguerra, Dr. Roderick Poblete, Dr. Antonio Bautista, Mr. Ray Angluben, Ms. Kate Lopez, Ms. Veronica Vitug, Ms. Emmalyn Tugas and representatives from the Epidemiology Bureau, Office for Health Laboratories, Centers for Health Development, Research Institute for Tropical Medicine, University of the Philippines, Philippine Dermatological Society, Culion Foundation, World Health Organization and UNICEF.

ACRONYMS

AFP	Acute flaccid paralysis
AO	Administrative Order
APP	Annual Procurement Plan
ASEAN	Association of Southeast Asian Nations
BD	Birth dose
BOD	Burden of disease
CESU	City Epidemiology Surveillance Unit
CHD	Center for Health Development
CHED	Commission on Higher Education
CPAB	Child protected at birth
CPG	Clinical Practice Guidelines
CRS	Congenital rubella syndrome
CSR	Corporate social responsibility
cVDPV	Circulating vaccine-derived poliovirus
DA	Department of Agriculture
DA-BAI	Department of Agriculture - Bureau of Animal Industry
DILG	Department of Interior and Local Government
DO	Department Order
DOH	Department of Health
DPCB	Disease Prevention and Control Bureau
DPRI	Drug price reference index
DRU	Disease reporting unit
EB	Epidemiology Bureau
EDCS	Epidemic Prone Disease Case Surveillance
EMTCT	Elimination of mother-to-child transmission

EO	Executive Order
EREID	Emerging and Re-Emerging Infectious Diseases
ESR	Event-based surveillance and response report
FDA	Food and Drug Administration
FHSIS	Field Health Services Information System
G2D	Grade-2 disability
GIDA	Geographically isolated and disadvantaged areas
HBsAg	Hepatitis B surface antigen
HBV	Hepatitis B virus
HCPN	Health Care Provider Network
HFAP	Health for All Policies
HHRDB	Health Human Resource Development Bureau
HIB	Haemophilus influenzae type B
HIS	Health information system
HIV	Human Immunodeficiency Virus
HRH	Human resources for health
HSRA	Health Sector Reform Agenda
HTA	Health technology assessment
HTAC	Health Technology Assessment Council
IACEH	Inter-Agency Committee on Environmental Health
IRS	Indoor residual spraying
KMITS	Knowledge Management and Information Technology Service
LB	Live birth
LDIs	Learning and development interventions
LF	Lymphatic Filariasis
LLIN	Long-lasting insecticide net
LPEP	Leprosy post-exposure prophylaxis
LQMS	Laboratory quality management system

MAH	Marketing Authorization Holder
MCV	Measles containing vaccines
MDA	Mass drug administration
MDEP	Multi-Disease Elimination Plan
MDT	Multi-Drug Therapy
MESU	Municipal Epidemiology Surveillance Unit
MMR	Measles, Mumps, and Rubella
MNTE	Maternal and Neonatal Tetanus
MTCT	Mother-to-Child Transmission
NEQAS	National External Quality Assessment Scheme
NPG	National Practice Guidelines
NRLs	National Reference Laboratories
OHG	Omnibus health guidelines
OHL	Office for Health Laboratories
PCPN	Primary Care Provider Network
PCT	Preventive chemotherapy
PEP	Post-exposure prophylaxis
PESU	Provincial Epidemiology Surveillance Unit
PHC	Primary Health Care
PHE	Public Health Emergencies
PhilCZ	Philippine Inter-Agency Committee on Zoonoses
PhilGEPS	Philippine Government Electronic Procurement System
PhilHealth	Philippine Health Insurance Corporation
PHLS	Philippine Health Laboratory System
PHTLs	Provincial Health Team Leads
PIDSR	Philippine Integrated Diseases Surveillance and Response
PIR	Program implementation review
PrEP	Pre-exposure prophylaxis

QA	Quality assurance
QI	Quality improvement
QMS	Quality management system
RA	Republic Act
RESU	Regional Epidemiology Surveillance Unit
RITM	Research Institute for Tropical Medicine
RPHLs	Regional Public Health Laboratories
SCH	Schistosomiasis
SCM	Supply chain management
SDG	Sustainable Development Goals
SDR	Single-dose rifampicin
SHF	Special Health Fund
SNLs	Subnational Reference Laboratories
STAG	Scientific and Technical Advisory Group
TAS	Transmission assessment survey
TWG	Technical Working Group
UHC	Universal Health Care
UNICEF	United Nations Children's Fund
UNOPS	United Nations Office for Project Services
USAID	US Agency for International Development
VPDs	Vaccine Preventable Diseases
WHO	World Health Organization

ABOUT THE DOCUMENT

Equitable use of limited resources is a challenge faced by public health programs including funding and human resources for health. By integrating health system components as an opportunity to address the problem, the Disease Prevention and Control Bureau (DPCB) integrated the strategies of selected vaccine preventable and infectious diseases to streamline efforts on disease elimination through the first Philippine Multi-Disease Elimination Plan (MDEP). The plan was developed by disease experts and health systems strengthening specialists through a series of consultations and workshops from July 2022 to July 2023.

The plan covers the years 2024-2030, in line with the Sustainable Development Goals (SDG). It shall be reviewed and updated periodically every three years by a technical working group (TWG), guided by a high level scientific and technical advisory group (STAG). The plan shall be used by implementers as a guide for planning and evaluation. Partners, researchers, the academe, and other interested individuals & organizations may use the document to understand the Department of Health's priority areas for support.

INTRODUCTION

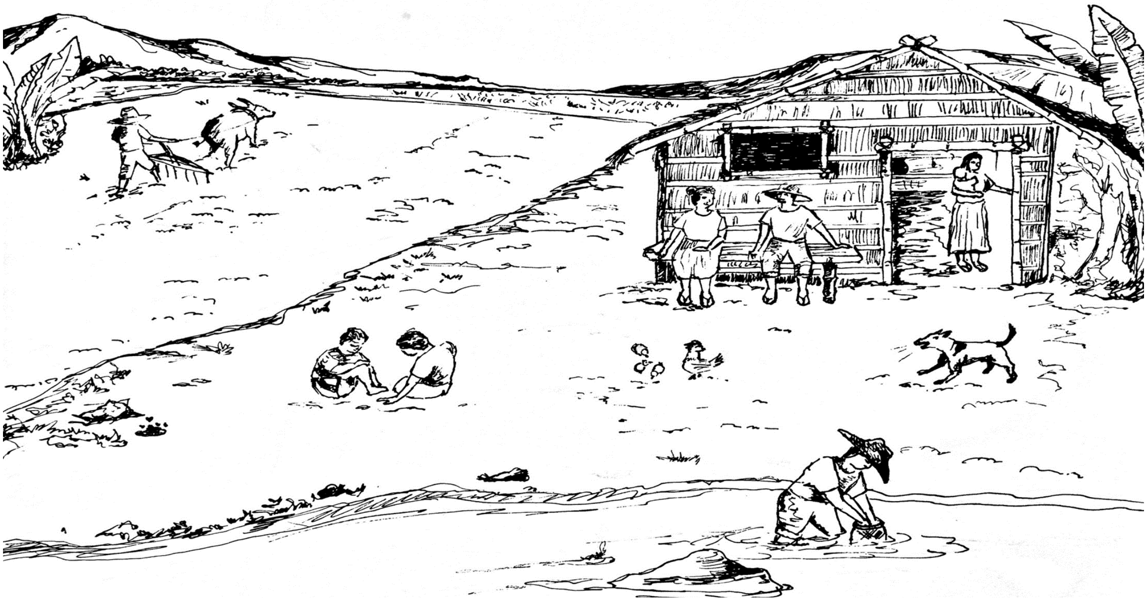

Disease elimination supports the attainment of SDG 3, of ensuring healthy lives and promoting well-being for all at all ages by reducing global maternal and neonatal mortality ratio through the (i) elimination of mother-to-child transmission of human immunodeficiency virus (HIV), Syphilis, and Hepatitis B, (ii) ending the epidemics of neglected tropical diseases and other communicable diseases, and (iii) achieving universal health coverage through financial risk protection, access to quality essential health care services, access to safe, effective, quality, and affordable essential medicines and vaccines for all.

There are challenges in eliminating these diseases in a devolved health care system set up. Many efforts were initiated including the development of the Health Sector Reform Agenda (HSRA), adoption of Primary Health Care (PHC) approach, the creation of the Universal Health Care (UHC) Law, and the latest with the issuance of the Department of Health's (DOH) 8-Point Agenda, not only for diseases for elimination but for other public health programs as well.

National public health programs of the DOH were historically organized by individual diseases, resulting in varied levels of success. In recent years, the DOH started exploring technical, managerial, and financial integration through a life stage approach, strengthening the coordination with other sectors such as environmental and animal health and recommending expansion on the inclusion of the primary care benefit package. The Department of Health is eager to test a new approach through an integrated service delivery framework that addresses the convergence of multiple

diseases. This approach strategically groups related diseases and identifies areas of potential collaboration resulting in a comprehensive service package. This optimizes the allocation of essential human, material and financial resources, ensuring their efficient utilization.

In 2022, the Disease Prevention and Control Bureau (DPCB) extensively reviewed 36 diseases for elimination and identified 13 priority diseases based on a set of criteria that included the review of disease burden and discussion on the feasibility of elimination or significant reduction of disease burden by 2030.

Table 1. MDEP diseases and 2022 status

Disease	Status as of 2022
**Leprosy**	Following global treatment protocols, the program adapted the multi-drug therapy (MDT) which resulted in the downward trend of prevalence rate from 7.2/10,000 cases in 1986 to 0.9/ 10,000 cases in 1998; leading to elimination of leprosy as a public health problem. However, the Philippines remains as number one for leprosy in the Association of Southeast Asian Nations (ASEAN) up to today.
**Lymphatic Filariasis (LF)**	In 2022, 44 out of the 46 provinces have stopped mass drug administration. The two remaining provinces are Sultan Kudarat and Zamboanga del Norte.
**Malaria**	In 2022, 81 out of 82 provinces reported zero indigenous cases and 66 provinces declared as Malaria free. Palawan is the only province reporting active transmission with 32% of its barangay (95 out of 292) with indigenous cases.
**Maternal and Neonatal Tetanus (MNT)**	By 2017, the Philippines achieved its validation for MNT Elimination. However, child protected at birth (CPAB) from tetanus coverage declined from 86% in 2018 to 60% in 2019 and 2020 which further declined in 2021 to 54%. The number of neonatal tetanus cases increased from 14 cases in 2021 to 33 cases in 2022.
**Measles**	In 2009, the immunization program implemented a two-dose measles containing vaccine: MCV 1 (monovalent measles at 9-11 months old) and MCV 2 (MMR at 12-15 months). Surveillance for measles and rubella cases is in place. Cases are reported regularly through the Epidemic Prone Disease Case Surveillance (EDCS). Despite the initiatives, data for routine measles coverage in the past 10 years showed the achievement below the 95% coverage target and the number of measles cases in 2022 is 370.

**Mother-to-Child Transmission (MTCT) of HIV, Syphilis, and Hepatitis B**	Out of the mothers screened from 2019-2022, the proportion of those positive for syphilis and hepatitis B ranges from 3%-6%. Screening of pregnant women for HIV also started in 2019. The accomplishment for Hepatitis B birth dose remains to be below 75% from 2011-2022.
**Poliomyelitis**	There is an 11% decline in the number of acute flaccid paralysis (AFP) cases reported in 2022 (580 AFP cases) as compared to the 2021 report. The Philippines was certified polio free in October 2000 and remains polio free until 2018. However, a vaccine derived poliomyelitis outbreak occurred in 2019.
**Rabies**	In the Philippines, a law was passed (RA 9482 also known as Anti-Rabies Act of 2007) to accelerate the control and elimination of human and animal rabies. The law mandates that a National Rabies Prevention and Control Program should be implemented through a multi-sectoral/multi-agency and chaired by the Department of Agriculture. Dogs are the principal reservoir of rabies in the Philippines. Animal Bite Treatment Center had been set up in strategic areas. These centers provide post exposure prophylaxis (human anti-rabies vaccine and Immunoglobulin). Individuals working in high-risk environments are given pre-exposure prophylaxis (PreP). A significant decline in the number of human rabies cases reported was noted in 2020. In 2022, Region 9 (51 cases), Region 6 (29 cases) and Region 11 (19 cases) reported the greatest number of cases. A total of 284 deaths due to rabies were reported in 2022.
**Rubella**	Immunization coverage below 95%, rubella cases below 100
**Schistosomiasis (SCH)**	An assessment of the program that covers the period of 2011-2017 showed that 12 regions, 28 provinces, 190 municipalities, 20 cities and 1,609 barangays are endemic to schistosomiasis. Focal survey showed a prevalence of 4% with 302 barangays with zero prevalence, 222 barangays with low prevalence, 435 barangays with moderate prevalence and 479 barangays with high prevalence.

**Yaws**	In 2017, the Philippines was declared the 14th country endemic for yaws. Information on the existence of Yaws in the Philippines came from 2 studies:<br>1) A cross sectional survey in elementary school in Liguasan Marsh done in 2017; 2) a case detection survey in Luzon and Visayas Island. The cross-sectional survey detected 4 children aged 5-10 years and confirmed secondary Yaws. Majority of serologically reactive cases (n=10) were adults without active yaws skin lesions (8 latent cases, 2 past/treated cases). The case detection survey was conducted in 5 remote villages (3 in Luzon and 2 in Visayas). Two indigenous communities were included in the study: Aetas of Quezon and Dumagat of Rizal. The study detected 19 cases among the 35 Aetas: 5 active cases (4 children, 1 adult); 2 latent cases (1 adult); 12 past cases (1 child, 11 adults) . Currently, Yaws is not included in the surveillance system. There is no program instituted for the prevention and control of Yaws.

The Department of Health aims to significantly reduce the burden of these diseases and achieve elimination by 2030, so alignment of activities among key stakeholders and standardization of the indicators of success were considered in the development of this plan.

Table 2. MDEP diseases and targets

Disease	Local Elimination Target	International Elimination Target
**Leprosy**	Zero new autochthonous cases	120 countries with zero new autochthonous cases<br><br>70% reduction in annual number of new cases detected<br><br>90% reduction in rate per million population of new cases with grade-2 disability (G2D)<br><br>90% reduction in rate per million children of new child cases with leprosy
**Lymphatic Filariasis**	National: All endemic provinces passed TAS 3<br><br>Sub-national: Endemic provinces passed TAS 1	Number of countries (58 =81%) validated for elimination as a public health problem (defined as infection sustained below transmission assessment survey threshold for at least four years after stopping mass drug administration availability of essential

		package of care in all areas of known patients)
**Malaria**	Zero Indigenous Malaria Cases for at least 5 years in all provinces<br><br>Sub national declaration	Reduce malaria mortality rates globally by at least 90% as compared with 2015<br>Reduce malaria case incidence globally by at least 90% as compared with 2015<br><br>Eliminate malaria from countries with transmission (35 countries)<br><br>Prevent re-establishment of malaria in all countries that are malaria free
**Maternal and Neonatal Tetanus**	<1 NT case per 1,000 live births per year in every province/city	Achieve MNT elimination (defined as <1 neonatal tetanus case/1000 live births in each district) in the Region; and<br><br>Maintain MNT elimination in every country and area.
**Measles**	Absence of endemic measles transmission in the country for ≥12 months in the presence of a well-performing surveillance system	Zero incidence of measles due to endemic measles virus infection
**Mother-to-Child Transmission of HIV, Syphilis, and Hepatitis B**	0% of HIV-exposed infants born in the past 12 months who are infected with HIV<br><br>< 50 neonatal syphilis per 100,000 live birth (LB)<br><br>HBV EMTCT: ≤0.1% HBsAg prevalence in <5-year-olds;<br><br>< 2% MTCT rate (where use of targeted HepB-BD)<br><br>Hepatitis B birth dose immunization coverage: > 95%	0% of HIV-exposed infants born in the past 12 months who are infected with HIV<br><br>Number of congenital syphilis cases per 100 000 live births per year = < 50 (2030)<br><br>Hepatitis B surface antigen (HBsAg) prevalence of 0.1% among children younger than 5 years old<br><br>Percentage of newborns who have benefitted from a timely birth dose of hepatitis vaccine and from other interventions to prevent the vertical (mother-to-child) transmission of hepatitis B virus

**Poliomyelitis**	Zero incidence of polio from any type of polio virus	Permanently interrupt all poliovirus transmission in endemic areas<br><br>Stop cVDPV transmission and prevent outbreaks in non-endemic countries<br><br>Zero incidence of polio due to any type of poliovirus infection (regional target)
**Rabies**	Zero indigenous human mediated rabies and dog rabies for at least 3 years	Zero human dog-mediated rabies deaths
**Rubella**	Absence of endemic rubella transmission in the Philippines for ≥12 months<br><br>Absence of congenital rubella syndrome (CRS) cases associated with endemic transmission in the presence of a well-performing surveillance system	Zero incidence of rubella due to endemic virus infection;<br><br>Zero cases of domestically acquired CRS.
**Schistosomiasis**	< 1% proportion of heavy intensity schistosomiasis infection<br><br>zero snail infection rate	Number of countries (78) validated for elimination as a public health problem (currently defined as < 1% proportion of heavy intensity schistosomiasis infection)
**Yaws**	Zero new autochthonous cases	194 countries (100%) certified free of transmission

The MDEP supports the DOH's sectoral primary health care strategic plan for 2023-2028 and the integrated disease prevention and control through primary care strategic plan 2023-2028. Shown below are the areas of alignment:

	SECTORAL PRIMARY HEALTH CARE STRATEGY 2023-2028			INTEGRATED DISEASE PREVENTION AND CONTROL THROUGH PRIMARY CARE STRATEGIC PLAN 2023-2028			
MDEP STRATEGIC PILLAR	Healthy and Safe Policies & Settings	Accessible, Comprehensive Primary Care for every life stage	Evidence and Data-in- formed Response at All Levels	Quality	Access	Self-sufficient Primary Care	Enabling Mechanism
Surveillance and Information Systems		✔		✔	✔		
Access to Laboratory Services		✔		✔	✔		
Service Delivery		✔		✔	✔		
Safe and Quality Medicines, Vaccines, and Technology		✔		✔	✔		✔
Human Resource and Capacity Building		✔		✔			✔
Environment and Social Determinants of Health	✔						✔
Stewardship and Finance		✔		✔	✔	✔	✔
Research		✔		✔			✔

VISION

Philippines with zero/reduced number of new infections of priority diseases for elimination through an effective health care system by 2030

GOALS

Goal 1: Eradication
Maintain zero indigenous case of polio and contribute to the global eradication of the disease.

Goal 2: Elimination of Infection
Achieve zero indigenous case of yaws, measles, rubella, malaria, leprosy and rabies.

Goal 3: Elimination of Disease as a Public Health Problem
Reduce the number of new infections of lymphatic filariasis, schistosomiasis, mother-to-child transmission of HIV, syphilis, and hepatitis B, and maternal and neonatal tetanus below the threshold of being considered a public health problem.

STRATEGIES AND MILESTONES

1 Surveillance and Information Systems

Box 1: Challenges/Gaps

- Legal mandates for reporting notifiable diseases does not include some of the diseases for eradication/elimination leading to data quality issues like late reports; incomplete reports. RA 11332 (Mandatory Reporting of Notifiable Diseases and Health Events of Public Health Concern) covers five priority diseases for eradication or elimination (poliomyelitis, measles, neonatal tetanus, rabies, malaria); AO 2021-0057 (Revised PIDSR Guidelines) does not specify certain diseases for elimination
- Data needs of diseases for elimination not in the present surveillance and information system – congenital syphilis, CRS, yaws
- Post validation surveillance for filariasis, and schistosomiasis not in place
- Diseases for elimination are captured by different information systems in place and may lead to inconsistency in the data reported, and double reporting
- Under reporting of cases are sometime observed (e.g. leprosy)
- Need to strengthen coordination across the human and animal disease surveillance and information system, case investigation (rabies, schistosomiasis)
- Monitoring system for non-canine rabies, and for syphilis not in place (case-based surveillance system for syphilis)
- Need for capacity development for core surveillance processes
- Inadequate proper maintenance of existing IS leading to weak functionality of systems (iClinicSys)
- Insufficient manpower to perform core function in IS management
- Inadequate utilization of the IS at the local level
- Inadequate data sharing mechanism among stakeholders for all priority diseases

Disease surveillance is the continuing, systematic collection, management, analysis, interpretation and timely dissemination of health-related data (human, animal and environmental) to enable planning, implementation and evaluation of disease control and prevention measures. Because the goal is to eradicate or eliminate these 13 infectious diseases, it is important to detect suspected cases early and track importation of cases (local and international) from endemic to non-endemic areas (re-emergence/re-introduction); investigate and validate reported suspect cases for immediate actions to mitigate the spread of infection; and measure trends and characterize the diseases for eradication/elimination. Surveillance data will illuminate changes in infectious and environmental agents which can be used in directing eradication/ elimination strategies. The Epidemiology Bureau (EB) leads and provides technical direction and guidance for all surveillance and response activities in the Philippines. At present, the country implements the Philippine Integrated Disease Surveillance and Response (PIDSR) framework, which encompasses Epidemic Prone Disease Case Surveillance (EDCS) and Event Based Surveillance and Response (ESR), monitoring both notifiable disease and other health related events of public health importance, to guide the implementation at all levels of the health care delivery system

in both public and private sectors. The EB is currently working on the preparations to implement the proposed reporting flow to eliminate the ladderized reporting of cases with the aim of having information at all levels as soon as they are encoded in the system, regardless of its entry point.

As cited in DOH Administrative Order No. 2018-0028 (Guidelines for the Inclusion and Delisting of Diseases, Syndromes, and Health Events in the List of Notifiable Diseases, Syndromes and Health Events of Public Health Concern), the current list of notifiable diseases shall undergo periodic assessment for inclusion or exclusion from the list based on the criteria set by the Technical Advisory Group for the Inclusion and Delisting for Notifiable Diseases, Syndromes, and Health Events of Public Health Concern (TAG-NDEPH).

In addition to disease surveillance, other programmatic health information systems (HIS) provide data needed to measure processes and activities critical to disease eradication and elimination such as human immunization coverage for vaccine preventable diseases. Data for immunization coverage for vaccine preventable diseases are being reported to the Field Health Services Information System (FHSIS). Various programmatic HIS which may lead to inconsistency of data reported by the different HIS, and too many HIS being implemented at the local level burden the implementers collecting the data.

On the animal health side, the Philippine Animal Health Information system, under the Department of Agriculture - Bureau of Animal Industry (DA-BAI), covers information on diseases and data for regulatory purposes. It mirrors the World Animal Health information system.

Component 1.1. A participatory, digitally transformed, and action-oriented system for public health surveillance to trigger timely and effective public health response

Vigilance in detecting all possible and suspect cases is important in disease eradication and elimination, but prompt action on confirming and implementing control measures to prevent spread or reintroduction of these diseases must follow detection. A surveillance and response framework provides direction for local health units on the flow of case investigation and response for detected suspect cases. Border control guidelines covering air, land, and sea transport systems both locally and internationally should be implemented at the local level to prevent re-introduction of cases in disease free areas. Proficient contact tracing systems should identify exposed and at-risk individuals. For zoonotic diseases, joint case investigation and data sharing with the DA-BAI and DENR at the local level should lead to holistic investigation of environmental, human and animal factors. Capacity building of local health staff on surveillance, data management and response action on each of the 13 diseases is

important. Provincial and regional epidemiology surveillance units (PESU/RESU) may provide technical support to the municipal and city epidemiology surveillance units (MESU/CESU) during investigation and response.

Desired Outcome

Responsive, participatory, and localized surveillance system for data needs and enables quick action at a local level

Milestones

2024

1. Inclusion of all thirteen diseases in the list of notifiable diseases and events of public health concern (NDEPH)
2. Veterinary Public Health Unit established within DOH

2025

1. Cadre-based training of public health workers to include use of surveillance data in public health decision-making
2. Inclusion of animal and environmental data for priority diseases in surveillance system dashboards

Component 1.2 Engagement of all stakeholders, especially the private sector, in mandatory reporting of notifiable diseases and events

Timely, complete, and accurate data from all disease reporting units is essential. All disease reporting units should be equipped to collect and report data for eradication and elimination as mandated by Republic Act (RA) 11332 on notifiable disease reporting and DOH Administrative Order (AO) 2021-0057 on the revised PIDSR guidelines. However, not all 13 diseases are being reported in the existing surveillance system. Also, RA 11332 and AO 2021-0057 did not include the following priority disease – Leprosy, Rabies, Filariasis, Schistosomiasis, MTCT – Hepatitis B, Syphilis and HIV in the mandatory reporting of notifiable diseases. With the on-going revision on PIDSR, these priority diseases should be included in the case-based surveillance or ESR to enhance the timely reporting and compliance of disease reporting units. At the Regional level, advocacy should be conducted to local government units on passing a resolution on mandatory reporting of the priority diseases. At the local level, strengthening of local Epidemiology and Surveillance Units shall improve the capacity of the system to receive, process, and flag signals related to the priority diseases. In addition to a policy endorsement of mandatory reporting, hardware and software resources should be in place in every disease reporting unit to ensure timely reporting. Human resource augmentation and capacity building for data management should be in place at the national, regional, and provincial levels. Logistic resources for a digitized system

(hardware, maintenance of the system) of reporting may be a burden at the local level due to limited resources. A regulatory mechanism can be explored to enforce compliance and maintenance of the HIS.

To maximize the use of participatory surveillance and to widen the scope of surveillance, engagement with communities, settings such as schools, workplaces, and correctional facilities, points of entry, and the private sector is paramount as an incentive mechanism to
complement enforcement of sanctions for failure to report.

Desired Outcome

100% functional and compliant DRUs in reporting of notifiable diseases for elimination

Milestones

2025

Joint DOH-DA-DILG Administrative Order and Operations Manual on integrated health information system disseminated to all 17 Centers for Health Development

2026 - 2030

100% of RESUs and PESUs / HUC ICC CESUs attaining targets for reporting rate for non-measles, non-rubella, and non-acute flaccid paralysis by 2030

Component 1.3 A comprehensive public health data strategy and information architecture that allows timely access and use of high-quality information (including burden of disease, risk factors, and supply chain management) for public health action

A multi-source (vector, human, animal health, environmental) and digitally transformed disease surveillance system should be in place to generate reliable information for the burden of disease and risk factors of priority diseases for elimination. Although various surveillance and information systems capture the 13 diseases for eradication and elimination, they only focus on human health data. EB is undergoing reassessment and revision of the public health surveillance framework and strategy. Priority activities under this include revision of the Philippine Integrated Diseases Surveillance and Response (PIDSR) framework, on-going changes in the surveillance information system

to add other diseases for elimination, and continuing capacity development for burden of disease (BOD) estimation.

Currently, there are two existing Inter-agency Committees, the Philippine Inter-Agency Committee on Zoonoses (PhilCZ) and the Inter-Agency Committee on Environmental Health (IACEH). Lessons learned in inter-agency linkages can be adapted in developing a multi-source disease surveillance. Managing different programmatic HIS requires resources (human and logistics) which can lead to delayed reports, data quality checks not done, and electronic systems not working at the facility level. EB and the Knowledge Management and Information Technology Service (KMITS) of the Department of Health is working on integrating the different programmatic HISs and ensuring functionality of the system at the local level. Additionally, to ensure that all data needed for elimination indicators are captured, the Epidemiology Bureau in coordination with DPCB is responsible for outlining all data needs of diseases for eradication/ elimination.

Desired Outcome

In the strengthening of disease surveillance and epidemic response, the EB aims to ensure that surveillance systems are digitally transformed while allowing a variety of data collection methods to cover areas with poor internet access

Milestones

2024

1. 1 dissemination forum on the result of the disease surveillance and health information system desk review with recommendations
2. Joint DOH-DA-DILG Administrative Order and Operations Manual on integrated health information system

2026

Multi-source surveillance platform designated (human, vector, animal, and environment)

2027

MDEP surveillance data used for immediate response and annual planning and budgeting and the years after

2 Access to Laboratory Services

Box 2: Challenges/Gaps

- Limited capacity for timely confirmatory testing for polio, measles, and rubella
- Insufficient animal laboratories for confirmatory testing of rabies
- Sustainability of the Subnational Laboratories for VPDs
- Other diseases for elimination need quality assessment and quality improvement systems

In disease eradication/elimination, timely collection, submission, processing and releasing of results is important since rapid disease detection mitigates transmission by instituting timely control actions. Thus, diagnostic services should be available and accessible to patients regardless of geographical location and financial capacity. DOH Department Order (DO) 2021-0421 creates the Office for Health Laboratories (OHL) which provides the overall direction, policies, programs, and plans including infrastructure, equipment, supplies and investments in the development of the Philippine Health Laboratory System (PHLS). In relation to its support to the MDEP, the gaps in Box 2 will be addressed through two components of this strategic pillar.

Component 2.1 Strengthening the public health laboratory network through the implementation of the Philippine Health Laboratory System

The Philippine Health Laboratory System is the overall system set up to deliver quality clinical and public health laboratory services in a timely, sustainable, and efficient manner to support the objectives of the Universal Health Care Act and respond to future public health emergencies. The PHLS Framework shall provide strategic direction, plans, policies, programs, and standards for the public health and clinical laboratories. The PHLS Framework shall strengthen the National Health Laboratory Network. Within this framework, laboratories are categorized into:

1. National Reference Laboratories (NRLs), by virtue of DO 2020-0820, the highest level of laboratory in the country performing complex procedures including end confirmatory testing not commonly performed by the lower level of laboratory. NRLs also train laboratory personnel and perform technical evaluation of In-Vitro Diagnostic Medical Devices. NRLs are the responsible entity for facilitating the National External Quality Assessment Scheme (NEQAS) to ensure compliance of quality standards for regulation and licensing of all laboratories in the Philippines.

2. Subnational Reference Laboratories (SNLs) are reference laboratories with a geographic subnational catchment area that perform complex procedures, including selected confirmatory testing, surveillance, research, training, and roll-out of Laboratory Quality Assurance Programs within their catchment areas.

3. Regional Public Health Laboratories (RPHLs) are laboratories providing research and limited diagnostic services for communicable and non-communicable diseases or other conditions of public health importance within their regional catchment.

The MDEP includes plans for the sustainability of Vaccine Preventable Diseases (VPD) Referral Laboratories that will help monitor and confirm VPDs, as well as shorten turn-around times for specimen transport.

Desired Outcome

Diseases for elimination are incorporated in the public health laboratory network through the implementation of the Philippine Health Laboratory System

Milestones

2024

1. Construction of Sub-National laboratories initiated (target is 3 years construction)
2. Sustainability plan for Vaccine Preventable Diseases (VPD) Referral Laboratories developed including human resource, training, and budget plans

2025

Implementation of the sustainability plan for VPD Referral Laboratories

2027

Training of selected laboratory personnel of SNL

2028

1. 100% of SNLs have the capacity to isolate/confirm priority diseases for elimination
2. Policies on cross-linking/collaboration and referral mechanism on services of VPD Referral Laboratories with the established SNL's under the Center for Health Development (CHD) have been developed

2029

100% of CHDs have Regional Public Health Laboratories

2030

PHLS institutionalized

Component 2.2 Ensure quality assurance mechanisms are in place for laboratory services

Laboratory quality can be defined by the accuracy, reliability, and timeliness of test results.   Errors in diagnosis may lead to unnecessary expenditures of repeated tests, loss of patients' and staff time, and mismanagement of patients. Processes involved in testing can be categorized into: pre-examination—selection of appropriate test based on clinical symptoms, specimen collection and transport; examination—processing of specimen; and post-examination—analysis and report (test result) release and record keeping. Each process should be carried out according to a quality management system. The essential components of a quality system in laboratories are a) management commitment and quality policy; b) quality standards; c) training of human resources; d) documentation; and e) assessment and accreditation. Although quality assurance (QA) and quality improvement (QI) processes are already in place for malaria and MTCT diseases, OHL together with WHO is developing the quality management system (QMS) for the national reference laboratories that will ensure the QI process covers other diseases for eradication/elimination. In addition, OHL prioritizes the following interventions to ensure implementation and maintenance of quality assurance mechanisms: 1) capacity development on QA/QI processes; 2) reinforcement of training for NRLs and capacitate SNLs in quality assurance; and 3) conduct proficiency testing for PHLS.

Desired Outcome

Accurate and timely laboratory confirmation and reporting, together with genotype information (for all applicable diseases)

Milestones

2024

1. Laboratory Quality Management System (including QA/QI) for identified diseases for elimination developed
2. Assessment of the whole quality of laboratory (LQMS)

2025

Reinforcement of training for NRLs and capacitate SNL in QA/QI

2027

Pilot implementation of Quality Assurance Programs (e.g proficiency testing) for selected laboratories in the PHLS

2028

Actual implementation of Quality Assurance Programs for all laboratories in the PHLS

Box 3: Challenges/Gaps

In the context of immunization services, challenges have been identified that hinder optimal immunization coverage. These challenges encompass:

- Inadequate dissemination of information to the intended audience, leading to vaccine hesitancy
- Accessibility issues stemming from geographical distances to vaccination sites, as well as access problems arising from scheduling conflicts
- Timely administration of crucial immunizations, including challenges related to the hepatitis B birth dose
- Complexities in setting accurate targets for immunizations initiatives

In the domain if vector-borne diseases, particularly malaria, the following issues have come to the forefront

- Failure to achieve targeted utilization rates for long-lasting insecticide nets, accompanied by concerns over quality assurance
- Insufficient supervisory mechanisms to ensure high-quality execution of spraying operations
- Erosion of support from local government units for vector control activities
- Scarcity of communication volunteers available for the effective implementation of vector-related interventions

For rabies control, the prevailing concern pertains to an inadequate budget allocation for dog rabies vaccines

In the realm of mass drug administration and preventive therapy, several deficiencies have been identified:

- Absence of clear policies for mass drug administration, particularly in the context of malaria prevention
- Lack of established guidelines for pre-transmission surveys related to filariasis control
- Question surrounding the sustainability of Post-exposure prophylaxis for newborns of HIV-infected mothers and pre-exposure prophylaxis for individuals with substantial risk factors
- Absence of mechanism for post-exposure immunoglobulin for newborns of mothers infected with Hepatitis B

Case management presents its own set of challenges, including varying capabilities among hospitals in managing human rabies cases. Additionally, instances have arisen where certain healthcare providers were unable to adhere to the established standards of care treatment, including appropriate follow-up for complications. The expansion of service delivery points for Hepatitis B management is currently pending, albeit with accompanying guidelines.

Access to screening and diagnostic tests faces multifaceted obstacles:

- Suboptimal coverage in screening pregnant women for HIV, syphilis, and hepatitis B
- Disruptions in the supply of test kits at primary healthcare facilities
- Limited availability of qualified laboratory staff (medical technologists), leading to non-specialized personnel performing laboratory tests
- Restricted access to laboratory services within geographically isolated and disadvantaged areas (GIDAs)

> Lastly, the engagement of the private sector demonstrates variance across thirteen diseases. Integration of services provided by the private sector has not been fully realized within the program's information system.

The implementation of critical activities focused on disease prevention, diagnosis, and management is ensured through well-defined service delivery packages. While various diseases are currently at different stages of elimination, it is feasible to identify strategic convergence points for service delivery. For instance, prenatal care serves as a platform for screening in the context of mother-to-child transmission (MTCT) of diseases. Additionally, prenatal care offers a setting for conducting catch-up activities to address immunization gaps.

The Omnibus Health Guidelines (OHG), organized according to different life stages, established uniform standards spanning the continuum of care. These guidelines ensure the provision of high-quality health services across diverse levels, ranging from local to national, and within varied healthcare settings, including primary healthcare facilities, hospitals, and both government and private healthcare sectors.

Component 3.1: Vaccination services

3.1.1 Routine and supplemental immunization

Clinical Practice Guidelines (CPGs) and operational guidelines provide actionable frameworks for immunization. The local government units implement a one-day-a-week immunization schedule. However, the challenge persists in achieving the objective of attaining a national coverage rate exceeding 95%. The coverage for the Hepatitis B birth dose remains notably low.

To counter vaccine hesitancy, the following strategies can be considered:

1. Customize vaccine-related messages (tailored communication) to address specific concerns of different groups.
2. Acknowledge and dispel misinformation while emphasizing the benefits of vaccination that will be given to families every facility/ home visit.

Furthermore, access issues, encompassing both geographic and service delivery dimensions due to conflicting clinic hours, warrant meticulous attention. A promising strategy includes integrating immunization activities into broader health service outreach initiatives, thereby addressing these access impediments. Collaborating with birthing clinics, irrespective of whether they belong to the public or private sector, holds promise in minimizing missed immunization opportunities particularly birthing

clinics operating round the clock, as they extend the possibility of rendering services on a flexible schedule, especially conducive for working mothers.

Digitalization of health service recording emerges as a transformative tool, poised to streamline tracking mechanisms for defaulters and referrals, enhancing overall program efficiency. The effectiveness of these strategies may vary depending on cultural, social, and geographical contexts.

3.1.2 Pre exposure prophylaxis for individuals at high risk for rabies

Pre-exposure prophylaxis (PrEP) entails the administration of rabies vaccination at no cost prior to any potential exposure to the rabies virus. This proactive approach is particularly directed toward individuals at an elevated risk of contracting rabies due to their professional roles and responsibilities. These roles include, but are not limited to, laboratory staff, veterinarians, animal handlers, vaccinators, and other individuals engaged in activities that involve direct or indirect exposure to the rabies virus.

PrEP holds significant value as a preventive strategy, acting as a safeguard against the potential transmission of the rabies virus in occupational settings where the risk of exposure is heightened. This preventive endeavor aligns with the broader spectrum of rabies control strategies, contributing to the collective efforts aimed at reducing the incidence and impact of rabies within vulnerable populations and the community at large.

3.1.3 Dog vaccination for rabies

Ninety-nine percent of human rabies transmission is attributed to bites from rabid dogs. Vaccinating dogs stands as a pivotal measure to interrupt the chain of rabies transmission, both between dogs and from dogs to humans.

However, the insufficiency of budget allocations for procuring rabies vaccines, dedicated to dog vaccination, is a challenge outlined in Box 3. Effective budget allocation for dog vaccines necessitates accurate forecasting of vaccine requirements, contingent upon the accurate registration of dogs. In light of these considerations, the exploration of biological banks emerges as a potential solution for ensuring the availability of safe, affordable, and high-quality dog vaccines. The collaboration between the DA and DOH, facilitated by international agencies, can pave the way for harnessing the resources of biological banks in this context.

A continued advocacy approach directed towards LGUs is pivotal in advocating for the budgetary allocation dedicated to anti-rabies vaccines for dogs. Additionally, the DA-BAI should forge collaborations with veterinary societies to access comprehensive

data concerning dog registration and vaccination practices conducted at private veterinary clinics.
The synthesis of these strategies underscores a comprehensive approach aimed at addressing the budgetary challenges, promoting vaccination awareness, and leveraging available resources to bolster dog vaccination initiatives in the pursuit of rabies elimination.

Desired Outcome (3.1.1-3.1.3)

1. 95% immunization coverage for vaccine preventable diseases
2. Zero outbreaks/incidence for vaccine preventable diseases

Milestones (3.1.1-3.1.3)

2024 - 2030

1. Incremental increase of immunization coverage by 2% per year for vaccine preventable diseases beginning at 2% from baseline, starting in 2024, and ending at 14% by 2030
2. Incremental decrease of outbreaks/incidence of vaccine preventable diseases decreased by 1% from baseline, starting in 2024, and ending at 7% by 2030

2025 - 2030

Incremental increase of 10% per year of rabies high burden areas having a functional rabies elimination task force, starting at 40% in 2025 and ending at 80% by 2030

2030

100% dog and cat anti-rabies vaccination coverage in 80% of the rabies high burden areas

Component 3.2. Vector control

3.2.1 Mosquito-borne diseases

Vector control plays a pivotal role in the drive to eliminate infections transmitted by mosquitoes. Within the spectrum of vector interventions, two prominent strategies take precedence: the deployment of long-lasting insecticide nets (LLINs) and indoor residual spraying (IRS). These interventions' implementation is rooted in a comprehensive synthesis of epidemiological and entomological data, ensuring their tailored applicability to each region's unique context.

In parallel, Box 3 outlines the prevailing challenges inherent to vector control interventions. A critical stride toward optimizing LLIN utilization, with a target rate of 98% juxtaposed against the current 94.53%, entails identifying the underlying factors driving this disparity.

Moreover, it is imperative to acknowledge that while IRS yields substantial dividends, a notable aftermath is the emergence of insecticide resistance, casting a shadow over the long-term efficacy of this approach. A rigorous initiative is underway—a comprehensive insecticide resistance monitoring effort conducted within sentinel sites in Palawan—to address this, spearheaded by the Research Institute for Tropical Medicine (RITM).

3.2.2 Snail control for Schistosomiasis

In the Philippines, a range of targeted control measures aimed at snails, notably the intermediate host snail *Oncomelania*, has been initiated as a pivotal component of the comprehensive strategy against *Schistosoma japonicum*, the causative agent of schistosomiasis. The collaborative endeavors of the DOH in conjunction with other governmental bodies and collaborative partners, encompass a range of strategic initiatives aimed at curbing the snail population and curtailing schistosomiasis transmission.
Of paramount significance is the implementation of comprehensive measures designed to restrain the intermediate host snails. This includes a repertoire of strategies such as habitat modification, biological control, and the judicious application of molluscicides. Notably, the Philippines employs niclosamide as the molluscicide of choice. Administered primarily within water bodies, including rice fields and irrigation canals—environments conducive to snail breeding—this intervention is strategically designed to diminish snail populations and decisively disrupt the transmission cycle.

In addition, with molluscicidal efforts, the deployment of environmental management strategies is manifest. This dimension encompasses measures designed to reconfigure snail habitats, rendering them less conducive to the propagation of intermediate host snails. Noteworthy tactics entail techniques like "block chain dragging" and "cementing of irrigation and drainage canal," which contribute to altering snail habitats and curtailing the viability of breeding sites.

Desired Outcome (3.2.1-3.2.2)

Integrated vector management in high at-risk population

Milestones (3.2.1-3.2.2)

2025

At least 80% of malaria, lymphatic filariasis, and schistosomiasis high burden endemic areas have updated vector maps

2026

100% of malaria, lymphatic filariasis, and schistosomiasis high burden endemic areas have updated vector maps

2027 - 2029

Incremental increase of 10% per year of malaria, lymphatic filariasis, and schistosomiasis high burden endemic areas with updated vector maps perform integrated vector management all-year round, beginning at 80% in 2027 and ending at 100% by 2029

Component 3.3 Mass drug administration (MDA) / preventive chemotherapy (PCT)

Preventive chemotherapy involves administering doses of medication at regular intervals to interrupt transmission and prevent development of disease. Success depends on the proper identification of endemic areas and subsequent administration of recommended medicines to either the entire eligible population or the most vulnerable subsets. To achieve optimal coverage, behavior change communication, close supervision, and monitoring should be in place. This strategy is also referred to as mass drug administration.

Currently, MDA is being implemented for lymphatic filariasis and schistosomiasis in the Philippines.

In 2018, the World Health Organization (WHO) endorsed the adoption of leprosy post-exposure prophylaxis (LPEP) using single-dose rifampicin (SDR) to reduce the incidence of new cases within endemic communities. In the Philippines, Administrative Order No. 2021-0004, titled "Updated Guidelines on the Treatment and Prevention of Leprosy in the Philippines," as well as the Philippine Leprosy Clinical Practice Guidelines, have already incorporated provisions for LPEP. However, the implementation of these guidelines has not yet been fully realized.

Depending on exposure frequency and duration, MDA could also be considered for malaria, administered prior to, during, and post-exposure to malaria transmission. The MDEP also includes plans to sustain and promote both pre-exposure and post-exposure

antiretroviral medications for newborns born to HIV-infected mothers. This initiative demonstrates the commitment to enhance prevention and management of mother-to-child transmission of HIV.

Desired Outcome

50% reduction of new cases from baseline

Milestones

2024

Rifampicin (for leprosy) and Azithromycin (for yaws) included in the Philippine National Formulary

2025 - 2030

Incremental increase of 2% per year for MDA, PreP, and PEP coverage beginning at 75% in 2025 and ending at 85% by 2030

Component 3.4 Intensified Case Management

Specialized care guidelines for tetanus and polio exist, but comprehensive policies for the case management of all 13 priority diseases should be prioritized. This includes service mapping and development of complication management strategies to ensure holistic and comprehensive healthcare provision for each disease. Intensified case management include will include processed for:

1. Prompt diagnosis for timely treatment. This necessitates robust communication to heighten awareness and proactive measures for disease detection.

2. Vigilant community engagement by elevating community awareness and orchestrating active case detection through surveys or contact tracing.

3. Correct treatment by facilitating access to safe, high-quality medicines and ensuring their administration under the guidance of trained healthcare professionals.

4. Comprehensive complication management including handling stigma and preventing disability, readily accessible to all patients.

Desired Outcome

50% improvement on disease outcome from baseline

Milestones

2025

All 13 diseases have standards of care

2026 - 2030

Incremental increase of 10% per year of standards of care cascaded to high burden areas beginning at 30% in 2026 and ending at 70% by 2030

Component 3.5 Ensure access to screening and diagnostic tests

Screening and diagnostic tests should be accessible and available to primary healthcare providers. While primary healthcare facilities are equipped to conduct fundamental laboratory tests, it is important for them to also understand the capabilities of the broader laboratory network within their locality. This understanding facilitates referral of specimens to designated testing facilities for further evaluation and analysis. This collaborative approach ensures that patients receive accurate and timely diagnosis, thereby optimizing healthcare outcomes.

Desired Outcome

50% improvement on screening and diagnostic test access from baseline

Milestones

2025

Screening and diagnostic tests included in the Primary Care Benefit Package

2026

Screening incorporated in the primary and secondary school enrollment

2027

Screening incorporated in annual medical examination

2028

Screening incorporated in pre-employment medical examination

Component 3.6 Health Care Provider Network

The MDEP framework is rooted in the adoption of a primary health care approach, emphasizing comprehensive health solutions in a single healthcare visit. By integrating the management of various health concerns, this approach optimizes healthcare delivery and enhances patient experience. The Health Care Provider Network (HCPN) functions as an interconnected network of public and private healthcare providers spanning primary to tertiary levels. The HCPN collaboratively addresses individuals' holistic well-being, efficiently attending to multiple health needs during one healthcare encounter. Under the UHC, there are three HCPN types based on ownership: Public HCPN, linking public providers in a province or city; Private HCPN, comprising private providers; and Mixed HCPN, involving both public and private providers. The structure includes two key components:

1. Primary Care Provider Network (PCPN): Serving as the foundation, the PCPN offers initial patient contact, coordinating primary care services and facilitating broader network collaboration.

2. Hospital Network: Providing secondary and tertiary healthcare, this segment enhances HCPN capabilities. The apex referral hospital adheres to DOH-set care and quality standards.

Each HCPN is connected to an apex referral hospital, while various facilities offer specialized care.

Desired Outcome

100% functional and efficient health care provider network for diseases for elimination

Milestones

2025

Specialists for each disease mapped

2026

Referral network established

2027 - 2029

Incremental increase of functional referral network by 20% per year starting at 60% in 2027 and ending at 100% by 2029

Component 3.7 Continuous Quality Improvement and Supportive Supervision

Quality of care stands as a cornerstone within the framework of universal health care. Its significance extends beyond the delivery of health services and includes empowerment of health practitioners, performance enhancement, and the fortification of health systems at large. In this context, the strategic intervention of supportive supervision assumes a pivotal role in elevating the standards of care provided.

In this context, supportive supervision, a facet of quality assurance, embodies a comprehensive process marked by guidance, assistance, training, and motivational measures directed towards health care personnel. The ultimate objective is the continuous refinement of their performance and ensuring the consistent delivery of high-quality services. Supportive supervision is a dynamic practice that transpires on-site, manifesting in both formal and informal settings. It takes shape during one-on-one meetings or peer discussions and assumes particular significance when health workers collectively review their own performance vis-à-vis established benchmarks.

Desired Outcome

50% improvement on disease outcome from baseline

Milestone

One (1) Program Implementation Review (PIR) conducted per year, from 2024-2030 (related to stewardship pillar)

4 Safe and Quality Medicines, Vaccines, and Technology

Box 4: Challenges/Gaps

- Administrative Order No. 2023-0002 or the Institutionalization of the Expanded National Practice Guidelines has yet to achieve widespread circulation
- Some diseases targeted for elimination do not have Clinical Practice Guidelines
- The process leading up to the recommendations from the Health Technology Assessment Council is protracted and intricate
- The availability of a local Marketing Authorization Holder (MAH) is contingent upon having a company holding authorization granted by local medical device regulatory authority
- Within the procurement system, the mechanisms in place are often convoluted, and adherence to designated timelines can be challenging
- Delays in procurement processes and transportation logistics frequently culminate in stockouts
- International procurement avenues are limited in their scope
- Drug Price Reference Index (DPRI) stipulates lower prices compared to prevalent local market rates, undermining cost-effectiveness
- Warehouse infrastructure and capacity at all levels remain insufficient
- Bottlenecks in delivery and distribution pipelines contribute to extended timelines.

Medical treatments, vaccines, and health technologies are thoroughly assessed and validated through health technology assessment (HTA) by the Health Technology Assessment Council (HTAC). The objective is to establish their appropriateness for application in disease elimination efforts spanning prevention, screening, diagnosis, treatment, and management. HTA also examines the clinical, economic, social, organizational, and ethical effects of these health technologies.

This strategic pillar also includes efficient supply chain management, ensuring the availability of accessible health commodities by upholding meticulous protocols in procurement and compliance with regulatory requirements.

This pillar stands as an indispensable enabler, fortifying disease elimination endeavors by facilitating the deployment of cutting-edge technologies that undergo rigorous assessment, ensuring they align with the broader objectives of enhancing health outcomes and minimizing societal burdens.

Component 4.1: Facilitate the process of standard setting and Clinical Practice Guidelines development

CPGs provide benchmarks of care, formulated based on the recommended actions, interventions, or processes delineated within the National Practice Guidelines (NPGs). Developed in collaboration with professional societies and academic institutions, CPGs undergo systematic evidence review based on the schedule identified by the Disease

Prevention and Control Bureau and assessment of benefits and risks. This meticulous process yields recommendations that equip healthcare practitioners with actionable insights for enhancing the quality of care across various clinical scenarios encompassing screening, diagnosis, management, and monitoring.

Desired Outcome

Standards of care determined based on the recommended actions, interventions, or processes in the NPGs
NPGs include OHG, DOH-approved CPGs, and other equivalent standard guidelines including interim public health and clinical guidance documents for Public Health Emergencies (PHE) and Emerging & Re-Emerging Infectious Diseases (EREID)

Milestones

2024

Mapping of MDEP 13 diseases in terms of current available standard of care

2025

Updated standard of care for priority diseases for elimination with existing standards (connected to component 3.4)

2027-2030

Priority diseases for elimination have regularly updated standard of care (connected to component 3.4)

Component 4.2 Ensure that regulatory and legal requirements on commodities are facilitated and enforced

Regulation involves government measures to control the quality, safety, and efficacy of health products and services. The National Health Insurance Act (RA 10606) mandates a rigorous process for health products, including medicines and vaccines, involving Health Technology Assessment, Health Technology Assessment Council (HTAC) recommendations, and Food and Drug Administration (FDA) certifications.

Technology appraisals are crucial to evaluate clinical and economic value, guiding decisions on their integration into the healthcare system. Administrative Order 2016-0034 outlines the Philippine National Formulary System guidelines, requiring thorough benefit-risk assessments for medicine inclusion based on safety, efficacy, cost-effectiveness, affordability, and public health relevance.

The creation of the FDA through RA 9711 empowers it to oversee drug registration and licensing.

Collaborating with development partners or research institutions can facilitate provisional usage of medicines and technologies during pilot phases, especially for diagnostic and patient-care innovations and health emergencies.

Government commitment, with national and international partners, is key to uphold these policies, ensuring safe and high-quality health technologies. This collective vigilance safeguards public health and integrates advanced health interventions seamlessly into the healthcare system.

Desired Outcome

All medicines, vaccines, and other health technologies that are registrable and requires HTA have authorization and HTAC recommendations and FDA certifications

Milestones

2030

95% of technologies submitted for HTA are timely and of high quality
95% of applications for permits and licenses to FDA processed within allowable timelines

Component 4.3 Ensure agile and responsive processes on the procurement of commodities

Box no. 4 highlights procurement challenges that cause stock-outs. Ensuring continuous availability of drugs and vaccines is vital for eliminating the 13 target diseases. Traditional procurement methods are time-consuming. Exploring agile procurement as an alternative model is promising. Agile methods focus on adaptability, collaboration, and continuous improvement. Strong stakeholder and supplier relationships are key for ensuring value. Involving stakeholders in solutions can resolve bottlenecks.

Supplier relationships yield competitive pricing, risk reduction, supply continuity, and innovation. Improved communication through supplier experience management fosters transparency and aligned goals. The government uses the Philippine Government Electronic Procurement System (PhilGEPS) platform for procurement services. To address gaps, commodities for treatment and prevention must be registered, included

in the Annual Procurement Plan (APP), and aligned with roles and timelines. Accurate commodity forecasting informs budget estimates.

Creating a comprehensive funding landscape considering internal, local government, and donor resources is crucial. Allocating funds for commodity augmentation until LGUs can fully procure is prudent. Exploring international procurement via development partner platforms like United Nations Office for Project Services (UNOPS), Wambo, United Nations Children's Fund (UNICEF) is also useful.

Desired Outcome

Available, accessible, and sufficient commodities with support from international procurement

Milestones

2024

Dedicated funding for multi-disease elimination program at all levels

2026

LGU pooled procurement

2030

LGU full procurement

Component 4.4 Prepositioning and uninterrupted supply of high-quality medicines and other commodities at the facility through supply chain management

Supply Chain Management (SCM) encompasses the intricate process of harmonizing supply and demand management, both internally within an organization and externally across all stakeholders and channels within the supply chain. This orchestration is aimed at ensuring seamless collaboration and efficiency among the various elements of the supply chain. An optimized supply chain management framework offers significant advantages to organizations, including the prevention of costly delays, mitigation of quality issues, and avoidance of potential legal complications.

In the year 2020, through funding from the US Agency for International Development (USAID), the DOH embarked on the development of the Procurement and Supply Chain Management Strategic Plan. This involved a comprehensive situational analysis to

comprehensively assess the current state of affairs. This critical assessment unveiled gaps within the existing supply chain management framework that required attention and remediation.

Desired Outcome

No stock outs of commodities

Milestones

2024

Assessment of LGU capacity on efficient and effective supply chain management

2026-2028

At least 80% of commodities are prepositioned with 10% buffer, increasing at 10% per year until 100% prepositioned with 10% buffer by 2028

2029

Maintained efficient and effective prepositioning with buffer and the years after

5 Human Resource and Capacity Building

Box 5: Challenges/Gaps

- Existing allied medical programs do not encompass all diseases targeted for eradication/elimination
- A scarcity of subject matter experts equipped to revise curriculum content
- Deficiency in subject matter experts for the development of comprehensive training manuals
- Inadequate funding allocated for capacity-building initiatives
- Insufficient human resources to facilitate training sessions
- Non-availability of and outdated training modules tailored for specific disease elimination (rabies, mother-to-child transmission, leprosy)
- Limited dissemination of clinical pathways (i.e. leprosy)

Human Resources for Health (HRH) are medical and allied professionals working as part of the healthcare system. From preventive to palliative care, HRH exists at all levels of healthcare, including in public and private sectors.

Uneven distribution of healthcare providers remains a challenge in the Philippines. Around 20% of active medical practitioners handle 70% of healthcare needs, with most concentrated in urban areas, leading to inequitable access. The Health Human Resource Development Bureau (HHRDB) addresses this through the National Health Workforce Support System, implementing programs to bolster HRH. HHRDB's strategic interventions contribute significantly to achieving equitable access to health care by augmenting and managing HRH.

Component 5.1 Align medical and allied health curriculum with the continuum of care of diseases for elimination

Ensuring an adequate and competent healthcare workforce is crucial for MDEP's success. Healthcare workers need to possess skills to manage the 13 priority diseases, including responding to resurgences. Devolution adds complexity, allowing local governments to hire healthcare workers without specific public health knowledge. Inconsistent onboarding and selection for DOH training by LGUs can lead to uneven proficiency nationwide, exacerbated by high turnover.

A solution is to update the pre-service curriculum so that future health workers are prepared to manage diseases for elimination before their entry in the workforce. This aligns with the DOH Academy's HHRDB framework, under platform 3 for partnering with higher education. Collaboration with around 15 universities is already established, with potential to integrate specialized disease skills into allied courses. Coordination

meetings involving the Commission on Higher Education (CHED), University Presidents, Chancellors, and Administrators can initiate this process.

Desired Outcome

Medical and allied health graduates equipped with diseases for elimination prevention, control, and management competencies

Milestones

2025

Meeting with CHED and College/University Administration

2026

Enhanced curriculum on diseases for elimination and maintained & updated in the years after

Component 5.2 Capability building and supportive supervision for primary care providers and facility-based health workers for effective delivery of services.

Ensuring healthcare personnel, both health care professionals and direct service providers, stay updated with evolving guidelines and technologies through continuous skill enhancement is important. Although disease-specific training manuals exist for conditions like Malaria, Leprosy, Polio, and MTCT-HIV, there's a critical need to modernize these resources. This ensures proficiency for effective elimination activities is embedded in educational materials. Moreover, the demand for training modules covering all priority diseases is evident. While some diseases have been addressed individually, an integrated approach calls for comprehensive modules covering disease clusters like morbidity management and disability prevention of leprosy and lymphatic filariasis. These modules can be integrated into the DOH Academy, utilizing blended learning. Healthcare workers engage in online didactic components followed by practical field training, creating a strong mix of theory and hands-on experience.

The DOH Academy's E-Learning Program provides convenient remote courses for HRH competency enhancement without compromising healthcare service delivery. Development partners like WHO and USAID offer specific courses, and other local and international partners can likewise contribute to enhance expertise in specific topics. Beyond initial training, ongoing supportive supervision enhances real-world application of skills. Regular feedback, guidance, and mentoring foster continuous improvement, bridging theory and competence. This comprehensive approach aligns with the goal of

sustaining a skilled healthcare workforce equipped to tackle the challenges posed by the 13 priority diseases.

Desired Outcome

HRH competent in preventing, controlling, and managing diseases for elimination

Milestones

2025

Learning gap identified

2026

Learning and development interventions (LDIs) developed

2027

LDI pilot tested

2028 - 2030

% of HRH capacitated, starting at 80% in 2028 and reaching 100% by 2030

6 Environment and Social Determinants of Health

Box 6: Challenges/Gaps

- Administrative Order No. 2023-0002 or the Institutionalization of the Expanded National Practice Guidelines has yet to achieve widespread circulation
- Some diseases targeted for elimination do not have Clinical Practice Guidelines
- The process leading up to the recommendations from the Health Technology Assessment Council is protracted and intricate
- The availability of a local Marketing Authorization Holder (MAH) is contingent upon having a company holding authorization granted by local medical device regulatory authority
- Within the procurement system, the mechanisms in place are often convoluted, and adherence to designated timelines can be challenging
- Delays in procurement processes and transportation logistics frequently culminate in stockouts
- International procurement avenues are limited in their scope
- Drug Price Reference Index (DPRI) stipulates lower prices compared to prevalent local market rates, undermining cost-effectiveness
- Warehouse infrastructure and capacity at all levels remain insufficient
- Bottlenecks in delivery and distribution pipelines contribute to extended timelines.

Social determinants include the conditions in which individuals are born, grow, live, work, and age, all of which impact their health status and health-seeking behaviors. A range of communication channels tailored to specific diseases or health programs have been employed to reach the public, but low health literacy and health-seeking behaviors persist.

Community engagement involves collaborative efforts with groups sharing geographic proximity, special interests, or similar situations to address issues that impact their well-being. This approach is a potent catalyst for effecting environmental and behavioral changes, often involving partnerships and coalitions to mobilize resources and influence systems. Community engagement takes various forms and includes a diverse range of partners, including organized groups, agencies, institutions, and individuals.

Viewed as a continuum of community involvement, community engagement spans health promotion, research, and policy-making domains.

Component 6.1 Improve health promotion activities directed towards diseases for elimination through social behavior change communication and demand generation activities

Health promotion efforts do not necessarily lead to immediate changes in health seeking behavior. In the context of the 13 priority diseases, addressing each disease individually through health promotion efforts can result in a multitude of messages and materials, often leading to information overload for the public. This highlights the need for a more streamlined and integrated approach in the form of a comprehensive communication plan for MDEP.

Developing an integrated communication plan requires a deep understanding of the epidemiological characteristics of each disease. By identifying common environmental and social factors across these diseases, it becomes possible to create messages and materials that are relevant to multiple conditions. Furthermore, tailoring health messages and promotional activities to at-risk groups can enhance the effectiveness of communication strategies, as messages can be crafted to align with the characteristics and preferences of the intended audience and work towards reducing stigma and discrimination.

Consider the example of an integrated communication plan aimed at preventing vector-borne diseases such as malaria and lymphatic filariasis. These diseases share common vectors and environmental risk factors, making it feasible to develop a unified message that emphasizes the importance of mosquito control and personal protective measures. By targeting both diseases with a single set of messages, the public can be better educated on effective preventive measures without being inundated with redundant information.

Forging partnerships with educational institutions, such as schools, can be a strategic approach. Integrating disease prevention measures into the school curriculum ensures that students are equipped with knowledge about health and hygiene from an early age.

Incentivizing and recognizing efforts in disease elimination can serve as a motivating factor for local government units. The incorporation of elimination targets into LGU scorecards can foster political commitment and financial support for elimination activities.

The integration of health promotion efforts within the MDEP requires a strategic and multi-faceted approach. By identifying common factors among diseases, tailoring messages to specific age groups, addressing stigma, collaborating with educational institutions, and implementing recognition and incentives, a comprehensive communication plan can effectively inform and engage the public, leading to improved

health seeking behavior and ultimately contributing to the successful elimination of the 13 priority diseases.

Desired Outcome

50% improvement on social behavior towards diseases for elimination from baseline

Milestones

2026

Two studies on social behavior towards diseases for elimination (client-focused and provider-focused)

2027

One multi-sectoral stakeholder meeting conducted

2028

Communication plan developed and disseminated to all 17 Centers for Health Development

Component 6.2 Strengthen multisectoral collaboration and coordination at all levels to address common environmental and social determinants of health

In the context of public health, a myriad of environmental, economic, and social factors exert influence over health behaviors and outcomes. Through the active engagement of multiple partners, each contributing their distinct resources, expertise, and perspectives, the complexity of these factors can be more effectively tackled.

The MDEP TWG will be composed of DPCB technical staff representing the diseases for elimination, representatives from other DOH offices, other sectors and academe. The TWG will be overseeing the implementation of the MDEP which includes activities addressing the environmental and social determinants affecting the 13 priority diseases.

The engagement of civil society organizations, academic institutions, and private sectors adds depth and diversity to the collaborative effort. Civil society organizations can mobilize communities and advocate for policies that prioritize health and well-being. Academic institutions can provide evidence-based research to guide decision-making, while the private sector can leverage innovation and resources to implement practical solutions.

The underlying principle of multisectoral collaboration is the recognition that challenges affecting health are often interconnected and multifaceted. A collaborative approach taps into the strengths of various sectors and harnesses their collective capacity to drive change. By aligning objectives, sharing knowledge, pooling resources, and implementing coordinated strategies, multisectoral collaboration becomes a powerful tool for addressing complex health issues and achieving meaningful and sustainable outcomes for the overall well-being of communities and populations.

Desired Outcome

Disease elimination prioritized and well-funded through multi-sectoral collaboration

Milestones

2026

Inclusion of diseases for elimination in various existing inter-agency and private sector councils/technical working groups, as appropriate

2024-2030

Annual stakeholders' meeting

7 Stewardship and Finance

Box 7: Challenges/Gaps

- Overlapping functions of oversight bodies
- Establishing permanent representation
- Scheduling conflicts
- Hesitations on the level of commitment
- Decision-making processes
- Unexplored territory
- Challenges in finding common ground
- Limited coverage in PhilHealth benefit package
- Insufficient funding for other diseases
- Lack of mechanisms for fund pooling

Stewardship involves leadership and governance. Every government must have strategic policy frameworks and direction combined with effective oversight, coalition building, regulation, attention to system design and accountability, practice judicious use of resources and sustainability of the plan.

Finance ensures that all Filipino citizens have access to a comprehensive set of health services without financial hardship.

Component 7.1 Stewardship through a multi-sectoral oversight committee

The 13 priority diseases may be grouped into vaccine preventable, vector-borne, neglected tropical disease and mother-to-child transmission. The prevention and control activities need involvement of other sectors (DA, academe, professional societies) and other bureaus within the DOH. A multi-sectoral oversight committee is necessary to establish processes, implement more effectively and efficiently monitor progress. A review and mapping of existing oversight committees is crucial to determine the possibility to adapt existing ones and learn from their success or failures. The MDEP TWG oversees the implementation of the MDEP. A scientific technical advisory group will be formed to provide technical advice and serve as external audit, composed of experts from international partners and professional societies. The MDEP TWG will work on the operations while the STAG will provide expert guidance based on evidence.

Desired Outcome

Multi-disease elimination guided by and overseen by functional groups with members various key sectors coming from both public and private organizations including societies

Milestones

2024

Joint AO establishing the diseases for elimination scientific technical advisory group (STAG) and technical working group (TWG)

2025-2030

Annual STAG meeting and quarterly TWG meetings

Component 7.2 Develop evidence-based policies

Developing evidence-based policies is a fundamental pillar within any disease elimination framework. While administrative orders and executive orders (EOs) have been employed as mechanisms for institutionalization, the prevailing trend has been the formulation of disease-specific AOs or EOs. However, the challenge lies in crafting policies that are not only evidence-based but also inter-sectoral, transcending the confines of single diseases to achieve holistic health outcomes.

To address this challenge, the Health for All Policies (HFAP) approach becomes paramount. This approach emphasizes identifying and maximizing co-benefits across different health domains, promoting synergy among sectors beyond healthcare. In the context of the MDEP, the TWG spearheads this endeavor by convening a panel of disease experts. This panel is entrusted with the task of crafting integrated and evidence-based policies that transcend singular diseases, fostering a comprehensive approach to health improvement.

A critical step in policy development is the meticulous mapping and review of existing CPGs within the NPGs. By conducting this review, policymakers can identify gaps and areas in need of updating or new guideline development. This process ensures that policies are aligned with the latest evidence and best practices in healthcare.

Desired Outcome

High quality, evidenced-based policies on diseases for elimination

Milestones

2024

Mapping of MDEP 13 diseases in terms of current available standard of care (part of component 4.1)

2025 - 2030

Annual stakeholder's meeting (integrated in the same event in component 6.2)

Component 7.3 Secure sufficient government and non-government financial resources in support of elimination strategies

This component involves a series of strategic approaches aimed at ensuring sustained government and non-government funding for the successful execution of disease elimination efforts.

A prominent strategy entails reinforcing the representation of the DOH in the development and formulation of the Special Health Fund (SHF). This entails working collaboratively with relevant stakeholders to advocate for increased LGU financing directed towards health services. By actively engaging in the development of this fund, the DOH aims to bolster financial support for health programs, thereby contributing to the effective implementation of disease elimination initiatives.

Another critical avenue for securing financial resources is by maximizing the utilization of PhilHealth packages through the HCPN. This involves leveraging the established network of healthcare providers to ensure that Philippine Health Insurance Corporation (PhilHealth) packages are effectively utilized, thereby channeling financial resources to the health services required for disease elimination. By optimizing the coverage and utilization of these packages, the program aims to create a sustainable funding stream that supports the comprehensive delivery of healthcare services.

Furthermore, achieving financial sustainability involves harmonizing government and partner funding dedicated to disease elimination efforts. By coordinating the allocation of funds from various sources, including both government and non-government entities, the program can ensure a more efficient and effective utilization of available resources. This alignment of financial support across stakeholders helps prevent duplication of efforts and optimizes the impact of each contribution towards the overarching goal of disease elimination.

Desired Outcome

1. Active involvement of Provincial Health Team Leads (PHTLs) in disease elimination
2. Reduction of patients' out-of-pocket expenses for services related to diseases for elimination
3. Secured and augmented funding for diseases for elimination from DOH up to LGU

Milestones

2024

1. Inventory of existing Philhealth packages, accredited facilities and existing health care provider network
2. Map of NGOs and private sector with corporate social responsibility (CSR) that can provide funding support

2025

1. Single line-item budget for diseases for elimination
2. 100% of PHTLs oriented on MDEP

2025 - 2030

Annual stakeholder's meeting (integrated in the same event in component 6.2 and 7.2)

2028

100% of LGUs have local ordinance on resource mobilization for diseases for elimination

2030

Diseases for elimination have Philhealth packages, provided that all have CPGs

8 Research

Box 8: Challenges/Gaps

- Fragmented processes in initiating research studies
- Fragmented research ecosystem
- Insufficient local research studies focusing on zoonotic diseases
- Inadequate high-quality research activities relevant to diseases for elimination aligned with the research agenda

Research forms the scientific foundation for shaping strategic directions and crafting policies based on solid evidence. This knowledge ensures that policies and strategies are well-informed, effective, and adaptable to the evolving nature of diseases and their surrounding environment.

Challenges in box 8 need to be addressed to ensure priority research is conducted and findings utilized for policymaking.

Component 8.1 Systematized identification of research needs

Moving away from the disjointed process of initiating research studies, the MDEP aims to evolve towards a unified approach that identifies research requirements spanning various bureaus and agencies. By embracing an integrated method for identifying research needs, the MDEP ensures that the collective expertise of various entities is harnessed to pinpoint the most pertinent research areas. Ultimately, transitioning to this integrated model empowers the MDEP to drive evidence-based strategies through a more cohesive and coordinated research effort.

Component 8.2 Strengthening of research that supports new innovations supporting diseases for elimination and eradication

Enhancing research efforts to bolster innovations involves generating novel ideas and solutions that are subjected to local research studies before being integrated into policies. By conducting rigorous research on these innovations, their efficacy, feasibility, and potential impact can be thoroughly assessed within the local context.

This approach not only ensures that new interventions are evidence-based but also promotes a seamless transition from research to policy implementation. Furthermore, the focus on innovations extends to animal health technologies, acknowledging the interconnectedness of human and animal health in disease transmission dynamics.

By strengthening research in this manner, the MDEP fosters a dynamic environment for continuous improvement and progress in advancing the goals of disease elimination and eradication.

Component 8.3 Propose, recommend, and conduct quality and representative surveys, studies, and serosurveys based on international standard methodologies

This component revolves around proposing, recommending, and conducting robust surveys, studies, and serosurveys using internationally recognized methodologies, ensuring the utilization of their outcomes. The focus is on enhancing the capabilities of relevant agencies, including national and local entities as well as academic institutions, to proficiently carry out diverse research endeavors.

The aim is to empower these stakeholders with the skills required to design, execute, and analyze various types of research, fostering a culture of evidence-based decision-making. By adhering to internationally accepted standards, the results generated from these endeavors gain credibility and reliability, contributing to informed policies and strategies for disease elimination and eradication.

Desired Outcome (8.1-8.3)

80% increase in the utilization of high quality local researches as evidence for policy, guidelines, or standards of care development from baseline

Milestones (8.1-8.3)

2024

1. Research agenda and operational plan to include capacity building (individual, and multi-center institutional) for research endorsed by the STAG
2. One public research forum to facilitate a review of past and ongoing researches and identify which research can be translated into policy already
3. Budget for 2025-2030 research included in the operational plan

2025

1. Approved research agenda for diseases for elimination
2. Compendium of research on diseases for elimination from the academe and other research institutions

2025-2030

1. Bi-annual research forum among academic and research institutions
2. 20% annual reduction on research gap
3. Translation of at least one research into policy per year

MONITORING AND EVALUATION

Monitoring and evaluation are management tools designed to assess the extent to which the program/project is attaining the expected outcomes (measurement of performance). Monitoring is defined as a continuing function that systematically collects data on specified indicators to provide management and relevant stakeholders of an on-going development intervention with indication of the extent of progress and achievement of objectives and progress in the use of allocated funds. Evaluation is the process of determining the worth or significance of development activity, policy, program to determine the relevance of objectives, the efficacy of design and implementation, the efficiency or resource use and sustainability of result. (World Bank) Other terminologies that are important in monitoring and evaluation:

- Reporting is the systematic provision of essential information at periodic intervals.
- Outputs are specific products and services that emerge from processing inputs
- Outcomes are changes in development conditions that the program aims to achieve
- Lesson learned is an instructive example based on experience that is applicable to a general situation rather than to specific circumstance.

Monitoring Tools and Mechanisms

Field visits

Field visits can be joint efforts —regional, provincial, local partners—other agencies (DA) and in some circumstances, international development partners, to validate reported results by observing the progress being made towards attainment of results (outcome and outputs) that are contributing to the goals of MDEP. An integrated monitoring checklist should be developed that covers areas and skills to be observed, data to be reviewed/validated and compliance to monitor. At the end of the field visit, a debrief meeting with the staff should be conducted to discuss findings, draw out difficulties/problems or challenges encountered by the health staff and provide recommendations. A written feedback report should be delivered to the health unit for filing (documentation of the field visit), which will also serve as the starting point of the next field visit.

Multi-sectoral meeting

Quarterly, one-day multi-sectoral meetings will be led by the MDEP TWG together with the STAG. The TWG chair initiates the multi-sectoral meeting and should ensure representation of all agencies, local and international partners. During the meeting, status of the indicators is presented against targets. Each strategic pillar will be discussed, including the status of work plan implementation, challenges encountered, actions taken, and potential risks assessment.

Performance Implementation Review

A performance implementation review (PIR) will be held annually. The purpose is to assess progress towards results and focus discussion on recurring problems/challenges identified during quarterly multi-sectoral meetings and to guide the review and adjustments of the work plan for the following year. One of the byproducts of the PIR will be the annual progress report.

Results Tracking

A separate monitoring team will be identified by the MDEP TWG. Progress on cross cutting indicators for health systems strengthening and impact indicators for each disease will be shared by the monitoring team to the MDEP TWG during the multi-sectoral meetings.

BUDGET

The budget below estimates the funding requirement from 2024-2030. It does not include budget requirements for the procurement of medicines, vaccines, test kits, and other commodities, and excludes the budget for construction of laboratories because funding for construction is incorporated in the strategic plan of the OHL.

Funding source is not indicated and is open for collaboration and co-funding among stakeholders.

Strategy	2024	2025	2026	2027	2028	2029	2030	TOTAL
**Surveillance and Information Systems**								
Small group meetings	10,000.00	10,000.00	10,000.00	10,000.00	10,000.00	10,000.00	10,000.00	70,000.00
Dissemination forum on the result of the disease surveillance and health information system desk review	500,000.00	-	-	-	-	-	-	500,000.00
Development and maintenance of a multi-source surveillance platform and service information system	-	1,500,000.00	5,000,000.00	1,500,000.00	1,500,000.00	1,500,000.00	1,500,000.00	12,500,000.00
Training on the use of the multi-source surveillance platform and service information system	-	-		3,000,000.00	3,000,000.00	3,000,000.00	3,000,000.00	12,000,000.00
Monitoring and supervisory visits	-	-			200,000.00	200,000.00	200,000.00	600,000.00
**TOTAL**	**510,000.00**	**1,510,000.00**	**5,010,000.00**	**4,510,000.00**	**4,710,000.00**	**4,710,000.00**	**4,710,000.00**	**25,670,000.00**

Strategy	2024	2025	2026	2027	2028	2029	2030	TOTAL
**Access to Laboratory Services**								
Development of sustainability plan for Vaccine Preventable Diseases (VPD) Referral Laboratories including human resource, training, and budget plans	2,000,000.00	-	-	-	-	-	-	2,000,000.00
Assistance in the implementation of the sustainability plan for VPD Referral Laboratories	-	1,000,000.00	1,000,000.00	-	-	-	-	2,000,000.00
Training of selected laboratory personnel of SNL	-	-	-	1,500,000.00	1,500,000.00	1,500,000.00	1,500,000.00	6,000,000.00
Development of policies on cross-linking/collaboration and referral mechanism on services of VPD Referral Laboratories with the established SNL's under the Center for Health Development (CHD)	-	-	1,000,000.00	-	-	-	-	1,000,000.00
Assessment of the whole quality of laboratory and development of Laboratory Quality Management System (including QA/QI) for identified diseases for elimination	2,000,000.00	-	-	-	-	-	-	2,000,000.00

Strategy	2024	2025	2026	2027	2028	2029	2030	TOTAL
Reinforcement trainings for NRLs and capacitate SNL in QA/QI	-	1,500,000.00	1,500,000.00	1,500,000.00	1,500,000.00	1,500,000.00	1,500,000.00	9,000,000.00
Pilot implementation of Quality Assurance Programs (e.g proficiency testing) for selected laboratories in the PHLS	-	-	-	3,000,000.00	-	-	-	3,000,000.00
Actual implementation of Quality Assurance Programs for all laboratories in the PHLS	-	-	-	-	10,000,000.00	10,000,000.00	10,000,000.00	30,000,000.00
Monitoring and supervisory visits	-	-			200,000.00	200,000.00	200,000.00	600,000.00
**TOTAL**	**4,000,000.00**	**2,500,000.00**	**3,500,000.00**	**6,000,000.00**	**13,200,000.00**	**13,200,000.00**	**13,200,000.00**	**55,600,000.00**

**Service Delivery**								
Small group meetings	10,000.00	10,000.00	10,000.00	10,000.00	10,000.00	10,000.00	10,000.00	70,000.00
Development of vector maps and regular updating	-	15,000,000.00	5,000,000.00	5,000,000.00	5,000,000.00	5,000,000.00	5,000,000.00	40,000,000.00
Review, updating, and dissemination/training on integrated vector management activities	-	-	-	5,000,000.00	3,000,000.00	3,000,000.00	3,000,000.00	14,000,000.00
Development and updating of standards of care for all 13 diseases	-	24,500,000.00	21,000,000.00	-	-	-	-	45,500,000.00

Strategy	2024	2025	2026	2027	2028	2029	2030	TOTAL
Cascading of the standards of care, with priority to high burden areas	-	-	3,000,000.00	4,000,000.00	5,000,000.00	6,000,000.00	7,000,000.00	25,000,000.00
Mapping of specialists for each disease	-	3,000,000.00	-	-	-	-	-	3,000,000.00
Establishment and maintenance of a functional referral network	-	-	3,000,000.00	3,000,000.00	3,000,000.00	3,000,000.00	3,000,000.00	15,000,000.00
Meetings with stakeholders to incorporate screening and diagnosis in the Primary Care Benefit Package, school enrollment, annual medical examination, and pre-employment	10,000.00	10,000.00	10,000.00	10,000.00	10,000.00	10,000.00	10,000.00	70,000.00
Development and orientation of integrated checklist	1,000,000.00	500,000.00						1,500,000.00
Monitoring and supervisory visits	-	-	200,000.00	-	200,000.00	-	200,000.00	600,000.00
**TOTAL**	**1,020,000.00**	**43,020,000.00**	**32,220,000.00**	**17,020,000.00**	**16,220,000.00**	**17,020,000.00**	**18,220,000.00**	**144,740,000.00**

Strategy	2024	2025	2026	2027	2028	2029	2030	TOTAL
**Safe and Quality Medicines, Vaccines, and Technology**								
Small group meetings	10,000.00	10,000.00	10,000.00	10,000.00	10,000.00	10,000.00	10,000.00	70,000.00
Assessment of LGU capacity on efficient and effective supply chain management	3,000,000.00	-	-	-	-	-	-	3,000,000.00
**TOTAL**	**3,010,000.00**	**10,000.00**	**10,000.00**	**10,000.00**	**10,000.00**	**10,000.00**	**10,000.00**	**3,070,000.00**
**Human Resource and Capacity Building**								
Small group meetings	10,000.00	10,000.00	10,000.00	10,000.00	10,000.00	10,000.00	10,000.00	70,000.00
Updating of pre-service curriculum, development of learning materials and monitoring & supervision	-	5,000,000.00	1,000,000.00	1,000,000.00	1,000,000.00	1,000,000.00	1,000,000.00	10,000,000.00
HRH learning gap analysis	-	2,500,000.00	-	-	-	-	-	2,500,000.00
Development of LDIs, pilot testing, and full dissemination	-	-	10,000,000.00	2,000,000.00	3,000,000.00	4,000,000.00	5,000,000.00	24,000,000.00
**TOTAL**	**10,000.00**	**7,510,000.00**	**11,010,000.00**	**3,010,000.00**	**4,010,000.00**	**5,010,000.00**	**6,010,000.00**	**36,570,000.00**
**Environment and Social Determinants of Health**								
Small group meetings	10,000.00	10,000.00	10,000.00	10,000.00	10,000.00	10,000.00	10,000.00	70,000.00
Two studies on social behavior towards diseases for elimination	-	-	5,000,000.00	-	-	-	-	5,000,000.00

Strategy	2024	2025	2026	2027	2028	2029	2030	TOTAL
Feedback and stakeholder meeting	-	-	-	3,000,000.00	-	-	-	3,000,000.00
Development, dissemination, and monitoring & supervision of communication plan to all 17 Centers for Health Development	-	-	-	-	3,000,000.00	3,000,000.00	3,000,000.00	9,000,000.00
**TOTAL**	**10,000.00**	**10,000.00**	**5,010,000.00**	**3,010,000.00**	**3,010,000.00**	**3,010,000.00**	**3,010,000.00**	**17,070,000.00**

Stewardship and Finance								
Annual STAG meeting and quarterly TWG meetings	1,000,000.00	1,000,000.00	1,000,000.00	1,000,000.00	1,000,000.00	1,000,000.00	1,000,000.00	7,000,000.00
Annual program implementation review	5,000,000.00	5,000,000.00	5,000,000.00	5,000,000.00	5,000,000.00	5,000,000.00	5,000,000.00	35,000,000.00
Inventory of existing Philhealth packages, accredited facilities and existing health care provider network	500,000.00	-	-	-	-	-	-	500,000.00
Development of annual report and learning stories		300,000.00	300,000.00	300,000.00	300,000.00	300,000.00	300,000.00	1,800,000.00
Map of NGOs and private sector with corporate social responsibility (CSR) that can provide funding support	500,000.00	-	-	-	-	-	-	500,000.00
**TOTAL**	**7,000,000.00**	**6,300,000.00**	**6,300,000.00**	**6,300,000.00**	**6,300,000.00**	**6,300,000.00**	**6,300,000.00**	**44,800,000.00**

Strategy	2024	2025	2026	2027	2028	2029	2030	TOTAL
**Research**								
Research forum	3,000,000.00	-	-	-	-	-	-	3,000,000.00
Development of a research agenda and research operational plan	3,000,000.00	-		-	-	-	-	3,000,000.00
Development of a compendium of researches on diseases for elimination	-	1,000,000.00	-	-	-	-	-	1,000,000.00
Annual research forum among academic and research institutions	-	1,500,000.00	1,500,000.00	1,500,000.00	1,500,000.00	1,500,000.00	1,500,000.00	9,000,000.00
**TOTAL**	**6,000,000.00**	**2,500,000.00**	**1,500,000.00**	**1,500,000.00**	**1,500,000.00**	**1,500,000.00**	**1,500,000.00**	**16,000,000.00**

REFERENCES

Ending the Neglect to Attain the Sustainable Development Goals: A Road Map for the Neglected Tropical Diseases 2021-2030. Geneva: World Health Organization, 2022, CC BY-NC-SA 3.0 IGO

Ending the Neglect to Attain the Sustainable Development Goals: A Strategic Framework for Integrated Control and Management of Skin-related Neglected Tropical Diseases 2021-2030. Geneva: World Health Organization, 2022 CC BY-NC-SA 3.0 IGO

Global Guidance on Criteria and Process for Validation: Elimination of Mother-to-Child Transmission of HIV, syphilis and Hepatitis B virus. Geneva: World Health Organization. 2022 CC BY-NC-SA 3.0 IGO

Global Health Sector Strategy on Sexually Transmitted Infections 2016-2021. Geneva: World Health Organization. 2016

Global Health Sector Strategy on respectively, HIV, viral hepatitis and sexually transmitted infections for the period 2022-2030. Geneva: World Health Organization. 2022 CC BY-NC-SA 3.0 IGO

Global Technical Strategy for malaria 2016-2030, 2021 Update. Geneva: World Health Organization. 2022 CC BY-NC-SA 3.0 IGO

Interruption of Transmission and elimination of Leprosy disease – Technical guidance. New Delhi: World Health Organization Regional Office for South East Asia, 2023

Measles and Rubella Strategic Framework 2022-2026: Delivering a Promise. Geneva: World Health Organization. 2022 CC BY-NC-SA 3.0 IGO

Polio Eradication Strategy 2022-2026: Delivering a promise. WHO, Geneva, 2021

Pan American Health Organization Integrated Sustainable Framework for the Elimination of Communicable Diseases in the Americas. Washington DC: PAHO, 2023

Polio Eradication Strategy 2022-2026: Delivering a promise. Geneva: World Health Organization, 2021 CC BY-NC-SA 3.0 IGO

Progress Towards Maternal and Neonatal Tetanus Elimination – Worldwide, 200-2018. WHO Weekly Epidemiologic Record, 1st May 2020 No. 18

Protecting All Against Tetanus: Guide to sustaining maternal and neonatal tetanus elimination (MNTE) and broadening tetanus protection for all populations. Geneva: World Health Organization, 2019 CC BY-NC-SA 3.0 IGO

Regional Framework for vaccine-preventable diseases and immunization in Western Pacific 2021-2030. Manila: WHO Regional Office for Western Pacific,2022 CC BY-NC-SA 3.0 IGO

Regional Framework for the Triple Elimination of MTCT

Towards Zero Leprosy: Global Leprosy (Hansen's Disease) Strategy 2021-2030. New Delhi, World Health Organization, Regional Office for South East Asia, 2017

Zero by 30: The Global Health Strategic Plan to End Human Death from Dog-Mediated Rabies by 2030. World Health Organization, Food and Agricultural Organization of the United Nation (FAO) and World Organization for Animal Health (OIE), 2018

Asia Pacific Strategy for Strengthening Health Laboratory Services 2010-2015. Regional Office for South East Asia and Regional office for the Pacific Region, WHO, 2010

Dayrit MM, Lagrada LP, Picazo OF, Pons MC, Villaverde MC. The Philippines Health System Review Vol. 8 No. 2, New Delhi: WHO Regional Health Office for South East Asia, 2018

Department of Health (2018). National Objectives for Health 2017-2022. Manila Philippines: DOH

FHSIS Annual Reports 2011-2022

Handbook on Monitoring and Evaluating Results. United Nations Development Program, NY,2002

Intersectoral Governance for Health in All Policies: Structure, Actions and Experience.

European Observatory on Health Systems and Policies, 2012

Laboratory Quality Management System Handbook Version1.1. WHO, 2011

Marquez, L and Kean L. Making Supervision Supportive and Sustainable: New Approaches to Old Problem. MAQ Paper, MSH and John Hopkins Center for Communication program, Nov. 4, 2022

Philippine National Health Accounts, Philippine Statistics Authority
Philippine Department of Health National Procurement and Supply Chain Management Strategic Plan, 2020

Principles of Community Engagement. Second Edition, NIH Publication No. 11-772, June 2011

Villaverde, MC; Ronquillo, KG, Espiritu NS II JMV (eds), Universal Health Care in the Philippines from Policy to Practice. Manila, Philippines, Department of Health. 2022

Malaria Transition, Elimination and Sustainability Plan (MTESP) 2023-2028

National Leprosy Control Program Medium Term Plan (2017-2020)

National Rabies Prevention and Control Program Strategic Plan 2020-2025

National Strategic Plan Towards Interruption of Schistosomiasis Transmission in the Philippines (2019-2025)

Abrigo M, Tam Z, Ortiz DA. Decentralization and Health in the Philippines: A Systematic Review of Empirical Evidences. Philippine Institute for Development Studies, Discussion Paper Series No. 2017-58

Chan JM, Chan H and Flores MJ, Status of Schistosomiasis in the Philippines: Prevalence, Control and Innovative Methods for detection and Elimination. DLSU Research Congress 2021. De la Salle University, Manila Philippines, July 2021

Diaz-Manalo P, Estrada MA, Baluyot D. Implications of the SC Ruling on the Madanas-Garcia IRA Case. Congressional Policy and Budget Research Department, House of Representatives, Issue No. 2, August 2021

Dofitas BL, Batac, MC, Richardus JH; Finding Yaws Among Indigenous People: Lessons from Case Detection Survey in Luzon and Visyas Island Group of the Philippines. Trop Med Hyg 2022

Dofitas BL, Kalin SP, Toledo CB, Richardus JH; Yaws in the Philippines: First Reported Cases since 1970. Infect Dis Poverty Jan 2020

Esperanza Cabral, MD, The Philippine Health Agenda 2016 to 2022. Philippine Journal of Internal Medicine, Vol. 54 No. 2, April-June 2016

Janet S. Cuenca. Fiscal Decentralization and Health Service Delivery: The Philippine case. Philippine Institute for Development Studies, June 2020

Lydia Leonardo et al, Schistosomiasis in the Philippines: Challenges and some successes in control. South East Asia Journal Tropical Med Public Health Vol 47 No 4, July 2016

M. Kron et al. Lymphatic Filariasis in the Philippines. Parasitology Today, Vol 16 No 8, 2000
WHA 54

www.who.int/Q&A/IHR

www.cdc.gov/globalhealth/security

www.dilg.gov.ph

Data4impactproject.org/prh/health-systems/policy-environment

www.who.int/initiatives/maternal-and-neonatal-tetanus eliminatio

Republic of the Philippines
Department of Health
