# Supplementary material for: Neglected Tropical Diseases Elimination in the Philippines: Challenges and Gaps
Source: Trop Med Infect Dis. 2026 Apr 17;11(4):106. doi: 10.3390/tropicalmed11040106 (PMC13120366; doi:10.3390/tropicalmed11040106)
Supplement: Supplementary file 1 [file tropicalmed-11-00106-s001.zip › Supplementary File S2. FHSIS 2020.pdf]

2020
ANNUAL REPORT

FIELD HEALTH SERVICES INFORMATION SYSTEM

SURVEYS, MONITORING AND EVALUATION DIVISION
EPIDEMIOLOGY BUREAU
DEPARTMENT OF HEALTH
SAN LAZARO COMPOUND, RIZAL AVENUE,
STA. CRUZ, MANILA

Republic of the Philippines
Department of Health

MARIA ROSARIO S. VERGEIRE, MD, MPH, CESO IV
OIC - Undersecretary of Health, Public Health Services Team

NESTOR F. SANTIAGO JR., MD, MPHC, MHSA, CESO II
Assistant Secretary of Health, Public Health Services Team

RODOLFO ANTONIO M. ALBORNOZ, MD, MPH, MDM, CESE
OIC-Director IV, Epidemiology Bureau

ALETHEA R. DE GUZMAN, MD, MCHM, PHSAE
OIC- Director III, Epidemiology Bureau

VITO G. ROQUE, JR., MD, PHSAE
Chief, Surveys, Monitoring and Evaluation Division

FHSIS UNIT STAFF

MARIEL A. DEJESA, MD, MPM
Medical Officer IV

Jose M. Hernaez
Information Systems Analyst III
Joel V. Cantero
Computer Programmer II
Hernan T. Tayer
Computer Programmer II
Julius T. Castro
Computer Programmer II
Lester C. Recio
Health Program Officer II

Denisse Lou M. Adriano
Senior Health Program Officer
Allan P. Ignacio
Statistician II
Dexter Jay B. Flores
Health Program Officer II
Karla Mae C. Ramirez
Health Program Officer II

FOREWORD

The Department of Health (DOH) presents the 2020 Field Health Services Information System (FHSIS) Annual Report. This report covers the data from the services rendered by the government to the public, demographic data and reported cases for notifiable diseases.

Relevant data is essential for health care providers and program managers in identifying valuable health interventions for the Filipinos and creation of policies and allocation of resources for health care by the decision-makers. Health data is also needed in monitoring and evaluating the effectiveness of interventions. Over the years, the FHSIS reports have been of great value for the health sector, academe, researchers and other organizations.

We are grateful for the hard work and contribution to this report of our health workers and staff from the barangay, municipal, city and provincial offices, our partners from the Local Government Units, the Centers for Health Development and the Epidemiology Bureau. Interventions and policies shall be derived from the content of the 2020 FHSIS Annual Report with the aim of the DOH to improve health outcomes and provide better health services advantageous to all Filipinos.

MARIA ROSARIO S. VERGEIRE, MD, MPH, CESO IV
OIC - Undersecretary of Health, Public Health Services Team

TABLE OF CONTENTS

Chapter 1 – Accomplishment of Public Health Programs

**1.A Family Planning Services**	
Summary Tables (by Method, Province/City, Age group and Region)	
1. No. of New Acceptors – Table 1.A.1	2
2. No. of Other Acceptors – Table 1.A.2	22
3. No. of Drop-outs – Table 1.A.3	42
4. No. of Current Users End – Table 1.A.4	62
5. No. of Unmet Needs – Table 1.A.5	82
Figures and Graphs (by Method and Age group)	
1. Percentage Distribution of New Acceptors (10-49 years old) – Figure 1.A.1	88
2. Percentage Distribution of Other Acceptors (10-49 years old) – Figure 1.A.2	88
3. Percentage Distribution of Drop-outs (10-49 years old) – Figure 1.A.3	89
4. Percentage Distribution of Current Users End (10-49 years old) – Figure 1.A.4	89
5. Percentage Distribution of Unmet Needs by Region – Figure 1.A.5	90
**1.B Maternal Care and Services**	
1.B.1 Prenatal Care	
Summary Tables (by Region, Province/City and Age group)	
1. Number and proportion of pregnant women with at least 4 prenatal check-ups – Table 1.B.1.1	91
2. Proportion of pregnant women according to their nutritional status (normal, low and high BMI) – Table 1.B.1.2	96
3. No. and proportion of women pregnant for the first time given at least 2 doses of Tetanus Diphtheria (Td) vaccination – Table 1.B.1.3	100
4. No. and proportion of Women pregnant for the 2nd or more times given at least 3 doses of Td vaccination (Td2 Plus) – Table 1.B.1.4	105
5. No. and proportion of pregnant women who completed the dose of Iron with folic acid supplementation – Table 1.B.1.5	110
6. No. and proportion of pregnant women who completed doses of calcium carbonate supplementation – Table 1.B.1.6	115
7. No. and proportion of pregnant women given iodine capsules – Table 1.B.1.7	120
8. No. and proportion of pregnant women given one dose of deworming tablet – Table 1.B.1.8	125
9. No. and proportion of pregnant women screened for syphilis – Table 1.B.1.9	130
10. No. and proportion of pregnant women tested positive for syphilis – Table 1.B.1.10	135
11. No. and proportion of pregnant women screened for Hepatitis B -Table 1.B.1.11	140
12. Proportion of pregnant woment tested positive for Hepatitis B – Table 1.B.1.12	145
13. No and proportion of pregnant women screened for HIV – Table 1.B.1.13	150
14. No and porportion of pregnant women tested for complete blood count (CBC) or Hemoglobin (Hgb) & Hemotocrit (Hct) count - Table 1.B.1.14	155

15. No. and proportion of pregnant women tested for complete blood count or Hgb & Hct count diagnosed with anemia – Table 1.B.1.15	160
16. No. and proportion of pregnant women screened for gestational diabetes – Table 1.B.1.16	165
17. No. and proportion of pregnant women tested positive for gestational diabetes – Table 1.B.1.17	169
Figures and Graphs (by Region)	
1. Pregnant women 10-14 yrs. old with 4 Prenatal Check-ups – Figure 1.B.1.1	175
2. Pregnant women 15-19 yrs. old with 4 Prenatal Check-ups – Figure 1.B.1.2	175
3. Pregnant women 20-49 yrs. old with 4 Prenatal Check-ups – Figure 1.B.1.3	176
4. Pregnant women (10-49 yrs. old) with 4 Prenatal Check-ups – Figure 1.B.1.4	176
5. Percentage distribution of Pregnant women seen during the 1st trimester with Normal BMI – Figure 1.B.1.5	177
6. Percentage distribution of Pregnant women seen during the 1st trimester with Low BMI – Figure 1.B.1.6	177
7. Percentage distribution of Pregnant women seen during the 1st trimester with High BMI – Figure 1.B.1.7	178
8. Percentage distribution of Women (10-14 yrs. old) pregnant for the 1st time given at least 2 doses of Tetanus Diphtheria (Td) vaccination – Figure 1.B.1.8	179
9. Percentage distribution of Women (15-19 yrs. old) pregnant for the 1st time given at least 2 doses of Td vaccination – Figure 1.B.1.9	179
10. Percentage distribution of Women (20-49 yrs. old) pregnant for the 1st time given at least 2 doses of Td vaccination – Figure 1.B.1.10	180
11. Percentage distribution of Women pregnant for the 1st time given at least 2 doses of Td vaccination by Age group – Figure 1.B.1.11	180
12. Percentage distribution of Women (10-14 yrs. old) pregnant for the 2nd time or more given at least 3 doses of Td vaccination – Figure 1.B.1.12	181
13. Percentage distribution of Women (15-19 yrs. old) pregnant for the 2nd time or more given at least 3 doses of Td vaccination – Figure 1.B.1.13	181
14. Percentage distribution of Women (20-49 yrs. old) pregnant for the 2nd time or more given at least 3 doses of Td vaccination – Figure 1.B.1.14	182
15. Percentage distribution of Women pregnant for the 2nd time or more given at least 3 doses of Td vaccination by Age group – Figure 1.B.1.15	182
16. Percentage distribution of pregnant women (10-14 yrs. old) who completed Iron with folic acid – Figure 1.B.1.16	183
17. Percentage distribution of pregnant women (15-19 yrs. old) who completed Iron with folic acid – Figure 1.B.1.17	183
18. Percentage distribution of pregnant women (20-49 yrs. old) who completed Iron with folic acid – Figure 1.B.1.18	184
19. Percentage distribution of pregnant women (10-49 yrs. old) who completed Iron with folic acid – Figure 1.B.1.19	184
20. Percentage distribution of pregnant women who completed Iron with folic acid by Age group – Figure 1.B.1.20	185
21. Percentage distribution of pregnant women (10-14 yrs. old) who completed Calcium Carbonate – Figure 1.B.1.21	186

22. Percentage distribution of pregnant women (15-19 yrs. old) who completed Calcium Carbonate – Figure 1.B.1.22	186
23. Percentage distribution of pregnant women (20-49 yrs. old) who completed Calcium Carbonate – Figure 1.B.1.23	187
24. Percentage distribution of pregnant women (10-49 yrs. old) who completed Calcium Carbonate – Figure 1.B.1.24	187

25. Percentage distribution of pregnant women who completed Calcium Carbonate by Age group– Figure 1.B.1.25	188
26. Percentage distribution of pregnant women (10-14 yrs. old) who completed Iodine – Figure 1.B.1.26	189
27. Percentage distribution of pregnant women (15-19 yrs. old) who completed Iodine – Figure 1.B.1.27	189
28. Percentage distribution of pregnant women (20-49 yrs. old) who completed Iodine – Figure 1.B.1.28	190
29. Percentage distribution of pregnant women (10-49 yrs. old) who completed Iodine – Figure 1.B.1.29	190
30. Percentage distribution of pregnant women who completed Iodine by Age group– Figure 1.B.1.30	191
31. Percentage distribution of pregnant women (10-14 yrs. old) given one dose of deworming tablet – Figure 1.B.1.31	192
32. Percentage distribution of pregnant women (15-19 yrs. old) given one dose of deworming tablet – Figure 1.B.1.32	192
33. Percentage distribution of pregnant women (20-49 yrs. old) given one dose of deworming tablet – Figure 1.B.1.33	193
34. Percentage distribution of pregnant women (10-49 yrs. old) given one dose of deworming tablet – Figure 1.B.1.34	193
35. Percentage distribution of pregnant women given one dose of deworming tablet by Age group – Figure 1.B.1.35	194
36. Percentage distribution of pregnant women (10-14 yrs. old) screened for syphilis – Figure 1.B.1.36	195
37. Percentage distribution of pregnant women (15-19 yrs. old) screened for syphilis – Figure 1.B.1.37	195
38. Percentage distribution of pregnant women (20-49 yrs. old) screened for syphilis – Figure 1.B.1.38	196
39. Percentage distribution of pregnant women (10-49) screened for syphilis – Figure 1.B.1.39	196
40. Percentage distribution of pregnant women screened for syphilis by Age group – Figure 1.B.1.40	197
41. Percentage distribution of pregnant women (10-14 yrs. old) tested positive for syphilis – Figure 1.B.1.41	198
42. Percentage distribution of pregnant women (15-19 yrs. old) tested positive for syphilis – Figure 1.B.1.42	198
43. Percentage distribution of pregnant women (20-49 yrs. old) tested positive for syphilis – Figure 1.B.1.43	199

44. Percentage distribution of pregnant women (10-49 yrs. old) tested positive for syphilis – Figure 1.B.1.44	199
45. Percentage distribution of pregnant women tested positive for syphilis by Age group – Figure 1.B.1.45	200
46. Percentage distribution of pregnant women (10-14 yrs. old) screened for Hepatitis B – Figure 1.B.1.46	201
**47.** Percentage distribution of pregnant women (15-19 yrs. old) screened for Hepatitis B – Figure 1.B.1.47	201
48. Percentage distribution of pregnant women (20-49 yrs. old) screened for Hepatitis B – Figure 1.B.1.48	202
49. Percentage distribution of pregnant women screened for Hepatitis B – Figure 1.B.1.49	202
50. Percentage distribution of pregnant women screened for Hepatitis B by Age group – Figure 1.B.1.50	203
51. Percentage distribution of pregnant women (10-14 yrs. old) tested positive for Hepatitis B – Figure 1.B.1.51	204
52. Percentage distribution of pregnant women (15-19 yrs. old) tested positive for Hepatitis B – Figure 1.B.1.52	204
53. Percentage distribution of pregnant women (20-49 yrs. old) tested positive for Hepatitis B – Figure 1.B.1.53	205
54. Percentage distribution of pregnant women (10-49 yrs. old) tested positive for Hepatitis B – Figure 1.B.1.54	205
55. Percentage distribution of pregnant women tested positive for Hepatitis B by Age group – Figure 1.B.1.55	206
56. Percentage distribution of pregnant women (10-14 yrs. old) screened for HIV – Figure 1.B.1.56	207
57. Percentage distribution of pregnant women (15-19 yrs. old) screened for HIV – Figure 1.B.1.57	207
58. Percentage distribution of pregnant women (20-49 yrs. old) screened for HIV – Figure 1.B.1.58	208
59. Percentage distribution of pregnant women (10-49 yrs. old) screened for HIV – Figure 1.B.1.59	208
60. Percentage distribution of pregnant women screened for HIV by Age group – Figure 1.B.1.60	209
61. Percentage distribution of pregnant women (10-14 yrs. old) tested for complete blood count (CBC) or Hemoglobin (Hgb) & Hemotocrit (Hct) count – Figure 1.B.1.61	210
62. Percentage distribution of pregnant women (15-19 yrs. old) tested for complete blood count (CBC) or Hemoglobin (Hgb) & Hemotocrit (Hct) count – Figure 1.B.1.62	210
63. Percentage distribution of pregnant women (20-49 yrs. old) tested for complete blood count (CBC) or Hemoglobin (Hgb) & Hemotocrit (Hct) count – Figure 1.B.1.63	211
64. Percentage distribution of pregnant women (10-49 yrs. old) tested for complete blood count (CBC) or Hemoglobin (Hgb) & Hemotocrit (Hct) count – Figure 1.B.1.64	211

65. Percentage distribution of pregnant women tested for complete blood count (CBC) or Hemoglobin (Hgb) & Hemotocrit (Hct) count by Age group – Figure 1.B.1.65 212
66. Percentage distribution of pregnant women (10-14 yrs. old) tested positive for complete blood count (CBC) or Hemoglobin (Hgb) & Hemotocrit (Hct) count – Figure 1.B.1.66 213
67. Percentage distribution of pregnant women (15-19 yrs. old) tested positive for complete blood count (CBC) or Hemoglobin (Hgb) & Hemotocrit (Hct) count – Figure 1.B.1.67 213
68. Percentage distribution of pregnant women (20-49 yrs. old) tested positive for complete blood count (CBC) or Hemoglobin (Hgb) & Hemotocrit (Hct) count – Figure 1.B.1.68 214
69. Percentage distribution of pregnant women tested positive for complete blood count (CBC) or Hemoglobin (Hgb) & Hemotocrit (Hct) count – Figure 1.B.1.69 214
70. Percentage distribution of pregnant women tested positive for complete blood count (CBC) or Hemoglobin (Hgb) & Hemotocrit (Hct) count by Age group – Figure 1.B.1.70 215
71. Percentage distribution of pregnant women (10-14 yrs. old) screened for Gestational Diabetes – Figure 1.B.1.71 216
72. Percentage distribution of pregnant women (15-19 yrs. old) screened for Gestational Diabetes – Figure 1.B.1.72 216
73. Percentage distribution of pregnant women (20-49 yrs. old) screened for Gestational Diabetes – Figure 1.B.1.73 217
74. Percentage distribution of pregnant women (10-49 yrs. old) screened for Gestational Diabetes – Figure 1.B.1.74 217
75. Percentage distribution of pregnant women screened for Gestational Diabetes by Age group – Figure 1.B.1.75 218
76. Percentage distribution of pregnant women (10-14 yrs. old) tested positive for Gestational Diabetes – Figure 1.B.1.76 219
77. Percentage distribution of pregnant women (15-19 yrs. old) tested positive for Gestational Diabetes – Figure 1.B.1.77 219
78. Percentage distribution of pregnant women (20-49 yrs. old) tested positive for Gestational Diabetes – Figure 1.B.1.78 220
79. Percentage distribution of pregnant women (10-49 yrs. old) tested positive for Gestational Diabetes – Figure 1.B.1.79 220
80. Percentage distribution of pregnant women tested positive for Gestational Diabetes by Age group – Figure 1.B.1.80 221

1.B.2 Intrapartum Care and Delivery Outcome

Summary Tables (by Region, Province/City and Age group)

1. Total Number of women who delivered a live baby or stillbirth/fetal death, deliveries attended by skilled health professionals and Facility Based delivery by Region – Table 1.B.2.1 222
2. No. and proportion of delivery by type, Vaginal and Cesarean – Table 1.B.2.2 225

3. No. and proportion of pregnancy outcome (Full term and Pre-term) – Table 1.B.2.3	230
4. No and proportion of pregnancy by outcome (Fetal death & Abortion) – Table 1.B.2.4	235
5. No. and Proportion of livebirths by birth weight – Table 1.B.2.5	240
Figures and Graphs	
1. Percentage of deliveries attended by Skilled Health Professional – Figure 1.B.2.1	245
2. Percentage of deliveries attended by Physician – Figure 1.B.2.2	245
3. Percentage of deliveries attended by Nurse – Figure 1.B.2.3	246
4. Percentage deliveries attended by Midwife – Figure 1.B.2.4	246
5. Facility Based Delivery - Figure 1.B.2.5	247
6. Facility Based Delivery by Ownership (Public and Private) – Figure 1.B.2.6	247
7. Facility Based Delivery by Ownership (Public) - Figure 1.B.2.7	248
8. Facility Based Delivery by Ownership (Private) - Figure 1.B.2.8	248
9. Percentage of Deliveries by Type (Vaginal) – Figure 1.B.2.9	249
10. Percentage of Deliveries by Type (Cesarean Section) – Figure 1.B.2.10	249
11. Percentage of Pregnancy outcome (Full term) – Figure 1.B.2.11	250
12. Percentage of Pregnancy outcome (Pre-term) – Figure 1.B.2.12	250
13. Percentage of Pregnancy outcome (Fetal Death) – Figure 1.B.2.13	251
14. Percentage Distribution of normal birth weight – Figure 1.B.2.14	252
15. Percentage Distribution of low birth weight – Figure 1.B.2.15	252
16. Percentage Distribution of unknown birth weight – Figure 1.B.2.16	253

1.B.3 Postpartum and Newborn Care	
Summary Tables (by Region, Province/City and Age group)	
1. No. and proportion of postpartum women together with their newborn who completed at least 2 postpartum check-ups – Table 1.B.3.1	254
2. Number and proportion of postpartum women who completed Iron with Folic Acid – Table 1.B.3.2	259
3. No. and proportion of postpartum women who completed Vitamin A supplementation – Table 1.B.3.3	264
Figures and Graphs	
1. Postpartum Women together with their Newborn who completed at least 2 Postpartum check-ups by Region - Figure 1.B.3.1	269
2. Postpartum Women together with their Newborn who completed at least 2 Postpartum check-ups by Age group and Region - Figure 1.B.3.2	269
3. Postpartum women who completed Iron with folic acid by Region – Figure 1.B.3.3	270
4. Postpartum women who completed Iron with folic acid by Age group and Region – Figure 1.B.3.4	270
5. Postpartum women who completed Vitamin A supplementation by Region – Figure 1.B.3.5	271
6. Postpartum women who completed Vitamin A supplementation by Age group and Region – Figure 1.B.3.6	271

1.C Child Care and Services

1.C.1 Immunization Services for Infants and Children

Summary Tables (by Region, Province/City and Age group)

1. No. and proportion of Child Protected at Birth (CPAB), newborn / infants vaccinated with BCG and Hepatitis B antigen within 24 hours after birth – Table 1.C.1.1	273
2. No. and proportion of Infants who completed 3 doses of DPT-HIB-HepB antigen – Table 1.C.1.2	276
3. Proportion of infants who completed 3 doses of Oral Polio vaccine (OPV) and Inactive Polio Vaccine (IPV) – Table 1.C.1.3	280
4. Proportion of infants who completed 3 doses of Pneumococcal Conjugate Vaccine (PCV) – Table 1.C.1.4	283
5. No. and proportion of Children vaccinated with 2 doses of Measles Containing Vaccine (MCV) – Table 1.C.1.5	287
6. No. and proportion of Fully-Immunized Children (FIC) and Completely Immunized Children (CIC) – Table 1.C.1.6	292

Figures and Graphs

1. Percentage distribution of Child Protected at Birth by Region – Figure 1.C.1.1	297
2. Percentage distribution of Infants given BCG by Region – Figure 1.C.1.2	297
3. Percentage distribution of newborn / infant given Hepatitis B1 Vaccine within 24 hours after birth by Region – Figure 1.C.1.3	298
4. Percentage distribution of Infants vaccinated with DPT-HIB-HepB 1 by Region – Figure 1.C.1.4	299
5. Percentage distribution of Infants vaccinated with DPT-Hib-HepB 2 by Region – Figure 1.C.1.5	299
6. Percentage distribution of Infants vaccinated with DPT-Hib-HepB 3 by Region – Figure 1.C.1.6	300
7. Percentage distribution of Infants who completed 3 doses of HIB-HepB antigen by Region – Figure 1.C.1.7	300
8. Percentage distribution of Infants given Oral Polio Vaccine 1 by Region – Figure 1.C.1.8	301
9. Percentage distribution of Infants given Oral Polio Vaccine 2 by Region – Figure 1.C.1.9	301
10. Percentage distribution of Infants given Oral Polio Vaccine 3 by Region – Figure 1.C.1.10	
11. Percentage distribution of Infants given Inactivated Polio Vaccine by Region – Figure 1.C.1.11	302
12. Percentage distribution of Infants who completed 3 dose of Oral Polio Vaccines by Region – Figure 1.C.1.12	302
13. Percentage distribution of Infants given Pneumococcal Conjugate Vaccine (PCV) 1 by Region – Figure 1.C.1.13	303
14. Percentage distribution of Infants given PCV 2 by Region – Figure 1.C.1.14	304
15. Percentage distribution of Infants given PCV 3 by Region – Figure 1.C.1.15	304
16. Percentage distribution of Infants who completed 3 doses of PCV's by Region – Figure 1.C.1.16	305
17. Percentage distribution of Children vaccinated with Measles Containing Vaccine 1 by Region – Figure 1.C.1.17	305

18. Percentage distribution of Children vaccinated with Measles Containing Vaccine 2 by Region – Figure 1.C.1.18 306 306
19. Percentage distribution of Children vaccinated with 2 dose of Measles Containing Vaccine by Region – Figure 1.C.1.19 307
20. Percentage of Fully Immunized Children (FIC) - Figure 1.C.1.20 308
21. Percentage of Completely Immunized Children (CIC) – Figure 1.C.1.21 308

1.C.2 Nutrition Services for Infants and Children

Summary Tables (by Region, Province/City and Age group)

1. No. and proportion of newborns who were initiated on breastfeeding immediately after birth lasting for at least 90 minutes, infants exclusively breastfed until 6th month and infants who continued breastfeeding and were introduced to complementary feeding beginning at 6 months of age – Table 1.C.2.1 309
2. No. and proportion of infants born preterm or with low birth given iron supplements and infants / children 6- 11 and 12-59 months old who completed Vitamin A supplementation – Table 1.C.2.2 313
3. No. and proportion of infants 6-11 months old and children 12-23 months old who completed Micronutrient Powder (MNP) supplementation – Table 1.C.2.3 316
4. No. and proportion of 0-59 months old who are Normal, Stunted and Overweight/obese – Table 1.C.2.4 320
5. No. and proportion of 0-59 months old who are Wasted-Mam, Wasted-Sam and Wasted – Table 1.C.2.5 324

Figures and Graphs

1. Percentage distribution of Newborns initiated on breastfeeding immediately after birth by Region – Figure 1.C.2.1 328
2. Percentage distribution of Infants Exclusively breastfed until 6th months by Region – Figure 1.C.2.2 328
3. Percentage distribution of infants who continued breastfeeding and were introduced to complementary feeding by Region – Figure 1.C.2.3 329
4. Percentage distribution of infants born preterm or with low birth given iron supplementation by Region – Figure 1.C.2.4 330
5. Percentage distribution of infants/children (6-11 mos. old) who completed Vitamin A supplementation – Figure 1.C.2.5 330
6. Percentage distribution of infants/children (12-59 mos. old) who completed Vitamin A supplementation – Figure 1.C.2.6 331
7. Percentage distribution of infants 6-11 months old who completed Micronutrient Powder (MNP) supplementation by Region – Figure 1.C.2.7 332
8. Percentage distribution of children 12-23 months old who completed Micronutrient Powder (MNP) supplementation by Region – Figure 1.C.2.8 332
9. Percentage of infants 6-11 months old and children 12-23 months old who completed Micronutrient Powder (MNP) supplementation – Figure 1.C.2.9 333
10. Percentage distribution of Children 0-59 mos. old whose Nutritional status is Normal by Region – Figure 1.C.2.10 334
11. Percentage distribution of Children 0-59 mos. old whose Nutritional status is stunted by Region – Figure 1.C.2.11 334
12. Percentage distribution of Children 0-59 mos. old whose Nutritional status is overweight/obese by Region – Figure 1.C.2.12
13. Percentage distribution of Children 0-59 mos. old whose Nutritional status is wasted by Region – Figure 1.C.2.13 335

14. Percentage distribution of Children 0-59 mos according to their Nutritional status by Region – Figure 1.C.2.14	335<br>336
**1.C.3 Deworming Services for Children and Adolescents**	
Summary Tables (by Region, Province/City and Age group)	
1. No and proportion of Children/Adolescents who completed 3 doses of de-worming tablet – Table 1.C.3.1	337
Figures and Graphs	
1. Percentage distribution of Children 1-4 yrs. old given 2 doses of deworming drugs by Region – Figure 1.C.3.1<br>2. Percentage distribution of Children 5-9 yrs. old given 2 doses of deworming drugs by Region – Figure 1.C.3.2<br>3. Percentage distribution of Children 10-19 yrs. old given 2 doses of deworming drugs by Region – Figure 1.C.3.3	341<br>341<br>342

**1.C.4 Management of Sick Infants and Children**	
Summary Tables (by Region, Province/City and Age group)	
1. No. and proportion of high risk infants and children with measles and/or persistent diarrhea who received Vitamin A capsule aside from routine supplementation – Table 1.C.4.1<br>2. No. and proportion of diarrhea cases seen (0-59 months old) who received oral rehydration and solution (ORS) and ORS with zinc drops or syrup and Pneumonia cases seen and received treatment – Table 1.C.4.2	343<br>347
Figures and Graphs	
1. Percentage distiribution of Sick Infants aged 6-11 mos. Vitamin A 100,000 IU by Region – Figure 1.C.4.1<br>2. Percentage distiribution of Sick Infants aged 12-59 mos. Vitamin A 200,000 IU by Region – Figure 1.C.4.2<br>3. Percentage distribution of children (0-59 mos.) with Diarrhea given ORS by Region – Figure 1.C.4.3<br>4. Percentage distribution of children (0-59 mos.) with Diarrhea given ORS with Zinc by Region – Figure 1.C.4.4<br>5. Percentage distribution of children (0-59 mos.) who are treated with Pneumonia by Region – Figure 1.C.4.5	351<br>351<br>352<br>352<br>353

1.D Oral Health Care and Services

Summary Tables (by Region, Province/City and Age group)	
1. No. and proportion of children 12-59 months old who are orally fit upon oral examination or after oral rehabilitation – Table 1.D.1	354
2. No. and proportion of clients 5 years old and above with new cases of Decayed-Missing Filled Teeth (DMFT) – Table 1.D.2	359
3. No. and proportion of infants 0-11 months old, 1-4 years old and 5-9 years old who received BOHC – Table 1.D.3	364
4. No. and proportion of adolescents (10-19 years old) and adults (20-59 years old) who received BOHC – Table 1.D.4	368
5. No. and proportion of senior citizen (60 years old and above) who received BOHC – Table 1.D.5	372
9. No. and proportion of pregnant women who received BOHC – Table 1.D.6	377
Figures and Graphs	
1. Percentage distribution of Children 12-59 months old who are orally fit upon oral examination by Region – Figure 1.D.1	382
2. Percentage distribution of Children 12-59 months old who are orally fit upon oral examination by Sex – Figure 1.D.2	382
3. Percentage distribution of children 5 years old and above with cases of Decayed, Missing Filled Teeth (DMFT) by Region – Figure 1.D.3	383
4. Percentage distribution of children 5 years old and above with cases of decayed, missing filled teeth by Region and Sex– Figure 1.D.4	383
5. Percentage distribution of children 5 years old and above with cases of decayed, missing filled teeth by Sex– Figure 1.D.5	384
6. Percentage distribution of infants 0-11 months old who received Basic Oral Health Care (BOHC) by Region – Figure 1.D.6	385
7. Percentage distribution of children 1-4 yrs. old who received BOHC by Region – Figure 1.D.7	385
8. Percentage distribution of children 5-9 yrs. old who received BOHC by Region – Figure 1.D.8	386
9. Percentage distribution of children who received BOHC by age group – Figure – 1.D.9	386
10. Percentage distribution of adolescent 10-14 yrs. old who received BOHC by Region – Figure 1.D.10	387
11. Percentage distribution of adolescent 16-19 yrs. old who received BOHC by Region – Figure 1.D.11	387
12. Percentage distribution of adult 20-49 yrs. old who received BOHC by Region – Figure 1.D.12	388
13. Percentage distribution of adolescent 10-19 yrs. old and adult 20-49 yrs. old who received BOHC by Region – Figure 1.D.13	388
14. Percentage distribution of Senior Citizen 60 yrs. old and above who received BOHC by Region – Figure 1.D.14	389
15. Percentage distribution of Pregnant women 10-14 yrs. old who received BOHC by Region – Figure 1.D.15	
16. Percentage distribution of Pregnant women 15-19 yrs. old who received BOHC by Region – Figure 1.D.16	390
17. Percentage distribution of Pregnant women 20-49 yrs. old who received BOHC by Region – Figure 1.D.17	390
	391

18. Percentage distribution of Pregnant women who received BOHC by Age group – Figure 1.D.18	391

**1.E Infectious Disease Prevention and Control Services**	
1.E.1 Filariasis Prevention and Control	
Summary Tables (by Region, Province/City and Age group)	
1. No. and Proportion of positive cases found, case detection rate, lymphatic filariasis cases and clinical Rate	392
Figures and Graphs	
1. Case detection rate by Region – Figure 1.E.1.1	398
2. Clinical Rate by Region – Figure 1.E.1.2	398
3. Case detection rate by Year – Figure 1.E.1.3	399
4. Clinical Rate (lymphatic Filariasis) by Year – Figure 1.E.1.4	399

1.E.2 Schistosomiasis Prevention and Control	
Summary Tables (by Region, Province/City and Age group)	
1. No. and Proportion of suspected schistosomiasis cases seen, acute clinically diagnosed cases seen and acute confirmed cases – Table 1.E.2.1	400
2. No. and proportion of chronic clinically diagnosed cases seen and confirmed chronic cases – Table 1.E.2.2	405
3. No. and proportion of chronic clinically diagnosed cases treated in the Health Facility and confirmed chronic cases referred to hospital facility – Table 1.E.2.3	410
Figures and Graphs	
1. Proportion of suspected cases seen by Region – Figure 1.E.2.1	415
2. Proportion of Acute clinically diagnosed cases seen by Region – Figure 1.E.2.2	415
3. Proportion of Acute confirmed cases by Region – Figure 1.E.2.3	416
4. Proportion of chronic clinically diagnosed cases seen – Figure 1.E.2.4	416
5. Proportion of chronic clinically diagnosed confirmed cases by Region – Figure 1.E.2.5	417
6. Proportion of chronic clinically diagnosed cases treated in the Health Facility by Region – Figure 1.E.2.6	417
7. Proportion of confirmed chronic cases referred to Hospital Facility by Region – Figure 1.E.2.7	418

1.E.3 Soil Transmitted Helminthiasis Prevention and Control	
Summary Tables (by Region, Province/City and Age group)	
1. No. and proportion of Pre School Age Children (PSAC) 1-4 yrs. old and School Age Children (SAC) 5-9 yrs. old who completed 2 doses of deworming tablet – Table 1.E.3.1	419
2. No. and proportion of Adolescents (10-19 yrs. old) and WRA (20-49 yrs.old) who completed 2 doses of deworming tablets – Table 1.E.3.2	424

3. No. and proportion of pregnant women, who completed 2 doses of deworming tablet – Table 1.E.3.3	429
Figures and Graphs	
1. Proportion of PSAC (1-4 yrs. old) who completed two (2) doses of deworming tablets by Region – Figure 1.E.3.1	434
2. Proportion of SAC (5-9 yrs. old) who completed two (2) doses of deworming tablets by Region – Figure 1.E.3.2	434
3. Proportion of Adolescent (10-19 yrs. old) who completed 2 doses of deworming tablets by Region – Figure 1.E.3.3	435
4. Proportion of WRA (20-49 yrs. old) who completed 2 doses of deworming tablets by Region – Figure 1.E.3.4	435
5. Proportion of pregnant women who completed 1 dose of deworming tablet – Figure 1.E.3.5	436

1.E.4 HIV-AIDS/STI Prevention and Control	
Summary Tables (by Region, Province/City and Age group)	
1. No. and proportion of pregnant women screened for Syphilis, positive for Syphilis and screened for HIV – Table 1.E.4.1	437
Figures and Graphs	
1. Proportion of pregnant women screened for syphilis by Region - Figure 1.E.4.1	442
2. Proportion of pregnant women tested positive for syphilis by Region – Figure 1.E.4.2	442
3. Proportion of pregnant women tested for HIV by Region – Figure 1.E.4.3	443

1.E.5 Tuberculosis Prevention and Control	
Summary Tables (by Region, Province/City and Age group	
1. Case Notification Rate (CNR), all forms – Table 1.E.5.1	444
2. Case Notification Rate (DRTB) – Table 1.E.5.2	449
3. Treatment Success Rate (TSR), DSTB-all forms – Table 1.E.5.3	454
4. Treatment Success Rate (TSR), MDRTB – Table 1.E.5.4	460
Figures and Graphs	
1. Case Notification Rate (CNR), all forms – Figure 1.E.5.1	465
2. Case Notification Rate (DRTB) – Figure 1.E.5.2	465
3. Treatment Success Rate (TSR), DSTB-all forms – Figure 1.E.5.3	466
4. Treatment Success Rate (TSR), MDRTB – Figure 1.E.5.4	466

1.E.6 Malaria Prevention and Control	
Summary Tables (by Region, Province/City and Age group	
1. No. and Rates of Probable/Clinically and confirmed Malaria Cases by Sex and Age group (<5 yrs. old and >=5 yrs. old) – Table 1.E.6.1	467
2. No. and Rates of Probable/Clinically and confirmed Malaria Deaths by Sex and Age group (<5 yrs. old and >=5 yrs. old) – Table 1.E.6.2	472
3. No. of confirmed Malaria Cases and Annual Parasite Incidence – Table 1.E.6.3	477

Figures and Graphs	
1. Morbidity rate among < 5 y/o of Malaria per 100,000/population by Region – Figure 1.E.6.1	482
2. Morbidity rate among >= 5 y/o of Malaria per 100,000/population by Region – Figure 1.E.6.2	482
3. Morbidity rate among < 5 y/o of Malaria per 100,000/population by Year – Figure 1.E.6.3	483
4. Morbidity rate among >= 5 y/o of Malaria per 100,000/population by Year – Figure 1.E.6.4	483
5. Mortality rate among < 5 y/o of Malaria per 100,000/population by Region – Figure 1.E.6.5	484
6. Mortality rate among >= 5 y/o of Malaria per 100,000/population by Region – Figure 1.E.6.6	484
7. Malaria parasite incidence by Region – Figure 1.E.6.7	485
8. Malaria parasite incidence by Year – Figure 1.E.6.8	485
1.E.7 Leprosy Prevention and Control	
Summary Tables (by Region, Province/City and Age group	
1. Leprosy cases undergoing treatment, Prevalence rate and Case detection rate – Table 1.E.7.1	486
Figures and Graphs	
1. Prevalence Rate by Region – Figure 1.E.7.1	491
2. Case Detection Rate by Region – Figure 1.E.7.2	491
3. Prevalence Rate by Year – Figure 1.E.7.3	492
4. Case Detection Rate by Region – Figure 1.E.7.4	492
1.E.8 Rabies	
Summary Tables (by Region, Province/City and Age group	
1. No. and proportion of deaths due to Rabies – Table 1.E.8.1	493
Figures and Graphs	
1. Proportion of death due to Rabies by Region – Figure 1.E.8.1	498
2. Proportion of death due to Rabies by Year – Figure 1.E.8.2	498

**1.F Non-Communicable Disease Prevention and Control Services**	
1.F.1 Lifestyle Related Diseases (Risk Assessment Using PhilPEN protocol)	
Summary Tables (by Region, Province/City and Age group)	
1. No. and proportion of adults age 20 years old and above who were risk assessed using the PhilPEN protocol – Table 1.F.1.1	499
2. No. and proportion of adults 20 years old and above who are current smokers, binge drinkers and overweight/obese based on the PhilPEN protocol – Table 1.F.1.2	504
Figures and Graphs	
1. Percentage distribution of adults aged 20 yrs. old and above who were risk-assessed using the PhilPEN protocol by Region – Figure 1.F.1.1	508
2. Percentage distribution of adults aged 20 yrs. old and above who are current smokers by Region – Figure 1.F.1.2	509

3. Percentage distribution of adults aged 20 yrs. old and above who are binge drinkers by Region – Figure 1.F.1.3	509
4. Percentage distribution of adults aged 20 yrs. old and above who are overweigh/obese by Region – Figure 1.F.1.4	510
5. Percentage distribution of adults aged 20 yrs. old and above who are current smokers, Binge drinkers and overweight/obese – Figure 1.F.1.5	510

1.F.2 Cancer Prevention and Control	
Summary Tables (by Region, Province/City and Age group)	
1. No. and proportion of newly-identified hypertensive and with Type 2 Diabetes Mellitus among 20 years old and above adults – Table 1.F.2.1	511
Figures and Graphs (by Region)	
1. Percentage distribution of women 20 yrs. old and above screened for cervical cancer – Figure 1.F.2.1	516
2. Percentage distribution of women 20 yrs. old and above found positive or suspected for cervical cancer – Figure 1.F.2.2	516
3. Percentage distribution of women 20 yrs. old and above screened for breast mass – Figure 1.F.2.3	517
4. Percentage distribution of women 20 yrs. old and above with suspicious breast mass – Figure 1.F.2.4	517
5. Percentage distribution of women 20 yrs. old and above screened and found positive/suspected for cervical cancer, screened for breast mass and with suspicious breast mass – Figure 1.F.2.5	518

1.F.3 Cardiovascular Disease and Diabetes Mellitus Prevention and Control	
Summary Tables (by Region, Province/City and Age group)	
1. No. and proportion of newly-identified hypertensive and with Type 2 Diabetes Mellitus among 20 years old and above adults – Table 1.F.3.1	519
Figures and Graphs (by Region)	
1. Percentage of newly identified 20 yrs. old and above adults with Hypertension by Sex – Figure 1.F.3.1	524
2. Percentage of newly identified 20 yrs. old and above adults with Type 2 Diabetes Mellitus by Sex – Figure 1.F.3.2	524
3. Percentage of newly identified 20 yrs. old and above adults with Hypertension – Figure 1.F.3.3	525
**4.** Percentage of newly identified 20 yrs. old and above adults with Type 2 Diabetes Mellitus – Figure 1.F.3.4	525
1.F.4 Blindness Prevention Program	
Summary Tables (by Region, Province/City and Age group)	
1. No. and proportion of Senior Citizens 60 years old and above screened for visual acuity and diagnosed with eye disease/s – Table 1.F.4.1	526

Figures and Graphs (by Region)	
1. *Percentage distribution of Senior Citizen (60 yrs. old and above) screened for visual acuity by Region – Figure 1.F.4.1*	531
2. *Percentage distribution of Senior Citizen (60 yrs. old and above) diagnosed with eye disease by Region – Figure 1.F.4.2*	531
3. *Percentage distribution of Senior Citizen (60 yrs. old and above) screened for visual acuity and diagnosed with eye disease – Figure 1.F.4.3*	532
**1.F.5 Immunization for Senior Citizens**	
Summary Tables (by Region, Province/City and Age group)	
1. No. and proportion of senior citizens 60 years old and above who received one (1) dose of pneumococcal polysaccharide vaccine (PPV) and one (1) dose of Influenza Vaccine – Table 1.F.5.1	533
Figures and Graphs (by Region)	
1. Proportion of senior citizens 60 years old and above who received one (1) dose of pneumococcal polysaccharide vaccine (PPV) – Figure 1.F.5.1	538
2. Proportion of senior citizens 60 years old and above who received one (1) dose of Influenza vaccine – Figure 1.F.5.2	538
3. Proportion of senior citizens 60 years old and above who received one (1) dose of pneumococcal polysaccharide vaccine (PPV) and Influenza Vaccine – Figure 1.F.5.3	539

**1.G Environmental Health and Sanitation Services**	
Summary Tables (by Region, Province/City and Age group)	
1. No. and proportion of households with access to basic safe water supply (Level I, II, III) and using safely managed drinking-water services – Table 1.G.1	540
2. No and proportion of households with Basic Sanitation Facility and using safely managed sanitation service – Table 1.G.2	544
3. No. and proportion of Industrial Establishments, Industrial Establishments issued with Sanitary Permit, Total No. of Barangay and No. of Barangays certified as Zero Open Defecation (ZOD) – Table 1.G.3	548
Figures and Graphs (by Region)	
1. Percentage of Households with access to basic safe water supply (level 1) – Figure 1.G.1	553
2. Percentage of Households with access to basic safe water supply (level 2) – Figure 1.G.2	553
3. Percentage of Households with access to basic safe water supply (level 3) – Figure 1.G.3	554
4. Percentage of Households with access to basic safe water supply (levels 1, 2 & 3) – Figure 1.G.4	554
5. Percentage of Households using Safely Manage Water Services – Figure 1.G.5	555
6. Percentage of Households with basic sanitation facility (Septic Tank) – Figure 1.G.6	556
7. Percentage of Households with basic sanitation facility (Community Sewerage System) – Figure 1.G.7	556
	557

8. Percentage of Households with basic sanitation facility (Ventilated improve Pit latrine) – Figure 1.G.8<br>9. Percentage of Households using safely manage sanitation service – Figure 1.G.9<br>10. Percentage of Industrial Establishment with Sanitary Permit – Figure 1.G.10<br>11. Percentage of Barangay certified as ZOD area – Figure 1.G.11	557<br>558<br>558

Program Summary	
**1. Family Planning**	560
**2. Maternal Care**	561
**3. Child Care**	567
**4. Oral Health**	574
**5. Non Communicable Diseases**	577
**6. Environmental**	580

Chapter 2 – Morbidity, Mortality and Natality

2.A.1 Top Ten Leading causes of Morbidity	
1. Top Ten Leading causes of Morbidity (Philippines) – Table 2.A.1.1<br>2. Top Ten Leading causes of Morbidity by Sex (Male) – Table 2.A.1.2<br>3. Top Ten Leading causes of Morbidity by Sex (Female) – Table 2.A.1.3	582<br>583<br>583
**2.A.2 Top Ten Leading causes of Morbidity by Region**	
1. Center for Health Development (CHD), Metro Manila – Table 2.A.2.1<br>2. CHD, Cordillera Administrative Region (CAR) – Table 2.A.2.2<br>3. CHD, Ilocos – Table 2.A.2.3<br>4. CHD, Cagayan Valley – Table 2.A.2.4<br>5. CHD, Central Luzon – Table 2.A.2.5<br>6. CHD, CALABARSON – Table 2.A.2.6<br>7. CHD, MIMAROPA – Table 2.A.2.7<br>8. CHD, Bicol – Table 2.A.2.8<br>9. CHD, Western Visayas – Table 2.A.2.9<br>10. CHD, Central Visayas – Table 2.A.2.10<br>11. CHD, Eastern Visayas – Table 2.A.2.11<br>12. CHD, Zamboanga Penisula – Table 2.A.2.12<br>13. CHD, Northern Mindanao – Table 2.A.2.13<br>14. CHD, Davao – Table 2.A.2.14<br>15. CHD, SOCCSARGEN – Table 2.A.2.15<br>16. CHD, Bangsamoro Autonomous Region in Muslim Mindanao (BARMM) – Table 2.A.2.16<br>17. CHD, CARAGA – Table 2.A.2.17	584<br>584<br>584<br>585<br>585<br>585<br>586<br>586<br>586<br>587<br>587<br>587<br>588<br>588<br>588<br>589<br>589

2.A Morbidity

Summary Tables

2.A.3 Morbidity Rate (by type of disease, sex and age group)	
1. Acute Bloody Diarrhea – Table 2.A.3.1	590
2. Acute Febrile Illness - Table 2.A.3.2	596
3. Acute Flaccid Paralysis – Table 2.A.3.3	602
4. Acute Hemorrhagic Fever – Table 2.A.3.4	608
5. Acute Lower Respiratory Tract Infection – Table 2.A.3.5	614
6. Animal Bites – Table 2.A.3.6	620
7. Acute Respiratory Infection (less than 5 years old) – Table 2.A.3.7	626
8. Acute Respiratory Infection (more than 5 years old) – Table 2.A.3.8	630
9. Acute Watery Diarrhea – Table 2.A.3.9	636
10. Bronchitis – Table 2.A.3.10	642
11. Cholera – Table 2.A.3.11	648
12. Chronic Obstructive Pulmonary Disease – Table 2.A.3.12	654
13. Diphtheria – Table 2.A.3.13	660
14. Diseases of the Heart – Table 2.A.3.14	666
15. Filariasis – Table 2.A.3.15	672
16. Fever of Unknown Origin – Table 2.A.3.16	678
17. Genital Ulcer – Table 2.A.3.17	684
18. Gonorrhea – Table 2.A.3.18	690
19. Hypertension – Table 2.A.3.19	696
20. Influenza Like Illness – Table 2.A.3.20	702
21. Influenza – Table 2.A.3.21	708
22. Leprosy – Table 2.A.3.22	714
23. Leptospirosis – Table 2.A.3.23	720
24. Malaria – Table 2.A.3.24	726
25. Measles – Table 2.A.3.25	732
26. Meningococcemia – Table 2.A.3.26	738
27. Neonatal Tetanus – Table 2.A.3.27	744
28. Non-Neonatal Tetanus – Table 2.A.3.28	749
29. Pneumonia – Table 2.A.3.29	755
30. Rabies – Table 2.A.3.30	761
31. Paralytic Shellfish Poisoning – Table 2.A.3.31	767
32. Schistosomiasis – Table 2.A.3.32	773
33. Skin Diseases – Table 2.A.3.33	779
34. Syphilis – Table 2.A.3.34	785
35. TB all Forms – Table 2.A.3.35	791
36. Typhoid Fever – Table 2.A.3.36	797
37. Urethral Discharge – Table 2.A.3.37	803
38. Urinary Tract Infection – Table 2.A.3.38	809
39. Viral Encephalitis – Table 2.A.3.39	815
40. Viral Hepatitis – Table 2.A.3.40	821
41. Viral Meningitis – Table 2.A.3.41	827
42. Whooping Cough – Table 2.A.3.42	833

Figures and Graphs (by Region, Rate per 100,000 population)	
1. Acute bloody diarrhea rate from the year 2016-2020 – Figure 2.A.3.1	839
2. Acute bloody diarrhea rate for the year 2020 – Figure 2.A.3.2	839
3. Acute febrile illness rate from the year 2016-2020 – Figure 2.A.3.3	840
4. Acute febrile illness rate for the year 2020 – Figure 2.A.3.4	840
5. Acute flaccid paralysis from the year 2016-2020 – Figure 2.A.3.5	841
6. Acute flaccid paralysis rate for the year 2020 – Figure 2.A.3.6	841
7. Acute hemorrhagic fever rate from the year 2016-2020 – Figure 2.A.3.7	842
8. Acute hemorrhagic fever rate for the year 2020 – Figure 2.A.3.8	842
9. Acute Lower Respiratory Tract Infection rate for the year 2019-2020 – Figure 2.A.3.9	843
10. Acute Lower Respiratory Tract Infection rate for year 2020 – Figure 2.A.3.10	843
11. Animal bites rate for the year 2019-2020 – Figure 2.A.3.11	844
12. Animal bites rate for the year 2020 – Figure 2.A.3.12	844
13. Acute respiratory infection (<5 yrs. old) rate for the year 2019-2020 – Figure 2.A.3.13	845
14. Acute respiratory infection (<5 years old) rate for the year 2020– Figure 2.A.3.14	845
15. Acute respiratory infection (>5 years old) rate for the year 2019-2020 – Figure 2.A.3.15	846
16. Acute respiratory infection (>5 years old) rate for the year 2020 – Figure 2.A.3.16	846
17. Acute watery diarrhea rate from the year 2016-2020 – Figure 2.A.3.17	847
18. Acute watery diarrhea rate for the year 2020 – Figure 2.A.3.18	847
19. Bronchitis rate from the year 2016-2020 – Figure 2.A.3.19	848
20. Bronchitis rate for the year 2020 – Figure 2.A.3.20	848
21. Cholera rate from the year 2016-2020 – Figure 2.A.3.21	849
22. Cholera rate for the year 2020 – Figure 2.A.3.22	849
23. Chronic Obstructive Pulmonary Disease (COPD) rate for the year 2019-2020 – Figure 2.A.3.23	850
24. Chronic Obstructive Pulmonary Disease rate for the 2020 – Figure 2.A.3.24	850
25. Diphtheria rate from the year 2016-2020 – Figure 2.A.3.25	851
26. Diphtheria rate for the year 2020 – Figure 2.A.3.26	851
27. Diseases of the heart rate from the year 2016-2020 – Figure 2.A.3.27	852
28. Diseases of the heart rate for the year 2020 – Figure 2.A.3.28	852
29. Filariasis rate from the year 2016-2020 – Figure 2.A.3.29	853
30. Filariasis rate for the year 2020 – Figure 2.A.3.30	853
31. Fever of unknown origin rate for the year 2019-2020 – Figure 2.A.3.31	854
32. Fever of unknown origin rate for the year 2020 – Figure 2.A.3.32	854
33. Genital ulcer rate for the year 2019-2020 – Figure 2.A.3.33	855
34. Genital ulcer rate for the year 2020 – Figure 2.A.3.34	855
35. Gonorrhea rate for the year 2019-2020 – Figure 2.A.3.35	856
36. Gonorrhea rate for the year 2020 – Figure 2.A.3.36	856
37. Hypertension rate from the year 2016-2020 – Figure 2.A.3.37	857
38. Hypertension rate for year 2020 – Figure 2.A.3.38	857
39. Influenza like illness rate for the year 2019-2020 – Figure 2.A.3.39	858
40. Influenza like illness rate for the year 2020 – Figure 2.A.3.40	858
41. Influenza rate from the year 2016-2020 – Figure 2.A.3.41	859
42. Influenza rate for the year 2020 – Figure 2.A.3.42	859
43. Leprosy rate from the year 2016-2020 – Figure 2.A.3.43	860
44. Leprosy rate for the year 2020 – Figure 2.A.3.44	860
45. Leptospirosis rate from the year 2016-2020 – Figure 2.A.3.45	861
46. Leptospirosis rate for the year 2020 – Figure 2.A.3.46	861
47. Malaria rate from the year 2016-2020 – Figure 2.A.3.47	862
48. Malaria rate for the 2020 – Figure 2.A.3.48	862
49. Measles rate from the year 2016-2020 – Figure 2.A.3.49	863

50. Measles rate for the year 2020 – Figure 2.A.3.50	863
51. Meningococcemia rate from the year 2016-2020 – Figure 2.A.3.51	864
52. Meningococcemia rate for the year 2020 – Figure 2.A.3.52	864
53. Neonatal Tetanus rate from the year 2016-2020 – Figure 2.A.3.53	865
54. Neonatal Tetanus rate for the year 2020 – Figure 2.A.3.54	865
55. Non-neonatal Tetanus rate from the year 2016-2020 – Figure 2.A.3.55	866
56. Non-neonatal Tetanus rate for the year 2020 – Figure 2.A.3.56	866
57. Pneumonia rate for the year 2019-2020 – Figure 2.A.3.57	867
58. Pneumonia rate for the year 2020 – Figure 2.A.3.58	867
59. Rabies (Human) rate from the year 2016-2020 – Figure 2.A.3.59	868
60. Rabies (Human) rate for the year 2020 – Figure 2.A.3.60	868
61. Paralytic Shellfish Poisoning rate from the year 2016-2020 – Figure 2.A.3.61	869
62. Paralytic Shellfish Poisoning rate for the year 2020 – Figure 2.A.3.62	869
63. Schistosomiasis rate from the year 2016-2020 – Figure 2.A.3.63	870
64. Schistosomiasis rate for the year 2020 – Figure 2.A.3.64	870
65. Skin disease rate for the year 2019-2020 – Figure 2.A.3.65	871
66. Skin disease rate for the year 2020 – Figure 2.A.3.66	871
67. Syphilis rate for the year 2019-2020 – Figure 2.A.3.67	872
68. Syphilis rate for the year 2020 – Figure 2.A.3.68	872
69. Tuberculosis all forms rate from the year 2016-2020 – Figure 2.A.3.69	873
70. Tuberculosis all forms rate for the year 2020 – Figure 2.A.3.70	873
71. Typhoid Fever rate from the year 2016-2020 – Figure 2.A.3.71	874
72. Typhoid Fever rate for the year 2020 – Figure 2.A.3.72	874
73. Urethral Discharge rate for the year 2019-2020 – Figure 2.A.3.73	875
74. Urethral Discharge rate for the year 2020 – Figure 2.A.3.74	875
75. Urinary tract infection rate from the year 2016-2020 – Figure 2.A.3.75	876
76. Urinary tract infection rate for the year 2020 – Figure 2.A.3.76	876
77. Viral encephalitis rate from the year 2016-2020 – Figure 2.A.3.77	877
78. Viral encephalitis rate for the year 2020 – Figure 2.A.3.78	877
79. Viral Hepatitis rate from the year 2016-2020 – Figure 2.A.3.79	878
80. Viral Hepatitis rate for the 2020 – Figure 2.A.3.80	878
81. Viral Meningitis rate from the year 2016-2020 – Figure 2.A.3.81	879
82. Viral Meningitis rate for the year 2020 – Figure 2.A.3.82	879
83. Whooping cough rate from the year 2016-2020 – Figure 2.A.3.83	880
84. Whooping cough rate for the year 2020 – Figure 2.A.3.84	880

**2.B Mortality and Natality**	
2.B.1 Mortality (by Province/City and Region)	
Summary Tables	
1. Total number of Deaths, Maternal Deaths, Under five yrs. and Rates – Table 2.B.1.1	881
2. Infant Deaths, Neonatal Deaths, Perinatal Deaths and Rates – Table 2.B.1.2	885
Figure and Graphs	
1. Mortality Rate per 1,000/population – Figure 2.B.1.1	888
2. Maternal mortality rate (MMR) per 100,000 livebirths – Figure 2.B.1.2	888
3. Under five mortality rate (UFMR) per 1,000 livebirths – Figure 2.B.1.3	889
4. Infant mortality rate (IMR) per 1,000 livebirths – Figure 2.B.1.4	890
5. Neonatal mortality rate (NMR) per 1,000 livebirths – Figure 2.B.1.5	890

6. Fetal mortality rate per 1,000 livebirths – Figure 2.B.1.6<br>7. Early neonatal mortality rate per 1,000 livebirths – Figure 2.B.1.7<br>8. Perinatal mortality rate (PMR) per 1,000 livebirths – Figure 2.B.1.8	891<br>891<br>892

2.B.2 Natality	
Summary Tables	
1. Number of Live births, Crude Birth Rate and Adolescent Birth Rate – Table 2.B.2.1	893
Figure and Graphs	
1. Crude Birth Rate (CBR) – Figure 2.B.2.1<br>2. Adolescent Birth Rate to Women 15-19 years old – Figure 2.B.2.2	897<br>897

**Chapter 2 – Summary (Morbidity, Mortality and Natality)**	
1. Morbidity	898
2. Mortality	898
3. Natality	902

Chapter 3– Demographic Data

**3.A Demographic Data**	
Summary Tables	
1. Projected Population for 2019 – Table 3.A.1<br>2. No. of Barangays, Health Centers, Barangay Health Stations and their Ratio to Population – Table 3.A.2<br>3. No. and Ratio of Physicians, Dentists and Public Health Nurses – Table 3.A.3<br>4. No. and Ratio of Midwives, Nutritionists and Medical Technologists – Table 3.A.4<br>5. No. and Ratio of Sanitary Engineers, Sanitary Inspectors and Active BHWs – Table 3.A.5<br><br>*Figure and Graphs*	904<br>907<br>911<br>915<br>919
1. Number of Physician per 20,000/population – Figure 3.A.1<br>2. Number of Dentist per 50,000/population – Figure 3.A.2<br>3. Number of Public Health Nurse per 10,000/population – Figure 3.A.3<br>4. Number of Rural Health Midwife per 5,000/population – Figure 3.A.4<br>5. Number of Nutritionist per 20,000/population – Figure 3.A.5<br>6. Number of Medical Technologist per 50,000/population – Figure 3.A.6<br>7. Number Sanitary Engineer per 20,000/population – Figure 3.A.7<br>8. Number of Sanitary Inspector per 20,000/population – Figure 3.A.8<br>9. Number of BHW per 20 Household – Figure 3.A.9	923<br>923<br>924<br>924<br>925<br>925<br>926<br>926<br>927

Acronyms and Accreditation

AOG	Age of Gestation
BCG	Bacillus Calmette–Guérin vaccine
BF	Breastfeed
BHS	Barangay Health Station
BMI	Body Mass Index
BHW	Barangay Health Worker
BOHC	Basic Oral Health Care
BTL	Bilateral Tubal Ligation
CHD	Center for Health Development
CHO	City Health Office/Officer
CMM	Cervical Mucus Method
COC	Combined Oral Contraceptive
CPAB	Child/Children Protected at Birth
CPR	Contraceptive Prevalence Rate
DMFT	Decay Missing Filled Teeth
DOH	Department of Health
DOH-CO	Department of Health - Central Office
DOH-EB	Department of Health - Epidemiology Bureau
DPCB	Disease Prevention and Control Bureau
DPT	Diphtheria, Pertussis, Tetanus,
DPT-HiB-Hep B	Diphtheria, Pertussis, Tetanus, Haemophilus influenzae type B, Hepatitis B vaccine
DRTB	Drug-Resistant Tuberculosis
DSTB	Drug-Susceptible Tuberculosis
EB	Epidemiology Bureau
FBDs	Facility-Based Deliveries
FHSIS	Field Health Services Information System
FIC	Fully Immunized Children
FP	Family Planning
HCs	Health Centers
HH	Household
HIV	Human Immunodeficiency Virus
HPDPB	Health Policy Development and Planning Bureau
ICV	Informed Choice and Voluntarism
IEC	Information, Education, Communication
ILIS	Integrated Leprosy Information System
IPV	Inactivated Polio Vaccine
ITIS	Integrated Tuberculosis Information System
ITR	Individual Treatment Record
LAM	Lactational Amenorrhea Method
LB	Live Birth
LBW	Low Birth Weight
LCEs	Local Chief Executives

LCR	Local Civil Registries
LGC	Local Government Code
LGU	Local Government Unit
LLIN	Long-Lasting Insecticide Nets
LRD	Lifestyle Related Disease
MAM	Moderate Acute Malnutrition
M and E	Monitoring and Evaluation
MCV	Measles-Containing Vaccine
MD	Medical Doctor
MDA	Mass Drug Administration
MFHSIS	Modified Field Health Service Information System
mFP	Modern Family Planning
MHC	Municipal Health Center
MHCs	Main Health Centers
MHO	Municipal Health Officer/Office
MMR	Maternal Mortality Ratio
MNCHN	Maternal-Newborn-Child Child Health and Nutrition
MNP	Micronutrient Powder
MOP	Manual of Procedures
MR	Measles and Rubella
MW	Midwife
NaRIS	National Rabies Information System
NBW	Normal Birth Weight
NCD/s	Non-Communicable Disease/s
NCDPC	National Center for Disease Prevention and Control
NDP/s	Nurse Deployment Programs
NFP	Natural Family Planning
NGO	Non-Governmental Organization
NHTS	National Household Targeting System
NOH	National Objectives for Health
NSCB	National Statistical Coordination Board
NSV	No-Scalpel Vasectomy
NTP	National Tuberculosis Program
OPD	Out Patient Department
OPV	Oral Polio Vaccine
ORS/ORT	Oral Rehydration Solution/Oral Rehydration Therapy
P/CHO	Provincial/City Health Officer
PCV	Pneumococcal Conjugate Vaccine
PhilMIS	Philippine Malaria Management Information System
PhilPEN	Philippine Protocol of Essential Non-Communicable Diseases Interventions
PHN	Public Health Nurse
PHO	Provincial Health Office/Officer
PNSDW	Philippine Drinking Water Standards
POP	Progestin Only Pills
PP	Post-partum
PPV	Pneumococcal Polysaccharide Vaccine

PSA	Philippine Statistics Authority
PSIC	Philippines Standard Industrial Classification
PSAC	Preschool-Aged Children
RDT	Rapid Diagnostic Test
RHM	Rural Health Midwife
RHMPP	Rural Health Midwives Placement Program
RHU	Rural Health Unit
RR	Respiratory Rate
RSI	Rural Sanitation Inspector
RUTF	Ready-to-Use Therapeutic Food
SAC	School Aged Children
SAM	Severe Acute Malnutrition
SBAs	Skilled Birth Attendants
SDM	Standard Days Method
SHPs	Skilled Health Professionals
STM	Symptothermal Method
SGD	Sustainable Development Goal/s
SI	Sanitation Inspector
STH	Soil-Transmitted Helminthiasis
STI	Sexually Transmitted Infection
UBW	Unknown Birth Weight
TBA	Traditional Birth Attendant
TCL	Target Client List
Td	Tetanus diphtheria
TWG	Technical Working Group
VIP	Ventilated Improved Pit
WRA	Women of Reproductive Age
ZOD	Zero Open Defecation

CHAPTER 1

FHSIS Annual Report 2020

Accomplishment of Public Health Programs

1. Family Planning
2. Maternal Care Services
3. Child Care Services
4. Oral Health Care
5. Infectious Disease Prevention and Control
6. Non-Communicable Disease Prevention and Control
7. Environmental Health and Sanitation Services

Table 1.A.1 - MODERN METHOD OF FAMILY PLANNING
New Acceptors
Annual Philippines, 2020

Area	Total Current Users	FSTR/BTL Age group 10-14	FSTR/BTL Age group 15-19	FSTR/BTL Age group 20-49	Total	MSTR/NSV Age group 10-14	MSTR/NSV Age group 15-19	MSTR/NSV Age group 20-49	Total
**PHILIPPINES**	**7,115,114**	**296**	**1,522**	**28,526**	**30,344**	**4**	**51**	**1,168**	**1,223**
**N C R**	**786,292**	**2**	**1**	**1,456**	**1,459**	**0**	**0**	**12**	**12**
Malabon	31,429	0	0	0	0	0	0	0	0
Navotas	22,416	0	0	43	43	0	0	0	0
Valenzuela City	60,865	0	0	90	90	0	0	1	1
Caloocan City	65,508	0	0	77	77	0	0	0	0
Marikina City	18,917	0	0	56	56	0	0	1	1
Pasig City	33,855	0	0	45	45	0	0	2	2
Pateros	2,995	0	0	12	12	0	0	0	0
Taguig	57,595	0	0	0	0	0	0	0	0
Quezon City	271,881	0	0	282	282	0	0	4	4
Makati City	8,979	0	0	63	63	0	0	1	1
Mandaluyong City	16,390	0	1	66	67	0	0	1	1
San Juan	5,866	0	0	1	1	0	0	0	0
Manila City	68,205	0	0	373	373	0	0	1	1
Las Piñas City	20,867	0	0	68	68	0	0	0	0
Muntinlupa City	33,645	0	0	27	27	0	0	0	0
Parañaque City	30,062	2	0	119	121	0	0	1	1
Pasay City	36,817	0	0	134	134	0	0	0	0
**C A R**	**120,779**	**0**	**0**	**256**	**256**	**0**	**0**	**0**	**0**
Abra	16,924	0	0	14	14	0	0	0	0
Apayao	11,643	0	0	3	3	0	0	0	0
Benguet	30,440	0	0	32	32	0	0	0	0
Ifugao	17,978	0	0	0	0	0	0	0	0
Kalinga	17,779	0	0	111	111	0	0	0	0
Mt. Province	11,955	0	0	27	27	0	0	0	0
Baguio City	14,060	0	0	69	69	0	0	0	0
**Region 1**	**460,308**	**0**	**9**	**354**	**363**	**0**	**0**	**2**	**2**
Ilocos Norte	41,436	0	0	28	28	0	0	1	1
Ilocos Sur	59,502	0	5	201	206	0	0	0	0
La Union	58,813	0	4	48	52	0	0	0	0
Pangasinan	226,567	0	0	4	4	0	0	1	1
Alaminos City	9,446	0	0	0	0	0	0	0	0
Candon City	7,299	0	0	2	2	0	0	0	0
Dagupan City	7,429	0	0	1	1	0	0	0	0
Laoag City	8,324	0	0	22	22	0	0	0	0
San Carlos City	11,747	0	0	0	0	0	0	0	0
San Fernando City	12,865	0	0	45	45	0	0	0	0
Urdaneta City	11,939	0	0	3	3	0	0	0	0
Vigan City	4,941	0	0	0	0	0	0	0	0
**Region 2**	**307,177**	**0**	**14**	**428**	**442**	**0**	**0**	**40**	**40**
Batanes	864	0	0	0	0	0	0	0	0
Cagayan	83,752	0	9	117	126	0	0	2	2
Isabela	105,390	0	4	57	61	0	0	0	0
Nueva Vizcaya	44,895	0	1	188	189	0	0	38	38
Quirino	18,556	0	0	19	19	0	0	0	0
Cauayan City	10,617	0	0	3	3	0	0	0	0
Ilagan City	14,066	0	0	32	32	0	0	0	0
Santiago City	15,258	0	0	2	2	0	0	0	0
Tuguegarao City	13,779	0	0	10	10	0	0	0	0
**Region 3**	**747,169**	**0**	**7**	**7,608**	**7,615**	**0**	**0**	**386**	**386**
Aurora	20,957	0	0	25	25	0	0	0	0
Bataan	44,561	0	0	90	90	0	0	0	0

Table 1.A.1 - MODERN METHOD OF FAMILY PLANNING
New Acceptors
Annual Philippines, 2020

Area	Total Current Users	FSTR/BTL			Total	MSTR/NSV			Total
		Age group				Age group			
		10-14	15-19	20-49		10-14	15-19	20-49	
Bulacan	138,968	0	2	837	839	0	0	312	312
Nueva Ecija	118,251	0	2	271	273	0	0	2	2
Pampanga	97,407	0	2	2,972	2,974	0	0	68	68
Tarlac	78,705	0	0	32	32	0	0	0	0
Zambales	37,319	0	0	2,333	2,333	0	0	2	2
Angeles City	21,284	0	0	199	199	0	0	0	0
Balanga City	4,693	0	0	4	4	0	0	0	0
Cabanatuan City	16,403	0	0	227	227	0	0	0	0
City of San Fernando	7,904	0	0	248	248	0	0	2	2
Gapan City	7,770	0	0	1	1	0	0	0	0
Mabalacat City	21,281	0	0	36	36	0	0	0	0
Malolos City	6,208	0	0	1	1	0	0	0	0
Meycauayan	8,403	0	0	1	1	0	0	0	0
Olongapo	9,362	0	1	278	279	0	0	0	0
Palayan City	3,344	0	0	0	0	0	0	0	0
San Jose City	12,893	0	0	0	0	0	0	0	0
San Jose del Monte City	72,493	0	0	43	43	0	0	0	0
Science City of Munoz	6,409	0	0	0	0	0	0	0	0
Tarlac City	12,554	0	0	10	10	0	0	0	0
**Region 4A**	**844,565**	**254**	**18**	**1,543**	**1,815**	**0**	**1**	**9**	**10**
Batangas	158,232	72	0	158	230	0	0	0	0
Cavite	83,830	0	0	140	140	0	0	1	1
Laguna	65,529	8	0	69	77	0	0	0	0
Quezon	106,906	0	1	206	207	0	1	4	5
Rizal	103,690	0	2	49	51	0	0	1	1
Antipolo City	37,890	0	0	0	0	0	0	0	0
Bacoor City	11,464	0	0	53	53	0	0	0	0
Batangas City	17,607	0	0	0	0	0	0	0	0
Biñan City	38,387	0	4	236	240	0	0	1	1
Cabuyao City	13,927	1	2	43	46	0	0	0	0
Calamba City	35,102	0	2	192	194	0	0	1	1
Cavite City	4,572	0	0	0	0	0	0	0	0
Dasmariñas City	42,608	0	1	20	21	0	0	0	0
General Trias City	11,506	0	0	14	14	0	0	0	0
Imus City	9,106	0	0	8	8	0	0	0	0
Lipa City	13,662	0	0	0	0	0	0	0	0
Lucena City	6,402	173	0	30	203	0	0	1	1
San Pablo City	10,199	0	0	0	0	0	0	0	0
San Pedro City	15,209	0	4	20	24	0	0	0	0
Santa Rosa City	33,075	0	2	264	266	0	0	0	0
Tagaytay City	7,989	0	0	39	39	0	0	0	0
Tanauan City	7,401	0	0	2	2	0	0	0	0
Tayabas City	5,399	0	0	0	0	0	0	0	0
Trece Martires City	4,872	0	0	0	0	0	0	0	0
**Region 4B**	**144,179**	**0**	**0**	**2,642**	**2,642**	**0**	**0**	**0**	**0**
Marinduque	14,040	0	0	60	60	0	0	0	0
Mindoro Occidental	32,450	0	0	1,028	1,028	0	0	0	0
Mindoro Oriental	58,295	0	0	31	31	0	0	0	0
Palawan	6,466	0	0	1,491	1,491	0	0	0	0
Romblon	4,160	0	0	4	4	0	0	0	0
Puerto Princesa City	28,768	0	0	28	28	0	0	0	0
**Region 5**	**397,155**	**0**	**2**	**251**	**253**	**0**	**0**	**3**	**3**
Albay	75,300	0	0	32	32	0	0	0	0
Camarines Norte	26,377	0	0	38	38	0	0	0	0
Camarines Sur	100,599	0	0	6	6	0	0	0	0
Catanduanes	24,369	0	0	8	8	0	0	0	0
Masbate	57,012	0	0	52	52	0	0	1	1
Sorsogon	60,469	0	2	42	44	0	0	1	1
Iriga City	9,974	0	0	4	4	0	0	0	0

Table 1.A.1 - MODERN METHOD OF FAMILY PLANNING
New Acceptors
Annual Philippines, 2020

Area	Total Current Users	FSTR/BTL			Total	MSTR/NSV			Total
		Age group				Age group			
		10-14	15-19	20-49		10-14	15-19	20-49	
Legaspi City	22,994	0	0	12	12	0	0	0	0
Naga City	20,061	0	0	57	57	0	0	1	1
**Region 6**	**580,884**	**0**	**43**	**2,128**	**2,171**	**0**	**0**	**36**	**36**
Aklan	41,264	0	1	572	573	0	0	0	0
Antique	47,572	0	0	80	80	0	0	2	2
Capiz	75,849	0	33	976	1,009	0	0	12	12
Guimaras	15,286	0	0	9	9	0	0	0	0
Iloilo	149,460	0	2	194	196	0	0	15	15
Negros Occidental	182,369	0	7	247	254	0	0	7	7
Bacolod City	25,422	0	0	33	33	0	0	0	0
Iloilo City	43,662	0	0	17	17	0	0	0	0
**Region 7**	**294,028**	**0**	**12**	**503**	**515**	**0**	**0**	**5**	**5**
Bohol	67,760	0	0	333	333	0	0	0	0
Cebu	71,864	0	0	28	28	0	0	0	0
Negros Oriental	58,940	0	12	21	33	0	0	0	0
Siquijor	8,043	0	0	30	30	0	0	0	0
Cebu City	29,645	0	0	64	64	0	0	5	5
Lapu-Lapu City	39,675	0	0	1	1	0	0	0	0
Mandaue City	18,101	0	0	26	26	0	0	0	0
**Region 8**	**202,207**	**0**	**0**	**0**	**0**	**0**	**0**	**0**	**0**
Biliran	10,480	0	0	0	0	0	0	0	0
Eastern Samar	41,040	0	0	0	0	0	0	0	0
Northern Leyte	0	0	0	0	0	0	0	0	0
Northern Samar	34,606	0	0	0	0	0	0	0	0
Southern Leyte	24,278	0	0	0	0	0	0	0	0
Western Samar	16,033	0	0	0	0	0	0	0	0
Calbayog City	8,908	0	0	0	0	0	0	0	0
Maasin City	2,705	0	0	0	0	0	0	0	0
Ormoc City	19,481	0	0	0	0	0	0	0	0
Tacloban City	44,676	0	0	0	0	0	0	0	0
**Region 9**	**271,392**	**0**	**0**	**4**	**4**	**0**	**0**	**0**	**0**
Zamboanga del Norte	86,000	0	0	0	0	0	0	0	0
Zamboanga del Sur	57,156	0	0	0	0	0	0	0	0
Zamboanga Sibugay	24,011	0	0	0	0	0	0	0	0
Dapitan City	7,405	0	0	0	0	0	0	0	0
Dipolog City	15,009	0	0	0	0	0	0	0	0
Isabela City	8,507	0	0	2	2	0	0	0	0
Pagadian City	17,446	0	0	0	0	0	0	0	0
Zamboanga City	55,858	0	0	2	2	0	0	0	0
**Region 10**	**518,887**	**0**	**9**	**367**	**376**	**4**	**44**	**53**	**101**
Bukidnon	111,273	0	0	71	71	4	43	8	55
Camiguin	6,349	0	0	6	6	0	0	0	0
Lanao del Norte	47,747	0	2	30	32	0	0	3	3
Misamis Occidental	24,432	0	6	12	18	0	0	0	0
Misamis Oriental	81,061	0	0	41	41	0	0	1	1
Cagayan de Oro City	140,501	0	0	112	112	0	0	0	0
El Salvador City	6,230	0	0	6	6	0	0	0	0
Gingoog City	10,166	0	0	0	0	0	0	0	0
Iligan City	27,571	0	0	54	54	0	0	0	0
Malaybalay City	19,999	0	0	0	0	0	0	0	0
Oroquieta City	6,341	0	0	0	0	0	0	0	0
Ozamis City	12,025	0	0	8	8	0	1	39	40
Tangub City	4,874	0	0	0	0	0	0	0	0
Valencia City	20,318	0	1	27	28	0	0	2	2

Table 1.A.1 - MODERN METHOD OF FAMILY PLANNING
New Acceptors
Annual Philippines, 2020

Area	Total Current Users	FSTR/BTL			Total	MSTR/NSV			Total
		Age group				Age group			
		10-14	15-19	20-49		10-14	15-19	20-49	
**Region 11**	**483,912**	**14**	**29**	**1,115**	**1,158**	**0**	**3**	**90**	**93**
Davao de Oro	81,390	6	2	115	123	0	0	3	3
Davao del Norte	99,809	2	2	244	248	0	0	1	1
Davao Oriental	48,937	1	2	69	72	0	0	1	1
Davao del Sur	63,208	3	2	84	89	0	0	3	3
Davao Occidental	31,727	0	4	24	28	0	0	0	0
Davao City	158,841	2	17	579	598	0	3	82	85
**Region 12**	**467,365**	**1**	**44**	**395**	**440**	**0**	**2**	**11**	**13**
North Cotabato	141,819	0	1	157	158	0	0	2	2
Sarangani	62,222	0	0	45	45	0	0	3	3
South Cotabato	95,537	1	22	122	145	0	2	5	7
Sultan Kudarat	88,018	0	21	54	75	0	0	0	0
					0				0
Cotabato City	30,729	0	0	7	7	0	0	0	0
Gen. Santos City	49,040	0	0	10	10	0	0	1	1
**B.A.R.M.M.**	**239,770**	**25**	**1,333**	**9,405**	**10,763**	**0**	**0**	**511**	**511**
Basilan	6,677	0	0	14	14	0	0	13	13
Lanao del Sur	66,116	0	0	14	14	0	0	0	0
Maguindanao	84,835	0	0	21	21	0	0	0	0
Sulu	49,455	0	57	0	57	0	0	0	0
Tawi-Tawi	27,758	0	2	0	2	0	0	0	0
					0				0
Lamitan City	0	25	1,274	9,346	10,645	0	0	498	498
Marawi City	4,929	0	0	10	10	0	0	0	0
**CARAGA**	**249,045**	**0**	**1**	**71**	**72**	**0**	**1**	**10**	**11**
Agusan del Norte	38,948	0	0	11	11	0	0	0	0
Agusan del Sur	80,275	0	0	13	13	0	0	10	10
Surigao del Norte	30,203	0	1	30	31	0	0	0	0
Surigao del Sur	39,690	0	0	7	7	0	0	0	0
Province of Dinagat	9,699	0	0	9	9	0	0	0	0
Bislig City	8,261	0	0	0	0	0	0	0	0
Butuan City	28,396	0	0	0	0	0	0	0	0
Surigao City	13,573	0	0	1	1	0	1	0	1

Table 1.A.1 - MODERN METHOD OF FAMILY PLANNING

New Acceptors

Annual Philippines, 2020

Area	CONDOM			Total	IUD-INTERVAL			Total	IUD-POSTPARTUM			Total
	Age group				Age group				Age group			
	10-14	15-19	20-49		10-14	15-19	20-49		10-14	15-19	20-49	
**PHILIPPINES**	**181**	**4,483**	**36,859**	**41,523**	**100**	**1,919**	**15,811**	**17,830**	**1,858**	**1,635**	**19,140**	**22,633**
**N C R**	**20**	**645**	**5,270**	**5,935**	**3**	**38**	**683**	**724**	**15**	**558**	**2,083**	**2,656**
Malabon	0	18	121	139	0	0	4	4	0	3	19	22
Navotas	0	138	305	443	0	3	29	32	0	2	1	3
Valenzuela City	0	7	176	183	0	0	13	13	0	1	9	10
Caloocan City	0	57	508	565	0	11	51	62	2	78	168	248
Marikina City	0	0	32	32	0	4	29	33	6	182	584	772
Pasig City	0	2	118	120	0	2	78	80	0	79	308	387
Pateros	0	1	23	24	0	0	2	2	0	0	0	0
Taguig	6	87	758	851	0	0	45	45	0	22	181	203
Quezon City	0	145	1,490	1,635	0	7	43	50	5	161	438	604
Makati City	0	6	101	107	0	0	13	13	0	3	25	28
Mandaluyong City	0	8	108	116	0	2	11	13	0	5	10	15
San Juan	0	0	13	13	0	0	28	28	0	0	0	0
Manila City	12	48	609	669	0	3	127	130	0	17	106	123
Las Piñas City	0	6	197	203	0	0	10	10	1	0	1	2
Muntinlupa City	0	8	93	101	0	3	57	60	0	0	5	5
Parañaque City	2	13	410	425	3	3	38	44	1	5	34	40
Pasay City	0	101	208	309	0	0	105	105	0	0	194	194
**C A R**	**1**	**25**	**259**	**285**	**0**	**6**	**29**	**35**	**0**	**13**	**5,485**	**5,498**
Abra	0	1	36	37	0	0	0	0	0	0	0	0
Apayao	0	5	15	20	0	1	0	1	0	3	11	14
Benguet	1	6	101	108	0	1	10	11	0	1	6	7
Ifugao	0	1	1	2	0	1	0	1	0	1	0	1
Kalinga	0	2	21	23	0	1	13	14	0	0	1	1
Mt. Province	0	2	13	15	0	0	2	2	0	7	8	15
Baguio City	0	8	72	80	0	2	4	6	0	1	5,459	5,460
**Region 1**	**0**	**64**	**927**	**991**	**0**	**49**	**268**	**317**	**1**	**7**	**115**	**123**
Ilocos Norte	0	4	304	308	0	0	8	8	0	3	95	98
Ilocos Sur	0	3	76	79	0	2	42	44	0	0	0	0
La Union	0	1	62	63	0	1	8	9	1	2	7	10
Pangasinan	0	20	299	319	0	41	180	221	0	0	0	0
Alaminos City	0	4	40	44	0	0	2	2	0	0	0	0
Candon City	0	0	0	0	0	0	0	0	0	0	0	0
Dagupan City	0	0	3	3	0	0	0	0	0	2	3	5
Laoag City	0	0	45	45	0	0	0	0	0	0	0	0
San Carlos City	0	1	26	27	0	1	22	23	0	0	0	0
San Fernando City	0	0	19	19	0	4	6	10	0	0	10	10
Urdaneta City	0	31	53	84	0	0	0	0	0	0	0	0
Vigan City	0	0	0	0	0	0	0	0	0	0	0	0
**Region 2**	**2**	**29**	**281**	**312**	**1**	**77**	**349**	**427**	**1**	**62**	**210**	**273**
Batanes	0	0	0	0	0	0	0	0	0	0	0	0
Cagayan	1	4	47	52	1	44	148	193	0	33	90	123
Isabela	1	13	110	124	0	8	39	47	1	19	68	88
Nueva Vizcaya	0	1	96	97	0	0	104	104	0	0	5	5
Quirino	0	8	15	23	0	0	6	6	0	0	0	0
Cauayan City	0	2	1	3	0	0	10	10	0	0	4	4
Ilagan City	0	1	3	4	0	22	3	25	0	0	1	1
Santiago City	0	0	0	0	0	0	4	4	0	0	0	0
Tuguegarao City	0	0	9	9	0	3	35	38	0	10	42	52
**Region 3**	**10**	**504**	**4,282**	**4,796**	**8**	**51**	**807**	**866**	**0**	**83**	**675**	**758**
Aurora	0	2	14	16	0	0	9	9	0	1	3	4
Bataan	6	41	188	235	0	0	16	16	0	1	14	15

Table 1.A.1 - MODERN METHOD OF FAMILY PLANNING

New Acceptors

Annual Philippines, 2020

Area	CONDOM			Total	IUD-INTERVAL			Total	IUD-POSTPARTUM			Total
	Age group				Age group				Age group			
	10-14	15-19	20-49		10-14	15-19	20-49		10-14	15-19	20-49	
Bulacan	0	50	598	648	0	6	199	205	0	6	167	173
Nueva Ecija	0	21	132	153	0	8	79	87	0	9	39	48
Pampanga	0	113	539	652	0	5	321	326	0	61	321	382
Tarlac	0	48	661	709	0	3	66	69	0	0	8	8
Zambales	0	8	225	233	0	0	14	14	0	1	30	31
Angeles City	0	4	35	39	0	0	4	4	0	0	11	11
Balanga City	0	0	2	2	0	0	0	0	0	0	0	0
Cabanatuan City	4	23	78	105	8	13	21	42	0	0	0	0
City of San Fernando	0	3	261	264	0	2	9	11	0	0	34	34
Gapan City	0	12	16	28	0	0	1	1	0	0	0	0
Mabalacat City	0	24	170	194	0	4	22	26	0	1	0	1
Malolos City	0	7	88	95	0	0	8	8	0	0	0	0
Meycauayan	0	8	91	99	0	0	1	1	0	0	0	0
Olongapo	0	3	105	108	0	0	0	0	0	1	26	27
Palayan City	0	0	4	4	0	1	3	4	0	0	0	0
San Jose City	0	4	12	16	0	0	2	2	0	2	5	7
San Jose del Monte City	0	80	895	975	0	0	7	7	0	0	0	0
Science City of Munoz	0	0	1	1	0	0	1	1	0	0	0	0
Tarlac City	0	53	167	220	0	9	24	33	0	0	17	17
**Region 4A**	**120**	**1,783**	**11,407**	**13,310**	**54**	**61**	**881**	**996**	**1,821**	**65**	**2,877**	**4,763**
Batangas	93	3	74	170	27	2	46	75	0	1	27	28
Cavite	0	9	147	156	0	4	67	71	0	6	7	13
Laguna	0	24	169	193	6	15	94	115	0	31	2,681	2,712
Quezon	1	7	371	379	0	26	217	243	1	2	17	20
Rizal	3	206	3,840	4,049	0	7	50	57	0	15	35	50
Antipolo City	0	0	0	0	0	0	0	0	0	0	0	0
Bacoor City	0	2	42	44	0	2	19	21	0	0	0	0
Batangas City	0	0	0	0	0	0	0	0	0	0	0	0
Biñan City	0	968	4,435	5,403	0	1	279	280	0	2	3	5
Cabuyao City	0	7	184	191	0	1	9	10	0	1	5	6
Calamba City	1	18	93	112	0	0	25	25	0	0	8	8
Cavite City	0	0	0	0	0	0	0	0	0	0	0	0
Dasmariñas City	0	10	912	922	0	3	21	24	0	0	1	1
General Trias City	0	0	31	31	0	0	9	9	0	3	0	3
Imus City	0	0	15	15	0	0	4	4	0	0	2	2
Lipa City	0	0	0	0	0	0	0	0	0	0	0	0
Lucena City	3	0	13	16	21	0	8	29	1,820	0	6	1,826
San Pablo City	0	0	0	0	0	0	0	0	0	0	0	0
San Pedro City	0	30	177	207	0	0	4	4	0	3	4	7
Santa Rosa City	19	496	756	1,271	0	0	21	21	0	1	21	22
Tagaytay City	0	0	5	5	0	0	0	0	0	0	3	3
Tanauan City	0	2	112	114	0	0	6	6	0	0	57	57
Tayabas City	0	0	0	0	0	0	0	0	0	0	0	0
Trece Martires City	0	1	31	32	0	0	2	2	0	0	0	0
**Region 4B**	**0**	**4**	**615**	**619**	**0**	**5**	**369**	**374**	**0**	**3**	**34**	**37**
Marinduque	0	0	19	19	0	0	8	8	0	0	0	0
Mindoro Occidental	0	1	6	7	0	2	100	102	0	1	2	3
Mindoro Oriental	0	2	99	101	0	3	103	106	0	2	24	26
Palawan	0	1	450	451	0	0	158	158	0	0	1	1
Romblon	0	0	1	1	0	0	0	0	0	0	0	0
Puerto Princesa City	0	0	40	40	0	0	0	0	0	0	7	7
**Region 5**	**1**	**102**	**2,172**	**2,275**	**0**	**8**	**145**	**153**	**0**	**17**	**83**	**100**
Albay	0	31	532	563	0	0	33	33	0	4	24	28
Camarines Norte	0	24	236	260	0	2	13	15	0	0	1	1
Camarines Sur	0	20	610	630	0	2	20	22	0	11	25	36
Catanduanes	0	2	17	19	0	0	3	3	0	0	1	1
Masbate	1	10	138	149	0	0	46	46	0	0	24	24
Sorsogon	0	11	43	54	0	2	10	12	0	1	3	4
Iriga City	0	2	64	66	0	0	1	1	0	0	1	1

Table 1.A.1 - MODERN METHOD OF FAMILY PLANNING
New Acceptors
Annual Philippines, 2020

Area	CONDOM			Total	IUD-INTERVAL			Total	IUD-POSTPARTUM			Total
	Age group				Age group				Age group			
	10-14	15-19	20-49		10-14	15-19	20-49		10-14	15-19	20-49	
Legaspi City	0	1	26	27	0	0	1	1	0	0	0	0
Naga City	0	1	506	507	0	2	18	20	0	1	4	5
**Region 6**	**2**	**151**	**2,014**	**2,167**	**3**	**93**	**1,961**	**2,057**	**0**	**99**	**2,936**	**3,035**
Aklan	0	81	909	990	0	6	385	391	0	0	1	1
Antique	1	7	146	154	0	0	33	33	0	0	12	12
Capiz	0	6	173	179	0	10	417	427	0	5	21	26
Guimaras	0	3	15	18	0	2	123	125	0	0	1	1
Iloilo	1	20	328	349	3	18	223	244	0	14	2,306	2,320
Negros Occidental	0	26	342	368	0	49	624	673	0	75	414	489
Bacolod City	0	7	66	73	0	6	121	127	0	3	170	173
Iloilo City	0	1	35	36	0	2	35	37	0	2	11	13
**Region 7**	**5**	**222**	**1,070**	**1,297**	**8**	**1,015**	**2,555**	**3,578**	**1**	**139**	**861**	**1,001**
Bohol	0	0	140	140	0	300	886	1,186	0	89	290	379
Cebu	0	17	295	312	0	20	282	302	1	19	274	294
Negros Oriental	0	138	216	354	5	657	683	1,345	0	1	16	17
Siquijor	0	3	78	81	0	3	483	486	0	1	148	149
Cebu City	0	5	141	146	0	13	146	159	0	20	51	71
Lapu-Lapu City	5	49	174	228	3	22	61	86	0	9	6	15
Mandaue City	0	10	26	36	0	0	14	14	0	0	76	76
**Region 8**	**0**	**0**	**0**	**0**	**0**	**19**	**186**	**205**	**0**	**11**	**41**	**52**
Biliran	0	0	0	0	0	0	8	8	0	0	2	2
Eastern Samar	0	0	0	0	0	0	45	45	0	0	3	3
Northern Leyte	0	0	0	0	0	0	0	0	0	0	0	0
Northern Samar	0	0	0	0	0	0	2	2	0	0	0	0
Southern Leyte	0	0	0	0	0	5	16	21	0	2	10	12
Western Samar	0	0	0	0	0	9	40	49	0	0	0	0
Calbayog City	0	0	0	0	0	0	10	10	0	0	1	1
Maasin City	0	0	0	0	0	3	17	20	0	0	6	6
Ormoc City	0	0	0	0	0	0	4	4	0	0	0	0
Tacloban City	0	0	0	0	0	2	44	46	0	9	19	28
**Region 9**	**0**	**1**	**48**	**49**	**0**	**3**	**266**	**269**	**0**	**10**	**36**	**46**
Zamboanga del Norte	0	0	0	0	0	0	0	0	0	0	0	0
Zamboanga del Sur	0	0	0	0	0	0	0	0	0	0	0	0
Zamboanga Sibugay	0	0	0	0	0	0	0	0	0	0	0	0
Dapitan City	0	1	0	1	0	1	0	1	0	1	0	1
Dipolog City	0	0	1	1	0	0	0	0	0	0	0	0
Isabela City	0	0	1	1	0	0	0	0	0	0	0	0
Pagadian City	0	0	6	6	0	2	10	12	0	9	36	45
Zamboanga City	0	0	40	40	0	0	256	256	0	0	0	0
**Region 10**	**6**	**331**	**3,570**	**3,907**	**4**	**205**	**4,682**	**4,891**	**4**	**121**	**1,158**	**1,283**
Bukidnon	3	31	248	282	0	39	186	225	0	10	46	56
Camiguin	0	0	4	4	0	1	1	2	0	0	5	5
Lanao del Norte	0	61	591	652	2	29	569	600	0	6	158	164
Misamis Occidental	3	28	158	189	0	10	50	60	0	0	2	2
Misamis Oriental	0	127	108	235	1	35	278	314	0	14	83	97
Cagayan de Oro City	0	20	128	148	1	40	1,396	1,437	1	31	607	639
El Salvador City	0	2	1	3	0	10	26	36	1	7	10	18
Gingoog City	0	22	282	304	0	4	130	134	0	6	114	120
Iligan City	0	4	48	52	0	18	78	96	2	45	106	153
Malaybalay City	0	0	6	6	0	0	5	5	0	0	5	5
Oroquieta City	0	0	0	0	0	0	5	5	0	0	0	0
Ozamis City	0	36	1,989	2,025	0	9	1,929	1,938	0	1	19	20
Tangub City	0	0	0	0	0	0	2	2	0	0	0	0
Valencia City	0	0	7	7	0	10	27	37	0	1	3	4

Table 1.A.1 - MODERN METHOD OF FAMILY PLANNING

New Acceptors

Annual Philippines, 2020

Area	CONDOM			Total	IUD-INTERVAL			Total	IUD-POSTPARTUM			Total
	Age group				Age group				Age group			
	10-14	15-19	20-49		10-14	15-19	20-49		10-14	15-19	20-49	
**Region 11**	**12**	**277**	**1,906**	**2,195**	**17**	**92**	**1,033**	**1,142**	**11**	**109**	**755**	**875**
Davao de Oro	2	18	122	142	1	11	105	117	2	12	48	62
Davao del Norte	1	11	198	210	3	9	131	143	3	31	174	208
Davao Oriental	0	10	91	101	2	3	44	49	1	2	32	35
Davao del Sur	2	32	100	134	4	31	132	167	0	18	115	133
Davao Occidental	1	5	45	51	1	10	23	34	0	7	18	25
Davao City	6	201	1,350	1,557	6	28	598	632	5	39	368	412
**Region 12**	**1**	**135**	**902**	**1,038**	**1**	**66**	**529**	**596**	**4**	**98**	**474**	**576**
North Cotabato	1	51	396	448	1	28	233	262	0	13	91	104
Sarangani	0	28	102	130	0	12	35	47	0	3	25	28
South Cotabato	0	44	236	280	0	20	116	136	0	40	115	155
Sultan Kudarat	0	9	44	53	0	4	47	51	4	15	54	73
				0				0				0
Cotabato City	0	2	88	90	0	0	56	56	0	22	127	149
Gen. Santos City	0	1	36	37	0	2	42	44	0	5	62	67
**B.A.R.M.M.**	**0**	**119**	**1,446**	**1,565**	**0**	**87**	**715**	**802**	**0**	**168**	**1,087**	**1,255**
Basilan	0	41	98	139	0	2	19	21	0	15	55	70
Lanao del Sur	0	11	728	739	0	1	6	7	0	0	2	2
Maguindanao	0	20	487	507	0	14	104	118	0	2	26	28
Sulu	0	25	0	25	0	1	0	1	0	0	0	0
Tawi-Tawi	0	16	2	18	0	0	140	140	0	0	0	0
				0				0				0
Lamitan City	0	4	24	28	0	69	445	514	0	151	1,004	1,155
Marawi City	0	2	107	109	0	0	1	1	0	0	0	0
**CARAGA**	**1**	**91**	**690**	**782**	**1**	**44**	**353**	**398**	**0**	**72**	**230**	**302**
Agusan del Norte	1	14	137	152	0	8	74	82	0	44	85	129
Agusan del Sur	0	17	178	195	1	13	101	115	0	4	40	44
Surigao del Norte	0	31	117	148	0	6	56	62	0	2	14	16
Surigao del Sur	0	11	116	127	0	7	52	59	0	17	64	81
Province of Dinagat	0	5	62	67	0	8	37	45	0	0	1	1
Bislig City	0	1	6	7	0	0	1	1	0	1	1	2
Butuan City	0	3	17	20	0	1	1	2	0	1	2	3
Surigao City	0	9	57	66	0	1	31	32	0	3	23	26

Table 1.A.1 - MODERN METHOD OF FAMILY PLANNING

New Acceptors

Annual Philippines, 2020

Area	PILLS-POP Age group 10-14	15-19	20-49	Total	PILLS-COC Age group 10-14	15-19	20-49	Total	INJECTABLES Age group 10-14	15-19	20-49	Total
**PHILIPPINES**	**1,016**	**9,360**	**47,032**	**57,408**	**255**	**14,596**	**171,120**	**185,971**	**564**	**22,082**	**147,420**	**170,066**
**N C R**	**17**	**1,744**	**8,744**	**10,505**	**43**	**1,195**	**12,032**	**13,270**	**70**	**3,464**	**23,576**	**27,110**
Malabon	0	66	411	477	0	39	362	401	2	252	933	1,187
Navotas	2	159	320	481	0	184	520	704	1	265	1,083	1,349
Valenzuela City	2	51	558	611	1	69	1,550	1,620	7	289	2,692	2,988
Caloocan City	2	126	563	691	2	125	939	1,066	1	533	2,888	3,422
Marikina City	0	31	216	247	0	15	300	315	3	200	1,138	1,341
Pasig City	0	50	330	380	0	61	673	734	0	147	1,752	1,899
Pateros	0	3	20	23	0	1	40	41	0	5	94	99
Taguig	0	63	731	794	0	48	643	691	0	195	2,173	2,368
Quezon City	2	466	1,659	2,127	1	159	2,105	2,265	1	372	2,359	2,732
Makati City	0	18	192	210	0	13	195	208	0	32	468	500
Mandaluyong City	0	14	134	148	0	12	150	162	0	21	301	322
San Juan	0	14	19	33	0	0	64	64	0	0	155	155
Manila City	5	430	1,904	2,339	25	127	1,346	1,498	20	192	2,404	2,616
Las Piñas City	0	92	673	765	1	56	1,058	1,115	0	169	1,478	1,647
Muntinlupa City	1	71	344	416	0	238	785	1,023	3	643	2,383	3,029
Parañaque City	3	29	352	384	13	48	751	812	32	149	842	1,023
Pasay City	0	61	318	379	0	0	551	551	0	0	433	433
**C A R**	**0**	**209**	**1,058**	**1,267**	**3**	**96**	**880**	**979**	**2**	**207**	**1,381**	**1,590**
Abra	0	30	145	175	0	23	165	188	0	29	169	198
Apayao	0	41	156	197	0	15	73	88	0	24	68	92
Benguet	0	73	254	327	2	32	305	339	1	51	380	432
Ifugao	0	0	6	6	0	1	5	6	0	2	9	11
Kalinga	0	16	70	86	0	10	90	100	0	7	76	83
Mt. Province	0	5	29	34	1	6	47	54	0	13	37	50
Baguio City	0	44	398	442	0	9	195	204	1	81	642	724
**Region 1**	**2**	**147**	**2,026**	**2,175**	**3**	**482**	**5,767**	**6,252**	**2**	**698**	**5,005**	**5,705**
Ilocos Norte	1	24	124	149	1	31	895	927	0	26	196	222
Ilocos Sur	0	41	939	980	0	60	1,797	1,857	0	58	693	751
La Union	1	14	63	78	0	27	261	288	0	98	552	650
Pangasinan	0	51	709	760	0	225	1,791	2,016	2	381	2,591	2,974
Alaminos City	0	1	15	16	0	75	166	241	0	44	126	170
Candon City	0	0	0	0	0	0	31	31	0	0	23	23
Dagupan City	0	2	17	19	0	3	100	103	0	25	170	195
Laoag City	0	0	23	23	0	0	69	69	0	0	10	10
San Carlos City	0	6	90	96	0	33	440	473	0	39	420	459
San Fernando City	0	0	0	0	0	6	133	139	0	1	73	74
Urdaneta City	0	8	46	54	2	22	83	107	0	26	150	176
Vigan City	0	0	0	0	0	0	1	1	0	0	1	1
**Region 2**	**1**	**346**	**1,718**	**2,065**	**6**	**381**	**3,755**	**4,142**	**5**	**466**	**3,403**	**3,874**
Batanes	0	0	0	0	0	0	1	1	0	1	0	1
Cagayan	0	93	414	507	4	99	1,875	1,978	2	94	527	623
Isabela	1	134	758	893	0	123	988	1,111	0	245	1,079	1,324
Nueva Vizcaya	0	44	141	185	0	26	231	257	0	31	183	214
Quirino	0	13	56	69	2	42	128	172	3	37	144	184
Cauayan City	0	4	11	15	0	7	59	66	0	17	43	60
Ilagan City	0	18	80	98	0	61	205	266	0	22	1,224	1,246
Santiago City	0	0	0	0	0	13	58	71	0	7	32	39
Tuguegarao City	0	40	258	298	0	10	210	220	0	12	171	183
**Region 3**	**2**	**834**	**4,482**	**5,318**	**3**	**1,685**	**18,402**	**20,090**	**10**	**3,512**	**22,368**	**25,890**
Aurora	0	6	35	41	0	7	139	146	0	27	88	115
Bataan	0	47	319	366	0	68	672	740	3	404	1,644	2,051

Table 1.A.1 - MODERN METHOD OF FAMILY PLANNING

New Acceptors

Annual Philippines, 2020

Area	PILLS-POP			Total	PILLS-COC			Total	INJECTABLES			Total
	Age group				Age group				Age group			
	10-14	15-19	20-49		10-14	15-19	20-49		10-14	15-19	20-49	
Bulacan	0	105	493	598	0	457	4,706	5,163	0	710	3,987	4,697
Nueva Ecija	0	182	769	951	0	182	1,574	1,756	0	426	2,515	2,941
Pampanga	1	135	631	767	0	172	2,238	2,410	1	221	2,106	2,328
Tarlac	0	81	748	829	0	113	2,205	2,318	0	177	2,084	2,261
Zambales	0	45	410	455	0	62	1,451	1,513	3	284	2,022	2,309
Angeles City	0	29	165	194	0	67	550	617	1	127	853	981
Balanga City	0	0	0	0	0	8	49	57	0	33	121	154
Cabanatuan City	1	51	175	227	0	43	363	406	0	73	318	391
City of San Fernando	0	29	118	147	0	48	512	560	0	52	481	533
Gapan City	0	19	92	111	0	26	207	233	0	35	241	276
Mabalacat City	0	10	25	35	0	47	533	580	0	156	919	1,075
Malolos City	0	0	0	0	0	6	180	186	0	9	263	272
Meycauayan	0	4	25	29	2	47	300	349	0	63	425	488
Olongapo	0	14	95	109	0	29	319	348	2	163	749	914
Palayan City	0	1	1	2	1	12	33	46	0	8	54	62
San Jose City	0	12	79	91	0	32	216	248	0	85	319	404
San Jose del Monte City	0	9	72	81	0	116	1,464	1,580	0	307	2,475	2,782
Science City of Munoz	0	4	18	22	0	0	42	42	0	13	44	57
Tarlac City	0	51	212	263	0	143	649	792	0	139	660	799
**Region 4A**	**709**	**1,303**	**1,303**	**3,315**	**68**	**2,420**	**38,441**	**40,929**	**186**	**2,396**	**18,505**	**21,087**
Batangas	672	35	35	742	0	32	4,131	4,163	76	96	982	1,154
Cavite	2	36	36	74	0	62	601	663	1	222	1,740	1,963
Laguna	1	77	77	155	24	82	3,336	3,442	0	90	945	1,035
Quezon	0	116	116	232	0	115	1,419	1,534	2	233	2,355	2,590
Rizal	2	51	51	104	1	149	6,470	6,620	31	317	1,560	1,908
Antipolo City	0	0	0	0	0	0	0	0	0	0	0	0
Bacoor City	0	3	3	6	12	230	2,143	2,385	51	63	545	659
Batangas City	22	5	5	32	0	3	145	148	0	0	0	0
Biñan City	0	670	670	1,340	0	948	4,991	5,939	0	679	3,921	4,600
Cabuyao City	0	35	35	70	0	52	2,669	2,721	0	83	639	722
Calamba City	0	56	56	112	3	12	622	637	0	31	846	877
Cavite City	0	0	0	0	0	0	0	0	0	0	0	0
Dasmariñas City	0	40	40	80	0	84	4,927	5,011	0	163	1,088	1,251
General Trias City	0	0	0	0	0	12	661	673	1	0	628	629
Imus City	0	3	3	6	20	8	432	460	8	28	247	283
Lipa City	0	0	0	0	0	0	0	0	0	0	0	0
Lucena City	3	0	0	3	0	0	0	0	9	0	455	464
San Pablo City	0	0	0	0	0	0	0	0	0	0	0	0
San Pedro City	1	99	99	199	0	61	690	751	0	19	249	268
Santa Rosa City	6	65	65	136	7	541	3,314	3,862	3	271	1,472	1,746
Tagaytay City	0	0	0	0	1	1	199	201	4	1	151	156
Tanauan City	0	8	8	16	0	4	780	784	0	16	163	179
Tayabas City	0	0	0	0	0	0	0	0	0	0	0	0
Trece Martires City	0	4	4	8	0	24	911	935	0	84	519	603
**Region 4B**	**1**	**59**	**577**	**637**	**0**	**65**	**6,617**	**6,682**	**1**	**123**	**4,884**	**5,008**
Marinduque	0	28	190	218	0	13	234	247	0	24	285	309
Mindoro Occidental	0	7	131	138	0	8	83	91	0	16	1,199	1,215
Mindoro Oriental	0	14	207	221	0	30	878	908	0	17	761	778
Palawan	1	10	35	46	0	14	5,184	5,198	1	66	1,850	1,917
Romblon	0	0	14	14	0	0	7	7	0	0	3	3
Puerto Princesa City	0	0	0	0	0	0	231	231	0	0	786	786
**Region 5**	**0**	**251**	**2,468**	**2,719**	**2**	**516**	**8,094**	**8,612**	**6**	**644**	**8,990**	**9,640**
Albay	0	58	1,026	1,084	0	83	1,487	1,570	0	154	3,715	3,869
Camarines Norte	0	32	154	186	0	64	490	554	1	94	695	790
Camarines Sur	0	88	844	932	0	173	2,634	2,807	2	218	2,590	2,810
Catanduanes	0	0	31	31	1	16	77	94	0	18	101	119
Masbate	0	43	160	203	1	128	1,591	1,720	3	73	892	968
Sorsogon	0	10	22	32	0	33	307	340	0	56	302	358
Iriga City	0	5	64	69	0	9	227	236	0	19	248	267

Table 1.A.1 - MODERN METHOD OF FAMILY PLANNING

New Acceptors

Annual Philippines, 2020

Area	PILLS-POP Age group			Total	PILLS-COC Age group			Total	INJECTABLES Age group			Total
	10-14	15-19	20-49		10-14	15-19	20-49		10-14	15-19	20-49	
Legaspi City	0	6	75	81	0	4	119	123	0	4	49	53
Naga City	0	9	92	101	0	6	1,162	1,168	0	8	398	406
**Region 6**	**134**	**456**	**4,889**	**5,479**	**10**	**1,730**	**18,747**	**20,487**	**5**	**1,597**	**10,983**	**12,585**
Aklan	0	47	234	281	2	166	5,454	5,622	1	404	954	1,359
Antique	0	17	174	191	0	47	899	946	0	139	1,365	1,504
Capiz	0	30	120	150	0	52	3,179	3,231	0	136	1,685	1,821
Guimaras	0	1	18	19	0	10	613	623	1	15	199	215
Iloilo	0	76	3,333	3,409	4	207	1,469	1,680	1	238	2,063	2,302
Negros Occidental	6	274	934	1,214	4	1,203	6,273	7,480	2	526	3,732	4,260
Bacolod City	128	11	65	204	0	31	271	302	0	103	701	804
Iloilo City	0	0	11	11	0	14	589	603	0	36	284	320
**Region 7**	**13**	**490**	**3,331**	**3,834**	**13**	**566**	**9,664**	**10,243**	**35**	**1,201**	**8,979**	**10,215**
Bohol	2	82	357	441	6	87	1,158	1,251	0	127	634	761
Cebu	1	74	1,152	1,227	1	54	2,307	2,362	0	233	1,672	1,905
Negros Oriental	0	74	226	300	1	211	905	1,117	2	233	1,647	1,882
Siquijor	0	27	228	255	0	43	3,491	3,534	0	68	2,172	2,240
Cebu City	0	62	467	529	0	54	1,347	1,401	3	239	1,673	1,915
Lapu-Lapu City	10	171	771	952	5	117	307	429	30	301	793	1,124
Mandaue City	0	0	130	130	0	0	149	149	0	0	388	388
**Region 8**	**0**	**0**	**0**	**0**	**0**	**0**	**0**	**0**	**0**	**0**	**0**	**0**
Biliran	0	0	0	0	0	0	0	0	0	0	0	0
Eastern Samar	0	0	0	0	0	0	0	0	0	0	0	0
Northern Leyte	0	0	0	0	0	0	0	0	0	0	0	0
Northern Samar	0	0	0	0	0	0	0	0	0	0	0	0
Southern Leyte	0	0	0	0	0	0	0	0	0	0	0	0
Western Samar	0	0	0	0	0	0	0	0	0	0	0	0
Calbayog City	0	0	0	0	0	0	0	0	0	0	0	0
Maasin City	0	0	0	0	0	0	0	0	0	0	0	0
Ormoc City	0	0	0	0	0	0	0	0	0	0	0	0
Tacloban City	0	0	0	0	0	0	0	0	0	0	0	0
**Region 9**	**10**	**37**	**540**	**587**	**0**	**152**	**512**	**664**	**0**	**30**	**525**	**555**
Zamboanga del Norte	0	0	0	0	0	0	0	0	0	0	0	0
Zamboanga del Sur	0	0	0	0	0	0	0	0	0	0	0	0
Zamboanga Sibugay	0	0	0	0	0	0	0	0	0	0	0	0
Dapitan City	0	3	2	5	0	1	66	67	0	1	10	11
Dipolog City	10	24	507	541	0	0	3	3	0	1	3	4
Isabela City	0	5	13	18	0	4	34	38	0	14	64	78
Pagadian City	0	1	11	12	0	1	49	50	0	0	0	0
Zamboanga City	0	4	7	11	0	146	360	506	0	14	448	462
**Region 10**	**22**	**638**	**3,606**	**4,266**	**13**	**1,282**	**26,117**	**27,412**	**18**	**1,213**	**11,955**	**13,186**
Bukidnon	1	150	341	492	1	177	901	1,079	0	171	732	903
Camiguin	0	0	5	5	0	2	20	22	0	2	29	31
Lanao del Norte	20	228	1,362	1,610	12	89	4,491	4,592	14	204	1,749	1,967
Misamis Occidental	0	22	184	206	0	54	301	355	0	29	292	321
Misamis Oriental	0	45	295	340	0	106	542	648	0	123	772	895
Cagayan de Oro City	1	51	427	479	0	128	3,271	3,399	0	173	2,456	2,629
El Salvador City	0	4	31	35	0	6	29	35	1	14	51	66
Gingoog City	0	35	199	234	0	48	338	386	1	74	222	297
Iligan City	0	30	199	229	0	62	661	723	2	88	745	835
Malaybalay City	0	10	16	26	0	5	66	71	0	5	136	141
Oroquieta City	0	9	28	37	0	5	29	34	0	2	14	16
Ozamis City	0	35	436	471	0	560	15,224	15,784	0	301	4,622	4,923
Tangub City	0	1	17	18	0	7	24	31	0	2	9	11
Valencia City	0	18	66	84	0	33	220	253	0	25	126	151

Table 1.A.1 - MODERN METHOD OF FAMILY PLANNING

New Acceptors

Annual Philippines, 2020

Area	PILLS-POP			Total	PILLS-COC			Total	INJECTABLES			Total
	Age group				Age group				Age group			
	10-14	15-19	20-49		10-14	15-19	20-49		10-14	15-19	20-49	
**Region 11**	**91**	**1,345**	**5,709**	**7,145**	**83**	**1,227**	**7,212**	**8,522**	**208**	**1,960**	**7,873**	**10,041**
Davao de Oro	18	269	744	1,031	17	154	996	1,167	12	239	877	1,128
Davao del Norte	9	207	917	1,133	46	138	1,284	1,468	20	330	1,490	1,840
Davao Oriental	12	70	622	704	6	59	1,100	1,165	5	81	438	524
Davao del Sur	21	234	581	836	7	109	625	741	150	346	954	1,450
Davao Occidental	1	47	74	122	2	80	304	386	8	325	655	988
Davao City	30	518	2,771	3,319	5	687	2,903	3,595	13	639	3,459	4,111
**Region 12**	**4**	**723**	**3,180**	**3,907**	**0**	**808**	**5,786**	**6,594**	**6**	**1,835**	**8,890**	**10,731**
North Cotabato	1	222	1,127	1,350	0	256	2,004	2,260	3	514	2,711	3,228
Sarangani	0	116	264	380	0	181	879	1,060	0	285	1,176	1,461
South Cotabato	3	251	734	988	0	211	1,448	1,659	3	592	2,385	2,980
Sultan Kudarat	0	61	689	750	0	99	599	698	0	278	1,188	1,466
				0				0				0
Cotabato City	0	51	196	247	0	27	604	631	0	78	959	1,037
Gen. Santos City	0	22	170	192	0	34	252	286	0	88	471	559
**B.A.R.M.M.**	**0**	**232**	**1,335**	**1,567**	**5**	**1,575**	**6,308**	**7,888**	**4**	**2,232**	**7,301**	**9,537**
Basilan	0	53	154	207	0	90	418	508	1	136	570	707
Lanao del Sur	0	42	308	350	0	47	1,487	1,534	0	54	1,300	1,354
Maguindanao	0	68	437	505	5	153	2,027	2,185	3	309	2,646	2,958
Sulu	0	0	0	0	0	486	0	486	0	680	0	680
Tawi-Tawi	0	0	140	140	0	520	0	520	0	699	101	800
				0				0				0
Lamitan City	0	64	259	323	0	275	2,259	2,534	0	350	2,563	2,913
Marawi City	0	5	37	42	0	4	117	121	0	4	121	125
**CARAGA**	**10**	**546**	**2,066**	**2,622**	**3**	**416**	**2,786**	**3,205**	**6**	**504**	**2,802**	**3,312**
Agusan del Norte	4	203	702	909	1	71	543	615	1	107	591	699
Agusan del Sur	3	107	429	539	1	124	649	774	3	126	682	811
Surigao del Norte	0	53	338	391	0	47	475	522	1	67	457	525
Surigao del Sur	1	76	291	368	1	82	480	563	1	59	299	359
Province of Dinagat	0	20	90	110	0	11	175	186	0	20	143	163
Bislig City	0	4	9	13	0	12	27	39	0	6	14	20
Butuan City	2	62	154	218	0	51	300	351	0	99	401	500
Surigao City	0	21	53	74	0	18	137	155	0	20	215	235

Table 1.A.1 - MODERN METHOD OF FAMILY PLANNING
New Acceptors
Annual Philippines, 2020

Area	IMPLANTS Age group			Total	NFP-CCM Age group			Total	NFP-BBT Age group			Total
	10-14	15-19	20-49		10-14	15-19	20-49		10-14	15-19	20-49	
**PHILIPPINES**	**672**	**5,252**	**30,577**	**36,501**	**7**	**209**	**2,248**	**2,464**	**6**	**298**	**1,423**	**1,727**
**N C R**	**34**	**1,333**	**5,033**	**6,400**	**0**	**8**	**46**	**54**	**2**	**123**	**649**	**774**
Malabon	0	19	47	66	0	0	0	0	0	0	0	0
Navotas	0	24	180	204	0	0	0	0	0	0	0	0
Valenzuela City	1	21	242	264	0	0	3	3	0	0	0	0
Caloocan City	5	86	296	387	0	0	0	0	0	0	0	0
Marikina City	1	31	213	245	0	2	0	2	0	0	0	0
Pasig City	0	35	381	416	0	0	0	0	0	0	0	0
Pateros	0	0	1	1	0	0	0	0	0	0	0	0
Taguig	0	0	69	69	0	0	0	0	0	0	0	0
Quezon City	9	303	541	853	0	0	0	0	0	3	18	21
Makati City	0	4	48	52	0	0	0	0	0	0	0	0
Mandaluyong City	1	8	68	77	0	6	43	49	0	0	0	0
San Juan	0	0	8	8	0	0	0	0	0	0	0	0
Manila City	17	739	2,396	3,152	0	0	0	0	2	120	631	753
Las Piñas City	0	43	203	246	0	0	0	0	0	0	0	0
Muntinlupa City	0	5	74	79	0	0	0	0	0	0	0	0
Parañaque City	0	7	67	74	0	0	0	0	0	0	0	0
Pasay City	0	8	199	207	0	0	0	0	0	0	0	0
**C A R**	**2**	**87**	**462**	**551**	**0**	**1**	**11**	**12**	**0**	**1**	**26**	**27**
Abra	0	9	80	89	0	1	11	12	0	0	17	17
Apayao	0	4	15	19	0	0	0	0	0	0	0	0
Benguet	0	15	45	60	0	0	0	0	0	0	1	1
Ifugao	1	0	3	4	0	0	0	0	0	0	0	0
Kalinga	0	5	32	37	0	0	0	0	0	1	6	7
Mt. Province	0	3	7	10	0	0	0	0	0	0	2	2
Baguio City	1	51	280	332	0	0	0	0	0	0	0	0
**Region 1**	**1**	**104**	**880**	**985**	**0**	**0**	**76**	**76**	**0**	**0**	**9**	**9**
Ilocos Norte	0	4	18	22	0	0	0	0	0	0	1	1
Ilocos Sur	0	8	116	124	0	0	3	3	0	0	4	4
La Union	0	11	70	81	0	0	71	71	0	0	0	0
Pangasinan	1	76	583	660	0	0	0	0	0	0	3	3
Alaminos City	0	0	14	14	0	0	0	0	0	0	0	0
Candon City	0	0	0	0	0	0	0	0	0	0	0	0
Dagupan City	0	0	5	5	0	0	0	0	0	0	1	1
Laoag City	0	0	0	0	0	0	0	0	0	0	0	0
San Carlos City	0	3	42	45	0	0	0	0	0	0	0	0
San Fernando City	0	2	28	30	0	0	0	0	0	0	0	0
Urdaneta City	0	0	4	4	0	0	0	0	0	0	0	0
Vigan City	0	0	0	0	0	0	2	2	0	0	0	0
**Region 2**	**1**	**79**	**356**	**436**	**0**	**0**	**0**	**0**	**0**	**0**	**1**	**1**
Batanes	0	0	0	0	0	0	0	0	0	0	0	0
Cagayan	1	25	129	155	0	0	0	0	0	0	0	0
Isabela	0	34	125	159	0	0	0	0	0	0	0	0
Nueva Vizcaya	0	13	76	89	0	0	0	0	0	0	1	1
Quirino	0	0	5	5	0	0	0	0	0	0	0	0
Cauayan City	0	1	4	5	0	0	0	0	0	0	0	0
Ilagan City	0	0	7	7	0	0	0	0	0	0	0	0
Santiago City	0	2	2	4	0	0	0	0	0	0	0	0
Tuguegarao City	0	4	8	12	0	0	0	0	0	0	0	0
**Region 3**	**4**	**388**	**3,047**	**3,439**	**0**	**5**	**28**	**33**	**0**	**17**	**99**	**116**
Aurora	0	5	22	27	0	0	0	0	0	0	4	4
Bataan	0	22	175	197	0	0	9	9	0	0	6	6

Table 1.A.1 - MODERN METHOD OF FAMILY PLANNING
New Acceptors
Annual Philippines, 2020

Area	IMPLANTS Age group			Total	NFP-CCM Age group			Total	NFP-BBT Age group			Total
	10-14	15-19	20-49		10-14	15-19	20-49		10-14	15-19	20-49	
Bulacan	0	67	563	630	0	0	1	1	0	0	0	0
Nueva Ecija	1	140	672	813	0	3	6	9	0	0	0	0
Pampanga	0	26	408	434	0	2	3	5	0	0	0	0
Tarlac	0	14	181	195	0	0	2	2	0	1	43	44
Zambales	0	26	342	368	0	0	7	7	0	1	0	1
Angeles City	0	17	250	267	0	0	0	0	0	0	0	0
Balanga City	0	4	5	9	0	0	0	0	0	0	0	0
Cabanatuan City	0	0	13	13	0	0	0	0	0	0	0	0
City of San Fernando	0	0	19	19	0	0	0	0	0	11	33	44
Gapan City	0	3	25	28	0	0	0	0	0	0	0	0
Mabalacat City	0	2	25	27	0	0	0	0	0	0	0	0
Malolos City	0	1	18	19	0	0	0	0	0	0	0	0
Meycauayan	0	7	22	29	0	0	0	0	0	0	0	0
Olongapo	3	43	141	187	0	0	0	0	0	0	0	0
Palayan City	0	0	0	0	0	0	0	0	0	0	0	0
San Jose City	0	2	13	15	0	0	0	0	0	0	0	0
San Jose del Monte City	0	4	73	77	0	0	0	0	0	0	0	0
Science City of Munoz	0	0	0	0	0	0	0	0	0	0	0	0
Tarlac City	0	5	80	85	0	0	0	0	0	4	13	17
**Region 4A**	**578**	**191**	**1,242**	**2,011**	**0**	**46**	**198**	**244**	**4**	**13**	**69**	**86**
Batangas	0	3	57	60	0	0	0	0	0	0	6	6
Cavite	0	14	95	109	0	0	32	32	0	0	0	0
Laguna	0	22	145	167	0	11	6	17	0	4	15	19
Quezon	0	2	124	126	0	0	4	4	0	0	4	4
Rizal	2	42	140	184	0	19	0	19	0	0	0	0
Antipolo City	0	0	0	0	0	0	0	0	0	0	0	0
Bacoor City	0	3	30	33	0	0	0	0	0	0	28	28
Batangas City	0	0	0	0	0	0	0	0	0	0	0	0
Biñan City	0	43	153	196	0	0	0	0	0	0	6	6
Cabuyao City	0	2	26	28	0	0	0	0	0	0	10	10
Calamba City	0	9	138	147	0	0	0	0	0	0	0	0
Cavite City	0	0	0	0	0	0	0	0	0	0	0	0
Dasmariñas City	0	10	50	60	0	0	0	0	0	1	0	1
General Trias City	0	0	22	22	0	1	0	1	0	3	0	3
Imus City	0	7	6	13	0	0	0	0	0	0	0	0
Lipa City	0	0	0	0	0	0	5	5	0	0	0	0
Lucena City	576	0	2	578	0	0	0	0	4	0	0	4
San Pablo City	0	0	0	0	0	0	0	0	0	0	0	0
San Pedro City	0	13	64	77	0	15	105	120	0	5	0	5
Santa Rosa City	0	6	100	106	0	0	0	0	0	0	0	0
Tagaytay City	0	0	86	86	0	0	0	0	0	0	0	0
Tanauan City	0	0	0	0	0	0	0	0	0	0	0	0
Tayabas City	0	0	0	0	0	0	46	46	0	0	0	0
Trece Martires City	0	15	4	19	0	0	0	0	0	0	0	0
**Region 4B**	**1**	**14**	**1,033**	**1,048**	**0**	**2**	**328**	**330**	**0**	**1**	**4**	**5**
Marinduque	1	7	87	95	0	0	0	0	0	0	0	0
Mindoro Occidental	0	5	56	61	0	2	9	11	0	1	0	1
Mindoro Oriental	0	0	119	119	0	0	310	310	0	0	2	2
Palawan	0	2	742	744	0	0	0	0	0	0	0	0
Romblon	0	0	16	16	0	0	9	9	0	0	2	2
Puerto Princesa City	0	0	13	13	0	0	0	0	0	0	0	0
**Region 5**	**2**	**184**	**1,892**	**2,078**	**7**	**23**	**342**	**372**	**0**	**9**	**56**	**65**
Albay	2	53	488	543	0	7	62	69	0	0	7	7
Camarines Norte	0	7	38	45	0	6	9	15	0	6	6	12
Camarines Sur	0	108	899	1,007	0	3	168	171	0	3	37	40
Catanduanes	0	6	51	57	0	0	0	0	0	0	0	0
Masbate	0	7	256	263	7	4	91	102	0	0	1	1
Sorsogon	0	1	26	27	0	3	10	13	0	0	0	0
Iriga City	0	1	62	63	0	0	0	0	0	0	3	3

Table 1.A.1 - MODERN METHOD OF FAMILY PLANNING
New Acceptors
Annual Philippines, 2020

Area	IMPLANTS Age group			Total	NFP-CCM Age group			Total	NFP-BBT Age group			Total
	10-14	15-19	20-49		10-14	15-19	20-49		10-14	15-19	20-49	
Legaspi City	0	1	23	24	0	0	0	0	0	0	0	0
Naga City	0	0	49	49	0	0	2	2	0	0	2	2
**Region 6**	**5**	**341**	**2,643**	**2,989**	**0**	**12**	**382**	**394**	**0**	**14**	**136**	**150**
Aklan	0	9	108	117	0	1	57	58	0	0	0	0
Antique	0	42	176	218	0	11	310	321	0	0	0	0
Capiz	1	20	471	492	0	0	10	10	0	4	24	28
Guimaras	0	1	3	4	0	0	0	0	0	0	6	6
Iloilo	2	79	736	817	0	0	5	5	0	1	23	24
Negros Occidental	2	171	911	1,084	0	0	0	0	0	9	64	73
Bacolod City	0	15	186	201	0	0	0	0	0	0	18	18
Iloilo City	0	4	52	56	0	0	0	0	0	0	1	1
**Region 7**	**4**	**247**	**2,068**	**2,319**	**0**	**0**	**0**	**0**	**0**	**9**	**13**	**22**
Bohol	0	93	718	811	0	0	0	0	0	0	0	0
Cebu	0	24	440	464	0	0	0	0	0	0	3	3
Negros Oriental	0	69	146	215	0	0	0	0	0	3	5	8
Siquijor	0	13	207	220	0	0	0	0	0	0	0	0
Cebu City	0	26	221	247	0	0	0	0	0	6	5	11
Lapu-Lapu City	4	22	73	99	0	0	0	0	0	0	0	0
Mandaue City	0	0	263	263	0	0	0	0	0	0	0	0
**Region 8**	**0**	**0**	**0**	**0**	**0**	**0**	**0**	**0**	**0**	**0**	**0**	**0**
Biliran	0	0	0	0	0	0	0	0	0	0	0	0
Eastern Samar	0	0	0	0	0	0	0	0	0	0	0	0
Northern Leyte	0	0	0	0	0	0	0	0	0	0	0	0
Northern Samar	0	0	0	0	0	0	0	0	0	0	0	0
Southern Leyte	0	0	0	0	0	0	0	0	0	0	0	0
Western Samar	0	0	0	0	0	0	0	0	0	0	0	0
Calbayog City	0	0	0	0	0	0	0	0	0	0	0	0
Maasin City	0	0	0	0	0	0	0	0	0	0	0	0
Ormoc City	0	0	0	0	0	0	0	0	0	0	0	0
Tacloban City	0	0	0	0	0	0	0	0	0	0	0	0
**Region 9**	**0**	**4**	**62**	**66**	**0**	**0**	**0**	**0**	**0**	**0**	**0**	**0**
Zamboanga del Norte	0	0	0	0	0	0	0	0	0	0	0	0
Zamboanga del Sur	0	0	0	0	0	0	0	0	0	0	0	0
Zamboanga Sibugay	0	0	0	0	0	0	0	0	0	0	0	0
Dapitan City	0	0	0	0	0	0	0	0	0	0	0	0
Dipolog City	0	0	0	0	0	0	0	0	0	0	0	0
Isabela City	0	4	22	26	0	0	0	0	0	0	0	0
Pagadian City	0	0	8	8	0	0	0	0	0	0	0	0
Zamboanga City	0	0	32	32	0	0	0	0	0	0	0	0
**Region 10**	**6**	**414**	**4,119**	**4,539**	**0**	**82**	**715**	**797**	**0**	**68**	**182**	**250**
Bukidnon	3	126	495	624	0	3	15	18	0	7	9	16
Camiguin	0	1	7	8	0	0	0	0	0	0	0	0
Lanao del Norte	2	35	592	629	0	0	15	15	0	12	12	24
Misamis Occidental	0	0	35	35	0	7	16	23	0	8	5	13
Misamis Oriental	0	48	178	226	0	0	4	4	0	0	0	0
Cagayan de Oro City	1	69	691	761	0	0	0	0	0	0	0	0
El Salvador City	0	16	60	76	0	0	0	0	0	0	0	0
Gingoog City	0	46	184	230	0	39	62	101	0	41	156	197
Iligan City	0	6	42	48	0	0	0	0	0	0	0	0
Malaybalay City	0	0	32	32	0	0	1	1	0	0	0	0
Oroquieta City	0	0	2	2	0	0	0	0	0	0	0	0
Ozamis City	0	66	1,795	1,861	0	33	602	635	0	0	0	0
Tangub City	0	0	1	1	0	0	0	0	0	0	0	0
Valencia City	0	1	5	6	0	0	0	0	0	0	0	0

Table 1.A.1 - MODERN METHOD OF FAMILY PLANNING

New Acceptors

Annual Philippines, 2020

Area	IMPLANTS Age group			Total	NFP-CCM Age group			Total	NFP-BBT Age group			Total
	10-14	15-19	20-49		10-14	15-19	20-49		10-14	15-19	20-49	
**Region 11**	**20**	**602**	**2,671**	**3,293**	**0**	**12**	**83**	**95**	**0**	**2**	**65**	**67**
Davao de Oro	4	61	273	338	0	0	1	1	0	0	0	0
Davao del Norte	0	125	507	632	0	6	10	16	0	0	28	28
Davao Oriental	2	7	55	64	0	0	0	0	0	0	5	5
Davao del Sur	3	86	305	394	0	0	2	2	0	0	2	2
Davao Occidental	0	56	131	187	0	0	0	0	0	0	0	0
Davao City	11	267	1,400	1,678	0	6	70	76	0	2	30	32
**Region 12**	**7**	**512**	**1,853**	**2,372**	**0**	**7**	**29**	**36**	**0**	**34**	**55**	**89**
North Cotabato	0	79	453	532	0	7	17	24	0	19	20	39
Sarangani	0	53	187	240	0	0	12	12	0	11	17	28
South Cotabato	7	194	416	617	0	0	0	0	0	0	0	0
Sultan Kudarat	0	146	430	576	0	0	0	0	0	0	1	1
				0				0				0
Cotabato City	0	20	245	265	0	0	0	0	0	4	17	21
Gen. Santos City	0	20	122	142	0	0	0	0	0	0	0	0
**B.A.R.M.M.**	**5**	**613**	**2,319**	**2,937**	**0**	**11**	**0**	**11**	**0**	**0**	**0**	**0**
Basilan	0	30	246	276	0	0	0	0	0	0	0	0
Lanao del Sur	0	4	97	101	0	0	0	0	0	0	0	0
Maguindanao	5	96	579	680	0	0	0	0	0	0	0	0
Sulu	0	98	0	98	0	0	0	0	0	0	0	0
Tawi-Tawi	0	248	51	299	0	11	0	11	0	0	0	0
				0				0				0
Lamitan City	0	137	1,314	1,451	0	0	0	0	0	0	0	0
Marawi City	0	0	32	32	0	0	0	0	0	0	0	0
**CARAGA**	**2**	**139**	**897**	**1,038**	**0**	**0**	**10**	**10**	**0**	**7**	**59**	**66**
Agusan del Norte	0	30	144	174	0	0	0	0	0	0	22	22
Agusan del Sur	1	49	140	190	0	0	0	0	0	6	16	22
Surigao del Norte	1	21	222	244	0	0	10	10	0	0	11	11
Surigao del Sur	0	7	94	101	0	0	0	0	0	0	0	0
Province of Dinagat	0	13	109	122	0	0	0	0	0	0	0	0
Bislig City	0	2	3	5	0	0	0	0	0	1	1	2
Butuan City	0	6	33	39	0	0	0	0	0	0	9	9
Surigao City	0	11	152	163	0	0	0	0	0	0	0	0

Table 1.A.1 - MODERN METHOD OF FAMILY PLANNING

New Acceptors

Annual Philippines, 2020

Area	NFP-STM Age group			Total	NFP-SDM Age group			Total	NFP-LAM Age group			Total	Total New Acceptors
	10-14	15-19	20-49		10-14	15-19	20-49		10-14	15-19	20-49		
**PHILIPPINES**	**192**	**37**	**516**	**745**	**407**	**314**	**9,084**	**9,805**	**1,517**	**59,427**	**338,891**	**399,835**	**978,075**
**N C R**	**0**	**0**	**0**	**0**	**0**	**12**	**105**	**117**	**146**	**8,568**	**66,576**	**75,290**	**144,306**
Malabon	0	0	0	0	0	0	4	4	3	197	668	868	3,168
Navotas	0	0	0	0	0	0	0	0	3	418	2,794	3,215	6,474
Valenzuela City	0	0	0	0	0	0	39	39	2	32	3,261	3,295	9,117
Caloocan City	0	0	0	0	0	0	2	2	11	853	4,332	5,196	11,716
Marikina City	0	0	0	0	0	0	1	1	0	100	448	548	3,593
Pasig City	0	0	0	0	0	0	1	1	2	143	2,743	2,888	6,952
Pateros	0	0	0	0	0	0	0	0	0	19	832	851	1,053
Taguig	0	0	0	0	0	0	0	0	7	972	11,741	12,720	17,741
Quezon City	0	0	0	0	0	12	27	39	14	1,761	10,380	12,155	22,767
Makati City	0	0	0	0	0	0	0	0	1	84	2,254	2,339	3,521
Mandaluyong City	0	0	0	0	0	0	0	0	2	244	2,443	2,689	3,659
San Juan	0	0	0	0	0	0	0	0	0	0	598	598	900
Manila City	0	0	0	0	0	0	1	1	27	718	7,324	8,069	19,724
Las Piñas City	0	0	0	0	0	0	0	0	1	360	3,563	3,924	7,980
Muntinlupa City	0	0	0	0	0	0	0	0	0	715	3,874	4,589	9,329
Parañaque City	0	0	0	0	0	0	0	0	73	812	5,073	5,958	8,882
Pasay City	0	0	0	0	0	0	30	30	0	1,140	4,248	5,388	7,730
**C A R**	**0**	**0**	**0**	**0**	**0**	**5**	**28**	**33**	**30**	**1,224**	**4,861**	**6,115**	**16,648**
Abra	0	0	0	0	0	4	3	7	1	262	1,298	1,561	2,298
Apayao	0	0	0	0	0	0	0	0	3	132	321	456	890
Benguet	0	0	0	0	0	0	10	10	1	222	1,068	1,291	2,618
Ifugao	0	0	0	0	0	1	5	6	3	172	551	726	763
Kalinga	0	0	0	0	0	0	6	6	20	251	960	1,231	1,699
Mt. Province	0	0	0	0	0	0	4	4	2	166	474	642	855
Baguio City	0	0	0	0	0	0	0	0	0	19	189	208	7,525
**Region 1**	**0**	**0**	**51**	**51**	**0**	**8**	**267**	**275**	**23**	**3,247**	**20,963**	**24,233**	**41,557**
Ilocos Norte	0	0	51	51	0	3	11	14	0	97	988	1,085	2,914
Ilocos Sur	0	0	0	0	0	1	91	92	0	340	4,139	4,479	8,619
La Union	0	0	0	0	0	1	11	12	7	436	1,812	2,255	3,569
Pangasinan	0	0	0	0	0	3	62	65	10	1,582	8,494	10,086	17,109
Alaminos City	0	0	0	0	0	0	0	0	0	380	1,984	2,364	2,851
Candon City	0	0	0	0	0	0	0	0	0	0	152	152	208
Dagupan City	0	0	0	0	0	0	0	0	2	103	806	911	1,243
Laoag City	0	0	0	0	0	0	0	0	0	43	1,270	1,313	1,482
San Carlos City	0	0	0	0	0	0	0	0	0	66	287	353	1,476
San Fernando City	0	0	0	0	0	0	92	92	0	14	389	403	822
Urdaneta City	0	0	0	0	0	0	0	0	4	162	556	722	1,150
Vigan City	0	0	0	0	0	0	0	0	0	24	86	110	114
**Region 2**	**0**	**0**	**0**	**0**	**0**	**1**	**25**	**26**	**38**	**2,253**	**7,536**	**9,827**	**21,865**
Batanes	0	0	0	0	0	0	1	1	0	16	50	66	69
Cagayan	0	0	0	0	0	0	0	0	12	600	2,486	3,098	6,857
Isabela	0	0	0	0	0	0	0	0	6	619	1,838	2,463	6,270
Nueva Vizcaya	0	0	0	0	0	0	24	24	14	421	1,392	1,827	3,030
Quirino	0	0	0	0	0	0	0	0	0	71	270	341	819
Cauayan City	0	0	0	0	0	0	0	0	4	138	318	460	626
Ilagan City	0	0	0	0	0	0	0	0	0	74	316	390	2,069
Santiago City	0	0	0	0	0	0	0	0	1	287	580	868	988
Tuguegarao City	0	0	0	0	0	1	0	1	1	27	286	314	1,137
**Region 3**	**0**	**0**	**9**	**9**	**0**	**5**	**149**	**154**	**29**	**4,580**	**30,689**	**35,298**	**104,768**
Aurora	0	0	0	0	0	0	3	3	2	198	410	610	1,000
Bataan	0	0	0	0	0	2	11	13	10	557	3,189	3,756	7,494

Table 1.A.1 - MODERN METHOD OF FAMILY PLANNING

New Acceptors

Annual Philippines, 2020

Area	NFP-STM			Total	NFP-SDM			Total	NFP-LAM			Total	Total New Acceptors
	Age group				Age group				Age group				
	10-14	15-19	20-49		10-14	15-19	20-49		10-14	15-19	20-49		
Bulacan	0	0	0	0	0	0	19	19	0	668	5,412	6,080	19,365
Nueva Ecija	0	0	0	0	0	0	0	0	0	605	2,904	3,509	10,542
Pampanga	0	0	0	0	0	3	54	57	1	264	3,380	3,645	14,048
Tarlac	0	0	0	0	0	0	47	47	0	135	2,450	2,585	9,099
Zambales	0	0	0	0	0	0	0	0	3	372	2,101	2,476	9,742
Angeles City	0	0	0	0	0	0	0	0	2	189	1,554	1,745	4,057
Balanga City	0	0	0	0	0	0	0	0	1	80	268	349	575
Cabanatuan City	0	0	0	0	0	0	0	0	2	281	1,181	1,464	2,875
City of San Fernando	0	0	0	0	0	0	0	0	1	125	1,052	1,178	3,040
Gapan City	0	0	0	0	0	0	0	0	4	89	490	583	1,261
Mabalacat City	0	0	0	0	0	0	0	0	0	258	1,774	2,032	4,006
Malolos City	0	0	0	0	0	0	0	0	0	3	17	20	601
Meycauayan	0	0	9	9	0	0	15	15	3	152	1,231	1,386	2,406
Olongapo	0	0	0	0	0	0	0	0	0	132	690	822	2,794
Palayan City	0	0	0	0	0	0	0	0	0	17	94	111	229
San Jose City	0	0	0	0	0	0	0	0	0	183	568	751	1,534
San Jose del Monte City	0	0	0	0	0	0	0	0	0	0	0	0	5,545
Science City of Munoz	0	0	0	0	0	0	0	0	0	0	0	0	123
Tarlac City	0	0	0	0	0	0	0	0	0	272	1,924	2,196	4,432
**Region 4A**	**192**	**1**	**104**	**297**	**406**	**19**	**433**	**858**	**788**	**5,720**	**52,950**	**59,458**	**149,179**
Batangas	0	0	0	0	310	0	358	668	681	170	2,487	3,338	10,634
Cavite	0	1	0	1	3	2	23	28	2	218	2,390	2,610	5,861
Laguna	0	0	2	2	91	16	36	143	8	484	1,489	1,981	10,058
Quezon	0	0	44	44	2	1	3	6	3	358	7,303	7,664	13,058
Rizal	0	0	3	3	0	0	0	0	3	533	2,441	2,977	16,023
Antipolo City	0	0	0	0	0	0	0	0	0	0	0	0	0
Bacoor City	0	0	0	0	0	0	0	0	25	247	6,462	6,734	9,963
Batangas City	0	0	0	0	0	0	0	0	0	0	0	0	180
Biñan City	0	0	0	0	0	0	0	0	1	1,736	4,723	6,460	24,470
Cabuyao City	0	0	0	0	0	0	0	0	3	237	10,219	10,459	14,263
Calamba City	0	0	0	0	0	0	0	0	25	630	3,524	4,179	6,292
Cavite City	0	0	0	0	0	0	0	0	0	0	0	0	0
Dasmariñas City	0	0	0	0	0	0	0	0	0	87	1,530	1,617	8,988
General Trias City	0	0	0	0	0	0	13	13	2	0	1,144	1,146	2,544
Imus City	0	0	0	0	0	0	0	0	0	12	725	737	1,528
Lipa City	0	0	0	0	0	0	0	0	0	0	0	0	5
Lucena City	192	0	0	192	0	0	0	0	10	0	127	137	3,453
San Pablo City	0	0	0	0	0	0	0	0	0	0	0	0	0
San Pedro City	0	0	0	0	0	0	0	0	2	384	3,017	3,403	5,065
Santa Rosa City	0	0	0	0	0	0	0	0	9	608	2,768	3,385	10,815
Tagaytay City	0	0	0	0	0	0	0	0	13	1	519	533	1,023
Tanauan City	0	0	0	0	0	0	0	0	1	5	842	848	2,006
Tayabas City	0	0	55	55	0	0	0	0	0	2	1,005	1,007	1,108
Trece Martires City	0	0	0	0	0	0	0	0	0	8	235	243	1,842
**Region 4B**	**0**	**0**	**0**	**0**	**0**	**2**	**1,081**	**1,083**	**34**	**1,180**	**6,257**	**7,471**	**25,936**
Marinduque	0	0	0	0	0	0	1	1	1	110	917	1,028	1,985
Mindoro Occidental	0	0	0	0	0	2	963	965	28	747	1,557	2,332	5,954
Mindoro Oriental	0	0	0	0	0	0	97	97	2	206	1,788	1,996	4,695
Palawan	0	0	0	0	0	0	5	5	3	103	1,479	1,585	11,596
Romblon	0	0	0	0	0	0	0	0	0	14	195	209	265
Puerto Princesa City	0	0	0	0	0	0	15	15	0	0	321	321	1,441
**Region 5**	**0**	**9**	**72**	**81**	**1**	**63**	**2,717**	**2,781**	**48**	**4,655**	**21,104**	**25,807**	**54,939**
Albay	0	0	8	8	0	16	1,049	1,065	4	413	3,318	3,735	12,606
Camarines Norte	0	0	3	3	0	20	122	142	1	563	1,981	2,545	4,606
Camarines Sur	0	9	61	70	0	8	440	448	24	1,455	6,030	7,509	16,488
Catanduanes	0	0	0	0	0	1	32	33	0	245	700	945	1,310
Masbate	0	0	0	0	0	10	873	883	12	1,210	4,814	6,036	10,448
Sorsogon	0	0	0	0	1	8	33	42	7	697	2,497	3,201	4,128
Iriga City	0	0	0	0	0	0	73	73	0	56	323	379	1,162

Table 1.A.1 - MODERN METHOD OF FAMILY PLANNING

New Acceptors

Annual Philippines, 2020

Area	NFP-STM			Total	NFP-SDM			Total	NFP-LAM			Total	Total New Acceptors
	Age group				Age group				Age group				
	10-14	15-19	20-49		10-14	15-19	20-49		10-14	15-19	20-49		
Legaspi City	0	0	0	0	0	0	0	0	0	9	163	172	493
Naga City	0	0	0	0	0	0	95	95	0	7	1,278	1,285	3,698
**Region 6**	**0**	**3**	**32**	**35**	**0**	**21**	**703**	**724**	**30**	**3,771**	**23,235**	**27,036**	**79,345**
Aklan	0	3	9	12	0	0	1	1	3	293	2,147	2,443	11,848
Antique	0	0	0	0	0	0	21	21	2	236	2,247	2,485	5,967
Capiz	0	0	0	0	0	0	2	2	1	169	1,855	2,025	9,412
Guimaras	0	0	0	0	0	0	3	3	1	65	615	681	1,704
Iloilo	0	0	0	0	0	0	60	60	3	731	5,822	6,556	17,977
Negros Occidental	0	0	23	23	0	21	616	637	19	2,100	9,460	11,579	28,141
Bacolod City	0	0	0	0	0	0	0	0	1	176	998	1,175	3,110
Iloilo City	0	0	0	0	0	0	0	0	0	1	91	92	1,186
**Region 7**	**0**	**0**	**0**	**0**	**0**	**0**	**452**	**452**	**98**	**3,752**	**19,577**	**23,427**	**56,908**
Bohol	0	0	0	0	0	0	0	0	0	308	1,469	1,777	7,079
Cebu	0	0	0	0	0	0	5	5	0	413	3,664	4,077	10,979
Negros Oriental	0	0	0	0	0	0	1	1	6	748	3,074	3,828	9,100
Siquijor	0	0	0	0	0	0	437	437	0	55	344	399	7,831
Cebu City	0	0	0	0	0	0	9	9	17	1,238	8,383	9,638	14,195
Lapu-Lapu City	0	0	0	0	0	0	0	0	75	937	2,482	3,494	6,428
Mandaue City	0	0	0	0	0	0	0	0	0	53	161	214	1,296
**Region 8**	**0**	**0**	**0**	**0**	**0**	**0**	**0**	**0**	**0**	**0**	**0**	**0**	**257**
Biliran	0	0	0	0	0	0	0	0	0	0	0	0	10
Eastern Samar	0	0	0	0	0	0	0	0	0	0	0	0	48
Northern Leyte	0	0	0	0	0	0	0	0	0	0	0	0	0
Northern Samar	0	0	0	0	0	0	0	0	0	0	0	0	2
Southern Leyte	0	0	0	0	0	0	0	0	0	0	0	0	33
Western Samar	0	0	0	0	0	0	0	0	0	0	0	0	49
Calbayog City	0	0	0	0	0	0	0	0	0	0	0	0	11
Maasin City	0	0	0	0	0	0	0	0	0	0	0	0	26
Ormoc City	0	0	0	0	0	0	0	0	0	0	0	0	4
Tacloban City	0	0	0	0	0	0	0	0	0	0	0	0	74
**Region 9**	**0**	**0**	**0**	**0**	**0**	**0**	**0**	**0**	**0**	**170**	**1,541**	**1,711**	**3,951**
Zamboanga del Norte	0	0	0	0	0	0	0	0	0	0	0	0	0
Zamboanga del Sur	0	0	0	0	0	0	0	0	0	0	0	0	0
Zamboanga Sibugay	0	0	0	0	0	0	0	0	0	0	0	0	0
Dapitan City	0	0	0	0	0	0	0	0	0	22	77	99	185
Dipolog City	0	0	0	0	0	0	0	0	0	0	0	0	549
Isabela City	0	0	0	0	0	0	0	0	0	59	196	255	418
Pagadian City	0	0	0	0	0	0	0	0	0	43	154	197	330
Zamboanga City	0	0	0	0	0	0	0	0	0	46	1,114	1,160	2,469
**Region 10**	**0**	**4**	**73**	**77**	**0**	**159**	**2,585**	**2,744**	**57**	**4,826**	**26,887**	**31,770**	**95,599**
Bukidnon	0	0	72	72	0	32	231	263	24	1,225	2,595	3,844	8,000
Camiguin	0	0	0	0	0	0	0	0	1	56	347	404	487
Lanao del Norte	0	0	0	0	0	0	20	20	2	213	2,690	2,905	13,213
Misamis Occidental	0	4	0	4	0	6	34	40	1	151	1,523	1,675	2,941
Misamis Oriental	0	0	0	0	0	56	171	227	8	415	2,021	2,444	5,472
Cagayan de Oro City	0	0	0	0	0	0	4	4	9	1,105	9,155	10,269	19,877
El Salvador City	0	0	0	0	0	0	0	0	0	75	154	229	504
Gingoog City	0	0	0	0	0	44	414	458	5	197	477	679	3,140
Iligan City	0	0	0	0	0	7	43	50	3	463	1,986	2,452	4,692
Malaybalay City	0	0	1	1	0	0	28	28	0	215	854	1,069	1,385
Oroquieta City	0	0	0	0	0	0	0	0	0	12	72	84	178
Ozamis City	0	0	0	0	0	9	1,618	1,627	4	403	4,169	4,576	33,908
Tangub City	0	0	0	0	0	4	0	4	0	57	203	260	327
Valencia City	0	0	0	0	0	1	22	23	0	239	641	880	1,475

Table 1.A.1 - MODERN METHOD OF FAMILY PLANNING

New Acceptors

Annual Philippines, 2020

Area	NFP-STM			Total	NFP-SDM			Total	NFP-LAM			Total	Total New Acceptors
	Age group				Age group				Age group				
	10-14	15-19	20-49		10-14	15-19	20-49		10-14	15-19	20-49		
**Region 11**	**0**	**2**	**118**	**120**	**0**	**9**	**185**	**194**	**100**	**3,033**	**12,931**	**16,064**	**51,004**
Davao de Oro	0	0	0	0	0	1	12	13	7	219	790	1,016	5,141
Davao del Norte	0	0	21	21	0	2	34	36	35	573	2,489	3,097	9,081
Davao Oriental	0	0	6	6	0	1	24	25	26	620	1,741	2,387	5,138
Davao del Sur	0	0	0	0	0	0	9	9	1	137	608	746	4,706
Davao Occidental	0	0	0	0	0	4	60	64	6	274	522	802	2,687
Davao City	0	2	91	93	0	1	46	47	25	1,210	6,781	8,016	24,251
**Region 12**	**0**	**14**	**51**	**65**	**0**	**1**	**41**	**42**	**49**	**5,113**	**16,669**	**21,831**	**48,330**
North Cotabato	0	14	51	65	0	0	17	17	25	841	3,576	4,442	12,931
Sarangani	0	0	0	0	0	0	2	2	0	1,254	2,452	3,706	7,142
South Cotabato	0	0	0	0	0	1	19	20	22	1,554	5,313	6,889	13,876
Sultan Kudarat	0	0	0	0	0	0	3	3	0	1,120	2,702	3,822	7,568
				0				0				0	
Cotabato City	0	0	0	0	0	0	0	0	2	162	1,714	1,878	4,381
Gen. Santos City	0	0	0	0	0	0	0	0	0	182	912	1,094	2,432
**B.A.R.M.M.**	**0**	**0**	**2**	**2**	**0**	**2**	**84**	**86**	**17**	**5,070**	**20,153**	**25,240**	**62,164**
Basilan	0	0	0	0	0	0	0	0	3	268	1,122	1,393	3,348
Lanao del Sur	0	0	2	2	0	0	60	60	2	525	9,545	10,072	14,235
Maguindanao	0	0	0	0	0	0	20	20	10	1,080	6,834	7,924	14,946
Sulu	0	0	0	0	0	0	0	0	0	852	0	852	2,199
Tawi-Tawi	0	0	0	0	0	2	0	2	0	2,009	620	2,629	4,561
				0				0				0	
Lamitan City	0	0	0	0	0	0	4	4	2	273	1,144	1,419	21,484
Marawi City	0	0	0	0	0	0	0	0	0	63	888	951	1,391
**CARAGA**	**0**	**4**	**4**	**8**	**0**	**7**	**229**	**236**	**30**	**2,265**	**6,962**	**9,257**	**21,319**
Agusan del Norte	0	0	0	0	0	0	27	27	6	261	755	1,022	3,842
Agusan del Sur	0	4	4	8	0	4	80	84	7	533	1,426	1,966	4,771
Surigao del Norte	0	0	0	0	0	0	64	64	1	176	1,162	1,339	3,363
Surigao del Sur	0	0	0	0	0	3	17	20	8	485	1,409	1,902	3,587
Province of Dinagat	0	0	0	0	0	0	14	14	0	67	379	446	1,163
Bislig City	0	0	0	0	0	0	0	0	5	136	234	375	464
Butuan City	0	0	0	0	0	0	0	0	2	540	1,087	1,629	2,771
Surigao City	0	0	0	0	0	0	27	27	1	67	510	578	1,358

Table 1.A.2 - MODERN METHOD OF FAMILY PLANNING
Other Acceptors
Annual Philippines, 2020

Area	Total Current Users	FSTR/BTL Age group 10-14	15-19	20-49	Total	MSTR/NSV Age group 10-14	15-19	20-49	Total
**PHILIPPINES**	**7,115,114**	**181**	**3,601**	**52,736**	**56,518**	**2**	**7**	**447**	**456**
**N C R**	**786,292**	**2**	**214**	**8,071**	**8,287**	**0**	**1**	**43**	**44**
Malabon	31,429	0	0	221	221	0	0	0	0
Navotas	22,416	0	1	30	31	0	0	0	0
Valenzuela City	60,865	0	0	1,764	1,764	0	0	19	19
Caloocan City	65,508	0	0	542	542	0	0	0	0
Marikina City	18,917	0	0	94	94	0	0	5	5
Pasig City	33,855	0	0	310	310	0	0	0	0
Pateros	2,995	0	0	14	14	0	0	0	0
Taguig	57,595	0	0	195	195	0	0	0	0
Quezon City	271,881	0	67	3,264	3,331	0	0	5	5
Makati City	8,979	0	0	123	123	0	0	2	2
Mandaluyong City	16,390	0	0	12	12	0	0	0	0
San Juan	5,866	0	0	0	0	0	0	0	0
Manila City	68,205	0	0	876	876	0	0	0	0
Las Piñas City	20,867	0	0	215	215	0	0	0	0
Muntinlupa City	33,645	0	0	55	55	0	0	0	0
Parañaque City	30,062	2	146	110	258	0	1	12	13
Pasay City	36,817	0	0	246	246	0	0	0	0
**C A R**	**120,779**	**0**	**1**	**1,538**	**1,539**	**0**	**0**	**15**	**15**
Abra	16,924	0	0	62	62	0	0	4	4
Apayao	11,643	0	0	55	55	0	0	0	0
Benguet	30,440	0	0	408	408	0	0	2	2
Ifugao	17,978	0	0	221	221	0	0	8	8
Kalinga	17,779	0	1	103	104	0	0	0	0
Mt. Province	11,955	0	0	296	296	0	0	0	0
Baguio City	14,060	0	0	393	393	0	0	1	1
**Region 1**	**460,308**	**0**	**8**	**2,424**	**2,432**	**0**	**0**	**15**	**15**
Ilocos Norte	41,436	0	0	141	141	0	0	0	0
Ilocos Sur	59,502	0	0	180	180	0	0	1	1
La Union	58,813	0	1	591	592	0	0	1	1
Pangasinan	226,567	0	7	757	764	0	0	3	3
Alaminos City	9,446	0	0	56	56	0	0	0	0
Candon City	7,299	0	0	0	0	0	0	0	0
Dagupan City	7,429	0	0	0	0	0	0	0	0
Laoag City	8,324	0	0	282	282	0	0	0	0
San Carlos City	11,747	0	0	88	88	0	0	0	0
San Fernando City	12,865	0	0	321	321	0	0	10	10
Urdaneta City	11,939	0	0	2	2	0	0	0	0
Vigan City	4,941	0	0	6	6	0	0	0	0
**Region 2**	**307,177**	**0**	**16**	**2,920**	**2,936**	**0**	**0**	**11**	**11**
Batanes	864	0	0	20	20	0	0	0	0
Cagayan	83,752	0	10	542	552	0	0	3	3
Isabela	105,390	0	3	975	978	0	0	5	5
Nueva Vizcaya	44,895	0	3	1,128	1,131	0	0	2	2
Quirino	18,556	0	0	90	90	0	0	1	1
Cauayan City	10,617	0	0	84	84	0	0	0	0
Ilagan City	14,066	0	0	29	29	0	0	0	0
Santiago City	15,258	0	0	13	13	0	0	0	0
Tuguegarao City	13,779	0	0	39	39	0	0	0	0
**Region 3**	**747,169**	**17**	**33**	**8,838**	**8,888**	**0**	**0**	**55**	**55**
Aurora	20,957	0	0	173	173	0	0	3	3
Bataan	44,561	0	6	85	91	0	0	2	2

Table 1.A.2 - MODERN METHOD OF FAMILY PLANNING
Other Acceptors
Annual Philippines, 2020

Area	Total Current Users	FSTR/BTL Age group 10-14	FSTR/BTL Age group 15-19	FSTR/BTL Age group 20-49	Total	MSTR/NSV Age group 10-14	MSTR/NSV Age group 15-19	MSTR/NSV Age group 20-49	Total
Bulacan	138,968	0	0	777	777	0	0	28	28
Nueva Ecija	118,251	0	23	2,365	2,388	0	0	0	0
Pampanga	97,407	0	3	2,159	2,162	0	0	11	11
Tarlac	78,705	0	0	407	407	0	0	6	6
Zambales	37,319	17	0	473	490	0	0	0	0
Angeles City	21,284	0	0	38	38	0	0	0	0
Balanga City	4,693	0	0	12	12	0	0	0	0
Cabanatuan City	16,403	0	0	818	818	0	0	0	0
City of San Fernando	7,904	0	0	46	46	0	0	3	3
Gapan City	7,770	0	0	224	224	0	0	0	0
Mabalacat City	21,281	0	0	9	9	0	0	0	0
Malolos City	6,208	0	0	806	806	0	0	1	1
Meycauayan	8,403	0	1	6	7	0	0	0	0
Olongapo	9,362	0	0	18	18	0	0	0	0
Palayan City	3,344	0	0	0	0	0	0	0	0
San Jose City	12,893	0	0	0	0	0	0	0	0
San Jose del Monte City	72,493	0	0	71	71	0	0	0	0
Science City of Munoz	6,409	0	0	22	22	0	0	0	0
Tarlac City	12,554	0	0	329	329	0	0	1	1
**Region 4A**	**844,565**	**160**	**80**	**14,512**	**14,752**	**0**	**0**	**36**	**36**
Batangas	158,232	64	0	3,128	3,192	0	0	24	24
Cavite	83,830	0	4	3,268	3,272	0	0	0	0
Laguna	65,529	0	12	1,100	1,112	0	0	4	4
Quezon	106,906	0	8	2,804	2,812	0	0	8	8
Rizal	103,690	0	0	1,784	1,784	0	0	0	0
Antipolo City	37,890	0	0	0	0	0	0	0	0
Bacoor City	11,464	0	0	96	96	0	0	0	0
Batangas City	17,607	0	0	0	0	0	0	0	0
Biñan City	38,387	0	0	148	148	0	0	0	0
Cabuyao City	13,927	40	0	32	72	0	0	0	0
Calamba City	35,102	12	24	244	280	0	0	0	0
Cavite City	4,572	0	0	0	0	0	0	0	0
Dasmariñas City	42,608	0	0	56	56	0	0	0	0
General Trias City	11,506	8	8	484	500	0	0	0	0
Imus City	9,106	0	0	460	460	0	0	0	0
Lipa City	13,662	0	0	0	0	0	0	0	0
Lucena City	6,402	4	0	60	64	0	0	0	0
San Pablo City	10,199	0	0	0	0	0	0	0	0
San Pedro City	15,209	0	12	116	128	0	0	0	0
Santa Rosa City	33,075	0	0	316	316	0	0	0	0
Tagaytay City	7,989	0	0	100	100	0	0	0	0
Tanauan City	7,401	32	12	56	100	0	0	0	0
Tayabas City	5,399	0	0	224	224	0	0	0	0
Trece Martires City	4,872	0	0	36	36	0	0	0	0
**Region 4B**	**144,179**	**0**	**0**	**1,523**	**1,523**	**0**	**0**	**1**	**1**
Marinduque	14,040	0	0	45	45	0	0	0	0
Mindoro Occidental	32,450	0	0	143	143	0	0	0	0
Mindoro Oriental	58,295	0	0	373	373	0	0	1	1
Palawan	6,466	0	0	818	818	0	0	0	0
Romblon	4,160	0	0	2	2	0	0	0	0
Puerto Princesa City	28,768	0	0	142	142	0	0	0	0
**Region 5**	**397,155**	**0**	**13**	**1,598**	**1,611**	**2**	**0**	**22**	**24**
Albay	75,300	0	5	187	192	0	0	1	1
Camarines Norte	26,377	0	6	141	147	2	0	5	7
Camarines Sur	100,599	0	0	10	10	0	0	0	0
Catanduanes	24,369	0	0	127	127	0	0	0	0
Masbate	57,012	0	0	112	112	0	0	0	0
Sorsogon	60,469	0	1	886	887	0	0	14	14
Iriga City	9,974	0	0	60	60	0	0	1	1

Table 1.A.2 - MODERN METHOD OF FAMILY PLANNING
Other Acceptors
Annual Philippines, 2020

Area	Total Current Users	FSTR/BTL Age group 10-14	15-19	20-49	Total	MSTR/NSV Age group 10-14	15-19	20-49	Total
Legaspi City	22,994	0	1	30	31	0	0	0	0
Naga City	20,061	0	0	45	45	0	0	1	1
**Region 6**	**580,884**	**1**	**88**	**2,430**	**2,519**	**0**	**1**	**68**	**69**
Aklan	41,264	0	0	81	81	0	0	11	11
Antique	47,572	0	81	793	874	0	0	23	23
Capiz	75,849	0	1	198	199	0	0	12	12
Guimaras	15,286	0	2	59	61	0	0	0	0
Iloilo	149,460	1	3	389	393	0	0	6	6
Negros Occidental	182,369	0	0	442	442	0	0	14	14
Bacolod City	25,422	0	0	213	213	0	0	0	0
Iloilo City	43,662	0	1	255	256	0	1	2	3
**Region 7**	**294,028**	**0**	**0**	**614**	**614**	**0**	**0**	**36**	**36**
Bohol	67,760	0	0	49	49	0	0	0	0
Cebu	71,864	0	0	81	81	0	0	1	1
Negros Oriental	58,940	0	0	87	87	0	0	8	8
Siquijor	8,043	0	0	7	7	0	0	0	0
Cebu City	29,645	0	0	296	296	0	0	26	26
Lapu-Lapu City	39,675	0	0	92	92	0	0	1	1
Mandaue City	18,101	0	0	2	2	0	0	0	0
**Region 8**	**202,207**	**0**	**2**	**733**	**735**	**0**	**0**	**4**	**4**
Biliran	10,480	0	0	62	62	0	0	0	0
Eastern Samar	41,040	0	2	25	27	0	0	0	0
Northern Leyte	0	0	0	0	0	0	0	0	0
Northern Samar	34,606	0	0	22	22	0	0	1	1
Southern Leyte	24,278	0	0	100	100	0	0	0	0
Western Samar	16,033	0	0	179	179	0	0	1	1
Calbayog City	8,908	0	0	123	123	0	0	0	0
Maasin City	2,705	0	0	8	8	0	0	0	0
Ormoc City	19,481	0	0	133	133	0	0	2	2
Tacloban City	44,676	0	0	81	81	0	0	0	0
**Region 9**	**271,392**	**0**	**0**	**26**	**26**	**0**	**0**	**0**	**0**
Zamboanga del Norte	86,000	0	0	0	0	0	0	0	0
Zamboanga del Sur	57,156	0	0	0	0	0	0	0	0
Zamboanga Sibugay	24,011	0	0	0	0	0	0	0	0
Dapitan City	7,405	0	0	0	0	0	0	0	0
Dipolog City	15,009	0	0	20	20	0	0	0	0
Isabela City	8,507	0	0	3	3	0	0	0	0
Pagadian City	17,446	0	0	0	0	0	0	0	0
Zamboanga City	55,858	0	0	3	3	0	0	0	0
**Region 10**	**518,887**	**0**	**6**	**2,145**	**2,151**	**0**	**0**	**23**	**23**
Bukidnon	111,273	0	2	606	608	0	0	4	4
Camiguin	6,349	0	0	41	41	0	0	0	0
Lanao del Norte	47,747	0	0	397	397	0	0	1	1
Misamis Occidental	24,432	0	0	7	7	0	0	0	0
Misamis Oriental	81,061	0	3	346	349	0	0	4	4
Cagayan de Oro City	140,501	0	1	243	244	0	0	2	2
El Salvador City	6,230	0	0	30	30	0	0	0	0
Gingoog City	10,166	0	0	30	30	0	0	0	0
Iligan City	27,571	0	0	50	50	0	0	0	0
Malaybalay City	19,999	0	0	193	193	0	0	9	9
Oroquieta City	6,341	0	0	0	0	0	0	0	0
Ozamis City	12,025	0	0	36	36	0	0	1	1
Tangub City	4,874	0	0	6	6	0	0	0	0
Valencia City	20,318	0	0	160	160	0	0	2	2

Table 1.A.2 - MODERN METHOD OF FAMILY PLANNING

Other Acceptors

Annual Philippines, 2020

Area	Total Current Users	FSTR/BTL			Total	MSTR/NSV			Total
		Age group				Age group			
		10-14	15-19	20-49		10-14	15-19	20-49	
**Region 11**	**483,912**	**0**	**89**	**1,675**	**1,764**	**0**	**2**	**67**	**69**
Davao de Oro	81,390	0	9	316	325	0	0	2	2
Davao del Norte	99,809	0	3	497	500	0	0	8	8
Davao Oriental	48,937	0	12	228	240	0	0	5	5
Davao del Sur	63,208	0	5	146	151	0	1	1	2
Davao Occidental	31,727	0	38	116	154	0	1	30	31
Davao City	158,841	0	22	372	394	0	0	21	21
**Region 12**	**467,365**	**1**	**19**	**1,873**	**1,893**	**0**	**2**	**27**	**29**
North Cotabato	141,819	0	16	391	407	0	0	16	16
Sarangani	62,222	0	0	196	196	0	1	1	2
South Cotabato	95,537	1	0	215	216	0	1	9	10
Sultan Kudarat	88,018	0	3	710	713	0	0	1	1
					0				0
Cotabato City	30,729	0	0	352	352	0	0	0	0
Gen. Santos City	49,040	0	0	9	9	0	0	0	0
**B.A.R.M.M.**	**239,770**	**0**	**3,022**	**303**	**3,325**	**0**	**0**	**4**	**4**
Basilan	6,677	0	0	128	128	0	0	0	0
Lanao del Sur	66,116	0	0	34	34	0	0	0	0
Maguindanao	84,835	0	0	122	122	0	0	4	4
Sulu	49,455	0	1,801	0	1,801	0	0	0	0
Tawi-Tawi	27,758	0	1,221	8	1,229	0	0	0	0
					0				0
Lamitan City	0	0	0	6	6	0	0	0	0
Marawi City	4,929	0	0	5	5	0	0	0	0
**CARAGA**	**249,045**	**0**	**10**	**1,513**	**1,523**	**0**	**1**	**20**	**21**
Agusan del Norte	38,948	0	2	233	235	0	0	0	0
Agusan del Sur	80,275	0	0	324	324	0	1	6	7
Surigao del Norte	30,203	0	3	94	97	0	0	3	3
Surigao del Sur	39,690	0	3	90	93	0	0	0	0
Province of Dinagat	9,699	0	1	14	15	0	0	10	10
Bislig City	8,261	0	0	64	64	0	0	0	0
Butuan City	28,396	0	1	689	690	0	0	1	1
Surigao City	13,573	0	0	5	5	0	0	0	0

Table 1.A.2 - MODERN METHOD OF FAMILY PLANNING
Other Acceptors
Annual Philippines, 2020

Area	CONDOM			Total	IUD-INTERVAL			Total	IUD-POSTPARTUM			Total
	Age group				Age group				Age group			
	10-14	15-19	20-49		10-14	15-19	20-49		10-14	15-19	20-49	
**PHILIPPINES**	**1,069**	**5,595**	**72,475**	**79,139**	**222**	**2,040**	**29,205**	**31,467**	**51**	**2,240**	**20,366**	**22,657**
**N C R**	**19**	**1,244**	**19,519**	**20,782**	**4**	**183**	**4,183**	**4,370**	**33**	**1,188**	**8,327**	**9,548**
Malabon	1	12	293	306	0	6	78	84	0	3	45	48
Navotas	1	7	201	209	0	3	38	41	0	0	2	2
Valenzuela City	0	1	731	732	0	3	250	253	0	26	181	207
Caloocan City	0	44	476	520	4	124	696	824	2	123	151	276
Marikina City	0	2	88	90	0	4	42	46	4	62	181	247
Pasig City	0	74	196	270	0	3	43	46	1	75	171	247
Pateros	0	0	8	8	0	0	14	14	0	0	0	0
Taguig	0	0	60	60	0	7	141	148	0	2	46	48
Quezon City	6	418	15,870	16,294	0	27	2,288	2,315	24	380	5,005	5,409
Makati City	0	17	358	375	0	4	84	88	0	3	82	85
Mandaluyong City	0	0	21	21	0	0	3	3	0	0	1	1
San Juan	0	0	0	0	0	0	0	0	0	0	0	0
Manila City	0	10	195	205	0	1	197	198	0	38	132	170
Las Piñas City	0	5	172	177	0	0	31	31	0	0	3	3
Muntinlupa City	0	6	175	181	0	0	12	12	0	0	0	0
Parañaque City	11	128	45	184	0	1	25	26	2	476	606	1,084
Pasay City	0	520	630	1,150	0	0	241	241	0	0	1,721	1,721
**C A R**	**0**	**81**	**1,459**	**1,540**	**1**	**48**	**466**	**515**	**0**	**31**	**270**	**301**
Abra	0	6	72	78	0	0	27	27	0	0	27	27
Apayao	0	6	48	54	0	1	18	19	0	3	28	31
Benguet	0	28	487	515	1	20	178	199	0	3	67	70
Ifugao	0	11	182	193	0	3	54	57	0	0	32	32
Kalinga	0	7	218	225	0	5	68	73	0	2	36	38
Mt. Province	0	7	151	158	0	6	41	47	0	23	53	76
Baguio City	0	16	301	317	0	13	80	93	0	0	27	27
**Region 1**	**0**	**43**	**1,392**	**1,435**	**0**	**42**	**832**	**874**	**0**	**7**	**275**	**282**
Ilocos Norte	0	4	79	83	0	0	28	28	0	0	17	17
Ilocos Sur	0	3	56	59	0	0	53	53	0	0	51	51
La Union	0	8	151	159	0	7	82	89	0	6	32	38
Pangasinan	0	21	856	877	0	30	571	601	0	0	0	0
Alaminos City	0	0	27	27	0	0	10	10	0	0	0	0
Candon City	0	0	0	0	0	0	0	0	0	0	136	136
Dagupan City	0	1	4	5	0	0	3	3	0	1	1	2
Laoag City	0	0	0	0	0	0	0	0	0	0	0	0
San Carlos City	0	1	21	22	0	4	39	43	0	0	0	0
San Fernando City	0	1	141	142	0	1	40	41	0	0	38	38
Urdaneta City	0	4	55	59	0	0	6	6	0	0	0	0
Vigan City	0	0	2	2	0	0	0	0	0	0	0	0
**Region 2**	**4**	**67**	**1,405**	**1,476**	**2**	**152**	**2,018**	**2,172**	**2**	**112**	**740**	**854**
Batanes	0	1	7	8	0	1	5	6	0	0	0	0
Cagayan	0	5	168	173	1	67	748	816	0	64	230	294
Isabela	2	41	415	458	0	54	285	339	2	30	229	261
Nueva Vizcaya	1	13	507	521	0	5	809	814	0	2	40	42
Quirino	0	6	96	102	0	1	18	19	0	0	4	4
Cauayan City	0	0	139	139	0	2	6	8	0	4	26	30
Ilagan City	1	1	53	55	0	0	6	6	0	0	1	1
Santiago City	0	0	8	8	1	22	46	69	0	0	0	0
Tuguegarao City	0	0	12	12	0	0	95	95	0	12	210	222
**Region 3**	**4**	**265**	**4,028**	**4,297**	**0**	**70**	**1,047**	**1,117**	**1**	**55**	**598**	**654**
Aurora	1	10	224	235	0	6	21	27	0	2	40	42
Bataan	0	25	265	290	0	2	53	55	1	4	88	93

Table 1.A.2 - MODERN METHOD OF FAMILY PLANNING
Other Acceptors
Annual Philippines, 2020

Area	CONDOM Age group 10-14	CONDOM Age group 15-19	CONDOM Age group 20-49	Total	IUD-INTERVAL Age group 10-14	IUD-INTERVAL Age group 15-19	IUD-INTERVAL Age group 20-49	Total	IUD-POSTPARTUM Age group 10-14	IUD-POSTPARTUM Age group 15-19	IUD-POSTPARTUM Age group 20-49	Total
Bulacan	0	31	577	608	0	19	145	164	0	24	64	88
Nueva Ecija	0	14	372	386	0	27	214	241	0	9	65	74
Pampanga	0	39	318	357	0	5	107	112	0	12	131	143
Tarlac	0	44	527	571	0	1	80	81	0	0	40	40
Zambales	0	6	192	198	0	1	33	34	0	2	133	135
Angeles City	0	2	17	19	0	0	123	123	0	0	4	4
Balanga City	0	0	15	15	0	0	1	1	0	0	0	0
Cabanatuan City	0	5	60	65	0	1	57	58	0	0	13	13
City of San Fernando	3	15	26	44	0	0	2	2	0	1	3	4
Gapan City	0	0	3	3	0	0	2	2	0	0	0	0
Mabalacat City	0	11	119	130	0	0	3	3	0	0	0	0
Malolos City	0	9	257	266	0	1	23	24	0	0	0	0
Meycauayan	0	9	57	66	0	0	0	0	0	1	8	9
Olongapo	0	0	47	47	0	0	0	0	0	0	5	5
Palayan City	0	1	4	5	0	0	8	8	0	0	0	0
San Jose City	0	0	13	13	0	0	15	15	0	0	1	1
San Jose del Monte City	0	24	795	819	0	0	40	40	0	0	0	0
Science City of Munoz	0	0	8	8	0	0	10	10	0	0	0	0
Tarlac City	0	20	132	152	0	7	110	117	0	0	3	3
**Region 4A**	**1,016**	**2,852**	**25,120**	**28,988**	**204**	**252**	**7,716**	**8,172**	**8**	**180**	**1,556**	**1,744**
Batangas	32	4	1,604	1,640	72	16	604	692	0	20	288	308
Cavite	0	44	3,628	3,672	4	32	1,088	1,124	0	20	44	64
Laguna	0	60	1,316	1,376	4	88	944	1,036	0	32	180	212
Quezon	0	48	5,236	5,284	0	72	3,620	3,692	0	4	216	220
Rizal	0	52	980	1,032	0	20	724	744	8	96	684	788
Antipolo City	0	0	0	0	0	0	0	0	0	0	0	0
Bacoor City	0	4	128	132	0	4	28	32	0	0	0	0
Batangas City	0	0	0	0	0	0	0	0	0	0	0	0
Biñan City	0	2,288	7,964	10,252	0	8	336	344	0	8	20	28
Cabuyao City	0	0	180	180	0	0	36	36	0	0	0	0
Calamba City	0	36	488	524	0	0	56	56	0	0	12	12
Cavite City	0	0	0	0	0	0	0	0	0	0	0	0
Dasmariñas City	0	48	484	532	0	0	0	0	0	0	16	16
General Trias City	0	0	424	424	0	0	68	68	0	0	28	28
Imus City	0	4	248	252	0	4	76	80	0	0	0	0
Lipa City	0	0	0	0	0	0	0	0	0	0	0	0
Lucena City	984	0	100	1,084	124	0	52	176	0	0	0	0
San Pablo City	0	0	0	0	0	0	0	0	0	0	0	0
San Pedro City	0	8	188	196	0	8	16	24	0	0	0	0
Santa Rosa City	0	256	1,836	2,092	0	0	20	20	0	0	24	24
Tagaytay City	0	0	60	60	0	0	0	0	0	0	16	16
Tanauan City	0	0	12	12	0	0	16	16	0	0	28	28
Tayabas City	0	0	224	224	0	0	32	32	0	0	0	0
Trece Martires City	0	0	20	20	0	0	0	0	0	0	0	0
**Region 4B**	**0**	**7**	**832**	**839**	**0**	**6**	**435**	**441**	**0**	**9**	**577**	**586**
Marinduque	0	0	36	36	0	0	16	16	0	0	0	0
Mindoro Occidental	0	1	169	170	0	4	63	67	0	3	13	16
Mindoro Oriental	0	4	191	195	0	2	252	254	0	6	520	526
Palawan	0	2	261	263	0	0	104	104	0	0	3	3
Romblon	0	0	0	0	0	0	0	0	0	0	0	0
Puerto Princesa City	0	0	175	175	0	0	0	0	0	0	41	41
**Region 5**	**10**	**143**	**3,654**	**3,807**	**0**	**20**	**406**	**426**	**0**	**5**	**197**	**202**
Albay	0	34	728	762	0	0	51	51	0	0	19	19
Camarines Norte	10	34	437	481	0	14	137	151	0	0	28	28
Camarines Sur	0	32	1,131	1,163	0	3	75	78	0	4	55	59
Catanduanes	0	3	149	152	0	0	3	3	0	0	24	24
Masbate	0	16	338	354	0	2	67	69	0	1	12	13
Sorsogon	0	22	536	558	0	1	56	57	0	0	59	59
Iriga City	0	1	80	81	0	0	5	5	0	0	0	0

Table 1.A.2 - MODERN METHOD OF FAMILY PLANNING
Other Acceptors
Annual Philippines, 2020

Area	CONDOM			Total	IUD-INTERVAL			Total	IUD-POSTPARTUM			Total
	Age group				Age group				Age group			
	10-14	15-19	20-49		10-14	15-19	20-49		10-14	15-19	20-49	
Legaspi City	0	0	32	32	0	0	4	4	0	0	0	0
Naga City	0	1	223	224	0	0	8	8	0	0	0	0
**Region 6**	**1**	**68**	**2,981**	**3,050**	**2**	**96**	**1,518**	**1,616**	**4**	**45**	**767**	**816**
Aklan	0	2	257	259	0	0	19	19	0	0	8	8
Antique	0	4	314	318	0	6	95	101	0	0	51	51
Capiz	0	11	170	181	0	0	121	121	0	1	20	21
Guimaras	0	1	104	105	0	2	12	14	0	0	1	1
Iloilo	1	22	1,028	1,051	1	19	401	421	1	12	225	238
Negros Occidental	0	11	666	677	0	21	496	517	0	16	307	323
Bacolod City	0	4	92	96	1	13	116	130	3	4	49	56
Iloilo City	0	13	350	363	0	35	258	293	0	12	106	118
**Region 7**	**0**	**42**	**1,700**	**1,742**	**0**	**261**	**1,767**	**2,028**	**0**	**125**	**822**	**947**
Bohol	0	0	358	358	0	160	459	619	0	86	109	195
Cebu	0	12	567	579	0	73	711	784	0	7	577	584
Negros Oriental	0	9	425	434	0	5	239	244	0	2	30	32
Siquijor	0	1	115	116	0	0	63	63	0	1	7	8
Cebu City	0	3	66	69	0	12	230	242	0	22	84	106
Lapu-Lapu City	0	17	168	185	0	11	65	76	0	6	12	18
Mandaue City	0	0	1	1	0	0	0	0	0	1	3	4
**Region 8**	**8**	**31**	**889**	**928**	**0**	**0**	**0**	**0**	**0**	**0**	**0**	**0**
Biliran	4	4	230	238	0	0	0	0	0	0	0	0
Eastern Samar	2	4	51	57	0	0	0	0	0	0	0	0
Northern Leyte	0	0	0	0	0	0	0	0	0	0	0	0
Northern Samar	0	0	36	36	0	0	0	0	0	0	0	0
Southern Leyte	0	2	155	157	0	0	0	0	0	0	0	0
Western Samar	0	0	97	97	0	0	0	0	0	0	0	0
Calbayog City	0	0	67	67	0	0	0	0	0	0	0	0
Maasin City	0	0	31	31	0	0	0	0	0	0	0	0
Ormoc City	0	9	194	203	0	0	0	0	0	0	0	0
Tacloban City	2	12	28	42	0	0	0	0	0	0	0	0
**Region 9**	**0**	**4**	**213**	**217**	**0**	**1**	**78**	**79**	**0**	**6**	**81**	**87**
Zamboanga del Norte	0	0	0	0	0	0	0	0	0	0	0	0
Zamboanga del Sur	0	0	0	0	0	0	0	0	0	0	0	0
Zamboanga Sibugay	0	0	0	0	0	0	0	0	0	0	0	0
Dapitan City	0	0	41	41	0	0	7	7	0	0	2	2
Dipolog City	0	4	81	85	0	0	0	0	0	1	42	43
Isabela City	0	0	8	8	0	0	0	0	0	0	0	0
Pagadian City	0	0	8	8	0	1	50	51	0	5	37	42
Zamboanga City	0	0	75	75	0	0	21	21	0	0	0	0
**Region 10**	**0**	**186**	**2,532**	**2,718**	**1**	**242**	**3,689**	**3,932**	**0**	**116**	**1,426**	**1,542**
Bukidnon	0	32	780	812	1	70	1,464	1,535	0	22	292	314
Camiguin	0	0	35	35	0	0	14	14	0	0	3	3
Lanao del Norte	0	9	389	398	0	47	654	701	0	0	83	83
Misamis Occidental	0	24	80	104	0	30	279	309	0	11	94	105
Misamis Oriental	0	17	317	334	0	19	475	494	0	22	168	190
Cagayan de Oro City	0	8	138	146	0	38	211	249	0	42	514	556
El Salvador City	0	0	17	17	0	6	74	80	0	5	22	27
Gingoog City	0	82	350	432	0	16	179	195	0	3	163	166
Iligan City	0	6	118	124	0	5	90	95	0	9	37	46
Malaybalay City	0	5	180	185	0	2	59	61	0	1	33	34
Oroquieta City	0	0	15	15	0	0	6	6	0	0	0	0
Ozamis City	0	0	29	29	0	0	13	13	0	0	1	1
Tangub City	0	1	13	14	0	0	6	6	0	0	0	0
Valencia City	0	2	71	73	0	9	165	174	0	1	16	17

Table 1.A.2 - MODERN METHOD OF FAMILY PLANNING
Other Acceptors
Annual Philippines, 2020

Area	CONDOM			Total	IUD-INTERVAL			Total	IUD-POSTPARTUM			Total
	Age group				Age group				Age group			
	10-14	15-19	20-49		10-14	15-19	20-49		10-14	15-19	20-49	
**Region 11**	**4**	**206**	**2,144**	**2,354**	**8**	**73**	**1,441**	**1,522**	**1**	**51**	**661**	**713**
Davao de Oro	0	13	460	473	0	6	287	293	0	3	75	78
Davao del Norte	0	16	477	493	7	27	419	453	0	14	176	190
Davao Oriental	0	8	195	203	1	3	157	161	0	2	54	56
Davao del Sur	0	3	200	203	0	15	176	191	0	0	63	63
Davao Occidental	0	6	105	111	0	9	193	202	0	0	38	38
Davao City	4	160	707	871	0	13	209	222	1	32	255	288
**Region 12**	**0**	**105**	**1,454**	**1,559**	**0**	**87**	**1,817**	**1,904**	**1**	**108**	**1,009**	**1,118**
North Cotabato	0	28	368	396	0	23	446	469	0	14	168	182
Sarangani	0	15	275	290	0	10	181	191	0	0	16	16
South Cotabato	0	36	340	376	0	25	270	295	0	33	172	205
Sultan Kudarat	0	17	366	383	0	20	372	392	0	20	298	318
				0				0				0
Cotabato City	0	3	43	46	0	8	530	538	1	36	343	380
Gen. Santos City	0	6	62	68	0	1	18	19	0	5	12	17
**B.A.R.M.M.**	**2**	**60**	**877**	**939**	**0**	**20**	**115**	**135**	**0**	**17**	**84**	**101**
Basilan	0	3	62	65	0	2	17	19	0	3	14	17
Lanao del Sur	2	2	468	472	0	2	8	10	0	0	5	5
Maguindanao	0	5	284	289	0	3	90	93	0	12	51	63
Sulu	0	26	0	26	0	11	0	11	0	0	0	0
Tawi-Tawi	0	22	4	26	0	0	0	0	0	0	0	0
				0				0				0
Lamitan City	0	0	0	0	0	2	0	2	0	2	14	16
Marawi City	0	2	59	61	0	0	0	0	0	0	0	0
**CARAGA**	**1**	**191**	**2,276**	**2,468**	**0**	**487**	**1,677**	**2,164**	**1**	**185**	**2,976**	**3,162**
Agusan del Norte	0	20	302	322	0	166	377	543	0	13	104	117
Agusan del Sur	1	106	763	870	0	252	511	763	0	16	1,895	1,911
Surigao del Norte	0	6	150	156	0	18	159	177	0	5	18	23
Surigao del Sur	0	43	436	479	0	33	240	273	1	18	357	376
Province of Dinagat	0	5	163	168	0	9	43	52	0	0	15	15
Bislig City	0	8	213	221	0	1	114	115	0	5	51	56
Butuan City	0	3	193	196	0	5	195	200	0	126	501	627
Surigao City	0	0	56	56	0	3	38	41	0	2	35	37

Table 1.A.2 - MODERN METHOD OF FAMILY PLANNING

Other Acceptors

Annual Philippines, 2020

Area	PILLS-POP			Total	PILLS-COC			Total	INJECTABLES			Total
	Age group				Age group				Age group			
	10-14	15-19	20-49		10-14	15-19	20-49		10-14	15-19	20-49	
**PHILIPPINES**	**906**	**9,119**	**61,022**	**71,047**	**312**	**24,334**	**385,142**	**409,788**	**779**	**23,604**	**289,459**	**313,842**
**N C R**	**60**	**1,725**	**12,488**	**14,273**	**71**	**3,415**	**75,407**	**78,893**	**89**	**2,813**	**35,912**	**38,814**
Malabon	0	29	341	370	0	66	1,075	1,141	2	130	1,526	1,658
Navotas	0	6	279	285	0	135	469	604	0	69	1,232	1,301
Valenzuela City	0	18	369	387	0	52	8,444	8,496	2	224	4,539	4,765
Caloocan City	1	44	595	640	2	176	2,203	2,381	2	519	5,690	6,211
Marikina City	0	21	133	154	0	23	600	623	3	145	785	933
Pasig City	0	62	179	241	0	82	598	680	1	135	1,340	1,476
Pateros	0	0	0	0	0	14	1,044	1,058	0	0	59	59
Taguig	0	7	105	112	2	706	11,324	12,032	0	27	583	610
Quezon City	58	954	7,674	8,686	7	420	27,868	28,295	4	667	13,021	13,692
Makati City	0	33	480	513	0	32	775	807	0	87	1,715	1,802
Mandaluyong City	0	6	37	43	0	4	116	120	0	3	150	153
San Juan	0	20	0	20	0	20	8	28	0	0	28	28
Manila City	0	110	1,139	1,249	21	648	8,757	9,426	2	142	1,273	1,417
Las Piñas City	1	21	264	286	1	52	782	835	1	57	794	852
Muntinlupa City	0	10	148	158	0	42	1,165	1,207	0	36	1,011	1,047
Parañaque City	0	23	202	225	38	474	4,907	5,419	72	568	552	1,192
Pasay City	0	361	543	904	0	469	5,272	5,741	0	4	1,614	1,618
**C A R**	**5**	**326**	**3,029**	**3,360**	**0**	**550**	**17,663**	**18,213**	**2**	**679**	**7,428**	**8,109**
Abra	2	50	344	396	0	25	794	819	0	58	469	527
Apayao	3	51	372	426	0	58	1,176	1,234	0	72	712	784
Benguet	0	84	563	647	0	152	9,493	9,645	0	188	1,409	1,597
Ifugao	0	42	528	570	0	76	2,542	2,618	0	85	1,287	1,372
Kalinga	0	56	388	444	0	78	1,365	1,443	1	63	1,066	1,130
Mt. Province	0	20	215	235	0	93	1,247	1,340	1	108	807	916
Baguio City	0	23	619	642	0	68	1,046	1,114	0	105	1,678	1,783
**Region 1**	**0**	**142**	**883**	**1,025**	**1**	**467**	**11,424**	**11,892**	**2**	**444**	**8,005**	**8,451**
Ilocos Norte	0	22	153	175	0	16	2,014	2,030	0	16	226	242
Ilocos Sur	0	87	384	471	0	59	741	800	0	32	347	379
La Union	0	32	341	373	0	70	1,632	1,702	0	144	1,899	2,043
Pangasinan	0	0	0	0	1	281	5,409	5,691	2	196	4,104	4,302
Alaminos City	0	0	0	0	0	1	63	64	0	23	81	104
Candon City	0	0	0	0	0	0	0	0	0	0	0	0
Dagupan City	0	1	5	6	0	2	55	57	0	4	67	71
Laoag City	0	0	0	0	0	0	0	0	0	0	9	9
San Carlos City	0	0	0	0	0	16	373	389	0	17	295	312
San Fernando City	0	0	0	0	0	10	790	800	0	2	762	764
Urdaneta City	0	0	0	0	0	8	345	353	0	10	208	218
Vigan City	0	0	0	0	0	4	2	6	0	0	7	7
**Region 2**	**3**	**525**	**4,963**	**5,491**	**4**	**1,017**	**17,718**	**18,739**	**8**	**1,061**	**9,634**	**10,703**
Batanes	0	3	16	19	0	2	55	57	0	25	159	184
Cagayan	1	92	877	970	0	177	3,812	3,989	2	129	1,970	2,101
Isabela	1	207	2,185	2,393	4	359	5,568	5,931	3	444	4,485	4,932
Nueva Vizcaya	1	120	1,241	1,362	0	172	5,873	6,045	3	179	1,459	1,641
Quirino	0	30	155	185	0	96	608	704	0	46	414	460
Cauayan City	0	17	95	112	0	74	524	598	0	38	355	393
Ilagan City	0	21	147	168	0	26	434	460	0	30	238	268
Santiago City	0	0	0	0	0	85	570	655	0	161	437	598
Tuguegarao City	0	35	247	282	0	26	274	300	0	9	117	126
**Region 3**	**0**	**495**	**5,382**	**5,877**	**13**	**1,893**	**26,359**	**28,265**	**14**	**2,393**	**23,103**	**25,510**
Aurora	0	42	265	307	0	64	1,187	1,251	1	73	896	970
Bataan	0	44	417	461	4	85	1,405	1,494	2	212	2,184	2,398

Table 1.A.2 - MODERN METHOD OF FAMILY PLANNING

Other Acceptors

Annual Philippines, 2020

Area	PILLS-POP			Total	PILLS-COC			Total	INJECTABLES			Total
	Age group				Age group				Age group			
	10-14	15-19	20-49		10-14	15-19	20-49		10-14	15-19	20-49	
Bulacan	0	72	939	1,011	0	435	4,746	5,181	9	553	4,292	4,854
Nueva Ecija	0	48	1,109	1,157	1	237	4,167	4,405	0	216	3,355	3,571
Pampanga	0	63	715	778	7	419	3,404	3,830	1	571	2,022	2,594
Tarlac	0	98	881	979	0	77	2,077	2,154	0	80	1,757	1,837
Zambales	0	22	253	275	1	96	1,518	1,615	0	142	2,867	3,009
Angeles City	0	5	67	72	0	21	292	313	0	15	243	258
Balanga City	0	0	1	1	0	16	185	201	0	38	481	519
Cabanatuan City	0	12	128	140	0	23	1,588	1,611	0	16	532	548
City of San Fernando	0	2	18	20	0	3	96	99	0	8	166	174
Gapan City	0	3	30	33	0	8	191	199	0	4	100	104
Mabalacat City	0	9	51	60	0	25	348	373	0	49	530	579
Malolos City	0	0	0	0	0	29	976	1,005	0	24	393	417
Meycauayan	0	0	11	11	0	45	205	250	0	27	245	272
Olongapo	0	7	83	90	0	59	398	457	1	68	797	866
Palayan City	0	0	5	5	0	13	134	147	0	12	93	105
San Jose City	0	7	76	83	0	33	305	338	0	43	248	291
San Jose del Monte City	0	10	85	95	0	50	1,316	1,366	0	90	1,067	1,157
Science City of Munoz	0	0	11	11	0	0	215	215	0	0	166	166
Tarlac City	0	51	237	288	0	155	1,606	1,761	0	152	669	821
**Region 4A**	**796**	**2,728**	**2,728**	**6,252**	**84**	**7,264**	**111,840**	**119,188**	**604**	**6,940**	**110,168**	**117,712**
Batangas	480	100	100	680	0	124	5,476	5,600	248	188	3,672	4,108
Cavite	4	68	68	140	4	288	13,124	13,416	4	564	12,144	12,712
Laguna	0	180	180	360	0	408	8,460	8,868	0	224	6,416	6,640
Quezon	0	528	528	1,056	0	1,360	46,760	48,120	12	1,740	34,728	36,480
Rizal	0	144	144	288	4	564	8,864	9,432	4	1,100	16,856	17,960
Antipolo City	0	0	0	0	0	0	0	0	0	0	0	0
Bacoor City	0	4	4	8	4	36	996	1,036	20	96	2,000	2,116
Batangas City	0	0	0	0	0	0	0	0	0	0	0	0
Biñan City	0	1,288	1,288	2,576	0	1,852	11,520	13,372	0	1,476	9,888	11,364
Cabuyao City	4	20	20	44	0	176	952	1,128	0	108	968	1,076
Calamba City	0	68	68	136	0	44	840	884	0	76	1,272	1,348
Cavite City	0	0	0	0	0	0	0	0	0	0	0	0
Dasmariñas City	0	80	80	160	0	140	2,008	2,148	0	156	4,272	4,428
General Trias City	0	44	44	88	0	68	3,652	3,720	0	136	4,904	5,040
Imus City	0	4	4	8	0	56	1,960	2,016	0	52	1,688	1,740
Lipa City	0	0	0	0	0	0	0	0	0	0	0	0
Lucena City	308	0	0	308	64	0	0	64	300	0	2,904	3,204
San Pablo City	0	0	0	0	0	0	0	0	0	0	0	0
San Pedro City	0	64	64	128	0	168	2,348	2,516	0	120	1,448	1,568
Santa Rosa City	0	112	112	224	0	1,916	2,880	4,796	0	784	1,216	2,000
Tagaytay City	0	4	4	8	8	8	1,564	1,580	16	24	1,604	1,644
Tanauan City	0	12	12	24	0	0	212	212	0	36	404	440
Tayabas City	0	8	8	16	0	4	28	32	0	16	2,876	2,892
Trece Martires City	0	0	0	0	0	52	196	248	0	44	908	952
**Region 4B**	**0**	**49**	**567**	**616**	**3**	**274**	**10,812**	**11,089**	**5**	**358**	**8,559**	**8,922**
Marinduque	0	0	51	51	0	0	246	246	0	7	136	143
Mindoro Occidental	0	25	169	194	1	162	2,395	2,558	3	190	2,517	2,710
Mindoro Oriental	0	14	293	307	1	88	2,812	2,901	2	88	1,630	1,720
Palawan	0	10	54	64	1	24	3,295	3,320	0	73	1,881	1,954
Romblon	0	0	0	0	0	0	9	9	0	0	3	3
Puerto Princesa City	0	0	0	0	0	0	2,055	2,055	0	0	2,392	2,392
**Region 5**	**5**	**317**	**3,693**	**4,015**	**1**	**937**	**20,368**	**21,306**	**3**	**1,129**	**16,684**	**17,816**
Albay	0	28	404	432	0	102	2,283	2,385	0	87	1,821	1,908
Camarines Norte	2	63	623	688	1	245	2,571	2,817	1	295	2,481	2,777
Camarines Sur	3	113	1,170	1,286	0	186	6,112	6,298	1	248	5,244	5,493
Catanduanes	0	7	213	220	0	44	908	952	0	107	1,166	1,273
Masbate	0	50	584	634	0	119	3,372	3,491	1	123	1,981	2,105
Sorsogon	0	43	374	417	0	211	3,753	3,964	0	231	3,363	3,594
Iriga City	0	9	143	152	0	25	478	503	0	33	498	531

Table 1.A.2 - MODERN METHOD OF FAMILY PLANNING
Other Acceptors
Annual Philippines, 2020

Area	PILLS-POP			Total	PILLS-COC			Total	INJECTABLES			Total
	Age group				Age group				Age group			
	10-14	15-19	20-49		10-14	15-19	20-49		10-14	15-19	20-49	
Legaspi City	0	1	30	31	0	2	55	57	0	5	19	24
Naga City	0	3	152	155	0	3	836	839	0	0	111	111
**Region 6**	**2**	**149**	**2,018**	**2,169**	**7**	**842**	**17,915**	**18,764**	**3**	**574**	**11,318**	**11,895**
Aklan	0	7	146	153	0	46	1,550	1,596	0	36	801	837
Antique	0	20	325	345	0	71	3,083	3,154	1	96	2,026	2,123
Capiz	0	2	87	89	0	22	919	941	0	34	702	736
Guimaras	0	9	72	81	1	28	544	573	0	12	279	291
Iloilo	0	28	513	541	6	194	2,507	2,707	2	96	1,593	1,691
Negros Occidental	0	63	695	758	0	384	7,019	7,403	0	226	4,527	4,753
Bacolod City	2	4	81	87	0	15	304	319	0	17	632	649
Iloilo City	0	16	99	115	0	82	1,989	2,071	0	57	758	815
**Region 7**	**1**	**210**	**1,608**	**1,819**	**0**	**259**	**8,358**	**8,617**	**1**	**475**	**8,048**	**8,524**
Bohol	0	42	334	376	0	50	1,281	1,331	0	116	1,314	1,430
Cebu	0	16	264	280	0	38	2,821	2,859	0	39	1,625	1,664
Negros Oriental	0	54	305	359	0	66	1,717	1,783	1	159	2,824	2,984
Siquijor	0	11	147	158	0	7	557	564	0	25	524	549
Cebu City	1	25	219	245	0	55	1,614	1,669	0	73	1,090	1,163
Lapu-Lapu City	0	62	295	357	0	43	338	381	0	63	557	620
Mandaue City	0	0	44	44	0	0	30	30	0	0	114	114
**Region 8**	**0**	**0**	**0**	**0**	**0**	**0**	**0**	**0**	**0**	**0**	**0**	**0**
Biliran	0	0	0	0	0	0	0	0	0	0	0	0
Eastern Samar	0	0	0	0	0	0	0	0	0	0	0	0
Northern Leyte	0	0	0	0	0	0	0	0	0	0	0	0
Northern Samar	0	0	0	0	0	0	0	0	0	0	0	0
Southern Leyte	0	0	0	0	0	0	0	0	0	0	0	0
Western Samar	0	0	0	0	0	0	0	0	0	0	0	0
Calbayog City	0	0	0	0	0	0	0	0	0	0	0	0
Maasin City	0	0	0	0	0	0	0	0	0	0	0	0
Ormoc City	0	0	0	0	0	0	0	0	0	0	0	0
Tacloban City	0	0	0	0	0	0	0	0	0	0	0	0
**Region 9**	**0**	**22**	**320**	**342**	**0**	**46**	**1,421**	**1,467**	**0**	**38**	**976**	**1,014**
Zamboanga del Norte	0	0	0	0	0	0	0	0	0	0	0	0
Zamboanga del Sur	0	0	0	0	0	0	0	0	0	0	0	0
Zamboanga Sibugay	0	0	0	0	0	0	0	0	0	0	0	0
Dapitan City	0	4	30	34	0	10	152	162	0	6	94	100
Dipolog City	0	12	133	145	0	19	340	359	0	29	383	412
Isabela City	0	4	134	138	0	7	152	159	0	2	89	91
Pagadian City	0	0	13	13	0	9	267	276	0	0	0	0
Zamboanga City	0	2	10	12	0	1	510	511	0	1	410	411
**Region 10**	**1**	**476**	**3,561**	**4,038**	**19**	**1,139**	**19,582**	**20,740**	**9**	**939**	**10,568**	**11,516**
Bukidnon	0	206	881	1,087	4	590	7,949	8,543	2	396	3,952	4,350
Camiguin	0	3	38	41	2	4	246	252	0	6	135	141
Lanao del Norte	0	107	1,058	1,165	4	122	3,302	3,428	1	217	1,769	1,987
Misamis Occidental	0	3	94	97	0	16	359	375	0	6	172	178
Misamis Oriental	0	41	552	593	2	66	1,240	1,308	1	35	831	867
Cagayan de Oro City	0	11	235	246	0	30	690	720	4	61	935	1,000
El Salvador City	0	8	43	51	0	5	118	123	0	24	162	186
Gingoog City	1	27	222	250	6	68	993	1,067	0	31	276	307
Iligan City	0	6	148	154	1	56	998	1,055	1	38	811	850
Malaybalay City	0	15	81	96	0	98	2,179	2,277	0	86	695	781
Oroquieta City	0	1	17	18	0	6	45	51	0	1	40	41
Ozamis City	0	11	43	54	0	5	133	138	0	10	149	159
Tangub City	0	24	22	46	0	8	91	99	0	5	30	35
Valencia City	0	13	127	140	0	65	1,239	1,304	0	23	611	634

Table 1.A.2 - MODERN METHOD OF FAMILY PLANNING

Other Acceptors

Annual Philippines, 2020

Area	PILLS-POP			Total	PILLS-COC			Total	INJECTABLES			Total
	Age group				Age group				Age group			
	10-14	15-19	20-49		10-14	15-19	20-49		10-14	15-19	20-49	
**Region 11**	**20**	**583**	**4,045**	**4,648**	**104**	**1,318**	**14,141**	**15,563**	**30**	**1,058**	**10,462**	**11,550**
Davao de Oro	0	79	804	883	2	201	3,154	3,357	2	124	1,758	1,884
Davao del Norte	9	61	865	935	40	205	2,716	2,961	13	173	2,075	2,261
Davao Oriental	1	77	454	532	49	193	2,021	2,263	7	147	1,265	1,419
Davao del Sur	1	49	497	547	3	152	1,763	1,918	2	139	1,526	1,667
Davao Occidental	6	45	168	219	10	220	1,859	2,089	2	217	1,820	2,039
Davao City	3	272	1,257	1,532	0	347	2,628	2,975	4	258	2,018	2,280
**Region 12**	**10**	**726**	**7,129**	**7,865**	**3**	**1,312**	**17,238**	**18,553**	**5**	**1,788**	**16,598**	**18,391**
North Cotabato	8	134	1,173	1,315	0	239	4,342	4,581	2	294	3,594	3,890
Sarangani	0	125	508	633	0	366	4,377	4,743	0	318	3,165	3,483
South Cotabato	2	167	870	1,039	2	302	3,126	3,430	3	551	3,507	4,061
Sultan Kudarat	0	252	2,862	3,114	0	289	3,368	3,657	0	428	3,898	4,326
				0				0				0
Cotabato City	0	19	1,577	1,596	1	67	1,182	1,250	0	116	1,436	1,552
Gen. Santos City	0	29	139	168	0	49	843	892	0	81	998	1,079
**B.A.R.M.M.**	**1**	**100**	**1,351**	**1,452**	**0**	**1,852**	**3,795**	**5,647**	**0**	**1,876**	**4,734**	**6,610**
Basilan	0	22	188	210	0	43	587	630	0	71	682	753
Lanao del Sur	0	20	580	600	0	24	932	956	0	34	1,039	1,073
Maguindanao	1	49	282	332	0	279	2,166	2,445	0	128	2,778	2,906
Sulu	0	0	0	0	0	846	0	846	0	1,072	0	1,072
Tawi-Tawi	0	0	224	224	0	656	0	656	0	565	78	643
				0				0				0
Lamitan City	0	8	32	40	0	4	36	40	0	4	98	102
Marawi City	0	1	45	46	0	0	74	74	0	2	59	61
**CARAGA**	**2**	**546**	**7,257**	**7,805**	**2**	**1,749**	**11,101**	**12,852**	**4**	**1,039**	**7,262**	**8,305**
Agusan del Norte	1	58	872	931	1	297	1,521	1,819	0	160	1,106	1,266
Agusan del Sur	0	228	3,817	4,045	1	766	3,048	3,815	0	453	2,133	2,586
Surigao del Norte	0	56	427	483	0	104	1,276	1,380	4	67	809	880
Surigao del Sur	1	92	1,357	1,450	0	386	2,660	3,046	0	219	1,388	1,607
Province of Dinagat	0	21	98	119	0	78	455	533	0	46	236	282
Bislig City	0	20	182	202	0	39	863	902	0	31	396	427
Butuan City	0	53	421	474	0	69	1,079	1,148	0	56	1,014	1,070
Surigao City	0	18	83	101	0	10	199	209	0	7	180	187

Table 1.A.2 - MODERN METHOD OF FAMILY PLANNING
Other Acceptors
Annual Philippines, 2020

Area	IMPLANTS Age group 10-14	15-19	20-49	Total	NFP-CCM Age group 10-14	15-19	20-49	Total	NFP-BBT Age group 10-14	15-19	20-49	Total
**PHILIPPINES**	**537**	**8,726**	**62,737**	**72,000**	**2,907**	**313**	**30,715**	**33,935**	**69**	**72**	**1,521**	**1,662**
**N C R**	**128**	**4,372**	**11,887**	**16,387**	**0**	**0**	**85**	**85**	**0**	**0**	**32**	**32**
Malabon	0	23	207	230	0	0	0	0	0	0	0	0
Navotas	0	6	115	121	0	0	0	0	0	0	0	0
Valenzuela City	56	1,208	3,755	5,019	0	0	52	52	0	0	32	32
Caloocan City	4	183	770	957	0	0	0	0	0	0	0	0
Marikina City	0	38	168	206	0	0	12	12	0	0	0	0
Pasig City	1	99	204	304	0	0	0	0	0	0	0	0
Pateros	0	0	5	5	0	0	0	0	0	0	0	0
Taguig	0	0	53	53	0	0	0	0	0	0	0	0
Quezon City	27	792	3,964	4,783	0	0	0	0	0	0	0	0
Makati City	0	54	86	140	0	0	0	0	0	0	0	0
Mandaluyong City	0	0	13	13	0	0	9	9	0	0	0	0
San Juan	0	0	6	6	0	0	0	0	0	0	0	0
Manila City	38	1,715	1,696	3,449	0	0	0	0	0	0	0	0
Las Piñas City	1	18	192	211	0	0	0	0	0	0	0	0
Muntinlupa City	0	0	19	19	0	0	0	0	0	0	0	0
Parañaque City	1	234	145	380	0	0	0	0	0	0	0	0
Pasay City	0	2	489	491	0	0	12	12	0	0	0	0
**C A R**	**1**	**153**	**2,029**	**2,183**	**0**	**32**	**744**	**776**	**0**	**5**	**87**	**92**
Abra	1	21	254	276	0	0	38	38	0	1	41	42
Apayao	0	36	179	215	0	0	0	0	0	0	0	0
Benguet	0	26	198	224	0	0	7	7	0	0	2	2
Ifugao	0	16	372	388	0	8	270	278	0	2	7	9
Kalinga	0	18	405	423	0	24	418	442	0	2	10	12
Mt. Province	0	12	109	121	0	0	11	11	0	0	16	16
Baguio City	0	24	512	536	0	0	0	0	0	0	11	11
**Region 1**	**0**	**134**	**2,190**	**2,324**	**0**	**1**	**177**	**178**	**0**	**4**	**13**	**17**
Ilocos Norte	0	0	46	46	0	1	32	33	0	1	5	6
Ilocos Sur	0	5	82	87	0	0	35	35	0	0	1	1
La Union	0	50	318	368	0	0	77	77	0	0	6	6
Pangasinan	0	72	1,545	1,617	0	0	0	0	0	1	0	1
Alaminos City	0	1	23	24	0	0	0	0	0	0	0	0
Candon City	0	0	0	0	0	0	5	5	0	0	0	0
Dagupan City	0	1	1	2	0	0	0	0	0	0	0	0
Laoag City	0	0	0	0	0	0	0	0	0	0	0	0
San Carlos City	0	3	28	31	0	0	0	0	0	0	1	1
San Fernando City	0	2	140	142	0	0	0	0	0	0	0	0
Urdaneta City	0	0	7	7	0	0	0	0	0	0	0	0
Vigan City	0	0	0	0	0	0	28	28	0	2	0	2
**Region 2**	**3**	**270**	**2,733**	**3,006**	**0**	**2**	**77**	**79**	**0**	**1**	**14**	**15**
Batanes	0	0	1	1	0	1	16	17	0	0	0	0
Cagayan	3	37	657	697	0	0	2	2	0	0	5	5
Isabela	0	158	1,126	1,284	0	0	1	1	0	0	7	7
Nueva Vizcaya	0	51	671	722	0	1	54	55	0	1	2	3
Quirino	0	0	43	43	0	0	4	4	0	0	0	0
Cauayan City	0	8	26	34	0	0	0	0	0	0	0	0
Ilagan City	0	2	61	63	0	0	0	0	0	0	0	0
Santiago City	0	12	139	151	0	0	0	0	0	0	0	0
Tuguegarao City	0	2	9	11	0	0	0	0	0	0	0	0
**Region 3**	**2**	**355**	**4,009**	**4,366**	**0**	**2**	**78**	**80**	**0**	**1**	**10**	**11**
Aurora	1	13	260	274	0	0	41	41	0	0	0	0
Bataan	0	55	211	266	0	0	7	7	0	0	0	0

Table 1.A.2 - MODERN METHOD OF FAMILY PLANNING
Other Acceptors
Annual Philippines, 2020

Area	IMPLANTS			Total	NFP-CCM			Total	NFP-BBT			Total
	Age group				Age group				Age group			
	10-14	15-19	20-49		10-14	15-19	20-49		10-14	15-19	20-49	
Bulacan	0	33	458	491	0	0	1	1	0	0	0	0
Nueva Ecija	0	92	1,034	1,126	0	0	1	1	0	0	0	0
Pampanga	1	94	692	787	0	2	23	25	0	1	10	11
Tarlac	0	1	171	172	0	0	0	0	0	0	0	0
Zambales	0	29	424	453	0	0	5	5	0	0	0	0
Angeles City	0	4	97	101	0	0	0	0	0	0	0	0
Balanga City	0	1	9	10	0	0	0	0	0	0	0	0
Cabanatuan City	0	0	57	57	0	0	0	0	0	0	0	0
City of San Fernando	0	0	5	5	0	0	0	0	0	0	0	0
Gapan City	0	0	13	13	0	0	0	0	0	0	0	0
Mabalacat City	0	0	13	13	0	0	0	0	0	0	0	0
Malolos City	0	19	177	196	0	0	0	0	0	0	0	0
Meycauayan	0	1	14	15	0	0	0	0	0	0	0	0
Olongapo	0	1	92	93	0	0	0	0	0	0	0	0
Palayan City	0	0	0	0	0	0	0	0	0	0	0	0
San Jose City	0	1	7	8	0	0	0	0	0	0	0	0
San Jose del Monte City	0	8	74	82	0	0	0	0	0	0	0	0
Science City of Munoz	0	0	4	4	0	0	0	0	0	0	0	0
Tarlac City	0	3	197	200	0	0	0	0	0	0	0	0
**Region 4A**	**336**	**704**	**8,504**	**9,544**	**2,904**	**4**	**23,996**	**26,904**	**64**	**4**	**724**	**792**
Batangas	20	28	488	536	0	0	20,220	20,220	0	0	0	0
Cavite	4	80	2,652	2,736	0	0	204	204	0	0	0	0
Laguna	8	108	1,228	1,344	0	0	704	704	0	0	40	40
Quezon	0	136	1,216	1,352	0	4	1,740	1,744	0	0	256	256
Rizal	8	264	1,364	1,636	0	0	0	0	0	0	8	8
Antipolo City	0	0	0	0	0	0	0	0	0	0	0	0
Bacoor City	0	4	44	48	0	0	0	0	0	0	0	0
Batangas City	0	0	0	0	0	0	0	0	0	0	0	0
Biñan City	0	0	60	60	0	0	0	0	0	0	0	0
Cabuyao City	0	0	56	56	0	0	0	0	0	0	0	0
Calamba City	0	12	248	260	0	0	0	0	0	0	0	0
Cavite City	0	0	0	0	0	0	0	0	0	0	0	0
Dasmariñas City	0	8	104	112	0	0	0	0	0	0	0	0
General Trias City	0	36	584	620	0	0	0	0	0	0	408	408
Imus City	0	16	72	88	0	0	0	0	0	0	0	0
Lipa City	0	0	0	0	0	0	0	0	0	0	0	0
Lucena City	296	0	0	296	2,904	0	968	3,872	64	0	4	68
San Pablo City	0	0	0	0	0	0	0	0	0	0	0	0
San Pedro City	0	4	120	124	0	0	0	0	0	4	4	8
Santa Rosa City	0	8	132	140	0	0	0	0	0	0	0	0
Tagaytay City	0	0	64	64	0	0	0	0	0	0	0	0
Tanauan City	0	0	8	8	0	0	0	0	0	0	0	0
Tayabas City	0	0	36	36	0	0	160	160	0	0	4	4
Trece Martires City	0	0	28	28	0	0	0	0	0	0	0	0
**Region 4B**	**0**	**37**	**1,697**	**1,734**	**0**	**27**	**350**	**377**	**0**	**0**	**1**	**1**
Marinduque	0	1	85	86	0	0	5	5	0	0	0	0
Mindoro Occidental	0	12	234	246	0	25	338	363	0	0	1	1
Mindoro Oriental	0	19	471	490	0	2	6	8	0	0	0	0
Palawan	0	5	581	586	0	0	1	1	0	0	0	0
Romblon	0	0	1	1	0	0	0	0	0	0	0	0
Puerto Princesa City	0	0	325	325	0	0	0	0	0	0	0	0
**Region 5**	**1**	**298**	**4,744**	**5,043**	**3**	**139**	**2,305**	**2,447**	**0**	**3**	**71**	**74**
Albay	1	34	510	545	0	0	247	247	0	0	16	16
Camarines Norte	0	23	355	378	0	5	25	30	0	0	6	6
Camarines Sur	0	107	1,804	1,911	3	3	229	235	0	2	39	41
Catanduanes	0	18	553	571	0	23	562	585	0	0	0	0
Masbate	0	64	619	683	0	6	547	553	0	1	4	5
Sorsogon	0	39	627	666	0	102	689	791	0	0	0	0
Iriga City	0	12	243	255	0	0	0	0	0	0	6	6

Table 1.A.2 - MODERN METHOD OF FAMILY PLANNING
Other Acceptors
Annual Philippines, 2020

Area	IMPLANTS Age group 10-14	15-19	20-49	Total	NFP-CCM Age group 10-14	15-19	20-49	Total	NFP-BBT Age group 10-14	15-19	20-49	Total
Legaspi City	0	1	4	5	0	0	0	0	0	0	0	0
Naga City	0	0	29	29	0	0	6	6	0	0	0	0
**Region 6**	**1**	**196**	**3,798**	**3,995**	**0**	**30**	**1,772**	**1,802**	**5**	**1**	**39**	**45**
Aklan	0	4	189	193	0	0	0	0	0	0	0	0
Antique	0	48	746	794	0	30	1,768	1,798	0	0	2	2
Capiz	0	9	249	258	0	0	0	0	0	0	14	14
Guimaras	0	0	32	32	0	0	0	0	0	0	0	0
Iloilo	1	48	944	993	0	0	0	0	0	1	23	24
Negros Occidental	0	62	1,347	1,409	0	0	4	4	0	0	0	0
Bacolod City	0	11	116	127	0	0	0	0	5	0	0	5
Iloilo City	0	14	175	189	0	0	0	0	0	0	0	0
**Region 7**	**0**	**119**	**2,331**	**2,450**	**0**	**0**	**0**	**0**	**0**	**0**	**20**	**20**
Bohol	0	51	369	420	0	0	0	0	0	0	0	0
Cebu	0	27	725	752	0	0	0	0	0	0	0	0
Negros Oriental	0	18	356	374	0	0	0	0	0	0	20	20
Siquijor	0	4	121	125	0	0	0	0	0	0	0	0
Cebu City	0	13	357	370	0	0	0	0	0	0	0	0
Lapu-Lapu City	0	6	202	208	0	0	0	0	0	0	0	0
Mandaue City	0	0	201	201	0	0	0	0	0	0	0	0
**Region 8**	**0**	**0**	**0**	**0**	**0**	**0**	**0**	**0**	**0**	**0**	**0**	**0**
Biliran	0	0	0	0	0	0	0	0	0	0	0	0
Eastern Samar	0	0	0	0	0	0	0	0	0	0	0	0
Northern Leyte	0	0	0	0	0	0	0	0	0	0	0	0
Northern Samar	0	0	0	0	0	0	0	0	0	0	0	0
Southern Leyte	0	0	0	0	0	0	0	0	0	0	0	0
Western Samar	0	0	0	0	0	0	0	0	0	0	0	0
Calbayog City	0	0	0	0	0	0	0	0	0	0	0	0
Maasin City	0	0	0	0	0	0	0	0	0	0	0	0
Ormoc City	0	0	0	0	0	0	0	0	0	0	0	0
Tacloban City	0	0	0	0	0	0	0	0	0	0	0	0
**Region 9**	**0**	**6**	**185**	**191**	**0**	**0**	**0**	**0**	**0**	**0**	**0**	**0**
Zamboanga del Norte	0	0	0	0	0	0	0	0	0	0	0	0
Zamboanga del Sur	0	0	0	0	0	0	0	0	0	0	0	0
Zamboanga Sibugay	0	0	0	0	0	0	0	0	0	0	0	0
Dapitan City	0	0	5	5	0	0	0	0	0	0	0	0
Dipolog City	0	2	53	55	0	0	0	0	0	0	0	0
Isabela City	0	0	11	11	0	0	0	0	0	0	0	0
Pagadian City	0	4	51	55	0	0	0	0	0	0	0	0
Zamboanga City	0	0	65	65	0	0	0	0	0	0	0	0
**Region 10**	**3**	**331**	**4,531**	**4,865**	**0**	**53**	**737**	**790**	**0**	**41**	**301**	**342**
Bukidnon	2	219	2,004	2,225	0	11	194	205	0	0	12	12
Camiguin	0	2	63	65	0	0	0	0	0	0	0	0
Lanao del Norte	0	7	423	430	0	0	0	0	0	0	0	0
Misamis Occidental	0	0	21	21	0	0	0	0	0	0	0	0
Misamis Oriental	0	25	542	567	0	0	109	109	0	0	11	11
Cagayan de Oro City	0	7	444	451	0	0	0	0	0	0	0	0
El Salvador City	1	14	147	162	0	0	0	0	0	0	0	0
Gingoog City	0	18	225	243	0	39	279	318	0	41	171	212
Iligan City	0	12	188	200	0	0	77	77	0	0	0	0
Malaybalay City	0	21	295	316	0	0	0	0	0	0	0	0
Oroquieta City	0	1	17	18	0	0	0	0	0	0	0	0
Ozamis City	0	4	82	86	0	0	0	0	0	0	4	4
Tangub City	0	1	0	1	0	0	0	0	0	0	0	0
Valencia City	0	0	80	80	0	3	78	81	0	0	103	103

Table 1.A.2 - MODERN METHOD OF FAMILY PLANNING

Other Acceptors

Annual Philippines, 2020

Area	IMPLANTS			Total	NFP-CCM			Total	NFP-BBT			Total
	Age group				Age group				Age group			
	10-14	15-19	20-49		10-14	15-19	20-49		10-14	15-19	20-49	
**Region 11**	**22**	**382**	**3,607**	**4,011**	**0**	**10**	**236**	**246**	**0**	**0**	**20**	**20**
Davao de Oro	0	64	667	731	0	3	32	35	0	0	1	1
Davao del Norte	19	116	860	995	0	0	18	18	0	0	3	3
Davao Oriental	0	43	433	476	0	1	41	42	0	0	11	11
Davao del Sur	3	36	452	491	0	1	2	3	0	0	0	0
Davao Occidental	0	48	450	498	0	0	0	0	0	0	1	1
Davao City	0	75	745	820	0	5	143	148	0	0	4	4
**Region 12**	**39**	**668**	**5,789**	**6,496**	**0**	**10**	**63**	**73**	**0**	**9**	**58**	**67**
North Cotabato	0	124	1,572	1,696	0	10	48	58	0	7	39	46
Sarangani	0	96	917	1,013	0	0	10	10	0	2	19	21
South Cotabato	0	222	916	1,138	0	0	0	0	0	0	0	0
Sultan Kudarat	39	193	1,644	1,876	0	0	1	1	0	0	0	0
				0				0				0
Cotabato City	0	20	597	617	0	0	1	1	0	0	0	0
Gen. Santos City	0	13	143	156	0	0	3	3	0	0	0	0
**B.A.R.M.M.**	**0**	**307**	**1,229**	**1,536**	**0**	**0**	**0**	**0**	**0**	**0**	**0**	**0**
Basilan	0	22	272	294	0	0	0	0	0	0	0	0
Lanao del Sur	0	2	165	167	0	0	0	0	0	0	0	0
Maguindanao	0	43	634	677	0	0	0	0	0	0	0	0
Sulu	0	69	0	69	0	0	0	0	0	0	0	0
Tawi-Tawi	0	156	62	218	0	0	0	0	0	0	0	0
				0				0				0
Lamitan City	0	14	80	94	0	0	0	0	0	0	0	0
Marawi City	0	1	16	17	0	0	0	0	0	0	0	0
**CARAGA**	**1**	**394**	**3,474**	**3,869**	**0**	**3**	**95**	**98**	**0**	**3**	**131**	**134**
Agusan del Norte	0	98	470	568	0	0	0	0	0	3	5	8
Agusan del Sur	0	112	1,214	1,326	0	0	8	8	0	0	0	0
Surigao del Norte	1	21	415	437	0	1	18	19	0	0	5	5
Surigao del Sur	0	40	379	419	0	0	19	19	0	0	121	121
Province of Dinagat	0	53	370	423	0	0	0	0	0	0	0	0
Bislig City	0	12	129	141	0	2	41	43	0	0	0	0
Butuan City	0	48	394	442	0	0	9	9	0	0	0	0
Surigao City	0	10	103	113	0	0	0	0	0	0	0	0

Table 1.A.2 - MODERN METHOD OF FAMILY PLANNING

Other Acceptors

Annual Philippines, 2020

Area	NFP-STM			Total	NFP-SDM			Total	NFP-LAM			Total	Total Other Acceptors
	Age group				Age group				Age group				
	10-14	15-19	20-49		10-14	15-19	20-49		10-14	15-19	20-49		
**PHILIPPINES**	**0**	**24**	**827**	**851**	**46**	**751**	**17,546**	**18,343**	**5,981**	**30,149**	**340,789**	**376,919**	**1,488,624**
**N C R**	**0**	**7**	**100**	**107**	**0**	**4**	**372**	**376**	**168**	**2,223**	**33,621**	**36,012**	**228,010**
Malabon	0	0	0	0	0	0	0	0	0	41	795	836	4,894
Navotas	0	0	0	0	0	0	0	0	1	42	794	837	3,431
Valenzuela City	0	0	89	89	0	1	206	207	4	40	2,909	2,953	24,975
Caloocan City	0	0	0	0	0	0	7	7	0	234	3,928	4,162	16,520
Marikina City	0	0	0	0	0	0	0	0	0	83	149	232	2,642
Pasig City	0	0	0	0	0	0	0	0	0	11	351	362	3,936
Pateros	0	0	0	0	0	0	0	0	0	3	36	39	1,197
Taguig	0	0	0	0	0	0	3	3	0	3	139	142	13,403
Quezon City	0	0	0	0	0	0	150	150	5	806	20,977	21,788	104,748
Makati City	0	0	0	0	0	0	0	0	1	127	2,233	2,361	6,296
Mandaluyong City	0	0	6	6	0	0	0	0	0	0	15	15	396
San Juan	0	0	0	0	0	0	0	0	0	0	0	0	82
Manila City	0	0	0	0	0	0	1	1	0	102	623	725	17,716
Las Piñas City	0	0	0	0	0	0	3	3	0	22	177	199	2,812
Muntinlupa City	0	0	0	0	0	0	0	0	0	44	22	66	2,745
Parañaque City	0	7	0	7	0	0	1	1	157	388	270	815	9,604
Pasay City	0	0	5	5	0	3	1	4	0	277	203	480	12,613
**C A R**	**0**	**0**	**38**	**38**	**0**	**24**	**1,248**	**1,272**	**2**	**492**	**9,102**	**9,596**	**47,549**
Abra	0	0	24	24	0	4	85	89	0	55	832	887	3,296
Apayao	0	0	0	0	0	1	3	4	0	33	615	648	3,470
Benguet	0	0	0	0	0	9	253	262	0	190	2,001	2,191	15,769
Ifugao	0	0	4	4	0	7	604	611	0	77	2,008	2,085	8,446
Kalinga	0	0	10	10	0	0	90	90	1	51	2,160	2,212	6,646
Mt. Province	0	0	0	0	0	3	206	209	1	76	1,207	1,284	4,709
Baguio City	0	0	0	0	0	0	7	7	0	10	279	289	5,213
**Region 1**	**0**	**0**	**18**	**18**	**0**	**14**	**816**	**830**	**2**	**764**	**17,962**	**18,728**	**48,501**
Ilocos Norte	0	0	5	5	0	2	19	21	0	54	984	1,038	3,865
Ilocos Sur	0	0	8	8	0	5	353	358	2	45	1,087	1,134	3,617
La Union	0	0	5	5	0	1	140	141	0	183	3,464	3,647	9,241
Pangasinan	0	0	0	0	0	4	105	109	0	425	10,415	10,840	24,805
Alaminos City	0	0	0	0	0	0	0	0	0	0	20	20	305
Candon City	0	0	0	0	0	0	0	0	0	0	60	60	201
Dagupan City	0	0	0	0	0	0	0	0	0	0	3	3	149
Laoag City	0	0	0	0	0	0	0	0	0	0	0	0	291
San Carlos City	0	0	0	0	0	0	0	0	0	20	1,318	1,338	2,224
San Fernando City	0	0	0	0	0	2	149	151	0	3	229	232	2,641
Urdaneta City	0	0	0	0	0	0	0	0	0	23	239	262	907
Vigan City	0	0	0	0	0	0	50	50	0	11	143	154	255
**Region 2**	**0**	**2**	**52**	**54**	**0**	**40**	**121**	**161**	**14**	**948**	**14,319**	**15,281**	**60,978**
Batanes	0	0	0	0	0	0	7	7	0	6	209	215	534
Cagayan	0	0	3	3	0	0	3	3	2	153	3,195	3,350	12,958
Isabela	0	0	0	0	0	0	3	3	9	370	4,129	4,508	21,100
Nueva Vizcaya	0	2	49	51	0	40	106	146	2	286	2,919	3,207	15,742
Quirino	0	0	0	0	0	0	2	2	0	29	510	539	2,153
Cauayan City	0	0	0	0	0	0	0	0	1	45	821	867	2,265
Ilagan City	0	0	0	0	0	0	0	0	0	10	401	411	1,461
Santiago City	0	0	0	0	0	0	0	0	0	39	1,981	2,020	3,514
Tuguegarao City	0	0	0	0	0	0	0	0	0	10	154	164	1,251
**Region 3**	**0**	**0**	**47**	**47**	**0**	**10**	**570**	**580**	**1**	**1,420**	**18,899**	**20,320**	**100,067**
Aurora	0	0	0	0	0	0	5	5	0	98	1,713	1,811	5,139
Bataan	0	0	0	0	0	4	32	36	0	71	1,075	1,146	6,339

Table 1.A.2 - MODERN METHOD OF FAMILY PLANNING
Other Acceptors
Annual Philippines, 2020

Area	NFP-STM Age group			Total	NFP-SDM Age group			Total	NFP-LAM Age group			Total	Total Other Acceptors
	10-14	15-19	20-49		10-14	15-19	20-49		10-14	15-19	20-49		
Bulacan	0	0	0	0	0	0	132	132	0	267	3,421	3,688	17,023
Nueva Ecija	0	0	0	0	0	0	33	33	0	167	2,928	3,095	16,477
Pampanga	0	0	47	47	0	5	333	338	0	198	1,676	1,874	13,069
Tarlac	0	0	0	0	0	0	7	7	0	86	1,501	1,587	7,841
Zambales	0	0	0	0	0	0	16	16	1	45	1,201	1,247	7,477
Angeles City	0	0	0	0	0	0	0	0	0	10	94	104	1,032
Balanga City	0	0	0	0	0	0	3	3	0	16	356	372	1,134
Cabanatuan City	0	0	0	0	0	0	0	0	0	114	1,479	1,593	4,903
City of San Fernando	0	0	0	0	0	0	0	0	0	16	147	163	560
Gapan City	0	0	0	0	0	0	0	0	0	4	83	87	665
Mabalacat City	0	0	0	0	0	0	0	0	0	94	735	829	1,996
Malolos City	0	0	0	0	0	0	0	0	0	4	150	154	2,869
Meycauayan	0	0	0	0	0	1	9	10	0	22	276	298	938
Olongapo	0	0	0	0	0	0	0	0	0	20	356	376	1,952
Palayan City	0	0	0	0	0	0	0	0	0	4	49	53	323
San Jose City	0	0	0	0	0	0	0	0	0	69	786	855	1,604
San Jose del Monte City	0	0	0	0	0	0	0	0	0	0	0	0	3,630
Science City of Munoz	0	0	0	0	0	0	0	0	0	0	0	0	436
Tarlac City	0	0	0	0	0	0	0	0	0	115	873	988	4,660
**Region 4A**	**0**	**0**	**260**	**260**	**40**	**40**	**1,862**	**1,942**	**5,660**	**8,744**	**88,632**	**103,036**	**439,322**
Batangas	0	0	0	0	40	4	60	104	1,672	2,648	14,360	18,680	55,784
Cavite	0	0	244	244	0	12	940	952	0	204	7,252	7,456	45,992
Laguna	0	0	8	8	0	4	256	260	4	528	8,140	8,672	30,636
Quezon	0	0	8	8	0	0	572	572	8	924	18,080	19,012	120,616
Rizal	0	0	0	0	0	0	16	16	0	564	11,280	11,844	45,532
Antipolo City	0	0	0	0	0	0	0	0	0	0	0	0	0
Bacoor City	0	0	0	0	0	0	0	0	0	64	1,328	1,392	4,860
Batangas City	0	0	0	0	0	0	0	0	0	0	0	0	0
Biñan City	0	0	0	0	0	0	0	0	0	1,068	4,664	5,732	43,876
Cabuyao City	0	0	0	0	0	20	0	20	4	248	900	1,152	3,764
Calamba City	0	0	0	0	0	0	18	18	0	228	2,512	2,740	6,258
Cavite City	0	0	0	0	0	0	0	0	0	0	0	0	0
Dasmariñas City	0	0	0	0	0	0	0	0	0	388	3,896	4,284	11,736
General Trias City	0	0	0	0	0	0	0	0	0	44	2,016	2,060	12,956
Imus City	0	0	0	0	0	0	0	0	0	60	1,600	1,660	6,304
Lipa City	0	0	0	0	0	0	0	0	0	0	0	0	0
Lucena City	0	0	0	0	0	0	0	0	3,904	0	424	4,328	13,464
San Pablo City	0	0	0	0	0	0	0	0	0	0	0	0	0
San Pedro City	0	0	0	0	0	0	0	0	0	80	2,128	2,208	6,900
Santa Rosa City	0	0	0	0	0	0	0	0	0	1,672	2,148	3,820	13,432
Tagaytay City	0	0	0	0	0	0	0	0	68	8	3,064	3,140	6,612
Tanauan City	0	0	0	0	0	0	0	0	0	0	108	108	948
Tayabas City	0	0	0	0	0	0	0	0	0	16	4,712	4,728	8,348
Trece Martires City	0	0	0	0	0	0	0	0	0	0	20	20	1,304
**Region 4B**	**0**	**0**	**0**	**0**	**0**	**0**	**113**	**113**	**14**	**677**	**11,105**	**11,796**	**38,038**
Marinduque	0	0	0	0	0	0	3	3	1	3	244	248	879
Mindoro Occidental	0	0	0	0	0	0	19	19	13	509	5,245	5,767	12,254
Mindoro Oriental	0	0	0	0	0	0	29	29	0	67	3,149	3,216	10,020
Palawan	0	0	0	0	0	0	40	40	0	98	1,779	1,877	9,030
Romblon	0	0	0	0	0	0	0	0	0	0	42	42	57
Puerto Princesa City	0	0	0	0	0	0	22	22	0	0	646	646	5,798
**Region 5**	**0**	**6**	**160**	**166**	**1**	**300**	**7,041**	**7,342**	**6**	**2,040**	**34,390**	**36,436**	**100,715**
Albay	0	0	2	2	1	3	376	380	1	112	2,857	2,970	9,910
Camarines Norte	0	0	0	0	0	32	588	620	1	278	3,781	4,060	12,190
Camarines Sur	0	6	132	138	0	54	1,800	1,854	2	379	11,651	12,032	30,598
Catanduanes	0	0	4	4	0	78	362	440	0	578	1,925	2,503	6,854
Masbate	0	0	5	5	0	93	1,922	2,015	2	329	5,256	5,587	15,626
Sorsogon	0	0	2	2	0	21	977	998	0	274	7,841	8,115	20,122
Iriga City	0	0	13	13	0	19	992	1,011	0	90	814	904	3,522

Table 1.A.2 - MODERN METHOD OF FAMILY PLANNING
Other Acceptors
Annual Philippines, 2020

Area	NFP-STM			Total	NFP-SDM			Total	NFP-LAM			Total	Total Other Acceptors
	Age group				Age group				Age group				
	10-14	15-19	20-49		10-14	15-19	20-49		10-14	15-19	20-49		
Legaspi City	0	0	2	2	0	0	1	1	0	0	14	14	201
Naga City	0	0	0	0	0	0	23	23	0	0	251	251	1,692
**Region 6**	**0**	**1**	**20**	**21**	**2**	**33**	**691**	**726**	**3**	**906**	**19,439**	**20,348**	**67,835**
Aklan	0	0	0	0	0	0	20	20	1	105	1,697	1,803	4,980
Antique	0	0	10	10	0	0	142	142	1	85	1,937	2,023	11,758
Capiz	0	0	0	0	0	0	1	1	0	12	672	684	3,257
Guimaras	0	0	0	0	0	0	9	9	0	31	1,142	1,173	2,340
Iloilo	0	0	9	9	2	15	345	362	0	206	3,747	3,953	12,389
Negros Occidental	0	1	1	2	0	18	148	166	1	402	9,403	9,806	26,274
Bacolod City	0	0	0	0	0	0	0	0	0	38	648	686	2,368
Iloilo City	0	0	0	0	0	0	26	26	0	27	193	220	4,469
**Region 7**	**0**	**0**	**0**	**0**	**0**	**3**	**186**	**189**	**2**	**849**	**11,791**	**12,642**	**39,628**
Bohol	0	0	0	0	0	0	42	42	0	215	1,205	1,420	6,240
Cebu	0	0	0	0	0	0	0	0	0	72	2,028	2,100	9,684
Negros Oriental	0	0	0	0	0	1	11	12	2	302	3,761	4,065	10,402
Siquijor	0	0	0	0	0	2	133	135	0	19	357	376	2,101
Cebu City	0	0	0	0	0	0	0	0	0	110	2,577	2,687	6,873
Lapu-Lapu City	0	0	0	0	0	0	0	0	0	131	1,766	1,897	3,835
Mandaue City	0	0	0	0	0	0	0	0	0	0	97	97	493
**Region 8**	**0**	**0**	**0**	**0**	**0**	**0**	**0**	**0**	**0**	**0**	**0**	**0**	**1,667**
Biliran	0	0	0	0	0	0	0	0	0	0	0	0	300
Eastern Samar	0	0	0	0	0	0	0	0	0	0	0	0	84
Northern Leyte	0	0	0	0	0	0	0	0	0	0	0	0	0
Northern Samar	0	0	0	0	0	0	0	0	0	0	0	0	59
Southern Leyte	0	0	0	0	0	0	0	0	0	0	0	0	257
Western Samar	0	0	0	0	0	0	0	0	0	0	0	0	277
Calbayog City	0	0	0	0	0	0	0	0	0	0	0	0	190
Maasin City	0	0	0	0	0	0	0	0	0	0	0	0	39
Ormoc City	0	0	0	0	0	0	0	0	0	0	0	0	338
Tacloban City	0	0	0	0	0	0	0	0	0	0	0	0	123
**Region 9**	**0**	**0**	**0**	**0**	**0**	**0**	**1**	**1**	**0**	**22**	**1,754**	**1,776**	**5,200**
Zamboanga del Norte	0	0	0	0	0	0	0	0	0	0	0	0	0
Zamboanga del Sur	0	0	0	0	0	0	0	0	0	0	0	0	0
Zamboanga Sibugay	0	0	0	0	0	0	0	0	0	0	0	0	0
Dapitan City	0	0	0	0	0	0	0	0	0	10	178	188	539
Dipolog City	0	0	0	0	0	0	0	0	0	0	0	0	1,119
Isabela City	0	0	0	0	0	0	1	1	0	5	146	151	562
Pagadian City	0	0	0	0	0	0	0	0	0	4	293	297	742
Zamboanga City	0	0	0	0	0	0	0	0	0	3	1,137	1,140	2,238
**Region 10**	**0**	**1**	**29**	**30**	**3**	**155**	**2,203**	**2,361**	**17**	**1,588**	**20,176**	**21,781**	**76,829**
Bukidnon	0	0	14	14	1	34	478	513	3	521	5,559	6,083	26,305
Camiguin	0	0	0	0	0	0	6	6	0	15	595	610	1,208
Lanao del Norte	0	0	4	4	0	0	32	32	3	101	2,124	2,228	10,854
Misamis Occidental	0	0	0	0	0	3	51	54	0	26	734	760	2,010
Misamis Oriental	0	0	0	0	0	3	220	223	3	97	2,215	2,315	7,364
Cagayan de Oro City	0	0	0	0	0	0	1	1	2	140	1,464	1,606	5,221
El Salvador City	0	0	0	0	0	0	2	2	0	24	386	410	1,088
Gingoog City	0	1	10	11	2	65	450	517	0	95	884	979	4,727
Iligan City	0	0	1	1	0	20	257	277	3	96	2,044	2,143	5,072
Malaybalay City	0	0	0	0	0	19	447	466	2	110	1,623	1,735	6,153
Oroquieta City	0	0	0	0	0	0	9	9	0	2	114	116	274
Ozamis City	0	0	0	0	0	2	137	139	0	68	738	806	1,466
Tangub City	0	0	0	0	0	8	0	8	0	193	154	347	562
Valencia City	0	0	0	0	0	1	113	114	1	100	1,542	1,643	4,525

Table 1.A.2 - MODERN METHOD OF FAMILY PLANNING
Other Acceptors
Annual Philippines, 2020

Area	NFP-STM			Total	NFP-SDM			Total	NFP-LAM			Total	Total Other Acceptors
	Age group				Age group				Age group				
	10-14	15-19	20-49		10-14	15-19	20-49		10-14	15-19	20-49		
**Region 11**	**0**	**3**	**21**	**24**	**0**	**20**	**512**	**532**	**64**	**1,055**	**9,552**	**10,671**	**53,687**
Davao de Oro	0	0	0	0	0	1	92	93	1	31	577	609	8,764
Davao del Norte	0	0	0	0	0	0	70	70	39	187	2,055	2,281	11,168
Davao Oriental	0	0	3	3	0	10	221	231	10	237	3,280	3,527	9,169
Davao del Sur	0	0	0	0	0	0	48	48	0	33	261	294	5,578
Davao Occidental	0	0	0	0	0	8	72	80	3	74	651	728	6,190
Davao City	0	3	18	21	0	1	9	10	11	493	2,728	3,232	12,818
**Region 12**	**0**	**1**	**34**	**35**	**0**	**4**	**201**	**205**	**19**	**2,412**	**21,269**	**23,700**	**81,888**
North Cotabato	0	1	32	33	0	0	74	74	0	219	3,205	3,424	16,587
Sarangani	0	0	1	1	0	1	24	25	0	463	4,845	5,308	15,932
South Cotabato	0	0	0	0	0	3	88	91	19	1,210	5,368	6,597	17,458
Sultan Kudarat	0	0	1	1	0	0	7	7	0	396	6,272	6,668	21,457
				0				0				0	
Cotabato City	0	0	0	0	0	0	8	8	0	80	917	997	7,337
Gen. Santos City	0	0	0	0	0	0	0	0	0	44	662	706	3,117
**B.A.R.M.M.**	**0**	**0**	**4**	**4**	**0**	**7**	**15**	**22**	**0**	**4,495**	**13,819**	**18,314**	**38,089**
Basilan	0	0	0	0	0	0	0	0	0	34	1,017	1,051	3,167
Lanao del Sur	0	0	4	4	0	0	11	11	0	142	4,739	4,881	8,213
Maguindanao	0	0	0	0	0	0	1	1	0	474	6,697	7,171	14,103
Sulu	0	0	0	0	0	0	0	0	0	1,538	0	1,538	5,363
Tawi-Tawi	0	0	0	0	0	7	0	7	0	2,281	661	2,942	5,945
				0				0				0	
Lamitan City	0	0	0	0	0	0	0	0	0	4	54	58	358
Marawi City	0	0	0	0	0	0	3	3	0	22	651	673	940
**CARAGA**	**0**	**3**	**44**	**47**	**0**	**97**	**1,594**	**1,691**	**9**	**1,514**	**14,959**	**16,482**	**60,621**
Agusan del Norte	0	0	0	0	0	0	69	69	0	160	1,665	1,825	7,703
Agusan del Sur	0	2	2	4	0	78	888	966	4	717	4,639	5,360	21,985
Surigao del Norte	0	0	0	0	0	6	245	251	1	36	868	905	4,816
Surigao del Sur	0	0	42	42	0	11	144	155	2	330	3,090	3,422	11,502
Province of Dinagat	0	0	0	0	0	2	48	50	0	48	266	314	1,981
Bislig City	0	0	0	0	0	0	185	185	1	50	1,124	1,175	3,531
Butuan City	0	0	0	0	0	0	0	0	1	149	3,141	3,291	8,148
Surigao City	0	1	0	1	0	0	15	15	0	24	166	190	955

Table 1.A.3 - MODERN METHOD OF FAMILY PLANNING
Drop Outs
Annual Philippines, 2020

Area	Total Current Users	FSTR/BTL Age group 10-14	15-19	20-49	Total	MSTR/NSV Age group 10-14	15-19	20-49	Total
**PHILIPPINES**	**7,115,114**	**199**	**2,195**	**92,442**	**94,836**	**0**	**12**	**2,556**	**2,568**
**N C R**	**786,292**	**0**	**147**	**9,896**	**10,043**	**0**	**1**	**200**	**201**
Malabon	31,429	0	0	73	73	0	0	0	0
Navotas	22,416	0	0	67	67	0	0	0	0
Valenzuela City	60,865	0	0	4,066	4,066	0	0	59	59
Caloocan City	65,508	0	4	177	181	0	0	3	3
Marikina City	18,917	0	0	393	393	0	0	4	4
Pasig City	33,855	0	0	70	70	0	0	0	0
Pateros	2,995	0	6	70	76	0	0	2	2
Taguig	57,595	0	46	449	495	0	0	0	0
Quezon City	271,881	0	0	1,527	1,527	0	0	22	22
Makati City	8,979	0	0	1	1	0	0	0	0
Mandaluyong City	16,390	0	0	91	91	0	0	0	0
San Juan	5,866	0	0	1	1	0	0	0	0
Manila City	68,205	0	0	2,156	2,156	0	1	102	103
Las Piñas City	20,867	0	0	145	145	0	0	0	0
Muntinlupa City	33,645	0	0	5	5	0	0	0	0
Parañaque City	30,062	0	1	174	175	0	0	3	3
Pasay City	36,817	0	90	431	521	0	0	5	5
**C A R**	**120,779**	**0**	**1**	**2,150**	**2,151**	**0**	**0**	**31**	**31**
Abra	16,924	0	0	539	539	0	0	4	4
Apayao	11,643	0	0	121	121	0	0	1	1
Benguet	30,440	0	0	406	406	0	0	13	13
Ifugao	17,978	0	0	416	416	0	0	12	12
Kalinga	17,779	0	1	234	235	0	0	0	0
Mt. Province	11,955	0	0	280	280	0	0	1	1
Baguio City	14,060	0	0	154	154	0	0	0	0
**Region 1**	**460,308**	**0**	**2**	**5,566**	**5,568**	**0**	**0**	**34**	**34**
Ilocos Norte	41,436	0	0	991	991	0	0	0	0
Ilocos Sur	59,502	0	1	1,386	1,387	0	0	2	2
La Union	58,813	0	0	662	662	0	0	1	1
Pangasinan	226,567	0	1	1,777	1,778	0	0	9	9
Alaminos City	9,446	0	0	9	9	0	0	0	0
Candon City	7,299	0	0	8	8	0	0	0	0
Dagupan City	7,429	0	0	21	21	0	0	0	0
Laoag City	8,324	0	0	49	49	0	0	0	0
San Carlos City	11,747	0	0	135	135	0	0	0	0
San Fernando City	12,865	0	0	292	292	0	0	14	14
Urdaneta City	11,939	0	0	222	222	0	0	8	8
Vigan City	4,941	0	0	14	14	0	0	0	0
**Region 2**	**307,177**	**0**	**62**	**4,381**	**4,443**	**0**	**0**	**69**	**69**
Batanes	864	0	0	15	15	0	0	0	0
Cagayan	83,752	0	5	948	953	0	0	14	14
Isabela	105,390	0	44	1,703	1,747	0	0	11	11
Nueva Vizcaya	44,895	0	3	1,083	1,086	0	0	43	43
Quirino	18,556	0	0	151	151	0	0	0	0
Cauayan City	10,617	0	10	127	137	0	0	0	0
Ilagan City	14,066	0	0	70	70	0	0	0	0
Santiago City	15,258	0	0	194	194	0	0	0	0
Tuguegarao City	13,779	0	0	90	90	0	0	1	1
**Region 3**	**747,169**	**0**	**187**	**12,792**	**12,979**	**0**	**1**	**505**	**506**
Aurora	20,957	0	4	166	170	0	0	1	1
Bataan	44,561	0	28	1,264	1,292	0	0	11	11

Table 1.A.3 - MODERN METHOD OF FAMILY PLANNING
Drop Outs
Annual Philippines, 2020

Area	Total Current Users	FSTR/BTL Age group			Total	MSTR/NSV Age group			Total
		10-14	15-19	20-49		10-14	15-19	20-49	
Bulacan	138,968	0	108	1,465	1,573	0	1	386	387
Nueva Ecija	118,251	0	23	2,597	2,620	0	0	3	3
Pampanga	97,407	0	3	2,492	2,495	0	0	82	82
Tarlac	78,705	0	0	580	580	0	0	11	11
Zambales	37,319	0	2	337	339	0	0	2	2
Angeles City	21,284	0	0	135	135	0	0	0	0
Balanga City	4,693	0	0	33	33	0	0	0	0
Cabanatuan City	16,403	0	0	634	634	0	0	0	0
City of San Fernando	7,904	0	0	136	136	0	0	2	2
Gapan City	7,770	0	18	281	299	0	0	0	0
Mabalacat City	21,281	0	0	29	29	0	0	0	0
Malolos City	6,208	0	0	279	279	0	0	4	4
Meycauayan	8,403	0	0	26	26	0	0	0	0
Olongapo	9,362	0	0	24	24	0	0	0	0
Palayan City	3,344	0	0	126	126	0	0	0	0
San Jose City	12,893	0	0	8	8	0	0	0	0
San Jose del Monte City	72,493	0	1	2,044	2,045	0	0	3	3
Science City of Munoz	6,409	0	0	91	91	0	0	0	0
Tarlac City	12,554	0	0	45	45	0	0	0	0
**Region 4A**	**844,565**	**172**	**144**	**22,593**	**22,909**	**0**	**0**	**116**	**116**
Batangas	158,232	136	0	6,836	6,972	0	0	0	0
Cavite	83,830	0	0	1,352	1,352	0	0	12	12
Laguna	65,529	8	40	3,272	3,320	0	0	32	32
Quezon	106,906	0	16	4,932	4,948	0	0	32	32
Rizal	103,690	0	44	932	976	0	0	20	20
Antipolo City	37,890	0	0	0	0	0	0	0	0
Bacoor City	11,464	0	0	65	65	0	0	4	4
Batangas City	17,607	0	0	0	0	0	0	0	0
Biñan City	38,387	0	0	24	24	0	0	8	8
Cabuyao City	13,927	4	0	340	344	0	0	0	0
Calamba City	35,102	0	40	1,144	1,184	0	0	0	0
Cavite City	4,572	0	0	0	0	0	0	0	0
Dasmariñas City	42,608	0	0	2,072	2,072	0	0	4	4
General Trias City	11,506	4	4	244	252	0	0	0	0
Imus City	9,106	0	0	100	100	0	0	0	0
Lipa City	13,662	0	0	0	0	0	0	0	0
Lucena City	6,402	20	0	124	144	0	0	0	0
San Pablo City	10,199	0	0	0	0	0	0	0	0
San Pedro City	15,209	0	0	4	4	0	0	4	4
Santa Rosa City	33,075	0	0	212	212	0	0	0	0
Tagaytay City	7,989	0	0	96	96	0	0	0	0
Tanauan City	7,401	0	0	240	240	0	0	0	0
Tayabas City	5,399	0	0	324	324	0	0	0	0
Trece Martires City	4,872	0	0	280	280	0	0	0	0
**Region 4B**	**144,179**	**0**	**0**	**1,082**	**1,082**	**0**	**0**	**14**	**14**
Marinduque	14,040	0	0	363	363	0	0	2	2
Mindoro Occidental	32,450	0	0	187	187	0	0	5	5
Mindoro Oriental	58,295	0	0	200	200	0	0	6	6
Palawan	6,466	0	0	139	139	0	0	0	0
Romblon	4,160	0	0	24	24	0	0	0	0
Puerto Princesa City	28,768	0	0	169	169	0	0	1	1
**Region 5**	**397,155**	**0**	**24**	**2,206**	**2,230**	**0**	**0**	**58**	**58**
Albay	75,300	0	13	207	220	0	0	2	2
Camarines Norte	26,377	0	4	139	143	0	0	5	5
Camarines Sur	100,599	0	4	506	510	0	0	7	7
Catanduanes	24,369	0	0	255	255	0	0	32	32
Masbate	57,012	0	0	150	150	0	0	5	5
Sorsogon	60,469	0	2	841	843	0	0	3	3
Iriga City	9,974	0	0	39	39	0	0	2	2

Table 1.A.3 - MODERN METHOD OF FAMILY PLANNING
Drop Outs
Annual Philippines, 2020

Area	Total Current Users	FSTR/BTL Age group 10-14	15-19	20-49	Total	MSTR/NSV Age group 10-14	15-19	20-49	Total
Legaspi City	22,994	0	1	12	13	0	0	0	0
Naga City	20,061	0	0	57	57	0	0	2	2
**Region 6**	**580,884**	**1**	**224**	**4,221**	**4,446**	**0**	**4**	**282**	**286**
Aklan	41,264	0	17	240	257	0	0	41	41
Antique	47,572	0	49	1,282	1,331	0	0	48	48
Capiz	75,849	0	2	250	252	0	0	18	18
Guimaras	15,286	0	0	95	95	0	1	1	2
Iloilo	149,460	1	22	963	986	0	2	24	26
Negros Occidental	182,369	0	134	1,006	1,140	0	0	130	130
Bacolod City	25,422	0	0	170	170	0	0	19	19
Iloilo City	43,662	0	0	215	215	0	1	1	2
**Region 7**	**294,028**	**0**	**0**	**2,395**	**2,395**	**0**	**0**	**183**	**183**
Bohol	67,760	0	0	1,181	1,181	0	0	36	36
Cebu	71,864	0	0	252	252	0	0	14	14
Negros Oriental	58,940	0	0	274	274	0	0	90	90
Siquijor	8,043	0	0	102	102	0	0	1	1
Cebu City	29,645	0	0	178	178	0	0	17	17
Lapu-Lapu City	39,675	0	0	408	408	0	0	25	25
Mandaue City	18,101	0	0	0	0	0	0	0	0
**Region 8**	**202,207**	**0**	**0**	**878**	**878**	**0**	**2**	**15**	**17**
Biliran	10,480	0	0	76	76	0	0	4	4
Eastern Samar	41,040	0	0	91	91	0	0	3	3
Northern Leyte	0	0	0	0	0	0	0	0	0
Northern Samar	34,606	0	0	39	39	0	0	0	0
Southern Leyte	24,278	0	0	165	165	0	2	3	5
Western Samar	16,033	0	0	123	123	0	0	0	0
Calbayog City	8,908	0	0	127	127	0	0	0	0
Maasin City	2,705	0	0	97	97	0	0	1	1
Ormoc City	19,481	0	0	132	132	0	0	2	2
Tacloban City	44,676	0	0	28	28	0	0	2	2
**Region 9**	**271,392**	**0**	**6**	**589**	**595**	**0**	**0**	**0**	**0**
Zamboanga del Norte	86,000	0	0	0	0	0	0	0	0
Zamboanga del Sur	57,156	0	0	0	0	0	0	0	0
Zamboanga Sibugay	24,011	0	0	0	0	0	0	0	0
Dapitan City	7,405	0	0	44	44	0	0	0	0
Dipolog City	15,009	0	6	45	51	0	0	0	0
Isabela City	8,507	0	0	2	2	0	0	0	0
Pagadian City	17,446	0	0	33	33	0	0	0	0
Zamboanga City	55,858	0	0	465	465	0	0	0	0
**Region 10**	**518,887**	**0**	**5**	**3,771**	**3,776**	**0**	**3**	**224**	**227**
Bukidnon	111,273	0	0	2,240	2,240	0	3	36	39
Camiguin	6,349	0	0	34	34	0	0	0	0
Lanao del Norte	47,747	0	0	241	241	0	0	0	0
Misamis Occidental	24,432	0	0	76	76	0	0	0	0
Misamis Oriental	81,061	0	4	455	459	0	0	43	43
Cagayan de Oro City	140,501	0	0	182	182	0	0	66	66
El Salvador City	6,230	0	0	37	37	0	0	0	0
Gingoog City	10,166	0	0	17	17	0	0	51	51
Iligan City	27,571	0	1	228	229	0	0	1	1
Malaybalay City	19,999	0	0	108	108	0	0	14	14
Oroquieta City	6,341	0	0	4	4	0	0	0	0
Ozamis City	12,025	0	0	43	43	0	0	2	2
Tangub City	4,874	0	0	0	0	0	0	0	0
Valencia City	20,318	0	0	106	106	0	0	11	11

Table 1.A.3 - MODERN METHOD OF FAMILY PLANNING

Drop Outs

Annual Philippines, 2020

Area	Total Current Users	FSTR/BTL			Total	MSTR/NSV			Total
		Age group				Age group			
		10-14	15-19	20-49		10-14	15-19	20-49	
**Region 11**	**483,912**	**1**	**7**	**4,273**	**4,281**	**0**	**0**	**189**	**189**
Davao de Oro	81,390	0	0	594	594	0	0	34	34
Davao del Norte	99,809	0	0	1,338	1,338	0	0	12	12
Davao Oriental	48,937	0	0	309	309	0	0	19	19
Davao del Sur	63,208	0	0	464	464	0	0	8	8
Davao Occidental	31,727	0	7	426	433	0	0	21	21
Davao City	158,841	1	0	1,142	1,143	0	0	95	95
**Region 12**	**467,365**	**0**	**41**	**2,363**	**2,404**	**0**	**1**	**69**	**70**
North Cotabato	141,819	0	17	596	613	0	0	15	15
Sarangani	62,222	0	10	354	364	0	1	8	9
South Cotabato	95,537	0	11	542	553	0	0	41	41
Sultan Kudarat	88,018	0	3	689	692	0	0	5	5
					0				0
Cotabato City	30,729	0	0	174	174	0	0	0	0
Gen. Santos City	49,040	0	0	8	8	0	0	0	0
**B.A.R.M.M.**	**239,770**	**25**	**1,341**	**10,122**	**11,488**	**0**	**0**	**498**	**498**
Basilan	6,677	0	0	138	138	0	0	0	0
Lanao del Sur	66,116	0	0	92	92	0	0	0	0
Maguindanao	84,835	0	25	532	557	0	0	0	0
Sulu	49,455	0	12	0	12	0	0	0	0
Tawi-Tawi	27,758	0	30	1	31	0	0	0	0
					0				0
Lamitan City	0	25	1,274	9,346	10,645	0	0	498	498
Marawi City	4,929	0	0	13	13	0	0	0	0
**CARAGA**	**249,045**	**0**	**4**	**3,164**	**3,168**	**0**	**0**	**69**	**69**
Agusan del Norte	38,948	0	0	558	558	0	0	6	6
Agusan del Sur	80,275	0	0	1,278	1,278	0	0	26	26
Surigao del Norte	30,203	0	2	321	323	0	0	3	3
Surigao del Sur	39,690	0	2	267	269	0	0	13	13
Province of Dinagat	9,699	0	0	56	56	0	0	10	10
Bislig City	8,261	0	0	46	46	0	0	0	0
Butuan City	28,396	0	0	43	43	0	0	0	0
Surigao City	13,573	0	0	595	595	0	0	11	11

Table 1.A.3 - MODERN METHOD OF FAMILY PLANNING
Drop Outs
Annual Philippines, 2020

Area	CONDOM			Total	IUD-INTERVAL			Total	IUD-POSTPARTUM			Total
	Age group				Age group				Age group			
	10-14	15-19	20-49		10-14	15-19	20-49		10-14	15-19	20-49	
**PHILIPPINES**	**378**	**7,390**	**119,488**	**127,256**	**143**	**1,720**	**64,793**	**66,656**	**1,837**	**1,009**	**19,449**	**22,295**
**N C R**	**16**	**1,553**	**43,451**	**45,020**	**7**	**251**	**9,173**	**9,431**	**1**	**259**	**6,468**	**6,728**
Malabon	0	23	532	555	0	3	62	65	0	2	32	34
Navotas	0	121	400	521	0	2	93	95	0	0	11	11
Valenzuela City	0	9	1,834	1,843	0	0	343	343	0	3	671	674
Caloocan City	0	40	839	879	0	1	141	142	0	64	830	894
Marikina City	0	2	521	523	0	5	259	264	0	3	595	598
Pasig City	0	38	359	397	0	3	136	139	0	92	622	714
Pateros	0	8	28	36	0	9	43	52	0	0	0	0
Taguig	0	26	538	564	0	36	572	608	0	0	18	18
Quezon City	0	350	27,217	27,567	0	23	3,776	3,799	1	85	2,283	2,369
Makati City	0	10	113	123	0	0	13	13	0	0	0	0
Mandaluyong City	0	0	60	60	0	0	20	20	0	5	0	5
San Juan	0	0	23	23	0	0	20	20	0	0	0	0
Manila City	14	371	8,017	8,402	2	24	344	370	0	4	85	89
Las Piñas City	0	20	372	392	0	0	32	32	0	0	0	0
Muntinlupa City	0	5	196	201	0	0	201	201	0	0	0	0
Parañaque City	0	29	354	383	0	19	1,052	1,071	0	1	5	6
Pasay City	2	501	2,048	2,551	5	126	2,066	2,197	0	0	1,316	1,316
**C A R**	**1**	**63**	**1,566**	**1,630**	**0**	**32**	**717**	**749**	**0**	**5**	**119**	**124**
Abra	0	6	106	112	0	0	15	15	0	0	9	9
Apayao	0	8	72	80	0	0	87	87	0	3	33	36
Benguet	1	15	611	627	0	8	301	309	0	1	26	27
Ifugao	0	5	239	244	0	1	56	57	0	0	27	27
Kalinga	0	7	140	147	0	3	90	93	0	1	10	11
Mt. Province	0	8	174	182	0	5	76	81	0	0	1	1
Baguio City	0	14	224	238	0	15	92	107	0	0	13	13
**Region 1**	**0**	**86**	**2,485**	**2,571**	**0**	**49**	**1,239**	**1,288**	**0**	**10**	**142**	**152**
Ilocos Norte	0	1	178	179	0	0	108	108	0	2	28	30
Ilocos Sur	0	32	311	343	0	1	173	174	0	0	33	33
La Union	0	3	280	283	0	12	152	164	0	5	33	38
Pangasinan	0	27	1,160	1,187	0	30	566	596	0	0	0	0
Alaminos City	0	0	22	22	0	0	4	4	0	0	0	0
Candon City	0	0	149	149	0	0	139	139	0	0	0	0
Dagupan City	0	1	62	63	0	0	6	6	0	3	6	9
Laoag City	0	0	28	28	0	0	0	0	0	0	0	0
San Carlos City	0	2	39	41	0	6	49	55	0	0	0	0
San Fernando City	0	4	223	227	0	0	41	41	0	0	42	42
Urdaneta City	0	16	32	48	0	0	1	1	0	0	0	0
Vigan City	0	0	1	1	0	0	0	0	0	0	0	0
**Region 2**	**39**	**290**	**1,257**	**1,586**	**0**	**128**	**2,692**	**2,820**	**1**	**82**	**1,437**	**1,520**
Batanes	0	2	11	13	0	1	3	4	0	0	0	0
Cagayan	0	4	202	206	0	61	920	981	0	41	192	233
Isabela	6	26	562	594	0	52	765	817	1	31	80	112
Nueva Vizcaya	33	255	263	551	0	1	290	291	0	0	1,059	1,059
Quirino	0	2	55	57	0	0	106	106	0	0	5	5
Cauayan City	0	1	114	115	0	0	143	143	0	1	8	9
Ilagan City	0	0	14	14	0	0	40	40	0	0	0	0
Santiago City	0	0	31	31	0	2	8	10	0	0	0	0
Tuguegarao City	0	0	5	5	0	11	417	428	0	9	93	102
**Region 3**	**4**	**935**	**10,341**	**11,280**	**8**	**123**	**2,656**	**2,787**	**0**	**76**	**908**	**984**
Aurora	0	2	177	179	0	7	89	96	0	2	23	25
Bataan	0	58	407	465	0	14	251	265	0	1	81	82

Table 1.A.3 - MODERN METHOD OF FAMILY PLANNING

Drop Outs

Annual Philippines, 2020

Area	CONDOM			Total	IUD-INTERVAL			Total	IUD-POSTPARTUM			Total
	Age group				Age group				Age group			
	10-14	15-19	20-49		10-14	15-19	20-49		10-14	15-19	20-49	
Bulacan	0	82	659	741	0	21	377	398	0	7	134	141
Nueva Ecija	0	13	675	688	0	3	342	345	0	7	53	60
Pampanga	0	42	1,575	1,617	0	8	383	391	0	59	450	509
Tarlac	0	86	550	636	0	2	272	274	0	0	10	10
Zambales	0	10	207	217	0	1	186	187	0	0	10	10
Angeles City	0	7	59	66	0	1	2	3	0	0	119	119
Balanga City	0	0	5	5	0	0	1	1	0	0	0	0
Cabanatuan City	4	27	160	191	8	13	21	42	0	0	3	3
City of San Fernando	0	5	101	106	0	0	0	0	0	0	0	0
Gapan City	0	0	1	1	0	5	5	10	0	0	11	11
Mabalacat City	0	10	264	274	0	0	32	32	0	0	0	0
Malolos City	0	3	104	107	0	0	86	86	0	0	0	0
Meycauayan	0	16	164	180	0	0	13	13	0	0	5	5
Olongapo	0	3	78	81	0	0	0	0	0	0	4	4
Palayan City	0	1	14	15	0	11	81	92	0	0	0	0
San Jose City	0	0	27	27	0	0	1	1	0	0	1	1
San Jose del Monte City	0	562	5,090	5,652	0	37	496	533	0	0	0	0
Science City of Munoz	0	0	7	7	0	0	4	4	0	0	0	0
Tarlac City	0	8	17	25	0	0	14	14	0	0	4	4
**Region 4A**	**251**	**2,847**	**30,628**	**33,726**	**112**	**292**	**10,692**	**11,096**	**1,824**	**140**	**4,444**	**6,408**
Batangas	126	61	3,753	3,940	100	4	968	1,072	0	9	387	396
Cavite	0	13	1,098	1,111	0	16	576	592	0	20	32	52
Laguna	0	123	2,075	2,198	12	68	1,700	1,780	0	68	3,036	3,104
Quezon	1	43	4,712	4,756	0	136	5,608	5,744	0	8	568	576
Rizal	4	440	1,888	2,332	0	44	632	676	4	28	220	252
Antipolo City	0	0	0	0	0	0	0	0	0	0	0	0
Bacoor City	0	3	393	396	0	4	100	104	0	0	0	0
Batangas City	0	0	0	0	0	0	0	0	0	0	0	0
Biñan City	0	1,072	6,630	7,702	0	0	140	140	0	0	8	8
Cabuyao City	0	12	852	864	0	8	104	112	0	0	12	12
Calamba City	0	4	476	480	0	0	244	244	0	0	4	4
Cavite City	0	0	0	0	0	0	0	0	0	0	0	0
Dasmariñas City	0	64	3,644	3,708	0	12	100	112	0	0	24	24
General Trias City	0	4	500	504	0	0	12	12	0	4	12	16
Imus City	0	0	259	259	0	0	24	24	0	0	0	0
Lipa City	0	0	0	0	0	0	0	0	0	0	0	0
Lucena City	100	0	64	164	0	0	64	64	1,820	0	0	1,820
San Pablo City	0	0	0	0	0	0	0	0	0	0	0	0
San Pedro City	0	0	84	84	0	0	20	20	0	0	0	0
Santa Rosa City	20	1,004	3,428	4,452	0	0	72	72	0	0	0	0
Tagaytay City	0	0	12	12	0	0	0	0	0	0	12	12
Tanauan City	0	0	311	311	0	0	44	44	0	3	129	132
Tayabas City	0	0	313	313	0	0	64	64	0	0	0	0
Trece Martires City	0	4	136	140	0	0	220	220	0	0	0	0
**Region 4B**	**1**	**4**	**754**	**759**	**0**	**2**	**442**	**444**	**0**	**3**	**63**	**66**
Marinduque	0	0	109	109	0	0	85	85	0	0	0	0
Mindoro Occidental	1	3	209	213	0	1	106	107	0	0	4	4
Mindoro Oriental	0	0	213	213	0	1	242	243	0	3	21	24
Palawan	0	1	44	45	0	0	6	6	0	0	2	2
Romblon	0	0	2	2	0	0	3	3	0	0	2	2
Puerto Princesa City	0	0	177	177	0	0	0	0	0	0	34	34
**Region 5**	**1**	**205**	**4,038**	**4,244**	**0**	**19**	**740**	**759**	**0**	**5**	**142**	**147**
Albay	0	73	606	679	0	3	71	74	0	0	27	27
Camarines Norte	0	41	382	423	0	6	181	187	0	0	12	12
Camarines Sur	0	45	1,418	1,463	0	5	178	183	0	5	47	52
Catanduanes	0	8	176	184	0	0	35	35	0	0	12	12
Masbate	1	17	361	379	0	2	139	141	0	0	15	15
Sorsogon	0	20	629	649	0	3	107	110	0	0	22	22
Iriga City	0	0	61	61	0	0	17	17	0	0	4	4

Table 1.A.3 - MODERN METHOD OF FAMILY PLANNING
Drop Outs
Annual Philippines, 2020

Area	CONDOM			Total	IUD-INTERVAL			Total	IUD-POSTPARTUM			Total
	Age group				Age group				Age group			
	10-14	15-19	20-49		10-14	15-19	20-49		10-14	15-19	20-49	
Legaspi City	0	1	55	56	0	0	0	0	0	0	1	1
Naga City	0	0	350	350	0	0	12	12	0	0	2	2
**Region 6**	**3**	**184**	**3,994**	**4,181**	**2**	**95**	**4,661**	**4,758**	**0**	**54**	**928**	**982**
Aklan	0	37	517	554	0	3	68	71	0	0	11	11
Antique	1	23	753	777	0	4	186	190	0	0	67	67
Capiz	0	2	256	258	0	7	318	325	0	1	13	14
Guimaras	0	11	184	195	0	1	38	39	0	1	6	7
Iloilo	2	10	598	610	2	14	2,782	2,798	0	4	135	139
Negros Occidental	0	63	1,203	1,266	0	43	1,017	1,060	0	45	544	589
Bacolod City	0	7	133	140	0	1	185	186	0	0	141	141
Iloilo City	0	31	350	381	0	22	67	89	0	3	11	14
**Region 7**	**0**	**88**	**3,950**	**4,038**	**0**	**213**	**3,662**	**3,875**	**0**	**65**	**928**	**993**
Bohol	0	0	676	676	0	177	1,490	1,667	0	52	342	394
Cebu	0	22	688	710	0	5	743	748	0	0	473	473
Negros Oriental	0	17	428	445	0	13	695	708	0	5	33	38
Siquijor	0	2	148	150	0	0	85	85	0	0	2	2
Cebu City	0	14	384	398	0	18	392	410	0	8	43	51
Lapu-Lapu City	0	33	1,612	1,645	0	0	257	257	0	0	35	35
Mandaue City	0	0	14	14	0	0	0	0	0	0	0	0
**Region 8**	**4**	**185**	**1,393**	**1,582**	**0**	**0**	**0**	**0**	**0**	**0**	**0**	**0**
Biliran	0	23	132	155	0	0	0	0	0	0	0	0
Eastern Samar	0	17	212	229	0	0	0	0	0	0	0	0
Northern Leyte	0	0	0	0	0	0	0	0	0	0	0	0
Northern Samar	0	0	86	86	0	0	0	0	0	0	0	0
Southern Leyte	0	54	152	206	0	0	0	0	0	0	0	0
Western Samar	4	18	269	291	0	0	0	0	0	0	0	0
Calbayog City	0	10	183	193	0	0	0	0	0	0	0	0
Maasin City	0	2	44	46	0	0	0	0	0	0	0	0
Ormoc City	0	2	209	211	0	0	0	0	0	0	0	0
Tacloban City	0	59	106	165	0	0	0	0	0	0	0	0
**Region 9**	**0**	**1**	**268**	**269**	**0**	**1**	**363**	**364**	**0**	**5**	**56**	**61**
Zamboanga del Norte	0	0	0	0	0	0	0	0	0	0	0	0
Zamboanga del Sur	0	0	0	0	0	0	0	0	0	0	0	0
Zamboanga Sibugay	0	0	0	0	0	0	0	0	0	0	0	0
Dapitan City	0	0	44	44	0	1	14	15	0	1	4	5
Dipolog City	0	1	69	70	0	0	0	0	0	1	42	43
Isabela City	0	0	16	16	0	0	4	4	0	0	0	0
Pagadian City	0	0	34	34	0	0	68	68	0	3	10	13
Zamboanga City	0	0	105	105	0	0	277	277	0	0	0	0
**Region 10**	**51**	**235**	**4,362**	**4,648**	**2**	**215**	**11,953**	**12,170**	**1**	**107**	**1,703**	**1,811**
Bukidnon	1	65	1,122	1,188	2	107	8,205	8,314	0	35	336	371
Camiguin	0	1	22	23	0	1	25	26	0	0	0	0
Lanao del Norte	0	77	569	646	0	1	307	308	0	1	25	26
Misamis Occidental	50	23	308	381	0	15	18	33	0	7	0	7
Misamis Oriental	0	15	370	385	0	22	1,132	1,154	0	10	258	268
Cagayan de Oro City	0	14	627	641	0	19	799	818	0	12	212	224
El Salvador City	0	1	26	27	0	21	193	214	0	3	4	7
Gingoog City	0	21	555	576	0	5	341	346	0	0	343	343
Iligan City	0	11	370	381	0	11	551	562	1	14	23	38
Malaybalay City	0	6	275	281	0	2	140	142	0	25	489	514
Oroquieta City	0	0	6	6	0	0	6	6	0	0	0	0
Ozamis City	0	0	29	29	0	3	29	32	0	0	0	0
Tangub City	0	0	0	0	0	0	1	1	0	0	0	0
Valencia City	0	1	83	84	0	8	206	214	0	0	13	13

Table 1.A.3 - MODERN METHOD OF FAMILY PLANNING

Drop Outs

Annual Philippines, 2020

Area	CONDOM			Total	IUD-INTERVAL			Total	IUD-POSTPARTUM			Total
	Age group				Age group				Age group			
	10-14	15-19	20-49		10-14	15-19	20-49		10-14	15-19	20-49	
**Region 11**	**3**	**151**	**4,000**	**4,154**	**7**	**66**	**4,123**	**4,196**	**3**	**26**	**442**	**471**
Davao de Oro	1	8	523	532	0	5	712	717	0	2	28	30
Davao del Norte	0	12	1,301	1,313	6	28	898	932	1	8	155	164
Davao Oriental	0	7	499	506	1	3	224	228	2	0	33	35
Davao del Sur	0	5	218	223	0	15	804	819	0	4	26	30
Davao Occidental	0	12	124	136	0	12	250	262	0	9	32	41
Davao City	2	107	1,335	1,444	0	3	1,235	1,238	0	3	168	171
**Region 12**	**1**	**146**	**2,503**	**2,650**	**5**	**152**	**4,064**	**4,221**	**7**	**145**	**928**	**1,080**
North Cotabato	1	40	652	693	0	42	2,235	2,277	0	20	192	212
Sarangani	0	17	408	425	0	4	245	249	0	5	10	15
South Cotabato	0	31	800	831	0	51	592	643	0	35	279	314
Sultan Kudarat	0	43	421	464	5	16	480	501	6	59	307	372
				0				0				0
Cotabato City	0	11	179	190	0	20	369	389	1	22	125	148
Gen. Santos City	0	4	43	47	0	19	143	162	0	4	15	19
**B.A.R.M.M.**	**1**	**240**	**1,795**	**2,036**	**0**	**0**	**0**	**0**	**0**	**0**	**0**	**0**
Basilan	0	13	173	186	0	0	0	0	0	0	0	0
Lanao del Sur	0	5	950	955	0	0	0	0	0	0	0	0
Maguindanao	0	33	495	528	0	0	0	0	0	0	0	0
Sulu	0	70	0	70	0	0	0	0	0	0	0	0
Tawi-Tawi	0	64	9	73	0	0	0	0	0	0	0	0
				0				0				0
Lamitan City	0	4	24	28	0	0	0	0	0	0	0	0
Marawi City	1	51	144	196	0	0	0	0	0	0	0	0
**CARAGA**	**2**	**177**	**2,703**	**2,882**	**0**	**82**	**7,616**	**7,698**	**0**	**27**	**741**	**768**
Agusan del Norte	1	13	481	495	0	13	635	648	0	7	410	417
Agusan del Sur	1	71	745	817	0	33	5,328	5,361	0	7	189	196
Surigao del Norte	0	43	417	460	0	15	294	309	0	4	27	31
Surigao del Sur	0	22	427	449	0	12	695	707	0	7	78	85
Province of Dinagat	0	5	112	117	0	2	115	117	0	0	7	7
Bislig City	0	7	166	173	0	6	161	167	0	2	9	11
Butuan City	0	13	146	159	0	1	36	37	0	0	6	6
Surigao City	0	3	209	212	0	0	352	352	0	0	15	15

Table 1.A.3 - MODERN METHOD OF FAMILY PLANNING
Drop Outs
Annual Philippines, 2020

Area	PILLS-POP			Total	PILLS-COC			Total	INJECTABLES			Total
	Age group				Age group				Age group			
	10-14	15-19	20-49		10-14	15-19	20-49		10-14	15-19	20-49	
**PHILIPPINES**	**1,593**	**10,315**	**103,996**	**115,904**	**345**	**20,381**	**430,486**	**451,212**	**778**	**23,617**	**378,937**	**403,332**
**N C R**	**39**	**3,434**	**50,285**	**53,758**	**13**	**2,612**	**86,594**	**89,219**	**46**	**4,615**	**70,445**	**75,106**
Malabon	0	66	488	554	0	183	1,752	1,935	1	208	2,105	2,314
Navotas	1	101	466	568	0	113	806	919	1	168	1,228	1,397
Valenzuela City	2	51	692	745	4	157	22,733	22,894	11	414	10,846	11,271
Caloocan City	2	142	745	889	1	259	4,418	4,678	4	869	7,549	8,422
Marikina City	0	43	508	551	0	58	1,685	1,743	0	324	3,402	3,726
Pasig City	0	10	175	185	0	29	1,070	1,099	0	143	1,715	1,858
Pateros	1	70	977	1,048	0	14	285	299	0	34	225	259
Taguig	0	10	11,227	11,237	0	14	373	387	9	403	3,787	4,199
Quezon City	4	501	4,991	5,496	0	516	40,231	40,747	3	723	21,622	22,348
Makati City	0	8	205	213	0	25	582	607	0	54	1,353	1,407
Mandaluyong City	0	5	92	97	0	12	356	368	0	19	378	397
San Juan	0	8	12	20	0	0	98	98	0	0	127	127
Manila City	23	1,554	18,025	19,602	7	632	8,116	8,755	9	697	9,343	10,049
Las Piñas City	0	85	798	883	0	81	1,585	1,666	3	148	2,146	2,297
Muntinlupa City	0	23	368	391	0	12	599	611	0	32	2,006	2,038
Parañaque City	0	55	4,910	4,965	1	41	362	404	2	99	1,100	1,201
Pasay City	6	702	5,606	6,314	0	466	1,543	2,009	3	280	1,513	1,796
**C A R**	**3**	**287**	**11,406**	**11,696**	**2**	**385**	**8,916**	**9,303**	**2**	**497**	**8,913**	**9,412**
Abra	0	47	361	408	0	33	1,015	1,048	0	46	579	625
Apayao	3	48	284	335	0	68	1,194	1,262	0	57	857	914
Benguet	0	71	8,486	8,557	2	90	1,919	2,011	0	106	2,275	2,381
Ifugao	0	25	994	1,019	0	45	1,608	1,653	0	41	1,267	1,308
Kalinga	0	20	339	359	0	44	1,237	1,281	1	54	930	985
Mt. Province	0	16	165	181	0	46	1,021	1,067	0	83	914	997
Baguio City	0	60	777	837	0	59	922	981	1	110	2,091	2,202
**Region 1**	**1**	**239**	**2,582**	**2,822**	**2**	**557**	**14,324**	**14,883**	**0**	**614**	**10,504**	**11,118**
Ilocos Norte	0	20	171	191	0	9	2,490	2,499	0	10	779	789
Ilocos Sur	0	122	942	1,064	0	82	1,917	1,999	0	101	1,549	1,650
La Union	1	16	313	330	0	75	1,786	1,861	0	158	1,997	2,155
Pangasinan	0	59	984	1,043	0	275	4,401	4,676	0	196	4,313	4,509
Alaminos City	0	8	16	24	0	0	608	608	0	40	118	158
Candon City	0	0	4	4	0	0	82	82	0	0	73	73
Dagupan City	0	4	16	20	0	29	390	419	0	30	251	281
Laoag City	0	0	23	23	0	0	338	338	0	0	8	8
San Carlos City	0	9	91	100	0	48	796	844	0	52	647	699
San Fernando City	0	0	0	0	0	22	1,282	1,304	0	4	532	536
Urdaneta City	0	1	22	23	2	10	164	176	0	22	190	212
Vigan City	0	0	0	0	0	7	70	77	0	1	47	48
**Region 2**	**6**	**359**	**2,512**	**2,877**	**72**	**976**	**17,654**	**18,702**	**9**	**836**	**12,904**	**13,749**
Batanes	0	2	16	18	0	1	47	48	1	15	177	193
Cagayan	1	90	371	462	3	169	3,853	4,025	2	169	2,278	2,449
Isabela	2	128	922	1,052	0	218	5,674	5,892	4	410	5,332	5,746
Nueva Vizcaya	3	98	967	1,068	68	498	4,447	5,013	2	121	2,045	2,168
Quirino	0	11	83	94	1	35	501	537	0	21	444	465
Cauayan City	0	3	11	14	0	14	842	856	0	33	669	702
Ilagan City	0	11	84	95	0	11	302	313	0	25	377	402
Santiago City	0	0	0	0	0	18	1,475	1,493	0	24	1,387	1,411
Tuguegarao City	0	16	58	74	0	12	513	525	0	18	195	213
**Region 3**	**0**	**537**	**4,311**	**4,848**	**5**	**2,983**	**41,564**	**44,552**	**7**	**3,583**	**34,495**	**38,085**
Aurora	0	27	183	210	0	50	962	1,012	1	58	738	797
Bataan	0	30	214	244	0	168	1,813	1,981	2	398	3,224	3,624

Table 1.A.3 - MODERN METHOD OF FAMILY PLANNING

Drop Outs

Annual Philippines, 2020

Area	PILLS-POP			Total	PILLS-COC			Total	INJECTABLES			Total
	Age group				Age group				Age group			
	10-14	15-19	20-49		10-14	15-19	20-49		10-14	15-19	20-49	
Bulacan	0	131	1,178	1,309	0	688	5,945	6,633	0	624	4,919	5,543
Nueva Ecija	0	55	717	772	0	306	5,193	5,499	0	467	4,571	5,038
Pampanga	0	134	824	958	1	228	6,259	6,488	1	339	3,880	4,220
Tarlac	0	45	313	358	0	354	4,195	4,549	0	174	1,943	2,117
Zambales	0	14	202	216	1	51	3,949	4,001	0	136	3,562	3,698
Angeles City	0	8	30	38	0	41	641	682	1	44	523	568
Balanga City	0	0	1	1	0	8	221	229	0	37	474	511
Cabanatuan City	0	19	84	103	0	60	783	843	0	46	380	426
City of San Fernando	0	14	80	94	0	13	322	335	0	11	323	334
Gapan City	0	9	15	24	0	55	172	227	0	57	143	200
Mabalacat City	0	5	49	54	0	20	290	310	0	33	379	412
Malolos City	0	0	0	0	0	16	739	755	0	53	556	609
Meycauayan	0	6	25	31	2	61	846	909	0	61	604	665
Olongapo	0	12	125	137	0	40	1,050	1,090	2	166	1,365	1,533
Palayan City	0	2	13	15	1	26	176	203	0	5	127	132
San Jose City	0	7	26	33	0	24	737	761	0	54	740	794
San Jose del Monte City	0	11	70	81	0	742	6,850	7,592	0	788	5,589	6,377
Science City of Munoz	0	0	0	0	0	3	171	174	0	0	103	103
Tarlac City	0	8	162	170	0	29	250	279	0	32	352	384
**Region 4A**	**1,460**	**2,412**	**2,412**	**6,284**	**116**	**5,385**	**112,153**	**117,654**	**664**	**6,044**	**136,248**	**142,956**
Batangas	1,204	20	20	1,244	0	160	10,424	10,584	288	140	6,276	6,704
Cavite	0	56	56	112	4	208	7,964	8,176	4	476	9,840	10,320
Laguna	0	112	112	224	24	488	12,012	12,524	0	332	7,568	7,900
Quezon	4	456	456	916	0	852	25,088	25,940	8	1,468	48,428	49,904
Rizal	4	92	92	188	0	560	16,020	16,580	52	1,180	21,312	22,544
Antipolo City	0	0	0	0	0	0	0	0	0	0	0	0
Bacoor City	0	4	4	8	16	276	3,292	3,584	72	304	3,768	4,144
Batangas City	0	0	0	0	0	0	0	0	0	0	0	0
Biñan City	0	1,316	1,316	2,632	0	1,384	6,388	7,772	0	852	5,588	6,440
Cabuyao City	8	156	156	320	0	188	3,800	3,988	0	180	2,440	2,620
Calamba City	8	60	60	128	4	52	1,476	1,532	0	40	2,268	2,308
Cavite City	0	0	0	0	0	0	0	0	0	0	0	0
Dasmariñas City	0	28	28	56	0	164	7,268	7,432	0	436	6,280	6,716
General Trias City	0	20	20	40	0	88	4,444	4,532	0	216	5,576	5,792
Imus City	0	40	40	80	20	40	2,504	2,564	8	92	2,632	2,732
Lipa City	0	0	0	0	0	0	0	0	0	0	0	0
Lucena City	232	0	0	232	32	0	0	32	232	0	3,840	4,072
San Pablo City	0	0	0	0	0	0	0	0	0	0	0	0
San Pedro City	0	40	40	80	0	128	1,360	1,488	0	8	472	480
Santa Rosa City	0	0	0	0	8	720	6,772	7,500	0	152	2,180	2,332
Tagaytay City	0	0	0	0	8	0	1,180	1,188	0	0	972	972
Tanauan City	0	4	4	8	0	1	1,045	1,046	0	4	1,636	1,640
Tayabas City	0	0	0	0	0	0	8	8	0	0	3,656	3,656
Trece Martires City	0	8	8	16	0	76	1,108	1,184	0	164	1,516	1,680
**Region 4B**	**0**	**25**	**380**	**405**	**1**	**84**	**7,987**	**8,072**	**2**	**112**	**6,626**	**6,740**
Marinduque	0	0	30	30	0	4	825	829	0	0	402	402
Mindoro Occidental	0	6	99	105	0	46	1,912	1,958	0	62	2,066	2,128
Mindoro Oriental	0	14	180	194	1	15	3,060	3,076	0	20	1,807	1,827
Palawan	0	5	70	75	0	19	551	570	2	30	654	686
Romblon	0	0	1	1	0	0	13	13	0	0	27	27
Puerto Princesa City	0	0	0	0	0	0	1,626	1,626	0	0	1,670	1,670
**Region 5**	**1**	**244**	**2,227**	**2,472**	**0**	**940**	**20,872**	**21,812**	**6**	**872**	**14,527**	**15,405**
Albay	0	33	272	305	0	151	1,883	2,034	0	87	1,712	1,799
Camarines Norte	0	54	264	318	0	224	2,703	2,927	1	214	2,585	2,800
Camarines Sur	1	67	851	919	0	261	6,746	7,007	2	243	5,164	5,409
Catanduanes	0	3	128	131	0	63	1,354	1,417	0	81	954	1,035
Masbate	0	47	425	472	0	99	3,640	3,739	3	77	1,064	1,144
Sorsogon	0	33	119	152	0	129	2,919	3,048	0	156	2,415	2,571
Iriga City	0	1	88	89	0	12	471	483	0	13	259	272

Table 1.A.3 - MODERN METHOD OF FAMILY PLANNING
Drop Outs
Annual Philippines, 2020

Area	PILLS-POP			Total	PILLS-COC			Total	INJECTABLES			Total
	Age group				Age group				Age group			
	10-14	15-19	20-49		10-14	15-19	20-49		10-14	15-19	20-49	
Legaspi City	0	5	49	54	0	1	116	117	0	1	85	86
Naga City	0	1	31	32	0	0	1,040	1,040	0	0	289	289
**Region 6**	**2**	**268**	**5,361**	**5,631**	**35**	**1,201**	**19,928**	**21,164**	**11**	**1,150**	**17,042**	**18,203**
Aklan	0	10	754	764	0	128	1,241	1,369	2	146	2,387	2,535
Antique	0	18	1,712	1,730	34	67	2,791	2,892	7	164	3,653	3,824
Capiz	0	6	71	77	0	59	969	1,028	0	43	1,200	1,243
Guimaras	0	5	126	131	1	40	482	523	1	47	247	295
Iloilo	0	40	884	924	0	87	5,935	6,022	1	46	1,465	1,512
Negros Occidental	0	170	1,276	1,446	0	686	6,930	7,616	0	567	6,607	7,174
Bacolod City	2	4	84	90	0	18	633	651	0	69	1,231	1,300
Iloilo City	0	15	454	469	0	116	947	1,063	0	68	252	320
**Region 7**	**0**	**244**	**2,590**	**2,834**	**5**	**545**	**13,596**	**14,146**	**3**	**628**	**13,152**	**13,783**
Bohol	0	66	442	508	2	155	1,767	1,924	0	130	1,340	1,470
Cebu	0	17	106	123	1	37	2,205	2,243	0	58	2,202	2,260
Negros Oriental	0	38	318	356	2	110	4,069	4,181	0	187	3,787	3,974
Siquijor	0	12	104	116	0	15	728	743	0	19	529	548
Cebu City	0	53	395	448	0	171	3,082	3,253	3	179	3,140	3,322
Lapu-Lapu City	0	58	1,158	1,216	0	57	1,722	1,779	0	55	2,073	2,128
Mandaue City	0	0	67	67	0	0	23	23	0	0	81	81
**Region 8**	**0**	**0**	**0**	**0**	**0**	**0**	**0**	**0**	**0**	**0**	**0**	**0**
Biliran	0	0	0	0	0	0	0	0	0	0	0	0
Eastern Samar	0	0	0	0	0	0	0	0	0	0	0	0
Northern Leyte	0	0	0	0	0	0	0	0	0	0	0	0
Northern Samar	0	0	0	0	0	0	0	0	0	0	0	0
Southern Leyte	0	0	0	0	0	0	0	0	0	0	0	0
Western Samar	0	0	0	0	0	0	0	0	0	0	0	0
Calbayog City	0	0	0	0	0	0	0	0	0	0	0	0
Maasin City	0	0	0	0	0	0	0	0	0	0	0	0
Ormoc City	0	0	0	0	0	0	0	0	0	0	0	0
Tacloban City	0	0	0	0	0	0	0	0	0	0	0	0
**Region 9**	**0**	**15**	**182**	**197**	**0**	**39**	**1,852**	**1,891**	**0**	**51**	**1,279**	**1,330**
Zamboanga del Norte	0	0	0	0	0	0	0	0	0	0	0	0
Zamboanga del Sur	0	0	0	0	0	0	0	0	0	0	0	0
Zamboanga Sibugay	0	0	0	0	0	0	0	0	0	0	0	0
Dapitan City	0	0	11	11	0	6	218	224	0	7	85	92
Dipolog City	0	7	24	31	0	9	397	406	0	22	355	377
Isabela City	0	6	102	108	0	4	82	86	0	7	92	99
Pagadian City	0	0	5	5	0	3	269	272	0	0	0	0
Zamboanga City	0	2	40	42	0	17	886	903	0	15	747	762
**Region 10**	**57**	**421**	**5,221**	**5,699**	**33**	**1,182**	**23,218**	**24,433**	**6**	**990**	**11,909**	**12,905**
Bukidnon	1	158	1,557	1,716	2	605	11,168	11,775	6	551	4,391	4,948
Camiguin	0	1	6	7	0	2	296	298	0	4	149	153
Lanao del Norte	1	60	1,727	1,788	0	156	1,793	1,949	0	82	1,394	1,476
Misamis Occidental	55	15	107	177	29	41	447	517	0	22	400	422
Misamis Oriental	0	28	328	356	0	38	2,443	2,481	0	27	1,347	1,374
Cagayan de Oro City	0	70	244	314	0	102	1,975	2,077	0	48	1,062	1,110
El Salvador City	0	4	37	41	0	14	244	258	0	18	272	290
Gingoog City	0	13	736	749	0	42	722	764	0	119	466	585
Iligan City	0	20	208	228	2	62	1,874	1,938	0	46	1,120	1,166
Malaybalay City	0	19	106	125	0	48	1,101	1,149	0	43	766	809
Oroquieta City	0	2	14	16	0	6	46	52	0	1	12	13
Ozamis City	0	0	4	4	0	1	222	223	0	0	63	63
Tangub City	0	0	13	13	0	3	56	59	0	0	22	22
Valencia City	0	31	134	165	0	62	831	893	0	29	445	474

Table 1.A.3 - MODERN METHOD OF FAMILY PLANNING

Drop Outs

Annual Philippines, 2020

Area	PILLS-POP			Total	PILLS-COC			Total	INJECTABLES			Total
	Age group				Age group				Age group			
	10-14	15-19	20-49		10-14	15-19	20-49		10-14	15-19	20-49	
**Region 11**	**16**	**551**	**4,316**	**4,883**	**56**	**881**	**23,340**	**24,277**	**18**	**1,030**	**13,209**	**14,257**
Davao de Oro	0	117	511	628	0	133	4,510	4,643	2	146	2,188	2,336
Davao del Norte	3	85	641	729	25	195	6,204	6,424	5	199	2,473	2,677
Davao Oriental	1	47	276	324	4	108	1,803	1,915	1	84	1,060	1,145
Davao del Sur	4	70	295	369	0	86	2,570	2,656	2	134	1,821	1,957
Davao Occidental	6	52	210	268	25	162	2,559	2,746	6	218	2,105	2,329
Davao City	2	180	2,383	2,565	2	197	5,694	5,893	2	249	3,562	3,813
**Region 12**	**2**	**810**	**6,522**	**7,334**	**4**	**1,751**	**19,091**	**20,846**	**3**	**1,890**	**18,979**	**20,872**
North Cotabato	1	194	1,010	1,205	0	501	5,235	5,736	1	573	4,683	5,257
Sarangani	0	159	468	627	0	311	3,067	3,378	0	194	2,865	3,059
South Cotabato	1	115	1,145	1,261	3	476	6,031	6,510	2	570	5,963	6,535
Sultan Kudarat	0	305	2,530	2,835	0	317	3,101	3,418	0	320	3,611	3,931
				0				0				0
Cotabato City	0	12	1,276	1,288	1	73	979	1,053	0	123	1,073	1,196
Gen. Santos City	0	25	93	118	0	73	678	751	0	110	784	894
**B.A.R.M.M.**	**0**	**0**	**0**	**0**	**0**	**0**	**0**	**0**	**0**	**0**	**0**	**0**
Basilan	0	0	0	0	0	0	0	0	0	0	0	0
Lanao del Sur	0	0	0	0	0	0	0	0	0	0	0	0
Maguindanao	0	0	0	0	0	0	0	0	0	0	0	0
Sulu	0	0	0	0	0	0	0	0	0	0	0	0
Tawi-Tawi	0	0	0	0	0	0	0	0	0	0	0	0
				0				0				0
Lamitan City	0	0	0	0	0	0	0	0	0	0	0	0
Marawi City	0	0	0	0	0	0	0	0	0	0	0	0
**CARAGA**	**6**	**469**	**3,689**	**4,164**	**1**	**860**	**19,397**	**20,258**	**1**	**705**	**8,705**	**9,411**
Agusan del Norte	4	189	1,452	1,645	1	121	3,236	3,358	0	125	1,476	1,601
Agusan del Sur	1	61	901	963	0	196	8,511	8,707	0	131	2,872	3,003
Surigao del Norte	0	71	492	563	0	95	1,252	1,347	1	89	1,086	1,176
Surigao del Sur	1	70	515	586	0	129	3,312	3,441	0	88	1,265	1,353
Province of Dinagat	0	8	57	65	0	22	607	629	0	15	350	365
Bislig City	0	8	40	48	0	23	680	703	0	22	317	339
Butuan City	0	37	150	187	0	263	1,429	1,692	0	224	832	1,056
Surigao City	0	25	82	107	0	11	370	381	0	11	507	518

Table 1.A.3 - MODERN METHOD OF FAMILY PLANNING

Drop Outs

Annual Philippines, 2020

Area	IMPLANTS			Total	NFP-CCM			Total	NFP-BBT			Total
	Age group				Age group				Age group			
	10-14	15-19	20-49		10-14	15-19	20-49		10-14	15-19	20-49	
**PHILIPPINES**	**912**	**4,383**	**74,864**	**80,159**	**4**	**329**	**33,678**	**34,011**	**0**	**100**	**1,494**	**1,594**
**N C R**	**21**	**778**	**11,704**	**12,503**	**0**	**11**	**273**	**284**	**0**	**44**	**569**	**613**
Malabon	1	32	385	418	0	0	0	0	0	0	0	0
Navotas	0	100	82	182	0	0	0	0	0	0	0	0
Valenzuela City	4	26	3,038	3,068	0	0	94	94	0	0	32	32
Caloocan City	5	33	708	746	0	0	0	0	0	0	0	0
Marikina City	0	4	574	578	0	0	9	9	0	0	0	0
Pasig City	1	0	247	248	0	0	0	0	0	0	0	0
Pateros	0	2	11	13	0	0	0	0	0	0	0	0
Taguig	2	44	304	350	0	0	0	0	0	0	0	0
Quezon City	2	115	891	1,008	0	0	3	3	0	3	18	21
Makati City	0	0	17	17	0	0	0	0	0	0	0	0
Mandaluyong City	0	9	60	69	0	4	11	15	0	0	0	0
San Juan	0	0	1	1	0	0	0	0	0	0	0	0
Manila City	5	318	3,929	4,252	0	4	28	32	0	41	519	560
Las Piñas City	0	43	587	630	0	0	1	1	0	0	0	0
Muntinlupa City	0	0	1	1	0	0	0	0	0	0	0	0
Parañaque City	0	1	229	230	0	0	0	0	0	0	0	0
Pasay City	1	51	640	692	0	3	127	130	0	0	0	0
**C A R**	**0**	**63**	**1,154**	**1,217**	**0**	**34**	**551**	**585**	**0**	**3**	**22**	**25**
Abra	0	5	135	140	0	1	10	11	0	1	8	9
Apayao	0	5	64	69	0	0	0	0	0	0	0	0
Benguet	0	19	181	200	0	0	10	10	0	0	3	3
Ifugao	0	10	284	294	0	8	211	219	0	0	1	1
Kalinga	0	5	203	208	0	25	274	299	0	2	10	12
Mt. Province	0	7	55	62	0	0	46	46	0	0	0	0
Baguio City	0	12	232	244	0	0	0	0	0	0	0	0
**Region 1**	**1**	**69**	**1,335**	**1,405**	**0**	**3**	**510**	**513**	**0**	**0**	**12**	**12**
Ilocos Norte	0	3	100	103	0	0	11	11	0	0	0	0
Ilocos Sur	0	0	78	78	0	0	31	31	0	0	4	4
La Union	0	21	128	149	0	0	169	169	0	0	7	7
Pangasinan	1	35	692	728	0	0	2	2	0	0	0	0
Alaminos City	0	0	55	55	0	0	0	0	0	0	0	0
Candon City	0	0	0	0	0	0	6	6	0	0	0	0
Dagupan City	0	4	118	122	0	0	0	0	0	0	0	0
Laoag City	0	0	0	0	0	0	0	0	0	0	0	0
San Carlos City	0	6	46	52	0	0	0	0	0	0	1	1
San Fernando City	0	0	76	76	0	0	0	0	0	0	0	0
Urdaneta City	0	0	40	40	0	0	0	0	0	0	0	0
Vigan City	0	0	2	2	0	3	291	294	0	0	0	0
**Region 2**	**2**	**201**	**2,112**	**2,315**	**0**	**2**	**186**	**188**	**0**	**0**	**7**	**7**
Batanes	0	0	1	1	0	0	9	9	0	0	0	0
Cagayan	0	17	249	266	0	0	82	82	0	0	7	7
Isabela	0	153	969	1,122	0	0	0	0	0	0	0	0
Nueva Vizcaya	2	18	747	767	0	2	90	92	0	0	0	0
Quirino	0	0	45	45	0	0	5	5	0	0	0	0
Cauayan City	0	8	23	31	0	0	0	0	0	0	0	0
Ilagan City	0	2	44	46	0	0	0	0	0	0	0	0
Santiago City	0	3	28	31	0	0	0	0	0	0	0	0
Tuguegarao City	0	0	6	6	0	0	0	0	0	0	0	0
**Region 3**	**0**	**393**	**5,479**	**5,872**	**0**	**5**	**1,002**	**1,007**	**0**	**1**	**30**	**31**
Aurora	0	8	390	398	0	2	595	597	0	0	17	17
Bataan	0	22	164	186	0	1	226	227	0	0	3	3

Table 1.A.3 - MODERN METHOD OF FAMILY PLANNING

Drop Outs

Annual Philippines, 2020

Area	IMPLANTS Age group			Total	NFP-CCM Age group			Total	NFP-BBT Age group			Total
	10-14	15-19	20-49		10-14	15-19	20-49		10-14	15-19	20-49	
Bulacan	0	46	697	743	0	0	0	0	0	0	0	0
Nueva Ecija	0	27	622	649	0	0	1	1	0	0	0	0
Pampanga	0	44	731	775	0	2	167	169	0	0	0	0
Tarlac	0	5	116	121	0	0	2	2	0	0	0	0
Zambales	0	12	460	472	0	0	9	9	0	0	0	0
Angeles City	0	2	29	31	0	0	0	0	0	0	0	0
Balanga City	0	1	15	16	0	0	0	0	0	0	0	0
Cabanatuan City	0	1	41	42	0	0	1	1	0	0	0	0
City of San Fernando	0	2	23	25	0	0	0	0	0	0	0	0
Gapan City	0	39	342	381	0	0	0	0	0	0	0	0
Mabalacat City	0	0	32	32	0	0	0	0	0	0	0	0
Malolos City	0	19	109	128	0	0	0	0	0	0	0	0
Meycauayan	0	3	88	91	0	0	1	1	0	0	0	0
Olongapo	0	3	76	79	0	0	0	0	0	0	0	0
Palayan City	0	0	0	0	0	0	0	0	0	0	0	0
San Jose City	0	0	9	9	0	0	0	0	0	0	0	0
San Jose del Monte City	0	150	1,283	1,433	0	0	0	0	0	0	0	0
Science City of Munoz	0	7	212	219	0	0	0	0	0	0	0	0
Tarlac City	0	2	40	42	0	0	0	0	0	1	10	11
**Region 4A**	**880**	**956**	**18,496**	**20,332**	**0**	**45**	**24,006**	**24,051**	**0**	**7**	**240**	**247**
Batangas	4	4	216	224	0	0	21,492	21,492	0	0	0	0
Cavite	0	52	1,532	1,584	0	0	84	84	0	0	0	0
Laguna	0	120	1,672	1,792	0	4	572	576	0	4	40	44
Quezon	0	164	3,400	3,564	0	0	1,008	1,008	0	0	136	136
Rizal	4	180	2,912	3,096	0	20	0	20	0	0	16	16
Antipolo City	0	0	0	0	0	0	0	0	0	0	0	0
Bacoor City	0	16	388	404	0	0	0	0	0	0	28	28
Batangas City	0	0	0	0	0	0	0	0	0	0	0	0
Biñan City	0	44	80	124	0	0	0	0	0	0	20	20
Cabuyao City	0	0	536	536	0	0	0	0	0	0	0	0
Calamba City	0	104	2,456	2,560	0	0	0	0	0	0	0	0
Cavite City	0	0	0	0	0	0	0	0	0	0	0	0
Dasmariñas City	0	88	2,656	2,744	0	0	0	0	0	0	0	0
General Trias City	0	36	688	724	0	1	0	1	0	3	0	3
Imus City	0	40	192	232	0	0	0	0	0	0	0	0
Lipa City	0	0	0	0	0	0	6	6	0	0	0	0
Lucena City	872	0	20	892	0	0	0	0	0	0	0	0
San Pablo City	0	0	0	0	0	0	0	0	0	0	0	0
San Pedro City	0	16	276	292	0	20	240	260	0	0	0	0
Santa Rosa City	0	64	196	260	0	0	0	0	0	0	0	0
Tagaytay City	0	0	864	864	0	0	0	0	0	0	0	0
Tanauan City	0	0	28	28	0	0	0	0	0	0	0	0
Tayabas City	0	0	184	184	0	0	604	604	0	0	0	0
Trece Martires City	0	28	200	228	0	0	0	0	0	0	0	0
**Region 4B**	**0**	**18**	**1,199**	**1,217**	**0**	**4**	**488**	**492**	**0**	**0**	**1**	**1**
Marinduque	0	0	54	54	0	0	132	132	0	0	0	0
Mindoro Occidental	0	6	107	113	0	3	290	293	0	0	1	1
Mindoro Oriental	0	3	123	126	0	0	8	8	0	0	0	0
Palawan	0	9	567	576	0	1	11	12	0	0	0	0
Romblon	0	0	5	5	0	0	47	47	0	0	0	0
Puerto Princesa City	0	0	343	343	0	0	0	0	0	0	0	0
**Region 5**	**0**	**153**	**3,225**	**3,378**	**1**	**107**	**3,279**	**3,387**	**0**	**1**	**156**	**157**
Albay	0	17	268	285	0	0	309	309	0	0	13	13
Camarines Norte	0	6	121	127	0	8	64	72	0	0	0	0
Camarines Sur	0	52	1,086	1,138	0	21	1,427	1,448	0	1	104	105
Catanduanes	0	11	368	379	0	43	452	495	0	0	0	0
Masbate	0	21	422	443	1	7	422	430	0	0	5	5
Sorsogon	0	43	837	880	0	28	583	611	0	0	5	5
Iriga City	0	3	60	63	0	0	13	13	0	0	26	26

Table 1.A.3 - MODERN METHOD OF FAMILY PLANNING
Drop Outs
Annual Philippines, 2020

Area	IMPLANTS Age group 10-14	15-19	20-49	Total	NFP-CCM Age group 10-14	15-19	20-49	Total	NFP-BBT Age group 10-14	15-19	20-49	Total
Legaspi City	0	0	9	9	0	0	0	0	0	0	0	0
Naga City	0	0	54	54	0	0	9	9	0	0	3	3
**Region 6**	**1**	**320**	**5,776**	**6,097**	**3**	**72**	**799**	**874**	**0**	**1**	**49**	**50**
Aklan	0	22	1,112	1,134	0	0	3	3	0	0	3	3
Antique	0	90	795	885	3	29	512	544	0	0	2	2
Capiz	0	0	367	367	0	0	26	26	0	1	33	34
Guimaras	0	15	93	108	0	4	8	12	0	0	0	0
Iloilo	0	32	1,226	1,258	0	0	192	192	0	0	4	4
Negros Occidental	1	121	1,915	2,037	0	39	58	97	0	0	5	5
Bacolod City	0	4	147	151	0	0	0	0	0	0	1	1
Iloilo City	0	36	121	157	0	0	0	0	0	0	1	1
**Region 7**	**1**	**160**	**3,034**	**3,195**	**0**	**0**	**14**	**14**	**0**	**5**	**8**	**13**
Bohol	0	106	700	806	0	0	2	2	0	0	0	0
Cebu	0	9	673	682	0	0	7	7	0	0	0	0
Negros Oriental	0	3	53	56	0	0	0	0	0	0	0	0
Siquijor	0	1	18	19	0	0	0	0	0	0	0	0
Cebu City	1	34	1,255	1,290	0	0	5	5	0	5	8	13
Lapu-Lapu City	0	7	253	260	0	0	0	0	0	0	0	0
Mandaue City	0	0	82	82	0	0	0	0	0	0	0	0
**Region 8**	**0**	**0**	**0**	**0**	**0**	**0**	**0**	**0**	**0**	**0**	**0**	**0**
Biliran	0	0	0	0	0	0	0	0	0	0	0	0
Eastern Samar	0	0	0	0	0	0	0	0	0	0	0	0
Northern Leyte	0	0	0	0	0	0	0	0	0	0	0	0
Northern Samar	0	0	0	0	0	0	0	0	0	0	0	0
Southern Leyte	0	0	0	0	0	0	0	0	0	0	0	0
Western Samar	0	0	0	0	0	0	0	0	0	0	0	0
Calbayog City	0	0	0	0	0	0	0	0	0	0	0	0
Maasin City	0	0	0	0	0	0	0	0	0	0	0	0
Ormoc City	0	0	0	0	0	0	0	0	0	0	0	0
Tacloban City	0	0	0	0	0	0	0	0	0	0	0	0
**Region 9**	**0**	**0**	**321**	**321**	**0**	**0**	**0**	**0**	**0**	**0**	**0**	**0**
Zamboanga del Norte	0	0	0	0	0	0	0	0	0	0	0	0
Zamboanga del Sur	0	0	0	0	0	0	0	0	0	0	0	0
Zamboanga Sibugay	0	0	0	0	0	0	0	0	0	0	0	0
Dapitan City	0	0	30	30	0	0	0	0	0	0	0	0
Dipolog City	0	0	17	17	0	0	0	0	0	0	0	0
Isabela City	0	0	15	15	0	0	0	0	0	0	0	0
Pagadian City	0	0	17	17	0	0	0	0	0	0	0	0
Zamboanga City	0	0	242	242	0	0	0	0	0	0	0	0
**Region 10**	**2**	**389**	**6,578**	**6,969**	**0**	**38**	**873**	**911**	**0**	**19**	**206**	**225**
Bukidnon	1	205	3,055	3,261	0	22	285	307	0	4	16	20
Camiguin	0	0	16	16	0	0	0	0	0	0	0	0
Lanao del Norte	0	50	477	527	0	0	62	62	0	12	0	12
Misamis Occidental	0	0	43	43	0	0	12	12	0	0	0	0
Misamis Oriental	0	8	488	496	0	5	243	248	0	0	5	5
Cagayan de Oro City	1	35	1,023	1,059	0	0	0	0	0	0	3	3
El Salvador City	0	0	21	21	0	0	0	0	0	0	0	0
Gingoog City	0	7	350	357	0	10	186	196	0	3	178	181
Iligan City	0	16	505	521	0	0	27	27	0	0	0	0
Malaybalay City	0	63	485	548	0	0	0	0	0	0	4	4
Oroquieta City	0	1	1	2	0	0	0	0	0	0	0	0
Ozamis City	0	3	33	36	0	0	13	13	0	0	0	0
Tangub City	0	0	0	0	0	0	0	0	0	0	0	0
Valencia City	0	1	81	82	0	1	45	46	0	0	0	0

Table 1.A.3 - MODERN METHOD OF FAMILY PLANNING

Drop Outs

Annual Philippines, 2020

Area	IMPLANTS			Total	NFP-CCM			Total	NFP-BBT			Total
	Age group				Age group				Age group			
	10-14	15-19	20-49		10-14	15-19	20-49		10-14	15-19	20-49	
**Region 11**	**3**	**199**	**4,980**	**5,182**	**0**	**4**	**428**	**432**	**0**	**0**	**59**	**59**
Davao de Oro	1	35	1,052	1,088	0	4	102	106	0	0	36	36
Davao del Norte	1	40	734	775	0	0	14	14	0	0	1	1
Davao Oriental	0	42	587	629	0	0	212	212	0	0	11	11
Davao del Sur	0	14	473	487	0	0	20	20	0	0	9	9
Davao Occidental	0	38	729	767	0	0	0	0	0	0	0	0
Davao City	1	30	1,405	1,436	0	0	80	80	0	0	2	2
**Region 12**	**0**	**568**	**5,395**	**5,963**	**0**	**3**	**179**	**182**	**0**	**18**	**96**	**114**
North Cotabato	0	162	1,460	1,622	0	3	165	168	0	8	21	29
Sarangani	0	19	595	614	0	0	1	1	0	3	8	11
South Cotabato	0	227	1,845	2,072	0	0	0	0	0	3	47	50
Sultan Kudarat	0	126	1,008	1,134	0	0	3	3	0	0	3	3
				0				0				0
Cotabato City	0	21	265	286	0	0	9	9	0	4	17	21
Gen. Santos City	0	13	222	235	0	0	1	1	0	0	0	0
**B.A.R.M.M.**	**0**	**0**	**0**	**0**	**0**	**0**	**0**	**0**	**0**	**0**	**0**	**0**
Basilan	0	0	0	0	0	0	0	0	0	0	0	0
Lanao del Sur	0	0	0	0	0	0	0	0	0	0	0	0
Maguindanao	0	0	0	0	0	0	0	0	0	0	0	0
Sulu	0	0	0	0	0	0	0	0	0	0	0	0
Tawi-Tawi	0	0	0	0	0	0	0	0	0	0	0	0
				0				0				0
Lamitan City	0	0	0	0	0	0	0	0	0	0	0	0
Marawi City	0	0	0	0	0	0	0	0	0	0	0	0
**CARAGA**	**1**	**116**	**4,076**	**4,193**	**0**	**1**	**1,090**	**1,091**	**0**	**1**	**39**	**40**
Agusan del Norte	0	37	870	907	0	0	25	25	0	0	22	22
Agusan del Sur	0	20	1,774	1,794	0	0	868	868	0	0	0	0
Surigao del Norte	1	5	369	375	0	1	24	25	0	0	6	6
Surigao del Sur	0	17	280	297	0	0	128	128	0	0	0	0
Province of Dinagat	0	9	403	412	0	0	0	0	0	0	0	0
Bislig City	0	5	67	72	0	0	33	33	0	1	2	3
Butuan City	0	16	264	280	0	0	9	9	0	0	9	9
Surigao City	0	7	49	56	0	0	3	3	0	0	0	0

Table 1.A.3 - MODERN METHOD OF FAMILY PLANNING
Drop Outs
Annual Philippines, 2020

Area	NFP-STM Age group 10-14	NFP-STM Age group 15-19	NFP-STM Age group 20-49	Total	NFP-SDM Age group 10-14	NFP-SDM Age group 15-19	NFP-SDM Age group 20-49	Total	NFP-LAM Age group 10-14	NFP-LAM Age group 15-19	NFP-LAM Age group 20-49	Total	Total New Acceptors
**PHILIPPINES**	**0**	**42**	**1,253**	**1,295**	**452**	**604**	**26,306**	**27,362**	**3,183**	**69,735**	**713,756**	**786,674**	**2,215,154**
**N C R**	**0**	**0**	**283**	**283**	**0**	**21**	**788**	**809**	**178**	**11,822**	**116,435**	**128,435**	**432,433**
Malabon	0	0	0	0	0	0	0	0	4	185	945	1,134	7,082
Navotas	0	0	0	0	0	0	0	0	4	553	4,217	4,774	8,534
Valenzuela City	0	0	89	89	0	4	352	356	4	32	9,863	9,899	55,433
Caloocan City	0	0	0	0	0	0	1	1	6	1,090	7,910	9,006	25,841
Marikina City	0	0	0	0	0	0	11	11	0	259	1,035	1,294	9,694
Pasig City	0	0	0	0	0	0	3	3	2	174	2,342	2,518	7,231
Pateros	0	0	0	0	0	0	0	0	0	18	884	902	2,687
Taguig	0	0	0	0	0	0	2	2	37	1,291	11,961	13,289	31,149
Quezon City	0	0	2	2	0	12	294	306	20	2,087	33,053	35,160	140,375
Makati City	0	0	0	0	0	0	0	0	2	100	3,514	3,616	5,997
Mandaluyong City	0	0	0	0	0	0	5	5	0	237	2,255	2,492	3,619
San Juan	0	0	0	0	0	0	0	0	0	0	798	798	1,088
Manila City	0	0	182	182	0	0	30	30	62	2,365	17,013	19,440	74,022
Las Piñas City	0	0	0	0	0	0	1	1	1	263	3,050	3,314	9,361
Muntinlupa City	0	0	0	0	0	0	0	0	0	143	2,051	2,194	5,642
Parañaque City	0	0	7	7	0	1	0	1	16	1,010	5,073	6,099	14,545
Pasay City	0	0	3	3	0	4	89	93	20	2,015	10,471	12,506	30,133
**C A R**	**0**	**0**	**9**	**9**	**0**	**17**	**876**	**893**	**6**	**1,271**	**13,905**	**15,182**	**53,007**
Abra	0	0	0	0	0	4	50	54	0	223	2,354	2,577	5,551
Apayao	0	0	0	0	0	1	4	5	1	122	857	980	3,890
Benguet	0	0	0	0	0	7	204	211	1	285	3,065	3,351	18,106
Ifugao	0	0	0	0	0	2	297	299	0	167	2,415	2,582	8,131
Kalinga	0	0	8	8	0	0	95	95	2	248	3,176	3,426	7,159
Mt. Province	0	0	1	1	0	3	220	223	2	194	1,673	1,869	4,991
Baguio City	0	0	0	0	0	0	6	6	0	32	365	397	5,179
**Region 1**	**0**	**0**	**28**	**28**	**0**	**34**	**1,217**	**1,251**	**13**	**2,692**	**38,585**	**41,290**	**82,935**
Ilocos Norte	0	0	10	10	0	5	127	132	0	103	1,775	1,878	6,921
Ilocos Sur	0	0	15	15	0	24	502	526	0	285	5,949	6,234	13,540
La Union	0	0	0	0	0	5	212	217	6	526	5,110	5,642	11,678
Pangasinan	0	0	0	0	0	0	180	180	5	1,371	18,770	20,146	34,854
Alaminos City	0	0	0	0	0	0	0	0	0	124	1,351	1,475	2,355
Candon City	0	0	2	2	0	0	0	0	0	10	228	238	701
Dagupan City	0	0	0	0	0	0	17	17	2	99	866	967	1,925
Laoag City	0	0	0	0	0	0	0	0	0	0	1,550	1,550	1,996
San Carlos City	0	0	0	0	0	0	0	0	0	91	1,611	1,702	3,629
San Fernando City	0	0	0	0	0	0	141	141	0	3	846	849	3,522
Urdaneta City	0	0	0	0	0	0	0	0	0	32	406	438	1,168
Vigan City	0	0	1	1	0	0	38	38	0	48	123	171	646
**Region 2**	**0**	**1**	**54**	**55**	**1**	**6**	**106**	**113**	**26**	**2,680**	**23,845**	**26,551**	**74,995**
Batanes	0	0	0	0	0	0	6	6	0	22	200	222	529
Cagayan	0	0	4	4	0	0	0	0	5	632	6,149	6,786	16,468
Isabela	0	0	0	0	0	0	7	7	11	925	6,261	7,197	24,297
Nueva Vizcaya	0	1	50	51	1	6	88	95	6	571	5,519	6,096	18,380
Quirino	0	0	0	0	0	0	5	5	0	61	727	788	2,258
Cauayan City	0	0	0	0	0	0	0	0	2	151	1,459	1,612	3,619
Ilagan City	0	0	0	0	0	0	0	0	0	70	814	884	1,864
Santiago City	0	0	0	0	0	0	0	0	1	224	2,432	2,657	5,827
Tuguegarao City	0	0	0	0	0	0	0	0	1	24	284	309	1,753
**Region 3**	**0**	**1**	**19**	**20**	**0**	**22**	**699**	**721**	**14**	**5,107**	**46,588**	**51,709**	**175,381**
Aurora	0	0	0	0	0	0	3	3	0	229	2,174	2,403	5,908
Bataan	0	1	13	14	0	8	114	122	3	557	3,560	4,120	12,636

Table 1.A.3 - MODERN METHOD OF FAMILY PLANNING

Drop Outs

Annual Philippines, 2020

Area	NFP-STM Age group			Total	NFP-SDM Age group			Total	NFP-LAM Age group			Total	Total New Acceptors
	10-14	15-19	20-49		10-14	15-19	20-49		10-14	15-19	20-49		
Bulacan	0	0	0	0	0	5	256	261	0	909	8,154	9,063	26,792
Nueva Ecija	0	0	0	0	0	0	0	0	0	867	5,548	6,415	22,090
Pampanga	0	0	3	3	0	7	185	192	1	252	4,866	5,119	23,018
Tarlac	0	0	1	1	0	0	76	76	0	292	4,607	4,899	13,634
Zambales	0	0	2	2	0	1	34	35	2	285	2,869	3,156	12,344
Angeles City	0	0	0	0	0	0	6	6	1	159	1,464	1,624	3,272
Balanga City	0	0	0	0	0	0	1	1	0	83	614	697	1,494
Cabanatuan City	0	0	0	0	0	0	0	0	1	371	2,388	2,760	5,045
City of San Fernando	0	0	0	0	0	0	0	0	0	50	933	983	2,015
Gapan City	0	0	0	0	0	0	0	0	4	106	924	1,034	2,187
Mabalacat City	0	0	0	0	0	0	0	0	0	304	1,960	2,264	3,407
Malolos City	0	0	0	0	0	0	0	0	0	5	157	162	2,130
Meycauayan	0	0	0	0	0	1	24	25	2	148	1,661	1,811	3,757
Olongapo	0	0	0	0	0	0	0	0	0	146	1,293	1,439	4,387
Palayan City	0	0	0	0	0	0	0	0	0	18	285	303	886
San Jose City	0	0	0	0	0	0	0	0	0	173	1,248	1,421	3,055
San Jose del Monte City	0	0	0	0	0	0	0	0	0	0	0	0	23,716
Science City of Munoz	0	0	0	0	0	0	0	0	0	0	35	35	633
Tarlac City	0	0	0	0	0	0	0	0	0	153	1,848	2,001	2,975
**Region 4A**	**0**	**0**	**156**	**156**	**444**	**60**	**5,624**	**6,128**	**2,652**	**16,580**	**198,224**	**217,456**	**609,519**
Batangas	0	0	0	0	352	4	3,020	3,376	2,376	2,844	25,532	30,752	86,756
Cavite	0	0	0	0	0	12	40	52	4	520	15,428	15,952	39,399
Laguna	0	0	4	4	92	36	1,272	1,400	4	1,324	12,668	13,996	48,894
Quezon	0	0	40	40	0	8	1,228	1,236	16	1,704	33,712	35,432	134,232
Rizal	0	0	24	24	0	0	0	0	0	2,060	22,392	24,452	71,176
Antipolo City	0	0	0	0	0	0	0	0	0	0	0	0	0
Bacoor City	0	0	0	0	0	0	0	0	32	572	10,180	10,784	19,521
Batangas City	0	0	0	0	0	0	0	0	0	0	0	0	0
Biñan City	0	0	0	0	0	0	0	0	0	3,120	12,628	15,748	40,618
Cabuyao City	0	0	0	0	0	0	0	0	8	760	13,312	14,080	22,876
Calamba City	0	0	0	0	0	0	0	0	32	1,088	8,520	9,640	18,080
Cavite City	0	0	0	0	0	0	0	0	0	0	0	0	0
Dasmariñas City	0	0	0	0	0	0	0	0	0	404	8,248	8,652	31,520
General Trias City	0	0	0	0	0	0	20	20	4	156	4,380	4,540	16,436
Imus City	0	0	0	0	0	0	0	0	0	36	2,940	2,976	8,967
Lipa City	0	0	0	0	0	0	0	0	0	0	0	0	6
Lucena City	0	0	0	0	0	0	0	0	104	0	956	1,060	8,480
San Pablo City	0	0	0	0	0	0	0	0	0	0	0	0	0
San Pedro City	0	0	0	0	0	0	32	32	0	668	7,372	8,040	10,784
Santa Rosa City	0	0	0	0	0	0	0	0	0	1,212	7,504	8,716	23,544
Tagaytay City	0	0	0	0	0	0	0	0	72	24	4,264	4,360	7,504
Tanauan City	0	0	0	0	0	0	0	0	0	8	1,136	1,144	4,593
Tayabas City	0	0	88	88	0	0	12	12	0	68	6,720	6,788	12,041
Trece Martires City	0	0	0	0	0	0	0	0	0	12	332	344	4,092
**Region 4B**	**0**	**0**	**36**	**36**	**0**	**1**	**211**	**212**	**20**	**1,368**	**16,578**	**17,966**	**37,506**
Marinduque	0	0	14	14	0	0	32	32	1	26	1,053	1,080	3,132
Mindoro Occidental	0	0	0	0	0	1	25	26	15	980	7,392	8,387	13,527
Mindoro Oriental	0	0	18	18	0	0	54	54	3	190	5,430	5,623	11,612
Palawan	0	0	0	0	0	0	38	38	1	161	1,360	1,522	3,671
Romblon	0	0	4	4	0	0	30	30	0	11	325	336	494
Puerto Princesa City	0	0	0	0	0	0	32	32	0	0	1,018	1,018	5,070
**Region 5**	**0**	**29**	**352**	**381**	**1**	**184**	**9,224**	**9,409**	**25**	**4,984**	**54,402**	**59,411**	**123,250**
Albay	0	1	27	28	1	7	306	314	3	376	5,266	5,645	11,734
Camarines Norte	0	0	2	2	0	34	621	655	0	759	5,596	6,355	14,026
Camarines Sur	0	28	303	331	0	55	5,627	5,682	12	1,504	18,881	20,397	44,651
Catanduanes	0	0	0	0	0	19	406	425	0	258	2,536	2,794	7,194
Masbate	0	0	0	0	0	37	964	1,001	5	1,150	9,605	10,760	18,684
Sorsogon	0	0	1	1	0	32	1,038	1,070	5	875	10,336	11,216	21,181
Iriga City	0	0	17	17	0	0	182	182	0	54	948	1,002	2,270

Table 1.A.3 - MODERN METHOD OF FAMILY PLANNING

Drop Outs

Annual Philippines, 2020

Area	NFP-STM			Total	NFP-SDM			Total	NFP-LAM			Total	Total New Acceptors
	Age group				Age group				Age group				
	10-14	15-19	20-49		10-14	15-19	20-49		10-14	15-19	20-49		
Legaspi City	0	0	0	0	0	0	1	1	0	7	246	253	590
Naga City	0	0	2	2	0	0	79	79	0	1	988	989	2,920
**Region 6**	**0**	**0**	**14**	**14**	**6**	**59**	**2,843**	**2,908**	**59**	**3,966**	**48,955**	**52,980**	**122,574**
Aklan	0	0	0	0	0	4	149	153	3	299	6,753	7,055	13,950
Antique	0	0	14	14	0	10	1,710	1,720	17	268	4,923	5,208	19,232
Capiz	0	0	0	0	0	0	1	1	0	97	2,295	2,392	6,035
Guimaras	0	0	0	0	0	0	12	12	0	151	1,841	1,992	3,411
Iloilo	0	0	0	0	0	23	574	597	3	596	10,452	11,051	26,119
Negros Occidental	0	0	0	0	6	22	371	399	35	2,329	20,945	23,309	46,268
Bacolod City	0	0	0	0	0	0	0	0	1	190	1,446	1,637	4,486
Iloilo City	0	0	0	0	0	0	26	26	0	36	300	336	3,073
**Region 7**	**0**	**0**	**7**	**7**	**0**	**0**	**434**	**434**	**21**	**3,175**	**33,107**	**36,303**	**82,213**
Bohol	0	0	0	0	0	0	275	275	0	804	3,069	3,873	12,812
Cebu	0	0	0	0	0	0	1	1	3	294	5,867	6,164	13,677
Negros Oriental	0	0	2	2	0	0	26	26	6	484	5,356	5,846	15,996
Siquijor	0	0	0	0	0	0	116	116	0	68	539	607	2,489
Cebu City	0	0	5	5	0	0	10	10	12	1,223	12,644	13,879	23,279
Lapu-Lapu City	0	0	0	0	0	0	6	6	0	302	5,337	5,639	13,398
Mandaue City	0	0	0	0	0	0	0	0	0	0	295	295	562
**Region 8**	**0**	**0**	**0**	**0**	**0**	**0**	**0**	**0**	**0**	**0**	**0**	**0**	**2,477**
Biliran	0	0	0	0	0	0	0	0	0	0	0	0	235
Eastern Samar	0	0	0	0	0	0	0	0	0	0	0	0	323
Northern Leyte	0	0	0	0	0	0	0	0	0	0	0	0	0
Northern Samar	0	0	0	0	0	0	0	0	0	0	0	0	125
Southern Leyte	0	0	0	0	0	0	0	0	0	0	0	0	376
Western Samar	0	0	0	0	0	0	0	0	0	0	0	0	414
Calbayog City	0	0	0	0	0	0	0	0	0	0	0	0	320
Maasin City	0	0	0	0	0	0	0	0	0	0	0	0	144
Ormoc City	0	0	0	0	0	0	0	0	0	0	0	0	345
Tacloban City	0	0	0	0	0	0	0	0	0	0	0	0	195
**Region 9**	**0**	**0**	**0**	**0**	**0**	**0**	**0**	**0**	**0**	**102**	**3,140**	**3,242**	**8,270**
Zamboanga del Norte	0	0	0	0	0	0	0	0	0	0	0	0	0
Zamboanga del Sur	0	0	0	0	0	0	0	0	0	0	0	0	0
Zamboanga Sibugay	0	0	0	0	0	0	0	0	0	0	0	0	0
Dapitan City	0	0	0	0	0	0	0	0	0	0	22	22	487
Dipolog City	0	0	0	0	0	0	0	0	0	0	0	0	995
Isabela City	0	0	0	0	0	0	0	0	0	48	285	333	663
Pagadian City	0	0	0	0	0	0	0	0	0	5	551	556	998
Zamboanga City	0	0	0	0	0	0	0	0	0	49	2,282	2,331	5,127
**Region 10**	**0**	**0**	**169**	**169**	**0**	**153**	**2,178**	**2,331**	**39**	**5,124**	**40,500**	**45,663**	**121,937**
Bukidnon	0	0	0	0	0	6	191	197	6	1,490	9,165	10,661	45,037
Camiguin	0	0	0	0	0	0	22	22	0	61	932	993	1,572
Lanao del Norte	0	0	4	4	0	1	156	157	6	420	4,160	4,586	11,782
Misamis Occidental	0	0	0	0	0	28	457	485	0	85	1,417	1,502	3,655
Misamis Oriental	0	0	3	3	0	25	370	395	2	478	5,317	5,797	13,464
Cagayan de Oro City	0	0	0	0	0	0	45	45	11	932	8,876	9,819	16,358
El Salvador City	0	0	0	0	0	0	7	7	0	90	592	682	1,584
Gingoog City	0	0	146	146	0	40	442	482	3	252	1,092	1,347	6,140
Iligan City	0	0	0	0	0	47	124	171	9	536	4,060	4,605	9,867
Malaybalay City	0	0	16	16	0	5	201	206	0	192	1,578	1,770	5,686
Oroquieta City	0	0	0	0	0	0	2	2	0	4	105	109	210
Ozamis City	0	0	0	0	0	0	16	16	2	144	593	739	1,200
Tangub City	0	0	0	0	0	0	45	45	0	57	268	325	465
Valencia City	0	0	0	0	0	1	100	101	0	383	2,345	2,728	4,917

Table 1.A.3 - MODERN METHOD OF FAMILY PLANNING

Drop Outs

Annual Philippines, 2020

Area	NFP-STM Age group 10-14	NFP-STM Age group 15-19	NFP-STM Age group 20-49	Total	NFP-SDM Age group 10-14	NFP-SDM Age group 15-19	NFP-SDM Age group 20-49	Total	NFP-LAM Age group 10-14	NFP-LAM Age group 15-19	NFP-LAM Age group 20-49	Total	Total New Acceptors
**Region 11**	**0**	**0**	**18**	**18**	**0**	**5**	**508**	**513**	**92**	**2,879**	**20,794**	**23,765**	**86,677**
Davao de Oro	0	0	4	4	0	1	24	25	3	147	1,347	1,497	12,270
Davao del Norte	0	0	7	7	0	0	89	89	58	590	4,660	5,308	19,783
Davao Oriental	0	0	0	0	0	2	337	339	15	654	5,181	5,850	11,522
Davao del Sur	0	0	0	0	0	0	18	18	1	137	866	1,004	8,064
Davao Occidental	0	0	0	0	0	2	29	31	13	294	1,263	1,570	8,604
Davao City	0	0	7	7	0	0	11	11	2	1,057	7,477	8,536	26,434
**Region 12**	**0**	**11**	**87**	**98**	**0**	**6**	**285**	**291**	**19**	**5,281**	**34,728**	**40,028**	**106,153**
North Cotabato	0	11	80	91	0	1	95	96	8	941	6,440	7,389	25,403
Sarangani	0	0	7	7	0	1	18	19	0	1,327	7,020	8,347	17,125
South Cotabato	0	0	0	0	0	4	118	122	9	1,184	8,073	9,266	28,198
Sultan Kudarat	0	0	0	0	0	0	30	30	0	1,374	9,231	10,605	23,993
				0				0				0	
Cotabato City	0	0	0	0	0	0	24	24	2	284	2,511	2,797	7,575
Gen. Santos City	0	0	0	0	0	0	0	0	0	171	1,453	1,624	3,859
**B.A.R.M.M.**	**0**	**0**	**0**	**0**	**0**	**0**	**0**	**0**	**0**	**0**	**0**	**0**	**14,022**
Basilan	0	0	0	0	0	0	0	0	0	0	0	0	324
Lanao del Sur	0	0	0	0	0	0	0	0	0	0	0	0	1,047
Maguindanao	0	0	0	0	0	0	0	0	0	0	0	0	1,085
Sulu	0	0	0	0	0	0	0	0	0	0	0	0	82
Tawi-Tawi	0	0	0	0	0	0	0	0	0	0	0	0	104
				0				0				0	
Lamitan City	0	0	0	0	0	0	0	0	0	0	0	0	11,171
Marawi City	0	0	0	0	0	0	0	0	0	0	0	0	209
**CARAGA**	**0**	**0**	**21**	**21**	**0**	**36**	**1,313**	**1,349**	**19**	**2,704**	**23,970**	**26,693**	**81,805**
Agusan del Norte	0	0	21	21	0	0	136	136	6	385	2,903	3,294	13,133
Agusan del Sur	0	0	0	0	0	34	517	551	2	671	6,269	6,942	30,506
Surigao del Norte	0	0	0	0	0	1	210	211	0	142	2,668	2,810	7,639
Surigao del Sur	0	0	0	0	0	1	209	210	3	556	4,669	5,228	12,766
Province of Dinagat	0	0	0	0	0	0	141	141	0	46	660	706	2,625
Bislig City	0	0	0	0	0	0	92	92	6	169	1,390	1,565	3,252
Butuan City	0	0	0	0	0	0	0	0	1	674	4,621	5,296	8,774
Surigao City	0	0	0	0	0	0	8	8	1	61	790	852	3,110

Table 1.A.4 - MODERN METHOD OF FAMILY PLANNING
Current User (Ending)
Annual Philippines, 2020

Area	Total Current Users	FSTR/BTL Age group 10-14	15-19	20-49	Total	MSTR/NSV Age group 10-14	15-19	20-49	Total
**PHILIPPINES**	**7,366,659**	**594**	**11,652**	**812,354**	**827,672**	**7**	**94**	**9,504**	**9,605**
**N C R**	**726,175**	**4**	**258**	**83,437**	**86,771**	**0**	**9**	**647**	**656**
Malabon	32,409	0	0	3,072	3,072	0	0	3	3
Navotas	23,787	0	2	527	529	0	4	4	8
Valenzuela City	39,524	0	0	6,322	6,322	0	0	68	68
Caloocan City	67,903	0	1	10,011	10,012	0	0	13	13
Marikina City	15,458	0	0	2,218	2,218	0	0	195	195
Pasig City	37,512	0	1	3,348	3,349	0	0	20	20
Pateros	2,558	0	1	360	361	0	1	4	5
Taguig	57,590	0	0	2,941	2,941	0	0	7	7
Quezon City	259,021	0	67	34,678	34,745	0	0	222	222
Makati City	12,799	0	0	1,267	1,267	0	0	8	8
Mandaluyong City	16,826	0	1	1,967	1,968	0	0	21	21
San Juan	5,760	0	0	257	257	0	0	10	10
Manila City	31,623	0	1	4,331	4,332	0	0	14	14
Las Piñas City	22,298	0	0	3,599	3,599	0	0	10	10
Muntinlupa City	40,077	0	1	3,146	3,147	0	1	12	13
Parañaque City	34,003	4	183	2,300	2,487	0	2	29	31
Pasay City	27,027	0	0	6,165	6,165	0	1	7	8
**C A R**	**131,969**	**0**	**0**	**30,637**	**30,637**	**0**	**0**	**153**	**153**
Abra	16,967	0	0	4,250	4,250	0	0	9	9
Apayao	12,113	0	0	1,928	1,928	0	0	8	8
Benguet	30,721	0	0	7,936	7,936	0	0	19	19
Ifugao	19,056	0	0	2,780	2,780	0	0	101	101
Kalinga	18,965	0	0	3,475	3,475	0	0	5	5
Mt. Province	12,528	0	0	4,671	4,671	0	0	3	3
Baguio City	21,619	0	0	5,597	5,597	0	0	8	8
**Region 1**	**467,431**	**0**	**129**	**72,096**	**72,225**	**0**	**0**	**111**	**111**
Ilocos Norte	41,294	0	9	8,539	8,548	0	0	16	16
Ilocos Sur	58,198	0	16	8,751	8,767	0	0	5	5
La Union	59,945	0	7	10,873	10,880	0	0	21	21
Pangasinan	233,627	0	29	31,437	31,466	0	0	43	43
Alaminos City	10,247	0	0	1,095	1,095	0	0	0	0
Candon City	7,007	0	0	894	894	0	0	0	0
Dagupan City	6,896	0	0	940	940	0	0	2	2
Laoag City	8,101	0	0	1,935	1,935	0	0	0	0
San Carlos City	11,818	0	0	2,487	2,487	0	0	0	0
San Fernando City	12,806	0	0	2,358	2,358	0	0	23	23
Urdaneta City	12,828	0	68	1,596	1,664	0	0	1	1
Vigan City	4,664	0	0	1,191	1,191	0	0	0	0
**Region 2**	**315,025**	**0**	**32**	**46,118**	**46,150**	**0**	**0**	**109**	**109**
Batanes	938	0	0	174	174	0	0	0	0
Cagayan	87,099	0	20	11,547	11,567	0	0	28	28
Isabela	108,463	0	3	16,737	16,740	0	0	22	22
Nueva Vizcaya	45,287	0	3	8,198	8,201	0	0	33	33
Quirino	19,270	0	0	2,470	2,470	0	0	7	7
Cauayan City	9,889	0	6	1,733	1,739	0	0	0	0
Ilagan City	15,732	0	0	1,366	1,366	0	0	0	0
Santiago City	13,933	0	0	1,483	1,483	0	0	0	0
Tuguegarao City	14,414	0	0	2,410	2,410	0	0	19	19
**Region 3**	**776,623**	**17**	**349**	**139,722**	**140,088**	**0**	**0**	**546**	**546**
Aurora	21,188	0	3	1,978	1,981	0	0	10	10
Bataan	45,758	0	7	3,724	3,731	0	0	6	6

Table 1.A.4 - MODERN METHOD OF FAMILY PLANNING

Current User (Ending)

Annual Philippines, 2020

Area	Total Current Users	FSTR/BTL Age group 10-14	FSTR/BTL Age group 15-19	FSTR/BTL Age group 20-49	Total	MSTR/NSV Age group 10-14	MSTR/NSV Age group 15-19	MSTR/NSV Age group 20-49	Total
Bulacan	148,564	0	6	26,964	26,970	0	0	373	373
Nueva Ecija	123,180	0	164	23,642	23,806	0	0	3	3
Pampanga	101,506	0	22	33,792	33,814	0	0	15	15
Tarlac	82,011	0	0	11,884	11,884	0	0	5	5
Zambales	42,194	17	2	7,467	7,486	0	0	9	9
Angeles City	23,101	0	0	5,177	5,177	0	0	71	71
Balanga City	4,908	0	0	925	925	0	0	3	3
Cabanatuan City	19,136	0	8	3,879	3,887	0	0	0	0
City of San Fernando	9,489	0	134	1,733	1,867	0	0	25	25
Gapan City	7,509	0	0	1,817	1,817	0	0	0	0
Mabalacat City	23,876	0	0	3,079	3,079	0	0	3	3
Malolos City	7,548	0	0	1,283	1,283	0	0	6	6
Meycauayan	7,990	0	1	381	382	0	0	5	5
Olongapo	9,721	0	2	1,588	1,590	0	0	3	3
Palayan City	3,010	0	0	544	544	0	0	0	0
San Jose City	12,976	0	0	790	790	0	0	2	2
San Jose del Monte City	57,952	0	0	6,158	6,158	0	0	5	5
Science City of Munoz	6,335	0	0	1,274	1,274	0	0	1	1
Tarlac City	18,671	0	0	1,643	1,643	0	0	1	1
**Region 4A**	**823,547**	**552**	**3,774**	**136,822**	**141,148**	**1**	**10**	**282**	**293**
Batangas	137,894	131	1,616	57,102	58,850	0	1	73	74
Cavite	96,284	30	376	16,017	16,424	0	1	34	35
Laguna	57,329	20	215	7,007	7,241	0	1	4	5
Quezon	106,348	22	266	8,337	8,626	0	2	-1	1
Rizal	94,069	25	268	12,534	12,827	0	1	33	34
Antipolo City	37,890	6	80	2,994	3,081	0	1	19	20
Bacoor City	6,766	4	44	1,735	1,783	0	0	1	1
Batangas City	17,787	7	81	3,056	3,144	0	0	4	4
Biñan City	66,115	3	35	1,531	1,569	0	0	-1	-1
Cabuyao City	9,078	41	46	1,373	1,459	0	0	2	2
Calamba City	29,572	23	121	4,359	4,502	0	0	7	7
Cavite City	4,572	3	36	1,363	1,402	0	0	2	2
Dasmariñas City	31,812	11	136	3,080	3,227	0	1	33	34
General Trias City	10,570	9	66	2,597	2,673	0	1	19	20
Imus City	7,971	2	28	1,416	1,446	0	0	3	3
Lipa City	13,661	6	79	2,972	3,058	0	0	0	0
Lucena City	14,839	158	10	333	501	0	0	4	4
San Pablo City	10,199	4	45	1,672	1,720	0	0	3	3
San Pedro City	16,390	2	40	1,037	1,079	0	0	11	11
Santa Rosa City	33,778	5	62	2,621	2,688	0	0	17	17
Tagaytay City	8,120	2	29	1,120	1,151	0	0	12	12
Tanauan City	5,762	35	47	1,143	1,225	0	0	2	2
Tayabas City	2,814	2	21	682	705	0	0	2	2
Trece Martires City	3,926	2	26	742	770	0	0	1	1
**Region 4B**	**170,647**	**0**	**0**	**18,126**	**18,126**	**0**	**0**	**72**	**72**
Marinduque	13,772	0	0	1,930	1,930	0	0	9	9
Mindoro Occidental	37,131	0	0	3,194	3,194	0	0	8	8
Mindoro Oriental	61,398	0	0	6,431	6,431	0	0	32	32
Palawan	23,421	0	0	3,090	3,090	0	0	0	0
Romblon	3,988	0	0	734	734	0	0	11	11
Puerto Princesa City	30,937	0	0	2,747	2,747	0	0	12	12
**Region 5**	**429,559**	**0**	**10**	**28,740**	**28,750**	**2**	**0**	**331**	**333**
Albay	86,082	0	0	4,767	4,767	0	0	28	28
Camarines Norte	29,147	0	2	1,413	1,415	2	0	12	14
Camarines Sur	103,034	0	2	5,934	5,936	0	0	23	23
Catanduanes	25,339	0	0	3,364	3,364	0	0	98	98
Masbate	64,402	0	1	2,475	2,476	0	0	28	28
Sorsogon	63,538	0	5	6,162	6,167	0	0	72	72
Iriga City	12,388	0	0	625	625	0	0	12	12

Table 1.A.4 - MODERN METHOD OF FAMILY PLANNING
Current User (Ending)
Annual Philippines, 2020

Area	Total Current Users	FSTR/BTL Age group			Total	MSTR/NSV Age group			Total
		10-14	15-19	20-49		10-14	15-19	20-49	
Legaspi City	23,098	0	0	1,854	1,854	0	0	0	0
Naga City	22,531	0	0	2,146	2,146	0	0	58	58
**Region 6**	**605,490**	**0**	**130**	**49,217**	**49,347**	**0**	**0**	**2,063**	**2,063**
Aklan	44,142	0	1	3,690	3,691	0	0	14	14
Antique	46,065	0	76	7,096	7,172	0	0	95	95
Capiz	82,483	0	42	5,676	5,718	0	0	359	359
Guimaras	15,919	0	2	1,282	1,284	0	0	7	7
Iloilo	153,707	0	0	12,889	12,889	0	0	306	306
Negros Occidental	190,516	0	7	11,847	11,854	0	0	1,173	1,173
Bacolod City	26,414	0	0	1,731	1,731	0	0	92	92
Iloilo City	46,244	0	2	5,006	5,008	0	0	17	17
**Region 7**	**308,351**	**0**	**12**	**21,434**	**21,446**	**0**	**0**	**1,117**	**1,117**
Bohol	68,267	0	0	9,332	9,332	0	0	240	240
Cebu	78,850	0	0	4,574	4,574	0	0	215	215
Negros Oriental	62,446	0	12	2,507	2,519	0	0	268	268
Siquijor	15,486	0	0	479	479	0	0	1	1
Cebu City	27,434	0	0	1,662	1,662	0	0	346	346
Lapu-Lapu City	36,540	0	0	2,545	2,545	0	0	46	46
Mandaue City	19,328	0	0	335	335	0	0	1	1
**Region 8**	**201,654**	**0**	**2**	**18,657**	**18,659**	**0**	**-2**	**353**	**351**
Biliran	10,555	0	0	1,127	1,127	0	0	66	66
Eastern Samar	40,849	0	2	5,122	5,124	0	0	110	110
Northern Leyte	0	0	0	0	0	0	0	0	0
Northern Samar	34,542	0	0	1,530	1,530	0	0	81	81
Southern Leyte	24,192	0	0	3,290	3,290	0	-2	49	47
Western Samar	15,945	0	0	982	982	0	0	1	1
Calbayog City	8,789	0	0	1,090	1,090	0	0	8	8
Maasin City	2,626	0	0	428	428	0	0	2	2
Ormoc City	19,478	0	0	1,437	1,437	0	0	15	15
Tacloban City	44,678	0	0	3,651	3,651	0	0	21	21
**Region 9**	**272,273**	**0**	**-2**	**14,360**	**14,358**	**0**	**0**	**182**	**182**
Zamboanga del Norte	86,000	0	0	4,327	4,327	0	0	90	90
Zamboanga del Sur	57,156	0	2	2,170	2,172	0	0	36	36
Zamboanga Sibugay	24,011	0	0	1,304	1,304	0	0	14	14
Dapitan City	7,642	0	0	765	765	0	0	12	12
Dipolog City	15,682	0	-6	930	924	0	0	2	2
Isabela City	8,824	0	2	886	888	0	0	4	4
Pagadian City	17,520	0	0	1,195	1,195	0	0	24	24
Zamboanga City	55,438	0	0	2,783	2,783	0	0	0	0
**Region 10**	**569,378**	**0**	**55**	**35,436**	**35,491**	**4**	**44**	**294**	**342**
Bukidnon	100,541	0	3	6,245	6,248	4	43	64	111
Camiguin	6,472	0	0	1,094	1,094	0	0	6	6
Lanao del Norte	60,032	0	2	1,266	1,268	0	0	8	8
Misamis Occidental	25,728	0	6	1,529	1,535	0	0	9	9
Misamis Oriental	80,433	0	-1	6,228	6,227	0	0	102	102
Cagayan de Oro City	149,241	0	1	9,880	9,881	0	0	-50	-50
El Salvador City	6,238	0	0	382	382	0	0	0	0
Gingoog City	11,893	0	16	885	901	0	0	-38	-38
Iligan City	27,468	0	3	1,785	1,788	0	0	24	24
Malaybalay City	21,851	0	0	1,735	1,735	0	0	37	37
Oroquieta City	6,583	0	0	813	813	0	0	2	2
Ozamis City	46,199	0	24	894	918	0	1	49	50
Tangub City	5,298	0	0	343	343	0	0	0	0
Valencia City	21,401	0	1	2,357	2,358	0	0	81	81

Table 1.A.4 - MODERN METHOD OF FAMILY PLANNING
Current User (Ending)
Annual Philippines, 2020

Area	Total Current Users	FSTR/BTL Age group 10-14	FSTR/BTL Age group 15-19	FSTR/BTL Age group 20-49	Total	MSTR/NSV Age group 10-14	MSTR/NSV Age group 15-19	MSTR/NSV Age group 20-49	Total
**Region 11**	**501,926**	**19**	**287**	**46,280**	**46,586**	**0**	**16**	**1,882**	**1,898**
Davao de Oro	83,025	6	58	7,903	7,967	0	0	179	179
Davao del Norte	100,275	2	93	11,437	11,532	0	0	611	611
Davao Oriental	51,722	1	14	5,571	5,586	0	1	133	134
Davao del Sur	65,428	3	7	4,987	4,997	0	11	77	88
Davao Occidental	32,000	6	53	4,391	4,450	0	1	66	67
Davao City	169,476	1	62	11,991	12,054	0	3	816	819
**Region 12**	**491,430**	**2**	**1,706**	**45,537**	**47,245**	**0**	**12**	**1,069**	**1,081**
North Cotabato	145,934	0	1,028	8,492	9,520	0	0	146	146
Sarangani	68,171	0	395	4,789	5,184	0	6	117	123
South Cotabato	98,673	2	100	10,595	10,697	0	6	535	541
Sultan Kudarat	93,050	0	121	9,517	9,638	0	0	173	173
Cotabato City	34,872	0	0	3,058	3,058	0	0	0	0
Gen. Santos City	50,730	0	62	9,086	9,148	0	0	98	98
**B.A.R.M.M.**	**326,001**	**0**	**4,903**	**6,426**	**11,329**	**0**	**3**	**72**	**75**
Basilan	12,868	0	0	349	349	0	0	13	13
Lanao del Sur	87,517	0	0	2,270	2,270	0	0	26	26
Maguindanao	112,799	0	0	3,474	3,474	0	0	33	33
Sulu	56,935	0	3,054	0	3,054	0	0	0	0
Tawi-Tawi	38,160	0	1,849	7	1,856	0	3	0	3
Lamitan City	10,671	0	0	6	6	0	0	0	0
Marawi City	7,051	0	0	320	320	0	0	0	0
**CARAGA**	**249,180**	**0**	**7**	**19,309**	**19,316**	**0**	**2**	**221**	**223**
Agusan del Norte	37,360	0	2	3,185	3,187	0	0	11	11
Agusan del Sur	76,525	0	0	6,081	6,081	0	1	62	63
Surigao del Norte	30,743	0	2	2,056	2,058	0	0	37	37
Surigao del Sur	42,013	0	1	3,144	3,145	0	0	63	63
Province of Dinagat	10,218	0	1	552	553	0	0	4	4
Bislig City	9,004	0	0	685	685	0	0	5	5
Butuan City	30,541	0	1	2,645	2,646	0	0	18	18
Surigao City	12,776	0	0	961	961	0	1	21	22

Table 1.A.4 - MODERN METHOD OF FAMILY PLANNING
Current User (Ending)
Annual Philippines, 2020

Area	CONDOM			Total	IUD-INTERVAL			Total	IUD-POSTPARTUM			Total
	Age group				Age group				Age group			
	10-14	15-19	20-49		10-14	15-19	20-49		10-14	15-19	20-49	
**PHILIPPINES**	**1,122**	**13,109**	**357,314**	**371,545**	**290**	**11,550**	**400,070**	**411,911**	**99**	**9,061**	**112,072**	**121,232**
**N C R**	**41**	**3,233**	**72,390**	**75,664**	**12**	**955**	**44,968**	**45,935**	**53**	**2,087**	**18,370**	**20,510**
Malabon	2	902	1,960	2,864	0	141	1,666	1,807	0	95	200	295
Navotas	1	229	1,656	1,886	0	6	441	447	0	4	47	51
Valenzuela City	0	1	782	783	0	16	461	477	0	32	678	710
Caloocan City	0	299	7,137	7,436	4	249	5,341	5,594	5	143	683	831
Marikina City	0	1	635	636	0	44	1,153	1,197	10	256	1,173	1,439
Pasig City	8	115	1,979	2,102	0	26	2,069	2,095	5	240	818	1,063
Pateros	1	1	178	180	1	1	360	362	0	0	0	0
Taguig	7	168	2,708	2,883	1	24	1,641	1,666	0	24	209	233
Quezon City	7	946	47,336	48,289	0	315	20,269	20,584	29	748	12,445	13,222
Makati City	0	23	614	637	0	10	468	478	0	7	144	151
Mandaluyong City	0	18	1,075	1,093	0	2	533	535	0	0	39	39
San Juan	0	0	158	158	0	0	1,358	1,358	0	0	0	0
Manila City	0	48	437	485	0	4	677	681	0	51	623	674
Las Piñas City	0	24	1,256	1,280	0	11	555	566	1	0	25	26
Muntinlupa City	0	54	1,060	1,114	1	68	3,228	3,297	0	7	52	59
Parañaque City	15	187	1,986	2,188	5	37	1,079	1,121	3	480	635	1,118
Pasay City	0	217	1,433	1,650	0	1	3,669	3,670	0	0	599	599
**C A R**	**0**	**61**	**5,381**	**5,442**	**1**	**24**	**6,305**	**6,330**	**0**	**41**	**6,096**	**6,137**
Abra	0	3	321	324	0	0	65	65	0	0	24	24
Apayao	0	6	136	142	0	2	353	355	0	4	15	19
Benguet	0	19	2,413	2,432	1	13	2,629	2,643	0	3	465	468
Ifugao	0	9	565	574	0	3	1,014	1,017	0	1	7	8
Kalinga	0	3	658	661	0	3	781	784	0	2	30	32
Mt. Province	0	3	513	516	0	3	516	519	0	30	61	91
Baguio City	0	18	775	793	0	0	947	947	0	1	5,494	5,495
**Region 1**	**0**	**427**	**19,393**	**19,820**	**0**	**345**	**10,453**	**10,798**	**1**	**28**	**534**	**563**
Ilocos Norte	0	17	1,444	1,461	0	15	507	522	0	6	123	129
Ilocos Sur	0	14	1,176	1,190	0	1	605	606	0	1	95	96
La Union	0	12	1,434	1,446	0	38	1,497	1,535	1	10	96	107
Pangasinan	0	317	12,983	13,300	0	241	6,759	7,000	0	0	0	0
Alaminos City	0	4	274	278	0	0	140	140	0	0	0	0
Candon City	0	0	955	955	0	0	0	0	0	0	136	136
Dagupan City	0	8	171	179	0	6	76	82	0	4	69	73
Laoag City	0	0	33	33	0	0	70	70	0	0	0	0
San Carlos City	0	0	367	367	0	7	388	395	0	0	0	0
San Fernando City	0	2	259	261	0	14	152	166	0	7	15	22
Urdaneta City	0	49	296	345	0	23	256	279	0	0	0	0
Vigan City	0	4	1	5	0	0	3	3	0	0	0	0
**Region 2**	**5**	**158**	**3,382**	**3,545**	**3**	**400**	**17,693**	**18,096**	**2**	**217**	**1,202**	**1,421**
Batanes	0	0	16	16	0	0	13	13	0	0	0	0
Cagayan	1	15	337	353	2	112	8,711	8,825	0	68	191	259
Isabela	2	52	1,177	1,231	0	112	3,243	3,355	2	80	456	538
Nueva Vizcaya	1	13	1,210	1,224	0	4	810	814	0	2	75	77
Quirino	0	19	263	282	0	3	728	731	0	0	4	4
Cauayan City	0	1	81	82	0	3	294	297	0	4	23	27
Ilagan City	1	54	106	161	0	27	905	932	0	4	127	131
Santiago City	0	0	54	54	1	20	388	409	0	0	0	0
Tuguegarao City	0	4	138	142	0	119	2,601	2,720	0	59	326	385
**Region 3**	**10**	**974**	**32,667**	**33,651**	**0**	**301**	**12,272**	**12,573**	**1**	**95**	**2,906**	**3,002**
Aurora	1	18	1,050	1,069	0	7	282	289	0	3	70	73
Bataan	6	79	2,359	2,444	0	25	537	562	1	11	197	209

Table 1.A.4 - MODERN METHOD OF FAMILY PLANNING
Current User (Ending)
Annual Philippines, 2020

Area	CONDOM			Total	IUD-INTERVAL			Total	IUD-POSTPARTUM			Total
	Age group				Age group				Age group			
	10-14	15-19	20-49		10-14	15-19	20-49		10-14	15-19	20-49	
Bulacan	0	52	2,915	2,967	0	27	3,487	3,514	0	29	1,331	1,360
Nueva Ecija	0	141	2,522	2,663	0	70	2,399	2,469	0	18	444	462
Pampanga	0	130	3,708	3,838	0	8	516	524	0	16	230	246
Tarlac	0	72	3,186	3,258	0	61	927	988	0	0	265	265
Zambales	0	16	1,045	1,061	0	2	595	597	0	6	162	168
Angeles City	0	16	601	617	0	5	271	276	0	0	56	56
Balanga City	0	2	99	101	0	0	39	39	0	0	0	0
Cabanatuan City	0	19	382	401	0	5	106	111	0	5	32	37
City of San Fernando	3	49	748	800	0	4	14	18	0	1	40	41
Gapan City	0	12	113	125	0	1	41	42	0	1	3	4
Mabalacat City	0	46	2,269	2,315	0	7	294	301	0	1	1	2
Malolos City	0	13	387	400	0	1	73	74	0	0	0	0
Meycauayan	0	37	383	420	0	0	15	15	0	1	26	27
Olongapo	0	1	621	622	0	0	50	50	0	1	27	28
Palayan City	0	0	17	17	0	1	181	182	0	0	0	0
San Jose City	0	9	176	185	0	1	121	122	0	2	6	8
San Jose del Monte City	0	124	9,243	9,367	0	20	1,839	1,859	0	0	0	0
Science City of Munoz	0	0	84	84	0	2	120	122	0	0	0	0
Tarlac City	0	138	759	897	0	54	365	419	0	0	16	16
**Region 4A**	**979**	**2,943**	**49,238**	**53,160**	**238**	**1,157**	**40,547**	**41,943**	**5**	**105**	**-11**	**99**
Batangas	14	128	4,741	4,882	9	141	4,449	4,600	0	12	-72	-60
Cavite	6	118	5,598	5,722	12	122	4,407	4,542	0	6	19	25
Laguna	11	93	4,367	4,470	12	209	5,853	6,073	0	-5	-175	-180
Quezon	6	84	3,598	3,688	20	211	7,557	7,788	1	-2	-335	-336
Rizal	9	-65	7,339	7,283	8	87	4,031	4,126	4	83	499	586
Antipolo City	3	42	1,574	1,619	3	32	1,203	1,238	0	0	0	0
Bacoor City	1	12	106	118	1	12	312	325	0	0	0	0
Batangas City	3	32	1,204	1,239	5	64	2,420	2,490	0	0	0	0
Biñan City	8	2,281	9,412	11,701	2	38	1,545	1,585	0	10	15	25
Cabuyao City	2	19	427	448	1	6	414	421	0	1	-7	-6
Calamba City	4	89	1,586	1,680	5	59	2,049	2,113	0	0	16	16
Cavite City	0	1	54	56	0	3	111	114	0	0	0	0
Dasmariñas City	5	50	-135	-80	5	48	2,050	2,103	0	0	-7	-7
General Trias City	1	4	260	265	1	10	427	437	0	-1	16	15
Imus City	1	14	382	397	0	6	118	124	0	0	2	2
Lipa City	2	27	1,022	1,052	3	34	1,271	1,308	0	0	0	0
Lucena City	887	3	144	1,034	145	1	40	186	0	0	6	6
San Pablo City	0	3	128	132	2	22	842	866	0	0	0	0
San Pedro City	1	54	889	945	1	22	535	558	0	3	4	7
Santa Rosa City	13	-79	5,656	5,590	1	11	388	400	0	1	45	46
Tagaytay City	1	9	380	389	0	2	61	63	0	0	7	7
Tanauan City	1	17	383	401	1	11	374	385	0	-3	-44	-47
Tayabas City	0	5	107	113	0	3	67	70	0	0	0	0
Trece Martires City	0	0	17	17	1	6	23	30	0	0	0	0
**Region 4B**	**0**	**11**	**4,812**	**4,823**	**0**	**49**	**4,858**	**4,907**	**0**	**19**	**4,143**	**4,162**
Marinduque	0	1	319	320	0	0	454	454	0	0	0	0
Mindoro Occidental	0	2	1,071	1,073	0	7	1,409	1,416	0	4	17	21
Mindoro Oriental	0	6	1,580	1,586	0	42	2,230	2,272	0	15	3,745	3,760
Palawan	0	2	816	818	0	0	347	347	0	0	2	2
Romblon	0	0	101	101	0	0	418	418	0	0	19	19
Puerto Princesa City	0	0	925	925	0	0	0	0	0	0	360	360
**Region 5**	**10**	**334**	**25,717**	**26,061**	**0**	**39**	**6,173**	**6,212**	**0**	**50**	**846**	**896**
Albay	0	86	7,906	7,992	0	0	704	704	0	7	93	100
Camarines Norte	10	52	1,535	1,597	0	14	1,240	1,254	0	25	28	53
Camarines Sur	0	51	4,470	4,521	0	8	1,462	1,470	0	15	169	184
Catanduanes	0	22	524	546	0	5	109	114	0	0	30	30
Masbate	0	27	1,649	1,676	0	6	1,757	1,763	0	1	103	104
Sorsogon	0	24	2,531	2,555	0	2	350	352	0	1	40	41
Iriga City	0	5	627	632	0	0	425	425	0	0	2	2

Table 1.A.4 - MODERN METHOD OF FAMILY PLANNING

Current User (Ending)

Annual Philippines, 2020

Area	CONDOM			Total	IUD-INTERVAL			Total	IUD-POSTPARTUM			Total
	Age group				Age group				Age group			
	10-14	15-19	20-49		10-14	15-19	20-49		10-14	15-19	20-49	
Legaspi City	0	63	2,088	2,151	0	2	104	106	0	0	65	65
Naga City	0	4	4,387	4,391	0	2	22	24	0	1	316	317
**Region 6**	**2**	**355**	**30,550**	**30,907**	**6**	**455**	**36,141**	**36,602**	**4**	**290**	**9,967**	**10,261**
Aklan	0	74	2,850	2,924	0	3	703	706	0	0	15	15
Antique	1	23	1,517	1,541	0	6	711	717	0	0	53	53
Capiz	0	46	4,349	4,395	0	37	8,198	8,235	0	5	28	33
Guimaras	0	5	932	937	0	1	352	353	0	0	11	11
Iloilo	1	54	8,687	8,742	3	104	8,625	8,732	1	22	2,396	2,419
Negros Occidental	0	105	7,037	7,142	2	183	13,152	13,337	0	208	5,106	5,314
Bacolod City	0	8	1,435	1,443	1	37	2,817	2,855	3	22	1,960	1,985
Iloilo City	0	40	3,743	3,783	0	84	1,583	1,667	0	33	398	431
**Region 7**	**5**	**307**	**14,970**	**15,282**	**8**	**1,698**	**37,830**	**39,536**	**1**	**330**	**7,294**	**7,625**
Bohol	0	0	4,168	4,168	0	568	13,296	13,864	0	174	1,900	2,074
Cebu	0	46	5,177	5,223	0	284	11,246	11,530	1	76	1,403	1,480
Negros Oriental	0	153	2,398	2,551	5	685	5,360	6,050	0	-1	393	392
Siquijor	0	3	403	406	0	3	940	943	0	3	301	304
Cebu City	0	21	680	701	0	111	2,722	2,833	0	55	533	588
Lapu-Lapu City	5	74	2,131	2,210	3	47	4,252	4,302	0	22	416	438
Mandaue City	0	10	13	23	0	0	14	14	0	1	2,348	2,349
**Region 8**	**29**	**44**	**8,901**	**8,974**	**0**	**155**	**12,700**	**12,855**	**0**	**54**	**1,150**	**1,204**
Biliran	29	40	617	686	0	11	534	545	0	0	52	52
Eastern Samar	2	0	3,324	3,326	0	14	657	671	0	2	262	264
Northern Leyte	0	0	0	0	0	0	0	0	0	0	0	0
Northern Samar	0	12	634	646	0	0	1,668	1,668	0	30	250	280
Southern Leyte	0	-34	847	813	0	19	3,399	3,418	0	12	352	364
Western Samar	-4	-17	113	92	0	17	814	831	0	0	0	0
Calbayog City	0	1	260	261	0	1	119	120	0	0	1	1
Maasin City	0	-1	111	110	0	5	362	367	0	0	10	10
Ormoc City	0	13	850	863	0	8	1,586	1,594	0	1	4	5
Tacloban City	2	30	2,145	2,177	0	80	3,561	3,641	0	9	219	228
**Region 9**	**1**	**115**	**10,614**	**10,730**	**0**	**74**	**12,860**	**12,934**	**5**	**110**	**17,754**	**17,869**
Zamboanga del Norte	1	12	4,294	4,307	0	5	225	230	0	2	8,194	8,196
Zamboanga del Sur	0	98	1,758	1,856	0	66	7,249	7,315	5	89	1,332	1,426
Zamboanga Sibugay	0	0	1,129	1,129	0	0	2,870	2,870	0	0	66	66
Dapitan City	0	1	623	624	0	0	20	20	0	0	871	871
Dipolog City	0	4	1,420	1,424	0	0	20	20	0	2	1,755	1,757
Isabela City	0	0	388	388	0	0	172	172	0	0	4	4
Pagadian City	0	0	584	584	0	3	2,304	2,307	0	17	932	949
Zamboanga City	0	0	418	418	0	0	0	0	0	0	4,600	4,600
**Region 10**	**25**	**789**	**24,648**	**25,462**	**6**	**1,646**	**62,092**	**63,744**	**9**	**1,318**	**16,015**	**17,342**
Bukidnon	3	60	2,990	3,053	2	292	12,323	12,617	2	80	823	905
Camiguin	0	2	274	276	0	1	740	741	0	1	38	39
Lanao del Norte	0	221	3,787	4,008	2	85	3,858	3,945	0	6	545	551
Misamis Occidental	22	119	1,529	1,670	0	35	2,553	2,588	0	17	370	387
Misamis Oriental	0	129	3,173	3,302	1	38	13,988	14,027	0	31	1,784	1,815
Cagayan de Oro City	0	60	6,420	6,480	1	913	17,970	18,884	3	1,041	11,187	12,231
El Salvador City	0	2	95	97	0	44	1,952	1,996	1	50	166	217
Gingoog City	0	117	555	672	0	33	563	596	0	27	39	66
Iligan City	0	31	1,630	1,661	0	139	3,077	3,216	3	85	267	355
Malaybalay City	0	0	879	879	0	0	-72	-72	0	-23	703	680
Oroquieta City	0	0	187	187	0	0	631	631	0	0	0	0
Ozamis City	0	46	2,659	2,705	0	11	2,556	2,567	0	1	25	26
Tangub City	0	1	71	72	0	1	71	72	0	0	0	0
Valencia City	0	1	399	400	0	54	1,882	1,936	0	2	68	70

Table 1.A.4 - MODERN METHOD OF FAMILY PLANNING

Current User (Ending)

Annual Philippines, 2020

Area	CONDOM			Total	IUD-INTERVAL			Total	IUD-POSTPARTUM			Total
	Age group				Age group				Age group			
	10-14	15-19	20-49		10-14	15-19	20-49		10-14	15-19	20-49	
**Region 11**	**14**	**843**	**19,251**	**20,108**	**18**	**592**	**41,610**	**42,220**	**13**	**212**	**5,189**	**5,414**
Davao de Oro	2	105	3,206	3,313	1	71	7,436	7,508	2	29	460	491
Davao del Norte	1	142	4,229	4,372	4	139	6,550	6,693	2	60	2,351	2,413
Davao Oriental	0	19	1,185	1,204	2	14	3,553	3,569	1	4	204	209
Davao del Sur	2	63	1,778	1,843	4	144	8,772	8,920	1	26	474	501
Davao Occidental	1	23	395	419	1	45	2,137	2,183	0	20	201	221
Davao City	8	491	8,458	8,957	6	179	13,162	13,347	7	73	1,499	1,579
**Region 12**	**0**	**1,181**	**15,603**	**16,784**	**-4**	**2,509**	**22,471**	**24,976**	**4**	**3,630**	**14,491**	**18,125**
North Cotabato	0	684	3,915	4,599	1	2,044	4,404	6,449	0	3,141	4,227	7,368
Sarangani	0	112	2,173	2,285	0	82	2,900	2,982	0	2	303	305
South Cotabato	0	158	3,433	3,591	0	185	6,332	6,517	2	166	1,506	1,674
Sultan Kudarat	0	99	1,983	2,082	-5	139	4,902	5,036	-2	181	4,112	4,291
Cotabato City	0	62	2,198	2,260	0	34	2,466	2,500	4	127	1,152	1,283
Gen. Santos City	0	66	1,901	1,967	0	25	1,467	1,492	0	13	3,191	3,204
**B.A.R.M.M.**	**1**	**1,134**	**9,941**	**11,076**	**0**	**621**	**5,259**	**5,880**	**0**	**212**	**1,999**	**2,211**
Basilan	0	34	403	437	0	4	173	177	0	18	96	114
Lanao del Sur	2	44	5,680	5,726	0	41	1,456	1,497	0	18	397	415
Maguindanao	0	60	3,003	3,063	0	64	3,024	3,088	0	23	488	511
Sulu	0	587	0	587	0	392	0	392	0	0	0	0
Tawi-Tawi	0	444	-3	441	0	49	140	189	0	0	0	0
Lamitan City	0	0	0	0	0	71	445	516	0	153	1,018	1,171
Marawi City	-1	-35	858	822	0	0	21	21	0	0	0	0
**CARAGA**	**0**	**200**	**9,856**	**10,056**	**2**	**530**	**25,838**	**26,370**	**1**	**263**	**4,127**	**4,391**
Agusan del Norte	0	32	1,433	1,465	0	173	5,362	5,535	0	51	1,114	1,165
Agusan del Sur	0	55	3,225	3,280	1	232	7,692	7,925	0	15	1,747	1,762
Surigao del Norte	0	22	933	955	1	18	2,282	2,301	0	3	21	24
Surigao del Sur	0	50	1,848	1,898	0	45	4,470	4,515	1	47	499	547
Province of Dinagat	0	7	770	777	0	15	1,040	1,055	0	0	9	9
Bislig City	0	9	604	613	0	18	1,212	1,230	0	10	126	136
Butuan City	0	18	916	934	0	21	1,778	1,799	0	132	563	695
Surigao City	0	7	127	134	0	8	2,002	2,010	0	5	48	53

Table 1.A.4 - MODERN METHOD OF FAMILY PLANNING

Current User (Ending)

Annual Philippines, 2020

Area	PILLS-POP			Total	PILLS-COC			Total	INJECTABLES			Total
	Age group				Age group				Age group			
	10-14	15-19	20-49		10-14	15-19	20-49		10-14	15-19	20-49	
**PHILIPPINES**	**1,063**	**30,422**	**653,161**	**684,645**	**412**	**91,233**	**2,155,549**	**2,247,194**	**1,079**	**103,285**	**1,379,327**	**1,483,691**
**N C R**	**101**	**4,768**	**28,451**	**33,320**	**112**	**6,685**	**149,812**	**156,609**	**154**	**11,670**	**142,404**	**154,228**
Malabon	0	1,054	1,951	3,005	0	1,286	5,810	7,096	3	1,664	6,412	8,079
Navotas	6	127	1,258	1,391	0	629	6,118	6,747	2	1,565	7,681	9,248
Valenzuela City	0	35	342	377	0	273	8,942	9,215	0	462	6,883	7,345
Caloocan City	1	173	1,357	1,531	4	526	8,479	9,009	2	1,191	18,305	19,498
Marikina City	0	63	476	539	0	37	2,553	2,590	6	377	3,833	4,216
Pasig City	0	167	986	1,153	1	289	7,990	8,280	2	843	11,815	12,660
Pateros	1	4	17	22	0	1	799	800	1	8	696	705
Taguig	15	895	2,918	3,828	2	740	11,594	12,336	0	490	14,072	14,562
Quezon City	58	1,376	12,001	13,435	9	1,417	64,235	65,661	4	2,010	33,881	35,895
Makati City	0	54	634	688	0	49	1,534	1,583	0	129	3,444	3,573
Mandaluyong City	0	43	1,205	1,248	0	46	2,788	2,834	0	96	3,471	3,567
San Juan	0	26	7	33	0	20	1,911	1,931	0	0	1,602	1,602
Manila City	0	73	524	597	39	144	1,996	2,179	19	282	4,510	4,811
Las Piñas City	2	87	1,000	1,089	2	155	4,700	4,857	1	279	3,745	4,025
Muntinlupa City	6	200	727	933	5	589	10,787	11,381	7	1,293	8,755	10,055
Parañaque City	11	334	2,333	2,678	50	481	5,296	5,827	107	981	7,008	8,096
Pasay City	1	57	715	773	0	3	4,280	4,283	0	0	6,291	6,291
**C A R**	**3**	**387**	**19,825**	**20,215**	**6**	**652**	**19,706**	**20,364**	**12**	**634**	**19,050**	**19,696**
Abra	2	83	725	810	5	352	6,253	6,610	10	148	1,642	1,800
Apayao	0	53	5,351	5,404	0	17	717	734	0	62	1,945	2,007
Benguet	0	86	741	827	0	94	7,879	7,973	1	133	4,534	4,668
Ifugao	1	33	3,966	4,000	0	54	1,858	1,912	0	73	2,938	3,011
Kalinga	0	74	5,654	5,728	0	64	569	633	0	21	3,170	3,191
Mt. Province	0	24	2,711	2,735	1	53	273	327	1	62	1,537	1,600
Baguio City	0	34	677	711	0	18	2,157	2,175	0	135	3,284	3,419
**Region 1**	**1**	**239**	**3,927**	**4,167**	**4**	**4,614**	**191,042**	**195,660**	**4**	**3,411**	**92,935**	**96,350**
Ilocos Norte	1	59	1,323	1,383	1	163	18,514	18,678	0	114	5,535	5,649
Ilocos Sur	0	107	2,252	2,359	0	334	22,907	23,241	0	252	10,116	10,368
La Union	0	70	525	595	0	217	22,321	22,538	0	485	14,844	15,329
Pangasinan	0	-8	-275	-283	3	2,177	99,059	101,239	4	1,943	48,410	50,357
Alaminos City	0	-7	-1	-8	0	1,274	2,673	3,947	0	390	1,391	1,781
Candon City	0	0	0	0	0	0	2,491	2,491	0	0	2,080	2,080
Dagupan City	0	2	48	50	0	32	2,585	2,617	0	66	1,585	1,651
Laoag City	0	0	18	18	0	0	2,670	2,670	0	0	2,059	2,059
San Carlos City	0	-3	-1	-4	0	16	4,793	4,809	0	22	2,373	2,395
San Fernando City	0	8	14	22	0	108	6,661	6,769	0	5	1,739	1,744
Urdaneta City	0	7	24	31	0	282	4,873	5,155	0	133	2,167	2,300
Vigan City	0	4	0	4	0	11	1,495	1,506	0	1	636	637
**Region 2**	**1**	**2,152**	**10,213**	**12,366**	**9**	**3,410**	**148,486**	**151,905**	**35**	**2,405**	**50,116**	**52,556**
Batanes	0	1	5	6	0	3	179	182	1	15	322	338
Cagayan	0	374	1,995	2,369	4	474	44,991	45,469	3	292	10,990	11,285
Isabela	0	359	3,045	3,404	4	742	55,109	55,855	2	785	16,992	17,779
Nueva Vizcaya	1	136	1,492	1,629	0	381	20,232	20,613	2	288	6,201	6,491
Quirino	0	41	210	251	1	323	9,068	9,392	27	145	4,418	4,590
Cauayan City	0	21	114	135	0	91	5,318	5,409	0	35	1,461	1,496
Ilagan City	0	1,133	2,782	3,915	0	1,138	2,177	3,315	0	640	4,466	5,106
Santiago City	0	0	0	0	0	105	5,383	5,488	0	162	4,006	4,168
Tuguegarao City	0	87	570	657	0	153	6,029	6,182	0	43	1,260	1,303
**Region 3**	**2**	**1,376**	**12,691**	**14,069**	**12**	**8,325**	**276,233**	**284,570**	**19**	**10,082**	**194,049**	**204,150**
Aurora	0	44	911	955	0	113	9,810	9,923	0	109	2,985	3,094
Bataan	0	77	670	747	4	490	12,669	13,163	4	1,311	17,244	18,559

Table 1.A.4 - MODERN METHOD OF FAMILY PLANNING

Current User (Ending)

Annual Philippines, 2020

Area	PILLS-POP			Total	PILLS-COC			Total	INJECTABLES			Total
	Age group				Age group				Age group			
	10-14	15-19	20-49		10-14	15-19	20-49		10-14	15-19	20-49	
Bulacan	0	171	2,179	2,350	0	1,509	53,406	54,915	9	2,065	36,378	38,452
Nueva Ecija	0	316	2,198	2,514	1	1,745	52,082	53,828	0	1,359	26,663	28,022
Pampanga	1	148	1,488	1,637	7	607	31,108	31,722	2	777	20,461	21,240
Tarlac	0	187	2,240	2,427	0	785	31,260	32,045	0	600	23,866	24,466
Zambales	0	70	643	713	0	325	12,673	12,998	3	633	12,639	13,275
Angeles City	0	31	251	282	0	344	8,021	8,365	0	331	4,757	5,088
Balanga City	0	0	1	1	0	67	1,438	1,505	0	117	1,555	1,672
Cabanatuan City	1	74	387	462	0	191	9,480	9,671	0	167	2,650	2,817
City of San Fernando	0	54	391	445	0	102	2,076	2,178	0	151	1,851	2,002
Gapan City	0	21	122	143	0	112	2,405	2,517	0	86	1,662	1,748
Mabalacat City	0	21	207	228	0	177	6,288	6,465	0	325	7,058	7,383
Malolos City	0	0	0	0	0	19	3,094	3,113	0	16	2,314	2,330
Meycauayan	0	5	59	64	0	248	2,787	3,035	0	121	2,211	2,332
Olongapo	0	22	186	208	0	119	2,145	2,264	1	142	2,411	2,554
Palayan City	0	0	22	22	0	26	1,585	1,611	0	34	484	518
San Jose City	0	17	154	171	0	124	5,582	5,706	0	202	4,255	4,457
San Jose del Monte City	0	10	118	128	0	545	18,258	18,803	0	898	16,712	17,610
Science City of Munoz	0	4	33	37	0	61	3,199	3,260	0	31	1,505	1,536
Tarlac City	0	104	431	535	0	616	6,867	7,483	0	607	4,388	4,995
**Region 4A**	**696**	**9,645**	**302,785**	**313,125**	**36**	**4,299**	**38,128**	**42,463**	**506**	**7,984**	**168,491**	**176,981**
Batangas	59	1,479	51,310	52,848	0	-4	-817	-821	75	625	16,437	17,137
Cavite	70	834	29,537	30,441	0	142	5,761	5,903	40	788	21,965	22,792
Laguna	52	771	23,641	24,464	0	2	-216	-214	20	232	9,176	9,428
Quezon	86	1,302	41,999	43,388	0	623	23,091	23,714	68	1,267	17,262	18,597
Rizal	72	1,013	34,233	35,317	5	153	-686	-528	50	1,061	28,011	29,122
Antipolo City	23	284	10,647	10,954	0	0	0	0	32	399	14,967	15,398
Bacoor City	6	78	2,803	2,887	0	-10	-153	-163	6	-62	1,878	1,822
Batangas City	37	196	7,166	7,399	0	3	145	148	6	73	2,746	2,825
Biñan City	40	1,138	19,258	20,436	0	1,416	10,123	11,539	20	1,544	17,278	18,842
Cabuyao City	10	76	6,527	6,613	0	40	-179	-139	5	67	1,258	1,329
Calamba City	19	400	12,683	13,103	-1	4	-14	-11	15	258	7,018	7,291
Cavite City	2	30	1,129	1,162	0	0	0	0	2	25	930	957
Dasmariñas City	26	416	12,257	12,700	0	60	-333	-273	15	73	6,202	6,290
General Trias City	7	111	3,283	3,401	0	-8	-131	-139	7	-9	2,621	2,619
Imus City	8	62	3,545	3,615	0	24	-112	-88	6	61	2,055	2,123
Lipa City	14	169	6,334	6,516	0	0	0	0	3	41	1,529	1,573
Lucena City	85	69	2,604	2,758	32	0	0	32	83	74	2,303	2,460
San Pablo City	9	107	4,019	4,135	0	0	0	0	4	55	2,069	2,129
San Pedro City	16	304	6,908	7,227	0	101	1,678	1,779	4	186	3,301	3,492
Santa Rosa City	36	543	13,911	14,490	-1	1,737	-578	1,158	11	1,001	4,197	5,210
Tagaytay City	7	95	3,418	3,521	1	9	583	593	24	71	2,494	2,588
Tanauan City	5	78	2,341	2,424	0	3	-53	-50	5	110	1,241	1,356
Tayabas City	4	52	1,667	1,723	0	4	20	24	3	50	496	549
Trece Martires City	3	38	1,565	1,606	0	0	-1	-1	2	-5	1,057	1,054
**Region 4B**	**1**	**108**	**1,957**	**2,066**	**4**	**533**	**71,250**	**71,787**	**6**	**835**	**36,545**	**37,386**
Marinduque	0	33	239	272	0	21	5,783	5,804	0	32	2,231	2,263
Mindoro Occidental	0	33	642	675	1	241	12,927	13,169	4	235	8,770	9,009
Mindoro Oriental	0	24	469	493	2	238	29,641	29,881	3	427	8,893	9,323
Palawan	1	18	165	184	1	33	10,200	10,234	-1	141	4,306	4,446
Romblon	0	0	442	442	0	0	595	595	0	0	570	570
Puerto Princesa City	0	0	0	0	0	0	12,104	12,104	0	0	11,775	11,775
**Region 5**	**4**	**633**	**15,738**	**16,375**	**6**	**2,637**	**154,179**	**156,822**	**4**	**2,653**	**76,592**	**79,249**
Albay	0	202	9,407	9,609	2	278	25,745	26,025	0	408	19,603	20,011
Camarines Norte	2	74	752	828	1	441	12,479	12,921	1	392	5,539	5,932
Camarines Sur	2	168	2,326	2,496	1	543	36,020	36,564	2	508	17,927	18,437
Catanduanes	0	4	129	133	1	170	4,575	4,746	0	440	4,508	4,948
Masbate	0	77	707	784	1	590	27,194	27,785	1	354	8,868	9,223
Sorsogon	0	55	1,075	1,130	0	384	23,720	24,104	0	389	11,139	11,528
Iriga City	0	13	215	228	0	50	3,767	3,817	0	62	1,599	1,661

Table 1.A.4 - MODERN METHOD OF FAMILY PLANNING
Current User (Ending)
Annual Philippines, 2020

Area	PILLS-POP			Total	PILLS-COC			Total	INJECTABLES			Total
	Age group				Age group				Age group			
	10-14	15-19	20-49		10-14	15-19	20-49		10-14	15-19	20-49	
Legaspi City	0	28	895	923	0	157	11,130	11,287	0	91	4,929	5,020
Naga City	0	12	232	244	0	24	9,549	9,573	0	9	2,480	2,489
**Region 6**	**134**	**670**	**8,588**	**9,392**	**24**	**5,088**	**255,637**	**260,749**	**8**	**3,692**	**113,007**	**116,707**
Aklan	0	81	1,688	1,769	2	279	15,751	16,032	1	519	11,776	12,296
Antique	0	39	569	608	0	220	14,342	14,562	1	365	11,695	12,061
Capiz	0	26	136	162	8	446	37,110	37,564	0	516	18,048	18,564
Guimaras	0	12	195	207	0	66	9,417	9,483	0	37	2,084	2,121
Iloilo	0	64	2,962	3,026	10	528	66,026	66,564	4	575	27,761	28,340
Negros Occidental	6	384	2,426	2,816	4	3,071	80,568	83,643	2	1,361	31,326	32,689
Bacolod City	128	24	341	493	0	154	6,111	6,265	0	97	4,490	4,587
Iloilo City	0	40	271	311	0	324	26,312	26,636	0	222	5,827	6,049
**Region 7**	**14**	**699**	**16,323**	**17,036**	**9**	**1,587**	**84,405**	**86,001**	**36**	**2,108**	**63,084**	**65,228**
Bohol	2	58	271	331	4	70	19,006	19,080	0	221	9,950	10,171
Cebu	1	103	1,773	1,877	0	168	26,619	26,787	0	414	14,852	15,266
Negros Oriental	0	125	1,187	1,312	0	507	21,016	21,523	4	555	16,586	17,145
Siquijor	0	41	438	479	0	71	6,685	6,756	0	124	4,240	4,364
Cebu City	1	89	803	893	0	561	5,692	6,253	2	359	3,987	4,348
Lapu-Lapu City	10	283	4,323	4,616	5	210	5,231	5,446	30	435	7,818	8,283
Mandaue City	0	0	7,528	7,528	0	0	156	156	0	0	5,651	5,651
**Region 8**	**0**	**430**	**13,326**	**13,756**	**3**	**1,094**	**62,865**	**63,962**	**1**	**1,079**	**34,524**	**35,604**
Biliran	0	15	132	147	1	62	4,402	4,465	0	46	1,315	1,361
Eastern Samar	0	6	30	36	2	211	14,559	14,772	0	171	8,198	8,369
Northern Leyte	0	0	0	0	0	0	0	0	0	0	0	0
Northern Samar	0	24	896	920	0	172	12,026	12,198	0	82	5,604	5,686
Southern Leyte	0	4	202	206	0	111	9,015	9,126	0	101	2,580	2,681
Western Samar	0	101	6,751	6,852	0	0	0	0	0	46	3,254	3,300
Calbayog City	0	45	2,109	2,154	0	16	2,082	2,098	1	50	1,959	2,010
Maasin City	0	1	134	135	0	3	914	917	0	0	270	270
Ormoc City	0	1	1,039	1,040	0	115	7,448	7,563	0	85	1,927	2,012
Tacloban City	0	233	2,033	2,266	0	404	12,419	12,823	0	498	9,417	9,915
**Region 9**	**10**	**261**	**108,849**	**109,120**	**1**	**225**	**12,584**	**12,810**	**0**	**284**	**44,969**	**45,253**
Zamboanga del Norte	0	12	39,167	39,179	1	34	1,116	1,151	0	46	15,151	15,197
Zamboanga del Sur	0	141	27,113	27,254	0	26	601	627	0	202	6,103	6,305
Zamboanga Sibugay	0	0	7,375	7,375	0	0	562	562	0	0	3,763	3,763
Dapitan City	0	7	3,657	3,664	0	5	0	5	0	0	591	591
Dipolog City	10	31	7,627	7,668	0	10	239	249	0	8	1,437	1,445
Isabela City	0	65	110	175	0	10	2,318	2,328	0	16	1,755	1,771
Pagadian City	0	1	243	244	0	10	6,483	6,493	0	12	2,892	2,904
Zamboanga City	0	4	23,557	23,561	0	130	1,265	1,395	0	0	13,277	13,277
**Region 10**	**-29**	**1,812**	**21,276**	**23,059**	**14**	**5,874**	**200,783**	**206,671**	**33**	**3,682**	**79,967**	**83,682**
Bukidnon	1	423	2,250	2,674	9	1,344	42,196	43,549	3	679	10,265	10,947
Camiguin	0	3	40	43	2	46	2,659	2,707	0	11	653	664
Lanao del Norte	20	474	4,178	4,672	16	916	22,896	23,828	15	927	11,577	12,519
Misamis Occidental	-53	81	1,129	1,157	-22	280	10,084	10,342	2	92	3,188	3,282
Misamis Oriental	0	68	4,182	4,250	2	156	25,469	25,627	1	147	10,137	10,285
Cagayan de Oro City	1	318	6,888	7,207	0	1,232	37,382	38,614	4	863	25,306	26,173
El Salvador City	0	24	171	195	0	54	1,503	1,557	1	58	1,017	1,076
Gingoog City	1	168	374	543	6	210	3,416	3,632	1	80	945	1,026
Iligan City	1	41	455	497	1	357	8,661	9,019	6	321	5,513	5,840
Malaybalay City	0	13	2	15	0	102	10,680	10,782	0	55	1,464	1,519
Oroquieta City	0	51	637	688	0	75	2,022	2,097	0	11	1,160	1,171
Ozamis City	0	54	603	657	0	743	20,243	20,986	0	400	6,211	6,611
Tangub City	0	25	30	55	0	64	3,030	3,094	0	19	415	434
Valencia City	0	69	337	406	0	295	10,542	10,837	0	19	2,116	2,135

Table 1.A.4 - MODERN METHOD OF FAMILY PLANNING
Current User (Ending)
Annual Philippines, 2020

Area	PILLS-POP			Total	PILLS-COC			Total	INJECTABLES			Total
	Age group				Age group				Age group			
	10-14	15-19	20-49		10-14	15-19	20-49		10-14	15-19	20-49	
**Region 11**	**95**	**2,396**	**23,057**	**25,548**	**153**	**7,377**	**199,619**	**207,149**	**240**	**5,502**	**74,362**	**80,104**
Davao de Oro	18	439	2,503	2,960	19	1,951	39,581	41,551	16	802	10,268	11,086
Davao del Norte	15	493	6,090	6,598	61	963	43,341	44,365	30	768	12,810	13,608
Davao Oriental	12	125	2,105	2,242	51	498	22,913	23,462	12	249	5,558	5,819
Davao del Sur	18	299	2,714	3,031	10	969	27,450	28,429	152	966	11,142	12,260
Davao Occidental	1	78	498	577	9	701	11,059	11,769	15	921	7,355	8,291
Davao City	31	962	9,147	10,140	3	2,295	55,275	57,573	15	1,796	27,229	29,040
**Region 12**	**15**	**3,351**	**38,342**	**41,708**	**8**	**5,666**	**150,280**	**155,954**	**8**	**7,978**	**94,496**	**102,482**
North Cotabato	10	1,009	20,449	21,468	1	1,751	40,326	42,078	4	2,049	27,611	29,664
Sarangani	0	745	3,267	4,012	3	1,588	25,839	27,430	0	1,400	12,905	14,305
South Cotabato	4	508	2,705	3,217	0	1,001	34,380	35,381	4	1,581	16,145	17,730
Sultan Kudarat	1	563	4,125	4,689	0	975	32,333	33,308	0	2,270	17,466	19,736
Cotabato City	0	113	1,312	1,425	4	220	10,651	10,875	0	394	9,316	9,710
Gen. Santos City	0	413	6,484	6,897	0	131	6,751	6,882	0	284	11,053	11,337
**B.A.R.M.M.**	**9**	**637**	**6,180**	**6,826**	**6**	**30,900**	**53,635**	**84,541**	**4**	**37,741**	**60,076**	**97,821**
Basilan	0	121	554	675	0	179	1,217	1,396	1	487	3,795	4,283
Lanao del Sur	8	144	2,608	2,760	0	247	22,093	22,340	0	352	18,531	18,883
Maguindanao	1	277	2,025	2,303	6	1,298	26,813	28,117	3	1,492	33,923	35,418
Sulu	0	0	0	0	0	17,513	0	17,513	0	23,910	0	23,910
Tawi-Tawi	0	0	364	364	0	11,365	0	11,365	0	11,122	179	11,301
Lamitan City	0	72	291	363	0	279	2,295	2,574	0	354	2,661	3,015
Marawi City	0	23	338	361	0	19	1,217	1,236	0	24	987	1,011
**CARAGA**	**6**	**858**	**21,633**	**22,497**	**5**	**2,267**	**86,905**	**89,177**	**9**	**1,545**	**34,660**	**36,214**
Agusan del Norte	1	122	1,756	1,879	1	341	13,173	13,515	1	212	5,218	5,431
Agusan del Sur	2	279	3,361	3,642	2	734	30,505	31,241	3	474	9,507	9,984
Surigao del Norte	0	63	13,220	13,283	0	122	1,584	1,706	4	90	4,902	4,996
Surigao del Sur	1	180	1,725	1,906	2	423	18,002	18,427	1	272	4,524	4,797
Province of Dinagat	0	37	181	218	0	71	3,524	3,595	0	66	1,353	1,419
Bislig City	0	24	268	292	0	110	3,571	3,681	0	53	754	807
Butuan City	2	136	1,072	1,210	0	380	12,114	12,494	0	333	5,419	5,752
Surigao City	0	17	50	67	0	86	4,432	4,518	0	45	2,983	3,028

Table 1.A.4 - MODERN METHOD OF FAMILY PLANNING
Current User (Ending)
Annual Philippines, 2020

Area	IMPLANTS			Total	NFP-CCM			Total	NFP-BBT			Total
	Age group				Age group				Age group			
	10-14	15-19	20-49		10-14	15-19	20-49		10-14	15-19	20-49	
**PHILIPPINES**	**470**	**30,193**	**404,152**	**434,815**	**2,925**	**1,007**	**40,101**	**44,034**	**110**	**407**	**4,607**	**5,125**
**N C R**	**189**	**7,977**	**59,859**	**68,025**	**3**	**6**	**558**	**567**	**2**	**122**	**632**	**756**
Malabon	9	540	2,333	2,882	0	0	0	0	0	0	0	0
Navotas	0	87	978	1,065	0	0	0	0	0	0	0	0
Valenzuela City	57	1,541	9,520	11,118	0	0	27	27	0	0	0	0
Caloocan City	11	646	6,929	7,586	0	0	11	11	0	0	0	0
Marikina City	1	110	1,631	1,742	0	2	3	5	0	0	0	0
Pasig City	2	215	2,877	3,094	0	0	11	11	0	0	0	0
Pateros	1	1	46	48	1	1	-1	1	0	0	0	0
Taguig	1	30	2,625	2,656	0	0	0	0	0	0	0	0
Quezon City	40	1,505	11,723	13,268	0	0	13	13	0	0	0	0
Makati City	0	78	1,063	1,141	0	0	0	0	0	0	0	0
Mandaluyong City	1	47	985	1,033	0	2	305	307	0	0	0	0
San Juan	0	0	78	78	0	0	0	0	0	0	0	0
Manila City	59	2,481	11,048	13,588	1	0	1	2	2	121	631	754
Las Piñas City	3	307	3,524	3,834	0	0	47	47	0	0	0	0
Muntinlupa City	1	87	1,478	1,566	0	0	0	0	0	0	0	0
Parañaque City	3	301	1,642	1,946	0	1	15	16	0	1	1	2
Pasay City	0	1	1,379	1,380	1	0	126	127	0	0	0	0
**C A R**	**5**	**226**	**7,207**	**7,438**	**0**	**1**	**2,190**	**2,191**	**0**	**4**	**127**	**131**
Abra	3	50	1,070	1,123	0	0	72	72	0	0	58	58
Apayao	0	38	650	688	0	0	3	3	0	0	0	0
Benguet	0	22	1,096	1,118	0	0	6	6	0	0	0	0
Ifugao	1	13	1,262	1,276	0	0	705	705	0	2	7	9
Kalinga	0	22	900	922	0	1	1,282	1,283	0	2	32	34
Mt. Province	0	14	335	349	0	0	119	119	0	0	19	19
Baguio City	1	67	1,894	1,962	0	0	3	3	0	0	11	11
**Region 1**	**0**	**847**	**13,668**	**14,515**	**0**	**2**	**1,955**	**1,957**	**0**	**12**	**95**	**107**
Ilocos Norte	0	18	518	536	0	1	363	364	0	1	7	8
Ilocos Sur	0	15	467	482	0	0	676	676	0	0	30	30
La Union	0	114	1,289	1,403	0	0	212	212	0	0	15	15
Pangasinan	0	434	9,343	9,777	0	0	4	4	0	9	14	23
Alaminos City	0	188	267	455	0	0	0	0	0	0	0	0
Candon City	0	0	4	4	0	0	55	55	0	0	24	24
Dagupan City	0	22	489	511	0	0	0	0	0	0	3	3
Laoag City	0	0	3	3	0	0	0	0	0	0	0	0
San Carlos City	0	8	506	514	0	0	0	0	0	0	0	0
San Fernando City	0	17	386	403	0	0	0	0	0	0	0	0
Urdaneta City	0	31	391	422	0	0	0	0	0	0	0	0
Vigan City	0	0	5	5	0	1	645	646	0	2	2	4
**Region 2**	**4**	**428**	**10,432**	**10,864**	**1**	**28**	**201**	**230**	**0**	**1**	**11**	**12**
Batanes	0	0	5	5	0	1	64	65	0	0	0	0
Cagayan	4	77	2,516	2,597	0	0	60	60	0	0	1	1
Isabela	0	215	4,911	5,126	0	0	5	5	0	0	7	7
Nueva Vizcaya	0	68	1,504	1,572	1	27	49	77	0	1	3	4
Quirino	0	0	396	396	0	0	23	23	0	0	0	0
Cauayan City	0	9	173	182	0	0	0	0	0	0	0	0
Ilagan City	0	30	312	342	0	0	0	0	0	0	0	0
Santiago City	0	18	453	471	0	0	0	0	0	0	0	0
Tuguegarao City	0	11	162	173	0	0	0	0	0	0	0	0
**Region 3**	**6**	**1,402**	**30,137**	**31,545**	**0**	**12**	**1,030**	**1,042**	**0**	**19**	**174**	**193**
Aurora	1	19	1,003	1,023	0	7	786	793	0	0	4	4
Bataan	0	98	1,766	1,864	0	0	74	74	0	0	76	76

Table 1.A.4 - MODERN METHOD OF FAMILY PLANNING

Current User (Ending)

Annual Philippines, 2020

Area	IMPLANTS			Total	NFP-CCM			Total	NFP-BBT			Total
	Age group				Age group				Age group			
	10-14	15-19	20-49		10-14	15-19	20-49		10-14	15-19	20-49	
Bulacan	0	193	6,927	7,120	0	0	3	3	0	0	4	4
Nueva Ecija	1	397	4,276	4,674	0	3	6	9	0	0	0	0
Pampanga	1	147	2,624	2,772	0	2	131	133	0	1	10	11
Tarlac	0	19	1,446	1,465	0	0	0	0	0	1	43	44
Zambales	0	117	2,283	2,400	0	0	30	30	0	1	0	1
Angeles City	0	57	1,289	1,346	0	0	0	0	0	2	1	3
Balanga City	0	25	180	205	0	0	0	0	0	0	0	0
Cabanatuan City	0	12	315	327	0	0	0	0	0	0	0	0
City of San Fernando	0	7	467	474	0	0	0	0	0	11	33	44
Gapan City	0	56	416	472	0	0	0	0	0	0	0	0
Mabalacat City	0	12	577	589	0	0	0	0	0	0	0	0
Malolos City	0	3	327	330	0	0	0	0	0	0	0	0
Meycauayan	0	25	370	395	0	0	0	0	0	0	0	0
Olongapo	3	114	1,078	1,195	0	0	0	0	0	0	0	0
Palayan City	0	0	0	0	0	0	0	0	0	0	0	0
San Jose City	0	16	274	290	0	0	0	0	0	0	0	0
San Jose del Monte City	0	56	3,966	4,022	0	0	0	0	0	0	0	0
Science City of Munoz	0	0	21	21	0	0	0	0	0	0	0	0
Tarlac City	0	29	532	561	0	0	0	0	0	3	3	6
**Region 4A**	**137**	**1,213**	**39,058**	**40,408**	**2,912**	**109**	**4,094**	**7,116**	**68**	**15**	**754**	**838**
Batangas	18	55	1,364	1,437	5	64	1,124	1,193	0	0	7	7
Cavite	18	219	7,872	8,109	0	4	303	307	0	2	83	85
Laguna	18	130	4,206	4,354	1	19	571	591	0	0	24	24
Quezon	6	49	771	827	0	10	945	955	0	0	133	133
Rizal	23	341	6,669	7,034	0	1	67	68	0	0	7	7
Antipolo City	3	34	1,271	1,308	0	2	90	93	0	0	0	0
Bacoor City	2	21	826	850	0	0	0	0	0	0	0	0
Batangas City	0	4	137	141	0	0	1	1	0	0	0	0
Biñan City	1	11	567	579	0	0	0	0	0	0	-9	-9
Cabuyao City	1	18	148	167	0	0	3	3	0	0	10	10
Calamba City	7	3	1,175	1,186	0	1	47	48	0	0	0	0
Cavite City	1	18	658	677	0	0	0	0	0	0	0	0
Dasmariñas City	23	210	8,020	8,253	0	0	11	11	0	2	22	24
General Trias City	3	33	1,142	1,177	0	0	5	5	0	0	408	408
Imus City	1	-3	427	426	0	0	0	0	0	0	0	0
Lipa City	0	3	125	129	0	0	-1	-1	0	0	0	0
Lucena City	0	1	32	33	2,904	0	968	3,872	68	0	4	72
San Pablo City	1	7	267	275	0	0	0	0	0	0	0	0
San Pedro City	3	36	1,206	1,245	0	-2	-5	-6	0	10	43	53
Santa Rosa City	4	-6	1,695	1,693	0	0	1	1	0	0	0	0
Tagaytay City	2	19	-6	14	0	0	0	0	0	0	0	0
Tanauan City	0	3	77	80	0	0	0	0	0	0	0	0
Tayabas City	0	4	3	7	1	10	-35	-25	0	1	23	24
Trece Martires City	1	2	405	409	0	0	0	0	0	0	0	0
**Region 4B**	**1**	**137**	**8,226**	**8,364**	**0**	**43**	**3,267**	**3,310**	**0**	**1**	**5**	**6**
Marinduque	1	10	658	669	0	0	191	191	0	0	1	1
Mindoro Occidental	0	60	1,399	1,459	0	41	2,157	2,198	0	1	0	1
Mindoro Oriental	0	51	2,197	2,248	0	2	606	608	0	0	2	2
Palawan	0	15	1,786	1,801	0	-1	36	35	0	0	0	0
Romblon	0	1	355	356	0	1	265	266	0	0	2	2
Puerto Princesa City	0	0	1,831	1,831	0	0	12	12	0	0	0	0
**Region 5**	**4**	**811**	**23,172**	**23,987**	**9**	**160**	**12,916**	**13,085**	**0**	**38**	**1,415**	**1,453**
Albay	4	123	2,642	2,769	0	30	2,221	2,251	0	27	959	986
Camarines Norte	0	38	906	944	0	4	36	40	0	6	12	18
Camarines Sur	0	297	8,643	8,940	3	13	2,472	2,488	0	4	238	242
Catanduanes	0	59	1,962	2,021	0	22	1,699	1,721	0	0	10	10
Masbate	0	192	4,803	4,995	6	13	1,781	1,800	0	1	1	2
Sorsogon	0	54	2,532	2,586	0	77	4,291	4,368	0	0	3	3
Iriga City	0	15	657	672	0	1	314	315	0	0	163	163

Table 1.A.4 - MODERN METHOD OF FAMILY PLANNING
Current User (Ending)
Annual Philippines, 2020

Area	IMPLANTS			Total	NFP-CCM			Total	NFP-BBT			Total
	Age group				Age group				Age group			
	10-14	15-19	20-49		10-14	15-19	20-49		10-14	15-19	20-49	
Legaspi City	0	31	601	632	0	0	44	44	0	0	0	0
Naga City	0	2	426	428	0	0	58	58	0	0	29	29
**Region 6**	**6**	**937**	**30,325**	**31,268**	**0**	**61**	**4,221**	**4,282**	**40**	**14**	**203**	**257**
Aklan	0	39	2,007	2,046	0	1	62	63	0	0	0	0
Antique	0	75	2,023	2,098	0	59	3,861	3,920	0	0	1	1
Capiz	1	55	2,728	2,784	0	0	193	193	0	3	5	8
Guimaras	0	8	402	410	0	0	0	0	0	0	6	6
Iloilo	3	167	8,372	8,542	0	0	40	40	0	2	114	116
Negros Occidental	2	445	11,022	11,469	0	0	35	35	0	9	59	68
Bacolod City	0	63	2,037	2,100	0	0	29	29	40	0	17	57
Iloilo City	0	85	1,734	1,819	0	1	1	2	0	0	1	1
**Region 7**	**5**	**793**	**24,119**	**24,917**	**0**	**0**	**157**	**157**	**0**	**4**	**67**	**71**
Bohol	0	139	7,271	7,410	0	0	7	7	0	0	0	0
Cebu	0	201	6,682	6,883	0	0	2	2	0	0	3	3
Negros Oriental	1	115	1,643	1,759	0	0	148	148	0	3	64	67
Siquijor	0	27	507	534	0	0	0	0	0	0	0	0
Cebu City	0	263	3,606	3,869	0	0	0	0	0	1	0	1
Lapu-Lapu City	4	48	1,447	1,499	0	0	0	0	0	0	0	0
Mandaue City	0	0	2,963	2,963	0	0	0	0	0	0	0	0
**Region 8**	**0**	**482**	**16,821**	**17,303**	**0**	**0**	**1,392**	**1,392**	**0**	**0**	**22**	**22**
Biliran	0	10	769	779	0	0	132	132	0	0	0	0
Eastern Samar	0	26	2,175	2,201	0	0	0	0	0	0	0	0
Northern Leyte	0	0	0	0	0	0	0	0	0	0	0	0
Northern Samar	0	153	4,515	4,668	0	0	7	7	0	0	9	9
Southern Leyte	0	31	1,794	1,825	0	0	94	94	0	0	13	13
Western Samar	0	19	1,522	1,541	0	0	1,057	1,057	0	0	0	0
Calbayog City	0	11	558	569	0	0	0	0	0	0	0	0
Maasin City	0	0	231	231	0	0	2	2	0	0	0	0
Ormoc City	0	38	2,154	2,192	0	0	3	3	0	0	0	0
Tacloban City	0	194	3,103	3,297	0	0	97	97	0	0	0	0
**Region 9**	**0**	**302**	**23,188**	**23,490**	**0**	**3**	**49**	**52**	**0**	**0**	**1**	**1**
Zamboanga del Norte	0	4	5,891	5,895	0	0	42	42	0	0	0	0
Zamboanga del Sur	0	261	6,792	7,053	0	0	0	0	0	0	0	0
Zamboanga Sibugay	0	0	4,910	4,910	0	0	3	3	0	0	0	0
Dapitan City	0	0	401	401	0	0	0	0	0	0	0	0
Dipolog City	0	2	410	412	0	0	0	0	0	0	0	0
Isabela City	0	28	1,871	1,899	0	0	0	0	0	0	0	0
Pagadian City	0	7	643	650	0	0	0	0	0	0	0	0
Zamboanga City	0	0	2,270	2,270	0	3	4	7	0	0	1	1
**Region 10**	**13**	**1,501**	**28,964**	**30,478**	**0**	**181**	**4,033**	**4,214**	**0**	**90**	**441**	**531**
Bukidnon	9	562	8,095	8,666	0	50	780	830	0	3	44	47
Camiguin	0	6	152	158	0	0	0	0	0	0	0	0
Lanao del Norte	2	115	2,336	2,453	0	0	76	76	0	0	14	14
Misamis Occidental	0	12	528	540	0	7	75	82	0	8	6	14
Misamis Oriental	0	76	2,752	2,828	0	-5	1,664	1,659	0	0	65	65
Cagayan de Oro City	0	517	8,048	8,565	0	0	0	0	0	0	-3	-3
El Salvador City	1	31	305	337	0	0	0	0	0	0	0	0
Gingoog City	0	80	513	593	0	83	382	465	0	79	192	271
Iligan City	1	43	1,442	1,486	0	0	79	79	0	0	1	1
Malaybalay City	0	-41	1,379	1,338	0	0	1	1	0	0	11	11
Oroquieta City	0	8	173	181	0	0	0	0	0	0	0	0
Ozamis City	0	91	2,453	2,544	0	44	798	842	0	0	4	4
Tangub City	0	1	3	4	0	0	0	0	0	0	0	0
Valencia City	0	0	785	785	0	2	178	180	0	0	107	107

Table 1.A.4 - MODERN METHOD OF FAMILY PLANNING

Current User (Ending)

Annual Philippines, 2020

Area	IMPLANTS			Total	NFP-CCM			Total	NFP-BBT			Total
	Age group				Age group				Age group			
	10-14	15-19	20-49		10-14	15-19	20-49		10-14	15-19	20-49	
**Region 11**	**43**	**1,958**	**34,989**	**36,990**	**0**	**124**	**2,393**	**2,517**	**0**	**2**	**395**	**397**
Davao de Oro	7	387	5,732	6,126	0	8	490	498	0	0	43	43
Davao del Norte	18	348	5,731	6,097	0	27	183	210	0	0	95	95
Davao Oriental	2	117	3,198	3,317	0	6	733	739	0	0	43	43
Davao del Sur	6	216	3,447	3,669	0	72	443	515	0	0	2	2
Davao Occidental	0	261	2,695	2,956	0	0	3	3	0	0	1	1
Davao City	10	629	14,186	14,825	0	11	541	552	0	2	211	213
**Region 12**	**50**	**3,180**	**27,495**	**30,725**	**0**	**161**	**855**	**1,016**	**0**	**75**	**95**	**170**
North Cotabato	0	1,636	8,645	10,281	0	152	540	692	0	27	85	112
Sarangani	1	224	3,045	3,270	0	7	96	103	0	21	50	71
South Cotabato	8	515	3,570	4,093	0	0	70	70	0	-2	-39	-41
Sultan Kudarat	40	641	7,313	7,994	0	2	76	78	0	2	-1	1
Cotabato City	1	73	1,549	1,623	0	0	56	56	0	0	0	0
Gen. Santos City	0	91	3,373	3,464	0	0	17	17	0	27	0	27
**B.A.R.M.M.**	**5**	**7,270**	**11,000**	**18,275**	**0**	**114**	**37**	**151**	**0**	**0**	**4**	**4**
Basilan	0	141	1,710	1,851	0	0	5	5	0	0	4	4
Lanao del Sur	0	10	2,426	2,436	0	0	10	10	0	0	0	0
Maguindanao	5	334	5,091	5,430	0	0	21	21	0	0	0	0
Sulu	0	4,030	0	4,030	0	0	0	0	0	0	0	0
Tawi-Tawi	0	2,601	113	2,714	0	114	0	114	0	0	0	0
Lamitan City	0	151	1,394	1,545	0	0	0	0	0	0	0	0
Marawi City	0	3	266	269	0	0	1	1	0	0	0	0
**CARAGA**	**2**	**729**	**15,492**	**16,223**	**0**	**2**	**753**	**755**	**0**	**10**	**166**	**176**
Agusan del Norte	0	117	2,372	2,489	0	0	75	75	0	3	5	8
Agusan del Sur	1	152	4,932	5,085	0	0	169	169	0	6	16	22
Surigao del Norte	1	102	1,537	1,640	0	0	282	282	0	1	20	21
Surigao del Sur	0	81	2,095	2,176	0	0	102	102	0	0	125	125
Province of Dinagat	0	65	1,531	1,596	0	0	0	0	0	0	0	0
Bislig City	0	31	408	439	0	2	123	125	0	0	0	0
Butuan City	0	165	1,917	2,082	0	0	2	2	0	0	0	0
Surigao City	0	16	700	716	0	0	0	0	0	0	0	0

Table 1.A.4 - MODERN METHOD OF FAMILY PLANNING
Current User (Ending)
Annual Philippines, 2020

Area	NFP-STM			Total	NFP-SDM			Total	NFP-LAM			Total
	Age group				Age group				Age group			
	10-14	15-19	20-49		10-14	15-19	20-49		10-14	15-19	20-49	
**PHILIPPINES**	**192**	**68**	**3,165**	**3,425**	**28**	**1,524**	**90,896**	**92,447**	**5,321**	**68,031**	**555,967**	**629,318**
**N C R**	**0**	**8**	**41**	**49**	**3**	**9**	**1,901**	**1,913**	**286**	**8,540**	**72,346**	**81,172**
Malabon	0	0	0	0	0	0	4	4	3	911	2,388	3,302
Navotas	0	0	0	0	0	0	3	3	0	489	1,923	2,412
Valenzuela City	0	0	0	0	0	7	4	11	2	90	2,979	3,071
Caloocan City	0	0	0	0	0	0	120	120	8	674	5,580	6,262
Marikina City	0	0	0	0	0	0	6	6	0	165	510	675
Pasig City	0	0	0	0	0	0	18	18	4	216	3,447	3,667
Pateros	0	0	0	0	0	0	0	0	1	5	68	74
Taguig	0	0	0	0	0	0	3	3	9	1,113	15,353	16,475
Quezon City	0	0	0	0	0	0	1,577	1,577	18	1,698	10,394	12,110
Makati City	0	0	0	0	0	0	0	0	0	118	3,155	3,273
Mandaluyong City	0	0	33	33	0	0	0	0	2	181	3,965	4,148
San Juan	0	0	0	0	0	0	10	10	0	0	323	323
Manila City	0	-1	1	0	1	1	1	3	1	260	3,242	3,503
Las Piñas City	0	0	0	0	0	0	31	31	4	271	2,659	2,934
Muntinlupa City	0	0	0	0	0	0	0	0	1	1,306	7,205	8,512
Parañaque City	0	8	0	8	1	0	58	59	233	1,043	7,150	8,426
Pasay City	0	1	7	8	1	1	66	68	0	0	2,005	2,005
**C A R**	**0**	**0**	**68**	**68**	**0**	**20**	**4,117**	**4,137**	**88**	**758**	**8,184**	**9,030**
Abra	0	0	24	24	0	7	173	180	62	238	1,318	1,618
Apayao	0	0	0	0	0	0	21	21	2	82	720	804
Benguet	0	0	0	0	0	2	792	794	0	127	1,710	1,837
Ifugao	0	0	9	9	0	11	1,932	1,943	3	120	1,588	1,711
Kalinga	0	0	35	35	0	0	431	431	20	117	1,614	1,751
Mt. Province	0	0	0	0	0	0	646	646	1	71	861	933
Baguio City	0	0	0	0	0	0	122	122	0	3	373	376
**Region 1**	**0**	**0**	**199**	**199**	**3**	**136**	**6,426**	**6,565**	**29**	**3,646**	**40,719**	**44,394**
Ilocos Norte	0	0	64	64	0	39	1,162	1,201	0	154	2,581	2,735
Ilocos Sur	0	0	60	60	3	78	3,192	3,273	5	480	6,560	7,045
La Union	0	0	10	10	0	2	779	781	2	429	4,642	5,073
Pangasinan	0	0	0	0	0	14	627	641	18	1,893	18,149	20,060
Alaminos City	0	0	0	0	0	0	0	0	0	256	2,303	2,559
Candon City	0	0	34	34	0	0	28	28	0	0	306	306
Dagupan City	0	0	0	0	0	0	35	35	0	63	690	753
Laoag City	0	0	0	0	0	0	0	0	0	43	1,270	1,313
San Carlos City	0	0	0	0	0	0	0	0	0	21	834	855
San Fernando City	0	0	0	0	0	3	493	496	0	43	499	542
Urdaneta City	0	0	0	0	0	0	52	52	4	251	2,324	2,579
Vigan City	0	0	31	31	0	0	58	58	0	13	561	574
**Region 2**	**0**	**1**	**111**	**112**	**0**	**41**	**527**	**568**	**32**	**1,616**	**15,443**	**17,091**
Batanes	0	0	0	0	0	0	32	32	0	8	99	107
Cagayan	0	0	2	2	0	0	142	142	13	465	3,664	4,142
Isabela	0	0	2	2	0	0	21	21	6	434	3,938	4,378
Nueva Vizcaya	0	1	107	108	0	40	289	329	10	166	3,939	4,115
Quirino	0	0	0	0	0	0	22	22	0	146	956	1,102
Cauayan City	0	0	0	0	0	0	2	2	3	74	443	520
Ilagan City	0	0	0	0	0	0	0	0	0	47	417	464
Santiago City	0	0	0	0	0	0	0	0	0	261	1,599	1,860
Tuguegarao City	0	0	0	0	0	1	19	20	0	15	388	403
**Region 3**	**0**	**0**	**64**	**64**	**0**	**12**	**3,935**	**3,947**	**16**	**4,235**	**42,932**	**47,183**
Aurora	0	0	0	0	0	1	74	75	2	252	1,645	1,899
Bataan	0	0	0	0	0	1	120	121	7	448	3,747	4,202

Table 1.A.4 - MODERN METHOD OF FAMILY PLANNING
Current User (Ending)
Annual Philippines, 2020

Area	NFP-STM			Total	NFP-SDM			Total	NFP-LAM			Total
	Age group				Age group				Age group			
	10-14	15-19	20-49		10-14	15-19	20-49		10-14	15-19	20-49	
Bulacan	0	0	0	0	0	1	2,669	2,670	0	499	7,367	7,866
Nueva Ecija	0	0	0	0	0	0	33	33	0	518	4,179	4,697
Pampanga	0	0	47	47	0	7	815	822	0	267	4,418	4,685
Tarlac	0	0	0	0	0	0	9	9	0	245	4,910	5,155
Zambales	0	0	5	5	0	0	45	45	2	365	3,039	3,406
Angeles City	0	0	0	0	0	0	79	79	1	207	1,533	1,741
Balanga City	0	0	0	0	0	0	21	21	1	78	357	436
Cabanatuan City	0	0	0	0	0	0	0	0	1	205	1,217	1,423
City of San Fernando	0	0	3	3	0	2	64	66	1	195	1,330	1,526
Gapan City	0	0	0	0	0	0	0	0	0	74	567	641
Mabalacat City	0	0	0	0	0	0	6	6	0	193	3,312	3,505
Malolos City	0	0	0	0	0	0	0	0	0	2	10	12
Meycauayan	0	0	9	9	0	0	0	0	1	170	1,135	1,306
Olongapo	0	0	0	0	0	0	0	0	0	82	1,125	1,207
Palayan City	0	0	0	0	0	0	0	0	0	12	104	116
San Jose City	0	0	0	0	0	0	0	0	0	136	1,109	1,245
San Jose del Monte City	0	0	0	0	0	0	0	0	0	0	0	0
Science City of Munoz	0	0	0	0	0	0	0	0	0	0	0	0
Tarlac City	0	0	0	0	0	0	0	0	0	287	1,828	2,115
**Region 4A**	**192**	**6**	**398**	**596**	**16**	**172**	**3,148**	**3,335**	**3,916**	**-639**	**-1,234**	**2,042**
Batangas	0	0	2	2	3	61	-304	-240	-9	148	-2,153	-2,014
Cavite	0	4	340	344	4	11	1,279	1,294	11	61	189	261
Laguna	0	0	15	15	2	24	503	529	16	-212	725	530
Quezon	0	0	21	21	5	25	539	568	10	-237	-1,394	-1,621
Rizal	0	0	-12	-12	0	1	68	69	19	-762	-1,121	-1,864
Antipolo City	0	0	0	0	0	2	84	86	9	106	3,979	4,094
Bacoor City	0	0	0	0	0	0	0	0	-3	-214	-639	-856
Batangas City	0	0	0	0	0	2	72	74	1	8	314	323
Biñan City	0	0	0	0	0	1	38	39	8	-229	31	-189
Cabuyao City	0	0	0	0	0	20	0	20	2	-243	-1,007	-1,249
Calamba City	0	0	2	2	1	7	268	275	-3	-176	-461	-639
Cavite City	0	0	0	0	0	0	2	2	0	5	194	200
Dasmariñas City	0	0	0	0	0	4	166	171	4	126	-771	-641
General Trias City	0	0	0	0	0	0	-3	-3	0	-85	-222	-307
Imus City	0	0	0	0	0	0	0	0	1	49	-126	-76
Lipa City	0	0	0	0	0	0	5	5	0	1	20	21
Lucena City	192	0	0	192	0	0	2	2	3,811	7	-130	3,688
San Pablo City	0	0	0	0	0	0	0	0	2	24	914	940
San Pedro City	0	1	30	31	1	12	429	442	6	-153	-325	-472
Santa Rosa City	0	0	0	0	0	0	1	1	17	1,171	1,296	2,485
Tagaytay City	0	0	0	0	0	0	0	0	10	-3	-224	-217
Tanauan City	0	0	0	0	0	0	0	0	1	2	-16	-13
Tayabas City	0	1	0	1	0	0	2	2	1	-33	-349	-380
Trece Martires City	0	0	0	0	0	0	0	0	0	-1	41	40
**Region 4B**	**0**	**0**	**0**	**0**	**0**	**3**	**1,699**	**1,702**	**35**	**1,031**	**12,870**	**13,936**
Marinduque	0	0	0	0	0	0	59	59	1	96	1,703	1,800
Mindoro Occidental	0	0	0	0	0	2	1,048	1,050	32	713	3,113	3,858
Mindoro Oriental	0	0	0	0	0	0	121	121	0	165	4,476	4,641
Palawan	0	0	0	0	0	1	257	258	2	50	2,154	2,206
Romblon	0	0	0	0	0	0	78	78	0	7	389	396
Puerto Princesa City	0	0	0	0	0	0	136	136	0	0	1,035	1,035
**Region 5**	**0**	**21**	**1,421**	**1,442**	**1**	**412**	**32,994**	**33,407**	**37**	**4,342**	**37,108**	**41,487**
Albay	0	8	251	259	0	38	4,051	4,089	5	373	6,114	6,492
Camarines Norte	0	0	3	3	0	27	1,300	1,327	2	362	2,437	2,801
Camarines Sur	0	13	814	827	0	83	10,936	11,019	17	1,045	8,825	9,887
Catanduanes	0	0	34	34	0	92	2,322	2,414	0	751	4,409	5,160
Masbate	0	0	5	5	0	110	6,503	6,613	11	1,107	6,030	7,148
Sorsogon	0	0	5	5	1	35	4,235	4,271	2	523	5,831	6,356
Iriga City	0	0	229	229	0	27	2,405	2,432	0	105	1,070	1,175

Table 1.A.4 - MODERN METHOD OF FAMILY PLANNING
Current User (Ending)
Annual Philippines, 2020

Area	NFP-STM			Total	NFP-SDM			Total	NFP-LAM			Total
	Age group				Age group				Age group			
	10-14	15-19	20-49		10-14	15-19	20-49		10-14	15-19	20-49	
Legaspi City	0	0	54	54	0	0	88	88	0	66	808	874
Naga City	0	0	26	26	0	0	1,154	1,154	0	10	1,584	1,594
**Region 6**	**0**	**4**	**87**	**91**	**2**	**77**	**6,661**	**6,740**	**24**	**3,397**	**43,403**	**46,824**
Aklan	0	3	23	26	0	0	275	275	2	297	3,986	4,285
Antique	0	0	10	10	0	0	317	317	7	211	2,692	2,910
Capiz	0	0	0	0	0	0	23	23	1	181	4,263	4,445
Guimaras	0	0	0	0	0	0	22	22	1	73	1,004	1,078
Iloilo	0	0	30	30	2	11	2,414	2,427	2	714	10,818	11,534
Negros Occidental	0	1	24	25	0	66	3,377	3,443	9	1,824	15,675	17,508
Bacolod City	0	0	0	0	0	0	38	38	2	54	4,683	4,739
Iloilo City	0	0	0	0	0	0	195	195	0	43	282	325
**Region 7**	**0**	**0**	**10**	**10**	**0**	**3**	**1,263**	**1,266**	**83**	**3,185**	**25,391**	**28,659**
Bohol	0	0	0	0	0	0	227	227	0	107	1,256	1,363
Cebu	0	0	0	0	0	0	8	8	-1	430	4,573	5,002
Negros Oriental	0	0	10	10	0	1	187	188	4	916	7,594	8,514
Siquijor	0	0	0	0	0	2	832	834	0	27	359	386
Cebu City	0	0	0	0	0	0	9	9	5	710	5,216	5,931
Lapu-Lapu City	0	0	0	0	0	0	0	0	75	942	6,138	7,155
Mandaue City	0	0	0	0	0	0	0	0	0	53	255	308
**Region 8**	**0**	**0**	**352**	**352**	**0**	**12**	**3,322**	**3,334**	**1**	**987**	**22,898**	**23,886**
Biliran	0	0	0	0	0	0	54	54	0	94	1,047	1,141
Eastern Samar	0	0	346	346	0	0	1,556	1,556	0	80	3,994	4,074
Northern Leyte	0	0	0	0	0	0	0	0	0	0	0	0
Northern Samar	0	0	0	0	0	1	71	72	1	139	6,637	6,777
Southern Leyte	0	0	0	0	0	0	172	172	0	211	1,932	2,143
Western Samar	0	0	0	0	0	0	0	0	0	49	1,240	1,289
Calbayog City	0	0	0	0	0	0	0	0	0	45	433	478
Maasin City	0	0	0	0	0	0	5	5	0	10	139	149
Ormoc City	0	0	0	0	0	6	942	948	0	137	1,669	1,806
Tacloban City	0	0	6	6	0	5	522	527	0	222	5,807	6,029
**Region 9**	**0**	**0**	**7**	**7**	**0**	**1**	**1,788**	**1,789**	**496**	**430**	**22,752**	**23,678**
Zamboanga del Norte	0	0	6	6	0	1	1,547	1,548	2	20	5,810	5,832
Zamboanga del Sur	0	0	0	0	0	0	0	0	0	264	2,848	3,112
Zamboanga Sibugay	0	0	0	0	0	0	183	183	493	0	1,339	1,832
Dapitan City	0	0	0	0	0	0	11	11	0	32	646	678
Dipolog City	0	0	0	0	0	0	9	9	1	16	1,755	1,772
Isabela City	0	0	0	0	0	0	38	38	0	29	1,128	1,157
Pagadian City	0	0	0	0	0	0	0	0	0	69	2,101	2,170
Zamboanga City	0	0	1	1	0	0	0	0	0	0	7,125	7,125
**Region 10**	**0**	**7**	**101**	**108**	**3**	**387**	**14,006**	**14,396**	**62**	**4,620**	**59,176**	**63,858**
Bukidnon	0	0	91	91	1	121	2,499	2,621	29	1,114	7,039	8,182
Camiguin	0	0	1	1	0	0	81	81	1	22	639	662
Lanao del Norte	0	0	0	0	0	1	163	164	2	559	5,965	6,526
Misamis Occidental	0	4	12	16	0	-9	348	339	3	201	3,563	3,767
Misamis Oriental	0	0	81	81	0	37	2,107	2,144	9	54	7,958	8,021
Cagayan de Oro City	0	0	0	0	0	0	264	264	2	1,025	19,968	20,995
El Salvador City	0	0	0	0	0	0	29	29	0	46	306	352
Gingoog City	0	3	-87	-84	2	154	1,574	1,730	2	228	1,290	1,520
Iligan City	0	0	2	2	0	41	941	982	7	329	2,182	2,518
Malaybalay City	0	0	1	1	0	18	2,539	2,557	2	142	2,224	2,368
Oroquieta City	0	0	0	0	0	0	262	262	0	45	506	551
Ozamis City	0	0	0	0	0	11	2,266	2,277	4	429	5,579	6,012
Tangub City	0	0	0	0	0	12	473	485	0	213	526	739
Valencia City	0	0	0	0	0	1	460	461	1	213	1,431	1,645

Table 1.A.4 - MODERN METHOD OF FAMILY PLANNING
Current User (Ending)
Annual Philippines, 2020

Area	NFP-STM			Total	NFP-SDM			Total	NFP-LAM			Total
	Age group				Age group				Age group			
	10-14	15-19	20-49		10-14	15-19	20-49		10-14	15-19	20-49	
**Region 11**	**0**	**5**	**216**	**221**	**0**	**35**	**2,903**	**2,938**	**98**	**2,588**	**27,150**	**29,836**
Davao de Oro	0	0	3	3	0	1	218	219	6	170	905	1,081
Davao del Norte	0	0	52	52	0	2	217	219	29	459	2,922	3,410
Davao Oriental	0	0	9	9	0	13	1,760	1,773	25	455	3,136	3,616
Davao del Sur	0	0	3	3	0	1	127	128	1	105	936	1,042
Davao Occidental	0	0	0	0	0	16	276	292	2	185	584	771
Davao City	0	5	149	154	0	2	305	307	35	1,214	18,667	19,916
**Region 12**	**0**	**8**	**11**	**19**	**0**	**20**	**790**	**810**	**72**	**7,087**	**43,176**	**50,335**
North Cotabato	0	4	10	14	0	11	118	129	22	1,690	11,702	13,414
Sarangani	0	4	0	4	0	0	173	173	1	1,336	6,587	7,924
South Cotabato	0	0	0	0	0	9	404	413	39	2,863	11,888	14,790
Sultan Kudarat	0	0	1	1	0	0	20	20	4	861	5,138	6,003
Cotabato City	0	0	0	0	0	0	75	75	6	140	1,861	2,007
Gen. Santos City	0	0	0	0	0	0	0	0	0	197	6,000	6,197
**B.A.R.M.M.**	**0**	**0**	**7**	**7**	**0**	**104**	**625**	**729**	**22**	**20,179**	**66,875**	**87,076**
Basilan	0	0	1	1	0	0	29	29	5	428	3,101	3,534
Lanao del Sur	0	0	6	6	0	0	467	467	4	929	29,748	30,681
Maguindanao	0	0	0	0	0	0	112	112	11	2,648	28,570	31,229
Sulu	0	0	0	0	0	8	0	8	0	7,329	112	7,441
Tawi-Tawi	0	0	0	0	0	96	0	96	0	8,436	1,281	9,717
Lamitan City	0	0	0	0	0	0	4	4	2	277	1,198	1,477
Marawi City	0	0	0	0	0	0	13	13	0	132	2,865	2,997
**CARAGA**	**0**	**8**	**72**	**80**	**0**	**80**	**4,791**	**4,871**	**24**	**2,029**	**16,778**	**18,831**
Agusan del Norte	0	0	13	13	0	0	348	348	1	237	2,001	2,239
Agusan del Sur	0	6	6	12	0	49	1,753	1,802	10	621	4,826	5,457
Surigao del Norte	0	0	9	9	0	7	1,316	1,323	2	123	1,983	2,108
Surigao del Sur	0	1	44	45	0	13	769	782	9	399	3,077	3,485
Province of Dinagat	0	0	0	0	0	1	245	246	0	85	661	746
Bislig City	0	0	0	0	0	10	291	301	0	35	655	690
Butuan City	0	0	0	0	0	0	22	22	2	466	2,419	2,887
Surigao City	0	1	0	1	0	0	47	47	0	63	1,156	1,219

Table 1.A.5 - MODERN FAMILY PLANNING
No. and proportion of WRA 15-49 yrs old with unmet needs
Philippines, 2020

Area	Estimated No. of WRA (TP x GR Factor)	Total No. of WRA (15-49 yrs old) with Unmet Needs	%
**PHILIPPINES**	**28,009,482**	**701,738**	**2.51**
**N C R**	**3,863,394**	**6,479**	**0.17**
Malabon	109,663	*	0.00
Navotas	74,843	785	1.05
Valenzuela City	186,137	389	0.21
Caloocan City	475,216	*	0.00
Marikina City	135,229	965	0.71
Pasig City	226,602	320	0.14
Pateros	19,155	*	0.00
Taguig	241,489	*	0.00
Quezon City	880,884	0	0.00
Makati City	174,790	2,286	1.31
Mandaluyong City	115,888	0	0.00
San Juan	36,655	*	0.00
Manila City	534,080		0.00
Las Piñas City	176,679	268	0.15
Muntinlupa City	151,360	1,466	0.97
Parañaque City	199,759	*	0.00
Pasay City	124,965	*	0.00
**C A R**	**471,501**	**9,233**	**1.96**
Abra	61,154	4,124	6.74
Apayao	30,662	288	0.94
Benguet	132,284	575	0.43
Ifugao	52,633	0	0.00
Kalinga	54,056	166	0.31
Mt. Province	38,329	905	2.36
Baguio City	102,383	3,175	3.10
**Region 1**	**1,310,980**	**0**	**0.00**
Ilocos Norte	127,383		0.00
Ilocos Sur	151,029		0.00
La Union	179,241		0.00
Pangasinan	611,121		0.00
Alaminos City	23,090		0.00
Candon City	15,914		0.00
Dagupan City	44,087		0.00
Laoag City	29,369		0.00

Table 1.A.5 - MODERN FAMILY PLANNING
No. and proportion of WRA 15-49 yrs old with unmet needs
Philippines, 2020

Area	Estimated No. of WRA (TP x GR Factor)	Total No. of WRA (15-49 yrs old) with Unmet Needs	%
San Carlos City	**48,543**		**0.00**
San Fernando City	**32,839**		**0.00**
Urdaneta City	**34,218**		**0.00**
Vigan City	**14,146**		**0.00**
**Region 2**	**916,734**	**48,808**	**5.32**
Batanes	**4,059**	0	**0.00**
Cagayan	**241,877**	240	**0.10**
Isabela	**351,716**	30,966	**8.80**
Nueva Vizcaya	**120,354**	21	**0.02**
Quirino	**48,596**	123	**0.25**
Cauayan City	**34,552**	865	**2.50**
Ilagan City	**38,830**	15,067	**38.80**
Santiago City	**35,970**	91	**0.25**
Tuguegarao City	**40,780**	1,435	**3.52**
**Region 3**	**3,231,594**	**23,644**	**0.73**
Aurora	**56,207**	608	**1.08**
Bataan	**191,690**	1,193	**0.62**
Bulacan	**668,037**	*	**0.00**
Nueva Ecija	**415,099**	15,836	**3.81**
Pampanga	**487,505**	*	**0.00**
Tarlac	**278,619**	*	**0.00**
Zambales	**164,524**	*	**0.00**
Angeles City	**122,314**	545	**0.45**
Balanga City	**27,708**	242	**0.87**
Cabanatuan City	**84,954**	687	**0.81**
City of San Fernando	**91,119**	679	**0.75**
Gapan City	**31,004**	*	**0.00**
Mabalacat City	**74,523**	41	**0.06**
Malolos City	**74,615**	*	**0.00**
Meycauayan	**61,890**	177	**0.29**
Olongapo	**64,893**	*	**0.00**
Palayan City	**11,536**	2,183	**18.92**
San Jose City	**39,278**	*	**0.00**
San Jose del Monte City	**169,940**	747	**0.44**
Science City of Munoz	**22,905**	*	**0.00**
Tarlac City	**93,234**	706	**0.76**
**Region 4A**	**4,330,534**	**50,028**	**1.16**
Batangas	**554,482**	11,106	**2.00**
Cavite	**436,635**	5,488	**1.26**

Table 1.A.5 - MODERN FAMILY PLANNING

No. and proportion of WRA 15-49 yrs old with unmet needs

Philippines, 2020

Area	Estimated No. of WRA (TP x GR Factor)	Total No. of WRA (15-49 yrs old) with Unmet Needs	%
Laguna	**302,960**	15,111	**4.99**
Quezon	**462,633**	9,565	**2.07**
Rizal	**636,082**	627	**0.10**
Antipolo City	**234,290**	*	**0.00**
Bacoor City	**191,299**	41	**0.02**
Batangas City	**98,409**	*	**0.00**
Biñan City	**101,588**	509	**0.50**
Cabuyao City	**94,180**	67	**0.07**
Calamba City	**138,638**	1,210	**0.87**
Cavite City	**32,743**	*	**0.00**
Dasmariñas City	**209,896**	990	**0.47**
General Trias City	**100,106**	627	**0.63**
Imus City	**128,606**	160	**0.12**
Lipa City	**99,154**	*	**0.00**
Lucena City	**70,112**	0	**0.00**
San Pablo City	**81,168**	0	**0.00**
San Pedro City	**99,387**	78	**0.08**
Santa Rosa City	**107,914**	0	**0.00**
Tagaytay City	**22,671**	32	**0.14**
Tanauan City	**51,713**	1,399	**2.71**
Tayabas City	**26,272**	0	**0.00**
Trece Martires City	**49,596**	3,018	**6.09**
**Region 4B**	**762,847**	**544**	**0.07**
Marinduque	**55,035**	272	**0.49**
Mindoro Occidental	**120,768**		**0.00**
Mindoro Oriental	**218,244**	5	**0.00**
Palawan	**230,838**	18	**0.01**
Romblon	**68,642**	193	**0.28**
		56	
Puerto Princesa City	**69,320**	0	**0.00**
**Region 5**	**1,462,355**	**12,784**	**0.87**
Albay	**291,272**	3,021	**1.04**
Camarines Norte	**149,133**	213	**0.14**
Camarines Sur	**414,568**	2,698	**0.65**
Catanduanes	**63,290**	207	**0.33**
Masbate	**215,721**	1,141	**0.53**
Sorsogon	**199,577**	3,226	**1.62**
Iriga City	**28,166**	790	**2.80**
Legaspi City	**51,226**	1,488	**2.90**
Naga City	**49,402**	0	**0.00**

Table 1.A.5 - MODERN FAMILY PLANNING
No. and proportion of WRA 15-49 yrs old with unmet needs
Philippines, 2020

Area	Estimated No. of WRA (TP x GR Factor)	Total No. of WRA (15-49 yrs old) with Unmet Needs	%
**Region 6**	**1,963,385**	**442,286**	**22.53**
Aklan	**151,522**	113,773	**75.09**
Antique	**143,015**	46,270	**32.35**
Capiz	**203,437**	12,305	**6.05**
Guimaras	**46,249**	7,602	**16.44**
Iloilo	**516,353**	16,420	**3.18**
Negros Occidental	**639,485**	166,308	**26.01**
Bacolod City	**143,877**	78,920	**54.85**
Iloilo City	**119,447**	688	**0.58**
**Region 7**	**2,008,836**	**9,681**	**0.48**
Bohol	**320,816**	1,932	**0.60**
Cebu	**836,834**	0	**0.00**
Negros Oriental	**345,210**	2,679	**0.78**
Siquijor	**23,807**	1,986	**8.34**
Cebu City	**262,702**	1,898	**0.72**
Lapu-Lapu City	**116,203**	1,186	**1.02**
Mandaue City	**103,264**	0	**0.00**
**Region 8**	**1,129,272**	**1,472**	**0.13**
Biliran	**42,282**		**0.00**
Eastern Samar	**117,267**		**0.00**
Northern Leyte	**393,264**		**0.00**
Northern Samar	**158,380**		**0.00**
Southern Leyte	**84,309**	733	**0.87**
Western Samar	**147,720**		**0.00**
Calbayog City	**45,510**		**0.00**
Maasin City	**21,458**	0	**0.00**
Ormoc City	**56,015**	712	**1.27**
Tacloban City	**63,067**	27	**0.04**
**Region 9**	**951,676**	**0**	**0.00**
Zamboanga del Norte	**201,010**	*	**0.00**
Zamboanga del Sur	**214,883**	*	**0.00**
Zamboanga Sibugay	**167,207**	*	**0.00**
Dapitan City	**20,755**	*	**0.00**
Dipolog City	**32,930**	*	**0.00**
Isabela City	**34,025**	*	**0.00**
Pagadian City	**52,703**	*	**0.00**

Table 1.A.5 - MODERN FAMILY PLANNING
No. and proportion of WRA 15-49 yrs old with unmet needs
Philippines, 2020

Area	Estimated No. of WRA (TP x GR Factor)	Total No. of WRA (15-49 yrs old) with Unmet Needs	%
Zamboanga City	**228,163**	*	**0.00**
**Region 10**	**1,264,275**	**25,345**	**2.00**
Bukidnon	**277,599**	1,460	**0.53**
Camiguin	**21,673**	97	**0.45**
Lanao del Norte	**186,841**	0	**0.00**
Misamis Occidental	**82,452**	4,208	**5.10**
Misamis Oriental	**198,005**	120	**0.06**
Cagayan de Oro City	**187,538**	18,699	**9.97**
El Salvador City	**13,929**	0	**0.00**
Gingoog City	**34,583**	0	**0.00**
Iligan City	**94,640**	548	**0.58**
Malaybalay City	**46,277**	0	**0.00**
Oroquieta City	**17,870**	0	**0.00**
Ozamis City	**35,814**	0	**0.00**
Tangub City	**15,911**	0	**0.00**
Valencia City	**51,143**	213	**0.42**
**Region 11**	**1,337,646**	**24,755**	**1.85**
Davao de Oro	**189,875**	*	**0.00**
Davao del Norte	**267,007**	*	**0.00**
Davao Oriental	**141,583**	20,938	**14.79**
Davao del Sur	**183,101**	*	**0.00**
Davao Occidental	**83,429**	218	**0.26**
Davao City	**472,651**	3,599	**0.76**
**Region 12**	**1,265,511**	**8,702**	**0.69**
North Cotabato	**384,855**	0	**0.00**
Sarangani	**145,901**	2,749	**1.88**
South Cotabato	**259,741**	1,608	**0.62**
Sultan Kudarat	**220,805**	0	**0.00**
Cotabato City	**85,517**	177	**0.21**
Gen. Santos City	**168,692**	4,168	**2.47**
**B.A.R.M.M.**	**1,075,649**	**0**	**0.00**
Basilan	**76,249**	0	**0.00**
Lanao del Sur	**243,380**		**0.00**
Maguindanao	**334,352**		**0.00**
Sulu	**236,060**		**0.00**
Tawi-Tawi	**106,416**		**0.00**

Table 1.A.5 - MODERN FAMILY PLANNING

No. and proportion of WRA 15-49 yrs old with unmet needs

Philippines, 2020

Area	Estimated No. of WRA (TP x GR Factor)	Total No. of WRA (15-49 yrs old) with Unmet Needs	%
Lamitan City	**20,978**	0	**0.00**
Marawi City	**58,214**	0	**0.00**
**CARAGA**	**663,293**	**37,977**	**5.73**
Agusan del Norte	**93,248**	677	**0.73**
Agusan del Sur	**175,453**	0	**0.00**
Surigao del Norte	**87,287**	0	**0.00**
Surigao del Sur	**123,630**	1,154	**0.93**
Province of Dinagat	**30,872**	0	**0.00**
Bislig City	**23,481**	4,895	**20.85**
Butuan City	**88,667**	2,952	**3.33**
Surigao City	**40,655**	28,299	**69.61**

Figure 1.A.1 - Percentage Distribution of New Acceptors of Family Planning by Method (10 - 49 yrs old) Philippines, 2020

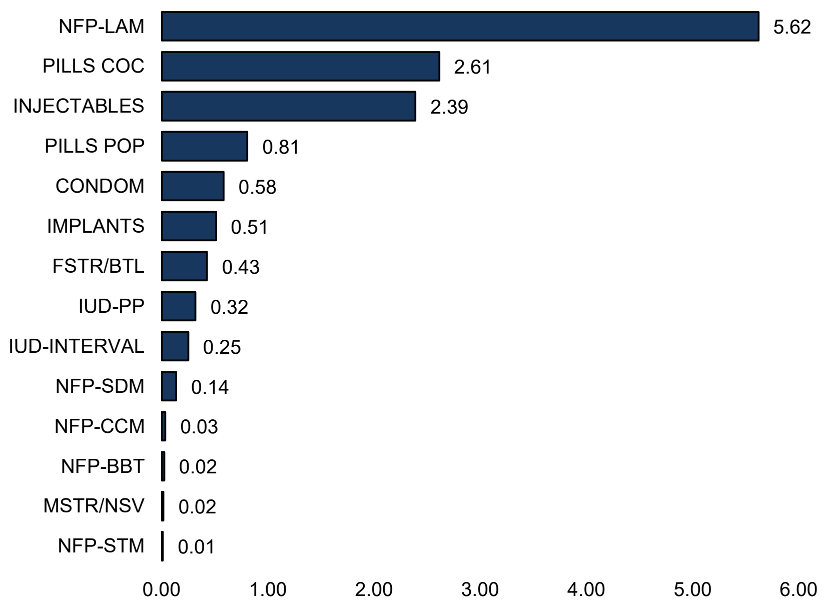

Figure 1.A.2 - Percentage Distribution of Other Acceptor of Family Planning by Method (10 - 49 yrs old) Philippines, 2020

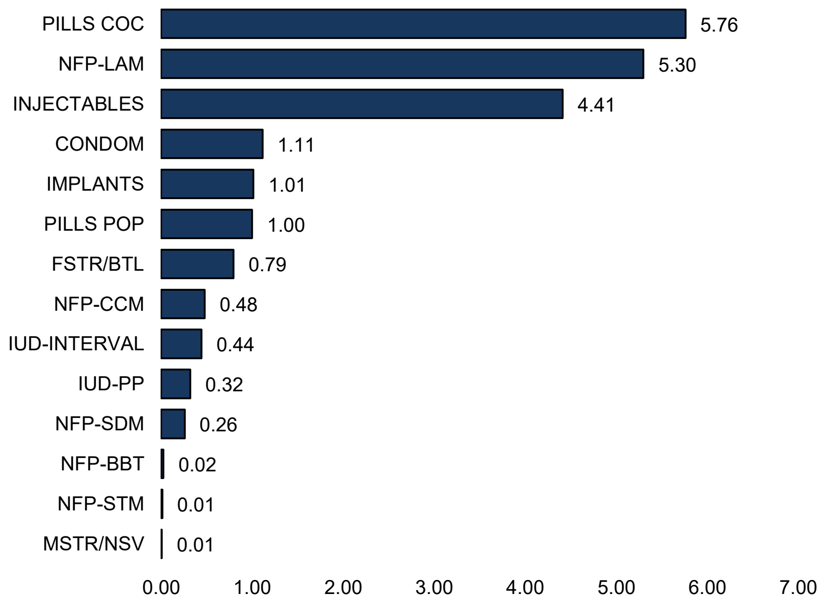

Figure 1.A.3 - Percentage Distribution of Drop-outs of Family Planning by Method (10 - 49 yrs old) Philippines, 2020

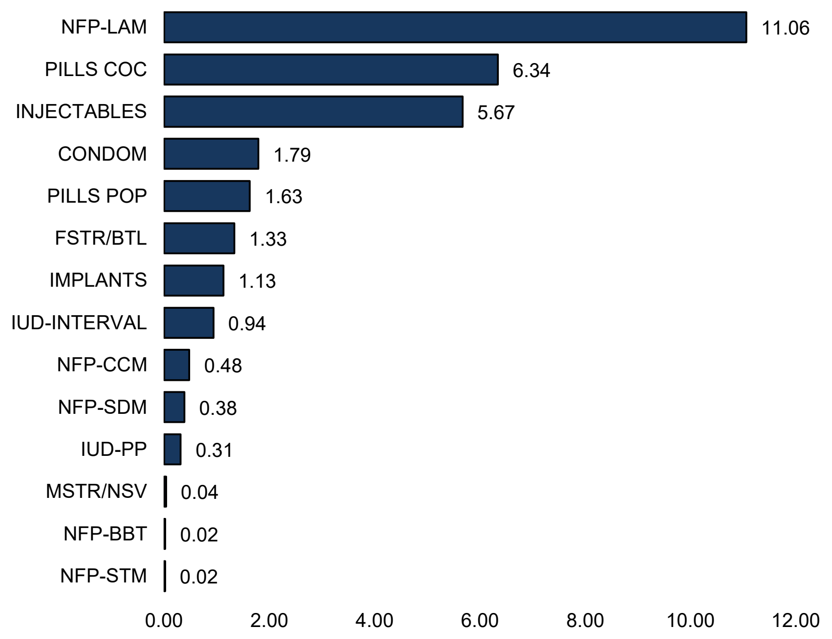

Table 1.A. 4 -Percentage Distribution of Current User Ending of Family Planning by Method (10 - 49 yrs old) Philippines, 2020

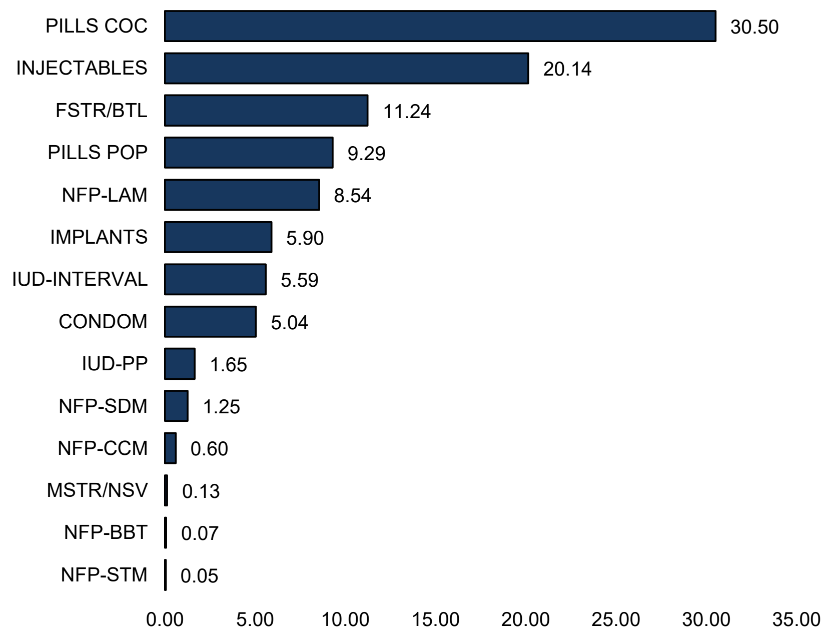

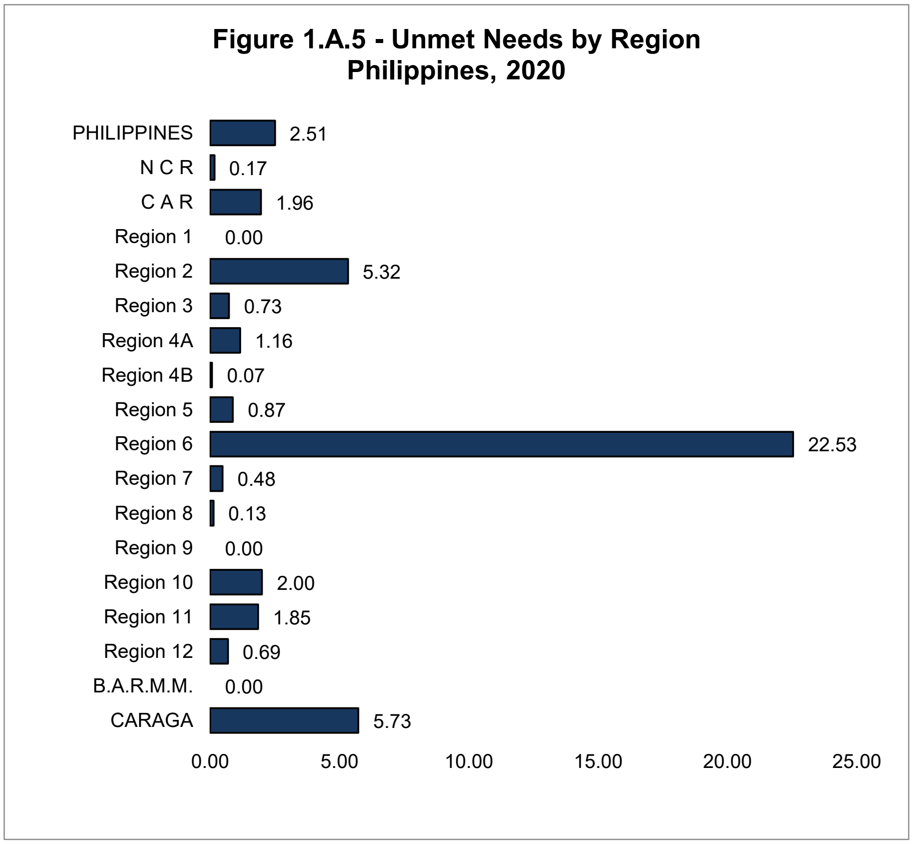
Figure 1.A.5 - Unmet Needs by Region
Philippines, 2020
PHILIPPINES
N C R
C A R
Region 1
Region 2
Region 3
Region 4A
Region 4B
Region 5
Region 6
Region 7
Region 8
Region 9
Region 10
Region 11
Region 12
B.A.R.M.M.
CARAGA
2.51
0.17
1.96
0.00
5.32
0.73
1.16
0.07
0.87
22.53
0.48
0.13
0.00
2.00
1.85
0.69
0.00
5.73
0.00
5.00
10.00
15.00
20.00
25.00

Table 1.B.1 - Prenatal Care

Number and proportion of Pregnant Women with at least 4 or more Prenatal Check-ups by Region, Province/city and age
Philippines, Annual 2020

Area	Eligible Pop.	Pregnant women with at least 4 prenatal check-ups							
		Age Group						Total	%
		10-14 yrs old		15-19 yrs old		20-49 yrs old			
		No.	%	No.	%	20-49	%		
PHILIPPINES	2,123,158	2,399	0.11	133,751	6.30	1,047,698	49.35	1,183,848	55.76
N C R	238,661	234	0.10	13,261	5.56	128,534	53.86	142,029	59.51
Malabon	6,775	10	0.15	359	5.30	2,370	34.98	2,739	40.43
Navotas	4,621	8	0.17	675	14.61	3,284	71.07	3,967	85.85
Valenzuela City	11,500	8	0.07	838	7.29	6,053	52.63	6,899	59.99
Caloocan City	29,363	55	0.19	2,652	9.03	23,140	78.81	25,847	88.03
Marikina City	8,354	4	0.05	375	4.49	5,627	67.36	6,006	71.89
Pasig City	13,996	15	0.11	638	4.56	9,572	68.39	10,225	73.06
Pateros	1,184	8	0.68	45	3.80	455	38.43	508	42.91
Taguig	14,918	28	0.19	1,137	7.62	9,072	60.81	10,237	68.62
Quezon City	54,413	25	0.05	2,883	5.30	38,494	70.74	41,402	76.09
Makati City	10,798	4	0.04	255	2.36	3,098	28.69	3,357	31.09
Mandaluyong City	7,160	5	0.07	362	5.06	3,314	46.28	3,681	51.41
San Juan	2,261	0	0.00	86	3.80	931	41.18	1,017	44.98
Manila City	32,980	11	0.03	662	2.01	4,327	13.12	5,000	15.16
Las Piñas City	10,914	11	0.10	583	5.34	5,478	50.19	6,072	55.63
Muntinlupa City	9,353	7	0.07	797	8.52	7,339	78.47	8,143	87.06
Parañaque City	12,343	34	0.28	563	4.56	2,287	18.53	2,884	23.37
Pasay City	7,728	1	0.01	351	4.54	3,693	47.79	4,045	52.34
C A R	35,099	15	0.04	1,769	5.04	17,131	48.81	18,915	53.89
Abra	4,309	1	0.02	257	5.96	2,019	46.86	2,277	52.84
Apayao	2,451	0	0.00	213	8.69	1,210	49.37	1,423	58.06
Benguet	9,188	3	0.03	376	4.09	4,359	47.44	4,738	51.57
Ifugao	4,396	3	0.07	256	5.82	2,574	58.55	2,833	64.44
Kalinga	4,617	2	0.04	333	7.21	3,124	67.66	3,459	74.92
Mt. Province	3,023	3	0.10	140	4.63	1,636	54.12	1,779	58.85
Baguio City	7,115	3	0.04	194	2.73	2,209	31.05	2,406	33.82
Region 1	97,261	73	0.08	6,120	6.29	59,140	60.81	65,333	67.17
Ilocos Norte	8,105	8	0.10	379	4.68	4,414	54.46	4,801	59.24
Ilocos Sur	9,330	2	0.02	555	5.95	6,906	74.02	7,463	79.99
La Union	11,511	16	0.14	712	6.19	6,503	56.49	7,231	62.82
Pangasinan	50,168	36	0.07	3,306	6.59	29,571	58.94	32,913	65.61
Alaminos City	1,894	0	0.00	159	8.39	1,694	89.44	1,853	97.84
Candon City	987	0	0.00	8	0.81	962	97.47	970	98.28
Dagupan City	3,620	2	0.06	303	8.37	1,606	44.36	1,911	52.79
Laoag City	1,870	0	0.00	64	3.42	1,725	92.25	1,789	95.67
San Carlos City	3,979	4	0.10	217	5.45	2,567	64.51	2,788	70.07
San Fernando City	2,115	1	0.05	137	6.48	1,358	64.21	1,496	70.73
Urdaneta City	2,806	4	0.14	229	8.16	1,496	53.31	1,729	61.62
Vigan City	876	0	0.00	51	5.82	338	38.58	389	44.41

Table 1.B.1 - Prenatal Care

Number and proportion of Pregnant Women with at least 4 or more Prenatal Check-ups by Region, Province/city and age
Philippines, Annual 2020

Area	Eligible Pop.	Pregnant women with at least 4 prenatal check-ups							
		Age Group						Total	%
		10-14 yrs old		15-19 yrs old		20-49 yrs old			
		No.	%	No.	%	20-49	%		
**Region 2**	**69,443**	**79**	**0.11**	**5,443**	**7.84**	**37,184**	**53.55**	**42,706**	**61.50**
Batanes	**340**	0	**0.00**	3	**0.88**	121	**35.59**	**124**	**36.47**
Cagayan	**17,971**	16	**0.09**	1,312	**7.30**	9,180	**51.08**	**10,508**	**58.47**
Isabela	**26,309**	34	**0.13**	1,873	**7.12**	12,426	**47.23**	**14,333**	**54.48**
Nueva Vizcaya	**9,573**	14	**0.15**	979	**10.23**	6,533	**68.24**	**7,526**	**78.62**
Quirino	**4,025**	3	**0.07**	374	**9.29**	2,987	**74.21**	**3,364**	**83.58**
Cauayan City	**2,590**	2	**0.08**	261	**10.08**	1,442	**55.68**	**1,705**	**65.83**
Ilagan City	**2,911**	2	**0.07**	245	**8.42**	1,474	**50.64**	**1,721**	**59.12**
Santiago City	**2,692**	6	**0.22**	336	**12.48**	2,278	**84.62**	**2,620**	**97.33**
Tuguegarao City	**3,032**	2	**0.07**	60	**1.98**	743	**24.51**	**805**	**26.55**
**Region 3**	**220,020**	**403**	**0.18**	**19,433**	**8.83**	**122,743**	**55.79**	**142,579**	**64.80**
Aurora	**4,770**	6	**0.13**	413	**8.66**	2,700	**56.60**	**3,119**	**65.39**
Bataan	**13,823**	32	**0.23**	1,223	**8.85**	7,575	**54.80**	**8,830**	**63.88**
Bulacan	**43,627**	65	**0.15**	3,575	**8.19**	21,167	**48.52**	**24,807**	**56.86**
Nueva Ecija	**28,782**	70	**0.24**	2,183	**7.58**	13,816	**48.00**	**16,069**	**55.83**
Pampanga	**31,359**	41	**0.13**	2,021	**6.44**	12,922	**41.21**	**14,984**	**47.78**
Tarlac	**19,765**	42	**0.21**	1,859	**9.41**	15,079	**76.29**	**16,980**	**85.91**
Zambales	**12,610**	11	**0.09**	498	**3.95**	3,236	**25.66**	**3,745**	**29.70**
Angeles City	**7,869**	9	**0.11**	756	**9.61**	4,186	**53.20**	**4,951**	**62.92**
Balanga City	**1,998**	3	**0.15**	124	**6.21**	738	**36.94**	**865**	**43.29**
Cabanatuan City	**5,896**	13	**0.22**	710	**12.04**	3,705	**62.84**	**4,428**	**75.10**
City of San Fernando	**5,863**	20	**0.34**	642	**10.95**	3,715	**63.36**	**4,377**	**74.65**
Gapan City	**2,153**	3	**0.14**	170	**7.90**	919	**42.68**	**1,092**	**50.72**
Mabalacat City	**4,793**	9	**0.19**	719	**15.00**	4,551	**94.95**	**5,279**	**110.14**
Malolos City	**4,880**	5	**0.10**	351	**7.19**	2,496	**51.15**	**2,852**	**58.44**
Meycauayan	**4,041**	21	**0.52**	661	**16.36**	3,159	**78.17**	**3,841**	**95.05**
Olongapo	**4,971**	7	**0.14**	388	**7.81**	2,036	**40.96**	**2,431**	**48.90**
Palayan City	**799**	1	**0.13**	71	**8.89**	358	**44.81**	**430**	**53.82**
San Jose City	**2,718**	16	**0.59**	376	**13.83**	1,844	**67.84**	**2,236**	**82.27**
San Jose del Monte City	**11,100**	16	**0.14**	1,458	**13.14**	12,003	**108.14**	**13,477**	**121.41**
Science City of Munoz	**1,586**	0	**0.00**	154	**9.71**	915	**57.69**	**1,069**	**67.40**
Tarlac City	**6,617**	13	**0.20**	1,081	**16.34**	5,623	**84.98**	**6,717**	**101.51**
**Region 4A**	**296,816**	**259**	**0.09**	**10,487**	**3.53**	**83,608**	**28.17**	**94,354**	**31.79**
Batangas	**38,441**	21	**0.05**	627	**1.63**	8,940	**23.26**	**9,588**	**24.94**
Cavite	**27,704**	45	**0.16**	1,146	**4.14**	10,190	**36.78**	**11,381**	**41.08**
Laguna	**19,727**	50	**0.25**	1,083	**5.49**	5,636	**28.57**	**6,769**	**34.31**
Quezon	**37,410**	16	**0.04**	661	**1.77**	5,209	**13.92**	**5,886**	**15.73**
Rizal	**44,791**	27	**0.06**	2,181	**4.87**	13,956	**31.16**	**16,164**	**36.09**
Antipolo City	**16,497**	16	**0.10**	1,085	**6.58**	9,866	**59.80**	**10,967**	**66.48**
Bacoor City	**12,142**	5	**0.04**	49	**0.40**	442	**3.64**	**496**	**4.08**
Batangas City	**6,823**	0	**0.00**	0	**0.00**	0	**0.00**	**0**	**0.00**
Biñan City	**6,607**	3	**0.05**	912	**13.80**	5,377	**81.38**	**6,292**	**95.23**
Cabuyao City	**6,130**	6	**0.10**	251	**4.09**	1,611	**26.28**	**1,868**	**30.47**

Table 1.B.1 - Prenatal Care

Number and proportion of Pregnant Women with at least 4 or more Prenatal Check-ups by Region, Province/city and age
Philippines, Annual 2020

Area	Eligible Pop.	Pregnant women with at least 4 prenatal check-ups							
		Age Group						Total	%
		10-14 yrs old		15-19 yrs old		20-49 yrs old			
		No.	%	No.	%	20-49	%		
Calamba City	9,028	13	0.14	974	10.79	7,725	85.57	8,712	96.50
Cavite City	2,075	0	0.00	0	0.00	0	0.00	0	0.00
Dasmariñas City	13,322	4	0.03	314	2.36	3,189	23.94	3,507	26.32
General Trias City	6,352	4	0.06	222	3.49	2,189	34.46	2,415	38.02
Imus City	8,156	0	0.00	43	0.53	297	3.64	340	4.17
Lipa City	6,880	0	0.00	0	0.00	0	0.00	0	0.00
Lucena City	5,672	0	0.00	0	0.00	0	0.00	0	0.00
San Pablo City	5,277	6	0.11	104	1.97	289	5.48	399	7.56
San Pedro City	6,467	3	0.05	87	1.35	385	5.95	475	7.34
Santa Rosa City	7,025	16	0.23	498	7.09	3,980	56.65	4,494	63.97
Tagaytay City	1,437	0	0.00	80	5.57	1,348	93.81	1,428	99.37
Tanauan City	3,584	14	0.39	100	2.79	2,036	56.81	2,150	59.99
Tayabas City	2,122	6	0.28	0	0.00	423	19.93	429	20.22
Trece Martires City	3,147	4	0.13	70	2.22	520	16.52	594	18.88
**Region 4B**	71,246	125	0.18	4,016	5.64	28,543	40.06	32,684	45.87
Marinduque	4,836	4	0.08	254	5.25	2,375	49.11	2,633	54.45
Mindoro Occidental	12,407	14	0.11	970	7.82	6,418	51.73	7,402	59.66
Mindoro Oriental	19,809	83	0.42	914	4.61	7,788	39.32	8,785	44.35
Palawan	21,375	20	0.09	1,766	8.26	10,438	48.83	12,224	57.19
Romblon	6,400	4	0.06	56	0.88	444	6.94	504	7.88
Puerto Princesa City	6,419	0	0.00	56	0.87	1,080	16.83	1,136	17.70
**Region 5**	136,116	66	0.05	6,930	5.09	65,026	47.77	72,022	52.91
Albay	23,737	3	0.01	1,045	4.40	13,041	54.94	14,089	59.35
Camarines Norte	14,283	7	0.05	1,045	7.32	7,025	49.18	8,077	56.55
Camarines Sur	38,796	11	0.03	1,718	4.43	16,942	43.67	18,671	48.13
Catanduanes	6,274	1	0.02	248	3.95	2,665	42.48	2,914	46.45
Masbate	22,521	31	0.14	1,426	6.33	9,712	43.12	11,169	49.59
Sorsogon	19,065	5	0.03	911	4.78	9,930	52.08	10,846	56.89
Iriga City	2,638	1	0.04	123	4.66	1,190	45.11	1,314	49.81
Legaspi City	4,174	5	0.12	221	5.29	2,905	69.60	3,131	75.01
Naga City	4,628	2	0.04	193	4.17	1,616	34.92	1,811	39.13
**Region 6**	146,526	126	0.09	8,420	5.75	68,577	46.80	77,123	52.63
Aklan	11,162	12	0.11	608	5.45	5,674	50.83	6,294	56.39
Antique	12,816	5	0.04	515	4.02	5,211	40.66	5,731	44.72
Capiz	13,986	15	0.11	648	4.63	6,109	43.68	6,772	48.42
Guimaras	3,085	6	0.19	311	10.08	2,080	67.42	2,397	77.70
Iloilo	36,541	26	0.07	1,911	5.23	18,931	51.81	20,868	57.11
Negros Occidental	49,368	46	0.09	3,695	7.48	24,479	49.58	28,220	57.16
Bacolod City	11,115	1	0.01	238	2.14	2,372	21.34	2,611	23.49
Iloilo City	8,453	15	0.18	494	5.84	3,721	44.02	4,230	50.04

Table 1.B.1 - Prenatal Care

Number and proportion of Pregnant Women with at least 4 or more Prenatal Check-ups by Region, Province/city and age
Philippines, Annual 2020

Area	Eligible Pop.	Pregnant women with at least 4 prenatal check-ups							
		Age Group						Total	%
		10-14 yrs old		15-19 yrs old		20-49 yrs old			
		No.	%	No.	%	20-49	%		
**Region 7**	**163,262**	**108**	**0.07**	**8,520**	**5.22**	**84,557**	**51.79**	**93,185**	**57.08**
Bohol	**27,312**	19	**0.07**	1,320	**4.83**	13,438	**49.20**	**14,777**	**54.10**
Cebu	**67,506**	42	**0.06**	3,390	**5.02**	32,516	**48.17**	**35,948**	**53.25**
Negros Oriental	**27,938**	17	**0.06**	1,741	**6.23**	11,976	**42.87**	**13,734**	**49.16**
Siquijor	**1,613**	0	**0.00**	84	**5.21**	820	**50.84**	**904**	**56.04**
Cebu City	**21,193**	24	**0.11**	1,337	**6.31**	10,741	**50.68**	**12,102**	**57.10**
Lapu-Lapu City	**9,372**	6	**0.06**	648	**6.91**	7,688	**82.03**	**8,342**	**89.01**
Mandaue City	**8,328**	0	**0.00**	0	**0.00**	7,378	**88.59**	**7,378**	**88.59**
**Region 8**	**102,619**	**82**	**0.08**	**5,010**	**4.88**	**45,334**	**44.18**	**50,426**	**49.14**
Biliran	**3,834**	1	**0.03**	222	**5.79**	1,783	**46.50**	**2,006**	**52.32**
Eastern Samar	**11,392**	6	**0.05**	643	**5.64**	5,390	**47.31**	**6,039**	**53.01**
Northern Leyte	**34,707**	29	**0.08**	1,331	**3.83**	14,196	**40.90**	**15,556**	**44.82**
Northern Samar	**15,370**	10	**0.07**	556	**3.62**	6,849	**44.56**	**7,415**	**48.24**
Southern Leyte	**6,451**	7	**0.11**	427	**6.62**	3,532	**54.75**	**3,966**	**61.48**
Western Samar	**14,305**	15	**0.10**	948	**6.63**	6,268	**43.82**	**7,231**	**50.55**
Calbayog City	**4,413**	1	**0.02**	208	**4.71**	1,565	**35.46**	**1,774**	**40.20**
Maasin City	**1,637**	0	**0.00**	59	**3.60**	560	**34.21**	**619**	**37.81**
Ormoc City	**4,941**	7	**0.14**	321	**6.50**	2,667	**53.98**	**2,995**	**60.62**
Tacloban City	**5,569**	6	**0.11**	295	**5.30**	2,524	**45.32**	**2,825**	**50.73**
**Region 9**	**80,051**	**59**	**0.07**	**6,024**	**7.53**	**42,377**	**52.94**	**48,460**	**60.54**
			**0.00**		**0.00**		**0.00**		**0.00**
Zamboanga del Norte	**17,249**	13	**0.08**	2,128	**12.34**	13,178	**76.40**	**15,319**	**88.81**
Zamboanga del Sur	**17,653**	26	**0.15**	1,064	**6.03**	5,799	**32.85**	**6,889**	**39.02**
Zamboanga Sibugay	**14,954**	7	**0.05**	857	**5.73**	5,220	**34.91**	**6,084**	**40.68**
Dapitan City	**1,784**	2	**0.11**	173	**9.70**	1,448	**81.17**	**1,623**	**90.98**
Dipolog City	**2,827**	0	**0.00**	291	**10.29**	2,149	**76.02**	**2,440**	**86.31**
Isabela City	**2,522**	1	**0.04**	255	**10.11**	1,529	**60.63**	**1,785**	**70.78**
Pagadian City	**4,325**	3	**0.07**	229	**5.29**	1,652	**38.20**	**1,884**	**43.56**
Zamboanga City	**18,737**	7	**0.04**	1,027	**5.48**	11,402	**60.85**	**12,436**	**66.37**
			**0.00**		**0.00**		**0.00**		**0.00**
**Region 10**	**101,411**	**216**	**0.21**	**10,722**	**10.57**	**63,935**	**63.05**	**74,873**	**73.83**
Bukidnon	**23,706**	93	**0.39**	3,410	**14.38**	14,439	**60.91**	**17,942**	**75.69**
Camiguin	**1,858**	0	**0.00**	61	**3.28**	606	**32.62**	**667**	**35.90**
Lanao del Norte	**14,960**	7	**0.05**	846	**5.66**	8,574	**57.31**	**9,427**	**63.01**
Misamis Occidental	**6,403**	2	**0.03**	408	**6.37**	3,978	**62.13**	**4,388**	**68.53**
Misamis Oriental	**15,131**	13	**0.09**	1,409	**9.31**	9,563	**63.20**	**10,985**	**72.60**
Cagayan de Oro City	**14,339**	29	**0.20**	1,605	**11.19**	11,563	**80.64**	**13,197**	**92.04**
El Salvador City	**1,065**	5	**0.47**	83	**7.79**	436	**40.94**	**524**	**49.20**
Gingoog City	**2,644**	0	**0.00**	398	**15.05**	2,041	**77.19**	**2,439**	**92.25**
Iligan City	**7,580**	59	**0.78**	1,224	**16.15**	4,782	**63.09**	**6,065**	**80.01**
Malaybalay City	**3,956**	3	**0.08**	286	**7.23**	1,637	**41.38**	**1,926**	**48.69**

Table 1.B.1 - Prenatal Care

Number and proportion of Pregnant Women with at least 4 or more Prenatal Check-ups by Region, Province/city and age
Philippines, Annual 2020

Area	Eligible Pop.	Pregnant women with at least 4 prenatal check-ups							
		Age Group						Total	%
		10-14 yrs old		15-19 yrs old		20-49 yrs old			
		No.	%	No.	%	20-49	%		
Oroquieta City	**1,389**	0	**0.00**	58	**4.18**	516	**37.15**	**574**	**41.32**
Ozamis City	**2,778**	4	**0.14**	370	**13.32**	2,012	**72.43**	**2,386**	**85.89**
Tangub City	**1,234**	0	**0.00**	91	**7.37**	1,055	**85.49**	**1,146**	**92.87**
Valencia City	**4,368**	1	**0.02**	473	**10.83**	2,733	**62.57**	**3,207**	**73.42**
**Region 11**	**107,247**	**313**	**0.29**	**8,652**	**8.07**	**55,112**	**51.39**	**64,077**	**59.75**
Compostela Valley	**15,562**	69	**0.44**	1,701	**10.93**	8,731	**56.10**	**10,501**	**67.48**
Davao del Norte	**21,326**	63	**0.30**	2,126	**9.97**	13,339	**62.55**	**15,528**	**72.81**
Davao Oriental	**13,007**	46	**0.35**	941	**7.23**	5,420	**41.67**	**6,407**	**49.26**
Davao del Sur	**14,151**	47	**0.33**	853	**6.03**	4,822	**34.08**	**5,722**	**40.44**
Davao Occidental	**6,670**	20	**0.30**	430	**6.45**	1,290	**19.34**	**1,740**	**26.09**
Davao City	**36,531**	68	**0.19**	2,601	**7.12**	21,510	**58.88**	**24,179**	**66.19**
**Region 12**	**104,552**	**135**	**0.13**	**10,100**	**9.66**	**58,609**	**56.06**	**68,844**	**65.85**
North Cotabato	**33,645**	42	**0.12**	2,770	**8.23**	16,298	**48.44**	**19,110**	**56.80**
Sarangani	**12,891**	25	**0.19**	1,850	**14.35**	7,291	**56.56**	**9,166**	**71.10**
South Cotabato	**21,113**	21	**0.10**	2,309	**10.94**	13,641	**64.61**	**15,971**	**75.65**
Sultan Kudarat	**17,359**	28	**0.16**	1,717	**9.89**	9,382	**54.05**	**11,127**	**64.10**
Cotabato City	**5,835**	3	**0.05**	291	**4.99**	4,378	**75.03**	**4,672**	**80.07**
Gen. Santos City	**13,709**	16	**0.12**	1,163	**8.48**	7,619	**55.58**	**8,798**	**64.18**
**BARMM**	**92,799**	**59**	**0.06**	**4,673**	**5.04**	**57,998**	**62.50**	**62,730**	**67.60**
Basilan	**7,541**	6	**0.08**	306	**4.06**	1,947	**25.82**	**2,259**	**29.96**
Lanao del Sur	**21,131**	6	**0.03**	1,020	**4.83**	19,574	**92.63**	**20,600**	**97.49**
Maguindanao	**31,128**	47	**0.15**	2,682	**8.62**	20,152	**64.74**	**22,881**	**73.51**
Sulu	**16,613**	0	**0.00**	0	**0.00**	7,902	**47.57**	**7,902**	**47.57**
Tawi-Tawi	**9,259**	0	**0.00**	0	**0.00**	5,362	**57.91**	**5,362**	**57.91**
Lamitan City	**2,074**	0	**0.00**	157	**7.57**	519	**25.02**	**676**	**32.59**
Marawi City	**5,053**	0	**0.00**	508	**10.05**	2,542	**50.31**	**3,050**	**60.36**
**CARAGA**	**60,029**	**47**	**0.08**	**4,171**	**6.95**	**29,290**	**48.79**	**33,508**	**55.82**
Agusan del Norte	**8,098**	6	**0.07**	533	**6.58**	3,557	**43.92**	**4,096**	**50.58**
Agusan del Sur	**17,592**	18	**0.10**	1,208	**6.87**	7,583	**43.10**	**8,809**	**50.07**
Surigao del Norte	**7,089**	2	**0.03**	499	**7.04**	4,047	**57.09**	**4,548**	**64.16**
Surigao del Sur	**11,482**	8	**0.07**	610	**5.31**	4,847	**42.21**	**5,465**	**47.60**
Province of Dinagat	**2,573**	4	**0.16**	133	**5.17**	1,014	**39.41**	**1,151**	**44.73**
Bislig City	**2,179**	2	**0.09**	155	**7.11**	1,268	**58.19**	**1,425**	**65.40**
Butuan City	**7,715**	3	**0.04**	787	**10.20**	5,014	**64.99**	**5,804**	**75.23**
Surigao City	**3,301**	4	**0.12**	246	**7.45**	1,960	**59.38**	**2,210**	**66.95**

Note: Put asterisk (*) for No Report and Zero (0) for No Case

Table 1.B.1.2 - Prenatal Care

Number and proportion of pregnant women according to their nutritional status by Region, Province/city and age group
Philippines, Annual 2020

Area	Eligible Pop.	Pregnant women seen during the 1st trimester according to their Body Mass Index (BMI)														
		Normal BMI					Low BMI					High BMI				
		Age Group			Total	%	Age Group			Total	%	Age Group			Total	%
		10-14	15-19	20-49			10-14	15-19	20-49			10-14	15-19	20-49		
**PHILIPPINES**	**2,123,158**	**2,163**	**97,182**	**637,772**	**737,117**	**34.72**	**613**	**10,563**	**39,296**	**50,472**	**2.38**	**294**	**7,821**	**79,208**	**87,323**	**4.11**
**N C R**	**238,661**	**109**	**5,266**	**45,251**	**50,626**	**21.21**	**55**	**975**	**2,813**	**3,843**	**1.61**	**16**	**478**	**5,431**	**5,925**	**2.48**
Malabon	**6,775**	8	308	1,560	**1,876**	**27.69**	4	179	342	**525**	**7.75**	1	92	867	**960**	**14.17**
Navotas	**4,621**	6	493	1,895	**2,394**	**51.81**	0	33	48	**81**	**1.75**	0	13	117	**130**	**2.81**
Valenzuela City	**11,500**	1	72	662	**735**	**6.39**	0	3	24	**27**	**0.23**	0	4	81	**85**	**0.74**
Caloocan City	**29,363**	12	1,027	7,480	**8,519**	**29.01**	15	209	516	**740**	**2.52**	0	60	657	**717**	**2.44**
Marikina City	**8,354**	0	69	450	**519**	**6.21**	0	11	29	**40**	**0.48**	0	19	264	**283**	**3.39**
Pasig City	**13,996**	11	296	1,996	**2,303**	**16.45**	1	81	229	**311**	**2.22**	1	39	560	**600**	**4.29**
Pateros	**1,184**	0	54	384	**438**	**36.99**	0	0	0	**0**	**0.00**	0	1	8	**9**	**0.76**
Taguig	**14,918**	24	158	933	**1,115**	**7.47**	24	21	142	**187**	**1.25**	8	19	171	**198**	**1.33**
Quezon City	**54,413**	18	1,263	19,083	**20,364**	**37.42**	3	180	571	**754**	**1.39**	1	49	595	**645**	**1.19**
Makati City	**10,798**	8	131	856	**995**	**9.21**	4	65	222	**291**	**2.69**	4	60	535	**599**	**5.55**
Mandaluyong City	**7,160**	1	134	1,445	**1,580**	**22.07**	0	9	68	**77**	**1.08**	0	4	141	**145**	**2.03**
San Juan	**2,261**	9	151	944	**1,104**	**48.83**	0	6	25	**31**	**1.37**	0	4	53	**57**	**2.52**
Manila City	**32,980**	3	226	1,219	**1,448**	**4.39**	0	28	77	**105**	**0.32**	0	29	239	**268**	**0.81**
Las Piñas City	**10,914**	4	341	3,298	**3,643**	**33.38**	1	75	289	**365**	**3.34**	1	57	875	**933**	**8.55**
Muntinlupa City	**9,353**	0	0	0	**0**	**0.00**	0	0	0	**0**	**0.00**	0	0	0	**0**	**0.00**
Parañaque City	**12,343**	4	473	2,251	**2,728**	**22.10**	2	43	218	**263**	**2.13**	0	26	231	**257**	**2.08**
Pasay City	**7,728**	0	70	795	**865**	**11.19**	1	32	13	**46**	**0.60**	0	2	37	**39**	**0.50**
**C A R**	**35,099**	**20**	**1,590**	**12,362**	**13,972**	**39.81**	**2**	**127**	**477**	**606**	**1.73**	**3**	**127**	**1,887**	**2,017**	**5.75**
Abra	**4,309**	1	273	1,566	**1,840**	**42.70**	0	32	58	**90**	**2.09**	0	12	122	**134**	**3.11**
Apayao	**2,451**	2	174	943	**1,119**	**45.65**	0	26	66	**92**	**3.75**	0	11	146	**157**	**6.41**
Benguet	**9,188**	4	372	3,198	**3,574**	**38.90**	0	14	77	**91**	**0.99**	1	32	550	**583**	**6.35**
Ifugao	**4,396**	5	236	2,049	**2,290**	**52.09**	0	17	133	**150**	**3.41**	0	20	223	**243**	**5.53**
Kalinga	**4,617**	3	253	2,033	**2,289**	**49.58**	0	17	65	**82**	**1.78**	1	14	283	**298**	**6.45**
Mt. Province	**3,023**	4	136	1,218	**1,358**	**44.92**	1	12	35	**48**	**1.59**	1	28	330	**359**	**11.88**
Baguio City	**7,115**	1	146	1,355	**1,502**	**21.11**	1	9	43	**53**	**0.74**	0	10	233	**243**	**3.42**
**Region 1**	**97,261**	**56**	**4,101**	**37,747**	**41,904**	**43.08**	**10**	**275**	**1,140**	**1,425**	**1.47**	**3**	**221**	**3,071**	**3,295**	**3.39**
Ilocos Norte	**8,105**	8	327	3,469	**3,804**	**46.93**	0	9	52	**61**	**0.75**	1	15	183	**199**	**2.46**
Ilocos Sur	**9,330**	6	385	4,810	**5,201**	**55.74**	1	20	133	**154**	**1.65**	2	41	888	**931**	**9.98**
La Union	**11,511**	11	558	4,659	**5,228**	**45.42**	0	66	223	**289**	**2.51**	0	21	302	**323**	**2.81**
Pangasinan	**50,168**	20	2,266	18,170	**20,456**	**40.77**	2	136	574	**712**	**1.42**	0	119	1,477	**1,596**	**3.18**
Alaminos City	**1,894**	0	132	1,268	**1,400**	**73.92**	0	0	1	**1**	**0.05**	0	0	2	**2**	**0.11**
Candon City	**987**	0	8	689	**697**	**70.62**	0	0	2	**2**	**0.20**	0	2	28	**30**	**3.04**
Dagupan City	**3,620**	0	60	436	**496**	**13.70**	0	4	7	**11**	**0.30**	0	2	2	**4**	**0.11**
Laoag City	**1,870**	0	64	1,725	**1,789**	**95.67**	0	0	0	**0**	**0.00**	0	0	0	**0**	**0.00**
San Carlos City	**3,979**	4	75	787	**866**	**21.76**	6	20	72	**98**	**2.46**	0	6	75	**81**	**2.04**
San Fernando City	**2,115**	2	90	909	**1,001**	**47.33**	0	2	14	**16**	**0.76**	0	1	13	**14**	**0.66**
Urdaneta City	**2,806**	3	114	658	**775**	**27.62**	1	16	49	**66**	**2.35**	0	12	76	**88**	**3.14**
Vigan City	**876**	2	22	167	**191**	**21.80**	0	2	13	**15**	**1.71**	0	2	25	**27**	**3.08**
**Region 2**	**69,443**	**71**	**4,264**	**26,014**	**30,349**	**43.70**	**14**	**581**	**1,766**	**2,361**	**3.40**	**8**	**283**	**3,016**	**3,307**	**4.76**
Batanes	**340**	0	4	106	**110**	**32.35**	0	1	5	**6**	**1.76**	0	3	41	**44**	**12.94**
Cagayan	**17,971**	11	1,182	7,367	**8,560**	**47.63**	3	95	327	**425**	**2.36**	2	64	619	**685**	**3.81**
Isabela	**26,309**	27	1,418	8,339	**9,784**	**37.19**	6	133	374	**513**	**1.95**	0	83	1,193	**1,276**	**4.85**
Nueva Vizcaya	**9,573**	15	669	3,931	**4,615**	**48.21**	3	266	816	**1,085**	**11.33**	3	87	516	**606**	**6.33**
Quirino	**4,025**	5	325	2,229	**2,559**	**63.58**	1	28	143	**172**	**4.27**	2	13	210	**225**	**5.59**
Cauayan City	**2,590**	2	203	1,032	**1,237**	**47.76**	0	5	25	**30**	**1.16**	0	10	80	**90**	**3.47**
Ilagan City	**2,911**	4	157	824	**985**	**33.84**	0	28	43	**71**	**2.44**	0	13	111	**124**	**4.26**
Santiago City	**2,692**	5	235	1,662	**1,902**	**70.65**	1	10	19	**30**	**1.11**	1	3	73	**77**	**2.86**
Tuguegarao City	**3,032**	2	71	524	**597**	**19.69**	0	15	14	**29**	**0.96**	0	7	173	**180**	**5.94**

Table 1.B.1.2 - Prenatal Care

Number and proportion of pregnant women according to their nutritional status by Region, Province/city and age group
Philippines, Annual 2020

Area	Eligible Pop.	Pregnant women seen during the 1st trimester according to their Body Mass Index (BMI)														
		Normal BMI					Low BMI					High BMI				
		Age Group			Total	%	Age Group			Total	%	Age Group			Total	%
		10-14	15-19	20-49			10-14	15-19	20-49			10-14	15-19	20-49		
**Region 3**	**220,020**	**284**	**13,575**	**78,038**	**91,897**	**41.77**	**56**	**1,234**	**5,051**	**6,341**	**2.88**	**14**	**789**	**7,465**	**8,268**	**3.76**
Aurora	**4,770**	6	437	2,149	**2,592**	**54.34**	1	38	91	**130**	**2.73**	0	7	100	**107**	**2.24**
Bataan	**13,823**	31	807	4,704	**5,542**	**40.09**	5	126	390	**521**	**3.77**	2	95	974	**1,071**	**7.75**
Bulacan	**43,627**	44	2,448	14,763	**17,255**	**39.55**	19	392	2,000	**2,411**	**5.53**	1	186	1,630	**1,817**	**4.16**
Nueva Ecija	**28,782**	55	1,901	10,091	**12,047**	**41.86**	7	215	700	**922**	**3.20**	2	163	1,365	**1,530**	**5.32**
Pampanga	**31,359**	15	1,147	7,256	**8,418**	**26.84**	5	153	656	**814**	**2.60**	3	115	1,004	**1,122**	**3.58**
Tarlac	**19,765**	16	1,004	8,031	**9,051**	**45.79**	4	76	624	**704**	**3.56**	2	31	430	**463**	**2.34**
Zambales	**12,610**	21	415	2,375	**2,811**	**22.29**	5	43	125	**173**	**1.37**	1	32	243	**276**	**2.19**
Angeles City	**7,869**	12	719	3,596	**4,327**	**54.99**	1	9	15	**25**	**0.32**	1	1	236	**238**	**3.02**
Balanga City	**1,998**	5	88	545	**638**	**31.93**	0	17	28	**45**	**2.25**	1	16	123	**140**	**7.01**
Cabanatuan City	**5,896**	6	270	1,387	**1,663**	**28.21**	0	9	52	**61**	**1.03**	0	11	132	**143**	**2.43**
City of San Fernando	**5,863**	17	469	2,489	**2,975**	**50.74**	3	7	30	**40**	**0.68**	0	5	27	**32**	**0.55**
Gapan City	**2,153**	3	118	638	**759**	**35.25**	0	3	6	**9**	**0.42**	0	3	8	**11**	**0.51**
Mabalacat City	**4,793**	3	362	2,276	**2,641**	**55.10**	1	30	38	**69**	**1.44**	0	36	233	**269**	**5.61**
Malolos City	**4,880**	10	316	1,854	**2,180**	**44.67**	1	28	44	**73**	**1.50**	0	40	389	**429**	**8.79**
Meycauayan	**4,041**	21	755	3,064	**3,840**	**95.03**	1	10	20	**31**	**0.77**	1	4	54	**59**	**1.46**
Olongapo	**4,971**	0	202	1,168	**1,370**	**27.56**	0	48	157	**205**	**4.12**	0	35	317	**352**	**7.08**
Palayan City	**799**	2	69	244	**315**	**39.42**	0	3	3	**6**	**0.75**	0	3	53	**56**	**7.01**
San Jose City	**2,718**	8	269	1,407	**1,684**	**61.96**	2	20	33	**55**	**2.02**	0	3	71	**74**	**2.72**
San Jose del Monte City	**11,100**	7	772	4,933	**5,712**	**51.46**	1	5	10	**16**	**0.14**	0	2	65	**67**	**0.60**
Science City of Munoz	**1,586**	0	102	528	**630**	**39.72**	0	0	0	**0**	**0.00**	0	1	3	**4**	**0.25**
Tarlac City	**6,617**	2	905	4,540	**5,447**	**82.32**	0	2	29	**31**	**0.47**	0	0	8	**8**	**0.12**
**Region 4A**	**296,816**	**156**	**7,026**	**48,254**	**55,436**	**18.68**	**64**	**844**	**6,159**	**7,067**	**2.38**	**16**	**1,312**	**9,763**	**11,091**	**3.74**
Batangas	**38,441**	10	353	4,601	**4,964**	**12.91**	1	43	320	**364**	**0.95**	0	13	87	**100**	**0.26**
Cavite	**27,704**	9	1,042	7,437	**8,488**	**30.64**	0	128	539	**667**	**2.41**	0	53	745	**798**	**2.88**
Laguna	**19,727**	23	533	2,789	**3,345**	**16.96**	6	151	1,571	**1,728**	**8.76**	2	425	2,331	**2,758**	**13.98**
Quezon	**37,410**	17	638	4,108	**4,763**	**12.73**	15	83	375	**473**	**1.26**	0	33	276	**309**	**0.83**
Rizal	**44,791**	18	1,128	6,312	**7,458**	**16.65**	1	66	294	**361**	**0.81**	0	29	491	**520**	**1.16**
Antipolo City	**16,497**	9	927	4,054	**4,990**	**30.25**	0	67	307	**374**	**2.27**	0	40	177	**217**	**1.32**
Bacoor City	**12,142**	0	27	117	**144**	**1.19**	0	3	21	**24**	**0.20**	0	3	34	**37**	**0.30**
Batangas City	**6,823**	0	0	0	**0**	**0.00**	0	0	0	**0**	**0.00**	0	0	0	**0**	**0.00**
Biñan City	**6,607**	3	720	4,505	**5,228**	**79.13**	0	26	203	**229**	**3.47**	0	54	173	**227**	**3.44**
Cabuyao City	**6,130**	3	118	1,232	**1,353**	**22.07**	0	24	703	**727**	**11.86**	0	231	1,585	**1,816**	**29.62**
Calamba City	**9,028**	9	464	3,352	**3,825**	**42.37**	9	104	359	**472**	**5.23**	5	58	714	**777**	**8.61**
Cavite City	**2,075**	0	0	0	**0**	**0.00**	0	0	0	**0**	**0.00**	0	0	0	**0**	**0.00**
Dasmariñas City	**13,322**	4	178	1,940	**2,122**	**15.93**	0	40	294	**334**	**2.51**	0	15	209	**224**	**1.68**
General Trias City	**6,352**	16	127	738	**881**	**13.87**	4	11	42	**57**	**0.90**	0	1	79	**80**	**1.26**
Imus City	**8,156**	0	32	260	**292**	**3.58**	0	3	22	**25**	**0.31**	0	8	67	**75**	**0.92**
Lipa City	**6,880**	0	0	0	**0**	**0.00**	0	0	0	**0**	**0.00**	0	0	0	**0**	**0.00**
Lucena City	**5,672**	0	0	0	**0**	**0.00**	0	0	0	**0**	**0.00**	0	0	0	**0**	**0.00**
San Pablo City	**5,277**	2	20	147	**169**	**3.20**	0	16	562	**578**	**10.95**	0	178	1,498	**1,676**	**31.76**
San Pedro City	**6,467**	1	38	250	**289**	**4.47**	1	12	237	**250**	**3.87**	0	39	340	**379**	**5.86**
Santa Rosa City	**7,025**	24	317	2,301	**2,642**	**37.61**	7	38	188	**233**	**3.32**	7	103	600	**710**	**10.11**
Tagaytay City	**1,437**	4	64	1,358	**1,426**	**99.23**	20	6	20	**46**	**3.20**	0	0	2	**2**	**0.14**
Tanauan City	**3,584**	2	70	1,491	**1,563**	**43.61**	0	7	62	**69**	**1.93**	0	15	79	**94**	**2.62**
Tayabas City	**2,122**	0	0	0	**0**	**0.00**	0	0	0	**0**	**0.00**	0	0	0	**0**	**0.00**
Trece Martires City	**3,147**	2	230	1,262	**1,494**	**47.47**	0	16	40	**56**	**1.78**	2	14	276	**292**	**9.28**
**Region 4B**	**71,246**	**78**	**1,899**	**12,980**	**14,957**	**20.99**	**12**	**202**	**520**	**734**	**1.03**	**4**	**87**	**909**	**1,000**	**1.40**
Marinduque	**4,836**	5	205	1,890	**2,100**	**43.42**	1	15	85	**101**	**2.09**	0	8	151	**159**	**3.29**
Mindoro Occidental	**12,407**	5	412	3,287	**3,704**	**29.85**	5	62	149	**216**	**1.74**	1	14	184	**199**	**1.60**
Mindoro Oriental	**19,809**	58	696	4,573	**5,327**	**26.89**	5	62	107	**174**	**0.88**	2	22	124	**148**	**0.75**
Palawan	**21,375**	8	497	2,418	**2,923**	**13.67**	1	51	148	**200**	**0.94**	1	34	313	**348**	**1.63**
Romblon	**6,400**	2	40	401	**443**	**6.92**	0	5	16	**21**	**0.33**	0	4	83	**87**	**1.36**
Puerto Princesa City	**6,419**	0	49	411	**460**	**7.17**	0	7	15	**22**	**0.34**	0	5	54	**59**	**0.92**
**Region 5**	**136,116**	**59**	**5,137**	**43,567**	**48,763**	**35.82**	**10**	**760**	**2,770**	**3,540**	**2.60**	**1**	**390**	**5,429**	**5,820**	**4.28**
Albay	**23,737**	8	603	8,885	**9,496**	**40.01**	2	80	313	**395**	**1.66**	0	23	612	**635**	**2.68**

Table 1.B.1.2 - Prenatal Care

Number and proportion of pregnant women according to their nutritional status by Region, Province/city and age group
Philippines, Annual 2020

Area	Eligible Pop.	Pregnant women seen during the 1st trimester according to their Body Mass Index (BMI)														
		Normal BMI					Low BMI					High BMI				
		Age Group			Total	%	Age Group			Total	%	Age Group			Total	%
		10-14	15-19	20-49			10-14	15-19	20-49			10-14	15-19	20-49		
Camarines Norte	**14,283**	7	881	4,814	**5,702**	**39.92**	1	134	391	**526**	**3.68**	0	81	795	**876**	**6.13**
Camarines Sur	**38,796**	9	1,315	11,256	**12,580**	**32.43**	3	217	896	**1,116**	**2.88**	0	115	1,293	**1,408**	**3.63**
Catanduanes	**6,274**	0	189	1,395	**1,584**	**25.25**	0	76	148	**224**	**3.57**	0	33	323	**356**	**5.67**
Masbate	**22,521**	27	1,007	6,404	**7,438**	**33.03**	3	95	256	**354**	**1.57**	1	68	666	**735**	**3.26**
Sorsogon	**19,065**	3	728	6,771	**7,502**	**39.35**	1	118	639	**758**	**3.98**	0	53	1,533	**1,586**	**8.32**
Iriga City	**2,638**	0	102	909	**1,011**	**38.32**	0	9	22	**31**	**1.18**	0	8	79	**87**	**3.30**
Legaspi City	**4,174**	3	198	2,374	**2,575**	**61.69**	0	20	72	**92**	**2.20**	0	7	52	**59**	**1.41**
Naga City	**4,628**	2	114	759	**875**	**18.91**	0	11	33	**44**	**0.95**	0	2	76	**78**	**1.69**
**Region 6**	**146,526**	**102**	**6,055**	**45,089**	**51,246**	**34.97**	**15**	**682**	**2,342**	**3,039**	**2.07**	**5**	**338**	**4,623**	**4,966**	**3.39**
Aklan	**11,162**	9	394	3,781	**4,184**	**37.48**	1	39	197	**237**	**2.12**	1	52	465	**518**	**4.64**
Antique	**12,816**	8	476	4,207	**4,691**	**36.60**	3	71	192	**266**	**2.08**	0	15	427	**442**	**3.45**
Capiz	**13,986**	3	436	3,757	**4,196**	**30.00**	0	20	110	**130**	**0.93**	0	21	384	**405**	**2.90**
Guimaras	**3,085**	1	159	1,524	**1,684**	**54.59**	0	30	85	**115**	**3.73**	0	11	335	**346**	**11.22**
Iloilo	**36,541**	25	1,354	12,389	**13,768**	**37.68**	1	126	584	**711**	**1.95**	1	58	708	**767**	**2.10**
Negros Occidental	**49,368**	45	2,635	16,180	**18,860**	**38.20**	10	328	982	**1,320**	**2.67**	2	117	1,664	**1,783**	**3.61**
Bacolod City	**11,115**	4	318	1,292	**1,614**	**14.52**	0	47	126	**173**	**1.56**	1	51	473	**525**	**4.72**
Iloilo City	**8,453**	7	283	1,959	**2,249**	**26.61**	0	21	66	**87**	**1.03**	0	13	167	**180**	**2.13**
**Region 7**	**163,262**	**104**	**5,904**	**44,693**	**50,701**	**31.05**	**7**	**584**	**2,149**	**2,740**	**1.68**	**6**	**555**	**7,700**	**8,261**	**5.06**
Bohol	**27,312**	8	778	7,157	**7,943**	**29.08**	0	72	317	**389**	**1.42**	1	59	1,018	**1,078**	**3.95**
Cebu	**67,506**	36	2,330	17,303	**19,669**	**29.14**	4	215	902	**1,121**	**1.66**	2	176	2,631	**2,809**	**4.16**
Negros Oriental	**27,938**	28	1,288	7,182	**8,498**	**30.42**	2	143	414	**559**	**2.00**	3	110	1,508	**1,621**	**5.80**
Siquijor	**1,613**	0	73	497	**570**	**35.34**	0	11	54	**65**	**4.03**	0	12	190	**202**	**12.52**
Cebu City	**21,193**	24	966	6,010	**7,000**	**33.03**	1	117	331	**449**	**2.12**	0	164	1,947	**2,111**	**9.96**
Lapu-Lapu City	**9,372**	8	469	5,268	**5,745**	**61.30**	0	26	131	**157**	**1.68**	0	34	406	**440**	**4.69**
Mandaue City	**8,328**	0	0	1,276	**1,276**	**15.32**	0	0	0	**0**	**0.00**	0	0	0	**0**	**0.00**
**Region 8**	**102,619**	**70**	**4,031**	**31,176**	**35,277**	**34.38**	**85**	**652**	**2,492**	**3,229**	**3.15**	**6**	**251**	**2,831**	**3,088**	**3.01**
Biliran	**3,834**	4	203	1,214	**1,421**	**37.06**	1	24	92	**117**	**3.05**	1	24	277	**302**	**7.88**
Eastern Samar	**11,392**	4	517	3,801	**4,322**	**37.94**	39	306	320	**665**	**5.84**	0	49	405	**454**	**3.99**
Northern Leyte	**34,707**	27	1,217	10,836	**12,080**	**34.81**	5	75	430	**510**	**1.47**	4	68	709	**781**	**2.25**
Northern Samar	**15,370**	6	436	4,619	**5,061**	**32.93**	3	56	541	**600**	**3.90**	0	25	321	**346**	**2.25**
Southern Leyte	**6,451**	4	259	1,797	**2,060**	**31.93**	1	47	175	**223**	**3.46**	0	23	322	**345**	**5.35**
Western Samar	**14,305**	13	534	3,658	**4,205**	**29.40**	1	54	382	**437**	**3.05**	0	26	260	**286**	**2.00**
Calbayog City	**4,413**	4	149	806	**959**	**21.73**	33	50	368	**451**	**10.22**	0	6	83	**89**	**2.02**
Maasin City	**1,637**	0	56	315	**371**	**22.66**	0	5	40	**45**	**2.75**	0	6	84	**90**	**5.50**
Ormoc City	**4,941**	6	362	2,060	**2,428**	**49.14**	2	28	82	**112**	**2.27**	1	21	351	**373**	**7.55**
Tacloban City	**5,569**	2	298	2,070	**2,370**	**42.56**	0	7	62	**69**	**1.24**	0	3	19	**22**	**0.40**
**Region 9**	**80,051**	**78**	**4,918**	**28,660**	**33,656**	**42.04**	**17**	**367**	**1,416**	**1,800**	**2.25**	**9**	**229**	**1,916**	**2,154**	**2.69**
Zamboanga del Norte	**17,249**	16	1,342	8,180	**9,538**	**55.30**	0	76	226	**302**	**1.75**	0	34	351	**385**	**2.23**
Zamboanga del Sur	**17,653**	28	911	5,287	**6,226**	**35.27**	15	167	615	**797**	**4.51**	7	64	502	**573**	**3.25**
Zamboanga Sibugay	**14,954**	12	857	4,145	**5,014**	**33.53**	0	38	249	**287**	**1.92**	0	52	303	**355**	**2.37**
Dapitan City	**1,784**	2	159	1,086	**1,247**	**69.90**	0	11	49	**60**	**3.36**	0	3	48	**51**	**2.86**
Dipolog City	**2,827**	2	280	1,702	**1,984**	**70.18**	0	17	54	**71**	**2.51**	0	15	153	**168**	**5.94**
Isabela City	**2,522**	3	263	1,159	**1,425**	**56.50**	0	11	21	**32**	**1.27**	0	10	70	**80**	**3.17**
Pagadian City	**4,325**	0	239	1,546	**1,785**	**41.27**	0	27	90	**117**	**2.71**	0	7	109	**116**	**2.68**
Zamboanga City	**18,737**	15	867	5,555	**6,437**	**34.35**	2	20	112	**134**	**0.72**	2	44	380	**426**	**2.27**
**Region 10**	**101,411**	**223**	**7,739**	**40,637**	**48,599**	**47.92**	**41**	**749**	**2,083**	**2,873**	**2.83**	**18**	**664**	**5,549**	**6,231**	**6.14**
Bukidnon	**23,706**	75	2,675	9,461	**12,211**	**51.51**	12	315	800	**1,127**	**4.75**	7	234	1,756	**1,997**	**8.42**
Camiguin	**1,858**	2	50	494	**546**	**29.39**	0	0	1	**1**	**0.05**	0	0	10	**10**	**0.54**
Lanao del Norte	**14,960**	16	855	6,791	**7,662**	**51.22**	2	37	145	**184**	**1.23**	6	26	159	**191**	**1.28**
Misamis Occidental	**6,403**	4	279	2,933	**3,216**	**50.23**	0	8	31	**39**	**0.61**	0	19	372	**391**	**6.11**
Misamis Oriental	**15,131**	88	1,191	6,966	**8,245**	**54.49**	20	68	287	**375**	**2.48**	0	38	370	**408**	**2.70**

Table 1.B.1.2 - Prenatal Care

Number and proportion of pregnant women according to their nutritional status by Region, Province/city and age group
Philippines, Annual 2020

Area	Eligible Pop.	Pregnant women seen during the 1st trimester according to their Body Mass Index (BMI)														
		Normal BMI					Low BMI					High BMI				
		Age Group			Total	%	Age Group			Total	%	Age Group			Total	%
		10-14	15-19	20-49			10-14	15-19	20-49			10-14	15-19	20-49		
Cagayan de Oro City	**14,339**	15	899	4,836	**5,750**	**40.10**	4	161	338	**503**	**3.51**	0	216	2,000	**2,216**	**15.45**
El Salvador City	**1,065**	1	57	302	**360**	**33.80**	2	5	5	**12**	**1.13**	0	4	38	**42**	**3.94**
Gingoog City	**2,644**	2	284	1,607	**1,893**	**71.60**	0	58	163	**221**	**8.36**	0	39	170	**209**	**7.90**
Iligan City	**7,580**	2	387	2,036	**2,425**	**31.99**	0	30	47	**77**	**1.02**	0	15	183	**198**	**2.61**
Malaybalay City	**3,956**	5	227	1,082	**1,314**	**33.22**	1	18	69	**88**	**2.22**	3	36	110	**149**	**3.77**
Oroquieta City	**1,389**	0	46	279	**325**	**23.40**	0	4	6	**10**	**0.72**	0	2	25	**27**	**1.94**
Ozamis City	**2,778**	2	322	1,749	**2,073**	**74.62**	0	6	10	**16**	**0.58**	0	0	16	**16**	**0.58**
Tangub City	**1,234**	0	0	88	**88**	**7.13**	0	0	88	**88**	**7.13**	0	0	0	**0**	**0.00**
Valencia City	**4,368**	11	467	2,013	**2,491**	**57.03**	0	39	93	**132**	**3.02**	2	35	340	**377**	**8.63**
**Region 11**	**107,247**	**371**	**8,720**	**42,624**	**51,715**	**48.22**	**147**	**1,212**	**3,644**	**5,003**	**4.66**	**47**	**1,107**	**10,827**	**11,981**	**11.17**
Compostela Valley	**15,562**	82	1,647	6,454	**8,183**	**52.58**	19	300	592	**911**	**5.85**	15	284	3,085	**3,384**	**21.75**
Davao del Norte	**21,326**	85	1,975	10,852	**12,912**	**60.55**	95	343	831	**1,269**	**5.95**	8	204	2,411	**2,623**	**12.30**
Davao Oriental	**13,007**	26	928	4,011	**4,965**	**38.17**	13	100	745	**858**	**6.60**	5	93	1,139	**1,237**	**9.51**
Davao del Sur	**14,151**	39	667	3,498	**4,204**	**29.71**	6	140	338	**484**	**3.42**	6	157	1,178	**1,341**	**9.48**
Davao Occidental	**6,670**	30	556	1,131	**1,717**	**25.74**	2	79	156	**237**	**3.55**	3	75	383	**461**	**6.91**
Davao City	**36,531**	109	2,947	16,678	**19,734**	**54.02**	12	250	982	**1,244**	**3.41**	10	294	2,631	**2,935**	**8.03**
**Region 12**	**104,552**	**164**	**9,753**	**45,919**	**55,836**	**53.41**	**20**	**826**	**2,052**	**2,898**	**2.77**	**13**	**479**	**5,097**	**5,589**	**5.35**
North Cotabato	**33,645**	37	2,644	12,759	**15,440**	**45.89**	3	154	415	**572**	**1.70**	5	129	1,032	**1,166**	**3.47**
Sarangani	**12,891**	37	1,706	5,830	**7,573**	**58.75**	4	126	238	**368**	**2.85**	4	82	944	**1,030**	**7.99**
South Cotabato	**21,113**	26	2,256	10,939	**13,221**	**62.62**	3	209	420	**632**	**2.99**	1	116	1,432	**1,549**	**7.34**
Sultan Kudarat	**17,359**	45	1,916	8,428	**10,389**	**59.85**	5	135	339	**479**	**2.76**	2	82	990	**1,074**	**6.19**
Cotabato City	**5,835**	0	291	3,547	**3,838**	**65.78**	1	10	50	**61**	**1.05**	0	7	103	**110**	**1.89**
Gen. Santos City	**13,709**	19	940	4,416	**5,375**	**39.21**	4	192	590	**786**	**5.73**	1	63	596	**660**	**4.81**
**BARMM**	**92,799**	**161**	**3,459**	**32,890**	**36,510**	**39.34**	**52**	**231**	**1,734**	**2,017**	**2.17**	**117**	**326**	**1,716**	**2,159**	**2.33**
Basilan	**7,541**	7	205	1,149	**1,361**	**18.05**	0	19	76	**95**	**1.26**	0	10	40	**50**	**0.66**
Lanao del Sur	**21,131**	5	833	17,344	**18,182**	**86.04**	1	7	31	**39**	**0.18**	0	2	53	**55**	**0.26**
Maguindanao	**31,128**	17	1,617	12,520	**14,154**	**45.47**	38	185	426	**649**	**2.08**	1	61	571	**633**	**2.03**
Sulu	**16,613**	0	0	0	**0**	**0.00**	0	0	0	**0**	**0.00**	0	0	0	**0**	**0.00**
Tawi-Tawi	**9,259**	0	0	0	**0**	**0.00**	0	0	1,033	**1,033**	**11.16**	0	0	683	**683**	**7.38**
Lamitan City	**2,074**	2	125	431	**558**	**26.90**	0	0	0	**0**	**0.00**	0	0	0	**0**	**0.00**
Marawi City	**5,053**	130	679	1,446	**2,255**	**44.63**	13	20	168	**201**	**3.98**	116	253	369	**738**	**14.61**
**CARAGA**	**60,029**	**57**	**3,745**	**21,871**	**25,673**	**42.77**	**6**	**262**	**688**	**956**	**1.59**	**8**	**185**	**1,978**	**2,171**	**3.62**
Agusan del Norte	**8,098**	9	611	3,198	**3,818**	**47.15**	2	36	84	**122**	**1.51**	0	26	243	**269**	**3.32**
Agusan del Sur	**17,592**	22	1,113	5,948	**7,083**	**40.26**	1	58	150	**209**	**1.19**	3	41	389	**433**	**2.46**
Surigao del Norte	**7,089**	3	421	2,677	**3,101**	**43.74**	0	24	73	**97**	**1.37**	2	17	156	**175**	**2.47**
Surigao del Sur	**11,482**	7	507	3,728	**4,242**	**36.94**	3	49	152	**204**	**1.78**	1	42	397	**440**	**3.83**
Province of Dinagat	**2,573**	1	143	861	**1,005**	**39.06**	0	11	26	**37**	**1.44**	0	5	76	**81**	**3.15**
Bislig City	**2,179**	5	124	845	**974**	**44.70**	0	11	21	**32**	**1.47**	1	12	163	**176**	**8.08**
Butuan City	**7,715**	7	537	2,909	**3,453**	**44.76**	0	24	69	**93**	**1.21**	0	9	226	**235**	**3.05**
Surigao City	**3,301**	3	289	1,705	**1,997**	**60.50**	0	49	113	**162**	**4.91**	1	33	328	**362**	**10.97**

Note: Put asterisk (*) for No Report and Zero (0) for No Case

Table 1.B.1.3 - Prenatal Care

Number and proportion of pregnant women given Tetanus Diphtheria (Td) Vaccination
Philippines, Annual 2020

Area	Eligible Pop.	Women pregnant for the 1st time given at least two (2) doses of Td Vaccine							
		Age Group						Total	%
		10-14 yrs old		15-19 yrs old		20-49 yrs old			
		No.	%	No.	%	No.	%		
**PHILIPPINES**	**2,123,158**	**2,601**	**0.12**	**111,010**	**5.23**	**431,357**	**20.32**	**544,968**	**25.67**
**N C R**	**238,661**	**213**	**0.09**	**8,599**	**3.60**	**53,006**	**22.21**	**61,818**	**25.90**
Malabon	**6,775**	10	**0.15**	290	**4.28**	1,118	**16.50**	**1,418**	**20.93**
Navotas	**4,621**	2	**0.04**	334	**7.23**	1,060	**22.94**	**1,396**	**30.21**
Valenzuela City	**11,500**	4	**0.03**	651	**5.66**	4,305	**37.43**	**4,960**	**43.13**
Caloocan City	**29,363**	29	**0.10**	1,751	**5.96**	10,999	**37.46**	**12,779**	**43.52**
Marikina City	**8,354**	4	**0.05**	280	**3.35**	2,595	**31.06**	**2,879**	**34.46**
Pasig City	**13,996**	3	**0.02**	319	**2.28**	4,500	**32.15**	**4,822**	**34.45**
Pateros	**1,184**	0	**0.00**	49	**4.14**	202	**17.06**	**251**	**21.20**
Taguig	**14,918**	10	**0.07**	689	**4.62**	3,120	**20.91**	**3,819**	**25.60**
Quezon City	**54,413**	28	**0.05**	1,809	**3.32**	10,324	**18.97**	**12,161**	**22.35**
Makati City	**10,798**	5	**0.05**	176	**1.63**	1,378	**12.76**	**1,559**	**14.44**
Mandaluyong City	**7,160**	4	**0.06**	208	**2.91**	1,260	**17.60**	**1,472**	**20.56**
San Juan	**2,261**	5	**0.22**	88	**3.89**	380	**16.81**	**473**	**20.92**
Manila City	**32,980**	4	**0.01**	860	**2.61**	4,867	**14.76**	**5,731**	**17.38**
Las Piñas City	**10,914**	10	**0.09**	401	**3.67**	2,470	**22.63**	**2,881**	**26.40**
Muntinlupa City	**9,353**	0	**0.00**	253	**2.71**	2,355	**25.18**	**2,608**	**27.88**
Parañaque City	**12,343**	94	**0.76**	307	**2.49**	961	**7.79**	**1,362**	**11.03**
Pasay City	**7,728**	1	**0.01**	134	**1.73**	1,112	**14.39**	**1,247**	**16.14**
**C A R**	**35,099**	**13**	**0.04**	**1,372**	**3.91**	**6,114**	**17.42**	**7,499**	**21.37**
Abra	**4,309**	3	**0.07**	220	**5.11**	878	**20.38**	**1,101**	**25.55**
Apayao	**2,451**	1	**0.04**	198	**8.08**	436	**17.79**	**635**	**25.91**
Benguet	**9,188**	4	**0.04**	243	**2.64**	1,523	**16.58**	**1,770**	**19.26**
Ifugao	**4,396**	1	**0.02**	179	**4.07**	1,012	**23.02**	**1,192**	**27.12**
Kalinga	**4,617**	0	**0.00**	261	**5.65**	968	**20.97**	**1,229**	**26.62**
Mt. Province	**3,023**	2	**0.07**	146	**4.83**	415	**13.73**	**563**	**18.62**
Baguio City	**7,115**	2	**0.03**	125	**1.76**	882	**12.40**	**1,009**	**14.18**
**Region 1**	**97,261**	**58**	**0.06**	**4,298**	**4.42**	**22,401**	**23.03**	**26,757**	**27.51**
Ilocos Norte	**8,105**	3	**0.04**	284	**3.50**	2,070	**25.54**	**2,357**	**29.08**
Ilocos Sur	**9,330**	9	**0.10**	328	**3.52**	2,545	**27.28**	**2,882**	**30.89**
La Union	**11,511**	11	**0.10**	628	**5.46**	3,074	**26.70**	**3,713**	**32.26**
Pangasinan	**50,168**	22	**0.04**	2,006	**4.00**	9,806	**19.55**	**11,834**	**23.59**
Alaminos City	**1,894**	0	**0.00**	320	**16.90**	1,002	**52.90**	**1,322**	**69.80**
Candon City	**987**	0	**0.00**	9	**0.91**	330	**33.43**	**339**	**34.35**
Dagupan City	**3,620**	2	**0.06**	182	**5.03**	420	**11.60**	**604**	**16.69**
Laoag City	**1,870**	0	**0.00**	44	**2.35**	1,398	**74.76**	**1,442**	**77.11**
San Carlos City	**3,979**	6	**0.15**	185	**4.65**	778	**19.55**	**969**	**24.35**
San Fernando City	**2,115**	4	**0.19**	104	**4.92**	487	**23.03**	**595**	**28.13**
Urdaneta City	**2,806**	1	**0.04**	170	**6.06**	361	**12.87**	**532**	**18.96**

Table 1.B.1.3 - Prenatal Care

Number and proportion of pregnant women given Tetanus Diphtheria (Td) Vaccination
Philippines, Annual 2020

Area	Eligible Pop.	Women pregnant for the 1st time given at least two (2) doses of Td Vaccine							
		Age Group						Total	%
		10-14 yrs old		15-19 yrs old		20-49 yrs old			
		No.	%	No.	%	No.	%		
Vigan City	876	0	0.00	38	4.34	130	14.84	168	19.18
**Region 2**	69,443	60	0.09	4,141	5.96	14,420	20.77	18,621	26.81
Batanes	340	0	0.00	16	4.71	71	20.88	87	25.59
Cagayan	17,971	9	0.05	1,120	6.23	3,503	19.49	4,632	25.77
Isabela	26,309	28	0.11	1,617	6.15	4,584	17.42	6,229	23.68
Nueva Vizcaya	9,573	8	0.08	422	4.41	2,312	24.15	2,742	28.64
Quirino	4,025	4	0.10	281	6.98	1,157	28.75	1,442	35.83
Cauayan City	2,590	5	0.19	192	7.41	699	26.99	896	34.59
Ilagan City	2,911	3	0.10	184	6.32	637	21.88	824	28.31
Santiago City	2,692	1	0.04	239	8.88	959	35.62	1,199	44.54
Tuguegarao City	3,032	2	0.07	70	2.31	498	16.42	570	18.80
			0.00		0.00		0.00		
**Region 3**	220,020	290	0.13	12,850	5.84	48,588	22.08	61,728	28.06
Aurora	4,770	3	0.06	348	7.30	918	19.25	1,269	26.60
Bataan	13,823	22	0.16	903	6.53	2,433	17.60	3,358	24.29
Bulacan	43,627	58	0.13	2,416	5.54	9,065	20.78	11,539	26.45
Nueva Ecija	28,782	34	0.12	1,549	5.38	4,933	17.14	6,516	22.64
Pampanga	31,359	37	0.12	1,167	3.72	5,439	17.34	6,643	21.18
Tarlac	19,765	25	0.13	1,277	6.46	5,816	29.43	7,118	36.01
Zambales	12,610	13	0.10	475	3.77	1,416	11.23	1,904	15.10
Angeles City	7,869	4	0.05	305	3.88	1,335	16.97	1,644	20.89
Balanga City	1,998	5	0.25	133	6.66	379	18.97	517	25.88
Cabanatuan City	5,896	10	0.17	364	6.17	1,031	17.49	1,405	23.83
City of San Fernando	5,863	23	0.39	392	6.69	1,510	25.75	1,925	32.83
Gapan City	2,153	0	0.00	119	5.53	313	14.54	432	20.07
Mabalacat City	4,793	0	0.00	354	7.39	1,573	32.82	1,927	40.20
Malolos City	4,880	3	0.06	232	4.75	844	17.30	1,079	22.11
Meycauayan	4,041	25	0.62	481	11.90	1,730	42.81	2,236	55.33
Olongapo	4,971	0	0.00	258	5.19	963	19.37	1,221	24.56
Palayan City	799	0	0.00	35	4.38	93	11.64	128	16.02
San Jose City	2,718	9	0.33	296	10.89	931	34.25	1,236	45.47
San Jose del Monte City	11,100	16	0.14	1,011	9.11	5,353	48.23	6,380	57.48
Science City of Munoz	1,586	1	0.06	110	6.94	239	15.07	350	22.07
Tarlac City	6,617	2	0.03	625	9.45	2,274	34.37	2,901	43.84
**Region 4A**	296,816	407	0.14	9,643	3.25	45,957	15.48	56,007	18.87
Batangas	38,441	9	0.02	398	1.04	3,856	10.03	4,263	11.09
Cavite	27,704	17	0.06	1,290	4.66	5,300	19.13	6,607	23.85
Laguna	19,727	315	1.60	2,061	10.45	5,879	29.80	8,255	41.85
Quezon	37,410	11	0.03	720	1.92	2,974	7.95	3,705	9.90
Rizal	44,791	12	0.03	1,260	2.81	4,033	9.00	5,305	11.84
Antipolo City	16,497	2	0.01	726	4.40	4,426	26.83	5,154	31.24
Bacoor City	12,142	0	0.00	48	0.40	315	2.59	363	2.99
Batangas City	6,823	0	0.00	0	0.00	0	0.00	0	0.00

Table 1.B.1.3 - Prenatal Care

Number and proportion of pregnant women given Tetanus Diphtheria (Td) Vaccination
Philippines, Annual 2020

Area	Eligible Pop.	Women pregnant for the 1st time given at least two (2) doses of Td Vaccine							
		Age Group						Total	%
		10-14 yrs old		15-19 yrs old		20-49 yrs old			
		No.	%	No.	%	No.	%		
Biñan City	**6,607**	0	**0.00**	769	**11.64**	3,199	**48.42**	**3,968**	**60.06**
Cabuyao City	**6,130**	2	**0.03**	273	**4.45**	2,680	**43.72**	**2,955**	**48.21**
Calamba City	**9,028**	8	**0.09**	462	**5.12**	2,438	**27.00**	**2,908**	**32.21**
Cavite City	**2,075**	0	**0.00**	0	**0.00**	0	**0.00**	**0**	**0.00**
Dasmariñas City	**13,322**	2	**0.02**	246	**1.85**	1,490	**11.18**	**1,738**	**13.05**
General Trias City	**6,352**	4	**0.06**	249	**3.92**	1,530	**24.09**	**1,783**	**28.07**
Imus City	**8,156**	0	**0.00**	57	**0.70**	245	**3.00**	**302**	**3.70**
Lipa City	**6,880**	0	**0.00**	0	**0.00**	0	**0.00**	**0**	**0.00**
Lucena City	**5,672**	0	**0.00**	0	**0.00**	0	**0.00**	**0**	**0.00**
San Pablo City	**5,277**	5	**0.09**	193	**3.66**	1,517	**28.75**	**1,715**	**32.50**
San Pedro City	**6,467**	2	**0.03**	60	**0.93**	1,115	**17.24**	**1,177**	**18.20**
Santa Rosa City	**7,025**	9	**0.13**	393	**5.59**	1,940	**27.62**	**2,342**	**33.34**
Tagaytay City	**1,437**	6	**0.42**	80	**5.57**	1,048	**72.93**	**1,134**	**78.91**
Tanauan City	**3,584**	1	**0.03**	56	**1.56**	1,035	**28.88**	**1,092**	**30.47**
Tayabas City	**2,122**	0	**0.00**	0	**0.00**	117	**5.51**	**117**	**5.51**
Trece Martires City	**3,147**	2	**0.06**	302	**9.60**	820	**26.06**	**1,124**	**35.72**
**Region 4B**	**71,246**	**40**	**0.06**	**2,066**	**2.90**	**8,877**	**12.46**	**10,983**	**15.42**
Marinduque	**4,836**	4	**0.08**	220	**4.55**	620	**12.82**	**844**	**17.45**
Mindoro Occidental	**12,407**	8	**0.06**	924	**7.45**	2,300	**18.54**	**3,232**	**26.05**
Mindoro Oriental	**19,809**	24	**0.12**	558	**2.82**	3,285	**16.58**	**3,867**	**19.52**
Palawan	**21,375**	0	**0.00**	288	**1.35**	724	**3.39**	**1,012**	**4.73**
Romblon	**6,400**	4	**0.06**	76	**1.19**	392	**6.13**	**472**	**7.38**
Puerto Princesa City	**6,419**	0	**0.00**	0	**0.00**	1,556	**24.24**	**1,556**	**24.24**
**Region 5**	**136,116**	**67**	**0.05**	**5,783**	**4.25**	**24,381**	**17.91**	**30,231**	**22.21**
Albay	**23,737**	3	**0.01**	783	**3.30**	4,632	**19.51**	**5,418**	**22.83**
Camarines Norte	**14,283**	11	**0.08**	871	**6.10**	2,322	**16.26**	**3,204**	**22.43**
Camarines Sur	**38,796**	7	**0.02**	1,405	**3.62**	6,713	**17.30**	**8,125**	**20.94**
Catanduanes	**6,274**	1	**0.02**	226	**3.60**	881	**14.04**	**1,108**	**17.66**
Masbate	**22,521**	36	**0.16**	1,484	**6.59**	4,469	**19.84**	**5,989**	**26.59**
Sorsogon	**19,065**	7	**0.04**	606	**3.18**	3,145	**16.50**	**3,758**	**19.71**
Iriga City	**2,638**	1	**0.04**	68	**2.58**	395	**14.97**	**464**	**17.59**
Legaspi City	**4,174**	0	**0.00**	141	**3.38**	1,065	**25.52**	**1,206**	**28.89**
Naga City	**4,628**	1	**0.02**	199	**4.30**	759	**16.40**	**959**	**20.72**
**Region 6**	**146,526**	**97**	**0.07**	**7,016**	**4.79**	**27,494**	**18.76**	**34,607**	**23.62**
Aklan	**11,162**	17	**0.15**	500	**4.48**	2,451	**21.96**	**2,968**	**26.59**
Antique	**12,816**	5	**0.04**	513	**4.00**	2,347	**18.31**	**2,865**	**22.35**
Capiz	**13,986**	2	**0.01**	446	**3.19**	2,324	**16.62**	**2,772**	**19.82**
Guimaras	**3,085**	2	**0.06**	173	**5.61**	791	**25.64**	**966**	**31.31**
Iloilo	**36,541**	13	**0.04**	1,327	**3.63**	7,400	**20.25**	**8,740**	**23.92**
Negros Occidental	**49,368**	45	**0.09**	3,333	**6.75**	9,290	**18.82**	**12,668**	**25.66**
Bacolod City	**11,115**	5	**0.04**	374	**3.36**	1,499	**13.49**	**1,878**	**16.90**
Iloilo City	**8,453**	8	**0.09**	350	**4.14**	1,392	**16.47**	**1,750**	**20.70**

Table 1.B.1.3 - Prenatal Care

Number and proportion of pregnant women given Tetanus Diphtheria (Td) Vaccination
Philippines, Annual 2020

Area	Eligible Pop.	Women pregnant for the 1st time given at least two (2) doses of Td Vaccine							
		Age Group						Total	%
		10-14 yrs old		15-19 yrs old		20-49 yrs old			
		No.	%	No.	%	No.	%		
**Region 7**	**163,262**	**127**	**0.08**	**7,846**	**4.81**	**30,524**	**18.70**	**38,497**	**23.58**
Bohol	**27,312**	26	**0.10**	1,201	**4.40**	4,816	**17.63**	**6,043**	**22.13**
Cebu	**67,506**	43	**0.06**	3,096	**4.59**	11,855	**17.56**	**14,994**	**22.21**
Negros Oriental	**27,938**	43	**0.15**	1,816	**6.50**	5,160	**18.47**	**7,019**	**25.12**
Siquijor	**1,613**	0	**0.00**	97	**6.01**	385	**23.87**	**482**	**29.88**
Cebu City	**21,193**	12	**0.06**	1,104	**5.21**	3,549	**16.75**	**4,665**	**22.01**
Lapu-Lapu City	**9,372**	3	**0.03**	532	**5.68**	2,943	**31.40**	**3,478**	**37.11**
Mandaue City	**8,328**	0	**0.00**	0	**0.00**	1,816	**21.81**	**1,816**	**21.81**
**Region 8**	**102,619**	**62**	**0.06**	**3,863**	**3.76**	**18,149**	**17.69**	**22,074**	**21.51**
Biliran	**3,834**	6	**0.16**	202	**5.27**	675	**17.61**	**883**	**23.03**
Eastern Samar	**11,392**	4	**0.04**	434	**3.81**	1,941	**17.04**	**2,379**	**20.88**
Northern Leyte	**34,707**	15	**0.04**	977	**2.81**	4,966	**14.31**	**5,958**	**17.17**
Northern Samar	**15,370**	7	**0.05**	563	**3.66**	3,730	**24.27**	**4,300**	**27.98**
Southern Leyte	**6,451**	11	**0.17**	269	**4.17**	1,374	**21.30**	**1,654**	**25.64**
Western Samar	**14,305**	9	**0.06**	611	**4.27**	1,964	**13.73**	**2,584**	**18.06**
Calbayog City	**4,413**	0	**0.00**	177	**4.01**	913	**20.69**	**1,090**	**24.70**
Maasin City	**1,637**	1	**0.06**	43	**2.63**	340	**20.77**	**384**	**23.46**
Ormoc City	**4,941**	9	**0.18**	326	**6.60**	1,018	**20.60**	**1,353**	**27.38**
Tacloban City	**5,569**	0	**0.00**	261	**4.69**	1,228	**22.05**	**1,489**	**26.74**
**Region 9**	**80,051**	**57**	**0.07**	**4,684**	**5.85**	**18,508**	**23.12**	**23,249**	**29.04**
Zamboanga del Norte	**17,249**	24	**0.14**	1,742	**10.10**	7,101	**41.17**	**8,867**	**51.41**
Zamboanga del Sur	**17,653**	8	**0.05**	850	**4.82**	2,411	**13.66**	**3,269**	**18.52**
Zamboanga Sibugay	**14,954**	9	**0.06**	595	**3.98**	1,660	**11.10**	**2,264**	**15.14**
Dapitan City	**1,784**	1	**0.06**	112	**6.28**	635	**35.59**	**748**	**41.93**
Dipolog City	**2,827**	1	**0.04**	253	**8.95**	1,540	**54.47**	**1,794**	**63.46**
Isabela City	**2,522**	2	**0.08**	182	**7.22**	876	**34.73**	**1,060**	**42.03**
Pagadian City	**4,325**	0	**0.00**	247	**5.71**	964	**22.29**	**1,211**	**28.00**
Zamboanga City	**18,737**	12	**0.06**	703	**3.75**	3,321	**17.72**	**4,036**	**21.54**
**Region 10**	**101,411**	**149**	**0.15**	**7,641**	**7.53**	**23,813**	**23.48**	**31,603**	**31.16**
Bukidnon	**23,706**	83	**0.35**	2,438	**10.28**	4,403	**18.57**	**6,924**	**29.21**
Camiguin	**1,858**	0	**0.00**	62	**3.34**	260	**13.99**	**322**	**17.33**
Lanao del Norte	**14,960**	4	**0.03**	621	**4.15**	4,528	**30.27**	**5,153**	**34.45**
Misamis Occidental	**6,403**	7	**0.11**	282	**4.40**	1,653	**25.82**	**1,942**	**30.33**
Misamis Oriental	**15,131**	14	**0.09**	1,049	**6.93**	3,767	**24.90**	**4,830**	**31.92**
Cagayan de Oro City	**14,339**	22	**0.15**	1,379	**9.62**	3,462	**24.14**	**4,863**	**33.91**
El Salvador City	**1,065**	3	**0.28**	105	**9.86**	170	**15.96**	**278**	**26.10**
Gingoog City	**2,644**	2	**0.08**	233	**8.81**	732	**27.69**	**967**	**36.57**
Iligan City	**7,580**	4	**0.05**	573	**7.56**	1,751	**23.10**	**2,328**	**30.71**
Malaybalay City	**3,956**	5	**0.13**	217	**5.49**	651	**16.46**	**873**	**22.07**

Table 1.B.1.3 - Prenatal Care

Number and proportion of pregnant women given Tetanus Diphtheria (Td) Vaccination
Philippines, Annual 2020

Area	Eligible Pop.	Women pregnant for the 1st time given at least two (2) doses of Td Vaccine							
		Age Group						Total	%
		10-14 yrs old		15-19 yrs old		20-49 yrs old			
		No.	%	No.	%	No.	%		
Oroquieta City	**1,389**	0	**0.00**	34	**2.45**	248	**17.85**	**282**	**20.30**
Ozamis City	**2,778**	4	**0.14**	247	**8.89**	724	**26.06**	**975**	**35.10**
Tangub City	**1,234**	0	**0.00**	83	**6.73**	636	**51.54**	**719**	**58.27**
Valencia City	**4,368**	1	**0.02**	318	**7.28**	828	**18.96**	**1,147**	**26.26**
**Region 11**	**107,247**	**474**	**0.44**	**9,284**	**8.66**	**23,644**	**22.05**	**33,402**	**31.14**
Compostela Valley	**15,562**	112	**0.72**	1,595	**10.25**	2,958	**19.01**	**4,665**	**29.98**
Davao del Norte	**21,326**	108	**0.51**	2,114	**9.91**	5,443	**25.52**	**7,665**	**35.94**
Davao Oriental	**13,007**	50	**0.38**	958	**7.37**	2,131	**16.38**	**3,139**	**24.13**
Davao del Sur	**14,151**	52	**0.37**	1,089	**7.70**	2,130	**15.05**	**3,271**	**23.11**
Davao Occidental	**6,670**	52	**0.78**	897	**13.45**	775	**11.62**	**1,724**	**25.85**
Davao City	**36,531**	100	**0.27**	2,631	**7.20**	10,207	**27.94**	**12,938**	**35.42**
**Region 12**	**104,552**	**150**	**0.14**	**8,611**	**8.24**	**28,680**	**27.43**	**37,441**	**35.81**
North Cotabato	**33,645**	40	**0.12**	2,158	**6.41**	7,955	**23.64**	**10,153**	**30.18**
Sarangani	**12,891**	25	**0.19**	1,631	**12.65**	3,479	**26.99**	**5,135**	**39.83**
South Cotabato	**21,113**	20	**0.09**	2,050	**9.71**	6,775	**32.09**	**8,845**	**41.89**
Sultan Kudarat	**17,359**	41	**0.24**	1,421	**8.19**	4,088	**23.55**	**5,550**	**31.97**
Cotabato City	**5,835**	1	**0.02**	232	**3.98**	3,069	**52.60**	**3,302**	**56.59**
Gen. Santos City	**13,709**	23	**0.17**	1,119	**8.16**	3,314	**24.17**	**4,456**	**32.50**
**BARMM**	**92,799**	**277**	**0.30**	**9,948**	**10.72**	**26,608**	**28.67**	**36,833**	**39.69**
Basilan	**7,541**	14	**0.19**	229	**3.04**	894	**11.86**	**1,137**	**15.08**
Lanao del Sur	**21,131**	8	**0.04**	643	**3.04**	11,953	**56.57**	**12,604**	**59.65**
Maguindanao	**31,128**	13	**0.04**	7,170	**23.03**	10,367	**33.30**	**17,550**	**56.38**
Sulu	**16,613**	0	**0.00**	0	**0.00**	0	**0.00**	**0**	**0.00**
Tawi-Tawi	**9,259**	0	**0.00**	1,077	**11.63**	1,733	**18.72**	**2,810**	**30.35**
Lamitan City	**2,074**	2	**0.10**	144	**6.94**	272	**13.11**	**418**	**20.15**
Marawi City	**5,053**	240	**4.75**	685	**13.56**	1,389	**27.49**	**2,314**	**45.79**
**CARAGA**	**60,029**	**60**	**0.10**	**3,365**	**5.61**	**10,193**	**16.98**	**13,618**	**22.69**
Agusan del Norte	**8,098**	15	**0.19**	462	**5.71**	1,514	**18.70**	**1,991**	**24.59**
Agusan del Sur	**17,592**	19	**0.11**	982	**5.58**	2,266	**12.88**	**3,267**	**18.57**
Surigao del Norte	**7,089**	11	**0.16**	346	**4.88**	1,433	**20.21**	**1,790**	**25.25**
Surigao del Sur	**11,482**	7	**0.06**	626	**5.45**	2,245	**19.55**	**2,878**	**25.07**
Province of Dinagat	**2,573**	3	**0.12**	110	**4.28**	368	**14.30**	**481**	**18.69**
Bislig City	**2,179**	1	**0.05**	121	**5.55**	270	**12.39**	**392**	**17.99**
Butuan City	**7,715**	3	**0.04**	534	**6.92**	1,513	**19.61**	**2,050**	**26.57**
Surigao City	**3,301**	1	**0.03**	184	**5.57**	584	**17.69**	**769**	**23.30**

Note: Put asterisk (*) for No Report and Zero (0) for No Case

Table 1.B.1.4 - Prenatal Care

Number and proportion of pregnant women given Tetanus Diphtheria (Td) Vaccination
Philippines, Annual 2020

Area	Eligible Pop.	Women pregnant for the 2nd or more times given at least three (3) doses of Td Vaccine							
		Age Group						Total	%
		10-14 yrs old		15-19 yrs old		20-49 yrs old			
		No.	%	No.	%	No.	%		
**PHILIPPINES**	**2,123,158**	**1,394**	**0.07**	**77,192**	**3.64**	**784,255**	**36.94**	**862,841**	**40.64**
**N C R**	**238,661**	**122**	**0.05**	**9,426**	**3.95**	**108,212**	**45.34**	**117,760**	**49.34**
Malabon	**6,775**	3	**0.04**	228	**3.37**	1,986	**29.31**	**2,217**	**32.72**
Navotas	**4,621**	2	**0.04**	312	**6.75**	1,513	**32.74**	**1,827**	**39.54**
Valenzuela City	**11,500**	1	**0.01**	518	**4.50**	4,681	**40.70**	**5,200**	**45.22**
Caloocan City	**29,363**	31	**0.11**	2,032	**6.92**	19,346	**65.89**	**21,409**	**72.91**
Marikina City	**8,354**	0	**0.00**	83	**0.99**	2,662	**31.86**	**2,745**	**32.86**
Pasig City	**13,996**	6	**0.04**	384	**2.74**	7,518	**53.72**	**7,908**	**56.50**
Pateros	**1,184**	0	**0.00**	66	**5.57**	528	**44.59**	**594**	**50.17**
Taguig	**14,918**	34	**0.23**	1,139	**7.64**	7,810	**52.35**	**8,983**	**60.22**
Quezon City	**54,413**	13	**0.02**	1,543	**2.84**	34,320	**63.07**	**35,876**	**65.93**
Makati City	**10,798**	4	**0.04**	184	**1.70**	3,268	**30.26**	**3,456**	**32.01**
Mandaluyong City	**7,160**	3	**0.04**	179	**2.50**	2,327	**32.50**	**2,509**	**35.04**
San Juan	**2,261**	1	**0.04**	68	**3.01**	594	**26.27**	**663**	**29.32**
Manila City	**32,980**	8	**0.02**	1,239	**3.76**	8,212	**24.90**	**9,459**	**28.68**
Las Piñas City	**10,914**	8	**0.07**	446	**4.09**	4,827	**44.23**	**5,281**	**48.39**
Muntinlupa City	**9,353**	4	**0.04**	350	**3.74**	3,260	**34.86**	**3,614**	**38.64**
Parañaque City	**12,343**	3	**0.02**	471	**3.82**	2,298	**18.62**	**2,772**	**22.46**
Pasay City	**7,728**	1	**0.01**	184	**2.38**	3,062	**39.62**	**3,247**	**42.02**
**C A R**	**35,099**	**7**	**0.02**	**1,004**	**2.86**	**11,950**	**34.05**	**12,961**	**36.93**
Abra	**4,309**	2	**0.05**	153	**3.55**	1,384	**32.12**	**1,539**	**35.72**
Apayao	**2,451**	0	**0.00**	81	**3.30**	924	**37.70**	**1,005**	**41.00**
Benguet	**9,188**	2	**0.02**	141	**1.53**	2,909	**31.66**	**3,052**	**33.22**
Ifugao	**4,396**	1	**0.02**	202	**4.60**	1,844	**41.95**	**2,047**	**46.57**
Kalinga	**4,617**	0	**0.00**	244	**5.28**	2,517	**54.52**	**2,761**	**59.80**
Mt. Province	**3,023**	2	**0.07**	121	**4.00**	1,150	**38.04**	**1,273**	**42.11**
Baguio City	**7,115**	0	**0.00**	62	**0.87**	1,222	**17.17**	**1,284**	**18.05**
**Region 1**	**97,261**	**28**	**0.03**	**3,169**	**3.26**	**42,566**	**43.76**	**45,763**	**47.05**
Ilocos Norte	**8,105**	3	**0.04**	184	**2.27**	3,100	**38.25**	**3,287**	**40.56**
Ilocos Sur	**9,330**	3	**0.03**	204	**2.19**	4,333	**46.44**	**4,540**	**48.66**
La Union	**11,511**	4	**0.03**	364	**3.16**	4,422	**38.42**	**4,790**	**41.61**
Pangasinan	**50,168**	17	**0.03**	1,629	**3.25**	22,229	**44.31**	**23,875**	**47.59**
Alaminos City	**1,894**	0	**0.00**	308	**16.26**	1,338	**70.64**	**1,646**	**86.91**
Candon City	**987**	0	**0.00**	1	**0.10**	505	**51.17**	**506**	**51.27**
Dagupan City	**3,620**	0	**0.00**	131	**3.62**	1,009	**27.87**	**1,140**	**31.49**
Laoag City	**1,870**	0	**0.00**	54	**2.89**	1,767	**94.49**	**1,821**	**97.38**
San Carlos City	**3,979**	0	**0.00**	48	**1.21**	1,629	**40.94**	**1,677**	**42.15**
San Fernando City	**2,115**	1	**0.05**	84	**3.97**	1,079	**51.02**	**1,164**	**55.04**
Urdaneta City	**2,806**	0	**0.00**	144	**5.13**	1,007	**35.89**	**1,151**	**41.02**

Table 1.B.1.4 - Prenatal Care

Number and proportion of pregnant women given Tetanus Diphtheria (Td) Vaccination
Philippines, Annual 2020

Area	Eligible Pop.	Women pregnant for the 2nd or more times given at least three (3) doses of Td Vaccine							
		Age Group						Total	%
		10-14 yrs old		15-19 yrs old		20-49 yrs old			
		No.	%	No.	%	No.	%		
Vigan City	**876**	0	**0.00**	18	**2.05**	148	**16.89**	**166**	**18.95**
**Region 2**	**69,443**	**32**	**0.05**	**2,752**	**3.96**	**27,709**	**39.90**	**30,493**	**43.91**
Batanes	**340**	0	**0.00**	2	**0.59**	84	**24.71**	**86**	**25.29**
Cagayan	**17,971**	2	**0.01**	532	**2.96**	6,697	**37.27**	**7,231**	**40.24**
Isabela	**26,309**	19	**0.07**	1,228	**4.67**	9,486	**36.06**	**10,733**	**40.80**
Nueva Vizcaya	**9,573**	3	**0.03**	281	**2.94**	4,231	**44.20**	**4,515**	**47.16**
Quirino	**4,025**	1	**0.02**	157	**3.90**	1,870	**46.46**	**2,028**	**50.39**
Cauayan City	**2,590**	5	**0.19**	204	**7.88**	1,140	**44.02**	**1,349**	**52.08**
Ilagan City	**2,911**	0	**0.00**	47	**1.61**	1,009	**34.66**	**1,056**	**36.28**
Santiago City	**2,692**	1	**0.04**	242	**8.99**	2,290	**85.07**	**2,533**	**94.09**
Tuguegarao City	**3,032**	1	**0.03**	59	**1.95**	902	**29.75**	**962**	**31.73**
**Region 3**	**220,020**	**212**	**0.10**	**10,623**	**4.83**	**91,939**	**41.79**	**102,774**	**46.71**
Aurora	**4,770**	1	**0.02**	113	**2.37**	1,764	**36.98**	**1,878**	**39.37**
Bataan	**13,823**	9	**0.07**	592	**4.28**	6,467	**46.78**	**7,068**	**51.13**
Bulacan	**43,627**	25	**0.06**	2,037	**4.67**	16,356	**37.49**	**18,418**	**42.22**
Nueva Ecija	**28,782**	16	**0.06**	1,269	**4.41**	10,703	**37.19**	**11,988**	**41.65**
Pampanga	**31,359**	20	**0.06**	1,185	**3.78**	9,253	**29.51**	**10,458**	**33.35**
Tarlac	**19,765**	9	**0.05**	958	**4.85**	9,878	**49.98**	**10,845**	**54.87**
Zambales	**12,610**	6	**0.05**	212	**1.68**	2,716	**21.54**	**2,934**	**23.27**
Angeles City	**7,869**	2	**0.03**	444	**5.64**	2,748	**34.92**	**3,194**	**40.59**
Balanga City	**1,998**	1	**0.05**	60	**3.00**	668	**33.43**	**729**	**36.49**
Cabanatuan City	**5,896**	2	**0.03**	138	**2.34**	2,139	**36.28**	**2,279**	**38.65**
City of San Fernando	**5,863**	56	**0.96**	409	**6.98**	2,759	**47.06**	**3,224**	**54.99**
Gapan City	**2,153**	0	**0.00**	59	**2.74**	510	**23.69**	**569**	**26.43**
Mabalacat City	**4,793**	1	**0.02**	461	**9.62**	2,949	**61.53**	**3,411**	**71.17**
Malolos City	**4,880**	10	**0.20**	257	**5.27**	1,737	**35.59**	**2,004**	**41.07**
Meycauayan	**4,041**	10	**0.25**	305	**7.55**	2,014	**49.84**	**2,329**	**57.63**
Olongapo	**4,971**	0	**0.00**	165	**3.32**	1,853	**37.28**	**2,018**	**40.60**
Palayan City	**799**	0	**0.00**	13	**1.63**	196	**24.53**	**209**	**26.16**
San Jose City	**2,718**	5	**0.18**	266	**9.79**	1,601	**58.90**	**1,872**	**68.87**
San Jose del Monte City	**11,100**	9	**0.08**	1,031	**9.29**	10,526	**94.83**	**11,566**	**104.20**
Science City of Munoz	**1,586**	1	**0.06**	138	**8.70**	806	**50.82**	**945**	**59.58**
Tarlac City	**6,617**	29	**0.44**	511	**7.72**	4,296	**64.92**	**4,836**	**73.08**
**Region 4A**	**296,816**	**73**	**0.02**	**7,468**	**2.52**	**73,246**	**24.68**	**80,787**	**27.22**
Batangas	**38,441**	6	**0.02**	258	**0.67**	6,483	**16.86**	**6,747**	**17.55**
Cavite	**27,704**	18	**0.06**	1,126	**4.06**	11,288	**40.75**	**12,432**	**44.87**
Laguna	**19,727**	8	**0.04**	902	**4.57**	6,552	**33.21**	**7,462**	**37.83**
Quezon	**37,410**	2	**0.01**	457	**1.22**	4,587	**12.26**	**5,046**	**13.49**
Rizal	**44,791**	10	**0.02**	1,045	**2.33**	9,324	**20.82**	**10,379**	**23.17**
Antipolo City	**16,497**	1	**0.01**	832	**5.04**	7,639	**46.31**	**8,472**	**51.35**
Bacoor City	**12,142**	0	**0.00**	64	**0.53**	422	**3.48**	**486**	**4.00**
Batangas City	**6,823**	0	**0.00**	0	**0.00**	0	**0.00**	**0**	**0.00**

Table 1.B.1.4 - Prenatal Care

Number and proportion of pregnant women given Tetanus Diphtheria (Td) Vaccination
Philippines, Annual 2020

Area	Eligible Pop.	Women pregnant for the 2nd or more times given at least three (3) doses of Td Vaccine							
		Age Group						Total	%
		10-14 yrs old		15-19 yrs old		20-49 yrs old			
		No.	%	No.	%	No.	%		
Biñan City	**6,607**	0	**0.00**	723	**10.94**	3,735	**56.53**	**4,458**	**67.47**
Cabuyao City	**6,130**	2	**0.03**	358	**5.84**	2,980	**48.61**	**3,340**	**54.49**
Calamba City	**9,028**	9	**0.10**	348	**3.85**	5,087	**56.35**	**5,444**	**60.30**
Cavite City	**2,075**	0	**0.00**	0	**0.00**	0	**0.00**	**0**	**0.00**
Dasmariñas City	**13,322**	1	**0.01**	194	**1.46**	2,216	**16.63**	**2,411**	**18.10**
General Trias City	**6,352**	1	**0.02**	213	**3.35**	2,444	**38.48**	**2,658**	**41.85**
Imus City	**8,156**	0	**0.00**	50	**0.61**	501	**6.14**	**551**	**6.76**
Lipa City	**6,880**	0	**0.00**	0	**0.00**	0	**0.00**	**0**	**0.00**
Lucena City	**5,672**	0	**0.00**	0	**0.00**	0	**0.00**	**0**	**0.00**
San Pablo City	**5,277**	2	**0.04**	295	**5.59**	927	**17.57**	**1,224**	**23.19**
San Pedro City	**6,467**	1	**0.02**	94	**1.45**	795	**12.29**	**890**	**13.76**
Santa Rosa City	**7,025**	5	**0.07**	288	**4.10**	2,994	**42.62**	**3,287**	**46.79**
Tagaytay City	**1,437**	4	**0.28**	34	**2.37**	1,356	**94.36**	**1,394**	**97.01**
Tanauan City	**3,584**	3	**0.08**	47	**1.31**	1,888	**52.68**	**1,938**	**54.07**
Tayabas City	**2,122**	0	**0.00**	0	**0.00**	248	**11.69**	**248**	**11.69**
Trece Martires City	**3,147**	0	**0.00**	140	**4.45**	1,780	**56.56**	**1,920**	**61.01**
**Region 4B**	**71,246**	**63**	**0.09**	**1,242**	**1.74**	**16,138**	**22.65**	**17,443**	**24.48**
									**0.00**
Marinduque	**4,836**	0	**0.00**	72	**1.49**	1,642	**33.95**	**1,714**	**35.44**
Mindoro Occidental	**12,407**	5	**0.04**	474	**3.82**	5,080	**40.94**	**5,559**	**44.81**
Mindoro Oriental	**19,809**	57	**0.29**	482	**2.43**	6,308	**31.84**	**6,847**	**34.57**
Palawan	**21,375**	1	**0.00**	198	**0.93**	1,942	**9.09**	**2,141**	**10.02**
Romblon	**6,400**	0	**0.00**	16	**0.25**	438	**6.84**	**454**	**7.09**
Puerto Princesa City	**6,419**	0	**0.00**	0	**0.00**	728	**11.34**	**728**	**11.34**
**Region 5**	**136,116**	**18**	**0.01**	**3,149**	**2.31**	**45,629**	**33.52**	**48,796**	**35.85**
Albay	**23,737**	3	**0.01**	297	**1.25**	8,321	**35.05**	**8,621**	**36.32**
Camarines Norte	**14,283**	2	**0.01**	545	**3.82**	6,014	**42.11**	**6,561**	**45.94**
Camarines Sur	**38,796**	3	**0.01**	573	**1.48**	10,330	**26.63**	**10,906**	**28.11**
Catanduanes	**6,274**	2	**0.03**	171	**2.73**	1,626	**25.92**	**1,799**	**28.67**
Masbate	**22,521**	6	**0.03**	890	**3.95**	8,159	**36.23**	**9,055**	**40.21**
Sorsogon	**19,065**	2	**0.01**	474	**2.49**	7,685	**40.31**	**8,161**	**42.81**
Iriga City	**2,638**	0	**0.00**	16	**0.61**	506	**19.18**	**522**	**19.79**
Legaspi City	**4,174**	0	**0.00**	54	**1.29**	1,683	**40.32**	**1,737**	**41.61**
Naga City	**4,628**	0	**0.00**	129	**2.79**	1,305	**28.20**	**1,434**	**30.99**
**Region 6**	**146,526**	**45**	**0.03**	**4,766**	**3.25**	**54,551**	**37.23**	**59,362**	**40.51**
Aklan	**11,162**	10	**0.09**	337	**3.02**	4,147	**37.15**	**4,494**	**40.26**
Antique	**12,816**	1	**0.01**	344	**2.68**	4,344	**33.90**	**4,689**	**36.59**
Capiz	**13,986**	0	**0.00**	290	**2.07**	4,403	**31.48**	**4,693**	**33.55**
Guimaras	**3,085**	3	**0.10**	187	**6.06**	1,850	**59.97**	**2,040**	**66.13**
Iloilo	**36,541**	10	**0.03**	1,017	**2.78**	13,882	**37.99**	**14,909**	**40.80**
Negros Occidental	**49,368**	19	**0.04**	2,180	**4.42**	21,139	**42.82**	**23,338**	**47.27**
Bacolod City	**11,115**	0	**0.00**	212	**1.91**	2,505	**22.54**	**2,717**	**24.44**
Iloilo City	**8,453**	2	**0.02**	199	**2.35**	2,281	**26.98**	**2,482**	**29.36**

Table 1.B.1.4 - Prenatal Care

Number and proportion of pregnant women given Tetanus Diphtheria (Td) Vaccination
Philippines, Annual 2020

Area	Eligible Pop.	Women pregnant for the 2nd or more times given at least three (3) doses of Td Vaccine							
		Age Group						Total	%
		10-14 yrs old		15-19 yrs old		20-49 yrs old			
		No.	%	No.	%	No.	%		
**Region 7**	**163,262**	**63**	**0.04**	**6,326**	**3.87**	**74,885**	**45.87**	**81,274**	**49.78**
Bohol	**27,312**	17	**0.06**	969	**3.55**	11,169	**40.89**	**12,155**	**44.50**
Cebu	**67,506**	19	**0.03**	2,186	**3.24**	29,464	**43.65**	**31,669**	**46.91**
Negros Oriental	**27,938**	19	**0.07**	1,539	**5.51**	12,916	**46.23**	**14,474**	**51.81**
Siquijor	**1,613**	1	**0.06**	121	**7.50**	885	**54.87**	**1,007**	**62.43**
Cebu City	**21,193**	7	**0.03**	1,107	**5.22**	9,931	**46.86**	**11,045**	**52.12**
Lapu-Lapu City	**9,372**	0	**0.00**	404	**4.31**	6,551	**69.90**	**6,955**	**74.21**
Mandaue City	**8,328**	0	**0.00**	0	**0.00**	3,969	**47.66**	**3,969**	**47.66**
**Region 8**	**102,619**	**15**	**0.01**	**1,961**	**1.91**	**30,636**	**29.85**	**32,612**	**31.78**
Biliran	**3,834**	0	**0.00**	113	**2.95**	1,602	**41.78**	**1,715**	**44.73**
Eastern Samar	**11,392**	0	**0.00**	306	**2.69**	3,649	**32.03**	**3,955**	**34.72**
Northern Leyte	**34,707**	7	**0.02**	676	**1.95**	10,666	**30.73**	**11,349**	**32.70**
Northern Samar	**15,370**	0	**0.00**	185	**1.20**	4,286	**27.89**	**4,471**	**29.09**
Southern Leyte	**6,451**	3	**0.05**	119	**1.84**	1,859	**28.82**	**1,981**	**30.71**
Western Samar	**14,305**	3	**0.02**	156	**1.09**	3,054	**21.35**	**3,213**	**22.46**
Calbayog City	**4,413**	2	**0.05**	130	**2.95**	1,440	**32.63**	**1,572**	**35.62**
Maasin City	**1,637**	0	**0.00**	14	**0.86**	244	**14.91**	**258**	**15.76**
Ormoc City	**4,941**	0	**0.00**	137	**2.77**	1,909	**38.64**	**2,046**	**41.41**
Tacloban City	**5,569**	0	**0.00**	125	**2.24**	1,927	**34.60**	**2,052**	**36.85**
**Region 9**	**80,051**	**40**	**0.05**	**3,749**	**4.68**	**37,246**	**46.53**	**41,035**	**51.26**
Zamboanga del Norte	**17,249**	12	**0.07**	1,069	**6.20**	10,085	**58.47**	**11,166**	**64.73**
Zamboanga del Sur	**17,653**	1	**0.01**	702	**3.98**	6,878	**38.96**	**7,581**	**42.94**
Zamboanga Sibugay	**14,954**	14	**0.09**	664	**4.44**	5,168	**34.56**	**5,846**	**39.09**
Dapitan City	**1,784**	2	**0.11**	69	**3.87**	721	**40.41**	**792**	**44.39**
Dipolog City	**2,827**	1	**0.04**	329	**11.64**	1,738	**61.48**	**2,068**	**73.15**
Isabela City	**2,522**	0	**0.00**	86	**3.41**	792	**31.40**	**878**	**34.81**
Pagadian City	**4,325**	0	**0.00**	278	**6.43**	2,446	**56.55**	**2,724**	**62.98**
Zamboanga City	**18,737**	10	**0.05**	552	**2.95**	9,418	**50.26**	**9,980**	**53.26**
**Region 10**	**101,411**	**90**	**0.09**	**4,508**	**4.45**	**37,952**	**37.42**	**42,550**	**41.96**
Bukidnon	**23,706**	60	**0.25**	1,751	**7.39**	10,156	**42.84**	**11,967**	**50.48**
Camiguin	**1,858**	0	**0.00**	29	**1.56**	543	**29.22**	**572**	**30.79**
Lanao del Norte	**14,960**	1	**0.01**	286	**1.91**	5,120	**34.22**	**5,407**	**36.14**
Misamis Occidental	**6,403**	8	**0.12**	182	**2.84**	2,755	**43.03**	**2,945**	**45.99**
Misamis Oriental	**15,131**	2	**0.01**	81	**0.54**	682	**4.51**	**765**	**5.06**
Cagayan de Oro City	**14,339**	11	**0.08**	766	**5.34**	6,930	**48.33**	**7,707**	**53.75**
El Salvador City	**1,065**	0	**0.00**	34	**3.19**	509	**47.79**	**543**	**50.99**
Gingoog City	**2,644**	1	**0.04**	244	**9.23**	1,375	**52.00**	**1,620**	**61.27**
Iligan City	**7,580**	2	**0.03**	399	**5.26**	3,311	**43.68**	**3,712**	**48.97**
Malaybalay City	**3,956**	2	**0.05**	151	**3.82**	1,327	**33.54**	**1,480**	**37.41**

Table 1.B.1.4 - Prenatal Care

Number and proportion of pregnant women given Tetanus Diphtheria (Td) Vaccination
Philippines, Annual 2020

Area	Eligible Pop.	Women pregnant for the 2nd or more times given at least three (3) doses of Td Vaccine							
		Age Group						Total	%
		10-14 yrs old		15-19 yrs old		20-49 yrs old			
		No.	%	No.	%	No.	%		
Oroquieta City	1,389	0	0.00	17	1.22	564	40.60	581	41.83
Ozamis City	2,778	2	0.07	161	5.80	1,451	52.23	1,614	58.10
Tangub City	1,234	0	0.00	65	5.27	1,009	81.77	1,074	87.03
Valencia City	4,368	1	0.02	342	7.83	2,220	50.82	2,563	58.68
**Region 11**	**107,247**	**164**	**0.15**	**3,081**	**2.87**	**40,695**	**37.95**	**43,940**	**40.97**
Compostela Valley	15,562	10	0.06	472	3.03	6,740	43.31	7,222	46.41
Davao del Norte	21,326	57	0.27	640	3.00	9,483	44.47	10,180	47.74
Davao Oriental	13,007	12	0.09	317	2.44	4,504	34.63	4,833	37.16
Davao del Sur	14,151	10	0.07	388	2.74	5,068	35.81	5,466	38.63
Davao Occidental	6,670	11	0.16	263	3.94	2,289	34.32	2,563	38.43
Davao City	36,531	64	0.18	1,001	2.74	12,611	34.52	13,676	37.44
**Region 12**	**104,552**	**95**	**0.09**	**6,408**	**6.13**	**42,889**	**41.02**	**49,392**	**47.24**
North Cotabato	33,645	25	0.07	1,571	4.67	11,395	33.87	12,991	38.61
Sarangani	12,891	11	0.09	864	6.70	5,377	41.71	6,252	48.50
South Cotabato	21,113	12	0.06	1,603	7.59	10,753	50.93	12,368	58.58
Sultan Kudarat	17,359	40	0.23	1,481	8.53	8,177	47.11	9,698	55.87
Cotabato City	5,835	0	0.00	102	1.75	1,462	25.06	1,564	26.80
Gen. Santos City	13,709	7	0.05	787	5.74	5,725	41.76	6,519	47.55
**BARMM**	**92,799**	**316**	**0.34**	**5,921**	**6.38**	**28,452**	**30.66**	**34,689**	**37.38**
Basilan	7,541	4	0.05	167	2.21	1,083	14.36	1,254	16.63
Lanao del Sur	21,131	0	0.00	329	1.56	12,981	61.43	13,310	62.99
Maguindanao	31,128	264	0.85	3,653	11.74	12,319	39.58	16,236	52.16
Sulu	16,613	0	0.00	0	0.00	0	0.00	0	0.00
Tawi-Tawi	9,259	0	0.00	1,378	14.88	1,014	10.95	2,392	25.83
Lamitan City	2,074	0	0.00	148	7.14	305	14.71	453	21.84
Marawi City	5,053	48	0.95	246	4.87	750	14.84	1,044	20.66
**CARAGA**	**60,029**	**11**	**0.02**	**1,639**	**2.73**	**19,560**	**32.58**	**21,210**	**35.33**
Agusan del Norte	8,098	3	0.04	175	2.16	1,941	23.97	2,119	26.17
Agusan del Sur	17,592	2	0.01	429	2.44	4,791	27.23	5,222	29.68
Surigao del Norte	7,089	0	0.00	147	2.07	2,483	35.03	2,630	37.10
Surigao del Sur	11,482	5	0.04	509	4.43	4,549	39.62	5,063	44.10
Province of Dinagat	2,573	1	0.04	30	1.17	673	26.16	704	27.36
Bislig City	2,179	0	0.00	63	2.89	924	42.40	987	45.30
Butuan City	7,715	0	0.00	165	2.14	2,872	37.23	3,037	39.36
Surigao City	3,301	0	0.00	121	3.67	1,327	40.20	1,448	43.87

Note: Put asterisk (*) for No Report and Zero (0) for No Case

Table 1.B.1.5 - Prenatal Care

No. of Pregnant women who Completed Iron with Folic Acid
Philippines, Annual 2020

Area	Eligible Pop.	Iron with Folic Acid Supplementation							
		Age Group						Total	%
		10-14 yrs old		15-19 yrs old		20-49 yrs old			
		No.	%	No.	%	No.	%		
**PHILIPPINES**	**2,123,158**	**2,288**	**0.11**	**130,477**	**6.15**	**948,630**	**44.68**	**1,081,395**	**50.93**
**N C R**	**238,661**	**234**	**0.10**	**14,298**	**5.99**	**103,577**	**43.40**	**118,109**	**49.49**
Malabon	**6,775**	15	**0.22**	649	**9.58**	3,457	**51.03**	**4,121**	**60.83**
Navotas	**4,621**	17	**0.37**	751	**16.25**	2,746	**59.42**	**3,514**	**76.04**
Valenzuela City	**11,500**	6	**0.05**	867	**7.54**	6,264	**54.47**	**7,137**	**62.06**
Caloocan City	**29,363**	41	**0.14**	2,639	**8.99**	20,265	**69.02**	**22,945**	**78.14**
Marikina City	**8,354**	7	**0.08**	364	**4.36**	4,688	**56.12**	**5,059**	**60.56**
Pasig City	**13,996**	26	**0.19**	869	**6.21**	8,145	**58.20**	**9,040**	**64.59**
Pateros	**1,184**	1	**0.08**	57	**4.81**	570	**48.14**	**628**	**53.04**
Taguig	**14,918**	53	**0.36**	2,150	**14.41**	12,655	**84.83**	**14,858**	**99.60**
Quezon City	**54,413**	8	**0.01**	774	**1.42**	5,821	**10.70**	**6,603**	**12.13**
Makati City	**10,798**	5	**0.05**	157	**1.45**	2,956	**27.38**	**3,118**	**28.88**
Mandaluyong City	**7,160**	4	**0.06**	321	**4.48**	3,044	**42.51**	**3,369**	**47.05**
San Juan	**2,261**	2	**0.09**	64	**2.83**	761	**33.66**	**827**	**36.58**
Manila City	**32,980**	15	**0.05**	1,565	**4.75**	10,159	**30.80**	**11,739**	**35.59**
Las Piñas City	**10,914**	8	**0.07**	643	**5.89**	5,126	**46.97**	**5,777**	**52.93**
Muntinlupa City	**9,353**	7	**0.07**	764	**8.17**	7,081	**75.71**	**7,852**	**83.95**
Parañaque City	**12,343**	15	**0.12**	1,001	**8.11**	4,061	**32.90**	**5,077**	**41.13**
Pasay City	**7,728**	4	**0.05**	663	**8.58**	5,778	**74.77**	**6,445**	**83.40**
**C A R**	**35,099**	**12**	**0.03**	**1,657**	**4.72**	**16,691**	**47.55**	**18,360**	**52.31**
Abra	**4,309**	1	**0.02**	268	**6.22**	1,951	**45.28**	**2,220**	**51.52**
Apayao	**2,451**	0	**0.00**	205	**8.36**	1,142	**46.59**	**1,347**	**54.96**
Benguet	**9,188**	3	**0.03**	382	**4.16**	4,560	**49.63**	**4,945**	**53.82**
Ifugao	**4,396**	1	**0.02**	200	**4.55**	2,447	**55.66**	**2,648**	**60.24**
Kalinga	**4,617**	2	**0.04**	274	**5.93**	2,897	**62.75**	**3,173**	**68.72**
Mt. Province	**3,023**	1	**0.03**	147	**4.86**	1,541	**50.98**	**1,689**	**55.87**
Baguio City	**7,115**	4	**0.06**	181	**2.54**	2,153	**30.26**	**2,338**	**32.86**
**Region 1**	**97,261**	**66**	**0.07**	**6,035**	**6.20**	**56,824**	**58.42**	**62,925**	**64.70**
Ilocos Norte	**8,105**	9	**0.11**	394	**4.86**	4,624	**57.05**	**5,027**	**62.02**
Ilocos Sur	**9,330**	2	**0.02**	480	**5.14**	6,449	**69.12**	**6,931**	**74.29**
La Union	**11,511**	13	**0.11**	792	**6.88**	6,702	**58.22**	**7,507**	**65.22**
Pangasinan	**50,168**	32	**0.06**	3,178	**6.33**	28,107	**56.03**	**31,317**	**62.42**
Alaminos City	**1,894**	0	**0.00**	171	**9.03**	1,632	**86.17**	**1,803**	**95.20**
Candon City	**987**	0	**0.00**	8	**0.81**	962	**97.47**	**970**	**98.28**
Dagupan City	**3,620**	1	**0.03**	351	**9.70**	1,547	**42.73**	**1,899**	**52.46**
Laoag City	**1,870**	0	**0.00**	64	**3.42**	1,725	**92.25**	**1,789**	**95.67**
San Carlos City	**3,979**	4	**0.10**	157	**3.95**	2,059	**51.75**	**2,220**	**55.79**
San Fernando City	**2,115**	1	**0.05**	129	**6.10**	1,273	**60.19**	**1,403**	**66.34**
Urdaneta City	**2,806**	4	**0.14**	272	**9.69**	1,412	**50.32**	**1,688**	**60.16**

Table 1.B.1.5 - Prenatal Care

No. of Pregnant women who Completed Iron with Folic Acid
Philippines, Annual 2020

Area	Eligible Pop.	Iron with Folic Acid Supplementation							
		Age Group						Total	%
		10-14 yrs old		15-19 yrs old		20-49 yrs old			
		No.	%	No.	%	No.	%		
Vigan City	876	0	0.00	39	4.45	332	37.90	371	42.35
**Region 2**	69,443	51	0.07	5,014	7.22	35,479	51.09	40,544	58.38
Batanes	340	0	0.00	10	2.94	144	42.35	154	45.29
Cagayan	17,971	7	0.04	1,194	6.64	8,538	47.51	9,739	54.19
Isabela	26,309	24	0.09	1,730	6.58	11,813	44.90	13,567	51.57
Nueva Vizcaya	9,573	10	0.10	870	9.09	5,712	59.67	6,592	68.86
Quirino	4,025	2	0.05	348	8.65	2,816	69.96	3,166	78.66
Cauayan City	2,590	2	0.08	232	8.96	1,540	59.46	1,774	68.49
Ilagan City	2,911	1	0.03	208	7.15	1,484	50.98	1,693	58.16
Santiago City	2,692	3	0.11	318	11.81	2,360	87.67	2,681	99.59
Tuguegarao City	3,032	2	0.07	104	3.43	1,072	35.36	1,178	38.85
**Region 3**	220,020	275	0.12	18,677	8.49	116,334	52.87	135,286	61.49
Aurora	4,770	6	0.13	352	7.38	2,501	52.43	2,859	59.94
Bataan	13,823	19	0.14	1,113	8.05	7,434	53.78	8,566	61.97
Bulacan	43,627	54	0.12	3,441	7.89	21,416	49.09	24,911	57.10
Nueva Ecija	28,782	35	0.12	2,181	7.58	13,494	46.88	15,710	54.58
Pampanga	31,359	31	0.10	1,662	5.30	11,812	37.67	13,505	43.07
Tarlac	19,765	34	0.17	1,729	8.75	13,443	68.01	15,206	76.93
Zambales	12,610	7	0.06	529	4.20	3,187	25.27	3,723	29.52
Angeles City	7,869	9	0.11	787	10.00	4,473	56.84	5,269	66.96
Balanga City	1,998	3	0.15	166	8.31	912	45.65	1,081	54.10
Cabanatuan City	5,896	10	0.17	659	11.18	3,504	59.43	4,173	70.78
City of San Fernando	5,863	9	0.15	613	10.46	3,404	58.06	4,026	68.67
Gapan City	2,153	1	0.05	194	9.01	990	45.98	1,185	55.04
Mabalacat City	4,793	2	0.04	618	12.89	4,136	86.29	4,756	99.23
Malolos City	4,880	8	0.16	357	7.32	2,503	51.29	2,868	58.77
Meycauayan	4,041	20	0.49	541	13.39	2,898	71.71	3,459	85.60
Olongapo	4,971	3	0.06	434	8.73	2,499	50.27	2,936	59.06
Palayan City	799	2	0.25	139	17.40	605	75.72	746	93.37
San Jose City	2,718	6	0.22	411	15.12	2,071	76.20	2,488	91.54
San Jose del Monte City	11,100	12	0.11	1,544	13.91	9,261	83.43	10,817	97.45
Science City of Munoz	1,586	0	0.00	110	6.94	651	41.05	761	47.98
Tarlac City	6,617	4	0.06	1,097	16.58	5,140	77.68	6,241	94.32
**Region 4A**	296,816	131	0.04	10,281	3.46	51,419	17.32	61,831	20.83
Batangas	38,441	11	0.03	314	0.82	3,922	10.20	4,247	11.05
Cavite	27,704	1	0.00	413	1.49	3,749	13.53	4,163	15.03
Laguna	19,727	27	0.14	3,730	18.91	3,902	19.78	7,659	38.82
Quezon	37,410	12	0.03	413	1.10	3,302	8.83	3,727	9.96
Rizal	44,791	22	0.05	1,474	3.29	11,609	25.92	13,105	29.26
Antipolo City	16,497	1	0.01	577	3.50	4,957	30.05	5,535	33.55
Bacoor City	12,142	0	0.00	11	0.09	84	0.69	95	0.78
Batangas City	6,823	0	0.00	0	0.00	0	0.00	0	0.00

Table 1.B.1.5 - Prenatal Care

No. of Pregnant women who Completed Iron with Folic Acid
Philippines, Annual 2020

Area	Eligible Pop.	Iron with Folic Acid Supplementation							
		Age Group						Total	%
		10-14 yrs old		15-19 yrs old		20-49 yrs old			
		No.	%	No.	%	No.	%		
Biñan City	**6,607**	7	**0.11**	873	**13.21**	4,641	**70.24**	**5,521**	**83.56**
Cabuyao City	**6,130**	1	**0.02**	353	**5.76**	2,287	**37.31**	**2,641**	**43.08**
Calamba City	**9,028**	10	**0.11**	644	**7.13**	2,105	**23.32**	**2,759**	**30.56**
Cavite City	**2,075**	0	**0.00**	0	**0.00**	0	**0.00**	**0**	**0.00**
Dasmariñas City	**13,322**	3	**0.02**	278	**2.09**	2,566	**19.26**	**2,847**	**21.37**
General Trias City	**6,352**	0	**0.00**	46	**0.72**	391	**6.16**	**437**	**6.88**
Imus City	**8,156**	0	**0.00**	0	**0.00**	0	**0.00**	**0**	**0.00**
Lipa City	**6,880**	0	**0.00**	0	**0.00**	0	**0.00**	**0**	**0.00**
Lucena City	**5,672**	0	**0.00**	0	**0.00**	414	**7.30**	**414**	**7.30**
San Pablo City	**5,277**	5	**0.09**	150	**2.84**	182	**3.45**	**337**	**6.39**
San Pedro City	**6,467**	0	**0.00**	340	**5.26**	650	**10.05**	**990**	**15.31**
Santa Rosa City	**7,025**	22	**0.31**	493	**7.02**	3,719	**52.94**	**4,234**	**60.27**
Tagaytay City	**1,437**	6	**0.42**	36	**2.51**	1,310	**91.16**	**1,352**	**94.08**
Tanauan City	**3,584**	1	**0.03**	26	**0.73**	605	**16.88**	**632**	**17.63**
Tayabas City	**2,122**	0	**0.00**	0	**0.00**	414	**19.51**	**414**	**19.51**
Trece Martires City	**3,147**	2	**0.06**	110	**3.50**	610	**19.38**	**722**	**22.94**
**Region 4B**	**71,246**	**120**	**0.17**	**2,722**	**3.82**	**22,903**	**32.15**	**25,745**	**36.14**
Marinduque	**4,836**	4	**0.08**	256	**5.29**	2,392	**49.46**	**2,652**	**54.84**
Mindoro Occidental	**12,407**	18	**0.15**	880	**7.09**	6,236	**50.26**	**7,134**	**57.50**
Mindoro Oriental	**19,809**	87	**0.44**	931	**4.70**	7,931	**40.04**	**8,949**	**45.18**
Palawan	**21,375**	9	**0.04**	532	**2.49**	3,230	**15.11**	**3,771**	**17.64**
Romblon	**6,400**	2	**0.03**	66	**1.03**	654	**10.22**	**722**	**11.28**
Puerto Princesa City	**6,419**	0	**0.00**	57	**0.89**	2,460	**38.32**	**2,517**	**39.21**
**Region 5**	**136,116**	**43**	**0.03**	**6,451**	**4.74**	**61,479**	**45.17**	**67,973**	**49.94**
Albay	**23,737**	2	**0.01**	924	**3.89**	12,572	**52.96**	**13,498**	**56.86**
Camarines Norte	**14,283**	6	**0.04**	932	**6.53**	6,941	**48.60**	**7,879**	**55.16**
Camarines Sur	**38,796**	10	**0.03**	1,435	**3.70**	15,285	**39.40**	**16,730**	**43.12**
Catanduanes	**6,274**	1	**0.02**	322	**5.13**	2,621	**41.78**	**2,944**	**46.92**
Masbate	**22,521**	11	**0.05**	1,443	**6.41**	9,110	**40.45**	**10,564**	**46.91**
Sorsogon	**19,065**	7	**0.04**	828	**4.34**	9,327	**48.92**	**10,162**	**53.30**
Iriga City	**2,638**	2	**0.08**	109	**4.13**	1,185	**44.92**	**1,296**	**49.13**
Legaspi City	**4,174**	2	**0.05**	216	**5.17**	2,758	**66.08**	**2,976**	**71.30**
Naga City	**4,628**	2	**0.04**	242	**5.23**	1,680	**36.30**	**1,924**	**41.57**
**Region 6**	**146,526**	**110**	**0.08**	**8,094**	**5.52**	**68,734**	**46.91**	**76,938**	**52.51**
Aklan	**11,162**	23	**0.21**	690	**6.18**	6,146	**55.06**	**6,859**	**61.45**
Antique	**12,816**	6	**0.05**	516	**4.03**	5,334	**41.62**	**5,856**	**45.69**
Capiz	**13,986**	3	**0.02**	486	**3.47**	4,991	**35.69**	**5,480**	**39.18**
Guimaras	**3,085**	3	**0.10**	212	**6.87**	2,116	**68.59**	**2,331**	**75.56**
Iloilo	**36,541**	21	**0.06**	1,668	**4.56**	17,789	**48.68**	**19,478**	**53.30**
Negros Occidental	**49,368**	40	**0.08**	3,625	**7.34**	25,814	**52.29**	**29,479**	**59.71**
Bacolod City	**11,115**	8	**0.07**	395	**3.55**	2,660	**23.93**	**3,063**	**27.56**
Iloilo City	**8,453**	6	**0.07**	502	**5.94**	3,884	**45.95**	**4,392**	**51.96**

Table 1.B.1.5 - Prenatal Care

No. of Pregnant women who Completed Iron with Folic Acid
Philippines, Annual 2020

Area	Eligible Pop.	Iron with Folic Acid Supplementation							
		Age Group						Total	%
		10-14 yrs old		15-19 yrs old		20-49 yrs old			
		No.	%	No.	%	No.	%		
**Region 7**	**163,262**	**89**	**0.05**	**8,276**	**5.07**	**80,935**	**49.57**	**89,300**	**54.70**
Bohol	**27,312**	17	**0.06**	1,108	**4.06**	12,679	**46.42**	**13,804**	**50.54**
Cebu	**67,506**	25	**0.04**	3,110	**4.61**	31,229	**46.26**	**34,364**	**50.91**
Negros Oriental	**27,938**	20	**0.07**	1,776	**6.36**	11,842	**42.39**	**13,638**	**48.82**
Siquijor	**1,613**	1	**0.06**	108	**6.70**	946	**58.65**	**1,055**	**65.41**
Cebu City	**21,193**	24	**0.11**	1,553	**7.33**	9,850	**46.48**	**11,427**	**53.92**
Lapu-Lapu City	**9,372**	2	**0.02**	621	**6.63**	7,807	**83.30**	**8,430**	**89.95**
Mandaue City	**8,328**	0	**0.00**	0	**0.00**	6,582	**79.03**	**6,582**	**79.03**
**Region 8**	**102,619**	**48**	**0.05**	**5,115**	**4.98**	**45,931**	**44.76**	**51,094**	**49.79**
Biliran	**3,834**	0	**0.00**	256	**6.68**	1,795	**46.82**	**2,051**	**53.50**
Eastern Samar	**11,392**	2	**0.02**	604	**5.30**	5,064	**44.45**	**5,670**	**49.77**
Northern Leyte	**34,707**	19	**0.05**	1,635	**4.71**	17,124	**49.34**	**18,778**	**54.10**
Northern Samar	**15,370**	8	**0.05**	552	**3.59**	5,998	**39.02**	**6,558**	**42.67**
Southern Leyte	**6,451**	6	**0.09**	418	**6.48**	3,069	**47.57**	**3,493**	**54.15**
Western Samar	**14,305**	8	**0.06**	843	**5.89**	6,061	**42.37**	**6,912**	**48.32**
Calbayog City	**4,413**	1	**0.02**	114	**2.58**	787	**17.83**	**902**	**20.44**
Maasin City	**1,637**	0	**0.00**	33	**2.02**	770	**47.04**	**803**	**49.05**
Ormoc City	**4,941**	4	**0.08**	364	**7.37**	2,925	**59.20**	**3,293**	**66.65**
Tacloban City	**5,569**	0	**0.00**	296	**5.32**	2,338	**41.98**	**2,634**	**47.30**
**Region 9**	**80,051**	**56**	**0.07**	**5,380**	**6.72**	**41,541**	**51.89**	**46,977**	**58.68**
Zamboanga del Norte	**17,249**	16	**0.09**	1,922	**11.14**	14,258	**82.66**	**16,196**	**93.90**
Zamboanga del Sur	**17,653**	13	**0.07**	656	**3.72**	4,217	**23.89**	**4,886**	**27.68**
Zamboanga Sibugay	**14,954**	11	**0.07**	799	**5.34**	5,410	**36.18**	**6,220**	**41.59**
Dapitan City	**1,784**	2	**0.11**	115	**6.45**	1,083	**60.71**	**1,200**	**67.26**
Dipolog City	**2,827**	0	**0.00**	343	**12.13**	2,239	**79.20**	**2,582**	**91.33**
Isabela City	**2,522**	4	**0.16**	286	**11.34**	1,614	**64.00**	**1,904**	**75.50**
Pagadian City	**4,325**	0	**0.00**	277	**6.40**	1,975	**45.66**	**2,252**	**52.07**
Zamboanga City	**18,737**	10	**0.05**	982	**5.24**	10,745	**57.35**	**11,737**	**62.64**
**Region 10**	**101,411**	**198**	**0.20**	**10,312**	**10.17**	**59,751**	**58.92**	**70,261**	**69.28**
Bukidnon	**23,706**	64	**0.27**	3,136	**13.23**	13,143	**55.44**	**16,343**	**68.94**
Camiguin	**1,858**	1	**0.05**	70	**3.77**	840	**45.21**	**911**	**49.03**
Lanao del Norte	**14,960**	7	**0.05**	672	**4.49**	8,108	**54.20**	**8,787**	**58.74**
Misamis Occidental	**6,403**	19	**0.30**	449	**7.01**	4,525	**70.67**	**4,993**	**77.98**
Misamis Oriental	**15,131**	13	**0.09**	1,339	**8.85**	9,243	**61.09**	**10,595**	**70.02**
Cagayan de Oro City	**14,339**	18	**0.13**	1,854	**12.93**	9,905	**69.08**	**11,777**	**82.13**
El Salvador City	**1,065**	4	**0.38**	103	**9.67**	532	**49.95**	**639**	**60.00**
Gingoog City	**2,644**	1	**0.04**	351	**13.28**	1,676	**63.39**	**2,028**	**76.70**
Iligan City	**7,580**	63	**0.83**	1,194	**15.75**	4,348	**57.36**	**5,605**	**73.94**
Malaybalay City	**3,956**	3	**0.08**	286	**7.23**	1,637	**41.38**	**1,926**	**48.69**

Table 1.B.1.5 - Prenatal Care

No. of Pregnant women who Completed Iron with Folic Acid
Philippines, Annual 2020

Area	Eligible Pop.	Iron with Folic Acid Supplementation							
		Age Group						Total	%
		10-14 yrs old		15-19 yrs old		20-49 yrs old			
		No.	%	No.	%	No.	%		
Oroquieta City	**1,389**	0	**0.00**	70	**5.04**	652	**46.94**	**722**	**51.98**
Ozamis City	**2,778**	3	**0.11**	243	**8.75**	1,349	**48.56**	**1,595**	**57.42**
Tangub City	**1,234**	0	**0.00**	78	**6.32**	1,001	**81.12**	**1,079**	**87.44**
Valencia City	**4,368**	2	**0.05**	467	**10.69**	2,792	**63.92**	**3,261**	**74.66**
**Region 11**	**107,247**	**659**	**0.61**	**10,136**	**9.45**	**60,742**	**56.64**	**71,537**	**66.70**
Compostela Valley	**15,562**	77	**0.49**	1,767	**11.35**	9,037	**58.07**	**10,881**	**69.92**
Davao del Norte	**21,326**	106	**0.50**	2,328	**10.92**	13,915	**65.25**	**16,349**	**76.66**
Davao Oriental	**13,007**	120	**0.92**	1,153	**8.86**	6,375	**49.01**	**7,648**	**58.80**
Davao del Sur	**14,151**	74	**0.52**	1,125	**7.95**	5,910	**41.76**	**7,109**	**50.24**
Davao Occidental	**6,670**	26	**0.39**	665	**9.97**	2,073	**31.08**	**2,764**	**41.44**
Davao City	**36,531**	256	**0.70**	3,098	**8.48**	23,432	**64.14**	**26,786**	**73.32**
**Region 12**	**104,552**	**111**	**0.11**	**9,467**	**9.05**	**56,051**	**53.61**	**65,629**	**62.77**
North Cotabato	**33,645**	37	**0.11**	2,732	**8.12**	16,264	**48.34**	**19,033**	**56.57**
Sarangani	**12,891**	36	**0.28**	2,046	**15.87**	7,731	**59.97**	**9,813**	**76.12**
South Cotabato	**21,113**	22	**0.10**	2,346	**11.11**	13,718	**64.97**	**16,086**	**76.19**
Sultan Kudarat	**17,359**	9	**0.05**	1,222	**7.04**	9,231	**53.18**	**10,462**	**60.27**
Cotabato City	**5,835**	1	**0.02**	170	**2.91**	3,195	**54.76**	**3,366**	**57.69**
Gen. Santos City	**13,709**	6	**0.04**	951	**6.94**	5,912	**43.12**	**6,869**	**50.11**
**BARMM**	**92,799**	**21**	**0.02**	**4,557**	**4.91**	**42,509**	**45.81**	**47,087**	**50.74**
Basilan	**7,541**	0	**0.00**	135	**1.79**	1,124	**14.91**	**1,259**	**16.70**
Lanao del Sur	**21,131**	6	**0.03**	676	**3.20**	15,632	**73.98**	**16,314**	**77.20**
Maguindanao	**31,128**	15	**0.05**	1,710	**5.49**	14,252	**45.79**	**15,977**	**51.33**
Sulu	**16,613**	0	**0.00**	1,229	**7.40**	4,838	**29.12**	**6,067**	**36.52**
Tawi-Tawi	**9,259**	0	**0.00**	0	**0.00**	4,103	**44.31**	**4,103**	**44.31**
Lamitan City	**2,074**	0	**0.00**	157	**7.57**	519	**25.02**	**676**	**32.59**
Marawi City	**5,053**	0	**0.00**	650	**12.86**	2,041	**40.39**	**2,691**	**53.26**
**CARAGA**	**60,029**	**64**	**0.11**	**4,005**	**6.67**	**27,730**	**46.19**	**31,799**	**52.97**
Agusan del Norte	**8,098**	9	**0.11**	649	**8.01**	3,752	**46.33**	**4,410**	**54.46**
Agusan del Sur	**17,592**	15	**0.09**	1,122	**6.38**	6,979	**39.67**	**8,116**	**46.13**
Surigao del Norte	**7,089**	13	**0.18**	425	**6.00**	3,542	**49.96**	**3,980**	**56.14**
Surigao del Sur	**11,482**	8	**0.07**	572	**4.98**	4,613	**40.18**	**5,193**	**45.23**
Province of Dinagat	**2,573**	4	**0.16**	115	**4.47**	1,039	**40.38**	**1,158**	**45.01**
Bislig City	**2,179**	5	**0.23**	155	**7.11**	1,304	**59.84**	**1,464**	**67.19**
Butuan City	**7,715**	6	**0.08**	726	**9.41**	4,609	**59.74**	**5,341**	**69.23**
Surigao City	**3,301**	4	**0.12**	241	**7.30**	1,892	**57.32**	**2,137**	**64.74**

Note: Put asterisk (*) for No Report and Zero (0) for No Case

Table 1.B.1.6 - Prenatal Care

No. of Pregnant women who Completed Calcium Carbonate
Philippines, Annual 2020

Area	Eligible Pop.	Calcium carbonate							
		Age Group						Total	%
		10-14 yrs old		15-19 yrs old		20-49 yrs old			
		No.	%	No.	%	No.	%		
PHILIPPINES	2,123,158	2,232	0.11	99,129	4.67	718,913	33.86	820,274	38.63
N C R	238,661	170	0.07	9,129	3.83	66,193	27.74	75,492	31.63
Malabon	6,775	6	0.09	473	6.98	2,649	39.10	3,128	46.17
Navotas	4,621	10	0.22	553	11.97	2,928	63.36	3,491	75.55
Valenzuela City	11,500	0	0.00	64	0.56	441	3.83	505	4.39
Caloocan City	29,363	44	0.15	2,354	8.02	17,440	59.39	19,838	67.56
Marikina City	8,354	4	0.05	267	3.20	1,877	22.47	2,148	25.71
Pasig City	13,996	13	0.09	489	3.49	5,402	38.60	5,904	42.18
Pateros	1,184	0	0.00	58	4.90	346	29.22	404	34.12
Taguig	14,918	57	0.38	2,055	13.78	11,367	76.20	13,479	90.35
Quezon City	54,413	5	0.01	684	1.26	6,569	12.07	7,258	13.34
Makati City	10,798	6	0.06	197	1.82	3,703	34.29	3,906	36.17
Mandaluyong City	7,160	3	0.04	69	0.96	636	8.88	708	9.89
San Juan	2,261	0	0.00	76	3.36	723	31.98	799	35.34
Manila City	32,980	9	0.03	450	1.36	2,528	7.67	2,987	9.06
Las Piñas City	10,914	1	0.01	234	2.14	1,903	17.44	2,138	19.59
Muntinlupa City	9,353	0	0.00	0	0.00	0	0.00	0	0.00
Parañaque City	12,343	8	0.06	542	4.39	2,172	17.60	2,722	22.05
Pasay City	7,728	4	0.05	564	7.30	5,509	71.29	6,077	78.64
C A R	35,099	12	0.03	1,510	4.30	14,274	40.67	15,796	45.00
Abra	4,309	0	0.00	217	5.04	1,712	39.73	1,929	44.77
Apayao	2,451	0	0.00	186	7.59	942	38.43	1,128	46.02
Benguet	9,188	2	0.02	291	3.17	3,677	40.02	3,970	43.21
Ifugao	4,396	1	0.02	170	3.87	1,813	41.24	1,984	45.13
Kalinga	4,617	4	0.09	499	10.81	4,042	87.55	4,545	98.44
Mt. Province	3,023	1	0.03	48	1.59	525	17.37	574	18.99
Baguio City	7,115	4	0.06	99	1.39	1,563	21.97	1,666	23.42
Region 1	97,261	64	0.07	5,765	5.93	54,537	56.07	60,366	62.07
Ilocos Norte	8,105	11	0.14	352	4.34	4,566	56.34	4,929	60.81
Ilocos Sur	9,330	2	0.02	472	5.06	5,881	63.03	6,355	68.11
La Union	11,511	10	0.09	762	6.62	6,568	57.06	7,340	63.77
Pangasinan	50,168	31	0.06	3,156	6.29	27,151	54.12	30,338	60.47
Alaminos City	1,894	0	0.00	148	7.81	1,701	89.81	1,849	97.62
Candon City	987	0	0.00	8	0.81	962	97.47	970	98.28
Dagupan City	3,620	1	0.03	255	7.04	1,387	38.31	1,643	45.39
Laoag City	1,870	0	0.00	64	3.42	1,725	92.25	1,789	95.67
San Carlos City	3,979	3	0.08	116	2.92	1,551	38.98	1,670	41.97
San Fernando City	2,115	2	0.09	118	5.58	1,250	59.10	1,370	64.78
Urdaneta City	2,806	4	0.14	272	9.69	1,462	52.10	1,738	61.94

Table 1.B.1.6 - Prenatal Care

No. of Pregnant women who Completed Calcium Carbonate
Philippines, Annual 2020

Area	Eligible Pop.	Calcium carbonate							
		Age Group						Total	%
		10-14 yrs old		15-19 yrs old		20-49 yrs old			
		No.	%	No.	%	No.	%		
Vigan City	876	0	0.00	42	4.79	333	38.01	375	42.81
**Region 2**	69,443	50	0.07	4,367	6.29	30,221	43.52	34,638	49.88
Batanes	340	0	0.00	0	0.00	19	5.59	19	5.59
Cagayan	17,971	5	0.03	729	4.06	5,267	29.31	6,001	33.39
Isabela	26,309	21	0.08	1,511	5.74	10,544	40.08	12,076	45.90
Nueva Vizcaya	9,573	11	0.11	884	9.23	5,698	59.52	6,593	68.87
Quirino	4,025	2	0.05	351	8.72	2,811	69.84	3,164	78.61
Cauayan City	2,590	1	0.04	196	7.57	1,257	48.53	1,454	56.14
Ilagan City	2,911	3	0.10	289	9.93	1,320	45.35	1,612	55.38
Santiago City	2,692	4	0.15	309	11.48	2,347	87.18	2,660	98.81
Tuguegarao City	3,032	3	0.10	98	3.23	958	31.60	1,059	34.93
**Region 3**	220,020	219	0.10	14,330	6.51	92,525	42.05	107,074	48.67
Aurora	4,770	2	0.04	307	6.44	2,085	43.71	2,394	50.19
Bataan	13,823	9	0.07	725	5.24	4,471	32.34	5,205	37.65
Bulacan	43,627	46	0.11	2,901	6.65	18,573	42.57	21,520	49.33
Nueva Ecija	28,782	49	0.17	1,949	6.77	11,804	41.01	13,802	47.95
Pampanga	31,359	15	0.05	1,329	4.24	9,783	31.20	11,127	35.48
Tarlac	19,765	25	0.13	1,148	5.81	8,969	45.38	10,142	51.31
Zambales	12,610	4	0.03	361	2.86	2,515	19.94	2,880	22.84
Angeles City	7,869	7	0.09	697	8.86	4,156	52.81	4,860	61.76
Balanga City	1,998	1	0.05	84	4.20	546	27.33	631	31.58
Cabanatuan City	5,896	3	0.05	383	6.50	2,096	35.55	2,482	42.10
City of San Fernando	5,863	13	0.22	474	8.08	2,683	45.76	3,170	54.07
Gapan City	2,153	2	0.09	175	8.13	939	43.61	1,116	51.83
Mabalacat City	4,793	2	0.04	367	7.66	2,214	46.19	2,583	53.89
Malolos City	4,880	3	0.06	201	4.12	1,471	30.14	1,675	34.32
Meycauayan	4,041	21	0.52	454	11.23	2,333	57.73	2,808	69.49
Olongapo	4,971	1	0.02	280	5.63	1,317	26.49	1,598	32.15
Palayan City	799	0	0.00	66	8.26	306	38.30	372	46.56
San Jose City	2,718	2	0.07	301	11.07	1,505	55.37	1,808	66.52
San Jose del Monte City	11,100	10	0.09	1,072	9.66	9,046	81.50	10,128	91.24
Science City of Munoz	1,586	0	0.00	97	6.12	621	39.16	718	45.27
Tarlac City	6,617	4	0.06	959	14.49	5,092	76.95	6,055	91.51
**Region 4A**	296,816	86	0.03	5,196	1.75	40,658	13.70	45,940	15.48
Batangas	38,441	3	0.01	183	0.48	2,157	5.61	2,343	6.10
Cavite	27,704	2	0.01	338	1.22	2,828	10.21	3,168	11.44
Laguna	19,727	26	0.13	499	2.53	5,488	27.82	6,013	30.48
Quezon	37,410	4	0.01	278	0.74	2,165	5.79	2,447	6.54
Rizal	44,791	14	0.03	969	2.16	6,154	13.74	7,137	15.93
Antipolo City	16,497	5	0.03	684	4.15	4,154	25.18	4,843	29.36
Bacoor City	12,142	0	0.00	6	0.05	48	0.40	54	0.44
Batangas City	6,823	0	0.00	0	0.00	0	0.00	0	0.00

Table 1.B.1.6 - Prenatal Care

No. of Pregnant women who Completed Calcium Carbonate
Philippines, Annual 2020

Area	Eligible Pop.	Calcium carbonate							
		Age Group						Total	%
		10-14 yrs old		15-19 yrs old		20-49 yrs old			
		No.	%	No.	%	No.	%		
Biñan City	**6,607**	4	**0.06**	715	**10.82**	3,638	**55.06**	**4,357**	**65.95**
Cabuyao City	**6,130**	3	**0.05**	338	**5.51**	2,141	**34.93**	**2,482**	**40.49**
Calamba City	**9,028**	8	**0.09**	184	**2.04**	2,512	**27.82**	**2,704**	**29.95**
Cavite City	**2,075**	0	**0.00**	0	**0.00**	0	**0.00**	**0**	**0.00**
Dasmariñas City	**13,322**	5	**0.04**	285	**2.14**	2,637	**19.79**	**2,927**	**21.97**
General Trias City	**6,352**	0	**0.00**	25	**0.39**	260	**4.09**	**285**	**4.49**
Imus City	**8,156**	0	**0.00**	0	**0.00**	0	**0.00**	**0**	**0.00**
Lipa City	**6,880**	0	**0.00**	0	**0.00**	0	**0.00**	**0**	**0.00**
Lucena City	**5,672**	0	**0.00**	0	**0.00**	0	**0.00**	**0**	**0.00**
San Pablo City	**5,277**	3	**0.06**	17	**0.32**	167	**3.16**	**187**	**3.54**
San Pedro City	**6,467**	0	**0.00**	56	**0.87**	526	**8.13**	**582**	**9.00**
Santa Rosa City	**7,025**	6	**0.09**	470	**6.69**	3,516	**50.05**	**3,992**	**56.83**
Tagaytay City	**1,437**	0	**0.00**	40	**2.78**	1,316	**91.58**	**1,356**	**94.36**
Tanauan City	**3,584**	3	**0.08**	23	**0.64**	413	**11.52**	**439**	**12.25**
Tayabas City	**2,122**	0	**0.00**	0	**0.00**	0	**0.00**	**0**	**0.00**
Trece Martires City	**3,147**	0	**0.00**	86	**2.73**	538	**17.10**	**624**	**19.83**
**Region 4B**	**71,246**	**141**	**0.20**	**1,811**	**2.54**	**13,360**	**18.75**	**15,312**	**21.49**
Marinduque	**4,836**	1	**0.02**	181	**3.74**	2,156	**44.58**	**2,338**	**48.35**
Mindoro Occidental	**12,407**	102	**0.82**	696	**5.61**	3,760	**30.31**	**4,558**	**36.74**
Mindoro Oriental	**19,809**	35	**0.18**	664	**3.35**	4,840	**24.43**	**5,539**	**27.96**
Palawan	**21,375**	2	**0.01**	223	**1.04**	896	**4.19**	**1,121**	**5.24**
Romblon	**6,400**	1	**0.02**	35	**0.55**	476	**7.44**	**512**	**8.00**
Puerto Princesa City	**6,419**	0	**0.00**	12	**0.19**	1,232	**19.19**	**1,244**	**19.38**
**Region 5**	**136,116**	**41**	**0.03**	**5,703**	**4.19**	**55,740**	**40.95**	**61,484**	**45.17**
Albay	**23,737**	1	**0.00**	912	**3.84**	11,578	**48.78**	**12,491**	**52.62**
Camarines Norte	**14,283**	5	**0.04**	781	**5.47**	5,949	**41.65**	**6,735**	**47.15**
Camarines Sur	**38,796**	11	**0.03**	1,319	**3.40**	14,373	**37.05**	**15,703**	**40.48**
Catanduanes	**6,274**	2	**0.03**	177	**2.82**	1,569	**25.01**	**1,748**	**27.86**
Masbate	**22,521**	9	**0.04**	1,191	**5.29**	8,275	**36.74**	**9,475**	**42.07**
Sorsogon	**19,065**	8	**0.04**	719	**3.77**	8,195	**42.98**	**8,922**	**46.80**
Iriga City	**2,638**	2	**0.08**	107	**4.06**	1,170	**44.35**	**1,279**	**48.48**
Legaspi City	**4,174**	1	**0.02**	210	**5.03**	2,749	**65.86**	**2,960**	**70.92**
Naga City	**4,628**	2	**0.04**	287	**6.20**	1,882	**40.67**	**2,171**	**46.91**
**Region 6**	**146,526**	**98**	**0.07**	**6,485**	**4.43**	**56,297**	**38.42**	**62,880**	**42.91**
Aklan	**11,162**	20	**0.18**	664	**5.95**	5,682	**50.90**	**6,366**	**57.03**
Antique	**12,816**	4	**0.03**	312	**2.43**	3,189	**24.88**	**3,505**	**27.35**
Capiz	**13,986**	3	**0.02**	418	**2.99**	4,228	**30.23**	**4,649**	**33.24**
Guimaras	**3,085**	3	**0.10**	200	**6.48**	2,036	**66.00**	**2,239**	**72.58**
Iloilo	**36,541**	25	**0.07**	1,457	**3.99**	15,702	**42.97**	**17,184**	**47.03**
Negros Occidental	**49,368**	32	**0.06**	2,708	**5.49**	20,290	**41.10**	**23,030**	**46.65**
Bacolod City	**11,115**	3	**0.03**	319	**2.87**	1,887	**16.98**	**2,209**	**19.87**
Iloilo City	**8,453**	8	**0.09**	407	**4.81**	3,283	**38.84**	**3,698**	**43.75**

Table 1.B.1.6 - Prenatal Care

No. of Pregnant women who Completed Calcium Carbonate
Philippines, Annual 2020

Area	Eligible Pop.	Calcium carbonate							
		Age Group						Total	%
		10-14 yrs old		15-19 yrs old		20-49 yrs old			
		No.	%	No.	%	No.	%		
**Region 7**	**163,262**	**37**	**0.02**	**4,763**	**2.92**	**43,081**	**26.39**	**47,881**	**29.33**
Bohol	**27,312**	0	**0.00**	467	**1.71**	4,989	**18.27**	**5,456**	**19.98**
Cebu	**67,506**	6	**0.01**	1,313	**1.95**	13,431	**19.90**	**14,750**	**21.85**
Negros Oriental	**27,938**	9	**0.03**	886	**3.17**	6,183	**22.13**	**7,078**	**25.33**
Siquijor	**1,613**	0	**0.00**	74	**4.59**	557	**34.53**	**631**	**39.12**
Cebu City	**21,193**	18	**0.08**	1,420	**6.70**	10,979	**51.80**	**12,417**	**58.59**
Lapu-Lapu City	**9,372**	4	**0.04**	603	**6.43**	6,942	**74.07**	**7,549**	**80.55**
Mandaue City	**8,328**	0	**0.00**	0	**0.00**	0	**0.00**	**0**	**0.00**
**Region 8**	**102,619**	**61**	**0.06**	**3,115**	**3.04**	**25,501**	**24.85**	**28,677**	**27.95**
Biliran	**3,834**	2	**0.05**	251	**6.55**	1,929	**50.31**	**2,182**	**56.91**
Eastern Samar	**11,392**	9	**0.08**	458	**4.02**	3,574	**31.37**	**4,041**	**35.47**
Northern Leyte	**34,707**	0	**0.00**	0	**0.00**	0	**0.00**	**0**	**0.00**
Northern Samar	**15,370**	22	**0.14**	464	**3.02**	5,335	**34.71**	**5,821**	**37.87**
Southern Leyte	**6,451**	9	**0.14**	411	**6.37**	3,001	**46.52**	**3,421**	**53.03**
Western Samar	**14,305**	8	**0.06**	757	**5.29**	5,337	**37.31**	**6,102**	**42.66**
Calbayog City	**4,413**	1	**0.02**	142	**3.22**	1,354	**30.68**	**1,497**	**33.92**
Maasin City	**1,637**	3	**0.18**	61	**3.73**	419	**25.60**	**483**	**29.51**
Ormoc City	**4,941**	4	**0.08**	263	**5.32**	2,156	**43.63**	**2,423**	**49.04**
Tacloban City	**5,569**	3	**0.05**	308	**5.53**	2,396	**43.02**	**2,707**	**48.61**
**Region 9**	**80,051**	**41**	**0.05**	**4,223**	**5.28**	**30,321**	**37.88**	**34,585**	**43.20**
Zamboanga del Norte	**17,249**	12	**0.07**	1,600	**9.28**	11,882	**68.89**	**13,494**	**78.23**
Zamboanga del Sur	**17,653**	9	**0.05**	578	**3.27**	3,544	**20.08**	**4,131**	**23.40**
Zamboanga Sibugay	**14,954**	7	**0.05**	681	**4.55**	4,525	**30.26**	**5,213**	**34.86**
Dapitan City	**1,784**	2	**0.11**	178	**9.98**	1,549	**86.83**	**1,729**	**96.92**
Dipolog City	**2,827**	0	**0.00**	251	**8.88**	1,622	**57.38**	**1,873**	**66.25**
Isabela City	**2,522**	3	**0.12**	160	**6.34**	1,073	**42.55**	**1,236**	**49.01**
Pagadian City	**4,325**	0	**0.00**	191	**4.42**	1,768	**40.88**	**1,959**	**45.29**
Zamboanga City	**18,737**	8	**0.04**	584	**3.12**	4,358	**23.26**	**4,950**	**26.42**
**Region 10**	**101,411**	**106**	**0.10**	**7,679**	**7.57**	**47,971**	**47.30**	**55,756**	**54.98**
Bukidnon	**23,706**	47	**0.20**	2,553	**10.77**	11,006	**46.43**	**13,606**	**57.39**
Camiguin	**1,858**	1	**0.05**	61	**3.28**	661	**35.58**	**723**	**38.91**
Lanao del Norte	**14,960**	4	**0.03**	540	**3.61**	6,314	**42.21**	**6,858**	**45.84**
Misamis Occidental	**6,403**	13	**0.20**	350	**5.47**	3,630	**56.69**	**3,993**	**62.36**
Misamis Oriental	**15,131**	12	**0.08**	1,180	**7.80**	7,800	**51.55**	**8,992**	**59.43**
Cagayan de Oro City	**14,339**	18	**0.13**	1,469	**10.24**	8,489	**59.20**	**9,976**	**69.57**
El Salvador City	**1,065**	1	**0.09**	95	**8.92**	602	**56.53**	**698**	**65.54**
Gingoog City	**2,644**	0	**0.00**	218	**8.25**	1,399	**52.91**	**1,617**	**61.16**
Iligan City	**7,580**	3	**0.04**	543	**7.16**	3,223	**42.52**	**3,769**	**49.72**
Malaybalay City	**3,956**	3	**0.08**	153	**3.87**	926	**23.41**	**1,082**	**27.35**

Table 1.B.1.6 - Prenatal Care

No. of Pregnant women who Completed Calcium Carbonate
Philippines, Annual 2020

Area	Eligible Pop.	Calcium carbonate							
		Age Group						Total	%
		10-14 yrs old		15-19 yrs old		20-49 yrs old			
		No.	%	No.	%	No.	%		
Oroquieta City	**1,389**	0	**0.00**	67	**4.82**	650	**46.80**	**717**	**51.62**
Ozamis City	**2,778**	3	**0.11**	270	**9.72**	1,554	**55.94**	**1,827**	**65.77**
Tangub City	**1,234**	0	**0.00**	79	**6.40**	979	**79.34**	**1,058**	**85.74**
Valencia City	**4,368**	1	**0.02**	101	**2.31**	738	**16.90**	**840**	**19.23**
**Region 11**	**107,247**	**298**	**0.28**	**9,079**	**8.47**	**56,974**	**53.12**	**66,351**	**61.87**
Compostela Valley	**15,562**	63	**0.40**	1,694	**10.89**	8,689	**55.83**	**10,446**	**67.13**
Davao del Norte	**21,326**	76	**0.36**	2,301	**10.79**	13,778	**64.61**	**16,155**	**75.75**
Davao Oriental	**13,007**	32	**0.25**	951	**7.31**	5,544	**42.62**	**6,527**	**50.18**
Davao del Sur	**14,151**	41	**0.29**	1,010	**7.14**	5,419	**38.29**	**6,470**	**45.72**
Davao Occidental	**6,670**	17	**0.25**	385	**5.77**	1,195	**17.92**	**1,597**	**23.94**
Davao City	**36,531**	69	**0.19**	2,738	**7.50**	22,349	**61.18**	**25,156**	**68.86**
**Region 12**	**104,552**	**96**	**0.09**	**7,034**	**6.73**	**37,998**	**36.34**	**45,128**	**43.16**
North Cotabato	**33,645**	26	**0.08**	2,232	**6.63**	13,203	**39.24**	**15,461**	**45.95**
Sarangani	**12,891**	30	**0.23**	1,248	**9.68**	4,489	**34.82**	**5,767**	**44.74**
South Cotabato	**21,113**	7	**0.03**	1,249	**5.92**	7,263	**34.40**	**8,519**	**40.35**
Sultan Kudarat	**17,359**	23	**0.13**	1,525	**8.79**	8,483	**48.87**	**10,031**	**57.79**
Cotabato City	**5,835**	0	**0.00**	27	**0.46**	315	**5.40**	**342**	**5.86**
Gen. Santos City	**13,709**	10	**0.07**	753	**5.49**	4,245	**30.97**	**5,008**	**36.53**
**BARMM**	**92,799**	**682**	**0.73**	**5,966**	**6.43**	**31,904**	**34.38**	**38,552**	**41.54**
Basilan	**7,541**	0	**0.00**	53	**0.70**	202	**2.68**	**255**	**3.38**
Lanao del Sur	**21,131**	9	**0.04**	818	**3.87**	16,959	**80.26**	**17,786**	**84.17**
Maguindanao	**31,128**	414	**1.33**	4,201	**13.50**	12,446	**39.98**	**17,061**	**54.81**
Sulu	**16,613**	0	**0.00**	0	**0.00**	0	**0.00**	**0**	**0.00**
Tawi-Tawi	**9,259**	0	**0.00**	0	**0.00**	0	**0.00**	**0**	**0.00**
Lamitan City	**2,074**	0	**0.00**	110	**5.30**	400	**19.29**	**510**	**24.59**
Marawi City	**5,053**	259	**5.13**	784	**15.52**	1,897	**37.54**	**2,940**	**58.18**
**CARAGA**	**60,029**	**30**	**0.05**	**2,974**	**4.95**	**21,358**	**35.58**	**24,362**	**40.58**
Agusan del Norte	**8,098**	1	**0.01**	180	**2.22**	876	**10.82**	**1,057**	**13.05**
Agusan del Sur	**17,592**	8	**0.05**	843	**4.79**	5,234	**29.75**	**6,085**	**34.59**
Surigao del Norte	**7,089**	2	**0.03**	320	**4.51**	2,849	**40.19**	**3,171**	**44.73**
Surigao del Sur	**11,482**	9	**0.08**	572	**4.98**	4,641	**40.42**	**5,222**	**45.48**
Province of Dinagat	**2,573**	0	**0.00**	114	**4.43**	907	**35.25**	**1,021**	**39.68**
Bislig City	**2,179**	6	**0.28**	185	**8.49**	1,415	**64.94**	**1,606**	**73.70**
Butuan City	**7,715**	2	**0.03**	553	**7.17**	3,813	**49.42**	**4,368**	**56.62**
Surigao City	**3,301**	2	**0.06**	207	**6.27**	1,623	**49.17**	**1,832**	**55.50**

Note: Put asterisk (*) for No Report and Zero (0) for No Case

Table 1.B.1.7 - Prenatal Care

No. of Pregnant women who Completed Iodine
Philippines, Annual 2020

Area	Eligible Pop.	Iodine Capsule							
		Age Group						Total	%
		10-14 yrs old		15-19 yrs old		20-49 yrs old			
		No.	%	No.	%	No.	%		
**PHILIPPINES**	**2,123,158**	**256**	**0.01**	**11,391**	**0.54**	**58,269**	**2.74**	**69,916**	**3.29**
**N C R**	**238,661**	**47**	**0.02**	**256**	**0.11**	**1,868**	**0.78**	**2,171**	**0.91**
Malabon	**6,775**	0	**0.00**	1	**0.01**	14	**0.21**	15	**0.22**
Navotas	**4,621**	2	**0.04**	40	**0.87**	132	**2.86**	174	**3.77**
Valenzuela City	**11,500**	0	**0.00**	0	**0.00**	0	**0.00**	0	**0.00**
Caloocan City	**29,363**	0	**0.00**	0	**0.00**	0	**0.00**	0	**0.00**
Marikina City	**8,354**	0	**0.00**	0	**0.00**	0	**0.00**	0	**0.00**
Pasig City	**13,996**	0	**0.00**	0	**0.00**	0	**0.00**	0	**0.00**
Pateros	**1,184**	0	**0.00**	0	**0.00**	0	**0.00**	0	**0.00**
Taguig	**14,918**	37	**0.25**	30	**0.20**	420	**2.82**	487	**3.26**
Quezon City	**54,413**	0	**0.00**	0	**0.00**	0	**0.00**	0	**0.00**
Makati City	**10,798**	8	**0.07**	88	**0.81**	817	**7.57**	913	**8.46**
Mandaluyong City	**7,160**	0	**0.00**	19	**0.27**	85	**1.19**	104	**1.45**
San Juan	**2,261**	0	**0.00**	0	**0.00**	0	**0.00**	0	**0.00**
Manila City	**32,980**	0	**0.00**	0	**0.00**	0	**0.00**	0	**0.00**
Las Piñas City	**10,914**	0	**0.00**	9	**0.08**	154	**1.41**	163	**1.49**
Muntinlupa City	**9,353**	0	**0.00**	0	**0.00**	0	**0.00**	0	**0.00**
Parañaque City	**12,343**	0	**0.00**	58	**0.47**	142	**1.15**	200	**1.62**
Pasay City	**7,728**	0	**0.00**	11	**0.14**	104	**1.35**	115	**1.49**
**C A R**	**35,099**	**6**	**0.02**	**698**	**1.99**	**7,051**	**20.09**	**7,755**	**22.09**
Abra	**4,309**	0	**0.00**	144	**3.34**	814	**18.89**	958	**22.23**
Apayao	**2,451**	0	**0.00**	63	**2.57**	369	**15.06**	432	**17.63**
Benguet	**9,188**	3	**0.03**	281	**3.06**	3,453	**37.58**	3,737	**40.67**
Ifugao	**4,396**	0	**0.00**	54	**1.23**	789	**17.95**	843	**19.18**
Kalinga	**4,617**	1	**0.02**	90	**1.95**	792	**17.15**	883	**19.12**
Mt. Province	**3,023**	1	**0.03**	35	**1.16**	325	**10.75**	361	**11.94**
Baguio City	**7,115**	1	**0.01**	31	**0.44**	509	**7.15**	541	**7.60**
**Region 1**	**97,261**	**4**	**0.00**	**626**	**0.64**	**5,816**	**5.98**	**6,446**	**6.63**
Ilocos Norte	**8,105**	1	**0.01**	29	**0.36**	548	**6.76**	578	**7.13**
Ilocos Sur	**9,330**	1	**0.01**	38	**0.41**	435	**4.66**	474	**5.08**
La Union	**11,511**	2	**0.02**	163	**1.42**	1,168	**10.15**	1,333	**11.58**
Pangasinan	**50,168**	0	**0.00**	350	**0.70**	2,386	**4.76**	2,736	**5.45**
Alaminos City	**1,894**	0	**0.00**	7	**0.37**	18	**0.95**	25	**1.32**
Candon City	**987**	0	**0.00**	8	**0.81**	422	**42.76**	430	**43.57**
Dagupan City	**3,620**	0	**0.00**	10	**0.28**	71	**1.96**	81	**2.24**
Laoag City	**1,870**	0	**0.00**	8	**0.43**	613	**32.78**	621	**33.21**
San Carlos City	**3,979**	0	**0.00**	10	**0.25**	54	**1.36**	64	**1.61**
San Fernando City	**2,115**	0	**0.00**	3	**0.14**	101	**4.78**	104	**4.92**
Urdaneta City	**2,806**	0	**0.00**	0	**0.00**	0	**0.00**	0	**0.00**

Table 1.B.1.7 - Prenatal Care

No. of Pregnant women who Completed Iodine
Philippines, Annual 2020

Area	Eligible Pop.	Iodine Capsule							
		Age Group						Total	%
		10-14 yrs old		15-19 yrs old		20-49 yrs old			
		No.	%	No.	%	No.	%		
Vigan City	876	0	0.00	0	0.00	0	0.00	0	0.00
**Region 2**	**69,443**	**0**	**0.00**	**108**	**0.16**	**892**	**1.28**	**1,000**	**1.44**
Batanes	340	0	0.00	0	0.00	0	0.00	0	0.00
Cagayan	17,971	0	0.00	19	0.11	144	0.80	163	0.91
Isabela	26,309	0	0.00	27	0.10	301	1.14	328	1.25
Nueva Vizcaya	9,573	0	0.00	33	0.34	126	1.32	159	1.66
Quirino	4,025	0	0.00	29	0.72	321	7.98	350	8.70
Cauayan City	2,590	0	0.00	0	0.00	0	0.00	0	0.00
Ilagan City	2,911	0	0.00	0	0.00	0	0.00	0	0.00
Santiago City	2,692	0	0.00	0	0.00	0	0.00	0	0.00
Tuguegarao City	3,032	0	0.00	0	0.00	0	0.00	0	0.00
**Region 3**	**220,020**	**10**	**0.00**	**595**	**0.27**	**4,669**	**2.12**	**5,274**	**2.40**
Aurora	4,770	0	0.00	1	0.02	10	0.21	11	0.23
Bataan	13,823	1	0.01	13	0.09	72	0.52	86	0.62
Bulacan	43,627	5	0.01	253	0.58	2,111	4.84	2,369	5.43
Nueva Ecija	28,782	0	0.00	135	0.47	1,158	4.02	1,293	4.49
Pampanga	31,359	1	0.00	42	0.13	286	0.91	329	1.05
Tarlac	19,765	0	0.00	52	0.26	550	2.78	602	3.05
Zambales	12,610	0	0.00	0	0.00	5	0.04	5	0.04
Angeles City	7,869	0	0.00	0	0.00	0	0.00	0	0.00
Balanga City	1,998	0	0.00	0	0.00	0	0.00	0	0.00
Cabanatuan City	5,896	0	0.00	0	0.00	0	0.00	0	0.00
City of San Fernando	5,863	1	0.02	41	0.70	132	2.25	174	2.97
Gapan City	2,153	0	0.00	5	0.23	24	1.11	29	1.35
Mabalacat City	4,793	0	0.00	0	0.00	3	0.06	3	0.06
Malolos City	4,880	0	0.00	0	0.00	0	0.00	0	0.00
Meycauayan	4,041	2	0.05	53	1.31	318	7.87	373	9.23
Olongapo	4,971	0	0.00	0	0.00	0	0.00	0	0.00
Palayan City	799	0	0.00	0	0.00	0	0.00	0	0.00
San Jose City	2,718	0	0.00	0	0.00	0	0.00	0	0.00
San Jose del Monte City	11,100	0	0.00	0	0.00	0	0.00	0	0.00
Science City of Munoz	1,586	0	0.00	0	0.00	0	0.00	0	0.00
Tarlac City	6,617	0	0.00	0	0.00	0	0.00	0	0.00
**Region 4A**	**296,816**	**7**	**0.00**	**2,485**	**0.84**	**1,250**	**0.42**	**3,742**	**1.26**
Batangas	38,441	0	0.00	6	0.02	66	0.17	72	0.19
Cavite	27,704	0	0.00	3	0.01	7	0.03	10	0.04
Laguna	19,727	1	0.01	2,166	10.98	166	0.84	2,333	11.83
Quezon	37,410	3	0.01	8	0.02	90	0.24	101	0.27
Rizal	44,791	1	0.00	31	0.07	281	0.63	313	0.70
Antipolo City	16,497	0	0.00	1	0.01	5	0.03	6	0.04
Bacoor City	12,142	0	0.00	0	0.00	0	0.00	0	0.00
Batangas City	6,823	0	0.00	0	0.00	0	0.00	0	0.00

Table 1.B.1.7 - Prenatal Care

No. of Pregnant women who Completed Iodine

Philippines, Annual 2020

Area	Eligible Pop.	Iodine Capsule							
		Age Group						Total	%
		10-14 yrs old		15-19 yrs old		20-49 yrs old			
		No.	%	No.	%	No.	%		
Biñan City	6,607	1	0.02	139	2.10	287	4.34	427	6.46
Cabuyao City	6,130	0	0.00	2	0.03	28	0.46	30	0.49
Calamba City	9,028	1	0.01	83	0.92	52	0.58	136	1.51
Cavite City	2,075	0	0.00	0	0.00	0	0.00	0	0.00
Dasmariñas City	13,322	0	0.00	19	0.14	96	0.72	115	0.86
General Trias City	6,352	0	0.00	0	0.00	0	0.00	0	0.00
Imus City	8,156	0	0.00	0	0.00	0	0.00	0	0.00
Lipa City	6,880	0	0.00	0	0.00	0	0.00	0	0.00
Lucena City	5,672	0	0.00	0	0.00	0	0.00	0	0.00
San Pablo City	5,277	0	0.00	7	0.13	0	0.00	7	0.13
San Pedro City	6,467	0	0.00	11	0.17	99	1.53	110	1.70
Santa Rosa City	7,025	0	0.00	9	0.13	38	0.54	47	0.67
Tagaytay City	1,437	0	0.00	0	0.00	0	0.00	0	0.00
Tanauan City	3,584	0	0.00	0	0.00	35	0.98	35	0.98
Tayabas City	2,122	0	0.00	0	0.00	0	0.00	0	0.00
Trece Martires City	3,147	0	0.00	0	0.00	0	0.00	0	0.00
**Region 4B**	71,246	9	0.01	51	0.07	259	0.36	319	0.45
Marinduque	4,836	0	0.00	0	0.00	6	0.12	6	0.12
Mindoro Occidental	12,407	2	0.02	11	0.09	67	0.54	80	0.64
Mindoro Oriental	19,809	6	0.03	32	0.16	135	0.68	173	0.87
Palawan	21,375	1	0.00	2	0.01	14	0.07	17	0.08
Romblon	6,400	0	0.00	6	0.09	16	0.25	22	0.34
Puerto Princesa City	6,419	0	0.00	0	0.00	21	0.33	21	0.33
**Region 5**	136,116	9	0.01	158	0.12	1,434	1.05	1,601	1.18
Albay	23,737	0	0.00	11	0.05	390	1.64	401	1.69
Camarines Norte	14,283	7	0.05	17	0.12	83	0.58	107	0.75
Camarines Sur	38,796	1	0.00	12	0.03	217	0.56	230	0.59
Catanduanes	6,274	0	0.00	0	0.00	32	0.51	32	0.51
Masbate	22,521	0	0.00	21	0.09	191	0.85	212	0.94
Sorsogon	19,065	0	0.00	5	0.03	8	0.04	13	0.07
Iriga City	2,638	0	0.00	2	0.08	24	0.91	26	0.99
Legaspi City	4,174	0	0.00	2	0.05	31	0.74	33	0.79
Naga City	4,628	1	0.02	88	1.90	458	9.90	547	11.82
**Region 6**	146,526	7	0.00	429	0.29	3,654	2.49	4,090	2.79
Aklan	11,162	0	0.00	13	0.12	92	0.82	105	0.94
Antique	12,816	0	0.00	61	0.48	603	4.71	664	5.18
Capiz	13,986	0	0.00	15	0.11	137	0.98	152	1.09
Guimaras	3,085	0	0.00	1	0.03	2	0.06	3	0.10
Iloilo	36,541	6	0.02	185	0.51	1,754	4.80	1,945	5.32
Negros Occidental	49,368	1	0.00	112	0.23	680	1.38	793	1.61
Bacolod City	11,115	0	0.00	38	0.34	340	3.06	378	3.40
Iloilo City	8,453	0	0.00	4	0.05	46	0.54	50	0.59

Table 1.B.1.7 - Prenatal Care

No. of Pregnant women who Completed Iodine
Philippines, Annual 2020

Area	Eligible Pop.	Iodine Capsule							
		Age Group						Total	%
		10-14 yrs old		15-19 yrs old		20-49 yrs old			
		No.	%	No.	%	No.	%		
**Region 7**	**163,262**	**0**	**0.00**	**197**	**0.12**	**1,214**	**0.74**	**1,411**	**0.86**
Bohol	**27,312**	0	**0.00**	43	**0.16**	132	**0.48**	175	**0.64**
Cebu	**67,506**	0	**0.00**	34	**0.05**	325	**0.48**	359	**0.53**
Negros Oriental	**27,938**	0	**0.00**	23	**0.08**	102	**0.37**	125	**0.45**
Siquijor	**1,613**	0	**0.00**	0	**0.00**	0	**0.00**	0	**0.00**
Cebu City	**21,193**	0	**0.00**	18	**0.08**	111	**0.52**	129	**0.61**
Lapu-Lapu City	**9,372**	0	**0.00**	79	**0.84**	544	**5.80**	623	**6.65**
Mandaue City	**8,328**	0	**0.00**	0	**0.00**	0	**0.00**	0	**0.00**
**Region 8**	**102,619**	**6**	**0.01**	**164**	**0.16**	**1,594**	**1.55**	**1,764**	**1.72**
Biliran	**3,834**	1	**0.03**	11	**0.29**	39	**1.02**	51	**1.33**
Eastern Samar	**11,392**	0	**0.00**	16	**0.14**	134	**1.18**	150	**1.32**
Northern Leyte	**34,707**	0	**0.00**	0	**0.00**	0	**0.00**	0	**0.00**
Northern Samar	**15,370**	3	**0.02**	77	**0.50**	1,066	**6.94**	1,146	**7.46**
Southern Leyte	**6,451**	0	**0.00**	12	**0.19**	62	**0.96**	74	**1.15**
Western Samar	**14,305**	2	**0.01**	39	**0.27**	242	**1.69**	283	**1.98**
Calbayog City	**4,413**	0	**0.00**	0	**0.00**	12	**0.27**	12	**0.27**
Maasin City	**1,637**	0	**0.00**	0	**0.00**	0	**0.00**	0	**0.00**
Ormoc City	**4,941**	0	**0.00**	0	**0.00**	0	**0.00**	0	**0.00**
Tacloban City	**5,569**	0	**0.00**	9	**0.16**	39	**0.70**	48	**0.86**
**Region 9**	**80,051**	**11**	**0.01**	**1,343**	**1.68**	**8,158**	**10.19**	**9,512**	**11.88**
Zamboanga del Norte	**17,249**	10	**0.06**	1,034	**5.99**	6,116	**35.46**	7,160	**41.51**
Zamboanga del Sur	**17,653**	0	**0.00**	23	**0.13**	107	**0.61**	130	**0.74**
Zamboanga Sibugay	**14,954**	0	**0.00**	52	**0.35**	338	**2.26**	390	**2.61**
Dapitan City	**1,784**	0	**0.00**	10	**0.56**	78	**4.37**	88	**4.93**
Dipolog City	**2,827**	1	**0.04**	204	**7.22**	1,438	**50.87**	1,643	**58.12**
Isabela City	**2,522**	0	**0.00**	2	**0.08**	8	**0.32**	10	**0.40**
Pagadian City	**4,325**	0	**0.00**	0	**0.00**	0	**0.00**	0	**0.00**
Zamboanga City	**18,737**	0	**0.00**	18	**0.10**	73	**0.39**	91	**0.49**
**Region 10**	**101,411**	**27**	**0.03**	**396**	**0.39**	**2,103**	**2.07**	**2,526**	**2.49**
Bukidnon	**23,706**	8	**0.03**	127	**0.54**	398	**1.68**	533	**2.25**
Camiguin	**1,858**	0	**0.00**	0	**0.00**	7	**0.38**	7	**0.38**
Lanao del Norte	**14,960**	1	**0.01**	64	**0.43**	287	**1.92**	352	**2.35**
Misamis Occidental	**6,403**	1	**0.02**	37	**0.58**	661	**10.32**	699	**10.92**
Misamis Oriental	**15,131**	0	**0.00**	112	**0.74**	334	**2.21**	446	**2.95**
Cagayan de Oro City	**14,339**	16	**0.11**	27	**0.19**	186	**1.30**	229	**1.60**
El Salvador City	**1,065**	0	**0.00**	1	**0.09**	54	**5.07**	55	**5.16**
Gingoog City	**2,644**	0	**0.00**	10	**0.38**	34	**1.29**	44	**1.66**
Iligan City	**7,580**	0	**0.00**	4	**0.05**	50	**0.66**	54	**0.71**
Malaybalay City	**3,956**	0	**0.00**	7	**0.18**	63	**1.59**	70	**1.77**

Table 1.B.1.7 - Prenatal Care

No. of Pregnant women who Completed Iodine
Philippines, Annual 2020

Area	Eligible Pop.	Iodine Capsule							
		Age Group						Total	%
		10-14 yrs old		15-19 yrs old		20-49 yrs old			
		No.	%	No.	%	No.	%		
Oroquieta City	**1,389**	0	**0.00**	0	**0.00**	0	**0.00**	0	**0.00**
Ozamis City	**2,778**	1	**0.04**	7	**0.25**	19	**0.68**	27	**0.97**
Tangub City	**1,234**	0	**0.00**	0	**0.00**	0	**0.00**	0	**0.00**
Valencia City	**4,368**	0	**0.00**	0	**0.00**	10	**0.23**	10	**0.23**
**Region 11**	**107,247**	**58**	**0.05**	**1,205**	**1.12**	**5,652**	**5.27**	**6,915**	**6.45**
Compostela Valley	**15,562**	8	**0.05**	61	**0.39**	285	**1.83**	354	**2.27**
Davao del Norte	**21,326**	6	**0.03**	165	**0.77**	961	**4.51**	1,132	**5.31**
Davao Oriental	**13,007**	0	**0.00**	25	**0.19**	180	**1.38**	205	**1.58**
Davao del Sur	**14,151**	13	**0.09**	73	**0.52**	262	**1.85**	348	**2.46**
Davao Occidental	**6,670**	20	**0.30**	397	**5.95**	951	**14.26**	1,368	**20.51**
Davao City	**36,531**	11	**0.03**	484	**1.32**	3,013	**8.25**	3,508	**9.60**
**Region 12**	**104,552**	**54**	**0.05**	**2,561**	**2.45**	**11,736**	**11.23**	**14,351**	**13.73**
North Cotabato	**33,645**	9	**0.03**	562	**1.67**	2,714	**8.07**	3,285	**9.76**
Sarangani	**12,891**	14	**0.11**	556	**4.31**	2,223	**17.24**	2,793	**21.67**
South Cotabato	**21,113**	6	**0.03**	384	**1.82**	1,766	**8.36**	2,156	**10.21**
Sultan Kudarat	**17,359**	17	**0.10**	771	**4.44**	3,587	**20.66**	4,375	**25.20**
Cotabato City	**5,835**	0	**0.00**	1	**0.02**	9	**0.15**	10	**0.17**
Gen. Santos City	**13,709**	8	**0.06**	287	**2.09**	1,437	**10.48**	1,732	**12.63**
**BARMM**	**92,799**	**0**	**0.00**	**30**	**0.03**	**266**	**0.29**	**296**	**0.32**
Basilan	**7,541**	0	**0.00**	0	**0.00**	0	**0.00**	0	**0.00**
Lanao del Sur	**21,131**	0	**0.00**	20	**0.09**	198	**0.94**	218	**1.03**
Maguindanao	**31,128**	0	**0.00**	10	**0.03**	68	**0.22**	78	**0.25**
Sulu	**16,613**	0	**0.00**	0	**0.00**	0	**0.00**	0	**0.00**
Tawi-Tawi	**9,259**	0	**0.00**	0	**0.00**	0	**0.00**	0	**0.00**
Lamitan City	**2,074**	0	**0.00**	0	**0.00**	0	**0.00**	0	**0.00**
Marawi City	**5,053**	0	**0.00**	0	**0.00**	0	**0.00**	0	**0.00**
**CARAGA**	**60,029**	**1**	**0.00**	**89**	**0.15**	**653**	**1.09**	**743**	**1.24**
Agusan del Norte	**8,098**	0	**0.00**	8	**0.10**	64	**0.79**	72	**0.89**
Agusan del Sur	**17,592**	0	**0.00**	56	**0.32**	462	**2.63**	518	**2.94**
Surigao del Norte	**7,089**	0	**0.00**	2	**0.03**	41	**0.58**	43	**0.61**
Surigao del Sur	**11,482**	1	**0.01**	14	**0.12**	48	**0.42**	63	**0.55**
Province of Dinagat	**2,573**	0	**0.00**	0	**0.00**	2	**0.08**	2	**0.08**
Bislig City	**2,179**	0	**0.00**	2	**0.09**	12	**0.55**	14	**0.64**
Butuan City	**7,715**	0	**0.00**	7	**0.09**	24	**0.31**	31	**0.40**
Surigao City	**3,301**	0	**0.00**	0	**0.00**	0	**0.00**	0	**0.00**

Note: Put asterisk (*) for No Report and Zero (0) for No Case

Table 1.B.1.8 - Prenatal Care

Number and proportion of Pregnant Women given one (1) dose of deworming tablet
Philippines, Annual 2020

Area	Eligible Pop.	Pregnant women given one (1) dose of Deworming Tablet							
		Age Group						Total	%
		10-14 yrs old		15-19 yrs old		20-49 yrs old			
		No.	%	No.	%	No.	%		
PHILIPPINES	2,123,158	520	0.02	29,807	1.40	211,087	9.94	241,414	11.37
N C R	238,661	22	0.01	2,193	0.92	23,245	9.74	25,460	10.67
Malabon	6,775	0	0.00	0	0.00	0	0.00	0	0.00
Navotas	4,621	0	0.00	8	0.17	28	0.61	36	0.78
Valenzuela City	11,500	0	0.00	0	0.00	0	0.00	0	0.00
Caloocan City	29,363	0	0.00	30	0.10	130	0.44	160	0.54
Marikina City	8,354	0	0.00	0	0.00	10	0.12	10	0.12
Pasig City	13,996	0	0.00	0	0.00	0	0.00	0	0.00
Pateros	1,184	0	0.00	32	2.70	268	22.64	300	25.34
Taguig	14,918	0	0.00	47	0.32	280	1.88	327	2.19
Quezon City	54,413	18	0.03	1,883	3.46	21,759	39.99	23,660	43.48
Makati City	10,798	4	0.04	74	0.69	217	2.01	295	2.73
Mandaluyong City	7,160	0	0.00	6	0.08	26	0.36	32	0.45
San Juan	2,261	0	0.00	0	0.00	0	0.00	0	0.00
Manila City	32,980	0	0.00	45	0.14	126	0.38	171	0.52
Las Piñas City	10,914	0	0.00	0	0.00	26	0.24	26	0.24
Muntinlupa City	9,353	0	0.00	0	0.00	0	0.00	0	0.00
Parañaque City	12,343	0	0.00	39	0.32	113	0.92	152	1.23
Pasay City	7,728	0	0.00	29	0.38	262	3.39	291	3.77
C A R	35,099	6	0.02	823	2.34	8,014	22.83	8,843	25.19
Abra	4,309	0	0.00	143	3.32	949	22.02	1,092	25.34
Apayao	2,451	0	0.00	189	7.71	992	40.47	1,181	48.18
Benguet	9,188	1	0.01	161	1.75	1,950	21.22	2,112	22.99
Ifugao	4,396	2	0.05	83	1.89	1,022	23.25	1,107	25.18
Kalinga	4,617	2	0.04	176	3.81	1,944	42.11	2,122	45.96
Mt. Province	3,023	0	0.00	14	0.46	74	2.45	88	2.91
Baguio City	7,115	1	0.01	57	0.80	1,083	15.22	1,141	16.04
Region 1	97,261	13	0.01	1,004	1.03	9,877	10.16	10,894	11.20
Ilocos Norte	8,105	1	0.01	115	1.42	1,293	15.95	1,409	17.38
Ilocos Sur	9,330	0	0.00	37	0.40	236	2.53	273	2.93
La Union	11,511	7	0.06	199	1.73	1,503	13.06	1,709	14.85
Pangasinan	50,168	3	0.01	470	0.94	4,799	9.57	5,272	10.51
Alaminos City	1,894	0	0.00	0	0.00	0	0.00	0	0.00
Candon City	987	0	0.00	1	0.10	260	26.34	261	26.44
Dagupan City	3,620	0	0.00	0	0.00	0	0.00	0	0.00
Laoag City	1,870	0	0.00	8	0.43	613	32.78	621	33.21
San Carlos City	3,979	0	0.00	34	0.85	324	8.14	358	9.00
San Fernando City	2,115	0	0.00	16	0.76	251	11.87	267	12.62
Urdaneta City	2,806	2	0.07	124	4.42	598	21.31	724	25.80

Table 1.B.1.8 - Prenatal Care

Number and proportion of Pregnant Women given one (1) dose of deworming tablet
Philippines, Annual 2020

Area	Eligible Pop.	Pregnant women given one (1) dose of Deworming Tablet							
		Age Group						Total	%
		10-14 yrs old		15-19 yrs old		20-49 yrs old			
		No.	%	No.	%	No.	%		
Vigan City	876	0	0.00	0	0.00	0	0.00	0	0.00
**Region 2**	69,443	11	0.02	843	1.21	5,523	7.95	6,377	9.18
Batanes	340	0	0.00	0	0.00	0	0.00	0	0.00
Cagayan	17,971	7	0.04	247	1.37	1,687	9.39	1,941	10.80
Isabela	26,309	2	0.01	236	0.90	1,301	4.95	1,539	5.85
Nueva Vizcaya	9,573	0	0.00	212	2.21	1,289	13.46	1,501	15.68
Quirino	4,025	0	0.00	26	0.65	254	6.31	280	6.96
Cauayan City	2,590	0	0.00	16	0.62	177	6.83	193	7.45
Ilagan City	2,911	0	0.00	7	0.24	68	2.34	75	2.58
Santiago City	2,692	2	0.07	33	1.23	140	5.20	175	6.50
Tuguegarao City	3,032	0	0.00	66	2.18	607	20.02	673	22.20
**Region 3**	220,020	17	0.01	1,139	0.52	11,330	5.15	12,486	5.67
Aurora	4,770	0	0.00	34	0.71	198	4.15	232	4.86
Bataan	13,823	1	0.01	31	0.22	220	1.59	252	1.82
Bulacan	43,627	6	0.01	197	0.45	1,592	3.65	1,795	4.11
Nueva Ecija	28,782	3	0.01	42	0.15	197	0.68	242	0.84
Pampanga	31,359	1	0.00	63	0.20	271	0.86	335	1.07
Tarlac	19,765	5	0.03	402	2.03	3,177	16.07	3,584	18.13
Zambales	12,610	0	0.00	65	0.52	341	2.70	406	3.22
Angeles City	7,869	0	0.00	32	0.41	163	2.07	195	2.48
Balanga City	1,998	0	0.00	0	0.00	0	0.00	0	0.00
Cabanatuan City	5,896	0	0.00	0	0.00	0	0.00	0	0.00
City of San Fernando	5,863	1	0.02	65	1.11	359	6.12	425	7.25
Gapan City	2,153	0	0.00	26	1.21	139	6.46	165	7.66
Mabalacat City	4,793	0	0.00	2	0.04	20	0.42	22	0.46
Malolos City	4,880	0	0.00	0	0.00	0	0.00	0	0.00
Meycauayan	4,041	0	0.00	32	0.79	145	3.59	177	4.38
Olongapo	4,971	0	0.00	60	1.21	508	10.22	568	11.43
Palayan City	799	0	0.00	12	1.50	47	5.88	59	7.38
San Jose City	2,718	0	0.00	0	0.00	0	0.00	0	0.00
San Jose del Monte City	11,100	0	0.00	2	0.02	3,326	29.96	3,328	29.98
Science City of Munoz	1,586	0	0.00	74	4.67	627	39.53	701	44.20
Tarlac City	6,617	0	0.00	0	0.00	0	0.00	0	0.00
**Region 4A**	296,816	2	0.00	298	0.10	6,139	2.07	6,439	2.17
Batangas	38,441	0	0.00	5	0.01	34	0.09	39	0.10
Cavite	27,704	0	0.00	48	0.17	213	0.77	261	0.94
Laguna	19,727	2	0.01	41	0.21	3,535	17.92	3,578	18.14
Quezon	37,410	0	0.00	19	0.05	169	0.45	188	0.50
Rizal	44,791	0	0.00	39	0.09	369	0.82	408	0.91
Antipolo City	16,497	0	0.00	4	0.02	10	0.06	14	0.08
Bacoor City	12,142	0	0.00	0	0.00	0	0.00	0	0.00
Batangas City	6,823	0	0.00	0	0.00	0	0.00	0	0.00

Table 1.B.1.8 - Prenatal Care

Number and proportion of Pregnant Women given one (1) dose of deworming tablet
Philippines, Annual 2020

Area	Eligible Pop.	Pregnant women given one (1) dose of Deworming Tablet							
		Age Group						Total	%
		10-14 yrs old		15-19 yrs old		20-49 yrs old			
		No.	%	No.	%	No.	%		
Biñan City	**6,607**	0	**0.00**	42	**0.64**	346	**5.24**	**388**	**5.87**
Cabuyao City	**6,130**	0	**0.00**	6	**0.10**	199	**3.25**	**205**	**3.34**
Calamba City	**9,028**	0	**0.00**	38	**0.42**	603	**6.68**	**641**	**7.10**
Cavite City	**2,075**	0	**0.00**	0	**0.00**	0	**0.00**	**0**	**0.00**
Dasmariñas City	**13,322**	0	**0.00**	26	**0.20**	355	**2.66**	**381**	**2.86**
General Trias City	**6,352**	0	**0.00**	5	**0.08**	20	**0.31**	**25**	**0.39**
Imus City	**8,156**	0	**0.00**	0	**0.00**	0	**0.00**	**0**	**0.00**
Lipa City	**6,880**	0	**0.00**	0	**0.00**	0	**0.00**	**0**	**0.00**
Lucena City	**5,672**	0	**0.00**	0	**0.00**	0	**0.00**	**0**	**0.00**
San Pablo City	**5,277**	0	**0.00**	2	**0.04**	14	**0.27**	**16**	**0.30**
San Pedro City	**6,467**	0	**0.00**	3	**0.05**	89	**1.38**	**92**	**1.42**
Santa Rosa City	**7,025**	0	**0.00**	15	**0.21**	108	**1.54**	**123**	**1.75**
Tagaytay City	**1,437**	0	**0.00**	0	**0.00**	0	**0.00**	**0**	**0.00**
Tanauan City	**3,584**	0	**0.00**	1	**0.03**	47	**1.31**	**48**	**1.34**
Tayabas City	**2,122**	0	**0.00**	0	**0.00**	0	**0.00**	**0**	**0.00**
Trece Martires City	**3,147**	0	**0.00**	4	**0.13**	28	**0.89**	**32**	**1.02**
**Region 4B**	**71,246**	**10**	**0.01**	**386**	**0.54**	**3,021**	**4.24**	**3,417**	**4.80**
Marinduque	**4,836**	0	**0.00**	52	**1.08**	540	**11.17**	**592**	**12.24**
Mindoro Occidental	**12,407**	1	**0.01**	40	**0.32**	360	**2.90**	**401**	**3.23**
Mindoro Oriental	**19,809**	7	**0.04**	189	**0.95**	1,462	**7.38**	**1,658**	**8.37**
Palawan	**21,375**	0	**0.00**	63	**0.29**	249	**1.16**	**312**	**1.46**
Romblon	**6,400**	2	**0.03**	42	**0.66**	410	**6.41**	**454**	**7.09**
Puerto Princesa City	**6,419**	0	**0.00**	0	**0.00**	0	**0.00**	**0**	**0.00**
**Region 5**	**136,116**	**16**	**0.01**	**3,359**	**2.47**	**27,123**	**19.93**	**30,498**	**22.41**
Albay	**23,737**	4	**0.02**	277	**1.17**	3,572	**15.05**	**3,853**	**16.23**
Camarines Norte	**14,283**	3	**0.02**	765	**5.36**	5,635	**39.45**	**6,403**	**44.83**
Camarines Sur	**38,796**	2	**0.01**	806	**2.08**	6,837	**17.62**	**7,645**	**19.71**
Catanduanes	**6,274**	1	**0.02**	238	**3.79**	1,494	**23.81**	**1,733**	**27.62**
Masbate	**22,521**	2	**0.01**	790	**3.51**	4,684	**20.80**	**5,476**	**24.32**
Sorsogon	**19,065**	2	**0.01**	365	**1.91**	3,744	**19.64**	**4,111**	**21.56**
Iriga City	**2,638**	0	**0.00**	78	**2.96**	796	**30.17**	**874**	**33.13**
Legaspi City	**4,174**	0	**0.00**	0	**0.00**	14	**0.34**	**14**	**0.34**
Naga City	**4,628**	2	**0.04**	40	**0.86**	347	**7.50**	**389**	**8.41**
**Region 6**	**146,526**	**38**	**0.03**	**2,407**	**1.64**	**19,372**	**13.22**	**21,817**	**14.89**
Aklan	**11,162**	4	**0.04**	261	**2.34**	2,128	**19.06**	**2,393**	**21.44**
Antique	**12,816**	9	**0.07**	328	**2.56**	2,838	**22.14**	**3,175**	**24.77**
Capiz	**13,986**	4	**0.03**	232	**1.66**	2,086	**14.91**	**2,322**	**16.60**
Guimaras	**3,085**	0	**0.00**	103	**3.34**	1,184	**38.38**	**1,287**	**41.72**
Iloilo	**36,541**	5	**0.01**	225	**0.62**	2,353	**6.44**	**2,583**	**7.07**
Negros Occidental	**49,368**	16	**0.03**	1,251	**2.53**	8,716	**17.66**	**9,983**	**20.22**
Bacolod City	**11,115**	0	**0.00**	6	**0.05**	62	**0.56**	**68**	**0.61**
Iloilo City	**8,453**	0	**0.00**	1	**0.01**	5	**0.06**	**6**	**0.07**

Table 1.B.1.8 - Prenatal Care

Number and proportion of Pregnant Women given one (1) dose of deworming tablet
Philippines, Annual 2020

Area	Eligible Pop.	Pregnant women given one (1) dose of Deworming Tablet							
		Age Group						Total	%
		10-14 yrs old		15-19 yrs old		20-49 yrs old			
		No.	%	No.	%	No.	%		
**Region 7**	**163,262**	**27**	**0.02**	**1,917**	**1.17**	**14,700**	**9.00**	**16,644**	**10.19**
Bohol	**27,312**	11	**0.04**	105	**0.38**	699	**2.56**	**815**	**2.98**
Cebu	**67,506**	5	**0.01**	355	**0.53**	3,537	**5.24**	**3,897**	**5.77**
Negros Oriental	**27,938**	3	**0.01**	323	**1.16**	2,096	**7.50**	**2,422**	**8.67**
Siquijor	**1,613**	0	**0.00**	67	**4.15**	453	**28.08**	**520**	**32.24**
Cebu City	**21,193**	8	**0.04**	916	**4.32**	6,240	**29.44**	**7,164**	**33.80**
Lapu-Lapu City	**9,372**	0	**0.00**	151	**1.61**	1,675	**17.87**	**1,826**	**19.48**
Mandaue City	**8,328**	0	**0.00**	0	**0.00**	0	**0.00**	**0**	**0.00**
**Region 8**	**102,619**	**21**	**0.02**	**1,160**	**1.13**	**9,243**	**9.01**	**10,424**	**10.16**
Biliran	**3,834**	1	**0.03**	215	**5.61**	1,464	**38.18**	**1,680**	**43.82**
Eastern Samar	**11,392**	8	**0.07**	227	**1.99**	1,610	**14.13**	**1,845**	**16.20**
Northern Leyte	**34,707**	0	**0.00**	0	**0.00**	0	**0.00**	**0**	**0.00**
Northern Samar	**15,370**	5	**0.03**	198	**1.29**	2,194	**14.27**	**2,397**	**15.60**
Southern Leyte	**6,451**	1	**0.02**	156	**2.42**	1,163	**18.03**	**1,320**	**20.46**
Western Samar	**14,305**	2	**0.01**	185	**1.29**	1,487	**10.39**	**1,674**	**11.70**
Calbayog City	**4,413**	0	**0.00**	22	**0.50**	229	**5.19**	**251**	**5.69**
Maasin City	**1,637**	0	**0.00**	0	**0.00**	0	**0.00**	**0**	**0.00**
Ormoc City	**4,941**	4	**0.08**	155	**3.14**	1,074	**21.74**	**1,233**	**24.95**
Tacloban City	**5,569**	0	**0.00**	2	**0.04**	22	**0.40**	**24**	**0.43**
**Region 9**	**80,051**	**21**	**0.03**	**1,334**	**1.67**	**9,036**	**11.29**	**10,391**	**12.98**
Zamboanga del Norte	**17,249**	5	**0.03**	425	**2.46**	2,957	**17.14**	**3,387**	**19.64**
Zamboanga del Sur	**17,653**	10	**0.06**	176	**1.00**	1,003	**5.68**	**1,189**	**6.74**
Zamboanga Sibugay	**14,954**	3	**0.02**	212	**1.42**	1,446	**9.67**	**1,661**	**11.11**
Dapitan City	**1,784**	2	**0.11**	178	**9.98**	1,451	**81.33**	**1,631**	**91.42**
Dipolog City	**2,827**	0	**0.00**	6	**0.21**	71	**2.51**	**77**	**2.72**
Isabela City	**2,522**	0	**0.00**	109	**4.32**	534	**21.17**	**643**	**25.50**
Pagadian City	**4,325**	0	**0.00**	6	**0.14**	82	**1.90**	**88**	**2.03**
Zamboanga City	**18,737**	1	**0.01**	222	**1.18**	1,492	**7.96**	**1,715**	**9.15**
**Region 10**	**101,411**	**43**	**0.04**	**2,591**	**2.55**	**12,545**	**12.37**	**15,179**	**14.97**
Bukidnon	**23,706**	34	**0.14**	1,430	**6.03**	5,799	**24.46**	**7,263**	**30.64**
Camiguin	**1,858**	0	**0.00**	13	**0.70**	150	**8.07**	**163**	**8.77**
Lanao del Norte	**14,960**	1	**0.01**	57	**0.38**	458	**3.06**	**516**	**3.45**
Misamis Occidental	**6,403**	0	**0.00**	75	**1.17**	1,029	**16.07**	**1,104**	**17.24**
Misamis Oriental	**15,131**	6	**0.04**	252	**1.67**	1,136	**7.51**	**1,394**	**9.21**
Cagayan de Oro City	**14,339**	0	**0.00**	3	**0.02**	31	**0.22**	**34**	**0.24**
El Salvador City	**1,065**	0	**0.00**	0	**0.00**	0	**0.00**	**0**	**0.00**
Gingoog City	**2,644**	0	**0.00**	0	**0.00**	8	**0.30**	**8**	**0.30**
Iligan City	**7,580**	2	**0.03**	420	**5.54**	2,075	**27.37**	**2,497**	**32.94**
Malaybalay City	**3,956**	0	**0.00**	16	**0.40**	137	**3.46**	**153**	**3.87**

Table 1.B.1.8 - Prenatal Care

Number and proportion of Pregnant Women given one (1) dose of deworming tablet
Philippines, Annual 2020

Area	Eligible Pop.	Pregnant women given one (1) dose of Deworming Tablet							
		Age Group						Total	%
		10-14 yrs old		15-19 yrs old		20-49 yrs old			
		No.	%	No.	%	No.	%		
Oroquieta City	1,389	0	0.00	0	0.00	0	0.00	0	0.00
Ozamis City	2,778	0	0.00	1	0.04	4	0.14	5	0.18
Tangub City	1,234	0	0.00	0	0.00	0	0.00	0	0.00
Valencia City	4,368	0	0.00	324	7.42	1,718	39.33	2,042	46.75
**Region 11**	**107,247**	**208**	**0.19**	**5,324**	**4.96**	**26,336**	**24.56**	**31,868**	**29.71**
Compostela Valley	15,562	50	0.32	1,204	7.74	5,683	36.52	6,937	44.58
Davao del Norte	21,326	48	0.23	1,232	5.78	6,342	29.74	7,622	35.74
Davao Oriental	13,007	22	0.17	624	4.80	3,094	23.79	3,740	28.75
Davao del Sur	14,151	13	0.09	327	2.31	1,455	10.28	1,795	12.68
Davao Occidental	6,670	39	0.58	638	9.57	1,563	23.43	2,240	33.58
Davao City	36,531	36	0.10	1,299	3.56	8,199	22.44	9,534	26.10
**Region 12**	**104,552**	**64**	**0.06**	**4,593**	**4.39**	**21,808**	**20.86**	**26,465**	**25.31**
North Cotabato	33,645	5	0.01	588	1.75	3,520	10.46	4,113	12.22
Sarangani	12,891	27	0.21	1,886	14.63	7,215	55.97	9,128	70.81
South Cotabato	21,113	10	0.05	908	4.30	4,445	21.05	5,363	25.40
Sultan Kudarat	17,359	22	0.13	967	5.57	5,297	30.51	6,286	36.21
Cotabato City	5,835	0	0.00	72	1.23	514	8.81	586	10.04
Gen. Santos City	13,709	0	0.00	172	1.25	817	5.96	989	7.21
**BARMM**	**92,799**	**0**	**0.00**	**110**	**0.12**	**1,633**	**1.76**	**1,743**	**1.88**
Basilan	7,541	0	0.00	27	0.36	134	1.78	161	2.13
Lanao del Sur	21,131	0	0.00	69	0.33	1,369	6.48	1,438	6.81
Maguindanao	31,128	0	0.00	14	0.04	130	0.42	144	0.46
Sulu	16,613	0	0.00	0	0.00	0	0.00	0	0.00
Tawi-Tawi	9,259	0	0.00	0	0.00	0	0.00	0	0.00
Lamitan City	2,074	0	0.00	0	0.00	0	0.00	0	0.00
Marawi City	5,053	0	0.00	0	0.00	0	0.00	0	0.00
**CARAGA**	**60,029**	**1**	**0.00**	**326**	**0.54**	**2,142**	**3.57**	**2,469**	**4.11**
Agusan del Norte	8,098	0	0.00	2	0.02	20	0.25	22	0.27
Agusan del Sur	17,592	0	0.00	68	0.39	357	2.03	425	2.42
Surigao del Norte	7,089	0	0.00	3	0.04	76	1.07	79	1.11
Surigao del Sur	11,482	1	0.01	184	1.60	1,187	10.34	1,372	11.95
Province of Dinagat	2,573	0	0.00	13	0.51	86	3.34	99	3.85
Bislig City	2,179	0	0.00	29	1.33	221	10.14	250	11.47
Butuan City	7,715	0	0.00	10	0.13	61	0.79	71	0.92
Surigao City	3,301	0	0.00	17	0.51	134	4.06	151	4.57

Note: Put asterisk (*) for No Report and Zero (0) for No Case

Table 1.B.1.9 - Prenatal Care

Number and proportion of pregnant women screened for Syphillis

Philippines, Annual 2020

Area	Eligible Pop.	Screened for Syphillis							
		Age Group						Total	%
		10-14 yrs old		15-19 yrs old		20-49 yrs old			
		No.	%	No.	%	No.	%		
**PHILIPPINES**	**2,123,158**	**8,251**	**0.39**	**44,134**	**2.08**	**340,718**	**16.05**	**393,103**	**18.52**
**N C R**	**238,661**	**204**	**0.09**	**8,572**	**3.59**	**75,920**	**31.81**	**84,696**	**35.49**
Malabon	**6,775**	12	**0.18**	327	**4.83**	1,758	**25.95**	2,097	**30.95**
Navotas	**4,621**	17	**0.37**	421	**9.11**	1,941	**42.00**	2,379	**51.48**
Valenzuela City	**11,500**	1	**0.01**	258	**2.24**	5,940	**51.65**	6,199	**53.90**
Caloocan City	**29,363**	16	**0.05**	1,119	**3.81**	7,124	**24.26**	8,259	**28.13**
								0	
Marikina City	**8,354**	3	**0.04**	189	**2.26**	1,164	**13.93**	1,356	**16.23**
Pasig City	**13,996**	10	**0.07**	524	**3.74**	6,719	**48.01**	7,253	**51.82**
Pateros	**1,184**	0	**0.00**	67	**5.66**	569	**48.06**	636	**53.72**
Taguig	**14,918**	33	**0.22**	518	**3.47**	7,333	**49.16**	7,884	**52.85**
Quezon City	**54,413**	48	**0.09**	2,208	**4.06**	20,841	**38.30**	23,097	**42.45**
Makati City	**10,798**	4	**0.04**	179	**1.66**	3,202	**29.65**	3,385	**31.35**
Mandaluyong City	**7,160**	3	**0.04**	226	**3.16**	2,233	**31.19**	2,462	**34.39**
San Juan	**2,261**	2	**0.09**	85	**3.76**	780	**34.50**	867	**38.35**
Manila City	**32,980**	23	**0.07**	1,188	**3.60**	5,967	**18.09**	7,178	**21.76**
Las Piñas City	**10,914**	4	**0.04**	203	**1.86**	2,019	**18.50**	2,226	**20.40**
Muntinlupa City	**9,353**	0	**0.00**	133	**1.42**	2,641	**28.24**	2,774	**29.66**
Parañaque City	**12,343**	21	**0.17**	598	**4.84**	3,147	**25.50**	3,766	**30.51**
Pasay City	**7,728**	7	**0.09**	329	**4.26**	2,542	**32.89**	2,878	**37.24**
**C A R**	**35,099**	**11**	**0.03**	**1,126**	**3.21**	**9,528**	**27.15**	**10,665**	**30.39**
Abra	**4,309**	0	**0.00**	63	**1.46**	392	**9.10**	455	**10.56**
Apayao	**2,451**	0	**0.00**	124	**5.06**	639	**26.07**	763	**31.13**
Benguet	**9,188**	3	**0.03**	308	**3.35**	2,725	**29.66**	3,036	**33.04**
Ifugao	**4,396**	0	**0.00**	104	**2.37**	1,177	**26.77**	1,281	**29.14**
Kalinga	**4,617**	3	**0.06**	145	**3.14**	1,153	**24.97**	1,301	**28.18**
Mt. Province	**3,023**	2	**0.07**	124	**4.10**	756	**25.01**	882	**29.18**
Baguio City	**7,115**	3	**0.04**	258	**3.63**	2,686	**37.75**	2,947	**41.42**
**Region 1**	**97,261**	**37**	**0.04**	**2,577**	**2.65**	**25,694**	**26.42**	**28,308**	**29.11**
Ilocos Norte	**8,105**	6	**0.07**	269	**3.32**	2,921	**36.04**	3,196	**39.43**
Ilocos Sur	**9,330**	3	**0.03**	360	**3.86**	3,762	**40.32**	4,125	**44.21**
La Union	**11,511**	13	**0.11**	498	**4.33**	3,660	**31.80**	4,171	**36.23**
Pangasinan	**50,168**	8	**0.02**	931	**1.86**	8,656	**17.25**	9,595	**19.13**
Alaminos City	**1,894**	0	**0.00**	77	**4.07**	1,029	**54.33**	1,106	**58.39**
Candon City	**987**	0	**0.00**	8	**0.81**	772	**78.22**	780	**79.03**
Dagupan City	**3,620**	2	**0.06**	100	**2.76**	960	**26.52**	1,062	**29.34**
Laoag City	**1,870**	0	**0.00**	59	**3.16**	1,758	**94.01**	1,817	**97.17**
San Carlos City	**3,979**	0	**0.00**	31	**0.78**	486	**12.21**	517	**12.99**
San Fernando City	**2,115**	0	**0.00**	96	**4.54**	790	**37.35**	886	**41.89**
Urdaneta City	**2,806**	5	**0.18**	127	**4.53**	800	**28.51**	932	**33.21**

Table 1.B.1.9 - Prenatal Care

Number and proportion of pregnant women screened for Syphillis

Philippines, Annual 2020

Area	Eligible Pop.	Screened for Syphillis							
		Age Group						Total	%
		10-14 yrs old		15-19 yrs old		20-49 yrs old			
		No.	%	No.	%	No.	%		
Vigan City	**876**	0	**0.00**	21	**2.40**	100	**11.42**	121	**13.81**
**Region 2**	**69,443**	**29**	**0.04**	**1,593**	**2.29**	**10,136**	**14.60**	**11,758**	**16.93**
Batanes	**340**	0	**0.00**	7	**2.06**	140	**41.18**	147	**43.24**
Cagayan	**17,971**	3	**0.02**	162	**0.90**	860	**4.79**	1,025	**5.70**
Isabela	**26,309**	20	**0.08**	665	**2.53**	3,931	**14.94**	4,616	**17.55**
Nueva Vizcaya	**9,573**	5	**0.05**	294	**3.07**	2,002	**20.91**	2,301	**24.04**
Quirino	**4,025**	0	**0.00**	160	**3.98**	1,116	**27.73**	1,276	**31.70**
Cauayan City	**2,590**	0	**0.00**	57	**2.20**	470	**18.15**	527	**20.35**
Ilagan City	**2,911**	1	**0.03**	139	**4.77**	943	**32.39**	1,083	**37.20**
Santiago City	**2,692**	0	**0.00**	66	**2.45**	395	**14.67**	461	**17.12**
Tuguegarao City	**3,032**	0	**0.00**	43	**1.42**	279	**9.20**	322	**10.62**
**Region 3**	**220,020**	**225**	**0.10**	**10,154**	**4.62**	**63,170**	**28.71**	**73,549**	**33.43**
Aurora	**4,770**	2	**0.04**	299	**6.27**	1,736	**36.39**	2,037	**42.70**
Bataan	**13,823**	18	**0.13**	784	**5.67**	5,406	**39.11**	6,208	**44.91**
Bulacan	**43,627**	52	**0.12**	1,549	**3.55**	9,441	**21.64**	11,042	**25.31**
Nueva Ecija	**28,782**	31	**0.11**	1,290	**4.48**	6,289	**21.85**	7,610	**26.44**
Pampanga	**31,359**	19	**0.06**	1,018	**3.25**	6,645	**21.19**	7,682	**24.50**
Tarlac	**19,765**	20	**0.10**	695	**3.52**	6,190	**31.32**	6,905	**34.94**
Zambales	**12,610**	5	**0.04**	398	**3.16**	1,947	**15.44**	2,350	**18.64**
Angeles City	**7,869**	3	**0.04**	241	**3.06**	1,311	**16.66**	1,555	**19.76**
Balanga City	**1,998**	6	**0.30**	159	**7.96**	765	**38.29**	930	**46.55**
Cabanatuan City	**5,896**	4	**0.07**	218	**3.70**	1,154	**19.57**	1,376	**23.34**
City of San Fernando	**5,863**	4	**0.07**	326	**5.56**	2,175	**37.10**	2,505	**42.73**
Gapan City	**2,153**	0	**0.00**	116	**5.39**	560	**26.01**	676	**31.40**
Mabalacat City	**4,793**	7	**0.15**	338	**7.05**	2,045	**42.67**	2,390	**49.86**
Malolos City	**4,880**	4	**0.08**	181	**3.71**	1,161	**23.79**	1,346	**27.58**
Meycauayan	**4,041**	16	**0.40**	311	**7.70**	1,504	**37.22**	1,831	**45.31**
Olongapo	**4,971**	0	**0.00**	226	**4.55**	1,204	**24.22**	1,430	**28.77**
Palayan City	**799**	2	**0.25**	33	**4.13**	156	**19.52**	191	**23.90**
San Jose City	**2,718**	10	**0.37**	123	**4.53**	645	**23.73**	778	**28.62**
San Jose del Monte City	**11,100**	14	**0.13**	1,187	**10.69**	9,889	**89.09**	11,090	**99.91**
Science City of Munoz	**1,586**	3	**0.19**	104	**6.56**	535	**33.73**	642	**40.48**
Tarlac City	**6,617**	5	**0.08**	558	**8.43**	2,412	**36.45**	2,975	**44.96**
**Region 4A**	**296,816**	**7,142**	**2.41**	**2,293**	**0.77**	**27,295**	**9.20**	**36,730**	**12.37**
Batangas	**38,441**	0	**0.00**	27	**0.07**	375	**0.98**	402	**1.05**
Cavite	**27,704**	4	**0.01**	237	**0.86**	2,228	**8.04**	2,469	**8.91**
Laguna	**19,727**	5,588	**28.33**	443	**2.25**	9,883	**50.10**	15,914	**80.67**
Quezon	**37,410**	2	**0.01**	158	**0.42**	1,030	**2.75**	1,190	**3.18**
Rizal	**44,791**	7	**0.02**	590	**1.32**	3,951	**8.82**	4,548	**10.15**
Antipolo City	**16,497**	0	**0.00**	192	**1.16**	719	**4.36**	911	**5.52**
Bacoor City	**12,142**	0	**0.00**	4	**0.03**	49	**0.40**	53	**0.44**
Batangas City	**6,823**	0	**0.00**	0	**0.00**	0	**0.00**	0	**0.00**

Table 1.B.1.9 - Prenatal Care

Number and proportion of pregnant women screened for Syphillis

Philippines, Annual 2020

Area	Eligible Pop.	Screened for Syphillis							
		Age Group						Total	%
		10-14 yrs old		15-19 yrs old		20-49 yrs old			
		No.	%	No.	%	No.	%		
Biñan City	**6,607**	1,531	**23.17**	72	**1.09**	2,214	**33.51**	3,817	**57.77**
Cabuyao City	**6,130**	0	**0.00**	0	**0.00**	0	**0.00**	0	**0.00**
Calamba City	**9,028**	3	**0.03**	83	**0.92**	1,417	**15.70**	1,503	**16.65**
Cavite City	**2,075**	0	**0.00**	0	**0.00**	0	**0.00**	0	**0.00**
Dasmariñas City	**13,322**	4	**0.03**	230	**1.73**	2,210	**16.59**	2,444	**18.35**
General Trias City	**6,352**	0	**0.00**	28	**0.44**	263	**4.14**	291	**4.58**
Imus City	**8,156**	0	**0.00**	0	**0.00**	0	**0.00**	0	**0.00**
Lipa City	**6,880**	0	**0.00**	0	**0.00**	0	**0.00**	0	**0.00**
Lucena City	**5,672**	0	**0.00**	0	**0.00**	0	**0.00**	0	**0.00**
San Pablo City	**5,277**	0	**0.00**	0	**0.00**	211	**4.00**	211	**4.00**
San Pedro City	**6,467**	0	**0.00**	0	**0.00**	0	**0.00**	0	**0.00**
Santa Rosa City	**7,025**	1	**0.01**	109	**1.55**	720	**10.25**	830	**11.81**
Tagaytay City	**1,437**	2	**0.14**	34	**2.37**	1,226	**85.32**	1,262	**87.82**
Tanauan City	**3,584**	0	**0.00**	18	**0.50**	327	**9.12**	345	**9.63**
Tayabas City	**2,122**	0	**0.00**	0	**0.00**	0	**0.00**	0	**0.00**
Trece Martires City	**3,147**	0	**0.00**	68	**2.16**	472	**15.00**	540	**17.16**
**Region 4B**	**71,246**	**64**	**0.09**	**776**	**1.09**	**5,199**	**7.30**	**6,039**	**8.48**
Marinduque	**4,836**	1	**0.02**	70	**1.45**	749	**15.49**	820	**16.96**
Mindoro Occidental	**12,407**	1	**0.01**	181	**1.46**	1,323	**10.66**	1,505	**12.13**
Mindoro Oriental	**19,809**	58	**0.29**	357	**1.80**	1,964	**9.91**	2,379	**12.01**
Palawan	**21,375**	2	**0.01**	105	**0.49**	589	**2.76**	696	**3.26**
Romblon	**6,400**	2	**0.03**	63	**0.98**	574	**8.97**	639	**9.98**
Puerto Princesa City	**6,419**	0	**0.00**	0	**0.00**	0	**0.00**	0	**0.00**
**Region 5**	**136,116**	**20**	**0.01**	**1,785**	**1.31**	**15,443**	**11.35**	**17,248**	**12.67**
Albay	**23,737**	9	**0.04**	271	**1.14**	4,126	**17.38**	4,406	**18.56**
Camarines Norte	**14,283**	0	**0.00**	220	**1.54**	1,474	**10.32**	1,694	**11.86**
Camarines Sur	**38,796**	3	**0.01**	150	**0.39**	1,520	**3.92**	1,673	**4.31**
Catanduanes	**6,274**	3	**0.05**	203	**3.24**	1,325	**21.12**	1,531	**24.40**
Masbate	**22,521**	2	**0.01**	593	**2.63**	3,252	**14.44**	3,847	**17.08**
Sorsogon	**19,065**	0	**0.00**	92	**0.48**	1,054	**5.53**	1,146	**6.01**
Iriga City	**2,638**	1	**0.04**	65	**2.46**	605	**22.93**	671	**25.44**
Legaspi City	**4,174**	2	**0.05**	134	**3.21**	1,706	**40.87**	1,842	**44.13**
Naga City	**4,628**	0	**0.00**	57	**1.23**	381	**8.23**	438	**9.46**
**Region 6**	**146,526**	**53**	**0.04**	**3,764**	**2.57**	**31,572**	**21.55**	**35,389**	**24.15**
Aklan	**11,162**	14	**0.13**	449	**4.02**	3,539	**31.71**	4,002	**35.85**
Antique	**12,816**	2	**0.02**	251	**1.96**	2,049	**15.99**	2,302	**17.96**
Capiz	**13,986**	3	**0.02**	274	**1.96**	2,420	**17.30**	2,697	**19.28**
Guimaras	**3,085**	1	**0.03**	178	**5.77**	1,579	**51.18**	1,758	**56.99**
Iloilo	**36,541**	15	**0.04**	1,197	**3.28**	12,069	**33.03**	13,281	**36.35**
Negros Occidental	**49,368**	6	**0.01**	609	**1.23**	4,298	**8.71**	4,913	**9.95**
Bacolod City	**11,115**	11	**0.10**	509	**4.58**	3,334	**30.00**	3,854	**34.67**
Iloilo City	**8,453**	1	**0.01**	297	**3.51**	2,284	**27.02**	2,582	**30.55**

Table 1.B.1.9 - Prenatal Care

Number and proportion of pregnant women screened for Syphillis
Philippines, Annual 2020

Area	Eligible Pop.	Screened for Syphillis							
		Age Group						Total	%
		10-14 yrs old		15-19 yrs old		20-49 yrs old			
		No.	%	No.	%	No.	%		
**Region 7**	**163,262**	**22**	**0.01**	**2,042**	**1.25**	**20,862**	**12.78**	**22,926**	**14.04**
Bohol	**27,312**	0	**0.00**	125	**0.46**	4,855	**17.78**	4,980	**18.23**
Cebu	**67,506**	3	**0.00**	370	**0.55**	3,239	**4.80**	3,612	**5.35**
Negros Oriental	**27,938**	5	**0.02**	188	**0.67**	1,236	**4.42**	1,429	**5.11**
Siquijor	**1,613**	0	**0.00**	109	**6.76**	922	**57.16**	1,031	**63.92**
Cebu City	**21,193**	14	**0.07**	1,000	**4.72**	6,629	**31.28**	7,643	**36.06**
Lapu-Lapu City	**9,372**	0	**0.00**	250	**2.67**	3,225	**34.41**	3,475	**37.08**
Mandaue City	**8,328**	0	**0.00**	0	**0.00**	756	**9.08**	756	**9.08**
**Region 8**	**102,619**	**6**	**0.01**	**968**	**0.94**	**7,424**	**7.23**	**8,398**	**8.18**
Biliran	**3,834**	5	**0.13**	248	**6.47**	1,480	**38.60**	1,733	**45.20**
Eastern Samar	**11,392**	0	**0.00**	140	**1.23**	943	**8.28**	1,083	**9.51**
Northern Leyte	**34,707**	0	**0.00**	0	**0.00**	0	**0.00**	0	**0.00**
Northern Samar	**15,370**	0	**0.00**	140	**0.91**	1,825	**11.87**	1,965	**12.78**
Southern Leyte	**6,451**	0	**0.00**	58	**0.90**	479	**7.43**	537	**8.32**
Western Samar	**14,305**	0	**0.00**	82	**0.57**	406	**2.84**	488	**3.41**
Calbayog City	**4,413**	0	**0.00**	18	**0.41**	150	**3.40**	168	**3.81**
Maasin City	**1,637**	0	**0.00**	0	**0.00**	0	**0.00**	0	**0.00**
Ormoc City	**4,941**	0	**0.00**	36	**0.73**	239	**4.84**	275	**5.57**
Tacloban City	**5,569**	1	**0.02**	246	**4.42**	1,902	**34.15**	2,149	**38.59**
**Region 9**	**80,051**	**2**	**0.00**	**525**	**0.66**	**3,306**	**4.13**	**3,833**	**4.79**
Zamboanga del Norte	**17,249**	0	**0.00**	92	**0.53**	459	**2.66**	551	**3.19**
Zamboanga del Sur	**17,653**	0	**0.00**	94	**0.53**	771	**4.37**	865	**4.90**
Zamboanga Sibugay	**14,954**	2	**0.01**	294	**1.97**	1,835	**12.27**	2,131	**14.25**
Dapitan City	**1,784**	0	**0.00**	30	**1.68**	130	**7.29**	160	**8.97**
Dipolog City	**2,827**	0	**0.00**	1	**0.04**	6	**0.21**	7	**0.25**
Isabela City	**2,522**	0	**0.00**	8	**0.32**	27	**1.07**	35	**1.39**
Pagadian City	**4,325**	0	**0.00**	6	**0.14**	72	**1.66**	78	**1.80**
Zamboanga City	**18,737**	0	**0.00**	0	**0.00**	6	**0.03**	6	**0.03**
**Region 10**	**101,411**	**43**	**0.04**	**1,408**	**1.39**	**7,525**	**7.42**	**8,976**	**8.85**
Bukidnon	**23,706**	24	**0.10**	316	**1.33**	973	**4.10**	1,313	**5.54**
Camiguin	**1,858**	0	**0.00**	34	**1.83**	414	**22.28**	448	**24.11**
Lanao del Norte	**14,960**	1	**0.01**	180	**1.20**	1,240	**8.29**	1,421	**9.50**
Misamis Occidental	**6,403**	0	**0.00**	22	**0.34**	204	**3.19**	226	**3.53**
Misamis Oriental	**15,131**	3	**0.02**	181	**1.20**	752	**4.97**	936	**6.19**
Cagayan de Oro City	**14,339**	8	**0.06**	313	**2.18**	1,978	**13.79**	2,299	**16.03**
El Salvador City	**1,065**	4	**0.38**	27	**2.54**	153	**14.37**	184	**17.28**
Gingoog City	**2,644**	0	**0.00**	0	**0.00**	0	**0.00**	0	**0.00**
Iligan City	**7,580**	0	**0.00**	12	**0.16**	90	**1.19**	102	**1.35**
Malaybalay City	**3,956**	1	**0.03**	106	**2.68**	536	**13.55**	643	**16.25**

Table 1.B.1.9 - Prenatal Care

Number and proportion of pregnant women screened for Syphillis

Philippines, Annual 2020

Area	Eligible Pop.	Screened for Syphillis							
		Age Group						Total	%
		10-14 yrs old		15-19 yrs old		20-49 yrs old			
		No.	%	No.	%	No.	%		
Oroquieta City	**1,389**	0	**0.00**	0	**0.00**	0	**0.00**	0	**0.00**
Ozamis City	**2,778**	2	**0.07**	196	**7.06**	1,057	**38.05**	1,255	**45.18**
Tangub City	**1,234**	0	**0.00**	0	**0.00**	0	**0.00**	0	**0.00**
Valencia City	**4,368**	0	**0.00**	21	**0.48**	128	**2.93**	149	**3.41**
**Region 11**	**107,247**	**332**	**0.31**	**2,723**	**2.54**	**16,137**	**15.05**	**19,192**	**17.90**
Compostela Valley	**15,562**	6	**0.04**	53	**0.34**	265	**1.70**	324	**2.08**
Davao del Norte	**21,326**	34	**0.16**	812	**3.81**	5,117	**23.99**	5,963	**27.96**
Davao Oriental	**13,007**	0	**0.00**	18	**0.14**	74	**0.57**	92	**0.71**
Davao del Sur	**14,151**	5	**0.04**	64	**0.45**	412	**2.91**	481	**3.40**
Davao Occidental	**6,670**	1	**0.01**	63	**0.94**	200	**3.00**	264	**3.96**
Davao City	**36,531**	286	**0.78**	1,713	**4.69**	10,069	**27.56**	12,068	**33.03**
**Region 12**	**104,552**	**45**	**0.04**	**2,752**	**2.63**	**14,861**	**14.21**	**17,658**	**16.89**
North Cotabato	**33,645**	0	**0.00**	196	**0.58**	1,426	**4.24**	1,622	**4.82**
Sarangani	**12,891**	10	**0.08**	550	**4.27**	2,255	**17.49**	2,815	**21.84**
South Cotabato	**21,113**	10	**0.05**	631	**2.99**	3,704	**17.54**	4,345	**20.58**
Sultan Kudarat	**17,359**	18	**0.10**	799	**4.60**	4,220	**24.31**	5,037	**29.02**
Cotabato City	**5,835**	1	**0.02**	24	**0.41**	215	**3.68**	240	**4.11**
Gen. Santos City	**13,709**	6	**0.04**	552	**4.03**	3,041	**22.18**	3,599	**26.25**
**BARMM**	**92,799**	**0**	**0.00**	**90**	**0.10**	**711**	**0.77**	**801**	**0.86**
Basilan	**7,541**	0	**0.00**	0	**0.00**	5	**0.07**	5	**0.07**
Lanao del Sur	**21,131**	0	**0.00**	0	**0.00**	112	**0.53**	112	**0.53**
Maguindanao	**31,128**	0	**0.00**	87	**0.28**	571	**1.83**	658	**2.11**
Sulu	**16,613**	0	**0.00**	0	**0.00**	0	**0.00**	0	**0.00**
Tawi-Tawi	**9,259**	0	**0.00**	0	**0.00**	0	**0.00**	0	**0.00**
Lamitan City	**2,074**	0	**0.00**	0	**0.00**	0	**0.00**	0	**0.00**
Marawi City	**5,053**	0	**0.00**	3	**0.06**	23	**0.46**	26	**0.51**
**CARAGA**	**60,029**	**16**	**0.03**	**986**	**1.64**	**5,935**	**9.89**	**6,937**	**11.56**
Agusan del Norte	**8,098**	2	**0.02**	57	**0.70**	334	**4.12**	393	**4.85**
Agusan del Sur	**17,592**	6	**0.03**	229	**1.30**	1,031	**5.86**	1,266	**7.20**
Surigao del Norte	**7,089**	1	**0.01**	80	**1.13**	439	**6.19**	520	**7.34**
Surigao del Sur	**11,482**	0	**0.00**	79	**0.69**	595	**5.18**	674	**5.87**
Province of Dinagat	**2,573**	0	**0.00**	60	**2.33**	340	**13.21**	400	**15.55**
Bislig City	**2,179**	0	**0.00**	13	**0.60**	120	**5.51**	133	**6.10**
Butuan City	**7,715**	3	**0.04**	291	**3.77**	1,884	**24.42**	2,178	**28.23**
Surigao City	**3,301**	4	**0.12**	177	**5.36**	1,192	**36.11**	1,373	**41.59**

Note: Put asterisk (*) for No Report and Zero (0) for No Case

Table 1.B.1.10 - Prenatal Care

Number and proportion of pregnant women tested positive for Syphillis
Philippines, Annual 2020

Area	Total No. of Screened for syphillis	Tested Positive for Syphillis							
		Age Group						Total	%
		10-14 yrs old		15-19 yrs old		20-49 yrs old			
		No.	%	No.	%	No.	%		
**PHILIPPINES**	**393,103**	**128**	**0.03**	**3,157**	**0.80**	**7,087**	**1.80**	**14,626**	**3.72**
**N C R**	**84,696**	**30**	**0.04**	**165**	**0.19**	**763**	**0.90**	**958**	**1.13**
Malabon	**2,097**	1	**0.05**	4	**0.19**	10	**0.48**	15	**0.72**
Navotas	**2,379**	0	**0.00**	19	**0.80**	50	**2.10**	69	**2.90**
Valenzuela City	**6,199**	0	**0.00**	0	**0.00**	9	**0.15**	9	**0.15**
Caloocan City	**8,259**	0	**0.00**	5	**0.06**	18	**0.22**	23	**0.28**
								0	
Marikina City	**1,356**	0	**0.00**	3	**0.22**	3	**0.22**	6	**0.44**
Pasig City	**7,253**	0	**0.00**	3	**0.04**	10	**0.14**	13	**0.18**
Pateros	**636**	0	**0.00**	0	**0.00**	0	**0.00**	0	**0.00**
Taguig	**7,884**	29	**0.37**	8	**0.10**	67	**0.85**	104	**1.32**
Quezon City	**23,097**	0	**0.00**	5	**0.02**	70	**0.30**	75	**0.32**
Makati City	**3,385**	0	**0.00**	0	**0.00**	5	**0.15**	5	**0.15**
Mandaluyong City	**2,462**	0	**0.00**	0	**0.00**	5	**0.20**	5	**0.20**
San Juan	**867**	0	**0.00**	0	**0.00**	0	**0.00**	0	**0.00**
Manila City	**7,178**	0	**0.00**	0	**0.00**	46	**0.64**	46	**0.64**
Las Piñas City	**2,226**	0	**0.00**	2	**0.09**	6	**0.27**	8	**0.36**
Muntinlupa City	**2,774**	0	**0.00**	1	**0.04**	102	**3.68**	103	**3.71**
Parañaque City	**3,766**	0	**0.00**	115	**3.05**	351	**9.32**	466	**12.37**
Pasay City	**2,878**	0	**0.00**	0	**0.00**	11	**0.38**	11	**0.38**
**C A R**	**10,665**	**0**	**0.00**	**6**	**0.06**	**58**	**0.54**	**64**	**0.60**
Abra	**455**	0	**0.00**	0	**0.00**	1	**0.22**	1	**0.22**
Apayao	**763**	0	**0.00**	3	**0.39**	6	**0.79**	9	**1.18**
Benguet	**3,036**	0	**0.00**	0	**0.00**	5	**0.16**	5	**0.16**
Ifugao	**1,281**	0	**0.00**	1	**0.08**	3	**0.23**	4	**0.31**
Kalinga	**1,301**	0	**0.00**	1	**0.08**	13	**1.00**	14	**1.08**
Mt. Province	**882**	0	**0.00**	0	**0.00**	0	**0.00**	0	**0.00**
Baguio City	**2,947**	0	**0.00**	1	**0.03**	30	**1.02**	31	**1.05**
**Region 1**	**28,308**	**0**	**0.00**	**12**	**0.04**	**161**	**0.57**	**173**	**0.61**
Ilocos Norte	**3,196**	0	**0.00**	1	**0.03**	11	**0.34**	12	**0.38**
Ilocos Sur	**4,125**	0	**0.00**	0	**0.00**	46	**1.12**	46	**1.12**
La Union	**4,171**	0	**0.00**	1	**0.02**	47	**1.13**	48	**1.15**
Pangasinan	**9,595**	0	**0.00**	10	**0.10**	52	**0.54**	62	**0.65**
Alaminos City	**1,106**	0	**0.00**	0	**0.00**	0	**0.00**	0	**0.00**
Candon City	**780**	0	**0.00**	0	**0.00**	0	**0.00**	0	**0.00**
Dagupan City	**1,062**	0	**0.00**	0	**0.00**	4	**0.38**	4	**0.38**
Laoag City	**1,817**	0	**0.00**	0	**0.00**	0	**0.00**	0	**0.00**
San Carlos City	**517**	0	**0.00**	0	**0.00**	1	**0.19**	1	**0.19**
San Fernando City	**886**	0	**0.00**	0	**0.00**	0	**0.00**	0	**0.00**
Urdaneta City	**932**	0	**0.00**	0	**0.00**	0	**0.00**	0	**0.00**

Table 1.B.1.10 - Prenatal Care

Number and proportion of pregnant women tested positive for Syphillis

Philippines, Annual 2020

Area	Total No. of Screened for syphillis	Tested Positive for Syphillis							
		Age Group						Total	%
		10-14 yrs old		15-19 yrs old		20-49 yrs old			
		No.	%	No.	%	No.	%		
Vigan City	121	0	0.00	0	0.00	0	0.00	0	0.00
**Region 2**	11,758	0	0.00	15	0.13	123	1.05	138	1.17
Batanes	147	0	0.00	0	0.00	2	1.36	2	1.36
Cagayan	1,025	0	0.00	2	0.20	6	0.59	8	0.78
Isabela	4,616	0	0.00	2	0.04	44	0.95	46	1.00
Nueva Vizcaya	2,301	0	0.00	7	0.30	21	0.91	28	1.22
Quirino	1,276	0	0.00	2	0.16	6	0.47	8	0.63
Cauayan City	527	0	0.00	0	0.00	0	0.00	0	0.00
Ilagan City	1,083	0	0.00	1	0.09	6	0.55	7	0.65
Santiago City	461	0	0.00	0	0.00	7	1.52	7	1.52
Tuguegarao City	322	0	0.00	1	0.31	31	9.63	32	9.94
**Region 3**	73,549	11	0.01	194	0.26	1,069	1.45	1,274	1.73
Aurora	2,037	0	0.00	2	0.10	16	0.79	18	0.88
Bataan	6,208	0	0.00	14	0.23	59	0.95	73	1.18
Bulacan	11,042	0	0.00	98	0.89	526	4.76	624	5.65
Nueva Ecija	7,610	4	0.05	36	0.47	164	2.16	204	2.68
Pampanga	7,682	0	0.00	15	0.20	153	1.99	168	2.19
Tarlac	6,905	0	0.00	6	0.09	64	0.93	70	1.01
Zambales	2,350	0	0.00	1	0.04	14	0.60	15	0.64
Angeles City	1,555	0	0.00	0	0.00	2	0.13	2	0.13
Balanga City	930	0	0.00	1	0.11	2	0.22	3	0.32
Cabanatuan City	1,376	0	0.00	0	0.00	0	0.00	0	0.00
City of San Fernando	2,505	0	0.00	1	0.04	3	0.12	4	0.16
Gapan City	676	0	0.00	0	0.00	0	0.00	0	0.00
Mabalacat City	2,390	0	0.00	4	0.17	13	0.54	17	0.71
Malolos City	1,346	0	0.00	0	0.00	2	0.15	2	0.15
Meycauayan	1,831	6	0.33	7	0.38	19	1.04	32	1.75
Olongapo	1,430	0	0.00	7	0.49	20	1.40	27	1.89
Palayan City	191	0	0.00	0	0.00	0	0.00	0	0.00
San Jose City	778	0	0.00	0	0.00	0	0.00	0	0.00
San Jose del Monte City	11,090	0	0.00	2	0.02	9	0.08	11	0.10
Science City of Munoz	642	0	0.00	0	0.00	3	0.47	3	0.47
Tarlac City	2,975	1	0.03	0	0.00	0	0.00	1	0.03
**Region 4A**	36,730	71	0.19	2,245	6.11	1,131	3.08	3,447	9.38
Batangas	402	0	0.00	0	0.00	2	0.50	2	0.50
Cavite	2,469	0	0.00	4	0.16	38	1.54	42	1.70
Laguna	15,914	8	0.05	1,478	9.29	25	0.16	1,511	9.49
Quezon	1,190	0	0.00	6	0.50	24	2.02	30	2.52
Rizal	4,548	63	1.39	191	4.20	13	0.29	267	5.87
Antipolo City	911	0	0.00	84	9.22	231	25.36	315	34.58
Bacoor City	53	0	0.00	0	0.00	0	0.00	0	0.00
Batangas City	0	0	0.00	0	0.00	0	0.00	0	0.00

Table 1.B.1.10 - Prenatal Care

Number and proportion of pregnant women tested positive for Syphillis
Philippines, Annual 2020

Area	Total No. of Screened for syphillis	Tested Positive for Syphillis							
		Age Group						Total	%
		10-14 yrs old		15-19 yrs old		20-49 yrs old			
		No.	%	No.	%	No.	%		
Biñan City	**3,817**	0	**0.00**	249	**6.52**	105	**2.75**	354	**9.27**
Cabuyao City	**0**	0	**0.00**	44	**0.00**	472	**0.00**	516	**0.00**
Calamba City	**1,503**	0	**0.00**	109	**7.25**	30	**2.00**	139	**9.25**
Cavite City	**0**	0	**0.00**	0	**0.00**	0	**0.00**	0	**0.00**
Dasmariñas City	**2,444**	0	**0.00**	15	**0.61**	89	**3.64**	104	**4.26**
General Trias City	**291**	0	**0.00**	0	**0.00**	0	**0.00**	0	**0.00**
Imus City	**0**	0	**0.00**	0	**0.00**	0	**0.00**	0	**0.00**
Lipa City	**0**	0	**0.00**	0	**0.00**	0	**0.00**	0	**0.00**
Lucena City	**0**	0	**0.00**	0	**0.00**	0	**0.00**	0	**0.00**
San Pablo City	**211**	0	**0.00**	35	**16.59**	0	**0.00**	35	**16.59**
San Pedro City	**0**	0	**0.00**	0	**0.00**	7	**0.00**	7	**0.00**
Santa Rosa City	**830**	0	**0.00**	30	**3.61**	78	**9.40**	108	**13.01**
Tagaytay City	**1,262**	0	**0.00**	0	**0.00**	6	**0.48**	6	**0.48**
Tanauan City	**345**	0	**0.00**	0	**0.00**	11	**3.19**	11	**3.19**
Tayabas City	**0**	0	**0.00**	0	**0.00**	0	**0.00**	0	**0.00**
Trece Martires City	**540**	0	**0.00**	0	**0.00**	0	**0.00**	0	**0.00**
**Region 4B**	**6,039**	**0**	**0.00**	**15**	**0.25**	**84**	**1.39**	**99**	**1.64**
Marinduque	**820**	0	**0.00**	0	**0.00**	0	**0.00**	0	**0.00**
Mindoro Occidental	**1,505**	0	**0.00**	5	**0.33**	26	**1.73**	31	**2.06**
Mindoro Oriental	**2,379**	0	**0.00**	3	**0.13**	22	**0.92**	25	**1.05**
Palawan	**696**	0	**0.00**	0	**0.00**	12	**1.72**	12	**1.72**
Romblon	**639**	0	**0.00**	7	**1.10**	24	**3.76**	31	**4.85**
Puerto Princesa City	**0**	0	**0.00**	0	**0.00**	0	**0.00**	0	**0.00**
**Region 5**	**17,248**	**0**	**0.00**	**22**	**0.13**	**289**	**1.68**	**4,565**	**26.47**
Albay	**4,406**	0	**0.00**	8	**0.18**	144	**3.27**	4,406	**100.00**
Camarines Norte	**1,694**	0	**0.00**	0	**0.00**	17	**1.00**	17	**1.00**
Camarines Sur	**1,673**	0	**0.00**	2	**0.12**	18	**1.08**	20	**1.20**
Catanduanes	**1,531**	0	**0.00**	0	**0.00**	4	**0.26**	4	**0.26**
Masbate	**3,847**	0	**0.00**	10	**0.26**	76	**1.98**	86	**2.24**
Sorsogon	**1,146**	0	**0.00**	0	**0.00**	10	**0.87**	10	**0.87**
Iriga City	**671**	0	**0.00**	0	**0.00**	5	**0.75**	5	**0.75**
Legaspi City	**1,842**	0	**0.00**	2	**0.11**	15	**0.81**	17	**0.92**
Naga City	**438**	0	**0.00**	0	**0.00**	0	**0.00**	0	**0.00**
**Region 6**	**35,389**	**2**	**0.01**	**88**	**0.25**	**462**	**1.31**	**552**	**1.56**
Aklan	**4,002**	0	**0.00**	6	**0.15**	55	**1.37**	61	**1.52**
Antique	**2,302**	0	**0.00**	5	**0.22**	41	**1.78**	46	**2.00**
Capiz	**2,697**	0	**0.00**	3	**0.11**	29	**1.08**	32	**1.19**
Guimaras	**1,758**	0	**0.00**	1	**0.06**	3	**0.17**	4	**0.23**
Iloilo	**13,281**	0	**0.00**	37	**0.28**	138	**1.04**	175	**1.32**
Negros Occidental	**4,913**	0	**0.00**	15	**0.31**	61	**1.24**	76	**1.55**
Bacolod City	**3,854**	2	**0.05**	18	**0.47**	109	**2.83**	129	**3.35**
Iloilo City	**2,582**	0	**0.00**	3	**0.12**	26	**1.01**	29	**1.12**

Table 1.B.1.10 - Prenatal Care

Number and proportion of pregnant women tested positive for Syphillis
Philippines, Annual 2020

Area	Total No. of Screened for syphillis	Tested Positive for Syphillis							
		Age Group						Total	%
		10-14 yrs old		15-19 yrs old		20-49 yrs old			
		No.	%	No.	%	No.	%		
**Region 7**	**22,926**	**0**	**0.00**	**76**	**0.33**	**870**	**3.79**	**946**	**4.13**
Bohol	**4,980**	0	**0.00**	0	**0.00**	9	**0.18**	9	**0.18**
Cebu	**3,612**	0	**0.00**	13	**0.36**	51	**1.41**	64	**1.77**
Negros Oriental	**1,429**	0	**0.00**	13	**0.91**	134	**9.38**	147	**10.29**
Siquijor	**1,031**	0	**0.00**	0	**0.00**	1	**0.10**	1	**0.10**
Cebu City	**7,643**	0	**0.00**	49	**0.64**	347	**4.54**	396	**5.18**
Lapu-Lapu City	**3,475**	0	**0.00**	1	**0.03**	13	**0.37**	14	**0.40**
Mandaue City	**756**	0	**0.00**	0	**0.00**	315	**41.67**	315	**41.67**
**Region 8**	**8,398**	**0**	**0.00**	**26**	**0.31**	**145**	**1.73**	**171**	**2.04**
Biliran	**1,733**	0	**0.00**	6	**0.35**	12	**0.69**	18	**1.04**
Eastern Samar	**1,083**	0	**0.00**	3	**0.28**	32	**2.95**	35	**3.23**
Northern Leyte	**0**	0	**0.00**	0	**0.00**	0	**0.00**	0	**0.00**
Northern Samar	**1,965**	0	**0.00**	3	**0.15**	39	**1.98**	42	**2.14**
Southern Leyte	**537**	0	**0.00**	1	**0.19**	6	**1.12**	7	**1.30**
Western Samar	**488**	0	**0.00**	7	**1.43**	19	**3.89**	26	**5.33**
Calbayog City	**168**	0	**0.00**	4	**2.38**	23	**13.69**	27	**16.07**
Maasin City	**0**	0	**0.00**	0	**0.00**	1	**0.00**	1	**0.00**
Ormoc City	**275**	0	**0.00**	1	**0.36**	3	**1.09**	4	**1.45**
Tacloban City	**2,149**	0	**0.00**	1	**0.05**	10	**0.47**	11	**0.51**
**Region 9**	**3,833**	**1**	**0.03**	**177**	**4.62**	**1,280**	**33.39**	**1,458**	**38.04**
Zamboanga del Norte	**551**	0	**0.00**	0	**0.00**	7	**1.27**	7	**1.27**
Zamboanga del Sur	**865**	0	**0.00**	59	**6.82**	555	**64.16**	614	**70.98**
Zamboanga Sibugay	**2,131**	1	**0.05**	92	**4.32**	592	**27.78**	685	**32.14**
Dapitan City	**160**	0	**0.00**	18	**11.25**	97	**60.63**	115	**71.88**
Dipolog City	**7**	0	**0.00**	0	**0.00**	1	**14.29**	1	**14.29**
Isabela City	**35**	0	**0.00**	8	**22.86**	27	**77.14**	35	**100.00**
Pagadian City	**78**	0	**0.00**	0	**0.00**	1	**1.28**	1	**1.28**
Zamboanga City	**6**	0	**0.00**	0	**0.00**	0	**0.00**	0	**0.00**
**Region 10**	**8,976**	**1**	**0.01**	**15**	**0.17**	**80**	**0.89**	**96**	**1.07**
Bukidnon	**1,313**	0	**0.00**	2	**0.15**	6	**0.46**	8	**0.61**
Camiguin	**448**	0	**0.00**	0	**0.00**	0	**0.00**	0	**0.00**
Lanao del Norte	**1,421**	0	**0.00**	2	**0.14**	2	**0.14**	4	**0.28**
Misamis Occidental	**226**	0	**0.00**	0	**0.00**	1	**0.44**	1	**0.44**
Misamis Oriental	**936**	0	**0.00**	2	**0.21**	25	**2.67**	27	**2.88**
Cagayan de Oro City	**2,299**	0	**0.00**	0	**0.00**	4	**0.17**	4	**0.17**
El Salvador City	**184**	0	**0.00**	0	**0.00**	0	**0.00**	0	**0.00**
Gingoog City	**0**	0	**0.00**	0	**0.00**	0	**0.00**	0	**0.00**
Iligan City	**102**	0	**0.00**	0	**0.00**	2	**1.96**	2	**1.96**
Malaybalay City	**643**	1	**0.16**	9	**1.40**	40	**6.22**	50	**7.78**

Table 1.B.1.10 - Prenatal Care

Number and proportion of pregnant women tested positive for Syphillis
Philippines, Annual 2020

Area	Total No. of Screened for syphillis	Tested Positive for Syphillis							
		Age Group						Total	%
		10-14 yrs old		15-19 yrs old		20-49 yrs old			
		No.	%	No.	%	No.	%		
Oroquieta City	0	0	0.00	0	0.00	0	0.00	0	0.00
Ozamis City	1,255	0	0.00	0	0.00	0	0.00	0	0.00
Tangub City	0	0	0.00	0	0.00	0	0.00	0	0.00
Valencia City	149	0	0.00	0	0.00	0	0.00	0	0.00
**Region 11**	**19,192**	**2**	**0.01**	**30**	**0.16**	**50**	**0.26**	**82**	**0.43**
Compostela Valley	324	0	0.00	0	0.00	1	0.31	1	0.31
Davao del Norte	5,963	0	0.00	1	0.02	3	0.05	4	0.07
Davao Oriental	92	0	0.00	0	0.00	0	0.00	0	0.00
Davao del Sur	481	0	0.00	0	0.00	4	0.83	4	0.83
Davao Occidental	264	0	0.00	8	3.03	11	4.17	19	7.20
Davao City	12,068	2	0.02	21	0.17	31	0.26	54	0.45
**Region 12**	**17,658**	**10**	**0.06**	**60**	**0.34**	**407**	**2.30**	**477**	**2.70**
North Cotabato	1,622	0	0.00	3	0.18	53	3.27	56	3.45
Sarangani	2,815	10	0.36	21	0.75	127	4.51	158	5.61
South Cotabato	4,345	0	0.00	12	0.28	58	1.33	70	1.61
Sultan Kudarat	5,037	0	0.00	5	0.10	38	0.75	43	0.85
Cotabato City	240	0	0.00	0	0.00	2	0.83	2	0.83
Gen. Santos City	3,599	0	0.00	19	0.53	129	3.58	148	4.11
**BARMM**	**801**	**0**	**0.00**	**0**	**0.00**	**0**	**0.00**	**0**	**0.00**
Basilan	5	0	0.00	0	0.00	0	0.00	0	0.00
Lanao del Sur	112	0	0.00	0	0.00	0	0.00	0	0.00
Maguindanao	658	0	0.00	0	0.00	0	0.00	0	0.00
Sulu	0	0	0.00	0	0.00	0	0.00	0	0.00
Tawi-Tawi	0	0	0.00	0	0.00	0	0.00	0	0.00
Lamitan City	0	0	0.00	0	0.00	0	0.00	0	0.00
Marawi City	26	0	0.00	0	0.00	0	0.00	0	0.00
**CARAGA**	**6,937**	**0**	**0.00**	**11**	**0.16**	**115**	**1.66**	**126**	**1.82**
Agusan del Norte	393	0	0.00	2	0.51	25	6.36	27	6.87
Agusan del Sur	1,266	0	0.00	4	0.32	19	1.50	23	1.82
Surigao del Norte	520	0	0.00	0	0.00	24	4.62	24	4.62
Surigao del Sur	674	0	0.00	2	0.30	13	1.93	15	2.23
Province of Dinagat	400	0	0.00	0	0.00	4	1.00	4	1.00
Bislig City	133	0	0.00	0	0.00	1	0.75	1	0.75
Butuan City	2,178	0	0.00	2	0.09	28	1.29	30	1.38
Surigao City	1,373	0	0.00	1	0.07	1	0.07	2	0.15

Note: Put asterisk (*) for No Report and Zero (0) for No Case

Table 1.B.1.11 - Prenatal Care

Number and proportion of pregnant women screened for Hepatitis B
Philippines, Annual 2020

Area	Eligible Pop.	Screened for Hepatitis B							
		Age Group						Total	%
		10-14 yrs old		15-19 yrs old		20-49 yrs old			
		No.	%	No.	%	No.	%		
**PHILIPPINES**	**2,123,158**	**1,004**	**0.05**	**53,477**	**2.52**	**396,220**	**18.66**	**450,701**	**21.23**
**N C R**	**238,661**	**140**	**0.06**	**7,052**	**2.95**	**64,044**	**26.83**	**71,236**	**29.85**
Malabon	**6,775**	11	**0.16**	294	**4.34**	1,653	**24.40**	**1,958**	**28.90**
Navotas	**4,621**	15	**0.32**	449	**9.72**	1,717	**37.16**	**2,181**	**47.20**
Valenzuela City	**11,500**	0	**0.00**	17	**0.15**	1,707	**14.84**	**1,724**	**14.99**
Caloocan City	**29,363**	15	**0.05**	1,142	**3.89**	7,353	**25.04**	**8,510**	**28.98**
Marikina City	**8,354**	0	**0.00**	140	**1.68**	1,108	**13.26**	**1,248**	**14.94**
Pasig City	**13,996**	11	**0.08**	521	**3.72**	6,772	**48.39**	**7,304**	**52.19**
Pateros	**1,184**	0	**0.00**	69	**5.83**	578	**48.82**	**647**	**54.65**
Taguig	**14,918**	9	**0.06**	321	**2.15**	5,008	**33.57**	**5,338**	**35.78**
Quezon City	**54,413**	39	**0.07**	2,181	**4.01**	20,049	**36.85**	**22,269**	**40.93**
Makati City	**10,798**	7	**0.06**	180	**1.67**	3,125	**28.94**	**3,312**	**30.67**
Mandaluyong City	**7,160**	1	**0.01**	201	**2.81**	1,975	**27.58**	**2,177**	**30.41**
San Juan	**2,261**	2	**0.09**	73	**3.23**	732	**32.38**	**807**	**35.69**
Manila City	**32,980**	13	**0.04**	574	**1.74**	3,395	**10.29**	**3,982**	**12.07**
Las Piñas City	**10,914**	4	**0.04**	212	**1.94**	2,174	**19.92**	**2,390**	**21.90**
Muntinlupa City	**9,353**	0	**0.00**	0	**0.00**	2,539	**27.15**	**2,539**	**27.15**
Parañaque City	**12,343**	8	**0.06**	519	**4.20**	2,833	**22.95**	**3,360**	**27.22**
Pasay City	**7,728**	5	**0.06**	159	**2.06**	1,326	**17.16**	**1,490**	**19.28**
**C A R**	**35,099**	**11**	**0.03**	**1,032**	**2.94**	**8,838**	**25.18**	**9,881**	**28.15**
Abra	**4,309**	0	**0.00**	47	**1.09**	340	**7.89**	**387**	**8.98**
Apayao	**2,451**	1	**0.04**	135	**5.51**	686	**27.99**	**822**	**33.54**
Benguet	**9,188**	4	**0.04**	300	**3.27**	2,709	**29.48**	**3,013**	**32.79**
Ifugao	**4,396**	0	**0.00**	125	**2.84**	1,287	**29.28**	**1,412**	**32.12**
Kalinga	**4,617**	3	**0.06**	90	**1.95**	708	**15.33**	**801**	**17.35**
Mt. Province	**3,023**	2	**0.07**	120	**3.97**	732	**24.21**	**854**	**28.25**
Baguio City	**7,115**	1	**0.01**	215	**3.02**	2,376	**33.39**	**2,592**	**36.43**
**Region 1**	**97,261**	**37**	**0.04**	**2,634**	**2.71**	**25,807**	**26.53**	**28,478**	**29.28**
Ilocos Norte	**8,105**	6	**0.07**	297	**3.66**	3,237	**39.94**	**3,540**	**43.68**
Ilocos Sur	**9,330**	5	**0.05**	385	**4.13**	3,872	**41.50**	**4,262**	**45.68**
La Union	**11,511**	10	**0.09**	386	**3.35**	3,234	**28.09**	**3,630**	**31.54**
Pangasinan	**50,168**	10	**0.02**	1,088	**2.17**	9,181	**18.30**	**10,279**	**20.49**
Alaminos City	**1,894**	0	**0.00**	76	**4.01**	942	**49.74**	**1,018**	**53.75**
Candon City	**987**	0	**0.00**	8	**0.81**	772	**78.22**	**780**	**79.03**
Dagupan City	**3,620**	1	**0.03**	77	**2.13**	780	**21.55**	**858**	**23.70**
Laoag City	**1,870**	0	**0.00**	59	**3.16**	1,758	**94.01**	**1,817**	**97.17**
San Carlos City	**3,979**	0	**0.00**	23	**0.58**	370	**9.30**	**393**	**9.88**
San Fernando City	**2,115**	0	**0.00**	98	**4.63**	828	**39.15**	**926**	**43.78**
Urdaneta City	**2,806**	5	**0.18**	116	**4.13**	728	**25.94**	**849**	**30.26**

Table 1.B.1.11 - Prenatal Care

Number and proportion of pregnant women screened for Hepatitis B

Philippines, Annual 2020

Area	Eligible Pop.	Screened for Hepatitis B							
		Age Group						Total	%
		10-14 yrs old		15-19 yrs old		20-49 yrs old			
		No.	%	No.	%	No.	%		
Vigan City	**876**	0	**0.00**	21	**2.40**	105	**11.99**	**126**	**14.38**
**Region 2**	**69,443**	**34**	**0.05**	**2,011**	**2.90**	**13,564**	**19.53**	**15,609**	**22.48**
Batanes	**340**	0	**0.00**	6	**1.76**	134	**39.41**	**140**	**41.18**
Cagayan	**17,971**	3	**0.02**	151	**0.84**	886	**4.93**	**1,040**	**5.79**
Isabela	**26,309**	20	**0.08**	805	**3.06**	5,226	**19.86**	**6,051**	**23.00**
Nueva Vizcaya	**9,573**	7	**0.07**	488	**5.10**	3,435	**35.88**	**3,930**	**41.05**
Quirino	**4,025**	1	**0.02**	229	**5.69**	1,689	**41.96**	**1,919**	**47.68**
Cauayan City	**2,590**	2	**0.08**	111	**4.29**	694	**26.80**	**807**	**31.16**
Ilagan City	**2,911**	1	**0.03**	143	**4.91**	972	**33.39**	**1,116**	**38.34**
Santiago City	**2,692**	0	**0.00**	78	**2.90**	515	**19.13**	**593**	**22.03**
Tuguegarao City	**3,032**	0	**0.00**	0	**0.00**	13	**0.43**	**13**	**0.43**
**Region 3**	**220,020**	**204**	**0.09**	**9,850**	**4.48**	**62,129**	**28.24**	**72,183**	**32.81**
Aurora	**4,770**	2	**0.04**	294	**6.16**	1,767	**37.04**	**2,063**	**43.25**
Bataan	**13,823**	19	**0.14**	842	**6.09**	5,901	**42.69**	**6,762**	**48.92**
Bulacan	**43,627**	57	**0.13**	2,124	**4.87**	12,373	**28.36**	**14,554**	**33.36**
Nueva Ecija	**28,782**	26	**0.09**	957	**3.32**	4,605	**16.00**	**5,588**	**19.41**
Pampanga	**31,359**	11	**0.04**	729	**2.32**	5,008	**15.97**	**5,748**	**18.33**
Tarlac	**19,765**	14	**0.07**	630	**3.19**	5,091	**25.76**	**5,735**	**29.02**
Zambales	**12,610**	2	**0.02**	489	**3.88**	2,663	**21.12**	**3,154**	**25.01**
Angeles City	**7,869**	1	**0.01**	95	**1.21**	594	**7.55**	**690**	**8.77**
Balanga City	**1,998**	5	**0.25**	164	**8.21**	797	**39.89**	**966**	**48.35**
Cabanatuan City	**5,896**	4	**0.07**	211	**3.58**	1,127	**19.11**	**1,342**	**22.76**
City of San Fernando	**5,863**	4	**0.07**	328	**5.59**	2,131	**36.35**	**2,463**	**42.01**
Gapan City	**2,153**	0	**0.00**	112	**5.20**	599	**27.82**	**711**	**33.02**
Mabalacat City	**4,793**	7	**0.15**	381	**7.95**	2,163	**45.13**	**2,551**	**53.22**
Malolos City	**4,880**	2	**0.04**	117	**2.40**	849	**17.40**	**968**	**19.84**
Meycauayan	**4,041**	16	**0.40**	347	**8.59**	1,677	**41.50**	**2,040**	**50.48**
Olongapo	**4,971**	0	**0.00**	223	**4.49**	1,154	**23.21**	**1,377**	**27.70**
Palayan City	**799**	2	**0.25**	32	**4.01**	151	**18.90**	**185**	**23.15**
San Jose City	**2,718**	10	**0.37**	117	**4.30**	576	**21.19**	**703**	**25.86**
San Jose del Monte City	**11,100**	14	**0.13**	1,188	**10.70**	10,513	**94.71**	**11,715**	**105.54**
Science City of Munoz	**1,586**	3	**0.19**	76	**4.79**	405	**25.54**	**484**	**30.52**
Tarlac City	**6,617**	5	**0.08**	394	**5.95**	1,985	**30.00**	**2,384**	**36.03**
**Region 4A**	**296,816**	**97**	**0.03**	**6,909**	**2.33**	**53,769**	**18.12**	**60,775**	**20.48**
Batangas	**38,441**	5	**0.01**	284	**0.74**	3,729	**9.70**	**4,018**	**10.45**
Cavite	**27,704**	18	**0.06**	797	**2.88**	7,980	**28.80**	**8,795**	**31.75**
Laguna	**19,727**	15	**0.08**	602	**3.05**	4,208	**21.33**	**4,825**	**24.46**
Quezon	**37,410**	16	**0.04**	811	**2.17**	5,142	**13.74**	**5,969**	**15.96**
Rizal	**44,791**	13	**0.03**	1,198	**2.67**	7,486	**16.71**	**8,697**	**19.42**
Antipolo City	**16,497**	4	**0.02**	484	**2.93**	1,668	**10.11**	**2,156**	**13.07**
Bacoor City	**12,142**	1	**0.01**	120	**0.99**	847	**6.98**	**968**	**7.97**
Batangas City	**6,823**	0	**0.00**	0	**0.00**	0	**0.00**	**0**	**0.00**

Table 1.B.1.11 - Prenatal Care

Number and proportion of pregnant women screened for Hepatitis B
Philippines, Annual 2020

Area	Eligible Pop.	Screened for Hepatitis B							
		Age Group						Total	%
		10-14 yrs old		15-19 yrs old		20-49 yrs old			
		No.	%	No.	%	No.	%		
Biñan City	**6,607**	3	**0.05**	553	**8.37**	4,093	**61.95**	**4,649**	**70.36**
Cabuyao City	**6,130**	1	**0.02**	278	**4.54**	3,105	**50.65**	**3,384**	**55.20**
Calamba City	**9,028**	4	**0.04**	247	**2.74**	2,229	**24.69**	**2,480**	**27.47**
Cavite City	**2,075**	1	**0.05**	32	**1.54**	157	**7.57**	**190**	**9.16**
Dasmariñas City	**13,322**	2	**0.02**	265	**1.99**	2,421	**18.17**	**2,688**	**20.18**
General Trias City	**6,352**	0	**0.00**	64	**1.01**	639	**10.06**	**703**	**11.07**
Imus City	**8,156**	0	**0.00**	91	**1.12**	893	**10.95**	**984**	**12.06**
Lipa City	**6,880**	0	**0.00**	0	**0.00**	0	**0.00**	**0**	**0.00**
Lucena City	**5,672**	0	**0.00**	0	**0.00**	0	**0.00**	**0**	**0.00**
San Pablo City	**5,277**	1	**0.02**	2	**0.04**	291	**5.51**	**294**	**5.57**
San Pedro City	**6,467**	2	**0.03**	169	**2.61**	1,179	**18.23**	**1,350**	**20.88**
Santa Rosa City	**7,025**	4	**0.06**	300	**4.27**	2,340	**33.31**	**2,644**	**37.64**
Tagaytay City	**1,437**	2	**0.14**	76	**5.29**	1,268	**88.24**	**1,346**	**93.67**
Tanauan City	**3,584**	1	**0.03**	52	**1.45**	1,460	**40.74**	**1,513**	**42.22**
Tayabas City	**2,122**	0	**0.00**	0	**0.00**	0	**0.00**	**0**	**0.00**
Trece Martires City	**3,147**	4	**0.13**	484	**15.38**	2,634	**83.70**	**3,122**	**99.21**
**Region 4B**	**71,246**	**78**	**0.11**	**1,089**	**1.53**	**7,074**	**9.93**	**8,241**	**11.57**
Marinduque	**4,836**	2	**0.04**	120	**2.48**	1,257	**25.99**	**1,379**	**28.52**
Mindoro Occidental	**12,407**	2	**0.02**	222	**1.79**	1,688	**13.61**	**1,912**	**15.41**
Mindoro Oriental	**19,809**	70	**0.35**	568	**2.87**	3,013	**15.21**	**3,651**	**18.43**
Palawan	**21,375**	2	**0.01**	107	**0.50**	499	**2.33**	**608**	**2.84**
Romblon	**6,400**	2	**0.03**	72	**1.13**	617	**9.64**	**691**	**10.80**
Puerto Princesa City	**6,419**	0	**0.00**	0	**0.00**	0	**0.00**	**0**	**0.00**
**Region 5**	**136,116**	**24**	**0.02**	**1,910**	**1.40**	**17,085**	**12.55**	**19,019**	**13.97**
Albay	**23,737**	11	**0.05**	298	**1.26**	4,376	**18.44**	**4,685**	**19.74**
Camarines Norte	**14,283**	0	**0.00**	249	**1.74**	1,869	**13.09**	**2,118**	**14.83**
Camarines Sur	**38,796**	3	**0.01**	205	**0.53**	1,886	**4.86**	**2,094**	**5.40**
Catanduanes	**6,274**	3	**0.05**	199	**3.17**	1,317	**20.99**	**1,519**	**24.21**
Masbate	**22,521**	2	**0.01**	533	**2.37**	2,919	**12.96**	**3,454**	**15.34**
Sorsogon	**19,065**	3	**0.02**	208	**1.09**	2,076	**10.89**	**2,287**	**12.00**
Iriga City	**2,638**	1	**0.04**	64	**2.43**	606	**22.97**	**671**	**25.44**
Legaspi City	**4,174**	1	**0.02**	142	**3.40**	1,952	**46.77**	**2,095**	**50.19**
Naga City	**4,628**	0	**0.00**	12	**0.26**	84	**1.82**	**96**	**2.07**
**Region 6**	**146,526**	**73**	**0.05**	**5,249**	**3.58**	**43,021**	**29.36**	**48,343**	**32.99**
Aklan	**11,162**	15	**0.13**	469	**4.20**	3,793	**33.98**	**4,277**	**38.32**
Antique	**12,816**	3	**0.02**	283	**2.21**	2,459	**19.19**	**2,745**	**21.42**
Capiz	**13,986**	1	**0.01**	345	**2.47**	3,316	**23.71**	**3,662**	**26.18**
Guimaras	**3,085**	2	**0.06**	188	**6.09**	1,618	**52.45**	**1,808**	**58.61**
Iloilo	**36,541**	17	**0.05**	1,358	**3.72**	13,899	**38.04**	**15,274**	**41.80**
Negros Occidental	**49,368**	25	**0.05**	1,730	**3.50**	12,038	**24.38**	**13,793**	**27.94**
Bacolod City	**11,115**	8	**0.07**	538	**4.84**	3,498	**31.47**	**4,044**	**36.38**
Iloilo City	**8,453**	2	**0.02**	338	**4.00**	2,400	**28.39**	**2,740**	**32.41**

Table 1.B.1.11 - Prenatal Care

Number and proportion of pregnant women screened for Hepatitis B

Philippines, Annual 2020

Area	Eligible Pop.	Screened for Hepatitis B							
		Age Group						Total	%
		10-14 yrs old		15-19 yrs old		20-49 yrs old			
		No.	%	No.	%	No.	%		
**Region 7**	**163,262**	**24**	**0.01**	**2,617**	**1.60**	**25,357**	**15.53**	**27,998**	**17.15**
Bohol	**27,312**	0	**0.00**	121	**0.44**	5,536	**20.27**	**5,657**	**20.71**
Cebu	**67,506**	0	**0.00**	314	**0.47**	2,681	**3.97**	**2,995**	**4.44**
Negros Oriental	**27,938**	10	**0.04**	712	**2.55**	5,352	**19.16**	**6,074**	**21.74**
Siquijor	**1,613**	0	**0.00**	78	**4.84**	804	**49.85**	**882**	**54.68**
Cebu City	**21,193**	13	**0.06**	999	**4.71**	6,717	**31.69**	**7,729**	**36.47**
Lapu-Lapu City	**9,372**	1	**0.01**	393	**4.19**	4,267	**45.53**	**4,661**	**49.73**
Mandaue City	**8,328**	0	**0.00**	0	**0.00**	0	**0.00**	**0**	**0.00**
**Region 8**	**102,619**	**12**	**0.01**	**1,443**	**1.41**	**11,342**	**11.05**	**12,797**	**12.47**
Biliran	**3,834**	2	**0.05**	232	**6.05**	1,359	**35.45**	**1,593**	**41.55**
Eastern Samar	**11,392**	1	**0.01**	124	**1.09**	943	**8.28**	**1,068**	**9.38**
Northern Leyte	**34,707**	0	**0.00**	0	**0.00**	0	**0.00**	**0**	**0.00**
Northern Samar	**15,370**	4	**0.03**	306	**1.99**	3,077	**20.02**	**3,387**	**22.04**
Southern Leyte	**6,451**	3	**0.05**	273	**4.23**	2,216	**34.35**	**2,492**	**38.63**
Western Samar	**14,305**	1	**0.01**	154	**1.08**	1,003	**7.01**	**1,158**	**8.10**
Calbayog City	**4,413**	0	**0.00**	24	**0.54**	132	**2.99**	**156**	**3.54**
Maasin City	**1,637**	0	**0.00**	0	**0.00**	0	**0.00**	**0**	**0.00**
Ormoc City	**4,941**	0	**0.00**	29	**0.59**	406	**8.22**	**435**	**8.80**
Tacloban City	**5,569**	1	**0.02**	301	**5.40**	2,206	**39.61**	**2,508**	**45.04**
**Region 9**	**80,051**	**0**	**0.00**	**198**	**0.25**	**1,294**	**1.62**	**1,492**	**1.86**
Zamboanga del Norte	**17,249**	0	**0.00**	76	**0.44**	536	**3.11**	**612**	**3.55**
Zamboanga del Sur	**17,653**	0	**0.00**	39	**0.22**	314	**1.78**	**353**	**2.00**
Zamboanga Sibugay	**14,954**	0	**0.00**	20	**0.13**	134	**0.90**	**154**	**1.03**
Dapitan City	**1,784**	0	**0.00**	27	**1.51**	131	**7.34**	**158**	**8.86**
Dipolog City	**2,827**	0	**0.00**	0	**0.00**	0	**0.00**	**0**	**0.00**
Isabela City	**2,522**	0	**0.00**	29	**1.15**	105	**4.16**	**134**	**5.31**
Pagadian City	**4,325**	0	**0.00**	5	**0.12**	25	**0.58**	**30**	**0.69**
Zamboanga City	**18,737**	0	**0.00**	2	**0.01**	49	**0.26**	**51**	**0.27**
**Region 10**	**101,411**	**41**	**0.04**	**3,791**	**3.74**	**18,996**	**18.73**	**22,828**	**22.51**
Bukidnon	**23,706**	11	**0.05**	576	**2.43**	2,377	**10.03**	**2,964**	**12.50**
Camiguin	**1,858**	0	**0.00**	36	**1.94**	401	**21.58**	**437**	**23.52**
Lanao del Norte	**14,960**	1	**0.01**	32	**0.21**	263	**1.76**	**296**	**1.98**
Misamis Occidental	**6,403**	0	**0.00**	32	**0.50**	344	**5.37**	**376**	**5.87**
Misamis Oriental	**15,131**	8	**0.05**	817	**5.40**	4,681	**30.94**	**5,506**	**36.39**
Cagayan de Oro City	**14,339**	15	**0.10**	1,509	**10.52**	6,840	**47.70**	**8,364**	**58.33**
El Salvador City	**1,065**	0	**0.00**	72	**6.76**	457	**42.91**	**529**	**49.67**
Gingoog City	**2,644**	2	**0.08**	135	**5.11**	609	**23.03**	**746**	**28.21**
Iligan City	**7,580**	0	**0.00**	78	**1.03**	508	**6.70**	**586**	**7.73**
Malaybalay City	**3,956**	1	**0.03**	207	**5.23**	960	**24.27**	**1,168**	**29.52**

Table 1.B.1.11 - Prenatal Care

Number and proportion of pregnant women screened for Hepatitis B
Philippines, Annual 2020

Area	Eligible Pop.	Screened for Hepatitis B							
		Age Group						Total	%
		10-14 yrs old		15-19 yrs old		20-49 yrs old			
		No.	%	No.	%	No.	%		
Oroquieta City	**1,389**	0	**0.00**	0	**0.00**	0	**0.00**	**0**	**0.00**
Ozamis City	**2,778**	2	**0.07**	204	**7.34**	1,117	**40.21**	**1,323**	**47.62**
Tangub City	**1,234**	0	**0.00**	0	**0.00**	0	**0.00**	**0**	**0.00**
Valencia City	**4,368**	1	**0.02**	93	**2.13**	439	**10.05**	**533**	**12.20**
**Region 11**	**107,247**	**161**	**0.15**	**2,805**	**2.62**	**16,323**	**15.22**	**19,289**	**17.99**
Compostela Valley	**15,562**	3	**0.02**	39	**0.25**	277	**1.78**	**319**	**2.05**
Davao del Norte	**21,326**	29	**0.14**	681	**3.19**	4,047	**18.98**	**4,757**	**22.31**
Davao Oriental	**13,007**	1	**0.01**	43	**0.33**	214	**1.65**	**258**	**1.98**
Davao del Sur	**14,151**	7	**0.05**	144	**1.02**	822	**5.81**	**973**	**6.88**
Davao Occidental	**6,670**	0	**0.00**	1	**0.01**	8	**0.12**	**9**	**0.13**
Davao City	**36,531**	121	**0.33**	1,897	**5.19**	10,955	**29.99**	**12,973**	**35.51**
**Region 12**	**104,552**	**49**	**0.05**	**3,348**	**3.20**	**18,034**	**17.25**	**21,431**	**20.50**
North Cotabato	**33,645**	10	**0.03**	797	**2.37**	4,543	**13.50**	**5,350**	**15.90**
Sarangani	**12,891**	17	**0.13**	947	**7.35**	3,904	**30.28**	**4,868**	**37.76**
South Cotabato	**21,113**	3	**0.01**	487	**2.31**	3,357	**15.90**	**3,847**	**18.22**
Sultan Kudarat	**17,359**	16	**0.09**	781	**4.50**	4,133	**23.81**	**4,930**	**28.40**
Cotabato City	**5,835**	0	**0.00**	48	**0.82**	312	**5.35**	**360**	**6.17**
Gen. Santos City	**13,709**	3	**0.02**	288	**2.10**	1,785	**13.02**	**2,076**	**15.14**
**BARMM**	**92,799**	**0**	**0.00**	**55**	**0.06**	**866**	**0.93**	**921**	**0.99**
Basilan	**7,541**	0	**0.00**	0	**0.00**	0	**0.00**	**0**	**0.00**
Lanao del Sur	**21,131**	0	**0.00**	1	**0.00**	336	**1.59**	**337**	**1.59**
Maguindanao	**31,128**	0	**0.00**	6	**0.02**	25	**0.08**	**31**	**0.10**
Sulu	**16,613**	0	**0.00**	0	**0.00**	0	**0.00**	**0**	**0.00**
Tawi-Tawi	**9,259**	0	**0.00**	0	**0.00**	0	**0.00**	**0**	**0.00**
Lamitan City	**2,074**	0	**0.00**	0	**0.00**	0	**0.00**	**0**	**0.00**
Marawi City	**5,053**	0	**0.00**	48	**0.95**	505	**9.99**	**553**	**10.94**
**CARAGA**	**60,029**	**19**	**0.03**	**1,484**	**2.47**	**8,677**	**14.45**	**10,180**	**16.96**
Agusan del Norte	**8,098**	3	**0.04**	223	**2.75**	1,186	**14.65**	**1,412**	**17.44**
Agusan del Sur	**17,592**	4	**0.02**	170	**0.97**	719	**4.09**	**893**	**5.08**
Surigao del Norte	**7,089**	2	**0.03**	177	**2.50**	1,380	**19.47**	**1,559**	**21.99**
Surigao del Sur	**11,482**	0	**0.00**	147	**1.28**	1,085	**9.45**	**1,232**	**10.73**
Province of Dinagat	**2,573**	1	**0.04**	51	**1.98**	400	**15.55**	**452**	**17.57**
Bislig City	**2,179**	0	**0.00**	1	**0.05**	24	**1.10**	**25**	**1.15**
Butuan City	**7,715**	5	**0.06**	486	**6.30**	2,376	**30.80**	**2,867**	**37.16**
Surigao City	**3,301**	4	**0.12**	229	**6.94**	1,507	**45.65**	**1,740**	**52.71**

Note: Put asterisk (*) for No Report and Zero (0) for No Case

Table 1.B.1.12 - Prenatal Care

Number and proportion of pregnant women tested positive for Hepatitis B
Philippines, Annual 2020

Area	Total No. of Screened for Hepatitis B	Tested Positive for Hepatitis B							
		Age Group						Total	%
		10-14 yrs old		15-19 yrs old		20-49 yrs old			
		No.	%	No.	%	No.	%		
**PHILIPPINES**	**450,701**	**53**	**0.01**	**1,394**	**0.31**	**12,277**	**2.72**	**13,724**	**3.05**
**N C R**	**71,236**	**15**	**0.02**	**128**	**0.18**	**1,106**	**1.55**	**1,249**	**1.75**
Malabon	**1,958**	0	**0.00**	3	**0.15**	15	**0.77**	**18**	**0.92**
Navotas	**2,181**	0	**0.00**	24	**1.10**	74	**3.39**	**98**	**4.49**
Valenzuela City	**1,724**	0	**0.00**	1	**0.06**	38	**2.20**	**39**	**2.26**
Caloocan City	**8,510**	1	**0.01**	19	**0.22**	87	**1.02**	**107**	**1.26**
Marikina City	**1,248**	0	**0.00**	0	**0.00**	4	**0.32**	**4**	**0.32**
Pasig City	**7,304**	0	**0.00**	2	**0.03**	51	**0.70**	**53**	**0.73**
Pateros	**647**	0	**0.00**	1	**0.15**	11	**1.70**	**12**	**1.85**
Taguig	**5,338**	14	**0.26**	23	**0.43**	155	**2.90**	**192**	**3.60**
Quezon City	**22,269**	0	**0.00**	40	**0.18**	385	**1.73**	**425**	**1.91**
Makati City	**3,312**	0	**0.00**	0	**0.00**	68	**2.05**	**68**	**2.05**
Mandaluyong City	**2,177**	0	**0.00**	0	**0.00**	15	**0.69**	**15**	**0.69**
San Juan	**807**	0	**0.00**	0	**0.00**	0	**0.00**	**0**	**0.00**
Manila City	**3,982**	0	**0.00**	1	**0.03**	13	**0.33**	**14**	**0.35**
Las Piñas City	**2,390**	0	**0.00**	3	**0.13**	41	**1.72**	**44**	**1.84**
Muntinlupa City	**2,539**	0	**0.00**	0	**0.00**	28	**1.10**	**28**	**1.10**
Parañaque City	**3,360**	0	**0.00**	10	**0.30**	94	**2.80**	**104**	**3.10**
Pasay City	**1,490**	0	**0.00**	1	**0.07**	27	**1.81**	**28**	**1.88**
**C A R**	**9,881**	**0**	**0.00**	**20**	**0.20**	**177**	**1.79**	**197**	**1.99**
Abra	**387**	0	**0.00**	0	**0.00**	2	**0.52**	**2**	**0.52**
Apayao	**822**	0	**0.00**	3	**0.36**	25	**3.04**	**28**	**3.41**
Benguet	**3,013**	0	**0.00**	3	**0.10**	15	**0.50**	**18**	**0.60**
Ifugao	**1,412**	0	**0.00**	4	**0.28**	21	**1.49**	**25**	**1.77**
Kalinga	**801**	0	**0.00**	1	**0.12**	18	**2.25**	**19**	**2.37**
Mt. Province	**854**	0	**0.00**	4	**0.47**	9	**1.05**	**13**	**1.52**
Baguio City	**2,592**	0	**0.00**	5	**0.19**	87	**3.36**	**92**	**3.55**
**Region 1**	**28,478**	**3**	**0.01**	**35**	**0.12**	**301**	**1.06**	**339**	**1.19**
Ilocos Norte	**3,540**	0	**0.00**	2	**0.06**	54	**1.53**	**56**	**1.58**
Ilocos Sur	**4,262**	0	**0.00**	8	**0.19**	111	**2.60**	**119**	**2.79**
La Union	**3,630**	2	**0.06**	14	**0.39**	38	**1.05**	**54**	**1.49**
Pangasinan	**10,279**	1	**0.01**	10	**0.10**	89	**0.87**	**100**	**0.97**
Alaminos City	**1,018**	0	**0.00**	0	**0.00**	0	**0.00**	**0**	**0.00**
Candon City	**780**	0	**0.00**	0	**0.00**	0	**0.00**	**0**	**0.00**
Dagupan City	**858**	0	**0.00**	0	**0.00**	6	**0.70**	**6**	**0.70**
Laoag City	**1,817**	0	**0.00**	0	**0.00**	0	**0.00**	**0**	**0.00**
San Carlos City	**393**	0	**0.00**	0	**0.00**	0	**0.00**	**0**	**0.00**
San Fernando City	**926**	0	**0.00**	1	**0.11**	3	**0.32**	**4**	**0.43**
Urdaneta City	**849**	0	**0.00**	0	**0.00**	0	**0.00**	**0**	**0.00**

Table 1.B.1.12 - Prenatal Care

Number and proportion of pregnant women tested positive for Hepatitis B

Philippines, Annual 2020

Area	Total No. of Screened for Hepatitis B	Tested Positive for Hepatitis B							
		Age Group						Total	%
		10-14 yrs old		15-19 yrs old		20-49 yrs old			
		No.	%	No.	%	No.	%		
Vigan City	126	0	0.00	0	0.00	0	0.00	0	0.00
**Region 2**	15,609	4	0.03	39	0.25	277	1.77	320	2.05
Batanes	140	0	0.00	1	0.71	3	2.14	4	2.86
Cagayan	1,040	0	0.00	1	0.10	9	0.87	10	0.96
Isabela	6,051	2	0.03	9	0.15	85	1.40	96	1.59
Nueva Vizcaya	3,930	1	0.03	22	0.56	125	3.18	148	3.77
Quirino	1,919	1	0.05	1	0.05	15	0.78	17	0.89
Cauayan City	807	0	0.00	1	0.12	0	0.00	1	0.12
Ilagan City	1,116	0	0.00	0	0.00	11	0.99	11	0.99
Santiago City	593	0	0.00	4	0.67	29	4.89	33	5.56
Tuguegarao City	13	0	0.00	0	0.00	0	0.00	0	0.00
**Region 3**	72,183	3	0.00	175	0.24	1,232	1.71	1,410	1.95
Aurora	2,063	0	0.00	2	0.10	33	1.60	35	1.70
Bataan	6,762	0	0.00	12	0.18	112	1.66	124	1.83
Bulacan	14,554	0	0.00	75	0.52	569	3.91	644	4.42
Nueva Ecija	5,588	0	0.00	11	0.20	64	1.15	75	1.34
Pampanga	5,748	0	0.00	25	0.43	129	2.24	154	2.68
Tarlac	5,735	0	0.00	11	0.19	70	1.22	81	1.41
Zambales	3,154	3	0.10	14	0.44	54	1.71	71	2.25
Angeles City	690	0	0.00	0	0.00	0	0.00	0	0.00
Balanga City	966	0	0.00	1	0.10	8	0.83	9	0.93
Cabanatuan City	1,342	0	0.00	0	0.00	3	0.22	3	0.22
City of San Fernando	2,463	0	0.00	0	0.00	10	0.41	10	0.41
Gapan City	711	0	0.00	0	0.00	1	0.14	1	0.14
Mabalacat City	2,551	0	0.00	0	0.00	10	0.39	10	0.39
Malolos City	968	0	0.00	0	0.00	7	0.72	7	0.72
Meycauayan	2,040	0	0.00	8	0.39	61	2.99	69	3.38
Olongapo	1,377	0	0.00	6	0.44	43	3.12	49	3.56
Palayan City	185	0	0.00	0	0.00	0	0.00	0	0.00
San Jose City	703	0	0.00	1	0.14	2	0.28	3	0.43
San Jose del Monte City	11,715	0	0.00	6	0.05	40	0.34	46	0.39
Science City of Munoz	484	0	0.00	3	0.62	10	2.07	13	2.69
Tarlac City	2,384	0	0.00	0	0.00	6	0.25	6	0.25
**Region 4A**	60,775	9	0.01	386	0.64	4,468	7.35	4,863	8.00
Batangas	4,018	0	0.00	6	0.15	34	0.85	40	1.00
Cavite	8,795	0	0.00	31	0.35	253	2.88	284	3.23
Laguna	4,825	0	0.00	138	2.86	1,759	36.46	1,897	39.32
Quezon	5,969	1	0.02	36	0.60	253	4.24	290	4.86
Rizal	8,697	0	0.00	29	0.33	202	2.32	231	2.66
Antipolo City	2,156	4	0.19	69	3.20	381	17.67	454	21.06
Bacoor City	968	0	0.00	12	1.24	40	4.13	52	5.37
Batangas City	0	0	0.00	0	0.00	0	0.00	0	0.00

Table 1.B.1.12 - Prenatal Care

Number and proportion of pregnant women tested positive for Hepatitis B
Philippines, Annual 2020

Area	Total No. of Screened for Hepatitis B	Tested Positive for Hepatitis B							
		Age Group						Total	%
		10-14 yrs old		15-19 yrs old		20-49 yrs old			
		No.	%	No.	%	No.	%		
Biñan City	4,649	0	0.00	9	0.19	138	2.97	147	3.16
Cabuyao City	3,384	0	0.00	18	0.53	768	22.70	786	23.23
Calamba City	2,480	0	0.00	2	0.08	130	5.24	132	5.32
Cavite City	190	0	0.00	1	0.53	5	2.63	6	3.16
Dasmariñas City	2,688	0	0.00	8	0.30	74	2.75	82	3.05
General Trias City	703	0	0.00	0	0.00	9	1.28	9	1.28
Imus City	984	4	0.41	1	0.10	21	2.13	26	2.64
Lipa City	0	0	0.00	0	0.00	0	0.00	0	0.00
Lucena City	0	0	0.00	0	0.00	0	0.00	0	0.00
San Pablo City	294	0	0.00	0	0.00	231	78.57	231	78.57
San Pedro City	1,350	0	0.00	4	0.30	50	3.70	54	4.00
Santa Rosa City	2,644	0	0.00	22	0.83	85	3.21	107	4.05
Tagaytay City	1,346	0	0.00	0	0.00	6	0.45	6	0.45
Tanauan City	1,513	0	0.00	0	0.00	13	0.86	13	0.86
Tayabas City	0	0	0.00	0	0.00	0	0.00	0	0.00
Trece Martires City	3,122	0	0.00	0	0.00	16	0.51	16	0.51
**Region 4B**	**8,241**	**1**	**0.01**	**26**	**0.32**	**191**	**2.32**	**218**	**2.65**
Marinduque	1,379	0	0.00	4	0.29	15	1.09	19	1.38
Mindoro Occidental	1,912	0	0.00	7	0.37	54	2.82	61	3.19
Mindoro Oriental	3,651	1	0.03	11	0.30	82	2.25	94	2.57
Palawan	608	0	0.00	3	0.49	18	2.96	21	3.45
Romblon	691	0	0.00	1	0.14	22	3.18	23	3.33
Puerto Princesa City	0	0	0.00	0	0.00	0	0.00	0	0.00
**Region 5**	**19,019**	**0**	**0.00**	**39**	**0.21**	**481**	**2.53**	**520**	**2.73**
Albay	4,685	0	0.00	1	0.02	115	2.45	116	2.48
Camarines Norte	2,118	0	0.00	3	0.14	47	2.22	50	2.36
Camarines Sur	2,094	0	0.00	2	0.10	52	2.48	54	2.58
Catanduanes	1,519	0	0.00	16	1.05	71	4.67	87	5.73
Masbate	3,454	0	0.00	16	0.46	114	3.30	130	3.76
Sorsogon	2,287	0	0.00	1	0.04	46	2.01	47	2.06
Iriga City	671	0	0.00	0	0.00	11	1.64	11	1.64
Legaspi City	2,095	0	0.00	0	0.00	25	1.19	25	1.19
Naga City	96	0	0.00	0	0.00	0	0.00	0	0.00
**Region 6**	**48,343**	**2**	**0.00**	**144**	**0.30**	**1,296**	**2.68**	**1,442**	**2.98**
Aklan	4,277	1	0.02	22	0.51	171	4.00	194	4.54
Antique	2,745	1	0.04	6	0.22	71	2.59	78	2.84
Capiz	3,662	0	0.00	16	0.44	167	4.56	183	5.00
Guimaras	1,808	0	0.00	1	0.06	33	1.83	34	1.88
Iloilo	15,274	0	0.00	21	0.14	369	2.42	390	2.55
Negros Occidental	13,793	0	0.00	34	0.25	287	2.08	321	2.33
Bacolod City	4,044	0	0.00	38	0.94	160	3.96	198	4.90
Iloilo City	2,740	0	0.00	6	0.22	38	1.39	44	1.61

Table 1.B.1.12 - Prenatal Care

Number and proportion of pregnant women tested positive for Hepatitis B
Philippines, Annual 2020

Area	Total No. of Screened for Hepatitis B	Tested Positive for Hepatitis B							
		Age Group						Total	%
		10-14 yrs old		15-19 yrs old		20-49 yrs old			
		No.	%	No.	%	No.	%		
**Region 7**	**27,998**	**0**	**0.00**	**104**	**0.37**	**842**	**3.01**	**946**	**3.38**
Bohol	**5,657**	0	**0.00**	18	**0.32**	141	**2.49**	**159**	**2.81**
Cebu	**2,995**	0	**0.00**	11	**0.37**	88	**2.94**	**99**	**3.31**
Negros Oriental	**6,074**	0	**0.00**	24	**0.40**	137	**2.26**	**161**	**2.65**
Siquijor	**882**	0	**0.00**	1	**0.11**	15	**1.70**	**16**	**1.81**
Cebu City	**7,729**	0	**0.00**	50	**0.65**	451	**5.84**	**501**	**6.48**
Lapu-Lapu City	**4,661**	0	**0.00**	0	**0.00**	10	**0.21**	**10**	**0.21**
Mandaue City	**0**	0	**0.00**	0	**0.00**	0	**0.00**	**0**	**0.00**
**Region 8**	**12,797**	**0**	**0.00**	**45**	**0.35**	**398**	**3.11**	**443**	**3.46**
Biliran	**1,593**	0	**0.00**	7	**0.44**	38	**2.39**	**45**	**2.82**
Eastern Samar	**1,068**	0	**0.00**	6	**0.56**	72	**6.74**	**78**	**7.30**
Northern Leyte	**0**	0	**0.00**	0	**0.00**	0	**0.00**	**0**	**0.00**
Northern Samar	**3,387**	0	**0.00**	9	**0.27**	77	**2.27**	**86**	**2.54**
Southern Leyte	**2,492**	0	**0.00**	4	**0.16**	64	**2.57**	**68**	**2.73**
Western Samar	**1,158**	0	**0.00**	10	**0.86**	75	**6.48**	**85**	**7.34**
Calbayog City	**156**	0	**0.00**	1	**0.64**	8	**5.13**	**9**	**5.77**
Maasin City	**0**	0	**0.00**	0	**0.00**	0	**0.00**	**0**	**0.00**
Ormoc City	**435**	0	**0.00**	1	**0.23**	15	**3.45**	**16**	**3.68**
Tacloban City	**2,508**	0	**0.00**	7	**0.28**	49	**1.95**	**56**	**2.23**
**Region 9**	**1,492**	**0**	**0.00**	**17**	**1.14**	**80**	**5.36**	**97**	**6.50**
Zamboanga del Norte	**612**	0	**0.00**	5	**0.82**	20	**3.27**	**25**	**4.08**
Zamboanga del Sur	**353**	0	**0.00**	8	**2.27**	42	**11.90**	**50**	**14.16**
Zamboanga Sibugay	**154**	0	**0.00**	1	**0.65**	2	**1.30**	**3**	**1.95**
Dapitan City	**158**	0	**0.00**	0	**0.00**	4	**2.53**	**4**	**2.53**
Dipolog City	**0**	0	**0.00**	0	**0.00**	0	**0.00**	**0**	**0.00**
Isabela City	**134**	0	**0.00**	1	**0.75**	7	**5.22**	**8**	**5.97**
Pagadian City	**30**	0	**0.00**	1	**3.33**	3	**10.00**	**4**	**13.33**
Zamboanga City	**51**	0	**0.00**	1	**1.96**	2	**3.92**	**3**	**5.88**
**Region 10**	**22,828**	**7**	**0.03**	**53**	**0.23**	**329**	**1.44**	**389**	**1.70**
Bukidnon	**2,964**	6	**0.20**	25	**0.84**	126	**4.25**	**157**	**5.30**
Camiguin	**437**	0	**0.00**	1	**0.23**	3	**0.69**	**4**	**0.92**
Lanao del Norte	**296**	0	**0.00**	2	**0.68**	8	**2.70**	**10**	**3.38**
Misamis Occidental	**376**	0	**0.00**	1	**0.27**	2	**0.53**	**3**	**0.80**
Misamis Oriental	**5,506**	0	**0.00**	6	**0.11**	34	**0.62**	**40**	**0.73**
Cagayan de Oro City	**8,364**	0	**0.00**	4	**0.05**	43	**0.51**	**47**	**0.56**
El Salvador City	**529**	0	**0.00**	6	**1.13**	22	**4.16**	**28**	**5.29**
Gingoog City	**746**	0	**0.00**	0	**0.00**	0	**0.00**	**0**	**0.00**
Iligan City	**586**	0	**0.00**	0	**0.00**	14	**2.39**	**14**	**2.39**
Malaybalay City	**1,168**	1	**0.09**	8	**0.68**	61	**5.22**	**70**	**5.99**

Table 1.B.1.12 - Prenatal Care

Number and proportion of pregnant women tested positive for Hepatitis B
Philippines, Annual 2020

Area	Total No. of Screened for Hepatitis B	Tested Positive for Hepatitis B							
		Age Group						Total	%
		10-14 yrs old		15-19 yrs old		20-49 yrs old			
		No.	%	No.	%	No.	%		
Oroquieta City	**0**	0	**0.00**	0	**0.00**	0	**0.00**	**0**	**0.00**
Ozamis City	**1,323**	0	**0.00**	0	**0.00**	14	**1.06**	**14**	**1.06**
Tangub City	**0**	0	**0.00**	0	**0.00**	0	**0.00**	**0**	**0.00**
Valencia City	**533**	0	**0.00**	0	**0.00**	2	**0.38**	**2**	**0.38**
**Region 11**	**19,289**	**7**	**0.04**	**26**	**0.13**	**161**	**0.83**	**194**	**1.01**
Compostela Valley	**319**	0	**0.00**	0	**0.00**	7	**2.19**	**7**	**2.19**
Davao del Norte	**4,757**	0	**0.00**	6	**0.13**	32	**0.67**	**38**	**0.80**
Davao Oriental	**258**	0	**0.00**	0	**0.00**	2	**0.78**	**2**	**0.78**
Davao del Sur	**973**	1	**0.10**	2	**0.21**	21	**2.16**	**24**	**2.47**
Davao Occidental	**9**	0	**0.00**	0	**0.00**	0	**0.00**	**0**	**0.00**
Davao City	**12,973**	6	**0.05**	18	**0.14**	99	**0.76**	**123**	**0.95**
**Region 12**	**21,431**	**2**	**0.01**	**107**	**0.50**	**602**	**2.81**	**711**	**3.32**
North Cotabato	**5,350**	1	**0.02**	29	**0.54**	194	**3.63**	**224**	**4.19**
Sarangani	**4,868**	1	**0.02**	53	**1.09**	208	**4.27**	**262**	**5.38**
South Cotabato	**3,847**	0	**0.00**	11	**0.29**	64	**1.66**	**75**	**1.95**
Sultan Kudarat	**4,930**	0	**0.00**	6	**0.12**	78	**1.58**	**84**	**1.70**
Cotabato City	**360**	0	**0.00**	1	**0.28**	3	**0.83**	**4**	**1.11**
Gen. Santos City	**2,076**	0	**0.00**	7	**0.34**	55	**2.65**	**62**	**2.99**
**BARMM**	**921**	**0**	**0.00**	**1**	**0.11**	**2**	**0.22**	**3**	**0.33**
Basilan	**0**	0	**0.00**	0	**0.00**	0	**0.00**	**0**	**0.00**
Lanao del Sur	**337**	0	**0.00**	0	**0.00**	0	**0.00**	**0**	**0.00**
Maguindanao	**31**	0	**0.00**	1	**3.23**	2	**6.45**	**3**	**9.68**
Sulu	**0**	0	**0.00**	0	**0.00**	0	**0.00**	**0**	**0.00**
Tawi-Tawi	**0**	0	**0.00**	0	**0.00**	0	**0.00**	**0**	**0.00**
Lamitan City	**0**	0	**0.00**	0	**0.00**	0	**0.00**	**0**	**0.00**
Marawi City	**553**	0	**0.00**	0	**0.00**	0	**0.00**	**0**	**0.00**
**CARAGA**	**10,180**	**0**	**0.00**	**49**	**0.48**	**334**	**3.28**	**383**	**3.76**
Agusan del Norte	**1,412**	0	**0.00**	10	**0.71**	73	**5.17**	**83**	**5.88**
Agusan del Sur	**893**	0	**0.00**	16	**1.79**	45	**5.04**	**61**	**6.83**
Surigao del Norte	**1,559**	0	**0.00**	3	**0.19**	61	**3.91**	**64**	**4.11**
Surigao del Sur	**1,232**	0	**0.00**	2	**0.16**	30	**2.44**	**32**	**2.60**
Province of Dinagat	**452**	0	**0.00**	2	**0.44**	18	**3.98**	**20**	**4.42**
Bislig City	**25**	0	**0.00**	0	**0.00**	2	**8.00**	**2**	**8.00**
Butuan City	**2,867**	0	**0.00**	10	**0.35**	88	**3.07**	**98**	**3.42**
Surigao City	**1,740**	0	**0.00**	6	**0.34**	17	**0.98**	**23**	**1.32**

Note: Put asterisk (*) for No Report and Zero (0) for No Case

Table 1.B.1.13 - Prenatal Care

Number and proportion of pregnant women screened for HIV
Philippines, Annual 2020

Area	Eligible Pop.	Screened for HIV							
		Age Group						Total	%
		10-14 yrs old		15-19 yrs old		20-49 yrs old			
		No.	%	No.	%	No.	%		
PHILIPPINES	2,123,158	586	0.03	33,286	1.57	229,725	10.82	263,597	12.42
N C R	238,661	159	0.07	7,769	3.26	67,952	28.47	75,880	31.79
Malabon	6,775	13	0.19	315	4.65	1,613	23.81	1,941	28.65
Navotas	4,621	15	0.32	417	9.02	1,712	37.05	2,144	46.40
Valenzuela City	11,500	1	0.01	135	1.17	6,001	52.18	6,137	53.37
Caloocan City	29,363	16	0.05	1,070	3.64	6,802	23.17	7,888	26.86
Marikina City	8,354	1	0.01	92	1.10	567	6.79	660	7.90
Pasig City	13,996	11	0.08	492	3.52	6,858	49.00	7,361	52.59
Pateros	1,184	0	0.00	34	2.87	210	17.74	244	20.61
Taguig	14,918	11	0.07	597	4.00	4,710	31.57	5,318	35.65
Quezon City	54,413	50	0.09	2,134	3.92	19,936	36.64	22,120	40.65
Makati City	10,798	6	0.06	177	1.64	2,751	25.48	2,934	27.17
Mandaluyong City	7,160	3	0.04	211	2.95	2,193	30.63	2,407	33.62
San Juan	2,261	2	0.09	81	3.58	788	34.85	871	38.52
Manila City	32,980	15	0.05	1,155	3.50	5,848	17.73	7,018	21.28
Las Piñas City	10,914	0	0.00	103	0.94	1,024	9.38	1,127	10.33
Muntinlupa City	9,353	0	0.00	0	0.00	2,476	26.47	2,476	26.47
Parañaque City	12,343	8	0.06	453	3.67	2,085	16.89	2,546	20.63
Pasay City	7,728	7	0.09	303	3.92	2,378	30.77	2,688	34.78
C A R	35,099	3	0.01	516	1.47	5,239	14.93	5,758	16.41
Abra	4,309	0	0.00	54	1.25	328	7.61	382	8.87
Apayao	2,451	0	0.00	13	0.53	69	2.82	82	3.35
Benguet	9,188	3	0.03	261	2.84	2,445	26.61	2,709	29.48
Ifugao	4,396	0	0.00	61	1.39	810	18.43	871	19.81
Kalinga	4,617	0	0.00	7	0.15	49	1.06	56	1.21
Mt. Province	3,023	0	0.00	4	0.13	83	2.75	87	2.88
Baguio City	7,115	0	0.00	116	1.63	1,455	20.45	1,571	22.08
Region 1	97,261	18	0.02	1,356	1.39	14,549	14.96	15,923	16.37
Ilocos Norte	8,105	2	0.02	103	1.27	1,268	15.64	1,373	16.94
Ilocos Sur	9,330	1	0.01	162	1.74	2,322	24.89	2,485	26.63
La Union	11,511	7	0.06	275	2.39	2,213	19.23	2,495	21.67
Pangasinan	50,168	2	0.00	417	0.83	3,355	6.69	3,774	7.52
Alaminos City	1,894	0	0.00	74	3.91	932	49.21	1,006	53.12
Candon City	987	0	0.00	8	0.81	460	46.61	468	47.42
Dagupan City	3,620	2	0.06	92	2.54	888	24.53	982	27.13
Laoag City	1,870	0	0.00	59	3.16	1,758	94.01	1,817	97.17
San Carlos City	3,979	0	0.00	6	0.15	125	3.14	131	3.29
San Fernando City	2,115	0	0.00	94	4.44	787	37.21	881	41.65
Urdaneta City	2,806	4	0.14	66	2.35	435	15.50	505	18.00

Table 1.B.1.13 - Prenatal Care

Number and proportion of pregnant women screened for HIV
Philippines, Annual 2020

Area	Eligible Pop.	Screened for HIV							
		Age Group						Total	%
		10-14 yrs old		15-19 yrs old		20-49 yrs old			
		No.	%	No.	%	No.	%		
Vigan City	**876**	0	**0.00**	0	**0.00**	6	**0.68**	**6**	**0.68**
**Region 2**	**69,443**	**10**	**0.01**	**792**	**1.14**	**5,172**	**7.45**	**5,974**	**8.60**
Batanes	**340**	0	**0.00**	1	**0.29**	35	**10.29**	**36**	**10.59**
Cagayan	**17,971**	1	**0.01**	106	**0.59**	566	**3.15**	**673**	**3.74**
Isabela	**26,309**	6	**0.02**	248	**0.94**	1,513	**5.75**	**1,767**	**6.72**
Nueva Vizcaya	**9,573**	1	**0.01**	148	**1.55**	921	**9.62**	**1,070**	**11.18**
Quirino	**4,025**	1	**0.02**	147	**3.65**	1,095	**27.20**	**1,243**	**30.88**
Cauayan City	**2,590**	0	**0.00**	44	**1.70**	397	**15.33**	**441**	**17.03**
Ilagan City	**2,911**	1	**0.03**	22	**0.76**	122	**4.19**	**145**	**4.98**
Santiago City	**2,692**	0	**0.00**	76	**2.82**	502	**18.65**	**578**	**21.47**
Tuguegarao City	**3,032**	0	**0.00**	0	**0.00**	21	**0.69**	**21**	**0.69**
**Region 3**	**220,020**	**173**	**0.08**	**7,971**	**3.62**	**50,177**	**22.81**	**58,321**	**26.51**
Aurora	**4,770**	2	**0.04**	293	**6.14**	1,723	**36.12**	**2,018**	**42.31**
Bataan	**13,823**	17	**0.12**	683	**4.94**	4,841	**35.02**	**5,541**	**40.09**
Bulacan	**43,627**	56	**0.13**	1,501	**3.44**	8,496	**19.47**	**10,053**	**23.04**
Nueva Ecija	**28,782**	21	**0.07**	686	**2.38**	3,216	**11.17**	**3,923**	**13.63**
Pampanga	**31,359**	11	**0.04**	727	**2.32**	4,674	**14.90**	**5,412**	**17.26**
Tarlac	**19,765**	4	**0.02**	237	**1.20**	2,460	**12.45**	**2,701**	**13.67**
Zambales	**12,610**	2	**0.02**	328	**2.60**	1,944	**15.42**	**2,274**	**18.03**
Angeles City	**7,869**	1	**0.01**	212	**2.69**	1,164	**14.79**	**1,377**	**17.50**
Balanga City	**1,998**	6	**0.30**	153	**7.66**	778	**38.94**	**937**	**46.90**
Cabanatuan City	**5,896**	4	**0.07**	217	**3.68**	1,155	**19.59**	**1,376**	**23.34**
City of San Fernando	**5,863**	4	**0.07**	311	**5.30**	2,018	**34.42**	**2,333**	**39.79**
Gapan City	**2,153**	0	**0.00**	111	**5.16**	569	**26.43**	**680**	**31.58**
Mabalacat City	**4,793**	5	**0.10**	332	**6.93**	1,832	**38.22**	**2,169**	**45.25**
Malolos City	**4,880**	5	**0.10**	169	**3.46**	1,043	**21.37**	**1,217**	**24.94**
Meycauayan	**4,041**	16	**0.40**	303	**7.50**	1,434	**35.49**	**1,753**	**43.38**
Olongapo	**4,971**	0	**0.00**	156	**3.14**	816	**16.42**	**972**	**19.55**
Palayan City	**799**	1	**0.13**	19	**2.38**	79	**9.89**	**99**	**12.39**
San Jose City	**2,718**	3	**0.11**	95	**3.50**	398	**14.64**	**496**	**18.25**
San Jose del Monte City	**11,100**	12	**0.11**	1,156	**10.41**	10,418	**93.86**	**11,586**	**104.38**
Science City of Munoz	**1,586**	2	**0.13**	38	**2.40**	231	**14.56**	**271**	**17.09**
Tarlac City	**6,617**	1	**0.02**	244	**3.69**	888	**13.42**	**1,133**	**17.12**
**Region 4A**	**296,816**	**47**	**0.02**	**5,490**	**1.85**	**17,071**	**5.75**	**22,608**	**7.62**
Batangas	**38,441**	0	**0.00**	120	**0.31**	1,448	**3.77**	**1,568**	**4.08**
Cavite	**27,704**	3	**0.01**	133	**0.48**	1,264	**4.56**	**1,400**	**5.05**
Laguna	**19,727**	12	**0.06**	2,998	**15.20**	1,014	**5.14**	**4,024**	**20.40**
Quezon	**37,410**	2	**0.01**	136	**0.36**	752	**2.01**	**890**	**2.38**
Rizal	**44,791**	9	**0.02**	741	**1.65**	4,640	**10.36**	**5,390**	**12.03**
Antipolo City	**16,497**	5	**0.03**	370	**2.24**	1,330	**8.06**	**1,705**	**10.34**
Bacoor City	**12,142**	0	**0.00**	3	**0.02**	39	**0.32**	**42**	**0.35**
Batangas City	**6,823**	0	**0.00**	0	**0.00**	0	**0.00**	**0**	**0.00**

Table 1.B.1.13 - Prenatal Care

Number and proportion of pregnant women screened for HIV

Philippines, Annual 2020

Area	Eligible Pop.	Screened for HIV							
		Age Group						Total	%
		10-14 yrs old		15-19 yrs old		20-49 yrs old			
		No.	%	No.	%	No.	%		
Biñan City	6,607	2	0.03	382	5.78	1,301	19.69	1,685	25.50
Cabuyao City	6,130	0	0.00	39	0.64	353	5.76	392	6.39
Calamba City	9,028	1	0.01	134	1.48	152	1.68	287	3.18
Cavite City	2,075	0	0.00	0	0.00	0	0.00	0	0.00
Dasmariñas City	13,322	3	0.02	217	1.63	2,184	16.39	2,404	18.05
General Trias City	6,352	0	0.00	22	0.35	242	3.81	264	4.16
Imus City	8,156	0	0.00	0	0.00	0	0.00	0	0.00
Lipa City	6,880	0	0.00	0	0.00	0	0.00	0	0.00
Lucena City	5,672	0	0.00	0	0.00	0	0.00	0	0.00
San Pablo City	5,277	0	0.00	35	0.66	220	4.17	255	4.83
San Pedro City	6,467	0	0.00	4	0.06	175	2.71	179	2.77
Santa Rosa City	7,025	8	0.11	119	1.69	718	10.22	845	12.03
Tagaytay City	1,437	2	0.14	28	1.95	1,124	78.22	1,154	80.31
Tanauan City	3,584	0	0.00	9	0.25	115	3.21	124	3.46
Tayabas City	2,122	0	0.00	0	0.00	0	0.00	0	0.00
Trece Martires City	3,147	0	0.00	0	0.00	0	0.00	0	0.00
**Region 4B**	71,246	23	0.03	386	0.54	3,827	5.37	4,236	5.95
Marinduque	4,836	1	0.02	40	0.83	470	9.72	511	10.57
Mindoro Occidental	12,407	0	0.00	38	0.31	379	3.05	417	3.36
Mindoro Oriental	19,809	20	0.10	183	0.92	1,087	5.49	1,290	6.51
Palawan	21,375	1	0.00	83	0.39	455	2.13	539	2.52
Romblon	6,400	1	0.02	42	0.66	449	7.02	492	7.69
Puerto Princesa City	6,419	0	0.00	0	0.00	987	15.38	987	15.38
**Region 5**	136,116	6	0.00	987	0.73	8,794	6.46	9,787	7.19
Albay	23,737	1	0.00	198	0.83	2,797	11.78	2,996	12.62
Camarines Norte	14,283	0	0.00	34	0.24	172	1.20	206	1.44
Camarines Sur	38,796	0	0.00	111	0.29	1,087	2.80	1,198	3.09
Catanduanes	6,274	0	0.00	87	1.39	583	9.29	670	10.68
Masbate	22,521	1	0.00	257	1.14	1,475	6.55	1,733	7.70
Sorsogon	19,065	0	0.00	36	0.19	267	1.40	303	1.59
Iriga City	2,638	1	0.04	60	2.27	579	21.95	640	24.26
Legaspi City	4,174	1	0.02	86	2.06	921	22.07	1,008	24.15
Naga City	4,628	2	0.04	118	2.55	913	19.73	1,033	22.32
**Region 6**	146,526	36	0.02	1,587	1.08	12,511	8.54	14,134	9.65
Aklan	11,162	11	0.10	229	2.05	1,760	15.77	2,000	17.92
Antique	12,816	1	0.01	96	0.75	972	7.58	1,069	8.34
Capiz	13,986	1	0.01	51	0.36	458	3.27	510	3.65
Guimaras	3,085	0	0.00	52	1.69	461	14.94	513	16.63
Iloilo	36,541	1	0.00	220	0.60	2,579	7.06	2,800	7.66
Negros Occidental	49,368	7	0.01	375	0.76	2,539	5.14	2,921	5.92
Bacolod City	11,115	13	0.12	330	2.97	2,045	18.40	2,388	21.48
Iloilo City	8,453	2	0.02	234	2.77	1,697	20.08	1,933	22.87

Table 1.B.1.13 - Prenatal Care

Number and proportion of pregnant women screened for HIV

Philippines, Annual 2020

Area	Eligible Pop.	Screened for HIV							
		Age Group						Total	%
		10-14 yrs old		15-19 yrs old		20-49 yrs old			
		No.	%	No.	%	No.	%		
**Region 7**	**163,262**	**18**	**0.01**	**1,954**	**1.20**	**15,939**	**9.76**	**17,911**	**10.97**
Bohol	**27,312**	0	**0.00**	89	**0.33**	768	**2.81**	**857**	**3.14**
Cebu	**67,506**	3	**0.00**	363	**0.54**	3,200	**4.74**	**3,566**	**5.28**
Negros Oriental	**27,938**	0	**0.00**	6	**0.02**	107	**0.38**	**113**	**0.40**
Siquijor	**1,613**	0	**0.00**	90	**5.58**	773	**47.92**	**863**	**53.50**
Cebu City	**21,193**	14	**0.07**	1,004	**4.74**	6,774	**31.96**	**7,792**	**36.77**
Lapu-Lapu City	**9,372**	1	**0.01**	402	**4.29**	4,317	**46.06**	**4,720**	**50.36**
Mandaue City	**8,328**	0	**0.00**	0	**0.00**	0	**0.00**	**0**	**0.00**
**Region 8**	**102,619**	**5**	**0.00**	**861**	**0.84**	**6,711**	**6.54**	**7,577**	**7.38**
Biliran	**3,834**	5	**0.13**	256	**6.68**	1,563	**40.77**	**1,824**	**47.57**
Eastern Samar	**11,392**	0	**0.00**	117	**1.03**	907	**7.96**	**1,024**	**8.99**
Northern Leyte	**34,707**	0	**0.00**	0	**0.00**	0	**0.00**	**0**	**0.00**
Northern Samar	**15,370**	0	**0.00**	146	**0.95**	1,966	**12.79**	**2,112**	**13.74**
Southern Leyte	**6,451**	0	**0.00**	74	**1.15**	559	**8.67**	**633**	**9.81**
Western Samar	**14,305**	0	**0.00**	69	**0.48**	404	**2.82**	**473**	**3.31**
Calbayog City	**4,413**	0	**0.00**	0	**0.00**	0	**0.00**	**0**	**0.00**
Maasin City	**1,637**	0	**0.00**	0	**0.00**	0	**0.00**	**0**	**0.00**
Ormoc City	**4,941**	0	**0.00**	68	**1.38**	421	**8.52**	**489**	**9.90**
Tacloban City	**5,569**	0	**0.00**	131	**2.35**	891	**16.00**	**1,022**	**18.35**
**Region 9**	**80,051**	**0**	**0.00**	**69**	**0.09**	**589**	**0.74**	**658**	**0.82**
Zamboanga del Norte	**17,249**	0	**0.00**	4	**0.02**	39	**0.23**	**43**	**0.25**
Zamboanga del Sur	**17,653**	0	**0.00**	18	**0.10**	140	**0.79**	**158**	**0.90**
Zamboanga Sibugay	**14,954**	0	**0.00**	44	**0.29**	375	**2.51**	**419**	**2.80**
Dapitan City	**1,784**	0	**0.00**	2	**0.11**	26	**1.46**	**28**	**1.57**
Dipolog City	**2,827**	0	**0.00**	0	**0.00**	0	**0.00**	**0**	**0.00**
Isabela City	**2,522**	0	**0.00**	0	**0.00**	0	**0.00**	**0**	**0.00**
Pagadian City	**4,325**	0	**0.00**	0	**0.00**	7	**0.16**	**7**	**0.16**
Zamboanga City	**18,737**	0	**0.00**	1	**0.01**	2	**0.01**	**3**	**0.02**
**Region 10**	**101,411**	**28**	**0.03**	**964**	**0.95**	**4,562**	**4.50**	**5,554**	**5.48**
Bukidnon	**23,706**	13	**0.05**	268	**1.13**	709	**2.99**	**990**	**4.18**
Camiguin	**1,858**	0	**0.00**	17	**0.91**	270	**14.53**	**287**	**15.45**
Lanao del Norte	**14,960**	1	**0.01**	71	**0.47**	523	**3.50**	**595**	**3.98**
Misamis Occidental	**6,403**	9	**0.14**	41	**0.64**	261	**4.08**	**311**	**4.86**
Misamis Oriental	**15,131**	0	**0.00**	70	**0.46**	336	**2.22**	**406**	**2.68**
Cagayan de Oro City	**14,339**	1	**0.01**	175	**1.22**	816	**5.69**	**992**	**6.92**
El Salvador City	**1,065**	1	**0.09**	16	**1.50**	67	**6.29**	**84**	**7.89**
Gingoog City	**2,644**	0	**0.00**	4	**0.15**	28	**1.06**	**32**	**1.21**
Iligan City	**7,580**	0	**0.00**	1	**0.01**	6	**0.08**	**7**	**0.09**
Malaybalay City	**3,956**	1	**0.03**	62	**1.57**	263	**6.65**	**326**	**8.24**

Table 1.B.1.13 - Prenatal Care

Number and proportion of pregnant women screened for HIV
Philippines, Annual 2020

Area	Eligible Pop.	Screened for HIV							
		Age Group						Total	%
		10-14 yrs old		15-19 yrs old		20-49 yrs old			
		No.	%	No.	%	No.	%		
Oroquieta City	1,389	0	0.00	0	0.00	0	0.00	0	0.00
Ozamis City	2,778	2	0.07	192	6.91	1,079	38.84	1,273	45.82
Tangub City	1,234	0	0.00	0	0.00	0	0.00	0	0.00
Valencia City	4,368	0	0.00	47	1.08	204	4.67	251	5.75
**Region 11**	107,247	33	0.03	1,110	1.03	7,029	6.55	8,172	7.62
Compostela Valley	15,562	1	0.01	30	0.19	118	0.76	149	0.96
Davao del Norte	21,326	9	0.04	223	1.05	1,484	6.96	1,716	8.05
Davao Oriental	13,007	0	0.00	12	0.09	42	0.32	54	0.42
Davao del Sur	14,151	5	0.04	66	0.47	396	2.80	467	3.30
Davao Occidental	6,670	0	0.00	5	0.07	13	0.19	18	0.27
Davao City	36,531	18	0.05	774	2.12	4,976	13.62	5,768	15.79
**Region 12**	104,552	8	0.01	738	0.71	4,333	4.14	5,079	4.86
North Cotabato	33,645	0	0.00	135	0.40	724	2.15	859	2.55
Sarangani	12,891	2	0.02	104	0.81	515	4.00	621	4.82
South Cotabato	21,113	4	0.02	318	1.51	1,929	9.14	2,251	10.66
Sultan Kudarat	17,359	0	0.00	29	0.17	151	0.87	180	1.04
Cotabato City	5,835	1	0.02	48	0.82	372	6.38	421	7.22
Gen. Santos City	13,709	1	0.01	104	0.76	642	4.68	747	5.45
**BARMM**	92,799	0	0.00	51	0.05	599	0.65	650	0.70
Basilan	7,541	0	0.00	0	0.00	0	0.00	0	0.00
Lanao del Sur	21,131	0	0.00	0	0.00	416	1.97	416	1.97
Maguindanao	31,128	0	0.00	0	0.00	1	0.00	1	0.00
Sulu	16,613	0	0.00	0	0.00	0	0.00	0	0.00
Tawi-Tawi	9,259	0	0.00	0	0.00	0	0.00	0	0.00
Lamitan City	2,074	0	0.00	0	0.00	0	0.00	0	0.00
Marawi City	5,053	0	0.00	51	1.01	182	3.60	233	4.61
**CARAGA**	60,029	19	0.03	685	1.14	4,671	7.78	5,375	8.95
Agusan del Norte	8,098	0	0.00	58	0.72	359	4.43	417	5.15
Agusan del Sur	17,592	11	0.06	87	0.49	395	2.25	493	2.80
Surigao del Norte	7,089	1	0.01	41	0.58	346	4.88	388	5.47
Surigao del Sur	11,482	0	0.00	60	0.52	482	4.20	542	4.72
Province of Dinagat	2,573	0	0.00	41	1.59	290	11.27	331	12.86
Bislig City	2,179	0	0.00	0	0.00	3	0.14	3	0.14
Butuan City	7,715	3	0.04	221	2.86	1,590	20.61	1,814	23.51
Surigao City	3,301	4	0.12	177	5.36	1,206	36.53	1,387	42.02

Note: Put asterisk (*) for No Report and Zero (0) for No Case

Table 1.B.1.14 - Prenatal Care

Number and proportion of pregnant women tested for CBC or Hemoglobin and Hematocrit Count

Philippines, Annual 2020

Area	Eligible Pop.	Tested for CBC/Hgb and Hct							
		Age Group						Total	%
		10-14 yrs old		15-19 yrs old		20-49 yrs old			
		No.	%	No.	%	No.	%		
**PHILIPPINES**	**2,123,158**	**1,690**	**0.08**	**75,087**	**3.54**	**517,483**	**24.37**	**594,260**	**27.99**
**N C R**	**238,661**	**155**	**0.06**	**7,164**	**3.00**	**56,653**	**23.74**	**63,972**	**26.80**
Malabon	**6,775**	11	**0.16**	285	**4.21**	1,554	**22.94**	**1,850**	**27.31**
Navotas	**4,621**	13	**0.28**	472	**10.21**	1,750	**37.87**	**2,235**	**48.37**
Valenzuela City	**11,500**	3	**0.03**	161	**1.40**	1,276	**11.10**	**1,440**	**12.52**
Caloocan City	**29,363**	8	**0.03**	852	**2.90**	6,092	**20.75**	**6,952**	**23.68**
Marikina City	**8,354**	0	**0.00**	160	**1.92**	1,303	**15.60**	**1,463**	**17.51**
Pasig City	**13,996**	9	**0.06**	554	**3.96**	7,222	**51.60**	**7,785**	**55.62**
Pateros	**1,184**	0	**0.00**	72	**6.08**	590	**49.83**	**662**	**55.91**
Taguig	**14,918**	22	**0.15**	231	**1.55**	1,579	**10.58**	**1,832**	**12.28**
Quezon City	**54,413**	37	**0.07**	2,243	**4.12**	18,320	**33.67**	**20,600**	**37.86**
Makati City	**10,798**	6	**0.06**	200	**1.85**	3,486	**32.28**	**3,692**	**34.19**
Mandaluyong City	**7,160**	4	**0.06**	186	**2.60**	2,072	**28.94**	**2,262**	**31.59**
San Juan	**2,261**	2	**0.09**	70	**3.10**	616	**27.24**	**688**	**30.43**
Manila City	**32,980**	11	**0.03**	446	**1.35**	2,520	**7.64**	**2,977**	**9.03**
Las Piñas City	**10,914**	12	**0.11**	272	**2.49**	2,489	**22.81**	**2,773**	**25.41**
Muntinlupa City	**9,353**	0	**0.00**	0	**0.00**	0	**0.00**	**0**	**0.00**
Parañaque City	**12,343**	10	**0.08**	666	**5.40**	3,211	**26.01**	**3,887**	**31.49**
Pasay City	**7,728**	7	**0.09**	294	**3.80**	2,573	**33.29**	**2,874**	**37.19**
**C A R**	**35,099**	**12**	**0.03**	**1,336**	**3.81**	**11,814**	**33.66**	**13,162**	**37.50**
Abra	**4,309**	0	**0.00**	77	**1.79**	539	**12.51**	**616**	**14.30**
Apayao	**2,451**	1	**0.04**	149	**6.08**	798	**32.56**	**948**	**38.68**
Benguet	**9,188**	3	**0.03**	342	**3.72**	3,162	**34.41**	**3,507**	**38.17**
Ifugao	**4,396**	0	**0.00**	140	**3.18**	1,616	**36.76**	**1,756**	**39.95**
Kalinga	**4,617**	4	**0.09**	243	**5.26**	1,984	**42.97**	**2,231**	**48.32**
Mt. Province	**3,023**	3	**0.10**	125	**4.13**	757	**25.04**	**885**	**29.28**
Baguio City	**7,115**	1	**0.01**	260	**3.65**	2,958	**41.57**	**3,219**	**45.24**
**Region 1**	**97,261**	**52**	**0.05**	**3,695**	**3.80**	**35,176**	**36.17**	**38,923**	**40.02**
Ilocos Norte	**8,105**	9	**0.11**	310	**3.82**	3,541	**43.69**	**3,860**	**47.62**
Ilocos Sur	**9,330**	5	**0.05**	418	**4.48**	4,381	**46.96**	**4,804**	**51.49**
La Union	**11,511**	14	**0.12**	590	**5.13**	4,448	**38.64**	**5,052**	**43.89**
Pangasinan	**50,168**	16	**0.03**	1,877	**3.74**	16,481	**32.85**	**18,374**	**36.62**
Alaminos City	**1,894**	0	**0.00**	77	**4.07**	960	**50.69**	**1,037**	**54.75**
Candon City	**987**	0	**0.00**	8	**0.81**	772	**78.22**	**780**	**79.03**
Dagupan City	**3,620**	1	**0.03**	75	**2.07**	884	**24.42**	**960**	**26.52**
Laoag City	**1,870**	0	**0.00**	49	**2.62**	1,238	**66.20**	**1,287**	**68.82**
San Carlos City	**3,979**	0	**0.00**	43	**1.08**	587	**14.75**	**630**	**15.83**
San Fernando City	**2,115**	2	**0.09**	101	**4.78**	897	**42.41**	**1,000**	**47.28**
Urdaneta City	**2,806**	5	**0.18**	125	**4.45**	882	**31.43**	**1,012**	**36.07**

Table 1.B.1.14 - Prenatal Care

Number and proportion of pregnant women tested for CBC or Hemoglobin and Hematocrit Count

Philippines, Annual 2020

Area	Eligible Pop.	Tested for CBC/Hgb and Hct							
		Age Group						Total	%
		10-14 yrs old		15-19 yrs old		20-49 yrs old			
		No.	%	No.	%	No.	%		
Vigan City	**876**	0	**0.00**	22	**2.51**	105	**11.99**	**127**	**14.50**
**Region 2**	**69,443**	**58**	**0.08**	**3,609**	**5.20**	**25,057**	**36.08**	**28,724**	**41.36**
Batanes	**340**	0	**0.00**	6	**1.76**	117	**34.41**	**123**	**36.18**
Cagayan	**17,971**	12	**0.07**	969	**5.39**	5,998	**33.38**	**6,979**	**38.83**
Isabela	**26,309**	27	**0.10**	1,098	**4.17**	7,270	**27.63**	**8,395**	**31.91**
Nueva Vizcaya	**9,573**	9	**0.09**	481	**5.02**	4,760	**49.72**	**5,250**	**54.84**
Quirino	**4,025**	4	**0.10**	486	**12.07**	3,191	**79.28**	**3,681**	**91.45**
Cauayan City	**2,590**	3	**0.12**	171	**6.60**	1,049	**40.50**	**1,223**	**47.22**
Ilagan City	**2,911**	1	**0.03**	145	**4.98**	1,102	**37.86**	**1,248**	**42.87**
Santiago City	**2,692**	0	**0.00**	76	**2.82**	645	**23.96**	**721**	**26.78**
Tuguegarao City	**3,032**	2	**0.07**	177	**5.84**	925	**30.51**	**1,104**	**36.41**
**Region 3**	**220,020**	**223**	**0.10**	**9,683**	**4.40**	**62,286**	**28.31**	**72,192**	**32.81**
Aurora	**4,770**	2	**0.04**	192	**4.03**	1,312	**27.51**	**1,506**	**31.57**
Bataan	**13,823**	26	**0.19**	843	**6.10**	5,775	**41.78**	**6,644**	**48.06**
Bulacan	**43,627**	46	**0.11**	1,877	**4.30**	11,374	**26.07**	**13,297**	**30.48**
Nueva Ecija	**28,782**	30	**0.10**	1,220	**4.24**	6,244	**21.69**	**7,494**	**26.04**
Pampanga	**31,359**	10	**0.03**	508	**1.62**	3,246	**10.35**	**3,764**	**12.00**
Tarlac	**19,765**	7	**0.04**	732	**3.70**	7,062	**35.73**	**7,801**	**39.47**
Zambales	**12,610**	2	**0.02**	277	**2.20**	1,797	**14.25**	**2,076**	**16.46**
Angeles City	**7,869**	0	**0.00**	280	**3.56**	1,456	**18.50**	**1,736**	**22.06**
Balanga City	**1,998**	7	**0.35**	170	**8.51**	944	**47.25**	**1,121**	**56.11**
Cabanatuan City	**5,896**	2	**0.03**	125	**2.12**	757	**12.84**	**884**	**14.99**
City of San Fernando	**5,863**	2	**0.03**	258	**4.40**	2,077	**35.43**	**2,337**	**39.86**
Gapan City	**2,153**	0	**0.00**	107	**4.97**	525	**24.38**	**632**	**29.35**
Mabalacat City	**4,793**	3	**0.06**	121	**2.52**	1,017	**21.22**	**1,141**	**23.81**
Malolos City	**4,880**	4	**0.08**	142	**2.91**	832	**17.05**	**978**	**20.04**
Meycauayan	**4,041**	19	**0.47**	351	**8.69**	1,335	**33.04**	**1,705**	**42.19**
Olongapo	**4,971**	0	**0.00**	209	**4.20**	1,146	**23.05**	**1,355**	**27.26**
Palayan City	**799**	1	**0.13**	19	**2.38**	96	**12.02**	**116**	**14.52**
San Jose City	**2,718**	0	**0.00**	133	**4.89**	596	**21.93**	**729**	**26.82**
San Jose del Monte City	**11,100**	12	**0.11**	1,174	**10.58**	10,415	**93.83**	**11,601**	**104.51**
Science City of Munoz	**1,586**	3	**0.19**	102	**6.43**	510	**32.16**	**615**	**38.78**
Tarlac City	**6,617**	47	**0.71**	843	**12.74**	3,770	**56.97**	**4,660**	**70.42**
**Region 4A**	**296,816**	**81**	**0.03**	**4,099**	**1.38**	**33,177**	**11.18**	**37,357**	**12.59**
Batangas	**38,441**	0	**0.00**	149	**0.39**	1,738	**4.52**	**1,887**	**4.91**
Cavite	**27,704**	6	**0.02**	344	**1.24**	2,981	**10.76**	**3,331**	**12.02**
Laguna	**19,727**	31	**0.16**	311	**1.58**	4,325	**21.92**	**4,667**	**23.66**
Quezon	**37,410**	4	**0.01**	445	**1.19**	3,170	**8.47**	**3,619**	**9.67**
Rizal	**44,791**	18	**0.04**	765	**1.71**	4,976	**11.11**	**5,759**	**12.86**
Antipolo City	**16,497**	3	**0.02**	563	**3.41**	1,898	**11.51**	**2,464**	**14.94**
Bacoor City	**12,142**	0	**0.00**	0	**0.00**	0	**0.00**	**0**	**0.00**
Batangas City	**6,823**	0	**0.00**	0	**0.00**	0	**0.00**	**0**	**0.00**

Table 1.B.1.14 - Prenatal Care

Number and proportion of pregnant women tested for CBC or Hemoglobin and Hematocrit Count

Philippines, Annual 2020

Area	Eligible Pop.	Tested for CBC/Hgb and Hct							
		Age Group						Total	%
		10-14 yrs old		15-19 yrs old		20-49 yrs old			
		No.	%	No.	%	No.	%		
Biñan City	6,607	5	0.08	335	5.07	3,154	47.74	3,494	52.88
Cabuyao City	6,130	0	0.00	255	4.16	2,461	40.15	2,716	44.31
Calamba City	9,028	4	0.04	66	0.73	825	9.14	895	9.91
Cavite City	2,075	0	0.00	0	0.00	0	0.00	0	0.00
Dasmariñas City	13,322	2	0.02	288	2.16	2,432	18.26	2,722	20.43
General Trias City	6,352	0	0.00	0	0.00	0	0.00	0	0.00
Imus City	8,156	0	0.00	0	0.00	0	0.00	0	0.00
Lipa City	6,880	0	0.00	0	0.00	0	0.00	0	0.00
Lucena City	5,672	0	0.00	0	0.00	0	0.00	0	0.00
San Pablo City	5,277	1	0.02	1	0.02	122	2.31	124	2.35
San Pedro City	6,467	0	0.00	65	1.01	458	7.08	523	8.09
Santa Rosa City	7,025	5	0.07	379	5.40	2,307	32.84	2,691	38.31
Tagaytay City	1,437	0	0.00	34	2.37	1,244	86.57	1,278	88.94
Tanauan City	3,584	0	0.00	19	0.53	360	10.04	379	10.57
Tayabas City	2,122	0	0.00	0	0.00	0	0.00	0	0.00
Trece Martires City	3,147	2	0.06	80	2.54	726	23.07	808	25.68
**Region 4B**	71,246	81	0.11	1,306	1.83	9,335	13.10	10,722	15.05
Marinduque	4,836	2	0.04	116	2.40	1,195	24.71	1,313	27.15
Mindoro Occidental	12,407	3	0.02	248	2.00	1,968	15.86	2,219	17.89
Mindoro Oriental	19,809	72	0.36	631	3.19	4,206	21.23	4,909	24.78
Palawan	21,375	2	0.01	191	0.89	1,174	5.49	1,367	6.40
Romblon	6,400	2	0.03	75	1.17	625	9.77	702	10.97
Puerto Princesa City	6,419	0	0.00	45	0.70	167	2.60	212	3.30
**Region 5**	136,116	74	0.05	3,296	2.42	28,342	20.82	31,712	23.30
Albay	23,737	36	0.15	639	2.69	7,084	29.84	7,759	32.69
Camarines Norte	14,283	4	0.03	514	3.60	3,332	23.33	3,850	26.96
Camarines Sur	38,796	5	0.01	316	0.81	3,443	8.87	3,764	9.70
Catanduanes	6,274	5	0.08	247	3.94	1,606	25.60	1,858	29.61
Masbate	22,521	2	0.01	613	2.72	3,633	16.13	4,248	18.86
Sorsogon	19,065	4	0.02	773	4.05	6,760	35.46	7,537	39.53
Iriga City	2,638	1	0.04	2	0.08	116	4.40	119	4.51
Legaspi City	4,174	17	0.41	191	4.58	2,362	56.59	2,570	61.57
Naga City	4,628	0	0.00	1	0.02	6	0.13	7	0.15
**Region 6**	146,526	80	0.05	6,369	4.35	50,269	34.31	56,718	38.71
Aklan	11,162	17	0.15	651	5.83	4,286	38.40	4,954	44.38
Antique	12,816	5	0.04	370	2.89	3,153	24.60	3,528	27.53
Capiz	13,986	1	0.01	404	2.89	3,877	27.72	4,282	30.62
Guimaras	3,085	2	0.06	198	6.42	1,856	60.16	2,056	66.65
Iloilo	36,541	14	0.04	1,443	3.95	15,011	41.08	16,468	45.07
Negros Occidental	49,368	32	0.06	2,440	4.94	16,395	33.21	18,867	38.22
Bacolod City	11,115	7	0.06	494	4.44	2,999	26.98	3,500	31.49
Iloilo City	8,453	2	0.02	369	4.37	2,692	31.85	3,063	36.24

Table 1.B.1.14 - Prenatal Care

Number and proportion of pregnant women tested for CBC or Hemoglobin and Hematocrit Count

Philippines, Annual 2020

Area	Eligible Pop.	Tested for CBC/Hgb and Hct							
		Age Group						Total	%
		10-14 yrs old		15-19 yrs old		20-49 yrs old			
		No.	%	No.	%	No.	%		
**Region 7**	**163,262**	**37**	**0.02**	**3,504**	**2.15**	**29,507**	**18.07**	**33,048**	**20.24**
Bohol	**27,312**	0	**0.00**	319	**1.17**	5,362	**19.63**	**5,681**	**20.80**
Cebu	**67,506**	2	**0.00**	511	**0.76**	4,246	**6.29**	**4,759**	**7.05**
Negros Oriental	**27,938**	17	**0.06**	1,123	**4.02**	7,791	**27.89**	**8,931**	**31.97**
Siquijor	**1,613**	0	**0.00**	65	**4.03**	575	**35.65**	**640**	**39.68**
Cebu City	**21,193**	16	**0.08**	1,021	**4.82**	6,884	**32.48**	**7,921**	**37.38**
Lapu-Lapu City	**9,372**	2	**0.02**	465	**4.96**	4,649	**49.61**	**5,116**	**54.59**
Mandaue City	**8,328**	0	**0.00**	0	**0.00**	0	**0.00**	**0**	**0.00**
**Region 8**	**102,619**	**29**	**0.03**	**2,192**	**2.14**	**16,672**	**16.25**	**18,893**	**18.41**
Biliran	**3,834**	5	**0.13**	269	**7.02**	1,609	**41.97**	**1,883**	**49.11**
Eastern Samar	**11,392**	10	**0.09**	210	**1.84**	1,533	**13.46**	**1,753**	**15.39**
Northern Leyte	**34,707**	0	**0.00**	0	**0.00**	0	**0.00**	**0**	**0.00**
Northern Samar	**15,370**	3	**0.02**	479	**3.12**	4,627	**30.10**	**5,109**	**33.24**
Southern Leyte	**6,451**	6	**0.09**	340	**5.27**	2,534	**39.28**	**2,880**	**44.64**
Western Samar	**14,305**	4	**0.03**	324	**2.26**	1,957	**13.68**	**2,285**	**15.97**
Calbayog City	**4,413**	0	**0.00**	21	**0.48**	156	**3.54**	**177**	**4.01**
Maasin City	**1,637**	0	**0.00**	38	**2.32**	410	**25.05**	**448**	**27.37**
Ormoc City	**4,941**	0	**0.00**	199	**4.03**	1,495	**30.26**	**1,694**	**34.28**
Tacloban City	**5,569**	1	**0.02**	312	**5.60**	2,351	**42.22**	**2,664**	**47.84**
**Region 9**	**80,051**	**6**	**0.01**	**1,447**	**1.81**	**9,162**	**11.45**	**10,615**	**13.26**
Zamboanga del Norte	**17,249**	1	**0.01**	376	**2.18**	2,060	**11.94**	**2,437**	**14.13**
Zamboanga del Sur	**17,653**	2	**0.01**	415	**2.35**	2,962	**16.78**	**3,379**	**19.14**
Zamboanga Sibugay	**14,954**	2	**0.01**	358	**2.39**	2,024	**13.53**	**2,384**	**15.94**
Dapitan City	**1,784**	0	**0.00**	29	**1.63**	119	**6.67**	**148**	**8.30**
Dipolog City	**2,827**	0	**0.00**	2	**0.07**	24	**0.85**	**26**	**0.92**
Isabela City	**2,522**	1	**0.04**	27	**1.07**	168	**6.66**	**196**	**7.77**
Pagadian City	**4,325**	0	**0.00**	205	**4.74**	1,586	**36.67**	**1,791**	**41.41**
Zamboanga City	**18,737**	0	**0.00**	35	**0.19**	219	**1.17**	**254**	**1.36**
**Region 10**	**101,411**	**173**	**0.17**	**6,232**	**6.15**	**33,671**	**33.20**	**40,076**	**39.52**
Bukidnon	**23,706**	124	**0.52**	2,077	**8.76**	8,308	**35.05**	**10,509**	**44.33**
Camiguin	**1,858**	1	**0.05**	47	**2.53**	432	**23.25**	**480**	**25.83**
Lanao del Norte	**14,960**	3	**0.02**	118	**0.79**	891	**5.96**	**1,012**	**6.76**
Misamis Occidental	**6,403**	3	**0.05**	246	**3.84**	2,180	**34.05**	**2,429**	**37.94**
Misamis Oriental	**15,131**	12	**0.08**	1,128	**7.45**	6,661	**44.02**	**7,801**	**51.56**
Cagayan de Oro City	**14,339**	15	**0.10**	1,089	**7.59**	7,257	**50.61**	**8,361**	**58.31**
El Salvador City	**1,065**	3	**0.28**	114	**10.70**	596	**55.96**	**713**	**66.95**
Gingoog City	**2,644**	2	**0.08**	222	**8.40**	993	**37.56**	**1,217**	**46.03**
Iligan City	**7,580**	1	**0.01**	188	**2.48**	1,299	**17.14**	**1,488**	**19.63**
Malaybalay City	**3,956**	4	**0.10**	251	**6.34**	1,181	**29.85**	**1,436**	**36.30**

Table 1.B.1.14 - Prenatal Care

Number and proportion of pregnant women tested for CBC or Hemoglobin and Hematocrit Count

Philippines, Annual 2020

Area	Eligible Pop.	Tested for CBC/Hgb and Hct							
		Age Group						Total	%
		10-14 yrs old		15-19 yrs old		20-49 yrs old			
		No.	%	No.	%	No.	%		
Oroquieta City	**1,389**	0	**0.00**	33	**2.38**	315	**22.68**	**348**	**25.05**
Ozamis City	**2,778**	2	**0.07**	239	**8.60**	1,254	**45.14**	**1,495**	**53.82**
Tangub City	**1,234**	0	**0.00**	11	**0.89**	40	**3.24**	**51**	**4.13**
Valencia City	**4,368**	3	**0.07**	469	**10.74**	2,264	**51.83**	**2,736**	**62.64**
**Region 11**	**107,247**	**460**	**0.43**	**9,971**	**9.30**	**52,023**	**48.51**	**62,454**	**58.23**
Compostela Valley	**15,562**	99	**0.64**	1,919	**12.33**	8,855	**56.90**	**10,873**	**69.87**
Davao del Norte	**21,326**	112	**0.53**	2,470	**11.58**	13,576	**63.66**	**16,158**	**75.77**
Davao Oriental	**13,007**	54	**0.42**	1,053	**8.10**	5,050	**38.83**	**6,157**	**47.34**
Davao del Sur	**14,151**	64	**0.45**	1,067	**7.54**	5,113	**36.13**	**6,244**	**44.12**
Davao Occidental	**6,670**	28	**0.42**	423	**6.34**	1,132	**16.97**	**1,583**	**23.73**
Davao City	**36,531**	103	**0.28**	3,039	**8.32**	18,297	**50.09**	**21,439**	**58.69**
**Region 12**	**104,552**	**105**	**0.10**	**7,056**	**6.75**	**36,647**	**35.05**	**43,808**	**41.90**
North Cotabato	**33,645**	24	**0.07**	2,074	**6.16**	11,531	**34.27**	**13,629**	**40.51**
Sarangani	**12,891**	29	**0.22**	1,538	**11.93**	6,313	**48.97**	**7,880**	**61.13**
South Cotabato	**21,113**	27	**0.13**	1,538	**7.28**	8,728	**41.34**	**10,293**	**48.75**
Sultan Kudarat	**17,359**	19	**0.11**	1,072	**6.18**	5,847	**33.68**	**6,938**	**39.97**
Cotabato City	**5,835**	0	**0.00**	86	**1.47**	562	**9.63**	**648**	**11.11**
Gen. Santos City	**13,709**	6	**0.04**	748	**5.46**	3,666	**26.74**	**4,420**	**32.24**
**BARMM**	**92,799**	**7**	**0.01**	**508**	**0.55**	**4,156**	**4.48**	**4,671**	**5.03**
Basilan	**7,541**	2	**0.03**	79	**1.05**	94	**1.25**	**175**	**2.32**
Lanao del Sur	**21,131**	2	**0.01**	185	**0.88**	2,699	**12.77**	**2,886**	**13.66**
Maguindanao	**31,128**	1	**0.00**	81	**0.26**	564	**1.81**	**646**	**2.08**
Sulu	**16,613**	0	**0.00**	0	**0.00**	0	**0.00**	**0**	**0.00**
Tawi-Tawi	**9,259**	0	**0.00**	0	**0.00**	0	**0.00**	**0**	**0.00**
Lamitan City	**2,074**	2	**0.10**	73	**3.52**	96	**4.63**	**171**	**8.24**
Marawi City	**5,053**	0	**0.00**	90	**1.78**	703	**13.91**	**793**	**15.69**
**CARAGA**	**60,029**	**57**	**0.09**	**3,620**	**6.03**	**23,536**	**39.21**	**27,213**	**45.33**
Agusan del Norte	**8,098**	9	**0.11**	551	**6.80**	3,050	**37.66**	**3,610**	**44.58**
Agusan del Sur	**17,592**	14	**0.08**	1,007	**5.72**	5,395	**30.67**	**6,416**	**36.47**
Surigao del Norte	**7,089**	2	**0.03**	339	**4.78**	2,880	**40.63**	**3,221**	**45.44**
Surigao del Sur	**11,482**	11	**0.10**	690	**6.01**	5,199	**45.28**	**5,900**	**51.38**
Province of Dinagat	**2,573**	4	**0.16**	132	**5.13**	1,037	**40.30**	**1,173**	**45.59**
Bislig City	**2,179**	5	**0.23**	164	**7.53**	1,308	**60.03**	**1,477**	**67.78**
Butuan City	**7,715**	7	**0.09**	447	**5.79**	2,880	**37.33**	**3,334**	**43.21**
Surigao City	**3,301**	5	**0.15**	290	**8.79**	1,787	**54.14**	**2,082**	**63.07**

Note: Put asterisk (*) for No Report and Zero (0) for No Case

Table 1.B.1.15 - Prenatal Care

Number and proportion of pregnant women tested for Complete Blood Count or Hemoglobin and Hematocrit Count

Philippines, Annual 2020

Area	Total No. tested for CBC or Hgb or Hct count	Tested for CBC/Hgb and Hct diagnosed with anemia							
		Age Group						Total	%
		10-14 yrs old		15-19 yrs old		20-49 yrs old			
		No.	%	No.	%	No.	%		
**PHILIPPINES**	**594,260**	**336**	**0.06**	**13,717**	**2.31**	**59,412**	**10.00**	**73,465**	**12.36**
**N C R**	**63,972**	**50**	**0.08**	**938**	**1.47**	**6,002**	**9.38**	**6,990**	**10.93**
Malabon	**1,850**	0	**0.00**	43	**2.32**	157	**8.49**	**200**	**10.81**
Navotas	**2,235**	1	**0.04**	74	**3.31**	275	**12.30**	**350**	**15.66**
Valenzuela City	**1,440**	0	**0.00**	8	**0.56**	61	**4.24**	**69**	**4.79**
Caloocan City	**6,952**	7	**0.10**	159	**2.29**	837	**12.04**	**1,003**	**14.43**
Marikina City	**1,463**	0	**0.00**	8	**0.55**	86	**5.88**	**94**	**6.43**
Pasig City	**7,785**	0	**0.00**	57	**0.73**	439	**5.64**	**496**	**6.37**
Pateros	**662**	0	**0.00**	6	**0.91**	12	**1.81**	**18**	**2.72**
Taguig	**1,832**	23	**1.26**	33	**1.80**	181	**9.88**	**237**	**12.94**
Quezon City	**20,600**	8	**0.04**	307	**1.49**	2,087	**10.13**	**2,402**	**11.66**
Makati City	**3,692**	5	**0.14**	69	**1.87**	637	**17.25**	**711**	**19.26**
Mandaluyong City	**2,262**	0	**0.00**	25	**1.11**	144	**6.37**	**169**	**7.47**
San Juan	**688**	0	**0.00**	1	**0.15**	5	**0.73**	**6**	**0.87**
Manila City	**2,977**	4	**0.13**	95	**3.19**	453	**15.22**	**552**	**18.54**
Las Piñas City	**2,773**	2	**0.07**	38	**1.37**	429	**15.47**	**469**	**16.91**
Muntinlupa City	**0**	0	**0.00**	0	**0.00**	0	**0.00**	**0**	**0.00**
Parañaque City	**3,887**	0	**0.00**	14	**0.36**	106	**2.73**	**120**	**3.09**
Pasay City	**2,874**	0	**0.00**	1	**0.03**	93	**3.24**	**94**	**3.27**
**C A R**	**13,162**	**2**	**0.02**	**155**	**1.18**	**1,111**	**8.44**	**1,268**	**9.63**
Abra	**616**	0	**0.00**	0	**0.00**	6	**0.97**	**6**	**0.97**
Apayao	**948**	0	**0.00**	16	**1.69**	35	**3.69**	**51**	**5.38**
Benguet	**3,507**	0	**0.00**	18	**0.51**	191	**5.45**	**209**	**5.96**
Ifugao	**1,756**	0	**0.00**	26	**1.48**	178	**10.14**	**204**	**11.62**
Kalinga	**2,231**	2	**0.09**	78	**3.50**	558	**25.01**	**638**	**28.60**
Mt. Province	**885**	0	**0.00**	12	**1.36**	56	**6.33**	**68**	**7.68**
Baguio City	**3,219**	0	**0.00**	5	**0.16**	87	**2.70**	**92**	**2.86**
**Region 1**	**38,923**	**8**	**0.02**	**304**	**0.78**	**2,352**	**6.04**	**2,664**	**6.84**
Ilocos Norte	**3,860**	0	**0.00**	8	**0.21**	83	**2.15**	**91**	**2.36**
Ilocos Sur	**4,804**	1	**0.02**	27	**0.56**	263	**5.47**	**291**	**6.06**
La Union	**5,052**	3	**0.06**	55	**1.09**	307	**6.08**	**365**	**7.22**
Pangasinan	**18,374**	4	**0.02**	148	**0.81**	1,256	**6.84**	**1,408**	**7.66**
Alaminos City	**1,037**	0	**0.00**	0	**0.00**	0	**0.00**	**0**	**0.00**
Candon City	**780**	0	**0.00**	1	**0.13**	31	**3.97**	**32**	**4.10**
Dagupan City	**960**	0	**0.00**	47	**4.90**	319	**33.23**	**366**	**38.13**
Laoag City	**1,287**	0	**0.00**	1	**0.08**	0	**0.00**	**1**	**0.08**
San Carlos City	**630**	0	**0.00**	11	**1.75**	55	**8.73**	**66**	**10.48**
San Fernando City	**1,000**	0	**0.00**	1	**0.10**	19	**1.90**	**20**	**2.00**
Urdaneta City	**1,012**	0	**0.00**	0	**0.00**	1	**0.10**	**1**	**0.10**

Table 1.B.1.15 - Prenatal Care

Number and proportion of pregnant women tested for Complete Blood Count or Hemoglobin and Hematocrit Count

Philippines, Annual 2020

Area	Total No. tested for CBC or Hgb or Hct count	Tested for CBC/Hgb and Hct diagnosed with anemia							
		Age Group						Total	%
		10-14 yrs old		15-19 yrs old		20-49 yrs old			
		No.	%	No.	%	No.	%		
Vigan City	127	0	0.00	5	3.94	18	14.17	23	18.11
**Region 2**	**28,724**	**6**	**0.02**	**469**	**1.63**	**2,162**	**7.53**	**2,637**	**9.18**
Batanes	123	0	0.00	0	0.00	1	0.81	1	0.81
Cagayan	6,979	3	0.04	92	1.32	548	7.85	643	9.21
Isabela	8,395	3	0.04	193	2.30	798	9.51	994	11.84
Nueva Vizcaya	5,250	0	0.00	75	1.43	361	6.88	436	8.30
Quirino	3,681	0	0.00	58	1.58	181	4.92	239	6.49
Cauayan City	1,223	0	0.00	15	1.23	58	4.74	73	5.97
Ilagan City	1,248	0	0.00	5	0.40	28	2.24	33	2.64
Santiago City	721	0	0.00	9	1.25	83	11.51	92	12.76
Tuguegarao City	1,104	0	0.00	22	1.99	104	9.42	126	11.41
**Region 3**	**72,192**	**20**	**0.03**	**952**	**1.32**	**4,735**	**6.56**	**5,707**	**7.91**
Aurora	1,506	0	0.00	11	0.73	72	4.78	83	5.51
Bataan	6,644	4	0.06	143	2.15	839	12.63	986	14.84
Bulacan	13,297	6	0.05	217	1.63	1,008	7.58	1,231	9.26
Nueva Ecija	7,494	3	0.04	122	1.63	456	6.08	581	7.75
Pampanga	3,764	2	0.05	29	0.77	199	5.29	230	6.11
Tarlac	7,801	0	0.00	49	0.63	314	4.03	363	4.65
Zambales	2,076	1	0.05	27	1.30	163	7.85	191	9.20
Angeles City	1,736	0	0.00	24	1.38	68	3.92	92	5.30
Balanga City	1,121	1	0.09	32	2.85	140	12.49	173	15.43
Cabanatuan City	884	0	0.00	0	0.00	4	0.45	4	0.45
City of San Fernando	2,337	1	0.04	47	2.01	166	7.10	214	9.16
Gapan City	632	0	0.00	5	0.79	2	0.32	7	1.11
Mabalacat City	1,141	1	0.09	41	3.59	536	46.98	578	50.66
Malolos City	978	0	0.00	2	0.20	24	2.45	26	2.66
Meycauayan	1,705	1	0.06	8	0.47	20	1.17	29	1.70
Olongapo	1,355	0	0.00	28	2.07	96	7.08	124	9.15
Palayan City	116	0	0.00	0	0.00	1	0.86	1	0.86
San Jose City	729	0	0.00	0	0.00	0	0.00	0	0.00
San Jose del Monte City	11,601	0	0.00	134	1.16	485	4.18	619	5.34
Science City of Munoz	615	0	0.00	0	0.00	0	0.00	0	0.00
Tarlac City	4,660	0	0.00	33	0.71	142	3.05	175	3.76
**Region 4A**	**37,357**	**9**	**0.02**	**2,647**	**7.09**	**2,425**	**6.49**	**5,081**	**13.60**
Batangas	1,887	0	0.00	2	0.11	24	1.27	26	1.38
Cavite	3,331	1	0.03	60	1.80	371	11.14	432	12.97
Laguna	4,667	3	0.06	2,201	47.16	446	9.56	2,650	56.78
Quezon	3,619	1	0.03	20	0.55	104	2.87	125	3.45
Rizal	5,759	1	0.02	17	0.30	228	3.96	246	4.27
Antipolo City	2,464	2	0.08	41	1.66	98	3.98	141	5.72
Bacoor City	0	0	0.00	0	0.00	0	0.00	0	0.00
Batangas City	0	0	0.00	1	0.00	17	0.00	18	0.00

Table 1.B.1.15 - Prenatal Care

Number and proportion of pregnant women tested for Complete Blood Count or Hemoglobin and Hematocrit Count

Philippines, Annual 2020

Area	Total No. tested for CBC or Hgb or Hct count	Tested for CBC/Hgb and Hct diagnosed with anemia							
		Age Group						Total	%
		10-14 yrs old		15-19 yrs old		20-49 yrs old			
		No.	%	No.	%	No.	%		
Biñan City	**3,494**	1	**0.03**	158	**4.52**	345	**9.87**	**504**	**14.42**
Cabuyao City	**2,716**	0	**0.00**	0	**0.00**	0	**0.00**	**0**	**0.00**
Calamba City	**895**	0	**0.00**	16	**1.79**	66	**7.37**	**82**	**9.16**
Cavite City	**0**	0	**0.00**	3	**0.00**	1	**0.00**	**4**	**0.00**
Dasmariñas City	**2,722**	0	**0.00**	34	**1.25**	376	**13.81**	**410**	**15.06**
General Trias City	**0**	0	**0.00**	0	**0.00**	0	**0.00**	**0**	**0.00**
Imus City	**0**	0	**0.00**	6	**0.00**	13	**0.00**	**19**	**0.00**
Lipa City	**0**	0	**0.00**	5	**0.00**	8	**0.00**	**13**	**0.00**
Lucena City	**0**	0	**0.00**	0	**0.00**	0	**0.00**	**0**	**0.00**
San Pablo City	**124**	0	**0.00**	7	**5.65**	3	**2.42**	**10**	**8.06**
San Pedro City	**523**	0	**0.00**	27	**5.16**	115	**21.99**	**142**	**27.15**
Santa Rosa City	**2,691**	0	**0.00**	35	**1.30**	131	**4.87**	**166**	**6.17**
Tagaytay City	**1,278**	0	**0.00**	14	**1.10**	62	**4.85**	**76**	**5.95**
Tanauan City	**379**	0	**0.00**	0	**0.00**	15	**3.96**	**15**	**3.96**
Tayabas City	**0**	0	**0.00**	0	**0.00**	0	**0.00**	**0**	**0.00**
Trece Martires City	**808**	0	**0.00**	0	**0.00**	2	**0.25**	**2**	**0.25**
**Region 4B**	**10,722**	**7**	**0.07**	**84**	**0.78**	**756**	**7.05**	**847**	**7.90**
Marinduque	**1,313**	0	**0.00**	2	**0.15**	47	**3.58**	**49**	**3.73**
Mindoro Occidental	**2,219**	1	**0.05**	7	**0.32**	71	**3.20**	**79**	**3.56**
Mindoro Oriental	**4,909**	6	**0.12**	44	**0.90**	408	**8.31**	**458**	**9.33**
Palawan	**1,367**	0	**0.00**	24	**1.76**	153	**11.19**	**177**	**12.95**
Romblon	**702**	0	**0.00**	7	**1.00**	77	**10.97**	**84**	**11.97**
Puerto Princesa City	**212**	0	**0.00**	0	**0.00**	0	**0.00**	**0**	**0.00**
**Region 5**	**31,712**	**5**	**0.02**	**411**	**1.30**	**2,837**	**8.95**	**3,253**	**10.26**
Albay	**7,759**	1	**0.01**	47	**0.61**	411	**5.30**	**459**	**5.92**
Camarines Norte	**3,850**	2	**0.05**	103	**2.68**	564	**14.65**	**669**	**17.38**
Camarines Sur	**3,764**	2	**0.05**	31	**0.82**	298	**7.92**	**331**	**8.79**
Catanduanes	**1,858**	0	**0.00**	30	**1.61**	194	**10.44**	**224**	**12.06**
Masbate	**4,248**	0	**0.00**	78	**1.84**	434	**10.22**	**512**	**12.05**
Sorsogon	**7,537**	0	**0.00**	102	**1.35**	784	**10.40**	**886**	**11.76**
Iriga City	**119**	0	**0.00**	1	**0.84**	3	**2.52**	**4**	**3.36**
Legaspi City	**2,570**	0	**0.00**	19	**0.74**	149	**5.80**	**168**	**6.54**
Naga City	**7**	0	**0.00**	0	**0.00**	0	**0.00**	**0**	**0.00**
**Region 6**	**56,718**	**6**	**0.01**	**606**	**1.07**	**3,770**	**6.65**	**4,382**	**7.73**
Aklan	**4,954**	1	**0.02**	50	**1.01**	328	**6.62**	**379**	**7.65**
Antique	**3,528**	0	**0.00**	49	**1.39**	272	**7.71**	**321**	**9.10**
Capiz	**4,282**	0	**0.00**	22	**0.51**	229	**5.35**	**251**	**5.86**
Guimaras	**2,056**	0	**0.00**	29	**1.41**	176	**8.56**	**205**	**9.97**
Iloilo	**16,468**	1	**0.01**	108	**0.66**	787	**4.78**	**896**	**5.44**
Negros Occidental	**18,867**	1	**0.01**	233	**1.23**	1,285	**6.81**	**1,519**	**8.05**
Bacolod City	**3,500**	3	**0.09**	99	**2.83**	567	**16.20**	**669**	**19.11**
Iloilo City	**3,063**	0	**0.00**	16	**0.52**	126	**4.11**	**142**	**4.64**

Table 1.B.1.15 - Prenatal Care

Number and proportion of pregnant women tested for Complete Blood Count or Hemoglobin and Hematocrit Count

Philippines, Annual 2020

Area	Total No. tested for CBC or Hgb or Hct count	Tested for CBC/Hgb and Hct diagnosed with anemia							
		Age Group						Total	%
		10-14 yrs old		15-19 yrs old		20-49 yrs old			
		No.	%	No.	%	No.	%		
**Region 7**	**33,048**	**5**	**0.02**	**411**	**1.24**	**2,764**	**8.36**	**3,180**	**9.62**
Bohol	**5,681**	0	**0.00**	12	**0.21**	434	**7.64**	**446**	**7.85**
Cebu	**4,759**	0	**0.00**	33	**0.69**	395	**8.30**	**428**	**8.99**
Negros Oriental	**8,931**	5	**0.06**	158	**1.77**	988	**11.06**	**1,151**	**12.89**
Siquijor	**640**	0	**0.00**	4	**0.63**	25	**3.91**	**29**	**4.53**
Cebu City	**7,921**	0	**0.00**	165	**2.08**	737	**9.30**	**902**	**11.39**
Lapu-Lapu City	**5,116**	0	**0.00**	39	**0.76**	185	**3.62**	**224**	**4.38**
Mandaue City	**0**	0	**0.00**	0	**0.00**	0	**0.00**	**0**	**0.00**
**Region 8**	**18,893**	**12**	**0.06**	**340**	**1.80**	**1,947**	**10.31**	**2,299**	**12.17**
Biliran	**1,883**	0	**0.00**	53	**2.81**	341	**18.11**	**394**	**20.92**
Eastern Samar	**1,753**	10	**0.57**	105	**5.99**	634	**36.17**	**749**	**42.73**
Northern Leyte	**0**	0	**0.00**	0	**0.00**	0	**0.00**	**0**	**0.00**
Northern Samar	**5,109**	0	**0.00**	24	**0.47**	231	**4.52**	**255**	**4.99**
Southern Leyte	**2,880**	0	**0.00**	29	**1.01**	115	**3.99**	**144**	**5.00**
Western Samar	**2,285**	2	**0.09**	69	**3.02**	321	**14.05**	**392**	**17.16**
Calbayog City	**177**	0	**0.00**	13	**7.34**	61	**34.46**	**74**	**41.81**
Maasin City	**448**	0	**0.00**	19	**4.24**	75	**16.74**	**94**	**20.98**
Ormoc City	**1,694**	0	**0.00**	24	**1.42**	139	**8.21**	**163**	**9.62**
Tacloban City	**2,664**	0	**0.00**	4	**0.15**	30	**1.13**	**34**	**1.28**
**Region 9**	**10,615**	**1**	**0.01**	**251**	**2.36**	**1,488**	**14.02**	**1,740**	**16.39**
Zamboanga del Norte	**2,437**	1	**0.04**	73	**3.00**	308	**12.64**	**382**	**15.68**
Zamboanga del Sur	**3,379**	0	**0.00**	79	**2.34**	496	**14.68**	**575**	**17.02**
Zamboanga Sibugay	**2,384**	0	**0.00**	45	**1.89**	326	**13.67**	**371**	**15.56**
Dapitan City	**148**	0	**0.00**	14	**9.46**	93	**62.84**	**107**	**72.30**
Dipolog City	**26**	0	**0.00**	1	**3.85**	8	**30.77**	**9**	**34.62**
Isabela City	**196**	0	**0.00**	7	**3.57**	55	**28.06**	**62**	**31.63**
Pagadian City	**1,791**	0	**0.00**	26	**1.45**	148	**8.26**	**174**	**9.72**
Zamboanga City	**254**	0	**0.00**	6	**2.36**	54	**21.26**	**60**	**23.62**
**Region 10**	**40,076**	**38**	**0.09**	**1,102**	**2.75**	**4,250**	**10.60**	**5,390**	**13.45**
Bukidnon	**10,509**	31	**0.29**	657	**6.25**	2,172	**20.67**	**2,860**	**27.21**
Camiguin	**480**	0	**0.00**	5	**1.04**	31	**6.46**	**36**	**7.50**
Lanao del Norte	**1,012**	2	**0.20**	17	**1.68**	203	**20.06**	**222**	**21.94**
Misamis Occidental	**2,429**	2	**0.08**	48	**1.98**	226	**9.30**	**276**	**11.36**
Misamis Oriental	**7,801**	0	**0.00**	33	**0.42**	200	**2.56**	**233**	**2.99**
Cagayan de Oro City	**8,361**	2	**0.02**	120	**1.44**	606	**7.25**	**728**	**8.71**
El Salvador City	**713**	0	**0.00**	11	**1.54**	51	**7.15**	**62**	**8.70**
Gingoog City	**1,217**	0	**0.00**	22	**1.81**	106	**8.71**	**128**	**10.52**
Iligan City	**1,488**	0	**0.00**	23	**1.55**	67	**4.50**	**90**	**6.05**
Malaybalay City	**1,436**	0	**0.00**	33	**2.30**	146	**10.17**	**179**	**12.47**

Table 1.B.1.15 - Prenatal Care

Number and proportion of pregnant women tested for Complete Blood Count or Hemoglobin and Hematocrit Count

Philippines, Annual 2020

Area	Total No. tested for CBC or Hgb or Hct count	Tested for CBC/Hgb and Hct diagnosed with anemia							
		Age Group						Total	%
		10-14 yrs old		15-19 yrs old		20-49 yrs old			
		No.	%	No.	%	No.	%		
Oroquieta City	**348**	0	**0.00**	0	**0.00**	0	**0.00**	**0**	**0.00**
Ozamis City	**1,495**	0	**0.00**	3	**0.20**	7	**0.47**	**10**	**0.67**
Tangub City	**51**	0	**0.00**	0	**0.00**	0	**0.00**	**0**	**0.00**
Valencia City	**2,736**	1	**0.04**	130	**4.75**	435	**15.90**	**566**	**20.69**
**Region 11**	**62,454**	**142**	**0.23**	**3,346**	**5.36**	**15,039**	**24.08**	**18,527**	**29.67**
Compostela Valley	**10,873**	26	**0.24**	650	**5.98**	2,671	**24.57**	**3,347**	**30.78**
Davao del Norte	**16,158**	36	**0.22**	775	**4.80**	3,436	**21.27**	**4,247**	**26.28**
Davao Oriental	**6,157**	12	**0.19**	346	**5.62**	1,441	**23.40**	**1,799**	**29.22**
Davao del Sur	**6,244**	21	**0.34**	388	**6.21**	1,555	**24.90**	**1,964**	**31.45**
Davao Occidental	**1,583**	7	**0.44**	105	**6.63**	310	**19.58**	**422**	**26.66**
Davao City	**21,439**	40	**0.19**	1,082	**5.05**	5,626	**26.24**	**6,748**	**31.48**
**Region 12**	**43,808**	**18**	**0.04**	**962**	**2.20**	**3,697**	**8.44**	**4,677**	**10.68**
North Cotabato	**13,629**	6	**0.04**	373	**2.74**	1,557	**11.42**	**1,936**	**14.21**
Sarangani	**7,880**	8	**0.10**	188	**2.39**	688	**8.73**	**884**	**11.22**
South Cotabato	**10,293**	3	**0.03**	137	**1.33**	357	**3.47**	**497**	**4.83**
Sultan Kudarat	**6,938**	0	**0.00**	102	**1.47**	421	**6.07**	**523**	**7.54**
Cotabato City	**648**	0	**0.00**	3	**0.46**	11	**1.70**	**14**	**2.16**
Gen. Santos City	**4,420**	1	**0.02**	159	**3.60**	663	**15.00**	**823**	**18.62**
**BARMM**	**4,671**	**0**	**0.00**	**13**	**0.28**	**113**	**2.42**	**126**	**2.70**
Basilan	**175**	0	**0.00**	0	**0.00**	0	**0.00**	**0**	**0.00**
Lanao del Sur	**2,886**	0	**0.00**	6	**0.21**	50	**1.73**	**56**	**1.94**
Maguindanao	**646**	0	**0.00**	7	**1.08**	63	**9.75**	**70**	**10.84**
Sulu	**0**	0	**0.00**	0	**0.00**	0	**0.00**	**0**	**0.00**
Tawi-Tawi	**0**	0	**0.00**	0	**0.00**	0	**0.00**	**0**	**0.00**
Lamitan City	**171**	0	**0.00**	0	**0.00**	0	**0.00**	**0**	**0.00**
Marawi City	**793**	0	**0.00**	0	**0.00**	0	**0.00**	**0**	**0.00**
**CARAGA**	**27,213**	**7**	**0.03**	**726**	**2.67**	**3,964**	**14.57**	**4,697**	**17.26**
Agusan del Norte	**3,610**	1	**0.03**	27	**0.75**	192	**5.32**	**220**	**6.09**
Agusan del Sur	**6,416**	4	**0.06**	250	**3.90**	1,182	**18.42**	**1,436**	**22.38**
Surigao del Norte	**3,221**	0	**0.00**	37	**1.15**	341	**10.59**	**378**	**11.74**
Surigao del Sur	**5,900**	1	**0.02**	167	**2.83**	994	**16.85**	**1,162**	**19.69**
Province of Dinagat	**1,173**	0	**0.00**	16	**1.36**	132	**11.25**	**148**	**12.62**
Bislig City	**1,477**	1	**0.07**	60	**4.06**	403	**27.29**	**464**	**31.42**
Butuan City	**3,334**	0	**0.00**	95	**2.85**	470	**14.10**	**565**	**16.95**
Surigao City	**2,082**	0	**0.00**	74	**3.55**	250	**12.01**	**324**	**15.56**

Note: Put asterisk (*) for No Report and Zero (0) for No Case

Tabe 1.B.1.16 - Prenatal Care

Number and proportion of pregnant women screened for Gestational Diabetes

Philippines, Annual 2020

Area	Eligible Pop.	Screened for Gestational Diabetes							
		Age Group						Total	%
		10-14 yrs old		15-19 yrs old		20-49 yrs old			
		No.	%	No.	%	No.	%		
**PHILIPPINES**	**2,123,158**	**377**	**0.02**	**21,569**	**1.02**	**150,996**	**7.11**	**172,942**	**8.15**
**N C R**	**238,661**	**59**	**0.02**	**2,613**	**1.09**	**30,728**	**12.88**	**33,400**	**13.99**
Malabon	**6,775**	0	**0.00**	28	**0.41**	259	**3.82**	**287**	**4.24**
Navotas	**4,621**	3	**0.06**	153	**3.31**	479	**10.37**	**635**	**13.74**
Valenzuela City	**11,500**	0	**0.00**	3	**0.03**	50	**0.43**	**53**	**0.46**
Caloocan City	**29,363**	0	**0.00**	113	**0.38**	1,350	**4.60**	**1,463**	**4.98**
Marikina City	**8,354**	0	**0.00**	56	**0.67**	585	**7.00**	**641**	**7.67**
Pasig City	**13,996**	2	**0.01**	182	**1.30**	3,338	**23.85**	**3,522**	**25.16**
Pateros	**1,184**	0	**0.00**	47	**3.97**	277	**23.40**	**324**	**27.36**
Taguig	**14,918**	3	**0.02**	38	**0.25**	168	**1.13**	**209**	**1.40**
Quezon City	**54,413**	20	**0.04**	1,218	**2.24**	17,685	**32.50**	**18,923**	**34.78**
Makati City	**10,798**	6	**0.06**	94	**0.87**	1,487	**13.77**	**1,587**	**14.70**
Mandaluyong City	**7,160**	3	**0.04**	58	**0.81**	629	**8.78**	**690**	**9.64**
San Juan	**2,261**	0	**0.00**	30	**1.33**	383	**16.94**	**413**	**18.27**
Manila City	**32,980**	8	**0.02**	268	**0.81**	1,568	**4.75**	**1,844**	**5.59**
Las Piñas City	**10,914**	2	**0.02**	30	**0.27**	450	**4.12**	**482**	**4.42**
Muntinlupa City	**9,353**	0	**0.00**	0	**0.00**	0	**0.00**	**0**	**0.00**
Parañaque City	**12,343**	10	**0.08**	221	**1.79**	1,340	**10.86**	**1,571**	**12.73**
Pasay City	**7,728**	2	**0.03**	74	**0.96**	680	**8.80**	**756**	**9.78**
**C A R**	**35,099**	**4**	**0.01**	**399**	**1.14**	**4,146**	**11.81**	**4,549**	**12.96**
Abra	**4,309**	0	**0.00**	29	**0.67**	190	**4.41**	**219**	**5.08**
Apayao	**2,451**	0	**0.00**	85	**3.47**	411	**16.77**	**496**	**20.24**
Benguet	**9,188**	1	**0.01**	70	**0.76**	801	**8.72**	**872**	**9.49**
Ifugao	**4,396**	0	**0.00**	7	**0.16**	264	**6.01**	**271**	**6.16**
Kalinga	**4,617**	0	**0.00**	18	**0.39**	244	**5.28**	**262**	**5.67**
Mt. Province	**3,023**	0	**0.00**	0	**0.00**	4	**0.13**	**4**	**0.13**
Baguio City	**7,115**	3	**0.04**	190	**2.67**	2,232	**31.37**	**2,425**	**34.08**
**Region 1**	**97,261**	**13**	**0.01**	**1,288**	**1.32**	**12,861**	**13.22**	**14,162**	**14.56**
Ilocos Norte	**8,105**	0	**0.00**	133	**1.64**	1,645	**20.30**	**1,778**	**21.94**
Ilocos Sur	**9,330**	3	**0.03**	292	**3.13**	3,018	**32.35**	**3,313**	**35.51**
La Union	**11,511**	4	**0.03**	129	**1.12**	1,162	**10.09**	**1,295**	**11.25**
Pangasinan	**50,168**	5	**0.01**	551	**1.10**	4,556	**9.08**	**5,112**	**10.19**
Alaminos City	**1,894**	0	**0.00**	26	**1.37**	179	**9.45**	**205**	**10.82**
Candon City	**987**	0	**0.00**	8	**0.81**	467	**47.32**	**475**	**48.13**
Dagupan City	**3,620**	0	**0.00**	5	**0.14**	45	**1.24**	**50**	**1.38**
Laoag City	**1,870**	0	**0.00**	16	**0.86**	709	**37.91**	**725**	**38.77**
San Carlos City	**3,979**	0	**0.00**	13	**0.33**	136	**3.42**	**149**	**3.74**
San Fernando City	**2,115**	0	**0.00**	78	**3.69**	697	**32.96**	**775**	**36.64**
Urdaneta City	**2,806**	1	**0.04**	37	**1.32**	247	**8.80**	**285**	**10.16**

Tabe 1.B.1.16 - Prenatal Care

Number and proportion of pregnant women screened for Gestational Diabetes

Philippines, Annual 2020

Area	Eligible Pop.	Screened for Gestational Diabetes							
		Age Group						Total	%
		10-14 yrs old		15-19 yrs old		20-49 yrs old			
		No.	%	No.	%	No.	%		
Vigan City	876	0	0.00	0	0.00	0	0.00	0	0.00
**Region 2**	69,443	6	0.01	696	1.00	5,515	7.94	6,217	8.95
Batanes	340	0	0.00	2	0.59	52	15.29	54	15.88
Cagayan	17,971	0	0.00	97	0.54	529	2.94	626	3.48
Isabela	26,309	0	0.00	261	0.99	2,464	9.37	2,725	10.36
Nueva Vizcaya	9,573	4	0.04	148	1.55	1,059	11.06	1,211	12.65
Quirino	4,025	0	0.00	66	1.64	527	13.09	593	14.73
Cauayan City	2,590	0	0.00	35	1.35	374	14.44	409	15.79
Ilagan City	2,911	1	0.03	26	0.89	71	2.44	98	3.37
Santiago City	2,692	1	0.04	61	2.27	435	16.16	497	18.46
Tuguegarao City	3,032	0	0.00	0	0.00	4	0.13	4	0.13
**Region 3**	220,020	41	0.02	2,608	1.19	19,679	8.94	22,328	10.15
Aurora	4,770	0	0.00	35	0.73	266	5.58	301	6.31
Bataan	13,823	5	0.04	245	1.77	1,806	13.07	2,056	14.87
Bulacan	43,627	2	0.00	366	0.84	2,529	5.80	2,897	6.64
Nueva Ecija	28,782	9	0.03	307	1.07	1,637	5.69	1,953	6.79
Pampanga	31,359	3	0.01	204	0.65	1,393	4.44	1,600	5.10
Tarlac	19,765	2	0.01	253	1.28	1,880	9.51	2,135	10.80
Zambales	12,610	2	0.02	57	0.45	325	2.58	384	3.05
Angeles City	7,869	1	0.01	21	0.27	509	6.47	531	6.75
Balanga City	1,998	0	0.00	3	0.15	59	2.95	62	3.10
Cabanatuan City	5,896	0	0.00	29	0.49	271	4.60	300	5.09
City of San Fernando	5,863	6	0.10	247	4.21	1,563	26.66	1,816	30.97
Gapan City	2,153	0	0.00	23	1.07	127	5.90	150	6.97
Mabalacat City	4,793	1	0.02	20	0.42	252	5.26	273	5.70
Malolos City	4,880	1	0.02	0	0.00	45	0.92	46	0.94
Meycauayan	4,041	1	0.02	46	1.14	217	5.37	264	6.53
Olongapo	4,971	0	0.00	73	1.47	540	10.86	613	12.33
Palayan City	799	0	0.00	0	0.00	0	0.00	0	0.00
San Jose City	2,718	0	0.00	0	0.00	2	0.07	2	0.07
San Jose del Monte City	11,100	8	0.07	650	5.86	6,154	55.44	6,812	61.37
Science City of Munoz	1,586	0	0.00	0	0.00	0	0.00	0	0.00
Tarlac City	6,617	0	0.00	29	0.44	104	1.57	133	2.01
**Region 4A**	296,816	41	0.01	5,742	1.93	15,800	5.32	21,583	7.27
Batangas	38,441	0	0.00	130	0.34	1,960	5.10	2,090	5.44
Cavite	27,704	11	0.04	141	0.51	1,730	6.24	1,882	6.79
Laguna	19,727	21	0.11	2,167	10.98	1,155	5.85	3,343	16.95
Quezon	37,410	0	0.00	64	0.17	557	1.49	621	1.66
Rizal	44,791	4	0.01	458	1.02	3,020	6.74	3,482	7.77
Antipolo City	16,497	0	0.00	36	0.22	156	0.95	192	1.16
Bacoor City	12,142	0	0.00	11	0.09	121	1.00	132	1.09
Batangas City	6,823	0	0.00	0	0.00	0	0.00	0	0.00

Tabe 1.B.1.16 - Prenatal Care

Number and proportion of pregnant women screened for Gestational Diabetes

Philippines, Annual 2020

Area	Eligible Pop.	Screened for Gestational Diabetes							
		Age Group						Total	%
		10-14 yrs old		15-19 yrs old		20-49 yrs old			
		No.	%	No.	%	No.	%		
Biñan City	**6,607**	1	**0.02**	354	**5.36**	1,943	**29.41**	**2,298**	**34.78**
Cabuyao City	**6,130**	0	**0.00**	705	**11.50**	1,187	**19.36**	**1,892**	**30.86**
Calamba City	**9,028**	0	**0.00**	216	**2.39**	386	**4.28**	**602**	**6.67**
Cavite City	**2,075**	0	**0.00**	1	**0.05**	6	**0.29**	**7**	**0.34**
Dasmariñas City	**13,322**	0	**0.00**	62	**0.47**	598	**4.49**	**660**	**4.95**
General Trias City	**6,352**	0	**0.00**	10	**0.16**	197	**3.10**	**207**	**3.26**
Imus City	**8,156**	2	**0.02**	6	**0.07**	75	**0.92**	**83**	**1.02**
Lipa City	**6,880**	0	**0.00**	0	**0.00**	0	**0.00**	**0**	**0.00**
Lucena City	**5,672**	0	**0.00**	0	**0.00**	0	**0.00**	**0**	**0.00**
San Pablo City	**5,277**	0	**0.00**	1,212	**22.97**	419	**7.94**	**1,631**	**30.91**
San Pedro City	**6,467**	0	**0.00**	25	**0.39**	103	**1.59**	**128**	**1.98**
Santa Rosa City	**7,025**	0	**0.00**	65	**0.93**	660	**9.40**	**725**	**10.32**
Tagaytay City	**1,437**	2	**0.14**	74	**5.15**	1,430	**99.51**	**1,506**	**104.80**
Tanauan City	**3,584**	0	**0.00**	5	**0.14**	95	**2.65**	**100**	**2.79**
Tayabas City	**2,122**	0	**0.00**	0	**0.00**	0	**0.00**	**0**	**0.00**
Trece Martires City	**3,147**	0	**0.00**	0	**0.00**	2	**0.06**	**2**	**0.06**
**Region 4B**	**71,246**	**13**	**0.02**	**257**	**0.36**	**1,915**	**2.69**	**2,185**	**3.07**
Marinduque	**4,836**	1	**0.02**	48	**0.99**	425	**8.79**	**474**	**9.80**
Mindoro Occidental	**12,407**	0	**0.00**	17	**0.14**	186	**1.50**	**203**	**1.64**
Mindoro Oriental	**19,809**	11	**0.06**	146	**0.74**	964	**4.87**	**1,121**	**5.66**
Palawan	**21,375**	1	**0.00**	26	**0.12**	155	**0.73**	**182**	**0.85**
Romblon	**6,400**	0	**0.00**	19	**0.30**	174	**2.72**	**193**	**3.02**
Puerto Princesa City	**6,419**	0	**0.00**	1	**0.02**	11	**0.17**	**12**	**0.19**
**Region 5**	**136,116**	**10**	**0.01**	**566**	**0.42**	**6,042**	**4.44**	**6,618**	**4.86**
Albay	**23,737**	8	**0.03**	148	**0.62**	2,075	**8.74**	**2,231**	**9.40**
Camarines Norte	**14,283**	0	**0.00**	15	**0.11**	197	**1.38**	**212**	**1.48**
Camarines Sur	**38,796**	0	**0.00**	159	**0.41**	1,405	**3.62**	**1,564**	**4.03**
Catanduanes	**6,274**	0	**0.00**	1	**0.02**	44	**0.70**	**45**	**0.72**
Masbate	**22,521**	0	**0.00**	101	**0.45**	691	**3.07**	**792**	**3.52**
Sorsogon	**19,065**	1	**0.01**	78	**0.41**	768	**4.03**	**847**	**4.44**
Iriga City	**2,638**	0	**0.00**	0	**0.00**	51	**1.93**	**51**	**1.93**
Legaspi City	**4,174**	1	**0.02**	57	**1.37**	681	**16.32**	**739**	**17.70**
Naga City	**4,628**	0	**0.00**	7	**0.15**	130	**2.81**	**137**	**2.96**
**Region 6**	**146,526**	**49**	**0.03**	**2,845**	**1.94**	**23,098**	**15.76**	**25,992**	**17.74**
Aklan	**11,162**	11	**0.10**	313	**2.80**	2,272	**20.35**	**2,596**	**23.26**
Antique	**12,816**	2	**0.02**	83	**0.65**	776	**6.05**	**861**	**6.72**
Capiz	**13,986**	1	**0.01**	150	**1.07**	1,430	**10.22**	**1,581**	**11.30**
Guimaras	**3,085**	1	**0.03**	152	**4.93**	1,292	**41.88**	**1,445**	**46.84**
Iloilo	**36,541**	8	**0.02**	752	**2.06**	7,397	**20.24**	**8,157**	**22.32**
Negros Occidental	**49,368**	15	**0.03**	839	**1.70**	5,951	**12.05**	**6,805**	**13.78**
Bacolod City	**11,115**	11	**0.10**	422	**3.80**	2,871	**25.83**	**3,304**	**29.73**
Iloilo City	**8,453**	0	**0.00**	134	**1.59**	1,109	**13.12**	**1,243**	**14.70**

Tabe 1.B.1.16 - Prenatal Care

Number and proportion of pregnant women screened for Gestational Diabetes

Philippines, Annual 2020

Area	Eligible Pop.	Screened for Gestational Diabetes							
		Age Group						Total	%
		10-14 yrs old		15-19 yrs old		20-49 yrs old			
		No.	%	No.	%	No.	%		
**Region 7**	**163,262**	**4**	**0.00**	**695**	**0.43**	**6,402**	**3.92**	**7,101**	**4.35**
Bohol	**27,312**	0	**0.00**	155	**0.57**	1,966	**7.20**	**2,121**	**7.77**
Cebu	**67,506**	0	**0.00**	100	**0.15**	796	**1.18**	**896**	**1.33**
Negros Oriental	**27,938**	0	**0.00**	51	**0.18**	405	**1.45**	**456**	**1.63**
Siquijor	**1,613**	0	**0.00**	11	**0.68**	46	**2.85**	**57**	**3.53**
Cebu City	**21,193**	4	**0.02**	298	**1.41**	2,231	**10.53**	**2,533**	**11.95**
Lapu-Lapu City	**9,372**	0	**0.00**	80	**0.85**	958	**10.22**	**1,038**	**11.08**
Mandaue City	**8,328**	0	**0.00**	0	**0.00**	0	**0.00**	**0**	**0.00**
**Region 8**	**102,619**	**5**	**0.00**	**311**	**0.30**	**2,570**	**2.50**	**2,886**	**2.81**
Biliran	**3,834**	0	**0.00**	23	**0.60**	173	**4.51**	**196**	**5.11**
Eastern Samar	**11,392**	4	**0.04**	41	**0.36**	204	**1.79**	**249**	**2.19**
Northern Leyte	**34,707**	0	**0.00**	0	**0.00**	0	**0.00**	**0**	**0.00**
Northern Samar	**15,370**	0	**0.00**	88	**0.57**	1,249	**8.13**	**1,337**	**8.70**
Southern Leyte	**6,451**	1	**0.02**	27	**0.42**	256	**3.97**	**284**	**4.40**
Western Samar	**14,305**	0	**0.00**	26	**0.18**	164	**1.15**	**190**	**1.33**
Calbayog City	**4,413**	0	**0.00**	68	**1.54**	48	**1.09**	**116**	**2.63**
Maasin City	**1,637**	0	**0.00**	0	**0.00**	4	**0.24**	**4**	**0.24**
Ormoc City	**4,941**	0	**0.00**	4	**0.08**	75	**1.52**	**79**	**1.60**
Tacloban City	**5,569**	0	**0.00**	34	**0.61**	397	**7.13**	**431**	**7.74**
**Region 9**	**80,051**	**1**	**0.00**	**112**	**0.14**	**806**	**1.01**	**919**	**1.15**
Zamboanga del Norte	**17,249**	1	**0.01**	46	**0.27**	343	**1.99**	**390**	**2.26**
Zamboanga del Sur	**17,653**	0	**0.00**	19	**0.11**	119	**0.67**	**138**	**0.78**
Zamboanga Sibugay	**14,954**	0	**0.00**	30	**0.20**	153	**1.02**	**183**	**1.22**
Dapitan City	**1,784**	0	**0.00**	0	**0.00**	0	**0.00**	**0**	**0.00**
Dipolog City	**2,827**	0	**0.00**	0	**0.00**	1	**0.04**	**1**	**0.04**
Isabela City	**2,522**	0	**0.00**	16	**0.63**	175	**6.94**	**191**	**7.57**
Pagadian City	**4,325**	0	**0.00**	1	**0.02**	3	**0.07**	**4**	**0.09**
Zamboanga City	**18,737**	0	**0.00**	0	**0.00**	12	**0.06**	**12**	**0.06**
**Region 10**	**101,411**	**10**	**0.01**	**966**	**0.95**	**5,059**	**4.99**	**6,035**	**5.95**
Bukidnon	**23,706**	3	**0.01**	174	**0.73**	767	**3.24**	**944**	**3.98**
Camiguin	**1,858**	0	**0.00**	24	**1.29**	268	**14.42**	**292**	**15.72**
Lanao del Norte	**14,960**	0	**0.00**	12	**0.08**	92	**0.61**	**104**	**0.70**
Misamis Occidental	**6,403**	2	**0.03**	36	**0.56**	178	**2.78**	**216**	**3.37**
Misamis Oriental	**15,131**	1	**0.01**	159	**1.05**	902	**5.96**	**1,062**	**7.02**
Cagayan de Oro City	**14,339**	2	**0.01**	220	**1.53**	1,493	**10.41**	**1,715**	**11.96**
El Salvador City	**1,065**	0	**0.00**	11	**1.03**	86	**8.08**	**97**	**9.11**
Gingoog City	**2,644**	0	**0.00**	14	**0.53**	116	**4.39**	**130**	**4.92**
Iligan City	**7,580**	0	**0.00**	7	**0.09**	71	**0.94**	**78**	**1.03**
Malaybalay City	**3,956**	0	**0.00**	13	**0.33**	99	**2.50**	**112**	**2.83**

Tabe 1.B.1.16 - Prenatal Care

Number and proportion of pregnant women screened for Gestational Diabetes

Philippines, Annual 2020

Area	Eligible Pop.	Screened for Gestational Diabetes							
		Age Group						Total	%
		10-14 yrs old		15-19 yrs old		20-49 yrs old			
		No.	%	No.	%	No.	%		
Oroquieta City	1,389	0	0.00	0	0.00	1	0.07	1	0.07
Ozamis City	2,778	2	0.07	296	10.66	974	35.06	1,272	45.79
Tangub City	1,234	0	0.00	0	0.00	2	0.16	2	0.16
Valencia City	4,368	0	0.00	0	0.00	10	0.23	10	0.23
**Region 11**	**107,247**	**96**	**0.09**	**982**	**0.92**	**6,720**	**6.27**	**7,798**	**7.27**
Compostela Valley	15,562	4	0.03	6	0.04	152	0.98	162	1.04
Davao del Norte	21,326	4	0.02	51	0.24	694	3.25	749	3.51
Davao Oriental	13,007	1	0.01	18	0.14	114	0.88	133	1.02
Davao del Sur	14,151	11	0.08	29	0.20	149	1.05	189	1.34
Davao Occidental	6,670	4	0.06	23	0.34	101	1.51	128	1.92
Davao City	36,531	72	0.20	855	2.34	5,510	15.08	6,437	17.62
**Region 12**	**104,552**	**17**	**0.02**	**1,047**	**1.00**	**5,674**	**5.43**	**6,738**	**6.44**
North Cotabato	33,645	1	0.00	171	0.51	1,181	3.51	1,353	4.02
Sarangani	12,891	6	0.05	473	3.67	1,927	14.95	2,406	18.66
South Cotabato	21,113	3	0.01	121	0.57	895	4.24	1,019	4.83
Sultan Kudarat	17,359	4	0.02	214	1.23	1,147	6.61	1,365	7.86
Cotabato City	5,835	0	0.00	7	0.12	83	1.42	90	1.54
Gen. Santos City	13,709	3	0.02	61	0.44	441	3.22	505	3.68
**BARMM**	**92,799**	**0**	**0.00**	**89**	**0.10**	**1,494**	**1.61**	**1,583**	**1.71**
Basilan	7,541	0	0.00	0	0.00	0	0.00	0	0.00
Lanao del Sur	21,131	0	0.00	29	0.14	865	4.09	894	4.23
Maguindanao	31,128	0	0.00	15	0.05	226	0.73	241	0.77
Sulu	16,613	0	0.00	0	0.00	0	0.00	0	0.00
Tawi-Tawi	9,259	0	0.00	0	0.00	0	0.00	0	0.00
Lamitan City	2,074	0	0.00	0	0.00	0	0.00	0	0.00
Marawi City	5,053	0	0.00	45	0.89	403	7.98	448	8.87
**CARAGA**	**60,029**	**8**	**0.01**	**353**	**0.59**	**2,487**	**4.14**	**2,848**	**4.74**
Agusan del Norte	8,098	0	0.00	13	0.16	208	2.57	221	2.73
Agusan del Sur	17,592	1	0.01	73	0.41	381	2.17	455	2.59
Surigao del Norte	7,089	0	0.00	37	0.52	249	3.51	286	4.03
Surigao del Sur	11,482	3	0.03	107	0.93	590	5.14	700	6.10
Province of Dinagat	2,573	1	0.04	10	0.39	72	2.80	83	3.23
Bislig City	2,179	0	0.00	6	0.28	63	2.89	69	3.17
Butuan City	7,715	1	0.01	43	0.56	385	4.99	429	5.56
Surigao City	3,301	2	0.06	64	1.94	539	16.33	605	18.33

Note: Put asterisk (*) for No Report and Zero (0) for No Case

Table 1.B.1.17 - Prenatal Care

Number and proportion of pregnant women tested positive for Gestational Diabetes

Philippines, Annual 2020

Area	Total No. of Screened for Gestational Diabetes	Tested positive for Gestational Diabetes							
		Age Group						Total	%
		10-14 yrs old		15-19 yrs old		20-49 yrs old			
		No.	%	No.	%	No.	%		
**PHILIPPINES**	**172,942**	**972**	**0.56**	**2,320**	**1.34**	**9,759**	**5.64**	**13,051**	**7.55**
**N C R**	**33,400**	**37**	**0.11**	**92**	**0.28**	**908**	**2.72**	**1,037**	**3.10**
Malabon	287	0	0.00	1	0.35	14	4.88	15	5.23
Navotas	635	0	0.00	0	0.00	45	7.09	45	7.09
Valenzuela City	53	0	0.00	0	0.00	6	11.32	6	11.32
Caloocan City	1,463	0	0.00	2	0.14	49	3.35	51	3.49
Marikina City	641	0	0.00	0	0.00	13	2.03	13	2.03
Pasig City	3,522	0	0.00	6	0.17	146	4.15	152	4.32
Pateros	324	0	0.00	1	0.31	0	0.00	1	0.31
Taguig	209	25	11.96	1	0.48	32	15.31	58	27.75
Quezon City	18,923	0	0.00	20	0.11	294	1.55	314	1.66
Makati City	1,587	11	0.69	40	2.52	139	8.76	190	11.97
Mandaluyong City	690	0	0.00	0	0.00	3	0.43	3	0.43
San Juan	413	0	0.00	0	0.00	0	0.00	0	0.00
Manila City	1,844	1	0.05	17	0.92	119	6.45	137	7.43
Las Piñas City	482	0	0.00	4	0.83	31	6.43	35	7.26
Muntinlupa City	0	0	0.00	0	0.00	0	0.00	0	0.00
Parañaque City	1,571	0	0.00	0	0.00	9	0.57	9	0.57
Pasay City	756	0	0.00	0	0.00	8	1.06	8	1.06
**C A R**	**4,549**	**0**	**0.00**	**0**	**0.00**	**90**	**1.98**	**90**	**1.98**
Abra	219	0	0.00	0	0.00	2	0.91	2	0.91
Apayao	496	0	0.00	0	0.00	4	0.81	4	0.81
Benguet	872	0	0.00	0	0.00	15	1.72	15	1.72
Ifugao	271	0	0.00	0	0.00	5	1.85	5	1.85
Kalinga	262	0	0.00	0	0.00	3	1.15	3	1.15
Mt. Province	4	0	0.00	0	0.00	0	0.00	0	0.00
Baguio City	2,425	0	0.00	0	0.00	61	2.52	61	2.52
**Region 1**	**14,162**	**0**	**0.00**	**31**	**0.22**	**328**	**2.32**	**359**	**2.53**
Ilocos Norte	1,778	0	0.00	8	0.45	82	4.61	90	5.06
Ilocos Sur	3,313	0	0.00	8	0.24	122	3.68	130	3.92
La Union	1,295	0	0.00	0	0.00	41	3.17	41	3.17
Pangasinan	5,112	0	0.00	15	0.29	56	1.10	71	1.39
Alaminos City	205	0	0.00	0	0.00	0	0.00	0	0.00
Candon City	475	0	0.00	0	0.00	19	4.00	19	4.00
Dagupan City	50	0	0.00	0	0.00	4	8.00	4	8.00
Laoag City	725	0	0.00	0	0.00	0	0.00	0	0.00
San Carlos City	149	0	0.00	0	0.00	3	2.01	3	2.01
San Fernando City	775	0	0.00	0	0.00	1	0.13	1	0.13
Urdaneta City	285	0	0.00	0	0.00	0	0.00	0	0.00

Table 1.B.1.17 - Prenatal Care

Number and proportion of pregnant women tested positive for Gestational Diabetes

Philippines, Annual 2020

Area	Total No. of Screened for Gestational Diabetes	Tested positive for Gestational Diabetes							
		Age Group						Total	%
		10-14 yrs old		15-19 yrs old		20-49 yrs old			
		No.	%	No.	%	No.	%		
Vigan City	0	0	0.00	0	0.00	0	0.00	0	0.00
**Region 2**	6,217	0	0.00	15	0.24	135	2.17	150	2.41
Batanes	54	0	0.00	0	0.00	1	1.85	1	1.85
Cagayan	626	0	0.00	0	0.00	18	2.88	18	2.88
Isabela	2,725	0	0.00	6	0.22	46	1.69	52	1.91
Nueva Vizcaya	1,211	0	0.00	3	0.25	30	2.48	33	2.73
Quirino	593	0	0.00	1	0.17	22	3.71	23	3.88
Cauayan City	409	0	0.00	1	0.24	3	0.73	4	0.98
Ilagan City	98	0	0.00	0	0.00	2	2.04	2	2.04
Santiago City	497	0	0.00	4	0.80	10	2.01	14	2.82
Tuguegarao City	4	0	0.00	0	0.00	3	75.00	3	75.00
**Region 3**	22,328	0	0.00	82	0.37	624	2.79	706	3.16
Aurora	301	0	0.00	0	0.00	4	1.33	4	1.33
Bataan	2,056	0	0.00	9	0.44	99	4.82	108	5.25
Bulacan	2,897	0	0.00	40	1.38	219	7.56	259	8.94
Nueva Ecija	1,953	0	0.00	2	0.10	20	1.02	22	1.13
Pampanga	1,600	0	0.00	14	0.88	45	2.81	59	3.69
Tarlac	2,135	0	0.00	7	0.33	62	2.90	69	3.23
Zambales	384	0	0.00	1	0.26	11	2.86	12	3.13
Angeles City	531	0	0.00	0	0.00	26	4.90	26	4.90
Balanga City	62	0	0.00	1	1.61	12	19.35	13	20.97
Cabanatuan City	300	0	0.00	0	0.00	0	0.00	0	0.00
City of San Fernando	1,816	0	0.00	6	0.33	76	4.19	82	4.52
Gapan City	150	0	0.00	0	0.00	0	0.00	0	0.00
Mabalacat City	273	0	0.00	0	0.00	19	6.96	19	6.96
Malolos City	46	0	0.00	0	0.00	0	0.00	0	0.00
Meycauayan	264	0	0.00	0	0.00	19	7.20	19	7.20
Olongapo	613	0	0.00	1	0.16	6	0.98	7	1.14
Palayan City	0	0	0.00	0	0.00	0	0.00	0	0.00
San Jose City	2	0	0.00	0	0.00	0	0.00	0	0.00
San Jose del Monte City	6,812	0	0.00	1	0.01	6	0.09	7	0.10
Science City of Munoz	0	0	0.00	0	0.00	0	0.00	0	0.00
Tarlac City	133	0	0.00	0	0.00	0	0.00	0	0.00
**Region 4A**	21,583	921	4.27	1,867	8.65	5,584	25.87	8,372	38.79
Batangas	2,090	0	0.00	1	0.05	16	0.77	17	0.81
Cavite	1,882	0	0.00	6	0.32	52	2.76	58	3.08
Laguna	3,343	365	10.92	1,330	39.78	3,351	100.24	5,046	150.94
Quezon	621	0	0.00	4	0.64	26	4.19	30	4.83
Rizal	3,482	0	0.00	3	0.09	56	1.61	59	1.69
Antipolo City	192	0	0.00	0	0.00	7	3.65	7	3.65
Bacoor City	132	0	0.00	2	1.52	4	3.03	6	4.55
Batangas City	0	0	0.00	0	0.00	0	0.00	0	0.00

Table 1.B.1.17 - Prenatal Care

Number and proportion of pregnant women tested positive for Gestational Diabetes

Philippines, Annual 2020

Area	Total No. of Screened for Gestational Diabetes	Tested positive for Gestational Diabetes							
		Age Group						Total	%
		10-14 yrs old		15-19 yrs old		20-49 yrs old			
		No.	%	No.	%	No.	%		
Biñan City	**2,298**	22	**0.96**	89	**3.87**	180	**7.83**	**291**	**12.66**
Cabuyao City	**1,892**	160	**8.46**	5	**0.26**	28	**1.48**	**193**	**10.20**
Calamba City	**602**	26	**4.32**	81	**13.46**	177	**29.40**	**284**	**47.18**
Cavite City	**7**	0	**0.00**	0	**0.00**	0	**0.00**	**0**	**0.00**
Dasmariñas City	**660**	0	**0.00**	2	**0.30**	25	**3.79**	**27**	**4.09**
General Trias City	**207**	0	**0.00**	1	**0.48**	10	**4.83**	**11**	**5.31**
Imus City	**83**	0	**0.00**	0	**0.00**	7	**8.43**	**7**	**8.43**
Lipa City	**0**	0	**0.00**	0	**0.00**	0	**0.00**	**0**	**0.00**
Lucena City	**0**	0	**0.00**	0	**0.00**	0	**0.00**	**0**	**0.00**
San Pablo City	**1,631**	346	**21.21**	337	**20.66**	1,520	**93.19**	**2,203**	**135.07**
San Pedro City	**128**	0	**0.00**	4	**3.13**	20	**15.63**	**24**	**18.75**
Santa Rosa City	**725**	2	**0.28**	2	**0.28**	65	**8.97**	**69**	**9.52**
Tagaytay City	**1,506**	0	**0.00**	0	**0.00**	0	**0.00**	**0**	**0.00**
Tanauan City	**100**	0	**0.00**	0	**0.00**	10	**10.00**	**10**	**10.00**
Tayabas City	**0**	0	**0.00**	0	**0.00**	0	**0.00**	**0**	**0.00**
Trece Martires City	**2**	0	**0.00**	0	**0.00**	30	**1,500.00**	**30**	**1,500.00**
**Region 4B**	**2,185**	**0**	**0.00**	**9**	**0.41**	**72**	**3.30**	**81**	**3.71**
Marinduque	**474**	0	**0.00**	0	**0.00**	3	**0.63**	**3**	**0.63**
Mindoro Occidental	**203**	0	**0.00**	9	**4.43**	26	**12.81**	**35**	**17.24**
Mindoro Oriental	**1,121**	0	**0.00**	0	**0.00**	6	**0.54**	**6**	**0.54**
Palawan	**182**	0	**0.00**	0	**0.00**	4	**2.20**	**4**	**2.20**
Romblon	**193**	0	**0.00**	0	**0.00**	28	**14.51**	**28**	**14.51**
Puerto Princesa City	**12**	0	**0.00**	0	**0.00**	5	**41.67**	**5**	**41.67**
**Region 5**	**6,618**	**1**	**0.02**	**11**	**0.17**	**277**	**4.19**	**289**	**4.37**
Albay	**2,231**	1	**0.04**	4	**0.18**	117	**5.24**	**122**	**5.47**
Camarines Norte	**212**	0	**0.00**	0	**0.00**	13	**6.13**	**13**	**6.13**
Camarines Sur	**1,564**	0	**0.00**	2	**0.13**	31	**1.98**	**33**	**2.11**
Catanduanes	**45**	0	**0.00**	0	**0.00**	0	**0.00**	**0**	**0.00**
Masbate	**792**	0	**0.00**	0	**0.00**	20	**2.53**	**20**	**2.53**
Sorsogon	**847**	0	**0.00**	4	**0.47**	56	**6.61**	**60**	**7.08**
Iriga City	**51**	0	**0.00**	0	**0.00**	1	**1.96**	**1**	**1.96**
Legaspi City	**739**	0	**0.00**	1	**0.14**	35	**4.74**	**36**	**4.87**
Naga City	**137**	0	**0.00**	0	**0.00**	4	**2.92**	**4**	**2.92**
**Region 6**	**25,992**	**1**	**0.00**	**48**	**0.18**	**467**	**1.80**	**516**	**1.99**
Aklan	**2,596**	0	**0.00**	3	**0.12**	56	**2.16**	**59**	**2.27**
Antique	**861**	1	**0.12**	6	**0.70**	57	**6.62**	**64**	**7.43**
Capiz	**1,581**	0	**0.00**	7	**0.44**	24	**1.52**	**31**	**1.96**
Guimaras	**1,445**	0	**0.00**	0	**0.00**	9	**0.62**	**9**	**0.62**
Iloilo	**8,157**	0	**0.00**	6	**0.07**	98	**1.20**	**104**	**1.27**
Negros Occidental	**6,805**	0	**0.00**	10	**0.15**	115	**1.69**	**125**	**1.84**
Bacolod City	**3,304**	0	**0.00**	16	**0.48**	80	**2.42**	**96**	**2.91**
Iloilo City	**1,243**	0	**0.00**	0	**0.00**	28	**2.25**	**28**	**2.25**

Table 1.B.1.17 - Prenatal Care

Number and proportion of pregnant women tested positive for Gestational Diabetes

Philippines, Annual 2020

Area	Total No. of Screened for Gestational Diabetes	Tested positive for Gestational Diabetes							
		Age Group						Total	%
		10-14 yrs old		15-19 yrs old		20-49 yrs old			
		No.	%	No.	%	No.	%		
**Region 7**	**7,101**	**0**	**0.00**	**38**	**0.54**	**262**	**3.69**	**300**	**4.22**
Bohol	**2,121**	0	**0.00**	3	**0.14**	97	**4.57**	**100**	**4.71**
Cebu	**896**	0	**0.00**	24	**2.68**	67	**7.48**	**91**	**10.16**
Negros Oriental	**456**	0	**0.00**	2	**0.44**	48	**10.53**	**50**	**10.96**
Siquijor	**57**	0	**0.00**	0	**0.00**	3	**5.26**	**3**	**5.26**
Cebu City	**2,533**	0	**0.00**	8	**0.32**	22	**0.87**	**30**	**1.18**
Lapu-Lapu City	**1,038**	0	**0.00**	1	**0.10**	25	**2.41**	**26**	**2.50**
Mandaue City	**0**	0	**0.00**	0	**0.00**	0	**0.00**	**0**	**0.00**
**Region 8**	**2,886**	**4**	**0.14**	**21**	**0.73**	**188**	**6.51**	**213**	**7.38**
Biliran	**196**	0	**0.00**	0	**0.00**	10	**5.10**	**10**	**5.10**
Eastern Samar	**249**	4	**1.61**	8	**3.21**	49	**19.68**	**61**	**24.50**
Northern Leyte	**0**	0	**0.00**	6	**0.00**	43	**0.00**	**49**	**0.00**
Northern Samar	**1,337**	0	**0.00**	4	**0.30**	38	**2.84**	**42**	**3.14**
Southern Leyte	**284**	0	**0.00**	1	**0.35**	25	**8.80**	**26**	**9.15**
Western Samar	**190**	0	**0.00**	1	**0.53**	13	**6.84**	**14**	**7.37**
Calbayog City	**116**	0	**0.00**	0	**0.00**	2	**1.72**	**2**	**1.72**
Maasin City	**4**	0	**0.00**	0	**0.00**	0	**0.00**	**0**	**0.00**
Ormoc City	**79**	0	**0.00**	0	**0.00**	7	**8.86**	**7**	**8.86**
Tacloban City	**431**	0	**0.00**	1	**0.23**	1	**0.23**	**2**	**0.46**
**Region 9**	**919**	**0**	**0.00**	**3**	**0.33**	**74**	**8.05**	**77**	**8.38**
Zamboanga del Norte	**390**	0	**0.00**	0	**0.00**	11	**2.82**	**11**	**2.82**
Zamboanga del Sur	**138**	0	**0.00**	0	**0.00**	10	**7.25**	**10**	**7.25**
Zamboanga Sibugay	**183**	0	**0.00**	1	**0.55**	18	**9.84**	**19**	**10.38**
Dapitan City	**0**	0	**0.00**	0	**0.00**	0	**0.00**	**0**	**0.00**
Dipolog City	**1**	0	**0.00**	0	**0.00**	0	**0.00**	**0**	**0.00**
Isabela City	**191**	0	**0.00**	1	**0.52**	30	**15.71**	**31**	**16.23**
Pagadian City	**4**	0	**0.00**	1	**25.00**	3	**75.00**	**4**	**100.00**
Zamboanga City	**12**	0	**0.00**	0	**0.00**	2	**16.67**	**2**	**16.67**
**Region 10**	**6,035**	**1**	**0.02**	**23**	**0.38**	**178**	**2.95**	**202**	**3.35**
Bukidnon	**944**	0	**0.00**	10	**1.06**	43	**4.56**	**53**	**5.61**
Camiguin	**292**	0	**0.00**	0	**0.00**	3	**1.03**	**3**	**1.03**
Lanao del Norte	**104**	0	**0.00**	0	**0.00**	23	**22.12**	**23**	**22.12**
Misamis Occidental	**216**	1	**0.46**	5	**2.31**	11	**5.09**	**17**	**7.87**
Misamis Oriental	**1,062**	0	**0.00**	3	**0.28**	12	**1.13**	**15**	**1.41**
Cagayan de Oro City	**1,715**	0	**0.00**	0	**0.00**	0	**0.00**	**0**	**0.00**
El Salvador City	**97**	0	**0.00**	0	**0.00**	2	**2.06**	**2**	**2.06**
Gingoog City	**130**	0	**0.00**	0	**0.00**	0	**0.00**	**0**	**0.00**
Iligan City	**78**	0	**0.00**	1	**1.28**	5	**6.41**	**6**	**7.69**
Malaybalay City	**112**	0	**0.00**	0	**0.00**	6	**5.36**	**6**	**5.36**

Table 1.B.1.17 - Prenatal Care

Number and proportion of pregnant women tested positive for Gestational Diabetes

Philippines, Annual 2020

Area	Total No. of Screened for Gestational Diabetes	Tested positive for Gestational Diabetes							
		Age Group						Total	%
		10-14 yrs old		15-19 yrs old		20-49 yrs old			
		No.	%	No.	%	No.	%		
Oroquieta City	1	0	0.00	0	0.00	0	0.00	0	0.00
Ozamis City	1,272	0	0.00	4	0.31	71	5.58	75	5.90
Tangub City	2	0	0.00	0	0.00	2	100.00	2	100.00
Valencia City	10	0	0.00	0	0.00	0	0.00	0	0.00
**Region 11**	7,798	0	0.00	33	0.42	134	1.72	167	2.14
Compostela Valley	162	0	0.00	1	0.62	15	9.26	16	9.88
Davao del Norte	749	0	0.00	1	0.13	27	3.60	28	3.74
Davao Oriental	133	0	0.00	0	0.00	5	3.76	5	3.76
Davao del Sur	189	0	0.00	0	0.00	8	4.23	8	4.23
Davao Occidental	128	0	0.00	0	0.00	8	6.25	8	6.25
Davao City	6,437	0	0.00	31	0.48	71	1.10	102	1.58
**Region 12**	6,738	7	0.10	22	0.33	237	3.52	266	3.95
North Cotabato	1,353	1	0.07	3	0.22	68	5.03	72	5.32
Sarangani	2,406	3	0.12	9	0.37	48	2.00	60	2.49
South Cotabato	1,019	0	0.00	0	0.00	26	2.55	26	2.55
Sultan Kudarat	1,365	0	0.00	10	0.73	84	6.15	94	6.89
Cotabato City	90	0	0.00	0	0.00	0	0.00	0	0.00
Gen. Santos City	505	3	0.59	0	0.00	11	2.18	14	2.77
**BARMM**	1,583	0	0.00	1	0.06	21	1.33	22	1.39
Basilan	0	0	0.00	0	0.00	0	0.00	0	0.00
Lanao del Sur	894	0	0.00	0	0.00	5	0.56	5	0.56
Maguindanao	241	0	0.00	1	0.41	16	6.64	17	7.05
Sulu	0	0	0.00	0	0.00	0	0.00	0	0.00
Tawi-Tawi	0	0	0.00	0	0.00	0	0.00	0	0.00
Lamitan City	0	0	0.00	0	0.00	0	0.00	0	0.00
Marawi City	448	0	0.00	0	0.00	0	0.00	0	0.00
**CARAGA**	2,848	0	0.00	24	0.84	180	6.32	204	7.16
Agusan del Norte	221	0	0.00	12	5.43	53	23.98	65	29.41
Agusan del Sur	455	0	0.00	1	0.22	23	5.05	24	5.27
Surigao del Norte	286	0	0.00	2	0.70	19	6.64	21	7.34
Surigao del Sur	700	0	0.00	6	0.86	43	6.14	49	7.00
Province of Dinagat	83	0	0.00	0	0.00	4	4.82	4	4.82
Bislig City	69	0	0.00	1	1.45	20	28.99	21	30.43
Butuan City	429	0	0.00	2	0.47	17	3.96	19	4.43
Surigao City	605	0	0.00	0	0.00	1	0.17	1	0.17

Note: Put asterisk (*) for No Report and Zero (0) for No Case

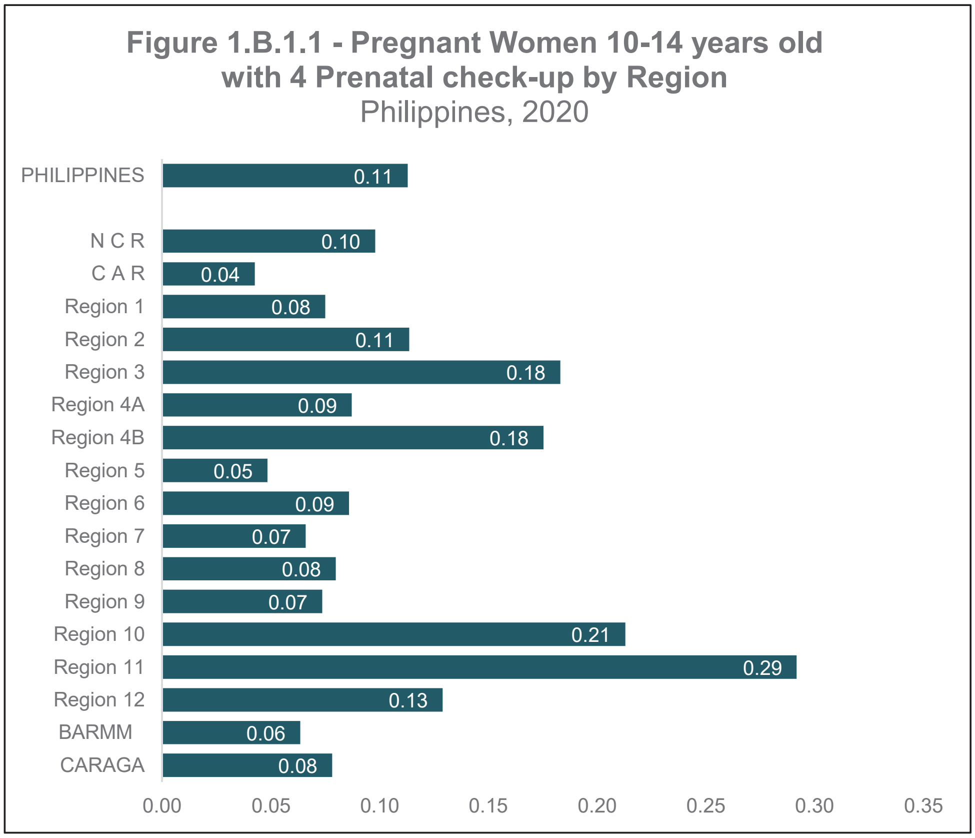
Figure 1.B.1.1 - Pregnant Women 10-14 years old with 4 Prenatal check-up by Region
Philippines, 2020
PHILIPPINES
0.11
N C R
0.10
C A R
0.04
Region 1
0.08
Region 2
0.11
Region 3
0.18
Region 4A
0.09
Region 4B
0.18
Region 5
0.05
Region 6
0.09
Region 7
0.07
Region 8
0.08
Region 9
0.07
Region 10
0.21
Region 11
0.29
Region 12
0.13
BARMM
0.06
CARAGA
0.08
0.00
0.05
0.10
0.15
0.20
0.25
0.30
0.35

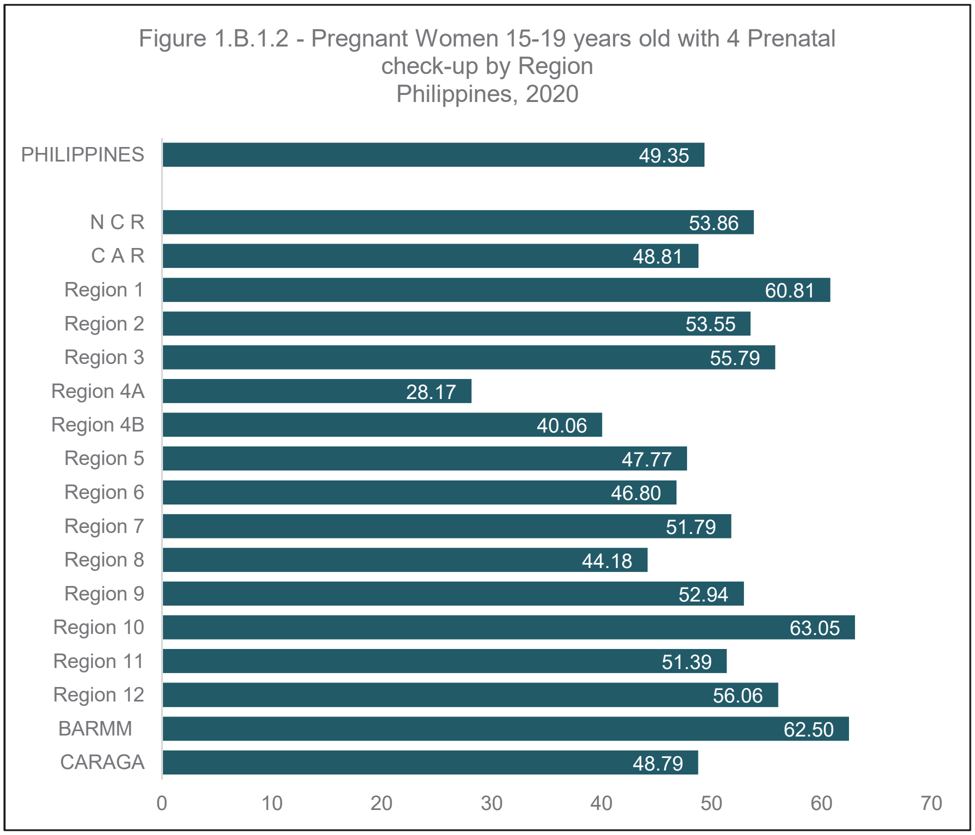
Figure 1.B.1.2 - Pregnant Women 15-19 years old with 4 Prenatal check-up by Region
Philippines, 2020
PHILIPPINES
49.35
N C R
53.86
C A R
48.81
Region 1
60.81
Region 2
53.55
Region 3
55.79
Region 4A
28.17
Region 4B
40.06
Region 5
47.77
Region 6
46.80
Region 7
51.79
Region 8
44.18
Region 9
52.94
Region 10
63.05
Region 11
51.39
Region 12
56.06
BARMM
62.50
CARAGA
48.79
0
10
20
30
40
50
60
70

Figure 1.B.1.3 - Pregnant Women 20-49 years old with 4 Prenatal check-up by Region
Philippines, 2020

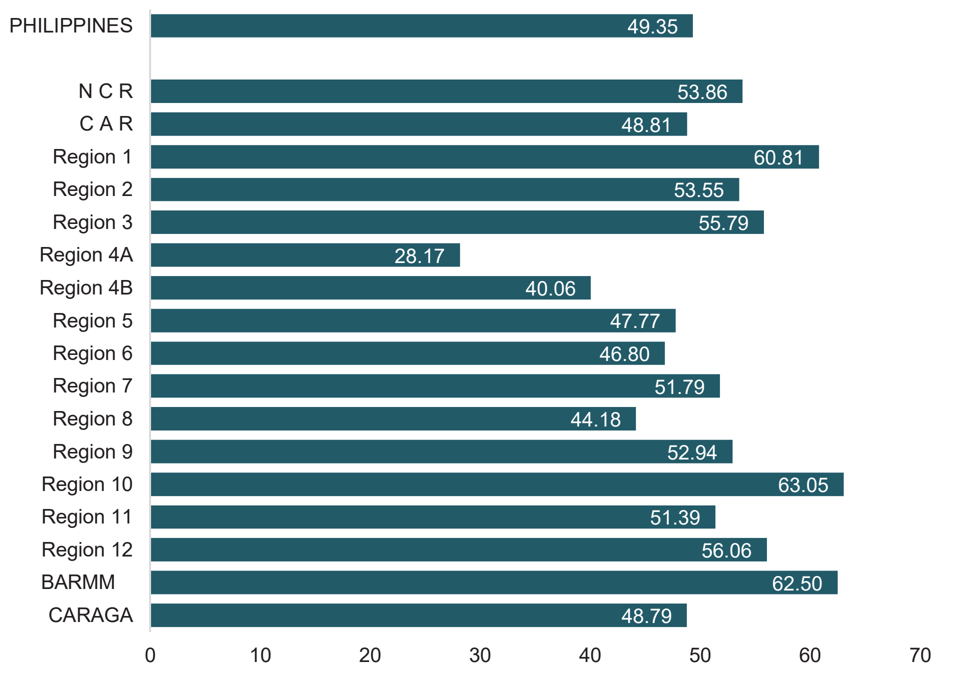

Figure 1.B.1.4 - Pregnant Women 10-49 years old with 4 Prenatal check-up by Region
Philippines, 2020

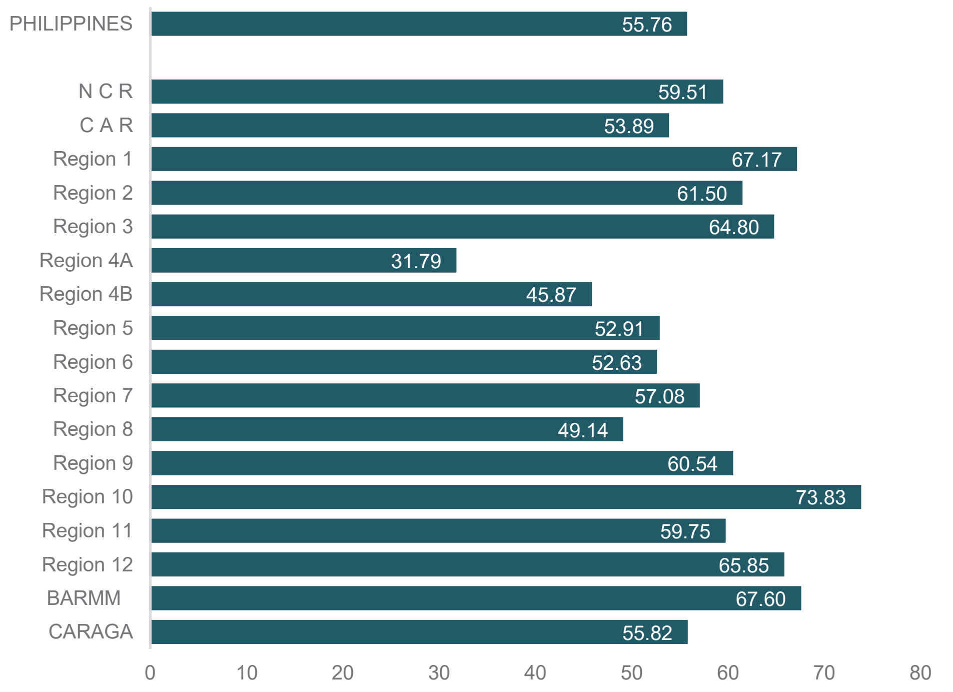

Figure 1.B.1.5 - Percentage Distribution of Pregnant women seen during the 1st Trimester with Normal BMI by Region
Philippines, 2020

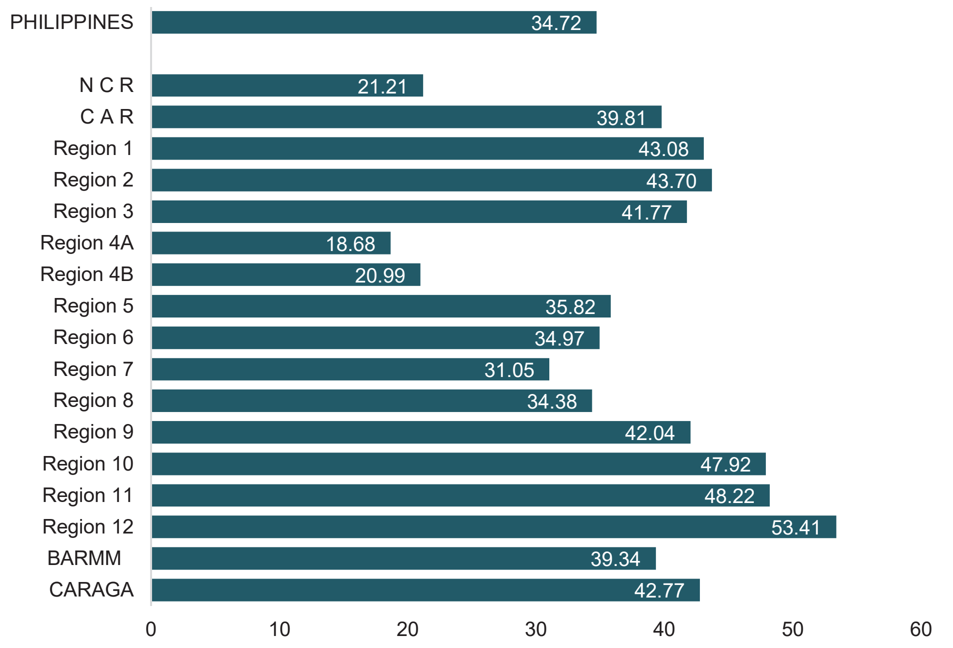

Figure 1.B.1.6 - Percentage Distribution of Pregnant women seen during the 1st Trimester with Low BMI by Region
Philippines, 2020

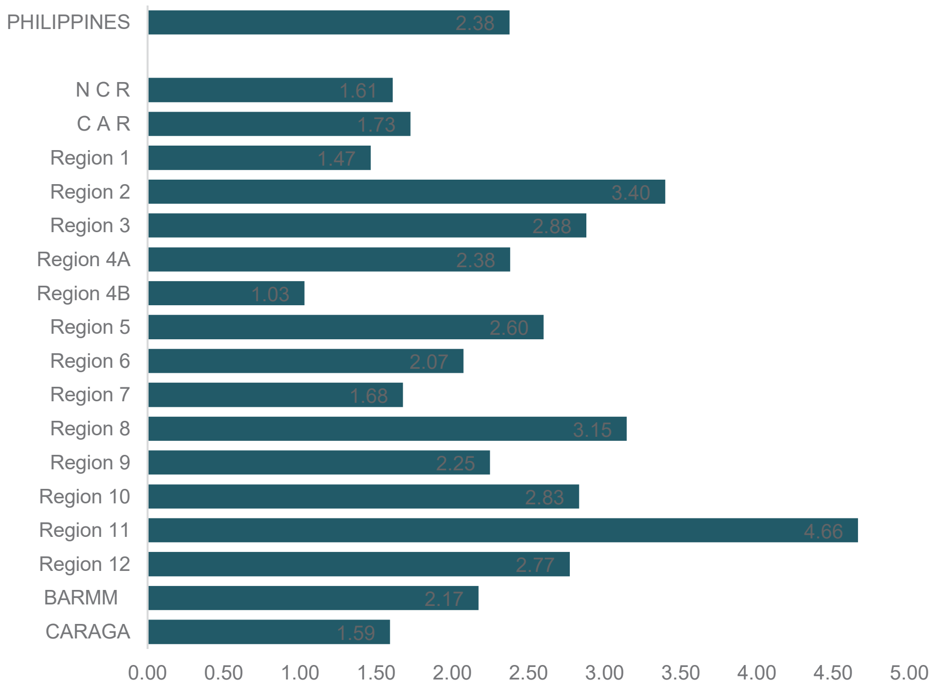

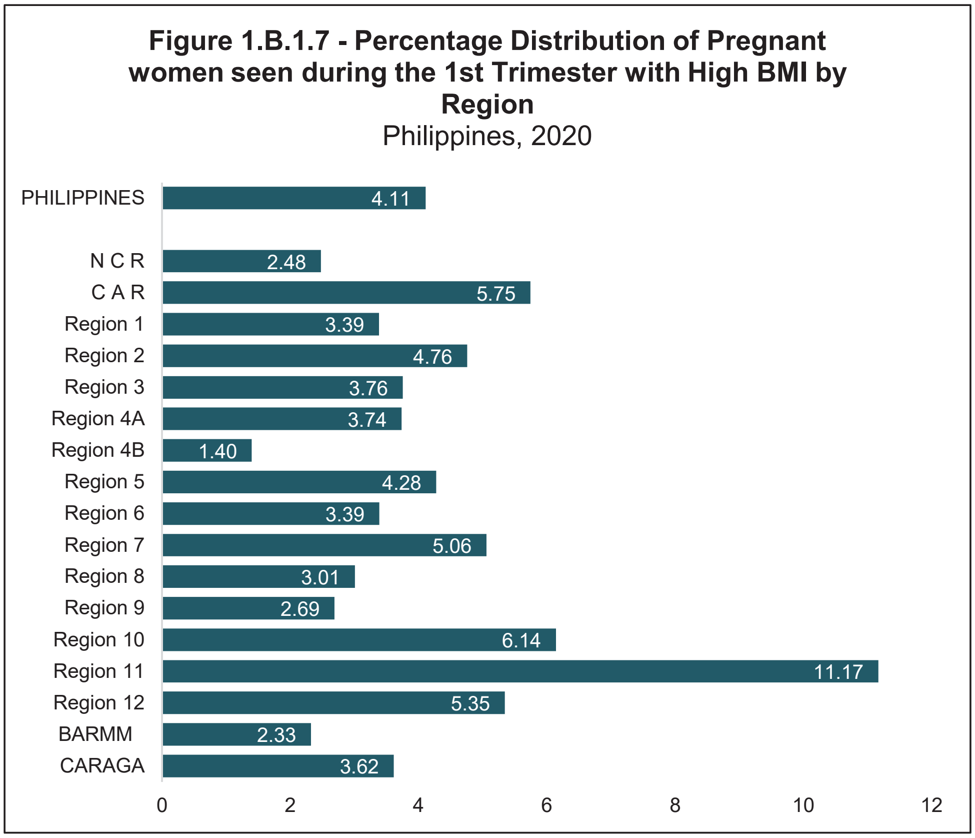
Figure 1.B.1.7 - Percentage Distribution of Pregnant women seen during the 1st Trimester with High BMI by Region
Philippines, 2020
PHILIPPINES 4.11
N C R 2.48
C A R 5.75
Region 1 3.39
Region 2 4.76
Region 3 3.76
Region 4A 3.74
Region 4B 1.40
Region 5 4.28
Region 6 3.39
Region 7 5.06
Region 8 3.01
Region 9 2.69
Region 10 6.14
Region 11 11.17
Region 12 5.35
BARMM 2.33
CARAGA 3.62
0
2
4
6
8
10
12

Figure 1.B.1.8 - Percentage Distribution of Women (10-14 yr. old) pregnant for the 1st time given at least 2 doses of Tetanus Diphtheria (Td) Vaccination by Region
Philippines, 2020

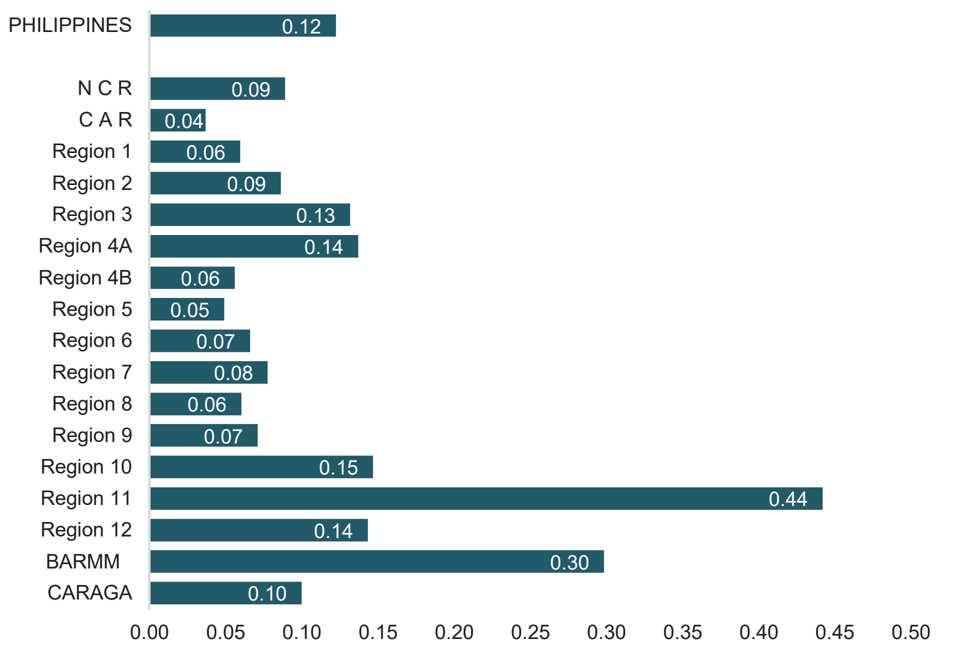

Figure 1.B.1.9 - Percentage Distribution of Women (15-19 yr. old) pregnant for the 1st time given at least 2 doses of Tetanus Diphtheria (Td) Vaccination by Region
Philippines, 2020

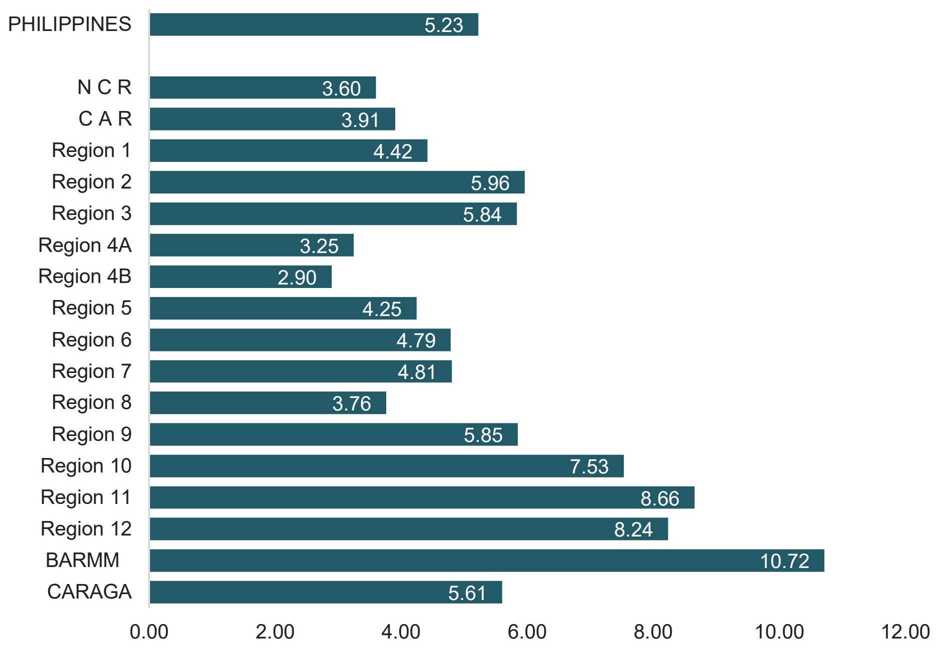

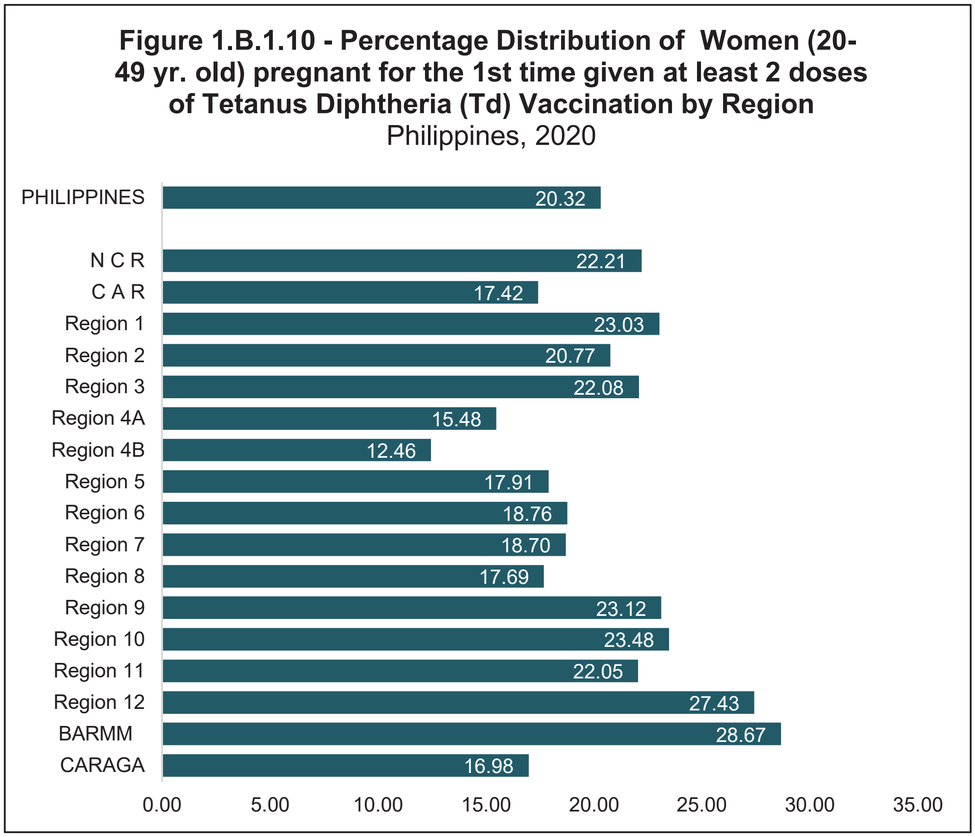
Figure 1.B.1.10 - Percentage Distribution of Women (20-49 yr. old) pregnant for the 1st time given at least 2 doses of Tetanus Diphtheria (Td) Vaccination by Region
Philippines, 2020
PHILIPPINES
20.32
N C R
22.21
C A R
17.42
Region 1
23.03
Region 2
20.77
Region 3
22.08
Region 4A
15.48
Region 4B
12.46
Region 5
17.91
Region 6
18.76
Region 7
18.70
Region 8
17.69
Region 9
23.12
Region 10
23.48
Region 11
22.05
Region 12
27.43
BARMM
28.67
CARAGA
16.98
0.00
5.00
10.00
15.00
20.00
25.00
30.00
35.00

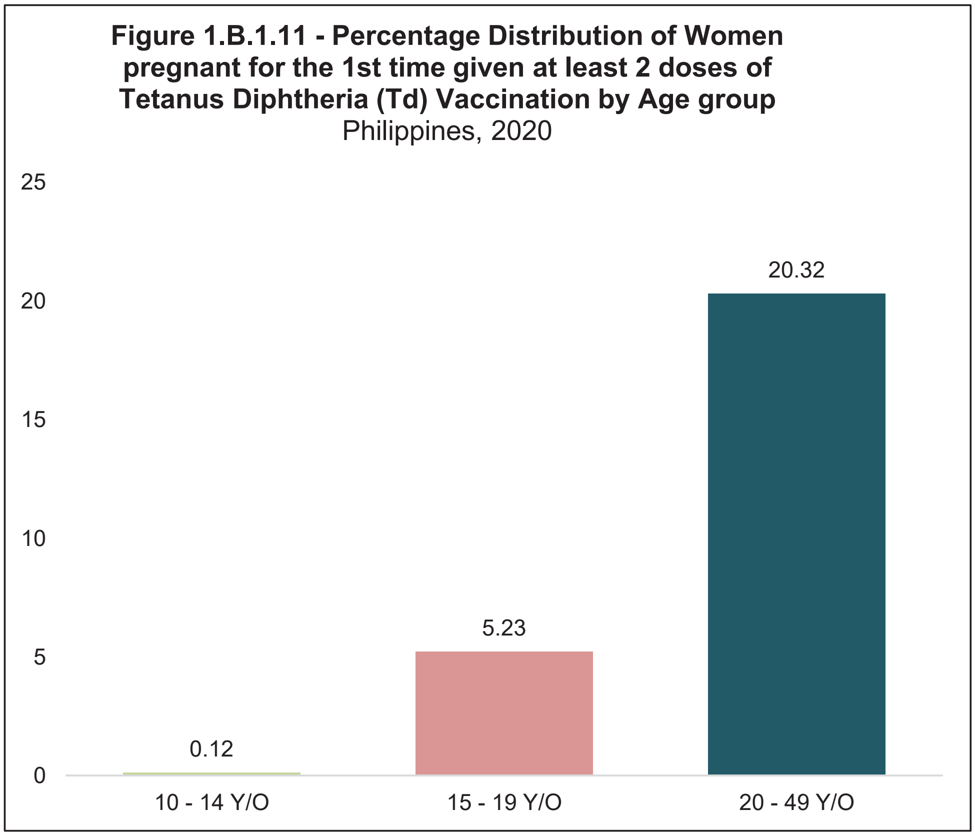
Figure 1.B.1.11 - Percentage Distribution of Women pregnant for the 1st time given at least 2 doses of Tetanus Diphtheria (Td) Vaccination by Age group
Philippines, 2020
25
20
15
10
5
0
0.12
5.23
20.32
10 - 14 Y/O
15 - 19 Y/O
20 - 49 Y/O

Figure 1.B.1.12 - Percentage Distribution of Women (10-14 yr old) pregnant for the 2nd time or more given at least 3 doses of Tetanus Diphtheria (Td) Vaccination by Region
Philippines, 2020

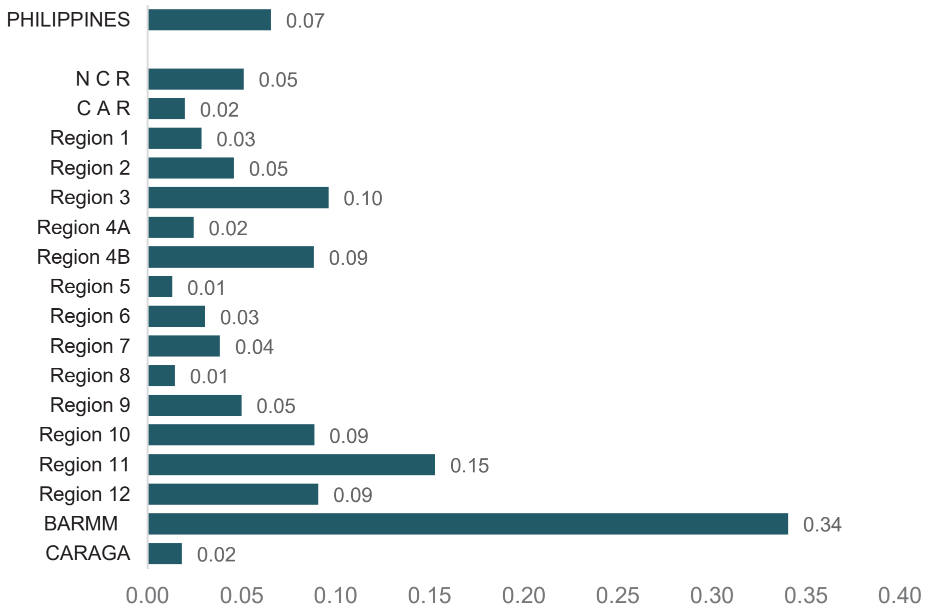

Figure 1.B.1.13 - Percentage Distribution of Women (15-19 yr old) pregnant for the 2nd time or more given at least 3 doses of Tetanus Diphtheria (Td) Vaccination by Region
Philippines, 2020

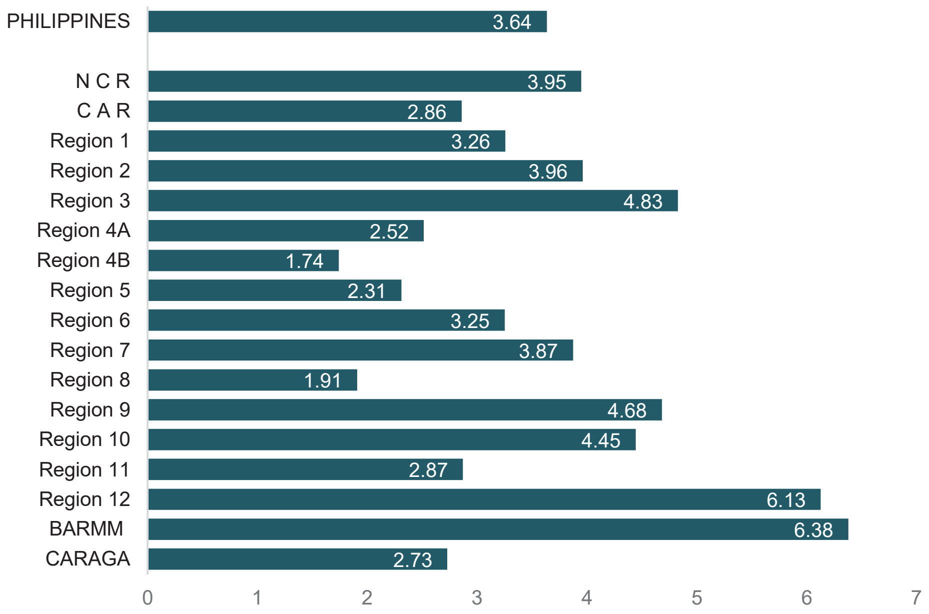

Figure 1.B.1.14 - Percentage Distribution of Women (20-49 yr old) pregnant for the 2nd time or more given at least 3 doses of Tetanus Diphtheria (Td) Vaccination by Region
Philippines, 2020

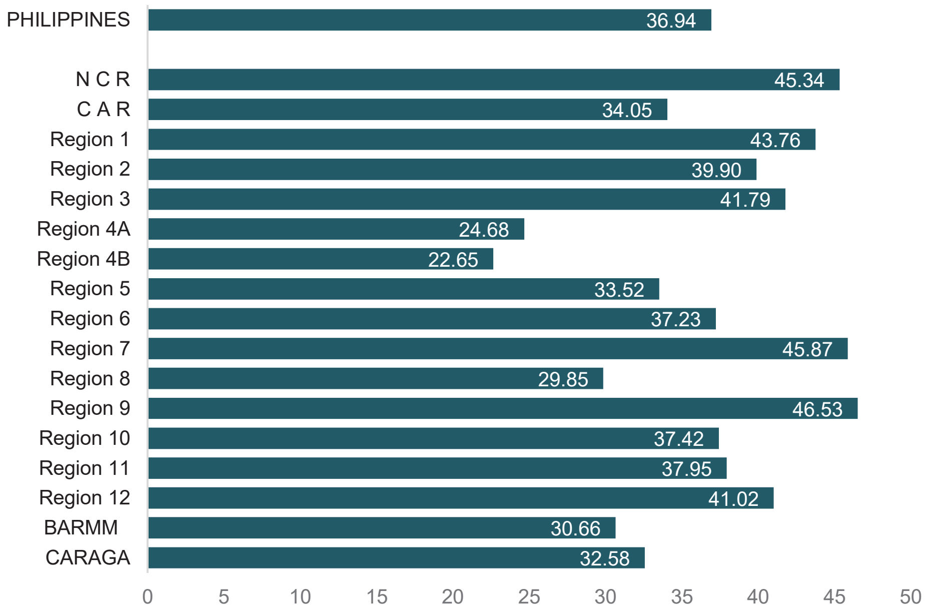

Figure 1.B.1.15- Percentage Distribution of Women (10-49 yrs.old) pregnant for the 2nd time or more given Tetanus Diphtheria (Td) Vaccination by Age group
Philippines, 2020

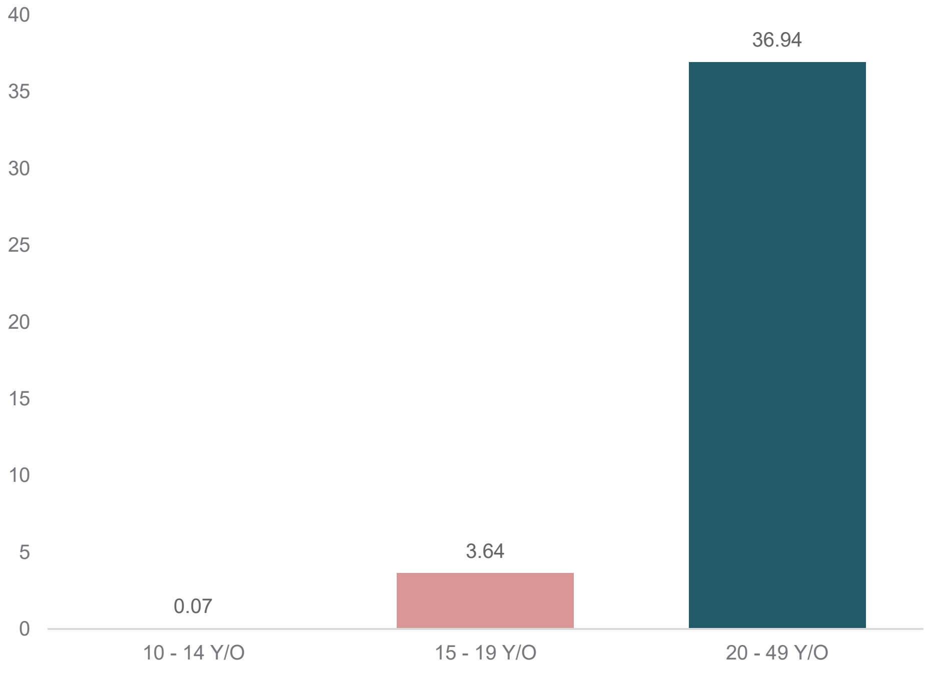

Figure 1.B.1.16 - Percentage Distribution of Pregnant Women (10 - 14 yrs. old) who completed Iron with Folic Acid by Region
Philippines, 2020

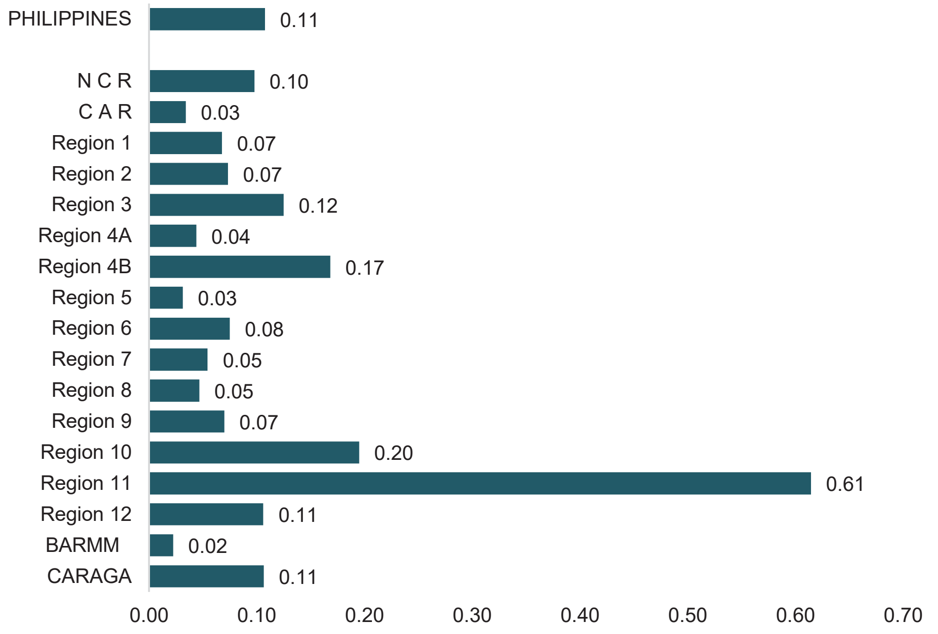

Figure 1.B.1.17 - Percentage Distribution of Pregnant Women (15 - 19 yrs. old) who completed Iron with Folic Acid by Region Philippines, 2020

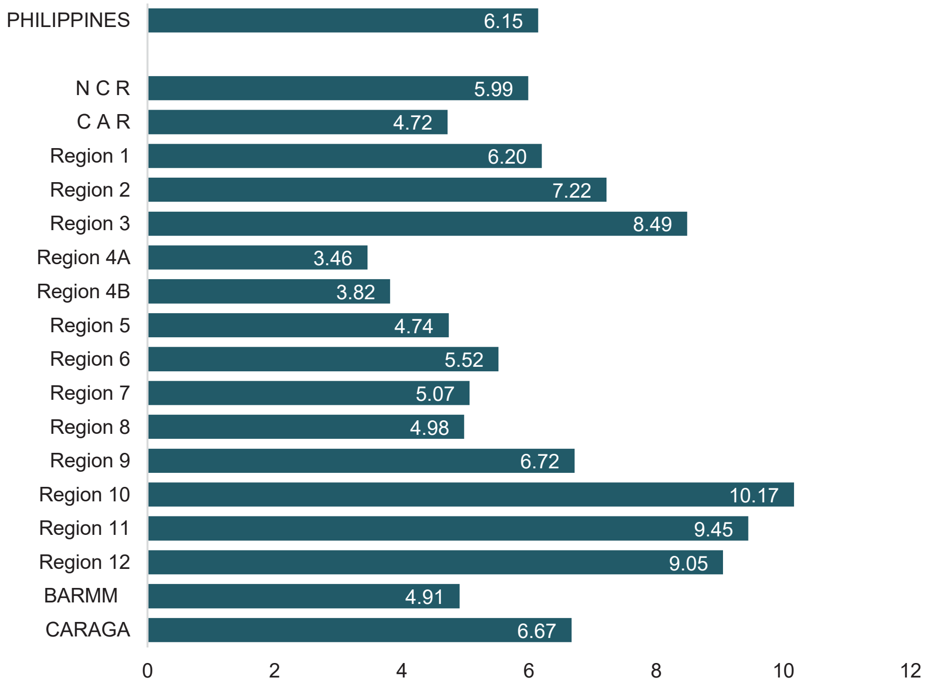

Figure 1.B.1.18 - Percentage Distribution of Pregnant Women (20 - 49 yrs. old) who completed Iron with Folic Acid by Region
Philippines, 2020

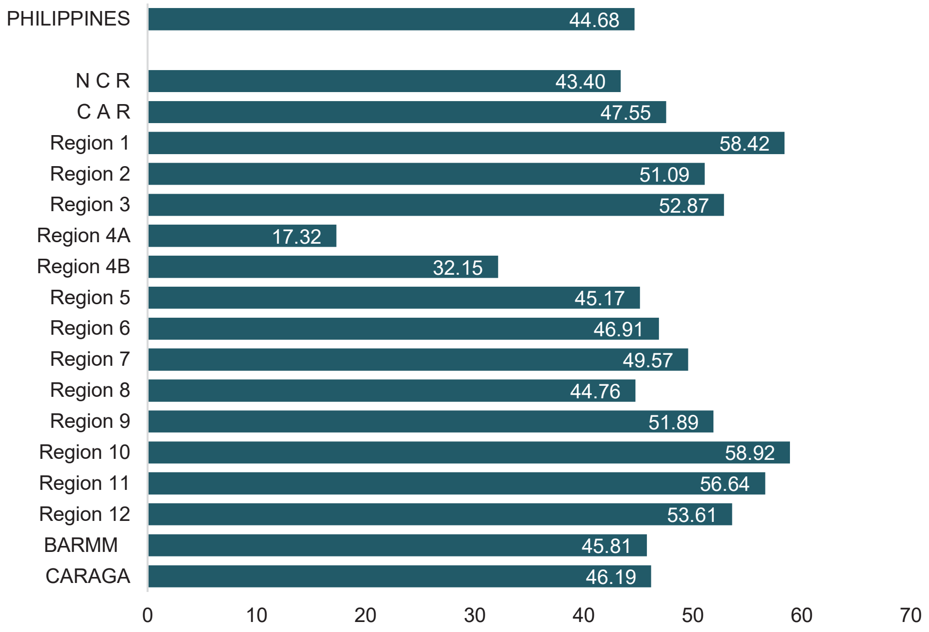

Figure 1.B.1.19 - Percentage Distribution of Pregnant Women (10 - 49 yrs. old) who completed Iron with Folic Acid by Region Philippines, 2020

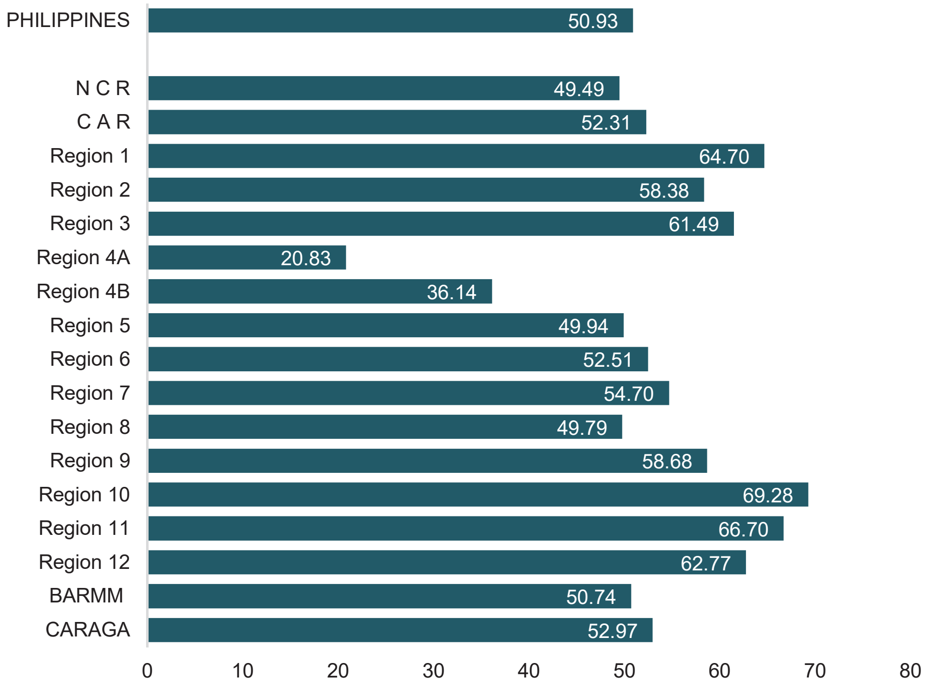

Figure 1.B.1.20 - Percentage Distribution of Pregnant Women (10-49 yrs. old) who completed Iron with Folic Acid by Age group
Philippines, 2020

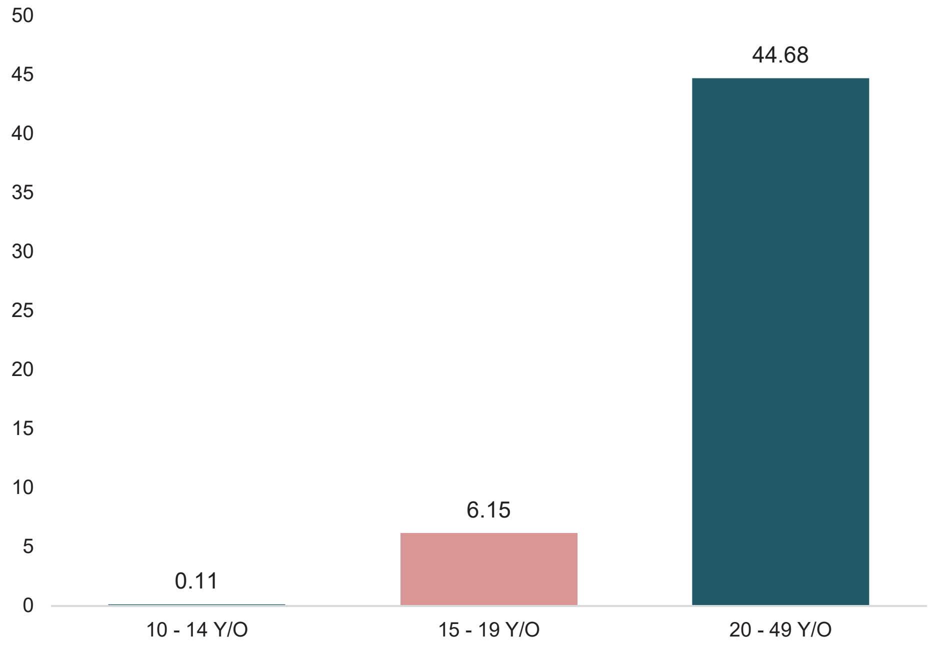

Figure 1.B.1.21 - Percentage Distribution of Pregnant Women (10-14 yrs. old) who completed Calcium Carbonate Supplementation by Region
Philippines, 2020

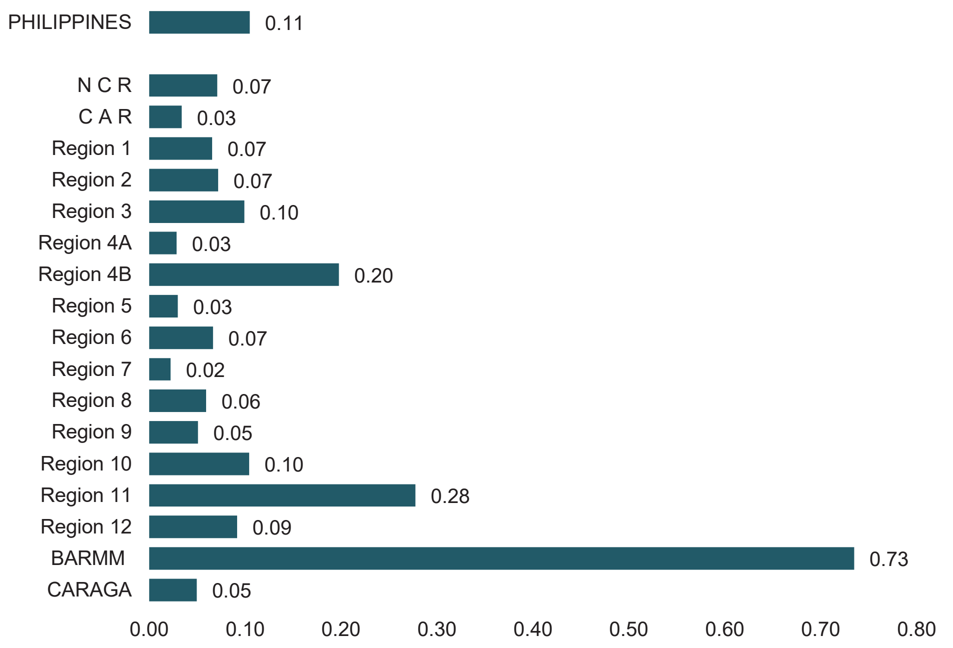

Figure 1.B.1.22 - Percentage Distribution of Pregnant Women (15-19 yrs. old) who completed Calcium Carbonate Supplementation by Region
Philippines, 2020

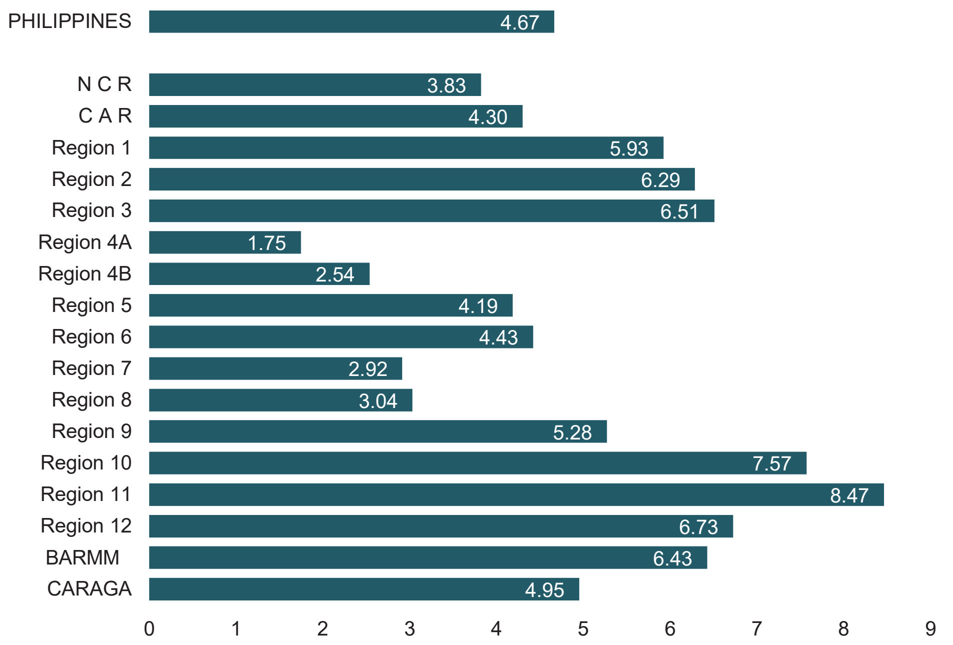

Figure 1.B.1.23 - Percentage Distribution of Pregnant Women (20-49 yrs. old) who completed Calcium Carbonate Supplementation by Region

Philippines, 2020

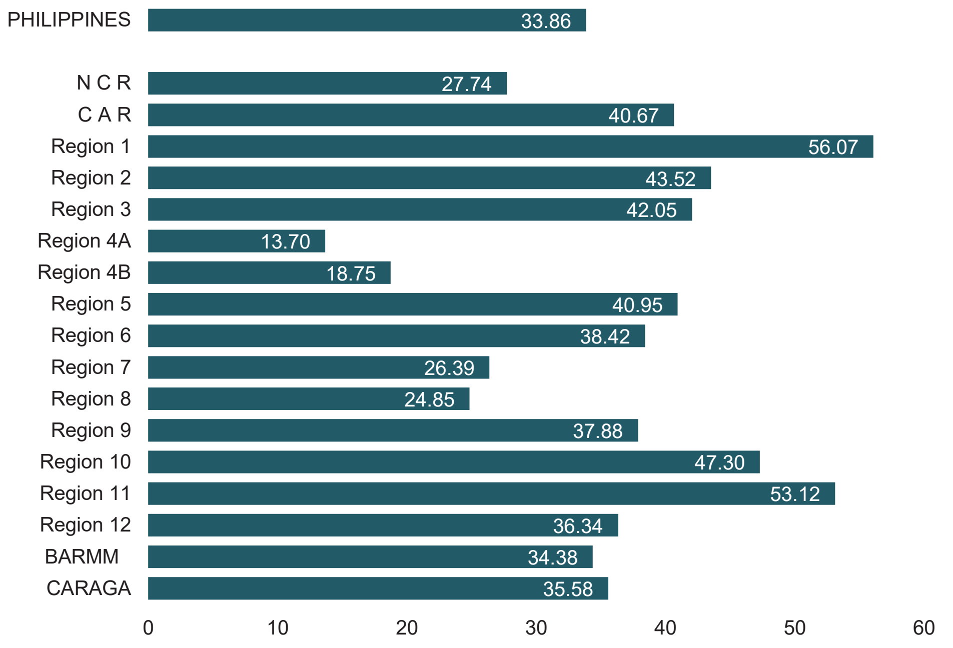

Figure 1.B.1.24 - Percentage Distribution of Pregnant Women (10-49 yrs. old) who completed Calcium Carbonate Supplementation by Region

Philippines, 2020

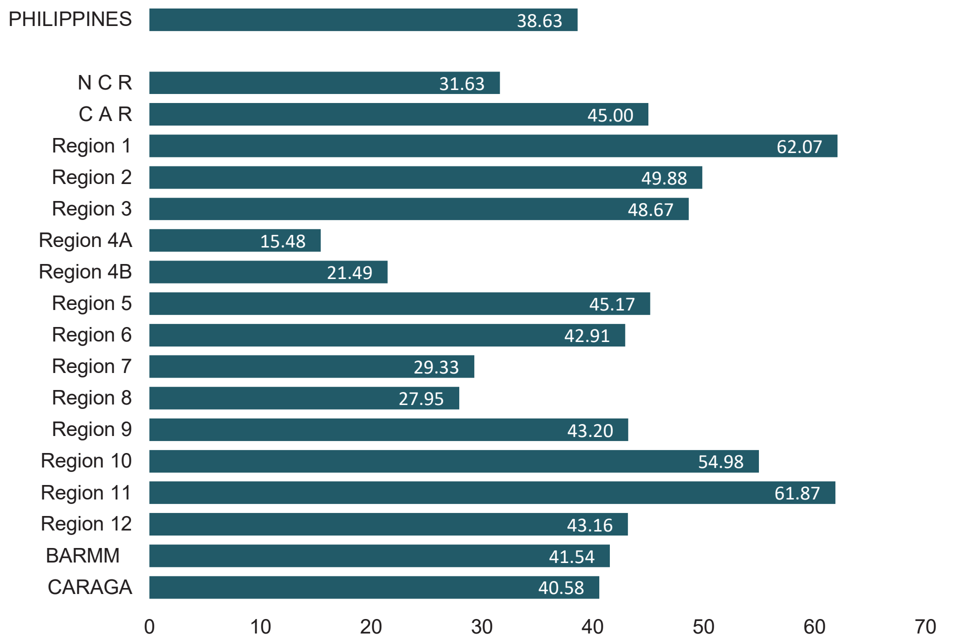

Figure 1.B.1.25 - Percentage Distribution of Pregnant Women (10-49 yrs. old) who complwtwd Calcium Carbonate Supplementation by Age group
Philippines, 2020

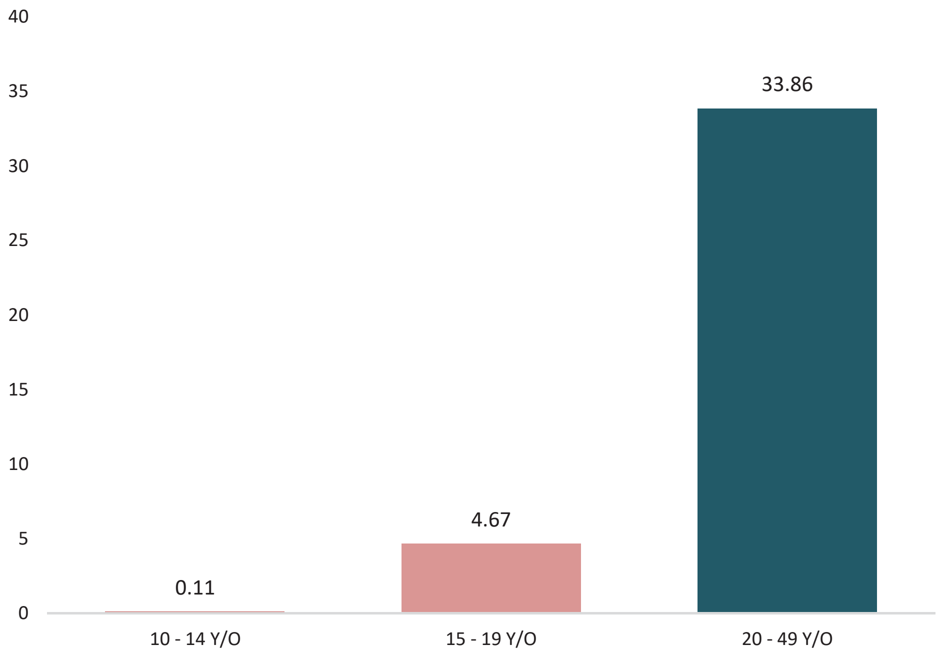

Figure 1.B.1.26 - Percentage Distribution of Pregnant Women (10-14 yrs. old) who completed Iodine by Region
Philippines, 2020

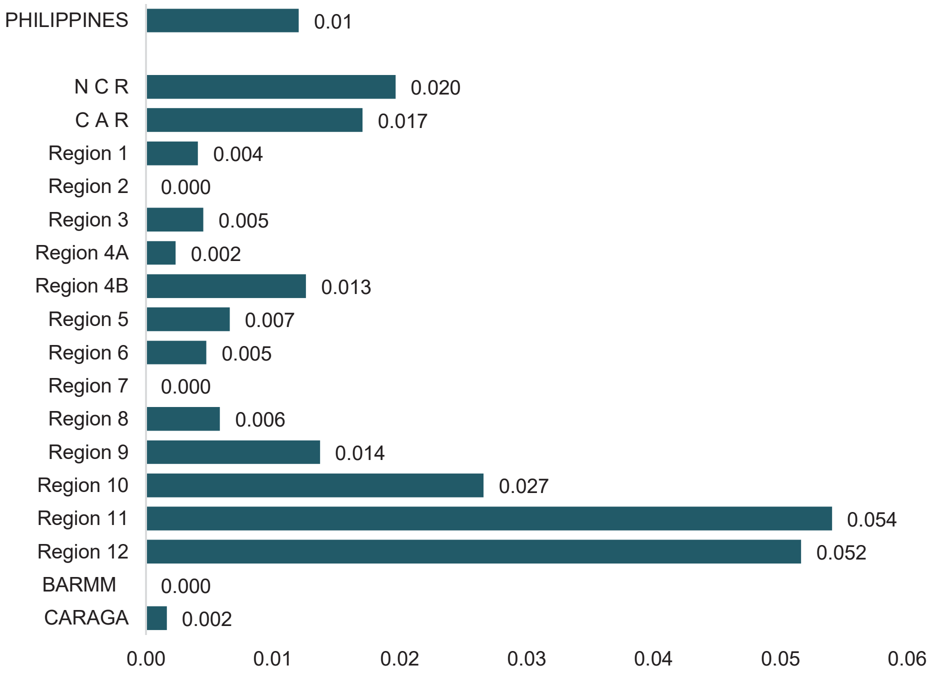

Figure 1.B.1.27 - Percentage Distribution of Pregnant Women (15-19 yrs. old) who completed Iodine by Region Philippines, 2020

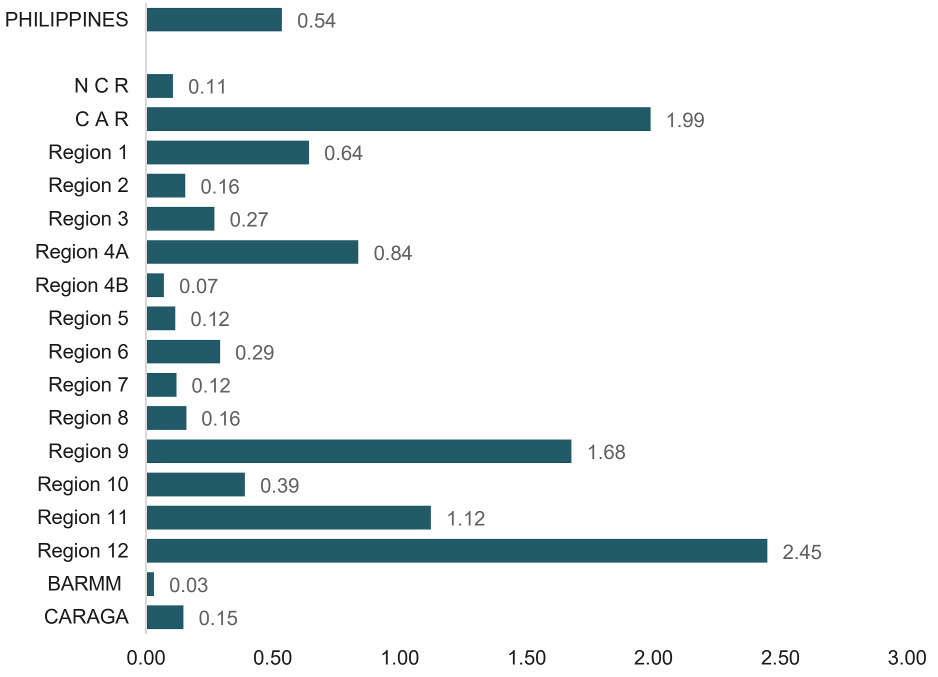

Figure 1.B.1.28 - Percentage Distribution of Pregnant Women (20-49 yrs. old) who completed Iodine by Region
Philippines, 2020

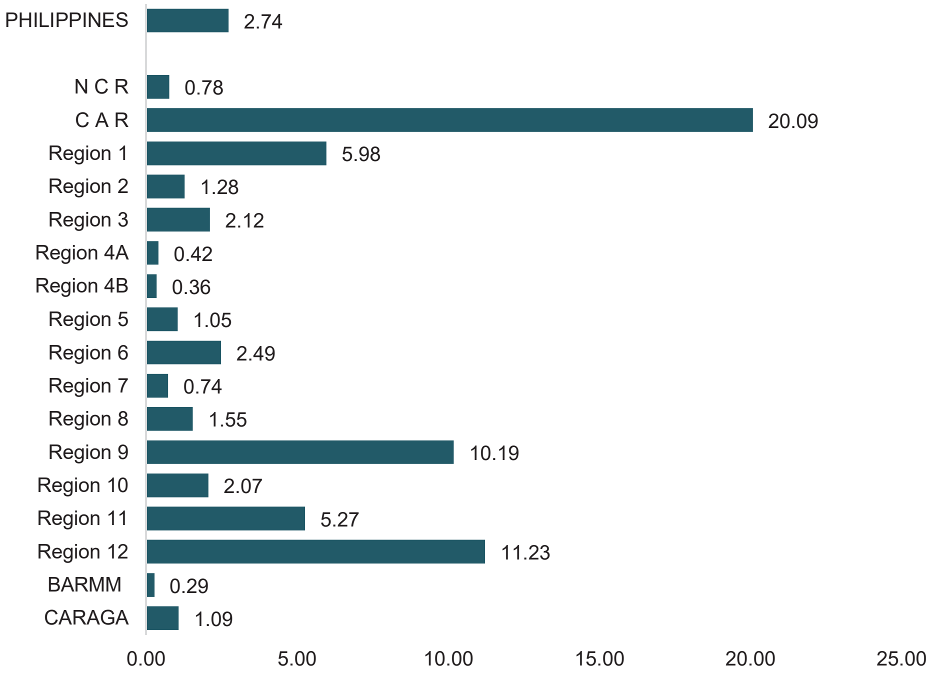

Figure 1.B.1.29 - Percentage Distribution of Pregnant Women (10-49 yrs. old) who completed Iodine by Region Philippines, 2020

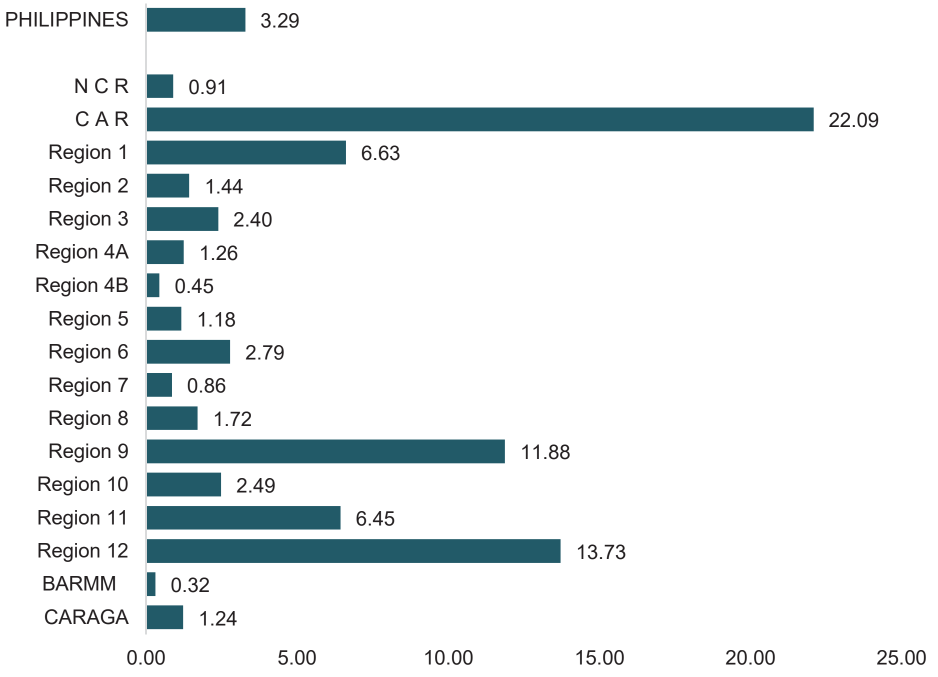

Figure 1.B.1.30 - Percentage Distribution of Pregnant Women (10-49 yrs. old) who completed Iodine by Age goup Philippines, 2020

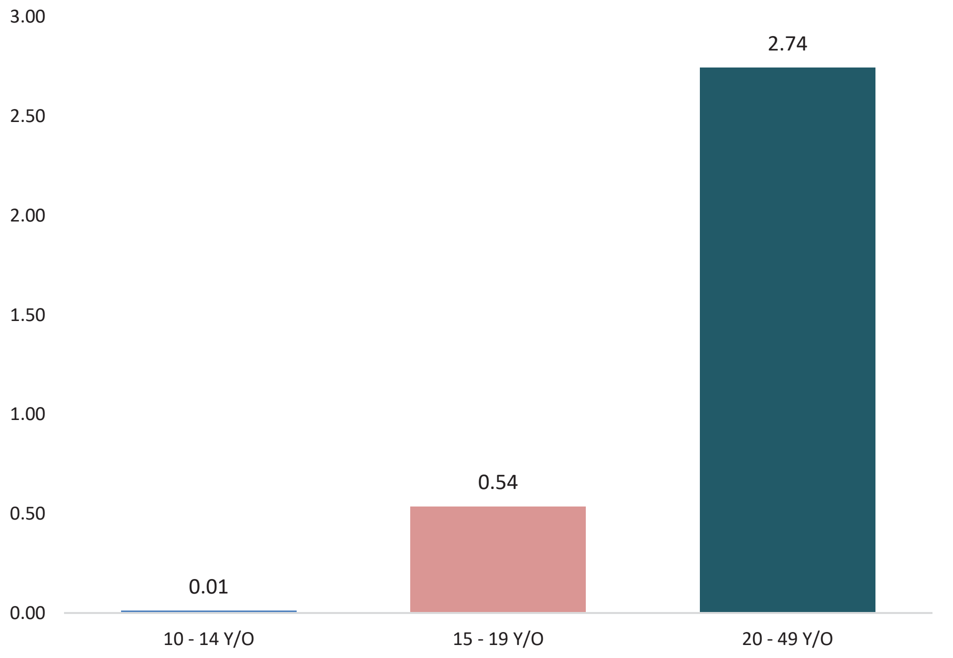

Figure 1.B.1.31 - Percentage Distribution of Pregnant Women (10-14 yrs. old) given one (1) dose of deworming tablet by Region
Philippines, 2020

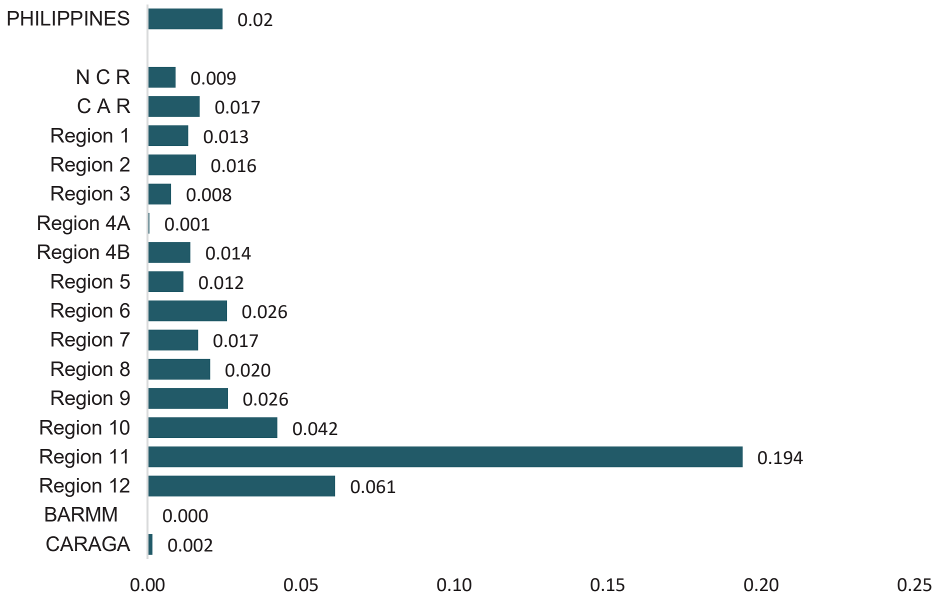

Figure 1.B.1.32 - Percentage Distribution of Pregnant Women (15-19 yrs. old) given one (1) dose of deworming tablet by Region
Philippines, 2020

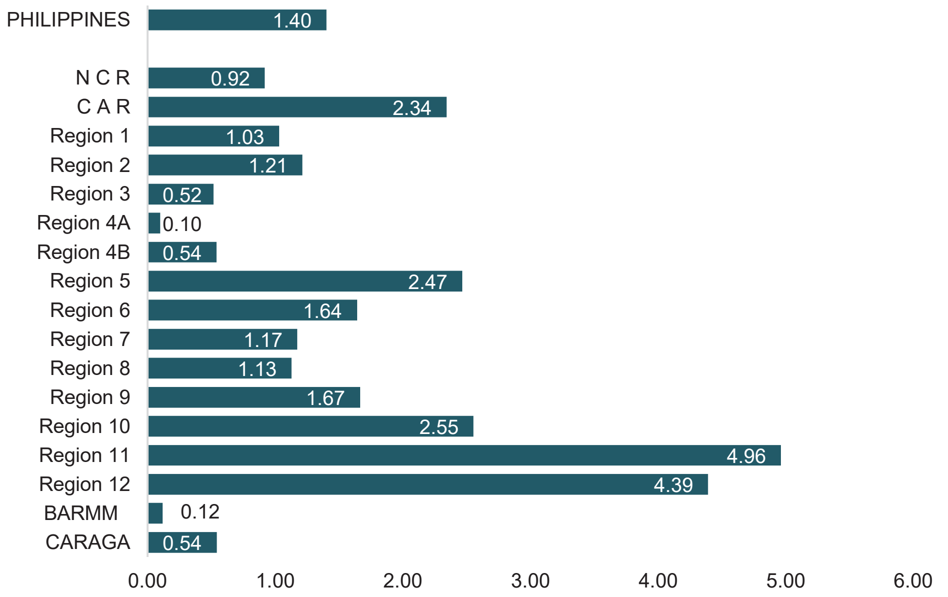

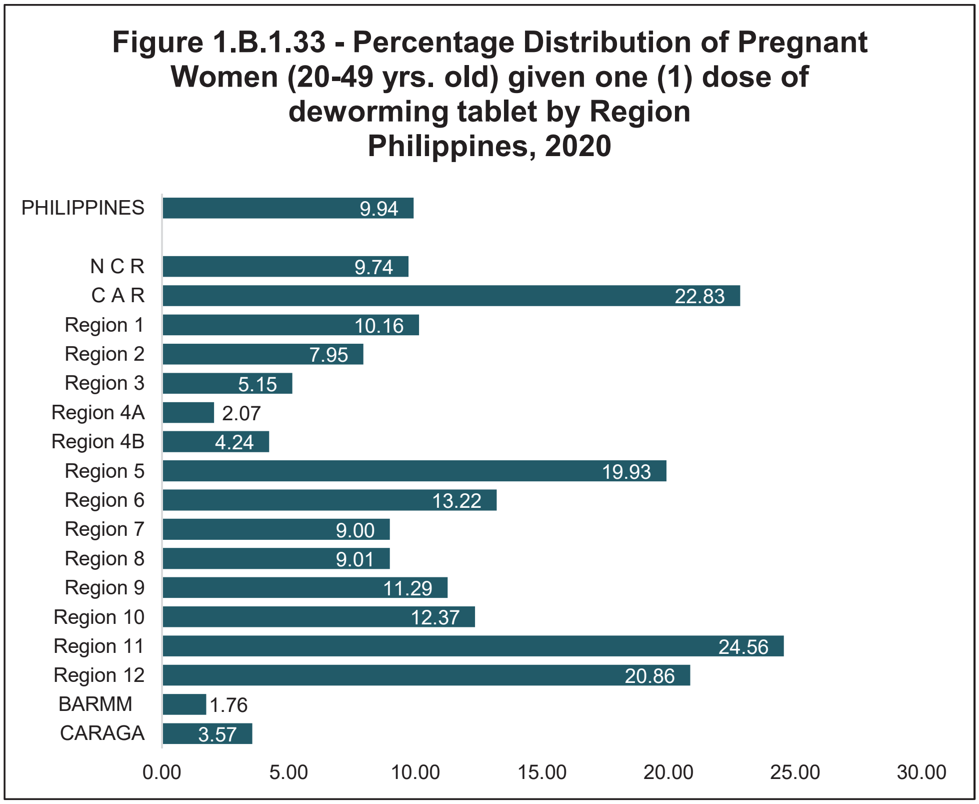
Figure 1.B.1.33 - Percentage Distribution of Pregnant Women (20-49 yrs. old) given one (1) dose of deworming tablet by Region
Philippines, 2020
PHILIPPINES
9.94
N C R
9.74
C A R
22.83
Region 1
10.16
Region 2
7.95
Region 3
5.15
Region 4A
2.07
Region 4B
4.24
Region 5
19.93
Region 6
13.22
Region 7
9.00
Region 8
9.01
Region 9
11.29
Region 10
12.37
Region 11
24.56
Region 12
20.86
BARMM
1.76
CARAGA
3.57
0.00
5.00
10.00
15.00
20.00
25.00
30.00

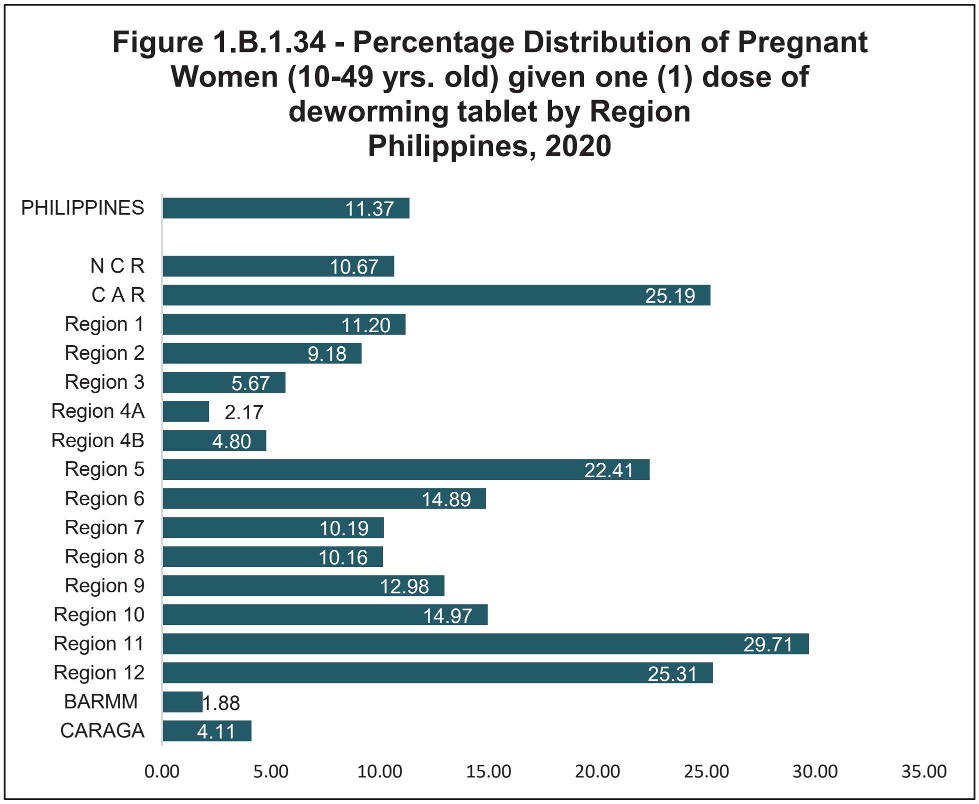
Figure 1.B.1.34 - Percentage Distribution of Pregnant Women (10-49 yrs. old) given one (1) dose of deworming tablet by Region
Philippines, 2020
PHILIPPINES
11.37
N C R
10.67
C A R
25.19
Region 1
11.20
Region 2
9.18
Region 3
5.67
Region 4A
2.17
Region 4B
4.80
Region 5
22.41
Region 6
14.89
Region 7
10.19
Region 8
10.16
Region 9
12.98
Region 10
14.97
Region 11
29.71
Region 12
25.31
BARMM
1.88
CARAGA
4.11
0.00
5.00
10.00
15.00
20.00
25.00
30.00
35.00

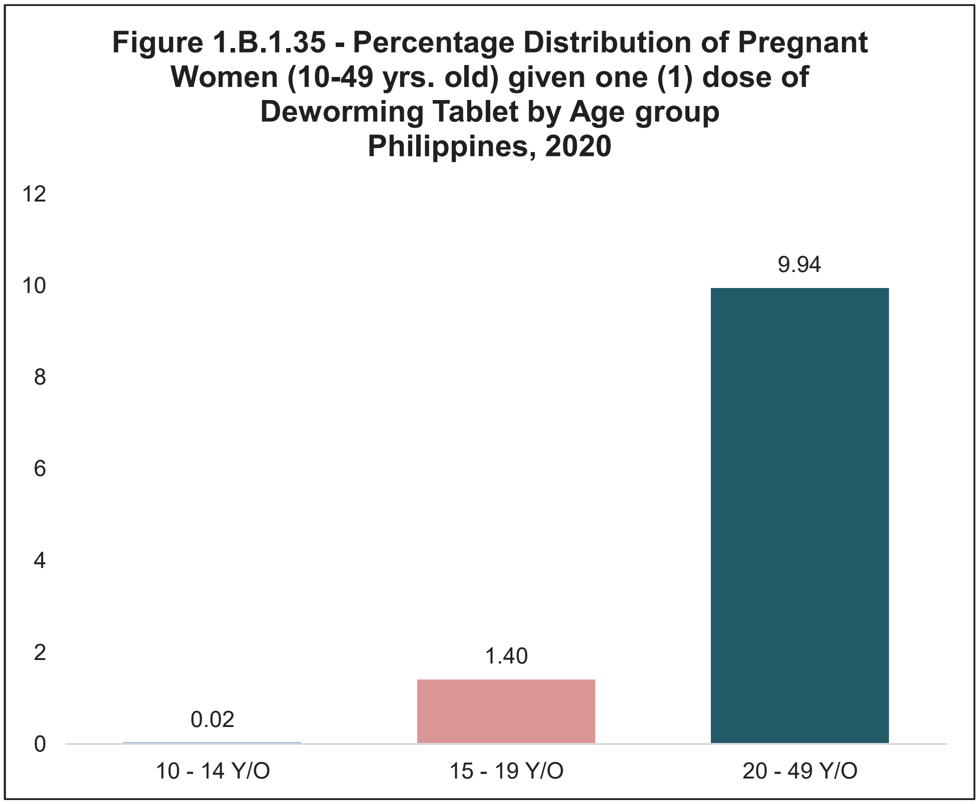
Figure 1.B.1.35 - Percentage Distribution of Pregnant Women (10-49 yrs. old) given one (1) dose of Deworming Tablet by Age group
Philippines, 2020
12
10
8
6
4
2
0
0.02
1.40
9.94
10 - 14 Y/O
15 - 19 Y/O
20 - 49 Y/O

Figure 1.B.1.36 - Percentage Distribution of Pregnant Women (10-14 yrs. old) screened for Syphillis by Region Philippines, 2020

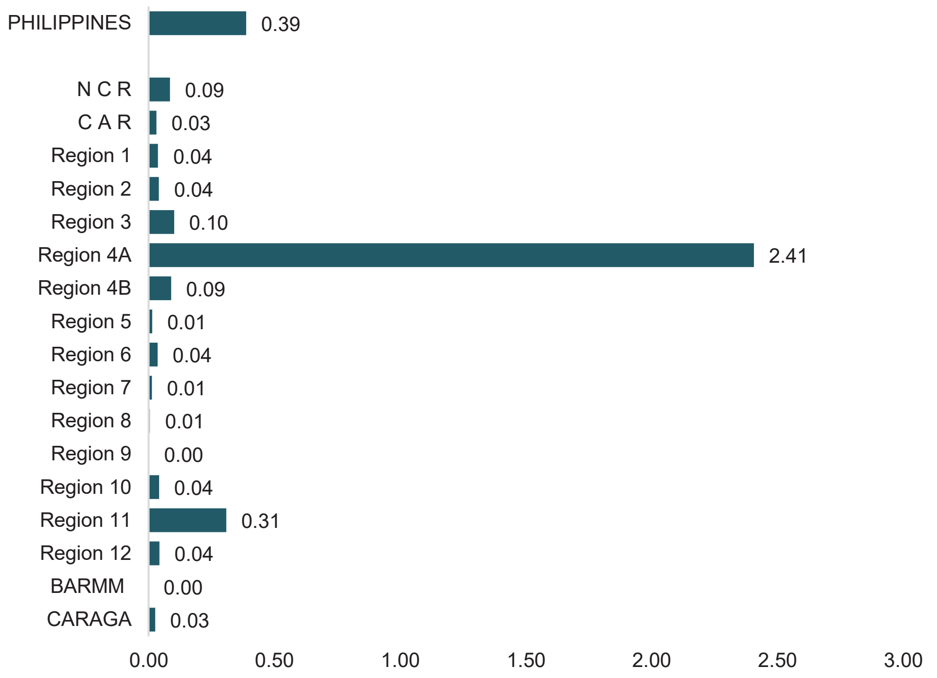

Figure 1.B.1.37 - Percentage Distribution of Pregnant Women (15-19 yrs. old) screened for Syphillis by Region Philippines, 2020

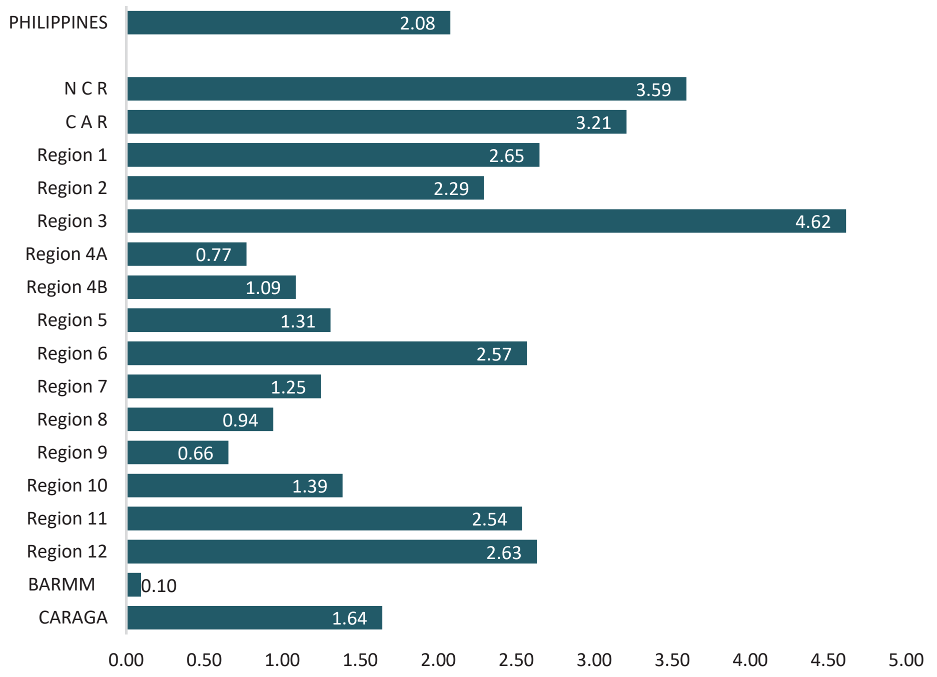

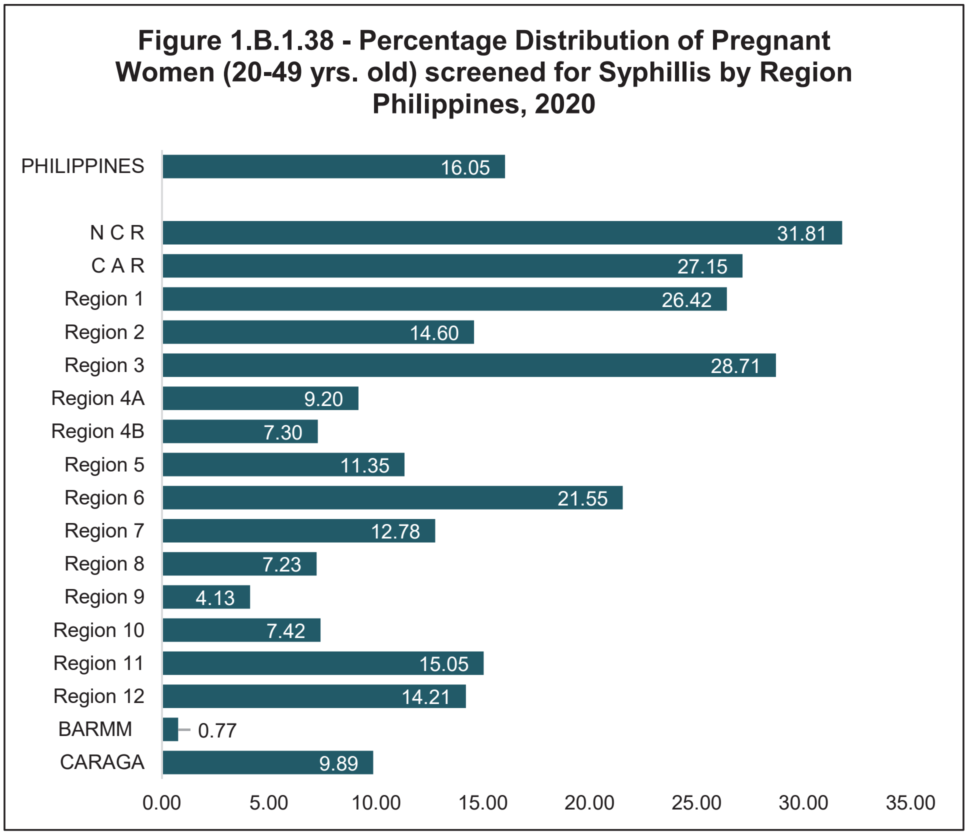
Figure 1.B.1.38 - Percentage Distribution of Pregnant Women (20-49 yrs. old) screened for Syphillis by Region Philippines, 2020
PHILIPPINES
16.05
N C R
31.81
C A R
27.15
Region 1
26.42
Region 2
14.60
Region 3
28.71
Region 4A
9.20
Region 4B
7.30
Region 5
11.35
Region 6
21.55
Region 7
12.78
Region 8
7.23
Region 9
4.13
Region 10
7.42
Region 11
15.05
Region 12
14.21
BARMM
0.77
CARAGA
9.89
0.00
5.00
10.00
15.00
20.00
25.00
30.00
35.00

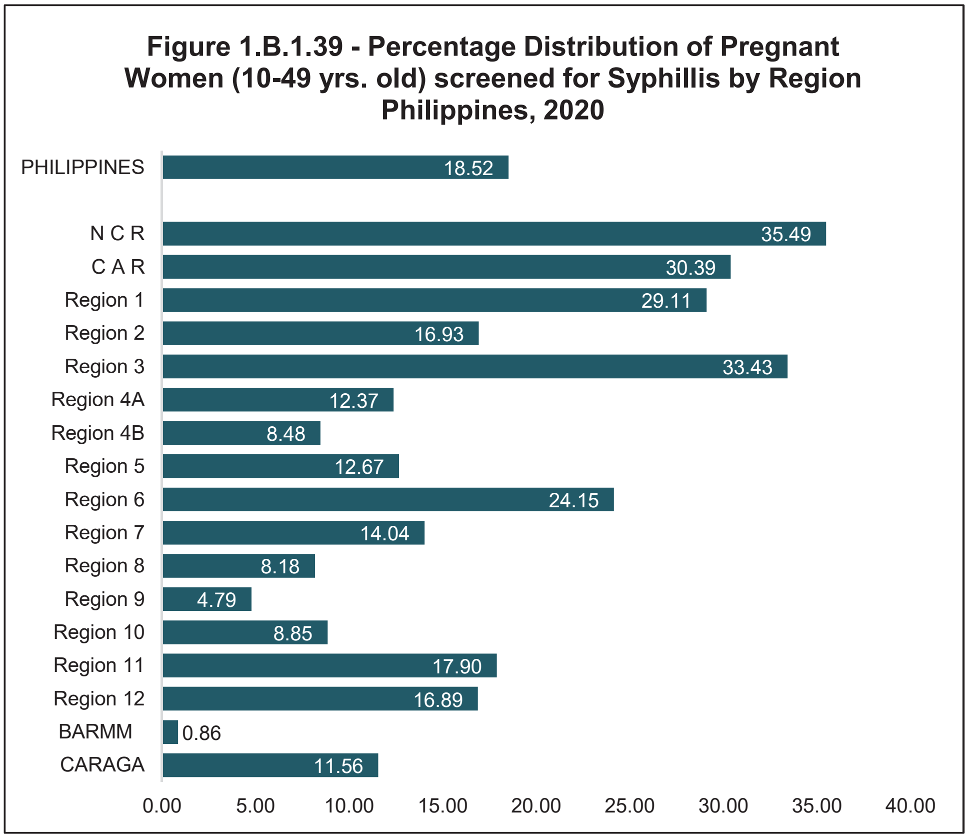
Figure 1.B.1.39 - Percentage Distribution of Pregnant Women (10-49 yrs. old) screened for Syphillis by Region Philippines, 2020
PHILIPPINES
18.52
N C R
35.49
C A R
30.39
Region 1
29.11
Region 2
16.93
Region 3
33.43
Region 4A
12.37
Region 4B
8.48
Region 5
12.67
Region 6
24.15
Region 7
14.04
Region 8
8.18
Region 9
4.79
Region 10
8.85
Region 11
17.90
Region 12
16.89
BARMM
0.86
CARAGA
11.56
0.00
5.00
10.00
15.00
20.00
25.00
30.00
35.00
40.00

Figure 1.B.1.40 - Percentage Distribution of Pregnant Women (10-49) screened for Syphillis by Age group Philippines, 2020

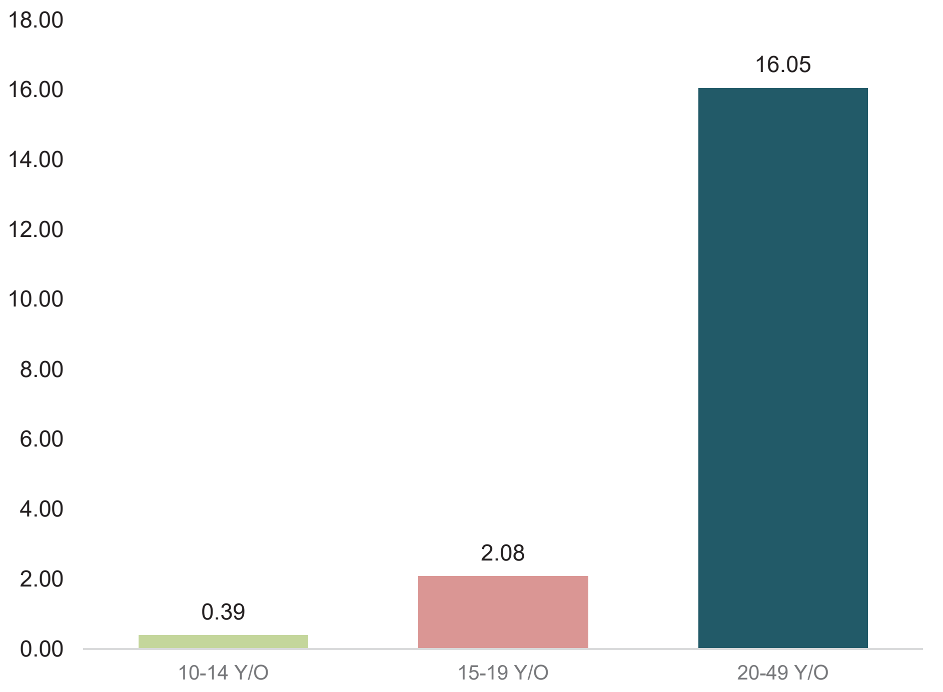

Figure 1.B.1.41 - Percentage Distribution of Pregnant Women Tested Positive for Syphillis (10 - 14 years old) by Region
Philippines, 2020

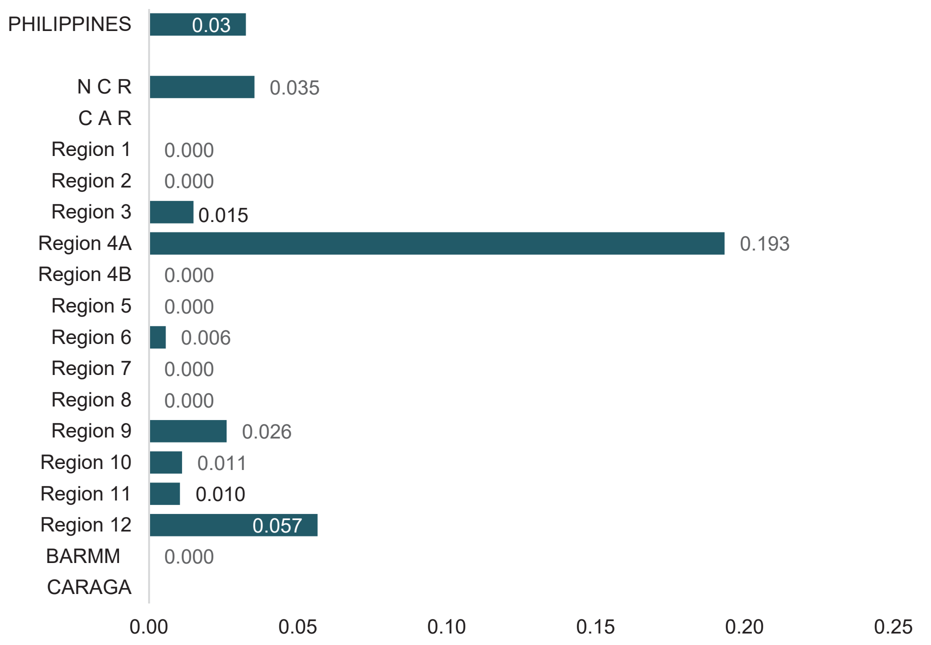

Figure 1.B.1.42 - Percentage Distribution of Pregnant Women Tested Positive for Syphillis (15 - 19 years old) by Region
Philippines, 2020

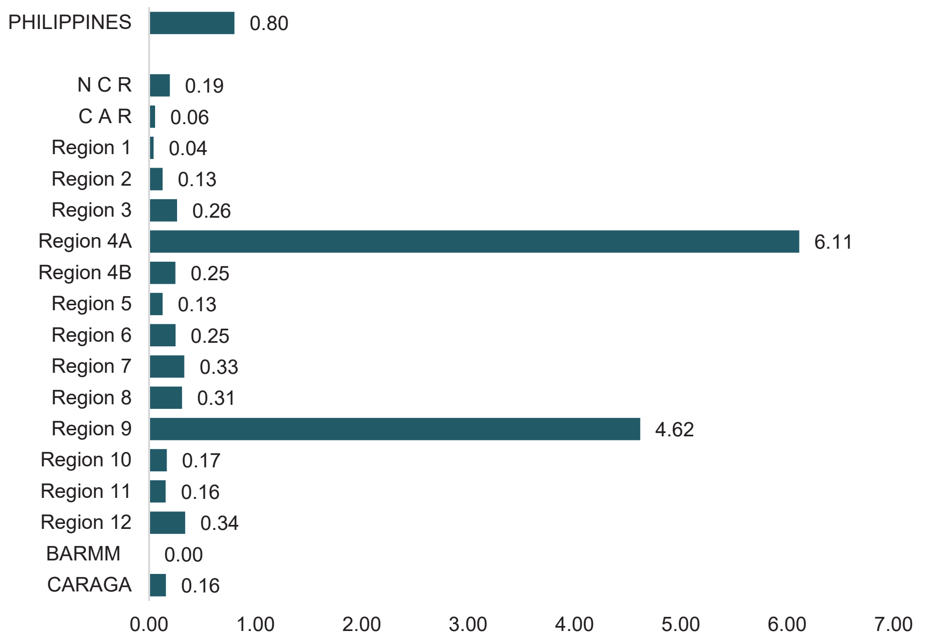

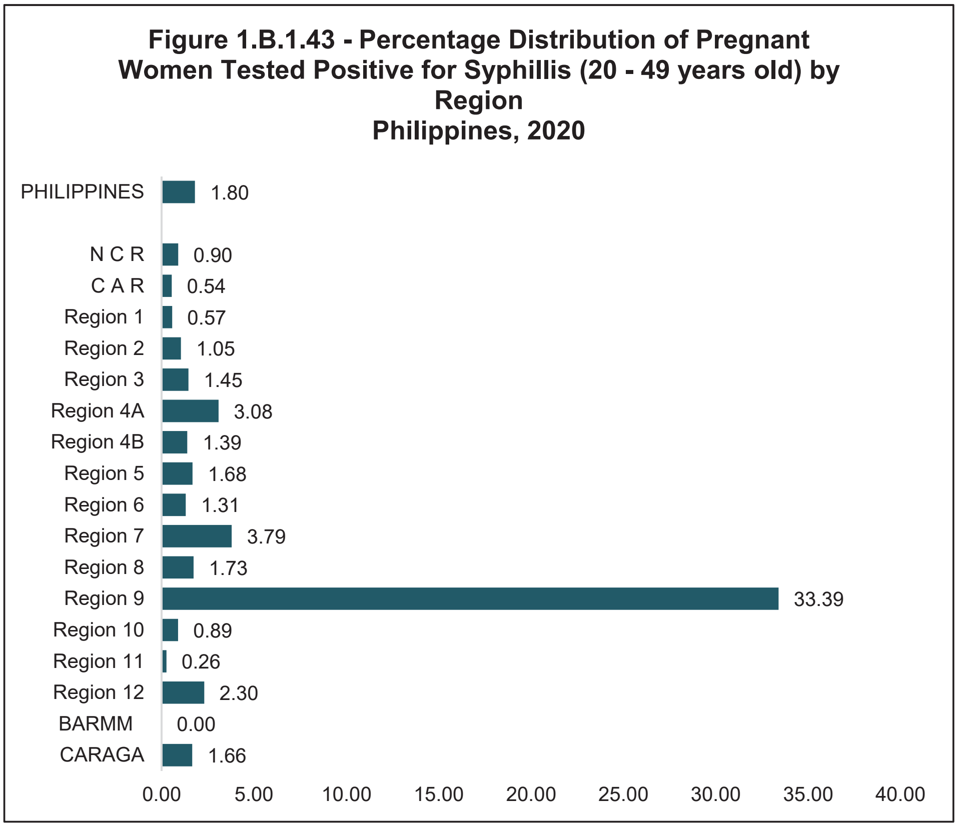
Figure 1.B.1.43 - Percentage Distribution of Pregnant Women Tested Positive for Syphillis (20 - 49 years old) by Region
Philippines, 2020
PHILIPPINES
1.80
N C R
0.90
C A R
0.54
Region 1
0.57
Region 2
1.05
Region 3
1.45
Region 4A
3.08
Region 4B
1.39
Region 5
1.68
Region 6
1.31
Region 7
3.79
Region 8
1.73
Region 9
33.39
Region 10
0.89
Region 11
0.26
Region 12
2.30
BARMM
0.00
CARAGA
1.66
0.00
5.00
10.00
15.00
20.00
25.00
30.00
35.00
40.00

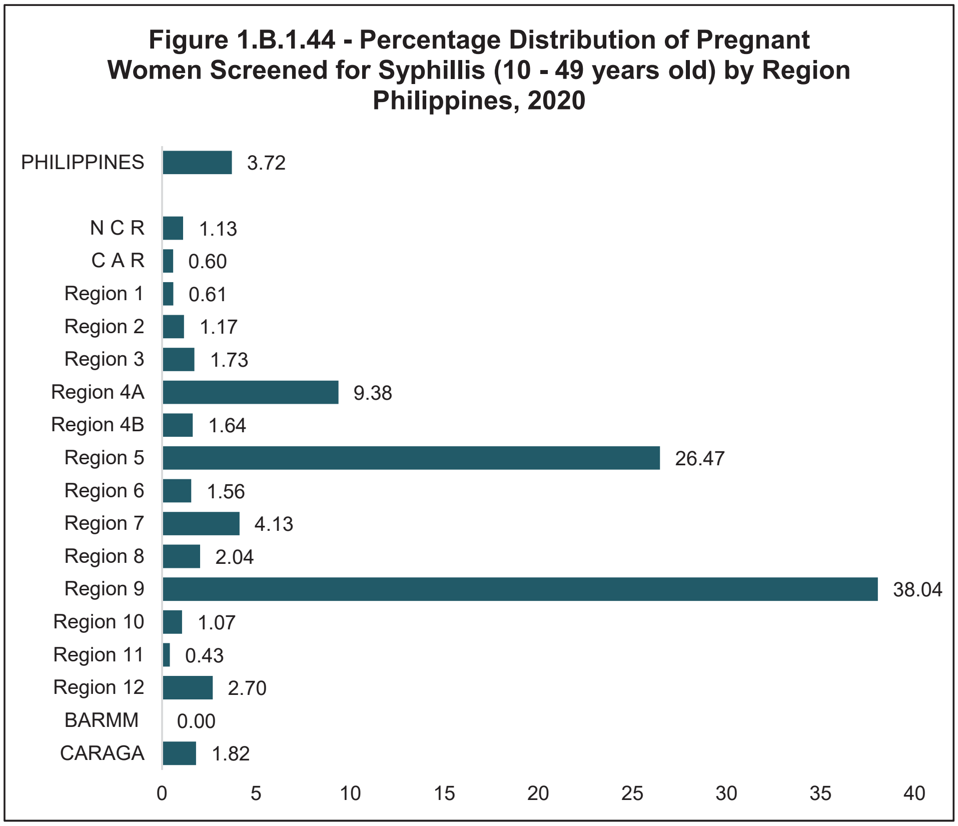
Figure 1.B.1.44 - Percentage Distribution of Pregnant Women Screened for Syphillis (10 - 49 years old) by Region
Philippines, 2020
PHILIPPINES
3.72
N C R
1.13
C A R
0.60
Region 1
0.61
Region 2
1.17
Region 3
1.73
Region 4A
9.38
Region 4B
1.64
Region 5
26.47
Region 6
1.56
Region 7
4.13
Region 8
2.04
Region 9
38.04
Region 10
1.07
Region 11
0.43
Region 12
2.70
BARMM
0.00
CARAGA
1.82
0
5
10
15
20
25
30
35
40

Figure 1.B.1.45 - Percentage Distribution of Pregnant Women (10-49 years old) Tested Positive for Syphillis by Age group Philippines, 2020

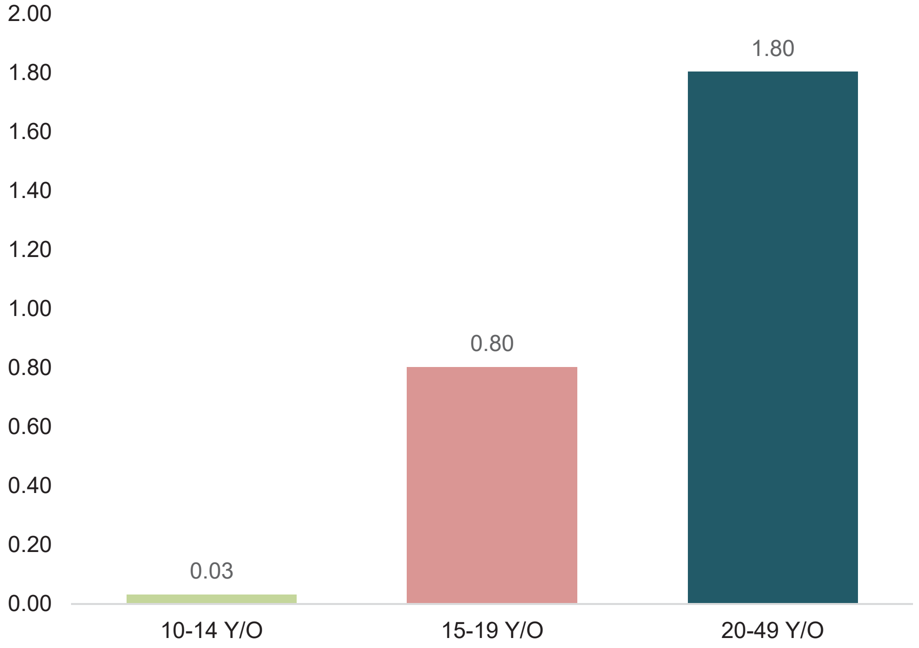

Figure 1.B.1.46 - Percentage Distribution of Pregnant Women (10-14 years old) screened for Hepatitis B by Region
Philippines, 2020

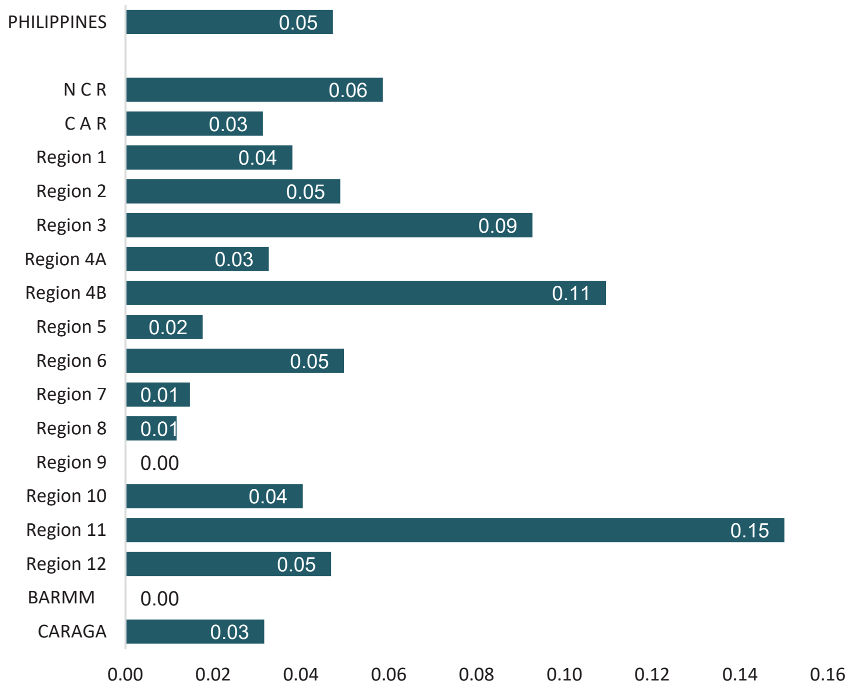

Figure 1.B.1.47 - Percentage Distribution of Pregnant Women (15-19 years old) screened for Hepatitis B by Region
Philippines, 2020

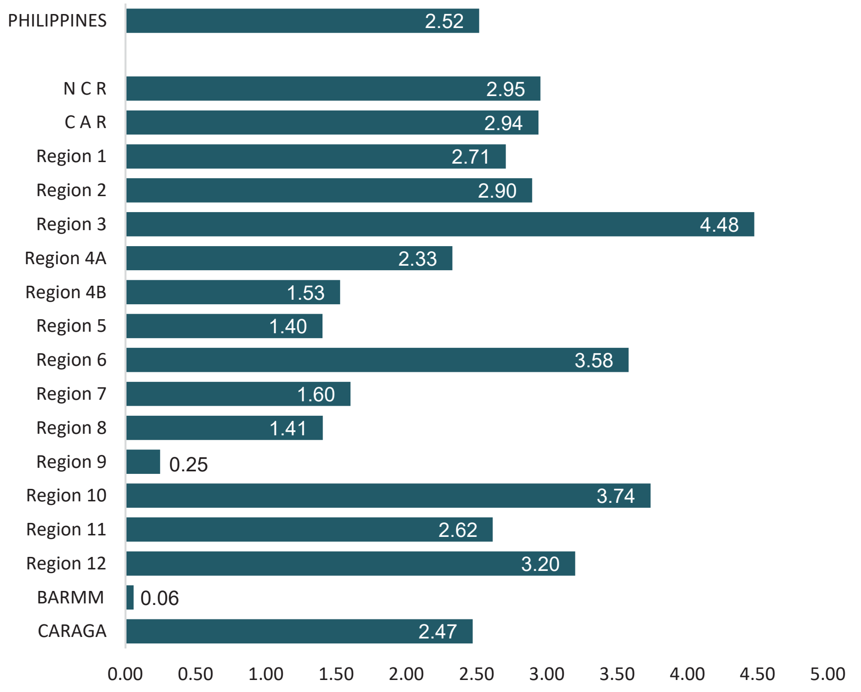

Figure 1.B.1.48 - Percentage Distribution of Pregnant Women (20-49 years old) screened for Hepatitis B by Region
Philippines, 2020

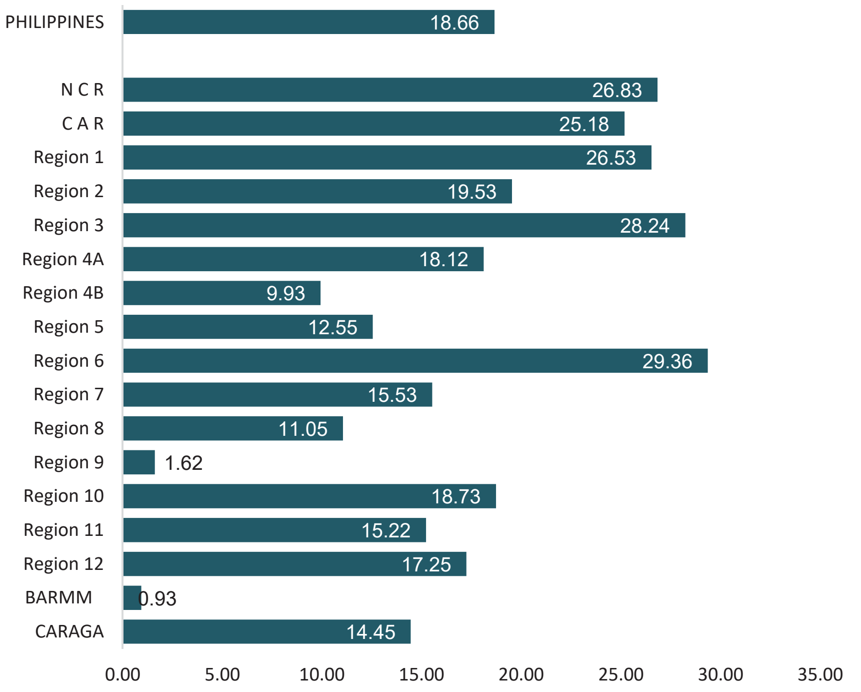

Figure 1.B.1.49 - Percentage Distribution of Pregnant Women (10 - 49 years old) screened for Hepatitis B by Region
Philippines, 2020

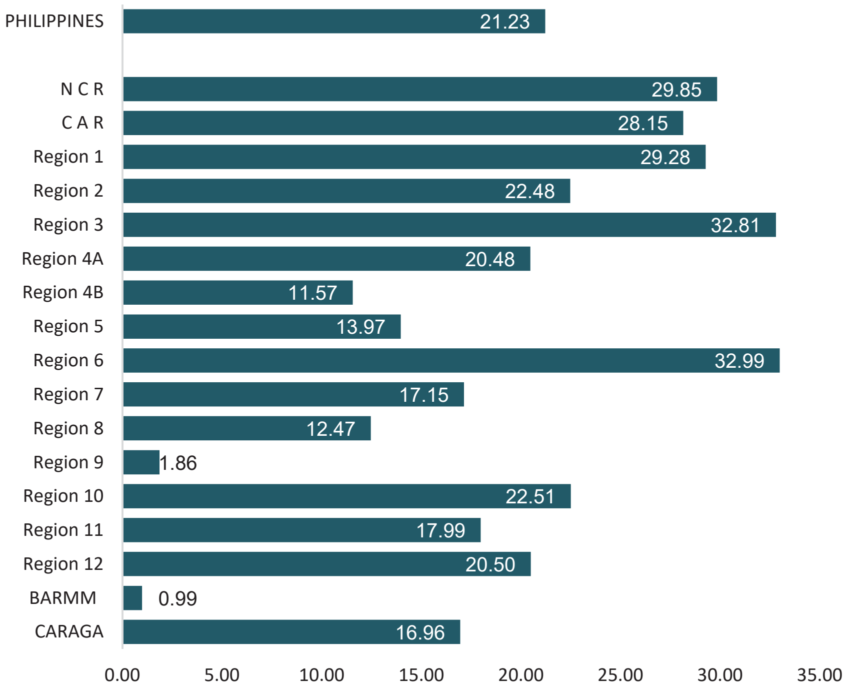

Figure 1.B.1.50 - Percentage Distribution of Pregnant Women (10-49 years old) screened for Hepatitis B by Age group
Philippines, 2020

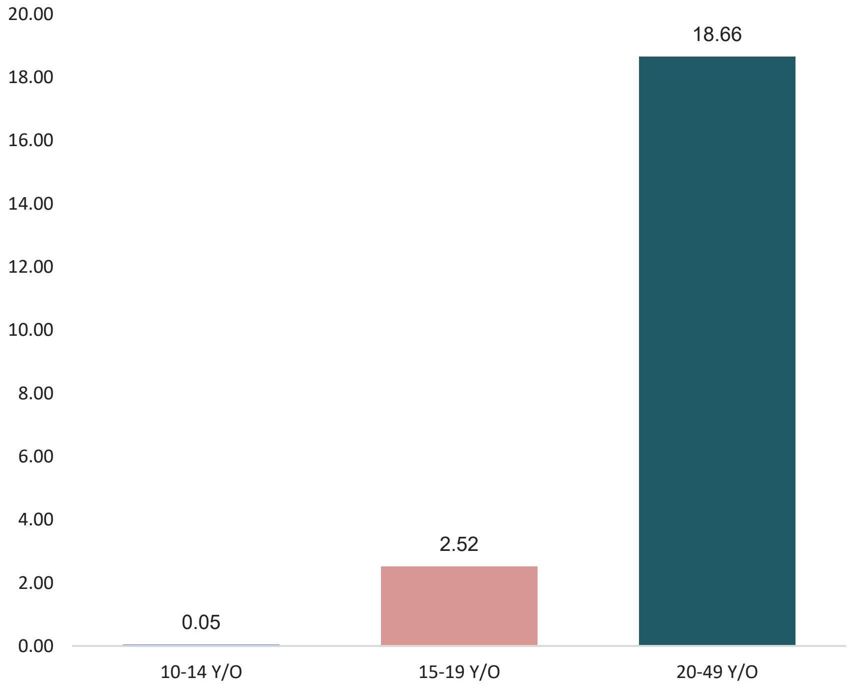

Figure 1.B.1.51 - Percentage Distribution of Pregnant Women (10-14 years old) Tested Positive for Hepatitis B by Region
Philippines, 2020

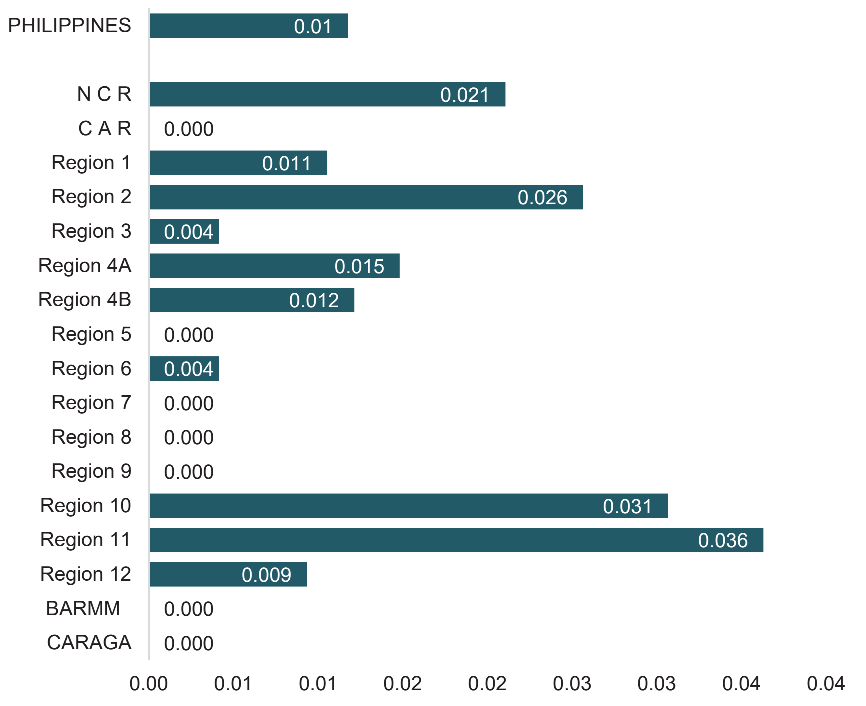

Figure 1.B.1.52 - Percentage Distribution of Pregnant Women (15 - 19 years old) Tested Positive for Hepatitis B by Region
Philippines, 2020

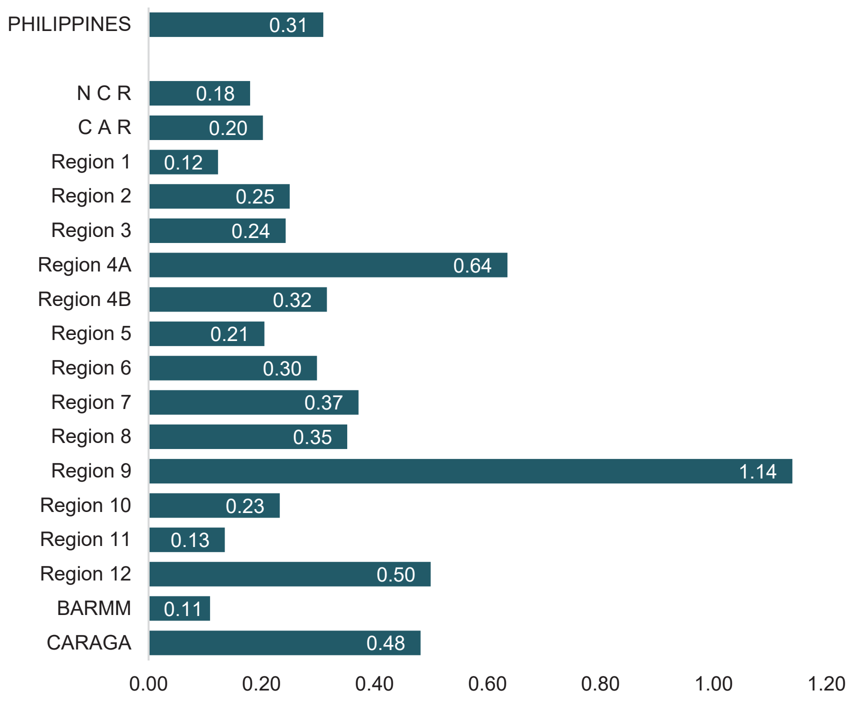

Figure 1.B.1.53 - Percentage Distribution of Pregnant Women (20 - 49 years old) Tested Positive for Hepatitis B by Region
Philippines, 2020

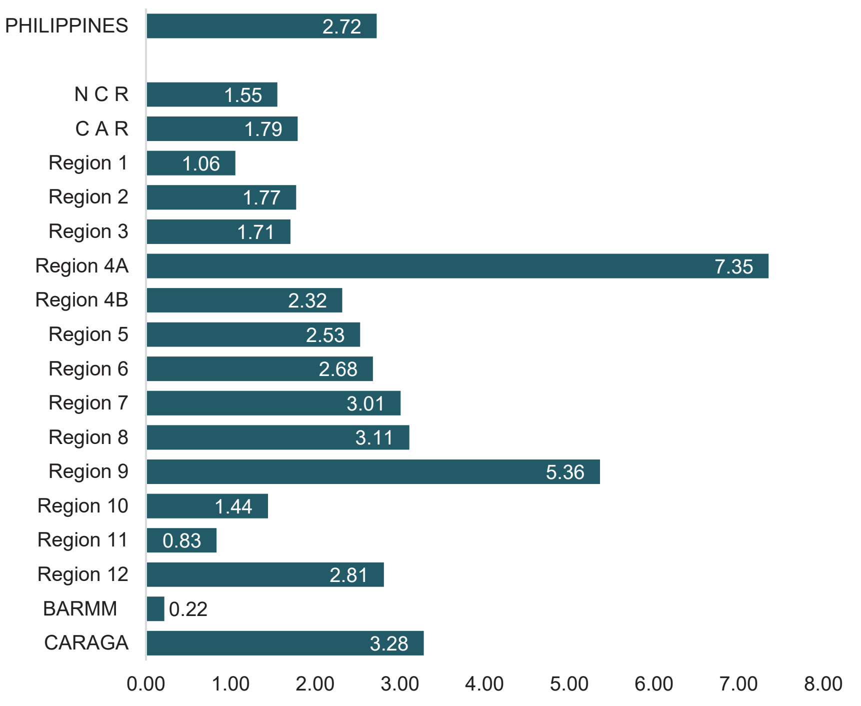

Figure 1.B.1.54 - Percentage Distribution of Pregnant Women (10 - 49 years old) Tested Positive for Hepatitis B by Region
Philippines, 2020

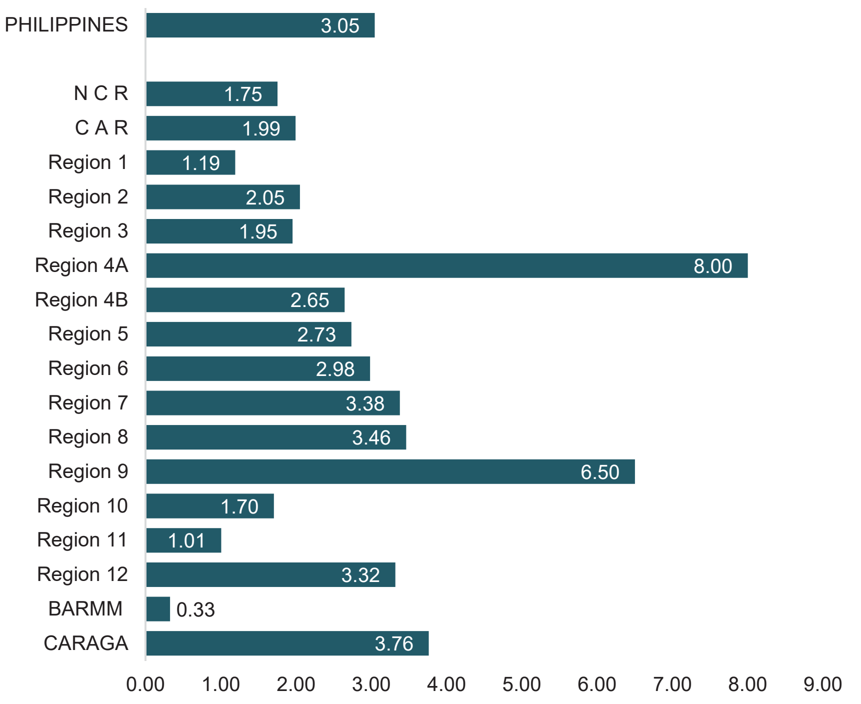

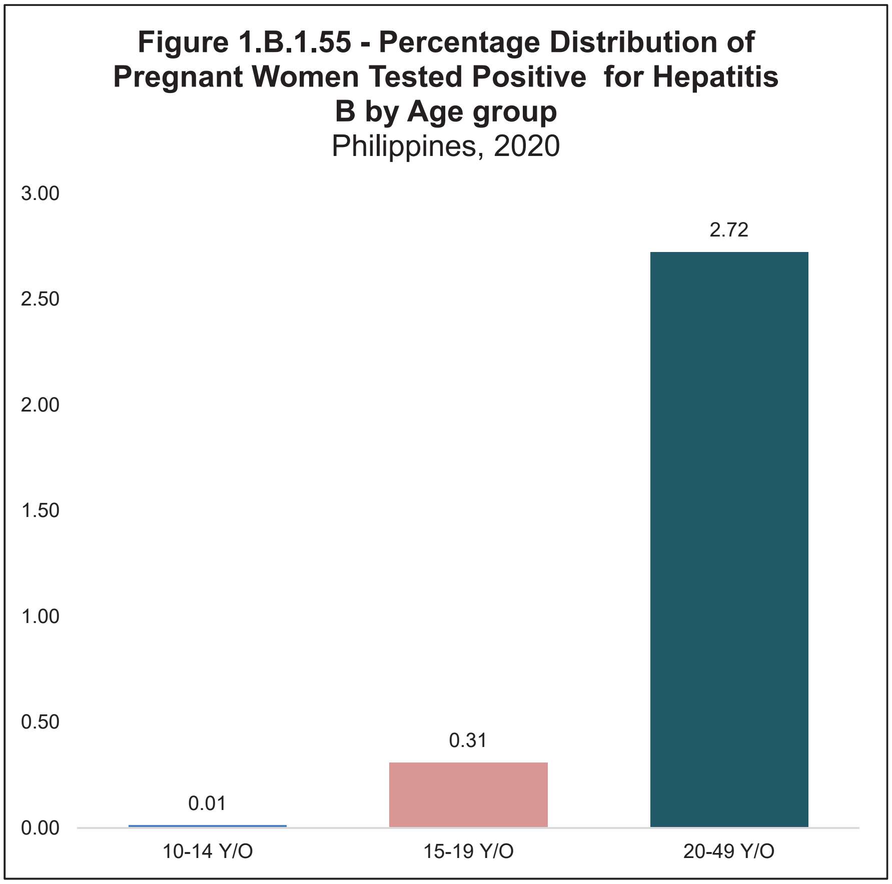
Figure 1.B.1.55 - Percentage Distribution of Pregnant Women Tested Positive for Hepatitis B by Age group
Philippines, 2020
3.00
2.50
2.00
1.50
1.00
0.50
0.00
2.72
0.31
0.01
10-14 Y/O
15-19 Y/O
20-49 Y/O

Figure 1.B.1.56 - Percentage Distribution of Pregnant Women (10-14 years old) screened for HIV by Region
Philippines, 2020

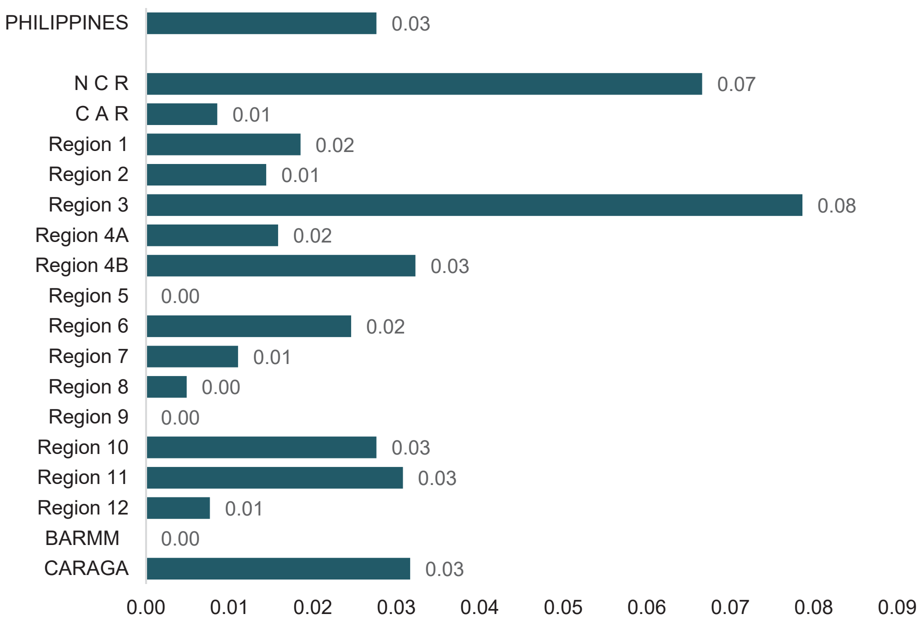

Figure 1.B.1.57 - Percentage Distribution of Pregnant Women (15-19 years old) screened for HIV by Region
Philippines, 2020

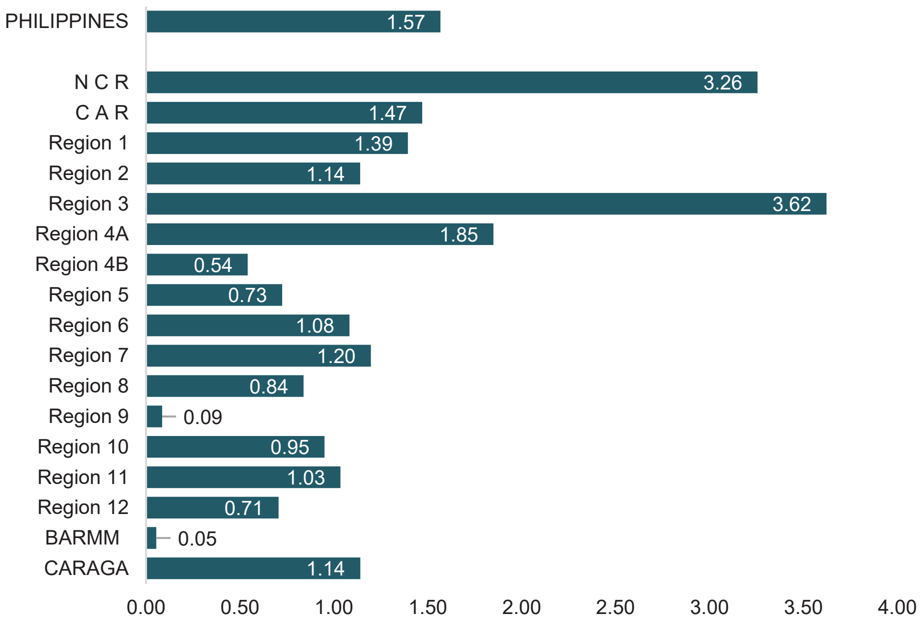

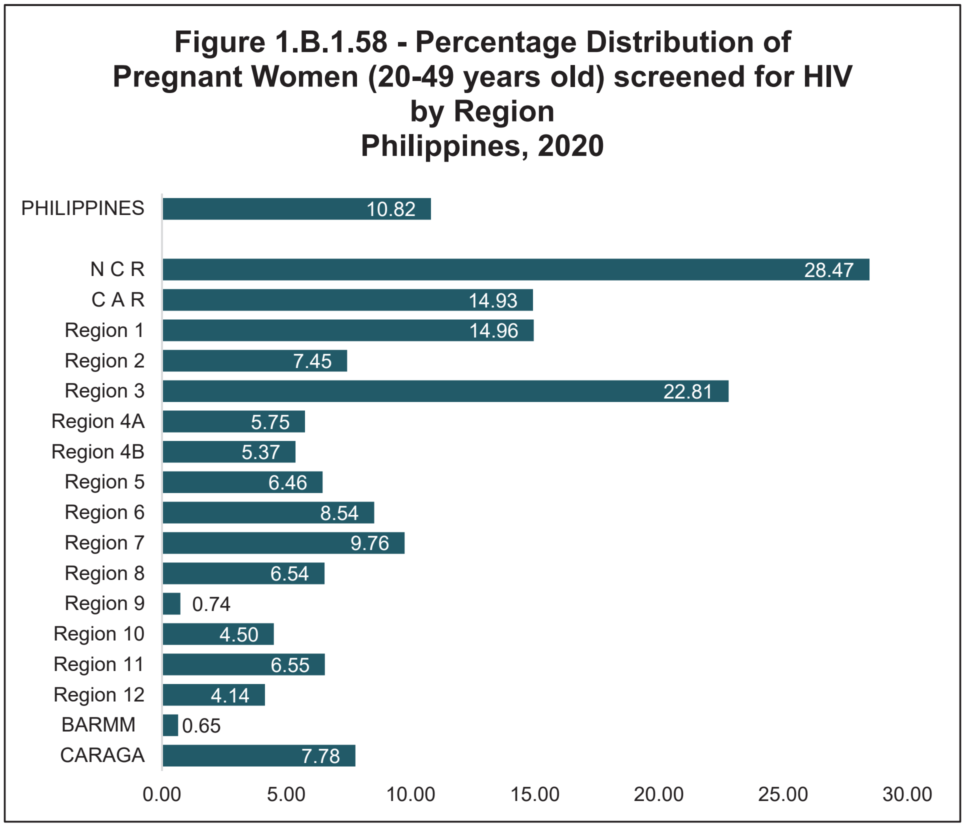
Figure 1.B.1.58 - Percentage Distribution of
Pregnant Women (20-49 years old) screened for HIV
by Region
Philippines, 2020
PHILIPPINES
10.82
N C R
28.47
C A R
14.93
Region 1
14.96
Region 2
7.45
Region 3
22.81
Region 4A
5.75
Region 4B
5.37
Region 5
6.46
Region 6
8.54
Region 7
9.76
Region 8
6.54
Region 9
0.74
Region 10
4.50
Region 11
6.55
Region 12
4.14
BARMM
0.65
CARAGA
7.78
0.00
5.00
10.00
15.00
20.00
25.00
30.00

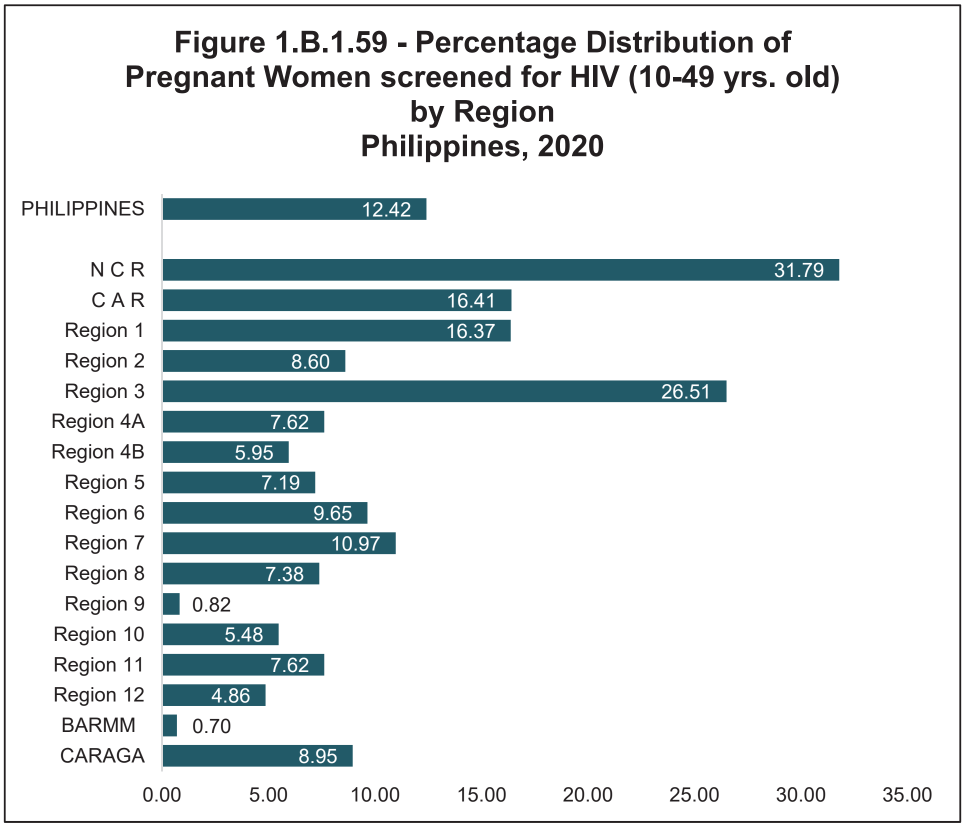
Figure 1.B.1.59 - Percentage Distribution of
Pregnant Women screened for HIV (10-49 yrs. old)
by Region
Philippines, 2020
PHILIPPINES
12.42
N C R
31.79
C A R
16.41
Region 1
16.37
Region 2
8.60
Region 3
26.51
Region 4A
7.62
Region 4B
5.95
Region 5
7.19
Region 6
9.65
Region 7
10.97
Region 8
7.38
Region 9
0.82
Region 10
5.48
Region 11
7.62
Region 12
4.86
BARMM
0.70
CARAGA
8.95
0.00
5.00
10.00
15.00
20.00
25.00
30.00
35.00

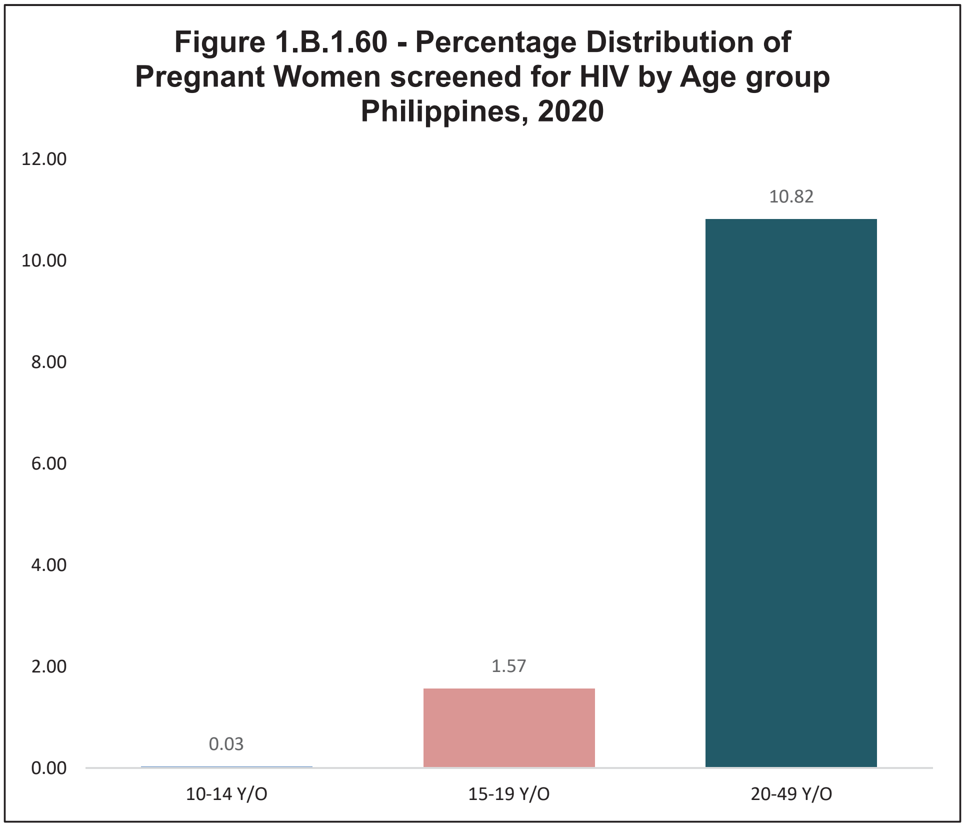
Figure 1.B.1.60 - Percentage Distribution of Pregnant Women screened for HIV by Age group Philippines, 2020
12.00
10.00
8.00
6.00
4.00
2.00
0.00
0.03
1.57
10.82
10-14 Y/O
15-19 Y/O
20-49 Y/O

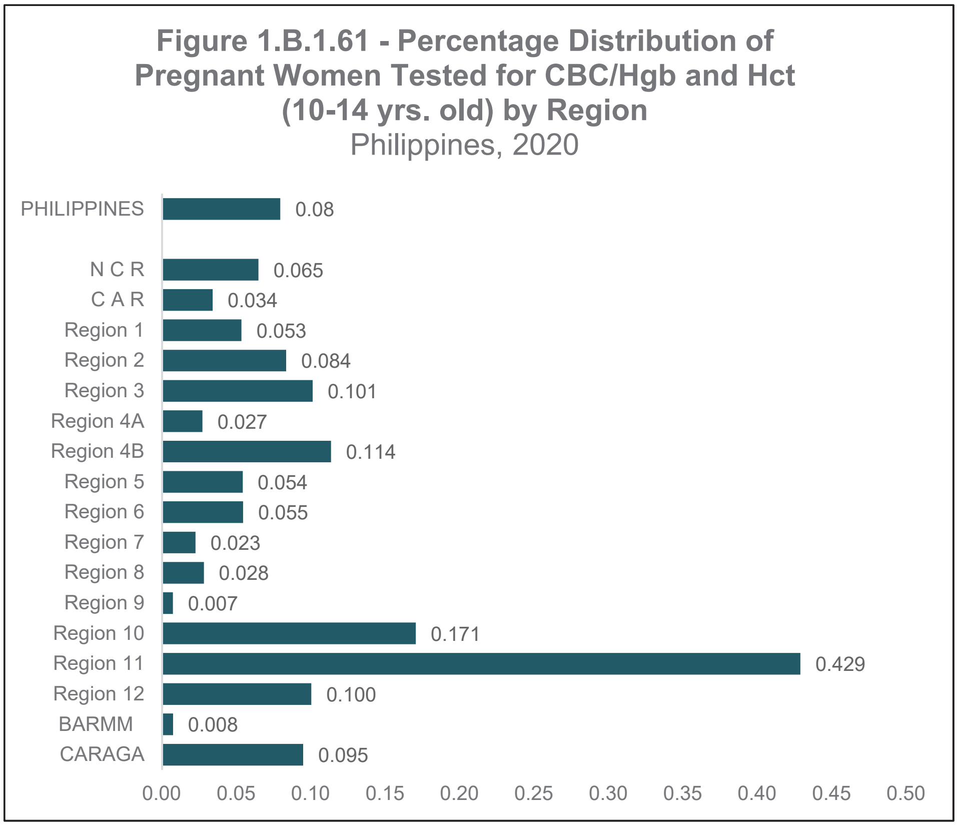
Figure 1.B.1.61 - Percentage Distribution of Pregnant Women Tested for CBC/Hgb and Hct (10-14 yrs. old) by Region
Philippines, 2020
PHILIPPINES 0.08
N C R 0.065
C A R 0.034
Region 1 0.053
Region 2 0.084
Region 3 0.101
Region 4A 0.027
Region 4B 0.114
Region 5 0.054
Region 6 0.055
Region 7 0.023
Region 8 0.028
Region 9 0.007
Region 10 0.171
Region 11 0.429
Region 12 0.100
BARMM 0.008
CARAGA 0.095
0.00 0.05 0.10 0.15 0.20 0.25 0.30 0.35 0.40 0.45 0.50

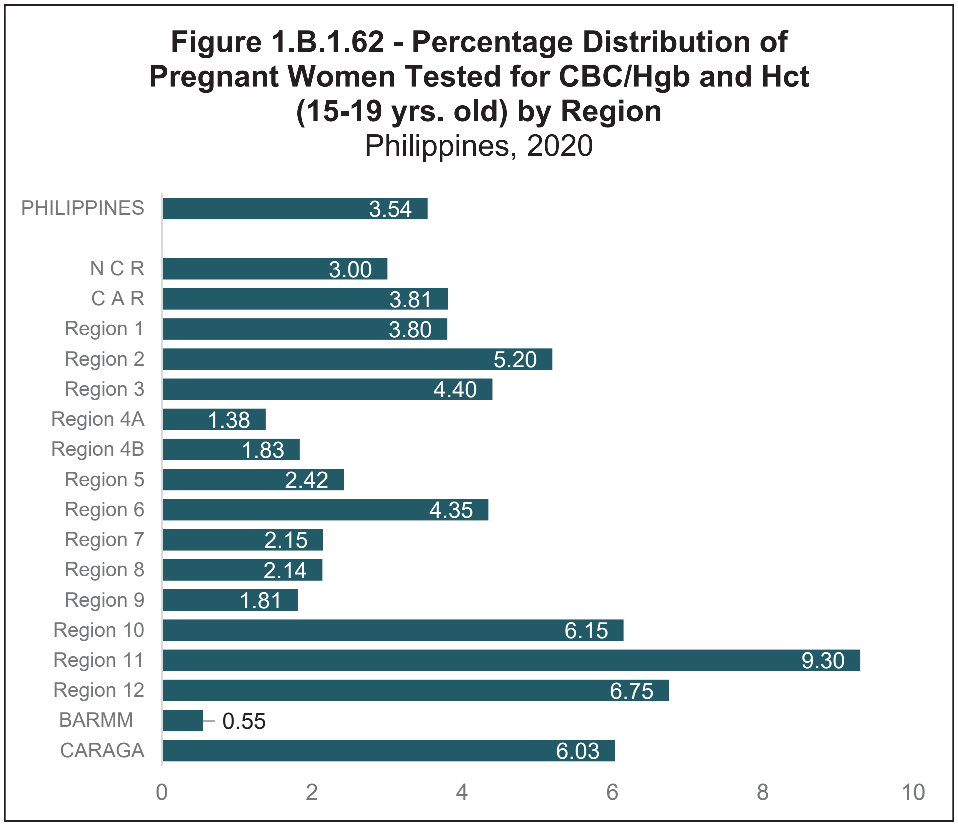
Figure 1.B.1.62 - Percentage Distribution of Pregnant Women Tested for CBC/Hgb and Hct (15-19 yrs. old) by Region
Philippines, 2020
PHILIPPINES 3.54
N C R 3.00
C A R 3.81
Region 1 3.80
Region 2 5.20
Region 3 4.40
Region 4A 1.38
Region 4B 1.83
Region 5 2.42
Region 6 4.35
Region 7 2.15
Region 8 2.14
Region 9 1.81
Region 10 6.15
Region 11 9.30
Region 12 6.75
BARMM 0.55
CARAGA 6.03
0 2 4 6 8 10

Figure 1.B.1.63 - Percentage Distribution of Pregnant Women Tested for CBC/Hgb and Hct (20-49 yrs. old) by Region
Philippines, 2020

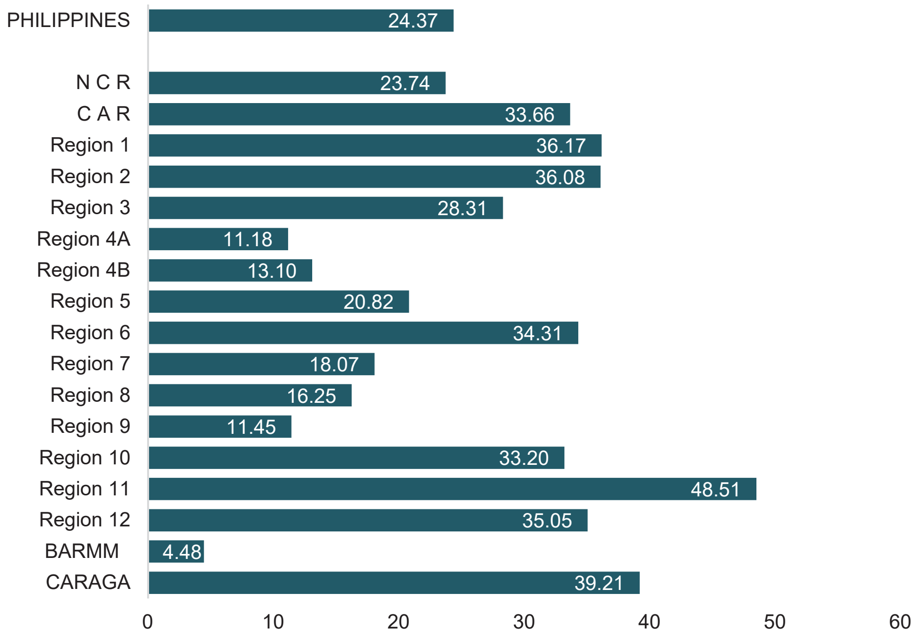

Figure 1.B.1.64 - Percentage Distribution of Pregnant Women Tested for CBC/Hgb and Hct (10-49 yrs. old) by Region Philippines, 2020

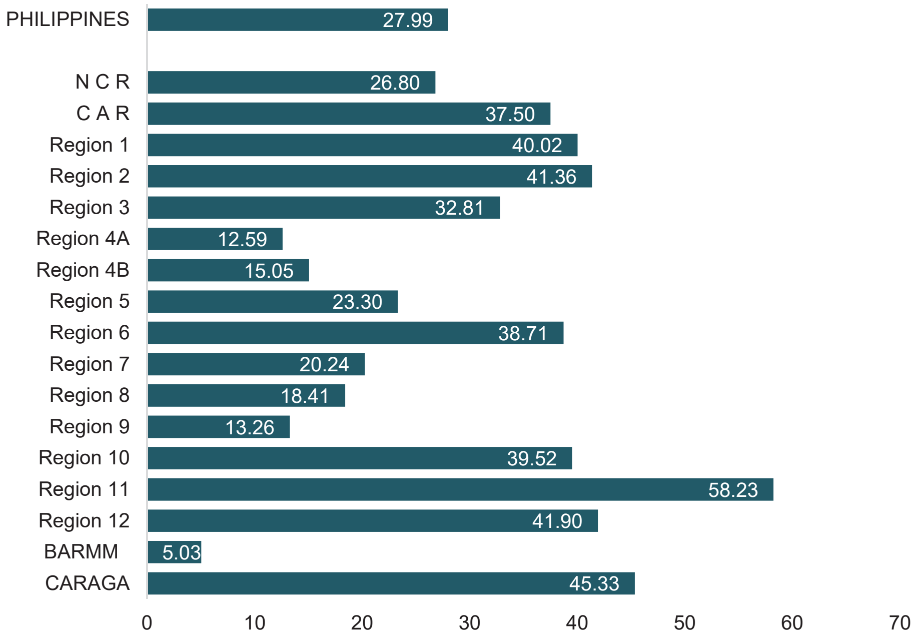

Figure 1.B.1.65 - Percentage Distribution of Pregnant Women Tested for CBC/Hgb and Hct by Age group Philippines, 2020

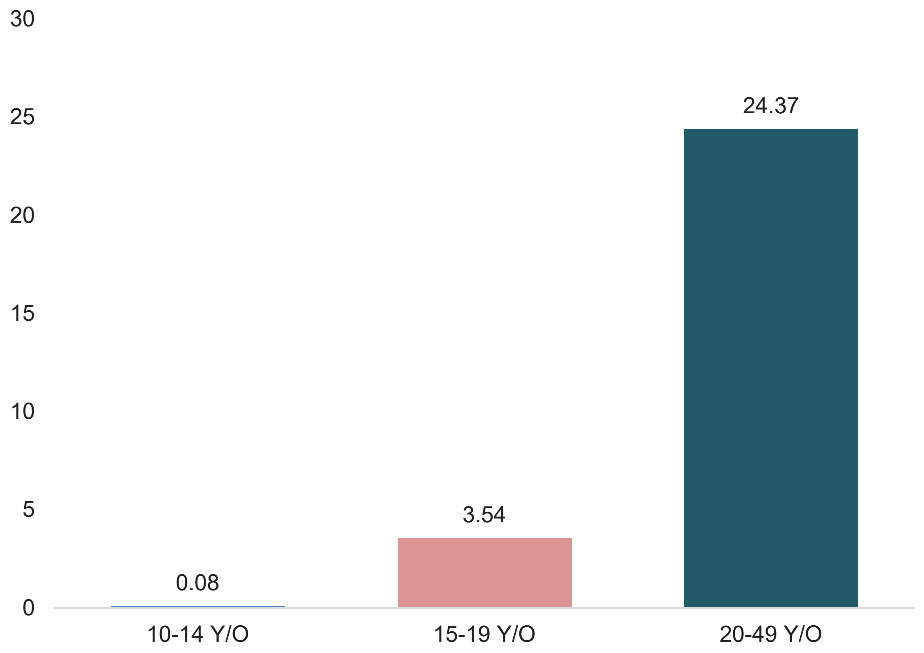

Figure 1.B.1.66 - Percentage Distribution of Pregnant Women Tested for CBC/Hgb and Hct diagnosed with anemia (10-14 yrs. old) by Region

Philippines, 2020

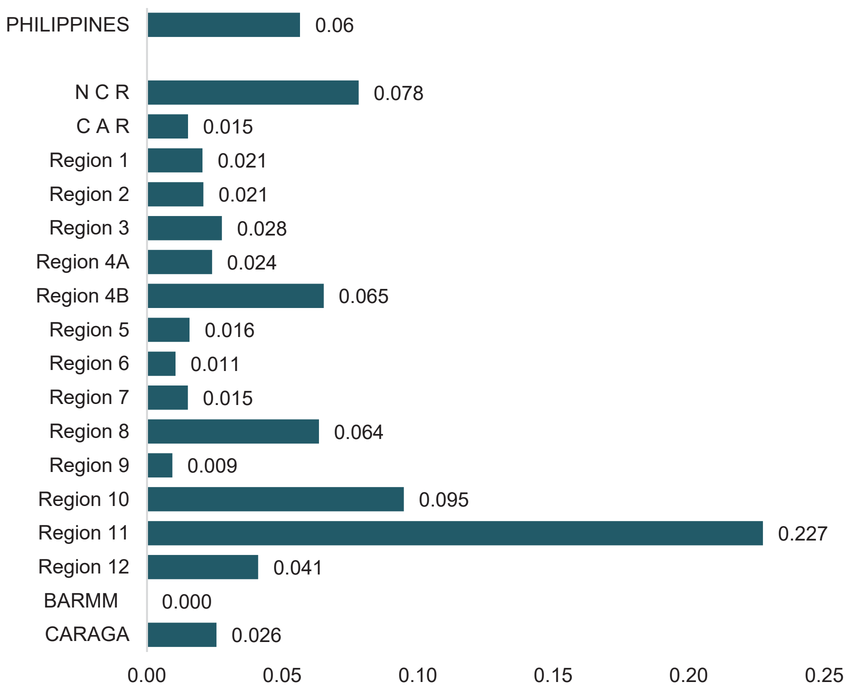

Figure 1.B.1.67 - Percentage Distribution of Pregnant Women Tested for CBC/Hgb and Hct diagnosed with anemia (15-19 yrs. old) by Region

Philippines, 2020

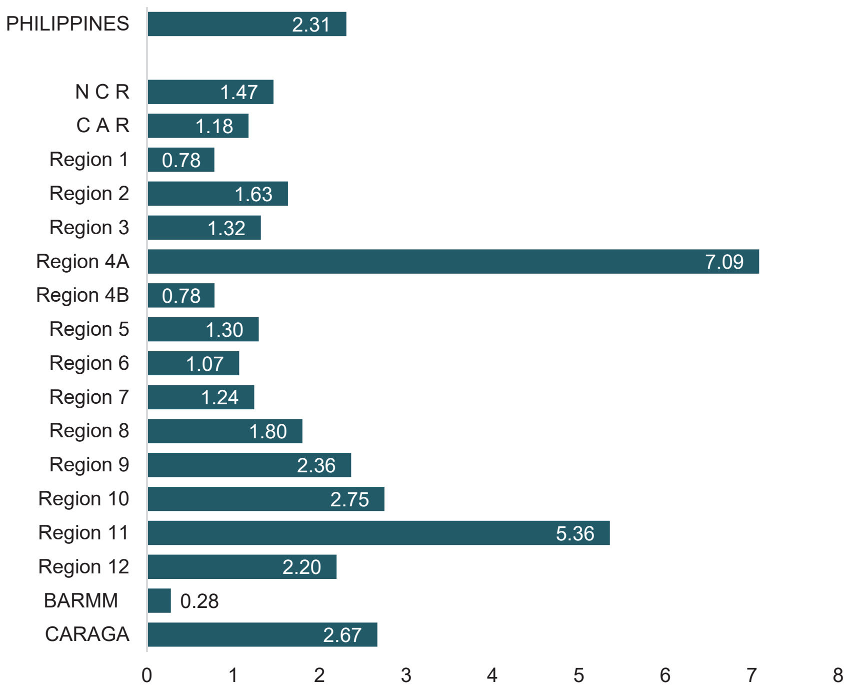

Figure 1.B.1.68 - Percentage Distribution of Pregnant Women Tested for CBC/Hgb and Hct diagnosed with anemia (20-49 yrs. old) by Region
Philippines, 2020

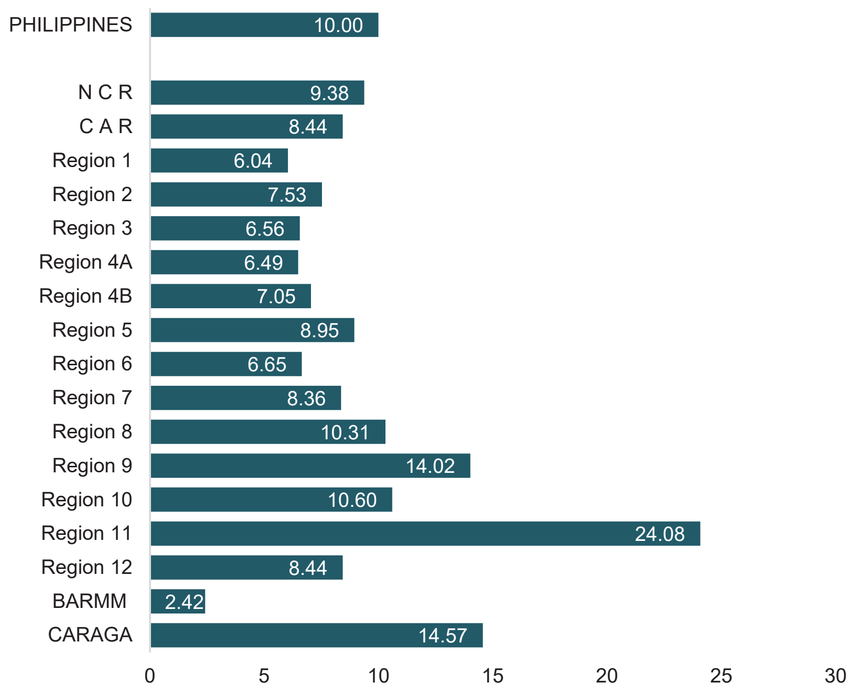

Figure 1.B.1.69 - Percentage Distribution of Pregnant Women Tested for CBC/Hgb and Hct diagnosed with anemia (10-49 yrs. old) by Region
Philippines, 2020

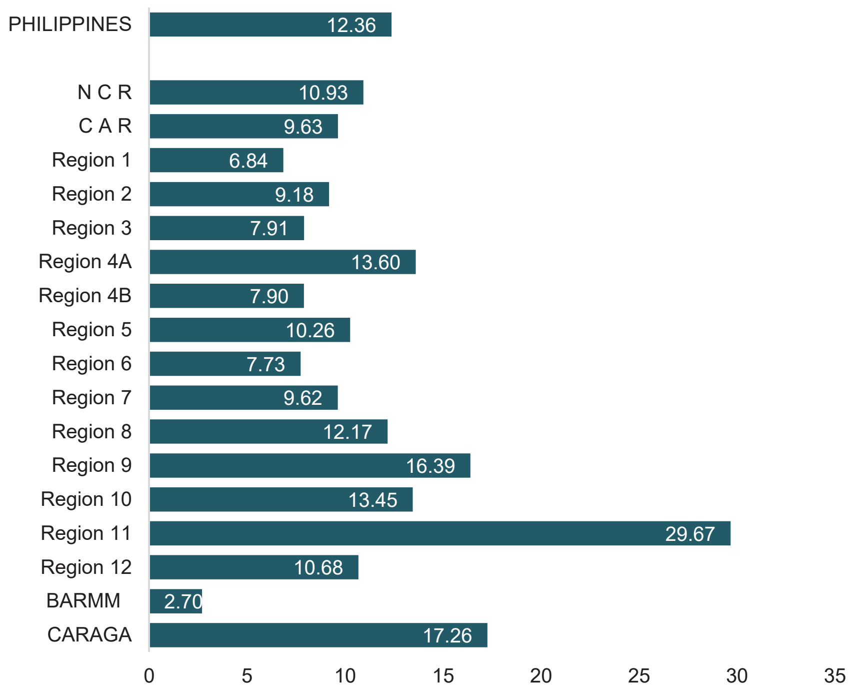

Figure 1.B.1.70 - Percentage Distribution of Pregnant Women Tested for CBC/Hgb and Hct diagnosed with anemia by Age group
Philippines, 2020

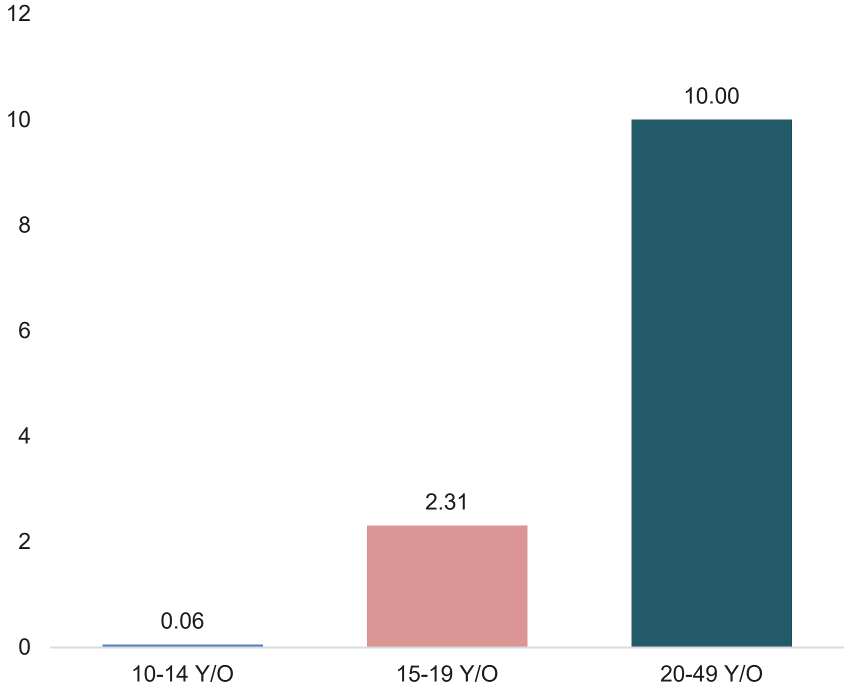

Figure 1.B.1.71 - Percentage Distribution of Pregnant Women Screened for Gestational Diabetes (10-14 yrs. old) by Region
Philippines, 2020

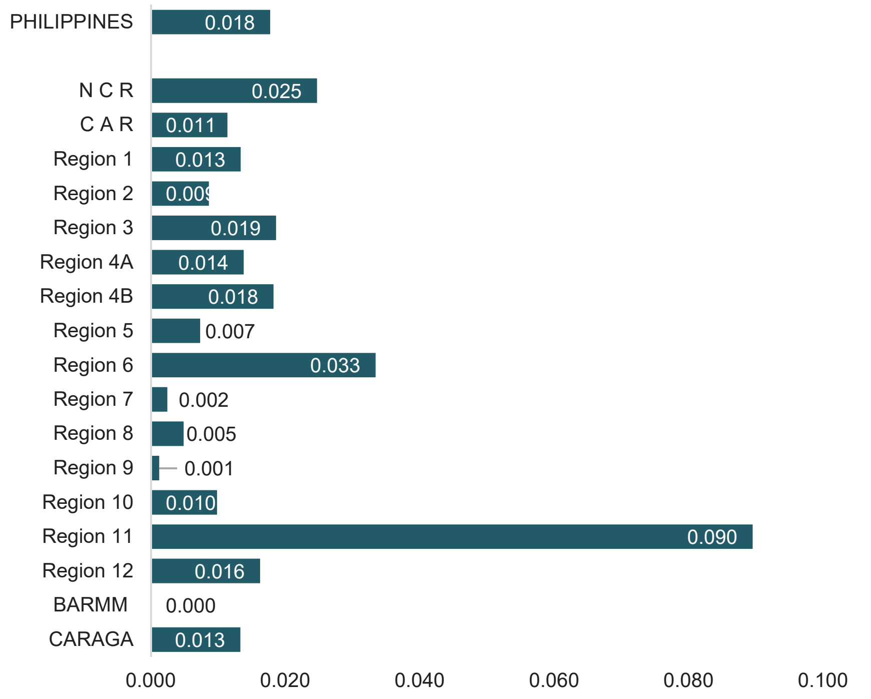

Figure 1.B.1.72 - Percentage Distribution of Pregnant Women Screened for Gestational Diabetes (15-19 yrs. old) by Region
Philippines, 2020

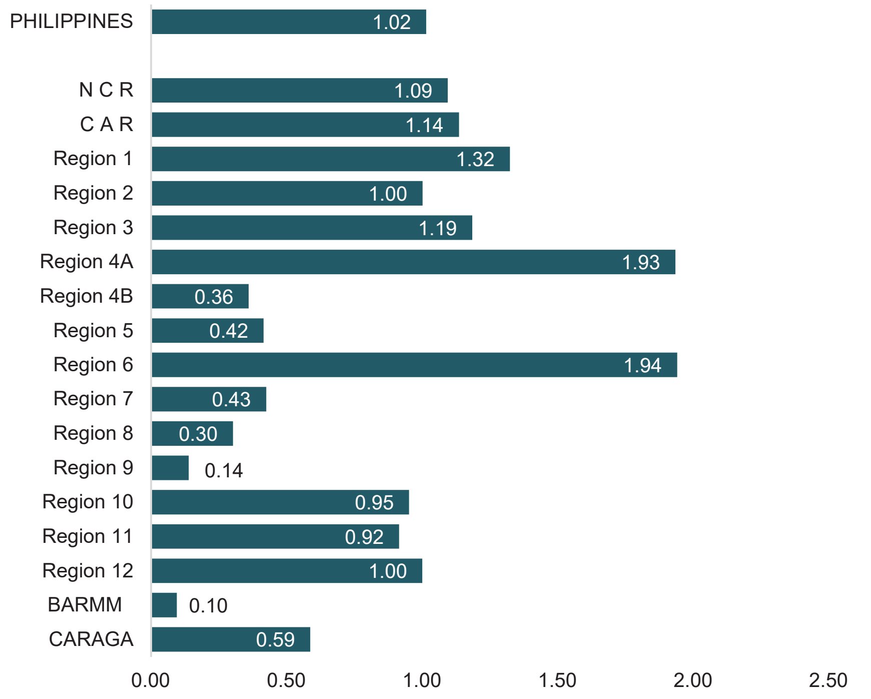

Figure 1.B.1.73 - Percentage Distribution of Pregnant Women Screened for Gestational Diabetes (20-49 yrs. old) by Region
Philippines, 2020

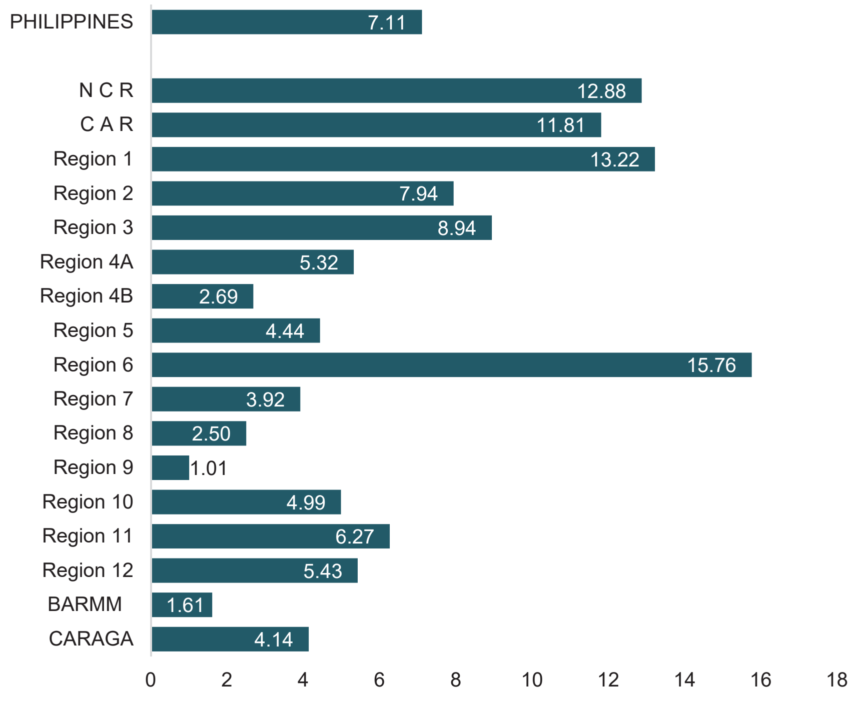

Figure 1.B.1.74 - Percentage Distribution of Pregnant Women Screened for Gestational Diabetes (10-49 yrs. old) by Region
Philippines, 2020

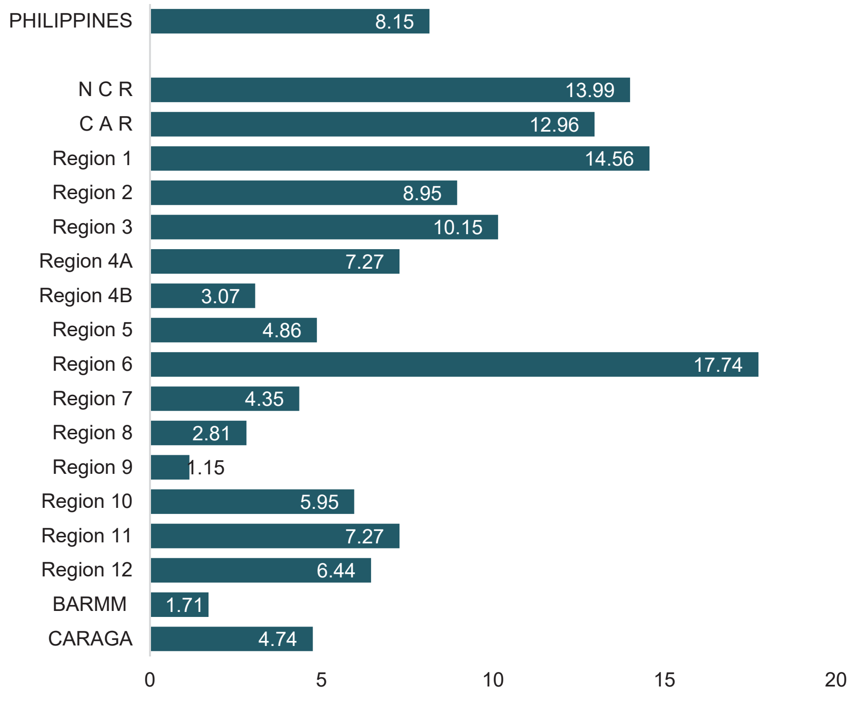

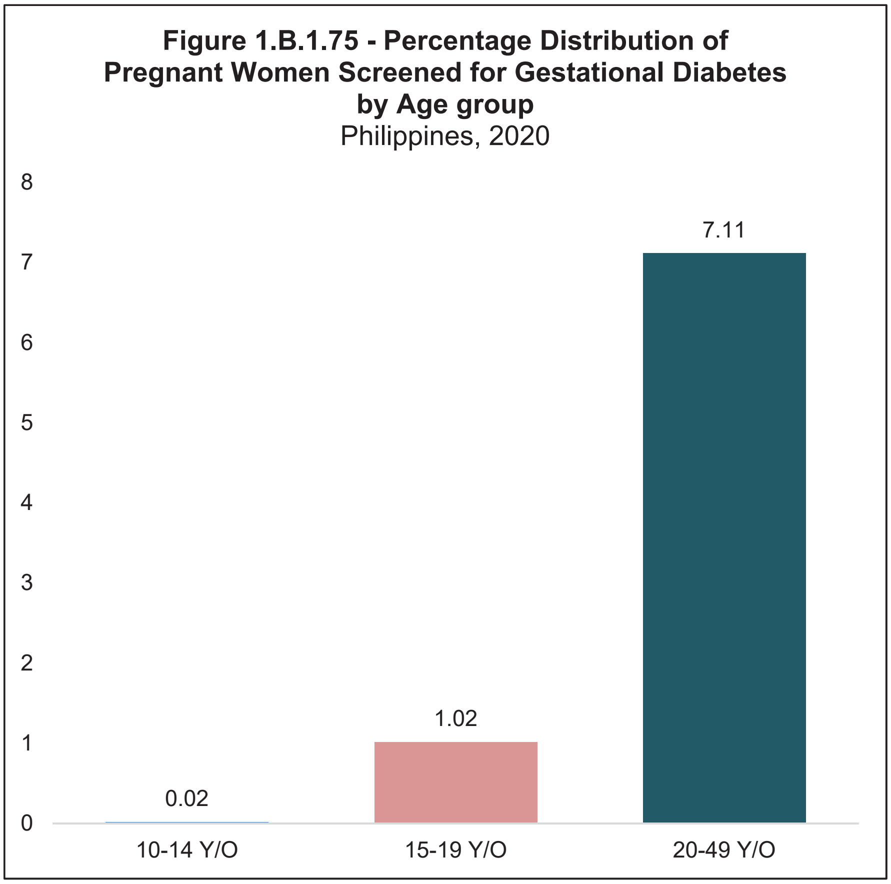
Figure 1.B.1.75 - Percentage Distribution of
Pregnant Women Screened for Gestational Diabetes
by Age group
Philippines, 2020
8
7
6
5
4
3
2
1
0
7.11
1.02
0.02
10-14 Y/O
15-19 Y/O
20-49 Y/O

Figure 1.B.1.76 - Percentage Distribution of Pregnant Women Tested Positive for Gestational Diabetes (10-14 yrs. old) by Region
Philippines, 2020

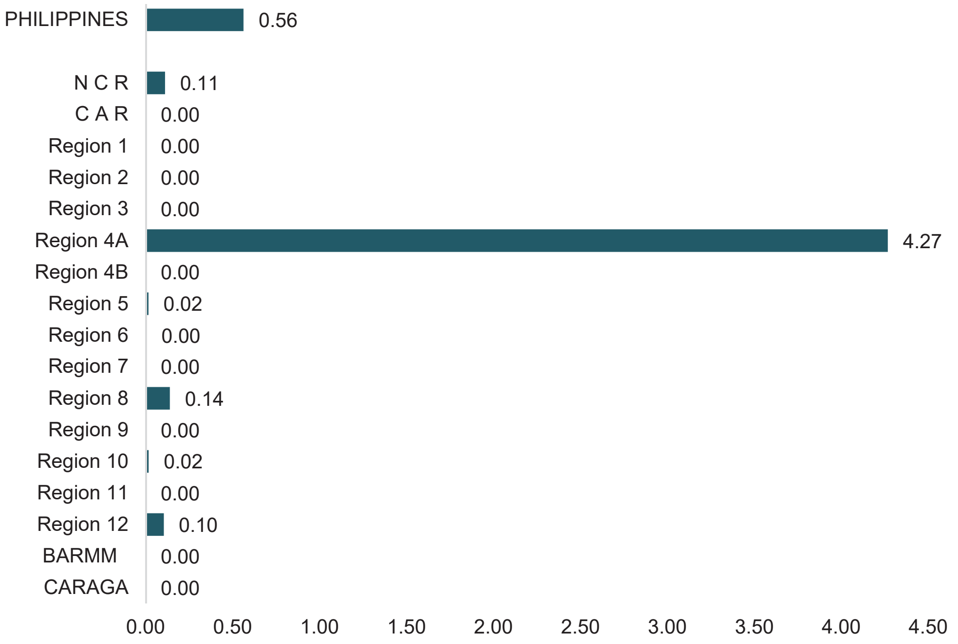

Figure 1.B.1.77 - Percentage Distribution of Pregnant Women Tested Positive for Gestational Diabetes (15-19 yrs. old) by Region
Philippines, 2020

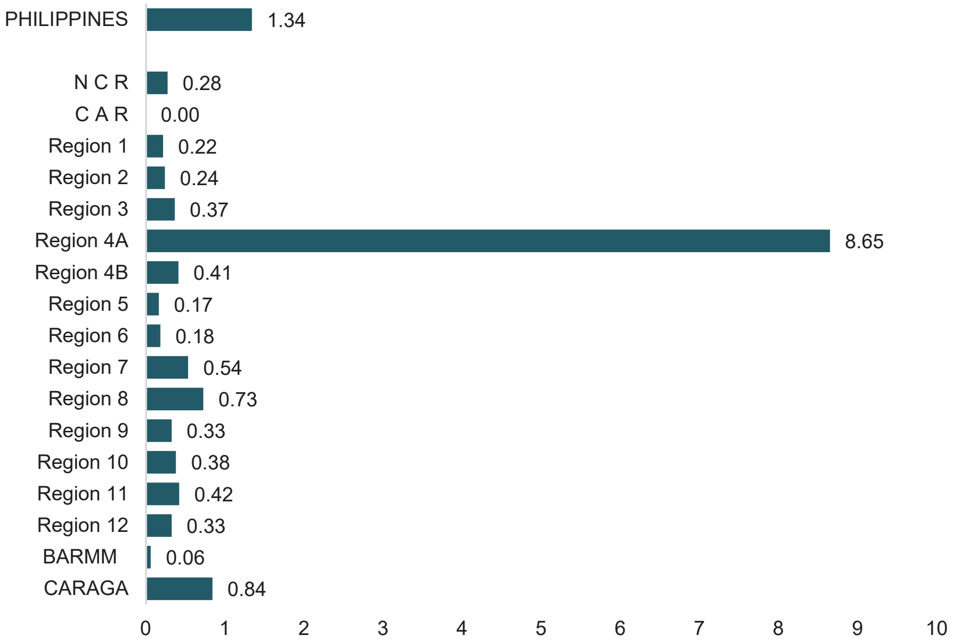

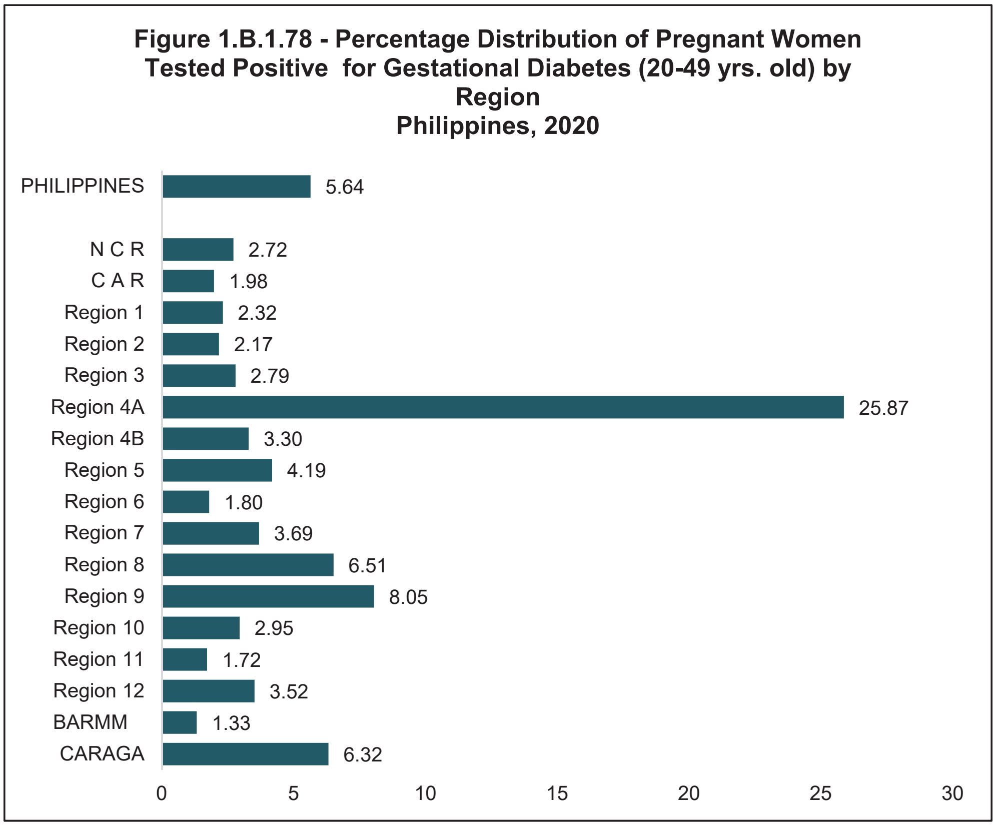
Figure 1.B.1.78 - Percentage Distribution of Pregnant Women Tested Positive for Gestational Diabetes (20-49 yrs. old) by Region
Philippines, 2020
PHILIPPINES 5.64
N C R 2.72
C A R 1.98
Region 1 2.32
Region 2 2.17
Region 3 2.79
Region 4A 25.87
Region 4B 3.30
Region 5 4.19
Region 6 1.80
Region 7 3.69
Region 8 6.51
Region 9 8.05
Region 10 2.95
Region 11 1.72
Region 12 3.52
BARMM 1.33
CARAGA 6.32
0 5 10 15 20 25 30

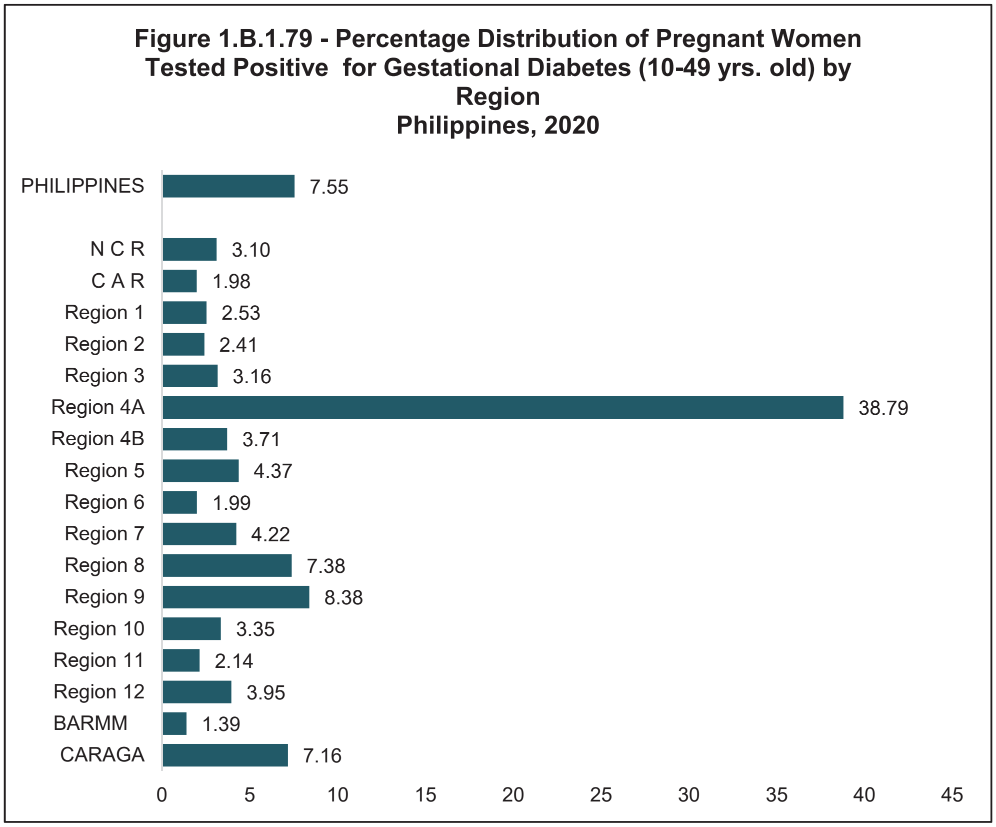
Figure 1.B.1.79 - Percentage Distribution of Pregnant Women Tested Positive for Gestational Diabetes (10-49 yrs. old) by Region
Philippines, 2020
PHILIPPINES 7.55
N C R 3.10
C A R 1.98
Region 1 2.53
Region 2 2.41
Region 3 3.16
Region 4A 38.79
Region 4B 3.71
Region 5 4.37
Region 6 1.99
Region 7 4.22
Region 8 7.38
Region 9 8.38
Region 10 3.35
Region 11 2.14
Region 12 3.95
BARMM 1.39
CARAGA 7.16
0 5 10 15 20 25 30 35 40 45

Figure 1.B.1.80 - Percentage Distribution of Pregnant Women Tested Positive for Gestational Diabetes by Age group Philippines, 2020

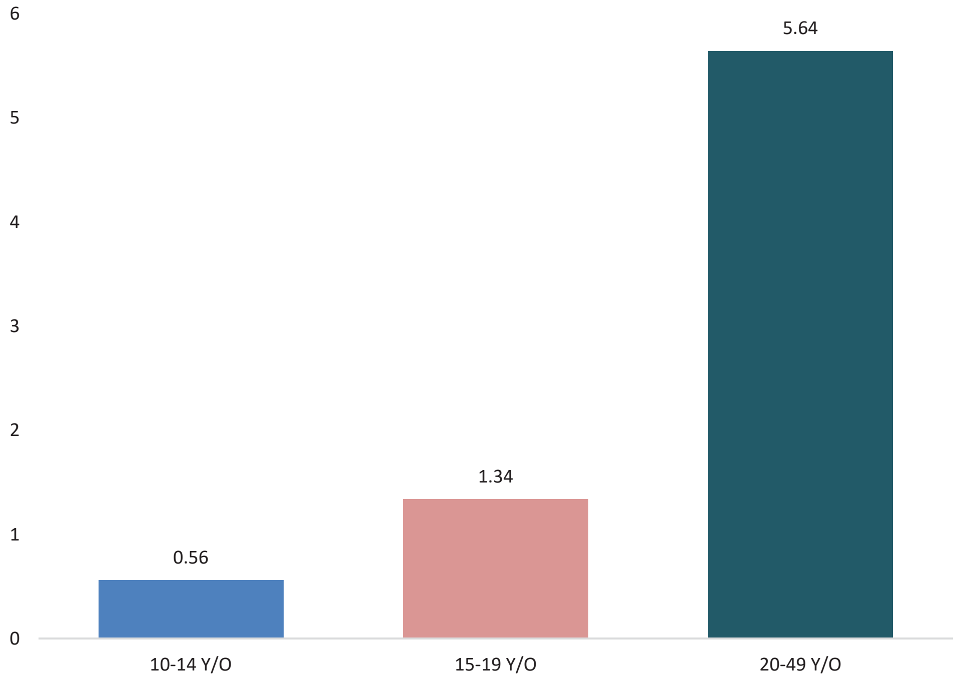

Table 1.B.2.1 - Intrapartum Care and Delivery Outcome

Total Number of women who delivered a live baby or stillbirth/fetal death, deliveries attended by skilled health professionals & deliveries in health facilities
Philippines, Annual 2020

Area	Total Deliveries				Skilled Health Professional								Facility Based Delivery					
	Total Number women who delivered				Deliveries attended by skilled health professional								Type of Health Facility				Total	%
	Age Group (in Years)			Total	MD	%	Nurses	%	Midwives	%	Total	%	Public	%	Private	%		
	10-14	15-19	20-49															
**PHILIPPINES**	**2,534**	**140,048**	**1,290,768**	**1,433,350**	**806,246**	**56.25**	**20,686**	**1.44**	**465,245**	**32.46**	**1,292,177**	**90.15**	**837,345**	**58.42**	**444,223**	**30.99**	**1,281,568**	**89.41**
**N C R**	**391**	**18,961**	**185,988**	**205,340**	**139,165**	**67.77**	**605**	**0.29**	**52,525**	**25.58**	**192,295**	**93.65**	**101,600**	**49.48**	**89,204**	**43.44**	**190,804**	**92.92**
Malabon	5	490	3,508	**4,003**	2,201	**54.98**	1	**0.02**	1,684	**42.07**	**3,886**	**97.08**	2,114	**52.81**	1,745	**43.59**	**3,859**	**96.40**
Navotas	2	530	3,733	**4,265**	2,253	**52.83**	0	**0.00**	2,009	**47.10**	**4,262**	**99.93**	2,384	**55.90**	1,862	**43.66**	**4,246**	**99.55**
Valenzuela City	12	357	5,335	**5,704**	3,973	**69.65**	27	**0.47**	1,489	**26.10**	**5,489**	**96.23**	3,696	**64.80**	1,646	**28.86**	**5,342**	**93.65**
Caloocan City	19	1,548	17,843	**19,410**	7,832	**40.35**	40	**0.21**	11,423	**58.85**	**19,295**	**99.41**	7,058	**36.36**	12,085	**62.26**	**19,143**	**98.62**
Marikina City	17	982	8,872	**9,871**	6,406	**64.90**	285	**2.89**	2,936	**29.74**	**9,627**	**97.53**	3,616	**36.63**	5,933	**60.11**	**9,549**	**96.74**
Pasig City	29	1,227	13,368	**14,624**	10,844	**74.15**	69	**0.47**	2,393	**16.36**	**13,306**	**90.99**	6,771	**46.30**	7,279	**49.77**	**14,050**	**96.07**
Pateros	0	39	1,296	**1,335**	761	**57.00**	0	**0.00**	573	**42.92**	**1,334**	**99.93**	0	**0.00**	1,325	**99.25**	**1,325**	**99.25**
Taguig	111	1,398	12,896	**14,405**	6,039	**41.92**	23	**0.16**	4,306	**29.89**	**10,368**	**71.98**	7,972	**55.34**	5,079	**35.26**	**13,051**	**90.60**
Quezon City	80	4,326	50,123	**54,529**	37,263	**68.34**	16	**0.03**	14,735	**27.02**	**52,014**	**95.39**	23,367	**42.85**	28,111	**51.55**	**51,478**	**94.40**
Makati City	8	361	6,527	**6,896**	6,092	**88.34**	4	**0.06**	626	**9.08**	**6,722**	**97.48**	3,451	**50.04**	3,258	**47.24**	**6,709**	**97.29**
Mandaluyong City	5	401	3,769	**4,175**	3,152	**75.50**	6	**0.14**	1,007	**24.12**	**4,165**	**99.76**	3,521	**84.34**	631	**15.11**	**4,152**	**99.45**
San Juan	0	66	1,416	**1,482**	1,422	**95.95**	40	**2.70**	14	**0.94**	**1,476**	**99.60**	500	**33.74**	963	**64.98**	**1,463**	**98.72**
Manila City	69	4,926	34,707	**39,702**	38,330	**96.54**	10	**0.03**	1,304	**3.28**	**39,644**	**99.85**	26,426	**66.56**	13,117	**33.04**	**39,543**	**99.60**
Las Piñas City	16	698	6,565	**7,279**	2,529	**34.74**	42	**0.58**	4,351	**59.77**	**6,922**	**95.10**	3,891	**53.46**	2,919	**40.10**	**6,810**	**93.56**
Muntinlupa City	7	846	6,273	**7,126**	2,380	**33.40**	4	**0.06**	1,177	**16.52**	**3,561**	**49.97**	0	**0.00**	0	**0.00**	**0**	**0.00**
Parañaque City	7	410	4,251	**4,668**	3,045	**65.23**	10	**0.21**	1,317	**28.21**	**4,372**	**93.66**	2,300	**49.27**	1,952	**41.82**	**4,252**	**91.09**
Pasay City	4	356	5,506	**5,866**	4,643	**79.15**	28	**0.48**	1,181	**20.13**	**5,852**	**99.76**	4,533	**77.28**	1,299	**22.14**	**5,832**	**99.42**
**C A R**	**20**	**2,404**	**23,890**	**26,314**	**22,674**	**86.17**	**518**	**1.97**	**2,587**	**9.83**	**25,779**	**97.97**	**19,540**	**74.26**	**5,253**	**19.96**	**24,793**	**94.22**
Abra	0	386	2,702	**3,088**	2,605	**84.36**	118	**3.82**	291	**9.42**	**3,014**	**97.60**	2,853	**92.39**	83	**2.69**	**2,936**	**95.08**
Apayao	4	377	1,963	**2,344**	2,171	**92.62**	22	**0.94**	91	**3.88**	**2,284**	**97.44**	1,743	**74.36**	153	**6.53**	**1,896**	**80.89**
Benguet	3	358	4,635	**4,996**	4,529	**90.65**	26	**0.52**	273	**5.46**	**4,828**	**96.64**	3,462	**69.30**	1,318	**26.38**	**4,780**	**95.68**
Ifugao	3	326	2,521	**2,850**	2,069	**72.60**	110	**3.86**	558	**19.58**	**2,737**	**96.04**	2,476	**86.88**	170	**5.96**	**2,646**	**92.84**
Kalinga	1	224	2,557	**2,782**	1,627	**58.48**	111	**3.99**	1,021	**36.70**	**2,759**	**99.17**	1,938	**69.66**	724	**26.02**	**2,662**	**95.69**
Mt. Province	1	296	2,551	**2,848**	2,377	**83.46**	131	**4.60**	252	**8.85**	**2,760**	**96.91**	2,416	**84.83**	70	**2.46**	**2,486**	**87.29**
Baguio City	8	437	6,961	**7,406**	7,296	**98.51**	0	**0.00**	101	**1.36**	**7,397**	**99.88**	4,652	**62.81**	2,735	**36.93**	**7,387**	**99.74**
**Region 1**	**60**	**4,999**	**50,436**	**55,495**	**44,004**	**79.29**	**138**	**0.25**	**11,187**	**20.16**	**55,329**	**99.70**	**41,782**	**75.29**	**13,453**	**24.24**	**55,235**	**99.53**
Ilocos Norte	9	468	5,199	**5,676**	5,400	**95.14**	9	**0.16**	267	**4.70**	**5,676**	**100.00**	4,820	**84.92**	848	**14.94**	**5,668**	**99.86**
Ilocos Sur	0	343	4,171	**4,514**	4,302	**95.30**	4	**0.09**	200	**4.43**	**4,506**	**99.82**	2,732	**60.52**	1,764	**39.08**	**4,496**	**99.60**
La Union	5	334	3,217	**3,556**	2,680	**75.37**	10	**0.28**	818	**23.00**	**3,508**	**98.65**	3,321	**93.39**	173	**4.87**	**3,494**	**98.26**
Pangasinan	11	1,438	14,459	**15,908**	9,175	**57.68**	76	**0.48**	6,631	**41.68**	**15,882**	**99.84**	13,392	**84.18**	2,456	**15.44**	**15,848**	**99.62**
Alaminos City	1	267	1,462	**1,730**	1,730	**100.00**	0	**0.00**	0	**0.00**	**1,730**	**100.00**	1,429	**82.60**	301	**17.40**	**1,730**	**100.00**
Candon City	0	8	1,010	**1,018**	949	**93.22**	5	**0.49**	64	**6.29**	**1,018**	**100.00**	222	**21.81**	796	**78.19**	**1,018**	**100.00**
Dagupan City	9	821	7,924	**8,754**	6,778	**77.43**	0	**0.00**	1,944	**22.21**	**8,722**	**99.63**	4,514	**51.56**	4,186	**47.82**	**8,700**	**99.38**
Laoag City	0	56	1,904	**1,960**	1,960	**100.00**	0	**0.00**	0	**0.00**	**1,960**	**100.00**	1,928	**98.37**	32	**1.63**	**1,960**	**100.00**
San Carlos City	4	257	2,626	**2,887**	2,272	**78.70**	3	**0.10**	560	**19.40**	**2,835**	**98.20**	1,444	**50.02**	1,385	**47.97**	**2,829**	**97.99**
San Fernando City	18	533	5,033	**5,584**	5,499	**98.48**	27	**0.48**	58	**1.04**	**5,584**	**100.00**	4,742	**84.92**	842	**15.08**	**5,584**	**100.00**
Urdaneta City	0	274	1,813	**2,087**	1,444	**69.19**	2	**0.10**	641	**30.71**	**2,087**	**100.00**	1,443	**69.14**	644	**30.86**	**2,087**	**100.00**
Vigan City	3	200	1,618	**1,821**	1,815	**99.67**	2	**0.11**	4	**0.22**	**1,821**	**100.00**	1,795	**98.57**	26	**1.43**	**1,821**	**100.00**
**Region 2**	**55**	**5,241**	**37,580**	**42,876**	**30,986**	**72.27**	**448**	**1.04**	**10,563**	**24.64**	**41,997**	**97.95**	**32,224**	**75.16**	**9,431**	**22.00**	**41,655**	**97.15**
Batanes	0	25	226	**251**	241	**96.02**	5	**1.99**	4	**1.59**	**250**	**99.60**	246	**98.01**	0	**0.00**	**246**	**98.01**
Cagayan	6	1,168	8,630	**9,804**	6,414	**65.42**	189	**1.93**	2,922	**29.80**	**9,525**	**97.15**	8,126	**82.88**	1,192	**12.16**	**9,318**	**95.04**
Isabela	18	1,596	10,883	**12,497**	8,348	**66.80**	203	**1.62**	3,717	**29.74**	**12,268**	**98.17**	7,633	**61.08**	4,546	**36.38**	**12,179**	**97.46**
Nueva Vizcaya	17	1,026	6,524	**7,567**	6,186	**81.75**	29	**0.38**	1,054	**13.93**	**7,269**	**96.06**	6,983	**92.28**	274	**3.62**	**7,257**	**95.90**
Quirino	1	391	2,634	**3,026**	2,739	**90.52**	14	**0.46**	237	**7.83**	**2,990**	**98.81**	2,923	**96.60**	61	**2.02**	**2,984**	**98.61**
Cauayan City	4	286	1,666	**1,956**	1,501	**76.74**	0	**0.00**	432	**22.09**	**1,933**	**98.82**	1,461	**74.69**	460	**23.52**	**1,921**	**98.21**
Ilagan City	4	268	2,523	**2,795**	2,745	**98.21**	0	**0.00**	49	**1.75**	**2,794**	**99.96**	2,046	**73.20**	737	**26.37**	**2,783**	**99.57**
Santiago City	4	351	2,583	**2,938**	1,177	**40.06**	3	**0.10**	1,756	**59.77**	**2,936**	**99.93**	1,409	**47.96**	1,524	**51.87**	**2,933**	**99.83**
Tuguegarao City	1	130	1,911	**2,042**	1,635	**80.07**	5	**0.24**	392	**19.20**	**2,032**	**99.51**	1,397	**68.41**	637	**31.19**	**2,034**	**99.61**
**Region 3**	**227**	**12,597**	**117,591**	**130,415**	**81,414**	**62.43**	**707**	**0.54**	**44,503**	**34.12**	**126,624**	**97.09**	**67,619**	**51.85**	**58,378**	**44.76**	**125,997**	**96.61**
Aurora	3	443	2,891	**3,337**	2,395	**71.77**	32	**0.96**	685	**20.53**	**3,112**	**93.26**	2,648	**79.35**	432	**12.95**	**3,080**	**92.30**
Bataan	11	757	5,538	**6,306**	4,123	**65.38**	41	**0.65**	1,997	**31.67**	**6,161**	**97.70**	4,681	**74.23**	1,361	**21.58**	**6,042**	**95.81**
Bulacan	27	1,765	18,594	**20,386**	7,846	**38.49**	107	**0.52**	11,413	**55.98**	**19,366**	**95.00**	2,885	**14.15**	16,531	**81.09**	**19,416**	**95.24**
Nueva Ecija	66	1,143	14,001	**15,210**	10,569	**69.49**	11	**0.07**	3,736	**24.56**	**14,316**	**94.12**	8,053	**52.95**	6,160	**40.50**	**14,213**	**93.45**
Pampanga	16	1,535	11,247	**12,798**	11,913	**93.08**	5	**0.04**	863	**6.74**	**12,781**	**99.87**	11,794	**92.16**	983	**7.68**	**12,777**	**99.84**
Tarlac	31	1,219	12,188	**13,438**	7,633	**56.80**	137	**1.02**	5,490	**40.85**	**13,260**	**98.68**	8,908	**66.29**	4,289	**31.92**	**13,197**	**98.21**
Zambales	6	794	6,658	**7,458**	5,004	**67.10**	12	**0.16**	2,442	**32.74**	**7,458**	**100.00**	7,180	**96.27**	278	**3.73**	**7,458**	**100.00**
Angeles City	7	553	6,808	**7,368**	3,913	**53.11**	2	**0.03**	3,401	**46.16**	**7,316**	**99.29**	1,089	**14.78**	6,182	**83.90**	**7,271**	**98.68**
Balanga City	5	204	1,239	**1,448**	1,175	**81.15**	14	**0.97**	251	**17.33**	**1,440**	**99.45**	964	**66.57**	475	**32.80**	**1,439**	**99.38**
Cabanatuan City	9	553	7,421	**7,983**	7,390	**92.57**	0	**0.00**	461	**5.77**	**7,851**	**98.35**	6,831	**85.57**	977	**12.24**	**7,808**	**97.81**
City of San Fernando	0	156	2,088	**2,244**	1,028	**45.81**	11	**0.49**	1,010	**45.01**	**2,049**	**91.31**	1,336	**59.54**	641	**28.57**	**1,977**	**88.10**
Gapan City	1	174	985	**1,160**	810	**69.83**	0	**0.00**	350	**30.17**	**1,160**	**100.00**	564	**48.62**	596	**51.38**	**1,160**	**100.00**
Mabalacat City	9	443	4,180	**4,632**	2,618	**56.52**	12	**0.26**	1,889	**40.78**	**4,519**	**97.56**	1,341	**28.95**	3,145	**67.90**	**4,486**	**96.85**
Malolos City	0	121	4,674	**4,795**	3,897	**81.27**	6	**0.13**	859	**17.91**	**4,762**	**99.31**	32	**0.67**	4,642	**96.81**	**4,674**	**97.48**
Meycauayan	0	190	2,346	**2,536**	457	**18.02**	0	**0.00**	2,047	**80.72**	**2,504**	**98.74**	108	**4.26**	2,378	**93.77**	**2,486**	**98.03**
Olongapo	8	356	3,871	**4,235**	2,625	**61.98**	35	**0.83**	912	**21.53**	**3,572**	**84.34**	1,875	**44.27**	1,661	**39.22**	**3,536**	**83.49**

Table 1.B.2.1 - Intrapartum Care and Delivery Outcome

Total Number of women who delivered a live baby or stillbirth/fetal death, deliveries attended by skilled health professionals & deliveries in health facilities

Philippines, Annual 2020

Area	Total Deliveries				Skilled Health Professional								Facility Based Delivery					
	Total Number women who delivered				Deliveries attended by skilled health professional								Type of Health Facility				Total	%
	Age Group (in Years)			Total	MD	%	Nurses	%	Midwives	%	Total	%	Public	%	Private	%		
	10-14	15-19	20-49															
Palayan City	0	0	188	**188**	0	**0.00**	0	**0.00**	168	**89.36**	**168**	**89.36**	168	**89.36**	0	**0.00**	**168**	**89.36**
San Jose City	9	342	1,851	**2,202**	877	**39.83**	154	**6.99**	1,146	**52.04**	**2,177**	**98.86**	1,500	**68.12**	672	**30.52**	**2,172**	**98.64**
San Jose del Monte City	14	938	5,663	**6,615**	3,722	**56.27**	0	**0.00**	2,892	**43.72**	**6,614**	**99.98**	2,610	**39.46**	3,997	**60.42**	**6,607**	**99.88**
Science City of Munoz	0	10	70	**80**	0	**0.00**	0	**0.00**	45	**56.25**	**45**	**56.25**	16	**20.00**	26	**32.50**	**42**	**52.50**
Tarlac City	5	901	5,090	**5,996**	3,419	**57.02**	128	**2.13**	2,446	**40.79**	**5,993**	**99.95**	3,036	**50.63**	2,952	**49.23**	**5,988**	**99.87**
**Region 4A**	**182**	**13,744**	**153,162**	**167,088**	**84,109**	**50.34**	**2,942**	**1.76**	**71,334**	**42.69**	**158,385**	**94.79**	**68,292**	**40.87**	**85,244**	**51.02**	**153,536**	**91.89**
Batangas	16	1,185	14,896	**16,097**	12,101	**75.18**	68	**0.42**	3,314	**20.59**	**15,483**	**96.19**	6,162	**38.28**	8,796	**54.64**	**14,958**	**92.92**
Cavite	29	1,932	21,329	**23,290**	8,843	**37.97**	118	**0.51**	13,207	**56.71**	**22,168**	**95.18**	6,179	**26.53**	15,481	**66.47**	**21,660**	**93.00**
Laguna	27	1,265	9,511	**10,803**	8,034	**74.37**	158	**1.46**	2,011	**18.62**	**10,203**	**94.45**	8,047	**74.49**	1,806	**16.72**	**9,853**	**91.21**
Quezon	19	1,466	14,168	**15,653**	8,045	**51.40**	164	**1.05**	5,383	**34.39**	**13,592**	**86.83**	9,845	**62.90**	3,514	**22.45**	**13,359**	**85.34**
Rizal	16	2,552	20,578	**23,146**	12,516	**54.07**	172	**0.74**	9,413	**40.67**	**22,101**	**95.49**	11,390	**49.21**	10,119	**43.72**	**21,509**	**92.93**
Antipolo City	22	355	9,574	**9,951**	5,701	**57.29**	124	**1.25**	3,040	**30.55**	**8,865**	**89.09**	6,169	**61.99**	2,126	**21.36**	**8,295**	**83.36**
Bacoor City	13	409	6,424	**6,846**	2,739	**40.01**	36	**0.53**	3,481	**50.85**	**6,256**	**91.38**	1,154	**16.86**	5,080	**74.20**	**6,234**	**91.06**
Batangas City	0	0	0	**0**	0	**0.00**	0	**0.00**	0	**0.00**	**0**	**0.00**	0	**0.00**	0	**0.00**	**0**	**0.00**
Biñan City	2	911	7,397	**8,310**	2,867	**34.50**	217	**2.61**	5,039	**60.64**	**8,123**	**97.75**	2,345	**28.22**	5,581	**67.16**	**7,926**	**95.38**
Cabuyao City	1	321	3,513	**3,835**	515	**13.43**	18	**0.47**	2,905	**75.75**	**3,438**	**89.65**	695	**18.12**	2,731	**71.21**	**3,426**	**89.34**
Calamba City	6	860	7,196	**8,062**	3,868	**47.98**	45	**0.56**	3,903	**48.41**	**7,816**	**96.95**	4,143	**51.39**	3,564	**44.21**	**7,707**	**95.60**
Cavite City	0	35	1,070	**1,105**	729	**65.97**	15	**1.36**	352	**31.86**	**1,096**	**99.19**	284	**25.70**	802	**72.58**	**1,086**	**98.28**
Dasmariñas City	4	596	7,971	**8,571**	3,723	**43.44**	368	**4.29**	4,480	**52.27**	**8,571**	**100.00**	1,854	**21.63**	6,704	**78.22**	**8,558**	**99.85**
General Trias City	2	109	1,142	**1,253**	532	**42.46**	15	**1.20**	592	**47.25**	**1,139**	**90.90**	128	**10.22**	1,082	**86.35**	**1,210**	**96.57**
Imus City	1	184	4,816	**5,001**	2,270	**45.39**	89	**1.78**	1,659	**33.17**	**4,018**	**80.34**	953	**19.06**	2,966	**59.31**	**3,919**	**78.36**
Lipa City	0	0	0	**0**	0	**0.00**	0	**0.00**	0	**0.00**	**0**	**0.00**	0	**0.00**	0	**0.00**	**0**	**0.00**
Lucena City	0	0	0	**0**	0	**0.00**	0	**0.00**	0	**0.00**	**0**	**0.00**	0	**0.00**	0	**0.00**	**0**	**0.00**
San Pablo City	0	28	301	**329**	448	**136.17**	1,307	**397.26**	177	**53.80**	**1,932**	**587.23**	30	**9.12**	421	**127.96**	**451**	**137.08**
San Pedro City	0	145	1,195	**1,340**	516	**38.51**	16	**1.19**	788	**58.81**	**1,320**	**98.51**	778	**58.06**	437	**32.61**	**1,215**	**90.67**
Santa Rosa City	7	764	7,514	**8,285**	3,607	**43.54**	3	**0.04**	4,016	**48.47**	**7,626**	**92.05**	3,273	**39.51**	4,302	**51.93**	**7,575**	**91.43**
Tagaytay City	0	84	2,736	**2,820**	1,344	**47.66**	0	**0.00**	1,462	**51.84**	**2,806**	**99.50**	72	**2.55**	2,702	**95.82**	**2,774**	**98.37**
Tanauan City	11	105	4,269	**4,385**	2,596	**59.20**	9	**0.21**	1,557	**35.51**	**4,162**	**94.91**	1,415	**32.27**	2,708	**61.76**	**4,123**	**94.03**
Tayabas City	0	0	1,594	**1,594**	357	**22.40**	0	**0.00**	1,221	**76.60**	**1,578**	**99.00**	946	**59.35**	632	**39.65**	**1,578**	**99.00**
Trece Martires City	6	438	5,968	**6,412**	2,758	**43.01**	0	**0.00**	3,334	**52.00**	**6,092**	**95.01**	2,430	**37.90**	3,690	**57.55**	**6,120**	**95.45**
**Region 4B**	**71**	**3,165**	**40,899**	**44,135**	**24,478**	**55.46**	**875**	**1.98**	**10,444**	**23.66**	**35,797**	**81.11**	**27,142**	**61.50**	**6,394**	**14.49**	**33,536**	**75.99**
Marinduque	2	358	2,912	**3,272**	2,763	**84.44**	71	**2.17**	384	**11.74**	**3,218**	**98.35**	3,215	**98.26**	3	**0.09**	**3,218**	**98.35**
Mindoro Occidental	23	1,001	6,951	**7,975**	4,203	**52.70**	77	**0.97**	1,856	**23.27**	**6,136**	**76.94**	5,608	**70.32**	490	**6.14**	**6,098**	**76.46**
Mindoro Oriental	21	367	11,892	**12,280**	9,101	**74.11**	144	**1.17**	1,791	**14.58**	**11,036**	**89.87**	6,816	**55.50**	4,210	**34.28**	**11,026**	**89.79**
Palawan	19	956	13,714	**14,689**	5,721	**38.95**	521	**3.55**	4,519	**30.76**	**10,761**	**73.26**	9,332	**63.53**	1,173	**7.99**	**10,505**	**71.52**
Romblon	6	94	2,071	**2,171**	930	**42.84**	21	**0.97**	74	**3.41**	**1,025**	**47.21**	1,836	**84.57**	135	**6.22**	**1,971**	**90.79**
Puerto Princesa City	0	389	3,359	**3,748**	1,760	**46.96**	41	**1.09**	1,820	**48.56**	**3,621**	**96.61**	335	**8.94**	383	**10.22**	**718**	**19.16**
**Region 5**	**56**	**7,828**	**71,785**	**79,669**	**30,602**	**38.41**	**6,467**	**8.12**	**37,503**	**47.07**	**74,572**	**93.60**	**49,225**	**61.79**	**24,878**	**31.23**	**74,103**	**93.01**
Albay	5	738	11,408	**12,151**	5,426	**44.65**	435	**3.58**	5,826	**47.95**	**11,687**	**96.18**	6,743	**55.49**	4,933	**40.60**	**11,676**	**96.09**
Camarines Norte	11	1,333	10,100	**11,444**	2,272	**19.85**	3,944	**34.46**	4,810	**42.03**	**11,026**	**96.35**	7,801	**68.17**	3,225	**28.18**	**11,026**	**96.35**
Camarines Sur	8	1,430	15,830	**17,268**	3,669	**21.25**	727	**4.21**	10,053	**58.22**	**14,449**	**83.68**	5,989	**34.68**	8,266	**47.87**	**14,255**	**82.55**
Catanduanes	1	478	4,100	**4,579**	2,889	**63.09**	318	**6.94**	1,310	**28.61**	**4,517**	**98.65**	3,805	**83.10**	558	**12.19**	**4,363**	**95.28**
Masbate	14	1,606	11,184	**12,804**	3,442	**26.88**	630	**4.92**	7,738	**60.43**	**11,810**	**92.24**	11,250	**87.86**	530	**4.14**	**11,780**	**92.00**
Sorsogon	11	1,663	11,568	**13,242**	8,538	**64.48**	385	**2.91**	4,182	**31.58**	**13,105**	**98.97**	10,264	**77.51**	2,764	**20.87**	**13,028**	**98.38**
Iriga City	1	174	1,982	**2,157**	924	**42.84**	5	**0.23**	1,187	**55.03**	**2,116**	**98.10**	197	**9.13**	1,918	**88.92**	**2,115**	**98.05**
Legaspi City	2	177	3,197	**3,376**	1,999	**59.21**	20	**0.59**	1,334	**39.51**	**3,353**	**99.32**	1,789	**52.99**	1,564	**46.33**	**3,353**	**99.32**
Naga City	3	229	2,416	**2,648**	1,443	**54.49**	3	**0.11**	1,063	**40.14**	**2,509**	**94.75**	1,387	**52.38**	1,120	**42.30**	**2,507**	**94.68**
**Region 6**	**94**	**6,235**	**78,538**	**84,867**	**56,138**	**66.15**	**499**	**0.59**	**24,371**	**28.72**	**81,008**	**95.45**	**58,557**	**69.00**	**21,532**	**25.37**	**80,089**	**94.37**
Aklan	8	570	6,597	**7,175**	5,275	**73.52**	47	**0.66**	1,210	**16.86**	**6,532**	**91.04**	5,033	**70.15**	923	**12.86**	**5,956**	**83.01**
Antique	14	841	7,908	**8,763**	6,103	**69.65**	17	**0.19**	2,025	**23.11**	**8,145**	**92.95**	7,765	**88.61**	327	**3.73**	**8,092**	**92.34**
Capiz	6	677	8,421	**9,104**	5,815	**63.87**	63	**0.69**	2,907	**31.93**	**8,785**	**96.50**	5,273	**57.92**	3,464	**38.05**	**8,737**	**95.97**
Guimaras	2	137	1,457	**1,596**	1,432	**89.72**	1	**0.06**	143	**8.96**	**1,576**	**98.75**	1,509	**94.55**	73	**4.57**	**1,582**	**99.12**
Iloilo	12	1,547	16,316	**17,875**	13,602	**76.10**	192	**1.07**	3,618	**20.24**	**17,412**	**97.41**	16,257	**90.95**	1,119	**6.26**	**17,376**	**97.21**
Negros Occidental	8	1,844	19,676	**21,528**	8,622	**40.05**	179	**0.83**	11,557	**53.68**	**20,358**	**94.57**	15,515	**72.07**	4,262	**19.80**	**19,777**	**91.87**
Bacolod City	2	140	8,684	**8,826**	7,179	**81.34**	0	**0.00**	1,621	**18.37**	**8,800**	**99.71**	3,108	**35.21**	5,661	**64.14**	**8,769**	**99.35**
Iloilo City	42	479	9,479	**10,000**	8,110	**81.10**	0	**0.00**	1,290	**12.90**	**9,400**	**94.00**	4,097	**40.97**	5,703	**57.03**	**9,800**	**98.00**
**Region 7**	**118**	**9,696**	**119,399**	**129,213**	**56,666**	**43.85**	**346**	**0.27**	**47,366**	**36.66**	**104,378**	**80.78**	**77,854**	**60.25**	**24,389**	**18.88**	**102,243**	**79.13**
Bohol	24	2,642	18,204	**20,870**	11,981	**57.41**	129	**0.62**	7,848	**37.60**	**19,958**	**95.63**	15,029	**72.01**	4,835	**23.17**	**19,864**	**95.18**
Cebu	34	2,645	52,115	**54,794**	13,219	**24.12**	66	**0.12**	21,554	**39.34**	**34,839**	**63.58**	28,386	**51.80**	6,445	**11.76**	**34,831**	**63.57**
Negros Oriental	21	2,116	18,157	**20,294**	11,730	**57.80**	71	**0.35**	6,092	**30.02**	**17,893**	**88.17**	15,468	**76.22**	2,329	**11.48**	**17,797**	**87.70**
Siquijor	2	125	1,133	**1,260**	1,024	**81.27**	0	**0.00**	235	**18.65**	**1,259**	**99.92**	1,184	**93.97**	75	**5.95**	**1,259**	**99.92**
Cebu City	37	1,845	14,496	**16,378**	10,500	**64.11**	66	**0.40**	5,666	**34.60**	**16,232**	**99.11**	10,339	**63.13**	5,459	**33.33**	**15,798**	**96.46**
Lapu-Lapu City	0	323	5,102	**5,425**	1,843	**33.97**	12	**0.22**	3,566	**65.73**	**5,421**	**99.93**	1,022	**18.84**	4,196	**77.35**	**5,218**	**96.18**
Mandaue City	0	0	10,192	**10,192**	6,369	**62.49**	2	**0.02**	2,405	**23.60**	**8,776**	**86.11**	6,426	**63.05**	1,050	**10.30**	**7,476**	**73.35**
**Region 8**	**78**	**6,451**	**56,038**	**62,567**	**36,074**	**57.66**	**1,060**	**1.69**	**20,396**	**32.60**	**57,530**	**91.95**	**42,944**	**68.64**	**14,179**	**22.66**	**57,123**	**91.30**
Biliran	2	370	2,764	**3,136**	1,436	**45.79**	98	**3.13**	1,577	**50.29**	**3,111**	**99.20**	3,077	**98.12**	18	**0.57**	**3,095**	**98.69**
Eastern Samar	6	715	5,813	**6,534**	4,923	**75.34**	84	**1.29**	1,181	**18.07**	**6,188**	**94.70**	4,033	**61.72**	2,157	**33.01**	**6,190**	**94.74**
Northern Leyte	13	1,929	17,773	**19,715**	9,583	**48.61**	91	**0.46**	8,118	**41.18**	**17,792**	**90.25**	13,009	**65.99**	4,505	**22.85**	**17,514**	**88.84**

Table 1.B.2.1 - Intrapartum Care and Delivery Outcome

Total Number of women who delivered a live baby or stillbirth/fetal death, deliveries attended by skilled health professionals & deliveries in health facilities
Philippines, Annual 2020

Area	Total Deliveries				Skilled Health Professional								Facility Based Delivery					
	Total Number women who delivered				Deliveries attended by skilled health professional								Type of Health Facility				Total	%
	Age Group (in Years)			Total	MD	%	Nurses	%	Midwives	%	Total	%	Public	%	Private	%		
	10-14	15-19	20-49															
Northern Samar	25	932	8,869	**9,826**	5,434	**55.30**	53	**0.54**	2,862	**29.13**	**8,349**	**84.97**	7,478	**76.10**	721	**7.34**	**8,199**	**83.44**
Southern Leyte	1	300	2,169	**2,470**	1,994	**80.73**	32	**1.30**	432	**17.49**	**2,458**	**99.51**	2,048	**82.91**	385	**15.59**	**2,433**	**98.50**
Western Samar	23	992	7,178	**8,193**	4,407	**53.79**	390	**4.76**	2,791	**34.07**	**7,588**	**92.62**	6,829	**83.35**	716	**8.74**	**7,545**	**92.09**
Calbayog City	0	204	1,926	**2,130**	1,207	**56.67**	26	**1.22**	360	**16.90**	**1,593**	**74.79**	1,244	**58.40**	419	**19.67**	**1,663**	**78.08**
Maasin City	0	234	2,391	**2,625**	2,504	**95.39**	80	**3.05**	0	**0.00**	**2,584**	**98.44**	1,533	**58.40**	1,086	**41.37**	**2,619**	**99.77**
Ormoc City	7	471	4,730	**5,208**	2,669	**51.25**	178	**3.42**	2,311	**44.37**	**5,158**	**99.04**	1,482	**28.46**	3,665	**70.37**	**5,147**	**98.83**
Tacloban City	1	304	2,425	**2,730**	1,917	**70.22**	28	**1.03**	764	**27.99**	**2,709**	**99.23**	2,211	**80.99**	507	**18.57**	**2,718**	**99.56**
**Region 9**	**51**	**6,585**	**49,351**	**55,987**	**28,142**	**50.27**	**994**	**1.78**	**16,916**	**30.21**	**46,052**	**82.25**	**40,101**	**71.63**	**5,623**	**10.04**	**45,724**	**81.67**
Zamboanga del Norte	14	2,096	14,019	**16,129**	7,005	**43.43**	161	**1.00**	4,593	**28.48**	**11,759**	**72.91**	14,046	**87.09**	107	**0.66**	**14,153**	**87.75**
Zamboanga del Sur	12	1,193	8,423	**9,628**	3,081	**32.00**	159	**1.65**	5,248	**54.51**	**8,488**	**88.16**	3,601	**37.40**	1,844	**19.15**	**5,445**	**56.55**
Zamboanga Sibugay	13	1,153	6,520	**7,686**	2,499	**32.51**	121	**1.57**	3,463	**45.06**	**6,083**	**79.14**	5,739	**74.67**	351	**4.57**	**6,090**	**79.23**
Dapitan City	3	180	1,511	**1,694**	1,337	**78.93**	30	**1.77**	65	**3.84**	**1,432**	**84.53**	1,648	**97.28**	29	**1.71**	**1,677**	**99.00**
Dipolog City	1	354	2,310	**2,665**	2,372	**89.01**	13	**0.49**	273	**10.24**	**2,658**	**99.74**	2,628	**98.61**	25	**0.94**	**2,653**	**99.55**
Isabela City	2	275	1,584	**1,861**	880	**47.29**	35	**1.88**	164	**8.81**	**1,079**	**57.98**	1,003	**53.90**	140	**7.52**	**1,143**	**61.42**
Pagadian City	1	293	2,594	**2,888**	1,471	**50.93**	0	**0.00**	1,312	**45.43**	**2,783**	**96.36**	1,500	**51.94**	1,283	**44.43**	**2,783**	**96.36**
Zamboanga City	5	1,041	12,390	**13,436**	9,497	**70.68**	475	**3.54**	1,798	**13.38**	**11,770**	**87.60**	9,936	**73.95**	1,844	**13.72**	**11,780**	**87.67**
**Region 10**	**186**	**11,536**	**71,630**	**83,352**	**52,578**	**63.08**	**1,260**	**1.51**	**23,152**	**27.78**	**76,990**	**92.37**	**58,213**	**69.84**	**13,312**	**15.97**	**71,525**	**85.81**
Bukidnon	84	3,842	15,940	**19,866**	10,721	**53.97**	136	**0.68**	7,309	**36.79**	**18,166**	**91.44**	15,810	**79.58**	2,324	**11.70**	**18,134**	**91.28**
Camiguin	0	126	1,184	**1,310**	1,091	**83.28**	3	**0.23**	210	**16.03**	**1,304**	**99.54**	1,168	**89.16**	136	**10.38**	**1,304**	**99.54**
Lanao del Norte	14	862	9,538	**10,414**	4,858	**46.65**	279	**2.68**	2,639	**25.34**	**7,776**	**74.67**	7,077	**67.96**	776	**7.45**	**7,853**	**75.41**
Misamis Occidental	5	397	4,181	**4,583**	2,627	**57.32**	178	**3.88**	1,516	**33.08**	**4,321**	**94.28**	3,736	**81.52**	453	**9.88**	**4,189**	**91.40**
Misamis Oriental	12	1,325	8,257	**9,594**	6,090	**63.48**	365	**3.80**	2,796	**29.14**	**9,251**	**96.42**	8,061	**84.02**	1,094	**11.40**	**9,155**	**95.42**
Cagayan de Oro City	48	2,204	15,352	**17,604**	13,075	**74.27**	91	**0.52**	4,256	**24.18**	**17,422**	**98.97**	9,379	**53.28**	2,933	**16.66**	**12,312**	**69.94**
El Salvador City	0	49	277	**326**	321	**98.47**	0	**0.00**	0	**0.00**	**321**	**98.47**	321	**98.47**	0	**0.00**	**321**	**98.47**
Gingoog City	3	388	1,847	**2,238**	1,172	**52.37**	130	**5.81**	908	**40.57**	**2,210**	**98.75**	1,656	**73.99**	582	**26.01**	**2,238**	**100.00**
Iligan City	9	873	6,252	**7,134**	6,278	**88.00**	0	**0.00**	497	**6.97**	**6,775**	**94.97**	4,179	**58.58**	2,545	**35.67**	**6,724**	**94.25**
Malaybalay City	4	412	2,100	**2,516**	1,266	**50.32**	35	**1.39**	983	**39.07**	**2,284**	**90.78**	1,313	**52.19**	862	**34.26**	**2,175**	**86.45**
Oroquieta City	1	102	820	**923**	762	**82.56**	0	**0.00**	159	**17.23**	**921**	**99.78**	761	**82.45**	160	**17.33**	**921**	**99.78**
Ozamis City	1	308	1,853	**2,162**	1,604	**74.19**	0	**0.00**	538	**24.88**	**2,142**	**99.07**	1,745	**80.71**	417	**19.29**	**2,162**	**100.00**
Tangub City	0	121	925	**1,046**	763	**72.94**	42	**4.02**	158	**15.11**	**963**	**92.07**	855	**81.74**	74	**7.07**	**929**	**88.81**
Valencia City	5	527	3,104	**3,636**	1,950	**53.63**	1	**0.03**	1,183	**32.54**	**3,134**	**86.19**	2,152	**59.19**	956	**26.29**	**3,108**	**85.48**
**Region 11**	**447**	**12,213**	**72,168**	**84,828**	**51,732**	**60.98**	**227**	**0.27**	**26,247**	**30.94**	**78,206**	**92.19**	**45,137**	**53.21**	**32,556**	**38.38**	**77,693**	**91.59**
Compostela Valley	62	2,015	9,964	**12,041**	8,381	**69.60**	70	**0.58**	2,620	**21.76**	**11,071**	**91.94**	9,139	**75.90**	1,939	**16.10**	**11,078**	**92.00**
Davao del Norte	154	3,017	17,249	**20,420**	13,292	**65.09**	48	**0.24**	6,077	**29.76**	**19,417**	**95.09**	11,797	**57.77**	7,231	**35.41**	**19,028**	**93.18**
Davao Oriental	39	1,251	7,093	**8,383**	6,771	**80.77**	52	**0.62**	1,223	**14.59**	**8,046**	**95.98**	7,658	**91.35**	142	**1.69**	**7,800**	**93.05**
Davao del Sur	55	1,525	7,908	**9,488**	5,808	**61.21**	22	**0.23**	2,965	**31.25**	**8,795**	**92.70**	2,303	**24.27**	6,557	**69.11**	**8,860**	**93.38**
Davao Occidental	34	1,043	3,331	**4,408**	1,951	**44.26**	10	**0.23**	934	**21.19**	**2,895**	**65.68**	1,833	**41.58**	999	**22.66**	**2,832**	**64.25**
Davao City	103	3,362	26,623	**30,088**	15,529	**51.61**	25	**0.08**	12,428	**41.31**	**27,982**	**93.00**	12,407	**41.24**	15,688	**52.14**	**28,095**	**93.38**
**Region 12**	**142**	**9,926**	**62,090**	**72,158**	**35,133**	**48.69**	**562**	**0.78**	**29,643**	**41.08**	**65,338**	**90.55**	**43,209**	**59.88**	**21,889**	**30.33**	**65,098**	**90.22**
North Cotabato	37	2,759	17,556	**20,352**	11,110	**54.59**	165	**0.81**	5,840	**28.69**	**17,115**	**84.09**	11,226	**55.16**	5,909	**29.03**	**17,135**	**84.19**
Sarangani	27	2,035	8,245	**10,307**	3,358	**32.58**	73	**0.71**	5,918	**57.42**	**9,349**	**90.71**	7,368	**71.49**	1,875	**18.19**	**9,243**	**89.68**
South Cotabato	35	2,488	14,356	**16,879**	10,917	**64.68**	111	**0.66**	4,825	**28.59**	**15,853**	**93.92**	11,252	**66.66**	4,599	**27.25**	**15,851**	**93.91**
Sultan Kudarat	37	1,797	10,438	**12,272**	5,096	**41.53**	44	**0.36**	6,174	**50.31**	**11,314**	**92.19**	8,667	**70.62**	2,599	**21.18**	**11,266**	**91.80**
Cotabato City	3	352	5,147	**5,502**	3,404	**61.87**	0	**0.00**	1,596	**29.01**	**5,000**	**90.88**	2,735	**49.71**	2,262	**41.11**	**4,997**	**90.82**
Gen. Santos City	3	495	6,348	**6,846**	1,248	**18.23**	169	**2.47**	5,290	**77.27**	**6,707**	**97.97**	1,961	**28.64**	4,645	**67.85**	**6,606**	**96.49**
**BARMM**	**306**	**4,661**	**65,693**	**70,660**	**11,246**	**15.92**	**2,727**	**3.86**	**21,848**	**30.92**	**35,821**	**50.69**	**37,062**	**52.45**	**9,328**	**13.20**	**46,390**	**65.65**
Basilan	63	342	2,755	**3,160**	205	**6.49**	423	**13.39**	1,049	**33.20**	**1,677**	**53.07**	1,024	**32.41**	74	**2.34**	**1,098**	**34.75**
Lanao del Sur	2	993	19,730	**20,725**	1,391	**6.71**	244	**1.18**	3,011	**14.53**	**4,646**	**22.42**	14,469	**69.81**	3,487	**16.83**	**17,956**	**86.64**
Maguindanao	27	2,949	22,531	**25,507**	6,969	**27.32**	1,218	**4.78**	7,470	**29.29**	**15,657**	**61.38**	11,162	**43.76**	4,981	**19.53**	**16,143**	**63.29**
Sulu	0	0	10,140	**10,140**	886	**8.74**	140	**1.38**	4,008	**39.53**	**5,034**	**49.64**	4,213	**41.55**	22	**0.22**	**4,235**	**41.77**
Tawi-Tawi	0	0	6,946	**6,946**	446	**6.42**	631	**9.08**	4,499	**64.77**	**5,576**	**80.28**	4,008	**57.70**	5	**0.07**	**4,013**	**57.77**
Lamitan City	0	178	924	**1,102**	1	**0.09**	0	**0.00**	1,098	**99.64**	**1,099**	**99.73**	1,040	**94.37**	1	**0.09**	**1,041**	**94.46**
Marawi City	214	199	2,667	**3,080**	1,348	**43.77**	71	**2.31**	713	**23.15**	**2,132**	**69.22**	1,146	**37.21**	758	**24.61**	**1,904**	**61.82**
**CARAGA**	**50**	**3,806**	**34,530**	**38,386**	**21,105**	**54.98**	**311**	**0.81**	**14,660**	**38.19**	**36,076**	**93.98**	**26,844**	**69.93**	**9,180**	**23.91**	**36,024**	**93.85**
Agusan del Norte	8	436	2,942	**3,386**	1,783	**52.66**	4	**0.12**	1,346	**39.75**	**3,133**	**92.53**	1,661	**49.05**	1,472	**43.47**	**3,133**	**92.53**
Agusan del Sur	12	856	11,097	**11,965**	4,342	**36.29**	20	**0.17**	6,260	**52.32**	**10,622**	**88.78**	8,051	**67.29**	2,571	**21.49**	**10,622**	**88.78**
Surigao del Norte	3	403	3,126	**3,532**	1,584	**44.85**	221	**6.26**	1,557	**44.08**	**3,362**	**95.19**	3,308	**93.66**	42	**1.19**	**3,350**	**94.85**
Surigao del Sur	13	809	5,652	**6,474**	4,970	**76.77**	8	**0.12**	1,125	**17.38**	**6,103**	**94.27**	5,977	**92.32**	98	**1.51**	**6,075**	**93.84**
Province of Dinagat	3	183	1,125	**1,311**	914	**69.72**	56	**4.27**	280	**21.36**	**1,250**	**95.35**	1,248	**95.19**	2	**0.15**	**1,250**	**95.35**
Bislig City	7	207	1,452	**1,666**	1,646	**98.80**	0	**0.00**	6	**0.36**	**1,652**	**99.16**	1,510	**90.64**	142	**8.52**	**1,652**	**99.16**
Butuan City	3	538	5,123	**5,664**	2,122	**37.46**	2	**0.04**	3,473	**61.32**	**5,597**	**98.82**	1,146	**20.23**	4,441	**78.41**	**5,587**	**98.64**
Surigao City	1	374	4,013	**4,388**	3,744	**85.32**	0	**0.00**	613	**13.97**	**4,357**	**99.29**	3,943	**89.86**	412	**9.39**	**4,355**	**99.25**

Note: Put asterisk (*) for No Report and Zero (0) for No Case

Table 1.B.2.2 - Intrapartum Care and Delivery Outcome

Number and proportion of delivery by type and age group
Philippines, Annual 2020

Area	Total number of Deliveries	Delivery by Type									
		Vaginal					Cesarean Section				
		Age Group in Year			Total	%	Age Group in Year			Total	%
		10-14	15-19	20-49			10-14	15-19	20-49		
**PHILIPPINES**	**1,433,350**	**1,780**	**121,639**	**1,173,433**	**1,296,852**	**90.48**	**333**	**8,830**	**127,335**	**136,498**	**9.52**
**N C R**	**205,340**	**143**	**8,873**	**188,420**	**197,436**	**96.15**	**43**	**491**	**7,370**	**7,904**	**3.85**
Malabon	4,003	5	464	3,159	**3,628**	**90.63**	0	24	351	**375**	**9.37**
Navotas	4,265	2	495	3,311	**3,808**	**89.28**	0	21	436	**457**	**10.72**
Valenzuela City	5,704	1	54	5,593	**5,648**	**99.02**	0	8	48	**56**	**0.98**
Caloocan City	19,410	17	1,464	16,896	**18,377**	**94.68**	1	63	969	**1,033**	**5.32**
Marikina City	9,871	0	0	9,871	**9,871**	**100.00**	0	0	0	**0**	**0.00**
Pasig City	14,624	3	132	14,250	**14,385**	**98.37**	0	11	228	**239**	**1.63**
Pateros	1,335	0	34	1,226	**1,260**	**94.38**	0	17	58	**75**	**5.62**
Taguig	14,405	4	296	13,622	**13,922**	**96.65**	42	109	332	**483**	**3.35**
Quezon City	54,529	80	4,209	47,604	**51,893**	**95.17**	0	116	2,520	**2,636**	**4.83**
Makati City	6,896	0	0	6,896	**6,896**	**100.00**	0	0	0	**0**	**0.00**
Mandaluyong City	4,175	5	358	3,416	**3,779**	**90.51**	0	22	374	**396**	**9.49**
San Juan	1,482	0	41	1,204	**1,245**	**84.01**	0	4	233	**237**	**15.99**
Manila City	39,702	0	0	39,702	**39,702**	**100.00**	0	0	0	**0**	**0.00**
Las Piñas City	7,279	16	624	5,986	**6,626**	**91.03**	0	38	615	**653**	**8.97**
Muntinlupa City	7,126	0	0	7,126	**7,126**	**100.00**	0	0	0	**0**	**0.00**
Parañaque City	4,668	7	383	3,426	**3,816**	**81.75**	0	27	825	**852**	**18.25**
Pasay City	5,866	3	319	5,132	**5,454**	**92.98**	0	31	381	**412**	**7.02**
**C A R**	**26,314**	**19**	**2,002**	**18,369**	**20,390**	**77.49**	**1**	**402**	**5,521**	**5,924**	**22.51**
Abra	3,088	0	294	1,933	**2,227**	**72.12**	0	92	769	**861**	**27.88**
Apayao	2,344	4	340	1,615	**1,959**	**83.58**	0	37	348	**385**	**16.42**
Benguet	4,996	3	307	4,148	**4,458**	**89.23**	0	51	487	**538**	**10.77**
Ifugao	2,850	3	278	2,121	**2,402**	**84.28**	0	48	400	**448**	**15.72**
Kalinga	2,782	1	207	2,197	**2,405**	**86.45**	0	17	360	**377**	**13.55**
Mt. Province	2,848	1	255	1,859	**2,115**	**74.26**	0	41	692	**733**	**25.74**
Baguio City	7,406	7	321	4,496	**4,824**	**65.14**	1	116	2,465	**2,582**	**34.86**
**Region 1**	**55,495**	**46**	**4,587**	**44,588**	**49,221**	**88.69**	**14**	**412**	**5,848**	**6,274**	**11.31**
Ilocos Norte	5,676	9	390	3,926	**4,325**	**76.20**	0	78	1,273	**1,351**	**23.80**
Ilocos Sur	4,514	0	292	3,377	**3,669**	**81.28**	0	51	794	**845**	**18.72**
La Union	3,556	5	324	3,021	**3,350**	**94.21**	0	10	196	**206**	**5.79**
Pangasinan	15,908	11	1,382	13,665	**15,058**	**94.66**	0	56	794	**850**	**5.34**
Alaminos City	1,730	1	257	1,310	**1,568**	**90.64**	0	10	152	**162**	**9.36**
Candon City	1,018	0	6	775	**781**	**76.72**	0	2	235	**237**	**23.28**
Dagupan City	8,754	9	821	7,919	**8,749**	**99.94**	0	0	5	**5**	**0.06**
Laoag City	1,960	0	55	1,731	**1,786**	**91.12**	0	1	173	**174**	**8.88**
San Carlos City	2,887	4	231	2,132	**2,367**	**81.99**	0	26	494	**520**	**18.01**
San Fernando City	5,584	6	404	3,925	**4,335**	**77.63**	12	129	1,108	**1,249**	**22.37**
Urdaneta City	2,087	0	263	1,657	**1,920**	**92.00**	0	11	156	**167**	**8.00**
Vigan City	1,821	1	162	1,150	**1,313**	**72.10**	2	38	468	**508**	**27.90**

Table 1.B.2.2 - Intrapartum Care and Delivery Outcome

Number and proportion of delivery by type and age group
Philippines, Annual 2020

Area	Total number of Deliveries	Delivery by Type: Vaginal – Age Group in Year 10-14	15-19	20-49	Vaginal Total	Vaginal %	Cesarean Section – Age Group in Year 10-14	15-19	20-49	Cesarean Total	Cesarean %
**Region 2**	**42,876**	**47**	**4,512**	**30,224**	**34,783**	**81.12**	**8**	**709**	**7,376**	**8,093**	**18.88**
Batanes	251	0	24	184	**208**	**82.87**	0	1	42	**43**	**17.13**
Cagayan	9,804	6	1,108	7,753	**8,867**	**90.44**	0	67	870	**937**	**9.56**
Isabela	12,497	14	1,298	8,095	**9,407**	**75.27**	4	298	2,788	**3,090**	**24.73**
Nueva Vizcaya	7,567	15	859	5,137	**6,011**	**79.44**	2	148	1,406	**1,556**	**20.56**
Quirino	3,026	1	331	2,090	**2,422**	**80.04**	0	59	545	**604**	**19.96**
Cauayan City	1,956	3	236	1,270	**1,509**	**77.15**	1	50	396	**447**	**22.85**
Ilagan City	2,795	4	243	2,250	**2,497**	**89.34**	0	23	275	**298**	**10.66**
Santiago City	2,938	4	300	2,137	**2,441**	**83.08**	0	44	453	**497**	**16.92**
Tuguegarao City	2,042	0	113	1,308	**1,421**	**69.59**	1	19	601	**621**	**30.41**
**Region 3**	**130,415**	**207**	**10,621**	**97,004**	**107,832**	**82.68**	**44**	**1,307**	**21,232**	**22,583**	**17.32**
Aurora	3,337	3	429	2,494	**2,926**	**87.68**	0	11	400	**411**	**12.32**
Bataan	6,306	11	735	5,207	**5,953**	**94.40**	0	22	331	**353**	**5.60**
Bulacan	20,386	81	1,626	18,679	**20,386**	**100.00**	0	0	0	**0**	**0.00**
Nueva Ecija	15,210	12	771	8,823	**9,606**	**63.16**	14	120	5,470	**5,604**	**36.84**
Pampanga	12,798	14	1,098	8,887	**9,999**	**78.13**	2	276	2,521	**2,799**	**21.87**
Tarlac	13,438	16	1,089	10,392	**11,497**	**85.56**	15	130	1,796	**1,941**	**14.44**
Zambales	7,458	8	605	4,737	**5,350**	**71.74**	2	213	1,893	**2,108**	**28.26**
Angeles City	7,368	7	534	6,339	**6,880**	**93.38**	0	19	469	**488**	**6.62**
Balanga City	1,448	2	153	743	**898**	**62.02**	3	53	494	**550**	**37.98**
Cabanatuan City	7,983	3	472	5,353	**5,828**	**73.01**	6	81	2,068	**2,155**	**26.99**
City of San Fernando	2,244	0	24	1,896	**1,920**	**85.56**	0	0	324	**324**	**14.44**
Gapan City	1,160	0	98	545	**643**	**55.43**	1	79	437	**517**	**44.57**
Mabalacat City	4,632	9	443	4,180	**4,632**	**100.00**	0	0	0	**0**	**0.00**
Malolos City	4,795	0	90	1,518	**1,608**	**33.53**	0	54	3,133	**3,187**	**66.47**
Meycauayan	2,536	0	175	2,237	**2,412**	**95.11**	0	15	109	**124**	**4.89**
Olongapo	4,235	7	309	3,817	**4,133**	**97.59**	0	5	97	**102**	**2.41**
Palayan City	188	0	0	188	**188**	**100.00**	0	0	0	**0**	**0.00**
San Jose City	2,202	8	256	1,322	**1,586**	**72.03**	1	79	536	**616**	**27.97**
San Jose del Monte City	6,615	14	878	4,879	**5,771**	**87.24**	0	60	784	**844**	**12.76**
Science City of Munoz	80	0	10	70	**80**	**100.00**	0	0	0	**0**	**0.00**
Tarlac City	5,996	12	826	4,698	**5,536**	**92.33**	0	90	370	**460**	**7.67**
**Region 4A**	**167,088**	**123**	**11,523**	**130,013**	**141,659**	**84.78**	**22**	**1,516**	**23,891**	**25,429**	**15.22**
Batangas	16,097	12	952	11,076	**12,040**	**74.80**	5	203	3,849	**4,057**	**25.20**
Cavite	23,290	14	1,632	18,058	**19,704**	**84.60**	2	165	3,419	**3,586**	**15.40**
Laguna	10,803	25	1,131	7,005	**8,161**	**75.54**	4	218	2,420	**2,642**	**24.46**
Quezon	15,653	12	1,326	12,525	**13,863**	**88.56**	3	70	1,717	**1,790**	**11.44**
Rizal	23,146	32	2,099	17,331	**19,462**	**84.08**	0	243	3,441	**3,684**	**15.92**
Antipolo City	9,951	0	418	8,562	**8,980**	**90.24**	1	106	864	**971**	**9.76**
Bacoor City	6,846	2	341	5,349	**5,692**	**83.14**	2	42	1,110	**1,154**	**16.86**
Batangas City	0	0	0	0	**0**	**0.00**	0	0	0	**0**	**0.00**
Biñan City	8,310	1	669	6,648	**7,318**	**88.06**	0	158	834	**992**	**11.94**
Cabuyao City	3,835	1	270	3,231	**3,502**	**91.32**	2	37	294	**333**	**8.68**
Calamba City	8,062	5	698	6,034	**6,737**	**83.56**	2	63	1,260	**1,325**	**16.44**
Cavite City	1,105	0	32	924	**956**	**86.52**	0	1	148	**149**	**13.48**

Table 1.B.2.2 - Intrapartum Care and Delivery Outcome

Number and proportion of delivery by type and age group
Philippines, Annual 2020

Area	Total number of Deliveries	Delivery by Type									
		Vaginal					Cesarean Section				
		Age Group in Year			Total	%	Age Group in Year			Total	%
		10-14	15-19	20-49			10-14	15-19	20-49		
Dasmariñas City	8,571	4	396	7,692	**8,092**	**94.41**	0	23	456	**479**	**5.59**
General Trias City	1,253	2	111	922	**1,035**	**82.60**	0	11	207	**218**	**17.40**
Imus City	5,001	0	126	4,017	**4,143**	**82.84**	0	19	839	**858**	**17.16**
Lipa City	0	0	0	0	**0**	**0.00**	0	0	0	**0**	**0.00**
Lucena City	0	0	0	0	**0**	**0.00**	0	0	0	**0**	**0.00**
San Pablo City	329	0	27	181	**208**	**63.22**	0	10	111	**121**	**36.78**
San Pedro City	1,340	0	94	987	**1,081**	**80.67**	0	25	234	**259**	**19.33**
Santa Rosa City	8,285	6	669	7,023	**7,698**	**92.91**	1	97	489	**587**	**7.09**
Tagaytay City	2,820	0	40	2,228	**2,268**	**80.43**	0	8	544	**552**	**19.57**
Tanauan City	4,385	1	60	3,556	**3,617**	**82.49**	0	11	757	**768**	**17.51**
Tayabas City	1,594	0	0	1,346	**1,346**	**84.44**	0	0	248	**248**	**15.56**
Trece Martires City	6,412	6	432	5,318	**5,756**	**89.77**	0	6	650	**656**	**10.23**
**Region 4B**	**44,135**	**64**	**3,029**	**37,039**	**40,132**	**90.93**	**7**	**139**	**3,857**	**4,003**	**9.07**
Marinduque	3,272	2	318	2,421	**2,741**	**83.77**	0	40	491	**531**	**16.23**
Mindoro Occidental	7,975	23	983	6,633	**7,639**	**95.79**	0	21	315	**336**	**4.21**
Mindoro Oriental	12,280	15	327	10,137	**10,479**	**85.33**	6	40	1,755	**1,801**	**14.67**
Palawan	14,689	19	923	12,804	**13,746**	**93.58**	0	33	910	**943**	**6.42**
Romblon	2,171	5	90	1,916	**2,011**	**92.63**	1	4	155	**160**	**7.37**
Puerto Princesa City	3,748	0	388	3,128	**3,516**	**93.81**	0	1	231	**232**	**6.19**
**Region 5**	**79,669**	**51**	**7,456**	**66,495**	**74,002**	**92.89**	**3**	**278**	**5,386**	**5,667**	**7.11**
Albay	12,151	3	742	10,814	**11,559**	**95.13**	1	9	582	**592**	**4.87**
Camarines Norte	11,444	10	1,264	8,828	**10,102**	**88.27**	1	66	1,275	**1,342**	**11.73**
Camarines Sur	17,268	8	1,428	15,690	**17,126**	**99.18**	0	2	140	**142**	**0.82**
Catanduanes	4,579	1	418	3,457	**3,876**	**84.65**	0	54	649	**703**	**15.35**
Masbate	12,804	14	1,605	11,071	**12,690**	**99.11**	0	0	114	**114**	**0.89**
Sorsogon	13,242	10	1,527	10,281	**11,818**	**89.25**	1	82	1,341	**1,424**	**10.75**
Iriga City	2,157	1	142	1,482	**1,625**	**75.34**	0	32	500	**532**	**24.66**
Legaspi City	3,376	2	124	2,764	**2,890**	**85.60**	0	13	473	**486**	**14.40**
Naga City	2,648	2	206	2,108	**2,316**	**87.46**	0	20	312	**332**	**12.54**
**Region 6**	**84,867**	**127**	**9,213**	**66,957**	**76,297**	**89.90**	**9**	**515**	**8,046**	**8,570**	**10.10**
Aklan	7,175	7	597	5,891	**6,495**	**90.52**	2	74	604	**680**	**9.48**
Antique	8,763	13	816	7,184	**8,013**	**91.44**	1	25	724	**750**	**8.56**
Capiz	9,104	6	632	7,684	**8,322**	**91.41**	1	39	742	**782**	**8.59**
Guimaras	1,596	3	166	1,234	**1,403**	**87.91**	0	5	188	**193**	**12.09**
Iloilo	17,875	24	1,233	15,170	**16,427**	**91.90**	2	54	1,392	**1,448**	**8.10**
Negros Occidental	21,528	60	4,788	13,581	**18,429**	**85.60**	2	223	2,874	**3,099**	**14.40**
Bacolod City	8,826	7	427	7,470	**7,904**	**89.55**	1	43	878	**922**	**10.45**
Iloilo City	10,000	7	554	8,743	**9,304**	**93.04**	0	52	644	**696**	**6.96**
**Region 7**	**129,213**	**193**	**7,941**	**113,500**	**121,634**	**94.13**	**6**	**204**	**7,369**	**7,579**	**5.87**
Bohol	20,870	20	1,797	18,045	**19,862**	**95.17**	1	26	981	**1,008**	**4.83**
Cebu	54,794	34	1,840	51,153	**53,027**	**96.78**	0	12	1,755	**1,767**	**3.22**
Negros Oriental	20,294	21	2,071	16,700	**18,792**	**92.60**	0	56	1,446	**1,502**	**7.40**

Table 1.B.2.2 - Intrapartum Care and Delivery Outcome

Number and proportion of delivery by type and age group
Philippines, Annual 2020

Area	Total number of Deliveries	Delivery by Type									
		Vaginal					Cesarean Section				
		Age Group in Year			Total	%	Age Group in Year			Total	%
		10-14	15-19	20-49			10-14	15-19	20-49		
Siquijor	1,260	2	121	1,018	**1,141**	**90.56**	0	4	115	**119**	**9.44**
Cebu City	16,378	116	1,792	13,453	**15,361**	**93.79**	5	64	948	**1,017**	**6.21**
Lapu-Lapu City	5,425	0	281	4,697	**4,978**	**91.76**	0	42	405	**447**	**8.24**
Mandaue City	10,192	0	39	8,434	**8,473**	**83.13**	0	0	1,719	**1,719**	**16.87**
**Region 8**	**62,567**	**65**	**5,653**	**51,973**	**57,691**	**92.21**	**12**	**331**	**4,533**	**4,876**	**7.79**
Biliran	3,136	2	352	2,542	**2,896**	**92.35**	0	19	221	**240**	**7.65**
Eastern Samar	6,534	4	622	4,964	**5,590**	**85.55**	3	78	863	**944**	**14.45**
Northern Leyte	19,715	10	1,539	17,761	**19,310**	**97.95**	0	5	400	**405**	**2.05**
Northern Samar	9,826	23	899	8,094	**9,016**	**91.76**	2	74	734	**810**	**8.24**
Southern Leyte	2,470	1	286	2,010	**2,297**	**93.00**	0	13	160	**173**	**7.00**
Western Samar	8,193	18	873	6,761	**7,652**	**93.40**	6	56	479	**541**	**6.60**
Calbayog City	2,130	0	190	1,800	**1,990**	**93.43**	0	16	124	**140**	**6.57**
Maasin City	2,625	0	212	1,748	**1,960**	**74.67**	0	26	639	**665**	**25.33**
Ormoc City	5,208	6	452	4,061	**4,519**	**86.77**	1	19	669	**689**	**13.23**
Tacloban City	2,730	1	228	2,232	**2,461**	**90.15**	0	25	244	**269**	**9.85**
**Region 9**	**55,987**	**44**	**6,180**	**47,605**	**53,829**	**96.15**	**3**	**192**	**1,963**	**2,158**	**3.85**
Zamboanga del Norte	16,129	16	2,069	13,668	**15,753**	**97.67**	0	44	332	**376**	**2.33**
Zamboanga del Sur	9,628	5	969	8,471	**9,445**	**98.10**	0	12	171	**183**	**1.90**
Zamboanga Sibugay	7,686	11	1,096	6,155	**7,262**	**94.48**	2	33	389	**424**	**5.52**
Dapitan City	1,694	3	170	1,424	**1,597**	**94.27**	0	10	87	**97**	**5.73**
Dipolog City	2,665	1	336	2,190	**2,527**	**94.82**	0	13	125	**138**	**5.18**
Isabela City	1,861	2	273	1,475	**1,750**	**94.04**	0	14	97	**111**	**5.96**
Pagadian City	2,888	2	282	2,457	**2,741**	**94.91**	0	8	139	**147**	**5.09**
Zamboanga City	13,436	4	985	11,765	**12,754**	**94.92**	1	58	623	**682**	**5.08**
**Region 10**	**83,352**	**139**	**9,859**	**68,573**	**78,571**	**94.26**	**13**	**474**	**4,294**	**4,781**	**5.74**
Bukidnon	19,866	72	3,561	14,565	**18,198**	**91.60**	12	219	1,437	**1,668**	**8.40**
Camiguin	1,310	0	140	1,046	**1,186**	**90.53**	0	4	120	**124**	**9.47**
Lanao del Norte	10,414	14	844	9,342	**10,200**	**97.95**	0	18	196	**214**	**2.05**
Misamis Occidental	4,583	3	362	3,955	**4,320**	**94.26**	0	22	241	**263**	**5.74**
Misamis Oriental	9,594	12	1,236	8,005	**9,253**	**96.45**	0	37	304	**341**	**3.55**
Cagayan de Oro City	17,604	20	1,099	15,843	**16,962**	**96.35**	0	39	603	**642**	**3.65**
El Salvador City	326	0	49	277	**326**	**100.00**	0	0	0	**0**	**0.00**
Gingoog City	2,238	2	361	1,642	**2,005**	**89.59**	1	26	206	**233**	**10.41**
Iligan City	7,134	6	885	5,791	**6,682**	**93.66**	0	25	427	**452**	**6.34**
Malaybalay City	2,516	3	396	1,937	**2,336**	**92.85**	0	17	163	**180**	**7.15**
Oroquieta City	923	1	95	720	**816**	**88.41**	0	7	100	**107**	**11.59**
Ozamis City	2,162	1	232	1,785	**2,018**	**93.34**	0	11	133	**144**	**6.66**
Tangub City	1,046	0	113	907	**1,020**	**97.51**	0	8	18	**26**	**2.49**
Valencia City	3,636	5	486	2,758	**3,249**	**89.36**	0	41	346	**387**	**10.64**
**Region 11**	**84,828**	**299**	**10,475**	**62,559**	**73,333**	**86.45**	**138**	**1,046**	**10,311**	**11,495**	**13.55**
Compostela Valley	12,041	52	1,847	8,720	**10,619**	**88.19**	6	137	1,279	**1,422**	**11.81**

Table 1.B.2.2 - Intrapartum Care and Delivery Outcome

Number and proportion of delivery by type and age group
Philippines, Annual 2020

Area	Total number of Deliveries	Delivery by Type									
		Vaginal					Cesarean Section				
		Age Group in Year			Total	%	Age Group in Year			Total	%
		10-14	15-19	20-49			10-14	15-19	20-49		
Davao del Norte	20,420	85	2,693	14,973	**17,751**	**86.93**	85	208	2,376	**2,669**	**13.07**
Davao Oriental	8,383	37	1,165	6,433	**7,635**	**91.08**	3	73	672	**748**	**8.92**
Davao del Sur	9,488	46	1,379	6,898	**8,323**	**87.72**	4	126	1,035	**1,165**	**12.28**
Davao Occidental	4,408	25	663	3,486	**4,174**	**94.69**	2	31	201	**234**	**5.31**
Davao City	30,088	54	2,728	22,049	**24,831**	**82.53**	38	471	4,748	**5,257**	**17.47**
**Region 12**	**72,158**	**136**	**9,363**	**55,055**	**64,554**	**89.46**	**9**	**548**	**7,047**	**7,604**	**10.54**
North Cotabato	20,352	36	2,555	15,582	**18,173**	**89.29**	3	199	1,977	**2,179**	**10.71**
Sarangani	10,307	26	1,991	7,838	**9,855**	**95.61**	2	41	409	**452**	**4.39**
South Cotabato	16,879	33	2,348	12,525	**14,906**	**88.31**	2	138	1,833	**1,973**	**11.69**
Sultan Kudarat	12,272	35	1,688	9,076	**10,799**	**88.00**	2	109	1,362	**1,473**	**12.00**
Cotabato City	5,502	3	320	4,078	**4,401**	**79.99**	0	27	1,074	**1,101**	**20.01**
Gen. Santos City	6,846	3	461	5,956	**6,420**	**93.78**	0	34	392	**426**	**6.22**
**BARMM**	**70,660**	**33**	**7,056**	**61,386**	**68,475**	**96.91**	**0**	**135**	**2,050**	**2,185**	**3.09**
Basilan	3,160	4	311	2,784	**3,099**	**98.07**	0	0	61	**61**	**1.93**
Lanao del Sur	20,725	2	1,105	19,228	**20,335**	**98.12**	0	24	366	**390**	**1.88**
Maguindanao	25,507	27	2,864	21,735	**24,626**	**96.55**	0	85	796	**881**	**3.45**
Sulu	10,140	0	2,254	7,514	**9,768**	**96.33**	0	0	372	**372**	**3.67**
Tawi-Tawi	6,946	0	0	6,911	**6,911**	**99.50**	0	0	35	**35**	**0.50**
Lamitan City	1,102	0	178	924	**1,102**	**100.00**	0	0	0	**0**	**0.00**
Marawi City	3,080	0	344	2,290	**2,634**	**85.52**	0	26	420	**446**	**14.48**
**CARAGA**	**38,386**	**44**	**3,296**	**33,673**	**37,013**	**96.42**	**1**	**131**	**1,241**	**1,373**	**3.58**
Agusan del Norte	3,386	6	395	2,982	**3,383**	**99.91**	0	0	3	**3**	**0.09**
Agusan del Sur	11,965	10	564	11,294	**11,868**	**99.19**	0	12	85	**97**	**0.81**
Surigao del Norte	3,532	3	375	3,141	**3,519**	**99.63**	0	0	13	**13**	**0.37**
Surigao del Sur	6,474	12	768	5,570	**6,350**	**98.08**	0	12	112	**124**	**1.92**
Province of Dinagat	1,311	3	181	1,120	**1,304**	**99.47**	0	0	7	**7**	**0.53**
Bislig City	1,666	6	202	1,331	**1,539**	**92.38**	1	6	120	**127**	**7.62**
Butuan City	5,664	3	503	4,319	**4,825**	**85.19**	0	35	804	**839**	**14.81**
Surigao City	4,388	1	308	3,916	**4,225**	**96.29**	0	66	97	**163**	**3.71**

Note: Put asterisk (*) for No Report and Zero (0) for No Case
Deliveries should be reported by place of occurrence.

Vaginal - deliveries by NSD, Vaccum and Forcep

Table 1.B.2.3 - Intrapartum Care and Delivery Outcome

A. Number and proportion of pregnancy outcome (Full term and Pre-term)
Philippines, Annual 2020

Area	Total number of Deliveries	Full term (37-42 weeks AOG)					Pre-term (22-36 weeks AOG)				
		Age Group in Year			Total	%	Age Group in Year			Total	%
		10-14	15-19	20-49			10-14	15-19	20-49		
**PHILIPPINES**	**1,433,350**	**1,995**	**121,166**	**1,060,424**	**1,183,585**	**82.57**	**116**	**2,234**	**10,812**	**13,162**	**0.92**
**N C R**	**205,340**	**162**	**8,601**	**96,049**	**104,812**	**51.04**	**37**	**45**	**260**	**342**	**0.17**
Malabon	4,003	5	486	3,494	**3,985**	**99.55**	0	4	13	**17**	**0.42**
Navotas	4,265	2	510	3,723	**4,235**	**99.30**	0	8	11	**19**	**0.45**
Valenzuela City	5,704	0	60	407	**467**	**8.19**	0	1	2	**3**	**0.05**
Caloocan City	19,410	31	1,506	17,851	**19,388**	**99.89**	0	7	15	**22**	**0.11**
Marikina City	9,871	0	0	0	**0**	**0.00**	0	0	0	**0**	**0.00**
Pasig City	14,624	3	134	1,862	**1,999**	**13.67**	0	1	12	**13**	**0.09**
Pateros	1,335	0	33	911	**944**	**70.71**	0	2	5	**7**	**0.52**
Taguig	14,405	19	129	1,393	**1,541**	**10.70**	34	3	30	**67**	**0.47**
Quezon City	54,529	79	4,309	49,817	**54,205**	**99.41**	2	9	55	**66**	**0.12**
Makati City	6,896	0	0	0	**0**	**0.00**	0	0	0	**0**	**0.00**
Mandaluyong City	4,175	5	379	3,770	**4,154**	**99.50**	0	2	16	**18**	**0.43**
San Juan	1,482	0	41	801	**842**	**56.82**	0	4	63	**67**	**4.52**
Manila City	39,702	0	0	0	**0**	**0.00**	0	0	0	**0**	**0.00**
Las Piñas City	7,279	16	661	6,569	**7,246**	**99.55**	0	1	14	**15**	**0.21**
Muntinlupa City	7,126	0	0	0	**0**	**0.00**	0	0	0	**0**	**0.00**
Parañaque City	4,668	0	0	0	**0**	**0.00**	0	0	0	**0**	**0.00**
Pasay City	5,866	2	353	5,451	**5,806**	**98.98**	1	3	24	**28**	**0.48**
**C A R**	**26,314**	**20**	**2,275**	**22,997**	**25,292**	**96.12**	**0**	**113**	**729**	**842**	**3.20**
Abra	3,088	0	371	2,608	**2,979**	**96.47**	0	13	58	**71**	**2.30**
Apayao	2,344	4	361	1,928	**2,293**	**97.82**	0	9	20	**29**	**1.24**
Benguet	4,996	3	335	4,448	**4,786**	**95.80**	0	23	165	**188**	**3.76**
Ifugao	2,850	3	323	2,510	**2,836**	**99.51**	0	2	4	**6**	**0.21**
Kalinga	2,782	1	217	2,520	**2,738**	**98.42**	0	6	23	**29**	**1.04**
Mt. Province	2,848	1	278	2,485	**2,764**	**97.05**	0	14	42	**56**	**1.97**
Baguio City	7,406	8	390	6,498	**6,896**	**93.11**	0	46	417	**463**	**6.25**
**Region 1**	**55,495**	**54**	**4,865**	**49,400**	**54,319**	**97.88**	**4**	**145**	**1,030**	**1,179**	**2.12**
Ilocos Norte	5,676	8	450	5,087	**5,545**	**97.69**	1	16	109	**126**	**2.22**
Ilocos Sur	4,514	0	326	4,123	**4,449**	**98.56**	0	17	47	**64**	**1.42**
La Union	3,556	4	330	3,191	**3,525**	**99.13**	1	4	26	**31**	**0.87**
Pangasinan	15,908	11	1,438	14,435	**15,884**	**99.85**	0	5	47	**52**	**0.33**
Alaminos City	1,730	1	267	1,444	**1,712**	**98.96**	0	0	8	**8**	**0.46**
Candon City	1,018	0	6	1,008	**1,014**	**99.61**	0	0	4	**4**	**0.39**
Dagupan City	8,754	7	828	7,909	**8,744**	**99.89**	0	4	23	**27**	**0.31**
Laoag City	1,960	0	56	1,900	**1,956**	**99.80**	0	0	3	**3**	**0.15**
San Carlos City	2,887	4	250	2,592	**2,846**	**98.58**	0	4	26	**30**	**1.04**
San Fernando City	5,584	16	505	4,770	**5,291**	**94.75**	2	29	245	**276**	**4.94**
Urdaneta City	2,087	0	272	1,810	**2,082**	**99.76**	0	2	4	**6**	**0.29**

Table 1.B.2.3 - Intrapartum Care and Delivery Outcome

A. Number and proportion of pregnancy outcome (Full term and Pre-term)

Philippines, Annual 2020

Area	Total number of Deliveries	Full term (37-42 weeks AOG)					Pre-term (22-36 weeks AOG)				
		Age Group in Year			Total	%	Age Group in Year			Total	%
		10-14	15-19	20-49			10-14	15-19	20-49		
Vigan City	1,821	3	137	1,131	**1,271**	**69.80**	0	64	488	**552**	**30.31**
**Region 2**	**42,876**	**53**	**4,986**	**37,021**	**42,060**	**98.10**	**1**	**107**	**461**	**569**	**1.33**
Batanes	251	0	23	208	**231**	**92.03**	0	2	12	**14**	**5.58**
Cagayan	9,804	6	1,128	8,495	**9,629**	**98.22**	0	14	88	**102**	**1.04**
Isabela	12,497	18	1,566	10,709	**12,293**	**98.37**	0	22	114	**136**	**1.09**
Nueva Vizcaya	7,567	16	964	6,337	**7,317**	**96.70**	0	41	153	**194**	**2.56**
Quirino	3,026	0	309	2,634	**2,943**	**97.26**	1	13	45	**59**	**1.95**
Cauayan City	1,956	4	280	1,630	**1,914**	**97.85**	0	5	23	**28**	**1.43**
Ilagan City	2,795	4	249	2,542	**2,795**	**100.00**	0	0	0	**0**	**0.00**
Santiago City	2,938	4	337	2,577	**2,918**	**99.32**	0	7	13	**20**	**0.68**
Tuguegarao City	2,042	1	130	1,889	**2,020**	**98.92**	0	3	13	**16**	**0.78**
**Region 3**	**130,415**	**257**	**12,207**	**109,424**	**121,888**	**93.46**	**2**	**154**	**910**	**1,066**	**0.82**
Aurora	3,337	3	394	2,779	**3,176**	**95.18**	0	21	90	**111**	**3.33**
Bataan	6,306	9	735	5,437	**6,181**	**98.02**	2	15	55	**72**	**1.14**
Bulacan	20,386	58	1,843	16,415	**18,316**	**89.85**	0	55	308	**363**	**1.78**
Nueva Ecija	15,210	51	1,046	10,146	**11,243**	**73.92**	0	8	41	**49**	**0.32**
Pampanga	12,798	15	1,269	10,797	**12,081**	**94.40**	0	4	36	**40**	**0.31**
Tarlac	13,438	31	1,212	12,131	**13,374**	**99.52**	0	6	50	**56**	**0.42**
Zambales	7,458	10	800	6,473	**7,283**	**97.65**	0	21	111	**132**	**1.77**
Angeles City	7,368	7	554	6,853	**7,414**	**100.62**	0	0	0	**0**	**0.00**
Balanga City	1,448	5	194	1,195	**1,394**	**96.27**	0	7	38	**45**	**3.11**
Cabanatuan City	7,983	9	549	7,392	**7,950**	**99.59**	0	3	18	**21**	**0.26**
City of San Fernando	2,244	0	23	1,073	**1,096**	**48.84**	0	0	102	**102**	**4.55**
Gapan City	1,160	5	171	975	**1,151**	**99.22**	0	2	8	**10**	**0.86**
Mabalacat City	4,632	9	443	4,180	**4,632**	**100.00**	0	0	0	**0**	**0.00**
Malolos City	4,795	1	227	4,540	**4,768**	**99.44**	0	0	26	**26**	**0.54**
Meycauayan	2,536	0	188	2,348	**2,536**	**100.00**	0	0	0	**0**	**0.00**
Olongapo	4,235	8	356	3,868	**4,232**	**99.93**	0	0	0	**0**	**0.00**
Palayan City	188	0	0	188	**188**	**100.00**	0	0	0	**0**	**0.00**
San Jose City	2,202	10	339	1,822	**2,171**	**98.59**	0	8	19	**27**	**1.23**
San Jose del Monte City	6,615	14	937	5,662	**6,613**	**99.97**	0	0	0	**0**	**0.00**
Science City of Munoz	80	0	7	63	**70**	**87.50**	0	0	0	**0**	**0.00**
Tarlac City	5,996	12	920	5,087	**6,019**	**100.38**	0	4	8	**12**	**0.20**
**Region 4A**	**167,088**	**115**	**11,488**	**143,666**	**155,269**	**92.93**	**21**	**181**	**988**	**1,190**	**0.71**
Batangas	16,097	17	1,070	14,251	**15,338**	**95.28**	0	15	71	**86**	**0.53**
Cavite	23,290	16	1,667	21,177	**22,860**	**98.15**	6	16	199	**221**	**0.95**
Laguna	10,803	11	588	5,251	**5,850**	**54.15**	0	11	55	**66**	**0.61**
Quezon	15,653	15	1,335	13,841	**15,191**	**97.05**	1	31	191	**223**	**1.42**
Rizal	23,146	16	2,202	20,968	**23,186**	**100.17**	9	20	122	**151**	**0.65**
Antipolo City	9,951	1	441	7,450	**7,892**	**79.31**	0	2	28	**30**	**0.30**
Bacoor City	6,846	4	341	6,074	**6,419**	**93.76**	0	0	11	**11**	**0.16**
Batangas City	0	0	0	0	**0**	**0.00**	0	0	0	**0**	**0.00**

Table 1.B.2.3 - Intrapartum Care and Delivery Outcome

A. Number and proportion of pregnancy outcome (Full term and Pre-term)
Philippines, Annual 2020

Area	Total number of Deliveries	Full term (37-42 weeks AOG)					Pre-term (22-36 weeks AOG)				
		Age Group in Year			Total	%	Age Group in Year			Total	%
		10-14	15-19	20-49			10-14	15-19	20-49		
Biñan City	8,310	0	579	7,406	**7,985**	**96.09**	0	18	34	**52**	**0.63**
Cabuyao City	3,835	1	293	3,418	**3,712**	**96.79**	0	13	44	**57**	**1.49**
Calamba City	8,062	14	723	6,976	**7,713**	**95.67**	0	17	65	**82**	**1.02**
Cavite City	1,105	0	32	1,067	**1,099**	**99.46**	0	0	0	**0**	**0.00**
Dasmariñas City	8,571	4	572	7,963	**8,539**	**99.63**	3	5	20	**28**	**0.33**
General Trias City	1,253	2	105	1,319	**1,426**	**113.81**	2	1	2	**5**	**0.40**
Imus City	5,001	0	138	3,870	**4,008**	**80.14**	0	0	39	**39**	**0.78**
Lipa City	0	0	0	0	**0**	**0.00**	0	0	0	**0**	**0.00**
Lucena City	0	0	0	0	**0**	**0.00**	0	0	0	**0**	**0.00**
San Pablo City	329	0	20	268	**288**	**87.54**	0	0	2	**2**	**0.61**
San Pedro City	1,340	0	109	1,239	**1,348**	**100.60**	0	2	1	**3**	**0.22**
Santa Rosa City	8,285	7	762	7,493	**8,262**	**99.72**	0	2	12	**14**	**0.17**
Tagaytay City	2,820	0	32	2,764	**2,796**	**99.15**	0	0	2	**2**	**0.07**
Tanauan City	4,385	1	61	3,441	**3,503**	**79.89**	0	6	28	**34**	**0.78**
Tayabas City	1,594	0	0	1,532	**1,532**	**96.11**	0	0	32	**32**	**2.01**
Trece Martires City	6,412	6	418	5,898	**6,322**	**98.60**	0	22	30	**52**	**0.81**
**Region 4B**	**44,135**	**49**	**2,950**	**29,933**	**32,932**	**74.62**	**3**	**86**	**229**	**318**	**0.72**
Marinduque	3,272	2	334	2,863	**3,199**	**97.77**	0	16	57	**73**	**2.23**
Mindoro Occidental	7,975	21	919	6,854	**7,794**	**97.73**	1	16	47	**64**	**0.80**
Mindoro Oriental	12,280	3	328	11,838	**12,169**	**99.10**	1	11	27	**39**	**0.32**
Palawan	14,689	20	878	4,421	**5,319**	**36.21**	1	40	81	**122**	**0.83**
Romblon	2,171	3	103	657	**763**	**35.15**	0	2	15	**17**	**0.78**
Puerto Princesa City	3,748	0	388	3,300	**3,688**	**98.40**	0	1	2	**3**	**0.08**
**Region 5**	**79,669**	**52**	**7,394**	**69,962**	**77,408**	**97.16**	**2**	**362**	**1,353**	**1,717**	**2.16**
Albay	12,151	4	736	11,175	**11,915**	**98.06**	0	6	171	**177**	**1.46**
Camarines Norte	11,444	9	1,127	9,411	**10,547**	**92.16**	1	189	627	**817**	**7.14**
Camarines Sur	17,268	8	1,417	15,678	**17,103**	**99.04**	0	9	89	**98**	**0.57**
Catanduanes	4,579	1	471	4,059	**4,531**	**98.95**	0	1	7	**8**	**0.17**
Masbate	12,804	13	1,578	11,024	**12,615**	**98.52**	0	19	43	**62**	**0.48**
Sorsogon	13,242	11	1,553	11,085	**12,649**	**95.52**	1	127	344	**472**	**3.56**
Iriga City	2,157	1	170	1,972	**2,143**	**99.35**	0	4	6	**10**	**0.46**
Legaspi City	3,376	2	125	3,188	**3,315**	**98.19**	0	2	39	**41**	**1.21**
Naga City	2,648	3	217	2,370	**2,590**	**97.81**	0	5	27	**32**	**1.21**
**Region 6**	**84,867**	**130**	**9,951**	**59,252**	**69,333**	**81.70**	**11**	**239**	**961**	**1,211**	**1.43**
Aklan	7,175	8	653	5,809	**6,470**	**90.17**	1	29	103	**133**	**1.85**
Antique	8,763	15	775	7,595	**8,385**	**95.69**	3	47	195	**245**	**2.80**
Capiz	9,104	7	668	6,698	**7,373**	**80.99**	0	4	35	**39**	**0.43**
Guimaras	1,596	3	167	1,014	**1,184**	**74.19**	0	4	50	**54**	**3.38**
Iloilo	17,875	24	1,770	14,956	**16,750**	**93.71**	2	19	125	**146**	**0.82**
Negros Occidental	21,528	58	4,843	13,957	**18,858**	**87.60**	3	104	329	**436**	**2.03**
Bacolod City	8,826	6	458	4,448	**4,912**	**55.65**	1	13	83	**97**	**1.10**
Iloilo City	10,000	9	617	4,775	**5,401**	**54.01**	1	19	41	**61**	**0.61**

Table 1.B.2.3 - Intrapartum Care and Delivery Outcome

A. Number and proportion of pregnancy outcome (Full term and Pre-term)
Philippines, Annual 2020

Area	Total number of Deliveries	Full term (37-42 weeks AOG)					Pre-term (22-36 weeks AOG)				
		Age Group in Year			Total	%	Age Group in Year			Total	%
		10-14	15-19	20-49			10-14	15-19	20-49		
**Region 7**	**129,213**	**154**	**5,063**	**56,680**	**61,897**	**47.90**	**9**	**65**	**428**	**502**	**0.39**
Bohol	20,870	12	1,298	13,379	**14,689**	**70.38**	2	4	77	**83**	**0.40**
Cebu	54,794	15	797	12,671	**13,483**	**24.61**	1	8	29	**38**	**0.07**
Negros Oriental	20,294	9	1,002	8,383	**9,394**	**46.29**	1	26	66	**93**	**0.46**
Siquijor	1,260	2	121	1,130	**1,253**	**99.44**	0	2	4	**6**	**0.48**
Cebu City	16,378	116	1,532	14,228	**15,876**	**96.93**	5	19	166	**190**	**1.16**
Lapu-Lapu City	5,425	0	313	4,948	**5,261**	**96.98**	0	6	86	**92**	**1.70**
Mandaue City	10,192	0	0	1,941	**1,941**	**19.04**	0	0	0	**0**	**0.00**
**Region 8**	**62,567**	**61**	**4,534**	**56,159**	**60,754**	**97.10**	**2**	**129**	**1,072**	**1,203**	**1.92**
Biliran	3,136	3	343	2,454	**2,800**	**89.29**	0	3	283	**286**	**9.12**
Eastern Samar	6,534	6	709	5,572	**6,287**	**96.22**	0	12	168	**180**	**2.75**
Northern Leyte	19,715	1	289	18,953	**19,243**	**97.61**	0	3	219	**222**	**1.13**
Northern Samar	9,826	20	854	8,521	**9,395**	**95.61**	2	68	219	**289**	**2.94**
Southern Leyte	2,470	1	297	2,157	**2,455**	**99.39**	0	1	3	**4**	**0.16**
Western Samar	8,193	22	905	7,169	**8,096**	**98.82**	0	24	51	**75**	**0.92**
Calbayog City	2,130	0	197	1,909	**2,106**	**98.87**	0	4	6	**10**	**0.47**
Maasin City	2,625	0	232	2,341	**2,573**	**98.02**	0	2	45	**47**	**1.79**
Ormoc City	5,208	7	459	4,628	**5,094**	**97.81**	0	9	62	**71**	**1.36**
Tacloban City	2,730	1	249	2,455	**2,705**	**99.08**	0	3	16	**19**	**0.70**
**Region 9**	**55,987**	**48**	**6,281**	**47,508**	**53,837**	**96.16**	**0**	**81**	**264**	**345**	**0.62**
Zamboanga del Norte	16,129	16	2,078	13,889	**15,983**	**99.09**	0	39	121	**160**	**0.99**
Zamboanga del Sur	9,628	5	930	7,059	**7,994**	**83.03**	0	5	30	**35**	**0.36**
Zamboanga Sibugay	7,686	14	1,156	6,589	**7,759**	**100.95**	0	13	45	**58**	**0.75**
Dapitan City	1,694	3	177	1,196	**1,376**	**81.23**	0	1	9	**10**	**0.59**
Dipolog City	2,665	1	343	2,283	**2,627**	**98.57**	0	5	13	**18**	**0.68**
Isabela City	1,861	2	268	1,575	**1,845**	**99.14**	0	5	9	**14**	**0.75**
Pagadian City	2,888	2	294	2,596	**2,892**	**100.14**	0	0	0	**0**	**0.00**
Zamboanga City	13,436	5	1,035	12,321	**13,361**	**99.44**	0	13	37	**50**	**0.37**
**Region 10**	**83,352**	**147**	**10,566**	**71,694**	**82,407**	**98.87**	**9**	**148**	**477**	**634**	**0.76**
Bukidnon	19,866	74	3,755	15,673	**19,502**	**98.17**	6	65	154	**225**	**1.13**
Camiguin	1,310	0	116	1,178	**1,294**	**98.78**	0	3	13	**16**	**1.22**
Lanao del Norte	10,414	14	934	9,428	**10,376**	**99.64**	0	2	37	**39**	**0.37**
Misamis Occidental	4,583	5	380	4,083	**4,468**	**97.49**	0	5	29	**34**	**0.74**
Misamis Oriental	9,594	12	1,262	8,283	**9,557**	**99.61**	0	2	35	**37**	**0.39**
Cagayan de Oro City	17,604	21	1,401	16,116	**17,538**	**99.63**	2	14	50	**66**	**0.37**
El Salvador City	326	0	49	277	**326**	**100.00**	0	0	0	**0**	**0.00**
Gingoog City	2,238	3	383	1,797	**2,183**	**97.54**	0	6	46	**52**	**2.32**
Iligan City	7,134	8	857	6,149	**7,014**	**98.32**	1	14	34	**49**	**0.69**
Malaybalay City	2,516	3	400	2,075	**2,478**	**98.49**	0	9	15	**24**	**0.95**

Table 1.B.2.3 - Intrapartum Care and Delivery Outcome

A. Number and proportion of pregnancy outcome (Full term and Pre-term)

Philippines, Annual 2020

Area	Total number of Deliveries	Full term (37-42 weeks AOG)					Pre-term (22-36 weeks AOG)				
		Age Group in Year			Total	%	Age Group in Year			Total	%
		10-14	15-19	20-49			10-14	15-19	20-49		
Oroquieta City	923	1	98	808	**907**	**98.27**	0	3	8	**11**	**1.19**
Ozamis City	2,162	1	303	1,851	**2,155**	**99.68**	0	5	3	**8**	**0.37**
Tangub City	1,046	0	113	910	**1,023**	**97.80**	0	8	15	**23**	**2.20**
Valencia City	3,636	5	515	3,066	**3,586**	**98.62**	0	12	38	**50**	**1.38**
**Region 11**	**84,828**	**384**	**11,788**	**71,798**	**83,970**	**98.99**	**12**	**193**	**653**	**858**	**1.01**
Compostela Valley	12,041	60	1,958	9,873	**11,891**	**98.75**	1	40	109	**150**	**1.25**
Davao del Norte	20,420	95	2,926	17,135	**20,156**	**98.71**	4	59	201	**264**	**1.29**
Davao Oriental	8,383	48	1,224	7,021	**8,293**	**98.93**	2	15	73	**90**	**1.07**
Davao del Sur	9,488	64	1,471	7,806	**9,341**	**98.45**	1	34	112	**147**	**1.55**
Davao Occidental	4,408	32	994	3,326	**4,352**	**98.73**	1	25	30	**56**	**1.27**
Davao City	30,088	85	3,215	26,637	**29,937**	**99.50**	3	20	128	**151**	**0.50**
**Region 12**	**72,158**	**136**	**9,715**	**61,198**	**71,049**	**98.46**	**2**	**113**	**547**	**662**	**0.92**
North Cotabato	20,352	35	2,713	17,342	**20,090**	**98.71**	1	33	138	**172**	**0.85**
Sarangani	10,307	27	1,989	8,130	**10,146**	**98.44**	0	17	46	**63**	**0.61**
South Cotabato	16,879	33	2,428	14,123	**16,584**	**98.25**	1	25	161	**187**	**1.11**
Sultan Kudarat	12,272	35	1,746	10,244	**12,025**	**97.99**	0	31	93	**124**	**1.01**
Cotabato City	5,502	3	351	5,065	**5,419**	**98.49**	0	1	70	**71**	**1.29**
Gen. Santos City	6,846	3	488	6,294	**6,785**	**99.11**	0	6	39	**45**	**0.66**
**BARMM**	**70,660**	**33**	**4,414**	**44,208**	**48,655**	**68.86**	**1**	**15**	**214**	**230**	**0.33**
Basilan	3,160	5	299	2,840	**3,144**	**99.49**	0	4	30	**34**	**1.08**
Lanao del Sur	20,725	2	990	20,079	**21,071**	**101.67**	0	2	22	**24**	**0.12**
Maguindanao	25,507	26	2,929	17,399	**20,354**	**79.80**	1	9	73	**83**	**0.33**
Sulu	10,140	0	0	0	**0**	**0.00**	0	0	0	**0**	**0.00**
Tawi-Tawi	6,946	0	0	0	**0**	**0.00**	0	0	0	**0**	**0.00**
Lamitan City	1,102	0	178	924	**1,102**	**100.00**	0	0	0	**0**	**0.00**
Marawi City	3,080	0	18	2,966	**2,984**	**96.88**	0	0	89	**89**	**2.89**
**CARAGA**	**38,386**	**140**	**4,088**	**33,475**	**37,703**	**98.22**	**0**	**58**	**236**	**294**	**0.77**
Agusan del Norte	3,386	7	422	2,947	**3,376**	**99.70**	0	2	2	**4**	**0.12**
Agusan del Sur	11,965	103	1,195	10,395	**11,693**	**97.73**	0	13	29	**42**	**0.35**
Surigao del Norte	3,532	3	398	3,082	**3,483**	**98.61**	0	1	19	**20**	**0.57**
Surigao del Sur	6,474	13	811	5,481	**6,305**	**97.39**	0	28	89	**117**	**1.81**
Province of Dinagat	1,311	3	172	1,111	**1,286**	**98.09**	0	10	8	**18**	**1.37**
Bislig City	1,666	7	202	1,427	**1,636**	**98.20**	0	1	13	**14**	**0.84**
Butuan City	5,664	3	514	5,025	**5,542**	**97.85**	0	3	71	**74**	**1.31**
Surigao City	4,388	1	374	4,007	**4,382**	**99.86**	0	0	5	**5**	**0.11**

Note: Put asterisk (*) for No Report and Zero (0) for No Case

Table 1.B.2.4 - Intrapartum Care and Delivery Outcome

B. Number and proportion of pregnancy outcome (Fetal Deaths and Abortion)
Philippines, Annual 2020

Area	Total number of Deliveries	Fetal Death					Abortion (Counts Only)			
		Age Group			Total	%	Age Group			Total
		10-14	15-19	20-49			10-14	15-19	20-49	
**PHILIPPINES**	**1,433,350**	**38**	**739**	**7,111**	**7,888**	**0.55**	**83**	**693**	**6,100**	**6,876**
**N C R**	**205,340**	**0**	**28**	**1,557**	**1,585**	**0.77**	**0**	**8**	**87**	**95**
Malabon	**4,003**	0	0	15	**15**	**0.37**	0	2	14	**16**
Navotas	**4,265**	0	0	20	**20**	**0.47**	0	0	2	**2**
Valenzuela City	**5,704**	0	0	41	**41**	**0.72**	0	0	0	**0**
Caloocan City	**19,410**	0	0	17	**17**	**0.09**	0	0	1	**1**
Marikina City	**9,871**	0	5	104	**109**	**1.10**	0	0	0	**0**
Pasig City	**14,624**	0	7	112	**119**	**0.81**	0	0	4	**4**
Pateros	**1,335**	0	0	0	**0**	**0.00**	0	1	2	**3**
Taguig	**14,405**	0	0	95	**95**	**0.66**	0	1	5	**6**
Quezon City	**54,529**	0	8	357	**365**	**0.67**	0	3	42	**45**
Makati City	**6,896**	0	3	56	**59**	**0.86**	0	0	0	**0**
Mandaluyong City	**4,175**	0	0	24	**24**	**0.57**	0	0	4	**4**
San Juan	**1,482**	0	0	24	**24**	**1.62**	0	0	0	**0**
Manila City	**39,702**	0	0	488	**488**	**1.23**	0	0	0	**0**
Las Piñas City	**7,279**	0	0	56	**56**	**0.77**	0	1	13	**14**
Muntinlupa City	**7,126**	0	0	53	**53**	**0.74**	0	0	0	**0**
Parañaque City	**4,668**	0	5	54	**59**	**1.26**	0	0	0	**0**
Pasay City	**5,866**	0	0	41	**41**	**0.70**	0	0	0	**0**
**C A R**	**26,314**	**0**	**16**	**164**	**180**	**0.68**	**1**	**35**	**722**	**758**
Abra	**3,088**	0	2	36	**38**	**1.23**	0	1	13	**14**
Apayao	**2,344**	0	7	15	**22**	**0.94**	1	2	22	**25**
Benguet	**4,996**	0	0	22	**22**	**0.44**	0	4	19	**23**
Ifugao	**2,850**	0	1	7	**8**	**0.28**	0	2	58	**60**
Kalinga	**2,782**	0	1	14	**15**	**0.54**	0	1	55	**56**
Mt. Province	**2,848**	0	4	24	**28**	**0.98**	0	4	101	**105**
Baguio City	**7,406**	0	1	46	**47**	**0.63**	0	21	454	**475**
**Region 1**	**55,495**	**0**	**15**	**235**	**250**	**0.45**	**0**	**12**	**160**	**172**
Ilocos Norte	**5,676**	0	4	22	**26**	**0.46**	0	2	20	**22**
Ilocos Sur	**4,514**	0	0	9	**9**	**0.20**	0	0	18	**18**
La Union	**3,556**	0	0	26	**26**	**0.73**	0	5	56	**61**
Pangasinan	**15,908**	0	2	15	**17**	**0.11**	0	3	46	**49**
Alaminos City	**1,730**	0	0	10	**10**	**0.58**	0	0	0	**0**
Candon City	**1,018**	0	0	6	**6**	**0.59**	0	0	0	**0**
Dagupan City	**8,754**	0	5	61	**66**	**0.75**	0	0	0	**0**
Laoag City	**1,960**	0	0	2	**2**	**0.10**	0	0	0	**0**
San Carlos City	**2,887**	0	4	23	**27**	**0.94**	0	0	0	**0**
San Fernando City	**5,584**	0	0	56	**56**	**1.00**	0	2	17	**19**
Urdaneta City	**2,087**	0	0	5	**5**	**0.24**	0	0	3	**3**

Table 1.B.2.4 - Intrapartum Care and Delivery Outcome

B. Number and proportion of pregnancy outcome (Fetal Deaths and Abortion)
Philippines, Annual 2020

Area	Total number of Deliveries	Fetal Death					Abortion (Counts Only)			
		Age Group			Total	%	Age Group			Total
		10-14	15-19	20-49			10-14	15-19	20-49	
Vigan City	**1,821**	0	0	0	**0**	**0.00**	0	0	0	**0**
**Region 2**	**42,876**	**3**	**29**	**215**	**247**	**0.58**	**2**	**56**	**649**	**707**
Batanes	**251**	0	0	6	**6**	**2.39**	0	1	13	**14**
Cagayan	**9,804**	0	9	64	**73**	**0.74**	0	5	77	**82**
Isabela	**12,497**	0	9	59	**68**	**0.54**	0	22	159	**181**
Nueva Vizcaya	**7,567**	3	4	49	**56**	**0.74**	2	24	325	**351**
Quirino	**3,026**	0	4	20	**24**	**0.79**	0	2	52	**54**
Cauayan City	**1,956**	0	3	11	**14**	**0.72**	0	1	7	**8**
Ilagan City	**2,795**	0	0	0	**0**	**0.00**	0	0	0	**0**
Santiago City	**2,938**	0	0	0	**0**	**0.00**	0	0	0	**0**
Tuguegarao City	**2,042**	0	0	6	**6**	**0.29**	0	1	16	**17**
**Region 3**	**130,415**	**1**	**32**	**215**	**248**	**0.19**	**4**	**19**	**229**	**252**
Aurora	**3,337**	0	5	45	**50**	**1.50**	0	1	22	**23**
Bataan	**6,306**	0	7	46	**53**	**0.84**	1	5	81	**87**
Bulacan	**20,386**	0	5	30	**35**	**0.17**	2	5	31	**38**
Nueva Ecija	**15,210**	0	1	10	**11**	**0.07**	0	0	6	**6**
Pampanga	**12,798**	1	1	17	**19**	**0.15**	1	1	15	**17**
Tarlac	**13,438**	0	1	7	**8**	**0.06**	0	1	15	**16**
Zambales	**7,458**	0	6	25	**31**	**0.42**	0	2	10	**12**
Angeles City	**7,368**	0	0	0	**0**	**0.00**	0	0	0	**0**
Balanga City	**1,448**	0	1	8	**9**	**0.62**	0	0	26	**26**
Cabanatuan City	**7,983**	0	1	11	**12**	**0.15**	0	1	0	**1**
City of San Fernando	**2,244**	0	0	0	**0**	**0.00**	0	0	0	**0**
Gapan City	**1,160**	0	0	1	**1**	**0.09**	0	0	0	**0**
Mabalacat City	**4,632**	0	0	0	**0**	**0.00**	0	0	0	**0**
Malolos City	**4,795**	0	0	4	**4**	**0.08**	0	0	2	**2**
Meycauayan	**2,536**	0	0	0	**0**	**0.00**	0	0	1	**1**
Olongapo	**4,235**	0	0	3	**3**	**0.07**	0	0	3	**3**
Palayan City	**188**	0	0	0	**0**	**0.00**	0	0	0	**0**
San Jose City	**2,202**	0	0	4	**4**	**0.18**	0	0	9	**9**
San Jose del Monte City	**6,615**	0	1	1	**2**	**0.03**	0	0	0	**0**
Science City of Munoz	**80**	0	0	0	**0**	**0.00**	0	3	7	**10**
Tarlac City	**5,996**	0	3	3	**6**	**0.10**	0	0	1	**1**
**Region 4A**	**167,088**	**14**	**50**	**464**	**528**	**0.32**	**24**	**56**	**359**	**439**
Batangas	**16,097**	1	7	60	**68**	**0.42**	0	6	35	**41**
Cavite	**23,290**	0	6	84	**90**	**0.39**	0	14	84	**98**
Laguna	**10,803**	0	3	21	**24**	**0.22**	7	7	20	**34**
Quezon	**15,653**	2	10	69	**81**	**0.52**	0	5	66	**71**
Rizal	**23,146**	10	16	114	**140**	**0.60**	9	10	11	**30**
						**0.00**				
Antipolo City	**9,951**	0	0	0	**0**	**0.00**	0	0	0	**0**
Bacoor City	**6,846**	0	0	0	**0**	**0.00**	0	0	0	**0**
Batangas City	**0**	0	0	0	**0**	**0.00**	0	0	0	**0**

Table 1.B.2.4 - Intrapartum Care and Delivery Outcome

B. Number and proportion of pregnancy outcome (Fetal Deaths and Abortion)
Philippines, Annual 2020

Area	Total number of Deliveries	Fetal Death					Abortion (Counts Only)			
		Age Group			Total	%	Age Group			Total
		10-14	15-19	20-49			10-14	15-19	20-49	
Biñan City	**8,310**	1	1	3	**5**	**0.06**	0	0	1	**1**
Cabuyao City	**3,835**	0	2	2	**4**	**0.10**	0	0	1	**1**
Calamba City	**8,062**	0	4	48	**52**	**0.65**	1	6	48	**55**
Cavite City	**1,105**	0	0	16	**16**	**1.45**	0	4	30	**34**
Dasmariñas City	**8,571**	0	0	15	**15**	**0.18**	0	1	19	**20**
General Trias City	**1,253**	0	0	6	**6**	**0.48**	0	0	10	**10**
Imus City	**5,001**	0	0	0	**0**	**0.00**	0	0	1	**1**
Lipa City	**0**	0	0	0	**0**	**0.00**	0	0	0	**0**
Lucena City	**0**	0	0	0	**0**	**0.00**	0	0	0	**0**
San Pablo City	**329**	0	1	2	**3**	**0.91**	7	3	7	**17**
San Pedro City	**1,340**	0	0	0	**0**	**0.00**	0	0	0	**0**
Santa Rosa City	**8,285**	0	0	9	**9**	**0.11**	0	0	7	**7**
Tagaytay City	**2,820**	0	0	4	**4**	**0.14**	0	0	4	**4**
Tanauan City	**4,385**	0	0	9	**9**	**0.21**	0	0	9	**9**
Tayabas City	**1,594**	0	0	0	**0**	**0.00**	0	0	0	**0**
Trece Martires City	**6,412**	0	0	2	**2**	**0.03**	0	0	6	**6**
**Region 4B**	**44,135**	**2**	**35**	**293**	**330**	**0.75**	**4**	**23**	**138**	**165**
Marinduque	**3,272**	0	3	44	**47**	**1.44**	1	0	21	**22**
Mindoro Occidental	**7,975**	0	10	82	**92**	**1.15**	1	7	42	**50**
Mindoro Oriental	**12,280**	1	3	41	**45**	**0.37**	0	1	15	**16**
Palawan	**14,689**	1	15	66	**82**	**0.56**	2	14	54	**70**
Romblon	**2,171**	0	1	2	**3**	**0.14**	0	1	6	**7**
Puerto Princesa City	**3,748**	0	3	58	**61**	**1.63**	0	0	0	**0**
**Region 5**	**79,669**	**3**	**63**	**478**	**544**	**0.68**	**2**	**85**	**877**	**964**
Albay	**12,151**	1	3	55	**59**	**0.49**	0	15	134	**149**
Camarines Norte	**11,444**	1	12	67	**80**	**0.70**	0	8	95	**103**
Camarines Sur	**17,268**	0	4	63	**67**	**0.39**	1	14	173	**188**
Catanduanes	**4,579**	0	1	39	**40**	**0.87**	0	4	16	**20**
Masbate	**12,804**	1	9	117	**127**	**0.99**	0	23	142	**165**
Sorsogon	**13,242**	0	31	90	**121**	**0.91**	1	16	232	**249**
Iriga City	**2,157**	0	0	4	**4**	**0.19**	0	0	10	**10**
Legaspi City	**3,376**	0	1	19	**20**	**0.59**	0	4	70	**74**
Naga City	**2,648**	0	2	24	**26**	**0.98**	0	1	5	**6**
**Region 6**	**84,867**	**1**	**74**	**472**	**547**	**0.64**	**4**	**57**	**506**	**567**
Aklan	**7,175**	0	2	25	**27**	**0.38**	0	2	37	**39**
Antique	**8,763**	0	12	71	**83**	**0.95**	1	5	22	**28**
Capiz	**9,104**	0	1	9	**10**	**0.11**	0	3	26	**29**
Guimaras	**1,596**	0	0	13	**13**	**0.81**	0	4	39	**43**
Iloilo	**17,875**	0	3	55	**58**	**0.32**	0	4	100	**104**
Negros Occidental	**21,528**	0	45	255	**300**	**1.39**	2	38	240	**280**
Bacolod City	**8,826**	1	9	18	**28**	**0.32**	1	1	25	**27**
Iloilo City	**10,000**	0	2	26	**28**	**0.28**	0	0	17	**17**

Table 1.B.2.4 - Intrapartum Care and Delivery Outcome

B. Number and proportion of pregnancy outcome (Fetal Deaths and Abortion)
Philippines, Annual 2020

Area	Total number of Deliveries	Fetal Death					Abortion (Counts Only)			
		Age Group			Total	%	Age Group			Total
		10-14	15-19	20-49			10-14	15-19	20-49	
**Region 7**	**129,213**	**0**	**47**	**273**	**320**	**0.25**	**0**	**10**	**109**	**119**
Bohol	**20,870**	0	20	64	**84**	**0.40**	0	7	65	**72**
Cebu	**54,794**	0	8	79	**87**	**0.16**	0	1	14	**15**
Negros Oriental	**20,294**	0	10	24	**34**	**0.17**	0	1	10	**11**
Siquijor	**1,260**	0	3	8	**11**	**0.87**	0	0	0	**0**
Cebu City	**16,378**	0	2	14	**16**	**0.10**	0	1	2	**3**
Lapu-Lapu City	**5,425**	0	4	56	**60**	**1.11**	0	0	7	**7**
Mandaue City	**10,192**	0	0	28	**28**	**0.27**	0	0	11	**11**
**Region 8**	**62,567**	**0**	**53**	**354**	**407**	**0.65**	**13**	**47**	**305**	**365**
Biliran	**3,136**	0	4	46	**50**	**1.59**	12	13	78	**103**
Eastern Samar	**6,534**	0	3	64	**67**	**1.03**	1	10	74	**85**
Northern Leyte	**19,715**	0	4	36	**40**	**0.20**	0	9	101	**110**
Northern Samar	**9,826**	0	31	111	**142**	**1.45**	0	2	41	**43**
Southern Leyte	**2,470**	0	1	10	**11**	**0.45**	0	0	3	**3**
Western Samar	**8,193**	0	8	14	**22**	**0.27**	0	11	2	**13**
Calbayog City	**2,130**	0	1	13	**14**	**0.66**	0	0	2	**2**
Maasin City	**2,625**	0	0	12	**12**	**0.46**	0	0	1	**1**
Ormoc City	**5,208**	0	0	43	**43**	**0.83**	0	0	0	**0**
Tacloban City	**2,730**	0	1	5	**6**	**0.22**	0	2	3	**5**
**Region 9**	**55,987**	**0**	**36**	**244**	**280**	**0.50**	**1**	**46**	**259**	**306**
Zamboanga del Norte	**16,129**	0	20	108	**128**	**0.79**	1	19	106	**126**
Zamboanga del Sur	**9,628**	0	4	17	**21**	**0.22**	0	7	17	**24**
Zamboanga Sibugay	**7,686**	0	8	59	**67**	**0.87**	0	13	97	**110**
Dapitan City	**1,694**	0	1	5	**6**	**0.35**	0	1	9	**10**
Dipolog City	**2,665**	0	2	14	**16**	**0.60**	0	1	2	**3**
Isabela City	**1,861**	0	0	8	**8**	**0.43**	0	3	3	**6**
Pagadian City	**2,888**	0	0	2	**2**	**0.07**	0	0	0	**0**
Zamboanga City	**13,436**	0	1	31	**32**	**0.24**	0	2	25	**27**
**Region 10**	**83,352**	**4**	**51**	**650**	**705**	**0.85**	**4**	**29**	**300**	**333**
Bukidnon	**19,866**	4	31	104	**139**	**0.70**	4	19	179	**202**
Camiguin	**1,310**	0	2	10	**12**	**0.92**	0	0	1	**1**
Lanao del Norte	**10,414**	0	0	3	**3**	**0.03**	0	0	0	**0**
Misamis Occidental	**4,583**	0	2	2	**4**	**0.09**	0	0	4	**4**
Misamis Oriental	**9,594**	0	1	12	**13**	**0.14**	0	0	2	**2**
Cagayan de Oro City	**17,604**	0	0	98	**98**	**0.56**	0	0	38	**38**
El Salvador City	**326**	0	0	0	**0**	**0.00**	0	0	0	**0**
Gingoog City	**2,238**	0	0	16	**16**	**0.71**	0	0	9	**9**
Iligan City	**7,134**	0	6	384	**390**	**5.47**	0	4	33	**37**
Malaybalay City	**2,516**	0	3	11	**14**	**0.56**	0	0	7	**7**

Table 1.B.2.4 - Intrapartum Care and Delivery Outcome

B. Number and proportion of pregnancy outcome (Fetal Deaths and Abortion)
Philippines, Annual 2020

Area	Total number of Deliveries	Fetal Death					Abortion (Counts Only)			
		Age Group			Total	%	Age Group			Total
		10-14	15-19	20-49			10-14	15-19	20-49	
Oroquieta City	**923**	0	2	5	**7**	**0.76**	0	2	3	**5**
Ozamis City	**2,162**	0	4	4	**8**	**0.37**	0	4	20	**24**
Tangub City	**1,046**	0	0	0	**0**	**0.00**	0	0	4	**4**
Valencia City	**3,636**	0	0	1	**1**	**0.03**	0	0	0	**0**
**Region 11**	**84,828**	**3**	**100**	**538**	**641**	**0.76**	**21**	**130**	**822**	**973**
Compostela Valley	**12,041**	0	14	87	**101**	**0.84**	0	20	144	**164**
Davao del Norte	**20,420**	2	27	155	**184**	**0.90**	15	45	295	**355**
Davao Oriental	**8,383**	0	16	74	**90**	**1.07**	1	15	120	**136**
Davao del Sur	**9,488**	0	10	89	**99**	**1.04**	1	16	103	**120**
Davao Occidental	**4,408**	1	22	70	**93**	**2.11**	1	19	68	**88**
Davao City	**30,088**	0	11	63	**74**	**0.25**	3	15	92	**110**
**Region 12**	**72,158**	**5**	**61**	**381**	**447**	**0.62**	**2**	**54**	**216**	**272**
North Cotabato	**20,352**	1	15	74	**90**	**0.44**	0	22	84	**106**
Sarangani	**10,307**	0	9	89	**98**	**0.95**	1	12	40	**53**
South Cotabato	**16,879**	1	16	91	**108**	**0.64**	1	10	42	**53**
Sultan Kudarat	**12,272**	2	20	101	**123**	**1.00**	0	9	48	**57**
Cotabato City	**5,502**	0	0	12	**12**	**0.22**	0	0	0	**0**
Gen. Santos City	**6,846**	1	1	14	**16**	**0.23**	0	1	2	**3**
**BARMM**	**70,660**	**0**	**20**	**220**	**240**	**0.34**	**1**	**8**	**188**	**197**
Basilan	**3,160**	0	11	53	**64**	**2.03**	0	1	6	**7**
Lanao del Sur	**20,725**	0	0	2	**2**	**0.01**	0	0	10	**10**
Maguindanao	**25,507**	0	9	89	**98**	**0.38**	1	7	57	**65**
Sulu	**10,140**	0	0	76	**76**	**0.75**	0	0	41	**41**
Tawi-Tawi	**6,946**	0	0	0	**0**	**0.00**	0	0	74	**74**
Lamitan City	**1,102**	0	0	0	**0**	**0.00**	0	0	0	**0**
Marawi City	**3,080**	0	0	0	**0**	**0.00**	0	0	0	**0**
**CARAGA**	**38,386**	**2**	**29**	**358**	**389**	**1.01**	**0**	**18**	**174**	**192**
Agusan del Norte	**3,386**	1	0	5	**6**	**0.18**	0	1	14	**15**
Agusan del Sur	**11,965**	0	11	219	**230**	**1.92**	0	0	37	**37**
Surigao del Norte	**3,532**	0	2	27	**29**	**0.82**	0	2	21	**23**
Surigao del Sur	**6,474**	0	7	45	**52**	**0.80**	0	11	58	**69**
Province of Dinagat	**1,311**	1	0	6	**7**	**0.53**	0	3	24	**27**
Bislig City	**1,666**	0	4	12	**16**	**0.96**	0	1	17	**18**
Butuan City	**5,664**	0	5	43	**48**	**0.85**	0	0	1	**1**
Surigao City	**4,388**	0	0	1	**1**	**0.02**	0	0	2	**2**

Note: Put asterisk (*) for No Report and Zero (0) for No Case

Table 1.B.2.5 - Intrapartum Care and Delivery Outcome

Number and proportion of livebirths by birth weight
Philippines, Annual 2020

Area	Total No. of Livebirths	NBW (≥ 2500 grams)		LBW (< 2500)		UBW	
		No.	%	No.	%	No.	%
**PHILIPPINES**	**1,433,557**	**1,311,951**	**91.52**	**88,513**	**6.17**	**33,093**	**2.31**
**N C R**	**200,958**	**178,549**	**88.85**	**21,479**	**10.69**	**930**	**0.46**
Malabon	**4,006**	3,780	**94.36**	142	**3.54**	84	**2.10**
Navotas	**4,245**	4,112	**96.87**	132	**3.11**	1	**0.02**
Valenzuela City	**5,556**	5,233	**94.19**	323	**5.81**	0	**0.00**
Caloocan City	**18,496**	17,563	**94.96**	919	**4.97**	14	**0.08**
Marikina City	**9,764**	8,634	**88.43**	1,130	**11.57**	0	**0.00**
Pasig City	**14,557**	12,528	**86.06**	1,979	**13.59**	50	**0.34**
Pateros	**1,335**	1,237	**92.66**	96	**7.19**	2	**0.15**
Taguig	**11,194**	10,058	**89.85**	1,065	**9.51**	71	**0.63**
Quezon City	**54,251**	46,635	**85.96**	7,485	**13.80**	131	**0.24**
Makati City	**6,834**	6,043	**88.43**	719	**10.52**	72	**1.05**
Mandaluyong City	**4,179**	4,023	**96.27**	156	**3.73**	0	**0.00**
San Juan	**1,482**	1,370	**92.44**	108	**7.29**	4	**0.27**
Manila City	**40,150**	34,109	**84.95**	6,041	**15.05**	0	**0.00**
Las Piñas City	**7,260**	6,984	**96.20**	127	**1.75**	149	**2.05**
Muntinlupa City	**7,089**	6,659	**93.93**	430	**6.07**	0	**0.00**
Parañaque City	**4,726**	3,899	**82.50**	475	**10.05**	352	**7.45**
Pasay City	**5,834**	5,682	**97.39**	152	**2.61**	0	**0.00**
**C A R**	**26,259**	**24,400**	**92.92**	**1,788**	**6.81**	**71**	**0.27**
Abra	**3,065**	2,875	**93.80**	167	**5.45**	23	**0.75**
Apayao	**2,355**	2,154	**91.46**	197	**8.37**	4	**0.17**
Benguet	**5,000**	4,630	**92.60**	334	**6.68**	36	**0.72**
Ifugao	**2,853**	2,744	**96.18**	105	**3.68**	4	**0.14**
Kalinga	**2,772**	2,635	**95.06**	135	**4.87**	2	**0.07**
Mt. Province	**2,823**	2,633	**93.27**	190	**6.73**	0	**0.00**
Baguio City	**7,391**	6,729	**91.04**	660	**8.93**	2	**0.03**
**Region 1**	**55,498**	**52,131**	**93.93**	**3,325**	**5.99**	**42**	**0.08**
Ilocos Norte	**5,671**	5,471	**96.47**	200	**3.53**	0	**0.00**
Ilocos Sur	**4,513**	4,348	**96.34**	163	**3.61**	2	**0.04**
La Union	**3,556**	3,385	**95.19**	171	**4.81**	0	**0.00**
Pangasinan	**15,936**	15,495	**97.23**	424	**2.66**	17	**0.11**
Alaminos City	**1,720**	1,720	**100.00**	0	**0.00**	0	**0.00**
Candon City	**1,018**	1,002	**98.43**	16	**1.57**	0	**0.00**
Dagupan City	**8,771**	7,391	**84.27**	1,359	**15.49**	21	**0.24**
Laoag City	**1,959**	1,958	**99.95**	1	**0.05**	0	**0.00**
San Carlos City	**2,876**	2,826	**98.26**	48	**1.67**	2	**0.07**
San Fernando City	**5,567**	4,881	**87.68**	686	**12.32**	0	**0.00**
Urdaneta City	**2,088**	2,013	**96.41**	75	**3.59**	0	**0.00**

Table 1.B.2.5 - Intrapartum Care and Delivery Outcome

Number and proportion of livebirths by birth weight

Philippines, Annual 2020

Area	Total No. of Livebirths	NBW (≥ 2500 grams)		LBW (< 2500)		UBW	
		No.	%	No.	%	No.	%
Vigan City	**1,823**	1,641	**90.02**	182	**9.98**	0	**0.00**
**Region 2**	**42,744**	**41,137**	**96.24**	**1,573**	**3.68**	**34**	**0.08**
Batanes	**246**	214	**86.99**	32	**13.01**	0	**0.00**
Cagayan	**9,758**	9,433	**96.67**	298	**3.05**	27	**0.28**
Isabela	**12,463**	12,143	**97.43**	317	**2.54**	3	**0.02**
Nueva Vizcaya	**7,520**	6,845	**91.02**	672	**8.94**	3	**0.04**
Quirino	**3,009**	2,890	**96.05**	119	**3.95**	0	**0.00**
Cauayan City	**1,954**	1,914	**97.95**	39	**2.00**	1	**0.05**
Ilagan City	**2,797**	2,797	**100.00**	0	**0.00**	0	**0.00**
Santiago City	**2,952**	2,914	**98.71**	38	**1.29**	0	**0.00**
Tuguegarao City	**2,045**	1,987	**97.16**	58	**2.84**	0	**0.00**
**Region 3**	**131,036**	**122,764**	**93.69**	**5,707**	**4.36**	**2,565**	**1.96**
Aurora	**3,217**	2,950	**91.70**	252	**7.83**	15	**0.47**
Bataan	**6,272**	5,947	**94.82**	303	**4.83**	22	**0.35**
Bulacan	**19,883**	17,869	**89.87**	1,465	**7.37**	549	**2.76**
Nueva Ecija	**15,082**	14,254	**94.51**	56	**0.37**	772	**5.12**
Pampanga	**12,800**	12,589	**98.35**	88	**0.69**	123	**0.96**
Tarlac	**13,439**	13,301	**98.97**	111	**0.83**	27	**0.20**
Zambales	**7,466**	6,824	**91.40**	587	**7.86**	55	**0.74**
Angeles City	**7,414**	6,794	**91.64**	620	**8.36**	0	**0.00**
Balanga City	**1,444**	1,365	**94.53**	77	**5.33**	2	**0.14**
Cabanatuan City	**8,036**	7,299	**90.83**	720	**8.96**	17	**0.21**
City of San Fernando	**2,219**	1,864	**84.00**	86	**3.88**	269	**12.12**
Gapan City	**1,162**	1,155	**99.40**	7	**0.60**	0	**0.00**
Mabalacat City	**4,632**	4,212	**90.93**	397	**8.57**	23	**0.50**
Malolos City	**4,797**	4,535	**94.54**	123	**2.56**	139	**2.90**
Meycauayan	**2,536**	2,533	**99.88**	0	**0.00**	3	**0.12**
Olongapo	**4,246**	3,462	**81.54**	245	**5.77**	539	**12.69**
Palayan City	**188**	188	**100.00**	0	**0.00**	0	**0.00**
San Jose City	**2,208**	2,113	**95.70**	85	**3.85**	10	**0.45**
San Jose del Monte City	**6,619**	6,322	**95.51**	297	**4.49**	0	**0.00**
Science City of Munoz	**70**	70	**100.00**	0	**0.00**	0	**0.00**
Tarlac City	**7,306**	7,118	**97.43**	188	**2.57**	0	**0.00**
**Region 4A**	**171,140**	**155,881**	**91.08**	**9,192**	**5.37**	**6,067**	**3.55**
Batangas	**15,862**	15,169	**95.63**	576	**3.63**	117	**0.74**
Cavite	**23,136**	21,454	**92.73**	1,152	**4.98**	530	**2.29**
Laguna	**12,308**	10,482	**85.16**	787	**6.39**	1,039	**8.44**
Quezon	**15,625**	14,436	**92.39**	697	**4.46**	492	**3.15**
Rizal	**23,951**	22,031	**91.98**	975	**4.07**	945	**3.95**
Antipolo City	**10,703**	9,303	**86.92**	1,130	**10.56**	270	**2.52**
Bacoor City	**6,609**	5,850	**88.52**	339	**5.13**	420	**6.35**
Batangas City	**0**	0	**0.00**	0	**0.00**	0	**0.00**

Table 1.B.2.5 - Intrapartum Care and Delivery Outcome

Number and proportion of livebirths by birth weight

Philippines, Annual 2020

Area	Total No. of Livebirths	NBW (≥ 2500 grams)		LBW (< 2500)		UBW	
		No.	%	No.	%	No.	%
Biñan City	**8,419**	7,998	**95.00**	258	**3.06**	163	**1.94**
Cabuyao City	**4,392**	3,694	**84.11**	146	**3.32**	552	**12.57**
Calamba City	**8,081**	7,532	**93.21**	397	**4.91**	152	**1.88**
Cavite City	**1,102**	1,056	**95.83**	42	**3.81**	4	**0.36**
Dasmariñas City	**8,571**	7,932	**92.54**	638	**7.44**	1	**0.01**
General Trias City	**1,410**	1,301	**92.27**	62	**4.40**	47	**3.33**
Imus City	**4,086**	3,760	**92.02**	191	**4.67**	135	**3.30**
Lipa City	**0**	0	**0.00**	0	**0.00**	0	**0.00**
Lucena City	**0**	0	**0.00**	0	**0.00**	0	**0.00**
San Pablo City	**2,037**	1,012	**49.68**	367	**18.02**	658	**32.30**
San Pedro City	**1,340**	1,281	**95.60**	51	**3.81**	8	**0.60**
Santa Rosa City	**8,276**	7,497	**90.59**	415	**5.01**	364	**4.40**
Tagaytay City	**2,778**	2,764	**99.50**	14	**0.50**	0	**0.00**
Tanauan City	**4,390**	4,102	**93.44**	230	**5.24**	58	**1.32**
Tayabas City	**1,594**	1,543	**96.80**	51	**3.20**	0	**0.00**
Trece Martires City	**6,470**	5,684	**87.85**	674	**10.42**	112	**1.73**
**Region 4B**	**43,771**	**39,312**	**89.81**	**3,475**	**7.94**	**984**	**2.25**
Marinduque	**3,247**	3,019	**92.98**	226	**6.96**	2	**0.06**
Mindoro Occidental	**7,924**	7,192	**90.76**	258	**3.26**	474	**5.98**
Mindoro Oriental	**12,260**	11,431	**93.24**	773	**6.31**	56	**0.46**
Palawan	**14,536**	12,262	**84.36**	1,931	**13.28**	343	**2.36**
Romblon	**2,101**	1,905	**90.67**	118	**5.62**	78	**3.71**
Puerto Princesa City	**3,703**	3,503	**94.60**	169	**4.56**	31	**0.84**
**Region 5**	**79,292**	**71,080**	**89.64**	**4,824**	**6.08**	**3,388**	**4.27**
Albay	**12,132**	11,374	**93.75**	595	**4.90**	163	**1.34**
Camarines Norte	**11,377**	10,110	**88.86**	1,226	**10.78**	41	**0.36**
Camarines Sur	**17,226**	14,531	**84.36**	820	**4.76**	1,875	**10.88**
Catanduanes	**4,550**	4,244	**93.27**	303	**6.66**	3	**0.07**
Masbate	**12,727**	11,565	**90.87**	655	**5.15**	507	**3.98**
Sorsogon	**13,129**	12,268	**93.44**	855	**6.51**	6	**0.05**
Iriga City	**2,156**	2,000	**92.76**	148	**6.86**	8	**0.37**
Legaspi City	**3,361**	2,446	**72.78**	151	**4.49**	764	**22.73**
Naga City	**2,634**	2,542	**96.51**	71	**2.70**	21	**0.80**
**Region 6**	**85,056**	**73,647**	**86.59**	**10,897**	**12.81**	**512**	**0.60**
Aklan	**7,095**	6,144	**86.60**	925	**13.04**	26	**0.37**
Antique	**8,727**	7,257	**83.16**	1,322	**15.15**	148	**1.70**
Capiz	**9,145**	8,294	**90.69**	830	**9.08**	21	**0.23**
Guimaras	**1,602**	1,462	**91.26**	140	**8.74**	0	**0.00**
Iloilo	**17,861**	16,230	**90.87**	1,549	**8.67**	82	**0.46**
Negros Occidental	**21,633**	18,362	**84.88**	3,130	**14.47**	141	**0.65**
Bacolod City	**8,899**	7,603	**85.44**	1,294	**14.54**	2	**0.02**
Iloilo City	**10,094**	8,295	**82.18**	1,707	**16.91**	92	**0.91**

Table 1.B.2.5 - Intrapartum Care and Delivery Outcome

Number and proportion of livebirths by birth weight
Philippines, Annual 2020

Area	Total No. of Livebirths	NBW (≥ 2500 grams)		LBW (< 2500)		UBW	
		No.	%	No.	%	No.	%
**Region 7**	**127,519**	**117,106**	**91.83**	**9,050**	**7.10**	**1,363**	**1.07**
Bohol	**19,885**	17,725	**89.14**	2,156	**10.84**	4	**0.02**
Cebu	**54,473**	51,252	**94.09**	2,908	**5.34**	313	**0.57**
Negros Oriental	**20,092**	17,739	**88.29**	1,315	**6.54**	1,038	**5.17**
Siquijor	**1,250**	1,102	**88.16**	148	**11.84**	0	**0.00**
Cebu City	**16,345**	15,628	**95.61**	717	**4.39**	0	**0.00**
Lapu-Lapu City	**5,358**	5,024	**93.77**	328	**6.12**	6	**0.11**
Mandaue City	**10,116**	8,636	**85.37**	1,478	**14.61**	2	**0.02**
**Region 8**	**60,669**	**56,137**	**92.53**	**3,019**	**4.98**	**1,513**	**2.49**
Biliran	**3,102**	2,794	**90.07**	308	**9.93**	0	**0.00**
Eastern Samar	**6,421**	6,095	**94.92**	153	**2.38**	173	**2.69**
Northern Leyte	**18,116**	17,322	**95.62**	501	**2.77**	293	**1.62**
Northern Samar	**9,702**	8,851	**91.23**	433	**4.46**	418	**4.31**
Southern Leyte	**2,460**	2,266	**92.11**	166	**6.75**	28	**1.14**
Western Samar	**8,158**	7,690	**94.26**	255	**3.13**	213	**2.61**
Calbayog City	**2,119**	1,661	**78.39**	74	**3.49**	384	**18.12**
Maasin City	**2,620**	2,232	**85.19**	388	**14.81**	0	**0.00**
Ormoc City	**5,243**	4,552	**86.82**	691	**13.18**	0	**0.00**
Tacloban City	**2,728**	2,674	**98.02**	50	**1.83**	4	**0.15**
**Region 9**	**61,984**	**57,302**	**92.45**	**1,413**	**2.28**	**3,269**	**5.27**
Zamboanga del Norte	**16,226**	14,861	**91.59**	408	**2.51**	957	**5.90**
Zamboanga del Sur	**9,922**	9,308	**93.81**	252	**2.54**	362	**3.65**
Zamboanga Sibugay	**9,057**	7,026	**77.58**	352	**3.89**	1,679	**18.54**
Dapitan City	**1,688**	1,612	**95.50**	70	**4.15**	6	**0.36**
Dipolog City	**2,645**	2,601	**98.34**	42	**1.59**	2	**0.08**
Isabela City	**1,855**	1,558	**83.99**	42	**2.26**	255	**13.75**
Pagadian City	**2,891**	2,813	**97.30**	78	**2.70**	0	**0.00**
Zamboanga City	**17,700**	17,523	**99.00**	169	**0.95**	8	**0.05**
**Region 10**	**82,736**	**76,958**	**93.02**	**3,962**	**4.79**	**1,816**	**2.19**
Bukidnon	**19,766**	18,516	**93.68**	996	**5.04**	254	**1.29**
Camiguin	**1,300**	1,275	**98.08**	23	**1.77**	2	**0.15**
Lanao del Norte	**10,411**	9,054	**86.97**	23	**0.22**	1,334	**12.81**
Misamis Occidental	**4,585**	4,441	**96.86**	60	**1.31**	84	**1.83**
Misamis Oriental	**9,589**	9,225	**96.20**	321	**3.35**	43	**0.45**
Cagayan de Oro City	**17,506**	15,506	**88.58**	2,000	**11.42**	0	**0.00**
El Salvador City	**326**	321	**98.47**	0	**0.00**	5	**1.53**
Gingoog City	**2,220**	2,150	**96.85**	30	**1.35**	40	**1.80**
Iligan City	**6,744**	6,545	**97.05**	186	**2.76**	13	**0.19**
Malaybalay City	**2,516**	2,419	**96.14**	97	**3.86**	0	**0.00**

Table 1.B.2.5 - Intrapartum Care and Delivery Outcome

Number and proportion of livebirths by birth weight
Philippines, Annual 2020

Area	Total No. of Livebirths	NBW (≥ 2500 grams)		LBW (< 2500)		UBW	
		No.	%	No.	%	No.	%
Oroquieta City	**917**	857	**93.46**	58	**6.32**	2	**0.22**
Ozamis City	**2,166**	2,156	**99.54**	10	**0.46**	0	**0.00**
Tangub City	**1,046**	997	**95.32**	27	**2.58**	22	**2.10**
Valencia City	**3,644**	3,496	**95.94**	131	**3.59**	17	**0.47**
**Region 11**	**84,121**	**77,928**	**92.64**	**3,813**	**4.53**	**2,380**	**2.83**
Compostela Valley	**11,860**	11,316	**95.41**	476	**4.01**	68	**0.57**
Davao del Norte	**20,226**	18,941	**93.65**	877	**4.34**	408	**2.02**
Davao Oriental	**8,249**	7,855	**95.22**	334	**4.05**	60	**0.73**
Davao del Sur	**9,376**	8,725	**93.06**	389	**4.15**	262	**2.79**
Davao Occidental	**4,275**	3,305	**77.31**	174	**4.07**	796	**18.62**
Davao City	**30,135**	27,786	**92.21**	1,563	**5.19**	786	**2.61**
**Region 12**	**71,992**	**67,634**	**93.95**	**2,097**	**2.91**	**2,261**	**3.14**
North Cotabato	**20,262**	18,664	**92.11**	646	**3.19**	952	**4.70**
Sarangani	**10,279**	9,862	**95.94**	94	**0.91**	323	**3.14**
South Cotabato	**16,880**	16,306	**96.60**	406	**2.41**	168	**1.00**
Sultan Kudarat	**12,234**	11,458	**93.66**	421	**3.44**	355	**2.90**
Cotabato City	**5,505**	5,069	**92.08**	126	**2.29**	310	**5.63**
Gen. Santos City	**6,832**	6,275	**91.85**	404	**5.91**	153	**2.24**
**BARMM**	**70,432**	**64,371**	**91.39**	**1,272**	**1.81**	**4,789**	**6.80**
Basilan	**3,125**	2,526	**80.83**	214	**6.85**	385	**12.32**
Lanao del Sur	**20,702**	19,612	**94.73**	58	**0.28**	1,032	**4.99**
Maguindanao	**25,491**	23,577	**92.49**	352	**1.38**	1,562	**6.13**
Sulu	**10,064**	9,138	**90.80**	212	**2.11**	714	**7.09**
Tawi-Tawi	**6,881**	6,493	**94.36**	154	**2.24**	234	**3.40**
Lamitan City	**1,102**	845	**76.68**	43	**3.90**	214	**19.42**
Marawi City	**3,067**	2,180	**71.08**	239	**7.79**	648	**21.13**
**CARAGA**	**38,350**	**35,614**	**92.87**	**1,627**	**4.24**	**1,109**	**2.89**
Agusan del Norte	**3,513**	3,214	**91.49**	171	**4.87**	128	**3.64**
Agusan del Sur	**11,887**	11,115	**93.51**	190	**1.60**	582	**4.90**
Surigao del Norte	**3,514**	3,231	**91.95**	183	**5.21**	100	**2.85**
Surigao del Sur	**6,439**	5,877	**91.27**	310	**4.81**	252	**3.91**
Province of Dinagat	**1,304**	1,216	**93.25**	81	**6.21**	7	**0.54**
Bislig City	**1,658**	1,616	**97.47**	34	**2.05**	8	**0.48**
Butuan City	**5,648**	5,350	**94.72**	276	**4.89**	22	**0.39**
Surigao City	**4,387**	3,995	**91.06**	382	**8.71**	10	**0.23**

Note: Put asterisk (*) for No Report and Zero (0) for No Case
Live births should be reported by place of occurrence.

NBW - Normal Birth Weight, LBW - Low Birth Weight
UBW - Unknown Birth Weight

Figure 1.B.2.1 - Deliveries attended by Skilled Health Professional Philippines, 2020

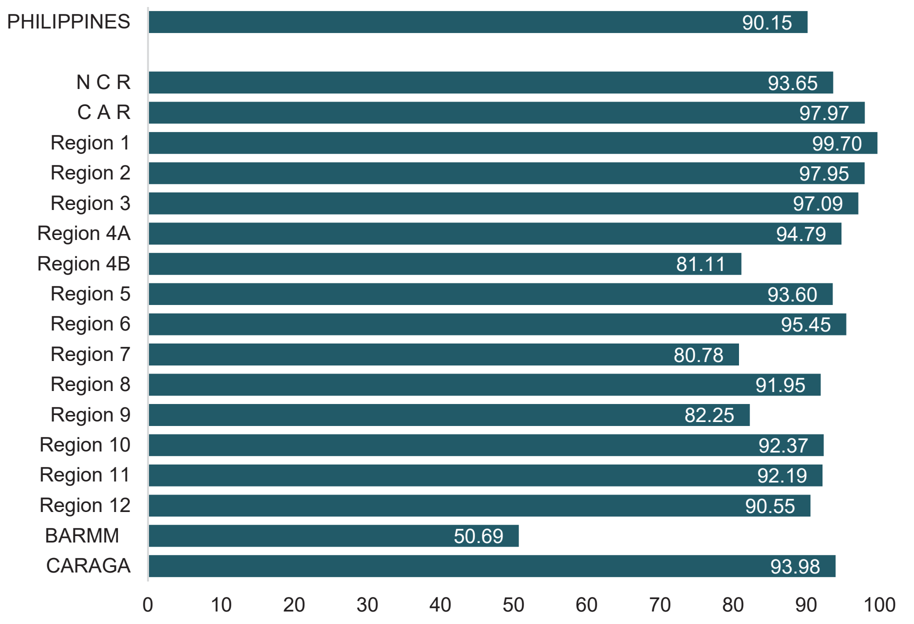

Figure 1.B.2.2 - Deliveries attended by Physicians Philippines, 2020

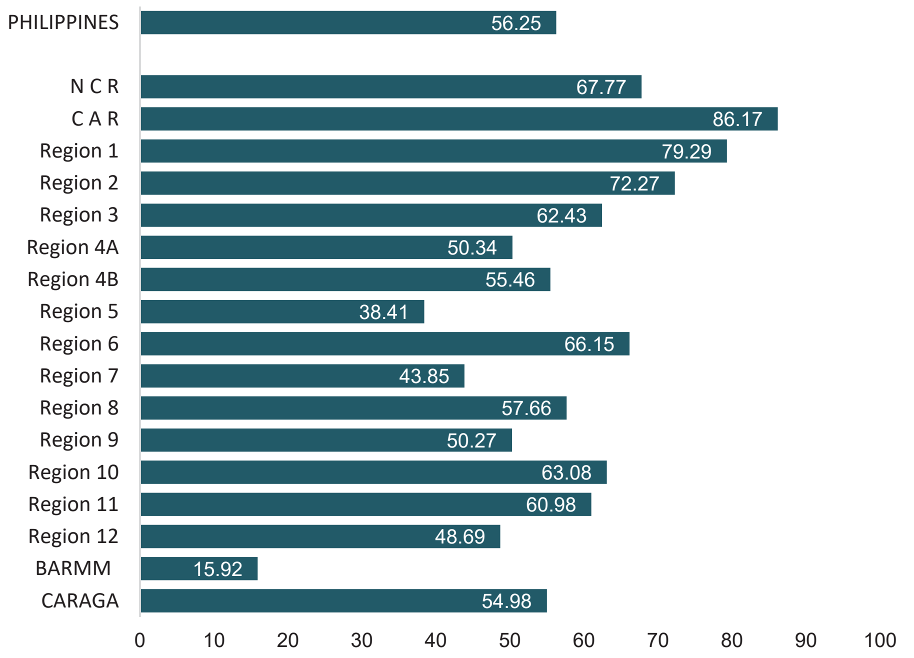

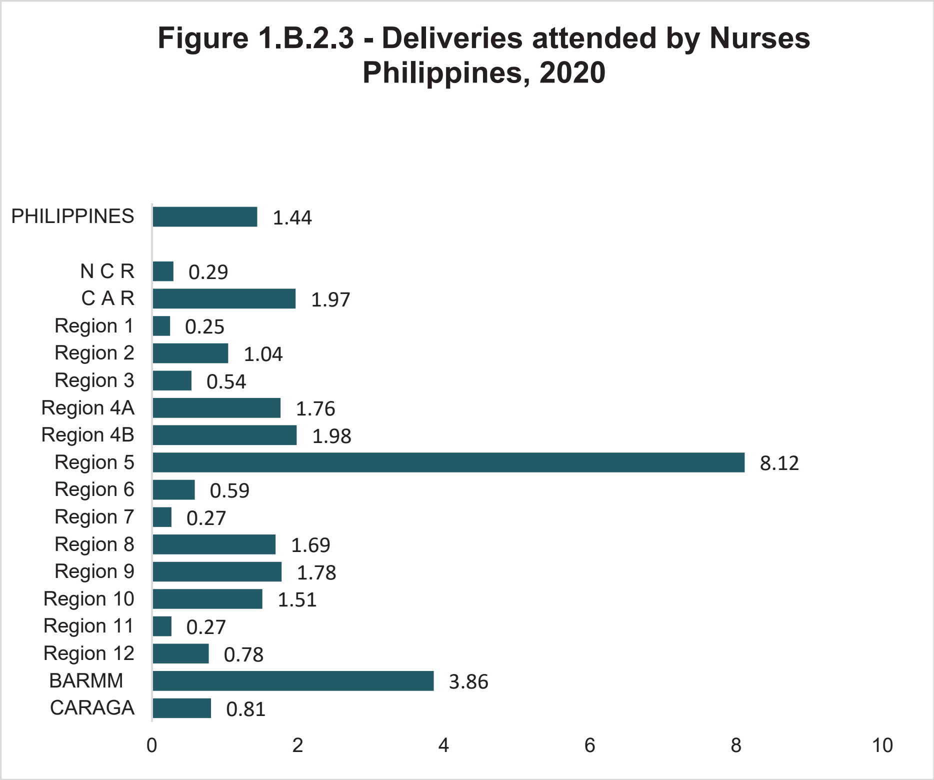
Figure 1.B.2.3 - Deliveries attended by Nurses
Philippines, 2020
PHILIPPINES
1.44
N C R
0.29
C A R
1.97
Region 1
0.25
Region 2
1.04
Region 3
0.54
Region 4A
1.76
Region 4B
1.98
Region 5
8.12
Region 6
0.59
Region 7
0.27
Region 8
1.69
Region 9
1.78
Region 10
1.51
Region 11
0.27
Region 12
0.78
BARMM
3.86
CARAGA
0.81
0
2
4
6
8
10

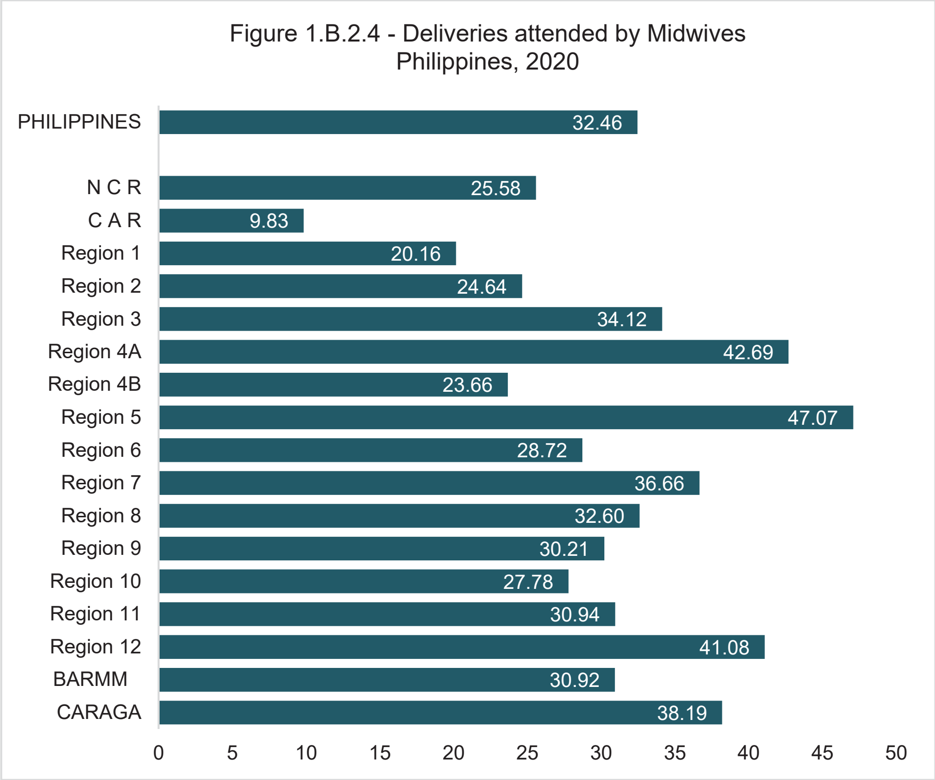
Figure 1.B.2.4 - Deliveries attended by Midwives
Philippines, 2020
PHILIPPINES
32.46
N C R
25.58
C A R
9.83
Region 1
20.16
Region 2
24.64
Region 3
34.12
Region 4A
42.69
Region 4B
23.66
Region 5
47.07
Region 6
28.72
Region 7
36.66
Region 8
32.60
Region 9
30.21
Region 10
27.78
Region 11
30.94
Region 12
41.08
BARMM
30.92
CARAGA
38.19
0
5
10
15
20
25
30
35
40
45
50

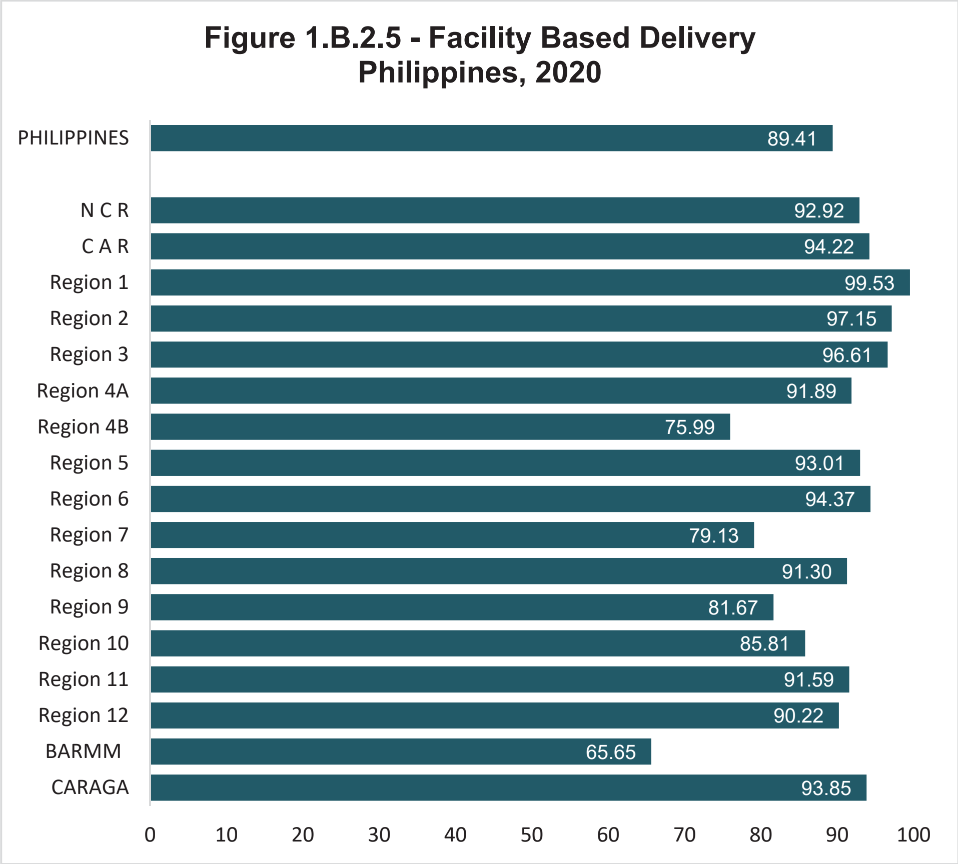
Figure 1.B.2.5 - Facility Based Delivery
Philippines, 2020
PHILIPPINES 89.41
N C R 92.92
C A R 94.22
Region 1 99.53
Region 2 97.15
Region 3 96.61
Region 4A 91.89
Region 4B 75.99
Region 5 93.01
Region 6 94.37
Region 7 79.13
Region 8 91.30
Region 9 81.67
Region 10 85.81
Region 11 91.59
Region 12 90.22
BARMM 65.65
CARAGA 93.85
0 10 20 30 40 50 60 70 80 90 100

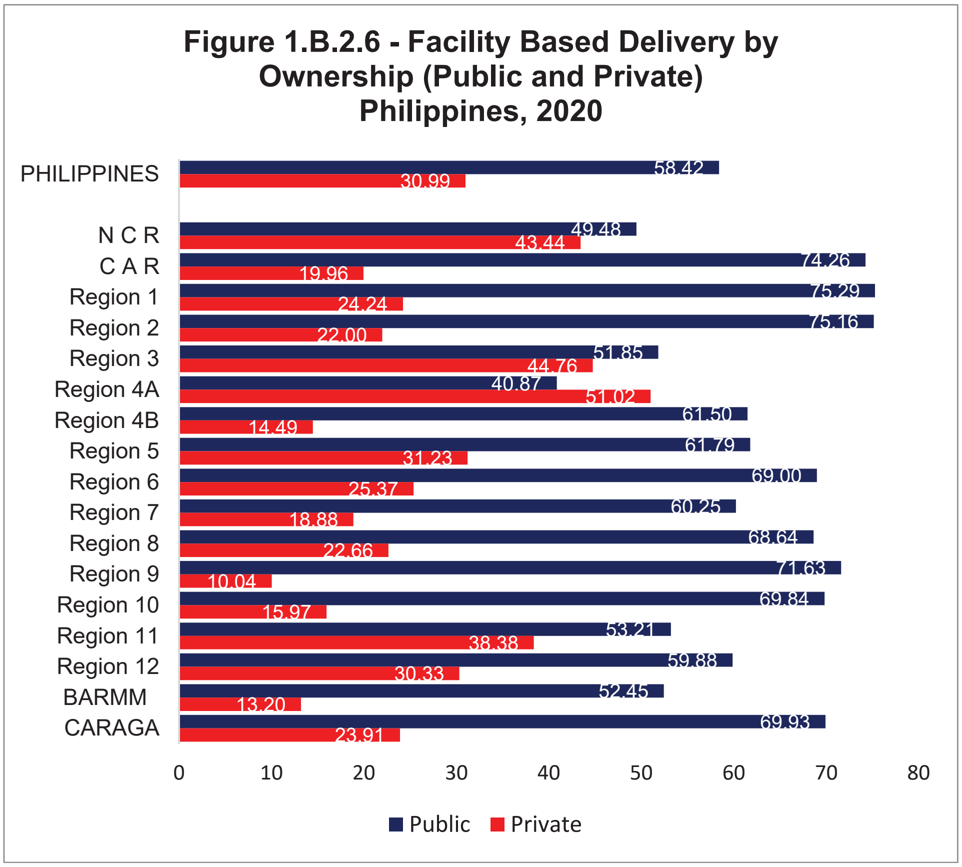
Figure 1.B.2.6 - Facility Based Delivery by Ownership (Public and Private)
Philippines, 2020
PHILIPPINES 58.42 30.99
N C R 49.48 43.44
C A R 74.26 19.96
Region 1 75.29 24.24
Region 2 75.16 22.00
Region 3 51.85 44.76
Region 4A 40.87 51.02
Region 4B 61.50 14.49
Region 5 61.79 31.23
Region 6 69.00 25.37
Region 7 60.25 18.88
Region 8 68.64 22.66
Region 9 71.63 10.04
Region 10 69.84 15.97
Region 11 53.21 38.38
Region 12 59.88 30.33
BARMM 52.45 13.20
CARAGA 69.93 23.91
0 10 20 30 40 50 60 70 80
Public
Private

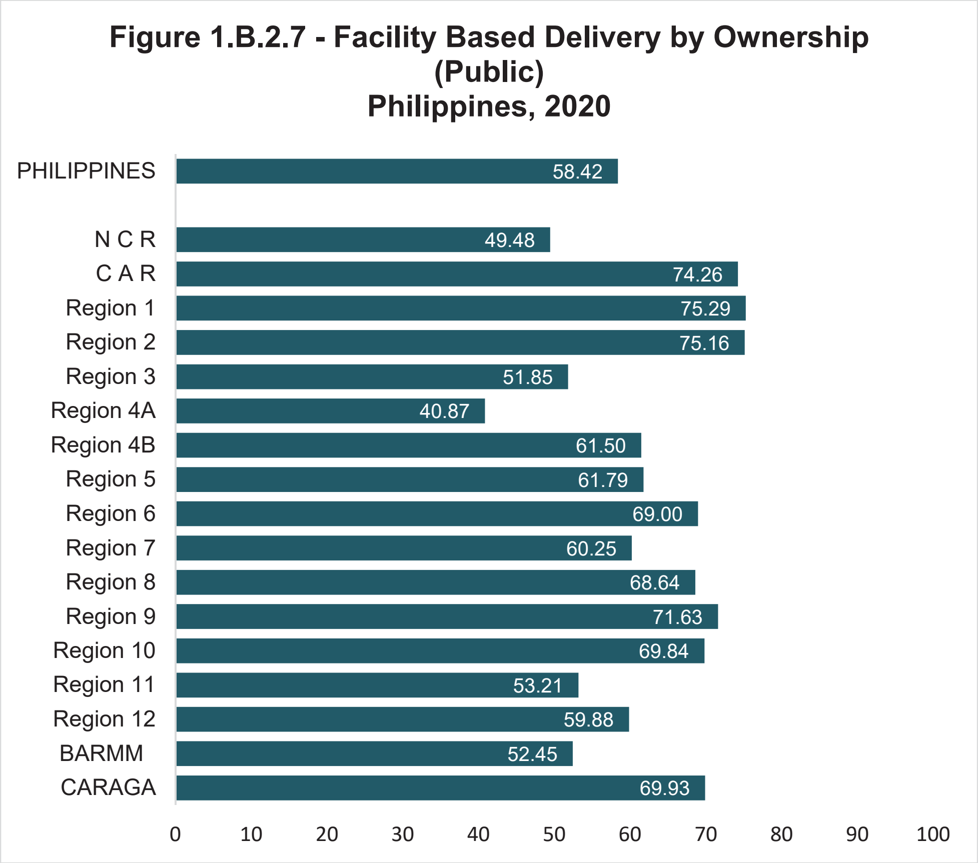
Figure 1.B.2.7 - Facility Based Delivery by Ownership (Public)
Philippines, 2020
PHILIPPINES 58.42
N C R 49.48
C A R 74.26
Region 1 75.29
Region 2 75.16
Region 3 51.85
Region 4A 40.87
Region 4B 61.50
Region 5 61.79
Region 6 69.00
Region 7 60.25
Region 8 68.64
Region 9 71.63
Region 10 69.84
Region 11 53.21
Region 12 59.88
BARMM 52.45
CARAGA 69.93
0 10 20 30 40 50 60 70 80 90 100

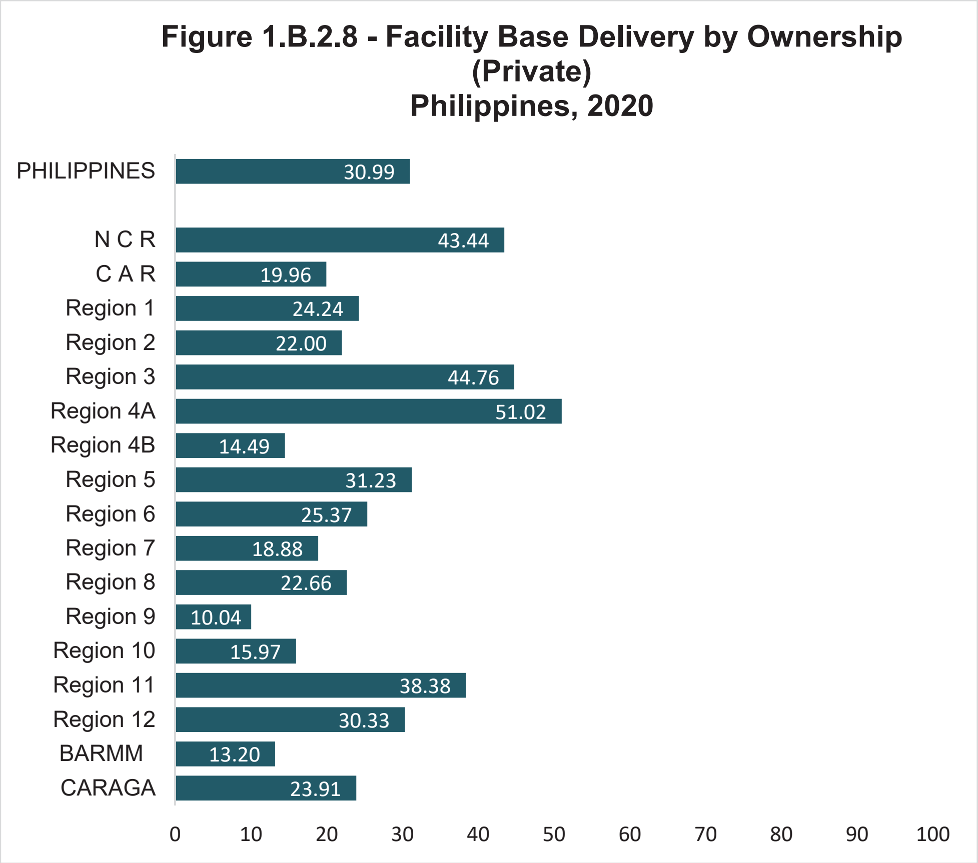
Figure 1.B.2.8 - Facility Base Delivery by Ownership (Private)
Philippines, 2020
PHILIPPINES 30.99
N C R 43.44
C A R 19.96
Region 1 24.24
Region 2 22.00
Region 3 44.76
Region 4A 51.02
Region 4B 14.49
Region 5 31.23
Region 6 25.37
Region 7 18.88
Region 8 22.66
Region 9 10.04
Region 10 15.97
Region 11 38.38
Region 12 30.33
BARMM 13.20
CARAGA 23.91
0 10 20 30 40 50 60 70 80 90 100

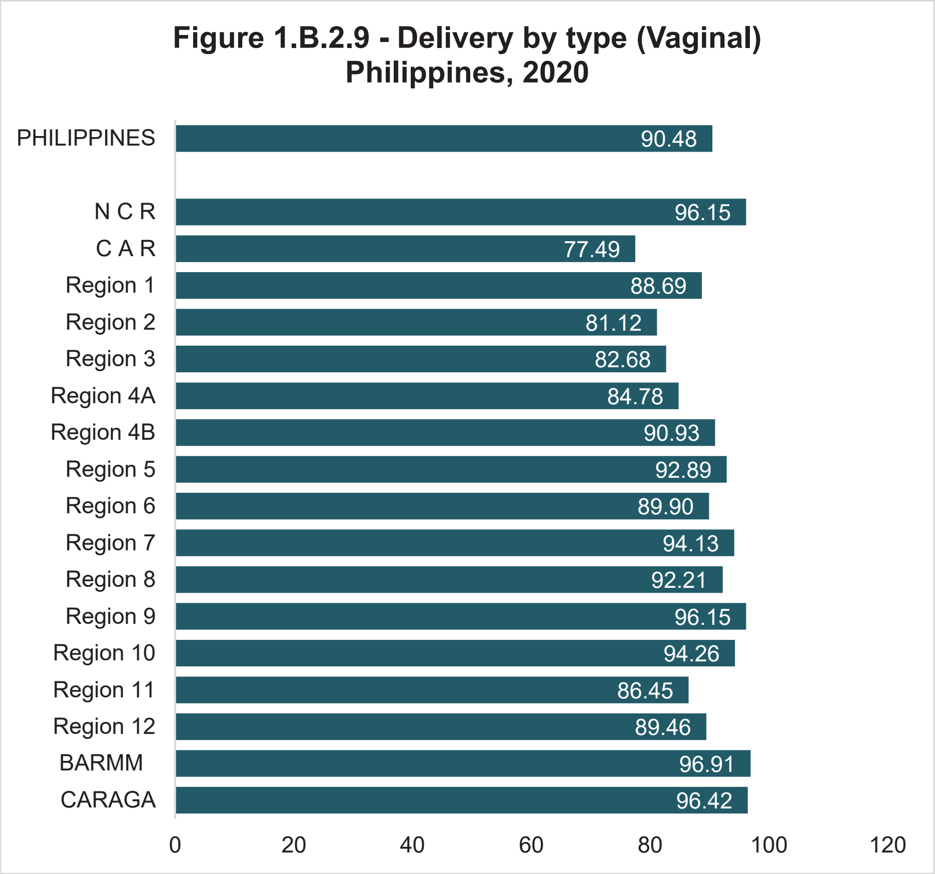
Figure 1.B.2.9 - Delivery by type (Vaginal)
Philippines, 2020
PHILIPPINES
90.48
N C R
96.15
C A R
77.49
Region 1
88.69
Region 2
81.12
Region 3
82.68
Region 4A
84.78
Region 4B
90.93
Region 5
92.89
Region 6
89.90
Region 7
94.13
Region 8
92.21
Region 9
96.15
Region 10
94.26
Region 11
86.45
Region 12
89.46
BARMM
96.91
CARAGA
96.42
0
20
40
60
80
100
120

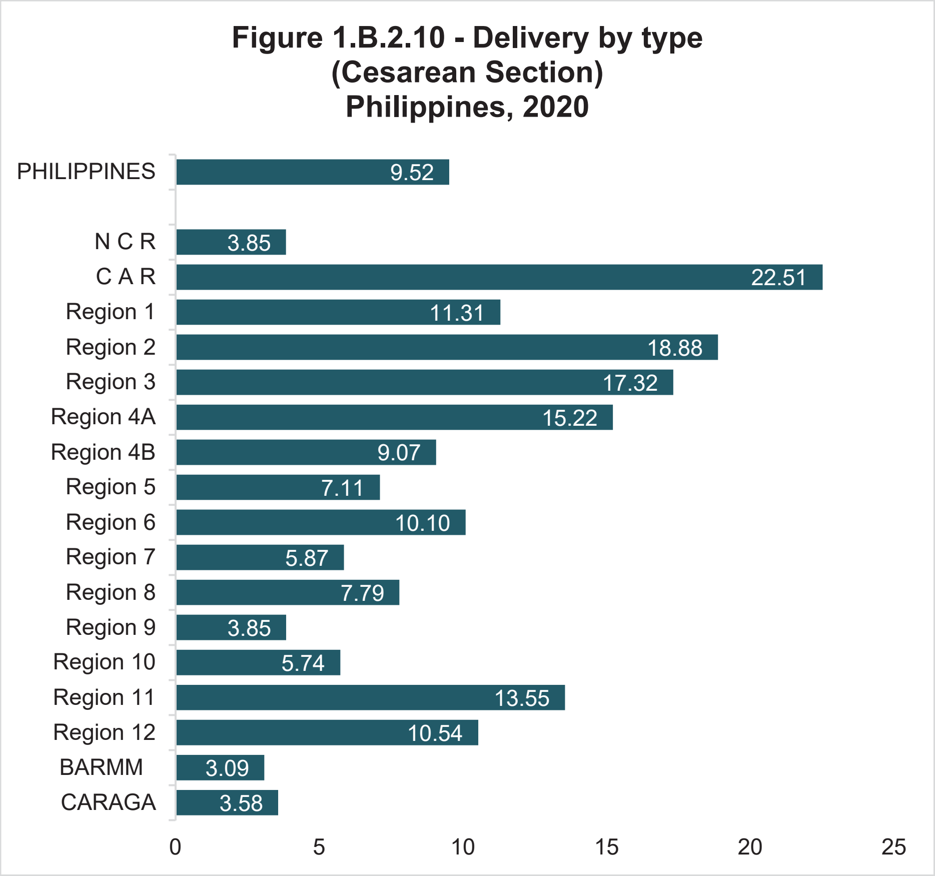
Figure 1.B.2.10 - Delivery by type
(Cesarean Section)
Philippines, 2020
PHILIPPINES
9.52
N C R
3.85
C A R
22.51
Region 1
11.31
Region 2
18.88
Region 3
17.32
Region 4A
15.22
Region 4B
9.07
Region 5
7.11
Region 6
10.10
Region 7
5.87
Region 8
7.79
Region 9
3.85
Region 10
5.74
Region 11
13.55
Region 12
10.54
BARMM
3.09
CARAGA
3.58
0
5
10
15
20
25

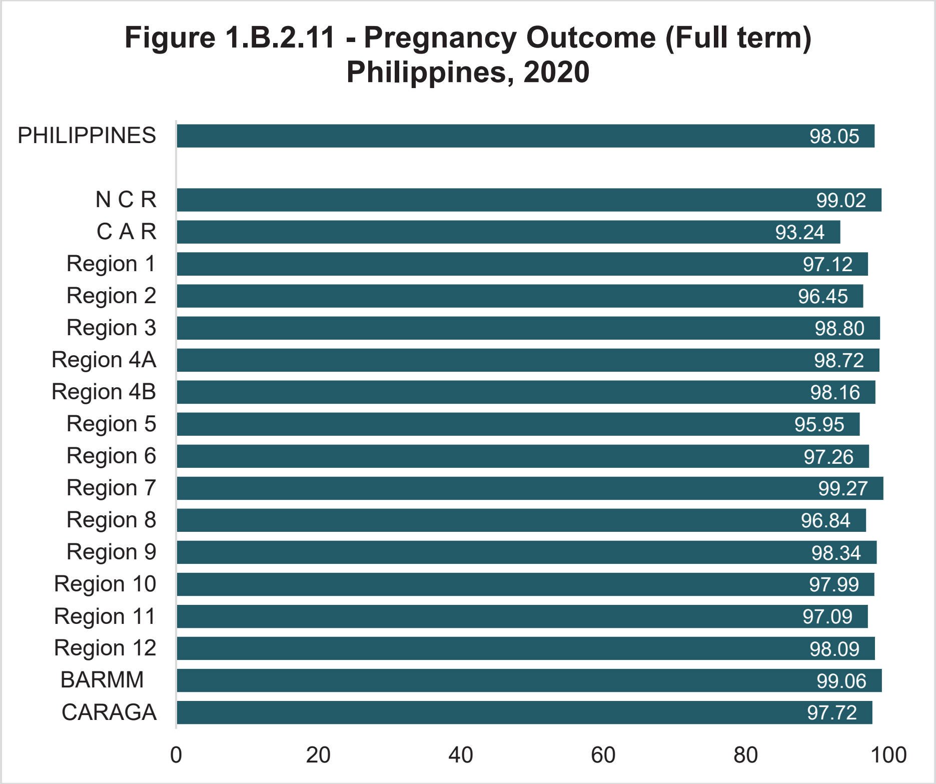
Figure 1.B.2.11 - Pregnancy Outcome (Full term)
Philippines, 2020
PHILIPPINES 98.05
N C R 99.02
C A R 93.24
Region 1 97.12
Region 2 96.45
Region 3 98.80
Region 4A 98.72
Region 4B 98.16
Region 5 95.95
Region 6 97.26
Region 7 99.27
Region 8 96.84
Region 9 98.34
Region 10 97.99
Region 11 97.09
Region 12 98.09
BARMM 99.06
CARAGA 97.72
0
20
40
60
80
100

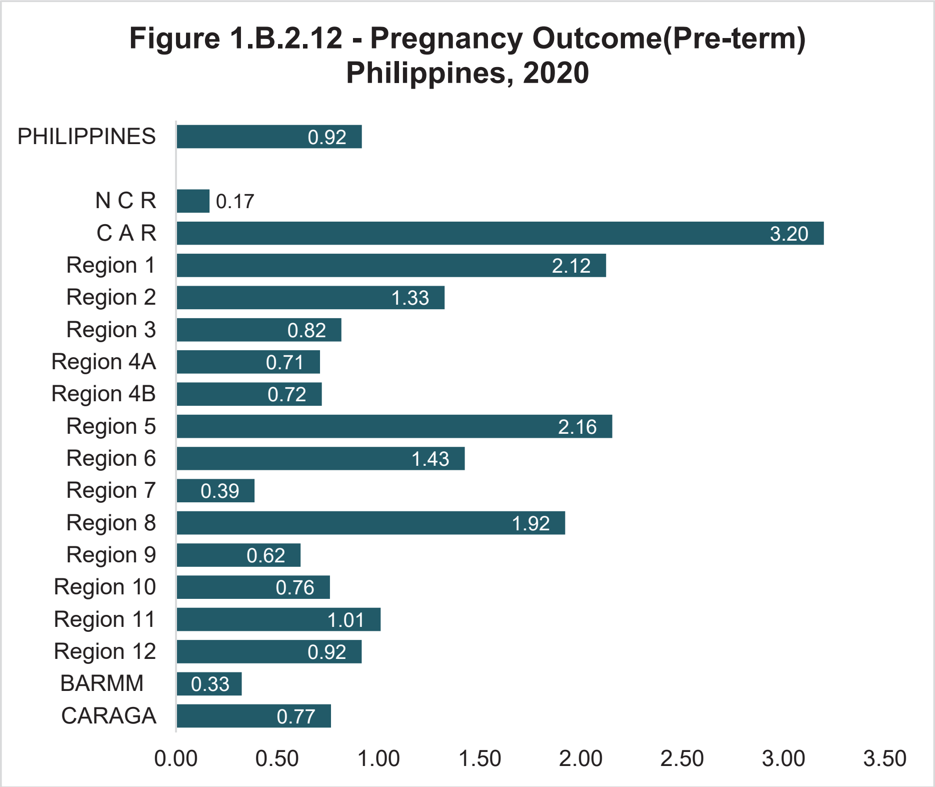
Figure 1.B.2.12 - Pregnancy Outcome(Pre-term)
Philippines, 2020
PHILIPPINES 0.92
N C R 0.17
C A R 3.20
Region 1 2.12
Region 2 1.33
Region 3 0.82
Region 4A 0.71
Region 4B 0.72
Region 5 2.16
Region 6 1.43
Region 7 0.39
Region 8 1.92
Region 9 0.62
Region 10 0.76
Region 11 1.01
Region 12 0.92
BARMM 0.33
CARAGA 0.77
0.00
0.50
1.00
1.50
2.00
2.50
3.00
3.50

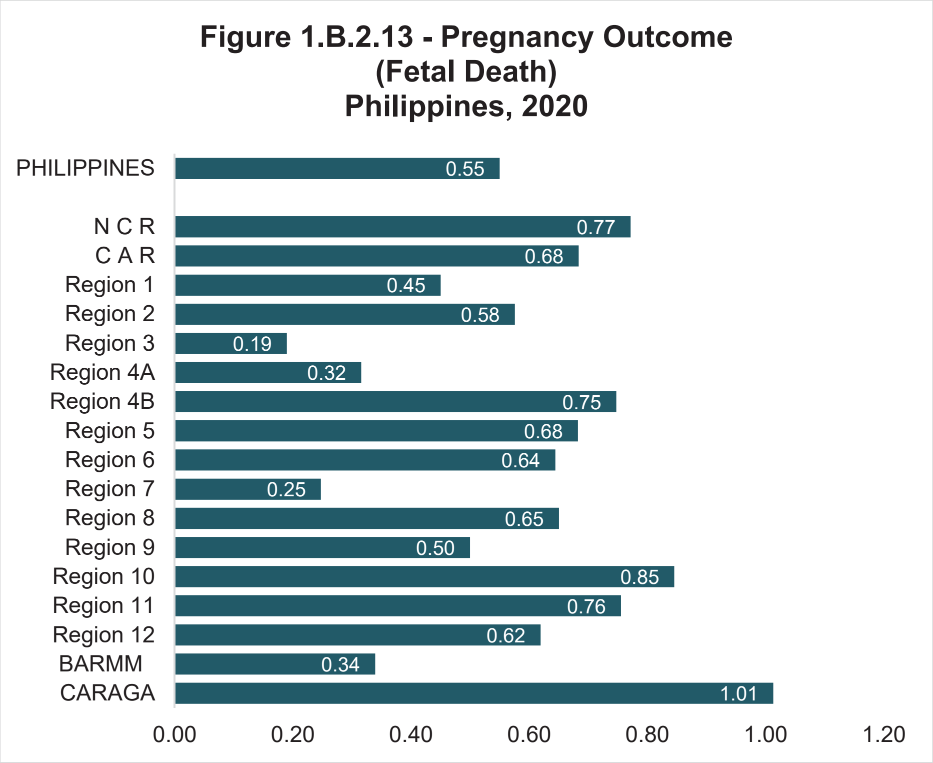
Figure 1.B.2.13 - Pregnancy Outcome
(Fetal Death)
Philippines, 2020
PHILIPPINES
0.55
N C R
0.77
C A R
0.68
Region 1
0.45
Region 2
0.58
Region 3
0.19
Region 4A
0.32
Region 4B
0.75
Region 5
0.68
Region 6
0.64
Region 7
0.25
Region 8
0.65
Region 9
0.50
Region 10
0.85
Region 11
0.76
Region 12
0.62
BARMM
0.34
CARAGA
1.01
0.00
0.20
0.40
0.60
0.80
1.00
1.20

Figure 1.B.2.14 - Percentage Distribution of Normal Birth Weight (≥ 2500 grams) by Region Philippines, 2020

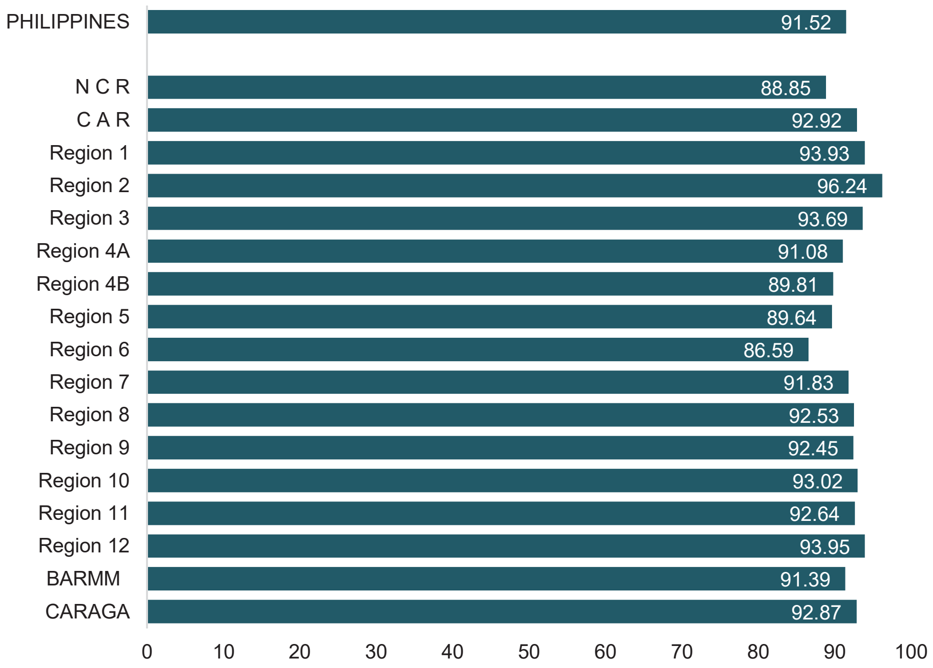

Figure 1.B.2.15 - Percentage Distribution of Low Birth Weight (< 2500) by Region Philippines, 2020

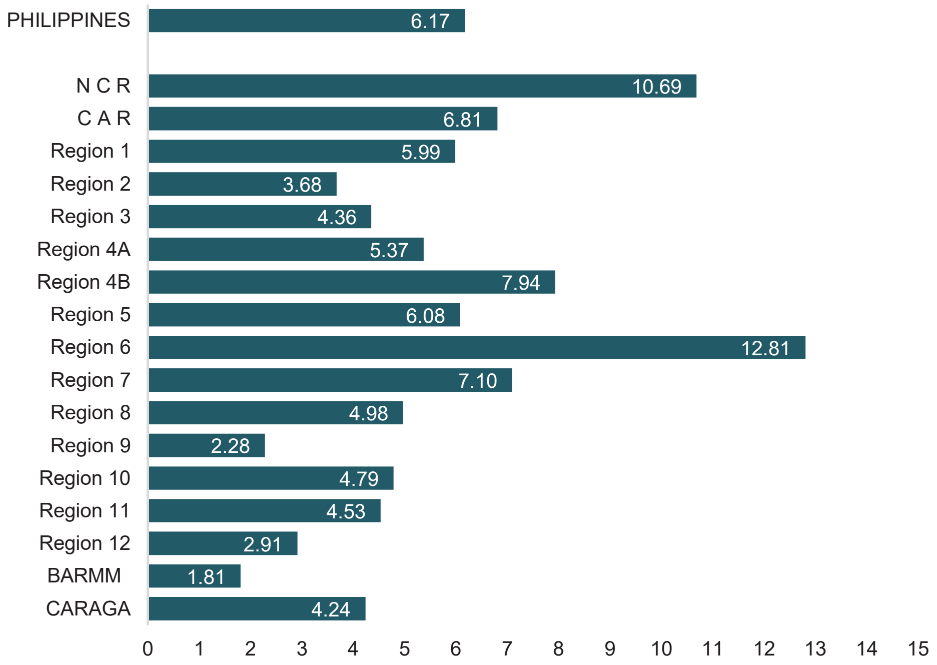

Figure 1.B.2.16 - Percentage Distribution of Unknown Birth Weight by Region Philippines, 2020

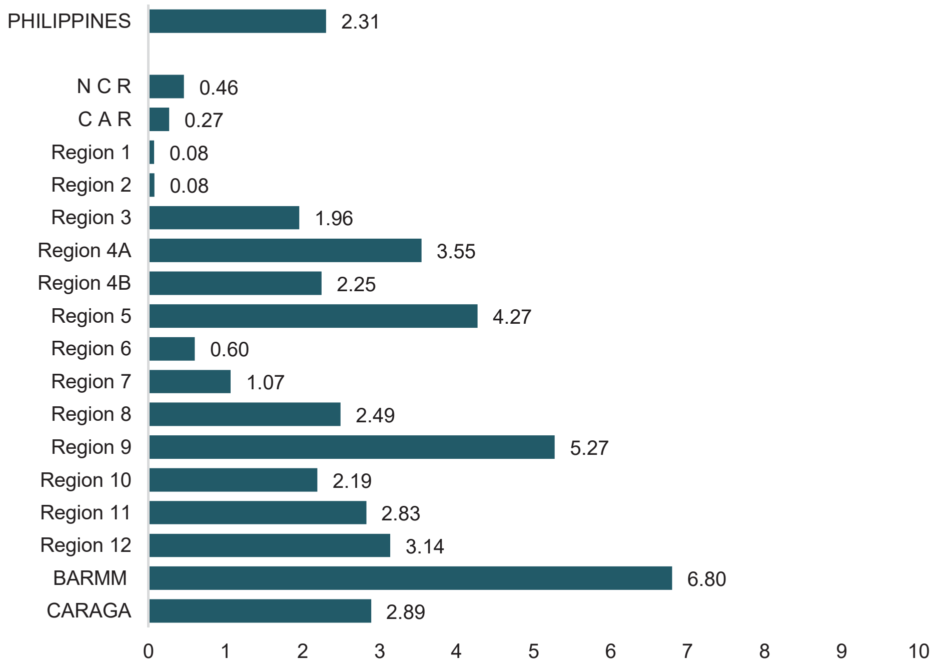

Table 1.B.3.1 - Postpartum and Newborn Care

Number and proportion of Postpartum women together with their newborn who completed at least 2 postpartum check-ups
Philippines, Annual 2020

Area	Eligible Pop.	Completed at least 2 postpartum check-ups							
		Age Group in Year						Total	%
		10 - 14		15-19		20-49			
		No.	%	No.	%	No.	%		
**PHILIPPINES**	**2,123,158**	**3,183**	**0.15**	**139,424**	**6.57**	**1,084,799**	**51.09**	**1,227,406**	**57.81**
**N C R**	**238,661**	**142**	**0.06**	**12,628**	**5.29**	**131,684**	**55.18**	**144,454**	**60.53**
Malabon	**6,775**	5	**0.07**	482	**7.11**	3,464	**51.13**	**3,951**	**58.32**
Navotas	**4,621**	6	**0.13**	553	**11.97**	3,289	**71.18**	**3,848**	**83.27**
Valenzuela City	**11,500**	7	**0.06**	644	**5.60**	5,910	**51.39**	**6,561**	**57.05**
Caloocan City	**29,363**	18	**0.06**	2,041	**6.95**	23,765	**80.94**	**25,824**	**87.95**
Marikina City	**8,354**	5	**0.06**	219	**2.62**	2,550	**30.52**	**2,774**	**33.21**
Pasig City	**13,996**	17	**0.12**	759	**5.42**	8,734	**62.40**	**9,510**	**67.95**
Pateros	**1,184**	0	**0.00**	87	**7.35**	776	**65.54**	**863**	**72.89**
Taguig	**14,918**	9	**0.06**	1,244	**8.34**	11,283	**75.63**	**12,536**	**84.03**
Quezon City	**54,413**	21	**0.04**	2,321	**4.27**	34,464	**63.34**	**36,806**	**67.64**
Makati City	**10,798**	7	**0.06**	330	**3.06**	4,082	**37.80**	**4,419**	**40.92**
Mandaluyong City	**7,160**	4	**0.06**	324	**4.53**	3,329	**46.49**	**3,657**	**51.08**
San Juan	**2,261**	1	**0.04**	116	**5.13**	1,116	**49.36**	**1,233**	**54.53**
Manila City	**32,980**	6	**0.02**	789	**2.39**	6,327	**19.18**	**7,122**	**21.59**
Las Piñas City	**10,914**	18	**0.16**	618	**5.66**	6,235	**57.13**	**6,871**	**62.96**
Muntinlupa City	**9,353**	9	**0.10**	858	**9.17**	7,853	**83.96**	**8,720**	**93.23**
Parañaque City	**12,343**	7	**0.06**	939	**7.61**	4,832	**39.15**	**5,778**	**46.81**
Pasay City	**7,728**	2	**0.03**	304	**3.93**	3,675	**47.55**	**3,981**	**51.51**
**C A R**	**35,099**	**18**	**0.05**	**2,326**	**6.63**	**20,479**	**58.35**	**22,823**	**65.02**
Abra	**4,309**	1	**0.02**	518	**12.02**	3,504	**81.32**	**4,023**	**93.36**
Apayao	**2,451**	2	**0.08**	225	**9.18**	1,289	**52.59**	**1,516**	**61.85**
Benguet	**9,188**	3	**0.03**	413	**4.49**	4,916	**53.50**	**5,332**	**58.03**
Ifugao	**4,396**	4	**0.09**	333	**7.58**	2,680	**60.96**	**3,017**	**68.63**
Kalinga	**4,617**	2	**0.04**	339	**7.34**	3,083	**66.77**	**3,424**	**74.16**
Mt. Province	**3,023**	3	**0.10**	258	**8.53**	2,190	**72.44**	**2,451**	**81.08**
Baguio City	**7,115**	3	**0.04**	240	**3.37**	2,817	**39.59**	**3,060**	**43.01**
**Region 1**	**97,261**	**51**	**0.05**	**5,962**	**6.13**	**58,535**	**60.18**	**64,548**	**66.37**
Ilocos Norte	**8,105**	8	**0.10**	451	**5.56**	5,124	**63.22**	**5,583**	**68.88**
Ilocos Sur	**9,330**	3	**0.03**	508	**5.44**	6,591	**70.64**	**7,102**	**76.12**
La Union	**11,511**	17	**0.15**	906	**7.87**	7,210	**62.64**	**8,133**	**70.65**
Pangasinan	**50,168**	11	**0.02**	2,828	**5.64**	27,366	**54.55**	**30,205**	**60.21**
Alaminos City	**1,894**	1	**0.05**	263	**13.89**	1,444	**76.24**	**1,708**	**90.18**
Candon City	**987**	0	**0.00**	8	**0.81**	910	**92.20**	**918**	**93.01**
Dagupan City	**3,620**	4	**0.11**	278	**7.68**	2,002	**55.30**	**2,284**	**63.09**
Laoag City	**1,870**	0	**0.00**	57	**3.05**	1,804	**96.47**	**1,861**	**99.52**
San Carlos City	**3,979**	4	**0.10**	251	**6.31**	2,580	**64.84**	**2,835**	**71.25**
San Fernando City	**2,115**	3	**0.14**	106	**5.01**	1,354	**64.02**	**1,463**	**69.17**
Urdaneta City	**2,806**	0	**0.00**	235	**8.37**	1,626	**57.95**	**1,861**	**66.32**
Vigan City	**876**	0	**0.00**	71	**8.11**	524	**59.82**	**595**	**67.92**
**Region 2**	**69,443**	**67**	**0.10**	**5,587**	**8.05**	**40,062**	**57.69**	**45,716**	**65.83**
Batanes	**340**	0	**0.00**	24	**7.06**	236	**69.41**	**260**	**76.47**
Cagayan	**17,971**	13	**0.07**	1,481	**8.24**	10,767	**59.91**	**12,261**	**68.23**
Isabela	**26,309**	25	**0.10**	1,842	**7.00**	12,777	**48.57**	**14,644**	**55.66**
Nueva Vizcaya	**9,573**	11	**0.11**	911	**9.52**	6,082	**63.53**	**7,004**	**73.16**

Table 1.B.3.1 - Postpartum and Newborn Care

Number and proportion of Postpartum women together with their newborn who completed at least 2 postpartum check-ups
Philippines, Annual 2020

Area	Eligible Pop.	Completed at least 2 postpartum check-ups							
		Age Group in Year						Total	%
		10 - 14		15-19		20-49			
		No.	%	No.	%	No.	%		
Quirino	**4,025**	8	**0.20**	424	**10.53**	3,033	**75.35**	**3,465**	**86.09**
Cauayan City	**2,590**	4	**0.15**	286	**11.04**	1,659	**64.05**	**1,949**	**75.25**
Ilagan City	**2,911**	1	**0.03**	148	**5.08**	1,342	**46.10**	**1,491**	**51.22**
Santiago City	**2,692**	4	**0.15**	344	**12.78**	2,290	**85.07**	**2,638**	**97.99**
Tuguegarao City	**3,032**	1	**0.03**	127	**4.19**	1,876	**61.87**	**2,004**	**66.09**
**Region 3**	**220,020**	**311**	**0.14**	**16,547**	**7.52**	**119,338**	**54.24**	**136,196**	**61.90**
Aurora	**4,770**	2	**0.04**	397	**8.32**	2,672	**56.02**	**3,071**	**64.38**
Bataan	**13,823**	30	**0.22**	1,161	**8.40**	8,466	**61.25**	**9,657**	**69.86**
Bulacan	**43,627**	92	**0.21**	3,379	**7.75**	22,125	**50.71**	**25,596**	**58.67**
Nueva Ecija	**28,782**	45	**0.16**	1,692	**5.88**	11,735	**40.77**	**13,472**	**46.81**
Pampanga	**31,359**	18	**0.06**	1,537	**4.90**	13,655	**43.54**	**15,210**	**48.50**
Tarlac	**19,765**	40	**0.20**	1,515	**7.67**	14,329	**72.50**	**15,884**	**80.36**
Zambales	**12,610**	5	**0.04**	530	**4.20**	4,150	**32.91**	**4,685**	**37.15**
Angeles City	**7,869**	7	**0.09**	458	**5.82**	3,330	**42.32**	**3,795**	**48.23**
Balanga City	**1,998**	4	**0.20**	206	**10.31**	1,196	**59.86**	**1,406**	**70.37**
Cabanatuan City	**5,896**	9	**0.15**	537	**9.11**	4,203	**71.29**	**4,749**	**80.55**
City of San Fernando	**5,863**	5	**0.09**	581	**9.91**	3,623	**61.79**	**4,209**	**71.79**
Gapan City	**2,153**	1	**0.05**	160	**7.43**	959	**44.54**	**1,120**	**52.02**
Mabalacat City	**4,793**	5	**0.10**	540	**11.27**	4,203	**87.69**	**4,748**	**99.06**
Malolos City	**4,880**	7	**0.14**	337	**6.91**	2,557	**52.40**	**2,901**	**59.45**
Meycauayan	**4,041**	7	**0.17**	418	**10.34**	2,473	**61.20**	**2,898**	**71.71**
Olongapo	**4,971**	4	**0.08**	282	**5.67**	2,631	**52.93**	**2,917**	**58.68**
Palayan City	**799**	0	**0.00**	2	**0.25**	209	**26.16**	**211**	**26.41**
San Jose City	**2,718**	11	**0.40**	352	**12.95**	1,849	**68.03**	**2,212**	**81.38**
San Jose del Monte City	**11,100**	14	**0.13**	1,432	**12.90**	8,870	**79.91**	**10,316**	**92.94**
Science City of Munoz	**1,586**	1	**0.06**	113	**7.12**	802	**50.57**	**916**	**57.76**
Tarlac City	**6,617**	4	**0.06**	918	**13.87**	5,301	**80.11**	**6,223**	**94.05**
**Region 4A**	**296,816**	**1,039**	**0.35**	**16,671**	**5.62**	**95,740**	**32.26**	**113,450**	**38.22**
Batangas	**38,441**	11	**0.03**	649	**1.69**	9,377	**24.39**	**10,037**	**26.11**
Cavite	**27,704**	19	**0.07**	1,498	**5.41**	16,275	**58.75**	**17,792**	**64.22**
Laguna	**19,727**	326	**1.65**	2,752	**13.95**	6,350	**32.19**	**9,428**	**47.79**
Quezon	**37,410**	12	**0.03**	861	**2.30**	7,640	**20.42**	**8,513**	**22.76**
Rizal	**44,791**	24	**0.05**	1,981	**4.42**	15,800	**35.27**	**17,805**	**39.75**
Antipolo City	**16,497**	2	**0.01**	541	**3.28**	5,992	**36.32**	**6,535**	**39.61**
Bacoor City	**12,142**	0	**0.00**	70	**0.58**	949	**7.82**	**1,019**	**8.39**
Batangas City	**6,823**	0	**0.00**	0	**0.00**	0	**0.00**	**0**	**0.00**
Biñan City	**6,607**	26	**0.39**	892	**13.50**	5,638	**85.33**	**6,556**	**99.23**
Cabuyao City	**6,130**	244	**3.98**	1,895	**30.91**	1,941	**31.66**	**4,080**	**66.56**
Calamba City	**9,028**	8	**0.09**	791	**8.76**	7,015	**77.70**	**7,814**	**86.55**
Cavite City	**2,075**	0	**0.00**	0	**0.00**	0	**0.00**	**0**	**0.00**
Dasmariñas City	**13,322**	3	**0.02**	326	**2.45**	4,470	**33.55**	**4,799**	**36.02**
General Trias City	**6,352**	3	**0.05**	203	**3.20**	2,130	**33.53**	**2,336**	**36.78**
Imus City	**8,156**	0	**0.00**	80	**0.98**	997	**12.22**	**1,077**	**13.21**
Lipa City	**6,880**	0	**0.00**	0	**0.00**	0	**0.00**	**0**	**0.00**
Lucena City	**5,672**	0	**0.00**	0	**0.00**	0	**0.00**	**0**	**0.00**
San Pablo City	**5,277**	307	**5.82**	1,964	**37.22**	243	**4.60**	**2,514**	**47.64**
San Pedro City	**6,467**	43	**0.66**	747	**11.55**	711	**10.99**	**1,501**	**23.21**
Santa Rosa City	**7,025**	6	**0.09**	545	**7.76**	4,260	**60.64**	**4,811**	**68.48**
Tagaytay City	**1,437**	0	**0.00**	44	**3.06**	1,372	**95.48**	**1,416**	**98.54**
Tanauan City	**3,584**	1	**0.03**	56	**1.56**	1,860	**51.90**	**1,917**	**53.49**
Tayabas City	**2,122**	0	**0.00**	0	**0.00**	435	**20.50**	**435**	**20.50**
Trece Martires City	**3,147**	4	**0.13**	776	**24.66**	2,285	**72.61**	**3,065**	**97.39**

Table 1.B.3.1 - Postpartum and Newborn Care

Number and proportion of Postpartum women together with their newborn who completed at least 2 postpartum check-ups
Philippines, Annual 2020

Area	Eligible Pop.	Completed at least 2 postpartum check-ups							
		Age Group in Year						Total	%
		10 - 14		15-19		20-49			
		No.	%	No.	%	No.	%		
**Region 4B**	**71,246**	**82**	**0.12**	**4,066**	**5.71**	**31,478**	**44.18**	**35,626**	**50.00**
Marinduque	**4,836**	3	**0.06**	332	**6.87**	2,840	**58.73**	**3,175**	**65.65**
Mindoro Occidental	**12,407**	25	**0.20**	1,089	**8.78**	7,159	**57.70**	**8,273**	**66.68**
Mindoro Oriental	**19,809**	9	**0.05**	618	**3.12**	9,108	**45.98**	**9,735**	**49.14**
Palawan	**21,375**	40	**0.19**	1,880	**8.80**	11,197	**52.38**	**13,117**	**61.37**
Romblon	**6,400**	5	**0.08**	89	**1.39**	647	**10.11**	**741**	**11.58**
Puerto Princesa City	**6,419**	0	**0.00**	58	**0.90**	527	**8.21**	**585**	**9.11**
**Region 5**	**136,116**	**91**	**0.07**	**7,846**	**5.76**	**71,364**	**52.43**	**79,301**	**58.26**
Albay	**23,737**	13	**0.05**	1,118	**4.71**	12,889	**54.30**	**14,020**	**59.06**
Camarines Norte	**14,283**	8	**0.06**	1,086	**7.60**	7,826	**54.79**	**8,920**	**62.45**
Camarines Sur	**38,796**	27	**0.07**	1,916	**4.94**	18,446	**47.55**	**20,389**	**52.55**
Catanduanes	**6,274**	4	**0.06**	451	**7.19**	3,733	**59.50**	**4,188**	**66.75**
Masbate	**22,521**	25	**0.11**	1,680	**7.46**	11,275	**50.06**	**12,980**	**57.64**
Sorsogon	**19,065**	9	**0.05**	1,163	**6.10**	11,201	**58.75**	**12,373**	**64.90**
Iriga City	**2,638**	1	**0.04**	91	**3.45**	1,089	**41.28**	**1,181**	**44.77**
Legaspi City	**4,174**	2	**0.05**	160	**3.83**	2,985	**71.51**	**3,147**	**75.40**
Naga City	**4,628**	2	**0.04**	181	**3.91**	1,920	**41.49**	**2,103**	**45.44**
**Region 6**	**146,526**	**209**	**0.14**	**9,272**	**6.33**	**76,134**	**51.96**	**85,615**	**58.43**
Aklan	**11,162**	18	**0.16**	665	**5.96**	5,989	**53.66**	**6,672**	**59.77**
Antique	**12,816**	11	**0.09**	663	**5.17**	6,484	**50.59**	**7,158**	**55.85**
Capiz	**13,986**	21	**0.15**	530	**3.79**	5,667	**40.52**	**6,218**	**44.46**
Guimaras	**3,085**	3	**0.10**	182	**5.90**	2,269	**73.55**	**2,454**	**79.55**
Iloilo	**36,541**	48	**0.13**	1,876	**5.13**	19,078	**52.21**	**21,002**	**57.48**
Negros Occidental	**49,368**	90	**0.18**	4,383	**8.88**	28,985	**58.71**	**33,458**	**67.77**
Bacolod City	**11,115**	7	**0.06**	367	**3.30**	2,935	**26.41**	**3,309**	**29.77**
Iloilo City	**8,453**	11	**0.13**	606	**7.17**	4,727	**55.92**	**5,344**	**63.22**
**Region 7**	**163,262**	**124**	**0.08**	**6,908**	**4.23**	**69,603**	**42.63**	**76,635**	**46.94**
Bohol	**27,312**	15	**0.05**	941	**3.45**	11,521	**42.18**	**12,477**	**45.68**
Cebu	**67,506**	39	**0.06**	1,420	**2.10**	14,603	**21.63**	**16,062**	**23.79**
Negros Oriental	**27,938**	34	**0.12**	2,023	**7.24**	15,415	**55.18**	**17,472**	**62.54**
Siquijor	**1,613**	1	**0.06**	136	**8.43**	1,082	**67.08**	**1,219**	**75.57**
Cebu City	**21,193**	32	**0.15**	1,869	**8.82**	14,489	**68.37**	**16,390**	**77.34**
Lapu-Lapu City	**9,372**	3	**0.03**	519	**5.54**	7,337	**78.29**	**7,859**	**83.86**
Mandaue City	**8,328**	0	**0.00**	0	**0.00**	5,156	**61.91**	**5,156**	**61.91**
**Region 8**	**102,619**	**119**	**0.12**	**6,147**	**5.99**	**51,119**	**49.81**	**57,385**	**55.92**
Biliran	**3,834**	2	**0.05**	307	**8.01**	2,332	**60.82**	**2,641**	**68.88**
Eastern Samar	**11,392**	7	**0.06**	674	**5.92**	5,587	**49.04**	**6,268**	**55.02**
Northern Leyte	**34,707**	27	**0.08**	2,142	**6.17**	19,257	**55.48**	**21,426**	**61.73**
Northern Samar	**15,370**	23	**0.15**	654	**4.26**	6,680	**43.46**	**7,357**	**47.87**
Southern Leyte	**6,451**	4	**0.06**	491	**7.61**	3,594	**55.71**	**4,089**	**63.39**
Western Samar	**14,305**	46	**0.32**	945	**6.61**	6,342	**44.33**	**7,333**	**51.26**
Calbayog City	**4,413**	0	**0.00**	120	**2.72**	1,128	**25.56**	**1,248**	**28.28**
Maasin City	**1,637**	0	**0.00**	76	**4.64**	730	**44.59**	**806**	**49.24**
Ormoc City	**4,941**	9	**0.18**	456	**9.23**	3,214	**65.05**	**3,679**	**74.46**

Table 1.B.3.1 - Postpartum and Newborn Care

Number and proportion of Postpartum women together with their newborn who completed at least 2 postpartum check-ups
Philippines, Annual 2020

Area	Eligible Pop.	Completed at least 2 postpartum check-ups							
		Age Group in Year						Total	%
		10 - 14		15-19		20-49			
		No.	%	No.	%	No.	%		
Tacloban City	**5,569**	1	**0.02**	282	**5.06**	2,255	**40.49**	**2,538**	**45.57**
**Region 9**	**80,051**	**82**	**0.10**	**6,284**	**7.85**	**47,135**	**58.88**	**53,501**	**66.83**
Zamboanga del Norte	**17,249**	17	**0.10**	2,117	**12.27**	14,403	**83.50**	**16,537**	**95.87**
Zamboanga del Sur	**17,653**	13	**0.07**	1,138	**6.45**	7,493	**42.45**	**8,644**	**48.97**
Zamboanga Sibugay	**14,954**	39	**0.26**	1,047	**7.00**	6,031	**40.33**	**7,117**	**47.59**
Dapitan City	**1,784**	3	**0.17**	191	**10.71**	1,432	**80.27**	**1,626**	**91.14**
Dipolog City	**2,827**	1	**0.04**	328	**11.60**	2,277	**80.54**	**2,606**	**92.18**
Isabela City	**2,522**	2	**0.08**	257	**10.19**	1,548	**61.38**	**1,807**	**71.65**
Pagadian City	**4,325**	2	**0.05**	222	**5.13**	1,885	**43.58**	**2,109**	**48.76**
Zamboanga City	**18,737**	5	**0.03**	984	**5.25**	12,066	**64.40**	**13,055**	**69.67**
**Region 10**	**101,411**	**244**	**0.24**	**10,135**	**9.99**	**63,035**	**62.16**	**73,414**	**72.39**
Bukidnon	**23,706**	82	**0.35**	3,384	**14.27**	14,128	**59.60**	**17,594**	**74.22**
Camiguin	**1,858**	0	**0.00**	127	**6.84**	1,177	**63.35**	**1,304**	**70.18**
Lanao del Norte	**14,960**	100	**0.67**	807	**5.39**	7,799	**52.13**	**8,706**	**58.20**
Misamis Occidental	**6,403**	4	**0.06**	379	**5.92**	4,031	**62.95**	**4,414**	**68.94**
Misamis Oriental	**15,131**	14	**0.09**	1,410	**9.32**	9,234	**61.03**	**10,658**	**70.44**
Cagayan de Oro City	**14,339**	22	**0.15**	1,316	**9.18**	10,030	**69.95**	**11,368**	**79.28**
El Salvador City	**1,065**	1	**0.09**	130	**12.21**	715	**67.14**	**846**	**79.44**
Gingoog City	**2,644**	1	**0.04**	350	**13.24**	2,058	**77.84**	**2,409**	**91.11**
Iligan City	**7,580**	8	**0.11**	773	**10.20**	5,150	**67.94**	**5,931**	**78.25**
Malaybalay City	**3,956**	5	**0.13**	412	**10.41**	2,097	**53.01**	**2,514**	**63.55**
Oroquieta City	**1,389**	1	**0.07**	95	**6.84**	812	**58.46**	**908**	**65.37**
Ozamis City	**2,778**	1	**0.04**	308	**11.09**	1,853	**66.70**	**2,162**	**77.83**
Tangub City	**1,234**	0	**0.00**	121	**9.81**	925	**74.96**	**1,046**	**84.76**
Valencia City	**4,368**	5	**0.11**	523	**11.97**	3,026	**69.28**	**3,554**	**81.36**
**Region 11**	**107,247**	**347**	**0.32**	**11,288**	**10.53**	**66,103**	**61.64**	**77,738**	**72.49**
Compostela Valley	**15,562**	52	**0.33**	1,933	**12.42**	9,555	**61.40**	**11,540**	**74.15**
Davao del Norte	**21,326**	105	**0.49**	2,825	**13.25**	16,190	**75.92**	**19,120**	**89.66**
Davao Oriental	**13,007**	34	**0.26**	1,221	**9.39**	6,700	**51.51**	**7,955**	**61.16**
Davao del Sur	**14,151**	44	**0.31**	1,411	**9.97**	7,216	**50.99**	**8,671**	**61.27**
Davao Occidental	**6,670**	29	**0.43**	775	**11.62**	2,307	**34.59**	**3,111**	**46.64**
Davao City	**36,531**	83	**0.23**	3,123	**8.55**	24,135	**66.07**	**27,341**	**74.84**
**Region 12**	**104,552**	**136**	**0.13**	**9,685**	**9.26**	**59,761**	**57.16**	**69,582**	**66.55**
North Cotabato	**33,645**	34	**0.10**	2,548	**7.57**	16,277	**48.38**	**18,859**	**56.05**
Sarangani	**12,891**	29	**0.22**	2,012	**15.61**	8,276	**64.20**	**10,317**	**80.03**
South Cotabato	**21,113**	38	**0.18**	2,585	**12.24**	14,830	**70.24**	**17,453**	**82.66**
Sultan Kudarat	**17,359**	30	**0.17**	1,761	**10.14**	10,288	**59.27**	**12,079**	**69.58**
Cotabato City	**5,835**	3	**0.05**	323	**5.54**	4,958	**84.97**	**5,284**	**90.56**
Gen. Santos City	**13,709**	2	**0.01**	456	**3.33**	5,132	**37.44**	**5,590**	**40.78**
**BARMM**	**92,799**	**50**	**0.05**	**3,683**	**3.97**	**52,912**	**57.02**	**56,645**	**61.04**
Basilan	**7,541**	35	**0.46**	221	**2.93**	2,249	**29.82**	**2,505**	**33.22**
Lanao del Sur	**21,131**	2	**0.01**	917	**4.34**	18,769	**88.82**	**19,688**	**93.17**
Maguindanao	**31,128**	13	**0.04**	2,016	**6.48**	15,178	**48.76**	**17,207**	**55.28**
Sulu	**16,613**	0	**0.00**	0	**0.00**	8,083	**48.65**	**8,083**	**48.65**
Tawi-Tawi	**9,259**	0	**0.00**	0	**0.00**	5,963	**64.40**	**5,963**	**64.40**

Table 1.B.3.1 - Postpartum and Newborn Care

Number and proportion of Postpartum women together with their newborn who completed at least 2 postpartum check-ups
Philippines, Annual 2020

Area	Eligible Pop.	Completed at least 2 postpartum check-ups							
		Age Group in Year						Total	%
		10 - 14		15-19		20-49			
		No.	%	No.	%	No.	%		
Lamitan City	**2,074**	0	**0.00**	178	**8.58**	924	**44.55**	**1,102**	**53.13**
Marawi City	**5,053**	0	**0.00**	351	**6.95**	1,746	**34.55**	**2,097**	**41.50**
**CARAGA**	**60,029**	**71**	**0.12**	**4,389**	**7.31**	**30,317**	**50.50**	**34,777**	**57.93**
Agusan del Norte	**8,098**	12	**0.15**	626	**7.73**	3,706	**45.76**	**4,344**	**53.64**
Agusan del Sur	**17,592**	20	**0.11**	1,052	**5.98**	7,244	**41.18**	**8,316**	**47.27**
Surigao del Norte	**7,089**	3	**0.04**	416	**5.87**	3,568	**50.33**	**3,987**	**56.24**
Surigao del Sur	**11,482**	20	**0.17**	848	**7.39**	6,156	**53.61**	**7,024**	**61.17**
Province of Dinagat	**2,573**	3	**0.12**	183	**7.11**	1,214	**47.18**	**1,400**	**54.41**
Bislig City	**2,179**	7	**0.32**	200	**9.18**	1,441	**66.13**	**1,648**	**75.63**
Butuan City	**7,715**	5	**0.06**	783	**10.15**	4,928	**63.88**	**5,716**	**74.09**
Surigao City	**3,301**	1	**0.03**	281	**8.51**	2,060	**62.41**	**2,342**	**70.95**

Note: Put asterisk (*) for No Report and Zero (0) for No Case

Table 1.B.3.2 -Postpartum and Newborn Care

Number and proportion of postpartum women who completed Iron with Folic Acid Supplementation
Philippines, Annual 2020

Area	Eligible Pop.	Iron with Folic Supplementation							
		Age Group in Year						Total	%
		10-14		15-19		20-49			
		No.	%	No.	%	No.	%		
**PHILIPPINES**	**2,123,158**	**2,648**	**0.12**	**125,036**	**5.89**	**989,459**	**46.60**	**1,117,143**	**52.62**
**N C R**	**238,661**	**129**	**0.05**	**11,813**	**4.95**	**106,246**	**44.52**	**118,188**	**49.52**
Malabon	**6,775**	5	**0.07**	486	**7.17**	3,482	**51.39**	**3,973**	**58.64**
Navotas	**4,621**	6	**0.13**	581	**12.57**	3,414	**73.88**	**4,001**	**86.58**
Valenzuela City	**11,500**	5	**0.04**	693	**6.03**	6,363	**55.33**	**7,061**	**61.40**
Caloocan City	**29,363**	20	**0.07**	2,320	**7.90**	22,273	**75.85**	**24,613**	**83.82**
Marikina City	**8,354**	5	**0.06**	331	**3.96**	3,427	**41.02**	**3,763**	**45.04**
Pasig City	**13,996**	22	**0.16**	580	**4.14**	6,273	**44.82**	**6,875**	**49.12**
Pateros	**1,184**	0	**0.00**	57	**4.81**	747	**63.09**	**804**	**67.91**
Taguig	**14,918**	10	**0.07**	1,387	**9.30**	12,519	**83.92**	**13,916**	**93.28**
Quezon City	**54,413**	7	**0.01**	557	**1.02**	6,805	**12.51**	**7,369**	**13.54**
Makati City	**10,798**	7	**0.06**	237	**2.19**	3,348	**31.01**	**3,592**	**33.27**
Mandaluyong City	**7,160**	5	**0.07**	328	**4.58**	3,327	**46.47**	**3,660**	**51.12**
San Juan	**2,261**	1	**0.04**	82	**3.63**	999	**44.18**	**1,082**	**47.85**
Manila City	**32,980**	10	**0.03**	1,772	**5.37**	11,293	**34.24**	**13,075**	**39.65**
Las Piñas City	**10,914**	9	**0.08**	658	**6.03**	6,474	**59.32**	**7,141**	**65.43**
Muntinlupa City	**9,353**	9	**0.10**	857	**9.16**	7,848	**83.91**	**8,714**	**93.17**
Parañaque City	**12,343**	6	**0.05**	580	**4.70**	3,955	**32.04**	**4,541**	**36.79**
Pasay City	**7,728**	2	**0.03**	307	**3.97**	3,699	**47.86**	**4,008**	**51.86**
**C A R**	**35,099**	**12**	**0.03**	**1,988**	**5.66**	**20,173**	**57.47**	**22,173**	**63.17**
Abra	**4,309**	1	**0.02**	488	**11.33**	3,426	**79.51**	**3,915**	**90.86**
Apayao	**2,451**	1	**0.04**	191	**7.79**	1,258	**51.33**	**1,450**	**59.16**
Benguet	**9,188**	1	**0.01**	413	**4.49**	5,147	**56.02**	**5,561**	**60.52**
Ifugao	**4,396**	3	**0.07**	215	**4.89**	2,375	**54.03**	**2,593**	**58.99**
Kalinga	**4,617**	0	**0.00**	272	**5.89**	3,189	**69.07**	**3,461**	**74.96**
Mt. Province	**3,023**	3	**0.10**	154	**5.09**	1,663	**55.01**	**1,820**	**60.21**
Baguio City	**7,115**	3	**0.04**	255	**3.58**	3,115	**43.78**	**3,373**	**47.41**
**Region 1**	**97,261**	**44**	**0.05**	**5,564**	**5.72**	**54,279**	**55.81**	**59,887**	**61.57**
Ilocos Norte	**8,105**	7	**0.09**	319	**3.94**	3,570	**44.05**	**3,896**	**48.07**
Ilocos Sur	**9,330**	3	**0.03**	483	**5.18**	6,597	**70.71**	**7,083**	**75.92**
La Union	**11,511**	16	**0.14**	757	**6.58**	6,260	**54.38**	**7,033**	**61.10**
Pangasinan	**50,168**	7	**0.01**	2,800	**5.58**	27,382	**54.58**	**30,189**	**60.18**
Alaminos City	**1,894**	1	**0.05**	267	**14.10**	1,445	**76.29**	**1,713**	**90.44**
Candon City	**987**	0	**0.00**	8	**0.81**	810	**82.07**	**818**	**82.88**
Dagupan City	**3,620**	3	**0.08**	289	**7.98**	1,948	**53.81**	**2,240**	**61.88**
Laoag City	**1,870**	0	**0.00**	19	**1.02**	601	**32.14**	**620**	**33.16**
San Carlos City	**3,979**	3	**0.08**	200	**5.03**	2,406	**60.47**	**2,609**	**65.57**
San Fernando City	**2,115**	4	**0.19**	117	**5.53**	1,106	**52.29**	**1,227**	**58.01**
Urdaneta City	**2,806**	0	**0.00**	235	**8.37**	1,626	**57.95**	**1,861**	**66.32**
Vigan City	**876**	0	**0.00**	70	**7.99**	528	**60.27**	**598**	**68.26**
**Region 2**	**69,443**	**58**	**0.08**	**4,942**	**7.12**	**37,334**	**53.76**	**42,334**	**60.96**
Batanes	**340**	0	**0.00**	12	**3.53**	237	**69.71**	**249**	**73.24**
Cagayan	**17,971**	9	**0.05**	1,226	**6.82**	9,904	**55.11**	**11,139**	**61.98**
Isabela	**26,309**	15	**0.06**	1,649	**6.27**	11,905	**45.25**	**13,569**	**51.58**
Nueva Vizcaya	**9,573**	20	**0.21**	800	**8.36**	5,622	**58.73**	**6,442**	**67.29**

Table 1.B.3.2 -Postpartum and Newborn Care

Number and proportion of postpartum women who completed Iron with Folic Acid Supplementation
Philippines, Annual 2020

Area	Eligible Pop.	Iron with Folic Acid Supplementation							
		Age Group in Year						Total	%
		10-14		15-19		20-49			
		No.	%	No.	%	No.	%		
Quirino	**4,025**	9	**0.22**	380	**9.44**	2,871	**71.33**	**3,260**	**80.99**
Cauayan City	**2,590**	2	**0.08**	244	**9.42**	1,659	**64.05**	**1,905**	**73.55**
Ilagan City	**2,911**	0	**0.00**	244	**8.38**	1,169	**40.16**	**1,413**	**48.54**
Santiago City	**2,692**	2	**0.07**	289	**10.74**	2,373	**88.15**	**2,664**	**98.96**
Tuguegarao City	**3,032**	1	**0.03**	98	**3.23**	1,594	**52.57**	**1,693**	**55.84**
**Region 3**	**220,020**	**285**	**0.13**	**16,031**	**7.29**	**111,812**	**50.82**	**128,128**	**58.23**
Aurora	**4,770**	2	**0.04**	358	**7.51**	2,623	**54.99**	**2,983**	**62.54**
Bataan	**13,823**	26	**0.19**	1,212	**8.77**	8,733	**63.18**	**9,971**	**72.13**
Bulacan	**43,627**	58	**0.13**	2,510	**5.75**	17,238	**39.51**	**19,806**	**45.40**
Nueva Ecija	**28,782**	48	**0.17**	1,701	**5.91**	11,886	**41.30**	**13,635**	**47.37**
Pampanga	**31,359**	29	**0.09**	1,609	**5.13**	12,479	**39.79**	**14,117**	**45.02**
Tarlac	**19,765**	39	**0.20**	1,394	**7.05**	14,439	**73.05**	**15,872**	**80.30**
Zambales	**12,610**	7	**0.06**	481	**3.81**	3,548	**28.14**	**4,036**	**32.01**
Angeles City	**7,869**	7	**0.09**	466	**5.92**	3,286	**41.76**	**3,759**	**47.77**
Balanga City	**1,998**	5	**0.25**	173	**8.66**	1,167	**58.41**	**1,345**	**67.32**
Cabanatuan City	**5,896**	13	**0.22**	476	**8.07**	3,777	**64.06**	**4,266**	**72.35**
City of San Fernando	**5,863**	4	**0.07**	596	**10.17**	3,656	**62.36**	**4,256**	**72.59**
Gapan City	**2,153**	2	**0.09**	157	**7.29**	1,026	**47.65**	**1,185**	**55.04**
Mabalacat City	**4,793**	1	**0.02**	482	**10.06**	3,627	**75.67**	**4,110**	**85.75**
Malolos City	**4,880**	3	**0.06**	296	**6.07**	2,418	**49.55**	**2,717**	**55.68**
Meycauayan	**4,041**	14	**0.35**	426	**10.54**	2,481	**61.40**	**2,921**	**72.28**
Olongapo	**4,971**	4	**0.08**	285	**5.73**	2,652	**53.35**	**2,941**	**59.16**
Palayan City	**799**	1	**0.13**	79	**9.89**	353	**44.18**	**433**	**54.19**
San Jose City	**2,718**	10	**0.37**	314	**11.55**	1,963	**72.22**	**2,287**	**84.14**
San Jose del Monte City	**11,100**	8	**0.07**	2,002	**18.04**	8,337	**75.11**	**10,347**	**93.22**
Science City of Munoz	**1,586**	0	**0.00**	95	**5.99**	832	**52.46**	**927**	**58.45**
Tarlac City	**6,617**	4	**0.06**	919	**13.89**	5,291	**79.96**	**6,214**	**93.91**
**Region 4A**	**296,816**	**864**	**0.29**	**14,027**	**4.73**	**94,337**	**31.78**	**109,228**	**36.80**
Batangas	**38,441**	15	**0.04**	545	**1.42**	9,170	**23.85**	**9,730**	**25.31**
Cavite	**27,704**	16	**0.06**	991	**3.58**	15,102	**54.51**	**16,109**	**58.15**
Laguna	**19,727**	315	**1.60**	2,642	**13.39**	6,144	**31.15**	**9,101**	**46.13**
Quezon	**37,410**	10	**0.03**	829	**2.22**	7,510	**20.07**	**8,349**	**22.32**
Rizal	**44,791**	16	**0.04**	1,471	**3.28**	12,066	**26.94**	**13,553**	**30.26**
Antipolo City	**16,497**	2	**0.01**	609	**3.69**	6,337	**38.41**	**6,948**	**42.12**
Bacoor City	**12,142**	0	**0.00**	52	**0.43**	865	**7.12**	**917**	**7.55**
Batangas City	**6,823**	0	**0.00**	0	**0.00**	0	**0.00**	**0**	**0.00**
Biñan City	**6,607**	18	**0.27**	930	**14.08**	5,839	**88.38**	**6,787**	**102.72**
Cabuyao City	**6,130**	263	**4.29**	1,885	**30.75**	1,933	**31.53**	**4,081**	**66.57**
Calamba City	**9,028**	10	**0.11**	757	**8.39**	7,268	**80.51**	**8,035**	**89.00**
Cavite City	**2,075**	0	**0.00**	0	**0.00**	0	**0.00**	**0**	**0.00**
Dasmariñas City	**13,322**	2	**0.02**	341	**2.56**	4,362	**32.74**	**4,705**	**35.32**
General Trias City	**6,352**	2	**0.03**	174	**2.74**	2,170	**34.16**	**2,346**	**36.93**
Imus City	**8,156**	0	**0.00**	25	**0.31**	778	**9.54**	**803**	**9.85**
Lipa City	**6,880**	0	**0.00**	0	**0.00**	0	**0.00**	**0**	**0.00**
Lucena City	**5,672**	0	**0.00**	0	**0.00**	0	**0.00**	**0**	**0.00**
San Pablo City	**5,277**	121	**2.29**	1,118	**21.19**	1,257	**23.82**	**2,496**	**47.30**
San Pedro City	**6,467**	48	**0.74**	692	**10.70**	652	**10.08**	**1,392**	**21.52**
Santa Rosa City	**7,025**	5	**0.07**	533	**7.59**	4,200	**59.79**	**4,738**	**67.44**
Tagaytay City	**1,437**	20	**1.39**	58	**4.04**	2,802	**194.99**	**2,880**	**200.42**
Tanauan City	**3,584**	1	**0.03**	33	**0.92**	1,939	**54.10**	**1,973**	**55.05**
Tayabas City	**2,122**	0	**0.00**	0	**0.00**	435	**20.50**	**435**	**20.50**
Trece Martires City	**3,147**	0	**0.00**	342	**10.87**	3,508	**111.47**	**3,850**	**122.34**

Table 1.B.3.2 -Postpartum and Newborn Care

Number and proportion of postpartum women who completed Iron with Folic Acid Supplementation
Philippines, Annual 2020

Area	Eligible Pop.	Iron with Folic Acid Supplementation							
		Age Group in Year						Total	%
		10-14		15-19		20-49			
		No.	%	No.	%	No.	%		
**Region 4B**	**71,246**	**67**	**0.09**	**2,774**	**3.89**	**22,372**	**31.40**	**25,213**	**35.39**
Marinduque	**4,836**	5	**0.10**	258	**5.33**	2,506	**51.82**	**2,769**	**57.26**
Mindoro Occidental	**12,407**	24	**0.19**	1,068	**8.61**	6,842	**55.15**	**7,934**	**63.95**
Mindoro Oriental	**19,809**	16	**0.08**	600	**3.03**	6,110	**30.84**	**6,726**	**33.95**
Palawan	**21,375**	19	**0.09**	723	**3.38**	4,714	**22.05**	**5,456**	**25.53**
Romblon	**6,400**	3	**0.05**	90	**1.41**	693	**10.83**	**786**	**12.28**
Puerto Princesa City	**6,419**	0	**0.00**	35	**0.55**	1,507	**23.48**	**1,542**	**24.02**
**Region 5**	**136,116**	**79**	**0.06**	**6,934**	**5.09**	**68,049**	**49.99**	**75,062**	**55.15**
Albay	**23,737**	14	**0.06**	1,107	**4.66**	14,966	**63.05**	**16,087**	**67.77**
Camarines Norte	**14,283**	4	**0.03**	1,000	**7.00**	7,517	**52.63**	**8,521**	**59.66**
Camarines Sur	**38,796**	21	**0.05**	1,585	**4.09**	16,779	**43.25**	**18,385**	**47.39**
Catanduanes	**6,274**	2	**0.03**	402	**6.41**	3,220	**51.32**	**3,624**	**57.76**
Masbate	**22,521**	23	**0.10**	1,397	**6.20**	10,131	**44.98**	**11,551**	**51.29**
Sorsogon	**19,065**	11	**0.06**	1,029	**5.40**	10,222	**53.62**	**11,262**	**59.07**
Iriga City	**2,638**	0	**0.00**	80	**3.03**	995	**37.72**	**1,075**	**40.75**
Legaspi City	**4,174**	3	**0.07**	162	**3.88**	2,649	**63.46**	**2,814**	**67.42**
Naga City	**4,628**	1	**0.02**	172	**3.72**	1,570	**33.92**	**1,743**	**37.66**
**Region 6**	**146,526**	**144**	**0.10**	**8,179**	**5.58**	**71,110**	**48.53**	**79,433**	**54.21**
Aklan	**11,162**	12	**0.11**	595	**5.33**	5,491	**49.19**	**6,098**	**54.63**
Antique	**12,816**	7	**0.05**	598	**4.67**	6,145	**47.95**	**6,750**	**52.67**
Capiz	**13,986**	10	**0.07**	439	**3.14**	4,882	**34.91**	**5,331**	**38.12**
Guimaras	**3,085**	1	**0.03**	136	**4.41**	2,164	**70.15**	**2,301**	**74.59**
Iloilo	**36,541**	42	**0.11**	1,611	**4.41**	17,999	**49.26**	**19,652**	**53.78**
Negros Occidental	**49,368**	58	**0.12**	3,899	**7.90**	27,467	**55.64**	**31,424**	**63.65**
Bacolod City	**11,115**	5	**0.04**	313	**2.82**	2,525	**22.72**	**2,843**	**25.58**
Iloilo City	**8,453**	9	**0.11**	588	**6.96**	4,437	**52.49**	**5,034**	**59.55**
**Region 7**	**163,262**	**82**	**0.05**	**5,855**	**3.59**	**65,449**	**40.09**	**71,386**	**43.72**
Bohol	**27,312**	16	**0.06**	949	**3.47**	11,464	**41.97**	**12,429**	**45.51**
Cebu	**67,506**	19	**0.03**	993	**1.47**	13,434	**19.90**	**14,446**	**21.40**
Negros Oriental	**27,938**	25	**0.09**	1,650	**5.91**	14,284	**51.13**	**15,959**	**57.12**
Siquijor	**1,613**	0	**0.00**	119	**7.38**	1,077	**66.77**	**1,196**	**74.15**
Cebu City	**21,193**	18	**0.08**	1,641	**7.74**	12,363	**58.34**	**14,022**	**66.16**
Lapu-Lapu City	**9,372**	4	**0.04**	503	**5.37**	7,736	**82.54**	**8,243**	**87.95**
Mandaue City	**8,328**	0	**0.00**	0	**0.00**	5,091	**61.13**	**5,091**	**61.13**
**Region 8**	**102,619**	**93**	**0.09**	**5,597**	**5.45**	**47,227**	**46.02**	**52,917**	**51.57**
Biliran	**3,834**	2	**0.05**	261	**6.81**	2,293	**59.81**	**2,556**	**66.67**
Eastern Samar	**11,392**	6	**0.05**	606	**5.32**	5,296	**46.49**	**5,908**	**51.86**
Northern Leyte	**34,707**	22	**0.06**	2,050	**5.91**	17,219	**49.61**	**19,291**	**55.58**
Northern Samar	**15,370**	21	**0.14**	601	**3.91**	6,430	**41.83**	**7,052**	**45.88**
Southern Leyte	**6,451**	3	**0.05**	414	**6.42**	3,527	**54.67**	**3,944**	**61.14**
Western Samar	**14,305**	33	**0.23**	917	**6.41**	6,173	**43.15**	**7,123**	**49.79**
Calbayog City	**4,413**	0	**0.00**	80	**1.81**	795	**18.01**	**875**	**19.83**
Maasin City	**1,637**	0	**0.00**	63	**3.85**	689	**42.09**	**752**	**45.94**
Ormoc City	**4,941**	6	**0.12**	356	**7.21**	2,740	**55.45**	**3,102**	**62.78**

Table 1.B.3.2 -Postpartum and Newborn Care

Number and proportion of postpartum women who completed Iron with Folic Acid Supplementation
Philippines, Annual 2020

Area	Eligible Pop.	Iron with Folic Acid Supplementation							
		Age Group in Year						Total	%
		10-14		15-19		20-49			
		No.	%	No.	%	No.	%		
Tacloban City	5,569	0	0.00	249	4.47	2,065	37.08	2,314	41.55
**Region 9**	80,051	85	0.11	6,056	7.57	48,296	60.33	54,437	68.00
Zamboanga del Norte	17,249	20	0.12	1,966	11.40	14,421	83.60	16,407	95.12
Zamboanga del Sur	17,653	8	0.05	1,222	6.92	8,328	47.18	9,558	54.14
Zamboanga Sibugay	14,954	41	0.27	877	5.86	5,617	37.56	6,535	43.70
Dapitan City	1,784	2	0.11	143	8.02	1,541	86.38	1,686	94.51
Dipolog City	2,827	0	0.00	319	11.28	2,249	79.55	2,568	90.84
Isabela City	2,522	4	0.16	255	10.11	1,546	61.30	1,805	71.57
Pagadian City	4,325	2	0.05	294	6.80	2,594	59.98	2,890	66.82
Zamboanga City	18,737	8	0.04	980	5.23	12,000	64.04	12,988	69.32
**Region 10**	101,411	125	0.12	9,433	9.30	60,322	59.48	69,880	68.91
Bukidnon	23,706	66	0.28	3,058	12.90	13,212	55.73	16,336	68.91
Camiguin	1,858	0	0.00	135	7.27	1,161	62.49	1,296	69.75
Lanao del Norte	14,960	13	0.09	660	4.41	7,194	48.09	7,867	52.59
Misamis Occidental	6,403	4	0.06	349	5.45	3,832	59.85	4,185	65.36
Misamis Oriental	15,131	12	0.08	1,265	8.36	8,778	58.01	10,055	66.45
Cagayan de Oro City	14,339	17	0.12	1,362	9.50	10,199	71.13	11,578	80.74
El Salvador City	1,065	1	0.09	131	12.30	729	68.45	861	80.85
Gingoog City	2,644	1	0.04	275	10.40	1,493	56.47	1,769	66.91
Iligan City	7,580	1	0.01	785	10.36	5,157	68.03	5,943	78.40
Malaybalay City	3,956	5	0.13	412	10.41	2,100	53.08	2,517	63.62
Oroquieta City	1,389	0	0.00	78	5.62	719	51.76	797	57.38
Ozamis City	2,778	1	0.04	308	11.09	1,853	66.70	2,162	77.83
Tangub City	1,234	0	0.00	121	9.81	925	74.96	1,046	84.76
Valencia City	4,368	4	0.09	494	11.31	2,970	67.99	3,468	79.40
**Region 11**	107,247	399	0.37	10,590	9.87	61,036	56.91	72,025	67.16
Compostela Valley	15,562	72	0.46	1,805	11.60	9,023	57.98	10,900	70.04
Davao del Norte	21,326	93	0.44	2,911	13.65	16,381	76.81	19,385	90.90
Davao Oriental	13,007	47	0.36	1,109	8.53	6,464	49.70	7,620	58.58
Davao del Sur	14,151	53	0.37	1,172	8.28	5,965	42.15	7,190	50.81
Davao Occidental	6,670	32	0.48	732	10.97	2,278	34.15	3,042	45.61
Davao City	36,531	102	0.28	2,861	7.83	20,925	57.28	23,888	65.39
**Region 12**	104,552	111	0.11	8,587	8.21	53,472	51.14	62,170	59.46
North Cotabato	33,645	34	0.10	2,230	6.63	14,596	43.38	16,860	50.11
Sarangani	12,891	25	0.19	1,840	14.27	7,786	60.40	9,651	74.87
South Cotabato	21,113	19	0.09	2,243	10.62	13,744	65.10	16,006	75.81
Sultan Kudarat	17,359	29	0.17	1,707	9.83	10,157	58.51	11,893	68.51
Cotabato City	5,835	1	0.02	179	3.07	2,955	50.64	3,135	53.73
Gen. Santos City	13,709	3	0.02	388	2.83	4,234	30.88	4,625	33.74
**BARMM**	92,799	13	0.01	2,781	3.00	40,362	43.49	43,156	46.50
Basilan	7,541	0	0.00	121	1.60	1,195	15.85	1,316	17.45
Lanao del Sur	21,131	2	0.01	610	2.89	16,119	76.28	16,731	79.18
Maguindanao	31,128	11	0.04	1,521	4.89	12,607	40.50	14,139	45.42
Sulu	16,613	0	0.00	0	0.00	5,434	32.71	5,434	32.71
Tawi-Tawi	9,259	0	0.00	0	0.00	2,719	29.37	2,719	29.37

Table 1.B.3.2 -Postpartum and Newborn Care

Number and proportion of postpartum women who completed Iron with Folic Acid Supplementation
Philippines, Annual 2020

Area	Eligible Pop.	Iron with Folic Acid Supplementation							
		Age Group in Year						Total	%
		10-14		15-19		20-49			
		No.	%	No.	%	No.	%		
Lamitan City	**2,074**	0	**0.00**	178	**8.58**	924	**44.55**	**1,102**	**53.13**
Marawi City	**5,053**	0	**0.00**	351	**6.95**	1,364	**26.99**	**1,715**	**33.94**
**CARAGA**	**60,029**	**58**	**0.10**	**3,885**	**6.47**	**27,583**	**45.95**	**31,526**	**52.52**
Agusan del Norte	**8,098**	11	**0.14**	557	**6.88**	3,504	**43.27**	**4,072**	**50.28**
Agusan del Sur	**17,592**	19	**0.11**	1,020	**5.80**	7,111	**40.42**	**8,150**	**46.33**
Surigao del Norte	**7,089**	3	**0.04**	358	**5.05**	3,156	**44.52**	**3,517**	**49.61**
Surigao del Sur	**11,482**	13	**0.11**	623	**5.43**	4,604	**40.10**	**5,240**	**45.64**
Province of Dinagat	**2,573**	2	**0.08**	177	**6.88**	1,138	**44.23**	**1,317**	**51.19**
Bislig City	**2,179**	5	**0.23**	177	**8.12**	1,453	**66.68**	**1,635**	**75.03**
Butuan City	**7,715**	4	**0.05**	722	**9.36**	4,659	**60.39**	**5,385**	**69.80**
Surigao City	**3,301**	1	**0.03**	251	**7.60**	1,958	**59.32**	**2,210**	**66.95**

Note: Put asterisk (*) for No Report and Zero (0) for No Case

Table 1.B.3.3 - Postpartum and Newborn Care

Number and proportion of postpartum women who completed Vitamin A Supplementation
Philippines, Annual 2020

Area	Eligible Pop.	Vitamin A Supplementation							
		Age Group in Year						Total	%
		10-14		15-19		20-49			
		No.	%	No.	%	No.	%		
**PHILIPPINES**	**2,123,158**	**2,962**	**0.14**	**127,675**	**6.01**	**995,696**	**46.90**	**1,127,541**	**53.11**
**N C R**	**238,661**	**157**	**0.07**	**12,456**	**5.22**	**126,041**	**52.81**	**138,747**	**58.14**
Malabon	**6,775**	5	**0.07**	454	**6.70**	3,322	**49.03**	**3,788**	**55.91**
Navotas	**4,621**	4	**0.09**	487	**10.54**	3,080	**66.65**	**3,582**	**77.51**
Valenzuela City	**11,500**	8	**0.07**	720	**6.26**	6,193	**53.85**	**6,927**	**60.24**
Caloocan City	**29,363**	16	**0.05**	1,877	**6.39**	21,530	**73.32**	**23,429**	**79.79**
Marikina City	**8,354**	5	**0.06**	298	**3.57**	2,732	**32.70**	**3,039**	**36.37**
Pasig City	**13,996**	23	**0.16**	511	**3.65**	5,576	**39.84**	**6,114**	**43.68**
Pateros	**1,184**	0	**0.00**	85	**7.18**	786	**66.39**	**878**	**74.17**
Taguig	**14,918**	5	**0.03**	297	**1.99**	5,080	**34.05**	**5,384**	**36.09**
Quezon City	**54,413**	26	**0.05**	2,802	**5.15**	38,317	**70.42**	**41,150**	**75.63**
Makati City	**10,798**	13	**0.12**	306	**2.83**	3,007	**27.85**	**3,329**	**30.83**
Mandaluyong City	**7,160**	4	**0.06**	339	**4.73**	3,381	**47.22**	**3,729**	**52.08**
San Juan	**2,261**	0	**0.00**	79	**3.49**	805	**35.60**	**887**	**39.25**
Manila City	**32,980**	11	**0.03**	1,639	**4.97**	10,843	**32.88**	**12,498**	**37.90**
Las Piñas City	**10,914**	19	**0.17**	622	**5.70**	6,272	**57.47**	**6,919**	**63.39**
Muntinlupa City	**9,353**	9	**0.10**	855	**9.14**	7,838	**83.80**	**8,711**	**93.14**
Parañaque City	**12,343**	7	**0.06**	901	**7.30**	4,761	**38.57**	**5,676**	**45.99**
Pasay City	**7,728**	2	**0.03**	184	**2.38**	2,518	**32.58**	**2,706**	**35.02**
**C A R**	**35,099**	**17**	**0.05**	**2,273**	**6.48**	**20,742**	**59.10**	**23,083**	**65.77**
Abra	**4,309**	2	**0.05**	479	**11.12**	3,384	**78.53**	**3,876**	**89.96**
Apayao	**2,451**	1	**0.04**	226	**9.22**	1,284	**52.39**	**1,520**	**62.03**
Benguet	**9,188**	6	**0.07**	448	**4.88**	5,135	**55.89**	**5,594**	**60.88**
Ifugao	**4,396**	2	**0.05**	333	**7.58**	2,742	**62.37**	**3,085**	**70.17**
Kalinga	**4,617**	0	**0.00**	299	**6.48**	3,064	**66.36**	**3,369**	**72.98**
Mt. Province	**3,023**	3	**0.10**	243	**8.04**	1,999	**66.13**	**2,253**	**74.53**
Baguio City	**7,115**	3	**0.04**	245	**3.44**	3,134	**44.05**	**3,385**	**47.58**
**Region 1**	**97,261**	**20**	**0.02**	**2,270**	**2.33**	**20,109**	**20.68**	**22,435**	**23.07**
Ilocos Norte	**8,105**	3	**0.04**	226	**2.79**	2,371	**29.25**	**2,603**	**32.11**
Ilocos Sur	**9,330**	2	**0.02**	204	**2.19**	2,897	**31.05**	**3,105**	**33.28**
La Union	**11,511**	8	**0.07**	389	**3.38**	3,113	**27.04**	**3,513**	**30.52**
Pangasinan	**50,168**	0	**0.00**	868	**1.73**	8,123	**16.19**	**8,993**	**17.93**
Alaminos City	**1,894**	0	**0.00**	239	**12.62**	745	**39.33**	**997**	**52.62**
Candon City	**987**	0	**0.00**	2	**0.20**	184	**18.64**	**186**	**18.87**
Dagupan City	**3,620**	3	**0.08**	127	**3.51**	891	**24.61**	**1,025**	**28.30**
Laoag City	**1,870**	0	**0.00**	9	**0.48**	81	**4.33**	**90**	**4.84**
San Carlos City	**3,979**	1	**0.03**	48	**1.21**	572	**14.38**	**622**	**15.64**
San Fernando City	**2,115**	3	**0.14**	93	**4.40**	652	**30.83**	**753**	**35.58**
Urdaneta City	**2,806**	0	**0.00**	56	**2.00**	387	**13.79**	**445**	**15.86**
Vigan City	**876**	0	**0.00**	9	**1.03**	93	**10.62**	**103**	**11.76**
**Region 2**	**69,443**	**81**	**0.12**	**5,641**	**8.12**	**39,126**	**56.34**	**44,927**	**64.70**
Batanes	**340**	0	**0.00**	24	**7.06**	234	**68.82**	**265**	**77.96**
Cagayan	**17,971**	12	**0.07**	1,567	**8.72**	9,656	**53.73**	**11,244**	**62.57**
Isabela	**26,309**	29	**0.11**	1,792	**6.81**	12,523	**47.60**	**14,351**	**54.55**
Nueva Vizcaya	**9,573**	21	**0.22**	836	**8.73**	6,775	**70.77**	**7,641**	**79.82**

Table 1.B.3.3 - Postpartum and Newborn Care

Number and proportion of postpartum women who completed Vitamin A Supplementation
Philippines, Annual 2020

Area	Eligible Pop.	Vitamin A Supplementation							
		Age Group in Year						Total	%
		10-14		15-19		20-49			
		No.	%	No.	%	No.	%		
Quirino	4,025	8	0.20	404	10.04	2,839	70.53	3,261	81.02
Cauayan City	2,590	6	0.23	286	11.04	1,664	64.25	1,967	75.96
Ilagan City	2,911	0	0.00	253	8.69	1,342	46.10	1,604	55.09
Santiago City	2,692	4	0.15	346	12.85	2,288	84.99	2,651	98.48
Tuguegarao City	3,032	1	0.03	133	4.39	1,805	59.53	1,943	64.10
**Region 3**	**220,020**	**309**	**0.14**	**15,389**	**6.99**	**113,463**	**51.57**	**129,334**	**58.78**
Aurora	4,770	3	0.06	383	8.03	2,517	52.77	2,911	61.03
Bataan	13,823	29	0.21	1,299	9.40	8,560	61.93	9,898	71.60
Bulacan	43,627	76	0.17	2,727	6.25	19,049	43.66	21,858	50.10
Nueva Ecija	28,782	33	0.11	1,308	4.54	9,804	34.06	11,150	38.74
Pampanga	31,359	20	0.06	1,379	4.40	11,386	36.31	12,789	40.78
Tarlac	19,765	39	0.20	1,203	6.09	12,096	61.20	13,344	67.51
Zambales	12,610	7	0.06	419	3.32	3,121	24.75	3,550	28.16
Angeles City	7,869	3	0.04	393	4.99	2,700	34.31	3,101	39.41
Balanga City	1,998	4	0.20	197	9.86	1,208	60.46	1,419	71.02
Cabanatuan City	5,896	7	0.12	521	8.84	3,889	65.96	4,426	75.07
City of San Fernando	5,863	5	0.09	647	11.04	3,543	60.43	4,206	71.74
Gapan City	2,153	1	0.05	64	2.97	446	20.72	514	23.87
Mabalacat City	4,793	5	0.10	521	10.87	3,992	83.29	4,529	94.49
Malolos City	4,880	12	0.25	529	10.84	4,497	92.15	5,049	103.46
Meycauayan	4,041	31	0.77	802	19.85	3,466	85.77	4,320	106.89
Olongapo	4,971	4	0.08	289	5.81	2,582	51.94	2,881	57.95
Palayan City	799	0	0.00	3	0.38	173	21.65	176	22.07
San Jose City	2,718	6	0.22	320	11.77	1,820	66.96	2,158	79.40
San Jose del Monte City	11,100	14	0.13	1,432	12.90	12,870	115.95	14,329	129.09
Science City of Munoz	1,586	0	0.00	72	4.54	866	54.60	943	59.43
Tarlac City	6,617	10	0.15	881	13.31	4,878	73.72	5,782	87.39
**Region 4A**	**296,816**	**990**	**0.33**	**15,369**	**5.18**	**97,804**	**32.95**	**114,334**	**38.52**
Batangas	38,441	15	0.04	427	1.11	6,100	15.87	6,543	17.02
Cavite	27,704	17	0.06	1,561	5.63	16,525	59.65	18,109	65.36
Laguna	19,727	299	1.52	2,773	14.06	12,271	62.20	15,359	77.86
Quezon	37,410	19	0.05	1,027	2.75	9,113	24.36	10,162	27.16
Rizal	44,791	22	0.05	1,406	3.14	11,488	25.65	12,919	28.84
Antipolo City	16,497	3	0.02	609	3.69	5,744	34.82	6,360	38.55
Bacoor City	12,142	0	0.00	79	0.65	892	7.35	972	8.00
Batangas City	6,823	0	0.00	0	0.00	0	0.00	0	0.00
Biñan City	6,607	18	0.27	574	8.69	4,130	62.51	4,731	71.61
Cabuyao City	6,130	219	3.57	1,776	28.97	1,867	30.46	3,895	63.53
Calamba City	9,028	12	0.13	753	8.34	6,937	76.84	7,710	85.41
Cavite City	2,075	0	0.00	0	0.00	0	0.00	0	0.00
Dasmariñas City	13,322	2	0.02	306	2.30	4,459	33.47	4,769	35.80
General Trias City	6,352	4	0.06	208	3.27	2,350	37.00	2,565	40.39
Imus City	8,156	0	0.00	55	0.67	869	10.65	925	11.34
Lipa City	6,880	0	0.00	0	0.00	0	0.00	0	0.00
Lucena City	5,672	0	0.00	0	0.00	0	0.00	0	0.00
San Pablo City	5,277	306	5.80	1,990	37.71	252	4.78	2,592	49.11
San Pedro City	6,467	37	0.57	807	12.48	759	11.74	1,616	24.99
Santa Rosa City	7,025	6	0.09	492	7.00	4,052	57.68	4,557	64.87
Tagaytay City	1,437	8	0.56	42	2.92	2,770	192.76	2,823	196.48
Tanauan City	3,584	1	0.03	54	1.51	2,139	59.68	2,196	61.26
Tayabas City	2,122	0	0.00	0	0.00	435	20.50	435	20.50
Trece Martires City	3,147	2	0.06	430	13.66	4,652	147.82	5,098	161.99

Table 1.B.3.3 - Postpartum and Newborn Care

Number and proportion of postpartum women who completed Vitamin A Supplementation
Philippines, Annual 2020

Area	Eligible Pop.	Vitamin A Supplementation							
		Age Group in Year						Total	%
		10-14		15-19		20-49			
		No.	%	No.	%	No.	%		
**Region 4B**	**71,246**	**57**	**0.08**	**2,833**	**3.98**	**22,264**	**31.25**	**25,178**	**35.34**
Marinduque	**4,836**	2	**0.04**	324	**6.70**	2,842	**58.77**	**3,175**	**65.65**
Mindoro Occidental	**12,407**	21	**0.17**	825	**6.65**	5,356	**43.17**	**6,209**	**50.04**
Mindoro Oriental	**19,809**	13	**0.07**	670	**3.38**	8,037	**40.57**	**8,723**	**44.04**
Palawan	**21,375**	16	**0.07**	859	**4.02**	4,831	**22.60**	**5,710**	**26.71**
Romblon	**6,400**	5	**0.08**	89	**1.39**	652	**10.19**	**747**	**11.68**
Puerto Princesa City	**6,419**	0	**0.00**	66	**1.03**	546	**8.51**	**613**	**9.55**
**Region 5**	**136,116**	**118**	**0.09**	**7,830**	**5.75**	**71,593**	**52.60**	**79,593**	**58.47**
Albay	**23,737**	18	**0.08**	1,203	**5.07**	14,990	**63.15**	**16,216**	**68.32**
Camarines Norte	**14,283**	8	**0.06**	1,054	**7.38**	7,706	**53.95**	**8,775**	**61.44**
Camarines Sur	**38,796**	29	**0.07**	1,763	**4.54**	17,400	**44.85**	**19,197**	**49.48**
Catanduanes	**6,274**	21	**0.33**	589	**9.39**	3,311	**52.77**	**3,931**	**62.65**
Masbate	**22,521**	27	**0.12**	1,637	**7.27**	11,224	**49.84**	**12,895**	**57.26**
Sorsogon	**19,065**	9	**0.05**	1,164	**6.11**	11,216	**58.83**	**12,395**	**65.02**
Iriga City	**2,638**	1	**0.04**	94	**3.56**	1,096	**41.55**	**1,195**	**45.28**
Legaspi City	**4,174**	2	**0.05**	171	**4.10**	3,004	**71.97**	**3,181**	**76.21**
Naga City	**4,628**	3	**0.06**	155	**3.35**	1,646	**35.57**	**1,807**	**39.05**
**Region 6**	**146,526**	**184**	**0.13**	**8,822**	**6.02**	**73,923**	**50.45**	**82,974**	**56.63**
Aklan	**11,162**	18	**0.16**	592	**5.30**	5,364	**48.06**	**5,979**	**53.57**
Antique	**12,816**	41	**0.32**	685	**5.34**	6,541	**51.04**	**7,273**	**56.75**
Capiz	**13,986**	3	**0.02**	481	**3.44**	5,142	**36.77**	**5,629**	**40.25**
Guimaras	**3,085**	3	**0.10**	190	**6.16**	2,297	**74.46**	**2,496**	**80.92**
Iloilo	**36,541**	40	**0.11**	1,814	**4.96**	18,883	**51.68**	**20,742**	**56.76**
Negros Occidental	**49,368**	59	**0.12**	4,132	**8.37**	28,391	**57.51**	**32,590**	**66.02**
Bacolod City	**11,115**	8	**0.07**	326	**2.93**	2,563	**23.06**	**2,900**	**26.09**
Iloilo City	**8,453**	12	**0.14**	602	**7.12**	4,742	**56.10**	**5,363**	**63.45**
**Region 7**	**163,262**	**101**	**0.06**	**6,476**	**3.97**	**66,076**	**40.47**	**72,688**	**44.52**
Bohol	**27,312**	15	**0.05**	907	**3.32**	10,823	**39.63**	**11,748**	**43.02**
Cebu	**67,506**	19	**0.03**	1,010	**1.50**	11,268	**16.69**	**12,299**	**18.22**
Negros Oriental	**27,938**	29	**0.10**	2,077	**7.43**	15,433	**55.24**	**17,547**	**62.81**
Siquijor	**1,613**	1	**0.06**	130	**8.06**	1,031	**63.92**	**1,170**	**72.54**
Cebu City	**21,193**	32	**0.15**	1,848	**8.72**	14,218	**67.09**	**16,107**	**76.00**
Lapu-Lapu City	**9,372**	5	**0.05**	504	**5.38**	6,937	**74.02**	**7,451**	**79.51**
Mandaue City	**8,328**	0	**0.00**	0	**0.00**	6,366	**76.44**	**6,366**	**76.44**
**Region 8**	**102,619**	**115**	**0.11**	**5,917**	**5.77**	**48,213**	**46.98**	**54,304**	**52.92**
Biliran	**3,834**	2	**0.05**	308	**8.03**	2,360	**61.55**	**2,678**	**69.85**
Eastern Samar	**11,392**	7	**0.06**	645	**5.66**	5,419	**47.57**	**6,077**	**53.34**
Northern Leyte	**34,707**	24	**0.07**	2,040	**5.88**	16,842	**48.53**	**18,912**	**54.49**
Northern Samar	**15,370**	23	**0.15**	697	**4.53**	6,810	**44.31**	**7,535**	**49.02**
Southern Leyte	**6,451**	4	**0.06**	467	**7.24**	3,647	**56.53**	**4,125**	**63.95**
Western Samar	**14,305**	38	**0.27**	922	**6.45**	6,216	**43.45**	**7,183**	**50.21**
Calbayog City	**4,413**	0	**0.00**	120	**2.72**	1,254	**28.42**	**1,377**	**31.20**
Maasin City	**1,637**	5	**0.31**	78	**4.76**	764	**46.67**	**852**	**52.05**
Ormoc City	**4,941**	8	**0.16**	453	**9.17**	3,205	**64.87**	**3,675**	**74.38**

Table 1.B.3.3 - Postpartum and Newborn Care

Number and proportion of postpartum women who completed Vitamin A Supplementation
Philippines, Annual 2020

Area	Eligible Pop.	Vitamin A Supplementation							
		Age Group in Year						Total	%
		10-14		15-19		20-49			
		No.	%	No.	%	No.	%		
Tacloban City	**5,569**	4	**0.07**	187	**3.36**	1,696	**30.45**	**1,890**	**33.95**
**Region 9**	**80,051**	**52**	**0.06**	**5,010**	**6.26**	**39,337**	**49.14**	**44,458**	**55.54**
Zamboanga del Norte	**17,249**	14	**0.08**	1,530	**8.87**	10,934	**63.39**	**12,487**	**72.39**
Zamboanga del Sur	**17,653**	12	**0.07**	820	**4.65**	5,655	**32.03**	**6,492**	**36.77**
Zamboanga Sibugay	**14,954**	12	**0.08**	738	**4.94**	4,610	**30.83**	**5,365**	**35.88**
Dapitan City	**1,784**	3	**0.17**	179	**10.03**	1,511	**84.70**	**1,703**	**95.47**
Dipolog City	**2,827**	0	**0.00**	261	**9.23**	1,838	**65.02**	**2,108**	**74.57**
Isabela City	**2,522**	3	**0.12**	264	**10.47**	1,433	**56.82**	**1,711**	**67.83**
Pagadian City	**4,325**	2	**0.05**	233	**5.39**	1,983	**45.85**	**2,223**	**51.41**
Zamboanga City	**18,737**	6	**0.03**	985	**5.26**	11,373	**60.70**	**12,369**	**66.02**
**Region 10**	**101,411**	**150**	**0.15**	**9,013**	**8.89**	**57,631**	**56.83**	**66,915**	**65.98**
Bukidnon	**23,706**	81	**0.34**	3,289	**13.87**	13,733	**57.93**	**17,117**	**72.21**
Camiguin	**1,858**	0	**0.00**	127	**6.84**	1,181	**63.56**	**1,315**	**70.77**
Lanao del Norte	**14,960**	13	**0.09**	693	**4.63**	7,475	**49.97**	**8,186**	**54.72**
Misamis Occidental	**6,403**	3	**0.05**	335	**5.23**	3,656	**57.10**	**3,999**	**62.46**
Misamis Oriental	**15,131**	13	**0.09**	1,110	**7.34**	6,945	**45.90**	**8,075**	**53.37**
Cagayan de Oro City	**14,339**	21	**0.15**	1,120	**7.81**	10,400	**72.53**	**11,549**	**80.54**
El Salvador City	**1,065**	1	**0.09**	131	**12.30**	728	**68.36**	**872**	**81.91**
Gingoog City	**2,644**	0	**0.00**	56	**2.12**	293	**11.08**	**351**	**13.28**
Iligan City	**7,580**	8	**0.11**	690	**9.10**	4,466	**58.92**	**5,173**	**68.25**
Malaybalay City	**3,956**	5	**0.13**	412	**10.41**	2,100	**53.08**	**2,528**	**63.89**
Oroquieta City	**1,389**	1	**0.07**	100	**7.20**	818	**58.89**	**926**	**66.69**
Ozamis City	**2,778**	1	**0.04**	308	**11.09**	1,853	**66.70**	**2,173**	**78.23**
Tangub City	**1,234**	0	**0.00**	121	**9.81**	925	**74.96**	**1,056**	**85.56**
Valencia City	**4,368**	3	**0.07**	521	**11.93**	3,058	**70.01**	**3,594**	**82.28**
**Region 11**	**107,247**	**368**	**0.34**	**11,564**	**10.78**	**66,462**	**61.97**	**78,465**	**73.16**
Compostela Valley	**15,562**	54	**0.35**	1,939	**12.46**	9,125	**58.64**	**11,131**	**71.53**
Davao del Norte	**21,326**	92	**0.43**	2,844	**13.34**	16,173	**75.84**	**19,123**	**89.67**
Davao Oriental	**13,007**	54	**0.42**	1,233	**9.48**	6,581	**50.60**	**7,878**	**60.57**
Davao del Sur	**14,151**	52	**0.37**	1,482	**10.47**	7,451	**52.65**	**8,996**	**63.57**
Davao Occidental	**6,670**	34	**0.51**	952	**14.27**	2,855	**42.80**	**3,856**	**57.81**
Davao City	**36,531**	82	**0.22**	3,114	**8.52**	24,277	**66.46**	**27,482**	**75.23**
**Region 12**	**104,552**	**132**	**0.13**	**8,837**	**8.45**	**53,603**	**51.27**	**62,623**	**59.90**
North Cotabato	**33,645**	30	**0.09**	2,062	**6.13**	13,420	**39.89**	**15,518**	**46.12**
Sarangani	**12,891**	30	**0.23**	1,914	**14.85**	7,859	**60.97**	**9,818**	**76.16**
South Cotabato	**21,113**	36	**0.17**	2,469	**11.69**	14,206	**67.29**	**16,723**	**79.21**
Sultan Kudarat	**17,359**	31	**0.18**	1,739	**10.02**	10,134	**58.38**	**11,914**	**68.63**
Cotabato City	**5,835**	3	**0.05**	292	**5.00**	4,617	**79.13**	**4,917**	**84.27**
Gen. Santos City	**13,709**	2	**0.01**	361	**2.63**	3,367	**24.56**	**3,733**	**27.23**
**BARMM**	**92,799**	**23**	**0.02**	**3,631**	**3.91**	**50,590**	**54.52**	**54,271**	**58.48**
Basilan	**7,541**	6	**0.08**	236	**3.13**	2,139	**28.36**	**2,384**	**31.62**
Lanao del Sur	**21,131**	3	**0.01**	885	**4.19**	17,232	**81.55**	**18,124**	**85.77**
Maguindanao	**31,128**	14	**0.04**	2,137	**6.87**	15,779	**50.69**	**17,937**	**57.62**
Sulu	**16,613**	0	**0.00**	0	**0.00**	9,549	**57.48**	**9,549**	**57.48**
Tawi-Tawi	**9,259**	0	**0.00**	0	**0.00**	3,815	**41.20**	**3,815**	**41.20**

Table 1.B.3.3 - Postpartum and Newborn Care

Number and proportion of postpartum women who completed Vitamin A Supplementation
Philippines, Annual 2020

Area	Eligible Pop.	Vitamin A Supplementation							
		Age Group in Year						Total	%
		10-14		15-19		20-49			
		No.	%	No.	%	No.	%		
Lamitan City	**2,074**	0	**0.00**	178	**8.58**	924	**44.55**	**1,111**	**53.55**
Marawi City	**5,053**	0	**0.00**	195	**3.86**	1,152	**22.80**	**1,351**	**26.73**
**CARAGA**	**60,029**	**88**	**0.15**	**4,344**	**7.24**	**28,719**	**47.84**	**33,212**	**55.33**
Agusan del Norte	**8,098**	22	**0.27**	636	**7.85**	3,612	**44.60**	**4,278**	**52.83**
Agusan del Sur	**17,592**	20	**0.11**	1,052	**5.98**	6,882	**39.12**	**7,960**	**45.25**
Surigao del Norte	**7,089**	10	**0.14**	332	**4.68**	2,589	**36.52**	**2,936**	**41.41**
Surigao del Sur	**11,482**	22	**0.19**	920	**8.01**	6,192	**53.93**	**7,142**	**62.20**
Province of Dinagat	**2,573**	2	**0.08**	169	**6.57**	1,148	**44.62**	**1,326**	**51.52**
Bislig City	**2,179**	7	**0.32**	197	**9.04**	1,454	**66.73**	**1,667**	**76.52**
Butuan City	**7,715**	5	**0.06**	771	**9.99**	4,851	**62.88**	**5,637**	**73.07**
Surigao City	**3,301**	0	**0.00**	267	**8.09**	1,991	**60.32**	**2,266**	**68.65**

Note: Put asterisk (*) for No Report and Zero (0) for No Case

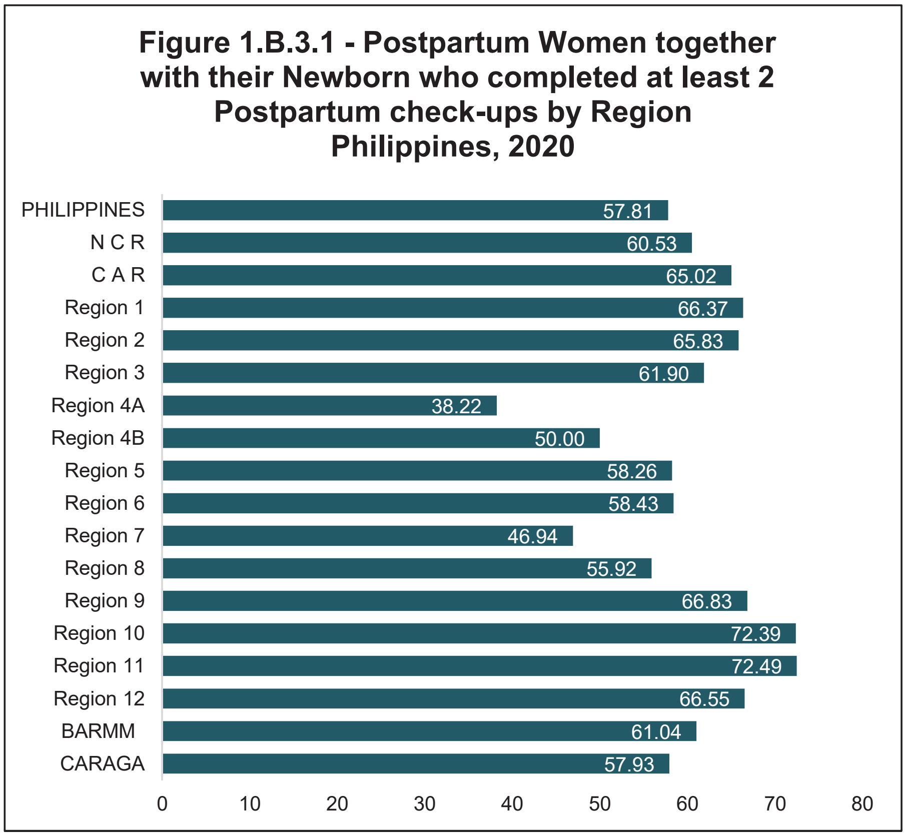
Figure 1.B.3.1 - Postpartum Women together with their Newborn who completed at least 2 Postpartum check-ups by Region Philippines, 2020
PHILIPPINES 57.81
N C R 60.53
C A R 65.02
Region 1 66.37
Region 2 65.83
Region 3 61.90
Region 4A 38.22
Region 4B 50.00
Region 5 58.26
Region 6 58.43
Region 7 46.94
Region 8 55.92
Region 9 66.83
Region 10 72.39
Region 11 72.49
Region 12 66.55
BARMM 61.04
CARAGA 57.93
0 10 20 30 40 50 60 70 80

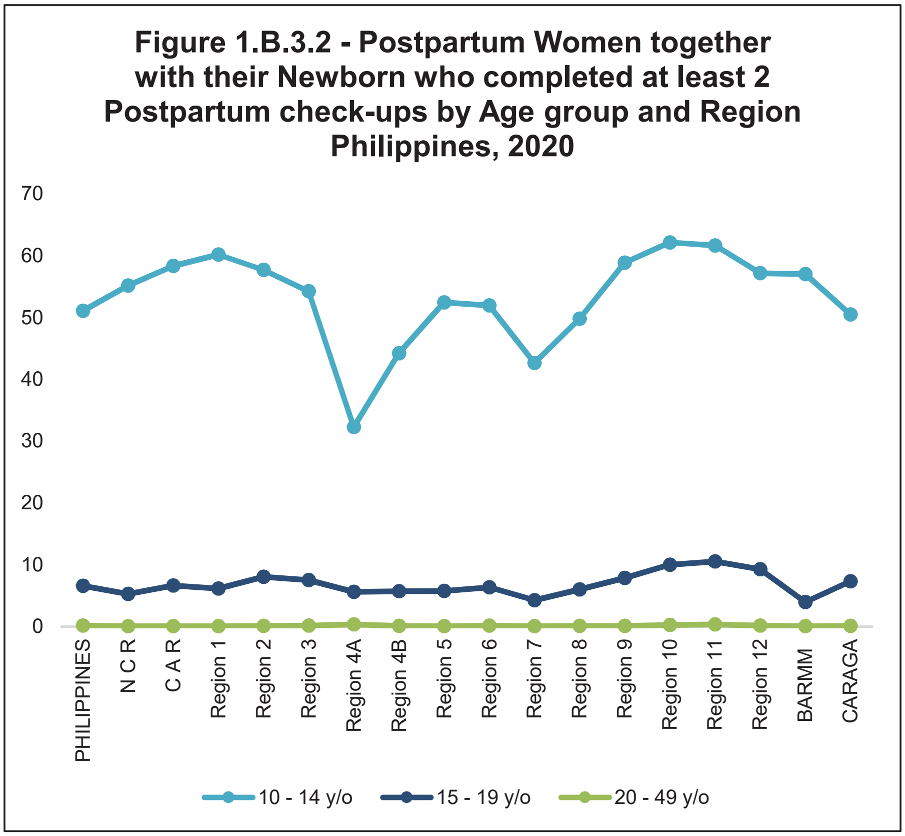
Figure 1.B.3.2 - Postpartum Women together with their Newborn who completed at least 2 Postpartum check-ups by Age group and Region Philippines, 2020
70
60
50
40
30
20
10
0
PHILIPPINES
N C R
C A R
Region 1
Region 2
Region 3
Region 4A
Region 4B
Region 5
Region 6
Region 7
Region 8
Region 9
Region 10
Region 11
Region 12
BARMM
CARAGA
10 - 14 y/o
15 - 19 y/o
20 - 49 y/o

Figure 1.B.3.3 - Postpartum Women who completed Iron with Folic Acid by Region Philippines, 2020

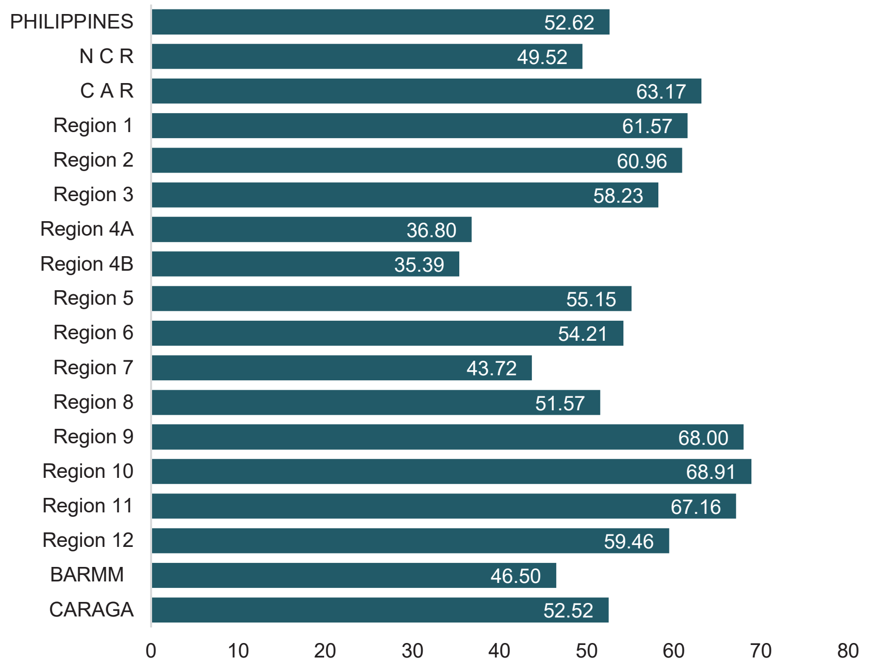

Figure 1.B.3.4 - Postpartum Women who completed Iron with Folic Acid by Age group and Region Philippines, 2020

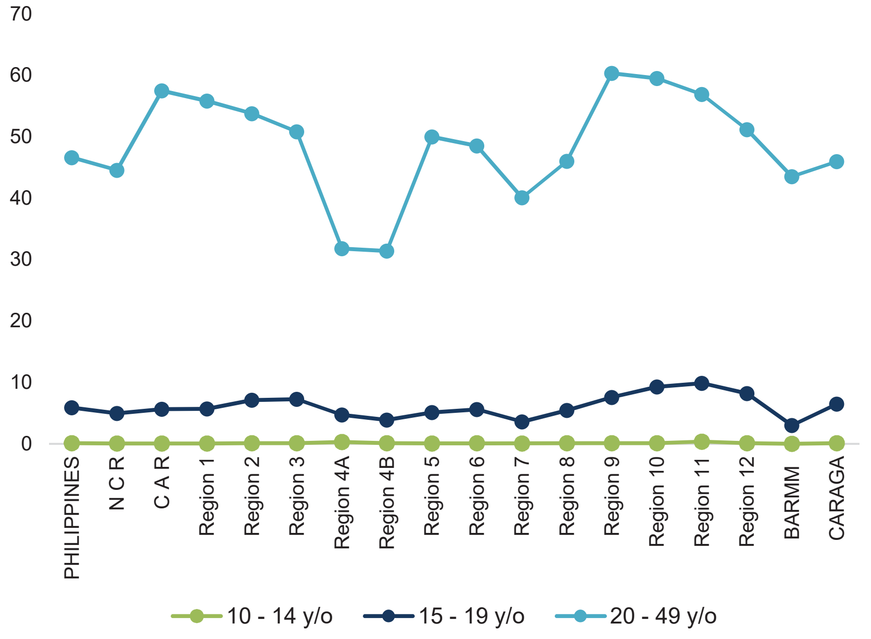

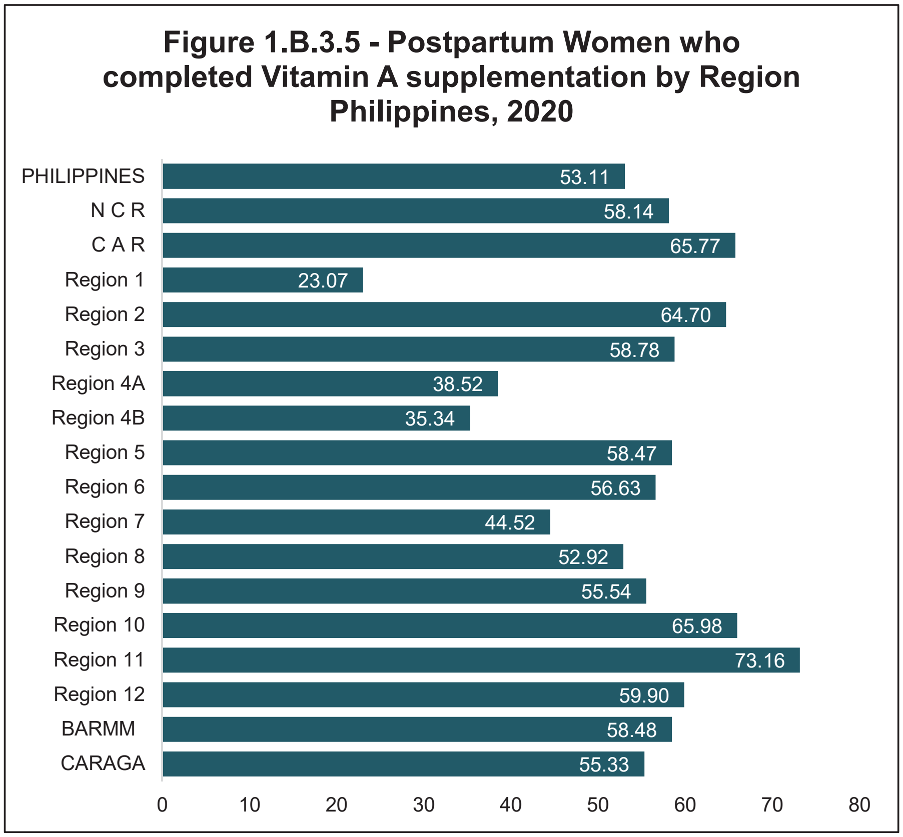
Figure 1.B.3.5 - Postpartum Women who completed Vitamin A supplementation by Region Philippines, 2020
PHILIPPINES
N C R
C A R
Region 1
Region 2
Region 3
Region 4A
Region 4B
Region 5
Region 6
Region 7
Region 8
Region 9
Region 10
Region 11
Region 12
BARMM
CARAGA
53.11
58.14
65.77
23.07
64.70
58.78
38.52
35.34
58.47
56.63
44.52
52.92
55.54
65.98
73.16
59.90
58.48
55.33
0
10
20
30
40
50
60
70
80

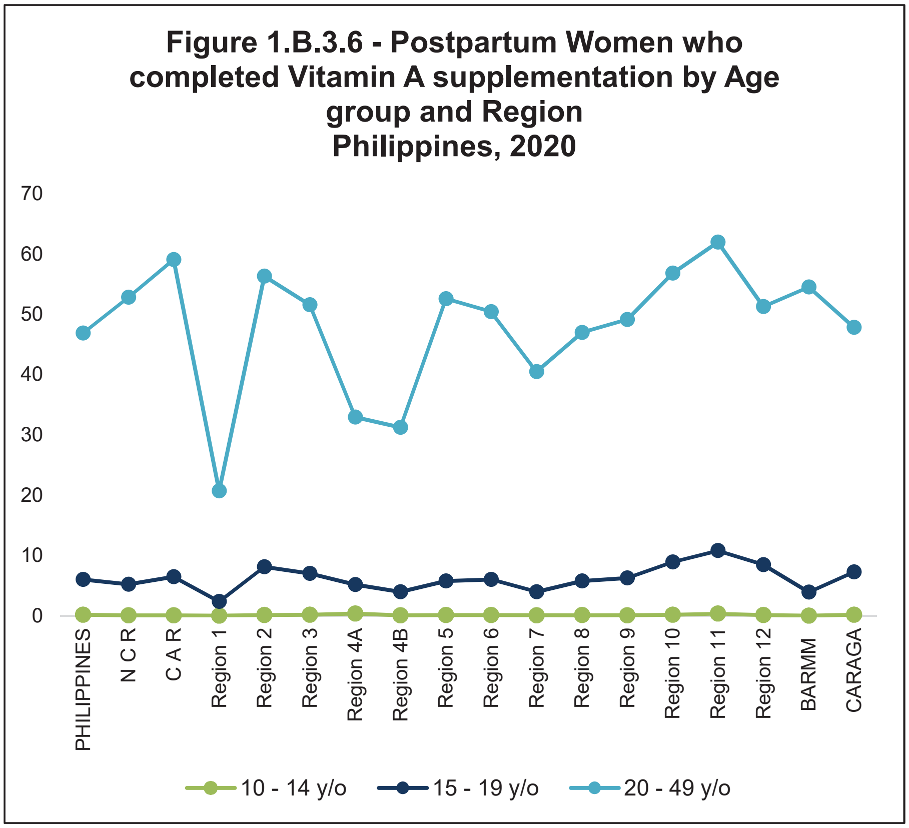
Figure 1.B.3.6 - Postpartum Women who completed Vitamin A supplementation by Age group and Region Philippines, 2020
70
60
50
40
30
20
10
0
PHILIPPINES
N C R
C A R
Region 1
Region 2
Region 3
Region 4A
Region 4B
Region 5
Region 6
Region 7
Region 8
Region 9
Region 10
Region 11
Region 12
BARMM
CARAGA
10 - 14 y/o
15 - 19 y/o
20 - 49 y/o

Table 1.C.1.1 - Immunization Services for Infants and Children

Number and Proportion of Children Protected at Birth (CPAB), Children Given BCG & Hepatitis B1 Vaccines
Philippines, Annual 2020

Area	Eligible Pop (Under 1 y.o.)	Child Protected at Birth (CPAB)				BCG				Hepa B1 (w/in 24 Hrs.) after birth			
		Male	Female	Total	%	Male	Female	Total	%	Male	Female	Total	%
**PHILIPPINES**	**2,123,158**	**646,574**	**631,648**	**1,278,222**	**60.20**	**733,996**	**709,400**	**1,443,396**	**67.98**	**607,157**	**598,171**	**1,205,328**	**56.77**
**N C R**	**238,661**	**81,834**	**79,161**	**160,995**	**67.46**	**99,843**	**96,515**	**196,358**	**82.27**	**90,867**	**87,952**	**178,819**	**74.93**
Malabon	6,775	2,942	3,008	5,950	87.82	2,672	2,727	5,399	79.69	2,520	2,515	5,035	74.32
Navotas	4,621	1,940	1,825	3,765	81.48	2,144	1,979	4,123	89.22	2,093	1,983	4,076	88.21
Valenzuela City	11,500	2,950	2,858	5,808	50.50	1,582	1,536	3,118	27.11	1,165	1,170	2,335	20.30
Caloocan City	29,363	9,283	8,949	18,232	62.09	12,578	12,257	24,835	84.58	11,223	10,938	22,161	75.47
Marikina City	8,354	2,783	2,781	5,564	66.60	4,274	4,080	8,354	100.00	4,061	3,937	7,998	95.74
Pasig City	13,996	4,085	3,553	7,638	54.57	6,730	6,285	13,015	92.99	6,173	5,781	11,954	85.41
Pateros	1,184	441	449	890	75.17	470	483	953	80.49	430	448	878	74.16
Taguig	14,918	3,818	3,859	7,677	51.46	7,006	6,967	13,973	93.67	5,901	5,613	11,514	77.18
Quezon City	54,413	22,442	22,506	44,948	82.61	25,145	25,165	50,310	92.46	23,721	23,828	47,549	87.39
Makati City	10,798	2,720	2,396	5,116	47.38	4,500	4,360	8,860	82.05	4,462	4,094	8,556	79.24
Mandaluyong City	7,160	629	702	1,331	18.59	1,274	1,244	2,518	35.17	473	418	891	12.44
San Juan	2,261	350	360	710	31.40	43	46	89	3.94	75	89	164	7.25
Manila City	32,980	17,052	15,928	32,980	100.00	17,212	15,768	32,980	100.00	16,924	16,056	32,980	100.00
Las Piñas City	10,914	2,986	3,005	5,991	54.89	3,607	3,573	7,180	65.79	2,481	2,401	4,882	44.73
Muntinlupa City	9,353	2,660	2,695	5,355	57.25	3,750	3,615	7,365	78.74	2,706	2,561	5,267	56.31
Parañaque City	12,343	2,625	2,289	4,914	39.81	3,938	3,508	7,446	60.33	3,656	3,331	6,987	56.61
Pasay City	7,728	2,128	1,998	4,126	53.39	2,918	2,922	5,840	75.57	2,803	2,789	5,592	72.36
**C A R**	**35,099**	**10,411**	**10,161**	**20,572**	**58.61**	**11,994**	**12,276**	**24,270**	**69.15**	**11,340**	**11,744**	**23,084**	**65.77**
Abra	4,309	1,160	1,241	2,401	55.72	1,099	1,795	2,894	67.16	1,060	1,752	2,812	65.26
Apayao	2,451	754	691	1,445	58.96	587	551	1,138	46.43	428	530	958	39.09
Benguet	9,188	3,079	3,020	6,099	66.38	2,454	2,219	4,673	50.86	2,424	2,152	4,576	49.80
Ifugao	4,396	1,272	1,186	2,458	55.91	1,494	1,419	2,913	66.26	1,195	1,137	2,332	53.05
Kalinga	4,617	1,639	1,596	3,235	70.07	1,484	1,425	2,909	63.01	1,355	1,305	2,660	57.61
Mt. Province	3,023	1,167	1,065	2,232	73.83	1,344	1,401	2,745	90.80	1,335	1,399	2,734	90.44
Baguio City	7,115	1,340	1,362	2,702	37.98	3,532	3,466	6,998	98.36	3,543	3,469	7,012	98.55
**Region 1**	**97,261**	**31,439**	**29,974**	**61,413**	**63.14**	**29,399**	**27,422**	**56,821**	**58.42**	**27,504**	**26,366**	**53,870**	**55.39**
Ilocos Norte	8,105	2,788	2,637	5,425	66.93	3,379	2,576	5,955	73.47	2,785	2,596	5,381	66.39
Ilocos Sur	9,330	3,717	3,628	7,345	78.72	3,659	3,658	7,317	78.42	3,718	3,636	7,354	78.82
La Union	11,511	3,958	3,805	7,763	67.44	4,012	3,832	7,844	68.14	4,001	3,827	7,828	68.00
Pangasinan	50,168	14,638	13,905	28,543	56.89	11,656	11,062	22,718	45.28	10,465	10,021	20,486	40.83
Alaminos City	1,894	923	799	1,722	90.92	923	799	1,722	90.92	923	799	1,722	90.92
Candon City	987	551	467	1,018	103.14	551	467	1,018	103.14	551	467	1,018	103.14
Dagupan City	3,620	747	774	1,521	42.02	837	784	1,621	44.78	765	764	1,529	42.24
Laoag City	1,870	985	975	1,960	104.81	986	976	1,962	104.92	987	977	1,964	105.03
San Carlos City	3,979	1,385	1,261	2,646	66.50	1,675	1,560	3,235	81.30	1,592	1,476	3,068	77.10
San Fernando City	2,115	761	744	1,505	71.16	754	740	1,494	70.64	752	837	1,589	75.13
Urdaneta City	2,806	853	841	1,694	60.37	853	841	1,694	60.37	853	841	1,694	60.37
Vigan City	876	133	138	271	30.94	114	127	241	27.51	112	125	237	27.05
**Region 2**	**69,443**	**23,482**	**23,018**	**46,500**	**66.96**	**23,282**	**22,316**	**45,598**	**65.66**	**22,383**	**21,497**	**43,880**	**63.19**
Batanes	340	124	113	237	69.71	121	125	246	72.35	122	120	242	71.18
Cagayan	17,971	5,896	5,780	11,676	64.97	5,333	5,004	10,337	57.52	5,102	4,878	9,980	55.53
Isabela	26,309	8,171	7,993	16,164	61.44	7,865	7,634	15,499	58.91	7,830	7,465	15,295	58.14
Nueva Vizcaya	9,573	3,246	3,120	6,366	66.50	3,877	3,662	7,539	78.75	3,433	3,304	6,737	70.38
Quirino	4,025	1,472	1,448	2,920	72.55	1,462	1,439	2,901	72.07	1,437	1,414	2,851	70.83
Cauayan City	2,590	948	920	1,868	72.12	1,181	1,101	2,282	88.11	1,176	1,095	2,271	87.68
Ilagan City	2,911	1,422	1,477	2,899	99.59	1,422	1,477	2,899	99.59	1,422	1,477	2,899	99.59
Santiago City	2,692	1,373	1,319	2,692	100.00	1,373	1,319	2,692	100.00	1,373	1,319	2,692	100.00
Tuguegarao City	3,032	830	848	1,678	55.34	648	555	1,203	39.68	488	425	913	30.11
**Region 3**	**220,020**	**69,164**	**68,550**	**137,714**	**62.59**	**75,171**	**77,220**	**152,391**	**69.26**	**54,796**	**60,820**	**115,616**	**52.55**
Aurora	4,770	1,401	1,317	2,718	56.98	712	5,154	5,866	122.98	562	4,947	5,509	115.49
Bataan	13,823	4,904	4,787	9,691	70.11	4,121	3,891	8,012	57.96	3,724	3,436	7,160	51.80

Table 1.C.1.1 - Immunization Services for Infants and Children

Number and Proportion of Children Protected at Birth (CPAB), Children Given BCG & Hepatitis B1 Vaccines
Philippines, Annual 2020

Area	Eligible Pop (Under 1 y.o.)	Child Protected at Birth (CPAB)				BCG				Hepa B1 (w/in 24 Hrs.) after birth			
		Male	Female	Total	%	Male	Female	Total	%	Male	Female	Total	%
Bulacan	43,627	11,473	11,381	22,854	52.38	13,680	13,505	27,185	62.31	10,932	10,562	21,494	49.27
Nueva Ecija	28,782	8,330	7,932	16,262	56.50	6,956	6,718	13,674	47.51	3,321	3,238	6,559	22.79
Pampanga	31,359	12,357	11,879	24,236	77.29	13,035	12,533	25,568	81.53	11,238	14,126	25,364	80.88
Tarlac	19,765	5,858	5,769	11,627	58.83	5,581	5,403	10,984	55.57	3,775	3,650	7,425	37.57
Zambales	12,610	2,803	2,976	5,779	45.83	4,870	4,652	9,522	75.51	3,197	3,625	6,822	54.10
Angeles City	7,869	1,777	1,929	3,706	47.10	1,753	1,752	3,505	44.54	141	133	274	3.48
Balanga City	1,998	553	555	1,108	55.46	312	318	630	31.53	306	317	623	31.18
Cabanatuan City	5,896	2,576	2,375	4,951	83.97	4,240	3,756	7,996	135.62	3,106	2,822	5,928	100.54
City of San Fernando	5,863	2,349	2,230	4,579	78.10	2,094	1,929	4,023	68.62	2,325	2,092	4,417	75.34
Gapan City	2,153	646	683	1,329	61.73	690	718	1,408	65.40	297	298	595	27.64
Mabalacat City	4,793	2,532	2,555	5,087	106.13	1,571	1,663	3,234	67.47	1,406	1,500	2,906	60.63
Malolos City	4,880	1,070	1,134	2,204	45.16	2,693	2,630	5,323	109.08	1,964	1,765	3,729	76.41
Meycauayan	4,041	1,410	1,499	2,909	71.99	1,764	1,763	3,527	87.28	1,494	1,450	2,944	72.85
Olongapo	4,971	1,405	1,388	2,793	56.19	819	699	1,518	30.54	203	195	398	8.01
Palayan City	799	302	349	651	81.48	185	180	365	45.68	41	62	103	12.89
San Jose City	2,718	1,050	986	2,036	74.91	1,124	1,095	2,219	81.64	1,069	987	2,056	75.64
San Jose del Monte City	11,100	3,271	3,436	6,707	60.42	5,339	5,329	10,668	96.11	2,860	2,848	5,708	51.42
Science City of Munoz	1,586	643	609	1,252	78.94	299	283	582	36.70	44	44	88	5.55
Tarlac City	6,617	2,454	2,781	5,235	79.11	3,333	3,249	6,582	99.47	2,791	2,723	5,514	83.33
**Region 4A**	**296,816**	**80,796**	**76,997**	**157,793**	**53.16**	**100,480**	**94,512**	**194,992**	**65.69**	**84,858**	**81,709**	**166,567**	**56.12**
Batangas	38,441	10,308	9,534	19,842	51.62	10,929	10,186	21,115	54.93	10,464	9,720	20,184	52.51
Cavite	27,704	12,321	11,903	24,224	87.44	13,730	13,147	26,877	97.01	12,538	11,928	24,466	88.31
Laguna	19,727	3,895	3,720	7,615	38.60	4,583	4,303	8,886	45.04	2,952	2,793	5,745	29.12
Quezon	37,410	10,065	9,349	19,414	51.90	12,243	11,276	23,519	62.87	9,594	8,952	18,546	49.57
Rizal	44,791	8,989	8,519	17,508	39.09	12,681	11,789	24,470	54.63	9,401	9,383	18,784	41.94
Antipolo City	16,497	3,255	3,308	6,563	39.78	5,015	4,844	9,859	59.76	3,060	2,949	6,009	36.42
Bacoor City	12,142	2,785	2,606	5,391	44.40	3,558	3,386	6,944	57.19	2,718	2,355	5,073	41.78
Batangas City	6,823	1,999	1,802	3,801	55.71	2,716	2,505	5,221	76.52	2,686	2,459	5,145	75.41
Biñan City	6,607	3,430	3,310	6,740	102.01	3,647	3,543	7,190	108.82	3,293	4,634	7,927	119.98
Cabuyao City	6,130	2,107	2,299	4,406	71.88	2,106	2,240	4,346	70.90	1,533	1,688	3,221	52.54
Calamba City	9,028	3,478	3,364	6,842	75.79	3,623	3,341	6,964	77.14	3,445	3,132	6,577	72.85
Cavite City	2,075	320	305	625	30.12	465	448	913	44.00	419	379	798	38.46
Dasmariñas City	13,322	2,018	1,875	3,893	29.22	2,623	2,439	5,062	38.00	2,543	2,379	4,922	36.95
General Trias City	6,352	1,575	1,583	3,158	49.72	2,616	2,508	5,124	80.67	2,372	2,282	4,654	73.27
Imus City	8,156	1,214	1,138	2,352	28.84	2,204	2,054	4,258	52.21	1,919	1,775	3,694	45.29
Lipa City	6,880	1,133	1,055	2,188	31.80	1,443	1,269	2,712	39.42	1,215	1,054	2,269	32.98
Lucena City	5,672	1,109	1,081	2,190	38.61	1,441	1,268	2,709	47.76	1,381	1,216	2,597	45.79
San Pablo City	5,277	1,499	1,361	2,860	54.20	2,166	1,916	4,082	77.35	1,946	1,790	3,736	70.80
San Pedro City	6,467	1,354	1,262	2,616	40.45	2,177	1,996	4,173	64.53	1,877	1,745	3,622	56.01
Santa Rosa City	7,025	2,035	2,019	4,054	57.71	2,496	2,473	4,969	70.73	2,093	2,103	4,196	59.73
Tagaytay City	1,437	1,490	1,328	2,818	196.10	1,482	1,316	2,798	194.71	1,472	1,314	2,786	193.88
Tanauan City	3,584	1,019	1,014	2,033	56.72	1,799	1,701	3,500	97.66	1,528	1,446	2,974	82.98
Tayabas City	2,122	788	796	1,584	74.65	1,015	930	1,945	91.66	957	887	1,844	86.90
Trece Martires City	3,147	2,610	2,466	5,076	161.30	3,722	3,634	7,356	233.75	3,452	3,346	6,798	216.02
**Region 4B**	**71,246**	**15,356**	**14,890**	**30,246**	**42.45**	**18,039**	**16,495**	**34,534**	**48.47**	**13,920**	**12,806**	**26,726**	**37.51**
Marinduque	4,836	1,331	1,299	2,630	54.38	1,432	1,362	2,794	57.78	1,424	1,305	2,729	56.43
Mindoro Occidental	12,407	3,701	3,515	7,216	58.16	4,174	3,959	8,133	65.55	3,239	2,936	6,175	49.77
Mindoro Oriental	19,809	4,445	4,461	8,906	44.96	5,575	4,679	10,254	51.76	4,242	3,948	8,190	41.34
Palawan	21,375	3,282	3,152	6,434	30.10	3,964	3,742	7,706	36.05	3,001	2,776	5,777	27.03
Romblon	6,400	1,120	1,043	2,163	33.80	1,255	1,180	2,435	38.05	1,034	935	1,969	30.77
Puerto Princesa City	6,419	1,477	1,420	2,897	45.13	1,639	1,573	3,212	50.04	980	906	1,886	29.38
**Region 5**	**136,116**	**32,521**	**32,050**	**64,571**	**47.44**	**43,221**	**40,870**	**84,091**	**61.78**	**34,348**	**32,991**	**67,339**	**49.47**
Albay	23,737	6,193	6,076	12,269	51.69	6,322	6,154	12,476	52.56	5,468	5,345	10,813	45.55
Camarines Norte	14,283	4,174	4,225	8,399	58.80	4,606	4,592	9,198	64.40	4,428	4,312	8,740	61.19
Camarines Sur	38,796	5,839	5,647	11,486	29.61	11,599	10,551	22,150	57.09	7,935	7,280	15,215	39.22
Catanduanes	6,274	1,807	1,746	3,553	56.63	1,881	1,846	3,727	59.40	1,851	1,732	3,583	57.11
Masbate	22,521	6,562	6,212	12,774	56.72	8,858	7,692	16,550	73.49	5,600	5,326	10,926	48.51
Sorsogon	19,065	5,416	5,448	10,864	56.98	6,619	6,668	13,287	69.69	6,144	5,926	12,070	63.31
Iriga City	2,638	374	374	748	28.35	561	504	1,065	40.37	252	219	471	17.85

Table 1.C.1.1 - Immunization Services for Infants and Children

Number and Proportion of Children Protected at Birth (CPAB), Children Given BCG & Hepatitis B1 Vaccines

Philippines, Annual 2020

Area	Eligible Pop (Under 1 y.o.)	Child Protected at Birth (CPAB)				BCG				Hepa B1 (w/in 24 Hrs.) after birth			
		Male	Female	Total	%	Male	Female	Total	%	Male	Female	Total	%
Legaspi City	4,174	1,192	1,384	2,576	61.72	1,407	1,570	2,977	71.32	1,326	1,558	2,884	69.09
Naga City	4,628	964	938	1,902	41.10	1,368	1,293	2,661	57.50	1,344	1,293	2,637	56.98
**Region 6**	**146,526**	**42,779**	**41,275**	**84,054**	**57.36**	**42,199**	**39,892**	**82,091**	**56.02**	**37,620**	**35,755**	**73,375**	**50.08**
Aklan	11,162	3,010	2,954	5,964	53.43	2,361	2,108	4,469	40.04	1,651	1,658	3,309	29.65
Antique	12,816	3,507	3,355	6,862	53.54	2,457	2,290	4,747	37.04	1,797	1,628	3,425	26.72
Capiz	13,986	3,154	3,022	6,176	44.16	4,568	4,289	8,857	63.33	4,430	4,234	8,664	61.95
Guimaras	3,085	1,255	1,204	2,459	79.71	1,177	1,080	2,257	73.16	1,275	1,130	2,405	77.96
Iloilo	36,541	10,164	9,893	20,057	54.89	8,844	8,481	17,325	47.41	7,673	7,509	15,182	41.55
Negros Occidental	49,368	16,585	15,994	32,579	65.99	18,202	17,395	35,597	72.11	16,766	15,965	32,731	66.30
Bacolod City	11,115	2,658	2,376	5,034	45.29	1,334	1,160	2,494	22.44	813	704	1,517	13.65
Iloilo City	8,453	2,446	2,477	4,923	58.24	3,256	3,089	6,345	75.06	3,215	2,927	6,142	72.66
**Region 7**	**163,262**	**48,650**	**47,370**	**96,020**	**58.81**	**57,513**	**55,048**	**112,561**	**68.95**	**44,073**	**43,272**	**87,345**	**53.50**
Bohol	27,312	9,562	9,100	18,662	68.33	7,234	7,001	14,235	52.12	9,665	9,261	18,926	69.30
Cebu	67,506	16,452	15,470	31,922	47.29	22,125	20,743	42,868	63.50	13,527	13,262	26,789	39.68
Negros Oriental	27,938	7,964	8,424	16,388	58.66	11,153	10,999	22,152	79.29	6,541	6,775	13,316	47.66
Siquijor	1,613	595	548	1,143	70.86	668	586	1,254	77.74	664	583	1,247	77.31
Cebu City	21,193	8,098	7,941	16,039	75.68	8,393	8,173	16,566	78.17	7,833	7,545	15,378	72.56
Lapu-Lapu City	9,372	3,666	3,610	7,276	77.64	4,158	4,023	8,181	87.29	3,566	3,428	6,994	74.63
Mandaue City	8,328	2,313	2,277	4,590	55.12	3,782	3,523	7,305	87.72	2,277	2,418	4,695	56.38
**Region 8**	**102,619**	**25,472**	**24,793**	**50,265**	**48.98**	**32,584**	**31,199**	**63,783**	**62.16**	**25,215**	**24,588**	**49,803**	**48.53**
Biliran	3,834	1,554	1,396	2,950	76.94	1,580	1,426	3,006	78.40	1,593	1,421	3,014	78.61
Eastern Samar	11,392	3,062	3,088	6,150	53.99	3,413	3,291	6,704	58.85	2,399	2,385	4,784	41.99
Northern Leyte	34,707	8,950	8,649	17,599	50.71	10,387	9,890	20,277	58.42	8,353	8,103	16,456	47.41
Northern Samar	15,370	2,855	2,892	5,747	37.39	5,413	5,160	10,573	68.79	3,629	3,650	7,279	47.36
Southern Leyte	6,451	1,728	1,740	3,468	53.76	2,084	2,057	4,141	64.19	1,761	1,794	3,555	55.11
Western Samar	14,305	3,069	2,925	5,994	41.90	4,467	4,316	8,783	61.40	2,778	2,562	5,340	37.33
Calbayog City	4,413	720	690	1,410	31.95	1,724	1,698	3,422	77.54	1,261	1,164	2,425	54.95
Maasin City	1,637	401	336	737	45.02	369	365	734	44.84	233	254	487	29.75
Ormoc City	4,941	1,824	1,695	3,519	71.22	1,829	1,630	3,459	70.01	1,831	1,629	3,460	70.03
Tacloban City	5,569	1,309	1,382	2,691	48.32	1,318	1,366	2,684	48.20	1,377	1,626	3,003	53.92
**Region 9**	**80,051**	**25,834**	**26,257**	**52,091**	**65.07**	**25,047**	**25,013**	**50,060**	**62.54**	**19,758**	**20,175**	**39,933**	**49.88**
Zamboanga del Norte	17,249	7,893	8,138	16,031	92.94	8,559	8,583	17,142	99.38	7,070	7,315	14,385	83.40
Zamboanga del Sur	17,653	3,955	3,830	7,785	44.10	4,045	4,219	8,264	46.81	3,106	3,268	6,374	36.11
Zamboanga Sibugay	14,954	3,457	3,657	7,114	47.57	3,385	3,336	6,721	44.94	2,994	2,992	5,986	40.03
Dapitan City	1,784	727	961	1,688	94.62	727	961	1,688	94.62	712	914	1,626	91.14
Dipolog City	2,827	1,134	1,133	2,267	80.19	1,288	1,193	2,481	87.76	1,172	1,122	2,294	81.15
Isabela City	2,522	688	663	1,351	53.57	1,051	1,013	2,064	81.84	536	475	1,011	40.09
Pagadian City	4,325	1,489	1,402	2,891	66.84	1,504	1,431	2,935	67.86	1,463	1,387	2,850	65.90
Zamboanga City	18,737	6,491	6,473	12,964	69.19	4,488	4,277	8,765	46.78	2,705	2,702	5,407	28.86
**Region 10**	**101,411**	**37,450**	**35,601**	**73,051**	**72.03**	**37,522**	**35,865**	**73,387**	**72.37**	**32,281**	**32,150**	**64,431**	**63.53**
Bukidnon	23,706	8,265	8,111	16,376	69.08	9,635	9,145	18,780	79.22	7,317	7,350	14,667	61.87
Camiguin	1,858	673	606	1,279	68.84	640	601	1,241	66.79	682	611	1,293	69.59
Lanao del Norte	14,960	4,275	4,424	8,699	58.15	4,565	4,556	9,121	60.97	5,086	5,098	10,184	68.07
Misamis Occidental	6,403	2,192	2,218	4,410	68.87	2,121	2,115	4,236	66.16	2,043	2,119	4,162	65.00
Misamis Oriental	15,131	6,543	5,268	11,811	78.06	6,115	5,666	11,781	77.86	5,406	5,190	10,596	70.03
Cagayan de Oro City	14,339	6,785	6,309	13,094	91.32	4,725	4,486	9,211	64.24	4,321	4,132	8,453	58.95
El Salvador City	1,065	172	152	324	30.42	450	457	907	85.16	433	465	898	84.32
Gingoog City	2,644	1,052	1,035	2,087	78.93	1,205	1,106	2,311	87.41	1,064	1,019	2,083	78.78
Iligan City	7,580	2,446	2,223	4,669	61.60	2,862	2,598	5,460	72.03	2,218	2,034	4,252	56.09
Malaybalay City	3,956	1,252	1,209	2,461	62.21	1,135	1,125	2,260	57.13	333	421	754	19.06
Oroquieta City	1,389	500	441	941	67.75	322	286	608	43.77	322	286	608	43.77
Ozamis City	2,778	1,147	1,129	2,276	81.93	1,119	1,109	2,228	80.20	1,120	1,109	2,229	80.24
Tangub City	1,234	552	494	1,046	84.76	597	612	1,209	97.97	345	575	920	74.55
Valencia City	4,368	1,596	1,982	3,578	81.91	2,031	2,003	4,034	92.35	1,591	1,741	3,332	76.28

Table 1.C.1.1 - Immunization Services for Infants and Children

Number and Proportion of Children Protected at Birth (CPAB), Children Given BCG & Hepatitis B1 Vaccines
Philippines, Annual 2020

Area	Eligible Pop (Under 1 y.o.)	Child Protected at Birth (CPAB)				BCG				Hepa B1 (w/in 24 Hrs.) after birth			
		Male	Female	Total	%	Male	Female	Total	%	Male	Female	Total	%
**Region 11**	**107,247**	**42,258**	**39,362**	**81,620**	**76.10**	**43,659**	**40,786**	**84,445**	**78.74**	**40,965**	**37,616**	**78,581**	**73.27**
Compostela Valley	15,562	6,296	5,674	11,970	76.92	6,279	5,812	12,091	77.70	6,381	5,586	11,967	76.90
Davao del Norte	21,326	10,022	9,583	19,605	91.93	10,193	9,736	19,929	93.45	10,133	9,435	19,568	91.76
Davao del Sur	13,007	4,045	3,663	7,708	59.26	4,454	4,010	8,464	65.07	3,979	3,576	7,555	58.08
Davao Oriental	14,151	4,808	4,473	9,281	65.59	4,676	4,344	9,020	63.74	4,953	4,663	9,616	67.95
Davao Occidental	6,670	2,023	1,819	3,842	57.60	2,210	2,010	4,220	63.27	1,419	1,249	2,668	40.00
Davao City	36,531	15,064	14,150	29,214	79.97	15,847	14,874	30,721	84.10	14,100	13,107	27,207	74.48
**Region 12**	**104,552**	**32,295**	**32,196**	**64,491**	**61.68**	**36,417**	**35,130**	**71,547**	**68.43**	**30,453**	**29,795**	**60,248**	**57.62**
North Cotabato	33,645	8,676	8,481	17,157	50.99	10,249	9,800	20,049	59.59	7,860	7,550	15,410	45.80
Sarangani	12,891	4,673	4,874	9,547	74.06	5,113	5,132	10,245	79.47	4,546	4,698	9,244	71.71
South Cotabato	21,113	8,476	8,554	17,030	80.66	8,613	8,363	16,976	80.41	8,029	7,914	15,943	75.51
Sultan Kudarat	17,359	5,846	5,461	11,307	65.14	6,462	6,063	12,525	72.15	5,710	5,312	11,022	63.49
Cotabato City	5,835	2,060	2,216	4,276	73.28	2,793	2,777	5,570	95.46	1,772	1,863	3,635	62.30
Gen. Santos City	13,709	2,564	2,610	5,174	37.74	3,187	2,995	6,182	45.09	2,536	2,458	4,994	36.43
**BARMM**	**92,799**	**29,069**	**31,718**	**60,787**	**65.50**	**35,257**	**37,398**	**72,655**	**78.29**	**24,068**	**26,233**	**50,301**	**54.20**
Basilan	7,541	1,032	1,128	2,160	28.64	1,731	1,911	3,642	48.30	590	721	1,311	17.38
Lanao del Sur	21,131	8,571	9,960	18,531	87.70	9,653	10,993	20,646	97.70	8,364	9,641	18,005	85.21
Maguindanao	31,128	11,007	11,430	22,437	72.08	14,175	13,933	28,108	90.30	7,548	7,754	15,302	49.16
Sulu	16,613	4,046	4,295	8,341	50.21	4,687	4,927	9,614	57.87	3,083	2,935	6,018	36.22
Tawi-Tawi	9,259	2,494	2,853	5,347	57.75	2,992	3,448	6,440	69.55	2,670	2,920	5,590	60.37
Lamitan City	2,074	544	577	1,121	54.05	644	699	1,343	64.75	577	596	1,173	56.56
Marawi City	5,053	1,375	1,475	2,850	56.40	1,375	1,487	2,862	56.64	1,236	1,666	2,902	57.43
**CARAGA**	**60,029**	**17,764**	**18,275**	**36,039**	**60.04**	**22,369**	**21,443**	**43,812**	**72.98**	**12,708**	**12,702**	**25,410**	**42.33**
Agusan del Norte	8,098	2,159	2,210	4,369	53.95	3,247	3,108	6,355	78.48	1,276	1,241	2,517	31.08
Agusan del Sur	17,592	4,823	4,764	9,587	54.50	6,941	6,697	13,638	77.52	4,026	3,899	7,925	45.05
Surigao del Norte	7,089	2,204	2,397	4,601	64.90	2,602	2,508	5,110	72.08	1,424	1,437	2,861	40.36
Surigao del Sur	11,482	3,209	3,185	6,394	55.69	3,915	3,816	7,731	67.33	2,681	2,677	5,358	46.66
Province of Dinagat	2,573	487	570	1,057	41.08	659	634	1,293	50.25	391	424	815	31.68
Bislig City	2,179	815	825	1,640	75.26	891	927	1,818	83.43	745	778	1,523	69.89
Butuan City	7,715	3,097	3,299	6,396	82.90	3,259	2,937	6,196	80.31	1,856	1,961	3,817	49.48
Surigao City	3,301	970	1,025	1,995	60.44	855	816	1,671	50.62	309	285	594	17.99

* - No Report
0 - No Cases

Table 1.C.1.2 - Immunization Services for Infants

Number and Proportion of Infants who completed 3 doses of HiB-HepB antigen

Philippines, Annual 2020

Area	Eligible Pop (Under 1 y.o.)	DPT-HiB-HepB 1				DPT-HiB-HepB 2				DPT-HiB-HepB 3			
		Male	Female	Total	%	Male	Female	Total	%	Male	Female	Total	%
**PHILIPPINES**	**2,123,158**	**850,892**	**800,260**	**1,651,152**	**77.77**	**831,647**	**788,693**	**1,620,340**	**76.32**	**813,738**	**774,469**	**1,588,207**	**74.80**
**N C R**	**238,661**	**89,255**	**85,685**	**174,940**	**73.30**	**82,047**	**78,540**	**160,587**	**67.29**	**76,801**	**74,488**	**151,289**	**63.39**
Malabon	6,775	3,102	3,028	6,130	90.48	2,906	2,818	5,724	84.49	2,809	2,641	5,450	80.44
Navotas	4,621	2,392	2,229	4,621	100.00	2,290	2,158	4,448	96.26	2,063	2,056	4,119	89.14
Valenzuela City	11,500	4,744	4,570	9,314	80.99	4,523	4,371	8,894	77.34	4,155	4,125	8,280	72.00
Caloocan City	29,363	11,136	10,881	22,017	74.98	10,433	9,343	19,776	67.35	8,826	8,504	17,330	59.02
Marikina City	8,354	3,735	3,581	7,316	87.57	3,360	3,271	6,631	79.38	3,216	3,133	6,349	76.00
Pasig City	13,996	5,564	5,086	10,650	76.09	5,195	4,889	10,084	72.05	5,073	4,810	9,883	70.61
Pateros	1,184	539	496	1,035	87.42	519	473	992	83.78	517	482	999	84.38
Taguig	14,918	7,078	6,708	13,786	92.41	6,599	6,271	12,870	86.27	6,444	6,069	12,513	83.88
Quezon City	54,413	16,798	16,422	33,220	61.05	15,064	14,878	29,942	55.03	14,389	14,223	28,612	52.58
Makati City	10,798	3,829	3,521	7,350	68.07	3,633	3,430	7,063	65.41	3,592	3,323	6,915	64.04
Mandaluyong City	7,160	2,141	2,022	4,163	58.14	2,060	1,997	4,057	56.66	2,140	1,987	4,127	57.64
San Juan	2,261	698	697	1,395	61.70	681	708	1,389	61.43	610	670	1,280	56.61
Manila City	32,980	11,839	11,610	23,449	71.10	10,528	10,290	20,818	63.12	9,547	9,546	19,093	57.89
Las Piñas City	10,914	4,293	3,988	8,281	75.88	3,973	3,679	7,652	70.11	3,688	3,541	7,229	66.24
Muntinlupa City	9,353	4,088	3,857	7,945	84.95	3,929	3,705	7,634	81.62	3,737	3,598	7,335	78.42
Parañaque City	12,343	4,554	4,250	8,804	71.33	4,098	3,772	7,870	63.76	3,711	3,542	7,253	58.76
Pasay City	7,728	2,725	2,739	5,464	70.70	2,256	2,487	4,743	61.37	2,284	2,238	4,522	58.51
**C A R**	**35,099**	**13,548**	**12,725**	**26,273**	**74.85**	**13,374**	**12,815**	**26,189**	**74.61**	**13,479**	**12,780**	**26,259**	**74.81**
Abra	4,309	1,579	1,533	3,112	72.22	1,652	1,602	3,254	75.52	1,638	1,652	3,290	76.35
Apayao	2,451	908	873	1,781	72.66	935	945	1,880	76.70	951	945	1,896	77.36
Benguet	9,188	3,276	3,055	6,331	68.91	3,167	3,117	6,284	68.39	3,239	3,136	6,375	69.38
Ifugao	4,396	1,795	1,561	3,356	76.34	1,747	1,526	3,273	74.45	1,792	1,519	3,311	75.32
Kalinga	4,617	2,183	1,963	4,146	89.80	2,145	1,983	4,128	89.41	2,116	1,987	4,103	88.87
Mt. Province	3,023	1,180	1,116	2,296	75.95	1,208	1,108	2,316	76.61	1,212	1,113	2,325	76.91
Baguio City	7,115	2,627	2,624	5,251	73.80	2,520	2,534	5,054	71.03	2,531	2,428	4,959	69.70
**Region 1**	**97,261**	**38,876**	**36,515**	**75,391**	**77.51**	**39,835**	**37,110**	**76,945**	**79.11**	**40,081**	**37,267**	**77,348**	**79.53**
Ilocos Norte	8,105	2,865	2,634	5,499	67.85	2,873	2,719	5,592	68.99	2,925	2,773	5,698	70.30
Ilocos Sur	9,330	3,892	3,676	7,568	81.11	4,030	3,850	7,880	84.46	4,096	3,851	7,947	85.18
La Union	11,511	4,520	4,360	8,880	77.14	4,568	4,328	8,896	77.28	4,613	4,353	8,966	77.89
Pangasinan	50,168	19,821	18,736	38,557	76.86	20,640	19,026	39,666	79.07	20,786	19,421	40,207	80.14
Alaminos City	1,894	1,404	972	2,376	125.45	1,414	941	2,355	124.34	1,382	925	2,307	121.81
Candon City	987	513	514	1,027	104.05	531	505	1,036	104.96	439	436	875	88.65
Dagupan City	3,620	1,224	1,096	2,320	64.09	1,198	1,104	2,302	63.59	1,168	1,066	2,234	61.71
Laoag City	1,870	973	982	1,955	104.55	993	993	1,986	106.20	969	1,016	1,985	106.15
San Carlos City	3,979	1,735	1,694	3,429	86.18	1,767	1,674	3,441	86.48	1,745	1,675	3,420	85.95
San Fernando City	2,115	764	686	1,450	68.56	707	885	1,592	75.27	760	706	1,466	69.31
Urdaneta City	2,806	973	1,002	1,975	70.38	920	940	1,860	66.29	1,020	903	1,923	68.53
Vigan City	876	192	163	355	40.53	194	145	339	38.70	178	142	320	36.53
**Region 2**	**69,443**	**28,046**	**26,138**	**54,184**	**78.03**	**27,685**	**26,600**	**54,285**	**78.17**	**28,009**	**26,274**	**54,283**	**78.17**
Batanes	340	130	132	262	77.06	124	136	260	76.47	124	130	254	74.71
Cagayan	17,971	7,685	7,114	14,799	82.35	7,957	7,395	15,352	85.43	7,913	7,286	15,199	84.58
Isabela	26,309	9,474	8,858	18,332	69.68	9,050	9,101	18,151	68.99	9,309	8,707	18,016	68.48
Nueva Vizcaya	9,573	4,004	3,613	7,617	79.57	3,940	3,649	7,589	79.28	3,881	3,708	7,589	79.28
Quirino	4,025	1,631	1,571	3,202	79.55	1,595	1,547	3,142	78.06	1,628	1,560	3,188	79.20
Cauayan City	2,590	1,297	1,107	2,404	92.82	1,157	1,038	2,195	84.75	1,323	1,138	2,461	95.02
Ilagan City	2,911	1,310	1,359	2,669	91.69	1,286	1,307	2,593	89.08	1,291	1,273	2,564	88.08
Santiago City	2,692	1,314	1,255	2,569	95.43	1,360	1,266	2,626	97.55	1,375	1,298	2,673	99.29
Tuguegarao City	3,032	1,201	1,129	2,330	76.85	1,216	1,161	2,377	78.40	1,165	1,174	2,339	77.14
**Region 3**	**220,020**	**102,490**	**96,644**	**199,134**	**90.51**	**101,701**	**96,673**	**198,374**	**90.16**	**101,625**	**96,860**	**198,485**	**90.21**
Aurora	4,770	1,978	1,906	3,884	81.43	2,003	1,951	3,954	82.89	2,035	1,954	3,989	83.63
Bataan	13,823	6,897	6,419	13,316	96.33	6,879	6,523	13,402	96.95	6,953	6,428	13,381	96.80

Table 1.C.1.2 - Immunization Services for Infants

Number and Proportion of Infants who completed 3 doses of HiB-HepB antigen
Philippines, Annual 2020

Area	Eligible Pop (Under 1 y.o.)	DPT-HiB-HepB 1				DPT-HiB-HepB 2				DPT-HiB-HepB 3			
		Male	Female	Total	%	Male	Female	Total	%	Male	Female	Total	%
Bulacan	43,627	18,771	17,565	36,336	83.29	18,509	17,435	35,944	82.39	18,188	17,027	35,215	80.72
Nueva Ecija	28,782	12,164	11,153	23,317	81.01	12,317	11,399	23,716	82.40	12,343	11,462	23,805	82.71
Pampanga	31,359	15,077	14,441	29,518	94.13	15,079	14,553	29,632	94.49	15,206	14,664	29,870	95.25
Tarlac	19,765	10,025	9,353	19,378	98.04	10,203	9,728	19,931	100.84	10,350	9,892	20,242	102.41
Zambales	12,610	4,981	4,609	9,590	76.05	4,854	4,812	9,666	76.65	4,758	4,846	9,604	76.16
Angeles City	7,869	3,994	3,760	7,754	98.54	3,981	3,738	7,719	98.09	3,990	3,725	7,715	98.04
Balanga City	1,998	856	1,298	2,154	107.81	858	798	1,656	82.88	838	801	1,639	82.03
Cabanatuan City	5,896	2,673	2,455	5,128	86.97	2,586	2,461	5,047	85.60	2,568	2,509	5,077	86.11
City of San Fernando	5,863	2,882	2,613	5,495	93.72	2,914	2,678	5,592	95.38	3,012	2,800	5,812	99.13
Gapan City	2,153	928	976	1,904	88.43	952	946	1,898	88.16	920	984	1,904	88.43
Mabalacat City	4,793	2,623	2,584	5,207	108.64	2,552	2,526	5,078	105.95	2,550	2,437	4,987	104.05
Malolos City	4,880	1,814	1,751	3,565	73.05	1,807	1,739	3,546	72.66	1,882	1,749	3,631	74.41
Meycauayan	4,041	1,851	1,796	3,647	90.25	1,816	1,725	3,541	87.63	1,834	1,735	3,569	88.32
Olongapo	4,971	1,890	1,638	3,528	70.97	1,766	1,707	3,473	69.87	1,705	1,656	3,361	67.61
Palayan City	799	371	368	739	92.49	386	392	778	97.37	398	408	806	100.88
San Jose City	2,718	1,439	1,249	2,688	98.90	1,396	1,270	2,666	98.09	1,391	1,281	2,672	98.31
San Jose del Monte City	11,100	6,966	6,577	13,543	122.01	6,553	6,156	12,709	114.50	6,447	6,336	12,783	115.16
Science City of Munoz	1,586	683	644	1,327	83.67	684	637	1,321	83.29	683	637	1,320	83.23
Tarlac City	6,617	3,627	3,489	7,116	107.54	3,606	3,499	7,105	107.37	3,574	3,529	7,103	107.34
**Region 4A**	**296,816**	**120,912**	**111,591**	**232,503**	**78.33**	**117,256**	**108,957**	**226,213**	**76.21**	**112,956**	**105,167**	**218,123**	**73.49**
Batangas	38,441	12,797	11,426	24,223	63.01	12,657	11,644	24,301	63.22	12,604	11,724	24,328	63.29
Cavite	27,704	15,812	14,835	30,647	110.62	15,804	14,717	30,521	110.17	15,339	14,286	29,625	106.93
Laguna	19,727	7,894	7,298	15,192	77.01	7,646	7,134	14,780	74.92	7,153	6,695	13,848	70.20
Quezon	37,410	14,906	13,675	28,581	76.40	14,245	13,047	27,292	72.95	13,347	12,014	25,361	67.79
Rizal	44,791	13,358	12,108	25,466	56.86	12,862	11,808	24,670	55.08	12,396	11,630	24,026	53.64
Antipolo City	16,497	8,350	7,983	16,333	99.01	7,954	7,756	15,710	95.23	7,470	7,292	14,762	89.48
Bacoor City	12,142	3,874	3,496	7,370	60.70	3,715	3,360	7,075	58.27	3,564	3,284	6,848	56.40
Batangas City	6,823	2,670	2,483	5,153	75.52	2,631	2,404	5,035	73.79	2,528	2,281	4,809	70.48
Biñan City	6,607	3,649	3,532	7,181	108.69	3,450	3,384	6,834	103.44	3,467	3,386	6,853	103.72
Cabuyao City	6,130	2,912	2,717	5,629	91.83	2,893	2,786	5,679	92.64	2,876	2,813	5,689	92.81
Calamba City	9,028	4,492	4,081	8,573	94.96	4,168	3,842	8,010	88.72	3,936	3,536	7,472	82.76
Cavite City	2,075	492	478	970	46.75	486	447	933	44.96	488	451	939	45.25
Dasmariñas City	13,322	3,505	3,221	6,726	50.49	3,438	3,147	6,585	49.43	3,285	3,089	6,374	47.85
General Trias City	6,352	3,356	3,199	6,555	103.20	3,348	3,177	6,525	102.72	3,295	3,091	6,386	100.54
Imus City	8,156	2,548	2,444	4,992	61.21	2,522	2,282	4,804	58.90	2,403	2,222	4,625	56.71
Lipa City	6,880	1,904	1,707	3,611	52.49	1,880	1,653	3,533	51.35	1,891	1,623	3,514	51.08
Lucena City	5,672	2,538	2,274	4,812	84.84	2,368	2,160	4,528	79.83	2,338	2,144	4,482	79.02
San Pablo City	5,277	2,188	1,895	4,083	77.37	2,062	1,882	3,944	74.74	1,977	1,802	3,779	71.61
San Pedro City	6,467	2,397	2,182	4,579	70.81	2,242	1,991	4,233	65.46	2,183	1,898	4,081	63.10
Santa Rosa City	7,025	3,390	3,331	6,721	95.67	3,245	3,139	6,384	90.88	3,195	3,047	6,242	88.85
Tagaytay City	1,437	1,496	1,276	2,772	192.90	1,570	1,286	2,856	198.75	1,506	1,228	2,734	190.26
Tanauan City	3,584	1,587	1,435	3,022	84.32	1,519	1,458	2,977	83.06	1,502	1,414	2,916	81.36
Tayabas City	2,122	933	881	1,814	85.49	903	877	1,780	83.88	867	853	1,720	81.06
Trece Martires City	3,147	3,864	3,634	7,498	238.26	3,648	3,576	7,224	229.55	3,346	3,364	6,710	213.22
**Region 4B**	**71,246**	**22,546**	**20,944**	**43,490**	**61.04**	**21,807**	**20,415**	**42,222**	**59.26**	**21,641**	**20,164**	**41,805**	**58.68**
Marinduque	4,836	1,758	1,529	3,287	67.97	1,838	1,568	3,406	70.43	1,748	1,523	3,271	67.64
Mindoro Occidental	12,407	4,856	4,465	9,321	75.13	4,603	4,363	8,966	72.27	4,568	4,241	8,809	71.00
Mindoro Oriental	19,809	7,454	6,911	14,365	72.52	7,133	6,705	13,838	69.86	7,138	6,616	13,754	69.43
Palawan	21,375	4,506	4,312	8,818	41.25	4,424	4,176	8,600	40.23	4,456	4,237	8,693	40.67
Romblon	6,400	1,276	1,224	2,500	39.06	1,276	1,222	2,498	39.03	1,243	1,237	2,480	38.75
Puerto Princesa City	6,419	2,696	2,503	5,199	80.99	2,533	2,381	4,914	76.55	2,488	2,310	4,798	74.75
**Region 5**	**136,116**	**53,195**	**49,188**	**102,383**	**75.22**	**52,103**	**49,693**	**101,796**	**74.79**	**52,243**	**48,518**	**100,761**	**74.03**
Albay	23,737	9,330	8,569	17,899	75.41	9,471	8,830	18,301	77.10	9,312	8,735	18,047	76.03
Camarines Norte	14,283	5,814	5,498	11,312	79.20	5,543	5,080	10,623	74.38	5,585	5,270	10,855	76.00
Camarines Sur	38,796	15,471	14,110	29,581	76.25	14,935	13,848	28,783	74.19	14,421	13,266	27,687	71.37
Catanduanes	6,274	2,214	2,054	4,268	68.03	2,198	2,063	4,261	67.92	2,155	2,028	4,183	66.67
Masbate	22,521	8,903	8,363	17,266	76.67	8,853	8,398	17,251	76.60	8,720	8,449	17,169	76.24
Sorsogon	19,065	7,219	6,770	13,989	73.38	6,703	7,192	13,895	72.88	7,563	6,630	14,193	74.45
Iriga City	2,638	893	786	1,679	63.65	870	792	1,662	63.00	886	800	1,686	63.91

Table 1.C.1.2 - Immunization Services for Infants

Number and Proportion of Infants who completed 3 doses of HiB-HepB antigen
Philippines, Annual 2020

Area	Eligible Pop (Under 1 y.o.)	DPT-HiB-HepB 1				DPT-HiB-HepB 2				DPT-HiB-HepB 3			
		Male	Female	Total	%	Male	Female	Total	%	Male	Female	Total	%
Legaspi City	4,174	1,574	1,469	3,043	72.90	1,765	1,908	3,673	88.00	1,933	1,762	3,695	88.52
Naga City	4,628	1,777	1,569	3,346	72.30	1,765	1,582	3,347	72.32	1,668	1,578	3,246	70.14
**Region 6**	**146,526**	**59,297**	**55,709**	**115,006**	**78.49**	**60,154**	**56,518**	**116,672**	**79.63**	**59,705**	**56,573**	**116,278**	**79.36**
Aklan	11,162	4,550	4,262	8,812	78.95	4,581	4,427	9,008	80.70	4,527	4,356	8,883	79.58
Antique	12,816	4,631	4,300	8,931	69.69	4,690	4,461	9,151	71.40	4,721	4,448	9,169	71.54
Capiz	13,986	5,284	5,085	10,369	74.14	5,373	5,153	10,526	75.26	5,402	5,064	10,466	74.83
Guimaras	3,085	1,325	1,312	2,637	85.48	1,359	1,340	2,699	87.49	1,399	1,359	2,758	89.40
Iloilo	36,541	13,984	13,296	27,280	74.66	14,612	13,753	28,365	77.63	14,263	13,645	27,908	76.37
Negros Occidental	49,368	21,703	20,211	41,914	84.90	21,682	20,175	41,857	84.79	21,741	20,682	42,423	85.93
Bacolod City	11,115	4,429	3,913	8,342	75.05	4,334	3,829	8,163	73.44	4,101	3,618	7,719	69.45
Iloilo City	8,453	3,391	3,330	6,721	79.51	3,523	3,380	6,903	81.66	3,551	3,401	6,952	82.24
**Region 7**	**163,262**	**70,332**	**65,886**	**136,218**	**83.44**	**69,210**	**65,380**	**134,590**	**82.44**	**67,642**	**64,402**	**132,044**	**80.88**
Bohol	27,312	11,116	10,202	21,318	78.05	10,725	10,151	20,876	76.44	10,913	10,207	21,120	77.33
Cebu	67,506	29,094	26,744	55,838	82.72	29,170	26,953	56,123	83.14	28,843	26,865	55,708	82.52
Negros Oriental	27,938	12,372	12,401	24,773	88.67	12,209	12,288	24,497	87.68	11,877	12,089	23,966	85.78
Siquijor	1,613	716	632	1,348	83.57	745	673	1,418	87.91	730	696	1,426	88.41
Cebu City	21,193	8,753	8,167	16,920	79.84	8,004	7,478	15,482	73.05	7,100	6,720	13,820	65.21
Lapu-Lapu City	9,372	4,868	4,586	9,454	100.87	4,874	4,604	9,478	101.13	4,692	4,491	9,183	97.98
Mandaue City	8,328	3,413	3,154	6,567	78.85	3,483	3,233	6,716	80.64	3,487	3,334	6,821	81.90
**Region 8**	**102,619**	**36,503**	**34,064**	**70,567**	**68.77**	**35,708**	**33,720**	**69,428**	**67.66**	**34,757**	**32,770**	**67,527**	**65.80**
Biliran	3,834	1,583	1,473	3,056	79.71	1,667	1,547	3,214	83.83	1,687	1,581	3,268	85.24
Eastern Samar	11,392	3,331	3,069	6,400	56.18	3,243	3,034	6,277	55.10	3,226	3,079	6,305	55.35
Northern Leyte	34,707	12,737	11,934	24,671	71.08	12,737	11,851	24,588	70.84	12,452	11,597	24,049	69.29
Northern Samar	15,370	5,280	4,898	10,178	66.22	5,136	4,934	10,070	65.52	4,934	4,578	9,512	61.89
Southern Leyte	6,451	2,289	2,167	4,456	69.07	2,427	2,265	4,692	72.73	2,397	2,204	4,601	71.32
Western Samar	14,305	5,133	4,799	9,932	69.43	4,727	4,701	9,428	65.91	4,559	4,470	9,029	63.12
Calbayog City	4,413	1,318	1,293	2,611	59.17	1,019	963	1,982	44.91	811	786	1,597	36.19
Maasin City	1,637	546	541	1,087	66.40	602	514	1,116	68.17	550	545	1,095	66.89
Ormoc City	4,941	2,053	1,902	3,955	80.04	2,021	1,901	3,922	79.38	2,104	1,901	4,005	81.06
Tacloban City	5,569	2,233	1,988	4,221	75.79	2,129	2,010	4,139	74.32	2,037	2,029	4,066	73.01
**Region 9**	**80,051**	**30,717**	**29,727**	**60,444**	**75.51**	**30,360**	**29,378**	**59,738**	**74.62**	**29,389**	**28,622**	**58,011**	**72.47**
Zamboanga del Norte	17,249	7,741	7,383	15,124	87.68	7,657	7,352	15,009	87.01	7,557	7,411	14,968	86.78
Zamboanga del Sur	17,653	6,511	6,382	12,893	73.04	6,631	6,394	13,025	73.78	6,479	6,308	12,787	72.44
Zamboanga Sibugay	14,954	4,223	4,129	8,352	55.85	4,257	4,156	8,413	56.26	4,164	4,149	8,313	55.59
Dapitan City	1,784	786	976	1,762	98.77	786	974	1,760	98.65	786	976	1,762	98.77
Dipolog City	2,827	1,344	1,313	2,657	93.99	1,318	1,265	2,583	91.37	1,280	1,250	2,530	89.49
Isabela City	2,522	1,157	1,127	2,284	90.56	1,137	1,161	2,298	91.12	1,176	1,196	2,372	94.05
Pagadian City	4,325	1,828	1,803	3,631	83.95	1,859	1,828	3,687	85.25	1,505	1,415	2,920	67.51
Zamboanga City	18,737	7,127	6,614	13,741	73.34	6,715	6,248	12,963	69.18	6,442	5,917	12,359	65.96
**Region 10**	**101,411**	**45,413**	**42,727**	**88,140**	**86.91**	**44,657**	**42,190**	**86,847**	**85.64**	**44,234**	**41,420**	**85,654**	**84.46**
Bukidnon	23,706	11,541	10,674	22,215	93.71	11,130	10,430	21,560	90.95	10,845	10,077	20,922	88.26
Camiguin	1,858	693	690	1,383	74.43	676	651	1,327	71.42	638	670	1,308	70.40
Lanao del Norte	14,960	5,146	5,038	10,184	68.07	5,206	5,175	10,381	69.39	5,489	5,152	10,641	71.13
Misamis Occidental	6,403	3,113	2,998	6,111	95.44	3,103	2,948	6,051	94.50	3,105	2,847	5,952	92.96
Misamis Oriental	15,131	6,624	6,408	13,032	86.13	6,800	6,351	13,151	86.91	6,769	6,479	13,248	87.56
Cagayan de Oro City	14,339	6,608	6,137	12,745	88.88	6,376	5,915	12,291	85.72	6,281	5,887	12,168	84.86
El Salvador City	1,065	458	457	915	85.92	474	446	920	86.38	447	455	902	84.69
Gingoog City	2,644	1,247	1,150	2,397	90.66	1,222	1,166	2,388	90.32	1,310	1,196	2,506	94.78
Iligan City	7,580	3,399	3,023	6,422	84.72	3,221	2,934	6,155	81.20	3,068	2,896	5,964	78.68
Malaybalay City	3,956	1,929	1,729	3,658	92.47	1,920	1,719	3,639	91.99	1,826	1,607	3,433	86.78
Oroquieta City	1,389	603	570	1,173	84.45	566	567	1,133	81.57	611	588	1,199	86.32
Ozamis City	2,778	1,327	1,218	2,545	91.61	1,353	1,330	2,683	96.58	1,355	1,124	2,479	89.24
Tangub City	1,234	638	571	1,209	97.97	631	577	1,208	97.89	616	604	1,220	98.87
Valencia City	4,368	2,087	2,064	4,151	95.03	1,979	1,981	3,960	90.66	1,874	1,838	3,712	84.98

Table 1.C.1.2 - Immunization Services for Infants

Number and Proportion of Infants who completed 3 doses of HiB-HepB antigen
Philippines, Annual 2020

Area	Eligible Pop (Under 1 y.o.)	DPT-HiB-HepB 1				DPT-HiB-HepB 2				DPT-HiB-HepB 3			
		Male	Female	Total	%	Male	Female	Total	%	Male	Female	Total	%
**Region 11**	**107,247**	**46,215**	**43,018**	**89,233**	**83.20**	**45,589**	**42,713**	**88,302**	**82.34**	**44,119**	**41,496**	**85,615**	**79.83**
Compostela Valley	15,562	6,757	6,283	13,040	83.79	6,692	6,372	13,064	83.95	6,555	6,245	12,800	82.25
Davao del Norte	21,326	10,464	10,001	20,465	95.96	10,286	9,855	20,141	94.44	9,844	9,360	19,204	90.05
Davao del Sur	13,007	4,905	4,479	9,384	72.15	4,951	4,588	9,539	73.34	4,933	4,654	9,587	73.71
Davao Oriental	14,151	5,647	5,242	10,889	76.95	5,720	5,225	10,945	77.34	5,597	5,177	10,774	76.14
Davao Occidental	6,670	2,593	2,388	4,981	74.68	2,574	2,442	5,016	75.20	2,413	2,296	4,709	70.60
Davao City	36,531	15,849	14,625	30,474	83.42	15,366	14,231	29,597	81.02	14,777	13,764	28,541	78.13
**Region 12**	**104,552**	**40,169**	**37,506**	**77,675**	**74.29**	**38,933**	**36,869**	**75,802**	**72.50**	**37,409**	**35,232**	**72,641**	**69.48**
North Cotabato	33,645	11,282	10,402	21,684	64.45	10,826	9,865	20,691	61.50	10,253	9,330	19,583	58.20
Sarangani	12,891	5,653	5,286	10,939	84.86	5,343	5,319	10,662	82.71	5,182	5,052	10,234	79.39
South Cotabato	21,113	8,911	8,432	17,343	82.14	9,006	8,588	17,594	83.33	8,955	8,474	17,429	82.55
Sultan Kudarat	17,359	6,943	6,499	13,442	77.44	6,910	6,484	13,394	77.16	6,749	6,398	13,147	75.74
Cotabato City	5,835	2,067	1,905	3,972	68.07	1,985	1,867	3,852	66.02	1,931	1,776	3,707	63.53
Gen. Santos City	13,709	5,313	4,982	10,295	75.10	4,863	4,746	9,609	70.09	4,339	4,202	8,541	62.30
**BARMM**	**92,799**	**28,867**	**29,238**	**58,105**	**62.61**	**27,264**	**28,307**	**55,571**	**59.88**	**26,262**	**30,093**	**56,355**	**60.73**
Basilan	7,541	1,793	1,825	3,618	47.98	1,599	1,678	3,277	43.46	1,379	1,485	2,864	37.98
Lanao del Sur	21,131	9,874	10,762	20,636	97.66	9,855	10,806	20,661	97.78	9,755	13,502	23,257	110.06
Maguindanao	31,128	14,074	13,437	27,511	88.38	13,469	13,402	26,871	86.32	12,861	12,767	25,628	82.33
Sulu	16,613	831	853	1,684	10.14	70	81	151	0.91	0	0	0	0.00
Tawi-Tawi	9,259	0	0	0	0.00	0	0	0	0.00	0	0	0	0.00
Lamitan City	2,074	663	672	1,335	64.37	660	666	1,326	63.93	600	637	1,237	59.64
Marawi City	5,053	1,632	1,689	3,321	65.72	1,611	1,674	3,285	65.01	1,667	1,702	3,369	66.67
**CARAGA**	**60,029**	**24,511**	**22,955**	**47,466**	**79.07**	**23,964**	**22,815**	**46,779**	**77.93**	**23,386**	**22,343**	**45,729**	**76.18**
Agusan del Norte	8,098	3,356	3,061	6,417	79.24	3,306	3,060	6,366	78.61	3,221	3,053	6,274	77.48
Agusan del Sur	17,592	6,950	6,367	13,317	75.70	6,689	6,376	13,065	74.27	6,587	6,222	12,809	72.81
Surigao del Norte	7,089	3,060	2,938	5,998	84.61	3,126	2,962	6,088	85.88	3,091	2,958	6,049	85.33
Surigao del Sur	11,482	4,598	4,325	8,923	77.71	4,397	4,324	8,721	75.95	4,247	4,213	8,460	73.68
Province of Dinagat	2,573	817	805	1,622	63.04	806	802	1,608	62.50	815	810	1,625	63.16
Bislig City	2,179	893	892	1,785	81.92	880	883	1,763	80.91	857	858	1,715	78.71
Butuan City	7,715	3,329	3,106	6,435	83.41	3,270	3,020	6,290	81.53	3,073	2,865	5,938	76.97
Surigao City	3,301	1,508	1,461	2,969	89.94	1,490	1,388	2,878	87.19	1,495	1,364	2,859	86.61

* - No Report
0 - No Cases

Table 1.C.1.3 - Immunization Services for Infants and Children

Number and Proportion of Infants who completed 3 doses of Oral Polio Vaccine (OPV) and Inactivated Polio Vaccine (IPV)
Philippines, Annual 2020

Area	Eligible Pop (Under 1 y.o.)	OPV 1				OPV 2				OPV 3				IPV			
		Male	Female	Total	%	Male	Female	Total	%	Male	Female	Total	%	Male	Female	Total	%
**PHILIPPINES**	**2,123,158**	**858,546**	**809,267**	**1,667,813**	**78.55**	**841,312**	**799,430**	**1,640,742**	**77.28**	**822,539**	**849,999**	**1,672,538**	**78.78**	**793,860**	**756,043**	**1,549,903**	**73.00**
**N C R**	**238,661**	**97,575**	**93,625**	**191,200**	**80.11**	**89,979**	**86,441**	**176,420**	**73.92**	**84,150**	**82,868**	**167,018**	**69.98**	**76,780**	**74,969**	**151,749**	**63.58**
Malabon	6,775	3,118	3,051	6,169	91.06	2,927	2,806	5,733	84.62	2,757	2,698	5,455	80.52	2,840	2,635	5,475	80.81
Navotas	4,621	2,389	2,232	4,621	100.00	2,286	2,165	4,451	96.32	2,044	2,059	4,103	88.79	1,807	1,823	3,630	78.55
Valenzuela City	11,500	4,865	4,687	9,552	83.06	4,699	4,553	9,252	80.45	4,372	4,378	8,750	76.09	1,855	1,785	3,640	31.65
Caloocan City	29,363	12,234	11,667	23,901	81.40	11,213	10,573	21,786	74.20	9,994	9,659	19,653	66.93	9,828	9,346	19,174	65.30
Marikina City	8,354	3,736	3,523	7,259	86.89	3,375	3,277	6,652	79.63	3,221	3,160	6,381	76.38	3,109	3,060	6,169	73.84
Pasig City	13,996	5,492	5,065	10,557	75.43	5,161	4,868	10,029	71.66	5,015	4,842	9,857	70.43	4,659	4,466	9,125	65.20
Pateros	1,184	538	496	1,034	87.33	522	473	995	84.04	519	477	996	84.12	520	456	976	82.43
Taguig	14,918	7,346	7,016	14,362	96.27	6,941	6,235	13,176	88.32	6,674	6,472	13,146	88.12	6,129	5,974	12,103	81.13
Quezon City	54,413	23,806	23,224	47,030	86.43	21,660	21,555	43,215	79.42	20,400	20,792	41,192	75.70	18,706	18,999	37,705	69.29
Makati City	10,798	3,859	3,538	7,397	68.50	3,652	3,447	7,099	65.74	3,595	3,389	6,984	64.68	3,379	3,047	6,426	59.51
Mandaluyong City	7,160	2,153	2,036	4,189	58.51	2,071	2,004	4,075	56.91	2,172	1,986	4,158	58.07	2,035	1,882	3,917	54.71
San Juan	2,261	698	697	1,395	61.70	681	708	1,389	61.43	610	670	1,280	56.61	608	676	1,284	56.79
Manila City	32,980	11,839	11,609	23,448	71.10	10,528	10,289	20,817	63.12	9,547	9,546	19,093	57.89	9,212	8,993	18,205	55.20
Las Piñas City	10,914	4,251	3,953	8,204	75.17	3,936	3,663	7,599	69.63	3,640	3,513	7,153	65.54	3,577	3,354	6,931	63.51
Muntinlupa City	9,353	4,063	3,894	7,957	85.07	3,899	3,681	7,580	81.04	3,724	3,562	7,286	77.90	2,871	2,747	5,618	60.07
Parañaque City	12,343	4,469	4,179	8,648	70.06	3,991	3,668	7,659	62.05	3,597	3,457	7,054	57.15	3,499	3,302	6,801	55.10
Pasay City	7,728	2,719	2,758	5,477	70.87	2,437	2,476	4,913	63.57	2,269	2,208	4,477	57.93	2,146	2,424	4,570	59.14
**C A R**	**35,099**	**13,508**	**12,691**	**26,199**	**74.64**	**13,439**	**12,804**	**26,243**	**74.77**	**13,594**	**12,980**	**26,574**	**75.71**	**12,921**	**12,281**	**25,202**	**71.80**
Abra	4,309	1,571	1,541	3,112	72.22	1,643	1,605	3,248	75.38	1,654	1,662	3,316	76.96	1,643	1,646	3,289	76.33
Apayao	2,451	885	895	1,780	72.62	945	939	1,884	76.87	989	969	1,958	79.89	933	916	1,849	75.44
Benguet	9,188	3,277	3,054	6,331	68.91	3,179	3,119	6,298	68.55	3,257	3,105	6,362	69.24	3,197	3,029	6,226	67.76
Ifugao	4,396	1,826	1,548	3,374	76.75	1,839	1,547	3,386	77.02	1,900	1,690	3,590	81.67	1,606	1,434	3,040	69.15
Kalinga	4,617	2,155	1,945	4,100	88.80	2,147	1,979	4,126	89.37	2,108	2,023	4,131	89.47	1,874	1,787	3,661	79.29
Mt. Province	3,023	1,185	1,092	2,277	75.32	1,185	1,085	2,270	75.09	1,188	1,089	2,277	75.32	1,188	1,052	2,240	74.10
Baguio City	7,115	2,609	2,616	5,225	73.44	2,501	2,530	5,031	70.71	2,498	2,442	4,940	69.43	2,480	2,417	4,897	68.83
**Region 1**	**97,261**	**38,906**	**36,265**	**75,171**	**77.29**	**41,002**	**38,392**	**79,394**	**81.63**	**39,989**	**37,251**	**77,240**	**79.42**	**39,821**	**37,073**	**76,894**	**79.06**
Ilocos Norte	8,105	2,868	2,639	5,507	67.95	2,884	2,745	5,629	69.45	2,929	2,774	5,703	70.36	2,923	2,764	5,687	70.17
Ilocos Sur	9,330	3,883	3,660	7,543	80.85	4,002	3,853	7,855	84.19	4,079	3,833	7,912	84.80	4,044	3,824	7,868	84.33
La Union	11,511	4,457	4,336	8,793	76.39	6,047	5,831	11,878	103.19	4,634	4,403	9,037	78.51	4,636	4,404	9,040	78.53
Pangasinan	50,168	19,917	18,588	38,505	76.75	20,403	19,017	39,420	78.58	20,758	19,418	40,176	80.08	20,712	19,333	40,045	79.82
Alaminos City	1,894	1,403	927	2,330	123.02	1,409	939	2,348	123.97	1,385	934	2,319	122.44	1,354	914	2,268	119.75
Candon City	987	513	514	1,027	104.05	531	505	1,036	104.96	439	436	875	88.65	439	436	875	88.65
Dagupan City	3,620	1,237	1,096	2,333	64.45	1,176	1,088	2,264	62.54	1,139	1,043	2,182	60.28	1,139	1,043	2,182	60.28
Laoag City	1,870	973	982	1,955	104.55	993	993	1,986	106.20	969	1,016	1,985	106.15	969	1,016	1,985	106.15
San Carlos City	3,979	1,716	1,674	3,390	85.20	1,710	1,657	3,367	84.62	1,703	1,656	3,359	84.42	1,658	1,623	3,281	82.46
San Fernando City	2,115	774	688	1,462	69.13	737	682	1,419	67.09	755	693	1,448	68.46	749	671	1,420	67.14
Urdaneta City	2,806	973	1,002	1,975	70.38	920	940	1,860	66.29	1,020	903	1,923	68.53	1,020	903	1,923	68.53
Vigan City	876	192	159	351	40.07	190	142	332	37.90	179	142	321	36.64	178	142	320	36.53
**Region 2**	**69,443**	**27,631**	**25,827**	**53,458**	**76.98**	**27,574**	**26,059**	**53,633**	**77.23**	**28,243**	**26,459**	**54,702**	**78.77**	**27,142**	**25,351**	**52,493**	**75.59**
Batanes	340	132	131	263	77.35	123	137	260	76.47	123	131	254	74.71	123	130	253	74.41
Cagayan	17,971	7,749	7,110	14,859	82.68	7,882	7,376	15,258	84.90	8,183	7,375	15,558	86.57	7,579	6,963	14,542	80.92
Isabela	26,309	9,098	8,530	17,628	67.00	8,999	8,560	17,559	66.74	9,208	8,703	17,911	68.08	8,936	8,376	17,312	65.80
Nueva Vizcaya	9,573	3,956	3,660	7,616	79.56	3,938	3,662	7,600	79.39	3,986	3,755	7,741	80.86	3,771	3,531	7,302	76.28
Quirino	4,025	1,614	1,551	3,165	78.63	1,592	1,547	3,139	77.99	1,640	1,602	3,242	80.55	1,587	1,543	3,130	77.76
Cauayan City	2,590	1,274	1,036	2,310	89.19	1,181	1,028	2,209	85.29	1,231	1,149	2,380	91.89	1,317	1,152	2,469	95.33
Ilagan City	2,911	1,303	1,365	2,668	91.65	1,302	1,320	2,622	90.07	1,282	1,275	2,557	87.84	1,305	1,267	2,572	88.35
Santiago City	2,692	1,307	1,315	2,622	97.40	1,361	1,261	2,622	97.40	1,363	1,292	2,655	98.63	1,304	1,221	2,525	93.80
Tuguegarao City	3,032	1,198	1,129	2,327	76.75	1,196	1,168	2,364	77.97	1,227	1,177	2,404	79.29	1,220	1,168	2,388	78.76
**Region 3**	**220,020**	**102,770**	**96,623**	**199,393**	**90.62**	**102,358**	**97,132**	**199,490**	**90.67**	**102,545**	**97,616**	**200,161**	**90.97**	**100,827**	**95,881**	**196,708**	**89.40**
Aurora	4,770	1,954	1,919	3,873	81.19	1,989	1,930	3,919	82.16	2,033	1,960	3,993	83.71	1,991	1,941	3,932	82.43
Bataan	13,823	6,833	6,407	13,240	95.78	6,869	6,463	13,332	96.45	6,905	6,383	13,288	96.13	6,919	6,370	13,289	96.14
Bulacan	43,627	19,327	18,118	37,445	85.83	18,916	17,852	36,768	84.28	18,658	17,708	36,366	83.36	17,979	17,010	34,989	80.20
Nueva Ecija	28,782	12,330	11,162	23,492	81.62	12,403	11,426	23,829	82.79	12,625	11,692	24,317	84.49	12,376	11,559	23,935	83.16
Pampanga	31,359	15,001	14,388	29,389	93.72	15,005	14,593	29,598	94.38	15,225	14,777	30,002	95.67	15,142	14,686	29,828	95.12
Tarlac	19,765	9,881	9,240	19,121	96.74	10,010	9,529	19,539	98.86	10,211	9,705	19,916	100.76	9,904	9,358	19,262	97.46
Zambales	12,610	5,090	4,811	9,901	78.52	5,037	4,828	9,865	78.23	4,958	4,759	9,717	77.06	4,734	4,477	9,211	73.05
Angeles City	7,869	4,106	3,866	7,972	101.31	4,094	3,858	7,952	101.05	4,096	3,799	7,895	100.33	4,077	3,764	7,841	99.64
Balanga City	1,998	841	798	1,639	82.03	858	799	1,657	82.93	843	802	1,645	82.33	867	803	1,670	83.58
Cabanatuan City	5,896	2,666	2,441	5,107	86.62	2,569	2,436	5,005	84.89	2,575	2,520	5,095	86.41	2,611	2,509	5,120	86.84
City of San Fernando	5,863	2,876	2,598	5,474	93.37	2,914	2,687	5,601	95.53	3,006	2,790	5,796	98.86	2,982	2,802	5,784	98.65
Gapan City	2,153	931	982	1,913	88.85	955	945	1,900	88.25	923	978	1,901	88.30	933	976	1,909	88.67
Mabalacat City	4,793	2,621	2,575	5,196	108.41	2,553	2,513	5,066	105.70	2,533	2,429	4,962	103.53	2,511	2,415	4,926	102.77
Malolos City	4,880	1,812	1,750	3,562	72.99	1,803	1,735	3,538	72.50	1,890	1,748	3,638	74.55	1,885	1,747	3,632	74.43
Meycauayan	4,041	1,821	1,807	3,628	89.78	1,792	1,667	3,459	85.60	1,790	1,655	3,445	85.25	1,782	1,677	3,459	85.60
Olongapo	4,971	1,880	1,632	3,512	70.65	1,761	1,713	3,474	69.89	1,694	1,680	3,374	67.87	1,673	1,623	3,296	66.30
Palayan City	799	365	393	758	94.87	392	392	784	98.12	389	408	797	99.75	389	408	797	99.75
San Jose City	2,718	1,449	1,226	2,675	98.42	1,387	1,290	2,677	98.49	1,393	1,288	2,681	98.64	1,377	1,320	2,697	99.23
San Jose del Monte City	11,100	6,672	6,315	12,987	117.00	6,724	6,323	13,047	117.54	6,487	6,361	12,848	115.75	6,432	6,312	12,744	114.81
Science City of Munoz	1,586	678	639	1,317	83.04	683	633	1,316	82.98	681	640	1,321	83.29	683	641	1,324	83.48

Table 1.C.1.3 - Immunization Services for Infants and Children

Number and Proportion of Infants who completed 3 doses of Oral Polio Vaccine (OPV) and Inactivated Polio Vaccine (IPV)
Philippines, Annual 2020

Area	Eligible Pop (Under 1 y.o.)	OPV 1				OPV 2				OPV 3				IPV			
		Male	Female	Total	%	Male	Female	Total	%	Male	Female	Total	%	Male	Female	Total	%
Tarlac City	6,617	3,636	3,556	7,192	108.69	3,644	3,520	7,164	108.27	3,630	3,534	7,164	108.27	3,580	3,483	7,063	106.74
**Region 4A**	**296,816**	**120,934**	**111,674**	**232,608**	**78.37**	**117,708**	**109,113**	**226,821**	**76.42**	**110,084**	**166,603**	**276,687**	**93.22**	**106,409**	**99,542**	**205,951**	**69.39**
Batangas	38,441	12,756	11,482	24,238	63.05	12,560	11,570	24,130	62.77	12,165	18,048	30,213	78.60	11,713	10,860	22,573	58.72
Cavite	27,704	15,780	14,768	30,548	110.27	15,719	14,669	30,388	109.69	14,929	22,432	37,361	134.86	15,131	14,259	29,390	106.09
Laguna	19,727	7,850	7,237	15,087	76.48	7,554	7,012	14,566	73.84	6,784	10,390	17,174	87.06	6,885	6,445	13,330	67.57
Quezon	37,410	15,194	13,878	29,072	77.71	14,944	13,513	28,457	76.07	13,696	20,910	34,606	92.50	11,731	10,809	22,540	60.25
Rizal	44,791	13,305	12,083	25,388	56.68	12,860	11,827	24,687	55.12	12,213	17,913	30,126	67.26	11,546	10,843	22,389	49.99
Antipolo City	16,497	8,346	7,985	16,331	98.99	7,915	7,716	15,631	94.75	7,255	10,845	18,100	109.72	7,006	6,755	13,761	83.42
Bacoor City	12,142	3,864	3,463	7,327	60.34	3,723	3,363	7,086	58.36	3,452	5,087	8,539	70.33	3,532	3,255	6,787	55.90
Batangas City	6,823	2,669	2,480	5,149	75.47	2,625	2,399	5,024	73.63	2,457	3,600	6,057	88.77	2,005	1,788	3,793	55.59
Biñan City	6,607	3,655	3,707	7,362	111.43	3,455	3,381	6,836	103.47	3,428	5,173	8,601	130.18	3,481	3,391	6,872	104.01
Cabuyao City	6,130	2,915	2,705	5,620	91.68	2,890	2,771	5,661	92.35	2,875	4,302	7,177	117.08	2,824	2,734	5,558	90.67
Calamba City	9,028	4,525	4,059	8,584	95.08	4,206	3,843	8,049	89.16	3,714	5,644	9,358	103.66	3,703	3,399	7,102	78.67
Cavite City	2,075	488	484	972	46.84	485	446	931	44.87	475	702	1,177	56.72	477	446	923	44.48
Dasmariñas City	13,322	3,416	3,124	6,540	49.09	3,414	3,111	6,525	48.98	3,037	4,788	7,825	58.74	2,800	2,622	5,422	40.70
General Trias City	6,352	3,347	3,156	6,503	102.38	3,327	3,181	6,508	102.46	3,217	5,083	8,300	130.67	3,276	3,070	6,346	99.91
Imus City	8,156	2,534	2,431	4,965	60.88	2,511	2,290	4,801	58.86	2,304	3,504	5,808	71.21	2,339	2,174	4,513	55.33
Lipa City	6,880	1,887	1,709	3,596	52.27	1,887	1,660	3,547	51.56	1,762	2,845	4,607	66.96	1,683	1,449	3,132	45.52
Lucena City	5,672	2,543	2,278	4,821	85.00	2,409	2,146	4,555	80.31	2,267	3,488	5,755	101.46	2,195	1,931	4,126	72.74
San Pablo City	5,277	2,158	1,900	4,058	76.90	2,055	1,881	3,936	74.59	2,005	3,094	5,099	96.63	2,030	1,806	3,836	72.69
San Pedro City	6,467	2,408	2,172	4,580	70.82	2,251	2,006	4,257	65.83	1,979	3,099	5,078	78.52	2,064	1,879	3,943	60.97
Santa Rosa City	7,025	3,363	3,285	6,648	94.63	3,243	3,133	6,376	90.76	3,090	4,717	7,807	111.13	2,851	2,780	5,631	80.16
Tagaytay City	1,437	1,576	1,290	2,866	199.44	1,592	1,300	2,892	201.25	1,306	2,092	3,398	236.46	1,514	1,308	2,822	196.38
Tanauan City	3,584	1,579	1,426	3,005	83.84	1,518	1,451	2,969	82.84	1,443	2,167	3,610	100.73	1,479	1,378	2,857	79.72
Tayabas City	2,122	922	888	1,810	85.30	909	862	1,771	83.46	877	1,396	2,273	107.12	902	875	1,777	83.74
Trece Martires City	3,147	3,854	3,684	7,538	239.53	3,656	3,582	7,238	230.00	3,354	5,284	8,638	274.48	3,242	3,286	6,528	207.44
**Region 4B**	**71,246**	**21,891**	**20,330**	**42,221**	**59.26**	**21,062**	**19,663**	**40,725**	**57.16**	**20,907**	**19,518**	**40,425**	**56.74**	**19,493**	**18,204**	**37,697**	**52.91**
Marinduque	4,836	1,747	1,519	3,266	67.54	1,826	1,571	3,397	70.24	1,714	1,539	3,253	67.27	1,628	1,471	3,099	64.08
Mindoro Occidental	12,407	4,648	4,360	9,008	72.60	4,330	4,011	8,341	67.23	4,281	3,999	8,280	66.74	4,076	3,761	7,837	63.17
Mindoro Oriental	19,809	7,342	6,792	14,134	71.35	7,062	6,644	13,706	69.19	7,123	6,573	13,696	69.14	6,772	6,303	13,075	66.01
Palawan	21,375	4,330	4,108	8,438	39.48	4,163	3,965	8,128	38.03	4,267	4,049	8,316	38.91	3,963	3,748	7,711	36.07
Romblon	6,400	1,269	1,172	2,441	38.14	1,268	1,216	2,484	38.81	1,250	1,226	2,476	38.69	1,215	1,179	2,394	37.41
Puerto Princesa City	6,419	2,555	2,379	4,934	76.87	2,413	2,256	4,669	72.74	2,272	2,132	4,404	68.61	1,839	1,742	3,581	55.79
**Region 5**	**136,116**	**53,441**	**50,987**	**104,428**	**76.72**	**53,226**	**49,628**	**102,854**	**75.56**	**53,013**	**49,724**	**102,737**	**75.48**	**50,975**	**48,530**	**99,505**	**73.10**
Albay	23,737	9,380	9,920	19,300	81.31	9,351	8,825	18,176	76.57	9,517	8,871	18,388	77.47	9,277	8,699	17,976	75.73
Camarines Norte	14,283	5,702	5,329	11,031	77.23	5,557	5,353	10,910	76.38	5,632	5,350	10,982	76.89	5,509	5,291	10,800	75.61
Camarines Sur	38,796	15,539	14,148	29,687	76.52	15,182	14,083	29,265	75.43	15,039	13,752	28,791	74.21	14,432	13,260	27,692	71.38
Catanduanes	6,274	2,188	2,052	4,240	67.58	2,174	2,042	4,216	67.20	2,167	2,010	4,177	66.58	2,097	1,985	4,082	65.06
Masbate	22,521	8,887	8,332	17,219	76.46	8,891	8,408	17,299	76.81	8,758	8,429	17,187	76.32	8,347	8,141	16,488	73.21
Sorsogon	19,065	7,064	6,943	14,007	73.47	7,462	6,713	14,175	74.35	7,342	6,958	14,300	75.01	6,922	7,030	13,952	73.18
Iriga City	2,638	931	809	1,740	65.96	884	838	1,722	65.28	931	847	1,778	67.40	927	816	1,743	66.07
Legaspi City	4,174	1,986	1,863	3,849	92.21	1,930	1,754	3,684	88.26	1,910	1,866	3,776	90.46	1,729	1,682	3,411	81.72
Naga City	4,628	1,764	1,591	3,355	72.49	1,795	1,612	3,407	73.62	1,717	1,641	3,358	72.56	1,735	1,626	3,361	72.62
**Region 6**	**146,526**	**59,582**	**55,828**	**115,410**	**78.76**	**59,818**	**56,582**	**116,400**	**79.44**	**59,830**	**56,755**	**116,585**	**79.57**	**59,059**	**56,061**	**115,120**	**78.57**
Aklan	11,162	4,524	4,304	8,828	79.09	4,604	4,410	9,014	80.76	4,614	4,449	9,063	81.20	4,578	4,399	8,977	80.42
Antique	12,816	4,597	4,289	8,886	69.34	4,710	4,430	9,140	71.32	4,742	4,438	9,180	71.63	4,585	4,301	8,886	69.34
Capiz	13,986	5,289	5,122	10,411	74.44	5,389	5,169	10,558	75.49	5,517	5,180	10,697	76.48	5,422	5,139	10,561	75.51
Guimaras	3,085	1,328	1,318	2,646	85.77	1,362	1,336	2,698	87.46	1,401	1,355	2,756	89.34	1,399	1,375	2,774	89.92
Iloilo	36,541	14,258	12,934	27,192	74.42	14,237	13,453	27,690	75.78	14,060	13,481	27,541	75.37	13,768	13,259	27,027	73.96
Negros Occidental	49,368	21,732	20,548	42,280	85.64	21,676	20,568	42,244	85.57	21,795	20,804	42,599	86.29	21,665	20,589	42,254	85.59
Bacolod City	11,115	4,451	3,921	8,372	75.32	4,313	3,839	8,152	73.34	4,149	3,650	7,799	70.17	4,102	3,630	7,732	69.56
Iloilo City	8,453	3,403	3,392	6,795	80.39	3,527	3,377	6,904	81.68	3,552	3,398	6,950	82.22	3,540	3,369	6,909	81.73
**Region 7**	**163,262**	**69,672**	**65,473**	**135,145**	**82.78**	**69,304**	**65,252**	**134,556**	**82.42**	**69,010**	**65,581**	**134,591**	**82.44**	**68,092**	**64,650**	**132,742**	**81.31**
Bohol	27,312	10,516	9,695	20,211	74.00	10,696	10,099	20,795	76.14	11,202	10,458	21,660	79.31	11,092	10,339	21,431	78.47
Cebu	67,506	28,981	26,844	55,825	82.70	28,976	26,741	55,717	82.54	29,019	27,021	56,040	83.01	28,842	26,715	55,557	82.30
Negros Oriental	27,938	12,409	12,314	24,723	88.49	12,375	12,333	24,708	88.44	12,482	12,673	25,155	90.04	12,201	12,401	24,602	88.06
Siquijor	1,613	719	633	1,352	83.82	735	670	1,405	87.10	731	701	1,432	88.78	727	696	1,423	88.22
Cebu City	21,193	8,748	8,269	17,017	80.30	8,165	7,565	15,730	74.22	7,372	6,940	14,312	67.53	7,221	6,846	14,067	66.38
Lapu-Lapu City	9,372	4,869	4,562	9,431	100.63	4,859	4,584	9,443	100.76	4,697	4,453	9,150	97.63	4,691	4,445	9,136	97.48
Mandaue City	8,328	3,430	3,156	6,586	79.08	3,498	3,260	6,758	81.15	3,507	3,335	6,842	82.16	3,318	3,208	6,526	78.36
**Region 8**	**102,619**	**36,202**	**33,471**	**69,673**	**67.89**	**34,940**	**32,601**	**67,541**	**65.82**	**33,979**	**31,833**	**65,812**	**64.13**	**33,002**	**31,106**	**64,108**	**62.47**
Biliran	3,834	1,592	1,479	3,071	80.10	1,667	1,525	3,192	83.26	1,656	1,551	3,207	83.65	1,657	1,545	3,202	83.52
Eastern Samar	11,392	3,674	3,347	7,021	61.63	3,461	3,247	6,708	58.88	3,430	3,179	6,609	58.01	3,320	3,179	6,499	57.05
Northern Leyte	34,707	12,184	11,458	23,642	68.12	12,211	11,233	23,444	67.55	11,763	10,868	22,631	65.21	11,424	10,596	22,020	63.45
Northern Samar	15,370	5,016	4,696	9,712	63.19	4,850	4,574	9,424	61.31	4,650	4,301	8,951	58.24	4,414	4,127	8,541	55.57
Southern Leyte	6,451	2,317	2,163	4,480	69.45	2,459	2,316	4,775	74.02	2,507	2,280	4,787	74.21	2,460	2,245	4,705	72.93
Western Samar	14,305	5,440	4,745	10,185	71.20	4,605	4,390	8,995	62.88	4,333	4,293	8,626	60.30	4,101	4,052	8,153	56.99
Calbayog City	4,413	1,195	1,198	2,393	54.23	996	945	1,941	43.98	884	861	1,745	39.54	891	860	1,751	39.68
Maasin City	1,637	540	533	1,073	65.55	582	521	1,103	67.38	562	543	1,105	67.50	552	544	1,096	66.95
Ormoc City	4,941	2,043	1,892	3,935	79.64	2,012	1,897	3,909	79.11	2,145	1,941	4,086	82.70	2,134	1,929	4,063	82.23

Table 1.C.1.3 - Immunization Services for Infants and Children

Number and Proportion of Infants who completed 3 doses of Oral Polio Vaccine (OPV) and Inactivated Polio Vaccine (IPV)
Philippines, Annual 2020

Area	Eligible Pop (Under 1 y.o.)	OPV 1				OPV 2				OPV 3				IPV			
		Male	Female	Total	%	Male	Female	Total	%	Male	Female	Total	%	Male	Female	Total	%
Tacloban City	5,569	2,201	1,960	4,161	74.72	2,097	1,953	4,050	72.72	2,049	2,016	4,065	72.99	2,049	2,029	4,078	73.23
**Region 9**	**80,051**	**29,651**	**28,942**	**58,593**	**73.19**	**29,252**	**28,327**	**57,579**	**71.93**	**29,097**	**28,309**	**57,406**	**71.71**	**28,254**	**27,693**	**55,947**	**69.89**
Zamboanga del Norte	17,249	7,423	7,049	14,472	83.90	7,376	7,096	14,472	83.90	7,229	7,038	14,267	82.71	6,684	6,527	13,211	76.59
Zamboanga del Sur	17,653	6,406	6,259	12,665	71.74	6,569	6,283	12,852	72.80	6,371	6,236	12,607	71.42	6,263	6,143	12,406	70.28
Zamboanga Sibugay	14,954	4,170	4,108	8,278	55.36	4,206	4,138	8,344	55.80	4,170	4,049	8,219	54.96	4,040	4,041	8,081	54.04
Dapitan City	1,784	786	976	1,762	98.77	786	974	1,760	98.65	786	974	1,760	98.65	753	980	1,733	97.14
Dipolog City	2,827	1,327	1,316	2,643	93.49	1,304	1,245	2,549	90.17	1,288	1,264	2,552	90.27	1,315	1,276	2,591	91.65
Isabela City	2,522	1,147	1,123	2,270	90.01	1,136	1,160	2,296	91.04	1,194	1,195	2,389	94.73	1,151	1,143	2,294	90.96
Pagadian City	4,325	1,471	1,752	3,223	74.52	1,488	1,459	2,947	68.14	1,854	1,857	3,711	85.80	1,858	1,874	3,732	86.29
Zamboanga City	18,737	6,921	6,359	13,280	70.88	6,387	5,972	12,359	65.96	6,205	5,696	11,901	63.52	6,190	5,709	11,899	63.51
**Region 10**	**101,411**	**45,696**	**42,408**	**88,104**	**86.88**	**45,750**	**43,506**	**89,256**	**88.01**	**46,923**	**44,920**	**91,843**	**90.57**	**44,994**	**42,211**	**87,205**	**85.99**
Bukidnon	23,706	11,620	10,511	22,131	93.36	11,399	10,632	22,031	92.93	11,350	10,654	22,004	92.82	10,080	9,424	19,504	82.27
Camiguin	1,858	690	688	1,378	74.17	687	669	1,356	72.98	700	689	1,389	74.76	716	681	1,397	75.19
Lanao del Norte	14,960	5,251	5,224	10,475	70.02	5,487	5,399	10,886	72.77	5,526	5,675	11,201	74.87	5,668	5,459	11,127	74.38
Misamis Occidental	6,403	3,096	2,889	5,985	93.47	3,132	2,975	6,107	95.38	3,221	3,070	6,291	98.25	3,234	3,071	6,305	98.47
Misamis Oriental	15,131	6,867	6,393	13,260	87.63	7,004	6,641	13,645	90.18	7,378	6,978	14,356	94.88	7,223	6,881	14,104	93.21
Cagayan de Oro City	14,339	6,625	6,078	12,703	88.59	6,457	6,321	12,778	89.11	6,881	6,810	13,691	95.48	6,786	6,185	12,971	90.46
El Salvador City	1,065	469	470	939	88.17	481	462	943	88.54	480	494	974	91.46	490	499	989	92.86
Gingoog City	2,644	1,221	1,140	2,361	89.30	1,217	1,174	2,391	90.43	1,286	1,181	2,467	93.31	1,209	1,208	2,417	91.41
Iligan City	7,580	3,231	2,924	6,155	81.20	3,199	2,910	6,109	80.59	3,121	2,883	6,004	79.21	3,007	2,748	5,755	75.92
Malaybalay City	3,956	1,996	1,732	3,728	94.24	1,986	1,742	3,728	94.24	2,111	1,893	4,004	101.21	1,865	1,662	3,527	89.16
Oroquieta City	1,389	604	566	1,170	84.23	597	593	1,190	85.67	678	661	1,339	96.40	622	614	1,236	88.98
Ozamis City	2,778	1,313	1,229	2,542	91.50	1,410	1,216	2,626	94.53	1,621	1,383	3,004	108.14	1,640	1,383	3,023	108.82
Tangub City	1,234	632	553	1,185	96.03	628	554	1,182	95.79	619	599	1,218	98.70	640	604	1,244	100.81
Valencia City	4,368	2,081	2,011	4,092	93.68	2,066	2,218	4,284	98.08	1,951	1,950	3,901	89.31	1,814	1,792	3,606	82.55
**Region 11**	**107,247**	**42,264**	**39,638**	**81,902**	**76.37**	**40,437**	**38,217**	**78,654**	**73.34**	**38,064**	**35,613**	**73,677**	**68.70**	**39,553**	**36,890**	**76,443**	**71.28**
Compostela Valley	15,562	6,304	5,916	12,220	78.52	6,077	5,719	11,796	75.80	5,810	5,524	11,334	72.83	5,969	5,698	11,667	74.97
Davao del Norte	21,326	9,778	9,355	19,133	89.72	9,328	9,006	18,334	85.97	8,801	8,353	17,154	80.44	9,499	8,962	18,461	86.57
Davao del Sur	13,007	4,450	4,115	8,565	65.85	4,357	4,070	8,427	64.79	4,277	3,913	8,190	62.97	4,454	4,122	8,576	65.93
Davao Oriental	14,151	4,983	4,642	9,625	68.02	5,026	4,662	9,688	68.46	4,824	4,432	9,256	65.41	5,458	4,982	10,440	73.78
Davao Occidental	6,670	2,374	2,205	4,579	68.65	2,350	2,239	4,589	68.80	2,023	1,898	3,921	58.79	1,894	1,784	3,678	55.14
Davao City	36,531	14,375	13,405	27,780	76.05	13,299	12,521	25,820	70.68	12,329	11,493	23,822	65.21	12,279	11,342	23,621	64.66
**Region 12**	**104,552**	**38,959**	**36,191**	**75,150**	**71.88**	**38,277**	**36,147**	**74,424**	**71.18**	**37,614**	**35,779**	**73,393**	**70.20**	**36,355**	**34,604**	**70,959**	**67.87**
North Cotabato	33,645	11,436	10,281	21,717	64.55	10,980	10,248	21,228	63.09	10,953	10,223	21,176	62.94	10,068	9,426	19,494	57.94
Sarangani	12,891	5,177	4,923	10,100	78.35	5,168	5,012	10,180	78.97	4,942	4,926	9,868	76.55	4,761	4,819	9,580	74.32
South Cotabato	21,113	8,737	8,279	17,016	80.59	8,879	8,440	17,319	82.03	8,881	8,371	17,252	81.71	8,732	8,194	16,926	80.17
Sultan Kudarat	17,359	6,899	6,438	13,337	76.83	6,931	6,377	13,308	76.66	6,809	6,493	13,302	76.63	6,737	6,357	13,094	75.43
Cotabato City	5,835	2,016	1,850	3,866	66.26	1,958	1,841	3,799	65.11	1,924	1,816	3,740	64.10	1,923	1,827	3,750	64.27
Gen. Santos City	13,709	4,694	4,420	9,114	66.48	4,361	4,229	8,590	62.66	4,105	3,950	8,055	58.76	4,134	3,981	8,115	59.19
**BARMM**	**92,799**	**35,558**	**36,462**	**72,020**	**77.61**	**35,010**	**36,851**	**71,861**	**77.44**	**33,799**	**35,522**	**69,321**	**74.70**	**28,591**	**30,130**	**58,721**	**63.28**
Basilan	7,541	1,248	1,216	2,464	32.67	1,075	1,106	2,181	28.92	965	958	1,923	25.50	617	670	1,287	17.07
Lanao del Sur	21,131	9,983	10,899	20,882	98.82	9,898	10,876	20,774	98.31	9,694	10,816	20,510	97.06	8,997	10,015	19,012	89.97
Maguindanao	31,128	14,645	14,122	28,767	92.42	14,537	14,406	28,943	92.98	13,703	13,706	27,409	88.05	11,489	11,244	22,733	73.03
Sulu	16,613	4,493	4,573	9,066	54.57	4,199	4,445	8,644	52.03	4,021	4,287	8,308	50.01	3,174	3,522	6,696	40.31
Tawi-Tawi	9,259	3,058	3,372	6,430	69.45	3,122	3,381	6,503	70.23	3,202	3,409	6,611	71.40	3,093	3,295	6,388	68.99
Lamitan City	2,074	692	683	1,375	66.30	638	657	1,295	62.44	629	655	1,284	61.91	501	602	1,103	53.18
Marawi City	5,053	1,439	1,597	3,036	60.08	1,541	1,980	3,521	69.68	1,585	1,691	3,276	64.83	720	782	1,502	29.72
**CARAGA**	**60,029**	**24,306**	**22,832**	**47,138**	**78.53**	**22,176**	**22,715**	**44,891**	**74.78**	**21,698**	**22,668**	**44,366**	**73.91**	**21,592**	**20,867**	**42,459**	**70.73**
Agusan del Norte	8,098	3,055	2,811	5,866	72.44	3,042	2,751	5,793	71.54	3,077	2,847	5,924	73.15	2,973	2,798	5,771	71.26
Agusan del Sur	17,592	6,865	6,395	13,260	75.38	4,940	6,349	11,289	64.17	4,762	6,339	11,101	63.10	5,905	5,711	11,616	66.03
Surigao del Norte	7,089	3,121	2,993	6,114	86.25	3,230	3,055	6,285	88.66	3,132	3,073	6,205	87.53	2,825	2,761	5,586	78.80
Surigao del Sur	11,482	4,604	4,368	8,972	78.14	4,569	4,540	9,109	79.33	4,594	4,481	9,075	79.04	4,091	4,027	8,118	70.70
Province of Dinagat	2,573	781	813	1,594	61.95	813	803	1,616	62.81	804	828	1,632	63.43	775	814	1,589	61.76
Bislig City	2,179	875	891	1,766	81.05	870	878	1,748	80.22	856	883	1,739	79.81	895	858	1,753	80.45
Butuan City	7,715	3,284	3,112	6,396	82.90	3,257	2,964	6,221	80.64	2,973	2,802	5,775	74.85	2,715	2,566	5,281	68.45
Surigao City	3,301	1,721	1,449	3,170	96.03	1,455	1,375	2,830	85.73	1,500	1,415	2,915	88.31	1,413	1,332	2,745	83.16

* - No Report

0 - No Cases

Table 1.C.1.4 - Immunization Services for Infants and Children

Number and Proportion of Infants who completed 3 doses of Pneumococcal Conjugate Vaccine (PCV)
Philippines, Annual 2020

Area	Eligible Pop (Under 1 y.o.)	PCV 1				PCV 2				PCV 3			
		Male	Female	Total	%	Male	Female	Total	%	Male	Female	Total	%
**PHILIPPINES**	**2,123,158**	**837,373**	**785,158**	**1,622,531**	**76.42**	**811,780**	**774,801**	**1,586,580**	**74.73**	**768,928**	**737,881**	**1,506,809**	**70.97**
**N C R**	**238,661**	**78,502**	**74,888**	**153,390**	**64.27**	**67,101**	**65,516**	**132,617**	**55.57**	**56,497**	**55,704**	**112,201**	**47.01**
Malabon	6,775	3,481	3,294	6,775	100.00	2,732	2,742	5,474	80.80	1,966	2,159	4,125	60.89
Navotas	4,621	2,392	2,229	4,621	100.00	2,322	2,299	4,621	100.00	1,825	1,801	3,626	78.47
Valenzuela City	11,500	4,217	4,147	8,364	72.73	3,794	3,694	7,488	65.11	3,252	3,246	6,498	56.50
Caloocan City	29,363	10,592	9,857	20,449	69.64	8,007	7,903	15,910	54.18	5,719	5,518	11,237	38.27
Marikina City	8,354	3,444	3,282	6,726	80.51	3,145	3,020	6,165	73.80	3,141	3,006	6,147	73.58
Pasig City	13,996	4,305	3,948	8,253	58.97	3,984	3,712	7,696	54.99	3,569	3,375	6,944	49.61
Pateros	1,184	179	234	413	34.88	93	116	209	17.65	15	16	31	2.62
Taguig	14,918	7,595	7,323	14,918	100.00	6,792	6,484	13,276	88.99	5,529	5,338	10,867	72.84
Quezon City	54,413	13,940	13,567	27,507	50.55	11,513	11,462	22,975	42.22	9,578	9,480	19,058	35.02
Makati City	10,798	3,422	3,207	6,629	61.39	2,712	2,667	5,379	49.81	2,250	2,198	4,448	41.19
Mandaluyong City	7,160	2,122	1,950	4,072	56.87	2,095	1,992	4,087	57.08	2,099	1,996	4,095	57.19
San Juan	2,261	646	619	1,265	55.95	606	611	1,217	53.83	573	617	1,190	52.63
Manila City	32,980	8,940	8,768	17,708	53.69	7,855	8,033	15,888	48.17	6,848	7,002	13,850	42.00
Las Piñas City	10,914	4,323	4,015	8,338	76.40	3,459	3,232	6,691	61.31	2,586	2,551	5,137	47.07
Muntinlupa City	9,353	3,262	3,138	6,400	68.43	2,898	2,742	5,640	60.30	2,937	2,768	5,705	61.00
Parañaque City	12,343	3,572	3,255	6,827	55.31	3,124	2,829	5,953	48.23	2,592	2,393	4,985	40.39
Pasay City	7,728	2,070	2,055	4,125	53.38	1,970	1,978	3,948	51.09	2,018	2,240	4,258	55.10
**C A R**	**35,099**	**13,463**	**12,609**	**26,072**	**74.28**	**13,292**	**12,635**	**25,927**	**73.87**	**13,411**	**12,680**	**26,091**	**74.34**
Abra	4,309	1,552	1,501	3,053	70.85	1,644	1,588	3,232	75.01	1,642	1,642	3,284	76.21
Apayao	2,451	918	883	1,801	73.48	939	914	1,853	75.60	937	923	1,860	75.89
Benguet	9,188	3,307	3,088	6,395	69.60	3,214	3,106	6,320	68.79	3,320	3,120	6,440	70.09
Ifugao	4,396	1,785	1,524	3,309	75.27	1,715	1,512	3,227	73.41	1,759	1,561	3,320	75.52
Kalinga	4,617	2,052	1,853	3,905	84.58	2,044	1,885	3,929	85.10	2,052	1,857	3,909	84.67
Mt. Province	3,023	1,199	1,108	2,307	76.31	1,178	1,091	2,269	75.06	1,156	1,052	2,208	73.04
Baguio City	7,115	2,650	2,652	5,302	74.52	2,558	2,539	5,097	71.64	2,545	2,525	5,070	71.26
**Region 1**	**97,261**	**39,140**	**36,744**	**75,884**	**78.02**	**40,082**	**37,561**	**77,643**	**79.83**	**40,176**	**37,598**	**77,774**	**79.96**
Ilocos Norte	8,105	2,869	2,651	5,520	68.11	2,866	2,744	5,610	69.22	2,932	2,790	5,722	70.60
Ilocos Sur	9,330	3,887	3,687	7,574	81.18	4,032	3,874	7,906	84.74	4,099	3,870	7,969	85.41
La Union	11,511	4,502	4,356	8,858	76.95	4,586	4,325	8,911	77.41	4,657	4,403	9,060	78.71
Pangasinan	50,168	20,263	18,952	39,215	78.17	20,958	19,552	40,510	80.75	20,954	19,607	40,561	80.85
Alaminos City	1,894	1,200	927	2,127	112.30	1,207	952	2,159	113.99	1,197	922	2,119	111.88
Candon City	987	513	514	1,027	104.05	523	505	1,028	104.15	442	425	867	87.84
Dagupan City	3,620	1,249	1,121	2,370	65.47	1,264	1,149	2,413	66.66	1,194	1,121	2,315	63.95
Laoag City	1,870	973	982	1,955	104.55	993	993	1,986	106.20	969	1,016	1,985	106.15
San Carlos City	3,979	1,755	1,701	3,456	86.86	1,789	1,685	3,474	87.31	1,754	1,698	3,452	86.76
San Fernando City	2,115	762	687	1,449	68.51	749	694	1,443	68.23	777	701	1,478	69.88
Urdaneta City	2,806	973	1,002	1,975	70.38	920	940	1,860	66.29	1,020	903	1,923	68.53
Vigan City	876	194	164	358	40.87	195	148	343	39.16	181	142	323	36.87
**Region 2**	**69,443**	**27,751**	**25,704**	**53,455**	**76.98**	**27,780**	**26,268**	**54,048**	**77.83**	**27,865**	**26,467**	**54,332**	**78.24**
Batanes	340	131	132	263	77.35	124	136	260	76.47	124	121	245	72.06
Cagayan	17,971	7,686	7,234	14,920	83.02	7,778	7,379	15,157	84.34	7,756	7,215	14,971	83.31
Isabela	26,309	9,157	8,419	17,576	66.81	9,271	8,764	18,035	68.55	9,328	9,052	18,380	69.86
Nueva Vizcaya	9,573	3,972	3,477	7,449	77.81	3,909	3,646	7,555	78.92	3,835	3,738	7,573	79.11
Quirino	4,025	1,606	1,582	3,188	79.20	1,591	1,569	3,160	78.51	1,586	1,457	3,043	75.60
Cauayan City	2,590	1,329	1,090	2,419	93.40	1,197	1,017	2,214	85.48	1,310	1,163	2,473	95.48
Ilagan City	2,911	1,337	1,360	2,697	92.65	1,297	1,310	2,607	89.56	1,299	1,244	2,543	87.36
Santiago City	2,692	1,315	1,254	2,569	95.43	1,367	1,266	2,633	97.81	1,372	1,300	2,672	99.26
Tuguegarao City	3,032	1,218	1,156	2,374	78.30	1,246	1,181	2,427	80.05	1,255	1,177	2,432	80.21
**Region 3**	**220,020**	**102,438**	**96,198**	**198,636**	**90.28**	**101,404**	**95,771**	**197,175**	**89.62**	**100,394**	**96,340**	**196,734**	**89.42**
Aurora	4,770	1,978	1,915	3,893	81.61	1,999	1,935	3,934	82.47	2,047	1,955	4,002	83.90
Bataan	13,823	6,937	6,468	13,405	96.98	6,938	6,555	13,493	97.61	6,888	6,445	13,333	96.46
Bulacan	43,627	18,716	17,443	36,159	82.88	17,974	16,869	34,843	79.87	17,347	16,674	34,021	77.98
Nueva Ecija	28,782	12,339	11,153	23,492	81.62	12,257	11,370	23,627	82.09	12,184	11,478	23,662	82.21
Pampanga	31,359	15,109	14,446	29,555	94.25	15,000	14,460	29,460	93.94	15,146	14,668	29,814	95.07
Tarlac	19,765	9,894	9,231	19,125	96.76	9,961	9,526	19,487	98.59	10,155	9,631	19,786	100.11

Table 1.C.1.4 - Immunization Services for Infants and Children

Number and Proportion of Infants who completed 3 doses of Pneumococcal Conjugate Vaccine (PCV)

Philippines, Annual 2020

Area	Eligible Pop (Under 1 y.o.)	PCV 1				PCV 2				PCV 3			
		Male	Female	Total	%	Male	Female	Total	%	Male	Female	Total	%
Zambales	12,610	4,905	4,863	9,768	77.46	5,083	4,456	9,539	75.65	4,588	4,624	9,212	73.05
Angeles City	7,869	4,087	3,824	7,911	100.53	4,048	3,732	7,780	98.87	4,109	3,829	7,938	100.88
Balanga City	1,998	846	788	1,634	81.78	863	783	1,646	82.38	832	811	1,643	82.23
Cabanatuan City	5,896	2,664	2,454	5,118	86.80	2,584	2,468	5,052	85.69	2,612	2,549	5,161	87.53
City of San Fernando	5,863	2,895	2,647	5,542	94.52	2,911	2,695	5,606	95.62	3,031	2,788	5,819	99.25
Gapan City	2,153	938	978	1,916	88.99	955	959	1,914	88.90	929	993	1,922	89.27
Mabalacat City	4,793	2,646	2,601	5,247	109.47	2,482	2,557	5,039	105.13	2,344	2,390	4,734	98.77
Malolos City	4,880	1,833	1,759	3,592	73.61	1,845	1,749	3,594	73.65	1,901	1,783	3,684	75.49
Meycauayan	4,041	1,899	1,818	3,717	91.98	1,860	1,773	3,633	89.90	1,881	1,805	3,686	91.22
Olongapo	4,971	1,919	1,701	3,620	72.82	1,802	1,700	3,502	70.45	1,703	1,677	3,380	67.99
Palayan City	799	387	383	770	96.37	382	406	788	98.62	387	399	786	98.37
San Jose City	2,718	1,517	1,319	2,836	104.34	1,461	1,347	2,808	103.31	1,424	1,272	2,696	99.19
San Jose del Monte C	11,100	6,600	6,308	12,908	116.29	6,684	6,294	12,978	116.92	6,571	6,462	13,033	117.41
Science City of Munoz	1,586	687	641	1,328	83.73	683	633	1,316	82.98	677	632	1,309	82.53
Tarlac City	6,617	3,642	3,458	7,100	107.30	3,632	3,504	7,136	107.84	3,638	3,475	7,113	107.50
**Region 4A**	**296,816**	**118,513**	**109,428**	**227,941**	**76.80**	**114,182**	**106,109**	**220,291**	**74.22**	**103,269**	**98,002**	**201,271**	**67.81**
Batangas	38,441	12,517	11,192	23,709	61.68	12,225	11,092	23,317	60.66	10,759	10,048	20,807	54.13
Cavite	27,704	16,274	15,120	31,394	113.32	16,515	15,543	32,058	115.72	16,094	15,360	31,454	113.54
Laguna	19,727	7,874	7,297	15,171	76.90	7,538	7,030	14,568	73.85	6,769	6,357	13,126	66.54
Quezon	37,410	14,470	13,302	27,772	74.24	13,643	12,529	26,172	69.96	11,900	11,136	23,036	61.58
Rizal	44,791	13,695	12,489	26,184	58.46	12,739	11,921	24,660	55.06	11,728	10,854	22,582	50.42
Antipolo City	16,497	8,108	7,803	15,911	96.45	7,748	7,489	15,237	92.36	6,850	6,717	13,567	82.24
Bacoor City	12,142	3,169	2,930	6,099	50.23	3,297	3,096	6,393	52.65	2,736	2,681	5,417	44.61
Batangas City	6,823	1,562	1,475	3,037	44.51	1,521	1,438	2,959	43.37	1,388	1,274	2,662	39.02
Biñan City	6,607	3,838	3,711	7,549	114.26	3,430	3,444	6,874	104.04	3,165	3,048	6,213	94.04
Cabuyao City	6,130	2,903	2,724	5,627	91.79	3,178	2,684	5,862	95.63	2,431	2,703	5,134	83.75
Calamba City	9,028	3,787	3,573	7,360	81.52	3,543	3,352	6,895	76.37	3,467	3,276	6,743	74.69
Cavite City	2,075	496	488	984	47.42	498	442	940	45.30	524	472	996	48.00
Dasmariñas City	13,322	3,568	3,274	6,842	51.36	3,460	3,109	6,569	49.31	3,116	2,881	5,997	45.02
General Trias City	6,352	3,655	3,360	7,015	110.44	3,769	3,423	7,192	113.22	3,783	3,669	7,452	117.32
Imus City	8,156	2,672	2,475	5,147	63.11	2,561	2,300	4,861	59.60	2,085	1,985	4,070	49.90
Lipa City	6,880	2,016	1,751	3,767	54.75	2,047	1,868	3,915	56.90	2,059	1,886	3,945	57.34
Lucena City	5,672	2,293	2,056	4,349	76.67	2,073	1,799	3,872	68.27	1,779	1,697	3,476	61.28
San Pablo City	5,277	1,957	1,709	3,666	69.47	1,791	1,692	3,483	66.00	1,717	1,553	3,270	61.97
San Pedro City	6,467	2,657	2,405	5,062	78.27	2,514	2,308	4,822	74.56	1,937	1,812	3,749	57.97
Santa Rosa City	7,025	3,220	2,969	6,189	88.10	2,718	2,531	5,249	74.72	2,226	2,194	4,420	62.92
Tagaytay City	1,437	1,658	1,436	3,094	215.31	1,708	1,452	3,160	219.90	1,728	1,356	3,084	214.61
Tanauan City	3,584	1,579	1,487	3,066	85.55	1,376	1,368	2,744	76.56	1,263	1,229	2,492	69.53
Tayabas City	2,122	675	668	1,343	63.29	642	633	1,275	60.08	463	508	971	45.76
Trece Martires City	3,147	3,870	3,734	7,604	241.63	3,648	3,566	7,214	229.23	3,302	3,306	6,608	209.98
**Region 4B**	**71,246**	**22,034**	**20,299**	**42,333**	**59.42**	**19,222**	**18,147**	**37,369**	**52.45**	**16,126**	**15,443**	**31,569**	**44.31**
Marinduque	4,836	2,108	1,844	3,952	81.72	1,603	1,437	3,040	62.86	1,325	1,208	2,533	52.38
Mindoro Occidental	12,407	3,599	3,394	6,993	56.36	2,821	2,686	5,507	44.39	2,231	2,184	4,415	35.58
Mindoro Oriental	19,809	7,876	7,236	15,112	76.29	6,940	6,467	13,407	67.68	5,863	5,509	11,372	57.41
Palawan	21,375	4,186	3,834	8,020	37.52	4,019	3,823	7,842	36.69	3,717	3,625	7,342	34.35
Romblon	6,400	1,803	1,698	3,501	54.70	1,792	1,751	3,543	55.36	1,585	1,502	3,087	48.23
Puerto Princesa City	6,419	2,462	2,293	4,755	74.08	2,047	1,983	4,030	62.78	1,405	1,415	2,820	43.93
**Region 5**	**136,116**	**54,663**	**50,037**	**104,700**	**76.92**	**53,241**	**50,974**	**104,215**	**76.56**	**51,636**	**49,420**	**101,056**	**74.24**
Albay	23,737	9,904	9,072	18,976	79.94	9,828	9,437	19,265	81.16	9,647	9,065	18,712	78.83
Camarines Norte	14,283	5,846	5,508	11,354	79.49	5,751	5,369	11,120	77.85	5,433	5,181	10,614	74.31
Camarines Sur	38,796	15,680	14,303	29,983	77.28	15,167	13,945	29,112	75.04	14,274	13,263	27,537	70.98
Catanduanes	6,274	2,213	2,079	4,292	68.41	2,211	2,079	4,290	68.38	2,221	2,059	4,280	68.22
Masbate	22,521	8,919	8,275	17,194	76.35	8,982	8,499	17,481	77.62	8,565	8,359	16,924	75.15
Sorsogon	19,065	7,627	6,630	14,257	74.78	6,885	7,476	14,361	75.33	7,205	7,496	14,701	77.11
Iriga City	2,638	908	802	1,710	64.82	882	832	1,714	64.97	833	801	1,634	61.94
Legaspi City	4,174	1,818	1,795	3,613	86.56	1,770	1,758	3,528	84.52	1,865	1,763	3,628	86.92
Naga City	4,628	1,748	1,573	3,321	71.76	1,765	1,579	3,344	72.26	1,593	1,433	3,026	65.38
**Region 6**	**146,526**	**59,739**	**55,870**	**115,609**	**78.90**	**60,180**	**56,820**	**117,000**	**79.85**	**59,792**	**56,204**	**115,996**	**79.16**
Aklan	11,162	4,573	4,330	8,903	79.76	4,572	4,731	9,303	83.35	4,590	4,343	8,933	80.03
Antique	12,816	4,617	4,303	8,920	69.60	4,720	4,430	9,150	71.40	4,678	4,397	9,075	70.81
Capiz	13,986	5,351	5,135	10,486	74.97	5,363	5,201	10,564	75.53	5,549	5,256	10,805	77.26

Table 1.C.1.4 - Immunization Services for Infants and Children

Number and Proportion of Infants who completed 3 doses of Pneumococcal Conjugate Vaccine (PCV)

Philippines, Annual 2020

Area	Eligible Pop (Under 1 y.o.)	PCV 1				PCV 2				PCV 3			
		Male	Female	Total	%	Male	Female	Total	%	Male	Female	Total	%
Guimaras	3,085	1,332	1,315	2,647	85.80	1,353	1,321	2,674	86.68	1,402	1,351	2,753	89.24
Iloilo	36,541	14,253	13,117	27,370	74.90	14,546	13,766	28,312	77.48	14,543	13,770	28,313	77.48
Negros Occidental	49,368	21,721	20,311	42,032	85.14	21,754	20,148	41,902	84.88	21,325	20,059	41,384	83.83
Bacolod City	11,115	4,462	3,949	8,411	75.67	4,332	3,827	8,159	73.41	4,158	3,666	7,824	70.39
Iloilo City	8,453	3,430	3,410	6,840	80.92	3,540	3,396	6,936	82.05	3,547	3,362	6,909	81.73
**Region 7**	**163,262**	**69,031**	**64,979**	**134,010**	**82.08**	**66,747**	**65,047**	**131,794**	**80.73**	**65,387**	**62,502**	**127,889**	**78.33**
Bohol	27,312	10,515	9,781	20,296	74.31	10,715	10,174	20,889	76.48	10,865	10,135	21,000	76.89
Cebu	67,506	28,938	26,839	55,777	82.63	29,018	26,914	55,932	82.85	27,876	26,144	54,020	80.02
Negros Oriental	27,938	12,078	12,043	24,121	86.34	12,042	12,194	24,236	86.75	11,365	11,651	23,016	82.38
Siquijor	1,613	710	625	1,335	82.77	728	662	1,390	86.17	706	671	1,377	85.37
Cebu City	21,193	8,705	8,177	16,882	79.66	6,140	7,539	13,679	64.54	6,680	6,475	13,155	62.07
Lapu-Lapu City	9,372	4,818	4,522	9,340	99.66	4,823	4,484	9,307	99.31	4,571	4,351	8,922	95.20
Mandaue City	8,328	3,267	2,992	6,259	75.16	3,281	3,080	6,361	76.38	3,324	3,075	6,399	76.84
**Region 8**	**102,619**	**36,825**	**34,190**	**71,015**	**69.20**	**35,994**	**33,984**	**69,978**	**68.19**	**35,119**	**33,791**	**68,910**	**67.15**
Biliran	3,834	1,563	1,465	3,028	78.98	1,669	1,536	3,205	83.59	1,677	1,639	3,316	86.49
Eastern Samar	11,392	4,045	3,443	7,488	65.73	3,589	3,406	6,995	61.40	3,595	3,450	7,045	61.84
Northern Leyte	34,707	12,471	11,663	24,134	69.54	12,359	11,713	24,072	69.36	12,260	11,656	23,916	68.91
Northern Samar	15,370	5,253	4,892	10,145	66.01	5,194	4,872	10,066	65.49	4,993	4,760	9,753	63.45
Southern Leyte	6,451	2,308	2,184	4,492	69.63	2,451	2,287	4,738	73.45	2,492	2,271	4,763	73.83
Western Samar	14,305	5,057	4,852	9,909	69.27	4,868	4,689	9,557	66.81	4,521	4,518	9,039	63.19
Calbayog City	4,413	1,290	1,289	2,579	58.44	978	958	1,936	43.87	806	852	1,658	37.57
Maasin City	1,637	559	534	1,093	66.77	601	518	1,119	68.36	554	546	1,100	67.20
Ormoc City	4,941	2,051	1,911	3,962	80.19	2,164	2,016	4,180	84.60	2,124	2,018	4,142	83.83
Tacloban City	5,569	2,228	1,957	4,185	75.15	2,121	1,989	4,110	73.80	2,097	2,081	4,178	75.02
**Region 9**	**80,051**	**28,621**	**27,418**	**56,039**	**70.00**	**27,835**	**27,065**	**54,900**	**68.58**	**27,040**	**26,646**	**53,686**	**67.06**
Zamboanga del Norte	17,249	7,555	7,098	14,653	84.95	7,521	7,153	14,674	85.07	7,424	7,143	14,567	84.45
Zamboanga del Sur	17,653	4,679	4,444	9,123	51.68	4,573	4,426	8,999	50.98	4,510	4,444	8,954	50.72
Zamboanga Sibugay	14,954	4,174	4,109	8,283	55.39	4,205	4,111	8,316	55.61	3,999	4,127	8,126	54.34
Dapitan City	1,784	786	976	1,762	98.77	786	974	1,760	98.65	787	973	1,760	98.65
Dipolog City	2,827	1,339	1,332	2,671	94.48	1,276	1,258	2,534	89.64	1,303	1,237	2,540	89.85
Isabela City	2,522	1,139	1,131	2,270	90.01	1,013	1,105	2,118	83.98	1,076	1,098	2,174	86.20
Pagadian City	4,325	1,822	1,810	3,632	83.98	1,858	1,844	3,702	85.60	1,785	1,799	3,584	82.87
Zamboanga City	18,737	7,127	6,518	13,645	72.82	6,603	6,194	12,797	68.30	6,156	5,825	11,981	63.94
**Region 10**	**101,411**	**46,358**	**42,530**	**88,888**	**87.65**	**44,694**	**42,078**	**86,772**	**85.56**	**43,561**	**41,720**	**85,281**	**84.09**
Bukidnon	23,706	12,415	10,589	23,004	97.04	11,047	10,370	21,417	90.34	10,604	10,079	20,683	87.25
Camiguin	1,858	683	667	1,350	72.66	667	655	1,322	71.15	653	672	1,325	71.31
Lanao del Norte	14,960	5,289	5,236	10,525	70.35	5,393	5,383	10,776	72.03	5,341	5,382	10,723	71.68
Misamis Occidental	6,403	3,207	3,014	6,221	97.16	3,207	3,102	6,309	98.53	3,297	3,202	6,499	101.50
Misamis Oriental	15,131	6,817	6,329	13,146	86.88	6,873	6,259	13,132	86.79	6,593	6,525	13,118	86.70
Cagayan de Oro City	14,339	6,419	5,992	12,411	86.55	6,244	5,852	12,096	84.36	6,119	5,719	11,838	82.56
El Salvador City	1,065	492	462	954	89.58	475	442	917	86.10	454	463	917	86.10
Gingoog City	2,644	1,210	1,191	2,401	90.81	1,219	1,159	2,378	89.94	1,292	1,177	2,469	93.38
Iligan City	7,580	3,273	2,993	6,266	82.66	3,187	2,881	6,068	80.05	2,938	2,699	5,637	74.37
Malaybalay City	3,956	1,927	1,688	3,615	91.38	1,931	1,706	3,637	91.94	1,938	1,737	3,675	92.90
Oroquieta City	1,389	608	565	1,173	84.45	570	563	1,133	81.57	605	595	1,200	86.39
Ozamis City	2,778	1,338	1,229	2,567	92.40	1,333	1,204	2,537	91.32	1,268	1,032	2,300	82.79
Tangub City	1,234	622	565	1,187	96.19	621	588	1,209	97.97	618	592	1,210	98.06
Valencia City	4,368	2,058	2,010	4,068	93.13	1,927	1,914	3,841	87.93	1,841	1,846	3,687	84.41
**Region 11**	**107,247**	**45,205**	**41,949**	**87,154**	**81.26**	**44,069**	**41,473**	**85,542**	**79.76**	**42,935**	**40,213**	**83,148**	**77.53**
Compostela Valley	15,562	6,650	5,968	12,618	81.08	6,488	6,053	12,541	80.59	6,429	5,885	12,314	79.13
Davao del Norte	21,326	10,418	9,899	20,317	95.27	10,214	9,885	20,099	94.25	9,913	9,560	19,473	91.31
Davao del Sur	13,007	4,962	4,444	9,406	72.31	4,885	4,654	9,539	73.34	4,937	4,654	9,591	73.74
Davao Oriental	14,151	5,600	5,130	10,730	75.83	5,641	5,173	10,814	76.42	5,558	5,101	10,659	75.32
Davao Occidental	6,670	2,420	2,309	4,729	70.90	2,482	2,331	4,813	72.16	2,406	2,290	4,696	70.40
Davao City	36,531	15,155	14,199	29,354	80.35	14,359	13,377	27,736	75.92	13,692	12,723	26,415	72.31

Table 1.C.1.4 - Immunization Services for Infants and Children

Number and Proportion of Infants who completed 3 doses of Pneumococcal Conjugate Vaccine (PCV)
Philippines, Annual 2020

Area	Eligible Pop (Under 1 y.o.)	PCV 1				PCV 2				PCV 3			
		Male	Female	Total	%	Male	Female	Total	%	Male	Female	Total	%
**Region 12**	**104,552**	**37,183**	**34,565**	**71,748**	**68.62**	**34,825**	**33,226**	**68,051**	**65.09**	**31,943**	**30,488**	**62,431**	**59.71**
North Cotabato	33,645	9,308	8,169	17,477	51.95	7,737	7,457	15,194	45.16	6,728	6,253	12,981	38.58
Sarangani	12,891	5,508	5,336	10,844	84.12	5,334	5,224	10,558	81.90	4,976	4,987	9,963	77.29
South Cotabato	21,113	8,937	8,515	17,452	82.66	8,906	8,419	17,325	82.06	8,424	8,102	16,526	78.27
Sultan Kudarat	17,359	6,888	6,432	13,320	76.73	6,902	6,410	13,312	76.69	6,656	6,341	12,997	74.87
Cotabato City	5,835	2,008	1,880	3,888	66.63	1,959	1,850	3,809	65.28	1,886	1,736	3,622	62.07
Gen. Santos City	13,709	4,534	4,233	8,767	63.95	3,987	3,866	7,853	57.28	3,273	3,069	6,342	46.26
**BARMM**	**92,799**	**33,771**	**35,045**	**68,816**	**74.16**	**37,402**	**39,732**	**77,133**	**83.12**	**30,610**	**32,374**	**62,984**	**67.87**
Basilan	7,541	1,597	1,598	3,195	42.37	1,319	1,318	2,637	34.97	1,107	1,115	2,222	29.47
Lanao del Sur	21,131	9,960	10,888	20,848	98.66	9,897	10,864	20,761	98.25	9,776	10,956	20,732	98.11
Maguindanao	31,128	12,329	11,961	24,290	78.03	11,309	12,283	23,592	75.79	9,968	9,979	19,947	64.08
Sulu	16,613	4,685	4,956	9,641	58.03	9,697	9,754	19,450	117.08	4,407	4,717	9,124	54.92
Tawi-Tawi	9,259	3,055	3,329	6,384	68.95	3,086	3,341	6,427	69.41	3,192	3,368	6,560	70.85
Lamitan City	2,074	607	643	1,250	60.27	551	591	1,142	55.06	526	582	1,108	53.42
Marawi City	5,053	1,538	1,670	3,208	63.49	1,543	1,581	3,124	61.82	1,634	1,657	3,291	65.13
**CARAGA**	**60,029**	**24,136**	**22,705**	**46,841**	**78.03**	**23,730**	**22,395**	**46,125**	**76.84**	**23,167**	**22,289**	**45,456**	**75.72**
Agusan del Norte	8,098	3,246	3,010	6,256	77.25	3,244	2,985	6,229	76.92	3,176	3,024	6,200	76.56
Agusan del Sur	17,592	6,641	6,202	12,843	73.00	6,466	6,069	12,535	71.25	6,241	6,107	12,348	70.19
Surigao del Norte	7,089	3,193	3,072	6,265	88.38	3,179	3,004	6,183	87.22	3,173	2,978	6,151	86.77
Surigao del Sur	11,482	4,570	4,281	8,851	77.09	4,423	4,349	8,772	76.40	4,355	4,301	8,656	75.39
Province of Dinagat	2,573	806	806	1,612	62.65	821	821	1,642	63.82	852	842	1,694	65.84
Bislig City	2,179	868	887	1,755	80.54	869	887	1,756	80.59	850	861	1,711	78.52
Butuan City	7,715	3,313	3,065	6,378	82.67	3,293	2,944	6,237	80.84	3,021	2,817	5,838	75.67
Surigao City	3,301	1,499	1,382	2,881	87.28	1,435	1,336	2,771	83.94	1,499	1,359	2,858	86.58

* - No Report
0 - No Cases

Table 1.C.1.5 - Immunization Services for Infants and Children

Number and Proportion of Children vaccinated with 2 doses of Measles Containing Vaccine (MCV)
Philippines, Annual 2020

Area	Eligible Pop (Under 1 y.o.)	MCV 1				MCV 2			
		Male	Female	Total	%	Male	Female	Total	%
**PHILIPPINES**	**2,123,158**	**839,829**	**789,176**	**1,629,005**	**76.73**	**799,060**	**755,153**	**1,554,213**	**73.20**
**N C R**	**238,661**	**83,868**	**79,548**	**163,416**	**68.47**	**70,520**	**67,278**	**137,798**	**57.74**
Malabon	6,775	2,427	2,252	4,679	69.06	2,639	2,536	5,175	76.38
Navotas	4,621	2,330	2,291	4,621	100.00	2,288	2,333	4,621	100.00
Valenzuela City	11,500	4,363	4,187	8,550	74.35	4,165	3,960	8,125	70.65
Caloocan City	29,363	10,279	9,601	19,880	67.70	8,048	7,383	15,431	52.55
Marikina City	8,354	2,923	2,728	5,651	67.64	2,415	2,209	4,624	55.35
Pasig City	13,996	5,137	5,096	10,233	73.11	3,873	3,862	7,735	55.27
Pateros	1,184	533	506	1,039	87.75	515	514	1,029	86.91
Taguig	14,918	6,346	5,884	12,230	81.98	6,022	5,266	11,288	75.67
Quezon City	54,413	20,274	19,576	39,850	73.24	16,627	16,374	33,001	60.65
Makati City	10,798	3,774	3,241	7,015	64.97	2,847	2,647	5,494	50.88
Mandaluyong City	7,160	1,972	1,889	3,861	53.92	1,872	1,828	3,700	51.68
San Juan	2,261	801	757	1,558	68.91	529	586	1,115	49.31
Manila City	32,980	9,316	8,913	18,229	55.27	6,970	6,588	13,558	41.11
Las Piñas City	10,914	3,710	3,520	7,230	66.25	3,148	3,097	6,245	57.22
Muntinlupa City	9,353	3,872	3,675	7,547	80.69	3,366	3,150	6,516	69.67
Parañaque City	12,343	3,531	3,375	6,906	55.95	3,277	3,157	6,434	52.13
Pasay City	7,728	2,280	2,057	4,337	56.12	1,919	1,788	3,707	47.97
**C A R**	**35,099**	**13,253**	**12,335**	**25,588**	**72.90**	**13,070**	**12,106**	**25,176**	**71.73**
Abra	4,309	1,629	1,581	3,210	74.50	1,707	1,609	3,316	76.96
Apayao	2,451	937	890	1,827	74.54	977	898	1,875	76.50
Benguet	9,188	3,242	3,069	6,311	68.69	3,185	2,976	6,161	67.05
Ifugao	4,396	1,774	1,501	3,275	74.50	1,784	1,584	3,368	76.62
Kalinga	4,617	2,236	2,017	4,253	92.12	2,286	2,059	4,345	94.11
Mt. Province	3,023	1,221	1,056	2,277	75.32	1,190	1,044	2,234	73.90
Baguio City	7,115	2,214	2,221	4,435	62.33	1,941	1,936	3,877	54.49
**Region 1**	**97,261**	**42,150**	**38,744**	**80,894**	**83.17**	**42,887**	**39,615**	**82,502**	**84.83**
Ilocos Norte	8,105	2,991	2,743	5,734	70.75	3,114	2,789	5,903	72.83
Ilocos Sur	9,330	4,290	3,852	8,142	87.27	4,565	4,137	8,702	93.27
La Union	11,511	5,130	4,711	9,841	85.49	4,909	4,603	9,512	82.63
Pangasinan	50,168	22,390	20,498	42,888	85.49	22,900	21,142	44,042	87.79
Alaminos City	1,894	1,118	955	2,073	109.45	1,234	984	2,218	117.11
Candon City	987	482	461	943	95.54	521	516	1,037	105.07
Dagupan City	3,620	1,123	1,027	2,150	59.39	950	867	1,817	50.19
Laoag City	1,870	961	989	1,950	104.28	982	1,002	1,984	106.10
San Carlos City	3,979	1,681	1,678	3,359	84.42	1,747	1,678	3,425	86.08
San Fernando City	2,115	812	795	1,607	75.98	776	744	1,520	71.87
Urdaneta City	2,806	1,014	893	1,907	67.96	990	995	1,985	70.74
Vigan City	876	158	142	300	34.25	199	158	357	40.75
**Region 2**	**69,443**	**28,246**	**26,148**	**54,394**	**78.33**	**28,673**	**26,353**	**55,026**	**79.24**
Batanes	340	107	120	227	66.76	116	128	244	71.76
Cagayan	17,971	7,967	7,268	15,235	84.78	8,260	7,541	15,801	87.92

Table 1.C.1.5 - Immunization Services for Infants and Children

Number and Proportion of Children vaccinated with 2 doses of Measles Containing Vaccine (MCV)
Philippines, Annual 2020

Area	Eligible Pop (Under 1 y.o.)	MCV 1				MCV 2			
		Male	Female	Total	%	Male	Female	Total	%
Isabela	26,309	9,220	8,605	17,825	67.75	9,332	8,516	17,848	67.84
Nueva Vizcaya	9,573	3,957	3,770	7,727	80.72	4,011	3,856	7,867	82.18
Quirino	4,025	1,730	1,559	3,289	81.71	1,794	1,618	3,412	84.77
Cauayan City	2,590	1,224	1,104	2,328	89.88	1,046	995	2,041	78.80
Ilagan City	2,911	1,339	1,354	2,693	92.51	1,440	1,386	2,826	97.08
Santiago City	2,692	1,473	1,219	2,692	100.00	1,426	1,266	2,692	100.00
Tuguegarao City	3,032	1,229	1,149	2,378	78.43	1,248	1,047	2,295	75.69
**Region 3**	**220,020**	**101,480**	**95,741**	**197,221**	**89.64**	**98,083**	**93,663**	**191,746**	**87.15**
Aurora	4,770	2,028	1,894	3,922	82.22	1,944	1,815	3,759	78.81
Bataan	13,823	6,907	6,407	13,314	96.32	6,602	6,031	12,633	91.39
Bulacan	43,627	17,888	16,734	34,622	79.36	17,015	16,412	33,427	76.62
Nueva Ecija	28,782	12,756	11,881	24,637	85.60	12,682	11,956	24,638	85.60
Pampanga	31,359	15,038	14,244	29,282	93.38	14,179	13,520	27,699	88.33
Tarlac	19,765	10,387	10,057	20,444	103.44	10,517	10,185	20,702	104.74
Zambales	12,610	4,771	4,667	9,438	74.85	4,498	4,369	8,867	70.32
Angeles City	7,869	3,945	3,784	7,729	98.22	3,827	3,682	7,509	95.43
Balanga City	1,998	873	855	1,728	86.49	882	905	1,787	89.44
Cabanatuan City	5,896	2,734	2,536	5,270	89.38	2,858	2,582	5,440	92.27
City of San Fernando	5,863	3,050	2,702	5,752	98.11	2,826	2,607	5,433	92.67
Gapan City	2,153	1,005	929	1,934	89.83	1,030	945	1,975	91.73
Mabalacat City	4,793	2,479	2,462	4,941	103.09	2,541	2,571	5,112	106.66
Malolos City	4,880	1,807	1,658	3,465	71.00	1,459	1,424	2,883	59.08
Meycauayan	4,041	1,870	1,759	3,629	89.80	1,873	1,692	3,565	88.22
Olongapo	4,971	1,717	1,629	3,346	67.31	1,534	1,467	3,001	60.37
Palayan City	799	412	368	780	97.62	363	386	749	93.74
San Jose City	2,718	1,462	1,245	2,707	99.60	1,382	1,226	2,608	95.95
San Jose del Monte City	11,100	6,362	6,190	12,552	113.08	6,198	6,157	12,355	111.31
Science City of Munoz	1,586	729	640	1,369	86.32	722	682	1,404	88.52
Tarlac City	6,617	3,260	3,100	6,360	96.12	3,151	3,049	6,200	93.70
**Region 4A**	**296,816**	**112,961**	**104,328**	**217,289**	**73.21**	**105,429**	**98,267**	**203,696**	**68.63**
Batangas	38,441	13,050	12,214	25,264	65.72	12,425	11,834	24,259	63.11
Cavite	27,704	15,529	14,392	29,921	108.00	14,934	13,836	28,770	103.85
Laguna	19,727	7,469	6,801	14,270	72.34	6,936	6,367	13,303	67.44
Quezon	37,410	14,044	12,894	26,938	72.01	13,442	12,270	25,712	68.73
Rizal	44,791	12,067	11,077	23,144	51.67	10,589	9,804	20,393	45.53
Antipolo City	16,497	6,760	6,148	12,908	78.24	5,778	5,409	11,187	67.81
Bacoor City	12,142	3,193	2,946	6,139	50.56	2,713	2,601	5,314	43.77
Batangas City	6,823	2,461	2,219	4,680	68.59	2,195	2,075	4,270	62.58
Biñan City	6,607	3,507	3,689	7,196	108.91	3,379	3,242	6,621	100.21
Cabuyao City	6,130	2,801	2,564	5,365	87.52	2,507	2,401	4,908	80.07
Calamba City	9,028	3,798	3,560	7,358	81.50	3,475	3,434	6,909	76.53
Cavite City	2,075	492	446	938	45.20	448	428	876	42.22
Dasmariñas City	13,322	3,333	3,093	6,426	48.24	3,285	2,976	6,261	47.00
General Trias City	6,352	3,377	2,959	6,336	99.75	3,080	2,791	5,871	92.43
Imus City	8,156	2,319	2,132	4,451	54.57	2,068	2,031	4,099	50.26
Lipa City	6,880	1,679	1,551	3,230	46.95	1,544	1,411	2,955	42.95
Lucena City	5,672	2,393	2,161	4,554	80.29	2,457	2,413	4,870	85.86
San Pablo City	5,277	2,081	1,866	3,947	74.80	1,865	1,714	3,579	67.82
San Pedro City	6,467	1,930	1,721	3,651	56.46	1,748	1,638	3,386	52.36

Table 1.C.1.5 - Immunization Services for Infants and Children

Number and Proportion of Children vaccinated with 2 doses of Measles Containing Vaccine (MCV)
Philippines, Annual 2020

Area	Eligible Pop (Under 1 y.o.)	MCV 1				MCV 2			
		Male	Female	Total	%	Male	Female	Total	%
Santa Rosa City	7,025	3,221	3,041	6,262	89.14	3,651	3,318	6,969	99.20
Tagaytay City	1,437	1,696	1,524	3,220	224.08	1,518	1,432	2,950	205.29
Tanauan City	3,584	1,473	1,311	2,784	77.68	1,451	1,285	2,736	76.34
Tayabas City	2,122	840	803	1,643	77.43	825	747	1,572	74.08
Trece Martires City	3,147	3,448	3,216	6,664	211.76	3,116	2,810	5,926	188.31
**Region 4B**	**71,246**	**20,957**	**19,354**	**40,311**	**56.58**	**20,410**	**18,923**	**39,333**	**55.21**
Marinduque	4,836	1,611	1,603	3,214	66.46	1,691	1,574	3,265	67.51
Mindoro Occidental	12,407	4,679	4,363	9,042	72.88	4,742	4,299	9,041	72.87
Mindoro Oriental	19,809	6,825	6,085	12,910	65.17	6,485	6,054	12,539	63.30
Palawan	21,375	4,270	4,080	8,350	39.06	4,331	4,070	8,401	39.30
Romblon	6,400	1,335	1,171	2,506	39.16	1,283	1,200	2,483	38.80
Puerto Princesa City	6,419	2,237	2,052	4,289	66.82	1,878	1,726	3,604	56.15
**Region 5**	**136,116**	**55,282**	**52,165**	**107,447**	**78.94**	**51,992**	**48,968**	**100,960**	**74.17**
Albay	23,737	10,315	9,677	19,992	84.22	10,277	9,761	20,038	84.42
Camarines Norte	14,283	6,159	5,513	11,672	81.72	5,855	5,380	11,235	78.66
Camarines Sur	38,796	16,188	14,707	30,895	79.63	14,181	13,301	27,482	70.84
Catanduanes	6,274	1,651	1,969	3,620	57.70	2,176	1,986	4,162	66.34
Masbate	22,521	9,138	8,782	17,920	79.57	7,851	7,573	15,424	68.49
Sorsogon	19,065	7,261	7,232	14,493	76.02	6,852	6,944	13,796	72.36
Iriga City	2,638	1,043	932	1,975	74.87	1,062	1,015	2,077	78.73
Legaspi City	4,174	1,887	1,898	3,785	90.68	2,155	1,671	3,826	91.66
Naga City	4,628	1,640	1,455	3,095	66.88	1,583	1,337	2,920	63.09
**Region 6**	**146,526**	**60,102**	**55,673**	**115,775**	**79.01**	**60,880**	**56,028**	**116,908**	**79.79**
Aklan	11,162	4,454	4,357	8,811	78.94	4,237	4,118	8,355	74.85
Antique	12,816	4,629	4,242	8,871	69.22	4,882	4,486	9,368	73.10
Capiz	13,986	5,392	5,049	10,441	74.65	5,172	4,840	10,012	71.59
Guimaras	3,085	1,436	1,339	2,775	89.95	1,477	1,384	2,861	92.74
Iloilo	36,541	14,229	13,142	27,371	74.90	14,850	13,440	28,290	77.42
Negros Occidental	49,368	22,166	20,378	42,544	86.18	22,129	20,255	42,384	85.85
Bacolod City	11,115	4,088	3,798	7,886	70.95	4,234	3,847	8,081	72.70
Iloilo City	8,453	3,708	3,368	7,076	83.71	3,899	3,658	7,557	89.40
**Region 7**	**163,262**	**69,096**	**64,515**	**133,611**	**81.84**	**68,057**	**64,332**	**132,389**	**81.09**
Bohol	27,312	12,354	11,173	23,527	86.14	11,969	11,027	22,996	84.20
Cebu	67,506	29,086	26,738	55,824	82.69	27,625	25,968	53,593	79.39
Negros Oriental	27,938	12,001	12,112	24,113	86.31	11,359	11,606	22,965	82.20
Siquijor	1,613	710	688	1,398	86.67	718	673	1,391	86.24
Cebu City	21,193	7,104	6,292	13,396	63.21	7,856	7,028	14,884	70.23
Lapu-Lapu City	9,372	4,422	4,110	8,532	91.04	5,094	4,816	9,910	105.74
Mandaue City	8,328	3,419	3,402	6,821	81.90	3,436	3,214	6,650	79.85
**Region 8**	**102,619**	**36,692**	**34,450**	**71,142**	**69.33**	**34,751**	**32,381**	**67,132**	**65.42**
Biliran	3,834	1,799	1,631	3,430	89.46	1,857	1,600	3,457	90.17
Eastern Samar	11,392	3,917	3,614	7,531	66.11	3,672	3,347	7,019	61.61

Table 1.C.1.5 - Immunization Services for Infants and Children

Number and Proportion of Children vaccinated with 2 doses of Measles Containing Vaccine (MCV)
Philippines, Annual 2020

Area	Eligible Pop (Under 1 y.o.)	MCV 1				MCV 2			
		Male	Female	Total	%	Male	Female	Total	%
Northern Leyte	34,707	12,720	11,930	24,650	71.02	11,822	10,995	22,817	65.74
Northern Samar	15,370	5,351	4,947	10,298	67.00	4,743	4,480	9,223	60.01
Southern Leyte	6,451	2,423	2,254	4,677	72.50	2,660	2,261	4,921	76.28
Western Samar	14,305	4,852	4,674	9,526	66.59	4,611	4,539	9,150	63.96
Calbayog City	4,413	969	1,056	2,025	45.89	926	947	1,873	42.44
Maasin City	1,637	619	498	1,117	68.23	558	477	1,035	63.23
Ormoc City	4,941	2,035	1,914	3,949	79.92	2,013	1,848	3,861	78.14
Tacloban City	5,569	2,007	1,932	3,939	70.73	1,889	1,887	3,776	67.80
**Region 9**	**80,051**	**29,083**	**28,406**	**57,489**	**71.82**	**27,817**	**27,274**	**55,091**	**68.82**
Zamboanga del Norte	17,249	7,293	6,984	14,277	82.77	7,246	7,082	14,328	83.07
Zamboanga del Sur	17,653	6,864	6,879	13,743	77.85	6,220	6,197	12,417	70.34
Zamboanga Sibugay	14,954	3,663	3,485	7,148	47.80	3,971	3,836	7,807	52.21
Dapitan City	1,784	764	1,002	1,766	98.99	763	1,000	1,763	98.82
Dipolog City	2,827	1,451	1,301	2,752	97.35	1,329	1,253	2,582	91.33
Isabela City	2,522	1,306	1,207	2,513	99.64	1,185	1,112	2,297	91.08
Pagadian City	4,325	1,678	1,819	3,497	80.86	1,691	1,680	3,371	77.94
Zamboanga City	18,737	6,064	5,729	11,793	62.94	5,412	5,114	10,526	56.18
**Region 10**	**101,411**	**44,814**	**42,155**	**86,969**	**85.76**	**41,923**	**39,402**	**81,325**	**80.19**
Bukidnon	23,706	11,048	10,321	21,369	90.14	9,752	8,857	18,609	78.50
Camiguin	1,858	659	652	1,311	70.56	639	663	1,302	70.08
Lanao del Norte	14,960	5,742	5,507	11,249	75.19	5,569	5,663	11,232	75.08
Misamis Occidental	6,403	2,876	2,766	5,642	88.11	2,441	2,396	4,837	75.54
Misamis Oriental	15,131	6,808	6,511	13,319	88.02	6,625	6,301	12,926	85.43
Cagayan de Oro City	14,339	6,666	6,258	12,924	90.13	6,147	5,697	11,844	82.60
El Salvador City	1,065	560	484	1,044	98.03	566	493	1,059	99.44
Gingoog City	2,644	1,260	1,168	2,428	91.83	1,396	1,114	2,510	94.93
Iligan City	7,580	3,065	2,836	5,901	77.85	2,983	2,860	5,843	77.08
Malaybalay City	3,956	1,926	1,712	3,638	91.96	1,725	1,496	3,221	81.42
Oroquieta City	1,389	578	549	1,127	81.14	528	550	1,078	77.61
Ozamis City	2,778	1,271	1,146	2,417	87.01	1,280	1,214	2,494	89.78
Tangub City	1,234	644	604	1,248	101.13	626	621	1,247	101.05
Valencia City	4,368	1,711	1,641	3,352	76.74	1,646	1,477	3,123	71.50
**Region 11**	**107,247**	**44,146**	**41,144**	**85,290**	**79.53**	**42,159**	**39,558**	**81,717**	**76.20**
Compostela Valley	15,562	6,874	6,174	13,048	83.85	6,788	6,103	12,891	82.84
Davao del Norte	21,326	10,079	9,685	19,764	92.68	10,532	10,019	20,551	96.37
Davao del Sur	13,007	4,959	4,681	9,640	74.11	4,551	4,329	8,880	68.27
Davao Oriental	14,151	5,453	4,944	10,397	73.47	4,926	4,577	9,503	67.15
Davao Occidental	6,670	2,234	2,176	4,410	66.12	2,071	1,931	4,002	60.00
Davao City	36,531	14,547	13,484	28,031	76.73	13,291	12,599	25,890	70.87
**Region 12**	**104,552**	**39,847**	**37,269**	**77,116**	**73.76**	**38,760**	**37,141**	**75,901**	**72.60**
North Cotabato	33,645	11,404	10,372	21,776	64.72	10,402	9,932	20,334	60.44
Sarangani	12,891	5,505	5,154	10,659	82.69	5,512	5,239	10,751	83.40
South Cotabato	21,113	9,060	8,628	17,688	83.78	9,177	8,679	17,856	84.57
Sultan Kudarat	17,359	7,094	6,606	13,700	78.92	7,231	7,045	14,276	82.24

Table 1.C.1.5 - Immunization Services for Infants and Children

Number and Proportion of Children vaccinated with 2 doses of Measles Containing Vaccine (MCV)
Philippines, Annual 2020

Area	Eligible Pop (Under 1 y.o.)	MCV 1				MCV 2			
		Male	Female	Total	%	Male	Female	Total	%
Cotabato City	5,835	2,173	2,028	4,201	72.00	2,243	2,246	4,489	76.93
Gen. Santos City	13,709	4,611	4,481	9,092	66.32	4,195	4,000	8,195	59.78
**BARMM**	**92,799**	**34,167**	**34,620**	**68,787**	**74.12**	**30,261**	**31,243**	**61,504**	**66.28**
Basilan	7,541	1,233	1,292	2,525	33.48	899	895	1,794	23.79
Lanao del Sur	21,131	9,799	10,429	20,228	95.73	9,630	10,174	19,804	93.72
Maguindanao	31,128	13,343	12,915	26,258	84.35	10,954	11,060	22,014	70.72
Sulu	16,613	4,109	4,292	8,401	50.57	3,648	3,944	7,592	45.70
Tawi-Tawi	9,259	3,326	3,322	6,648	71.80	2,852	2,899	5,751	62.11
Lamitan City	2,074	563	623	1,186	57.18	512	586	1,098	52.94
Marawi City	5,053	1,794	1,747	3,541	70.08	1,766	1,685	3,451	68.30
**CARAGA**	**60,029**	**23,685**	**22,581**	**46,266**	**77.07**	**23,388**	**22,621**	**46,009**	**76.64**
Agusan del Norte	8,098	3,135	3,028	6,163	76.11	3,223	3,152	6,375	78.72
Agusan del Sur	17,592	6,342	6,012	12,354	70.23	6,062	5,954	12,016	68.30
Surigao del Norte	7,089	3,136	2,944	6,080	85.77	2,994	2,923	5,917	83.47
Surigao del Sur	11,482	4,664	4,439	9,103	79.28	4,322	4,183	8,505	74.07
Province of Dinagat	2,573	802	776	1,578	61.33	816	776	1,592	61.87
Bislig City	2,179	903	879	1,782	81.78	902	812	1,714	78.66
Butuan City	7,715	3,352	3,203	6,555	84.96	3,684	3,507	7,191	93.21
Surigao City	3,301	1,351	1,300	2,651	80.31	1,385	1,314	2,699	81.76

* - No Report
0 - No Cases

Table 1.C.1.6 - Immunization Services for Infants and Children

Number and Proportion of Fully Immunized Children & Completely Immunized Children
Philippines, Annual 2020

Area	Eligible Pop (Under 1 y.o.)	FIC				CIC			
		Male	Female	Total	%	Male	Female	Total	%
**PHILIPPINES**	**2,123,158**	**710,163**	**673,609**	**1,383,772**	**65.18**	**158,396**	**151,923**	**310,319**	**14.62**
**N C R**	**238,661**	**72,386**	**69,154**	**141,540**	**59.31**	**19,572**	**20,193**	**39,765**	**16.66**
Malabon	6,775	2,547	2,563	5,110	75.42	2,523	2,455	4,978	73.48
Navotas	4,621	2,260	2,361	4,621	100.00	228	212	440	9.52
Valenzuela City	11,500	4,273	4,084	8,357	72.67	1,430	1,360	2,790	24.26
Caloocan City	29,363	9,251	8,665	17,916	61.02	2,259	2,024	4,283	14.59
Marikina City	8,354	2,129	1,952	4,081	48.85	357	316	673	8.06
Pasig City	13,996	5,037	5,009	10,046	71.78	741	729	1,470	10.50
Pateros	1,184	505	488	993	83.87	22	14	36	3.04
Taguig	14,918	5,818	5,572	11,390	76.35	2,467	2,384	4,851	32.52
Quezon City	54,413	14,352	13,698	28,050	51.55	4,972	5,071	10,043	18.46
Makati City	10,798	3,679	3,172	6,851	63.45	217	164	381	3.53
Mandaluyong City	7,160	1,744	1,710	3,454	48.24	227	223	450	6.28
San Juan	2,261	808	776	1,584	70.06	56	62	118	5.22
Manila City	32,980	8,390	7,923	16,313	49.46	1,917	1,809	3,726	11.30
Las Piñas City	10,914	3,202	3,115	6,317	57.88	782	772	1,554	14.24
Muntinlupa City	9,353	3,589	3,470	7,059	75.47	346	271	617	6.60
Parañaque City	12,343	3,036	2,926	5,962	48.30	716	2,045	2,761	22.37
Pasay City	7,728	1,766	1,670	3,436	44.46	312	282	594	7.69
**C A R**	**35,099**	**12,363**	**11,473**	**23,836**	**67.91**	**1,155**	**1,106**	**2,261**	**6.44**
Abra	4,309	1,672	1,581	3,253	75.49	35	31	66	1.53
Apayao	2,451	903	853	1,756	71.64	32	26	58	2.37
Benguet	9,188	2,979	2,780	5,759	62.68	199	180	379	4.12
Ifugao	4,396	1,619	1,487	3,106	70.66	126	129	255	5.80
Kalinga	4,617	2,244	2,028	4,272	92.53	78	61	139	3.01
Mt. Province	3,023	1,126	991	2,117	70.03	25	23	48	1.59
Baguio City	7,115	1,820	1,753	3,573	50.22	660	656	1,316	18.50
**Region 1**	**97,261**	**38,724**	**35,810**	**74,534**	**76.63**	**4,724**	**4,383**	**9,107**	**9.36**
Ilocos Norte	8,105	3,114	2,789	5,903	72.83	2	3	5	0.06
Ilocos Sur	9,330	4,528	4,103	8,631	92.51	31	26	57	0.61
La Union	11,511	4,069	3,769	7,838	68.09	884	862	1,746	15.17
Pangasinan	50,168	19,903	18,437	38,340	76.42	3,192	2,947	6,139	12.24
Alaminos City	1,894	1,221	957	2,178	114.99	1	1	2	0.11
Candon City	987	521	516	1,037	105.07	2	2	4	0.41
Dagupan City	3,620	702	680	1,382	38.18	461	409	870	24.03
Laoag City	1,870	982	1,002	1,984	106.10	0	0	0	0.00
San Carlos City	3,979	1,664	1,598	3,262	81.98	136	120	256	6.43
San Fernando City	2,115	773	740	1,513	71.54	3	4	7	0.33
Urdaneta City	2,806	988	995	1,983	70.67	0	0	0	0.00
Vigan City	876	259	224	483	55.14	12	9	21	2.40

Table 1.C.1.6 - Immunization Services for Infants and Children

Number and Proportion of Fully Immunized Children & Completely Immunized Children
Philippines, Annual 2020

Area	Eligible Pop (Under 1 y.o.)	FIC				CIC			
		Male	Female	Total	%	Male	Female	Total	%
**Region 2**	**69,443**	**26,582**	**24,529**	**51,111**	**73.60**	**2,476**	**2,048**	**4,524**	**6.51**
Batanes	340	115	127	242	71.18	1	0	1	0.29
Cagayan	17,971	7,725	7,094	14,819	82.46	406	389	795	4.42
Isabela	26,309	8,337	7,632	15,969	60.70	771	697	1,468	5.58
Nueva Vizcaya	9,573	3,662	3,538	7,200	75.21	388	335	723	7.55
Quirino	4,025	1,878	1,700	3,578	88.89	38	27	65	1.61
Cauayan City	2,590	996	968	1,964	75.83	254	298	552	21.31
Ilagan City	2,911	1,456	1,373	2,829	97.18	302	39	341	11.71
Santiago City	2,692	1,245	1,120	2,365	87.85	185	151	336	12.48
Tuguegarao City	3,032	1,168	977	2,145	70.75	131	112	243	8.01
**Region 3**	**220,020**	**88,214**	**85,054**	**173,268**	**78.75**	**21,663**	**20,450**	**42,113**	**19.14**
Aurora	4,770	1,944	1,818	3,762	78.87	241	192	433	9.08
Bataan	13,823	6,097	5,698	11,795	85.33	1,088	831	1,919	13.88
Bulacan	43,627	13,481	13,051	26,532	60.82	3,684	3,442	7,126	16.33
Nueva Ecija	28,782	9,905	9,451	19,356	67.25	3,034	2,665	5,699	19.80
Pampanga	31,359	13,777	13,534	27,311	87.09	5,185	5,181	10,366	33.06
Tarlac	19,765	9,519	9,279	18,798	95.11	1,058	1,009	2,067	10.46
Zambales	12,610	3,884	3,858	7,742	61.40	1,026	1,091	2,117	16.79
Angeles City	7,869	3,579	3,479	7,058	89.69	766	707	1,473	18.72
Balanga City	1,998	802	840	1,642	82.18	77	63	140	7.01
Cabanatuan City	5,896	2,433	2,265	4,698	79.68	429	346	775	13.14
City of San Fernando	5,863	2,763	2,544	5,307	90.52	386	369	755	12.88
Gapan City	2,153	953	833	1,786	82.95	187	187	374	17.37
Mabalacat City	4,793	2,307	2,356	4,663	97.29	230	225	455	9.49
Malolos City	4,880	1,444	1,423	2,867	58.75	287	258	545	11.17
Meycauayan	4,041	1,733	1,591	3,324	82.26	708	635	1,343	33.23
Olongapo	4,971	1,956	1,740	3,696	74.35	1,720	1,747	3,467	69.74
Palayan City	799	339	345	684	85.61	74	80	154	19.27
San Jose City	2,718	1,262	1,142	2,404	88.45	152	167	319	11.74
San Jose del Monte C	11,100	5,984	5,940	11,924	107.42	707	666	1,373	12.37
Science City of Muno:	1,586	666	621	1,287	81.15	56	62	118	7.44
Tarlac City	6,617	3,386	3,246	6,632	100.23	568	527	1,095	16.55
**Region 4A**	**296,816**	**84,466**	**79,057**	**163,523**	**55.09**	**17,235**	**15,869**	**33,104**	**11.15**
Batangas	38,441	11,338	10,662	22,000	57.23	2,430	2,420	4,850	12.62
Cavite	27,704	12,173	11,406	23,579	85.11	1,493	1,348	2,841	10.25
Laguna	19,727	5,172	4,793	9,965	50.51	1,195	1,043	2,238	11.34
Quezon	37,410	9,592	8,873	18,465	49.36	2,272	1,976	4,248	11.36
Rizal	44,791	7,394	6,813	14,207	31.72	1,473	1,315	2,788	6.22
Antipolo City	16,497	5,287	4,935	10,222	61.96	1,149	1,073	2,222	13.47
Bacoor City	12,142	1,977	1,921	3,898	32.10	370	320	690	5.68
Batangas City	6,823	1,715	1,565	3,280	48.07	212	200	412	6.04
Biñan City	6,607	3,447	3,380	6,827	103.33	811	686	1,497	22.66
Cabuyao City	6,130	2,368	2,227	4,595	74.96	807	758	1,565	25.53
Calamba City	9,028	2,687	2,639	5,326	58.99	279	277	556	6.16
Cavite City	2,075	382	367	749	36.10	5	4	9	0.43
Dasmariñas City	13,322	2,941	2,711	5,652	42.43	686	626	1,312	9.85

Table 1.C.1.6 - Immunization Services for Infants and Children

Number and Proportion of Fully Immunized Children & Completely Immunized Children
Philippines, Annual 2020

Area	Eligible Pop (Under 1 y.o.)	FIC				CIC			
		Male	Female	Total	%	Male	Female	Total	%
General Trias City	6,352	2,268	2,060	4,328	68.14	581	531	1,112	17.51
Imus City	8,156	1,808	1,761	3,569	43.76	377	335	712	8.73
Lipa City	6,880	1,095	1,024	2,119	30.80	515	479	994	14.45
Lucena City	5,672	1,844	1,812	3,656	64.46	764	731	1,495	26.36
San Pablo City	5,277	1,151	1,022	2,173	41.18	205	193	398	7.54
San Pedro City	6,467	1,133	1,055	2,188	33.83	312	287	599	9.26
Santa Rosa City	7,025	3,529	3,166	6,695	95.30	431	448	879	12.51
Tagaytay City	1,437	1,502	1,430	2,932	204.04	24	4	28	1.95
Tanauan City	3,584	1,084	977	2,061	57.51	541	510	1,051	29.32
Tayabas City	2,122	605	612	1,217	57.35	39	37	76	3.58
Trece Martires City	3,147	1,974	1,846	3,820	121.39	264	268	532	16.90
**Region 4B**	**71,246**	**19,264**	**17,919**	**37,183**	**52.19**	**2,222**	**2,044**	**4,266**	**5.99**
Marinduque	4,836	1,517	1,423	2,940	60.79	115	112	227	4.69
Mindoro Occidental	12,407	4,344	4,017	8,361	67.39	490	420	910	7.33
Mindoro Oriental	19,809	6,111	5,591	11,702	59.07	1,004	959	1,963	9.91
Palawan	21,375	4,264	3,977	8,241	38.55	304	266	570	2.67
Romblon	6,400	1,216	1,174	2,390	37.34	55	51	106	1.66
Puerto Princesa City	6,419	1,812	1,737	3,549	55.29	254	236	490	7.63
**Region 5**	**136,116**	**39,342**	**36,730**	**76,072**	**55.89**	**17,231**	**16,646**	**33,877**	**24.89**
Albay	23,737	6,823	6,633	13,456	56.69	3,717	3,518	7,235	30.48
Camarines Norte	14,283	4,664	4,233	8,897	62.29	1,739	1,687	3,426	23.99
Camarines Sur	38,796	10,268	9,650	19,918	51.34	5,604	5,328	10,932	28.18
Catanduanes	6,274	2,078	1,915	3,993	63.64	198	206	404	6.44
Masbate	22,521	5,332	5,195	10,527	46.74	3,422	3,319	6,741	29.93
Sorsogon	19,065	6,360	5,610	11,970	62.79	1,198	1,211	2,409	12.64
Iriga City	2,638	777	709	1,486	56.33	322	304	626	23.73
Legaspi City	4,174	1,592	1,567	3,159	75.68	722	785	1,507	36.10
Naga City	4,628	1,448	1,218	2,666	57.61	309	288	597	12.90
**Region 6**	**146,526**	**53,946**	**50,116**	**104,062**	**71.02**	**8,930**	**8,703**	**17,633**	**12.03**
Aklan	11,162	4,009	3,886	7,895	70.73	811	849	1,660	14.87
Antique	12,816	4,318	3,977	8,295	64.72	384	377	761	5.94
Capiz	13,986	4,427	4,133	8,560	61.20	999	1,240	2,239	16.01
Guimaras	3,085	1,292	1,218	2,510	81.36	193	189	382	12.38
Iloilo	36,541	14,178	12,948	27,126	74.23	1,171	1,059	2,230	6.10
Negros Occidental	49,368	18,912	17,578	36,490	73.91	4,165	3,864	8,029	16.26
Bacolod City	11,115	3,040	2,857	5,897	53.05	1,067	1,006	2,073	18.65
Iloilo City	8,453	3,770	3,519	7,289	86.23	140	119	259	3.06
**Region 7**	**163,262**	**53,135**	**50,958**	**104,093**	**63.76**	**19,455**	**18,503**	**37,958**	**23.25**
Bohol	27,312	8,327	7,739	16,066	58.82	3,666	3,377	7,043	25.79
Cebu	67,506	21,009	20,110	41,119	60.91	7,450	6,833	14,283	21.16
Negros Oriental	27,938	9,389	9,373	18,762	67.16	2,405	2,544	4,949	17.71
Siquijor	1,613	667	637	1,304	80.84	52	42	94	5.83

Table 1.C.1.6 - Immunization Services for Infants and Children

Number and Proportion of Fully Immunized Children & Completely Immunized Children
Philippines, Annual 2020

Area	Eligible Pop (Under 1 y.o.)	FIC				CIC			
		Male	Female	Total	%	Male	Female	Total	%
Cebu City	21,193	5,824	5,505	11,329	53.46	2,134	1,855	3,989	18.82
Lapu-Lapu City	9,372	4,150	3,969	8,119	86.63	968	1,139	2,107	22.48
Mandaue City	8,328	3,769	3,625	7,394	88.78	2,780	2,713	5,493	65.96
**Region 8**	**102,619**	**31,418**	**29,532**	**60,950**	**59.39**	**5,795**	**5,347**	**11,142**	**10.86**
Biliran	3,834	1,566	1,384	2,950	76.94	342	308	650	16.95
Eastern Samar	11,392	3,118	2,939	6,057	53.17	927	898	1,825	16.02
Northern Leyte	34,707	11,510	10,781	22,291	64.23	1,378	1,202	2,580	7.43
Northern Samar	15,370	3,545	3,356	6,901	44.90	1,586	1,509	3,095	20.14
Southern Leyte	6,451	2,484	2,131	4,615	71.54	184	137	321	4.98
Western Samar	14,305	4,239	4,227	8,466	59.18	764	725	1,489	10.41
Calbayog City	4,413	849	877	1,726	39.11	136	83	219	4.96
Maasin City	1,637	514	399	913	55.77	97	101	198	12.10
Ormoc City	4,941	1,765	1,620	3,385	68.51	251	247	498	10.08
Tacloban City	5,569	1,828	1,818	3,646	65.47	130	137	267	4.79
**Region 9**	**80,051**	**24,536**	**24,049**	**48,585**	**60.69**	**6,894**	**6,591**	**13,485**	**16.85**
Zamboanga del Norte	17,249	6,414	6,274	12,688	73.56	1,697	1,553	3,250	18.84
Zamboanga del Sur	17,653	4,898	4,909	9,807	55.55	1,800	1,753	3,553	20.13
Zamboanga Sibugay	14,954	3,941	3,807	7,748	51.81	1,066	1,005	2,071	13.85
Dapitan City	1,784	765	1,001	1,766	98.99	22	44	66	3.70
Dipolog City	2,827	1,105	1,019	2,124	75.13	290	297	587	20.76
Isabela City	2,522	1,101	1,032	2,133	84.58	323	324	647	25.65
Pagadian City	4,325	1,452	1,430	2,882	66.64	269	270	539	12.46
Zamboanga City	18,737	4,860	4,577	9,437	50.37	1,427	1,345	2,772	14.79
**Region 10**	**101,411**	**38,830**	**36,949**	**75,779**	**74.72**	**6,407**	**6,261**	**12,668**	**12.49**
Bukidnon	23,706	8,897	8,400	17,297	72.96	1,608	1,400	3,008	12.69
Camiguin	1,858	485	484	969	52.15	55	45	100	5.38
Lanao del Norte	14,960	5,176	5,142	10,318	68.97	869	869	1,738	11.62
Misamis Occidental	6,403	2,651	2,633	5,284	82.52	624	578	1,202	18.77
Misamis Oriental	15,131	6,136	5,850	11,986	79.21	846	827	1,673	11.06
Cagayan de Oro City	14,339	5,721	5,315	11,036	76.96	1,198	1,064	2,262	15.78
El Salvador City	1,065	391	322	713	66.95	175	171	346	32.49
Gingoog City	2,644	1,301	1,208	2,509	94.89	205	585	790	29.88
Iligan City	7,580	2,701	2,605	5,306	70.00	44	47	91	1.20
Malaybalay City	3,956	1,404	1,200	2,604	65.82	533	467	1,000	25.28
Oroquieta City	1,389	516	547	1,063	76.53	12	3	15	1.08
Ozamis City	2,778	1,295	1,123	2,418	87.04	9	8	17	0.61
Tangub City	1,234	619	631	1,250	101.30	28	25	53	4.29
Valencia City	4,368	1,537	1,489	3,026	69.28	201	172	373	8.54
**Region 11**	**107,247**	**40,909**	**37,899**	**78,808**	**73.48**	**8,087**	**7,423**	**15,510**	**14.46**
Compostela Valley	15,562	6,610	5,943	12,553	80.66	560	490	1,050	6.75
Davao del Norte	21,326	10,202	9,523	19,725	92.49	692	639	1,331	6.24
Davao del Sur	13,007	4,790	4,502	9,292	71.44	804	711	1,515	11.65
Davao Oriental	14,151	4,726	4,263	8,989	63.52	1,057	950	2,007	14.18

Table 1.C.1.6 - Immunization Services for Infants and Children

Number and Proportion of Fully Immunized Children & Completely Immunized Children
Philippines, Annual 2020

Area	Eligible Pop (Under 1 y.o.)	FIC				CIC			
		Male	Female	Total	%	Male	Female	Total	%
Davao Occidental	6,670	1,943	1,791	3,734	55.98	421	318	739	11.08
Davao City	36,531	12,638	11,877	24,515	67.11	4,553	4,315	8,868	24.28
**Region 12**	**104,552**	**34,126**	**32,948**	**67,074**	**64.15**	**5,722**	**5,335**	**11,057**	**10.58**
North Cotabato	33,645	8,642	8,242	16,884	50.18	2,266	2,222	4,488	13.34
Sarangani	12,891	5,019	4,857	9,876	76.61	613	515	1,128	8.75
South Cotabato	21,113	8,308	7,976	16,284	77.13	958	816	1,774	8.40
Sultan Kudarat	17,359	6,373	6,228	12,601	72.59	908	856	1,764	10.16
Cotabato City	5,835	2,027	2,015	4,042	69.27	216	231	447	7.66
Gen. Santos City	13,709	3,757	3,630	7,387	53.88	761	695	1,456	10.62
**BARMM**	**92,799**	**28,775**	**29,235**	**58,010**	**62.51**	**7,972**	**8,207**	**16,179**	**17.43**
Basilan	7,541	933	1,051	1,984	26.31	442	407	849	11.26
Lanao del Sur	21,131	9,682	10,097	19,779	93.60	1,981	2,084	4,065	19.24
Maguindanao	31,128	9,321	9,064	18,385	59.06	3,156	3,288	6,444	20.70
Sulu	16,613	3,166	3,284	6,450	38.83	1,746	1,820	3,566	21.47
Tawi-Tawi	9,259	3,428	3,426	6,854	74.03	91	92	183	1.98
Lamitan City	2,074	427	510	937	45.18	117	102	219	10.56
Marawi City	5,053	1,818	1,803	3,621	71.66	439	414	853	16.88
**CARAGA**	**60,029**	**23,147**	**22,197**	**45,344**	**75.54**	**2,856**	**2,814**	**5,670**	**9.45**
Agusan del Norte	8,098	2,944	2,914	5,858	72.34	305	265	570	7.04
Agusan del Sur	17,592	7,406	7,069	14,475	82.28	777	790	1,567	8.91
Surigao del Norte	7,089	2,793	2,724	5,517	77.82	289	294	583	8.22
Surigao del Sur	11,482	3,473	3,353	6,826	59.45	1,079	1,006	2,085	18.16
Province of Dinagat	2,573	777	731	1,508	58.61	51	64	115	4.47
Bislig City	2,179	894	803	1,697	77.88	28	37	65	2.98
Butuan City	7,715	3,536	3,354	6,890	89.31	245	277	522	6.77
Surigao City	3,301	1,324	1,249	2,573	77.95	82	81	163	4.94

* - No Report
0 - No Cases

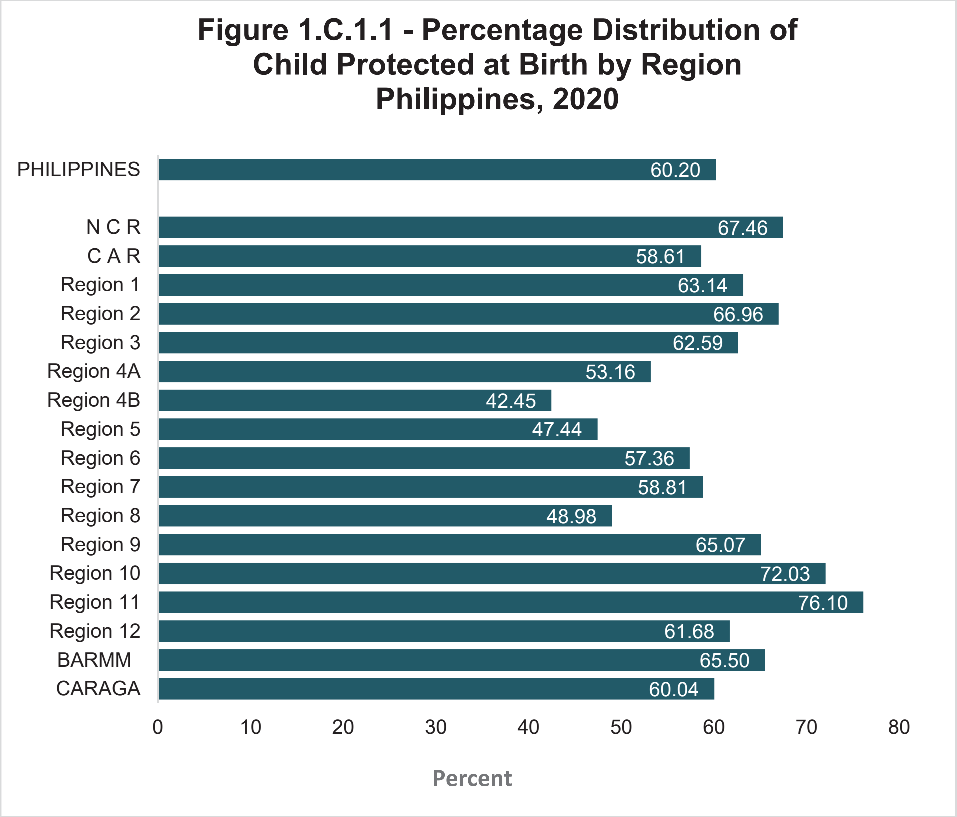
Figure 1.C.1.1 - Percentage Distribution of
Child Protected at Birth by Region
Philippines, 2020
PHILIPPINES
60.20
N C R
67.46
C A R
58.61
Region 1
63.14
Region 2
66.96
Region 3
62.59
Region 4A
53.16
Region 4B
42.45
Region 5
47.44
Region 6
57.36
Region 7
58.81
Region 8
48.98
Region 9
65.07
Region 10
72.03
Region 11
76.10
Region 12
61.68
BARMM
65.50
CARAGA
60.04
0
10
20
30
40
50
60
70
80
Percent

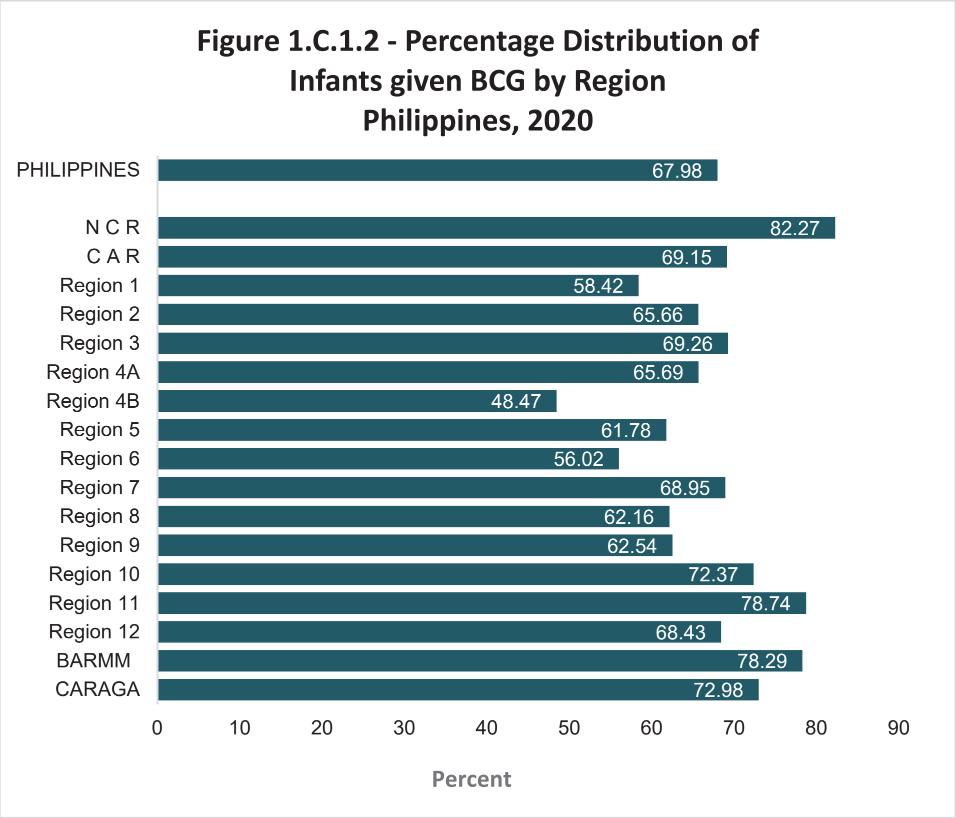
Figure 1.C.1.2 - Percentage Distribution of
Infants given BCG by Region
Philippines, 2020
PHILIPPINES
67.98
N C R
82.27
C A R
69.15
Region 1
58.42
Region 2
65.66
Region 3
69.26
Region 4A
65.69
Region 4B
48.47
Region 5
61.78
Region 6
56.02
Region 7
68.95
Region 8
62.16
Region 9
62.54
Region 10
72.37
Region 11
78.74
Region 12
68.43
BARMM
78.29
CARAGA
72.98
0
10
20
30
40
50
60
70
80
90
Percent

Figure 1.C.1.3 - Percentage Distribution of Infants given Hepatitis B1 Vaccine within 24 hours after birth by Region Philippines, 2020

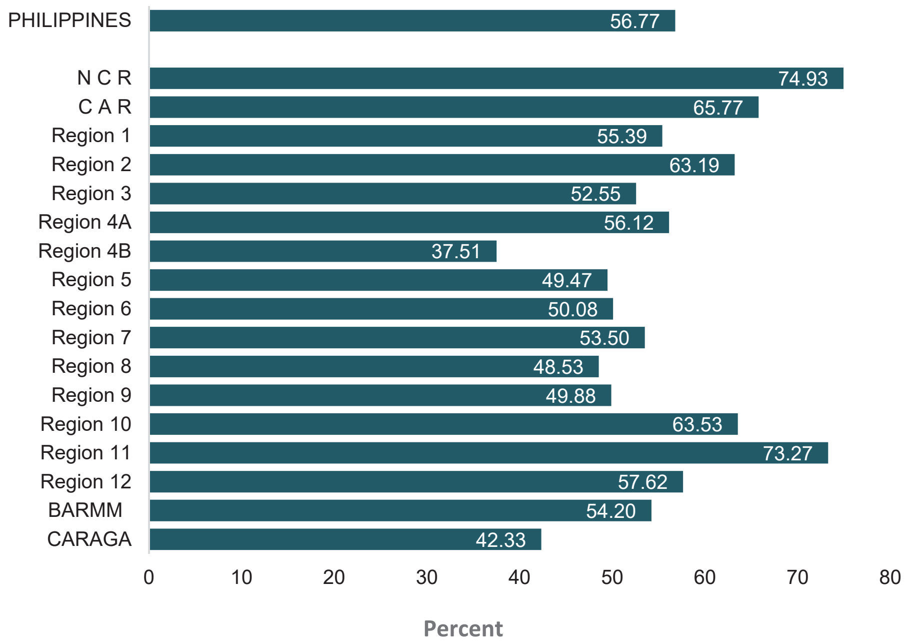

Figure 1.C.1.4 - Percentage Distribution of Infants vaccinated with DPT-HiB-HepB 1 by Region Philippines, 2020

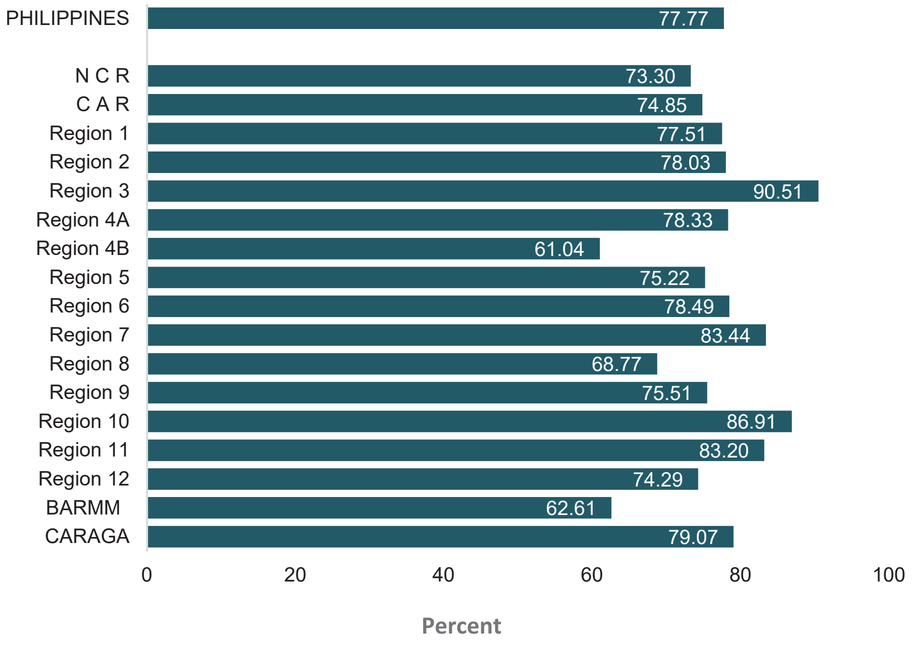

Figure 1.C.1.5 - Percentage Distribution of Infants vaccinated with DPT-HiB-HepB 2 by Region Philippines, 2020

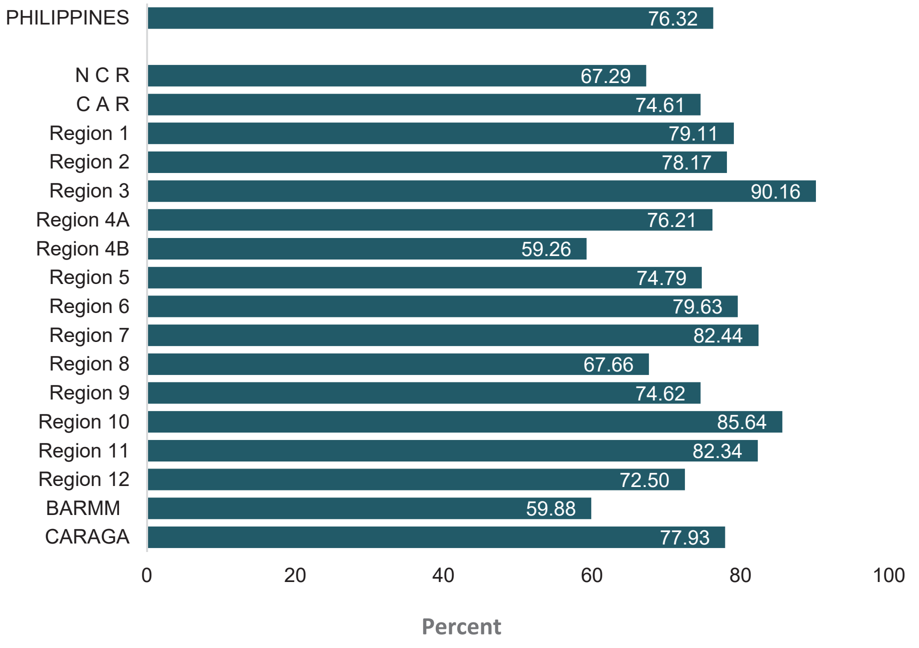

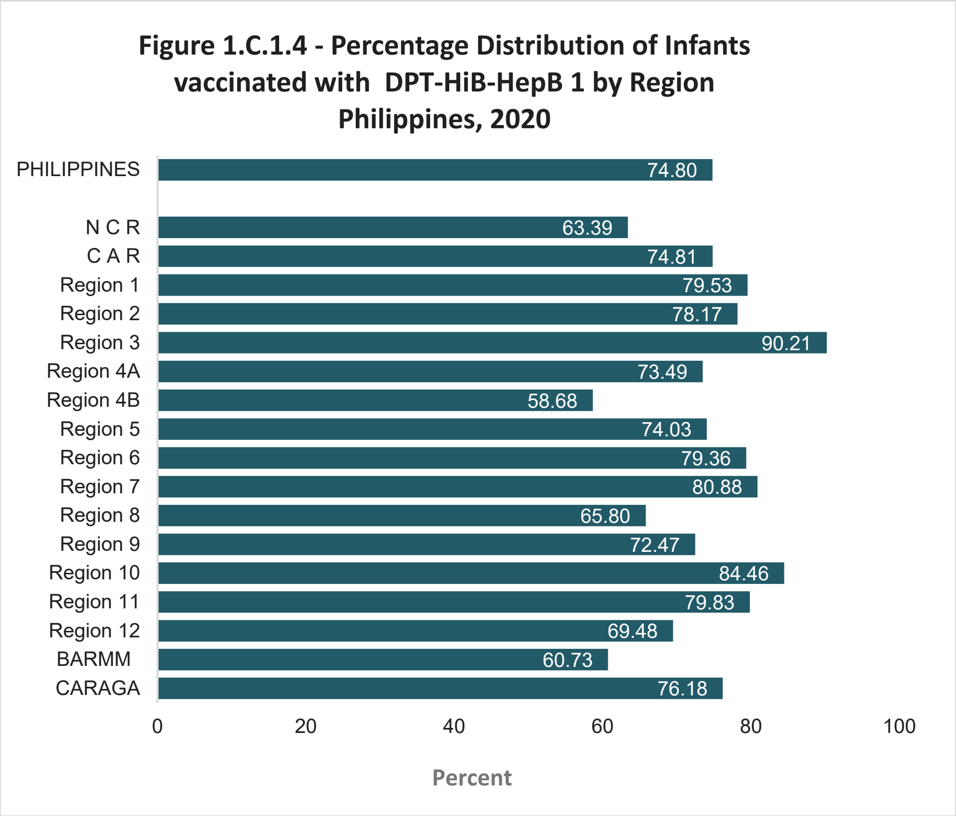
Figure 1.C.1.4 - Percentage Distribution of Infants vaccinated with DPT-HiB-HepB 1 by Region Philippines, 2020
PHILIPPINES
N C R
C A R
Region 1
Region 2
Region 3
Region 4A
Region 4B
Region 5
Region 6
Region 7
Region 8
Region 9
Region 10
Region 11
Region 12
BARMM
CARAGA
74.80
63.39
74.81
79.53
78.17
90.21
73.49
58.68
74.03
79.36
80.88
65.80
72.47
84.46
79.83
69.48
60.73
76.18
0
20
40
60
80
100
Percent

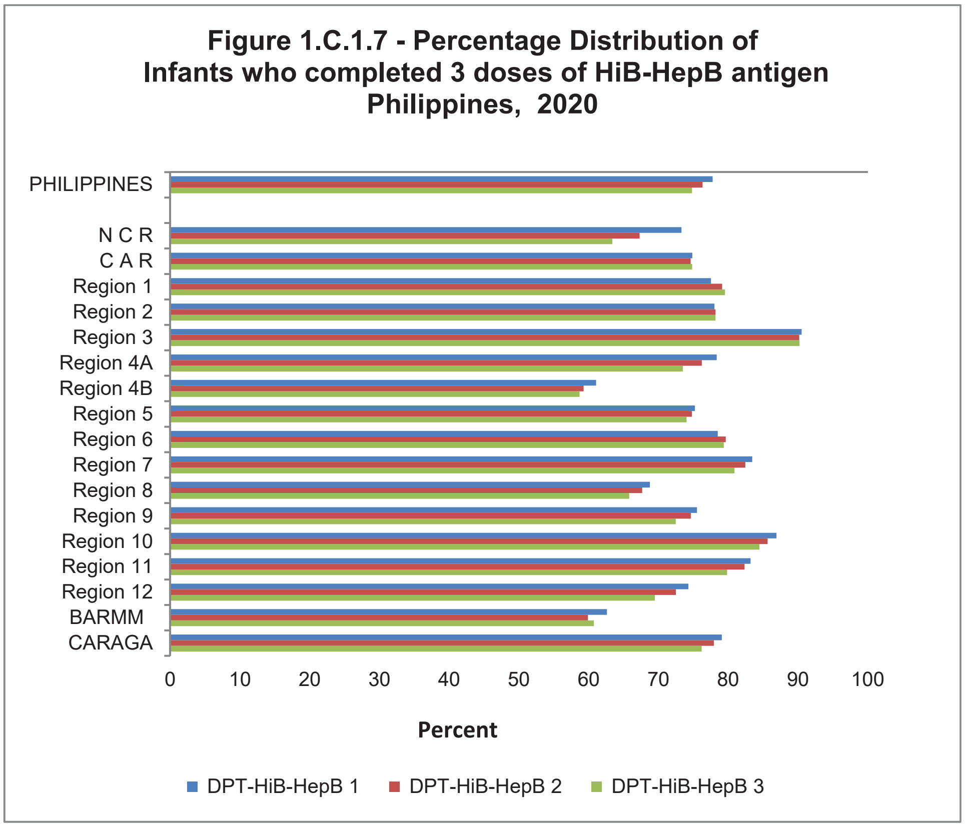
Figure 1.C.1.7 - Percentage Distribution of Infants who completed 3 doses of HiB-HepB antigen Philippines, 2020
PHILIPPINES
N C R
C A R
Region 1
Region 2
Region 3
Region 4A
Region 4B
Region 5
Region 6
Region 7
Region 8
Region 9
Region 10
Region 11
Region 12
BARMM
CARAGA
0
10
20
30
40
50
60
70
80
90
100
Percent
DPT-HiB-HepB 1
DPT-HiB-HepB 2
DPT-HiB-HepB 3

Figure 1.C.1.8 - Percentage Distribution of Infants given Oral Polio Vaccine 1 by Region Philippines, 2020

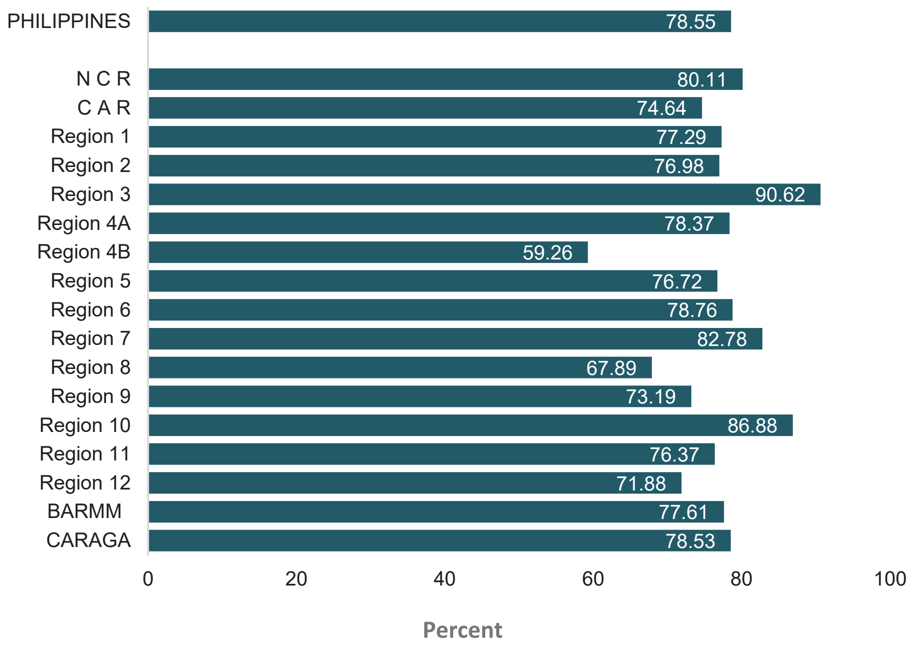

Figure 1.C.1.9 - Percentage Distribution of Infants given Oral Polio Vaccine 2 by Region Philippines, 2020

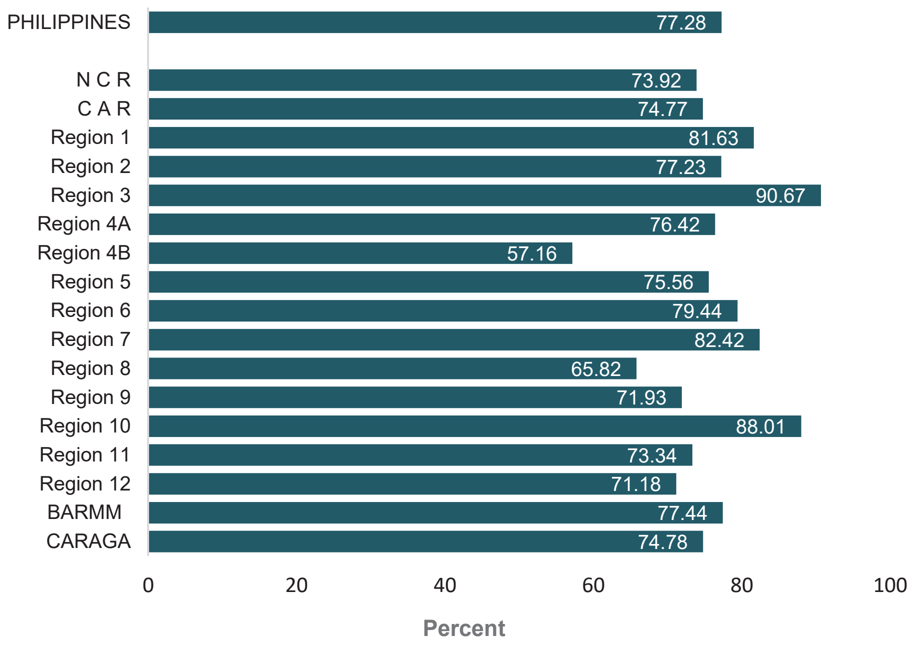

Figure 1.C.1.10 - Percentage Distribution of Infants given Oral Polio Vaccine 3 by Region Philippines, 2020

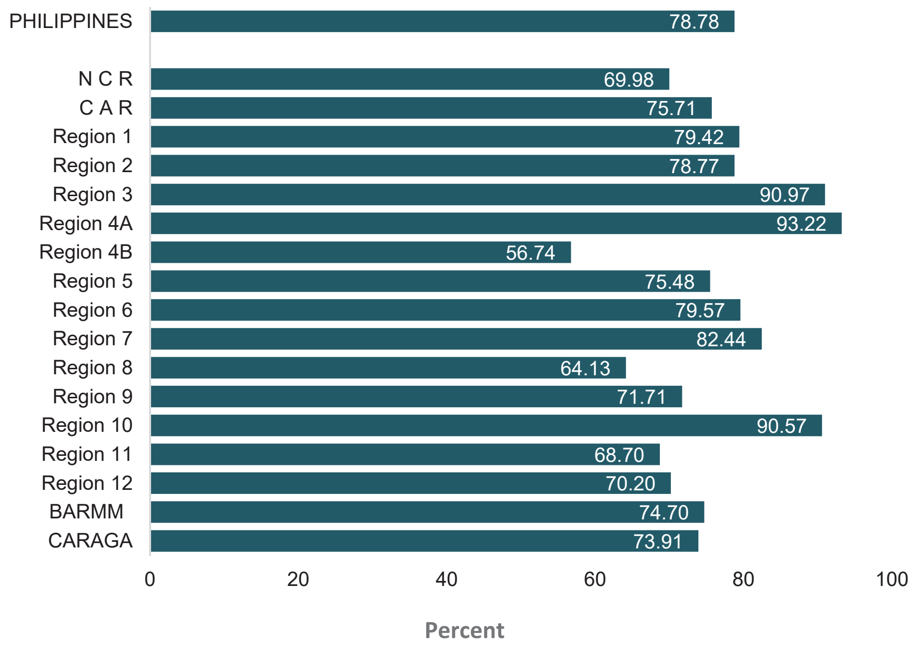

Figure 1.C.1.11 - Percentage Distribution of Infants given Inactivated Polio Vaccine by Region Philippines, 2020

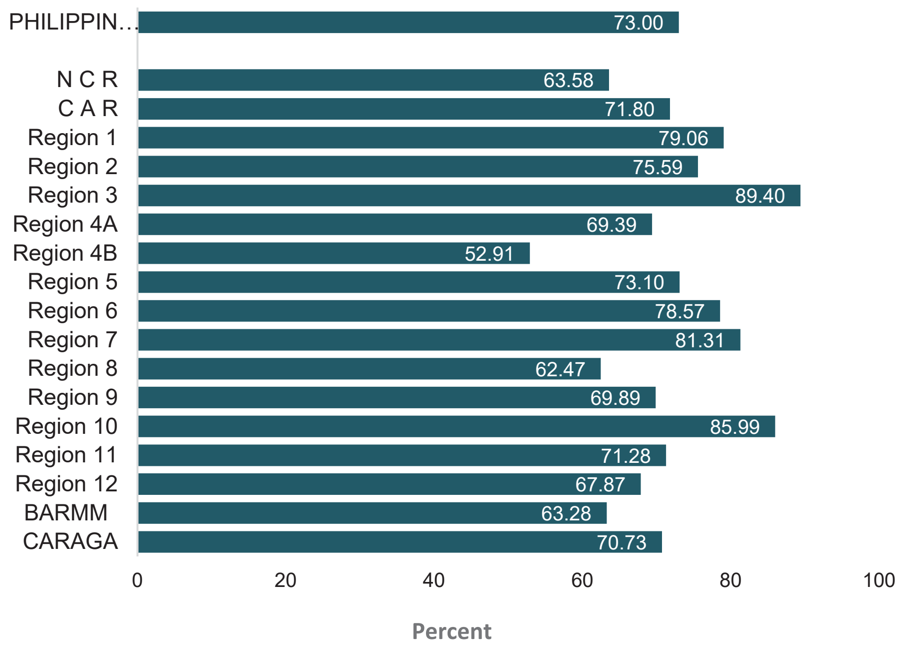

Figure 1.C.1.12 - Percentage Distribution of Infants who completed 3 doses of Oral Polio Vaccines Philippines, 2020

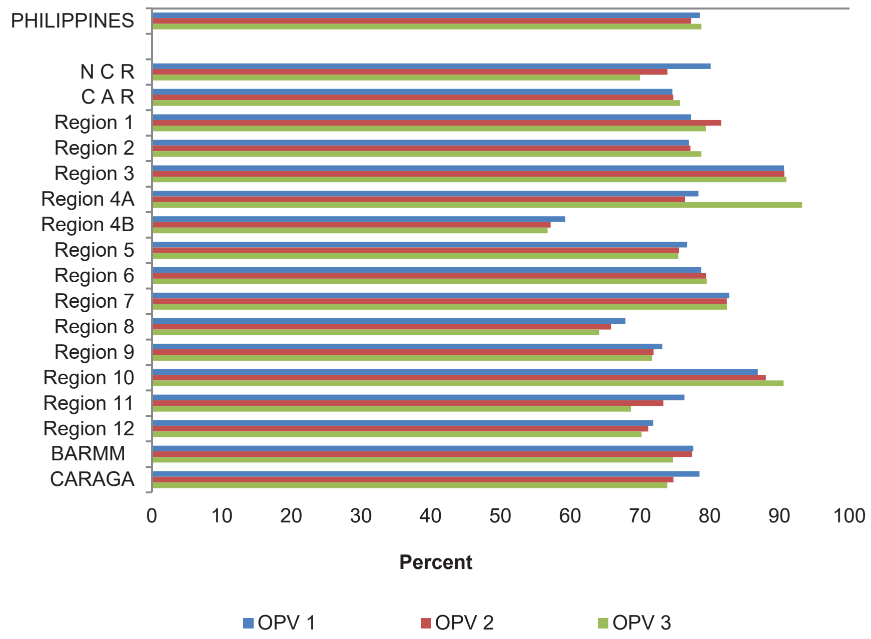

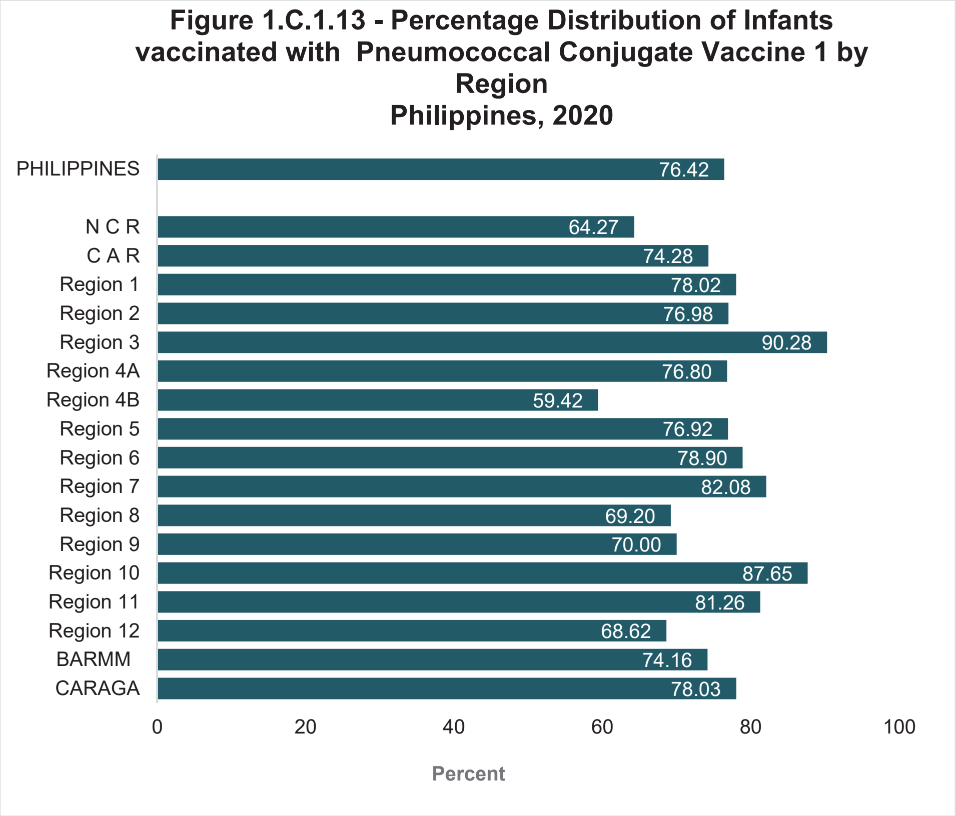
Figure 1.C.1.13 - Percentage Distribution of Infants vaccinated with Pneumococcal Conjugate Vaccine 1 by Region
Philippines, 2020
PHILIPPINES
N C R
C A R
Region 1
Region 2
Region 3
Region 4A
Region 4B
Region 5
Region 6
Region 7
Region 8
Region 9
Region 10
Region 11
Region 12
BARMM
CARAGA
76.42
64.27
74.28
78.02
76.98
90.28
76.80
59.42
76.92
78.90
82.08
69.20
70.00
87.65
81.26
68.62
74.16
78.03
0
20
40
60
80
100
Percent

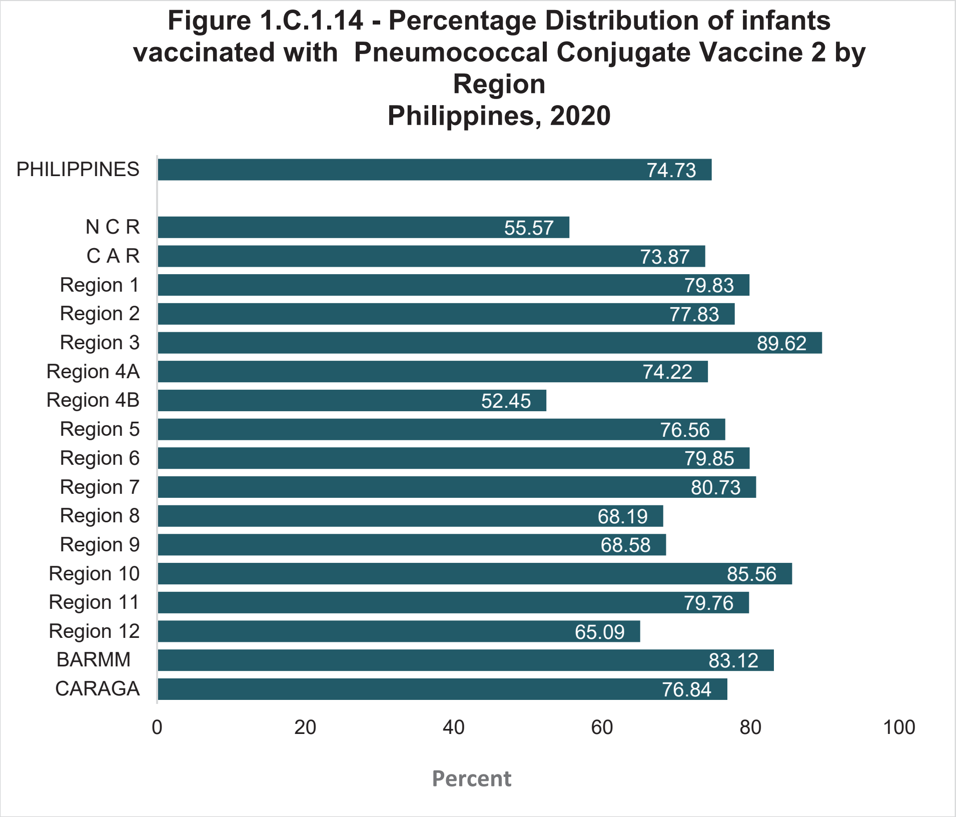
Figure 1.C.1.14 - Percentage Distribution of infants vaccinated with Pneumococcal Conjugate Vaccine 2 by Region
Philippines, 2020
PHILIPPINES
N C R
C A R
Region 1
Region 2
Region 3
Region 4A
Region 4B
Region 5
Region 6
Region 7
Region 8
Region 9
Region 10
Region 11
Region 12
BARMM
CARAGA
74.73
55.57
73.87
79.83
77.83
89.62
74.22
52.45
76.56
79.85
80.73
68.19
68.58
85.56
79.76
65.09
83.12
76.84
0
20
40
60
80
100
Percent

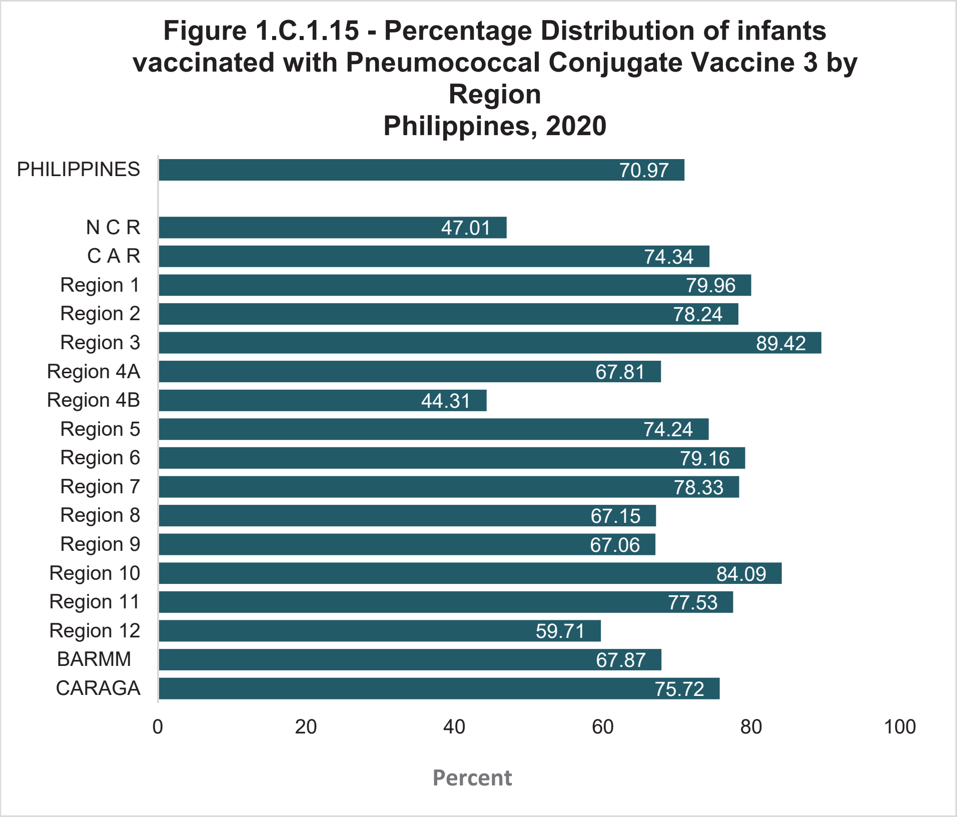
Figure 1.C.1.15 - Percentage Distribution of infants vaccinated with Pneumococcal Conjugate Vaccine 3 by Region
Philippines, 2020
PHILIPPINES
N C R
C A R
Region 1
Region 2
Region 3
Region 4A
Region 4B
Region 5
Region 6
Region 7
Region 8
Region 9
Region 10
Region 11
Region 12
BARMM
CARAGA
70.97
47.01
74.34
79.96
78.24
89.42
67.81
44.31
74.24
79.16
78.33
67.15
67.06
84.09
77.53
59.71
67.87
75.72
0
20
40
60
80
100
Percent

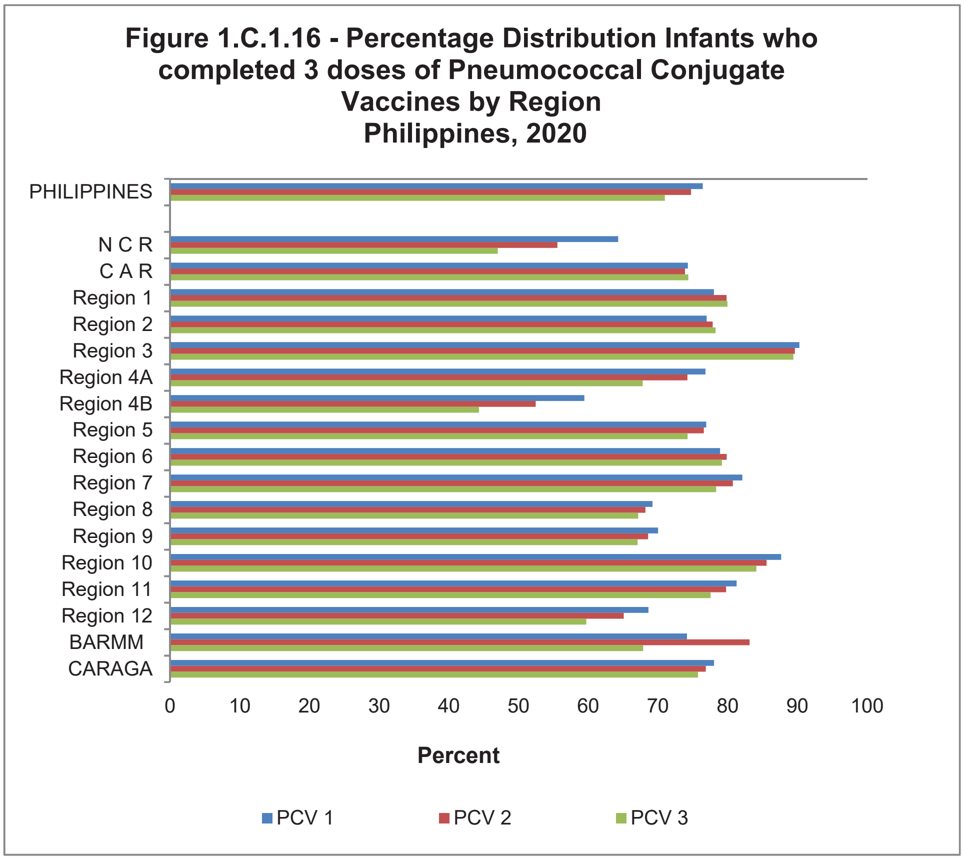
Figure 1.C.1.16 - Percentage Distribution Infants who completed 3 doses of Pneumococcal Conjugate Vaccines by Region
Philippines, 2020
PHILIPPINES
N C R
C A R
Region 1
Region 2
Region 3
Region 4A
Region 4B
Region 5
Region 6
Region 7
Region 8
Region 9
Region 10
Region 11
Region 12
BARMM
CARAGA
0
10
20
30
40
50
60
70
80
90
100
Percent
PCV 1
PCV 2
PCV 3

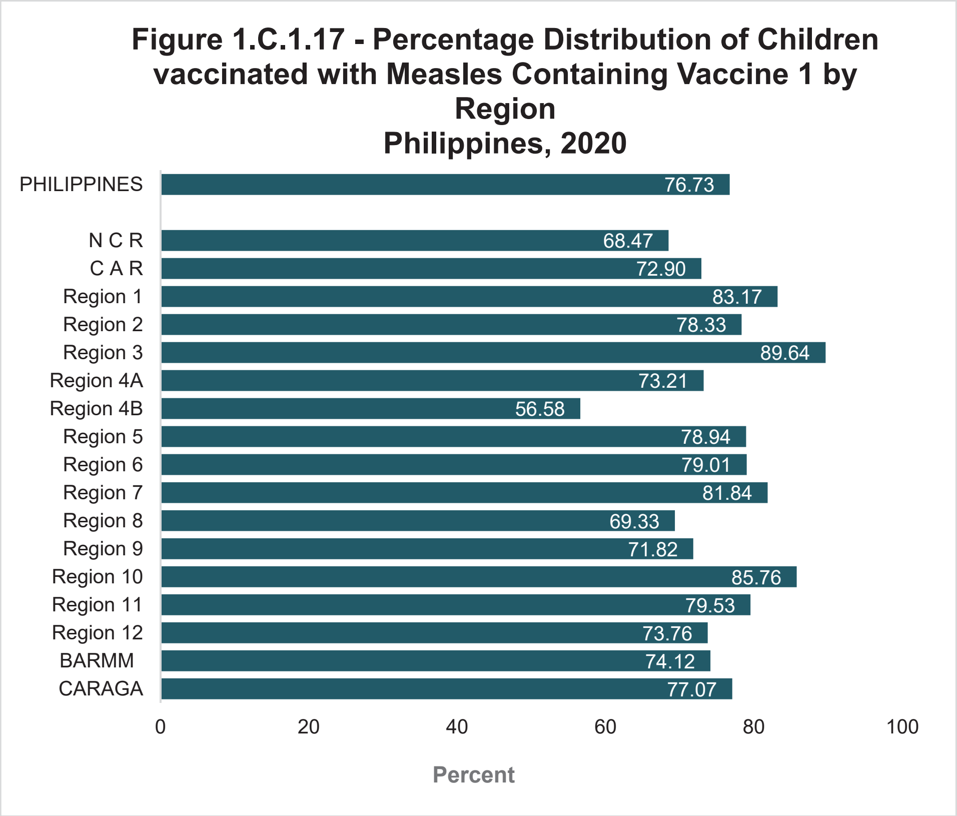
Figure 1.C.1.17 - Percentage Distribution of Children vaccinated with Measles Containing Vaccine 1 by Region
Philippines, 2020
PHILIPPINES 76.73
N C R 68.47
C A R 72.90
Region 1 83.17
Region 2 78.33
Region 3 89.64
Region 4A 73.21
Region 4B 56.58
Region 5 78.94
Region 6 79.01
Region 7 81.84
Region 8 69.33
Region 9 71.82
Region 10 85.76
Region 11 79.53
Region 12 73.76
BARMM 74.12
CARAGA 77.07
0
20
40
60
80
100
Percent

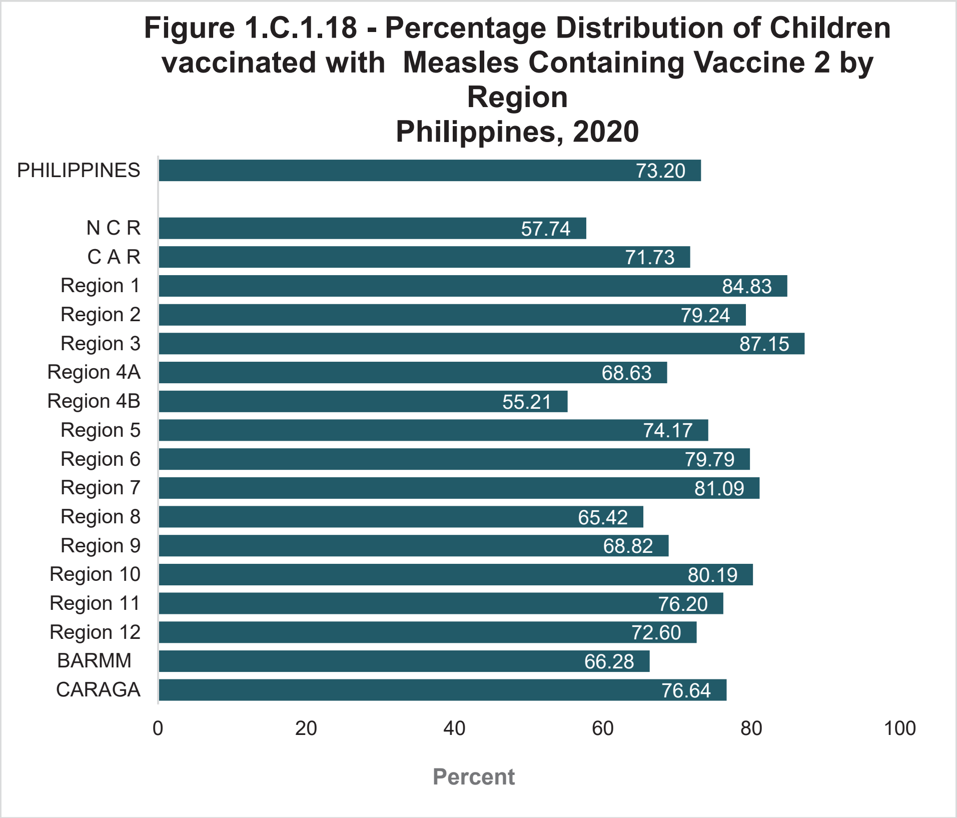
Figure 1.C.1.18 - Percentage Distribution of Children vaccinated with Measles Containing Vaccine 2 by Region
Philippines, 2020
PHILIPPINES 73.20
N C R 57.74
C A R 71.73
Region 1 84.83
Region 2 79.24
Region 3 87.15
Region 4A 68.63
Region 4B 55.21
Region 5 74.17
Region 6 79.79
Region 7 81.09
Region 8 65.42
Region 9 68.82
Region 10 80.19
Region 11 76.20
Region 12 72.60
BARMM 66.28
CARAGA 76.64
0
20
40
60
80
100
Percent

Figure 1.C.1.19 - Percentage Distribution of Children vaccinated with 2 doses of Measles Containing Vaccine Philippines, 2020

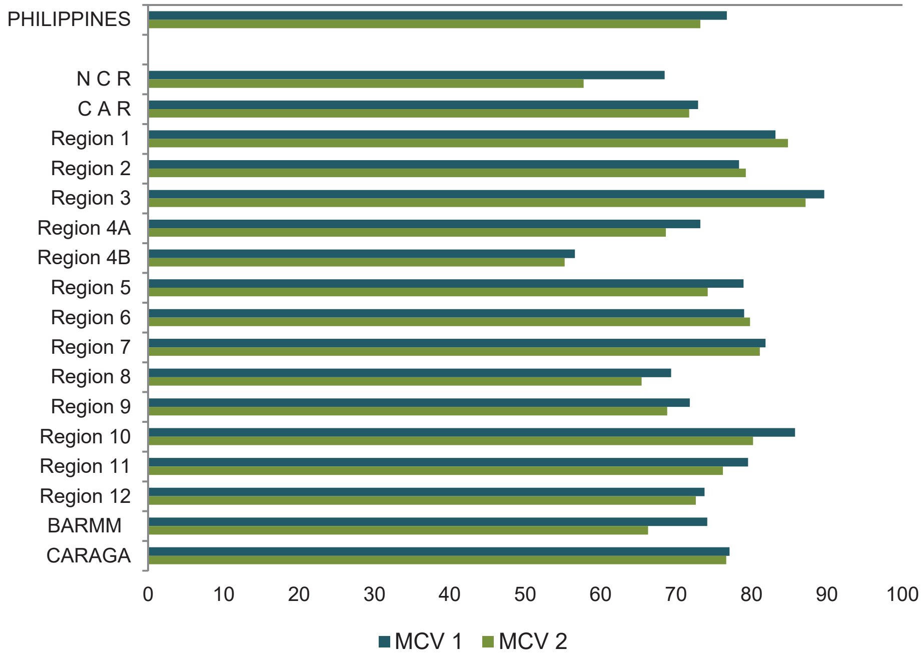

Figure 1.C.1.20- Percentage Distribution of Fully Immunized Children by Region Philippines, 2020

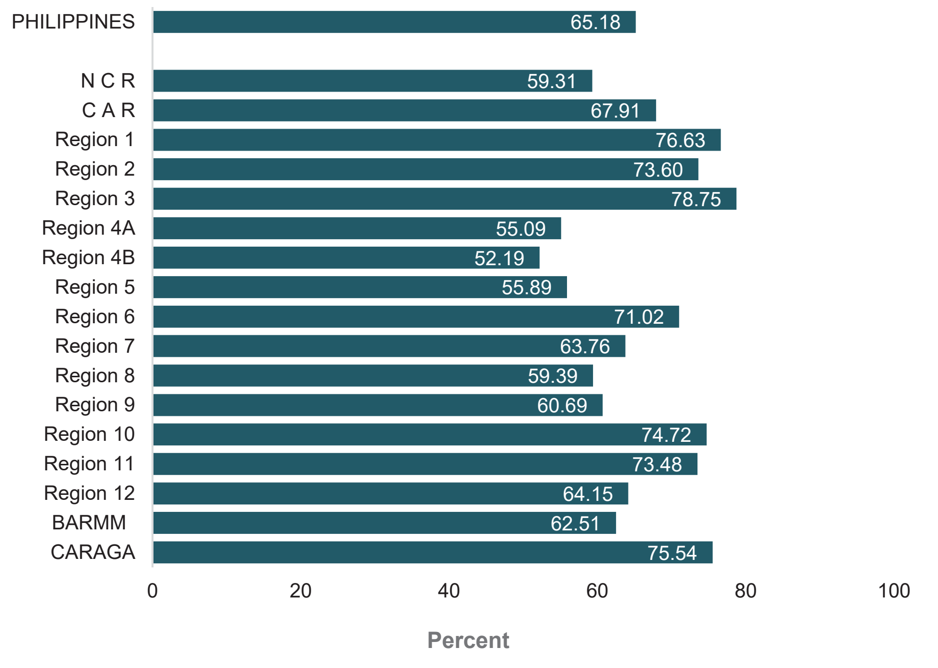

Figure 1.C.1.21 - Percentage Distribution of Completely Immunized Children by Region Philippines, 2020

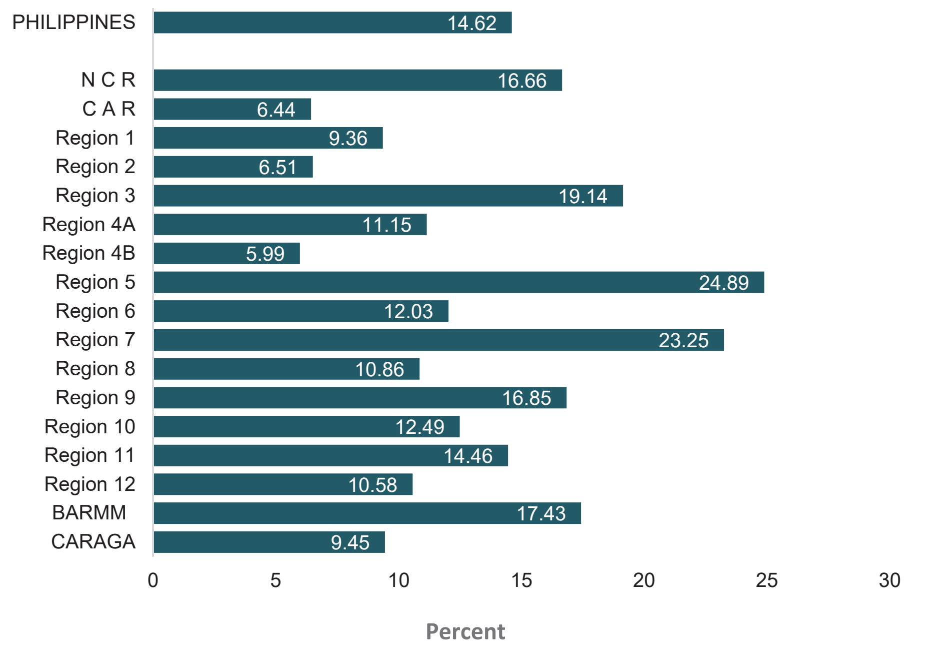

Table 1.C.2.1 - Nutrition Services for Infants and Children

Numbers and Proportion of newborns initiated on breastfeeding immediately after birth lasting for at least 90 minutes, infants exclusively breastfed until 6th month, and infants who continued breastfeeding and were introduced to complementary feeding beginning at 6 months of age

Philippines, Annual 2020

Area	Live births	Newborns initiated on breastfeeding immediately after birth				Eligible Pop (Under 1 y.o.)	Infants Exclusively Breastfed until 6th month				E.Pop (6-11 Mos.)	Infants who continued breastfeeding and were introduced to complementary feeding			
		Male	Female	Total	%		Male	Female	Total	%		Male	Female	Total	%
**PHILIPPINES**	**1,418,039**	**546,469**	**560,896**	**1,107,365**	**78.09**	**2,123,158**	**588,590**	**584,650**	**1,173,240**	**55.26**	**1,061,536**	**527,023**	**525,742**	**1,052,765**	**99.17**
**N C R**	**200,958**	**58,203**	**60,210**	**118,413**	**58.92**	**238,661**	**64,406**	**65,610**	**130,016**	**54.48**	**119,342**	**46,406**	**44,341**	**90,747**	**76.04**
Malabon	4,006	1,336	1,387	2,723	67.97	6,775	2,157	2,161	4,318	63.73	3,385	1,644	1,718	3,362	99.32
Navotas	4,245	1,500	1,478	2,978	70.15	4,621	1,907	1,888	3,795	82.13	2,312	1,138	1,174	2,312	100.00
Valenzuela City	5,556	848	786	1,634	29.41	11,500	3,436	3,360	6,796	59.10	5,752	998	840	1,838	31.95
Caloocan City	18,496	4,861	5,029	9,890	53.47	29,363	3,575	3,515	7,090	24.15	14,674	3,498	3,577	7,075	48.21
Marikina City	9,764	1,984	2,057	4,041	41.39	8,354	1,591	1,515	3,106	37.18	4,177	1,823	1,722	3,545	84.87
Pasig City	14,557	2,047	2,141	4,188	28.77	13,996	1,772	1,804	3,576	25.55	7,001	1,987	1,963	3,950	56.42
Pateros	1,335	434	468	902	67.57	1,184	389	369	758	64.02	590	318	272	590	100.00
Taguig	11,194	1,294	1,380	2,674	23.89	14,918	6,491	7,406	13,897	93.16	7,458	1,564	1,550	3,114	41.75
Quezon City	54,251	13,091	13,770	26,861	49.51	54,413	18,180	18,339	36,519	67.11	27,208	13,651	13,558	27,209	100.00
Makati City	6,834	2,161	2,042	4,203	61.50	10,798	1,679	1,679	3,358	31.10	5,399	2,015	1,917	3,932	72.83
Mandaluyong City	4,179	1,031	988	2,019	48.31	7,160	953	940	1,893	26.44	3,579	919	924	1,843	51.49
San Juan	1,482	324	424	748	50.47	2,261	303	306	609	26.93	1,132	257	267	524	46.29
Manila City	40,150	17,866	19,032	36,898	91.90	32,980	11,785	12,727	24,512	74.32	16,497	7,605	6,450	14,055	85.20
Las Piñas City	7,260	3,246	3,152	6,398	88.13	10,914	3,043	2,915	5,958	54.59	5,461	2,598	2,609	5,207	95.35
Muntinlupa City	7,089	2,231	2,188	4,419	62.34	9,353	3,827	3,477	7,304	78.09	4,677	2,561	2,116	4,677	100.00
Parañaque City	4,726	2,291	2,214	4,505	95.32	12,343	1,970	1,944	3,914	31.71	6,173	2,381	2,257	4,638	75.13
Pasay City	5,834	1,658	1,674	3,332	57.11	7,728	1,348	1,265	2,613	33.81	3,867	1,449	1,427	2,876	74.37
**C A R**	**26,259**	**12,024**	**12,447**	**24,471**	**93.19**	**35,099**	**10,444**	**10,036**	**20,480**	**58.35**	**17,552**	**10,027**	**9,613**	**19,640**	**111.90**
Abra	3,065	1,363	1,670	3,033	98.96	4,309	1,221	1,170	2,391	55.49	2,153	1,205	1,139	2,344	108.87
Apayao	2,355	906	949	1,855	78.77	2,451	750	761	1,511	61.65	1,219	732	744	1,476	121.08
Benguet	5,000	2,392	2,508	4,900	98.00	9,188	2,782	2,600	5,382	58.58	4,584	2,720	2,533	5,253	114.59
Ifugao	2,853	1,370	1,272	2,642	92.60	4,396	1,150	1,062	2,212	50.32	2,199	1,067	1,004	2,071	94.18
Kalinga	2,772	1,365	1,393	2,758	99.49	4,617	1,866	1,769	3,635	78.73	2,314	1,830	1,724	3,554	153.59
Mt. Province	2,823	1,269	1,346	2,615	92.63	3,023	1,043	903	1,946	64.37	1,517	857	753	1,610	106.13
Baguio City	7,391	3,359	3,309	6,668	90.22	7,115	1,632	1,771	3,403	47.83	3,566	1,616	1,716	3,332	93.44
**Region 1**	**55,498**	**27,267**	**26,508**	**53,775**	**96.90**	**97,261**	**36,514**	**34,957**	**71,471**	**73.48**	**48,625**	**33,440**	**32,070**	**65,510**	**134.72**
Ilocos Norte	5,671	2,655	2,632	5,287	93.23	8,105	2,730	2,627	5,357	66.10	4,054	2,576	2,585	5,161	127.31
Ilocos Sur	4,513	2,202	2,232	4,434	98.25	9,330	3,741	3,725	7,466	80.02	4,655	2,828	2,797	5,625	120.84
La Union	3,556	1,722	1,654	3,376	94.94	11,511	4,296	4,155	8,451	73.42	5,763	4,464	4,271	8,735	151.57
Pangasinan	15,936	7,618	8,043	15,661	98.27	50,168	19,092	18,138	37,230	74.21	25,081	17,250	16,551	33,801	134.77
Alaminos City	1,720	921	799	1,720	100.00	1,894	976	825	1,801	95.09	949	972	825	1,797	189.36
Candon City	1,018	550	458	1,008	99.02	987	469	402	871	88.25	491	502	370	872	177.60
Dagupan City	8,771	4,636	4,135	8,771	100.00	3,620	750	795	1,545	42.68	1,809	679	718	1,397	77.22
Laoag City	1,959	945	935	1,880	95.97	1,870	881	914	1,795	95.99	934	605	615	1,220	130.62
San Carlos City	2,876	1,474	1,360	2,834	98.54	3,979	1,339	1,277	2,616	65.75	1,990	1,317	1,255	2,572	129.25
San Fernando City	5,567	2,855	2,695	5,550	99.69	2,115	787	704	1,491	70.50	1,055	710	649	1,359	128.82
Urdaneta City	2,088	847	841	1,688	80.84	2,806	942	920	1,862	66.36	1,407	1,022	981	2,003	142.36
Vigan City	1,823	842	724	1,566	85.90	876	511	475	986	112.56	437	515	453	968	221.51
**Region 2**	**42,744**	**19,753**	**19,876**	**39,629**	**92.71**	**69,443**	**21,521**	**21,302**	**42,823**	**61.67**	**34,688**	**15,998**	**15,991**	**31,989**	**92.22**
Batanes	246	122	119	241	97.97	340	90	100	190	55.88	171	80	87	167	97.66
Cagayan	9,758	4,848	4,794	9,642	98.81	17,971	6,370	6,113	12,483	69.46	8,970	5,206	5,078	10,284	114.65
Isabela	12,463	6,121	6,169	12,290	98.61	26,309	6,453	6,312	12,765	48.52	13,158	5,394	5,277	10,671	81.10
Nueva Vizcaya	7,520	2,784	2,794	5,578	74.18	9,573	3,196	3,256	6,452	67.40	4,784	2,323	2,462	4,785	100.02
Quirino	3,009	1,281	1,216	2,497	82.98	4,025	1,270	1,352	2,622	65.14	2,005	873	775	1,648	82.19
															0.00
Cauayan City	1,954	977	972	1,949	99.74	2,590	760	797	1,557	60.12	1,289	555	583	1,138	88.29
Ilagan City	2,797	1,226	1,370	2,596	92.81	2,911	1,037	1,100	2,137	73.41	1,451	512	695	1,207	83.18
Santiago City	2,952	1,462	1,490	2,952	100.00	2,692	1,351	1,334	2,685	99.74	1,343	401	378	779	58.00
Tuguegarao City	2,045	932	952	1,884	92.13	3,032	994	938	1,932	63.72	1,517	654	656	1,310	86.35
**Region 3**	**131,036**	**55,916**	**58,196**	**114,112**	**87.08**	**220,020**	**57,970**	**57,019**	**114,989**	**52.26**	**109,998**	**50,594**	**49,397**	**99,991**	**90.90**
Aurora	3,217	1,324	1,290	2,614	81.26	4,770	1,627	1,645	3,272	68.60	2,383	1,477	1,383	2,860	120.02
Bataan	6,272	4,642	4,588	9,230	147.16	13,823	5,085	4,880	9,965	72.09	6,915	4,378	4,142	8,520	123.21
Bulacan	19,883	10,199	10,850	21,049	105.86	43,627	7,890	7,858	15,748	36.10	21,799	7,477	7,364	14,841	68.08
Nueva Ecija	15,082	4,235	4,362	8,597	57.00	28,782	7,629	7,106	14,735	51.20	14,389	5,768	5,445	11,213	77.93
Pampanga	12,800	5,149	5,249	10,398	81.23	31,359	4,851	4,997	9,848	31.40	15,673	4,372	4,479	8,851	56.47
Tarlac	13,439	5,583	6,091	11,674	86.87	19,765	7,410	7,020	14,430	73.01	9,896	6,057	5,626	11,683	118.06
Zambales	7,466	3,141	3,036	6,177	82.74	12,610	2,696	2,483	5,179	41.07	6,309	2,118	2,016	4,134	65.53
Angeles City	7,414	640	704	1,344	18.13	7,869	1,456	1,962	3,418	43.44	3,937	1,528	1,707	3,235	82.17

Table 1.C.2.1 - Nutrition Services for Infants and Children

Numbers and Proportion of newborns initiated on breastfeeding immediately after birth lasting for at least 90 minutes, infants exclusively breastfed until 6th month, and infants who continued breastfeeding and were introduced to complementary feeding beginning at 6 months of age

Philippines, Annual 2020

Area	Live births	Newborns initiated on breastfeeding immediately after birth				Eligible Pop (Under 1 y.o.)	Infants Exclusively Breastfed until 6th month				E.Pop (6-11 Mos.)	Infants who continued breastfeeding and were introduced to complementary feeding			
		Male	Female	Total	%		Male	Female	Total	%		Male	Female	Total	%
Balanga City	1,444	701	679	1,380	95.57	1,998	632	606	1,238	61.96	999	615	590	1,205	120.62
Cabanatuan City	8,036	4,180	3,831	8,011	99.69	5,896	1,471	1,460	2,931	49.71	2,946	1,650	1,682	3,332	113.10
City of San Fernando	2,219	290	336	626	28.21	5,863	2,282	2,150	4,432	75.59	2,930	1,866	1,800	3,666	125.12
Gapan City	1,162	509	544	1,053	90.62	2,153	712	709	1,421	66.00	1,077	661	682	1,343	124.70
Mabalacat City	4,632	1,535	1,732	3,267	70.53	4,793	1,431	1,486	2,917	60.86	2,396	1,209	1,264	2,473	103.21
Malolos City	4,797	1,128	1,299	2,427	50.59	4,880	887	828	1,715	35.14	2,436	788	736	1,524	62.56
Meycauayan	2,536	1,409	1,561	2,970	117.11	4,041	1,589	1,626	3,215	79.56	2,019	1,500	1,493	2,993	148.24
Olongapo	4,246	1,409	1,395	2,804	66.04	4,971	768	739	1,507	30.32	2,485	617	581	1,198	48.21
Palayan City	188	84	93	177	94.15	799	319	320	639	79.97	399	328	329	657	164.66
San Jose City	2,208	1,106	1,002	2,108	95.47	2,718	1,411	1,315	2,726	100.29	1,367	1,010	926	1,936	141.62
San Jose del Monte City	6,619	6,035	6,233	12,268	185.35	11,100	4,762	4,589	9,351	84.24	5,545	4,325	4,140	8,465	152.66
Science City of Munoz	70	32	38	70	100.00	1,586	639	585	1,224	77.18	794	640	584	1,224	154.16
Tarlac City	7,306	2,585	3,283	5,868	80.32	6,617	2,423	2,655	5,078	76.74	3,304	2,210	2,428	4,638	140.38
**Region 4A**	**171,140**	**65,240**	**67,279**	**132,519**	**77.43**	**296,816**	**62,034**	**60,076**	**122,110**	**41.14**	**148,421**	**66,586**	**64,264**	**130,850**	**88.16**
Batangas	15,862	7,304	7,095	14,399	90.78	38,441	9,109	8,481	17,590	45.76	19,225	9,905	9,304	19,209	99.92
Cavite	23,136	9,570	9,996	19,566	84.57	27,704	4,509	4,515	9,024	32.57	13,848	6,021	6,109	12,130	87.59
Laguna	12,308	4,613	4,645	9,258	75.22	19,727	5,105	4,878	9,983	50.61	9,871	5,046	4,682	9,728	98.55
Quezon	15,625	5,902	5,690	11,592	74.19	37,410	9,791	8,976	18,767	50.17	18,698	10,701	10,122	20,823	111.36
Rizal	23,951	9,282	9,466	18,748	78.28	44,791	6,468	6,613	13,081	29.20	22,402	6,330	6,396	12,726	56.81
Antipolo City	10,703	830	901	1,731	16.17	16,497	2,445	2,415	4,860	29.46	8,249	1,645	1,659	3,304	40.05
Bacoor City	6,609	2,068	2,341	4,409	66.71	12,142	1,608	1,597	3,205	26.40	6,071	983	1,215	2,198	36.20
Batangas City	0	0	0	0	0.00	6,823	737	677	1,414	20.72	3,414	2,161	1,964	4,125	120.83
Biñan City	8,419	3,822	4,024	7,846	93.19	6,607	3,448	3,591	7,039	106.54	3,305	3,001	3,055	6,056	183.24
Cabuyao City	4,392	2,115	2,446	4,561	103.85	6,130	2,012	2,104	4,116	67.15	3,066	1,779	1,837	3,616	117.94
Calamba City	8,081	3,569	3,672	7,241	89.61	9,028	3,891	3,848	7,739	85.72	4,511	3,629	3,523	7,152	158.55
Cavite City	1,102	187	233	420	38.11	2,075	91	108	199	9.59	1,036	109	131	240	23.17
Dasmariñas City	8,571	2,142	2,379	4,521	52.75	13,322	1,854	1,978	3,832	28.76	6,659	2,114	2,115	4,229	63.51
General Trias City	1,410	1,377	1,438	2,815	199.65	6,352	748	714	1,462	23.02	3,180	977	941	1,918	60.31
Imus City	4,086	1,393	1,512	2,905	71.10	8,156	906	930	1,836	22.51	4,081	753	812	1,565	38.35
Lipa City	0	0	0	0	0.00	6,880	118	126	244	3.55	3,435	1,640	1,519	3,159	91.97
Lucena City	0	0	0	0	0.00	5,672	1,127	999	2,126	37.48	2,835	1,747	1,505	3,252	114.71
San Pablo City	2,037	1,410	1,402	2,812	138.05	5,277	960	891	1,851	35.08	2,648	737	670	1,407	53.13
San Pedro City	1,340	1,378	1,374	2,752	205.37	6,467	1,311	1,274	2,585	39.97	3,233	1,211	1,139	2,350	72.69
Santa Rosa City	8,276	1,837	2,366	4,203	50.79	7,025	2,029	1,997	4,026	57.31	3,512	2,078	1,961	4,039	115.01
Tagaytay City	2,778	1,482	1,322	2,804	100.94	1,437	1,606	1,384	2,990	208.07	722	1,570	1,358	2,928	405.54
Tanauan City	4,390	1,074	1,144	2,218	50.52	3,584	350	349	699	19.50	1,788	648	618	1,266	70.81
Tayabas City	1,594	781	801	1,582	99.25	2,122	851	785	1,636	77.10	1,057	849	785	1,634	154.59
Trece Martires City	6,470	3,104	3,032	6,136	94.84	3,147	960	846	1,806	57.39	1,575	952	844	1,796	114.03
**Region 4B**	**43,771**	**13,760**	**13,314**	**27,074**	**61.85**	**71,246**	**13,999**	**13,486**	**27,485**	**38.58**	**35,617**	**12,308**	**11,748**	**24,056**	**67.54**
Marinduque	3,247	1,686	1,552	3,238	99.72	4,836	1,364	1,254	2,618	54.14	2,425	1,384	1,267	2,651	109.32
Mindoro Occidental	7,924	3,544	3,333	6,877	86.79	12,407	4,106	3,948	8,054	64.91	6,206	4,268	4,061	8,329	134.21
Mindoro Oriental	12,260	4,616	4,431	9,047	73.79	19,809	4,361	4,294	8,655	43.69	9,906	3,037	2,979	6,016	60.73
Palawan	14,536	2,617	2,719	5,336	36.71	21,375	3,295	3,030	6,325	29.59	10,665	2,957	2,779	5,736	53.78
Romblon	2,101	662	656	1,318	62.73	6,400	583	651	1,234	19.28	3,208	662	662	1,324	41.27
Puerto Princesa City	3,703	635	623	1,258	33.97	6,419	290	309	599	9.33	3,207	0	0	0	0.00
**Region 5**	**79,292**	**32,147**	**32,772**	**64,919**	**81.87**	**136,116**	**35,656**	**35,183**	**70,839**	**52.04**	**68,083**	**34,772**	**33,682**	**68,454**	**100.54**
Albay	12,132	5,057	5,015	10,072	83.02	23,737	5,888	5,769	11,657	49.11	11,865	4,981	4,986	9,967	84.00
Camarines Norte	11,377	5,507	5,465	10,972	96.44	14,283	4,663	4,599	9,262	64.85	7,140	4,336	4,096	8,432	118.10
Camarines Sur	17,226	7,356	7,075	14,431	83.77	38,796	11,886	11,792	23,678	61.03	19,419	10,982	10,905	21,887	112.71
Catanduanes	4,550	1,875	1,973	3,848	84.57	6,274	1,961	2,032	3,993	63.64	3,136	1,768	1,819	3,587	114.38
Masbate	12,727	4,384	5,453	9,837	77.29	22,521	7,294	7,083	14,377	63.84	11,264	4,765	4,409	9,174	81.45
Sorsogon	13,129	4,963	4,777	9,740	74.19	19,065	748	850	1,598	8.38	9,543	4,784	4,660	9,444	98.96
Iriga City	2,156	565	559	1,124	52.13	2,638	736	692	1,428	54.13	1,317	815	490	1,305	99.09
Legaspi City	3,361	1,126	1,159	2,285	67.99	4,174	1,389	1,299	2,688	64.40	2,086	1,303	1,314	2,617	125.46
Naga City	2,634	1,314	1,296	2,610	99.09	4,628	1,091	1,067	2,158	46.63	2,313	1,038	1,003	2,041	88.24
**Region 6**	**96,092**	**38,856**	**38,606**	**77,462**	**80.61**	**146,526**	**31,347**	**30,629**	**61,976**	**42.30**	**73,242**	**29,406**	**28,514**	**57,920**	**79.08**
Aklan	6,953	2,950	3,026	5,976	85.95	11,162	3,195	3,295	6,490	58.14	5,577	3,318	3,291	6,609	118.50
Antique	8,727	3,356	3,181	6,537	74.91	12,816	3,074	2,967	6,041	47.14	6,396	2,937	2,789	5,726	89.52
Capiz	7,412	3,353	3,327	6,680	90.12	13,986	3,503	3,450	6,953	49.71	6,993	3,230	3,190	6,420	91.81
Guimaras	2,474	1,246	1,144	2,390	96.60	3,085	1,138	1,032	2,170	70.34	1,548	1,136	1,103	2,239	144.64
Iloilo	20,578	8,723	8,848	17,571	85.39	36,541	9,992	9,641	19,633	53.73	18,256	9,004	8,835	17,839	97.72
Negros Occidental	38,413	15,376	15,649	31,025	80.77	49,368	7,459	7,503	14,962	30.31	24,696	6,962	6,584	13,546	54.85
Bacolod City	6,112	1,362	904	2,266	37.07	11,115	980	695	1,675	15.07	5,550	893	811	1,704	30.70
Iloilo City	5,423	2,490	2,527	5,017	92.51	8,453	2,006	2,046	4,052	47.94	4,226	1,926	1,911	3,837	90.80

Table 1.C.2.1 - Nutrition Services for Infants and Children

Numbers and Proportion of newborns initiated on breastfeeding immediately after birth lasting for at least 90 minutes, infants exclusively breastfed until 6th month, and infants who continued breastfeeding and were introduced to complementary feeding beginning at 6 months of age

Philippines, Annual 2020

Area	Live births	Newborns initiated on breastfeeding immediately after birth				Eligible Pop (Under 1 y.o.)	Infants Exclusively Breastfed until 6th month				E.Pop (6-11 Mos.)	Infants who continued breastfeeding and were introduced to complementary feeding			
		Male	Female	Total	%		Male	Female	Total	%		Male	Female	Total	%
**Region 7**	**111,658**	**41,418**	**41,483**	**82,901**	**74.25**	**163,262**	**52,360**	**50,513**	**102,873**	**63.01**	**81,628**	**51,125**	**50,304**	**101,429**	**124.26**
Bohol	19,885	7,744	6,897	14,641	73.63	27,312	7,975	7,433	15,408	56.41	13,659	8,324	8,137	16,461	120.51
Cebu	35,946	14,954	14,847	29,801	82.90	67,506	22,619	21,064	43,683	64.71	33,732	21,411	19,800	41,211	122.17
Negros Oriental	20,092	5,064	5,805	10,869	54.10	27,938	9,812	10,555	20,367	72.90	13,975	10,238	11,185	21,423	153.30
Siquijor	1,250	659	578	1,237	98.96	1,613	651	572	1,223	75.82	812	491	437	928	114.29
Cebu City	16,345	7,857	8,242	16,099	98.49	21,193	5,228	5,146	10,374	48.95	10,591	4,595	4,556	9,151	86.40
Lapu-Lapu City	9,110	3,707	3,721	7,428	81.54	9,372	4,000	3,752	7,752	82.71	4,688	3,403	3,243	6,646	141.77
Mandaue City	9,030	1,433	1,393	2,826	31.30	8,328	2,075	1,991	4,066	48.82	4,171	2,663	2,946	5,609	134.48
**Region 8**	**60,669**	**17,972**	**18,654**	**36,626**	**60.37**	**102,619**	**21,762**	**21,306**	**43,068**	**41.97**	**51,272**	**18,125**	**18,016**	**36,141**	**70.49**
Biliran	3,102	1,417	1,307	2,724	87.81	3,834	1,198	1,132	2,330	60.77	1,909	989	915	1,904	99.74
Eastern Samar	6,421	2,425	2,801	5,226	81.39	11,392	2,446	2,411	4,857	42.64	5,709	2,426	2,474	4,900	85.83
Northern Leyte	18,116	3,267	3,158	6,425	35.47	34,707	4,304	4,059	8,363	24.10	17,351	3,369	3,246	6,615	38.12
Northern Samar	9,702	2,838	3,262	6,100	62.87	15,370	3,975	4,169	8,144	52.99	7,661	3,487	3,659	7,146	93.28
Southern Leyte	2,460	902	966	1,868	75.93	6,451	1,789	1,864	3,653	56.63	3,223	1,589	1,635	3,224	100.03
Western Samar	8,158	3,121	2,977	6,098	74.75	14,305	3,593	3,521	7,114	49.73	7,148	3,236	3,171	6,407	89.63
	0	0	0				0	0				0	0		
Calbayog City	2,119	720	781	1,501	70.84	4,413	831	707	1,538	34.85	2,200	668	583	1,251	56.86
Maasin City	2,620	255	331	586	22.37	1,637	283	271	554	33.84	822	254	242	496	60.34
Ormoc City	5,243	1,786	1,699	3,485	66.47	4,941	1,968	1,838	3,806	77.03	2,469	1,246	1,219	2,465	99.84
Tacloban City	2,728	1,241	1,372	2,613	95.78	5,569	1,375	1,334	2,709	48.64	2,780	861	872	1,733	62.34
**Region 9**	**54,911**	**20,772**	**23,358**	**44,130**	**80.37**	**80,051**	**20,949**	**22,438**	**43,387**	**54.20**	**40,010**	**8,380**	**8,911**	**17,291**	**43.22**
Zamboanga del Norte	15,986	7,294	8,122	15,416	96.43	17,249	6,929	7,131	14,060	81.51	8,617	1,258	1,268	2,526	29.31
Zamboanga del Sur	9,546	3,356	3,660	7,016	73.50	17,653	3,797	4,019	7,816	44.28	8,824	1,101	1,166	2,267	25.69
Zamboanga Sibugay	7,654	2,888	3,738	6,626	86.57	14,954	2,773	3,168	5,941	39.73	7,476	2,464	2,845	5,309	71.01
Dapitan City	1,736	624	851	1,475	84.97	1,784	478	564	1,042	58.41	888	76	110	186	20.95
Dipolog City	2,648	1,301	1,313	2,614	98.72	2,827	1,125	1,179	2,304	81.50	1,411	156	143	299	21.19
Isabela City	1,897	687	678	1,365	71.96	2,522	662	737	1,399	55.47	1,256	637	680	1,317	104.86
Pagadian City	2,891	1,358	1,278	2,636	91.18	4,325	1,261	1,272	2,533	58.57	2,167	518	575	1,093	50.44
Zamboanga City	12,553	3,264	3,718	6,982	55.62	18,737	3,924	4,368	8,292	44.25	9,371	2,170	2,124	4,294	45.82
**Region 10**	**82,736**	**33,909**	**36,648**	**70,557**	**85.28**	**101,411**	**40,214**	**40,242**	**80,456**	**79.34**	**50,699**	**38,665**	**38,361**	**77,026**	**151.93**
Bukidnon	19,766	8,003	8,713	16,716	84.57	23,706	9,406	9,522	18,928	79.84	11,844	8,799	8,876	17,675	149.23
Camiguin	1,300	667	624	1,291	99.31	1,858	567	531	1,098	59.10	929	637	566	1,203	129.49
Lanao del Norte	10,411	3,606	4,317	7,923	76.10	14,960	4,379	4,553	8,932	59.71	7,492	3,943	4,183	8,126	108.46
Misamis Occidental	4,585	1,594	2,095	3,689	80.46	6,403	2,825	3,174	5,999	93.69	3,189	2,629	3,127	5,756	180.50
Misamis Oriental	9,589	4,232	4,863	9,095	94.85	15,131	5,272	5,439	10,711	70.79	7,567	5,199	5,120	10,319	136.37
Cagayan de Oro City	17,506	6,316	6,279	12,595	71.95	14,339	7,276	6,650	13,926	97.12	7,168	8,107	7,115	15,222	212.36
El Salvador City	326	172	153	325	99.69	1,065	410	439	849	79.72	535	407	435	842	157.38
Gingoog City	2,220	1,031	1,013	2,044	92.07	2,644	1,082	1,093	2,175	82.26	1,326	1,082	1,072	2,154	162.44
Iligan City	6,744	3,517	3,238	6,755	100.16	7,580	3,531	3,472	7,003	92.39	3,790	3,446	3,399	6,845	180.61
Malaybalay City	2,516	1,165	1,182	2,347	93.28	3,956	1,181	1,064	2,245	56.75	1,976	448	378	826	41.80
Oroquieta City	917	445	459	904	98.58	1,389	362	384	746	53.71	693	332	351	683	98.56
Ozamis City	2,166	1,040	1,179	2,219	102.45	2,778	1,527	1,680	3,207	115.44	1,389	1,435	1,581	3,016	217.13
Tangub City	1,046	549	494	1,043	99.71	1,234	593	435	1,028	83.31	620	576	541	1,117	180.16
Valencia City	3,644	1,572	2,039	3,611	99.09	4,368	1,803	1,806	3,609	82.62	2,181	1,625	1,617	3,242	148.65
**Region 11**	**84,001**	**39,575**	**36,945**	**76,520**	**91.09**	**107,247**	**36,047**	**33,716**	**69,763**	**65.05**	**53,626**	**37,996**	**35,772**	**73,768**	**137.56**
Compostela Valley	11,860	6,324	5,736	12,060	101.69	15,562	6,012	5,452	11,464	73.67	7,781	6,179	5,681	11,860	152.42
Davao del Norte	20,226	9,744	9,310	19,054	94.21	21,326	8,011	7,779	15,790	74.04	10,661	8,580	8,314	16,894	158.47
Davao del Sur	9,256	3,503	3,199	6,702	72.41	13,007	3,562	3,232	6,794	52.23	6,508	4,108	3,690	7,798	119.82
Davao Oriental	8,249	4,130	3,770	7,900	95.77	14,151	4,609	4,250	8,859	62.60	7,065	4,953	4,531	9,484	134.24
Davao Occidental	4,275	1,228	1,079	2,307	53.96	6,670	1,698	1,585	3,283	49.22	3,347	1,669	1,581	3,250	97.10
Davao City	30,135	14,646	13,851	28,497	94.56	36,531	12,155	11,418	23,573	64.53	18,264	12,507	11,975	24,482	134.05
**Region 12**	**68,492**	**32,465**	**33,206**	**65,671**	**95.88**	**104,552**	**37,900**	**39,009**	**76,909**	**73.56**	**52,301**	**36,629**	**45,390**	**82,019**	**156.82**
North Cotabato	17,482	8,700	8,563	17,263	98.75	33,645	11,670	11,054	22,724	67.54	16,840	10,954	19,992	30,946	183.76
Sarangani	10,279	4,720	5,418	10,138	98.63	12,891	5,629	5,762	11,391	88.36	6,446	5,237	5,454	10,691	165.85
South Cotabato	16,880	8,214	8,500	16,714	99.02	21,113	8,514	8,521	17,035	80.68	10,554	7,225	7,236	14,461	137.02
Sultan Kudarat	12,234	5,890	5,465	11,355	92.82	17,359	6,879	6,693	13,572	78.18	8,690	8,973	8,580	17,553	201.99
Cotabato City	5,505	2,716	2,718	5,434	98.71	5,835	1,313	1,333	2,646	45.35	2,920	1,511	1,314	2,825	96.75
Gen. Santos City	6,112	2,225	2,542	4,767	77.99	13,709	3,895	5,646	9,541	69.60	6,851	2,729	2,814	5,543	80.91

Table 1.C.2.1 - Nutrition Services for Infants and Children

Numbers and Proportion of newborns initiated on breastfeeding immediately after birth lasting for at least 90 minutes, infants exclusively breastfed until 6th month, and infants who continued breastfeeding and were introduced to complementary feeding beginning at 6 months of age

Philippines, Annual 2020

Area	Live births	Newborns initiated on breastfeeding immediately after birth				Eligible Pop (Under 1 y.o.)	Infants Exclusively Breastfed until 6th month				E.Pop (6-11 Mos.)	Infants who continued breastfeeding and were introduced to complementary feeding			
		Male	Female	Total	%		Male	Female	Total	%		Male	Female	Total	%
**BARMM**	**70,432**	**22,188**	**25,071**	**47,259**	**67.10**	**92,799**	**27,603**	**30,385**	**57,988**	**62.49**	**46,406**	**20,654**	**22,697**	**43,351**	**93.42**
Basilan	3,125	889	959	1,848	59.14	7,541	607	751	1,358	18.01	3,765	669	785	1,454	38.62
Lanao del Sur	20,702	8,374	10,053	18,427	89.01	21,131	8,793	10,447	19,240	91.05	10,552	8,520	9,894	18,414	174.51
Maguindanao	25,491	9,955	10,910	20,865	81.85	31,128	10,468	10,933	21,401	68.75	15,563	8,975	9,379	18,354	117.93
Sulu	10,064	807	907	1,714	17.03	16,613	3,518	3,656	7,174	43.18	8,328	815	914	1,729	20.76
Tawi-Tawi	6,881	862	911	1,773	25.77	9,259	2,785	3,019	5,804	62.68	4,635	975	958	1,933	41.70
Lamitan City	1,102	649	656	1,305	118.42	2,074	541	628	1,169	56.36	1,032	507	561	1,068	103.49
Marawi City	3,067	652	675	1,327	43.27	5,053	891	951	1,842	36.45	2,531	193	206	399	15.76
**CARAGA**	**38,350**	**15,004**	**16,323**	**31,327**	**81.69**	**60,029**	**17,864**	**18,743**	**36,607**	**60.98**	**30,026**	**15,912**	**16,671**	**32,583**	**108.52**
Agusan del Norte	3,513	1,276	1,319	2,595	73.87	8,098	2,327	2,419	4,746	58.61	4,058	2,230	2,261	4,491	110.67
Agusan del Sur	11,887	3,211	4,008	7,219	60.73	17,592	5,202	5,528	10,730	60.99	8,786	4,849	5,018	9,867	112.30
Surigao del Norte	3,514	1,478	1,847	3,325	94.62	7,089	2,063	2,142	4,205	59.32	3,552	1,563	1,648	3,211	90.40
Surigao del Sur	6,439	2,995	3,081	6,076	94.36	11,482	3,446	3,647	7,093	61.77	5,740	3,237	3,425	6,662	116.06
Province of Dinagat	1,304	639	665	1,304	100.00	2,573	595	625	1,220	47.42	1,289	572	664	1,236	95.89
Bislig City	1,658	813	845	1,658	100.00	2,179	702	691	1,393	63.93	1,088	549	583	1,132	104.04
Butuan City	5,648	2,798	2,830	5,628	99.65	7,715	2,528	2,663	5,191	67.28	3,855	1,960	2,087	4,047	104.98
Surigao City	4,387	1,794	1,728	3,522	80.28	3,301	1,001	1,028	2,029	61.47	1,658	952	985	1,937	116.83

* - No Report

0 - No Cases

Table 1.C.2.2- Nutrition Services for Infants and Children

Number of Infants born preterm or with low birth weight given iron supplements and infants 6-11 mos. old and children 12-59 mos. old given Vitamin A supplementation
Philippines, Annual 2020

Area	Total No. of live births with low birth weight	Infants preterm or with LBW (< 2500 grams) given Iron				Eligible Pop (6-11 months)	Infants 6-11 mos. old Given Vitamin A				Eligible Pop (12-59 months)	Children 12-59 mos. Old Given Vitamin A			
		Male	Female	Total	%		Male	Female	Total	%		Male	Female	Total	%
**PHILIPPINES**	**88,513**	**12,211**	**13,412**	**25,623**	**28.95**	**1,061,536**	**700,037**	**688,643**	**1,388,680**	**130.82**	**8,941,598**	**2,657,271**	**2,700,009**	**5,357,280**	**59.91**
**NCR**	**21,479**	**1,689**	**1,796**	**3,485**	**16.23**	**119,342**	**109,489**	**116,539**	**226,028**	**189.40**	**959,072**	**255,244**	**311,786**	**567,030**	**59.12**
Malabon	142	38	37	75	52.82	3,385	2,855	2,709	5,564	164.37	27,220	4,252	4,233	8,485	31.17
Navotas	132	55	55	110	83.33	2,312	3,001	3,138	6,139	265.53	18,580	3,839	3,803	7,642	41.13
Valenzuela City	323	3	2	5	1.55	5,752	2,490	2,300	4,790	83.28	46,210	11,546	9,908	21,454	46.43
Caloocan City	919	31	34	65	7.07	14,674	8,339	7,877	16,216	110.51	117,972	38,349	36,457	74,806	63.41
Marikina City	1,130	96	70	166	14.69	4,177	2,427	2,296	4,723	113.07	33,571	1,439	1,438	2,877	8.57
Pasig City	1,979	17	14	31	1.57	7,001	4,245	4,255	8,500	121.41	56,251	3,801	3,634	7,435	13.22
Pateros	96	1	2	3	3.13	590	563	489	1,052	178.31	4,755	1,106	1,293	2,399	50.45
Taguig	1,065	407	458	865	81.22	7,458	11,722	12,430	24,152	323.84	59,949	32,426	33,452	65,878	109.89
Quezon City	7,485	349	359	708	9.46	27,208	26,179	26,338	52,517	193.02	218,674	42,545	43,950	86,495	39.55
Makati City	719	69	107	176	24.48	5,399	2,916	2,789	5,705	105.67	43,390	14,966	14,101	29,067	66.99
Mandaluyong City	156	14	18	32	20.51	3,579	1,510	1,473	2,983	83.35	28,766	3,239	3,201	6,440	22.39
San Juan	108	25	14	39	36.11	1,132	646	605	1,251	110.51	9,101	1,219	1,116	2,335	25.66
Manila City	6,041	377	414	791	13.09	16,497	31,179	38,882	70,061	424.69	132,587	52,736	111,490	164,226	123.86
Las Piñas City	127	18	18	36	28.35	5,461	4,512	4,280	8,792	161.00	43,860	7,794	7,597	15,391	35.09
Muntinlupa City	430	113	113	226	52.56	4,677	2,610	2,638	5,248	112.21	37,574	19,546	19,876	39,422	104.92
Parañaque City	475	0	0	0	0.00	6,173	2,320	2,175	4,495	72.82	49,589	12,188	12,026	24,214	48.83
Pasay City	152	76	81	157	103.29	3,867	1,975	1,865	3,840	99.30	31,023	4,253	4,211	8,464	27.28
**C A R**	**1,788**	**174**	**209**	**383**	**21.42**	**17,552**	**8,845**	**8,449**	**17,294**	**98.53**	**146,939**	**45,786**	**43,371**	**89,157**	**60.68**
Abra	167	31	33	64	38.32	2,153	1,082	1,070	2,152	99.95	19,128	6,877	6,307	13,184	68.93
Apayao	197	25	35	60	30.46	1,219	592	573	1,165	95.57	10,841	6,400	6,615	13,015	120.05
Benguet	334	24	21	45	13.47	4,584	2,317	2,146	4,463	97.36	36,459	11,072	10,040	21,112	57.91
Ifugao	105	4	7	11	10.48	2,199	1,087	1,030	2,117	96.27	19,116	7,400	6,977	14,377	75.21
Kalinga	135	24	47	71	52.59	2,314	1,188	1,126	2,314	100.00	19,738	4,668	4,668	9,336	47.30
Mt. Province	190	30	22	52	27.37	1,517	792	726	1,518	100.07	13,439	3,952	3,638	7,590	56.48
Baguio City	660	36	44	80	12.12	3,566	1,787	1,778	3,565	99.97	28,218	5,417	5,126	10,543	37.36
**Region 1**	**3,325**	**317**	**340**	**657**	**19.76**	**48,625**	**37,872**	**37,388**	**75,260**	**154.78**	**407,689**	**139,622**	**136,894**	**276,516**	**67.83**
Ilocos Norte	200	49	41	90	45.00	4,054	5,531	7,373	12,904	318.30	33,003	16,793	16,307	33,100	100.29
Ilocos Sur	163	37	49	86	52.76	4,655	5,359	4,913	10,272	220.67	40,744	22,219	19,864	42,083	103.29
La Union	171	30	38	68	39.77	5,763	6,088	5,600	11,688	202.81	48,703	32,484	30,930	63,414	130.21
Pangasinan	424	150	155	305	71.93	25,081	13,333	12,478	25,811	102.91	209,264	40,510	40,523	81,033	38.72
Alaminos City	0	1	0	1	0.00	949	1,426	1,119	2,545	268.18	7,908	5,169	5,083	10,252	129.64
Candon City	16	2	2	4	25.00	491	641	566	1,207	245.82	4,292	794	791	1,585	36.93
Dagupan City	1,359	14	21	35	2.58	1,809	1,493	1,348	2,841	157.05	15,094	7,649	7,835	15,484	102.58
Laoag City	1	2	1	3	300.00	934	802	1,081	1,883	201.61	7,609	4,064	6,161	10,225	134.38
San Carlos City	48	8	9	17	35.42	1,990	967	897	1,864	93.67	16,616	1,161	1,132	2,293	13.80
San Fernando City	686	19	16	35	5.10	1,055	1,536	1,389	2,925	277.25	8,922	8,369	7,872	16,241	182.03
Urdaneta City	75	3	6	9	12.00	1,407	519	479	998	70.93	11,718	225	219	444	3.79
Vigan City	182	2	2	4	2.20	437	177	145	322	73.68	3,816	185	177	362	9.49
**Region 2**	**1,573**	**383**	**436**	**819**	**52.07**	**34,688**	**17,243**	**16,295**	**33,538**	**96.68**	**291,567**	**122,849**	**119,761**	**242,610**	**83.21**
Batanes	32	1	2	3	9.38	171	23	23	46	26.90	1,518	671	636	1,307	86.10
Cagayan	298	82	89	171	57.38	8,970	5,170	5,043	10,213	113.86	75,523	36,088	33,706	69,794	92.41
Isabela	317	96	104	200	63.09	13,158	6,298	5,509	11,807	89.73	110,057	49,603	47,225	96,828	87.98
Nueva Vizcaya	672	35	47	82	12.20	4,784	1,957	2,144	4,101	85.72	40,344	17,710	20,484	38,194	94.67
Quirino	119	20	22	42	35.29	2,005	960	1,025	1,985	99.00	17,186	4,618	4,349	8,967	52.18
Cauayan City	39	10	18	28	71.79	1,289	665	624	1,289	100.00	10,804	1,919	1,956	3,875	35.87
Ilagan City	0	132	141	273	0.00	1,451	632	626	1,258	86.70	12,151	1,650	1,563	3,213	26.44
Santiago City	38	3	11	14	36.84	1,343	687	635	1,322	98.44	11,254	4,891	4,513	9,404	83.56
Tuguegarao City	58	4	2	6	10.34	1,517	851	666	1,517	100.00	12,730	5,699	5,329	11,028	86.63
**Region 3**	**5,707**	**897**	**898**	**1,795**	**31.45**	**109,998**	**80,537**	**78,285**	**158,822**	**144.39**	**912,991**	**275,987**	**264,804**	**540,791**	**59.23**
Aurora	252	15	12	27	10.71	2,383	2,268	2,186	4,454	186.91	20,464	10,305	10,041	20,346	99.42
Bataan	303	51	46	97	32.01	6,915	5,733	5,497	11,230	162.40	54,850	27,467	25,750	53,217	97.02
Bulacan	1,465	241	256	497	33.92	21,799	14,262	13,840	28,102	128.91	180,961	35,898	34,779	70,677	39.06
Nueva Ecija	56	144	119	263	469.64	14,389	9,696	9,315	19,011	132.12	121,573	40,191	38,891	79,082	65.05
Pampanga	88	105	117	222	252.27	15,673	7,948	7,774	15,722	100.31	130,856	18,694	17,589	36,283	27.73
Tarlac	111	42	60	102	91.89	9,896	4,789	4,782	9,571	96.72	81,757	39,718	40,583	80,301	98.22
Zambales	587	81	65	146	24.87	6,309	2,323	2,362	4,685	74.26	51,197	13,256	12,729	25,985	50.75
Angeles City	620	6	6	12	1.94	3,937	3,661	3,584	7,245	184.02	32,828	3,713	3,830	7,543	22.98
Balanga City	77	25	32	57	74.03	999	628	681	1,309	131.03	7,927	3,274	3,107	6,381	80.50
Cabanatuan City	720	41	41	82	11.39	2,946	2,442	2,303	4,745	161.07	24,884	3,595	3,323	6,918	27.80
City of San Fernando	86	21	18	39	45.35	2,930	3,490	3,188	6,678	227.92	24,459	13,566	9,052	22,618	92.47
Gapan City	7	5	1	6	85.71	1,077	858	856	1,714	159.15	9,083	5,636	5,671	11,307	124.49
Mabalacat City	397	5	7	12	3.02	2,396	3,672	3,673	7,345	306.55	20,000	9,458	9,799	19,257	96.29
Malolos City	123	25	17	42	34.15	2,436	1,447	1,441	2,888	118.56	20,216	6,629	6,599	13,228	65.43
Meycauayan	0	4	7	11	0.00	2,019	1,809	1,769	3,578	177.22	16,766	3,377	3,265	6,642	39.62
Olongapo	245	14	20	34	13.88	2,485	1,685	1,598	3,283	132.11	20,191	2,338	2,275	4,613	22.85
Palayan City	0	0	0	0	0.00	399	200	180	380	95.24	3,377	3,930	3,297	7,227	214.01
San Jose City	85	1	1	2	2.35	1,367	1,899	1,739	3,638	266.13	11,500	12,668	12,320	24,988	217.29

Table 1.C.2.2- Nutrition Services for Infants and Children

Number of Infants born preterm or with low birth weight given iron supplements and infants 6-11 mos. old and children 12-59 mos. old given Vitamin A supplementation
Philippines, Annual 2020

Area	Total No. of live births with low birth weight	Infants preterm or with LBW (< 2500 grams) given Iron				Eligible Pop (6-11 months)	Infants 6-11 mos. old Given Vitamin A				Eligible Pop (12-59 months)	Children 12-59 mos. Old Given Vitamin A			
		Male	Female	Total	%		Male	Female	Total	%		Male	Female	Total	%
San Jose del Monte City	297	55	60	115	38.72	5,545	7,769	7,671	15,440	278.45	46,033	14,061	13,696	27,757	60.30
Science City of Munoz	0	0	0	0	0.00	794	375	352	727	91.56	6,708	3,058	3,066	6,124	91.29
Tarlac City	188	16	13	29	15.43	3,304	3,583	3,494	7,077	214.19	27,361	5,155	5,142	10,297	37.63
**Region 4A**	**9,192**	**621**	**755**	**1,376**	**14.97**	**148,421**	**85,754**	**80,862**	**166,616**	**112.26**	**1,227,422**	**223,784**	**216,164**	**439,948**	**35.84**
Batangas	576	24	15	39	6.77	19,225	7,296	6,751	14,047	73.07	162,024	41,044	39,103	80,147	49.47
Cavite	1,152	111	140	251	21.79	13,848	9,430	8,805	18,235	131.68	113,921	21,718	20,224	41,942	36.82
Laguna	787	56	89	145	18.42	9,871	6,652	6,326	12,978	131.48	80,610	17,000	16,041	33,041	40.99
Quezon	697	94	59	153	21.95	18,698	11,794	10,960	22,754	121.69	158,632	38,569	37,369	75,938	47.87
Rizal	975	89	119	208	21.33	22,402	10,193	9,709	19,902	88.84	181,864	17,855	17,454	35,309	19.42
Antipolo City	1,130	6	12	18	1.59	8,249	6,748	6,549	13,297	161.20	66,984	17,707	18,083	35,790	53.43
Bacoor City	339	2	0	2	0.59	6,071	867	777	1,644	27.08	49,915	708	729	1,437	2.88
Batangas City	0	0	0	0	0.00	3,414	2,359	2,141	4,500	131.81	28,751	3,437	3,177	6,614	23.00
Biñan City	258	28	83	111	43.02	3,305	4,110	3,983	8,093	244.87	27,031	11,559	12,240	23,799	88.04
Cabuyao City	146	36	37	73	50.00	3,066	1,577	1,589	3,166	103.26	25,061	2,869	2,471	5,340	21.31
Calamba City	397	50	53	103	25.94	4,511	4,345	4,053	8,398	186.17	36,888	6,575	6,156	12,731	34.51
Cavite City	42	0	0	0	0.00	1,036	0	0	0	0.00	8,541	0	0	0	0.00
Dasmariñas City	638	20	29	49	7.68	6,659	3,146	3,060	6,206	93.20	54,762	8,347	7,689	16,036	29.28
General Trias City	62	9	14	23	37.10	3,180	2,248	1,984	4,232	133.08	26,122	3,267	2,977	6,244	23.90
Imus City	191	15	10	25	13.09	4,081	1,086	1,075	2,161	52.95	33,554	2,186	2,136	4,322	12.88
Lipa City	0	0	0	0	0.00	3,435	412	420	832	24.22	28,975	591	604	1,195	4.12
Lucena City	0	7	6	13	0.00	2,835	2,254	2,118	4,372	154.22	24,048	5,481	5,312	10,793	44.88
San Pablo City	367	1	2	3	0.82	2,648	1,492	1,576	3,068	115.86	21,595	2,232	2,160	4,392	20.34
San Pedro City	51	31	29	60	117.65	3,233	1,087	1,011	2,098	64.89	26,445	1,791	1,672	3,463	13.10
Santa Rosa City	415	5	14	19	4.58	3,512	2,712	2,555	5,267	149.97	28,715	3,111	2,880	5,991	20.86
Tagaytay City	14	2	2	4	28.57	722	1,826	1,600	3,426	474.52	5,917	12,164	12,112	24,276	410.28
Tanauan City	230	1	6	7	3.04	1,788	1,200	1,111	2,311	129.25	15,111	1,077	1,005	2,082	13.78
Tayabas City	51	0	2	2	3.92	1,057	0	1	1	0.09	9,016	0	0	0	0.00
Trece Martires City	674	34	34	68	10.09	1,575	2,920	2,708	5,628	357.33	12,940	4,496	4,570	9,066	70.06
**Region 4B**	**3,475**	**1,264**	**1,404**	**2,668**	**76.78**	**35,617**	**17,306**	**16,480**	**33,786**	**94.86**	**298,390**	**89,114**	**84,525**	**173,639**	**58.19**
Marinduque	226	31	40	71	31.42	2,425	2,185	1,991	4,176	172.21	21,709	9,762	8,903	18,665	85.98
Mindoro Occidental	258	16	8	24	9.30	6,206	4,080	3,902	7,982	128.62	50,349	24,341	23,295	47,636	94.61
Mindoro Oriental	773	1,153	1,287	2,440	315.65	9,906	6,002	5,832	11,834	119.46	83,165	36,095	33,975	70,070	84.25
Palawan	1,931	34	33	67	3.47	10,665	3,981	3,732	7,713	72.32	88,284	14,207	13,931	28,138	31.87
Romblon	118	26	30	56	47.46	3,208	740	661	1,401	43.67	28,368	4,558	4,293	8,851	31.20
Puerto Princesa City	169	4	6	10	5.92	3,207	318	362	680	21.20	26,515	151	128	279	1.05
**Region 5**	**4,824**	**1,017**	**1,282**	**2,299**	**47.66**	**68,083**	**43,488**	**40,034**	**83,522**	**122.68**	**586,320**	**227,406**	**222,374**	**449,780**	**76.71**
Albay	595	99	119	218	36.64	11,865	9,677	9,094	18,771	158.20	103,227	53,577	52,768	106,345	103.02
Camarines Norte	1,226	110	141	251	20.47	7,140	4,255	3,895	8,150	114.15	59,893	32,749	35,401	68,150	113.79
Camarines Sur	820	92	106	198	24.15	19,419	14,800	12,505	27,305	140.61	166,213	47,762	45,283	93,045	55.98
Catanduanes	303	98	114	212	69.97	3,136	2,700	2,513	5,213	166.23	26,561	9,881	9,457	19,338	72.81
Masbate	655	161	154	315	48.09	11,264	3,658	3,647	7,305	64.85	99,437	29,211	28,073	57,284	57.61
Sorsogon	855	210	205	415	48.54	9,543	4,551	4,845	9,396	98.46	81,734	28,708	26,934	55,642	68.08
Iriga City	148	21	26	47	31.76	1,317	672	637	1,309	99.39	11,291	6,643	6,395	13,038	115.47
Legaspi City	151	222	410	632	418.54	2,086	2,277	2,048	4,325	207.33	18,151	15,434	14,875	30,309	166.98
Naga City	71	4	7	11	15.49	2,313	898	850	1,748	75.57	19,813	3,441	3,188	6,629	33.46
**Region 6**	**10,897**	**523**	**582**	**1,105**	**10.14**	**73,242**	**35,763**	**40,947**	**76,710**	**104.73**	**623,733**	**220,028**	**247,099**	**467,127**	**74.89**
Aklan	925	43	54	97	10.49	5,577	4,983	4,815	9,798	175.69	47,850	13,247	12,571	25,818	53.96
Antique	1,322	59	103	162	12.25	6,396	3,372	3,190	6,562	102.60	53,822	20,922	19,430	40,352	74.97
Capiz	830	25	41	66	7.95	6,993	3,251	3,141	6,392	91.41	60,081	30,031	29,021	59,052	98.29
Guimaras	140	49	42	91	65.00	1,548	1,231	1,191	2,422	156.46	14,501	6,267	6,130	12,397	85.49
Iloilo	1,549	61	64	125	8.07	18,256	5,121	12,356	17,477	95.73	153,611	53,618	87,989	141,607	92.19
Negros Occidental	3,130	211	216	427	13.64	24,696	12,361	11,657	24,018	97.25	210,882	74,149	71,625	145,774	69.13
Bacolod City	1,294	58	52	110	8.50	5,550	2,262	1,565	3,827	68.95	47,451	4,105	3,056	7,161	15.09
Iloilo City	1,707	17	10	27	1.58	4,226	3,182	3,032	6,214	147.04	35,535	17,689	17,277	34,966	98.40
**Region 7**	**9,050**	**1,082**	**1,050**	**2,132**	**23.56**	**81,628**	**49,926**	**47,471**	**97,397**	**119.32**	**652,415**	**146,650**	**152,747**	**299,397**	**45.89**
Bohol	2,156	36	29	65	3.01	13,659	8,292	8,020	16,312	119.42	115,081	36,939	36,673	73,612	63.97
Cebu	2,908	729	675	1,404	48.28	33,732	20,424	19,282	39,706	117.71	263,210	60,701	57,496	118,197	44.91
Negros Oriental	1,315	93	114	207	15.74	13,975	5,561	5,351	10,912	78.08	115,018	27,340	24,892	52,232	45.41
Siquijor	148	7	7	14	9.46	812	788	691	1,479	182.14	7,440	2,389	2,359	4,748	63.82
Cebu City	717	147	156	303	42.26	10,591	7,069	6,642	13,711	129.46	82,633	9,931	22,539	32,470	39.29
Lapu-Lapu City	328	53	53	106	32.32	4,688	4,937	4,697	9,634	205.50	36,553	5,433	5,197	10,630	29.08
Mandaue City	1,478	17	16	33	2.23	4,171	2,855	2,788	5,643	135.29	32,480	3,917	3,591	7,508	23.12
**Region 8**	**3,019**	**850**	**993**	**1,843**	**61.05**	**51,272**	**29,294**	**21,463**	**50,757**	**99.00**	**433,112**	**107,293**	**103,258**	**210,551**	**48.61**
Biliran	308	273	295	568	184.42	1,909	946	957	1,903	99.69	16,820	3,894	3,605	7,499	44.58
Eastern Samar	153	47	46	93	60.78	5,709	2,657	3,023	5,680	99.49	46,281	11,179	11,376	22,555	48.73
Northern Leyte	501	95	106	201	40.12	17,351	10,739	6,300	17,039	98.20	143,608	33,854	30,621	64,475	44.90
Northern Samar	433	92	127	219	50.58	7,661	4,850	2,810	7,660	99.99	67,406	24,268	24,078	48,346	71.72
Southern Leyte	166	87	116	203	122.29	3,223	1,805	1,415	3,220	99.91	28,790	8,794	8,445	17,239	59.88
Western Samar	255	153	163	316	123.92	7,148	3,868	3,250	7,118	99.58	60,698	12,168	11,977	24,145	39.78

Table 1.C.2.2- Nutrition Services for Infants and Children

Number of Infants born preterm or with low birth weight given iron supplements and infants 6-11 mos. old and children 12-59 mos. old given Vitamin A supplementation
Philippines, Annual 2020

Area	Total No. of live births with low birth weight	Infants preterm or with LBW (< 2500 grams) given Iron				Eligible Pop (6-11 months)	Infants 6-11 mos. old Given Vitamin A				Eligible Pop (12-59 months)	Children 12-59 mos. Old Given Vitamin A			
		Male	Female	Total	%		Male	Female	Total	%		Male	Female	Total	%
Calbayog City	74	45	31	76	102.70	2,200	1,260	820	2,080	94.55	18,707	1,227	1,136	2,363	12.63
Maasin City	388	2	10	12	3.09	822	450	370	820	99.76	7,329	1,424	1,264	2,688	36.68
Ormoc City	691	52	88	140	20.26	2,469	1,219	1,243	2,462	99.72	20,450	7,417	7,627	15,044	73.56
Tacloban City	50	4	11	15	30.00	2,780	1,500	1,275	2,775	99.82	23,023	3,068	3,129	6,197	26.92
**Region 9**	**1,413**	**646**	**636**	**1,282**	**90.73**	**40,010**	**31,300**	**31,231**	**62,531**	**156.29**	**339,485**	**83,882**	**84,213**	**168,095**	**49.51**
Zamboanga del Norte	408	101	123	224	54.90	8,617	11,115	11,086	22,201	257.64	74,872	30,620	31,117	61,737	82.46
Zamboanga del Sur	252	17	32	49	19.44	8,824	4,827	5,168	9,995	113.27	72,143	13,276	13,501	26,777	37.12
Zamboanga Sibugay	352	174	166	340	96.59	7,476	4,707	4,756	9,463	126.58	66,111	22,600	21,865	44,465	67.26
Dapitan City	70	13	19	32	45.71	888	793	731	1,524	171.62	7,736	2,503	2,530	5,033	65.06
Dipolog City	42	2	2	4	9.52	1,411	1,841	1,808	3,649	258.61	12,264	4,665	4,844	9,509	77.54
Isabela City	42	36	69	105	250.00	1,256	870	829	1,699	135.27	12,059	1,015	1,054	2,069	17.16
Pagadian City	78	275	193	468	600.00	2,167	1,756	1,777	3,533	163.04	17,694	2,839	2,857	5,696	32.19
Zamboanga City	169	28	32	60	35.50	9,371	5,391	5,076	10,467	111.70	76,606	6,364	6,445	12,809	16.72
**Region 10**	**3,962**	**720**	**781**	**1,501**	**37.88**	**50,699**	**37,628**	**39,866**	**77,494**	**152.85**	**434,937**	**212,455**	**213,091**	**425,546**	**97.84**
Bukidnon	996	128	139	267	26.81	11,844	11,490	11,696	23,186	195.76	100,578	51,837	50,815	102,652	102.06
Camiguin	23	1	4	5	21.74	929	880	820	1,700	182.99	8,000	5,627	5,425	11,052	138.15
Lanao del Norte	23	257	270	527	2291.30	7,492	5,827	6,101	11,928	159.21	67,507	18,311	19,664	37,975	56.25
Misamis Occidental	60	18	25	43	71.67	3,189	1,627	1,751	3,378	105.93	27,424	14,104	14,600	28,704	104.67
Misamis Oriental	321	48	59	107	33.33	7,567	6,397	6,317	12,714	168.02	63,311	46,966	46,319	93,285	147.34
Cagayan de Oro City	2,000	26	20	46	2.30	7,168	3,674	5,511	9,185	128.14	59,976	35,923	37,699	73,622	122.75
El Salvador City	0	10	8	18	0.00	535	117	113	230	42.99	4,456	55	54	109	2.45
Gingoog City	30	28	34	62	206.67	1,326	879	885	1,764	133.03	11,052	6,358	6,408	12,766	115.51
Iligan City	186	113	137	250	134.41	3,790	2,248	2,199	4,447	117.34	34,195	3,841	3,820	7,661	22.40
Malaybalay City	97	19	16	35	36.08	1,976	1,470	1,459	2,929	148.23	16,766	8,244	8,061	16,305	97.25
Oroquieta City	58	3	3	6	10.34	693	785	762	1,547	223.23	5,942	4,781	4,513	9,294	156.41
Ozamis City	10	2	3	5	50.00	1,389	363	357	720	51.84	11,909	2,948	2,784	5,732	48.13
Tangub City	27	13	8	21	77.78	620	908	840	1,748	281.94	5,293	2,741	2,579	5,320	100.51
Valencia City	131	54	55	109	83.21	2,181	963	1,055	2,018	92.53	18,528	10,719	10,350	21,069	113.71
**Region 11**	**3,813**	**760**	**751**	**1,511**	**39.63**	**53,626**	**44,659**	**41,833**	**86,492**	**161.29**	**448,208**	**225,406**	**209,886**	**435,292**	**97.12**
Compostela Valley	476	92	77	169	35.50	7,781	8,636	7,717	16,353	210.17	68,129	46,438	41,382	87,820	128.90
Davao del Norte	877	224	245	469	53.48	10,661	9,600	9,206	18,806	176.40	87,663	56,597	53,066	109,663	125.10
Davao del Sur	334	73	58	131	39.22	6,508	4,641	4,543	9,184	141.12	56,161	19,806	19,063	38,869	69.21
Davao Oriental	389	71	58	129	33.16	7,065	6,108	5,586	11,694	165.52	57,348	30,305	27,984	58,289	101.64
Davao Occidental	174	24	17	41	23.56	3,347	1,935	1,784	3,719	111.11	30,859	7,149	6,794	13,943	45.18
Davao City	1,563	276	296	572	36.60	18,264	13,739	12,997	26,736	146.39	148,048	65,111	61,597	126,708	85.59
**Region 12**	**2,097**	**475**	**642**	**1,117**	**53.27**	**52,301**	**23,328**	**23,108**	**46,436**	**88.79**	**437,544**	**133,095**	**136,561**	**269,656**	**61.63**
North Cotabato	646	97	236	333	51.55	16,840	7,113	6,784	13,897	82.52	139,074	41,784	43,888	85,672	61.60
Sarangani	94	29	52	81	86.17	6,446	2,961	3,034	5,995	93.00	54,603	32,687	33,041	65,728	120.37
South Cotabato	406	124	176	300	73.89	10,554	4,697	4,845	9,542	90.41	85,230	23,225	22,557	45,782	53.72
Sultan Kudarat	421	172	133	305	72.45	8,690	4,284	4,265	8,549	98.38	74,909	15,298	17,123	32,421	43.28
Cotabato City	126	0	0	0	0.00	2,920	1,536	1,382	2,918	99.93	28,378	14,772	14,823	29,595	104.29
Gen. Santos City	404	53	45	98	24.26	6,851	2,737	2,798	5,535	80.79	55,350	5,329	5,129	10,458	18.89
**BARMM**	**1,272**	**379**	**431**	**810**	**63.68**	**46,406**	**24,676**	**25,676**	**50,352**	**108.50**	**483,196**	**41,924**	**44,987**	**86,911**	**17.99**
Basilan	214	1	0	1	0.47	3,765	656	772	1,428	37.93	35,490	643	659	1,302	3.67
Lanao del Sur	58	142	220	362	624.14	10,552	7,012	7,639	14,651	138.85	110,328	15,302	17,313	32,615	29.56
Maguindanao	352	180	170	350	99.43	15,563	11,943	12,049	23,992	154.16	148,947	12,021	12,394	24,415	16.39
Sulu	212	14	18	32	15.09	8,328	1,775	1,733	3,508	42.12	107,280	7,093	7,570	14,663	13.67
Tawi-Tawi	154	18	11	29	18.83	4,635	1,148	1,100	2,248	48.50	44,992	1,386	1,321	2,707	6.02
Lamitan City	43	23	12	35	81.40	1,032	269	319	588	56.98	9,764	652	537	1,189	12.18
Marawi City	239	1	0	1	0.42	2,531	1,873	2,064	3,937	155.55	26,395	4,827	5,193	10,020	37.96
**CARAGA**	**1,627**	**414**	**426**	**840**	**51.63**	**30,026**	**22,929**	**22,716**	**45,645**	**152.02**	**258,578**	**106,746**	**108,488**	**215,234**	**83.24**
Agusan del Norte	171	66	69	135	78.95	4,058	3,213	3,099	6,312	155.54	34,103	13,460	13,119	26,579	77.94
Agusan del Sur	190	200	225	425	223.68	8,786	5,337	5,425	10,762	122.49	76,814	28,677	31,360	60,037	78.16
Surigao del Norte	183	18	12	30	16.39	3,552	2,020	2,081	4,101	115.46	31,554	12,657	12,964	25,621	81.20
Surigao del Sur	310	50	54	104	33.55	5,740	4,701	4,775	9,476	165.09	48,534	22,033	22,125	44,158	90.98
Province of Dinagat	81	19	22	41	50.62	1,289	735	698	1,433	111.17	11,231	6,119	5,730	11,849	105.50
Bislig City	34	17	8	25	73.53	1,088	665	745	1,410	129.60	9,222	4,609	4,515	9,124	98.94
Butuan City	276	12	18	30	10.87	3,855	4,959	4,691	9,650	250.32	32,429	17,034	16,570	33,604	103.62
Surigao City	382	32	18	50	13.09	1,658	1,299	1,202	2,501	150.84	14,691	2,157	2,105	4,262	29.01

Note: Put asterisk (*) for No Report and Zero (0) for No Case

Table 1.C.2.3 - Nutrition Services for Infants and Children

Number and Proportion of infants 6-11 mos. old and children 12-23 mos. old who completed Micronutrient Powder supplementation

Philippines, Annual 2020

Area	Eligible Pop (6-11 mos.)	Infants 6-11 mos. old who completed MNP supplementation				Eligible Pop (12-23 mos.)	Children 12-23 mos. old who completed MNP supplementation			
		Male	Female	Total	%		Male	Female	Total	%
**PHILIPPINES**	**1,061,536**	**130,618**	**129,077**	**259,695**	**24.46**	**2,138,446**	**195,605**	**197,193**	**392,798**	**18.37**
**N C R**	**119,342**	**20,129**	**20,368**	**40,497**	**33.93**	**236,097**	**31,890**	**32,870**	**64,760**	**27.43**
Malabon	3,385	1,656	1,621	3,277	96.81	6,699	2,376	2,392	4,768	71.17
Navotas	2,312	823	905	1,728	74.74	4,573	730	782	1,512	33.06
Valenzuela City	5,752	223	261	484	8.41	11,373	662	623	1,285	11.30
Caloocan City	14,674	2,555	2,649	5,204	35.46	29,045	4,373	4,243	8,616	29.66
Marikina City	4,177	210	199	409	9.79	8,264	192	199	391	4.73
Pasig City	7,001	673	699	1,372	19.60	13,846	688	676	1,364	9.85
Pateros	590	167	165	332	56.27	1,170	315	327	642	54.87
Taguig	7,458	3,458	3,504	6,962	93.35	14,752	5,117	5,455	10,572	71.66
Quezon City	27,208	551	547	1,098	4.04	53,837	651	621	1,272	2.36
Makati City	5,399	2,502	2,351	4,853	89.89	10,681	2,473	2,340	4,813	45.06
Mandaluyong City	3,579	57	65	122	3.41	7,086	38	50	88	1.24
San Juan	1,132	528	604	1,132	100.00	2,239	1,268	971	2,239	100.00
Manila City	16,497	1,620	1,635	3,255	19.73	32,646	3,645	4,886	8,531	26.13
Las Piñas City	5,461	174	170	344	6.30	10,798	326	333	659	6.10
Muntinlupa City	4,677	2,297	2,380	4,677	100.00	9,249	4,568	4,681	9,249	100.00
Parañaque City	6,173	1,405	1,357	2,762	44.74	12,206	3,251	3,107	6,358	52.09
Pasay City	3,867	1,230	1,256	2,486	64.29	7,633	1,217	1,184	2,401	31.46
**C A R**	**17,552**	**848**	**833**	**1,681**	**9.58**	**35,870**	**1,962**	**1,932**	**3,894**	**10.86**
Abra	2,153	201	176	377	17.51	4,426	324	331	655	14.80
Apayao	1,219	47	42	89	7.30	2,477	123	162	285	11.51
Benguet	4,584	277	314	591	12.89	9,317	714	700	1,414	15.18
Ifugao	2,199	121	101	222	10.10	4,700	88	65	153	3.26
Kalinga	2,314	153	140	293	12.66	4,717	290	259	549	11.64
Mt. Province	1,517	23	27	50	3.30	3,033	58	51	109	3.59
Baguio City	3,566	26	33	59	1.65	7,200	365	364	729	10.13
**Region 1**	**48,625**	**9,064**	**8,483**	**17,547**	**36.09**	**97,450**	**11,851**	**11,191**	**23,042**	**23.64**
Ilocos Norte	4,054	801	839	1,640	40.45	7,826	1,807	1,726	3,533	45.14
Ilocos Sur	4,655	1,191	1,210	2,401	51.58	9,605	2,221	2,179	4,400	45.81
La Union	5,763	860	842	1,702	29.53	11,504	1,786	1,632	3,418	29.71
Pangasinan	25,081	3,411	3,201	6,612	26.36	50,347	3,203	3,077	6,280	12.47
Alaminos City	949	1,200	940	2,140	225.50	1,897	873	683	1,556	82.02
Candon City	491	613	547	1,160	236.25	1,015	691	629	1,320	130.05
Dagupan City	1,809	707	638	1,345	74.35	3,629	926	884	1,810	49.88
Laoag City	934	35	33	68	7.28	1,810	60	67	127	7.02
San Carlos City	1,990	5	6	11	0.55	3,998	9	6	15	0.38
San Fernando City	1,055	77	100	177	16.78	2,107	80	65	145	6.88
Urdaneta City	1,407	135	104	239	16.99	2,816	163	215	378	13.42
Vigan City	437	29	23	52	11.90	896	32	28	60	6.70
**Region 2**	**34,688**	**6,819**	**6,565**	**13,384**	**38.58**	**70,242**	**9,396**	**9,133**	**18,529**	**26.38**
Batanes	171	11	5	16	9.36	337	24	14	38	11.28
Cagayan	8,970	1,449	1,479	2,928	32.64	18,384	1,896	1,909	3,805	20.70
Isabela	13,158	2,537	2,466	5,003	38.02	26,265	3,747	3,663	7,410	28.21
Nueva Vizcaya	4,784	986	949	1,935	40.45	9,742	854	744	1,598	16.40
Quirino	2,005	268	273	541	26.98	4,254	385	281	666	15.66
Cauayan City	1,289	269	240	509	39.49	2,582	349	365	714	27.65
Ilagan City	1,451	248	211	459	31.63	2,903	250	280	530	18.26
Santiago City	1,343	648	587	1,235	91.96	2,688	1,289	1,275	2,564	95.39
Tuguegarao City	1,517	403	355	758	49.97	3,087	602	602	1,204	39.00

Table 1.C.2.3 - Nutrition Services for Infants and Children

Number and Proportion of infants 6-11 mos. old and children 12-23 mos. old who completed Micronutrient Powder supplementation

Philippines, Annual 2020

Area	Eligible Pop (6-11 mos.)	Infants 6-11 mos. old who completed MNP supplementation				Eligible Pop (12-23 mos.)	Children 12-23 mos. old who completed MNP supplementation			
		Male	Female	Total	%		Male	Female	Total	%
**Region 3**	**109,998**	**14,083**	**14,052**	**28,135**	**25.58**	**218,289**	**19,055**	**18,962**	**38,017**	**17.42**
Aurora	2,383	314	306	620	26.02	4,940	658	643	1,301	26.34
Bataan	6,915	988	853	1,841	26.62	13,349	1,847	1,541	3,388	25.38
Bulacan	21,799	2,408	2,401	4,809	22.06	43,168	3,163	3,115	6,278	14.54
Nueva Ecija	14,389	1,345	1,327	2,672	18.57	28,958	1,095	1,085	2,180	7.53
Pampanga	15,673	850	844	1,694	10.81	30,964	1,237	1,241	2,478	8.00
Tarlac	9,896	1,834	1,961	3,795	38.35	19,559	3,718	3,817	7,535	38.52
Zambales	6,309	362	368	730	11.57	12,588	418	440	858	6.82
Angeles City	3,937	1,023	1,036	2,059	52.30	7,768	966	1,010	1,976	25.44
Balanga City	999	92	100	192	19.22	1,931	123	132	255	13.21
Cabanatuan City	2,946	342	332	674	22.88	5,926	356	371	727	12.27
City of San Fernando	2,930	309	285	594	20.27	5,791	283	299	582	10.05
Gapan City	1,077	108	125	233	21.63	2,166	176	163	339	15.65
Mabalacat City	2,396	879	923	1,802	75.21	4,741	957	988	1,945	41.03
Malolos City	2,436	0	0	0	0.00	4,821	28	19	47	0.97
Meycauayan	2,019	703	750	1,453	71.97	4,001	1,025	1,069	2,094	52.34
Olongapo	2,485	323	335	658	26.48	4,965	262	262	524	10.55
Palayan City	399	70	69	139	34.84	800	167	176	343	42.88
San Jose City	1,367	319	303	622	45.50	2,739	557	548	1,105	40.34
San Jose del Monte City	5,545	750	733	1,483	26.74	10,980	954	956	1,910	17.40
Science City of Munoz	794	0	0	0	0.00	1,595	0	0	0	0.00
Tarlac City	3,304	1,064	1,001	2,065	62.50	6,539	1,065	1,087	2,152	32.91
**Region 4A**	**148,421**	**10,342**	**10,073**	**20,415**	**13.75**	**295,101**	**13,069**	**12,746**	**25,815**	**8.75**
Batangas	19,225	209	202	411	2.14	38,682	574	553	1,127	2.91
Cavite	13,848	252	292	544	3.93	27,229	612	595	1,207	4.43
Laguna	9,871	648	605	1,253	12.69	19,491	813	891	1,704	8.74
Quezon	18,698	773	755	1,528	8.17	38,069	1,261	1,257	2,518	6.61
Rizal	22,402	1,088	1,003	2,091	9.33	44,134	805	841	1,646	3.73
Antipolo City	8,249	258	324	582	7.06	16,253	499	526	1,025	6.31
Bacoor City	6,071	767	755	1,522	25.07	11,928	536	513	1,049	8.79
Batangas City	3,414	0	0	0	0.00	6,872	0	0	0	0.00
Biñan City	3,305	1,957	1,868	3,825	115.73	6,537	3,503	3,317	6,820	104.33
Cabuyao City	3,066	477	541	1,018	33.20	6,061	532	513	1,045	17.24
Calamba City	4,511	620	548	1,168	25.89	8,922	566	515	1,081	12.12
Cavite City	1,036	2	7	9	0.87	2,047	9	4	13	0.64
Dasmariñas City	6,659	676	672	1,348	20.24	13,089	608	638	1,246	9.52
General Trias City	3,180	156	146	302	9.50	6,245	75	86	161	2.58
Imus City	4,081	45	47	92	2.25	8,020	19	24	43	0.54
Lipa City	3,435	0	0	0	0.00	6,919	5	4	9	0.13
Lucena City	2,835	11	12	23	0.81	5,773	34	33	67	1.16
San Pablo City	2,648	168	160	328	12.39	5,220	8	7	15	0.29
San Pedro City	3,233	403	395	798	24.68	6,394	509	502	1,011	15.81
Santa Rosa City	3,512	431	405	836	23.80	6,937	735	619	1,354	19.52
Tagaytay City	722	174	194	368	50.97	1,414	966	932	1,898	134.23
Tanauan City	1,788	19	19	38	2.13	3,607	14	30	44	1.22
Tayabas City	1,057	0	0	0	0.00	2,164	0	0	0	0.00
Trece Martires City	1,575	1,208	1,123	2,331	148.00	3,094	386	346	732	23.66
**Region 4B**	**35,617**	**883**	**852**	**1,735**	**4.87**	**71,270**	**1,166**	**1,061**	**2,227**	**3.12**
Marinduque	2,425	178	170	348	14.35	4,908	152	106	258	5.26
Mindoro Occidental	6,206	162	116	278	4.48	12,500	138	144	282	2.26
Mindoro Oriental	9,906	170	181	351	3.54	19,592	40	44	84	0.43
Palawan	10,665	373	385	758	7.11	21,317	836	767	1,603	7.52
Romblon	3,208	0	0	0	0.00	6,551	0	0	0	0.00
Puerto Princesa City	3,207	0	0	0	0.00	6,402	0	0	0	0.00
**Region 5**	**68,083**	**8,035**	**7,489**	**15,524**	**22.80**	**139,190**	**12,114**	**12,068**	**24,182**	**17.37**
Albay	11,865	1,040	983	2,023	17.05	24,401	3,328	3,298	6,626	27.15
Camarines Norte	7,140	3,807	3,305	7,112	99.61	13,958	1,007	986	1,993	14.28

Table 1.C.2.3 - Nutrition Services for Infants and Children

Number and Proportion of infants 6-11 mos. old and children 12-23 mos. old who completed Micronutrient Powder supplementation

Philippines, Annual 2020

Area	Eligible Pop (6-11 mos.)	Infants 6-11 mos. old who completed MNP supplementation				Eligible Pop (12-23 mos.)	Children 12-23 mos. old who completed MNP supplementation			
		Male	Female	Total	%		Male	Female	Total	%
Camarines Sur	19,419	336	304	640	3.30	39,520	611	584	1,195	3.02
Catanduanes	3,136	477	447	924	29.46	6,451	207	218	425	6.59
Masbate	11,264	889	1,036	1,925	17.09	23,758	2,547	2,796	5,343	22.49
Sorsogon	9,543	1,373	1,274	2,647	27.74	19,423	4,183	3,953	8,136	41.89
Iriga City	1,317	65	85	150	11.39	2,683	112	106	218	8.13
Legaspi City	2,086	0	0	0	0.00	4,285	0	0	0	0.00
Naga City	2,313	48	55	103	4.45	4,711	119	127	246	5.22
**Region 6**	**73,242**	**14,740**	**14,233**	**28,973**	**39.56**	**147,764**	**19,258**	**19,450**	**38,708**	**26.20**
Aklan	5,577	993	1,009	2,002	35.90	11,443	2,463	2,302	4,765	41.64
Antique	6,396	994	922	1,916	29.96	12,831	1,920	1,989	3,909	30.47
Capiz	6,993	2,368	2,439	4,807	68.74	14,265	203	210	413	2.90
Guimaras	1,548	54	48	102	6.59	3,340	204	219	423	12.66
Iloilo	18,256	5,732	5,211	10,943	59.94	36,511	7,873	7,780	15,653	42.87
Negros Occidental	24,696	4,210	4,229	8,439	34.17	49,734	6,069	6,356	12,425	24.98
Bacolod City	5,550	389	375	764	13.77	11,193	526	594	1,120	10.01
Iloilo City	4,226	0	0	0	0.00	8,447	0	0	0	0.00
**Region 7**	**81,628**	**5,504**	**5,156**	**10,660**	**13.06**	**159,649**	**9,083**	**9,400**	**18,483**	**11.58**
Bohol	13,659	774	741	1,515	11.09	27,336	1,945	1,974	3,919	14.34
Cebu	33,732	2,506	2,236	4,742	14.06	65,183	4,201	4,056	8,257	12.67
Negros Oriental	13,975	380	390	770	5.51	27,829	1,317	1,759	3,076	11.05
Siquijor	812	0	0	0	0.00	1,728	0	0	0	0.00
Cebu City	10,591	405	351	756	7.14	20,471	333	361	694	3.39
Lapu-Lapu City	4,688	1,412	1,401	2,813	60.00	9,056	1,261	1,237	2,498	27.58
Mandaue City	4,171	27	37	64	1.53	8,046	26	13	39	0.48
**Region 8**	**51,272**	**9,139**	**8,718**	**17,857**	**34.83**	**102,450**	**12,214**	**12,342**	**24,556**	**23.97**
Biliran	1,909	1,137	752	1,889	98.95	4,061	221	189	410	10.10
Eastern Samar	5,709	270	342	612	10.72	10,982	1,117	1,192	2,309	21.03
Northern Leyte	17,351	1,074	1,042	2,116	12.20	34,016	1,053	1,033	2,086	6.13
Northern Samar	7,661	3,466	3,430	6,896	90.01	15,705	4,707	4,752	9,459	60.23
Southern Leyte	3,223	11	22	33	1.02	6,814	295	249	544	7.98
Western Samar	7,148	1,874	1,785	3,659	51.19	14,395	3,878	4,034	7,912	54.96
Calbayog City	2,200	1,094	1,101	2,195	99.77	4,442	448	421	869	19.56
Maasin City	822	6	11	17	2.07	1,728	40	10	50	2.89
Ormoc City	2,469	83	126	209	8.46	4,845	191	198	389	8.03
Tacloban City	2,780	124	107	231	8.31	5,462	264	264	528	9.67
**Region 9**	**40,010**	**3,997**	**4,349**	**8,346**	**20.86**	**80,319**	**9,245**	**10,002**	**19,247**	**23.96**
Zamboanga del Norte	8,617	2,452	2,664	5,116	59.37	17,491	4,247	4,354	8,601	49.17
Zamboanga del Sur	8,824	223	334	557	6.31	17,289	747	836	1,583	9.16
Zamboanga Sibugay	7,476	322	314	636	8.51	15,434	1,242	1,330	2,572	16.66
Dapitan City	888	96	141	237	26.69	1,813	330	538	868	47.88
Dipolog City	1,411	432	365	797	56.48	2,868	1,337	1,400	2,737	95.43
Isabela City	1,256	10	13	23	1.83	2,841	57	59	116	4.08
Pagadian City	2,167	0	0	0	0.00	4,239	0	0	0	0.00
Zamboanga City	9,371	462	518	980	10.46	18,344	1,285	1,485	2,770	15.10
**Region 10**	**50,699**	**5,659**	**5,997**	**11,656**	**22.99**	**104,156**	**10,758**	**11,200**	**21,958**	**21.08**
Bukidnon	11,844	1,457	1,425	2,882	24.33	24,361	3,605	3,898	7,503	30.80
Camiguin	929	23	16	39	4.20	1,869	81	85	166	8.88
Lanao del Norte	7,492	950	1,090	2,040	27.23	15,741	1,637	1,914	3,551	22.56
Misamis Occidental	3,189	563	605	1,168	36.63	6,556	677	734	1,411	21.52
Misamis Oriental	7,567	388	381	769	10.16	15,305	483	409	892	5.83
Cagayan de Oro City	7,168	522	579	1,101	15.36	14,512	1,060	1,082	2,142	14.76

Table 1.C.2.3 - Nutrition Services for Infants and Children

Number and Proportion of infants 6-11 mos. old and children 12-23 mos. old who completed Micronutrient Powder supplementation
Philippines, Annual 2020

Area	Eligible Pop (6-11 mos.)	Infants 6-11 mos. old who completed MNP supplementation				Eligible Pop (12-23 mos.)	Children 12-23 mos. old who completed MNP supplementation			
		Male	Female	Total	%		Male	Female	Total	%
El Salvador City	535	19	15	34	6.36	1,079	67	42	109	10.10
Gingoog City	1,326	540	568	1,108	83.56	2,677	1,484	1,395	2,879	107.55
Iligan City	3,790	540	536	1,076	28.39	7,972	471	472	943	11.83
Malaybalay City	1,976	95	182	277	14.02	4,061	133	134	267	6.57
Oroquieta City	693	0	2	2	0.29	1,420	257	219	476	33.52
Ozamis City	1,389	38	56	94	6.77	2,851	92	113	205	7.19
Tangub City	620	199	116	315	50.81	1,265	155	167	322	25.45
Valencia City	2,181	325	426	751	34.43	4,487	556	536	1,092	24.34
**Region 11**	**53,626**	**5,239**	**4,993**	**10,232**	**19.08**	**108,114**	**10,232**	**9,994**	**20,226**	**18.71**
Compostela Valley	7,781	427	411	838	10.77	16,183	1,625	1,669	3,294	20.35
Davao del Norte	10,661	716	728	1,444	13.54	21,337	1,411	1,481	2,892	13.55
Davao del Sur	6,508	477	450	927	14.24	13,238	847	782	1,629	12.31
Davao Oriental	7,065	1,014	926	1,940	27.46	14,005	2,190	1,945	4,135	29.53
Davao Occidental	3,347	152	144	296	8.84	7,172	312	286	598	8.34
Davao City	18,264	2,453	2,334	4,787	26.21	36,179	3,847	3,831	7,678	21.22
**Region 12**	**52,301**	**9,052**	**9,337**	**18,389**	**35.16**	**105,056**	**13,322**	**13,447**	**26,769**	**25.48**
North Cotabato	16,840	2,667	2,903	5,570	33.08	33,423	4,662	4,948	9,610	28.75
Sarangani	6,446	3,148	3,055	6,203	96.23	13,278	5,056	4,878	9,934	74.82
South Cotabato	10,554	283	308	591	5.60	20,826	472	459	931	4.47
Sultan Kudarat	8,690	1,730	1,861	3,591	41.32	17,812	1,917	1,895	3,812	21.40
Cotabato City	2,920	38	27	65	2.23	6,187	36	49	85	1.37
Gen. Santos City	6,851	1,186	1,183	2,369	34.58	13,530	1,179	1,218	2,397	17.72
**BARMM**	**46,406**	**3,041**	**3,294**	**6,335**	**13.65**	**105,834**	**1,673**	**1,771**	**3,444**	**3.25**
Basilan	3,765	20	23	43	1.14	7,862	20	29	49	0.62
Lanao del Sur	10,552	2,401	2,631	5,032	47.69	24,775	1,067	1,133	2,200	8.88
Maguindanao	15,563	556	567	1,123	7.22	34,175	488	497	985	2.88
Sulu	8,328	44	51	95	1.14	21,182	81	88	169	0.80
Tawi-Tawi	4,635	1	0	1	0.02	9,764	0	0	0	0.00
Lamitan City	1,032	19	22	41	3.97	2,154	17	24	41	1.90
Marawi City	2,531	0	0	0	0.00	5,922	0	0	0	0.00
**CARAGA**	**30,026**	**4,044**	**4,285**	**8,329**	**27.74**	**61,595**	**9,317**	**9,624**	**18,941**	**30.75**
Agusan del Norte	4,058	363	388	751	18.51	8,190	1,557	1,782	3,339	40.77
Agusan del Sur	8,786	992	1,099	2,091	23.80	18,410	1,402	1,502	2,904	15.77
Surigao del Norte	3,552	475	565	1,040	29.28	7,503	1,431	1,423	2,854	38.04
Surigao del Sur	5,740	898	931	1,829	31.86	11,386	1,980	2,081	4,061	35.67
Province of Dinagat	1,289	129	130	259	20.09	2,654	363	297	660	24.87
Bislig City	1,088	181	211	392	36.03	2,161	275	283	558	25.82
Butuan City	3,855	776	729	1,505	39.04	7,797	2,072	2,006	4,078	52.30
Surigao City	1,658	230	232	462	27.86	3,494	237	250	487	13.94

Note: Put asterisk (*) for No Report and Zero (0) for No Case

Table 1.C.2.4 - Nutrition Services for Infants and Children

Number and Proportion of children 0-59 mos. old whose nutritional status are normal, stunted and overweight/obese

Philippines, Annual 2020

Area	Eligible Pop (0-59 mos)	Normal				Stunted				Overweight/obese			
		Male	Female	Total	%	Male	Female	Total	%	Male	Female	Total	%
**PHILIPPINES**	**11,064,817**	**2,280,688**	**2,165,326**	**4,446,014**	**40.18**	**177,279**	**150,745**	**328,024**	**2.96**	**58,559**	**50,607**	**109,166**	**0.99**
**N C R**	**1,197,748**	**395,757**	**383,071**	**778,828**	**65.02**	**5,820**	**4,677**	**10,497**	**0.88**	**2,921**	**2,523**	**5,444**	**0.45**
Malabon	34,000	6,873	6,583	13,456	39.58	499	427	926	2.72	124	98	222	0.65
Navotas	23,205	6,283	6,291	12,574	54.19	93	74	167	0.72	100	86	186	0.80
Valenzuela City	57,709	19,263	18,172	37,435	64.87	321	269	590	1.02	207	151	358	0.62
Caloocan City	147,324	46,948	46,741	93,689	63.59	624	492	1,116	0.76	542	463	1,005	0.68
Marikina City	41,926	13,515	12,710	26,225	62.55	168	115	283	0.67	179	136	315	0.75
Pasig City	70,248	18,115	17,461	35,576	50.64	324	246	570	0.81	152	116	268	0.38
Pateros	5,939	1,552	1,647	3,199	53.86	50	43	93	1.57	53	21	74	1.25
Taguig	74,867	46,646	46,850	93,496	124.88	92	81	173	0.23	33	23	56	0.07
Quezon City	273,090	80,878	81,444	162,322	59.44	526	445	971	0.36	307	302	609	0.22
Makati City	54,190	12,392	11,049	23,441	43.26	122	107	229	0.42	204	155	359	0.66
Mandaluyong City	35,926	16,253	15,574	31,827	88.59	86	59	145	0.40	17	15	32	0.09
San Juan	11,365	3,944	3,308	7,252	63.81	59	44	103	0.91	31	35	66	0.58
Manila City	165,590	48,371	44,998	93,369	56.39	1,411	1,135	2,546	1.54	431	390	821	0.50
Las Piñas City	54,776	20,360	19,025	39,385	71.90	514	403	917	1.67	125	135	260	0.47
Muntinlupa City	46,924	23,572	22,155	45,727	97.45	197	142	339	0.72	85	100	185	0.39
Parañaque City	61,930	15,062	14,363	29,425	47.51	412	322	734	1.19	152	146	298	0.48
Pasay City	38,739	15,730	14,700	30,430	78.55	322	273	595	1.54	179	151	330	0.85
**C A R**	**182,036**	**68,657**	**64,300**	**132,957**	**73.04**	**7,242**	**6,160**	**13,402**	**7.36**	**1,990**	**1,486**	**3,476**	**1.91**
Abra	23,449	7,712	7,166	14,878	63.45	1,579	1,292	2,871	12.24	376	290	666	2.84
Apayao	13,294	4,691	4,258	8,949	67.32	857	683	1,540	11.58	229	186	415	3.12
Benguet	45,641	17,111	16,187	33,298	72.96	1,031	981	2,012	4.41	411	277	688	1.51
Ifugao	23,514	9,163	8,574	17,737	75.43	644	577	1,221	5.19	190	112	302	1.28
Kalinga	24,345	9,466	8,864	18,330	75.29	1,682	1,372	3,054	12.54	486	398	884	3.63
Mt. Province	16,466	6,221	5,717	11,938	72.50	1,135	952	2,087	12.67	200	131	331	2.01
Baguio City	35,327	14,293	13,534	27,827	78.77	314	303	617	1.75	98	92	190	0.54
**Region 1**	**504,897**	**40,328**	**37,248**	**77,576**	**15.36**	**2,658**	**2,293**	**4,951**	**0.98**	**1,062**	**772**	**1,834**	**0.36**
Ilocos Norte	41,142	*	*	0	0.00	*	*	0	0.00	*	*	0	0.00
Ilocos Sur	50,073	19,462	18,115	37,577	75.04	1,607	1,321	2,928	5.85	348	232	580	1.16
La Union	60,215	16,392	14,983	31,375	52.10	906	836	1,742	2.89	527	393	920	1.53
Pangasinan	259,360	*	*	0	0.00	*	*	0	0.00	*	*	0	0.00
Alaminos City	9,800	*	*	0	0.00	*	*	0	0.00	*	*	0	0.00
Candon City	5,277	2,400	2,278	4,678	88.65	93	84	177	3.35	38	35	73	1.38
Dagupan City	18,708	*	*	0	0.00	*	*	0	0.00	*	*	0	0.00
Laoag City	9,483	*	*	0	0.00	*	*	0	0.00	*	*	0	0.00
San Carlos City	20,603	*	*	0	0.00	*	*	0	0.00	*	*	0	0.00
San Fernando City	11,026	*	*	0	0.00	*	*	0	0.00	147	107	254	2.30
Urdaneta City	14,521	*	*	0	0.00	*	*	0	0.00	*	*	0	0.00
Vigan City	4,689	2,074	1,872	3,946	84.15	52	52	104	2.22	2	5	7	0.15
**Region 2**	**361,003**	**0**	**0**	**0**	**0.00**	**0**	**0**	**0**	**0.00**	**0**	**0**	**0**	**0.00**
Batanes	1,859	*	*	0	0.00	*	*	0	0.00	*	*	0	0.00
Cagayan	93,452	*	*	0	0.00	*	*	0	0.00	*	*	0	0.00
Isabela	136,381	*	*	0	0.00	*	*	0	0.00	*	*	0	0.00
Nueva Vizcaya	49,932	*	*	0	0.00	*	*	0	0.00	*	*	0	0.00
Quirino	21,219	*	*	0	0.00	*	*	0	0.00	*	*	0	0.00
Cauayan City	13,395	*	*	0	0.00	*	*	0	0.00	*	*	0	0.00
Ilagan City	15,055	*	*	0	0.00	*	*	0	0.00	*	*	0	0.00
Santiago City	13,944	*	*	0	0.00	*	*	0	0.00	*	*	0	0.00
Tuguegarao City	15,766	*	*	0	0.00	*	*	0	0.00	*	*	0	0.00
**Region 3**	**1,133,017**	**323,277**	**311,214**	**634,491**	**56.00**	**19,541**	**16,835**	**36,376**	**3.21**	**12,582**	**11,108**	**23,690**	**2.09**
Aurora	25,236	8,201	8,722	16,923	67.06	1,169	1,026	2,195	8.70	170	162	332	1.32
Bataan	68,669	33,558	31,434	64,992	94.65	1,719	1,512	3,231	4.71	1,190	999	2,189	3.19
Bulacan	224,595	40,891	38,511	79,402	35.35	1,610	1,191	2,801	1.25	1,034	830	1,864	0.83
Nueva Ecija	150,372	22,953	21,797	44,750	29.76	3,389	3,058	6,447	4.29	2,210	1,914	4,124	2.74
Pampanga	162,203	107,138	101,047	208,185	128.35	5,962	5,043	11,005	6.78	3,204	2,894	6,098	3.76
Tarlac	101,537	17,190	16,812	34,002	33.49	952	782	1,734	1.71	996	879	1,875	1.85
Zambales	63,798	2,783	2,693	5,476	8.58	471	447	918	1.44	169	150	319	0.50

Table 1.C.2.4 - Nutrition Services for Infants and Children

Number and Proportion of children 0-59 mos. old whose nutritional status are normal, stunted and overweight/obese

Philippines, Annual 2020

Area	Eligible Pop (0-59 mos)	Normal				Stunted				Overweight/obese			
		Male	Female	Total	%	Male	Female	Total	%	Male	Female	Total	%
Angeles City	40,695	0	0	0	0.00	0	0	0	0.00	0	0	0	0.00
Balanga City	9,926	3,992	3,918	7,910	79.69	147	127	274	2.76	150	111	261	2.63
Cabanatuan City	30,776	14,463	17,369	31,832	103.43	129	122	251	0.82	136	145	281	0.91
City of San Fernando	30,316	10	15	25	0.08	6	8	14	0.05	5	10	15	0.05
Gapan City	11,232	5,422	4,748	10,170	90.54	299	271	570	5.07	207	174	381	3.39
Mabalacat City	24,796	11,920	12,284	24,204	97.61	181	204	385	1.55	178	199	377	1.52
Malolos City	25,089	6,309	6,007	12,316	49.09	968	866	1,834	7.31	563	479	1,042	4.15
Meycauayan	20,809	3,842	3,749	7,591	36.48	8	11	19	0.09	5	5	10	0.05
Olongapo	25,163	11,183	10,203	21,386	84.99	169	139	308	1.22	125	119	244	0.97
Palayan City	4,180	1,577	1,579	3,156	75.50	186	167	353	8.44	82	68	150	3.59
San Jose City	14,230	461	434	895	6.29	0	0	0	0.00	475	448	923	6.49
San Jose del Monte City	57,133	27,185	26,292	53,477	93.60	2,020	1,747	3,767	6.59	1,618	1,471	3,089	5.41
Science City of Munoz	8,292	4,199	3,600	7,799	94.05	156	114	270	3.26	65	51	116	1.40
Tarlac City	33,970	0	0	0	0.00	0	0	0	0.00	0	0	0	0.00
**Region 4A**	**1,524,327**	**0**	**0**	**0**	**0.00**	**0**	**0**	**0**	**0.00**	**26**	**18**	**44**	**0.00**
Batangas	200,478	0	0	0	0.00	0	0	0	0.00	0	0	0	0.00
Cavite	141,654	0	0	0	0.00	0	0	0	0.00	26	18	44	0.03
Laguna	100,330	0	0	0	0.00	0	0	0	0.00	0	0	0	0.00
Quezon	196,065	0	0	0	0.00	0	0	0	0.00	0	0	0	0.00
Rizal	226,655	0	0	0	0.00	0	0	0	0.00	0	0	0	0.00
Antipolo City	83,485	0	0	0	0.00	0	0	0	0.00	0	0	0	0.00
Bacoor City	62,060	0	0	0	0.00	0	0	0	0.00	0	0	0	0.00
Batangas City	35,565	0	0	0	0.00	0	0	0	0.00	0	0	0	0.00
Biñan City	33,648	0	0	0	0.00	0	0	0	0.00	0	0	0	0.00
Cabuyao City	31,190	0	0	0	0.00	0	0	0	0.00	0	0	0	0.00
Calamba City	45,918	0	0	0	0.00	0	0	0	0.00	0	0	0	0.00
Cavite City	10,620	0	0	0	0.00	0	0	0	0.00	0	0	0	0.00
Dasmariñas City	68,089	0	0	0	0.00	0	0	0	0.00	0	0	0	0.00
General Trias City	32,479	0	0	0	0.00	0	0	0	0.00	0	0	0	0.00
Imus City	41,725	0	0	0	0.00	0	0	0	0.00	0	0	0	0.00
Lipa City	35,846	0	0	0	0.00	0	0	0	0.00	0	0	0	0.00
Lucena City	29,712	0	0	0	0.00	0	0	0	0.00	0	0	0	0.00
San Pablo City	26,882	0	0	0	0.00	0	0	0	0.00	0	0	0	0.00
San Pedro City	32,915	0	0	0	0.00	0	0	0	0.00	0	0	0	0.00
Santa Rosa City	35,736	0	0	0	0.00	0	0	0	0.00	0	0	0	0.00
Tagaytay City	7,355	0	0	0	0.00	0	0	0	0.00	0	0	0	0.00
Tanauan City	18,699	0	0	0	0.00	0	0	0	0.00	0	0	0	0.00
Tayabas City	11,136	0	0	0	0.00	0	0	0	0.00	0	0	0	0.00
Trece Martires City	16,085	0	0	0	0.00	0	0	0	0.00	0	0	0	0.00
**Region 4B**	**369,642**	**87,456**	**79,911**	**167,367**	**45.28**	**11,343**	**8,451**	**19,794**	**5.35**	**2,175**	**1,706**	**3,881**	**1.05**
Marinduque	26,548	27,870	24,048	51,918	195.56	3,035	2,298	5,333	20.09	670	497	1,167	4.40
Mindoro Occidental	62,744	26,437	25,060	51,497	82.07	4,778	3,909	8,687	13.85	538	419	957	1.53
Mindoro Oriental	102,984	3,817	3,624	7,441	7.23	456	429	885	0.86	85	64	149	0.14
Palawan	109,653	16,118	14,640	30,758	28.05	709	628	1,337	1.22	117	116	233	0.21
Romblon	34,781	2,380	2,105	4,485	12.89	316	332	648	1.86	93	51	144	0.41
Puerto Princesa City	32,932	10,834	10,434	21,268	64.58	2,049	855	2,904	8.82	672	559	1,231	3.74
**Region 5**	**722,518**	**221,119**	**205,808**	**426,927**	**59.09**	**33,566**	**28,023**	**61,589**	**8.52**	**7,998**	**6,417**	**14,415**	**2.00**
Albay	126,974	27,012	24,858	51,870	40.85	3,292	2,857	6,149	4.84	1,228	964	2,192	1.73
Camarines Norte	74,181	26,657	24,849	51,506	69.43	4,194	3,364	7,558	10.19	680	510	1,190	1.60
Camarines Sur	205,033	62,466	58,236	120,702	58.87	11,241	9,864	21,105	10.29	2,275	1,844	4,119	2.01
Catanduanes	32,856	11,335	10,756	22,091	67.24	2,443	2,090	4,533	13.80	419	292	711	2.16
Masbate	121,967	32,607	30,609	63,216	51.83	5,807	4,622	10,429	8.55	1,005	819	1,824	1.50
Sorsogon	100,821	36,378	34,075	70,453	69.88	6,076	4,775	10,851	10.76	1,904	1,467	3,371	3.34
Iriga City	13,932	5,104	4,861	9,965	71.53	24	24	48	0.34	1	2	3	0.02
Legaspi City	22,322	9,127	8,001	17,128	76.73	220	180	400	1.79	227	368	595	2.67
Naga City	24,432	10,433	9,563	19,996	81.84	269	247	516	2.11	259	151	410	1.68
**Region 6**	**770,279**	**332,848**	**304,926**	**637,774**	**82.80**	**23,614**	**19,407**	**43,021**	**5.59**	**8,761**	**8,530**	**17,291**	**2.24**
Aklan	59,005	23,393	21,904	45,297	76.77	1,704	1,423	3,127	5.30	528	443	971	1.65
Antique	66,616	20,825	21,838	42,663	64.04	3,041	2,376	5,417	8.13	624	599	1,223	1.84
Capiz	74,073	31,497	30,421	61,918	83.59	1,712	1,287	2,999	4.05	1,085	858	1,943	2.62
Guimaras	17,596	7,867	7,481	15,348	87.22	113	84	197	1.12	250	195	445	2.53
Iloilo	190,173	85,791	80,970	166,761	87.69	8,120	6,742	14,862	7.81	3,008	2,611	5,619	2.95
Negros Occidental	260,257	111,409	104,627	216,036	83.01	6,116	5,261	11,377	4.37	1,707	1,348	3,055	1.17

Table 1.C.2.4 - Nutrition Services for Infants and Children

Number and Proportion of children 0-59 mos. old whose nutritional status are normal, stunted and overweight/obese

Philippines, Annual 2020

Area	Eligible Pop (0-59 mos)	Normal				Stunted				Overweight/obese			
		Male	Female	Total	%	Male	Female	Total	%	Male	Female	Total	%
Bacolod City	58,565	31,308	17,827	49,135	83.90	2,249	1,751	4,000	6.83	1,035	2,004	3,039	5.19
Iloilo City	43,994	20,758	19,858	40,616	92.32	559	483	1,042	2.37	524	472	996	2.26
**Region 7**	**815,641**	**37,155**	**43,152**	**80,307**	**9.85**	**4,176**	**4,150**	**8,326**	**1.02**	**1,015**	**916**	**1,931**	**0.24**
Bohol	142,369	2,186	2,206	4,392	3.08	0	0	0	0.00	91	60	151	0.11
Cebu	330,719	3,629	3,169	6,798	2.06	211	197	408	0.12	57	31	88	0.03
Negros Oriental	142,955	30,304	36,764	67,068	46.92	3,892	3,848	7,740	5.41	836	810	1,646	1.15
Siquijor	9,051	526	549	1,075	11.88	42	72	114	1.26	23	12	35	0.39
Cebu City	103,819	510	464	974	0.94	31	33	64	0.06	8	3	11	0.01
Lapu-Lapu City	45,926	0	0	0	0.00	0	0	0	0.00	0	0	0	0.00
Mandaue City	40,802	0	0	0	0.00	0	0	0	0.00	0	0	0	0.00
**Region 8**	**535,655**	**64,307**	**63,222**	**127,529**	**23.81**	**11,915**	**10,658**	**22,573**	**4.21**	**3,671**	**3,247**	**6,918**	**1.29**
Biliran	20,662	7,470	6,952	14,422	69.80	1,414	1,142	2,556	12.37	352	279	631	3.05
Eastern Samar	57,642	6,648	8,862	15,510	26.91	1,066	1,453	2,519	4.37	453	482	935	1.62
Northern Leyte	178,281	3,094	2,984	6,078	3.41	645	607	1,252	0.70	378	378	756	0.42
Northern Samar	82,757	25,003	23,546	48,549	58.66	4,603	3,896	8,499	10.27	1,172	1,049	2,221	2.68
Southern Leyte	35,207	3,798	3,608	7,406	21.04	727	639	1,366	3.88	325	240	565	1.60
Western Samar	75,044	1,292	1,160	2,452	3.27	493	455	948	1.26	88	56	144	0.19
Calbayog City	23,119	4,303	4,120	8,423	36.43	811	674	1,485	6.42	281	263	544	2.35
Maasin City	8,966	2,994	2,666	5,660	63.13	209	164	373	4.16	49	45	94	1.05
Ormoc City	25,396	7,742	7,521	15,263	60.10	1,930	1,607	3,537	13.93	560	448	1,008	3.97
Tacloban City	28,581	1,963	1,803	3,766	13.18	17	21	38	0.13	13	7	20	0.07
**Region 9**	**419,501**	**38,782**	**39,479**	**78,261**	**18.66**	**2,475**	**2,355**	**4,830**	**1.15**	**431**	**460**	**891**	**0.21**
Zamboanga del Norte	92,110	16,340	16,509	32,849	35.66	568	561	1,129	1.23	89	96	185	0.20
Zamboanga del Sur	89,792	5,327	5,554	10,881	12.12	1,361	1,267	2,628	2.93	231	192	423	0.47
Zamboanga Sibugay	81,062	3,068	4,394	7,462	9.21	325	310	635	0.78	15	85	100	0.12
Dapitan City	9,507	3,729	3,318	7,047	74.12	119	86	205	2.16	27	18	45	0.47
Dipolog City	15,087	7,789	7,165	14,954	99.12	33	30	63	0.42	1	2	3	0.02
Isabela City	14,580	0	0	0	0.00	41	74	115	0.79	42	36	78	0.53
Pagadian City	22,024	0	0	0	0.00	0	0	0	0.00	0	0	0	0.00
Zamboanga City	95,339	2,529	2,539	5,068	5.32	28	27	55	0.06	26	31	57	0.06
**Region 10**	**536,376**	**125,405**	**111,577**	**236,982**	**44.18**	**7,239**	**5,874**	**13,113**	**2.44**	**3,676**	**3,356**	**7,032**	**1.31**
Bukidnon	124,287	57,687	53,543	111,230	89.49	3,999	3,360	7,359	5.92	2,790	2,709	5,499	4.42
Camiguin	9,865	3,753	3,483	7,236	73.35	411	342	753	7.63	105	98	203	2.06
Lanao del Norte	82,485	4,548	4,174	8,722	10.57	286	224	510	0.62	63	66	129	0.16
Misamis Occidental	33,811	4,968	4,889	9,857	29.15	160	125	285	0.84	20	12	32	0.09
Misamis Oriental	78,469	9,233	964	10,197	12.99	173	56	229	0.29	95	12	107	0.14
Cagayan de Oro City	74,313	3,121	2,943	6,064	8.16	17	14	31	0.04	12	9	21	0.03
El Salvador City	5,517	2,655	2,418	5,073	91.95	28	43	71	1.29	17	11	28	0.51
Gingoog City	13,703	0	0	0	0.00	0	0	0	0.00	0	0	0	0.00
Iligan City	41,779	12,203	11,256	23,459	56.15	745	570	1,315	3.15	217	186	403	0.96
Malaybalay City	20,713	8,074	7,564	15,638	75.50	1,098	900	1,998	9.65	227	193	420	2.03
Oroquieta City	7,326	1,263	1,205	2,468	33.69	135	81	216	2.95	46	24	70	0.96
Ozamis City	14,688	8,454	8,436	16,890	114.99	109	97	206	1.40	39	27	66	0.45
Tangub City	6,525	*	*	0	0.00	*	*	0	0.00	*	*	0	0.00
Valencia City	22,895	9,446	10,702	20,148	88.00	78	62	140	0.61	45	9	54	0.24
**Region 11**	**555,461**	**295,318**	**280,962**	**576,280**	**103.75**	**17,739**	**15,041**	**32,780**	**5.90**	**5,515**	**4,375**	**9,890**	**1.78**
Compostela Valley	83,688	35,649	33,841	69,490	83.03	2,113	1,968	4,081	4.88	510	371	881	1.05
Davao del Norte	108,999	82,353	76,877	159,230	146.08	3,594	2,991	6,585	6.04	1,459	1,227	2,686	2.46
Davao del Sur	69,170	35,213	32,875	68,088	98.44	3,067	2,368	5,435	7.86	867	665	1,532	2.21
Davao Oriental	71,494	36,791	36,738	73,529	102.85	2,692	2,238	4,930	6.90	1,187	892	2,079	2.91
Davao Occidental	37,541	13,521	13,089	26,610	70.88	1,997	1,767	3,764	10.03	462	424	886	2.36
Davao City	184,569	91,791	87,542	179,333	97.16	4,276	3,709	7,985	4.33	1,030	796	1,826	0.99
**Region 12**	**542,112**	**155,333**	**146,679**	**302,012**	**55.71**	**20,662**	**18,562**	**39,224**	**7.24**	**4,105**	**3,491**	**7,596**	**1.40**
North Cotabato	172,722	94,267	88,759	183,026	105.97	8,318	7,266	15,584	9.02	1,943	1,730	3,673	2.13
Sarangani	67,497	22,835	22,219	45,054	66.75	3,146	3,042	6,188	9.17	420	330	750	1.11

Table 1.C.2.4 - Nutrition Services for Infants and Children

Number and Proportion of children 0-59 mos. old whose nutritional status are normal, stunted and overweight/obese

Philippines, Annual 2020

Area	Eligible Pop (0-59 mos)	Normal				Stunted				Overweight/obese			
		Male	Female	Total	%	Male	Female	Total	%	Male	Female	Total	%
South Cotabato	106,332	0	0	0	0.00	2,495	2,460	4,955	4.66	0	0	0	0.00
Sultan Kudarat	92,288	0	0	0	0.00	4,146	3,704	7,850	8.51	765	638	1,403	1.52
Cotabato City	34,220	7,027	6,740	13,767	40.23	582	570	1,152	3.37	102	75	177	0.52
Gen. Santos City	69,053	31,204	28,961	60,165	87.13	1,975	1,520	3,495	5.06	875	718	1,593	2.31
**BARMM**	**576,008**	**0**	**0**	**0**	**0.00**	**6**	**10**	**16**	**0.00**	**0**	**0**	**0**	**0.00**
Basilan	43,019	0	0	0	0.00	0	0	0	0.00	0	0	0	0.00
Lanao del Sur	131,469	0	0	0	0.00	0	0	0	0.00	0	0	0	0.00
Maguindanao	180,080	0	0	0	0.00	0	0	0	0.00	0	0	0	0.00
Sulu	123,904	0	0	0	0.00	0	0	0	0.00	0	0	0	0.00
Tawi-Tawi	54,261	0	0	0	0.00	0	0	0	0.00	0	0	0	0.00
Lamitan City	11,832	0	0	0	0.00	0	0	0	0.00	0	0	0	0.00
Marawi City	31,443	0	0	0	0.00	6	10	16	0.05	0	0	0	0.00
**CARAGA**	**318,596**	**94,946**	**93,777**	**188,723**	**59.24**	**9,283**	**8,249**	**17,532**	**5.50**	**2,631**	**2,202**	**4,833**	**1.52**
Agusan del Norte	42,202	26,922	27,092	54,014	127.99	2,267	2,130	4,397	10.42	1,005	913	1,918	4.54
Agusan del Sur	94,397	16,454	16,027	32,481	34.41	1,824	1,563	3,387	3.59	477	397	874	0.93
Surigao del Norte	38,645	7,777	8,279	16,056	41.55	761	764	1,525	3.95	250	214	464	1.20
Surigao del Sur	60,014	22,812	22,867	45,679	76.11	3,266	2,746	6,012	10.02	663	488	1,151	1.92
Province of Dinagat	13,805	3,229	3,038	6,267	45.40	214	217	431	3.12	126	71	197	1.43
Bislig City	11,399	2,533	2,572	5,105	44.78	777	659	1,436	12.60	66	40	106	0.93
Butuan City	40,128	14,717	13,315	28,032	69.86	152	147	299	0.75	37	72	109	0.27
Surigao City	18,006	502	587	1,089	6.05	22	23	45	0.25	7	7	14	0.08

Note: Put asterisk (*) for No Report and Zero (0) for No Case

Table 1.C.2.5 - Nutrition Services for Infants and Children

Number and Proportion of children 0-59 mos. old whose nutritional status are Wasted-MAM, Wasted-SAM and Wasted
Philippines, Annual 2020

Area	Eligible Pop (0-59 mos)	Wasted-MAM				Wasted-SAM				Wasted			
		Male	Female	Total	%	Male	Female	Total	%	Male	Female	Total	%
**PHILIPPINES**	**11,064,817**	**36,697**	**36,863**	**73,560**	**0.66**	**13,947**	**10,924**	**24,871**	**0.22**	**52,212**	**48,967**	**101,179**	**0.91**
**N C R**	**1,197,748**	**0**	**0**	**0**	**0.00**	**0**	**0**	**0**	**0.00**	**0**	**0**	**0**	**0.00**
Malabon	34,000	*	*	0	0.00	*	*	0	0.00	*	*	0	0.00
Navotas	23,205	*	*	0	0.00	*	*	0	0.00	*	*	0	0.00
Valenzuela City	57,709	*	*	0	0.00	*	*	0	0.00	*	*	0	0.00
Caloocan City	147,324	*	*	0	0.00	*	*	0	0.00	*	*	0	0.00
Marikina City	41,926	*	*	0	0.00	*	*	0	0.00	*	*	0	0.00
Pasig City	70,248	*	*	0	0.00	*	*	0	0.00	*	*	0	0.00
Pateros	5,939	*	*	0	0.00	*	*	0	0.00	*	*	0	0.00
Taguig	74,867	*	*	0	0.00	*	*	0	0.00	*	*	0	0.00
Quezon City	273,090	*	*	0	0.00	*	*	0	0.00	*	*	0	0.00
Makati City	54,190	*	*	0	0.00	*	*	0	0.00	*	*	0	0.00
Mandaluyong City	35,926	*	*	0	0.00	*	*	0	0.00	*	*	0	0.00
San Juan	11,365	*	*	0	0.00	*	*	0	0.00	*	*	0	0.00
Manila City	165,590	*	*	0	0.00	*	*	0	0.00	*	*	0	0.00
Las Piñas City	54,776	*	*	0	0.00	*	*	0	0.00	*	*	0	0.00
Muntinlupa City	46,924	*	*	0	0.00	*	*	0	0.00	*	*	0	0.00
Parañaque City	61,930	*	*	0	0.00	*	*	0	0.00	*	*	0	0.00
Pasay City	38,739	*	*	0	0.00	*	*	0	0.00	*	*	0	0.00
**C A R**	**182,036**	**960**	**7,852**	**8,812**	**4.84**	**380**	**320**	**700**	**0.38**	**1,340**	**8,172**	**9,512**	**5.23**
Abra	23,449	261	202	463	1.97	115	91	206	0.88	376	293	669	2.85
Apayao	13,294	148	122	270	2.03	43	43	86	0.65	191	165	356	2.68
Benguet	45,641	84	69	153	0.34	29	20	49	0.11	113	89	202	0.44
Ifugao	23,514	52	7,166	7,218	30.70	17	20	37	0.16	69	7,186	7,255	30.85
Kalinga	24,345	314	216	530	2.18	133	114	247	1.01	447	330	777	3.19
Mt. Province	16,466	71	49	120	0.73	33	23	56	0.34	104	72	176	1.07
Baguio City	35,327	30	28	58	0.16	10	9	19	0.05	40	37	77	0.22
**Region 1**	**504,897**	**657**	**523**	**1,180**	**0.23**	**51**	**49**	**100**	**0.02**	**708**	**572**	**1,280**	**0.25**
Ilocos Norte	41,142	*	*	0	0.00	*	*	0	0.00	0	0	0	0.00
Ilocos Sur	50,073	374	286	660	1.32	0	0	0	0.00	374	286	660	1.32
La Union	60,215	209	180	389	0.65	38	33	71	0.12	247	213	460	0.76
Pangasinan	259,360	*	*	0	0.00			0	0.00	0	0	0	0.00
Alaminos City	9,800	*	*	0	0.00	*	*	0	0.00	0	0	0	0.00
Candon City	5,277	18	12	30	0.57	0	0	0	0.00	18	12	30	0.57
Dagupan City	18,708	*	*	0	0.00	*	*	0	0.00	0	0	0	0.00
Laoag City	9,483	*	*	0	0.00	*	*	0	0.00	0	0	0	0.00
San Carlos City	20,603	*	*	0	0.00	*	*	0	0.00	0	0	0	0.00
San Fernando City	11,026	47	42	89	0.81	13	16	29	0.26	60	58	118	1.07
Urdaneta City	14,521	*	*	0	0.00	*	*	0	0.00	0	0	0	0.00
Vigan City	4,689	9	3	12	0.26	0	0	0	0.00	9	3	12	0.26
**Region 2**	**361,003**	**0**	**0**	**0**	**0.00**	**0**	**0**	**0**	**0.00**	**0**	**0**	**0**	**0.00**
Batanes	1,859	*	*	0	0.00	*	*	0	0.00	*	*	0	0.00
Cagayan	93,452	*	*	0	0.00	*	*	0	0.00	*	*	0	0.00
Isabela	136,381	*	*	0	0.00	*	*	0	0.00	*	*	0	0.00
Nueva Vizcaya	49,932	*	*	0	0.00	*	*	0	0.00	*	*	0	0.00
Quirino	21,219	*	*	0	0.00	*	*	0	0.00	*	*	0	0.00
Cauayan City	13,395	*	*	0	0.00	*	*	0	0.00	*	*	0	0.00
Ilagan City	15,055	*	*	0	0.00	*	*	0	0.00	*	*	0	0.00
Santiago City	13,944	*	*	0	0.00	*	*	0	0.00	*	*	0	0.00
Tuguegarao City	15,766	*	*	0	0.00	*	*	0	0.00	*	*	0	0.00
**Region 3**	**1,133,017**	**6,340**	**5,165**	**11,505**	**1.02**	**2,716**	**1,962**	**4,678**	**0.41**	**10,624**	**8,307**	**18,931**	**1.67**
Aurora	25,236	247	169	416	1.65	232	89	321	1.27	479	258	737	2.92
Bataan	68,669	735	640	1,375	2.00	201	198	399	0.58	936	838	1,774	2.58
Bulacan	224,595	31	27	58	0.03	0	0	0	0.00	877	604	1,481	0.66

Table 1.C.2.5 - Nutrition Services for Infants and Children

Number and Proportion of children 0-59 mos. old whose nutritional status are Wasted-MAM, Wasted-SAM and Wasted
Philippines, Annual 2020

Area	Eligible Pop (0-59 mos)	Wasted-MAM				Wasted-SAM				Wasted			
		Male	Female	Total	%	Male	Female	Total	%	Male	Female	Total	%
Nueva Ecija	150,372	0	0	0	0.00	0	0	0	0.00	612	499	1,111	0.74
Pampanga	162,203	3,105	2,537	5,642	3.48	1,627	1,234	2,861	1.76	4,732	3,771	8,503	5.24
Tarlac	101,537	538	397	935	0.92	0	0	0	0.00	538	397	935	0.92
Zambales	63,798	54	51	105	0.16	49	39	88	0.14	103	90	193	0.30
Angeles City	40,695	0	0	0	0.00	0	0	0	0.00	0	0	0	0.00
Balanga City	9,926	71	51	122	1.23	21	20	41	0.41	92	71	163	1.64
Cabanatuan City	30,776	136	158	294	0.96	0	0	0	0.00	136	158	294	0.96
City of San Fernando	30,316	6	8	14	0.05	0	0	0	0.00	6	8	14	0.05
Gapan City	11,232	126	67	193	1.72	45	24	69	0.61	171	91	262	2.33
Mabalacat City	24,796	0	0	0	0.00	0	0	0	0.00	110	104	214	0.86
Malolos City	25,089	0	0	0	0.00	0	0	0	0.00	0	0	0	0.00
Meycauayan	20,809	4	5	9	0.04	0	0	0	0.00	4	5	9	0.04
Olongapo	25,163	0	0	0	0.00	0	0	0	0.00	0	0	0	0.00
Palayan City	4,180	0	0	0	0.00	0	0	0	0.00	0	0	0	0.00
San Jose City	14,230	461	434	895	6.29	0	0	0	0.00	461	434	895	6.29
San Jose del Monte City	57,133	701	530	1,231	2.15	510	335	845	1.48	1,211	865	2,076	3.63
Science City of Munoz	8,292	125	91	216	2.60	31	23	54	0.65	156	114	270	3.26
Tarlac City	33,970	0	0	0	0.00	0	0	0	0.00	0	0	0	0.00
**Region 4A**	**1,524,327**	**0**	**0**	**0**	**0.00**	**0**	**0**	**0**	**0.00**	**0**	**0**	**0**	**0.00**
Batangas	200,478	*	*	0	0.00	*	*	0	0.00	*	*	0	0.00
Cavite	141,654	*	*	0	0.00	*	*	0	0.00	*	*	0	0.00
Laguna	100,330	*	*	0	0.00	*	*	0	0.00	*	*	0	0.00
Quezon	196,065	*	*	0	0.00	*	*	0	0.00	*	*	0	0.00
Rizal	226,655	*	*	0	0.00	*	*	0	0.00	*	*	0	0.00
Antipolo City	83,485	*	*	0	0.00	*	*	0	0.00	*	*	0	0.00
Bacoor City	62,060	*	*	0	0.00	*	*	0	0.00	*	*	0	0.00
Batangas City	35,565	*	*	0	0.00	*	*	0	0.00	*	*	0	0.00
Biñan City	33,648	*	*	0	0.00	*	*	0	0.00	*	*	0	0.00
Cabuyao City	31,190	*	*	0	0.00	*	*	0	0.00	*	*	0	0.00
Calamba City	45,918	*	*	0	0.00	*	*	0	0.00	*	*	0	0.00
Cavite City	10,620	*	*	0	0.00	*	*	0	0.00	*	*	0	0.00
Dasmariñas City	68,089	*	*	0	0.00	*	*	0	0.00	*	*	0	0.00
General Trias City	32,479	*	*	0	0.00	*	*	0	0.00	*	*	0	0.00
Imus City	41,725	*	*	0	0.00	*	*	0	0.00	*	*	0	0.00
Lipa City	35,846	*	*	0	0.00	*	*	0	0.00	*	*	0	0.00
Lucena City	29,712	*	*	0	0.00	*	*	0	0.00	*	*	0	0.00
San Pablo City	26,882	*	*	0	0.00	*	*	0	0.00	*	*	0	0.00
San Pedro City	32,915	*	*	0	0.00	*	*	0	0.00	*	*	0	0.00
Santa Rosa City	35,736	*	*	0	0.00	*	*	0	0.00	*	*	0	0.00
Tagaytay City	7,355	*	*	0	0.00	*	*	0	0.00	*	*	0	0.00
Tanauan City	18,699	*	*	0	0.00	*	*	0	0.00	*	*	0	0.00
Tayabas City	11,136	*	*	0	0.00	*	*	0	0.00	*	*	0	0.00
Trece Martires City	16,085	*	*	0	0.00	*	*	0	0.00	*	*	0	0.00
**Region 4B**	**369,642**	**1,852**	**1,527**	**3,379**	**0.91**	**813**	**580**	**1,393**	**0.38**	**2,665**	**2,107**	**4,772**	**1.29**
Marinduque	26,548	737	678	1,415	5.33	253	181	434	1.63	990	859	1,849	6.96
Mindoro Occidental	62,744	785	622	1,407	2.24	220	162	382	0.61	1,005	784	1,789	2.85
Mindoro Oriental	102,984	0	0	0	0.00	0	0	0	0.00	0	0	0	0.00
Palawan	109,653	273	189	462	0.42	61	31	92	0.08	334	220	554	0.51
Romblon	34,781	57	38	95	0.27	59	47	106	0.30	116	85	201	0.58
Puerto Princesa City	32,932	0	0	0	0.00	220	159	379	1.15	220	159	379	1.15
**Region 5**	**722,518**	**5,250**	**4,142**	**9,392**	**1.30**	**1,944**	**1,425**	**3,369**	**0.47**	**7,194**	**5,567**	**12,761**	**1.77**
Albay	126,974	709	506	1,215	0.96	195	106	301	0.24	904	612	1,516	1.19
Camarines Norte	74,181	440	326	766	1.03	110	88	198	0.27	550	414	964	1.30
Camarines Sur	205,033	827	772	1,599	0.78	333	264	597	0.29	1,160	1,036	2,196	1.07
Catanduanes	32,856	368	263	631	1.92	69	76	145	0.44	437	339	776	2.36
Masbate	121,967	1,265	969	2,234	1.83	548	401	949	0.78	1,813	1,370	3,183	2.61
Sorsogon	100,821	1,378	1,111	2,489	2.47	585	405	990	0.98	1,963	1,516	3,479	3.45
Iriga City	13,932	29	22	51	0.37	3	2	5	0.04	32	24	56	0.40
Legaspi City	22,322	112	81	193	0.86	56	52	108	0.48	168	133	301	1.35
Naga City	24,432	122	92	214	0.88	45	31	76	0.31	167	123	290	1.19

Table 1.C.2.5 - Nutrition Services for Infants and Children

Number and Proportion of children 0-59 mos. old whose nutritional status are Wasted-MAM, Wasted-SAM and Wasted
Philippines, Annual 2020

Area	Eligible Pop (0-59 mos)	Wasted-MAM				Wasted-SAM				Wasted			
		Male	Female	Total	%	Male	Female	Total	%	Male	Female	Total	%
**Region 6**	**770,279**	**5,567**	**4,350**	**9,917**	**1.29**	**2,124**	**1,636**	**3,760**	**0.49**	**7,691**	**5,986**	**13,677**	**1.78**
Aklan	59,005	595	482	1,077	1.83	249	184	433	0.73	844	666	1,510	2.56
Antique	66,616	500	323	823	1.24	133	104	237	0.36	633	427	1,060	1.59
Capiz	74,073	465	327	792	1.07	463	318	781	1.05	928	645	1,573	2.12
Guimaras	17,596	90	70	160	0.91	23	14	37	0.21	113	84	197	1.12
Iloilo	190,173	1,624	1,175	2,799	1.47	444	367	811	0.43	2,068	1,542	3,610	1.90
Negros Occidental	260,257	2,114	1,833	3,947	1.52	742	584	1,326	0.51	2,856	2,417	5,273	2.03
Bacolod City	58,565	16	17	33	0.06	3	15	18	0.03	19	32	51	0.09
Iloilo City	43,994	163	123	286	0.65	67	50	117	0.27	230	173	403	0.92
**Region 7**	**815,641**	**104**	**117**	**221**	**0.03**	**56**	**35**	**91**	**0.01**	**160**	**152**	**312**	**0.04**
Bohol	142,369	47	45	92	0.06	28	19	47	0.03	75	64	139	0.10
Cebu	330,719	50	48	98	0.03	27	15	42	0.01	77	63	140	0.04
Negros Oriental	142,955	0	0	0	0.00	0	0	0	0.00	0	0	0	0.00
Siquijor	9,051	5	17	22	0.24	0	0	0	0.00	5	17	22	0.24
Cebu City	103,819	2	7	9	0.01	1	1	2	0.00	3	8	11	0.01
Lapu-Lapu City	45,926	0	0	0	0.00	0	0	0	0.00	0	0	0	0.00
Mandaue City	40,802	0	0	0	0.00	0	0	0	0.00	0	0	0	0.00
**Region 8**	**535,655**	**2,594**	**2,476**	**5,070**	**0.95**	**1,836**	**1,467**	**3,303**	**0.62**	**4,430**	**3,943**	**8,373**	**1.56**
Biliran	20,662	330	271	601	2.91	96	72	168	0.81	426	343	769	3.72
Eastern Samar	57,642	245	315	560	0.97	172	186	358	0.62	417	501	918	1.59
Northern Leyte	178,281	222	218	440	0.25	157	130	287	0.16	379	348	727	0.41
Northern Samar	82,757	922	923	1,845	2.23	1,035	810	1,845	2.23	1,957	1,733	3,690	4.46
Southern Leyte	35,207	210	179	389	1.10	16	16	32	0.09	226	195	421	1.20
Western Samar	75,044	65	83	148	0.20	55	49	104	0.14	120	132	252	0.34
Calbayog City	23,119	205	138	343	1.48	144	82	226	0.98	349	220	569	2.46
Maasin City	8,966	24	24	48	0.54	0	0	0	0.00	24	24	48	0.54
Ormoc City	25,396	370	322	692	2.72	161	121	282	1.11	531	443	974	3.84
Tacloban City	28,581	1	3	4	0.01	0	1	1	0.00	1	4	5	0.02
**Region 9**	**419,501**	**44**	**63**	**107**	**0.03**	**0**	**0**	**0**	**0.00**	**44**	**63**	**107**	**0.03**
Zamboanga del Norte	92,110	0	0	0	0.00	0	0	0	0.00	0	0	0	0.00
Zamboanga del Sur	89,792	4	9	13	0.01	0	0	0	0.00	4	9	13	0.01
Zamboanga Sibugay	81,062	0	0	0	0.00	0	0	0	0.00	0	0	0	0.00
Dapitan City	9,507	0	0	0	0.00	0	0	0	0.00	0	0	0	0.00
Dipolog City	15,087	0	0	0	0.00	0	0	0	0.00	0	0	0	0.00
Isabela City	14,580	40	54	94	0.64	nd	nd	0	0.00	40	54	94	0.64
Pagadian City	22,024	0	0	0	0.00	0	0	0	0.00	0	0	0	0.00
Zamboanga City	95,339	0	0	0	0.00	0	0	0	0.00	0	0	0	0.00
**Region 10**	**536,376**	**1,240**	**896**	**2,136**	**0.40**	**312**	**277**	**589**	**0.11**	**1,552**	**1,173**	**2,725**	**0.51**
Bukidnon	124,287	305	284	589	0.47	124	119	243	0.20	429	403	832	0.67
Camiguin	9,865	10	8	18	0.18	19	9	28	0.28	29	17	46	0.47
Lanao del Norte	82,485	0	0	0	0.00	0	0	0	0.00	0	0	0	0.00
Misamis Occidental	33,811	0	0	0	0.00	0	0	0	0.00	0	0	0	0.00
Misamis Oriental	78,469	*	*	0	0.00	*	*	0	0.00	0	0	0	0.00
Cagayan de Oro City	74,313	14	7	21	0.03	1	3	4	0.01	15	10	25	0.03
El Salvador City	5,517	23	11	34	0.62	15	15	30	0.54	38	26	64	1.16
Gingoog City	13,703	7	8	15	0.11	0	1	1	0.01	7	9	16	0.12
Iligan City	41,779	395	274	669	1.60	153	130	283	0.68	548	404	952	2.28
Malaybalay City	20,713	486	304	790	3.81	0	0	0	0.00	486	304	790	3.81
Oroquieta City	7,326	0	0	0	0.00	0	0	0	0.00	0	0	0	0.00
Ozamis City	14,688	0	0	0	0.00	0	0	0	0.00	0	0	0	0.00
Tangub City	6,525	*	*	0	0.00	*	*	0	0.00	0	0	0	0.00
Valencia City	22,895	0	0	0	0.00	0	0	0	0.00	0	0	0	0.00

Table 1.C.2.5 - Nutrition Services for Infants and Children

Number and Proportion of children 0-59 mos. old whose nutritional status are Wasted-MAM, Wasted-SAM and Wasted
Philippines, Annual 2020

Area	Eligible Pop (0-59 mos)	Wasted-MAM				Wasted-SAM				Wasted			
		Male	Female	Total	%	Male	Female	Total	%	Male	Female	Total	%
**Region 11**	**555,461**	**3,911**	**3,296**	**7,207**	**1.30**	**1,020**	**903**	**1,923**	**0.35**	**4,931**	**4,199**	**9,130**	**1.64**
Compostela Valley	83,688	374	329	703	0.84	99	78	177	0.21	473	407	880	1.05
Davao del Norte	108,999	900	718	1,618	1.48	94	93	187	0.17	994	811	1,805	1.66
Davao del Sur	69,170	642	502	1,144	1.65	214	185	399	0.58	856	687	1,543	2.23
Davao Oriental	71,494	721	609	1,330	1.86	249	212	461	0.64	970	821	1,791	2.51
Davao Occidental	37,541	769	662	1,431	3.81	321	293	614	1.64	1,090	955	2,045	5.45
Davao City	184,569	505	476	981	0.53	43	42	85	0.05	548	518	1,066	0.58
**Region 12**	**542,112**	**6,085**	**4,615**	**10,700**	**1.97**	**1,722**	**1,537**	**3,259**	**0.60**	**7,807**	**6,152**	**13,959**	**2.57**
North Cotabato	172,722	2,860	2,373	5,233	3.03	823	790	1,613	0.93	3,683	3,163	6,846	3.96
Sarangani	67,497	52	45	97	0.14	50	46	96	0.14	102	91	193	0.29
South Cotabato	106,332	394	400	794	0.75	85	77	162	0.15	479	477	956	0.90
Sultan Kudarat	92,288	1,712	1,408	3,120	3.38	0	0	0	0.00	1,712	1,408	3,120	3.38
Cotabato City	34,220	145	98	243	0.71	145	98	243	0.71	290	196	486	1.42
Gen. Santos City	69,053	922	291	1,213	1.76	619	526	1,145	1.66	1,541	817	2,358	3.41
**BARMM**	**576,008**	**2**	**2**	**4**	**0.00**	**0**	**0**	**0**	**0.00**	**2**	**2**	**4**	**0.00**
Basilan	43,019	0	0	0	0.00	0	0	0	0.00	0	0	0	0.00
Lanao del Sur	131,469	0	0	0	0.00	0	0	0	0.00	0	0	0	0.00
Maguindanao	180,080	0	0	0	0.00	0	0	0	0.00	0	0	0	0.00
Sulu	123,904	0	0	0	0.00	0	0	0	0.00	0	0	0	0.00
Tawi-Tawi	54,261	0	0	0	0.00	0	0	0	0.00	0	0	0	0.00
Lamitan City	11,832	0	0	0	0.00	0	0	0	0.00	0	0	0	0.00
Marawi City	31,443	2	2	4	0.01	0	0	0	0.00	2	2	4	0.01
**CARAGA**	**318,596**	**2,091**	**1,839**	**3,930**	**1.23**	**973**	**733**	**1,706**	**0.54**	**3,064**	**2,572**	**5,636**	**1.77**
Agusan del Norte	42,202	471	426	897	2.13	243	153	396	0.94	714	579	1,293	3.06
Agusan del Sur	94,397	471	426	897	0.95	243	153	396	0.42	714	579	1,293	1.37
Surigao del Norte	38,645	115	124	239	0.62	64	74	138	0.36	179	198	377	0.98
Surigao del Sur	60,014	713	588	1,301	2.17	282	242	524	0.87	995	830	1,825	3.04
Province of Dinagat	13,805	32	47	79	0.57	1	13	14	0.10	33	60	93	0.67
Bislig City	11,399	233	184	417	3.66	135	90	225	1.97	368	274	642	5.63
Butuan City	40,128	53	39	92	0.23	5	7	12	0.03	58	46	104	0.26
Surigao City	18,006	3	5	8	0.04	0	1	1	0.01	3	6	9	0.05

Note: Put asterisk (*) for No Report and Zero (0) for No Case

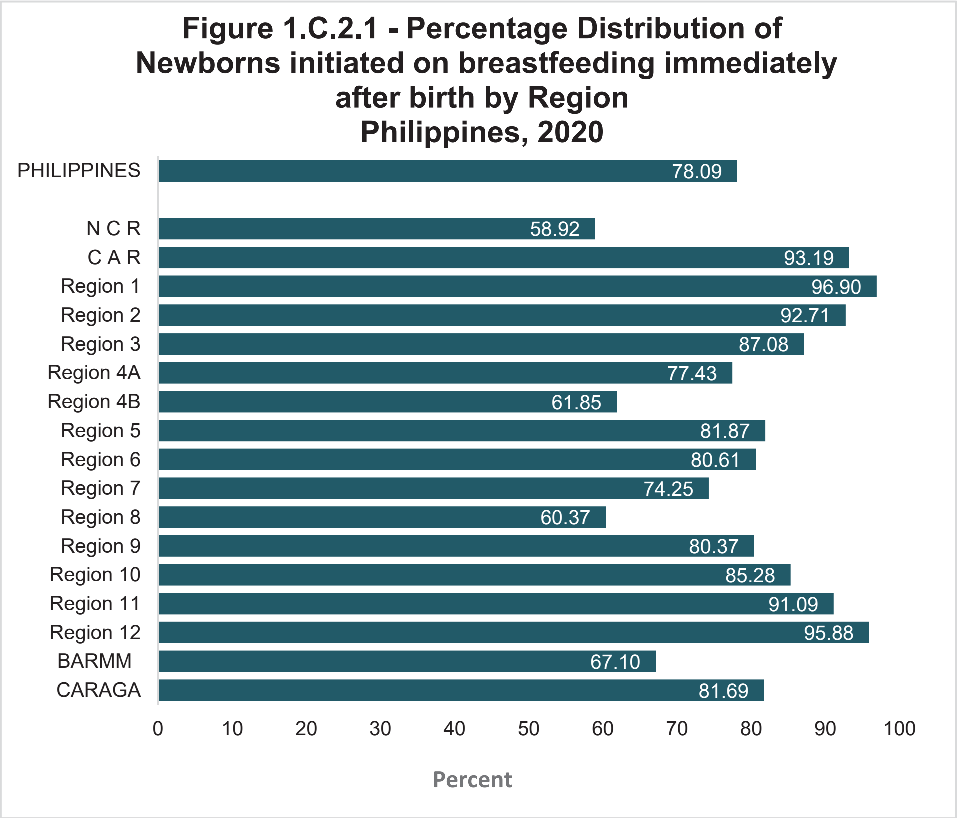
Figure 1.C.2.1 - Percentage Distribution of Newborns initiated on breastfeeding immediately after birth by Region Philippines, 2020
PHILIPPINES
78.09
N C R
58.92
C A R
93.19
Region 1
96.90
Region 2
92.71
Region 3
87.08
Region 4A
77.43
Region 4B
61.85
Region 5
81.87
Region 6
80.61
Region 7
74.25
Region 8
60.37
Region 9
80.37
Region 10
85.28
Region 11
91.09
Region 12
95.88
BARMM
67.10
CARAGA
81.69
0
10
20
30
40
50
60
70
80
90
100
Percent

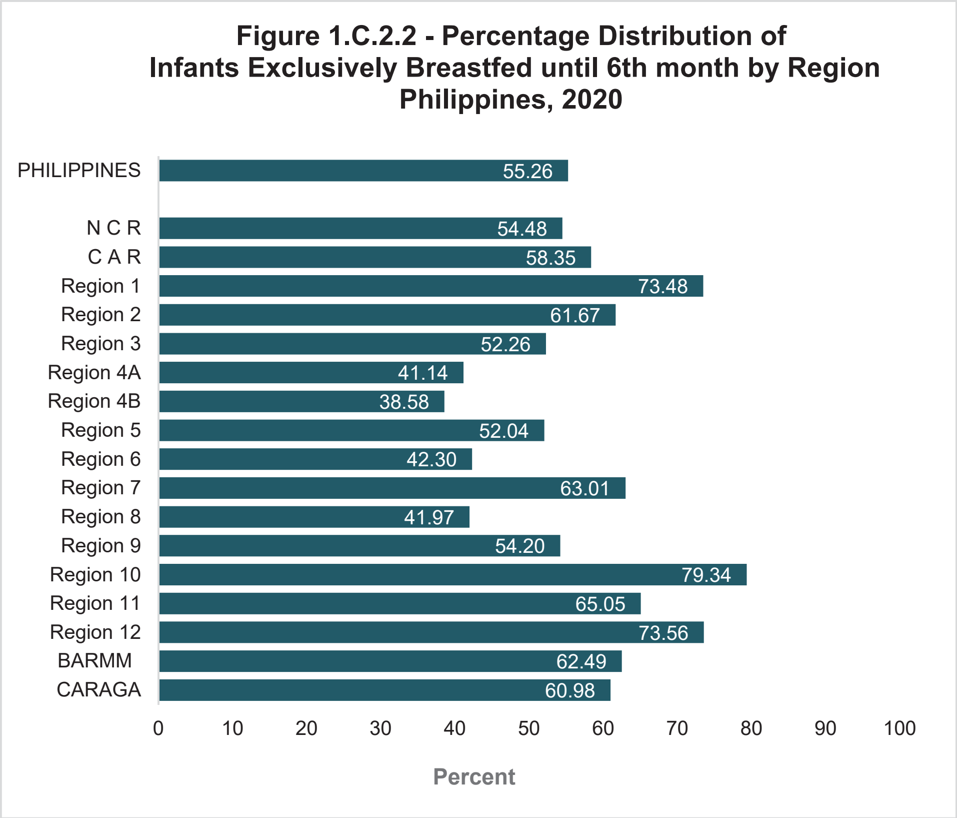
Figure 1.C.2.2 - Percentage Distribution of Infants Exclusively Breastfed until 6th month by Region Philippines, 2020
PHILIPPINES
55.26
N C R
54.48
C A R
58.35
Region 1
73.48
Region 2
61.67
Region 3
52.26
Region 4A
41.14
Region 4B
38.58
Region 5
52.04
Region 6
42.30
Region 7
63.01
Region 8
41.97
Region 9
54.20
Region 10
79.34
Region 11
65.05
Region 12
73.56
BARMM
62.49
CARAGA
60.98
0
10
20
30
40
50
60
70
80
90
100
Percent

Figure 1.C.2.3 - Percentage Distribution of Infants who continued breastfeeding and were introduced to complementary feeding beginning at 6th months by Region Philippines, 2020

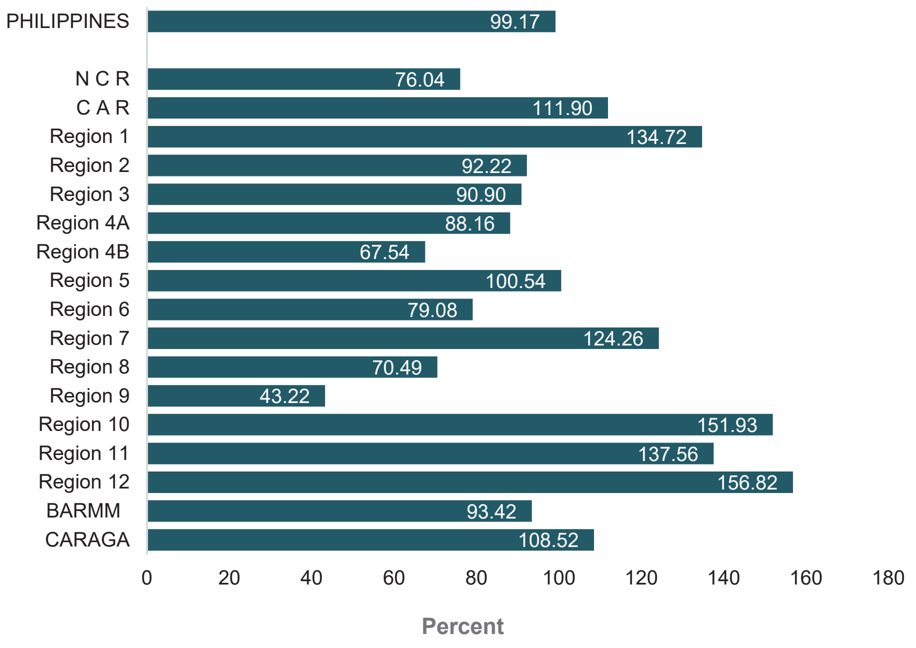

Figure 1.C.2.4 - Percentage of Infants preterm or with LBW (< 2500 grams) given Iron by Region Philippines, 2020

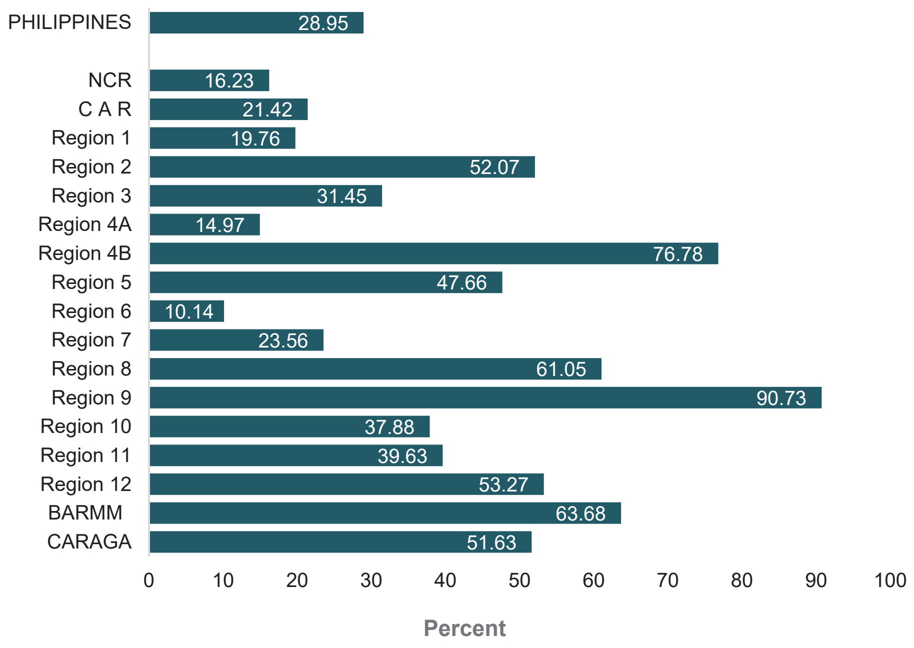

Figure 1.C.2.5 - Percentage Distribution of Infants 6-11 mos. old Given Vitamin A by Region Philippines, 2020

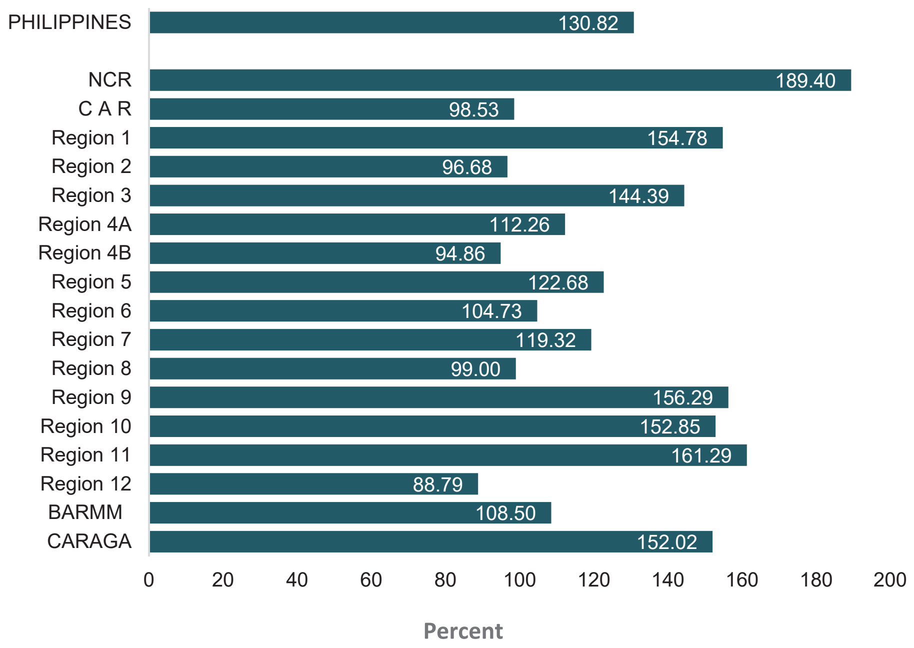

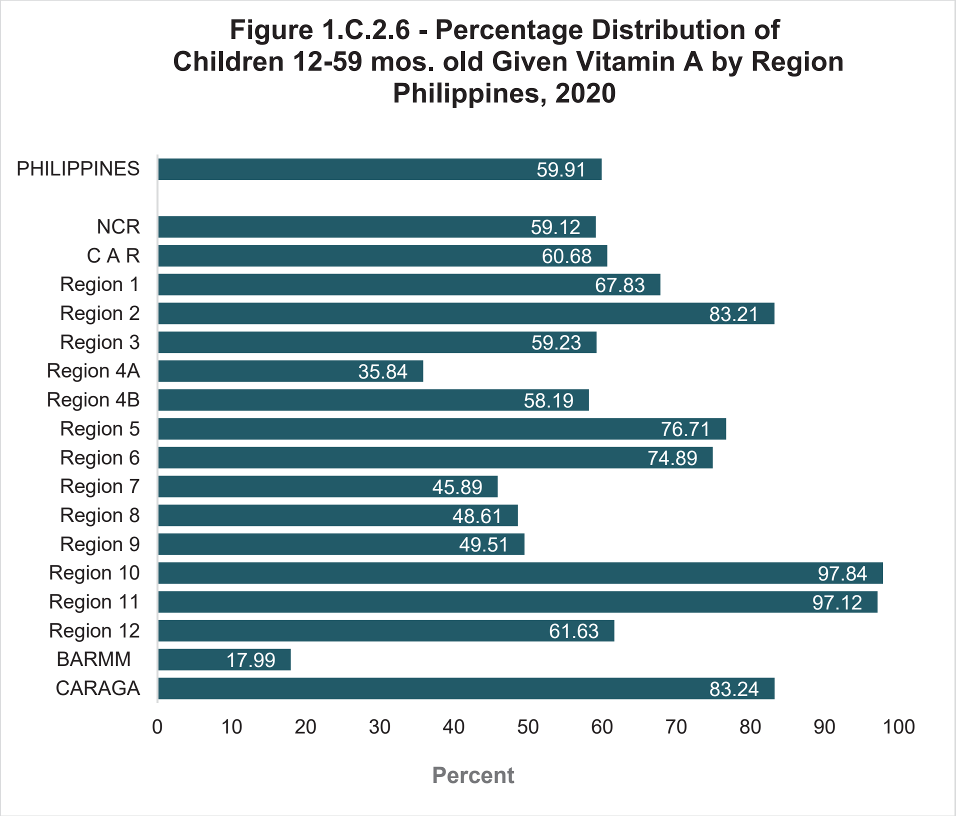
Figure 1.C.2.6 - Percentage Distribution of
Children 12-59 mos. old Given Vitamin A by Region
Philippines, 2020
PHILIPPINES
59.91
NCR
59.12
C A R
60.68
Region 1
67.83
Region 2
83.21
Region 3
59.23
Region 4A
35.84
Region 4B
58.19
Region 5
76.71
Region 6
74.89
Region 7
45.89
Region 8
48.61
Region 9
49.51
Region 10
97.84
Region 11
97.12
Region 12
61.63
BARMM
17.99
CARAGA
83.24
0
10
20
30
40
50
60
70
80
90
100
Percent

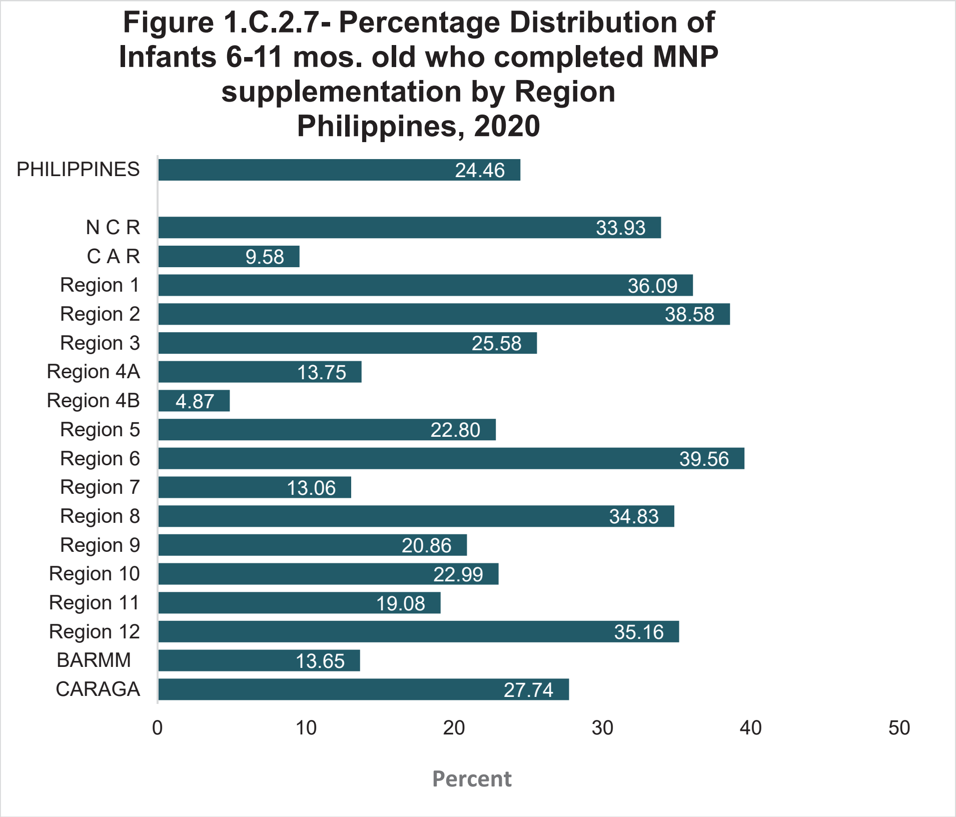
Figure 1.C.2.7- Percentage Distribution of Infants 6-11 mos. old who completed MNP supplementation by Region Philippines, 2020
PHILIPPINES 24.46
N C R 33.93
C A R 9.58
Region 1 36.09
Region 2 38.58
Region 3 25.58
Region 4A 13.75
Region 4B 4.87
Region 5 22.80
Region 6 39.56
Region 7 13.06
Region 8 34.83
Region 9 20.86
Region 10 22.99
Region 11 19.08
Region 12 35.16
BARMM 13.65
CARAGA 27.74
0
10
20
30
40
50
Percent

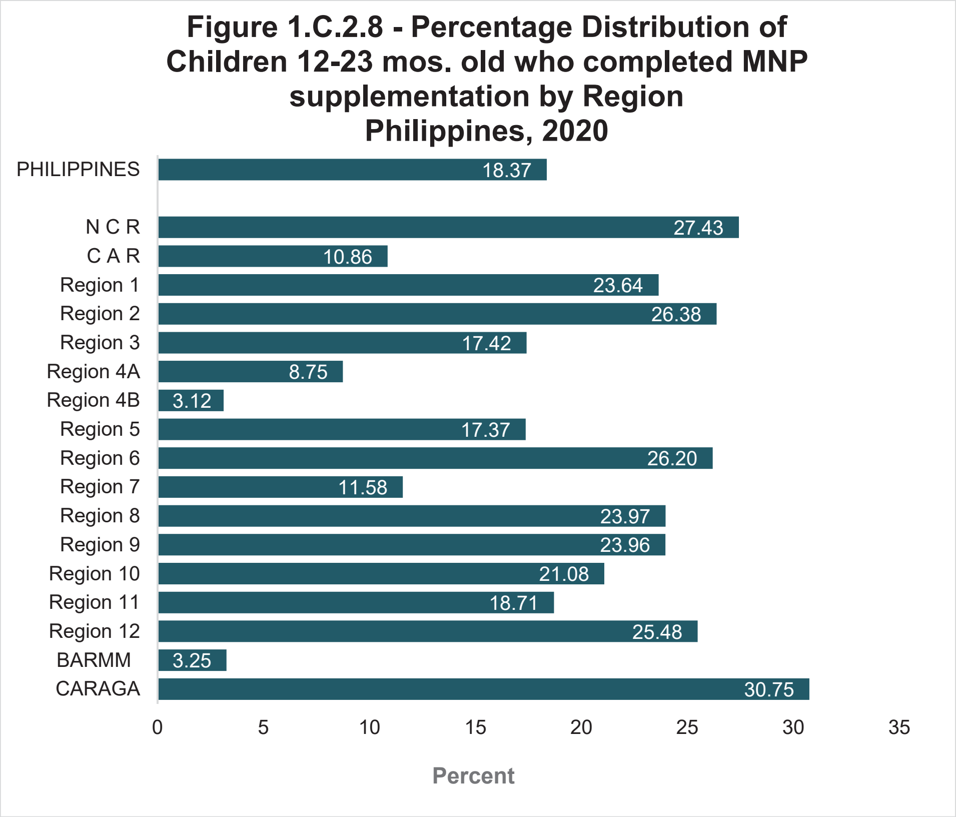
Figure 1.C.2.8 - Percentage Distribution of Children 12-23 mos. old who completed MNP supplementation by Region Philippines, 2020
PHILIPPINES 18.37
N C R 27.43
C A R 10.86
Region 1 23.64
Region 2 26.38
Region 3 17.42
Region 4A 8.75
Region 4B 3.12
Region 5 17.37
Region 6 26.20
Region 7 11.58
Region 8 23.97
Region 9 23.96
Region 10 21.08
Region 11 18.71
Region 12 25.48
BARMM 3.25
CARAGA 30.75
0
5
10
15
20
25
30
35
Percent

Figure 1.C.2.9 - Percentage of Infants 6-11 and Children 12-23 months old who completed MNP supplementation Philippines, 2020

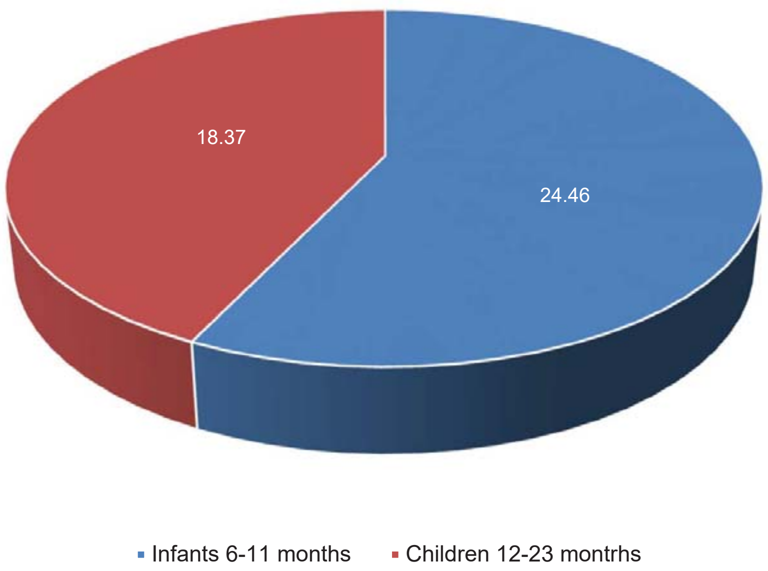

Figure 1.C.2.10- Percentage Distribution of Children 0-59 mos. old whose Nutritional status is Normal by Region Philippines, 2020

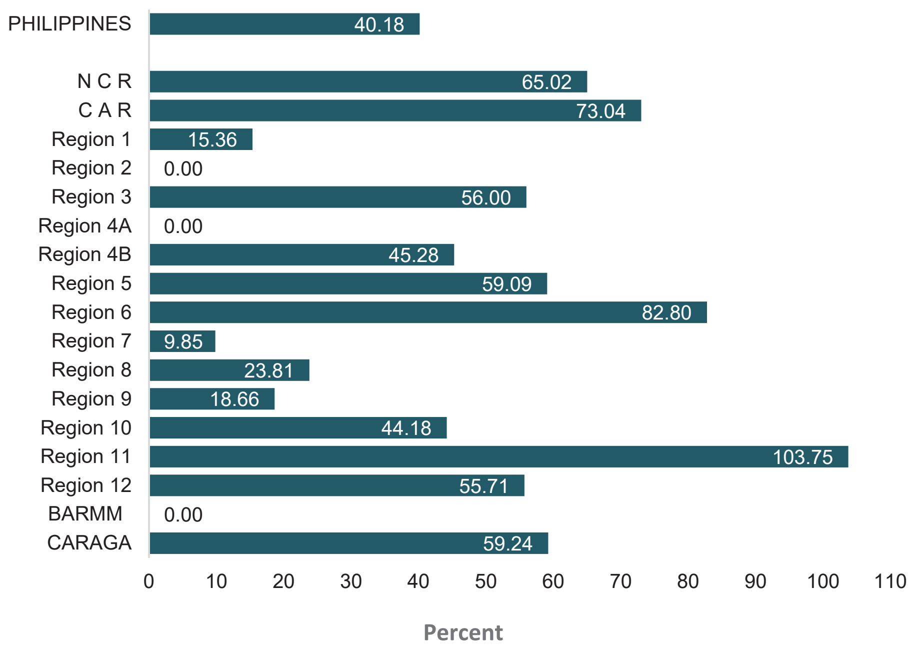

Figure 1.C.2.11 - Percentage Distribution of Children 0-59 mos. old whose Nutritional status is stunted by Region Philippines, 2020

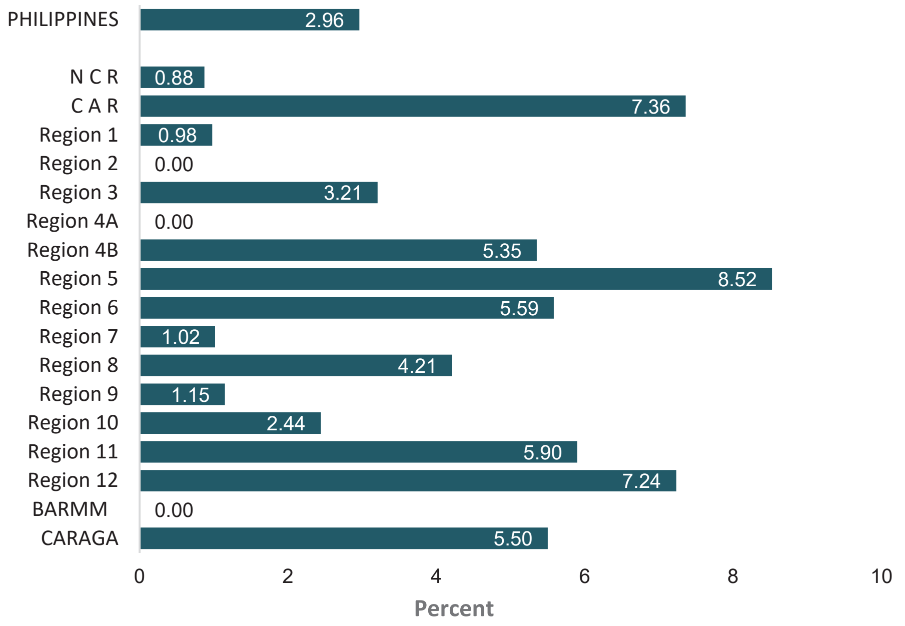

Figure 1.C.2.12 - Percentage Distribution of Children 0-59 mos. old whose nutritional status is Overweight/obese by Region Philippines, 2020

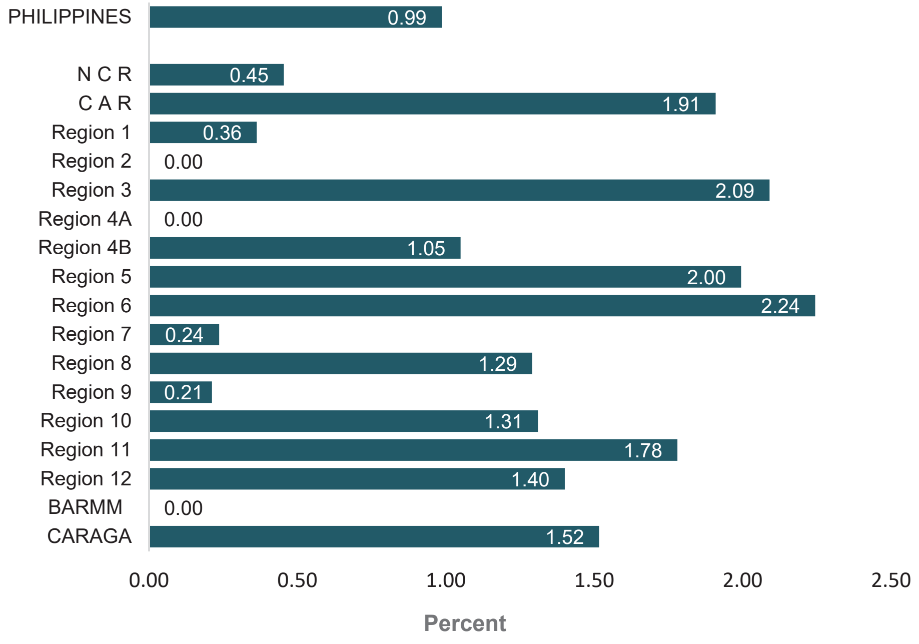

Figure 1.C.2.13 - Percentage Distribution of Children 0-59 mos. old whose nutritional status is Wasted by Region Philippines, 2020

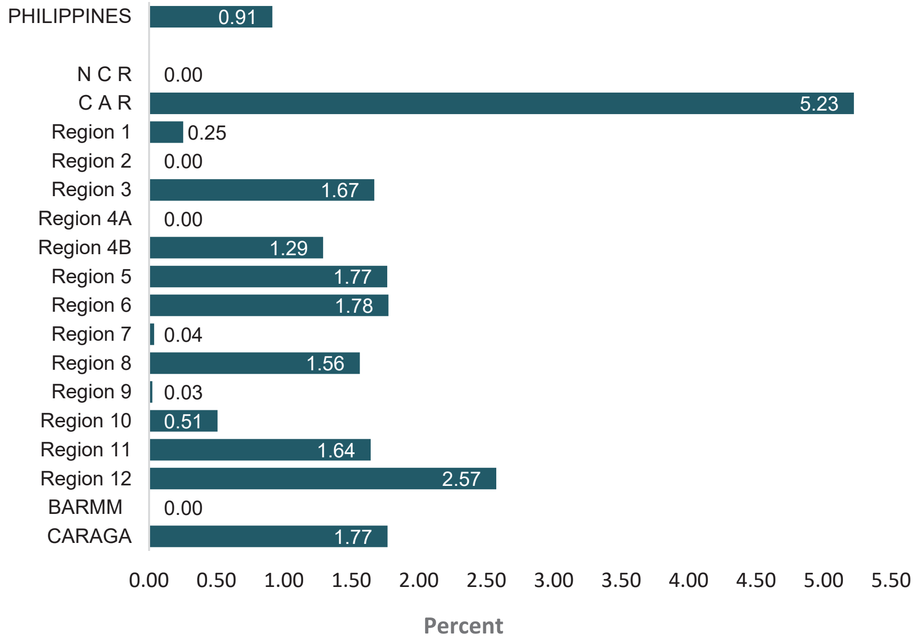

Figure 1.C.2.14 - Percentage of Children 0-59 mos. old according to their Nutritional status Philippines, 2020

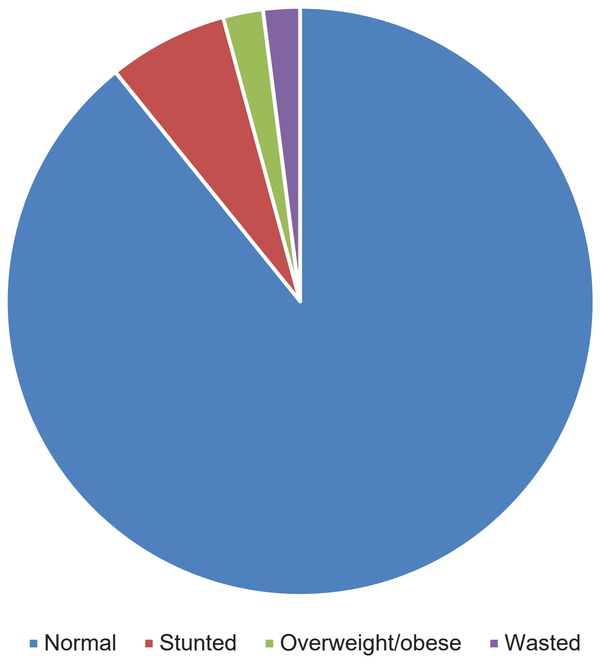

Table 1.C.3.1 -Deworming Services for Children and Adolescents

Number and Proportion of Children/Adolescents who completed 2 doses of deworming tablet
Philippines, Annual 2020

Area	Eligible Pop (1-4 years old)	No. of children 1-4 y/o given 2 doses of deworming drugs				Eligible Pop (5-9 years old)	No. of children 5-9 y/o given 2 doses of deworming drugs				Eligible Pop (10-19 years old)	No. of children 10-19 y/o given 2 doses of deworming drugs			
		Male	Female	Total	%		Male	Female	Total	%		Male	Female	Total	%
**PHILIPPINES**	**8,941,598**	**2,280,440**	**2,195,485**	**4,475,925**	**50.06**	**11,089,133**	**1,501,172**	**1,392,240**	**2,893,412**	**26.09**	**21,139,332**	**1,942,137**	**1,796,232**	**3,738,369**	**17.68**
**N C R**	**959,072**	**204,435**	**253,075**	**457,510**	**47.70**	**1,218,972**	**116,086**	**136,978**	**253,064**	**20.76**	**2,436,251**	**72,074**	**76,463**	**148,537**	**6.10**
Malabon	27,220	5,628	5,223	10,851	39.86	34,600	938	960	1,898	5.49	69,154	160	144	304	0.44
Navotas	18,580	1,599	1,495	3,094	16.65	23,615	791	732	1,523	6.45	47,197	533	516	1,049	2.22
Valenzuela City	46,210	2,428	2,768	5,196	11.24	58,728	0	0	0	0.00	117,378	0	0	0	0.00
Caloocan City	117,972	30,419	29,681	60,100	50.94	149,936	8,500	8,077	16,577	11.06	299,669	6,206	6,226	12,432	4.15
Marikina City	33,571	834	890	1,724	5.14	42,668	284	270	554	1.30	85,275	239	271	510	0.60
Pasig City	56,251	5,426	4,632	10,058	17.88	71,496	789	796	1,585	2.22	142,894	113	115	228	0.16
Pateros	4,755	813	974	1,787	37.58	6,046	342	366	708	11.71	12,078	254	261	515	4.26
Taguig	59,949	28,367	31,582	59,949	100.00	76,193	34,344	33,715	68,059	89.32	152,281	32,620	34,730	67,350	44.23
Quezon City	218,674	35,059	37,733	72,792	33.29	277,938	6,712	6,894	13,606	4.90	555,484	3,929	4,431	8,360	1.50
Makati City	43,390	7,732	7,535	15,267	35.19	55,151	2,232	2,154	4,386	7.95	110,220	1,034	899	1,933	1.75
Mandaluyong City	28,766	4,204	4,106	8,310	28.89	36,563	93	124	217	0.59	73,078	24	24	48	0.07
San Juan	9,101	0	0	0	0.00	11,563	0	0	0	0.00	23,116	0	0	0	0.00
Manila City	132,587	44,421	88,166	132,587	100.00	168,516	47,115	68,941	116,056	68.87	336,793	13,365	15,765	29,130	8.65
Las Piñas City	43,860	5,901	6,197	12,098	27.58	55,745	2,170	2,120	4,290	7.70	111,417	1,574	1,604	3,178	2.85
Muntinlupa City	37,574	18,581	18,993	37,574	100.00	47,757	8,916	9,025	17,941	37.57	95,448	10,204	9,721	19,925	20.88
Parañaque City	49,589	9,907	10,086	19,993	40.32	63,027	2,596	2,539	5,135	8.15	125,966	1,650	1,635	3,285	2.61
Pasay City	31,023	3,116	3,014	6,130	19.76	39,430	264	265	529	1.34	78,803	169	121	290	0.37
**C A R**	**146,939**	**36,839**	**34,496**	**71,335**	**48.55**	**180,835**	**17,962**	**16,989**	**34,951**	**19.33**	**350,174**	**27,355**	**26,556**	**53,911**	**15.40**
Abra	19,128	6,196	5,878	12,074	63.12	23,939	1,814	1,776	3,590	15.00	46,972	2,150	2,061	4,211	8.96
Apayao	10,841	4,001	3,606	7,607	70.17	13,208	762	790	1,552	11.75	24,918	406	591	997	4.00
Benguet	36,459	8,388	7,698	16,086	44.12	44,545	1,044	1,034	2,078	4.66	88,915	3,823	3,567	7,390	8.31
Ifugao	19,116	5,618	5,327	10,945	57.26	23,298	6,451	5,958	12,409	53.26	43,099	9,851	9,342	19,193	44.53
Kalinga	19,738	4,391	4,299	8,690	44.03	24,377	2,365	2,203	4,568	18.74	45,412	4,025	4,243	8,268	18.21
Mt. Province	13,439	4,003	3,620	7,623	56.72	16,997	4,262	3,928	8,190	48.18	32,035	5,789	5,340	11,129	34.74
Baguio City	28,218	4,242	4,068	8,310	29.45	34,471	1,264	1,300	2,564	7.44	68,823	1,311	1,412	2,723	3.96
**Region 1**	**407,689**	**94,050**	**91,673**	**185,723**	**45.56**	**518,508**	**46,582**	**43,873**	**90,455**	**17.45**	**997,577**	**70,683**	**70,768**	**141,451**	**14.18**
Ilocos Norte	33,003	18,239	17,473	35,712	108.21	42,073	1,864	1,634	3,498	8.31	86,137	2,753	2,739	5,492	6.38
Ilocos Sur	40,744	14,402	13,745	28,147	69.08	52,587	9,557	9,189	18,746	35.65	105,735	15,525	14,290	29,815	28.20
La Union	48,703	28,197	26,541	54,738	112.39	62,338	22,810	20,703	43,513	69.80	126,283	34,236	35,997	70,233	55.62
Pangasinan	209,264	20,431	20,243	40,674	19.44	264,918	6,218	6,325	12,543	4.73	494,145	8,285	8,043	16,328	3.30
Alaminos City	7,908	2,512	3,349	5,861	74.11	10,011	0	0	0	0.00	18,674	11	4	15	0.08
Candon City	4,292	4,709	4,642	9,351	217.87	5,545	3,216	3,233	6,449	116.30	11,144	5,989	5,934	11,923	106.99
Dagupan City	15,094	2,125	2,143	4,268	28.28	19,112	1,370	1,307	2,677	14.01	35,647	1,902	1,877	3,779	10.60
Laoag City	7,609	1,117	1,204	2,321	30.50	9,700	0	0	0	0.00	19,860	0	0	0	0.00
San Carlos City	16,616	1	1	2	0.01	21,044	0	2	2	0.01	39,247	4	2	6	0.02
San Fernando City	8,922	647	658	1,305	14.63	11,423	510	452	962	8.42	23,132	749	661	1,410	6.10
Urdaneta City	11,718	0	0	0	0.00	14,833	0	0	0	0.00	27,670	4	4	8	0.03
Vigan City	3,816	1,670	1,674	3,344	87.63	4,924	1,037	1,028	2,065	41.94	9,903	1,225	1,217	2,442	24.66
**Region 2**	**291,567**	**85,155**	**78,703**	**163,858**	**56.20**	**364,952**	**25,034**	**23,436**	**48,470**	**13.28**	**685,244**	**26,405**	**24,362**	**50,767**	**7.41**
Batanes	1,518	314	247	561	36.96	1,857	39	47	86	4.63	3,361	0	0	0	0.00
Cagayan	75,523	30,125	28,259	58,384	77.31	94,659	5,253	5,076	10,329	10.91	180,347	7,493	6,784	14,277	7.92
Isabela	110,057	28,204	26,570	54,774	49.77	138,714	12,317	11,919	24,236	17.47	259,726	13,902	12,697	26,599	10.24
Nueva Vizcaya	40,344	12,300	10,449	22,749	56.39	49,227	2,576	2,557	5,133	10.43	92,577	1,898	1,762	3,660	3.95
Quirino	17,186	3,100	2,498	5,598	32.57	21,388	1,915	874	2,789	13.04	38,117	751	576	1,327	3.48
Cauayan City	10,804	884	990	1,874	17.35	13,629	538	541	1,079	7.92	25,513	146	137	283	1.11
Ilagan City	12,151	2,008	2,041	4,049	33.32	15,323	217	477	694	4.53	28,669	263	400	663	2.31
Santiago City	11,254	3,127	2,834	5,961	52.97	14,181	403	340	743	5.24	26,564	697	604	1,301	4.90
Tuguegarao City	12,730	5,093	4,815	9,908	77.83	15,974	1,776	1,605	3,381	21.17	30,370	1,255	1,402	2,657	8.75
**Region 3**	**912,991**	**351,446**	**232,252**	**583,698**	**63.93**	**1,158,742**	**280,653**	**154,839**	**435,492**	**37.58**	**2,307,413**	**363,184**	**185,090**	**548,274**	**23.76**
Aurora	20,464	7,895	7,607	15,502	75.75	24,766	4,492	4,245	8,737	35.28	45,843	6,317	6,116	12,433	27.12
Bataan	54,850	28,533	27,318	55,851	101.82	69,111	9,315	8,874	18,189	26.32	135,886	6,455	6,273	12,728	9.37
Bulacan	180,961	36,472	37,612	74,084	40.94	232,440	39,027	37,679	76,706	33.00	466,981	52,334	53,817	106,151	22.73
Nueva Ecija	121,573	24,447	23,778	48,225	39.67	154,631	12,957	12,286	25,243	16.32	301,882	9,298	9,007	18,305	6.06
Pampanga	130,856	124,859	4,327	129,186	98.72	166,792	125,224	3,855	129,079	77.39	341,826	179,819	254	180,073	52.68
Tarlac	81,757	21,332	25,319	46,651	57.06	103,427	14,038	13,946	27,984	27.06	205,636	14,261	15,420	29,681	14.43
Zambales	51,197	9,435	9,205	18,640	36.41	62,518	9,726	8,965	18,691	29.90	119,635	13,938	14,051	27,989	23.40
Angeles City	32,828	3,011	2,844	5,855	17.84	41,853	2,456	2,360	4,816	11.51	85,762	1,799	1,888	3,687	4.30
Balanga City	7,927	3,900	3,852	7,752	97.79	9,987	0	0	0	0.00	19,646	0	0	0	0.00
Cabanatuan City	24,884	8,696	7,984	16,680	67.03	31,642	3,687	3,340	7,027	22.21	61,790	3,908	3,739	7,647	12.38
City of San Fernando	24,459	3,166	3,675	6,841	27.97	31,176	2,714	3,862	6,576	21.09	63,892	1,923	2,177	4,100	6.42
Gapan City	9,083	5,685	5,554	11,239	123.74	11,551	2,037	2,087	4,124	35.70	22,550	854	931	1,785	7.92
Mabalacat City	20,000	8,737	8,359	17,096	85.48	25,495	7,473	7,318	14,791	58.02	52,256	11,631	11,492	23,123	44.25
Malolos City	20,216	10,055	9,394	19,449	96.21	25,961	11,646	10,846	22,492	86.64	52,154	18,679	18,163	36,842	70.64

Table 1.C.3.1 -Deworming Services for Children and Adolescents

Number and Proportion of Children/Adolescents who completed 2 doses of deworming tablet

Philippines, Annual 2020

Area	Eligible Pop (1-4 years old)	No. of children 1-4 y/o given 2 doses of deworming drugs				Eligible Pop (5-9 years old)	No. of children 5-9 y/o given 2 doses of deworming drugs				Eligible Pop (10-19 years old)	No. of children 10-19 y/o given 2 doses of deworming drugs			
		Male	Female	Total	%		Male	Female	Total	%		Male	Female	Total	%
Meycauayan	16,766	3,149	3,063	6,212	37.05	21,533	4,051	3,968	8,019	37.24	43,264	4,484	4,585	9,069	20.96
Olongapo	20,191	2,771	2,547	5,318	26.34	24,655	1,892	1,809	3,701	15.01	47,187	1,870	1,831	3,701	7.84
Palayan City	3,377	3,596	3,614	7,210	213.50	4,299	1,798	1,807	3,605	83.86	8,392	0	0	0	0.00
San Jose City	11,500	4,638	3,950	8,588	74.68	14,635	2,011	1,930	3,941	26.93	28,571	2,124	1,787	3,911	13.69
San Jose del Monte C	46,033	27,644	28,655	56,299	122.30	59,129	17,468	17,114	34,582	58.49	118,790	22,409	22,008	44,417	37.39
Science City of Munoz	6,708	6,180	6,246	12,426	185.24	8,533	37	25	62	0.73	16,661	30	31	61	0.37
Tarlac City	27,361	7,245	7,349	14,594	53.34	34,608	8,604	8,523	17,127	49.49	68,809	11,051	11,520	22,571	32.80
**Region 4A**	**1,227,422**	**176,897**	**170,634**	**347,531**	**28.31**	**1,534,044**	**127,291**	**118,684**	**245,975**	**16.03**	**3,029,605**	**137,569**	**132,529**	**270,098**	**8.92**
Batangas	162,024	31,134	29,987	61,121	37.72	198,436	24,341	23,767	48,108	24.24	393,518	26,110	26,617	52,727	13.40
Cavite	113,921	31,012	24,627	55,639	48.84	144,040	23,963	19,418	43,381	30.12	291,469	32,751	28,140	60,891	20.89
Laguna	80,610	12,551	11,673	24,224	30.05	100,562	11,131	10,247	21,378	21.26	198,309	12,753	13,076	25,829	13.02
Quezon	158,632	15,896	15,706	31,602	19.92	200,155	9,913	9,700	19,613	9.80	380,865	13,179	12,842	26,021	6.83
Rizal	181,864	12,661	13,633	26,294	14.46	227,086	9,287	8,588	17,875	7.87	446,436	4,910	5,023	9,933	2.22
Antipolo City	66,984	1,931	3,697	5,628	8.40	83,642	169	192	361	0.43	164,439	0	0	0	0.00
Bacoor City	49,915	2,668	2,552	5,220	10.46	63,114	545	418	963	1.53	127,690	678	592	1,270	0.99
Batangas City	28,751	0	0	0	0.00	35,217	0	0	0	0.00	69,849	0	0	0	0.00
Biñan City	27,031	13,872	15,368	29,240	108.17	33,723	10,243	9,850	20,093	59.58	66,498	18,803	17,359	36,162	54.38
Cabuyao City	25,061	2,875	2,754	5,629	22.46	31,259	1,839	1,640	3,479	11.13	61,652	1,295	1,207	2,502	4.06
Calamba City	36,888	3,848	4,028	7,876	21.35	46,023	3,208	2,712	5,920	12.86	90,755	2,320	1,786	4,106	4.52
Cavite City	8,541	481	534	1,015	11.88	10,795	284	338	622	5.76	21,848	260	391	651	2.98
Dasmariñas City	54,762	7,950	7,671	15,621	28.53	69,250	1,149	1,023	2,172	3.14	140,114	962	920	1,882	1.34
General Trias City	26,122	2,525	2,378	4,903	18.77	33,027	1,394	1,305	2,699	8.17	66,826	1,351	1,376	2,727	4.08
Imus City	33,554	7,156	6,621	13,777	41.06	42,432	2,495	2,383	4,878	11.50	85,845	2,772	2,754	5,526	6.44
Lipa City	28,975	0	0	0	0.00	35,488	0	0	0	0.00	70,370	0	0	0	0.00
Lucena City	24,048	0	0	0	0.00	30,332	0	0	0	0.00	57,721	0	0	0	0.00
San Pablo City	21,595	5,724	5,365	11,089	51.35	26,932	2,408	2,461	4,869	18.08	53,131	1,920	2,018	3,938	7.41
San Pedro City	26,445	2,136	1,931	4,067	15.38	32,991	371	322	693	2.10	65,056	582	717	1,299	2.00
Santa Rosa City	28,715	11,345	10,289	21,634	75.34	35,818	8,886	7,890	16,776	46.84	70,644	8,036	7,810	15,846	22.43
Tagaytay City	5,917	0	0	0	0.00	7,481	10,500	10,994	21,494	287.31	15,129	0	0	0	0.00
Tanauan City	15,111	478	432	910	6.02	18,509	309	274	583	3.15	36,706	253	259	512	1.39
Tayabas City	9,016	0	0	0	0.00	11,368	0	0	0	0.00	21,628	0	0	0	0.00
Trece Martires City	12,940	10,654	11,388	22,042	170.34	16,364	4,856	5,162	10,018	61.22	33,107	8,634	9,642	18,276	55.20
**Region 4B**	**298,390**	**71,009**	**65,473**	**136,482**	**45.74**	**370,516**	**34,491**	**32,204**	**66,695**	**18.00**	**668,760**	**44,578**	**42,299**	**86,877**	**12.99**
Marinduque	21,709	11,635	10,552	22,187	102.20	27,424	2,282	2,103	4,385	15.99	49,620	1,167	1,061	2,228	4.49
Mindoro Occidental	50,349	15,784	14,825	30,609	60.79	62,012	5,223	4,765	9,988	16.11	109,972	8,547	8,058	16,605	15.10
Mindoro Oriental	83,165	23,564	21,106	44,670	53.71	104,232	15,187	13,936	29,123	27.94	186,936	21,043	20,130	41,173	22.03
Palawan	88,284	13,502	12,880	26,382	29.88	109,150	7,753	7,256	15,009	13.75	199,006	8,437	8,021	16,458	8.27
Romblon	28,368	2,580	2,392	4,972	17.53	34,915	1,623	1,554	3,177	9.10	63,462	3,177	2,945	6,122	9.65
Puerto Princesa City	26,515	3,944	3,718	7,662	28.90	32,783	2,423	2,590	5,013	15.29	59,764	2,207	2,084	4,291	7.18
**Region 5**	**586,320**	**157,415**	**150,001**	**307,416**	**52.43**	**709,108**	**157,048**	**147,110**	**304,158**	**42.89**	**1,309,410**	**225,083**	**218,851**	**443,934**	**33.90**
Albay	103,227	46,468	43,980	90,448	87.62	128,530	54,729	51,964	106,693	83.01	245,184	72,190	68,584	140,774	57.42
Camarines Norte	59,893	24,604	24,375	48,979	81.78	71,010	22,882	21,847	44,729	62.99	128,951	36,350	35,156	71,506	55.45
Camarines Sur	166,213	2,597	2,467	5,064	3.05	202,028	3,060	2,968	6,028	2.98	372,689	5,137	4,807	9,944	2.67
Catanduanes	26,561	15,324	14,310	29,634	111.57	31,863	13,320	12,365	25,685	80.61	58,075	21,879	23,439	45,318	78.03
Masbate	99,437	22,863	21,500	44,363	44.61	117,083	25,053	23,162	48,215	41.18	209,911	38,275	36,458	74,733	35.60
Sorsogon	81,734	29,106	27,328	56,434	69.05	98,190	24,023	21,677	45,700	46.54	181,745	37,449	35,142	72,591	39.94
Iriga City	11,291	3,613	3,425	7,038	62.33	13,728	1,739	1,741	3,480	25.35	25,325	1,213	3,043	4,256	16.81
Legaspi City	18,151	7,374	7,282	14,656	80.74	22,600	6,841	6,490	13,331	58.99	43,117	9,510	8,972	18,482	42.86
Naga City	19,813	5,466	5,334	10,800	54.51	24,076	5,401	4,896	10,297	42.77	44,413	3,080	3,250	6,330	14.25
**Region 6**	**623,733**	**189,154**	**210,177**	**399,331**	**64.02**	**781,067**	**114,573**	**128,850**	**243,423**	**31.17**	**1,506,147**	**166,015**	**189,745**	**355,760**	**23.62**
Aklan	47,850	28,725	25,676	54,401	113.69	60,473	18,550	16,566	35,116	58.07	114,290	31,278	28,480	59,758	52.29
Antique	53,822	20,246	18,452	38,698	71.90	67,343	23,245	21,994	45,239	67.18	122,416	38,939	37,073	76,012	62.09
Capiz	60,081	18,354	19,155	37,509	62.43	74,216	9,167	9,480	18,647	25.13	150,172	10,553	10,974	21,527	14.33
Guimaras	14,501	6,061	5,785	11,846	81.69	17,641	3,469	3,056	6,525	36.99	33,820	4,297	3,809	8,106	23.97
Iloilo	153,611	53,243	79,865	133,108	86.65	193,219	35,551	53,326	88,877	46.00	379,815	55,001	82,507	137,508	36.20
Negros Occidental	210,882	35,347	34,322	69,669	33.04	264,052	4,023	4,543	8,566	3.24	504,299	5,479	5,813	11,292	2.24
Bacolod City	47,451	24,910	24,718	49,628	104.59	59,410	19,808	19,211	39,019	65.68	113,475	19,623	20,230	39,853	35.12
Iloilo City	35,535	2,268	2,204	4,472	12.58	44,713	760	674	1,434	3.21	87,860	845	859	1,704	1.94
**Region 7**	**652,415**	**128,951**	**124,921**	**253,872**	**38.91**	**814,449**	**35,563**	**34,128**	**69,691**	**8.56**	**1,537,252**	**26,131**	**17,698**	**43,829**	**2.85**
Bohol	115,081	32,005	30,998	63,003	54.75	145,394	1,764	1,575	3,339	2.30	272,769	238	162	400	0.15
Cebu	263,210	39,093	38,455	77,548	29.46	327,915	15,819	15,512	31,331	9.55	616,905	15,033	15,076	30,109	4.88
Negros Oriental	115,018	37,292	35,926	73,218	63.66	142,678	11,080	10,492	21,572	15.12	273,837	8,384	0	8,384	3.06
Siquijor	7,440	0	0	0	0.00	9,519	0	0	0	0.00	18,299	0	0	0	0.00
Cebu City	82,633	15,512	14,389	29,901	36.19	102,939	5,320	4,973	10,293	10.00	193,655	1,319	1,206	2,525	1.30
Lapu-Lapu City	36,553	4,841	4,886	9,727	26.61	45,536	1,580	1,576	3,156	6.93	85,664	1,157	1,254	2,411	2.81
Mandaue City	32,480	208	267	475	1.46	40,468	0	0	0	0.00	76,123	0	0	0	0.00

Table 1.C.3.1 -Deworming Services for Children and Adolescents

Number and Proportion of Children/Adolescents who completed 2 doses of deworming tablet
Philippines, Annual 2020

Area	Eligible Pop (1-4 years old)	No. of children 1-4 y/o given 2 doses of deworming drugs				Eligible Pop (5-9 years old)	No. of children 5-9 y/o given 2 doses of deworming drugs				Eligible Pop (10-19 years old)	No. of children 10-19 y/o given 2 doses of deworming drugs			
		Male	Female	Total	%		Male	Female	Total	%		Male	Female	Total	%
**Region 8**	**433,112**	**92,791**	**86,657**	**179,448**	**41.43**	**525,349**	**38,035**	**38,187**	**76,222**	**14.51**	**988,361**	**47,771**	**47,612**	**95,383**	**9.65**
Biliran	16,820	9,453	7,176	16,629	98.86	19,961	8,594	7,978	16,572	83.02	36,618	9,878	11,478	21,356	58.32
Eastern Samar	46,281	9,374	8,781	18,155	39.23	56,723	3,658	3,676	7,334	12.93	106,296	3,804	3,464	7,268	6.84
Northern Leyte	143,608	30,628	28,919	59,547	41.46	174,787	3,315	3,199	6,514	3.73	330,139	1,589	1,569	3,158	0.96
Northern Samar	67,406	16,522	16,799	33,321	49.43	80,514	13,906	15,220	29,126	36.18	149,469	23,158	22,146	45,304	30.31
Southern Leyte	28,790	8,589	8,008	16,597	57.65	36,435	3,967	3,837	7,804	21.42	68,795	4,403	4,027	8,430	12.25
Western Samar	60,698	6,116	5,864	11,980	19.74	72,410	2,322	2,171	4,493	6.20	137,277	3,555	3,509	7,064	5.15
Calbayog City	18,707	385	367	752	4.02	22,321	66	102	168	0.75	42,291	79	53	132	0.31
Maasin City	7,329	121	103	224	3.06	9,270	0	0	0	0.00	17,505	15	9	24	0.14
Ormoc City	20,450	9,415	8,572	17,987	87.96	24,892	1,858	1,682	3,540	14.22	47,029	1,000	972	1,972	4.19
Tacloban City	23,023	2,188	2,068	4,256	18.49	28,036	349	322	671	2.39	52,942	290	385	675	1.27
**Region 9**	**339,485**	**102,040**	**111,649**	**213,689**	**62.95**	**413,615**	**103,025**	**121,167**	**224,192**	**54.20**	**780,103**	**178,095**	**195,007**	**373,102**	**47.83**
Zamboanga del Norte	74,872	38,874	43,612	82,486	110.17	90,365	43,238	49,338	92,576	102.45	169,312	75,062	81,082	156,144	92.22
Zamboanga del Sur	72,143	18,614	19,621	38,235	53.00	89,016	19,314	24,316	43,630	49.01	171,317	33,043	44,883	77,926	45.49
Zamboanga Sibugay	66,111	24,040	25,804	49,844	75.39	79,622	20,118	19,800	39,918	50.13	143,445	33,272	32,390	65,662	45.78
Dapitan City	7,736	85	78	163	2.11	9,334	0	0	0	0.00	17,483	0	0	0	0.00
Dipolog City	12,264	5,513	6,017	11,530	94.02	14,803	4,375	10,555	14,930	100.86	27,734	15,565	15,547	31,112	112.18
Isabela City	12,059	31	36	67	0.56	14,129	131	152	283	2.00	26,882	0	0	0	0.00
Pagadian City	17,694	1,937	2,032	3,969	22.43	21,831	2,054	1,959	4,013	18.38	42,023	3,298	3,033	6,331	15.07
Zamboanga City	76,606	12,946	14,449	27,395	35.76	94,515	13,795	15,047	28,842	30.52	181,907	17,855	18,072	35,927	19.75
**Region 10**	**434,937**	**158,898**	**167,880**	**326,778**	**75.13**	**529,358**	**147,383**	**144,694**	**292,077**	**55.18**	**997,830**	**234,779**	**242,883**	**477,662**	**47.87**
Bukidnon	100,578	45,065	49,015	94,080	93.54	121,110	56,030	54,878	110,908	91.58	225,350	77,358	79,488	156,846	69.60
Camiguin	8,000	1,349	1,232	2,581	32.26	9,619	1,915	1,811	3,726	38.74	17,959	3,587	3,364	6,951	38.70
Lanao del Norte	67,507	31,343	32,608	63,951	94.73	82,121	23,216	25,222	48,438	58.98	153,822	57,732	64,820	122,552	79.67
Misamis Occidental	27,424	9,936	10,327	20,263	73.89	34,240	11,458	11,003	22,461	65.60	65,971	15,252	15,533	30,785	46.66
Misamis Oriental	63,311	36,735	35,704	72,439	114.42	77,205	35,920	31,406	67,326	87.20	146,905	41,268	38,765	80,033	54.48
Cagayan de Oro City	59,976	7,637	10,149	17,786	29.66	73,139	0	0	0	0.00	139,147	0	0	0	0.00
El Salvador City	4,456	1,946	2,379	4,325	97.06	5,434	2,664	3,258	5,922	108.98	10,336	3,568	4,361	7,929	76.71
Gingoog City	11,052	3,122	5,690	8,812	79.73	13,494	1,221	1,003	2,224	16.48	25,656	2,121	1,999	4,120	16.06
Iligan City	34,195	10,673	11,360	22,033	64.43	41,600	8,768	9,558	18,326	44.05	77,917	24,049	24,531	48,580	62.35
Malaybalay City	16,766	6,251	4,540	10,791	64.36	20,189	2,780	2,922	5,702	28.24	37,566	4,890	5,154	10,044	26.74
Oroquieta City	5,942	2,099	1,909	4,008	67.45	7,418	354	323	677	9.13	14,299	391	311	702	4.91
Ozamis City	11,909	548	582	1,130	9.49	14,869	618	854	1,472	9.90	28,656	1,270	1,158	2,428	8.47
Tangub City	5,293	0	0	0	0.00	6,607	0	0	0	0.00	12,731	0	0	0	0.00
Valencia City	18,528	2,194	2,385	4,579	24.71	22,313	2,439	2,456	4,895	21.94	41,515	3,293	3,399	6,692	16.12
**Region 11**	**448,208**	**179,537**	**167,944**	**347,481**	**77.53**	**556,337**	**39,654**	**37,282**	**76,936**	**13.83**	**1,037,244**	**33,957**	**32,306**	**66,263**	**6.39**
Compostela Valley	68,129	35,593	31,905	67,498	99.07	83,118	10,096	9,494	19,590	23.57	152,579	9,852	8,837	18,689	12.25
Davao del Norte	87,663	41,084	38,322	79,406	90.58	109,372	10,092	8,862	18,954	17.33	202,917	10,530	9,747	20,277	9.99
Davao del Sur	56,161	16,026	15,108	31,134	55.44	67,926	5,668	5,464	11,132	16.39	124,424	3,534	3,177	6,711	5.39
Davao Oriental	57,348	21,134	18,670	39,804	69.41	71,687	3,721	3,557	7,278	10.15	136,340	3,085	3,028	6,113	4.48
Davao Occidental	30,859	5,706	5,018	10,724	34.75	39,153	2,481	2,638	5,119	13.07	69,065	2,996	3,519	6,515	9.43
Davao City	148,048	59,994	58,921	118,915	80.32	185,081	7,596	7,267	14,863	8.03	351,919	3,960	3,998	7,958	2.26
**Region 12**	**437,544**	**129,413**	**128,865**	**258,278**	**59.03**	**538,919**	**113,647**	**112,411**	**226,058**	**41.95**	**1,002,796**	**128,758**	**139,539**	**268,297**	**26.75**
North Cotabato	139,074	41,442	41,609	83,051	59.72	171,738	8,301	8,069	16,370	9.53	314,112	6,152	6,239	12,391	3.94
Sarangani	54,603	16,991	17,237	34,228	62.69	66,685	31,208	31,122	62,330	93.47	125,021	28,616	29,847	58,463	46.76
South Cotabato	85,230	24,480	23,785	48,265	56.63	103,762	15,486	15,460	30,946	29.82	195,419	16,714	16,818	33,532	17.16
Sultan Kudarat	74,909	25,119	26,086	51,205	68.36	94,222	33,956	33,399	67,355	71.49	175,410	53,755	54,909	108,664	61.95
Cotabato City	28,378	7,794	7,469	15,263	53.78	35,115	17,281	16,786	34,067	97.02	65,916	12,804	11,982	24,786	37.60
Gen. Santos City	55,350	13,587	12,679	26,266	47.45	67,397	7,415	7,575	14,990	22.24	126,918	10,717	19,744	30,461	24.00
**BARMM**	**483,196**	**3,198**	**3,360**	**6,558**	**1.36**	**566,299**	**3,250**	**3,658**	**6,908**	**1.22**	**946,794**	**4,620**	**4,485**	**9,105**	**0.96**
Basilan	35,490	29	29	58	0.16	41,356	21	14	35	0.08	69,446	60	52	112	0.16
Lanao del Sur	110,328	0	0	0	0.00	128,049	0	0	0	0.00	206,497	0	0	0	0.00
Maguindanao	148,947	340	387	727	0.49	177,629	310	293	603	0.34	301,147	308	341	649	0.22
Sulu	107,280	0	0	0	0.00	123,774	0	0	0	0.00	208,263	0	0	0	0.00
Tawi-Tawi	44,992	0	0	0	0.00	53,485	0	0	0	0.00	92,942	0	0	0	0.00
Lamitan City	9,764	453	420	873	8.94	11,374	487	508	995	8.75	19,104	664	621	1,285	6.73
Marawi City	26,395	2,376	2,524	4,900	18.56	30,632	2,432	2,843	5,275	17.22	49,395	3,588	3,471	7,059	14.29
**CARAGA**	**258,578**	**119,212**	**117,725**	**236,937**	**91.63**	**308,063**	**100,895**	**97,750**	**198,645**	**64.48**	**558,371**	**155,080**	**150,039**	**305,119**	**54.64**
Agusan del Norte	34,103	7,541	7,474	15,015	44.03	40,882	7,170	6,582	13,752	33.64	75,453	11,592	11,353	22,945	30.41
Agusan del Sur	76,814	41,014	40,982	81,996	106.75	88,260	17,224	15,769	32,993	37.38	151,814	25,678	24,570	50,248	33.10
Surigao del Norte	31,554	10,691	10,390	21,081	66.81	38,709	16,399	15,647	32,046	82.79	73,380	25,314	24,048	49,362	67.27
Surigao del Sur	48,534	23,903	24,541	48,444	99.81	58,122	26,283	26,274	52,557	90.43	105,770	40,096	40,515	80,611	76.21
Province of Dinagat	11,231	4,730	4,348	9,078	80.83	14,160	5,044	5,167	10,211	72.11	25,959	8,002	8,949	16,951	65.30

Table 1.C.3.1 -Deworming Services for Children and Adolescents

Number and Proportion of Children/Adolescents who completed 2 doses of deworming tablet

Philippines, Annual 2020

Area	Eligible Pop (1-4 years old)	No. of children 1-4 y/o given 2 doses of deworming drugs				Eligible Pop (5-9 years old)	No. of children 5-9 y/o given 2 doses of deworming drugs				Eligible Pop (10-19 years old)	No. of children 10-19 y/o given 2 doses of deworming drugs			
		Male	Female	Total	%		Male	Female	Total	%		Male	Female	Total	%
Bislig City	9,222	2,667	2,542	5,209	56.48	11,041	2,099	2,002	4,101	37.14	20,089	4,400	4,090	8,490	42.26
Butuan City	32,429	25,650	24,520	50,170	154.71	38,862	21,049	20,661	41,710	107.33	71,736	30,893	28,388	59,281	82.64
Surigao City	14,691	3,016	2,928	5,944	40.46	18,027	5,627	5,648	11,275	62.55	34,170	9,105	8,126	17,231	50.43

Note: Put asterisk (*) for No Report and Zero (0) for No Case

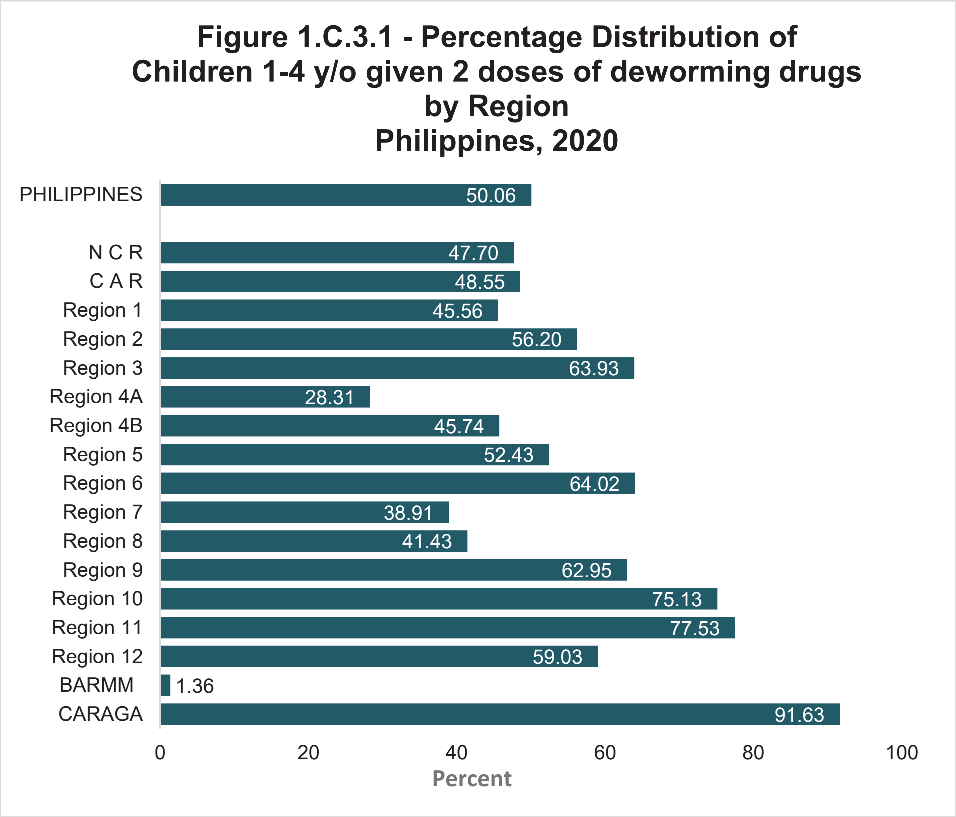
Figure 1.C.3.1 - Percentage Distribution of
Children 1-4 y/o given 2 doses of deworming drugs
by Region
Philippines, 2020
PHILIPPINES
50.06
N C R
47.70
C A R
48.55
Region 1
45.56
Region 2
56.20
Region 3
63.93
Region 4A
28.31
Region 4B
45.74
Region 5
52.43
Region 6
64.02
Region 7
38.91
Region 8
41.43
Region 9
62.95
Region 10
75.13
Region 11
77.53
Region 12
59.03
BARMM
1.36
CARAGA
91.63
0
20
40
60
80
100
Percent

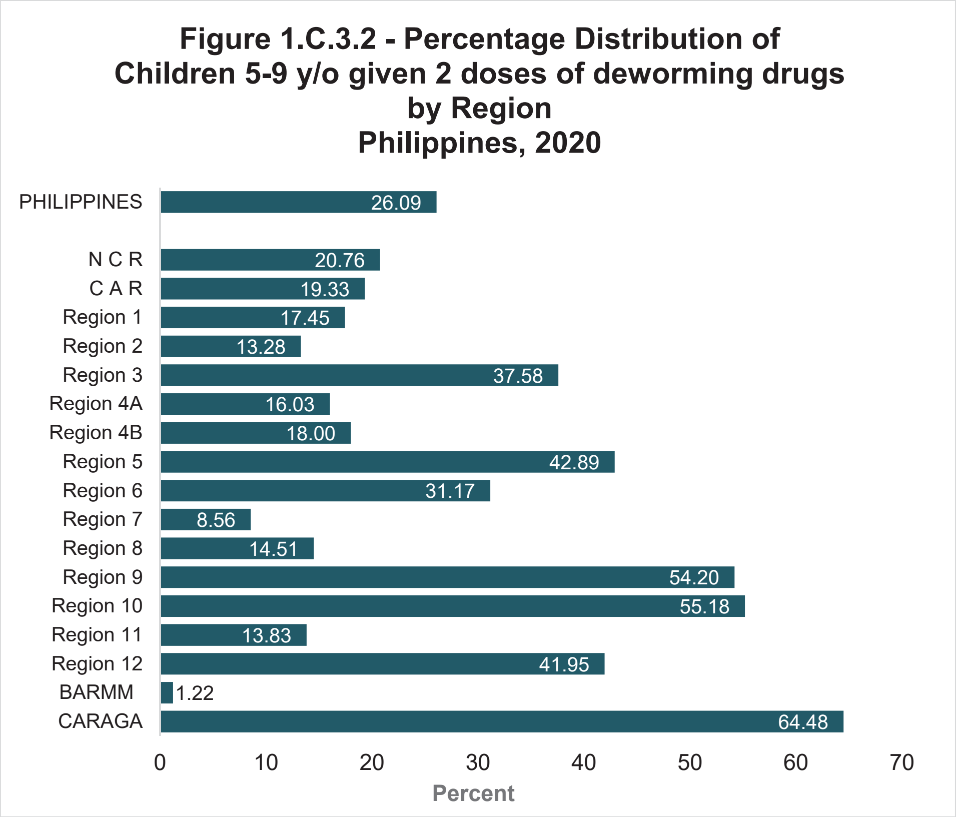
Figure 1.C.3.2 - Percentage Distribution of
Children 5-9 y/o given 2 doses of deworming drugs
by Region
Philippines, 2020
PHILIPPINES
26.09
N C R
20.76
C A R
19.33
Region 1
17.45
Region 2
13.28
Region 3
37.58
Region 4A
16.03
Region 4B
18.00
Region 5
42.89
Region 6
31.17
Region 7
8.56
Region 8
14.51
Region 9
54.20
Region 10
55.18
Region 11
13.83
Region 12
41.95
BARMM
1.22
CARAGA
64.48
0
10
20
30
40
50
60
70
Percent

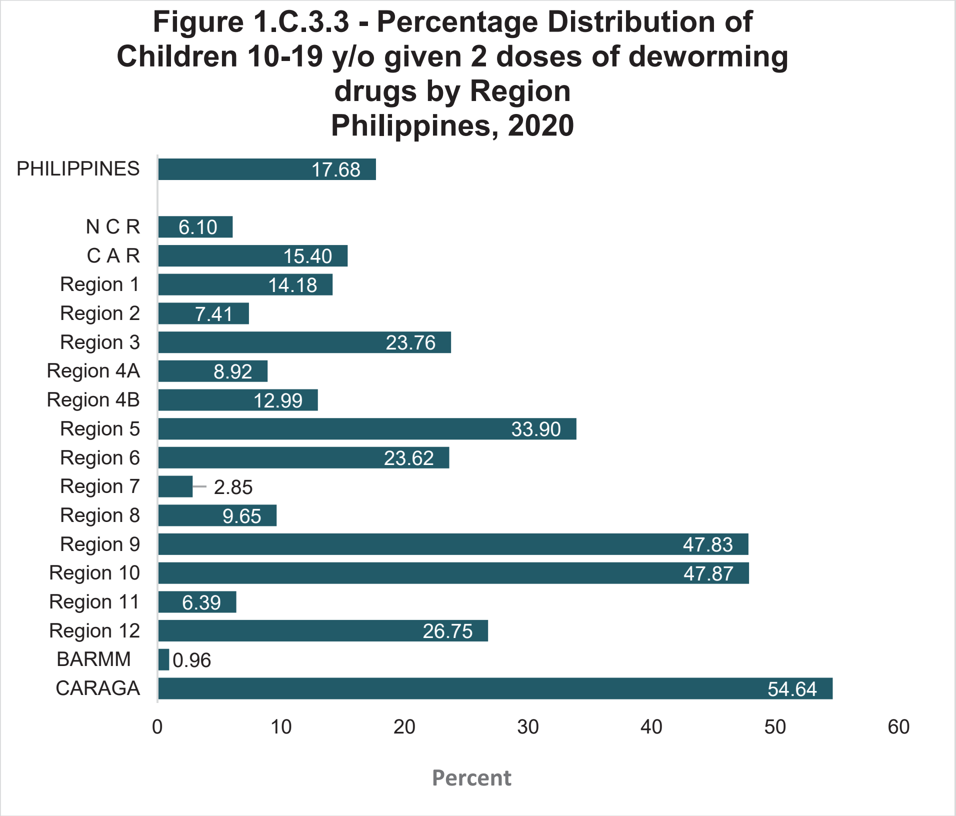
Figure 1.C.3.3 - Percentage Distribution of Children 10-19 y/o given 2 doses of deworming drugs by Region
Philippines, 2020
PHILIPPINES
17.68
N C R
6.10
C A R
15.40
Region 1
14.18
Region 2
7.41
Region 3
23.76
Region 4A
8.92
Region 4B
12.99
Region 5
33.90
Region 6
23.62
Region 7
2.85
Region 8
9.65
Region 9
47.83
Region 10
47.87
Region 11
6.39
Region 12
26.75
BARMM
0.96
CARAGA
54.64
0
10
20
30
40
50
60
Percent

Table 1.C.4.1 - Management of Sick Infants and Children

Number of high risk infants and children with measles and/or persistent diarrhea who received Vitamin A capsule aside from routine supplementation

Philippines, Annual 2020

Area	Sick Infants aged 6-11 mos.							Sick Children aged 12-59 mos.						
	Seen			given Vitamin A 100,000 IU				Seen			given Vitamin A 200,000 IU			
	Male	Female	Total	Male	Female	Total	%	Male	Female	Total	Male	Female	Total	%
**PHILIPPINES**	**71,543**	**68,636**	**140,179**	**38,189**	**37,739**	**75,928**	**54.17**	**146,165**	**139,745**	**285,910**	**75,549**	**74,759**	**150,308**	**52.57**
**N C R**	**6,875**	**6,803**	**13,678**	**5,024**	**5,040**	**10,064**	**73.58**	**12,931**	**12,072**	**25,003**	**7,956**	**7,845**	**15,801**	**63.20**
Malabon	87	76	163	30	25	55	33.74	91	68	159	20	22	42	26.42
Navotas	563	545	1,108	455	423	878	79.24	903	734	1,637	426	457	883	53.94
Valenzuela City	247	254	501	38	28	66	13.17	499	445	944	80	67	147	15.57
Caloocan City	431	380	811	248	245	493	60.79	613	608	1,221	613	608	1,221	100.00
Marikina City	8	1	9	8	1	9	100.00	29	41	70	29	39	68	97.14
Pasig City	0	0	0	0	0	0	0.00	0	1	1	0	1	1	100.00
Pateros	27	16	43	15	10	25	58.14	52	46	98	16	12	28	28.57
Taguig	1,071	1,039	2,110	534	504	1,038	49.19	4,158	3,624	7,782	1,404	1,287	2,691	34.58
Quezon City	452	501	953	441	479	920	96.54	630	659	1,289	600	634	1,234	95.73
							0.00							0.00
Makati City	65	47	112	38	36	74	66.07	126	128	254	104	112	216	85.04
Mandaluyong City	21	12	33	20	11	31	93.94	25	16	41	21	15	36	87.80
San Juan	15	8	23	15	8	23	100.00	49	43	92	49	43	92	100.00
Manila City	3,550	3,599	7,149	3,036	3,108	6,144	85.94	5,012	5,052	10,064	4,208	4,222	8,430	83.76
							0.00							0.00
Las Piñas City	10	14	24	10	14	24	100.00	16	8	24	16	8	24	100.00
Muntinlupa City	81	76	157	52	52	104	66.24	141	116	257	127	103	230	89.49
Parañaque City	237	232	469	74	93	167	35.61	573	466	1,039	229	198	427	41.10
Pasay City	10	3	13	10	3	13	100.00	14	17	31	14	17	31	100.00
**C A R**	**300**	**303**	**603**	**238**	**251**	**489**	**81.09**	**629**	**607**	**1,236**	**446**	**424**	**870**	**70.39**
Abra	150	155	305	124	137	261	85.57	406	381	787	314	300	614	78.02
Apayao	15	13	28	14	11	25	89.29	16	11	27	15	11	26	96.30
Benguet	15	14	29	13	12	25	86.21	21	17	38	10	8	18	47.37
Ifugao	71	74	145	48	51	99	68.28	151	158	309	82	77	159	51.46
Kalinga	35	36	71	34	35	69	97.18	16	25	41	16	24	40	97.56
Mt. Province	13	9	22	5	3	8	36.36	18	11	29	8	1	9	31.03
Baguio City	1	2	3	0	2	2	66.67	1	4	5	1	3	4	80.00
**Region 1**	**2,720**	**2,413**	**5,133**	**1,902**	**1,751**	**3,653**	**71.17**	**6,191**	**5,759**	**11,950**	**4,075**	**3,759**	**7,834**	**65.56**
Ilocos Norte	203	196	399	178	181	359	89.97	453	399	852	373	308	681	79.93
Ilocos Sur	535	503	1,038	444	403	847	81.60	1,476	1,360	2,836	1,084	985	2,069	72.95
La Union	94	66	160	10	7	17	10.63	343	326	669	22	23	45	6.73
Pangasinan	1,526	1,308	2,834	929	834	1,763	62.21	3,357	3,130	6,487	2,109	1,962	4,071	62.76
Alaminos City	106	95	201	106	95	201	100.00	136	147	283	136	147	283	100.00
Candon City	41	54	95	40	47	87	91.58	68	78	146	58	65	123	84.25
Dagupan City	18	8	26	8	3	11	42.31	86	75	161	36	35	71	44.10
Laoag City	4	3	7	2	3	5	71.43	2	4	6	2	3	5	83.33
San Carlos City	102	98	200	102	98	200	100.00	163	160	323	163	160	323	100.00
San Fernando City	5	4	9	3	4	7	77.78	20	15	35	17	11	28	80.00
Urdaneta City	86	78	164	80	76	156	95.12	87	65	152	75	60	135	88.82
Vigan City	0	0	0	0	0	0	0.00	0	0	0	0	0	0	0.00
**Region 2**	**3,795**	**3,484**	**7,279**	**2,111**	**1,961**	**4,072**	**55.94**	**8,220**	**7,488**	**15,708**	**3,893**	**3,531**	**7,424**	**47.26**
Batanes	0	0	0	0	0	0	0.00	0	0	0	0	0	0	0.00
Cagayan	648	610	1,258	247	258	505	40.14	1,858	1,578	3,436	543	435	978	28.46
Isabela	2,218	2,055	4,273	1,086	1,017	2,103	49.22	4,742	4,464	9,206	2,043	1,906	3,949	42.90
Nueva Vizcaya	157	164	321	127	131	258	80.37	355	309	664	207	206	413	62.20
Quirino	212	163	375	108	84	192	51.20	350	323	673	200	181	381	56.61
Cauayan City	480	426	906	480	413	893	98.57	809	740	1,549	796	732	1,528	98.64
Ilagan City	80	66	146	63	58	121	82.88	101	73	174	99	70	169	97.13
Santiago City	0	0	0	0	0	0	0.00	0	0	0	0	0	0	0.00
Tuguegarao City	0	0	0	0	0	0	0.00	5	1	6	5	1	6	100.00
**Region 3**	**10,935**	**10,324**	**21,259**	**6,165**	**6,127**	**12,292**	**57.82**	**25,454**	**24,387**	**49,841**	**14,972**	**14,652**	**29,624**	**59.44**
Aurora	185	169	354	55	54	109	30.79	397	368	765	127	119	246	32.16
Bataan	555	525	1,080	137	108	245	22.69	1,259	1,246	2,505	340	325	665	26.55
Bulacan	3,011	2,672	5,683	1,059	1,053	2,112	37.16	6,700	6,191	12,891	2,101	2,011	4,112	31.90
Nueva Ecija	875	750	1,625	399	371	770	47.38	2,509	2,281	4,790	1,204	1,149	2,353	49.12
Pampanga	363	355	718	320	329	649	90.39	447	427	874	380	383	763	87.30

Table 1.C.4.1 - Management of Sick Infants and Children

Number of high risk infants and children with measles and/or persistent diarrhea who received Vitamin A capsule aside from routine supplementation

Philippines, Annual 2020

Area	Sick Infants aged 6-11 mos.							Sick Children aged 12-59 mos.						
	Seen			given Vitamin A 100,000 IU				Seen			given Vitamin A 200,000 IU			
	Male	Female	Total	Male	Female	Total	%	Male	Female	Total	Male	Female	Total	%
Tarlac	1,329	1,197	2,526	1,027	980	2,007	79.45	2,287	2,249	4,536	1,488	1,405	2,893	63.78
Zambales	588	655	1,243	241	288	529	42.56	872	859	1,731	386	388	774	44.71
Angeles City	406	454	860	396	433	829	96.40	543	530	1,073	509	497	1,006	93.76
Balanga City	0	5	5	0	5	5	100.00	2	0	2	0	0	0	0.00
Cabanatuan City	52	46	98	46	40	86	87.76	56	54	110	57	52	109	99.09
City of San Fernando	426	374	800	299	268	567	70.88	572	579	1,151	324	364	688	59.77
Gapan City	85	84	169	69	71	140	82.84	134	167	301	61	56	117	38.87
Mabalacat City	66	71	137	2	4	6	4.38	120	105	225	10	12	22	9.78
Malolos City	23	21	44	2	8	10	22.73	27	37	64	5	2	7	10.94
Meycauayan	1,715	1,821	3,536	990	1,107	2,097	59.30	2,677	2,577	5,254	1,366	1,395	2,761	52.55
Olongapo	102	100	202	37	25	62	30.69	231	200	431	50	37	87	20.19
Palayan City	5	6	11	5	6	11	100.00	29	28	57	25	28	53	92.98
San Jose City	13	19	32	13	19	32	100.00	47	44	91	47	44	91	100.00
San Jose del Monte City	10	8	18	10	8	18	100.00	29	31	60	29	31	60	100.00
Science City of Munoz	778	624	1,402	778	624	1,402	100.00	6,130	6,006	12,136	6,130	6,006	12,136	100.00
Tarlac City	348	368	716	280	326	606	84.64	386	408	794	333	348	681	85.77
**Region 4A**	**10,675**	**9,563**	**20,238**	**3,199**	**3,130**	**6,329**	**31.27**	**23,554**	**21,659**	**45,213**	**6,336**	**6,959**	**13,295**	**29.41**
Batangas	299	249	548	62	63	125	22.81	852	734	1,586	235	236	471	29.70
Cavite	2,865	2,599	5,464	300	316	616	11.27	5,750	5,373	11,123	529	464	993	8.93
Laguna	448	398	846	149	133	282	33.33	972	872	1,844	234	245	479	25.98
Quezon	1,503	1,229	2,732	441	366	807	29.54	2,723	2,655	5,378	1,314	1,614	2,928	54.44
Rizal	474	413	887	244	215	459	51.75	1,229	1,103	2,332	498	435	933	40.01
Antipolo City	748	819	1,567	640	759	1,399	89.28	1,489	1,519	3,008	1,132	1,137	2,269	75.43
Bacoor City	877	804	1,681	196	185	381	22.67	2,099	1,886	3,985	226	316	542	13.60
Batangas City	3	2	5	1	1	2	40.00	6	9	15	5	6	11	73.33
Biñan City	150	120	270	126	103	229	84.81	340	263	603	254	237	491	81.43
Cabuyao City	100	88	188	29	14	43	22.87	263	171	434	57	130	187	43.09
Calamba City	666	597	1,263	207	219	426	33.73	1,608	1,350	2,958	312	574	886	29.95
Cavite City	78	69	147	0	1	1	0.68	149	163	312	0	0	0	0.00
Dasmariñas City	343	345	688	259	256	515	74.85	1,016	913	1,929	740	760	1,500	77.76
General Trias City	487	371	858	34	25	59	6.88	1,201	1,074	2,275	31	27	58	2.55
Imus City	952	823	1,775	408	371	779	43.89	2,205	1,951	4,156	587	602	1,189	28.61
Lipa City	9	6	15	6	5	11	73.33	8	9	17	7	7	14	82.35
Lucena City	180	166	346	37	38	75	21.68	470	397	867	122	114	236	27.22
San Pablo City	11	8	19	7	8	15	78.95	5	2	7	0	0	0	0.00
San Pedro City	4	5	9	4	5	9	100.00	10	8	18	5	9	14	77.78
Santa Rosa City	149	126	275	17	21	38	13.82	206	186	392	14	22	36	9.18
Tagaytay City	322	314	636	32	26	58	9.12	934	996	1,930	34	24	58	3.01
Tanauan City	3	8	11	0	0	0	0.00	14	21	35	0	0	0	0.00
Tayabas City	4	4	8	0	0	0	0.00	5	4	9	0	0	0	0.00
Trece Martires City	0	0	0	0	0	0	0.00	0	0	0	0	0	0	0.00
**Region 4B**	**876**	**832**	**1,708**	**375**	**372**	**747**	**43.74**	**3,213**	**3,116**	**6,329**	**1,160**	**1,191**	**2,351**	**37.15**
Marinduque	101	94	195	101	94	195	100.00	188	212	400	187	211	398	99.50
Mindoro Occidental	290	254	544	36	40	76	13.97	827	774	1,601	69	79	148	9.24
Mindoro Oriental	257	266	523	132	131	263	50.29	1,119	1,095	2,214	380	378	758	34.24
Palawan	132	131	263	106	107	213	80.99	750	762	1,512	524	523	1,047	69.25
Romblon	0	0	0	0	0	0	0.00	1	0	1	0	0	0	0.00
Puerto Princesa City	96	87	183	0	0	0	0.00	328	273	601	0	0	0	0.00
**Region 5**	**5,617**	**5,621**	**11,238**	**2,503**	**2,504**	**5,007**	**44.55**	**12,229**	**11,783**	**24,012**	**4,904**	**4,665**	**9,569**	**39.85**
Albay	1,745	1,744	3,489	691	725	1,416	40.58	4,399	4,286	8,685	1,369	1,352	2,721	31.33
Camarines Norte	605	592	1,197	322	350	672	56.14	1,063	940	2,003	675	566	1,241	61.96
Camarines Sur	685	661	1,346	304	296	600	44.58	1,678	1,600	3,278	676	675	1,351	41.21
Catanduanes	44	37	81	27	27	54	66.67	100	102	202	49	35	84	41.58
Masbate	549	548	1,097	271	249	520	47.40	774	759	1,533	508	508	1,016	66.28
Sorsogon	1,285	1,386	2,671	593	582	1,175	43.99	2,998	3,003	6,001	1,109	1,007	2,116	35.26
Iriga City	89	84	173	46	48	94	54.34	134	150	284	96	111	207	72.89
Legaspi City	533	492	1,025	184	160	344	33.56	929	800	1,729	287	289	576	33.31
Naga City	82	77	159	65	67	132	83.02	154	143	297	135	122	257	86.53
**Region 6**	**4,254**	**4,083**	**8,337**	**2,434**	**2,366**	**4,800**	**57.57**	**8,116**	**7,801**	**15,917**	**4,240**	**4,264**	**8,504**	**53.43**
Aklan	796	791	1,587	489	488	977	61.56	1,216	1,210	2,426	767	798	1,565	64.51

Table 1.C.4.1 - Management of Sick Infants and Children

Number of high risk infants and children with measles and/or persistent diarrhea who received Vitamin A capsule aside from routine supplementation

Philippines, Annual 2020

Area	Sick Infants aged 6-11 mos.							Sick Children aged 12-59 mos.						
	Seen			given Vitamin A 100,000 IU				Seen			given Vitamin A 200,000 IU			
	Male	Female	Total	Male	Female	Total	%	Male	Female	Total	Male	Female	Total	%
Antique	404	446	850	190	159	349	41.06	757	736	1,493	235	227	462	30.94
Capiz	95	112	207	69	81	150	72.46	90	95	185	74	84	158	85.41
Guimaras	16	8	24	14	8	22	91.67	13	24	37	6	18	24	64.86
Iloilo	1,363	1,330	2,693	762	784	1,546	57.41	2,823	2,541	5,364	1,445	1,433	2,878	53.65
Negros Occidental	1,313	1,238	2,551	765	763	1,528	59.90	2,896	2,904	5,800	1,585	1,577	3,162	54.52
Bacolod City	59	25	84	44	15	59	70.24	50	35	85	35	23	58	68.24
Iloilo City	208	133	341	101	68	169	49.56	271	256	527	93	104	197	37.38
**Region 7**	**1,432**	**1,327**	**2,759**	**749**	**692**	**1,441**	**52.23**	**1,485**	**1,490**	**2,975**	**1,111**	**1,044**	**2,155**	**72.44**
Bohol	252	257	509	132	96	228	44.79	212	210	422	212	210	422	100.00
Cebu	568	503	1,071	153	123	276	25.77	297	302	599	297	302	599	100.00
Negros Oriental	69	83	152	38	47	85	55.92	177	221	398	126	134	260	65.33
Siquijor	42	43	85	0	0	0	0.00	183	198	381	0	0	0	0.00
Cebu City	154	166	320	107	112	219	68.44	233	209	442	161	131	292	66.06
Lapu-Lapu City	190	130	320	190	130	320	100.00	228	183	411	228	183	411	100.00
Mandaue City	157	145	302	129	184	313	103.64	155	167	322	87	84	171	53.11
**Region 8**	**4,603**	**4,644**	**9,247**	**1,759**	**1,816**	**3,575**	**38.66**	**8,809**	**8,046**	**16,855**	**3,440**	**3,186**	**6,626**	**39.31**
Biliran	167	612	779	51	222	273	35.04	270	347	617	86	77	163	26.42
Eastern Samar	552	497	1,049	207	183	390	37.18	1,446	1,303	2,749	310	292	602	21.90
Northern Leyte	986	894	1,880	416	363	779	41.44	1,960	1,771	3,731	816	679	1,495	40.07
Northern Samar	1,269	1,144	2,413	560	541	1,101	45.63	2,569	2,220	4,789	1,104	974	2,078	43.39
Southern Leyte	102	81	183	34	36	70	38.25	247	220	467	83	78	161	34.48
Western Samar	847	793	1,640	396	374	770	46.95	1,777	1,725	3,502	852	911	1,763	50.34
Calbayog City	3	1	4	3	1	4	100.00	3	1	4	3	1	4	100.00
Maasin City	464	385	849	0	0	0	0.00	21	13	34	19	11	30	88.24
Ormoc City	7	8	15	7	8	15	100.00	31	22	53	31	22	53	100.00
Tacloban City	206	229	435	85	88	173	39.77	485	424	909	136	141	277	30.47
**Region 9**	**3,766**	**3,824**	**7,590**	**2,538**	**2,553**	**5,091**	**67.08**	**6,072**	**6,391**	**12,463**	**3,826**	**4,240**	**8,066**	**64.72**
Zamboanga del Norte	2,091	2,193	4,284	1,443	1,436	2,879	67.20	3,261	3,437	6,698	2,113	2,411	4,524	67.54
Zamboanga del Sur	261	265	526	195	239	434	82.51	539	574	1,113	350	401	751	67.48
Zamboanga Sibugay	437	475	912	227	259	486	53.29	887	1,022	1,909	490	587	1,077	56.42
Dapitan City	100	93	193	63	76	139	72.02	171	136	307	160	128	288	93.81
Dipolog City	45	30	75	36	21	57	76.00	32	28	60	22	16	38	63.33
Isabela City	127	108	235	116	100	216	91.91	248	241	489	151	139	290	59.30
Pagadian City	0	0	0	0	0	0	0.00	0	0	0	0	0	0	0.00
Zamboanga City	705	660	1,365	458	422	880	64.47	934	953	1,887	540	558	1,098	58.19
**Region 10**	**6,692**	**6,455**	**13,147**	**3,337**	**3,350**	**6,687**	**50.86**	**12,758**	**12,346**	**25,104**	**7,590**	**7,148**	**14,738**	**58.71**
Bukidnon	1,622	1,646	3,268	738	746	1,484	45.41	2,436	2,482	4,918	1,008	1,015	2,023	41.13
Camiguin	9	6	15	0	0	0	0.00	17	16	33	3	2	5	15.15
Lanao del Norte	963	900	1,863	568	668	1,236	66.34	1,375	1,401	2,776	1,031	1,043	2,074	74.71
Misamis Occidental	374	353	727	258	298	556	76.48	523	667	1,190	321	432	753	63.28
Misamis Oriental	1,889	1,676	3,565	426	362	788	22.10	2,661	2,450	5,111	760	576	1,336	26.14
Cagayan de Oro City	152	172	324	106	100	206	63.58	298	305	603	140	139	279	46.27
El Salvador City	0	0	0	0	0	0	0.00	0	0	0	0	0	0	0.00
Gingoog City	218	215	433	184	130	314	72.52	450	472	922	182	167	349	37.85
Iligan City	549	566	1,115	482	488	970	87.00	1,071	893	1,964	991	826	1,817	92.52
Malaybalay City	282	284	566	103	67	170	30.04	467	435	902	122	63	185	20.51
Oroquieta City	0	0	0	0	0	0	0.00	0	0	0	0	0	0	0.00
Ozamis City	363	357	720	309	315	624	86.67	2,948	2,784	5,732	2,833	2,681	5,514	96.20
Tangub City	4	4	8	4	4	8	100.00	3	3	6	3	3	6	100.00
Valencia City	267	276	543	159	172	331	60.96	509	438	947	196	201	397	41.92
**Region 11**	**971**	**802**	**1,773**	**838**	**679**	**1,517**	**85.56**	**1,725**	**1,450**	**3,175**	**1,468**	**1,246**	**2,714**	**85.48**
Compostela Valley	38	32	70	33	22	55	78.57	87	83	170	77	64	141	82.94
Davao del Norte	128	107	235	112	99	211	89.79	217	182	399	184	158	342	85.71
Davao del Sur	264	249	513	233	209	442	86.16	481	447	928	432	399	831	89.55
Davao Oriental	85	80	165	82	78	160	96.97	147	145	292	128	127	255	87.33
Davao Occidental	20	14	34	12	7	19	55.88	36	18	54	22	13	35	64.81

Table 1.C.4.1 - Management of Sick Infants and Children

Number of high risk infants and children with measles and/or persistent diarrhea who received Vitamin A capsule aside from routine supplementation

Philippines, Annual 2020

Area	Sick Infants aged 6-11 mos.							Sick Children aged 12-59 mos.						
	Seen			given Vitamin A 100,000 IU				Seen			given Vitamin A 200,000 IU			
	Male	Female	Total	Male	Female	Total	%	Male	Female	Total	Male	Female	Total	%
Davao City	436	320	756	366	264	630	83.33	757	575	1,332	625	485	1,110	83.33
**Region 12**	**4,190**	**4,207**	**8,397**	**2,754**	**2,789**	**5,543**	**66.01**	**8,446**	**8,697**	**17,143**	**6,298**	**6,408**	**12,706**	**74.12**
North Cotabato	2,135	2,236	4,371	814	900	1,714	39.21	3,746	3,987	7,733	1,750	1,839	3,589	46.41
Sarangani	974	945	1,919	974	945	1,919	100.00	3,401	3,425	6,826	3,401	3,425	6,826	100.00
South Cotabato	621	565	1,186	565	515	1,080	91.06	779	785	1,564	663	686	1,349	86.25
Sultan Kudarat	244	274	518	244	274	518	100.00	234	249	483	234	249	483	100.00
Cotabato City	2	3	5	2	3	5	100.00	0	0	0	0	0	0	0.00
Gen. Santos City	214	184	398	155	152	307	77.14	286	251	537	250	209	459	85.47
**BARMM**	**1,461**	**1,527**	**2,988**	**1,332**	**1,438**	**2,770**	**92.70**	**2,316**	**2,508**	**4,824**	**2,066**	**2,381**	**4,447**	**92.18**
Basilan	453	532	985	441	524	965	97.97	527	677	1,204	394	582	976	81.06
Lanao del Sur	519	525	1,044	416	452	868	83.14	1,017	1,119	2,136	931	1,115	2,046	95.79
Maguindanao	15	12	27	14	11	25	92.59	36	33	69	32	29	61	88.41
Sulu	23	21	44	22	22	44	100.00	26	16	42	25	15	40	95.24
Tawi-Tawi	28	29	57	28	28	56	98.25	45	59	104	45	58	103	99.04
Lamitan City	24	26	50	12	19	31	62.00	67	38	105	51	27	78	74.29
Marawi City	399	382	781	399	382	781	100.00	598	566	1,164	588	555	1,143	98.20
**CARAGA**	**2,381**	**2,424**	**4,805**	**931**	**920**	**1,851**	**38.52**	**4,017**	**4,145**	**8,162**	**1,768**	**1,816**	**3,584**	**43.91**
Agusan del Norte	256	254	510	158	147	305	59.80	431	459	890	243	275	518	58.20
Agusan del Sur	978	968	1,946	395	357	752	38.64	1,595	1,597	3,192	829	854	1,683	52.73
Surigao del Norte	421	507	928	85	99	184	19.83	864	897	1,761	133	130	263	14.93
Surigao del Sur	224	212	436	100	105	205	47.02	344	338	682	177	160	337	49.41
Province of Dinagat	191	159	350	20	9	29	8.29	237	258	495	46	41	87	17.58
Bislig City	57	39	96	41	24	65	67.71	141	135	276	118	103	221	80.07
Butuan City	115	118	233	46	62	108	46.35	168	196	364	74	84	158	43.41
Surigao City	139	167	306	86	117	203	66.34	237	265	502	148	169	317	63.15

Note: Put asterisk (*) for No Report and Zero (0) for No Case

Table 1.C.4.2 - Management of Sick Infants and Children

Number and proportion of diarrhea cases 0-59 mos. old who received Oral Rehydration Salt (ORS) solution and ORS with Zinc drops/syrup and Pneumonia cases among children 0-59 mos. old who received treatment

Philippines, Annual 2020

Area	Children 0-59 mos. old with Diarrhea											Children 0-59 mos. old with Pneumonia						
	Seen			Given ORS				Given ORS w/ zinc				Seen			Received treatment			
	Male	Female	Total	Male	Female	Total	%	Male	Female	Total	%	Male	Female	Total	Male	Female	Total	%
**PHILIPPINES**	**53,424**	**49,321**	**102,745**	**33,640**	**30,964**	**64,604**	**62.88**	**31,083**	**28,496**	**59,579**	**57.99**	**34,085**	**31,325**	**65,410**	**32,821**	**30,373**	**63,194**	**96.61**
**N C R**	**5,147**	**4,645**	**9,792**	**2,406**	**2,295**	**4,701**	**48.01**	**2,739**	**2,350**	**5,089**	**51.97**	**2,859**	**2,661**	**5,520**	**2,816**	**2,607**	**5,423**	**98.24**
Malabon	65	58	123	0	0	0	0.00	65	58	123	100.00	27	33	60	27	33	60	100.00
Navotas	276	257	533	164	145	309	57.97	112	112	224	42.03	63	47	110	63	47	110	100.00
Valenzuela City	490	386	876	269	183	452	51.60	221	203	424	48.40	277	256	533	243	229	472	88.56
Caloocan City	104	83	187	32	24	56	29.95	72	59	131	70.05	78	48	126	72	45	117	92.86
Marikina City	67	76	143	44	35	79	55.24	23	41	64	44.76	8	1	9	8	1	9	100.00
Pasig City	197	172	369	71	72	143	38.75	126	100	226	61.25	52	32	84	52	32	84	100.00
Pateros	27	22	49	0	0	0	0.00	27	22	49	100.00	3	3	6	3	3	6	100.00
Taguig	470	401	871	200	156	356	40.87	270	245	515	59.13	90	92	182	87	88	175	96.15
Quezon City	572	555	1,127	296	305	601	53.33	274	250	524	46.50	549	464	1,013	549	447	996	98.32
Makati City	342	259	601	23	20	43	7.15	319	239	558	92.85	113	90	203	113	89	202	99.51
Mandaluyong City	110	84	194	73	51	124	63.92	37	33	70	36.08	147	144	291	147	144	291	100.00
San Juan	53	41	94	53	41	94	100.00	0	0	0	0.00	29	19	48	29	19	48	100.00
Manila City	1,532	1,559	3,091	1,065	1,172	2,237	72.37	467	387	854	27.63	1,235	1,282	2,517	1,235	1,280	2,515	99.92
Las Piñas City	400	339	739	0	0	0	0.00	400	339	739	100.00	98	81	179	98	81	179	100.00
Muntinlupa City	247	197	444	18	13	31	6.98	229	184	413	93.02	65	43	108	65	43	108	100.00
Parañaque City	141	113	254	98	78	176	69.29	43	35	78	30.71	12	18	30	12	18	30	100.00
Pasay City	54	43	97	0	0	0	0.00	54	43	97	100.00	13	8	21	13	8	21	100.00
**C A R**	**2,209**	**1,899**	**4,108**	**689**	**569**	**1,258**	**30.62**	**1,502**	**1,312**	**2,814**	**68.50**	**947**	**762**	**1,709**	**939**	**759**	**1,698**	**99.36**
Abra	289	257	546	64	55	119	21.79	221	198	419	76.74	116	110	226	115	110	225	99.56
Apayao	98	77	175	16	10	26	14.86	80	65	145	82.86	26	16	42	26	16	42	100.00
Benguet	316	269	585	54	29	83	14.19	261	240	501	85.64	120	110	230	120	110	230	100.00
Ifugao	523	399	922	231	175	406	44.03	292	223	515	55.86	234	182	416	232	181	413	99.28
Kalinga	607	525	1,132	140	126	266	23.50	459	393	852	75.27	132	101	233	132	101	233	100.00
Mt. Province	293	264	557	182	174	356	63.91	109	85	194	34.83	186	133	319	183	132	315	98.75
Baguio City	83	108	191	2	0	2	1.05	80	108	188	98.43	133	110	243	131	109	240	98.77
**Region 1**	**2,504**	**2,030**	**4,534**	**1,254**	**976**	**2,230**	**49.18**	**1,192**	**994**	**2,186**	**48.21**	**959**	**896**	**1,855**	**937**	**876**	**1,813**	**97.74**
Ilocos Norte	143	130	273	22	24	46	16.85	115	100	215	78.75	86	69	155	86	69	155	100.00
Ilocos Sur	426	350	776	85	57	142	18.30	341	290	631	81.31	153	140	293	153	140	293	100.00
La Union	154	109	263	57	41	98	37.26	97	68	165	62.74	147	128	275	143	121	264	96.00
Pangasinan	1,252	1,004	2,256	828	665	1,493	66.18	372	288	660	29.26	446	396	842	429	384	813	96.56
Alaminos City	42	37	79	34	29	63	79.75	8	8	16	20.25	17	16	33	17	16	33	100.00
Candon City	45	45	90	26	26	52	57.78	19	19	38	42.22	18	21	39	17	20	37	94.87
Dagupan City	64	50	114	0	0	0	0.00	64	50	114	100.00	26	20	46	26	20	46	100.00
Laoag City	5	2	7	2	0	2	28.57	3	2	5	71.43	2	1	3	2	1	3	100.00
San Carlos City	320	269	589	181	121	302	51.27	139	148	287	48.73	41	77	118	41	77	118	100.00
San Fernando City	15	6	21	3	0	3	14.29	12	6	18	85.71	12	11	23	12	11	23	100.00
Urdaneta City	20	14	34	2	0	2	5.88	18	14	32	94.12	0	0	0	0	0	0	0.00
Vigan City	18	14	32	14	13	27	84.38	4	1	5	15.63	11	17	28	11	17	28	100.00
**Region 2**	**2,723**	**2,320**	**5,043**	**2,106**	**1,796**	**3,902**	**77.37**	**2,067**	**1,837**	**3,904**	**77.41**	**1,613**	**1,322**	**2,935**	**1,554**	**1,284**	**2,838**	**96.70**
Batanes	4	0	4	4	0	4	100.00	0	0	0	0.00	1	3	4	1	3	4	100.00
Cagayan	509	440	949	422	371	793	83.56	311	304	615	64.81	231	154	385	194	135	329	85.45
Isabela	1,421	1,239	2,660	1,343	1,157	2,500	93.98	1,113	998	2,111	79.36	1,265	1,084	2,349	1,249	1,073	2,322	98.85
Nueva Vizcaya	283	268	551	186	176	362	65.70	206	207	413	74.95	39	29	68	34	24	58	85.29
Quirino	74	68	142	60	49	109	76.76	30	31	61	42.96	7	2	9	7	0	7	77.78
Cauayan City	300	237	537	0	0	0	0.00	300	237	537	100.00	2	3	5	2	3	5	100.00
Ilagan City	95	42	137	86	41	127	92.70	72	35	107	78.10	31	20	51	31	20	51	100.00
Santiago City	0	0	0	0	0	0	0.00	0	0	0	0.00	0	0	0	0	0	0	0.00
Tuguegarao City	37	26	63	5	2	7	11.11	35	25	60	95.24	37	27	64	36	26	62	96.88
**Region 3**	**8,198**	**7,810**	**16,008**	**5,294**	**5,060**	**10,354**	**64.68**	**4,230**	**3,973**	**8,203**	**51.24**	**3,587**	**3,357**	**6,944**	**3,556**	**3,309**	**6,865**	**98.86**
Aurora	91	71	162	87	65	152	93.83	46	36	82	50.62	65	54	119	62	54	116	97.48
Bataan	924	845	1,769	460	405	865	48.90	457	437	894	50.54	272	236	508	271	232	503	99.02
Bulacan	2,026	1,976	4,002	1,586	1,579	3,165	79.09	925	811	1,736	43.38	261	204	465	250	196	446	95.91
Nueva Ecija	739	603	1,342	624	517	1,141	85.02	510	418	928	69.15	2,586	2,483	5,069	2,582	2,480	5,062	99.86
Pampanga	358	312	670	304	256	560	83.58	54	56	110	16.42	99	98	197	94	82	176	89.34
Tarlac	1,181	1,093	2,274	1,058	1,001	2,059	90.55	132	101	233	10.25	95	90	185	95	90	185	100.00
Zambales	153	149	302	92	101	193	63.91	93	96	189	62.58	8	16	24	5	5	10	41.67
Angeles City	333	335	668	240	259	499	74.70	256	263	519	77.69	37	45	82	37	45	82	100.00
Balanga City	26	15	41	1	0	1	2.44	25	15	40	97.56	9	2	11	9	2	11	100.00
Cabanatuan City	199	192	391	129	127	256	65.47	72	63	135	34.53	28	23	51	28	23	51	100.00
City of San Fernando	88	96	184	60	70	130	70.65	50	28	78	42.39	14	6	20	14	6	20	100.00
Gapan City	203	225	428	71	82	153	35.75	147	161	308	71.96	6	6	12	6	6	12	100.00

Table 1.C.4.2 - Management of Sick Infants and Children

Number and proportion of diarrhea cases 0-59 mos. old who received Oral Rehydration Salt (ORS) solution and ORS with Zinc drops/syrup and Pneumonia cases among children 0-59 mos. old who received treatment

Philippines, Annual 2020

Area	Children 0-59 mos. old with Diarrhea											Children 0-59 mos. old with Pneumonia						
	Seen			Given ORS				Given ORS w/ zinc				Seen			Received treatment			
	Male	Female	Total	Male	Female	Total	%	Male	Female	Total	%	Male	Female	Total	Male	Female	Total	%
Mabalacat City	24	28	52	4	2	6	11.54	20	26	46	88.46	7	6	13	7	6	13	100.00
Malolos City	41	31	72	31	27	58	80.56	26	25	51	70.83	6	6	12	6	6	12	100.00
Meycauayan	632	657	1,289	262	264	526	40.81	343	365	708	54.93	14	15	29	11	10	21	72.41
Olongapo	33	25	58	5	5	10	17.24	33	22	55	94.83	7	3	10	7	3	10	100.00
Palayan City	35	35	70	3	6	9	12.86	34	35	69	98.57	44	39	83	44	39	83	100.00
San Jose City	4	5	9	4	5	9	100.00	4	5	9	100.00	1	3	4	1	3	4	100.00
San Jose del Monte City	547	567	1,114	71	71	142	12.75	476	496	972	87.25	3	4	7	3	4	7	100.00
Science City of Munoz	88	56	144	2	1	3	2.08	88	56	144	100.00	13	5	18	13	5	18	100.00
Tarlac City	473	494	967	200	217	417	43.12	439	458	897	92.76	12	13	25	11	12	23	92.00
**Region 4A**	**3,857**	**3,305**	**7,162**	**2,958**	**2,631**	**5,589**	**78.04**	**2,286**	**1,959**	**4,245**	**59.27**	**1,123**	**918**	**2,041**	**1,058**	**853**	**1,911**	**93.63**
Batangas	218	162	380	158	113	271	71.32	108	80	188	49.47	109	112	221	108	112	220	99.55
Cavite	735	598	1,333	385	377	762	57.16	452	377	829	62.19	96	59	155	87	48	135	87.10
Laguna	205	152	357	126	108	234	65.55	121	90	211	59.10	56	31	87	32	9	41	47.13
Quezon	539	453	992	511	429	940	94.76	325	265	590	59.48	582	479	1,061	578	469	1,047	98.68
Rizal	346	315	661	336	307	643	97.28	275	258	533	80.64	50	35	85	48	35	83	97.65
Antipolo City	182	187	369	184	186	370	100.27	172	157	329	89.16	78	92	170	78	91	169	99.41
Bacoor City	146	132	278	76	75	151	54.32	126	106	232	83.45	6	5	11	5	4	9	81.82
Batangas City	31	19	50	13	7	20	40.00	30	19	49	98.00	7	8	15	7	8	15	100.00
Biñan City	317	324	641	278	292	570	88.92	154	152	306	47.74	5	4	9	5	4	9	100.00
Cabuyao City	120	103	223	113	97	210	94.17	96	92	188	84.30	8	12	20	1	2	3	15.00
Calamba City	279	215	494	272	202	474	95.95	144	138	282	57.09	14	9	23	10	8	18	78.26
Cavite City	9	14	23	8	14	22	95.65	2	3	5	21.74	0	0	0	0	0	0	0.00
Dasmariñas City	137	169	306	80	68	148	48.37	33	33	66	21.57	7	6	13	1	1	2	15.38
General Trias City	43	37	80	35	31	66	82.50	11	12	23	28.75	33	26	59	31	26	57	96.61
Imus City	170	119	289	128	96	224	77.51	35	23	58	20.07	1	2	3	0	0	0	0.00
Lipa City	0	0	0	0	0	0	0.00	0	0	0	0.00	0	0	0	0	0	0	0.00
Lucena City	36	32	68	36	34	70	102.94	27	27	54	79.41	6	3	9	2	2	4	44.44
San Pablo City	4	5	9	4	5	9	100.00	0	1	1	11.11	0	0	0	0	0	0	0.00
San Pedro City	16	15	31	6	8	14	45.16	6	6	12	38.71	0	0	0	0	0	0	0.00
Santa Rosa City	76	61	137	71	58	129	94.16	8	4	12	8.76	0	0	0	0	0	0	0.00
Tagaytay City	6	8	14	4	6	10	71.43	6	8	14	100.00	0	0	0	0	0	0	0.00
Tanauan City	10	10	20	8	8	16	80.00	3	2	5	25.00	0	0	0	0	0	0	0.00
Tayabas City	47	32	79	47	32	79	100.00	0	0	0	0.00	61	34	95	61	34	95	100.00
Trece Martires City	185	143	328	79	78	157	47.87	152	106	258	78.66	4	1	5	4	0	4	80.00
**Region 4B**	**698**	**607**	**1,305**	**596**	**521**	**1,117**	**85.59**	**444**	**356**	**800**	**61.30**	**196**	**165**	**361**	**191**	**167**	**358**	**99.17**
Marinduque	60	54	114	57	52	109	95.61	26	27	53	46.49	65	45	110	65	45	110	100.00
Mindoro Occidental	232	175	407	178	137	315	77.40	140	106	246	60.44	11	6	17	10	8	18	105.88
Mindoro Oriental	158	169	327	127	132	259	79.20	86	68	154	47.09	43	41	84	41	41	82	97.62
Palawan	243	206	449	229	197	426	94.88	191	155	346	77.06	43	37	80	41	37	78	97.50
Romblon	4	3	7	4	3	7	100.00	1	0	1	14.29	1	0	1	1	0	1	100.00
Puerto Princesa City	1	0	1	1	0	1	100.00	0	0	0	0.00	33	36	69	33	36	69	100.00
**Region 5**	**3,210**	**2,970**	**6,180**	**1,978**	**1,912**	**3,890**	**62.94**	**1,734**	**1,568**	**3,302**	**53.43**	**1,083**	**895**	**1,978**	**864**	**714**	**1,578**	**79.78**
Albay	633	602	1,235	496	466	962	77.89	405	405	810	65.59	196	178	374	173	163	336	89.84
Camarines Norte	463	420	883	398	367	765	86.64	271	275	546	61.83	111	93	204	103	81	184	90.20
Camarines Sur	869	719	1,588	329	312	641	40.37	526	395	921	58.00	315	214	529	289	200	489	92.44
Catanduanes	113	122	235	79	76	155	65.96	49	61	110	46.81	113	92	205	105	92	197	96.10
Masbate	439	447	886	248	275	523	59.03	225	207	432	48.76	233	207	440	97	84	181	41.14
Sorsogon	380	354	734	246	219	465	63.35	143	135	278	37.87	77	82	159	62	66	128	80.50
Iriga City	61	64	125	44	43	87	69.60	17	21	38	30.40	1	0	1	0	0	0	0.00
Legaspi City	77	70	147	66	57	123	83.67	28	18	46	31.29	11	5	16	9	4	13	81.25
Naga City	175	172	347	72	97	169	48.70	70	51	121	34.87	26	24	50	26	24	50	100.00
**Region 6**	**1,895**	**1,798**	**3,693**	**1,540**	**1,384**	**2,924**	**79.18**	**1,241**	**1,112**	**2,353**	**63.72**	**2,826**	**2,532**	**5,358**	**2,731**	**2,479**	**5,210**	**97.24**
Aklan	217	210	427	204	193	397	92.97	102	98	200	46.84	118	102	220	118	101	219	99.55
Antique	105	145	250	135	66	201	80.40	66	46	112	44.80	571	504	1,075	563	497	1,060	98.60
Capiz	77	87	164	24	27	51	31.10	27	27	54	32.93	204	168	372	204	168	372	100.00
Guimaras	1	3	4	0	2	2	50.00	1	3	4	100.00	3	6	9	3	5	8	88.89
Iloilo	268	218	486	243	195	438	90.12	150	137	287	59.05	783	710	1,493	713	684	1,397	93.57
Negros Occidental	1,133	1,061	2,194	846	839	1,685	76.80	864	772	1,636	74.57	992	943	1,935	982	926	1,908	98.60
Bacolod City	45	34	79	40	25	65	82.28	26	21	47	59.49	8	3	11	2	3	5	45.45
Iloilo City	49	40	89	48	37	85	95.51	5	8	13	14.61	147	96	243	146	95	241	99.18
**Region 7**	**3,248**	**2,999**	**6,247**	**2,151**	**1,834**	**3,985**	**63.79**	**2,343**	**2,148**	**4,491**	**71.89**	**4,422**	**4,402**	**8,824**	**4,371**	**4,343**	**8,714**	**98.75**
Bohol	1,803	1,524	3,327	1,781	1,498	3,279	98.56	1,098	857	1,955	58.76	1,232	1,365	2,597	1,232	1,365	2,597	100.00
Cebu	366	352	718	242	222	464	64.62	236	233	469	65.32	935	910	1,845	909	885	1,794	97.24
Negros Oriental	643	753	1,396	0	0	0	0.00	630	740	1,370	98.14	1,684	1,653	3,337	1,665	1,632	3,297	98.80
Siquijor	23	36	59	4	2	6	10.17	18	34	52	88.14	58	45	103	58	45	103	100.00

Table 1.C.4.2 - Management of Sick Infants and Children

Number and proportion of diarrhea cases 0-59 mos. old who received Oral Rehydration Salt (ORS) solution and ORS with Zinc drops/syrup and Pneumonia cases among children 0-59 mos. old who received treatment

Philippines, Annual 2020

Area	Children 0-59 mos. old with Diarrhea											Children 0-59 mos. old with Pneumonia						
	Seen			Given ORS				Given ORS w/ zinc				Seen			Received treatment			
	Male	Female	Total	Male	Female	Total	%	Male	Female	Total	%	Male	Female	Total	Male	Female	Total	%
Cebu City	164	125	289	96	84	180	62.28	135	97	232	80.28	303	260	563	297	247	544	96.63
Lapu-Lapu City	240	198	438	19	17	36	8.22	221	181	402	91.78	204	163	367	204	163	367	100.00
Mandaue City	9	11	20	9	11	20	100.00	5	6	11	55.00	6	6	12	6	6	12	100.00
**Region 8**	**1,808**	**1,565**	**3,373**	**1,483**	**1,286**	**2,769**	**82.09**	**1,421**	**1,205**	**2,626**	**77.85**	**2,417**	**2,192**	**4,609**	**2,376**	**2,167**	**4,543**	**98.57**
Biliran	139	123	262	82	83	165	62.98	94	78	172	65.65	109	106	215	103	102	205	95.35
Eastern Samar	194	156	350	163	142	305	87.14	147	113	260	74.29	564	559	1,123	560	556	1,116	99.38
Northern Leyte	395	370	765	356	334	690	90.20	276	251	527	68.89	509	436	945	502	431	933	98.73
Northern Samar	487	397	884	461	369	830	93.89	395	313	708	80.09	298	271	569	286	262	548	96.31
Southern Leyte	50	42	92	11	6	17	18.48	39	35	74	80.43	81	54	135	80	53	133	98.52
Western Samar	467	430	897	374	325	699	77.93	404	375	779	86.85	631	583	1,214	623	582	1,205	99.26
Calbayog City	13	3	16	13	3	16	100.00	9	3	12	75.00	4	0	4	4	0	4	100.00
Maasin City	2	3	5	1	1	2	40.00	2	3	5	100.00	0	0	0	0	0	0	0.00
Ormoc City	39	16	55	0	0	0	0.00	39	16	55	100.00	130	106	236	130	106	236	100.00
Tacloban City	22	25	47	22	23	45	95.74	16	18	34	72.34	91	77	168	88	75	163	97.02
**Region 9**	**2,354**	**2,288**	**4,642**	**1,870**	**1,829**	**3,699**	**79.69**	**1,729**	**1,693**	**3,422**	**73.72**	**1,252**	**1,235**	**2,487**	**1,169**	**1,146**	**2,315**	**93.08**
Zamboanga del Norte	1,096	1,168	2,264	1,025	1,078	2,103	92.89	965	996	1,961	86.62	905	908	1,813	857	861	1,718	94.76
Zamboanga del Sur	212	181	393	164	139	303	77.10	28	31	59	15.01	117	108	225	110	104	214	95.11
Zamboanga Sibugay	245	219	464	130	115	245	52.80	102	107	209	45.04	103	102	205	83	81	164	80.00
Dapitan City	36	52	88	21	31	52	59.09	24	27	51	57.95	5	7	12	5	7	12	100.00
Dipolog City	23	22	45	23	17	40	88.89	13	13	26	57.78	0	0	0	0	0	0	0.00
Isabela City	352	288	640	290	259	549	85.78	263	217	480	75.00	77	80	157	75	74	149	94.90
Pagadian City	0	0	0	0	0	0	0.00	0	0	0	0.00	0	0	0	0	0	0	0.00
Zamboanga City	390	358	748	217	190	407	54.41	334	302	636	85.03	45	30	75	39	19	58	77.33
**Region 10**	**2,422**	**2,404**	**4,826**	**2,208**	**2,167**	**4,375**	**90.65**	**1,756**	**1,725**	**3,481**	**72.13**	**2,662**	**2,424**	**5,086**	**2,564**	**2,327**	**4,891**	**96.17**
Bukidnon	689	688	1,377	674	674	1,348	97.89	573	564	1,137	82.57	1,121	852	1,973	1,075	815	1,890	95.79
Camiguin	5	6	11	1	0	1	9.09	4	6	10	90.91	22	23	45	22	23	45	100.00
Lanao del Norte	329	336	665	304	308	612	92.03	218	238	456	68.57	231	244	475	222	233	455	95.79
Misamis Occidental	138	174	312	136	172	308	98.72	137	173	310	99.36	166	306	472	149	281	430	91.10
Misamis Oriental	388	346	734	326	288	614	83.65	250	210	460	62.67	332	311	643	317	298	615	95.65
Cagayan de Oro City	176	178	354	175	174	349	98.59	71	53	124	35.03	236	207	443	233	203	436	98.42
El Salvador City	15	14	29	15	14	29	100.00	14	12	26	89.66	1	0	1	1	0	1	100.00
Gingoog City	63	65	128	31	29	60	46.88	30	28	58	45.31	31	25	56	28	22	50	89.29
Iligan City	189	167	356	167	147	314	88.20	95	73	168	47.19	47	42	89	46	37	83	93.26
Malaybalay City	101	81	182	98	79	177	97.25	78	65	143	78.57	69	63	132	68	63	131	99.24
Oroquieta City	0	0	0	0	0	0	0.00	0	0	0	0.00	0	0	0	0	0	0	0.00
Ozamis City	206	249	455	177	194	371	81.54	225	230	455	100.00	185	177	362	182	178	360	99.45
Tangub City	1	0	1	1	0	1	100.00	1	0	1	100.00	0	0	0	0	0	0	0.00
Valencia City	122	100	222	103	88	191	86.04	60	73	133	59.91	221	174	395	221	174	395	100.00
**Region 11**	**1,194**	**1,076**	**2,270**	**413**	**350**	**763**	**33.61**	**743**	**668**	**1,411**	**62.16**	**1,319**	**1,045**	**2,364**	**1,253**	**990**	**2,243**	**94.88**
Compostela Valley	100	86	186	67	54	121	65.05	31	29	60	32.26	151	128	279	150	124	274	98.21
Davao del Norte	292	257	549	69	40	109	19.85	216	209	425	77.41	349	257	606	325	242	567	93.56
Davao del Sur	256	238	494	26	23	49	9.92	224	202	426	86.23	322	269	591	319	266	585	98.98
Davao Oriental	175	141	316	20	13	33	10.44	152	125	277	87.66	207	154	361	203	152	355	98.34
Davao Occidental	96	85	181	44	36	80	44.20	43	40	83	45.86	7	10	17	7	10	17	100.00
Davao City	275	269	544	187	184	371	68.20	77	63	140	25.74	283	227	510	249	196	445	87.25
**Region 12**	**3,300**	**3,191**	**6,491**	**2,301**	**2,207**	**4,508**	**69.45**	**812**	**789**	**1,601**	**24.66**	**2,827**	**2,627**	**5,454**	**2,598**	**2,489**	**5,087**	**93.27**
North Cotabato	915	956	1,871	423	450	873	46.66	305	309	614	32.82	1,075	1,025	2,100	1,012	997	2,009	95.67
Sarangani	974	940	1,914	822	831	1,653	86.36	152	109	261	13.64	379	330	709	239	238	477	67.28
South Cotabato	708	635	1,343	515	448	963	71.71	193	187	380	28.29	881	781	1,662	855	764	1,619	97.41
Sultan Kudarat	528	496	1,024	370	314	684	66.80	158	182	340	33.20	352	362	714	352	362	714	100.00
Cotabato City	20	26	46	18	25	43	93.48	2	1	3	6.52	99	82	181	99	82	181	100.00
Gen. Santos City	155	138	293	153	139	292	99.66	2	1	3	1.02	41	47	88	41	46	87	98.86
**BARMM**	**7,108**	**6,943**	**14,051**	**3,325**	**3,094**	**6,419**	**45.68**	**4,257**	**4,271**	**8,528**	**60.69**	**2,882**	**2,793**	**5,675**	**2,760**	**2,778**	**5,538**	**97.59**
Basilan	662	601	1,263	426	393	819	64.85	305	290	595	47.11	253	232	485	247	226	473	97.53
Lanao del Sur	1,065	1,118	2,183	788	879	1,667	76.36	599	614	1,213	55.57	408	434	842	364	392	756	89.79
Maguindanao	3,143	3,120	6,263	623	569	1,192	19.03	2,508	2,550	5,058	80.76	1,322	1,286	2,608	1,321	1,379	2,700	103.53
Sulu	953	991	1,944	746	578	1,324	68.11	436	480	916	47.12	293	281	574	278	266	544	94.77
Tawi-Tawi	657	535	1,192	124	103	227	19.04	399	331	730	61.24	410	398	808	354	353	707	87.50
Lamitan City	32	15	47	22	9	31	65.96	10	6	16	34.04	26	18	44	26	18	44	100.00
Marawi City	596	563	1,159	596	563	1,159	100.00	0	0	0	0.00	170	144	314	170	144	314	100.00

Table 1.C.4.2 - Management of Sick Infants and Children

Number and proportion of diarrhea cases 0-59 mos. old who received Oral Rehydration Salt (ORS) solution and ORS with Zinc drops/syrup and Pneumonia cases among children 0-59 mos. old who received treatment

Philippines, Annual 2020

Area	Children 0-59 mos. old with Diarrhea											Children 0-59 mos. old with Pneumonia						
	Seen			Given ORS				Given ORS w/ zinc				Seen			Received treatment			
	Male	Female	Total	Male	Female	Total	%	Male	Female	Total	%	Male	Female	Total	Male	Female	Total	%
**CARAGA**	**1,549**	**1,471**	**3,020**	**1,068**	**1,053**	**2,121**	**70.23**	**587**	**536**	**1,123**	**37.19**	**1,111**	**1,099**	**2,210**	**1,084**	**1,085**	**2,169**	**98.14**
Agusan del Norte	252	209	461	75	59	134	29.07	178	151	329	71.37	2	1	3	2	1	3	100.00
Agusan del Sur	749	721	1,470	597	577	1,174	79.86	188	177	365	24.83	416	336	752	407	334	741	98.54
Surigao del Norte	108	129	237	97	119	216	91.14	42	43	85	35.86	185	189	374	175	187	362	96.79
Surigao del Sur	200	190	390	123	132	255	65.38	90	93	183	46.92	444	511	955	436	501	937	98.12
Province of Dinagat	89	66	155	42	35	77	49.68	47	31	78	50.32	18	15	33	18	15	33	100.00
Bislig City	20	25	45	10	13	23	51.11	10	15	25	55.56	2	2	4	2	2	4	100.00
Butuan City	77	80	157	74	77	151	96.18	3	3	6	3.82	2	5	7	2	5	7	100.00
Surigao City	54	51	105	50	41	91	86.67	29	23	52	49.52	42	40	82	42	40	82	100.00

Note: Put asterisk (*) for No Report and Zero (0) for No Case

Figure 1.C.4.1 - Percentage Distribution of Sick Infants aged 6-11 mos. given Vitamin A 100,000 IU by Region Philippines, 2020

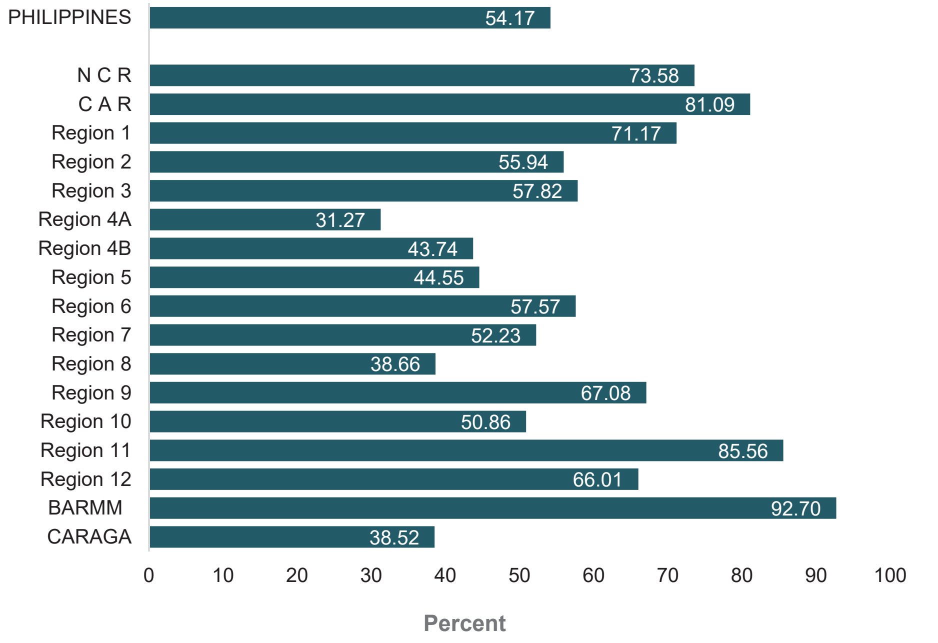

Figure 1.C.4.2 - Percentage Distribution of Sick Children aged 12-59 mos. given Vitamin A 200,000 IU by Region Philippines, 2020

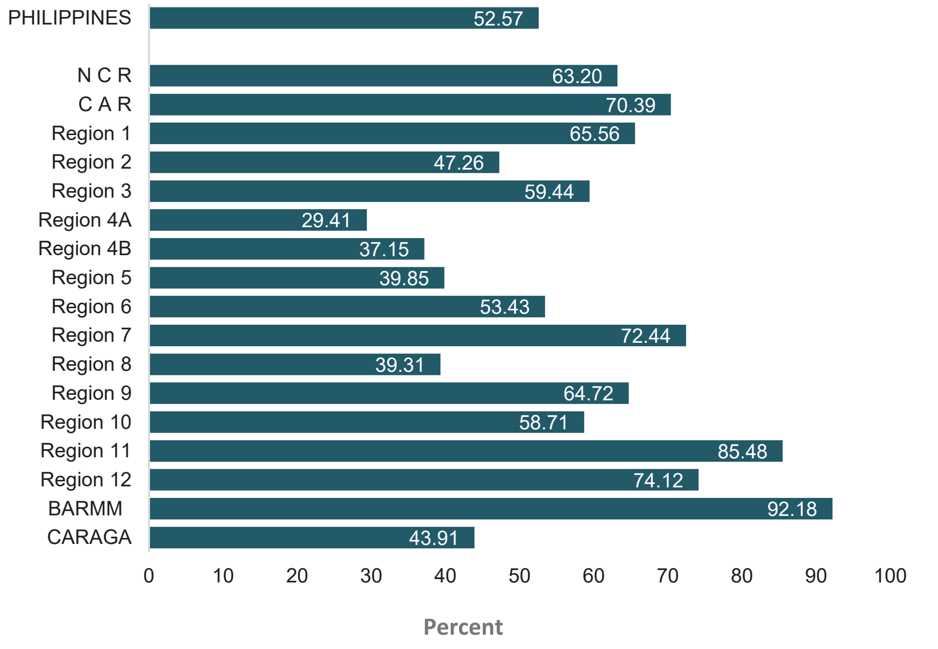

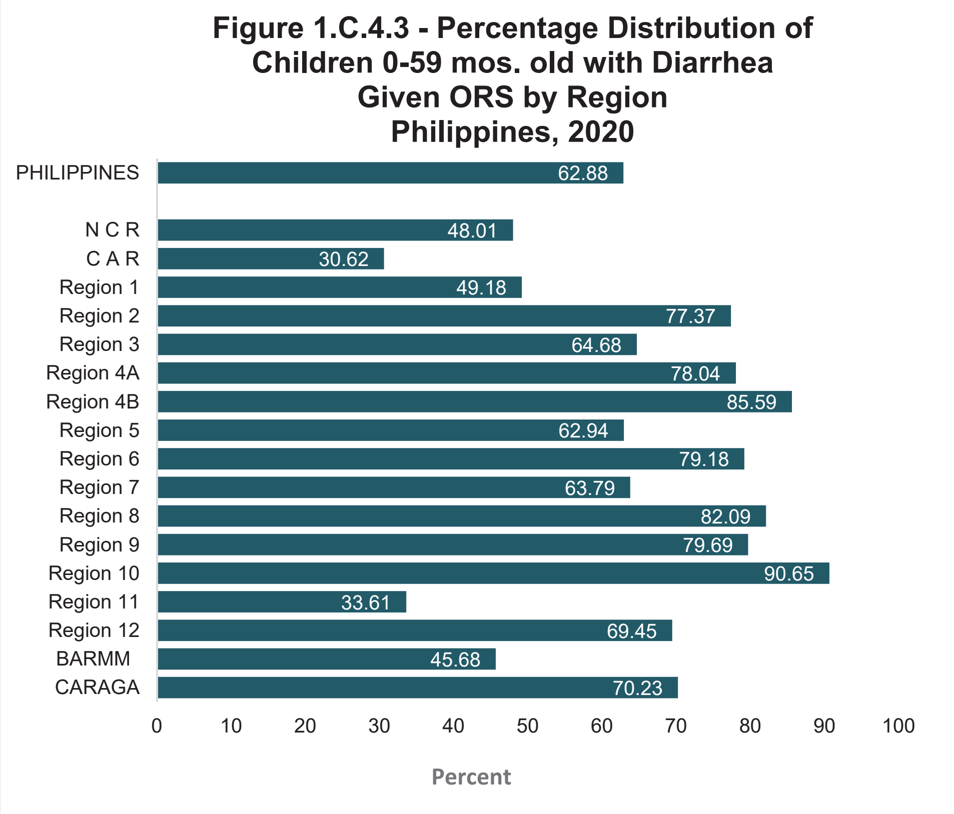
Figure 1.C.4.3 - Percentage Distribution of Children 0-59 mos. old with Diarrhea Given ORS by Region Philippines, 2020
PHILIPPINES
62.88
N C R
48.01
C A R
30.62
Region 1
49.18
Region 2
77.37
Region 3
64.68
Region 4A
78.04
Region 4B
85.59
Region 5
62.94
Region 6
79.18
Region 7
63.79
Region 8
82.09
Region 9
79.69
Region 10
90.65
Region 11
33.61
Region 12
69.45
BARMM
45.68
CARAGA
70.23
0
10
20
30
40
50
60
70
80
90
100
Percent

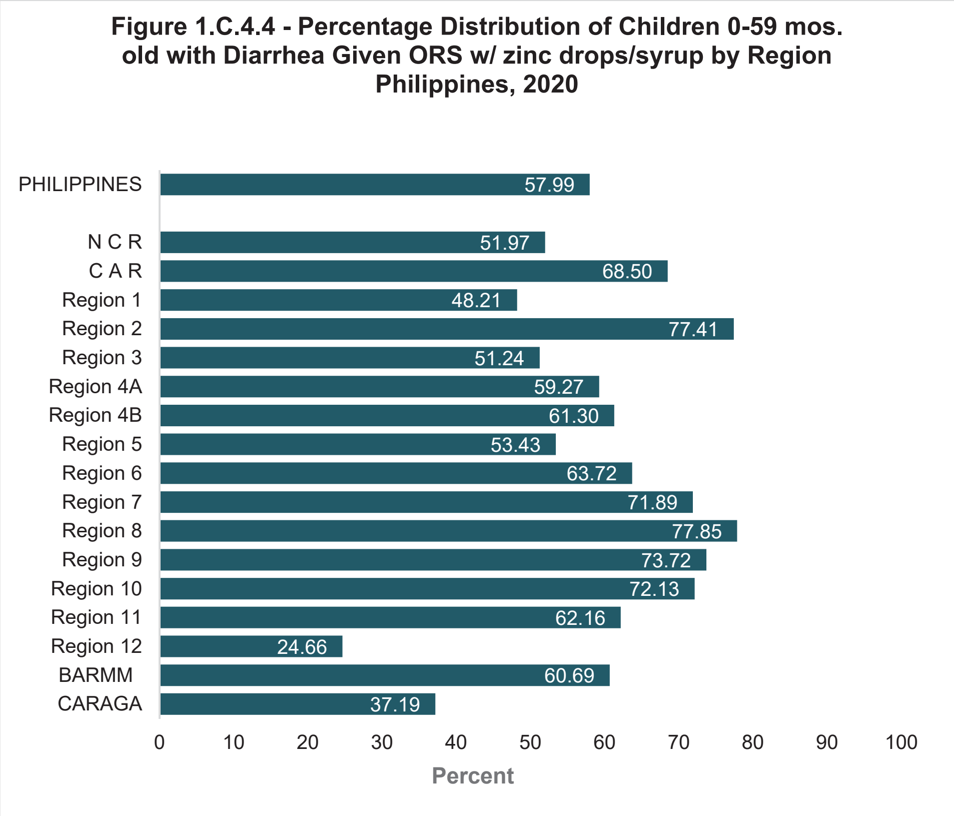
Figure 1.C.4.4 - Percentage Distribution of Children 0-59 mos. old with Diarrhea Given ORS w/ zinc drops/syrup by Region Philippines, 2020
PHILIPPINES
57.99
N C R
51.97
C A R
68.50
Region 1
48.21
Region 2
77.41
Region 3
51.24
Region 4A
59.27
Region 4B
61.30
Region 5
53.43
Region 6
63.72
Region 7
71.89
Region 8
77.85
Region 9
73.72
Region 10
72.13
Region 11
62.16
Region 12
24.66
BARMM
60.69
CARAGA
37.19
0
10
20
30
40
50
60
70
80
90
100
Percent

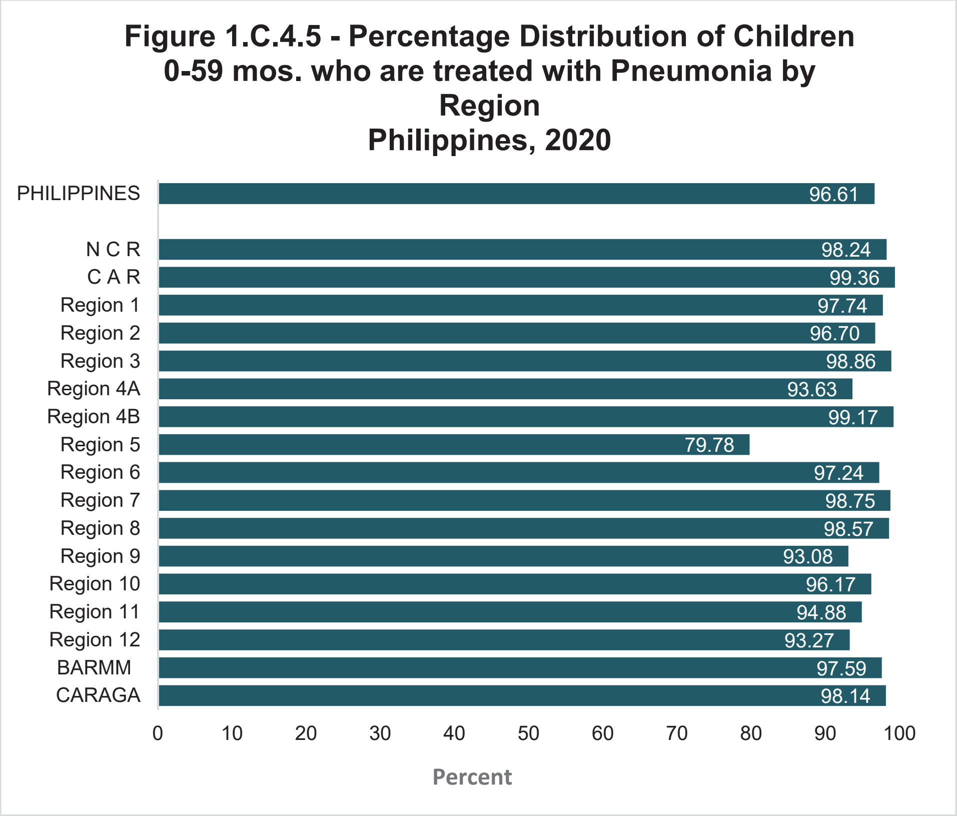
Figure 1.C.4.5 - Percentage Distribution of Children 0-59 mos. who are treated with Pneumonia by Region
Philippines, 2020
PHILIPPINES
96.61
N C R
98.24
C A R
99.36
Region 1
97.74
Region 2
96.70
Region 3
98.86
Region 4A
93.63
Region 4B
99.17
Region 5
79.78
Region 6
97.24
Region 7
98.75
Region 8
98.57
Region 9
93.08
Region 10
96.17
Region 11
94.88
Region 12
93.27
BARMM
97.59
CARAGA
98.14
0
10
20
30
40
50
60
70
80
90
100
Percent

Table 1.D.1 Oral Health Care

Number and proportion of children 12-59 months old who are orally fit upon oral examination or after oral rehabilitation
Philippines, Annual 2020

Area	Eligible Pop. (12 - 59 mos. x 20%)	Orally fit		Total	%
		Male	Female		
**PHILIPPINES**	**1,788,320**	**139,314**	**144,796**	**284,110**	**15.89**
**N C R**	**191,814**	**33,893**	**34,275**	**68,168**	**35.54**
Malabon	**5,444**	737	730	**1,467**	**26.95**
Navotas	**3,716**	2,731	3,053	**5,784**	**155.65**
Valenzuela City	**9,242**	1,479	1,497	**2,976**	**32.20**
Caloocan City	**23,594**	1,773	1,601	**3,374**	**14.30**
Marikina City	**6,714**	1,192	1,137	**2,329**	**34.69**
Pasig City	**11,250**	1,286	1,584	**2,870**	**25.51**
Pateros	**951**	209	238	**447**	**47.00**
Taguig	**11,990**	740	680	**1,420**	**11.84**
Quezon City	**43,735**	15,545	15,243	**30,788**	**70.40**
Makati City	**8,678**	2,244	2,334	**4,578**	**52.75**
Mandaluyong City	**5,753**	1,838	1,893	**3,731**	**64.85**
San Juan	**1,820**	343	291	**634**	**34.83**
Manila City	**26,517**	1,956	2,185	**4,141**	**15.62**
Las Piñas City	**8,772**	485	461	**946**	**10.78**
Muntinlupa City	**7,515**	263	258	**521**	**6.93**
Parañaque City	**9,918**	636	596	**1,232**	**12.42**
Pasay City	**6,205**	436	494	**930**	**14.99**
**C A R**	**29,388**	**3,661**	**4,059**	**7,720**	**26.27**
Abra	**3,826**	381	402	**783**	**20.47**
Apayao	**2,168**	691	724	**1,415**	**65.26**
Benguet	**7,292**	1,389	1,632	**3,021**	**41.43**
Ifugao	**3,823**	211	272	**483**	**12.63**
Kalinga	**3,948**	365	375	**740**	**18.75**
Mt. Province	**2,688**	306	320	**626**	**23.29**
Baguio City	**5,644**	318	334	**652**	**11.55**
**Region 1**	**81,538**	**7,605**	**8,325**	**15,930**	**19.54**
Ilocos Norte	**6,601**	1,033	1,234	**2,267**	**34.35**
Ilocos Sur	**8,149**	2,035	1,999	**4,034**	**49.50**
La Union	**9,741**	781	758	**1,539**	**15.80**
Pangasinan	**41,853**	2,452	2,952	**5,404**	**12.91**
Alaminos City	**1,582**	109	191	**300**	**18.97**
Candon City	**858**	57	57	**114**	**13.28**
Dagupan City	**3,019**	416	507	**923**	**30.58**
Laoag City	**1,522**	308	227	**535**	**35.16**
San Carlos City	**3,323**	270	271	**541**	**16.28**
San Fernando City	**1,784**	7	11	**18**	**1.01**
Urdaneta City	**2,344**	9	5	**14**	**0.60**
Vigan City	**763**	128	113	**241**	**31.58**

Table 1.D.1 Oral Health Care

Number and proportion of children 12-59 months old who are orally fit upon oral examination or after oral rehabilitation
Philippines, Annual 2020

Area	Eligible Pop. (12 - 59 mos. x 20%)	Orally fit		Total	%
		Male	Female		
**Region 2**	**58,313**	**5,543**	**6,143**	**11,686**	**20.04**
Batanes	**304**	141	114	**255**	**83.99**
Cagayan	**15,105**	900	1,123	**2,023**	**13.39**
Isabela	**22,011**	1,325	1,516	**2,841**	**12.91**
Nueva Vizcaya	**8,069**	1,053	1,096	**2,149**	**26.63**
Quirino	**3,437**	185	231	**416**	**12.10**
Cauayan City	**2,161**	738	930	**1,668**	**77.19**
Ilagan City	**2,430**	857	823	**1,680**	**69.13**
Santiago City	**2,251**	341	300	**641**	**28.48**
Tuguegarao City	**2,546**	3	10	**13**	**0.51**
**Region 3**	**182,598**	**11,627**	**12,602**	**24,229**	**13.27**
Aurora	**4,093**	341	327	**668**	**16.32**
Bataan	**10,970**	818	807	**1,625**	**14.81**
Bulacan	**36,192**	2,710	2,599	**5,309**	**14.67**
Nueva Ecija	**24,315**	1,321	1,580	**2,901**	**11.93**
Pampanga	**26,171**	168	189	**357**	**1.36**
Tarlac	**16,351**	3,128	3,514	**6,642**	**40.62**
Zambales	**10,239**	311	365	**676**	**6.60**
Angeles City	**6,566**	64	64	**128**	**1.95**
Balanga City	**1,585**	178	211	**389**	**24.54**
Cabanatuan City	**4,977**	732	812	**1,544**	**31.02**
City of San Fernando	**4,892**	351	394	**745**	**15.23**
Gapan City	**1,817**	18	19	**37**	**2.04**
Mabalacat City	**4,000**	112	132	**244**	**6.10**
Malolos City	**4,043**	285	335	**620**	**15.33**
Meycauayan	**3,353**	188	247	**435**	**12.97**
Olongapo	**4,038**	64	76	**140**	**3.47**
Palayan City	**675**	89	93	**182**	**26.95**
San Jose City	**2,300**	191	190	**381**	**16.57**
San Jose del Monte City	**9,207**	95	119	**214**	**2.32**
Science City of Munoz	**1,342**	229	282	**511**	**38.09**
Tarlac City	**5,472**	234	247	**481**	**8.79**
**Region 4A**	**245,484**	**15,703**	**15,774**	**31,477**	**12.82**
Batangas	**32,405**	3,076	3,041	**6,117**	**18.88**
Cavite	**22,784**	190	199	**389**	**1.71**
Laguna	**16,122**	2,582	2,484	**5,066**	**31.42**
Quezon	**31,726**	1,820	1,820	**3,640**	**11.47**
Rizal	**36,373**	2,862	3,033	**5,895**	**16.21**
Antipolo City	**13,397**	0	0	**0**	**0.00**
Bacoor City	**9,983**	90	115	**205**	**2.05**
Batangas City	**5,750**	230	233	**463**	**8.05**
Biñan City	**5,406**	638	610	**1,248**	**23.08**
Cabuyao City	**5,012**	109	91	**200**	**3.99**

Table 1.D.1 Oral Health Care

Number and proportion of children 12-59 months old who are orally fit upon oral examination or after oral rehabilitation
Philippines, Annual 2020

Area	Eligible Pop. (12 - 59 mos. x 20%)	Orally fit		Total	%
		Male	Female		
Calamba City	**7,378**	770	701	**1,471**	**19.94**
Cavite City	**1,708**	1	0	**1**	**0.06**
Dasmariñas City	**10,952**	0	0	**0**	**0.00**
General Trias City	**5,224**	0	0	**0**	**0.00**
Imus City	**6,711**	1,074	1,040	**2,114**	**31.50**
Lipa City	**5,795**	355	359	**714**	**12.32**
Lucena City	**4,810**	37	20	**57**	**1.19**
San Pablo City	**4,319**	66	68	**134**	**3.10**
San Pedro City	**5,289**	0	0	**0**	**0.00**
Santa Rosa City	**5,743**	147	179	**326**	**5.68**
Tagaytay City	**1,183**	378	328	**706**	**59.66**
Tanauan City	**3,022**	272	355	**627**	**20.75**
Tayabas City	**1,803**	486	574	**1,060**	**58.78**
Trece Martires City	**2,588**	520	524	**1,044**	**40.34**
**Region 4B**	**59,678**	**946**	**1,063**	**2,009**	**3.37**
Marinduque	**4,342**	392	401	**793**	**18.26**
Mindoro Occidental	**10,070**	102	122	**224**	**2.22**
Mindoro Oriental	**16,633**	295	349	**644**	**3.87**
Palawan	**17,657**	52	66	**118**	**0.67**
Romblon	**5,674**	44	46	**90**	**1.59**
Puerto Princesa City	**5,303**	61	79	**140**	**2.64**
**Region 5**	**117,264**	**19,473**	**19,667**	**39,140**	**33.38**
Albay	**20,645**	2,755	3,113	**5,868**	**28.42**
Camarines Norte	**11,979**	2,386	2,342	**4,728**	**39.47**
Camarines Sur	**33,243**	4,376	4,485	**8,861**	**26.66**
Catanduanes	**5,312**	1,426	1,491	**2,917**	**54.91**
Masbate	**19,887**	2,260	2,288	**4,548**	**22.87**
Sorsogon	**16,347**	4,453	4,207	**8,660**	**52.98**
Iriga City	**2,258**	83	97	**180**	**7.97**
Legaspi City	**3,630**	1,565	1,466	**3,031**	**83.49**
Naga City	**3,963**	169	178	**347**	**8.76**
**Region 6**	**124,747**	**9,915**	**10,382**	**20,297**	**16.27**
Aklan	**9,570**	102	125	**227**	**2.37**
Antique	**10,764**	36	39	**75**	**0.70**
Capiz	**12,016**	516	644	**1,160**	**9.65**
Guimaras	**2,900**	17	30	**47**	**1.62**
Iloilo	**30,722**	7,924	8,246	**16,170**	**52.63**
Negros Occidental	**42,176**	589	568	**1,157**	**2.74**
Bacolod City	**9,490**	109	82	**191**	**2.01**
Iloilo City	**7,107**	622	648	**1,270**	**17.87**

Table 1.D.1 Oral Health Care

Number and proportion of children 12-59 months old who are orally fit upon oral examination or after oral rehabilitation
Philippines, Annual 2020

Area	Eligible Pop. (12 - 59 mos. x 20%)	Orally fit		Total	%
		Male	Female		
**Region 7**	**130,483**	**4,631**	**4,642**	**9,273**	**7.11**
Bohol	**23,016**	2,490	2,361	**4,851**	**21.08**
Cebu	**52,642**	262	294	**556**	**1.06**
Negros Oriental	**23,004**	248	295	**543**	**2.36**
Siquijor	**1,488**	266	247	**513**	**34.48**
Cebu City	**16,527**	1,317	1,383	**2,700**	**16.34**
Lapu-Lapu City	**7,311**	48	62	**110**	**1.50**
Mandaue City	**6,496**	0	0	**0**	**0.00**
**Region 8**	**86,622**	**2,231**	**2,421**	**4,652**	**5.37**
Biliran	**3,364**	84	91	**175**	**5.20**
Eastern Samar	**9,256**	310	359	**669**	**7.23**
Northern Leyte	**28,722**	1,044	1,106	**2,150**	**7.49**
Northern Samar	**13,481**	90	115	**205**	**1.52**
Southern Leyte	**5,758**	6	10	**16**	**0.28**
Western Samar	**12,140**	103	118	**221**	**1.82**
Calbayog City	**3,741**	63	73	**136**	**3.64**
Maasin City	**1,466**	145	143	**288**	**19.65**
Ormoc City	**4,090**	367	396	**763**	**18.66**
Tacloban City	**4,605**	19	10	**29**	**0.63**
**Region 9**	**67,897**	**3,687**	**4,088**	**7,775**	**11.45**
Zamboanga del Norte	**14,974**	801	901	**1,702**	**11.37**
Zamboanga del Sur	**14,429**	1,621	1,831	**3,452**	**23.92**
Zamboanga Sibugay	**13,222**	879	929	**1,808**	**13.67**
Dapitan City	**1,547**	251	271	**522**	**33.74**
Dipolog City	**2,453**	63	68	**131**	**5.34**
Isabela City	**2,412**	0	0	**0**	**0.00**
Pagadian City	**3,539**	72	88	**160**	**4.52**
Zamboanga City	**15,321**	0	0	**0**	**0.00**
**Region 10**	**86,987**	**3,694**	**3,989**	**7,683**	**8.83**
Bukidnon	**20,116**	526	701	**1,227**	**6.10**
Camiguin	**1,600**	215	189	**404**	**25.25**
Lanao del Norte	**13,501**	210	236	**446**	**3.30**
Misamis Occidental	**5,485**	417	528	**945**	**17.23**
Misamis Oriental	**12,662**	594	606	**1,200**	**9.48**
Cagayan de Oro City	**11,995**	67	49	**116**	**0.97**
El Salvador City	**891**	0	0	**0**	**0.00**
Gingoog City	**2,210**	271	282	**553**	**25.02**
Iligan City	**6,839**	6	6	**12**	**0.18**
Malaybalay City	**3,353**	854	820	**1,674**	**49.92**
Oroquieta City	**1,188**	0	0	**0**	**0.00**
Ozamis City	**2,382**	342	331	**673**	**28.26**

Table 1.D.1 Oral Health Care

Number and proportion of children 12-59 months old who are orally fit upon oral examination or after oral rehabilitation
Philippines, Annual 2020

Area	Eligible Pop. (12 - 59 mos. x 20%)	Orally fit		Total	%
		Male	Female		
Tangub City	**1,059**	69	87	**156**	**14.74**
Valencia City	**3,706**	123	154	**277**	**7.48**
**Region 11**	**89,642**	**4,520**	**4,640**	**9,160**	**10.22**
Compostela Valley	**13,626**	1,062	1,263	**2,325**	**17.06**
Davao del Norte	**17,533**	1,003	1,121	**2,124**	**12.11**
Davao Oriental	**11,232**	734	653	**1,387**	**12.35**
Davao del Sur	**11,470**	449	387	**836**	**7.29**
Davao Occidental	**6,172**	146	183	**329**	**5.33**
Davao City	**29,610**	1,126	1,033	**2,159**	**7.29**
**Region 12**	**87,509**	**3,955**	**4,300**	**8,255**	**9.43**
North Cotabato	**27,815**	1,965	1,989	**3,954**	**14.22**
Sarangani	**10,921**	465	517	**982**	**8.99**
South Cotabato	**17,046**	386	398	**784**	**4.60**
Sultan Kudarat	**14,982**	741	948	**1,689**	**11.27**
Cotabato City	**5,676**	61	77	**138**	**2.43**
Gen. Santos City	**11,070**	337	371	**708**	**6.40**
**BARMM**	**96,639**	**4,575**	**4,708**	**9,283**	**9.61**
Basilan	**7,098**	0	0	**0**	**0.00**
Lanao del Sur	**22,066**	902	1,052	**1,954**	**8.86**
Maguindanao	**29,789**	2,140	2,266	**4,406**	**14.79**
Sulu	**21,456**	833	544	**1,377**	**6.42**
Tawi-Tawi	**8,998**	144	164	**308**	**3.42**
Lamitan City	**1,953**	535	632	**1,167**	**59.76**
Marawi City	**5,279**	21	50	**71**	**1.34**
**CARAGA**	**51,716**	**3,655**	**3,718**	**7,373**	**14.26**
Agusan del Norte	**6,821**	171	196	**367**	**5.38**
Agusan del Sur	**15,363**	374	436	**810**	**5.27**
Surigao del Norte	**6,311**	94	84	**178**	**2.82**
Surigao del Sur	**9,707**	1,010	1,108	**2,118**	**21.82**
Province of Dinagat	**2,246**	404	390	**794**	**35.35**
Bislig City	**1,844**	446	433	**879**	**47.66**
Butuan City	**6,486**	484	491	**975**	**15.03**
Surigao City	**2,938**	672	580	**1,252**	**42.61**

Note: Put asterisk (*) for No Report and Zero (0) for No Case

Table 1.D.2 - Oral Health Care

Number and Proportion of children 5 years old and above with cases of decayed, missing, filled teeth (DMFT)
Philippines, Annual 2020

Area	Total Examined	Cases of 5 y/o and above with DMFT		Total with DMFT	%
		Male	Female		
PHILIPPINES	2,855,449	455,833	668,792	1,124,625	39.39
N C R	454,081	157,976	249,060	407,036	89.64
Malabon	22,865	10,305	12,334	22,639	99.01
Navotas	13,256	5,732	6,522	12,254	92.44
Valenzuela City	2,701	1,116	1,585	2,701	100.00
Caloocan City	20,817	6,183	10,634	16,817	80.78
Marikina City	18,807	6,583	8,807	15,390	81.83
Pasig City	17,034	6,915	6,719	13,634	80.04
Pateros	774	325	449	774	100.00
Taguig	1,788	848	940	1,788	100.00
Quezon City	124,526	45,707	67,519	113,226	90.93
Makati City	10,425	3,921	6,404	10,325	99.04
Mandaluyong City	32,952	9,206	13,346	22,552	68.44
San Juan	3,232	1,085	1,147	2,232	69.06
Manila City	12,920	2,923	9,797	12,720	98.45
Las Piñas City	2,258	1,143	1,115	2,258	100.00
Muntinlupa City	167,008	54,668	100,340	155,008	92.81
Parañaque City	1,096	533	563	1,096	100.00
Pasay City	1,622	783	839	1,622	100.00
C A R	57,385	10,433	19,552	29,985	52.25
Abra	2,736	1,066	1,565	2,631	96.16
Apayao	2,951	1,139	1,250	2,389	80.96
Benguet	8,011	2,385	3,913	6,298	78.62
Ifugao	2,151	621	1,429	2,050	95.30
Kalinga	5,359	1,778	3,045	4,823	90.00
Mt. Province	11,267	210	368	578	5.13
Baguio City	24,910	3,234	7,982	11,216	45.03
Region 1	126,893	44,577	56,946	101,523	80.01
Ilocos Norte	5,963	1,505	2,035	3,540	59.37
Ilocos Sur	32,043	10,190	11,701	21,891	68.32
La Union	6,236	221	223	444	7.12
Pangasinan	66,805	26,832	36,508	63,340	94.81
Alaminos City	5,197	2,389	2,745	5,134	98.79
Candon City	294	133	161	294	100.00
Dagupan City	2,396	401	796	1,197	49.96
Laoag City	0	235	262	497	0.00
San Carlos City	1,433	465	525	990	69.09
San Fernando City	2,109	50	50	100	4.74
Urdaneta City	2,631	833	1,583	2,416	91.83

Table 1.D.2 - Oral Health Care

Number and Proportion of children 5 years old and above with cases of decayed, missing, filled teeth (DMFT)
Philippines, Annual 2020

Area	Total Examined	Cases of 5 y/o and above with DMFT		Total with DMFT	%
		Male	Female		
Vigan City	1,786	1,323	357	1,680	94.06
**Region 2**	**56,051**	**22,537**	**33,514**	**56,051**	**100.00**
Batanes	1	0	1	1	100.00
Cagayan	3,901	1,498	2,403	3,901	100.00
Isabela	11,079	4,634	6,445	11,079	100.00
Nueva Vizcaya	21,601	8,206	13,395	21,601	100.00
Quirino	1,735	793	942	1,735	100.00
Cauayan City	7,381	3,299	4,082	7,381	100.00
Ilagan City	9,853	3,993	5,860	9,853	100.00
Santiago City	482	106	376	482	100.00
Tuguegarao City	18	8	10	18	100.00
**Region 3**	**84,447**	**32,564**	**48,073**	**80,637**	**95.49**
Aurora	1,258	538	720	1,258	100.00
Bataan	1,316	547	769	1,316	100.00
Bulacan	6,658	2,398	4,260	6,658	100.00
Nueva Ecija	6,993	1,936	2,555	4,491	64.22
Pampanga	26,931	10,324	16,607	26,931	100.00
Tarlac	11,496	4,758	6,738	11,496	100.00
Zambales	11,156	4,825	6,188	11,013	98.72
Angeles City	0	0	0	0	0.00
Balanga City	1,228	533	695	1,228	100.00
Cabanatuan City	2,394	755	1,639	2,394	100.00
City of San Fernando	2,294	840	1,627	2,467	107.54
Gapan City	116	31	85	116	100.00
Mabalacat City	1,422	271	398	669	47.05
Malolos City	381	160	207	367	96.33
Meycauayan	1,186	332	324	656	55.31
Olongapo	6	3	3	6	100.00
Palayan City	397	140	257	397	100.00
San Jose City	1,178	530	648	1,178	100.00
San Jose del Monte City	1,106	567	539	1,106	100.00
Science City of Munoz	967	390	577	967	100.00
Tarlac City	5,964	2,686	3,237	5,923	99.31
**Region 4A**	**119,484**	**51,202**	**58,093**	**109,295**	**91.47**
Batangas	91,274	42,606	43,424	86,030	94.25
Cavite	2,126	746	1,033	1,779	83.68
Laguna	3,303	665	1,925	2,590	78.41
Quezon	2,495	806	1,102	1,908	76.47
Rizal	8,123	2,578	4,210	6,788	83.57
Antipolo City	0	0	0	0	0.00
Bacoor City	180	74	106	180	100.00
Batangas City	0	0	0	0	0.00

Table 1.D.2 - Oral Health Care

Number and Proportion of children 5 years old and above with cases of decayed, missing, filled teeth (DMFT)
Philippines, Annual 2020

Area	Total Examined	Cases of 5 y/o and above with DMFT		Total with DMFT	%
		Male	Female		
Biñan City	929	355	501	856	92.14
Cabuyao City	0	0	0	0	0.00
Calamba City	3,282	910	1,779	2,689	81.93
Cavite City	12	3	4	7	58.33
Dasmariñas City	0	0	0	0	0.00
General Trias City	542	115	342	457	84.32
Imus City	2,387	457	1,377	1,834	76.83
Lipa City	0	0	0	0	0.00
Lucena City	209	91	108	199	95.22
San Pablo City	0	0	0	0	0.00
San Pedro City	0	0	0	0	0.00
Santa Rosa City	2,950	1,233	1,220	2,453	83.15
Tagaytay City	0	0	0	0	0.00
Tanauan City	1,670	562	962	1,524	91.26
Tayabas City	2	1	0	1	50.00
Trece Martires City	0	0	0	0	0.00
**Region 4B**	**3,686**	**1,235**	**2,394**	**3,629**	**98.45**
Marinduque	874	319	518	837	95.77
Mindoro Occidental	330	158	172	330	100.00
Mindoro Oriental	30	12	18	30	100.00
Palawan	179	53	106	159	88.83
Romblon	71	31	40	71	100.00
Puerto Princesa City	2,202	662	1,540	2,202	100.00
**Region 5**	**1,571,592**	**32,968**	**58,688**	**91,656**	**5.83**
Albay	41,754	3,131	5,717	8,848	21.19
Camarines Norte	84,299	3,510	4,789	8,299	9.84
Camarines Sur	108,451	8,040	17,977	26,017	23.99
Catanduanes	730,344	9,333	14,628	23,961	3.28
Masbate	390,157	3,721	5,218	8,939	2.29
Sorsogon	14,882	3,215	6,692	9,907	66.57
Iriga City	16,109	1,252	2,325	3,577	22.20
Legaspi City	3,293	664	1,220	1,884	57.21
Naga City	182,303	102	122	224	0.12
**Region 6**	**124,952**	**28,789**	**38,729**	**67,518**	**54.04**
Aklan	1,069	918	1,585	2,503	234.14
Antique	0	0	0	0	0.00
Capiz	16,656	3,856	5,924	9,780	58.72
Guimaras	1,631	707	827	1,534	94.05
Iloilo	72,959	14,030	19,202	33,232	45.55
Negros Occidental	11,537	1,891	3,246	5,137	44.53
Bacolod City	12,501	3,070	3,662	6,732	53.85
Iloilo City	8,599	4,317	4,283	8,600	100.01

Table 1.D.2 - Oral Health Care

Number and Proportion of children 5 years old and above with cases of decayed, missing, filled teeth (DMFT)
Philippines, Annual 2020

Area	Total Examined	Cases of 5 y/o and above with DMFT		Total with DMFT	%
		Male	Female		
**Region 7**	**30,364**	**10,878**	**19,386**	**30,264**	**99.67**
Bohol	16,618	6,335	10,283	16,618	100.00
Cebu	1,901	637	1,264	1,901	100.00
Negros Oriental	760	310	350	660	86.84
Siquijor	2,014	787	1,227	2,014	100.00
Cebu City	8,796	2,692	6,104	8,796	100.00
Lapu-Lapu City	275	117	158	275	100.00
Mandaue City	0	0	0	0	0.00
**Region 8**	**62,746**	**4,870**	**8,407**	**13,277**	**21.16**
Biliran	16,244	286	313	599	3.69
Eastern Samar	5,251	191	207	398	7.58
Northern Leyte	12,236	3,456	6,784	10,240	83.69
Northern Samar	1,331	139	202	341	25.62
Southern Leyte	677	17	18	35	5.17
Western Samar	6,273	78	54	132	2.10
Calbayog City	2,319	16	23	39	1.68
Maasin City	7,467	220	360	580	7.77
Ormoc City	8,948	318	303	621	6.94
Tacloban City	2,000	149	143	292	14.60
**Region 9**	**20,093**	**9,075**	**11,394**	**20,469**	**101.87**
Zamboanga del Norte	5,413	2,144	3,269	5,413	100.00
Zamboanga del Sur	6,761	3,125	3,603	6,728	99.51
Zamboanga Sibugay	4,232	2,236	2,405	4,641	109.66
Dapitan City	442	232	210	442	100.00
Dipolog City	2,606	1,008	1,598	2,606	100.00
Isabela City	321	167	154	321	100.00
Pagadian City	318	163	155	318	100.00
Zamboanga City	0	0	0	0	0.00
**Region 10**	**26,198**	**9,231**	**12,832**	**22,063**	**84.22**
Bukidnon	5,054	1,224	2,113	3,337	66.03
Camiguin	1,714	698	769	1,467	85.59
Lanao del Norte	5,108	1,168	1,769	2,937	57.50
Misamis Occidental	2,356	1,016	1,340	2,356	100.00
Misamis Oriental	2,596	1,086	1,510	2,596	100.00
Cagayan de Oro City	25	13	12	25	100.00
El Salvador City	8	3	5	8	100.00
Gingoog City	362	192	170	362	100.00
Iligan City	0	0	0	0	0.00
Malaybalay City	472	204	268	472	100.00

Table 1.D.2 - Oral Health Care

Number and Proportion of children 5 years old and above with cases of decayed, missing, filled teeth (DMFT)
Philippines, Annual 2020

Area	Total Examined	Cases of 5 y/o and above with DMFT		Total with DMFT	%
		Male	Female		
Oroquieta City	0	0	0	0	0.00
Ozamis City	5,873	2,471	3,402	5,873	100.00
Tangub City	310	163	147	310	100.00
Valencia City	2,320	993	1,327	2,320	100.00
**Region 11**	**12,165**	**3,955**	**6,104**	**10,059**	**82.69**
Compostela Valley	2,566	1,172	1,235	2,407	93.80
Davao del Norte	2,507	1,114	1,334	2,448	97.65
Davao Oriental	1,723	888	793	1,681	97.56
Davao del Sur	674	286	311	597	88.58
Davao Occidental	450	186	222	408	90.67
Davao City	4,245	309	2,209	2,518	59.32
**Region 12**	**36,857**	**14,661**	**20,921**	**35,582**	**96.54**
North Cotabato	12,433	5,608	6,825	12,433	100.00
Sarangani	8,713	3,851	4,862	8,713	100.00
South Cotabato	4,609	1,166	2,168	3,334	72.34
Sultan Kudarat	6,626	2,578	4,048	6,626	100.00
Cotabato City	3,280	1,327	1,953	3,280	100.00
Gen. Santos City	1,196	131	1,065	1,196	100.00
**BARMM**	**21,182**	**9,657**	**10,210**	**19,867**	**93.79**
Basilan	0	0	0	0	0.00
Lanao del Sur	7,908	3,921	3,987	7,908	100.00
Maguindanao	13,049	5,646	6,088	11,734	89.92
Sulu	0	0	0	0	0.00
Tawi-Tawi	0	0	0	0	0.00
Lamitan City	0	0	0	0	0.00
Marawi City	225	90	135	225	100.00
**CARAGA**	**47,273**	**11,225**	**14,489**	**25,714**	**54.39**
Agusan del Norte	15,271	810	1,572	2,382	15.60
Agusan del Sur	9,494	3,959	4,118	8,077	85.07
Surigao del Norte	492	206	286	492	100.00
Surigao del Sur	8,658	3,478	5,175	8,653	99.94
Province of Dinagat	2,001	551	1,010	1,561	78.01
Bislig City	768	371	397	768	100.00
Butuan City	1,363	700	663	1,363	100.00
Surigao City	9,226	1,150	1,268	2,418	26.21

Note: Put asterisk (*) for No Report and Zero (0) for No Case

Table 1.D.3 - Oral Health Care

Number and proportion of infants 0-11 months old, 1-4 years old and 5-9 years old who received Basic Oral Health Care (BOHC)
Philippines, Annual 2020

Area	Infants 0-11 months old who received BOHC					Infants 1-4 years old who received BOHC					Children 5-9 years old who received BOHC				
	Eligible Pop. (0 - 11 mos. x 30%)	Male	Female	Total	%	Eligible Pop. (1 - 4 yrs. old x 30%)	Male	Female	Total	%	Eligible Pop. (5 - 9 yrs. old x 30%)	Male	Female	Total	%
**PHILIPPINES**	**636,947**	**102,312**	**103,557**	**205,869**	**32.32**	**2,682,479**	**351,766**	**364,609**	**716,375**	**26.71**	**3,326,740**	**228,324**	**234,239**	**462,563**	**13.90**
**N C R**	**71,598**	**26,423**	**25,890**	**52,313**	**73.06**	**287,722**	**52,394**	**53,446**	**105,840**	**36.79**	**365,692**	**45,938**	**47,427**	**93,365**	**25.53**
Malabon	**2,033**	925	1,067	**1,992**	**98.01**	**8,166**	2,766	2,753	**5,519**	**67.59**	**10,380**	4,372	5,305	**9,677**	**93.23**
Navotas	**1,386**	1,001	208	**1,209**	**87.21**	**5,574**	2,245	2,504	**4,749**	**85.20**	**7,085**	2,907	3,271	**6,178**	**87.20**
Valenzuela City	**3,450**	1,735	1,667	**3,402**	**98.61**	**13,863**	2,473	2,610	**5,083**	**36.67**	**17,618**	2,900	3,171	**6,071**	**34.46**
Caloocan City	**8,809**	1,165	1,152	**2,317**	**26.30**	**35,392**	3,709	4,023	**7,732**	**21.85**	**44,981**	1,007	1,113	**2,120**	**4.71**
Marikina City	**2,506**	1,361	1,046	**2,407**	**96.04**	**10,071**	1,335	1,296	**2,631**	**26.12**	**12,800**	3,473	3,348	**6,821**	**53.29**
Pasig City	**4,199**	1,675	1,647	**3,322**	**79.12**	**16,875**	1,767	1,768	**3,535**	**20.95**	**21,449**	3,081	3,402	**6,483**	**30.23**
Pateros	**355**	85	87	**172**	**48.42**	**1,427**	316	267	**583**	**40.87**	**1,814**	246	314	**560**	**30.87**
Taguig	**4,475**	589	619	**1,208**	**26.99**	**17,985**	7,027	6,669	**13,696**	**76.15**	**22,858**	7,896	7,680	**15,576**	**68.14**
Quezon City	**16,324**	7,679	8,274	**15,953**	**97.73**	**65,602**	17,512	17,874	**35,386**	**53.94**	**83,381**	5,091	5,228	**10,319**	**12.38**
Makati City	**3,239**	1,101	973	**2,074**	**64.02**	**13,017**	2,816	2,969	**5,785**	**44.44**	**16,545**	7,979	7,555	**15,534**	**93.89**
Mandaluyong City	**2,148**	1,093	1,026	**2,119**	**98.65**	**8,630**	2,304	2,385	**4,689**	**54.33**	**10,969**	354	388	**742**	**6.76**
San Juan	**678**	416	258	**674**	**99.37**	**2,730**	744	678	**1,422**	**52.08**	**3,469**	560	531	**1,091**	**31.45**
Manila City	**9,894**	4,060	4,066	**8,126**	**82.13**	**39,776**	2,436	2,705	**5,141**	**12.92**	**50,555**	400	412	**812**	**1.61**
Las Piñas City	**3,274**	1,563	1,586	**3,149**	**96.18**	**13,158**	884	860	**1,744**	**13.25**	**16,724**	729	695	**1,424**	**8.51**
Muntinlupa City	**2,806**	1,247	1,531	**2,778**	**99.01**	**11,272**	1,801	1,788	**3,589**	**31.84**	**14,327**	2,288	2,216	**4,504**	**31.44**
Parañaque City	**3,703**	630	573	**1,203**	**32.49**	**14,877**	1,005	980	**1,985**	**13.34**	**18,908**	740	783	**1,523**	**8.05**
Pasay City	**2,318**	98	110	**208**	**8.97**	**9,307**	1,254	1,317	**2,571**	**27.62**	**11,829**	1,915	2,015	**3,930**	**33.22**
**C A R**	**10,530**	**2,706**	**2,711**	**5,417**	**51.44**	**44,082**	**9,033**	**8,974**	**18,007**	**40.85**	**54,251**	**8,725**	**9,503**	**18,228**	**33.60**
Abra	**1,293**	306	259	**565**	**43.71**	**5,738**	1,395	1,420	**2,815**	**49.06**	**7,182**	727	740	**1,467**	**20.43**
Apayao	**735**	191	169	**360**	**48.96**	**3,252**	1,551	1,682	**3,233**	**99.41**	**3,962**	1,215	1,208	**2,423**	**61.15**
Benguet	**2,756**	1,349	1,373	**2,722**	**98.75**	**10,938**	3,043	2,903	**5,946**	**54.36**	**13,364**	3,525	4,072	**7,597**	**56.85**
Ifugao	**1,319**	36	38	**74**	**5.61**	**5,735**	433	380	**813**	**14.18**	**6,989**	651	714	**1,365**	**19.53**
Kalinga	**1,385**	275	308	**583**	**42.09**	**5,921**	743	790	**1,533**	**25.89**	**7,313**	1,280	1,363	**2,643**	**36.14**
Mt. Province	**907**	136	149	**285**	**31.43**	**4,032**	1,039	979	**2,018**	**50.05**	**5,099**	729	740	**1,469**	**28.81**
Baguio City	**2,135**	413	415	**828**	**38.79**	**8,465**	829	820	**1,649**	**19.48**	**10,341**	598	666	**1,264**	**12.22**
**Region 1**	**29,178**	**4,282**	**5,208**	**9,490**	**32.52**	**122,307**	**16,514**	**17,685**	**34,199**	**27.96**	**155,552**	**16,899**	**18,206**	**35,105**	**22.57**
Ilocos Norte	**2,432**	714	815	**1,529**	**62.88**	**9,901**	1,914	2,241	**4,155**	**41.97**	**12,622**	2,409	2,681	**5,090**	**40.33**
Ilocos Sur	**2,799**	477	506	**983**	**35.12**	**12,223**	3,181	3,142	**6,323**	**51.73**	**15,776**	3,890	3,923	**7,813**	**49.52**
La Union	**3,453**	379	379	**758**	**21.95**	**14,611**	2,179	2,085	**4,264**	**29.18**	**18,701**	1,603	1,549	**3,152**	**16.85**
Pangasinan	**15,050**	1,853	2,732	**4,585**	**30.46**	**62,779**	5,699	6,572	**12,271**	**19.55**	**79,475**	6,671	7,596	**14,267**	**17.95**
Alaminos City	**568**	88	71	**159**	**27.98**	**2,372**	512	630	**1,142**	**48.14**	**3,003**	661	796	**1,457**	**48.51**
Candon City	**296**	15	13	**28**	**9.46**	**1,288**	183	185	**368**	**28.58**	**1,664**	108	112	**220**	**13.23**
Dagupan City	**1,086**	203	201	**404**	**37.20**	**4,528**	811	899	**1,710**	**37.76**	**5,734**	580	564	**1,144**	**19.95**
Laoag City	**561**	82	87	**169**	**30.12**	**2,283**	308	227	**535**	**23.44**	**2,910**	235	262	**497**	**17.08**
San Carlos City	**1,194**	320	304	**624**	**52.27**	**4,985**	509	495	**1,004**	**20.14**	**6,313**	469	500	**969**	**15.35**
San Fernando City	**635**	1	1	**2**	**0.32**	**2,677**	704	671	**1,375**	**51.37**	**3,427**	83	82	**165**	**4.81**
Urdaneta City	**842**	0	0	**0**	**0.00**	**3,515**	9	5	**14**	**0.40**	**4,450**	30	25	**55**	**1.24**
Vigan City	**263**	150	99	**249**	**94.75**	**1,145**	505	533	**1,038**	**90.67**	**1,477**	160	116	**276**	**18.68**
**Region 2**	**20,833**	**5,033**	**4,882**	**9,915**	**47.59**	**87,470**	**14,686**	**15,320**	**30,006**	**34.30**	**109,486**	**16,248**	**12,374**	**28,622**	**26.14**
Batanes	**102**	0	0	**0**	**0.00**	**455**	10	13	**23**	**5.05**	**557**	287	257	**544**	**97.65**
Cagayan	**5,391**	397	457	**854**	**15.84**	**22,657**	1,871	2,176	**4,047**	**17.86**	**28,398**	1,884	2,006	**3,890**	**13.70**
Isabela	**7,893**	2,009	2,063	**4,072**	**51.59**	**33,017**	4,875	5,071	**9,946**	**30.12**	**41,614**	2,843	3,046	**5,889**	**14.15**
Nueva Vizcaya	**2,872**	1,506	1,330	**2,836**	**98.75**	**12,103**	3,038	2,855	**5,893**	**48.69**	**14,768**	6,747	2,643	**9,390**	**63.58**
Quirino	**1,208**	19	17	**36**	**2.98**	**5,156**	592	645	**1,237**	**23.99**	**6,416**	566	560	**1,126**	**17.55**
Cauayan City	**777**	363	414	**777**	**100.00**	**3,241**	1,283	1,583	**2,866**	**88.42**	**4,089**	1,408	1,512	**2,920**	**71.42**
Ilagan City	**873**	292	238	**530**	**60.69**	**3,645**	1,802	1,814	**3,616**	**99.20**	**4,597**	1,054	1,038	**2,092**	**45.51**
Santiago City	**808**	447	360	**807**	**99.93**	**3,376**	1,013	987	**2,000**	**59.24**	**4,254**	1,434	1,293	**2,727**	**64.10**
Tuguegarao City	**910**	0	3	**3**	**0.33**	**3,819**	202	176	**378**	**9.90**	**4,792**	25	19	**44**	**0.92**
**Region 3**	**66,006**	**10,655**	**10,840**	**21,495**	**32.57**	**273,897**	**46,438**	**48,174**	**94,612**	**34.54**	**347,623**	**22,192**	**22,314**	**44,506**	**12.80**
Aurora	**1,431**	242	288	**530**	**37.04**	**6,139**	1,081	1,131	**2,212**	**36.03**	**7,430**	548	525	**1,073**	**14.44**
Bataan	**4,147**	163	154	**317**	**7.64**	**16,455**	2,936	3,076	**6,012**	**36.54**	**20,733**	540	534	**1,074**	**5.18**
Bulacan	**13,088**	2,466	2,332	**4,798**	**36.66**	**54,288**	9,478	9,656	**19,134**	**35.25**	**69,732**	3,272	3,384	**6,656**	**9.55**
Nueva Ecija	**8,635**	716	609	**1,325**	**15.35**	**36,472**	3,870	3,928	**7,798**	**21.38**	**46,389**	2,629	2,745	**5,374**	**11.58**
Pampanga	**9,408**	1,380	1,557	**2,937**	**31.22**	**39,257**	4,594	4,678	**9,272**	**23.62**	**50,038**	2,031	1,891	**3,922**	**7.84**
Tarlac	**5,930**	1,823	1,956	**3,779**	**63.73**	**24,527**	9,751	10,082	**19,833**	**80.86**	**31,028**	5,403	5,347	**10,750**	**34.65**
Zambales	**3,783**	254	157	**411**	**10.86**	**15,359**	959	895	**1,854**	**12.07**	**18,755**	1,579	1,459	**3,038**	**16.20**
Angeles City	**2,361**	0	0	**0**	**0.00**	**9,848**	503	556	**1,059**	**10.75**	**12,556**	0	0	**0**	**0.00**
Balanga City	**599**	127	108	**235**	**39.21**	**2,378**	625	601	**1,226**	**51.55**	**2,996**	178	189	**367**	**12.25**

Table 1.D.3 - Oral Health Care

Number and proportion of infants 0-11 months old, 1-4 years old and 5-9 years old who received Basic Oral Health Care (BOHC)
Philippines, Annual 2020

Area	Infants 0-11 months old who received BOHC					Infants 1-4 years old who received BOHC					Children 5-9 years old who received BOHC				
	Eligible Pop. (0 - 11 mos. x 30%)	Male	Female	Total	%	Eligible Pop. (1 - 4 yrs. old x 30%)	Male	Female	Total	%	Eligible Pop. (5 - 9 yrs. old x 30%)	Male	Female	Total	%
Cabanatuan City	**1,769**	106	102	**208**	**11.76**	**7,465**	1,729	1,898	**3,627**	**48.59**	**9,493**	668	718	**1,386**	**14.60**
City of San Fernando	**1,759**	311	296	**607**	**34.51**	**7,338**	858	944	**1,802**	**24.56**	**9,353**	3	4	**7**	**0.07**
Gapan City	**646**	97	140	**237**	**36.69**	**2,725**	833	909	**1,742**	**63.93**	**3,465**	26	22	**48**	**1.39**
Mabalacat City	**1,438**	50	46	**96**	**6.68**	**6,000**	817	869	**1,686**	**28.10**	**7,649**	338	335	**673**	**8.80**
Malolos City	**1,464**	475	531	**1,006**	**68.72**	**6,065**	1,241	1,306	**2,547**	**42.00**	**7,788**	294	350	**644**	**8.27**
Meycauayan	**1,212**	57	70	**127**	**10.48**	**5,030**	611	604	**1,215**	**24.16**	**6,460**	435	435	**870**	**13.47**
Olongapo	**1,491**	6	6	**12**	**0.80**	**6,057**	92	74	**166**	**2.74**	**7,397**	34	35	**69**	**0.93**
Palayan City	**240**	4	4	**8**	**3.34**	**1,013**	165	165	**330**	**32.57**	**1,290**	40	56	**96**	**7.44**
San Jose City	**815**	408	407	**815**	**99.95**	**3,450**	783	926	**1,709**	**49.54**	**4,391**	883	912	**1,795**	**40.88**
San Jose del Monte City	**3,330**	1,600	1,707	**3,307**	**99.31**	**13,810**	3,701	4,049	**7,750**	**56.12**	**17,739**	2,606	2,627	**5,233**	**29.50**
Science City of Munoz	**476**	38	33	**71**	**14.92**	**2,012**	773	807	**1,580**	**78.51**	**2,560**	215	275	**490**	**19.14**
Tarlac City	**1,985**	332	337	**669**	**33.70**	**8,208**	1,038	1,020	**2,058**	**25.07**	**10,382**	470	471	**941**	**9.06**
**Region 4A**	**89,045**	**13,179**	**12,895**	**26,074**	**29.28**	**368,227**	**77,053**	**79,947**	**157,000**	**42.64**	**460,213**	**12,532**	**13,461**	**25,993**	**5.65**
Batangas	**11,532**	1,092	1,093	**2,185**	**18.95**	**48,607**	8,763	8,863	**17,626**	**36.26**	**59,531**	5,234	5,236	**10,470**	**17.59**
Cavite	**8,311**	76	82	**158**	**1.90**	**34,176**	1,584	1,681	**3,265**	**9.55**	**43,212**	1,127	1,109	**2,236**	**5.17**
Laguna	**5,918**	117	99	**216**	**3.65**	**24,183**	28,490	28,488	**56,978**	**235.61**	**30,169**	671	716	**1,387**	**4.60**
Quezon	**11,223**	504	526	**1,030**	**9.18**	**47,590**	2,935	3,387	**6,322**	**13.28**	**60,047**	700	673	**1,373**	**2.29**
Rizal	**13,437**	7,713	7,536	**15,249**	**113.48**	**54,559**	2,876	3,732	**6,608**	**12.11**	**68,126**	2,575	2,532	**5,107**	**7.50**
Antipolo City	**4,949**	0	0	**0**	**0.00**	**20,095**	0	0	**0**	**0.00**	**25,093**	0	0	**0**	**0.00**
Bacoor City	**3,643**	2	3	**5**	**0.14**	**14,975**	809	837	**1,646**	**10.99**	**18,934**	216	239	**455**	**2.40**
Batangas City	**2,047**	0	0	**0**	**0.00**	**8,625**	2,167	2,112	**4,279**	**49.61**	**10,565**	0	0	**0**	**0.00**
Biñan City	**1,982**	1,362	1,300	**2,662**	**134.30**	**8,109**	968	1,136	**2,104**	**25.95**	**10,117**	621	825	**1,446**	**14.29**
Cabuyao City	**1,839**	0	0	**0**	**0.00**	**7,518**	0	0	**0**	**0.00**	**9,378**	0	0	**0**	**0.00**
Calamba City	**2,708**	1	1	**2**	**0.07**	**11,066**	21,139	22,445	**43,584**	**393.84**	**13,807**	29	43	**72**	**0.52**
Cavite City	**623**	1	2	**3**	**0.48**	**2,562**	18	24	**42**	**1.64**	**3,239**	26	39	**65**	**2.01**
Dasmariñas City	**3,997**	0	0	**0**	**0.00**	**16,429**	15	19	**34**	**0.21**	**20,775**	0	0	**0**	**0.00**
General Trias City	**1,906**	26	18	**44**	**2.31**	**7,837**	583	515	**1,098**	**14.01**	**9,908**	0	0	**0**	**0.00**
Imus City	**2,447**	1,884	1,817	**3,701**	**151.26**	**10,066**	220	220	**440**	**4.37**	**12,730**	126	185	**311**	**2.44**
Lipa City	**2,064**	0	0	**0**	**0.00**	**8,693**	524	493	**1,017**	**11.70**	**10,646**	9	4	**13**	**0.12**
Lucena City	**1,702**	4	2	**6**	**0.35**	**7,214**	72	79	**151**	**2.09**	**9,100**	98	107	**205**	**2.25**
San Pablo City	**1,583**	0	0	**0**	**0.00**	**6,479**	573	672	**1,245**	**19.22**	**8,080**	0	0	**0**	**0.00**
San Pedro City	**1,940**	0	0	**0**	**0.00**	**7,934**	0	0	**0**	**0.00**	**9,897**	0	0	**0**	**0.00**
Santa Rosa City	**2,108**	318	340	**658**	**31.22**	**8,615**	2,704	2,716	**5,420**	**62.92**	**10,745**	196	895	**1,091**	**10.15**
Tagaytay City	**431**	36	36	**72**	**16.70**	**1,775**	890	834	**1,724**	**97.12**	**2,244**	0	0	**0**	**0.00**
Tanauan City	**1,075**	2	4	**6**	**0.56**	**4,533**	436	495	**931**	**20.54**	**5,553**	163	152	**315**	**5.67**
Tayabas City	**637**	41	36	**77**	**12.10**	**2,705**	119	135	**254**	**9.39**	**3,410**	3	14	**17**	**0.50**
Trece Martires City	**944**	0	0	**0**	**0.00**	**3,882**	1,168	1,064	**2,232**	**57.50**	**4,909**	738	692	**1,430**	**29.13**
**Region 4B**	**21,374**	**759**	**745**	**1,504**	**7.04**	**89,517**	**2,288**	**2,363**	**4,651**	**5.20**	**111,155**	**2,331**	**2,388**	**4,719**	**4.25**
Marinduque	**1,451**	136	162	**298**	**20.54**	**6,513**	586	544	**1,130**	**17.35**	**8,227**	408	404	**812**	**9.87**
Mindoro Occidental	**3,722**	105	126	**231**	**6.21**	**15,105**	560	570	**1,130**	**7.48**	**18,604**	320	320	**640**	**3.44**
Mindoro Oriental	**5,943**	494	438	**932**	**15.68**	**24,950**	999	1,083	**2,082**	**8.34**	**31,270**	1,510	1,587	**3,097**	**9.90**
Palawan	**6,413**	0	0	**0**	**0.00**	**26,485**	52	66	**118**	**0.45**	**32,745**	12	10	**22**	**0.07**
Romblon	**1,920**	15	9	**24**	**1.25**	**8,510**	56	61	**117**	**1.37**	**10,475**	41	43	**84**	**0.80**
Puerto Princesa City	**1,926**	9	10	**19**	**0.99**	**7,955**	35	39	**74**	**0.93**	**9,835**	40	24	**64**	**0.65**
**Region 5**	**40,835**	**5,270**	**5,082**	**10,352**	**25.35**	**175,896**	**44,823**	**44,618**	**89,441**	**50.85**	**212,732**	**19,358**	**19,707**	**39,065**	**18.36**
Albay	**7,121**	117	143	**260**	**3.65**	**30,968**	6,053	6,678	**12,731**	**41.11**	**38,559**	1,909	2,019	**3,928**	**10.19**
Camarines Norte	**4,285**	509	610	**1,119**	**26.11**	**17,968**	5,120	4,986	**10,106**	**56.24**	**21,303**	4,116	4,515	**8,631**	**40.52**
Camarines Sur	**11,639**	2,177	1,963	**4,140**	**35.57**	**49,864**	12,777	12,240	**25,017**	**50.17**	**60,608**	4,914	4,916	**9,830**	**16.22**
Catanduanes	**1,882**	701	688	**1,389**	**73.80**	**7,968**	3,293	3,146	**6,439**	**80.81**	**9,559**	1,466	1,445	**2,911**	**30.45**
Masbate	**6,756**	680	610	**1,290**	**19.09**	**29,831**	4,429	4,519	**8,948**	**30.00**	**35,125**	2,464	2,467	**4,931**	**14.04**
Sorsogon	**5,720**	1,009	1,007	**2,016**	**35.25**	**24,520**	9,836	9,700	**19,536**	**79.67**	**29,457**	3,683	3,441	**7,124**	**24.18**
Iriga City	**791**	37	29	**66**	**8.34**	**3,387**	219	288	**507**	**14.97**	**4,118**	153	176	**329**	**7.99**
Legaspi City	**1,252**	26	22	**48**	**3.83**	**5,445**	2,748	2,664	**5,412**	**99.39**	**6,780**	511	531	**1,042**	**15.37**
Naga City	**1,388**	14	10	**24**	**1.73**	**5,944**	348	397	**745**	**12.53**	**7,223**	142	197	**339**	**4.69**
**Region 6**	**43,958**	**7,264**	**7,751**	**15,015**	**34.16**	**187,120**	**18,120**	**18,826**	**36,946**	**19.74**	**234,320**	**18,218**	**17,416**	**35,634**	**15.21**
Aklan	**3,349**	370	460	**830**	**24.79**	**14,355**	369	399	**768**	**5.35**	**18,142**	587	632	**1,219**	**6.72**
Antique	**3,845**	23	20	**43**	**1.12**	**16,147**	89	127	**216**	**1.34**	**20,203**	158	201	**359**	**1.78**
Capiz	**4,196**	578	723	**1,301**	**31.01**	**18,024**	1,434	1,622	**3,056**	**16.95**	**22,265**	1,711	1,890	**3,601**	**16.17**
Guimaras	**926**	25	30	**55**	**5.94**	**4,350**	1,576	1,712	**3,288**	**75.58**	**5,292**	309	332	**641**	**12.11**
Iloilo	**10,962**	4,669	4,876	**9,545**	**87.07**	**46,083**	11,180	11,446	**22,626**	**49.10**	**57,966**	9,602	8,455	**18,057**	**31.15**
Negros Occidental	**14,810**	307	318	**625**	**4.22**	**63,265**	1,420	1,452	**2,872**	**4.54**	**79,216**	2,934	2,868	**5,802**	**7.32**
Bacolod City	**3,335**	72	89	**161**	**4.83**	**14,235**	648	628	**1,276**	**8.96**	**17,823**	792	755	**1,547**	**8.68**
Iloilo City	**2,536**	1,220	1,235	**2,455**	**96.81**	**10,661**	1,404	1,440	**2,844**	**26.68**	**13,414**	2,125	2,283	**4,408**	**32.86**

Table 1.D.3 - Oral Health Care

Number and proportion of infants 0-11 months old, 1-4 years old and 5-9 years old who received Basic Oral Health Care (BOHC)
Philippines, Annual 2020

Area	Infants 0-11 months old who received BOHC					Infants 1-4 years old who received BOHC					Children 5-9 years old who received BOHC				
	Eligible Pop. (0 - 11 mos. x 30%)	Male	Female	Total	%	Eligible Pop. (1 - 4 yrs. old x 30%)	Male	Female	Total	%	Eligible Pop. (5 - 9 yrs. old x 30%)	Male	Female	Total	%
**Region 7**	**48,979**	**6,586**	**6,673**	**13,259**	**27.07**	**195,725**	**14,075**	**15,101**	**29,176**	**14.91**	**244,335**	**11,313**	**12,459**	**23,772**	**9.73**
Bohol	**8,194**	1,662	1,633	**3,295**	**40.21**	**34,524**	4,561	4,741	**9,302**	**26.94**	**43,618**	4,784	5,331	**10,115**	**23.19**
Cebu	**20,252**	1,074	1,175	**2,249**	**11.11**	**78,963**	2,654	2,736	**5,390**	**6.83**	**98,375**	1,571	1,680	**3,251**	**3.30**
Negros Oriental	**8,381**	1,025	864	**1,889**	**22.54**	**34,505**	1,225	1,350	**2,575**	**7.46**	**42,803**	750	885	**1,635**	**3.82**
Siquijor	**484**	35	28	**63**	**13.02**	**2,232**	817	922	**1,739**	**77.91**	**2,856**	287	267	**554**	**19.40**
Cebu City	**6,358**	1,701	1,825	**3,526**	**55.46**	**24,790**	4,115	4,511	**8,626**	**34.80**	**30,882**	2,086	2,281	**4,367**	**14.14**
Lapu-Lapu City	**2,812**	18	26	**44**	**1.56**	**10,966**	128	153	**281**	**2.56**	**13,661**	102	103	**205**	**1.50**
Mandaue City	**2,498**	1,071	1,122	**2,193**	**87.78**	**9,744**	575	688	**1,263**	**12.96**	**12,140**	1,733	1,912	**3,645**	**30.02**
**Region 8**	**30,786**	**2,024**	**2,099**	**4,123**	**13.39**	**129,934**	**5,719**	**6,196**	**11,915**	**9.17**	**157,605**	**5,321**	**5,910**	**11,231**	**7.13**
Biliran	**1,150**	1	1	**2**	**0.17**	**5,046**	30	42	**72**	**1.43**	**5,988**	128	160	**288**	**4.81**
Eastern Samar	**3,418**	204	255	**459**	**13.43**	**13,884**	681	769	**1,450**	**10.44**	**17,017**	752	889	**1,641**	**9.64**
Northern Leyte	**10,412**	857	906	**1,763**	**16.93**	**43,082**	1,915	1,949	**3,864**	**8.97**	**52,436**	1,524	1,637	**3,161**	**6.03**
Northern Samar	**4,611**	66	58	**124**	**2.69**	**20,222**	252	276	**528**	**2.61**	**24,154**	421	426	**847**	**3.51**
Southern Leyte	**1,935**	11	12	**23**	**1.19**	**8,637**	80	93	**173**	**2.00**	**10,931**	68	95	**163**	**1.49**
Western Samar	**4,292**	38	39	**77**	**1.79**	**18,209**	488	646	**1,134**	**6.23**	**21,723**	1,446	1,641	**3,087**	**14.21**
Calbayog City	**1,324**	42	54	**96**	**7.25**	**5,612**	266	305	**571**	**10.17**	**6,696**	314	359	**673**	**10.05**
Maasin City	**491**	0	0	**0**	**0.00**	**2,199**	540	553	**1,093**	**49.71**	**2,781**	112	126	**238**	**8.56**
Ormoc City	**1,482**	732	721	**1,453**	**98.02**	**6,135**	1,338	1,451	**2,789**	**45.46**	**7,468**	425	414	**839**	**11.24**
Tacloban City	**1,671**	73	53	**126**	**7.54**	**6,907**	129	112	**241**	**3.49**	**8,411**	131	163	**294**	**3.50**
**Region 9**	**24,015**	**909**	**953**	**1,862**	**7.75**	**101,846**	**5,378**	**5,509**	**10,887**	**10.69**	**124,085**	**6,190**	**6,754**	**12,944**	**10.43**
Zamboanga del Norte	**5,175**	249	277	**526**	**10.16**	**22,462**	2,292	2,232	**4,524**	**20.14**	**27,110**	2,480	2,391	**4,871**	**17.97**
Zamboanga del Sur	**5,296**	374	400	**774**	**14.62**	**21,643**	1,318	1,503	**2,821**	**13.03**	**26,705**	1,934	2,314	**4,248**	**15.91**
Zamboanga Sibugay	**4,486**	225	202	**427**	**9.52**	**19,833**	1,350	1,314	**2,664**	**13.43**	**23,887**	951	974	**1,925**	**8.06**
Dapitan City	**535**	7	16	**23**	**4.30**	**2,321**	251	271	**522**	**22.49**	**2,800**	233	211	**444**	**15.86**
Dipolog City	**848**	0	0	**0**	**0.00**	**3,679**	55	67	**122**	**3.32**	**4,441**	15	12	**27**	**0.61**
Isabela City	**757**	0	0	**0**	**0.00**	**3,618**	0	0	**0**	**0.00**	**4,239**	0	0	**0**	**0.00**
Pagadian City	**1,298**	54	58	**112**	**8.63**	**5,308**	112	122	**234**	**4.41**	**6,549**	577	852	**1,429**	**21.82**
Zamboanga City	**5,621**	0	0	**0**	**0.00**	**22,982**	0	0	**0**	**0.00**	**28,355**	0	0	**0**	**0.00**
**Region 10**	**30,423**	**2,824**	**2,758**	**5,582**	**18.35**	**130,481**	**13,650**	**15,704**	**29,354**	**22.50**	**158,807**	**9,007**	**9,562**	**18,569**	**11.69**
Bukidnon	**7,112**	396	258	**654**	**9.20**	**30,173**	1,739	2,141	**3,880**	**12.86**	**36,333**	1,337	1,338	**2,675**	**7.36**
Camiguin	**557**	17	16	**33**	**5.92**	**2,400**	461	401	**862**	**35.92**	**2,886**	330	360	**690**	**23.91**
Lanao del Norte	**4,488**	5	8	**13**	**0.29**	**20,252**	633	650	**1,283**	**6.34**	**24,636**	318	333	**651**	**2.64**
Misamis Occidental	**1,921**	405	506	**911**	**47.43**	**8,227**	1,641	2,040	**3,681**	**44.74**	**10,272**	2,129	2,487	**4,616**	**44.94**
Misamis Oriental	**4,539**	600	604	**1,204**	**26.52**	**18,993**	2,312	2,163	**4,475**	**23.56**	**23,162**	1,768	1,701	**3,469**	**14.98**
Cagayan de Oro City	**4,302**	75	93	**168**	**3.91**	**17,993**	2,237	3,361	**5,598**	**31.11**	**21,942**	189	228	**417**	**1.90**
El Salvador City	**320**	18	14	**32**	**10.02**	**1,337**	53	77	**130**	**9.72**	**1,630**	259	281	**540**	**33.12**
Gingoog City	**793**	331	342	**673**	**84.85**	**3,316**	1,215	1,271	**2,486**	**74.98**	**4,048**	633	680	**1,313**	**32.43**
Iligan City	**2,274**	1	2	**3**	**0.13**	**10,259**	64	64	**128**	**1.25**	**12,480**	205	198	**403**	**3.23**
Malaybalay City	**1,187**	577	503	**1,080**	**91.00**	**5,030**	1,363	1,366	**2,729**	**54.26**	**6,057**	479	484	**963**	**15.90**
Oroquieta City	**417**	0	0	**0**	**0.00**	**1,783**	2	2	**4**	**0.22**	**2,225**	5	5	**10**	**0.45**
Ozamis City	**833**	206	233	**439**	**52.68**	**3,573**	831	939	**1,770**	**49.54**	**4,461**	547	633	**1,180**	**26.45**
Tangub City	**370**	156	139	**295**	**79.69**	**1,588**	334	327	**661**	**41.63**	**1,982**	481	431	**912**	**46.01**
Valencia City	**1,310**	37	40	**77**	**5.88**	**5,558**	765	902	**1,667**	**29.99**	**6,694**	327	403	**730**	**10.91**
**Region 11**	**32,174**	**1,033**	**1,199**	**2,232**	**6.94**	**134,462**	**3,611**	**3,763**	**7,374**	**5.48**	**166,901**	**4,480**	**5,276**	**9,756**	**5.85**
Compostela Valley	**4,669**	141	188	**329**	**7.05**	**20,439**	692	658	**1,350**	**6.61**	**24,935**	971	956	**1,927**	**7.73**
Davao del Norte	**6,398**	202	220	**422**	**6.60**	**26,299**	667	814	**1,481**	**5.63**	**32,812**	994	1,155	**2,149**	**6.55**
Davao Oriental	**3,902**	136	161	**297**	**7.61**	**16,848**	488	522	**1,010**	**5.99**	**20,378**	585	736	**1,321**	**6.48**
Davao del Sur	**4,245**	108	153	**261**	**6.15**	**17,204**	282	348	**630**	**3.66**	**21,506**	345	452	**797**	**3.71**
Davao Occidental	**2,001**	62	94	**156**	**7.80**	**9,258**	154	252	**406**	**4.39**	**11,746**	169	319	**488**	**4.15**
Davao City	**10,959**	384	383	**767**	**7.00**	**44,414**	1,328	1,169	**2,497**	**5.62**	**55,524**	1,416	1,658	**3,074**	**5.54**
**Region 12**	**31,366**	**5,659**	**5,905**	**11,564**	**36.87**	**131,263**	**10,146**	**11,252**	**21,398**	**16.30**	**161,676**	**8,822**	**9,737**	**18,559**	**11.48**
North Cotabato	**10,094**	2,295	2,418	**4,713**	**46.69**	**41,722**	3,622	3,986	**7,608**	**18.23**	**51,521**	5,126	5,662	**10,788**	**20.94**
Sarangani	**3,867**	514	504	**1,018**	**26.32**	**16,381**	1,658	1,658	**3,316**	**20.24**	**20,006**	1,149	1,202	**2,351**	**11.75**
South Cotabato	**6,334**	504	466	**970**	**15.31**	**25,569**	878	1,037	**1,915**	**7.49**	**31,129**	753	947	**1,700**	**5.46**
Sultan Kudarat	**5,208**	218	275	**493**	**9.47**	**22,473**	741	1,028	**1,769**	**7.87**	**28,267**	941	1,117	**2,058**	**7.28**
Cotabato City	**1,751**	203	196	**399**	**22.79**	**8,513**	1,643	1,784	**3,427**	**40.25**	**10,535**	415	369	**784**	**7.44**
Gen. Santos City	**4,113**	1,925	2,046	**3,971**	**96.55**	**16,605**	1,604	1,759	**3,363**	**20.25**	**20,219**	438	440	**878**	**4.34**

Table 1.D.3 - Oral Health Care

Number and proportion of infants 0-11 months old, 1-4 years old and 5-9 years old who received Basic Oral Health Care (BOHC)
Philippines, Annual 2020

Area	Infants 0-11 months old who received BOHC					Infants 1-4 years old who received BOHC					Children 5-9 years old who received BOHC				
	Eligible Pop. (0 - 11 mos. x 30%)	Male	Female	Total	%	Eligible Pop. (1 - 4 yrs. old x 30%)	Male	Female	Total	%	Eligible Pop. (5 - 9 yrs. old x 30%)	Male	Female	Total	%
**BARMM**	**27,840**	**3,341**	**3,361**	**6,702**	**24.07**	**144,959**	**8,949**	**9,289**	**18,238**	**12.58**	**169,890**	**9,735**	**10,026**	**19,761**	**11.63**
Basilan	**2,262**	0	0	**0**	**0.00**	**10,647**	0	0	**0**	**0.00**	**12,407**	0	0	**0**	**0.00**
Lanao del Sur	**6,339**	757	767	**1,524**	**24.04**	**33,098**	3,858	4,267	**8,125**	**24.55**	**38,415**	4,040	4,404	**8,444**	**21.98**
Maguindanao	**9,338**	2,548	2,516	**5,064**	**54.23**	**44,684**	4,839	4,822	**9,661**	**21.62**	**53,289**	5,448	5,386	**10,834**	**20.33**
Sulu	**4,984**	0	0	**0**	**0.00**	**32,184**	0	0	**0**	**0.00**	**37,132**	0	0	**0**	**0.00**
Tawi-Tawi	**2,778**	0	0	**0**	**0.00**	**13,498**	0	0	**0**	**0.00**	**16,046**	0	0	**0**	**0.00**
Lamitan City	**622**	0	0	**0**	**0.00**	**2,929**	0	0	**0**	**0.00**	**3,412**	0	0	**0**	**0.00**
Marawi City	**1,516**	36	78	**114**	**7.52**	**7,919**	252	200	**452**	**5.71**	**9,190**	247	236	**483**	**5.26**
**CARAGA**	**18,009**	**4,365**	**4,605**	**8,970**	**49.81**	**77,573**	**8,889**	**8,442**	**17,331**	**22.34**	**92,419**	**11,015**	**11,719**	**22,734**	**24.60**
Agusan del Norte	**2,429**	396	509	**905**	**37.25**	**10,231**	650	783	**1,433**	**14.01**	**12,265**	607	729	**1,336**	**10.89**
Agusan del Sur	**5,278**	403	492	**895**	**16.96**	**23,044**	1,456	1,654	**3,110**	**13.50**	**26,478**	4,415	4,740	**9,155**	**34.58**
Surigao del Norte	**2,127**	336	344	**680**	**31.97**	**9,466**	370	368	**738**	**7.80**	**11,613**	374	441	**815**	**7.02**
Surigao del Sur	**3,445**	1,192	1,249	**2,441**	**70.86**	**14,560**	2,535	2,485	**5,020**	**34.48**	**17,437**	2,925	2,918	**5,843**	**33.51**
Province of Dinagat	**772**	312	384	**696**	**90.17**	**3,369**	921	900	**1,821**	**54.05**	**4,248**	463	515	**978**	**23.02**
Bislig City	**654**	375	263	**638**	**97.60**	**2,767**	967	332	**1,299**	**46.95**	**3,312**	226	225	**451**	**13.62**
Butuan City	**2,315**	884	862	**1,746**	**75.44**	**9,729**	687	706	**1,393**	**14.32**	**11,659**	1,300	1,407	**2,707**	**23.22**
Surigao City	**990**	467	502	**969**	**97.85**	**4,407**	1,303	1,214	**2,517**	**57.11**	**5,408**	705	744	**1,449**	**26.79**

Note: Put asterisk (*) for No Report and Zero (0) for No Case

Table 1.D.4 - Oral Health Care

Number and proportion of adolescents 10-19 years old and adults 20 -59 years old who received Basic Oral Health Care (BOHC)
Philippines, Annual 2020

Area	Adolescents 10-14 years old who received BOHC					Adolescents 15-19 years old who received BOHC					Adults 20-59 years old who received BOHC				
	Eligible Pop. (10 - 14 yrs. old x 30%)	Male	Female	Total	%	Eligible Pop. (15 - 19 yrs. old x 30%)	Male	Female	Total	%	Eligible Pop. (20 - 59 yrs. old x 30%)	Male	Female	Total	%
**PHILIPPINES**	**3,239,507**	**180,351**	**203,588**	**383,939**	**11.85**	**3,102,298**	**118,710**	**144,886**	**263,596**	**8.50**	**16,803,163**	**282,974**	**404,207**	**687,181**	**4.09**
**N C R**	**370,174**	**34,669**	**36,912**	**71,581**	**19.34**	**360,694**	**23,762**	**27,127**	**50,889**	**14.11**	**2,322,720**	**34,984**	**49,878**	**84,862**	**3.65**
Malabon	**10,508**	911	1,070	**1,981**	**18.85**	**10,239**	1,190	1,314	**2,504**	**24.45**	**65,931**	2,402	1,425	**3,827**	**5.80**
Navotas	**7,171**	2,590	2,711	**5,301**	**73.93**	**6,988**	2,637	2,953	**5,590**	**80.00**	**44,996**	3,185	3,510	**6,695**	**14.88**
Valenzuela City	**17,836**	1,202	1,249	**2,451**	**13.74**	**17,378**	1,278	1,399	**2,677**	**15.40**	**111,907**	3,590	5,443	**9,033**	**8.07**
Caloocan City	**45,533**	742	869	**1,611**	**3.54**	**44,370**	1,451	1,948	**3,399**	**7.66**	**285,708**	2,324	3,772	**6,096**	**2.13**
Marikina City	**12,956**	3,373	3,604	**6,977**	**53.85**	**12,626**	280	267	**547**	**4.33**	**81,302**	634	1,025	**1,659**	**2.04**
Pasig City	**21,712**	1,926	2,049	**3,975**	**18.31**	**21,156**	1,985	1,736	**3,721**	**17.59**	**136,237**	3,624	4,358	**7,982**	**5.86**
Pateros	**1,835**	162	142	**304**	**16.56**	**1,788**	169	152	**321**	**17.95**	**11,515**	218	308	**526**	**4.57**
Taguig	**23,140**	92	87	**179**	**0.77**	**22,546**	99	143	**242**	**1.07**	**145,187**	756	1,148	**1,904**	**1.31**
Quezon City	**84,401**	4,917	5,554	**10,471**	**12.41**	**82,243**	5,044	6,355	**11,399**	**13.86**	**529,597**	6,524	12,267	**18,791**	**3.55**
Makati City	**16,748**	4,652	4,563	**9,215**	**55.02**	**16,318**	1,815	2,028	**3,843**	**23.55**	**105,086**	4,300	5,028	**9,328**	**8.88**
Mandaluyong City	**11,105**	5,142	5,474	**10,616**	**95.60**	**10,818**	429	434	**863**	**7.98**	**69,674**	906	1,524	**2,430**	**3.49**
San Juan	**3,512**	603	542	**1,145**	**32.60**	**3,423**	129	167	**296**	**8.65**	**22,038**	476	927	**1,403**	**6.37**
Manila City	**51,171**	441	466	**907**	**1.77**	**49,856**	616	730	**1,346**	**2.70**	**321,090**	759	1,111	**1,870**	**0.58**
Las Piñas City	**16,929**	2,396	2,533	**4,929**	**29.12**	**16,497**	1,708	2,027	**3,735**	**22.64**	**106,222**	505	942	**1,447**	**1.36**
Muntinlupa City	**14,503**	3,838	4,222	**8,060**	**55.58**	**14,131**	3,042	3,331	**6,373**	**45.10**	**91,001**	3,027	4,947	**7,974**	**8.76**
Parañaque City	**19,139**	776	804	**1,580**	**8.26**	**18,651**	329	407	**736**	**3.95**	**120,097**	731	853	**1,584**	**1.32**
Pasay City	**11,976**	906	973	**1,879**	**15.69**	**11,666**	1,561	1,736	**3,297**	**28.26**	**75,131**	1,023	1,290	**2,313**	**3.08**
**C A R**	**52,577**	**3,142**	**4,745**	**7,887**	**15.00**	**52,487**	**4,769**	**7,605**	**12,374**	**23.58**	**282,909**	**10,653**	**20,888**	**31,541**	**11.15**
Abra	**7,176**	217	264	**481**	**6.70**	**6,921**	437	546	**983**	**14.20**	**37,895**	812	1,324	**2,136**	**5.64**
Apayao	**3,816**	394	481	**875**	**22.93**	**3,658**	219	306	**525**	**14.35**	**18,701**	446	1,183	**1,629**	**8.71**
Benguet	**12,891**	1,188	2,159	**3,347**	**25.96**	**13,787**	2,388	3,748	**6,136**	**44.51**	**78,425**	6,028	10,243	**16,271**	**20.75**
Ifugao	**6,674**	166	293	**459**	**6.88**	**6,258**	424	742	**1,166**	**18.63**	**31,442**	781	1,751	**2,532**	**8.05**
Kalinga	**7,052**	582	666	**1,248**	**17.70**	**6,572**	577	1,013	**1,590**	**24.19**	**32,540**	821	2,030	**2,851**	**8.76**
Mt. Province	**4,990**	195	292	**487**	**9.76**	**4,619**	263	507	**770**	**16.67**	**23,207**	416	889	**1,305**	**5.62**
Baguio City	**9,978**	400	590	**990**	**9.92**	**10,672**	461	743	**1,204**	**11.28**	**60,699**	1,349	3,468	**4,817**	**7.94**
**Region 1**	**154,902**	**6,696**	**7,204**	**13,900**	**8.97**	**144,398**	**16,498**	**19,228**	**35,726**	**24.74**	**806,139**	**51,684**	**54,829**	**106,513**	**13.21**
Ilocos Norte	**12,722**	419	468	**887**	**6.97**	**13,127**	2,316	2,842	**5,158**	**39.29**	**80,212**	4,258	5,577	**9,835**	**12.26**
Ilocos Sur	**16,007**	3,366	3,494	**6,860**	**42.86**	**15,715**	3,203	3,490	**6,693**	**42.59**	**94,832**	15,307	17,547	**32,854**	**34.64**
La Union	**18,819**	625	681	**1,306**	**6.94**	**19,069**	297	381	**678**	**3.56**	**111,516**	699	1,643	**2,342**	**2.10**
Pangasinan	**78,523**	1,819	2,061	**3,880**	**4.94**	**69,737**	8,252	9,728	**17,980**	**25.78**	**370,805**	18,168	23,175	**41,343**	**11.15**
Alaminos City	**2,968**	0	0	**0**	**0.00**	**2,635**	1,232	1,371	**2,603**	**98.79**	**14,010**	10,762	3,041	**13,803**	**98.52**
Candon City	**1,686**	72	64	**136**	**8.07**	**1,655**	95	110	**205**	**12.38**	**9,994**	159	175	**334**	**3.34**
Dagupan City	**5,665**	117	143	**260**	**4.59**	**5,030**	126	229	**355**	**7.06**	**26,749**	385	699	**1,084**	**4.05**
Laoag City	**2,933**	0	0	**0**	**0.00**	**3,024**	315	309	**624**	**20.63**	**18,492**	256	385	**641**	**3.47**
San Carlos City	**6,237**	0	0	**0**	**0.00**	**5,537**	400	451	**851**	**15.37**	**29,451**	629	940	**1,569**	**5.33**
San Fernando City	**3,448**	87	83	**170**	**4.93**	**3,493**	90	145	**235**	**6.73**	**20,432**	440	885	**1,325**	**6.49**
Urdaneta City	**4,396**	25	43	**68**	**1.55**	**3,905**	10	12	**22**	**0.56**	**20,763**	131	214	**345**	**1.66**
Vigan City	**1,499**	166	167	**333**	**22.21**	**1,471**	162	160	**322**	**21.89**	**8,884**	490	548	**1,038**	**11.68**
**Region 2**	**106,469**	**6,865**	**7,766**	**14,631**	**13.74**	**99,102**	**6,019**	**8,021**	**14,040**	**14.17**	**568,298**	**14,378**	**26,161**	**40,539**	**7.13**
Batanes	**578**	62	137	**199**	**34.44**	**430**	96	119	**215**	**49.98**	**2,618**	276	409	**685**	**26.16**
Cagayan	**27,528**	993	1,032	**2,025**	**7.36**	**26,588**	846	1,231	**2,077**	**7.81**	**149,624**	1,910	3,892	**5,802**	**3.88**
Isabela	**40,486**	2,013	2,225	**4,238**	**10.47**	**37,427**	1,896	2,377	**4,273**	**11.42**	**218,728**	3,624	6,561	**10,185**	**4.66**
Nueva Vizcaya	**14,416**	1,083	1,388	**2,471**	**17.14**	**13,350**	953	1,545	**2,498**	**18.71**	**73,953**	2,915	6,379	**9,294**	**12.57**
Quirino	**6,227**	245	297	**542**	**8.70**	**5,208**	66	139	**205**	**3.94**	**30,127**	95	332	**427**	**1.42**
Cauayan City	**3,976**	422	449	**871**	**21.91**	**3,675**	450	504	**954**	**25.96**	**21,488**	1,731	2,053	**3,784**	**17.61**
Ilagan City	**4,471**	1,018	1,058	**2,076**	**46.44**	**4,133**	943	1,164	**2,107**	**50.99**	**24,149**	1,778	2,671	**4,449**	**18.42**
Santiago City	**4,139**	998	1,135	**2,133**	**51.53**	**3,827**	729	899	**1,628**	**42.54**	**22,367**	1,935	3,530	**5,465**	**24.43**
Tuguegarao City	**4,648**	31	45	**76**	**1.64**	**4,464**	40	43	**83**	**1.86**	**25,245**	114	334	**448**	**1.77**
**Region 3**	**345,216**	**4,905**	**5,697**	**10,602**	**3.07**	**347,009**	**8,895**	**10,356**	**19,251**	**5.55**	**1,971,635**	**17,819**	**26,712**	**44,531**	**2.26**
Aurora	**7,033**	321	359	**680**	**9.67**	**6,719**	372	403	**775**	**11.53**	**33,859**	1,237	1,254	**2,491**	**7.36**
Bataan	**20,412**	164	183	**347**	**1.70**	**20,359**	214	178	**392**	**1.93**	**116,958**	538	949	**1,487**	**1.27**
Bulacan	**69,174**	1,052	1,346	**2,398**	**3.47**	**70,910**	1,119	1,395	**2,514**	**3.55**	**405,206**	2,714	4,209	**6,923**	**1.71**
Nueva Ecija	**45,997**	141	189	**330**	**0.72**	**44,568**	1,647	1,873	**3,520**	**7.90**	**256,084**	993	1,791	**2,784**	**1.09**
Pampanga	**50,807**	576	647	**1,223**	**2.41**	**51,749**	174	199	**373**	**0.72**	**294,496**	1,161	1,517	**2,678**	**0.91**
Tarlac	**30,649**	0	0	**0**	**0.00**	**31,037**	1,959	2,269	**4,228**	**13.62**	**173,095**	2,743	3,895	**6,638**	**3.83**
Zambales	**18,052**	372	422	**794**	**4.40**	**17,835**	551	569	**1,120**	**6.28**	**100,585**	521	784	**1,305**	**1.30**
Angeles City	**12,747**	134	196	**330**	**2.59**	**12,983**	113	189	**302**	**2.33**	**73,888**	683	782	**1,465**	**1.98**
Balanga City	**2,950**	92	86	**178**	**6.03**	**2,942**	74	87	**161**	**5.47**	**16,907**	286	448	**734**	**4.34**

Table 1.D.4 - Oral Health Care

Number and proportion of adolescents 10-19 years old and adults 20 -59 years old who received Basic Oral Health Care (BOHC)
Philippines, Annual 2020

Area	Adolescents 10-14 years old who received BOHC					Adolescents 15-19 years old who received BOHC					Adults 20-59 years old who received BOHC				
	Eligible Pop. (10 - 14 yrs. old x 30%)	Male	Female	Total	%	Eligible Pop. (15 - 19 yrs. old x 30%)	Male	Female	Total	%	Eligible Pop. (20 - 59 yrs. old x 30%)	Male	Female	Total	%
Cabanatuan City	9,413	61	57	118	1.25	9,122	26	33	59	0.65	52,412	63	189	252	0.48
City of San Fernando	9,497	3	3	6	0.06	9,672	68	79	147	1.52	55,046	0	0	0	0.00
Gapan City	3,436	7	7	14	0.41	3,330	2	2	4	0.12	19,129	49	131	180	0.94
Mabalacat City	7,767	0	0	0	0.00	7,911	69	76	145	1.83	45,019	188	310	498	1.11
Malolos City	7,729	55	64	119	1.54	7,922	163	249	412	5.20	45,261	363	591	954	2.11
Meycauayan	6,410	529	616	1,145	17.86	6,569	172	160	332	5.05	37,541	1,413	1,945	3,358	8.94
Olongapo	7,120	13	24	37	0.52	7,036	24	26	50	0.71	39,673	41	70	111	0.28
Palayan City	1,280	12	12	24	1.88	1,239	9	26	35	2.82	7,118	39	98	137	1.92
San Jose City	4,353	200	218	418	9.60	4,218	835	1,000	1,835	43.51	24,234	1,013	1,384	2,397	9.89
San Jose del Monte City	17,599	937	1,052	1,989	11.30	18,040	832	1,069	1,901	10.54	103,074	2,613	4,934	7,547	7.32
Science City of Munoz	2,538	20	39	59	2.32	2,460	46	82	128	5.20	14,130	103	223	326	2.31
Tarlac City	10,256	216	177	393	3.83	10,388	426	392	818	7.87	57,923	1,058	1,208	2,266	3.91
**Region 4A**	455,339	77,076	86,238	163,314	35.87	453,561	13,656	14,889	28,545	6.29	2,585,001	44,014	52,482	96,496	3.73
Batangas	58,697	12,457	13,333	25,790	43.94	59,366	6,297	6,261	12,558	21.15	332,633	29,126	28,933	58,059	17.45
Cavite	43,330	258	377	635	1.47	44,114	755	952	1,707	3.87	259,122	2,105	3,585	5,690	2.20
Laguna	29,868	24,012	26,268	50,280	168.34	29,624	218	278	496	1.67	181,115	614	844	1,458	0.81
Quezon	58,810	3,050	3,417	6,467	11.00	55,452	1,329	1,452	2,781	5.02	278,820	3,190	4,323	7,513	2.69
Rizal	66,981	1,886	2,071	3,957	5.91	66,951	1,675	1,995	3,670	5.48	377,970	3,816	5,514	9,330	2.47
Antipolo City	24,671	0	0	0	0.00	24,661	0	0	0	0.00	139,218	0	0	0	0.00
Bacoor City	18,982	160	203	363	1.91	19,327	191	254	445	2.30	113,526	526	1,150	1,676	1.48
Batangas City	10,417	1,513	3,872	5,385	51.69	10,534	0	0	0	0.00	59,034	0	0	0	0.00
Biñan City	10,017	488	528	1,016	10.14	9,933	627	790	1,417	14.27	60,730	1,089	2,871	3,960	6.52
Cabuyao City	9,286	0	0	0	0.00	9,209	0	0	0	0.00	56,302	31	152	183	0.33
Calamba City	13,670	28,078	30,537	58,615	428.79	13,556	139	177	316	2.33	82,878	585	988	1,573	1.90
Cavite City	3,250	165	225	390	12.00	3,308	165	253	418	12.63	19,432	293	574	867	4.46
Dasmariñas City	20,828	0	0	0	0.00	21,209	0	0	0	0.00	124,565	0	16	16	0.01
General Trias City	9,934	14	22	36	0.36	10,114	224	299	523	5.17	59,409	10	28	38	0.06
Imus City	12,763	68	71	139	1.09	12,994	79	150	229	1.76	76,321	312	693	1,005	1.32
Lipa City	10,496	824	908	1,732	16.50	10,614	0	2	2	0.02	59,483	0	0	0	0.00
Lucena City	8,912	41	35	76	0.85	8,406	42	56	98	1.17	42,257	156	356	512	1.21
San Pablo City	8,002	2,416	2,763	5,179	64.72	7,933	0	0	0	0.00	48,520	0	0	0	0.00
San Pedro City	9,800	0	0	0	0.00	9,718	0	0	0	0.00	59,414	0	0	0	0.00
Santa Rosa City	10,640	77	86	163	1.53	10,552	83	82	165	1.56	64,511	166	231	397	0.62
Tagaytay City	2,249	502	514	1,016	45.18	2,290	562	528	1,090	47.61	13,452	33	62	95	0.71
Tanauan City	5,475	174	170	344	6.28	5,538	153	203	356	6.43	31,024	380	637	1,017	3.28
Tayabas City	3,341	77	92	169	5.06	3,150	171	297	468	14.86	15,833	524	619	1,143	7.22
Trece Martires City	4,921	816	746	1,562	31.74	5,010	946	860	1,806	36.05	29,433	1,058	906	1,964	6.67
**Region 4B**	106,516	1,462	1,785	3,247	3.05	94,106	1,284	1,575	2,859	3.04	451,859	2,300	3,805	6,105	1.35
Marinduque	7,992	204	238	442	5.53	6,891	175	206	381	5.53	33,279	326	641	967	2.91
Mindoro Occidental	17,878	198	171	369	2.06	15,113	167	146	313	2.07	72,363	265	329	594	0.82
Mindoro Oriental	29,938	674	796	1,470	4.91	26,146	589	693	1,282	4.90	129,383	1,572	2,532	4,104	3.17
Palawan	31,109	71	76	147	0.47	28,591	66	70	136	0.48	134,946	29	66	95	0.07
Romblon	10,256	40	32	72	0.70	8,777	34	27	61	0.70	41,363	9	9	18	0.04
Puerto Princesa City	9,343	275	472	747	8.00	8,588	253	433	686	7.99	40,525	99	228	327	0.81
**Region 5**	204,866	4,838	6,542	11,380	5.55	187,955	6,055	8,923	14,978	7.97	860,750	13,508	30,353	43,861	5.10
Albay	37,757	853	998	1,851	4.90	35,794	689	825	1,514	4.23	172,727	3,147	3,074	6,221	3.60
Camarines Norte	20,067	1,241	1,686	2,927	14.59	18,625	938	1,310	2,248	12.07	88,903	1,588	3,183	4,771	5.37
Camarines Sur	58,603	393	675	1,068	1.82	53,206	1,050	1,836	2,886	5.42	243,255	2,326	12,371	14,697	6.04
Catanduanes	9,218	181	191	372	4.04	8,201	730	797	1,527	18.62	37,707	993	2,070	3,063	8.12
Masbate	32,835	902	1,482	2,384	7.26	30,140	1,144	1,837	2,981	9.89	124,626	2,718	4,235	6,953	5.58
Sorsogon	28,783	836	1,010	1,846	6.41	25,738	1,066	1,594	2,660	10.34	117,639	2,060	4,165	6,225	5.29
Iriga City	3,982	140	162	302	7.58	3,616	164	161	325	8.99	16,529	283	543	826	5.00
Legaspi City	6,638	36	36	72	1.08	6,294	172	388	560	8.90	30,377	134	280	414	1.36
Naga City	6,984	256	302	558	7.99	6,341	102	175	277	4.37	28,987	259	432	691	2.38
**Region 6**	228,123	9,061	10,495	19,556	8.57	223,697	7,797	9,985	17,782	7.95	1,207,783	19,710	28,203	47,913	3.97
Aklan	17,354	142	180	322	1.86	16,931	404	451	855	5.05	92,770	581	1,032	1,613	1.74
Antique	19,856	131	147	278	1.40	16,868	0	0	0	0.00	88,592	267	493	760	0.86
Capiz	21,974	817	1,007	1,824	8.30	23,077	1,009	1,535	2,544	11.02	125,093	1,858	3,015	4,873	3.90
Guimaras	5,055	150	167	317	6.27	5,089	109	197	306	6.01	28,977	253	414	667	2.30
Iloilo	56,774	5,073	6,012	11,085	19.52	57,152	3,789	4,811	8,600	15.05	318,253	7,814	11,924	19,738	6.20
Negros Occidental	76,715	1,108	1,217	2,325	3.03	74,576	1,595	1,996	3,591	4.82	392,226	4,993	6,952	11,945	3.05
Bacolod City	17,263	1,227	1,293	2,520	14.60	16,778	425	538	963	5.74	88,248	3,227	3,321	6,548	7.42
Iloilo City	13,134	413	472	885	6.74	13,227	466	457	923	6.98	73,624	717	1,052	1,769	2.40

Table 1.D.4 - Oral Health Care

Number and proportion of adolescents 10-19 years old and adults 20 -59 years old who received Basic Oral Health Care (BOHC)
Philippines, Annual 2020

Area	Adolescents 10-14 years old who received BOHC					Adolescents 15-19 years old who received BOHC					Adults 20-59 years old who received BOHC				
	Eligible Pop. (10 - 14 yrs. old x 30%)	Male	Female	Total	%	Eligible Pop. (15 - 19 yrs. old x 30%)	Male	Female	Total	%	Eligible Pop. (20 - 59 yrs. old x 30%)	Male	Female	Total	%
**Region 7**	**237,165**	**3,291**	**3,918**	**7,209**	**3.04**	**224,019**	**3,004**	**4,506**	**7,510**	**3.35**	**1,213,067**	**6,697**	**14,305**	**21,002**	**1.73**
Bohol	**43,611**	2,215	2,522	**4,737**	**10.86**	**38,224**	1,648	2,363	**4,011**	**10.49**	**197,066**	2,166	4,726	**6,892**	**3.50**
Cebu	**94,582**	196	290	**486**	**0.51**	**90,497**	212	383	**595**	**0.66**	**500,968**	614	1,451	**2,065**	**0.41**
Negros Oriental	**41,602**	473	513	**986**	**2.37**	**40,545**	419	514	**933**	**2.30**	**211,220**	798	1,273	**2,071**	**0.98**
Siquijor	**2,877**	83	134	**217**	**7.54**	**2,614**	116	175	**291**	**11.13**	**15,162**	326	632	**958**	**6.32**
Cebu City	**29,690**	213	319	**532**	**1.79**	**28,407**	435	811	**1,246**	**4.39**	**157,268**	1,349	4,503	**5,852**	**3.72**
Lapu-Lapu City	**13,133**	111	140	**251**	**1.91**	**12,566**	174	260	**434**	**3.45**	**69,566**	1,444	1,720	**3,164**	**4.55**
Mandaue City	**11,670**	0	0	**0**	**0.00**	**11,167**	0	0	**0**	**0.00**	**61,818**	0	0	**0**	**0.00**
**Region 8**	**151,698**	**1,694**	**2,077**	**3,771**	**2.49**	**144,802**	**2,526**	**3,143**	**5,669**	**3.92**	**676,983**	**4,410**	**7,648**	**12,058**	**1.78**
Biliran	**5,619**	50	52	**102**	**1.82**	**5,361**	90	177	**267**	**4.98**	**25,441**	246	312	**558**	**2.19**
Eastern Samar	**16,487**	143	173	**316**	**1.92**	**15,403**	352	492	**844**	**5.48**	**70,663**	727	1,228	**1,955**	**2.77**
Northern Leyte	**50,048**	329	416	**745**	**1.49**	**48,992**	524	670	**1,194**	**2.44**	**237,805**	1,133	2,819	**3,952**	**1.66**
Northern Samar	**23,234**	173	275	**448**	**1.93**	**21,605**	204	309	**513**	**2.37**	**91,349**	307	501	**808**	**0.88**
Southern Leyte	**10,700**	54	60	**114**	**1.07**	**9,936**	101	115	**216**	**2.17**	**52,063**	172	229	**401**	**0.77**
Western Samar	**21,198**	701	793	**1,494**	**7.05**	**19,987**	364	501	**865**	**4.33**	**87,458**	272	261	**533**	**0.61**
Calbayog City	**6,531**	99	117	**216**	**3.31**	**6,156**	143	166	**309**	**5.02**	**26,951**	378	490	**868**	**3.22**
Maasin City	**2,724**	14	28	**42**	**1.54**	**2,530**	14	14	**28**	**1.11**	**13,250**	61	92	**153**	**1.15**
Ormoc City	**7,132**	0	0	**0**	**0.00**	**6,978**	702	665	**1,367**	**19.59**	**33,872**	1,035	1,635	**2,670**	**7.88**
Tacloban City	**8,026**	131	163	**294**	**3.66**	**7,855**	32	34	**66**	**0.84**	**38,133**	79	81	**160**	**0.42**
**Region 9**	**118,305**	**2,991**	**3,662**	**6,653**	**5.62**	**115,703**	**4,052**	**6,117**	**10,169**	**8.79**	**560,134**	**8,045**	**12,860**	**20,905**	**3.73**
Zamboanga del Norte	**25,762**	1,155	1,402	**2,557**	**9.93**	**25,019**	411	481	**892**	**3.57**	**120,750**	366	611	**977**	**0.81**
Zamboanga del Sur	**25,622**	1,705	2,090	**3,795**	**14.81**	**25,766**	1,350	2,146	**3,496**	**13.57**	**125,708**	3,986	6,061	**10,047**	**7.99**
Zamboanga Sibugay	**22,460**	46	67	**113**	**0.50**	**20,570**	527	753	**1,280**	**6.22**	**98,189**	951	1,675	**2,626**	**2.67**
Dapitan City	**2,662**	58	74	**132**	**4.96**	**2,585**	59	75	**134**	**5.18**	**12,467**	125	172	**297**	**2.38**
Dipolog City	**4,220**	27	29	**56**	**1.33**	**4,100**	0	0	**0**	**0.00**	**19,781**	0	0	**0**	**0.00**
Isabela City	**4,082**	0	0	**0**	**0.00**	**3,984**	355	516	**871**	**21.86**	**18,919**	572	1,120	**1,692**	**8.94**
Pagadian City	**6,285**	0	0	**0**	**0.00**	**6,320**	1,350	2,146	**3,496**	**55.32**	**30,834**	2,045	3,221	**5,266**	**17.08**
Zamboanga City	**27,212**	0	0	**0**	**0.00**	**27,360**	0	0	**0**	**0.00**	**133,486**	0	0	**0**	**0.00**
**Region 10**	**151,518**	**7,595**	**9,266**	**16,861**	**11.13**	**147,848**	**6,383**	**7,428**	**13,811**	**9.34**	**761,187**	**17,079**	**21,411**	**38,490**	**5.06**
Bukidnon	**34,075**	568	745	**1,313**	**3.85**	**33,537**	514	760	**1,274**	**3.80**	**169,165**	746	1,478	**2,224**	**1.31**
Camiguin	**2,803**	83	151	**234**	**8.35**	**2,585**	91	121	**212**	**8.20**	**13,445**	215	420	**635**	**4.72**
Lanao del Norte	**23,400**	278	260	**538**	**2.30**	**22,746**	348	369	**717**	**3.15**	**106,990**	524	656	**1,180**	**1.10**
Misamis Occidental	**10,121**	2,773	3,398	**6,171**	**60.97**	**9,674**	2,932	3,343	**6,275**	**64.87**	**51,014**	9,542	11,303	**20,845**	**40.86**
Misamis Oriental	**22,248**	367	339	**706**	**3.17**	**21,830**	440	395	**835**	**3.83**	**120,407**	796	1,007	**1,803**	**1.50**
Cagayan de Oro City	**21,071**	234	302	**536**	**2.54**	**20,677**	0	0	**0**	**0.00**	**114,046**	328	375	**703**	**0.62**
El Salvador City	**1,565**	19	16	**35**	**2.24**	**1,536**	5	10	**15**	**0.98**	**8,470**	93	99	**192**	**2.27**
Gingoog City	**3,886**	971	914	**1,885**	**48.51**	**3,813**	648	703	**1,351**	**35.43**	**21,031**	423	412	**835**	**3.97**
Iligan City	**11,853**	53	74	**127**	**1.07**	**11,522**	47	85	**132**	**1.15**	**54,194**	280	456	**736**	**1.36**
Malaybalay City	**5,679**	125	149	**274**	**4.82**	**5,589**	170	250	**420**	**7.51**	**28,199**	381	804	**1,185**	**4.20**
Oroquieta City	**2,193**	7	9	**16**	**0.73**	**2,096**	6	7	**13**	**0.62**	**11,056**	57	46	**103**	**0.93**
Ozamis City	**4,394**	861	1,305	**2,166**	**49.29**	**4,200**	64	194	**258**	**6.14**	**22,157**	1,395	1,933	**3,328**	**15.02**
Tangub City	**1,952**	227	194	**421**	**21.56**	**1,866**	578	495	**1,073**	**57.50**	**9,846**	1,356	1,044	**2,400**	**24.38**
Valencia City	**6,278**	1,029	1,410	**2,439**	**38.85**	**6,177**	540	696	**1,236**	**20.01**	**31,165**	943	1,378	**2,321**	**7.45**
**Region 11**	**160,882**	**1,730**	**2,068**	**3,798**	**2.36**	**150,293**	**1,586**	**1,869**	**3,455**	**2.30**	**810,996**	**13,698**	**18,079**	**31,777**	**3.92**
Compostela Valley	**23,867**	266	347	**613**	**2.57**	**21,910**	202	279	**481**	**2.20**	**118,249**	1,877	2,980	**4,857**	**4.11**
Davao del Norte	**31,481**	357	402	**759**	**2.41**	**29,395**	327	363	**690**	**2.35**	**163,812**	3,870	4,124	**7,994**	**4.88**
Davao Oriental	**19,418**	187	241	**428**	**2.20**	**17,912**	180	226	**406**	**2.27**	**86,534**	1,579	2,178	**3,757**	**4.34**
Davao del Sur	**20,954**	218	246	**464**	**2.21**	**19,941**	198	232	**430**	**2.16**	**109,531**	1,356	1,654	**3,010**	**2.75**
Davao Occidental	**11,063**	114	142	**256**	**2.31**	**9,657**	83	113	**196**	**2.03**	**50,127**	752	866	**1,618**	**3.23**
Davao City	**54,100**	588	690	**1,278**	**2.36**	**51,477**	596	656	**1,252**	**2.43**	**282,744**	4,264	6,277	**10,541**	**3.73**
**Region 12**	**154,587**	**6,264**	**6,459**	**12,723**	**8.23**	**146,246**	**5,702**	**6,694**	**12,396**	**8.48**	**746,014**	**9,923**	**17,075**	**26,998**	**3.62**
North Cotabato	**48,632**	4,289	4,013	**8,302**	**17.07**	**45,592**	3,725	3,714	**7,439**	**16.32**	**227,065**	5,932	6,939	**12,871**	**5.67**
Sarangani	**19,194**	488	502	**990**	**5.16**	**18,314**	419	574	**993**	**5.42**	**86,020**	624	1,273	**1,897**	**2.21**
South Cotabato	**29,890**	452	506	**958**	**3.21**	**28,739**	666	866	**1,532**	**5.33**	**154,965**	576	1,245	**1,821**	**1.18**
Sultan Kudarat	**27,120**	644	1,120	**1,764**	**6.50**	**25,503**	529	884	**1,413**	**5.54**	**130,478**	1,310	2,612	**3,922**	**3.01**
Cotabato City	**10,339**	306	210	**516**	**4.99**	**9,435**	256	265	**521**	**5.52**	**46,843**	298	331	**629**	**1.34**
Gen. Santos City	**19,412**	85	108	**193**	**0.99**	**18,664**	107	391	**498**	**2.67**	**100,643**	1,183	4,675	**5,858**	**5.82**

Table 1.D.4 - Oral Health Care

Number and proportion of adolescents 10-19 years old and adults 20 -59 years old who received Basic Oral Health Care (BOHC)
Philippines, Annual 2020

Area	Adolescents 10-14 years old who received BOHC					Adolescents 15-19 years old who received BOHC					Adults 20-59 years old who received BOHC				
	Eligible Pop. (10 - 14 yrs. old x 30%)	Male	Female	Total	%	Eligible Pop. (15 - 19 yrs. old x 30%)	Male	Female	Total	%	Eligible Pop. (20 - 59 yrs. old x 30%)	Male	Female	Total	%
**BARMM**	**153,776**	**3,112**	**3,524**	**6,636**	**4.32**	**130,266**	**3,059**	**3,104**	**6,163**	**4.73**	**576,476**	**8,197**	**8,464**	**16,661**	**2.89**
Basilan	**11,174**	0	0	**0**	**0.00**	**9,664**	0	0	**0**	**0.00**	**40,725**	0	0	**0**	**0.00**
Lanao del Sur	**33,528**	203	237	**440**	**1.31**	**28,430**	317	360	**677**	**2.38**	**127,275**	3,622	3,905	**7,527**	**5.91**
Maguindanao	**48,295**	2,164	2,470	**4,634**	**9.60**	**42,049**	2,165	2,136	**4,301**	**10.23**	**182,855**	3,980	3,868	**7,848**	**4.29**
Sulu	**34,672**	602	618	**1,220**	**3.52**	**27,797**	569	597	**1,166**	**4.19**	**125,722**	440	406	**846**	**0.67**
Tawi-Tawi	**15,013**	0	0	**0**	**0.00**	**12,870**	0	0	**0**	**0.00**	**58,253**	0	0	**0**	**0.00**
Lamitan City	**3,074**	0	0	**0**	**0.00**	**2,658**	0	0	**0**	**0.00**	**11,206**	0	0	**0**	**0.00**
Marawi City	**8,020**	143	199	**342**	**4.26**	**6,799**	8	11	**19**	**0.28**	**30,440**	155	285	**440**	**1.45**
**CARAGA**	**87,394**	**4,960**	**5,230**	**10,190**	**11.66**	**80,113**	**3,663**	**4,316**	**7,979**	**9.96**	**401,210**	**5,875**	**11,054**	**16,929**	**4.22**
Agusan del Norte	**11,532**	187	208	**395**	**3.43**	**11,101**	218	263	**481**	**4.33**	**56,262**	472	1,298	**1,770**	**3.15**
Agusan del Sur	**24,174**	748	777	**1,525**	**6.31**	**21,371**	1,338	1,294	**2,632**	**12.32**	**105,047**	1,377	1,992	**3,369**	**3.21**
Surigao del Norte	**11,546**	163	200	**363**	**3.14**	**10,468**	168	228	**396**	**3.78**	**52,994**	414	538	**952**	**1.80**
Surigao del Sur	**16,475**	2,013	2,079	**4,092**	**24.84**	**15,253**	867	1,086	**1,953**	**12.80**	**75,427**	1,331	2,458	**3,789**	**5.02**
Province of Dinagat	**4,196**	281	279	**560**	**13.35**	**3,589**	219	328	**547**	**15.24**	**18,979**	539	1,060	**1,599**	**8.43**
Bislig City	**3,130**	202	283	**485**	**15.50**	**2,897**	144	186	**330**	**11.39**	**14,327**	459	715	**1,174**	**8.19**
Butuan City	**10,965**	1,042	1,131	**2,173**	**19.82**	**10,559**	498	715	**1,213**	**11.49**	**53,495**	852	2,510	**3,362**	**6.28**
Surigao City	**5,377**	324	273	**597**	**11.10**	**4,875**	211	216	**427**	**8.76**	**24,680**	431	483	**914**	**3.70**

Note: Put asterisk (*) for No Report and Zero (0) for No Case

1.D.5 - Oral Health Care

Number and proportion of senior citizens 60 years old and above who received Basic Oral Health Care (BOHC)

Philippines, Annual 2020

Area	Senior citizens 60 years old and above who received BOHC				
	Eligible Pop. 60 yrs. old and above x 30%)	Male	Female	Total	%
**PHILIPPINES**	**2,840,531**	**131,197**	**131,364**	**262,561**	**9.24**
**N C R**	**362,789**	**16,023**	**20,369**	**36,392**	**10.03**
Malabon	**10,298**	680	775	**1,455**	**14.13**
Navotas	**7,028**	1,501	1,679	**3,180**	**45.25**
Valenzuela City	**17,478**	1,196	1,713	**2,909**	**16.64**
Caloocan City	**44,625**	733	905	**1,638**	**3.67**
Marikina City	**12,698**	597	732	**1,329**	**10.47**
Pasig City	**21,279**	1,546	1,563	**3,109**	**14.61**
Pateros	**1,799**	34	66	**100**	**5.56**
Taguig	**22,676**	105	118	**223**	**0.98**
Quezon City	**82,719**	3,981	5,408	**9,389**	**11.35**
Makati City	**16,415**	684	910	**1,594**	**9.71**
Mandaluyong City	**10,883**	375	565	**940**	**8.64**
San Juan	**3,442**	566	667	**1,233**	**35.82**
Manila City	**50,152**	1,236	1,550	**2,786**	**5.56**
Las Piñas City	**16,592**	676	1,062	**1,738**	**10.48**
Muntinlupa City	**14,214**	1,314	1,778	**3,092**	**21.75**
Parañaque City	**18,758**	417	425	**842**	**4.49**
Pasay City	**11,735**	382	453	**835**	**7.12**
**C A R**	**45,497**	**3,237**	**5,056**	**8,293**	**18.23**
Abra	**8,024**	464	619	**1,083**	**13.50**
Apayao	**3,043**	155	243	**398**	**13.08**
Benguet	**11,343**	1,601	2,956	**4,557**	**40.17**
Ifugao	**4,782**	287	404	**691**	**14.45**
Kalinga	**5,287**	338	412	**750**	**14.19**
Mt. Province	**4,239**	155	161	**316**	**7.45**
Baguio City	**8,779**	237	261	**498**	**5.67**
**Region 1**	**168,802**	**10,678**	**11,331**	**22,009**	**13.04**
Ilocos Norte	**18,529**	1,125	668	**1,793**	**9.68**
Ilocos Sur	**21,746**	3,688	3,802	**7,490**	**34.44**
La Union	**23,827**	211	290	**501**	**2.10**
Pangasinan	**73,661**	4,419	4,985	**9,404**	**12.77**
Alaminos City	**2,783**	592	714	**1,306**	**46.92**
Candon City	**2,292**	16	53	**69**	**3.01**
Dagupan City	**5,314**	84	108	**192**	**3.61**
Laoag City	**4,271**	78	85	**163**	**3.82**
San Carlos City	**5,850**	285	412	**697**	**11.91**
San Fernando City	**4,367**	89	116	**205**	**4.69**
Urdaneta City	**4,124**	11	24	**35**	**0.85**

1.D.5 - Oral Health Care

Number and proportion of senior citizens 60 years old and above who received Basic Oral Health Care (BOHC)

Philippines, Annual 2020

Area	Senior citizens 60 years old and above who received BOHC				
	Eligible Pop. 60 yrs. old and above x 30%)	Male	Female	Total	%
Vigan City	**2,038**	80	74	**154**	**7.56**
**Region 2**	**105,683**	**7,245**	**10,282**	**17,527**	**16.58**
Batanes	**619**	47	72	**119**	**19.23**
Cagayan	**30,144**	1,235	1,599	**2,834**	**9.40**
Isabela	**39,264**	1,616	2,419	**4,035**	**10.28**
Nueva Vizcaya	**13,401**	1,260	1,826	**3,086**	**23.03**
Quirino	**5,035**	28	41	**69**	**1.37**
Cauayan City	**3,857**	513	626	**1,139**	**29.53**
Ilagan City	**4,335**	940	1,321	**2,261**	**52.16**
Santiago City	**4,015**	1,584	2,351	**3,935**	**98.01**
Tuguegarao City	**5,013**	22	27	**49**	**0.98**
**Region 3**	**342,735**	**6,251**	**8,473**	**14,724**	**4.30**
Aurora	**5,651**	249	274	**523**	**9.26**
Bataan	**20,082**	120	171	**291**	**1.45**
Bulacan	**67,570**	421	563	**984**	**1.46**
Nueva Ecija	**46,853**	361	873	**1,234**	**2.63**
Pampanga	**49,988**	219	241	**460**	**0.92**
Tarlac	**31,945**	1,498	1,686	**3,184**	**9.97**
Zambales	**18,019**	274	284	**558**	**3.10**
Angeles City	**12,541**	116	122	**238**	**1.90**
Balanga City	**2,903**	45	28	**73**	**2.52**
Cabanatuan City	**9,588**	39	202	**241**	**2.51**
City of San Fernando	**9,343**	33	25	**58**	**0.62**
Gapan City	**3,500**	3	3	**6**	**0.17**
Mabalacat City	**7,641**	53	53	**106**	**1.39**
Malolos City	**7,547**	73	110	**183**	**2.42**
Meycauayan	**6,260**	161	258	**419**	**6.69**
Olongapo	**7,108**	7	10	**17**	**0.24**
Palayan City	**1,302**	11	14	**25**	**1.92**
San Jose City	**4,434**	181	226	**407**	**9.18**
San Jose del Monte City	**17,188**	1,936	2,785	**4,721**	**27.47**
Science City of Munoz	**2,586**	38	78	**116**	**4.49**
Tarlac City	**10,689**	413	467	**880**	**8.23**
**Region 4A**	**405,801**	**54,752**	**35,664**	**90,416**	**22.28**
Batangas	**57,502**	7,260	7,212	**14,472**	**25.17**
Cavite	**38,543**	447	612	**1,059**	**2.75**
Laguna	**27,375**	24,510	12,464	**36,974**	**135.07**
Quezon	**49,161**	1,679	2,064	**3,743**	**7.61**
Rizal	**55,527**	618	726	**1,344**	**2.42**
Antipolo City	**20,452**	0	0	**0**	**0.00**
Bacoor City	**16,886**	177	257	**434**	**2.57**
Batangas City	**10,205**	133	205	**338**	**3.31**

1.D.5 - Oral Health Care

Number and proportion of senior citizens 60 years old and above who received Basic Oral Health Care (BOHC)

Philippines, Annual 2020

Area	Senior citizens 60 years old and above who received BOHC				
	Eligible Pop. 60 yrs. old and above x 30%)	Male	Female	Total	%
Biñan City	**9,178**	358	593	**951**	**10.36**
Cabuyao City	**8,510**	0	0	**0**	**0.00**
Calamba City	**12,525**	16,757	7,136	**23,893**	**190.77**
Cavite City	**2,890**	59	99	**158**	**5.47**
Dasmariñas City	**18,530**	18	15	**33**	**0.18**
General Trias City	**8,836**	342	613	**955**	**10.81**
Imus City	**11,354**	168	286	**454**	**4.00**
Lipa City	**10,282**	771	919	**1,690**	**16.44**
Lucena City	**7,451**	39	28	**67**	**0.90**
San Pablo City	**7,333**	276	1,292	**1,568**	**21.38**
San Pedro City	**8,979**	0	0	**0**	**0.00**
Santa Rosa City	**9,749**	84	94	**178**	**1.83**
Tagaytay City	**2,002**	478	438	**916**	**45.76**
Tanauan City	**5,363**	87	107	**194**	**3.62**
Tayabas City	**2,792**	95	104	**199**	**7.13**
Trece Martires City	**4,379**	396	400	**796**	**18.18**
**Region 4B**	**77,929**	**635**	**797**	**1,432**	**1.84**
Marinduque	**7,955**	171	222	**393**	**4.94**
Mindoro Occidental	**11,754**	66	69	**135**	**1.15**
Mindoro Oriental	**22,762**	296	328	**624**	**2.74**
Palawan	**20,262**	18	27	**45**	**0.22**
Romblon	**9,110**	1	4	**5**	**0.05**
Puerto Princesa City	**6,086**	83	147	**230**	**3.78**
**Region 5**	**157,121**	**4,218**	**7,170**	**11,388**	**7.25**
Albay	**33,269**	1,233	2,297	**3,530**	**10.61**
Camarines Norte	**15,608**	411	617	**1,028**	**6.59**
Camarines Sur	**43,277**	554	1,209	**1,763**	**4.07**
Catanduanes	**7,344**	528	771	**1,299**	**17.69**
Masbate	**21,815**	781	1,161	**1,942**	**8.90**
Sorsogon	**21,858**	608	928	**1,536**	**7.03**
Iriga City	**2,941**	44	99	**143**	**4.86**
Legaspi City	**5,851**	10	22	**32**	**0.55**
Naga City	**5,158**	49	66	**115**	**2.23**
**Region 6**	**246,448**	**6,569**	**9,036**	**15,605**	**6.33**
Aklan	**18,778**	137	228	**365**	**1.94**
Antique	**18,222**	75	92	**167**	**0.92**
Capiz	**26,300**	677	958	**1,635**	**6.22**
Guimaras	**5,864**	100	136	**236**	**4.02**
Iloilo	**67,786**	4,029	5,522	**9,551**	**14.09**
Negros Occidental	**76,587**	952	1,262	**2,214**	**2.89**
Bacolod City	**17,231**	323	500	**823**	**4.78**
Iloilo City	**15,680**	276	338	**614**	**3.92**

1.D.5 - Oral Health Care

Number and proportion of senior citizens 60 years old and above who received Basic Oral Health Care (BOHC)

Philippines, Annual 2020

Area	Senior citizens 60 years old and above who received BOHC				
	Eligible Pop. 60 yrs. old and above x 30%)	Male	Female	Total	%
**Region 7**	**223,830**	**2,695**	**3,340**	**6,035**	**2.70**
Bohol	**44,302**	1,879	2,228	**4,107**	**9.27**
Cebu	**84,246**	165	244	**409**	**0.49**
Negros Oriental	**42,825**	273	345	**618**	**1.44**
Siquijor	**3,915**	79	115	**194**	**4.95**
Cebu City	**26,448**	175	285	**460**	**1.74**
Lapu-Lapu City	**11,699**	124	123	**247**	**2.11**
Mandaue City	**10,395**	0	0	**0**	**0.00**
**Region 8**	**130,928**	**1,723**	**2,299**	**4,022**	**3.07**
Biliran	**5,199**	39	78	**117**	**2.25**
Eastern Samar	**14,186**	238	290	**528**	**3.72**
Northern Leyte	**46,393**	532	731	**1,263**	**2.72**
Northern Samar	**15,457**	85	161	**246**	**1.59**
Southern Leyte	**12,069**	44	45	**89**	**0.74**
Western Samar	**15,674**	404	488	**892**	**5.69**
Calbayog City	**4,829**	103	157	**260**	**5.38**
Maasin City	**3,069**	8	13	**21**	**0.68**
Ormoc City	**6,610**	265	332	**597**	**9.03**
Tacloban City	**7,441**	5	4	**9**	**0.12**
**Region 9**	**90,719**	**1,211**	**1,596**	**2,807**	**3.09**
Zamboanga del Norte	**21,677**	153	226	**379**	**1.75**
Zamboanga del Sur	**19,996**	676	906	**1,582**	**7.91**
Zamboanga Sibugay	**14,395**	159	225	**384**	**2.67**
Dapitan City	**2,239**	13	39	**52**	**2.32**
Dipolog City	**3,550**	4	1	**5**	**0.14**
Isabela City	**2,722**	40	32	**72**	**2.65**
Pagadian City	**4,906**	166	167	**333**	**6.79**
Zamboanga City	**21,233**	0	0	**0**	**0.00**
**Region 10**	**124,872**	**3,949**	**5,003**	**8,952**	**7.17**
Bukidnon	**24,983**	295	433	**728**	**2.91**
Camiguin	**3,056**	18	25	**43**	**1.41**
Lanao del Norte	**15,584**	101	392	**493**	**3.16**
Misamis Occidental	**10,811**	1,381	1,764	**3,145**	**29.09**
Misamis Oriental	**20,365**	296	434	**730**	**3.58**
Cagayan de Oro City	**19,295**	104	131	**235**	**1.22**
El Salvador City	**1,434**	10	27	**37**	**2.58**
Gingoog City	**3,559**	460	485	**945**	**26.55**
Iligan City	**7,893**	31	32	**63**	**0.80**
Malaybalay City	**4,166**	45	46	**91**	**2.18**

1.D.5 - Oral Health Care

Number and proportion of senior citizens 60 years old and above who received Basic Oral Health Care (BOHC)

Philippines, Annual 2020

Area	Senior citizens 60 years old and above who received BOHC				
	Eligible Pop. 60 yrs. old and above x 30%)	Male	Female	Total	%
Oroquieta City	**2,343**	12	11	**23**	**0.98**
Ozamis City	**4,694**	438	464	**902**	**19.22**
Tangub City	**2,085**	236	201	**437**	**20.96**
Valencia City	**4,603**	522	558	**1,080**	**23.46**
**Region 11**	**131,565**	**698**	**859**	**1,557**	**1.18**
Compostela Valley	**18,168**	97	123	**220**	**1.21**
Davao del Norte	**26,742**	150	163	**313**	**1.17**
Davao Oriental	**15,172**	70	98	**168**	**1.11**
Davao del Sur	**17,774**	90	119	**209**	**1.18**
Davao Occidental	**7,825**	39	56	**95**	**1.21**
Davao City	**45,884**	252	300	**552**	**1.20**
**Region 12**	**104,805**	**2,638**	**3,259**	**5,897**	**5.63**
North Cotabato	**33,509**	1,525	1,493	**3,018**	**9.01**
Sarangani	**11,355**	217	323	**540**	**4.76**
South Cotabato	**22,430**	192	217	**409**	**1.82**
Sultan Kudarat	**17,516**	311	461	**772**	**4.41**
Cotabato City	**5,428**	100	219	**319**	**5.88**
Gen. Santos City	**14,567**	293	546	**839**	**5.76**
**BARMM**	**51,785**	**6,553**	**3,998**	**10,551**	**20.37**
Basilan	**4,102**	0	0	**0**	**0.00**
Lanao del Sur	**10,430**	3,872	1,031	**4,903**	**47.01**
Maguindanao	**16,406**	1,402	1,701	**3,103**	**18.91**
Sulu	**11,576**	1,068	1,053	**2,121**	**18.32**
Tawi-Tawi	**5,648**	211	213	**424**	**7.51**
Lamitan City	**1,129**	0	0	**0**	**0.00**
Marawi City	**2,495**	0	0	**0**	**0.00**
**CARAGA**	**69,222**	**2,122**	**2,832**	**4,954**	**7.16**
Agusan del Norte	**9,793**	176	267	**443**	**4.52**
Agusan del Sur	**15,109**	546	690	**1,236**	**8.18**
Surigao del Norte	**9,886**	143	186	**329**	**3.33**
Surigao del Sur	**13,847**	292	482	**774**	**5.59**
Province of Dinagat	**4,048**	288	397	**685**	**16.92**
Bislig City	**2,629**	332	330	**662**	**25.18**
Butuan City	**9,309**	232	346	**578**	**6.21**
Surigao City	**4,601**	113	134	**247**	**5.37**

Note: Put asterisk (*) for No Report and Zero (0) for No Case

Table 1.D.6 - Oral Health Care

Number and proportion of pregnant women 10-14, 15-19 and 20-49 years old who received Basic Oral Health Care (BOHC)
Philippines, Annual 2020

Area	Eligible Pop.	Pregnant Women 10-14 y/o received BOHC		Pregnant Women 15-19 y/o received BOHC		Pregnant Women 20-49 y/o received BOHC	
		No.	%	No.	%	No.	%
**PHILIPPINES**	**2,123,158**	**28,949**	**1.36**	**45,024**	**2.12**	**193,335**	**9.11**
**N C R**	**238,661**	**370**	**0.16**	**8,285**	**3.47**	**42,916**	**17.98**
Malabon	6,775	72	1.06	2,157	31.84	3,636	53.67
Navotas	4,621	6	0.13	123	2.66	325	7.03
Valenzuela City	11,500	19	0.17	564	4.90	2,764	24.03
Caloocan City	29,363	40	0.14	679	2.31	1,694	5.77
Marikina City	8,354	8	0.10	192	2.30	1,378	16.50
Pasig City	13,996	0	0.00	205	1.46	1,092	7.80
Pateros	1,184	0	0.00	49	4.14	93	7.85
Taguig	14,918	5	0.03	21	0.14	97	0.65
Quezon City	54,413	17	0.03	1,518	2.79	11,927	21.92
Makati City	10,798	22	0.20	533	4.94	4,608	42.67
Mandaluyong City	7,160	22	0.31	367	5.13	2,572	35.92
San Juan	2,261	1	0.04	41	1.81	367	16.23
Manila City	32,980	57	0.17	492	1.49	2,604	7.90
Las Piñas City	10,914	4	0.04	280	2.57	1,696	15.54
Muntinlupa City	9,353	7	0.07	585	6.25	3,872	41.40
Parañaque City	12,343	63	0.51	355	2.88	1,237	10.02
Pasay City	7,728	27	0.35	124	1.60	2,954	38.22
**C A R**	**35,099**	**91**	**0.26**	**839**	**2.39**	**6,035**	**17.19**
Abra	4,309	5	0.12	129	2.99	989	22.95
Apayao	2,451	1	0.04	69	2.82	473	19.30
Benguet	9,188	24	0.26	261	2.84	1,547	16.84
Ifugao	4,396	0	0.00	41	0.93	595	13.54
Kalinga	4,617	59	1.28	94	2.04	739	16.01
Mt. Province	3,023	1	0.03	15	0.50	222	7.34
Baguio City	7,115	1	0.01	230	3.23	1,470	20.66
**Region 1**	**97,261**	**76**	**0.08**	**1,743**	**1.79**	**14,202**	**14.60**
Ilocos Norte	8,105	36	0.44	131	1.62	717	8.85
Ilocos Sur	9,330	23	0.25	543	5.82	2,451	26.27
La Union	11,511	2	0.02	121	1.05	775	6.73
Pangasinan	50,168	13	0.03	696	1.39	7,496	14.94
Alaminos City	1,894	0	0.00	0	0.00	730	38.54
Candon City	987	0	0.00	0	0.00	109	11.04
Dagupan City	3,620	0	0.00	123	3.40	619	17.10
Laoag City	1,870	0	0.00	0	0.00	0	0.00
San Carlos City	3,979	0	0.00	61	1.53	880	22.12
San Fernando City	2,115	2	0.09	46	2.17	306	14.47
Urdaneta City	2,806	0	0.00	0	0.00	0	0.00

Table 1.D.6 - Oral Health Care

Number and proportion of pregnant women 10-14, 15-19 and 20-49 years old who received Basic Oral Health Care (BOHC)
Philippines, Annual 2020

Area	Eligible Pop.	Pregnant Women 10-14 y/o received BOHC		Pregnant Women 15-19 y/o received BOHC		Pregnant Women 20-49 y/o received BOHC	
		No.	%	No.	%	No.	%
Vigan City	876	0	0.00	22	2.51	119	13.58
**Region 2**	**69,443**	**117**	**0.17**	**2,782**	**4.01**	**14,170**	**20.41**
Batanes	340	0	0.00	6	1.76	32	9.41
Cagayan	17,971	5	0.03	733	4.08	2,003	11.15
Isabela	26,309	15	0.06	486	1.85	2,941	11.18
Nueva Vizcaya	9,573	20	0.21	437	4.56	2,544	26.57
Quirino	4,025	0	0.00	30	0.75	179	4.45
Cauayan City	2,590	4	0.15	262	10.12	1,507	58.19
Ilagan City	2,911	65	2.23	353	12.13	1,908	65.54
Santiago City	2,692	8	0.30	448	16.64	2,813	104.49
Tuguegarao City	3,032	0	0.00	27	0.89	243	8.01
**Region 3**	**220,020**	**564**	**0.26**	**4,945**	**2.25**	**19,692**	**8.95**
Aurora	4,770	5	0.10	165	3.46	783	16.42
Bataan	13,823	3	0.02	118	0.85	1,009	7.30
Bulacan	43,627	442	1.01	1,635	3.75	3,550	8.14
Nueva Ecija	28,782	14	0.05	456	1.58	1,841	6.40
Pampanga	31,359	3	0.01	148	0.47	1,057	3.37
Tarlac	19,765	40	0.20	556	2.81	3,361	17.00
Zambales	12,610	0	0.00	78	0.62	296	2.35
Angeles City	7,869	0	0.00	132	1.68	581	7.38
Balanga City	1,998	0	0.00	45	2.25	210	10.51
Cabanatuan City	5,896	0	0.00	122	2.07	214	3.63
City of San Fernando	5,863	0	0.00	0	0.00	0	0.00
Gapan City	2,153	0	0.00	1	0.05	20	0.93
Mabalacat City	4,793	2	0.04	15	0.31	78	1.63
Malolos City	4,880	8	0.16	119	2.44	460	9.43
Meycauayan	4,041	3	0.07	36	0.89	227	5.62
Olongapo	4,971	0	0.00	26	0.52	110	2.21
Palayan City	799	0	0.00	0	0.00	0	0.00
San Jose City	2,718	5	0.18	234	8.61	608	22.37
San Jose del Monte City	11,100	21	0.19	938	8.45	4,479	40.35
Science City of Munoz	1,586	0	0.00	29	1.83	170	10.72
Tarlac City	6,617	18	0.27	92	1.39	638	9.64
**Region 4A**	**296,816**	**23,612**	**7.96**	**39**	**0.01**	**1,174**	**0.40**
Batangas	38,441	5,298	13.78	0	0.00	595	1.55
Cavite	27,704	526	1.90	0	0.00	0	0.00
Laguna	19,727	467	2.37	0	0.00	0	0.00
Quezon	37,410	5,680	15.18	39	0.10	579	1.55
Rizal	44,791	4,288	9.57	0	0.00	0	0.00
Antipolo City	16,497	0	0.00	0	0.00	0	0.00
Bacoor City	12,142	291	2.40	0	0.00	0	0.00
Batangas City	6,823	650	9.53	0	0.00	0	0.00

Table 1.D.6 - Oral Health Care

Number and proportion of pregnant women 10-14, 15-19 and 20-49 years old who received Basic Oral Health Care (BOHC)
Philippines, Annual 2020

Area	Eligible Pop.	Pregnant Women 10-14 y/o received BOHC		Pregnant Women 15-19 y/o received BOHC		Pregnant Women 20-49 y/o received BOHC	
		No.	%	No.	%	No.	%
Biñan City	6,607	914	13.83	0	0.00	0	0.00
Cabuyao City	6,130	0	0.00	0	0.00	0	0.00
Calamba City	9,028	267	2.96	0	0.00	0	0.00
Cavite City	2,075	159	7.66	0	0.00	0	0.00
Dasmariñas City	13,322	0	0.00	0	0.00	0	0.00
General Trias City	6,352	248	3.90	0	0.00	0	0.00
Imus City	8,156	1,013	12.42	0	0.00	0	0.00
Lipa City	6,880	648	9.42	0	0.00	0	0.00
Lucena City	5,672	248	4.37	0	0.00	0	0.00
San Pablo City	5,277	0	0.00	0	0.00	0	0.00
San Pedro City	6,467	0	0.00	0	0.00	0	0.00
Santa Rosa City	7,025	551	7.84	0	0.00	0	0.00
Tagaytay City	1,437	418	29.09	0	0.00	0	0.00
Tanauan City	3,584	142	3.96	0	0.00	0	0.00
Tayabas City	2,122	1,252	59.00	0	0.00	0	0.00
Trece Martires City	3,147	552	17.54	0	0.00	0	0.00
**Region 4B**	**71,246**	**0**	**0.00**	**121**	**0.17**	**2,710**	**3.80**
Marinduque	4,836	0	0.00	53	1.10	398	8.23
Mindoro Occidental	12,407	0	0.00	31	0.25	189	1.52
Mindoro Oriental	19,809	0	0.00	2	0.01	1,942	9.80
Palawan	21,375	0	0.00	0	0.00	0	0.00
Romblon	6,400	0	0.00	1	0.02	0	0.00
Puerto Princesa City	6,419	0	0.00	34	0.53	181	2.82
**Region 5**	**136,116**	**686**	**0.50**	**4,659**	**3.42**	**11,114**	**8.17**
Albay	23,737	56	0.24	410	1.73	1,081	4.55
Camarines Norte	14,283	129	0.90	780	5.46	1,783	12.48
Camarines Sur	38,796	147	0.38	531	1.37	1,208	3.11
Catanduanes	6,274	13	0.21	295	4.70	836	13.32
Masbate	22,521	118	0.52	997	4.43	1,905	8.46
Sorsogon	19,065	223	1.17	1,597	8.38	4,029	21.13
Iriga City	2,638	0	0.00	19	0.72	130	4.93
Legaspi City	4,174	0	0.00	27	0.65	122	2.92
Naga City	4,628	0	0.00	3	0.06	20	0.43
**Region 6**	**146,526**	**147**	**0.10**	**2,462**	**1.68**	**13,862**	**9.46**
Aklan	11,162	0	0.00	85	0.76	533	4.78
Antique	12,816	1	0.01	0	0.00	457	3.57
Capiz	13,986	88	0.63	456	3.26	1,639	11.72
Guimaras	3,085	5	0.16	22	0.71	121	3.92
Iloilo	36,541	24	0.07	882	2.41	5,022	13.74
Negros Occidental	49,368	29	0.06	705	1.43	3,563	7.22
Bacolod City	11,115	0	0.00	312	2.81	1,278	11.50
Iloilo City	8,453	0	0.00	0	0.00	1,249	14.78

Table 1.D.6 - Oral Health Care

Number and proportion of pregnant women 10-14, 15-19 and 20-49 years old who received Basic Oral Health Care (BOHC)
Philippines, Annual 2020

Area	Eligible Pop.	Pregnant Women 10-14 y/o received BOHC		Pregnant Women 15-19 y/o received BOHC		Pregnant Women 20-49 y/o received BOHC	
		No.	%	No.	%	No.	%
**Region 7**	**163,262**	**154**	**0.09**	**894**	**0.55**	**5,235**	**3.21**
Bohol	27,312	0	0.00	437	1.60	1,149	4.21
Cebu	67,506	0	0.00	107	0.16	999	1.48
Negros Oriental	27,938	3	0.01	350	1.25	631	2.26
Siquijor	1,613	151	9.36	0	0.00	55	3.41
Cebu City	21,193	0	0.00	0	0.00	2,395	11.30
Lapu-Lapu City	9,372	0	0.00	0	0.00	6	0.06
Mandaue City	8,328	0	0.00	0	0.00	0	0.00
**Region 8**	**102,619**	**36**	**0.04**	**820**	**0.80**	**9,399**	**9.16**
Biliran	3,834	3	0.08	84	2.19	428	11.16
Eastern Samar	11,392	29	0.25	71	0.62	1,018	8.94
Northern Leyte	34,707	0	0.00	472	1.36	4,026	11.60
Northern Samar	15,370	4	0.03	128	0.83	915	5.95
Southern Leyte	6,451	0	0.00	0	0.00	82	1.27
Western Samar	14,305	0	0.00	0	0.00	203	1.42
Calbayog City	4,413	0	0.00	0	0.00	229	5.19
Maasin City	1,637	0	0.00	7	0.43	61	3.73
Ormoc City	4,941	0	0.00	0	0.00	2,081	42.12
Tacloban City	5,569	0	0.00	58	1.04	356	6.39
**Region 9**	**80,051**	**1,637**	**2.04**	**598**	**0.75**	**2,476**	**3.09**
Zamboanga del Norte	17,249	1,499	8.69	34	0.20	215	1.25
Zamboanga del Sur	17,653	78	0.44	394	2.23	1,791	10.15
Zamboanga Sibugay	14,954	4	0.03	86	0.58	313	2.09
Dapitan City	1,784	0	0.00	0	0.00	17	0.95
Dipolog City	2,827	0	0.00	6	0.21	6	0.21
Isabela City	2,522	0	0.00	0	0.00	0	0.00
Pagadian City	4,325	56	1.29	78	1.80	134	3.10
Zamboanga City	18,737	0	0.00	0	0.00	0	0.00
**Region 10**	**101,411**	**129**	**0.13**	**3,445**	**3.40**	**10,460**	**10.31**
Bukidnon	23,706	34	0.14	374	1.58	1,145	4.83
Camiguin	1,858	0	0.00	32	1.72	172	9.26
Lanao del Norte	14,960	9	0.06	143	0.96	569	3.80
Misamis Occidental	6,403	12	0.19	888	13.87	2,186	34.14
Misamis Oriental	15,131	35	0.23	303	2.00	1,815	12.00
Cagayan de Oro City	14,339	0	0.00	111	0.77	331	2.31
El Salvador City	1,065	26	2.44	66	6.20	215	20.19
Gingoog City	2,644	0	0.00	227	8.59	354	13.39
Iligan City	7,580	0	0.00	1	0.01	0	0.00
Malaybalay City	3,956	13	0.33	170	4.30	848	21.44

Table 1.D.6 - Oral Health Care

Number and proportion of pregnant women 10-14, 15-19 and 20-49 years old who received Basic Oral Health Care (BOHC)
Philippines, Annual 2020

Area	Eligible Pop.	Pregnant Women 10-14 y/o received BOHC		Pregnant Women 15-19 y/o received BOHC		Pregnant Women 20-49 y/o received BOHC	
		No.	%	No.	%	No.	%
Oroquieta City	1,389	0	0.00	0	0.00	23	1.66
Ozamis City	2,778	0	0.00	124	4.46	970	34.92
Tangub City	1,234	0	0.00	87	7.05	625	50.65
Valencia City	4,368	0	0.00	919	21.04	1,207	27.63
**Region 11**	**107,247**	**238**	**0.22**	**6,685**	**6.23**	**13,406**	**12.50**
Compostela Valley	15,562	35	0.22	1,108	7.12	2,336	15.01
Davao del Norte	21,326	44	0.21	1,362	6.39	3,077	14.43
Davao Oriental	13,007	28	0.22	915	7.03	1,622	12.47
Davao del Sur	14,151	29	0.20	876	6.19	842	5.95
Davao Occidental	6,670	21	0.31	281	4.21	433	6.49
Davao City	36,531	81	0.22	2,143	5.87	5,096	13.95
**Region 12**	**104,552**	**850**	**0.81**	**4,392**	**4.20**	**15,300**	**14.63**
North Cotabato	33,645	315	0.94	1,256	3.73	2,077	6.17
Sarangani	12,891	13	0.10	394	3.06	1,642	12.74
South Cotabato	21,113	12	0.06	381	1.80	2,406	11.40
Sultan Kudarat	17,359	11	0.06	569	3.28	3,251	18.73
Cotabato City	5,835	469	8.04	857	14.69	1,326	22.72
Gen. Santos City	13,709	30	0.22	935	6.82	4,598	33.54
**BARMM**	**92,799**	**122**	**0.13**	**610**	**0.66**	**3,222**	**3.47**
Basilan	7,541	0	0.00	0	0.00	0	0.00
Lanao del Sur	21,131	46	0.22	195	0.92	733	3.47
Maguindanao	31,128	76	0.24	403	1.29	1,727	5.55
Sulu	16,613	0	0.00	0	0.00	345	2.08
Tawi-Tawi	9,259	0	0.00	0	0.00	313	3.38
Lamitan City	2,074	0	0.00	0	0.00	0	0.00
Marawi City	5,053	0	0.00	12	0.24	104	2.06
**CARAGA**	**60,029**	**120**	**0.20**	**1,705**	**2.84**	**7,962**	**13.26**
Agusan del Norte	8,098	10	0.12	116	1.43	481	5.94
Agusan del Sur	17,592	43	0.24	372	2.11	1,419	8.07
Surigao del Norte	7,089	10	0.14	83	1.17	509	7.18
Surigao del Sur	11,482	9	0.08	502	4.37	2,369	20.63
Province of Dinagat	2,573	2	0.08	121	4.70	947	36.81
Bislig City	2,179	10	0.46	157	7.21	297	13.63
Butuan City	7,715	4	0.05	133	1.72	798	10.34
Surigao City	3,301	32	0.97	221	6.69	1,142	34.60

Note: Put asterisk (*) for No Report and Zero (0) for No Case

Figure 1.D.1 - Percentage Distribution of children 12-59 months old who are orally fit upon oral examination by Region
Philippines, 2020

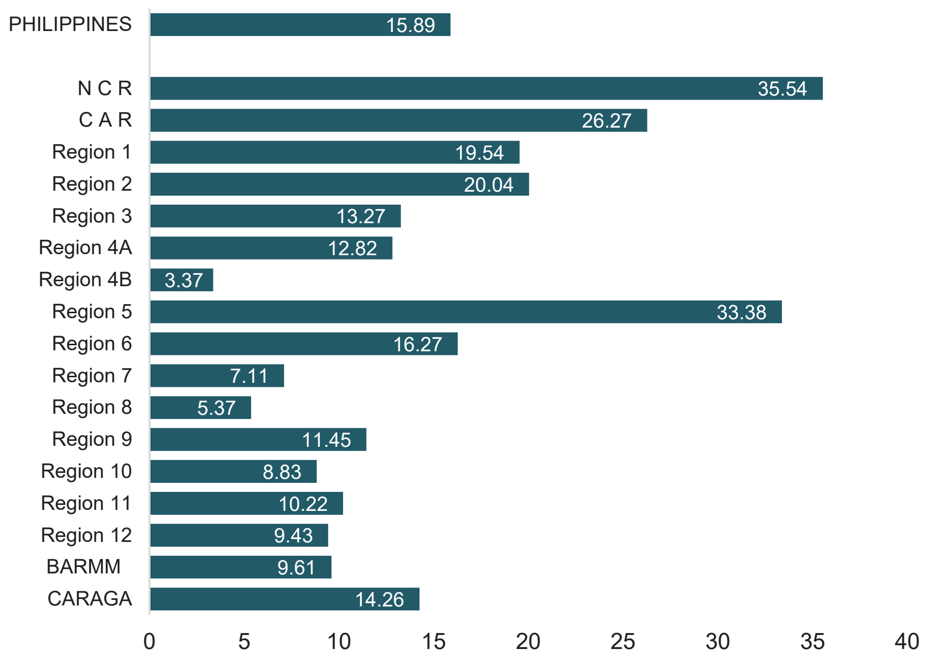

Figure 1.D.2 - Percentage Distribution of children 12-59 months old who are orally fit upon oral examination or after oral rehabilitation by sex Philippines, 2020

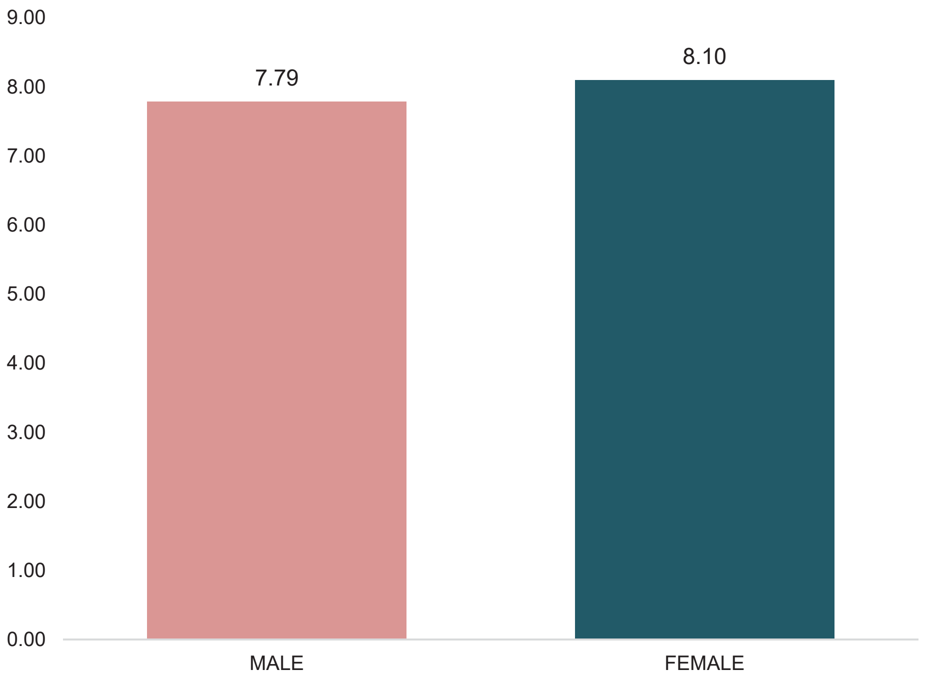

Figure 1.D.3 - Percentage Distribution of children 5 years old and above with cases of decayed, missing, filled teeth (DMFT) by Region Philippines, 2020

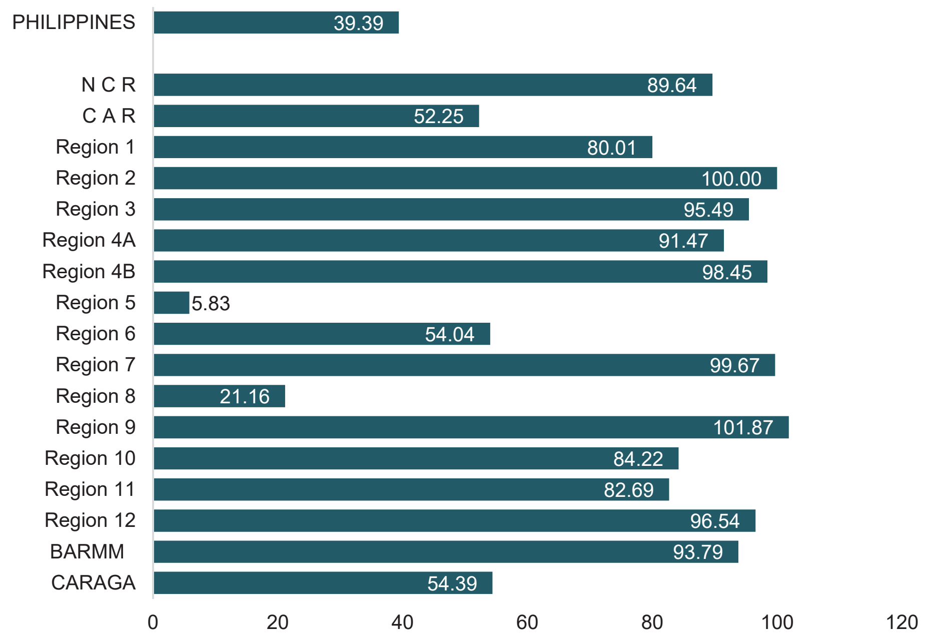

Figure 1.D.4 - Percentage Distribution of children 5 years old and above with cases of decayed, missing, filled teeth (DMFT) by sex and Region Philippines, 2020

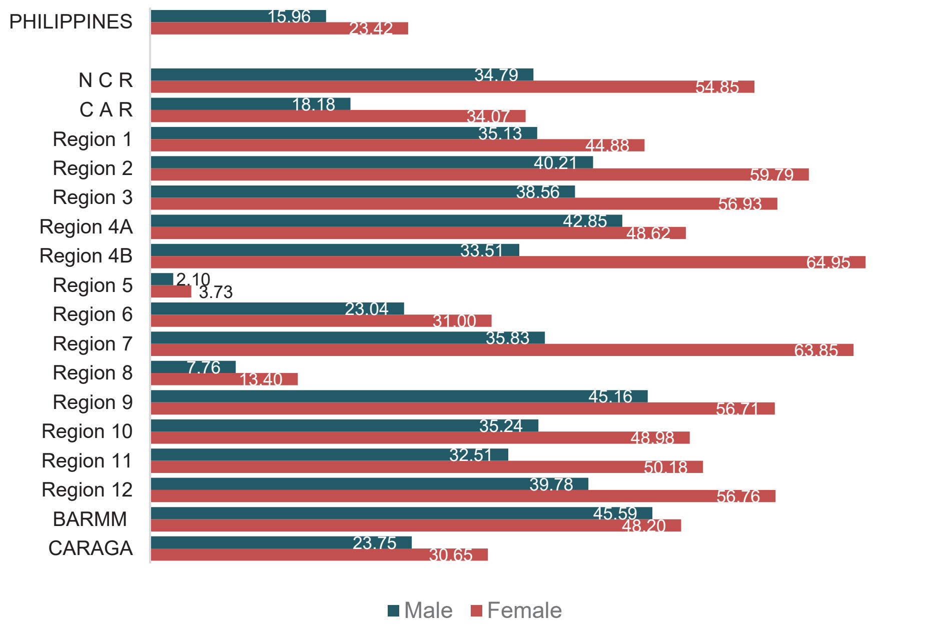

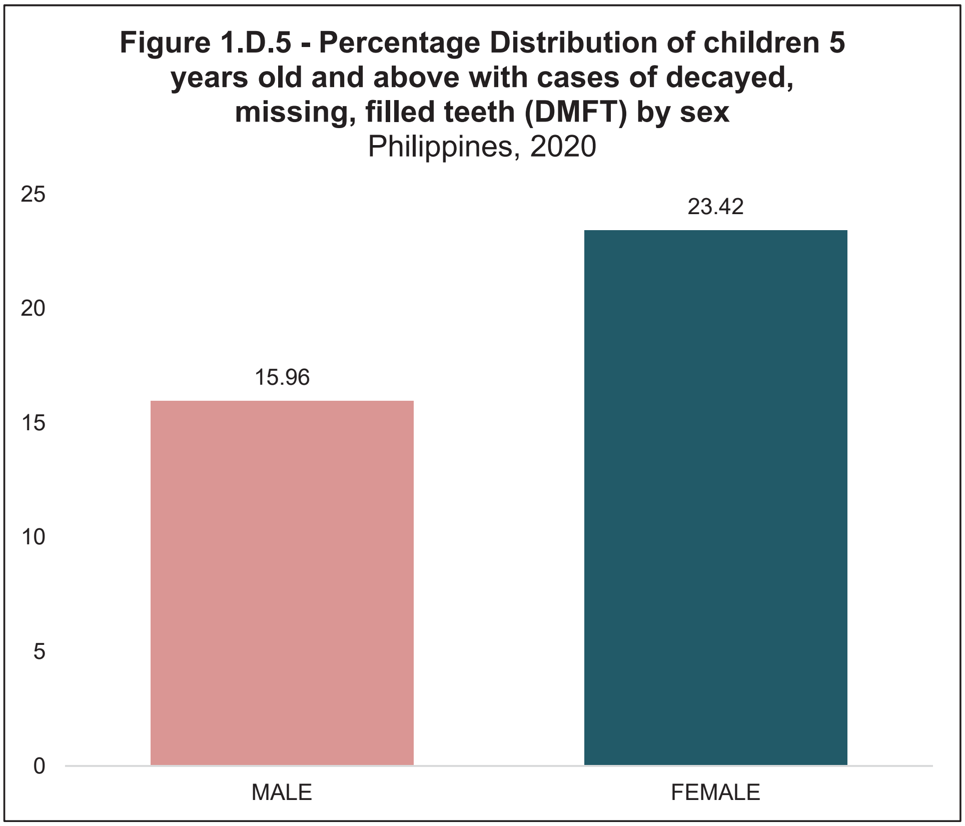
Figure 1.D.5 - Percentage Distribution of children 5 years old and above with cases of decayed, missing, filled teeth (DMFT) by sex
Philippines, 2020
25
20
15
10
5
0
15.96
23.42
MALE
FEMALE

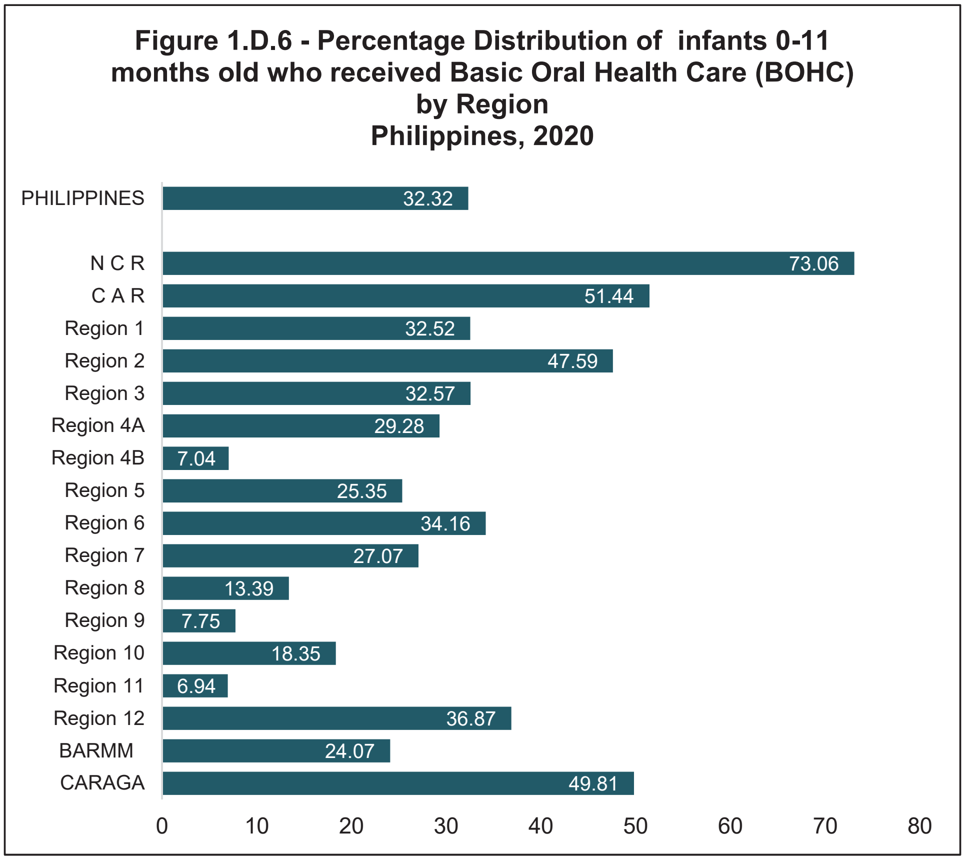
Figure 1.D.6 - Percentage Distribution of infants 0-11 months old who received Basic Oral Health Care (BOHC) by Region
Philippines, 2020
PHILIPPINES
32.32
N C R
73.06
C A R
51.44
Region 1
32.52
Region 2
47.59
Region 3
32.57
Region 4A
29.28
Region 4B
7.04
Region 5
25.35
Region 6
34.16
Region 7
27.07
Region 8
13.39
Region 9
7.75
Region 10
18.35
Region 11
6.94
Region 12
36.87
BARMM
24.07
CARAGA
49.81
0
10
20
30
40
50
60
70
80

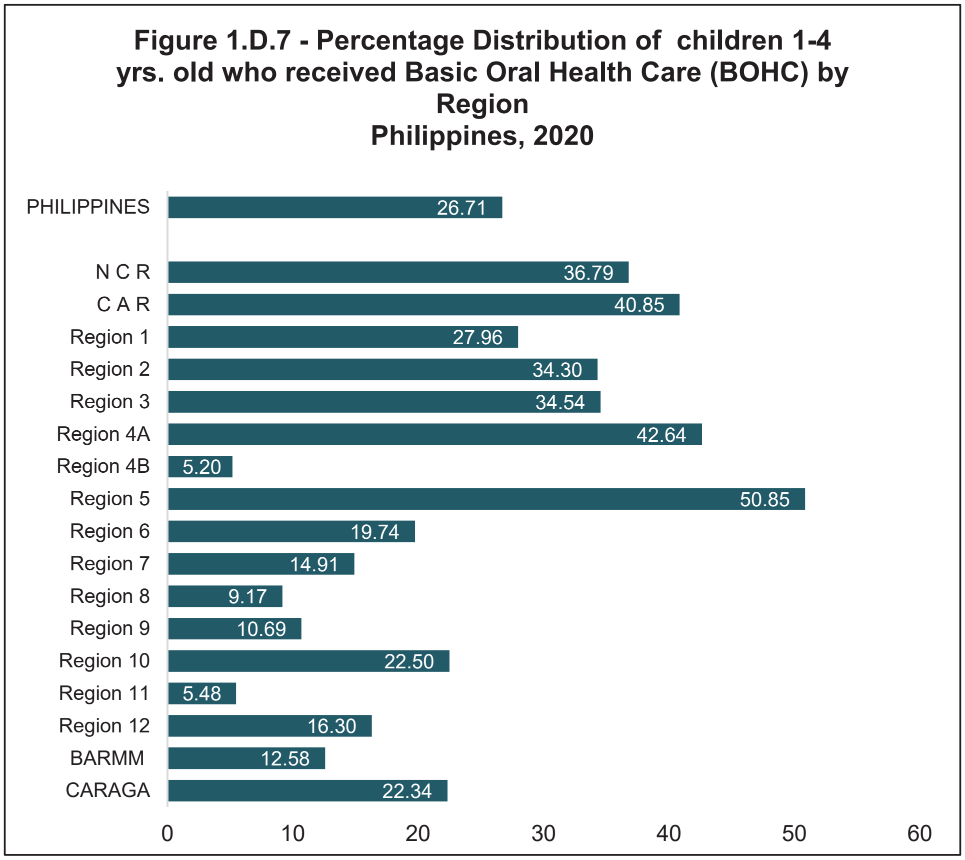
Figure 1.D.7 - Percentage Distribution of children 1-4 yrs. old who received Basic Oral Health Care (BOHC) by Region
Philippines, 2020
PHILIPPINES
26.71
N C R
36.79
C A R
40.85
Region 1
27.96
Region 2
34.30
Region 3
34.54
Region 4A
42.64
Region 4B
5.20
Region 5
50.85
Region 6
19.74
Region 7
14.91
Region 8
9.17
Region 9
10.69
Region 10
22.50
Region 11
5.48
Region 12
16.30
BARMM
12.58
CARAGA
22.34
0
10
20
30
40
50
60

Figure 1.D.8 - Percentage Distribution of children 5-9 yrs. old who received Basic Oral Health Care (BOHC) by Region Philippines, 2020

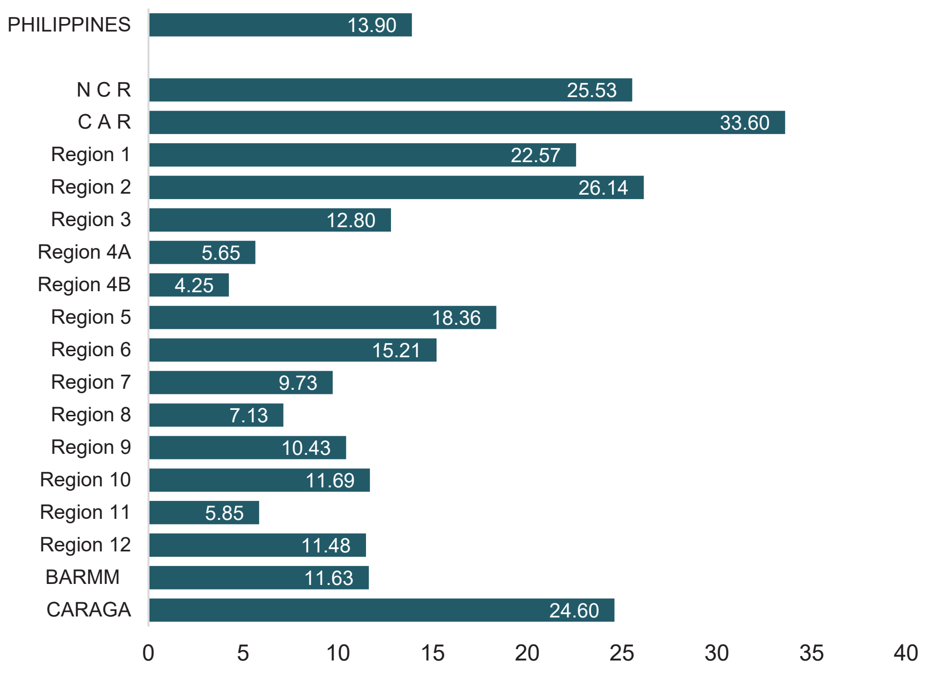

Figure 1.D.9 - Percentage Distribution of children who received Basic Oral Health Care (BOHC) by Age group Philippines, 2020

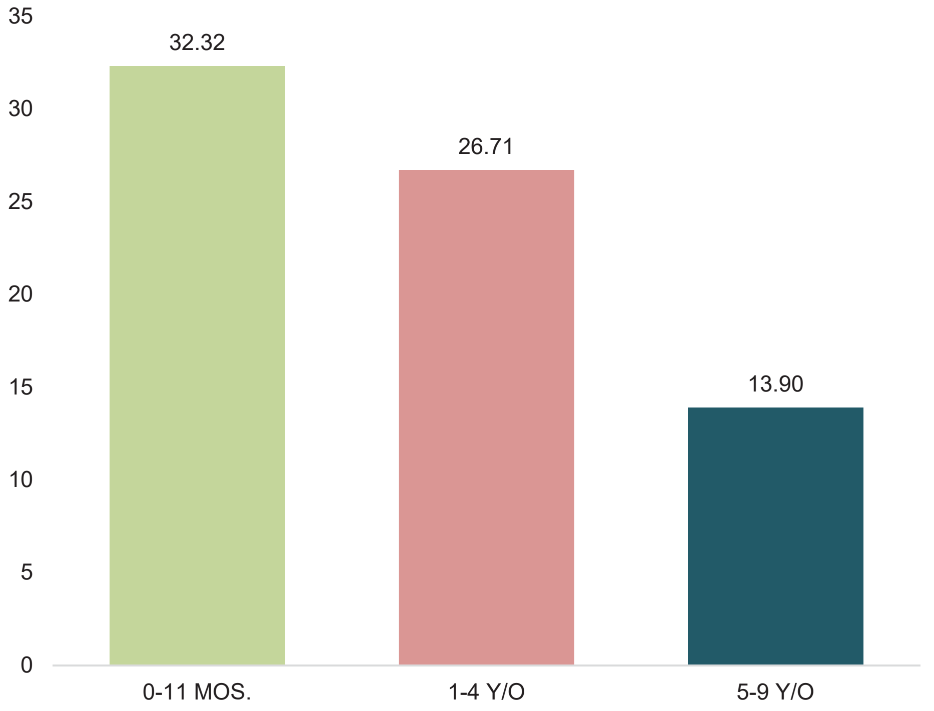

Figure 1.D.10 - Percentage Distribution of adolescent 10-14 years old who received Basic Oral Health Care (BOHC) by Region Philippines, 2020

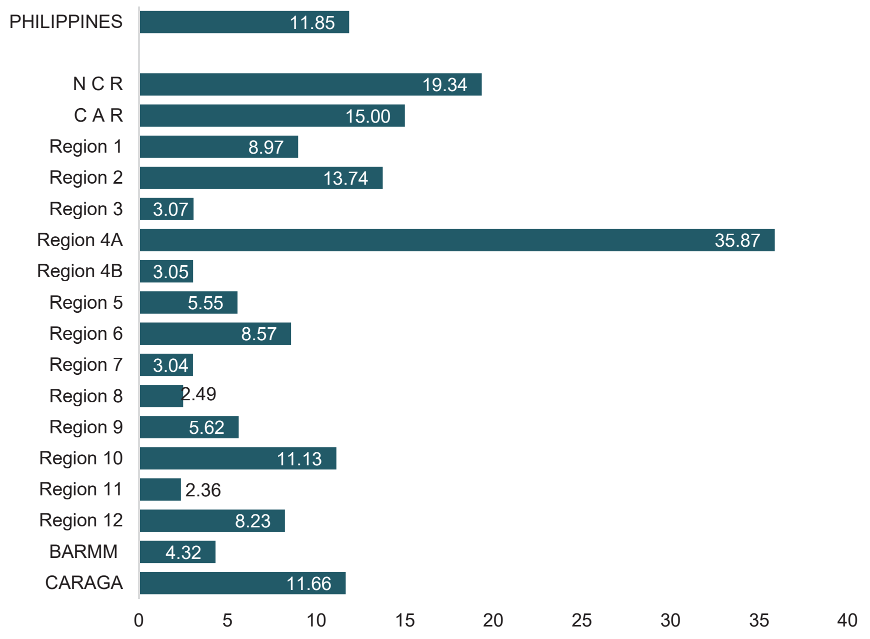

Figure 1.D.11 - Percentage Distribution of adolescent 15-19 years old who received Basic Oral Health Care (BOHC) by Region Philippines, 2020

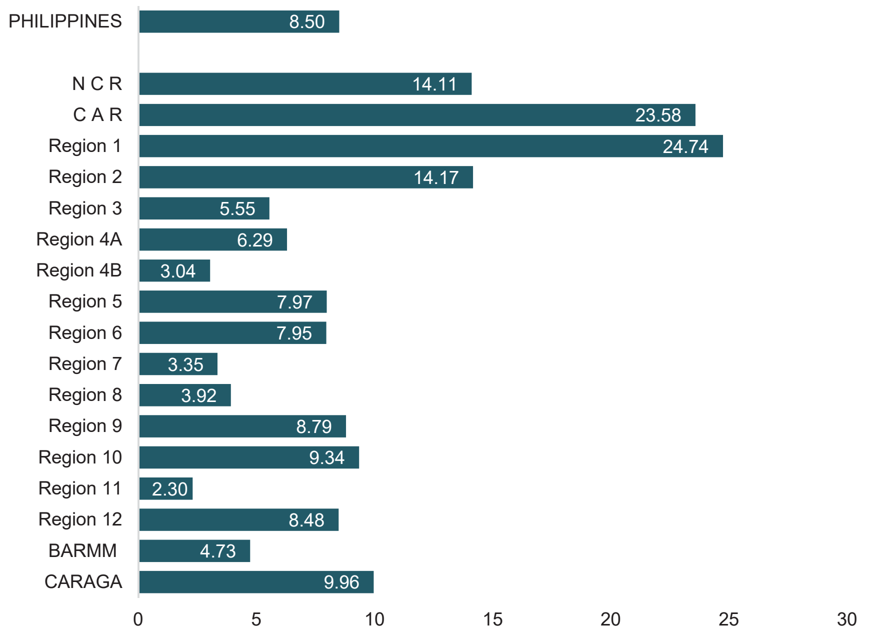

Figure 1.D.12 - Percentage Distribution of adult 20-49 years old who received Basic Oral Health Care (BOHC) by Region Philippines, 2020

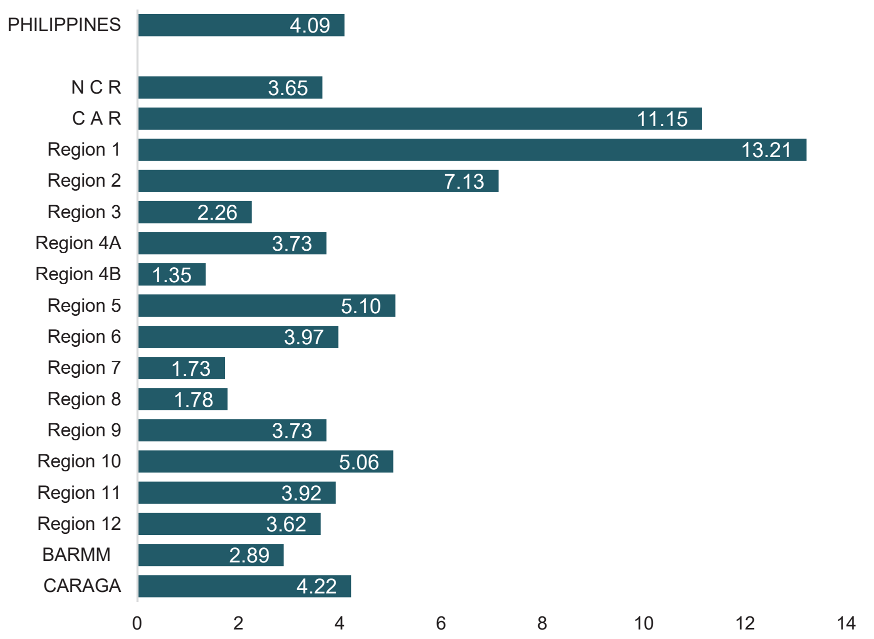

Figure 1.D.13 - Percentage Distribution of adolescent (10-19 yrs. old) and adult (20- 49 yrs. old) who received Basic Oral Health Care (BOHC) Philippines, 2020

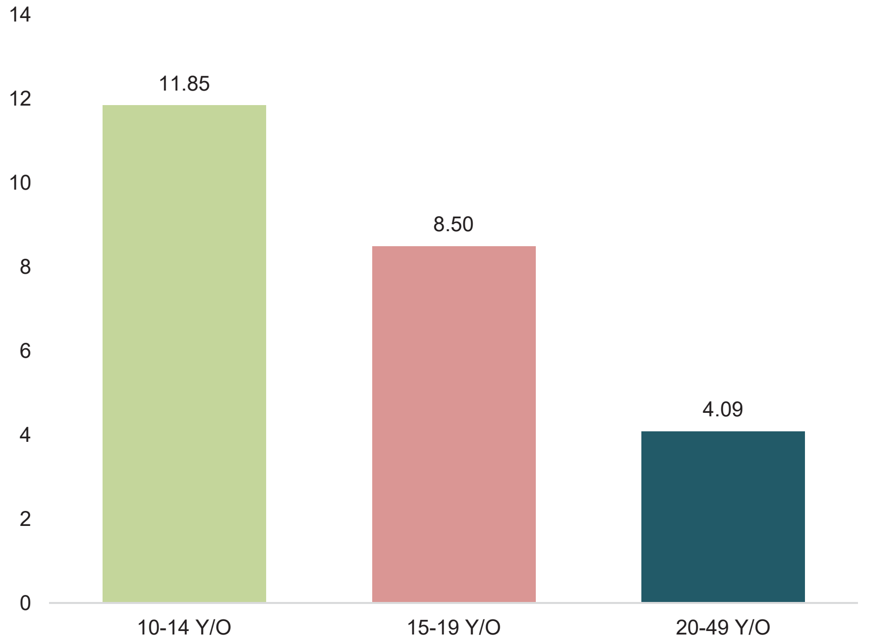

Figure 1.D.14 - Percentage Distribution of Senior Citizen 60 yrs. old and above who received BOHC by Region
Philippines, 2020

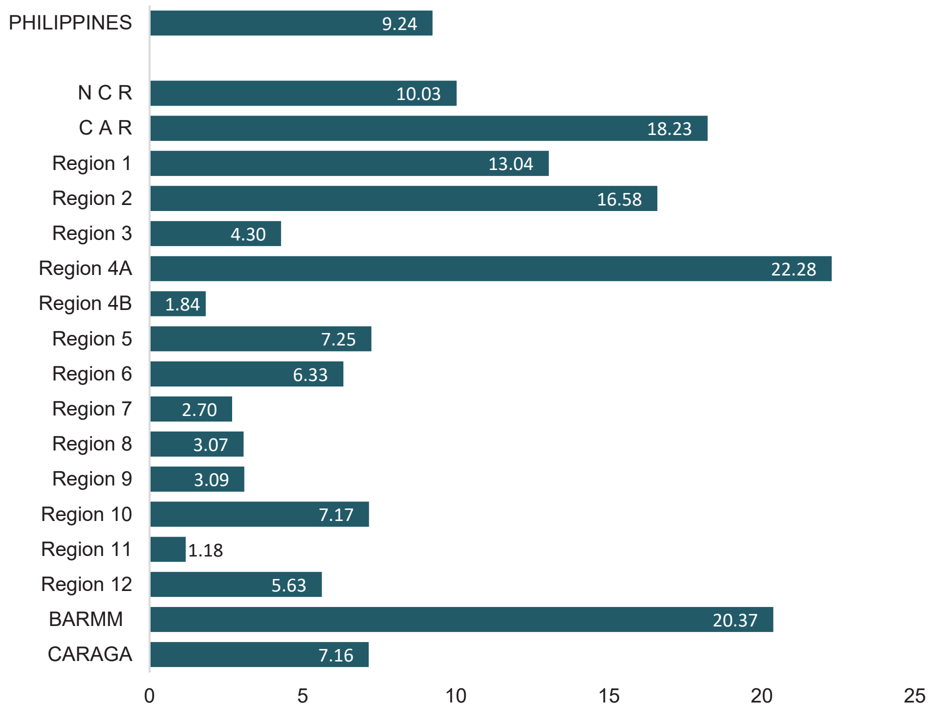

Figure 1.D.15 - Percentage Distribution of Pregnant women 10-14 yrs. old who received Basic Oral Health Care (BOHC) by Region
Philippines, 2020

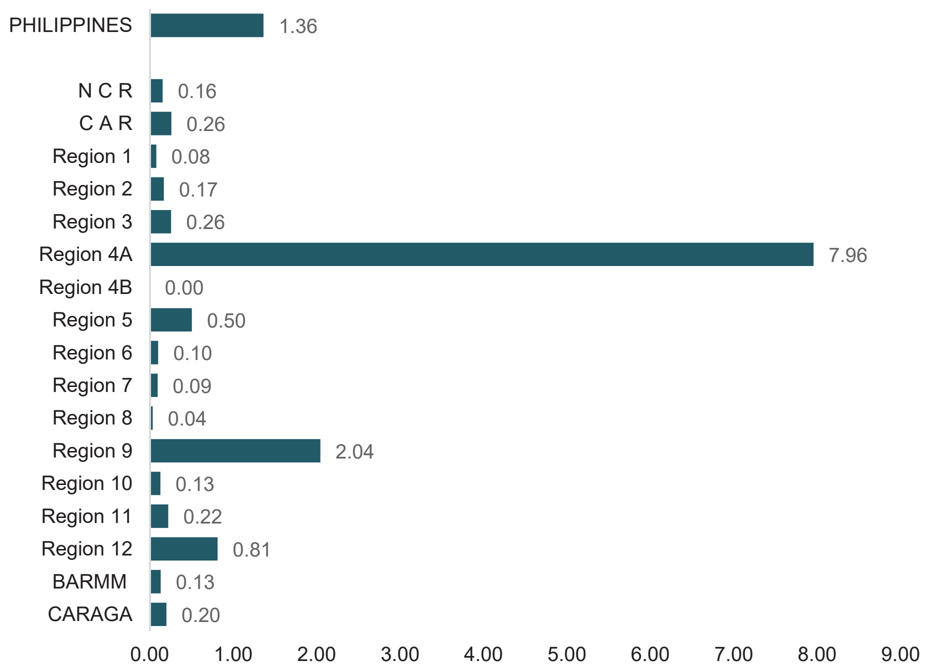

Figure 1.D.16 - Percentage Distribution of Pregnant women 15-19 yrs. old who received Basic Oral Health Care (BOHC) by Region
Philippines, 2020

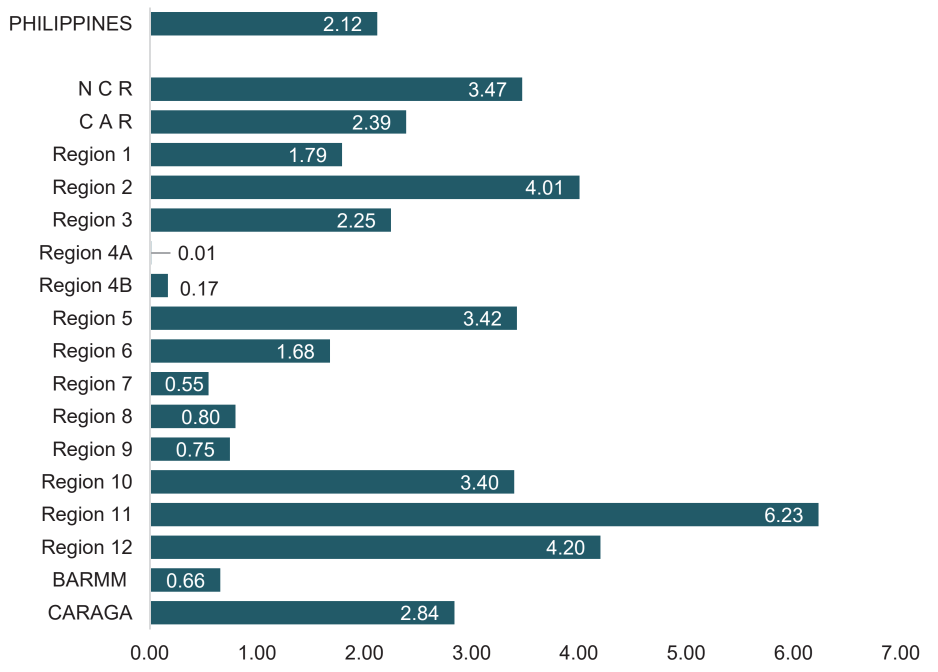

Figure 1.D.17 - Percentage Distribution of Pregnant women 20-49 yrs. old who received Basic Oral Health Care (BOHC) by Region
Philippines, 2020

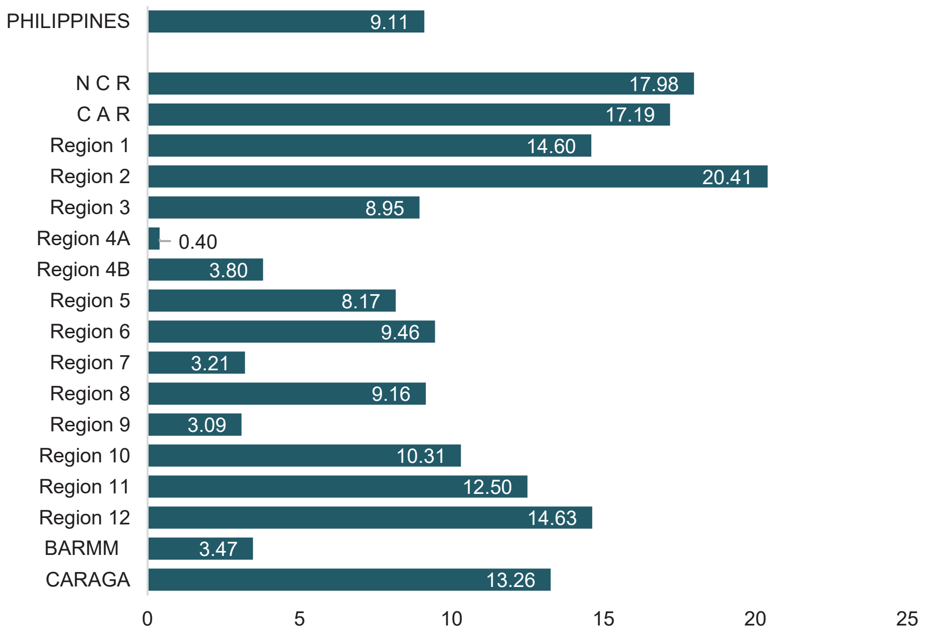

Figure 1.D.18 - Percentage Distribution of Pregnant women who received Basic Oral Health Care (BOHC) by Age group
Philippines, 2020

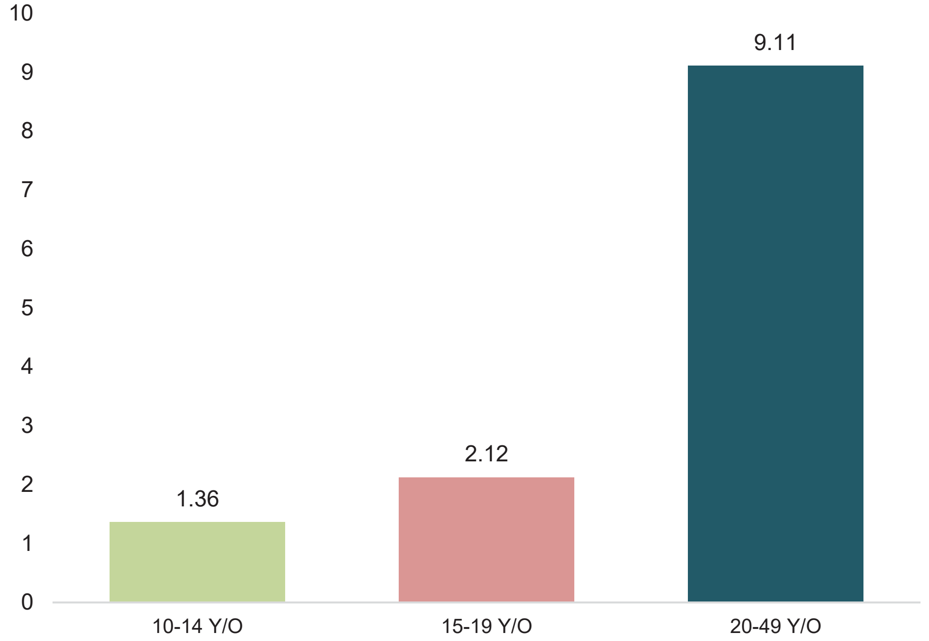

Table 1.E.1.1 - Filariasis Prevention and Control

Case Detection Rate (CDR) and Clinical Rate of Lymphatic Filariasis

PHILIPPINES, Annual 2020

Area	Total Examined for lymphatic filariasis	Number positive cases found				Lymphatic filariasis cases examined with disabilities/deformities			
		Male	Female	Total	Case Det. Rate	Male	Female	Total	Clinical Rate
**PHILIPPINES**	**9,237**	**183**	**189**	**372**	**4.03**	**4**	**0**	**4**	**1.08**
**N C R**	**0**	**0**	**0**	**0**	**0.00**	**0**	**0**	**0**	**0**
Malabon City	0	0	0	0	0.00	0	0	0	0.00
Navotas City	0	0	0	0	0.00	0	0	0	0.00
Valenzuela City	0	0	0	0	0.00	0	0	0	0.00
Caloocan City	0	0	0	0	0.00	0	0	0	0.00
Marikina City	0	0	0	0	0.00	0	0	0	0.00
Pasig City	0	0	0	0	0.00	0	0	0	0.00
Pateros	0	0	0	0	0.00	0	0	0	0.00
Taguig City	0	0	0	0	0.00	0	0	0	0.00
Quezon City	0	0	0	0	0.00	0	0	0	0.00
Makati City	0	0	0	0	0.00	0	0	0	0.00
Mandaluyong City	0	0	0	0	0.00	0	0	0	0.00
San Juan City	0	0	0	0	0.00	0	0	0	0.00
Manila City	0	0	0	0	0.00	0	0	0	0.00
Las Piñas City	0	0	0	0	0.00	0	0	0	0.00
Muntinlupa City	0	0	0	0	0.00	0	0	0	0.00
Parañaque City	0	0	0	0	0.00	0	0	0	0.00
Pasay City	0	0	0	0	0.00	0	0	0	0.00
**C A R**	**0**	**0**	**0**	**0**	**0.00**	**0**	**0**	**0**	**0.00**
Abra	0	0	0	0	0.00	0	0	0	0.00
Apayao	0	0	0	0	0.00	0	0	0	0.00
Benguet	0	0	0	0	0.00	0	0	0	0.00
Ifugao	0	0	0	0	0.00	0	0	0	0.00
Kalinga	0	0	0	0	0.00	0	0	0	0.00
Mt. Province	0	0	0	0	0.00	0	0	0	0.00
Baguio City	0	0	0	0	0.00	0	0	0	0.00
**Region 1**	**0**	**0**	**0**	**0**	**0.00**	**0**	**0**	**0**	**0.00**
Ilocos Norte	0	0	0	0	0.00	0	0	0	0.00
Ilocos Sur	0	0	0	0	0.00	0	0	0	0.00
La Union	0	0	0	0	0.00	0	0	0	0.00
Pangasinan	0	0	0	0	0.00	0	0	0	0.00
Alaminos City	0	0	0	0	0.00	0	0	0	0.00
Candon City	0	0	0	0	0.00	0	0	0	0.00
Dagupan City	0	0	0	0	0.00	0	0	0	0.00

Table 1.E.1.1 - Filariasis Prevention and Control
Case Detection Rate (CDR) and Clinical Rate of Lymphatic Filariasis
PHILIPPINES, Annual 2020

Area	Total Examined for lymphatic filariasis	Number positive cases found				Lymphatic filariasis cases examined with disabilities/deformities			
		Male	Female	Total	Case Det. Rate	Male	Female	Total	Clinical Rate
Laoag City	0	0	0	0	0.00	0	0	0	0.00
San Carlos City	0	0	0	0	0.00	0	0	0	0.00
San Fernando City	0	0	0	0	0.00	0	0	0	0.00
Urdaneta City	0	0	0	0	0.00	0	0	0	0.00
Vigan City	0	0	0	0	0.00	0	0	0	0.00
**Region 2**	**0**	**0**	**0**	**0**	**0.00**	**0**	**0**	**0**	**0.00**
Batanes	0	0	0	0	0.00	0	0	0	0.00
Cagayan	0	0	0	0	0.00	0	0	0	0.00
Isabela	0	0	0	0	0.00	0	0	0	0.00
Nueva Vizcaya	0	0	0	0	0.00	0	0	0	0.00
Quirino	0	0	0	0	0.00	0	0	0	0.00
Cauayan City	0	0	0	0	0.00	0	0	0	0.00
Ilagan City	0	0	0	0	0.00	0	0	0	0.00
Santiago City	0	0	0	0	0.00	0	0	0	0.00
Tuguegarao City	0	0	0	0	0.00	0	0	0	0.00
**Region 3**	**0**	**0**	**0**	**0**	**0.00**	**0**	**0**	**0**	**0.00**
Aurora	0	0	0	0	0.00	0	0	0	0.00
Bataan	0	0	0	0	0.00	0	0	0	0.00
Bulacan	0	0	0	0	0.00	0	0	0	0.00
Nueva Ecija	0	0	0	0	0.00	0	0	0	0.00
Pampanga	0	0	0	0	0.00	0	0	0	0.00
Tarlac	0	0	0	0	0.00	0	0	0	0.00
Zambales	0	0	0	0	0.00	0	0	0	0.00
Angeles City	0	0	0	0	0.00	0	0	0	0.00
Balanga City	0	0	0	0	0.00	0	0	0	0.00
Cabanatuan City	0	0	0	0	0.00	0	0	0	0.00
City of San Fernando	0	0	0	0	0.00	0	0	0	0.00
Gapan City	0	0	0	0	0.00	0	0	0	0.00
Mabalacat City	0	0	0	0	0.00	0	0	0	0.00
Malolos City	0	0	0	0	0.00	0	0	0	0.00
Meycauayan City	0	0	0	0	0.00	0	0	0	0.00
Olongapo City	0	0	0	0	0.00	0	0	0	0.00
Palayan City	0	0	0	0	0.00	0	0	0	0.00
San Jose City	0	0	0	0	0.00	0	0	0	0.00
San Jose del Monte City	0	0	0	0	0.00	0	0	0	0.00
Science City of Munoz	0	0	0	0	0.00	0	0	0	0.00
Tarlac City	0	0	0	0	0.00	0	0	0	0.00
**Region 4A**	**0**	**0**	**0**	**0**	**0.00**	**0**	**0**	**0**	**0.00**
Batangas	0	0	0	0	0.00	0	0	0	0.00

Table 1.E.1.1 - Filariasis Prevention and Control

Case Detection Rate (CDR) and Clinical Rate of Lymphatic Filariasis

PHILIPPINES, Annual 2020

Area	Total Examined for lymphatic filariasis	Number positive cases found				Lymphatic filariasis cases examined with disabilities/deformities			
		Male	Female	Total	Case Det. Rate	Male	Female	Total	Clinical Rate
Cavite	0	0	0	0	0.00	0	0	0	0.00
Laguna	0	0	0	0	0.00	0	0	0	0.00
Quezon	0	0	0	0	0.00	0	0	0	0.00
Rizal	0	0	0	0	0.00	0	0	0	0.00
Antipolo City	0	0	0	0	0.00	0	0	0	0.00
Bacoor City	0	0	0	0	0.00	0	0	0	0.00
Batangas City	0	0	0	0	0.00	0	0	0	0.00
Biñan City	0	0	0	0	0.00	0	0	0	0.00
Cabuyao City	0	0	0	0	0.00	0	0	0	0.00
Calamba City	0	0	0	0	0.00	0	0	0	0.00
Cavite City	0	0	0	0	0.00	0	0	0	0.00
Dasmariñas City	0	0	0	0	0.00	0	0	0	0.00
General Trias City	0	0	0	0	0.00	0	0	0	0.00
Imus City	0	0	0	0	0.00	0	0	0	0.00
Lipa City	0	0	0	0	0.00	0	0	0	0.00
Lucena City	0	0	0	0	0.00	0	0	0	0.00
San Pablo City	0	0	0	0	0.00	0	0	0	0.00
San Pedro City	0	0	0	0	0.00	0	0	0	0.00
Santa Rosa City	0	0	0	0	0.00	0	0	0	0.00
Tagaytay City	0	0	0	0	0.00	0	0	0	0.00
Tanauan City	0	0	0	0	0.00	0	0	0	0.00
Tayabas City	0	0	0	0	0.00	0	0	0	0.00
Trece Martires City	0	0	0	0	0.00	0	0	0	0.00
**Region 4B**	**2,237**	**82**	**60**	**142**	**6.35**	**0**	**0**	**0**	**0.00**
Marinduque	0	0	0	0	0.00	0	0	0	0.00
Mindoro Occidental	0	0	0	0	0.00	0	0	0	0.00
Mindoro Oriental	2237	82	60	142	6.35	0	0	0	0.00
Palawan	0	0	0	0	0.00	0	0	0	0.00
Romblon	0	0	0	0	0.00	0	0	0	0.00
Puerto Princesa City	0	0	0	0	0.00	0	0	0	0.00
**Region 5**	**102**	**5**	**1**	**6**	**5.88**	**1**	**0**	**1**	**16.67**
Albay	0	0	0	0	0.00	0	0	0	0.00
Camarines Norte	102	5	1	6	5.88	1	0	1	16.67
Camarines Sur	0	0	0	0	0.00	0	0	0	0.00
Catanduanes	0	0	0	0	0.00	0	0	0	0.00
Masbate	0	0	0	0	0.00	0	0	0	0.00
Sorsogon	0	0	0	0	0.00	0	0	0	0.00
Iriga City	0	0	0	0	0.00	0	0	0	0.00
Legaspi City	0	0	0	0	0.00	0	0	0	0.00
Naga City	0	0	0	0	0.00	0	0	0	0.00

Table 1.E.1.1 - Filariasis Prevention and Control
Case Detection Rate (CDR) and Clinical Rate of Lymphatic Filariasis
PHILIPPINES, Annual 2020

Area	Total Examined for lymphatic filariasis	Number positive cases found				Lymphatic filariasis cases examined with disabilities/deformities			
		Male	Female	Total	Case Det. Rate	Male	Female	Total	Clinical Rate
**Region 6**	**2,030**	**0**	**4**	**4**	**0.20**	**0**	**0**	**0**	**0.00**
Aklan	1571	0	4	4	0.25	0	0	0	0.00
Antique	342	0	0	0	0.00	0	0	0	0.00
Capiz	0	0	0	0	0.00	0	0	0	0.00
Guimaras	0	0	0	0	0.00	0	0	0	0.00
Iloilo	117	0	0	0	0.00	0	0	0	0.00
Negros Occidental	0	0	0	0	0.00	0	0	0	0.00
Bacolod City	0	0	0	0	0.00	0	0	0	0.00
Iloilo City	0	0	0	0	0.00	0	0	0	0.00
**Region 7**	**0**	**0**	**0**	**0**	**0.00**	**0**	**0**	**0**	**0.00**
Bohol	0	0	0	0	0.00	0	0	0	0.00
Cebu	0	0	0	0	0.00	0	0	0	0.00
Negros Oriental	0	0	0	0	0.00	0	0	0	0.00
Siquijor	0	0	0	0	0.00	0	0	0	0.00
Cebu City	0	0	0	0	0.00	0	0	0	0.00
Lapu-Lapu City	0	0	0	0	0.00	0	0	0	0.00
Mandaue City	0	0	0	0	0.00	0	0	0	0.00
**Region 8**	**2,360**	**20**	**21**	**41**	**1.74**	**3**	**0**	**3**	**7.32**
Biliran	0	0	0	0	0.00	0	0	0	0.00
Eastern Samar	0	0	0	0	0.00	0	0	0	0.00
Northern Leyte	0	0	0	0	0.00	0	0	0	0.00
Northern Samar	2,360	20	21	41	1.74	3	0	3	7.32
Southern Leyte	0	0	0	0	0.00	0	0	0	0.00
Western Samar	0	0	0	0	0.00	0	0	0	0.00
Calbayog City	0	0	0	0	0.00	0	0	0	0.00
Maasin City	0	0	0	0	0.00	0	0	0	0.00
Ormoc City	0	0	0	0	0.00	0	0	0	0.00
Tacloban City	0	0	0	0	0.00	0	0	0	0.00
**Region 9**	**0**	**0**	**0**	**0**	**0.00**	**0**	**0**	**0**	**0.00**
Zamboanga del Norte	0	0	0	0	0.00	0	0	0	0.00
Zamboanga del Sur	0	0	0	0	0.00	0	0	0	0.00
Zamboanga Sibugay	0	0	0	0	0.00	0	0	0	0.00
Dapitan City	0	0	0	0	0.00	0	0	0	0.00
Dipolog City	0	0	0	0	0.00	0	0	0	0.00
Isabela City	0	0	0	0	0.00	0	0	0	0.00

Table 1.E.1.1 - Filariasis Prevention and Control

Case Detection Rate (CDR) and Clinical Rate of Lymphatic Filariasis

PHILIPPINES, Annual 2020

Area	Total Examined for lymphatic filariasis	Number positive cases found				Lymphatic filariasis cases examined with disabilities/deformities			
		Male	Female	Total	Case Det. Rate	Male	Female	Total	Clinical Rate
Pagadian City	0	0	0	0	0.00	0	0	0	0.00
Zamboanga City	0	0	0	0	0.00	0	0	0	0.00
**Region 10**	**0**	**0**	**0**	**0**	**0.00**	**0**	**0**	**0**	**0.00**
Bukidnon	0	0	0	0	0.00	0	0	0	0.00
Camiguin	0	0	0	0	0.00	0	0	0	0.00
Lanao del Norte	0	0	0	0	0.00	0	0	0	0.00
Misamis Occidental	0	0	0	0	0.00	0	0	0	0.00
Misamis Oriental	0	0	0	0	0.00	0	0	0	0.00
Cagayan de Oro City	0	0	0	0	0.00	0	0	0	0.00
El Salvador City	0	0	0	0	0.00	0	0	0	0.00
Gingoog City	0	0	0	0	0.00	0	0	0	0.00
Iligan City	0	0	0	0	0.00	0	0	0	0.00
Malaybalay City	0	0	0	0	0.00	0	0	0	0.00
Oroquieta City	0	0	0	0	0.00	0	0	0	0.00
Ozamis City	0	0	0	0	0.00	0	0	0	0.00
Tangub City	0	0	0	0	0.00	0	0	0	0.00
Valencia City	0	0	0	0	0.00	0	0	0	0.00
**Region 11**	**0**	**0**	**0**	**0**	**0.00**	**0**	**0**	**0**	**0.00**
Compostela Valley	0	0	0	0	0.00	0	0	0	0.00
Davao del Norte	0	0	0	0	0.00	0	0	0	0.00
Davao del Sur	0	0	0	0	0.00	0	0	0	0.00
Davao Oriental	0	0	0	0	0.00	0	0	0	0.00
Davao Occidental	0	0	0	0	0.00	0	0	0	0.00
Davao City	0	0	0	0	0.00	0	0	0	0.00
**Region 12**	**1,820**	**65**	**75**	**140**	**7.69**	**0**	**0**	**0**	**0.00**
North Cotabato	0	0	0	0	0.00	0	0	0	0.00
Sarangani	0	0	0	0	0.00	0	0	0	0.00
South Cotabato	0	0	0	0	0.00	0	0	0	0.00
Sultan Kudarat	1820	65	75	140	7.69	0	0	0	0.00
Cotabato City	0	0	0	0	0.00	0	0	0	0.00
Gen. Santos City	0	0	0	0	0.00	0	0	0	0.00
**BARMM**	**0**	**0**	**0**	**0**	**0.00**	**0**	**0**	**0**	**0.00**
Basilan	0	0	0	0	0.00	0	0	0	0.00
Lanao del Sur	0	0	0	0	0.00	0	0	0	0.00
Maguindanao	0	0	0	0	0.00	0	0	0	0.00
Sulu	0	0	0	0	0.00	0	0	0	0.00

Table 1.E.1.1 - Filariasis Prevention and Control

Case Detection Rate (CDR) and Clinical Rate of Lymphatic Filariasis

PHILIPPINES, Annual 2020

Area	Total Examined for lymphatic filariasis	Number positive cases found				Lymphatic filariasis cases examined with disabilities/deformities			
		Male	Female	Total	Case Det. Rate	Male	Female	Total	Clinical Rate
Tawi-Tawi	0	0	0	0	0.00	0	0	0	0.00
Lamitan City	0	0	0	0	0.00	0	0	0	0.00
Marawi City	0	0	0	0	0.00	0	0	0	0.00
**CARAGA**	**688**	**11**	**28**	**39**	**5.67**	**0**	**0**	**0**	**0.00**
	`								
Agusan del Norte	558	9	27	36	6.45	0	0	0	0.00
Agusan del Sur	5	2	1	3	60.00	0	0	0	0.00
Surigao del Norte	121	0	0	0	0.00	0	0	0	0.00
Surigao del Sur	2	0	0	0	0.00	0	0	0	0.00
Province of Dinagat	0	0	0	0	0.00	0	0	0	0.00
Bislig City	0	0	0	0	0.00	0	0	0	0.00
Butuan City	2	0	0	0	0.00	0	0	0	0.00
Surigao City	0	0	0	0	0.00	0	0	0	0.00

Legend:

0 = No Case

** = No Report*

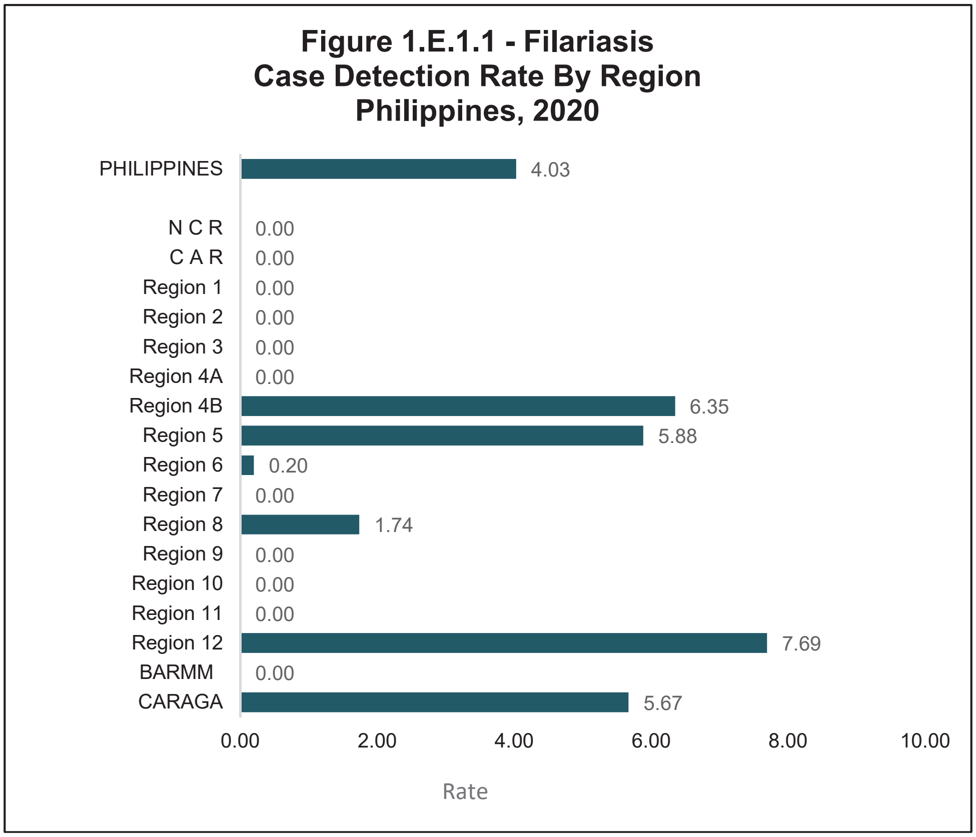
Figure 1.E.1.1 - Filariasis
Case Detection Rate By Region
Philippines, 2020
PHILIPPINES
4.03
N C R
0.00
C A R
0.00
Region 1
0.00
Region 2
0.00
Region 3
0.00
Region 4A
0.00
Region 4B
6.35
Region 5
5.88
Region 6
0.20
Region 7
0.00
Region 8
1.74
Region 9
0.00
Region 10
0.00
Region 11
0.00
Region 12
7.69
BARMM
0.00
CARAGA
5.67
0.00
2.00
4.00
6.00
8.00
10.00
Rate

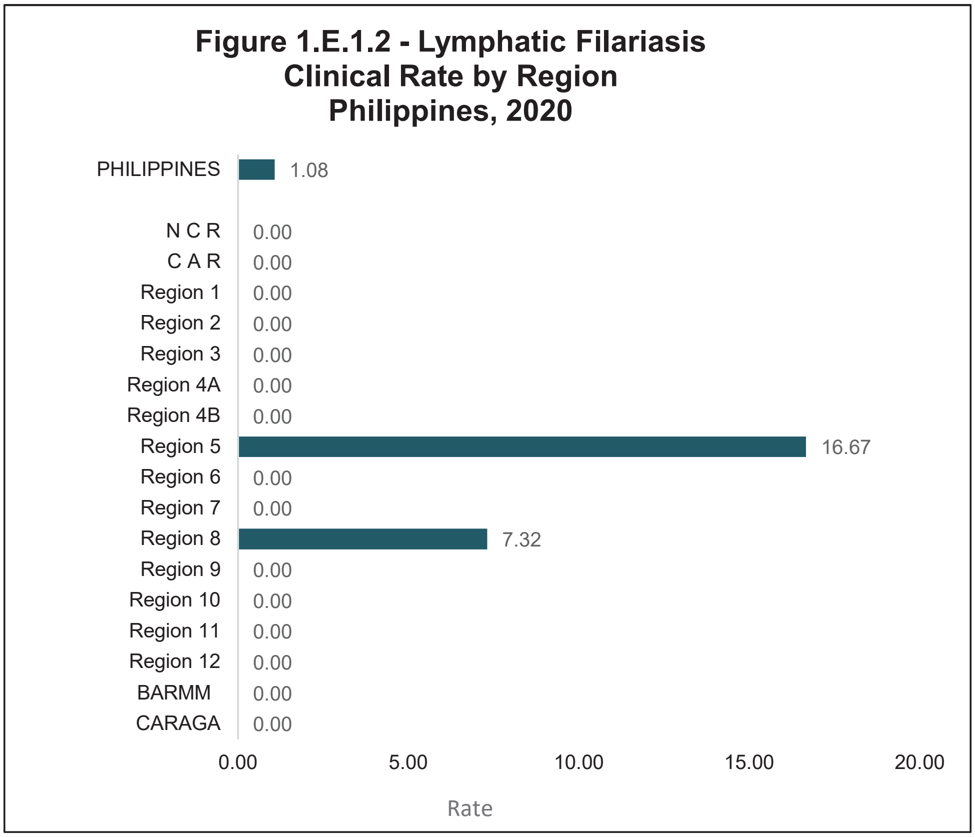
Figure 1.E.1.2 - Lymphatic Filariasis
Clinical Rate by Region
Philippines, 2020
PHILIPPINES
1.08
N C R
0.00
C A R
0.00
Region 1
0.00
Region 2
0.00
Region 3
0.00
Region 4A
0.00
Region 4B
0.00
Region 5
16.67
Region 6
0.00
Region 7
0.00
Region 8
7.32
Region 9
0.00
Region 10
0.00
Region 11
0.00
Region 12
0.00
BARMM
0.00
CARAGA
0.00
0.00
5.00
10.00
15.00
20.00
Rate

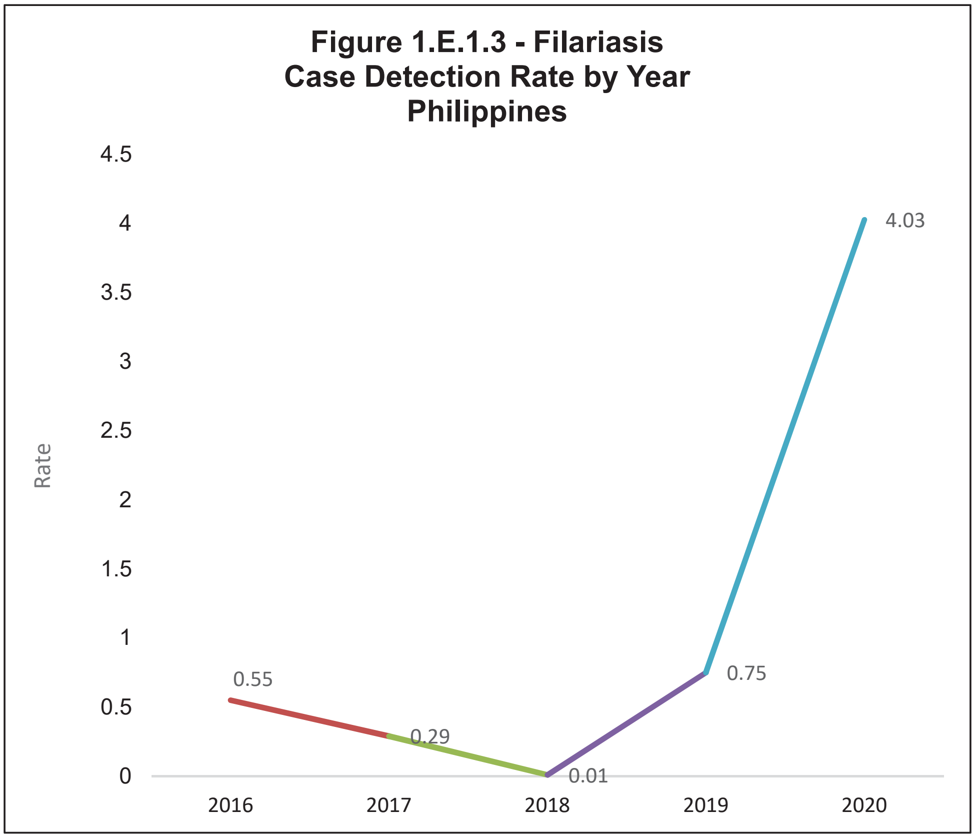
Figure 1.E.1.3 - Filariasis
Case Detection Rate by Year
Philippines
4.5
4
3.5
3
2.5
2
1.5
1
0.5
0
Rate
0.55
0.29
0.01
0.75
4.03
2016
2017
2018
2019
2020

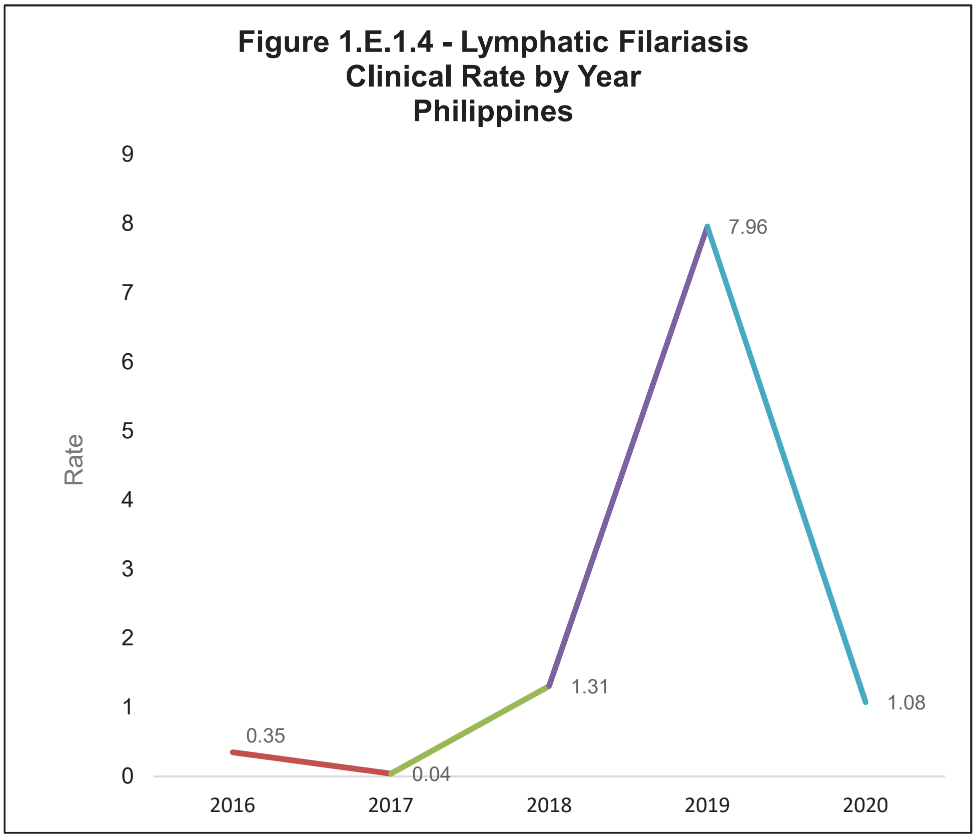
Figure 1.E.1.4 - Lymphatic Filariasis
Clinical Rate by Year
Philippines
9
8
7
6
5
4
3
2
1
0
Rate
0.35
0.04
1.31
7.96
1.08
2016
2017
2018
2019
2020

Table 1.E.2.1 - Schistosomiasis Prevention and Control

No. and Proportion of Suspected Schistosomiasis cases seen, Acute clinically diagnosed cases seen and Acute Confirmed Cases by Stool Examination

PHILIPPINES, Annual 2020

Area	Total patients seen	Number of Suspected Cases Seen				No. of Acute Clinically Diagnosed Cases Seen				No. of Acute Cases Confirmed Positive by Stool Examination			
		Male	Female	Total	%	Male	Female	Total	%	Male	Female	Total	%
**PHILIPPINES**	**34,704**	**4,505**	**3,417**	**7,922**	**22.83**	**1,704**	**1,359**	**3,063**	**38.66**	**228**	**130**	**358**	**11.69**
**N C R**	**0**	**0**	**0**	**0**	**0.00**	**0**	**0**	**0**	**0.00**	**0**	**0**	**0**	**0.00**
Malabon City	0	0	0	0	0.00	0	0	0	0.00	0	0	0	0.00
Navotas City	0	0	0	0	0.00	0	0	0	0.00	0	0	0	0.00
Valenzuela City	0	0	0	0	0.00	0	0	0	0.00	0	0	0	0.00
Caloocan City	0	0	0	0	0.00	0	0	0	0.00	0	0	0	0.00
Marikina City	0	0	0	0	0.00	0	0	0	0.00	0	0	0	0.00
Pasig City	0	0	0	0	0.00	0	0	0	0.00	0	0	0	0.00
Pateros	0	0	0	0	0.00	0	0	0	0.00	0	0	0	0.00
Taguig City	0	0	0	0	0.00	0	0	0	0.00	0	0		0.00
Quezon City	0	0	0	0	0.00	0	0	0	0.00	0	0	0	0.00
Makati City	0	0	0	0	0.00	0	0	0	0.00	0	0	0	0.00
Mandaluyong City	0	0	0	0	0.00	0	0	0	0.00	0	0	0	0.00
San Juan City	0	0	0	0	0.00	0	0	0	0.00	0	0	0	0.00
Manila City	0	0	0	0	0.00	0	0	0	0.00	0	0	0	0.00
Las Piñas City	0	0	0	0	0.00	0	0	0	0.00	0	0	0	0.00
Muntinlupa City	0	0	0	0	0.00	0	0	0	0.00	0	0	0	0.00
Parañaque City	0	0	0	0	0.00	0	0	0	0.00	0	0	0	0.00
Pasay City	0	0	0	0	0.00	0	0	0	0.00	0	0	0	0.00
**C A R**	0	0	0	**0**	0.00	0	0	**0**	0.00	0	0	**0**	0.00
Abra	0	0	0	0	0.00	0	0	0	0.00	0	0	0	0.00
Apayao	0	0	0	0	0.00	0	0	0	0.00	0	0	0	0.00
Benguet	0	0	0	0	0.00	0	0	0	0.00	0	0	0	0.00
Ifugao	0	0	0	0	0.00	0	0	0	0.00	0	0	0	0.00
Kalinga	0	0	0	0	0.00	0	0	0	0.00	0	0	0	0.00
Mt. Province	0	0	0	0	0.00	0	0	0	0.00	0	0	0	0.00
Baguio City	0	0	0	0	0.00	0	0	0	0.00	0	0	0	0.00
**Region 1**	0	0	0	**0**	0.00	0	0	**0**	0.00	0	0	**0**	0.00
Ilocos Norte	0	0	0	0	0.00	0	0	0	0.00	0	0	0	0.00
Ilocos Sur	0	0	0	0	0.00	0	0	0	0.00	0	0	0	0.00
La Union	0	0	0	0	0.00	0	0	0	0.00	0	0	0	0.00
Pangasinan	0	0	0	0	0.00	0	0	0	0.00	0	0	0	0.00
Alaminos City	0	0	0	0	0.00	0	0	0	0.00	0	0	0	0.00
Candon City	0	0	0	0	0.00	0	0	0	0.00	0	0	0	0.00
Dagupan City	0	0	0	0	0.00	0	0	0	0.00	0	0	0	0.00
Laoag City	0	0	0	0	0.00	0	0	0	0.00	0	0	0	0.00
San Carlos City	0	0	0	0	0.00	0	0	0	0.00	0	0	0	0.00
San Fernando City	0	0	0	0	0.00	0	0	0	0.00	0	0	0	0.00
Urdaneta City	0	0	0	0	0.00	0	0	0	0.00	0	0	0	0.00
Vigan City	0	0	0	0	0.00	0	0	0	0.00	0	0	0	0.00
**Region 2**	0	0	0	**0**	0.00	0	0	**0**	0.00	0	0	**0**	0.00
Batanes	0	0	0	0	0.00	0	0	0	0.00	0	0	0	0.00

Table 1.E.2.1 - Schistosomiasis Prevention and Control

No. and Proportion of Suspected Schistosomiasis cases seen, Acute clinically diagnosed cases seen and Acute Confirmed Cases by Stool Examination

PHILIPPINES, Annual 2020

Area	Total patients seen	Number of Suspected Cases Seen				No. of Acute Clinically Diagnosed Cases Seen				No. of Acute Cases Confirmed Positive by Stool Examination			
		Male	Female	Total	%	Male	Female	Total	%	Male	Female	Total	%
Cagayan	0	0	0	0	0.00	0	0	0	0.00	0	0	0	0.00
Isabela	0	0	0	0	0.00	0	0	0	0.00	0	0	0	0.00
Nueva Vizcaya	0	0	0	0	0.00	0	0	0	0.00	0	0	0	0.00
Quirino	0	0	0	0	0.00	0	0	0	0.00	0	0	0	0.00
Cauayan City	0	0	0	0	0.00	0	0	0	0.00	0	0	0	0.00
Ilagan City	0	0	0	0	0.00	0	0	0	0.00	0	0	0	0.00
Santiago City	0	0	0	0	0.00	0	0	0	0.00	0	0	0	0.00
Tuguegarao City	0	0	0	0	0.00	0	0	0	0.00	0	0	0	0.00
**Region 3**	249	0	0	**0**	0.00	0	0	**0**	0.00	0	0	**0**	0.00
Aurora	0	0	0	0	0.00	0	0	0	0.00	0	0	0	0.00
Bataan	0	0	0	0	0.00	0	0	0	0.00	0	0	0	0.00
Bulacan	0	0	0	0	0.00	0	0	0	0.00	0	0	0	0.00
Nueva Ecija	0	0	0	0	0.00	0	0	0	0.00	0	0	0	0.00
Pampanga	0	0	0	0	0.00	0	0	0	0.00	0	0	0	0.00
Tarlac	0	0	0	0	0.00	0	0	0	0.00	0	0	0	0.00
Zambales	0	0	0	0	0.00	0	0	0	0.00	0	0	0	0.00
Angeles City	0	0	0	0	0.00	0	0	0	0.00	0	0	0	0.00
Balanga City	0	0	0	0	0.00	0	0	0	0.00	0	0	0	0.00
Cabanatuan City	0	0	0	0	0.00	0	0	0	0.00	0	0	0	0.00
City of San Fernando	0	0	0	0	0.00	0	0	0	0.00	0	0	0	0.00
Gapan City	0	0	0	0	0.00	0	0	0	0.00	0	0	0	0.00
Mabalacat City	0	0	0	0	0.00	0	0	0	0.00	0	0	0	0.00
Malolos City	0	0	0	0	0.00	0	0	0	0.00	0	0	0	0.00
Meycauayan City	0	0	0	0	0.00	0	0	0	0.00	0	0	0	0.00
Olongapo City	249	0	0	0	0.00	0	0	0	0.00	0	0	0	0.00
Palayan City	0	0	0	0	0.00	0	0	0	0.00	0	0	0	0.00
San Jose City	0	0	0	0	0.00	0	0	0	0.00	0	0	0	0.00
San Jose del Monte City	0	0	0	0	0.00	0	0	0	0.00	0	0	0	0.00
Science City of Munoz	0	0	0	0	0.00	0	0	0	0.00	0	0	0	0.00
Tarlac City	0	0	0	0	0.00	0	0	0	0.00	0	0	0	0.00
**Region 4A**	367	2	5	7	1.91	0	0	0	0.00	3	2	5	0.00
Batangas	138	2	5	7	5.07	0	0	0	0.00	3	2	5	0.00
Cavite	27	0	0	0	0.00	0	0	0	0.00	0	0	0	0.00
Laguna	128	0	0	0	0.00	0	0	0	0.00	0	0	0	0.00
Quezon	5	0	0	0	0.00	0	0	0	0.00	0	0	0	0.00
Rizal	3	0	0	0	0.00	0	0	0	0.00	0	0	0	0.00
Antipolo City	0	0	0	0	0.00	0	0	0	0.00	0	0	0	0.00
Bacoor City	0	0	0	0	0.00	0	0	0	0.00	0	0	0	0.00
Batangas City	0	0	0	0	0.00	0	0	0	0.00	0	0	0	0.00
Biñan City	0	0	0	0	0.00	0	0	0	0.00	0	0	0	0.00
Cabuyao City	0	0	0	0	0.00	0	0	0	0.00	0	0	0	0.00
Calamba City	3	0	0	0	0.00	0	0	0	0.00	0	0	0	0.00
Cavite City	0	0	0	0	0.00	0	0	0	0.00	0	0	0	0.00
Dasmariñas City	0	0	0	0	0.00	0	0	0	0.00	0	0	0	0.00
General Trias City	63	0	0	0	0.00	0	0	0	0.00	0	0	0	0.00
Imus City	0	0	0	0	0.00	0	0	0	0.00	0	0	0	0.00
Lipa City	0	0	0	0	0.00	0	0	0	0.00	0	0	0	0.00
Lucena City	0	0	0	0	0.00	0	0	0	0.00	0	0	0	0.00

Table 1.E.2.1 - Schistosomiasis Prevention and Control

No. and Proportion of Suspected Schistosomiasis cases seen, Acute clinically diagnosed cases seen and Acute Confirmed Cases by Stool Examination

PHILIPPINES, Annual 2020

Area	Total patients seen	Number of Suspected Cases Seen				No. of Acute Clinically Diagnosed Cases Seen				No. of Acute Cases Confirmed Positive by Stool Examination			
		Male	Female	Total	%	Male	Female	Total	%	Male	Female	Total	%
San Pablo City	0	0	0	0	0.00	0	0	0	0.00	0	0	0	0.00
San Pedro City	0	0	0	0	0.00	0	0	0	0.00	0	0	0	0.00
Santa Rosa City	0	0	0	0	0.00	0	0	0	0.00	0	0	0	0.00
Tagaytay City	0	0	0	0	0.00	0	0	0	0.00	0	0	0	0.00
Tanauan City	0	0	0	0	0.00	0	0	0	0.00	0	0	0	0.00
Tayabas City	0	0	0	0	0.00	0	0	0	0.00	0	0	0	0.00
Trece Martires City	0	0	0	0	0.00	0	0	0	0.00	0	0	0	0.00
**Region 4B**	0	0	0	0	0.00	0	0	0	0.00	0	0	0	0.00
Marinduque	0	0	0	0	0.00	0	0	0	0.00	0	0	0	0.00
Mindoro Occidental	0	0	0	0	0.00	0	0	0	0.00	0	0	0	0.00
Mindoro Oriental	0	0	0	0	0.00	0	0	0	0.00	0	0	0	0.00
Palawan	0	0	0	0	0.00	0	0	0	0.00	0	0	0	0.00
Romblon	0	0	0	0	0.00	0	0	0	0.00	0	0	0	0.00
Puerto Princesa City	0	0	0	0	0.00	0	0	0	0.00	0	0	0	0.00
**Region 5**	97	32	10	**42**	43.30	2	1	**3**	7.14	2	1	**3**	100.00
Albay	46	0	0	0	0.00	0	0	0	0.00	0	0	0	0.00
Camarines Norte	0	0	0	0	0.00	0	0	0	0.00	0	0	0	0.00
Camarines Sur	0	0	0	0	0.00	0	0	0	0.00	0	0	0	0.00
Catanduanes	0	0	0	0	0.00	0	0	0	0.00	0	0	0	0.00
Masbate	0	0	0	0	0.00	0	0	0	0.00	0	0	0	0.00
Sorsogon	51	32	10	42	82.35	2	1	3	7.14	2	1	3	100.00
Iriga City	0	0	0	0	0.00	0	0	0	0.00	0	0	0	0.00
Legaspi City	0	0	0	0	0.00	0	0	0	0.00	0	0	0	0.00
Naga City	0	0	0	0	0.00	0	0	0	0.00	0	0	0	0.00
**Region 6**	0	0	0	**0**	0.00	0	0	**0**	0.00	0	0	**0**	0.00
Aklan	0	0	0	0	0.00	0	0	0	0.00	0	0	0	0.00
Antique	0	0	0	0	0.00	0	0	0	0.00	0	0	0	0.00
Capiz	0	0	0	0	0.00	0	0	0	0.00	0	0	0	0.00
Guimaras	0	0	0	0	0.00	0	0	0	0.00	0	0	0	0.00
Iloilo	0	0	0	0	0.00	0	0	0	0.00	0	0	0	0.00
Negros Occidental	0	0	0	0	0.00	0	0	0	0.00	0	0	0	0.00
Bacolod City	0	0	0	0	0.00	0	0	0	0.00	0	0	0	0.00
Bago City	0	0	0	0	0.00	0	0	0	0.00	0	0	0	0.00
**Region 7**	0	0	0	0	0.00	0	0	0	0.00	0	0	0	0.00
Bohol	0	0	0	0	0.00	0	0	0	0.00	0	0	0	0.00
Cebu	0	0	0	0	0.00	0	0	0	0.00	0	0	0	0.00
Negros Oriental	0	0	0	0	0.00	0	0	0	0.00	0	0	0	0.00
Siquijor	0	0	0	0	0.00	0	0	0	0.00	0	0	0	0.00
Cebu City	0	0	0	0	0.00	0	0	0	0.00	0	0	0	0.00
Lapu-Lapu City	0	0	0	0	0.00	0	0	0	0.00	0	0	0	0.00
Mandaue City	0	0	0	0	0.00	0	0	0	0.00	0	0	0	0.00

Table 1.E.2.1 - Schistosomiasis Prevention and Control

No. and Proportion of Suspected Schistosomiasis cases seen, Acute clinically diagnosed cases seen and Acute Confirmed Cases by Stool Examination

PHILIPPINES, Annual 2020

Area	Total patients seen	Number of Suspected Cases Seen				No. of Acute Clinically Diagnosed Cases Seen				No. of Acute Cases Confirmed Positive by Stool Examination			
		Male	Female	Total	%	Male	Female	Total	%	Male	Female	Total	%
**Region 8**	1,874	933	941	1,874	100.00	0	0	0	0.00	0	0	0	0.00
Biliran	0	0	0	0	0.00	0	0	0	0.00	0	0	0	0.00
Eastern Samar	1,874	933	941	1,874	100.00	0	0	0	0.00	0	0	0	0.00
Northern Leyte	0	0	0	0	0.00	0	0	0	0.00	0	0	0	0.00
Northern Samar	0	0	0	0	0.00	0	0	0	0.00	0	0	0	0.00
Southern Leyte	0	0	0	0	0.00	0	0	0	0.00	0	0	0	0.00
Western Samar	0	0	0	0	0.00	0	0	0	0.00	0	0	0	0.00
Calbayog City	0	0	0	0	0.00	0	0	0	0.00	0	0	0	0.00
Maasin City	0	0	0	0	0.00	0	0	0	0.00	0	0	0	0.00
Ormoc City	0	0	0	0	0.00	0	0	0	0.00	0	0	0	0.00
Tacloban City	0	0	0	0	0.00	0	0	0	0.00	0	0	0	0.00
**Region 9**	0	0	0	0	0.00	0	0	0	0.00	0	0	0	0.00
Zamboanga del Norte	0	0	0	0	0.00	0	0	0	0.00	0	0	0	0.00
Zamboanga del Sur	0	0	0	0	0.00	0	0	0	0.00	0	0	0	0.00
Zamboanga Sibugay	0	0	0	0	0.00	0	0	0	0.00	0	0	0	0.00
Dapitan City	0	0	0	0	0.00	0	0	0	0.00	0	0	0	0.00
Dipolog City	0	0	0	0	0.00	0	0	0	0.00	0	0	0	0.00
Isabela City	0	0	0	0	0.00	0	0	0	0.00	0	0	0	0.00
Pagadian City	0	0	0	0	0.00	0	0	0	0.00	0	0	0	0.00
Zamboanga City	0	0	0	0	0.00	0	0	0	0.00	0	0	0	0.00
**Region 10**	11,096	346	210	556	5.01	146	66	212	38.13	115	52	167	78.77
Bukidnon	191	88	98	186	97.38	18	7	25	13.44	9	6	15	60.00
Camiguin	0	0	0	0	0.00	0	0	0	0.00	0	0	0	0.00
Lanao del Norte	16	8	8	16	100.00	5	5	10	62.50	4	2	6	60.00
Misamis Occidental	10,354	0	0	0	0.00	0	0	0	0.00	0	0	0	0.00
Misamis Oriental	163	2	1	3	1.84	2	0	2	66.67	1	0	1	50.00
Cagayan de Oro City	0	0	0	0	0.00	0	0	0	0.00	0	0	0	0.00
El Salvador City	0	0	0	0	0.00	0	0	0	0.00	0	0	0	0.00
Gingoog City	0	0	0	0	0.00	0	0	0	0.00	0	0	0	0.00
Iligan City	0	0	0	0	0.00	0	0	0	0.00	0	0	0	0.00
Malaybalay City	85	65	20	85	100.00	16	3	19	22.35	3	0	3	15.79
Oroquieta City	5	4	1	5	100.00	1	0	1	20.00	0	0	0	0.00
Ozamis City	0	0	0	0	0.00	0	0	0	0.00	0	0	0	0.00
Tangub City	36	14	1	15	41.67	0	0	0	0.00	14	1	15	0.00
Valencia City	246	165	81	246	100.00	104	51	155	63.01	84	43	127	81.94
**Region 11**	8,813	1,433	1,196	2,629	29.83	1,423	1,185	2,608	99.20	21	8	29	1.11
Compostela Valley	2,795	1,264	815	2,079	74.38	1,263	815	2,078	99.95	13	6	19	0.91
Davao del Norte	1,438	169	381	550	38.25	157	366	523	95.09	8	2	10	1.91
Davao del Sur	1,401	0	0	0	0.00	0	0	0	0.00	0	0	0	0.00
Davao Oriental	3,179	0	0	0	0.00	3	4	7	0.00	0	0	0	0.00
Davao Occidental	0	0	0	0	0.00	0	0	0	0.00	0	0	0	0.00
Davao City	0	0	0	0	0.00	0	0	0	0.00	0	0	0	0.00

Table 1.E.2.1 - Schistosomiasis Prevention and Control

No. and Proportion of Suspected Schistosomiasis cases seen, Acute clinically diagnosed cases seen and Acute Confirmed Cases by Stool Examination

PHILIPPINES, Annual 2020

Area	Total patients seen	Number of Suspected Cases Seen				No. of Acute Clinically Diagnosed Cases Seen				No. of Acute Cases Confirmed Positive by Stool Examination			
		Male	Female	Total	%	Male	Female	Total	%	Male	Female	Total	%
**Region 12**	1,120	320	476	796	71.07	9	20	29	3.64	4	0	4	13.79
North Cotabato	730	203	212	415	56.85	1	0	1	0.24	1	0	1	100.00
Sarangani	0	0	0	0	0.00	0	0	0	0.00	0	0	0	0.00
South Cotabato	372	104	259	363	97.58	8	20	28	7.71	3	0	3	10.71
Sultan Kudarat	0	0	0	0	0.00	0	0	0	0.00	0	0	0	0.00
Cotabato City	0	0	0	0	0.00	0	0	0	0.00	0	0	0	0.00
Gen. Santos City	18	13	5	18	100.00	0	0	0	0.00	0	0	0	0.00
**BARMM**	0	4	2	6	0.00	4	2	6	100.00	0	0	0	0.00
Basilan	0	0	0	0	0.00	0	0	0	0.00	0	0	0	0.00
Lanao del Sur	0	0	0	0	0.00	0	0	0	0.00	0	0	0	0.00
Maguindanao	0	4	2	6	0.00	4	2	6	100.00	0	0	0	0.00
Sulu	0	0	0	0	0.00	0	0	0	0.00	0	0	0	0.00
Tawi-Tawi	0	0	0	0	0.00	0	0	0	0.00	0	0	0	0.00
Lamitan City	0	0	0	0	0.00	0	0	0	0.00	0	0	0	0.00
Marawi City	0	0	0	0	0.00	0	0	0	0.00	0	0	0	0.00
**CARAGA**	11,088	1,435	577	2,012	18.15	120	85	205	10.19	83	67	150	73.17
Agusan del Norte	1,549	729	8	737	47.58	6	6	12	1.63	5	6	11	91.67
Agusan del Sur	9,186	621	527	1,148	12.50	79	64	143	12.46	63	52	115	80.42
Surigao del Norte	160	69	37	106	66.25	22	10	32	30.19	12	8	20	62.50
Surigao del Sur	0	0	0	0	0.00	0	0	0	0.00	0	0	0	0.00
Province of Dinagat	0	0	0	0	0.00	0	0	0	0.00	0	0	0	0.00
Bislig City	0	0	0	0	0.00	0	0	0	0.00	0	0	0	0.00
Butuan City	193	16	5	21	10.88	13	5	18	85.71	3	1	4	22.22
Surigao City	0	0	0	0	0.00	0	0	0	0.00	0	0	0	0.00

Legend:

0 = No Case

** = No Report*

Table 1.E.2.2 - Schistosomiasis Prevention and Control

No. and Proportion of Chronic Clinically Diagnosed Cases Seen and Chronic Cllinically diagnosed cases confirmed by stool examinantion

PHILIPPINES, Annual 2020

Area	No. of Clinically Diagnosed Chronic Cases Seen				No. of Chronic Clinically Diagnosed Cases confirmed by Stool Examination			
	Male	Female	Total	%	Male	Female	Total	%
**PHILIPPINES**	**272**	**146**	**418**	5.28	**204**	**111**	315	75.36
**N C R**	0	0	0	0.00	0	0	0	0.00
Malabon City	0	0	0	0.00	0	0	0	0.00
Navotas City	0	0	0	0.00	0	0	0	0.00
Valenzuela City	0	0	0	0.00	0	0	0	0.00
Caloocan City	0	0	0	0.00	0	0	0	0.00
Marikina City	0	0	0	0.00	0	0	0	0.00
Pasig City	0	0	0	0.00	0	0	0	0.00
Pateros	0	0	0	0.00	0	0	0	0.00
Taguig City	0	0	0	0.00	0	0	0	0.00
Quezon City	0	0	0	0.00	0	0	0	0.00
Makati City	0	0	0	0.00	0	0	0	0.00
Mandaluyong City	0	0	0	0.00	0	0	0	0.00
San Juan City	0	0	0	0.00	0	0	0	0.00
Manila City	0	0	0	0.00	0	0	0	0.00
Las Piñas City	0	0	0	0.00	0	0	0	0.00
Muntinlupa City	0	0	0	0.00	0	0	0	0.00
Parañaque City	0	0	0	0.00	0	0	0	0.00
Pasay City	0	0	0	0.00	0	0	0	0.00
**C A R**	0	0	0	0.00	0	0	0	0.00
Abra	0	0	0	0.00	0	0	0	0.00
Apayao	0	0	0	0.00	0	0	0	0.00
Benguet	0	0	0	0.00	0	0	0	0.00
Ifugao	0	0	0	0.00	0	0	0	0.00
Kalinga	0	0	0	0.00	0	0	0	0.00
Mt. Province	0	0	0	0.00	0	0	0	0.00
Baguio City	0	0	0	0.00	0	0	0	0.00
**Region 1**	0	0	0	0.00	0	0	0	0.00
Ilocos Norte	0	0	0	0.00	0	0	0	0.00
Ilocos Sur	0	0	0	0.00	0	0	0	0.00
La Union	0	0	0	0.00	0	0	0	0.00
Pangasinan	0	0	0	0.00	0	0	0	0.00
Alaminos City	0	0	0	0.00	0	0	0	0.00
Candon City	0	0	0	0.00	0	0	0	0.00
Dagupan City	0	0	0	0.00	0	0	0	0.00
Laoag City	0	0	0	0.00	0	0	0	0.00
San Carlos City	0	0	0	0.00	0	0	0	0.00
San Fernando City	0	0	0	0.00	0	0	0	0.00
Urdaneta City	0	0	0	0.00	0	0	0	0.00
Vigan City	0	0	0	0.00	0	0	0	0.00

Table 1.E.2.2 - Schistosomiasis Prevention and Control

No. and Proportion of Chronic Clinically Diagnosed Cases Seen and Chronic Cllinically diagnosed cases confirmed by stool examinantion

PHILIPPINES, Annual 2020

Area	No. of Clinically Diagnosed Chronic Cases Seen				No. of Chronic Clinically Diagnosed Cases confirmed by Stool Examination			
	Male	Female	Total	%	Male	Female	Total	%
**Region 2**	0	0	0	0.00	0	0	0	0.00
							0	0.00
Batanes	0	0	0	0.00	0	0	0	0.00
Cagayan	0	0	0	0.00	0	0	0	0.00
Isabela	0	0	0	0.00	0	0	0	0.00
Nueva Vizcaya	0	0	0	0.00	0	0	0	0.00
Quirino	0	0	0	0.00	0	0	0	0.00
Cauayan City	0	0	0	0.00	0	0	0	0.00
Ilagan City	0	0	0	0.00	0	0	0	0.00
Santiago City	0	0	0	0.00	0	0	0	0.00
Tuguegarao City	0	0	0	0.00	0	0	0	0.00
**Region 3**	0	0	0	0.00	0	0	0	0.00
Aurora	0	0	0	0.00	0	0	0	0.00
Bataan	0	0	0	0.00	0	0	0	0.00
Bulacan	0	0	0	0.00	0	0	0	0.00
Nueva Ecija	0	0	0	0.00	0	0	0	0.00
Pampanga	0	0	0	0.00	0	0	0	0.00
Tarlac	0	0	0	0.00	0	0	0	0.00
Zambales	0	0	0	0.00	0	0	0	0.00
Angeles City	0	0	0	0.00	0	0	0	0.00
Balanga City	0	0	0	0.00	0	0	0	0.00
Cabanatuan City	0	0	0	0.00	0	0	0	0.00
City of San Fernando	0	0	0	0.00	0	0	0	0.00
Gapan City	0	0	0	0.00	0	0	0	0.00
Mabalacat City	0	0	0	0.00	0	0	0	0.00
Malolos City	0	0	0	0.00	0	0	0	0.00
Meycauayan City	0	0	0	0.00	0	0	0	0.00
Olongapo City	0	0	0	0.00	0	0	0	0.00
Palayan City	0	0	0	0.00	0	0	0	0.00
San Jose City	0	0	0	0.00	0	0	0	0.00
San Jose del Monte Cit	0	0	0	0.00	0	0	0	0.00
Science City of Munoz	0	0	0	0.00	0	0	0	0.00
Tarlac City	0	0	0	0.00	0	0	0	0.00
**Region 4A**	1	1	2	28.57	2	0	2	100.00
Batangas	1	1	2	28.57	1	0	1	50.00
Cavite	0	0	0	0.00	0	0	0	0.00
Laguna	0	0	0	0.00	0	0	0	0.00
Quezon	0	0	0	0.00	0	0	0	0.00
Rizal	0	0	0	0.00	0	0	0	0.00
Antipolo City	0	0	0	0.00	0	0	0	0.00
Bacoor City	0	0	0	0.00	1	0	1	0.00
Batangas City	0	0	0	0.00	0	0	0	0.00
Biñan City	0	0	0	0.00	0	0	0	0.00
Cabuyao City	0	0	0	0.00	0	0	0	0.00
Calamba City	0	0	0	0.00	0	0	0	0.00
Cavite City	0	0	0	0.00	0	0	0	0.00

Table 1.E.2.2 - Schistosomiasis Prevention and Control

No. and Proportion of Chronic Clinically Diagnosed Cases Seen and Chronic Cllinically diagnosed cases confirmed by stool examinantion

PHILIPPINES, Annual 2020

Area	No. of Clinically Diagnosed Chronic Cases Seen				No. of Chronic Clinically Diagnosed Cases confirmed by Stool Examination			
	Male	Female	Total	%	Male	Female	Total	%
Dasmariñas City	0	0	0	0.00	0	0	0	0.00
General Trias City	0	0	0	0.00	0	0	0	0.00
Imus City	0	0	0	0.00	0	0	0	0.00
Lipa City	0	0	0	0.00	0	0	0	0.00
Lucena City	0	0	0	0.00	0	0	0	0.00
San Pablo City	0	0	0	0.00	0	0	0	0.00
San Pedro City	0	0	0	0.00	0	0	0	0.00
Santa Rosa City	0	0	0	0.00	0	0	0	0.00
Tagaytay City	0	0	0	0.00	0	0	0	0.00
Tanauan City	0	0	0	0.00	0	0	0	0.00
Tayabas City	0	0	0	0.00	0	0	0	0.00
Trece Martires City	0	0	0	0.00	0	0	0	0.00
**Region 4B**	0	0	0	0.00	0	0	0	0.00
Marinduque	0	0	0	0.00	0	0	0	0.00
Mindoro Occidental	0	0	0	0.00	0	0	0	0.00
Mindoro Oriental	0	0	0	0.00	0	0	0	0.00
Palawan	0	0	0	0.00	0	0	0	0.00
Romblon	0	0	0	0.00	0	0	0	0.00
Puerto Princesa City	0	0	0	0.00	0	0	0	0.00
**Region 5**	1	0	1	2.38	1	0	1	100.00
Albay	0	0	0	0.00	0	0	0	0.00
Camarines Norte	0	0	0	0.00	0	0	0	0.00
Camarines Sur	0	0	0	0.00	0	0	0	0.00
Catanduanes	0	0	0	0.00	0	0	0	0.00
Masbate	0	0	0	0.00	0	0	0	0.00
Sorsogon	1	0	1	2.38	1	0	1	100.00
Iriga City	0	0	0	0.00	0	0	0	0.00
Legaspi City	0	0	0	0.00	0	0	0	0.00
Naga City	0	0	0	0.00	0	0	0	0.00
**Region 6**	0	0	0	0.00	0	0	0	0.00
Aklan	0	0	0	0.00	0	0	0	0.00
Antique	0	0	0	0.00	0	0	0	0.00
Capiz	0	0	0	0.00	0	0	0	0.00
Guimaras	0	0	0	0.00	0	0	0	0.00
Iloilo	0	0	0	0.00	0	0	0	0.00
Negros Occidental	0	0	0	0.00	0	0	0	0.00
Bacolod City	0	0	0	0.00	0	0	0	0.00
Bago City	0	0	0	0.00	0	0	0	0.00
**Region 7**	0	0	0	0.00	0	0	0	0.00
Bohol	0	0	0	0.00	0	0	0	0.00
Cebu	0	0	0	0.00	0	0	0	0.00
Negros Oriental	0	0	0	0.00	0	0	0	0.00

Table 1.E.2.2 - Schistosomiasis Prevention and Control

No. and Proportion of Chronic Clinically Diagnosed Cases Seen and Chronic Cllinically diagnosed cases confirmed by stool examinantion

PHILIPPINES, Annual 2020

Area	No. of Clinically Diagnosed Chronic Cases Seen				No. of Chronic Clinically Diagnosed Cases confirmed by Stool Examination			
	Male	Female	Total	%	Male	Female	Total	%
Siquijor	0	0	0	0.00	0	0	0	0.00
				0.00				
Cebu City	0	0	0	0.00	0	0	0	0.00
Lapu-Lapu City	0	0	0	0.00	0	0	0	0.00
Mandaue City	0	0	0	0.00	0	0	0	0.00
**Region 8**	0	0	0	0.00	0	0	0	0.00
Biliran	0	0	0	0.00	0	0	0	0.00
Eastern Samar	0	0	0	0.00	0	0	0	0.00
Northern Leyte	0	0	0	0.00	0	0	0	0.00
Northern Samar	0	0	0	0.00	0	0	0	0.00
Southern Leyte	0	0	0	0.00	0	0	0	0.00
Western Samar	0	0	0	0.00	0	0	0	0.00
Calbayog City	0	0	0	0.00	0	0	0	0.00
Maasin City	0	0	0	0.00	0	0	0	0.00
Ormoc City	0	0	0	0.00	0	0	0	0.00
Tacloban City	0	0	0	0.00	0	0	0	0.00
**Region 9**	0	0	0	0.00	0	0	0	0.00
Zamboanga del Norte	0	0	0	0.00	0	0	0	0.00
Zamboanga del Sur	0	0	0	0.00	0	0	0	0.00
Zamboanga Sibugay	0	0	0	0.00	0	0	0	0.00
Dapitan City	0	0	0	0.00	0	0	0	0.00
Dipolog City	0	0	0	0.00	0	0	0	0.00
Isabela City	0	0	0	0.00	0	0	0	0.00
Pagadian City	0	0	0	0.00	0	0	0	0.00
Zamboanga City	0	0	0	0.00	0	0	0	0.00
**Region 10**	167	78	245	44.06	117	49	166	67.76
Bukidnon	12	3	15	8.06	12	3	15	100.00
Camiguin	0	0	0	0.00	0	0	0	0.00
Lanao del Norte	3	1	4	25.00	3	1	4	100.00
Misamis Occidental	0	0	0	0.00	0	0	0	0.00
Misamis Oriental	0	0	0	0.00	0	0	0	0.00
Cagayan de Oro City	0	0	0	0.00	0	0	0	0.00
El Salvador City	0	0	0	0.00	0	0	0	0.00
Gingoog City	0	0	0	0.00	0	0	0	0.00
Iligan City	0	0	0	0.00	0	0	0	0.00
Malaybalay City	0	0	0	0.00	0	0	0	0.00
Oroquieta City	4	1	5	100.00	4	1	5	100.00
Ozamis City	0	0	0	0.00	0	0	0	0.00
Tangub City	14	1	15	100.00	14	1	15	100.00
Valencia City	134	72	206	83.74	84	43	127	61.65
**Region 11**	13	4	17	0.65	11	4	15	88.24
Compostela Valley	6	3	9	0.43	6	3	9	100.00

Table 1.E.2.2 - Schistosomiasis Prevention and Control

No. and Proportion of Chronic Clinically Diagnosed Cases Seen and Chronic Cllinically diagnosed cases confirmed by stool examinantion

PHILIPPINES, Annual 2020

Area	No. of Clinically Diagnosed Chronic Cases Seen				No. of Chronic Clinically Diagnosed Cases confirmed by Stool Examination			
	Male	Female	Total	%	Male	Female	Total	%
Davao del Norte	6	1	7	1.27	4	1	5	71.43
Davao del Sur	0	0	0	0.00	0	0	0	0.00
Davao Oriental	1	0	1	0.00	1	0	1	100.00
Davao Occidental	0	0	0	0.00	0	0	0	0.00
							0	0.00
Davao City	0	0	0	0.00	0	0	0	0.00
**Region 12**	7	0	7	0.88	4	0	4	57.14
North Cotabato	0	0	0	0.00	0	0	0	0.00
Sarangani	0	0	0	0.00	0	0	0	0.00
South Cotabato	7	0	7	1.93	4	0	4	57.14
Sultan Kudarat	0	0	0	0.00	0	0	0	0.00
Cotabato City	0	0	0	0.00	0	0	0	0.00
Gen. Santos City	0	0	0	0.00	0	0	0	0.00
**BARMM**	0	0	0	0.00	0	0	0	0.00
							0	0.00
Basilan	0	0	0	0.00	0	0	0	0.00
Lanao del Sur	0	0	0	0.00	0	0	0	0.00
Maguindanao	0	0	0	0.00	0	0	0	0.00
Sulu	0	0	0	0.00	0	0	0	0.00
Tawi-Tawi	0	0	0	0.00	0	0	0	0.00
Lamitan City	0	0	0	0.00	0	0	0	0.00
Marawi City	0	0	0	0.00	0	0	0	0.00
**CARAGA**	83	63	146	7.26	69	58	127	86.99
Agusan del Norte	12	5	17	2.3	0	2	2	11.76
Agusan del Sur	39	37	76	6.62	38	37	75	98.68
Surigao del Norte	32	21	53	50.00	31	19	50	94.34
Surigao del Sur	0	0	0	0.00	0	0	0	0.00
Province of Dinagat	0	0	0	0.00	0	0	0	0.00
Bislig City	0	0	0	0.00	0	0	0	0.00
Butuan City	0	0	0	0.00	0	0	0	0.00
Surigao City	0	0	0	0.00	0	0	0	0.00

Legend:
0 = No Case
** = No Report*

Table 1.E.2.3 - Schistosomiasis Prevention and Control

No. and Proportion of Chronic Clinically Diagnosed cases treated in the Health Facility and Confirmed Chronic Cases referred to a Hospital Facility
PHILIPPINES, Annual 2020

Area	No. of Chronic Clinically Diagnosed cases treated in the Health Facility				No. of Confirmed Chronic Cases referred to Hospital Facility			
	Male	Female	Total	%	Male	Female	Total	%
**PHILIPPINES**	**271**	**139**	**410**	**60.92**	**11**	**1**	**12**	**3.81**
**N C R**	**0**	**0**	**0**	**0.00**	**0**	**0**	**0**	**0.00**
Malabon City	0	0	0	0.00	0	0	0	0.00
Navotas City	0	0	0	0.00	0	0	0	0.00
Valenzuela City	0	0	0	0.00	0	0	0	0.00
Caloocan City	0	0	0	0.00	0	0	0	0.00
Marikina City	0	0	0	0.00	0	0	0	0.00
Pasig City	0	0	0	0.00	0	0	0	0.00
Pateros	0	0	0	0.00	0	0	0	0.00
Taguig City	0	0	0	0.00	0	0	0	0.00
Quezon City	0	0	0	0.00	0	0	0	0.00
Makati City	0	0	0	0.00	0	0	0	0.00
Mandaluyong City	0	0	0	0.00	0	0	0	0.00
San Juan City	0	0	0	0.00	0	0	0	0.00
Manila City	0	0	0	0.00	0	0	0	0.00
Las Piñas City	0	0	0	0.00	0	0	0	0.00
Muntinlupa City	0	0	0	0.00	0	0	0	0.00
Parañaque City	0	0	0	0.00	0	0	0	0.00
Pasay City	0	0	0	0.00	0	0	0	0.00
**C A R**	**0**	**0**	**0**	**0.00**	**0**	**0**	**0**	**0.00**
Abra	0	0	0	0.00	0	0	0	0.00
Apayao	0	0	0	0.00	0	0	0	0.00
Benguet	0	0	0	0.00	0	0	0	0.00
Ifugao	0	0	0	0.00	0	0	0	0.00
Kalinga	0	0	0	0.00	0	0	0	0.00
Mt. Province	0	0	0	0.00	0	0	0	0.00
Baguio City	0	0	0	0.00	0	0	0	0.00
**Region 1**	**0**	**0**	**0**	**0.00**	**0**	**0**	**0**	**0.00**
Ilocos Norte	0	0	0	0.00	0	0	0	0.00
Ilocos Sur	0	0	0	0.00	0	0	0	0.00
La Union	0	0	0	0.00	0	0	0	0.00
Pangasinan	0	0	0	0.00	0	0	0	0.00
Alaminos City	0	0	0	0.00	0	0	0	0.00
Candon City	0	0	0	0.00	0	0	0	0.00
Dagupan City	0	0	0	0.00	0	0	0	0.00
Laoag City	0	0	0	0.00	0	0	0	0.00
San Carlos City	0	0	0	0.00	0	0	0	0.00
San Fernando City	0	0	0	0.00	0	0	0	0.00
Urdaneta City	0	0	0	0.00	0	0	0	0.00
Vigan City	0	0	0	0.00	0	0	0	0.00

Table 1.E.2.3 - Schistosomiasis Prevention and Control

No. and Proportion of Chronic Clinically Diagnosed cases treated in the Health Facility and Confirmed Chronic Cases referred to a Hospital Facility
PHILIPPINES, Annual 2020

Area	No. of Chronic Clinically Diagnosed cases treated in the Health Facility				No. of Confirmed Chronic Cases referred to Hospital Facility			
	Male	Female	Total	%	Male	Female	Total	%
**Region 2**	**0**	**0**	**0**	**0.00**	**0**	**0**	**0**	**0.00**
Batanes	0	0	0	0.00	0	0	0	0.00
Cagayan	0	0	0	0.00	0	0	0	0.00
Isabela	0	0	0	0.00	0	0	0	0.00
Nueva Vizcaya	0	0	0	0.00	0	0	0	0.00
Quirino	0	0	0	0.00	0	0	0	0.00
Cauayan City	0	0	0	0.00	0	0	0	0.00
Ilagan City	0	0	0	0.00	0	0	0	0.00
Santiago City	0	0	0	0.00	0	0	0	0.00
Tuguegarao City	0	0	0	0.00	0	0	0	0.00
**Region 3**	**0**	**0**	**0**	**0.00**	**0**	**0**	**0**	**0.00**
Aurora	0	0	0	0.00	0	0	0	0.00
Bataan	0	0	0	0.00	0	0	0	0.00
Bulacan	0	0	0	0.00	0	0	0	0.00
Nueva Ecija	0	0	0	0.00	0	0	0	0.00
Pampanga	0	0	0	0.00	0	0	0	0.00
Tarlac	0	0	0	0.00	0	0	0	0.00
Zambales	0	0	0	0.00	0	0	0	0.00
Angeles City	0	0	0	0.00	0	0	0	0.00
Balanga City	0	0	0	0.00	0	0	0	0.00
Cabanatuan City	0	0	0	0.00	0	0	0	0.00
City of San Fernando	0	0	0	0.00	0	0	0	0.00
Gapan City	0	0	0	0.00	0	0	0	0.00
Mabalacat City	0	0	0	0.00	0	0	0	0.00
Malolos City	0	0	0	0.00	0	0	0	0.00
Meycauayan City	0	0	0	0.00	0	0	0	0.00
Olongapo City	0	0	0	0.00	0	0	0	0.00
Palayan City	0	0	0	0.00	0	0	0	0.00
San Jose City	0	0	0	0.00	0	0	0	0.00
San Jose del Monte C	0	0	0	0.00	0	0	0	0.00
Science City of Muno	0	0	0	0.00	0	0	0	0.00
Tarlac City	0	0	0	0.00	0	0	0	0.00
**Region 4A**	**0**	**0**	**0**	**0.00**	**0**	**0**	**0**	**0.00**
Batangas	0	0	0	0.00	0	0	0	0.00
Cavite	0	0	0	0.00	0	0	0	0.00
Laguna	0	0	0	0.00	0	0	0	0.00
Quezon	0	0	0	0.00	0	0	0	0.00
Rizal	0	0	0	0.00	0	0	0	0.00
Antipolo City	0	0	0	0.00	0	0	0	0.00
Bacoor City	0	0	0	0.00	0	0	0	0.00
Batangas City	0	0	0	0.00	0	0	0	0.00
Biñan City	0	0	0	0.00	0	0	0	0.00
Cabuyao City	0	0	0	0.00	0	0	0	0.00
Calamba City	0	0	0	0.00	0	0	0	0.00
Cavite City	0	0	0	0.00	0	0	0	0.00

Table 1.E.2.3 - Schistosomiasis Prevention and Control

No. and Proportion of Chronic Clinically Diagnosed cases treated in the Health Facility
and Confirmed Chronic Cases referred to a Hospital Facility
PHILIPPINES, Annual 2020

Area	No. of Chronic Clinically Diagnosed cases treated in the Health Facility				No. of Confirmed Chronic Cases referred to Hospital Facility			
	Male	Female	Total	%	Male	Female	Total	%
Dasmariñas City	0	0	0	0.00	0	0	0	0.00
General Trias City	0	0	0	0.00	0	0	0	0.00
Imus City	0	0	0	0.00	0	0	0	0.00
Lipa City	0	0	0	0.00	0	0	0	0.00
Lucena City	0	0	0	0.00	0	0	0	0.00
San Pablo City	0	0	0	0.00	0	0	0	0.00
San Pedro City	0	0	0	0.00	0	0	0	0.00
Santa Rosa City	0	0	0	0.00	0	0	0	0.00
Tagaytay City	0	0	0	0.00	0	0	0	0.00
Tanauan City	0	0	0	0.00	0	0	0	0.00
Tayabas City	0	0	0	0.00	0	0	0	0.00
Trece Martires City	0	0	0	0.00	0	0	0	0.00
**Region 4B**	**0**	**0**	**0**	**0.00**	**0**	**0**	**0**	**0.00**
Marinduque	0	0	0	0.00	0	0	0	0.00
Mindoro Occidental	0	0	0	0.00	0	0	0	0.00
Mindoro Oriental	0	0	0	0.00	0	0	0	0.00
Palawan	0	0	0	0.00	0	0	0	0.00
Romblon	0	0	0	0.00	0	0	0	0.00
Puerto Princesa City	0	0	0	0.00	0	0	0	0.00
**Region 5**	**3**	**1**	**4**	**100.00**	**0**	**0**	**0**	**0.00**
Albay	0	0	0	0.00	0	0	0	0.00
Camarines Norte	0	0	0	0.00	0	0	0	0.00
Camarines Sur	0	0	0	0.00	0	0	0	0.00
Catanduanes	0	0	0	0.00	0	0	0	0.00
Masbate	0	0	0	0.00	0	0	0	0.00
Sorsogon	3	1	4	100.00	0	0	0	0.00
Iriga City	0	0	0	0.00	0	0	0	0.00
Legaspi City	0	0	0	0.00	0	0	0	0.00
Naga City	0	0	0	0.00	0	0	0	0.00
**Region 6**	**0**	**0**	**0**	**0.00**	**0**	**0**	**0**	**0.00**
Aklan	0	0	0	0.00	0	0	0	0.00
Antique	0	0	0	0.00	0	0	0	0.00
Capiz	0	0	0	0.00	0	0	0	0.00
Guimaras	0	0	0	0.00	0	0	0	0.00
Iloilo	0	0	0	0.00	0	0	0	0.00
Negros Occidental	0	0	0	0.00	0	0	0	0.00
Bacolod City	0	0	0	0.00	0	0	0	0.00
Bago City	0	0	0	0.00	0	0	0	0.00
**Region 7**	**0**	**0**	**0**	**0.00**	**0**	**0**	**0**	**0.00**
Bohol	0	0	0	0.00	0	0	0	0.00
Cebu	0	0	0	0.00	0	0	0	0.00
Negros Oriental	0	0	0	0.00	0	0	0	0.00

Table 1.E.2.3 - Schistosomiasis Prevention and Control

No. and Proportion of Chronic Clinically Diagnosed cases treated in the Health Facility and Confirmed Chronic Cases referred to a Hospital Facility

PHILIPPINES, Annual 2020

Area	No. of Chronic Clinically Diagnosed cases treated in the Health Facility				No. of Confirmed Chronic Cases referred to Hospital Facility			
	Male	Female	Total	%	Male	Female	Total	%
Siquijor	0	0	0	0.00	0	0	0	0.00
Cebu City	0	0	0	0.00	0	0	0	0.00
Lapu-Lapu City	0	0	0	0.00	0	0	0	0.00
Mandaue City	0	0	0	0.00	0	0	0	0.00
**Region 8**	**0**	**0**	**0**	**0.00**	**0**	**0**	**0**	**0.00**
Biliran	0	0	0	0.00	0	0	0	0.00
Eastern Samar	0	0	0	0.00	0	0	0	0.00
Northern Leyte	0	0	0	0.00	0	0	0	0.00
Northern Samar	0	0	0	0.00	0	0	0	0.00
Southern Leyte	0	0	0	0.00	0	0	0	0.00
Western Samar	0	0	0	0.00	0	0	0	0.00
Calbayog City	0	0	0	0.00	0	0	0	0.00
Maasin City	0	0	0	0.00	0	0	0	0.00
Ormoc City	0	0	0	0.00	0	0	0	0.00
Tacloban City	0	0	0	0.00	0	0	0	0.00
**Region 9**	**0**	**0**	**0**	**0.00**	**0**	**0**	**0**	**0.00**
Zamboanga del Norte	0	0	0	0.00	0	0	0	0.00
Zamboanga del Sur	0	0	0	0.00	0	0	0	0.00
Zamboanga Sibugay	0	0	0	0.00	0	0	0	0.00
Dapitan City	0	0	0	0.00	0	0	0	0.00
Dipolog City	0	0	0	0.00	0	0	0	0.00
Isabela City	0	0	0	0.00	0	0	0	0.00
Pagadian City	0	0	0	0.00	0	0	0	0.00
Zamboanga City	0	0	0	0.00	0	0	0	0.00
**Region 10**	**193**	**86**	**279**	**83.78**	**4**	**1**	**5**	**3.01**
Bukidnon	12	3	15	50.00	0	0	0	0.00
Camiguin	0	0	0	0.00	0	0	0	0.00
Lanao del Norte	0	0	0	0.00	0	0	0	0.00
Misamis Occidental	0	0	0	0.00	0	0	0	0.00
Misamis Oriental	0	0	0	0.00	0	0	0	0.00
Cagayan de Oro City	0	0	0	0.00	0	0	0	0.00
El Salvador City	0	0	0	0.00	0	0	0	0.00
Gingoog City	0	0	0	0.00	0	0	0	0.00
Iligan City	0	0	0	0.00	0	0	0	0.00
Malaybalay City	0	0	0	0.00	0	0	0	0.00
Oroquieta City	2	1	3	60.00	4	1	5	100.00
Ozamis City	0	0	0	0.00	0	0	0	0.00
Tangub City	14	1	15	50.00	0	0	0	0.00
Valencia City	165	81	246	96.85	0	0	0	0.00
**Region 11**	**9**	**6**	**15**	**34.09**	**1**	**0**	**1**	**6.67**
Compostela Valley	2	2	4	14.29	0	0	0	0.00

Table 1.E.2.3 - Schistosomiasis Prevention and Control

No. and Proportion of Chronic Clinically Diagnosed cases treated in the Health Facility
and Confirmed Chronic Cases referred to a Hospital Facility
PHILIPPINES, Annual 2020

Area	No. of Chronic Clinically Diagnosed cases treated in the Health Facility				No. of Confirmed Chronic Cases referred to Hospital Facility			
	Male	Female	Total	%	Male	Female	Total	%
Davao del Norte	7	4	11	73.33	1	0	1	20.00
Davao del Sur	0	0	0	0.00	0	0	0	0.00
Davao Oriental	0	0	0	0.00	0	0	0	0.00
Davao Occidental	0	0	0	0.00	0	0	0	0.00
Davao City	0	0	0	0.00	0	0	0	0.00
**Region 12**	**0**	**0**	**0**	**0.00**	**0**	**0**	**0**	**0.00**
North Cotabato	0	0	0	0.00	0	0	0	0.00
Sarangani	0	0	0	0.00	0	0	0	0.00
South Cotabato	0	0	0	0.00	0	0	0	0.00
Sultan Kudarat	0	0	0	0.00	0	0	0	0.00
Cotabato City	0	0	0	0.00	0	0	0	0.00
Gen. Santos City	0	0	0	0.00	0	0	0	0.00
**BARMM**	**0**	**0**	**0**	**0.00**	**0**	**0**	**0**	**0.00**
Basilan	0	0	0	0.00	0	0	0	0.00
Lanao del Sur	0	0	0	0.00	0	0	0	0.00
Maguindanao	0	0	0	0.00	0	0	0	0.00
Sulu	0	0	0	0.00	0	0	0	0.00
Tawi-Tawi	0	0	0	0.00	0	0	0	0.00
				0.00				
Lamitan City	0	0	0	0.00	0	0	0	0.00
Marawi City	0	0	0	0.00	0	0	0	0.00
**CARAGA**	**66**	**46**	**112**	**40.43**	**6**	**0**	**6**	**4.72**
Agusan del Norte	12	5	17	130.77	4	0	4	200.00
Agusan del Sur	44	40	84	44.21	1	0	1	1.33
Surigao del Norte	10	1	11	15.71	1	0	1	2.00
Surigao del Sur	0	0	0	0.00	0	0	0	0.00
Province of Dinagat	0	0	0	0.00	0	0	0	0.00
Bislig City	0	0	0	0.00	0	0	0	0.00
Butuan City	0	0	0	0.00	0	0	0	0.00
Surigao City	0	0	0	0.00	0	0	0	0.00

Legend:
0 = No Case
** = No Report*

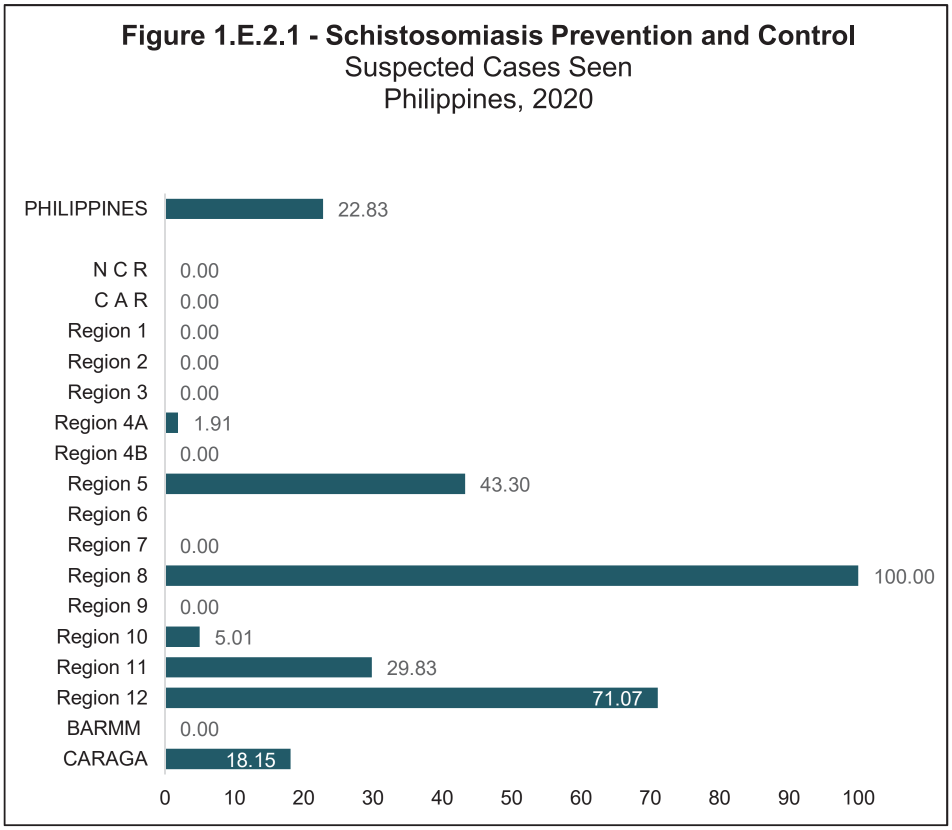
Figure 1.E.2.1 - Schistosomiasis Prevention and Control
Suspected Cases Seen
Philippines, 2020
PHILIPPINES
22.83
N C R
C A R
Region 1
Region 2
Region 3
Region 4A
Region 4B
Region 5
Region 6
Region 7
Region 8
Region 9
Region 10
Region 11
Region 12
BARMM
CARAGA
0.00
0.00
0.00
0.00
0.00
1.91
0.00
43.30
0.00
100.00
0.00
5.01
29.83
71.07
0.00
18.15
0
10
20
30
40
50
60
70
80
90
100

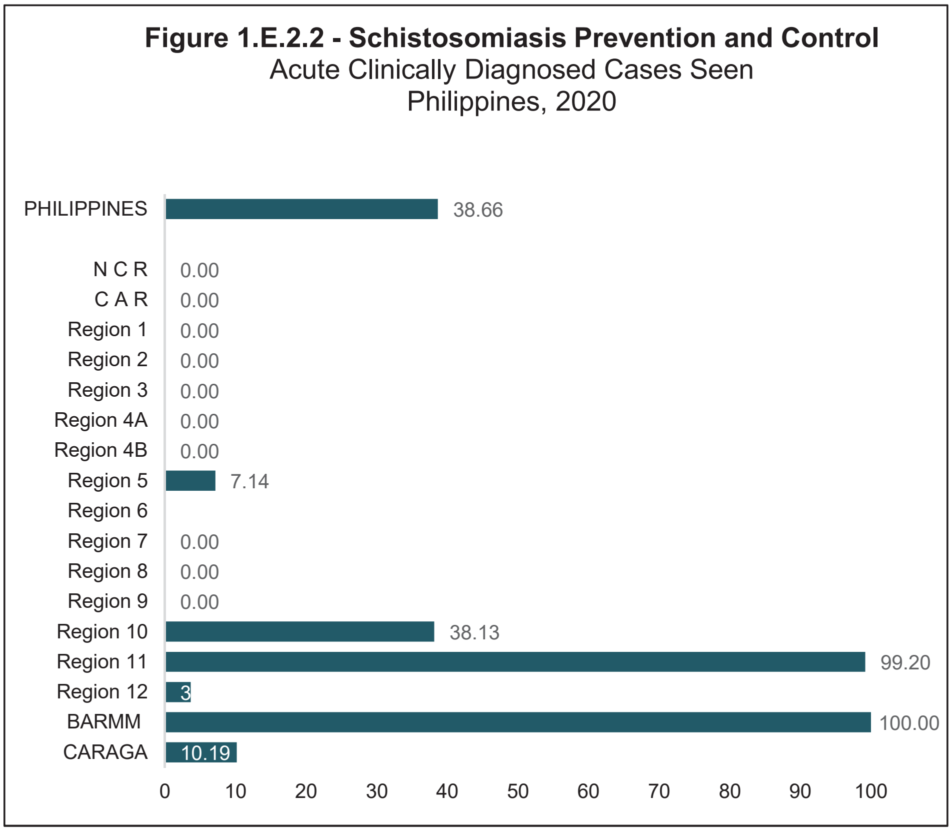
Figure 1.E.2.2 - Schistosomiasis Prevention and Control
Acute Clinically Diagnosed Cases Seen
Philippines, 2020
PHILIPPINES
38.66
N C R
C A R
Region 1
Region 2
Region 3
Region 4A
Region 4B
Region 5
Region 6
Region 7
Region 8
Region 9
Region 10
Region 11
Region 12
BARMM
CARAGA
0.00
0.00
0.00
0.00
0.00
0.00
0.00
7.14
0.00
0.00
0.00
38.13
99.20
3
100.00
10.19
0
10
20
30
40
50
60
70
80
90
100

Figure 1.E.2.3 - Schistosomiasis Prevention and Control
Acute Confirmed Cases by Stool Examination
Philippines, 2020

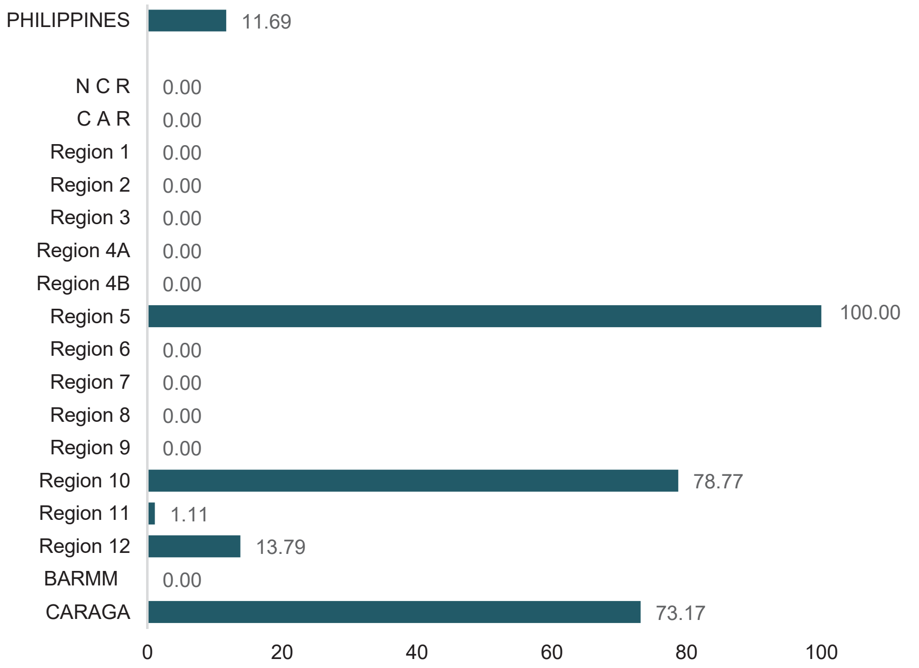

Table 1.E.2.4 - Schistosomiasis Prevention and Control
Proportion Chronic Clinically Diagnosed Cases Seen
Philippines, 2020

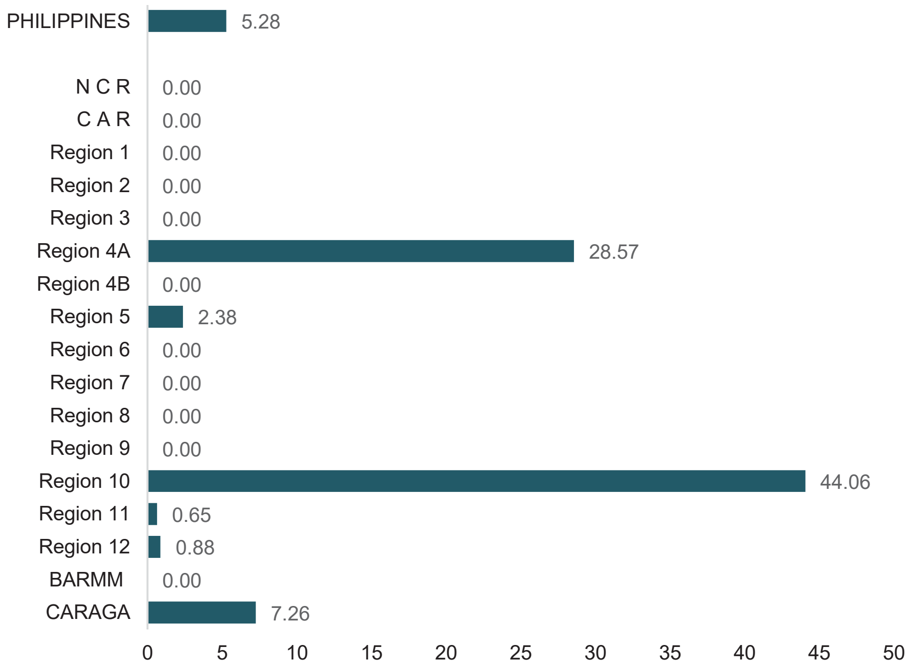

Table 1.E.2.5 - Schistosomiasis Prevention and Control
Proportion of Chronic Cllinically diagnosed cases confirmed
by stool examinantion
Philippines, 2020

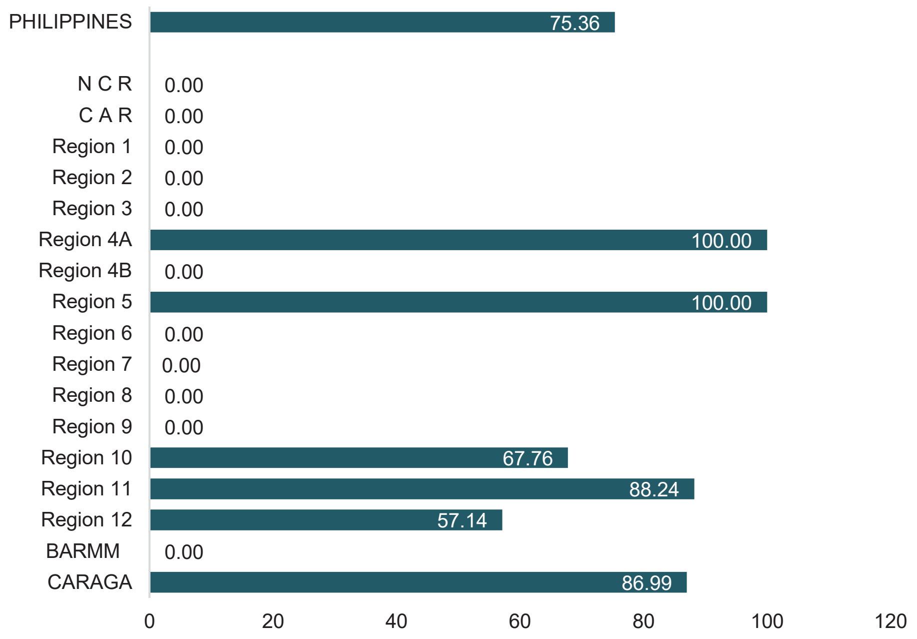

Table 1.E.2.6 - Schistosomiasis Prevention and Control
Proportion of Chronic Clinically Diagnosed cases treated in
the Health Facility
Philippines, 2020

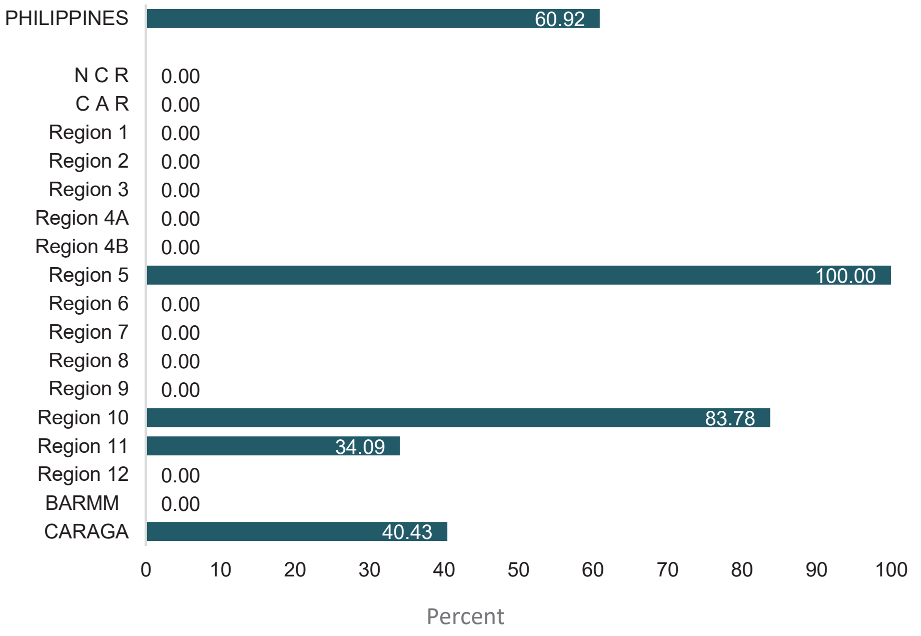

Table 1.E.2.7 - Schistosomiasis Prevention and Control
Proportion of Confirmed Chronic Cases referred to Hospital Facility
Philippines, 2020

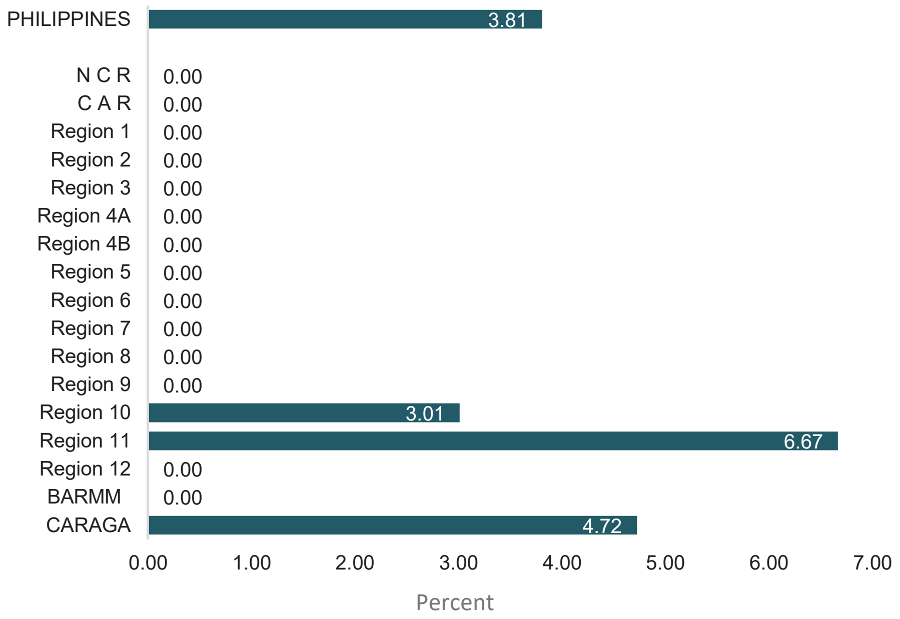

Table 1.E.3.1 - Soil Transmitted Helminthiasis Prevention and Control

No. and Proportion of PSAC [1](1-4) and SAC [2](5-9) years old who completed 2 doses of deworming tablets

PHILIPPINES, Annual 2020

Area	Elig. Pop	Number PSAC [1] (1-4 yrs. Old) who completed 2 doses of deworming tablets				Elig. Pop	Number SAC [2] (5-9 yrs. Old) who completed 2 doses of deworming tablets			
		Male	Female	Total	%		Male	Female	Total	%
**PHILIPPINES**	**8,941,598**	**1,932,724**	**1,854,347**	**3,787,071**	**42.35**	**11,089,133**	**1,305,274**	**1,208,093**	**2,513,367**	**22.67**
**NCR**	**959,072**	**204,435**	**253,075**	**457,510**	**47.70**	**1,218,972**	**116,086**	**136,978**	**253,064**	**20.76**
Malabon City	27,220	5,628	5,223	10,851	39.86	34,600	938	960	1,898	5.49
Navotas City	18,580	1,599	1,495	3,094	16.65	23,615	791	732	1,523	6.45
Valenzuela City	46,210	2,428	2,768	5,196	11.24	58,728	0	0	0	0.00
Caloocan City	117,972	30,419	29,681	60,100	50.94	149,936	8,500	8,077	16,577	11.06
Marikina City	33,571	834	890	1,724	5.14	42,668	284	270	554	1.30
Pasig City	56,251	5,426	4,632	10,058	17.88	71,496	789	796	1,585	2.22
Pateros	4,755	813	974	1,787	37.58	6,046	342	366	708	11.71
Taguig City	59,949	28,367	31,582	59,949	100.00	76,193	34,344	33,715	68,059	89.32
Quezon City	218,674	35,059	37,733	72,792	33.29	277,938	6,712	6,894	13,606	4.90
Makati City	43,390	7,732	7,535	15,267	35.19	55,151	2,232	2,154	4,386	7.95
Mandaluyong City	28,766	4,204	4,106	8,310	28.89	36,563	93	124	217	0.59
San Juan City	9,101	0	0	0	0.00	11,563	0	0	0	0.00
Manila City	132,587	44,421	88,166	132,587	100.00	168,516	47,115	68,941	116,056	68.87
Las Piñas City	43,860	5,901	6,197	12,098	27.58	55,745	2,170	2,120	4,290	7.70
Muntinlupa City	37,574	18,581	18,993	37,574	100.00	47,757	8,916	9,025	17,941	37.57
Parañaque City	49,589	9,907	10,086	19,993	40.32	63,027	2,596	2,539	5,135	8.15
Pasay City	31,023	3,116	3,014	6,130	19.76	39,430	264	265	529	1.34
**CAR**	**146,939**	**36,839**	**34,496**	**71,335**	**48.55**	**180,835**	**17,962**	**16,989**	**34,951**	**19.33**
Abra	19,128	6,196	5,878	12,074	63.12	23,939	1,814	1,776	3,590	18.77
Apayao	10,841	4,001	3,606	7,607	70.17	13,208	762	790	1,552	14.32
Benguet	36,459	8,388	7,698	16,086	44.12	44,545	1,044	1,034	2,078	5.70
Ifugao	19,116	5,618	5,327	10,945	57.26	23,298	6,451	5,958	12,409	64.91
Kalinga	19,738	4,391	4,299	8,690	44.03	24,377	2,365	2,203	4,568	23.14
Mt. Province	13,439	4,003	3,620	7,623	56.72	16,997	4,262	3,928	8,190	60.94
Baguio City	28,218	4,242	4,068	8,310	29.45	34,471	1,264	1,300	2,564	9.09
**Region 1**	**407,689**	**93,281**	**90,947**	**184,228**	**45.19**	**518,508**	**46,581**	**43,870**	**90,451**	**17.44**
Ilocos Norte	33,003	17,471	16,747	34,218	103.68	42,073	1,863	1,631	3,494	8.30
Ilocos Sur	40,744	14,402	13,745	28,147	69.08	52,587	9,557	9,189	18,746	35.65
La Union	48,703	28,197	26,541	54,738	112.39	62,338	22,810	20,703	43,513	69.80
Pangasinan	209,264	20,431	20,243	40,674	19.44	264,918	6,218	6,325	12,543	4.73
Alaminos City	7,908	2,512	3,349	5,861	74.11	10,011	0	0	0	0.00
Candon City	4,292	4,709	4,642	9,351	217.87	5,545	3,216	3,233	6,449	116.30
Dagupan City	15,094	2,124	2,143	4,267	28.27	19,112	1,370	1,307	2,677	14.01
Laoag City	7,609	1,117	1,204	2,321	30.50	9,700	0	0	0	0.00
San Carlos City	16,616	1	1	2	0.01	21,044	0	2	2	0.01
San Fernando City	8,922	647	658	1,305	14.63	11,423	510	452	962	8.42
Urdaneta City	11,718	0	0	0	0.00	14,833	0	0	0	0.00
Vigan City	3,816	1,670	1,674	3,344	87.63	4,924	1,037	1,028	2,065	41.94
**Region 2**	**291,567**	**85,155**	**78,703**	**163,858**	**56.20**	**364,952**	**25,034**	**23,436**	**48,470**	**13.28**
Batanes	1,518	314	247	561	36.96	1,857	39	47	86	4.63
Cagayan	86,777	30,125	28,259	58,384	67.28	108,840	5,253	5,076	10,329	9.49
Isabela	98,803	28,204	26,570	54,774	55.44	124,533	12,317	11,919	24,236	19.46
Nueva Vizcaya	40,344	12,300	10,449	22,749	56.39	49,227	2,576	2,557	5,133	10.43
Quirino	17,186	3,100	2,498	5,598	32.57	21,388	1,915	874	2,789	13.04
Cauayan City	10,804	884	990	1,874	17.35	13,629	538	541	1,079	7.92
Ilagan City	12,151	2,008	2,041	4,049	33.32	15,323	217	477	694	4.53

Table 1.E.3.1 - Soil Transmitted Helminthiasis Prevention and Control

No. and Proportion of PSAC [1](1-4) and SAC [2](5-9) years old who completed 2 doses of deworming tablets

PHILIPPINES, Annual 2020

Area	Elig. Pop	Number PSAC [1] (1-4 yrs. Old) who completed 2 doses of deworming tablets				Elig. Pop	Number SAC [2] (5-9 yrs. Old) who completed 2 doses of deworming tablets			
		Male	Female	Total	%		Male	Female	Total	%
Santiago City	11,254	3,127	2,834	5,961	52.97	14,181	403	340	743	5.24
Tuguegarao City	12,730	5,093	4,815	9,908	77.83	15,974	1,776	1,605	3,381	21.17
**Region 3**	**912,991**	**351,446**	**232,252**	**583,698**	**63.93**	**1,158,742**	**280,653**	**154,839**	**435,492**	**37.58**
Aurora	20,464	7,895	7,607	15,502	75.75	24,766	4,492	4,245	8,737	35.28
Bataan	54,850	28,533	27,318	55,851	101.82	69,111	9,315	8,874	18,189	26.32
Bulacan	180,961	36,472	37,612	74,084	40.94	232,440	39,027	37,679	76,706	33.00
Nueva Ecija	121,573	24,447	23,778	48,225	39.67	154,631	12,957	12,286	25,243	16.32
Pampanga	130,856	124,859	4,327	129,186	98.72	166,792	125,224	3,855	129,079	77.39
Tarlac	81,757	21,332	25,319	46,651	57.06	103,427	14,038	13,946	27,984	27.06
Zambales	51,197	9,435	9,205	18,640	36.41	62,518	9,726	8,965	18,691	29.90
Angeles City	32,828	3,011	2,844	5,855	17.84	41,853	2,456	2,360	4,816	11.51
Balanga City	7,927	3,900	3,852	7,752	97.79	9,987	0	0	0	0.00
Cabanatuan City	24,884	8,696	7,984	16,680	67.03	31,642	3,687	3,340	7,027	22.21
City of San Fernando	24,459	3,166	3,675	6,841	27.97	31,176	2,714	3,862	6,576	21.09
Gapan City	9,083	5,685	5,554	11,239	123.74	11,551	2,037	2,087	4,124	35.70
Mabalacat City	20,000	8,737	8,359	17,096	85.48	25,495	7,473	7,318	14,791	58.02
Malolos City	20,216	10,055	9,394	19,449	96.21	25,961	11,646	10,846	22,492	86.64
Meycauayan City	16,766	3,149	3,063	6,212	37.05	21,533	4,051	3,968	8,019	37.24
Olongapo City	20,191	2,771	2,547	5,318	26.34	24,655	1,892	1,809	3,701	15.01
Palayan City	3,377	3,596	3,614	7,210	213.50	4,299	1,798	1,807	3,605	83.86
San Jose City	11,500	4,638	3,950	8,588	74.68	14,635	2,011	1,930	3,941	26.93
San Jose del Monte Ci	46,033	27,644	28,655	56,299	122.30	59,129	17,468	17,114	34,582	58.49
Science City of Munoz	6,708	6,180	6,246	12,426	185.24	8,533	37	25	62	0.73
Tarlac City	27,361	7,245	7,349	14,594	53.34	34,608	8,604	8,523	17,127	49.49
**Region 4A**	**1,227,422**	**19,117**	**17,303**	**36,420**	**2.97**	**1,534,044**	**17,437**	**15,566**	**33,003**	**2.15**
Batangas	162,024	0	0	0	0.00	198,436	0	0	0	0.00
Cavite	113,921	920	796	1,716	1.51	144,040	0	0	0	0.00
Laguna	80,610	1,533	1,386	2,919	3.62	100,562	1,836	1,619	3,455	3.44
Quezon	158,632	3,397	2,498	5,895	3.72	200,155	213	202	415	0.21
Rizal	181,864	0	0	0	0.00	227,086	2,347	2,211	4,558	2.01
Antipolo City	66,984	130	138	268	0.40	83,642	0	0	0	0.00
Bacoor City	49,915	0	0	0	0.00	63,114	0	0	0	0.00
Batangas City	28,751	0	0	0	0.00	35,217	0	0	0	0.00
Biñan City	27,031	7,343	6,823	14,166	52.41	33,723	6,677	5,907	12,584	37.32
Cabuyao City	25,061	1,377	1,300	2,677	10.68	31,259	816	578	1,394	4.46
Calamba City	36,888	1,417	1,557	2,974	8.06	46,023	2,171	2,087	4,258	9.25
Cavite City	8,541	0	0	0	0.00	10,795	0	0	0	0.00
Dasmariñas City	54,762	105	102	207	0.38	69,250	65	60	125	0.18
General Trias City	26,122	0	0	0	0.00	33,027	0	0	0	0.00
Imus City	33,554	0	0	0	0.00	42,432	0	0	0	0.00
Lipa City	28,975	0	0	0	0.00	35,488	0	0	0	0.00
Lucena City	24,048	0	0	0	0.00	30,332	0	0	0	0.00
San Pablo City	21,595	1	1	2	0.01	26,932	0	0	0	0.00
San Pedro City	26,445	2,815	2,614	5,429	20.53	32,991	0	0	0	0.00
Santa Rosa City	28,715	79	88	167	0.58	35,818	3,312	2,902	6,214	17.35
Tagaytay City	5,917	0	0	0	0.00	7,481	0	0	0	0.00
Tanauan City	15,111	0	0	0	0.00	18,509	0	0	0	0.00
Tayabas City	9,016	0	0	0	0.00	11,368	0	0	0	0.00
Trece Martires City	12,940	0	0	0	0.00	16,364	0	0	0	0.00
**Region 4B**	**298,390**	**74,027**	**68,109**	**142,136**	**47.63**	**370,516**	**34,851**	**32,621**	**67,472**	**18.21**
Marinduque	21,709	11,635	10,552	22,187	102.20	27,424	2,282	2,103	4,385	15.99
Mindoro Occidental	50,349	19,762	18,409	38,171	75.81	62,012	6,570	6,067	12,637	20.38
Mindoro Oriental	83,165	23,564	21,106	44,670	53.71	104,232	15,187	13,936	29,123	27.94
Palawan	88,284	12,542	11,932	24,474	27.72	109,150	6,766	6,371	13,137	12.04

Table 1.E.3.1 - Soil Transmitted Helminthiasis Prevention and Control

No. and Proportion of PSAC [1](1-4) and SAC [2](5-9) years old who completed 2 doses of deworming tablets

PHILIPPINES, Annual 2020

Area	Elig. Pop	Number PSAC [1] (1-4 yrs. Old) who completed 2 doses of deworming tablets				Elig. Pop	Number SAC [2] (5-9 yrs. Old) who completed 2 doses of deworming tablets			
		Male	Female	Total	%		Male	Female	Total	%
Romblon	28,368	2,580	2,392	4,972	17.53	34,915	1,623	1,554	3,177	9.10
Puerto Princesa City	26,515	3,944	3,718	7,662	28.90	32,783	2,423	2,590	5,013	15.29
**Region 5**	**586,320**	**157,415**	**150,001**	**307,416**	**52.43**	**709,108**	**157,048**	**147,110**	**304,158**	**42.89**
Albay	103,227	46,468	43,980	90,448	87.62	128,530	54,729	51,964	106,693	83.01
Camarines Norte	59,893	24,604	24,375	48,979	81.78	71,010	22,882	21,847	44,729	62.99
Camarines Sur	166,213	2,597	2,467	5,064	3.05	202,028	3,060	2,968	6,028	2.98
Catanduanes	26,561	15,324	14,310	29,634	111.57	31,863	13,320	12,365	25,685	80.61
Masbate	99,437	22,863	21,500	44,363	44.61	117,083	25,053	23,162	48,215	41.18
Sorsogon	81,734	29,106	27,328	56,434	69.05	98,190	24,023	21,677	45,700	46.54
Iriga City	11,291	3,613	3,425	7,038	62.33	13,728	1,739	1,741	3,480	25.35
Legaspi City	18,151	7,374	7,282	14,656	80.74	22,600	6,841	6,490	13,331	58.99
Naga City	19,813	5,466	5,334	10,800	54.51	24,076	5,401	4,896	10,297	42.77
**Region 6**	**623,733**	**189,154**	**210,177**	**399,331**	**64.02**	**781,067**	**114,573**	**128,850**	**243,423**	**31.17**
Aklan	47,850	28,725	25,676	54,401	113.69	60,473	18,550	16,566	35,116	58.07
Antique	53,822	20,246	18,452	38,698	71.90	67,343	23,245	21,994	45,239	67.18
Capiz	60,081	18,354	19,155	37,509	62.43	74,216	9,167	9,480	18,647	25.13
Guimaras	14,501	6,061	5,785	11,846	81.69	17,641	3,469	3,056	6,525	36.99
Iloilo	153,611	53,243	79,865	133,108	86.65	193,219	35,551	53,326	88,877	46.00
Negros Occidental	210,882	35,347	34,322	69,669	33.04	264,052	4,023	4,543	8,566	3.24
Bacolod City	47,451	24,910	24,718	49,628	104.59	59,410	19,808	19,211	39,019	65.68
Iloilo City	35,535	2,268	2,204	4,472	12.58	44,713	760	674	1,434	3.21
**Region 7**	**652,415**	**89,858**	**86,912**	**176,770**	**27.09**	**814,449**	**22,845**	**22,416**	**45,261**	**5.56**
Bohol	115,081	32,005	30,998	63,003	54.75	145,394	1,764	1,575	3,339	2.30
Cebu	263,210	23,499	22,405	45,904	17.44	327,915	7,462	7,257	14,719	4.49
Negros Oriental	115,018	11,107	11,402	22,509	19.57	142,678	2,651	3,093	5,744	4.03
Siquijor	7,440	2,686	2,565	5,251	70.58	9,519	4,068	3,942	8,010	84.15
Cebu City	82,633	15,512	14,389	29,901	36.19	102,939	5,320	4,973	10,293	10.00
Lapu-Lapu City	36,553	4,841	4,886	9,727	26.61	45,536	1,580	1,576	3,156	6.93
Mandaue City	32,480	208	267	475	1.46	40,468	0	0	0	0.00
**Region 8**	**433,112**	**91,023**	**84,931**	**175,954**	**40.63**	**525,349**	**37,695**	**37,897**	**75,592**	**14.39**
Biliran	16,820	9,453	7,176	16,629	98.86	19,961	8,594	7,978	16,572	83.02
Eastern Samar	46,281	9,374	8,781	18,155	39.23	56,723	3,658	3,676	7,334	12.93
Northern Leyte	143,608	28,860	27,193	56,053	39.03	174,787	2,975	2,909	5,884	3.37
Northern Samar	67,406	16,522	16,799	33,321	49.43	80,514	13,906	15,220	29,126	36.18
Southern Leyte	28,790	8,589	8,008	16,597	57.65	36,435	3,967	3,837	7,804	21.42
Western Samar	60,698	6,116	5,864	11,980	19.74	72,410	2,322	2,171	4,493	6.20
Calbayog City	18,707	385	367	752	4.02	22,321	66	102	168	0.75
Maasin City	7,329	121	103	224	3.06	9,270	0	0	0	0.00
Ormoc City	20,450	9,415	8,572	17,987	87.96	24,892	1,858	1,682	3,540	14.22
Tacloban City	23,023	2,188	2,068	4,256	18.49	28,036	349	322	671	2.39
**Region 9**	**339,485**	**138,421**	**142,890**	**281,311**	**82.86**	**413,615**	**119,569**	**139,485**	**210,503**	**50.89**
Zamboanga del Norte	74,872	53,583	54,454	108,037	144.30	90,365	43,242	49,342	92,584	102.46
Zamboanga del Sur	72,143	25,553	26,315	51,868	71.90	89,016	21,856	29,504	2,809	3.16
Zamboanga Sibugay	66,111	29,348	27,724	57,072	86.33	79,622	26,171	27,272	53,443	67.12
Dapitan City	7,736	4,085	4,142	8,227	106.35	9,334	4,565	5,267	9,832	105.34
Dipolog City	12,264	9,113	12,017	21,130	172.29	14,803	7,375	10,555	17,930	121.12

Table 1.E.3.1 - Soil Transmitted Helminthiasis Prevention and Control

No. and Proportion of PSAC [1](1-4) and SAC [2](5-9) years old who completed 2 doses of deworming tablets

PHILIPPINES, Annual 2020

Area	Elig. Pop	Number PSAC [1] (1-4 yrs. Old) who completed 2 doses of deworming tablets				Elig. Pop	Number SAC [2] (5-9 yrs. Old) who completed 2 doses of deworming tablets			
		Male	Female	Total	%		Male	Female	Total	%
Isabela City	12,059	31	36	67	0.56	14,129	131	152	283	2.00
Pagadian City	17,694	1,937	2,032	3,969	22.43	21,831	2,054	1,959	4,013	18.38
Zamboanga City	76,606	14,771	16,170	30,941	40.39	94,515	14,175	15,434	29,609	31.33
**Region 10**	**434,937**	**151,973**	**161,406**	**313,379**	**72.05**	**529,358**	**137,525**	**135,449**	**272,974**	**51.57**
Bukidnon	100,578	45,065	49,015	94,080	93.54	121,110	56,030	54,878	110,908	91.58
Camiguin	8,000	0	0	0	0.00	9,619	0	0	0	0.00
Lanao del Norte	67,507	28,979	29,774	58,753	87.03	82,121	19,246	20,881	40,127	48.86
Misamis Occidental	27,424	6,724	7,919	14,643	53.39	34,240	7,485	7,912	15,397	44.97
Misamis Oriental	63,311	36,735	35,704	72,439	114.42	77,205	35,920	31,406	67,326	87.20
Cagayan de Oro City	59,976	7,637	10,149	17,786	29.66	73,139	0	0	0	0.00
El Salvador City	4,456	1,946	2,379	4,325	97.06	5,434	2,664	3,256	5,920	108.94
Gingoog City	11,052	3,122	5,690	8,812	79.73	13,494	1,221	1,003	2,224	16.48
Iligan City	34,195	10,673	11,360	22,033	64.43	41,600	8,768	9,558	18,326	44.05
Malaybalay City	16,766	6,251	4,540	10,791	64.36	20,189	2,780	2,922	5,702	28.24
Oroquieta City	5,942	2,099	1,909	4,008	67.45	7,418	354	323	677	9.13
Ozamis City	11,909	548	582	1,130	9.49	14,869	618	854	1,472	9.90
Tangub City	5,293	0	0	0	0.00	6,607	0	0	0	0.00
Valencia City	18,528	2,194	2,385	4,579	24.71	22,313	2,439	2,456	4,895	21.94
**Region 11**	**448,208**	**64,860**	**60,114**	**124,974**	**27.88**	**556,337**	**22,512**	**20,682**	**43,194**	**7.76**
Compostela Valley	68,129	9,286	8,760	18,046	26.49	83,118	5,588	5,213	10,801	12.99
Davao del Norte	87,663	26,889	25,388	52,277	59.63	109,372	7,764	6,820	14,584	13.33
Davao del Sur	56,161	9,406	8,586	17,992	32.04	67,926	3,041	3,062	6,103	8.98
Davao Oriental	57,348	10,147	9,481	19,628	34.23	71,687	3,946	3,802	7,748	10.81
Davao Occidental	30,859	3,216	2,187	5,403	17.51	39,153	1,204	1,135	2,339	5.97
Davao City	148,048	5,916	5,712	11,628	7.85	185,081	969	650	1,619	0.87
**Region 12**	**437,544**	**66,382**	**65,176**	**131,558**	**30.07**	**538,919**	**53,909**	**54,072**	**107,981**	**20.04**
North Cotabato	139,074	12,038	11,990	24,028	17.28	171,738	4,018	3,870	7,888	4.59
Sarangani	54,603	16,990	16,841	33,831	61.96	66,685	14,509	14,302	28,811	43.20
South Cotabato	85,230	9,122	8,679	17,801	20.89	103,762	2,695	3,017	5,712	5.50
Sultan Kudarat	74,909	11,054	11,204	22,258	29.71	94,222	11,546	11,432	22,978	24.39
Cotabato City	28,378	7,794	7,469	15,263	53.78	35,115	17,281	16,786	34,067	97.02
Gen. Santos City	55,350	9,384	8,993	18,377	33.20	67,397	3,860	4,665	8,525	12.65
**BARMM**	**483,196**	**126**	**130**	**256**	**0.05**	**566,299**	**99**	**83**	**182**	**0.03**
Basilan	35,490	0	0	0	0.00	41,356	0	0	0	0.00
Lanao del Sur	110,328	0	0	0	0.00	128,049	0	0	0	0.00
Maguindanao	148,947	126	130	256	0.17	177,629	99	83	182	0.10
Sulu	107,280	0	0	0	0.00	123,774	0	0	0	0.00
Tawi-Tawi	44,992	0	0	0	0.00	53,485	0	0	0	0.00
Lamitan City	9,764	0	0	0	0.00	11,374	0	0	0	0.00
Marawi City	26,395	0	0	0	0.00	30,632	0	0	0	0.00
**CARAGA**	**258,578**	**119,212**	**117,725**	**236,937**	**91.63**	**308,063**	**100,895**	**97,750**	**198,645**	**64.48**
Agusan del Norte	34,103	7,541	7,474	15,015	44.03	40,882	7,170	6,582	13,752	33.64
Agusan del Sur	76,814	41,014	40,982	81,996	106.75	88,260	17,224	15,769	32,993	37.38
Surigao del Norte	31,554	10,691	10,390	21,081	66.81	38,709	16,399	15,647	32,046	82.79
Surigao del Sur	48,534	23,903	24,541	48,444	99.81	58,122	26,283	26,274	52,557	90.43
Province of Dinagat	11,231	4,730	4,348	9,078	80.83	14,160	5,044	5,167	10,211	72.11
Bislig City	9,222	2,667	2,542	5,209	56.48	11,041	2,099	2,002	4,101	37.14

Table 1.E.3.1 - Soil Transmitted Helminthiasis Prevention and Control

No. and Proportion of PSAC [1](1-4) and SAC [2](5-9) years old who completed 2 doses of deworming tablets

PHILIPPINES, Annual 2020

Area	Elig. Pop	Number PSAC [1] (1-4 yrs. Old) who completed 2 doses of deworming tablets				Elig. Pop	Number SAC [2] (5-9 yrs. Old) who completed 2 doses of deworming tablets			
		Male	Female	Total	%		Male	Female	Total	%
Butuan City	32,429	25,650	24,520	50,170	154.71	38,862	21,049	20,661	41,710	107.33
Surigao City	14,691	3,016	2,928	5,944	40.46	18,027	5,627	5,648	11,275	62.55

Legend:
0 = No Case
** = No Report*

PSAC [1] - Pre School Age Children
SAC [2] - School Age Children

Table 1.E.3.2 - Soil Transmitted Helminthiasis Prevention and Control

No. and Proportion of Adolecent (10-19 Years Old) and WRA (20-49 years old) who completed 2 doses of deworming tablets
PHILIPPINES, Annual 2020

Area	Elig. Pop (TP x GR factor)	No. of Adolecents (10-19 yrs. Old) who completed 2 doses of deworming tablets				Elig. Pop (TP x GR factor)	No of WRA (20 - 49 yrs. Old) who completed 2 doses of deworming tablets	
		Male	Female	Total	%		Female	%
**PHILIPPINES**	**21,139,331**	**1,694,251**	**1,580,867**	**3,275,118**	**15.49**	**22,947,249**	**131,937**	0.57
**N C R**	2,436,251	71,905	76,342	148,247	6.09	3,261,045	9,526	0.29
Malabon City	69,154	160	144	304	0.44	92,565	0	0.00
Navotas City	47,197	533	516	1,049	2.22	63,173	43	0.07
Valenzuela City	117,378	0	0	0	0.00	157,116	0	0.00
Caloocan City	299,669	6,206	6,226	12,432	4.15	401,132	8,269	2.06
Marikina City	85,275	239	271	510	0.60	114,144	639	0.56
Pasig City	142,894	113	115	228	0.16	191,274	0	0.00
Pateros	12,078	254	261	515	4.26	16,167	249	1.54
Taguig City	152,281	32,620	34,730	67,350	44.23	203,838	14	0.01
Quezon City	555,484	3,929	4,431	8,360	1.50	743,545	0	0.00
Makati City	110,220	1,034	899	1,933	1.75	147,537	265	0.18
Mandaluyong City	73,078	24	24	48	0.07	97,822	0	0.00
San Juan City	23,116	0	0	0	0.00	30,942	0	0.00
Manila City	336,793	13,365	15,765	29,130	8.65	450,806	0	0.00
Las Piñas City	111,417	1,574	1,604	3,178	2.85	149,130	0	0.00
Muntinlupa City	95,448	10,204	9,721	19,925	20.88	127,763	0	0.00
Parañaque City	125,966	1,650	1,635	3,285	2.61	168,614	47	0.03
Pasay City	78,803	0	0	0	0.00	105,477	0	0.00
**C A R**	350,174	27,355	26,556	53,911	15.40	385,390	0	0.00
Abra	46,972	2,150	2,061	4,211	8.96	50,073	0	0.00
Apayao	24,918	406	591	997	4.00	24,763	0	0.00
Benguet	88,915	3,823	3,567	7,390	8.31	109,134	0	0.000
Ifugao	43,099	9,851	9,342	19,193	44.53	42,449	0	0.00
Kalinga	45,412	4,025	4,243	8,268	18.21	43,607	0	0.00
Mt. Province	32,035	5,789	5,340	11,129	34.74	30,898	0	0.00
Baguio City	68,823	1,311	1,412	2,723	3.96	84,466	0	0.00
**Region 1**	997,577	70,683	70,780	141,463	14.18	1,078,994	9,568	0.89
Ilocos Norte	86,137	2,753	2,739	5,492	6.38	106,386	961	0.90
Ilocos Sur	105,735	15,525	14,302	29,827	28.21	125,769	240	0.19
La Union	126,283	34,236	35,997	70,233	55.62	148,536	1,503	1.01
Pangasinan	494,145	8,285	8,043	16,328	3.30	499,073	4,799	0.96
Alaminos City	18,674	11	4	15	0.08	18,858	0	0.00
Candon City	11,144	5,989	5,934	11,923	106.99	13,252	260	1.96
Dagupan City	35,647	1,902	1,877	3,779	10.60	36,004	19	0.05
Laoag City	19,860	0	0	0	0.00	24,532	613	2.50
San Carlos City	39,247	4	2	6	0.02	39,643	324	0.82
San Fernando City	23,132	749	661	1,410	6.10	27,216	251	0.92
Urdaneta City	27,670	4	4	8	0.03	27,942	598	2.14
Vigan City	9,903	1,225	1,217	2,442	24.66	11,783	0	0.00

Table 1.E.3.2 - Soil Transmitted Helminthiasis Prevention and Control

No. and Proportion of Adolecent (10-19 Years Old) and WRA (20-49 years old) who completed 2 doses of deworming tablets

PHILIPPINES, Annual 2020

Area	Elig. Pop (TP x GR factor)	No. of Adolecents (10-19 yrs. Old) who completed 2 doses of deworming tablets				Elig. Pop (TP x GR factor)	No of WRA (20 - 49 yrs. Old) who completed 2 doses of deworming tablets	
		Male	Female	Total	%		Female	%
**Region 2**	685,244	4,591	4,153	8,744	1.28	756,585	3,232	0.43
Batanes	3,361	0	0	0	0.00	3,357	0	0.00
Cagayan	206,911	632	295	927	0.45	228,870	2,457	1.07
Isabela	233,162	2,802	2,665	5,467	2.34	261,507	377	0.14
Nueva Vizcaya	92,577	0	0	0	0.00	98,476	0	0.00
Quirino	38,117	21	20	41	0.11	40,216	352	0.88
Cauayan City	25,513	84	76	160	0.63	28,614	46	0.16
Ilagan City	28,669	259	396	655	2.28	32,165	0	0.00
Santiago City	26,564	697	604	1,301	4.90	29,790	0	0.00
Tuguegarao City	30,370	96	97	193	0.64	33,590	0	0.00
**Region 3**	2,307,413	363,184	185,090	548,274	23.76	2,668,783	3,332	0.12
Aurora	45,843	6,317	6,116	12,433	27.12	45,403	190	0.42
Bataan	135,886	6,455	6,273	12,728	9.37	158,946	0	0.00
Bulacan	466,981	52,334	53,817	106,151	22.73	552,523	0	0.00
Nueva Ecija	301,882	9,298	9,007	18,305	6.06	343,036	162	0.05
Pampanga	341,826	179,819	254	180,073	52.68	403,249	212	0.05
Tarlac	205,636	14,261	15,420	29,681	14.43	228,584	0	0.00
Zambales	119,635	13,938	14,051	27,989	23.40	135,674	243	0.18
Angeles City	85,762	1,799	1,888	3,687	4.30	101,172	137	0.14
Balanga City	19,646	0	0	0	0.00	22,975	0	0.00
Cabanatuan City	61,790	3,908	3,739	7,647	12.38	70,204	0	0.00
City of San Fernando	63,892	1,923	2,177	4,100	6.42	75,369	714	0.95
Gapan City	22,550	854	931	1,785	7.92	25,624	0	0.00
Mabalacat City	52,256	11,631	11,492	23,123	44.25	61,641	0	0.00
Malolos City	52,154	18,679	18,163	36,842	70.64	61,715	0	0.00
Meycauayan City	43,264	4,484	4,585	9,069	20.96	51,187	282	0.55
Olongapo City	47,187	1,870	1,831	3,701	7.84	53,515	1,392	2.60
Palayan City	8,392	0	0	0	0.00	9,534	0	0.00
San Jose City	28,571	2,124	1,787	3,911	13.69	32,460	0	0.00
San Jose del Monte C	118,790	22,409	22,008	44,417	37.39	140,552	0	0.00
Science City of Muno	16,661	30	31	61	0.37	18,930	0	0.00
Tarlac City	68,809	11,051	11,520	22,571	32.80	76,490	0	0.00
**Region 4A**	3,029,605	91,074	89,138	180,212	5.95	3,589,926	3,061	0.09
Batangas	393,518	9,253	8,800	18,053	4.59	457,791	0	0.00
Cavite	291,469	17,905	17,256	35,161	12.06	364,314	0	0.00
Laguna	198,309	12,625	12,970	25,595	12.91	254,470	2,059	0.81
Quezon	380,865	3,049	2,956	6,005	1.58	373,121	0	0.00
Rizal	446,436	4,910	5,023	9,933	2.22	526,422	105	0.02
Antipolo City	164,439	0	0	0	0.00	193,897	0	0.00
Bacoor City	127,690	0	0	0	0.00	159,618	0	0.00
Batangas City	69,849	0	0	0	0.00	81,242	0	0.00
Biñan City	66,498	18,803	17,359	36,162	54.38	85,324	485	0.57
Cabuyao City	61,652	638	552	1,190	1.93	79,106	10	0.01
Calamba City	90,755	2,320	1,786	4,106	4.52	116,451	333	0.29
Cavite City	21,848	0	0	0	0.00	27,319	0	0.00

Table 1.E.3.2 - Soil Transmitted Helminthiasis Prevention and Control

No. and Proportion of Adolecent (10-19 Years Old) and WRA (20-49 years old) who completed 2 doses of deworming tablets
PHILIPPINES, Annual 2020

Area	Elig. Pop (TP x GR factor)	No. of Adolecents (10-19 yrs. Old) who completed 2 doses of deworming tablets				Elig. Pop (TP x GR factor)	No of WRA (20 - 49 yrs. Old) who completed 2 doses of deworming tablets	
		Male	Female	Total	%		Female	%
Dasmariñas City	140,114	962	920	1,882	1.34	175,134	0	0.00
General Trias City	66,826	1,351	1,376	2,727	4.08	83,524	37	0.04
Imus City	85,845	313	316	629	0.73	107,304	0	0.00
Lipa City	70,370	0	0	0	0.00	81,861	0	0.00
Lucena City	57,721	0	0	0	0.00	56,550	0	0.00
San Pablo City	53,131	1,920	2,018	3,938	7.41	68,173	0	0.00
San Pedro City	65,056	111	107	218	0.34	83,478	0	0.00
Santa Rosa City	70,644	8,036	7,810	15,846	22.43	90,639	23	0.03
Tagaytay City	15,129	0	0	0	0.00	18,918	0	0.00
Tanauan City	36,706	244	247	491	1.34	42,697	9	0.02
Tayabas City	21,628	0	0	0	0.00	21,194	0	0.00
Trece Martires City	33,107	8,634	9,642	18,276	55.20	41,379	0	0.00
**Region 4B**	668,760	31,997	30,612	62,609	9.36	610,488	0	0.00
Marinduque	49,620	1,167	1,061	2,228	4.49	44,068	0	0.00
Mindoro Occidental	109,972	7,580	7,337	14,917	13.56	96,732	0	0.00
Mindoro Oriental	186,936	21,043	20,130	41,173	22.03	175,816	0	0.00
Palawan	199,006	0	0	0	0.00	184,013	0	0.00
Romblon	63,462	0	0	0	0.00	54,594	0	0.00
Puerto Princesa City	59,764	2,207	2,084	4,291	7.18	55,265	0	0.00
**Region 5**	1,309,409	225,083	218,851	443,934	33.90	1,159,920	13,748	1.19
Albay	245,184	72,190	68,584	140,774	57.42	233,665	310	0.13
Camarines Norte	128,951	36,350	35,156	71,506	55.45	118,757	717	0.60
Camarines Sur	372,689	5,137	4,807	9,944	2.67	329,089	11,975	3.64
Catanduanes	58,075	21,879	23,439	45,318	78.03	50,259	0	0.00
Masbate	209,911	38,275	36,458	74,733	35.60	167,561	654	0.39
Sorsogon	181,744	37,449	35,142	72,591	39.94	157,920	92	0.06
Iriga City	25,325	1,213	3,043	4,256	16.81	22,362	0	0.00
Legaspi City	43,117	9,510	8,972	18,482	42.86	41,091	0	0.00
Naga City	44,413	3,080	3,250	6,330	14.25	39,216	0	0.00
**Region 6**	1,506,147	166,015	189,745	355,760	23.62	1,601,371	15,420	0.96
Aklan	114,290	31,278	28,480	59,758	52.29	123,932	1,853	1.50
Antique	122,416	38,939	37,073	76,012	62.09	116,176	312	0.27
Capiz	150,172	10,553	10,974	21,527	14.33	165,608	3,283	1.98
Guimaras	33,820	4,297	3,809	8,106	23.97	38,055	35	0.09
Iloilo	379,815	55,001	82,507	137,508	36.20	423,715	1,751	0.41
Negros Occidental	504,299	5,479	5,813	11,292	2.24	519,071	7,901	1.52
Bacolod City	113,475	19,623	20,230	39,853	35.12	116,791	285	0.24
Iloilo City	87,860	845	859	1,704	1.94	98,023	0	0.00
**Region 7**	1,537,252	20,993	19,721	40,714	2.65	1,644,844	701	0.04
Bohol	272,769	193	130	323	0.12	260,015	72	0.03
Cebu	616,905	7,649	7,678	15,327	2.48	689,040	0	0.00
Negros Oriental	273,837	3,011	2,233	5,244	1.92	279,099	28	0.01

Table 1.E.3.2 - Soil Transmitted Helminthiasis Prevention and Control

No. and Proportion of Adolecent (10-19 Years Old) and WRA (20-49 years old) who completed 2 doses of deworming tablets
PHILIPPINES, Annual 2020

Area	Elig. Pop (TP x GR factor)	No. of Adolecents (10-19 yrs. Old) who completed 2 doses of deworming tablets				Elig. Pop (TP x GR factor)	No of WRA (20 - 49 yrs. Old) who completed 2 doses of deworming tablets	
		Male	Female	Total	%		Female	%
Siquijor	18,299	7,664	7,220	14,884	81.34	19,676	0	0.00
Cebu City	193,655	1,319	1,206	2,525	1.30	216,307	110	0.05
Lapu-Lapu City	85,664	1,157	1,254	2,411	2.81	95,682	491	0.51
Mandaue City	76,123	0	0	0	0.00	85,025	0	0.00
**Region 8**	988,361	40,573	40,603	81,176	8.21	897,101	4,281	0.48
Biliran	36,618	9,878	11,478	21,356	58.32	33,877	1,344	3.97
Eastern Samar	106,296	2,267	1,957	4,224	3.97	92,769	136	0.15
Northern Leyte	330,139	0	0	0	0.00	314,255	0	0.00
Northern Samar	149,469	22,120	20,777	42,897	28.70	123,764	12	0.01
Southern Leyte	68,795	1,392	1,354	2,746	3.99	68,424	2,573	3.76
Western Samar	137,277	3,548	3,635	7,183	5.23	115,768	0	0.00
Calbayog City	42,291	63	36	99	0.23	35,676	0	0.00
Maasin City	17,505	15	9	24	0.14	17,411	0	0.00
Ormoc City	47,029	1,000	972	1,972	4.19	44,765	216	0.48
Tacloban City	52,942	290	385	675	1.27	50,392	0	0.00
**Region 9**	780,103	169,080	206,796	375,876	48.18	762,178	0	0.00
Zamboanga del Norte	169,312	75,064	81,083	156,147	92.22	160,619	0	0.00
Zamboanga del Sur	171,317	37,389	61,993	99,382	58.01	172,413	0	0.00
Zamboanga Sibugay	143,445	44,458	44,466	88,924	61.99	133,605	0	0.00
Dapitan City	17,483	0	0	0	0.00	16,587	0	0.00
Dipolog City	27,734	12,165	19,247	31,412	113.26	26,308	0	0.00
Isabela City	26,882	0	0	0	0.00	27,296	0	0.00
Pagadian City	42,023	4	7	11	0.03	42,284	0	0.00
Zamboanga City	181,907	17,919	18,148	36,067	19.83	183,066	0	0.00
**Region 10**	997,830	202,747	209,507	412,254	41.32	1,022,340	32,686	3.20
Bukidnon	225,350	69,668	70,148	139,816	62.04	223,317	508	0.23
Camiguin	17,959	0	0	0	0.00	17,494	0	0.00
Lanao del Norte	153,822	41,106	48,832	89,938	58.47	148,997	0	0.00
Misamis Occidental	65,971	11,133	10,866	21,999	33.35	66,744	2,998	4.49
Misamis Oriental	146,905	41,268	38,765	80,033	54.48	162,218	0	0.00
Cagayan de Oro City	139,147	0	0	0	0.00	153,645	0	0.00
El Salvador City	10,336	3,568	4,361	7,929	76.71	11,413	0	0.00
Gingoog City	25,656	2,121	1,999	4,120	16.06	28,336	0	0.00
Iligan City	77,917	24,049	24,531	48,580	62.35	75,474	29,180	38.66
Malaybalay City	37,566	4,880	5,137	10,017	26.67	37,223	0	0.00
Oroquieta City	14,299	391	311	702	4.91	14,466	0	0.00
Ozamis City	28,656	1,270	1,158	2,428	8.47	28,991	0	0.00
Tangub City	12,731	0	0	0	0.00	12,882	0	0.00
Valencia City	41,515	3,293	3,399	6,692	16.12	41,140	0	0.00
**Region 11**	1,037,244	19,795	18,981	38,776	3.74	1,091,253	4,527	0.41
Compostela Valley	152,579	4,606	4,126	8,732	5.72	154,829	357	0.23

Table 1.E.3.2 - Soil Transmitted Helminthiasis Prevention and Control

No. and Proportion of Adolecent (10-19 Years Old) and WRA (20-49 years old) who completed 2 doses of deworming tablets

PHILIPPINES, Annual 2020

Area	Elig. Pop (TP x GR factor)	No. of Adolecents (10-19 yrs. Old) who completed 2 doses of deworming tablets				Elig. Pop (TP x GR factor)	No of WRA (20 - 49 yrs. Old) who completed 2 doses of deworming tablets	
		Male	Female	Total	%		Female	%
Davao del Norte	202,917	9,162	8,748	17,910	8.83	219,579	146	0.07
Davao del Sur	124,424	2,299	2,203	4,502	3.62	113,002	303	0.27
Davao Oriental	136,340	2,580	2,489	5,069	3.72	149,745	3,528	2.36
Davao Occidental	69,065	909	882	1,791	2.59	67,542	193	0.29
Davao City	351,919	239	533	772	0.22	386,556	0	0.00
**Region 12**	1,002,796	38,435	47,983	86,418	8.62	1,024,918	28,124	2.74
North Cotabato	314,112	528	652	1,180	0.38	309,705	0	0.00
Sarangani	125,021	1,737	1,790	3,527	2.82	116,287	2,104	1.81
South Cotabato	195,419	1,722	1,867	3,589	1.84	212,483	290	0.14
Sultan Kudarat	175,410	17,145	17,191	34,336	19.57	179,126	25,703	14.35
Cotabato City	65,916	12,804	11,982	24,786	37.60	69,314	0	0.00
Gen. Santos City	126,918	4,499	14,501	19,000	14.97	138,003	27	0.02
**ARMM**	946,794	59	60	119	0.01	858,357	0	0.00
Basilan	69,446	0	0	0	0.00	60,288	0	0.00
Lanao del Sur	206,497	0	0	0	0.00	194,896	0	0.00
Maguindanao	301,147	59	60	119	0.04	265,992	0	0.00
Sulu	208,263	0	0	0	0.00	188,991	0	0.00
Tawi-Tawi	92,942	0	0	0	0.00	84,987	0	0.00
Lamitan City	19,104	0	0	0	0.00	16,589	0	0.00
Marawi City	49,395	0	0	0	0.00	46,614	0	0.00
**CARAGA**	558,371	150,682	145,949	296,631	53.12	533,756	3,731	0.70
Agusan del Norte	75,453	11,592	11,353	22,945	30.41	75,115	13	0.02
Agusan del Sur	151,814	25,678	24,570	50,248	33.10	140,690	1	0.00
Surigao del Norte	73,380	25,314	24,048	49,362	67.27	70,549	3,717	5.27
Surigao del Sur	105,770	40,096	40,515	80,611	76.21	99,164	0	0.00
Province of Dinagat	25,959	8,004	8,949	16,953	65.31	25,137	0	0.00
								0.00
Bislig City	20,089	0	0	0	0.00	18,834	0	0.00
Butuan City	71,736	30,893	28,388	59,281	82.64	71,415	0	0.00
Surigao City	34,170	9,105	8,126	17,231	50.43	32,852	0	0.00

Legend:

0 = No Case

** = No Report*

PSAC [1] - Pre School Age Children

SAC [2] - School Age Children

Table 1.E.3.3 -Soil Transmitted Helminthiasis Prevention and Control

No. and Proportion of Pregnant Women who completed 1 dose of deworming tablet

Philippines, Annual 2020

Area	Elig. Pop	Pregnant Women who completed 1 dose of deworming tablet	
		No.	%
**PHILIPPINES**	**2,123,157**	**236,085**	11.12
**N C R**	**238,661**	**25,460**	**10.67**
Malabon City	6,775	0	0.00
Navotas City	4,621	36	0.78
Valenzuela City	11,500	0	0.00
Caloocan City	29,363	160	0.54
Marikina City	8,354	10	0.12
Pasig City	13,996	0	0.00
Pateros	1,184	300	25.34
Taguig City	14,918	327	2.19
Quezon City	54,413	23,660	43.48
Makati City	10,798	295	2.73
Mandaluyong City	7,160	32	0.45
San Juan City	2,261	0	0.00
Manila City	32,980	171	0.52
Las Piñas City	10,914	26	0.24
Muntinlupa City	9,353	0	0.00
Parañaque City	12,343	152	1.23
Pasay City	7,728	291	3.77
**C A R**	**35,099**	**8,843**	**25.19**
Abra	4,309	1,092	25.34
Apayao	2,451	1,181	48.18
Benguet	9,188	2,112	22.99
Ifugao	4,396	1,107	25.18
Kalinga	4,617	2,122	45.96
Mt. Province	3,023	88	2.91
Baguio City	7,115	1,141	16.04
**Region 1**	**97,261**	**10,897**	**11.20**
Ilocos Norte	8,105	1,389	17.14
Ilocos Sur	9,330	277	2.97
La Union	11,511	1,709	14.85
Pangasinan	50,168	5,272	10.51
Alaminos City	1,894	0	0.00
Candon City	987	261	26.44
Dagupan City	3,620	19	0.52
Laoag City	1,870	621	33.21
San Carlos City	3,979	358	9.00
San Fernando City	2,115	267	12.62
Urdaneta City	2,806	724	25.80
Vigan City	876	0	0.00

Table 1.E.3.3 -Soil Transmitted Helminthiasis Prevention and Control

No. and Proportion of Pregnant Women who completed 1 dose of deworming tablet

Philippines, Annual 2020

Area	Elig. Pop	Pregnant Women who completed 1 dose of deworming tablet	
		No.	%
**Region 2**	**69,443**	**6,377**	**9.18**
Batanes	340	0	0.00
Cagayan	20,663	1,941	9.39
Isabela	23,617	1,539	6.52
Nueva Vizcaya	9,573	1,501	15.68
Quirino	4,025	280	4.80
			0.00
Cauayan City	2,590	193	0.00
Ilagan City	2,911	75	2.58
Santiago City	2,692	175	6.50
Tuguegarao City	3,032	673	22.20
**Region 3**	**220,020**	**12,486**	**5.67**
Aurora	4,770	232	4.86
Bataan	13,823	252	1.82
Bulacan	43,627	1,795	4.11
Nueva Ecija	28,782	242	0.84
Pampanga	31,359	335	1.07
Tarlac	19,765	3,584	18.13
Zambales	12,610	406	3.22
Angeles City	7,869	195	2.48
Balanga City	1,998	0	0.00
Cabanatuan City	5,896	0	0.00
City of San Fernando	5,863	425	7.25
Gapan City	2,153	165	7.66
Mabalacat City	4,793	22	0.46
Malolos City	4,880	0	0.00
Meycauayan City	4,041	177	4.38
Olongapo City	4,971	568	11.43
Palayan City	799	59	7.38
San Jose City	2,718	0	0.00
San Jose del Monte City	11,100	3,328	29.98
Science City of Munoz	1,586	701	44.20
Tarlac City	6,617	0	0.00
**Region 4A**	**296,816**	**7,959**	**2.68**
Batangas	38,441	51	0.13
Cavite	27,704	500	1.81
Laguna	19,727	1,339	6.79
Quezon	37,410	286	0.77
Rizal	44,791	516	1.15
Antipolo City	16,497	14	0.08
Bacoor City	12,142	0	0.00
Batangas City	6,823	0	0.00
Biñan City	6,607	1,858	28.13
Cabuyao City	6,130	714	11.65
Calamba City	9,028	1,455	16.12
Cavite City	2,075	0	0.00
Dasmariñas City	13,322	387	2.91
General Trias City	6,352	126	1.98

Table 1.E.3.3 -Soil Transmitted Helminthiasis Prevention and Control

No. and Proportion of Pregnant Women who completed 1 dose of deworming tablet

Philippines, Annual 2020

Area	Elig. Pop	Pregnant Women who completed 1 dose of deworming tablet	
		No.	%
Imus City	8,156	65	0.79
Lipa City	6,880	0	0.00
Lucena City	5,672	0	0.00
San Pablo City	5,277	25	0.48
San Pedro City	6,467	29	0.45
Santa Rosa City	7,025	160	2.28
Tagaytay City	1,437	0	0.00
Tanauan City	3,584	246	6.85
Tayabas City	2,122	0	0.00
Trece Martires City	3,147	187	5.94
**Region 4B**	**71,246**	**3,582**	**5.03**
Marinduque	4,836	592	12.24
Mindoro Occidental	12,407	672	5.42
Mindoro Oriental	19,809	1,658	8.37
Palawan	21,375	206	0.96
Romblon	6,400	454	7.09
Puerto Princesa City	6,419	0	0.00
**Region 5**	**136,115**	**30,498**	**22.41**
Albay	23,737	3,853	16.23
Camarines Norte	14,283	6,403	44.83
Camarines Sur	38,796	7,645	19.71
Catanduanes	6,274	1,733	27.62
Masbate	22,521	5,476	24.32
Sorsogon	19,064	4,111	21.56
Iriga City	2,638	874	33.13
Legaspi City	4,174	14	0.34
Naga City	4,628	389	8.41
**Region 6**	**146,526**	**21,817**	**14.89**
Aklan	11,162	2,393	21.44
Antique	12,816	3,175	24.77
Capiz	13,986	2,322	16.60
Guimaras	3,085	1,287	41.72
Iloilo	36,541	2,583	7.07
Negros Occidental	49,368	9,983	20.22
Bacolod City	11,115	68	0.61
Iloilo City	8,453	6	0.07
**Region 7**	**163,262**	**12,241**	**7.50**
Bohol	27,312	770	2.82
Cebu	67,506	3,897	5.77
Negros Oriental	27,938	40	0.14
Siquijor	1,613	496	30.75
Cebu City	21,193	5,223	24.64
Lapu-Lapu City	9,372	1,815	19.37

Table 1.E.3.3 -Soil Transmitted Helminthiasis Prevention and Control

No. and Proportion of Pregnant Women who completed 1 dose of deworming tablet

Philippines, Annual 2020

Area	Elig. Pop	Pregnant Women who completed 1 dose of deworming tablet	
		No.	%
Mandaue City	8,328	0	0.00
**Region 8**	**102,619**	**10,076**	**9.82**
Biliran	3,834	1,680	43.82
Eastern Samar	11,392	2,260	19.84
Northern Leyte	34,707	0	0.00
Northern Samar	15,370	2,126	13.83
Southern Leyte	6,451	1,131	17.53
Western Samar	14,305	1,416	9.90
Calbayog City	4,413	206	4.67
Maasin City	1,637	0	0.00
Ormoc City	4,941	1,233	24.95
Tacloban City	5,569	24	0.43
**Region 9**	**80,051**	**9,536**	**11.91**
Zamboanga del Norte	17,249	3,387	19.64
Zamboanga del Sur	17,653	0	0.00
Zamboanga Sibugay	14,954	2,002	13.39
Dapitan City	1,784	1,631	91.42
Dipolog City	2,827	77	2.72
Isabela City	2,522	643	25.50
Pagadian City	4,325	81	1.87
Zamboanga City	18,737	1,715	9.15
**Region 10**	**101,411**	**14,400**	**14.20**
Bukidnon	23,706	7,263	30.64
Camiguin	1,858	50	2.69
Lanao del Norte	14,960	239	1.60
Misamis Occidental	6,403	1,104	17.24
Misamis Oriental	15,131	1,394	9.21
Cagayan de Oro City	14,339	34	0.24
El Salvador City	1,065	0	0.00
Gingoog City	2,644	8	0.30
Iligan City	7,580	1,991	26.27
Malaybalay City	3,956	176	4.45
Oroquieta City	1,389	0	0.00
Ozamis City	2,778	241	8.68
Tangub City	1,234	0	0.00
Valencia City	4,368	1,900	43.50
**Region 11**	**107,247**	**31,135**	**29.03**
Compostela Valley	15,562	6,575	42.25
Davao del Norte	21,326	7,253	34.01
Davao del Sur	13,007	3,762	28.92
Davao Oriental	14,151	1,816	12.83
Davao Occidental	6,670	2,195	32.91
Davao City	36,531	9,534	26.10

Table 1.E.3.3 -Soil Transmitted Helminthiasis Prevention and Control

No. and Proportion of Pregnant Women who completed 1 dose of deworming tablet

Philippines, Annual 2020

Area	Elig. Pop	Pregnant Women who completed 1 dose of deworming tablet	
		No.	%
**Region 12**	**104,552**	**26,465**	**25.31**
North Cotabato	33,645	4,113	12.22
Sarangani	12,891	9,128	70.81
South Cotabato	21,113	5,363	25.40
Sultan Kudarat	17,359	6,286	36.21
Cotabato City	5,835	586	10.04
Gen. Santos City	13,709	989	7.21
**BARMM**	**92,799**	**1,844**	**1.99**
Basilan	7,541	0	0.00
Lanao del Sur	21,131	0	0.00
Maguindanao	31,128	1,844	5.92
Sulu	16,613	0	0.00
Tawi-Tawi	9,259	0	0.00
Lamitan City	2,074	0	0.00
Marawi City	5,053	0	0.00
**CARAGA**	**60,029**	**2,469**	**4.11**
Agusan del Norte	8,098	22	0.27
Agusan del Sur	17,592	425	2.42
Surigao del Norte	7,089	79	1.11
Surigao del Sur	11,482	1,372	11.95
Province of Dinagat	2,573	99	3.85
Bislig City	2,179	250	11.47
Butuan City	7,715	71	0.92
Surigao City	3,301	151	4.57

Legend:
0 = No Case
** = No Report*

Figure 1.E.3.1 - Soil Transmitted Helminthiasis Prevention & Control
Proportion of PSAC (1-4 yrs. Old) who completed 2 doses of deworming tablets
Philippines, 2020

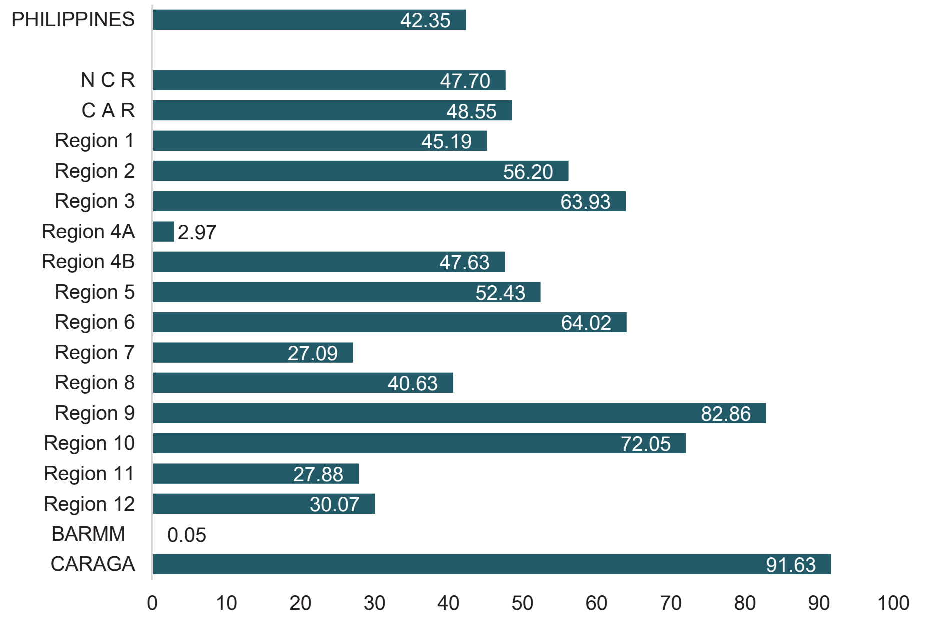

Figure 1.E.3.2 - Soil Transmitted Helminthiasis Prevention & Control
Proportion of SAC (5-9 yrs. Old) who completed 2 doses of deworming tablets
Philippines, 2020

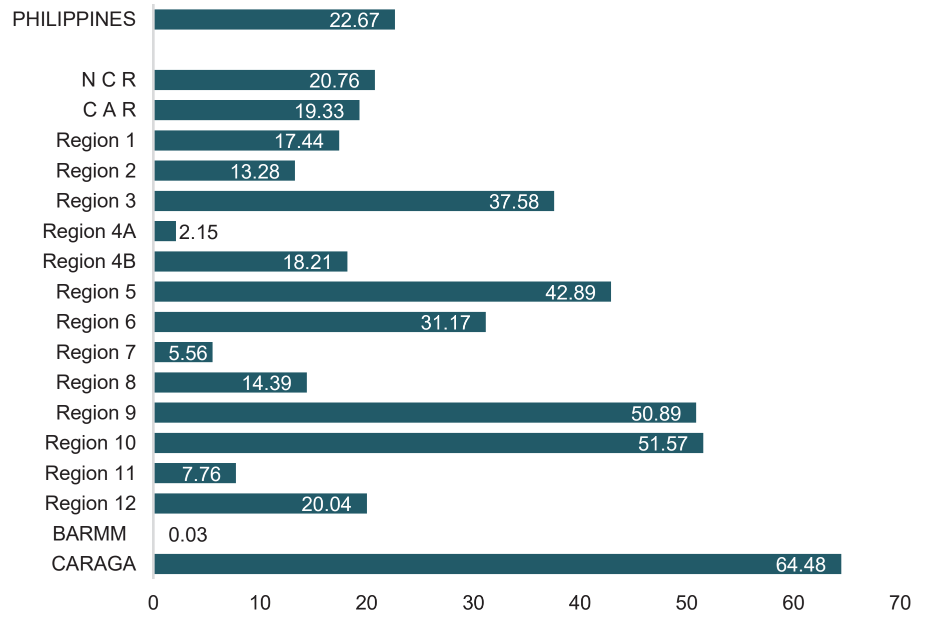

Figure 1.E.3.3 - Soil Transmitted Helminthiasis Prevention & Control
Proportion of Adolecents (10-19 yrs. Old) who completed 2 doses of deworming tablets
Philippines, 2020

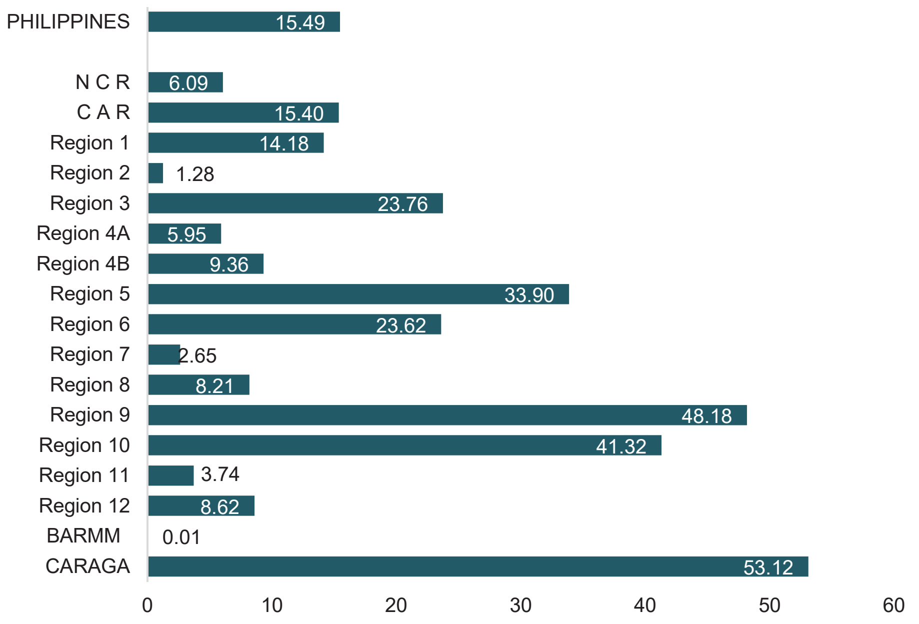

Figure 1.E.3.4 - Soil Transmitted Helminthiasis Prevention & Control
Proportion of WRA (20 - 49 yrs. Old) who completed 2 doses of deworming tablets
Philippines, 2020

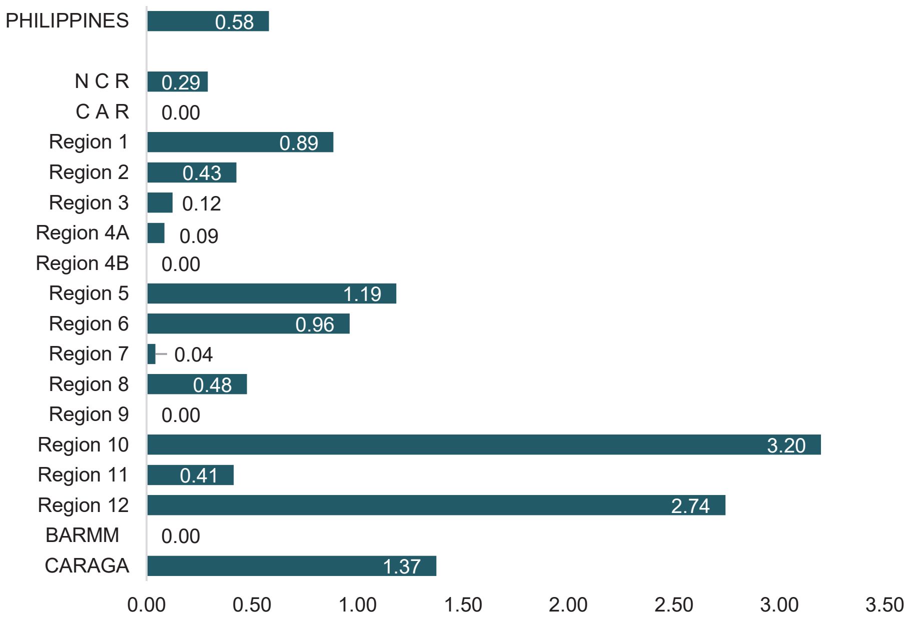

Figure 1.E.3.5 - Soil Transmitted Helminthiasis Prevention & Control
Proportion of Pregnant Women who completed 1 dose of deworming tablet
Philippines, 2020

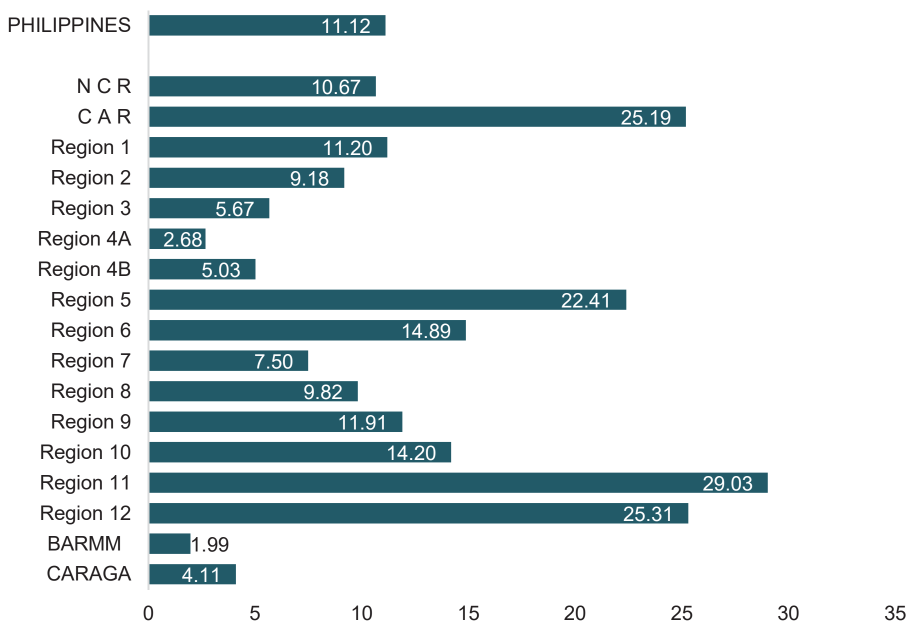

Table 1.E.4.1 - HIV-AIDS/STI Prevention and Control Services

Proportion of Pregnant Women Screened for Screened Syphilis, Positive for Syphilis and Screened for HIV

Philippines, Annual 2020

Area	Eligible Pop. (Total Pop x Multiplier)	Pregnant Women					
		Screened for Syphilis	%	Positive for Sphilis	%	Screened for HIV	%
**PHILIPPINES**	**2,123,157**	**392,807**	**18.50**	**7,021**	**1.79**	**266,297**	**12.54**
**NCR**	**238,661**	**84,696**	**35.49**	**958**	**1.13**	**75,880**	**31.79**
Malabon City	6,775	2,097	30.95	15	0.72	1,941	28.65
Navotas City	4,621	2,379	51.48	69	2.90	2,144	46.40
Valenzuela City	11,500	6,199	53.90	9	0.15	6,137	53.37
Caloocan City	29,363	8,259	28.13	23	0.28	7,888	26.86
Marikina City	8,354	1,356	16.23	6	0.44	660	7.90
Pasig City	13,996	7,253	51.82	13	0.18	7,361	52.59
Pateros	1,184	636	53.72	0	0.00	244	20.61
Taguig City	14,918	7,884	52.85	104	1.32	5,318	35.65
Quezon City	54,413	23,097	42.45	75	0.32	22,120	40.65
Makati City	10,798	3,385	31.35	5	0.15	2,934	27.17
Mandaluyong City	7,160	2,462	34.39	5	0.20	2,407	33.62
San Juan City	2,261	867	38.35	0	0.00	871	38.52
Manila City	32,980	7,178	21.76	46	0.64	7,018	21.28
Las Piñas City	10,914	2,226	20.40	8	0.36	1,127	10.33
Muntinlupa City	9,353	2,774	29.66	103	3.71	2,476	26.47
Parañaque City	12,343	3,766	30.51	466	12.37	2,546	20.63
Pasay City	7,728	2,878	37.24	11	0.38	2,688	34.78
**CAR**	**35,099**	**10,665**	**30.39**	**64**	**0.60**	**5,758**	**16.41**
Abra	4,309	455	10.56	1	0.22	382	8.87
Apayao	2,451	763	31.13	9	1.18	82	3.35
Benguet	9,188	3,036	33.04	5	0.16	2,709	29.48
Ifugao	4,396	1,281	29.14	4	0.31	871	19.81
Kalinga	4,617	1,301	28.18	14	1.08	56	1.21
Mt. Province	3,023	882	29.18	0	0.00	87	2.88
Baguio City	7,115	2,947	41.42	31	1.05	1,571	22.08
**Region 1**	**97,261**	**28,351**	**29.15**	**216**	**0.76**	**16,073**	**16.53**
Ilocos Norte	8,105	3,141	38.75	12	0.38	1,366	16.85
Ilocos Sur	9,330	4,233	45.37	89	2.10	2,400	25.72
La Union	11,511	4,171	36.23	48	1.15	2,495	21.67
Pangasinan	50,168	9,595	19.13	62	0.65	3,774	7.52
Alaminos City	1,894	1,106	58.39	0	0.00	1,006	53.12
Candon City	987	780	79.03	0	0.00	468	47.42
Dagupan City	3,620	1,052	29.06	4	0.38	982	27.13
Laoag City	1,870	1,817	97.17	0	0.00	1,817	97.17
San Carlos City	3,979	517	12.99	1	0.19	131	3.29
San Fernando City	2,115	886	41.89	0	0.00	881	41.65
Urdaneta City	2,806	932	33.21	0	0.00	747	26.62
Vigan City	876	121	13.81	0	0.00	6	0.68
**Region 2**	**69,443**	**11,758**	**16.93**	**138**	**1.17**	**5,745**	**8.27**
Batanes	340	147	43.24	2	1.36	36	10.59
Cagayan	20,663	1,025	4.96	8	0.78	444	2.15
Isabela	23,617	4,616	19.55	46	1.00	1,767	7.48

Table 1.E.4.1 - HIV-AIDS/STI Prevention and Control Services

Proportion of Pregnant Women Screened for Screened Syphilis, Positive for Syphilis and Screened for HIV

Philippines, Annual 2020

Area	Eligible Pop. (Total Pop x Multiplier)	Pregnant Women					
		Screened for Syphilis	%	Positive for Sphilis	%	Screened for HIV	%
Nueva Vizcaya	9,573	2,301	24.04	28	1.22	1,070	11.18
Quirino	4,025	1,276	31.70	8	0.63	1,243	30.88
Cauayan City	2,590	527	20.35	0	0.00	441	17.03
Ilagan City	2,911	1,083	37.20	7	0.65	145	4.98
Santiago City	2,692	461	17.12	7	1.52	578	21.47
Tuguegarao City	3,032	322	10.62	32	9.94	21	0.69
**Region 3**	**220,020**	**74,169**	**33.71**	**1,274**	**1.72**	**58,321**	**26.51**
Aurora	4,770	2,037	42.70	18	0.88	2,018	42.31
Bataan	13,823	6,208	44.91	73	1.18	5,541	40.09
Bulacan	43,627	11,042	25.31	624	5.65	10,053	23.04
Nueva Ecija	28,782	7,610	26.44	204	2.68	3,923	13.63
Pampanga	31,359	7,682	24.50	168	2.19	5,412	17.26
Tarlac	19,765	6,905	34.94	70	1.01	2,701	13.67
Zambales	12,610	2,350	18.64	15	0.64	2,274	18.03
Angeles City	7,869	1,555	19.76	2	0.13	1,377	17.50
Balanga City	1,998	930	46.55	3	0.32	937	46.90
Cabanatuan City	5,896	1,376	23.34	0	0.00	1,376	23.34
City of San Fernando	5,863	2,505	42.73	4	0.16	2,333	39.79
Gapan City	2,153	676	31.40	0	0.00	680	31.58
Mabalacat City	4,793	2,390	49.86	17	0.71	2,169	45.25
Malolos City	4,880	1,346	27.58	2	0.15	1,217	24.94
Meycauayan City	4,041	1,831	45.31	32	1.75	1,753	43.38
Olongapo City	4,971	1,430	28.77	27	1.89	972	19.55
Palayan City	799	191	23.90	0	0.00	99	12.39
San Jose City	2,718	778	28.62	0	0.00	496	18.25
San Jose del Monte City	11,100	11,710	105.50	11	0.09	11,586	104.38
Science City of Munoz	1,586	642	40.48	3	0.47	271	17.09
Tarlac City	6,617	2,975	44.96	1	0.03	1,133	17.12
**Region 4A**	**296,816**	**51,568**	**17.37**	**2,085**	**4.04**	**32,420**	**10.92**
Batangas	38,441	501	1.30	2	0.40	1,859	4.84
Cavite	27,704	6,318	22.81	283	4.48	3,568	12.88
Laguna	19,727	6,488	32.89	250	3.85	2,641	13.39
Quezon	37,410	1,590	4.25	46	2.89	1,682	4.50
Rizal	44,791	7,307	16.31	118	1.61	8,657	19.33
Antipolo City	16,497	1,143	6.93	484	42.34	2,471	14.98
Bacoor City	12,142	235	1.94	2	0.85	127	1.05
Batangas City	6,823	*	0.00	*	0.00	*	0.00
Biñan City	6,607	3,108	47.04	138	4.44	3,390	51.31
Cabuyao City	6,130	7,068	115.30	251	3.55	499	8.14
Calamba City	9,028	1,106	12.25	32	2.89	463	5.13
Cavite City	2,075	*	0.00	*	0.00	701	33.78
Dasmariñas City	13,322	2,450	18.39	104	4.24	2,410	18.09
General Trias City	6,352	624	9.82	3	0.48	423	6.66
Imus City	8,156	409	5.01	50	12.22	483	5.92
Lipa City	6,880	*	0.00	*	0.00	*	0.00
Lucena City	5,672	*	0.00	*	0.00	*	0.00
San Pablo City	5,277	211	4.00	37	17.54	38	0.72
San Pedro City	6,467	6,352	98.22	54	0.85	253	3.91
Santa Rosa City	7,025	1,010	14.38	178	17.62	946	13.47
Tagaytay City	1,437	1,952	135.84	6	0.31	1,360	94.64
Tanauan City	3,584	1,364	38.06	33	2.42	439	12.25
Tayabas City	2,122	*	0.00	*	0.00	*	0.00

Table 1.E.4.1 - HIV-AIDS/STI Prevention and Control Services

Proportion of Pregnant Women Screened for Screened Syphilis, Positive for Syphilis and Screened for HIV

Philippines, Annual 2020

Area	Eligible Pop. (Total Pop x Multiplier)	Pregnant Women					
		Screened for Syphilis	%	Positive for Sphilis	%	Screened for HIV	%
Trece Martires City	3,147	2,332	74.10	14	0.60	10	0.32
**Region 4B**	**71,246**	**7,115**	**9.99**	**89**	**1.25**	**4,599**	**6.46**
Marinduque	4,836	820	16.96	0	0.00	511	10.57
Mindoro Occidental	12,407	1,487	11.99	31	2.08	377	3.04
Mindoro Oriental	19,809	2,379	12.01	25	1.05	1,290	6.51
Palawan	21,375	803	3.76	0	0.00	942	4.41
Romblon	6,400	639	9.98	31	4.85	492	7.69
Puerto Princesa City	6,419	987	15.38	2	0.20	987	15.38
**Region 5**	**136,115**	**17,248**	**12.67**	**286**	**1.66**	**9,367**	**6.88**
Albay	23,737	4,406	18.56	152	3.45	2,995	12.62
Camarines Norte	14,283	1,694	11.86	17	1.00	206	1.44
Camarines Sur	38,796	1,673	4.31	20	1.20	779	2.01
Catanduanes	6,274	1,531	24.40	4	0.26	670	10.68
Masbate	22,521	3,847	17.08	61	1.59	1,733	7.70
Sorsogon	19,064	1,146	6.01	10	0.87	303	1.59
Iriga City	2,638	671	25.44	5	0.75	640	24.26
Legaspi City	4,174	1,842	44.13	17	0.92	1,008	24.15
Naga City	4,628	438	9.46	0	0.00	1,033	22.32
**Region 6**	**146,526**	**35,389**	**24.15**	**552**	**1.56**	**14,134**	**9.65**
Aklan	11,162	4,002	35.85	61	1.52	2,000	17.92
Antique	12,816	2,302	17.96	46	2.00	1,069	8.34
Capiz	13,986	2,697	19.28	32	1.19	510	3.65
Guimaras	3,085	1,758	56.99	4	0.23	513	16.63
Iloilo	36,541	13,281	36.35	173	1.30	2,800	7.66
Negros Occidental	49,368	4,913	9.95	78	1.59	2,921	5.92
Bacolod City	11,115	3,854	34.67	129	3.35	2,388	21.48
Iloilo City	8,453	2,582	30.55	29	1.12	1,933	22.87
**Region 7**	**163,262**	**19,783**	**12.12**	**384**	**1.94**	**14,489**	**8.87**
Bohol	27,312	4,980	18.23	9	0.18	857	3.14
Cebu	67,506	3,612	5.35	64	1.77	3,566	5.28
Negros Oriental	27,938	1,095	3.92	34	3.11	360	1.29
Siquijor	1,613	1,031	63.92	1	0.10	616	38.19
Cebu City	21,193	5,590	26.38	262	4.69	5,645	26.64
Lapu-Lapu City	9,372	3,475	37.08	14	0.40	3,445	36.76
Mandaue City	8,328	0	0.00	0	0.00	0	0.00
**Region 8**	**102,619**	**10,380**	**10.12**	**213**	**2.05**	**8,842**	**8.62**
Biliran	3,834	1,733	45.20	18	1.04	1,824	47.57
Eastern Samar	11,392	1,226	10.76	37	3.02	1,217	10.68
Northern Leyte	34,707	2,057	5.93	78	3.79	1,329	3.83
Northern Samar	15,370	1,851	12.04	42	2.27	1,964	12.78
Southern Leyte	6,451	537	8.32	7	1.30	633	9.81
Western Samar	14,305	411	2.87	15	3.65	364	2.54
Calbayog City	4,413	141	3.20	0	0.00	0	0.00
Maasin City	1,637	0	0.00	1	0.00	0	0.00

Table 1.E.4.1 - HIV-AIDS/STI Prevention and Control Services
Proportion of Pregnant Women Screened for Screened Syphilis, Positive for Syphilis and Screened for HIV
Philippines, Annual 2020

Area	Eligible Pop. (Total Pop x Multiplier)	Pregnant Women					
		Screened for Syphilis	%	Positive for Sphilis	%	Screened for HIV	%
Ormoc City	4,941	275	5.57	4	1.45	489	9.90
Tacloban City	5,569	2,149	38.59	11	0.51	1,022	18.35
**Region 9**	**80,051**	**4,218**	**5.27**	**15**	**0.36**	**574**	**0.72**
Zamboanga del Norte	17,249	518	3.00	2	0.39	263	1.52
Zamboanga del Sur	17,653	1,011	5.73	5	0.49	159	0.90
Zamboanga Sibugay	14,954	2,188	14.63	5	0.23	13	0.09
Dapitan City	1,784	160	8.97	0	0.00	26	1.46
Dipolog City	2,827	7	0.25	0	0.00	0	0.00
Isabela City	2,522	37	1.47	0	0.00	37	1.47
Pagadian City	4,325	77	1.78	0	0.00	7	0.16
Zamboanga City	18,737	220	1.17	3	1.36	69	0.37
**Region 10**	**101,411**	**8,940**	**8.82**	**103**	**1.15**	**5,254**	**5.18**
Bukidnon	23,706	1,313	5.54	8	0.61	990	4.18
Camiguin	1,858	396	21.31	0	0.00	288	15.50
Lanao del Norte	14,960	1,421	9.50	4	0.28	464	3.10
Misamis Occidental	6,403	253	3.95	1	0.40	181	2.83
Misamis Oriental	15,131	936	6.19	27	2.88	406	2.68
Cagayan de Oro City	14,339	2,299	16.03	4	0.17	992	6.92
El Salvador City	1,065	184	17.28	0	0.00	84	7.89
Gingoog City	2,644	*	0.00	-	0.00	14	0.53
Iligan City	7,580	102	1.35	2	1.96	6	0.08
Malaybalay City	3,956	611	15.44	57	9.33	318	8.04
Oroquieta City	1,389	0	0.00	0	0.00	0	0.00
Ozamis City	2,778	1,276	45.93	0	0.00	1,274	45.86
Tangub City	1,234	0	0.00	0	0.00	0	0.00
Valencia City	4,368	149	3.41	0	0.00	237	5.43
**Region 11**	**107,247**	**7,714**	**7.19**	**98**	**1.27**	**5,640**	**5.26**
Compostela Valley	15,562	189	1.21	0	0.00	132	0.85
Davao del Norte	21,326	1,684	7.90	2	0.12	1,702	7.98
Davao del Sur	13,007	89	0.68	0	0.00	89	0.68
Davao Oriental	14,151	74	0.52	2	2.70	81	0.57
Davao Occidental	6,670	52	0.78	10	19.23	13	0.19
Davao City	36,531	5,626	15.40	84	1.49	3,623	9.92
**Region 12**	**104,552**	**14,648**	**14.01**	**428**	**2.92**	**3,913**	**3.74**
North Cotabato	33,645	1,038	3.09	55	5.30	587	1.74
Sarangani	12,891	2,778	21.55	151	5.44	488	3.79
South Cotabato	21,113	3,510	16.62	61	1.74	1,849	8.76
Sultan Kudarat	17,359	5,037	29.02	43	0.85	180	1.04
Cotabato City	5,835	246	4.22	1	0.41	371	6.36
Gen. Santos City	13,709	2,039	14.87	117	5.74	438	3.19
**BARMM**	**92,799**	**5**	**0.01**	**0**	**0.00**	**0**	**0.00**
Basilan	7,541	5	0.07	0	0.00	0	0.00
Lanao del Sur	21,131	*	0.00	*	0.00	*	0.00
Maguindanao	31,128	*	0.00	*	0.00	*	0.00
Sulu	16,613	*	0.00	*	0.00	*	0.00

Table 1.E.4.1 - HIV-AIDS/STI Prevention and Control Services

Proportion of Pregnant Women Screened for Screened Syphilis, Positive for Syphilis and Screened for HIV

Philippines, Annual 2020

Area	Eligible Pop. (Total Pop x Multiplier)	Pregnant Women					
		Screened for Syphilis	%	Positive for Sphilis	%	Screened for HIV	%
Tawi-Tawi	9,259	*	0.00	*	0.00	*	0.00
Lamitan City	2,074	*	0.00	*	0.00	*	0.00
Marawi City	5,053	*	0.00	*	0.00	*	0.00
**CARAGA**	**60,029**	**6,160**	**10.26**	**118**	**1.92**	**5,288**	**8.81**
Agusan del Norte	8,098	393	4.85	27	6.87	417	5.15
Agusan del Sur	17,592	1,266	7.20	23	1.82	493	2.80
Surigao del Norte	7,089	520	7.34	24	4.62	388	5.47
Surigao del Sur	11,482	440	3.83	7	1.59	455	3.96
Province of Dinagat	2,573	317	12.32	4	1.26	331	12.86
Bislig City	2,179	120	5.51	1	0.83	3	0.14
Butuan City	7,715	2,178	28.23	30	1.38	1,814	23.51
Surigao City	3,301	926	28.05	2	0.22	1,387	42.02

Legend:
0 = No Case
** = No Report*

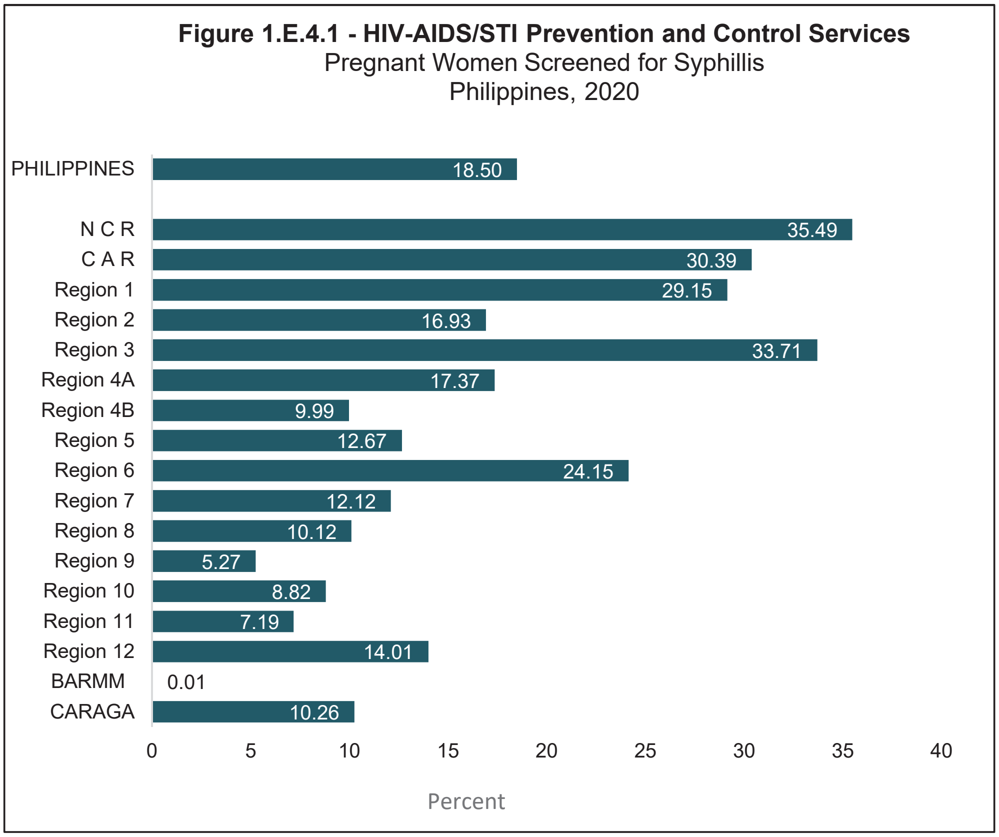
Figure 1.E.4.1 - HIV-AIDS/STI Prevention and Control Services
Pregnant Women Screened for Syphillis
Philippines, 2020
PHILIPPINES 18.50
N C R 35.49
C A R 30.39
Region 1 29.15
Region 2 16.93
Region 3 33.71
Region 4A 17.37
Region 4B 9.99
Region 5 12.67
Region 6 24.15
Region 7 12.12
Region 8 10.12
Region 9 5.27
Region 10 8.82
Region 11 7.19
Region 12 14.01
BARMM 0.01
CARAGA 10.26
0
5
10
15
20
25
30
35
40
Percent

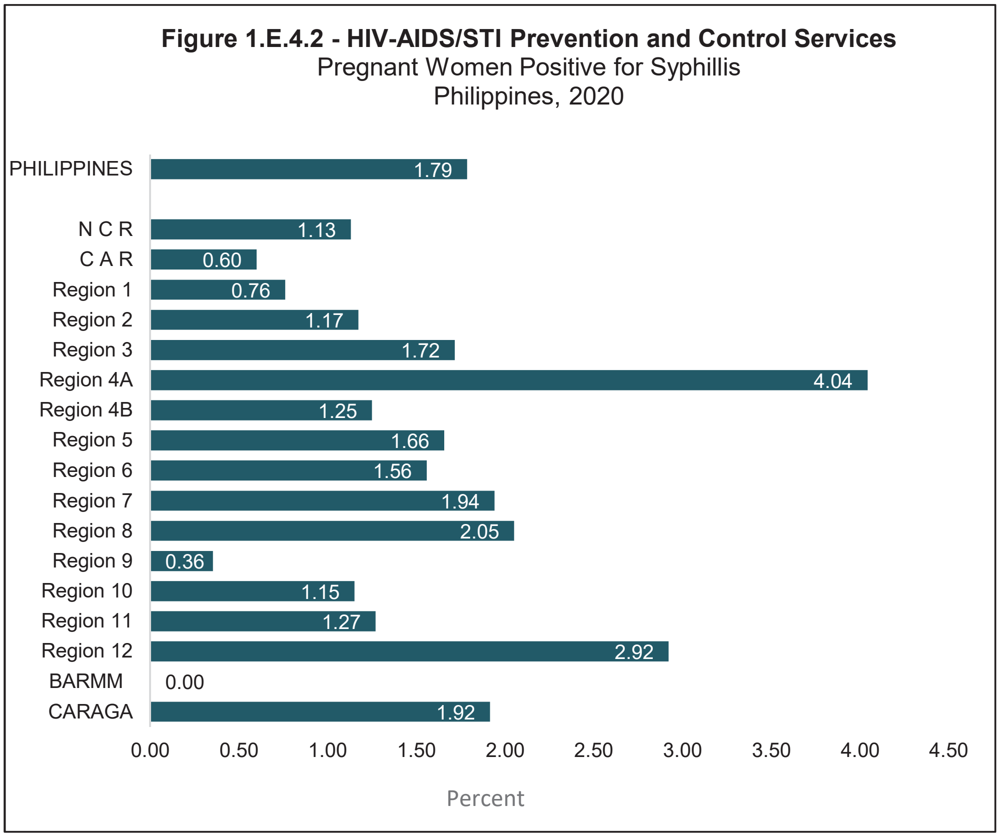
Figure 1.E.4.2 - HIV-AIDS/STI Prevention and Control Services
Pregnant Women Positive for Syphillis
Philippines, 2020
PHILIPPINES 1.79
N C R 1.13
C A R 0.60
Region 1 0.76
Region 2 1.17
Region 3 1.72
Region 4A 4.04
Region 4B 1.25
Region 5 1.66
Region 6 1.56
Region 7 1.94
Region 8 2.05
Region 9 0.36
Region 10 1.15
Region 11 1.27
Region 12 2.92
BARMM 0.00
CARAGA 1.92
0.00
0.50
1.00
1.50
2.00
2.50
3.00
3.50
4.00
4.50
Percent

Figure 1.E.4.3 - HIV-AIDS/STI Prevention and Control Services
Pregnant Women Screened for HIV
Philippines, 2020

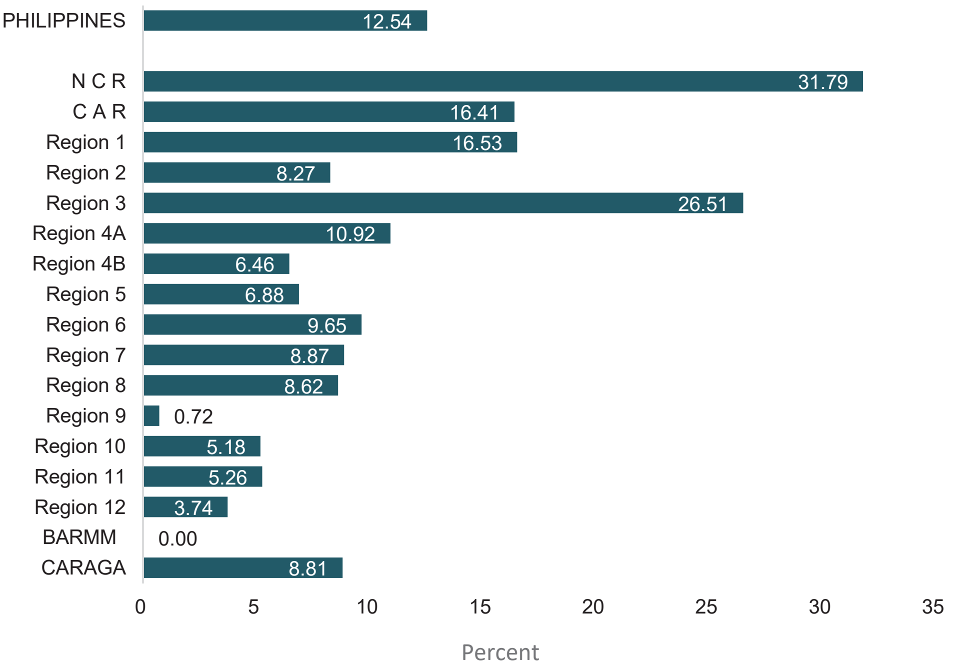

Table 1.E.5.1 - Tuberculosis Case Notification Rate (All forms)
Philippines, Annual 2020

Area	Total Population	Notified TB cases all forms			CNR (All Forms)
		Male	Female	Total	
**PHILIPPINES**	**108,020,395**	**171,387**	**85,154**	**256,541**	**237.49**
**No Location**		220	84	304	
**N C R**	**13,694,816**	**22,451**	**12,103**	**34,554**	**252.31**
Malabon City	373,284	770	458	1,228	328.97
Navotas City	248,638	443	238	681	273.89
Valenzuela City	653,305	1,539	835	2,374	363.38
Caloocan City	1,651,493	2,729	1,448	4,177	252.92
Marikina City	469,181	1,276	699	1,975	420.95
Pasig City	822,854	1,446	784	2,230	271.01
Pateros	63,376	49	37	86	135.70
Taguig City	947,362	1,734	1,048	2,782	293.66
Quezon City	3,065,597	4,161	2,397	6,558	213.92
Makati City	624,587	905	654	1,559	249.60
Mandaluyong City	438,677	915	412	1,327	302.50
San Juan City	122,505	209	84	293	239.17
Manila City	1,880,120	3,151	1,570	4,721	251.10
Las Piñas City	614,860	760	368	1,128	183.46
Muntinlupa City	556,831	946	372	1,318	236.70
Parañaque City	728,176	667	331	998	137.05
Pasay City	433,969	751	368	1,119	257.85
**C A R**	**1,805,792**	**1,585**	**734**	**2,319**	**128.42**
Abra	246,062	272	114	386	156.87
Apayao	124,308	160	86	246	197.90
Benguet	477,579	174	68	242	50.67
Ifugao	212,001	182	102	284	133.96
Kalinga	221,317	307	160	467	211.01
Mt. Province	154,891	117	47	164	105.88
Baguio City	369,634	373	157	530	143.39
**Region 1**	**5,243,726**	**6,806**	**3,249**	**10,055**	**191.75**
Ilocos Norte	497,711	420	208	628	126.18
Ilocos Sur	595,267	660	284	944	158.58
La Union	694,488	715	362	1,077	155.08
Pangasinan	2,485,875	3,276	1,628	4,904	197.27
Alaminos City	93,926	144	69	213	226.77
Candon City	62,742	28	8	36	57.38
Dagupan City	179,324	401	165	566	315.63
Laoag City	114,758	96	25	121	105.44
San Carlos City	197,438	322	149	471	238.56
San Fernando City	127,244	336	185	521	409.45
Urdaneta City	139,191	308	125	433	311.08
Vigan City	55,762	100	41	141	252.86

Table 1.E.5.1 - Tuberculosis Case Notification Rate (All forms)
Philippines, Annual 2020

Area	Total Population	Notified TB cases all forms			CNR (All Forms)
		Male	Female	Total	
**Region 2**	**3,626,877**	**5,223**	**2,616**	**7,839**	**216.14**
Batanes	17,740	29	14	43	242.39
Cagayan	1,097,028	1,530	757	2,287	208.47
Isabela	1,244,626	1,728	914	2,642	212.27
Nueva Vizcaya	476,796	557	265	822	172.40
Quirino	198,628	276	114	390	196.35
Cauayan City	136,196	138	88	226	165.94
Ilagan City	153,068	246	124	370	241.72
Santiago City	141,776	405	190	595	419.67
Tuguegarao City	161,018	314	150	464	288.17
**Region 3**	**12,099,325**	**22,511**	**11,545**	**34,056**	**281.47**
Aurora	224,648	471	223	694	308.93
Bataan	716,595	1,260	565	1,825	254.68
Bulacan	2,464,952	4,575	2,368	6,943	281.67
Nueva Ecija	1,585,623	2,436	1,318	3,754	236.75
Pampanga	1,779,362	2,917	1,495	4,412	247.95
Tarlac	1,078,598	1,704	864	2,568	238.09
Zambales	630,172	1,409	771	2,180	345.94
Angeles City	446,436	724	379	1,103	247.07
Balanga City	103,578	360	167	527	508.80
Cabanatuan City	324,532	791	417	1,208	372.23
City of San Fernando	332,586	782	356	1,138	342.17
Gapan City	118,442	204	106	310	261.73
Mabalacat City	272,003	552	320	872	320.58
Malolos City	275,321	509	240	749	272.05
Meycauayan City	228,365	416	243	659	288.57
Olongapo City	248,550	676	357	1,033	415.61
Palayan City	44,069	80	17	97	220.11
San Jose City	150,049	22	22	44	29.32
San Jose del Monte City	627,032	1,320	681	2,001	319.12
Science City of Munoz	87,495	133	89	222	253.73
Tarlac City	360,918	1,170	547	1,717	475.73
**Region 4A**	**15,937,325**	**29,827**	**16,498**	**46,325**	**290.67**
Batangas	2,040,324	2,826	1,485	4,311	211.29
Cavite	1,560,675	2,398	1,294	3,692	236.56
Laguna	1,092,719	2,679	1,708	4,387	401.48
Quezon	1,845,487	3,028	1,626	4,654	252.18
Rizal	2,355,368	4,732	2,517	7,249	307.77
Antipolo City	867,558	1,628	844	2,472	284.94
Bacoor City	683,759	698	401	1,099	160.73
Batangas City	362,106	825	398	1,223	337.75
Biñan City	366,406	795	472	1,267	345.79
Cabuyao City	339,689	616	371	987	290.56
Calamba City	500,037	1,300	631	1,931	386.17
Cavite City	117,039	313	166	479	409.27
Dasmariñas City	750,256	1,228	764	1,992	265.51

Table 1.E.5.1 - Tuberculosis Case Notification Rate (All forms)
Philippines, Annual 2020

Area	Total Population	Notified TB cases all forms			CNR (All Forms)
		Male	Female	Total	
General Trias City	357,816	414	229	643	179.70
Imus City	459,686	721	482	1,203	261.70
Lipa City	364,864	686	395	1,081	296.28
Lucena City	279,688	996	580	1,576	563.48
San Pablo City	292,735	803	410	1,213	414.37
San Pedro City	358,463	784	434	1,218	339.78
Santa Rosa City	389,223	1,112	634	1,746	448.59
Tagaytay City	81,036	138	62	200	246.81
Tanauan City	190,306	335	174	509	267.46
Tayabas City	104,816	187	120	307	292.89
Trece Martires City	177,270	585	301	886	499.80
**Region 4B**	**3,138,950**	**5,756**	**3,074**	**8,830**	**281.30**
Marinduque	239,634	471	251	722	301.29
Mindoro Occidental	514,763	1,108	515	1,623	315.29
Mindoro Oriental	890,416	1,527	831	2,358	264.82
Palawan	918,681	1,445	873	2,318	252.32
Romblon	299,553	549	287	836	279.08
Puerto Princesa City	275,902	656	317	973	352.66
**Region 5**	**6,094,462**	**10,771**	**5,326**	**16,097**	**264.13**
Albay	1,172,705	1,973	993	2,966	252.92
Camarines Norte	615,349	1,148	385	1,533	249.13
Camarines Sur	1,731,531	3,170	1,646	4,816	278.14
Catanduanes	272,452	453	250	703	258.03
Masbate	937,990	1,526	873	2,399	255.76
Sorsogon	834,217	1,418	644	2,062	247.18
Iriga City	117,651	218	97	315	267.74
Legaspi City	206,226	391	207	598	289.97
Naga City	206,340	474	231	705	341.67
**Region 6**	**7,877,110**	**15,749**	**7,201**	**22,950**	**291.35**
Aklan	605,792	967	391	1,358	224.17
Antique	610,348	1,203	549	1,752	287.05
Capiz	794,010	1,205	562	1,767	222.54
Guimaras	183,846	325	153	478	260.00
Iloilo	2,035,320	3,448	1,536	4,984	244.88
Negros Occidental	2,593,413	5,690	2,622	8,312	320.50
Bacolod City	583,509	1,568	673	2,241	384.06
Iloilo City	470,872	1,343	715	2,058	437.06
**Region 7**	**7,879,119**	**10,750**	**4,833**	**15,583**	**197.78**
Bohol	1,358,850	1,835	920	2,755	202.75
Cebu	3,179,974	3,723	1,626	5,349	168.21
Negros Oriental	1,408,244	2,296	1,010	3,306	234.76
Siquijor	99,819	156	53	209	209.38

Table 1.E.5.1 - Tuberculosis Case Notification Rate (All forms)
Philippines, Annual 2020

Area	Total Population	Notified TB cases all forms			CNR (All Forms)
		Male	Female	Total	
Cebu City	998,264	1,353	590	1,943	194.64
Lapu-Lapu City	441,577	752	355	1,107	250.69
Mandaue City	392,391	635	279	914	232.93
**Region 8**	**4,711,363**	5,860	2,940	**8,800**	186.78
Biliran	179,339	313	151	464	258.73
Eastern Samar	497,883	502	276	778	156.26
Northern Leyte	1,619,930	1,907	793	2,700	166.67
Northern Samar	666,747	801	438	1,239	185.83
Southern Leyte	350,279	408	183	591	168.72
Western Samar	624,949	618	353	971	155.37
Calbayog City	192,578	260	149	409	212.38
Maasin City	89,146	121	75	196	219.87
Ormoc City	230,739	268	153	421	182.46
Tacloban City	259,774	662	369	1,031	396.88
**Region 9**	**3,805,545**	**5,477**	**2,268**	**7,745**	**203.52**
Zamboanga del Norte	831,128	793	349	1,142	137.40
Zamboanga del Sur	847,502	1,090	439	1,529	180.41
Zamboanga Sibugay	671,808	843	355	1,198	178.32
Dapitan City	85,816	97	55	152	177.12
Dipolog City	136,151	179	70	249	182.89
Isabela City	125,373	105	72	177	141.18
Pagadian City	207,862	253	136	389	187.14
Zamboanga City	899,906	2,117	792	2,909	323.26
**Region 10**	**5,004,949**	**6,084**	**2,701**	**8,785**	**175.53**
Bukidnon	1,116,543	724	277	1,001	89.65
Camiguin	92,126	109	41	150	162.82
Lanao del Norte	723,628	791	445	1,236	170.81
Misamis Occidental	341,195	424	202	626	183.47
Misamis Oriental	768,704	931	388	1,319	171.59
Cagayan de Oro City	728,088	1,309	573	1,882	258.49
El Salvador City	54,076	108	50	158	292.18
Gingoog City	134,263	167	83	250	186.20
Iligan City	366,543	752	304	1,056	288.10
Malaybalay City	186,116	162	64	226	121.43
Oroquieta City	73,935	93	60	153	206.94
Ozamis City	148,198	226	106	332	224.02
Tangub City	65,841	72	38	110	167.07
Valencia City	205,692	216	70	286	139.04

Table 1.E.5.1 - Tuberculosis Case
Notification Rate (All forms)
Philippines, Annual 2020

Area	Total Population	Notified TB cases all forms			CNR (All Forms)
		Male	Female	Total	
**Region 11**	**5,237,160**	**7,161**	**2,675**	**9,836**	**187.81**
Compostela Valley	774,793	953	305	1,258	162.37
Davao del Norte	1,072,302	1,700	580	2,280	212.63
Davao del Sur	687,961	815	268	1,083	157.42
Davao Oriental	591,886	828	299	1,127	190.41
Davao Occidental	334,284	304	134	438	131.03
Davao City	1,775,934	2,561	1,089	3,650	205.53
**Region 12**	**4,900,137**	**8,230**	**3,661**	**11,891**	**242.67**
North Cotabato	1,506,217	2,210	1,013	3,223	213.98
Sarangani	580,709	725	386	1,111	191.32
South Cotabato	986,496	1,709	679	2,388	242.07
Sultan Kudarat	864,172	1,392	721	2,113	244.51
Cotabato City	321,850	532	303	835	259.44
Gen. Santos City	640,692	1,662	559	2,221	346.66
**BARMM**	**4,234,144**	**2,322**	**1,485**	3,807	**89.91**
Basilan	307,767	170	132	302	98.13
Lanao del Sur	918,076	577	458	1,035	112.74
Maguindanao	1,380,136	1,054	554	1,608	116.51
Sulu	914,152	214	157	371	40.58
Tawi-Tawi	409,746	121	70	191	46.61
Lamitan City	84,679	91	43	134	158.25
Marawi City	219,588	95	71	166	75.60
**CARAGA**	**2,729,595**	**4,604**	**2,161**	**6,765**	**248**
Agusan del Norte	374,612	642	246	888	237.05
Agusan del Sur	735,532	1,093	490	1,583	215.22
Surigao del Norte	354,346	662	294	956	269.79
Surigao del Sur	518,074	998	491	1,489	287.41
Province of Dinagat	127,413	133	74	207	162.46
Bislig City	98,402	241	151	392	398.37
Butuan City	356,183	416	216	632	177.44
Surigao City	165,033	419	199	618	374.47

Legend:
0 = No Case
** = No Report*

Table 1.E.5.2 - Tuberculosis
Case Notification Rate (DRTB)
Philippines, Annual 2020

Area	Registered Bacteriologically Confirmed Drug Resistant TB			Estimated MDR among New & Retreatment			CNR (DRTB)
	Male	Female	Total	Male	Female	Total	
**PHILIPPINES**	**3,381**	**1,438**	**4,819**	**0**	**0**	**10,286**	46.85
No Location	**35**	**17**	**52**			**16**	**325.00**
**N C R**	**652**	**299**	**951**	**0**	**0**	**1,631**	**58.31**
Malabon City	24	13	37			69	53.62
Navotas City	30	11	41			49	83.67
Valenzuela City	29	14	43			122	35.25
Caloocan City	49	24	73			192	38.02
Marikina City	43	23	66			86	76.74
Pasig City	36	23	59			99	59.60
Pateros	1	0	1			3	33.33
Taguig City	51	17	68			128	53.13
Quezon City	152	72	224			273	82.05
Makati City	20	8	28			69	40.58
Mandaluyong City	22	7	29			69	42.03
San Juan City	7	1	8			13	61.54
Manila City	111	54	165			242	68.18
Las Piñas City	13	7	20			41	48.78
Muntinlupa City	33	4	37			72	51.39
Parañaque City	14	14	28			52	53.85
Pasay City	17	7	24			52	46.15
**C A R**	**22**	**8**	**30**	**0**	**0**	**72**	**41.67**
Abra	4	0	4			12	33.33
Apayao	3	2	5			6	83.33
Benguet	0	0	0			6	0.00
Ifugao	1	1	2			8	25.00
Kalinga	1	2	3			18	16.67
Mt. Province	0	1	1			7	14.29
Baguio City	13	2	15			15	100.00
**Region 1**	**144**	**67**	**211**	**0**	**0**	**322**	**65.53**
Ilocos Norte	4	1	5			23	21.74
Ilocos Sur	9	3	12			30	40.00
La Union	9	2	11			30	36.67
Pangasinan	6	3	9			129	6.98
Alaminos City	12	5	17			8	212.50
Candon City	0	0	0			2	0.00
Dagupan City	34	10	44			28	157.14
Laoag City	0	0	0			3	0.00
San Carlos City	21	23	44			20	220.00
San Fernando City	16	4	20			26	76.92
Urdaneta City	29	16	45			17	264.71
Vigan City	4	0	4			6	66.67

Table 1.E.5.2 - Tuberculosis
Case Notification Rate (DRTB)
Philippines, Annual 2020

Area	Registered Bacteriologically Confirmed Drug Resistant TB			Estimated MDR among New & Retreatment			CNR (DRTB)
	Male	Female	Total	Male	Female	Total	
**Region 2**	**69**	**28**	**97**	**0**	**0**	**240**	**40.42**
Batanes	0	0	0			2	0.00
Cagayan	8	2	10			60	16.67
Isabela	0	0	0			85	0.00
Nueva Vizcaya	6	4	10			25	40.00
Quirino	3	2	5			13	38.46
Cauayan City	0	0	0			10	0.00
Ilagan City	0	0	0			10	0.00
Santiago City	28	4	32			17	188.24
Tuguegarao City	24	16	40			18	222.22
**Region 3**	**468**	**212**	**680**	**0**	**0**	**1,356**	**50.15**
Aurora	10	1	11			27	40.74
Bataan	11	5	16			75	21.33
Bulacan	87	43	130			260	50.00
Nueva Ecija	29	12	41			122	33.61
Pampanga	39	19	58			177	32.77
Tarlac	33	15	48			105	45.71
Zambales	29	6	35			102	34.31
Angeles City	16	12	28			42	66.67
Balanga City	19	10	29			24	120.83
Cabanatuan City	28	14	42			33	127.27
City of San Fernando	25	15	40			45	88.89
Gapan City	0	0	0			19	0.00
Mabalacat City	22	6	28			34	82.35
Malolos City	42	15	57			34	167.65
Meycauayan City	0	0	0			31	0.00
Olongapo City	11	7	18			84	21.43
Palayan City	0	0	0			4	0.00
San Jose City	0	0	0			1	0.00
San Jose del Monte City	38	22	60			65	92.31
Science City of Munoz	0	0	0			6	0.00
Tarlac City	29	10	39			66	59.09
**Region 4A**	**563**	**255**	**818**	**0**	**0**	**1,790**	**45.70**
Batangas	35	11	46			158	29.11
Cavite	11	6	17			130	13.08
Laguna	39	16	55			178	30.90
Quezon	41	15	56			180	31.11
Rizal	112	55	167			323	51.70
Antipolo City	45	22	67			109	61.47
Bacoor City	17	7	24			39	61.54
Batangas City	13	9	22			46	47.83
Biñan City	0	0	0			51	0.00
Cabuyao City	0	0	0			31	0.00
Calamba City	31	6	37			76	48.68
Cavite City	0	0	0			21	0.00

Table 1.E.5.2 - Tuberculosis
Case Notification Rate (DRTB)
Philippines, Annual 2020

Area	Registered Bacteriologically Confirmed Drug Resistant TB			Estimated MDR among New & Retreatment			CNR (DRTB)
	Male	Female	Total	Male	Female	Total	
Dasmariñas City	35	10	45			83	54.22
General Trias City	0	0	0			26	0.00
Imus City	17	12	29			38	76.32
Lipa City	31	7	38			32	118.75
Lucena City	26	14	40			84	47.62
San Pablo City	0	0	0			32	0.00
San Pedro City	0	0	0			33	0.00
Santa Rosa City	59	31	90			55	163.64
Tagaytay City	10	5	15			8	187.50
Tanauan City	0	0	0			12	0.00
Tayabas City	0	0	0			15	0.00
Trece Martires City	41	29	70			30	233.33
**Region 4B**	**103**	**43**	**146**	**0**	**0**	**364**	**40.11**
Marinduque	12	4	16			37	43.24
Mindoro Occidental	21	6	27			76	35.53
Mindoro Oriental	38	14	52			96	54.17
Palawan	11	9	20			84	23.81
Romblon	12	3	15			37	40.54
Puerto Princesa City	9	7	16			34	47.06
**Region 5**	**279**	**104**	**383**	**0**	**0**	**682**	**56.16**
Albay	23	6	29			144	20.14
Camarines Norte	49	11	60			55	109.09
Camarines Sur	15	4	19			201	9.45
Catanduanes	15	4	19			29	65.52
Masbate	28	13	41			91	45.05
Sorsogon	34	10	44			81	54.32
Iriga City	0	0	0			10	0.00
Legaspi City	45	16	61			36	169.44
Naga City	70	40	110			35	314.29
**Region 6**	**278**	**85**	**363**	**0**	**0**	**1,085**	**33.46**
Aklan	19	9	28			61	45.90
Antique	21	7	28			70	40.00
Capiz	24	2	26			54	48.15
Guimaras	0	0	0			16	0.00
Iloilo	30	9	39			218	17.89
Negros Occidental	110	33	143			428	33.41
Bacolod City	26	10	36			139	25.90
Iloilo City	48	15	63			99	63.64
**Region 7**	**197**	**74**	**271**	**0**	**0**	**595**	**45.55**
Bohol	31	20	51			107	47.66
Cebu	0	0	0			167	0.00
Negros Oriental	33	6	39			151	25.83

Table 1.E.5.2 - Tuberculosis
Case Notification Rate (DRTB)
Philippines, Annual 2020

Area	Registered Bacteriologically Confirmed Drug Resistant TB			Estimated MDR among New & Retreatment			CNR (DRTB)
	Male	Female	Total	Male	Female	Total	
Siquijor	0	0	0			10	0.00
Cebu City	67	25	92			76	121.05
Lapu-Lapu City	0	0	0			45	0.00
Mandaue City	66	23	89			39	228.21
**Region 8**	76	37	**113**	0	0	**343**	**32.94**
Biliran	0	0	0			14	0.00
Eastern Samar	7	6	13			35	37.14
Northern Leyte	33	14	47			104	45.19
Northern Samar	8	5	13			47	27.66
Southern Leyte	0	0	0			20	0.00
Western Samar	0	0	0			41	0.00
Calbayog City	13	3	16			16	100.00
Maasin City	15	9	24			10	240.00
Ormoc City	0	0	0			19	0.00
Tacloban City	0	0	0			37	0.00
**Region 9**	**64**	**29**	**93**	**0**	**0**	**290**	**32.07**
Zamboanga del Norte	0	0	0			37	0.00
Zamboanga del Sur	15	6	21			48	43.75
Zamboanga Sibugay	12	8	20			51	39.22
Dapitan City	11	5	16			8	200.00
Dipolog City	0	0	0			9	0.00
Isabela City	2	1	3			6	50.00
Pagadian City	0	0	0			10	0.00
Zamboanga City	24	9	33			121	27.27
**Region 10**	**89**	**42**	**131**	**0**	**0**	**309**	**42.39**
Bukidnon	0	1	1			33	3.03
Camiguin	0	0	0			6	0.00
Lanao del Norte	15	7	22			44	50.00
Misamis Occidental	0	0	0			17	0.00
Misamis Oriental	0	1	1			48	2.08
Cagayan de Oro City	25	11	36			58	62.07
El Salvador City	0	0	0			9	0.00
Gingoog City	5	2	7			10	70.00
Iligan City	19	9	28			38	73.68
Malaybalay City	13	3	16			10	160.00
Oroquieta City	4	6	10			7	142.86
Ozamis City	8	2	10			15	66.67
Tangub City	0	0	0			5	0.00
Valencia City	0	0	0			9	0.00
**Region 11**	**125**	**41**	**166**	**0**	**0**	**339**	**48.97**
Compostela Valley	0	0	0			41	0.00

Table 1.E.5.2 - Tuberculosis
Case Notification Rate (DRTB)
Philippines, Annual 2020

Area	Registered Bacteriologically Confirmed Drug Resistant TB			Estimated MDR among New & Retreatment			CNR (DRTB)
	Male	Female	Total	Male	Female	Total	
Davao del Norte	59	13	72			86	83.72
Davao del Sur	14	3	17			33	51.52
Davao Oriental	6	6	12			50	24.00
Davao Occidental	3	0	3			14	21.43
Davao City	43	19	62			115	53.91
**Region 12**	**132**	**40**	**172**	**0**	**0**	**483**	**35.61**
North Cotabato	39	7	46			132	34.85
Sarangani	3	1	4			57	7.02
South Cotabato	21	11	32			62	51.61
Sultan Kudarat	21	5	26			77	33.77
Cotabato City	16	12	28			31	90.32
Gen. Santos City	32	4	36			124	29.03
**BARMM**	**20**	**19**	39	**0**	**0**	**137**	**28.47**
Basilan	0	0	0			9	0.00
Lanao del Sur	0	0	0			29	0.00
Maguindanao	10	2	12			67	17.91
Sulu	4	3	7			10	70.00
Tawi-Tawi	0	0	0			5	0.00
Lamitan City	0	0	0			6	0.00
Marawi City	6	14	20			11	181.82
**CARAGA**	**65**	**38**	**103**	**0**	**0**	**232**	**44.40**
Agusan del Norte	0	0	0			25	0.00
Agusan del Sur	13	10	23			51	45.10
Surigao del Norte	2	3	5			31	16.13
Surigao del Sur	13	7	20			50	40.00
Province of Dinagat	2	2	4			6	66.67
Bislig City	9	6	15			20	75.00
Butuan City	12	6	18			27	66.67
Surigao City	14	4	18			22	81.82

Legend:
0 = No Case
** = No Report*

Table 1.E.5.3 - Tuberculosis

Treatment Success Rate DSTB - All Forms

Philippines, Annual 2020

Area	TB all forms cured and Completely Treated			Registered TB all forms Registered during Specified Period			Treatment Success Rate
	Male	Female	Total	Male	Female	Total	
**PHILIPPINES**	**192,847**	**102,853**	**295,700**	**209,911**	**118,314**	**328,225**	**90.09**
**N C R**	**29,773**	**17,981**	**47,754**	**33,127**	**19,617**	**52,744**	**90.54**
Malabon City	1,172	701	1,873	1,317	818	2,135	87.73
Navotas City	739	406	1,145	803	436	1,239	92.41
Valenzuela City	1,991	1,313	3,304	2,188	1,413	3,601	91.75
Caloocan City	2,854	1,771	4,625	3,255	1,954	5,209	88.79
Marikina City	1,318	721	2,039	1,456	807	2,263	90.10
Pasig City	1,625	1,078	2,703	1,878	1,210	3,088	87.53
Pateros	124	63	187	134	69	203	92.12
Taguig City	2,327	1,405	3,732	2,443	1,463	3,906	95.55
Quezon City	6,103	3,972	10,075	6,842	4,298	11,140	90.44
Makati City	1,155	764	1,919	1,278	848	2,126	90.26
Mandaluyong City	1,265	723	1,988	1,434	788	2,222	89.47
San Juan City	158	99	257	189	118	307	83.71
Manila City	4,384	2,502	6,886	4,861	2,746	7,607	90.52
Las Piñas City	932	559	1,491	1,052	602	1,654	90.15
Muntinlupa City	1,317	571	1,888	1,515	645	2,160	87.41
Parañaque City	1,218	730	1,948	1,269	747	2,016	96.63
Pasay City	1,091	603	1,694	1,213	655	1,868	90.69
**C A R**	**1,731**	**830**	**2,561**	**1,920**	**923**	**2,843**	**90.08**
Abra	326	122	448	354	133	487	91.99
Apayao	185	88	273	186	91	277	98.56
Benguet	193	83	276	226	93	319	86.52
Ifugao	203	104	307	224	112	336	91.37
Kalinga	358	166	524	381	176	557	94.08
Mt. Province	145	51	196	152	55	207	94.69
Baguio City	321	216	537	397	263	660	81.36
**Region 1**	**8,657**	**4,721**	**13,378**	**9,559**	**5,161**	**14,720**	**90.88**
Ilocos Norte	559	258	817	696	321	1,017	80.33
Ilocos Sur	790	410	1,200	1,004	521	1,525	78.69
La Union	988	578	1,566	1,061	622	1,683	93.05
Pangasinan	4,406	2,385	6,791	4,515	2,436	6,951	97.70
Alaminos City	215	111	326	215	111	326	100.00
Candon City	0	0	0	90	50	140	64.29
Dagupan City	431	266	697	507	285	792	88.01
Laoag City	109	39	148	156	58	214	69.16

Table 1.E.5.3 - Tuberculosis
Treatment Success Rate DSTB - All Forms
Philippines, Annual 2020

Area	TB all forms cured and Completely Treated			Registered TB all forms Registered during Specified Period			Treatment Success Rate
	Male	Female	Total	Male	Female	Total	
San Carlos City	395	225	620	410	235	645	96.12
San Fernando City	380	234	614	457	278	735	83.54
Urdaneta City	280	159	439	304	165	469	93.60
Vigan City	104	56	160	144	79	223	71.75
**Region 2**	**5,776**	**3,183**	**8,959**	**6,183**	**3,353**	**9,536**	**93.95**
Batanes	22	19	41	24	19	43	95.35
Cagayan	1,847	970	2,817	1,940	1,013	2,953	95.39
Isabela	1,876	1,056	2,932	2,005	1,098	3,103	94.49
Nueva Vizcaya	650	379	1,029	714	417	1,131	90.98
Quirino	263	126	389	305	143	448	86.83
Cauayan City	178	106	284	211	118	329	86.32
Ilagan City	278	163	441	298	166	464	95.04
Santiago City	375	199	574	382	204	586	97.95
Tuguegarao City	287	165	452	304	175	479	94.36
**Region 3**	**21,279**	**11,588**	**25,997**	**24,018**	**12,892**	**29,107**	**89.32**
Aurora	463	237	700	601	298	899	77.86
Bataan	1,511	917	2,428	1,614	966	2,580	94.11
Bulacan	4,353	2,281	6,634	5,114	2,669	7,783	85.24
Nueva Ecija	2,197	1,185	3,382	2,538	1,316	3,854	87.75
Pampanga	2,624	1,375	3,999	2,948	1,515	4,463	89.60
Tarlac	1,901	1,043	2,944	1,933	1,073	3,006	97.94
Zambales	1,494	989	2,483	1,772	1,160	2,932	84.69
Angeles City	634	325	959	675	341	1,016	94.39
Balanga City	324	188	512	355	191	546	93.77
Cabanatuan City	519	327	846	540	339	879	96.25
City of San Fernando	566	256	822	580	262	842	97.62
Gapan City	178	110	288	187	120	307	93.81
Mabalacat City	445	231	676	517	269	786	86.01
Malolos City	386	192	578	555	230	785	73.63
Meycauayan City	477	259	736	650	345	995	73.97
Olongapo City	615	352	967	703	409	1,112	86.96
Palayan City	120	40	160	138	48	186	86.02
San Jose City	256	136	392	320	164	484	80.99
San Jose del Monte City	1,199	665	1,864	1,227	688	1,915	97.34
Science City of Munoz	95	63	158	106	67	173	91.33
Tarlac City	922	417	1,339	945	422	1,367	97.95
**Region 4A**	**28,751**	**16,368**	**45,119**	**31,058**	**17,494**	**48,552**	**92.93**
Batangas	3,868	2,325	6,193	4,176	2,492	6,668	92.88
Cavite	2,635	1,553	4,188	2,907	1,688	4,595	91.14
Laguna	2170	1242	3,412	2326	1329	3,655	93.35

Table 1.E.5.3 - Tuberculosis
Treatment Success Rate DSTB - All Forms
Philippines, Annual 2020

Area	TB all forms cured and Completely Treated			Registered TB all forms Registered during Specified Period			Treatment Success Rate
	Male	Female	Total	Male	Female	Total	
Quezon	3082	1685	4,767	3334	1789	5,123	93.05
Rizal	4360	2481	6,841	4729	2670	7,399	92.46
Antipolo City	1600	1052	2,652	1768	1148	2,916	90.95
Bacoor City	706	450	1,156	824	498	1,322	87.44
Batangas City	711	340	1,051	792	367	1,159	90.68
Biñan City	1151	624	1,775	1168	643	1,811	98.01
Cabuyao City	449	227	676	509	264	773	87.45
Calamba City	1472	665	2,137	1481	670	2,151	99.35
Cavite City	195	121	316	218	128	346	91.33
Dasmariñas City	1370	794	2,164	1488	850	2,338	92.56
General Trias City	546	338	884	578	356	934	94.65
Imus City	681	438	1,119	721	458	1,179	94.91
Lipa City	512	271	783	523	279	802	97.63
Lucena City	592	384	976	626	393	1,019	95.78
San Pablo City	382	187	569	440	219	659	86.34
San Pedro City	475	248	723	504	261	765	94.51
Santa Rosa City	498	265	763	531	275	806	94.67
Tagaytay City	216	98	314	235	102	337	93.18
Tanauan City	396	178	574	430	189	619	92.73
Tayabas City	205	103	308	231	109	340	90.59
Trece Martires City	479	299	778	519	317	836	93.06
**Region 4B**	**6,766**	**3,763**	**10,529**	**7,720**	**4,261**	**11,981**	**87.88**
Marinduque	644	383	1,027	728	415	1,143	89.85
Mindoro Occidental	1056	564	1,620	1155	615	1,770	91.53
Mindoro Oriental	1979	1132	3,111	2184	1229	3,413	91.15
Palawan	1707	1116	2,823	2078	1346	3,424	82.45
Romblon	556	273	829	715	353	1,068	77.62
Puerto Princesa City	824	295	1,119	860	303	1,163	96.22
**Region 5**	**11,552**	**5,987**	**17,539**	**12,615**	**6,477**	**19,092**	**91.87**
Albay	2,414	1,187	3,601	2,517	1,215	3,732	96.49
Camarines Norte	1,077	454	1,531	1,185	503	1,688	90.70
Camarines Sur	3,334	1,870	5,204	3,689	2,057	5,746	90.57
Catanduanes	444	261	705	501	295	796	88.57
Masbate	1,758	893	2,651	1,927	964	2,891	91.70
Sorsogon	1,334	687	2,021	1,477	765	2,242	90.14
							0.00
Iriga City	272	146	418	299	155	454	92.07
Legaspi City	450	257	707	494	276	770	91.82
Naga City	469	232	701	526	247	773	90.69

Table 1.E.5.3 - Tuberculosis

Treatment Success Rate DSTB - All Forms

Philippines, Annual 2020

Area	TB all forms cured and Completely Treated			Registered TB all forms Registered during Specified Period			Treatment Success Rate
	Male	Female	Total	Male	Female	Total	
**Region 6**	**20,141**	**9,600**	**29,741**	**21,648**	**10,255**	**31,903**	**93.22**
Aklan	1,140	515	1,655	1,292	566	1,858	89.07
Antique	1,590	776	2,366	1,700	820	2,520	93.89
Capiz	1,708	813	2,521	1,819	853	2,672	94.35
Guimaras	385	213	598	403	217	620	96.45
Iloilo	4,172	2,014	6,186	4,393	2,097	6,490	95.32
Negros Occidental	7,636	3,529	11,165	8,291	3,846	12,137	91.99
Bacolod City	2,039	917	2,956	2,147	966	3,113	94.96
Iloilo City	1,471	823	2,294	1,603	890	2,493	92.02
**Region 7**	**13,019**	**6,249**	**19,268**	**14,559**	**6,850**	**21,409**	**90.00**
Bohol	1,903	948	2,851	2,050	1,010	3,060	93.17
Cebu	4,616	2,060	6,676	5,086	2,257	7,343	90.92
Negros Oriental	2,478	1,219	3,697	2,715	1,300	4,015	92.08
Siquijor	196	70	266	209	73	282	94.33
Cebu City	1,782	901	2,683	2,145	1,018	3,163	84.82
Lapu-Lapu City	1,125	601	1,726	1,285	691	1,976	87.35
Mandaue City	919	450	1,369	1,069	501	1,570	87.20
**Region 8**	**6,476**	**3,335**	**9,811**	**7,434**	**3,780**	**11,214**	**87.49**
Biliran	362	188	550	378	193	571	96.32
Eastern Samar	634	341	975	694	373	1,067	91.38
Northern Leyte	2,086	1,019	3,105	2,361	1,139	3,500	88.71
Northern Samar	865	446	1,311	973	498	1,471	89.12
Southern Leyte	485	207	692	536	235	771	89.75
Western Samar	669	366	1,035	745	394	1,139	90.87
Calbayog City	295	173	468	363	207	570	82.11
Maasin City	116	51	167	124	54	178	93.82
Ormoc City	331	191	522	520	298	818	63.81
Tacloban City	633	353	986	740	389	1,129	87.33
**Region 9**	**6,101**	**2,729**	**8,830**	**6,611**	**2,924**	**9,535**	**92.61**
Zamboanga del Norte	1079	446	1,525	1141	469	1,610	94.72
Zamboanga del Sur	1155	542	1,697	1286	589	1,875	90.51
Zamboanga Sibugay	778	332	1,110	886	387	1,273	87.20
Dapitan City	151	52	203	163	56	219	92.69
Dipolog City	227	101	328	252	109	361	90.86
Isabela City	173	109	282	178	109	287	98.26
Pagadian City	339	175	514	362	188	550	93.45

Table 1.E.5.3 - Tuberculosis

Treatment Success Rate DSTB - All Forms

Philippines, Annual 2020

Area	TB all forms cured and Completely Treated			Registered TB all forms Registered during Specified Period			Treatment Success Rate
	Male	Female	Total	Male	Female	Total	
Zamboanga City	2199	972	3,171	2343	1017	3,360	94.38
**Region 10**	**7,713**	**3,667**	**11,380**	**8,339**	**3,979**	**12,318**	**92.39**
Bukidnon	909	467	1,376	984	513	1,497	91.92
Camiguin	126	41	167	137	42	179	93.30
Lanao del Norte	1,030	579	1,609	1,056	596	1,652	97.40
Misamis Occidental	586	225	811	621	232	853	95.08
Misamis Oriental	1,260	571	1,831	1,306	599	1,905	96.12
			0				
Cagayan de Oro City	1,532	743	2,275	1,708	824	2,532	89.85
El Salvador City	135	69	204	151	72	223	91.48
Gingoog City	255	104	359	271	113	384	93.49
Iligan City	794	404	1,198	848	431	1,279	93.67
Malaybalay City	223	84	307	287	116	403	76.18
Oroquieta City	156	58	214	170	63	233	91.85
Ozamis City	386	187	573	398	193	591	96.95
Tangub City	57	28	85	107	65	172	49.42
Valencia City	264	107	371	295	120	415	89.40
**Region 11**	**7,663**	**3,205**	**10,868**	**8,404**	**3,466**	**11,870**	**91.56**
Davao de Oro	1,089	345	1,434	1,151	363	1,514	94.72
Davao del Norte	1,729	624	2,353	1,937	684	2,621	89.77
Davao del Sur	920	447	1,367	965	475	1,440	94.93
Davao Occidental	307	138	445	337	147	484	91.94
Davao Oriental	860	344	1,204	919	373	1,292	93.19
Davao City	2,758	1,307	4,065	3,095	1,424	4,519	89.95
**Region 12**	**9,286**	**4,786**	**14,072**	**10,096**	**5,126**	**15,222**	**92.45**
North Cotabato	2,447	1,279	3,726	2,724	1,403	4,127	90.28
Sarangani	919	495	1,414	984	523	1,507	93.83
South Cotabato	1,995	843	2,838	2,125	875	3,000	94.60
Sultan Kudarat	2,053	1,148	3,201	2,125	1,182	3,307	96.79
Cotabato City	645	414	1,059	742	455	1,197	88.47
Gen. Santos City	1,227	607	1,834	1,396	688	2,084	88.00
**BARMM**	**2,578**	**1,928**	**4,506**	**3,349**	**2,411**	**5,760**	**78.23**
Basilan	229	200	429	269	230	499	85.97
Lanao del Sur	785	720	1,505	852	763	1,615	93.19
Maguindanao	1,248	737	1,985	1,299	765	2,064	96.17
Sulu	6	10	16	351	250	601	2.66
Tawi-Tawi	0	0	0	205	107	312	0.00

Table 1.E.5.3 - Tuberculosis
Treatment Success Rate DSTB - All Forms
Philippines, Annual 2020

Area	TB all forms cured and Completely Treated			Registered TB all forms Registered during Specified Period			Treatment Success Rate
	Male	Female	Total	Male	Female	Total	
Lamitan City	137	96	233	152	104	256	91.02
Marawi City	173	165	338	221	192	413	81.84
**CARAGA**	**5,585**	**2,933**	**8,518**	**6,101**	**3,162**	**9,263**	**91.96**
Agusan del Norte	788	382	1,170	884	423	1,307	89.52
Agusan del Sur	1,413	697	2,110	1,514	747	2,261	93.32
Surigao del Norte	695	358	1,053	751	380	1,131	93.10
Surigao del Sur	995	539	1,534	1,100	583	1,683	91.15
Province of Dinagat	264	141	405	271	149	420	96.43
Bislig City	326	199	525	339	203	542	96.86
Butuan City	621	315	936	686	335	1,021	91.67
Surigao City	483	302	785	556	342	898	87.42

Legend:
0 = No Case
** = No Report*

Table 1.E.5.4 - Tuberculosis
Treatment Success Rate - MDRTB
Philippines, Annual 2020

Area	Registered Bacteriology Confirmed Drug Resistant			Registered Bacteriologically Confirmed Drug Resistant Specified Period			Treatment Success Rate
	Male	Female	Total	Male	Female	Total	
**PHILIPPINES**	**2,792**	**1,202**	**3,994**	**4,190**	**1,755**	**5,945**	67.18
**N C R**	701	312	**1,013**	1,119	502	**1,621**	62.49
Malabon City	20	14	34	38	20	58	58.62
Navotas City	16	12	28	31	19	50	56.00
Valenzuela City	21	13	34	50	19	69	49.28
Caloocan City	88	44	132	88	61	149	88.59
Marikina City	34	11	45	52	19	71	63.38
Pasig City	38	20	58	64	30	94	61.70
Pateros	0	0	0	0	0	0	0.00
Taguig City	45	13	58	81	21	102	56.86
Quezon City	160	70	230	245	114	359	64.07
Makati City	30	19	49	51	25	76	64.47
Mandaluyong City	22	7	29	31	13	44	65.91
San Juan City	12	2	14	13	4	17	82.35
Manila City	119	50	169	227	94	321	52.65
Las Piñas City	21	13	34	35	20	55	61.82
Muntinlupa City	38	8	46	53	16	69	66.67
Parañaque City	20	6	26	32	11	43	60.47
Pasay City	17	10	27	28	16	44	61.36
**C A R**	20	11	**31**	33	16	**49**	63.27
Abra	2	3	5	6	4	10	50.00
Apayao	3	0	3	4	1	5	60.00
Benguet	0	0	0	0	0	0	0.00
Ifugao	0	0	0	2	0	2	0.00
Kalinga	4	1	5	5	2	7	71.43
Mt. Province	1	1	2	1	1	2	100.00
						0	
Baguio City	10	6	16	15	8	23	69.57
**Region 1**	142	46	**188**	177	56	**233**	80.69
Ilocos Norte	9	7	16	10	8	18	88.89
Ilocos Sur	14	1	15	15	2	17	88.24
La Union	1	2	3	1	2	3	100.00
Pangasinan	0	0	0	0	0	0	0.00
Alaminos City	9	1	10	9	3	12	83.33
Candon City	0	0	0	0	0	0	0.00
Dagupan City	48	15	63	67	17	84	75.00
Laoag City	0	0	0	0	0	0	0.00
San Carlos City	7	5	12	11	5	16	75.00
San Fernando City	29	6	35	32	9	41	85.37
Urdaneta City	22	8	30	29	9	38	78.95
Vigan City	3	1	4	3	1	4	100.00

Table 1.E.5.4 - Tuberculosis
Treatment Success Rate - MDRTB
Philippines, Annual 2020

Area	Registered Bacteriology Confirmed Drug Resistant			Registered Bacteriologically Confirmed Drug Resistant Specified Period			Treatment Success Rate
	Male	Female	Total	Male	Female	Total	
**Region 2**	69	15	**84**	92	17	**109**	77.06
Batanes	0	0	0	0	0	0	0.00
Cagayan	14	1	15	15	1	16	93.75
Isabela	0	0	0	0	0	0	0.00
Nueva Vizcaya	2	2	4	2	2	4	100.00
Quirino	4	1	5	4	1	5	100.00
Cauayan City	0	0	0	0	0	0	0.00
Ilagan City	0		0	0	0	0	0.00
Santiago City	30	7	37	40	7	47	78.72
Tuguegarao City	19	4	23	31	6	37	62.16
**Region 3**	332	151	**0**	550	230	**0**	0.00
Aurora	9	1	10	11	2	0	0.00
Bataan	6	2	8	14	3	0	0.00
Bulacan	40	10	50	63	22	0	0.00
Nueva Ecija	25	13	38	36	13	0	0.00
Pampanga	5	1	6	7	2	0	0.00
Tarlac	13	10	23	22	13	8	78.00
Zambales	26	11	37	39	17	0	0.00
Angeles City	6	2	8	11	2	0	0.00
Balanga City	19	5	24	35	13	0	0.00
Cabanatuan City	15	12	27	31	18	0	0.00
City of San Fernando	41	12	53	70	18	0	0.00
Gapan City	0	0	0	0	0	0	0.00
Mabalacat City	25	12	37	31	18	0	0.00
Malolos City	49	25	74	83	37	0	0.00
Meycauayan City	0	0	0	0	0	0	0.00
Olongapo City	16	7	23	30	8	0	0.00
Palayan City	0	0	0	0	0	0	0.00
San Jose City	7	3	10	14	6	0	0.00
San Jose del Monte City	14	13	27	27	21	0	0.00
Science City of Munoz	0	0	0	0	0	0	0.00
Tarlac City	16	12	28	26	17	0	0.00
**Region 4A**	402	212	**614**	633	311	**944**	65.04
Batangas	24	18	42	37	23	60	70.00
Cavite	1	2	3	5	3	8	37.50
Laguna	50	19	69	72	27	99	69.70
Quezon	27	21	48	40	27	67	71.64
Rizal	68	32	100	110	51	161	62.11
Antipolo City	20	18	38	38	29	67	56.72
Bacoor City	11	1	12	18	1	19	63.16
Batangas City	19	13	32	32	16	48	66.67
Biñan City	0	0	0	0	0	0	0.00
Cabuyao City	0	0	0	0	0	0	0.00
Calamba City	41	11	52	48	13	61	85.25
Cavite City	0	0	0	0	0	0	0.00

Table 1.E.5.4 - Tuberculosis

Treatment Success Rate - MDRTB

Philippines, Annual 2020

Area	Registered Bacteriology Confirmed Drug Resistant			Registered Bacteriologically Confirmed Drug Resistant Specified Period			Treatment Success Rate
	Male	Female	Total	Male	Female	Total	
Dasmariñas City	29	16	45	53	34	87	51.72
General Trias City	0	0	0	0	0	0	0.00
Imus City	6	5	11	15	6	21	52.38
Lipa City	17	10	27	30	13	43	62.79
Lucena City	23	11	42	42	15	57	73.68
San Pablo City	0	0	0	0	0	0	0.00
San Pedro City	0	0	0	0	0	0	0.00
Santa Rosa City	28	19	47	39	26	65	72.31
Tagaytay City	4	3	7	7	3	10	70.00
Tanauan City	0	0	0	0	0	0	0.00
Tayabas City	0	0	0	0	0	0	0.00
Trece Martires City	34	13	47	47	24	71	66.20
**Region 4B**	115	39	**154**	158	52	**210**	73.33
Marinduque	10	2	12	16	4	20	60.00
Mindoro Occidental	20	8	28	25	8	33	84.85
Mindoro Oriental	40	11	51	52	14	66	77.27
Palawan	20	11	31	25	14	39	79.49
Romblon	9	1	10	13	1	14	71.43
Puerto Princesa City	16	6	22	27	11	38	57.89
**Region 5**	220	76	**296**	284	113	**397**	74.56
Albay	1	0	1	2	0	2	50.00
Camarines Norte	39	16	55	50	18	68	80.88
Camarines Sur	60	22	82	79	34	113	72.57
Catanduanes	4	5	9	10	9	19	47.37
Masbate	25	10	35	31	13	44	79.55
Sorsogon	34	7	41	38	9	47	87.23
Iriga City	0	0	0	0	0	0	0.00
Legaspi City	57	16	73	74	30	104	70.19
Naga City	0	0	0	0	0	0	0.00
**Region 6**	240	97	**337**	325	119	**444**	75.90
Aklan	18	10	28	31	14	45	62.22
Antique	15	9	24	17	9	26	92.31
Capiz	14	5	19	21	8	29	65.52
Guimaras	0	0	0	0	0	0	0.00
Iloilo	26	13	39	33	15	48	81.25
Negros Occidental	92	37	129	120	43	163	79.14
			0			0	
Bacolod City	35	8	43	50	11	61	70.49
Iloilo City	40	15	55	53	19	72	76.39
**Region 7**	124	50	**174**	200	68	**268**	64.93
Bohol	15	12	27	25	16	41	65.85
Cebu	13	6	19	24	7	31	61.29
Negros Oriental	20	7	27	26	9	35	77.14
Siquijor	0	0	0	0	0	0	0.00

Table 1.E.5.4 - Tuberculosis
Treatment Success Rate - MDRTB
Philippines, Annual 2020

Area	Registered Bacteriology Confirmed Drug Resistant			Registered Bacteriologically Confirmed Drug Resistant Specified Period			Treatment Success Rate
	Male	Female	Total	Male	Female	Total	
Cebu City	44	14	58	64	17	81	71.60
Lapu-Lapu City	0	0	0	0	0	0	0.00
Mandaue City	32	11	43	61	19	80	53.75
**Region 8**	71	35	**0**	91	42	**0**	0.00
Biliran	0	0	0	0	0	0	0.00
Eastern Samar	12	8	20	16	8	24	83.33
Northern Leyte	40	17	57	55	22	77	74.03
Northern Samar	6	3	9	7	4	11	81.82
Southern Leyte	13	7	20	13	8	21	95.24
Western Samar	0	0	0	0	0	0	0.00
Calbayog City	0	0	0	0	0	0	0.00
Maasin City	0	0	0	0	0	0	0.00
Ormoc City	0	0	0	0	0	0	0.00
Tacloban City	0	0	0	0	0	0	0.00
**Region 9**	62	17	**79**	76	25	**101**	78.22
Zamboanga del Norte	0	0	0	0	0	0	0.00
Zamboanga del Sur	18	2	20	20	3	23	86.96
Zamboanga Sibugay	12	6	18	16	8	24	75.00
						0	
Dapitan City	12	5	17	14	7	21	80.95
Dipolog City	0	0	0	0	0	0	0.00
Isabela City	0	1	1	0	2	2	50.00
Pagadian City	0	0	0	0	0	0	0.00
Zamboanga City	20	3	23	26	5	31	74.19
**Region 10**	85	39	**124**	121	53	**174**	71.26
Bukidnon	2	3	5	5	3	8	62.50
Camiguin	0	0	0	0	0	0	0.00
Lanao del Norte	9	5	14	17	6	23	60.87
Misamis Occidental	0	0	0	0	0	0	0.00
Misamis Oriental	0	0	0	0	0	0	0.00
			0				
Cagayan de Oro City	26	16	42	28	19	47	89.36
El Salvador City	0	0	0	0	0	0	0.00
Gingoog City	3	4	7	3	5	8	87.50
Iligan City	22	9	31	34	15	49	63.27
Malaybalay City	9	2	11	11	3	14	78.57
Oroquieta City	1	0	1	3	1	4	25.00
Ozamis City	13		13	20	1	21	61.90
Tangub City	0	0	0	0	0	0	0.00
Valencia City	0	0	0	0	0	0	0.00
**Region 11**	58	22	**80**	92	29	**121**	66.12
Compostela Valley	0	0	0	0	0	0	0.00
Davao del Norte	11	7	18	21	8	29	62.07
Davao del Sur	7	2	9	13	4	17	52.94

Table 1.E.5.4 - Tuberculosis
Treatment Success Rate - MDRTB
Philippines, Annual 2020

Area	Registered Bacteriology Confirmed Drug Resistant			Registered Bacteriologically Confirmed Drug Resistant Specified Period			Treatment Success Rate
	Male	Female	Total	Male	Female	Total	
Davao Occidental	4		4	5	0	5	80.00
Davao Oriental	5	2	7	8	3	11	63.64
						0	
Davao City	31	11	42	45	14	59	71.19
**Region 12**	65	38	**103**	102	49	**151**	68.21
North Cotabato	13	4	17	19	5	24	70.83
Sarangani	0	1	1	1	1	2	50.00
South Cotabato	19	6	25	29	14	43	58.14
Sultan Kudarat	5	2	7	11	2	13	53.85
Cotabato City	11	12	23	21	14	35	65.71
Gen. Santos City	17	13	30	21	13	34	88.24
**BARMM**	16	13	29	43	31	74	39.19
Basilan	0	0	0	0	0	0	0.00
Lanao del Sur	0	0	0	11	8	19	0.00
Maguindanao	1		1	5	1	6	16.67
Sulu	4	2	6	10	4	14	42.86
Tawi-Tawi	1	1	2	3	3	6	33.33
Lamitan City	0	0	0	0	0	0	0.00
Marawi City	10	10	20	14	15	29	68.97
**CARAGA**	70	29	**99**	94	42	**136**	72.79
Agusan del Norte	9	6	15	11	10	21	71.43
Agusan del Sur	0	0	0	0	0	0	0.00
Surigao del Norte	15	9	24	21	10	31	77.42
Surigao del Sur	30	10	40	41	17	58	68.97
Province of Dinagat			0	0		0	0.00
Bislig City	0	0	0	0	0	0	0.00
Butuan City	16	4	20	21	5	26	76.92
Surigao City	0	0	0	0	0	0	0.00

Legend:
0 = No Case
** = No Report*

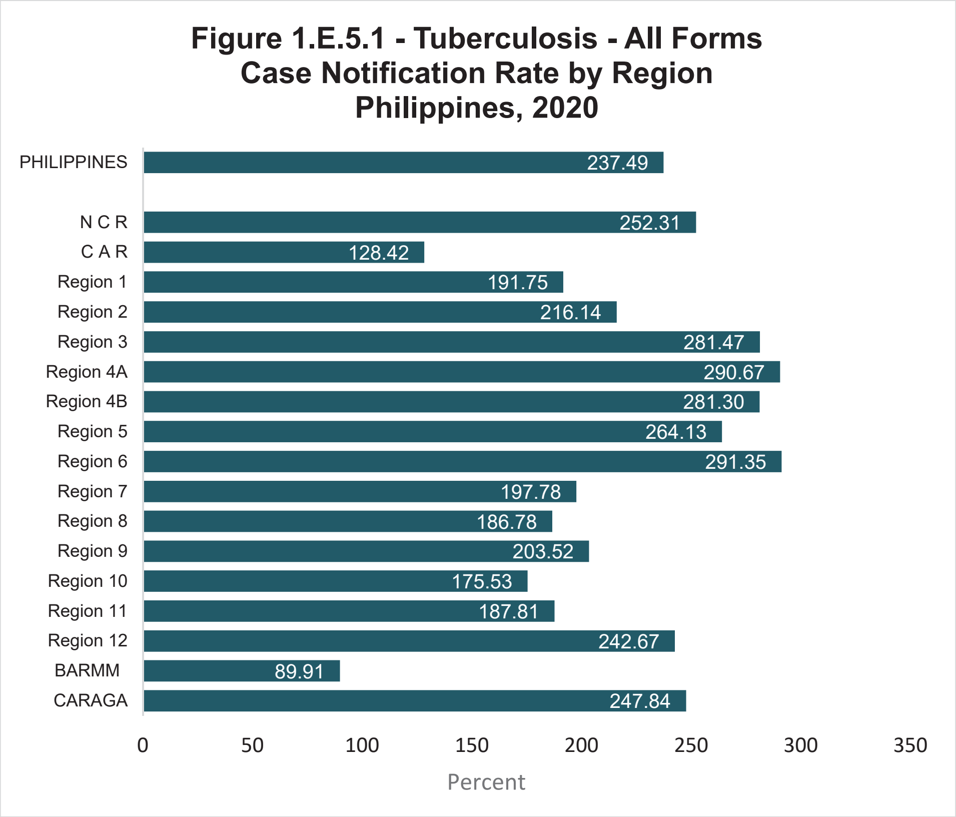
Figure 1.E.5.1 - Tuberculosis - All Forms
Case Notification Rate by Region
Philippines, 2020
PHILIPPINES 237.49
N C R 252.31
C A R 128.42
Region 1 191.75
Region 2 216.14
Region 3 281.47
Region 4A 290.67
Region 4B 281.30
Region 5 264.13
Region 6 291.35
Region 7 197.78
Region 8 186.78
Region 9 203.52
Region 10 175.53
Region 11 187.81
Region 12 242.67
BARMM 89.91
CARAGA 247.84
0 50 100 150 200 250 300 350
Percent

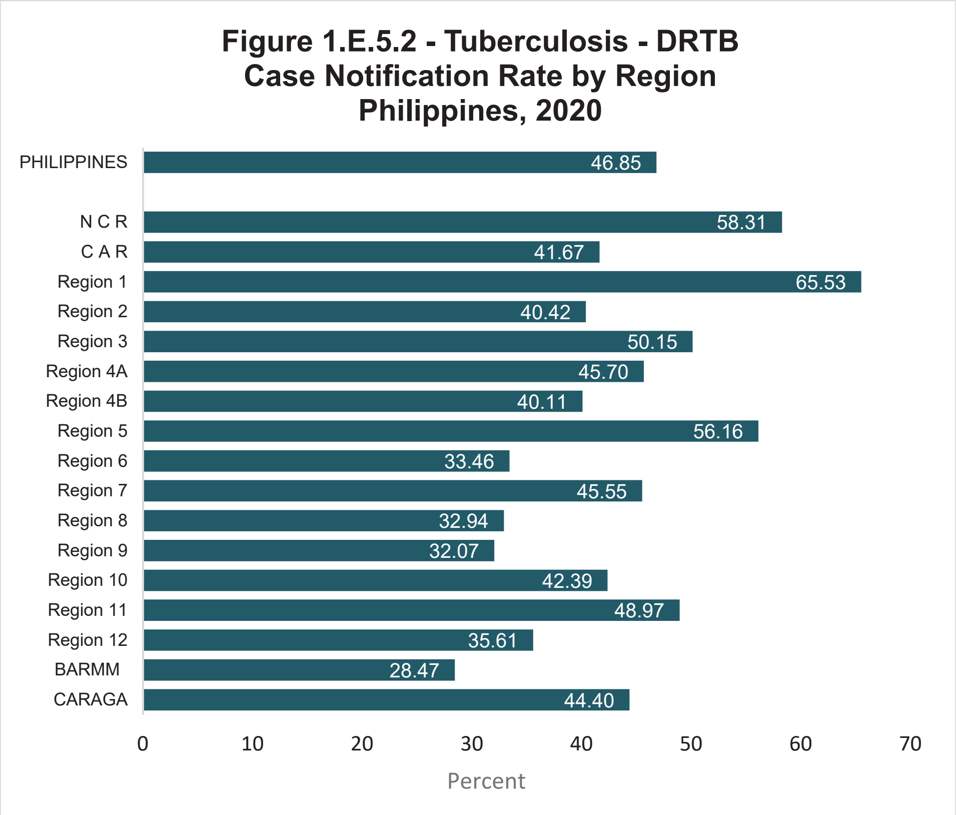
Figure 1.E.5.2 - Tuberculosis - DRTB
Case Notification Rate by Region
Philippines, 2020
PHILIPPINES 46.85
N C R 58.31
C A R 41.67
Region 1 65.53
Region 2 40.42
Region 3 50.15
Region 4A 45.70
Region 4B 40.11
Region 5 56.16
Region 6 33.46
Region 7 45.55
Region 8 32.94
Region 9 32.07
Region 10 42.39
Region 11 48.97
Region 12 35.61
BARMM 28.47
CARAGA 44.40
0 10 20 30 40 50 60 70
Percent

Figure 1.E.5.3 - Tuberculosis
DSTB - All Forms
Treatment Success Rate by Region
Philippines, 2020

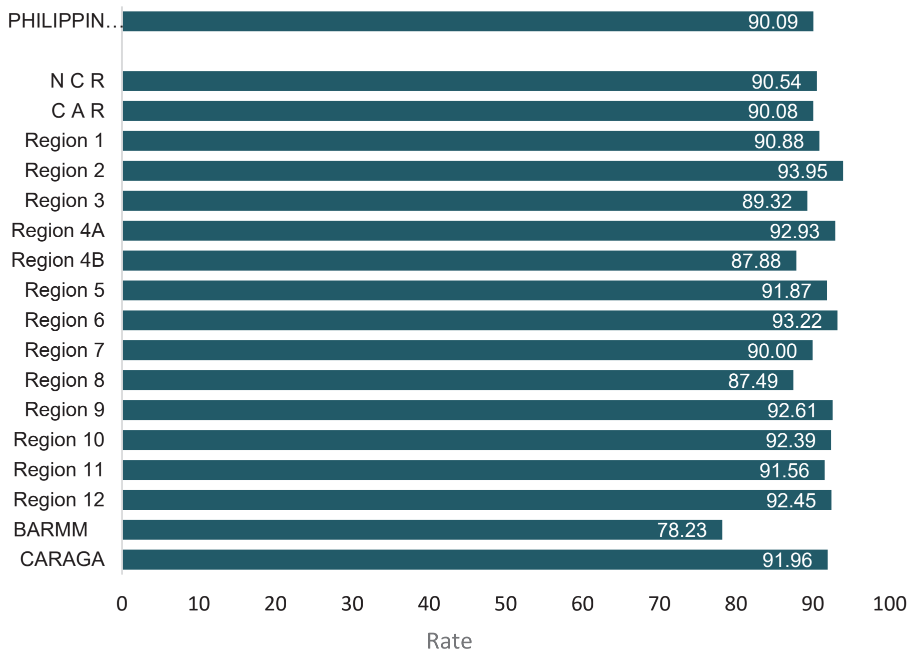

Figure 1.E.5.4 - Tuberculosis
MDRTB
Treatment Success Rate by Region
Philippines, 2020

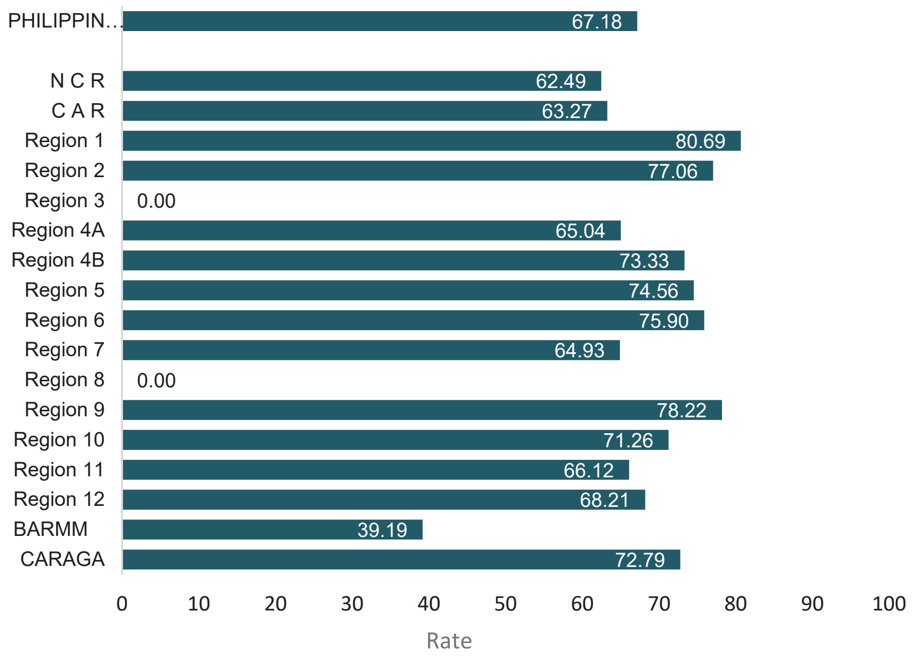

Table 1.E.6.1 - Malaria

Probable/Clinically and Confirmed Malaria Cases Disaggregated by Age group and Sex Philippines, Annual 2020

Area	Population	Probable/Clinically and Confirmed Malaria Cases							
		<5 yrs Old			Morbidity Rate	>=5 yrs Old			Morbidity Rate
		Male	Female	Total		Male	Female	Total	
**PHILIPPINES**	**110,198,654**	**6**	**9**	**15**	**0.01**	**42**	**25**	**67**	**0.06**
**N C R**	**13,966,223**	**0**	**0**	**0**	**0.00**	**0**	**0**	**0**	**0.00**
Malabon City	396,434	0	0	0	0.00	0	0	0	0.00
Navotas City	270,559	0	0	0	0.00	0	0	0	0.00
Valenzuela City	672,888	0	0	0	0.00	0	0	0	0.00
Caloocan City	1,717,928	0	0	0	0.00	0	0	0	0.00
Marikina City	488,857	0	0	0	0.00	0	0	0	0.00
Pasig City	819,171	0	0	0	0.00	0	0	0	0.00
Pateros	69,239	0	0	0	0.00	0	0	0	0.00
Taguig City	872,980	0	0	0	0.00	0	0	0	0.00
Quezon City	3,184,407	0	0	0	0.00	0	0	0	0.00
Makati City	631,867	0	0	0	0.00	0	0	0	0.00
Mandaluyong City	418,942	0	0	0	0.00	0	0	0	0.00
San Juan City	132,512	0	0	0	0.00	0	0	0	0.00
Manila City	1,930,696	0	0	0	0.00	0	0	0	0.00
Las Piñas City	638,694	0	0	0	0.00	0	0	0	0.00
Muntinlupa City	547,173	0	0	0	0.00	0	0	0	0.00
Parañaque City	722,126	0	0	0	0.00	0	0	0	0.00
Pasay City	451,750	0	0	0	0.00	0	0	0	0.00
**C A R**	**1,822,719**	**0**	**0**	**0**	**0.00**	**1**	**0**	**1**	**0.05**
Abra	248,468	0	0	0	0.00	0	0	0	0.00
Apayao	124,728	0	0	0	0.00	0	0	0	0.00
Benguet	483,934	0	0	0	0.00	1	0	1	0.21
Ifugao	212,063	0	0	0	0.00	0	0	0	0.00
Kalinga	221,563	0	0	0	0.00	0	0	0	0.00
Mt. Province	157,413	0	0	0	0.00	0	0	0	0.00
Baguio City	374,550	0	0	0	0.00	0	0	0	0.00
**Region 1**	**5,313,223**	**0**	**0**	**0**	**0.00**	**1**	**0**	**1**	**0.02**
Ilocos Norte	501,276	0	0	0	0.00	0	0	0	0.00
Ilocos Sur	600,674	0	0	0	0.00	0	0	0	0.00
La Union	705,988	0	0	0	0.00	1	0	1	0.14
Pangasinan	2,522,027	0	0	0	0.00	0	0	0	0.00
Alaminos City	95,291	0	0	0	0.00	0	0	0	0.00
Candon City	63,312	0	0	0	0.00	0	0	0	0.00
Dagupan City	181,929	0	0	0	0.00	0	0	0	0.00
Laoag City	115,582	0	0	0	0.00	0	0	0	0.00
San Carlos City	200,310	0	0	0	0.00	0	0	0	0.00
San Fernando City	129,350	0	0	0	0.00	0	0	0	0.00
Urdaneta City	141,215	0	0	0	0.00	0	0	0	0.00
Vigan City	56,269	0	0	0	0.00	0	0	0	0.00
**Region 2**	**3,693,664**	**0**	**0**	**0**	**0.00**	**0**	**0**	**0**	**0.00**
Batanes	18,013	0	0	0	0.00	0	0	0	0.00
Cagayan	1,121,561	0	0	0	0.00	0	0	0	0.00
Isabela	1,264,006	0	0	0	0.00	0	0	0	0.00

Table 1.E.6.1 - Malaria

Probable/Clinically and Confirmed Malaria Cases Disaggregated by Age group and Sex

Philippines, Annual 2020

Area	Population	Probable/Clinically and Confirmed Malaria Cases							
		<5 yrs Old			Morbidity Rate	>=5 yrs Old			Morbidity Rate
		Male	Female	Total		Male	Female	Total	
Nueva Vizcaya	488,238	0	0	0	0.00	0	0	0	0.00
Quirino	199,473	0	0	0	0.00	0	0	0	0.00
Cauayan City	138,317	0	0	0	0.00	0	0	0	0.00
Ilagan City	155,452	0	0	0	0.00	0	0	0	0.00
Santiago City	143,982	0	0	0	0.00	0	0	0	0.00
Tuguegarao City	164,622	0	0	0	0.00	0	0	0	0.00
**Region 3**	**12,513,847**	**2**	**0**	**2**	**0.02**	**0**	**1**	**1**	**0.01**
Aurora	229,841	0	0	0	0.00	0	0	0	0.00
Bataan	742,197	0	0	0	0.00	0	0	0	0.00
Bulacan	2,542,502	0	0	0	0.00	0	0	0	0.00
Nueva Ecija	1,646,738	0	0	0	0.00	0	0	0	0.00
Pampanga	1,850,369	0	0	0	0.00	0	0	0	0.00
Tarlac	1,106,339	0	0	0	0.00	0	0	0	0.00
Zambales	650,180	2	0	2	0.31	0	0	0	0.00
		0	0	0	0.00	0	0	0	0.00
Angeles City	464,253	0	0	0	0.00	0	0	0	0.00
Balanga City	107,277	0	0	0	0.00	0	0	0	0.00
Cabanatuan City	337,037	0	0	0	0.00	0	0	0	0.00
City of San Fernando	345,857	0	0	0	0.00	0	0	0	0.00
Gapan City	123,009	0	0	0	0.00	0	0	0	0.00
Mabalacat City	282,858	0	0	0	0.00	0	0	0	0.00
Malolos City	283,977	0	0	0	0.00	0	0	0	0.00
Meycauayan City	235,549	0	0	0	0.00	0	0	0	0.00
Olongapo City	256,443	0	0	0	0.00	0	1	1	0.39
Palayan City	45,768	0	0	0	0.00	0	0	0	0.00
San Jose City	155,831	0	0	0	0.00	0	0	0	0.00
San Jose del Monte City	646,756	0	0	0	0.00	0	0	0	0.00
Science City of Munoz	90,867	0	0	0	0.00	0	0	0	0.00
Tarlac City	370,199	0	0	0	0.00	0	0	0	0.00
**Region 4A**	**16,361,107**	**1**	**0**	**1**	**0.01**	**1**	**0**	**1**	**0.01**
Batangas	2,144,028	0	0	0	0.00	0	0	0	0.00
Cavite	1,604,676	0	0	0	0.00	0	0	0	0.00
Laguna	1,111,798	0	0	0	0.00	0	0	0	0.00
Quezon	1,890,167	0	0	0	0.00	0	0	0	0.00
Rizal	2,386,862	1	0	1	0.04	1	0	1	0.04
Antipolo City	879,159	0	0	0	0.00	0	0	0	0.00
Bacoor City	703,029	0	0	0	0.00	0	0	0	0.00
Batangas City	380,515	0	0	0	0.00	0	0	0	0.00
Biñan City	372,804	0	0	0	0.00	0	0	0	0.00
Cabuyao City	345,623	0	0	0	0.00	0	0	0	0.00
Calamba City	508,770	0	0	0	0.00	0	0	0	0.00
Cavite City	120,342	0	0	0	0.00	0	0	0	0.00
Dasmariñas City	771,409	0	0	0	0.00	0	0	0	0.00
General Trias City	367,906	0	0	0	0.00	0	0	0	0.00
Imus City	472,646	0	0	0	0.00	0	0	0	0.00
Lipa City	383,404	0	0	0	0.00	0	0	0	0.00
Lucena City	286,460	0	0	0	0.00	0	0	0	0.00
San Pablo City	297,847	0	0	0	0.00	0	0	0	0.00
San Pedro City	364,721	0	0	0	0.00	0	0	0	0.00
Santa Rosa City	396,019	0	0	0	0.00	0	0	0	0.00
Tagaytay City	83,320	0	0	0	0.00	0	0	0	0.00
Tanauan City	199,978	0	0	0	0.00	0	0	0	0.00

Table 1.E.6.1 - Malaria

Probable/Clinically and Confirmed Malaria Cases Disaggregated by Age group and Sex

Philippines, Annual 2020

Area	Population	Probable/Clinically and Confirmed Malaria Cases							
		<5 yrs Old			Morbidity Rate	>=5 yrs Old			Morbidity Rate
		Male	Female	Total		Male	Female	Total	
Tayabas City	107,355	0	0	0	0.00	0	0	0	0.00
Trece Martires City	182,269	0	0	0	0.00	0	0	0	0.00
**Region 4B**	**3,212,211**	**3**	**9**	**12**	**0.37**	**39**	**24**	**63**	**1.96**
Marinduque	242,420	0	0	0	0.00	0	0	0	0.00
Mindoro Occidental	519,990	0	0	0	0.00	0	0	0	0.00
Mindoro Oriental	911,307	0	0	0	0.00	7	5	12	1.32
Palawan	950,209	0	0	0	0.00	2	0	2	0.21
Romblon	302,917	0	0	0	0.00	0	0	0	0.00
		0	0	0	0.00	0	0	0	0.00
Puerto Princesa City	285,368	3	9	12	4.21	30	19	49	17.17
**Region 5**	**6,192,924**	**0**	**0**	**0**	**0.00**	**0**	**0**	**0**	**0.00**
Albay	1,199,362	0	0	0	0.00	0	0	0	0.00
Camarines Norte	629,402	0	0	0	0.00	0	0	0	0.00
Camarines Sur	1,750,680	0	0	0	0.00	0	0	0	0.00
Catanduanes	275,043	0	0	0	0.00	0	0	0	0.00
Masbate	944,945	0	0	0	0.00	0	0	0	0.00
Sorsogon	855,000	0	0	0	0.00	0	0	0	0.00
Iriga City	118,952	0	0	0	0.00	0	0	0	0.00
Legaspi City	210,918	0	0	0	0.00	0	0	0	0.00
Naga City	208,622	0	0	0	0.00	0	0	0	0.00
**Region 6**	**7,970,551**	**0**	**0**	**0**	**0.00**	**0**	**0**	**0**	**0.00**
Aklan	610,948	0	0	0	0.00	0	0	0	0.00
Antique	617,788	0	0	0	0.00	0	0	0	0.00
Capiz	811,948	0	0	0	0.00	0	0	0	0.00
Guimaras	187,011	0	0	0	0.00	0	0	0	0.00
Iloilo	2,069,653	0	0	0	0.00	0	0	0	0.00
Negros Occidental	2,607,671	0	0	0	0.00	0	0	0	0.00
Bacolod City	586,715	0	0	0	0.00	0	0	0	0.00
Iloilo City	478,817	0	0	0	0.00	0	0	0	0.00
**Region 7**	**8,054,772**	**0**	**0**	**0**	**0.00**	**0**	**0**	**0**	**0.00**
Bohol	1,374,101	0	0	0	0.00	0	0	0	0.00
Cebu	3,276,440	0	0	0	0.00	0	0	0	0.00
Negros Oriental	1,415,196	0	0	0	0.00	0	0	0	0.00
Siquijor	101,227	0	0	0	0.00	0	0	0	0.00
Cebu City	1,028,542	0	0	0	0.00	0	0	0	0.00
Lapu-Lapu City	454,972	0	0	0	0.00	0	0	0	0.00
Mandaue City	404,294	0	0	0	0.00	0	0	0	0.00
**Region 8**	**4,803,767**	**0**	**0**	**0**	**0.00**	**0**	**0**	**0**	**0.00**
Biliran	180,717	0	0	0	0.00	0	0	0	0.00
Eastern Samar	511,290	0	0	0	0.00	0	0	0	0.00
Northern Leyte	1,656,424	0	0	0	0.00	0	0	0	0.00
Northern Samar	675,196	0	0	0	0.00	0	0	0	0.00
Southern Leyte	358,038	0	0	0	0.00	0	0	0	0.00
Western Samar	634,040	0	0	0	0.00	0	0	0	0.00

Table 1.E.6.1 - Malaria

Probable/Clinically and Confirmed Malaria Cases Disaggregated by Age group and Sex

Philippines, Annual 2020

Area	Population	Probable/Clinically and Confirmed Malaria Cases							
		<5 yrs Old			Morbidity Rate	>=5 yrs Old			Morbidity Rate
		Male	Female	Total		Male	Female	Total	
Calbayog City	195,379	0	0	0	0.00	0	0	0	0.00
Maasin City	91,120	0	0	0	0.00	0	0	0	0.00
Ormoc City	235,937	0	0	0	0.00	0	0	0	0.00
Tacloban City	265,626	0	0	0	0.00	0	0	0	0.00
**Region 9**	**3,809,540**	**0**	**0**	**0**	**0.00**	**0**	**0**	**0**	**0.00**
Zamboanga del Norte	831,480	0	0	0	0.00	0	0	0	0.00
Zamboanga del Sur	840,050	0	0	0	0.00	0	0	0	0.00
Zamboanga Sibugay	687,546	0	0	0	0.00	0	0	0	0.00
Dapitan City	85,853	0	0	0	0.00	0	0	0	0.00
Dipolog City	136,208	0	0	0	0.00	0	0	0	0.00
Isabela City	130,380	0	0	0	0.00	0	0	0	0.00
Pagadian City	206,034	0	0	0	0.00	0	0	0	0.00
Zamboanga City	891,989	0	0	0	0.00	0	0	0	0.00
**Region 10**	**5,074,563**	**0**	**0**	**0**	**0.00**	**0**	**0**	**0**	**0.00**
Bukidnon	1,130,220	0	0	0	0.00	0	0	0	0.00
Camiguin	93,118	0	0	0	0.00	0	0	0	0.00
Lanao del Norte	735,928	0	0	0	0.00	0	0	0	0.00
Misamis Occidental	342,462	0	0	0	0.00	0	0	0	0.00
Misamis Oriental	782,065	0	0	0	0.00	0	0	0	0.00
Cagayan de Oro City	740,740	0	0	0	0.00	0	0	0	0.00
El Salvador City	55,016	0	0	0	0.00	0	0	0	0.00
Gingoog City	136,593	0	0	0	0.00	0	0	0	0.00
Iligan City	372,772	0	0	0	0.00	0	0	0	0.00
Malaybalay City	188,395	0	0	0	0.00	0	0	0	0.00
Oroquieta City	74,210	0	0	0	0.00	0	0	0	0.00
Ozamis City	148,747	0	0	0	0.00	0	0	0	0.00
Tangub City	66,085	0	0	0	0.00	0	0	0	0.00
Valencia City	208,212	0	0	0	0.00	0	0	0	0.00
**Region 11**	**5,371,254**	**0**	**0**	**0**	**0.00**	**0**	**0**	**0**	**0.00**
Compostela Valley	780,742	0	0	0	0.00	0	0	0	0.00
Davao del Norte	1,063,436	0	0	0	0.00	0	0	0	0.00
Davao del Sur	607,848	0	0	0	0.00	0	0	0	0.00
Davao Oriental	719,362	0	0	0	0.00	0	0	0	0.00
Davao Occidental	342,874	0	0	0	0.00	0	0	0	0.00
Davao City	1,856,992	0	0	0	0.00	0	0	0	0.00
**Region 12**	**4,985,878**	**0**	**0**	**0**	**0.00**	**0**	**0**	**0**	**0.00**
North Cotabato	1,553,081	0	0	0	0.00	0	0	0	0.00
Sarangani	590,746	0	0	0	0.00	0	0	0	0.00
South Cotabato	1,011,240	0	0	0	0.00	0	0	0	0.00
Sultan Kudarat	862,787	0	0	0	0.00	0	0	0	0.00
Cotabato City	311,264	0	0	0	0.00	0	0	0	0.00
Gen. Santos City	656,760	0	0	0	0.00	0	0	0	0.00
**BARMM**	**4,270,193**	**0**	**0**	**0**	**0.00**	**0**	**0**	**0**	**0.00**
Basilan	310,153	0	0	0	0.00	0	0	0	0.00

Table 1.E.6.1 - Malaria

Probable/Clinically and Confirmed Malaria Cases Disaggregated by Age group and Sex

Philippines, Annual 2020

Area	Population	Probable/Clinically and Confirmed Malaria Cases							
		<5 yrs Old			Morbidity Rate	>=5 yrs Old			Morbidity Rate
		Male	Female	Total		Male	Female	Total	
Lanao del Sur	942,654	0	0	0	0.00	0	0	0	0.00
Maguindanao	1,355,941	0	0	0	0.00	0	0	0	0.00
Sulu	932,940	0	0	0	0.00	0	0	0	0.00
Tawi-Tawi	417,702	0	0	0	0.00	0	0	0	0.00
Lamitan City	85,335	0	0	0	0.00	0	0	0	0.00
Marawi City	225,468	0	0	0	0.00	0	0	0	0.00
**CARAGA**	**2,782,218**	**0**	**0**	**0**	**0.00**	**0**	**0**	**0**	**0.00**
Agusan del Norte	382,946	0	0	0	0.00	0	0	0	0.00
Agusan del Sur	741,035	0	0	0	0.00	0	0	0	0.00
Surigao del Norte	366,639	0	0	0	0.00	0	0	0	0.00
Surigao del Sur	525,627	0	0	0	0.00	0	0	0	0.00
Province of Dinagat	131,274	0	0	0	0.00	0	0	0	0.00
Bislig City	99,839	0	0	0	0.00	0	0	0	0.00
Butuan City	364,103	0	0	0	0.00	0	0	0	0.00
Surigao City	170,755	0	0	0	0.00	0	0	0	0.00

Legend:
0 = No Case
** = No Report*

Table 1.E.6.2 - Malaria

No. and Rates of Probable/Clinically and confirmed Malaria Deaths by Sex and Age group

PHILIPPINES, Annual 2020

Area	Population	Lab. Confirmed Malaria Death (< 5 years of age)				Lab. Confirmed Malaria Death (>=5 years of age)			
		Male	Female	Total	Mortality Rate	Male	Female	Total	Mortality Rate
**PHILIPPINES**	**108,020,395**	**28**	**17**	**45**	**0.04**	**0**	**0**	**0**	**0.00**
**N C R**	**13,694,816**	**0**	**0**	**0**	**0.00**	**0**	**0**	**0**	**0.00**
Malabon City	373,284	0	0	0	0.00	0	0	0	0.00
Navotas City	248,638	0	0	0	0.00	0	0	0	0.00
Valenzuela City	653,305	0	0	0	0.00	0	0	0	0.00
Caloocan City	1,651,493	0	0	0	0.00	0	0	0	0.00
Marikina City	469,181	0	0	0	0.00	0	0	0	0.00
Pasig City	822,854	0	0	0	0.00	0	0	0	0.00
Pateros	63,376	0	0	0	0.00	0	0	0	0.00
Taguig City	947,362	0	0	0	0.00	0	0	0	0.00
Quezon City	3,065,597	0	0	0	0.00	0	0	0	0.00
Makati City	624,587	0	0	0	0.00	0	0	0	0.00
Mandaluyong City	438,677	0	0	0	0.00	0	0	0	0.00
San Juan City	122,505	0	0	0	0.00	0	0	0	0.00
Manila City	1,880,120	0	0	0	0.00	0	0	0	0.00
Las Piñas City	614,860	0	0	0	0.00	0	0	0	0.00
Muntinlupa City	556,831	0	0	0	0.00	0	0	0	0.00
Parañaque City	728,176	0	0	0	0.00	0	0	0	0.00
Pasay City	433,969	0	0	0	0.00	0	0	0	0.00
**C A R**	**1,805,792**	**0**	**0**	**0**	**0.00**	**0**	**0**	**0**	**0.00**
Abra	246,062	0	0	0	0.00	0	0	0	0.00
Apayao	124,308	0	0	0	0.00	0	0	0	0.00
Benguet	477,579	0	0	0	0.00	0	0	0	0.00
Ifugao	212,001	0	0	0	0.00	0	0	0	0.00
Kalinga	221,317	0	0	0	0.00	0	0	0	0.00
Mt. Province	154,891	0	0	0	0.00	0	0	0	0.00
Baguio City	369,634	0	0	0	0.00	0	0	0	0.00
**Region 1**	**5,243,726**	1	1	2	0.04	0	0	0	0.00
Ilocos Norte	497,711	0	0	0	0.00	0	0	0	0.00
Ilocos Sur	595,267	0	0	0	0.00	0	0	0	0.00
La Union	694,488	1	1	2	0.29	0	0	0	0.00
Pangasinan	2,485,875	0	0	0	0.00	0	0	0	0.00
Alaminos City	93,926	0	0	0	0.00	0	0	0	0.00
Candon City	62,742	0	0	0	0.00	0	0	0	0.00
Dagupan City	179,324	0	0	0	0.00	0	0	0	0.00
Laoag City	114,758	0	0	0	0.00	0	0	0	0.00
San Carlos City	197,438	0	0	0	0.00	0	0	0	0.00
San Fernando City	127,244	0	0	0	0.00	0	0	0	0.00
Urdaneta City	139,191	0	0	0	0.00	0	0	0	0.00
Vigan City	55,762	0	0	0	0.00	0	0	0	0.00

Table 1.E.6.2 - Malaria

No. and Rates of Probable/Clinically and confirmed Malaria Deaths by Sex and Age group

PHILIPPINES, Annual 2020

Area	Population	Lab. Confirmed Malaria Death (< 5 years of age)				Lab. Confirmed Malaria Death (>=5 years of age)			
		Male	Female	Total	Mortality Rate	Male	Female	Total	Mortality Rate
**Region 2**	**3,626,877**	0	0	0	0.00	0	0	0	0.00
Batanes	17,740	0	0	0	0.00	0	0	0	0.00
Cagayan	1,097,028	0	0	0	0.00	0	0	0	0.00
Isabela	1,244,626	0	0	0	0.00	0	0	0	0.00
Nueva Vizcaya	476,796	0	0	0	0.00	0	0	0	0.00
Quirino	198,628	0	0	0	0.00	0	0	0	0.00
Cauayan City	136,196	0	0	0	0.00	0	0	0	0.00
Ilagan City	153,068	0	0	0	0.00	0	0	0	0.00
Santiago City	141,776	0	0	0	0.00	0	0	0	0.00
Tuguegarao City	161,018	0	0	0	0.00	0	0	0	0.00
**Region 3**	**12,099,325**	**27**	**15**	42	0.35	**0**	**0**	0	0.00
Aurora	224,648	27	15	42	18.70	0	0	0	0.00
Bataan	716,595	0	0	0	0.00	0	0	0	0.00
Bulacan	2,464,952	0	0	0	0.00	0	0	0	0.00
Nueva Ecija	1,585,623	0	0	0	0.00	0	0	0	0.00
Pampanga	1,779,362	0	0	0	0.00	0	0	0	0.00
Tarlac	1,078,598	0	0	0	0.00	0	0	0	0.00
Zambales	630,172	0	0	0	0.00	0	0	0	0.00
		0	0	0	0.00	0	0	0	0.00
Angeles City	446,436	0	0	0	0.00	0	0	0	0.00
Balanga City	103,578	0	0	0	0.00	0	0	0	0.00
Cabanatuan City	324,532	0	0	0	0.00	0	0	0	0.00
City of San Fernando	332,586	0	0	0	0.00	0	0	0	0.00
Gapan City	118,442	0	0	0	0.00	0	0	0	0.00
Mabalacat City	272,003	0	0	0	0.00	0	0	0	0.00
Malolos City	275,321	0	0	0	0.00	0	0	0	0.00
Meycauayan City	228,365	0	0	0	0.00	0	0	0	0.00
Olongapo City	248,550	0	0	0	0.00	0	0	0	0.00
Palayan City	44,069	0	0	0	0.00	0	0	0	0.00
San Jose City	150,049	0	0	0	0.00	0	0	0	0.00
San Jose del Monte City	627,032	0	0	0	0.00	0	0	0	0.00
Science City of Munoz	87,495	0	0	0	0.00	0	0	0	0.00
Tarlac City	360,918	0	0	0	0.00	0	0	0	0.00
**Region 4A**	**15,937,325**	**0**	**1**	1	0.01	**0**	**0**	0	0.00
Batangas	2,040,324	0	0	0	0.00	0	0	0	0.00
Cavite	1,560,675	0	1	1	0.06	0	0	0	0.00
Laguna	1,092,719	0	0	0	0.00	0	0	0	0.00
Quezon	1,845,487	0	0	0	0.00	0	0	0	0.00
Rizal	2,355,368	0	0	0	0.00	0	0	0	0.00
Antipolo City	867,558	0	0	0	0.00	0	0	0	0.00
Bacoor City	683,759	0	0	0	0.00	0	0	0	0.00
Batangas City	362,106	0	0	0	0.00	0	0	0	0.00
Biñan City	366,406	0	0	0	0.00	0	0	0	0.00
Cabuyao City	339,689	0	0	0	0.00	0	0	0	0.00
Calamba City	500,037	0	0	0	0.00	0	0	0	0.00
Cavite City	117,039	0	0	0	0.00	0	0	0	0.00
Dasmariñas City	750,256	0	0	0	0.00	0	0	0	0.00

Table 1.E.6.2 - Malaria

No. and Rates of Probable/Clinically and confirmed Malaria Deaths by Sex and Age group

PHILIPPINES, Annual 2020

Area	Population	Lab. Confirmed Malaria Death (< 5 years of age)				Lab. Confirmed Malaria Death (>=5 years of age)			
		Male	Female	Total	Mortality Rate	Male	Female	Total	Mortality Rate
General Trias City	357,816	0	0	0	0.00	0	0	0	0.00
Imus City	459,686	0	0	0	0.00	0	0	0	0.00
Lipa City	364,864	0	0	0	0.00	0	0	0	0.00
Lucena City	279,688	0	0	0	0.00	0	0	0	0.00
San Pablo City	292,735	0	0	0	0.00	0	0	0	0.00
San Pedro City	358,463	0	0	0	0.00	0	0	0	0.00
Santa Rosa City	389,223	0	0	0	0.00	0	0	0	0.00
Tagaytay City	81,036	0	0	0	0.00	0	0	0	0.00
Tanauan City	190,306	0	0	0	0.00	0	0	0	0.00
Tayabas City	104,816	0	0	0	0.00	0	0	0	0.00
Trece Martires City	177,270	0	0	0	0.00	0	0	0	0.00
**Region 4B**	**3,138,950**	**0**	**0**	0	0.00	**0**	**0**	0	0.00
Marinduque	239,634	0	0	0	0.00	0	0	0	0.00
Mindoro Occidental	514,763	0	0	0	0.00	0	0	0	0.00
Mindoro Oriental	890,416	0	0	0	0.00	0	0	0	0.00
Palawan	918,681	0	0	0	0.00	0	0	0	0.00
Romblon	299,553	0	0	0	0.00	0	0	0	0.00
		0	0	0	0.00	0	0	0	0.00
Puerto Princesa City	275,902	0	0	0	0.00	0	0	0	0.00
**Region 5**	**6,094,462**	**0**	**0**	0	0.00	**0**	**0**	0	0.00
Albay	1,172,705	0	0	0	0.00	0	0	0	0.00
Camarines Norte	615,349	0	0	0	0.00	0	0	0	0.00
Camarines Sur	1,731,531	0	0	0	0.00	0	0	0	0.00
Catanduanes	272,452	0	0	0	0.00	0	0	0	0.00
Masbate	937,990	0	0	0	0.00	0	0	0	0.00
Sorsogon	834,217	0	0	0	0.00	0	0	0	0.00
Iriga City	117,651	0	0	0	0.00	0	0	0	0.00
Legaspi City	206,226	0	0	0	0.00	0	0	0	0.00
Naga City	206,340	0	0	0	0.00	0	0	0	0.00
**Region 6**	**7,877,110**	**0**	**0**	0	0.00	**0**	**0**	0	0.00
Aklan	605,792	0	0	0	0.00	0	0	0	0.00
Antique	610,348	0	0	0	0.00	0	0	0	0.00
Capiz	794,010	0	0	0	0.00	0	0	0	0.00
Guimaras	183,846	0	0	0	0.00	0	0	0	0.00
Iloilo	2,035,320	0	0	0	0.00	0	0	0	0.00
Negros Occidental	2,593,413	0	0	0	0.00	0	0	0	0.00
Bacolod City	583,509	0	0	0	0.00	0	0	0	0.00
Iloilo City	470,872	0	0	0	0.00	0	0	0	0.00
**Region 7**	**7,879,119**	**0**	**0**	0	0.00	**0**	**0**	0	0.00
Bohol	1,358,850	0	0	0	0.00	0	0	0	0.00
Cebu	3,179,974	0	0	0	0.00	0	0	0	0.00
Negros Oriental	1,408,244	0	0	0	0.00	0	0	0	0.00
Siquijor	99,819	0	0	0	0.00	0	0	0	0.00

Table 1.E.6.2 - Malaria

No. and Rates of Probable/Clinically and confirmed Malaria Deaths by Sex and Age group

PHILIPPINES, Annual 2020

Area	Population	Lab. Confirmed Malaria Death (< 5 years of age)				Lab. Confirmed Malaria Death (>=5 years of age)			
		Male	Female	Total	Mortality Rate	Male	Female	Total	Mortality Rate
Cebu City	998,264	0	0	0	0.00	0	0	0	0.00
Lapu-Lapu City	441,577	0	0	0	0.00	0	0	0	0.00
Mandaue City	392,391	0	0	0	0.00	0	0	0	0.00
**Region 8**	**4,711,363**	**0**	**0**	0	0.00	**0**	**0**	0	0.00
Biliran	179,339	0	0	0	0.00	0	0	0	0.00
Eastern Samar	497,883	0	0	0	0.00	0	0	0	0.00
Northern Leyte	1,619,930	0	0	0	0.00	0	0	0	0.00
Northern Samar	666,747	0	0	0	0.00	0	0	0	0.00
Southern Leyte	350,279	0	0	0	0.00	0	0	0	0.00
Western Samar	624,949	0	0	0	0.00	0	0	0	0.00
Calbayog City	192,578	0	0	0	0.00	0	0	0	0.00
Maasin City	89,146	0	0	0	0.00	0	0	0	0.00
Ormoc City	230,739	0	0	0	0.00	0	0	0	0.00
Tacloban City	259,774	0	0	0	0.00	0	0	0	0.00
**Region 9**	**3,805,545**	**0**	**0**	0	0.00	**0**	**0**	0	0.00
Zamboanga del Norte	831,128	0	0	0	0.00	0	0	0	0.00
Zamboanga del Sur	847,502	0	0	0	0.00	0	0	0	0.00
Zamboanga Sibugay	671,808	0	0	0	0.00	0	0	0	0.00
Dapitan City	85,816	0	0	0	0.00	0	0	0	0.00
Dipolog City	136,151	0	0	0	0.00	0	0	0	0.00
Isabela City	125,373	0	0	0	0.00	0	0	0	0.00
Pagadian City	207,862	0	0	0	0.00	0	0	0	0.00
Zamboanga City	899,906	0	0	0	0.00	0	0	0	0.00
**Region 10**	**5,004,949**	**0**	**0**	**0**	**0.00**	**0**	**0**	**0**	**0.00**
Bukidnon	1,116,543	0	0	0	0.00	0	0	0	0.00
Camiguin	92,126	0	0	0	0.00	0	0	0	0.00
Lanao del Norte	723,628	0	0	0	0.00	0	0	0	0.00
Misamis Occidental	341,195	0	0	0	0.00	0	0	0	0.00
Misamis Oriental	768,704	0	0	0	0.00	0	0	0	0.00
Cagayan de Oro City	728,088	0	0	0	0.00	0	0	0	0.00
El Salvador City	54,076	0	0	0	0.00	0	0	0	0.00
Gingoog City	134,263	0	0	0	0.00	0	0	0	0.00
Iligan City	366,543	0	0	0	0.00	0	0	0	0.00
Malaybalay City	186,116	0	0	0	0.00	0	0	0	0.00
Oroquieta City	73,935	0	0	0	0.00	0	0	0	0.00
Ozamis City	148,198	0	0	0	0.00	0	0	0	0.00
Tangub City	65,841	0	0	0	0.00	0	0	0	0.00
Valencia City	205,692	0	0	0	0.00	0	0	0	0.00
**Region 11**	**5,237,160**	**0**	**0**	**0**	**0.00**	**0**	**0**	**0**	**0.00**
Compostela Valley	774,793	0	0	0	0.00	0	0	0	0.00
Davao del Norte	1,072,302	0	0	0	0.00	0	0	0	0.00
Davao del Sur	687,961	0	0	0	0.00	0	0	0	0.00
Davao Oriental	591,886	0	0	0	0.00	0	0	0	0.00

Table 1.E.6.2 - Malaria

No. and Rates of Probable/Clinically and confirmed Malaria Deaths by Sex and Age group

PHILIPPINES, Annual 2020

Area	Population	Lab. Confirmed Malaria Death (< 5 years of age)				Lab. Confirmed Malaria Death (>=5 years of age)			
		Male	Female	Total	Mortality Rate	Male	Female	Total	Mortality Rate
Davao Occidental	334,284	0	0	0	0.00	0	0	0	0.00
Davao City	1,775,934	0	0	0	0.00	0	0	0	0.00
**Region 12**	**4,900,137**	**0**	**0**	**0**	**0.00**	**0**	**0**	**0**	**0.00**
North Cotabato	1,506,217	0	0	0	0.00	0	0	0	0.00
Sarangani	580,709	0	0	0	0.00	0	0	0	0.00
South Cotabato	986,496	0	0	0	0.00	0	0	0	0.00
Sultan Kudarat	864,172	0	0	0	0.00	0	0	0	0.00
Cotabato City	321,850	0	0	0	0.00	0	0	0	0.00
Gen. Santos City	640,692	0	0	0	0.00	0	0	0	0.00
**BARMM**	**4,234,144**	**0**	**0**	**0**	**0.00**	**0**	**0**	**0**	**0.00**
Basilan	307,767	0	0	0	0.00	0	0	0	0.00
Lanao del Sur	918,076	0	0	0	0.00	0	0	0	0.00
Maguindanao	1,380,136	0	0	0	0.00	0	0	0	0.00
Sulu	914,152	0	0	0	0.00	0	0	0	0.00
Tawi-Tawi	409,746	0	0	0	0.00	0	0	0	0.00
Lamitan City	84,679	0	0	0	0.00	0	0	0	0.00
Marawi City	219,588	0	0	0	0.00	0	0	0	0.00
**CARAGA**	**2,729,595**	**0**	**0**	**0**	**0.00**	**0**	**0**	**0**	**0.00**
Agusan del Norte	374,612	0	0	0	0.00	0	0	0	0.00
Agusan del Sur	735,532	0	0	0	0.00	0	0	0	0.00
Surigao del Norte	354,346	0	0	0	0.00	0	0	0	0.00
Surigao del Sur	518,074	0	0	0	0.00	0	0	0	0.00
Province of Dinagat	127,413	0	0	0	0.00	0	0	0	0.00
Bislig City	98,402	0	0	0	0.00	0	0	0	0.00
Butuan City	356,183	0	0	0	0.00	0	0	0	0.00
Surigao City	165,033	0	0	0	0.00	0	0	0	0.00

Legend:
0 = No Case
** = No Report*

Table 1.E.6.3 - Malaria

No. of confirmed Malaria Cases and Parasite Incidence

Philippines, Annual 2020

Area	Population at Risk	Confirmed Malaria Cases			Annual Parasite Incidence
		Male	Female	Total	
**PHILIPPINES**	**639,994**	**108**	**98**	**206**	**0.32**
**NCR**	**0**	**0**	**0**	**0**	**0.00**
Malabon City	0	0	0	0	0.00
Navotas City	0	0	0	0	0.00
Valenzuela City	0	0	0	0	0.00
Caloocan City	0	0	0	0	0.00
Marikina City	0	0	0	0	0.00
Pasig City	0	0	0	0	0.00
Pateros	0	0	0	0	0.00
Taguig City	0	0	0	0	0.00
Quezon City	0	0	0	0	0.00
Makati City	0	0	0	0	0.00
Mandaluyong City	0	0	0	0	0.00
San Juan City	0	0	0	0	0.00
Manila City	0	0	0	0	0.00
Las Piñas City	0	0	0	0	0.00
Muntinlupa City	0	0	0	0	0.00
Parañaque City	0	0	0	0	0.00
Pasay City	0	0	0	0	0.00
**CAR**	**0**	**0**	**0**	**0**	**0.00**
Abra	0	0	0	0	0.00
Apayao	0	0	0	0	0.00
Benguet	0	0	0	0	0.00
Ifugao	0	0	0	0	0.00
Kalinga	0	0	0	0	0.00
Mt. Province	0	0	0	0	0.00
Baguio City	0	0	0	0	0.00
**Region 1**	**168,229**	**2**	**0**	**2**	**0.01**
Ilocos Norte	0	0	0	0	0.00
Ilocos Sur	140,249	0	0	0	0.00
La Union	60	2	0	2	33.33
Pangasinan	0	0	0	0	0.00
Alaminos City	0	0	0	0	0.00
Candon City	14,782	0	0	0	0.00
Dagupan City	0	0	0	0	0.00
Laoag City	0	0	0	0	0.00
San Carlos City	0	0	0	0	0.00
San Fernando City	0	0	0	0	0.00
Urdaneta City	0	0	0	0	0.00
Vigan City	13,138	0	0	0	0.00

Table 1.E.6.3 - Malaria
No. of confirmed Malaria Cases and Parasite Incidence
Philippines, Annual 2020

Area	Population at Risk	Confirmed Malaria Cases			Annual Parasite Incidence
		Male	Female	Total	
**Region 2**	**0**	**0**	**0**	**0**	**0.00**
Batanes	0	0	0	0	0.00
Cagayan	0	0	0	0	0.00
Isabela	0	0	0	0	0.00
Nueva Vizcaya	0	0	0	0	0.00
Quirino	0	0	0	0	0.00
Cauayan City	0	0	0	0	0.00
Ilagan City	0	0	0	0	0.00
Santiago City	0	0	0	0	0.00
Tuguegarao City	0	0	0	0	0.00
**Region 3**	**0**	**0**	**0**	**0**	**0.00**
Aurora	0	0	0	0	0.00
Bataan	0	0	0	0	0.00
Bulacan	0	0	0	0	0.00
Nueva Ecija	0	0	0	0	0.00
Pampanga	0	0	0	0	0.00
Tarlac	0	0	0	0	0.00
Zambales	0	0	0	0	0.00
Angeles City	0	0	0	0	0.00
Balanga City	0	0	0	0	0.00
Cabanatuan City	0	0	0	0	0.00
City of San Fernando	0	0	0	0	0.00
Gapan City	0	0	0	0	0.00
Mabalacat City	0	0	0	0	0.00
Malolos City	0	0	0	0	0.00
Meycauayan City	0	0	0	0	0.00
Olongapo City	0	0	0	0	0.00
Palayan City	0	0	0	0	0.00
San Jose City	0	0	0	0	0.00
San Jose del Monte City	0	0	0	0	0.00
Science City of Munoz	0	0	0	0	0.00
Tarlac City	0	0	0	0	0.00
**Region 4A**	**0**	**1**	**0**	**1**	**0.00**
Batangas	0	0	0	0	0.00
Cavite	0	0	0	0	0.00
Laguna	0	0	0	0	0.00
Quezon	0	0	0	0	0.00
Rizal	0	1	0	1	0.00
Antipolo City	0	0	0	0	0.00
Bacoor City	0	0	0	0	0.00
Batangas City	0	0	0	0	0.00
Biñan City	0	0	0	0	0.00
Cabuyao City	0	0	0	0	0.00
Calamba City	0	0	0	0	0.00
Cavite City	0	0	0	0	0.00
Dasmariñas City	0	0	0	0	0.00

Table 1.E.6.3 - Malaria
No. of confirmed Malaria Cases and Parasite Incidence
Philippines, Annual 2020

Area	Population at Risk	Confirmed Malaria Cases			Annual Parasite Incidence
		Male	Female	Total	
General Trias City	0	0	0	0	0.00
Imus City	0	0	0	0	0.00
Lipa City	0	0	0	0	0.00
Lucena City	0	0	0	0	0.00
San Pablo City	0	0	0	0	0.00
San Pedro City	0	0	0	0	0.00
Santa Rosa City	0	0	0	0	0.00
Tagaytay City	0	0	0	0	0.00
Tanauan City	0	0	0	0	0.00
Tayabas City	0	0	0	0	0.00
Trece Martires City	0	0	0	0	0.00
**Region 4B**	**0**	**33**	**28**	**61**	**0.00**
Marinduque	0	0	0	0	0.00
Mindoro Occidental	0	0	0	0	0.00
Mindoro Oriental	0	0	0	0	0.00
Palawan	0	0	0	0	0.00
Romblon	0	0	0	0	0.00
Puerto Princesa City	0	33	28	61	0.00
**Region 5**	**0**	**0**	**0**	**0**	**0.00**
Albay	0	0	0	0	0.00
Camarines Norte	0	0	0	0	0.00
Camarines Sur	0	0	0	0	0.00
Catanduanes	0	0	0	0	0.00
Masbate	0	0	0	0	0.00
Sorsogon	0	0	0	0	0.00
Iriga City	0	0	0	0	0.00
Legaspi City	0	0	0	0	0.00
Naga City	0	0	0	0	0.00
**Region 6**	**0**	**0**	**0**	**0**	**0.00**
Aklan	0	0	0	0	0.00
Antique	0	0	0	0	0.00
Capiz	0	0	0	0	0.00
Guimaras	0	0	0	0	0.00
Iloilo	0	0	0	0	0.00
Negros Occidental	0	0	0	0	0.00
Bacolod City	0	0	0	0	0.00
Iloilo City	0	0	0	0	0.00
**Region 7**	**0**	**0**	**0**	**0**	**0.00**
Bohol	0	0	0	0	0.00
Cebu	0	0	0	0	0.00
Negros Oriental	0	0	0	0	0.00
Siquijor	0	0	0	0	0.00

Table 1.E.6.3 - Malaria
No. of confirmed Malaria Cases and Parasite Incidence
Philippines, Annual 2020

Area	Population at Risk	Confirmed Malaria Cases			Annual Parasite Incidence
		Male	Female	Total	
Cebu City	0	0	0	0	0.00
Lapu-Lapu City	0	0	0	0	0.00
Mandaue City	0	0	0	0	0.00
**Region 8**	**0**	**0**	**0**	**0**	**0.00**
Biliran	0	0	0	0	0.00
Eastern Samar	0	0	0	0	0.00
Northern Leyte	0	0	0	0	0.00
Northern Samar	0	0	0	0	0.00
Southern Leyte	0	0	0	0	0.00
Western Samar	0	0	0	0	0.00
Calbayog City	0	0	0	0	0.00
Maasin City	0	0	0	0	0.00
Ormoc City	0	0	0	0	0.00
Tacloban City	0	0	0	0	0.00
**Region 9**	**0**	**0**	**0**	**0**	**0.00**
Zamboanga del Norte	0	0	0	0	0.00
Zamboanga del Sur	0	0	0	0	0.00
Zamboanga Sibugay	0	0	0	0	0.00
Dapitan City	0	0	0	0	0.00
Dipolog City	0	0	0	0	0.00
Isabela City	0	0	0	0	0.00
Pagadian City	0	0	0	0	0.00
Zamboanga City	0	0	0	0	0.00
**Region 10**	**0**	**0**	**0**	**0**	**0.00**
Bukidnon	0	0	0	0	0.00
Camiguin	0	0	0	0	0.00
Lanao del Norte	0	0	0	0	0.00
Misamis Occidental	0	0	0	0	0.00
Misamis Oriental	0	0	0	0	0.00
Cagayan de Oro City	0	0	0	0	0.00
El Salvador City	0	0	0	0	0.00
Gingoog City	0	0	0	0	0.00
Iligan City	0	0	0	0	0.00
Malaybalay City	0	0	0	0	0.00
Oroquieta City	0	0	0	0	0.00
Ozamis City	0	0	0	0	0.00
Tangub City	0	0	0	0	0.00
Valencia City	0	0	0	0	0.00
**Region 11**	**324,872**	**0**	**0**	**0**	**0.00**
Compostela Valley	0	0	0	0	0.00
Davao del Norte	136,528	0	0	0	0.00
Davao del Sur	0	0	0	0	0.00
Davao Oriental	0	0	0	0	0.00

Table 1.E.6.3 - Malaria
No. of confirmed Malaria Cases and Parasite Incidence
Philippines, Annual 2020

Area	Population at Risk	Confirmed Malaria Cases			Annual Parasite Incidence
		Male	Female	Total	
Davao Occidental	0	0	0	0	0.00
Davao City	188,344	0	0	0	0.00
**Region 12**	**146,893**	**72**	**70**	**142**	**0.97**
North Cotabato	0	0	0	0	0.00
Sarangani	0	0	0	0	0.00
South Cotabato	0	0	0	0	0.00
Sultan Kudarat	146,893	72	70	142	0.97
Cotabato City	0	0	0	0	0.00
Gen. Santos City	0	0	0	0	0.00
**BARMM**	**0**	**0**	**0**	**0**	**0.00**
Basilan	0	0	0	0	0.00
Lanao del Sur	0	0	0	0	0.00
Maguindanao	0	0	0	0	0.00
Sulu	0	0	0	0	0.00
Tawi-Tawi	0	0	0	0	0.00
Lamitan City	0	0	0	0	0.00
Marawi City	0	0	0	0	0.00
**CARAGA**	**0**	**0**	**0**	**0**	**0.00**
Agusan del Norte	0	0	0	0	0.00
Agusan del Sur	0	0	0	0	0.00
Surigao del Norte	0	0	0	0	0.00
Surigao del Sur	0	0	0	0	0.00
Province of Dinagat	0	0	0	0	0.00
Bislig City	0	0	0	0	0.00
Butuan City	0	0	0	0	0.00
Surigao City	0	0	0	0	0.00

Legend:
0 = No Case
** = No Report*

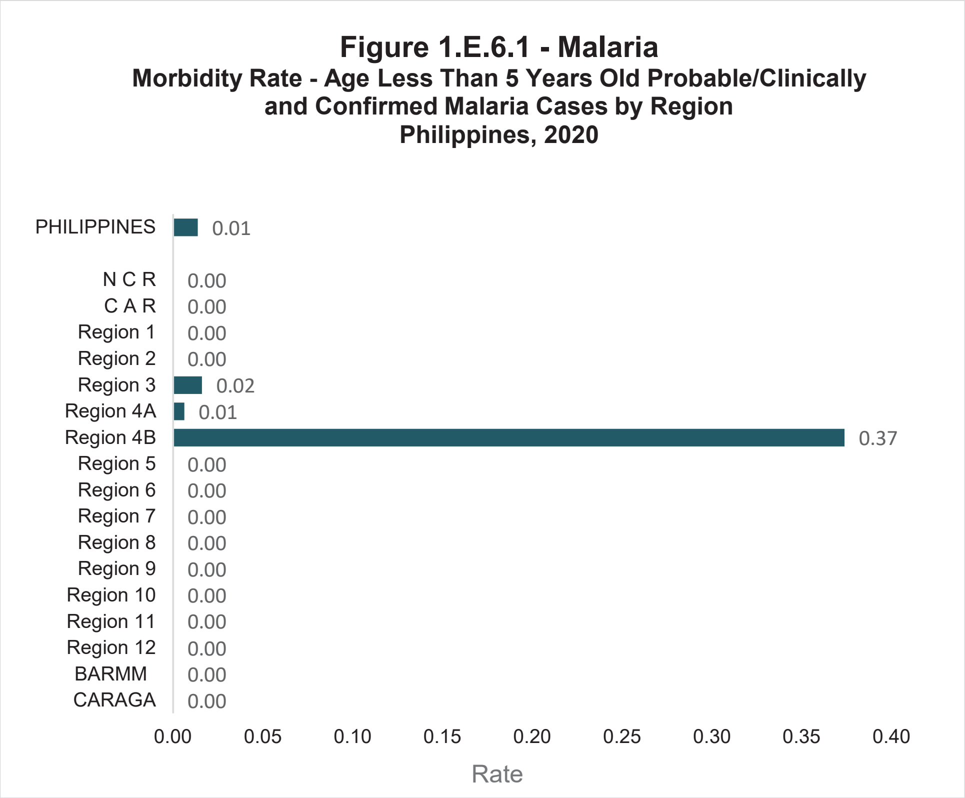
Figure 1.E.6.1 - Malaria
Morbidity Rate - Age Less Than 5 Years Old Probable/Clinically and Confirmed Malaria Cases by Region
Philippines, 2020
PHILIPPINES 0.01
N C R 0.00
C A R 0.00
Region 1 0.00
Region 2 0.00
Region 3 0.02
Region 4A 0.01
Region 4B 0.37
Region 5 0.00
Region 6 0.00
Region 7 0.00
Region 8 0.00
Region 9 0.00
Region 10 0.00
Region 11 0.00
Region 12 0.00
BARMM 0.00
CARAGA 0.00
0.00 0.05 0.10 0.15 0.20 0.25 0.30 0.35 0.40
Rate

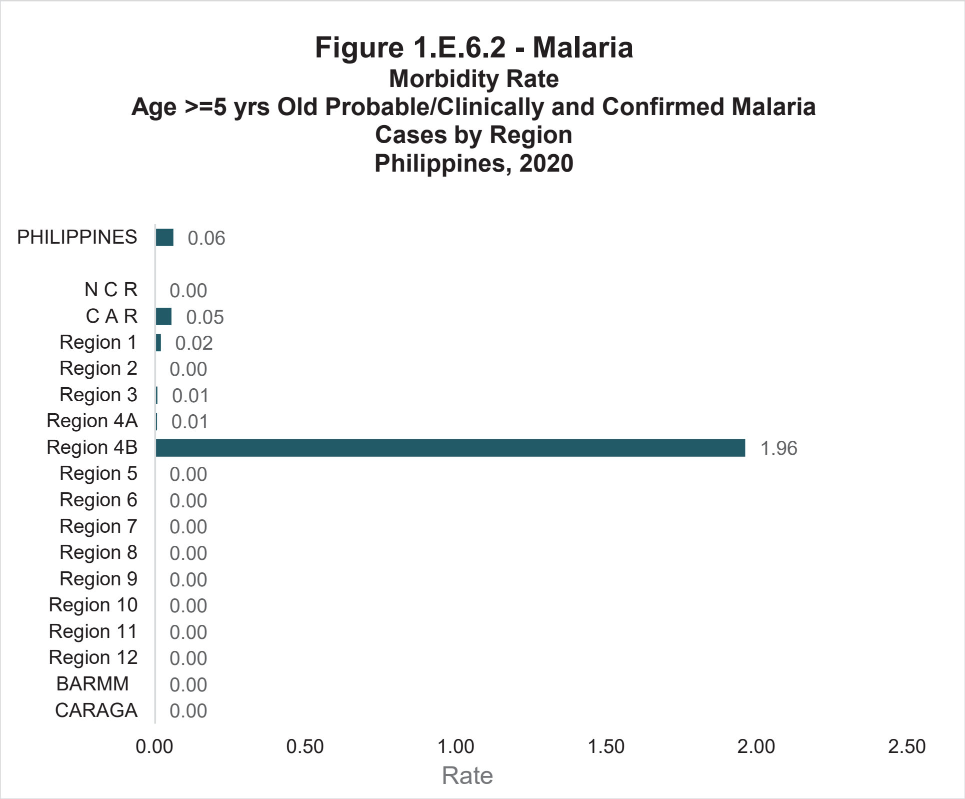
Figure 1.E.6.2 - Malaria
Morbidity Rate
Age >=5 yrs Old Probable/Clinically and Confirmed Malaria Cases by Region
Philippines, 2020
PHILIPPINES 0.06
N C R 0.00
C A R 0.05
Region 1 0.02
Region 2 0.00
Region 3 0.01
Region 4A 0.01
Region 4B 1.96
Region 5 0.00
Region 6 0.00
Region 7 0.00
Region 8 0.00
Region 9 0.00
Region 10 0.00
Region 11 0.00
Region 12 0.00
BARMM 0.00
CARAGA 0.00
0.00 0.50 1.00 1.50 2.00 2.50
Rate

Figure 1.E.6.3 - Malaria
Morbidity Rate
Annual Age Less Than 5 Years old Probable/Clinically and Confirmed Malaria Cases
Philippines, 2019 vs 2020

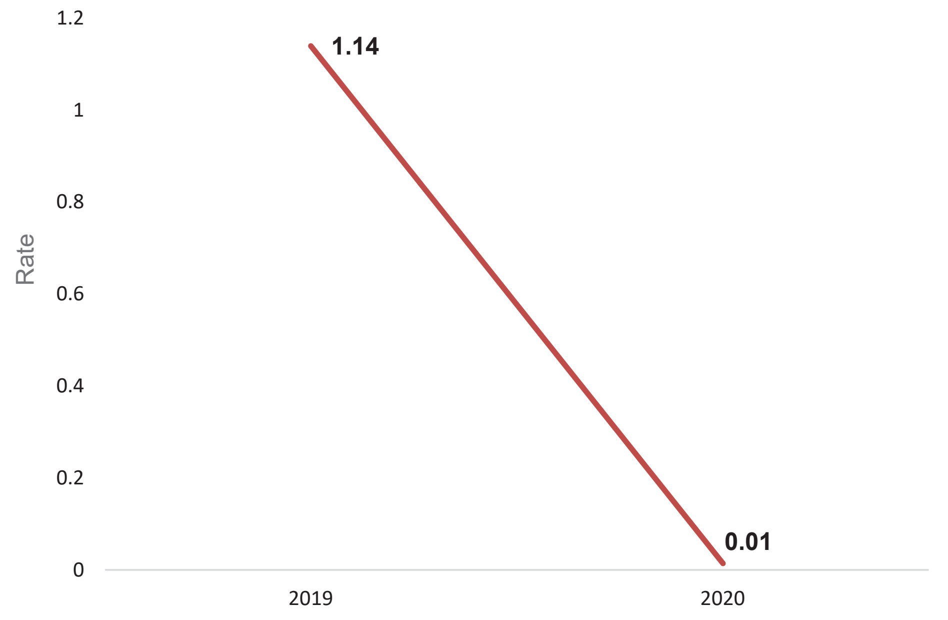

Figure 1.E.6.4 - Malaria
Morbidity Rate
Annual Age >=5 yrs Old Probable/Clinically and Confirmed Malaria Cases
Philippines, 2019 vs 2020

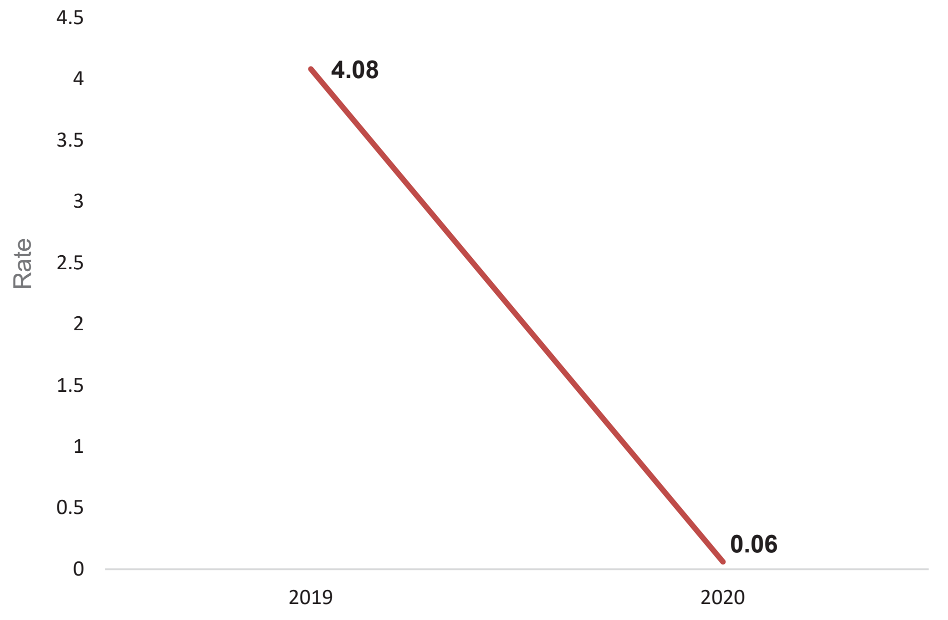

Figure 1.E.6.5 - Malaria
Mortality Rate - Age < 5 Years Old
Laboratory Confirmed Malaria Cases by Region
Philippines, 2020

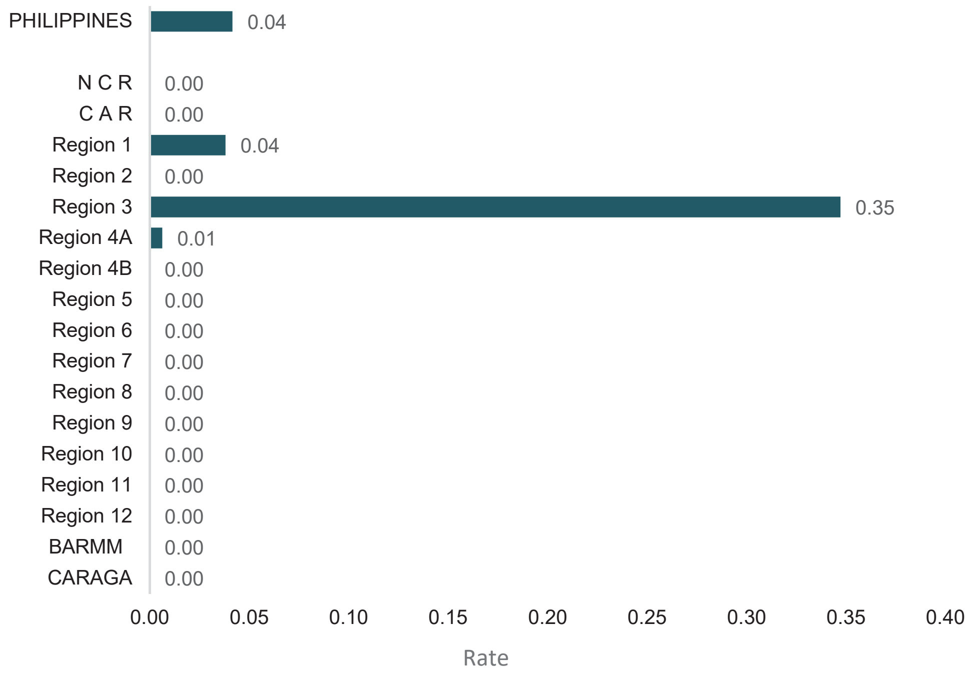

Figure 1.E.6.6 - Malaria
Mortality Rate Age >=5 Years Old
Laboratory Confirmed Malaria Cases by Region
Philippines, 2020

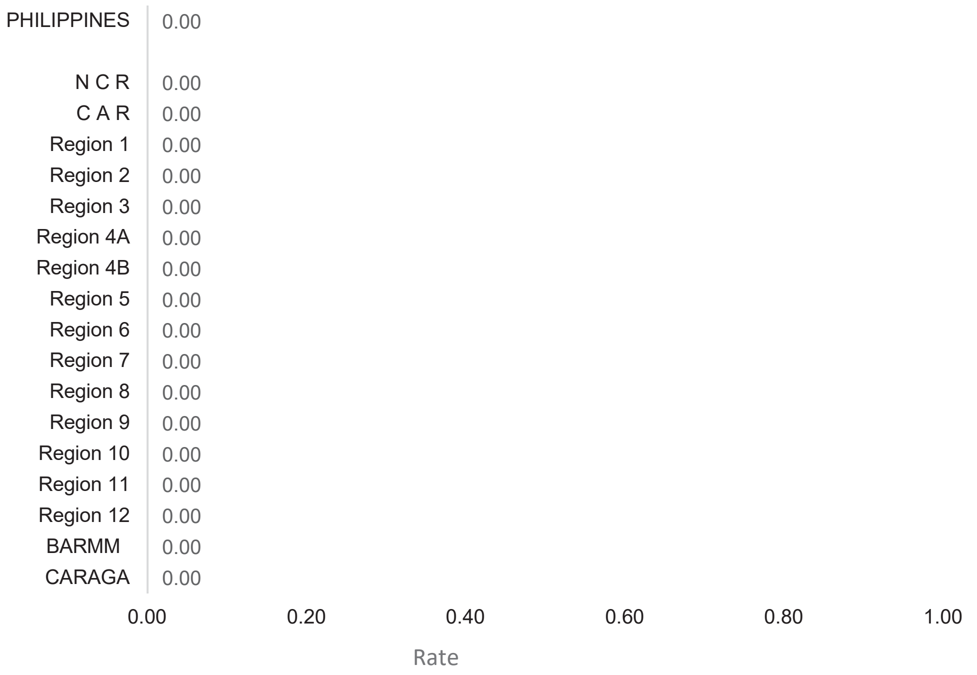

Figure 1.E.6.7 - Malaria
Annual Parasite Incidence by Region
Philippines, 2020

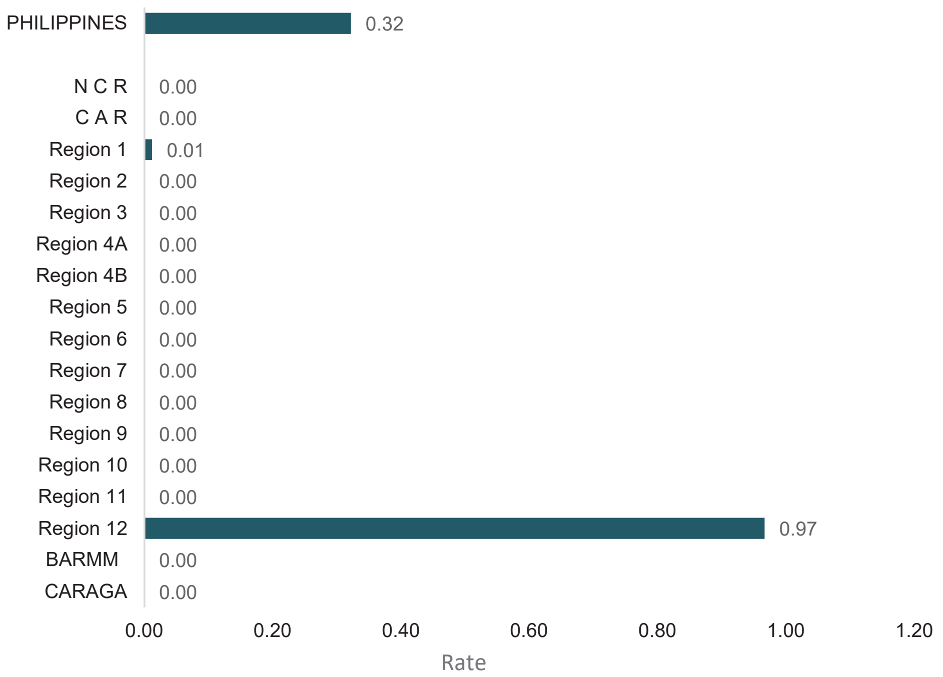

Figure 1.E.6.8 - Malaria
Annual Parasite Incidence by Year
Philippines, 2019 vs 2020

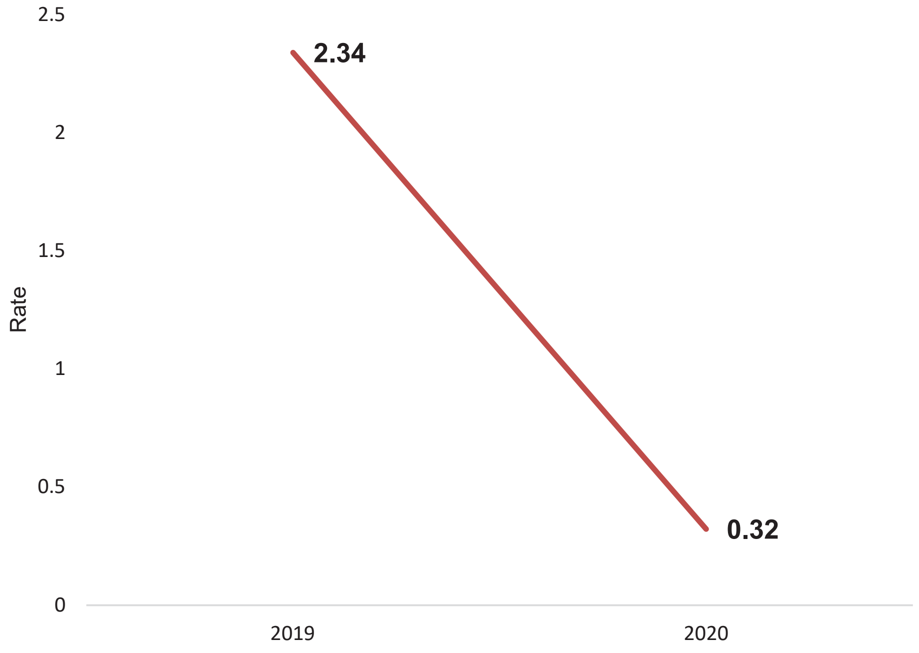

Figure 1.E.7.1 - Leprosy

Prevalence and Detection Rate

PHILIPPINES, Annual 2020

Area	Eligible Pop. (Total Pop x Multiplier)	Leprosy Cases undergoing Treatment				Newly Detected Case			
		Male	Female	Total	Prev. Rate	Male	Female	Total	Case Detection Rate
**PHILIPPINES**	**108,771,978**	**3,081**	**1,407**	**4488**	**0.41**	**497**	**187**	**684**	**0.63**
**N C R**	**13,804,656**	**44**	**11**	**55**	**0.04**	**1**	**1**	**2**	**0.01**
Malabon City	391,850	0	0	0	0.00	0	0	0	0.00
Navotas City	267,429	0	0	0	0.00	0	0	0	0.00
Valenzuela City	665,103	0	0	0	0.00	0	0	0	0.00
Caloocan City	1,698,054	0	0	0	0.00	0	0	0	0.00
Marikina City	483,201	1	0	1	0.02	0	0	0	0.00
Pasig City	809,696	15	3	18	0.22	0	0	0	0.00
Pateros	68,438	0	0	0	0.00	0	0	0	0.00
Taguig City	862,885	14	7	21	0.24	0	0	0	0.00
Quezon City	3,147,572	3	1	4	0.01	0	1	1	0.03
Makati City	624,559	0	0	0	0.00	0	0	0	0.00
Mandaluyong City	414,095	2	0	2	0.05	1	0	1	0.24
San Juan City	130,978	0	0	0	0.00	0	0	0	0.00
Manila City	1,908,353	9	0	9	0.05	0	0	0	0.00
Las Piñas City	631,305	0	0	0	0.00	0	0	0	0.00
Muntinlupa City	540,842	0	0	0	0.00	0	0	0	0.00
Parañaque City	713,774	0	0	0	0.00	0	0	0	0.00
Pasay City	446,522	0	0	0	0.00	0	0	0	0.00
**C A R**	**1,807,738**	**40**	**15**	**55**	**0.30**	**12**	**4**	**16**	**0.89**
Abra	247,402	25	9	34	1.37	6	2	8	3.23
Apayao	123,898	12	1	13	1.05	1	0	1	0.81
Benguet	478,334	2	0	2	0.04	1	0	1	0.21
Ifugao	210,669	0	0	0	0.00	1	0	1	0.47
Kalinga	220,229	0	0	0	0.00	0	0	0	0.00
Mt. Province	156,988	0	0	0	0.00	0	1	1	0.64
Baguio City	370,218	1	5	6	0.16	3	1	4	1.08
**Region 1**	**5,270,807**	**229**	**84**	**313**	**0.59**	**44**	**17**	**61**	**1.16**
Ilocos Norte	498,491	15	11	26	0.52	1	1	2	0.40
Ilocos Sur	596,972	35	11	46	0.77	11	3	14	2.35
La Union	699,954	0	0	0	0.00	0	0	0	0.00
Pangasinan	2,500,029	140	43	183	0.73	23	8	31	1.24
Alaminos City	94,460	2	3	5	0.53	0	0	0	0.00
Candon City	62,916	6	3	9	1.43	3	2	5	7.95
Dagupan City	180,345	7	6	13	0.72	0	1	1	0.55
Laoag City	114,931	0	0	0	0.00	0	0	0	0.00
San Carlos City	198,565	24	7	31	1.56	6	2	8	4.03
San Fernando City	128,239	0	0	0	0.00	0	0	0	0.00
Urdaneta City	139,982	0	0	0	0.00	0	0	0	0.00
Vigan City	55,923	0	0	0	0.00	0	0	0	0.00

Figure 1.E.7.1 - Leprosy
Prevalence and Detection Rate
PHILIPPINES, Annual 2020

Area	Eligible Pop. (Total Pop x Multiplier)	Leprosy Cases undergoing Treatment				Newly Detected Case			
		Male	Female	Total	Prev. Rate	Male	Female	Total	Case Detection Rate
**Region 2**	**3,657,741**	**151**	**43**	194	0.53	**21**	**4**	25	0.68
Batanes	17,875	0	0	0	0.00	1	0	1	5.59
Cagayan	1,110,345	32	3	35	0.32	1	0	1	0.09
Isabela	1,252,107	66	32	98	0.78	6	2	8	0.64
Nueva Vizcaya	482,893	10	0	10	0.21	3	1	4	0.83
Quirino	197,918	2	4	6	0.30	1	0	1	0.51
Cauayan City	137,012	22	3	25	1.82	6	1	7	5.11
Ilagan City	153,990	2	0	2	0.13	1	0	1	0.65
Santiago City	142,627	1	0	1	0.07	1	0	1	0.70
Tuguegarao City	162,974	16	1	17	1.04	1	0	1	0.61
**Region 3**	**12,313,718**	**849**	**516**	1,365	1.11	**40**	**11**	51	0.41
Aurora	227,530	3	2	5	0.22	1	0	1	0.44
Bataan	730,487	0	0	0	0.00	2	0	2	0.27
Bulacan	2,499,919	8	1	9	0.04	3	0	3	0.12
Nueva Ecija	1,616,656	73	24	97	0.60	10	5	15	0.93
Pampanga	1,819,117	19	5	24	0.13	2	1	3	0.16
Tarlac	1,094,044	0	0	0	0.00	0	0	0	0.00
Zambales	641,311	17	8	25	0.39	0	0	0	0.00
Angeles City	456,412	98	22	120	2.63	5	1	6	1.31
Balanga City	105,590	0	0	0	0.00	0	0	0	0.00
Cabanatuan City	330,882	64	48	112	3.38	1	0	1	0.30
City of San Fernando	340,016	6	1	7	0.21	0	0	0	0.00
Gapan City	120,759	1	0	1	0.08	0	0	0	0.00
Mabalacat City	278,080	0	0	0	0.00	0	0	0	0.00
Malolos City	279,229	0	0	0	0.00	0	0	0	0.00
Meycauayan City	231,606	308	320	628	27.12	0	0	0	0.00
Olongapo City	252,943	0	0	0	0.00	2	0	2	0.79
Palayan City	44,930	0	0	0	0.00	0	0	0	0.00
San Jose City	152,983	49	25	74	4.84	8	0	8	5.23
San Jose del Monte City	635,927	0	0	0	0.00	0	0	0	0.00
Science City of Munoz	89,206	0	0	0	0.00	0	0	0	0.00
Tarlac City	366,091	203	60	263	7.18	6	4	10	2.73
**Region 4A**	**16,057,299**	**56**	**20**	76	0.05	**6**	**6**	12	0.07
Batangas	2,092,860	1	0	1	0.00	0	0	0	0.00
Cavite	1,569,376	3	9	12	0.08	0	2	2	0.13
Laguna	1,094,168	1	0	1	0.01	1	4	5	0.46
Quezon	1,870,352	31	8	39	0.21	3	0	3	0.16
Rizal	2,345,161	12	2	14	0.06	0	0	0	0.00
Antipolo City	863,800	0	0	0	0.00	0	0	0	0.00
Bacoor City	687,569	0	0	0	0.00	0	0	0	0.00
Batangas City	371,423	0	0	0	0.00	0	0	0	0.00
Biñan City	366,892	0	0	0	0.00	0	0	0	0.00
Cabuyao City	340,140	0	0	0	0.00	0	0	0	0.00
Calamba City	500,702	7	1	8	0.16	0	0	0	0.00
Cavite City	117,690	0	0	0	0.00	0	0	0	0.00

Figure 1.E.7.1 - Leprosy

Prevalence and Detection Rate

PHILIPPINES, Annual 2020

Area	Eligible Pop. (Total Pop x Multiplier)	Leprosy Cases undergoing Treatment				Newly Detected Case			
		Male	Female	Total	Prev. Rate	Male	Female	Total	Case Detection Rate
Dasmariñas City	754,432	0	0	0	0.00	0	0	0	0.00
General Trias City	359,813	0	0	0	0.00	0	0	0	0.00
Imus City	462,245	1	0	1	0.02	0	0	0	0.00
Lipa City	374,252	0	0	0	0.00	0	0	0	0.00
Lucena City	283,457	0	0	0	0.00	2	0	2	0.71
San Pablo City	293,122	0	0	0	0.00	0	0	0	0.00
San Pedro City	358,941	0	0	0	0.00	0	0	0	0.00
Santa Rosa City	389,738	0	0	0	0.00	0	0	0	0.00
Tagaytay City	81,484	0	0	0	0.00	0	0	0	0.00
Tanauan City	195,198	0	0	0	0.00	0	0	0	0.00
Tayabas City	106,225	0	0	0	0.00	0	0	0	0.00
Trece Martires City	178,259	0	0	0	0.00	0	0	0	0.00
**Region 4B**	**3,174,859**	**27**	**22**	**49**	0.15	**5**	**5**	**10**	0.31
Marinduque	241,037	3	3	6	0.25	0	2	2	0.83
Mindoro Occidental	515,134	5	8	13	0.25	1	2	3	0.58
Mindoro Oriental	901,270	7	1	8	0.09	4	0	4	0.44
Palawan	935,159	0	0	0	0.00	0	0	0	0.00
Romblon	301,410	1	0	1	0.03	0	0	0	0.00
Puerto Princesa City	280,849	11	10	21	0.75	0	1	1	0.36
**Region 5**	**6,133,836**	**62**	**36**	**98**	0.16	**8**	**9**	**17**	0.28
Albay	1,187,296	19	5	24	0.20	1	2	3	0.25
Camarines Norte	622,515	4	6	10	0.16	2	2	4	0.64
Camarines Sur	1,734,879	0	0	0	0.00	0	0	0	0.00
Catanduanes	272,940	3	0	3	0.11	0	1	1	0.37
Masbate	937,079	12	5	17	0.18	4	2	6	0.64
Sorsogon	845,717	18	20	38	0.45	1	2	3	0.35
Iriga City	117,879	5	0	5	0.42	0	0	0	0.00
Legaspi City	208,794	0	0	0	0.00	0	0	0	0.00
Naga City	206,737	1	0	1	0.05	0	0	0	0.00
**Region 6**	**7,904,899**	**90**	**59**	149	0.19	**12**	**7**	19	0.24
Aklan	605,584	21	18	39	0.64	2	0	2	0.33
Antique	612,454	6	1	7	0.11	5	1	6	0.98
Capiz	803,083	7	2	9	0.11	0	1	1	0.12
Guimaras	185,174	1	0	1	0.05	0	0	0	0.00
Iloilo	2,049,963	16	6	22	0.11	2	1	3	0.15
Negros Occidental	2,591,346	23	25	48	0.19	3	3	6	0.23
Bacolod City	583,045	16	7	23	0.39	0	1	1	0.17
Iloilo City	474,250	0	0	0	0.00	0	0	0	0.00
**Region 7**	**7,957,046**	**250**	**137**	387	0.49	**27**	**3**	30	0.38
Bohol	1,365,108	0	0	0	0.00	0	0	0	0.00
Cebu	3,226,286	115	89	204	0.63	9	1	10	0.31
Negros Oriental	1,406,275	75	29	104	0.74	10	0	10	0.71
Siquijor	100,456	3	2	5	0.50	0	0	0	0.00

Figure 1.E.7.1 - Leprosy

Prevalence and Detection Rate

PHILIPPINES, Annual 2020

Area	Eligible Pop. (Total Pop x Multiplier)	Leprosy Cases undergoing Treatment				Newly Detected Case			
		Male	Female	Total	Prev. Rate	Male	Female	Total	Case Detection Rate
Cebu City	1,012,802	23	0	23	0.23	3	0	3	0.30
Lapu-Lapu City	448,009	34	17	51	1.14	5	2	7	1.56
Mandaue City	398,110	0	0	0	0.00	0	0	0	0.00
**Region 8**	**4,742,337**	**398**	**216**	614	1.29	**74**	**25**	99	2.09
Biliran	179,362	29	4	33	1.84	4	0	4	2.23
Eastern Samar	503,495	22	18	40	0.79	3	3	6	1.19
Northern Leyte	1,630,537	106	70	176	1.08	23	6	29	1.78
Northern Samar	668,766	87	44	131	1.96	13	1	14	2.09
Southern Leyte	354,211	8	7	15	0.42	5	2	7	1.98
Western Samar	628,438	66	26	92	1.46	15	2	17	2.71
Calbayog City	193,658	17	28	45	2.32	4	6	10	5.16
Maasin City	90,143	3	0	3	0.33	0	0	0	0.00
Ormoc City	232,252	25	9	34	1.46	2	1	3	1.29
Tacloban City	261,475	35	10	45	1.72	5	4	9	3.44
**Region 9**	**3,782,761**	**341**	**99**	440	1.16	**105**	**44**	149	3.94
Zamboanga del Norte	826,547	95	27	122	1.48	68	26	94	11.37
Zamboanga del Sur	835,832	4	0	4	0.05	5	5	10	1.20
Zamboanga Sibugay	679,404	34	49	83	1.22	5	3	8	1.18
Dapitan City	85,344	18	8	26	3.05	18	8	26	30.46
Dipolog City	135,400	32	0	32	2.36	1	0	1	0.74
Isabela City	127,728	13	2	15	1.17	4	0	4	3.13
Pagadian City	204,997	3	0	3	0.15	0	0	0	0.00
Zamboanga City	887,509	142	13	155	1.75	4	2	6	0.68
**Region 10**	**5,017,051**	**370**	**117**	487	0.97	**43**	**21**	64	1.28
Bukidnon	1,117,904	93	50	143	1.28	10	12	22	1.97
Camiguin	92,431	3	0	3	0.32	1	0	1	1.08
Lanao del Norte	727,012	28	2	30	0.41	4	1	5	0.69
Misamis Occidental	340,103	9	3	12	0.35	1	2	3	0.88
Misamis Oriental	771,837	67	17	84	1.09	12	1	13	1.68
Cagayan de Oro City	731,056	75	15	90	1.23	6	1	7	0.96
El Salvador City	54,299	10	0	10	1.84	2	0	2	3.68
Gingoog City	134,810	12	3	15	1.11	0	0	0	0.00
Iligan City	368,258	21	15	36	0.98	1	3	4	1.09
Malaybalay City	186,340	13	4	17	0.91	2	0	2	1.07
Oroquieta City	73,699	6	3	9	1.22	0	0	0	0.00
Ozamis City	147,725	4	1	5	0.34	1	0	1	0.68
Tangub City	65,632	2	0	2	0.30	0	0	0	0.00
Valencia City	205,945	27	4	31	1.51	3	1	4	1.94
**Region 11**	**5,290,869**	**0**	**0**	230	0.43	**39**	**12**	51	0.96
Davao de Oro	774,104	Without Sex Disaggregation		15	0.19	2	0	2	0.26
Davao del Norte	1,056,458			53	0.50	6	3	9	0.85
Davao del Sur	600,530			25	0.42	8	2	10	1.67

Figure 1.E.7.1 - Leprosy

Prevalence and Detection Rate

PHILIPPINES, Annual 2020

Area	Eligible Pop. (Total Pop x Multiplier)	Leprosy Cases undergoing Treatment				Newly Detected Case			
		Male	Female	Total	Prev. Rate	Male	Female	Total	Case Detection Rate
Davao Oriental	703,867	Without Sex Disaggregation		39	0.55	2	5	7	0.99
Davao Occidental	338,925			62	1.83	18	0	18	5.31
Davao City	1,816,985			36	0.20	3	2	5	0.28
**Region 12**	**4,919,936**	**69**	**19**	88	0.18	**38**	**12**	50	1.02
North Cotabato	1,527,149	19	1	20	0.13	5	0	5	0.33
Sarangani	583,791	0	0	0	0.00	0	0	0	0.00
South Cotabato	996,843	24	3	27	0.27	2	2	4	0.40
Sultan Kudarat	855,242	14	3	17	0.20	12	4	16	1.87
Cotabato City	309,498	4	5	9	0.29	2	1	3	0.97
Gen. Santos City	647,413	8	7	15	0.23	17	5	22	3.40
**BARMM**	**4,183,316**	**23**	**4**	27	0.06	**8**	**3**	11	0.26
Basilan	303,263	0	0	0	0.00	0	0	0	0.00
Lanao del Sur	925,035	0	0	0	0.00	0	0	0	0.00
Maguindanao	1,323,050	11	1	12	0.09	3	1	4	0.30
Sulu	913,593	8	3	11	0.12	5	2	7	0.77
Tawi-Tawi	413,690	0	0	0	0.00	0	0	0	0.00
Lamitan City	83,435	4	0	4	0.48	0	0	0	0.00
Marawi City	221,250	0	0	0	0.00	0	0	0	0.00
**CARAGA**	**2,753,109**	**82**	**9**	91	0.33	**14**	**3**	17	0.62
Agusan del Norte	378,703	13	0	13	0.34	1	0	1	0.26
Agusan del Sur	734,983	32	2	34	0.46	3	0	3	0.41
Surigao del Norte	360,328	8	0	8	0.22	8	3	11	3.05
Surigao del Sur	521,485	17	4	21	0.40	2	0	2	0.38
Province of Dinagat	130,672	3	1	4	0.31	0	0	0	0.00
Bislig City	99,046	5	0	5	0.50	0	0	0	0.00
Butuan City	360,073	4	2	6	0.17	0	0	0	0.00
Surigao City	167,819	0	0	0	0.00	0	0	0	0.00

Legend:

0 = No Case

** = No Report*

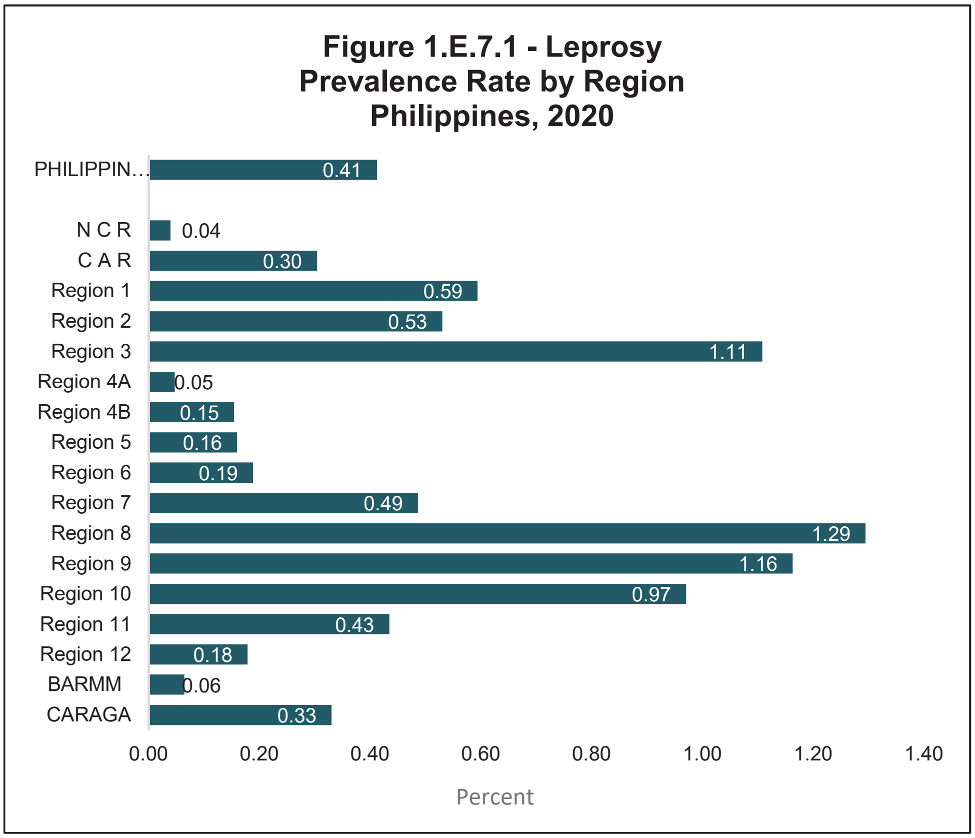
Figure 1.E.7.1 - Leprosy
Prevalence Rate by Region
Philippines, 2020
PHILIPPIN...
N C R
C A R
Region 1
Region 2
Region 3
Region 4A
Region 4B
Region 5
Region 6
Region 7
Region 8
Region 9
Region 10
Region 11
Region 12
BARMM
CARAGA
0.41
0.04
0.30
0.59
0.53
1.11
0.05
0.15
0.16
0.19
0.49
1.29
1.16
0.97
0.43
0.18
0.06
0.33
0.00
0.20
0.40
0.60
0.80
1.00
1.20
1.40
Percent

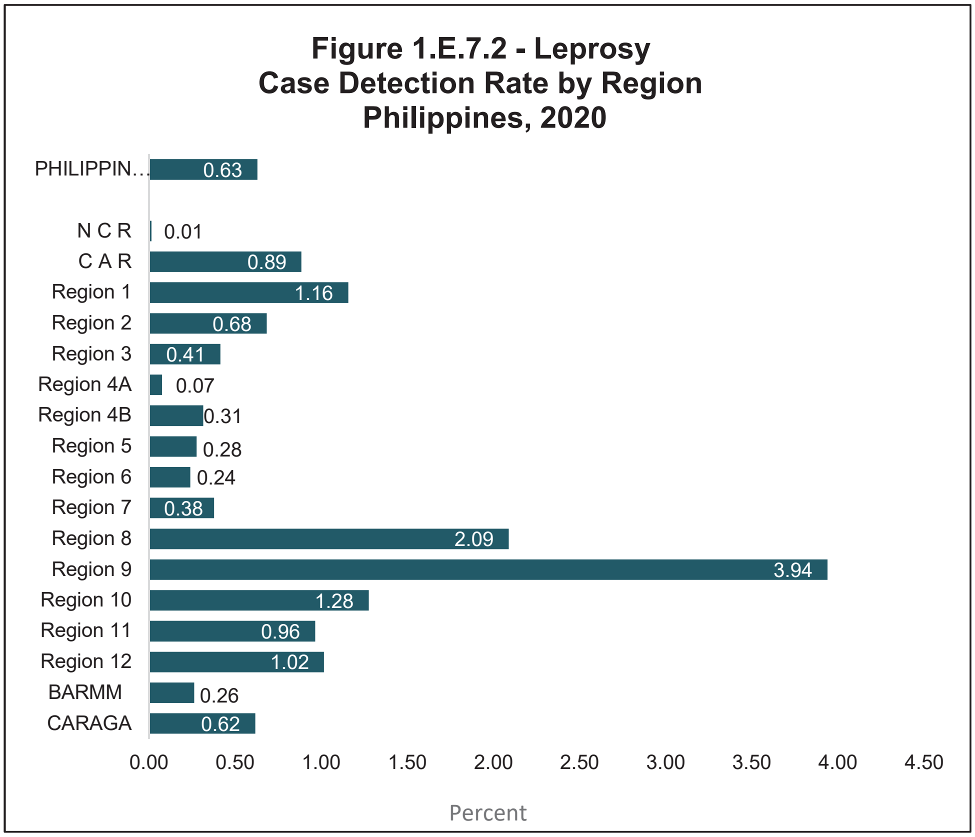
Figure 1.E.7.2 - Leprosy
Case Detection Rate by Region
Philippines, 2020
PHILIPPIN...
N C R
C A R
Region 1
Region 2
Region 3
Region 4A
Region 4B
Region 5
Region 6
Region 7
Region 8
Region 9
Region 10
Region 11
Region 12
BARMM
CARAGA
0.63
0.01
0.89
1.16
0.68
0.41
0.07
0.31
0.28
0.24
0.38
2.09
3.94
1.28
0.96
1.02
0.26
0.62
0.00
0.50
1.00
1.50
2.00
2.50
3.00
3.50
4.00
4.50
Percent

**Figure 1.E.7.3 - Leprosy
Annual Prevalence Rate by Year
Philippines, 2019 vs 2020**

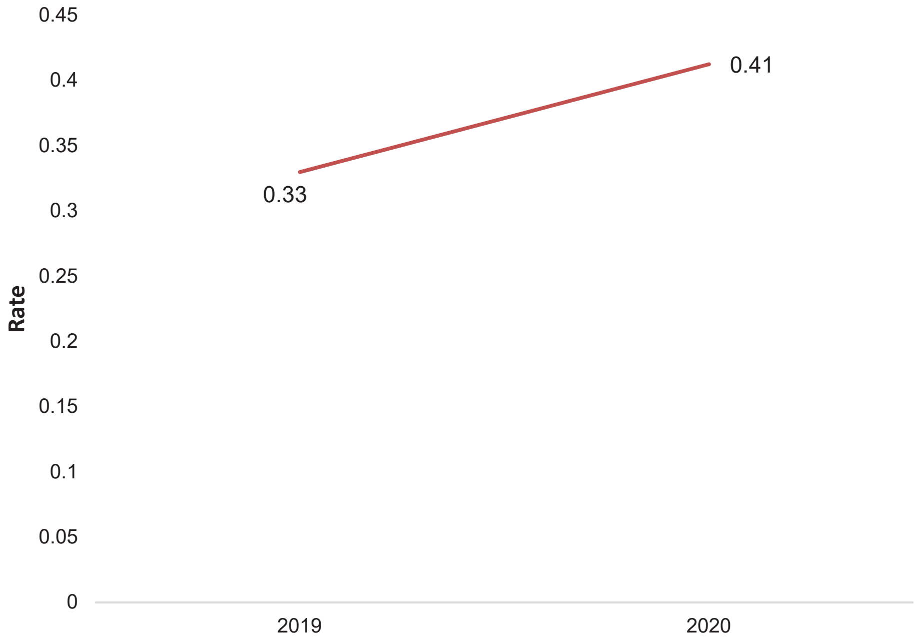

**Figure 1.E.7.4 - Leprosy
Annual Case Detection Rate by Year
Philippines, 2019 vs 2020**

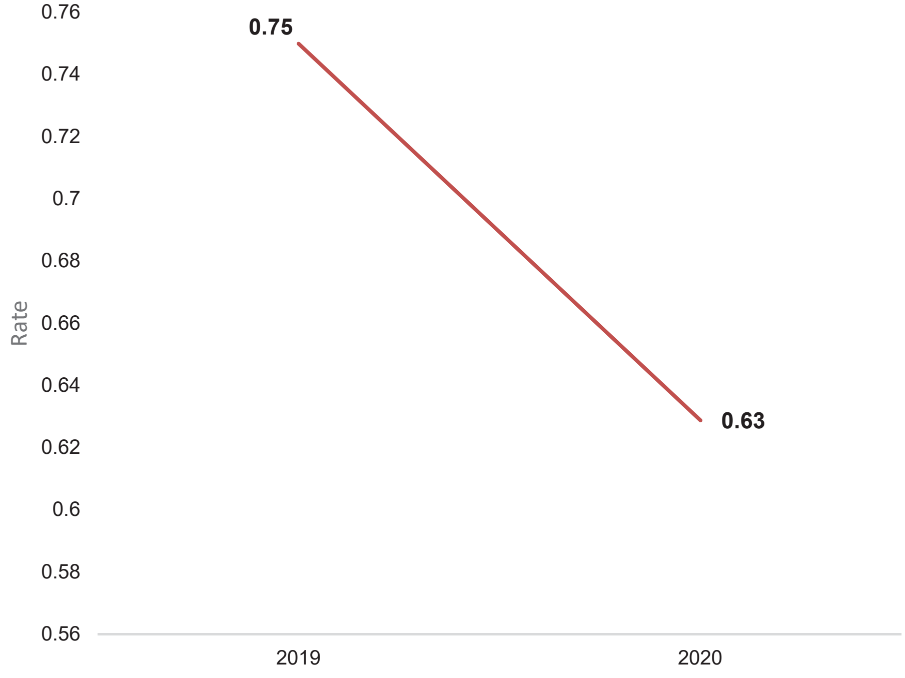

Table 1.E.8.1 - Rabies
Number and Proportion of Deaths Due to Rabies
PHILIPPINES, Annual 2020

Area	Animal Bites			No. of Deaths due to rabies			Rate
	Male	Female	Total	Male	Female	Total	
**PHILIPPINES**	**311,263**	**307,708**	**618,971**	**152**	**87**	**239**	**0.04**
**N C R**	**48,537**	**52,185**	**100,722**	**4**	**2**	**6**	**0.01**
Malabon City	1,970	2,024	3,994	0	0	0	0.00
Navotas City	1,304	1,283	2,587	1	0	1	0.04
Valenzuela City	6,174	6,374	12,548	0	0	0	0.00
Caloocan City	4,240	4,180	8,420	2	0	2	0.02
Marikina City	2,119	2,437	4,556	0	0	0	0.00
Pasig City	5,581	6,526	12,107	0	0	0	0.00
Pateros	449	503	952	0	0	0	0.00
Taguig City	11,236	11,557	22,793	0	0	0	0.00
Quezon City	2,694	3,111	5,805	0	0	0	0.00
Makati City	2,076	2,580	4,656	0	0	0	0.00
Mandaluyong City	3	0	3	0	0	0	0.00
San Juan City	1,067	1,215	2,282	0	0	0	0.00
Manila City	1,595	1,592	3,187	0	2	2	0.06
Las Piñas City	1,615	1,638	3,253	0	0	0	0.00
Muntinlupa City	4,651	5,287	9,938	1	0	1	0.01
Parañaque City	1,243	1,308	2,551	0	0	0	0.00
Pasay City	520	570	1,090	0	0	0	0.00
**C A R**	**15,241**	**13,322**	**28,563**	**0**	**1**	**1**	**0.00**
Abra	1,711	1,474	3,185	0	0	0	0.00
Apayao	1,406	1,131	2,537	0	1	1	0.04
Benguet	2,711	2,225	4,936	0	0	0	0.00
Ifugao	1,889	1,507	3,396	0	0	0	0.00
Kalinga	1,699	1,399	3,098	0	0	0	0.00
Mt. Province	1,424	1,156	2,580	0	0	0	0.00
Baguio City	4,401	4,430	8,831	0	0	0	0.00
**Region 1**	**15,260**	**14,743**	**30,003**	**9**	**4**	**13**	**0.04**
Ilocos Norte	703	635	1,338	0	0	0	0.00
Ilocos Sur	269	200	469	2	0	2	0.43
La Union	6,334	5,973	12,307	0	0	0	0.00
Pangasinan	2,975	2,904	5,879	6	4	10	0.17
Alaminos City	2	0	2	1	0	1	50.00
Candon City	0	0	0	0	0	0	0.00
Dagupan City	1,919	1,818	3,737	0	0	0	0.00
Laoag City	0	0	0	0	0	0	0.00
San Carlos City	1,888	1,833	3,721	0	0	0	0.00
San Fernando City	1,166	1,374	2,540	0	0	0	0.00
Urdaneta City	0	0	0	0	0	0	0.00
Vigan City	4	6	10	0	0	0	0.00

Table 1.E.8.1 - Rabies
Number and Proportion of Deaths Due to Rabies
PHILIPPINES, Annual 2020

Area	Animal Bites			No. of Deaths due to rabies			Rate
	Male	Female	Total	Male	Female	Total	
**Region 2**	**19,510**	**18,097**	**37,607**	**6**	**3**	**9**	**0.02**
Batanes	1	6	7	0	0	0	0.00
Cagayan	3,548	3,241	6,789	1	0	1	0.01
Isabela	5,118	4,915	10,033	0	0	0	0.00
Nueva Vizcaya	5,394	5,314	10,708	5	3	8	0.07
Quirino	2,488	1,889	4,377	0	0	0	0.00
Cauayan City	705	693	1,398	0	0	0	0.00
Ilagan City	820	718	1,538	0	0	0	0.00
Santiago City	1,436	1,321	2,757	0	0	0	0.00
Tuguegarao City	0	0	0	0	0	0	0.00
**Region 3**	**24,710**	**25,183**	**49,893**	**5**	**0**	**5**	**0.01**
Aurora	1,154	1,113	2,267	1	0	1	0.04
Bataan	2,145	2,055	4,200	0	0	0	0.00
Bulacan	2,227	2,605	4,832	0	0	0	0.00
Nueva Ecija	6,487	6,514	13,001	2	0	2	0.02
Pampanga	185	189	374	0	0	0	0.00
Tarlac	0	0	0	0	0	0	0.00
Zambales	846	851	1,697	2	0	2	0.12
Angeles City	988	1,026	2,014	0	0	0	0.00
Balanga City	960	963	1,923	0	0	0	0.00
Cabanatuan City	974	1,035	2,009	0	0	0	0.00
City of San Fernando	364	367	731	0	0	0	0.00
Gapan City	58	43	101	0	0	0	0.00
Mabalacat City	0	0	0	0	0	0	0.00
Malolos City	0	0	0	0	0	0	0.00
Meycauayan City	251	281	532	0	0	0	0.00
Olongapo City	575	661	1,236	0	0	0	0.00
Palayan City	15	11	26	0	0	0	0.00
San Jose City	1,423	1,039	2,462	0	0	0	0.00
San Jose del Monte City	2,714	2,901	5,615	0	0	0	0.00
Science City of Munoz	1,027	1,050	2,077	0	0	0	0.00
Tarlac City	2,317	2,479	4,796	0	0	0	0.00
**Region 4A**	**17,791**	**18,532**	**36,323**	**42**	**38**	**80**	**0.22**
Batangas	529	471	1,000	1	0	1	0.10
Cavite	942	996	1,938	0	0	0	0.00
Laguna	3,143	3,306	6,449	4	1	5	0.08
Quezon	414	451	865	1	0	1	0.12
Rizal	5,235	5,327	10,562	6	4	10	0.09
Antipolo City	0	0	0	0	0	0	0.00
Bacoor City	1,181	1,299	2,480	0	0	0	0.00
Batangas City	0	0	0	0	0	0	0.00
Biñan City	716	790	1,506	1	0	1	0.07

Table 1.E.8.1 - Rabies
Number and Proportion of Deaths Due to Rabies
PHILIPPINES, Annual 2020

Area	Animal Bites			No. of Deaths due to rabies			Rate
	Male	Female	Total	Male	Female	Total	
Cabuyao City	5	6	11	0	0	0	0.00
Calamba City	1,432	1,459	2,891	27	33	60	2.08
Cavite City	0	0	0	0	0	0	0.00
Dasmariñas City	16	9	25	0	0	0	0.00
General Trias City	118	101	219	1	0	1	0.46
Imus City	6	4	10	0	0	0	0.00
Lipa City	0	0	0	0	0	0	0.00
Lucena City	111	125	236	0	0	0	0.00
San Pablo City	374	412	786	1	0	1	0.13
San Pedro City	0	0	0	0	0	0	0.00
Santa Rosa City	1,397	1,580	2,977	0	0	0	0.00
Tagaytay City	0	0	0	0	0	0	0.00
Tanauan City	1,440	1,454	2,894	0	0	0	0.00
Tayabas City	0	0	0	0	0	0	0.00
Trece Martires City	732	742	1,474	0	0	0	0.00
**Region 4B**	**7,296**	**7,218**	**14,514**	**6**	**5**	**11**	**0.08**
Marinduque	942	1,078	2,020	0	0	0	0.00
Mindoro Occidental	481	391	872	0	0	0	0.00
Mindoro Oriental	4,774	4,769	9,543	6	5	11	0.12
Palawan	0	0	0	0	0	0	0.00
Romblon	16	19	35	0	0	0	0.00
Puerto Princesa City	1,083	961	2,044	0	0	0	0.00
**Region 5**	**11,556**	**10,569**	**22,125**	**24**	**14**	**38**	**0.17**
Albay	351	233	584	2	0	2	0.34
Camarines Norte	350	307	657	11	8	19	2.89
Camarines Sur	2,103	1,921	4,024	3	3	6	0.15
Catanduanes	1,701	1,512	3,213	0	0	0	0.00
Masbate	131	124	255	2	0	2	0.78
Sorsogon	4,010	3,591	7,601	6	2	8	0.11
Iriga City	1,229	1,190	2,419	0	0	0	0.00
Legaspi City	147	140	287	0	1	1	0.35
Naga City	1,534	1,551	3,085	0	0	0	0.00
**Region 6**	**53,109**	**54,096**	**107,205**	**13**	**3**	**16**	**0.01**
Aklan	3,773	3,330	7,103	1	0	1	0.01
Antique	3,925	3,846	7,771	1	1	2	0.03
Capiz	5,760	6,291	12,051	2	0	2	0.02
Guimaras	1,435	1,490	2,925	1	0	1	0.03
Iloilo	17,091	18,217	35,308	6	1	7	0.02
Negros Occidental	9,956	8,989	18,945	1	1	2	0.01
Bacolod City	4,665	4,565	9,230	0	0	0	0.00
Bago City	6,504	7,368	13,872	1	0	1	0.01
**Region 7**	**7,714**	**7,481**	**15,195**	**2**	**1**	**3**	**0.02**
Bohol	0	0	0	0	0	0	0.00

Table 1.E.8.1 - Rabies
Number and Proportion of Deaths Due to Rabies
PHILIPPINES, Annual 2020

Area	Animal Bites			No. of Deaths due to rabies			Rate
	Male	Female	Total	Male	Female	Total	
Cebu	0	0	0	0	0	0	0.00
Negros Oriental	2,579	2,417	4,996	1	1	2	0.04
Siquijor	0	0	0	0	0	0	0.00
Cebu City	0	0	0	0	0	0	0.00
Lapu-Lapu City	5,135	5,064	10,199	1	0	1	0.01
Mandaue City	0	0	0	0	0	0	0.00
**Region 8**	**17,228**	**15,609**	**32,837**	**4**	**1**	**5**	**0.02**
Biliran	577	486	1,063	0	0	0	0.00
Eastern Samar	1,928	1,797	3,725	0	1	1	0.03
Northern Leyte	3,529	3,091	6,620	1	0	1	0.02
Northern Samar	3,734	3,551	7,285	1	0	1	0.01
Southern Leyte	1,198	1,250	2,448	0	0	0	0.00
Western Samar	1,306	1,111	2,417	1	0	1	0.04
Calbayog City	1,741	1,548	3,289	0	0	0	0.00
Maasin City	752	659	1,411	0	0	0	0.00
Ormoc City	1,337	1,259	2,596	0	0	0	0.00
Tacloban City	1,126	857	1,983	1	0	1	0.05
**Region 9**	**26,040**	**26,201**	**52,241**	**6**	**1**	**7**	**0.01**
							0.00
Zamboanga del Norte	5,379	5,442	10,821	1	0	1	0.01
Zamboanga del Sur	5,217	5,030	10,247	1	1	2	0.02
Zamboanga Sibugay	4,580	4,522	9,102	2	0	2	0.02
Dapitan City	981	1,034	2,015	1	0	1	0.05
Dipolog City	616	667	1,283	1	0	1	0.08
Isabela City	750	712	1,462	0	0	0	0.00
Pagadian City	1,260	1,158	2,418	0	0	0	0.00
Zamboanga City	7,257	7,636	14,893	0	0	0	0.00
**Region 10**	**14,427**	**13,753**	**28,180**	**11**	**3**	**14**	**0.05**
Bukidnon	555	551	1,106	5	1	6	0.54
Camiguin	486	469	955	0	0	0	0.00
Lanao del Norte	1,262	1,113	2,375	2	0	2	0.08
Misamis Occidental	44	47	91	0	1	1	1.10
Misamis Oriental	813	730	1,543	1	1	2	0.13
Cagayan de Oro City	3,087	3,569	6,656	1	0	1	0.02
El Salvador City	676	526	1,202	0	0	0	0.00
Gingoog City	1,289	1,145	2,434	0	0	0	0.00
Iligan City	1,625	1,564	3,189	2	0	2	0.06
Malaybalay City	1,679	1,462	3,141	0	0	0	0.00
Oroquieta City	718	664	1,382	0	0	0	0.00

Table 1.E.8.1 - Rabies
Number and Proportion of Deaths Due to Rabies
PHILIPPINES, Annual 2020

Area	Animal Bites			No. of Deaths due to rabies			Rate
	Male	Female	Total	Male	Female	Total	
Ozamis City	597	528	1,125	0	0	0	0.00
Tangub City	0	0	0	0	0	0	0.00
Valencia City	1,596	1,385	2,981	0	0	0	0.00
**Region 11**	**10,442**	**10,305**	**20,747**	**8**	**3**	**11**	**0.05**
Compostela Valley	1,240	1,117	2,357	0	0	0	0.00
Davao del Norte	2,810	2,716	5,526	2	1	3	0.05
Davao del Sur	939	884	1,823	3	0	3	0.16
Davao Oriental	993	963	1,956	1	2	3	0.15
Davao Occidental	799	615	1,414	2	0	2	0.14
Davao City	3,661	4,010	7,671	0	0	0	0.00
**Region 12**	**13,228**	**11,822**	**25,050**	**9**	**3**	**12**	**0.05**
North Cotabato	3,390	3,000	6,390	4	1	5	0.08
Sarangani	0	0	0	0	0	0	0.00
South Cotabato	3,823	3,400	7,223	5	2	7	0.10
Sultan Kudarat	5,544	4,968	10,512	0	0	0	0.00
Cotabato City	0	0	0	0	0	0	0.00
Gen. Santos City	471	454	925	0	0	0	0.00
**BARMM**	**507**	**405**	**912**	**0**	**0**	**0**	**0.00**
Basilan	0	0	0	0	0	0	0.00
Lanao del Sur	251	206	457	0	0	0	0.00
Maguindanao	234	177	411	0	0	0	0.00
Sulu	22	22	44	0	0	0	0.00
Tawi-Tawi	0	0	0	0	0	0	0.00
Lamitan City	0	0	0	0	0	0	0.00
Marawi City	0	0	0	0	0	0	0.00
**CARAGA**	**8,667**	**8,187**	**16,854**	**3**	**5**	**8**	**0.05**
Agusan del Norte	323	272	595	0	1	1	0.17
Agusan del Sur	2,872	2,563	5,435	0	4	4	0.07
Surigao del Norte	1,476	1,497	2,973	0	0	0	0.00
Surigao del Sur	2,110	2,038	4,148	0	0	0	0.00
Province of Dinagat	76	83	159	0	0	0	0.00
Bislig City	484	514	998	0	0	0	0.00
Butuan City	267	212	479	2	0	2	0.42
Surigao City	1,059	1,008	2,067	1	0	1	0.05

Legend:
0 = No Case
** = No Report*

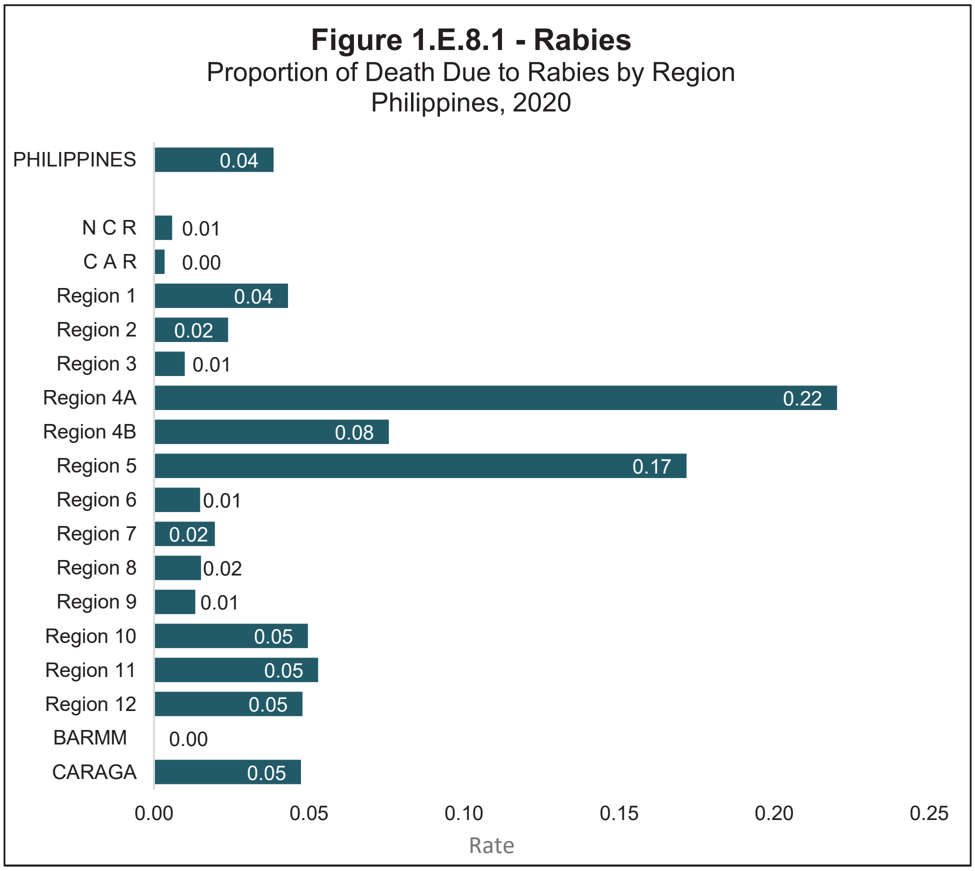
Figure 1.E.8.1 - Rabies
Proportion of Death Due to Rabies by Region
Philippines, 2020
PHILIPPINES
0.04
N C R
0.01
C A R
0.00
Region 1
0.04
Region 2
0.02
Region 3
0.01
Region 4A
0.22
Region 4B
0.08
Region 5
0.17
Region 6
0.01
Region 7
0.02
Region 8
0.02
Region 9
0.01
Region 10
0.05
Region 11
0.05
Region 12
0.05
BARMM
0.00
CARAGA
0.05
0.00
0.05
0.10
0.15
0.20
0.25
Rate

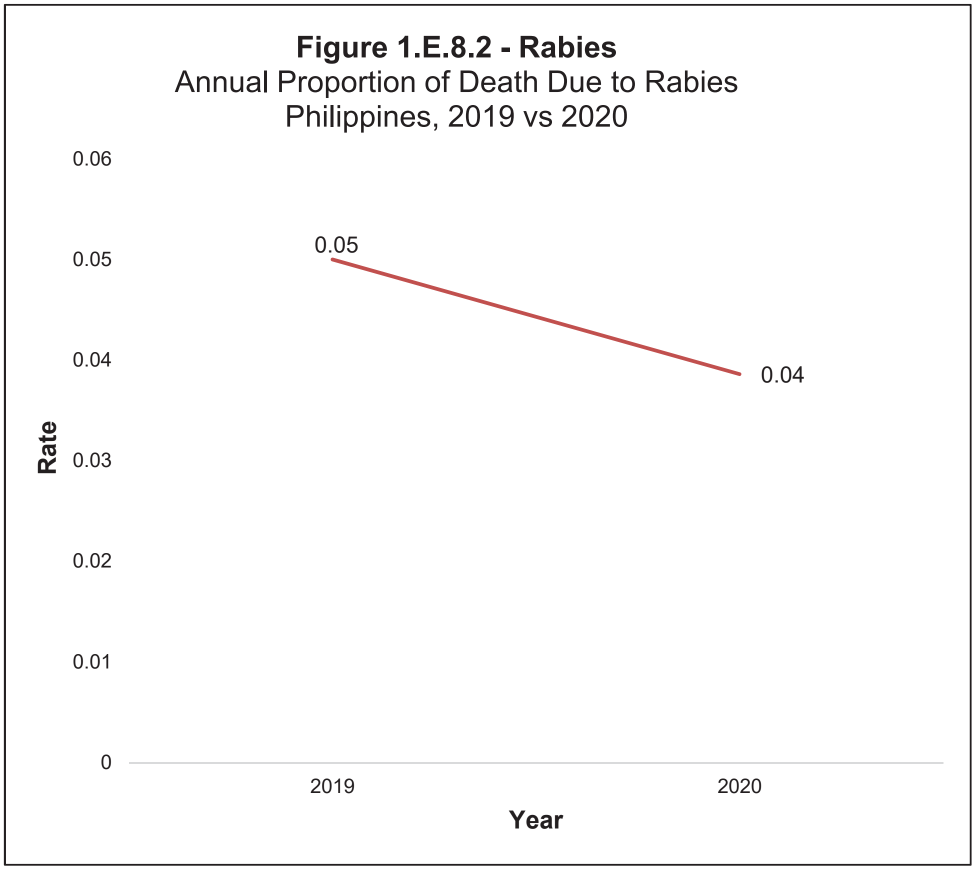
Figure 1.E.8.2 - Rabies
Annual Proportion of Death Due to Rabies
Philippines, 2019 vs 2020
0.06
0.05
0.04
0.03
0.02
0.01
0
Rate
0.05
0.04
2019
2020
Year

Table 1.F.1.1 - Lifestyle Related Diseases (Risk Assessment)

Number and proportion of adults ages 20 yrs old and above who were risk-assessed using the PhilPEN protocol
Philippines, Annual 2020

Area	Eligible Pop. (20 y/o and above)	Adults who were risk-assessed			
		Male	Female	Total	%
**PHILIPPINES**	**65,479,046**	**1,890,564**	**1,439,192**	**3,329,756**	**5.09**
**N C R**	**8,951,742**	**60,058**	**85,120**	**145,178**	**1.62**
Malabon	**254,096**	2,164	2,449	**4,613**	**1.82**
Navotas	**173,418**	4,732	6,720	**11,452**	**6.60**
Valenzuela City	**431,289**	9,084	8,384	**17,468**	**4.05**
Caloocan City	**1,101,115**	4,821	15,506	**20,327**	**1.85**
Marikina City	**313,336**	1,842	2,029	**3,871**	**1.24**
Pasig City	**525,053**	5,521	5,435	**10,956**	**2.09**
Pateros	**44,379**	327	480	**807**	**1.82**
Taguig	**559,548**	2,909	3,345	**6,254**	**1.12**
Quezon City	**2,041,074**	7,484	13,392	**20,876**	**1.02**
Makati City	**405,000**	2,901	3,602	**6,503**	**1.61**
Mandaluyong City	**268,524**	2,284	3,131	**5,415**	**2.02**
San Juan	**84,933**	1,196	1,903	**3,099**	**3.65**
Manila City	**1,237,490**	7,294	7,308	**14,602**	**1.18**
Las Piñas City	**409,373**	1,227	1,432	**2,659**	**0.65**
Muntinlupa City	**350,711**	1,901	2,767	**4,668**	**1.33**
Parañaque City	**462,854**	2,637	4,306	**6,943**	**1.50**
Pasay City	**289,549**	1,734	2,931	**4,665**	**1.61**
**C A R**	**1,094,725**	**40,672**	**46,790**	**87,462**	**7.99**
Abra	**153,072**	12,690	8,728	**21,418**	**13.99**
Apayao	**72,487**	9,103	12,899	**22,002**	**30.35**
Benguet	**299,234**	7,774	9,947	**17,721**	**5.92**
Ifugao	**120,751**	1,667	2,431	**4,098**	**3.39**
Kalinga	**126,091**	3,508	2,688	**6,196**	**4.91**
Mt. Province	**91,489**	3,215	3,784	**6,999**	**7.65**
Baguio City	**231,601**	2,715	6,313	**9,028**	**3.90**
**Region 1**	**3,249,853**	**133,244**	**136,565**	**269,809**	**8.30**
Ilocos Norte	**329,151**	9,732	11,685	**21,417**	**6.51**
Ilocos Sur	**388,606**	19,894	20,848	**40,742**	**10.48**
La Union	**451,139**	21,407	26,689	**48,096**	**10.66**
Pangasinan	**1,481,559**	63,332	57,847	**121,179**	**8.18**
Alaminos City	**55,983**	4,104	3,992	**8,096**	**14.46**
Candon City	**40,959**	193	120	**313**	**0.76**
Dagupan City	**106,876**	155	172	**327**	**0.31**
Laoag City	**75,886**	825	1,087	**1,912**	**2.52**
San Carlos City	**117,674**	1,364	1,123	**2,487**	**2.11**
San Fernando City	**82,656**	2,589	3,417	**6,006**	**7.27**
Urdaneta City	**82,960**	9,389	9,550	**18,939**	**22.83**
Vigan City	**36,404**	260	35	**295**	**0.81**

Table 1.F.1.1 - Lifestyle Related Diseases (Risk Assessment)

Number and proportion of adults ages 20 yrs old and above who were risk-assessed using the PhilPEN protocol
Philippines, Annual 2020

Area	Eligible Pop. (20 y/o and above)	Adults who were risk-assessed			
		Male	Female	Total	%
**Region 2**	**2,246,619**	**171,025**	**206,003**	**377,028**	**16.78**
Batanes	**10,792**	1,891	1,963	**3,854**	**35.71**
Cagayan	**599,258**	41,719	43,504	**85,223**	**14.22**
Isabela	**859,969**	40,243	60,837	**101,080**	**11.75**
Nueva Vizcaya	**291,164**	25,376	29,734	**55,110**	**18.93**
Quirino	**117,211**	19,706	22,601	**42,307**	**36.09**
Cauayan City	**84,477**	2,129	3,134	**5,263**	**6.23**
Ilagan City	**94,949**	4,641	6,386	**11,027**	**11.61**
Santiago City	**87,943**	34,210	36,098	**70,308**	**79.95**
Tuguegarao City	**100,856**	1,110	1,746	**2,856**	**2.83**
**Region 3**	**7,714,630**	**98,212**	**116,345**	**214,557**	**2.78**
Aurora	**131,698**	1,006	1,345	**2,351**	**1.79**
Bataan	**456,821**	9,114	10,981	**20,095**	**4.40**
Bulacan	**1,575,910**	9,377	10,452	**19,829**	**1.26**
Nueva Ecija	**1,009,810**	1,374	1,627	**3,001**	**0.30**
Pampanga	**1,148,278**	14,528	12,336	**26,864**	**2.34**
Tarlac	**683,470**	16,585	25,013	**41,598**	**6.09**
Zambales	**395,348**	11,106	11,451	**22,557**	**5.71**
Angeles City	**288,101**	1,273	1,085	**2,358**	**0.82**
Balanga City	**66,030**	695	798	**1,493**	**2.26**
Cabanatuan City	**206,673**	5,581	7,618	**13,199**	**6.39**
City of San Fernando	**214,629**	2,386	2,896	**5,282**	**2.46**
Gapan City	**75,430**	240	131	**371**	**0.49**
Mabalacat City	**175,533**	7,657	16,393	**24,050**	**13.70**
Malolos City	**176,026**	1,732	2,265	**3,997**	**2.27**
Meycauayan	**145,999**	514	558	**1,072**	**0.73**
Olongapo	**155,937**	839	966	**1,805**	**1.16**
Palayan City	**28,064**	80	123	**203**	**0.72**
San Jose City	**95,556**	3,338	1,049	**4,387**	**4.59**
San Jose del Monte City	**400,885**	8,534	6,725	**15,259**	**3.81**
Science City of Munoz	**55,721**	1,828	2,228	**4,056**	**7.28**
Tarlac City	**228,711**	425	305	**730**	**0.32**
**Region 4A**	**9,969,297**	**153,532**	**26,523**	**180,055**	**1.81**
Batangas	**1,300,450**	93,499	4,399	**97,898**	**7.53**
Cavite	**992,217**	5,871	851	**6,722**	**0.68**
Laguna	**694,950**	1,758	872	**2,630**	**0.38**
Quezon	**1,093,251**	22,777	7,593	**30,370**	**2.78**
Rizal	**1,444,977**	1,225	1,061	**2,286**	**0.16**
Antipolo City	**532,235**	0	0	**0**	**0.00**
Bacoor City	**434,705**	32	9	**41**	**0.01**
Batangas City	**230,788**	0	0	**0**	**0.00**
Biñan City	**233,028**	251	117	**368**	**0.16**
Cabuyao City	**216,038**	753	538	**1,291**	**0.60**

Table 1.F.1.1 - Lifestyle Related Diseases (Risk Assessment)

Number and proportion of adults ages 20 yrs old and above who were risk-assessed using the PhilPEN protocol

Philippines, Annual 2020

Area	Eligible Pop. (20 y/o and above)	Adults who were risk-assessed			
		Male	Female	Total	%
Calamba City	**318,012**	9,395	3,671	**13,066**	**4.11**
Cavite City	**74,399**	0	0	**0**	**0.00**
Dasmariñas City	**476,986**	14,178	6,031	**20,209**	**4.24**
General Trias City	**227,483**	546	224	**770**	**0.34**
Imus City	**292,240**	537	182	**719**	**0.25**
Lipa City	**232,552**	0	0	**0**	**0.00**
Lucena City	**165,688**	0	0	**0**	**0.00**
San Pablo City	**186,174**	72	37	**109**	**0.06**
San Pedro City	**227,978**	0	0	**0**	**0.00**
Santa Rosa City	**247,541**	163	57	**220**	**0.09**
Tagaytay City	**51,518**	0	0	**0**	**0.00**
Tanauan City	**121,290**	641	291	**932**	**0.77**
Tayabas City	**62,095**	60	76	**136**	**0.22**
Trece Martires City	**112,702**	1,774	514	**2,288**	**2.03**
**Region 4B**	**1,765,968**	**72,245**	**7,582**	**79,827**	**4.52**
Marinduque	**137,441**	66,719	5,143	**71,862**	**52.29**
Mindoro Occidental	**280,390**	1,656	92	**1,748**	**0.62**
Mindoro Oriental	**507,140**	849	985	**1,834**	**0.36**
Palawan	**517,380**	3,021	1,362	**4,383**	**0.85**
Romblon	**168,240**	0	0	**0**	**0.00**
Puerto Princesa City	**155,377**	0	0	**0**	**0.00**
**Region 5**	**3,392,866**	**185,506**	**186,475**	**371,981**	**10.96**
Albay	**686,653**	48,319	30,582	**78,901**	**11.49**
Camarines Norte	**348,352**	18,352	19,256	**37,608**	**10.80**
Camarines Sur	**955,127**	67,791	80,858	**148,649**	**15.56**
Catanduanes	**150,152**	5,512	8,169	**13,681**	**9.11**
Masbate	**488,133**	8,787	5,175	**13,962**	**2.86**
Sorsogon	**464,988**	27,262	29,758	**57,020**	**12.26**
Iriga City	**64,897**	5,181	7,223	**12,404**	**19.11**
Legaspi City	**120,750**	2,179	3,050	**5,229**	**4.33**
Naga City	**113,814**	2,123	2,404	**4,527**	**3.98**
**Region 6**	**4,847,394**	**176,248**	**175,548**	**351,796**	**7.26**
Aklan	**371,812**	16,785	20,634	**37,419**	**10.06**
Antique	**356,032**	19,504	20,825	**40,329**	**11.33**
Capiz	**504,627**	13,317	13,208	**26,525**	**5.26**
Guimaras	**116,130**	13,771	14,937	**28,708**	**24.72**
Iloilo	**1,286,783**	47,439	42,802	**90,241**	**7.01**
Negros Occidental	**1,562,708**	49,666	45,066	**94,732**	**6.06**
Bacolod City	**351,603**	6,653	4,782	**11,435**	**3.25**
Iloilo City	**297,699**	9,113	13,294	**22,407**	**7.53**

Table 1.F.1.1 - Lifestyle Related Diseases (Risk Assessment)

Number and proportion of adults ages 20 yrs old and above who were risk-assessed using the PhilPEN protocol
Philippines, Annual 2020

Area	Eligible Pop. (20 y/o and above)	Adults who were risk-assessed			
		Male	Female	Total	%
**Region 7**	**4,789,695**	**22,046**	**28,517**	**50,563**	**1.06**
Bohol	**804,555**	4,547	4,627	**9,174**	**1.14**
Cebu	**1,950,745**	8,247	13,417	**21,664**	**1.11**
Negros Oriental	**846,838**	3,238	4,049	**7,287**	**0.86**
Siquijor	**63,580**	1,624	2,287	**3,911**	**6.15**
Cebu City	**612,379**	1,288	1,556	**2,844**	**0.46**
Lapu-Lapu City	**270,885**	3,102	2,581	**5,683**	**2.10**
Mandaue City	**240,713**	0	0	**0**	**0.00**
					**0.00**
**Region 8**	**2,692,992**	**57,863**	**66,826**	**124,689**	**4.63**
Biliran	**102,127**	2,989	3,624	**6,613**	**6.48**
Eastern Samar	**282,853**	5,296	5,269	**10,565**	**3.74**
Northern Leyte	**947,307**	0	0	**0**	**0.00**
Northern Samar	**356,043**	7,576	8,416	**15,992**	**4.49**
Southern Leyte	**213,764**	22,003	24,179	**46,182**	**21.60**
Western Samar	**343,724**	15,496	18,941	**34,437**	**10.02**
Calbayog City	**105,917**	1,260	2,085	**3,345**	**3.16**
Maasin City	**54,399**	974	1,249	**2,223**	**4.09**
Ormoc City	**134,941**	1,523	2,230	**3,753**	**2.78**
Tacloban City	**151,917**	746	833	**1,579**	**1.04**
**Region 9**	**2,169,543**	**55,189**	**49,631**	**104,820**	**4.83**
Zamboanga del Norte	**474,764**	23,328	14,403	**37,731**	**7.95**
Zamboanga del Sur	**485,696**	15,461	26,279	**41,740**	**8.59**
Zamboanga Sibugay	**375,291**	12,229	3,574	**15,803**	**4.21**
Dapitan City	**49,022**	331	441	**772**	**1.57**
Dipolog City	**77,771**	774	604	**1,378**	**1.77**
Isabela City	**72,140**	240	208	**448**	**0.62**
Pagadian City	**119,125**	119	132	**251**	**0.21**
Zamboanga City	**515,734**	2,707	3,990	**6,697**	**1.30**
**Region 10**	**2,953,540**	**236,796**	**43,772**	**280,568**	**9.50**
Bukidnon	**647,185**	111,879	14,047	**125,926**	**19.46**
Camiguin	**54,998**	1,729	297	**2,026**	**3.68**
Lanao del Norte	**408,579**	12,960	3,435	**16,395**	**4.01**
Misamis Occidental	**206,073**	996	1,207	**2,203**	**1.07**
Misamis Oriental	**469,264**	23,807	4,624	**28,431**	**6.06**
Cagayan de Oro City	**444,470**	67,589	13,871	**81,460**	**18.33**
El Salvador City	**33,014**	46	39	**85**	**0.26**
Gingoog City	**81,963**	0	0	**0**	**0.00**
Iligan City	**206,959**	2,752	1,377	**4,129**	**2.00**
Malaybalay City	**107,877**	11,603	835	**12,438**	**11.53**
Oroquieta City	**44,656**	0	0	**0**	**0.00**
Ozamis City	**89,510**	0	0	**0**	**0.00**

Table 1.F.1.1 - Lifestyle Related Diseases (Risk Assessment)

Number and proportion of adults ages 20 yrs old and above who were risk-assessed using the PhilPEN protocol
Philippines, Annual 2020

Area	Eligible Pop. (20 y/o and above)	Adults who were risk-assessed			
		Male	Female	Total	%
Tangub City	**39,768**	60	54	**114**	**0.29**
Valencia City	**119,224**	3,375	3,986	**7,361**	**6.17**
**Region 11**	**3,141,861**	**264,230**	**142,878**	**407,108**	**12.96**
Compostela Valley	**454,730**	77,980	57,320	**135,300**	**29.75**
Davao del Norte	**635,179**	73,616	40,366	**113,982**	**17.94**
Davao Oriental	**339,021**	35,724	25,555	**61,279**	**18.08**
Davao del Sur	**424,344**	58,559	11,864	**70,423**	**16.60**
Davao Occidental	**193,169**	11,346	1,729	**13,075**	**6.77**
Davao City	**1,095,418**	7,005	6,044	**13,049**	**1.19**
**Region 12**	**2,836,047**	**80,385**	**82,033**	**162,418**	**5.73**
North Cotabato	**868,551**	24,595	28,394	**52,989**	**6.10**
Sarangani	**324,585**	18,602	9,476	**28,078**	**8.65**
South Cotabato	**591,315**	5,596	5,872	**11,468**	**1.94**
Sultan Kudarat	**493,320**	27,804	32,996	**60,800**	**12.32**
Cotabato City	**174,239**	2,562	3,652	**6,214**	**3.57**
Gen. Santos City	**384,037**	1,226	1,643	**2,869**	**0.75**
**BARMM**	**2,094,186**	**15,343**	**15,352**	**30,695**	**1.47**
Basilan	**149,438**	0	0	**0**	**0.00**
Lanao del Sur	**459,008**	4,116	4,378	**8,494**	**1.85**
Maguindanao	**664,198**	6,776	4,889	**11,665**	**1.76**
Sulu	**457,647**	0	0	**0**	**0.00**
Tawi-Tawi	**212,998**	0	0	**0**	**0.00**
Lamitan City	**41,114**	0	0	**0**	**0.00**
Marawi City	**109,783**	4,451	6,085	**10,536**	**9.60**
**CARAGA**	**1,568,088**	**67,970**	**23,232**	**91,202**	**5.82**
Agusan del Norte	**220,179**	10,281	3,093	**13,374**	**6.07**
Agusan del Sur	**400,516**	16,036	3,919	**19,955**	**4.98**
Surigao del Norte	**209,600**	9,221	2,273	**11,494**	**5.48**
Surigao del Sur	**297,578**	12,903	6,051	**18,954**	**6.37**
Province of Dinagat	**76,752**	1,755	392	**2,147**	**2.80**
Bislig City	**56,515**	2,361	1,603	**3,964**	**7.01**
Butuan City	**209,337**	14,159	5,029	**19,188**	**9.17**
Surigao City	**97,611**	1,254	872	**2,126**	**2.18**

[1] *Based on the PhilPEN protocol*

Note: Put asterisk (*) for No Report and Zero (0) for No Case

Table 1.F.1.2 - Lifestyle Related Diseases (Tobacco Use, Harmful Use of Alcohol, Unhealthy Diet)

Number and proportion of adults ages 20 yrs old and above who are current smokers, binge drinkers and overweight or obese
Philippines, Annual 2020

Area	Total No. of clients ≥ 20 y/o risk-assesed[1]	Current Smokers				Binge Drinkers				Overweight or obese			
		Male	Female	Total	%	Male	Female	Total	%	Male	Female	Total	%
**PHILIPPINES**	3,329,756	1,053,637	204,195	1,257,832	37.78	829,555	185,221	1,014,776	30.48	187,603	228,797	416,400	12.51
**N C R**	145,178	11,883	6,748	18,631	12.83	13,274	7,861	21,135	14.56	22,685	32,087	54,772	37.73
Malabon	4,613	320	295	615	13.33	309	284	593	12.85	995	920	1,915	41.51
Navotas	11,452	1,136	450	1,586	13.85	1,538	663	2,201	19.22	2,114	2,836	4,950	43.22
Valenzuela City	17,468	633	585	1,218	6.97	1,303	1,201	2,504	14.33	2,199	2,127	4,326	24.77
Caloocan City	20,327	1,190	853	2,043	10.05	1,389	1,491	2,880	14.17	1,712	5,306	7,018	34.53
Marikina City	3,871	710	130	840	21.70	488	127	615	15.89	1,142	1,152	2,294	59.26
Pasig City	10,956	796	450	1,246	11.37	1,051	302	1,353	12.35	1,590	1,777	3,367	30.73
Pateros	807	88	39	127	15.74	95	52	147	18.22	168	280	448	55.51
Taguig	6,254	575	137	712	11.38	783	318	1,101	17.60	1,434	1,498	2,932	46.88
Quezon City	20,876	1,808	1,302	3,110	14.90	1,990	1,229	3,219	15.42	2,417	4,262	6,679	31.99
Makati City	6,503	688	527	1,215	18.68	392	158	550	8.46	1,821	2,917	4,738	72.86
Mandaluyong City	5,415	638	411	1,049	19.37	758	370	1,128	20.83	1,112	1,454	2,566	47.39
San Juan	3,099	310	143	453	14.62	321	300	621	20.04	750	1,128	1,878	60.60
Manila City	14,602	779	747	1,526	10.45	739	736	1,475	10.10	2,083	2,169	4,252	29.12
Las Piñas City	2,659	373	138	511	19.22	403	141	544	20.46	516	652	1,168	43.93
Muntinlupa City	4,668	739	49	788	16.88	628	18	646	13.84	1,287	1,357	2,644	56.64
Parañaque City	6,943	623	284	907	13.06	657	362	1,019	14.68	581	981	1,562	22.50
Pasay City	4,665	477	208	685	14.68	430	109	539	11.55	764	1,271	2,035	43.62
**C A R**	87,462	13,302	3,627	16,929	19.36	14,743	3,601	18,344	20.97	6,825	11,999	18,824	21.52
Abra	21,418	5,439	1,404	6,843	31.95	4,953	1,233	6,186	28.88	1,347	2,326	3,673	17.15
Apayao	22,002	2,901	894	3,795	17.25	4,210	1,221	5,431	24.68	741	1,249	1,990	9.04
Benguet	17,721	1,776	286	2,062	11.64	1,647	395	2,042	11.52	1,709	2,834	4,543	25.64
Ifugao	4,098	207	79	286	6.98	329	70	399	9.74	230	255	485	11.84
Kalinga	6,196	1,518	526	2,044	32.99	1,763	223	1,986	32.05	390	584	974	15.72
Mt. Province	6,999	894	124	1,018	14.54	1,187	75	1,262	18.03	1,122	1,699	2,821	40.31
Baguio City	9,028	567	314	881	9.76	654	384	1,038	11.50	1,286	3,052	4,338	48.05
**Region 1**	269,809	42,797	11,834	54,631	20.25	41,676	12,311	53,987	20.01	17,789	20,787	38,576	14.30
Ilocos Norte	21,417	2,757	716	3,473	16.22	2,090	651	2,741	12.80	1,314	1,778	3,092	14.44
Ilocos Sur	40,742	11,507	1,744	13,251	32.52	8,670	1,096	9,766	23.97	3,271	3,961	7,232	17.75
La Union	48,096	7,434	2,158	9,592	19.94	8,840	2,748	11,588	24.09	4,387	5,601	9,988	20.77
Pangasinan	121,179	15,731	5,159	20,890	17.24	16,127	5,959	22,086	18.23	6,042	6,612	12,654	10.44
Alaminos City	8,096	329	259	588	7.26	156	92	248	3.06	121	74	195	2.41
Candon City	313	74	9	83	26.52	46	4	50	15.97	98	45	143	45.69
Dagupan City	327	130	35	165	50.46	115	9	124	37.92	26	15	41	12.54
Laoag City	1,912	193	23	216	11.30	141	12	153	8.00	77	122	199	10.41
San Carlos City	2,487	701	380	1,081	43.47	728	391	1,119	44.99	294	474	768	30.88
San Fernando City	6,006	826	527	1,353	22.53	1,942	705	2,647	44.07	527	591	1,118	18.61
Urdaneta City	18,939	2,861	793	3,654	19.29	2,821	644	3,465	18.30	1,632	1,512	3,144	16.60
Vigan City	295	254	31	285	96.61	0	0	0	0.00	0	2	2	0.68
**Region 2**	377,028	54,572	11,172	65,744	17.44	58,600	11,368	69,968	18.56	22,802	24,478	47,280	12.54
Batanes	3,854	434	117	551	14.30	510	337	847	21.98	1,308	1,227	2,535	65.78
Cagayan	85,223	16,548	2,337	18,885	22.16	17,179	2,021	19,200	22.53	4,589	5,185	9,774	11.47
Isabela	101,080	13,762	2,973	16,735	16.56	12,784	2,217	15,001	14.84	7,892	7,468	15,360	15.20
Nueva Vizcaya	55,110	5,043	1,795	6,838	12.41	5,878	1,240	7,118	12.92	1,430	1,889	3,319	6.02
Quirino	42,307	7,164	1,232	8,396	19.85	7,005	1,551	8,556	20.22	2,107	3,024	5,131	12.13
Cauayan City	5,263	105	15	120	2.28	104	11	115	2.19	35	37	72	1.37
Ilagan City	11,027	2,174	611	2,785	25.26	2,612	652	3,264	29.60	229	242	471	4.27
Santiago City	70,308	9,026	2,027	11,053	15.72	12,101	3,292	15,393	21.89	4,795	4,789	9,584	13.63
Tuguegarao City	2,856	316	65	381	13.34	427	47	474	16.60	417	617	1,034	36.20
**Region 3**	214,557	30,084	15,408	45,492	21.20	25,770	13,969	39,739	18.52	10,284	16,140	26,424	12.32
Aurora	2,351	161	70	231	9.83	273	150	423	17.99	124	212	336	14.29
Bataan	20,095	3,107	697	3,804	18.93	1,546	312	1,858	9.25	1,098	1,957	3,055	15.20
Bulacan	19,829	2,234	846	3,080	15.53	2,165	1,180	3,345	16.87	1,124	1,526	2,650	13.36
Nueva Ecija	3,001	397	127	524	17.46	97	31	128	4.27	50	29	79	2.63
Pampanga	26,864	1,037	253	1,290	4.80	881	166	1,047	3.90	463	662	1,125	4.19
Tarlac	41,598	4,227	5,892	10,119	24.33	3,647	4,680	8,327	20.02	1,569	3,124	4,693	11.28
Zambales	22,557	4,676	1,475	6,151	27.27	4,129	1,140	5,269	23.36	1,678	1,820	3,498	15.51
Angeles City	2,358	771	425	1,196	50.72	593	373	966	40.97	329	337	666	28.24
Balanga City	1,493	340	55	395	26.46	264	63	327	21.90	130	193	323	21.63
Cabanatuan City	13,199	2,317	820	3,137	23.77	2,861	1,323	4,184	31.70	285	324	609	4.61

Table 1.F.1.2 - Lifestyle Related Diseases (Tobacco Use, Harmful Use of Alcohol, Unhealthy Diet)

Number and proportion of adults ages 20 yrs old and above who are current smokers, binge drinkers and overweight or obese
Philippines, Annual 2020

Area	Total No. of clients ≥ 20 y/o risk-assesed[1]	Current Smokers				Binge Drinkers				Overweight or obese			
		Male	Female	Total	%	Male	Female	Total	%	Male	Female	Total	%
City of San Fernando	5,282	952	539	1,491	28.23	558	313	871	16.49	197	297	494	9.35
Gapan City	371	128	50	178	47.98	82	20	102	27.49	53	54	107	28.84
Mabalacat City	24,050	2,333	992	3,325	13.83	2,586	1,533	4,119	17.13	1,814	3,998	5,812	24.17
Malolos City	3,997	511	314	825	20.64	419	326	745	18.64	119	131	250	6.25
Meycauayan	1,072	234	106	340	31.72	196	81	277	25.84	52	46	98	9.14
Olongapo	1,805	155	43	198	10.97	75	36	111	6.15	76	85	161	8.92
Palayan City	203	135	39	174	85.71	98	36	134	66.01	23	25	48	23.65
San Jose City	4,387	1,719	454	2,173	49.53	1,704	406	2,110	48.10	72	136	208	4.74
San Jose del Monte City	15,259	3,854	2,078	5,932	38.88	3,194	1,728	4,922	32.26	909	1,018	1,927	12.63
Science City of Munoz	4,056	553	58	611	15.06	237	29	266	6.56	11	10	21	0.52
Tarlac City	730	243	75	318	43.56	165	43	208	28.49	108	156	264	36.16
**Region 4A**	180,055	85,215	10,828	96,043	53.34	62,156	9,758	71,914	39.94	6,095	7,864	13,959	7.75
Batangas	97,898	52,762	1,818	54,580	55.75	39,656	1,610	41,266	42.15	1,072	1,207	2,279	2.33
Cavite	6,722	4,498	396	4,894	72.81	1,170	112	1,282	19.07	203	382	585	8.70
Laguna	2,630	878	359	1,237	47.03	645	205	850	32.32	273	383	656	24.94
Quezon	30,370	12,455	1,956	14,411	47.45	8,952	3,932	12,884	42.42	1,462	2,341	3,803	12.52
Rizal	2,286	658	447	1,105	48.34	264	200	464	20.30	348	545	893	39.06
Antipolo City	0	*	*	0	0.00	*	*	0	0.00	*	*	0	0.00
Bacoor City	41	30	7	37	90.24	*	*	0	0.00	2	2	4	9.76
Batangas City	0	*	*	0	0.00	*	*	0	0.00	*	*	0	0.00
Biñan City	368	156	70	226	61.41	79	24	103	27.99	17	25	42	11.41
Cabuyao City	1,291	476	395	871	67.47	230	78	308	23.86	58	79	137	10.61
Calamba City	13,066	5,772	2,068	7,840	60.00	3,082	1,079	4,161	31.85	526	648	1,174	8.99
Cavite City	0	*	*	0	0.00	0	0	0	0.00	0	0	0	0.00
Dasmariñas City	20,209	5,572	2,691	8,263	40.89	6,548	2,132	8,680	42.95	1,792	1,779	3,571	17.67
General Trias City	770	317	60	377	48.96	164	75	239	31.04	75	95	170	22.08
Imus City	719	302	101	403	56.05	211	51	262	36.44	26	34	60	8.34
Lipa City	0	*	*	0	0.00	*	*	0	0.00	*	*	0	0.00
Lucena City	0	*	*	0	0.00	*	*	0	0.00	*	*	0	0.00
San Pablo City	109	42	9	51	46.79	15	5	20	18.35	16	25	41	37.61
San Pedro City	0	*	*	0	0.00	*	*	0	0.00	*	*	0	0.00
Santa Rosa City	220	79	23	102	46.36	63	17	80	36.36	22	20	42	19.09
Tagaytay City	0	*	*	0	0.00	*	*	0	0.00	*	*	0	0.00
Tanauan City	932	268	124	392	42.06	282	80	362	38.84	87	112	199	21.35
Tayabas City	136	*	*	0	0.00	21	14	35	25.74	62	101	163	119.85
Trece Martires City	2,288	950	304	1,254	54.81	774	144	918	40.12	54	86	140	6.12
**Region 4B**	79,827	41,741	1,626	43,367	54.33	70,844	5,311	76,155	95.40	881	1,081	1,962	2.46
Marinduque	71,862	39,045	1,449	40,494	56.35	66,719	5,143	71,862	100.00	621	794	1,415	1.97
Mindoro Occidental	1,748	1,575	26	1,601	91.59	1,628	11	1,639	93.76	19	30	49	2.80
Mindoro Oriental	1,834	*	*	0	0.00	*	*	0	0.00	*	*	0	0.00
Palawan	4,383	1,121	151	1,272	29.02	2,497	157	2,654	60.54	241	257	498	11.36
Romblon	0	*	*	0	0.00	*	*	0	0.00	*	*	0	0.00
Puerto Princesa City	0	*	*	0	0.00	*	*	0	0.00	*	*	0	0.00
**Region 5**	371,981	66,471	12,960	79,431	21.35	50,471	15,955	66,426	17.86	18,263	28,956	47,219	12.69
Albay	78,901	13,290	2,156	15,446	19.58	7,452	1,312	8,764	11.11	3,649	3,328	6,977	8.84
Camarines Norte	37,608	6,461	2,410	8,871	23.59	5,303	3,116	8,419	22.39	2,610	4,091	6,701	17.82
Camarines Sur	148,649	23,366	3,614	26,980	18.15	18,534	6,234	24,768	16.66	5,224	9,942	15,166	10.20
Catanduanes	13,681	2,612	256	2,868	20.96	2,632	696	3,328	24.33	804	1,220	2,024	14.79
Masbate	13,962	6,124	1,723	7,847	56.20	3,414	1,044	4,458	31.93	945	2,245	3,190	22.85
Sorsogon	57,020	11,584	1,965	13,549	23.76	10,131	2,605	12,736	22.34	3,151	5,156	8,307	14.57
Iriga City	12,404	1,184	124	1,308	10.54	1,398	380	1,778	14.33	1,246	2,142	3,388	27.31
Legaspi City	5,229	899	356	1,255	24.00	693	262	955	18.26	268	363	631	12.07
Naga City	4,527	951	356	1,307	28.87	914	306	1,220	26.95	366	469	835	18.44
**Region 6**	351,796	74,545	17,346	91,891	26.12	60,833	12,460	73,293	20.83	12,846	12,862	25,708	7.31
Aklan	37,419	12,247	4,519	16,766	44.81	5,772	1,015	6,787	18.14	993	1,248	2,241	5.99
Antique	40,329	8,289	1,602	9,891	24.53	6,815	848	7,663	19.00	753	886	1,639	4.06
Capiz	26,525	4,015	192	4,207	15.86	5,642	318	5,960	22.47	1,555	1,519	3,074	11.59
Guimaras	28,708	4,610	489	5,099	17.76	2,314	271	2,585	9.00	870	973	1,843	6.42
Iloilo	90,241	17,341	3,918	21,259	23.56	13,902	3,954	17,856	19.79	4,625	3,240	7,865	8.72
Negros Occidental	94,732	20,026	4,217	24,243	25.59	19,468	3,461	22,929	24.20	2,824	3,299	6,123	6.46
Bacolod City	11,435	5,864	1,743	7,607	66.52	5,314	2,246	7,560	66.11	541	998	1,539	13.46
Iloilo City	22,407	2,153	666	2,819	12.58	1,606	347	1,953	8.72	685	699	1,384	6.18
**Region 7**	50,563	10,914	2,475	13,389	26.48	11,081	2,767	13,848	27.39	4,031	6,532	10,563	20.89
Bohol	9,174	1,443	232	1,675	18.26	1,479	249	1,728	18.84	743	914	1,657	18.06
Cebu	21,664	4,753	1,007	5,760	26.59	5,719	1,470	7,189	33.18	2,351	3,823	6,174	28.50

Table 1.F.1.2 - Lifestyle Related Diseases (Tobacco Use, Harmful Use of Alcohol, Unhealthy Diet)

Number and proportion of adults ages 20 yrs old and above who are current smokers, binge drinkers and overweight or obese
Philippines, Annual 2020

Area	Total No. of clients ≥ 20 y/o risk-assesed[1]	Current Smokers				Binge Drinkers				Overweight or obese			
		Male	Female	Total	%	Male	Female	Total	%	Male	Female	Total	%
Negros Oriental	7,287	1,742	382	2,124	29.15	1,508	212	1,720	23.60	342	896	1,238	16.99
Siquijor	3,911	885	132	1,017	26.00	323	86	409	10.46	141	292	433	11.07
Cebu City	2,844	764	366	1,130	39.73	573	333	906	31.86	205	283	488	17.16
Lapu-Lapu City	5,683	1,327	356	1,683	29.61	1,479	417	1,896	33.36	249	324	573	10.08
Mandaue City	0	0	0	0	0.00	0	0	0	0.00	0	0	0	0.00
**Region 8**	124,689	33,218	6,959	40,177	32.22	36,586	16,081	52,667	42.24	7,297	8,406	15,703	12.59
Biliran	6,613	1,914	335	2,249	34.01	1,941	1,049	2,990	45.21	579	836	1,415	21.40
Eastern Samar	10,565	11,604	1,432	13,036	123.39	17,679	8,006	25,685	243.11	691	728	1,419	13.43
Northern Leyte	0	*	*	0	0.00	*	*	0	0.00	*	*	0	0.00
Northern Samar	15,992	8,243	1,299	9,542	59.67	6,749	2,314	9,063	56.67	1,119	807	1,926	12.04
Southern Leyte	46,182	7,068	1,875	8,943	19.36	7,157	2,958	10,115	21.90	3,979	4,606	8,585	18.59
Western Samar	34,437	3,364	1,049	4,413	12.81	2,514	1,290	3,804	11.05	488	950	1,438	4.18
Calbayog City	3,345	275	108	383	11.45	315	248	563	16.83	220	366	586	17.52
Maasin City	2,223	572	763	1,335	60.05	0	0	0	0.00	186	75	261	11.74
Ormoc City	3,753	123	55	178	4.74	187	172	359	9.57	35	38	73	1.95
Tacloban City	1,579	55	43	98	6.21	44	44	88	5.57	0	0	0	0.00
**Region 9**	104,820	77,285	20,673	97,958	93.45	38,212	18,077	56,289	53.70	6,540	11,599	18,139	17.30
Zamboanga del Norte	37,731	57,207	9,422	66,629	176.59	29,663	6,384	36,047	95.54	4,165	3,764	7,929	21.01
Zamboanga del Sur	41,740	9,972	10,085	20,057	48.05	6,561	11,157	17,718	42.45	1,622	6,835	8,457	20.26
Zamboanga Sibugay	15,803	8,921	442	9,363	59.25	821	265	1,086	6.87	406	522	928	5.87
Dapitan City	772	18	0	18	2.33	0	0	0	0.00	8	6	14	1.81
Dipolog City	1,378	386	469	855	62.05	582	90	672	48.77	87	98	185	13.43
Isabela City	448	101	61	162	36.16	46	41	87	19.42	24	33	57	12.72
Pagadian City	251	18	12	30	11.95	26	1	27	10.76	4	5	9	3.59
Zamboanga City	6,697	662	182	844	12.60	513	139	652	9.74	224	336	560	8.36
**Region 10**	280,568	207,012	30,183	237,195	84.54	170,648	27,740	198,388	70.71	12,385	10,443	22,828	8.14
Bukidnon	125,926	96,291	10,068	106,359	84.46	79,062	7,110	86,172	68.43	3,182	3,489	6,671	5.30
Camiguin	2,026	826	37	863	42.60	692	66	758	37.41	156	113	269	13.28
Lanao del Norte	16,395	23,128	3,780	26,908	164.12	11,493	2,605	14,098	85.99	2,143	1,862	4,005	24.43
Misamis Occidental	2,203	445	213	658	29.87	209	80	289	13.12	87	90	177	8.03
Misamis Oriental	28,431	3,830	417	4,247	14.94	3,758	789	4,547	15.99	344	469	813	2.86
Cagayan de Oro City	81,460	67,589	13,871	81,460	100.00	61,673	15,177	76,850	94.34	4,927	2,895	7,822	9.60
El Salvador City	85	*	*	0	0.00	*	*	0	0.00	*	*	0	0.00
Gingoog City	0	*	*	0	0.00	*	*	0	0.00	*	*	0	0.00
Iligan City	4,129	2,417	810	3,227	78.15	2,186	971	3,157	76.46	352	385	737	17.85
Malaybalay City	12,438	11,119	749	11,868	95.42	10,194	646	10,840	87.15	387	393	780	6.27
Oroquieta City	0	*	*	0	0.00	*	*	0	0.00	*	*	0	0.00
Ozamis City	0	*	*	0	0.00	*	*	0	0.00	*	*	0	0.00
Tangub City	114	*	*	0	0.00	*	*	0	0.00	5	6	11	9.65
Valencia City	7,361	1,367	238	1,605	21.80	1,381	296	1,677	22.78	802	741	1,543	20.96
**Region 11**	407,108	146,526	26,925	173,451	42.61	83,177	10,641	93,818	23.04	25,849	19,045	44,894	11.03
Compostela Valley	135,300	53,471	5,087	58,558	43.28	27,639	2,638	30,277	22.38	15,460	8,151	23,611	17.45
Davao del Norte	113,982	47,424	8,175	55,599	48.78	18,995	1,687	20,682	18.14	3,183	2,323	5,506	4.83
Davao Oriental	61,279	22,902	6,518	29,420	48.01	12,494	2,968	15,462	25.23	3,253	4,242	7,495	12.23
Davao del Sur	70,423	17,106	5,758	22,864	32.47	19,291	1,881	21,172	30.06	1,218	1,038	2,256	3.20
Davao Occidental	13,075	1,308	142	1,450	11.09	2,754	48	2,802	21.43	948	802	1,750	13.38
Davao City	13,049	4,315	1,245	5,560	42.61	2,004	1,419	3,423	26.23	1,787	2,489	4,276	32.77
**Region 12**	162,418	87,801	8,659	96,460	59.39	45,537	6,197	51,734	31.85	6,891	9,262	16,153	9.95
North Cotabato	52,989	53,963	4,508	58,471	110.35	26,984	2,484	29,468	55.61	2,753	3,887	6,640	12.53
Sarangani	28,078	20,288	2,486	22,774	81.11	8,652	2,164	10,816	38.52	677	867	1,544	5.50
South Cotabato	11,468	5,311	499	5,810	50.66	3,577	457	4,034	35.18	612	500	1,112	9.70
Sultan Kudarat	60,800	7,600	1,059	8,659	14.24	5,929	1,006	6,935	11.41	2,394	3,234	5,628	9.26
Cotabato City	6,214	447	60	507	8.16	180	35	215	3.46	375	620	995	16.01
Gen. Santos City	2,869	192	47	239	8.33	215	51	266	9.27	80	154	234	8.16
**BARMM**	30,695	9,108	2,678	11,786	38.40	1,176	122	1,298	4.23	560	729	1,289	4.20
Basilan	0	1,496	246	1,742	0.00	461	59	520	0.00	12	15	27	0.00
Lanao del Sur	8,494	4,840	1,893	6,733	79.27	227	19	246	2.90	401	426	827	9.74
Maguindanao	11,665	2,772	539	3,311	28.38	488	44	532	4.56	147	288	435	3.73
Sulu	0	*	*	0	0.00	*	*	0	0.00	*	*	0	0.00
Tawi-Tawi	0	*	*	0	0.00	*	*	0	0.00	*	*	0	0.00

Table 1.F.1.2 - Lifestyle Related Diseases (Tobacco Use, Harmful Use of Alcohol, Unhealthy Diet)

Number and proportion of adults ages 20 yrs old and above who are current smokers, binge drinkers and overweight or obese
Philippines, Annual 2020

Area	Total No. of clients ≥ 20 y/o risk-assesed[1]	Current Smokers				Binge Drinkers				Overweight or obese			
		Male	Female	Total	%	Male	Female	Total	%	Male	Female	Total	%
Lamitan City	**0**	*	*	**0**	**0.00**	*	*	**0**	**0.00**	*	*	**0**	**0.00**
Marawi City	**10,536**	*	*	**0**	**0.00**	*	*	**0**	**0.00**	*	*	**0**	**0.00**
**CARAGA**	**91,202**	**61,163**	**14,094**	**75,257**	**82.52**	**44,771**	**11,002**	**55,773**	**61.15**	**5,580**	**6,527**	**12,107**	**13.27**
Agusan del Norte	**13,374**	10,073	2,888	**12,961**	**96.91**	3,253	2,089	**5,342**	**39.94**	3,477	3,546	**7,023**	**52.51**
Agusan del Sur	**19,955**	15,297	2,385	**17,682**	**88.61**	12,809	2,247	**15,056**	**75.45**	350	244	**594**	**2.98**
Surigao del Norte	**11,494**	9,457	1,201	**10,658**	**92.73**	6,410	1,282	**7,692**	**66.92**	333	389	**722**	**6.28**
Surigao del Sur	**18,954**	7,305	1,667	**8,972**	**47.34**	9,671	3,818	**13,489**	**71.17**	495	598	**1,093**	**5.77**
Province of Dinagat	**2,147**	1,755	193	**1,948**	**90.73**	1,586	322	**1,908**	**88.87**	401	278	**679**	**31.63**
Bislig City	**3,964**	2,453	749	**3,202**	**80.78**	1,417	533	**1,950**	**49.19**	303	1,127	**1,430**	**36.07**
Butuan City	**19,188**	14,109	4,923	**19,032**	**99.19**	9,083	586	**9,669**	**50.39**	126	142	**268**	**1.40**
Surigao City	**2,126**	714	88	**802**	**37.72**	542	125	**667**	**31.37**	95	203	**298**	**14.02**

[1] Based on the PhilPEN protocol

Note: Put asterisk (*) for No Report and Zero (0) for No Case

Figure 1.F.1.1 - Percentage Distribution of Adult ages 20 yrs old and above who were risk-assessed using the PhilPEN protocol by Region
Philippines, 2020

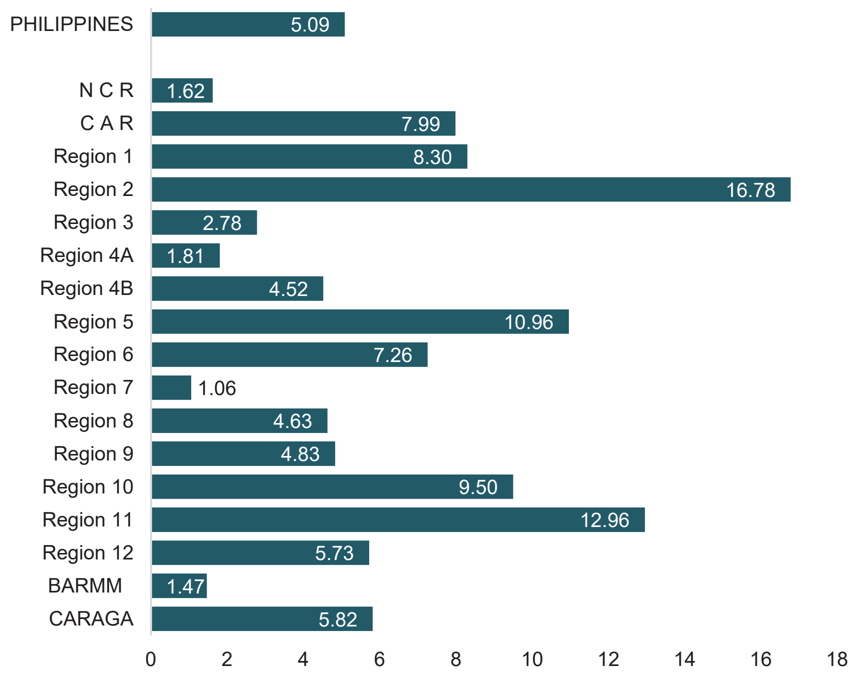

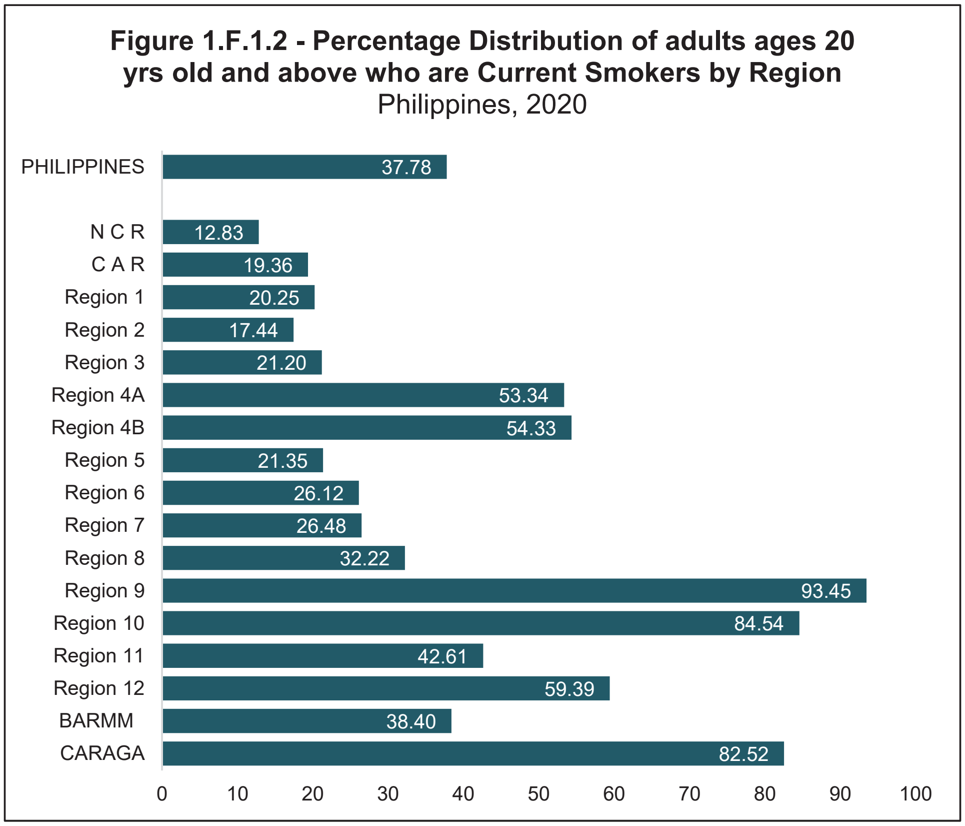
Figure 1.F.1.2 - Percentage Distribution of adults ages 20 yrs old and above who are Current Smokers by Region
Philippines, 2020
PHILIPPINES
37.78
N C R
12.83
C A R
19.36
Region 1
20.25
Region 2
17.44
Region 3
21.20
Region 4A
53.34
Region 4B
54.33
Region 5
21.35
Region 6
26.12
Region 7
26.48
Region 8
32.22
Region 9
93.45
Region 10
84.54
Region 11
42.61
Region 12
59.39
BARMM
38.40
CARAGA
82.52
0
10
20
30
40
50
60
70
80
90
100

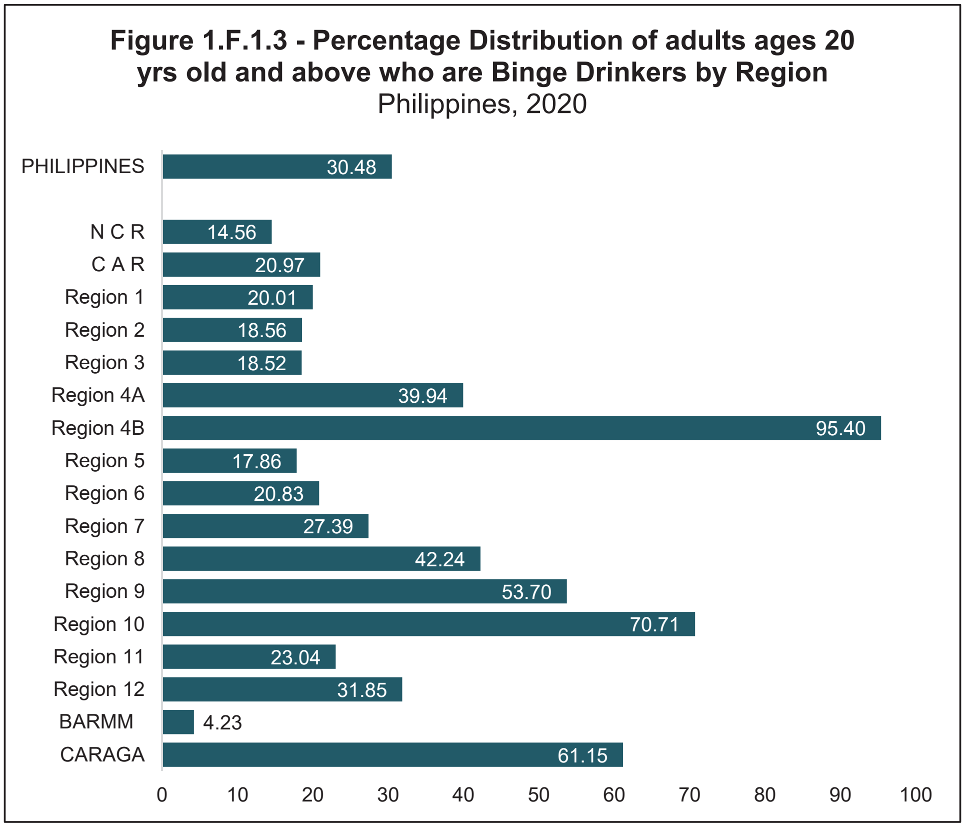
Figure 1.F.1.3 - Percentage Distribution of adults ages 20 yrs old and above who are Binge Drinkers by Region
Philippines, 2020
PHILIPPINES
30.48
N C R
14.56
C A R
20.97
Region 1
20.01
Region 2
18.56
Region 3
18.52
Region 4A
39.94
Region 4B
95.40
Region 5
17.86
Region 6
20.83
Region 7
27.39
Region 8
42.24
Region 9
53.70
Region 10
70.71
Region 11
23.04
Region 12
31.85
BARMM
4.23
CARAGA
61.15
0
10
20
30
40
50
60
70
80
90
100

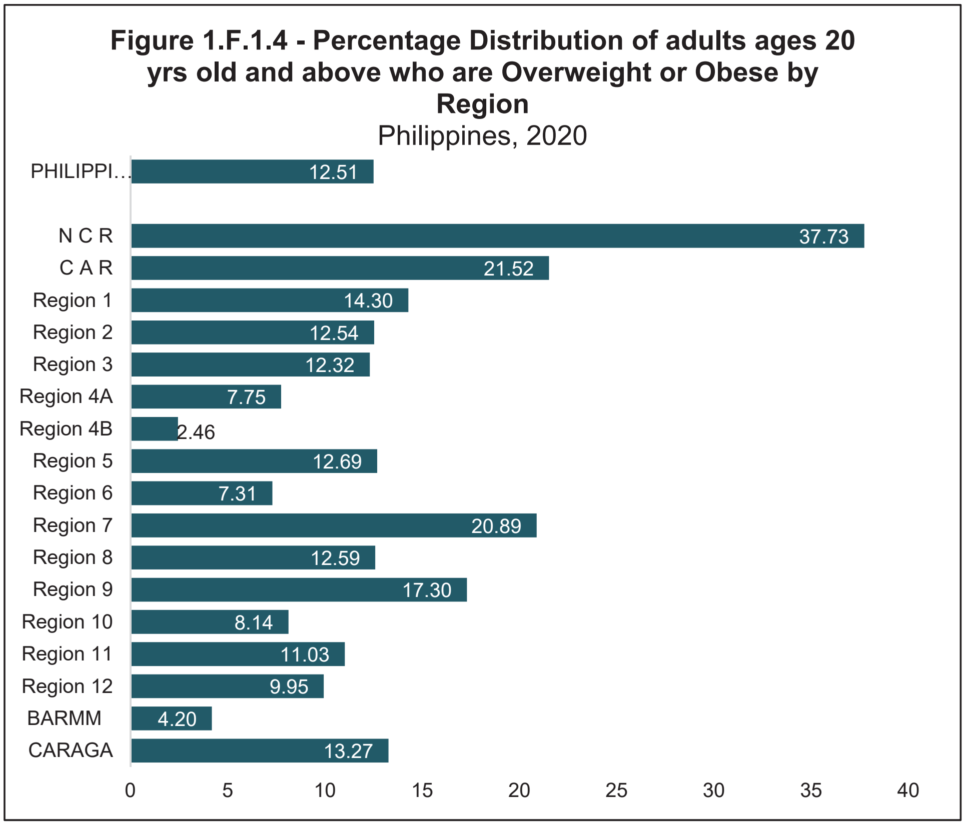
Figure 1.F.1.4 - Percentage Distribution of adults ages 20 yrs old and above who are Overweight or Obese by Region
Philippines, 2020
PHILIPPI...
12.51
N C R
37.73
C A R
21.52
Region 1
14.30
Region 2
12.54
Region 3
12.32
Region 4A
7.75
Region 4B
2.46
Region 5
12.69
Region 6
7.31
Region 7
20.89
Region 8
12.59
Region 9
17.30
Region 10
8.14
Region 11
11.03
Region 12
9.95
BARMM
4.20
CARAGA
13.27
0
5
10
15
20
25
30
35
40

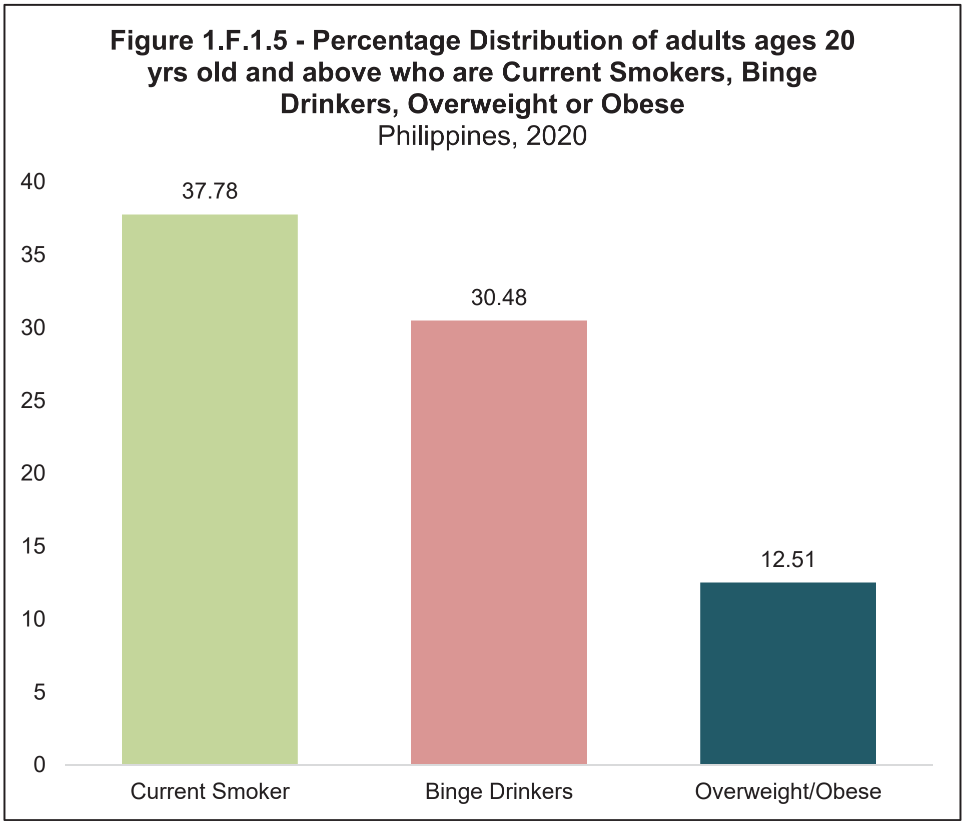
Figure 1.F.1.5 - Percentage Distribution of adults ages 20 yrs old and above who are Current Smokers, Binge Drinkers, Overweight or Obese
Philippines, 2020
40
35
30
25
20
15
10
5
0
37.78
30.48
12.51
Current Smoker
Binge Drinkers
Overweight/Obese

Table 1.G.2.1 - Cancer Prevention and Control (Cervical and Breast Cancer Screening)

Number and proportion of women ages 20 yrs old and above screened for Cervical and Breast Cancer
Philippines, Annual 2020

Area	Eligible Pop. (20 y/o and above female)	Women screened for Cervical Cancer		Women found positive or suspected for Cervical Cancer		Women screened for breast mass		Women with suspicious breast mass	
		Total	%	Total	%	Total	%	Total	%
**PHILIPPINES**	**32,789,368**	**40,420**	**0.12**	**2,285**	**5.65**	**185,763**	**0.57**	**5,058**	**2.72**
**N C R**	**4,614,005**	**9,565**	**0.21**	**254**	**2.66**	**12,329**	**0.27**	**425**	**3.45**
Malabon	**130,969**	1,849	**1.41**	0	**0.00**	0	**0.00**	0	**0.00**
Navotas	**89,385**	600	**0.67**	0	**0.00**	112	**0.13**	5	**4.46**
Valenzuela City	**222,303**	0	**0.00**	0	**0.00**	0	**0.00**	0	**0.00**
Caloocan City	**567,554**	1,407	**0.25**	101	**7.18**	1,816	**0.32**	44	**2.42**
Marikina City	**161,503**	173	**0.11**	0	**0.00**	351	**0.22**	16	**4.56**
Pasig City	**270,628**	21	**0.01**	1	**4.76**	25	**0.01**	23	**92.00**
Pateros	**22,874**	97	**0.42**	0	**0.00**	57	**0.25**	0	**0.00**
Taguig	**288,409**	528	**0.18**	92	**17.42**	470	**0.16**	65	**13.83**
Quezon City	**1,052,030**	2,238	**0.21**	0	**0.00**	1,698	**0.16**	1	**0.06**
Makati City	**208,750**	798	**0.38**	58	**7.27**	1,601	**0.77**	96	**6.00**
Mandaluyong City	**138,405**	100	**0.07**	0	**0.00**	404	**0.29**	1	**0.25**
San Juan	**43,779**	0	**0.00**	0	**0.00**	0	**0.00**	0	**0.00**
Manila City	**637,835**	324	**0.05**	1	**0.31**	3,491	**0.55**	121	**3.47**
Las Piñas City	**211,003**	43	**0.02**	1	**2.33**	437	**0.21**	50	**11.44**
Muntinlupa City	**180,767**	874	**0.48**	0	**0.00**	42	**0.02**	3	**7.14**
Parañaque City	**238,570**	12	**0.01**	0	**0.00**	380	**0.16**	0	**0.00**
Pasay City	**149,241**	501	**0.34**	0	**0.00**	1,445	**0.97**	0	**0.00**
**C A R**	**539,059**	**886**	**0.16**	**25**	**2.82**	**15,745**	**2.92**	**287**	**1.82**
Abra	**75,509**	2	**0.00**	2	**100.00**	782	**1.04**	28	**3.58**
Apayao	**35,013**	2	**0.01**	2	**100.00**	3,079	**8.79**	46	**1.49**
Benguet	**149,077**	307	**0.21**	3	**0.98**	5,556	**3.73**	146	**2.63**
Ifugao	**58,363**	5	**0.01**	0	**0.00**	184	**0.32**	10	**5.43**
Kalinga	**61,245**	0	**0.00**	0	**0.00**	725	**1.18**	21	**2.90**
Mt. Province	**44,473**	77	**0.17**	1	**1.30**	963	**2.17**	16	**1.66**
Baguio City	**115,379**	493	**0.43**	17	**3.45**	4,456	**3.86**	20	**0.45**
**Region 1**	**1,635,297**	**156**	**0.01**	**4**	**2.56**	**8,798**	**0.54**	**153**	**1.74**
Ilocos Norte	**166,415**	7	**0.00**	0	**0.00**	593	**0.36**	8	**1.35**
Ilocos Sur	**196,115**	11	**0.01**	3	**27.27**	1,907	**0.97**	54	**2.83**
La Union	**227,005**	52	**0.02**	1	**1.92**	2,977	**1.31**	10	**0.34**
Pangasinan	**744,177**	35	**0.00**	0	**0.00**	873	**0.12**	70	**8.02**
Alaminos City	**28,120**	40	**0.14**	0	**0.00**	1,789	**6.36**	4	**0.22**
Candon City	**20,670**	0	**0.00**	0	**0.00**	2	**0.01**	0	**0.00**
Dagupan City	**53,682**	0	**0.00**	0	**0.00**	316	**0.59**	1	**0.32**
Laoag City	**38,366**	0	**0.00**	0	**0.00**	0	**0.00**	0	**0.00**
San Carlos City	**59,109**	8	**0.01**	0	**0.00**	335	**0.57**	3	**0.90**
San Fernando City	**41,594**	0	**0.00**	0	**0.00**	3	**0.01**	3	**100.00**
Urdaneta City	**41,671**	0	**0.00**	0	**0.00**	0	**0.00**	0	**0.00**

Table 1.G.2.1 - Cancer Prevention and Control (Cervical and Breast Cancer Screening)

Number and proportion of women ages 20 yrs old and above screened for Cervical and Breast Cancer
Philippines, Annual 2020

Area	Eligible Pop. (20 y/o and above female)	Women screened for Cervical Cancer		Women found positive or suspected for Cervical Cancer		Women screened for breast mass		Women with suspicious breast mass	
		Total	%	Total	%	Total	%	Total	%
Vigan City	**18,373**	3	**0.02**	0	**0.00**	3	**0.02**	0	**0.00**
**Region 2**	**1,113,591**	**2,939**	**0.26**	**24**	**0.82**	**23,161**	**2.08**	**127**	**0.55**
Batanes	**5,357**	0	**0.00**	0	**0.00**	0	**0.00**	0	**0.00**
Cagayan	**299,121**	2,130	**0.71**	11	**0.52**	8,940	**2.99**	27	**0.30**
Isabela	**425,629**	97	**0.02**	1	**1.03**	992	**0.23**	39	**3.93**
Nueva Vizcaya	**143,485**	265	**0.18**	12	**4.53**	2,895	**2.02**	17	**0.59**
Quirino	**57,376**	1	**0.00**	0	**0.00**	988	**1.72**	2	**0.20**
Cauayan City	**41,812**	2	**0.00**	0	**0.00**	108	**0.26**	0	**0.00**
Ilagan City	**46,998**	0	**0.00**	0	**0.00**	87	**0.19**	13	**14.94**
Santiago City	**43,525**	192	**0.44**	0	**0.00**	9,151	**21.02**	29	**0.32**
Tuguegarao City	**50,288**	252	**0.50**	0	**0.00**	0	**0.00**	0	**0.00**
**Region 3**	**3,865,273**	**6,890**	**0.18**	**372**	**5.40**	**16,146**	**0.42**	**547**	**3.39**
Aurora	**64,497**	13	**0.02**	13	**100.00**	1	**0.00**	1	**100.00**
Bataan	**229,554**	40	**0.02**	0	**0.00**	2,568	**1.12**	89	**3.47**
Bulacan	**795,517**	302	**0.04**	8	**2.65**	148	**0.02**	59	**39.86**
Nueva Ecija	**504,010**	0	**0.00**	0	**0.00**	0	**0.00**	0	**0.00**
Pampanga	**576,293**	0	**0.00**	0	**0.00**	495	**0.09**	3	**0.61**
Tarlac	**339,141**	79	**0.02**	16	**20.25**	175	**0.05**	2	**1.14**
Zambales	**196,464**	3	**0.00**	1	**33.33**	1,303	**0.66**	6	**0.46**
Angeles City	**144,590**	441	**0.31**	2	**0.45**	1,791	**1.24**	4	**0.22**
Balanga City	**33,181**	110	**0.33**	9	**8.18**	613	**1.85**	10	**1.63**
Cabanatuan City	**103,155**	0	**0.00**	0	**0.00**	0	**0.00**	0	**0.00**
City of San Fernando	**107,718**	7	**0.01**	2	**28.57**	11	**0.01**	1	**9.09**
Gapan City	**37,650**	31	**0.08**	0	**0.00**	330	**0.88**	41	**12.42**
Mabalacat City	**88,095**	10	**0.01**	0	**0.00**	7,763	**8.81**	0	**0.00**
Malolos City	**88,859**	95	**0.11**	0	**0.00**	179	**0.20**	0	**0.00**
Meycauayan	**73,701**	1	**0.00**	0	**0.00**	3	**0.00**	0	**0.00**
Olongapo	**77,492**	58	**0.07**	2	**3.45**	21	**0.03**	6	**28.57**
Palayan City	**14,007**	5	**0.04**	2	**40.00**	27	**0.19**	0	**0.00**
San Jose City	**47,691**	0	**0.00**	0	**0.00**	0	**0.00**	0	**0.00**
San Jose del Monte City	**202,364**	5,646	**2.79**	317	**5.61**	574	**0.28**	325	**56.62**
Science City of Munoz	**27,810**	8	**0.03**	0	**0.00**	3	**0.01**	0	**0.00**
Tarlac City	**113,484**	41	**0.04**	0	**0.00**	141	**0.12**	0	**0.00**
**Region 4A**	**5,066,418**	**1,429**	**0.03**	**133**	**9.31**	**3,993**	**0.08**	**152**	**3.81**
Batangas	**657,859**	8	**0.00**	8	**100.00**	102	**0.02**	8	**7.84**
Cavite	**509,580**	17	**0.00**	1	**5.88**	20	**0.00**	22	**110.00**
Laguna	**355,685**	28	**0.01**	1	**3.57**	92	**0.03**	8	**8.70**
Quezon	**542,561**	308	**0.06**	26	**8.44**	2,162	**0.40**	53	**2.45**
Rizal	**733,011**	225	**0.03**	17	**7.56**	601	**0.08**	20	**3.33**
Antipolo City	**269,993**	0	**0.00**	0	**0.00**	154	**0.06**	1	**0.65**
Bacoor City	**223,251**	3	**0.00**	0	**0.00**	25	**0.01**	1	**4.00**
Batangas City	**116,749**	0	**0.00**	0	**0.00**	0	**0.00**	0	**0.00**

Table 1.G.2.1 - Cancer Prevention and Control (Cervical and Breast Cancer Screening)

Number and proportion of women ages 20 yrs old and above screened for Cervical and Breast Cancer
Philippines, Annual 2020

Area	Eligible Pop. (20 y/o and above female)	Women screened for Cervical Cancer		Women found positive or suspected for Cervical Cancer		Women screened for breast mass		Women with suspicious breast mass	
		Total	%	Total	%	Total	%	Total	%
Biñan City	**119,266**	82	**0.07**	1	**1.22**	82	**0.07**	7	**8.54**
Cabuyao City	**110,572**	78	**0.07**	2	**2.56**	61	**0.06**	4	**6.56**
Calamba City	**162,763**	120	**0.07**	60	**50.00**	100	**0.06**	11	**11.00**
Cavite City	**38,208**	0	**0.00**	0	**0.00**	0	**0.00**	0	**0.00**
Dasmariñas City	**244,969**	397	**0.16**	0	**0.00**	276	**0.11**	9	**3.26**
General Trias City	**116,829**	0	**0.00**	7	**0.00**	0	**0.00**	0	**0.00**
Imus City	**150,087**	12	**0.01**	0	**0.00**	45	**0.03**	0	**0.00**
Lipa City	**117,636**	0	**0.00**	0	**0.00**	0	**0.00**	0	**0.00**
Lucena City	**82,229**	0	**0.00**	0	**0.00**	0	**0.00**	0	**0.00**
San Pablo City	**95,287**	0	**0.00**	0	**0.00**	2	**0.00**	0	**0.00**
San Pedro City	**116,682**	0	**0.00**	0	**0.00**	0	**0.00**	0	**0.00**
Santa Rosa City	**126,694**	25	**0.02**	0	**0.00**	105	**0.08**	0	**0.00**
Tagaytay City	**26,459**	0	**0.00**	0	**0.00**	0	**0.00**	0	**0.00**
Tanauan City	**61,357**	0	**0.00**	0	**0.00**	62	**0.10**	5	**8.06**
Tayabas City	**30,811**	104	**0.34**	8	**7.69**	104	**0.34**	3	**2.88**
Trece Martires City	**57,880**	22	**0.04**	2	**9.09**	0	**0.00**	0	**0.00**
**Region 4B**	**866,413**	**36**	**0.00**	**3**	**8.33**	**122**	**0.01**	**122**	**100.00**
Marinduque	**69,186**	35	**0.05**	3	**8.57**	25	**0.04**	25	**100.00**
Mindoro Occidental	**135,701**	0	**0.00**	0	**0.00**	1	**0.00**	1	**100.00**
Mindoro Oriental	**252,517**	0	**0.00**	0	**0.00**	0	**0.00**	0	**0.00**
Palawan	**249,849**	1	**0.00**	0	**0.00**	96	**0.04**	96	**100.00**
Romblon	**84,126**	0	**0.00**	0	**0.00**	0	**0.00**	0	**0.00**
Puerto Princesa City	**75,034**	0	**0.00**	0	**0.00**	0	**0.00**	0	**0.00**
**Region 5**	**1,684,097**	**688**	**0.04**	**81**	**11.77**	**4,815**	**0.29**	**286**	**5.94**
Albay	**343,919**	0	**0.00**	0	**0.00**	0	**0.00**	0	**0.00**
Camarines Norte	**171,591**	11	**0.01**	0	**0.00**	865	**0.50**	67	**7.75**
Camarines Sur	**473,776**	254	**0.05**	4	**1.57**	714	**0.15**	86	**12.04**
Catanduanes	**73,989**	0	**0.00**	0	**0.00**	51	**0.07**	1	**1.96**
Masbate	**240,917**	111	**0.05**	0	**0.00**	136	**0.06**	19	**13.97**
Sorsogon	**230,782**	10	**0.00**	4	**40.00**	561	**0.24**	79	**14.08**
Iriga City	**32,193**	14	**0.04**	0	**0.00**	1,055	**3.28**	9	**0.85**
Legaspi City	**60,477**	12	**0.02**	0	**0.00**	8	**0.01**	3	**37.50**
Naga City	**56,453**	276	**0.49**	73	**26.45**	1,425	**2.52**	22	**1.54**
**Region 6**	**2,410,308**	**1,512**	**0.06**	**154**	**10.19**	**33,694**	**1.40**	**337**	**1.00**
Aklan	**184,625**	3	**0.00**	2	**66.67**	44	**0.02**	7	**15.91**
Antique	**174,795**	20	**0.01**	0	**0.00**	1,591	**0.91**	27	**1.70**
Capiz	**251,481**	25	**0.01**	3	**12.00**	1,471	**0.58**	9	**0.61**
Guimaras	**56,763**	0	**0.00**	0	**0.00**	14,324	**25.23**	32	**0.22**
Iloilo	**645,091**	134	**0.02**	28	**20.90**	3,867	**0.60**	55	**1.42**
Negros Occidental	**774,132**	1,171	**0.15**	118	**10.08**	5,226	**0.68**	164	**3.14**
Bacolod City	**174,179**	95	**0.05**	2	**2.11**	314	**0.18**	4	**1.27**
Iloilo City	**149,242**	64	**0.04**	1	**1.56**	6,857	**4.59**	39	**0.57**

Table 1.G.2.1 - Cancer Prevention and Control (Cervical and Breast Cancer Screening)

Number and proportion of women ages 20 yrs old and above screened for Cervical and Breast Cancer
Philippines, Annual 2020

Area	Eligible Pop. (20 y/o and above female)	Women screened for Cervical Cancer		Women found positive or suspected for Cervical Cancer		Women screened for breast mass		Women with suspicious breast mass	
		Total	%	Total	%	Total	%	Total	%
**Region 7**	**2,390,909**	**989**	**0.04**	**77**	**7.79**	**13,439**	**0.56**	**1,155**	**8.59**
Bohol	**399,802**	0	**0.00**	0	**0.00**	153	**0.04**	28	**18.30**
Cebu	**978,019**	834	**0.09**	72	**8.63**	9,646	**0.99**	323	**3.35**
Negros Oriental	**417,913**	41	**0.01**	0	**0.00**	56	**0.01**	11	**19.64**
Siquijor	**31,657**	12	**0.04**	0	**0.00**	121	**0.38**	0	**0.00**
Cebu City	**307,022**	102	**0.03**	5	**4.90**	1,856	**0.60**	774	**41.70**
Lapu-Lapu City	**135,812**	0	**0.00**	0	**0.00**	1,607	**1.18**	19	**1.18**
Mandaue City	**120,684**	0	**0.00**	0	**0.00**	0	**0.00**	0	**0.00**
**Region 8**	**1,315,053**	**73**	**0.01**	**9**	**12.33**	**1,545**	**0.12**	**62**	**4.01**
Biliran	**50,482**	0	**0.00**	0	**0.00**	141	**0.28**	3	**2.13**
Eastern Samar	**136,595**	1	**0.00**	0	**0.00**	20	**0.01**	5	**25.00**
Northern Leyte	**462,888**	0	**0.00**	0	**0.00**	0	**0.00**	0	**0.00**
Northern Samar	**174,587**	47	**0.03**	4	**8.51**	30	**0.02**	17	**56.67**
Southern Leyte	**105,767**	13	**0.01**	5	**38.46**	1,330	**1.26**	13	**0.98**
Western Samar	**166,382**	12	**0.01**	0	**0.00**	24	**0.01**	24	**100.00**
Calbayog City	**51,268**	0	**0.00**	0	**0.00**	0	**0.00**	0	**0.00**
Maasin City	**26,915**	0	**0.00**	0	**0.00**	0	**0.00**	0	**0.00**
Ormoc City	**65,937**	0	**0.00**	0	**0.00**	0	**0.00**	0	**0.00**
Tacloban City	**74,232**	0	**0.00**	0	**0.00**	0	**0.00**	0	**0.00**
**Region 9**	**1,071,821**	**120**	**0.01**	**7**	**5.83**	**3,289**	**0.31**	**164**	**4.99**
Zamboanga del Norte	**232,262**	111	**0.05**	5	**4.50**	968	**0.42**	109	**11.26**
Zamboanga del Sur	**241,697**	0	**0.00**	0	**0.00**	601	**0.25**	28	**4.66**
Zamboanga Sibugay	**182,846**	1	**0.00**	1	**100.00**	645	**0.35**	11	**1.71**
Dapitan City	**23,983**	0	**0.00**	0	**0.00**	0	**0.00**	0	**0.00**
Dipolog City	**38,044**	0	**0.00**	0	**0.00**	12	**0.03**	2	**16.67**
Isabela City	**37,068**	0	**0.00**	0	**0.00**	237	**0.64**	0	**0.00**
Pagadian City	**59,278**	0	**0.00**	0	**0.00**	0	**0.00**	0	**0.00**
Zamboanga City	**256,643**	8	**0.00**	1	**12.50**	826	**0.32**	14	**1.69**
**Region 10**	**1,451,065**	**511**	**0.04**	**34**	**6.65**	**1,894**	**0.13**	**201**	**10.61**
Bukidnon	**309,024**	45	**0.01**	0	**0.00**	162	**0.05**	40	**24.69**
Camiguin	**27,158**	25	**0.09**	0	**0.00**	74	**0.27**	2	**2.70**
Lanao del Norte	**204,773**	19	**0.01**	1	**5.26**	97	**0.05**	8	**8.25**
Misamis Occidental	**102,293**	50	**0.05**	0	**0.00**	46	**0.04**	1	**2.17**
Misamis Oriental	**232,335**	282	**0.12**	27	**9.57**	819	**0.35**	80	**9.77**
Cagayan de Oro City	**220,057**	42	**0.02**	3	**7.14**	595	**0.27**	42	**7.06**
El Salvador City	**16,345**	28	**0.17**	0	**0.00**	43	**0.26**	6	**13.95**
Gingoog City	**40,578**	14	**0.03**	0	**0.00**	0	**0.00**	0	**0.00**
Iligan City	**103,730**	3	**0.00**	1	**33.33**	48	**0.05**	12	**25.00**
Malaybalay City	**51,511**	0	**0.00**	0	**0.00**	1	**0.00**	1	**100.00**

Table 1.G.2.1 - Cancer Prevention and Control (Cervical and Breast Cancer Screening)

Number and proportion of women ages 20 yrs old and above screened for Cervical and Breast Cancer
Philippines, Annual 2020

Area	Eligible Pop. (20 y/o and above female)	Women screened for Cervical Cancer		Women found positive or suspected for Cervical Cancer		Women screened for breast mass		Women with suspicious breast mass	
		Total	%	Total	%	Total	%	Total	%
Oroquieta City	**22,164**	0	**0.00**	0	**0.00**	0	**0.00**	0	**0.00**
Ozamis City	**44,429**	3	**0.01**	2	**66.67**	8	**0.02**	8	**100.00**
Tangub City	**19,740**	0	**0.00**	0	**0.00**	1	**0.01**	1	**100.00**
Valencia City	**56,928**	0	**0.00**	0	**0.00**	0	**0.00**	0	**0.00**
**Region 11**	**1,537,276**	**12,969**	**0.84**	**893**	**6.89**	**26,503**	**1.72**	**541**	**2.04**
Compostela Valley	**215,889**	5,217	**2.42**	19	**0.36**	7,505	**3.48**	151	**2.01**
Davao del Norte	**309,694**	1,219	**0.39**	718	**58.90**	4,408	**1.42**	72	**1.63**
Davao Oriental	**163,166**	1,199	**0.73**	121	**10.09**	6,763	**4.14**	103	**1.52**
Davao del Sur	**210,643**	3,658	**1.74**	2	**0.05**	1,701	**0.81**	45	**2.65**
Davao Occidental	**94,121**	1,100	**1.17**	27	**2.45**	251	**0.27**	10	**3.98**
Davao City	**543,763**	576	**0.11**	6	**1.04**	5,875	**1.08**	160	**2.72**
**Region 12**	**1,396,805**	**901**	**0.06**	**187**	**20.75**	**16,453**	**1.18**	**343**	**2.08**
North Cotabato	**425,819**	133	**0.03**	11	**8.27**	1,628	**0.38**	138	**8.48**
Sarangani	**156,092**	640	**0.41**	171	**26.72**	5,364	**3.44**	143	**2.67**
South Cotabato	**292,601**	3	**0.00**	2	**66.67**	1,917	**0.66**	31	**1.62**
Sultan Kudarat	**241,653**	96	**0.04**	3	**3.13**	4,079	**1.69**	11	**0.27**
Cotabato City	**90,608**	4	**0.00**	0	**0.00**	132	**0.15**	12	**9.09**
Gen. Santos City	**190,032**	25	**0.01**	0	**0.00**	3,333	**1.75**	8	**0.24**
**BARMM**	**1,067,766**	**34**	**0.00**	**0**	**0.00**	**2,090**	**0.20**	**110**	**5.26**
Basilan	**76,301**	0	**0.00**	0	**0.00**	0	**0.00**	0	**0.00**
Lanao del Sur	**238,159**	4	**0.00**	0	**0.00**	2,056	**0.86**	93	**4.52**
Maguindanao	**332,100**	0	**0.00**	0	**0.00**	0	**0.00**	0	**0.00**
Sulu	**236,012**	0	**0.00**	0	**0.00**	0	**0.00**	0	**0.00**
Tawi-Tawi	**107,243**	0	**0.00**	0	**0.00**	0	**0.00**	0	**0.00**
Lamitan City	**20,990**	0	**0.00**	0	**0.00**	0	**0.00**	0	**0.00**
Marawi City	**56,961**	30	**0.05**	0	**0.00**	34	**0.06**	17	**50.00**
**CARAGA**	**764,212**	**722**	**0.09**	**28**	**3.88**	**1,747**	**0.23**	**46**	**2.63**
Agusan del Norte	**107,998**	18	**0.02**	1	**5.56**	280	**0.26**	7	**2.50**
Agusan del Sur	**191,940**	175	**0.09**	5	**2.86**	147	**0.08**	8	**5.44**
Surigao del Norte	**103,445**	38	**0.04**	3	**7.89**	23	**0.02**	7	**30.43**
Surigao del Sur	**144,481**	14	**0.01**	4	**28.57**	108	**0.07**	13	**12.04**
Province of Dinagat	**38,053**	148	**0.39**	5	**3.38**	110	**0.29**	5	**4.55**
Bislig City	**27,437**	84	**0.31**	6	**7.14**	130	**0.47**	1	**0.77**
Butuan City	**102,684**	75	**0.07**	0	**0.00**	68	**0.07**	2	**2.94**
Surigao City	**48,174**	170	**0.35**	4	**2.35**	881	**1.83**	3	**0.34**

Note: Put asterisk (*) for No Report and Zero (0) for No Case

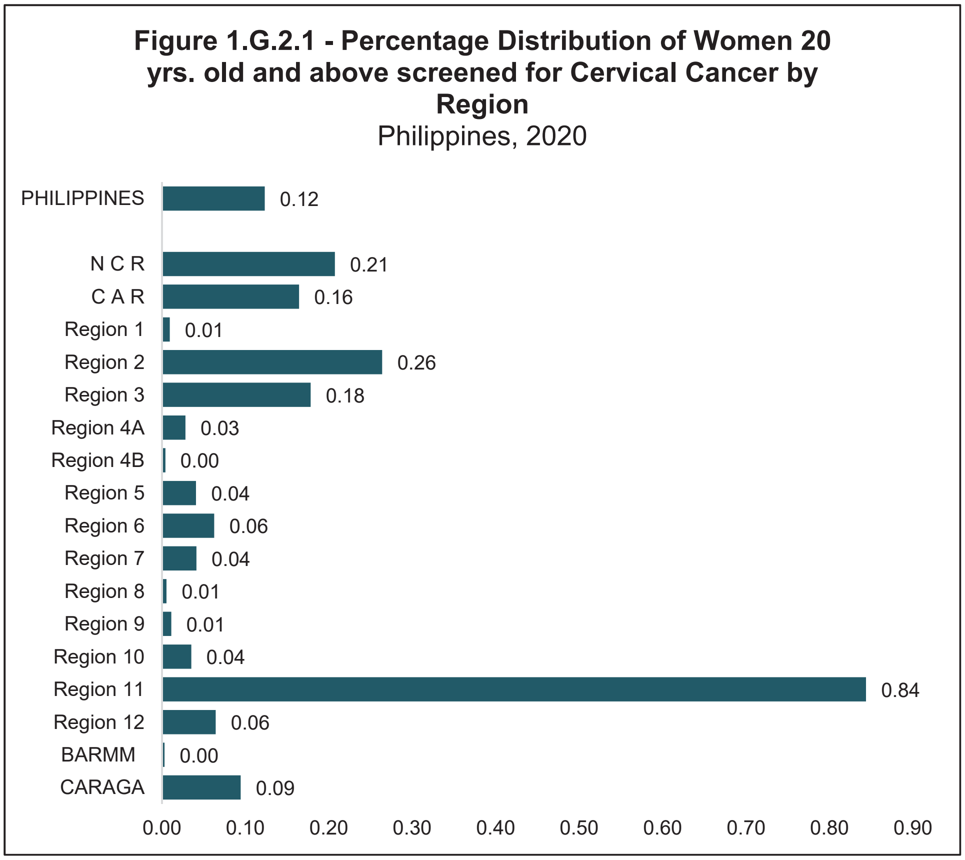
Figure 1.G.2.1 - Percentage Distribution of Women 20 yrs. old and above screened for Cervical Cancer by Region
Philippines, 2020
PHILIPPINES
0.12
N C R
0.21
C A R
0.16
Region 1
0.01
Region 2
0.26
Region 3
0.18
Region 4A
0.03
Region 4B
0.00
Region 5
0.04
Region 6
0.06
Region 7
0.04
Region 8
0.01
Region 9
0.01
Region 10
0.04
Region 11
0.84
Region 12
0.06
BARMM
0.00
CARAGA
0.09
0.00
0.10
0.20
0.30
0.40
0.50
0.60
0.70
0.80
0.90

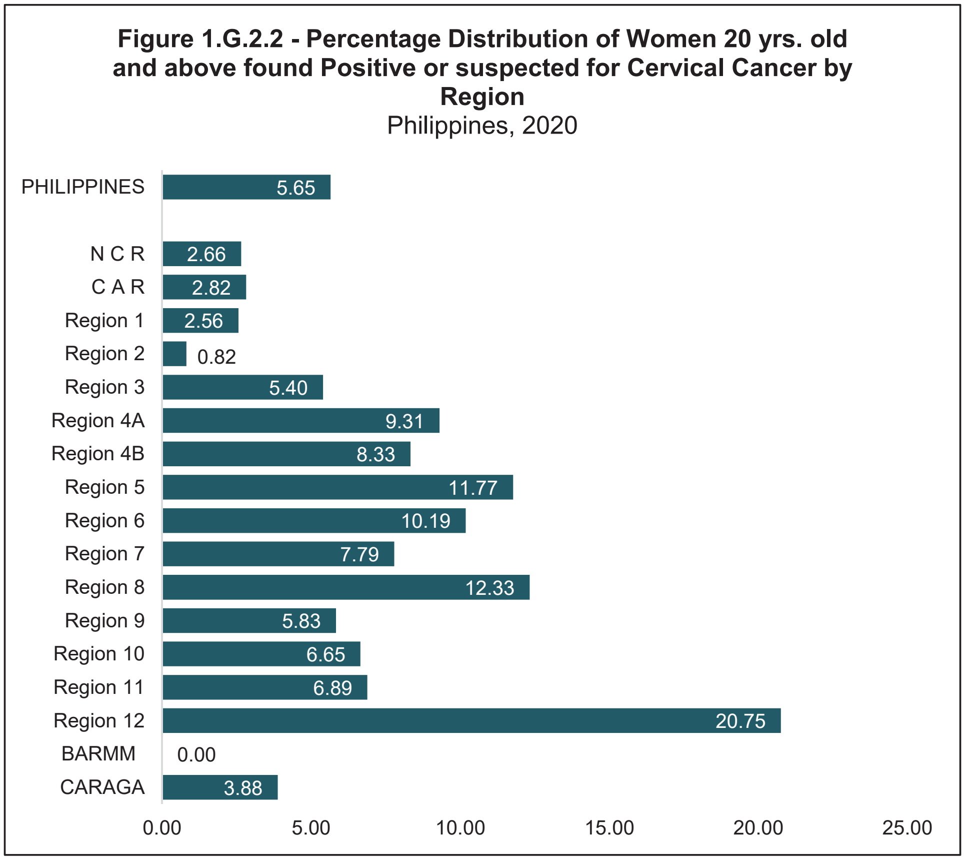
Figure 1.G.2.2 - Percentage Distribution of Women 20 yrs. old and above found Positive or suspected for Cervical Cancer by Region
Philippines, 2020
PHILIPPINES
5.65
N C R
2.66
C A R
2.82
Region 1
2.56
Region 2
0.82
Region 3
5.40
Region 4A
9.31
Region 4B
8.33
Region 5
11.77
Region 6
10.19
Region 7
7.79
Region 8
12.33
Region 9
5.83
Region 10
6.65
Region 11
6.89
Region 12
20.75
BARMM
0.00
CARAGA
3.88
0.00
5.00
10.00
15.00
20.00
25.00

Figure 1.G.2.3 - Percentage Distribution of Women 20 yrs. old and above Screened for Breast Mass by Region
Philippines, 2020

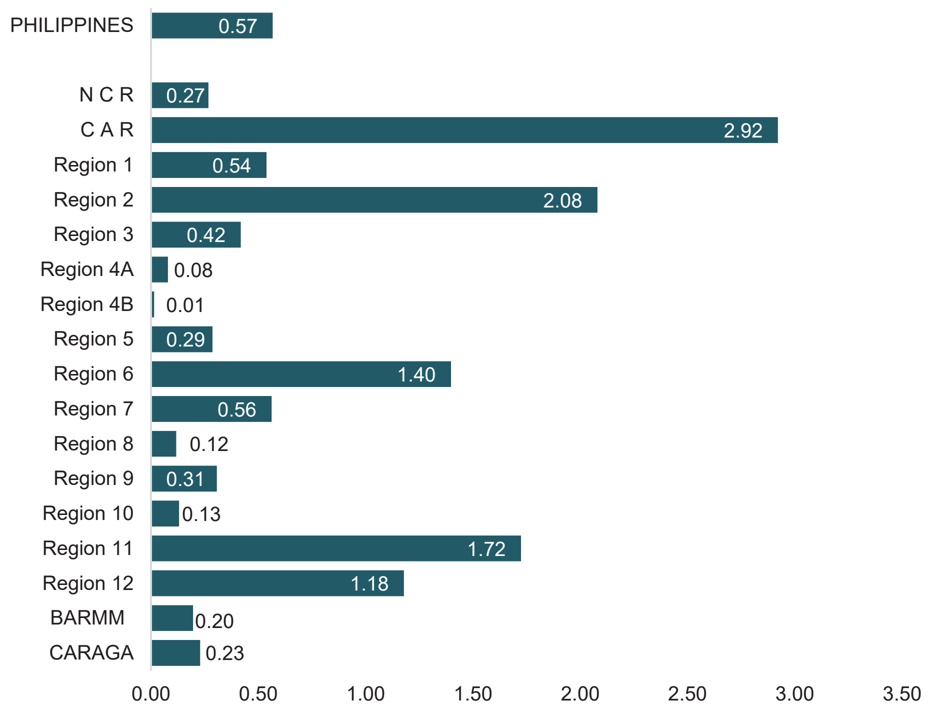

Figure 1.G.2.4 - Percentage Distribution of Women 20 yrs. old and above with suspicious Breast Mass by Region
Philippines, 2020

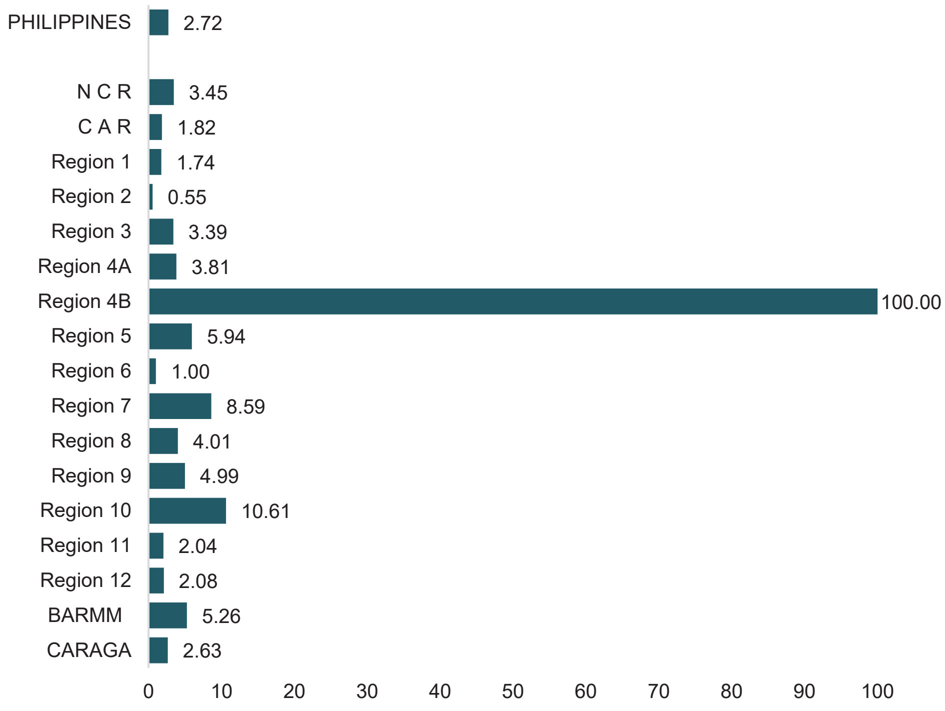

Figure 1.G.2.5 - Percentage Distribution of Women 20 yrs. old and above screened for Cervical Cancer, found Positive or suspected for Cervical Cancer, screened for Breast mass and women with suspicious breast mass
Philippines, 2020

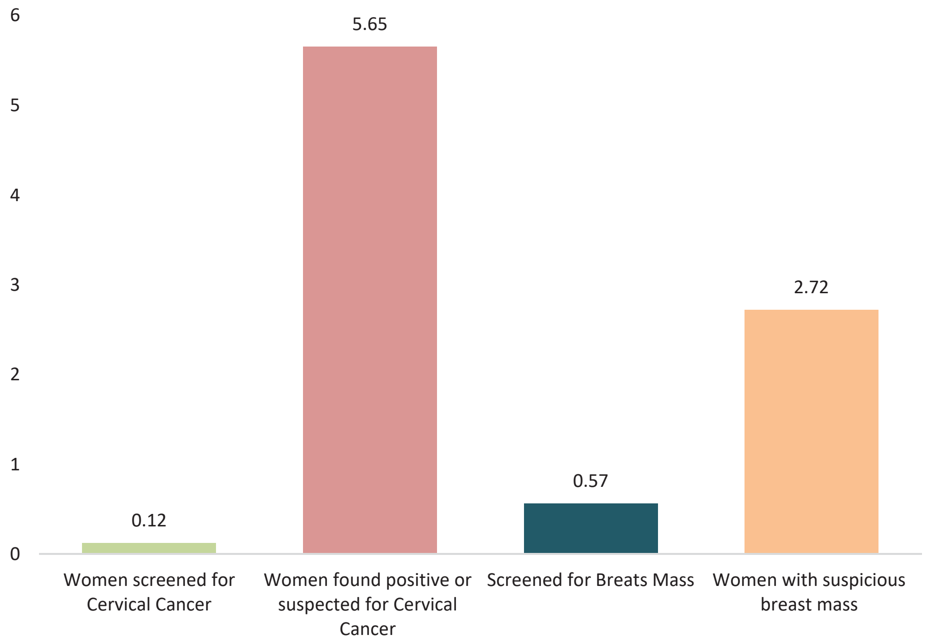

Table 1.F.3.1 -Cardiovascular Disease and Diabetes Mellitus Prevention and Control

Number and proportion of newly-identified 20 yrs old and above adults with Hypertension and Type 2 Diabetes
Philippines, Annual 2020

Area	Total No. of clients ≥ 20 y/o risk-assesed[1]	Newly identified Hypertensive Adults				Newly identified with Type 2 Diabetes Mellitus			
		Male	Female	Total	%	Male	Female	Total	%
**PHILIPPINES**	**3,279,731**	**161,358**	**199,437**	**360,795**	**11.00**	**32,896**	**42,509**	**75,405**	**2.30**
**N C R**	**145,178**	**13,477**	**15,903**	**29,380**	**20.24**	**3,239**	**4,255**	**7,494**	**5.16**
Malabon	4,613	1,161	1,108	**2,269**	**49.19**	436	399	**835**	**18.10**
Navotas	11,452	843	894	**1,737**	**15.17**	106	154	**260**	**2.27**
Valenzuela City	17,468	1,192	1,102	**2,294**	**13.13**	310	303	**613**	**3.51**
Caloocan City	20,327	1,293	2,053	**3,346**	**16.46**	337	572	**909**	**4.47**
Marikina City	3,871	536	451	**987**	**25.50**	81	88	**169**	**4.37**
Pasig City	10,956	841	1,203	**2,044**	**18.66**	264	317	**581**	**5.30**
Pateros	807	118	139	**257**	**31.85**	20	20	**40**	**4.96**
Taguig	6,254	421	537	**958**	**15.32**	103	424	**527**	**8.43**
Quezon City	20,876	1,446	2,276	**3,722**	**17.83**	256	391	**647**	**3.10**
Makati City	6,503	639	816	**1,455**	**22.37**	318	423	**741**	**11.39**
Mandaluyong City	5,415	323	367	**690**	**12.74**	63	86	**149**	**2.75**
San Juan	3,099	57	77	**134**	**4.32**	4	3	**7**	**0.23**
Manila City	14,602	2,720	2,721	**5,441**	**37.26**	476	499	**975**	**6.68**
Las Piñas City	2,659	779	848	**1,627**	**61.19**	195	239	**434**	**16.32**
Muntinlupa City	4,668	598	568	**1,166**	**24.98**	92	139	**231**	**4.95**
Parañaque City	6,943	271	326	**597**	**8.60**	94	97	**191**	**2.75**
Pasay City	4,665	239	417	**656**	**14.06**	84	101	**185**	**3.97**
**C A R**	**87,462**	**4,755**	**5,919**	**10,674**	**12.20**	**511**	**762**	**1,273**	**1.46**
Abra	21,418	891	1,067	**1,958**	**9.14**	83	128	**211**	**0.99**
Apayao	22,002	139	134	**273**	**1.24**	15	21	**36**	**0.16**
Benguet	17,721	1,619	2,078	**3,697**	**20.86**	188	272	**460**	**2.60**
Ifugao	4,098	173	170	**343**	**8.37**	12	20	**32**	**0.78**
Kalinga	6,196	896	1,078	**1,974**	**31.86**	62	96	**158**	**2.55**
Mt. Province	6,999	251	260	**511**	**7.30**	35	58	**93**	**1.33**
Baguio City	9,028	786	1,132	**1,918**	**21.25**	116	167	**283**	**3.13**
**Region 1**	**269,809**	**21,940**	**22,957**	**44,897**	**16.64**	**3,098**	**4,023**	**7,121**	**2.64**
Ilocos Norte	21,417	1,384	1,474	**2,858**	**13.34**	173	235	**408**	**1.91**
Ilocos Sur	40,742	1,219	1,147	**2,366**	**5.81**	197	325	**522**	**1.28**
La Union	48,096	1,886	2,000	**3,886**	**8.08**	345	415	**760**	**1.58**
Pangasinan	121,179	16,170	16,903	**33,073**	**27.29**	2,057	2,623	**4,680**	**3.86**
Alaminos City	8,096	77	73	**150**	**1.85**	14	9	**23**	**0.28**
Candon City	313	56	44	**100**	**31.95**	13	20	**33**	**10.54**
Dagupan City	327	68	66	**134**	**40.98**	25	24	**49**	**14.98**
Laoag City	1,912	274	276	**550**	**28.77**	51	95	**146**	**7.64**
San Carlos City	2,487	372	326	**698**	**28.07**	143	153	**296**	**11.90**
San Fernando City	6,006	324	489	**813**	**13.54**	66	112	**178**	**2.96**
Urdaneta City	18,939	68	124	**192**	**1.01**	5	3	**8**	**0.04**

Table 1.F.3.1 -Cardiovascular Disease and Diabetes Mellitus Prevention and Control
Number and proportion of newly-identified 20 yrs old and above adults with Hypertension and Type 2 Diabetes
Philippines, Annual 2020

Area	Total No. of clients ≥ 20 y/o risk-assesed[1]	Newly identified Hypertensive Adults				Newly identified with Type 2 Diabetes Mellitus			
		Male	Female	Total	%	Male	Female	Total	%
Vigan City	295	42	35	**77**	**26.10**	9	9	**18**	**6.10**
**Region 2**	**377,028**	**6,034**	**7,314**	**13,348**	**3.54**	**837**	**1,015**	**1,852**	**0.49**
Batanes	3,854	28	17	**45**	**1.17**	3	9	**12**	**0.31**
Cagayan	85,223	1,304	1,715	**3,019**	**3.54**	202	267	**469**	**0.55**
Isabela	101,080	2,486	2,957	**5,443**	**5.38**	209	319	**528**	**0.52**
Nueva Vizcaya	55,110	1,018	1,197	**2,215**	**4.02**	195	200	**395**	**0.72**
Quirino	42,307	490	517	**1,007**	**2.38**	66	57	**123**	**0.29**
Cauayan City	5,263	274	397	**671**	**12.75**	22	31	**53**	**1.01**
Ilagan City	11,027	171	218	**389**	**3.53**	49	40	**89**	**0.81**
Santiago City	70,308	200	180	**380**	**0.54**	77	78	**155**	**0.22**
Tuguegarao City	2,856	63	116	**179**	**6.27**	14	14	**28**	**0.98**
**Region 3**	**214,557**	**11,014**	**14,496**	**25,510**	**11.89**	**3,268**	**4,561**	**7,829**	**3.65**
Aurora	2,351	553	868	**1,421**	**60.44**	80	122	**202**	**8.59**
Bataan	20,095	894	1,162	**2,056**	**10.23**	175	198	**373**	**1.86**
Bulacan	19,829	1,897	2,491	**4,388**	**22.13**	818	1,107	**1,925**	**9.71**
Nueva Ecija	3,001	215	261	**476**	**15.86**	110	151	**261**	**8.70**
Pampanga	26,864	1,496	1,988	**3,484**	**12.97**	334	470	**804**	**2.99**
Tarlac	41,598	1,286	1,924	**3,210**	**7.72**	395	671	**1,066**	**2.56**
Zambales	22,557	871	1,141	**2,012**	**8.92**	239	256	**495**	**2.19**
Angeles City	2,358	506	591	**1,097**	**46.52**	102	140	**242**	**10.26**
Balanga City	1,493	251	309	**560**	**37.51**	65	87	**152**	**10.18**
Cabanatuan City	13,199	321	512	**833**	**6.31**	100	155	**255**	**1.93**
City of San Fernando	5,282	399	440	**839**	**15.88**	147	226	**373**	**7.06**
Gapan City	371	111	84	**195**	**52.56**	14	33	**47**	**12.67**
Mabalacat City	24,050	294	392	**686**	**2.85**	112	133	**245**	**1.02**
Malolos City	3,997	293	516	**809**	**20.24**	104	162	**266**	**6.65**
Meycauayan	1,072	395	451	**846**	**78.92**	128	166	**294**	**27.43**
Olongapo	1,805	217	237	**454**	**25.15**	39	55	**94**	**5.21**
Palayan City	203	63	106	**169**	**83.25**	11	10	**21**	**10.34**
San Jose City	4,387	69	83	**152**	**3.46**	17	29	**46**	**1.05**
San Jose del Monte City	15,259	588	676	**1,264**	**8.28**	187	259	**446**	**2.92**
Science City of Munoz	4,056	11	10	**21**	**0.52**	14	24	**38**	**0.94**
Tarlac City	730	284	254	**538**	**73.70**	77	107	**184**	**25.21**
**Region 4A**	**180,055**	**3,966**	**2,495**	**6,461**	**3.59**	**562**	**1,038**	**1,600**	**0.89**
Batangas	97,898	172	83	**255**	**0.26**	7	14	**21**	**0.02**
Cavite	6,722	319	207	**526**	**7.83**	49	82	**131**	**1.95**
Laguna	2,630	444	374	**818**	**31.10**	95	277	**372**	**14.14**
Quezon	30,370	332	197	**529**	**1.74**	12	49	**61**	**0.20**
Rizal	2,286	564	300	**864**	**37.80**	60	124	**184**	**8.05**
Antipolo City	*	*	*	**0**	**0.00**	*	*	**0**	**0.00**
Bacoor City	41	*	*	**0**	**0.00**	*	*	**0**	**0.00**
Batangas City	*	*	*	**0**	**0.00**	*	*	**0**	**0.00**

Table 1.F.3.1 -Cardiovascular Disease and Diabetes Mellitus Prevention and Control

Number and proportion of newly-identified 20 yrs old and above adults with Hypertension and Type 2 Diabetes
Philippines, Annual 2020

Area	Total No. of clients ≥ 20 y/o risk-assesed[1]	Newly identified Hypertensive Adults				Newly identified with Type 2 Diabetes Mellitus			
		Male	Female	Total	%	Male	Female	Total	%
Biñan City	368	90	131	**221**	**60.05**	17	36	**53**	**14.40**
Cabuyao City	1,291	221	214	**435**	**33.69**	51	62	**113**	**8.75**
Calamba City	13,066	925	400	**1,325**	**10.14**	132	244	**376**	**2.88**
Cavite City	*	*	*	**0**	**0.00**	*	*	**0**	**0.00**
Dasmariñas City	20,209	713	489	**1,202**	**5.95**	100	107	**207**	**1.02**
General Trias City	770	27	41	**68**	**8.83**	11	20	**31**	**4.03**
Imus City	719	*	*	**0**	**0.00**	*	*	**0**	**0.00**
Lipa City	*	*	*	**0**	**0.00**	*	*	**0**	**0.00**
Lucena City	*	*	*	**0**	**0.00**	*	*	**0**	**0.00**
San Pablo City	109	58	37	**95**	**87.16**	26	21	**47**	**43.12**
San Pedro City	*	*	*	**0**	**0.00**	*	*	**0**	**0.00**
Santa Rosa City	220	40	11	**51**	**23.18**	2	2	**4**	**1.82**
Tagaytay City	*	*	*	**0**	**0.00**	*	*	**0**	**0.00**
Tanauan City	932	*	*	**0**	**0.00**	*	*	**0**	**0.00**
Tayabas City	136	0	0	**0**	**0.00**	0	0	**0**	**0.00**
Trece Martires City	2,288	61	11	**72**	**3.15**	0	0	**0**	**0.00**
**Region 4B**	**79,827**	**6,557**	**12,340**	**18,897**	**23.67**	**1,018**	**1,765**	**2,783**	**3.49**
Marinduque	71,862	5,359	10,786	**16,145**	**22.47**	837	1,510	**2,347**	**3.27**
Mindoro Occidental	1,748	99	94	**193**	**11.04**	22	19	**41**	**2.35**
Mindoro Oriental	1,834	799	978	**1,777**	**96.89**	123	178	**301**	**16.41**
Palawan	4,383	300	482	**782**	**17.84**	36	58	**94**	**2.14**
Romblon	*	*	*	**0**	**0.00**	*	*	**0**	**0.00**
Puerto Princesa City	*	*	*	**0**	**0.00**	*	*	**0**	**0.00**
**Region 5**	**371,981**	**13,484**	**19,918**	**33,402**	**8.98**	**2,355**	**2,732**	**5,087**	**1.37**
Albay	78,901	2,337	3,377	**5,714**	**7.24**	285	331	**616**	**0.78**
Camarines Norte	37,608	1,409	2,214	**3,623**	**9.63**	163	237	**400**	**1.06**
Camarines Sur	148,649	2,720	3,787	**6,507**	**4.38**	284	389	**673**	**0.45**
Catanduanes	13,681	402	561	**963**	**7.04**	36	55	**91**	**0.67**
Masbate	13,962	2,474	5,035	**7,509**	**53.78**	834	917	**1,751**	**12.54**
Sorsogon	57,020	2,461	2,545	**5,006**	**8.78**	527	521	**1,048**	**1.84**
Iriga City	12,404	360	588	**948**	**7.64**	38	50	**88**	**0.71**
Legaspi City	5,229	608	977	**1,585**	**30.31**	54	90	**144**	**2.75**
Naga City	4,527	713	834	**1,547**	**34.17**	134	142	**276**	**6.10**
**Region 6**	**351,796**	**26,274**	**34,673**	**60,947**	**17.32**	**7,101**	**8,949**	**16,050**	**4.56**
Aklan	37,419	1,845	2,529	**4,374**	**11.69**	270	639	**909**	**2.43**
Antique	40,329	4,298	6,535	**10,833**	**26.86**	1,222	1,711	**2,933**	**7.27**
Capiz	26,525	1,515	1,940	**3,455**	**13.03**	205	365	**570**	**2.15**
Guimaras	28,708	794	1,211	**2,005**	**6.98**	130	379	**509**	**1.77**
Iloilo	90,241	10,783	12,602	**23,385**	**25.91**	3,403	3,229	**6,632**	**7.35**
Negros Occidental	94,732	2,357	3,098	**5,455**	**5.76**	506	703	**1,209**	**1.28**
Bacolod City	11,435	345	541	**886**	**7.75**	53	94	**147**	**1.29**
Iloilo City	22,407	4,337	6,217	**10,554**	**47.10**	1,312	1,829	**3,141**	**14.02**

Table 1.F.3.1 -Cardiovascular Disease and Diabetes Mellitus Prevention and Control

Number and proportion of newly-identified 20 yrs old and above adults with Hypertension and Type 2 Diabetes
Philippines, Annual 2020

Area	Total No. of clients ≥ 20 y/o risk-assesed[1]	Newly identified Hypertensive Adults				Newly identified with Type 2 Diabetes Mellitus			
		Male	Female	Total	%	Male	Female	Total	%
**Region 7**	**50,563**	**5,138**	**6,225**	**11,363**	**22.47**	**907**	**1,155**	**2,062**	**4.08**
Bohol	9,174	1,199	1,078	**2,277**	**24.82**	49	77	**126**	**1.37**
Cebu	21,664	1,789	2,424	**4,213**	**19.45**	233	272	**505**	**2.33**
Negros Oriental	7,287	395	523	**918**	**12.60**	179	240	**419**	**5.75**
Siquijor	3,911	216	296	**512**	**13.09**	64	88	**152**	**3.89**
Cebu City	2,844	770	1,057	**1,827**	**64.24**	183	250	**433**	**15.23**
Lapu-Lapu City	5,683	769	847	**1,616**	**28.44**	199	228	**427**	**7.51**
Mandaue City	*	*	*	**0**	**0.00**	*	*	**0**	**0.00**
**Region 8**	**124,689**	**4,224**	**5,814**	**10,038**	**8.05**	**771**	**1,032**	**1,803**	**1.45**
Biliran	6,613	287	345	**632**	**9.56**	76	72	**148**	**2.24**
Eastern Samar	10,565	553	688	**1,241**	**11.75**	102	179	**281**	**2.66**
Northern Leyte	*	*	*	**0**	**0.00**	*	*	**0**	**0.00**
Northern Samar	15,992	435	586	**1,021**	**6.38**	69	97	**166**	**1.04**
Southern Leyte	46,182	845	1,006	**1,851**	**4.01**	159	236	**395**	**0.86**
Western Samar	34,437	654	1,144	**1,798**	**5.22**	93	118	**211**	**0.61**
Calbayog City	3,345	677	937	**1,614**	**48.25**	210	277	**487**	**14.56**
Maasin City	2,223	153	153	**306**	**13.77**	19	9	**28**	**1.26**
Ormoc City	3,753	484	766	**1,250**	**33.31**	29	31	**60**	**1.60**
Tacloban City	1,579	136	189	**325**	**20.58**	14	13	**27**	**1.71**
**Region 9**	**54,795**	**1,523**	**1,380**	**2,903**	**5.30**	**273**	**383**	**656**	**1.20**
Zamboanga del Norte	39,881	*	*	**0**	**0.00**	*	*	**0**	**0.00**
Zamboanga del Sur	*	*	*	**0**	**0.00**	*	*	**0**	**0.00**
Zamboanga Sibugay	12,316	1,405	1,288	**2,693**	**21.87**	158	174	**332**	**2.70**
Dapitan City	772	*	*	**0**	**0.00**	*	*	**0**	**0.00**
Dipolog City	1,378	*	*	**0**	**0.00**	*	*	**0**	**0.00**
Isabela City	448	*	*	**0**	**0.00**	*	*	**0**	**0.00**
Pagadian City	*	118	92	**210**	**0.00**	115	209	**324**	**0.00**
Zamboanga City	*	*	*	**0**	**0.00**	*	*	**0**	**0.00**
**Region 10**	**280,568**	**8,558**	**6,091**	**14,649**	**5.22**	**1,488**	**1,508**	**2,996**	**1.07**
Bukidnon	125,926	1,892	1,501	**3,393**	**2.69**	103	210	**313**	**0.25**
Camiguin	2,026	110	100	**210**	**10.37**	23	30	**53**	**2.62**
Lanao del Norte	16,395	2,814	1,679	**4,493**	**27.40**	313	346	**659**	**4.02**
Misamis Occidental	2,203	159	125	**284**	**12.89**	37	54	**91**	**4.13**
Misamis Oriental	28,431	1,798	681	**2,479**	**8.72**	751	575	**1,326**	**4.66**
Cagayan de Oro City	81,460	625	708	**1,333**	**1.64**	87	96	**183**	**0.22**
El Salvador City	85	44	40	**84**	**98.82**	4	4	**8**	**9.41**
Gingoog City	*	0	0	**0**	**0.00**	0	0	**0**	**0.00**
Iligan City	4,129	629	651	**1,280**	**31.00**	83	108	**191**	**4.63**
Malaybalay City	12,438	46	64	**110**	**0.88**	1	5	**6**	**0.05**

Table 1.F.3.1 -Cardiovascular Disease and Diabetes Mellitus Prevention and Control

Number and proportion of newly-identified 20 yrs old and above adults with Hypertension and Type 2 Diabetes
Philippines, Annual 2020

Area	Total No. of clients ≥ 20 y/o risk-assesed[1]	Newly identified Hypertensive Adults				Newly identified with Type 2 Diabetes Mellitus			
		Male	Female	Total	%	Male	Female	Total	%
Oroquieta City	*	*	*	**0**	**0.00**	*	*	**0**	**0.00**
Ozamis City	0	146	185	**331**	**0.00**	15	21	**36**	**0.00**
Tangub City	114	41	45	**86**	**75.44**	9	8	**17**	**14.91**
Valencia City	7,361	254	312	**566**	**7.69**	62	51	**113**	**1.54**
**Region 11**	**407,108**	**19,315**	**24,850**	**44,165**	**10.85**	**4,129**	**5,071**	**9,200**	**2.26**
Compostela Valley	135,300	5,995	7,197	**13,192**	**9.75**	801	1,163	**1,964**	**1.45**
Davao del Norte	113,982	2,941	3,549	**6,490**	**5.69**	792	870	**1,662**	**1.46**
Davao Oriental	61,279	3,789	5,537	**9,326**	**15.22**	692	1,053	**1,745**	**2.85**
Davao del Sur	70,423	3,901	5,344	**9,245**	**13.13**	1,049	1,055	**2,104**	**2.99**
Davao Occidental	13,075	378	443	**821**	**6.28**	79	116	**195**	**1.49**
Davao City	13,049	2,311	2,780	**5,091**	**39.01**	716	814	**1,530**	**11.73**
**Region 12**	**162,418**	**8,861**	**12,269**	**21,130**	**13.01**	**2,054**	**2,398**	**4,452**	**2.74**
North Cotabato	52,989	3,176	4,212	**7,388**	**13.94**	665	915	**1,580**	**2.98**
Sarangani	28,078	1,838	2,402	**4,240**	**15.10**	319	441	**760**	**2.71**
South Cotabato	11,468	1,121	1,245	**2,366**	**20.63**	153	168	**321**	**2.80**
Sultan Kudarat	60,800	1,927	2,375	**4,302**	**7.08**	268	395	**663**	**1.09**
Cotabato City	6,214	330	484	**814**	**13.10**	58	88	**146**	**2.35**
Gen. Santos City	2,869	469	1,551	**2,020**	**70.41**	591	391	**982**	**34.23**
**BARMM**	**30,695**	**2,108**	**2,806**	**4,914**	**16.01**	**444**	**501**	**945**	**3.08**
Basilan	0	45	68	**113**	**0.00**	10	8	**18**	**0.00**
Lanao del Sur	8,494	922	1,036	**1,958**	**23.05**	200	220	**420**	**4.94**
Maguindanao	11,665	585	698	**1,283**	**11.00**	106	133	**239**	**2.05**
Sulu	*	*	*	**0**	**0.00**	*	*	**0**	**0.00**
Tawi-Tawi	*	*	*	**0**	**0.00**	*	*	**0**	**0.00**
Lamitan City	*	*	*	**0**	**0.00**	*	*	**0**	**0.00**
Marawi City	10,536	556	1,004	**1,560**	**14.81**	128	140	**268**	**2.54**
**CARAGA**	**91,202**	**4,130**	**3,987**	**8,117**	**8.90**	**841**	**1,361**	**2,202**	**2.41**
Agusan del Norte	13,374	1,581	1,037	**2,618**	**19.58**	416	615	**1,031**	**7.71**
Agusan del Sur	19,955	374	583	**957**	**4.80**	23	71	**94**	**0.47**
Surigao del Norte	11,494	546	502	**1,048**	**9.12**	123	182	**305**	**2.65**
Surigao del Sur	18,954	929	1,146	**2,075**	**10.95**	150	257	**407**	**2.15**
Province of Dinagat	2,147	155	155	**310**	**14.44**	20	47	**67**	**3.12**
Bislig City	3,964	26	26	**52**	**1.31**	33	77	**110**	**2.77**
Butuan City	19,188	309	330	**639**	**3.33**	35	59	**94**	**0.49**
Surigao City	2,126	210	208	**418**	**19.66**	41	53	**94**	**4.42**

[1] *Total adults 20 yrs old and above who were risk-assessed using the PhilPEN protocol*

Note: Put asterisk (*) for No Report and Zero (0) for No Case

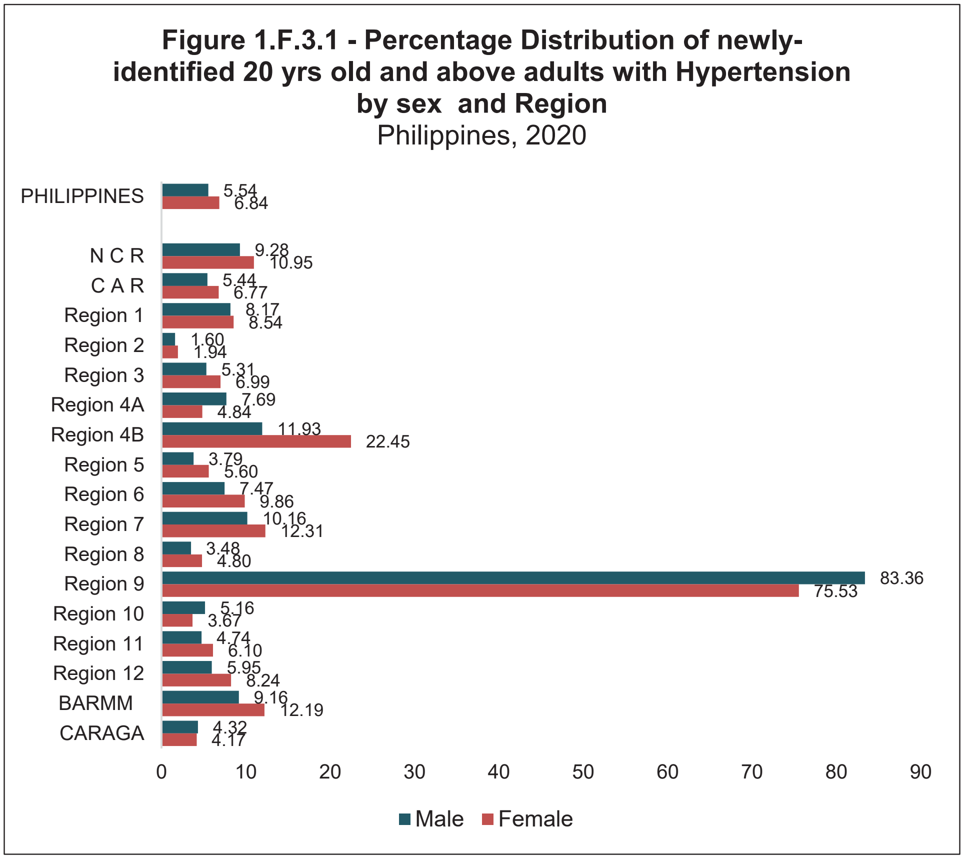
Figure 1.F.3.1 - Percentage Distribution of newly-identified 20 yrs old and above adults with Hypertension by sex and Region
Philippines, 2020
PHILIPPINES 5.54 6.84
N C R 9.28 10.95
C A R 5.44 6.77
Region 1 8.17 8.54
Region 2 1.60 1.94
Region 3 5.31 6.99
Region 4A 7.69 4.84
Region 4B 11.93 22.45
Region 5 3.79 5.60
Region 6 7.47 9.86
Region 7 10.16 12.31
Region 8 3.48 4.80
Region 9 83.36 75.53
Region 10 5.16 3.67
Region 11 4.74 6.10
Region 12 5.95 8.24
BARMM 9.16 12.19
CARAGA 4.32 4.17
0 10 20 30 40 50 60 70 80 90
Male Female

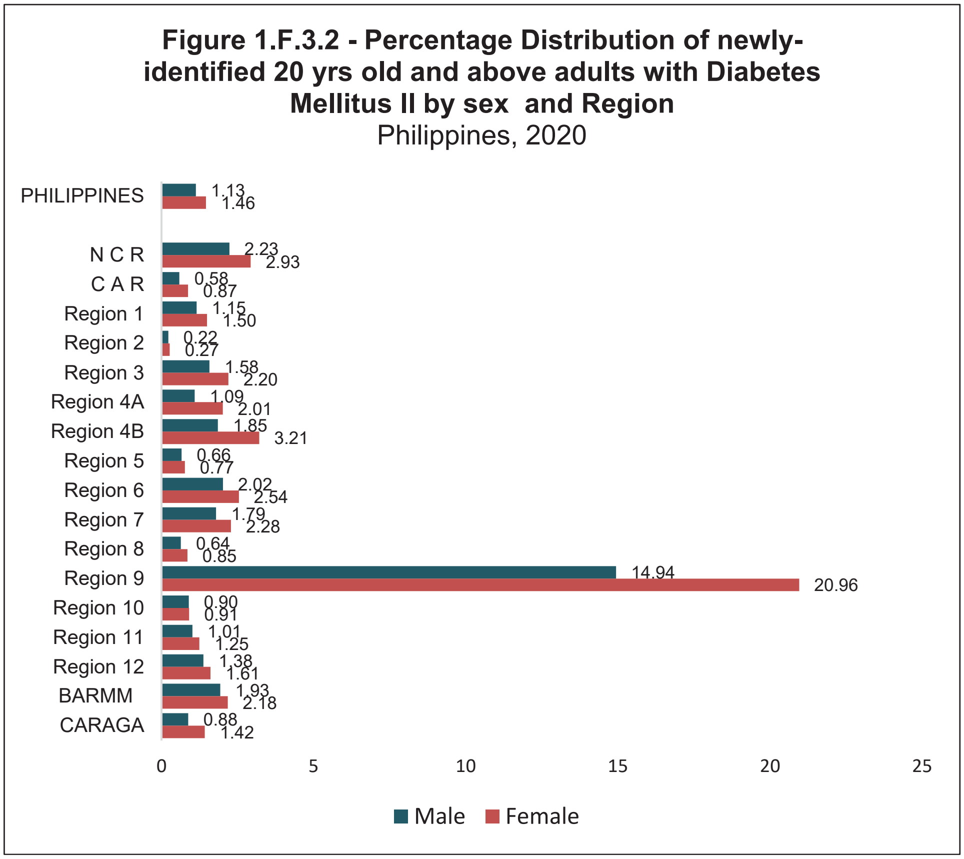
Figure 1.F.3.2 - Percentage Distribution of newly-identified 20 yrs old and above adults with Diabetes Mellitus II by sex and Region
Philippines, 2020
PHILIPPINES 1.13 1.46
N C R 2.23 2.93
C A R 0.58 0.87
Region 1 1.15 1.50
Region 2 0.22 0.27
Region 3 1.58 2.20
Region 4A 1.09 2.01
Region 4B 1.85 3.21
Region 5 0.66 0.77
Region 6 2.02 2.54
Region 7 1.79 2.28
Region 8 0.64 0.85
Region 9 14.94 20.96
Region 10 0.90 0.91
Region 11 1.01 1.25
Region 12 1.38 1.61
BARMM 1.93 2.18
CARAGA 0.88 1.42
0 5 10 15 20 25
Male Female

Figure 1.F.3.3 - Percentage Distribution of newly-identified 20 yrs old and above adults with Hypertension by and Region
Philippines, 2020

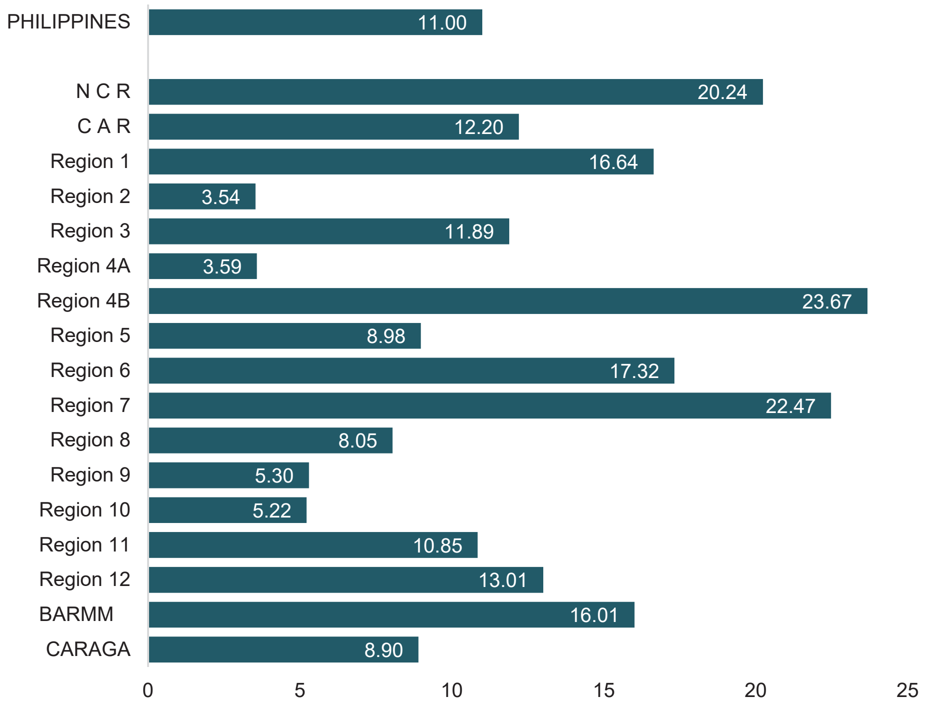

Figure 1.F.3.4 - Percentage Distribution of newly-identified 20 yrs old and above adults with Diabetes Mellitus II by and Region
Philippines, 2020

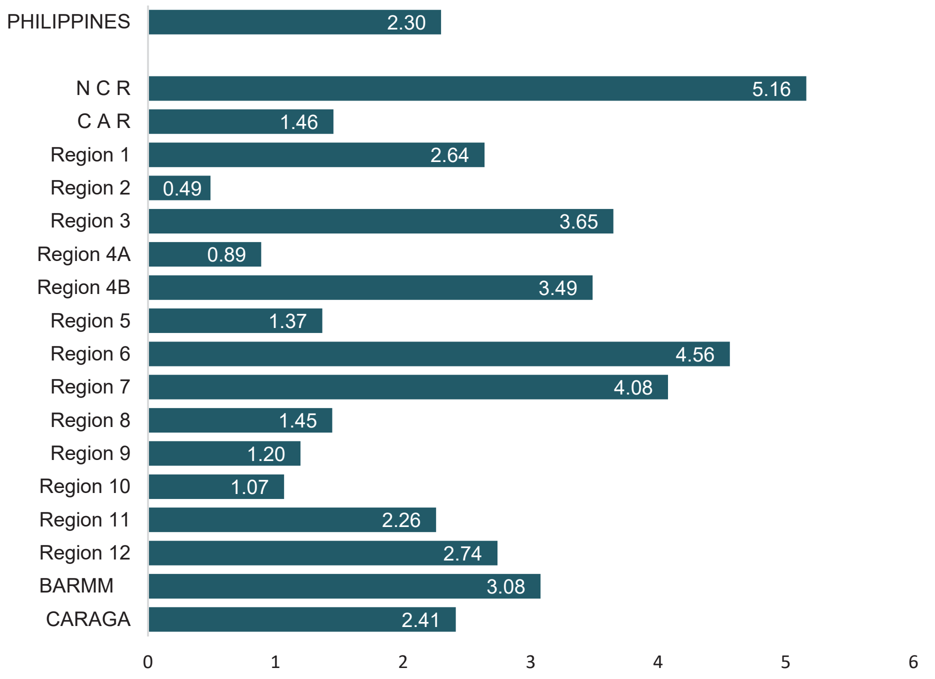

Table 1.F.4.1 - Blindness Prevention Program

Number and proportion of senior citizens 60 yrs old and above screened visual acuity and diagnosed with eye diseases
Philippines, Annual 2020

Area	Eligible Pop.(60 y/o and above)	Screened for Visual Acuity				Diagnosed with eye diseases			
		Male	Female	Total	%	Male	Female	Total	%
**PHILIPPINES**	**9,468,436**	**42,962**	**51,690**	**94,652**	**1.00**	**6,342**	**9,207**	**15,549**	**16.43**
**N C R**	**1,209,298**	**1,023**	**1,543**	**2,566**	**0.21**	**170**	**273**	**443**	**17.26**
Malabon	34,328	0	0	**0**	**0.00**	0	0	**0**	**0.00**
Navotas	23,426	15	22	**37**	**0.16**	4	3	**7**	**18.92**
Valenzuela City	58,259	38	46	**84**	**0.14**	6	6	**12**	**14.29**
Caloocan City	148,749	531	725	**1,256**	**0.84**	49	56	**105**	**8.36**
Marikina City	42,328	0	0	**0**	**0.00**	0	0	**0**	**0.00**
Pasig City	70,931	57	105	**162**	**0.23**	0	24	**24**	**14.81**
Pateros	5,996	22	69	**91**	**1.52**	9	13	**22**	**24.18**
Taguig	75,587	0	2	**2**	**0.00**	0	2	**2**	**100.00**
Quezon City	275,729	238	351	**589**	**0.21**	29	32	**61**	**10.36**
Makati City	54,715	31	59	**90**	**0.16**	31	59	**90**	**100.00**
Mandaluyong City	36,277	26	49	**75**	**0.21**	14	34	**48**	**64.00**
San Juan	11,474	0	0	**0**	**0.00**	0	0	**0**	**0.00**
Manila City	167,174	0	0	**0**	**0.00**	0	0	**0**	**0.00**
Las Piñas City	55,306	14	16	**30**	**0.05**	12	13	**25**	**83.33**
Muntinlupa City	47,379	13	29	**42**	**0.09**	11	25	**36**	**85.71**
Parañaque City	62,525	4	10	**14**	**0.02**	0	0	**0**	**0.00**
Pasay City	39,115	34	60	**94**	**0.24**	5	6	**11**	**11.70**
**C A R**	**151,658**	**819**	**1,272**	**2,091**	**1.38**	**212**	**382**	**594**	**28.41**
Abra	26,748	39	54	**93**	**0.35**	6	7	**13**	**13.98**
Apayao	10,142	209	365	**574**	**5.66**	62	116	**178**	**31.01**
Benguet	37,810	178	280	**458**	**1.21**	56	89	**145**	**31.66**
Ifugao	15,940	127	124	**251**	**1.57**	28	48	**76**	**30.28**
Kalinga	17,624	42	78	**120**	**0.68**	24	40	**64**	**53.33**
Mt. Province	14,130	86	180	**266**	**1.88**	24	73	**97**	**36.47**
Baguio City	29,264	138	191	**329**	**1.12**	12	9	**21**	**6.38**
**Region 1**	**562,673**	**1,902**	**2,825**	**4,727**	**0.84**	**432**	**519**	**951**	**20.12**
Ilocos Norte	61,764	8	6	**14**	**0.02**	0	1	**1**	**7.14**
Ilocos Sur	72,485	878	1,508	**2,386**	**3.29**	28	41	**69**	**2.89**
La Union	79,423	358	477	**835**	**1.05**	12	33	**45**	**5.39**
Pangasinan	245,538	382	494	**876**	**0.36**	296	358	**654**	**74.66**
Alaminos City	9,278	186	258	**444**	**4.79**	44	29	**73**	**16.44**
Candon City	7,639	45	25	**70**	**0.92**	9	12	**21**	**30.00**
Dagupan City	17,712	4	3	**7**	**0.04**	4	3	**7**	**100.00**
Laoag City	14,238	0	0	**0**	**0.00**	0	0	**0**	**0.00**
San Carlos City	19,501	7	9	**16**	**0.08**	7	8	**15**	**93.75**
San Fernando City	14,555	34	45	**79**	**0.54**	32	34	**66**	**83.54**
Urdaneta City	13,748	0	0	**0**	**0.00**	0	0	**0**	**0.00**
Vigan City	6,792	0	0	**0**	**0.00**	0	0	**0**	**0.00**
**Region 2**	**352,277**	**4,731**	**6,370**	**11,101**	**3.15**	**521**	**798**	**1,319**	**11.88**
Batanes	2,063	0	0	**0**	**0.00**	0	0	**0**	**0.00**
Cagayan	100,481	1,139	1,337	**2,476**	**2.46**	81	150	**231**	**9.33**
Isabela	130,881	1,234	1,606	**2,840**	**2.17**	147	207	**354**	**12.46**

Table 1.F.4.1 - Blindness Prevention Program

Number and proportion of senior citizens 60 yrs old and above screened visual acuity and diagnosed with eye diseases
Philippines, Annual 2020

Area	Eligible Pop.(60 y/o and above)	Screened for Visual Acuity				Diagnosed with eye diseases			
		Male	Female	Total	%	Male	Female	Total	%
Nueva Vizcaya	44,670	294	449	**743**	**1.66**	43	82	**125**	**16.82**
Quirino	16,784	124	185	**309**	**1.84**	43	43	**86**	**27.83**
Cauayan City	12,856	0	0	**0**	**0.00**	0	0	**0**	**0.00**
Ilagan City	14,450	18	22	**40**	**0.28**	8	9	**17**	**42.50**
Santiago City	13,383	1,451	2,079	**3,530**	**26.38**	110	186	**296**	**8.39**
Tuguegarao City	16,709	471	692	**1,163**	**6.96**	89	121	**210**	**18.06**
**Region 3**	**1,142,449**	**481**	**2,201**	**2,682**	**0.23**	**165**	**786**	**951**	**35.46**
Aurora	18,836	1	1	**2**	**0.01**	0	1	**1**	**50.00**
Bataan	66,939	59	65	**124**	**0.19**	17	22	**39**	**31.45**
Bulacan	225,233	70	58	**128**	**0.06**	43	30	**73**	**57.03**
Nueva Ecija	156,175	15	20	**35**	**0.02**	15	10	**25**	**71.43**
Pampanga	166,626	19	33	**52**	**0.03**	2	1	**3**	**5.77**
Tarlac	106,482	128	1,825	**1,953**	**1.83**	2	592	**594**	**30.41**
Zambales	60,063	58	68	**126**	**0.21**	8	8	**16**	**12.70**
Angeles City	41,802	12	28	**40**	**0.10**	5	8	**13**	**32.50**
Balanga City	9,675	4	9	**13**	**0.13**	0	0	**0**	**0.00**
Cabanatuan City	31,961	1	1	**2**	**0.01**	1	1	**2**	**100.00**
City of San Fernando	31,143	12	14	**26**	**0.08**	9	17	**26**	**100.00**
Gapan City	11,665	5	7	**12**	**0.10**	1	2	**3**	**25.00**
Mabalacat City	25,469	1	7	**8**	**0.03**	1	7	**8**	**100.00**
Malolos City	25,157	8	10	**18**	**0.07**	0	2	**2**	**11.11**
Meycauayan	20,867	33	8	**41**	**0.20**	12	5	**17**	**41.46**
Olongapo	23,694	12	14	**26**	**0.11**	0	2	**2**	**7.69**
Palayan City	4,339	9	14	**23**	**0.53**	0	1	**1**	**4.35**
San Jose City	14,780	0	0	**0**	**0.00**	0	0	**0**	**0.00**
San Jose del Monte City	57,294	10	13	**23**	**0.04**	5	7	**12**	**52.17**
Science City of Munoz	8,620	24	6	**30**	**0.35**	3	1	**4**	**13.33**
Tarlac City	35,629	0	0	**0**	**0.00**	41	69	**110**	**0.00**
**Region 4A**	**1,352,670**	**2,140**	**2,128**	**4,268**	**0.32**	**0**	**0**	**0**	**0.00**
Batangas	191,674	73	117	**190**	**0.10**	*	*	**0**	**0.00**
Cavite	128,478	58	147	**205**	**0.16**	*	*	**0**	**0.00**
Laguna	91,249	259	66	**325**	**0.36**	*	*	**0**	**0.00**
Quezon	163,869	666	680	**1,346**	**0.82**	*	*	**0**	**0.00**
Rizal	185,089	32	71	**103**	**0.06**	*	*	**0**	**0.00**
Antipolo City	68,173	0	0	**0**	**0.00**	*	*	**0**	**0.00**
Bacoor City	56,285	0	0	**0**	**0.00**	*	*	**0**	**0.00**
Batangas City	34,017	0	0	**0**	**0.00**	*	*	**0**	**0.00**
Biñan City	30,593	21	17	**38**	**0.12**	*	*	**0**	**0.00**
Cabuyao City	28,366	91	123	**214**	**0.75**	*	*	**0**	**0.00**
Calamba City	41,749	148	196	**344**	**0.82**	*	*	**0**	**0.00**
Cavite City	9,633	535	634	**1,169**	**12.14**	*	*	**0**	**0.00**
Dasmariñas City	61,765	16	24	**40**	**0.06**	*	*	**0**	**0.00**
General Trias City	29,452	29	28	**57**	**0.19**	*	*	**0**	**0.00**
Imus City	37,846	0	0	**0**	**0.00**	*	*	**0**	**0.00**
Lipa City	34,274	0	0	**0**	**0.00**	*	*	**0**	**0.00**
Lucena City	24,837	0	0	**0**	**0.00**	*	*	**0**	**0.00**
San Pablo City	24,443	3	3	**6**	**0.02**	*	*	**0**	**0.00**
San Pedro City	29,929	194	7	**201**	**0.67**	*	*	**0**	**0.00**
Santa Rosa City	32,498	0	0	**0**	**0.00**	*	*	**0**	**0.00**
Tagaytay City	6,672	5	3	**8**	**0.12**	*	*	**0**	**0.00**
Tanauan City	17,877	10	12	**22**	**0.12**	*	*	**0**	**0.00**

Table 1.F.4.1 - Blindness Prevention Program

Number and proportion of senior citizens 60 yrs old and above screened visual acuity and diagnosed with eye diseases
Philippines, Annual 2020

Area	Eligible Pop.(60 y/o and above)	Screened for Visual Acuity				Diagnosed with eye diseases			
		Male	Female	Total	%	Male	Female	Total	%
Tayabas City	9,307	0	0	**0**	**0.00**	*	*	**0**	**0.00**
Trece Martires City	14,595	0	0	**0**	**0.00**	*	*	**0**	**0.00**
**Region 4B**	**259,762**	**186**	**160**	**346**	**0.13**	**80**	**68**	**148**	**42.77**
Marinduque	26,515	0	0	**0**	**0.00**	0	0	**0**	**0.00**
Mindoro Occidental	39,181	0	3	**3**	**0.01**	0	3	**3**	**100.00**
Mindoro Oriental	75,874	172	124	**296**	**0.39**	80	65	**145**	**48.99**
Palawan	67,539	14	33	**47**	**0.07**	0	0	**0**	**0.00**
Romblon	30,365	0	0	**0**	**0.00**	0	0	**0**	**0.00**
									**0.00**
Puerto Princesa City	20,288	0	0	**0**	**0.00**	0	0	**0**	**0.00**
**Region 5**	**523,737**	**1,658**	**2,551**	**4,209**	**0.80**	**391**	**766**	**1,157**	**27.49**
Albay	110,897	417	548	**965**	**0.87**	80	167	**247**	**25.60**
Camarines Norte	52,028	285	331	**616**	**1.18**	58	92	**150**	**24.35**
Camarines Sur	144,256	272	563	**835**	**0.58**	43	119	**162**	**19.40**
Catanduanes	24,480	148	262	**410**	**1.67**	46	78	**124**	**30.24**
Masbate	72,716	136	180	**316**	**0.43**	28	123	**151**	**47.78**
Sorsogon	72,860	233	441	**674**	**0.93**	43	61	**104**	**15.43**
Iriga City	9,802	17	32	**49**	**0.50**	6	6	**12**	**24.49**
Legaspi City	19,504	51	64	**115**	**0.59**	11	10	**21**	**18.26**
Naga City	17,194	99	130	**229**	**1.33**	76	110	**186**	**81.22**
**Region 6**	**821,493**	**2,599**	**3,374**	**5,973**	**0.73**	**501**	**724**	**1,225**	**20.51**
Aklan	62,594	39	49	**88**	**0.14**	10	21	**31**	**35.23**
Antique	60,739	119	132	**251**	**0.41**	51	74	**125**	**49.80**
Capiz	87,668	403	565	**968**	**1.10**	128	197	**325**	**33.57**
Guimaras	19,545	2	8	**10**	**0.05**	0	0	**0**	**0.00**
Iloilo	225,954	743	1,027	**1,770**	**0.78**	29	44	**73**	**4.12**
Negros Occidental	255,291	1,283	1,579	**2,862**	**1.12**	281	387	**668**	**23.34**
Bacolod City	57,435	4	6	**10**	**0.02**	2	0	**2**	**20.00**
Iloilo City	52,267	6	8	**14**	**0.03**	0	1	**1**	**7.14**
**Region 7**	**746,101**	**247**	**288**	**535**	**0.07**	**108**	**135**	**243**	**45.42**
Bohol	147,672	0	0	**0**	**0.00**	0	0	**0**	**0.00**
Cebu	280,821	65	70	**135**	**0.05**	56	76	**132**	**97.78**
Negros Oriental	142,750	44	51	**95**	**0.07**	4	8	**12**	**12.63**
Siquijor	13,051	17	30	**47**	**0.36**	4	3	**7**	**14.89**
Cebu City	88,161	15	9	**24**	**0.03**	10	7	**17**	**70.83**
Lapu-Lapu City	38,995	99	115	**214**	**0.55**	27	28	**55**	**25.70**
Mandaue City	34,651	7	13	**20**	**0.06**	7	13	**20**	**100.00**
**Region 8**	**436,426**	**465**	**595**	**1,060**	**0.24**	**140**	**262**	**402**	**37.92**
Biliran	17,330	156	246	**402**	**2.32**	0	2	**2**	**0.50**
Eastern Samar	47,288	86	147	**233**	**0.49**	1	1	**2**	**0.86**
Northern Leyte	154,643	4	5	**9**	**0.01**	0	0	**0**	**0.00**
Northern Samar	51,523	153	57	**210**	**0.41**	80	51	**131**	**62.38**
Southern Leyte	40,229	24	24	**48**	**0.12**	11	24	**35**	**72.92**
Western Samar	52,248	42	116	**158**	**0.30**	46	64	**110**	**69.62**

Table 1.F.4.1 - Blindness Prevention Program

Number and proportion of senior citizens 60 yrs old and above screened visual acuity and diagnosed with eye diseases
Philippines, Annual 2020

Area	Eligible Pop.(60 y/o and above)	Screened for Visual Acuity				Diagnosed with eye diseases			
		Male	Female	Total	%	Male	Female	Total	%
Calbayog City	16,096	0	0	**0**	**0.00**	0	0	**0**	**0.00**
Maasin City	10,231	0	0	**0**	**0.00**	1	40	**41**	**0.00**
Ormoc City	22,034	0	0	**0**	**0.00**	1	80	**81**	**0.00**
Tacloban City	24,804	0	0	**0**	**0.00**	0	0	**0**	**0.00**
**Region 9**	**302,395**	**169**	**189**	**358**	**0.12**	**8**	**12**	**20**	**5.59**
Zamboanga del Norte	72,258	*	*	**0**	**0.00**	*	*	**0**	**0.00**
Zamboanga del Sur	66,654	*	*	**0**	**0.00**	*	*	**0**	**0.00**
Zamboanga Sibugay	47,984	54	66	**120**	**0.25**	8	12	**20**	**16.67**
Dapitan City	7,462	0	0	**0**	**0.00**	*	*	**0**	**0.00**
Dipolog City	11,834	115	123	**238**	**2.01**	*	*	**0**	**0.00**
Isabela City	9,073	*	*	**0**	**0.00**	*	*	**0**	**0.00**
Pagadian City	16,353	*	*	**0**	**0.00**	*	*	**0**	**0.00**
Zamboanga City	70,777	*	*	**0**	**0.00**	*	*	**0**	**0.00**
**Region 10**	**416,240**	**677**	**713**	**1,390**	**0.33**	**318**	**416**	**734**	**52.81**
Bukidnon	83,276	96	118	**214**	**0.26**	57	75	**132**	**61.68**
Camiguin	10,186	48	47	**95**	**0.93**	27	37	**64**	**67.37**
Lanao del Norte	51,948	61	26	**87**	**0.17**	29	51	**80**	**91.95**
Misamis Occidental	36,038	70	66	**136**	**0.38**	47	54	**101**	**74.26**
Misamis Oriental	67,884	63	68	**131**	**0.19**	29	54	**83**	**63.36**
Cagayan de Oro City	64,318	209	212	**421**	**0.65**	39	26	**65**	**15.44**
El Salvador City	4,779	7	7	**14**	**0.29**	0	1	**1**	**7.14**
Gingoog City	11,864	0	0	**0**	**0.00**	0	0	**0**	**0.00**
Iligan City	26,310	14	21	**35**	**0.13**	12	19	**31**	**88.57**
Malaybalay City	13,886	28	34	**62**	**0.45**	28	30	**58**	**93.55**
Oroquieta City	7,811	0	0	**0**	**0.00**	0	0	**0**	**0.00**
Ozamis City	15,647	69	90	**159**	**1.02**	45	57	**102**	**64.15**
Tangub City	6,950	3	7	**10**	**0.14**	3	6	**9**	**90.00**
Valencia City	15,343	9	17	**26**	**0.17**	2	6	**8**	**30.77**
**Region 11**	**438,550**	**23,310**	**24,232**	**47,542**	**10.84**	**2,704**	**3,087**	**5,791**	**12.18**
Compostela Valley	60,559	4,431	4,995	**9,426**	**15.56**	162	169	**331**	**3.51**
Davao del Norte	89,141	2,514	2,648	**5,162**	**5.79**	413	548	**961**	**18.62**
Davao Oriental	50,574	7,328	7,095	**14,423**	**28.52**	499	619	**1,118**	**7.75**
Davao del Sur	59,247	4,217	3,848	**8,065**	**13.61**	539	495	**1,034**	**12.82**
Davao Occidental	26,083	3,870	4,304	**8,174**	**31.34**	715	752	**1,467**	**17.95**
Davao City	152,946	950	1,342	**2,292**	**1.50**	376	504	**880**	**38.39**
**Region 12**	**349,350**	**1,143**	**1,534**	**2,677**	**0.77**	**340**	**555**	**895**	**33.43**
North Cotabato	111,695	111	182	**293**	**0.26**	75	121	**196**	**66.89**
Sarangani	37,850	625	904	**1,529**	**4.04**	96	238	**334**	**21.84**
South Cotabato	74,767	146	122	**268**	**0.36**	71	113	**184**	**68.66**
Sultan Kudarat	58,386	200	247	**447**	**0.77**	91	75	**166**	**37.14**
Cotabato City	18,094	12	21	**33**	**0.18**	7	8	**15**	**45.45**
Gen. Santos City	48,558	49	58	**107**	**0.22**	0	0	**0**	**0.00**
**BARMM**	**172,617**	**219**	**339**	**558**	**0.32**	**153**	**259**	**412**	**73.84**
Basilan	13,674	0	0	**0**	**0.00**	0	0	**0**	**0.00**

Table 1.F.4.1 - Blindness Prevention Program

Number and proportion of senior citizens 60 yrs old and above screened visual acuity and diagnosed with eye diseases
Philippines, Annual 2020

Area	Eligible Pop.(60 y/o and above)	Screened for Visual Acuity				Diagnosed with eye diseases			
		Male	Female	Total	%	Male	Female	Total	%
Lanao del Sur	34,766	1	2	**3**	**0.01**	1	2	**3**	**100.00**
Maguindanao	54,685	29	58	**87**	**0.16**	18	45	**63**	**72.41**
Sulu	38,585	33	37	**70**	**0.18**	0	1	**1**	**1.43**
Tawi-Tawi	18,828	0	0	**0**	**0.00**	0	0	**0**	**0.00**
Lamitan City	3,764	0	0	**0**	**0.00**	0	0	**0**	**0.00**
Marawi City	8,315	156	242	**398**	**4.79**	134	211	**345**	**86.68**
**CARAGA**	**230,740**	**1,193**	**1,376**	**2,569**	**1.11**	**99**	**165**	**264**	**10.28**
Agusan del Norte	32,642	94	100	**194**	**0.59**	13	46	**59**	**30.41**
Agusan del Sur	50,364	73	76	**149**	**0.30**	21	30	**51**	**34.23**
Surigao del Norte	32,952	59	71	**130**	**0.39**	12	16	**28**	**21.54**
Surigao del Sur	46,156	587	665	**1,252**	**2.71**	31	41	**72**	**5.75**
Province of Dinagat	13,493	346	417	**763**	**5.65**	15	21	**36**	**4.72**
Bislig City	8,764	5	7	**12**	**0.14**	5	6	**11**	**91.67**
Butuan City	31,031	8	11	**19**	**0.06**	2	3	**5**	**26.32**
Surigao City	15,338	21	29	**50**	**0.33**	0	2	**2**	**4.00**

Note: Put asterisk (*) for No Report and Zero (0) for No Case

Figure 1.F.4.1 - Percentage Distribution of senior citizens 60 yrs old and above screened visual acuity by Region
Philippines, 2020

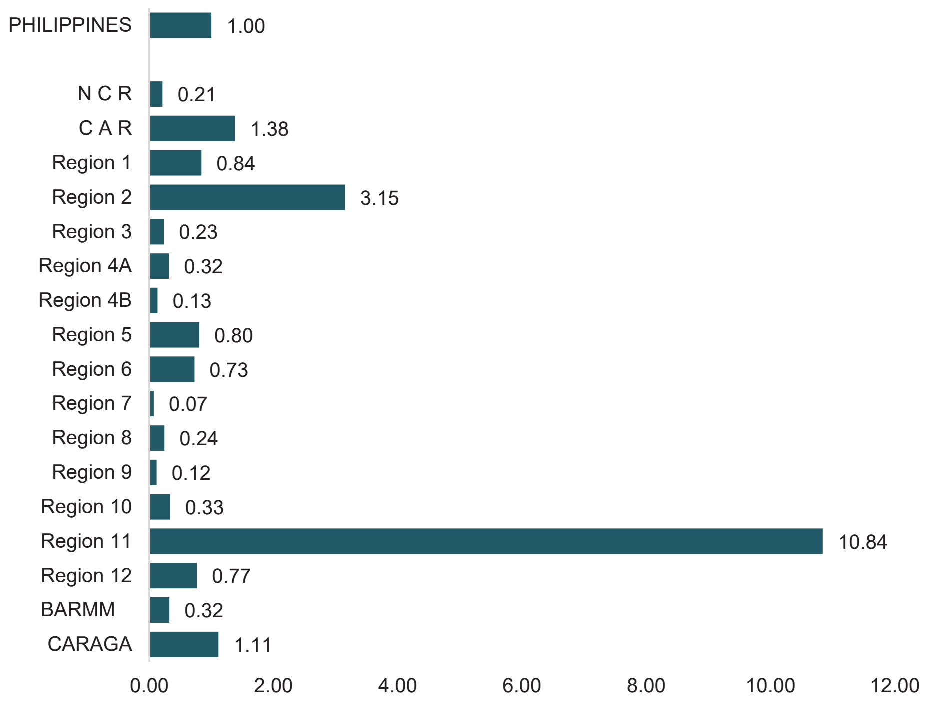

Figure 1.F.4.2 - Percentage Distribution of senior citizens 60 yrs old and above Diagnosed with eye diseases by Region
Philippines, 2020

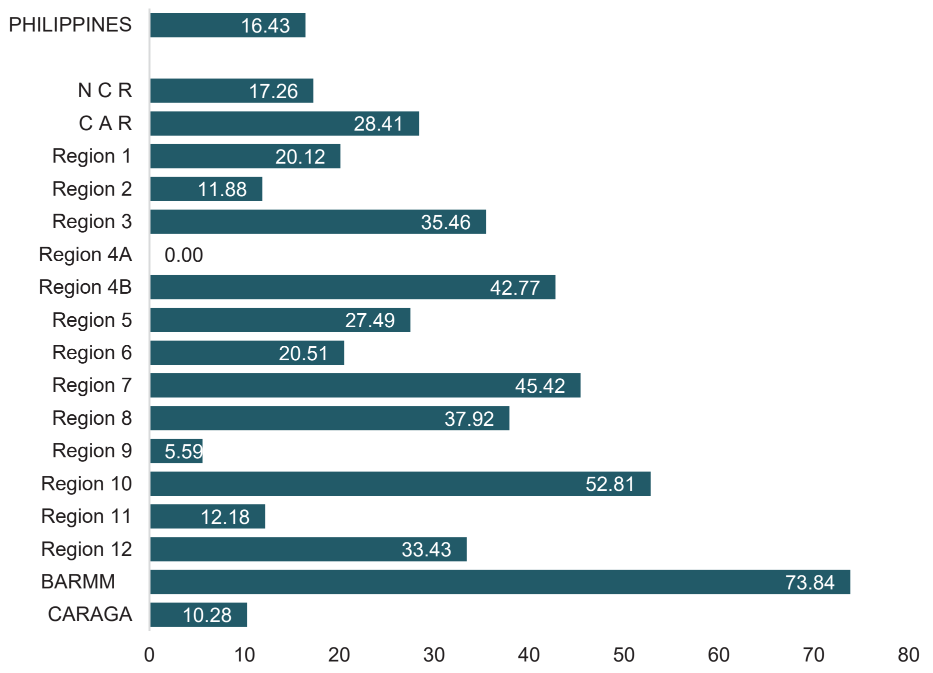

Figure 1.F.4.3 - Percentage Distribution of senior citizens 60 yrs old and above screened visual acuity and diagnosed with eye diseases
Philippines, 2020

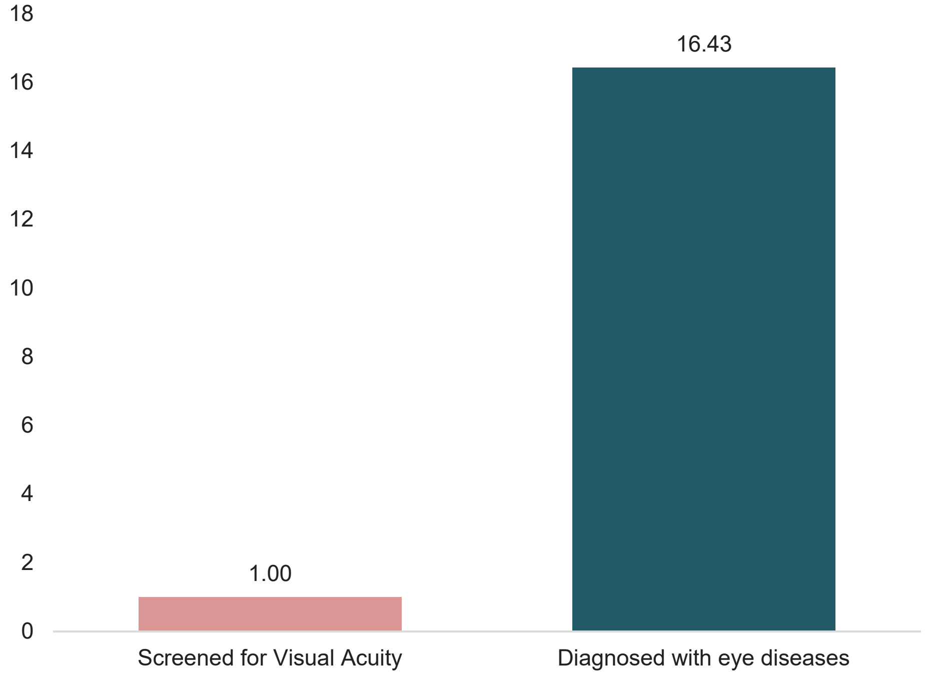

Table 1.F.5.1 - Immunization for Senior Citizen

Number and proportion of senior citizen 60 yrs old and above who received one (1) dose of Pneumococcal Polysaccharide Vaccine (PPV) and one (1) dose of Influenza Vaccine
Philippines, Annual 2020

Area	Eligible Pop. (60 y/o and above)	Received one (1) dose of PPV				Received one (1) dose of Influenza Vaccine			
		Male	Female	Total	%	Male	Female	Total	%
**PHILIPPINES**	9,468,436	87,113	123,221	210,334	2.22	213,398	321,241	534,639	5.65
**N C R**	1,209,298	7,900	10,459	18,359	1.52	28,379	40,848	69,227	5.72
Malabon	34,328	0	0	0	0.00	0	0	0	0.00
Navotas	23,426	152	189	341	1.46	400	576	976	4.17
Valenzuela City	58,259	0	0	0	0.00	0	0	0	0.00
Caloocan City	148,749	311	651	962	0.65	5,081	7,401	12,482	8.39
Marikina City	42,328	662	893	1,555	3.67	1,305	1,892	3,197	7.55
Pasig City	70,931	2,074	2,323	4,397	6.20	1,783	3,040	4,823	6.80
Pateros	5,996	68	130	198	3.30	136	261	397	6.62
Taguig	75,587	3	14	17	0.02	621	591	1,212	1.60
Quezon City	275,729	1,371	2,119	3,490	1.27	8,336	12,951	21,287	7.72
Makati City	54,715	747	1,117	1,864	3.41	1,562	2,372	3,934	7.19
Mandaluyong City	36,277	253	357	610	1.68	483	703	1,186	3.27
San Juan	11,474	424	473	897	7.82	192	310	502	4.38
Manila City	167,174	525	546	1,071	0.64	4,290	4,466	8,756	5.24
Las Piñas City	55,306	1,123	1,429	2,552	4.61	2,423	3,868	6,291	11.37
Muntinlupa City	47,379	19	7	26	0.05	51	28	79	0.17
Parañaque City	62,525	0	13	13	0.02	1,078	1,573	2,651	4.24
Pasay City	39,115	168	198	366	0.94	638	816	1,454	3.72
**C A R**	151,658	3,355	5,725	9,080	5.99	7,369	12,383	19,752	13.02
Abra	26,748	935	1,589	2,524	9.44	1,595	2,661	4,256	15.91
Apayao	10,142	361	687	1,048	10.33	349	599	948	9.35
Benguet	37,810	380	660	1,040	2.75	1,268	2,323	3,591	9.50
Ifugao	15,940	229	331	560	3.51	393	510	903	5.66
Kalinga	17,624	171	294	465	2.64	547	921	1,468	8.33
Mt. Province	14,130	393	944	1,337	9.46	737	1,924	2,661	18.83
Baguio City	29,264	886	1,220	2,106	7.20	2,480	3,445	5,925	20.25
**Region 1**	562,673	4,212	5,889	10,101	1.80	9,716	15,173	24,889	4.42
Ilocos Norte	61,764	438	725	1,163	1.88	1,664	2,746	4,410	7.14
Ilocos Sur	72,485	827	1,161	1,988	2.74	1,043	1,777	2,820	3.89
La Union	79,423	771	1,047	1,818	2.29	1,019	1,535	2,554	3.22
Pangasinan	245,538	1,744	2,367	4,111	1.67	4,604	7,059	11,663	4.75
Alaminos City	9,278	50	50	100	1.08	0	0	0	0.00
Candon City	7,639	0	0	0	0.00	0	0	0	0.00
Dagupan City	17,712	228	362	590	3.33	365	647	1,012	5.71
Laoag City	14,238	0	0	0	0.00	0	0	0	0.00
San Carlos City	19,501	43	81	124	0.64	487	668	1,155	5.92
San Fernando City	14,555	111	96	207	1.42	37	45	82	0.56
Urdaneta City	13,748	0	0	0	0.00	497	696	1,193	8.68

Table 1.F.5.1 - Immunization for Senior Citizen

Number and proportion of senior citizen 60 yrs old and above who received one (1) dose of Pneumococcal Polysaccharide Vaccine (PPV) and one (1) dose of Influenza Vaccine
Philippines, Annual 2020

Area	Eligible Pop. (60 y/o and above)	Received one (1) dose of PPV				Received one (1) dose of Influenza Vaccine			
		Male	Female	Total	%	Male	Female	Total	%
Vigan City	6,792	0	0	0	0.00	0	0	0	0.00
**Region 2**	352,277	7,068	10,415	17,483	4.96	17,077	26,619	43,696	12.40
Batanes	2,063	35	106	141	6.83	134	274	408	19.78
Cagayan	100,481	1,603	2,579	4,182	4.16	4,454	7,033	11,487	11.43
Isabela	130,881	2,628	3,669	6,297	4.81	6,008	9,597	15,605	11.92
Nueva Vizcaya	44,670	735	1,100	1,835	4.11	1,275	2,366	3,641	8.15
Quirino	16,784	360	499	859	5.12	435	616	1,051	6.26
Cauayan City	12,856	516	674	1,190	9.26	1,552	1,531	3,083	23.98
Ilagan City	14,450	277	434	711	4.92	831	1,297	2,128	14.73
Santiago City	13,383	536	699	1,235	9.23	1,422	2,242	3,664	27.38
Tuguegarao City	16,709	378	655	1,033	6.18	966	1,663	2,629	15.73
**Region 3**	1,142,449	6,769	8,152	14,921	1.31	22,639	32,205	54,844	4.80
Aurora	18,836	23	44	67	0.36	309	413	722	3.83
Bataan	66,939	499	682	1,181	1.76	1,878	2,813	4,691	7.01
Bulacan	225,233	436	260	696	0.31	840	853	1,693	0.75
Nueva Ecija	156,175	1,423	2,076	3,499	2.24	5,419	7,423	12,842	8.22
Pampanga	166,626	729	843	1,572	0.94	1,381	2,319	3,700	2.22
Tarlac	106,482	0	0	0	0.00	0	0	0	0.00
Zambales	60,063	104	232	336	0.56	258	400	658	1.10
Angeles City	41,802	497	540	1,037	2.48	480	556	1,036	2.48
Balanga City	9,675	97	133	230	2.38	2,544	3,596	6,140	63.46
Cabanatuan City	31,961	21	28	49	0.15	562	1,083	1,645	5.15
City of San Fernando	31,143	32	61	93	0.30	563	638	1,201	3.86
Gapan City	11,665	157	136	293	2.51	415	562	977	8.38
Mabalacat City	25,469	0	0	0	0.00	0	0	0	0.00
Malolos City	25,157	2	10	12	0.05	178	272	450	1.79
Meycauayan	20,867	302	327	629	3.01	850	1,101	1,951	9.35
Olongapo	23,694	82	113	195	0.82	338	539	877	3.70
Palayan City	4,339	18	15	33	0.76	164	239	403	9.29
San Jose City	14,780	259	72	331	2.24	231	136	367	2.48
San Jose del Monte City	57,294	625	866	1,491	2.60	4,573	6,762	11,335	19.78
Science City of Munoz	8,620	1,237	1,357	2,594	30.09	294	315	609	7.06
Tarlac City	35,629	226	357	583	1.64	1,362	2,185	3,547	9.96
**Region 4A**	1,352,670	3,249	5,223	8,472	0.63	12,892	20,399	33,291	2.46
Batangas	191,674	140	168	308	0.16	1,030	1,687	2,717	1.42
Cavite	128,478	519	693	1,212	0.94	857	1,239	2,096	1.63
Laguna	91,249	35	73	108	0.12	531	1,076	1,607	1.76
Quezon	163,869	68	184	252	0.15	482	1,079	1,561	0.95
Rizal	185,089	529	1,044	1,573	0.85	1,589	2,737	4,326	2.34
Antipolo City	68,173	0	0	0	0.00	0	53	53	0.08
Bacoor City	56,285	0	2	2	0.00	10	13	23	0.04
Batangas City	34,017	0	0	0	0.00	0	0	0	0.00

Table 1.F.5.1 - Immunization for Senior Citizen

Number and proportion of senior citizen 60 yrs old and above who received one (1) dose of Pneumococcal Polysaccharide Vaccine (PPV) and one (1) dose of Influenza Vaccine
Philippines, Annual 2020

Area	Eligible Pop. (60 y/o and above)	Received one (1) dose of PPV				Received one (1) dose of Influenza Vaccine			
		Male	Female	Total	%	Male	Female	Total	%
Biñan City	30,593	223	364	587	1.92	954	1,358	2,312	7.56
Cabuyao City	28,366	187	198	385	1.36	1,529	1,599	3,128	11.03
Calamba City	41,749	841	1,410	2,251	5.39	673	1,006	1,679	4.02
Cavite City	9,633	0	0	0	0.00	0	0	0	0.00
Dasmariñas City	61,765	281	421	702	1.14	668	1,045	1,713	2.77
General Trias City	29,452	19	38	57	0.19	433	678	1,111	3.77
Imus City	37,846	0	0	0	0.00	581	660	1,241	3.28
Lipa City	34,274	0	0	0	0.00	0	0	0	0.00
Lucena City	24,837	0	0	0	0.00	0	0	0	0.00
San Pablo City	24,443	15	26	41	0.17	317	650	967	3.96
San Pedro City	29,929	0	0	0	0.00	0	0	0	0.00
Santa Rosa City	32,498	140	148	288	0.89	1,214	1,507	2,721	8.37
Tagaytay City	6,672	0	0	0	0.00	0	0	0	0.00
Tanauan City	17,877	2	6	8	0.04	1,263	2,467	3,730	20.86
Tayabas City	9,307	114	190	304	3.27	257	597	854	9.18
Trece Martires City	14,595	136	258	394	2.70	504	948	1,452	9.95
**Region 4B**	**259,762**	**1,237**	**1,905**	**3,142**	**1.21**	**1,451**	**2,776**	**4,227**	**1.63**
Marinduque	26,515	370	643	1,013	3.82	1,277	2,495	3,772	14.23
Mindoro Occidental	39,181	326	412	738	1.88	142	214	356	0.91
Mindoro Oriental	75,874	497	767	1,264	1.67	9	10	19	0.03
Palawan	67,539	44	83	127	0.19	23	57	80	0.12
Romblon	30,365	0	0	0	0.00	0	0	0	0.00
Puerto Princesa City	20,288	0	0	0	0.00	0	0	0	0.00
**Region 5**	**523,737**	**6,377**	**11,726**	**18,103**	**3.46**	**15,692**	**28,249**	**43,941**	**8.39**
Albay	110,897	1,560	2,948	4,508	4.07	3,222	6,096	9,318	8.40
Camarines Norte	52,028	520	775	1,295	2.49	2,406	4,589	6,995	13.44
Camarines Sur	144,256	1,096	2,074	3,170	2.20	2,805	6,180	8,985	6.23
Catanduanes	24,480	377	667	1,044	4.26	1,292	2,228	3,520	14.38
Masbate	72,716	178	254	432	0.59	1,604	1,740	3,344	4.60
Sorsogon	72,860	1,981	3,781	5,762	7.91	3,514	5,964	9,478	13.01
Iriga City	9,802	220	483	703	7.17	181	417	598	6.10
Legaspi City	19,504	350	527	877	4.50	612	952	1,564	8.02
Naga City	17,194	95	217	312	1.81	56	83	139	0.81
**Region 6**	**821,493**	**12,891**	**18,308**	**31,199**	**3.80**	**26,943**	**40,868**	**67,811**	**8.25**
Aklan	62,594	895	1,375	2,270	3.63	1,498	2,557	4,055	6.48
Antique	60,739	1,712	2,442	4,154	6.84	2,699	4,060	6,759	11.13
Capiz	87,668	1,014	1,342	2,356	2.69	4,004	5,965	9,969	11.37
Guimaras	19,545	551	733	1,284	6.57	532	943	1,475	7.55
Iloilo	225,954	5,025	6,422	11,447	5.07	8,228	11,169	19,397	8.58
Negros Occidental	255,291	2,341	3,790	6,131	2.40	8,028	12,706	20,734	8.12
Bacolod City	57,435	297	631	928	1.62	410	991	1,401	2.44
Iloilo City	52,267	1,056	1,573	2,629	5.03	1,544	2,477	4,021	7.69

Table 1.F.5.1 - Immunization for Senior Citizen

Number and proportion of senior citizen 60 yrs old and above who received one (1) dose of Pneumococcal Polysaccharide Vaccine (PPV) and one (1) dose of Influenza Vaccine
Philippines, Annual 2020

Area	Eligible Pop. (60 y/o and above)	Received one (1) dose of PPV				Received one (1) dose of Influenza Vaccine			
		Male	Female	Total	%	Male	Female	Total	%
**Region 7**	**746,101**	**5,781**	**7,038**	**12,819**	**1.72**	**9,137**	**13,378**	**22,515**	**3.02**
Bohol	**147,672**	4,382	**4,897**	**9,279**	**6.28**	3,896	**5,410**	**9,306**	**6.30**
Cebu	**280,821**	202	**289**	**491**	**0.17**	1,718	**2,464**	**4,182**	**1.49**
Negros Oriental	**142,750**	248	**342**	**590**	**0.41**	1,170	**1,868**	**3,038**	**2.13**
Siquijor	**13,051**	114	**189**	**303**	**2.32**	108	**221**	**329**	**2.52**
Cebu City	**88,161**	674	**1,077**	**1,751**	**1.99**	1,362	**2,162**	**3,524**	**4.00**
Lapu-Lapu City	**38,995**	161	**244**	**405**	**1.04**	883	**1,253**	**2,136**	**5.48**
Mandaue City	**34,651**	0	**0**	**0**	**0.00**	0	**0**	**0**	**0.00**
**Region 8**	**436,426**	**3,429**	**4,887**	**8,316**	**1.91**	**4,589**	**7,946**	**12,535**	**2.87**
Biliran	**17,330**	419	**655**	**1,074**	**6.20**	541	**867**	**1,408**	**8.12**
Eastern Samar	**47,288**	29	**58**	**87**	**0.18**	522	**1,340**	**1,862**	**3.94**
Northern Leyte	**154,643**	0	**0**	**0**	**0.00**	0	**0**	**0**	**0.00**
Northern Samar	**51,523**	268	**316**	**584**	**1.13**	638	**1,201**	**1,839**	**3.57**
Southern Leyte	**40,229**	306	**420**	**726**	**1.80**	1,020	**1,620**	**2,640**	**6.56**
Western Samar	**52,248**	72	**116**	**188**	**0.36**	214	**339**	**553**	**1.06**
Calbayog City	**16,096**	132	**327**	**459**	**2.85**	0	**0**	**0**	**0.00**
Maasin City	**10,231**	258	**435**	**693**	**6.77**	53	**60**	**113**	**1.10**
Ormoc City	**22,034**	1,942	**2,555**	**4,497**	**20.41**	1,601	**2,519**	**4,120**	**18.70**
Tacloban City	**24,804**	3	**5**	**8**	**0.03**	0	**0**	**0**	**0.00**
**Region 9**	**302,395**	**1,326**	**1,604**	**2,930**	**0.97**	**3,093**	**3,793**	**6,886**	**2.28**
Zamboanga del Norte	**72,258**	*	*	**0**	**0.00**	*	*	**0**	**0.00**
Zamboanga del Sur	**66,654**	*	*	**0**	**0.00**	*	*	**0**	**0.00**
Zamboanga Sibugay	**47,984**	675	**869**	**1,544**	**3.22**	2,134	**2,562**	**4,696**	**9.79**
Dapitan City	**7,462**	651	**735**	**1,386**	**18.57**	959	**1,231**	**2,190**	**29.35**
Dipolog City	**11,834**	*	*	**0**	**0.00**	*	*	**0**	**0.00**
Isabela City	**9,073**	*	*	**0**	**0.00**	*	*	**0**	**0.00**
Pagadian City	**16,353**	*	*	**0**	**0.00**	*	*	**0**	**0.00**
Zamboanga City	**70,777**	*	*	**0**	**0.00**	*	*	**0**	**0.00**
**Region 10**	**416,240**	**7,432**	**10,661**	**18,093**	**4.35**	**10,509**	**14,968**	**25,477**	**6.12**
Bukidnon	**83,276**	1,063	**1,627**	**2,690**	**3.23**	1,741	**2,680**	**4,421**	**5.31**
Camiguin	**10,186**	32	**65**	**97**	**0.95**	699	**1,043**	**1,742**	**17.10**
Lanao del Norte	**51,948**	1,038	**1,159**	**2,197**	**4.23**	1,335	**1,707**	**3,042**	**5.86**
Misamis Occidental	**36,038**	0	**0**	**0**	**0.00**	0	**0**	**0**	**0.00**
Misamis Oriental	**67,884**	587	**968**	**1,555**	**2.29**	1,161	**1,848**	**3,009**	**4.43**
Cagayan de Oro City	**64,318**	2,658	**4,044**	**6,702**	**10.42**	1,353	**1,889**	**3,242**	**5.04**
El Salvador City	**4,779**	67	**70**	**137**	**2.87**	151	**261**	**412**	**8.62**
Gingoog City	**11,864**	227	**344**	**571**	**4.81**	1,150	**1,380**	**2,530**	**21.33**
Iligan City	**26,310**	667	**1,007**	**1,674**	**6.36**	2,127	**3,346**	**5,473**	**20.80**
Malaybalay City	**13,886**	640	**847**	**1,487**	**10.71**	114	**172**	**286**	**2.06**

Table 1.F.5.1 - Immunization for Senior Citizen

Number and proportion of senior citizen 60 yrs old and above who received one (1) dose of Pneumococcal Polysaccharide Vaccine (PPV) and one (1) dose of Influenza Vaccine
Philippines, Annual 2020

Area	Eligible Pop. (60 y/o and above)	Received one (1) dose of PPV				Received one (1) dose of Influenza Vaccine			
		Male	Female	Total	%	Male	Female	Total	%
Oroquieta City	7,811	0	0	0	0.00	0	0	0	0.00
Ozamis City	15,647	153	255	408	2.61	1	1	2	0.01
Tangub City	6,950	29	91	120	1.73	95	146	241	3.47
Valencia City	15,343	271	184	455	2.97	582	495	1,077	7.02
**Region 11**	**438,550**	**2,556**	**3,187**	**5,743**	**1.31**	**12,623**	**17,572**	**30,195**	**6.89**
Compostela Valley	60,559	63	87	150	0.25	1,271	1,701	2,972	4.91
Davao del Norte	89,141	415	616	1,031	1.16	2,625	3,664	6,289	7.06
Davao Oriental	50,574	880	1,236	2,116	4.18	1,956	2,813	4,769	9.43
Davao del Sur	59,247	530	554	1,084	1.83	2,151	2,825	4,976	8.40
Davao Occidental	26,083	50	41	91	0.35	107	142	249	0.95
Davao City	152,946	618	653	1,271	0.83	4,513	6,427	10,940	7.15
**Region 12**	**349,350**	**8,044**	**11,199**	**19,243**	**5.51**	**17,764**	**24,677**	**42,441**	**12.15**
North Cotabato	111,695	1,977	2,749	4,726	4.23	5,280	7,361	12,641	11.32
Sarangani	37,850	1,749	2,288	4,037	10.67	2,185	3,022	5,207	13.76
South Cotabato	74,767	1,727	2,726	4,453	5.96	5,550	7,692	13,242	17.71
Sultan Kudarat	58,386	2,366	3,157	5,523	9.46	4,050	5,558	9,608	16.46
Cotabato City	18,094	92	126	218	1.20	277	326	603	3.33
Gen. Santos City	48,558	133	153	286	0.59	422	718	1,140	2.35
**BARMM**	**172,617**	**1,596**	**2,294**	**3,890**	**2.25**	**3,521**	**4,913**	**8,434**	**4.89**
Basilan	13,674	0	0	0	0.00	0	0	0	0.00
Lanao del Sur	34,766	80	143	223	0.64	110	166	276	0.79
Maguindanao	54,685	1,201	1,656	2,857	5.22	3,022	4,252	7,274	13.30
Sulu	38,585	0	0	0	0.00	0	0	0	0.00
Tawi-Tawi	18,828	0	0	0	0.00	0	0	0	0.00
Lamitan City	3,764	0	0	0	0.00	0	0	0	0.00
Marawi City	8,315	315	495	810	9.74	389	495	884	10.63
**CARAGA**	**230,740**	**3,891**	**4,549**	**8,440**	**3.66**	**10,004**	**14,474**	**24,478**	**10.61**
Agusan del Norte	32,642	187	320	507	1.55	1,187	1,836	3,023	9.26
Agusan del Sur	50,364	102	113	215	0.43	677	916	1,593	3.16
Surigao del Norte	32,952	853	1,074	1,927	5.85	2,823	3,138	5,961	18.09
Surigao del Sur	46,156	883	651	1,534	3.32	2,045	3,291	5,336	11.56
Province of Dinagat	13,493	162	222	384	2.85	183	359	542	4.02
Bislig City	8,764	501	635	1,136	12.96	235	310	545	6.22
Butuan City	31,031	460	638	1,098	3.54	1,295	1,959	3,254	10.49
Surigao City	15,338	743	896	1,639	10.69	1,559	2,665	4,224	27.54

Note: Put asterisk (*) for No Report and Zero (0) for No Case

Figure 1.F.5.1 - Percentage Distribution of senior citizen 60 yrs old and above who received one (1) dose of Pneumococcal Polysaccharide Vaccine (PPV) by Region
Philippines, 2020

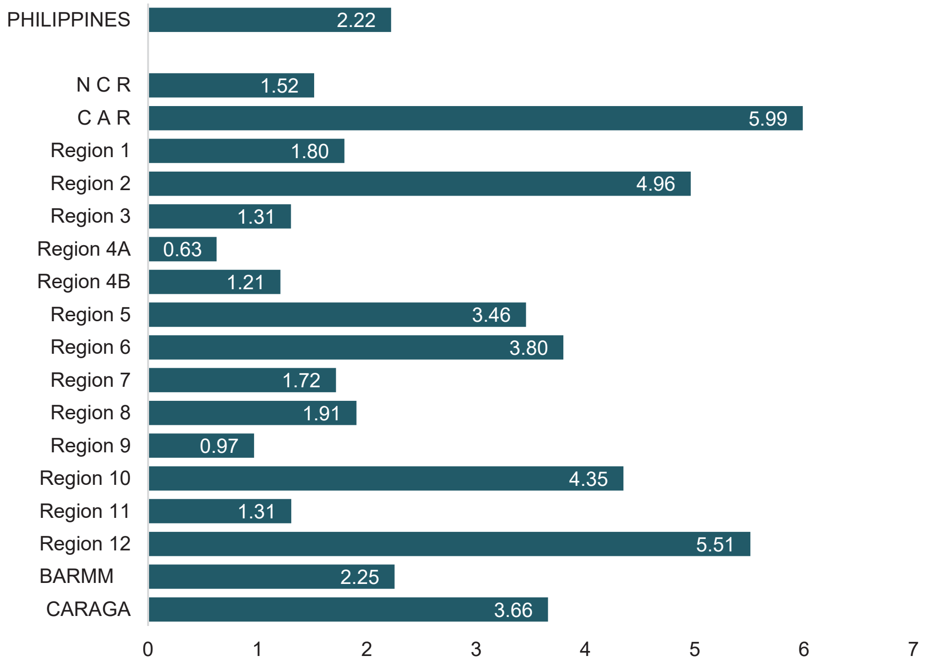

Figure 1.F.5.2 - Percentage Distribution of senior citizen 60 yrs old and above who received one (1) dose of Influenza Vaccine by Region
Philippines, 2020

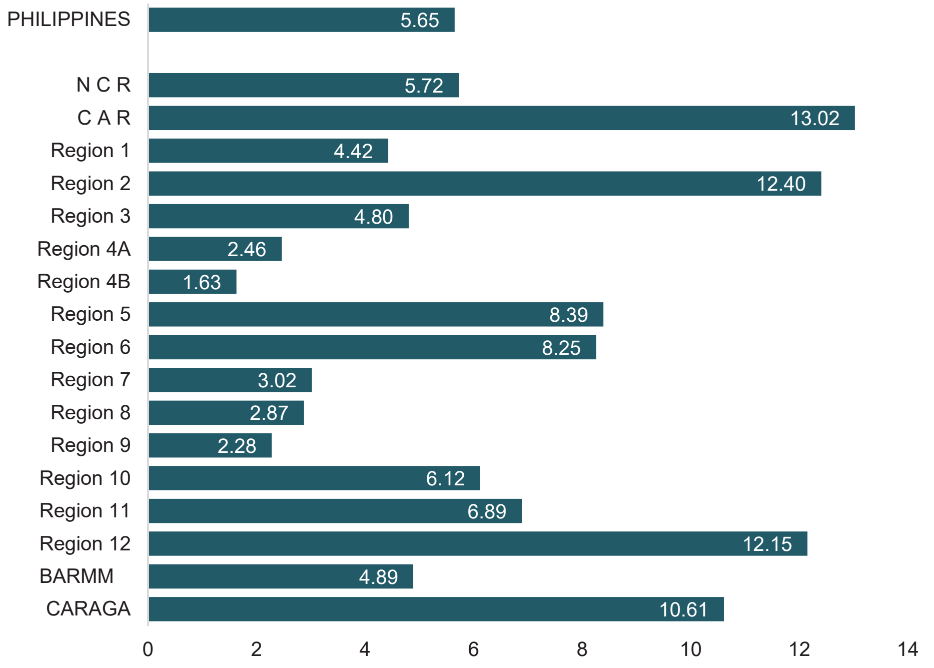

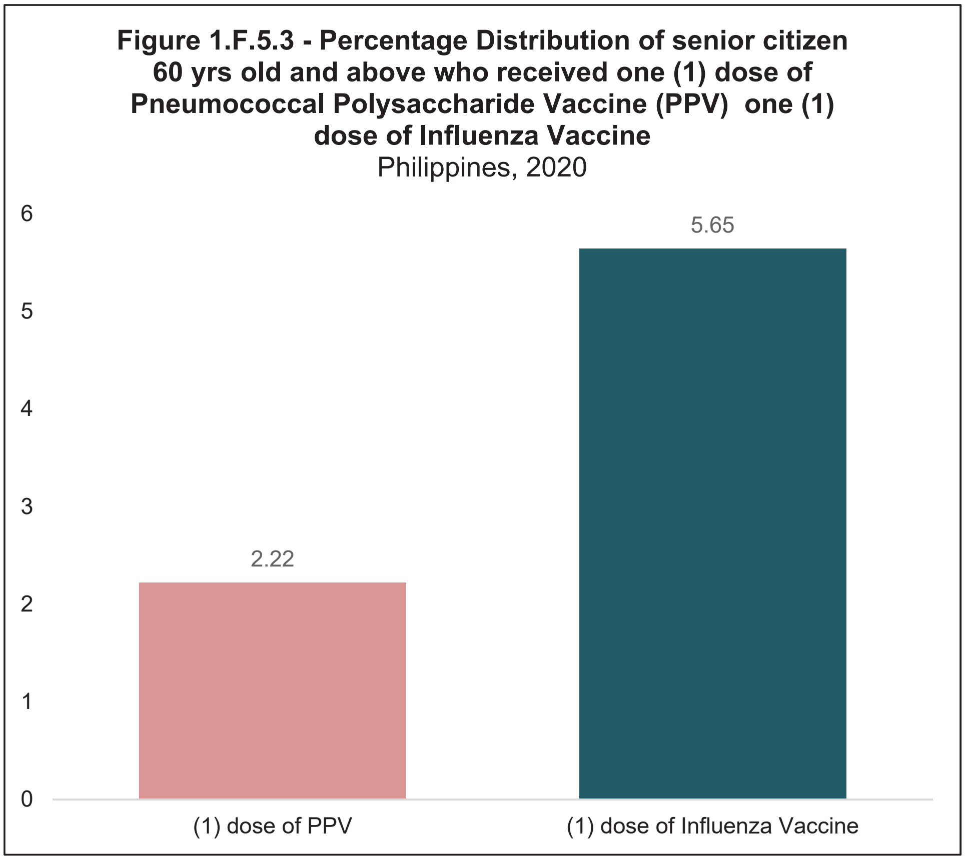
Figure 1.F.5.3 - Percentage Distribution of senior citizen 60 yrs old and above who received one (1) dose of Pneumococcal Polysaccharide Vaccine (PPV) one (1) dose of Influenza Vaccine
Philippines, 2020
6
5
4
3
2
1
0
2.22
5.65
(1) dose of PPV
(1) dose of Influenza Vaccine

ENVIRONMENTAL HEALTH AND SANITATION SERVICES

Proportion and Total No. of Households (HHs) with access to basic safe water supply and manage drinking water services

Philippines, 2020

Annual

Area	Projected No. of HouseHolds	HHs with Access to Basic Safe Water Supply								HHs using Safely Managed Drinking Water Services	
		Level 1		Level 2		Level 3		Total	%		
		Total	%	Total	%	Total	%			Total	%
**PHILIPPINES**	**24,843,795**	**4,940,891**	**26.5**	**2,824,872**	**15.2**	**10,863,653**	**58.3**	**18,629,417**	**75.0**	**12,658,690**	**51.0**
**N C R**	**3,342,508**	**1,474**	**0.1**	**29,942**	**1.1**	**2,684,947**	**98.8**	**2,716,363**	**81.3**	**2,622,782**	**78.5**
Malabon	92,713	0	0.0	0	0.0	75,045	100.0	75,045	80.9	64,352	69.4
Navotas	65,365	0	0.0	0	0.0	49,030	100.0	49,030	75.0	49,030	75.0
Valenzuela City	164,353	0	0.0	0	0.0	139,082	100.0	139,082	84.6	139,082	84.6
Caloocan City	395,109	0	0.0	0	0.0	339,661	100.0	339,661	86.0	289,213	73.2
Marikina City	105,746	0	0.0	40	0.04	105,707	100.0	105,747	100.0	105,747	100.0
Pasig City	194,203	0	0.0	0	0.0	159,507	100.0	159,507	82.1	159,507	82.1
Pateros	15,257	0	0.0	1,004	6.2	15,257	93.8	16,261	106.6	13,736	90.0
Taguig	213,535	0	0.0	0	0.0	136,301	100.0	136,301	63.8	136,301	63.8
Quezon City	736,452	0	0.0	14	0.002	633,359	100.0	633,373	86.0	633,359	86.0
Makati City	166,096	0	0.0	1,516	0.9	164,177	99.1	165,693	99.8	167,337	100.7
Mandaluyong City	109,983	0	0.0	0	0.0	4,850	100.0	4,850	4.4	0	0.0
San Juan	31,059	0	0.0	0	0.0	23,706	100.0	23,706	76.3	23,706	76.3
Manila City	471,028	0	0.0	26,686	7.0	354,546	93.0	381,232	80.9	354,546	75.3
Las Piñas City	152,462	0	0.0	9	0.0	152,453	100.0	152,462	100.0	152,453	100.0
Muntinlupa City	137,282	1,474	2.6	673	1.2	55,302	96.3	57,449	41.8	57,449	41.8
Parañaque City	175,369	0	0.0	0	0.0	175,369	100.0	175,369	100.0	175,369	100.0
Pasay City	116,496	0	0.0	0	0.0	101,595	100.0	101,595	87.2	101,595	87.2
**C A R**	**418,155**	**89,325**	**24.6**	**72,177**	**19.8**	**202,127**	**55.6**	**363,629**	**87.0**	**228,275**	**54.6**
Abra	54,656	8,104	23.2	9,129	26.2	17,665	50.6	34,898	63.9	21,695	39.7
Apayao	26,695	15,060	64.4	4,916	21.0	3,396	14.5	23,372	87.6	1,139	4.3
Benguet	114,949	23,842	24.4	9,536	9.8	64,302	65.8	97,680	46.0	69,278	60.3
Ifugao	45,021	16,292	37.1	21,688	49.4	5,928	13.5	43,908	97.5	36,148	80.3
Kalinga	43,750	16,626	35.7	14,313	30.7	15,652	33.6	46,591	106.5	23,961	54.8
Mt. Province	35,729	5,491	15.1	5,562	15.3	25,273	69.6	36,326	101.7	6,133	17.2
Baguio City	97,355	3,910	4.8	7,033	8.7	69,911	86.5	80,854	83.1	69,921	71.8
**Region 1**	**1,210,488**	**647,515**	**55.0**	**55,406**	**4.7**	**473,894**	**40.3**	**1,176,815**	**97.2**	**877,227**	**72.5**
Ilocos Norte	118,240	29,292	25.6	10,417	9.1	74,924	65.4	114,633	96.9	98,672	83.5
Ilocos Sur	136,614	74,978	60.0	9,725	7.8	40,315	32.2	125,018	91.5	39,834	29.2
La Union	160,547	113,423	71.6	17,593	11.1	27,446	17.3	158,462	98.7	67,443	42.0
Pangasinan	572,036	336,513	60.2	17,400	3.1	205,410	36.7	559,323	97.8	492,551	86.1
Alaminos City	22,767	0	0.0	0	0.0	21,495	100.0	21,495	94.4	21,495	94.4
Candon City	14,385	12,148	82.9	72	0.5	2,427	16.6	14,647	101.8	2,536	17.6
Dagupan City	40,457	0	0.0	0	0.0	41,160	100.0	41,160	101.7	41,160	101.7
Laoag City	26,317	2,171	8.9	0	0.0	22,116	91.1	24,287	92.3	24,287	92.3
San Carlos City	44,344	33,323	75.1	93	0.2	10,928	24.6	44,344	100.0	42,470	95.8
San Fernando City	30,155	19,012	63.0	106	0.4	11,036	36.6	30,154	100.0	14,503	48.1
Urdaneta City	32,118	21,233	68.3	0	0.0	9,873	31.7	31,106	96.8	25,512	79.4
Vigan City	12,508	5,422	44.5	0	0.0	6,764	55.5	12,186	97.4	6,764	54.1
**Region 2**	**854,610**	**319,826**	**59.9**	**47,521**	**8.9**	**166,313**	**31.2**	**533,660**	**62.4**	**277,079**	**32.4**
Batanes	4,975	13	0.3	0	0.0	4,962	99.7	4,975	100.0	0	0.0
Cagayan	253,805	89,896	66.9	17,652	13.1	26,897	20.0	134,445	53.0	75,067	29.6
Isabela	293,529	109,885	67.6	8,059	5.0	44,639	27.5	162,583	55.4	74,973	25.5
Nueva Vizcaya	119,190	37,281	56.9	8,924	13.6	19,324	29.5	65,529	55.0	21,470	18.0
Quirino	45,625	25,133	55.1	12,266	26.9	8,226	18.0	45,625	100.0	5,700	12.5
Cauayan City	32,509	18,085	59.9	420	1.4	11,710	38.8	30,215	92.9	30,213	92.9
Ilagan City	36,758	18,901	70.8	200	0.7	7,581	28.4	26,682	72.6	26,682	72.6
Santiago City	32,358	18,100	55.9	0	0.0	14,258	44.1	32,358	100.0	14,258	44.1
Tuguegarao City	35,861	2,532	8.1	0	0.0	28,716	91.9	31,248	87.1	28,716	80.1

ENVIRONMENTAL HEALTH AND SANITATION SERVICES

Proportion and Total No. of Households (HHs) with access to basic safe water supply and manage drinking water services

Philippines, 2020

Annual

Area	Projected No. of HouseHolds	HHs with Access to Basic Safe Water Supply								HHs using Safely Managed Drinking Water Services	
		Level 1		Level 2		Level 3		Total	%		
		Total	%	Total	%	Total	%			Total	%
**Region 3**	**2,823,964**	**624,325**	**32.2**	**76,204**	**3.9**	**1,237,408**	**63.9**	**1,937,937**	**68.6**	**1,324,575**	**46.9**
Aurora	52,610	21,095	47.3	9,633	21.6	13,835	31.0	44,563	84.7	25,727	48.9
Bataan	167,936	35,544	21.6	13,898	8.4	115,080	69.9	164,522	98.0	164,522	98.0
Bulacan	581,278	44,994	22.0	661	0.3	158,680	77.7	204,335	35.2	167,433	28.8
Nueva Ecija	375,215	208,596	58.4	28,613	8.0	119,889	33.6	357,098	95.2	178,913	47.7
Pampanga	393,208	70,117	25.9	6,353	2.3	194,044	71.7	270,514	68.8	57,313	14.6
Tarlac	253,419	104,211	50.6	1,927	0.9	99,794	48.5	205,932	81.3	173,136	68.3
Zambales	154,027	48,968	57.6	7,100	8.4	28,878	34.0	84,946	55.2	67,986	44.1
Angeles City	107,406	4,207	4.5	1,402	1.5	87,871	94.0	93,480	87.0	93,138	86.7
Balanga City	23,838	839	74.0	295	26.0	0	0.0	1,134	4.8	16,833	70.6
Cabanatuan City	75,105	23,011	32.0	0	0.0	48,848	68.0	71,859	95.7	48,848	65.0
City of San Fernando	76,304	0	0.0	0	0.0	0	0.0	0	0.0	0	0.0
Gapan City	27,277	4,359	62.7	66	0.9	2,526	36.3	6,951	25.5	7,187	26.3
Mabalacat City	62,847	443	3.4	0	0.0	12,686	96.6	13,129	20.9	13,234	21.1
Malolos City	64,702	2,928	11.1	0	0.0	23,455	88.9	26,383	40.8	29,573	45.7
Meycauayan	58,247	628	1.1	0	0.0	57,270	98.9	57,898	99.4	57,253	98.3
Olongapo	61,628	2,615	4.4	0	0.0	57,198	95.6	59,813	97.1	0	0.0
Palayan City	10,362	734	8.8	0	0.0	7,647	91.2	8,381	80.9	7,647	73.8
San Jose City	36,028	10,929	35.7	0	0.0	19,704	64.3	30,633	85.0	30,619	85.0
San Jose del Monte City	140,346	0	0.0	1,276	0.9	137,860	99.1	139,136	99.1	139,136	99.1
Science City of Munoz	21,072	6,369	30.0	4,980	23.5	9,852	46.5	21,201	100.6	3,786	18.0
Tarlac City	81,109	33,738	44.4	0	0.0	42,291	55.6	76,029	93.7	42,291	52.1
**Region 4A**	**3,796,575**	**251,983**	**14.1**	**121,954**	**6.8**	**1,412,726**	**79.1**	**1,786,663**	**47.1**	**1,024,875**	**27.0**
Batangas	479,201	51,093	24.5	8,576	4.1	149,125	71.4	208,794	43.6	150,324	31.4
Cavite	374,847	51,342	15.0	4,867	1.4	285,287	83.5	341,496	91.1	92,938	24.8
Laguna	263,057	26,905	25.9	12,344	11.9	64,514	62.2	103,763	39.4	32,299	12.3
Quezon	429,215	85,513	45.4	48,433	25.7	54,228	28.8	188,174	43.8	113,099	26.4
Rizal	537,641	2,227	1.3	1,510	0.9	166,592	97.8	170,329	31.7	163,235	30.4
		0		0		0				0	
Antipolo City	190,127	9,374	5.3	28,166	16.0	138,059	78.6	175,599	92.4	161,888	85.1
Bacoor City	171,121	2,703	100.0	0	0.0	0	0.0	2,703	1.6	0	0.0
Batangas City	88,409	0	0.0	0	0.0	0	0.0	0	0.0	0	0.0
Biñan City	95,820	321	2.5	0	0.0	12,626	97.5	12,947	13.5	6,959	7.3
Cabuyao City	90,086	0	0.0	0	0.0	0	0.0	0	0.0	0	0.0
Calamba City	136,966	3,941	13.3	1,746	5.9	23,856	80.8	29,543	21.6	0	0.0
Cavite City	29,850	0	0.0	0	0.0	0	0.0	0	0.0	0	0.0
Dasmariñas City	169,581	298	0.3	0	0.0	107,895	99.7	108,193	63.8	86,962	51.3
General Trias City	92,259	915	1.0	293	0.3	87,075	98.6	88,283	95.7	85,601	92.8
Imus City	111,780	1,392	1.3	0	0.0	109,098	98.7	110,490	98.8	0	0.0
Lipa City	83,393	0	0.0	0	0.0	0	0.0	0	0.0	0	0.0
Lucena City	64,000	2,769	4.3	10,891	17.0	50,328	78.7	63,988	100.0	0	0.0
San Pablo City	68,933	0	0.0	0	0.0	0	0.0	0	0.0	0	0.0
San Pedro City	80,595	2,799	4.4	0	0.0	61,095	95.6	63,894	79.3	63,407	78.7
Santa Rosa City	111,749	7,456	14.7	0	0.0	43,270	85.3	50,726	45.4	2,485	2.2
Tagaytay City	18,843	0	0.0	0	0.0	0	0.0	0	0.0	0	0.0
Tanauan City	43,147	516	1.2	0	0.0	41,816	98.8	42,332	98.1	42,183	97.8
Tayabas City	25,409	2,419	9.5	5,128	20.2	17,862	70.3	25,409	100.0	23,495	92.5
Trece Martires City	40,546	0	0.0	0	0.0	0	0.0	0	0.0	0	0.0
**Region 4B**	**735,729**	**215,885**	**46.3**	**73,944**	**15.9**	**176,087**	**37.8**	**465,916**	**63.3**	**121,413**	**16.5**
Marinduque	56,138	14,185	25.7	15,431	27.9	25,621	46.4	55,237	98.4	17,488	31.2
Mindoro Occidental	119,031	56,589	59.5	11,854	12.5	26,642	28.0	95,085	79.9	57,271	48.1
Mindoro Oriental	204,292	112,857	55.0	38,939	19.0	53,314	26.0	205,110	100.4	20,176	9.9
Palawan	215,709	27,236	29.2	5,753	6.2	60,191	64.6	93,180	43.2	12,004	5.6
Romblon	70,681	3,496	83.5	373	8.9	316	7.6	4,185	5.9	4,185	5.9
Puerto Princesa City	69,878	1,522	11.6	1,594	12.2	10,003	76.2	13,119	18.8	10,289	14.7
**Region 5**	**1,290,761**	**311,934**	**28.9**	**299,115**	**27.7**	**467,786**	**43.4**	**1,078,835**	**83.6**	**586,765**	**45.5**
Albay	250,331	73,730	30.4	72,679	30.0	96,170	39.6	242,579	96.9	157,151	62.8

ENVIRONMENTAL HEALTH AND SANITATION SERVICES

Proportion and Total No. of Households (HHs) with access to basic safe water supply and manage drinking water services

Philippines, 2020

Annual

Area	Projected No. of HouseHolds	HHs with Access to Basic Safe Water Supply								HHs using Safely Managed Drinking Water Services	
		Level 1		Level 2		Level 3		Total	%		
		Total	%	Total	%	Total	%			Total	%
Camarines Norte	139,090	24,349	19.2	41,479	32.7	61,099	48.1	126,927	91.3	97,951	70.4
Camarines Sur	355,666	92,704	34.2	79,254	29.3	98,807	36.5	270,765	76.1	68,165	19.2
Catanduanes	56,744	12,770	22.9	13,617	24.4	29,368	52.7	55,755	98.3	21,457	37.8
Masbate	197,899	48,624	46.0	30,063	28.4	27,097	25.6	105,784	53.5	82,819	41.8
Sorsogon	178,551	44,000	24.8	51,976	29.3	81,552	45.9	177,528	99.4	66,420	37.2
Iriga City	23,143	4,091	18.6	3,562	16.2	14,287	65.1	21,940	94.8	15,681	67.8
Legaspi City	44,497	11,231	34.2	5,423	16.5	16,228	49.4	32,882	73.9	32,881	73.9
Naga City	44,840	435	1.0	1,062	2.4	43,178	96.6	44,675	99.6	44,240	98.7
**Region 6**	**1,795,882**	**762,159**	**44.9**	**253,158**	**14.9**	**681,434**	**40.2**	**1,696,751**	**94.5**	**840,518**	**46.8**
Aklan	141,390	52,350	39.8	22,849	17.4	56,320	42.8	131,519	93.0	67,957	48.1
Antique	136,830	44,760	31.3	55,858	39.1	42,294	29.6	142,912	104.4	76,065	55.6
Capiz	190,227	102,141	55.0	17,680	9.5	65,818	35.5	185,639	97.6	64,765	34.0
Guimaras	43,100	28,314	58.8	2,174	4.5	17,671	36.7	48,159	111.7	15,368	35.7
Iloilo	462,422	265,175	62.1	57,935	13.6	104,033	24.4	427,143	92.4	135,717	29.3
Negros Occidental	585,785	231,140	42.9	92,730	17.2	214,666	39.9	538,536	91.9	313,186	53.5
Bacolod City	134,663	10,012	8.2	3,932	3.2	107,906	88.6	121,850	90.5	94,734	70.3
Iloilo City	101,465	28,267	28.0	0	0.0	72,726	72.0	100,993	99.5	72,726	71.7
**Region 7**	**1,837,947**	**248,512**	**16.6**	**423,850**	**28.4**	**820,447**	**55.0**	**1,492,809**	**81.2**	**1,306,192**	**71.1**
Bohol	300,900	51,900	22.6	28,480	12.4	149,499	65.0	229,879	76.4	209,064	69.5
Cebu	737,529	136,622	18.6	162,860	22.2	435,510	59.3	734,992	99.7	671,125	91.0
Negros Oriental	323,413	48,852	18.5	106,162	40.2	109,382	41.4	264,396	81.8	197,534	61.1
Siquijor	23,947	1,121	4.7	6,654	27.7	16,261	67.7	24,036	100.4	19,586	81.8
Cebu City	237,756	3,069	7.8	12,044	30.6	24,289	61.6	39,402	16.6	9,805	4.1
Lapu-Lapu City	109,782	590	0.6	104,480	99.4	0	0.0	105,070	95.7	104,044	94.8
Mandaue City	104,620	6,358	6.7	3,170	3.3	85,506	90.0	95,034	90.8	95,034	90.8
**Region 8**	**1,057,790**	**254,150**	**26.7**	**327,457**	**34.5**	**368,672**	**38.8**	**950,279**	**89.8**	**697,360**	**65.9**
Biliran	40,465	462	1.1	12,584	31.2	27,342	67.7	40,388	99.8	35,011	86.5
Eastern Samar	115,011	31,570	28.1	31,896	28.4	49,037	43.6	112,503	97.8	97,776	85.0
Northern Leyte	385,952	88,694	28.3	112,371	35.9	112,371	35.9	313,436	81.2	230,039	59.6
Northern Samar	134,252	67,453	50.6	57,650	43.2	8,236	6.2	133,339	99.3	107,516	80.1
Southern Leyte	78,381	8,448	10.8	17,825	22.7	52,108	66.5	78,381	100.0	58,368	74.5
Western Samar	133,888	44,627	36.8	39,038	32.2	37,563	31.0	121,228	90.5	26,431	19.7
Calbayog City	40,620	140	0.5	5,127	20.1	20,197	79.3	25,464	62.7	21,000	51.7
Maasin City	19,759	3,386	17.1	5,968	30.2	10,405	52.7	19,759	100.0	13,389	67.8
Ormoc City	54,513	4,771	8.8	19,180	35.2	30,556	56.1	54,507	100.0	54,304	99.6
Tacloban City	54,949	4,599	9.0	25,818	50.4	20,857	40.7	51,274	93.3	53,526	97.4
**Region 9**	**836,189**	**114,582**	**19.5**	**162,232**	**27.7**	**309,514**	**52.8**	**586,328**	**70.1**	**204,843**	**24.5**
Zamboanga del Norte	183,423	46,370	28.4	48,126	29.5	68,625	42.1	163,121	88.9	69,214	37.7
Zamboanga del Sur	180,993	23,201	23.2	40,879	40.8	36,033	36.0	100,113	55.3	50,239	27.8
Zamboanga Sibugay	151,327	15,988	38.2	11,464	27.4	14,353	34.3	41,805	27.6	36,276	24.0
Dapitan City	18,847	2,084	12.5	4,285	25.8	10,256	61.7	16,625	88.2	14,653	77.7
Dipolog City	31,338	0	0.0	1,296	4.0	30,760	96.0	32,056	102.3	30,496	97.3
Isabela City	26,106	2,222	10.7	8,756	42.2	9,780	47.1	20,758	79.5	0	0.0
Pagadian City	42,905	3,190	10.9	9,485	32.3	16,650	56.8	29,325	68.3	3,965	9.2
Zamboanga City	201,250	21,527	11.8	37,941	20.8	123,057	67.4	182,525	90.7	0	0.0
**Region 10**	**1,122,320**	**182,866**	**17.2**	**277,896**	**26.1**	**605,461**	**56.8**	**1,066,223**	**95.0**	**827,708**	**73.7**
Bukidnon	241,792	38,310	17.4	78,504	35.7	102,867	46.8	219,681	90.9	163,878	67.8
Camiguin	21,023	120	0.6	1,895	9.2	18,611	90.2	20,626	98.1	13,582	64.6
Lanao del Norte	151,237	56,418	39.6	40,153	28.2	45,819	32.2	142,390	94.2	118,043	78.1
Misamis Occidental	78,311	15,094	20.5	24,875	33.7	33,777	45.8	73,746	94.2	51,193	65.4
Misamis Oriental	174,536	31,808	19.3	45,028	27.4	87,596	53.3	164,432	94.2	154,187	88.3

ENVIRONMENTAL HEALTH AND SANITATION SERVICES

Proportion and Total No. of Households (HHs) with access to basic safe water supply and manage drinking water services

Philippines, 2020

Annual

Area	Projected No. of HouseHolds	HHs with Access to Basic Safe Water Supply								HHs using Safely Managed Drinking Water Services	
		Level 1		Level 2		Level 3		Total	%		
		Total	%	Total	%	Total	%			Total	%
Cagayan de Oro City	177,954	5,432	3.1	13,316	7.6	157,621	89.4	176,369	99.1	157,621	88.6
El Salvador City	12,940	17	0.1	853	6.6	12,070	93.3	12,940	100.0	5,865	45.3
Gingoog City	28,773	2,800	9.9	10,870	38.3	14,719	51.8	28,389	98.7	28,389	98.7
Iligan City	83,193	1,156	1.7	25,923	37.1	42,858	61.3	69,937	84.1	67,434	81.1
Malaybalay City	42,220	6,904	15.8	14,376	33.0	22,316	51.2	43,596	103.3	33,342	79.0
Oroquieta City	17,400	960	5.5	3,984	22.8	12,524	71.7	17,468	100.4	8,224	47.3
Ozamis City	31,827	10,420	31.5	4,122	12.4	18,571	56.1	33,113	104.0	694	2.2
Tangub City	13,628	2,966	20.0	6,287	42.4	5,588	37.7	14,841	108.9	0	0.0
Valencia City	47,486	10,461	21.5	7,710	15.8	30,524	62.7	48,695	102.5	25,256	53.2
**Region 11**	**1,281,403**	**300,627**	**25.4**	**223,339**	**18.9**	**658,267**	**55.7**	**1,182,233**	**92.3**	**762,662**	**59.5**
Compostela Valley	178,642	68,404	40.6	52,559	31.2	47,424	28.2	168,387	94.3	122,534	68.6
Davao del Norte	244,701	123,078	56.9	25,055	11.6	68,273	31.5	216,406	88.4	49,161	20.1
Davao Oriental	138,613	44,109	31.3	31,520	22.4	65,235	46.3	140,864	101.6	41,828	30.2
Davao del Sur	177,223	45,073	33.3	46,793	34.6	43,446	32.1	135,312	76.4	119,765	67.6
Davao Occidental	83,115	17,390	28.1	35,833	58.0	8,604	13.9	61,827	74.4	67,278	80.9
Davao City	459,109	2,573	0.6	31,579	6.9	425,285	92.6	459,437	100.1	362,096	78.9
**Region 12**	**1,141,452**	**345,053**	**45.2**	**171,557**	**22.5**	**247,031**	**32.3**	**763,641**	**66.9**	**496,921**	**43.5**
North Cotabato	356,294	115,063	41.1	68,318	24.4	96,504	34.5	279,885	78.6	228,729	64.2
Sarangani	133,555	51,283	51.4	29,623	29.7	18,776	18.8	99,682	74.6	87,836	65.8
South Cotabato	237,980	33,059	33.7	33,666	34.4	31,274	31.9	97,999	41.2	116,194	48.8
Sultan Kudarat	194,915	126,877	72.3	22,383	12.8	26,156	14.9	175,416	90.0	38,127	19.6
Cotabato City	61,091	3,018	8.2	7,899	21.4	26,035	70.5	36,952	60.5	26,035	42.6
Gen. Santos City	157,617	15,753	21.4	9,668	13.1	48,286	65.5	73,707	46.8	0	0.0
**B.A.R.M.M.**	**686,781**	**195,190**	**59.6**	**73,468**	**22.4**	**58,676**	**17.9**	**327,335**	**47.7**	**112,681**	**16.4**
Basilan	50,019	3,199	51.3	3,042	48.7	0	0.0	6,241	12.5	0	0.0
Lanao del Sur	143,274	9,880	26.6	15,104	40.7	12,104	32.6	37,089	25.9	610	0.4
Maguindanao	218,181	104,693	66.5	33,137	21.0	19,604	12.5	157,434	72.2	112,071	51.4
Sulu	153,404	34,225	56.1	7,355	12.1	19,442	31.9	61,022	39.8	0	0.0
Tawi-Tawi	71,684	39,350	81.2	7,264	15.0	1,848	3.8	48,462	67.6	0	0.0
Lamitan City	16,830	0	0.0	0	0.0	0	0.0	0	0.0	0	0.0
Marawi City	33,389	3,843	22.5	7,566	44.3	5,678	33.2	17,087	51.2	0	0.0
**CARAGA**	**611,241**	**75,485**	**15.0**	**135,652**	**26.9**	**292,863**	**58.1**	**504,000**	**82.5**	**346,814**	**56.7**
Agusan del Norte	83,144	10,454	23.5	10,911	24.5	23,194	52.1	44,559	53.6	23,209	27.9
Agusan del Sur	161,620	28,549	26.8	34,693	32.5	43,411	40.7	106,653	66.0	77,115	47.7
Surigao del Norte	79,817	8,103	10.2	30,774	38.8	40,521	51.0	79,398	99.5	60,164	75.4
Surigao del Sur	113,747	20,818	18.5	30,534	27.2	60,971	54.3	112,323	98.7	53,628	47.1
Province of Dinagat	29,395	3,213	12.5	9,585	37.2	12,948	50.3	25,746	87.6	16,395	55.8
Bislig City	22,464	879	4.4	3,813	19.0	15,343	76.6	20,035	89.2	15,343	68.3
Butuan City	81,831	2,142	2.7	2,343	2.9	75,790	94.4	80,275	98.1	80,275	98.1
Surigao City	39,223	1,327	3.8	12,999	37.1	20,685	59.1	35,011	89.3	20,685	52.7

Legend:
0 = No Case
** = No Report*

Table 1.G.2 - ENVIRONMENTAL HEALTH AND SANITATION SERVICES
Proportion of Households (HHs) with basic sanitation facility and using Safely Managed Sanitation Service
Philippines, Annual 2020

Area	Projected No. of HHs	HH with Basic Sanitation Facility									HHs using Safely Managed Sanitation Service	
		Total	%	Pour/Flush Toilet								
				Septic Tank		Community sewer/sewerage system		Ventilated improved Pit Latrine (VIP)				
				No.	%	No.	%	No.	%		No.	%
**PHILIPPINES**	**24,817,689**	**16,883,478**	**68.03**	**15,407,118**	**91.26**	**671,114**	**3.97**	**805,246**	**4.77**		**11,180,632**	**45.05**
**N C R**	**3,342,508**	**2,217,584**	**66.34**	**2,006,348**	**90.47**	**211,094**	**9.52**	**142**	**0.01**		**2,224,807**	**66.56**
Malabon	92,713	70,730	76.29	60,036	84.88	10,694	15.12	0	0.00		67,541	72.85
Navotas	65,365	48,744	74.57	48,602	99.71	0	0.00	142	0.29		48,602	74.35
Valenzuela City	164,353	138,326	84.16	138,326	100.00	0	0.00	0	0.00		138,326	84.16
Caloocan City	395,109	289,213	73.20	289,213	100.00	0	0.00	0	0.00		289,213	73.20
Marikina City	105,746	87,758	82.99	86,858	98.97	900	1.03	0	0.00		105,747	100.00
Pasig City	194,203	159,403	82.08	159,403	100.00	0	0.00	0	0.00		159,505	82.13
Pateros	15,257	15,247	99.93	15,247	100.00	0	0.00	0	0.00		14,189	93.00
Taguig	213,535	157,788	73.89	157,788	100.00	0	0.00	0	0.00		157,788	73.89
Quezon City	736,452	629,256	85.44	629,256	100.00	0	0.00	0	0.00		629,256	85.44
Makati City	166,096	162,167	97.63	148,942	91.84	13,225	8.16	0	0.00		156,383	94.15
Mandaluyong City	109,983	695	0.63	695	100.00	0	0.00	0	0.00		0	0.00
San Juan	31,059	23,706	76.33	23,706	100.00	0	0.00	0	0.00		23,706	76.33
Manila City	471,028	285,924	60.70	100,074	35.00	185,850	65.00	0	0.00		285,924	60.70
Las Piñas City	152,462	0	0.00	0	0.00	0	0.00	0	0.00		0	0.00
Muntinlupa City	137,282	46,359	33.77	45,934	99.08	425	0.92	0	0.00		46,359	33.77
Parañaque City	175,369	0	0.00	0	0.00	0	0.00	0	0.00		0	0.00
Pasay City	116,496	102,268	87.79	102,268	100.00	0	0.00	0	0.00		102,268	87.79
**C A R**	**418,155**	**349,562**	**83.60**	**302,023**	**86.40**	**15,260**	**4.37**	**32,279**	**9.23**		**306,587**	**73.32**
Abra	54,656	33,689	61.64	31,184	92.56	24	0.07	2,481	7.36		25,888	47.37
Apayao	26,695	24,110	90.32	19,392	80.43	0	0.00	4,718	19.57		22,760	85.26
Benguet	114,949	106,466	92.62	91,399	85.85	1,263	1.19	13,804	12.97		83,381	72.54
Ifugao	45,021	40,051	88.96	33,659	84.04	0	0.00	6,392	15.96		27,888	61.94
Kalinga	43,750	32,474	74.23	29,059	89.48	0	0.00	3,415	10.52		35,022	80.05
Mt. Province	35,729	32,786	91.76	31,384	95.72	0	0.00	1,402	4.28		31,835	89.10
Baguio City	97,355	79,986	82.16	65,946	82.45	13,973	17.47	67	0.08		79,813	81.98
**Region 1**	**1,210,488**	**1,115,758**	**92.17**	**1,101,899**	**98.76**	**5,342**	**0.48**	**8,517**	**0.76**		**908,422**	**75.05**
Ilocos Norte	144,557	81,223	56.19	81,004	99.73	0	0.00	219	0.27		101,265	70.05
Ilocos Sur	163,507	117,457	71.84	117,451	99.99	0	0.00	6	0.01		116,321	71.14
La Union	190,702	155,460	81.52	146,905	94.50	263	0.17	8,292	5.33		16,678	8.75
Pangasinan	711,722	544,835	76.55	544,835	100.00	0	0.00	0	0.00		498,417	70.03
Alaminos City	22,767	20,954	92.04	20,954	100.00	0	0.00	0	0.00		20,734	91.07
Candon City	14,385	13,966	97.09	13,966	100.00	0	0.00	0	0.00		13,966	97.09
Dagupan City	40,457	40,593	100.34	40,593	100.00	0	0.00	0	0.00		39,141	96.75
Laoag City	26,317	24,287	92.29	24,287	100.00	0	0.00	0	0.00		24,287	92.29
San Carlos City	44,344	42,791	96.50	42,791	100.00	0	0.00	0	0.00		24,256	54.70
San Fernando City	30,155	31,110	103.17	26,031	83.67	5,079	16.33	0	0.00		16,637	55.17
Urdaneta City	32,118	31,088	96.79	31,088	100.00	0	0.00	0	0.00		24,726	76.98
Vigan City	12,508	11,994	95.89	11,994	100.00	0	0.00	0	0.00		11,994	95.89
**Region 2**	**854,610**	**486,389**	**56.91**	**455,349**	**93.62**	**7,639**	**1.57**	**23,401**	**4.81**		**360,925**	**42.23**
Batanes	4,975	4,975	100.00	4,975	100.00	0	0.00	0	0.00		4,975	100.00
Cagayan	289,666	105,285	36.35	83,784	79.58	4,420	4.20	17,081	16.22		66,215	22.86
Isabela	395,154	146,729	37.13	141,571	96.48	3,219	2.19	1,939	1.32		66,215	16.76
Nueva Vizcaya	119,190	70,717	59.33	69,616	98.44	0	0.00	1,101	1.56		72,092	60.48
Quirino	45,625	45,158	98.98	41,878	92.74	0	0.00	3,280	7.26		38,645	84.70
Cauayan City	32,509	30,243	93.03	30,243	100.00	0	0.00	0	0.00		30,243	93.03
Ilagan City	36,758	25,360	68.99	25,360	100.00	0	0.00	0	0.00		25,360	68.99
Santiago City	32,358	28,464	87.97	28,464	100.00	0	0.00	0	0.00		28,464	87.97
Tuguegarao City	35,861	29,458	82.14	29,458	100.00	0	0.00	0	0.00		28,716	80.08

Table 1.G.2 - ENVIRONMENTAL HEALTH AND SANITATION SERVICES
Proportion of Households (HHs) with basic sanitation facility and using Safely Managed Sanitation Service
Philippines, Annual 2020

Area	Projected No. of HHs	HH with Basic Sanitation Facility: Total	%	Pour/Flush Toilet: Septic Tank No.	Septic Tank %	Community sewer/sewerage system No.	Community sewer/sewerage system %	Ventilated improved Pit Latrine (VIP) No.	VIP %	HHs using Safely Managed Sanitation Service No.	%
**Region 3**	**2,823,964**	**1,704,439**	**60.36**	**1,659,902**	**97.39**	**22,411**	**1.31**	**22,126**	**1.30**	**1,011,002**	**35.80**
Aurora	52,610	45,413	86.32	43,494	95.77	614	1.35	1,305	2.87	30,001	57.03
Bataan	191,774	162,713	84.85	162,713	100.00	0	0.00	0	0.00	143,301	74.72
Bulacan	844,573	166,817	19.75	166,817	100.00	0	0.00	0	0.00	109,722	12.99
Nueva Ecija	545,059	229,972	42.19	216,572	94.17	0	0.00	13,400	5.83	178,388	32.73
Pampanga	639,765	313,436	48.99	308,454	98.41	3,617	1.15	1,365	0.44	45,259	7.07
Tarlac	334,528	178,051	53.22	177,697	99.80	0	0.00	354	0.20	160,485	47.97
Zambales	215,655	59,115	27.41	50,081	84.72	7,000	11.84	2,034	3.44	11,601	5.38
Angeles City	107,406	93,138	86.72	93,138	100.00	0	0.00	0	0.00	0	0.00
Balanga City	23,838	23,635	99.15	12,455	52.70	11,180	47.30	0	0.00	0	0.00
Cabanatuan City	75,105	57,586	76.67	56,323	97.81	0	0.00	1,263	2.19	70,135	93.38
City of San Fernando	76,304	0	0.00	0	0.00	0	0.00	0	0.00	0	0.00
Gapan City	27,277	6,682	24.50	6,682	100.00	0	0.00	0	0.00	6,682	24.50
Mabalacat City	62,847	13,210	21.02	12,916	97.77	0	0.00	294	2.23	12,251	19.49
Malolos City	64,702	24,289	37.54	24,289	100.00	0	0.00	0	0.00	24,279	37.52
Meycauayan	58,247	58,013	99.60	58,013	100.00	0	0.00	0	0.00	58,013	99.60
Olongapo	61,628	49,936	81.03	49,714	99.56	0	0.00	222	0.44	0	0.00
Palayan City	10,362	9,568	92.34	9,568	100.00	0	0.00	0	0.00	0	0.00
San Jose City	36,028	30,943	85.89	30,943	100.00	0	0.00	0	0.00	0	0.00
San Jose del Monte City	140,346	139,136	99.14	139,136	100.00	0	0.00	0	0.00	139,136	99.14
Science City of Munoz	21,072	21,911	103.98	20,022	91.38	0	0.00	1,889	8.62	874	4.15
Tarlac City	81,109	20,875	25.74	20,875	100.00	0	0.00	0	0.00	20,875	25.74
**Region 4A**	**3,796,575**	**2,085,587**	**54.93**	**1,814,994**	**87.03**	**129,952**	**6.23**	**140,641**	**6.74**	**0**	**0.00**
Batangas	694,150	198,959	28.66	190,938	95.97	4,507	2.27	3,514	1.77	0	0.00
Cavite	1,008,827	290,094	28.76	289,693	99.86	401	0.14	0	0.00	0	0.00
Laguna	847,206	95,955	11.33	85,086	88.67	9,342	9.74	1,527	1.59	0	0.00
Quezon	518,624	185,038	35.68	181,477	98.08	979	0.53	2,582	1.40	0	0.00
Rizal	727,768	236,678	32.52	166,691	70.43	69,987	29.57	0	0.00	0	0.00
Antipolo City	190,127	173,155	91.07	173,155	100.00	0	0.00	0	0.00	0	0.00
Bacoor City	171,121	166,973	97.58	166,798	99.90	175	0.10	0	0.00	0	0.00
Batangas City	88,409	0	0.00	0	0.00	0	0.00	0	0.00	0	0.00
Biñan City	95,820	7,583	7.91	7,125	93.96	458	6.04	0	0.00	0	0.00
Cabuyao City	90,086	0	0.00	0	0.00	0	0.00	0	0.00	0	0.00
Calamba City	136,966	28,710	20.96	28,345	98.73	359	1.25	6	0.02	0	0.00
Cavite City	29,850	0	0.00	0	0.00	0	0.00	0	0.00	0	0.00
Dasmariñas City	169,581	107,829	63.59	107,829	100.00	0	0.00	0	0.00	0	0.00
General Trias City	92,259	171,202	185.57	85,601	50.00	0	0.00	85,601	50.00	0	0.00
Imus City	111,780	109,151	97.65	109,151	100.00	0	0.00	0	0.00	0	0.00
Lipa City	83,393	0	0.00	0	0.00	0	0.00	0	0.00	0	0.00
Lucena City	64,000	49,198	76.87	49,198	100.00	0	0.00	0	0.00	0	0.00
San Pablo City	68,933	0	0.00	0	0.00	0	0.00	0	0.00	0	0.00
San Pedro City	80,595	62,729	77.83	62,729	100.00	0	0.00	0	0.00	0	0.00
Santa Rosa City	111,749	51,972	46.51	50,127	96.45	1,561	3.00	284	0.55	0	0.00
Tagaytay City	18,843	0	0.00	0	0.00	0	0.00	0	0.00	0	0.00
Tanauan City	43,147	126,549	293.30	42,183	33.33	42,183	33.33	42,183	33.33	0	0.00
Tayabas City	25,409	23,812	93.71	18,868	79.24	0	0.00	4,944	20.76	0	0.00
Trece Martires City	40,546	0	0.00	0	0.00	0	0.00	0	0.00	0	0.00
**Region 4B**	**735,729**	**436,248**	**59.29**	**419,359**	**96.13**	**3,903**	**0.89**	**12,986**	**2.98**	**145,166**	**19.73**
Marinduque	56,138	52,725	93.92	52,725	100.00	0	0.00	0	0.00	52,725	93.92
Mindoro Occidental	119,031	72,384	60.81	72,384	100.00	0	0.00	0	0.00	51,195	43.01
Mindoro Oriental	204,292	178,651	87.45	178,651	100.00	0	0.00	0	0.00	20,828	10.20
Palawan	285,587	120,159	42.07	103,689	86.29	3,903	3.25	12,567	10.46	7,976	2.79
Romblon	70,681	4,155	5.88	4,150	99.88	0	0.00	5	0.12	4,155	5.88
Puerto Princesa City	69,878	8,174	11.70	7,760	94.94	0	0.00	414	5.06	8,287	11.86
**Region 5**	**1,290,761**	**963,372**	**74.64**	**913,446**	**94.82**	**15,087**	**1.57**	**34,839**	**3.62**	**706,844**	**54.76**
Albay	294,828	216,388	73.39	210,676	97.36	579	0.27	5,133	2.37	170,785	57.93

Table 1.G.2 - ENVIRONMENTAL HEALTH AND SANITATION SERVICES
Proportion of Households (HHs) with basic sanitation facility and using Safely Managed Sanitation Service
Philippines, Annual 2020

Area	Projected No. of HHs	HH with Basic Sanitation Facility									HHs using Safely Managed Sanitation Service	
		Total	%	Pour/Flush Toilet								
				Septic Tank		Community sewer/sewerage system		Ventilated improved Pit Latrine (VIP)				
				No.	%	No.	%	No.	%		No.	%
Camarines Norte	139,090	123,542	88.82	119,134	96.43	1,561	1.26	2,847	2.30		112,285	80.73
Camarines Sur	423,649	208,324	49.17	193,956	93.10	4,884	2.34	9,484	4.55		87,241	20.59
Catanduanes	56,744	50,985	89.85	50,774	99.59	143	0.28	68	0.13		44,210	77.91
Masbate	197,899	104,335	52.72	90,401	86.64	5,973	5.72	7,961	7.63		79,916	40.38
Sorsogon	178,551	154,959	86.79	154,431	99.66	421	0.27	107	0.07		117,139	65.61
Iriga City	23,143	20,590	88.97	9,825	47.72	1,526	7.41	9,239	44.87		17,712	76.53
Legaspi City	44,497	39,574	88.94	39,574	100.00	0	0.00	0	0.00		32,881	73.89
Naga City	44,840	44,675	99.63	44,675	100.00	0	0.00	0	0.00		44,675	99.63
**Region 6**	**1,795,882**	**1,539,550**	**85.73**	**1,402,111**	**91.07**	**36,890**	**2.40**	**100,549**	**6.53**		**1,022,102**	**56.91**
Aklan	141,390	129,983	91.93	101,067	77.75	13,125	10.10	15,791	12.15		49,353	34.91
Antique	136,830	114,164	83.43	112,415	98.47	602	0.53	1,147	1.00		106,921	78.14
Capiz	190,227	172,311	90.58	133,271	77.34	0	0.00	39,040	22.66		150,732	79.24
Guimaras	43,100	46,165	107.11	45,056	97.60	0	0.00	1,109	2.40		42,127	97.74
Iloilo	462,422	412,949	73.23	365,038	88.40	23,163	5.61	24,748	5.99		137,037	29.63
Negros Occidental	585,785	448,070	62.19	432,657	96.56	0	0.00	15,413	3.44		338,582	57.80
Bacolod City	134,663	119,957	89.08	116,656	97.25	0	0.00	3,301	2.75		101,399	75.30
Iloilo City	101,465	95,951	94.57	95,951	100.00	0	0.00	0	0.00		95,951	94.57
**Region 7**	**1,837,947**	**1,553,736**	**84.54**	**1,486,627**	**95.68**	**39,933**	**2.57**	**27,176**	**1.75**		**914,265**	**49.74**
Bohol	300,900	222,536	73.96	222,490	99.98	10	0.00	36	0.02		85,354	28.37
Cebu	737,529	714,232	96.84	666,991	93.39	37,203	5.21	10,038	1.41		502,665	68.16
Negros Oriental	323,413	284,465	87.96	270,323	95.03	170	0.06	13,972	4.91		123,876	38.30
Siquijor	23,947	23,318	97.37	21,120	90.57	0	0.00	2,198	9.43		21,586	90.14
Cebu City	237,756	127,272	53.53	123,790	97.26	2,550	2.00	932	0.73		91,198	38.36
Lapu-Lapu City	109,782	89,586	81.60	89,586	100.00	0	0.00	0	0.00		89,586	81.60
Mandaue City	104,620	92,327	88.25	92,327	100.00	0	0.00	0	0.00		0	0.00
**Region 8**	**1,057,790**	**665,176**	**62.88**	**473,579**	**71.20**	**74,609**	**11.22**	**116,988**	**17.59**		**636,992**	**60.22**
Biliran	40,465	34,125	84.33	34,125	100.00	0	0.00	0	0.00		30,412	75.16
Eastern Samar	115,011	7,782	6.77	7,604	97.71	110	1.41	68	0.87		25,611	22.27
Northern Leyte	495,414	297,466	60.04	252,104	84.75	18,971	6.38	26,391	8.87		163,697	33.04
Northern Samar	134,252	48,254	35.94	47,053	97.51	371	0.77	830	1.72		131,921	98.26
Southern Leyte	98,140	74,461	75.87	0	0.00	0	0.00	74,461	100.00		74,461	75.87
Western Samar	174,508	67,433	38.64	60,605	89.87	6,348	9.41	480	0.71		93,682	53.68
Calbayog City	40,620	8,039	19.79	8,039	100.00	0	0.00	0	0.00		5,000	12.31
Maasin City	19,759	17,726	89.71	0	0.00	17,726	100.00	0	0.00		17,726	89.71
Ormoc City	54,513	54,411	99.81	43,916	80.71	0	0.00	10,495	19.29		51,592	94.64
Tacloban City	54,949	55,479	100.96	20,133	36.29	31,083	56.03	4,263	7.68		42,890	78.05
**Region 9**	**810,083**	**484,828**	**59.85**	**411,625**	**84.90**	**19,297**	**3.98**	**53,906**	**11.12**		**401,429**	**49.55**
Zamboanga del Norte	233,608	101,058	43.26	93,077	92.10	326	0.32	7,655	7.57		98,068	41.98
Zamboanga del Sur	425,148	95,168	22.38	59,283	62.29	13,938	14.65	21,947	23.06		59,475	13.99
Zamboanga Sibugay	151,327	44,776	29.59	35,084	78.35	5,033	11.24	4,659	10.41		31,891	21.07
Dapitan City	18,847	14,710	78.05	14,710	100.00	0	0.00	0	0.00		30	0.16
Dipolog City	31,338	30,355	96.86	30,355	100.00	0	0.00	0	0.00		30,355	96.86
Isabela City	26,106	7,996	30.63	4,347	54.36	0	0.00	3,649	45.64		13,405	51.35
Pagadian City	42,905	27,144	63.27	11,148	41.07	0	0.00	15,996	58.93		4,584	10.68
Zamboanga City	201,250	163,621	81.30	163,621	100.00	0	0.00	0	0.00		163,621	81.30
**Region 10**	**1,122,320**	**1,003,570**	**89.42**	**903,942**	**90.07**	**40,299**	**4.02**	**59,329**	**5.91**		**534,094**	**47.59**
Bukidnon	331,498	238,498	71.95	198,744	83.33	26,747	11.21	13,007	5.45		112,959	34.08
Camiguin	21,023	19,137	91.03	19,136	99.99	0	0.00	1	0.01		19,282	91.72
Lanao del Norte	234,430	124,487	53.10	99,168	79.66	12,340	9.91	12,979	10.43		83,475	35.61
Misamis Occidental	141,166	69,414	49.17	58,266	83.94	0	0.00	11,148	16.06		57,353	40.63
Misamis Oriental	394,203	157,346	39.91	147,245	93.58	629	0.40	9,472	6.02		33,056	8.39

Table 1.G.2 - ENVIRONMENTAL HEALTH AND SANITATION SERVICES

Proportion of Households (HHs) with basic sanitation facility and using Safely Managed Sanitation Service

Philippines, Annual 2020

Area	Projected No. of HHs	HH with Basic Sanitation Facility								HHs using Safely Managed Sanitation Service	
		Total	%	Pour/Flush Toilet							
				Septic Tank		Community sewer/sewerage system		Ventilated improved Pit Latrine (VIP)			
				No.	%	No.	%	No.	%	No.	%
Cagayan de Oro City	177,954	168,273	94.56	166,666	99.05	0	0.00	1,607	0.95	168,273	94.56
El Salvador City	12,940	11,617	89.78	11,465	98.69	0	0.00	152	1.31	0	0.00
Gingoog City	28,773	27,098	94.18	27,098	100.00	0	0.00	0	0.00	0	0.00
Iligan City	83,193	56,438	67.84	55,940	99.12	2	0.00	496	0.88	20	0.02
Malaybalay City	42,220	45,001	106.59	35,468	78.82	0	0.00	9,533	21.18	34,521	81.76
Oroquieta City	17,400	13,911	79.95	13,911	100.00	0	0.00	0	0.00	13,072	75.13
Ozamis City	31,827	24,188	76.00	24,188	100.00	0	0.00	0	0.00	0	0.00
Tangub City	13,628	1,776	13.03	581	32.71	581	32.71	614	34.57	12,000	88.05
Valencia City	47,486	46,386	97.68	46,066	99.31	0	0.00	320	0.69	83	0.17
**Region 11**	**1,281,403**	**700,208**	**54.64**	**686,321**	**98.02**	**3,038**	**0.43**	**10,849**	**1.55**	**944,093**	**73.68**
Compostela Valley	178,642	147,939	82.81	135,688	91.72	2,960	2.00	9,291	6.28	139,742	78.22
Davao del Norte	244,701	0	0.00	0	0.00	0	0.00	0	0.00	81,542	33.32
Davao Oriental	138,613	0	0.00	0	0.00	0	0.00	0	0.00	125,369	90.45
Davao del Sur	636,332	127,303	20.01	125,667	98.71	78	0.06	1,558	1.22	127,299	20.01
Davao Occidental	83,115	0	0.00	0	0.00	0	0.00	0	0.00	45,942	55.28
Davao City	459,109	424,966	92.56	424,966	100.00	0	0.00	0	0.00	424,199	92.40
**Region 12**	**1,141,452**	**655,879**	**57.46**	**576,583**	**87.91**	**31,344**	**4.78**	**47,952**	**7.31**	**420,146**	**36.81**
North Cotabato	417,385	199,148	47.71	168,696	84.71	2,158	1.08	28,294	14.21	150,275	36.00
Sarangani	133,555	93,142	69.74	84,429	90.65	6,268	6.73	2,445	2.63	81,679	61.16
South Cotabato	395,597	95,275	24.08	80,811	84.82	8,902	9.34	5,562	5.84	63,743	16.11
Sultan Kudarat	194,915	130,830	67.12	119,357	91.23	0	0.00	11,473	8.77	119,357	61.24
Cotabato City	61,091	59,742	97.79	52,645	88.12	7,008	11.73	89	0.15	5,092	8.34
Gen. Santos City	157,617	77,742	49.32	70,645	90.87	7,008	9.01	89	0.11	0	0.00
**BARMM**	**686,781**	**435,036**	**63.34**	**328,680**	**75.55**	**9,844**	**2.26**	**96,512**	**22.18**	**305,378**	**44.47**
Basilan	66,849	15,466	23.14	11,940	77.20	3,526	22.80	0	0.00	0	0.00
Lanao del Sur	176,663	62,284	35.26	37,746	60.60	0	0.00	24,538	39.40	9,984	5.65
Maguindanao	218,181	315,242	144.49	236,950	75.16	6,318	2.00	71,974	22.83	236,826	108.55
Sulu	153,404	0	0.00	0	0.00	0	0.00	0	0.00	0	0.00
Tawi-Tawi	71,684	0	0.00	0	0.00	0	0.00	0	0.00	0	0.00
Lamitan City	16,830	0	0.00	0	0.00	0	0.00	0	0.00	0	0.00
Marawi City	33,389	42,044	125.92	42,044	100.00	0	0.00	0	0.00	58,568	175.41
**CARAGA**	**611,241**	**486,556**	**79.60**	**464,330**	**95.43**	**5,172**	**1.11**	**17,054**	**3.51**	**338,380**	**55.36**
Agusan del Norte	164,975	36,597	22.18	35,398	96.72	1,055	2.88	144	0.39	24,327	14.75
Agusan del Sur	161,620	124,243	76.87	113,689	91.51	4,115	3.31	6,439	5.18	51,069	31.60
Surigao del Norte	119,040	73,963	62.13	72,561	98.10	2	0.00	1,400	1.89	65,635	55.14
Surigao del Sur	136,211	101,438	74.47	93,204	91.88	0	0.00	8,234	8.12	80,112	58.81
Province of Dinagat	29,395	22,642	77.03	22,642	100.00	0	0.00	0	0.00	0	0.00
Bislig City	22,464	18,195	81.00	17,358	95.40	0	0.00	837	4.60	16,640	74.07
Butuan City	81,831	77,649	94.89	77,649	100.00	0	0.00	0	0.00	69,586	85.04
Surigao City	39,223	31,829	81.15	31,829	100.00	0	0.00	0	0.00	31,011	79.06

Legend:
0 = No Case
** = No Report*

Table 1.G.3 - ENVIRONMENTAL HEALTH AND SANITATION SERVICES
Percentage of Industrial Establishment with Sanitary Permit and Barangays Certified as ZOD
Philippines, Annual 2020

Area	Total No. of Industrial Establishment	Industrial Establishment with Sanitary Permit		Total No. of Barangays	No. of Barangays Certified as ZOD areas	
		No.	%		No.	%
**PHILIPPINES**	**352,044**	**278,061**	**78.98**	**42,134**	**11,138**	**26.43**
**N C R**	**1,594**	**936**	**58.72**	**1,709**	**356**	**20.83**
Malabon	*	0	0.00	21	21	100.00
Navotas	*	0	0.00	18	18	100.00
Valenzuela City	*	0	0.00	33	22	66.67
Caloocan City	579	579	100.00	188	188	100.00
Marikina City	*	0	0.00	16	16	100.00
Pasig City	*	0	0.00	30	30	100.00
Pateros	15	15	100.00	10	0	0.00
Taguig	1,000	342	34.20	28	28	100.00
Quezon City	*	0	0.00	142	0	0.00
Makati City	*	0	0.00	33	33	100.00
Mandaluyong City	*	0	0.00	27	0	0.00
San Juan	*	0	0.00	21	0	0.00
Manila City	*	0	0.00	896	0	0.00
Las Piñas City	*	0	0.00	20	0	0.00
Muntinlupa City	*	0	0.00	9	0	0.00
Parañaque City	*	0	0.00	16	0	0.00
Pasay City	*	0	0.00	201	0	0.00
**C A R**	**11,391**	**9,737**	**85.48**	**1,185**	**315**	**26.58**
Abra	943	943	100.00	303	30	9.90
Apayao	228	228	100.00	134	0	0.00
Benguet	5,356	5,344	99.78	140	0	0.00
Ifugao	171	107	62.57	179	97	54.19
Kalinga	*	0	0.00	157	61	38.85
Mt. Province	3,671	2,847	77.55	144	119	82.64
Baguio City	1,022	268	26.22	128	8	6.25
**Region 1**	**48,224**	**43,036**	**89.24**	**3,265**	**2,263**	**69.31**
Ilocos Norte	8,027	7,877	98.13	477	213	44.65
Ilocos Sur	10,079	9,808	97.31	687	361	52.55
La Union	4,519	2,978	65.90	517	497	96.13
Pangasinan	18,480	16,330	88.37	1,174	885	75.38
Alaminos City	184	184	100.00	39	39	100.00
Candon City	1,701	1,629	95.77	42	32	76.19
Dagupan City	493	493	100.00	31	12	38.71
Laoag City	906	906	100.00	80	72	90.00
San Carlos City	870	700	80.46	86	48	55.81
San Fernando City	1,585	1,066	67.26	59	59	100.00
Urdaneta City	564	249	44.15	34	14	41.18

Table 1.G.3 - ENVIRONMENTAL HEALTH AND SANITATION SERVICES
Percentage of Industrial Establishment with Sanitary Permit and Barangays Certified as ZOD
Philippines, Annual 2020

Area	Total No. of Industrial Establishment	Industrial Establishment with Sanitary Permit		Total No. of Barangays	No. of Barangays Certified as ZOD areas	
		No.	%		No.	%
Vigan City	816	816	100.00	39	31	79.49
**Region 2**	**32,757**	**32,757**	**100.00**	**2,311**	**255**	**11.03**
Batanes	0	0	0.00	29	0	0.00
Cagayan	5,288	5,288	100.00	771	44	5.71
Isabela	23,445	23,445	100.00	862	70	8.12
Nueva Vizcaya	3,787	3,787	100.00	275	9	3.27
Quirino	172	172	100.00	132	41	31.06
Cauayan City	35	35	100.00	65	0	0.00
Ilagan City	16	16	100.00	91	91	100.00
Santiago City	10	10	100.00	37	0	0.00
Tuguegarao City	4	4	100.00	49	0	0.00
**Region 3**	**38,723**	**36,272**	**93.67**	**3,139**	**434**	**13.83**
Aurora	2,584	2,421	93.69	151	0	0.00
Bataan	37	37	100.00	212	0	0.00
Bulacan	2,187	2,187	100.00	433	0	0.00
Nueva Ecija	1,099	850	77.34	680	292	42.94
Pampanga	20,312	19,023	93.65	443	0	0.00
Tarlac	896	861	96.09	435	1	0.23
Zambales	1,762	1,648	93.53	230	45	19.57
Angeles City	965	965	100.00	33	0	0.00
Balanga City	112	112	100.00	25	3	12.00
Cabanatuan City	221	7	3.17	89	89	100.00
City of San Fernando	*	0	0.00	35	0	0.00
Gapan City	27	27	100.00	23	0	0.00
Mabalacat City	201	201	100.00	27	0	0.00
Malolos City	1,158	308	26.60	51	0	0.00
Meycauayan	191	191	100.00	26	0	0.00
Olongapo	*	0	0.00	17	0	0.00
Palayan City	21	21	100.00	19	0	0.00
San Jose City	484	418	86.36	38	0	0.00
San Jose del Monte City	6,331	6,331	100.00	59	0	0.00
Science City of Munoz	*	529	0.00	37	0	0.00
Tarlac City	135	135	100.00	76	4	5.26
**Region 4A**	**12,285**	**10,031**	**81.65**	**4,028**	**798**	**19.81**
Batangas	4,083	3,185	78.01	853	0	0.00
Cavite	1,243	1,066	85.76	420	14	3.33
Laguna	2,754	2,092	75.96	460	25	5.43
Quezon	2,972	2,648	89.10	1,143	751	65.70
Rizal	1,002	865	86.33	178	0	0.00
Antipolo City	*	0	0.00	16	0	0.00
Bacoor City	*	0	0.00	73	0	0.00
Batangas City	*	0	0.00	105	0	0.00

Table 1.G.3 - ENVIRONMENTAL HEALTH AND SANITATION SERVICES
Percentage of Industrial Establishment with Sanitary Permit and Barangays Certified as ZOD
Philippines, Annual 2020

Area	Total No. of Industrial Establishment	Industrial Establishment with Sanitary Permit		Total No. of Barangays	No. of Barangays Certified as ZOD areas	
		No.	%		No.	%
Biñan City	*	0	0.00	24	0	0.00
Cabuyao City	*	0	0.00	18	0	0.00
Calamba City	*	0	0.00	54	0	0.00
Cavite City	*	0	0.00	84	0	0.00
Dasmariñas City	*	0	0.00	75	0	0.00
General Trias City	*	0	0.00	33	0	0.00
Imus City	*	0	0.00	97	0	0.00
Lipa City	*	0	0.00	72	0	0.00
Lucena City	*	0	0.00	33	0	0.00
San Pablo City	*	0	0.00	84	0	0.00
San Pedro City	102	46	45.10	27	0	0.00
Santa Rosa City	*	0	0.00	18	0	0.00
Tagaytay City	93	93	100.00	34	8	23.53
Tanauan City	36	36	100.00	48	0	0.00
Tayabas City	*	0	0.00	66	0	0.00
Trece Martires City	*	0	0.00	13	0	0.00
**Region 4B**	**2,767**	**2,767**	**100.00**	**1,459**	**6**	**0.41**
Marinduque	266	266	100.00	218	0	0.00
Mindoro Occidental	1,917	1,917	100.00	163	3	1.84
Mindoro Oriental	446	446	100.00	426	2	0.47
Palawan	138	138	100.00	367	0	0.00
Romblon	0	0	0.00	219	1	0.46
Puerto Princesa City	0	0	0.00	66	0	0.00
**Region 5**	**8,792**	**7,768**	**88.35**	**3,471**	**737**	**21.23**
Albay	114	48	42.11	650	576	88.62
Camarines Norte	76	76	100.00	282	2	0.71
Camarines Sur	*	0	0.00	1,000	22	2.20
Catanduanes	262	249	95.04	315	0	0.00
Masbate	5,406	5,386	99.63	550	36	6.55
Sorsogon	2,318	1,682	72.56	541	95	17.56
Iriga City	575	296	51.48	36	0	0.00
Legaspi City	30	20	66.67	70	6	8.57
Naga City	11	11	100.00	27	0	0.00
**Region 6**	**19,549**	**14,769**	**75.55**	**4,051**	**2,010**	**49.62**
Aklan	1,019	948	93.03	327	83	25.38
Antique	577	553	95.84	590	219	37.12
Capiz	1,369	1,369	100.00	473	389	82.24
Guimaras	441	433	98.19	98	47	47.96
Iloilo	6,978	6,695	95.94	1,721	1,236	71.82
Negros Occidental	2,349	1,972	83.95	601	36	5.99
Bacolod City	1,428	1,377	96.43	61	0	0.00
Iloilo City	5,388	1,422	26.39	180	0	0.00

Table 1.G.3 - ENVIRONMENTAL HEALTH AND SANITATION SERVICES

Percentage of Industrial Establishment with Sanitary Permit and Barangays Certified as ZOD

Philippines, Annual 2020

Area	Total No. of Industrial Establishment	Industrial Establishment with Sanitary Permit		Total No. of Barangays	No. of Barangays Certified as ZOD areas	
		No.	%		No.	%
**Region 7**	**106,410**	**80,373**	**75.53**	**3,018**	**643**	**21.31**
Bohol	83,192	74,599	89.67	1,109	0	0.00
Cebu	2,806	2,806	100.00	1,066	169	15.85
Negros Oriental	17,497	74	0.42	557	358	64.27
Siquijor	800	800	100.00	134	102	76.12
Cebu City	*	0	0.00	80	0	0.00
Lapu-Lapu City	553	532	96.20	30	12	40.00
Mandaue City	1,562	1,562	100.00	42	2	4.76
**Region 8**	**13,841**	**7,611**	**54.99**	**4,390**	**1,478**	**33.67**
Biliran	1,013	921	90.92	132	92	69.70
Eastern Samar	2,191	2,191	100.00	597	34	5.70
Northern Leyte	2,699	2,161	80.07	1,393	625	44.87
Northern Samar	*	0	0.00	569	180	31.63
Southern Leyte	864	745	86.23	430	360	83.72
Western Samar	5,521	932	16.88	794	78	9.82
Calbayog City	*	0	0.00	157	20	12.74
Maasin City	587	262	44.63	70	63	90.00
Ormoc City	253	114	45.06	110	26	23.64
Tacloban City	713	285	39.97	138	0	0.00
**Region 9**	**0**	**0**	**0.00**	**1,906**	**331**	**17.37**
Zamboanga del Norte	*	0	0.00	622	33	5.31
Zamboanga del Sur	*	0	0.00	628	287	45.70
Zamboanga Sibugay	*	0	0.00	389	0	0.00
Dapitan City	*	0	0.00	49	0	0.00
Dipolog City	*	0	0.00	21	11	52.38
Isabela City	*	0	0.00	45	0	0.00
Pagadian City	*	0	0.00	54	0	0.00
Zamboanga City	*	0	0.00	98	0	0.00
**Region 10**	**26,792**	**14,898**	**55.61**	**2,022**	**421**	**20.82**
Bukidnon	721	621	86.13	387	44	11.37
Camiguin	57	48	84.21	58	51	87.93
Lanao del Norte	3,704	3,704	100.00	462	90	19.48
Misamis Occidental	139	109	78.42	337	146	43.32
Misamis Oriental	983	982	99.90	330	13	3.94
Cagayan de Oro City	16,793	5,048	30.06	80	0	0.00
El Salvador City	112	112	100.00	15	5	33.33
Gingoog City	2,940	2,940	100.00	79	19	24.05
Iligan City	15	8	53.33	44	2	4.55
Malaybalay City	1,112	1,112	100.00	46	0	0.00

Table 1.G.3 - ENVIRONMENTAL HEALTH AND SANITATION SERVICES

Percentage of Industrial Establishment with Sanitary Permit and Barangays Certified as ZOD

Philippines, Annual 2020

Area	Total No. of Industrial Establishment	Industrial Establishment with Sanitary Permit		Total No. of Barangays	No. of Barangays Certified as ZOD areas	
		No.	%		No.	%
Oroquieta City	28	26	92.86	47	44	93.62
Ozamis City	89	89	100.00	51	7	13.73
Tangub City	*	0	0.00	55	0	0.00
Valencia City	99	99	100.00	31	0	0.00
**Region 11**	**1,778**	**1,077**	**60.57**	**1,177**	**142**	**12.06**
Compostela Valley	*	0	0.00	252	21	8.33
Davao del Norte	*	0	0.00	223	24	10.76
Davao Oriental	388	136	35.05	232	36	15.52
Davao del Sur	791	645	81.54	183	31	16.94
Davao Occidental	392	161	41.07	105	30	28.57
Davao City	207	135	65.22	182	0	0.00
**Region 12**	**22,528**	**12,791**	**56.78**	**1,202**	**394**	**32.78**
North Cotabato	2,977	2,977	100.00	544	175	32.17
Sarangani	413	413	100.00	141	86	60.99
South Cotabato	886	886	100.00	199	11	5.53
Sultan Kudarat	1,586	1,322	83.35	255	122	47.84
Cotabato City	4,937	2,975	60.26	37	0	0.00
Gen. Santos City	11,729	4,218	35.96	26	0	0.00
**BARMM**	**3**	**3**	**100.00**	**2,490**	**271**	**10.88**
Basilan	*	0	0.00	165	0	0.00
Lanao del Sur	*	0	0.00	1,063	30	2.82
Maguindanao	3	3	100.00	508	241	47.44
Sulu	*	0	0.00	410	0	0.00
Tawi-Tawi	*	0	0.00	203	0	0.00
Lamitan City	*	0	0.00	45	0	0.00
Marawi City	*	0	0.00	96	0	0.00
**CARAGA**	**4,610**	**3,235**	**70.17**	**1,311**	**284**	**21.66**
Agusan del Norte	1,749	710	40.59	167	11	6.59
Agusan del Sur	505	385	76.24	314	18	5.73
Surigao del Norte	10	10	100.00	281	92	32.74
Surigao del Sur	1,949	1,733	88.92	285	144	50.53
Province of Dinagat	319	319	100.00	100	7	7.00
Bislig City	58	58	100.00	24	0	0.00
Butuan City	15	15	100.00	86	0	0.00
Surigao City	5	5	100.00	54	12	22.22

Legend:
0 = No Case
** = No Report*

Figure 1.G.1 - Percentage of Household with access to basic safe water supply (level 1) by Region Philippines, 2020

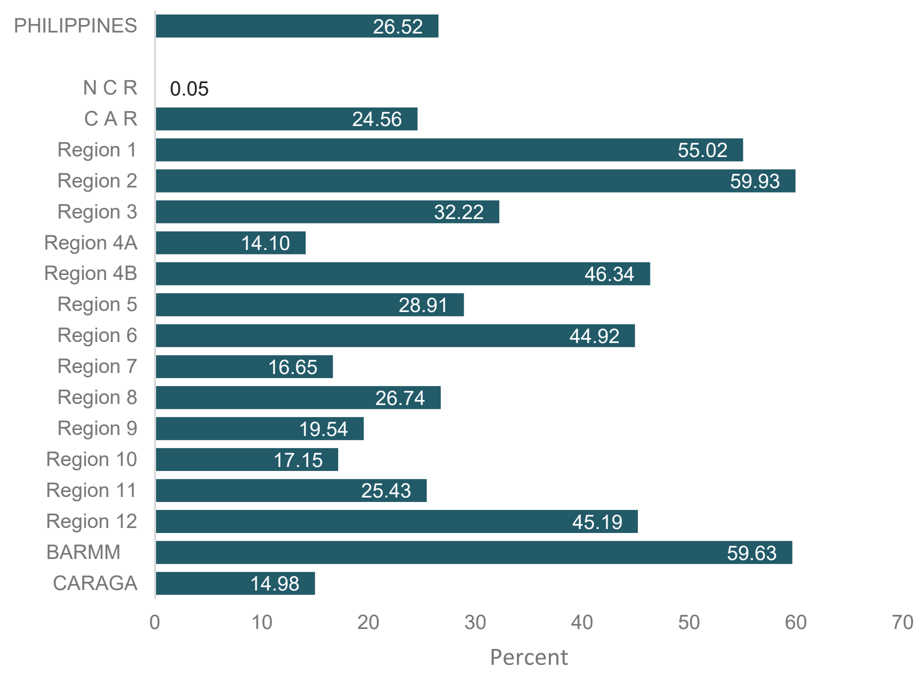

Figure 1.G.2 - Percentage of Household with access to basic safe water supply (level 2) by Region Philippines, 2020

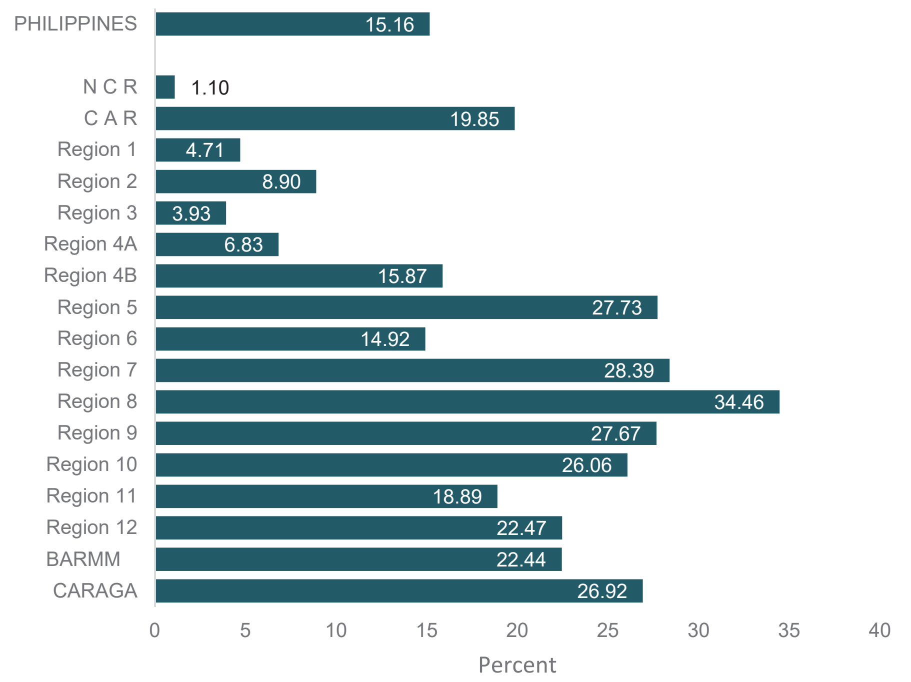

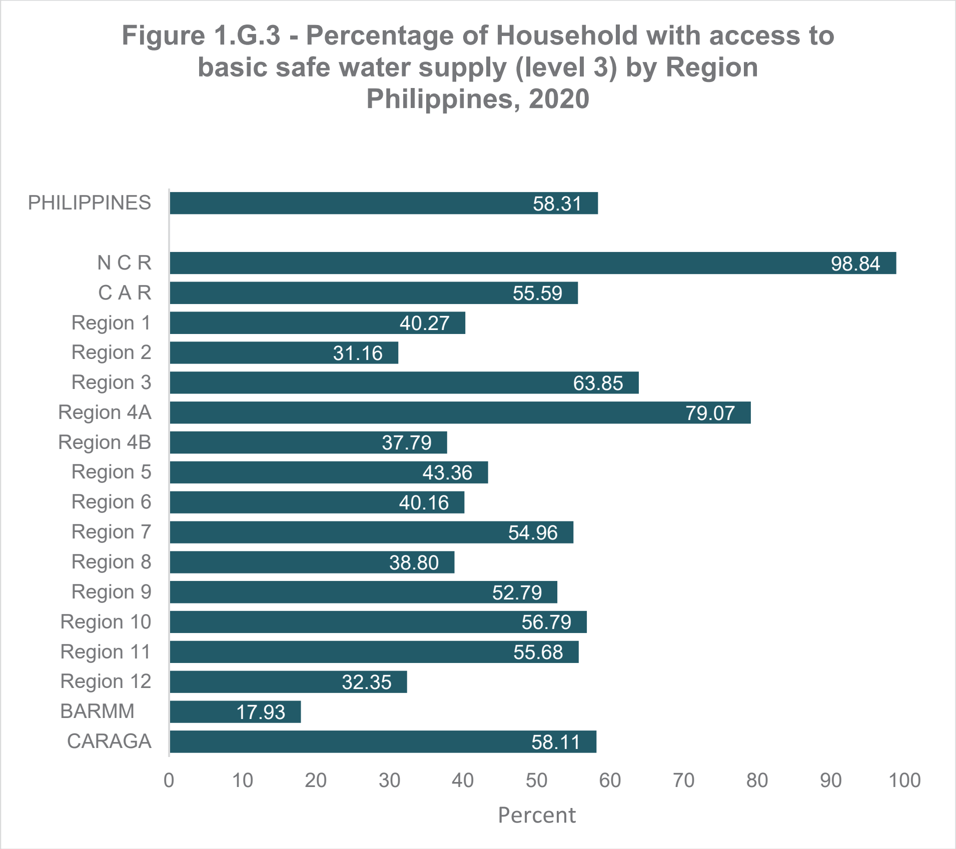
Figure 1.G.3 - Percentage of Household with access to basic safe water supply (level 3) by Region Philippines, 2020
PHILIPPINES 58.31
N C R 98.84
C A R 55.59
Region 1 40.27
Region 2 31.16
Region 3 63.85
Region 4A 79.07
Region 4B 37.79
Region 5 43.36
Region 6 40.16
Region 7 54.96
Region 8 38.80
Region 9 52.79
Region 10 56.79
Region 11 55.68
Region 12 32.35
BARMM 17.93
CARAGA 58.11
0 10 20 30 40 50 60 70 80 90 100
Percent

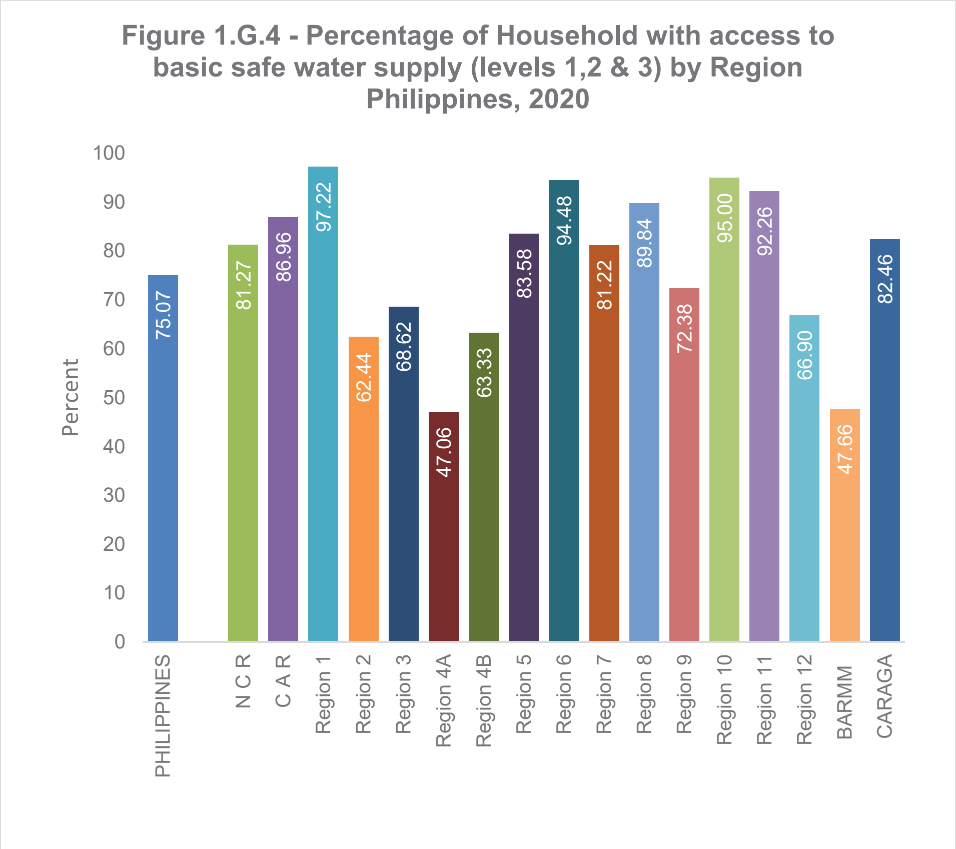
Figure 1.G.4 - Percentage of Household with access to basic safe water supply (levels 1,2 & 3) by Region Philippines, 2020
100 90 80 70 60 50 40 30 20 10 0
Percent
PHILIPPINES 75.07
N C R 81.27
C A R 86.96
Region 1 97.22
Region 2 62.44
Region 3 68.62
Region 4A 47.06
Region 4B 63.33
Region 5 83.58
Region 6 94.48
Region 7 81.22
Region 8 89.84
Region 9 72.38
Region 10 95.00
Region 11 92.26
Region 12 66.90
BARMM 47.66
CARAGA 82.46

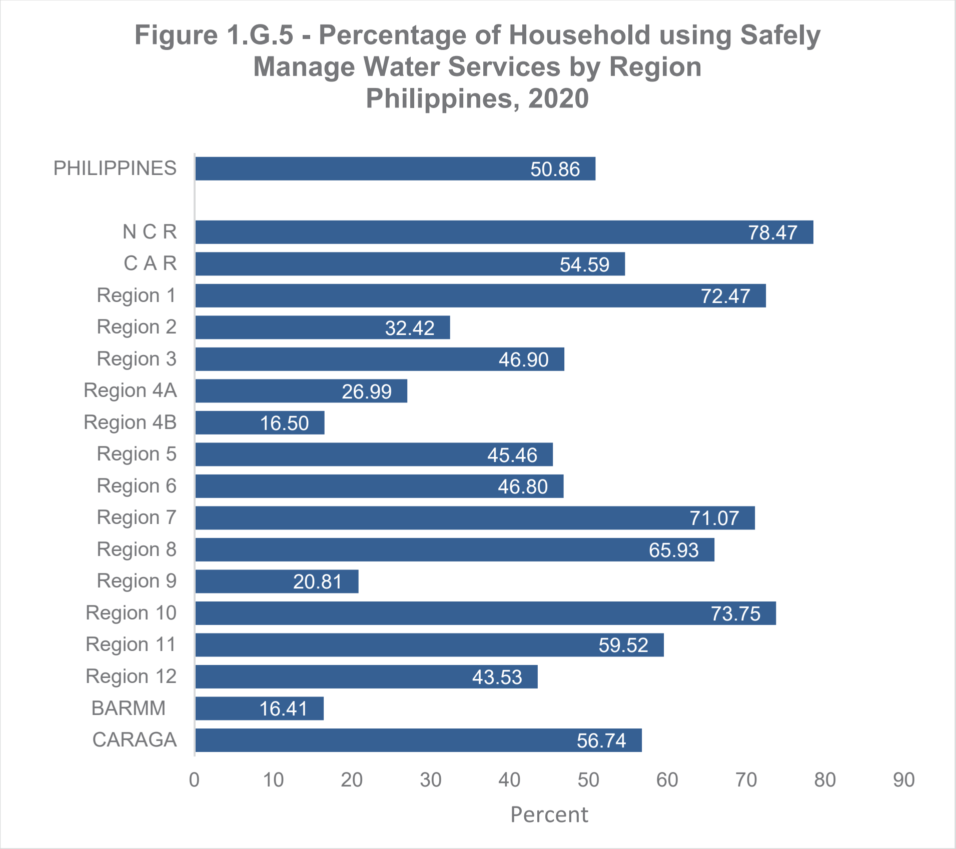
Figure 1.G.5 - Percentage of Household using Safely Manage Water Services by Region
Philippines, 2020
PHILIPPINES
50.86
N C R
78.47
C A R
54.59
Region 1
72.47
Region 2
32.42
Region 3
46.90
Region 4A
26.99
Region 4B
16.50
Region 5
45.46
Region 6
46.80
Region 7
71.07
Region 8
65.93
Region 9
20.81
Region 10
73.75
Region 11
59.52
Region 12
43.53
BARMM
16.41
CARAGA
56.74
0
10
20
30
40
50
60
70
80
90
Percent

Figure 1.G.6 - Percentage of Household with basic Sanitation Facility (Septic Tank) by Region Philippines, 2020

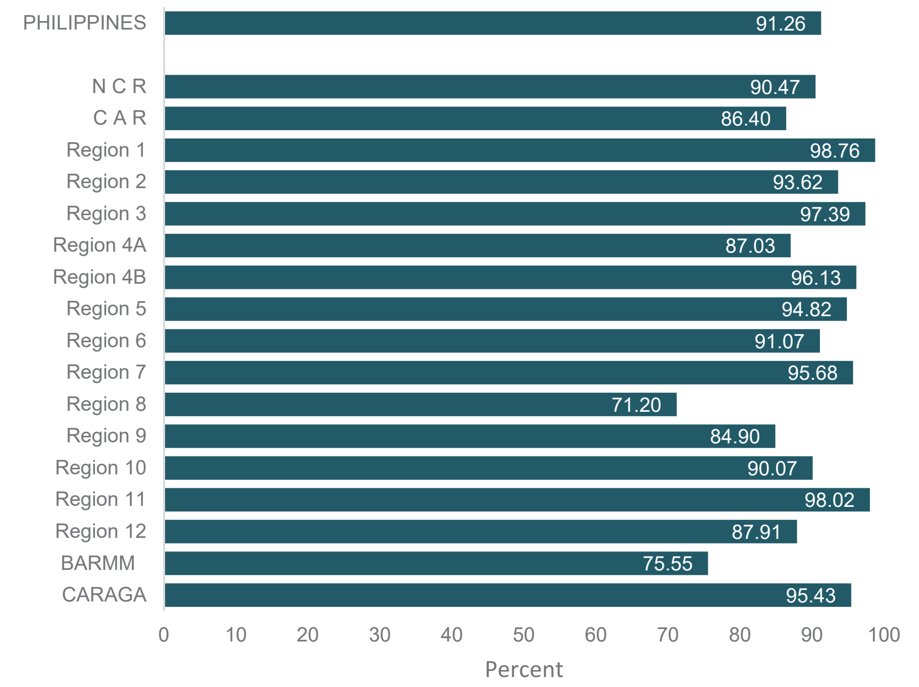

Figure 1.G.7 - Percentage of Household with basic Sanitation Facility (Community Sewerage System) by Region
Philippines, 2020

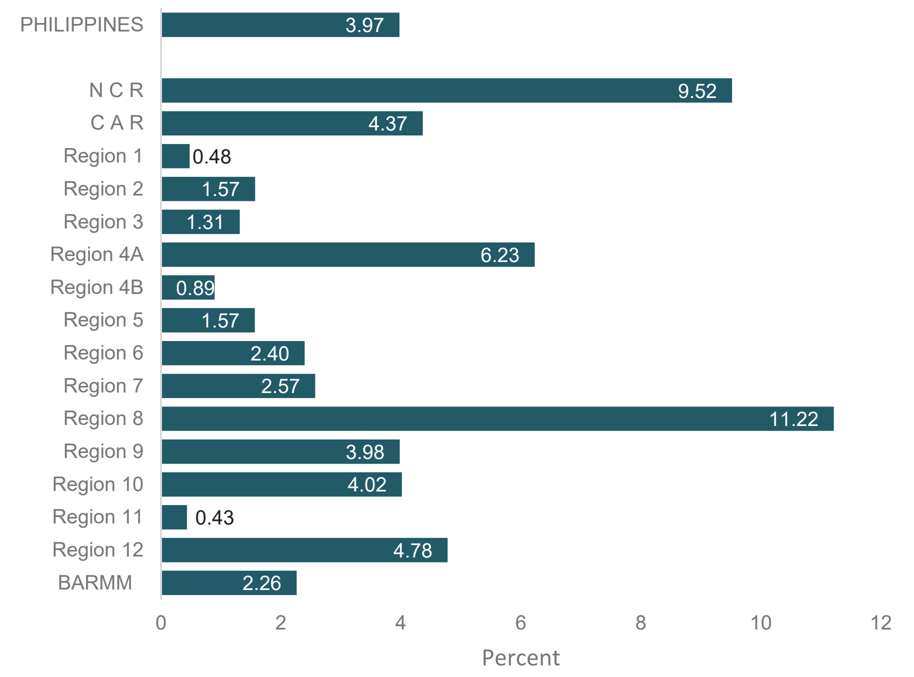

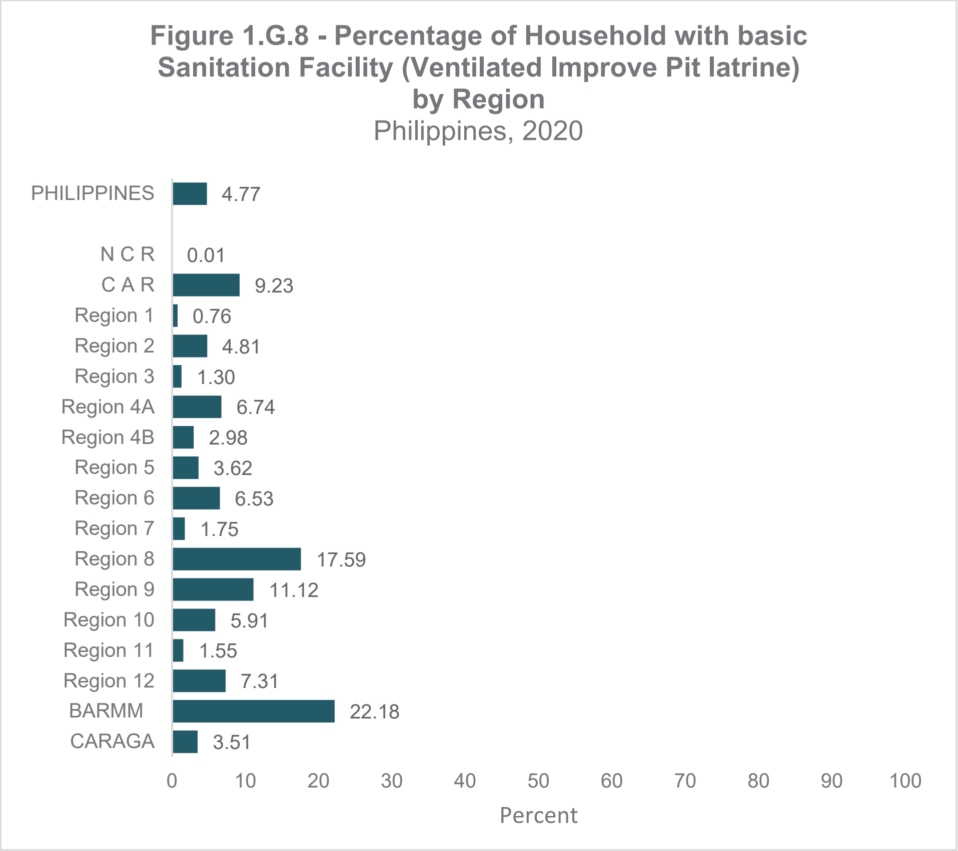
Figure 1.G.8 - Percentage of Household with basic Sanitation Facility (Ventilated Improve Pit latrine) by Region
Philippines, 2020
PHILIPPINES 4.77
N C R 0.01
C A R 9.23
Region 1 0.76
Region 2 4.81
Region 3 1.30
Region 4A 6.74
Region 4B 2.98
Region 5 3.62
Region 6 6.53
Region 7 1.75
Region 8 17.59
Region 9 11.12
Region 10 5.91
Region 11 1.55
Region 12 7.31
BARMM 22.18
CARAGA 3.51
0 10 20 30 40 50 60 70 80 90 100
Percent

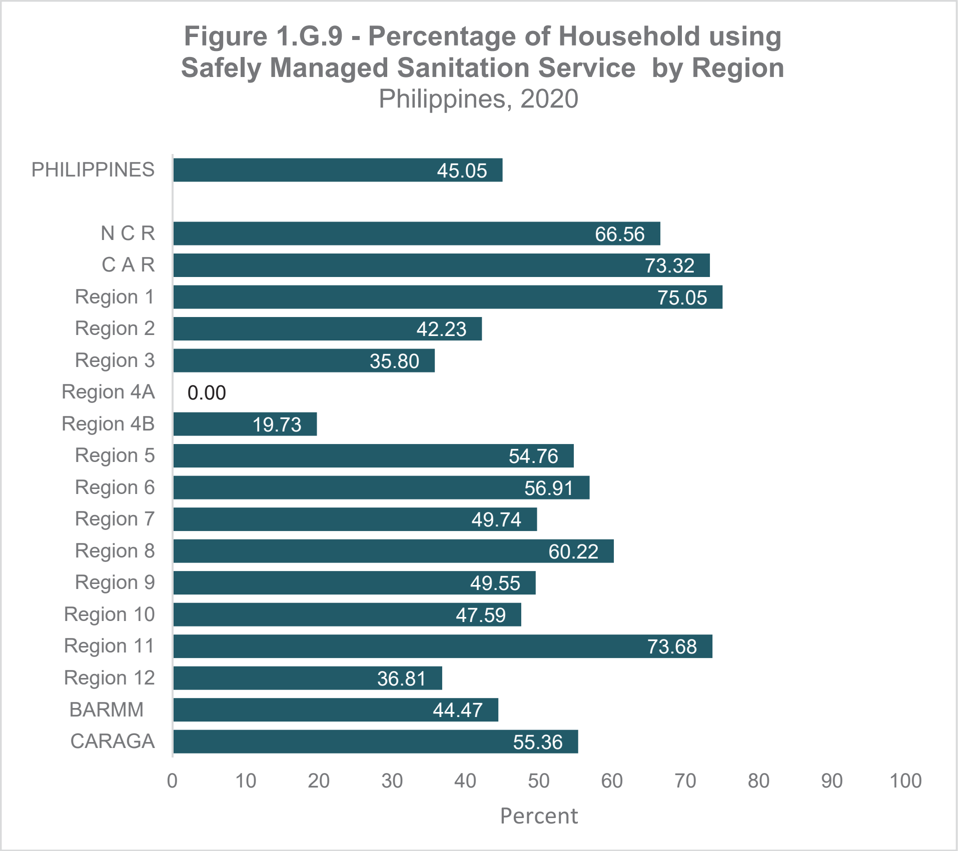
Figure 1.G.9 - Percentage of Household using Safely Managed Sanitation Service by Region
Philippines, 2020
PHILIPPINES 45.05
N C R 66.56
C A R 73.32
Region 1 75.05
Region 2 42.23
Region 3 35.80
Region 4A 0.00
Region 4B 19.73
Region 5 54.76
Region 6 56.91
Region 7 49.74
Region 8 60.22
Region 9 49.55
Region 10 47.59
Region 11 73.68
Region 12 36.81
BARMM 44.47
CARAGA 55.36
0 10 20 30 40 50 60 70 80 90 100
Percent

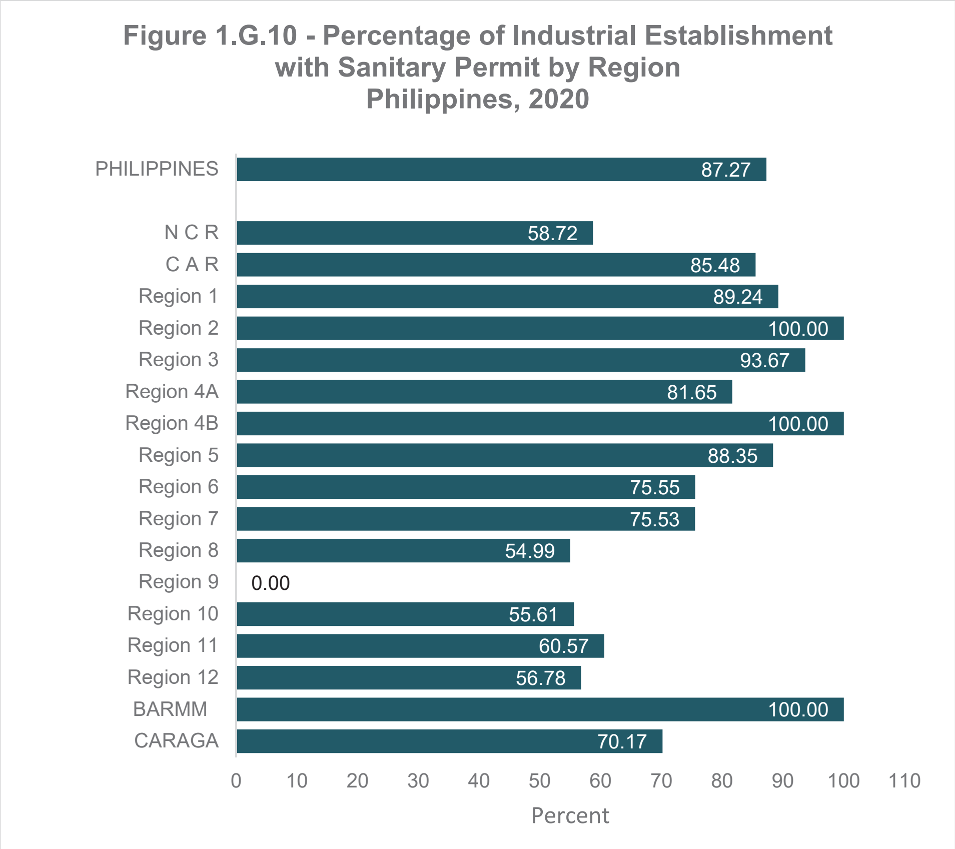
Figure 1.G.10 - Percentage of Industrial Establishment with Sanitary Permit by Region
Philippines, 2020
PHILIPPINES 87.27
N C R 58.72
C A R 85.48
Region 1 89.24
Region 2 100.00
Region 3 93.67
Region 4A 81.65
Region 4B 100.00
Region 5 88.35
Region 6 75.55
Region 7 75.53
Region 8 54.99
Region 9 0.00
Region 10 55.61
Region 11 60.57
Region 12 56.78
BARMM 100.00
CARAGA 70.17
0 10 20 30 40 50 60 70 80 90 100 110
Percent

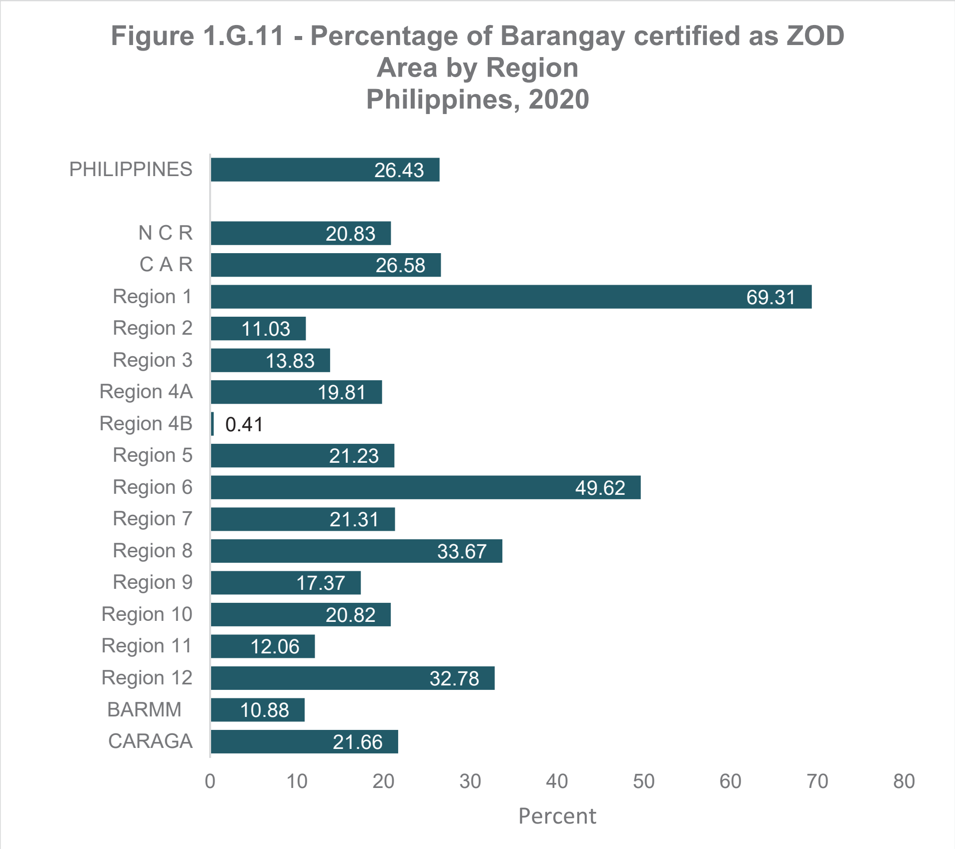
Figure 1.G.11 - Percentage of Barangay certified as ZOD Area by Region
Philippines, 2020
PHILIPPINES 26.43
N C R 20.83
C A R 26.58
Region 1 69.31
Region 2 11.03
Region 3 13.83
Region 4A 19.81
Region 4B 0.41
Region 5 21.23
Region 6 49.62
Region 7 21.31
Region 8 33.67
Region 9 17.37
Region 10 20.82
Region 11 12.06
Region 12 32.78
BARMM 10.88
CARAGA 21.66
0 10 20 30 40 50 60 70 80
Percent

CHAPTER 1

2020 FHSIS Annual Report

Program Summary

1.A Family Planning

In 2020, there is an increase in the number of new acceptors except for Pills-POP which has a 36% decrease in the number of new acceptors. Among the top 5 family planning methods with the highest % increase in new acceptors are: 1) MSTR/NSV with 413% increase; 2) IUD-Postpartum and NFP-STM at 375% increase; 3) PILLS-COC with 246% increase; 4) NFP-BBT with 191% increase and 5) FSTR/BTL with 172% increase.

Table 1.A.1 - Number of New Acceptors by Type of Family Planning Method
Philippines, 2019 vs. 2020

Method	New Acceptors		% Change
	2019	2020	
FSTR/BTL	16,855	45,898	172% ↑
MSTR/NSV	430	2,205	413% ↑
CONDOM	35,676	51,478	44% ↑
IUD-INTERVAL	15,637	38,551	147% ↑
IUD-POSTPARTUM	6,443	30,580	375% ↑
PILLS-POP	97,462	62,727	36% ↓
PILLS-COC	79,830	275,988	246% ↑
INJECTABLES	150,654	192,593	28% ↑
IMPLANTS	45,555	47,759	5% ↑
NFP-CCM	1,788	3,976	122% ↑
NFP-BBT	781	2,276	191% ↑
NFP-STM	424	2,012	375% ↑
NFP-SDM	5,336	10,267	92% ↑
NFP-LAM	399,195	457,231	15% ↑

There is an increase in the number of current users by type of family planning method in 2020 except for FSTR/BTL and Condom with 1% decrease. The family planning method with the most number of current users are PILLS-COC with 411% increase and IUD-Postpartum with 322% increase.

Table 1.A.2 - Number of Current Users by Type of Family Planning Method
Philippines, 2019 vs. 2020

Method	Current Users		% Change
	2019	2020	
FSTR/BTL	1,002,083	995,656	1% ↓
MSTR/NSV	15,041	15,040	0% ↑
CONDOM	402,602	399,696	1% ↓
IUD-INTERVAL	551,953	555,991	1% ↑
IUD-POSTPARTUM	10,210	43,037	322% ↑
PILLS-POP	2,846,552	2,866,232	1% ↑
PILLS-COC	61,893	316,554	411% ↑
INJECTABLES	1,456,906	1,561,599	7% ↑
IMPLANTS	433,558	474,217	9% ↑
NFP-CCM	52,960	56,304	6% ↑
NFP-BBT	3,440	5,728	67% ↑
NFP-STM	3,392	4,937	46% ↑
NFP-SDM	98,943	99,785	1% ↑
NFP-LAM	677,835	691,144	2% ↑

Table 1.A.3 - Number of Drop Outs by Type of Family Planning Method
Philippines, 2019 vs. 2020

Method	Drop Outs		%
	2019	2020	Change
FSTR/BTL	71,687	108,089	**51%** ↑
MSTR/NSV	3,208	3,319	**3%** ↑
CONDOM	122,656	139,770	**14%** ↑
IUD-INTERVAL	62,817	71,980	**15%** ↑
IUD-POSTPARTUM	12,004	24,661	**105%** ↑
PILLS-POP	368,775	122,223	**67%** ↓
PILLS-COC	167,283	490,016	**193%** ↑
INJECTABLES	300,141	436,008	**45%** ↑
IMPLANTS	44,521	91,178	**105%** ↑
NFP-CCM	14,382	34,879	**143%** ↑
NFP-BBT	940	1,729	**84%** ↑
NFP-STM	1,179	1,327	**13%** ↑
NFP-SDM	20,288	28,472	**40%** ↑
NFP-LAM	701,175	871,007	**24%** ↑

For the number of drop-outs by type of family planning method, an increase was noted in all types of the method except for PILLS-POP with a 67% decrease in drop-outs. The type of family planning with the most number of drop-outs are: 1) PILLS-COC with a 193% increase; 2) NFP-CCM with 143% increase; 3) IUD-Postpartum and Implants with 105% increase; 4) NFP-BBT with 84% increase; and 5) FSTR/BTL with 51% increase.

Figure 1.A.1 - Proportion of Women of Reproductive Age with Unmet Need for Modern FP (MFP) by Region
Philippines, 2020

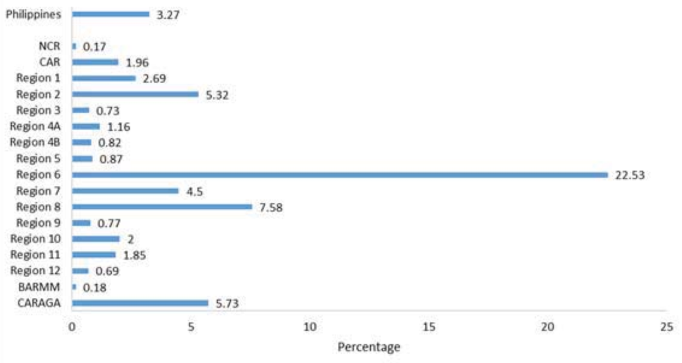

The proportion of women of reproductive age with unmet need is 3.27% in 2020. The regions with the highest unmet need are Region 6 with 22.53%, followed by Region 8 with 7.58%, and CARAGA with 5.73%, while the regions with the least unmet need are NCR with 0.17%, followed by BARMM with 0.18%, and Region 12 with 0.69%.

1.B Maternal Care

- **Prenatal Care**

Figure 1.B.1 shows that 55.76% of pregnant women in 2020 had 4 or more prenatal or antenatal checkups (4ANC) availed as follows: at least one during the 1st trimester, at least one during the 2nd trimester, and at least two during the 3rd or last trimester of pregnancy. A decrease was noted for the proportion of 4ANC from 55.41% in 2016 to 52.55% in 2018 while an abrupt increase was noted in 2019 at 58.37%. Out of the pregnant women who had 4ANC in 2020, 1,047,698 (49.35%) were aged 20-49 years old, while 133,751 (6.30%) were adolescents aged 15-19 years old and 2,399 (0.11%) were from the 10-14 years old

Figure 1.B.1 - Proportion of Pregnant Women with at least 4 Prenatal Check-ups
Philippines, 2016-2020

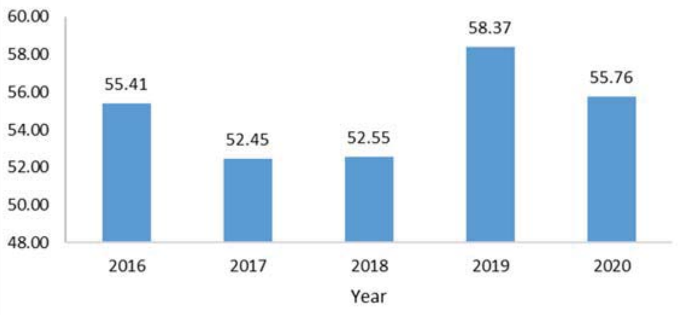

In 2020, the top 3 regions with the highest proportion of pregnant women (10-49 years old) who have had at least 4 prenatal check-ups were from Region 10 at 73.83%, followed by BARMM at 67.60% and Region 1 at 67.17%. Meanwhile, the lowest proportion of pregnant women who have had at least 4 prenatal check-ups were Regions 4A and 4B at 31.79% and 45.87%, respectively.

Figure 1.B.2 - Pregnant Women 10-49 years old with 4 Prenatal check-up by Region
Philippines, 2020

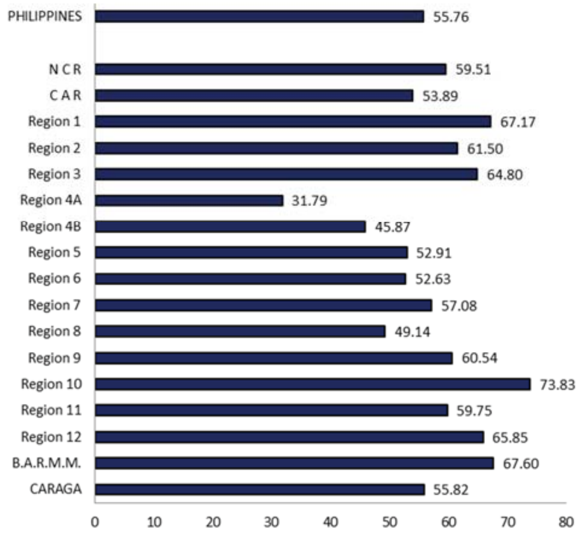

Pregnant women have been getting both tetanus and diphtheria toxoids (Td) and tetanus toxoid (TT) vaccines to prevent neonatal tetanus. In 2020, 66.31% of the estimated number of pregnant women in the country were given sufficient doses of Td vaccine. Of this, 544,968 (25.67%) of pregnant women were vaccinated with Td (2 doses) during their first pregnancy and 862,841 (40.64%) were vaccinated with Td (at least 3 doses) during their 2nd and succeeding pregnancies.

In the 20-49 years old age group, 20.32% were given 2 doses of Tetanus Diphtheria Vaccine on their 1st pregnancy and 36.94% were given at least 3 doses of Tetanus Diphtheria Vaccine on their 2nd and succeeding pregnancies.

Figure 1.B.3 - Proportion of Pregnant Women for the 1st, 2nd and more times given Tetanus Diphtheria Vaccine by Region
Philippines, 2020

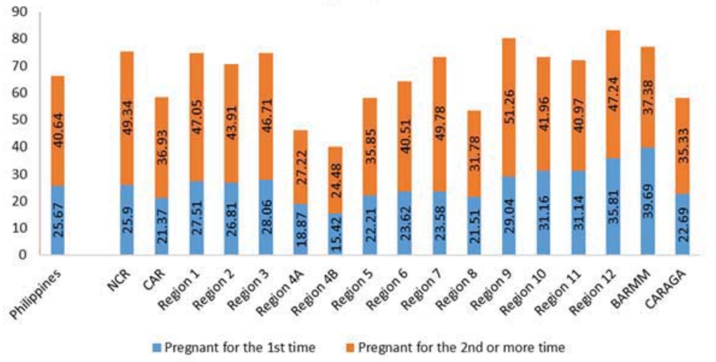

Figure 1.B.4 - Proportion of Pregnant Women given Tetanus Diphtheria Vaccine by Age Group
Philippines, 2020

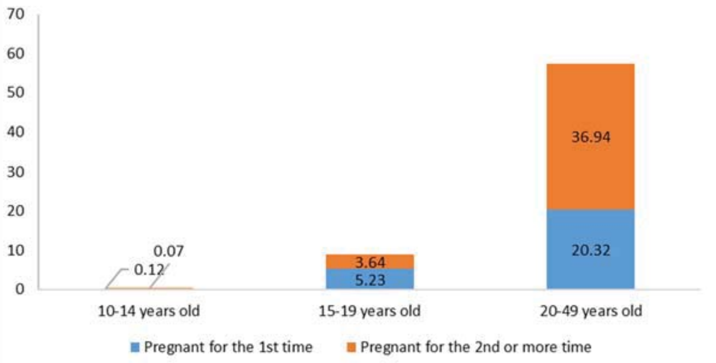

Figure 1.B.5 illustrates the increase both in the proportion of pregnant women who completed Iron and Folic Acid supplementation from 51.66% in 2016 to 57.75% in 2019 and on the proportion of postpartum women who completed Iron and Folic Acid supplementation from 51.25% in 2016 to 59.72% in 2019. A steep decline was seen in 2020 at 50.93% for pregnant women who completed Iron and Folic Acid supplementation and 52.62% for postpartum women. Complete supplementation means that these pregnant women received 60 mg of elemental iron with 400 mcg folic acid once daily for six (6) months and the same dose of iron and folic acid supplementation given to postpartum women for 3 months after delivery.

Figure 1.B.5 - Proportion of Pregnant and Postpartum Women who completed doses of Iron and Folic Acid
Philippines, 2016 - 2020

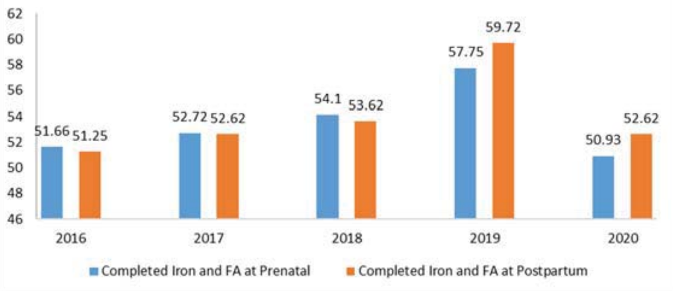

- **Intrapartum Care**

Skilled health professionals are competent maternal and newborn health personnel who are educated, trained, and regulated to national and international standards. They are competent in managing labor and delivery to ensure a positive childbirth experience for women.

In addition, they are competent in identifying and managing, or referring women and/or newborn infants with health complications. *(figo.org)*

Figure 1.B.6 - Proportion of Deliveries by Skilled Health Professionals
Philippines, 2016-2020

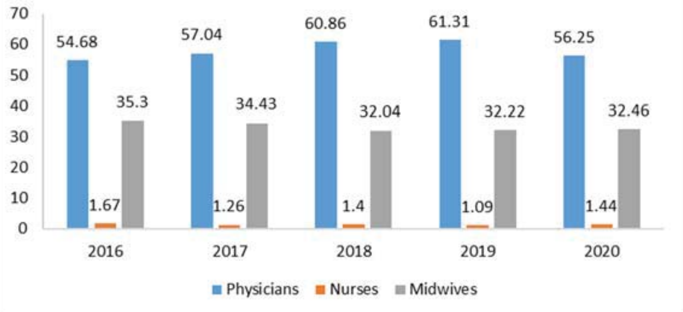

The proportion of deliveries by physicians had a steady increase from 2016 to 2019 and a decline from 2019 to 2020 with 61.31% and 56.25% respectively. For the deliveries by nurses and midwives, not much change was seen from 2016 to 2020.

Of the total deliveries in 2020, 1,292,177 (90.15%) were delivered by Skilled Health Professionals; 806,246 (56.25%) were attended by physicians while 20,686 (1.44%) were delivered by nurses and 465,245 (32.46%) were delivered by midwives.

Figure 1.B.7- Deliveries Attended by Skilled Health Professionals
Philippines, 2020

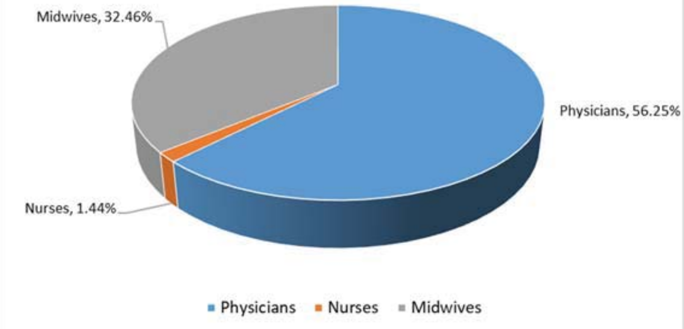

The regions with the highest percentage of deliveries attended by skilled health professional are Region 1 with 55,329 (99.70%) deliveries followed by CAR with 25,779 (97.97%) deliveries. The lowest percentage of deliveries by skilled health professional was recorded in BARMM and Region 7 with 35,821 (50.69%) and 104,378 (80.78%) deliveries, respectively.

Figure 1.B.8 - Deliveries attended by Skilled Health Professional
Philippines, 2020

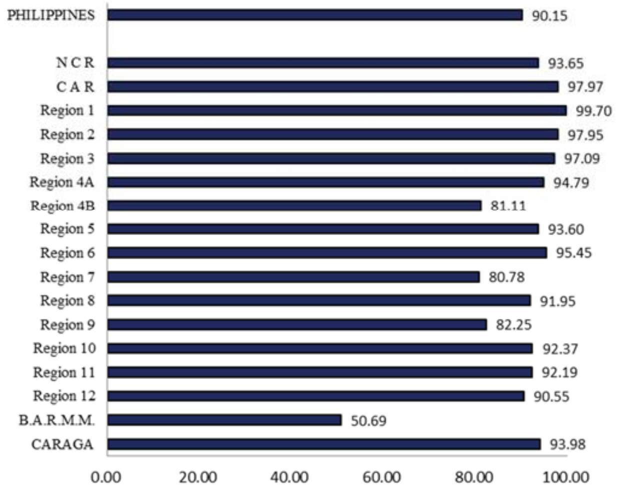

An increasing trend was seen from 2016 to 2018 with 89.14% and 92.53% respectively. But a decreasing trend was seen from 92.53% in 2018 to 89.41% in 2020.

Figure 1.B.9 - Facility Based Delivery
Philippines, 2016-2020

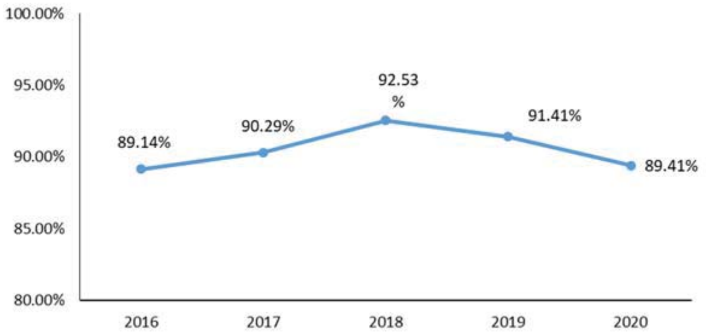

As for the deliveries in the health facility, Region 1 has the highest births in the health facilities with 99.53% followed by Region 2 with 97.15% and Region 3 with 96.61%. The lowest recorded facility delivery is BARMM with 65.65%.

Figure 1.B.10 - Facility Based Delivery
Philippines, 2020

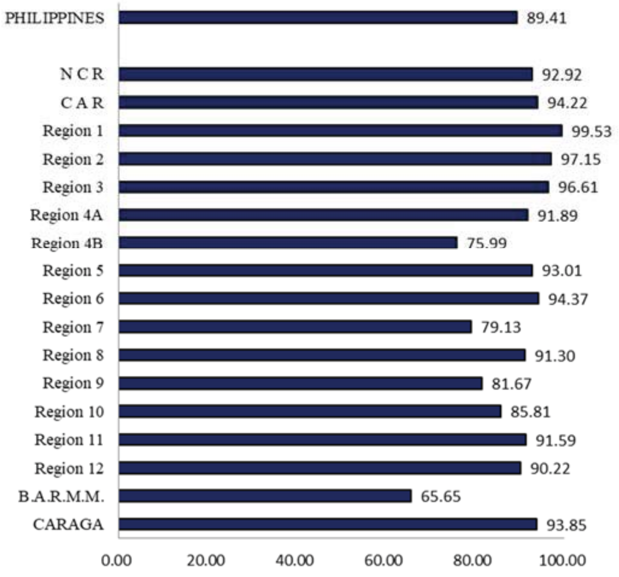

Birth weight is an important indicator of a newborn's health status. Neonates born with low birth weight generally have higher rates of morbidity and mortality. A newborn's weight at birth is strongly associated with mortality risk during the first year and, to a lesser degree, with developmental problems in childhood and the risk of various diseases in adulthood. Epidemiological analyses often regard birthweight as the causal pathway to these health outcomes. *(https://academic.oup.com/ije/article/30/6/1233/651751)*

Among neonates with reported birth weight in 2020, 91.52% were classified as having normal birthweight or with a birth weight ≥ 2,500 grams. Figure 1.B.11 shows a slight increase in the percentage of neonates with normal birth weight from 2019 to 2020. Neonates with low birth weight (≤ 2,500 grams) remained at less than 10% of the total live births at 7.15% in 2019 and 6.17% in 2020. Births with no reported weight decreased from 2.62% in 2019 to 2.31% in 2020.

Figure 1.B.11 - Percentage of Livebirths by Weight
Philippines, 2019 vs 2020

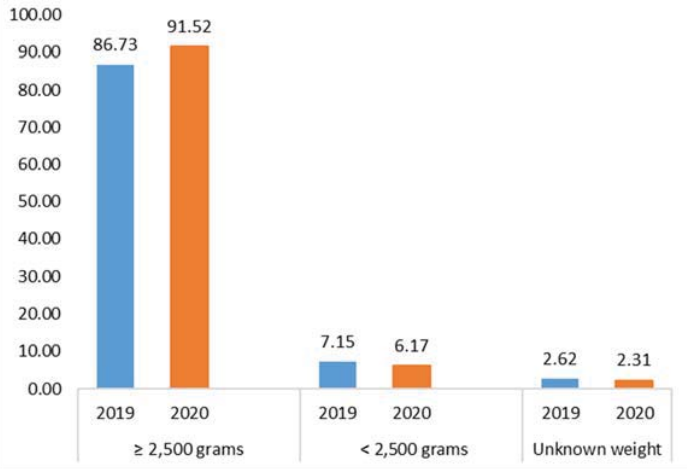

- **Postpartum Care**

From 2016 to 2018, there was a decrease in the proportion of postpartum women with at least 2 postpartum check-ups followed by a 7.45% increase from 2018 to 2019, and from 2019 to 2020, there was a 4.37% decrease in the proportion of postpartum women with at least 2 postpartum check-ups.

Figure 1.B.12 - Proportion of Postpartum Women with at least 2 Postpartum Check-ups
Philippines, 2016 - 2020

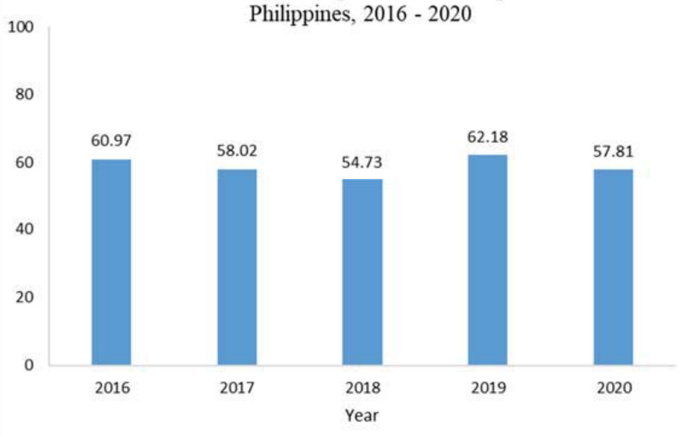

Among women aged 10-49 years old who gave birth in 2020, 57.81% completed two postpartum (2PP) check-ups together with their newborn. The majority of the postpartum women belonged to the 20-49 age group at 51.09% followed by the 15-19 age group at 6.57% (Table 1.B.1). The first check-up was done within 24 hours or before the mother was discharged from the birthing facility and the second check-up was within seven (7) days after delivery.

Table 1.B.1 - Number and Proportion of Postpartum Women with at least 2 Postpartum Check-ups
Philippines, 2020

Age Group	No.	%
10 - 14	3,183	0.15%
15 - 19	139,424	6.57%
20 - 49	1,084,799	51.09%

In 2020, the regions with the highest proportion of women with 2 postpartum check-ups were recorded in Region 11 at 72.49%, followed by Region 10 at 72.39% and Region 9 at 66.83%. The three (3) regions with the least proportion of women with 2 postpartum check-ups were recorded in Region 4A at 38.22%, Region 7 at 46.94%, and Region 4B at 50%.

Figure 1.B.13 - Postpartum Women together with their Newborn who completed at least 2 Postpartum Check-ups by Region
Philippines, 2020

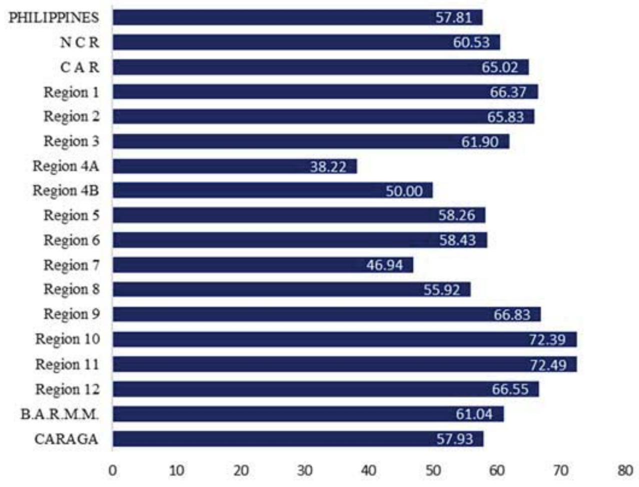

For this 5-year period, there was an average increase in the proportion of postpartum women who completed an Iron with Folic Acid Supplementation from 2016 to 2019 at 51.25% and 59.72%, respectively, and 7.1% decline was seen from 2019 to 2020.

Figure 1.B.14 - Proportion of Postpartum Women who Completed Iron with Folic Acid Supplementation
Philippines, 2016 - 2020

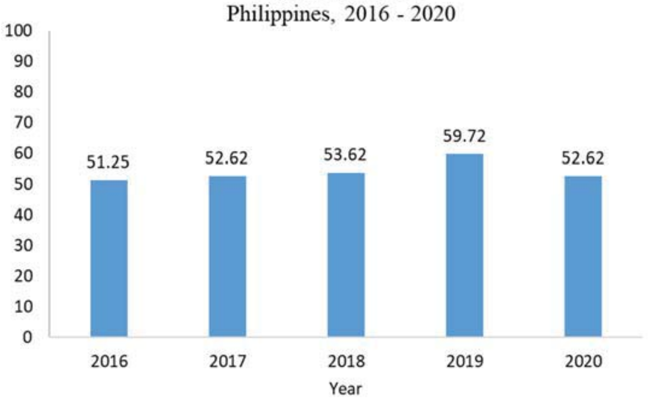

There was an average increase in the proportion of postpartum women who completed Vitamin A supplementation from 55.55% in 2016 to 59.93% in 2019. In this 5-year period, 2017 and 2020 have recorded the lowest percentage of vitamin A supplementation among postpartum women at 52.49% and 53.11%, respectively.

Figure 1.B.15 - Proportion of Postpartum Women Given Vitamin A Supplementation
Philippines, 2016-2020

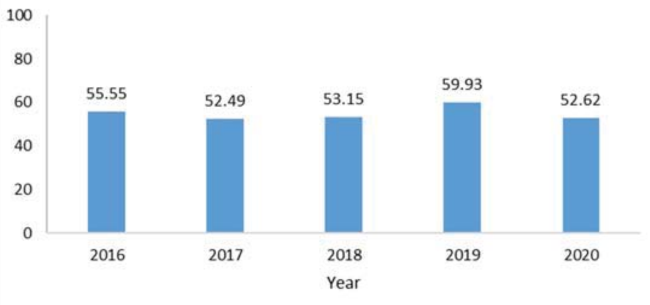

1.C Child Care

- **Immunization Services for Infants and Children**

Fully Immunized Child is an infant who received one dose of BCG, three doses each of OPV, DPT, and Hepatitis B vaccines, and one dose of measles vaccine before reaching one year of age. In the Philippines, there were 1,383,772 (65.18%) fully immunized children (FIC) in 2020. This was 29.82% below the national program target of 95%. Figure 1.C.1 shows that in 5 years, the annual proportion of fully immunized children has not achieved the desired immunization program coverage and it has declined from 2016 (80.20%) to 2018 (73.62%) with a slight increase from 2018 to 2020 (79.8%).

Figure 1.C.1 - Percentage of FIC + CIC versus the Program Targets
Philippines, 2016 - 2020

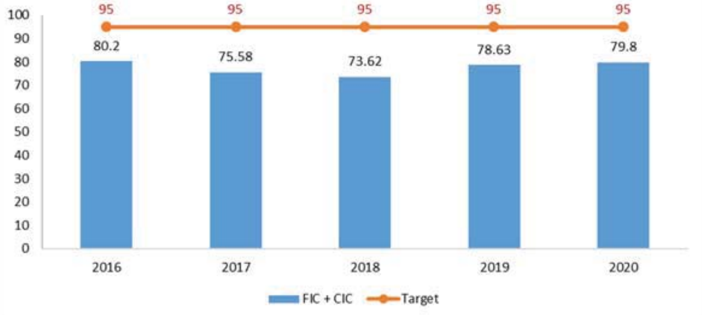

There was an increased percentage of completely immunized children (CIC) or children (13-23 months) who were completely immunized outside the recommended vaccination schedule from 9.55% in 2019 to 14.62% in 2020. In figure 1.C.2, the top 3 regions with the highest percentage distribution of Fully Immunized Children were Region 3 at 78.75%, followed by Region 1 at 76.63% and CARAGA at 75.54% while Region 4B has the lowest percentage at 52.19%. Moreover, all regions have FIC percentages of more than 50%. For CIC, Region 5 recorded the highest percentage at 24.89% followed by Region 7 at 23.25%. While Region 4B has the lowest percentage at 5.99%.

Figure 1.C.2 - Percentage Distribution of FIC and CIC by Region
Philippines, 2020

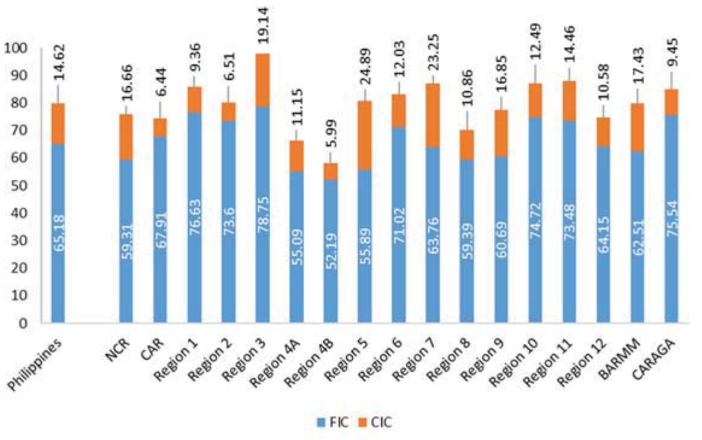

A minimal increase was observed from 2016 to 2018 in the proportion of children protected at birth (CPAB). However, there was an abrupt decrease from 2018 at 85.98% to 2019 and 2020 at 60.32% and 60.20%, respectively.

Figure 1.C.3 - Proportion of Children Protected at Birth
Philippines, 2016-2020

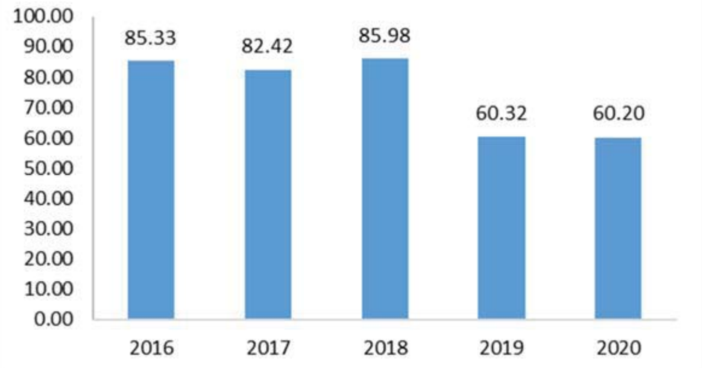

In the Philippines, the proportion of infants given Hepatitis B vaccine slightly decreased from 52.12% in 2016 to 49.90% in 2018, followed by an increase in 2019 at 59.93% and a minimal decrease in 2020 at 56.77%. The same changes were noted in the proportion of infants given BCG vaccine with a decrease from 69.78% in 2016 to 60.58% in 2018, an increase in 2019 at 70.48%, and a slight decrease in 2020 at 67.98%.

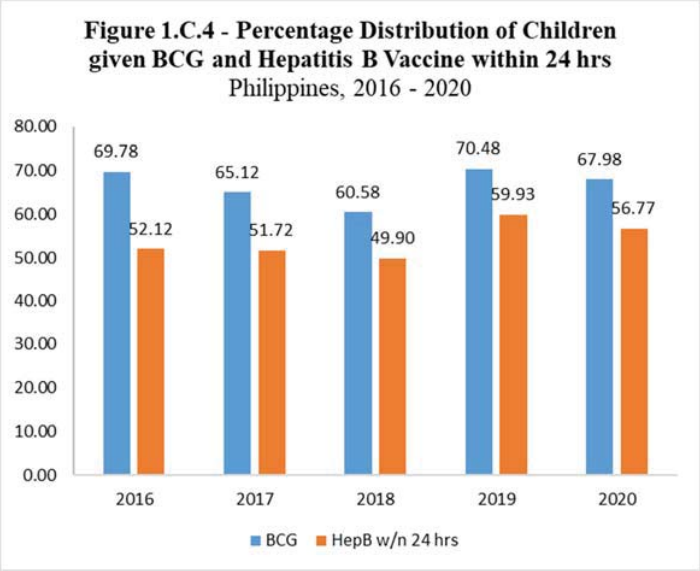

In 2020, the region with the highest percentage distribution of infants given BCG Vaccine was recorded in the NCR at 82.27% followed by Region 11 at 78.74% while the region with the lowest percentage distribution of infants given BCG vaccine was Region 4B at 48.47%, Region 6 at 56.02%, and Region 1 at 58.42%.

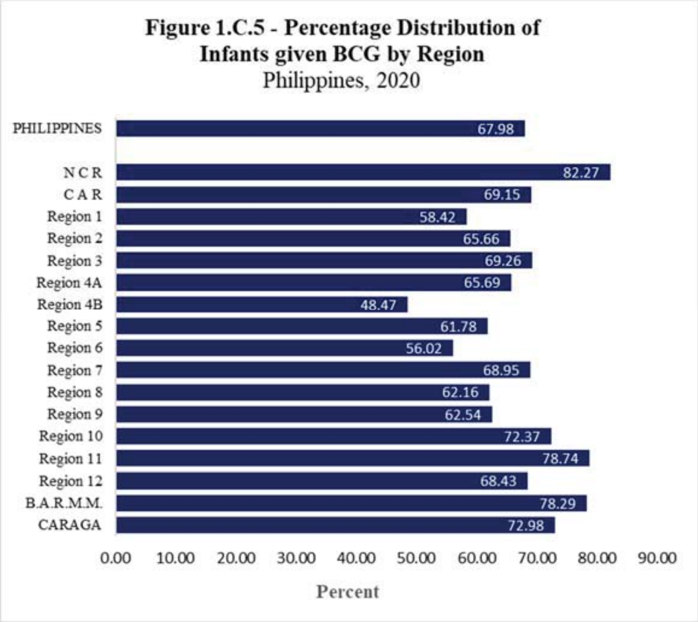

The region with the highest percentage of infants given Hepatitis B vaccine within 24 hours after birth was recorded in NCR at 74.93% and followed by Region 11 at 73.27%. There were 5 regions with less than 50% coverage of infants in the eligible estimated population who were given Hepatitis B vaccine within the first 24 hours of life namely: Region 4B at 37.51%, CARAGA at 42.33%, Region 8 at 48.53%, Region 5 at 49.47% and Region 9 at 49.88%.

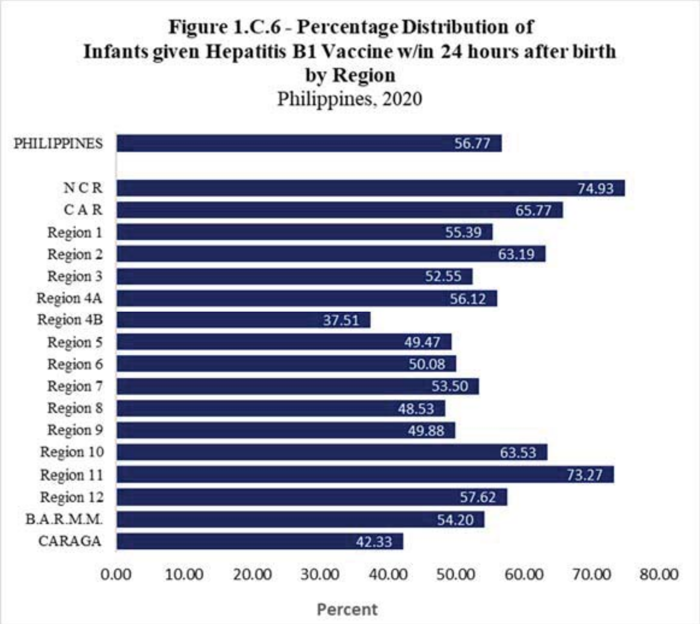

Diphtheria, Pertussis, Tetanus, (DPT), Hepatitis B, and *Haemophilus influenza type B* (Hib) vaccines are part of the Expanded Program on Immunization in the Philippines. For the proportion of infants vaccinated with complete doses of Pentavalent or DPT-Hib-HepB, the highest increase was noted in 2016 with 84.86% for the 3rd dose and lowest in 2018 with 65.10%. A gradual increase in the proportion of infants vaccinated with complete pentavalent vaccines was seen from 2018 to 2020 at 65.10% and 74.80% respectively.

Figure 1.C.7 - Proportion of Children given DPT-Hib-HepB Vaccine
Philippines, 2016-2020

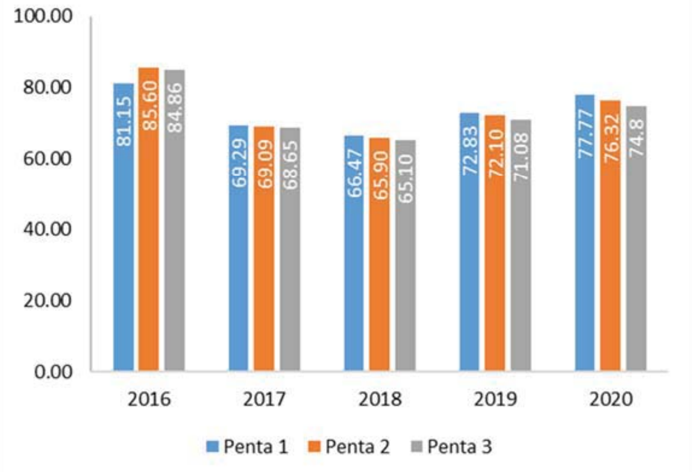

In 2020, the top 3 regions with the highest proportion of infants vaccinated with DPT-Hib-HepB3 are Region 3 at 90.21%, Region 10 at 84.46%, and Region 7 at 80.88%.

For the proportion of infants vaccinated with DPT-Hib-HepB2, the top 3 regions are Region 3 at 90.16%, Region 10 at 85.64%, and Region 7 at 82.44%.

The top 3 regions for the proportion of infants vaccinated with DPT-Hib-HepB1 are Region 3 at 90.51%, Region 10 at 86.91%, and Region 7 at 83.44%.

Figure 1.C.8 - Percentage Distribution of Infants who completed 3 doses of HiB-HepB antigen
Philippines, 2020

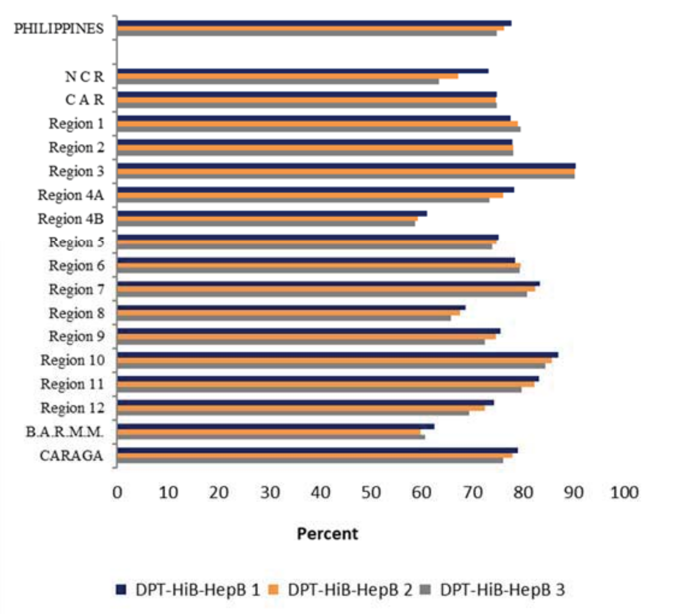

As shown in Figure 1.C.9, the proportion of infants vaccinated with OPV 1, 2, and 3 has a relative decline from 2016 to 2018. It was also observed that the coverage rates decreased for each subsequent dose. There was an increase in the proportion of infants vaccinated with OPV 1, 2, and 3 in 2019 and 2020 and recorded the highest coverage in the 5-year period.

Figure 1.C.9 - Proportion of Children given Oral Polio Vaccine (OPV)
Philippines, 2016 - 2020

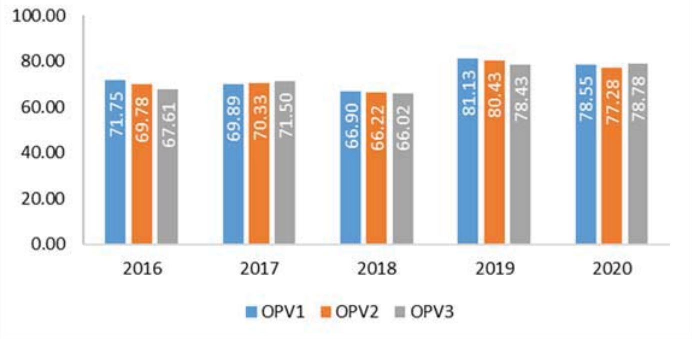

The region with the highest proportion of infants given OPV 1, 2, and 3 was recorded in Region 3 with 90.62%, 90.62%, and 90.97%, respectively. This figure also shows that all that all regions, except Region 4B and Region 8, have the percentage distribution of infants given OPV 1, 2, and 3 of more than 70%.

Figure 1.C.10 - Percentage Distribution of Infants who completed 3 doses of Oral Polio Vaccines
Philippines, 2020

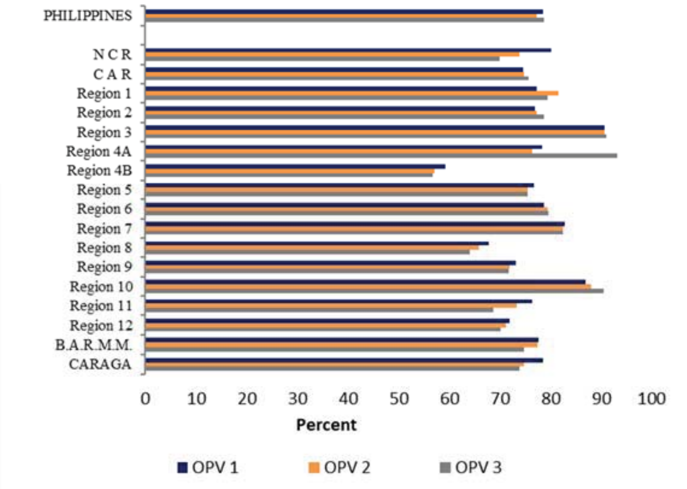

There were 1,549,903 (73.00%) infants in the Philippines vaccinated with Inactivated Polio Vaccine (IPV). The region with the highest percentage distribution of infants given one dose of IPV was recorded in Region 3 at 89.40%, followed by Region 10 at 85.99% then Region 7 at 81.31%. The rest of the regions have recorded at least 50% coverage of infants vaccinated with IPV.

Figure 1.C.11 - Percentage Distribution of Infants given Inactivated Polio Vaccine by Region
Philippines, 2020

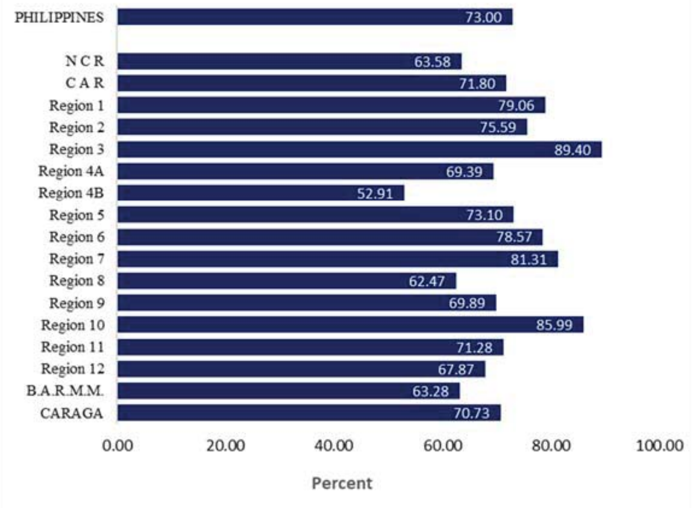

The proportion of infants who completed 3 doses of Pneumococcal Conjugate Vaccine (PCV) steadily increased from 2016 to 2020, Over the 5-year period, 2020 has the highest percentage of infants who were given complete dose of PCV having 76.42% for PCV1, 74.73% for PCV2 and 70.97% for PCV3. It was also observed that the coverage rates decreased for each subsequent dose in 5 years.

Figure 1.C.12 - Proportion of Children Vaccinated with PCV 1, 2 and 3
Philippines, 2016-2020

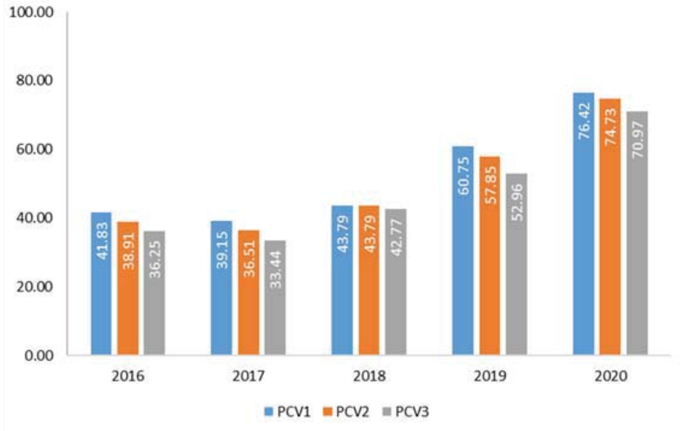

Table 1.C.1 - Percentage Distribution of Infants given MCV1 and MCV2
Philippines 2020

Vaccine	Male	Female	Total	%
MCV1	839,829	789,176	1,629,005	76.73
MCV2	799,060	755,153	1,554,213	73.20

As for the proportion of infants vaccinated with two (2) doses of Measles Containing Vaccine (MCV), a total of 1,629,005 (76.73%) children were vaccinated with MCV1 and 1,554,213 (73.20%) with MCV 2. More males were vaccinated in both doses (Table 1.C.1). Region 3 has the highest percentage distribution of infants vaccinated with MCV 1 and MCV 2 at 89.64% and 87.15% respectively followed by Region 1 at 83.17% for MCV1 and 84.83% for MCV2. In both doses, percentage distribution of more than 50% was observed for all regions.

Figure 1.C.13 - Percentage Distribution of Children vaccinated with 2 doses of Measles Containing Vaccine
Philippines, 2020

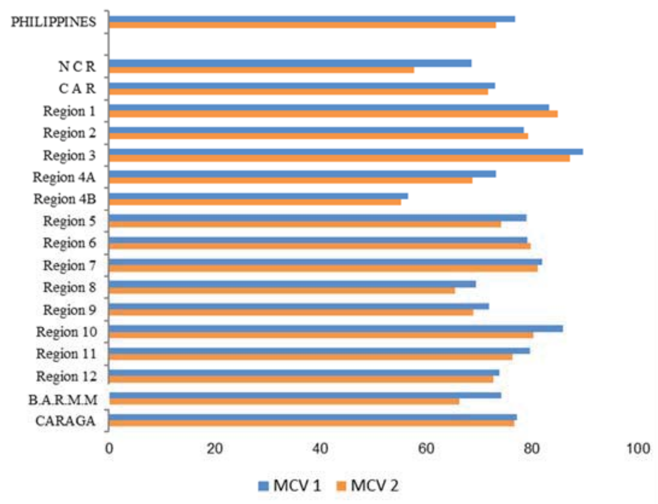

Figure 1.C.14 - Percentage Distribution of Newborns initiated on breastfeeding immediately after birth by Region
Philippines, 2020

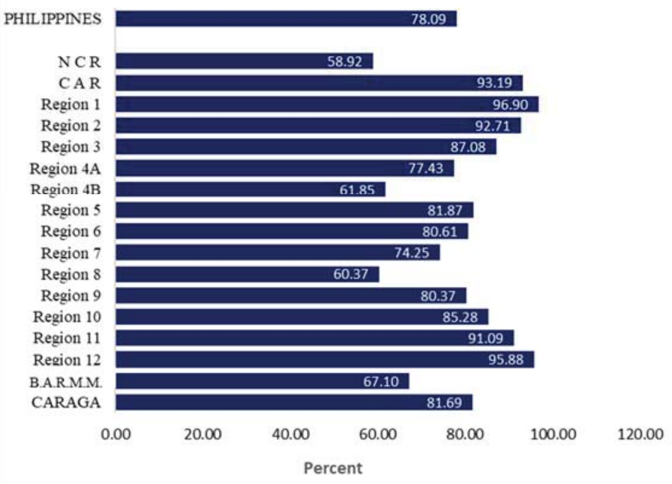

- **Nutrition Services for Infants and Children**

In the Philippines, the number of newborns initiated on breastfeeding immediately after birth reached 1,107,365 or 78.09%. The regions with the highest percentage distribution of newborns initiated on breastfeeding immediately after birth are Region 1 at 96.90%, followed by Region 12 at 95.88%, and CAR at 93.19%. Among the regions reported, NCR has the lowest percentage distribution at 58.92%, followed by Region 8 at 60.37%.

Figure 1.C.15 - Proportion of Infants Exclusively Breastfed until 6 months
Philippines, 2016-2020

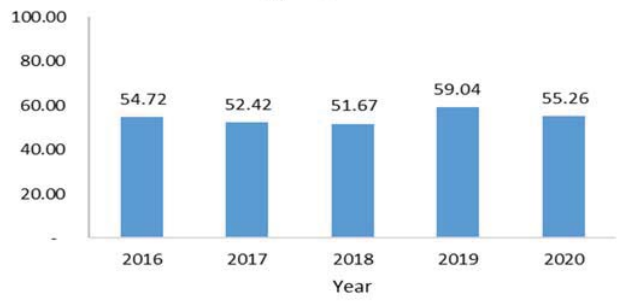

In 2020, the percentage distribution of infants who exclusively breastfed within the first six (6) months after birth was at 55.26%. A decreasing trend was observed from 2016 to 2018 at 54.72% and 51.67%, respectively. An increase was noted in 2019 at 59.04% with a slight decrease of 3.78% in 2020.

In 2020, the top three (3) regions with the highest percentage of infants exclusively breastfed within the first six (6) months after birth were Region 10 at 79.34%, followed by Region 12 at 73.56% then Region 1 at 73.48%.

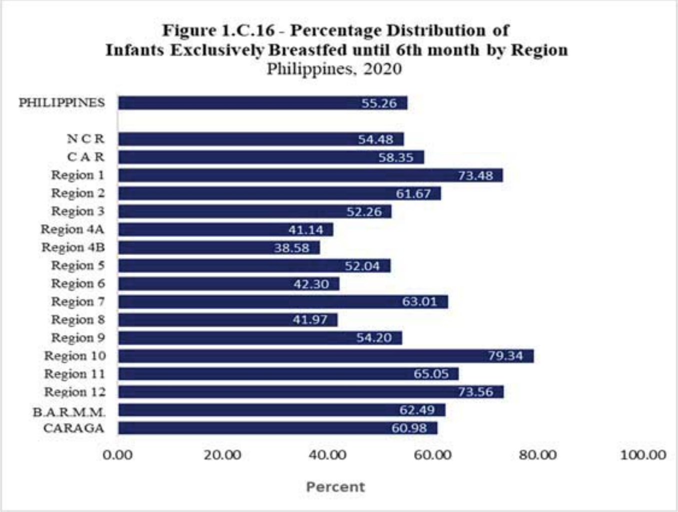

For the proportion of infants born preterm or with low birth weight (LBW) who were given iron supplementation, the regions with the highest percentage are Region 9 at 90.73%, followed by Region 4B at 76.78% and BARMM at 63.68%. Meanwhile, Region 6 has recorded the lowest percentage distribution at 10.14%.

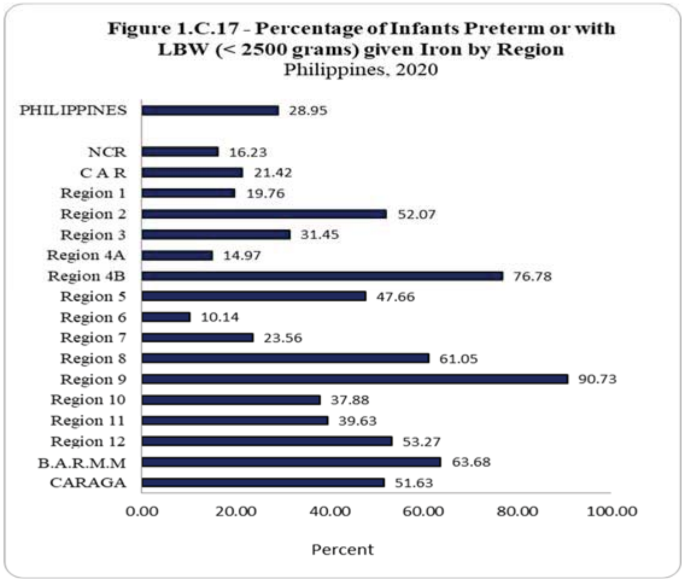

- **Management of Sick Infants and Children**

The regions with the highest percentage of sick infants aged 6-11 months who were given Vitamin A supplementation were BARMM at 92.70%, followed by Region 11 at 85.56% and CAR at 81.09%. Five regions had Vitamin A supplementation coverage for 6-11 months that were lower than 50%. These are Region 5 at 44.55%, Region 4B at 43.74%, Region 8 at 38.66%, CARAGA at 38.52% and Region 4A at 31.27%.

Table 1.C.2 - Proportion of Sick Infants (6-11 mos.) and Children (12-59 mos.) given Vitamin A
Philippines, 2020

Region	Vitamin A Supplementation 6-11 months (%)	Vitamin A Supplementation 12-59 months (%)
**Philippines**	**54.17**	**52.57**
NCR	73.58	63.2
CAR	81.09	70.39
Region 1	71.17	65.56
Region 2	55.94	47.26
Region 3	57.82	59.44
Region 4A	31.27	29.41
Region 4B	43.74	37.15
Region 5	44.55	39.85
Region 6	57.57	53.43
Region 7	52.23	72.44
Region 8	38.66	39.31
Region 9	67.08	64.72
Region 10	50.86	58.71
Region 11	85.56	85.48
Region 12	66.01	74.12
BARMM	92.7	92.18
CARAGA	38.52	43.91

In 2020, there are 54.17% of sick infants aged 6-11 months who were given Vitamin A while in 2016 it was at 48.99%. This figure shows that the coverage in the provision of Vitamin A for sick infants aged 6-11 months had a steady increase from 2016 to 2019 and a slight decrease in 2020. The sick children aged 12-59 months who were given Vitamin A in the health facility reached 52.57% in 2020. Compared to the sick children of the same age in 2016, only 40.34% were given vitamin A.

Figure 1.C.18 - Proportion of Sick Infants and Children given Vitamin A
Philippines, 2016-2020

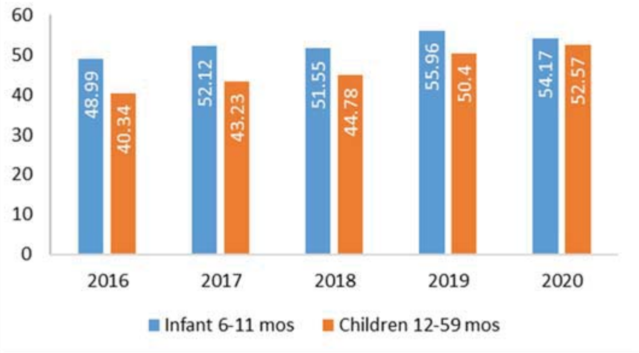

Figure 1.C.19 shows a steady decrease in the proportion of children aged 0-59 months with diarrhea who were given Oral Rehydration Salt Solution (ORS) alone from 2016 to 2020. The proportion of children aged 0-59 months given ORS with Zinc drops/syrup shows an increase from 2016 at 47.74% to 57.99% in 2020.

Figure 1.C.19 - Proportion of Children (0-59mos) with Diarrhea given ORS and ORS with Zinc drops/syrup
Philippines, 2016-2020

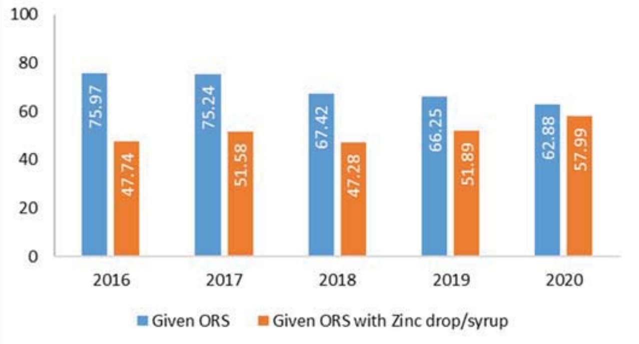

The regions with the highest percentage of children aged 0-59 months old with diarrhea but were given ORS are Region 10 at 90.65%, followed by Region 4B at 85.59% then Region 8 at 82.09%. As for children aged 0-59 months with diarrhea but were given ORS and Zinc, Region 8 has reported the highest coverage, accounted for to 77.85% while Region 12 has the lowest reported coverage of 24.66%.

Figure 1.C.20 - Proportion of Children (0-59mos) with Diarrhea given ORS and ORS with Zinc drops/syrup by Region
Philippines, 2020

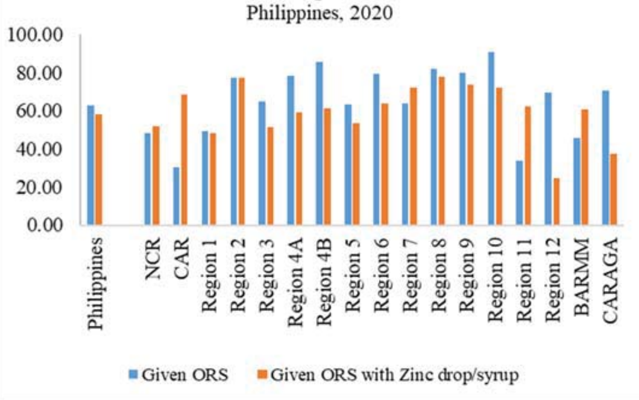

Figure 1.C.21 shows that in 2020, the proportion of children aged 0-59 months with pneumonia who received treatment is 96.61%. There is a minimal decrease from 2016 to 2020. It was also noted that the treatment coverage over the 5-year period remained more than 95%.

Figure 1.C.21 - Proportion of Diarrhea cases 0-59 mos old with Pneumonia who received treatment
Philippines, 2020

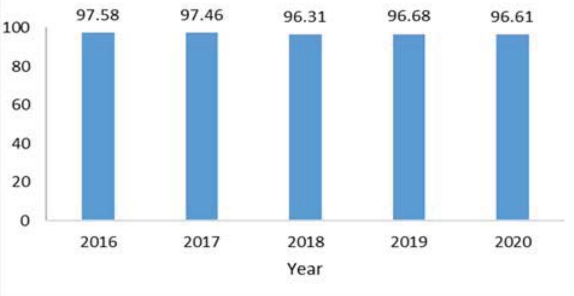

The regions with the highest reported percentage distribution of children 0-59 months who are treated for pneumonia are CAR at 99.36%, Region 4B at 99.17% and Region 3 at 98.86%. This is close to the program target of 100% children with pneumonia treated. The lowest proportion of treatment coverage among children 0-59 months with pneumonia is Region 5 at 79.78%. The rest of the regions have a coverage of more than 93%.

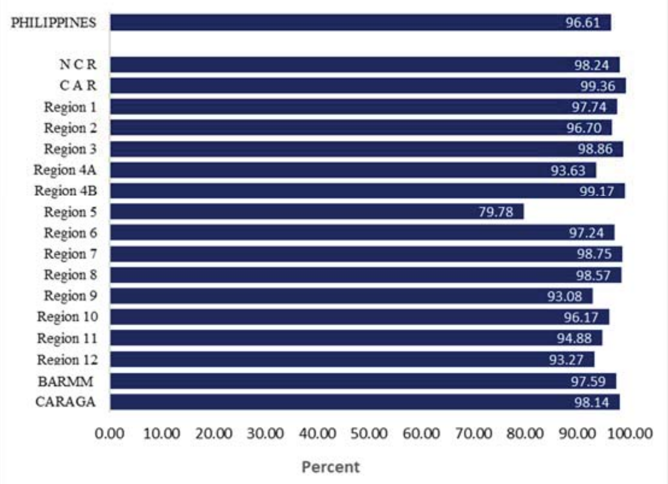

Figure 1.C.22 - Percentage Distribution of Children 0-59 mos. who are treated with Pneumonia by Region
Philippines, 2020

1.D Oral Health

In 2020, a total of 284,110 (or 15.89%) children aged 12-59 months old were orally fit upon oral examination or after oral rehabilitation among the service target (20%) of the total estimated number of 12-59 months old children. Among the regions reported, NCR with 35.54% has the highest proportion of orally fit 12-59 months old children while Region 4B with 3.37% has the lowest proportion.

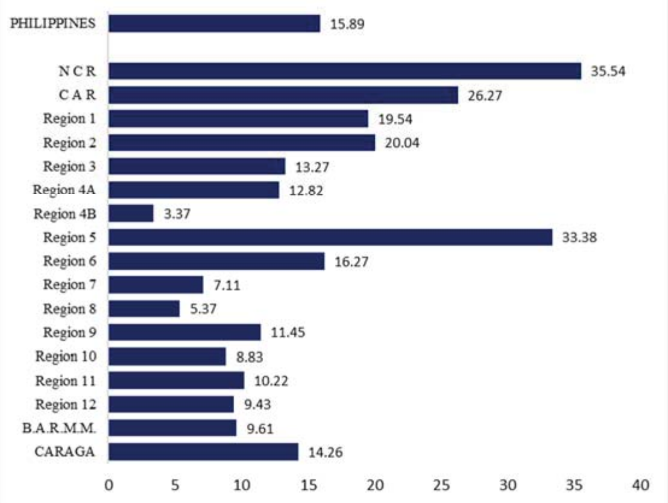

Figure 1.D.1 - Percentage Distribution of children 12-59 months old who are orally fit upon oral examination by Region
Philippines, 2020

Figure 1.D.2 shows that 32.32% of infants aged 0-11 months old received Basic Oral Health Care (BOHC) among the service target (30%) the estimated number of 0-11 months old infants in 17 regions. Among the regions reported, NCR with 73.06 has the highest proportion of infants who received BOHC, followed by CAR with 51.44%, and then CARAGA with 49.81%.

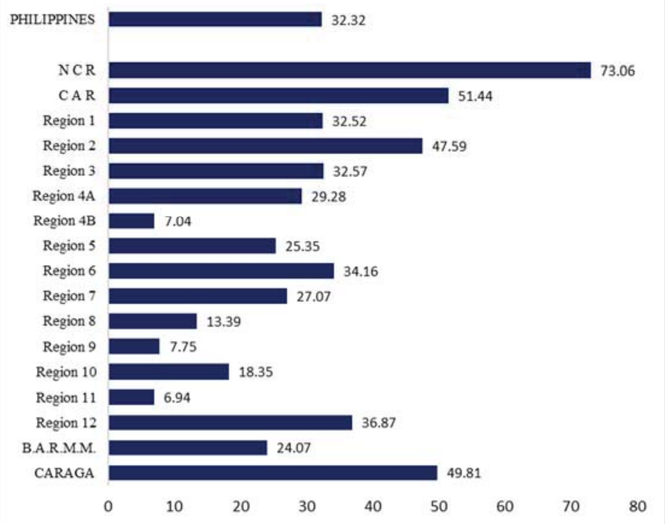

Figure 1.D.2 - Percentage Distribution of Infants 0-11 months old who received Basic Oral Health Care (BOHC) by Region
Philippines, 2020

For children 1-4 years old who received BOHC, the region with the highest percentage distribution of BOHC is Region 5 with 50.85% while the region with the lowest percentage distribution is Region 4B with 5.20%.

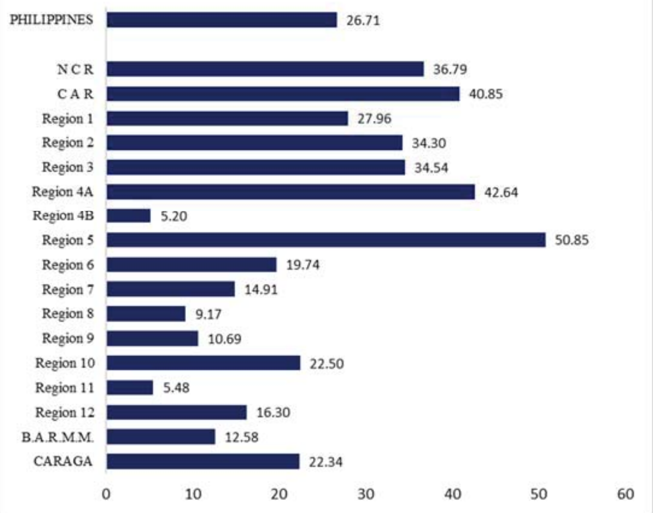

Figure 1.D.3 - Percentage Distribution of Children 1-4 yrs. old who received Basic Oral Health Care (BOHC) by Region
Philippines, 2020

Among the regions that were reported, CAR has the highest proportion of school-aged children 5-9 years old who received BOHC at 33.60%, followed by Region 2 with 26.14%. The region with less than 5% service coverage for BOHC is Region 4B with 4.25%.

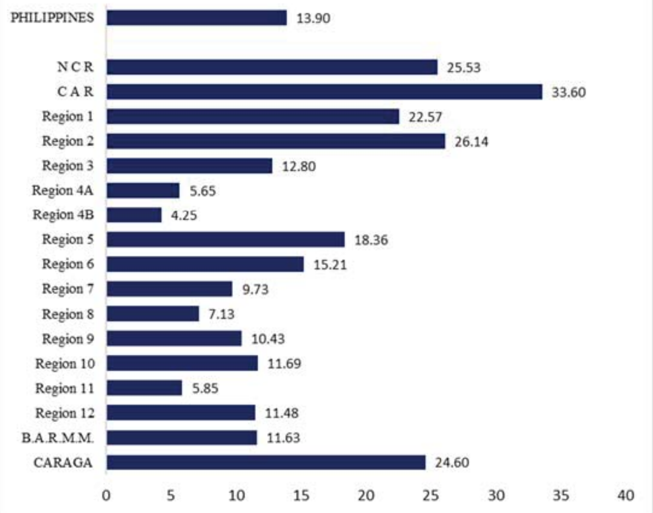

Figure 1.D.4 - Percentage Distribution of Children 5-9 yrs. old who received Basic Oral Health Care (BOHC) by Region
Philippines, 2020

Among the 10-14 years old age group, the region with the highest percentage distribution of the said age group of adolescents who received Basic Oral Health Care (BOHC) is Region 4A at 35.87%, and the regions with the lowest percentage distribution are Region 11 at 2.36%, Region 8 at 2.49%, Region 7 at 3.04%, Region 4B at 3.05%, and Region 3 at 3.07%.

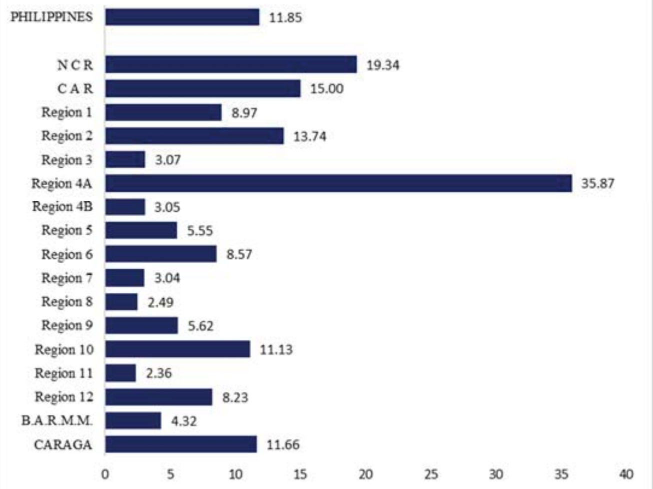

Figure 1.D.5 - Percentage Distribution of Adolescent 10-14 years old who received Basic Oral Health Care (BOHC) by Region
Philippines, 2020

The regions with the highest percentage distribution of 15-19 years old adolescents who received Basic Oral Health Care are Region 1 with 24.74% and CAR with 23.58%. The regions with the lowest percentage are Region 11 with 2.30%, Region 4B with 3.04%, Region 7 with 3.35%, Region 8 with 3.92%, and BARMM with 4.73%.

Figure 1.D.6 - Percentage Distribution of Adolescent 15-19 years old who received Basic Oral Health Care (BOHC) by Region
Philippines, 2020

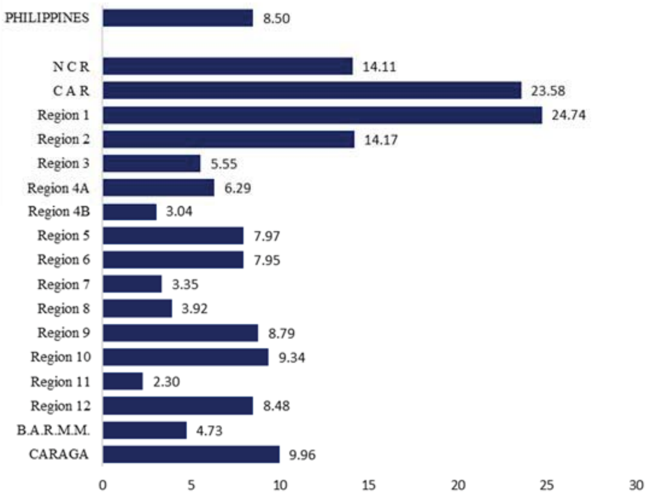

The regions with the highest percentage distribution of 20-49 years old adults who received Basic Oral Health Care are Region 1 at 13.21%, CAR at 11.15%, and Region 2 at 7.13%. The regions with the lowest percentage distribution are Region 4B at 1.35%, Region 7 at 1.73%, Region 8 at 1.78%, Region 3 at 2.26% and BARMM at 2.89%.

Figure 1.D.7 - Percentage Distribution of Adult 20-49 years old who received Basic Oral Health Care (BOHC) by Region Philippines, 2020

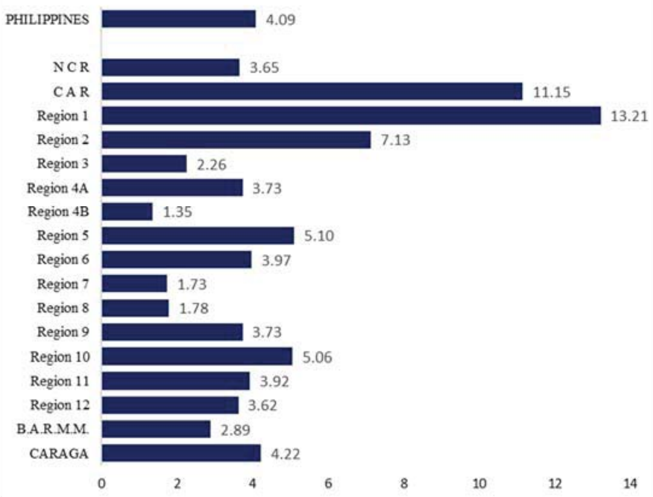

As for the percentage distribution of senior citizen 60 years old and above who received BOHC, the regions with the highest percentage distribution are Region 4A at 22.38%, BARMM at 20.37%, CAR at 18.23%, Region 2 at 16.58%, and Region 1 at 13.04% while the regions with the lowest percentage distribution are Region 11 at 1.18%, Region 4B at 1.84%, Region 7 at 2.70%, Region 8 at 3.07% and Region 9 at 3.09%.

Figure 1.D.8 - Percentage Distribution of Senior Citizens 60 yrs. old and above who received Basic Oral Health Care (BOHC) by Region
Philippines, 2020

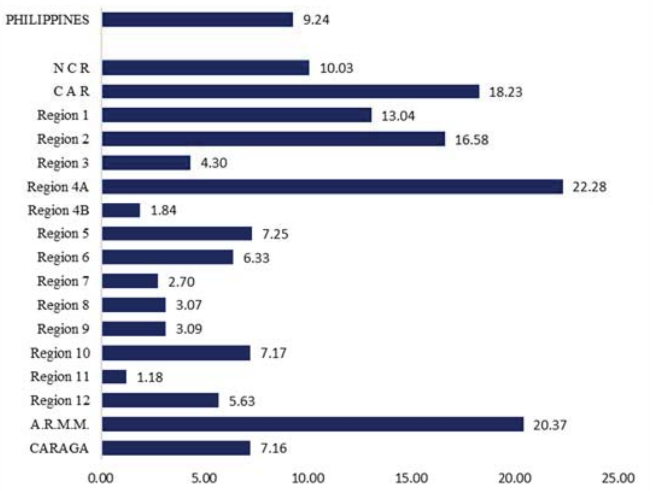

As for the percentage distribution of Pregnant Women who received Basic Oral Health Care, the 20-49 years old age group availed most of the services at 9.11%.

Figure 1.D.9 - Percentage Distribution of Pregnant women who received Basic Oral Health Care (BOHC) by Age group
Philippines, 2020

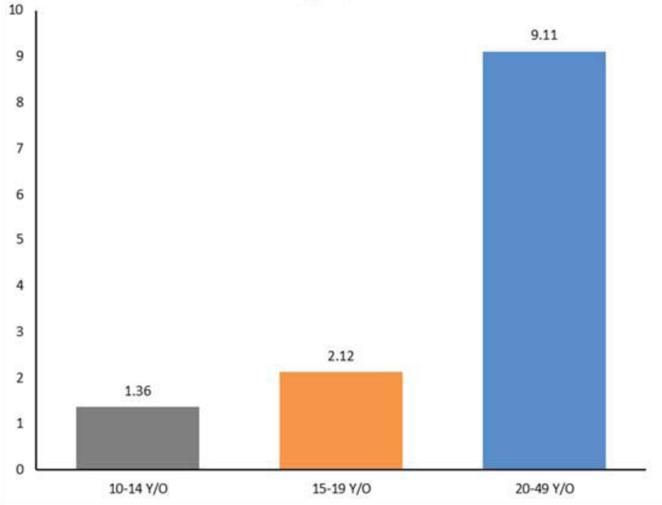

1.F Non-Communicable Disease Prevention and Control Service

From the estimated number of adults aged 20 years old and above, 3,329,756 (5.09%) were risk-assessed using the PhilPEN protocol. The region with the highest number of adults risk-assessed is Region 2 which covers 16.78%% of the total number of adults who were risk-assessed.

Figure 1.F.1 - Percentage Distribution of Adult ages 20 yrs old and above who were risk-assessed using the PhilPEN protocol by Region
Philippines, 2020

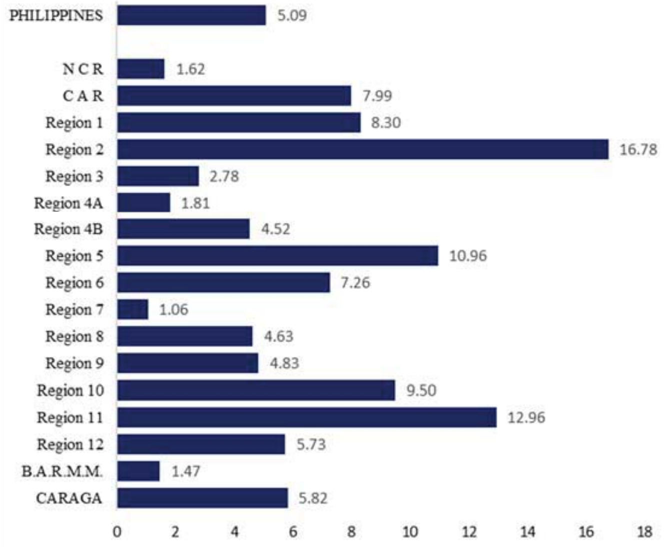

Out of the 5.09% of 20 years old and above who were risk assesed, 37.78% are current smokers. The risk assessment also showed that 30.48% are binge drinkers and 12.51% have a Body Mass Index (BMI) of ≥ 25 or are obese.

Figure 1.F.2 - Proportion of Adults 20 years old and above who were Risked Assessed as Current Smokers, Binge Drinkers and Overweight/Obese
Philippines, 2020

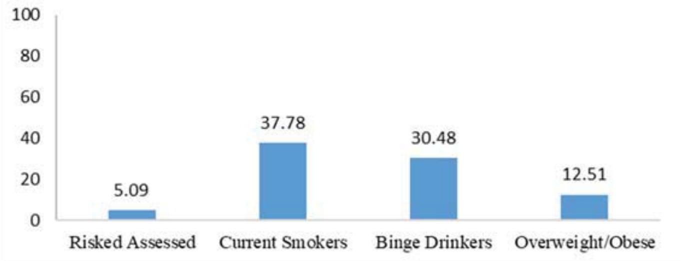

The highest percentage distribution of current smokers among 20 years old and above is Region 9 with 93.45%, followed by Region 10 with 84.54%. NCR has the least percentage distribution of adults who are current smokers at 12.83%. Among the proportion of binge drinkers, the regions with the highest percentage distribution are Region 4B with 95.40%, Region 10 with 70.71%, and CARAGA with 61.15% while BARMM has the lowest percentage at 4.23%. As for the overweight and obese, NCR has the highest percentage distribution at 37.73%, and Region 4B with the lowest percentage distribution at 2.46%

Table 1.F.1 - Proportion of Adults 20 years old and above who were Current Smokers, Binge Drinkers and Overweight/Obese by Region
Philippines 2020

Region	Current Smokers %	Binge Drinkers %	Overweight or Obese %
**Philippines**	37.78	30.48	12.51
**NCR**	12.83	14.56	37.73
**CAR**	19.36	20.97	21.52
**Region 1**	20.25	20.01	14.3
**Region 2**	17.44	18.56	12.54
**Region 3**	21.2	18.52	12.32
**Region 4A**	53.34	39.94	7.75
**Region 4B**	54.33	95.4	2.46
**Region 5**	21.35	17.86	12.69
**Region 6**	26.12	20.83	7.31
**Region 7**	26.48	27.39	20.89
**Region 8**	32.22	42.24	12.59
**Region 9**	93.45	53.7	17.3
**Region 10**	84.54	70.71	8.14
**Region 11**	42.61	23.04	11.03
**Region 12**	59.39	31.85	9.95
**BARMM**	38.4	4.23	4.2
**CARAGA**	82.52	61.15	13.27

Among the adults aged 20 years old and above who were risk assessed, there were 11.00% newly identified hypertensive adults while 2.30% were newly identified Type 2 diabetes mellitus. Among the regions, NCR has the highest percentage distribution of newly identified hypertensive adults with 5.16%, followed by Region 6 with 4.56%. For newly identified Type 2 diabetes mellitus, Region 4B has the highest percentage distribution with 23.67%, followed by Region 7 with 22.47% among those who were risk-assessed.

Figure 1.F.3 - Proportion of Newly Identified Adults 20 years old and above with Hypertension and Type II Diabetes Mellitus
Philippines, 2020

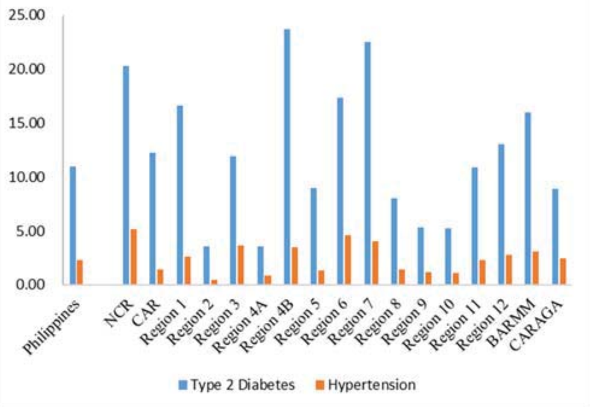

There were 40,420 women aged 20 years old and above screened for cervical cancer, this is less than 1% of the estimated number of women aged 20 years old and above. Among the regions that were reported, Region 12 has the highest proportion of women found positive or suspected for cervical cancer with 20.75% followed by Region 8 with 12.33%. For women screened for breast mass, Region 4B has the highest number of women with suspicious breast mass with 100% followed by Region 10 with 10.61%.

Figure 1.F.4 - Proportion of Women 20 years ond and above Found Positive/Suspected for Cervical Cancer and with Suspicious Breast Mass
Philippines, 2020

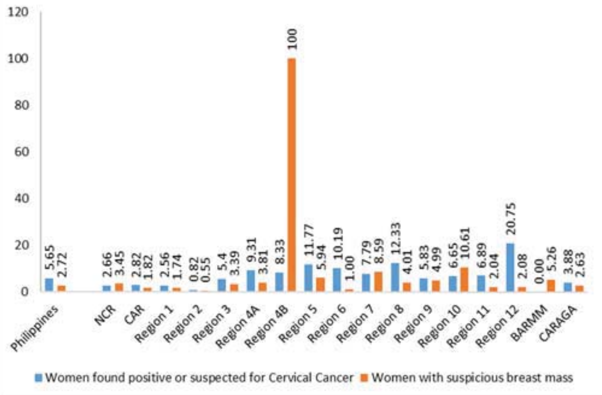

There were 94,652 senior citizens aged 60 years old and above screened for visual acuity. Among the regions that reported, Region 11 has the highest proportion of senior citizen that was screened for visual acuity with 10.84% followed by Region 2 with 3.15%. Out of those who were screened, 16.43% were diagnosed to have eye diseases and the region with the highest percentage distribution is BARMM with 73.84% and Region 4A having no senior citizen with eye diseases.

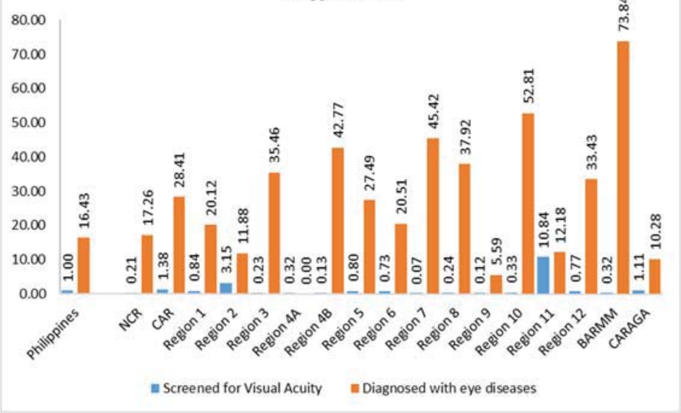

Figure 1.F.5 - Proportion of Senior Citizens 60 yrs old and above Screened Visual for Acuity and Diagnosed with Eye Diseases
Philippines, 2020

A total of 210,334 senior citizens aged 60 years old and above received one dose of pneumococcal polysaccharide vaccine (PPV) or only about 2.22% of the estimated number of senior citizens, while 5.65% of them received one dose of influenza vaccine. From the regions that reported, CAR has the highest percentage distribution of senior citizens who received PPV accounted for 5.99% while CAR has the highest percentage distribution of senior citizens who received influenza vaccine with 13.02%. Region 4A recorded the lowest percentage distribution of senior citizens vaccinated with PPV at 0.63% and Region 9 at 0.97%, Region 4B and Region 9 for influenza vaccine with 1.63% and 2.28% respectively.

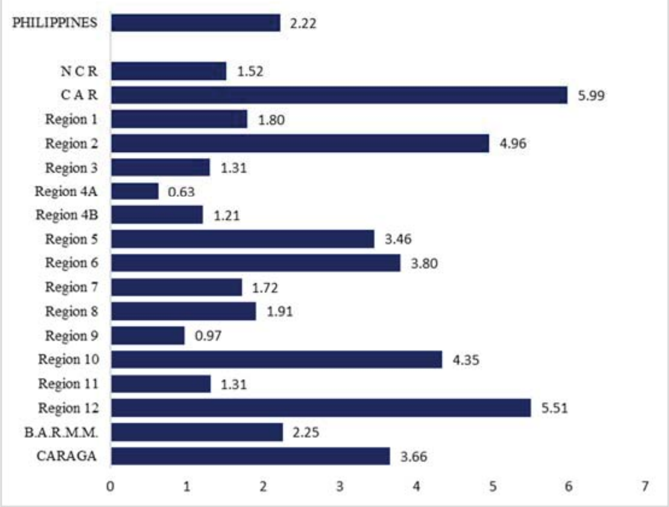

Figure 1.F.6 - Percentage Distribution of Senior Citizens 60 yrs old and above who received one (1) dose of Pneumococcal Polysaccharide Vaccine (PPV) by Region
Philippines, 2020

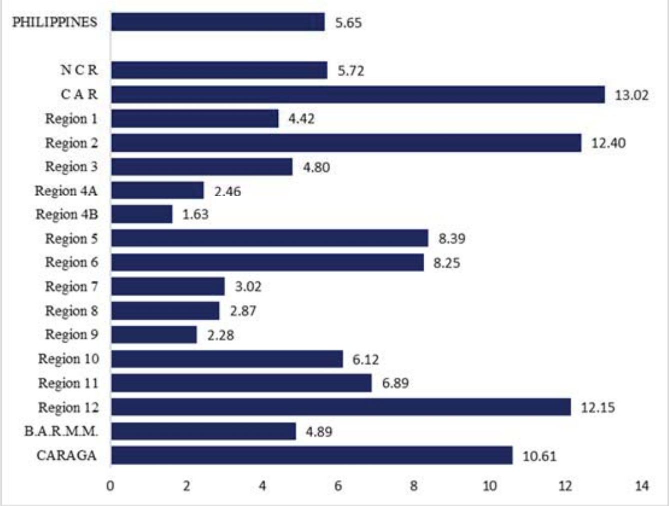

Figure 1.F.7 - Percentage Distribution of Senior Citizens 60 yrs old and above who received one (1) dose of Influenza Vaccine by Region
Philippines, 2020

1.G Environmental Health and Sanitation Services

The percentage of households that have access to basic safe water supply is 74.94% in 2020. A steady decrease is observed from 2016 to 2019 and a minimal increase in 2020 while for households with basic sanitation facilities a steep decrease was noted in 2019. As for households with satisfactory solid waste management, a decrease of 27.56% from 14,085,985 in 2015 to 10,203,622 in 2019,

Figure 1.G.1 Proportion of Households with Access to Basic Safe Water Supply and Basic Sanitation Facility
Philippines, 2016-2020

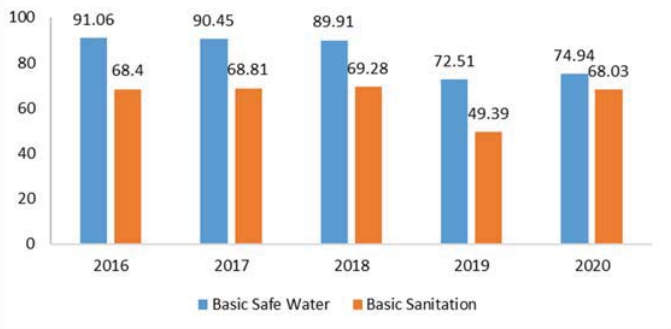

The regions with the proportion of households using safely managed drinking services are NCR at 78.47%, Region 10 at 73.75%, and Region 1 at 72.47%. For the safely managed sanitation services, Region 1 has the highest percentage distribution at 75.05% followed by Region 11 at 73.68% and CAR with 73.32%.

Figure 1.G.2 Proportion of Households using Safely Managed Drinking Water Services and Safely Managed Sanitation Services
Philippines, 2020

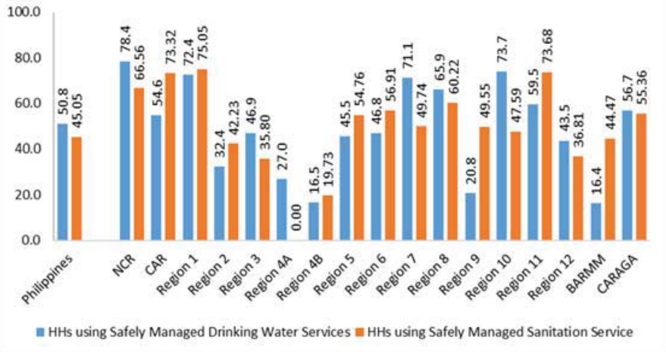

In 2020, the percentage of industrial establishments with sanitary permits reached 87.27%. Three regions achieved 100% percentage distribution namely, Region 2, Region 4B, and BARMM.

Figure 1.G.3 - Percentage of Industrial Establishment with Sanitary Permits by Region
Philippines, 2020

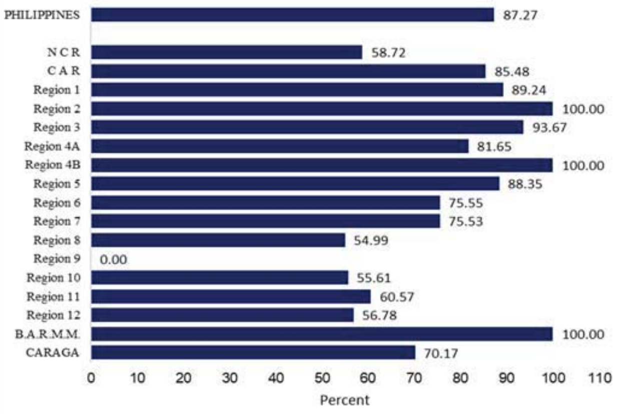

The Philippines registered 26.43% of barangays certified as Zero Open Defecation (ZOD) area while the regions with the highest percentage distribution are Region 1 with 69.31% and Region 6 with 49.62% while the regions with the lowest percentage distribution are Region 4B with 0.41%, BARMM with 10.88% and Region 2 with 11.03%.

Figure 1.G.4 - Percentage of Barangay certified as Zero Open Defecation (ZOD) Area by Region
Philippines, 2020

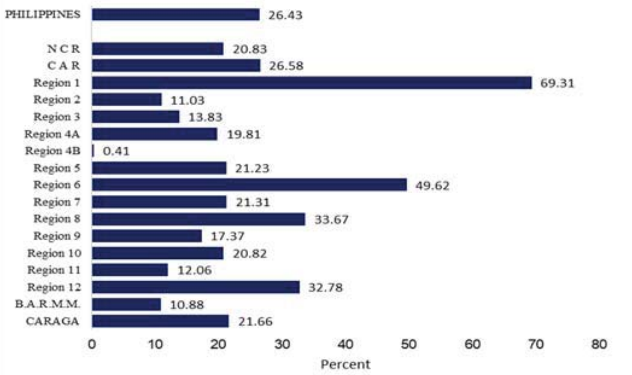

CHAPTER 2

FHSIS Annual Report 2020

Morbidity, Mortality and Natality

1. Philippines

Table 2A.1.1 - Ten Leading Causes of Morbidity

Philippines, 2020

	Disease	Number of Case	Rate per 100,000 population
1	Acute Respiratory Infection	710,151	652.88
2	Hypertension	445,834	409.88
3	Urinary Tract Infection	154,841	142.35
4	Animal Bites	149,815	137.73
5	Skin Disease	119,110	109.50
6	Acute Lower Respiratory Tract Infection	97,507	89.64
7	Pneumonia	87,537	80.48
8	Acute Watery Diarrhea	56,710	52.14
9	Bronchitis	55,657	51.17
10	TB All Forms	46,917	43.13

2. Ten Leading Causes of Morbidity by Gender

Table 2A.1.2 - Male
Philippines, 2020

	Disease	Number of case	Rate per 100,000 population
1	Acute Respiratory Infection	328,809	598.53
2	Hypertension	185,207	337.13
3	Animal Bites	76,867	139.92
	Urinary Tract Infection	51,806	94.30
4	Acute Lower Respiratory Tract Infection	46,083	83.89
6	Pneumonia	43,453	79.10
7	TB All Forms	28,923	52.65
8	Acute Watery Diarrhea	28,122	51.19
9	Bronchitis	26,645	48.50
10	Influenza-Like Illness	20,822	37.90

Table 2A.1.3 - Female
Philippines, 2020

	Disease	Number of case	Rate per 100,000 population
1	Acute Respiratory Infection	381,342	708.34
2	Hypertension	260,627	484.11
3	Urinary Tract Infection	103,035	191.39
4	Animal Bites	72,948	135.50
5	Acute Lower Respiratory Tract Infection	51,424	95.52
6	Pneumonia	44,084	81.89
7	Bronchitis	29,012	53.89
8	Acute Watery Diarrhea	28,588	53.10
9	Diseases of the Heart	26,621	49.45
10	Influenza-Like Illness	22,589	41.96

3. Ten Leading Causes of Morbidity by Region 2020

Table 2A.2.1 - CHD - METRO MANILA

	Disease	Number of case	Rate per 100,000 population
1	Hypertension	102,458	742.20
2	Acute Respiratory Infection	39,360	285.12
3	Urinary Tract Infection	17,070	123.65
4	Skin Disease	15,503	112.30
5	TB All Forms	5,691	41.23
6	Animal Bites	5,436	39.38
7	Pneumonia	5,296	38.36
8	Bronchitis	1,532	11.10
9	Influenza	1,410	10.21
10	Acute Lower Respiratory Tract Infection	1,102	7.98

2020 FHSIS

Table 2A.2.2 - CHD - Cordillera Administrative Region (CAR)

	Disease	Number of case	Rate per 100,000 population
1	Acute Respiratory Infection	73,643	4,073.77
2	Hypertension	26,343	1,457.24
3	Urinary Tract Infection	16,587	917.56
4	Skin Disease	15,013	830.49
5	Acute Watery Diarrhea	8,912	492.99
6	Fever of Unknown Origin	7,664	423.96
7	Animal Bites	6,985	386.39
8	Bronchitis	5,959	329.64
9	Pneumonia	3,906	216.07
10	Chronic Obstructive Pulmonary Disease	1,995	110.36

2020 FHSIS

Table 2A.2.3 - CHD - ILOCOS

	Disease	Number of case	Rate per 100,000 population
1	Acute Respiratory Infection	36,682	695.95
2	Skin Disease	15,013	284.83
3	Acute Lower Respiratory Tract Infection	4,935	93.63
4	Hypertension	3,542	67.20
5	Bronchitis	3,114	59.08
6	Urinary Tract Infection	2,618	49.67
7	Influenza-Like Illness	2,207	41.87
8	Animal Bites	1,674	31.76
9	Pneumonia	1,608	30.51
10	Acute Watery Diarrhea	1,384	26.26

2020 FHSIS

Table 2A.2.4 - CHD - CAGAYAN VALLEY

	Disease	Number of case	Rate per 100,000 population
1	Acute Respiratory Infection	142,624	3,899.24
2	Hypertension	32,628	892.03
3	Skin Disease	18,311	500.61
4	Urinary Tract Infection	17,474	477.73
5	Animal Bites	13,207	361.07
6	Acute Lower Respiratory Tract Infection	10,032	274.27
7	Acute Watery Diarrhea	8,324	227.57
8	Pneumonia	7,188	196.51
9	TB All Forms	4,470	122.21
10	Bronchitis	3,599	98.39

2020 FHSIS

Table 2A.2.5 - CHD - CENTRAL LUZON

	Disease	Number of case	Rate per 100,000 population
1	Acute Respiratory Infection	32,032	260.13
2	Acute Lower Respiratory Tract Infection	8,435	68.50
3	Hypertension	2,643	21.46
4	Urinary Tract Infection	2,092	16.99
5	Animal Bites	1,971	16.01
6	Skin Disease	1,749	14.20
7	Acute Watery Diarrhea	1,142	9.27
8	TB All Forms	913	7.41
9	Influenza-Like Illness	675	5.48
10	Diseases of the Heart	525	4.26

2020 FHSIS

Table 2A.2.6 - CHD -CALABARZON

	Disease	Number of case	Rate per 100,000 population
1	Acute Respiratory Infection	67,819	422.36
2	Hypertension	31,333	195.13
3	Diseases of the Heart	31,109	193.74
4	Acute Lower Respiratory Tract Infection	23,193	144.44
5	Bronchitis	17,045	106.15
6	Animal Bites	16,455	102.48
7	Acute Febrile Illness	13,725	85.48
8	Urinary Tract Infection	12,482	77.73
9	Acute Watery Diarrhea	11,974	74.57
10	Skin Disease	9,660	60.16

2020 FHSIS

Table 2A.2.7 - CHD - MIMAROPA

	Disease	Number of case	Rate per 100,000 population
1	Acute Respiratory Infection	32,108	1,011.32
2	Hypertension	11,827	372.52
3	Urinary Tract Infection	7,693	242.31
4	Animal Bites	6,384	201.08
5	Pneumonia	3,164	99.66
6	Skin Disease	3,072	96.76
7	Acute Watery Diarrhea	2,925	92.13
8	TB All Forms	1,806	56.88
9	Acute Lower Respiratory Tract Infection	1,313	41.36
10	Influenza	1,261	39.72

2020 FHSIS

Table 2A.2.8 - CHD - BICOL

	Disease	Number of case	Rate per 100,000 population
1	Acute Respiratory Infection	113,167	1,844.96
2	Hypertension	86,841	1,415.77
3	Fever Of Unknown Origin	21,199	345.61
4	Urinary Tract Infection	16,319	266.05
5	Skin Disease	12,507	203.90
6	Acute Lower Respiratory Tract Infection	11,780	192.05
7	Acute Febrile Illness	7,764	126.58
8	Animal Bites	6,298	102.68
9	TB All Forms	5,871	95.71
10	Influenza-Like Illness	5,101	83.16

2020 FHSIS

Table 2A.2.9 - CHD - WESTERN VISAYAS

	Disease	Number of case	Rate per 100,000 population
1	Animal Bites	44,121	558.15
2	Hypertension	44,020	556.87
3	Acute Respiratory Infection	42,880	542.45
4	Urinary Tract Infection	25,512	322.74
5	Pneumonia	18,934	239.52
6	TB All Forms	14,201	179.65
7	Skin Disease	13,894	175.76
8	Bronchitis	6,885	87.10
9	Acute Lower Respiratory Tract Infection	3,989	50.46
10	Influenza	3,826	48.40

2020 FHSIS

Table 2A.2.10 - CHD - CENTRAL VISAYAS

	Disease	Number of case	Rate per 100,000 population
1	Hypertension	10,395	130.64
2	Pneumonia	8,622	108.36
3	Acute Lower Respiratory Tract Infection	8,152	102.45
4	Animal Bites	6,821	85.72
5	Bronchitis	2,934	36.87
6	Skin Disease	2,925	36.76
7	Urinary Tract Infection	2,036	25.59
8	Acute Febrile Illness	1,186	14.91
9	Acute Watery Diarrhea	1,129	14.19
10	Fever of Unknown Origin	862	10.83

2020 FHSIS

Table 2A.2.11 - CHD - EASTERN VISAYAS

	Disease	Number of case	Rate per 100,000 population
1	Hypertension	16,868	355.69
2	Acute Respiratory Infection	15,187	320.24
3	Urinary Tract Infection	6,221	131.18
4	Skin Disease	5,369	113.21
5	Pneumonia	5,019	105.83
6	Bronchitis	3,145	66.32
7	Acute Febrile Illness	3,026	63.81
8	Acute Watery Diarrhea	2,692	56.77
9	Diseases of the Heart	1,935	40.80
10	Animal Bites	1,904	40.15

2020 FHSIS

Table 2A.2.12 - CHD - ZAMBOANGA PENINSULA

	Disease	Number of case	Rate per 100,000 population
1	Acute Hemorrhagic Fever	2,130	56.31
2	Acute Bloody Diarrhea	698	18.45
3	Influenza-Like Illness	396	10.47
4	Pneumonia	364	9.62
5	Typhoid and Paratyphoid Fever	342	9.04
6	Bronchitis	324	8.57
7	Influenza	250	6.61
8	Hypertension	237	6.27
9	Measles	175	4.63
10	Viral Hepatitis	143	3.78

2020 FHSIS

Table 2A.2.13 - CHD - NORTHERN MINDANAO

	Disease	Number of case	Rate per 100,000 population
1	Hypertension	47,485	946.47
2	Acute Respiratory Infection	33,325	664.23
3	Animal Bites	21,657	431.67
4	Skin Disease	15,606	311.06
5	Urinary Tract Infection	14,447	287.96
6	Acute Febrile Illness	7,339	146.28
7	Pneumonia	7,034	140.20
8	Acute Lower Respiratory Tract Infection	6,983	139.19
9	Bronchitis	6,539	130.34
10	Influenza-Like Illness	6,234	124.26

2020 FHSIS

Table 2A.2.14 - CHD - DAVAO

	Disease	Number of case	Rate per 100,000 population
1	Acute Respiratory Infection	21,025	397.38
2	Hypertension	11,151	210.76
3	Influenza-Like Illness	8,455	159.80
4	Urinary Tract Infection	8,085	152.81
5	Pneumonia	7,649	144.57
6	Acute Lower Respiratory Tract Infection	5,876	111.06
7	Skin Disease	3,587	67.80
8	Animal Bites	3,014	56.97
9	Acute Watery Diarrhea	2,827	53.43
10	Acute Hemorrhagic Fever	2,682	50.69

2020 FHSIS

Table 4A.2.15 - CHD - SOCCSARGEN

	Disease	Number of case	Rate per 100,000 population
1	Acute Respiratory Infection	23,724	482.20
2	Animal Bites	10,194	207.20
3	Acute Hemorrhagic Fever	9,229	187.58
4	Pneumonia	5,294	107.60
5	Acute Febrile Illness	4,655	94.62
6	Acute Lower Respiratory Tract Infection	4,254	86.46
7	Urinary Tract Infection	3,659	74.37
8	Skin Disease	3,587	72.91
9	Influenza	2,338	47.52
10	Acute Watery Diarrhea	2,174	44.19

2020 FHSIS

Table 4A.2.16 -Bangsamoro Autonomous Region for Muslim Mindanao (BARMM)

	Disease	Number of case	Rate per 100,000 population
1	Hypertension	4,294	102.65
2	Urinary Tract Infection	1,793	42.86
3	Tb All Forms	1,642	39.25
4	Pneumonia	989	23.64
5	Acute Lower Respiratory Tract Infection	125	2.99
6	Acute Watery Diarrhea	98	2.34
7	Bronchitis	64	1.53
8	Acute Respiratory Infection	26	0.62
9			
10			

2020 FHSIS

Table 4A.2.17 - CARAGA Region

	Disease	Number of case	Rate per 100,000 population
1	Acute Respiratory Infection	35,600	1,293.08
2	Hypertension	13,458	488.83
3	Animal Bites	3,518	127.78
4	Acute Lower Respiratory Tract Infection	3,260	118.41
5	Influenza	3,037	110.31
6	Influenza-Like Illness	2,494	90.59
7	Pneumonia	1,762	64.00
8	Acute Watery Diarrhea	1,162	42.21
9	Bronchitis	864	31.38
10	Urinary Tract Infection	740	26.88

2020 FHSIS

Table 2.A.3.1 - Acute Bloody Diarrhea
by Age group, Sex, Region, Province and City
Philippines, Annual 2020

Region/Province/City	0 - 6 days		7 - 28 days		29 days - 11 mos.		1 - 4 yrs.		5 - 9 yrs.		10 -14 yrs.		15 - 19 yrs.		20 - 24 yrs.		25 - 29 yrs.		30 - 34 yrs.	
	M	F	M	F	M	F	M	F	M	F	M	F	M	F	M	F	M	F	M	F
**PHILIPPINES**	**4**	**8**	**7**	**8**	**248**	**187**	**554**	**484**	**227**	**225**	**131**	**108**	**73**	**109**	**78**	**101**	**83**	**84**	**62**	**107**
**METRO MANILA**	**0**	**0**	**0**	**0**	**0**	**0**	**0**	**2**	**1**	**0**	**0**	**0**	**0**	**0**	**0**	**0**	**0**	**0**	**0**	**0**
Malabon City	0	0	0	0	0	0	0	0	0	0	0	0	0	0	0	0	0	0	0	0
Navotas City	0	0	0	0	0	0	0	0	0	0	0	0	0	0	0	0	0	0	0	0
Valenzuela City	0	0	0	0	0	0	0	0	0	0	0	0	0	0	0	0	0	0	0	0
Caloocan City	0	0	0	0	0	0	0	0	0	0	0	0	0	0	0	0	0	0	0	0
Marikina City	0	0	0	0	0	0	0	1	0	0	0	0	0	0	0	0	0	0	0	0
Pasig City	0	0	0	0	0	0	0	0	0	0	0	0	0	0	0	0	0	0	0	0
Pateros	0	0	0	0	0	0	0	0	0	0	0	0	0	0	0	0	0	0	0	0
Taguig City	0	0	0	0	0	0	0	0	0	0	0	0	0	0	0	0	0	0	0	0
Quezon City	0	0	0	0	0	0	0	1	0	0	0	0	0	0	0	0	0	0	0	0
Makati City	0	0	0	0	0	0	0	0	0	0	0	0	0	0	0	0	0	0	0	0
Mandaluyong City	0	0	0	0	0	0	0	0	1	0	0	0	0	0	0	0	0	0	0	0
San Juan City	0	0	0	0	0	0	0	0	0	0	0	0	0	0	0	0	0	0	0	0
Manila City	0	0	0	0	0	0	0	0	0	0	0	0	0	0	0	0	0	0	0	0
Las Piñas City	0	0	0	0	0	0	0	0	0	0	0	0	0	0	0	0	0	0	0	0
Muntinlupa City	0	0	0	0	0	0	0	0	0	0	0	0	0	0	0	0	0	0	0	0
Parañaque City	0	0	0	0	0	0	0	0	0	0	0	0	0	0	0	0	0	0	0	0
Pasay City	0	0	0	0	0	0	0	0	0	0	0	0	0	0	0	0	0	0	0	0
**CAR**	**0**	**0**	**0**	**1**	**25**	**20**	**51**	**53**	**29**	**29**	**14**	**12**	**2**	**8**	**4**	**5**	**6**	**3**	**4**	**13**
Abra	0	0	0	0	3	1	1	2	0	0	1	0	0	0	0	0	0	0	0	0
Apayao	0	0	0	0	0	0	0	1	0	0	0	0	0	1	0	0	0	0	0	0
Benguet	0	0	0	1	5	3	1	5	3	1	1	0	0	0	0	0	1	0	1	0
Ifugao	0	0	0	0	11	8	26	28	14	23	8	5	2	2	4	1	5	1	3	8
Kalinga	0	0	0	0	3	6	7	8	9	2	1	6	0	3	0	2	0	2	0	5
Mt. Province	0	0	0	0	2	1	7	5	3	2	0	0	0	0	0	0	0	0	0	0
Baguio City	0	0	0	0	1	1	9	4	0	1	3	1	0	2	0	2	0	0	0	0
**ILOCOS**	**0**	**0**	**0**	**0**	**0**	**0**	**8**	**3**	**5**	**4**	**2**	**1**	**1**	**3**	**0**	**0**	**2**	**0**	**0**	**0**
Ilocos Note	0	0	0	0	0	0	2	1	0	0	0	0	0	0	0	0	0	0	0	0
Ilocos Sur	0	0	0	0	0	0	5	0	2	1	2	1	1	1	0	0	0	0	0	0
La Union	0	0	0	0	0	0	0	0	0	0	0	0	0	0	0	0	0	0	0	0
Pangasinan	0	0	0	0	0	0	0	2	2	2	0	0	0	2	0	0	2	0	0	0
Alaminos City	0	0	0	0	0	0	0	0	0	0	0	0	0	0	0	0	0	0	0	0
Candon City	0	0	0	0	0	0	0	0	0	0	0	0	0	0	0	0	0	0	0	0
Dagupan City	0	0	0	0	0	0	0	0	0	0	0	0	0	0	0	0	0	0	0	0
Laoag City	0	0	0	0	0	0	1	0	1	1	0	0	0	0	0	0	0	0	0	0
San Carlos City	0	0	0	0	0	0	0	0	0	0	0	0	0	0	0	0	0	0	0	0
San Fernando City	0	0	0	0	0	0	0	0	0	0	0	0	0	0	0	0	0	0	0	0
Urdaneta City	0	0	0	0	0	0	0	0	0	0	0	0	0	0	0	0	0	0	0	0
Vigan City	0	0	0	0	0	0	0	0	0	0	0	0	0	0	0	0	0	0	0	0
**CAGAYAN VALLEY**	**0**	**0**	**0**	**0**	**3**	**6**	**8**	**13**	**24**	**25**	**14**	**12**	**12**	**5**	**4**	**11**	**7**	**11**	**2**	**7**
Batanes	0	0	0	0	0	0	0	0	0	0	0	0	0	0	0	0	0	0	0	0
Cagayan	0	0	0	0	0	0	0	3	1	1	0	0	8	0	1	3	0	4	0	0
Isabela	0	0	0	0	1	1	2	2	1	1	1	1	1	1	0	1	2	0	0	2
Nueva Vizcaya	0	0	0	0	1	4	3	2	2	5	0	0	0	0	0	0	0	0	0	0
Quirino	0	0	0	0	0	0	0	1	20	16	10	10	3	2	3	5	4	5	1	5
Cauayan City	0	0	0	0	0	0	0	0	0	0	0	0	0	0	0	0	0	0	0	0
Ilagan City	0	0	0	0	0	0	0	0	0	0	0	0	0	0	0	0	0	0	0	0
Santiago City	0	0	0	0	1	1	3	5	0	2	3	1	0	2	0	2	1	2	1	0
Tuguegarao City	0	0	0	0	0	0	0	0	0	0	0	0	0	0	0	0	0	0	0	0
**CENTRAL LUZON**	**0**	**0**	**0**	**0**	**2**	**3**	**8**	**8**	**4**	**3**	**2**	**0**	**2**	**2**	**1**	**3**	**2**	**0**	**0**	**5**
Aurora	0	0	0	0	0	0	0	0	0	0	0	0	0	0	0	0	0	0	0	0
Bataan	0	0	0	0	0	0	0	0	0	0	0	0	0	0	0	0	0	0	0	0
Bulacan	0	0	0	0	0	0	0	0	0	0	0	0	0	0	0	0	0	0	0	0
Nueva Ecija	0	0	0	0	0	0	0	0	0	0	0	0	0	0	0	0	0	0	0	0
Pampanga	0	0	0	0	0	0	0	0	0	0	0	0	0	0	0	0	0	0	0	0
Tarlac	0	0	0	0	0	0	0	0	0	0	0	0	0	0	0	0	0	0	0	0
Zambales	0	0	0	0	0	0	0	0	0	0	0	0	0	0	0	0	0	0	0	0
Angeles City	0	0	0	0	0	0	2	2	0	2	0	0	1	0	0	2	0	0	0	1
Balanga City	0	0	0	0	1	1	2	3	0	0	1	0	0	0	0	0	0	0	0	0
Cabanatuan City	0	0	0	0	0	0	0	0	0	0	0	0	0	0	0	0	0	0	0	0
City of San Fernando	0	0	0	0	0	1	0	0	2	0	0	0	0	0	0	0	0	0	0	0
Gapan City	0	0	0	0	0	0	0	0	0	0	0	0	0	0	0	0	0	0	0	0
Mabalacat City	0	0	0	0	0	0	0	0	0	0	0	0	0	0	0	0	0	0	0	0
Malolos City	0	0	0	0	0	0	0	0	0	0	0	0	0	0	0	0	0	0	0	0
Meycauayan City	0	0	0	0	0	0	0	0	0	0	0	0	0	0	0	0	0	0	0	0
Olongapo City	0	0	0	0	0	0	0	0	0	0	0	0	0	0	0	0	0	0	0	0
Palayan City	0	0	0	0	0	0	0	0	0	0	0	0	0	0	0	0	0	0	0	0
San Jose City	0	0	0	0	0	0	0	0	0	0	0	0	0	0	0	0	0	0	0	0
San Jose del Monte City	0	0	0	0	1	0	3	1	2	0	0	0	1	1	1	1	0	0	0	1
Science City of Munoz	0	0	0	0	0	0	0	0	0	0	0	0	0	0	0	0	0	0	0	0
Tarlac City	0	0	0	0	0	1	1	2	0	1	1	0	0	1	0	0	2	0	0	3

Table 2.A.3.1 - Acute Bloody Diarrhea
by Age group, Sex, Region, Province and City
Philippines, Annual 2020

Region/Province/City	0 - 6 days		7 - 28 days		29 days - 11 mos.		1 - 4 yrs.		5 - 9 yrs.		10 -14 yrs.		15 - 19 yrs.		20 - 24 yrs.		25 - 29 yrs.		30 - 34 yrs.	
	M	F	M	F	M	F	M	F	M	F	M	F	M	F	M	F	M	F	M	F
**CALABARZON**	**0**	**0**	**0**	**0**	**0**	**0**	**0**	**0**	**0**	**0**	**0**	**0**	**0**	**0**	**0**	**0**	**0**	**0**	**0**	**0**
Batangas	0	0	0	0	0	0	0	0	0	0	0	0	0	0	0	0	0	0	0	0
Cavite	0	0	0	0	0	0	0	0	0	0	0	0	0	0	0	0	0	0	0	0
Laguna	0	0	0	0	0	0	0	0	0	0	0	0	0	0	0	0	0	0	0	0
Quezon	0	0	0	0	0	0	0	0	0	0	0	0	0	0	0	0	0	0	0	0
Rizal	0	0	0	0	0	0	0	0	0	0	0	0	0	0	0	0	0	0	0	0
Antipolo City	0	0	0	0	0	0	0	0	0	0	0	0	0	0	0	0	0	0	0	0
Bacoor City	0	0	0	0	0	0	0	0	0	0	0	0	0	0	0	0	0	0	0	0
Batangas City	0	0	0	0	0	0	0	0	0	0	0	0	0	0	0	0	0	0	0	0
Biñan City	0	0	0	0	0	0	0	0	0	0	0	0	0	0	0	0	0	0	0	0
Cabuyao City	0	0	0	0	0	0	0	0	0	0	0	0	0	0	0	0	0	0	0	0
Calamba City	0	0	0	0	0	0	0	0	0	0	0	0	0	0	0	0	0	0	0	0
Cavite City	0	0	0	0	0	0	0	0	0	0	0	0	0	0	0	0	0	0	0	0
Dasmariñas City	0	0	0	0	0	0	0	0	0	0	0	0	0	0	0	0	0	0	0	0
General Trias City	0	0	0	0	0	0	0	0	0	0	0	0	0	0	0	0	0	0	0	0
Imus City	0	0	0	0	0	0	0	0	0	0	0	0	0	0	0	0	0	0	0	0
Lipa City	0	0	0	0	0	0	0	0	0	0	0	0	0	0	0	0	0	0	0	0
Lucena City	0	0	0	0	0	0	0	0	0	0	0	0	0	0	0	0	0	0	0	0
San Pablo City	0	0	0	0	0	0	0	0	0	0	0	0	0	0	0	0	0	0	0	0
San Pedro City	0	0	0	0	0	0	0	0	0	0	0	0	0	0	0	0	0	0	0	0
Santa Rosa City	0	0	0	0	0	0	0	0	0	0	0	0	0	0	0	0	0	0	0	0
Tagaytay City	0	0	0	0	0	0	0	0	0	0	0	0	0	0	0	0	0	0	0	0
Tanauan City	0	0	0	0	0	0	0	0	0	0	0	0	0	0	0	0	0	0	0	0
Tayabas City	0	0	0	0	0	0	0	0	0	0	0	0	0	0	0	0	0	0	0	0
Trece Martires City	0	0	0	0	0	0	0	0	0	0	0	0	0	0	0	0	0	0	0	0
**MIMAROPA**	**0**	**0**	**0**	**0**	**10**	**10**	**21**	**22**	**12**	**10**	**4**	**7**	**1**	**4**	**3**	**3**	**1**	**1**	**3**	**2**
Marinduque	0	0	0	0	2	1	2	2	2	0	0	1	0	0	0	0	0	1	0	1
Mindoro Occidental	0	0	0	0	1	3	5	8	4	7	2	2	0	0	1	0	0	0	1	1
Mindoro Oriental	0	0	0	0	0	0	4	4	1	0	0	1	0	0	1	1	0	0	0	0
Palawan	0	0	0	0	0	0	0	0	0	0	0	0	0	0	0	0	0	0	0	0
Romblon	0	0	0	0	7	6	10	8	5	3	2	2	1	3	1	2	1	0	1	0
Puerto Princesa City	0	0	0	0	0	0	0	0	0	0	0	1	0	1	0	0	0	0	1	0
**BICOL**	**2**	**2**	**0**	**0**	**17**	**12**	**70**	**53**	**26**	**22**	**13**	**6**	**2**	**8**	**2**	**5**	**10**	**7**	**2**	**12**
Albay	1	1	0	0	7	4	15	27	10	7	3	4	0	0	1	3	1	0	2	6
Camarines Norte	0	0	0	0	1	1	9	3	4	1	1	0	0	1	1	0	4	2	0	2
Camarines Sur	1	1	0	0	9	7	37	21	9	10	3	1	2	4	0	2	4	4	0	4
Catanduanes	0	0	0	0	0	0	1	0	0	0	0	1	0	1	0	0	1	0	0	0
Masbate	0	0	0	0	0	0	2	0	0	0	4	0	0	2	0	0	0	0	0	0
Sorsogon	0	0	0	0	0	0	2	0	1	1	2	0	0	0	0	0	0	1	0	0
Iriga City	0	0	0	0	0	0	0	0	0	0	0	0	0	0	0	0	0	0	0	0
Legaspi City	0	0	0	0	0	0	4	2	2	3	0	0	0	0	0	0	0	0	0	0
Naga City	0	0	0	0	0	0	0	0	0	0	0	0	0	0	0	0	0	0	0	0
[truncated: 2,042,739 more chars]
