# Supplementary material for: Neglected Tropical Diseases Elimination in the Philippines: Challenges and Gaps
Source: Trop Med Infect Dis. 2026 Apr 17;11(4):106. doi: 10.3390/tropicalmed11040106 (PMC13120366; doi:10.3390/tropicalmed11040106)
Supplement: Supplementary file 1 [file tropicalmed-11-00106-s001.zip › Supplementary File S4. FHSIS 2022.pdf]

FIELD HEALTH SERVICES INFORMATION SYSTEM

ANNUAL REPORT 2022

Monitoring and Evaluation Division
Epidemiology Bureau
Department of Health,
San Lazaro Compound, Sta. Cruz, Manila

8651-7800 loc. 2928/2953

fhsis@doh.gov.ph

Republic of the Philippines
Department of Health
OFFICE OF THE SECRETARY

MESSAGE

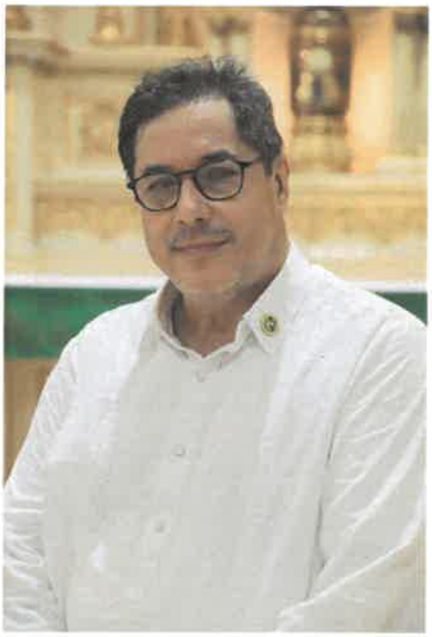

As one Department of Health, we are proud to present the 2022 Field Health Services Information System (FHSIS) Annual Report. This report covers the data on public health services, demographics, and morbidity that are captured in public health facilities.

Indeed, relevant FHSIS data is essential for health care providers, program managers, and other stakeholders in crafting new policies and guidelines, prioritization and allocation of resources, and monitoring and evaluating the effectiveness of interventions for Filipinos. Over the years, the FHSIS reports have been of great value to the health sector, academe, researchers, and other organizations.

In essence, the FHSIS provides our local governments the opportunity to determine priorities for public health - making implemented programs, campaigns, and other efforts in communities all the more effective. These are done through constant data validation and quarterly monitoring visits.

Likewise, the DOH, in its commitment to oversee the overall well-being of every Filipino, also banners its newly-adopted "Sulong Kalusugan" Health Sector Strategy (HSS) for 2023 to 2028. With the help of this program, we are now able to give Filipinos access to improved health facilities with expanded services and offers. The timely release of this data also creates an avenue for healthcare workers to excel in their field and experience eased day-to-day workload.

We are grateful for the hard work and contribution of our health workers and staff from the barangay, municipal, city and provincial offices, our partners from Local Government Units, Centers for Health Development, Ministry of Health-Bangsamoro Autonomous Region in Muslim Mindanao, Disease Prevention and Control Bureau, and Epidemiology Bureau to this report. It is hoped that the data integrity of the 2022 FHSIS Annual Report will improve the health outcomes and provide quality health care and services to all Juan and Juana.

Together, let us all work hand in hand to provide every Filipino an improved state of living, allow them to create healthier life choices, and transform their communities to healthier settings - all geared towards a healthier Pilipinas.

Sa Healthy Pilipinas... bawat buhay mahalaga!

TEODORO J. HERBOSA, MD
Secretary, Department of Health

Republic of the Philippines
Department of Health

MARIA ROSARIO SINGH-VERGEIRE, MD, MPH, CESO II
Undersecretary of Health, Public Health Services Team

BEVERLY LORRAINE C. HO, MD, MPH
Assistant Secretary of Health, Public Health Services Team

GLORIA NENITA V. VELASCO, MD, DipEpi, MScPH
Director III, Epidemiology Bureau

VITO G. ROQUE JR., MD, PHSAE
Medical Officer V, Monitoring and Evaluation Division

FHSIS UNIT STAFF

MARIEL A. DEJESA, MD, MPM
Medical Officer IV

Jose M. Hernaez
Information Systems Analyst III
Karla Mae C. Ramirez
Senior Health Program Officer
Joel V. Cantero
Computer Programmer II
Hernan T. Tayer
Computer Programmer II
Julius T. Castro
Computer Programmer II

Theresa D. Timbang
Supervising Health Program Officer
Rhency M. Legaspi
Supervising Health Program Officer
Allan P. Ignacio
Statistician II
Dexter Jay B. Flores
Health Program Officer II

TABLE OF CONTENTS

Field Health Services Information System

2022 Annual Report

CHAPTER 1 – MAIN REPORT ANALYSIS

	**1.A Family Planning (FP) Services**	
	2022 FP Key Findings	**13**
Figure 1.A.1	Current Users of Modern FP Methods, Philippines, 2018-2022	**13**
Table 1.A.1	Modern Contraception Use by Method Type, Philippines, 2022	**14**
Figure 1.A.2	New Acceptors of Modern FP Methods, Philippines, 2018-2022	**14**
Table 1.A.2	New Acceptors of Modern Contraception by Method Type, Philippines, 2022	**15**
Table 1.A.3	Unmet Need for Modern FP, Philippines, 2019-2022	**15**
Figure 1.A.3	Unmet Need for Modern FP, by Region, 2022	**16**
Figure 1.A.4	Adolescent Birth Rate, Philippines, 2019-2022	**17**
Table 1.A.4	Adolescent Birth Rate, by Region, 2022	**17**
	**1.B Maternal Health Indicators**	
	2022 Key Findings	**18**
1.B.1 Prenatal Care		
Figure 1.B.1.1	Prenatal Care Indicators, Philippines, 2018-2022	**18**
Figure 1.B.1.2	Prenatal Care Indicators, by Region, 2022	**18**
1.B.2 Intrapartum Care and Delivery Outcomes		
Figure 1.B.2.1	Facility-Based Deliveries and Skilled Birth Attendance, Philippines, 2018-2022	**20**
Figure 1.B.2.2	Facility-Based Deliveries and Skilled Birth Attendance, by Region, 2022	**21**
Table 1.B.2.1	Count and Proportion of Live Births with Low Birth Weight (<2500g), Philippines, 2018-2022	**21**
Figure 1.B.2.3	Proportion of Live Births with Low Birth Weight (<2500g), by Region, 2022	**22**
1.B.3 Postnatal Care		
Figure 1.B.3.1	Women with at least 2 Postnatal Check-ups, Philippines, 2018-2022	**23**
Figure 1.B.3.2	Women with at least 2 Postnatal Check-ups, by Region, 2022	**23**
1.B.4 Maternal Mortality		
Figure 1.B.4.1	Maternal Mortality, Philippines, 2018-2022	**24**
Figure 1.B.4.2	Maternal Mortality, by Region, 2022	**25**
	**1.C Child Health Indicators**	
	2022 Key Findings	**26**
1.C.1 Immunization Services for Infants and Children		
Figure 1.C.1.1	Fully Immunized Children (FIC) and Completely Immunized Children (CIC), Philippines, 2018-2022	**28**
Figure 1.C.1.2	FIC and CIC, by Region, 2022	**28**
Figure 1.C.1.3	Immunization in Children, Philippines, 2018-2022	**29**
Figure 1.C.1.4	Immunization in Children, by Region, 2022	**30**
1.C.2 Nutrition Services for Infants and Children		
Figure 1.C.2.1	Immediate Breastfeeding and Exclusive Breastfeeding, by Region, 2022	**31**
Figure 1.C.2.2	Supplementation in Children, by Region, 2022	**32**
1.C.3 Management of Sick Infants and Children		
Figure 1.C.3.1	Management of Sick Children, Philippines, 2019-2022	**33**
Figure 1.C.3.2	Management of Sick Children, by Region, 2022	**34**

1.C.4 Child Mortality		
Figure 1.C.4.1	Child Mortality, by Region, 2019-2022	**35**
Figure 1.C.4.2	Under-five Mortality Rate, by Region, 2022	**36**
Table 1.C.4.1	Under-five, Infant, and Neonatal Deaths, by Region, 2022	**37**
	**1.D Oral Health Care and Services**	
	2022 Key Findings	**38**
Figure 1.D.1	Basic Oral Health Care (BOHC) Coverage Among Different Age Groups, Philippines, 2019-2022	**39**
Figure 1.D.2	BOHC Coverage among Pregnant Women (10-49 years old), Philippines, 2019-2022	**40**
Figure 1.D.3	BOHC Coverage among Infants and Children, by Region, 2022	**40**
Figure 1.D.4	BOHC Coverage among Adolescents and Adults, by Region, 2022	**41**
Figure 1.D.5	BOHC Coverage among Seniors 60 years old and above, by Region, 2022	**41**
Figure 1.D.6	BOHC Coverage among Pregnant Women, by Region, 2022	**42**
	**1.E Non-Communicable Disease Prevention and Control Services**	
	2022 Key Findings	**43**
Figure 1.E.1	Percentage of Adults 20 years old and above who were PhilPEN Risk Assessed and Newly Identified as Hypertensives or with Type 2 Diabetes Mellitus, Philippines, 2019-2022	**45**
Figure 1.E.2	Percentage of Female Adults 20 years old and above who were Screened for Cervical Cancer and Breast Mass, Philippines, 2019-2022	**46**
Figure 1.E.3	Percentage of Seniors 60 years old and above who were Vaccinated with PPV or Influenza vaccine, Philippines, 2019-2022	**47**
Figure 1.E.4	Percentage of Adults 20 years old and above who were PhilPEN Risk Assessed, by Region, 2022	**47**
Figure 1.E.5	Percentage of PhilPEN Risk Assessed Adults 20 years old and above Newly Identified as Hypertensive or with Type 2 Diabetes Mellitus by Region, 2022	**48**
Figure 1.E.6	Percentage of Female Adults 20 years old and above who were Screened for Cervical Cancer and Breast Mass, by Region, 2022	**49**
Figure 1.E.7	Percentage of Screened for Cervical Cancer and Breast Mass found with Suspicious Cervical Cancer or Breast Mass, by Region, 2022	**49**
Figure 1.E.8	Percentage of Seniors 60 years old and above who were Vaccinated with Pneumococcal Polysaccharide Vaccine (PPV) or Influenza Vaccine, by Region, 2022	**50**
	**1.F Environmental Health and Sanitation Services**	
	2022 Key Findings	**51**
Figure 1.F.1	Percentage of Household (HH) with access to Basic Safe Water Supply (BSWS) and using Safely Managed Drinking Water Services (SMDWS), Philippines, 2019-2022	**52**
Figure 1.F.2	Percentage of HH with access to Basic Sanitation Facility (BSF) and using Safely Managed Sanitation Services (SMSS), Philippines, 2019-2022	**53**
Figure 1.F.3	Percentage of Barangays given the Zero Open Defecation (ZOD) Certification, Philippines, 2019-2022	**53**
Figure 1.F.4	Percentage of HH with access to BSWS and using SMDWS, by Region, 2022	**54**
Figure 1.F.5	Percentage of HH with access to BSF and using SMSS, by Region, 2022	**54**
Figure 1.F.6	Percentage of Barangays given the ZOD Certification, by Region, 2022	**55**
	**1.G Morbidity**	
Table 1.G	Causes of Mordbidity Ranking, Philippines 2019-2022	**56**
	**1.H Demographic Data**	
	2022 Key Findings	**57**
Figure 1.H.1	Population to Health Facility Ratio, Philippines, 2019-2022	**59**
Figure 1.H.2	Population to Health Care Worker Ratio, Philippines, 2019-2022	**59**
Figure 1.H.3	Population to Health Center (Municipal Health Center, City Health Center, and Rural Health Unit) Ratio, by Region, 2022	**60**

CHAPTER 2 – SUMMARY TABLES AND GRAPHS

2.A Family Planning Services

Table 2.A.1	New Acceptors	**62**
Table 2.A.2	Other Acceptors	**82**
Table 2.A.3	Drop-outs	**102**
Table 2.A.4	Current Users	**122**
Table 2.A.5	Unmet Needs	**142**

2.B Maternal Care and Services

2.B.1 Prenatal Care

Table 2.B.1.1	Women who gave birth with at least 4 or more Prenatal Check-ups	**147**
Table 2.B.1.2	Pregnant Women seen according to their Nutritional Status	**152**
Table 2.B.1.3	Pregnant Women for the 1st time given at least 2 doses of Tetanus Diphtheria (Td) vaccination	**155**
Table 2.B.1.4	Pregnant Women for the 2nd or more times given at least 3 doses of Td vaccination (Td2 Plus)	**160**
Table 2.B.1.5	Pregnant Women who completed Iron with Folic Acid Supplementation	**165**
Table 2.B.1.6	Pregnant Women who completed doses of Calcium Carbonate Supplementation	**169**
Table 2.B.1.7	Pregnant Women given Iodine capsules	**174**
Table 2.B.1.8	Pregnant Women given 1 dose of deworming tablet	**178**
Table 2.B.1.9	Pregnant Women screened for Syphilis	**183**
Table 2.B.1.10	Pregnant Women tested positive for Syphilis	**188**
Table 2.B.1.11	Pregnant Women screened for Hepatitis B	**192**
Table 2.B.1.12	Pregnant Women tested positive for Hepatitis B	**196**
Table 2.B.1.13	Pregnant Women screened for HIV	**200**
Table 2.B.1.14	Pregnant Women tested for Complete Blood Count (CBC) or Hemoglobin (Hgb) & Hematocrit (Hct) Count	**204**
Table 2.B.1.15	Pregnant Women tested for Complete Blood Count or Hgb & Hct count diagnosed with Anemia	**208**
Table 2.B.1.16	Pregnant Women screened for Gestational Diabetes	**212**
Table 2.B.1.17	Pregnant Women tested positive for Gestational Diabetes	**216**

2.B.2 Intrapartum Care and Delivery Outcome

Table 2.B.2.1	Total Number of Women who Delivered a Live Baby or Stillbirth/Fetal Death<br>Deliveries Attended by Skilled Health Professionals<br>Facility Based Delivery	**220**
Table 2.B.2.2	Delivery by Type (Vaginal and Cesarean)	**223**
Table 2.B.2.3	Pregnancy by Outcome (Full Term and Pre-term)	**228**
Table 2.B.2.4	Pregnancy by Outcome (Fetal Deaths and Abortion)	**233**
Table 2.B.2.5	Live Births by Birth Weight	**238**

2.B.3 Postpartum and Newborn Care

Table 2.B.3.1	Postpartum Women together with their Newborn who completed at least 2 Postpartum Check-ups	**243**
Table 2.B.3.2	Postpartum Women who completed Iron with Folic Acid	**248**
Table 2.B.3.3	Postpartum Women who completed Vitamin A supplementation	**252**

2.C Child Care and Services

2.C.1 Immunization Services for Infants and Children

Table 2.C.1.1	Children Protected at Birth (CPAB)<br>Newborn / Infants Vaccinated with BCG<br>Newborn Vaccinated with Hepatitis B antigen	**256**

Table 2.C.1.2	3 doses of DPT-HiB-HepB antigen	**260**
Table 2.C.1.3	3 doses of Oral Polio Vaccine (OPV)	**263**
	2 doses of Inactivated Polio Vaccine (IPV)	**266**
Table 2.C.1.4	3 doses of Pneumococcal Conjugate Vaccine (PCV)	**269**
Table 2.C.1.5	2 doses of Measles Containing Vaccine (MCV)	**273**
Table 2.C.1.6	Fully Immunized Children (FIC)	**277**
	Completely Immunized Children (CIC)	
2.C.2 Nutrition Services for Infants and Children		
Table 2.C.2.1	Initiated on Breastfeeding immediately after birth lasting for at least 90 minutes	**281**
	Infants Exclusively Breastfed until 5th month and 29 days	
	Infants who Continued Breastfeeding and were Introduced to Complementary Feeding beginning at 6 months of age	
Table 2.C.2.2	Infants Born Preterm or with Low Birth Weight Given Iron Supplements	
	Infants/Children 6-11 and 12-59 months old who completed Vitamin A Supplementation	**284**
Table 2.C.2.3	Infants 6-11 months old and Children 12-23 months old who completed Micronutrient Powder (MNP) Supplementation	**287**
Table 2.C.2.4	Children 0-59 months old whose Nutritional Status are Normal, Stunted and Overweight/Obese	**292**
Table 2.C.2.5	Children 0-59 months old whose Nutritional Status are Wasted-MAM, Wasted-SAM and Wasted	**296**
2.C.3 Deworming Services for Children and Adolescents		
Table 2.C.3.1	Children/Adolescents who completed 2 doses of Deworming Tablet	**300**
2.C.4 Management of Sick Infants and Children		
Table 2.C.4.1	High Risk Infants and Children with Measles and/or Persistent Diarrhea who received Vitamin A capsule aside from routine supplementation	**303**
Table 2.C.4.2	Diarrhea cases seen (0-59 months old) who received Oral Rehydration Salt Solution (ORS) and ORS with Zinc drops or syrup	
	Pneumonia cases seen (0-59 months old) and received treatment	**307**
**2.D Oral Health Care and Services**		
Table 2.D.1	Children 12-59 months old who are orally fit upon oral examination or after oral rehabilitation	**311**
Table 2.D.2	Children 5 years old and above with new cases of Decayed-Missing Filled Teeth (DMFT)	**316**
Table 2.D.3	Basic Oral Health Care (BOHC) Infants 0-11 months	**321**
Table 2.D.4	BOHC Children 1-4 years old	**326**
Table 2.D.5	BOHC Children 5-9 years old	**331**
Table 2.D.6	BOHC Adolescents 10-14 years old	**336**
Table 2.D.7	BOHC Adolescents 15-19 years old	**341**
Table 2.D.8	BOHC Adolescents 20-59 years old	**346**
Table 2.D.9	BOHC Senior Citizen 60 years old and above	**351**
Table 2.D.10	BOHC Pregnant Women (10-14, 15-19, and 20-49 years old)	**356**
**2.E Infectious Disease Prevention and Control Services**		
2.E.1 Filariasis Prevention and Control		
Table 2.E.1.1	Positive Cases Found and Case Detection Rate	
	Lymphatic Filariasis Cases and Clinical Rate	**361**
2.E.2 Schistosomiasis Prevention and Control		
Table 2.E.2.1	Suspected Schistosomiasis Cases Seen	**366**
	Acute Clinically Diagnosed Cases Seen	
	Acute Confirmed Cases	

Table 2.E.2.2	Chronic Clinically Diagnosed Cases Seen<br>Confirmed Chronic Cases	**372**
Table 2.E.2.3	Chronic Clinically Diagnosed Cases Treated in the Health Facility<br>Confirmed Chronic Cases referred to a Hospital Facility	**378**
2.E.3 Soil Transmitted Helminthiasis Prevention and Control		
Table 2.E.3.1	Preschool-Aged Children (PSAC) who completed 2 doses of Deworming Tablets<br><br>School Aged Children (SAC) who completed 2 doses of Deworming Tablets	**383**
Table 2.E.3.2	Adolescents 10-19 years old who completed 2 doses of Deworming Tablet<br>Women of Reproductive Age (WRA) 20-49 years old who completed 2 doses of Deworming Tablet	**388**
Table 2.E.3.3	Pregnant Women who completed 1 dose of Deworming Tablet	**394**
2.E.4 HIV-AIDS/STI Prevention and Control		
Table 2.E.4.1	Pregnant Women Screened for Syphilis<br>Pregnant Women Positive for Syphilis<br>Pregnant Women Screened for HIV	**398**
2.E.5 Leprosy Prevention and Control		
Table 2.E.5.1	Leprosy Cases Undergoing Treatment<br>Prevalence Rate<br>Case Detection Rate	**403**
2.E.6 Rabies		
Table 2.E.6.1	Deaths due to Rabies	**407**
2.E.7 Tuberculosis Prevention and Control *(source: Integrated Tuberculosis Information System)*		
Table 2.E.7.1	Case Notification Rate (CNR), all forms	**412**
Table 2.E.7.2	Case Notification Rate (DRTB)	**417**
Table 2.E.7.3	Treatment Success Rate (TSR), DSTB-all forms	**423**
Table 2.E.7.4	Treatment Success Rate (TSR), MDRTB	**428**
**2.F Non-Communicable Disease Prevention and Control Services**		
2.F.1 Lifestyle Related Diseases (Risk Assessment Using PhilPEN protocol)		
Table 2.F.1.1	Adults Age 20 years old and above who were Risk-assessed using the PhilPEN Protocol	**433**
Table 2.F.1.2	Adults 20 years old and above who are Current Smokers based on the PhilPEN Protocol<br>Adults 20 years old and above who are Binge Drinkers based on the PhilPEN Protocol<br><br>Adults 20 years old and above who are Overweight/Obese based on the PhilPEN Protocol	**438**
2.F.2 Cancer Prevention and Control		
Table 2.F.2.1	Women 20 years old and above Screened for Cervical Cancer<br>Women 20 years old and above Found Positive or Suspected for Cervical Cancer<br><br>Women 20 years old and above Screened for Breast Mass<br>Women 20 years old and above with Suspicious Breast Mass	**441**
2.F.3 Cardiovascular Disease and Diabetes Mellitus Prevention and Control		
Table 2.F.3.1	Newly-identified Hypertensive among 20 years old and above adults<br>Newly-identified Type 2 Diabetes Mellitus among 20 years old and above adults	**445**
2.F.4 Blindness Prevention Program		
Table 2.F.4.1	Senior Citizens 60 years old and above Screened for Visual Acuity<br>Senior Citizens 60 years old and above Diagnosed with Eye Disease/s	**449**
2.F.5 Immunization for Senior Citizens		
Table 2.F.5.1	1 dose of Pneumococcal Polysaccharide Vaccine (PPV)<br>1 dose of Influenza Vaccine	**453**

2.G Environmental Health and Sanitation Services

Table 2.G.1	Households with Access to Basic Safe Water Supply (Level I, II, III)<br>Households using Safely Managed Drinking-Water Services	**857**
Table 2.G.2	Households with Basic Sanitation Facility<br>Households using Safely Managed Sanitation Service	**461**
Table 2.G.3	Industrial Establishments and Industrial Establishments Issued with Sanitary Permit<br><br>Number of Barangays and Barangays Certified as Zero Open Defecation (ZOD)	**465**

2.H Morbidity

2.H.1 Top Ten Leading Causes of Morbidity

Table 2.H.1.1	Top Ten Leading Causes of Morbidity	**470**

2.H.2 Morbidity Rate (by Type of Disease, Sex and Age Group)

Table 2.H.2.1	Acute Bloody Diarrhea	**471**
Table 2.H.2.2	Acute Febrile Illness	**477**
Table 2.H.2.3	Acute Flaccid Paralysis	**483**
Table 2.H.2.4	Acute Hemorrhagic Fever	**489**
Table 2.H.2.5	Acute Lower Respiratory Tract Infection	**495**
Table 2.H.2.6	Acute Respiratory Infection (less than 5 years old)	**501**
Table 2.H.2.7	Acute Respiratory Infection (more than 5 years old)	**505**
Table 2.H.2.8	Acute Watery Diarrhea	**511**
Table 2.H.2.9	Animal Bites	**517**
Table 2.H.2.10	Bronchitis	**523**
Table 2.H.2.11	Cholera	**529**
Table 2.H.2.12	Chronic Obstructive Pulmonary Disease	**535**
Table 2.H.2.13	Diphtheria	**541**
Table 2.H.2.14	Diseases of the Heart	**547**
Table 2.H.2.15	Filariasis	**553**
Table 2.H.2.16	Fever of Unknown Origin	**559**
Table 2.H.2.17	Genital Ulcer	**565**
Table 2.H.2.18	Gonorrhea	**571**
Table 2.H.2.19	Hypertension	**577**
Table 2.H.2.20	Influenza-Like Illness	**583**
Table 2.H.2.21	Influenza	**589**
Table 2.H.2.22	Leprosy	**595**
Table 2.H.2.23	Leptospirosis	**601**
Table 2.H.2.24	Malaria	**607**
Table 2.H.2.25	Measles	**613**
Table 2.H.2.26	Meningococcal Infection	**619**
Table 2.H.2.27	Neonatal Tetanus	**625**
Table 2.H.2.28	Non-Neonatal Tetanus	**629**
Table 2.H.2.29	Pneumonia	**635**
Table 2.H.2.30	Rabies (Human)	**641**
Table 2.H.2.31	Paralytic Shellfish Poisoning	**647**
Table 2.H.2.32	Schistosomiasis	**653**
Table 2.H.2.33	Skin Diseases	**659**
Table 2.H.2.34	Syphilis	**665**
Table 2.H.2.35	Tuberculosis (All Forms)	**671**
Table 2.H.2.36	Typhoid and Paratyphoid Fever	**677**
Table 2.H.2.37	Urethral Discharge	**683**
Table 2.H.2.38	Urinary Tract Infection	**689**
Table 2.H.2.39	Viral Encephalitis	**695**
Table 2.H.2.40	Viral Hepatitis	**701**
Table 2.H.2.41	Viral Meningitis	**707**
Table 2.H.2.42	Whooping Cough	**713**

Figures and Graphs

Figure 2.H.2.1	Acute Bloody Diarrhea, 2018-2022	**719**
Figure 2.H.2.2	Acute Bloody Diarrhea, 2022	**719**
Figure 2.H.2.3	Acute Febrile Illness, 2018-2022	**720**
Figure 2.H.2.4	Acute Febrile Illness, 2022	**720**
Figure 2.H.2.5	Acute Flaccid Paralysis, 2018-2022	**721**
Figure 2.H.2.6	Acute Flaccid Paralysis, 2022	**721**
Figure 2.H.2.7	Acute Hemorrhagic Fever, 2018-2022	**722**
Figure 2.H.2.8	Acute Hemorrhagic Fever, 2022	**722**
Figure 2.H.2.9	Acute Lower Respiratory Tract Infection, 2019-2022	**723**
Figure 2.H.2.10	Acute Lower Respiratory Tract Infection, 2022	**723**
Figure 2.H.2.11	Acute Respiratory Infection (<5 years old), 2019-2022	**724**
Figure 2.H.2.12	Acute Respiratory Infection (<5 years old), 2022	**724**
Figure 2.H.2.13	Acute Respiratory Infection (>5 years old), 2019-2022	**725**
Figure 2.H.2.14	Acute Respiratory Infection (>5 years old), 2022	**725**
Figure 2.H.2.15	Acute Watery Diarrhea, 2018-2022	**726**
Figure 2.H.2.16	Acute Watery Diarrhea, 2022	**726**
Figure 2.H.2.17	Animal Bites, 2019-2022	**727**
Figure 2.H.2.18	Animal Bites, 2022	**727**
Figure 2.H.2.19	Bronchitis, 2018-2022	**728**
Figure 2.H.2.20	Bronchitis, 2022	**728**
Figure 2.H.2.21	Cholera, 2018-2022	**729**
Figure 2.H.2.22	Cholera, 2022	**729**
Figure 2.H.2.23	Chronic Obstructive Pulmonary Disease, 2019-2022	**730**
Figure 2.H.2.24	Chronic Obstructive Pulmonary Disease, 2022	**730**
Figure 2.H.2.25	Diphtheria, 2018-2022	**731**
Figure 2.H.2.26	Diphtheria, 2022	**731**
Figure 2.H.2.27	Diseases of the Heart, 2018-2022	**732**
Figure 2.H.2.28	Diseases of the Heart, 2022	**732**
Figure 2.H.2.29	Filariasis, 2018-2022	**733**
Figure 2.H.2.30	Filariasis, 2022	**733**
Figure 2.H.2.31	Fever of Unknown Origin, 2019-2022	**734**
Figure 2.H.2.32	Fever of Unknown Origin, 2022	**734**
Figure 2.H.2.33	Genital Ulcer, 2019-2022	**735**
Figure 2.H.2.34	Genital Ulcer, 2022	**735**
Figure 2.H.2.35	Gonorrhea, 2019-2022	**736**
Figure 2.H.2.36	Gonorrhea, 2022	**736**
Figure 2.H.2.37	Hypertension, 2018-2022	**737**
Figure 2.H.2.38	Hypertension, 2022	**737**
Figure 2.H.2.39	Influenza-Like Illness, 2019-2022	**738**
Figure 2.H.2.40	Influenza-Like Illness, 2022	**738**
Figure 2.H.2.41	Influenza, 2018-2022	**739**
Figure 2.H.2.42	Influenza, 2022	**739**
Figure 2.H.2.43	Leprosy, 2018-2022	**740**
Figure 2.H.2.44	Leprosy, 2022	**740**
Figure 2.H.2.45	Leptospirosis, 2018-2022	**741**
Figure 2.H.2.46	Leptospirosis, 2022	**741**
Figure 2.H.2.47	Malaria, 2018-2022	**742**
Figure 2.H.2.48	Malaria, 2022	**742**
Figure 2.H.2.49	Measles, 2018-2022	**743**
Figure 2.H.2.50	Measles, 2022	**743**
Figure 2.H.2.51	Meningococcemia, 2018-2022	**744**
Figure 2.H.2.52	Meningococcemia, 2022	**744**
Figure 2.H.2.53	Neonatal Tetanus, 2018-2022	**745**
Figure 2.H.2.54	Neonatal Tetanus, 2022	**745**

Figure 2.H.2.55	Non-Neonatal Tetanus, 2018-2022	746
Figure 2.H.2.56	Non-Neonatal Tetanus, 2022	746
Figure 2.H.2.57	Pneumonia, 2019-2022	747
Figure 2.H.2.58	Pneumonia, 2022	747
Figure 2.H.2.59	Rabies (Human), 2018-2022	748
Figure 2.H.2.60	Rabies (Human), 2022	748
Figure 2.H.2.61	Paralytic Shellfish Poisoning, 2018-2022	749
Figure 2.H.2.62	Paralytic Shellfish Poisoning, 2022	749
Figure 2.H.2.63	Schistosomiasis, 2018-2022	750
Figure 2.H.2.64	Schistosomiasis, 2022	750
Figure 2.H.2.65	Skin Diseases, 2019-2022	751
Figure 2.H.2.66	Skin Diseases, 2022	751
Figure 2.H.2.67	Syphilis, 2019-2022	752
Figure 2.H.2.68	Syphilis, 2022	752
Figure 2.H.2.69	Tuberculosis (All Forms), 2018-2022	753
Figure 2.H.2.70	Tuberculosis (All Forms), 2022	753
Figure 2.H.2.71	Typhoid and Paratyphoid Fever, 2018-2022	754
Figure 2.H.2.72	Typhoid and Paratyphoid Fever, 2022	754
Figure 2.H.2.73	Urethral Discharge, 2019-2022	755
Figure 2.H.2.74	Urethral Discharge, 2022	755
Figure 2.H.2.75	Urinary Tract Infection, 2018-2022	756
Figure 2.H.2.76	Urinary Tract Infection, 2022	756
Figure 2.H.2.77	Viral Encephalitis, 2018-2022	757
Figure 2.H.2.78	Viral Encephalitis, 2022	757
Figure 2.H.2.79	Viral Hepatitis, 2018-2022	758
Figure 2.H.2.80	Viral Hepatitis, 2022	758
Figure 2.H.2.81	Viral Meningitis, 2018-2022	759
Figure 2.H.2.82	Viral Meningitis, 2022	759
Figure 2.H.2.83	Whooping Cough, 2018-2022	760
Figure 2.H.2.84	Whooping Cough, 2022	760
**2.I Mortality**		
Table 2.I.1	Maternal Mortality Ratio (MMR)	761
	Under Five Mortality Rate (UFMR)	
Table 2.I.2	Infant Mortality Rate (IMR)	765
	Neonatal Mortality Rate (NMR)	
	Perinatal Mortality Rate (PMR)	
**2.J Natality**		
Table 2.J.1	Live Births	769
	Crude Birth Rate	
	Adolescent Birth Rate	
**2.K Demographic Data**		
Table 2.K.1	Projected Population and Number of Households	772
Table 2.K.2	Number and Ratio of Barangays	777
	Number and Ratio of Health Centers (HC)	
	Number and Ratio of Barangay Health Stations (BHS)	
Table 2.K.3	Number and Ratio of Physicians	780
	Number and Ratio of Dentists	
	Number and Ratio of Public Health Nurses (PHN)	
	Number and Ratio of Midwives	
	Number and Ratio of Nutritionists	
Table 2.K.4	Number and Ratio of Medical Technologists	786
	Number and Ratio of Sanitary Engineers	
	Number and Ratio of Sanitary Inspectors	
	Number and Ratio of Active Barangay Health Workers (BHWs)	

CHAPTER I

MAIN REPORT ANALYSIS

1.A Family Planning Services

2022 KEY FINDINGS

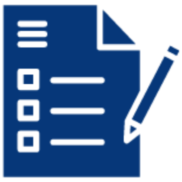

8,372,559

women of aged 10 to 49 years were using modern methods of family planning

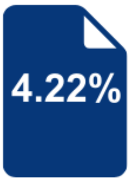

1,186,993

estimated WRA had an unmet need for modern family planning

- The most commonly used family planning method is combined-oral-contraceptive pills. Around 35% of the total current users were using pills-COC, followed by injectables (20%).
- Of the 990,201 new acceptors of modern family planning methods, 40% were using lactational amenorrhea. Compared to 2021, an increase in the new acceptors of implants (88% higher) and condoms (70% higher) was observed in 2022.
- A total of 123,362 live births were reported among women aged 15 to 19 years, corresponding to an adolescent birth rate of 24.36 births per 1,000 women.

The Philippines commits to full and effective implementation of the country's Universal Health Care (UHC) Law, along with the Reproductive Health (RH) Law, that will guarantee all Filipinos equitable and inclusive access to quality and affordable health care, goods, and services, including family planning (FP).

The Philippines is currently in the stage of transitioning from low to high modern contraceptive use and has an opportunity for rapid growth. During this stage, it is important that there are no barriers in accessing family planning by ensuring contraceptive availability, high-quality services, and continued demand generation. This chapter presents the service statistics for family planning provided by public health facilities.

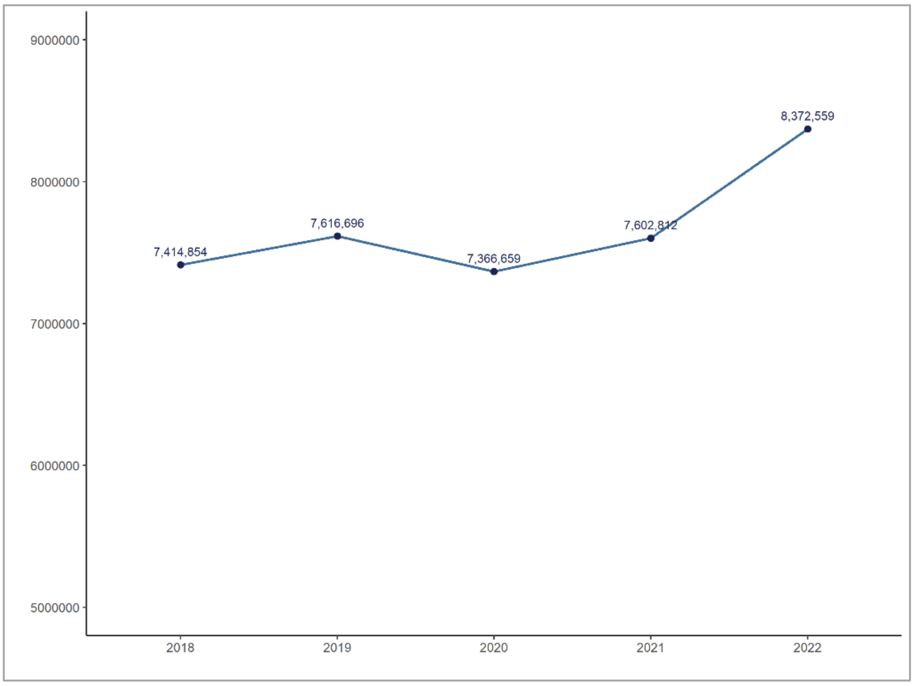

***Figure 1.A.1:** Current Users of Modern Family Planning Methods, Philippines, 2018-2022*

In 2022, 8,372,559 women aged 10 to 49 years were using modern methods of family planning *(Figure 1.A.1)*. This is the highest number of current modern method users reported in the FHSIS in the last five years and is 10% higher than in 2021.

1.A Family Planning Services

Modern methods of family planning include bilateral tubal ligation (BTL), no-scalpel vasectomy (NSV), condoms, intrauterine devices (IUD, interval and postpartum), oral pills (combined-oral-contraceptives [COC] and progestin-only [POP]), injectables, implants, and natural family planning methods (NFP) namely cervical mucus method (CCM), basal body temperature (BBT), symptothermal method (STM), standard days method (SDM), and lactational amenorrhea (LAM).

Oral pills, specifically combined-oral-contraceptives or COCs, remain to be the most commonly used modern family planning method, accounting for 35% of the total current users. This was followed by injectables with 1,702,295 women using them or 20% of total current users *(Table 1.A.1).*

Notably, a 28.97% increase in the number of users of implants was observed from 2021 to 2022. This result is encouraging as implants are short-acting reversible family planning method that are more effective in preventing pregnancies compared to pills. According to the World Health Organization (WHO), oral pills, as commonly used by women, may lead to 7 pregnancies per 100 women while it is only 0.1 pregnancies per 100 women for implants. Meanwhile, a decrease in the users of CCM, NSV, BBT, and STM was reported.

New acceptors of modern family planning methods are defined as clients using a contraceptive method for the first time or have never accepted any modern family planning method. In 2022, around 990,201 women were reported as new acceptors of modern family planning method *(Figure 1.A.2)*. This is a 21.4% increase from 2021, although it is still lower than the number of new acceptors in 2018.

Table 1.A.1: *Modern Contraception Use by Method Type, Philippines, 2022*

Method	2022	Percent	2021 Comparison	
			2021	% change
PILLS COC	2,929,378	34.99%	2,660,391	10.11%
INJECTABLES	1,702,295	20.33%	1,531,638	11.14%
FSTR/BTL	819,597	9.79%	783,073	4.66%
NFP-LAM	671,595	8.02%	606,265	10.78%
IMPLANTS	636,689	7.60%	493,679	28.97%
IUD-INTERVAL	445,713	5.32%	426,502	4.50%
PILLS POP	423,170	5.05%	408,534	3.58%
CONDOM	412,406	4.93%	372,201	10.80%
IUD-PP	146,484	1.75%	135,261	8.30%
NFP-SDM	118,646	1.42%	114,500	3.62%
NFP-CCM	49,437	0.59%	50,418	-1.95%
MSTR/NSV	9,393	0.11%	11,577	-18.86%
NFP-BBT	4,199	0.05%	4,254	-1.29%
NFP-STM	3,557	0.04%	4,519	-21.29%
**Total**	**8,372,559**	**100%**	**7,602,812**	**10.12%**

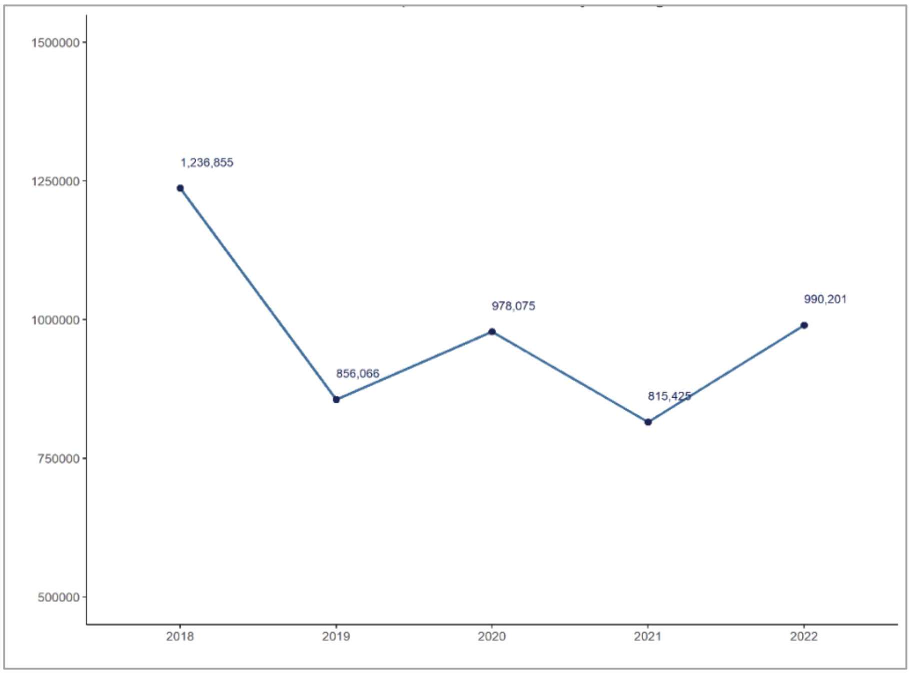

Figure 1.A.2: *New Acceptors of Modern Family Planning Methods, Philippines, 2018-2022*

1.A Family Planning Services

Forty-one percent (41.21%) of the reported new acceptors of modern methods were using lactational amenorrhea (NFP-LAM) which is the temporary postnatal infertility that occurs when a woman is not menstruating and is fully breastfeeding. It is important to keep track of these women to properly guide them in transitioning to other modern methods once the temporary infertility has passed. Injectables and pills-COC comprised 17.74% and 13.29% of the new acceptors, respectively *(Table 1.A.2).*

Table 1.A.2: *New Acceptors of Modern Contraception by Method Type, Philippines, 2022*

Method	2022	Percent	2021 Comparison	
			2021	% change
NFP-LAM	408,032	41.21%	340,641	19.78%
INJECTABLES	175,664	17.74%	132,582	32.49%
PILLS COC	131,606	13.29%	147,171	-10.58%
PILLS POP	87,609	8.85%	67,406	29.97%
IMPLANTS	84,913	8.58%	45,069	88.41%
CONDOM	48,188	4.87%	28,338	70.05%
FSTR/BTL	18,094	1.83%	15,317	18.13%
IUD-PP	16,701	1.69%	11,573	44.31%
IUD-INTERVAL	11,057	1.12%	11,708	-5.56%
NFP-SDM	4,549	0.46%	8,155	-44.22%
NFP-CCM	1,908	0.19%	4,628	-58.77%
NFP-BBT	979	0.10%	1,045	-6.32%
NFP-STM	493	0.05%	1,350	-63.48%
MSTR/NSV	408	0.04%	442	-7.69%
**Total**	**990,201**	**100%**	**815,425**	**21.43%**

As with the data on current users of modern family planning, the increase in the usage of implants was also reflected among new acceptors. Specifically, there were 84,913 new acceptors of contraceptive implants in 2022, which is 88.41% higher than in 2021. A 70% increase in the number of new acceptors of condoms was also noted in 2022, while a decrease in all other natural family planning methods (except LAM) is observed.

Aside from tracking the provision of family planning commodities, it is also vital to look at women who wish to delay or prevent pregnancies but are not using modern family planning. This group is termed as women with unmet need for modern family planning and includes those that are currently using traditional family planning methods (e.g., withdrawal) but wish to transition to modern methods. Since the intention to delay or limit childbearing is already present, it is important to reach this group to determine and address the reason for the non-use of modern methods. After all, reproductive health entails that people have the capability and the freedom if and when to have children. Furthermore, unintended pregnancies have been shown to be associated with poor health outcomes for both the mother and the child, as well as with social and economic repercussions.

Despite the reported increase in the number of current users of modern family planning, the number of women with unmet need for modern family planning is still increasing, indicating a possible increase in the overall demand for modern contraception in the country. In 2022, around 1,186,993 WRA were found to have unmet need for modern contraception, which was a 27.5% increase from 2021 *(Table 1.A.3).*

Table 1.A.3: *Unmet Need for Modern Family Planning, Philippines, 2019-2022*

	2019	2020	2021	2022
No. of women with unmet need for modern family planning	504,219	844,752	930,909	1,186,993
% among estimated women of reproductive age (15-49 y.o)	1.80	3.02	3.28	4.22

1.A Family Planning Services

Looking at regional data would show that majority or 47% of the reported number of women with unmet need for modern family planning was in Region 11. Around 554,265 women in Region 11 had an unmet need for modern FP, which translates to 40.3% of the estimated total WRA in the region. Region 6 and Region 4A also had higher number of women with unmet need for FP, relative to the remaining regions. Meanwhile, Region 9, Region 1, and Region 2 had the lowest number of women with unmet need for modern FP *(Figure 1.A.3)*.

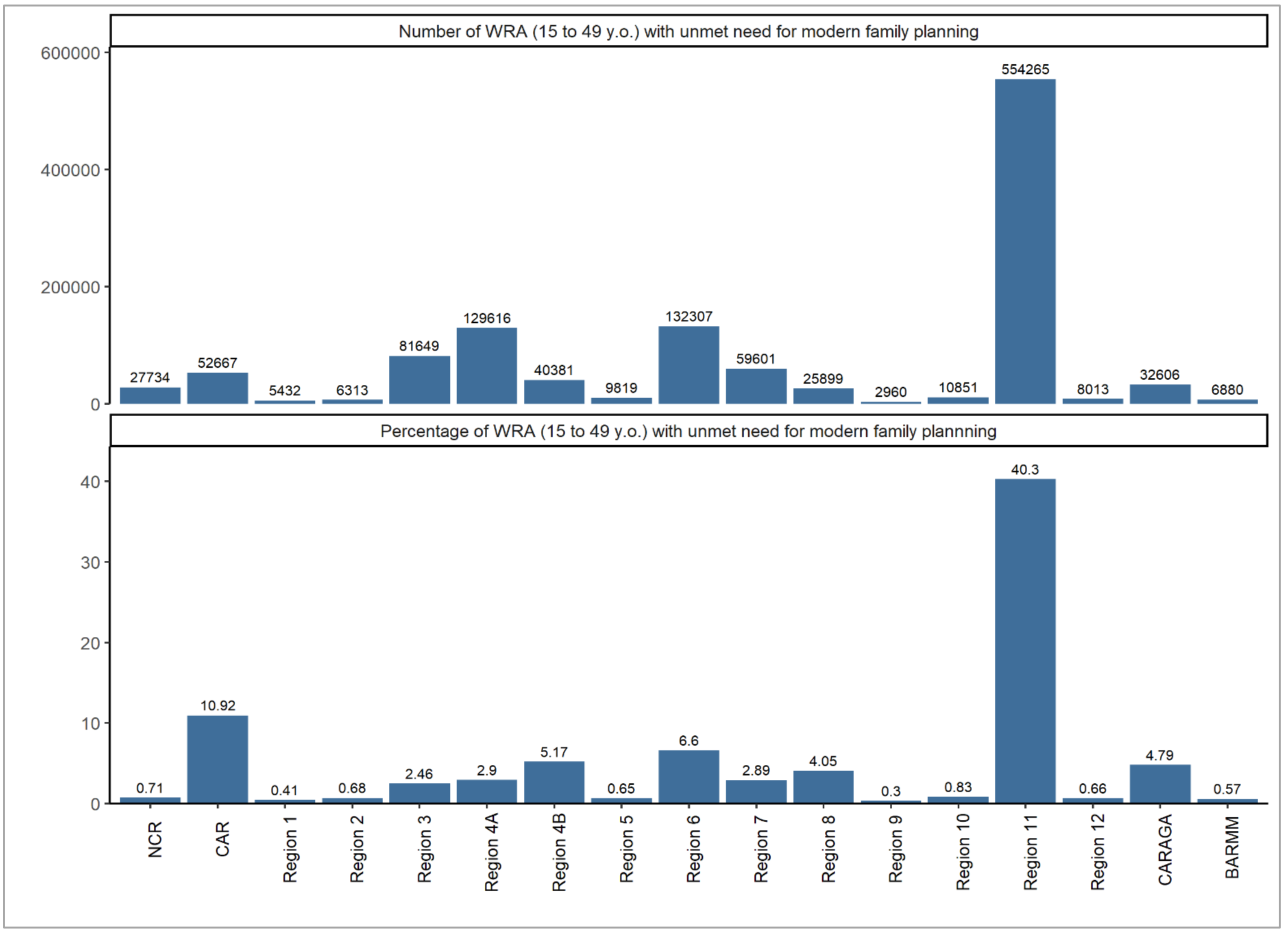

Figure 1.A.3: *Unmet Need for Modern Family Planning, by Region, 2022*

SDG Target 3.7 aims to "ensure universal access to sexual and reproductive health-care services, including for family planning, information and education, and the integration of reproductive health into national strategies and programmes". In line with this, the Philippine adolescent (aged 15 to 19 years) birth rate per 1,000 women is aimed to be lowered to 30.3 come 2030. Although the official sources for monitoring this SGD target are the PSA and National Demographic and Health Surveys (NDHS), FHSIS also has data on live births in the 15 to 19 age group that is useful for local government unit (LGU) performance monitoring.

Early pregnancies among adolescent women come with increased health risks for mothers and newborns. Babies born to mothers under 20 years of age face higher risks of low birth weight, preterm delivery and severe neonatal conditions, while young mothers have higher risks of eclampsia, puerperal endometritis and systemic infections. In addition to health risks, adolescent pregnancies may also come with social and economic consequences. Early pregnancies among adolescent women are preventable by ensuring information and services on reproductive health, including family planning, are made accessible to the population.

1.A Family Planning Services

In 2022, a total of 123,362 live births among women aged 15 to 19 years were recorded in the FHSIS. The number of live births among this age group has fluctuated in recent years, averaging to around 119,416 live births per year from 2020 to 2022. Likewise, the ABR in 2022 is 24.36 births per 1,000 women, which is a 9.19% increase compared to the ABR in 2021 *(Figure 1.A.4)*.

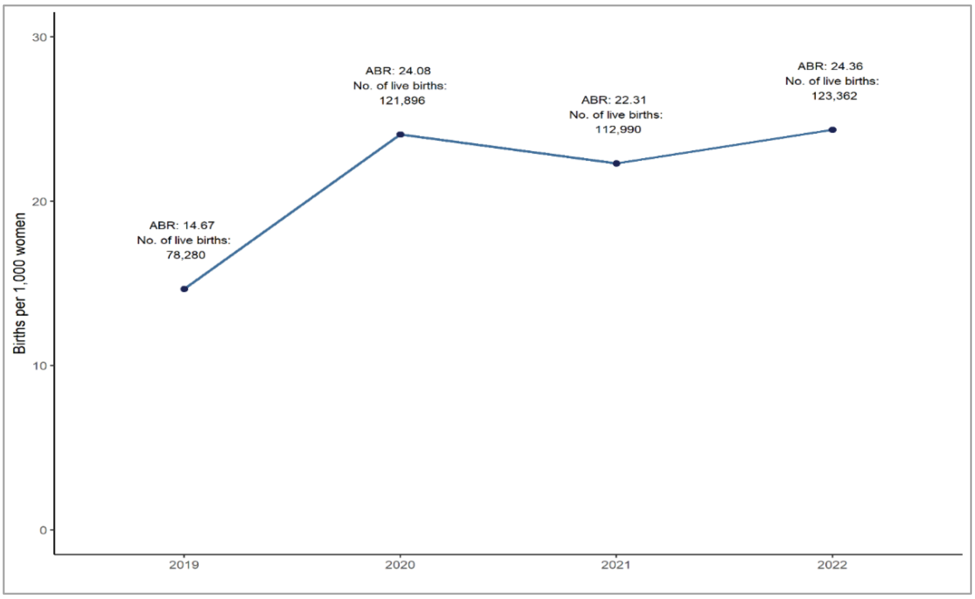

Figure 1.A.4: *Adolescent Birth Rate, Philippines, 2019-2022*

In terms of absolute counts, NCR, Region 4A, Region 3, and Region 10 had the most recorded live births among adolescent women aged 15 to 19 years in 2022. Cumulatively, the four (4) aforementioned regions comprised around 40% of the total adolescent live births in the country *(Table 1.A.4).*

Looking at the ABRs, which take into account the estimated number of 15 to 19 year old women in the population, Region 10 had the highest ABR of 41.66 births per 1,000 women. This was followed by Region 12 with an ABR of 41.15 and Region 11 with an ABR of 38.56. Meanwhile, Region 7 recorded the lowest ABR of 11.99 births per 1,000 women *(Table 1.A.4)*.

Table 1.A.4: *Adolescent Birth Rate, by Region, 2022*

Region	No. of live births among women aged 15-19 years	ABR per 1,000 women
PHILIPPINES	123,362	24.36
NCR	15,342	26.28
CAR	2,057	24.31
Region 1	3,850	16.55
Region 2	4,379	27.55
Region 3	11,525	20.21
Region 4A	13,514	17.94
Region 4B	4,957	32.65
Region 5	8,757	28.97
Region 6	6,032	16.71
Region 7	4,380	11.99
Region 8	5,085	21.95
Region 9	5,116	27.08
Region 10	10,145	41.66
Region 11	9,538	38.56
Region 12	9,259	41.15
BARMM	5,189	22.14
CARAGA	4,237	32.55

1.B Maternal Health Indicators

2022 KEY FINDINGS

- 80.7% of women who gave birth had at least four (4) antenatal care visits.
- 37.46% of women with repeat pregnancy and 23.56% of women who were pregnant for the first time completed Tetanus-Diphtheria vaccination.
- Almost 93% of the total deliveries were assisted by skilled health professionals, while 91.69% occurred in health facilities.
- Among postpartum women, 88.69% had at least two (2) postnatal check-ups.
- In 2022, a total of 914 maternal deaths were reported, leading to an MMR of 64.68 deaths per 100,000 live births.

1.B.1 Prenatal Care

Formula:

Women who gave birth and had at least 4ANC check-ups

Numerator: Number of pregnant women who gave birth with at least 4 or more antenatal check-ups
Denominator: Total number of deliveries

Women pregnant for the first time given Tetanus Diphtheria (Td) Vaccination

Numerator: Number of pregnant women for the first time given at least 2 doses of Td vaccine
Denominator: Eligible population under 1

Pregnant women with repeat pregnancy given Tetanus Diphtheria (Td) Vaccination

Numerator: Number of pregnant women for the 2nd or more times given at least 3 doses of Td vaccine (Td2 Plus)
Denominator: Eligible population under 1

Maternal Health Services are vital to provide women access to quality healthcare for a safer pregnancy and delivery. These services aim to promote the health and well-being of mothers of a Filipino family. Our goal is to reduce the maternal mortality ratio (MMR) of 70 deaths per 100,000 live births by 2030 and consequently reduce newborn deaths. The Philippines is committed in providing women full access to health services towards making their pregnancy and childbirth safer guided by the Universal Health Care. Our target outcomes for antenatal care, facility-based delivery, and postnatal care shall be maintained at 95% or higher.

This chapter presents data on prenatal care including antenatal care visits and Tetanus-Diphtheria vaccination, intrapartum care and delivery outcomes, and postnatal care. Getting timely and regular care before childbirth is vital in ensuring the safety and health of both the mother and the child. Prenatal care is essential in detecting and managing underlying conditions of the mothers, as well as in providing them with essential supplements and vaccines.

1.B Maternal Health Indicators

In the Philippines, the proportion of women who had at least four (4) antenatal care (ANC) check-ups among those who have given birth has decreased compared to previous years. In 2022, this stood at 80.7% and is lower than its value during the pre-pandemic years 2018-2019 *(Figure 1.B.1.1)*. While this is lower than the target of 95%, it is important to note that this report does not include deliveries that occurred in private hospitals.

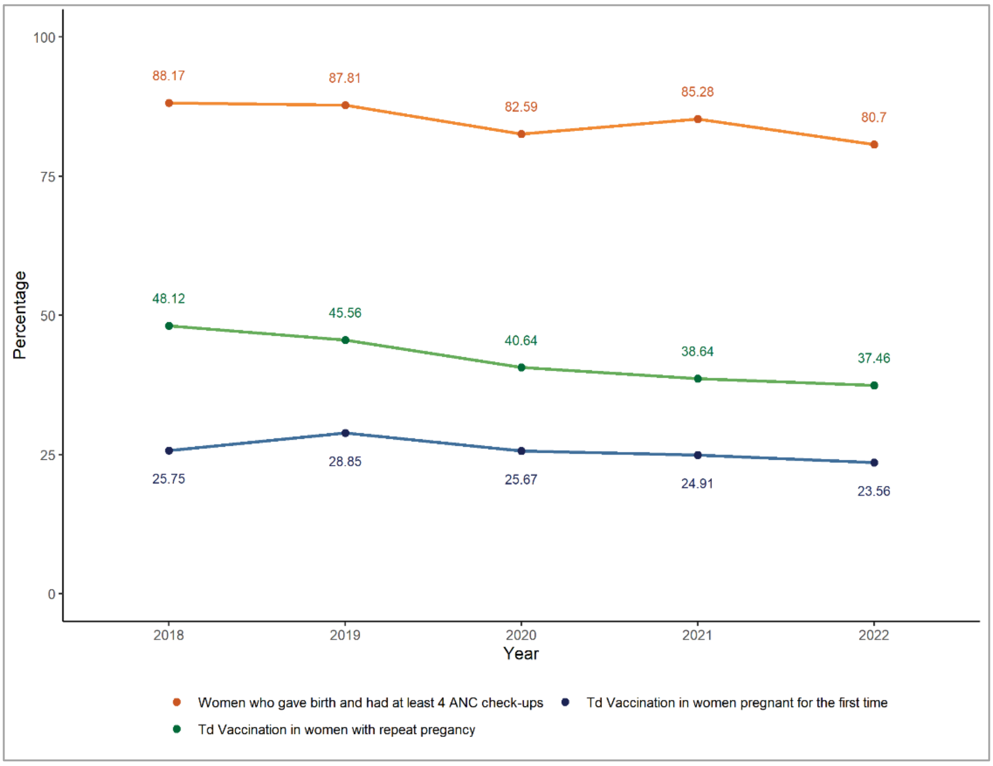

Figure 1.B.1.1: *Prenatal Care Indicators, Philippines, 2018-2022*

Meanwhile, vaccination against Tetanus-Diphtheria (Td) is higher in women with repeat pregnancy (37.46%) than those who were pregnant for the first time (23.56%). Nonetheless, the decreasing trend in the recent years is observed in both groups *(Figure 1.B.1.1)*. Increasing Td coverage in pregnant women is vital as the antibodies developed through vaccination not only protect the mother, but also offers protection for the child.

Looking at the regions, Regions 1, 2, and 3 have reached the 95% target for ANC check-ups in 2022, while Regions 10 and 12 have more than 90% of women with at least four (4) ANC check-ups. The accomplishments of the remaining regions for the said indicator range from 67% to 83%. Td vaccination is consistently higher in women with repeat pregnancies across all regions, with Regions 5 and 8 reporting the lowest coverage in both groups *(Figure 1.B.1.2).*

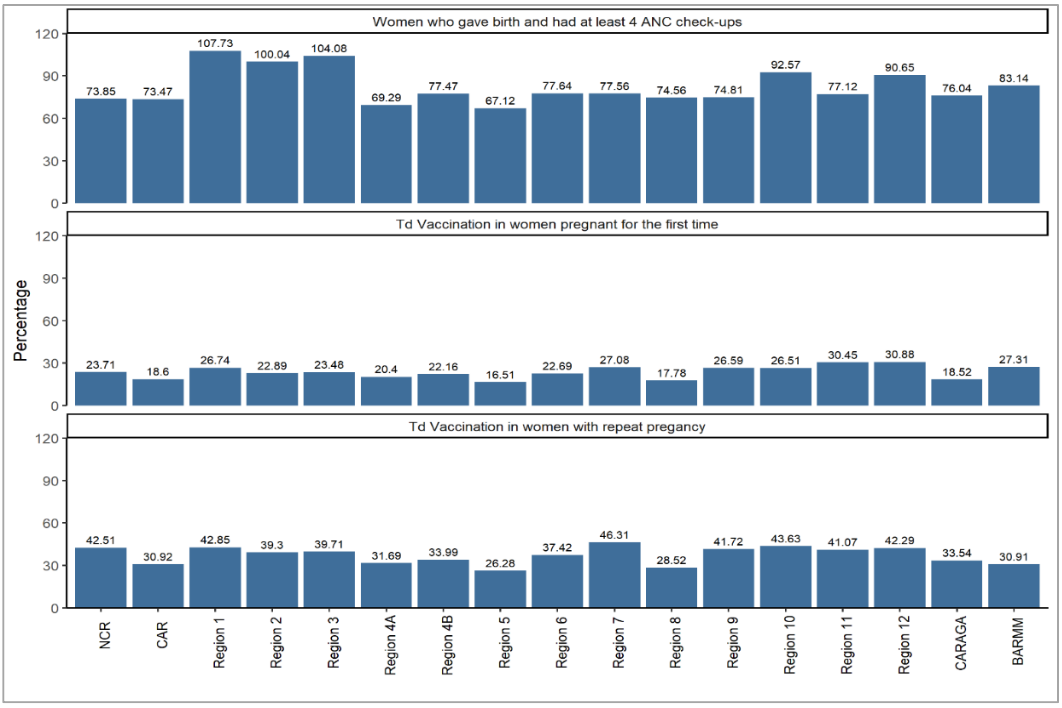

Figure 1.B.1.2: *Prenatal Care Indicators, by Region, 2022*

1.B Maternal Health Indicators

1.B.2 Intrapartum Care and Delivery Outcomes

Formula:
Facility-based Deliveries
Numerator: Number of deliveries in health facilities (clinics, BHS, RHUs, lying-ins, hospitals, etc.)
Denominator: Total number of deliveries

Births attended by Skilled Health Professionals
Numerator: Number of deliveries attended by skilled health professionals (licensed midwives, doctors or nurses)
Denominator: Total number of deliveries

A positive childbirth experience is an important component of maternal health care. Globally, over a third of maternal deaths occur during labor and childbirth. Ensuring that births are conducted by skilled professionals in facilities equipped with necessary tools is essential in preventing and/or managing complications that may arise during labor and childbirth.

This chapter presents data on facility-based deliveries and deliveries attended by skilled health professionals. Additionally, data on low birth weights is also provided.

Despite the slight decrease in the proportion of deliveries occurring in facilities and attended by skilled health professionals (midwives, doctors, nurses) in 2020, which may be attributed to the onset of the COVID-19 pandemic, both indicators have been increasing since 2021. In 2022, among the 1,419,070 deliveries reported to the FHSIS, 1,301,149 or 91.69% occurred in health facilities including clinics, barangay health stations (BHS), rural health units (RHUs), lying-ins, birthing clinics, hospitals, and DOH-licensed ambulance. *(Figure 1.B.2.1).*

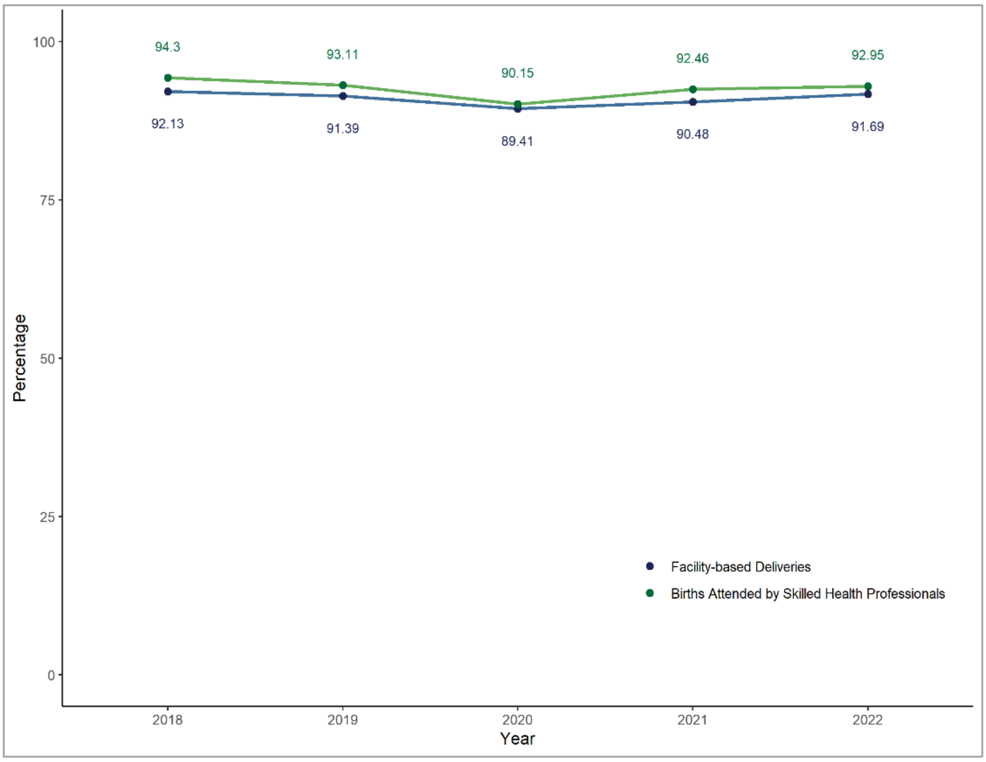

Figure 1.B.2.1: *Facility-Based Deliveries and Skilled Birth Attendance, Philippines, 2018-2022*

Furthermore, 1,319,081 deliveries or 92.95% of the total deliveries were assisted by skilled health professionals *(Figure 1.B.2.1)*. More than half or 57.61% of the total deliveries were assisted by doctors, 33.98% were assisted by midwives, and the remaining 1.36% were assisted by nurses. It is important for births to be assisted by skilled health professionals inside health facilities to ensure that any complications that may arise during childbirth are managed and proper referrals are conducted, if needed.

1.B Maternal Health Indicators

In 2022, 12 out the 17 regions in the country have achieved more than 90% of deliveries occurring in health facilities, while 14 regions have more than 90% of their births being assisted by skilled health professionals. Region 1 had the highest accomplishment in both indicators while BARMM had the lowest *(Figure 1.B.2.2).*

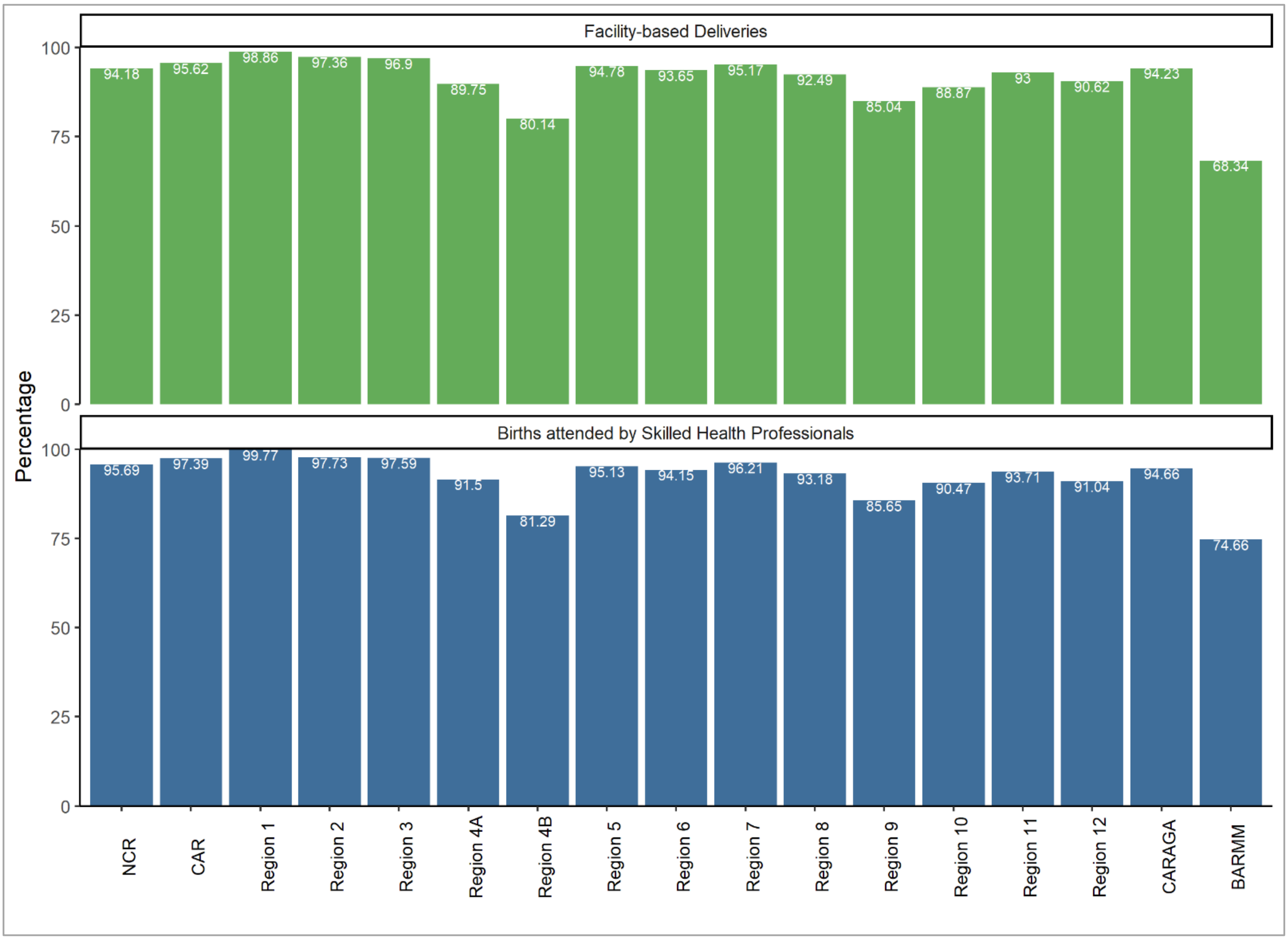

Figure 1.B.2.2: *Facility-Based Deliveries and Skilled Birth Attendance, by Region, 2022*

Table 1.B.2.1: *Count and Proportion of Live Births with Low Birth Weight (<2500g), Philippines, 2018-2022*

	2018	2019	2020	2021	2022
No. of live births with low birth weight	126,996	108,342	88,513	68,902	88,879
% of total live births	7.41	7.41	6.17	5.53	6.29

Additionally, of the 1,413,107 live births in 2022, 6.29% resulted in low birth weight or weighed less than 2500 grams. In terms of absolute counts, this corresponds to 88,879 live births which has generally decreased compared to 2018. However, comparing it to 2021 data would result to an increase in the number of births with low birth weight, partly due to an increase in the total live births in 2022 *(Table 1.B.2.1).*

1.B Maternal Health Indicators

Regionally, the percentage of live births with low birth weight is highest in NCR (11.91%) and lowest in BARMM (1.25%). Furthermore, NCR with 22,261 live births with low birth weight contributes around 25% to the total count in the country, followed by Region 4A with 9,916 live births with low birth weight *(Figure 1.B.2.3).*

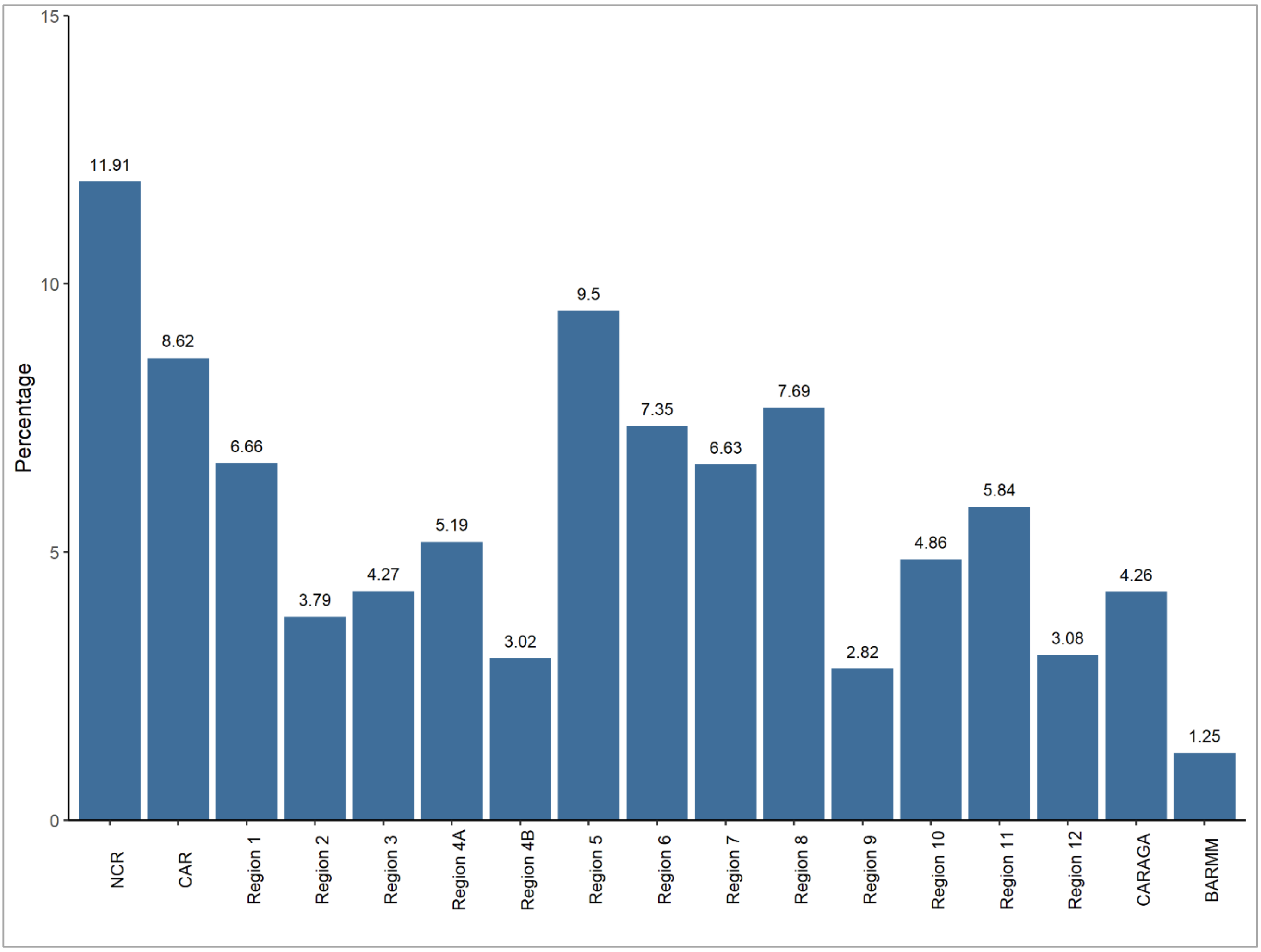

Figure 1.B.2.3: *Proportion of Live Births with Low Birth Weight (<2500g), by Region, 2022*

1.B.3 Postnatal Care

Formula:
Women together with their newborn who completed at least 2 postpartum check-ups

Numerator: Number of postpartum women and their newborn who completed at least 2 postnatal check-ups

Denominator: Total number of deliveries

Care does not stop after childbirth. Most maternal and infant deaths occur in the first six weeks after delivery, and these could be prevented majority of the time. Therefore, utmost care and monitoring shall be provided during the postpartum phase to prevent and manage complications, to ensure health of the mother and the child, as well as to promote birth planning.

1.B Maternal Health Indicators

This report monitors the percentage of postpartum women who have completed at least two (2) postnatal check-ups. The first check-up shall be within 24 hours after delivery, while the second check-up is conducted within seven (7) days after delivery. Women who had stillbirths shall still have at least 2 check-ups after delivery.

The proportion of women with at least two postnatal check-ups has decreased since 2018. Although it increased in 2021, a 2% decrease was observed in 2022.

In 2022, 88.69% or 1,258,619 postpartum women had at least two (2) postnatal checkups *(Figure 1.B.3.1).*

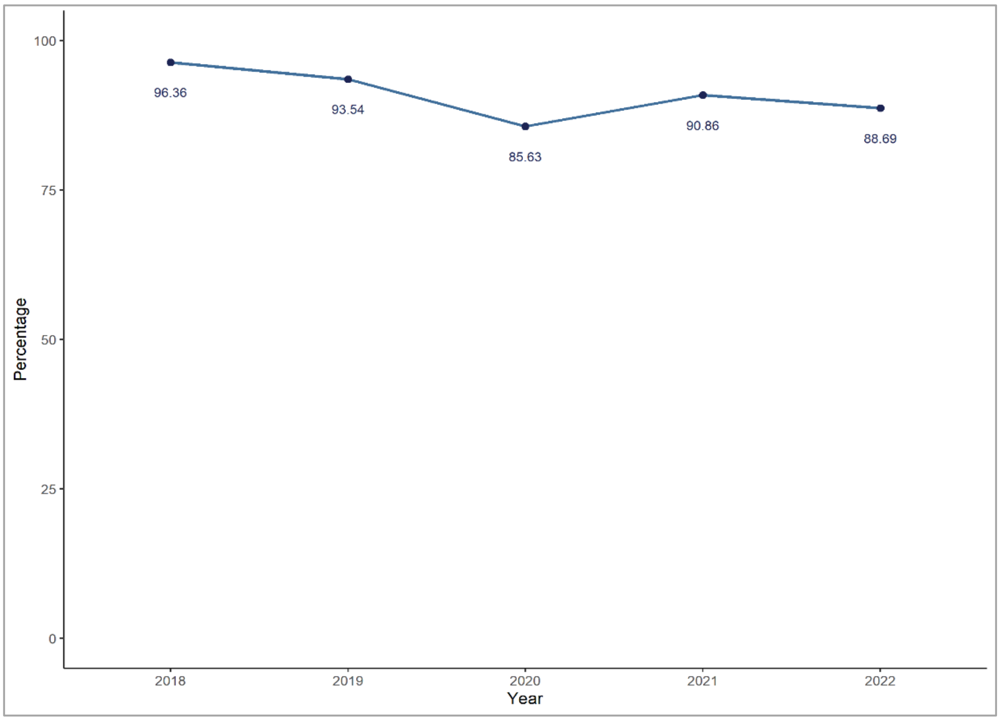

Figure 1.B.3.1: *Women with at least 2 Postnatal Check-ups, Philippines, 2018-2022*

Looking at the regional disaggregation, Regions 1, 2, 3, and 12 have reached more than 95% of postpartum women completing at least two (2) postnatal check-ups *(Figure 1.B.3.2).* Notably, Regions 1, 2, and 3 also had the highest percentage of women with at least 4 ANC check-ups, and percentage of deliveries occurring in health facilities and assisted by skilled health professionals.

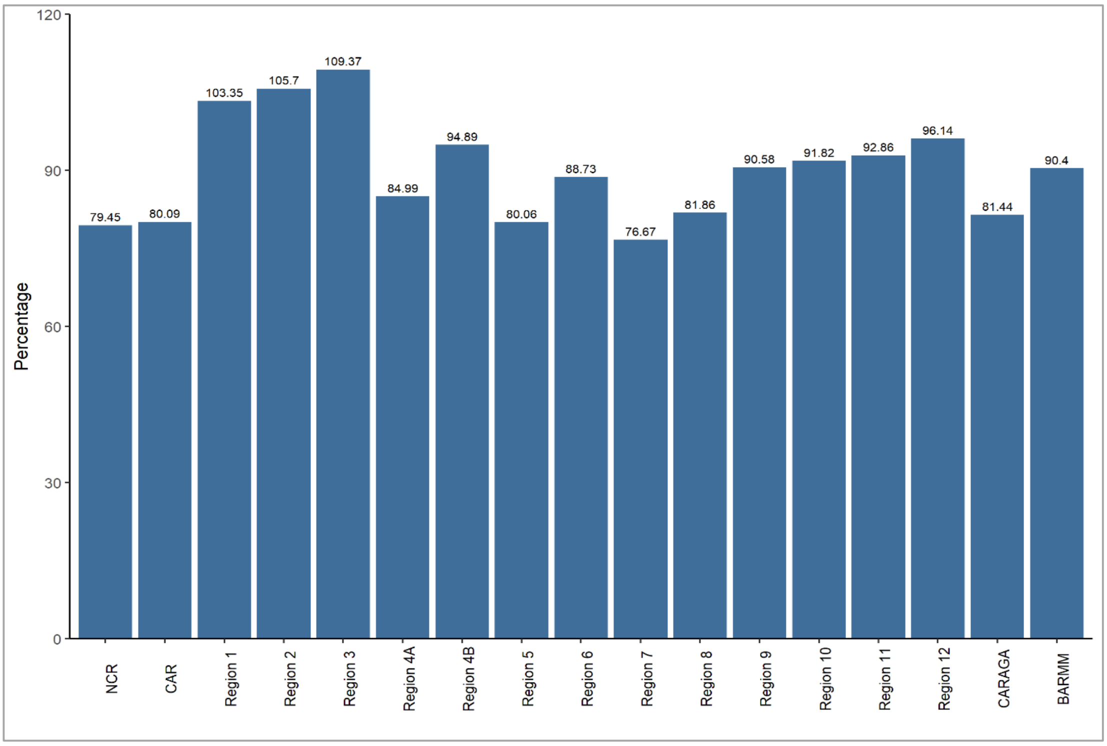

Figure 1.B.3.2: *Women with at least 2 Postnatal Check-ups, by Region, 2022*

1.B Maternal Health Indicators

1.B.4 Maternal Mortality

Formula:
Maternal Mortality Ratio per 100,000 live births
Numerator: Number of maternal deaths
Denominator: Total number of live births

Maternal health services during pregnancy, and during and after childbirth ultimately aim to prevent and/or manage complications and eliminate maternal and child mortality. The maternal mortality ratio (MMR) is a widely used indicator to measure how well maternal health programs are performing. Maternal death is defined as the death of woman while pregnant or within 42 days of termination of pregnancy, irrespective of the duration and the site of the pregnancy, from any cause related to or aggravated by the pregnancy or its management, but not from accidental or incidental causes. Under the Sustainable Development Goal 3 (SDG 3) is to reduce the global MMR to less than 70 per 100,000 live births.

In 2022, there were 914 maternal deaths recorded in the FHSIS, which was a 13.7% decrease from the maternal deaths reported in 2021. As a result, the maternal mortality ratio per 100,000 live births in 2022 stood at 64.68, the lowest since 2020 *(Figure 1.B.4.1).* Note, however, that live births that occurred in private hospitals are not included in this report.

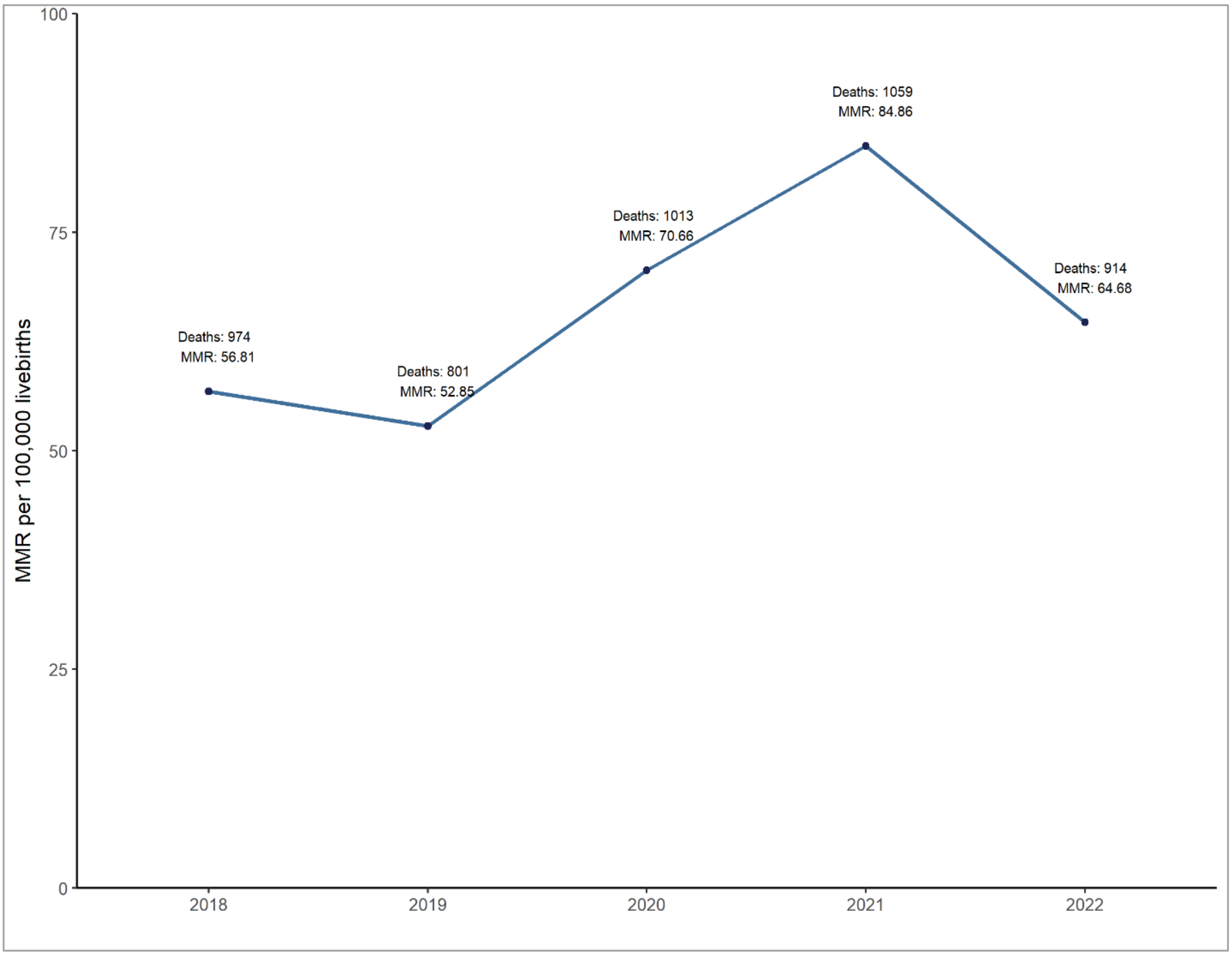

Figure 1.B.4.1: *Maternal Mortality, Philippines, 2018-2022*

1.B Maternal Health Indicators

In terms of absolute mortality counts, around 19% of the maternal deaths in 2022 were reported in NCR. However, accounting for total live births, Region 5 posed the highest MMR with 100.07 deaths per 100,000 live births, followed by Region 11 with 96.49, and NCR with 95.21. On the other hand, Region 9 had the lowest MMR at 16.28 per 100,000 live births, reporting only eight (8) maternal deaths *(Figure 1.B.4.2).*

While this report does not include data on the causes of death and where in the pregnancy, delivery, or postpartum phase these occurred, further strengthening of maternal programs is essential in preventing deaths, especially since the impact of these programs is heavily translated to child health.

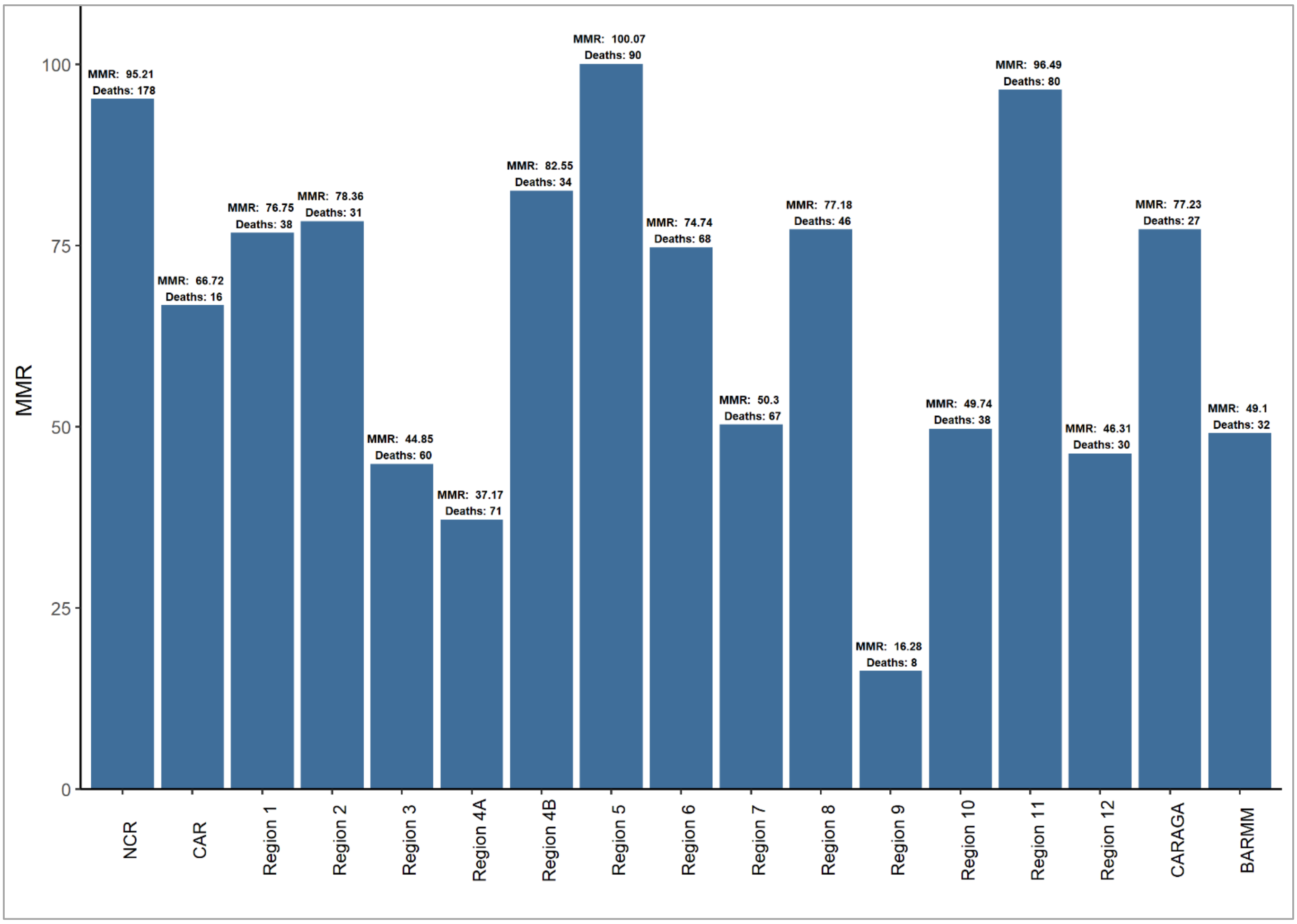

Figure 1.B.4.2: *Maternal Mortality, by Region, 2022*

1.C Child Health Indicators

2022 KEY FINDINGS

59.92%
Fully-Immunized Children (FIC)

12.84%
Completely Immunized Children (CIC)

81.98%
Initiated on breastfeeding immediately after birth

51.93%
Exclusively breastfed until 5th month and 29 days

63.16%
Children aged 0-59 months old with diarrhea were given Oral Rehydration Solution (ORS)

51.06%
Sick infants aged 6-11 months given Vitamin A 100,000 IU

43.94%
Sick infants aged 12-59 months were given Vitamin A 200,000 IU

1,413,107
Total number of Live Births

8,451
Total number of Fetal Deaths

Under Five Mortality Rate (UFMR): **14.00** per 1,000 live births

Infant Mortality Rate (IMR): **10.36** per 1,000 live births

Neonatal Mortality Rate (NMR): **6.64** per 1,000 live births

Perinatal Mortality Rate (PMR): **10.44** per 1,000 live births plus stillbirths

1.C Child Health Indicators

Health is a critical and fundamental element in children's welfare. Information on children's health plays an important role in assessing their immunity and overall health status. Consequently, these data serve as a basis for assessing programs as well as for decision-making and resource allocation.

This chapter presents data on childhood immunization, nutrition services, management of sick children, and mortality.

1.C.1 Immunization Services for Infants and Children

Formula:

Bacillus Calmette–Guérin (BCG)

Numerator: Number of newborn / infants vaccinated with BCG antigen

Denominator: Eligible population 0-11 months

Children Protected at Birth (CPAB)

Numerator: Number of newborns whose mothers pregnant for the first time who have received at least 2 doses of TT/Td vaccination at least a month prior to delivery PLUS number of newborns whose mothers pregnant for the 2nd or more times have received at least 3 doses of TT/Td

Denominator: Eligible population 0-11 months

Oral Polio Vaccine (OPV)

Numerator: Number of infants vaccinated with OPV 1, 2, 3

Denominator: Eligible population 0-11 months

Pneumococcal Conjugate Vaccine (PCV)

Numerator: Number of infants vaccinated with PCV 1, 2, 3

Denominator: Eligible population 0-11 months

Fully-Immunized Children (FIC)

Numerator: Number of FIC

Denominator: Eligible population 0-12 months

Hepatitis B within 24 hours after birth

Numerator: Number of newborn vaccinated with birth dose of HepB antigen within 24 hours

Denominator: Eligible population 0-11 months

DPT-HiB-HepB

Numerator: Number of infants vaccinated with DPT-HiB-HepB 1, 2, 3

Denominator: Eligible population 0-11 months

Inactivated Polio Vaccine (IPV) 1

Numerator: Number of infants vaccinated with IPV1

Denominator: Eligible population 0-11 months

Measles Containing Vaccine (MCV) 2

Numerator: Number of children vaccinated with MCV2

Denominator: Eligible Population 0-12 months

Completely Immunized Children (CIC)

Numerator: Number of CIC

Denominator: Eligible population 13-23 months

Immunization protects us, including children, from life-threatening diseases. Routine vaccines given to children in the Philippines include: BCG for Tuberculosis, Pentavalent for Diphtheria, Pertussis (whooping cough), Tetanus, Haemophilus Influenzae type B and Hepatitis B (DPT-HiB-HepB), Oral Polio and Inactivated Polio, PCV, and MCV. Moreover, doses against neonatal tetanus are also given to pregnant mothers to provide additional protection to children.

1.C Child Health Indicators

For vaccines to be effective, timely immunization is of importance. A child is considered fully immunized if they received 1 dose of BCG, 3 doses of OPV, 3 doses of DPT-HiB-HepB vaccines and 2 doses of MCV by 12 months (MCV1 at 9 months and MCV2 at 12 months). Those who missed any of the routine vaccines could still complete the prescribed doses before they reach two years old and are tagged as CIC.

Nationally, the proportion of FIC has been decreasing over the years. In 2022, 1,384,472 or 59.92% of children aged 0-12 months were considered as FIC, which was a 5% decrease from the previous year. Meanwhile, an additional 252,699 or 12.84% of children aged 13 to 23 months were CIC *(Figure 1.C.1.1)*.

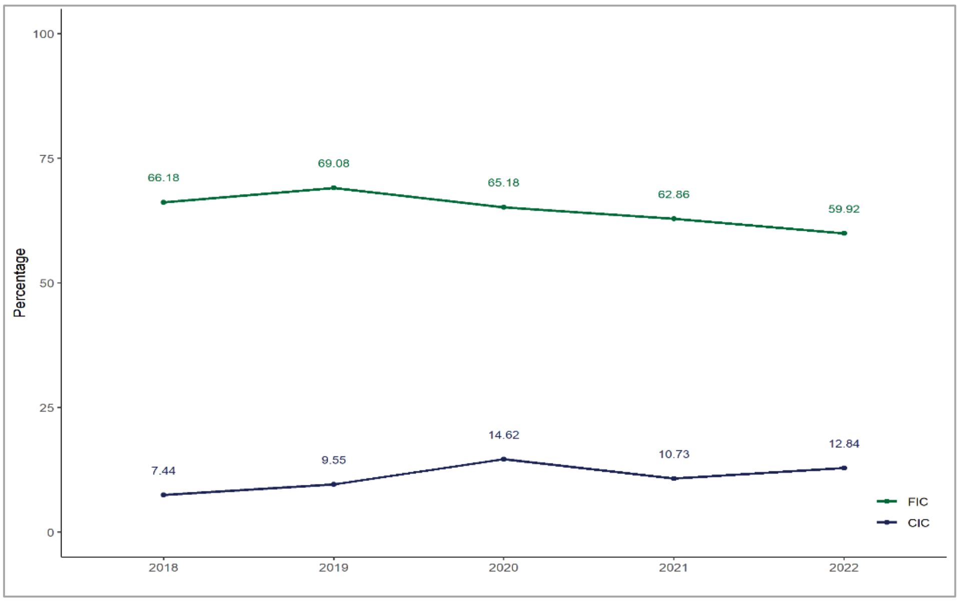

Figure 1.C.1.1: *FIC and CIC, Philippines, 2018-2022*

The latest data reveals some important information about immunization coverage in different regions of the country. In NCR, about 72.14% of children have received all the necessary vaccines, making it the region with the highest proportion of FIC. This means that a large number of children in NCR are protected against vaccine-preventable diseases. On the other hand, Region 8 has the lowest percentage of FIC at only 46.20%. This indicates that a lower proportion of children in this region have completed their immunization schedule, leaving them more vulnerable to diseases *(Figure 1.C.1.2)*.

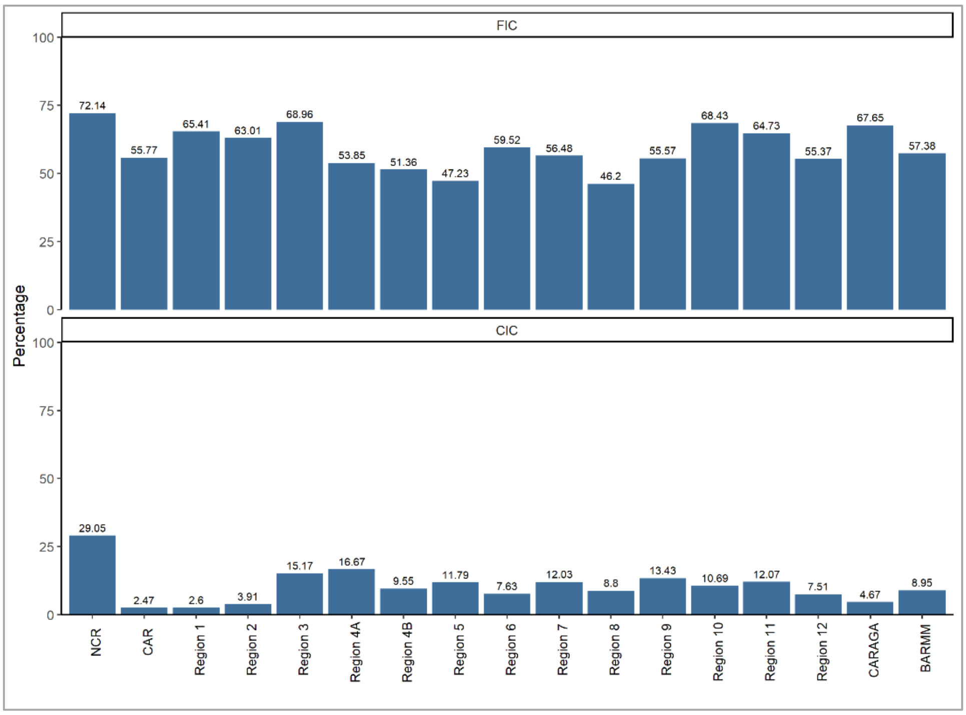

Figure 1.C.1.2: *FIC and CIC, by Region, 2022*

Looking at the percentage of CIC, NCR still has the highest coverage at 29.05%. This means that nearly one-third of children in NCR have received the full set of recommended vaccines before reaching two years of age, in addition to the FIC. Unfortunately, CAR, with only 55.77% FIC, has the lowest CIC rate of 2.47% *(Figure 1.C.1.2)*.

1.C Child Health Indicators

The analysis of the FHSIS five-year trend on immunization on children reveals varying coverage rates for different vaccines. The BCG vaccine had fluctuating coverage, with the highest in 2019 (70.48%) and the lowest in 2021 (58.91%). The percentage of CPAB against neonatal tetanus was high in 2018 (85.98%) but declined thereafter. Hepatitis B vaccine coverage remained stagnant, ranging from 49% to 59%. In 2022, the proportion of children with complete doses of DPT-HiB-HepB, OPV, and IPV in the Philippines are relatively similar, with coverages of 74.64%, 75.79%, and 74.05%, respectively. The PCV3 coverage showed a generally increasing trend, reaching 74.32% in 2022. Lastly, the MCV2 coverage in 2022 was 65.42%, a 4.54% decrease compared to 2021 *(Figure 1.C.1.3)*. These data indicate that while a significant proportion of children in the country have received these vaccines, these still fall short for the target of 95% coverage.

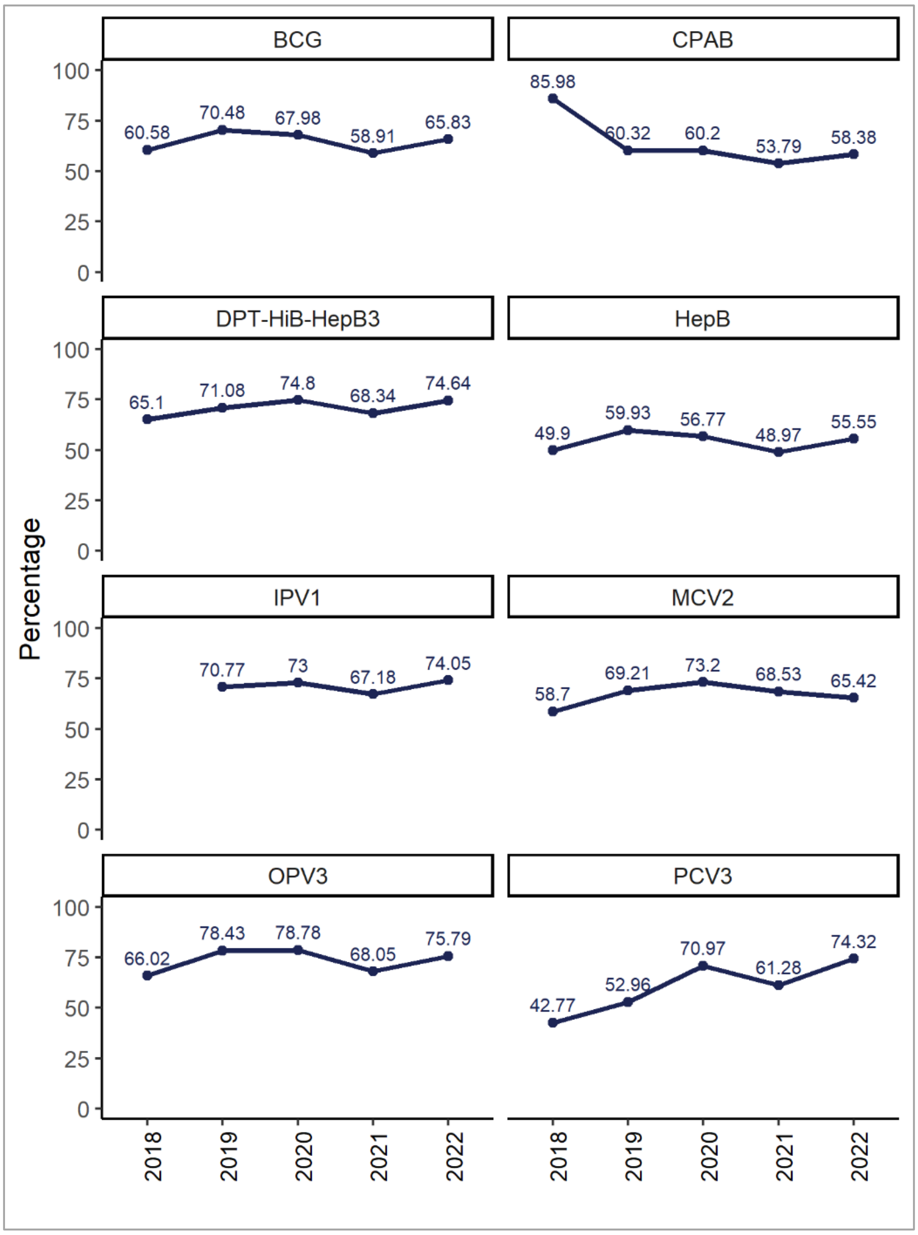

Figure 1.C.1.3: *Immunization in Children, Philippines, 2018-2022*

1.C Child Health Indicators

As shown in Figure 1.C.1.4, Region 3 has highest coverage on IPV1 with 87.80% and on the complete doses of DPT-HiB-HepB (85.76%), OPV (86.75%) and PCV (87.66%). On the other hand, Region 11 leads the highest coverage on BCG (79.91%) and CPAB (73.02%). Regions 4B and Region 5 were consistently among the five regions with lowest coverages across all vaccines, while NCR is among the top three regions with highest coverages across all vaccines.

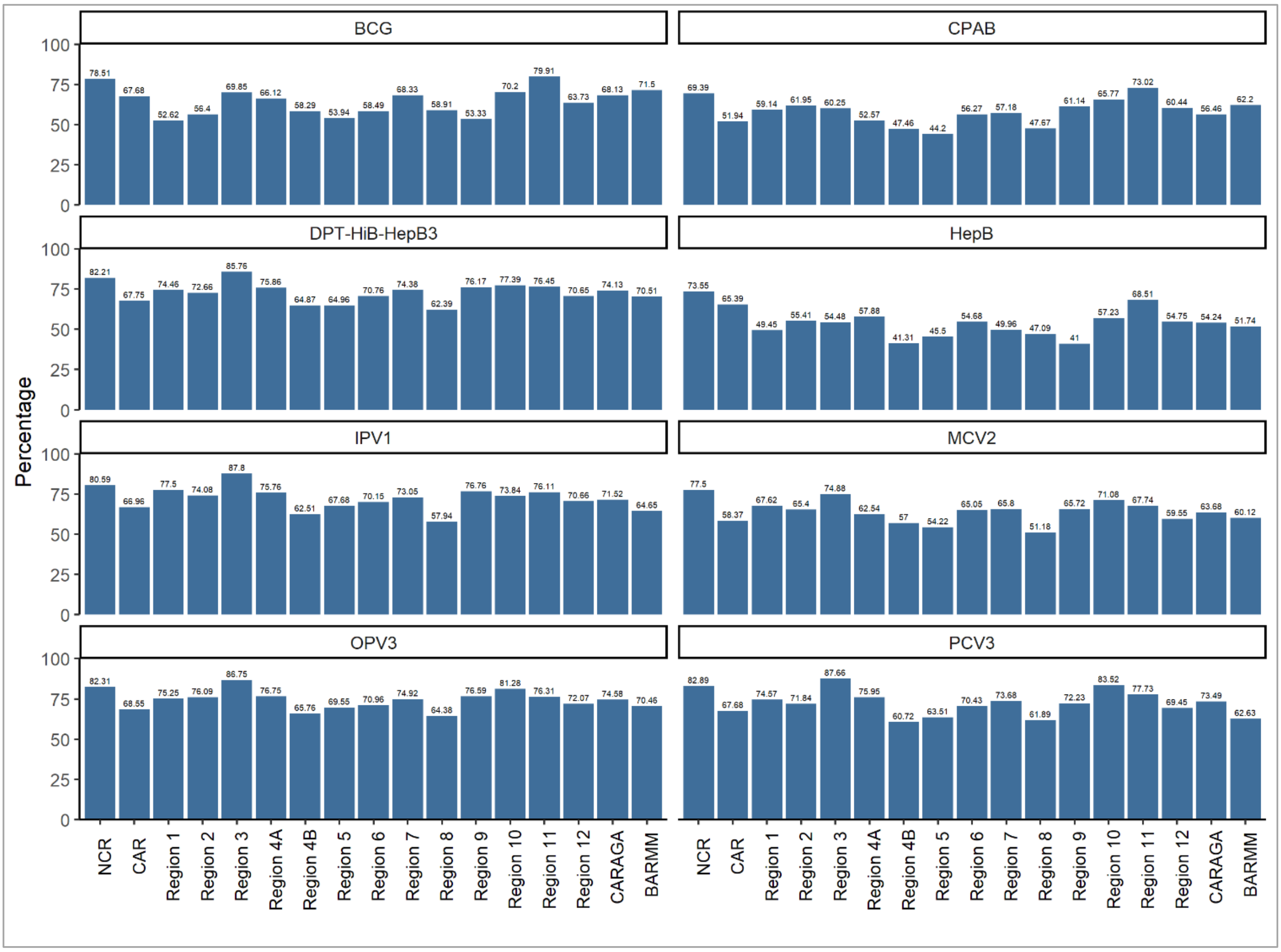

Figure 1.C.1.4: *Immunization in Children, by Region, 2022*

Overall, the data underscores the importance of addressing regional variations in National Immunization Program (NIP) coverage to ensure equitable access to vaccines for all children across the Philippines. Efforts should be made to strengthen immunization programs, raise awareness, and improve healthcare strategies in regions with lower coverage rates.

1.C.2 Nutrition Services for Infants and Children

Formula:

Initiated Breastfeeding

Numerator: Number of newborns who were initiated on breastfeeding immediately after birth lasting for at least 90 minutes

Denominator: Total number of live births

Exclusively Breastfed

Numerator: Total number of infants age 5 months and 29 days old who have been exclusively breastfed from birth until 5th month and 1 day before the child turns 6 months

Denominator: Eligible population under 1

1.C Child Health Indicators

Formula:

Vitamin A Supplementation 6-11 months

Numerator: Number of Infant 6-11 months old who completed 1 dose of Vitamin A
Denominator: Eligible population 6-11 months

Vitamin A Supplementation 12-59 months

Numerator: Number of children with ages 12-59 months old who completed 2 doses Vitamin A
Denominator: Eligible population 12-59 months

Iron Supplementation

Numerator: Number of preterm infants and/or infants with low birth weight (less than 2500 grams) given iron supplement starting 1 month until 3 months
Denominator: Total number of live births with low birth weight

Breastfeeding is one key and impactful practice to ensure child health and survival. Breast milk contains all nutrients that infants need in their first months of life, promoting optimal child growth and development. Moreover, breast milk contains antibodies that can help protect infants from childhood diseases. The World Health Organization (WHO) and the United Nations International Children's Emergency Fund (UNICEF) recommend that children initiate breastfeeding within the first hour of birth and be exclusively breastfed for the first 6 months of life.

The percentage of newborns initiated on breastfeeding immediately after birth varies across regions. The highest percentage is observed in Region 1 with 98.25%, followed by CAR with 96.71%, and CARAGA with 94.93%. The lowest percentages are seen in NCR with 70.88%, Region 7 with 72.50%, and Region 8 with 76.97% *(Figure 1.C.2.1)*.

For children who were exclusively breastfed up to 5th month and 29 days, Figure 1.C.2.1 shows that Region 10 has the highest percentage at 67.83% coverage, followed by Region 1 with 65.45% and CARAGA with 62.83%. On the other hand, Region 4A has the lowest percentage with 38.96%, followed by Region 6 with 40.91% and Region 8 with 41.59%.

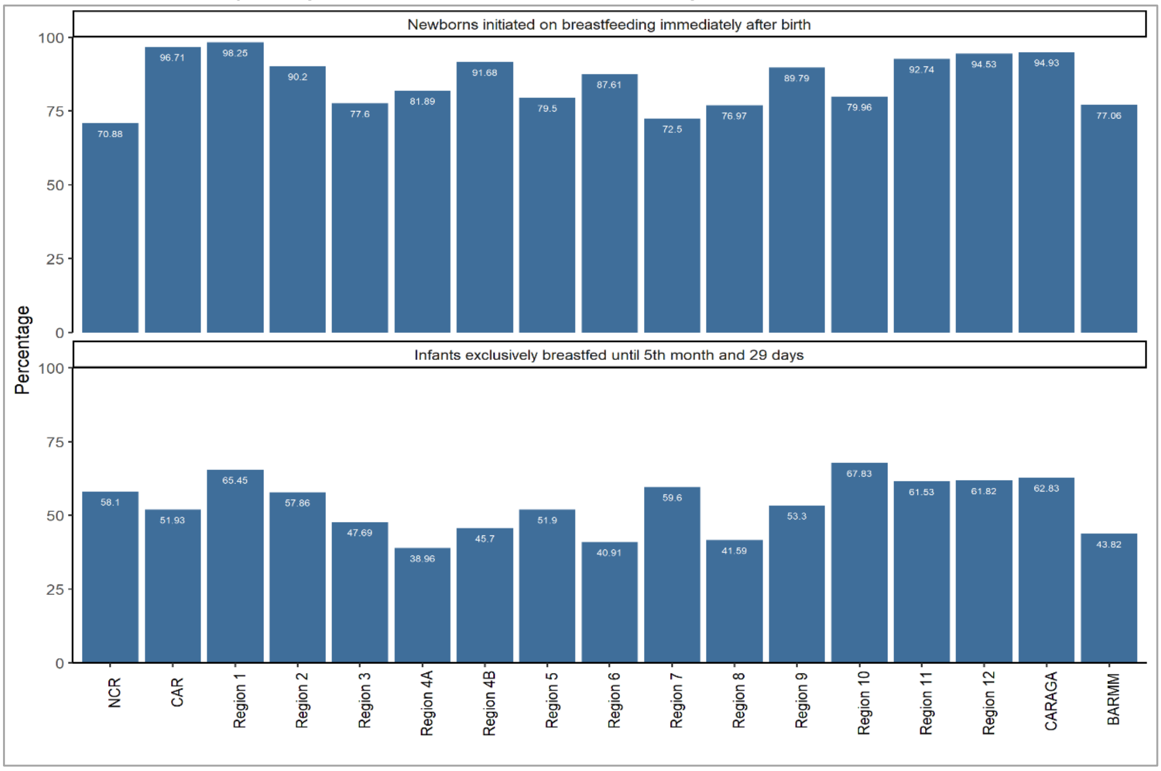

Figure 1.C.2.1: *Immediate Breastfeeding and Exclusive Breastfeeding, by Region, 2022*

1.C Child Health Indicators

In conclusion, the analysis reveals regional variations in both the initiation of breastfeeding and exclusive breastfeeding practices. Barriers to the implementation of both practices shall be identified to properly come up with targeted interventions to improve breastfeeding rates, provide appropriate support, and raise awareness about the benefits of breastfeeding for newborns and infants.

The Vitamin A deficiency is a major public health problem affecting an estimated 190 million preschool-age children, mostly from the WHO regions of Africa and South-East Asia. Infants and children have increased Vitamin A requirements to promote rapid growth and to help combat infections. Inadequate intakes of Vitamin A at this age could lead to Vitamin A deficiency, which, when severe, may cause visual impairment (night blindness) or increase the risk of illness and mortality from childhood infections such as measles and those causing diarrhea[1]. Moreover, iron, a mineral necessary to carry oxygen in hemoglobin, is an essential nutrient for development and cell growth in the immune and neural systems, and in muscle metabolism. Iron deficiency in children under-two years of age can pose significant and irreversible effects on brain development, which consequently leads to negative consequences on learning and school performance later in life.

Figure 1.C.2.2 indicates that iron supplementation on infants who were preterm or with low birth weight (LBW) has national coverage of 21.34%. Region 4B stands out with the highest coverage of 77.65%, while the Region 7 has the lowest coverage at 11.89%. The data shows that Vitamin A supplementation for infants aged 6 to 11 months has a coverage of over 100% in most regions, except for Regions 1, 2, 7, and 8. It is important to note that the denominator used to calculate coverage is an estimate and may not precisely reflect the actual number of eligible children. Additionally, since the report only includes services provided by public health facilities, a child who received Vitamin A supplementation multiple times may be reported more than once in the data.

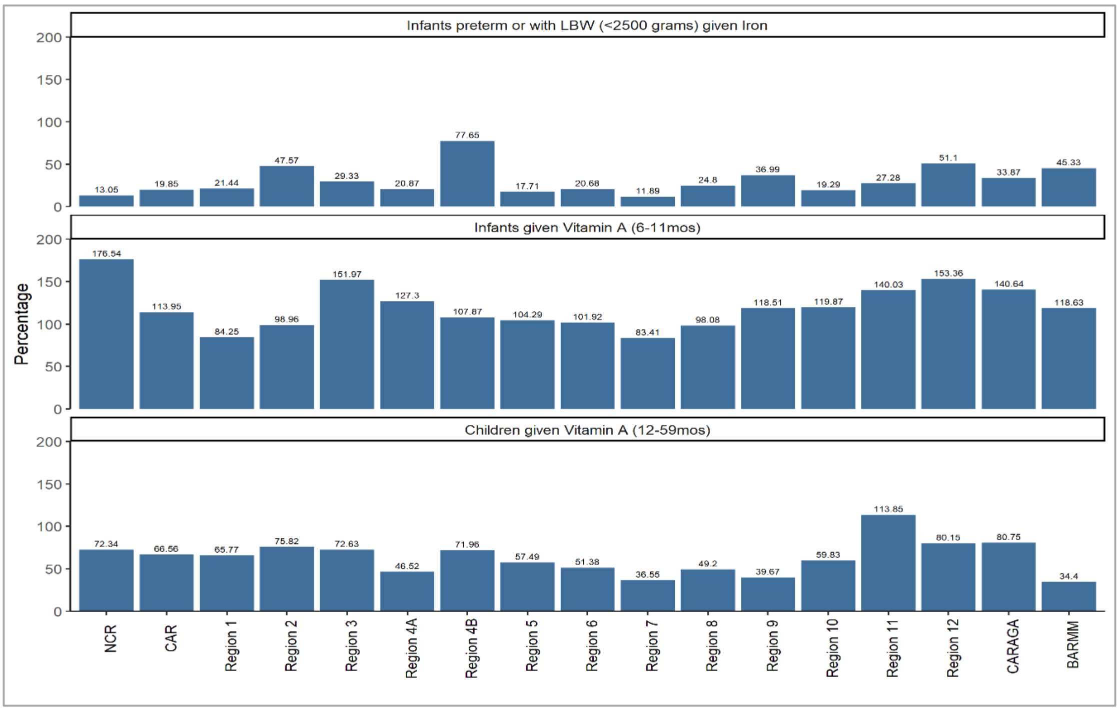

Figure 1.C.2.2: *Supplementation in Children, by Region, 2022*

[1] *WHO Guidelines: Vitamin A supplementation in infants and children 6–59 months of age Geneva, World Health Organization, 2011*

1.C Child Health Indicators

1.C.3 Management of Sick Infants and Children

Formula:

Diarrhea Cases who received Oral Rehydration Salt Solution (ORS) and ORS with Zinc

Numerator: Number of diarrhea cases 0-59 months old who received ORS or ORS with Zinc

Denominator: Number of acute diarrhea cases 0-59 months old seen

Pneumonia cases and received treatment

Numerator: Number of pneumonia cases 0-59 months old who received treatment

Denominator: Number of pneumonia cases 0-59 months old seen

Children with Measles and/or Persistent Diarrhea who received Vitamin A capsule

Numerator: Number of sick infants who received Vitamin A capsule aside from routine supplementation (6-11 and 12-59 months)

Denominator: Total number of sick infants seen (6-11 and 12-59 months)

Globally, over 80% of the under five deaths are due to neonatal conditions and infectious diseases like pneumonia, diarrhea, malaria, measles and meningitis, often compounded by malnutrition. Most childhood deaths can be prevented with effective interventions that are feasible for implementation, even in resource constrained settings[2]. Management of sick children is a cost-effective health intervention. The WHO and UNICEF designed the Integrated Management of Childhood Illness (IMCI) which aims to strengthen prevention and management of common childhood illnesses and support children's healthy growth and development. The DOH Administrative Order No. 119 s. 2003 dated 2 December 2003, "Updated Guidelines on Micronutrient Supplementation (Vitamin A, Iron, and Iodine)", provides for the supplementation of Vitamin A to high risk children to help reestablish body reserves drained by chronic or repeated infectious diseases and protects the children against severity of subsequent infection.

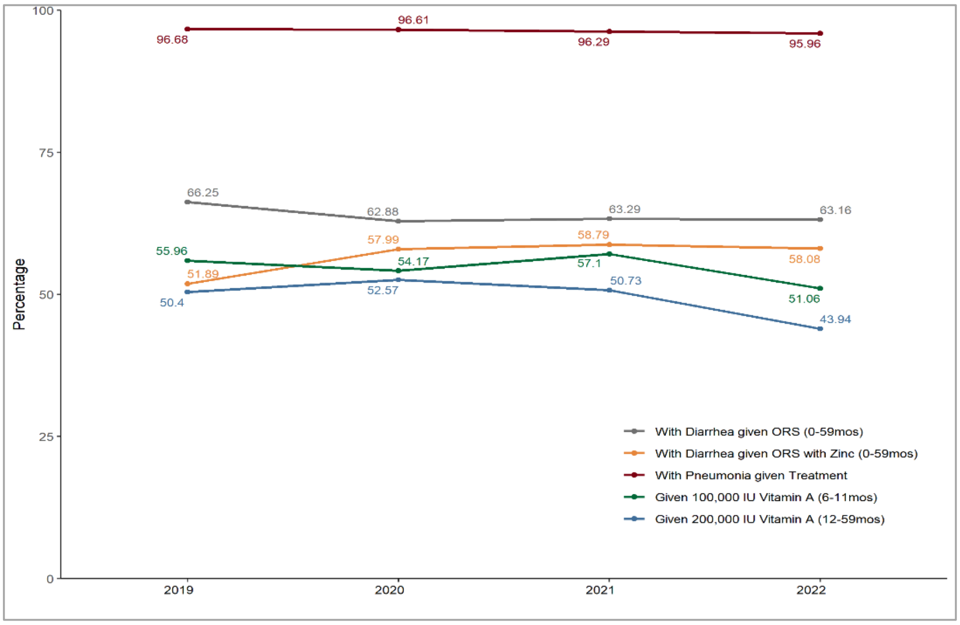

Figure 1.C.3.1: *Management of Sick Children, Philippines, 2019-2022*

The percentage of sick infants aged 6 to 11 months given 100,000 IU of Vitamin A fluctuated from 2019 to 2022, with a peak of 57.10% in 2021 which then declined to 51.06% in 2022. Similarly, the proportion of sick infants aged 12 to 59 months given 200,000 IU of Vitamin A has further decreased from 50.73% in 2021 to only 43.94% in 2022. On the other hand, the percentage of children with diarrhea aged 0-59 months old that were given ORS or ORS with Zinc remained relatively stagnant since 2020, with a coverage of 63.16% and 58.08%, respectively, in 2022. Notably, the highest coverage was the provision of pneumonia treatment among children aged 0-59 months old, which has stayed at roughly 96% in recent years *(Figure 1.C.3.1)*.

---

[2] *https://www.who.int/teams/maternal-newborn-child-adolescent-health-and-ageing/child-health/integrated-management-of-childhood-illness*

1.C Child Health Indicators

These findings highlight the need for sustained efforts to improve provision of Vitamin A and ORS to sick infants and to continue the successful service provision of treatment to children with pneumonia.

There are noticeable differences in how essential healthcare services for children are provided in different regions. For example, Region 4B has the highest rate of service delivery of ORS to children with diarrhea at 89.22%, while Region 9 has the lowest rate at 19.47%. Similarly, in terms of administering ORS with Zinc to children being treated for diarrhea, Region 2 has the highest rate at 80.49%, while Region 12 has the lowest rate at 30.86% *(Figure 1.C.3.2)*. These variations implicate the presence of gaps in access to healthcare, available resources, and the quality of infrastructure across regions.

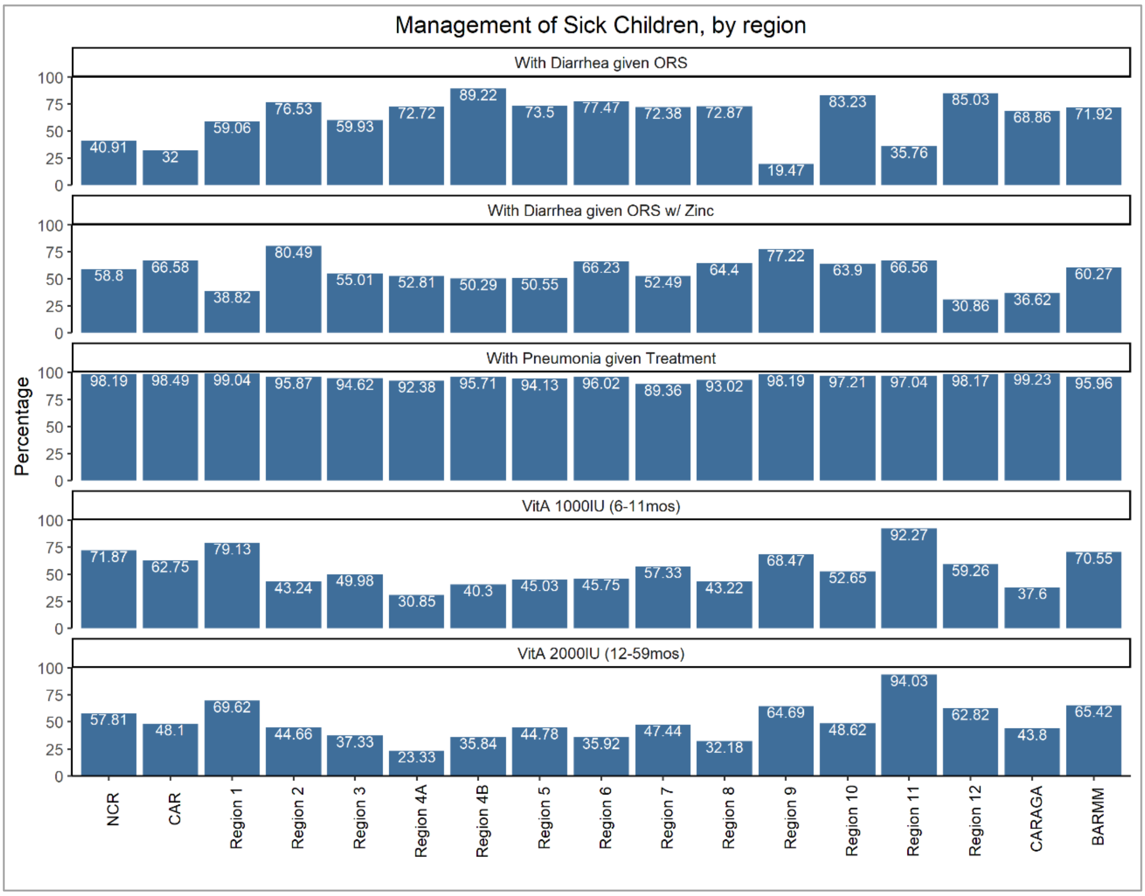

Figure 1.C.3.2: *Management of Sick Children, by Region, 2022*

Furthermore, provision of Vitamin A to 6-59 months old children with measles and/or persistent diarrhea in Region 11 consistently shows higher service delivery rates compared to the rest of the regions, while Region 4A consistently exhibits lower service provision rates. Lastly, treatment of pneumonia is generally high across regions with CARAGA having the highest treatment provision rate of 99.23%, while Region 7 has the lowest rate of 89.36% *(Figure 1.C.3.2)*.

These findings indicate the importance of understanding the reasons behind these regional variations in service delivery. Factors like availability and access to healthcare resources, limitations in health infrastructure, and variations in regional healthcare strategies could all be contributing factors. It is crucial to conduct further assessments to identify the specific reasons behind these variations and to implement the best practices from well-performing regions.

1.C Child Health Indicators

1.C.4 Child Mortality

Article 24 of the United Nations Convention on the Rights of the Child specifically obliges all States to take appropriate measures to diminish infant and child mortality. Consequently, the Sustainable Development Goals aim to end preventable deaths in children under-five years of age globally by 2030. Specifically, the under-five mortality rate is targeted to be lowered to 25 deaths per 1,000 live births, while neonatal mortality is aimed to be as low as 12 deaths per 1,000 live births. In most economies, a child has a very good chance of reaching the fifth birthday if he or she survives the first year of life[3].

Monitoring mortality in children is essential in assessing the effectiveness of the existing child care programs. As the health of the child is heavily dependent on the mother's health, indicators of child mortality may also be linked to maternal health care programs. This chapter presents data on under-five mortality, as well as infant (under 1 year old), neonatal (0 to 28 days), and perinatal mortality.

Nationally, the under-five mortality rate computed from deaths and live births reported in the FHSIS has been increasing since 2021. The same pattern is observed for infant mortality ratio and neonatal mortality. On the other hand, the perinatal mortality, which includes stillbirths and early neonatal deaths, has been consistently increasing since 2019.

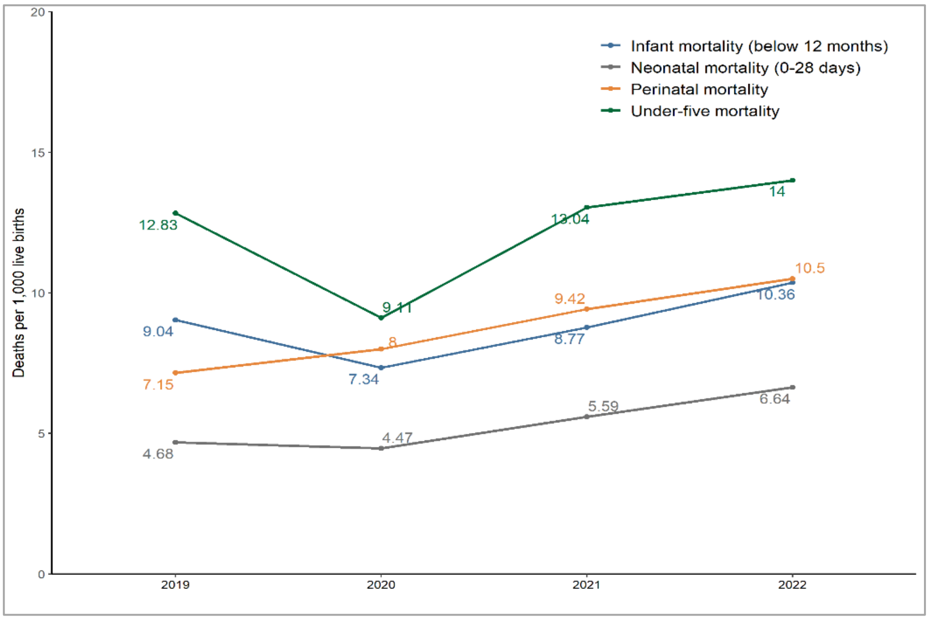

Figure 1.C.4.1: *Child Mortality, by Region, 2019-2022*

In 2022, there were 19,784 deaths among the under-five population. This translates to an under-five mortality rate of 14.00 deaths per 1,000 live births, which is a 7.36% increase compared to the previous year *(Figure 1.C.4.1)*. While this rate is already below the Sustainable Development Goals (SDG) target, it is important to note that this is based on mortality data collected by the municipal and city health offices. Thus, this information may be different from the reports of the Philippine Statistics Authority (PSA) which is the government agency mandated to release official data on vital statistics. Nonetheless, monitoring this in the FHSIS is beneficial in the assessment of the programs being implemented by the government, as well as for planning and resource allocation.

Furthermore, 14,645 deaths of the total under-five deaths were among infants aged below 1 year old, which corresponds to an infant mortality ratio of 10.36 deaths per 1,000 live births *(Figure 1.C.4.1)*. Further disaggregation shows that 9,381 of these deaths occurred in the neonatal period or the first 28 days of life. In summary, the majority or approximately 7 out of 10

---

[3] *https://www.adb.org/sites/default/files/publication/27727/goal-04.pdf*

under-five deaths happen before the child reaches 1 year old, while roughly 4 out of 10 deaths occur in the neonatal period *(Table 1.C.4.1)*.

These trends highlight the need for continued attention and efforts to address child mortality in the Philippines. It is crucial to understand the underlying factors contributing to these deaths and implement targeted interventions to reduce child mortality and improve the well-being of children in the country. Further analysis and consideration of other factors, such as causes of deaths, access to health care, and socio-economic conditions, would provide a more comprehensive understanding of the situation and guide effective strategies to end preventable deaths in children.

Regional data show that under-five mortality ratios range from as low as 1.57 deaths to 30.67 deaths for every 1,000 live births. Region 5 with 2,758 under-five deaths recorded the highest mortality rate. Meanwhile, Region 9 had the lowest number of under-five deaths (77 deaths) and under-five mortality rate (1.57 deaths per 1,000 live births) *(Figure 1.C.4.2)*. Further investigation of the recorded deaths, especially in Region 5, Region 1, and NCR, may be conducted to gain more insights on why these deaths occurred to inform response and policy-making.

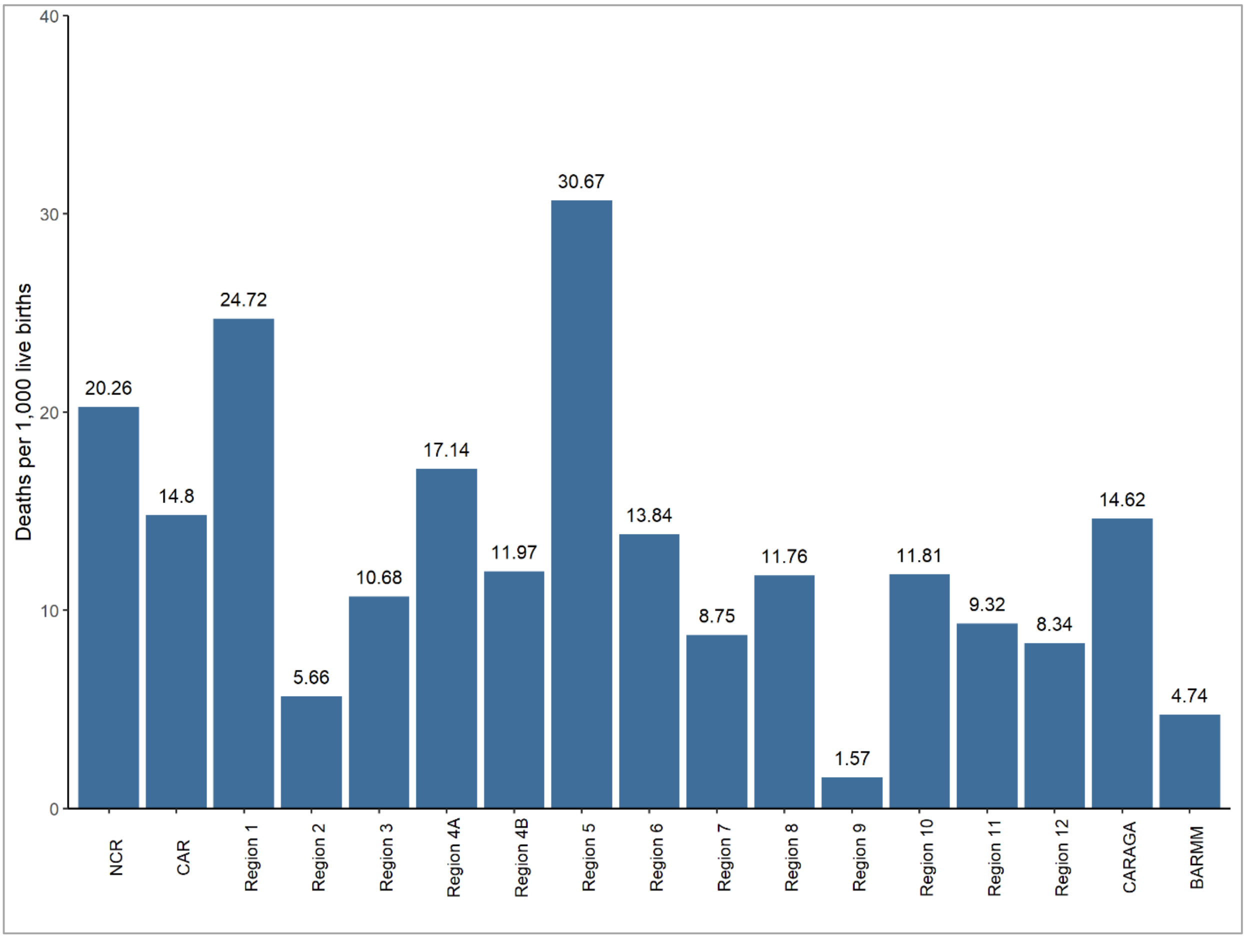

Figure 1.C.4.2: *Under-five Mortality Rate, by Region, 2022*

1.C Child Health Indicators

Evidently, more than half of the under-five deaths in all regions were infant deaths or deaths of children less than 1 year old. In NCR, CAR, and BARMM, approximately 8 out of 10 under-five deaths were among the under 1 age group. Furthermore, in CAR, Region 1, Region 5, and Region 9, more than half of their reported under-five deaths were among neonates or infants aged 0 to 28 days. Although this report does not include data on the causes of deaths, previous data from the PSA suggest that most infant deaths were due to conditions originating in the perinatal period, infectious and parasitic diseases, and diseases of the respiratory system. These conditions are often preventable, thus the need to strengthen relevant maternal and child care services, including nutrition and immunization.

Table 1.C.4.1: *Under-five, Infant, and Neonatal Deaths, by Region, 2022*

Region	Under-five (U5) deaths	Infant deaths		Neonatal deaths	
		Count	% of U5 deaths	Count	% of U5 deaths
PHILIPPINES	19,784	14,645	74.02%	9,381	47.42%
NCR	3,788	3,249	85.77%	1,831	48.34%
CAR	355	304	85.63%	237	66.76%
Region 1	1,224	872	71.24%	630	51.47%
Region 2	224	133	59.38%	105	46.88%
Region 3	1,429	1,066	74.60%	690	48.29%
Region 4A	3,275	2,258	68.95%	1,556	47.51%
Region 4B	493	328	66.53%	191	38.74%
Region 5	2,758	1,988	72.08%	1,595	57.83%
Region 6	1,259	818	64.97%	506	40.19%
Region 7	1,166	937	80.36%	428	36.71%
Region 8	701	548	78.17%	334	47.65%
Region 9	77	66	85.71%	58	75.32%
Region 10	902	459	50.89%	281	31.15%
Region 11	773	623	80.60%	374	48.38%
Region 12	540	383	70.93%	259	47.96%
BARMM	309	258	83.50%	92	29.77%
CARAGA	511	355	69.47%	214	41.88%

1.D Oral Health Care and Services

2022 KEY FINDINGS

Basic Oral Health Care (BOHC) Coverage Among Specified Age

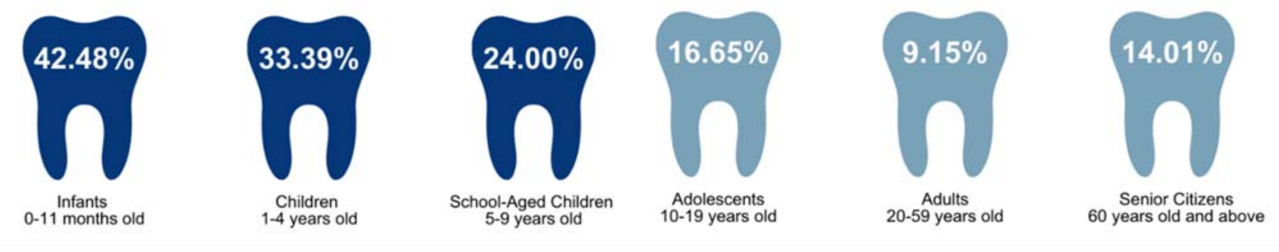

BOHC Coverage Among Pregnant Women in Specified Age Groups

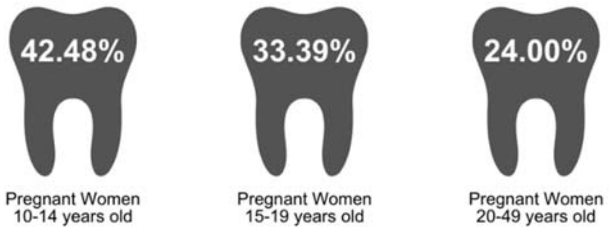

Formula:
BOHC proportion specified age groups
Numerator: Total number of clients per specified age groups who received BOHC
Denominator: Eligible population of the specified age group x 30%

BOHC proportion among pregnant women
Numerator: Total number of pregnant women in specified age groups who received BOHC
Denominator: Eligible population of under 1 year old

The Philippines enacted Republic Act 11223, or the Universal Health Care (UHC) Act, which aims to provide affordable, quality, and comprehensive health services to encourage Filipinos to obtain oral health services. Good oral health is fundamental to overall health and well-being. In 2007, the Department of Health issued Administrative Order 2007-0007 providing for Guidelines in the Implementation of Oral Health Programs for Public Health Services. The program provides preventive, curative, and promotive dental health care to Filipinos through a lifecycle approach. A package of essential BOHC was established to provide a continuum of quality care. FHSIS monitors and tracks the provision of BOHC at the RHU/MHC levels which are targeted at 100% among the target population of the specified age groups and 50% of the target population among pregnant women. Preventive oral health habits such as BOHC developed early in life can lead to better overall oral health throughout a person's life.

This chapter presents data on the proportion of clients provided with BOHC across different lifecycle stages including during pregnancy.

1.D Oral Health Care and Services

Nationally, across different lifecycle stages, the trend of BOHC coverage is generally increasing from 2019-2022. The BOHC coverage among infants in 2022 is at its highest in recent years. Between 2019 and 2022, BOHC coverage among infants increased from 20.86% to 42.48%. However, rates of progress for BOHC coverage would need to accelerate to achieve the targets of 100% among the target population (30% of the estimated age group population) and 50% of the pregnant women target population.

In 2022, the highest BOHC coverage is among 0-11 months old infants (42.48%) and lowest among adults 20-50 years old (9.15%). As most adults are part of the workforce, one strategy to possibly increase the coverage among this age group is to collaborate with labor agencies both in the government and private sectors to advocate for BOHC. The BOHC coverage among older Filipinos (60 years old and above) was only 14.01% of the target elderly population *(Figure 1.D.1)*. There is an increased risk of some oral health problems among the elderly as teeth become weaker with age. Thus, with the low coverage of BOHC among this group, oral health problems among the elderly are imminent.

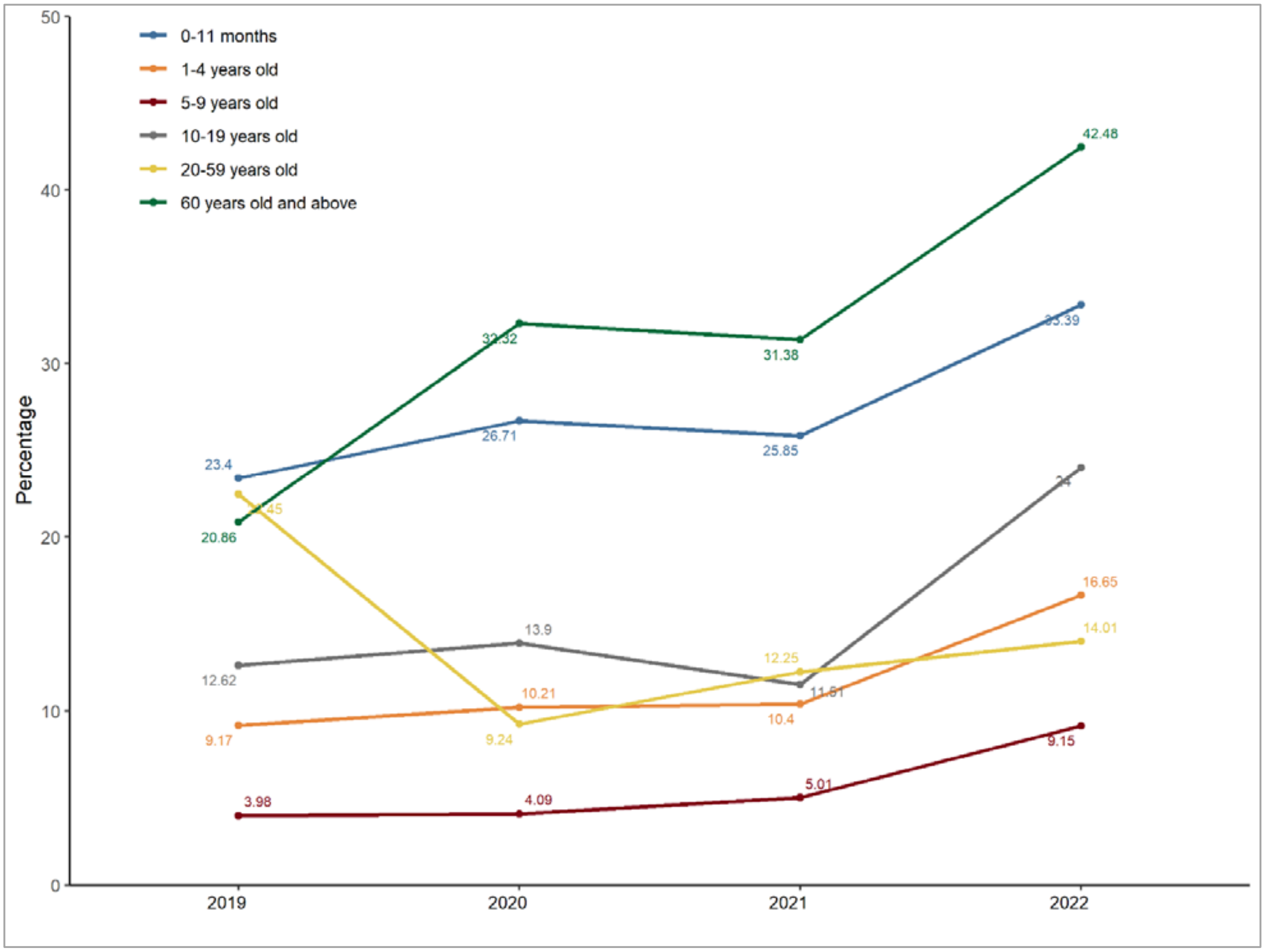

Figure 1.D.1: *BOHC Coverage among different Age Groups, Philippines, 2019-2022*

1.D Oral Health Care and Services

Likewise, the 18.34% BOHC coverage among pregnant women aged 10-49 years old did not reach the target of 50% coverage *(Figure 1.D.2)*. The low coverage may pose a public health concern as poor oral health during pregnancy can lead to poor health outcomes for the mother and the baby as pregnancy may make women more prone to oral diseases. Advocacy campaigns should be strengthened to encourage Filipinos to practice good oral health care and avail of the BOHC services for every lifecycle stage, starting from infancy to old age and during pregnancy.

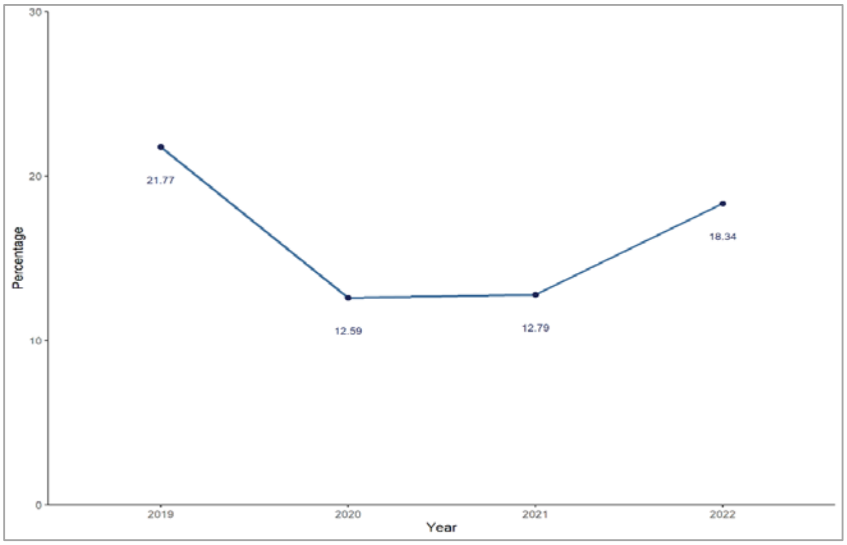

Figure 1.D.2: *BOHC Coverage among Pregnant Women (10-49 years old), Philippines, 2019-2022*

In 2022, regional data showed that BOHC coverage among infants is highest in NCR (89.70%) and lowest in Region 4B (10.76%). Among the 17 regions, seven (7) regions (NCR, 2, 3, 4A, 6, 7, 12) had at least one province/city with 100% BOHC coverage among infants. However, there were also provinces/cities with 0% coverage and these areas have to exert more efforts to advocate for BOHC especially among infants as it would impact the overall oral health throughout their life. For children aged 1-4 years old, NCR had the highest BOHC coverage (70.60% of the target population) and while Region 11 had the lowest (6.41%). Among school-aged children (5-9 years old), BOHC coverage was highest in CAR (48.76%) and lowest again in Region 11 (3.46%). Other regions with less than 10% coverage were Regions 4B (8.21%) and Region 8 with 8.99% *(Figure 1.D.3)*.

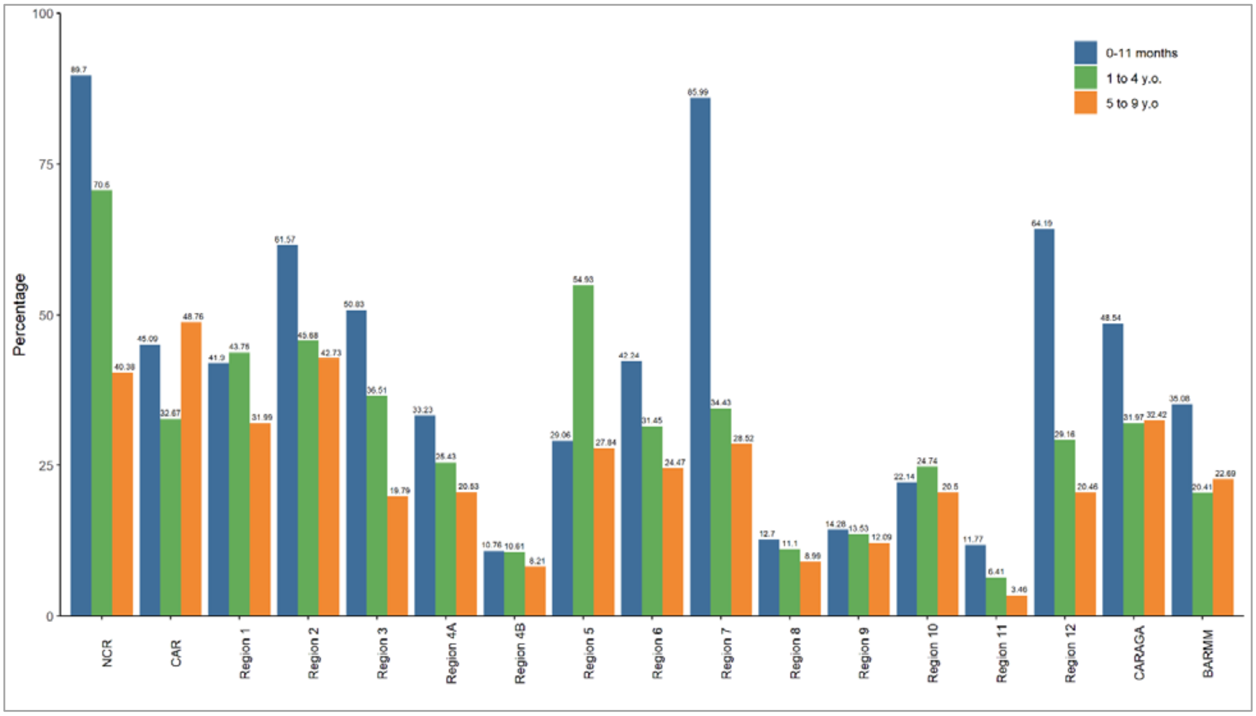

Figure 1.D.3: *BOHC Coverage among Infants and Children, by Region, 2022*

1.D Oral Health Care and Services

Among the adolescents (10-19 years old), BOHC coverage was highest in NCR (30.96%) and lowest in Region 11 (4.02%). Among the adults aged 20-59 years old, BOHC was availed by 16.47% in Region 1 (highest) and only 0.73% in Region 11 (lowest) *(Figure 1.D.4)*. The BOHC coverage among the elderly (60 years old and above) was highest in BARMM (34.37%) and lowest in Region 11 with 1.70% *(Figure 1.D.5)*.

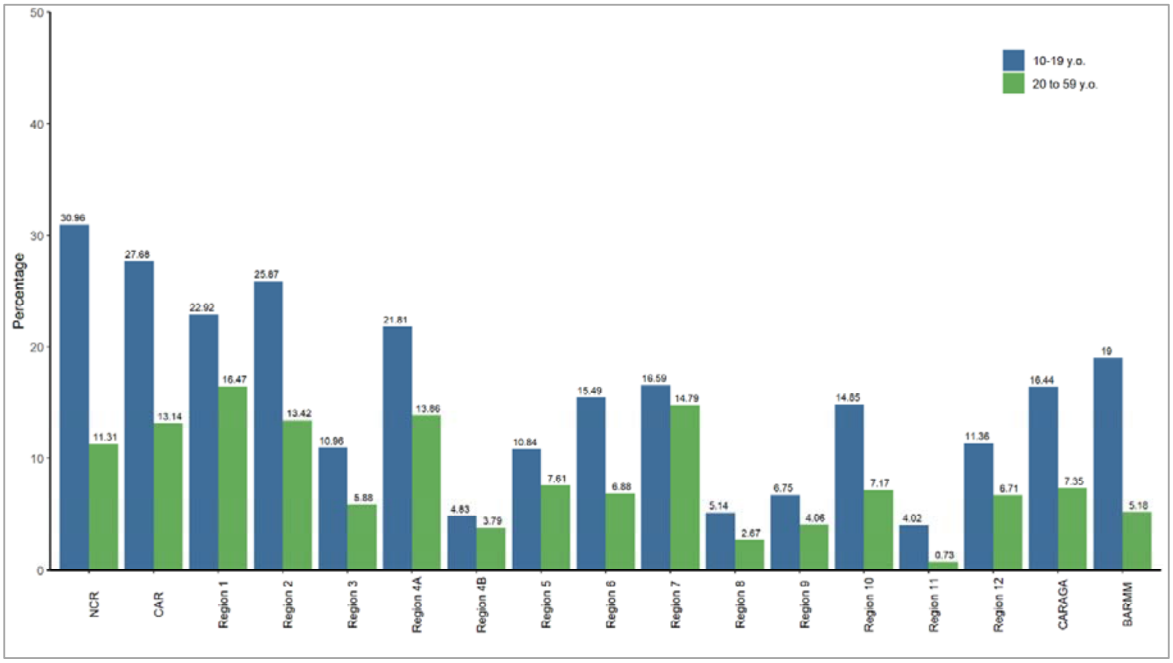

Figure 1.D.4: *BOHC Coverage among Adolescents and Adults, by Region, 2022*

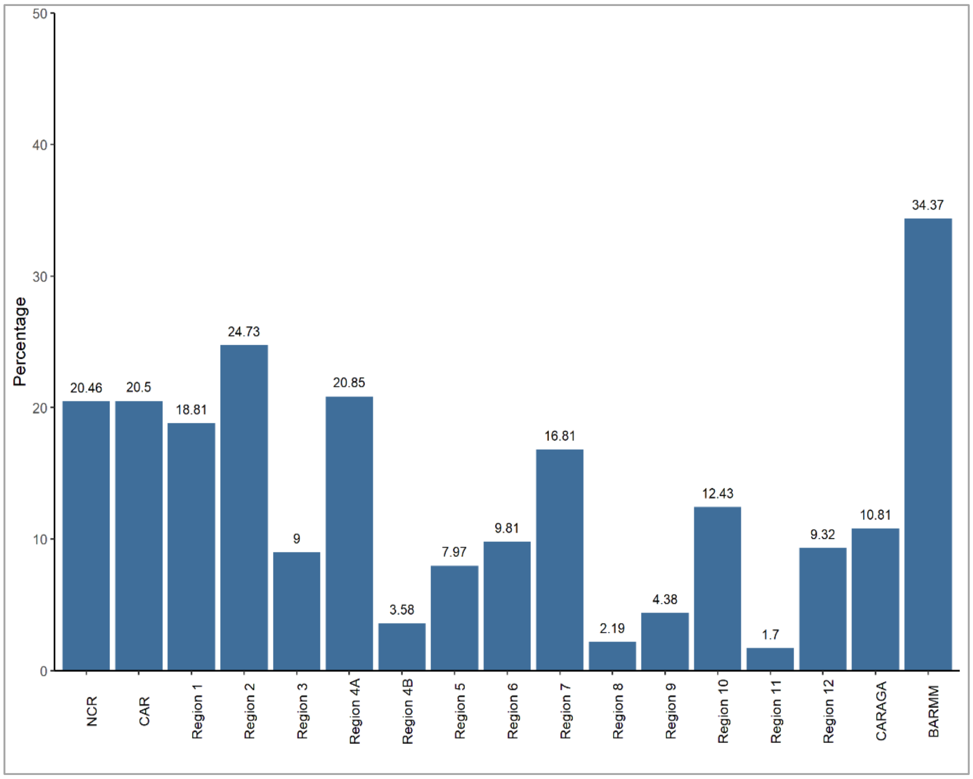

Figure 1.D.5: *BOHC Coverage among Seniors 60 years old and above, by Region, 2022*

1.D Oral Health Care and Services

For the vulnerable group of pregnant women aged 10-49 years old, the highest BOHC coverage was in NCR at 39.20% and lowest in Region 11 (3.45%). Overall, regional data in 2022 showed that all regions still have a lot to do to achieve the 100% BOHC target coverage particularly Region 11 with the lowest coverage reported among pregnant women and in all lifecycle stages except among infants *(Figure 1.D.6)*.

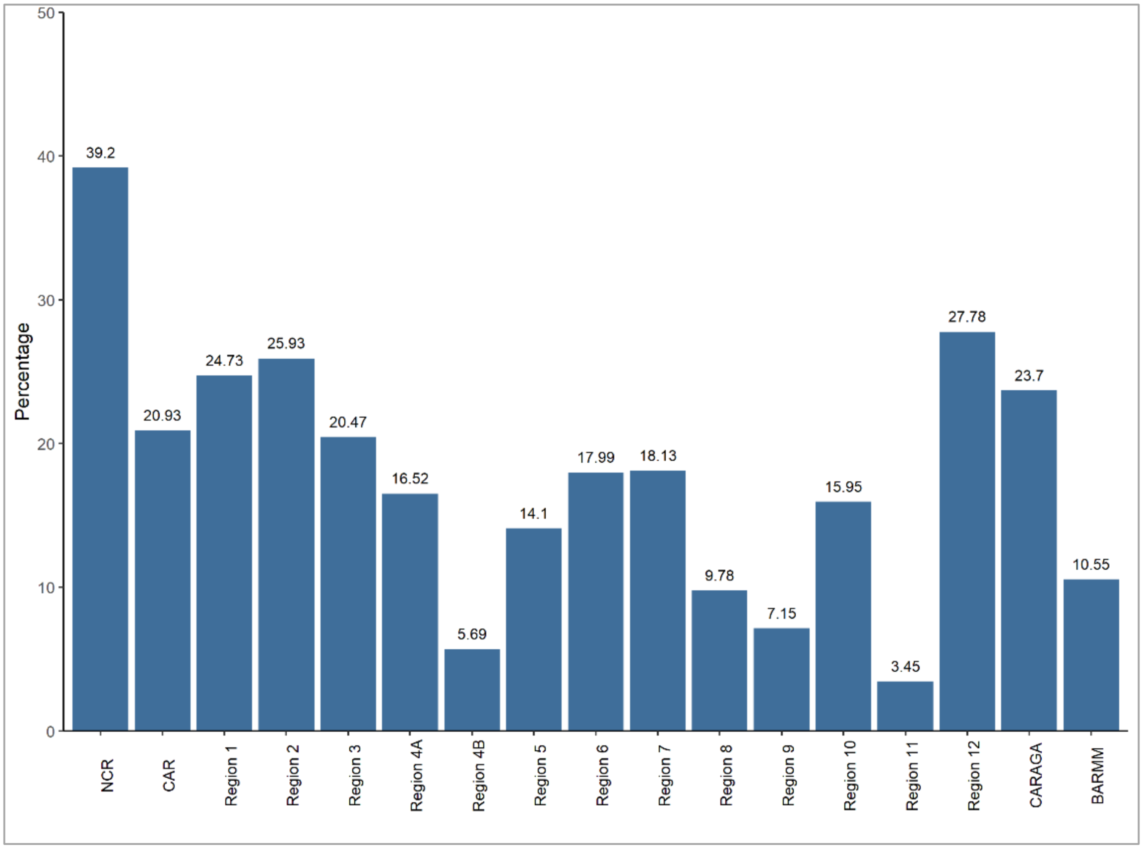

Figure 1.D.6: *BOHC Coverage among Pregnant Women, by Region, 2022*

1.E Non-Communicable Diseases Prevention and Control Services

2022 KEY FINDINGS

Lifestyle Related Diseases
Hypertension and Diabetes Mellitus Type 2

68,116,194
Estimated 20 years old and above population (both sexes)

10.57%
20 years old and above adults risk assessed using PhilPEN protocol

8.61%
Newly-identified Hypertensive among those risk assessed

2.22%
Newly-identified Diabetes Mellitus Type 2 among those risk assessed

Cancer Prevention and Control
Cervical Cancer and Breast Mass Screening and Detection

34,111,514
Estimated 20 years old and above population (female)

0.39%
Screened for Cervical Cancer among the 20 years old and above female

2.22%
Screened for Breast Mass among the 20 years old and above female

4.06%
Found positive or suspect with Cervical Cancer among those screened

1.68%
Found with suspicious Breast Mass among those screened

1.E Non-Communicable Diseases Prevention and Control Services

Non-communicable diseases (NCDs) are conditions that tend to be of long duration and are the result of a combination of genetic, physiological, environmental, and behavioral factors. The rise of NCDs including Cardiovascular Diseases (CVDs) is largely attributed to the high prevalence of risk factors which are considered modifiable– namely smoking, unhealthy diet, physical inactivity, alcohol use, and obesity. The early detection, screening, and treatment of NCDs, as well as palliative care, are key components of the response to NCDs[1]. The Department of Health (DOH) Administrative Order (AO) No. 2012-0029 dated 04 December 2012 prescribes the implementing guidelines on the institutionalization of the Philippine Package of Essential NCD Interventions (PhilPEN). PhilPEN is a protocol for the Integrated Management of Hypertension and Diabetes in primary healthcare settings adapted from the WHOPEN. Specifically, the AO mandates the Local Government Units (LGUs) to adopt and implement the PhilPEN and provide services and products in primary healthcare facilities and hospitals in their localities.

Likewise, the most cost-effective long-term strategy for the control of cancer is prevention. Among women, cervical and breast cancers are the most preventable types of cancer. Regular screening can help prevent cervical and breast cancers or identify the conditions early when they are most treatable.

On the other hand, the elderly population is especially vulnerable to developing serious complications from diseases that are vaccine-preventable such as influenza and pneumococcal infections due to age-related impairment of the immune system which is worsened by decreased physical activity and poor nutrition. In recognition of this, the Republic Act 9994, or the “Expanded Senior Citizens Act”, was signed, mandating the DOH to provide for free influenza and pneumococcal immunization of indigent senior citizens.

The FHSIS monitors indicators on the NCD Program including the adoption of PhilPEN which reflects the ability of the local health system in integrating risk assessment in the routine delivery of health care and properly classifying those who have found to have risk. Early identification of risk factors among the 20 years old and above population could lead to responsive/appropriate preventive measures. Further, for the elderly, FHSIS tracks the provision of Pneumococcal Polysaccharide Vaccine (PPV) and Influenza vaccine.

This chapter presents data on NCD Program indicators including the adoption of PhilPEN and provision of PPV and Influenza vaccine among the elderly.

---

[1] *Noncommunicable diseases - World Health Organization (WHO) https://www.who.int › Newsroom › Fact sheets › Detail Sept. 16, 2022)*

1.E Non-Communicable Diseases Prevention and Control Services

Indicators on NCDs were included in the FHSIS monitoring only in 2019 and data from 2019 to 2022 showed a steady but slow increment in the percentage of Filipinos aged 20 years old and above who were risk assessed. In 2022, only 10.57% were risk assessed using the PhilPEN protocol among the target population which is much lower than the 70% target. Among those risk assessed, 8.61% were newly identified hypertensives and 2.22% were newly diagnosed with Diabetes Mellitus Type 2 *(Figure 1.E.1)*.

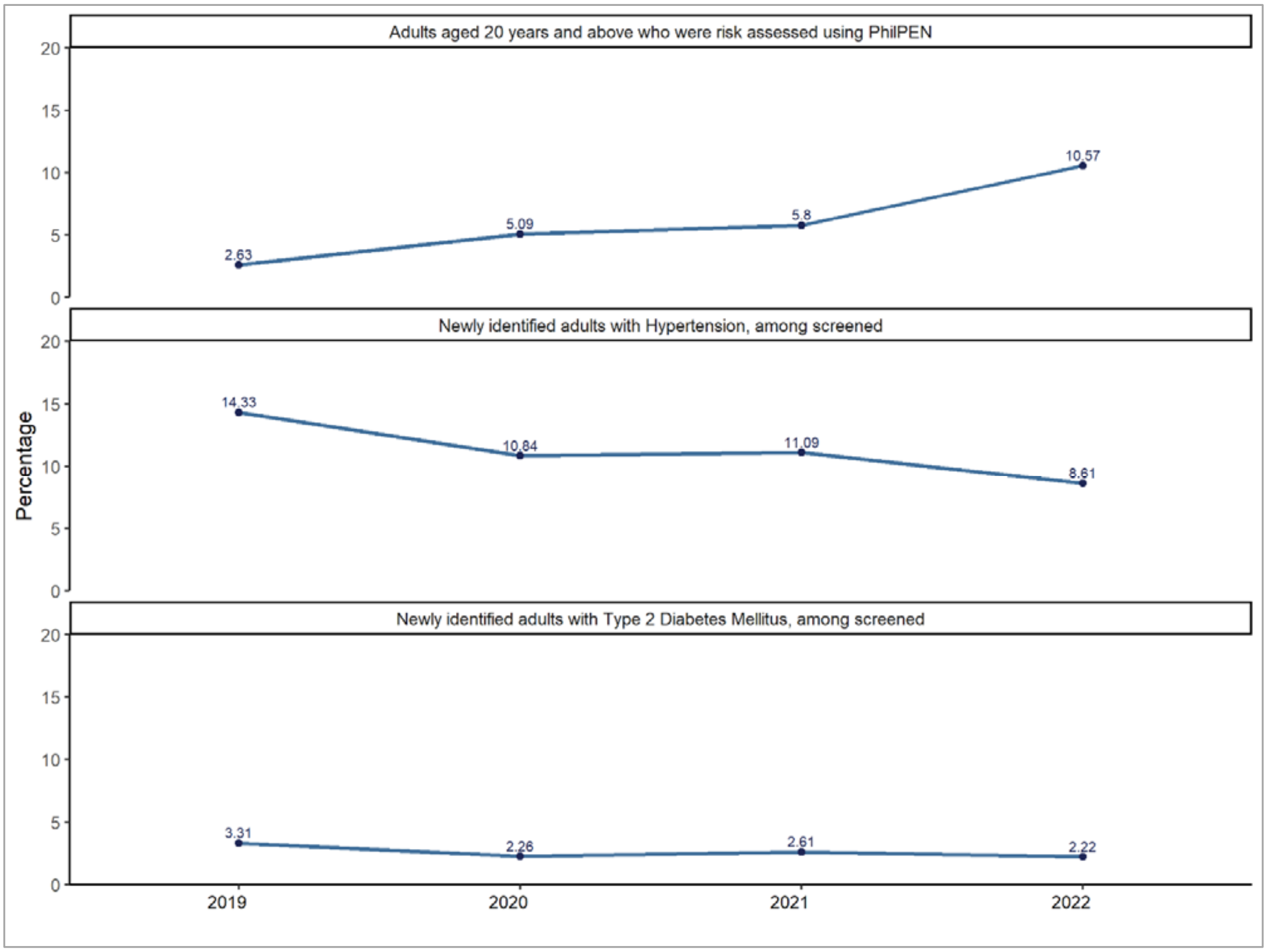

Figure 1.E.1: *Percentage of Adults 20 years old and above who were PhilPEN Risk Assessed and Newly Identified as Hypertensives or with Type 2 Diabetes Mellitus, Philippines, 2019-2022*

1.E Non-Communicable Diseases Prevention and Control Services

For the Cancer Prevention and Control Program, screening for cervical cancer among women aged 20 years old and above was at 0.71% in 2019 and dropped to 0.12% in 2020, then steadily increased in 2021 and 2022 at 0.22% and 0.39%, respectively. However, the 0.39% screening coverage was extremely below the 70% target set for 2022. Among those screened, 4.06% were found positive or suspected for cervical cancer *(Figure 1.E.2)*.

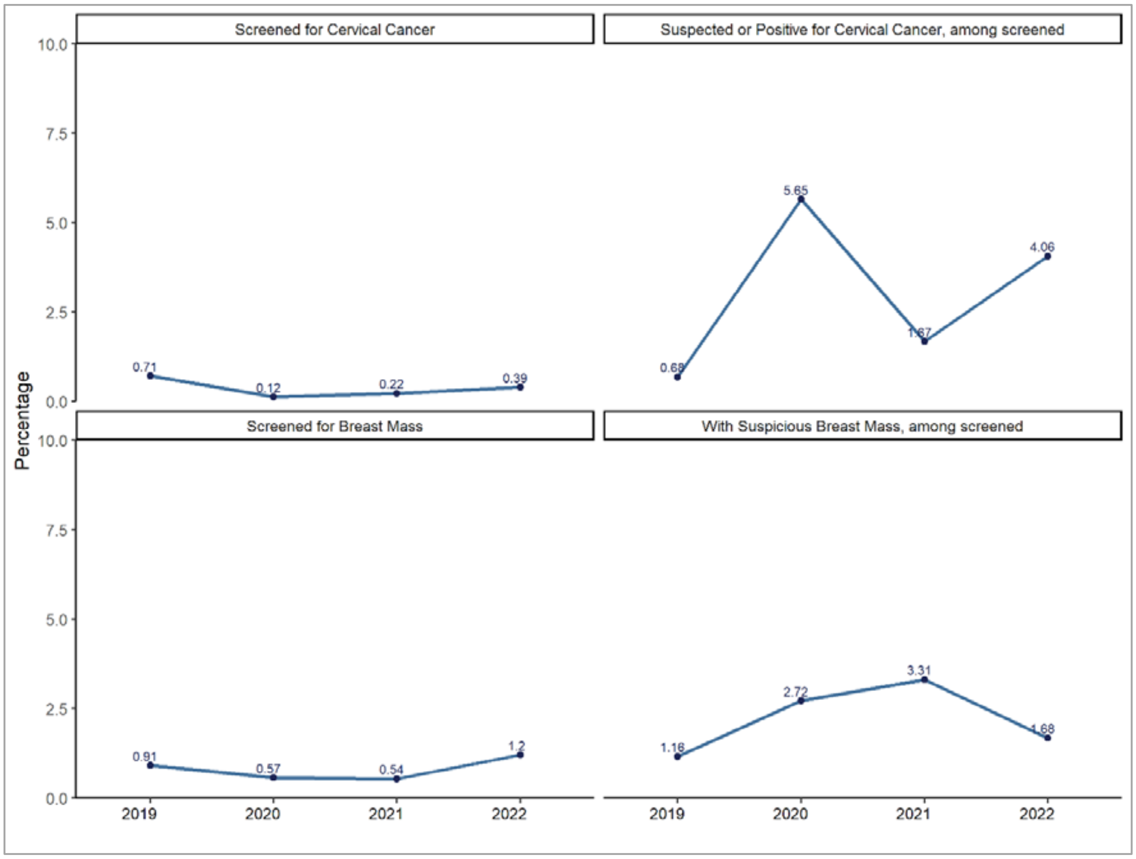

Figure 1.E.2: *Percentage of Female Adults 20 years old and above who were Screened for Cervical Cancer and Breast Mass, Philippines, 2019-2022*

Similarly, breast cancer screening among the same target population was at 0.91% in 2019 but decreased to 0.57% in 2020 and 0.54% in 2021. In 2022, it was registered at 1.20%. Among those screened in 2022, there were 1.68% detected with a suspicious breast mass *(Figure 1.E.2)*. Health promotion activities on the importance and availability of screening for cervical and breast cancer need to be strengthened to break possible barriers such as lack of knowledge, fear, and poor health-seeking behavior among others that could be preventing the target population from having the screening.

In 2019, RA No. 11215, National Integrated Cancer Control Act was enacted. The Act provides for the establishment of the National Integrated Cancer Control Program aiming to decrease the overall mortality and impact of all adult and child cancer and lessen the incidence of preventable cancer in adults and children by scaling up essential programs and increasing investments for the prevention of cancer, better screening, prompt and accurate diagnosis, timely and optimal treatment among others.

1.E Non-Communicable Diseases Prevention and Control Services

The Immunization Program for senior citizens (60 years old and above) for PPV showed an increasing trend from 2019 to 2020 but declined in 2021 and 2022. As for the Influenza vaccine, a gradual increase was noted from 2019 at 2.64% to 8.81% in 2022 *(Figure 1.E.3)*.

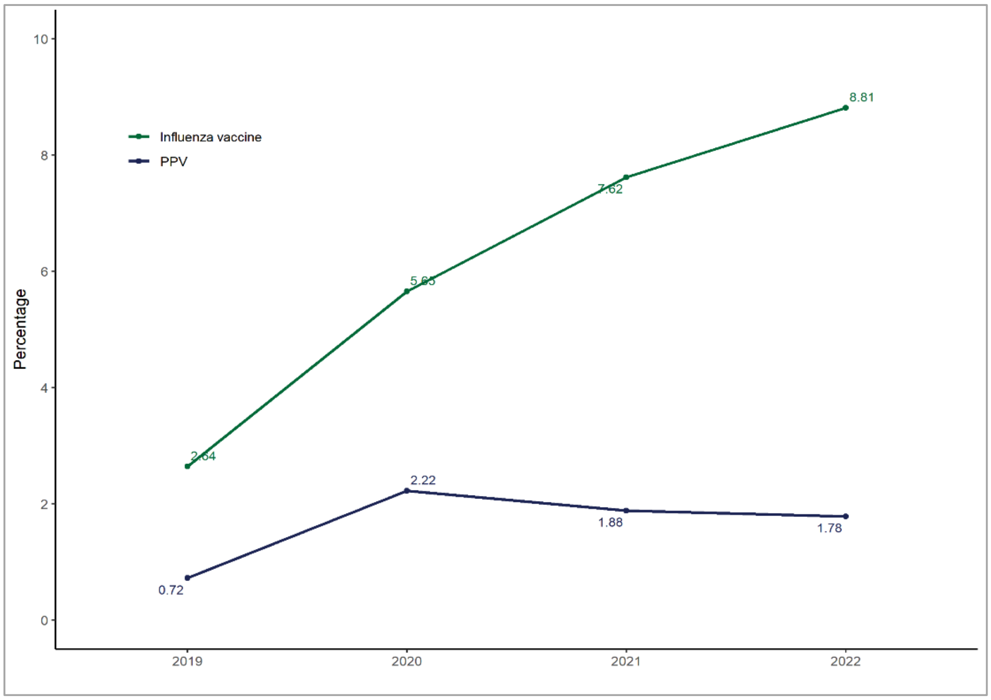

Figure 1.E.3: *Percentage of Seniors 60 years old and above who were Vaccinated with PPV or Influenza vaccine, Philippines, 2019-2022*

For risk assessment using the PhilPEN protocol, FHSIS reports in 2022 showed that none of the 17 regions met the 70% target. The highest reported was 31.09% in Region 2, while BARMM had the lowest coverage at 2.75% *(Figure 1.E.4)*.

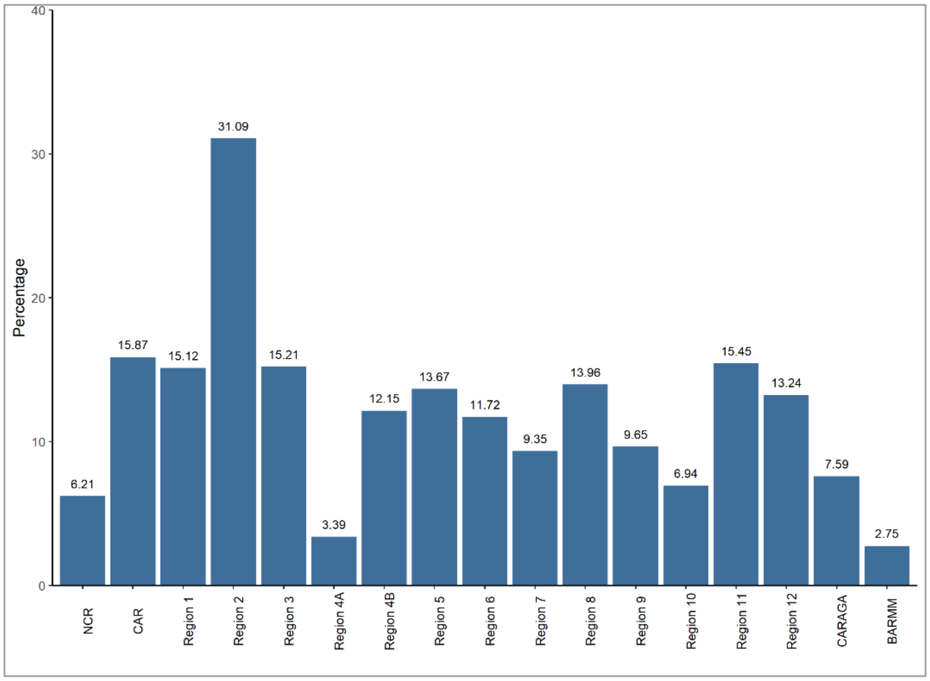

Figure 1.E.4: *Percentage of Adults 20 years old and above who were PhilPEN Risk Assessed, by Region, 2022*

1.E Non-Communicable Diseases Prevention and Control Services

There were 20 newly identified hypertensives per 100 individuals who were risk assessed in Region 4A (highest) and only 3 out of 100 in Region 2 (lowest). Similarly, newly identified Type 2 Diabetes Mellitus among those who were risk assessed was also highest in Region 4A (6.21%) and also lowest in Region 2 with 0.46% *(Figure 1.E.5)*.

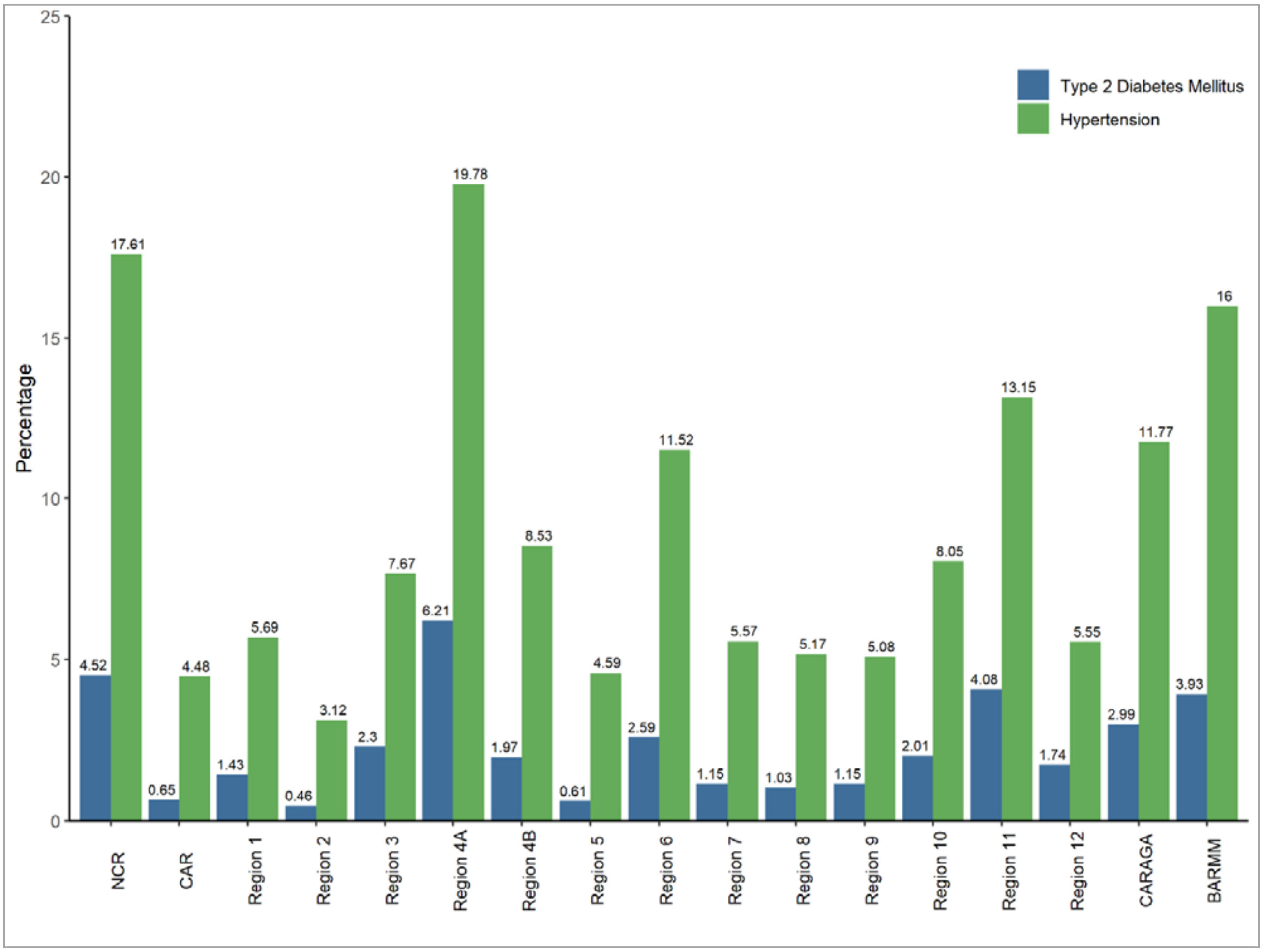

Figure 1.E.5: *Percentage of PhilPEN Risk Assessed Adults 20 years old and above Newly Identified as Hypertensive or with Type 2 Diabetes Mellitus by Region, 2022*

1.E Non-Communicable Diseases Prevention and Control Services

For cervical cancer screening, all regions had less than 1.0% of their eligible population screened except for Region 11 at 3.69 *(Figure 1.E.6)*. All regions were far below the target of 70% of the eligible population being screened for cervical cancer. Among those screened, Region 6 had the highest (9.55%) detected positive or suspected for cervical cancer *(Figure 1.E.7)*. Screening for breast mass among women aged 20 years old and above also seemed to be unpopular with all regions registering less than 5% women screened among their eligible population. The highest screening coverage was 4.08% in CAR and lowest in Region 8 at 0.13% *(Figure 1.E.6)*. Among those screened for breast mass, the region with the highest percentage of detected suspicious breast mass was Region 4B at 9.67% and the lowest in BARMM at 0.69% *(Figure 1.E.7)*. Treatment of cancer at an early stage increases the chance of survival, thus early detection through screening is very vital in reducing both cancer incidence and mortality.

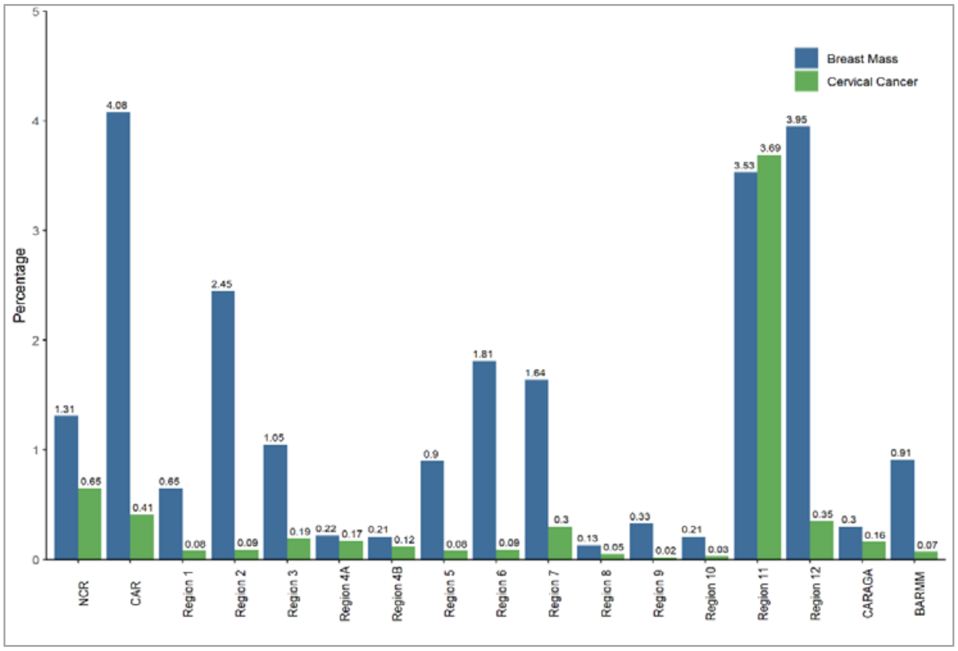

Figure 1.E.6: *Percentage of Female Adults 20 years old and above who were Screened for Cervical Cancer and Breast Mass, by Region, 2022*

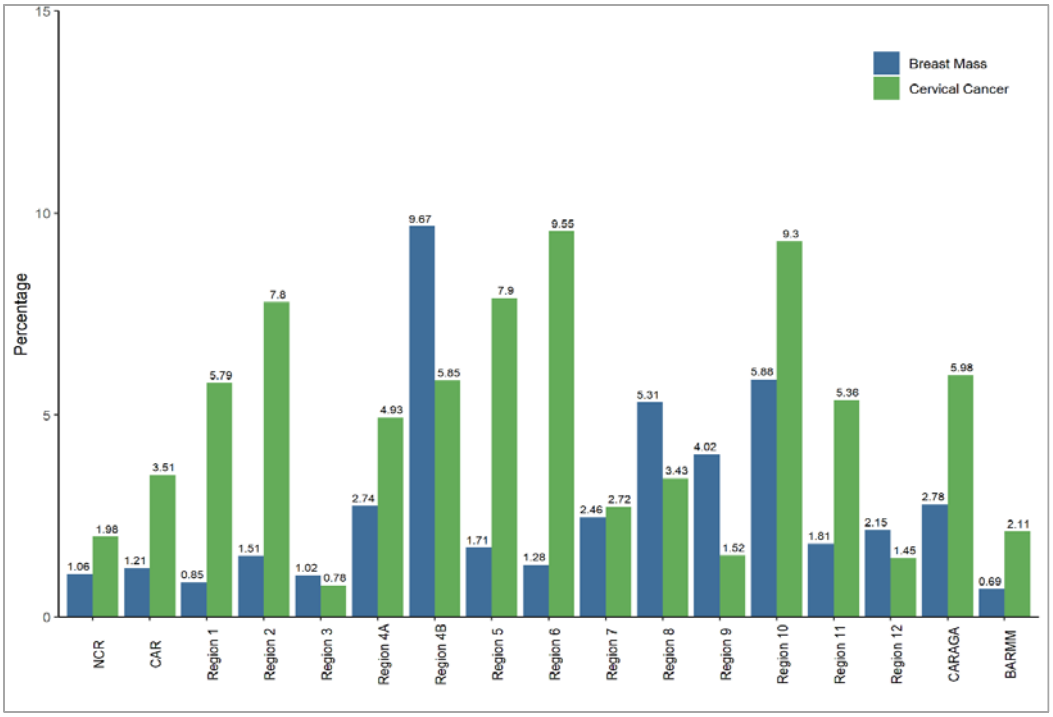

Figure 1.E.7: *Percentage of Screened for Cervical Cancer and Breast Mass found with Suspicious Cervical Cancer or Breast Mass, by Region, 2022*

1.E Non-Communicable Diseases Prevention and Control Services

Immunization coverage among seniors for PPV and Influenza Vaccines in 2022 were also low for all regions. The highest coverage was reported in Region 1 at 2.86% for PPV and in Region 3 at 16.28% for Influenza. The lowest coverage was in Region 4B (0.45%) for PPV and in Region 7 (2.49%) for Influenza *(Figure 1.E.8)*. New strategies to reach out to seniors for immunization must be considered particularly for vaccine-preventable diseases.

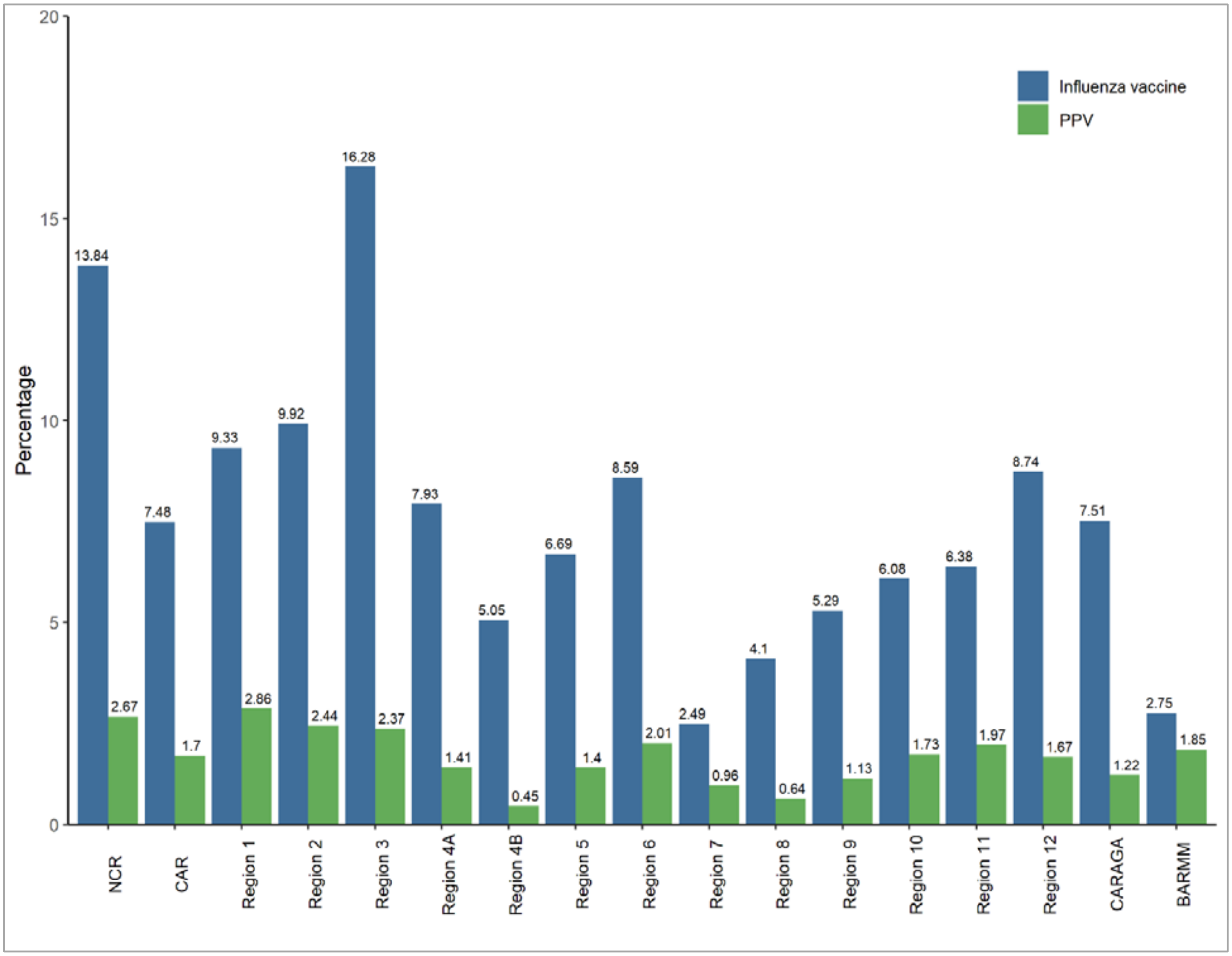

Figure 1.E.8: *Percentage of Seniors 60 years old and above who were Vaccinated with PPV or Influenza Vaccine, by Region, 2022*

1.F Environmental Health and Sanitation Services

2022 KEY FINDINGS

Access and Use of Safe Water and Sanitation Facilities

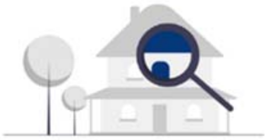

25,481,993
Estimated number of Households (HH)

88.74%
HH with access to Basic Safe Water Supply (BSWS)

83.64%
HH with Basic Sanitation Facility (BSF)

64.82%
HH using Safely Managed Drinking Water Services (SMDWS)

66.10%
HH using Safely Managed Sanitation Services (SMSS)

Zero Open Defecation (ZOD) Certification of Barangays

42,034
Total number of Barangays

32.32%
Barangays certified as ZOD

Formula:

Basic Safe Water Supply proportion
Numerator: Total number of HH with access to BSWS
Denominator: Estimated total number of HH

Basic Sanitation Facility proportion
Numerator: Total number of HH with BSF
Denominator: Estimated total number of HH

Safely Managed Drinking Water Services proportion
Numerator: Total number of HH using SMDWS
Denominator: Estimated total number of HH

Safely Managed Sanitation Services proportion
Numerator: Total number of HH using SMSS
Denominator: Estimated total number of HH

ZOD proportion
Numerator: Total number of barangays certified as ZOD area
Denominator: Total number of barangays

1.F Environmental Health and Sanitation Services

The environment is inevitably linked to health, thus, unhealthy environmental conditions contribute to the exposure pathways for both communicable and noncommunicable diseases. In 2010, the UN General Assembly explicitly recognized the human right to water and sanitation. Everyone has the right to sufficient, continuous, safe, acceptable, physically accessible and affordable water for personal and domestic use.1 Accordingly, the Department of Health through Administrative Order No. 2010-0021 issued on 25 June 2010 declared sustainable sanitation as a national policy and program priority. Further, Administrative Order No. 2019-0054, "Guidelines on the Implementation of the Philippine Approach to Sustainable Sanitation (PhATSS)" was issued in 2019. The AO aims to contribute to the reduction of risks and diseases related to environmental sanitation through the provision of the implementing guidelines to operationalize the national policy on sustainable sanitation and achieve the Sustainable Development Goal 6 (SDG 6) on sanitation. Specifically, SDG 6 calls for ensuring universal access to safe and affordable drinking water, sanitation, and hygiene (WASH), and ending open defecation. The FHSIS continues to track and monitor indicators related to the achievement of SDG 6 targets.

This chapter presents data on environmental and health sanitation services particularly indicators on SDG 6 targets.

The estimated total number of HH gradually increased from 2019 to 2022. In 2022, there were 25,481,993 estimated total number of HH registering a 3.68 percent increase from the 24,576,545 in 2019. Similarly, there was a steady increment in the percentage of HH having access to basic safe water supply (BSWS) in the same period, from 72.61% in 2019 to 88.74% in 2022 *(Figure 1.F.1)*. However, there was a substantial gap between the proportion of HH with access to BSWS and those using safely managed drinking water services (SMDWS). The use of SMDWS in a HH is considered based on the location, availability, microbiological validation, and result of water testing of the water source to which the HH has access.

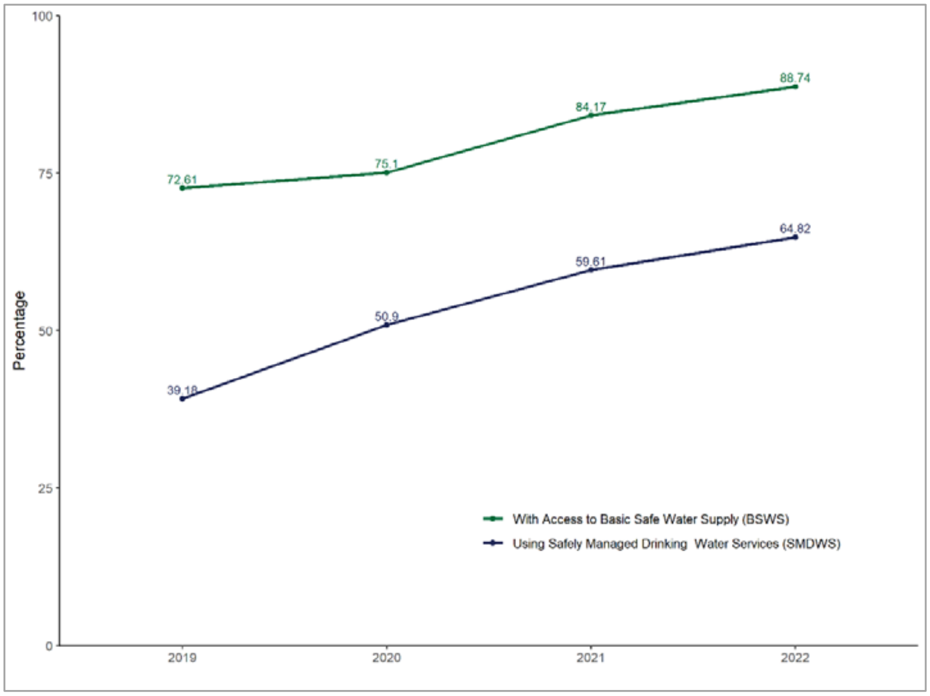

Figure 1.F.1: *Percentage of HH with access to BSWS and using SMDWS, Philippines, 2019-2022*

[1] *https://www.who.int/news-room/fact-sheets/detail/drinking-water*

1.F Environmental Health and Sanitation Services

The presence of basic sanitation facilities (BSF) in 2022 was evident in 83.64% of the HH which is 69.06% higher than the 49.47% reported in 2019 *(Figure 1.F.2)*. The presence of BSF must be complemented with the safe disposal/ treatment of excreta/ sewage before a HH can be considered to be using Safely Managed Sanitary Services (SMSS). In spite of the improvement in the presence of BSF, a lot is still to be done to improve the use of SMSS. As such, strategies need to be developed to address challenges in the safe disposal/ treatment of excreta/sewage that would increase the use of SMSS in every HH.

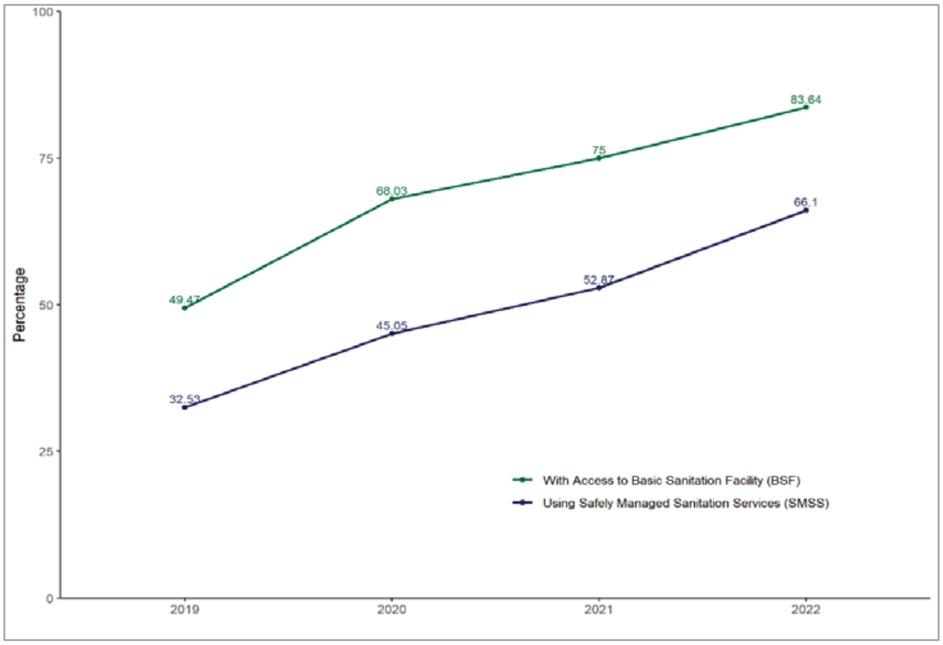

Figure 1.F.2: *Percentage of HH with access to BSF and using SMSS, Philippines, 2019-2022*

As to the target of having all barangays to be ZOD certified, better strategies must be developed and implemented since in 2022, there were only 32.32% ZOD certified barangays reported, which is 44.93 percent increase from the 22.3% reported in 2019 *(Figure 1.F.3)*.

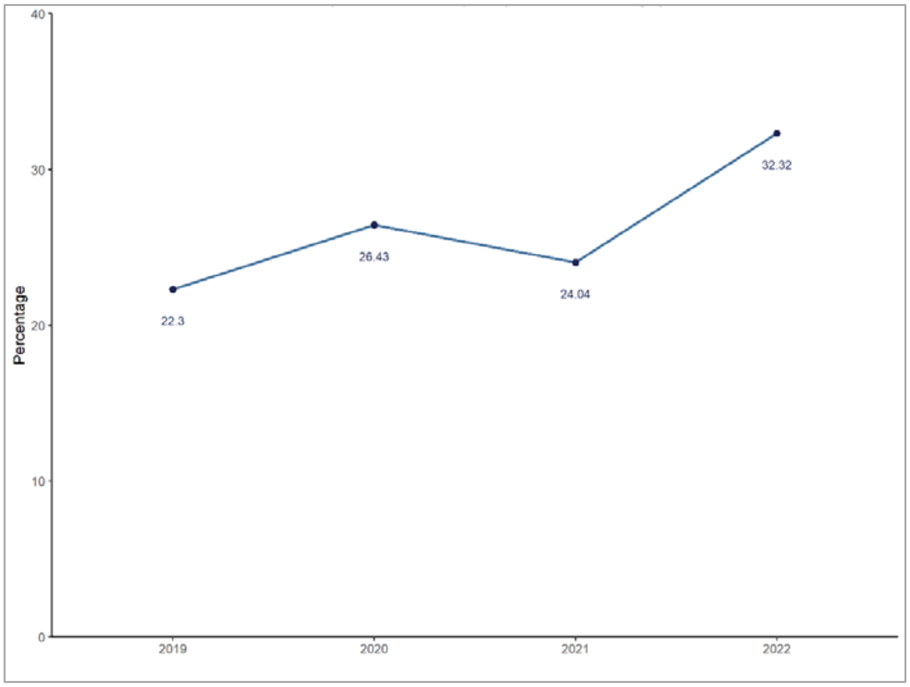

Figure 1.F.3: *Percentage of Barangays given the ZOD Certification, Philippines, 2019-2022*

1.F Environmental Health and Sanitation Services

In 2022, 12 out of the 17 regions had 90% or more HH with access to BSWS. CAR registered the highest percentage at 99.39% while Region 7 had the lowest at 46.93%. Consequently, SMDWS were used in at least 50% of HH in 12 regions, with NCR reporting the highest at 92.77%, while BARMM had the lowest at 18.2% *(Figure 1.F.4)*. Notably, there were regions with a high percentage of HH with access to BSWS and yet low in the use of SMDWS.

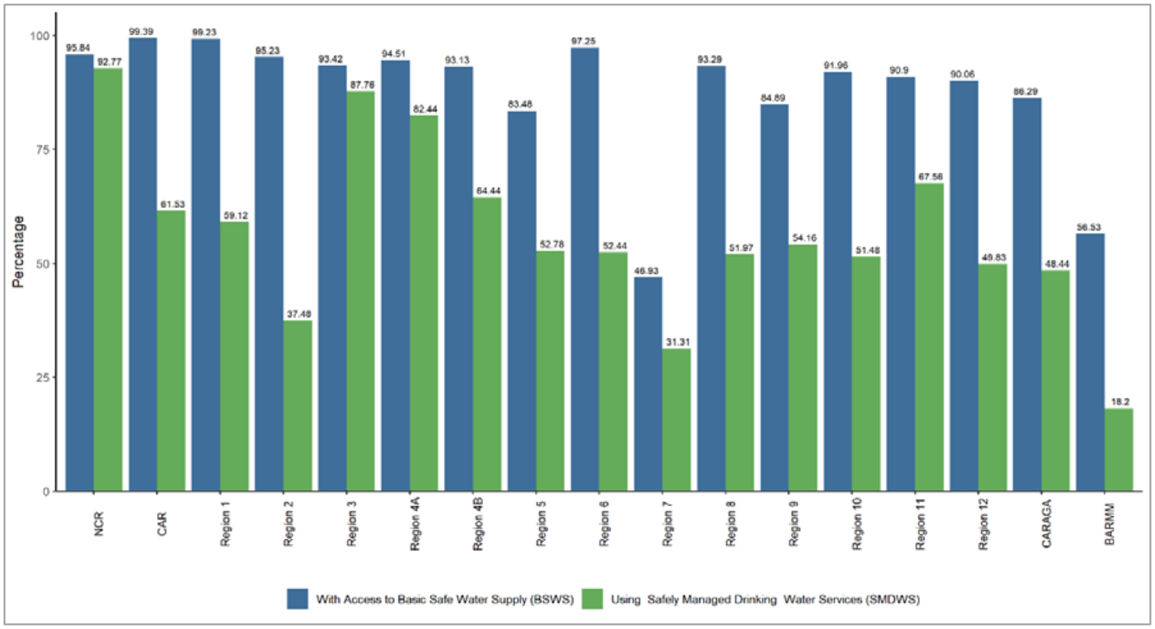

Figure 1.F.4: *Percentage of HH with access to BSWS and using SMDWS, by Region, 2022*

Twelve (12) out of the 17 regions in the country had at least 80% of HH with BSF. The highest percentage of HH with BSF is Region 1 (97.06%) and lowest in BARMM (37.21%). Likewise, 12 of the 17 regions had 50% or more HH using SMSS. The percentage of SMSS use in the HH was highest in NCR (92.45%) and lowest in BARMM (8.75%). However, there were regions with a high percentage of HH with BSF yet low in using SMSS, like Region 1 with 97.06% of HH with BSF but only 34.05% were using SMSS *(Figure 1.F.5)*.

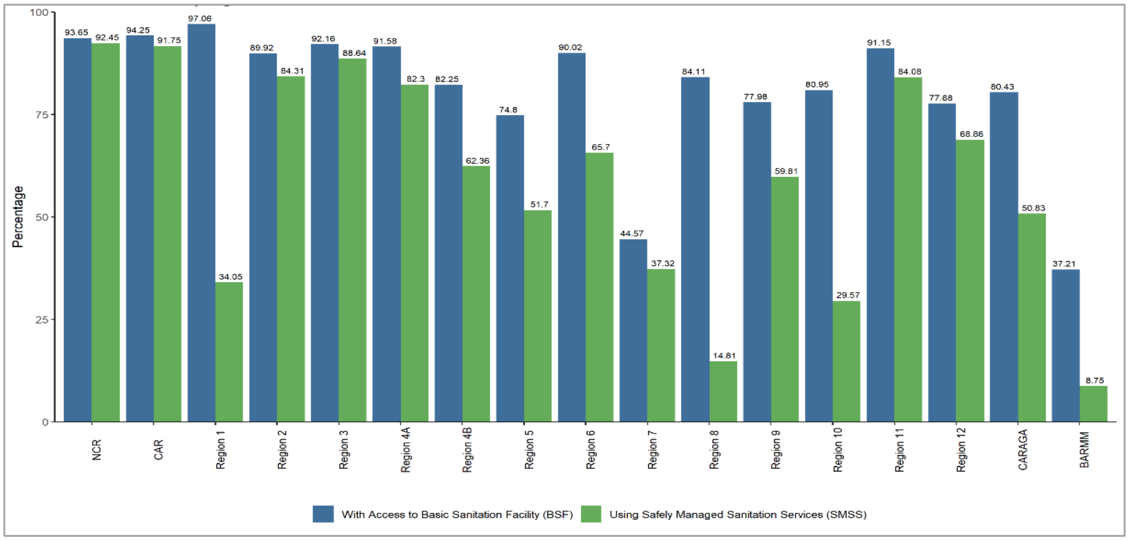

Figure 1.F.5: *Percentage of HH with access to BSF and using SMSS, by Region, 2022*

1.F Environmental Health and Sanitation Services

The target to end open defecation is promising in Region 1 which reported 84.30% barangays that were certified as ZOD areas and Region 6 with 69.46% ZOD-certified areas. The rest of the regions had less than 50% of barangays certified as ZOD areas, with as low as 6.0% in Region 4A *(Figure 1.F.6)*.

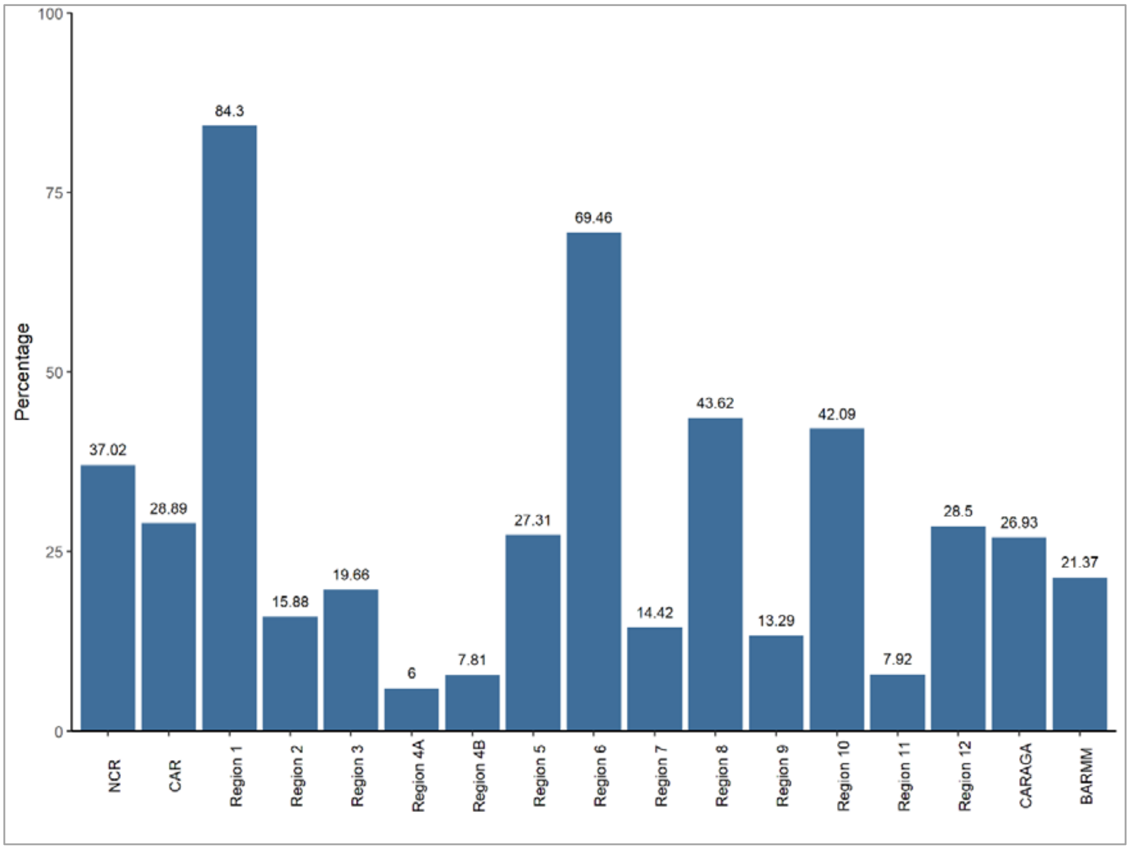

Figure 1.F.6: *Percentage of Barangays given the ZOD Certification, by Region, 2022*

1.G Morbidity

Causes of Morbidity Ranking
Philippines, 2019-2022

2019	2020	2021	2022	Causes of Morbidity
1	1	1	1	Acute Respiratory Infection
2	2	2	2	Hypertension
8	4	3	3	Animal Bites
3	3	4	4	Urinary Tract Infection
4	6	7	5	Acute Lower Respiratory Tract Infection
7	5	5	6	Skin Diseases
6	7	6	7	Pneumonia
>10	>10	10	8	Diseases of the Heart
>10	10	8	9	TB All Forms
>10	>10	9	10	Fever of Unknown Origin

Rank	Causes of Morbidity	Number of case	Rate per 100,000 population
1	Acute Respiratory Infection	3,080,648	2761.12
2	Hypertension	668,871	599.50
3	Animal Bites	503,167	450.98
4	Urinary Tract Infection	347,855	311.78
5	Acute Lower Tract Respiratory Infection	282,092	252.83
6	Skin Diseases	211,724	189.76
7	Pneumonia	201,798	180.87
8	Diseases of the Heart	155,147	139.06
9	TB All Forms	119,558	107.16
10	Fever of Unknown Origin	95,257	85.38

The Causes of Morbidity ranking above showed Acute Respiratory Infection and Hypertension consistently at the top 1 and 2 ranks, respectively from 2019 to 2022. The Animal Bites which ranked 8 in 2019 climbed up to rank 4 in 2020 and stayed at rank 3 from 2021 to 2022. Urinary Tract Infection which ranked 3 in 2019 to 2020, slide down to rank 4 in 2021 to 2022. Diseases of the heart which was beyond top 10 rank in 2019 and 2020, gradually went up to rank 9 in 2021 and rank 8 in 2022.

1.H Demographics

2022 KEY FINDINGS

Population, Household (HH), and Barangay

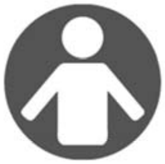

111,572,254
Estimated Total Population

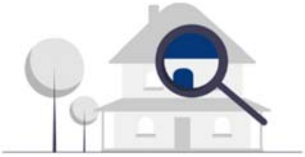

25,481,993
Estimated Total Number of Household

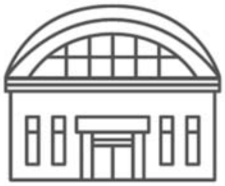

42,034
Reported Total Number of Barangay

Population and Health Facility

Health Center (HC) and Barangay Health Station (BHS)

41,477:1
Population to HC Ratio

4,725:1
Population to BHS Ratio

Population and Public Health Workers

26,014:1
Population to Physician Ratio

57,630:1
Population to Dentist Ratio

4,911:1
Population to Public Health Nurse Ratio

5,074:1
Population to Midwife Ratio

165,783:1
Population to Nutritionist Ratio

35,657:1
Population to Medical Technologist Ratio

2,936,112:1
Population to Sanitary Engineer Ratio

271,015:1
Population to Sanitary Inspector Ratio

1.H Demographics

Formula:

Population to Barangay Ratio
Numerator: Total number of barangays
Denominator: Estimated total population

Population to HC Ratio
Numerator: Total number of HC
Denominator: Estimated total population

Population to Public Health Worker Ratio
Numerator: Total number of public health worker (per group)
Denominator: Estimated total population

Population to BHS
Numerator: Total number of BHS
Denominator: Estimated total population

HH to Active Barangay Health Worker (BHW) Ratio
Numerator: Total number of active BHW
Denominator: Estimated total number of HH

The recommended population to health workers ratio:

Physician/Doctor	20,000:1	Nutritionist	20,000:1
Dentist	50,000:1	Medical Technologist	50,000:1
Public Health Nurse	10,000:1	Sanitary Engineer	-
Midwife	5,000:1	Sanitary Inspector	20,000:1

The FHSIS continues to track and monitor community-based or population-based data, specifically demographic data that are only reported on an annual basis. The Annual Report on demographic data is the official health report of the barangay/municipality. Demographic data includes information about the area in terms of its total catchment population, HH, barangays, number of existing health facilities (HC and BHS) and healthcare personnel. The provision of quality healthcare services depends greatly on the equitable distribution of healthcare facilities and workers.

At the Local Government Unit (LGU) level, DOH sets ideal distribution ratios of health professionals with the population working in a primary health care facility that are used as a norm by both the DOH and the LGUs in identifying staffing needs at the primary care facilities. Additionally, the National Human Resources for Health Master Plan (NHRHMP) 2020-2040 was developed by the Department of Health and the Human Resources for Health Network Philippines to guide the management and development of the human resources for health (HRH) of the Philippines according to the goals of Universal Health Care.[1]

This chapter presents demographic data on population and its ratio to health facility and healthcare personnel.

[1] *https://hhrdb.doh.gov.ph/hrh-network/*

1.H Demographics

Demographic data from 2019-2022 showed a 3.29% change in the estimated population or an average of 0.82% annual change. A decreasing trend in the number of barangays from 2020 (42,134) to 2022 (42,034) was also noted. The increasing trend in the number of BHS from 2020 (20,663) to 2022 (23,614) resulted in a decrease in the population to BHS ratio. The increasing trend in the number of HC from 2019 (2,434) to 2021 (3,073) was halted in 2022 (2,690) resulting to 41,477 population to 1 HC ratio *(Figure 1.H.1).*

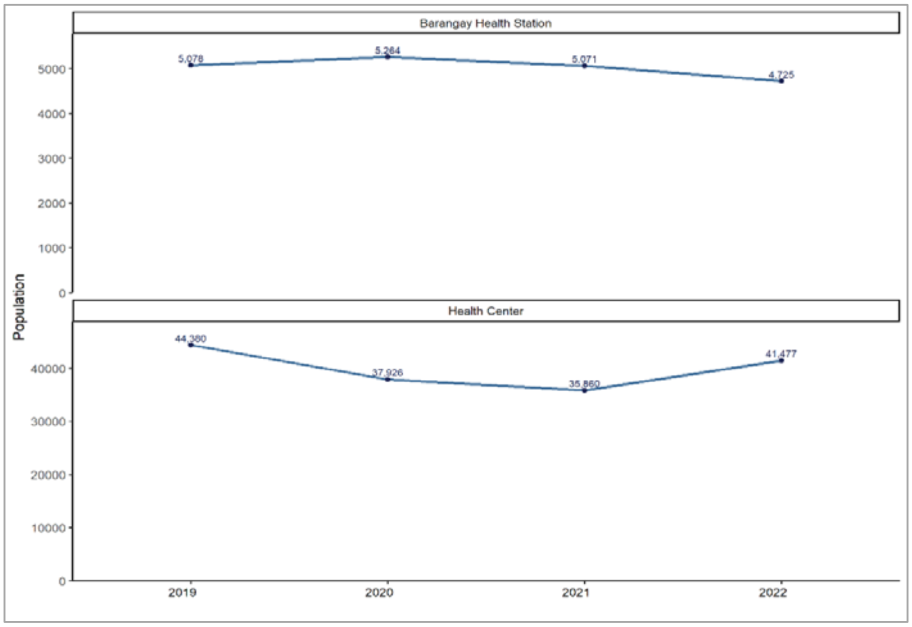

Figure 1.H.1: *Population to Health Facility Ratio, Philippines, 2019-2022*

Likewise, an improved population-to-public health care worker ratio was noted from 2019 to 2022. This was a result of a generally increasing trend in the number of public health workers, although the recommended ratios of groups of health workers to population have yet to be reached since 2019 except for public health nurses. The population-to-nutritionist ratio remains to be very far from the recommended ratio since 2019. Similarly, the number of sanitary engineers are quite few for the population, with a 1,928,936 population to 1 sanitary engineer in 2019 and 2,936,112:1 in 2022. An improvement in the ratio of a HH to active BHW has been noted from 2019 to 2022, but still far from the recommended 20 HH to 1 active BHW. The population growth simply means more people needing healthcare services and healthcare services cannot be effectively delivered without an adequate and competent health care workforce and facilities. With the NHRHMP 2020-2040, it is hoped that the challenges for HRH will be addressed.

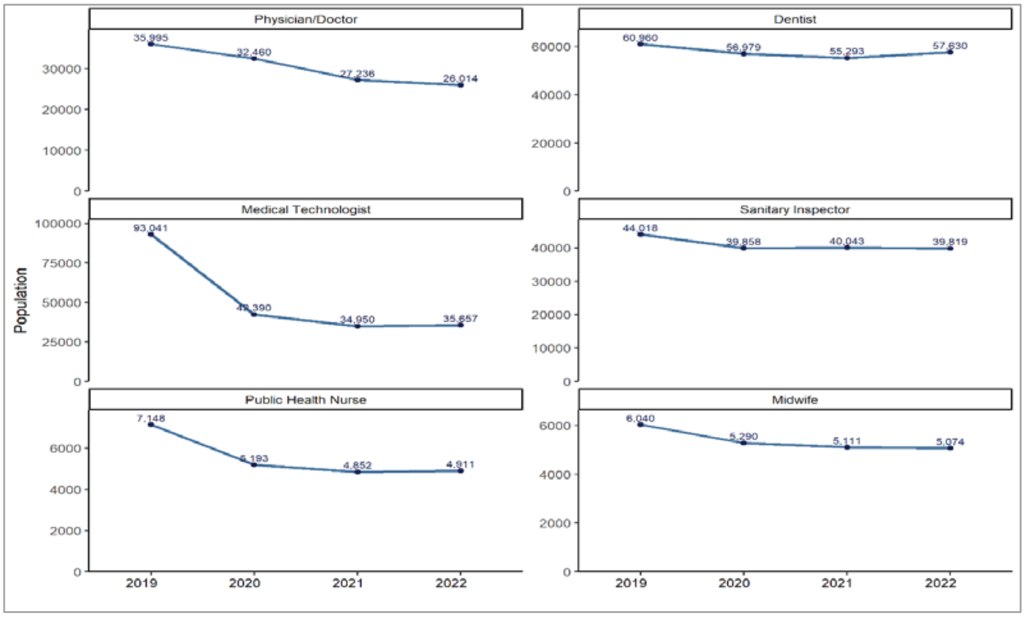

Figure 1.H.2: *Population to Health Care Worker Ratio, Philippines, 2019-2022*

1.H Demographics

In 2022, Region 4A had the highest estimated total population with 16,654,975 and CAR had the least population at 1,836,857 individuals. Similarly, Region 4A had the highest number of HH (3,868,352) and CAR had the lowest (421,691). Meanwhile, the total number of barangays was highest in Region 8 (4,390) and lowest in CARAGA (1,311). On average, NCR had the most population in a barangay (8,257) and Region 8 had the least (1,108).

The number of HC was highest in NCR (466 HC) with a 30,298 population to 1 HC ratio and lowest in Region 11 (52 HC) with 104,836 to 1 HC ratio. As to the number of BHS, Region 4A had the most with 2,811 BHS and a 5,925:1 population to BHS ratio, while NCR had the least with only 34 BHS resulting in a 415,255:1 population to BHS ratio *(Figure 1.H.3)*.

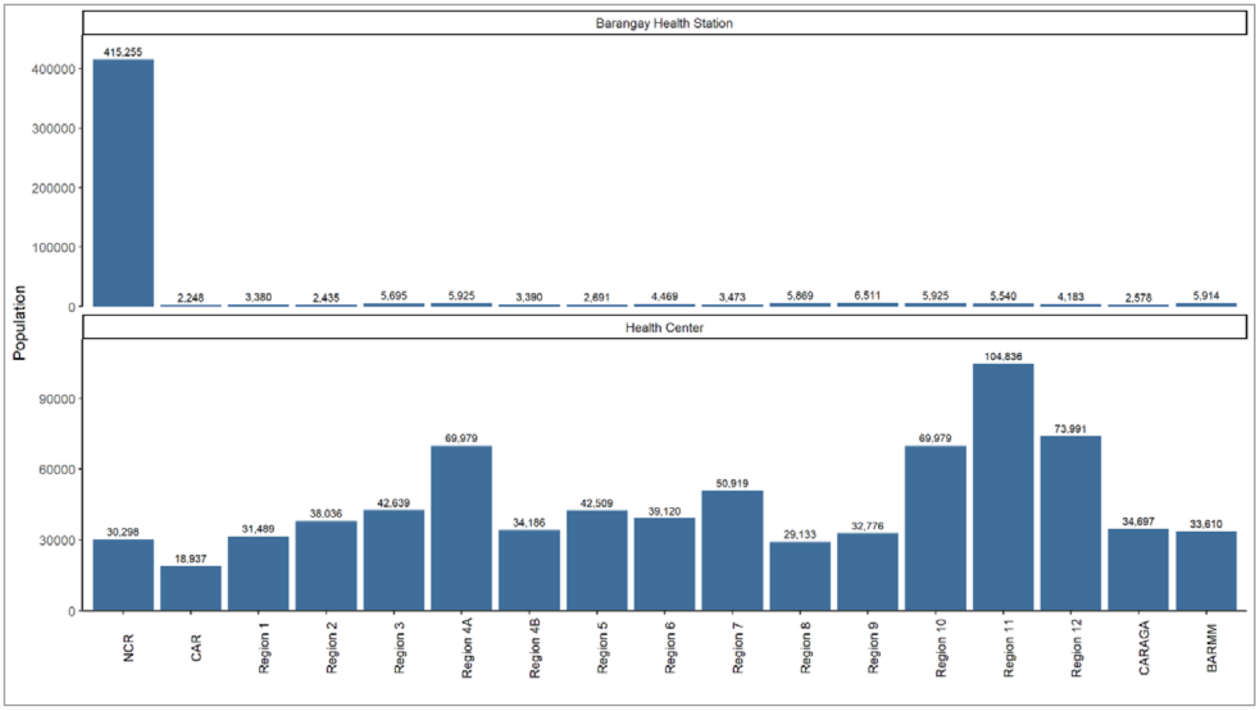

Figure 1.H.3: *Population to BHS and HC (Municipal HC, City HC, and Rural Health Unit) Ratio, by Region, 2022*

Only NCR and CAR reportedly met the recommended population to physician ratio with 15,297:1 and 16,852:1 ratios, respectively. Region 11, with only 134 reported total number of LGU and DOH hired physicians, had 1 physician for every 40,682 population. In terms of the number of dentists, only NCR and CAR among the 17 regions were within the 1 dentist for every 50,000 population recommended ratio with 24,726:1 and 45,921:1 ratios, respectively. All regions met the recommended ratio of 1 PHN for every 10,000 population. On the other hand, NCR, Regions 3, 4A, and 11 had to increase the number of midwives to reach the recommended target of 1 midwife for every 5,000 population. Across all regions, there is a critical need to increase the number of nutritionists attending to the nutrition-related health care services of the population to meet the recommended ratio. For the medical technologist manpower requirement, only Region 4A and BARMM did not reach the recommended ratio of 1 medical technologist per 50,000 population. Notably, there were 3 regions (Regions 6, 9, and BARMM) with no sanitary engineer and only CAR met the 20,000 population to 1 sanitary inspector target ratio. Lastly, the household to active barangay health worker ratio was highest in NCR and lowest in Region 5.

CHAPTER II

SUMMARY TABLES AND GRAPHS

Table 2.A.1. MODERN METHOD OF FAMILY PLANNING
New Acceptors
Philippines, 2022

Area	Total Current Users	FSTR/BTL Age group 10-14	15-19	20-49	Total	MSTR/NSV Age group 10-14	15-19	20-49	Total
**PHILIPPINES**	**8,019,000**	**3**	**125**	**17,966**	**18,094**	**0**	**9**	**399**	**408**
**N C R**	**753,263**	**0**	**2**	**2,499**	**2,501**	**0**	**0**	**83**	**83**
Malabon	31,679	0	0	5	5	0	0	0	0
Navotas	23,423	0	0	21	21	0	0	1	1
Valenzuela City	39,922	0	0	73	73	0	0	3	3
Caloocan City	68,156	0	0	32	32	0	0	0	0
Marikina City	17,020	0	0	17	17	0	0	5	5
Pasig City	42,380	0	0	475	475	0	0	12	12
Pateros	2,674	0	0	14	14	0	0	1	1
Taguig	61,050	0	0	10	10	0	0	0	0
Quezon City	272,854	0	0	260	260	0	0	1	1
Makati City	16,005	0	0	152	152	0	0	12	12
Mandaluyong City	17,504	0	2	141	143	0	0	15	15
San Juan	5,796	0	0	1	1	0	0	0	0
Manila City	27,488	0	0	787	787	0	0	31	31
Las Piñas City	20,363	0	0	75	75	0	0	0	0
Muntinlupa City	40,839	0	0	200	200	0	0	2	2
Parañaque City	37,239	0	0	201	201	0	0	0	0
Pasay City	28,871	0	0	35	35	0	0	0	0
**C A R**	**147,908**	**0**	**0**	**257**	**257**	**0**	**0**	**53**	**53**
Abra	17,362	0	0	0	0	0	0	0	0
Apayao	23,877	0	0	3	3	0	0	0	0
Benguet	31,732	0	0	68	68	0	0	2	2
Ifugao	20,003	0	0	0	0	0	0	0	0
Kalinga	19,143	0	0	109	109	0	0	0	0
Mt. Province	13,215	0	0	27	27	0	0	0	0
Baguio City	22,576	0	0	50	50	0	0	51	51
**Region 1**	**477,257**	**0**	**2**	**439**	**441**	**0**	**0**	**1**	**1**
Ilocos Norte	41,393	0	0	0	0	0	0	0	0
Ilocos Sur	62,434	0	0	248	248	0	0	0	0
La Union	59,808	0	0	23	23	0	0	0	0
Pangasinan	238,991	0	0	124	124	0	0	1	1
Alaminos City	10,832	0	0	3	3	0	0	0	0
Candon City	7,006	0	0	10	10	0	0	0	0
Dagupan City	6,976	0	0	7	7	0	0	0	0
Laoag City	8,253	0	0	0	0	0	0	0	0
San Carlos City	11,178	0	0	0	0	0	0	0	0
San Fernando City	13,027	0	2	24	26	0	0	0	0
Urdaneta City	12,909	0	0	0	0	0	0	0	0
Vigan City	4,450	0	0	0	0	0	0	0	0
**Region 2**	**318,442**	**0**	**4**	**374**	**378**	**0**	**0**	**0**	**0**
Batanes	960	0	0	0	0	0	0	0	0
Cagayan	88,783	0	0	77	77	0	0	0	0
Isabela	109,565	0	4	106	110	0	0	0	0
Nueva Vizcaya	46,874	0	0	149	149	0	0	0	0
Quirino	19,341	0	0	4	4	0	0	0	0
Cauayan City	10,017	0	0	15	15	0	0	0	0
Ilagan City	15,480	0	0	19	19	0	0	0	0
Santiago City	12,758	0	0	0	0	0	0	0	0
Tuguegarao City	14,664	0	0	4	4	0	0	0	0

Table 2.A.1. MODERN METHOD OF FAMILY PLANNING
New Acceptors
Philippines, 2022

Area	Total Current Users	FSTR/BTL Age group 10-14	15-19	20-49	Total	MSTR/NSV Age group 10-14	15-19	20-49	Total
**Region 3**	**831,669**	**0**	**13**	**5,987**	**6,000**	**0**	**1**	**16**	**17**
Aurora	22,728	0	0	7	7	0	0	0	0
Bataan	53,599	0	0	42	42	0	0	5	5
Bulacan	156,537	0	0	2,573	2,573	0	0	1	1
Nueva Ecija	135,568	0	2	307	309	0	0	0	0
Pampanga	114,189	0	11	1,963	1,974	0	1	4	5
Tarlac	88,617	0	0	66	66	0	0	2	2
Zambales	43,500	0	0	427	427	0	0	1	1
Angeles City	24,153	0	0	115	115	0	0	0	0
Balanga City	4,623	0	0	3	3	0	0	0	0
Cabanatuan City	20,017	0	0	25	25	0	0	0	0
City of San Fernando	11,292	0	0	100	100	0	0	1	1
Gapan City	7,573	0	0	5	5	0	0	0	0
Mabalacat City	21,462	0	0	107	107	0	0	1	1
Malolos City	7,988	0	0	0	0	0	0	0	0
Meycauayan	8,375	0	0	7	7	0	0	0	0
Olongapo	10,879	0	0	94	94	0	0	0	0
Palayan City	2,864	0	0	0	0	0	0	0	0
San Jose City	12,941	0	0	5	5	0	0	0	0
San Jose del Monte Cit	56,123	0	0	63	63	0	0	1	1
Science City of Munoz	6,280	0	0	0	0	0	0	0	0
Tarlac City	22,361	0	0	78	78	0	0	0	0
**Region 4A**	**866,447**	**0**	**20**	**1,856**	**1,876**	**0**	**0**	**18**	**18**
Batangas	129,569	0	0	127	127	0	0	0	0
Cavite	83,857	0	15	297	312	0	0	0	0
Laguna	74,923	0	0	57	57	0	0	3	3
Quezon	97,255	0	0	135	135	0	0	5	5
Rizal	111,121	0	0	242	242	0	0	5	5
Antipolo City	32,668	0	0	296	296	0	0	0	0
Bacoor City	10,282	0	0	61	61	0	0	3	3
Batangas City	17,852	0	0	0	0	0	0	0	0
Biñan City	84,449	0	0	104	104	0	0	1	1
Cabuyao City	18,581	0	0	20	20	0	0	0	0
Calamba City	30,526	0	0	294	294	0	0	1	1
Cavite City	4,111	0	0	6	6	0	0	0	0
Dasmariñas City	40,305	0	0	21	21	0	0	0	0
General Trias City	7,331	0	0	3	3	0	0	0	0
Imus City	8,271	0	0	38	38	0	0	0	0
Lipa City	8,190	0	0	21	21	0	0	0	0
Lucena City	6,718	0	0	4	4	0	0	0	0
San Pablo City	12,520	0	0	21	21	0	0	0	0
San Pedro City	17,276	0	1	58	59	0	0	0	0
Santa Rosa City	47,684	0	4	44	48	0	0	0	0
Tagaytay City	8,795	0	0	4	4	0	0	0	0
Tanauan City	6,741	0	0	3	3	0	0	0	0
Tayabas City	4,078	0	0	0	0	0	0	0	0
Trece Martires City	3,344	0	0	0	0	0	0	0	0
**Region 4B**	**271,491**	**0**	**20**	**656**	**676**	**0**	**0**	**2**	**2**
Marinduque	14,336	0	0	85	85	0	0	0	0
Mindoro Occidental	47,186	0	0	16	16	0	0	0	0
Mindoro Oriental	65,781	0	9	357	366	0	0	2	2
Palawan	88,744	0	11	129	140	0	0	0	0
Romblon	22,330	0	0	50	50	0	0	0	0
Puerto Princesa City	33,114	0	0	19	19	0	0	0	0
**Region 5**	**501,196**	**0**	**3**	**457**	**460**	**0**	**0**	**8**	**8**
Albay	97,609	0	2	43	45	0	0	2	2
Camarines Norte	44,713	0	0	63	63	0	0	0	0

Table 2.A.1. MODERN METHOD OF FAMILY PLANNING
New Acceptors
Philippines, 2022

Area	Total Current Users	FSTR/BTL Age group 10-14	15-19	20-49	Total	MSTR/NSV Age group 10-14	15-19	20-49	Total
Camarines Sur	106,529	0	0	0	0	0	0	0	0
Catanduanes	24,595	0	1	123	124	0	0	0	0
Masbate	87,357	0	0	11	11	0	0	6	6
Sorsogon	76,074	0	0	147	147	0	0	0	0
Iriga City	12,491	0	0	7	7	0	0	0	0
Legaspi City	23,050	0	0	25	25	0	0	0	0
Naga City	28,778	0	0	38	38	0	0	0	0
**Region 6**	**612,103**	**0**	**4**	**1,307**	**1,311**	**0**	**5**	**131**	**136**
Aklan	44,365	0	0	133	133	0	0	0	0
Antique	47,618	0	0	202	202	0	0	1	1
Capiz	76,500	0	0	24	24	0	0	7	7
Guimaras	16,308	0	0	14	14	0	0	0	0
Iloilo	157,750	0	0	34	34	0	0	1	1
Negros Occidental	193,157	0	4	598	602	0	5	122	127
Bacolod City	25,916	0	0	294	294	0	0	0	0
Iloilo City	50,489	0	0	8	8	0	0	0	0
**Region 7**	**547,072**	**0**	**0**	**616**	**616**	**0**	**0**	**13**	**13**
Bohol	81,628	0	0	103	103	0	0	0	0
Cebu	274,919	0	0	187	187	0	0	9	9
Negros Oriental	90,879	0	0	171	171	0	0	1	1
Siquijor	8,892	0	0	45	45	0	0	0	0
Cebu City	32,674	0	0	88	88	0	0	3	3
Lapu-Lapu City	35,065	0	0	17	17	0	0	0	0
Mandaue City	23,015	0	0	5	5	0	0	0	0
**Region 8**	**349,783**	**0**	**0**	**365**	**365**	**0**	**0**	**6**	**6**
Biliran	11,512	0	0	18	18	0	0	0	0
Eastern Samar	41,802	0	0	46	46	0	0	0	0
Northern Leyte	118,358	0	0	118	118	0	0	1	1
Northern Samar	48,444	0	0	10	10	0	0	1	1
Southern Leyte	26,721	0	0	49	49	0	0	4	4
Western Samar	24,584	0	0	50	50	0	0	0	0
Calbayog City	8,315	0	0	7	7	0	0	0	0
Maasin City	5,078	0	0	41	41	0	0	0	0
Ormoc City	20,494	0	0	0	0	0	0	0	0
Tacloban City	44,475	0	0	26	26	0	0	0	0
**Region 9**	**325,087**	**0**	**0**	**339**	**339**	**0**	**0**	**0**	**0**
Zamboanga del Norte	93,748	0	0	0	0	0	0	0	0
Zamboanga del Sur	73,802	0	0	0	0	0	0	0	0
Zamboanga Sibugay	42,410	0	0	2	2	0	0	0	0
Dapitan City	7,472	0	0	0	0	0	0	0	0
Dipolog City	17,962	0	0	0	0	0	0	0	0
Isabela City	8,228	0	0	0	0	0	0	0	0
Pagadian City	18,316	0	0	0	0	0	0	0	0
Zamboanga City	63,149	0	0	337	337	0	0	0	0
**Region 10**	**489,677**	**0**	**7**	**459**	**466**	**0**	**2**	**7**	**9**
Bukidnon	114,313	0	3	157	160	0	1	2	3
Camiguin	6,418	0	1	12	13	0	0	2	2
Lanao del Norte	63,113	0	3	89	92	0	1	3	4
Misamis Occidental	27,464	0	0	26	26	0	0	0	0
Misamis Oriental	75,691	0	0	50	50	0	0	0	0
Cagayan de Oro City	61,925	0	0	41	41	0	0	0	0

Table 2.A.1. MODERN METHOD OF FAMILY PLANNING
New Acceptors
Philippines, 2022

Area	Total Current Users	FSTR/BTL Age group			Total	MSTR/NSV Age group			Total
		10-14	15-19	20-49		10-14	15-19	20-49	
El Salvador City	6,345	0	0	9	9	0	0	0	0
Gingoog City	28,686	0	0	0	0	0	0	0	0
Iligan City	28,662	0	0	46	46	0	0	0	0
Malaybalay City	18,648	0	0	6	6	0	0	0	0
Oroquieta City	6,937	0	0	2	2	0	0	0	0
Ozamis City	25,480	0	0	4	4	0	0	0	0
Tangub City	4,130	0	0	11	11	0	0	0	0
Valencia City	21,865	0	0	6	6	0	0	0	0
**Region 11**	**528,307**	**3**	**39**	**1,182**	**1,224**	**0**	**0**	**43**	**43**
Davao de Oro	85,230	0	33	89	122	0	0	1	1
Davao del Norte	103,058	0	0	390	390	0	0	16	16
Davao Oriental	76,678	0	3	104	107	0	0	0	0
Davao del Sur	31,942	0	0	19	19	0	0	1	1
Davao Occidental	53,567	0	0	55	55	0	0	0	0
Davao City	177,832	3	3	525	531	0	0	25	25
**Region 12**	**435,275**	**0**	**5**	**520**	**525**	**0**	**1**	**11**	**12**
North Cotabato	137,970	0	1	320	321	0	0	10	10
Sarangani	65,032	0	4	50	54	0	1	0	1
South Cotabato	92,506	0	0	101	101	0	0	1	1
Sultan Kudarat	95,199	0	0	38	38	0	0	0	0
					0				0
Gen. Santos City	44,568	0	0	11	11	0	0	0	0
**BARMM**	**295,125**	**0**	**0**	**120**	**120**	**0**	**0**	**0**	**0**
Basilan	10,783	0	0	2	2	0	0	0	0
Lanao del Sur	30,652	0	0	1	1	0	0	0	0
Maguindanao	91,298	0	0	73	73	0	0	0	0
Sulu	92,228	0	0	28	28	0	0	0	0
Tawi-Tawi	28,444	0	0	5	5	0	0	0	0
					0				0
Lamitan City	5,882	0	0	0	0	0	0	0	0
Marawi City	5,206	0	0	3	3	0	0	0	0
Cotabato City	30,632	0	0	8	8	0	0	0	0
**CARAGA**	**268,898**	**0**	**6**	**533**	**539**	**0**	**0**	**7**	**7**
Agusan del Norte	39,895	0	3	480	483	0	0	0	0
Agusan del Sur	83,282	0	0	4	4	0	0	0	0
Surigao del Norte	33,804	0	0	8	8	0	0	6	6
Surigao del Sur	42,911	0	0	0	0	0	0	0	0
Province of Dinagat	10,370	0	1	1	2	0	0	1	1
Bislig City	9,590	0	0	0	0	0	0	0	0
Butuan City	34,962	0	2	40	42	0	0	0	0
Surigao City	14,084	0	0	0	0	0	0	0	0

Table 2.A.1. MODERN METHOD OF FAMILY PLANNING
New Acceptors
Philippines, 2022

Area	CONDOM			Total	IUD-INTERVAL			Total	IUD-POSTPARTUM			Total
	Age group				Age group				Age group			
	10-14	15-19	20-49		10-14	15-19	20-49		10-14	15-19	20-49	
**PHILIPPINES**	56	4,805	43,327	48,188	16	940	10,101	11,057	110	2,698	13,893	16,701
**N C R**	3	810	13,275	14,088	0	101	1,036	1,137	48	863	4,010	4,921
Malabon	0	34	295	329	0	0	7	7	0	2	3	5
Navotas	0	88	219	307	0	0	2	2	0	1	0	1
Valenzuela City	0	6	347	353	0	0	1	1	0	0	8	8
Caloocan City	0	52	606	658	0	4	18	22	13	137	283	433
Marikina City	0	2	27	29	0	0	4	4	2	100	527	629
Pasig City	0	11	504	515	0	30	330	360	3	183	793	979
Pateros	0	0	30	30	0	0	16	16	0	0	2	2
Taguig	2	94	2,150	2,246	0	6	63	69	0	4	40	44
Quezon City	0	166	2,392	2,558	0	19	42	61	23	185	883	1,091
Makati City	0	11	693	704	0	1	127	128	0	41	563	604
Mandaluyong City	0	39	582	621	0	5	41	46	4	154	458	616
San Juan	0	1	9	10	0	0	36	36	0	0	0	0
Manila City	1	114	3,152	3,267	0	31	154	185	2	24	242	268
Las Piñas City	0	25	531	556	0	2	23	25	1	26	77	104
Muntinlupa City	0	5	118	123	0	0	40	40	0	0	0	0
Parañaque City	0	64	1,207	1,271	0	3	27	30	0	6	13	19
Pasay City	0	98	413	511	0	0	105	105	0	0	118	118
**C A R**	0	76	267	343	1	34	38	73	0	5	14	19
Abra	0	66	119	185	0	0	0	0	0	1	1	2
Apayao	0	2	10	12	0	0	1	1	0	2	1	3
Benguet	0	2	44	46	0	3	17	20	0	0	2	2
Ifugao	0	0	3	3	0	0	1	1	0	0	0	0
Kalinga	0	0	5	5	0	0	10	10	0	0	2	2
Mt. Province	0	1	6	7	1	1	3	5	0	0	1	1
Baguio City	0	5	80	85	0	30	6	36	0	2	7	9
**Region 1**	1	52	637	690	2	38	214	254	0	7	47	54
Ilocos Norte	0	2	13	15	0	4	12	16	0	0	1	1
Ilocos Sur	0	14	102	116	0	1	33	34	0	0	13	13
La Union	0	0	8	8	0	8	17	25	0	1	4	5
Pangasinan	1	34	403	438	2	21	131	154	0	0	0	0
Alaminos City	0	0	2	2	0	0	0	0	0	0	0	0
Candon City	0	0	0	0	0	0	0	0	0	0	0	0
Dagupan City	0	0	33	33	0	1	1	2	0	2	6	8
Laoag City	0	0	0	0	0	0	0	0	0	0	0	0
San Carlos City	0	0	11	11	0	0	2	2	0	0	0	0
San Fernando City	0	2	62	64	0	3	18	21	0	4	23	27
Urdaneta City	0	0	3	3	0	0	0	0	0	0	0	0
Vigan City	0	0	0	0	0	0	0	0	0	0	0	0
**Region 2**	0	7	125	132	2	28	331	361	0	21	128	149
Batanes	0	0	0	0	0	0	0	0	0	0	0	0
Cagayan	0	0	26	26	2	18	107	127	0	4	33	37
Isabela	0	6	38	44	0	5	37	42	0	6	16	22
Nueva Vizcaya	0	0	49	49	0	2	83	85	0	2	42	44
Quirino	0	1	6	7	0	0	8	8	0	0	1	1
Cauayan City	0	0	1	1	0	1	24	25	0	0	0	0
Ilagan City	0	0	4	4	0	0	5	5	0	0	8	8
Santiago City	0	0	0	0	0	0	0	0	0	0	0	0
Tuguegarao City	0	0	1	1	0	2	67	69	0	9	28	37

Table 2.A.1. MODERN METHOD OF FAMILY PLANNING
New Acceptors
Philippines, 2022

Area	CONDOM			Total	IUD-INTERVAL			Total	IUD-POSTPARTUM			Total
	Age group				Age group				Age group			
	10-14	15-19	20-49		10-14	15-19	20-49		10-14	15-19	20-49	
**Region 3**	**6**	**587**	**5,975**	**6,568**	**0**	**27**	**496**	**523**	**8**	**199**	**2,034**	**2,241**
Aurora	0	2	5	7	0	0	0	0	0	0	2	2
Bataan	3	53	344	400	0	5	18	23	0	4	20	24
Bulacan	0	63	642	705	0	1	155	156	0	2	79	81
Nueva Ecija	0	35	221	256	0	5	59	64	8	174	600	782
Pampanga	0	33	818	851	0	2	82	84	0	11	1,276	1,287
Tarlac	0	68	797	865	0	3	48	51	0	0	1	1
Zambales	2	11	485	498	0	0	13	13	0	0	14	14
Angeles City	0	1	15	16	0	0	4	4	0	0	2	2
Balanga City	0	1	2	3	0	0	0	0	0	0	0	0
Cabanatuan City	0	7	57	64	0	1	12	13	0	0	0	0
City of San Fernando	0	138	773	911	0	0	7	7	0	0	6	6
Gapan City	0	0	23	23	0	0	1	1	0	1	2	3
Mabalacat City	1	18	286	305	0	5	13	18	0	5	9	14
Malolos City	0	0	32	32	0	0	0	0	0	0	0	0
Meycauayan	0	15	177	192	0	0	0	0	0	0	0	0
Olongapo	0	0	75	75	0	3	18	21	0	1	13	14
Palayan City	0	0	5	5	0	0	0	0	0	0	0	0
San Jose City	0	13	49	62	0	0	0	0	0	0	2	2
San Jose del Monte Cit	0	64	925	989	0	0	30	30	0	0	0	0
Science City of Munoz	0	0	3	3	0	0	0	0	0	0	0	0
Tarlac City	0	65	241	306	0	2	36	38	0	1	8	9
**Region 4A**	**4**	**1,946**	**8,098**	**10,048**	**0**	**54**	**887**	**941**	**10**	**71**	**695**	**776**
Batangas	0	33	111	144	0	0	85	85	0	5	27	32
Cavite	1	15	285	301	0	17	110	127	0	3	17	20
Laguna	0	4	56	60	0	8	48	56	0	13	23	36
Quezon	0	26	206	232	0	11	113	124	0	8	37	45
Rizal	2	25	263	290	0	5	86	91	1	24	75	100
Antipolo City	0	0	49	49	0	0	260	260	0	0	408	408
Bacoor City	0	2	54	56	0	2	13	15	0	2	10	12
Batangas City	0	7	84	91	0	0	3	3	0	0	6	6
Biñan City	0	1,574	5,295	6,869	0	0	39	39	0	2	11	13
Cabuyao City	0	54	277	331	0	2	42	44	0	0	4	4
Calamba City	0	10	425	435	0	0	17	17	0	1	23	24
Cavite City	0	0	6	6	0	0	2	2	0	1	3	4
Dasmariñas City	0	18	207	225	0	6	27	33	0	7	3	10
General Trias City	0	0	2	2	0	0	1	1	0	0	0	0
Imus City	0	1	40	41	0	0	12	12	0	0	0	0
Lipa City	0	0	10	10	0	0	1	1	0	0	0	0
Lucena City	1	5	15	21	0	0	0	0	0	0	0	0
San Pablo City	0	1	8	9	0	1	4	5	0	2	6	8
San Pedro City	0	17	125	142	0	2	7	9	0	0	1	1
Santa Rosa City	0	154	571	725	0	0	12	12	9	1	30	40
Tagaytay City	0	0	2	2	0	0	0	0	0	0	6	6
Tanauan City	0	0	3	3	0	0	1	1	0	2	5	7
Tayabas City	0	0	0	0	0	0	0	0	0	0	0	0
Trece Martires City	0	0	4	4	0	0	4	4	0	0	0	0
**Region 4B**	**2**	**27**	**237**	**266**	**0**	**11**	**98**	**109**	**0**	**41**	**277**	**318**
Marinduque	0	0	15	15	0	0	7	7	0	0	1	1
Mindoro Occidental	0	3	31	34	0	2	0	2	0	0	0	0
Mindoro Oriental	0	7	68	75	0	0	50	50	0	37	217	254
Palawan	2	11	63	76	0	8	31	39	0	1	40	41
Romblon	0	2	37	39	0	0	5	5	0	2	16	18
Puerto Princesa City	0	4	23	27	0	1	5	6	0	1	3	4
**Region 5**	**0**	**71**	**1,626**	**1,697**	**0**	**21**	**203**	**224**	**0**	**31**	**142**	**173**
Albay	0	15	335	350	0	0	6	6	0	0	25	25
Camarines Norte	0	16	176	192	0	16	125	141	0	14	61	75

Table 2.A.1. MODERN METHOD OF FAMILY PLANNING
New Acceptors
Philippines, 2022

Area	CONDOM			Total	IUD-INTERVAL			Total	IUD-POSTPARTUM			Total
	Age group				Age group				Age group			
	10-14	15-19	20-49		10-14	15-19	20-49		10-14	15-19	20-49	
Camarines Sur	0	16	325	341	0	1	9	10	0	5	18	23
Catanduanes	0	2	31	33	0	1	0	1	0	0	3	3
Masbate	0	9	145	154	0	1	26	27	0	1	10	11
Sorsogon	0	7	38	45	0	0	0	0	0	7	15	22
Iriga City	0	1	38	39	0	0	4	4	0	3	1	4
Legaspi City	0	2	19	21	0	0	2	2	0	0	2	2
Naga City	0	3	519	522	0	2	31	33	0	1	7	8
**Region 6**	**0**	**191**	**2,014**	**2,205**	**2**	**93**	**1,023**	**1,118**	**7**	**163**	**1,046**	**1,216**
Aklan	0	12	391	403	0	0	15	15	0	0	3	3
Antique	0	4	79	83	0	0	13	13	0	0	110	110
Capiz	0	3	77	80	0	4	60	64	0	0	25	25
Guimaras	0	1	4	5	0	0	4	4	0	0	8	8
Iloilo	0	80	959	1,039	0	14	465	479	0	25	301	326
Negros Occidental	0	87	406	493	0	53	296	349	2	75	223	300
Bacolod City	0	3	86	89	2	21	119	142	0	21	149	170
Iloilo City	0	1	12	13	0	1	51	52	5	42	227	274
**Region 7**	**1**	**126**	**2,124**	**2,251**	**2**	**183**	**1,905**	**2,090**	**12**	**487**	**1,924**	**2,423**
Bohol	0	10	224	234	0	35	352	387	1	145	879	1,025
Cebu	1	66	1,102	1,169	2	124	989	1,115	11	323	956	1,290
Negros Oriental	0	15	278	293	0	3	228	231	0	4	28	32
Siquijor	0	0	17	17	0	3	6	9	0	1	0	1
Cebu City	0	13	348	361	0	16	273	289	0	12	39	51
Lapu-Lapu City	0	20	83	103	0	2	10	12	0	0	4	4
Mandaue City	0	2	72	74	0	0	47	47	0	2	18	20
**Region 8**	**1**	**44**	**552**	**597**	**0**	**22**	**281**	**303**	**0**	**26**	**159**	**185**
Biliran	0	3	25	28	0	0	5	5	0	0	11	11
Eastern Samar	0	3	90	93	0	6	16	22	0	3	12	15
Northern Leyte	1	22	130	153	0	0	73	73	0	8	44	52
Northern Samar	0	2	57	59	0	9	10	19	0	1	6	7
Southern Leyte	0	0	45	45	0	0	5	5	0	0	1	1
Western Samar	0	2	23	25	0	4	51	55	0	1	21	22
Calbayog City	0	2	27	29	0	0	4	4	0	0	2	2
Maasin City	0	1	31	32	0	0	60	60	0	0	6	6
Ormoc City	0	0	6	6	0	0	0	0	0	1	8	9
Tacloban City	0	9	118	127	0	3	57	60	0	12	48	60
**Region 9**	**0**	**45**	**320**	**365**	**0**	**33**	**221**	**254**	**5**	**280**	**1,271**	**1,556**
Zamboanga del Norte	0	10	72	82	0	5	94	99	0	2	7	9
Zamboanga del Sur	0	3	22	25	0	10	15	25	0	20	35	55
Zamboanga Sibugay	0	17	75	92	0	13	53	66	0	7	16	23
Dapitan City	0	0	2	2	0	0	0	0	0	0	0	0
Dipolog City	0	0	0	0	0	0	0	0	0	0	0	0
Isabela City	0	1	6	7	0	0	0	0	0	0	0	0
Pagadian City	0	0	7	7	0	2	9	11	0	19	60	79
Zamboanga City	0	14	136	150	0	3	50	53	5	232	1,153	1,390
**Region 10**	**2**	**258**	**2,309**	**2,569**	**3**	**122**	**738**	**863**	**5**	**292**	**592**	**889**
Bukidnon	1	29	135	165	3	71	211	285	1	31	60	92
Camiguin	0	0	0	0	0	0	0	0	0	0	0	0
Lanao del Norte	0	51	1,252	1,303	0	12	104	116	0	14	137	151
Misamis Occidental	0	41	287	328	0	0	27	27	0	2	16	18
Misamis Oriental	0	7	95	102	0	6	79	85	0	15	60	75
Cagayan de Oro City	0	29	192	221	0	18	146	164	3	132	183	318

Table 2.A.1. MODERN METHOD OF FAMILY PLANNING
New Acceptors
Philippines, 2022

Area	CONDOM			Total	IUD-INTERVAL			Total	IUD-POSTPARTUM			Total
	Age group				Age group				Age group			
	10-14	15-19	20-49		10-14	15-19	20-49		10-14	15-19	20-49	
El Salvador City	0	0	1	1	0	1	13	14	0	11	11	22
Gingoog City	1	0	0	1	0	1	71	72	0	59	17	76
Iligan City	0	23	172	195	0	3	32	35	1	27	108	136
Malaybalay City	0	1	5	6	0	1	3	4	0	0	0	0
Oroquieta City	0	2	1	3	0	0	2	2	0	0	0	0
Ozamis City	0	26	68	94	0	0	0	0	0	0	0	0
Tangub City	0	15	39	54	0	0	0	0	0	0	0	0
Valencia City	0	34	62	96	0	9	50	59	0	1	0	1
**Region 11**	**32**	**305**	**2,137**	**2,474**	**1**	**82**	**868**	**951**	**10**	**67**	**624**	**701**
Davao de Oro	2	7	80	89	0	17	74	91	3	2	8	13
Davao del Norte	3	30	316	349	1	12	236	249	1	17	110	128
Davao Oriental	0	10	107	117	0	19	99	118	2	9	40	51
Davao del Sur	0	11	24	35	0	5	13	18	1	3	4	8
Davao Occidental	0	0	22	22	0	1	5	6	0	1	3	4
Davao City	27	247	1,588	1,862	0	28	441	469	3	35	459	497
**Region 12**	**2**	**101**	**1,445**	**1,548**	**2**	**50**	**1,401**	**1,453**	**2**	**54**	**473**	**529**
North Cotabato	0	49	885	934	1	16	1,149	1,166	0	7	331	338
Sarangani	0	19	73	92	0	20	104	124	0	2	7	9
South Cotabato	2	14	212	228	0	8	55	63	0	6	42	48
Sultan Kudarat	0	11	108	119	0	1	39	40	1	16	30	47
				0				0				0
Gen. Santos City	0	8	167	175	1	5	54	60	1	23	63	87
**BARMM**	**1**	**95**	**1,703**	**1,799**	**0**	**7**	**80**	**87**	**0**	**13**	**133**	**146**
Basilan	0	8	42	50	0	0	0	0	0	0	0	0
Lanao del Sur	1	21	639	661	0	1	16	17	0	3	4	7
Maguindanao	0	45	584	629	0	1	11	12	0	2	28	30
Sulu	0	12	92	104	0	0	4	4	0	0	11	11
Tawi-Tawi	0	0	16	16	0	0	0	0	0	0	0	0
				0				0				0
Lamitan City	0	1	2	3	0	5	24	29	0	3	31	34
Marawi City	0	1	114	115	0	0	0	0	0	0	1	1
Cotabato City	0	7	214	221	0	0	25	25	0	5	58	63
**CARAGA**	**1**	**64**	**483**	**548**	**1**	**34**	**281**	**316**	**3**	**78**	**324**	**405**
Agusan del Norte	0	15	119	134	0	6	56	62	1	19	89	109
Agusan del Sur	0	15	89	104	1	14	72	87	0	17	55	72
Surigao del Norte	0	6	106	112	0	1	53	54	0	8	56	64
Surigao del Sur	1	14	63	78	0	8	37	45	2	21	51	74
Province of Dinagat	0	5	28	33	0	2	21	23	0	1	6	7
Bislig City	0	4	21	25	0	2	0	2	0	1	3	4
Butuan City	0	4	52	56	0	0	7	7	0	11	54	65
Surigao City	0	1	5	6	0	1	35	36	0	0	10	10

Table 2.A.1. MODERN METHOD OF FAMILY PLANNING
New Acceptors
Philippines, 2022

Area	PILLS-POP			Total	PILLS-COC			Total	INJECTABLES			Total
	Age group				Age group				Age group			
	10-14	15-19	20-49		10-14	15-19	20-49		10-14	15-19	20-49	
**PHILIPPINES**	**231**	**11,582**	**75,796**	**87,609**	**180**	**10,909**	**120,517**	**131,606**	**271**	**19,671**	**155,722**	**175,664**
**N C R**	**34**	**1,448**	**13,199**	**14,681**	**23**	**1,081**	**18,693**	**19,797**	**70**	**3,255**	**30,055**	**33,380**
Malabon	1	40	312	353	1	29	345	375	1	185	773	959
Navotas	0	69	179	248	0	120	397	517	1	238	842	1,081
Valenzuela City	0	75	894	969	0	67	2,657	2,724	1	216	2,785	3,002
Caloocan City	0	170	873	1,043	1	148	1,057	1,206	11	507	2,831	3,349
Marikina City	0	11	204	215	0	8	276	284	1	128	1,152	1,281
Pasig City	0	39	753	792	1	77	2,127	2,205	3	241	2,506	2,750
Pateros	0	2	22	24	0	8	118	126	0	1	166	167
Taguig	2	108	1,304	1,414	5	65	1,632	1,702	2	262	3,125	3,389
Quezon City	2	255	2,402	2,659	0	136	2,886	3,022	3	417	3,181	3,601
Makati City	0	22	710	732	0	7	732	739	0	34	1,566	1,600
Mandaluyong City	0	56	293	349	0	97	512	609	0	78	797	875
San Juan	0	1	49	50	0	1	68	69	0	5	198	203
Manila City	28	363	2,840	3,231	15	131	2,646	2,792	47	463	4,493	5,003
Las Piñas City	0	50	916	966	0	53	1,076	1,129	0	172	2,085	2,257
Muntinlupa City	0	53	295	348	0	54	387	441	0	96	1,438	1,534
Parañaque City	1	84	462	547	0	77	729	806	0	212	1,297	1,509
Pasay City	0	50	691	741	0	3	1,048	1,051	0	0	820	820
**C A R**	**2**	**126**	**769**	**897**	**0**	**58**	**636**	**694**	**2**	**163**	**1,242**	**1,407**
Abra	0	16	83	99	0	2	146	148	0	11	81	92
Apayao	2	31	58	91	0	9	62	71	0	19	69	88
Benguet	0	26	185	211	0	27	214	241	0	27	290	317
Ifugao	0	2	8	10	0	1	16	17	0	2	12	14
Kalinga	0	9	39	48	0	6	57	63	2	6	62	70
Mt. Province	0	7	26	33	0	1	16	17	0	3	50	53
Baguio City	0	35	370	405	0	12	125	137	0	95	678	773
**Region 1**	**0**	**66**	**1,071**	**1,137**	**2**	**355**	**5,641**	**5,998**	**2**	**315**	**3,824**	**4,141**
Ilocos Norte	0	10	210	220	0	6	912	918	0	8	283	291
Ilocos Sur	0	35	532	567	0	60	1,314	1,374	2	51	636	689
La Union	0	7	175	182	0	11	146	157	0	38	330	368
Pangasinan	0	0	0	0	2	235	2,662	2,899	0	172	1,947	2,119
Alaminos City	0	0	0	0	0	10	35	45	0	5	43	48
Candon City	0	0	17	17	0	0	38	38	0	0	37	37
Dagupan City	0	4	16	20	0	15	96	111	0	18	235	253
Laoag City	0	0	27	27	0	0	10	10	0	0	5	5
San Carlos City	0	0	0	0	0	9	232	241	0	7	171	178
San Fernando City	0	10	94	104	0	4	140	144	0	12	90	102
Urdaneta City	0	0	0	0	0	5	49	54	0	4	41	45
Vigan City	0	0	0	0	0	0	7	7	0	0	6	6
**Region 2**	**3**	**270**	**1,325**	**1,598**	**0**	**218**	**2,004**	**2,222**	**7**	**270**	**1,439**	**1,716**
Batanes	0	0	0	0	0	0	1	1	0	0	0	0
Cagayan	2	78	417	497	0	59	830	889	2	64	442	508
Isabela	0	99	385	484	0	93	609	702	2	137	488	627
Nueva Vizcaya	1	23	80	104	0	22	219	241	3	31	224	258
Quirino	0	9	35	44	0	10	27	37	0	3	40	43
Cauayan City	0	6	135	141	0	19	153	172	0	12	37	49
Ilagan City	0	32	87	119	0	7	38	45	0	14	58	72
Santiago City	0	0	0	0	0	2	7	9	0	0	9	9
Tuguegarao City	0	23	186	209	0	6	120	126	0	9	141	150

Table 2.A.1. MODERN METHOD OF FAMILY PLANNING
New Acceptors
Philippines, 2022

Area	PILLS-POP			Total	PILLS-COC			Total	INJECTABLES			Total
	Age group				Age group				Age group			
	10-14	15-19	20-49		10-14	15-19	20-49		10-14	15-19	20-49	
**Region 3**	**13**	**1,220**	**6,745**	**7,978**	**8**	**1,654**	**19,479**	**21,141**	**24**	**3,077**	**25,539**	**28,640**
Aurora	0	2	39	41	0	3	23	26	0	5	35	40
Bataan	6	274	821	1,101	1	71	932	1,004	2	270	2,019	2,291
Bulacan	0	182	977	1,159	1	351	5,380	5,732	2	755	6,042	6,799
Nueva Ecija	0	97	556	653	3	172	1,097	1,272	2	283	1,733	2,018
Pampanga	2	99	904	1,005	0	147	2,053	2,200	2	259	2,930	3,191
Tarlac	2	54	391	447	1	213	2,499	2,713	1	185	2,108	2,294
Zambales	1	32	278	311	0	32	941	973	1	208	1,297	1,506
Angeles City	0	30	165	195	1	24	554	579	1	63	1,170	1,234
Balanga City	0	0	16	16	0	13	54	67	1	27	117	145
Cabanatuan City	1	57	218	276	1	75	484	560	4	105	588	697
City of San Fernando	0	84	325	409	0	32	436	468	3	73	614	690
Gapan City	0	8	56	64	0	13	160	173	1	37	226	264
Mabalacat City	0	9	116	125	0	42	489	531	0	151	1,108	1,259
Malolos City	0	2	27	29	0	12	121	133	0	10	236	246
Meycauayan	0	38	255	293	0	39	332	371	0	44	330	374
Olongapo	0	17	191	208	0	42	516	558	2	126	916	1,044
Palayan City	0	2	19	21	0	25	106	131	0	10	47	57
San Jose City	0	24	62	86	0	31	144	175	0	66	296	362
San Jose del Monte Cit	1	65	756	822	0	129	1,555	1,684	2	233	2,224	2,459
Science City of Munoz	0	3	15	18	0	6	37	43	0	13	100	113
Tarlac City	0	141	558	699	0	182	1,566	1,748	0	154	1,403	1,557
**Region 4A**	**20**	**2,201**	**12,512**	**14,733**	**21**	**2,201**	**17,116**	**19,338**	**42**	**3,380**	**25,449**	**28,871**
Batangas	2	155	1,247	1,404	0	69	1,054	1,123	17	132	1,520	1,669
Cavite	0	48	374	422	1	66	1,166	1,233	6	219	2,489	2,714
Laguna	0	82	398	480	0	44	459	503	0	126	995	1,121
Quezon	4	120	542	666	12	98	1,143	1,253	11	410	2,035	2,456
Rizal	2	135	674	811	7	112	1,208	1,327	3	449	3,139	3,591
Antipolo City	0	0	638	638	0	0	159	159	0	0	1,488	1,488
Bacoor City	0	8	89	97	0	17	196	213	0	99	967	1,066
Batangas City	0	20	98	118	0	26	416	442	0	57	424	481
Biñan City	0	1,228	5,254	6,482	0	1,403	6,541	7,944	0	1,186	6,165	7,351
Cabuyao City	0	71	482	553	0	77	765	842	0	103	609	712
Calamba City	0	75	669	744	0	53	1,091	1,144	0	38	1,080	1,118
Cavite City	0	0	11	11	0	5	38	43	0	30	86	116
Dasmariñas City	0	39	259	298	0	48	1,043	1,091	0	80	1,284	1,364
General Trias City	0	22	65	87	0	8	99	107	1	49	287	337
Imus City	0	6	107	113	0	14	158	172	0	44	288	332
Lipa City	0	9	49	58	0	10	90	100	0	11	176	187
Lucena City	1	9	40	50	0	10	103	113	1	40	383	424
San Pablo City	0	7	54	61	0	15	135	150	2	36	259	297
San Pedro City	0	76	408	484	0	27	432	459	0	43	347	390
Santa Rosa City	11	85	985	1,081	1	82	656	739	1	206	1,132	1,339
Tagaytay City	0	0	15	15	0	0	27	27	0	0	28	28
Tanauan City	0	2	35	37	0	5	19	24	0	9	111	120
Tayabas City	0	0	0	0	0	0	0	0	0	0	0	0
Trece Martires City	0	4	19	23	0	12	118	130	0	13	157	170
**Region 4B**	**43**	**321**	**973**	**1,337**	**4**	**188**	**1,586**	**1,778**	**0**	**778**	**2,949**	**3,727**
Marinduque	0	17	121	138	0	0	145	145	0	24	198	222
Mindoro Occidental	0	10	48	58	0	16	98	114	0	19	83	102
Mindoro Oriental	0	53	200	253	1	33	567	601	0	76	652	728
Palawan	41	217	450	708	3	130	475	608	0	530	1,482	2,012
Romblon	0	6	65	71	0	2	222	224	0	34	187	221
Puerto Princesa City	2	18	89	109	0	7	79	86	0	95	347	442
**Region 5**	**1**	**245**	**2,088**	**2,334**	**0**	**278**	**4,709**	**4,987**	**0**	**344**	**4,084**	**4,428**
Albay	0	37	438	475	0	51	810	861	0	72	1,231	1,303
Camarines Norte	0	70	256	326	0	40	694	734	0	68	428	496

Table 2.A.1. MODERN METHOD OF FAMILY PLANNING
New Acceptors
Philippines, 2022

Area	PILLS-POP			Total	PILLS-COC			Total	INJECTABLES			Total
	Age group				Age group				Age group			
	10-14	15-19	20-49		10-14	15-19	20-49		10-14	15-19	20-49	
Camarines Sur	0	66	706	772	0	74	1,146	1,220	0	89	1,113	1,202
Catanduanes	0	0	10	10	0	1	21	22	0	9	43	52
Masbate	1	47	411	459	0	68	531	599	0	56	494	550
Sorsogon	0	7	43	50	0	15	136	151	0	23	161	184
Iriga City	0	6	81	87	0	7	131	138	0	4	83	87
Legaspi City	0	7	74	81	0	15	86	101	0	5	79	84
Naga City	0	5	69	74	0	7	1,154	1,161	0	18	452	470
**Region 6**	**6**	**830**	**4,683**	**5,519**	**6**	**639**	**7,170**	**7,815**	**11**	**948**	**8,879**	**9,838**
Aklan	2	38	568	608	0	38	546	584	0	93	862	955
Antique	0	39	292	331	0	26	594	620	0	96	858	954
Capiz	1	48	303	352	1	24	545	570	0	75	774	849
Guimaras	0	10	51	61	0	5	115	120	0	4	61	65
Iloilo	1	122	1,450	1,573	4	118	1,583	1,705	2	224	2,915	3,141
Negros Occidental	2	552	1,861	2,415	1	385	3,088	3,474	2	388	2,578	2,968
Bacolod City	0	20	142	162	0	33	410	443	7	63	652	722
Iloilo City	0	1	16	17	0	10	289	299	0	5	179	184
**Region 7**	**8**	**782**	**5,549**	**6,339**	**3**	**564**	**8,627**	**9,194**	**13**	**1,563**	**13,546**	**15,122**
Bohol	0	98	700	798	0	55	897	952	1	159	1,591	1,751
Cebu	6	415	2,638	3,059	2	285	5,031	5,318	5	686	6,456	7,147
Negros Oriental	1	109	563	673	1	120	891	1,012	5	304	1,760	2,069
Siquijor	0	6	48	54	0	1	24	25	0	13	78	91
Cebu City	0	113	1,145	1,258	0	87	1,545	1,632	2	331	2,753	3,086
Lapu-Lapu City	0	24	145	169	0	8	81	89	0	27	259	286
Mandaue City	1	17	310	328	0	8	158	166	0	43	649	692
**Region 8**	**1**	**156**	**862**	**1,019**	**1**	**166**	**2,595**	**2,762**	**5**	**307**	**2,932**	**3,244**
Biliran	0	11	45	56	0	11	187	198	0	13	86	99
Eastern Samar	0	42	110	152	1	37	351	389	3	83	536	622
Northern Leyte	1	26	314	341	0	44	829	873	1	81	740	822
Northern Samar	0	22	66	88	0	21	310	331	0	42	350	392
Southern Leyte	0	1	32	33	0	11	259	270	1	10	368	379
Western Samar	0	10	46	56	0	5	149	154	0	20	152	172
Calbayog City	0	6	64	70	0	18	157	175	0	33	334	367
Maasin City	0	3	35	38	0	0	100	100	0	6	99	105
Ormoc City	0	16	50	66	0	6	41	47	0	10	47	57
Tacloban City	0	19	100	119	0	13	212	225	0	9	220	229
**Region 9**	**1**	**217**	**879**	**1,097**	**1**	**234**	**1,730**	**1,965**	**6**	**492**	**3,058**	**3,556**
Zamboanga del Norte	0	20	137	157	0	28	175	203	1	63	404	468
Zamboanga del Sur	0	44	120	164	1	24	205	230	1	55	160	216
Zamboanga Sibugay	0	49	154	203	0	39	198	237	0	110	496	606
Dapitan City	0	2	0	2	0	7	7	14	0	3	22	25
Dipolog City	0	0	0	0	0	0	0	0	0	0	0	0
Isabela City	0	12	38	50	0	16	184	200	0	25	316	341
Pagadian City	0	2	72	74	0	4	75	79	0	7	105	112
Zamboanga City	1	88	358	447	0	116	886	1,002	4	229	1,555	1,788
**Region 10**	**9**	**561**	**9,309**	**9,879**	**2**	**700**	**4,456**	**5,158**	**5**	**512**	**6,416**	**6,933**
Bukidnon	2	163	316	481	2	98	937	1,037	3	137	553	693
Camiguin	0	0	0	0	0	0	4	4	0	1	7	8
Lanao del Norte	4	125	7,536	7,665	0	285	1,688	1,973	0	74	3,821	3,895
Misamis Occidental	0	26	289	315	0	55	317	372	0	21	326	347
Misamis Oriental	0	43	233	276	0	33	364	397	0	46	339	385
Cagayan de Oro City	0	36	250	286	0	31	377	408	2	79	634	715

Table 2.A.1. MODERN METHOD OF FAMILY PLANNING
New Acceptors
Philippines, 2022

Area	PILLS-POP			Total	PILLS-COC			Total	INJECTABLES			Total
	Age group				Age group				Age group			
	10-14	15-19	20-49		10-14	15-19	20-49		10-14	15-19	20-49	
El Salvador City	0	5	18	23	0	0	21	21	0	8	52	60
Gingoog City	0	2	27	29	0	3	13	16	0	4	21	25
Iligan City	3	81	438	522	0	30	300	330	0	63	425	488
Malaybalay City	0	16	32	48	0	6	26	32	0	13	29	42
Oroquieta City	0	1	8	9	0	0	7	7	0	1	12	13
Ozamis City	0	1	9	10	0	15	39	54	0	14	53	67
Tangub City	0	10	28	38	0	25	34	59	0	0	11	11
Valencia City	0	52	125	177	0	119	329	448	0	51	133	184
**Region 11**	**64**	**1,231**	**7,138**	**8,433**	**69**	**974**	**8,409**	**9,452**	**49**	**1,442**	**6,620**	**8,111**
Davao de Oro	16	192	677	885	2	71	570	643	9	170	633	812
Davao del Norte	4	202	962	1,168	1	123	1,366	1,490	8	242	1,155	1,405
Davao Oriental	8	250	1,740	1,998	47	184	2,555	2,786	4	323	767	1,094
Davao del Sur	1	31	76	108	1	52	300	353	11	174	386	571
Davao Occidental	4	62	288	354	1	26	374	401	5	50	291	346
Davao City	31	494	3,395	3,920	17	518	3,244	3,779	12	483	3,388	3,883
**Region 12**	**17**	**1,046**	**4,467**	**5,530**	**28**	**812**	**8,671**	**9,511**	**17**	**1,434**	**8,621**	**10,072**
North Cotabato	6	474	2,662	3,142	18	327	5,113	5,458	2	523	4,413	4,938
Sarangani	0	169	338	507	0	168	1,288	1,456	0	229	913	1,142
South Cotabato	7	137	792	936	9	149	1,100	1,258	9	264	1,354	1,627
Sultan Kudarat	1	163	262	426	1	65	510	576	5	162	666	833
				0				0				0
Gen. Santos City	3	103	413	519	0	103	660	763	1	256	1,275	1,532
**BARMM**	**1**	**337**	**2,152**	**2,490**	**5**	**595**	**6,552**	**7,152**	**13**	**1,121**	**9,507**	**10,641**
Basilan	0	41	168	209	1	62	369	432	1	111	650	762
Lanao del Sur	0	39	501	540	0	19	752	771	0	58	926	984
Maguindanao	1	119	686	806	0	214	2,433	2,647	8	574	3,585	4,167
Sulu	0	116	343	459	4	195	1,142	1,341	1	125	1,576	1,702
Tawi-Tawi	0	2	45	47	0	40	437	477	0	65	585	650
				0				0				0
Lamitan City	0	7	25	32	0	21	115	136	3	44	273	320
Marawi City	0	1	112	113	0	1	127	128	0	0	131	131
Cotabato City	0	12	272	284	0	43	1,177	1,220	0	144	1,781	1,925
**CARAGA**	**8**	**525**	**2,075**	**2,608**	**7**	**192**	**2,443**	**2,642**	**5**	**270**	**1,562**	**1,837**
Agusan del Norte	2	138	571	711	1	33	403	437	4	88	387	479
Agusan del Sur	3	109	357	469	0	27	256	283	0	60	272	332
Surigao del Norte	0	48	324	372	0	24	586	610	1	31	271	303
Surigao del Sur	1	86	247	334	1	27	570	598	0	25	164	189
Province of Dinagat	2	16	118	136	0	11	142	153	0	17	109	126
Bislig City	0	12	30	42	0	8	15	23	0	3	6	9
Butuan City	0	103	349	452	5	53	354	412	0	29	192	221
Surigao City	0	13	79	92	0	9	117	126	0	17	161	178

Table 2.A.1. MODERN METHOD OF FAMILY PLANNING
New Acceptors
Philippines, 2022

Area	IMPLANTS Age group			Total	NFP-CCM Age group			Total	NFP-BBT Age group			Total
	10-14	15-19	20-49		10-14	15-19	20-49		10-14	15-19	20-49	
**PHILIPPINES**	**264**	**12,607**	**72,042**	**84,913**	**12**	**224**	**1,672**	**1,908**	**32**	**117**	**830**	**979**
**N C R**	**69**	**2,714**	**15,079**	**17,862**	**1**	**1**	**37**	**39**	**0**	**7**	**21**	**28**
Malabon	1	64	226	291	0	0	0	0	0	0	0	0
Navotas	1	65	260	326	0	0	0	0	0	0	0	0
Valenzuela City	0	89	770	859	0	0	0	0	0	0	0	0
Caloocan City	2	353	1,314	1,669	0	0	0	0	0	0	0	0
Marikina City	5	168	839	1,012	0	0	0	0	0	0	0	0
Pasig City	1	133	1,587	1,721	0	0	0	0	0	0	1	1
Pateros	0	0	33	33	0	0	0	0	0	0	0	0
Taguig	1	43	671	715	0	0	0	0	0	0	0	0
Quezon City	24	460	1,926	2,410	1	1	0	2	0	6	5	11
Makati City	0	10	498	508	0	0	0	0	0	0	0	0
Mandaluyong City	2	60	726	788	0	0	31	31	0	0	1	1
San Juan	0	4	88	92	0	0	0	0	0	0	0	0
Manila City	27	1,006	4,520	5,553	0	0	0	0	0	1	14	15
Las Piñas City	5	237	891	1,133	0	0	0	0	0	0	0	0
Muntinlupa City	0	9	310	319	0	0	0	0	0	0	0	0
Parañaque City	0	13	249	262	0	0	0	0	0	0	0	0
Pasay City	0	0	171	171	0	0	6	6	0	0	0	0
**C A R**	**2**	**186**	**941**	**1,129**	**0**	**0**	**8**	**8**	**0**	**0**	**7**	**7**
Abra	0	5	68	73	0	0	2	2	0	0	0	0
Apayao	0	13	31	44	0	0	0	0	0	0	0	0
Benguet	0	21	124	145	0	0	0	0	0	0	0	0
Ifugao	0	0	1	1	0	0	0	0	0	0	0	0
Kalinga	0	16	63	79	0	0	0	0	0	0	0	0
Mt. Province	2	13	41	56	0	0	5	5	0	0	0	0
Baguio City	0	118	613	731	0	0	1	1	0	0	7	7
**Region 1**	**2**	**155**	**1,516**	**1,673**	**0**	**1**	**58**	**59**	**0**	**10**	**177**	**187**
Ilocos Norte	1	7	142	150	0	0	3	3	0	0	0	0
Ilocos Sur	0	16	157	173	0	0	1	1	0	0	0	0
La Union	0	22	215	237	0	1	5	6	0	0	0	0
Pangasinan	1	64	788	853	0	0	9	9	0	6	97	103
Alaminos City	0	16	32	48	0	0	0	0	0	0	0	0
Candon City	0	0	5	5	0	0	0	0	0	0	0	0
Dagupan City	0	14	22	36	0	0	0	0	0	0	0	0
Laoag City	0	0	1	1	0	0	0	0	0	0	0	0
San Carlos City	0	1	33	34	0	0	0	0	0	0	0	0
San Fernando City	0	13	102	115	0	0	0	0	0	0	0	0
Urdaneta City	0	2	19	21	0	0	0	0	0	4	80	84
Vigan City	0	0	0	0	0	0	40	40	0	0	0	0
**Region 2**	**2**	**102**	**457**	**561**	**0**	**0**	**1**	**1**	**0**	**0**	**0**	**0**
Batanes	0	0	0	0	0	0	0	0	0	0	0	0
Cagayan	0	61	181	242	0	0	0	0	0	0	0	0
Isabela	2	27	139	168	0	0	1	1	0	0	0	0
Nueva Vizcaya	0	8	67	75	0	0	0	0	0	0	0	0
Quirino	0	1	10	11	0	0	0	0	0	0	0	0
Cauayan City	0	4	22	26	0	0	0	0	0	0	0	0
Ilagan City	0	1	7	8	0	0	0	0	0	0	0	0
Santiago City	0	0	1	1	0	0	0	0	0	0	0	0
Tuguegarao City	0	0	30	30	0	0	0	0	0	0	0	0

Table 2.A.1. MODERN METHOD OF FAMILY PLANNING
New Acceptors
Philippines, 2022

Area	IMPLANTS			Total	NFP-CCM			Total	NFP-BBT			Total
	Age group				Age group				Age group			
	10-14	15-19	20-49		10-14	15-19	20-49		10-14	15-19	20-49	
**Region 3**	**21**	**1,269**	**7,216**	**8,506**	**0**	**1**	**9**	**10**	**0**	**5**	**31**	**36**
Aurora	0	4	13	17	0	0	0	0	0	2	3	5
Bataan	2	350	443	795	0	0	0	0	0	2	7	9
Bulacan	0	95	1,509	1,604	0	0	0	0	0	0	0	0
Nueva Ecija	16	468	1,158	1,642	0	1	3	4	0	0	0	0
Pampanga	0	53	1,234	1,287	0	0	1	1	0	1	18	19
Tarlac	0	22	345	367	0	0	2	2	0	0	0	0
Zambales	2	42	437	481	0	0	0	0	0	0	0	0
Angeles City	1	22	368	391	0	0	1	1	0	0	0	0
Balanga City	0	15	28	43	0	0	0	0	0	0	0	0
Cabanatuan City	0	1	22	23	0	0	0	0	0	0	0	0
City of San Fernando	0	4	115	119	0	0	0	0	0	0	2	2
Gapan City	0	2	16	18	0	0	2	2	0	0	0	0
Mabalacat City	0	7	128	135	0	0	0	0	0	0	0	0
Malolos City	0	0	29	29	0	0	0	0	0	0	0	0
Meycauayan	0	12	28	40	0	0	0	0	0	0	0	0
Olongapo	0	78	311	389	0	0	0	0	0	0	1	1
Palayan City	0	0	0	0	0	0	0	0	0	0	0	0
San Jose City	0	4	18	22	0	0	0	0	0	0	0	0
San Jose del Monte Cit	0	74	825	899	0	0	0	0	0	0	0	0
Science City of Munoz	0	1	2	3	0	0	0	0	0	0	0	0
Tarlac City	0	15	187	202	0	0	0	0	0	0	0	0
**Region 4A**	**18**	**679**	**6,237**	**6,934**	**9**	**1**	**46**	**56**	**9**	**8**	**117**	**134**
Batangas	0	16	169	185	0	0	0	0	0	0	28	28
Cavite	2	101	917	1,020	0	0	42	42	0	0	1	1
Laguna	3	71	470	544	0	0	2	2	0	0	0	0
Quezon	0	38	366	404	0	0	0	0	0	3	6	9
Rizal	0	130	989	1,119	0	0	0	0	0	2	1	3
Antipolo City	0	0	102	102	0	0	0	0	0	0	20	20
Bacoor City	1	28	158	187	0	0	0	0	0	0	0	0
Batangas City	0	0	2	2	0	0	0	0	0	0	0	0
Biñan City	0	21	562	583	0	0	0	0	0	0	0	0
Cabuyao City	0	3	165	168	0	0	1	1	0	2	22	24
Calamba City	2	106	999	1,107	0	0	0	0	0	0	14	14
Cavite City	0	11	72	83	0	0	0	0	0	0	0	0
Dasmariñas City	0	19	432	451	0	0	0	0	0	0	21	21
General Trias City	0	6	19	25	0	0	0	0	0	1	0	1
Imus City	0	10	89	99	0	1	1	2	0	0	0	0
Lipa City	0	2	29	31	0	0	0	0	0	0	0	0
Lucena City	0	1	11	12	0	0	0	0	0	0	0	0
San Pablo City	0	12	27	39	0	0	0	0	0	0	2	2
San Pedro City	0	17	95	112	0	0	0	0	0	0	0	0
Santa Rosa City	9	74	487	570	9	0	0	9	9	0	2	11
Tagaytay City	0	1	5	6	0	0	0	0	0	0	0	0
Tanauan City	1	6	29	36	0	0	0	0	0	0	0	0
Tayabas City	0	0	0	0	0	0	0	0	0	0	0	0
Trece Martires City	0	6	43	49	0	0	0	0	0	0	0	0
**Region 4B**	**1**	**531**	**1,981**	**2,513**	**0**	**8**	**105**	**113**	**0**	**4**	**25**	**29**
Marinduque	0	28	207	235	0	0	0	0	0	0	0	0
Mindoro Occidental	1	11	59	71	0	3	52	55	0	0	0	0
Mindoro Oriental	0	49	251	300	0	0	0	0	0	0	16	16
Palawan	0	416	1,231	1,647	0	3	16	19	0	4	9	13
Romblon	0	18	186	204	0	2	37	39	0	0	0	0
Puerto Princesa City	0	9	47	56	0	0	0	0	0	0	0	0
**Region 5**	**1**	**263**	**2,261**	**2,525**	**2**	**187**	**542**	**731**	**0**	**9**	**62**	**71**
Albay	0	38	412	450	0	2	55	57	0	1	27	28
Camarines Norte	0	34	202	236	0	0	1	1	0	0	5	5

Table 2.A.1. MODERN METHOD OF FAMILY PLANNING
New Acceptors
Philippines, 2022

Area	IMPLANTS			Total	NFP-CCM			Total	NFP-BBT			Total
	Age group				Age group				Age group			
	10-14	15-19	20-49		10-14	15-19	20-49		10-14	15-19	20-49	
Camarines Sur	1	142	1,254	1,397	0	0	23	23	0	4	12	16
Catanduanes	0	11	57	68	0	0	0	0	0	0	2	2
Masbate	0	1	26	27	2	182	426	610	0	2	1	3
Sorsogon	0	19	46	65	0	3	35	38	0	2	8	10
Iriga City	0	12	112	124	0	0	0	0	0	0	4	4
Legaspi City	0	0	35	35	0	0	0	0	0	0	0	0
Naga City	0	6	117	123	0	0	2	2	0	0	3	3
**Region 6**	**17**	**610**	**4,093**	**4,720**	**0**	**2**	**149**	**151**	**20**	**2**	**6**	**28**
Aklan	0	15	125	140	0	0	0	0	0	0	0	0
Antique	1	36	216	253	0	1	133	134	0	0	0	0
Capiz	0	16	251	267	0	0	0	0	0	0	0	0
Guimaras	0	4	14	18	0	0	0	0	0	0	0	0
Iloilo	0	121	1,051	1,172	0	1	16	17	0	0	1	1
Negros Occidental	8	284	1,802	2,094	0	0	0	0	0	0	0	0
Bacolod City	8	128	551	687	0	0	0	0	20	2	5	27
Iloilo City	0	6	83	89	0	0	0	0	0	0	0	0
**Region 7**	**10**	**786**	**6,738**	**7,534**	**0**	**0**	**7**	**7**	**0**	**32**	**134**	**166**
Bohol	3	148	1,626	1,777	0	0	0	0	0	0	3	3
Cebu	5	384	3,162	3,551	0	0	2	2	0	22	90	112
Negros Oriental	1	40	362	403	0	0	5	5	0	7	25	32
Siquijor	0	4	15	19	0	0	0	0	0	0	0	0
Cebu City	1	156	1,213	1,370	0	0	0	0	0	3	16	19
Lapu-Lapu City	0	12	61	73	0	0	0	0	0	0	0	0
Mandaue City	0	42	299	341	0	0	0	0	0	0	0	0
**Region 8**	**4**	**198**	**1,351**	**1,553**	**0**	**0**	**49**	**49**	**0**	**1**	**11**	**12**
Biliran	0	12	42	54	0	0	7	7	0	0	0	0
Eastern Samar	0	22	129	151	0	0	39	39	0	0	0	0
Northern Leyte	3	56	445	504	0	0	0	0	0	1	5	6
Northern Samar	0	18	144	162	0	0	0	0	0	0	0	0
Southern Leyte	0	20	154	174	0	0	1	1	0	0	0	0
Western Samar	0	20	84	104	0	0	1	1	0	0	0	0
Calbayog City	0	3	23	26	0	0	0	0	0	0	0	0
Maasin City	0	3	62	65	0	0	1	1	0	0	6	6
Ormoc City	0	6	29	35	0	0	0	0	0	0	0	0
Tacloban City	1	38	239	278	0	0	0	0	0	0	0	0
**Region 9**	**7**	**952**	**3,110**	**4,069**	**0**	**2**	**12**	**14**	**0**	**0**	**0**	**0**
Zamboanga del Norte	3	215	561	779	0	0	0	0	0	0	0	0
Zamboanga del Sur	2	199	458	659	0	0	0	0	0	0	0	0
Zamboanga Sibugay	0	228	681	909	0	2	12	14	0	0	0	0
Dapitan City	0	3	4	7	0	0	0	0	0	0	0	0
Dipolog City	0	0	0	0	0	0	0	0	0	0	0	0
Isabela City	0	13	78	91	0	0	0	0	0	0	0	0
Pagadian City	0	5	32	37	0	0	0	0	0	0	0	0
Zamboanga City	2	289	1,296	1,587	0	0	0	0	0	0	0	0
**Region 10**	**25**	**702**	**4,191**	**4,918**	**0**	**16**	**333**	**349**	**1**	**18**	**116**	**135**
Bukidnon	3	331	1,388	1,722	0	7	105	112	0	2	49	51
Camiguin	0	1	2	3	0	0	0	0	0	1	12	13
Lanao del Norte	1	71	922	994	0	2	51	53	0	0	16	16
Misamis Occidental	0	8	131	139	0	0	0	0	0	0	0	0
Misamis Oriental	1	69	412	482	0	1	16	17	1	8	11	20
Cagayan de Oro City	1	164	1,032	1,197	0	0	0	0	0	0	0	0

Table 2.A.1. MODERN METHOD OF FAMILY PLANNING
New Acceptors
Philippines, 2022

Area	IMPLANTS			Total	NFP-CCM			Total	NFP-BBT			Total
	Age group				Age group				Age group			
	10-14	15-19	20-49		10-14	15-19	20-49		10-14	15-19	20-49	
El Salvador City	1	17	62	80	0	0	0	0	0	0	0	0
Gingoog City	0	4	3	7	0	0	110	110	0	2	1	3
Iligan City	18	4	99	121	0	0	0	0	0	2	13	15
Malaybalay City	0	18	55	73	0	0	0	0	0	2	2	4
Oroquieta City	0	0	8	8	0	0	0	0	0	0	0	0
Ozamis City	0	1	17	18	0	0	0	0	0	1	7	8
Tangub City	0	0	5	5	0	0	0	0	0	0	0	0
Valencia City	0	14	55	69	0	6	51	57	0	0	5	5
**Region 11**	**42**	**1,306**	**6,110**	**7,458**	**0**	**4**	**83**	**87**	**0**	**3**	**62**	**65**
Davao de Oro	5	132	420	557	0	0	31	31	0	0	32	32
Davao del Norte	7	238	1,256	1,501	0	2	9	11	0	0	5	5
Davao Oriental	10	124	483	617	0	0	13	13	0	0	0	0
Davao del Sur	5	93	195	293	0	0	0	0	0	0	0	0
Davao Occidental	1	22	154	177	0	2	10	12	0	0	0	0
Davao City	14	697	3,602	4,313	0	0	20	20	0	3	25	28
**Region 12**	**19**	**989**	**5,113**	**6,121**	**0**	**1**	**167**	**168**	**2**	**7**	**34**	**43**
North Cotabato	2	247	2,340	2,589	0	0	5	5	0	1	24	25
Sarangani	0	99	771	870	0	1	153	154	0	0	0	0
South Cotabato	10	324	868	1,202	0	0	6	6	2	2	6	10
Sultan Kudarat	4	144	321	469	0	0	3	3	0	0	0	0
				0				0				0
Gen. Santos City	3	175	813	991	0	0	0	0	0	4	4	8
**BARMM**	**10**	**508**	**3,292**	**3,810**	**0**	**0**	**2**	**2**	**0**	**8**	**13**	**21**
Basilan	4	100	361	465	0	0	0	0	0	0	0	0
Lanao del Sur	1	15	245	261	0	0	2	2	0	0	2	2
Maguindanao	3	215	1,126	1,344	0	0	0	0	0	4	1	5
Sulu	1	63	540	604	0	0	0	0	0	0	0	0
Tawi-Tawi	1	51	505	557	0	0	0	0	0	0	0	0
				0				0				0
Lamitan City	0	35	119	154	0	0	0	0	0	0	0	0
Marawi City	0	1	27	28	0	0	0	0	0	0	0	0
Cotabato City	0	28	369	397	0	0	0	0	0	4	10	14
**CARAGA**	**14**	**657**	**2,356**	**3,027**	**0**	**0**	**64**	**64**	**0**	**3**	**14**	**17**
Agusan del Norte	4	118	460	582	0	0	0	0	0	0	2	2
Agusan del Sur	7	228	662	897	0	0	64	64	0	2	3	5
Surigao del Norte	0	71	438	509	0	0	0	0	0	0	7	7
Surigao del Sur	1	97	223	321	0	0	0	0	0	1	1	2
Province of Dinagat	0	31	161	192	0	0	0	0	0	0	1	1
Bislig City	0	4	12	16	0	0	0	0	0	0	0	0
Butuan City	2	82	217	301	0	0	0	0	0	0	0	0
Surigao City	0	26	183	209	0	0	0	0	0	0	0	0

Table 2.A.1. MODERN METHOD OF FAMILY PLANNING
New Acceptors
Philippines, 2022

Area	NFP-STM			Total	NFP-SDM			Total	NFP-LAM			Total
	Age group				Age group				Age group			
	10-14	15-19	20-49		10-14	15-19	20-49		10-14	15-19	20-49	
**PHILIPPINES**	**9**	**57**	**427**	**493**	**4**	**1,288**	**3,257**	**4,549**	**1,078**	**58,016**	**348,938**	**408,032**
**N C R**	**0**	**0**	**58**	**58**	**0**	**9**	**100**	**109**	**188**	**7,668**	**66,924**	**74,780**
Malabon	0	0	0	0	0	0	0	0	10	210	607	827
Navotas	0	0	0	0	0	0	0	0	4	330	1,774	2,108
Valenzuela City	0	0	0	0	0	0	1	1	3	123	2,792	2,918
Caloocan City	0	0	0	0	0	2	5	7	3	832	3,935	4,770
Marikina City	0	0	0	0	0	0	0	0	1	77	578	656
Pasig City	0	0	0	0	0	0	13	13	3	366	4,870	5,239
Pateros	0	0	0	0	0	0	0	0	0	16	727	743
Taguig	0	0	0	0	0	0	3	3	20	1,021	11,210	12,251
Quezon City	0	0	0	0	0	0	24	24	10	1,436	10,017	11,463
Makati City	0	0	0	0	0	0	0	0	1	100	2,738	2,839
Mandaluyong City	0	0	49	49	0	0	11	11	0	325	3,341	3,666
San Juan	0	0	0	0	0	0	0	0	0	38	785	823
Manila City	0	0	9	9	0	2	23	25	52	1,061	9,788	10,901
Las Piñas City	0	0	0	0	0	0	0	0	3	313	3,840	4,156
Muntinlupa City	0	0	0	0	0	0	0	0	0	239	2,688	2,927
Parañaque City	0	0	0	0	0	5	20	25	78	939	5,987	7,004
Pasay City	0	0	0	0	0	0	0	0	0	242	1,247	1,489
**C A R**	**0**	**1**	**3**	**4**	**0**	**0**	**31**	**31**	**18**	**966**	**4,038**	**5,022**
Abra	0	0	0	0	0	0	13	13	2	207	789	998
Apayao	0	0	0	0	0	0	2	2	5	165	484	654
Benguet	0	0	0	0	0	0	2	2	2	135	960	1,097
Ifugao	0	0	0	0	0	0	6	6	5	140	497	642
Kalinga	0	1	3	4	0	0	1	1	0	189	745	934
Mt. Province	0	0	0	0	0	0	7	7	4	114	384	502
Baguio City	0	0	0	0	0	0	0	0	0	16	179	195
**Region 1**	**0**	**4**	**1**	**5**	**0**	**14**	**105**	**119**	**33**	**2,296**	**18,080**	**20,409**
Ilocos Norte	0	0	0	0	0	0	0	0	0	76	1,471	1,547
Ilocos Sur	0	4	0	4	0	12	78	90	6	292	3,207	3,505
La Union	0	0	0	0	0	0	1	1	6	357	1,625	1,988
Pangasinan	0	0	1	1	0	2	9	11	18	1,218	7,770	9,006
Alaminos City	0	0	0	0	0	0	0	0	1	68	1,244	1,313
Candon City	0	0	0	0	0	0	0	0	0	0	399	399
Dagupan City	0	0	0	0	0	0	0	0	1	99	688	788
Laoag City	0	0	0	0	0	0	0	0	0	2	935	937
San Carlos City	0	0	0	0	0	0	0	0	0	56	200	256
San Fernando City	0	0	0	0	0	0	17	17	0	8	193	201
Urdaneta City	0	0	0	0	0	0	0	0	1	66	199	266
Vigan City	0	0	0	0	0	0	0	0	0	54	149	203
**Region 2**	**0**	**0**	**0**	**0**	**0**	**0**	**7**	**7**	**39**	**1,744**	**7,189**	**8,972**
Batanes	0	0	0	0	0	0	1	1	0	7	84	91
Cagayan	0	0	0	0	0	0	0	0	10	460	2,368	2,838
Isabela	0	0	0	0	0	0	2	2	9	415	1,524	1,948
Nueva Vizcaya	0	0	0	0	0	0	4	4	11	308	1,290	1,609
Quirino	0	0	0	0	0	0	0	0	1	96	264	361
Cauayan City	0	0	0	0	0	0	0	0	2	129	276	407
Ilagan City	0	0	0	0	0	0	0	0	0	79	534	613
Santiago City	0	0	0	0	0	0	0	0	6	233	634	873
Tuguegarao City	0	0	0	0	0	0	0	0	0	17	215	232

Table 2.A.1. MODERN METHOD OF FAMILY PLANNING
New Acceptors
Philippines, 2022

Area	NFP-STM			Total	NFP-SDM			Total	NFP-LAM			Total
	Age group				Age group				Age group			
	10-14	15-19	20-49		10-14	15-19	20-49		10-14	15-19	20-49	
**Region 3**	0	9	6	15	0	6	209	215	47	4,058	27,739	31,844
Aurora	0	0	0	0	0	0	5	5	4	184	367	555
Bataan	0	0	0	0	0	0	0	0	8	403	2,751	3,162
Bulacan	0	0	0	0	0	6	28	34	1	675	5,228	5,904
Nueva Ecija	0	0	0	0	0	0	4	4	5	451	2,292	2,748
Pampanga	0	0	0	0	0	0	154	154	11	257	3,424	3,692
Tarlac	0	0	1	1	0	0	12	12	0	363	3,822	4,185
Zambales	0	0	0	0	0	0	0	0	9	221	844	1,074
Angeles City	0	0	0	0	0	0	1	1	2	93	1,231	1,326
Balanga City	0	0	0	0	0	0	0	0	1	68	150	219
Cabanatuan City	0	0	0	0	0	0	0	0	2	271	1,293	1,566
City of San Fernando	0	9	5	14	0	0	1	1	2	101	696	799
Gapan City	0	0	0	0	0	0	0	0	0	57	382	439
Mabalacat City	0	0	0	0	0	0	0	0	0	286	1,718	2,004
Malolos City	0	0	0	0	0	0	0	0	0	2	15	17
Meycauayan	0	0	0	0	0	0	0	0	0	97	774	871
Olongapo	0	0	0	0	0	0	4	4	0	47	379	426
Palayan City	0	0	0	0	0	0	0	0	0	14	157	171
San Jose City	0	0	0	0	0	0	0	0	2	198	500	700
San Jose del Monte Cit	0	0	0	0	0	0	0	0	0	0	0	0
Science City of Munoz	0	0	0	0	0	0	0	0	0	0	0	0
Tarlac City	0	0	0	0	0	0	0	0	0	270	1,716	1,986
**Region 4A**	9	0	90	99	2	1,140	189	1,331	139	4,948	38,812	43,899
Batangas	0	0	4	4	0	9	20	29	68	298	3,280	3,646
Cavite	0	0	0	0	0	1	10	11	7	299	4,058	4,364
Laguna	0	0	0	0	0	3	7	10	2	290	1,113	1,405
Quezon	0	0	0	0	2	1,127	4	1,133	15	535	2,582	3,132
Rizal	0	0	0	0	0	0	110	110	13	853	5,704	6,570
Antipolo City	0	0	86	86	0	0	8	8	0	5	2,110	2,115
Bacoor City	0	0	0	0	0	0	0	0	0	198	2,472	2,670
Batangas City	0	0	0	0	0	0	2	2	0	7	14	21
Biñan City	0	0	0	0	0	0	6	6	0	905	4,096	5,001
Cabuyao City	0	0	0	0	0	0	0	0	1	377	3,324	3,702
Calamba City	0	0	0	0	0	0	12	12	6	220	1,347	1,573
Cavite City	0	0	0	0	0	0	0	0	0	30	66	96
Dasmariñas City	0	0	0	0	0	0	0	0	0	159	2,631	2,790
General Trias City	0	0	0	0	0	0	0	0	0	44	316	360
Imus City	0	0	0	0	0	0	0	0	0	44	235	279
Lipa City	0	0	0	0	0	0	3	3	0	0	0	0
Lucena City	0	0	0	0	0	0	0	0	0	4	4	8
San Pablo City	0	0	0	0	0	0	0	0	8	179	868	1,055
San Pedro City	0	0	0	0	0	0	0	0	6	111	1,113	1,230
Santa Rosa City	9	0	0	9	0	0	0	0	12	270	2,771	3,053
Tagaytay City	0	0	0	0	0	0	0	0	0	14	313	327
Tanauan City	0	0	0	0	0	0	0	0	0	2	53	55
Tayabas City	0	0	0	0	0	0	0	0	1	101	326	428
Trece Martires City	0	0	0	0	0	0	7	7	0	3	16	19
**Region 4B**	0	0	1	1	0	1	35	36	47	2,345	8,330	10,722
Marinduque	0	0	0	0	0	0	0	0	1	26	345	372
Mindoro Occidental	0	0	0	0	0	0	0	0	11	867	1,479	2,357
Mindoro Oriental	0	0	1	1	0	0	24	24	13	315	1,610	1,938
Palawan	0	0	0	0	0	1	6	7	17	890	2,828	3,735
Romblon	0	0	0	0	0	0	1	1	3	190	1,931	2,124
Puerto Princesa City	0	0	0	0	0	0	4	4	2	57	137	196
**Region 5**	0	32	178	210	0	33	1,324	1,357	66	4,220	18,432	22,718
Albay	0	1	2	3	0	3	433	436	8	374	3,549	3,931
Camarines Norte	0	29	116	145	0	13	55	68	13	492	1,918	2,423

Table 2.A.1. MODERN METHOD OF FAMILY PLANNING
New Acceptors
Philippines, 2022

Area	NFP-STM			Total	NFP-SDM			Total	NFP-LAM			Total
	Age group				Age group				Age group			
	10-14	15-19	20-49		10-14	15-19	20-49		10-14	15-19	20-49	
Camarines Sur	0	2	59	61	0	1	102	103	16	1,167	5,078	6,261
Catanduanes	0	0	0	0	0	0	60	60	4	142	471	617
Masbate	0	0	0	0	0	14	480	494	14	1,275	3,530	4,819
Sorsogon	0	0	0	0	0	0	100	100	9	706	2,190	2,905
Iriga City	0	0	0	0	0	2	24	26	2	51	301	354
Legaspi City	0	0	0	0	0	0	1	1	0	9	182	191
Naga City	0	0	1	1	0	0	69	69	0	4	1,213	1,217
**Region 6**	**0**	**0**	**14**	**14**	**0**	**6**	**131**	**137**	**54**	**3,875**	**23,137**	**27,066**
Aklan	0	0	0	0	0	0	2	2	1	240	2,064	2,305
Antique	0	0	0	0	0	0	14	14	3	178	1,564	1,745
Capiz	0	0	0	0	0	0	0	0	3	121	1,367	1,491
Guimaras	0	0	0	0	0	1	0	1	1	141	559	701
Iloilo	0	0	1	1	0	0	38	38	19	790	6,572	7,381
Negros Occidental	0	0	13	13	0	5	72	77	26	2,280	10,178	12,484
Bacolod City	0	0	0	0	0	0	5	5	1	125	815	941
Iloilo City	0	0	0	0	0	0	0	0	0	0	18	18
**Region 7**	**0**	**0**	**0**	**0**	**0**	**1**	**117**	**118**	**79**	**5,538**	**37,860**	**43,477**
Bohol	0	0	0	0	0	0	38	38	8	347	1,774	2,129
Cebu	0	0	0	0	0	1	71	72	32	2,634	20,311	22,977
Negros Oriental	0	0	0	0	0	0	4	4	21	1,218	5,127	6,366
Siquijor	0	0	0	0	0	0	4	4	0	59	185	244
Cebu City	0	0	0	0	0	0	0	0	18	1,189	9,338	10,545
Lapu-Lapu City	0	0	0	0	0	0	0	0	0	89	886	975
Mandaue City	0	0	0	0	0	0	0	0	0	2	239	241
**Region 8**	**0**	**3**	**10**	**13**	**0**	**19**	**97**	**116**	**13**	**1,680**	**9,532**	**11,225**
Biliran	0	0	0	0	0	0	0	0	1	70	356	427
Eastern Samar	0	0	0	0	0	4	68	72	1	180	1,275	1,456
Northern Leyte	0	0	1	1	0	9	27	36	5	493	3,309	3,807
Northern Samar	0	0	0	0	0	0	0	0	1	177	971	1,149
Southern Leyte	0	3	9	12	0	0	0	0	3	51	300	354
Western Samar	0	0	0	0	0	6	2	8	0	163	763	926
Calbayog City	0	0	0	0	0	0	0	0	1	156	889	1,046
Maasin City	0	0	0	0	0	0	0	0	0	10	160	170
Ormoc City	0	0	0	0	0	0	0	0	0	260	607	867
Tacloban City	0	0	0	0	0	0	0	0	1	120	902	1,023
**Region 9**	**0**	**0**	**0**	**0**	**0**	**0**	**2**	**2**	**52**	**3,311**	**8,655**	**12,018**
Zamboanga del Norte	0	0	0	0	0	0	1	1	22	922	2,045	2,989
Zamboanga del Sur	0	0	0	0	0	0	0	0	7	322	538	867
Zamboanga Sibugay	0	0	0	0	0	0	1	1	1	365	1,091	1,457
Dapitan City	0	0	0	0	0	0	0	0	1	118	225	344
Dipolog City	0	0	0	0	0	0	0	0	4	154	394	552
Isabela City	0	0	0	0	0	0	0	0	0	113	565	678
Pagadian City	0	0	0	0	0	0	0	0	0	48	344	392
Zamboanga City	0	0	0	0	0	0	0	0	17	1,269	3,453	4,739
**Region 10**	**0**	**7**	**59**	**66**	**2**	**52**	**652**	**706**	**57**	**4,290**	**17,681**	**22,028**
Bukidnon	0	6	27	33	2	35	447	484	18	1,378	2,868	4,264
Camiguin	0	0	0	0	0	0	0	0	2	63	223	288
Lanao del Norte	0	1	2	3	0	1	70	71	13	395	3,356	3,764
Misamis Occidental	0	0	0	0	0	0	2	2	1	79	1,072	1,152
Misamis Oriental	0	0	1	1	0	0	62	62	11	490	2,228	2,729
Cagayan de Oro City	0	0	0	0	0	0	5	5	6	636	4,220	4,862

Table 2.A.1. MODERN METHOD OF FAMILY PLANNING
New Acceptors
Philippines, 2022

Area	NFP-STM			Total	NFP-SDM			Total	NFP-LAM			Total
	Age group				Age group				Age group			
	10-14	15-19	20-49		10-14	15-19	20-49		10-14	15-19	20-49	
El Salvador City	0	0	0	0	0	0	1	1	1	52	131	184
Gingoog City	0	0	26	26	0	0	23	23	0	73	139	212
Iligan City	0	0	2	2	0	10	25	35	2	363	1,655	2,020
Malaybalay City	0	0	0	0	0	4	4	8	3	336	696	1,035
Oroquieta City	0	0	0	0	0	0	0	0	0	12	64	76
Ozamis City	0	0	0	0	0	2	6	8	0	84	265	349
Tangub City	0	0	1	1	0	0	0	0	0	28	166	194
Valencia City	0	0	0	0	0	0	7	7	0	301	598	899
**Region 11**	**0**	**1**	**6**	**7**	**0**	**2**	**59**	**61**	**133**	**2,570**	**21,240**	**23,943**
Davao de Oro	0	0	0	0	0	0	0	0	10	164	536	710
Davao del Norte	0	0	0	0	0	1	14	15	37	453	2,209	2,699
Davao Oriental	0	0	0	0	0	0	20	20	8	155	9,112	9,275
Davao del Sur	0	0	0	0	0	0	0	0	5	201	296	502
Davao Occidental	0	0	0	0	0	0	9	9	17	575	1,572	2,164
Davao City	0	1	6	7	0	1	16	17	56	1,022	7,515	8,593
**Region 12**	**0**	**0**	**0**	**0**	**0**	**0**	**65**	**65**	**50**	**3,826**	**13,710**	**17,586**
North Cotabato	0	0	0	0	0	0	1	1	11	806	4,103	4,920
Sarangani	0	0	0	0	0	0	13	13	0	1,250	2,612	3,862
South Cotabato	0	0	0	0	0	0	51	51	22	886	2,983	3,891
Sultan Kudarat	0	0	0	0	0	0	0	0	16	531	1,586	2,133
				0				0				0
Gen. Santos City	0	0	0	0	0	0	0	0	1	353	2,426	2,780
**BARMM**	**0**	**0**	**1**	**1**	**0**	**0**	**14**	**14**	**32**	**2,710**	**19,951**	**22,693**
Basilan	0	0	0	0	0	0	0	0	2	112	446	560
Lanao del Sur	0	0	1	1	0	0	12	12	3	471	6,458	6,932
Maguindanao	0	0	0	0	0	0	1	1	20	1,322	6,096	7,438
Sulu	0	0	0	0	0	0	0	0	0	163	1,396	1,559
Tawi-Tawi	0	0	0	0	0	0	0	0	0	209	1,448	1,657
				0				0				0
Lamitan City	0	0	0	0	0	0	0	0	4	109	356	469
Marawi City	0	0	0	0	0	0	1	1	0	33	779	812
Cotabato City	0	0	0	0	0	0	0	0	3	291	2,972	3,266
**CARAGA**	**0**	**0**	**0**	**0**	**0**	**5**	**120**	**125**	**31**	**1,971**	**7,628**	**9,630**
Agusan del Norte	0	0	0	0	0	0	0	0	4	213	1,686	1,903
Agusan del Sur	0	0	0	0	0	0	20	20	7	435	1,102	1,544
Surigao del Norte	0	0	0	0	0	3	45	48	2	144	1,055	1,201
Surigao del Sur	0	0	0	0	0	0	5	5	1	398	1,091	1,490
Province of Dinagat	0	0	0	0	0	0	23	23	0	98	486	584
Bislig City	0	0	0	0	0	0	0	0	7	155	314	476
Butuan City	0	0	0	0	0	2	26	28	10	435	1,293	1,738
Surigao City	0	0	0	0	0	0	1	1	0	93	601	694

Table 2.A.2. MODERN METHOD OF FAMILY PLANNING
Other Acceptors
Philippines, 2022

Area	Total Current Users	FSTR/BTL Age group			Total	MSTR/NSV Age group			Total
		10-14	15-19	20-49		10-14	15-19	20-49	
**PHILIPPINES**	**8,019,000**	**2**	**259**	**72,145**	**72,406**	**1**	**28**	**746**	**775**
**N C R**	**753,263**	**0**	**3**	**8,283**	**8,286**	**0**	**0**	**105**	**105**
Malabon	31,679	0	0	291	291	0	0	1	1
Navotas	23,423	0	0	68	68	0	0	1	1
Valenzuela City	39,922	0	0	1,462	1,462	0	0	56	56
Caloocan City	68,156	0	2	899	901	0	0	2	2
Marikina City	17,020	0	0	61	61	0	0	0	0
Pasig City	42,380	0	0	395	395	0	0	7	7
Pateros	2,674	0	0	124	124	0	0	2	2
Taguig	61,050	0	0	477	477	0	0	4	4
Quezon City	272,854	0	1	2,754	2,755	0	0	22	22
Makati City	16,005	0	0	47	47	0	0	2	2
Mandaluyong City	17,504	0	0	101	101	0	0	0	0
San Juan	5,796	0	0	0	0	0	0	0	0
Manila City	27,488	0	0	472	472	0	0	2	2
Las Piñas City	20,363	0	0	331	331	0	0	5	5
Muntinlupa City	40,839	0	0	56	56	0	0	0	0
Parañaque City	37,239	0	0	325	325	0	0	1	1
Pasay City	28,871	0	0	420	420	0	0	0	0
**C A R**	**147,908**	**0**	**0**	**4,937**	**4,937**	**0**	**1**	**44**	**45**
Abra	17,362	0	0	729	729	0	0	0	0
Apayao	23,877	0	0	494	494	0	0	0	0
Benguet	31,732	0	0	551	551	0	1	38	39
Ifugao	20,003	0	0	276	276	0	0	6	6
Kalinga	19,143	0	0	137	137	0	0	0	0
Mt. Province	13,215	0	0	505	505	0	0	0	0
Baguio City	22,576	0	0	2,245	2,245	0	0	0	0
**Region 1**	**477,257**	**0**	**3**	**3,713**	**3,716**	**0**	**16**	**6**	**22**
Ilocos Norte	41,393	0	2	1,031	1,033	0	0	0	0
Ilocos Sur	62,434	0	0	290	290	0	0	0	0
La Union	59,808	0	0	522	522	0	0	5	5
Pangasinan	238,991	0	1	1,347	1,348	0	0	1	1
Alaminos City	10,832	0	0	4	4	0	0	0	0
Candon City	7,006	0	0	0	0	0	0	0	0
Dagupan City	6,976	0	0	16	16	0	0	0	0
Laoag City	8,253	0	0	21	21	0	16	0	16
San Carlos City	11,178	0	0	145	145	0	0	0	0
San Fernando City	13,027	0	0	16	16	0	0	0	0
Urdaneta City	12,909	0	0	289	289	0	0	0	0
Vigan City	4,450	0	0	32	32	0	0	0	0
**Region 2**	**318,442**	**0**	**49**	**3,102**	**3,151**	**0**	**0**	**14**	**14**
Batanes	960	0	0	8	8	0	0	0	0
Cagayan	88,783	0	44	811	855	0	0	0	0
Isabela	109,565	0	3	847	850	0	0	0	0
Nueva Vizcaya	46,874	0	1	943	944	0	0	14	14
Quirino	19,341	0	1	174	175	0	0	0	0
Cauayan City	10,017	0	0	139	139	0	0	0	0
Ilagan City	15,480	0	0	66	66	0	0	0	0
Santiago City	12,758	0	0	78	78	0	0	0	0
Tuguegarao City	14,664	0	0	36	36	0	0	0	0

Table 2.A.2. MODERN METHOD OF FAMILY PLANNING
Other Acceptors
Philippines, 2022

Area	Total Current Users	FSTR/BTL Age group			Total	MSTR/NSV Age group			Total
		10-14	15-19	20-49		10-14	15-19	20-49	
**Region 3**	**831,669**	**2**	**8**	**16,638**	**16,648**	**0**	**0**	**45**	**45**
Aurora	22,728	0	0	337	337	0	0	1	1
Bataan	53,599	0	0	682	682	0	0	12	12
Bulacan	156,537	0	0	1,096	1,096	0	0	1	1
Nueva Ecija	135,568	0	2	3,278	3,280	0	0	0	0
Pampanga	114,189	0	0	4,839	4,839	0	0	18	18
Tarlac	88,617	2	6	3,074	3,082	0	0	3	3
Zambales	43,500	0	0	671	671	0	0	0	0
Angeles City	24,153	0	0	638	638	0	0	5	5
Balanga City	4,623	0	0	15	15	0	0	0	0
Cabanatuan City	20,017	0	0	475	475	0	0	0	0
City of San Fernando	11,292	0	0	98	98	0	0	0	0
Gapan City	7,573	0	0	33	33	0	0	0	0
Mabalacat City	21,462	0	0	221	221	0	0	0	0
Malolos City	7,988	0	0	692	692	0	0	4	4
Meycauayan	8,375	0	0	28	28	0	0	0	0
Olongapo	10,879	0	0	33	33	0	0	0	0
Palayan City	2,864	0	0	0	0	0	0	0	0
San Jose City	12,941	0	0	25	25	0	0	0	0
San Jose del Monte City	56,123	0	0	51	51	0	0	1	1
Science City of Munoz	6,280	0	0	37	37	0	0	0	0
Tarlac City	22,361	0	0	315	315	0	0	0	0
**Region 4A**	**866,447**	**0**	**50**	**6,186**	**6,236**	**0**	**0**	**41**	**41**
Batangas	129,569	0	0	801	801	0	0	1	1
Cavite	83,857	0	8	1,224	1,232	0	0	7	7
Laguna	74,923	0	0	636	636	0	0	3	3
Quezon	97,255	0	29	923	952	0	0	1	1
Rizal	111,121	0	0	926	926	0	0	3	3
Antipolo City	32,668	0	5	64	69	0	0	1	1
Bacoor City	10,282	0	0	33	33	0	0	0	0
Batangas City	17,852	0	0	22	22	0	0	0	0
Biñan City	84,449	0	0	40	40	0	0	0	0
Cabuyao City	18,581	0	0	80	80	0	0	0	0
Calamba City	30,526	0	0	224	224	0	0	1	1
Cavite City	4,111	0	0	74	74	0	0	1	1
Dasmariñas City	40,305	0	0	62	62	0	0	0	0
General Trias City	7,331	0	0	96	96	0	0	0	0
Imus City	8,271	0	0	103	103	0	0	3	3
Lipa City	8,190	0	8	83	91	0	0	0	0
Lucena City	6,718	0	0	75	75	0	0	13	13
San Pablo City	12,520	0	0	272	272	0	0	0	0
San Pedro City	17,276	0	0	51	51	0	0	0	0
Santa Rosa City	47,684	0	0	86	86	0	0	5	5
Tagaytay City	8,795	0	0	94	94	0	0	1	1
Tanauan City	6,741	0	0	30	30	0	0	0	0
Tayabas City	4,078	0	0	36	36	0	0	0	0
Trece Martires City	3,344	0	0	151	151	0	0	1	1
**Region 4B**	**271,491**	**0**	**52**	**4,221**	**4,273**	**0**	**9**	**154**	**163**
Marinduque	14,336	0	0	624	624	0	0	3	3
Mindoro Occidental	47,186	0	0	138	138	0	0	0	0
Mindoro Oriental	65,781	0	2	2,046	2,048	0	9	59	68
Palawan	88,744	0	5	1,132	1,137	0	0	88	88
Romblon	22,330	0	10	128	138	0	0	0	0
Puerto Princesa City	33,114	0	35	153	188	0	0	4	4
**Region 5**	**501,196**	**0**	**22**	**1,931**	**1,953**	**0**	**1**	**43**	**44**
Albay	97,609	0	9	636	645	0	1	10	11

Table 2.A.2. MODERN METHOD OF FAMILY PLANNING
Other Acceptors
Philippines, 2022

Area	Total Current Users	FSTR/BTL Age group 10-14	15-19	20-49	Total	MSTR/NSV Age group 10-14	15-19	20-49	Total
Camarines Norte	44,713	0	0	109	109	0	0	17	17
Camarines Sur	106,529	0	0	3	3	0	0	0	0
Catanduanes	24,595	0	10	321	331	0	0	7	7
Masbate	87,357	0	1	240	241	0	0	5	5
Sorsogon	76,074	0	2	559	561	0	0	2	2
Iriga City	12,491	0	0	23	23	0	0	1	1
Legaspi City	23,050	0	0	8	8	0	0	0	0
Naga City	28,778	0	0	32	32	0	0	1	1
**Region 6**	**612,103**	**0**	**11**	**9,427**	**9,438**	**0**	**0**	**48**	**48**
Aklan	44,365	0	1	74	75	0	0	0	0
Antique	47,618	0	2	225	227	0	0	7	7
Capiz	76,500	0	0	129	129	0	0	4	4
Guimaras	16,308	0	0	91	91	0	0	0	0
Iloilo	157,750	0	0	1,521	1,521	0	0	11	11
Negros Occidental	193,157	0	3	3,495	3,498	0	0	22	22
Bacolod City	25,916	0	0	1,475	1,475	0	0	0	0
Iloilo City	50,489	0	5	2,417	2,422	0	0	4	4
**Region 7**	**547,072**	**0**	**4**	**1,989**	**1,993**	**0**	**0**	**30**	**30**
Bohol	81,628	0	0	44	44	0	0	0	0
Cebu	274,919	0	0	1,557	1,557	0	0	20	20
Negros Oriental	90,879	0	2	230	232	0	0	3	3
Siquijor	8,892	0	0	0	0	0	0	0	0
Cebu City	32,674	0	2	155	157	0	0	4	4
Lapu-Lapu City	35,065	0	0	1	1	0	0	3	3
Mandaue City	23,015	0	0	2	2	0	0	0	0
**Region 8**	**349,783**	**0**	**11**	**1,563**	**1,574**	**0**	**1**	**31**	**32**
Biliran	11,512	0	0	58	58	0	0	22	22
Eastern Samar	41,802	0	6	124	130	0	0	2	2
Northern Leyte	118,358	0	0	179	179	0	0	0	0
Northern Samar	48,444	0	5	51	56	0	1	0	1
Southern Leyte	26,721	0	0	698	698	0	0	0	0
Western Samar	24,584	0	0	124	124	0	0	0	0
Calbayog City	8,315	0	0	86	86	0	0	0	0
Maasin City	5,078	0	0	25	25	0	0	0	0
Ormoc City	20,494	0	0	213	213	0	0	7	7
Tacloban City	44,475	0	0	5	5	0	0	0	0
**Region 9**	**325,087**	**0**	**0**	**69**	**69**	**0**	**0**	**0**	**0**
Zamboanga del Norte	93,748	0	0	2	2	0	0	0	0
Zamboanga del Sur	73,802	0	0	18	18	0	0	0	0
Zamboanga Sibugay	42,410	0	0	33	33	0	0	0	0
Dapitan City	7,472	0	0	0	0	0	0	0	0
Dipolog City	17,962	0	0	0	0	0	0	0	0
Isabela City	8,228	0	0	0	0	0	0	0	0
Pagadian City	18,316	0	0	0	0	0	0	0	0
Zamboanga City	63,149	0	0	16	16	0	0	0	0
**Region 10**	**489,677**	**0**	**4**	**3,067**	**3,071**	**0**	**0**	**47**	**47**
Bukidnon	114,313	0	1	748	749	0	0	3	3
Camiguin	6,418	0	2	86	88	0	0	0	0
Lanao del Norte	63,113	0	0	40	40	0	0	3	3
Misamis Occidental	27,464	0	0	66	66	0	0	0	0
Misamis Oriental	75,691	0	1	826	827	0	0	2	2

Table 2.A.2. MODERN METHOD OF FAMILY PLANNING
Other Acceptors
Philippines, 2022

Area	Total Current Users	FSTR/BTL Age group			Total	MSTR/NSV Age group			Total
		10-14	15-19	20-49		10-14	15-19	20-49	
Cagayan de Oro City	61,925	0	0	443	443	0	0	1	1
El Salvador City	6,345	0	0	42	42	0	0	0	0
Gingoog City	28,686	0	0	273	273	0	0	35	35
Iligan City	28,662	0	0	85	85	0	0	0	0
Malaybalay City	18,648	0	0	288	288	0	0	3	3
Oroquieta City	6,937	0	0	11	11	0	0	0	0
Ozamis City	25,480	0	0	8	8	0	0	0	0
Tangub City	4,130	0	0	32	32	0	0	0	0
Valencia City	21,865	0	0	119	119	0	0	0	0
**Region 11**	**528,307**	**0**	**20**	**2,427**	**2,447**	**1**	**0**	**70**	**71**
Davao de Oro	85,230	0	4	462	466	0	0	3	3
Davao del Norte	103,058	0	2	769	771	0	0	10	10
Davao Oriental	76,678	0	1	103	104	0	0	0	0
Davao del Sur	31,942	0	0	188	188	0	0	0	0
Davao Occidental	53,567	0	5	348	353	0	0	39	39
Davao City	177,832	0	8	557	565	1	0	18	19
**Region 12**	**435,275**	**0**	**8**	**1,905**	**1,913**	**0**	**0**	**44**	**44**
North Cotabato	137,970	0	2	768	770	0	0	6	6
Sarangani	65,032	0	1	277	278	0	0	1	1
South Cotabato	92,506	0	0	490	490	0	0	29	29
Sultan Kudarat	95,199	0	2	282	284	0	0	1	1
									0
Gen. Santos City	44,568	0	3	88	91	0	0	7	7
**BARMM**	**295,125**	**0**	**0**	**579**	**579**	**0**	**0**	**1**	**1**
Basilan	10,783	0	0	9	9	0	0	0	0
Lanao del Sur	30,652	0	0	150	150	0	0	1	1
Maguindanao	91,298	0	0	109	109	0	0	0	0
Sulu	92,228	0	0	49	49	0	0	0	0
Tawi-Tawi	28,444	0	0	104	104	0	0	0	0
Lamitan City	5,882	0	0	32	32	0	0	0	0
Marawi City	5,206	0	0	21	21	0	0	0	0
Cotabato City	30,632	0	0	105	105	0	0	0	0
**CARAGA**	**268,898**	**0**	**14**	**2,108**	**2,122**	**0**	**0**	**23**	**23**
Agusan del Norte	39,895	0	0	215	215	0	0	1	1
Agusan del Sur	83,282	0	1	409	410	0	0	4	4
Surigao del Norte	33,804	0	0	328	328	0	0	8	8
Surigao del Sur	42,911	0	1	934	935	0	0	9	9
Province of Dinagat	10,370	0	12	41	53	0	0	0	0
Bislig City	9,590	0	0	66	66	0	0	0	0
Butuan City	34,962	0	0	96	96	0	0	1	1
Surigao City	14,084	0	0	19	19	0	0	0	0

Table 2.A.2. MODERN METHOD OF FAMILY PLANNING
Other Acceptors
Philippines, 2022

Area	CONDOM			Total	IUD-INTERVAL			Total	IUD-POSTPARTUM			Total
	Age group				Age group				Age group			
	10-14	15-19	20-49		10-14	15-19	20-49		10-14	15-19	20-49	
**PHILIPPINES**	**39**	**3,373**	**67,646**	**71,058**	**89**	**1,278**	**32,174**	**33,541**	**50**	**1,809**	**19,844**	**21,703**
**N C R**	**8**	**1,190**	**23,121**	**24,319**	**1**	**212**	**3,244**	**3,457**	**1**	**235**	**4,897**	**5,133**
Malabon	0	25	390	415	0	6	112	118	0	1	23	24
Navotas	3	114	204	321	0	13	60	73	0	0	3	3
Valenzuela City	0	5	738	743	0	4	330	334	0	1	99	100
Caloocan City	0	26	663	689	1	74	532	607	0	37	544	581
Marikina City	0	3	140	143	0	0	14	14	0	0	59	59
Pasig City	1	9	256	266	0	5	266	271	0	15	201	216
Pateros	0	2	67	69	0	0	139	139	0	0	2	2
Taguig	0	3	200	203	0	20	323	343	1	6	87	94
Quezon City	1	640	17,622	18,263	0	55	928	983	0	146	2,894	3,040
Makati City	0	5	282	287	0	2	43	45	0	2	26	28
Mandaluyong City	0	44	134	178	0	4	44	48	0	9	210	219
San Juan	0	0	14	14	0	0	1	1	0	0	0	0
Manila City	2	59	495	556	0	21	153	174	0	6	60	66
Las Piñas City	0	7	197	204	0	1	65	66	0	2	93	95
Muntinlupa City	0	1	22	23	0	0	16	16	0	0	0	0
Parañaque City	1	5	674	680	0	7	28	35	0	10	50	60
Pasay City	0	242	1,023	1,265	0	0	190	190	0	0	546	546
**C A R**	**5**	**101**	**2,465**	**2,571**	**0**	**17**	**558**	**575**	**6**	**43**	**679**	**728**
Abra	0	37	878	915	0	0	0	0	0	0	0	0
Apayao	0	3	110	113	0	2	117	119	6	31	378	415
Benguet	0	9	640	649	0	7	223	230	0	5	101	106
Ifugao	0	4	135	139	0	3	27	30	0	0	50	50
Kalinga	4	27	205	236	0	4	94	98	0	2	39	41
Mt. Province	0	3	125	128	0	1	47	48	0	3	19	22
Baguio City	1	18	372	391	0	0	50	50	0	2	92	94
**Region 1**	**0**	**39**	**1,251**	**1,290**	**1**	**41**	**717**	**759**	**1**	**16**	**117**	**134**
Ilocos Norte	0	2	42	44	0	1	4	5	0	3	10	13
Ilocos Sur	0	2	78	80	0	0	17	17	1	0	10	11
La Union	0	3	78	81	0	12	112	124	0	11	91	102
Pangasinan	0	26	817	843	1	23	467	491	0	0	0	0
Alaminos City	0	0	4	4	0	0	47	47	0	0	0	0
Candon City	0	0	0	0	0	0	0	0	0	0	0	0
Dagupan City	0	0	6	6	0	0	3	3	0	0	1	1
Laoag City	0	0	0	0	0	0	0	0	0	0	0	0
San Carlos City	0	0	27	27	0	4	54	58	0	0	0	0
San Fernando City	0	5	37	42	0	1	11	12	0	2	5	7
Urdaneta City	0	1	136	137	0	0	1	1	0	0	0	0
Vigan City	0	0	26	26	0	0	1	1	0	0	0	0
**Region 2**	**0**	**27**	**687**	**714**	**1**	**83**	**1,212**	**1,296**	**1**	**33**	**398**	**432**
Batanes	0	0	7	7	0	0	1	1	0	0	0	0
Cagayan	0	0	75	75	0	48	508	556	1	15	119	135
Isabela	0	5	187	192	0	11	115	126	0	3	35	38
Nueva Vizcaya	0	19	298	317	1	18	357	376	0	9	140	149
Quirino	0	0	43	43	0	0	28	28	0	0	16	16
Cauayan City	0	1	12	13	0	2	27	29	0	0	24	24
Ilagan City	0	0	40	40	0	0	46	46	0	0	2	2
Santiago City	0	2	13	15	0	2	16	18	0	0	0	0
Tuguegarao City	0	0	12	12	0	2	114	116	0	6	62	68

Table 2.A.2. MODERN METHOD OF FAMILY PLANNING
Other Acceptors
Philippines, 2022

Area	CONDOM			Total	IUD-INTERVAL			Total	IUD-POSTPARTUM			Total
	Age group				Age group				Age group			
	10-14	15-19	20-49		10-14	15-19	20-49		10-14	15-19	20-49	
**Region 3**	**7**	**380**	**9,525**	**9,912**	**54**	**22**	**1,417**	**1,493**	**0**	**21**	**735**	**756**
Aurora	0	13	284	297	0	0	59	59	0	1	41	42
Bataan	0	2	149	151	0	0	10	10	0	0	6	6
Bulacan	0	83	1,648	1,731	0	3	306	309	0	1	199	200
Nueva Ecija	0	78	1,818	1,896	0	2	432	434	0	1	60	61
Pampanga	1	38	1,226	1,265	54	3	219	276	0	9	134	143
Tarlac	0	54	512	566	0	0	73	73	0	0	9	9
Zambales	6	21	482	509	0	2	40	42	0	1	17	18
Angeles City	0	0	188	188	0	5	50	55	0	0	213	213
Balanga City	0	0	13	13	0	0	0	0	0	0	2	2
Cabanatuan City	0	1	111	112	0	1	33	34	0	2	9	11
City of San Fernando	0	11	542	553	0	1	2	3	0	4	20	24
Gapan City	0	0	11	11	0	0	0	0	0	0	1	1
Mabalacat City	0	11	745	756	0	1	14	15	0	2	22	24
Malolos City	0	3	204	207	0	2	51	53	0	0	0	0
Meycauayan	0	9	34	43	0	0	1	1	0	0	0	0
Olongapo	0	0	92	92	0	0	0	0	0	0	0	0
Palayan City	0	0	7	7	0	0	2	2	0	0	0	0
San Jose City	0	1	49	50	0	0	0	0	0	0	0	0
San Jose del Monte City	0	20	1,205	1,225	0	0	71	71	0	0	0	0
Science City of Munoz	0	0	5	5	0	2	29	31	0	0	0	0
Tarlac City	0	35	200	235	0	0	25	25	0	0	2	2
**Region 4A**	**7**	**365**	**5,069**	**5,441**	**4**	**130**	**2,314**	**2,448**	**5**	**121**	**966**	**1,092**
Batangas	2	29	927	958	0	3	228	231	0	4	71	75
Cavite	0	31	584	615	0	9	410	419	0	5	87	92
Laguna	0	10	262	272	0	52	260	312	1	8	95	104
Quezon	5	22	659	686	2	17	734	753	2	4	96	102
Rizal	0	13	508	521	1	30	319	350	0	25	123	148
Antipolo City	0	20	225	245	0	0	24	24	0	1	28	29
Bacoor City	0	1	27	28	0	1	10	11	0	0	1	1
Batangas City	0	1	120	121	0	1	29	30	1	69	346	416
Biñan City	0	91	671	762	0	1	3	4	0	0	2	2
Cabuyao City	0	42	90	132	0	3	8	11	0	0	2	2
Calamba City	0	2	111	113	0	0	49	49	1	0	10	11
Cavite City	0	6	16	22	0	0	2	2	0	1	4	5
Dasmariñas City	0	42	228	270	0	2	44	46	0	1	26	27
General Trias City	0	2	28	30	0	0	14	14	0	0	11	11
Imus City	0	2	87	89	0	0	16	16	0	0	5	5
Lipa City	0	0	7	7	0	0	10	10	0	0	3	3
Lucena City	0	2	84	86	0	0	9	9	0	0	1	1
San Pablo City	0	3	56	59	0	3	112	115	0	1	31	32
San Pedro City	0	1	20	21	0	0	0	0	0	1	1	2
Santa Rosa City	0	44	192	236	0	0	1	1	0	0	2	2
Tagaytay City	0	0	103	103	0	0	6	6	0	0	4	4
Tanauan City	0	0	12	12	0	0	11	11	0	0	11	11
Tayabas City	0	1	43	44	0	0	0	0	0	1	5	6
Trece Martires City	0	0	9	9	1	8	15	24	0	0	1	1
**Region 4B**	**2**	**84**	**1,917**	**2,003**	**0**	**31**	**2,217**	**2,248**	**0**	**25**	**687**	**712**
Marinduque	0	0	103	103	0	0	95	95	0	0	5	5
Mindoro Occidental	0	5	150	155	0	5	58	63	0	0	9	9
Mindoro Oriental	0	14	465	479	0	18	934	952	0	13	312	325
Palawan	1	35	924	960	0	5	490	495	0	9	118	127
Romblon	0	1	62	63	0	0	84	84	0	2	138	140
Puerto Princesa City	1	29	213	243	0	3	556	559	0	1	105	106
**Region 5**	**1**	**97**	**3,483**	**3,581**	**0**	**28**	**628**	**656**	**0**	**30**	**655**	**685**
Albay	0	24	881	905	0	1	141	142	0	7	76	83

Table 2.A.2. MODERN METHOD OF FAMILY PLANNING
Other Acceptors
Philippines, 2022

Area	CONDOM Age group 10-14	15-19	20-49	Total	IUD-INTERVAL Age group 10-14	15-19	20-49	Total	IUD-POSTPARTUM Age group 10-14	15-19	20-49	Total
Camarines Norte	0	13	386	399	0	17	233	250	0	4	95	99
Camarines Sur	0	15	902	917	0	4	60	64	0	3	67	70
Catanduanes	0	13	186	199	0	0	20	20	0	0	5	5
Masbate	1	13	377	391	0	0	135	135	0	16	363	379
Sorsogon	0	19	541	560	0	3	18	21	0	0	24	24
Iriga City	0	0	16	16	0	0	3	3	0	0	19	19
Legaspi City	0	0	22	22	0	0	1	1	0	0	1	1
Naga City	0	0	172	172	0	3	17	20	0	0	5	5
**Region 6**	**0**	**166**	**3,372**	**3,538**	**1**	**123**	**3,279**	**3,403**	**12**	**405**	**3,216**	**3,633**
Aklan	0	2	178	180	0	0	21	21	0	0	40	40
Antique	0	4	175	179	0	1	36	37	0	0	4	4
Capiz	0	1	128	129	0	3	91	94	0	0	3	3
Guimaras	0	0	162	162	0	0	15	15	0	0	3	3
Iloilo	0	50	1,121	1,171	1	13	568	582	2	178	1,389	1,569
Negros Occidental	0	77	727	804	0	67	2,132	2,199	2	131	1,052	1,185
Bacolod City	0	4	76	80	0	5	60	65	0	3	24	27
Iloilo City	0	28	805	833	0	34	356	390	8	93	701	802
**Region 7**	**1**	**73**	**2,837**	**2,911**	**2**	**104**	**3,398**	**3,504**	**8**	**373**	**2,786**	**3,167**
Bohol	0	10	602	612	0	15	606	621	0	93	677	770
Cebu	1	30	1,193	1,224	2	87	1,900	1,989	8	273	1,957	2,238
Negros Oriental	0	21	535	556	0	1	435	436	0	5	70	75
Siquijor	0	0	40	40	0	0	51	51	0	0	7	7
Cebu City	0	4	147	151	0	1	252	253	0	2	71	73
Lapu-Lapu City	0	8	93	101	0	0	7	7	0	0	0	0
Mandaue City	0	0	227	227	0	0	147	147	0	0	4	4
**Region 8**	**1**	**131**	**1,322**	**1,454**	**2**	**43**	**1,346**	**1,391**	**2**	**20**	**275**	**297**
Biliran	0	11	54	65	0	3	46	49	0	0	25	25
Eastern Samar	0	2	154	156	0	6	350	356	0	1	3	4
Northern Leyte	0	46	191	237	0	14	355	369	1	11	59	71
Northern Samar	0	9	199	208	0	1	66	67	0	1	23	24
Southern Leyte	1	42	39	82	2	13	140	155	1	4	59	64
Western Samar	0	5	181	186	0	3	131	134	0	3	49	52
Calbayog City	0	2	76	78	0	3	41	44	0	0	9	9
Maasin City	0	0	23	23	0	0	52	52	0	0	6	6
Ormoc City	0	13	394	407	0	0	164	164	0	0	26	26
Tacloban City	0	1	11	12	0	0	1	1	0	0	16	16
**Region 9**	**0**	**49**	**1,414**	**1,463**	**0**	**60**	**1,745**	**1,805**	**1**	**93**	**1,046**	**1,140**
Zamboanga del Norte	0	14	481	495	0	7	585	592	0	3	122	125
Zamboanga del Sur	0	6	292	298	0	24	645	669	1	51	543	595
Zamboanga Sibugay	0	21	229	250	0	13	314	327	0	16	102	118
Dapitan City	0	0	11	11	0	1	3	4	0	0	1	1
Dipolog City	0	3	63	66	0	1	35	36	0	0	0	0
Isabela City	0	0	12	12	0	0	1	1	0	0	0	0
Pagadian City	0	4	105	109	0	10	87	97	0	16	208	224
Zamboanga City	0	1	221	222	0	4	75	79	0	7	70	77
**Region 10**	**3**	**156**	**3,055**	**3,214**	**17**	**192**	**5,058**	**5,267**	**3**	**200**	**944**	**1,147**
Bukidnon	1	33	699	733	1	101	1,620	1,722	0	31	193	224
Camiguin	0	1	14	15	0	2	18	20	0	1	5	6
Lanao del Norte	0	22	544	566	0	0	127	127	0	9	79	88
Misamis Occidental	0	2	100	102	0	1	13	14	0	0	2	2
Misamis Oriental	2	16	404	422	0	37	2,517	2,554	2	45	108	155

Table 2.A.2. MODERN METHOD OF FAMILY PLANNING
Other Acceptors
Philippines, 2022

Area	CONDOM Age group			Total	IUD-INTERVAL Age group			Total	IUD-POSTPARTUM Age group			Total
	10-14	15-19	20-49		10-14	15-19	20-49		10-14	15-19	20-49	
Cagayan de Oro City	0	6	59	65	0	14	134	148	1	103	228	332
El Salvador City	0	1	22	23	0	7	77	84	0	4	58	62
Gingoog City	0	1	151	152	16	10	154	180	0	1	186	187
Iligan City	0	7	132	139	0	11	111	122	0	6	70	76
Malaybalay City	0	0	262	262	0	6	145	151	0	0	3	3
Oroquieta City	0	0	13	13	0	0	7	7	0	0	0	0
Ozamis City	0	0	36	36	0	0	1	1	0	0	0	0
Tangub City	0	37	533	570	0	0	5	5	0	0	9	9
Valencia City	0	30	86	116	0	3	129	132	0	0	3	3
**Region 11**	**3**	**137**	**1,924**	**2,064**	**1**	**43**	**1,720**	**1,764**	**6**	**28**	**601**	**635**
Davao de Oro	1	5	181	187	0	6	227	233	0	0	24	24
Davao del Norte	0	28	538	566	0	6	536	542	0	2	136	138
Davao Oriental	0	5	113	118	1	5	202	208	0	1	18	19
Davao del Sur	0	3	27	30	0	7	311	318	0	1	15	16
Davao Occidental	0	18	162	180	0	0	119	119	0	4	79	83
Davao City	2	78	903	983	0	19	325	344	6	20	329	355
**Region 12**	**0**	**140**	**1,966**	**2,106**	**3**	**57**	**1,324**	**1,384**	**0**	**58**	**542**	**600**
North Cotabato	0	28	724	752	3	18	542	563	0	9	177	186
Sarangani	0	5	206	211	0	3	141	144	0	0	27	27
South Cotabato	0	38	516	554	0	27	374	401	0	10	149	159
Sultan Kudarat	0	21	289	310	0	7	171	178	0	13	128	141
				0				0				0
Gen. Santos City	0	48	231	279	0	2	96	98	0	26	61	87
**BARMM**	**0**	**65**	**2,058**	**2,123**	**2**	**11**	**255**	**268**	**0**	**32**	**438**	**470**
Basilan	0	6	49	55	0	0	1	1	0	0	16	16
Lanao del Sur	0	22	911	933	0	0	21	21	0	0	19	19
Maguindanao	0	30	432	462	0	6	65	71	0	2	147	149
Sulu	0	2	121	123	0	2	101	103	0	0	10	10
Tawi-Tawi	0	0	269	269	0	0	1	1	0	0	14	14
Lamitan City	0	0	1	1	0	1	21	22	0	0	16	16
Marawi City	0	4	240	244	0	0	3	3	0	0	1	1
Cotabato City	0	1	35	36	2	2	42	46	0	30	215	245
**CARAGA**	**1**	**173**	**2,180**	**2,354**	**0**	**81**	**1,742**	**1,823**	**4**	**76**	**862**	**942**
Agusan del Norte	1	16	202	219	0	12	164	176	0	5	124	129
Agusan del Sur	0	21	418	439	0	15	454	469	0	5	223	228
Surigao del Norte	0	115	813	928	0	30	610	640	2	23	183	208
Surigao del Sur	0	9	331	340	0	20	266	286	1	6	108	115
Province of Dinagat	0	1	68	69	0	0	26	26	0	0	40	40
Bislig City	0	6	174	180	0	1	103	104	0	3	45	48
Butuan City	0	3	149	152	0	2	60	62	1	3	49	53
Surigao City	0	2	25	27	0	1	59	60	0	31	90	121

Table 2.A.2. MODERN METHOD OF FAMILY PLANNING
Other Acceptors
Philippines, 2022

Area	PILLS-POP			Total	PILLS-COC			Total	INJECTABLES			Total
	Age group				Age group				Age group			
	10-14	15-19	20-49		10-14	15-19	20-49		10-14	15-19	20-49	
**PHILIPPINES**	**130**	**9,150**	**101,308**	**110,588**	**244**	**16,611**	**387,852**	**404,707**	**228**	**17,783**	**287,396**	**305,407**
**N C R**	**12**	**1,145**	**16,305**	**17,462**	**24**	**1,752**	**59,639**	**61,415**	**42**	**2,110**	**47,304**	**49,456**
Malabon	0	39	392	431	0	98	1,409	1,507	0	132	2,024	2,156
Navotas	0	64	198	262	0	113	397	510	0	164	828	992
Valenzuela City	0	28	903	931	0	145	5,536	5,681	0	201	4,023	4,224
Caloocan City	0	85	963	1,048	0	124	2,579	2,703	0	288	6,384	6,672
Marikina City	0	5	125	130	0	5	398	403	0	21	441	462
Pasig City	0	14	499	513	0	84	1,210	1,294	0	66	2,266	2,332
Pateros	0	0	7	7	0	0	669	669	0	0	140	140
Taguig	1	5	248	254	0	22	1,160	1,182	2	46	1,300	1,348
Quezon City	2	700	9,400	10,102	3	833	38,952	39,788	1	761	20,123	20,885
Makati City	0	4	168	172	0	4	485	489	0	17	668	685
Mandaluyong City	1	55	207	263	0	77	363	440	0	63	588	651
San Juan	0	5	182	187	0	1	27	28	0	7	59	66
Manila City	7	66	1,265	1,338	16	93	1,563	1,672	37	111	3,084	3,232
Las Piñas City	0	19	220	239	0	36	936	972	2	77	1,157	1,236
Muntinlupa City	1	4	51	56	5	8	620	633	0	13	725	738
Parañaque City	0	47	323	370	0	62	992	1,054	0	143	1,042	1,185
Pasay City	0	5	1,154	1,159	0	47	2,343	2,390	0	0	2,452	2,452
**C A R**	**9**	**405**	**4,087**	**4,501**	**6**	**469**	**18,598**	**19,073**	**22**	**697**	**9,246**	**9,965**
Abra	0	43	382	425	0	49	1,440	1,489	0	22	528	550
Apayao	1	89	802	892	1	86	6,261	6,348	1	105	1,292	1,398
Benguet	0	30	573	603	0	40	2,157	2,197	0	53	1,529	1,582
Ifugao	2	54	509	565	1	57	3,349	3,407	2	62	1,063	1,127
Kalinga	2	58	531	591	4	148	2,880	3,032	6	148	1,066	1,220
Mt. Province	0	50	514	564	0	29	970	999	0	51	741	792
Baguio City	4	81	776	861	0	60	1,541	1,601	13	256	3,027	3,296
**Region 1**	**1**	**64**	**651**	**716**	**1**	**513**	**12,672**	**13,186**	**1**	**370**	**7,725**	**8,096**
Ilocos Norte	0	8	160	168	0	10	934	944	0	6	370	376
Ilocos Sur	0	33	227	260	0	54	720	774	0	27	335	362
La Union	1	19	218	238	0	42	1,710	1,752	1	67	1,245	1,313
Pangasinan	0	0	0	0	1	348	6,909	7,258	0	252	4,980	5,232
Alaminos City	0	0	0	0	0	12	342	354	0	4	130	134
Candon City	0	0	0	0	0	0	0	0	0	0	0	0
Dagupan City	0	0	14	14	0	0	92	92	0	1	88	89
Laoag City	0	0	0	0	0	0	0	0	0	0	0	0
San Carlos City	0	0	0	0	0	10	401	411	0	5	336	341
San Fernando City	0	4	32	36	0	3	67	70	0	4	37	41
Urdaneta City	0	0	0	0	0	27	1,323	1,350	0	0	69	69
Vigan City	0	0	0	0	0	7	174	181	0	4	135	139
**Region 2**	**2**	**258**	**3,418**	**3,678**	**0**	**622**	**14,753**	**15,375**	**7**	**474**	**8,351**	**8,832**
Batanes	0	0	7	7	0	1	62	63	0	9	170	179
Cagayan	0	53	738	791	0	112	4,195	4,307	1	80	1,850	1,931
Isabela	2	51	759	812	0	131	3,693	3,824	4	181	3,056	3,241
Nueva Vizcaya	0	99	1,278	1,377	0	274	3,884	4,158	1	69	1,570	1,640
Quirino	0	15	179	194	0	11	605	616	0	34	393	427
Cauayan City	0	15	187	202	0	32	609	641	0	27	295	322
Ilagan City	0	19	147	166	0	22	421	443	0	7	320	327
Santiago City	0	0	0	0	0	34	708	742	1	65	597	663
Tuguegarao City	0	6	123	129	0	5	576	581	0	2	100	102

Table 2.A.2. MODERN METHOD OF FAMILY PLANNING
Other Acceptors
Philippines, 2022

Area	PILLS-POP Age group			Total	PILLS-COC Age group			Total	INJECTABLES Age group			Total
	10-14	15-19	20-49		10-14	15-19	20-49		10-14	15-19	20-49	
**Region 3**	**5**	**834**	**8,656**	**9,495**	**9**	**1,884**	**43,869**	**45,762**	**15**	**1,907**	**34,888**	**36,810**
Aurora	0	22	309	331	0	95	2,086	2,181	1	68	941	1,010
Bataan	0	45	1,061	1,106	0	44	1,138	1,182	1	122	2,722	2,845
Bulacan	0	248	1,598	1,846	0	472	12,939	13,411	0	482	6,247	6,729
Nueva Ecija	0	71	1,401	1,472	0	470	5,894	6,364	3	171	5,167	5,341
Pampanga	4	90	1,154	1,248	3	260	6,331	6,594	7	286	4,041	4,334
Tarlac	0	93	694	787	0	92	2,257	2,349	0	88	2,861	2,949
Zambales	0	23	433	456	6	113	3,419	3,538	0	190	2,234	2,424
Angeles City	0	6	110	116	0	8	1,773	1,781	1	16	2,570	2,587
Balanga City	0	7	27	34	0	8	222	230	0	35	531	566
Cabanatuan City	1	29	220	250	0	38	1,399	1,437	2	36	909	947
City of San Fernando	0	22	152	174	0	16	273	289	0	12	216	228
Gapan City	0	20	114	134	0	13	178	191	0	7	160	167
Mabalacat City	0	5	54	59	0	34	1,352	1,386	0	62	1,854	1,916
Malolos City	0	12	283	295	0	24	585	609	0	17	389	406
Meycauayan	0	43	140	183	0	28	145	173	0	19	134	153
Olongapo	0	19	182	201	0	27	616	643	0	50	1,003	1,053
Palayan City	0	0	2	2	0	8	128	136	0	2	86	88
San Jose City	0	12	51	63	0	10	201	211	0	34	308	342
San Jose del Monte City	0	21	449	470	0	50	1,453	1,503	0	124	1,926	2,050
Science City of Munoz	0	5	40	45	0	24	560	584	0	24	260	284
Tarlac City	0	41	182	223	0	50	920	970	0	62	329	391
**Region 4A**	**32**	**751**	**8,304**	**9,087**	**85**	**1,320**	**31,472**	**32,877**	**55**	**1,864**	**36,810**	**38,729**
Batangas	22	73	1,487	1,582	45	117	3,196	3,358	15	86	2,335	2,436
Cavite	0	38	803	841	3	182	5,832	6,017	4	186	5,188	5,378
Laguna	1	44	347	392	0	100	2,226	2,326	1	108	1,837	1,946
Quezon	6	103	1,187	1,296	34	212	6,039	6,285	29	309	6,981	7,319
Rizal	0	83	751	834	0	166	4,282	4,448	3	444	7,482	7,929
Antipolo City	0	23	612	635	0	43	413	456	0	57	1,994	2,051
Bacoor City	0	4	93	97	0	6	324	330	0	32	882	914
Batangas City	0	2	67	69	0	17	1,039	1,056	0	26	810	836
Biñan City	0	129	895	1,024	0	125	1,432	1,557	0	118	1,244	1,362
Cabuyao City	1	51	121	173	0	43	262	305	0	54	286	340
Calamba City	0	42	188	230	0	43	527	570	0	31	476	507
Cavite City	0	1	13	14	0	5	127	132	0	18	182	200
Dasmariñas City	0	34	330	364	0	77	969	1,046	0	56	1,333	1,389
General Trias City	1	7	202	210	2	7	582	591	2	21	782	805
Imus City	1	5	136	142	0	12	359	371	0	46	671	717
Lipa City	0	0	10	10	0	2	38	40	0	2	62	64
Lucena City	0	52	281	333	1	43	864	908	0	85	1,296	1,381
San Pablo City	0	4	83	87	0	29	1,287	1,316	0	23	533	556
San Pedro City	0	30	403	433	0	43	663	706	0	42	592	634
Santa Rosa City	0	10	108	118	0	30	249	279	0	74	550	624
Tagaytay City	0	0	53	53	0	1	260	261	0	1	266	267
Tanauan City	0	2	24	26	0	0	34	34	0	5	129	134
Tayabas City	0	13	59	72	0	8	212	220	1	34	542	577
Trece Martires City	0	1	51	52	0	9	256	265	0	6	357	363
**Region 4B**	**21**	**967**	**12,873**	**13,861**	**64**	**1,644**	**26,369**	**28,077**	**0**	**2,632**	**22,364**	**24,996**
Marinduque	0	8	2,850	2,858	0	1	628	629	0	10	562	572
Mindoro Occidental	0	85	1,293	1,378	6	127	3,331	3,464	0	117	2,242	2,359
Mindoro Oriental	6	111	1,907	2,024	34	121	10,965	11,120	0	203	3,212	3,415
Palawan	10	470	4,619	5,099	20	1,174	9,628	10,822	0	1,551	14,196	15,747
Romblon	0	11	406	417	2	9	721	732	0	35	691	726
Puerto Princesa City	5	282	1,798	2,085	2	212	1,096	1,310	0	716	1,461	2,177
**Region 5**	**8**	**459**	**8,080**	**8,547**	**1**	**535**	**20,844**	**21,380**	**3**	**661**	**13,999**	**14,663**
Albay	0	36	1,062	1,098	0	125	4,585	4,710	0	84	3,133	3,217

Table 2.A.2. MODERN METHOD OF FAMILY PLANNING
Other Acceptors
Philippines, 2022

Area	PILLS-POP Age group 10-14	15-19	20-49	Total	PILLS-COC Age group 10-14	15-19	20-49	Total	INJECTABLES Age group 10-14	15-19	20-49	Total
Camarines Norte	0	125	1,132	1,257	0	69	1,495	1,564	1	133	1,441	1,575
Camarines Sur	0	111	2,223	2,334	0	94	4,199	4,293	0	125	3,315	3,440
Catanduanes	0	8	105	113	1	22	1,092	1,115	0	75	1,655	1,730
Masbate	8	113	2,181	2,302	0	120	5,632	5,752	2	104	1,793	1,899
Sorsogon	0	61	1,244	1,305	0	93	3,296	3,389	0	135	2,338	2,473
Iriga City	0	2	34	36	0	2	99	101	0	1	127	128
Legaspi City	0	3	61	64	0	3	79	82	0	2	42	44
Naga City	0	0	38	38	0	7	367	374	0	2	155	157
**Region 6**	**1**	**647**	**4,981**	**5,629**	**4**	**778**	**21,793**	**22,575**	**6**	**890**	**13,017**	**13,913**
Aklan	0	11	302	313	2	18	1,123	1,143	2	50	864	916
Antique	0	21	469	490	0	47	1,649	1,696	0	39	1,516	1,555
Capiz	0	7	252	259	0	23	1,327	1,350	0	28	628	656
Guimaras	0	12	158	170	0	14	848	862	0	6	267	273
Iloilo	0	313	1,472	1,785	0	132	5,563	5,695	0	226	2,832	3,058
Negros Occidental	0	241	2,092	2,333	1	370	8,915	9,286	0	502	5,701	6,203
Bacolod City	1	9	102	112	1	46	493	540	4	27	701	732
Iloilo City	0	33	134	167	0	128	1,875	2,003	0	12	508	520
**Region 7**	**1**	**346**	**4,283**	**4,630**	**3**	**434**	**17,111**	**17,548**	**5**	**754**	**16,425**	**17,184**
Bohol	1	45	722	768	0	46	2,149	2,195	0	102	2,288	2,390
Cebu	0	136	1,621	1,757	2	147	7,262	7,411	2	280	5,548	5,830
Negros Oriental	0	116	1,040	1,156	1	184	5,091	5,276	3	229	5,345	5,577
Siquijor	0	3	107	110	0	6	347	353	0	21	380	401
Cebu City	0	27	571	598	0	31	1,658	1,689	0	106	1,993	2,099
Lapu-Lapu City	0	16	89	105	0	14	91	105	0	10	219	229
Mandaue City	0	3	133	136	0	6	513	519	0	6	652	658
**Region 8**	**0**	**355**	**3,667**	**4,022**	**10**	**577**	**15,995**	**16,582**	**9**	**775**	**8,779**	**9,563**
Biliran	0	5	97	102	0	9	518	527	0	19	367	386
Eastern Samar	0	4	69	73	0	72	963	1,035	1	33	1,662	1,696
Northern Leyte	0	90	613	703	3	125	2,275	2,403	0	105	1,074	1,179
Northern Samar	0	12	319	331	0	26	886	912	0	37	1,454	1,491
Southern Leyte	0	51	1,017	1,068	3	146	5,105	5,254	6	354	629	989
Western Samar	0	10	151	161	0	31	1,214	1,245	0	53	817	870
Calbayog City	0	118	827	945	4	50	2,057	2,111	2	49	857	908
Maasin City	0	2	25	27	0	0	173	173	0	1	55	56
Ormoc City	0	63	534	597	0	115	2,710	2,825	0	121	1,738	1,859
Tacloban City	0	0	15	15	0	3	94	97	0	3	126	129
**Region 9**	**0**	**252**	**3,419**	**3,671**	**1**	**583**	**12,192**	**12,776**	**3**	**632**	**12,068**	**12,703**
Zamboanga del Norte	0	28	686	714	1	151	2,766	2,918	1	182	2,786	2,969
Zamboanga del Sur	0	49	812	861	0	177	4,137	4,314	0	137	3,173	3,310
Zamboanga Sibugay	0	75	888	963	0	76	1,718	1,794	0	111	2,236	2,347
Dapitan City	0	7	35	42	0	5	154	159	0	17	243	260
Dipolog City	0	26	238	264	0	20	532	552	0	56	821	877
Isabela City	0	3	32	35	0	9	146	155	0	10	293	303
Pagadian City	0	11	246	257	0	15	411	426	0	39	558	597
Zamboanga City	0	53	482	535	0	130	2,328	2,458	2	80	1,958	2,040
**Region 10**	**6**	**749**	**4,242**	**4,997**	**7**	**1,578**	**24,936**	**26,521**	**14**	**953**	**9,881**	**10,848**
Bukidnon	3	311	1,399	1,713	6	736	9,342	10,084	2	371	3,439	3,812
Camiguin	0	0	7	7	0	3	167	170	0	0	76	76
Lanao del Norte	0	26	403	429	1	161	1,791	1,953	0	93	1,043	1,136
Misamis Occidental	0	5	122	127	0	8	390	398	0	9	232	241
Misamis Oriental	3	196	631	830	0	132	5,764	5,896	10	249	1,737	1,996

Table 2.A.2. MODERN METHOD OF FAMILY PLANNING
Other Acceptors
Philippines, 2022

Area	PILLS-POP Age group			Total	PILLS-COC Age group			Total	INJECTABLES Age group			Total
	10-14	15-19	20-49		10-14	15-19	20-49		10-14	15-19	20-49	
Cagayan de Oro City	0	19	95	114	0	25	563	588	0	23	371	394
El Salvador City	0	6	53	59	0	7	248	255	0	14	195	209
Gingoog City	0	11	395	406	0	56	1,388	1,444	0	33	454	487
Iligan City	0	45	284	329	0	49	592	641	1	23	672	696
Malaybalay City	0	41	424	465	0	99	2,152	2,251	1	40	738	779
Oroquieta City	0	0	18	18	0	0	66	66	0	1	26	27
Ozamis City	0	0	16	16	0	1	84	85	0	1	84	85
Tangub City	0	8	123	131	0	83	861	944	0	14	136	150
Valencia City	0	81	272	353	0	218	1,528	1,746	0	82	678	760
**Region 11**	**12**	**526**	**4,993**	**5,531**	**10**	**1,402**	**18,547**	**19,959**	**13**	**814**	**9,389**	**10,216**
Davao de Oro	0	73	939	1,012	0	203	3,493	3,696	2	83	1,448	1,533
Davao del Norte	0	85	1,033	1,118	2	332	5,109	5,443	1	183	2,143	2,327
Davao Oriental	0	57	519	576	3	193	1,586	1,782	1	88	1,063	1,152
Davao del Sur	1	34	187	222	0	192	2,420	2,612	1	104	1,053	1,158
Davao Occidental	3	101	580	684	0	206	2,860	3,066	1	114	1,260	1,375
Davao City	8	176	1,735	1,919	5	276	3,079	3,360	7	242	2,422	2,671
**Region 12**	**4**	**550**	**4,606**	**5,160**	**5**	**1,277**	**20,088**	**21,370**	**17**	**1,154**	**12,697**	**13,868**
North Cotabato	3	148	1,511	1,662	0	283	5,573	5,856	12	291	3,511	3,814
Sarangani	0	97	769	866	0	204	3,576	3,780	0	232	2,254	2,486
South Cotabato	0	101	914	1,015	2	344	5,095	5,441	0	247	3,068	3,315
Sultan Kudarat	0	104	714	818	2	300	4,348	4,650	2	213	2,096	2,311
				0				0				0
Gen. Santos City	1	100	698	799	1	146	1,496	1,643	3	171	1,768	1,942
**BARMM**	**9**	**293**	**3,251**	**3,553**	**3**	**589**	**13,825**	**14,417**	**7**	**720**	**17,750**	**18,477**
Basilan	0	17	146	163	1	23	486	510	2	59	734	795
Lanao del Sur	0	36	846	882	0	38	1,367	1,405	0	56	1,730	1,786
Maguindanao	0	107	677	784	0	184	3,554	3,738	2	278	4,635	4,915
Sulu	9	69	625	703	1	121	6,011	6,133	0	112	8,016	8,128
Tawi-Tawi	0	3	118	121	0	125	918	1,043	1	80	900	981
Lamitan City	0	4	29	33	0	9	149	158	1	38	323	362
Marawi City	0	10	404	414	0	4	289	293	0	3	277	280
Cotabato City	0	47	406	453	1	85	1,051	1,137	1	94	1,135	1,230
**CARAGA**	**7**	**549**	**5,492**	**6,048**	**11**	**654**	**15,149**	**15,814**	**9**	**376**	**6,703**	**7,088**
Agusan del Norte	4	36	524	564	0	88	1,418	1,506	3	72	827	902
Agusan del Sur	0	126	1,086	1,212	1	143	2,455	2,599	0	54	897	951
Surigao del Norte	2	160	1,407	1,569	0	158	4,612	4,770	4	100	2,739	2,843
Surigao del Sur	0	79	1,003	1,082	8	81	2,855	2,944	2	54	1,041	1,097
Province of Dinagat	0	8	105	113	0	12	371	383	0	22	214	236
Bislig City	1	36	370	407	2	33	874	909	0	9	123	132
Butuan City	0	86	839	925	0	110	2,283	2,393	0	54	695	749
Surigao City	0	18	158	176	0	29	281	310	0	11	167	178

Table 2.A.2. MODERN METHOD OF FAMILY PLANNING
Other Acceptors
Philippines, 2022

Area	IMPLANTS			Total	NFP-CCM			Total	NFP-BBT			Total
	Age group				Age group				Age group			
	10-14	15-19	20-49		10-14	15-19	20-49		10-14	15-19	20-49	
**PHILIPPINES**	**145**	**11,130**	**154,908**	**166,183**	**5**	**1,196**	**10,735**	**11,936**	**31**	**81**	**1,394**	**1,506**
**N C R**	**12**	**1,320**	**21,890**	**23,222**	**0**	**0**	**229**	**229**	**0**	**4**	**10**	**14**
Malabon	0	106	827	933	0	0	0	0	0	0	0	0
Navotas	2	87	326	415	0	0	1	1	0	0	0	0
Valenzuela City	0	107	2,724	2,831	0	0	0	0	0	0	0	0
Caloocan City	0	243	2,936	3,179	0	0	38	38	0	0	0	0
Marikina City	0	22	640	662	0	0	0	0	0	0	0	0
Pasig City	0	29	1,339	1,368	0	0	0	0	0	0	0	0
Pateros	0	0	167	167	0	0	0	0	0	0	0	0
Taguig	1	18	453	472	0	0	0	0	0	0	0	0
Quezon City	4	435	7,562	8,001	0	0	0	0	0	0	0	0
Makati City	0	8	188	196	0	0	0	0	0	0	0	0
Mandaluyong City	0	30	533	563	0	0	100	100	0	4	3	7
San Juan	0	0	20	20	0	0	0	0	0	0	0	0
Manila City	4	157	2,224	2,385	0	0	0	0	0	0	4	4
Las Piñas City	1	51	1,054	1,106	0	0	0	0	0	0	0	0
Muntinlupa City	0	4	120	124	0	0	0	0	0	0	0	0
Parañaque City	0	23	300	323	0	0	0	0	0	0	3	3
Pasay City	0	0	477	477	0	0	90	90	0	0	0	0
**C A R**	**11**	**234**	**4,093**	**4,338**	**0**	**83**	**920**	**1,003**	**0**	**2**	**108**	**110**
Abra	0	26	334	360	0	18	247	265	0	0	16	16
Apayao	2	33	548	583	0	0	1	1	0	0	0	0
Benguet	0	12	688	700	0	0	1	1	0	0	17	17
Ifugao	0	25	325	350	0	2	195	197	0	0	6	6
Kalinga	1	33	511	545	0	63	372	435	0	2	69	71
Mt. Province	0	31	377	408	0	0	104	104	0	0	0	0
Baguio City	8	74	1,310	1,392	0	0	0	0	0	0	0	0
**Region 1**	**1**	**196**	**3,141**	**3,338**	**0**	**6**	**966**	**972**	**0**	**0**	**114**	**114**
Ilocos Norte	0	9	115	124	0	3	50	53	0	0	0	0
Ilocos Sur	0	7	163	170	0	0	25	25	0	0	0	0
La Union	1	43	614	658	0	1	228	229	0	0	9	9
Pangasinan	0	134	1,880	2,014	0	1	8	9	0	0	5	5
Alaminos City	0	0	238	238	0	0	0	0	0	0	0	0
Candon City	0	0	0	0	0	0	0	0	0	0	0	0
Dagupan City	0	1	7	8	0	0	0	0	0	0	0	0
Laoag City	0	0	0	0	0	0	0	0	0	0	0	0
San Carlos City	0	0	57	57	0	0	0	0	0	0	0	0
San Fernando City	0	2	38	40	0	0	0	0	0	0	0	0
Urdaneta City	0	0	27	27	0	0	0	0	0	0	0	0
Vigan City	0	0	2	2	0	1	655	656	0	0	100	100
**Region 2**	**3**	**168**	**3,339**	**3,510**	**0**	**1**	**139**	**140**	**0**	**2**	**1**	**3**
Batanes	0	0	0	0	0	1	19	20	0	0	0	0
Cagayan	2	35	731	768	0	0	6	6	0	0	0	0
Isabela	0	61	878	939	0	0	2	2	0	0	0	0
Nueva Vizcaya	1	39	1,289	1,329	0	0	112	112	0	2	1	3
Quirino	0	14	142	156	0	0	0	0	0	0	0	0
Cauayan City	0	1	65	66	0	0	0	0	0	0	0	0
Ilagan City	0	5	64	69	0	0	0	0	0	0	0	0
Santiago City	0	12	133	145	0	0	0	0	0	0	0	0
Tuguegarao City	0	1	37	38	0	0	0	0	0	0	0	0

Table 2.A.2. MODERN METHOD OF FAMILY PLANNING
Other Acceptors
Philippines, 2022

Area	IMPLANTS Age group			Total	NFP-CCM Age group			Total	NFP-BBT Age group			Total
	10-14	15-19	20-49		10-14	15-19	20-49		10-14	15-19	20-49	
**Region 3**	**11**	**560**	**10,560**	**11,131**	**0**	**33**	**101**	**134**	**0**	**5**	**132**	**137**
Aurora	0	45	550	595	0	0	35	35	0	2	2	4
Bataan	0	60	1,320	1,380	0	0	0	0	0	0	0	0
Bulacan	0	115	1,429	1,544	0	0	0	0	0	0	0	0
Nueva Ecija	1	106	3,187	3,294	0	1	3	4	0	0	0	0
Pampanga	10	60	946	1,016	0	30	44	74	0	3	116	119
Tarlac	0	26	790	816	0	0	0	0	0	0	0	0
Zambales	0	48	757	805	0	0	0	0	0	0	1	1
Angeles City	0	7	297	304	0	0	4	4	0	0	8	8
Balanga City	0	7	98	105	0	0	0	0	0	0	0	0
Cabanatuan City	0	5	72	77	0	0	0	0	0	0	0	0
City of San Fernando	0	2	38	40	0	2	15	17	0	0	2	2
Gapan City	0	1	16	17	0	0	0	0	0	0	0	0
Mabalacat City	0	12	97	109	0	0	0	0	0	0	0	0
Malolos City	0	1	170	171	0	0	0	0	0	0	0	0
Meycauayan	0	1	30	31	0	0	0	0	0	0	0	0
Olongapo	0	1	45	46	0	0	0	0	0	0	3	3
Palayan City	0	0	0	0	0	0	0	0	0	0	0	0
San Jose City	0	1	23	24	0	0	0	0	0	0	0	0
San Jose del Monte City	0	62	603	665	0	0	0	0	0	0	0	0
Science City of Munoz	0	0	23	23	0	0	0	0	0	0	0	0
Tarlac City	0	0	69	69	0	0	0	0	0	0	0	0
**Region 4A**	**21**	**654**	**10,703**	**11,378**	**0**	**98**	**730**	**828**	**30**	**3**	**92**	**125**
Batangas	3	20	408	431	0	82	569	651	30	0	16	46
Cavite	0	160	2,339	2,499	0	0	34	34	0	2	17	19
Laguna	9	134	1,507	1,650	0	1	45	46	0	0	0	0
Quezon	3	34	893	930	0	0	7	7	0	0	5	5
Rizal	3	129	2,322	2,454	0	0	0	0	0	1	0	1
Antipolo City	0	8	223	231	0	0	73	73	0	0	0	0
Bacoor City	0	4	85	89	0	0	0	0	0	0	0	0
Batangas City	0	21	157	178	0	0	0	0	0	0	0	0
Biñan City	0	9	86	95	0	0	2	2	0	0	0	0
Cabuyao City	1	5	127	133	0	0	0	0	0	0	0	0
Calamba City	1	35	531	567	0	0	0	0	0	0	0	0
Cavite City	0	13	71	84	0	0	0	0	0	0	0	0
Dasmariñas City	0	28	409	437	0	0	0	0	0	0	11	11
General Trias City	0	7	126	133	0	0	0	0	0	0	0	0
Imus City	0	9	226	235	0	0	0	0	0	0	0	0
Lipa City	0	4	59	63	0	0	0	0	0	0	0	0
Lucena City	0	3	111	114	0	0	0	0	0	0	0	0
San Pablo City	1	8	85	94	0	0	0	0	0	0	18	18
San Pedro City	0	4	148	152	0	15	0	15	0	0	25	25
Santa Rosa City	0	8	353	361	0	0	0	0	0	0	0	0
Tagaytay City	0	0	254	254	0	0	0	0	0	0	0	0
Tanauan City	0	1	55	56	0	0	0	0	0	0	0	0
Tayabas City	0	0	18	18	0	0	0	0	0	0	0	0
Trece Martires City	0	10	110	120	0	0	0	0	0	0	0	0
**Region 4B**	**0**	**880**	**10,563**	**11,443**	**1**	**29**	**1,298**	**1,328**	**0**	**10**	**154**	**164**
Marinduque	0	9	484	493	0	0	141	141	0	0	0	0
Mindoro Occidental	0	111	1,839	1,950	0	17	335	352	0	1	13	14
Mindoro Oriental	0	78	1,419	1,497	0	2	199	201	0	5	106	111
Palawan	0	564	5,806	6,370	0	5	572	577	0	2	34	36
Romblon	0	28	453	481	1	5	46	52	0	0	1	1
Puerto Princesa City	0	90	562	652	0	0	5	5	0	2	0	2
**Region 5**	**8**	**439**	**8,593**	**9,040**	**4**	**830**	**3,997**	**4,831**	**0**	**6**	**91**	**97**
Albay	0	48	1,116	1,164	0	2	189	191	0	1	1	2

Table 2.A.2. MODERN METHOD OF FAMILY PLANNING
Other Acceptors
Philippines, 2022

Area	IMPLANTS Age group			Total	NFP-CCM Age group			Total	NFP-BBT Age group			Total
	10-14	15-19	20-49		10-14	15-19	20-49		10-14	15-19	20-49	
Camarines Norte	0	81	715	796	0	0	236	236	0	0	8	8
Camarines Sur	1	149	3,327	3,477	0	2	209	211	0	1	14	15
Catanduanes	0	51	1,158	1,209	0	13	378	391	0	2	14	16
Masbate	6	29	870	905	4	793	2,030	2,827	0	0	40	40
Sorsogon	1	78	1,203	1,282	0	20	955	975	0	1	7	8
Iriga City	0	2	142	144	0	0	0	0	0	1	7	8
Legaspi City	0	0	16	16	0	0	0	0	0	0	0	0
Naga City	0	1	46	47	0	0	0	0	0	0	0	0
**Region 6**	**4**	**707**	**8,754**	**9,465**	**0**	**19**	**333**	**352**	**0**	**0**	**70**	**70**
Aklan	0	17	237	254	0	0	0	0	0	0	9	9
Antique	0	44	828	872	0	19	315	334	0	0	0	0
Capiz	0	17	280	297	0	0	0	0	0	0	6	6
Guimaras	0	1	154	155	0	0	0	0	0	0	0	0
Iloilo	2	195	2,202	2,399	0	0	3	3	0	0	29	29
Negros Occidental	1	350	3,578	3,929	0	0	14	14	0	0	26	26
Bacolod City	1	46	1,085	1,132	0	0	0	0	0	0	0	0
Iloilo City	0	37	390	427	0	0	1	1	0	0	0	0
**Region 7**	**7**	**594**	**10,634**	**11,235**	**0**	**7**	**311**	**318**	**0**	**0**	**32**	**32**
Bohol	1	87	2,311	2,399	0	0	0	0	0	0	0	0
Cebu	6	371	5,337	5,714	0	1	5	6	0	0	0	0
Negros Oriental	0	77	1,096	1,173	0	6	306	312	0	0	7	7
Siquijor	0	3	107	110	0	0	0	0	0	0	0	0
Cebu City	0	42	1,101	1,143	0	0	0	0	0	0	25	25
Lapu-Lapu City	0	1	73	74	0	0	0	0	0	0	0	0
Mandaue City	0	13	609	622	0	0	0	0	0	0	0	0
**Region 8**	**3**	**313**	**4,184**	**4,500**	**0**	**9**	**333**	**342**	**0**	**1**	**145**	**146**
Biliran	0	35	262	297	0	0	58	58	0	1	5	6
Eastern Samar	0	20	565	585	0	0	136	136	0	0	33	33
Northern Leyte	1	39	533	573	0	0	0	0	0	0	0	0
Northern Samar	1	17	484	502	0	0	0	0	0	0	2	2
Southern Leyte	1	101	564	666	0	0	90	90	0	0	101	101
Western Samar	0	15	426	441	0	0	49	49	0	0	2	2
Calbayog City	0	8	80	88	0	0	0	0	0	0	0	0
Maasin City	0	5	86	91	0	0	0	0	0	0	0	0
Ormoc City	0	67	1,099	1,166	0	9	0	9	0	0	2	2
Tacloban City	0	6	85	91	0	0	0	0	0	0	0	0
**Region 9**	**9**	**964**	**12,834**	**13,807**	**0**	**0**	**39**	**39**	**0**	**0**	**0**	**0**
Zamboanga del Norte	2	200	3,157	3,359	0	0	3	3	0	0	0	0
Zamboanga del Sur	2	426	4,906	5,334	0	0	0	0	0	0	0	0
Zamboanga Sibugay	1	161	2,297	2,459	0	0	36	36	0	0	0	0
Dapitan City	0	9	68	77	0	0	0	0	0	0	0	0
Dipolog City	1	64	417	482	0	0	0	0	0	0	0	0
Isabela City	0	3	79	82	0	0	0	0	0	0	0	0
Pagadian City	0	12	352	364	0	0	0	0	0	0	0	0
Zamboanga City	3	89	1,558	1,650	0	0	0	0	0	0	0	0
**Region 10**	**26**	**1,228**	**11,362**	**12,616**	**0**	**55**	**788**	**843**	**1**	**34**	**192**	**227**
Bukidnon	9	741	5,011	5,761	0	15	144	159	0	3	18	21
Camiguin	0	3	36	39	0	0	0	0	0	1	67	68
Lanao del Norte	0	46	1,096	1,142	0	0	7	7	0	0	11	11
Misamis Occidental	0	15	76	91	0	0	0	0	0	0	0	0
Misamis Oriental	11	212	2,515	2,738	0	25	283	308	1	25	48	74

Table 2.A.2. MODERN METHOD OF FAMILY PLANNING
Other Acceptors
Philippines, 2022

Area	IMPLANTS			Total	NFP-CCM			Total	NFP-BBT			Total
	Age group				Age group				Age group			
	10-14	15-19	20-49		10-14	15-19	20-49		10-14	15-19	20-49	
Cagayan de Oro City	1	45	468	514	0	0	0	0	0	0	0	0
El Salvador City	1	16	244	261	0	0	0	0	0	0	0	0
Gingoog City	4	15	138	157	0	11	218	229	0	0	18	18
Iligan City	0	0	184	184	0	0	70	70	0	0	0	0
Malaybalay City	0	99	1,095	1,194	0	0	1	1	0	0	1	1
Oroquieta City	0	0	5	5	0	0	0	0	0	0	0	0
Ozamis City	0	11	74	85	0	0	0	0	0	0	5	5
Tangub City	0	0	16	16	0	0	0	0	0	0	0	0
Valencia City	0	25	404	429	0	4	65	69	0	5	24	29
**Region 11**	**15**	**546**	**7,152**	**7,713**	**0**	**5**	**128**	**133**	**0**	**0**	**75**	**75**
Davao de Oro	2	72	1,231	1,305	0	0	79	79	0	0	1	1
Davao del Norte	3	102	1,776	1,881	0	0	8	8	0	0	0	0
Davao Oriental	0	56	610	666	0	0	0	0	0	0	0	0
Davao del Sur	1	80	793	874	0	0	0	0	0	0	0	0
Davao Occidental	2	76	826	904	0	0	27	27	0	0	50	50
Davao City	7	160	1,916	2,083	0	5	14	19	0	0	24	24
**Region 12**	**4**	**1,089**	**10,640**	**11,733**	**0**	**4**	**178**	**182**	**0**	**5**	**25**	**30**
North Cotabato	1	196	2,590	2,787	0	1	110	111	0	0	12	12
Sarangani	0	132	1,851	1,983	0	0	3	3	0	0	0	0
South Cotabato	2	361	3,001	3,364	0	3	50	53	0	5	4	9
Sultan Kudarat	1	234	1,582	1,817	0	0	15	15	0	0	5	5
				0				0				0
Gen. Santos City	0	166	1,616	1,782	0	0	0	0	0	0	4	4
**BARMM**	**2**	**357**	**5,094**	**5,453**	**0**	**0**	**0**	**0**	**0**	**0**	**2**	**2**
Basilan	1	64	608	673	0	0	0	0	0	0	0	0
Lanao del Sur	0	8	368	376	0	0	0	0	0	0	0	0
Maguindanao	1	97	1,196	1,294	0	0	0	0	0	0	2	2
Sulu	0	41	1,235	1,276	0	0	0	0	0	0	0	0
Tawi-Tawi	0	52	865	917	0	0	0	0	0	0	0	0
Lamitan City	0	33	252	285	0	0	0	0	0	0	0	0
Marawi City	0	0	80	80	0	0	0	0	0	0	0	0
Cotabato City	0	62	490	552	0	0	0	0	0	0	0	0
**CARAGA**	**8**	**881**	**11,372**	**12,261**	**0**	**17**	**245**	**262**	**0**	**9**	**151**	**160**
Agusan del Norte	0	142	1,017	1,159	0	0	2	2	0	0	0	0
Agusan del Sur	3	284	3,157	3,444	0	16	186	202	0	7	25	32
Surigao del Norte	4	178	2,984	3,166	0	0	14	14	0	0	96	96
Surigao del Sur	1	123	2,258	2,382	0	0	3	3	0	2	30	32
Province of Dinagat	0	25	407	432	0	0	0	0	0	0	0	0
Bislig City	0	18	251	269	0	1	23	24	0	0	0	0
Butuan City	0	82	1,046	1,128	0	0	17	17	0	0	0	0
Surigao City	0	29	252	281	0	0	0	0	0	0	0	0

Table 2.A.2. MODERN METHOD OF FAMILY PLANNING
Other Acceptors
Philippines, 2022

Area	NFP-STM			Total	NFP-SDM			Total	NFP-LAM			Total
	Age group				Age group				Age group			
	10-14	15-19	20-49		10-14	15-19	20-49		10-14	15-19	20-49	
**PHILIPPINES**	0	67	1,121	1,188	1	966	21,473	22,440	376	19,311	347,482	367,169
**N C R**	0	0	9	9	0	8	188	196	37	1,552	37,690	39,279
Malabon	0	0	0	0	0	0	1	1	0	37	873	910
Navotas	0	0	0	0	0	0	0	0	3	75	574	652
Valenzuela City	0	0	0	0	0	0	0	0	0	67	2,183	2,250
Caloocan City	0	0	0	0	0	6	12	18	0	225	4,010	4,235
Marikina City	0	0	0	0	0	0	1	1	0	5	151	156
Pasig City	0	0	0	0	0	0	0	0	0	8	353	361
Pateros	0	0	0	0	0	0	0	0	0	0	64	64
Taguig	0	0	0	0	0	0	1	1	0	22	709	731
Quezon City	0	0	0	0	0	0	72	72	0	829	23,375	24,204
Makati City	0	0	0	0	0	0	0	0	0	3	131	134
Mandaluyong City	0	0	0	0	0	0	88	88	0	37	106	143
San Juan	0	0	0	0	0	0	0	0	0	2	17	19
Manila City	0	0	9	9	0	0	6	6	27	70	573	670
Las Piñas City	0	0	0	0	0	0	1	1	0	11	159	170
Muntinlupa City	0	0	0	0	0	0	0	0	0	6	54	60
Parañaque City	0	0	0	0	0	2	3	5	7	44	219	270
Pasay City	0	0	0	0	0	0	3	3	0	111	4,139	4,250
**C A R**	0	1	18	19	0	28	2,313	2,341	6	465	9,610	10,081
Abra	0	1	15	16	0	2	121	123	0	56	1,307	1,363
Apayao	0	0	0	0	0	0	7	7	1	66	962	1,029
Benguet	0	0	0	0	0	9	1,122	1,131	0	79	2,001	2,080
Ifugao	0	0	0	0	0	15	604	619	1	44	1,687	1,732
Kalinga	0	0	0	0	0	0	130	130	3	151	2,022	2,176
Mt. Province	0	0	3	3	0	2	319	321	1	63	1,268	1,332
Baguio City	0	0	0	0	0	0	10	10	0	6	363	369
**Region 1**	0	0	129	129	0	25	605	630	7	737	18,583	19,327
Ilocos Norte	0	0	1	1	0	0	191	191	0	31	1,093	1,124
Ilocos Sur	0	0	0	0	0	22	130	152	0	60	1,091	1,151
La Union	0	0	28	28	0	0	93	93	4	191	3,739	3,934
Pangasinan	0	0	0	0	0	3	172	175	3	375	10,889	11,267
Alaminos City	0	0	0	0	0	0	0	0	0	7	63	70
Candon City	0	0	0	0	0	0	0	0	0	0	0	0
Dagupan City	0	0	0	0	0	0	0	0	0	2	10	12
Laoag City	0	0	0	0	0	0	0	0	0	0	0	0
San Carlos City	0	0	0	0	0	0	0	0	0	24	1,157	1,181
San Fernando City	0	0	0	0	0	0	16	16	0	2	29	31
Urdaneta City	0	0	0	0	0	0	0	0	0	11	262	273
Vigan City	0	0	100	100	0	0	3	3	0	34	250	284
**Region 2**	0	3	86	89	0	0	207	207	19	645	12,668	13,332
Batanes	0	0	0	0	0	0	0	0	0	4	155	159
Cagayan	0	0	0	0	0	0	0	0	0	97	2,667	2,764
Isabela	0	0	0	0	0	0	23	23	1	114	2,908	3,023
Nueva Vizcaya	0	3	86	89	0	0	184	184	17	259	3,043	3,319
Quirino	0	0	0	0	0	0	0	0	0	37	461	498
Cauayan City	0	0	0	0	0	0	0	0	1	56	715	772
Ilagan City	0	0	0	0	0	0	0	0	0	21	663	684
Santiago City	0	0	0	0	0	0	0	0	0	50	1,916	1,966
Tuguegarao City	0	0	0	0	0	0	0	0	0	7	140	147

Table 2.A.2. MODERN METHOD OF FAMILY PLANNING
Other Acceptors
Philippines, 2022

Area	NFP-STM Age group 10-14	NFP-STM Age group 15-19	NFP-STM Age group 20-49	Total	NFP-SDM Age group 10-14	NFP-SDM Age group 15-19	NFP-SDM Age group 20-49	Total	NFP-LAM Age group 10-14	NFP-LAM Age group 15-19	NFP-LAM Age group 20-49	Total
**Region 3**	**0**	**0**	**8**	**8**	**0**	**9**	**1,165**	**1,174**	**17**	**1,249**	**19,737**	**21,003**
Aurora	0	0	0	0	0	0	0	0	0	85	1,684	1,769
Bataan	0	0	0	0	0	0	0	0	1	40	737	778
Bulacan	0	0	0	0	0	4	479	483	1	249	3,664	3,914
Nueva Ecija	0	0	0	0	0	2	3	5	10	113	2,585	2,708
Pampanga	0	0	0	0	0	1	615	616	0	130	2,669	2,799
Tarlac	0	0	0	0	0	0	0	0	3	105	1,823	1,931
Zambales	0	0	0	0	0	0	8	8	1	55	1,406	1,462
Angeles City	0	0	5	5	0	2	51	53	0	10	252	262
Balanga City	0	0	0	0	0	0	1	1	0	14	379	393
Cabanatuan City	0	0	0	0	0	0	0	0	0	46	1,302	1,348
City of San Fernando	0	0	3	3	0	0	0	0	1	25	210	236
Gapan City	0	0	0	0	0	0	0	0	0	14	127	141
Mabalacat City	0	0	0	0	0	0	8	8	0	102	730	832
Malolos City	0	0	0	0	0	0	0	0	0	0	131	131
Meycauayan	0	0	0	0	0	0	0	0	0	26	214	240
Olongapo	0	0	0	0	0	0	0	0	0	5	235	240
Palayan City	0	0	0	0	0	0	0	0	0	1	67	68
San Jose City	0	0	0	0	0	0	0	0	0	29	633	662
San Jose del Monte City	0	0	0	0	0	0	0	0	0	0	1	1
Science City of Munoz	0	0	0	0	0	0	0	0	0	0	0	0
Tarlac City	0	0	0	0	0	0	0	0	0	200	888	1,088
**Region 4A**	**0**	**0**	**19**	**19**	**0**	**27**	**408**	**435**	**45**	**1,482**	**30,446**	**31,973**
Batangas	0	0	0	0	0	22	168	190	17	127	5,197	5,341
Cavite	0	0	3	3	0	0	68	68	2	99	2,959	3,060
Laguna	0	0	1	1	0	3	43	46	0	112	1,932	2,044
Quezon	0	0	14	14	0	2	109	111	15	228	3,901	4,144
Rizal	0	0	0	0	0	0	6	6	6	352	6,375	6,733
Antipolo City	0	0	0	0	0	0	0	0	0	86	1,553	1,639
Bacoor City	0	0	0	0	0	0	0	0	0	30	490	520
Batangas City	0	0	0	0	0	0	12	12	0	19	825	844
Biñan City	0	0	0	0	0	0	0	0	0	53	552	605
Cabuyao City	0	0	0	0	0	0	0	0	0	54	261	315
Calamba City	0	0	0	0	0	0	2	2	0	25	462	487
Cavite City	0	0	0	0	0	0	0	0	0	20	76	96
Dasmariñas City	0	0	0	0	0	0	0	0	0	79	1,159	1,238
General Trias City	0	0	0	0	0	0	0	0	4	21	616	641
Imus City	0	0	0	0	0	0	0	0	0	11	437	448
Lipa City	0	0	0	0	0	0	0	0	0	0	0	0
Lucena City	0	0	0	0	0	0	0	0	0	5	60	65
San Pablo City	0	0	0	0	0	0	0	0	0	58	784	842
San Pedro City	0	0	0	0	0	0	0	0	0	22	591	613
Santa Rosa City	0	0	0	0	0	0	0	0	0	41	550	591
Tagaytay City	0	0	1	1	0	0	0	0	1	17	659	677
Tanauan City	0	0	0	0	0	0	0	0	0	0	47	47
Tayabas City	0	0	0	0	0	0	0	0	0	23	933	956
Trece Martires City	0	0	0	0	0	0	0	0	0	0	27	27
**Region 4B**	**0**	**0**	**62**	**62**	**0**	**14**	**392**	**406**	**67**	**1,526**	**20,122**	**21,715**
Marinduque	0	0	0	0	0	0	18	18	3	10	661	674
Mindoro Occidental	0	0	11	11	0	4	111	115	10	275	5,852	6,137
Mindoro Oriental	0	0	18	18	0	0	43	43	21	260	4,292	4,573
Palawan	0	0	22	22	0	7	56	63	24	815	7,586	8,425
Romblon	0	0	6	6	0	3	99	102	5	78	1,238	1,321
Puerto Princesa City	0	0	5	5	0	0	65	65	4	88	493	585
**Region 5**	**0**	**7**	**430**	**437**	**1**	**318**	**6,629**	**6,948**	**13**	**1,626**	**37,938**	**39,577**
Albay	0	0	4	4	0	9	635	644	4	103	3,394	3,501

Table 2.A.2. MODERN METHOD OF FAMILY PLANNING
Other Acceptors
Philippines, 2022

Area	NFP-STM			Total	NFP-SDM			Total	NFP-LAM			Total
	Age group				Age group				Age group			
	10-14	15-19	20-49		10-14	15-19	20-49		10-14	15-19	20-49	
Camarines Norte	0	1	203	204	0	4	322	326	4	233	4,113	4,350
Camarines Sur	0	6	126	132	0	25	1,980	2,005	1	353	11,185	11,539
Catanduanes	0	0	33	33	0	2	573	575	2	154	2,796	2,952
Masbate	0	0	64	64	1	259	2,242	2,502	0	423	8,047	8,470
Sorsogon	0	0	0	0	0	19	711	730	2	350	7,867	8,219
Iriga City	0	0	0	0	0	0	144	144	0	8	225	233
Legaspi City	0	0	0	0	0	0	0	0	0	2	39	41
Naga City	0	0	0	0	0	0	22	22	0	0	272	272
**Region 6**	**0**	**0**	**16**	**16**	**0**	**42**	**1,701**	**1,743**	**6**	**929**	**19,211**	**20,146**
Aklan	0	0	2	2	0	0	44	44	0	49	1,282	1,331
Antique	0	0	0	0	0	0	37	37	0	36	1,414	1,450
Capiz	0	0	0	0	0	0	0	0	0	16	718	734
Guimaras	0	0	0	0	0	0	27	27	0	20	1,032	1,052
Iloilo	0	0	14	14	0	8	791	799	1	211	3,797	4,009
Negros Occidental	0	0	0	0	0	34	797	831	5	519	10,328	10,852
Bacolod City	0	0	0	0	0	0	1	1	0	60	501	561
Iloilo City	0	0	0	0	0	0	4	4	0	18	139	157
**Region 7**	**0**	**0**	**0**	**0**	**0**	**11**	**123**	**134**	**3**	**817**	**22,396**	**23,216**
Bohol	0	0	0	0	0	0	18	18	0	129	2,284	2,413
Cebu	0	0	0	0	0	8	27	35	2	309	8,763	9,074
Negros Oriental	0	0	0	0	0	1	58	59	0	201	6,583	6,784
Siquijor	0	0	0	0	0	2	20	22	0	13	354	367
Cebu City	0	0	0	0	0	0	0	0	1	132	3,594	3,727
Lapu-Lapu City	0	0	0	0	0	0	0	0	0	32	603	635
Mandaue City	0	0	0	0	0	0	0	0	0	1	215	216
**Region 8**	**0**	**0**	**58**	**58**	**0**	**9**	**433**	**442**	**9**	**1,025**	**10,285**	**11,319**
Biliran	0	0	50	50	0	2	167	169	0	69	577	646
Eastern Samar	0	0	4	4	0	0	111	111	6	68	806	880
Northern Leyte	0	0	0	0	0	0	12	12	1	140	2,248	2,389
Northern Samar	0	0	3	3	0	7	55	62	1	62	1,288	1,351
Southern Leyte	0	0	0	0	0	0	5	5	1	463	903	1,367
Western Samar	0	0	1	1	0	0	45	45	0	102	1,696	1,798
Calbayog City	0	0	0	0	0	0	0	0	0	12	263	275
Maasin City	0	0	0	0	0	0	2	2	0	4	90	94
Ormoc City	0	0	0	0	0	0	36	36	0	96	2,116	2,212
Tacloban City	0	0	0	0	0	0	0	0	0	9	298	307
**Region 9**	**0**	**0**	**0**	**0**	**0**	**34**	**484**	**518**	**10**	**1,397**	**23,061**	**24,468**
Zamboanga del Norte	0	0	0	0	0	33	462	495	5	355	5,832	6,192
Zamboanga del Sur	0	0	0	0	0	0	0	0	1	221	3,156	3,378
Zamboanga Sibugay	0	0	0	0	0	1	21	22	1	135	2,498	2,634
Dapitan City	0	0	0	0	0	0	0	0	0	24	600	624
Dipolog City	0	0	0	0	0	0	1	1	1	55	1,322	1,378
Isabela City	0	0	0	0	0	0	0	0	0	14	381	395
Pagadian City	0	0	0	0	0	0	0	0	1	221	3,156	3,378
Zamboanga City	0	0	0	0	0	0	0	0	1	372	6,116	6,489
**Region 10**	**0**	**56**	**260**	**316**	**0**	**403**	**4,257**	**4,660**	**31**	**1,880**	**20,873**	**22,784**
Bukidnon	0	55	168	223	0	37	674	711	10	833	6,902	7,745
Camiguin	0	0	0	0	0	0	4	4	0	21	518	539
Lanao del Norte	0	1	5	6	0	0	9	9	14	201	2,422	2,637
Misamis Occidental	0	0	3	3	0	3	184	187	0	31	676	707
Misamis Oriental	0	0	57	57	0	11	347	358	1	300	2,343	2,644

Table 2.A.2. MODERN METHOD OF FAMILY PLANNING

Other Acceptors

Philippines, 2022

Area	NFP-STM			Total	NFP-SDM			Total	NFP-LAM			Total
	Age group				Age group				Age group			
	10-14	15-19	20-49		10-14	15-19	20-49		10-14	15-19	20-49	
Cagayan de Oro City	0	0	0	0	0	0	0	0	1	77	931	1,009
El Salvador City	0	0	5	5	0	0	10	10	0	6	273	279
Gingoog City	0	0	7	7	0	13	605	618	1	69	882	952
Iligan City	0	0	0	0	0	15	122	137	4	83	1,827	1,914
Malaybalay City	0	0	0	0	0	17	920	937	0	102	1,710	1,812
Oroquieta City	0	0	0	0	0	0	9	9	0	7	104	111
Ozamis City	0	0	0	0	0	0	54	54	0	28	609	637
Tangub City	0	0	12	12	0	302	1,104	1,406	0	9	279	288
Valencia City	0	0	3	3	0	5	215	220	0	113	1,397	1,510
**Region 11**	**0**	**0**	**7**	**7**	**0**	**4**	**382**	**386**	**75**	**850**	**9,122**	**10,047**
Davao de Oro	0	0	0	0	0	1	9	10	0	28	501	529
Davao del Norte	0	0	0	0	0	0	51	51	3	143	1,903	2,049
Davao Oriental	0	0	3	3	0	0	15	15	0	14	210	224
Davao del Sur	0	0	0	0	0	1	45	46	1	92	728	821
Davao Occidental	0	0	1	1	0	2	203	205	5	358	3,712	4,075
Davao City	0	0	3	3	0	0	59	59	66	215	2,068	2,349
**Region 12**	**0**	**0**	**18**	**18**	**0**	**1**	**238**	**239**	**16**	**1,332**	**15,261**	**16,609**
North Cotabato	0	0	16	16	0	0	27	27	2	131	2,737	2,870
Sarangani	0	0	0	0	0	1	38	39	0	421	4,850	5,271
South Cotabato	0	0	1	1	0	0	109	109	5	343	3,280	3,628
Sultan Kudarat	0	0	0	0	0	0	63	63	6	283	2,562	2,851
				0				0				0
Gen. Santos City	0	0	1	1	0	0	1	1	3	154	1,832	1,989
**BARMM**	**0**	**0**	**0**	**0**	**0**	**2**	**73**	**75**	**1**	**678**	**23,886**	**24,565**
Basilan	0	0	0	0	0	0	7	7	0	19	708	727
Lanao del Sur	0	0	0	0	0	0	13	13	0	121	6,836	6,957
Maguindanao	0	0	0	0	0	2	9	11	0	272	7,223	7,495
Sulu	0	0	0	0	0	0	0	0	0	63	5,195	5,258
Tawi-Tawi	0	0	0	0	0	0	44	44	0	111	2,160	2,271
Lamitan City	0	0	0	0	0	0	0	0	0	17	238	255
Marawi City	0	0	0	0	0	0	0	0	0	41	1,083	1,124
Cotabato City	0	0	0	0	0	0	0	0	1	34	443	478
**CARAGA**	**0**	**0**	**1**	**1**	**0**	**31**	**1,875**	**1,906**	**14**	**1,121**	**16,593**	**17,728**
Agusan del Norte	0	0	0	0	0	1	19	20	3	90	1,270	1,363
Agusan del Sur	0	0	1	1	0	13	193	206	8	258	3,658	3,924
Surigao del Norte	0	0	0	0	0	16	1,414	1,430	2	239	2,912	3,153
Surigao del Sur	0	0	0	0	0	0	82	82	0	161	3,846	4,007
Province of Dinagat	0	0	0	0	0	1	49	50	0	18	265	283
Bislig City	0	0	0	0	0	0	49	49	0	142	899	1,041
Butuan City	0	0	0	0	0	0	62	62	1	198	3,531	3,730
Surigao City	0	0	0	0	0	0	7	7	0	15	212	227

Table 2.A.3. MODERN METHOD OF FAMILY PLANNING
Drop Outs
Philippines, 2022

Area	Total Current Users	FSTR/BTL Age group			Total	MSTR/NSV Age group			Total
		10-14	15-19	20-49		10-14	15-19	20-49	
**PHILIPPINES**	**8,019,000**	**5**	**891**	**93,966**	**94,862**	**1**	**70**	**3,464**	**3,535**
**N C R**	**753,263**	**0**	**186**	**6,533**	**6,719**	**0**	**0**	**76**	**76**
Malabon	31,679	0	0	448	448	0	0	1	1
Navotas	23,423	0	0	59	59	0	0	1	1
Valenzuela City	39,922	0	0	2,148	2,148	0	0	39	39
Caloocan City	68,156	0	2	1,113	1,115	0	0	4	4
Marikina City	17,020	0	0	40	40	0	0	0	0
Pasig City	42,380	0	0	65	65	0	0	8	8
Pateros	2,674	0	0	104	104	0	0	1	1
Taguig	61,050	0	0	17	17	0	0	0	0
Quezon City	272,854	0	0	1,271	1,271	0	0	9	9
Makati City	16,005	0	0	0	0	0	0	0	0
Mandaluyong City	17,504	0	0	43	43	0	0	0	0
San Juan	5,796	0	0	30	30	0	0	1	1
Manila City	27,488	0	0	98	98	0	0	3	3
Las Piñas City	20,363	0	0	559	559	0	0	4	4
Muntinlupa City	40,839	0	0	18	18	0	0	0	0
Parañaque City	37,239	0	184	45	229	0	0	1	1
Pasay City	28,871	0	0	475	475	0	0	4	4
**C A R**	**147,908**	**0**	**0**	**3,707**	**3,707**	**0**	**1**	**38**	**39**
Abra	17,362	0	0	653	653	0	0	2	2
Apayao	23,877	0	0	1,654	1,654	0	0	2	2
Benguet	31,732	0	0	454	454	0	1	20	21
Ifugao	20,003	0	0	177	177	0	0	11	11
Kalinga	19,143	0	0	364	364	0	0	2	2
Mt. Province	13,215	0	0	313	313	0	0	0	0
Baguio City	22,576	0	0	92	92	0	0	1	1
**Region 1**	**477,257**	**0**	**10**	**6,046**	**6,056**	**0**	**0**	**23**	**23**
Ilocos Norte	41,393	0	2	927	929	0	0	1	1
Ilocos Sur	62,434	0	0	567	567	0	0	1	1
La Union	59,808	0	6	1,112	1,118	0	0	14	14
Pangasinan	238,991	0	2	2,093	2,095	0	0	5	5
Alaminos City	10,832	0	0	405	405	0	0	0	0
Candon City	7,006	0	0	3	3	0	0	0	0
Dagupan City	6,976	0	0	98	98	0	0	0	0
Laoag City	8,253	0	0	206	206	0	0	0	0
San Carlos City	11,178	0	0	243	243	0	0	0	0
San Fernando City	13,027	0	0	24	24	0	0	2	2
Urdaneta City	12,909	0	0	271	271	0	0	0	0
Vigan City	4,450	0	0	97	97	0	0	0	0
**Region 2**	**318,442**	**0**	**3**	**4,442**	**4,445**	**0**	**0**	**4**	**4**
Batanes	960	0	0	8	8	0	0	0	0
Cagayan	88,783	0	0	1,049	1,049	0	0	0	0
Isabela	109,565	0	0	1,441	1,441	0	0	0	0
Nueva Vizcaya	46,874	0	3	1,229	1,232	0	0	0	0
Quirino	19,341	0	0	119	119	0	0	0	0
Cauayan City	10,017	0	0	181	181	0	0	0	0
Ilagan City	15,480	0	0	151	151	0	0	0	0
Santiago City	12,758	0	0	131	131	0	0	0	0
Tuguegarao City	14,664	0	0	133	133	0	0	4	4

Table 2.A.3. MODERN METHOD OF FAMILY PLANNING
Drop Outs
Philippines, 2022

Area	Total Current Users	FSTR/BTL Age group			Total	MSTR/NSV Age group			Total
		10-14	15-19	20-49		10-14	15-19	20-49	
**Region 3**	**831,669**	**2**	**65**	**19,810**	**19,877**	**0**	**35**	**712**	**747**
Aurora	22,728	0	3	212	215	0	0	13	13
Bataan	53,599	0	7	270	277	0	0	6	6
Bulacan	156,537	0	4	2,991	2,995	0	0	348	348
Nueva Ecija	135,568	0	9	3,067	3,076	0	0	1	1
Pampanga	114,189	0	12	6,901	6,913	0	30	239	269
Tarlac	88,617	2	7	617	626	0	0	6	6
Zambales	43,500	0	0	1,249	1,249	0	0	85	85
Angeles City	24,153	0	0	581	581	0	0	3	3
Balanga City	4,623	0	0	8	8	0	0	0	0
Cabanatuan City	20,017	0	5	681	686	0	0	0	0
City of San Fernando	11,292	0	11	298	309	0	5	8	13
Gapan City	7,573	0	0	168	168	0	0	0	0
Mabalacat City	21,462	0	0	834	834	0	0	0	0
Malolos City	7,988	0	0	377	377	0	0	0	0
Meycauayan	8,375	0	0	75	75	0	0	0	0
Olongapo	10,879	0	0	19	19	0	0	0	0
Palayan City	2,864	0	0	81	81	0	0	0	0
San Jose City	12,941	0	0	29	29	0	0	0	0
San Jose del Monte City	56,123	0	0	966	966	0	0	1	1
Science City of Munoz	6,280	0	0	98	98	0	0	1	1
Tarlac City	22,361	0	7	288	295	0	0	1	1
**Region 4A**	**866,447**	**0**	**62**	**9,974**	**10,036**	**0**	**0**	**55**	**55**
Batangas	129,569	0	0	1,041	1,041	0	0	2	2
Cavite	83,857	0	15	2,295	2,310	0	0	8	8
Laguna	74,923	0	2	542	544	0	0	9	9
Quezon	97,255	0	28	753	781	0	0	2	2
Rizal	111,121	0	2	2,265	2,267	0	0	5	5
Antipolo City	32,668	0	0	148	148	0	0	1	1
Bacoor City	10,282	0	0	200	200	0	0	0	0
Batangas City	17,852	0	0	349	349	0	0	2	2
Biñan City	84,449	0	0	130	130	0	0	0	0
Cabuyao City	18,581	0	0	161	161	0	0	1	1
Calamba City	30,526	0	0	227	227	0	0	1	1
Cavite City	4,111	0	0	109	109	0	0	0	0
Dasmariñas City	40,305	0	0	429	429	0	0	3	3
General Trias City	7,331	0	0	238	238	0	0	1	1
Imus City	8,271	0	0	175	175	0	0	1	1
Lipa City	8,190	0	0	155	155	0	0	0	0
Lucena City	6,718	0	0	166	166	0	0	11	11
San Pablo City	12,520	0	0	158	158	0	0	0	0
San Pedro City	17,276	0	0	5	5	0	0	0	0
Santa Rosa City	47,684	0	15	246	261	0	0	5	5
Tagaytay City	8,795	0	0	36	36	0	0	3	3
Tanauan City	6,741	0	0	57	57	0	0	0	0
Tayabas City	4,078	0	0	62	62	0	0	0	0
Trece Martires City	3,344	0	0	27	27	0	0	0	0
**Region 4B**	**271,491**	**0**	**78**	**6,165**	**6,243**	**0**	**9**	**1,297**	**1,306**
Marinduque	14,336	0	0	325	325	0	0	6	6
Mindoro Occidental	47,186	0	0	262	262	0	0	3	3
Mindoro Oriental	65,781	0	3	1,719	1,722	0	9	1,277	1,286
Palawan	88,744	0	65	3,121	3,186	0	0	6	6
Romblon	22,330	0	10	273	283	0	0	5	5
Puerto Princesa City	33,114	0	0	465	465	0	0	0	0
**Region 5**	**501,196**	**0**	**254**	**3,818**	**4,072**	**0**	**1**	**122**	**123**
Albay	97,609	0	221	504	725	0	1	30	31
Camarines Norte	44,713	0	1	242	243	0	0	17	17

Table 2.A.3. MODERN METHOD OF FAMILY PLANNING
Drop Outs
Philippines, 2022

Area	Total Current Users	FSTR/BTL Age group 10-14	15-19	20-49	Total	MSTR/NSV Age group 10-14	15-19	20-49	Total
Camarines Sur	106,529	0	18	743	761	0	0	3	3
Catanduanes	24,595	0	10	610	620	0	0	30	30
Masbate	87,357	0	0	223	223	0	0	9	9
Sorsogon	76,074	0	4	1,278	1,282	0	0	28	28
Iriga City	12,491	0	0	108	108	0	0	3	3
Legaspi City	23,050	0	0	34	34	0	0	0	0
Naga City	28,778	0	0	76	76	0	0	2	2
**Region 6**	**612,103**	**0**	**8**	**4,861**	**4,869**	**0**	**0**	**205**	**205**
Aklan	44,365	0	0	275	275	0	0	0	0
Antique	47,618	0	0	748	748	0	0	10	10
Capiz	76,500	0	0	402	402	0	0	31	31
Guimaras	16,308	0	1	157	158	0	0	0	0
Iloilo	157,750	0	0	1,439	1,439	0	0	28	28
Negros Occidental	193,157	0	2	1,420	1,422	0	0	133	133
Bacolod City	25,916	0	0	180	180	0	0	0	0
Iloilo City	50,489	0	5	240	245	0	0	3	3
**Region 7**	**547,072**	**0**	**0**	**1,807**	**1,807**	**0**	**0**	**173**	**173**
Bohol	81,628	0	0	566	566	0	0	39	39
Cebu	274,919	0	0	587	587	0	0	40	40
Negros Oriental	90,879	0	0	398	398	0	0	53	53
Siquijor	8,892	0	0	54	54	0	0	0	0
Cebu City	32,674	0	0	173	173	0	0	41	41
Lapu-Lapu City	35,065	0	0	10	10	0	0	0	0
Mandaue City	23,015	0	0	19	19	0	0	0	0
**Region 8**	**349,783**	**0**	**0**	**11,458**	**11,458**	**0**	**0**	**217**	**217**
Biliran	11,512	0	0	120	120	0	0	5	5
Eastern Samar	41,802	0	0	3,184	3,184	0	0	150	150
Northern Leyte	118,358	0	0	263	263	0	0	2	2
Northern Samar	48,444	0	0	691	691	0	0	31	31
Southern Leyte	26,721	0	0	952	952	0	0	2	2
Western Samar	24,584	0	0	2,863	2,863	0	0	7	7
Calbayog City	8,315	0	0	187	187	0	0	0	0
Maasin City	5,078	0	0	124	124	0	0	3	3
Ormoc City	20,494	0	0	230	230	0	0	2	2
Tacloban City	44,475	0	0	2,844	2,844	0	0	15	15
**Region 9**	**325,087**	**0**	**0**	**818**	**818**	**0**	**0**	**1**	**1**
Zamboanga del Norte	93,748	0	0	276	276	0	0	0	0
Zamboanga del Sur	73,802	0	0	159	159	0	0	1	1
Zamboanga Sibugay	42,410	0	0	127	127	0	0	0	0
Dapitan City	7,472	0	0	42	42	0	0	0	0
Dipolog City	17,962	0	0	79	79	0	0	0	0
Isabela City	8,228	0	0	18	18	0	0	0	0
Pagadian City	18,316	0	0	0	0	0	0	0	0
Zamboanga City	63,149	0	0	117	117	0	0	0	0
**Region 10**	**489,677**	**3**	**126**	**3,678**	**3,807**	**0**	**21**	**120**	**141**
Bukidnon	114,313	0	7	1,340	1,347	0	0	25	25
Camiguin	6,418	0	3	114	117	0	0	0	0
Lanao del Norte	63,113	0	8	137	145	0	1	6	7
Misamis Occidental	27,464	0	7	38	45	0	0	3	3
Misamis Oriental	75,691	0	2	496	498	0	0	9	9
Cagayan de Oro City	61,925	0	13	629	642	0	0	0	0

Table 2.A.3. MODERN METHOD OF FAMILY PLANNING
Drop Outs
Philippines, 2022

Area	Total Current Users	FSTR/BTL Age group 10-14	FSTR/BTL Age group 15-19	FSTR/BTL Age group 20-49	Total	MSTR/NSV Age group 10-14	MSTR/NSV Age group 15-19	MSTR/NSV Age group 20-49	Total
El Salvador City	6,345	0	0	37	37	0	0	0	0
Gingoog City	28,686	2	54	291	347	0	0	4	4
Iligan City	28,662	0	0	102	102	0	0	4	4
Malaybalay City	18,648	0	0	220	220	0	0	17	17
Oroquieta City	6,937	0	0	1	1	0	0	0	0
Ozamis City	25,480	0	24	27	51	0	20	50	70
Tangub City	4,130	0	4	26	30	0	0	0	0
Valencia City	21,865	1	4	220	225	0	0	2	2
**Region 11**	**528,307**	**0**	**80**	**4,184**	**4,264**	**1**	**3**	**162**	**166**
Davao de Oro	85,230	0	0	737	737	0	0	40	40
Davao del Norte	103,058	0	0	1,078	1,078	0	0	27	27
Davao Oriental	76,678	0	3	317	320	0	0	9	9
Davao del Sur	31,942	0	0	515	515	0	0	18	18
Davao Occidental	53,567	0	24	373	397	0	0	7	7
Davao City	177,832	0	53	1,164	1,217	1	3	61	65
**Region 12**	**435,275**	**0**	**12**	**2,797**	**2,809**	**0**	**0**	**206**	**206**
North Cotabato	137,970	0	6	1,229	1,235	0	0	27	27
Sarangani	65,032	0	4	173	177	0	0	3	3
South Cotabato	92,506	0	0	939	939	0	0	111	111
Sultan Kudarat	95,199	0	2	422	424	0	0	62	62
					0				0
Gen. Santos City	44,568	0	0	34	34	0	0	3	3
**BARMM**	**295,125**	**0**	**0**	**1,875**	**1,875**	**0**	**0**	**2**	**2**
Basilan	10,783	0	0	51	51	0	0	0	0
Lanao del Sur	30,652	0	0	119	119	0	0	0	0
Maguindanao	91,298	0	0	645	645	0	0	2	2
Sulu	92,228	0	0	399	399	0	0	0	0
Tawi-Tawi	28,444	0	0	411	411	0	0	0	0
					0				0
Lamitan City	5,882	0	0	61	61	0	0	0	0
Marawi City	5,206	0	0	76	76	0	0	0	0
Cotabato City	30,632	0	0	113	113	0	0	0	0
**CARAGA**	**268,898**	**0**	**7**	**1,993**	**2,000**	**0**	**0**	**51**	**51**
Agusan del Norte	39,895	0	3	517	520	0	0	2	2
Agusan del Sur	83,282	0	1	302	303	0	0	15	15
Surigao del Norte	33,804	0	0	443	443	0	0	8	8
Surigao del Sur	42,911	0	1	466	467	0	0	20	20
Province of Dinagat	10,370	0	0	42	42	0	0	1	1
Bislig City	9,590	0	0	88	88	0	0	0	0
Butuan City	34,962	0	2	79	81	0	0	4	4
Surigao City	14,084	0	0	56	56	0	0	1	1

Table 2.A.3. MODERN METHOD OF FAMILY PLANNING
Drop Outs
Philippines, 2022

Area	CONDOM			Total	IUD-INTERVAL			Total	IUD-POSTPARTUM			Total
	Age group				Age group				Age group			
	10-14	15-19	20-49		10-14	15-19	20-49		10-14	15-19	20-49	
**PHILIPPINES**	45	5,972	97,398	103,415	40	3,081	53,209	56,330	50	1,900	22,719	24,669
**N C R**	6	1,256	28,604	29,866	3	197	4,564	4,764	13	281	2,554	2,848
Malabon	0	38	467	505	0	33	409	442	0	1	14	15
Navotas	1	53	147	201	0	11	115	126	0	2	4	6
Valenzuela City	0	8	1,107	1,115	0	5	348	353	0	1	243	244
Caloocan City	0	267	4,992	5,259	2	103	681	786	9	155	642	806
Marikina City	0	2	53	55	0	0	7	7	0	0	32	32
Pasig City	0	17	370	387	0	11	212	223	0	57	342	399
Pateros	0	1	53	54	0	0	45	45	0	0	0	0
Taguig	0	18	716	734	0	0	29	29	0	0	23	23
Quezon City	3	403	17,811	18,217	0	23	1,667	1,690	1	47	824	872
Makati City	0	17	333	350	0	3	37	40	0	0	3	3
Mandaluyong City	0	38	121	159	0	3	93	96	0	1	20	21
San Juan	0	0	21	21	0	0	69	69	0	0	0	0
Manila City	1	39	554	594	0	0	30	30	0	0	46	46
Las Piñas City	0	20	523	543	0	1	116	117	3	14	32	49
Muntinlupa City	0	1	175	176	0	0	210	210	0	0	0	0
Parañaque City	1	10	235	246	1	4	24	29	0	3	7	10
Pasay City	0	324	926	1,250	0	0	472	472	0	0	322	322
**C A R**	4	44	1,489	1,537	0	25	1,155	1,180	6	29	332	367
Abra	0	4	78	82	0	0	19	19	0	0	1	1
Apayao	0	5	94	99	0	9	410	419	6	25	143	174
Benguet	0	10	458	468	0	10	246	256	0	1	55	56
Ifugao	0	1	154	155	0	3	62	65	0	0	14	14
Kalinga	4	16	280	300	0	2	197	199	0	3	60	63
Mt. Province	0	5	143	148	0	1	79	80	0	0	8	8
Baguio City	0	3	282	285	0	0	142	142	0	0	51	51
**Region 1**	1	135	2,530	2,666	1	130	1,191	1,322	0	16	85	101
Ilocos Norte	0	0	91	91	0	2	7	9	0	4	14	18
Ilocos Sur	0	4	147	151	0	0	35	35	0	0	19	19
La Union	0	3	668	671	0	32	263	295	0	10	41	51
Pangasinan	1	62	1,285	1,348	1	60	526	587	0	0	0	0
Alaminos City	0	4	123	127	0	0	16	16	0	0	0	0
Candon City	0	0	17	17	0	0	0	0	0	0	4	4
Dagupan City	0	6	19	25	0	4	4	8	0	1	4	5
Laoag City	0	0	0	0	0	0	0	0	0	0	0	0
San Carlos City	0	2	81	83	0	4	48	52	0	0	0	0
San Fernando City	0	4	33	37	0	5	32	37	0	1	3	4
Urdaneta City	0	50	59	109	0	23	260	283	0	0	0	0
Vigan City	0	0	7	7	0	0	0	0	0	0	0	0
**Region 2**	0	31	1,073	1,104	1	132	1,969	2,102	1	87	328	416
Batanes	0	0	5	5	0	0	1	1	0	0	0	0
Cagayan	0	1	123	124	0	63	873	936	1	56	112	169
Isabela	0	9	218	227	0	26	309	335	0	21	82	103
Nueva Vizcaya	0	9	534	543	1	13	296	310	0	3	77	80
Quirino	0	4	57	61	0	0	65	65	0	0	0	0
Cauayan City	0	1	17	18	0	3	124	127	0	2	25	27
Ilagan City	0	0	38	38	0	0	46	46	0	0	0	0
Santiago City	0	6	38	44	0	7	83	90	0	0	0	0
Tuguegarao City	0	1	43	44	0	20	172	192	0	5	32	37

Table 2.A.3. MODERN METHOD OF FAMILY PLANNING
Drop Outs
Philippines, 2022

Area	CONDOM			Total	IUD-INTERVAL			Total	IUD-POSTPARTUM			Total
	Age group				Age group				Age group			
	10-14	15-19	20-49		10-14	15-19	20-49		10-14	15-19	20-49	
**Region 3**	5	768	12,158	12,931	1	111	2,166	2,278	0	106	1,651	1,757
Aurora	1	5	501	507	0	3	79	82	0	1	22	23
Bataan	0	31	338	369	0	0	53	53	0	0	5	5
Bulacan	0	47	876	923	0	24	557	581	0	5	266	271
Nueva Ecija	0	97	2,755	2,852	0	2	412	414	0	16	109	125
Pampanga	0	64	1,778	1,842	0	11	208	219	0	78	1,118	1,196
Tarlac	0	146	1,561	1,707	0	1	160	161	0	0	33	33
Zambales	2	25	257	284	0	1	41	42	0	3	9	12
Angeles City	0	2	116	118	0	3	54	57	0	0	34	34
Balanga City	0	0	20	20	0	0	1	1	0	0	0	0
Cabanatuan City	0	15	255	270	0	3	35	38	0	1	20	21
City of San Fernando	2	38	289	329	0	2	9	11	0	0	15	15
Gapan City	0	12	20	32	0	0	3	3	0	0	6	6
Mabalacat City	0	9	696	705	0	1	27	28	0	0	1	1
Malolos City	0	3	226	229	0	1	35	36	0	0	0	0
Meycauayan	0	42	131	173	0	0	0	0	0	1	8	9
Olongapo	0	0	90	90	0	0	2	2	0	0	0	0
Palayan City	0	1	7	8	0	1	8	9	0	0	0	0
San Jose City	0	1	31	32	0	0	0	0	0	0	0	0
San Jose del Monte City	0	65	1,809	1,874	0	2	312	314	0	0	0	0
Science City of Munoz	0	0	32	32	0	2	33	35	0	0	0	0
Tarlac City	0	165	370	535	1	54	137	192	0	1	5	6
**Region 4A**	6	1,279	9,477	10,762	1	112	3,539	3,652	7	161	1,483	1,651
Batangas	0	16	889	905	0	6	240	246	0	1	89	90
Cavite	6	31	811	848	0	8	525	533	2	10	103	115
Laguna	0	38	461	499	1	21	379	401	1	21	90	112
Quezon	0	30	921	951	0	25	765	790	0	6	109	115
Rizal	0	36	724	760	0	17	730	747	1	47	254	302
Antipolo City	0	0	61	61	0	0	35	35	0	1	70	71
Bacoor City	0	1	113	114	0	2	36	38	0	0	15	15
Batangas City	0	4	307	311	0	3	75	78	3	56	317	376
Biñan City	0	534	1,882	2,416	0	0	117	117	0	1	59	60
Cabuyao City	0	12	344	356	0	4	114	118	0	0	245	245
Calamba City	0	17	192	209	0	0	102	102	0	0	17	17
Cavite City	0	1	13	14	0	1	5	6	0	0	2	2
Dasmariñas City	0	31	500	531	0	12	133	145	0	9	34	43
General Trias City	0	1	55	56	0	3	32	35	0	0	4	4
Imus City	0	0	102	102	0	1	22	23	0	0	8	8
Lipa City	0	0	118	118	0	0	44	44	0	0	1	1
Lucena City	0	10	134	144	0	0	12	12	0	0	0	0
San Pablo City	0	8	51	59	0	0	66	66	0	3	15	18
San Pedro City	0	37	180	217	0	2	27	29	0	4	0	4
Santa Rosa City	0	467	1,485	1,952	0	3	43	46	0	0	2	2
Tagaytay City	0	0	7	7	0	0	4	4	0	0	30	30
Tanauan City	0	2	61	63	0	0	11	11	0	1	18	19
Tayabas City	0	2	51	53	0	1	7	8	0	1	1	2
Trece Martires City	0	1	15	16	0	3	15	18	0	0	0	0
**Region 4B**	5	82	1,889	1,976	0	39	2,078	2,117	1	63	3,661	3,725
Marinduque	0	1	89	90	0	0	101	101	0	0	5	5
Mindoro Occidental	0	8	233	241	0	8	265	273	0	2	4	6
Mindoro Oriental	0	27	423	450	0	19	806	825	0	55	3,119	3,174
Palawan	5	42	895	942	0	9	496	505	1	4	113	118
Romblon	0	2	79	81	0	3	289	292	0	2	71	73
Puerto Princesa City	0	2	170	172	0	0	121	121	0	0	349	349
**Region 5**	5	566	6,583	7,154	0	64	1,819	1,883	0	41	608	649
Albay	0	419	1,356	1,775	0	20	279	299	0	1	38	39
Camarines Norte	0	27	391	418	0	33	586	619	0	4	111	115

Table 2.A.3. MODERN METHOD OF FAMILY PLANNING
Drop Outs
Philippines, 2022

Area	CONDOM			Total	IUD-INTERVAL			Total	IUD-POSTPARTUM			Total
	Age group				Age group				Age group			
	10-14	15-19	20-49		10-14	15-19	20-49		10-14	15-19	20-49	
Camarines Sur	3	38	1,733	1,774	0	8	294	302	0	15	89	104
Catanduanes	0	14	320	334	0	1	28	29	0	0	23	23
Masbate	2	39	895	936	0	0	323	323	0	18	319	337
Sorsogon	0	22	1,264	1,286	0	1	232	233	0	2	21	23
Iriga City	0	3	124	127	0	1	54	55	0	1	1	2
Legaspi City	0	0	91	91	0	0	6	6	0	0	1	1
Naga City	0	4	409	413	0	0	17	17	0	0	5	5
**Region 6**	**0**	**150**	**4,884**	**5,034**	**2**	**128**	**3,270**	**3,400**	**2**	**106**	**2,627**	**2,735**
Aklan	0	15	341	356	0	1	69	70	0	0	4	4
Antique	0	11	408	419	0	5	108	113	0	0	6	6
Capiz	0	10	498	508	0	19	711	730	0	1	15	16
Guimaras	0	3	125	128	0	0	60	60	0	0	5	5
Iloilo	0	54	1,656	1,710	0	16	773	789	0	11	152	163
Negros Occidental	0	43	1,470	1,513	2	60	1,280	1,342	2	79	2,246	2,327
Bacolod City	0	2	108	110	0	0	158	158	0	2	192	194
Iloilo City	0	12	278	290	0	27	111	138	0	13	7	20
**Region 7**	**2**	**256**	**6,175**	**6,433**	**3**	**147**	**5,573**	**5,723**	**7**	**192**	**1,895**	**2,094**
Bohol	0	20	797	817	0	36	1,562	1,598	0	34	350	384
Cebu	2	177	3,896	4,075	3	65	2,002	2,070	7	146	1,444	1,597
Negros Oriental	0	28	784	812	0	33	1,490	1,523	0	8	57	65
Siquijor	0	1	59	60	0	3	99	102	0	1	2	3
Cebu City	0	11	418	429	0	9	301	310	0	3	35	38
Lapu-Lapu City	0	16	88	104	0	1	12	13	0	0	0	0
Mandaue City	0	3	133	136	0	0	107	107	0	0	7	7
**Region 8**	**1**	**162**	**3,485**	**3,648**	**2**	**79**	**2,826**	**2,907**	**1**	**124**	**1,214**	**1,339**
Biliran	0	5	125	130	1	1	41	43	0	3	9	12
Eastern Samar	1	28	1,162	1,191	1	4	77	82	0	9	89	98
Northern Leyte	0	15	193	208	0	51	546	597	0	53	308	361
Northern Samar	0	19	192	211	0	2	128	130	0	8	70	78
Southern Leyte	0	7	82	89	0	4	91	95	1	22	65	88
Western Samar	0	9	227	236	0	3	289	292	0	2	174	176
Calbayog City	0	3	88	91	0	0	19	19	0	0	1	1
Maasin City	0	1	64	65	0	5	177	182	0	5	28	33
Ormoc City	0	13	417	430	0	8	330	338	0	12	33	45
Tacloban City	0	62	935	997	0	1	1,128	1,129	0	10	437	447
**Region 9**	**0**	**77**	**1,967**	**2,044**	**0**	**101**	**2,699**	**2,800**	**1**	**132**	**798**	**931**
Zamboanga del Norte	0	28	572	600	0	27	689	716	0	12	128	140
Zamboanga del Sur	0	9	524	533	0	34	727	761	0	81	312	393
Zamboanga Sibugay	0	23	350	373	0	23	497	520	1	11	43	55
Dapitan City	0	0	53	53	0	1	34	35	0	0	9	9
Dipolog City	0	5	148	153	0	0	86	86	0	0	2	2
Isabela City	0	0	32	32	0	0	2	2	0	0	1	1
Pagadian City	0	0	0	0	0	0	0	0	0	0	0	0
Zamboanga City	0	12	288	300	0	16	664	680	0	28	303	331
**Region 10**	**0**	**350**	**4,810**	**5,160**	**18**	**1,417**	**9,305**	**10,740**	**1**	**243**	**2,182**	**2,426**
Bukidnon	0	33	1,268	1,301	0	153	4,275	4,428	0	59	439	498
Camiguin	0	0	41	41	0	0	22	22	0	1	3	4
Lanao del Norte	0	131	570	701	2	71	837	910	0	30	375	405
Misamis Occidental	0	10	198	208	0	0	53	53	0	3	6	9
Misamis Oriental	0	39	924	963	16	1,003	1,300	2,319	0	22	366	388
Cagayan de Oro City	0	23	913	936	0	78	1,401	1,479	1	90	773	864

Table 2.A.3. MODERN METHOD OF FAMILY PLANNING
Drop Outs
Philippines, 2022

Area	CONDOM			Total	IUD-INTERVAL			Total	IUD-POSTPARTUM			Total
	Age group				Age group				Age group			
	10-14	15-19	20-49		10-14	15-19	20-49		10-14	15-19	20-49	
El Salvador City	0	2	34	36	0	13	381	394	0	22	134	156
Gingoog City	0	60	155	215	0	23	125	148	0	0	38	38
Iligan City	0	7	206	213	0	47	218	265	0	15	44	59
Malaybalay City	0	3	302	305	0	9	265	274	0	0	0	0
Oroquieta City	0	1	3	4	0	0	7	7	0	0	0	0
Ozamis City	0	9	63	72	0	0	21	21	0	0	0	0
Tangub City	0	2	23	25	0	0	5	5	0	0	2	2
Valencia City	0	30	110	140	0	20	395	415	0	1	2	3
**Region 11**	**10**	**248**	**3,511**	**3,769**	**1**	**100**	**4,105**	**4,206**	**6**	**86**	**898**	**990**
Davao de Oro	2	7	374	383	0	6	722	728	0	11	84	95
Davao del Norte	6	24	829	859	1	31	859	891	2	22	300	324
Davao Oriental	0	2	192	194	0	16	450	466	1	2	85	88
Davao del Sur	0	11	114	125	0	15	283	298	0	0	22	22
Davao Occidental	0	12	236	248	0	2	387	389	0	2	30	32
Davao City	2	192	1,766	1,960	0	30	1,404	1,434	3	49	377	429
**Region 12**	**0**	**176**	**2,428**	**2,604**	**3**	**126**	**2,827**	**2,956**	**0**	**64**	**916**	**980**
North Cotabato	0	34	850	884	3	29	1,314	1,346	0	10	237	247
Sarangani	0	8	227	235	0	17	189	206	0	2	8	10
South Cotabato	0	62	805	867	0	46	707	753	0	27	448	475
Sultan Kudarat	0	43	334	377	0	10	512	522	0	18	162	180
				0				0				0
Gen. Santos City	0	29	212	241	0	24	105	129	0	7	61	68
**BARMM**	**0**	**154**	**3,517**	**3,671**	**2**	**41**	**676**	**719**	**0**	**50**	**666**	**716**
Basilan	0	22	88	110	0	0	30	30	0	9	19	28
Lanao del Sur	0	17	852	869	0	0	82	82	0	1	6	7
Maguindanao	0	82	1,237	1,319	0	13	307	320	0	9	255	264
Sulu	0	6	536	542	0	4	108	112	0	0	51	51
Tawi-Tawi	0	0	70	70	0	0	0	0	0	0	0	0
				0				0				0
Lamitan City	0	0	3	3	0	13	47	60	0	19	231	250
Marawi City	0	19	480	499	0	0	5	5	0	0	0	0
Cotabato City	0	8	251	259	2	11	97	110	0	12	104	116
**CARAGA**	**0**	**238**	**2,818**	**3,056**	**2**	**132**	**3,447**	**3,581**	**4**	**119**	**821**	**944**
Agusan del Norte	0	28	361	389	0	29	697	726	0	19	108	127
Agusan del Sur	0	56	635	691	1	43	786	830	1	44	193	238
Surigao del Norte	0	105	855	960	0	28	826	854	3	24	210	237
Surigao del Sur	0	24	531	555	1	19	665	685	0	8	129	137
Province of Dinagat	0	5	135	140	0	7	165	172	0	0	9	9
Bislig City	0	17	180	197	0	2	163	165	0	4	52	56
Butuan City	0	0	87	87	0	0	78	78	0	8	21	29
Surigao City	0	3	34	37	0	4	67	71	0	12	99	111

Table 2.A.3. MODERN METHOD OF FAMILY PLANNING
Drop Outs
Philippines, 2022

Area	PILLS-POP			Total	PILLS-COC			Total	INJECTABLES			Total
	Age group				Age group				Age group			
	10-14	15-19	20-49		10-14	15-19	20-49		10-14	15-19	20-49	
**PHILIPPINES**	203	13,423	128,009	141,635	227	25,033	450,099	475,359	292	28,496	375,030	403,818
**N C R**	26	1,778	17,842	19,646	25	2,330	59,515	61,870	74	3,671	56,957	60,702
Malabon	1	93	759	853	0	67	1,421	1,488	0	162	1,165	1,327
Navotas	0	89	372	461	0	119	571	690	0	220	1,005	1,225
Valenzuela City	0	119	1,580	1,699	0	187	9,122	9,309	1	280	5,462	5,743
Caloocan City	2	258	1,254	1,514	2	524	4,609	5,135	1	839	12,733	13,573
Marikina City	0	39	339	378	0	23	816	839	1	54	1,038	1,093
Pasig City	0	28	472	500	0	128	1,431	1,559	1	195	2,029	2,225
Pateros	0	4	12	16	0	7	271	278	0	1	127	128
Taguig	0	54	761	815	0	75	1,698	1,773	0	167	2,180	2,347
Quezon City	0	492	7,137	7,629	1	765	32,989	33,755	1	881	19,820	20,702
Makati City	0	20	358	378	0	18	497	515	0	48	974	1,022
Mandaluyong City	0	42	108	150	0	51	245	296	0	60	290	350
San Juan	0	2	79	81	0	2	283	285	0	0	204	204
Manila City	23	259	2,070	2,352	21	112	1,288	1,421	19	243	2,682	2,944
Las Piñas City	0	79	1,130	1,209	1	97	2,213	2,311	3	338	3,074	3,415
Muntinlupa City	0	23	167	190	0	13	647	660	1	59	2,427	2,487
Parañaque City	0	65	300	365	0	93	664	757	46	124	1,169	1,339
Pasay City	0	112	944	1,056	0	49	750	799	0	0	578	578
**C A R**	4	374	7,460	7,838	6	391	11,397	11,794	7	399	9,287	9,693
Abra	0	50	374	424	0	24	792	816	0	40	498	538
Apayao	1	70	1,799	1,870	1	74	3,967	4,042	1	85	1,413	1,499
Benguet	0	66	652	718	0	78	1,531	1,609	0	78	1,386	1,464
Ifugao	1	48	1,439	1,488	1	53	1,340	1,394	0	29	1,026	1,055
Kalinga	2	90	2,098	2,190	4	94	1,505	1,603	6	54	1,509	1,569
Mt. Province	0	30	361	391	0	51	1,040	1,091	0	40	801	841
Baguio City	0	20	737	757	0	17	1,222	1,239	0	73	2,654	2,727
**Region 1**	1	122	1,143	1,266	3	2,143	13,060	15,206	1	1,135	9,059	10,195
Ilocos Norte	0	27	423	450	0	22	1,457	1,479	0	12	344	356
Ilocos Sur	0	44	346	390	3	79	768	850	0	52	589	641
La Union	1	45	295	341	0	89	2,972	3,061	1	193	2,686	2,880
Pangasinan	0	0	0	0	0	563	5,516	6,079	0	376	3,747	4,123
Alaminos City	0	0	0	0	0	1,242	396	1,638	0	382	156	538
Candon City	0	0	0	0	0	0	39	39	0	0	25	25
Dagupan City	0	2	18	20	0	16	220	236	0	40	128	168
Laoag City	0	0	14	14	0	0	66	66	0	0	10	10
San Carlos City	0	0	0	0	0	36	929	965	0	22	668	690
San Fernando City	0	4	47	51	0	2	73	75	0	4	65	69
Urdaneta City	0	0	0	0	0	92	422	514	0	53	395	448
Vigan City	0	0	0	0	0	2	202	204	0	1	246	247
**Region 2**	3	504	7,364	7,871	2	935	14,202	15,139	3	734	9,384	10,121
Batanes	0	1	5	6	0	0	51	51	0	6	179	185
Cagayan	2	156	808	966	0	162	3,415	3,577	1	99	1,755	1,855
Isabela	1	154	1,534	1,689	0	208	4,802	5,010	1	251	2,970	3,222
Nueva Vizcaya	0	111	1,842	1,953	2	453	2,262	2,717	0	135	1,334	1,469
Quirino	0	18	154	172	0	43	569	612	0	35	427	462
Cauayan City	0	9	220	229	0	14	765	779	0	27	329	356
Ilagan City	0	22	2,682	2,704	0	5	1,418	1,423	1	124	1,666	1,791
Santiago City	0	0	0	0	0	40	665	705	0	45	503	548
Tuguegarao City	0	33	119	152	0	10	255	265	0	12	221	233

Table 2.A.3. MODERN METHOD OF FAMILY PLANNING
Drop Outs
Philippines, 2022

Area	PILLS-POP			Total	PILLS-COC			Total	INJECTABLES			Total
	Age group				Age group				Age group			
	10-14	15-19	20-49		10-14	15-19	20-49		10-14	15-19	20-49	
**Region 3**	8	1,405	9,921	11,334	6	2,896	48,808	51,710	38	4,109	44,488	48,635
Aurora	0	32	400	432	0	62	1,253	1,315	1	73	755	829
Bataan	4	277	1,354	1,635	0	96	1,689	1,785	3	293	3,153	3,449
Bulacan	0	201	1,448	1,649	0	627	10,724	11,351	9	1,227	10,077	11,313
Nueva Ecija	0	193	1,158	1,351	0	242	8,461	8,703	3	334	8,206	8,543
Pampanga	4	149	2,440	2,593	4	303	5,513	5,820	8	398	4,100	4,506
Tarlac	0	76	736	812	0	311	4,699	5,010	4	283	3,968	4,255
Zambales	0	31	333	364	0	129	1,497	1,626	3	279	2,631	2,913
Angeles City	0	25	175	200	0	104	1,219	1,323	0	113	858	971
Balanga City	0	5	25	30	0	30	275	305	2	61	598	661
Cabanatuan City	0	64	353	417	1	92	2,186	2,279	4	113	1,106	1,223
City of San Fernando	0	24	169	193	1	109	975	1,085	0	50	382	432
Gapan City	0	21	51	72	0	42	479	521	0	22	259	281
Mabalacat City	0	7	52	59	0	56	1,040	1,096	0	58	1,252	1,310
Malolos City	0	0	19	19	0	30	992	1,022	0	27	624	651
Meycauayan	0	32	110	142	0	92	956	1,048	0	55	661	716
Olongapo	0	18	180	198	0	28	669	697	1	81	1,074	1,156
Palayan City	0	2	8	10	0	7	237	244	0	26	152	178
San Jose City	0	15	58	73	0	32	609	641	0	53	751	804
San Jose del Monte City	0	39	338	377	0	163	2,245	2,408	0	299	2,327	2,626
Science City of Munoz	0	7	54	61	0	29	356	385	0	38	307	345
Tarlac City	0	187	460	647	0	312	2,734	3,046	0	226	1,247	1,473
**Region 4A**	12	1,654	14,796	16,462	19	2,682	44,986	47,687	43	3,747	48,140	51,930
Batangas	2	109	2,012	2,123	1	106	4,147	4,254	29	106	2,455	2,590
Cavite	2	71	1,115	1,188	3	201	6,949	7,153	7	378	7,538	7,923
Laguna	2	97	555	654	0	159	2,618	2,777	0	169	2,032	2,201
Quezon	2	186	2,272	2,460	12	241	6,896	7,149	6	512	8,288	8,806
Rizal	2	182	1,474	1,658	1	336	5,491	5,828	0	748	8,442	9,190
Antipolo City	0	23	474	497	0	25	629	654	0	57	1,668	1,725
Bacoor City	0	8	103	111	0	39	1,270	1,309	0	154	2,169	2,323
Batangas City	0	23	194	217	0	42	1,891	1,933	0	50	1,087	1,137
Biñan City	0	571	2,270	2,841	0	609	2,262	2,871	0	652	2,526	3,178
Cabuyao City	1	51	1,909	1,961	0	82	1,784	1,866	0	61	850	911
Calamba City	0	90	437	527	0	29	713	742	0	18	814	832
Cavite City	0	5	15	20	0	12	181	193	0	19	224	243
Dasmariñas City	0	46	434	480	0	101	1,730	1,831	0	158	1,895	2,053
General Trias City	0	25	175	200	0	18	1,048	1,066	0	35	1,257	1,292
Imus City	0	14	161	175	0	17	954	971	0	56	1,116	1,172
Lipa City	0	2	153	155	0	6	462	468	0	3	210	213
Lucena City	1	31	228	260	1	122	1,675	1,798	0	245	2,410	2,655
San Pablo City	0	11	71	82	0	46	590	636	1	70	622	693
San Pedro City	0	73	466	539	0	18	534	552	0	42	213	255
Santa Rosa City	0	9	105	114	1	441	2,112	2,554	0	121	588	709
Tagaytay City	0	0	15	15	0	0	256	256	0	0	185	185
Tanauan City	0	11	59	70	0	6	203	209	0	21	313	334
Tayabas City	0	13	31	44	0	11	265	276	0	25	504	529
Trece Martires City	0	3	68	71	0	15	326	341	0	47	734	781
**Region 4B**	48	540	11,879	12,467	68	796	29,640	30,504	6	1,496	21,779	23,281
Marinduque	0	31	810	841	0	21	2,902	2,923	0	31	439	470
Mindoro Occidental	0	50	932	982	3	120	3,230	3,353	0	184	2,754	2,938
Mindoro Oriental	3	113	7,470	7,586	33	167	5,900	6,100	0	222	3,392	3,614
Palawan	42	303	2,134	2,479	22	442	13,185	13,649	5	940	10,113	11,058
Romblon	0	10	351	361	9	21	1,315	1,345	1	47	752	800
Puerto Princesa City	3	33	182	218	1	25	3,108	3,134	0	72	4,329	4,401
**Region 5**	10	601	6,578	7,189	5	1,833	36,442	38,280	7	1,505	22,195	23,707
Albay	0	94	1,423	1,517	1	697	5,811	6,509	0	382	4,340	4,722
Camarines Norte	0	126	605	731	1	136	2,637	2,774	0	161	1,556	1,717

Table 2.A.3. MODERN METHOD OF FAMILY PLANNING
Drop Outs
Philippines, 2022

Area	PILLS-POP			Total	PILLS-COC			Total	INJECTABLES			Total
	Age group				Age group				Age group			
	10-14	15-19	20-49		10-14	15-19	20-49		10-14	15-19	20-49	
Camarines Sur	0	220	2,383	2,603	2	327	9,604	9,933	6	254	5,619	5,879
Catanduanes	0	14	427	441	0	152	1,376	1,528	0	176	2,039	2,215
Masbate	10	57	588	655	1	172	4,985	5,158	1	167	3,280	3,448
Sorsogon	0	79	924	1,003	0	319	10,297	10,616	0	330	4,636	4,966
Iriga City	0	4	56	60	0	17	519	536	0	22	296	318
Legaspi City	0	5	160	165	0	2	277	279	0	5	129	134
Naga City	0	2	12	14	0	11	936	947	0	8	300	308
**Region 6**	**7**	**640**	**6,582**	**7,229**	**14**	**1,423**	**22,872**	**24,309**	**9**	**1,333**	**20,223**	**21,565**
Aklan	1	64	408	473	1	58	1,363	1,422	0	71	1,296	1,367
Antique	0	49	455	504	0	72	1,918	1,990	0	202	3,027	3,229
Capiz	1	27	277	305	8	63	1,611	1,682	0	62	1,679	1,741
Guimaras	0	15	135	150	0	32	566	598	0	34	377	411
Iloilo	0	59	1,433	1,492	1	197	5,439	5,637	0	145	3,568	3,713
Negros Occidental	5	400	3,513	3,918	4	813	10,379	11,196	9	695	8,585	9,289
Bacolod City	0	13	275	288	0	51	846	897	0	55	1,280	1,335
Iloilo City	0	13	86	99	0	137	750	887	0	69	411	480
**Region 7**	**1**	**531**	**4,816**	**5,348**	**7**	**811**	**19,560**	**20,378**	**8**	**1,346**	**19,181**	**20,535**
Bohol	0	78	406	484	0	91	2,923	3,014	1	107	2,127	2,235
Cebu	1	216	1,983	2,200	4	254	7,058	7,316	5	436	6,165	6,606
Negros Oriental	0	130	1,057	1,187	3	345	6,937	7,285	1	469	6,998	7,468
Siquijor	0	7	97	104	0	18	435	453	0	24	312	336
Cebu City	0	83	1,192	1,275	0	93	1,886	1,979	1	288	3,309	3,598
Lapu-Lapu City	0	16	78	94	0	10	171	181	0	16	210	226
Mandaue City	0	1	3	4	0	0	150	150	0	6	60	66
**Region 8**	**1**	**520**	**5,429**	**5,950**	**12**	**919**	**18,809**	**19,740**	**8**	**861**	**12,952**	**13,821**
Biliran	0	36	500	536	0	67	608	675	0	60	662	722
Eastern Samar	0	33	1,210	1,243	3	181	1,897	2,081	5	121	1,059	1,185
Northern Leyte	0	13	343	356	3	91	2,089	2,183	0	74	1,111	1,185
Northern Samar	0	66	114	180	3	58	1,362	1,423	3	61	946	1,010
Southern Leyte	1	6	124	131	3	46	1,745	1,794	0	40	1,319	1,359
Western Samar	0	8	175	183	0	52	1,281	1,333	0	39	769	808
Calbayog City	0	37	1,398	1,435	0	32	1,542	1,574	0	43	692	735
Maasin City	0	7	72	79	0	5	132	137	0	6	111	117
Ormoc City	0	87	511	598	0	127	2,838	2,965	0	130	1,899	2,029
Tacloban City	0	227	982	1,209	0	260	5,315	5,575	0	287	4,384	4,671
**Region 9**	**3**	**399**	**5,551**	**5,953**	**5**	**1,105**	**17,693**	**18,803**	**6**	**1,034**	**13,527**	**14,567**
Zamboanga del Norte	1	57	3,046	3,104	1	223	3,794	4,018	2	218	2,582	2,802
Zamboanga del Sur	0	75	928	1,003	2	297	5,431	5,730	1	197	3,518	3,716
Zamboanga Sibugay	0	97	640	737	1	139	2,591	2,731	2	214	2,786	3,002
Dapitan City	0	9	32	41	0	6	276	282	0	11	203	214
Dipolog City	1	15	85	101	1	21	807	829	0	24	591	615
Isabela City	0	11	58	69	0	43	482	525	0	54	529	583
Pagadian City	0	0	0	0	0	0	0	0	0	0	0	0
Zamboanga City	1	135	762	898	0	376	4,312	4,688	1	316	3,318	3,635
**Region 10**	**28**	**1,039**	**6,184**	**7,251**	**15**	**1,971**	**36,833**	**38,819**	**18**	**1,497**	**14,501**	**16,016**
Bukidnon	3	427	1,668	2,098	11	1,054	13,668	14,733	3	513	4,693	5,209
Camiguin	0	0	5	5	0	6	190	196	0	9	83	92
Lanao del Norte	25	277	1,524	1,826	3	241	9,444	9,688	14	540	1,256	1,810
Misamis Occidental	0	5	155	160	0	17	321	338	1	12	276	289
Misamis Oriental	0	73	583	656	1	85	2,821	2,907	0	57	1,690	1,747
Cagayan de Oro City	0	63	788	851	0	121	4,022	4,143	0	174	3,103	3,277

Table 2.A.3. MODERN METHOD OF FAMILY PLANNING
Drop Outs
Philippines, 2022

Area	PILLS-POP Age group 10-14	15-19	20-49	Total	PILLS-COC Age group 10-14	15-19	20-49	Total	INJECTABLES Age group 10-14	15-19	20-49	Total
El Salvador City	0	8	49	57	0	20	426	446	0	23	518	541
Gingoog City	0	3	496	499	0	25	1,051	1,076	0	7	468	475
Iligan City	0	19	139	158	0	56	658	714	0	31	662	693
Malaybalay City	0	77	423	500	0	132	2,460	2,592	0	43	733	776
Oroquieta City	0	0	12	12	0	0	26	26	0	0	21	21
Ozamis City	0	0	4	4	0	5	90	95	0	8	101	109
Tangub City	0	3	55	58	0	10	28	38	0	8	51	59
Valencia City	0	84	283	367	0	199	1,628	1,827	0	72	846	918
**Region 11**	**31**	**1,062**	**7,451**	**8,544**	**12**	**1,379**	**19,450**	**20,841**	**37**	**1,475**	**14,219**	**15,731**
Davao de Oro	3	229	1,421	1,653	2	137	3,511	3,650	12	134	2,049	2,195
Davao del Norte	5	174	1,514	1,693	1	251	5,003	5,255	5	284	3,403	3,692
Davao Oriental	4	94	478	576	1	132	1,743	1,876	3	197	1,443	1,643
Davao del Sur	0	56	308	364	0	212	1,925	2,137	6	312	2,131	2,449
Davao Occidental	6	72	650	728	2	155	2,089	2,246	3	71	1,155	1,229
Davao City	13	437	3,080	3,530	6	492	5,179	5,677	8	477	4,038	4,523
**Region 12**	**7**	**961**	**5,434**	**6,402**	**7**	**1,620**	**18,419**	**20,046**	**5**	**1,959**	**16,349**	**18,313**
North Cotabato	3	341	2,117	2,461	2	456	6,552	7,010	1	688	5,543	6,232
Sarangani	0	171	552	723	0	329	3,087	3,416	0	160	1,999	2,159
South Cotabato	0	183	1,282	1,465	2	330	4,627	4,959	1	494	4,721	5,216
Sultan Kudarat	3	146	999	1,148	2	323	2,290	2,615	3	330	1,686	2,019
				0				0				0
Gen. Santos City	1	120	484	605	1	182	1,863	2,046	0	287	2,400	2,687
**BARMM**	**3**	**541**	**5,343**	**5,887**	**9**	**918**	**24,467**	**25,394**	**11**	**1,424**	**31,794**	**33,229**
Basilan	0	92	316	408	0	180	1,307	1,487	2	279	1,041	1,322
Lanao del Sur	0	61	957	1,018	0	32	1,906	1,938	0	62	2,097	2,159
Maguindanao	1	231	1,513	1,745	1	320	5,191	5,512	2	560	6,257	6,819
Sulu	0	80	1,597	1,677	2	198	8,487	8,687	5	275	14,928	15,208
Tawi-Tawi	1	28	189	218	5	89	6,082	6,176	0	43	5,608	5,651
				0				0				0
Lamitan City	1	13	155	169	0	34	420	454	1	113	785	899
Marawi City	0	11	293	304	0	20	572	592	0	16	435	451
Cotabato City	0	25	323	348	1	45	502	548	1	76	643	720
**CARAGA**	**10**	**752**	**4,236**	**4,998**	**12**	**881**	**13,946**	**14,839**	**11**	**771**	**10,995**	**11,777**
Agusan del Norte	7	213	1,068	1,288	3	117	1,669	1,789	4	178	1,490	1,672
Agusan del Sur	0	109	480	589	1	180	2,559	2,740	1	170	2,282	2,453
Surigao del Norte	2	168	1,375	1,545	0	180	4,742	4,922	5	149	3,674	3,828
Surigao del Sur	1	122	666	789	0	180	3,017	3,197	0	117	1,649	1,766
Province of Dinagat	0	23	101	124	1	27	501	529	0	46	536	582
Bislig City	0	10	136	146	2	41	613	656	0	23	455	478
Butuan City	0	95	339	434	5	145	641	791	1	72	638	711
Surigao City	0	12	71	83	0	11	204	215	0	16	271	287

Table 2.A.3. MODERN METHOD OF FAMILY PLANNING
Drop Outs
Philippines, 2022

Area	IMPLANTS Age group			Total	NFP-CCM Age group			Total	NFP-BBT Age group			Total
	10-14	15-19	20-49		10-14	15-19	20-49		10-14	15-19	20-49	
**PHILIPPINES**	186	9,575	122,836	132,597	8	1,086	14,658	15,752	1	150	2,684	2,835
**N C R**	71	2,024	19,412	21,507	1	1	121	123	0	32	358	390
Malabon	0	124	1,035	1,159	0	0	0	0	0	0	0	0
Navotas	1	41	221	263	0	0	2	2	0	0	0	0
Valenzuela City	44	353	4,663	5,060	0	0	0	0	0	0	0	0
Caloocan City	7	404	3,213	3,624	0	0	28	28	0	0	0	0
Marikina City	0	14	255	269	0	0	0	0	0	0	0	0
Pasig City	1	82	1,014	1,097	0	0	0	0	0	0	0	0
Pateros	0	0	11	11	0	0	0	0	0	0	0	0
Taguig	0	0	184	184	0	0	0	0	0	0	0	0
Quezon City	0	283	2,682	2,965	1	1	0	2	0	6	5	11
Makati City	1	41	336	378	0	0	0	0	0	0	0	0
Mandaluyong City	0	11	107	118	0	0	90	90	0	3	3	6
San Juan	0	0	38	38	0	0	0	0	0	0	0	0
Manila City	17	525	3,343	3,885	0	0	0	0	0	23	350	373
Las Piñas City	0	139	1,475	1,614	0	0	0	0	0	0	0	0
Muntinlupa City	0	0	300	300	0	0	0	0	0	0	0	0
Parañaque City	0	7	168	175	0	0	0	0	0	0	0	0
Pasay City	0	0	367	367	0	0	1	1	0	0	0	0
**C A R**	2	158	2,692	2,852	0	121	1,484	1,605	0	4	115	119
Abra	0	18	248	266	0	18	203	221	0	0	43	43
Apayao	0	12	462	474	0	0	2	2	0	0	0	0
Benguet	0	11	358	369	0	0	6	6	0	0	7	7
Ifugao	0	12	270	282	0	2	156	158	0	2	13	15
Kalinga	1	16	395	412	0	101	973	1,074	0	2	52	54
Mt. Province	1	10	195	206	0	0	144	144	0	0	0	0
Baguio City	0	79	764	843	0	0	0	0	0	0	0	0
**Region 1**	1	345	2,280	2,626	0	13	838	851	0	17	300	317
Ilocos Norte	0	5	61	66	0	1	30	31	0	0	0	0
Ilocos Sur	0	4	136	140	0	0	107	107	0	3	5	8
La Union	1	59	321	381	0	2	215	217	0	0	15	15
Pangasinan	0	46	1,250	1,296	0	0	0	0	0	10	2	12
Alaminos City	0	182	18	200	0	0	0	0	0	0	0	0
Candon City	0	0	0	0	0	0	1	1	0	0	0	0
Dagupan City	0	8	21	29	0	0	0	0	0	0	0	0
Laoag City	0	0	0	0	0	0	0	0	0	0	0	0
San Carlos City	0	6	141	147	0	0	0	0	0	0	0	0
San Fernando City	0	5	21	26	0	0	0	0	0	0	0	0
Urdaneta City	0	28	311	339	0	0	0	0	0	4	80	84
Vigan City	0	2	0	2	0	10	485	495	0	0	198	198
**Region 2**	1	165	2,306	2,472	0	1	79	80	0	2	2	4
Batanes	0	0	1	1	0	1	19	20	0	0	0	0
Cagayan	1	60	557	618	0	0	18	18	0	0	0	0
Isabela	0	57	872	929	0	0	1	1	0	0	0	0
Nueva Vizcaya	0	26	510	536	0	0	34	34	0	2	2	4
Quirino	0	6	52	58	0	0	7	7	0	0	0	0
Cauayan City	0	4	65	69	0	0	0	0	0	0	0	0
Ilagan City	0	4	137	141	0	0	0	0	0	0	0	0
Santiago City	0	7	86	93	0	0	0	0	0	0	0	0
Tuguegarao City	0	1	26	27	0	0	0	0	0	0	0	0

Table 2.A.3. MODERN METHOD OF FAMILY PLANNING
Drop Outs
Philippines, 2022

Area	IMPLANTS Age group			Total	NFP-CCM Age group			Total	NFP-BBT Age group			Total
	10-14	15-19	20-49		10-14	15-19	20-49		10-14	15-19	20-49	
**Region 3**	**13**	**550**	**9,214**	**9,777**	**0**	**41**	**802**	**843**	**0**	**23**	**192**	**215**
Aurora	0	13	299	312	0	5	535	540	0	4	11	15
Bataan	0	32	542	574	0	0	0	0	0	0	0	0
Bulacan	0	89	2,112	2,201	0	0	2	2	0	0	0	0
Nueva Ecija	4	145	2,223	2,372	0	15	9	24	0	0	0	0
Pampanga	9	87	981	1,077	0	18	216	234	0	8	150	158
Tarlac	0	50	507	557	0	0	2	2	0	0	0	0
Zambales	0	49	445	494	0	0	4	4	0	1	2	3
Angeles City	0	24	259	283	0	0	0	0	0	2	1	3
Balanga City	0	6	38	44	0	0	0	0	0	0	0	0
Cabanatuan City	0	7	98	105	0	0	0	0	0	0	0	0
City of San Fernando	0	1	48	49	0	2	15	17	0	6	24	30
Gapan City	0	7	93	100	0	0	1	1	0	0	0	0
Mabalacat City	0	2	64	66	0	0	0	0	0	0	0	0
Malolos City	0	2	80	82	0	0	0	0	0	0	0	0
Meycauayan	0	0	73	73	0	1	18	19	0	0	0	0
Olongapo	0	2	147	149	0	0	0	0	0	0	1	1
Palayan City	0	0	0	0	0	0	0	0	0	0	0	0
San Jose City	0	1	40	41	0	0	0	0	0	0	0	0
San Jose del Monte City	0	20	994	1,014	0	0	0	0	0	0	0	0
Science City of Munoz	0	0	14	14	0	0	0	0	0	0	0	0
Tarlac City	0	13	157	170	0	0	0	0	0	2	3	5
**Region 4A**	**7**	**641**	**11,358**	**12,006**	**0**	**17**	**1,975**	**1,992**	**0**	**15**	**501**	**516**
Batangas	0	7	261	268	0	1	1,790	1,791	0	0	22	22
Cavite	2	77	1,714	1,793	0	0	26	26	0	2	11	13
Laguna	1	69	624	694	0	0	79	79	0	1	2	3
Quezon	0	8	561	569	0	0	8	8	0	3	0	3
Rizal	3	160	2,663	2,826	0	0	0	0	0	0	9	9
Antipolo City	0	1	104	105	0	0	0	0	0	0	0	0
Bacoor City	0	27	416	443	0	0	0	0	0	0	0	0
Batangas City	0	11	56	67	0	0	0	0	0	0	0	0
Biñan City	0	13	55	68	0	0	2	2	0	0	0	0
Cabuyao City	0	1	87	88	0	0	0	0	0	1	0	1
Calamba City	1	15	551	567	0	0	0	0	0	0	14	14
Cavite City	0	1	142	143	0	0	0	0	0	0	0	0
Dasmariñas City	0	192	2,506	2,698	0	0	13	13	0	0	54	54
General Trias City	0	16	387	403	0	0	0	0	0	0	0	0
Imus City	0	11	226	237	0	1	0	1	0	0	0	0
Lipa City	0	0	44	44	0	0	0	0	0	0	0	0
Lucena City	0	4	122	126	0	0	0	0	0	0	0	0
San Pablo City	0	11	124	135	0	0	0	0	0	0	17	17
San Pedro City	0	13	268	281	0	15	0	15	0	0	28	28
Santa Rosa City	0	2	83	85	0	0	0	0	0	3	343	346
Tagaytay City	0	0	123	123	0	0	0	0	0	0	0	0
Tanauan City	0	0	23	23	0	0	0	0	0	5	1	6
Tayabas City	0	0	8	8	0	0	57	57	0	0	0	0
Trece Martires City	0	2	210	212	0	0	0	0	0	0	0	0
**Region 4B**	**0**	**539**	**9,247**	**9,786**	**1**	**76**	**1,249**	**1,326**	**0**	**15**	**141**	**156**
Marinduque	0	11	227	238	0	0	204	204	0	0	0	0
Mindoro Occidental	0	37	992	1,029	0	13	240	253	0	1	11	12
Mindoro Oriental	0	64	1,436	1,500	0	2	202	204	0	5	106	111
Palawan	0	387	5,897	6,284	0	60	533	593	0	6	23	29
Romblon	0	35	478	513	1	1	59	61	0	1	1	2
Puerto Princesa City	0	5	217	222	0	0	11	11	0	2	0	2
**Region 5**	**12**	**408**	**6,545**	**6,965**	**6**	**742**	**5,470**	**6,218**	**0**	**13**	**343**	**356**
Albay	1	76	747	824	0	9	242	251	0	1	78	79
Camarines Norte	0	30	267	297	0	0	20	20	0	0	3	3

Table 2.A.3. MODERN METHOD OF FAMILY PLANNING
Drop Outs
Philippines, 2022

Area	IMPLANTS Age group			Total	NFP-CCM Age group			Total	NFP-BBT Age group			Total
	10-14	15-19	20-49		10-14	15-19	20-49		10-14	15-19	20-49	
Camarines Sur	2	163	2,743	2,908	3	8	1,490	1,501	0	8	197	205
Catanduanes	1	49	461	511	0	20	622	642	0	0	4	4
Masbate	7	39	1,198	1,244	3	622	697	1,322	0	0	17	17
Sorsogon	1	43	864	908	0	83	2,348	2,431	0	4	26	30
Iriga City	0	7	139	146	0	0	51	51	0	0	16	16
Legaspi City	0	0	16	16	0	0	0	0	0	0	0	0
Naga City	0	1	110	111	0	0	0	0	0	0	2	2
**Region 6**	**0**	**477**	**9,165**	**9,642**	**0**	**17**	**539**	**556**	**0**	**0**	**73**	**73**
Aklan	0	1	277	278	0	0	0	0	0	0	0	0
Antique	0	65	762	827	0	17	506	523	0	0	3	3
Capiz	0	18	492	510	0	0	15	15	0	0	0	0
Guimaras	0	7	90	97	0	0	0	0	0	0	1	1
Iloilo	0	83	1,962	2,045	0	0	7	7	0	0	30	30
Negros Occidental	0	247	3,995	4,242	0	0	10	10	0	0	39	39
Bacolod City	0	42	1,434	1,476	0	0	0	0	0	0	0	0
Iloilo City	0	14	153	167	0	0	1	1	0	0	0	0
**Region 7**	**7**	**570**	**9,343**	**9,920**	**0**	**0**	**91**	**91**	**0**	**0**	**30**	**30**
Bohol	0	103	2,918	3,021	0	0	5	5	0	0	0	0
Cebu	5	342	3,991	4,338	0	0	2	2	0	0	0	0
Negros Oriental	2	54	1,054	1,110	0	0	84	84	0	0	5	5
Siquijor	0	2	110	112	0	0	0	0	0	0	0	0
Cebu City	0	65	1,103	1,168	0	0	0	0	0	0	25	25
Lapu-Lapu City	0	1	32	33	0	0	0	0	0	0	0	0
Mandaue City	0	3	135	138	0	0	0	0	0	0	0	0
**Region 8**	**0**	**159**	**4,853**	**5,012**	**0**	**1**	**160**	**161**	**0**	**4**	**59**	**63**
Biliran	0	11	92	103	0	0	9	9	0	1	1	2
Eastern Samar	0	6	220	226	0	0	13	13	0	3	25	28
Northern Leyte	0	18	765	783	0	0	61	61	0	0	0	0
Northern Samar	0	16	585	601	0	0	4	4	0	0	0	0
Southern Leyte	0	7	426	433	0	1	46	47	0	0	0	0
Western Samar	0	10	337	347	0	0	8	8	0	0	4	4
Calbayog City	0	2	189	191	0	0	0	0	0	0	0	0
Maasin City	0	11	95	106	0	0	18	18	0	0	18	18
Ormoc City	0	31	681	712	0	0	1	1	0	0	11	11
Tacloban City	0	47	1,463	1,510	0	0	0	0	0	0	0	0
**Region 9**	**10**	**565**	**5,024**	**5,599**	**0**	**0**	**94**	**94**	**0**	**0**	**1**	**1**
Zamboanga del Norte	4	142	1,312	1,458	0	0	50	50	0	0	0	0
Zamboanga del Sur	4	264	1,822	2,090	0	0	4	4	0	0	1	1
Zamboanga Sibugay	1	100	890	991	0	0	40	40	0	0	0	0
Dapitan City	0	3	122	125	0	0	0	0	0	0	0	0
Dipolog City	1	8	90	99	0	0	0	0	0	0	0	0
Isabela City	0	6	141	147	0	0	0	0	0	0	0	0
Pagadian City	0	0	0	0	0	0	0	0	0	0	0	0
Zamboanga City	0	42	647	689	0	0	0	0	0	0	0	0
**Region 10**	**18**	**731**	**7,268**	**8,017**	**0**	**43**	**1,018**	**1,061**	**1**	**13**	**235**	**249**
Bukidnon	11	571	3,909	4,491	0	34	483	517	0	8	24	32
Camiguin	0	0	32	32	0	0	0	0	0	2	61	63
Lanao del Norte	2	30	747	779	0	1	38	39	0	0	21	21
Misamis Occidental	0	8	114	122	0	0	4	4	0	0	0	0
Misamis Oriental	0	39	530	569	0	3	260	263	1	0	94	95
Cagayan de Oro City	0	26	748	774	0	0	0	0	0	0	19	19

Table 2.A.3. MODERN METHOD OF FAMILY PLANNING
Drop Outs
Philippines, 2022

Area	IMPLANTS Age group			Total	NFP-CCM Age group			Total	NFP-BBT Age group			Total
	10-14	15-19	20-49		10-14	15-19	20-49		10-14	15-19	20-49	
El Salvador City	1	11	65	77	0	0	0	0	0	0	0	0
Gingoog City	4	13	134	151	0	0	57	57	0	0	0	0
Iligan City	0	1	219	220	0	0	14	14	0	0	0	0
Malaybalay City	0	24	463	487	0	1	4	5	0	0	4	4
Oroquieta City	0	1	19	20	0	0	0	0	0	0	0	0
Ozamis City	0	0	40	40	0	0	1	1	0	0	5	5
Tangub City	0	0	0	0	0	0	0	0	0	0	0	0
Valencia City	0	7	248	255	0	4	157	161	0	3	7	10
**Region 11**	**24**	**571**	**6,643**	**7,238**	**0**	**9**	**390**	**399**	**0**	**2**	**108**	**110**
Davao de Oro	9	82	1,265	1,356	0	1	86	87	0	0	40	40
Davao del Norte	3	108	1,314	1,425	0	0	42	42	0	0	4	4
Davao Oriental	0	34	476	510	0	1	3	4	0	0	4	4
Davao del Sur	3	123	759	885	0	0	1	1	0	0	0	0
Davao Occidental	0	34	711	745	0	4	157	161	0	0	0	0
Davao City	9	190	2,118	2,317	0	3	101	104	0	2	60	62
**Region 12**	**8**	**796**	**5,451**	**6,255**	**0**	**1**	**125**	**126**	**0**	**5**	**24**	**29**
North Cotabato	3	174	1,991	2,168	0	0	95	95	0	0	8	8
Sarangani	0	39	425	464	0	1	6	7	0	0	0	0
South Cotabato	4	191	1,248	1,443	0	0	4	4	0	5	14	19
Sultan Kudarat	1	298	1,059	1,358	0	0	20	20	0	0	0	0
				0				0				0
Gen. Santos City	0	94	728	822	0	0	0	0	0	0	2	2
**BARMM**	**4**	**419**	**6,706**	**7,129**	**0**	**0**	**11**	**11**	**0**	**4**	**14**	**18**
Basilan	1	42	475	518	0	0	2	2	0	0	0	0
Lanao del Sur	0	6	361	367	0	0	3	3	0	0	2	2
Maguindanao	0	168	1,281	1,449	0	0	3	3	0	0	2	2
Sulu	3	104	2,538	2,645	0	0	0	0	0	0	0	0
Tawi-Tawi	0	8	1,335	1,343	0	0	3	3	0	0	0	0
				0				0				0
Lamitan City	0	79	461	540	0	0	0	0	0	0	0	0
Marawi City	0	1	94	95	0	0	0	0	0	0	0	0
Cotabato City	0	11	161	172	0	0	0	0	0	4	10	14
**CARAGA**	**8**	**457**	**5,329**	**5,794**	**0**	**3**	**212**	**215**	**0**	**1**	**188**	**189**
Agusan del Norte	0	87	596	683	0	0	4	4	0	0	3	3
Agusan del Sur	0	59	741	800	0	2	102	104	0	1	3	4
Surigao del Norte	7	156	2,433	2,596	0	0	18	18	0	0	67	67
Surigao del Sur	1	65	695	761	0	0	52	52	0	0	114	114
Province of Dinagat	0	14	211	225	0	0	0	0	0	0	0	0
Bislig City	0	34	125	159	0	1	36	37	0	0	1	1
Butuan City	0	38	423	461	0	0	0	0	0	0	0	0
Surigao City	0	4	105	109	0	0	0	0	0	0	0	0

Table 2.A.3. MODERN METHOD OF FAMILY PLANNING
Drop Outs
Philippines, 2022

Area	NFP-STM Age group 10-14	NFP-STM Age group 15-19	NFP-STM Age group 20-49	Total	NFP-SDM Age group 10-14	NFP-SDM Age group 15-19	NFP-SDM Age group 20-49	Total	NFP-LAM Age group 10-14	NFP-LAM Age group 15-19	NFP-LAM Age group 20-49	Total
**PHILIPPINES**	13	170	2,597	2,780	13	752	37,346	38,111	1,088	69,100	661,363	731,551
**N C R**	0	0	9	9	0	2	164	166	151	8,236	93,026	101,413
Malabon	0	0	0	0	0	0	5	5	6	216	1,511	1,733
Navotas	0	0	0	0	0	0	10	10	7	493	2,722	3,222
Valenzuela City	0	0	0	0	0	0	6	6	0	190	5,573	5,763
Caloocan City	0	0	0	0	0	0	5	5	4	1,000	7,262	8,266
Marikina City	0	0	0	0	0	0	0	0	0	42	393	435
Pasig City	0	0	0	0	0	0	9	9	0	136	2,628	2,764
Pateros	0	0	0	0	0	0	0	0	0	9	754	763
Taguig	0	0	0	0	0	0	0	0	6	728	9,737	10,471
Quezon City	0	0	0	0	0	0	100	100	5	2,138	31,810	33,953
Makati City	0	0	0	0	0	0	0	0	1	163	2,506	2,670
Mandaluyong City	0	0	0	0	0	0	10	10	2	327	2,792	3,121
San Juan	0	0	0	0	0	0	0	0	0	23	773	796
Manila City	0	0	9	9	0	0	15	15	2	735	6,761	7,498
Las Piñas City	0	0	0	0	0	0	1	1	2	339	4,475	4,816
Muntinlupa City	0	0	0	0	0	0	0	0	0	138	2,366	2,504
Parañaque City	0	0	0	0	0	2	3	5	116	1,222	6,098	7,436
Pasay City	0	0	0	0	0	0	0	0	0	337	4,865	5,202
**C A R**	0	1	22	23	0	18	1,243	1,261	32	1,209	13,338	14,579
Abra	0	0	0	0	0	3	184	187	1	217	1,852	2,070
Apayao	0	0	0	0	0	0	8	8	3	215	1,506	1,724
Benguet	0	0	0	0	0	10	364	374	2	220	2,811	3,033
Ifugao	0	0	1	1	0	4	371	375	1	142	2,059	2,202
Kalinga	0	1	20	21	0	0	94	94	24	259	2,930	3,213
Mt. Province	0	0	1	1	0	1	198	199	1	146	1,648	1,795
Baguio City	0	0	0	0	0	0	24	24	0	10	532	542
**Region 1**	0	11	273	284	0	13	983	996	25	2,745	38,264	41,034
Ilocos Norte	0	0	1	1	0	5	97	102	0	85	1,555	1,640
Ilocos Sur	0	0	44	44	0	5	116	121	5	300	4,361	4,666
La Union	0	0	3	3	0	0	472	472	5	499	6,187	6,691
Pangasinan	0	11	27	38	0	3	186	189	12	1,158	16,954	18,124
Alaminos City	0	0	0	0	0	0	0	0	0	253	3,168	3,421
Candon City	0	0	0	0	0	0	1	1	0	0	154	154
Dagupan City	0	0	0	0	0	0	5	5	3	85	708	796
Laoag City	0	0	0	0	0	0	0	0	0	24	1,005	1,029
San Carlos City	0	0	0	0	0	0	0	0	0	81	1,219	1,300
San Fernando City	0	0	0	0	0	0	53	53	0	13	272	285
Urdaneta City	0	0	0	0	0	0	52	52	0	152	2,133	2,285
Vigan City	0	0	198	198	0	0	1	1	0	95	548	643
**Region 2**	0	3	121	124	0	1	249	250	37	2,034	18,713	20,784
Batanes	0	0	0	0	0	0	6	6	0	19	250	269
Cagayan	0	0	0	0	0	0	1	1	1	480	4,719	5,200
Isabela	0	0	0	0	0	0	2	2	3	420	3,921	4,344
Nueva Vizcaya	0	3	121	124	0	1	224	225	23	466	4,048	4,537
Quirino	0	0	0	0	0	0	12	12	1	95	830	926
Cauayan City	0	0	0	0	0	0	0	0	3	157	901	1,061
Ilagan City	0	0	0	0	0	0	0	0	1	100	903	1,004
Santiago City	0	0	0	0	0	0	0	0	5	281	2,795	3,081
Tuguegarao City	0	0	0	0	0	0	4	4	0	16	346	362

Table 2.A.3. MODERN METHOD OF FAMILY PLANNING
Drop Outs
Philippines, 2022

Area	NFP-STM			Total	NFP-SDM			Total	NFP-LAM			Total
	Age group				Age group				Age group			
	10-14	15-19	20-49		10-14	15-19	20-49		10-14	15-19	20-49	
**Region 3**	**0**	**0**	**13**	**13**	**0**	**11**	**715**	**726**	**43**	**5,244**	**49,535**	**54,822**
Aurora	0	0	0	0	0	1	36	37	4	300	2,514	2,818
Bataan	0	0	0	0	0	0	4	4	5	430	3,570	4,005
Bulacan	0	0	0	0	0	1	471	472	1	946	9,316	10,263
Nueva Ecija	0	0	0	0	0	1	4	5	3	555	5,010	5,568
Pampanga	0	0	7	7	0	4	111	115	12	383	6,108	6,503
Tarlac	0	0	1	1	0	0	26	26	1	460	6,363	6,824
Zambales	0	0	0	0	0	0	7	7	4	367	2,729	3,100
Angeles City	0	0	2	2	0	0	10	10	1	97	1,499	1,597
Balanga City	0	0	0	0	0	0	5	5	2	82	577	661
Cabanatuan City	0	0	0	0	0	0	0	0	2	334	2,856	3,192
City of San Fernando	0	0	3	3	0	4	29	33	2	138	1,050	1,190
Gapan City	0	0	0	0	0	0	0	0	0	63	367	430
Mabalacat City	0	0	0	0	0	0	12	12	0	317	2,240	2,557
Malolos City	0	0	0	0	0	0	0	0	0	2	157	159
Meycauayan	0	0	0	0	0	0	0	0	1	136	1,223	1,360
Olongapo	0	0	0	0	0	0	0	0	0	58	588	646
Palayan City	0	0	0	0	0	0	0	0	0	12	156	168
San Jose City	0	0	0	0	0	0	0	0	5	208	1,252	1,465
San Jose del Monte City	0	0	0	0	0	0	0	0	0	0	1	1
Science City of Munoz	0	0	0	0	0	0	0	0	0	0	0	0
Tarlac City	0	0	0	0	0	0	0	0	0	356	1,959	2,315
**Region 4A**	**0**	**2**	**161**	**163**	**0**	**52**	**560**	**612**	**110**	**5,408**	**62,800**	**68,318**
Batangas	0	0	0	0	0	2	119	121	23	427	8,819	9,269
Cavite	0	1	159	160	0	2	75	77	7	368	7,027	7,402
Laguna	0	0	0	0	0	4	101	105	3	342	3,130	3,475
Quezon	0	0	1	1	0	0	126	126	37	611	5,617	6,265
Rizal	0	0	0	0	0	0	2	2	23	930	10,697	11,650
Antipolo City	0	0	1	1	0	0	0	0	0	91	2,450	2,541
Bacoor City	0	0	0	0	0	0	0	0	0	197	2,832	3,029
Batangas City	0	0	0	0	0	0	12	12	0	24	953	977
Biñan City	0	0	0	0	0	0	23	23	0	706	2,374	3,080
Cabuyao City	0	0	0	0	0	9	0	9	1	303	3,760	4,064
Calamba City	0	0	0	0	0	2	3	5	4	211	1,421	1,636
Cavite City	0	0	0	0	0	0	0	0	0	19	86	105
Dasmariñas City	0	1	0	1	0	33	93	126	0	259	3,991	4,250
General Trias City	0	0	0	0	0	0	0	0	0	37	796	833
Imus City	0	0	0	0	0	0	0	0	0	54	644	698
Lipa City	0	0	0	0	0	0	2	2	0	0	2	2
Lucena City	0	0	0	0	0	0	0	0	0	5	156	161
San Pablo City	0	0	0	0	0	0	0	0	9	223	1,595	1,827
San Pedro City	0	0	0	0	0	0	4	4	2	182	1,776	1,960
Santa Rosa City	0	0	0	0	0	0	0	0	0	266	2,249	2,515
Tagaytay City	0	0	0	0	0	0	0	0	0	7	1,105	1,112
Tanauan City	0	0	0	0	0	0	0	0	0	2	103	105
Tayabas City	0	0	0	0	0	0	0	0	1	137	1,197	1,335
Trece Martires City	0	0	0	0	0	0	0	0	0	7	20	27
**Region 4B**	**12**	**0**	**54**	**66**	**6**	**30**	**10,387**	**10,423**	**138**	**3,438**	**31,284**	**34,860**
Marinduque	0	0	0	0	0	0	23	23	0	94	1,246	1,340
Mindoro Occidental	0	0	5	5	0	2	7,597	7,599	31	1,131	7,206	8,368
Mindoro Oriental	0	0	7	7	0	0	22	22	25	546	7,008	7,579
Palawan	12	0	30	42	3	25	2,524	2,552	68	1,346	11,574	12,988
Romblon	0	0	7	7	3	3	142	148	12	265	3,113	3,390
Puerto Princesa City	0	0	5	5	0	0	79	79	2	56	1,137	1,195
**Region 5**	**0**	**13**	**911**	**924**	**2**	**350**	**13,371**	**13,723**	**68**	**5,604**	**54,504**	**60,176**
Albay	0	0	37	37	1	8	678	687	15	480	6,781	7,276
Camarines Norte	0	1	215	216	0	38	786	824	8	656	5,309	5,973

Table 2.A.3. MODERN METHOD OF FAMILY PLANNING
Drop Outs
Philippines, 2022

Area	NFP-STM			Total	NFP-SDM			Total	NFP-LAM			Total
	Age group				Age group				Age group			
	10-14	15-19	20-49		10-14	15-19	20-49		10-14	15-19	20-49	
Camarines Sur	0	12	504	516	0	85	6,130	6,215	27	1,568	15,992	17,587
Catanduanes	0	0	1	1	0	0	781	781	2	388	3,685	4,075
Masbate	0	0	61	61	0	204	3,686	3,890	12	1,495	10,391	11,898
Sorsogon	0	0	7	7	1	8	756	765	4	877	9,823	10,704
Iriga City	0	0	86	86	0	7	422	429	0	127	923	1,050
Legaspi City	0	0	0	0	0	0	9	9	0	9	227	236
Naga City	0	0	0	0	0	0	123	123	0	4	1,373	1,377
**Region 6**	**0**	**0**	**49**	**49**	**0**	**32**	**1,016**	**1,048**	**57**	**4,287**	**42,349**	**46,693**
Aklan	0	0	0	0	0	0	13	13	3	239	3,179	3,421
Antique	0	0	0	0	0	0	62	62	2	211	3,297	3,510
Capiz	0	0	0	0	0	0	4	4	3	90	2,339	2,432
Guimaras	0	0	0	0	0	0	29	29	1	115	1,686	1,802
Iloilo	0	0	12	12	0	5	207	212	14	772	9,547	10,333
Negros Occidental	0	0	37	37	0	27	684	711	34	2,708	20,846	23,588
Bacolod City	0	0	0	0	0	0	3	3	0	140	1,299	1,439
Iloilo City	0	0	0	0	0	0	14	14	0	12	156	168
**Region 7**	**0**	**0**	**0**	**0**	**0**	**4**	**345**	**349**	**61**	**4,653**	**47,722**	**52,436**
Bohol	0	0	0	0	0	0	101	101	13	474	4,175	4,662
Cebu	0	0	0	0	0	0	62	62	16	1,612	18,248	19,876
Negros Oriental	0	0	0	0	0	2	134	136	21	1,329	11,713	13,063
Siquijor	0	0	0	0	0	2	48	50	0	44	466	510
Cebu City	0	0	0	0	0	0	0	0	11	1,051	11,387	12,449
Lapu-Lapu City	0	0	0	0	0	0	0	0	0	140	1,495	1,635
Mandaue City	0	0	0	0	0	0	0	0	0	3	238	241
**Region 8**	**0**	**84**	**527**	**611**	**0**	**31**	**2,481**	**2,512**	**17**	**2,471**	**20,063**	**22,551**
Biliran	0	0	100	100	0	0	37	37	3	133	1,005	1,141
Eastern Samar	0	5	276	281	0	17	1,894	1,911	0	312	2,346	2,658
Northern Leyte	0	0	1	1	0	0	27	27	5	390	4,505	4,900
Northern Samar	0	0	0	0	0	2	2	4	4	246	2,004	2,254
Southern Leyte	0	0	0	0	0	0	7	7	2	122	1,302	1,426
Western Samar	0	0	0	0	0	0	46	46	0	261	2,115	2,376
Calbayog City	0	0	0	0	0	0	0	0	1	101	1,085	1,187
Maasin City	0	0	0	0	0	0	18	18	2	17	220	239
Ormoc City	0	79	150	229	0	12	449	461	0	391	3,040	3,431
Tacloban City	0	0	0	0	0	0	1	1	0	498	2,441	2,939
**Region 9**	**0**	**0**	**0**	**0**	**2**	**13**	**341**	**356**	**50**	**4,009**	**27,725**	**31,784**
Zamboanga del Norte	0	0	0	0	2	13	326	341	25	1,164	7,718	8,907
Zamboanga del Sur	0	0	0	0	0	0	0	0	5	570	4,033	4,608
Zamboanga Sibugay	0	0	0	0	0	0	13	13	2	455	3,325	3,782
Dapitan City	0	0	0	0	0	0	2	2	1	136	818	955
Dipolog City	0	0	0	0	0	0	0	0	4	203	1,846	2,053
Isabela City	0	0	0	0	0	0	0	0	0	108	789	897
Pagadian City	0	0	0	0	0	0	0	0	0	0	0	0
Zamboanga City	0	0	0	0	0	0	0	0	13	1,373	9,196	10,582
**Region 10**	**0**	**55**	**229**	**284**	**3**	**140**	**2,766**	**2,909**	**79**	**5,667**	**40,904**	**46,650**
Bukidnon	0	55	98	153	2	43	839	884	23	2,089	9,972	12,084
Camiguin	0	0	0	0	0	0	3	3	2	45	624	671
Lanao del Norte	0	0	5	5	0	0	27	27	17	678	6,664	7,359
Misamis Occidental	0	0	0	0	0	0	35	35	0	80	1,158	1,238
Misamis Oriental	0	0	119	119	0	33	308	341	14	523	6,131	6,668
Cagayan de Oro City	0	0	0	0	0	0	0	0	17	818	5,627	6,462

Table 2.A.3. MODERN METHOD OF FAMILY PLANNING
Drop Outs
Philippines, 2022

Area	NFP-STM			Total	NFP-SDM			Total	NFP-LAM			Total
	Age group				Age group				Age group			
	10-14	15-19	20-49		10-14	15-19	20-49		10-14	15-19	20-49	
El Salvador City	0	0	5	5	0	0	10	10	0	76	515	591
Gingoog City	0	0	0	0	1	3	210	214	0	120	865	985
Iligan City	0	0	2	2	0	33	334	367	3	408	3,242	3,653
Malaybalay City	0	0	0	0	0	28	871	899	3	360	2,328	2,691
Oroquieta City	0	0	0	0	0	0	4	4	0	0	146	146
Ozamis City	0	0	0	0	0	0	8	8	0	103	812	915
Tangub City	0	0	0	0	0	0	49	49	0	17	285	302
Valencia City	0	0	0	0	0	0	68	68	0	350	2,535	2,885
**Region 11**	**0**	**0**	**40**	**40**	**0**	**0**	**598**	**598**	**94**	**3,202**	**22,297**	**25,593**
Davao de Oro	0	0	0	0	0	0	41	41	7	115	1,018	1,140
Davao del Norte	0	0	10	10	0	0	58	58	10	445	3,983	4,438
Davao Oriental	0	0	0	0	0	0	12	12	3	124	803	930
Davao del Sur	0	0	0	0	0	0	89	89	9	326	1,064	1,399
Davao Occidental	0	0	1	1	0	0	290	290	17	837	5,213	6,067
Davao City	0	0	29	29	0	0	108	108	48	1,355	10,216	11,619
**Region 12**	**0**	**0**	**21**	**21**	**0**	**8**	**172**	**180**	**61**	**4,628**	**26,948**	**31,637**
North Cotabato	0	0	16	16	0	0	46	46	9	1,149	7,110	8,268
Sarangani	0	0	0	0	0	0	2	2	0	1,054	5,676	6,730
South Cotabato	0	0	5	5	0	8	101	109	23	1,226	6,572	7,821
Sultan Kudarat	0	0	0	0	0	0	21	21	22	735	4,080	4,837
				0				0				0
Gen. Santos City	0	0	0	0	0	0	2	2	7	464	3,510	3,981
**BARMM**	**1**	**0**	**125**	**126**	**0**	**0**	**122**	**122**	**36**	**3,414**	**49,294**	**52,744**
Basilan	1	0	124	125	0	0	7	7	3	194	1,391	1,588
Lanao del Sur	0	0	1	1	0	0	80	80	3	544	13,540	14,087
Maguindanao	0	0	0	0	0	0	13	13	10	1,619	15,486	17,115
Sulu	0	0	0	0	0	0	0	0	1	274	7,288	7,563
Tawi-Tawi	0	0	0	0	0	0	14	14	15	239	5,464	5,718
				0				0				0
Lamitan City	0	0	0	0	0	0	0	0	0	118	660	778
Marawi City	0	0	0	0	0	0	2	2	0	140	2,082	2,222
Cotabato City	0	0	0	0	0	0	6	6	4	286	3,383	3,673
**CARAGA**	**0**	**1**	**42**	**43**	**0**	**47**	**1,833**	**1,880**	**29**	**2,851**	**22,597**	**25,477**
Agusan del Norte	0	0	0	0	0	3	58	61	5	322	3,002	3,329
Agusan del Sur	0	0	2	2	0	22	261	283	7	651	4,636	5,294
Surigao del Norte	0	0	0	0	0	17	1,126	1,143	4	364	3,840	4,208
Surigao del Sur	0	0	40	40	0	5	262	267	3	583	4,040	4,626
Province of Dinagat	0	0	0	0	0	0	50	50	0	81	613	694
Bislig City	0	0	0	0	0	0	67	67	5	128	1,067	1,200
Butuan City	0	0	0	0	0	0	2	2	5	636	4,518	5,159
Surigao City	0	1	0	1	0	0	7	7	0	86	881	967

Table 2.A.4. MODERN METHOD OF FAMILY PLANNING
Current User (Ending)
Philippines, 2022

Area	Total Current Users	FSTR/BTL Age group			Total	MSTR/NSV Age group			Total
		10-14	15-19	20-49		10-14	15-19	20-49	
**PHILIPPINES**	**8,372,559**	**3**	**929**	**815,771**	**819,597**	**0**	**55**	**9,338**	**9,393**
**N C R**	**859,210**	**0**	**82**	**89,554**	**92,530**	**0**	**7**	**770**	**777**
Malabon	33,641	0	0	2,894	2,894	0	0	4	4
Navotas	25,067	0	1	644	645	0	4	5	9
Valenzuela City	37,966	0	0	6,219	6,219	0	0	93	93
Caloocan City	61,903	0	1	8,606	8,607	0	0	10	10
Marikina City	20,095	0	0	2,261	2,261	0	0	199	199
Pasig City	55,229	0	1	4,476	4,477	0	0	28	28
Pateros	3,813	0	0	366	366	0	0	6	6
Taguig	71,609	0	0	3,711	3,711	0	1	30	31
Quezon City	306,956	0	68	37,093	37,161	0	0	231	231
Makati City	20,752	0	0	1,668	1,668	0	0	24	24
Mandaluyong City	23,665	0	9	2,139	2,148	0	0	33	33
San Juan	5,890	0	0	233	233	0	0	9	9
Manila City	50,873	0	1	6,078	6,079	0	0	43	43
Las Piñas City	20,551	0	0	3,429	3,429	0	0	11	11
Muntinlupa City	41,934	0	1	3,491	3,492	0	1	14	15
Parañaque City	42,632	0	0	2,969	2,969	0	1	30	31
Pasay City	36,634	0	0	6,171	6,171	0	0	0	0
**C A R**	**161,545**	**0**	**0**	**32,395**	**32,395**	**0**	**0**	**191**	**191**
Abra	19,903	0	0	3,895	3,895	0	0	3	3
Apayao	24,278	0	0	1,061	1,061	0	0	1	1
Benguet	34,934	0	0	8,130	8,130	0	0	35	35
Ifugao	21,809	0	0	2,955	2,955	0	0	87	87
Kalinga	18,022	0	0	3,111	3,111	0	0	3	3
Mt. Province	13,937	0	0	5,223	5,223	0	0	4	4
Baguio City	28,662	0	0	8,020	8,020	0	0	58	58
**Region 1**	**481,911**	**0**	**37**	**68,263**	**68,300**	**0**	**16**	**86**	**102**
Ilocos Norte	43,457	0	9	7,893	7,902	0	0	15	15
Ilocos Sur	64,800	0	10	8,603	8,613	0	0	4	4
La Union	55,686	0	1	10,155	10,156	0	0	7	7
Pangasinan	249,456	0	15	30,063	30,078	0	0	36	36
Alaminos City	6,797	0	0	700	700	0	0	0	0
Candon City	7,268	0	0	892	892	0	0	0	0
Dagupan City	7,085	0	0	854	854	0	0	2	2
Laoag City	7,945	0	0	1,641	1,641	0	16	0	16
San Carlos City	10,640	0	0	2,329	2,329	0	0	0	0
San Fernando City	13,496	0	2	2,373	2,375	0	0	21	21
Urdaneta City	11,143	0	0	1,642	1,642	0	0	1	1
Vigan City	4,138	0	0	1,118	1,118	0	0	0	0
**Region 2**	**320,396**	**0**	**80**	**44,513**	**44,593**	**0**	**0**	**104**	**104**
Batanes	945	0	0	170	170	0	0	0	0
Cagayan	91,699	0	64	11,303	11,367	0	0	26	26
Isabela	109,482	0	14	15,857	15,871	0	0	21	21
Nueva Vizcaya	49,739	0	0	8,123	8,123	0	0	36	36
Quirino	19,516	0	1	2,490	2,491	0	0	7	7
Cauayan City	10,214	0	0	1,694	1,694	0	0	0	0
Ilagan City	10,918	0	1	1,271	1,272	0	0	0	0
Santiago City	12,585	0	0	1,325	1,325	0	0	0	0
Tuguegarao City	15,298	0	0	2,280	2,280	0	0	14	14

Table 2.A.4. MODERN METHOD OF FAMILY PLANNING

Current User (Ending)

Philippines, 2022

Area	Total Current Users	FSTR/BTL Age group			Total	MSTR/NSV Age group			Total
		10-14	15-19	20-49		10-14	15-19	20-49	
**Region 3**	**884,246**	**0**	**12**	**157,833**	**157,845**	**0**	**0**	**182**	**182**
Aurora	22,956	0	0	2,119	2,119	0	0	2	2
Bataan	58,445	0	0	7,442	7,442	0	0	19	19
Bulacan	170,180	0	0	30,053	30,053	0	0	29	29
Nueva Ecija	137,145	0	0	27,876	27,876	0	0	0	0
Pampanga	121,828	0	1	39,018	39,019	0	0	6	6
Tarlac	92,168	0	1	14,297	14,298	0	0	7	7
Zambales	48,549	0	2	7,599	7,601	0	0	9	9
Angeles City	29,054	0	0	5,877	5,877	0	0	74	74
Balanga City	4,743	0	0	882	882	0	0	1	1
Cabanatuan City	19,701	0	1	3,671	3,672	0	0	0	0
City of San Fernando	12,777	0	5	1,655	1,660	0	0	8	8
Gapan City	7,646	0	0	1,676	1,676	0	0	0	0
Mabalacat City	24,619	0	0	2,197	2,197	0	0	1	1
Malolos City	8,467	0	0	1,685	1,685	0	0	10	10
Meycauayan	7,760	0	0	324	324	0	0	5	5
Olongapo	13,066	0	2	2,179	2,181	0	0	3	3
Palayan City	2,854	0	0	461	461	0	0	0	0
San Jose City	12,647	0	0	809	809	0	0	2	2
San Jose del Monte City	59,526	0	0	4,708	4,708	0	0	6	6
Science City of Munoz	6,498	0	0	1,196	1,196	0	0	0	0
Tarlac City	23,617	0	0	2,109	2,109	0	0	0	0
**Region 4A**	**910,368**	**0**	**32**	**97,313**	**97,345**	**0**	**0**	**248**	**248**
Batangas	131,424	0	0	16,295	16,295	0	0	19	19
Cavite	85,159	0	8	12,427	12,435	0	0	33	33
Laguna	77,425	0	0	13,276	13,276	0	0	33	33
Quezon	101,428	0	3	9,438	9,441	0	0	19	19
Rizal	114,489	0	0	10,446	10,446	0	0	39	39
Antipolo City	37,911	0	5	3,050	3,055	0	0	8	8
Bacoor City	9,103	0	0	1,547	1,547	0	0	8	8
Batangas City	17,143	0	0	2,177	2,177	0	0	2	2
Biñan City	109,509	0	0	2,044	2,044	0	0	7	7
Cabuyao City	16,692	0	0	1,429	1,429	0	0	0	0
Calamba City	34,891	0	2	4,668	4,670	0	0	4	4
Cavite City	4,273	0	0	1,242	1,242	0	0	3	3
Dasmariñas City	38,842	0	0	4,080	4,080	0	0	7	7
General Trias City	6,657	0	0	1,535	1,535	0	0	0	0
Imus City	7,925	0	0	1,106	1,106	0	0	5	5
Lipa City	7,687	0	8	1,511	1,519	0	0	0	0
Lucena City	5,002	0	0	177	177	0	0	3	3
San Pablo City	13,867	0	0	3,431	3,431	0	0	2	2
San Pedro City	18,925	0	4	1,054	1,058	0	0	15	15
Santa Rosa City	49,034	0	2	2,399	2,401	0	0	24	24
Tagaytay City	9,160	0	0	1,225	1,225	0	0	10	10
Tanauan City	6,491	0	0	1,286	1,286	0	0	3	3
Tayabas City	4,061	0	0	739	739	0	0	2	2
Trece Martires City	3,270	0	0	731	731	0	0	2	2
**Region 4B**	**266,333**	**0**	**50**	**22,230**	**22,280**	**0**	**0**	**228**	**228**
Marinduque	15,205	0	0	2,219	2,219	0	0	16	16
Mindoro Occidental	40,816	0	0	2,277	2,277	0	0	6	6
Mindoro Oriental	63,083	0	9	5,667	5,676	0	0	65	65
Palawan	93,326	0	6	6,108	6,114	0	0	86	86
Romblon	22,228	0	0	3,503	3,503	0	0	40	40
Puerto Princesa City	31,675	0	35	2,456	2,491	0	0	15	15
**Region 5**	**484,139**	**0**	**15**	**29,742**	**29,757**	**0**	**0**	**313**	**313**
Albay	97,127	0	3	6,095	6,098	0	0	68	68

Table 2.A.4. MODERN METHOD OF FAMILY PLANNING
Current User (Ending)
Philippines, 2022

Area	Total Current Users	FSTR/BTL			Total	MSTR/NSV			Total
		Age group				Age group			
		10-14	15-19	20-49		10-14	15-19	20-49	
Camarines Norte	46,841	0	7	1,697	1,704	0	0	7	7
Camarines Sur	96,167	0	0	4,681	4,681	0	0	17	17
Catanduanes	23,049	0	1	3,294	3,295	0	0	75	75
Masbate	91,518	0	1	2,905	2,906	0	0	38	38
Sorsogon	65,058	0	3	6,472	6,475	0	0	46	46
Iriga City	11,234	0	0	486	486	0	0	5	5
Legaspi City	22,901	0	0	1,858	1,858	0	0	0	0
Naga City	30,244	0	0	2,254	2,254	0	0	57	57
**Region 6**	**639,939**	**0**	**12**	**53,468**	**53,480**	**0**	**5**	**1,967**	**1,972**
Aklan	46,162	0	2	3,584	3,586	0	0	3	3
Antique	47,022	0	2	6,681	6,683	0	0	82	82
Capiz	75,514	0	0	4,174	4,174	0	0	345	345
Guimaras	16,676	0	0	1,249	1,249	0	0	6	6
Iloilo	169,693	0	0	12,736	12,736	0	0	284	284
Negros Occidental	199,968	0	7	14,387	14,394	0	5	1,151	1,156
Bacolod City	28,243	0	0	3,447	3,447	0	0	83	83
Iloilo City	56,661	0	1	7,210	7,211	0	0	13	13
**Region 7**	**597,007**	**0**	**31**	**38,444**	**38,475**	**0**	**1**	**1,442**	**1,443**
Bohol	86,129	0	5	9,966	9,971	0	0	210	210
Cebu	309,013	0	2	20,543	20,545	0	0	662	662
Negros Oriental	90,628	0	22	3,202	3,224	0	0	211	211
Siquijor	9,078	0	0	469	469	0	0	1	1
Cebu City	39,810	0	2	1,834	1,836	0	1	304	305
Lapu-Lapu City	35,757	0	0	2,176	2,176	0	0	54	54
Mandaue City	26,592	0	0	254	254	0	0	0	0
**Region 8**	**332,964**	**0**	**11**	**20,404**	**20,415**	**0**	**1**	**277**	**278**
Biliran	11,240	0	0	1,144	1,144	0	0	35	35
Eastern Samar	35,729	0	6	2,142	2,148	0	0	41	41
Northern Leyte	122,333	0	0	9,703	9,703	0	0	91	91
Northern Samar	49,055	0	5	1,304	1,309	0	1	47	48
Southern Leyte	32,164	0	0	1,354	1,354	0	0	8	8
Western Samar	22,586	0	0	355	355	0	0	23	23
Calbayog City	9,165	0	0	1,121	1,121	0	0	8	8
Maasin City	5,112	0	0	567	567	0	0	1	1
Ormoc City	19,622	0	0	1,393	1,393	0	0	17	17
Tacloban City	25,958	0	0	1,321	1,321	0	0	6	6
**Region 9**	**339,030**	**0**	**5**	**12,964**	**12,969**	**0**	**0**	**45**	**45**
Zamboanga del Norte	93,987	0	0	3,391	3,391	0	0	3	3
Zamboanga del Sur	75,821	0	2	1,955	1,957	0	0	7	7
Zamboanga Sibugay	44,632	0	0	1,922	1,922	0	0	13	13
Dapitan City	7,286	0	0	645	645	0	0	0	0
Dipolog City	18,153	0	0	782	782	0	0	0	0
Isabela City	8,304	0	2	584	586	0	0	2	2
Pagadian City	24,559	0	0	1,139	1,139	0	0	20	20
Zamboanga City	66,288	0	1	2,546	2,547	0	0	0	0
**Region 10**	**497,673**	**0**	**19**	**31,816**	**31,835**	**0**	**9**	**780**	**789**
Bukidnon	109,755	0	16	6,347	6,363	0	1	49	50
Camiguin	6,535	0	0	1,082	1,082	0	0	8	8
Lanao del Norte	67,645	0	0	1,162	1,162	0	0	8	8
Misamis Occidental	29,624	0	0	1,516	1,516	0	0	17	17
Misamis Oriental	81,691	0	0	6,656	6,656	0	0	49	49

Table 2.A.4. MODERN METHOD OF FAMILY PLANNING
Current User (Ending)
Philippines, 2022

Area	Total Current Users	FSTR/BTL			Total	MSTR/NSV			Total
		Age group				Age group			
		10-14	15-19	20-49		10-14	15-19	20-49	
Cagayan de Oro City	54,303	0	0	4,585	4,585	0	6	479	485
El Salvador City	5,699	0	0	406	406	0	0	0	0
Gingoog City	30,222	0	0	2,448	2,448	0	2	75	77
Iligan City	30,536	0	3	1,783	1,786	0	0	16	16
Malaybalay City	19,283	0	0	1,572	1,572	0	0	6	6
Oroquieta City	7,083	0	0	809	809	0	0	2	2
Ozamis City	25,713	0	0	908	908	0	0	0	0
Tangub City	7,498	0	0	326	326	0	0	0	0
Valencia City	22,086	0	0	2,216	2,216	0	0	71	71
**Region 11**	**559,876**	**3**	**304**	**45,548**	**45,855**	**0**	**12**	**1,761**	**1,773**
Davao de Oro	86,149	0	98	7,413	7,511	0	0	127	127
Davao del Norte	107,592	0	97	11,462	11,559	0	0	597	597
Davao Oriental	91,109	0	12	4,822	4,834	0	11	63	74
Davao del Sur	31,833	0	53	3,979	4,032	0	1	48	49
Davao Occidental	55,738	0	-5	5,554	5,549	0	0	133	133
Davao City	187,455	3	49	12,318	12,370	0	0	793	793
**Region 12**	**471,130**	**0**	**183**	**42,037**	**42,220**	**0**	**3**	**741**	**744**
North Cotabato	151,206	0	13	10,397	10,410	0	0	124	124
Sarangani	74,273	0	160	4,316	4,476	0	3	97	100
South Cotabato	96,310	0	3	9,654	9,657	0	0	370	370
Sultan Kudarat	99,744	0	2	9,309	9,311	0	0	93	93
Gen. Santos City	49,597	0	5	8,361	8,366	0	0	57	57
**BARMM**	**282,441**	**0**	**33**	**8,803**	**8,836**	**0**	**0**	**23**	**23**
Basilan	10,543	0	0	298	298	0	0	0	0
Lanao del Sur	32,654	0	0	1,375	1,375	0	0	9	9
Maguindanao	92,272	0	6	2,702	2,708	0	0	16	16
Sulu	82,939	0	27	1,274	1,301	0	0	-2	-2
Tawi-Tawi	18,014	0	0	301	301	0	0	0	0
Lamitan City	5,009	0	0	220	220	0	0	0	0
Marawi City	4,752	0	0	248	248	0	0	0	0
Cotabato City	36,258	0	0	2,385	2,385	0	0	0	0
**CARAGA**	**284,351**	**0**	**23**	**20,444**	**20,467**	**0**	**1**	**180**	**181**
Agusan del Norte	40,460	0	3	3,402	3,405	0	0	7	7
Agusan del Sur	86,938	0	3	6,824	6,827	0	0	48	48
Surigao del Norte	35,422	0	2	1,848	1,850	0	0	32	32
Surigao del Sur	45,885	0	1	3,476	3,477	0	0	51	51
Province of Dinagat	10,768	0	13	383	396	0	0	2	2
Bislig City	10,166	0	0	767	767	0	0	5	5
Butuan City	39,815	0	1	2,702	2,703	0	0	15	15
Surigao City	14,897	0	0	1,042	1,042	0	1	20	21

Table 2.A.4. MODERN METHOD OF FAMILY PLANNING
Current User (Ending)
Philippines, 2022

Area	CONDOM			Total	IUD-INTERVAL			Total	IUD-POSTPARTUM			Total
	Age group				Age group				Age group			
	10-14	15-19	20-49		10-14	15-19	20-49		10-14	15-19	20-49	
**PHILIPPINES**	**179**	**16,930**	**395,297**	**412,406**	**159**	**8,258**	**437,296**	**445,713**	**293**	**11,132**	**135,059**	**146,484**
**N C R**	**29**	**4,282**	**81,349**	**85,660**	**3**	**1,048**	**42,283**	**43,334**	**108**	**3,383**	**28,209**	**31,700**
Malabon	1	926	2,197	3,124	0	110	1,347	1,457	0	98	234	332
Navotas	3	395	1,999	2,397	0	7	363	370	0	3	46	49
Valenzuela City	0	8	841	849	0	0	372	372	0	29	529	558
Caloocan City	0	109	2,677	2,786	1	146	3,603	3,750	5	78	716	799
Marikina City	0	8	634	642	0	46	1,162	1,208	23	509	2,408	2,940
Pasig City	9	120	2,425	2,554	0	44	2,409	2,453	14	476	1,842	2,332
Pateros	0	1	226	227	0	0	442	442	0	0	4	4
Taguig	9	340	5,126	5,475	1	69	2,165	2,235	2	30	359	391
Quezon City	5	1,469	50,578	52,052	0	397	19,082	19,479	53	1,246	16,943	18,242
Makati City	0	22	1,502	1,524	0	15	716	731	0	75	943	1,018
Mandaluyong City	0	121	1,700	1,821	0	11	519	530	7	198	886	1,091
San Juan	0	1	117	118	0	0	1,328	1,328	0	0	0	0
Manila City	2	164	3,542	3,708	0	67	919	986	3	89	949	1,041
Las Piñas City	0	27	1,008	1,035	0	10	429	439	0	49	360	409
Muntinlupa City	0	65	1,084	1,149	1	68	2,907	2,976	0	7	57	64
Parañaque City	0	254	3,549	3,803	0	58	1,139	1,197	1	496	707	1,204
Pasay City	0	252	2,144	2,396	0	0	3,381	3,381	0	0	1,226	1,226
**C A R**	**1**	**199**	**6,675**	**6,875**	**1**	**64**	**5,792**	**5,857**	**0**	**36**	**6,523**	**6,559**
Abra	0	105	1,231	1,336	0	0	40	40	0	6	42	48
Apayao	0	3	171	174	0	0	281	281	0	9	280	289
Benguet	0	17	2,735	2,752	0	19	2,500	2,519	0	4	519	523
Ifugao	0	10	543	553	0	3	970	973	0	1	53	54
Kalinga	0	15	530	545	0	6	685	691	0	4	42	46
Mt. Province	0	3	516	519	1	6	442	449	0	8	63	71
Baguio City	1	46	949	996	0	30	874	904	0	4	5,524	5,528
**Region 1**	**1**	**465**	**19,647**	**20,113**	**8**	**349**	**10,298**	**10,655**	**1**	**42**	**638**	**681**
Ilocos Norte	0	20	1,671	1,691	0	19	525	544	0	6	125	131
Ilocos Sur	0	57	1,514	1,571	0	2	543	545	1	2	110	113
La Union	0	14	857	871	0	27	1,364	1,391	0	18	171	189
Pangasinan	1	364	13,299	13,664	8	279	7,144	7,431	0	0	0	0
Alaminos City	0	0	156	156	0	0	31	31	0	0	0	0
Candon City	0	0	1,043	1,043	0	0	2	2	0	0	129	129
Dagupan City	0	1	181	182	0	1	73	74	0	5	69	74
Laoag City	0	0	0	0	0	0	64	64	0	0	0	0
San Carlos City	0	0	324	324	0	9	388	397	0	0	0	0
San Fernando City	0	9	346	355	0	12	159	171	0	11	34	45
Urdaneta City	0	0	211	211	0	0	3	3	0	0	0	0
Vigan City	0	0	45	45	0	0	2	2	0	0	0	0
**Region 2**	**4**	**146**	**3,342**	**3,492**	**4**	**392**	**17,259**	**17,655**	**1**	**209**	**1,434**	**1,644**
Batanes	0	0	21	21	0	0	11	11	0	0	0	0
Cagayan	1	12	306	319	4	134	8,402	8,540	0	38	263	301
Isabela	2	50	1,321	1,373	0	103	3,031	3,134	1	77	433	511
Nueva Vizcaya	0	12	1,118	1,130	0	13	1,127	1,140	0	13	152	165
Quirino	0	14	259	273	0	0	704	704	0	0	19	19
Cauayan City	0	0	61	61	0	3	209	212	0	1	23	24
Ilagan City	1	54	113	168	0	27	900	927	0	4	138	142
Santiago City	0	1	39	40	0	10	271	281	0	0	0	0
Tuguegarao City	0	3	104	107	0	102	2,604	2,706	0	76	406	482

Table 2.A.4. MODERN METHOD OF FAMILY PLANNING
Current User (Ending)
Philippines, 2022

Area	CONDOM Age group 10-14	15-19	20-49	Total	IUD-INTERVAL Age group 10-14	15-19	20-49	Total	IUD-POSTPARTUM Age group 10-14	15-19	20-49	Total
**Region 3**	**21**	**1,334**	**38,351**	**39,706**	**54**	**213**	**11,943**	**12,210**	**30**	**1,183**	**10,363**	**11,576**
Aurora	0	41	982	1,023	0	5	266	271	0	2	83	85
Bataan	10	117	2,608	2,735	0	34	481	515	1	19	506	526
Bulacan	0	188	5,058	5,246	0	5	3,522	3,527	0	29	1,615	1,644
Nueva Ecija	0	184	2,718	2,902	0	55	2,284	2,339	29	1,062	3,119	4,210
Pampanga	1	89	4,505	4,595	54	10	806	870	0	21	4,007	4,028
Tarlac	0	120	3,752	3,872	0	64	895	959	0	5	211	216
Zambales	6	33	1,715	1,754	0	6	601	607	0	5	216	221
Angeles City	0	15	676	691	0	3	275	278	0	2	229	231
Balanga City	0	3	97	100	0	0	31	31	0	0	3	3
Cabanatuan City	0	8	361	369	0	5	121	126	0	3	26	29
City of San Fernando	3	167	1,692	1,862	0	5	22	27	0	10	63	73
Gapan City	0	0	144	144	0	0	33	33	0	2	2	4
Mabalacat City	1	57	2,386	2,444	0	11	248	259	0	9	32	41
Malolos City	0	7	433	440	0	2	92	94	0	0	0	0
Meycauayan	0	25	514	539	0	0	20	20	0	0	18	18
Olongapo	0	1	720	721	0	3	76	79	0	12	115	127
Palayan City	0	0	28	28	0	0	160	160	0	0	0	0
San Jose City	0	19	275	294	0	1	109	110	0	2	10	12
San Jose del Monte City	0	114	8,661	8,775	0	4	1,454	1,458	0	0	0	0
Science City of Munoz	0	0	59	59	0	0	98	98	0	0	0	0
Tarlac City	0	146	967	1,113	0	0	349	349	0	0	108	108
**Region 4A**	**42**	**5,273**	**53,528**	**58,843**	**16**	**450**	**29,816**	**30,282**	**24**	**460**	**11,691**	**12,175**
Batangas	10	310	8,157	8,477	0	19	2,480	2,499	0	20	1,498	1,518
Cavite	2	44	3,115	3,161	4	45	3,084	3,133	0	16	272	288
Laguna	0	99	4,039	4,138	1	135	4,884	5,020	0	62	4,341	4,403
Quezon	5	56	2,394	2,455	9	49	8,169	8,227	2	12	368	382
Rizal	5	129	4,360	4,494	1	88	2,720	2,809	2	110	950	1,062
Antipolo City	0	20	2,080	2,100	0	0	1,291	1,291	0	0	381	381
Bacoor City	0	3	231	234	0	10	243	253	0	5	53	58
Batangas City	0	9	1,049	1,058	0	5	554	559	1	179	2,229	2,409
Biñan City	0	3,785	15,341	19,126	0	15	1,377	1,392	0	6	94	100
Cabuyao City	1	156	964	1,121	0	4	347	351	0	1	16	17
Calamba City	0	46	1,666	1,712	0	5	588	593	1	3	130	134
Cavite City	0	9	73	82	0	2	56	58	0	3	21	24
Dasmariñas City	0	131	1,923	2,054	0	35	1,449	1,484	0	12	381	393
General Trias City	0	2	64	66	0	0	147	147	0	2	15	17
Imus City	0	3	378	381	0	0	64	64	0	1	35	36
Lipa City	0	1	349	350	0	1	283	284	0	0	56	56
Lucena City	1	1	82	84	0	0	15	15	0	0	2	2
San Pablo City	0	5	206	211	0	10	833	843	0	11	438	449
San Pedro City	0	65	626	691	0	13	226	239	0	4	15	19
Santa Rosa City	18	399	5,529	5,946	0	6	459	465	18	3	136	157
Tagaytay City	0	0	260	260	0	0	10	10	0	0	118	118
Tanauan City	0	0	416	416	0	1	281	282	0	10	136	146
Tayabas City	0	0	181	181	0	0	82	82	0	0	4	4
Trece Martires City	0	0	45	45	1	7	174	182	0	0	2	2
**Region 4B**	**1**	**87**	**6,903**	**6,991**	**0**	**48**	**6,692**	**6,740**	**0**	**25**	**1,645**	**1,670**
Marinduque	0	0	320	320	0	0	394	394	0	0	1	1
Mindoro Occidental	0	7	983	990	0	10	1,133	1,143	0	0	19	19
Mindoro Oriental	0	8	1,728	1,736	0	24	2,860	2,884	0	13	565	578
Palawan	0	40	2,239	2,279	0	7	1,096	1,103	0	8	58	66
Romblon	0	1	604	605	0	3	769	772	0	2	888	890
Puerto Princesa City	1	31	1,029	1,061	0	4	440	444	0	2	114	116
**Region 5**	**10**	**475**	**29,109**	**29,594**	**0**	**129**	**6,515**	**6,644**	**0**	**86**	**1,184**	**1,270**
Albay	0	172	8,479	8,651	0	11	1,027	1,038	0	13	415	428

Table 2.A.4. MODERN METHOD OF FAMILY PLANNING
Current User (Ending)
Philippines, 2022

Area	CONDOM			Total	IUD-INTERVAL			Total	IUD-POSTPARTUM			Total
	Age group				Age group				Age group			
	10-14	15-19	20-49		10-14	15-19	20-49		10-14	15-19	20-49	
Camarines Norte	10	58	2,465	2,533	0	88	1,404	1,492	0	41	94	135
Camarines Sur	0	55	4,176	4,231	0	7	1,120	1,127	0	16	222	238
Catanduanes	0	6	474	480	0	0	50	50	0	0	51	51
Masbate	0	65	2,610	2,675	0	6	1,857	1,863	0	2	209	211
Sorsogon	0	34	2,435	2,469	0	3	233	236	0	6	58	64
Iriga City	0	9	568	577	0	2	338	340	0	3	21	24
Legaspi City	0	65	2,025	2,090	0	2	101	103	0	0	71	71
Naga City	0	11	5,877	5,888	0	10	385	395	0	5	43	48
**Region 6**	**2**	**575**	**30,865**	**31,442**	**9**	**562**	**36,505**	**37,076**	**24**	**796**	**12,047**	**12,867**
Aklan	0	53	2,981	3,034	0	2	666	668	0	0	51	51
Antique	1	14	1,341	1,356	0	1	627	628	0	0	136	136
Capiz	0	31	3,625	3,656	0	26	6,960	6,986	0	1	64	65
Guimaras	0	3	994	997	0	0	297	297	0	1	13	14
Iloilo	1	157	8,915	9,073	6	141	8,787	8,934	4	227	4,150	4,381
Negros Occidental	0	247	6,834	7,081	0	259	14,043	14,302	4	367	4,260	4,631
Bacolod City	0	6	1,470	1,476	3	60	3,040	3,103	3	49	1,959	2,011
Iloilo City	0	64	4,705	4,769	0	73	2,085	2,158	13	151	1,414	1,578
**Region 7**	**2**	**341**	**26,720**	**27,063**	**4**	**1,210**	**74,356**	**75,570**	**19**	**1,300**	**13,436**	**14,755**
Bohol	0	35	5,222	5,257	1	195	12,430	12,626	1	397	4,345	4,743
Cebu	1	75	12,645	12,721	3	733	43,365	44,101	18	780	7,561	8,359
Negros Oriental	1	104	3,158	3,263	0	115	7,763	7,878	0	24	314	338
Siquijor	0	3	409	412	0	3	478	481	0	1	110	111
Cebu City	0	25	987	1,012	0	127	3,677	3,804	0	73	733	806
Lapu-Lapu City	0	95	2,120	2,215	0	35	4,136	4,171	0	22	274	296
Mandaue City	0	4	2,179	2,183	0	2	2,507	2,509	0	3	99	102
**Region 8**	**2**	**329**	**12,355**	**12,686**	**2**	**246**	**23,688**	**23,936**	**2**	**275**	**3,385**	**3,662**
Biliran	0	11	317	328	0	16	585	601	0	0	71	71
Eastern Samar	0	45	2,722	2,767	0	12	679	691	0	3	42	45
Northern Leyte	1	108	3,896	4,005	0	76	12,026	12,102	1	83	1,289	1,373
Northern Samar	0	23	1,388	1,411	0	26	1,991	2,017	0	15	384	399
Southern Leyte	1	8	442	451	2	31	762	795	1	41	569	611
Western Samar	0	14	828	842	0	26	3,108	3,134	0	16	260	276
Calbayog City	0	6	293	299	0	1	110	111	0	0	6	6
Maasin City	0	0	145	145	0	1	1,290	1,291	0	1	38	39
Ormoc City	0	11	915	926	0	3	1,291	1,294	0	1	35	36
Tacloban City	0	103	1,409	1,512	0	54	1,846	1,900	0	115	691	806
**Region 9**	**15**	**193**	**11,179**	**11,387**	**0**	**345**	**26,523**	**26,868**	**9**	**978**	**11,293**	**12,280**
Zamboanga del Norte	0	62	3,801	3,863	0	52	7,418	7,470	0	18	364	382
Zamboanga del Sur	15	16	2,340	2,371	0	89	8,032	8,121	1	205	3,345	3,551
Zamboanga Sibugay	0	71	1,669	1,740	0	94	4,050	4,144	0	58	407	465
Dapitan City	0	8	484	492	0	19	551	570	0	5	148	153
Dipolog City	0	0	1,303	1,303	0	2	1,720	1,722	0	57	1,580	1,637
Isabela City	0	10	181	191	0	0	102	102	0	0	4	4
Pagadian City	0	11	729	740	0	60	2,125	2,185	0	129	1,308	1,437
Zamboanga City	0	15	672	687	0	29	2,525	2,554	8	506	4,137	4,651
**Region 10**	**30**	**806**	**20,562**	**21,398**	**22**	**1,363**	**50,996**	**52,381**	**16**	**1,037**	**10,876**	**11,929**
Bukidnon	2	101	2,589	2,692	4	548	11,794	12,346	5	132	921	1,058
Camiguin	0	5	232	237	0	3	734	737	0	2	39	41
Lanao del Norte	0	172	5,379	5,551	0	29	3,191	3,220	0	29	590	619
Misamis Occidental	11	143	2,088	2,242	0	28	2,178	2,206	0	10	145	155
Misamis Oriental	2	67	2,640	2,709	2	164	14,732	14,898	1	105	1,342	1,448

Table 2.A.4. MODERN METHOD OF FAMILY PLANNING
Current User (Ending)
Philippines, 2022

Area	CONDOM			Total	IUD-INTERVAL			Total	IUD-POSTPARTUM			Total
	Age group				Age group				Age group			
	10-14	15-19	20-49		10-14	15-19	20-49		10-14	15-19	20-49	
Cagayan de Oro City	0	60	1,674	1,734	0	224	7,359	7,583	4	464	6,624	7,092
El Salvador City	0	0	92	92	0	30	1,571	1,601	2	55	130	187
Gingoog City	15	38	1,465	1,518	16	161	2,654	2,831	0	110	512	622
Iligan City	0	72	1,724	1,796	0	101	2,992	3,093	4	125	508	637
Malaybalay City	0	1	603	604	0	43	760	803	0	-1	-20	-21
Oroquieta City	0	1	205	206	0	0	633	633	0	0	0	0
Ozamis City	0	33	760	793	0	2	610	612	0	0	0	0
Tangub City	0	50	631	681	0	0	63	63	0	0	15	15
Valencia City	0	63	480	543	0	30	1,725	1,755	0	6	70	76
**Region 11**	**3**	**1,158**	**20,560**	**21,721**	**21**	**679**	**38,973**	**39,673**	**25**	**264**	**6,200**	**6,489**
Davao de Oro	6	115	3,038	3,159	1	100	6,753	6,854	7	22	430	459
Davao del Norte	-2	199	4,277	4,474	4	135	6,267	6,406	2	78	2,403	2,483
Davao Oriental	2	132	1,823	1,957	8	173	8,463	8,644	2	43	453	498
Davao del Sur	1	23	308	332	1	59	2,155	2,215	1	13	151	165
Davao Occidental	0	54	1,102	1,156	0	8	2,985	2,993	0	8	367	375
Davao City	-4	635	10,012	10,643	7	204	12,350	12,561	13	100	2,396	2,509
**Region 12**	**10**	**639**	**13,052**	**13,701**	**12**	**583**	**27,036**	**27,631**	**20**	**420**	**9,357**	**9,797**
North Cotabato	0	180	4,516	4,696	1	199	12,209	12,409	0	89	1,703	1,792
Sarangani	4	142	2,222	2,368	0	68	2,593	2,661	0	13	146	159
South Cotabato	2	108	3,012	3,122	0	142	5,495	5,637	1	108	1,985	2,094
Sultan Kudarat	0	149	2,328	2,477	4	115	4,280	4,399	9	108	4,069	4,186
Gen. Santos City	4	60	974	1,038	7	59	2,459	2,525	10	102	1,454	1,566
**BARMM**	**4**	**287**	**10,019**	**10,310**	**1**	**115**	**3,643**	**3,759**	**5**	**263**	**2,502**	**2,770**
Basilan	0	8	298	306	0	0	36	36	0	0	46	46
Lanao del Sur	1	70	3,225	3,296	0	23	759	782	0	4	220	224
Maguindanao	3	105	2,707	2,815	0	16	1,410	1,426	0	26	467	493
Sulu	0	31	422	453	0	-2	309	307	3	0	128	131
Tawi-Tawi	0	0	462	462	0	0	1	1	0	0	14	14
Lamitan City	0	4	23	27	1	51	248	300	0	88	371	459
Marawi City	0	6	711	717	0	0	15	15	0	1	2	3
Cotabato City	0	63	2,171	2,234	0	27	865	892	2	144	1,254	1,400
**CARAGA**	**2**	**341**	**11,081**	**11,424**	**2**	**462**	**24,978**	**25,442**	**9**	**375**	**4,276**	**4,660**
Agusan del Norte	1	55	1,546	1,602	0	173	4,454	4,627	2	67	1,304	1,373
Agusan del Sur	0	56	3,383	3,439	1	140	8,845	8,986	1	35	1,158	1,194
Surigao del Norte	0	95	1,358	1,453	1	32	1,983	2,016	0	16	182	198
Surigao del Sur	1	55	1,765	1,821	0	53	3,890	3,943	5	54	542	601
Province of Dinagat	0	14	722	736	0	11	887	898	0	1	61	62
Bislig City	0	3	641	644	0	19	1,113	1,132	0	9	139	148
Butuan City	0	54	1,525	1,579	0	24	1,747	1,771	1	157	745	903
Surigao City	0	9	141	150	0	10	2,059	2,069	0	36	145	181

Table 2.A.4. MODERN METHOD OF FAMILY PLANNING
Current User (Ending)
Philippines, 2022

Area	PILLS-POP			Total	PILLS-COC			Total	INJECTABLES			Total
	Age group				Age group				Age group			
	10-14	15-19	20-49		10-14	15-19	20-49		10-14	15-19	20-49	
**PHILIPPINES**	706	38,236	384,228	423,170	678	80,847	2,847,853	2,929,378	975	88,573	1,612,747	1,702,295
**N C R**	113	6,026	46,259	52,398	57	7,532	176,807	184,396	145	14,077	167,531	181,753
Malabon	0	1,088	2,109	3,197	1	1,366	6,961	8,328	5	1,663	6,510	8,178
Navotas	6	144	1,240	1,390	0	704	6,497	7,201	1	1,761	8,379	10,141
Valenzuela City	0	33	444	477	0	367	8,052	8,419	1	560	7,038	7,599
Caloocan City	0	311	2,550	2,861	3	380	8,366	8,749	12	1,146	18,304	19,462
Marikina City	0	63	757	820	0	33	2,237	2,270	6	502	4,738	5,246
Pasig City	3	221	2,177	2,401	4	362	10,265	10,631	10	1,040	16,219	17,269
Pateros	0	0	51	51	0	4	1,338	1,342	0	3	928	931
Taguig	18	1,028	4,314	5,360	16	799	12,496	13,311	4	744	16,101	16,849
Quezon City	65	1,895	19,099	21,059	12	1,718	78,020	79,750	17	2,701	37,781	40,499
Makati City	0	56	1,796	1,852	0	44	2,803	2,847	0	133	5,585	5,718
Mandaluyong City	1	161	1,556	1,718	0	187	3,865	4,052	0	259	4,728	4,987
San Juan	0	4	152	156	0	3	1,718	1,721	0	12	1,747	1,759
Manila City	12	253	2,762	3,027	11	232	4,313	4,556	82	600	8,298	8,980
Las Piñas City	0	85	1,160	1,245	0	112	3,821	3,933	1	244	4,242	4,487
Muntinlupa City	7	257	1,080	1,344	10	656	11,650	12,316	5	1,423	7,859	9,287
Parañaque City	1	420	3,110	3,531	0	561	6,774	7,335	1	1,286	9,074	10,361
Pasay City	0	7	1,902	1,909	0	4	7,631	7,635	0	0	10,000	10,000
**C A R**	11	657	10,813	11,481	7	1,113	45,304	46,424	36	1,285	21,581	22,902
Abra	2	110	928	1,040	5	447	7,137	7,589	15	223	2,064	2,302
Apayao	3	128	978	1,109	0	239	16,817	17,056	2	174	2,171	2,347
Benguet	0	90	1,075	1,165	0	84	9,081	9,165	1	131	5,256	5,388
Ifugao	2	67	2,892	2,961	2	75	4,287	4,364	2	139	3,138	3,279
Kalinga	0	79	3,208	3,287	0	124	2,983	3,107	2	135	2,942	3,079
Mt. Province	0	35	413	448	0	63	2,640	2,703	1	88	1,620	1,709
Baguio City	4	148	1,319	1,471	0	81	2,359	2,440	13	395	4,390	4,798
**Region 1**	2	311	5,999	6,312	9	3,673	200,575	204,257	7	3,054	97,008	100,069
Ilocos Norte	1	63	1,820	1,884	1	165	18,698	18,864	0	116	6,037	6,153
Ilocos Sur	0	154	3,193	3,347	3	472	25,627	26,102	2	322	11,155	11,479
La Union	1	65	772	838	0	160	21,279	21,439	1	361	13,634	13,996
Pangasinan	0	0	0	0	5	2,383	105,776	108,164	4	2,000	52,613	54,617
Alaminos City	0	0	0	0	0	50	2,722	2,772	0	20	1,427	1,447
Candon City	0	0	21	21	0	0	2,425	2,425	0	0	2,050	2,050
Dagupan City	0	5	56	61	0	36	2,504	2,540	0	86	1,908	1,994
Laoag City	0	0	13	13	0	0	2,787	2,787	0	0	2,055	2,055
San Carlos City	0	0	0	0	0	40	4,348	4,388	0	39	2,039	2,078
San Fernando City	0	24	124	148	0	125	6,873	6,998	0	24	1,831	1,855
Urdaneta City	0	0	0	0	0	232	6,001	6,233	0	82	1,876	1,958
Vigan City	0	0	0	0	0	10	1,535	1,545	0	4	383	387
**Region 2**	8	2,314	10,026	12,348	7	3,628	152,463	156,098	48	2,585	50,222	52,855
Batanes	0	0	3	3	0	2	196	198	0	16	320	336
Cagayan	1	385	2,876	3,262	3	476	47,283	47,762	6	393	11,839	12,238
Isabela	5	400	2,882	3,287	3	817	56,179	56,999	10	888	17,425	18,323
Nueva Vizcaya	1	175	2,221	2,397	0	432	22,003	22,435	4	300	6,674	6,978
Quirino	0	51	352	403	1	341	9,210	9,552	27	146	4,364	4,537
Cauayan City	0	37	281	318	0	141	5,298	5,439	0	58	1,444	1,502
Ilagan City	1	1,160	420	1,581	0	1,171	881	2,052	0	566	3,102	3,668
Santiago City	0	0	0	0	0	96	4,827	4,923	1	172	3,758	3,931
Tuguegarao City	0	106	991	1,097	0	152	6,586	6,738	0	46	1,296	1,342

Table 2.A.4. MODERN METHOD OF FAMILY PLANNING
Current User (Ending)
Philippines, 2022

Area	PILLS-POP Age group 10-14	15-19	20-49	Total	PILLS-COC Age group 10-14	15-19	20-49	Total	INJECTABLES Age group 10-14	15-19	20-49	Total
**Region 3**	**13**	**3,009**	**26,429**	**29,451**	**28**	**10,041**	**292,683**	**302,752**	**36**	**12,755**	**220,239**	**233,030**
Aurora	0	47	1,036	1,083	0	181	11,338	11,519	0	154	3,556	3,710
Bataan	4	251	2,002	2,257	6	546	13,333	13,885	7	1,548	19,894	21,449
Bulacan	0	500	4,144	4,644	1	1,808	62,025	63,834	2	2,312	40,194	42,508
Nueva Ecija	0	491	4,780	5,271	5	2,336	50,000	52,341	2	1,759	27,120	28,881
Pampanga	3	244	3,052	3,299	1	859	31,368	32,228	3	1,138	25,278	26,419
Tarlac	2	245	2,832	3,079	8	914	34,383	35,305	6	759	25,949	26,714
Zambales	1	108	1,200	1,309	6	350	15,521	15,877	3	911	14,084	14,998
Angeles City	0	70	449	519	1	274	9,248	9,523	2	342	7,938	8,282
Balanga City	0	5	55	60	0	66	1,417	1,483	1	135	1,515	1,651
Cabanatuan City	2	191	823	1,016	0	244	9,027	9,271	2	225	3,415	3,642
City of San Fernando	0	173	1,131	1,304	0	130	2,940	3,070	3	226	2,534	2,763
Gapan City	0	27	296	323	0	87	2,224	2,311	1	109	1,909	2,019
Mabalacat City	0	28	241	269	0	217	6,360	6,577	0	505	8,392	8,897
Malolos City	0	14	291	305	0	31	2,982	3,013	0	25	2,380	2,405
Meycauayan	0	97	495	592	0	250	2,295	2,545	0	126	1,962	2,088
Olongapo	0	47	416	463	0	149	2,993	3,142	2	267	3,339	3,608
Palayan City	0	2	16	18	0	52	1,531	1,583	0	28	472	500
San Jose City	0	68	244	312	0	171	5,136	5,307	0	344	4,240	4,584
San Jose del Monte City	1	111	1,669	1,781	0	493	18,243	18,736	2	863	18,966	19,831
Science City of Munoz	0	4	40	44	0	62	3,347	3,409	0	41	1,612	1,653
Tarlac City	0	286	1,217	1,503	0	821	6,972	7,793	0	938	5,490	6,428
**Region 4A**	**78**	**5,363**	**75,255**	**80,696**	**173**	**9,704**	**262,288**	**272,165**	**122**	**10,915**	**204,787**	**215,824**
Batangas	24	598	12,481	13,103	45	610	40,315	40,970	5	704	19,198	19,907
Cavite	6	101	1,772	1,879	17	441	28,931	29,389	21	686	21,678	22,385
Laguna	1	211	5,560	5,772	2	488	20,434	20,924	2	410	9,964	10,376
Quezon	9	341	5,014	5,364	56	710	35,919	36,685	54	997	26,655	27,706
Rizal	1	406	4,318	4,725	18	895	32,171	33,084	12	2,191	39,287	41,490
Antipolo City	0	0	11,313	11,313	0	18	324	342	0	0	12,033	12,033
Bacoor City	0	27	417	444	0	39	1,475	1,514	0	147	2,735	2,882
Batangas City	0	52	217	269	0	62	6,677	6,739	0	139	2,941	3,080
Biñan City	0	2,479	14,086	16,565	0	3,320	29,502	32,822	0	2,865	22,382	25,247
Cabuyao City	0	164	2,891	3,055	0	245	4,356	4,601	1	322	3,038	3,361
Calamba City	4	300	2,848	3,152	5	411	11,548	11,964	2	207	7,173	7,382
Cavite City	0	0	31	31	0	36	1,187	1,223	0	85	924	1,009
Dasmariñas City	0	122	1,025	1,147	0	341	10,364	10,705	0	398	9,304	9,702
General Trias City	2	34	396	432	2	32	1,633	1,667	3	81	1,681	1,765
Imus City	1	13	464	478	0	38	2,594	2,632	0	95	2,297	2,392
Lipa City	0	15	325	340	0	25	3,512	3,537	0	21	1,315	1,336
Lucena City	0	35	102	137	0	53	1,648	1,701	1	139	2,658	2,798
San Pablo City	0	18	171	189	0	106	4,472	4,578	1	120	2,286	2,407
San Pedro City	1	166	4,696	4,863	0	189	5,032	5,221	0	164	3,454	3,618
Santa Rosa City	29	229	6,669	6,927	28	1,571	13,082	14,681	19	986	6,981	7,986
Tagaytay City	0	0	73	73	0	1	3,865	3,866	0	1	2,498	2,499
Tanauan City	0	30	230	260	0	31	1,648	1,679	0	64	2,144	2,208
Tayabas City	0	16	87	103	0	7	907	914	1	48	1,040	1,089
Trece Martires City	0	6	69	75	0	35	692	727	0	45	1,121	1,166
**Region 4B**	**18**	**1,009**	**12,585**	**13,612**	**17**	**1,832**	**98,178**	**100,027**	**0**	**2,808**	**59,918**	**62,726**
Marinduque	0	36	2,461	2,497	0	6	4,415	4,421	0	48	2,563	2,611
Mindoro Occidental	0	97	1,348	1,445	7	335	15,785	16,127	0	274	8,063	8,337
Mindoro Oriental	4	106	1,978	2,088	2	265	30,865	31,132	0	267	10,906	11,173
Palawan	10	476	4,478	4,964	7	1,017	30,125	31,149	0	1,417	24,035	25,452
Romblon	0	27	615	642	0	15	6,157	6,172	0	63	3,589	3,652
Puerto Princesa City	4	267	1,705	1,976	1	194	10,831	11,026	0	739	10,762	11,501
**Region 5**	**1**	**1,011**	**15,800**	**16,812**	**3**	**2,856**	**188,558**	**191,417**	**5**	**2,362**	**74,266**	**76,633**
Albay	0	158	2,482	2,640	0	563	38,152	38,715	1	460	18,887	19,348

Table 2.A.4. MODERN METHOD OF FAMILY PLANNING
Current User (Ending)
Philippines, 2022

Area	PILLS-POP			Total	PILLS-COC			Total	INJECTABLES			Total
	Age group				Age group				Age group			
	10-14	15-19	20-49		10-14	15-19	20-49		10-14	15-19	20-49	
Camarines Norte	0	204	2,052	2,256	1	489	20,955	21,445	2	486	6,693	7,181
Camarines Sur	1	208	3,789	3,998	0	449	35,159	35,608	0	424	14,724	15,148
Catanduanes	0	0	127	127	1	22	4,326	4,349	0	118	4,319	4,437
Masbate	0	264	3,685	3,949	1	840	39,623	40,464	2	412	9,876	10,290
Sorsogon	0	82	1,636	1,718	0	224	23,769	23,993	0	271	10,079	10,350
Iriga City	0	25	386	411	0	55	3,634	3,689	0	45	1,446	1,491
Legaspi City	0	34	883	917	0	175	10,979	11,154	0	98	4,931	5,029
Naga City	0	36	760	796	0	39	11,961	12,000	0	48	3,311	3,359
**Region 6**	**136**	**1,922**	**16,053**	**18,111**	**21**	**4,878**	**265,590**	**270,489**	**26**	**4,519**	**118,047**	**122,592**
Aklan	3	85	2,351	2,439	3	292	16,356	16,651	4	625	12,499	13,128
Antique	0	77	1,144	1,221	0	218	15,115	15,333	0	287	11,879	12,166
Capiz	0	80	1,228	1,308	3	373	35,089	35,465	0	513	17,863	18,376
Guimaras	0	18	328	346	0	70	10,098	10,168	0	35	2,054	2,089
Iloilo	2	505	4,507	5,014	13	653	69,803	70,469	8	965	31,126	32,099
Negros Occidental	2	1,054	5,672	6,728	1	2,749	82,551	85,301	3	1,742	31,259	33,004
Bacolod City	129	44	477	650	1	198	5,937	6,136	11	172	4,705	4,888
Iloilo City	0	59	346	405	0	325	30,641	30,966	0	180	6,662	6,842
**Region 7**	**16**	**1,987**	**21,419**	**23,422**	**3**	**2,873**	**174,044**	**176,920**	**19**	**4,714**	**117,611**	**122,344**
Bohol	1	176	2,332	2,509	0	202	24,355	24,557	1	495	13,090	13,586
Cebu	9	953	9,163	10,125	1	1,096	91,266	92,363	4	1,917	54,844	56,765
Negros Oriental	4	302	2,005	2,311	2	808	34,004	34,814	11	1,163	24,104	25,278
Siquijor	0	30	402	432	0	24	3,341	3,365	0	74	2,548	2,622
Cebu City	1	218	1,948	2,167	0	542	7,427	7,969	3	668	7,175	7,846
Lapu-Lapu City	0	260	4,862	5,122	0	170	5,196	5,366	0	311	8,507	8,818
Mandaue City	1	48	707	756	0	31	8,455	8,486	0	86	7,343	7,429
**Region 8**	**3**	**799**	**13,598**	**14,400**	**7**	**2,160**	**126,628**	**128,795**	**15**	**2,319**	**55,752**	**58,086**
Biliran	0	26	300	326	0	47	4,886	4,933	0	53	1,200	1,253
Eastern Samar	0	68	1,146	1,214	1	263	13,847	14,111	4	266	7,406	7,676
Northern Leyte	1	279	6,143	6,423	0	761	45,076	45,837	2	706	18,510	19,218
Northern Samar	2	69	1,959	2,030	0	232	17,675	17,907	0	181	9,684	9,865
Southern Leyte	0	42	1,209	1,251	2	233	13,657	13,892	7	431	5,318	5,756
Western Samar	0	31	377	408	0	64	9,871	9,935	0	155	3,101	3,256
Calbayog City	0	105	262	367	4	74	3,990	4,068	2	76	1,983	2,061
Maasin City	0	3	178	181	0	1	1,636	1,637	0	7	477	484
Ormoc City	0	81	668	749	0	158	9,027	9,185	0	122	1,784	1,906
Tacloban City	0	95	1,356	1,451	0	327	6,963	7,290	0	322	6,289	6,611
**Region 9**	**10**	**822**	**12,668**	**13,500**	**8**	**2,548**	**118,283**	**120,839**	**16**	**2,698**	**59,741**	**62,455**
Zamboanga del Norte	1	146	5,213	5,360	2	694	33,119	33,815	2	743	17,592	18,337
Zamboanga del Sur	0	136	1,487	1,623	0	396	30,207	30,603	1	308	10,181	10,490
Zamboanga Sibugay	0	166	2,172	2,338	6	472	12,859	13,337	3	473	8,236	8,712
Dapitan City	0	20	76	96	0	69	3,527	3,596	0	63	834	897
Dipolog City	9	39	877	925	0	12	7,839	7,851	1	50	1,621	1,672
Isabela City	0	68	384	452	0	74	1,994	2,068	0	117	2,063	2,180
Pagadian City	0	95	1,252	1,347	0	309	6,033	6,342	0	252	4,029	4,281
Zamboanga City	0	152	1,207	1,359	0	522	22,705	23,227	9	692	15,185	15,886
**Region 10**	**19**	**2,368**	**25,360**	**27,747**	**34**	**6,059**	**170,275**	**176,368**	**45**	**3,453**	**64,947**	**68,445**
Bukidnon	8	637	3,280	3,925	0	1,696	42,838	44,534	11	732	9,848	10,591
Camiguin	0	2	37	39	2	45	2,649	2,696	0	2	686	688
Lanao del Norte	0	393	10,836	11,229	7	797	17,991	18,795	1	598	15,945	16,544
Misamis Occidental	2	133	1,674	1,809	2	333	11,763	12,098	2	81	3,199	3,282
Misamis Oriental	1	271	2,111	2,383	1	566	28,565	29,132	10	402	9,399	9,811

Table 2.A.4. MODERN METHOD OF FAMILY PLANNING
Current User (Ending)
Philippines, 2022

Area	PILLS-POP			Total	PILLS-COC			Total	INJECTABLES			Total
	Age group				Age group				Age group			
	10-14	15-19	20-49		10-14	15-19	20-49		10-14	15-19	20-49	
Cagayan de Oro City	1	82	947	1,030	0	342	12,668	13,010	2	369	7,857	8,228
El Salvador City	0	32	196	228	0	39	1,297	1,336	0	71	748	819
Gingoog City	1	268	1,353	1,622	1	548	8,350	8,899	0	248	3,051	3,299
Iligan City	6	199	1,387	1,592	2	379	9,436	9,817	6	394	6,077	6,477
Malaybalay City	0	34	280	314	0	173	9,191	9,364	1	84	953	1,038
Oroquieta City	0	58	689	747	0	109	2,190	2,299	0	22	1,206	1,228
Ozamis City	0	76	601	677	0	491	10,574	11,065	2	297	3,809	4,108
Tangub City	0	40	1,414	1,454	0	110	2,044	2,154	0	39	152	191
Valencia City	0	143	555	698	19	431	10,719	11,169	10	114	2,017	2,141
**Region 11**	**188**	**4,107**	**31,632**	**35,927**	**188**	**9,053**	**223,155**	**232,396**	**291**	**6,994**	**78,295**	**85,580**
Davao de Oro	34	670	3,492	4,196	20	2,255	42,175	44,450	19	1,008	10,155	11,182
Davao del Norte	24	805	7,631	8,460	73	1,223	44,889	46,185	40	1,042	13,188	14,270
Davao Oriental	43	728	5,277	6,048	59	1,390	39,297	40,746	165	1,424	11,972	13,561
Davao del Sur	3	100	497	600	10	803	12,054	12,867	20	1,017	6,431	7,468
Davao Occidental	15	250	2,276	2,541	9	621	26,038	26,668	17	347	6,690	7,054
Davao City	69	1,554	12,459	14,082	17	2,761	58,702	61,480	30	2,156	29,859	32,045
**Region 12**	**60**	**3,639**	**34,330**	**38,029**	**94**	**6,756**	**162,633**	**169,483**	**128**	**7,698**	**86,535**	**94,361**
North Cotabato	6	1,001	7,760	8,767	16	1,862	60,662	62,540	13	1,646	28,924	30,583
Sarangani	10	878	3,375	4,263	13	1,802	30,558	32,373	17	1,661	12,800	14,478
South Cotabato	7	449	3,481	3,937	10	1,365	38,050	39,425	12	1,272	15,624	16,908
Sultan Kudarat	4	982	17,540	18,526	5	1,185	23,863	25,053	18	2,319	18,796	21,133
Gen. Santos City	33	329	2,174	2,536	50	542	9,500	10,092	68	800	10,391	11,259
**BARMM**	**15**	**1,170**	**10,233**	**11,418**	**8**	**3,466**	**80,081**	**83,555**	**21**	**4,554**	**101,299**	**105,874**
Basilan	0	59	430	489	2	176	2,897	3,075	2	275	3,390	3,667
Lanao del Sur	4	121	2,081	2,206	0	149	8,012	8,161	0	252	7,911	8,163
Maguindanao	2	373	2,662	3,037	1	1,137	25,032	26,170	9	2,013	34,401	36,423
Sulu	9	453	2,947	3,409	5	1,393	24,010	25,408	2	1,106	36,274	37,382
Tawi-Tawi	0	2	111	113	0	150	6,063	6,213	1	140	5,699	5,840
Lamitan City	0	34	75	109	0	142	959	1,101	7	198	1,444	1,649
Marawi City	0	22	628	650	0	11	875	886	0	13	839	852
Cotabato City	0	106	1,299	1,405	0	308	12,233	12,541	0	557	11,341	11,898
**CARAGA**	**15**	**1,722**	**15,769**	**17,506**	**14**	**2,675**	**110,308**	**112,997**	**15**	**1,783**	**34,968**	**36,766**
Agusan del Norte	5	298	2,505	2,808	0	320	14,363	14,683	7	275	5,466	5,748
Agusan del Sur	6	488	3,484	3,978	4	760	34,476	35,240	1	502	9,847	10,350
Surigao del Norte	1	167	3,319	3,487	0	269	14,234	14,503	5	197	4,387	4,589
Surigao del Sur	0	266	2,745	3,011	10	391	18,919	19,320	2	226	3,874	4,102
Province of Dinagat	2	52	384	438	0	81	3,653	3,734	0	57	1,337	1,394
Bislig City	1	88	691	780	0	122	3,932	4,054	0	37	437	474
Butuan City	0	317	2,347	2,664	0	583	15,670	16,253	0	419	6,389	6,808
Surigao City	0	46	294	340	0	149	5,061	5,210	0	70	3,231	3,301

Table 2.A.4. MODERN METHOD OF FAMILY PLANNING
Current User (Ending)
Philippines, 2022

Area	IMPLANTS			Total	NFP-CCM			Total	NFP-BBT			Total
	Age group				Age group				Age group			
	10-14	15-19	20-49		10-14	15-19	20-49		10-14	15-19	20-49	
**PHILIPPINES**	**696**	**43,368**	**592,625**	**636,689**	**46**	**1,225**	**48,166**	**49,437**	**125**	**328**	**3,746**	**4,199**
**N C R**	**222**	**9,926**	**77,925**	**88,073**	**0**	**2**	**558**	**560**	**2**	**32**	**16**	**50**
Malabon	1	574	2,010	2,585	0	0	0	0	0	0	0	0
Navotas	3	219	1,339	1,561	0	0	0	0	0	0	0	0
Valenzuela City	8	1,499	8,924	10,431	0	0	1	1	0	0	0	0
Caloocan City	2	775	7,480	8,257	0	0	21	21	0	0	0	0
Marikina City	10	355	3,055	3,420	0	2	1	3	0	0	0	0
Pasig City	6	380	5,477	5,863	0	0	11	11	0	0	1	1
Pateros	0	1	243	244	0	0	0	0	0	0	0	0
Taguig	4	80	3,557	3,641	0	0	0	0	0	0	0	0
Quezon City	74	2,386	20,646	23,106	0	0	13	13	0	0	0	0
Makati City	0	60	1,582	1,642	0	0	0	0	0	0	0	0
Mandaluyong City	2	116	2,299	2,417	0	0	359	359	0	1	1	2
San Juan	0	4	165	169	0	0	0	0	0	0	0	0
Manila City	101	2,574	12,412	15,087	0	0	0	0	2	31	11	44
Las Piñas City	8	461	3,239	3,708	0	0	0	0	0	0	0	0
Muntinlupa City	1	112	1,755	1,868	0	0	0	0	0	0	0	0
Parañaque City	2	330	2,095	2,427	0	0	0	0	0	0	3	3
Pasay City	0	0	1,647	1,647	0	0	152	152	0	0	0	0
**C A R**	**15**	**612**	**10,217**	**10,844**	**0**	**73**	**2,074**	**2,147**	**0**	**2**	**181**	**183**
Abra	3	75	1,103	1,181	0	0	224	224	0	0	108	108
Apayao	2	54	761	817	0	0	1	1	0	0	0	0
Benguet	0	40	1,446	1,486	0	0	2	2	0	0	10	10
Ifugao	0	51	1,328	1,379	0	1	779	780	0	0	0	0
Kalinga	0	66	1,221	1,287	0	72	967	1,039	0	2	51	53
Mt. Province	1	48	781	830	0	0	100	100	0	0	5	5
Baguio City	9	278	3,577	3,864	0	0	1	1	0	0	7	7
**Region 1**	**2**	**995**	**17,386**	**18,383**	**0**	**7**	**2,025**	**2,032**	**0**	**10**	**303**	**313**
Ilocos Norte	1	33	799	833	0	5	396	401	0	1	22	23
Ilocos Sur	0	39	651	690	0	0	598	598	0	2	23	25
La Union	0	134	2,016	2,150	0	0	338	338	0	0	10	10
Pangasinan	1	691	11,791	12,483	0	1	21	22	0	7	118	125
Alaminos City	0	24	537	561	0	0	0	0	0	0	0	0
Candon City	0	0	9	9	0	0	43	43	0	0	24	24
Dagupan City	0	28	508	536	0	0	0	0	0	0	4	4
Laoag City	0	0	5	5	0	0	0	0	0	0	0	0
San Carlos City	0	7	333	340	0	0	0	0	0	0	0	0
San Fernando City	0	37	620	657	0	0	0	0	0	0	0	0
Urdaneta City	0	2	112	114	0	0	0	0	0	0	0	0
Vigan City	0	0	5	5	0	1	629	630	0	0	102	102
**Region 2**	**7**	**582**	**11,317**	**11,906**	**0**	**0**	**277**	**277**	**0**	**8**	**51**	**59**
Batanes	0	0	2	2	0	0	78	78	0	0	0	0
Cagayan	4	143	2,929	3,076	0	0	12	12	0	1	16	17
Isabela	2	281	4,593	4,876	0	0	8	8	0	6	35	41
Nueva Vizcaya	1	84	2,327	2,412	0	0	162	162	0	1	0	1
Quirino	0	9	463	472	0	0	17	17	0	0	0	0
Cauayan City	0	6	204	210	0	0	0	0	0	0	0	0
Ilagan City	0	31	155	186	0	0	0	0	0	0	0	0
Santiago City	0	19	474	493	0	0	0	0	0	0	0	0
Tuguegarao City	0	9	170	179	0	0	0	0	0	0	0	0

Table 2.A.4. MODERN METHOD OF FAMILY PLANNING
Current User (Ending)
Philippines, 2022

Area	IMPLANTS			Total	NFP-CCM			Total	NFP-BBT			Total
	Age group				Age group				Age group			
	10-14	15-19	20-49		10-14	15-19	20-49		10-14	15-19	20-49	
**Region 3**	**48**	**3,226**	**42,015**	**45,289**	**0**	**33**	**389**	**422**	**0**	**6**	**52**	**58**
Aurora	1	58	1,376	1,435	0	0	217	217	0	0	4	4
Bataan	14	642	4,511	5,167	0	0	39	39	0	3	16	19
Bulacan	0	330	7,749	8,079	0	0	7	7	0	0	4	4
Nueva Ecija	22	1,159	7,621	8,802	0	1	0	1	0	0	0	0
Pampanga	1	145	4,662	4,808	0	32	94	126	0	3	7	10
Tarlac	1	52	2,177	2,230	0	0	0	0	0	0	0	0
Zambales	3	154	3,123	3,280	0	0	24	24	0	0	0	0
Angeles City	1	80	1,851	1,932	0	0	5	5	0	0	8	8
Balanga City	0	41	214	255	0	0	0	0	0	0	0	0
Cabanatuan City	0	7	251	258	0	0	0	0	0	0	0	0
City of San Fernando	0	7	366	373	0	0	1	1	0	0	10	10
Gapan City	0	32	281	313	0	0	2	2	0	0	0	0
Mabalacat City	0	30	621	651	0	0	0	0	0	0	0	0
Malolos City	0	2	512	514	0	0	0	0	0	0	0	0
Meycauayan	0	39	344	383	0	0	0	0	0	0	0	0
Olongapo	5	227	1,342	1,574	0	0	0	0	0	0	3	3
Palayan City	0	0	0	0	0	0	0	0	0	0	0	0
San Jose City	0	20	274	294	0	0	0	0	0	0	0	0
San Jose del Monte City	0	169	4,062	4,231	0	0	0	0	0	0	0	0
Science City of Munoz	0	1	38	39	0	0	0	0	0	0	0	0
Tarlac City	0	31	640	671	0	0	0	0	0	0	0	0
**Region 4A**	**63**	**2,171**	**45,897**	**48,131**	**27**	**109**	**5,744**	**5,880**	**57**	**10**	**311**	**378**
Batangas	3	56	1,370	1,429	0	81	3,834	3,915	30	0	24	54
Cavite	5	270	6,659	6,934	0	0	188	188	0	0	7	7
Laguna	12	341	8,072	8,425	0	13	453	466	0	0	0	0
Quezon	3	102	2,832	2,937	0	0	446	446	0	0	156	156
Rizal	5	412	6,613	7,030	0	0	11	11	0	3	3	6
Antipolo City	0	7	706	713	0	0	588	588	0	0	58	58
Bacoor City	1	58	494	553	0	0	0	0	0	0	0	0
Batangas City	0	37	393	430	0	0	0	0	0	0	0	0
Biñan City	0	110	1,489	1,599	0	0	0	0	0	0	1	1
Cabuyao City	2	26	611	639	0	0	1	1	0	2	22	24
Calamba City	3	225	3,522	3,750	0	0	0	0	0	0	0	0
Cavite City	0	40	391	431	0	0	0	0	0	0	0	0
Dasmariñas City	0	225	6,172	6,397	0	0	5	5	0	0	1	1
General Trias City	0	22	399	421	0	0	0	0	0	1	0	1
Imus City	0	16	453	469	0	0	1	1	0	0	0	0
Lipa City	0	8	252	260	0	0	0	0	0	0	0	0
Lucena City	0	0	32	32	0	0	0	0	0	0	0	0
San Pablo City	1	27	607	635	0	0	0	0	0	0	11	11
San Pedro City	0	31	953	984	0	15	101	116	0	4	26	30
Santa Rosa City	27	129	2,799	2,955	27	0	0	27	27	0	2	29
Tagaytay City	0	2	627	629	0	0	0	0	0	0	0	0
Tanauan City	1	7	139	147	0	0	0	0	0	0	0	0
Tayabas City	0	1	50	51	0	0	116	116	0	0	0	0
Trece Martires City	0	19	262	281	0	0	0	0	0	0	0	0
**Region 4B**	**2**	**1,201**	**25,087**	**26,290**	**1**	**70**	**5,381**	**5,452**	**0**	**2**	**146**	**148**
Marinduque	0	43	1,219	1,262	0	0	15	15	0	0	1	1
Mindoro Occidental	2	164	2,965	3,131	0	48	2,317	2,365	0	1	76	77
Mindoro Oriental	0	141	2,788	2,929	0	0	318	318	0	0	32	32
Palawan	0	718	14,528	15,246	0	15	703	718	0	1	31	32
Romblon	0	41	1,369	1,410	1	7	2,022	2,030	0	0	6	6
Puerto Princesa City	0	94	2,218	2,312	0	0	6	6	0	0	0	0
**Region 5**	**3**	**1,356**	**32,591**	**33,950**	**17**	**471**	**16,196**	**16,684**	**0**	**11**	**350**	**361**
Albay	2	243	5,217	5,462	0	5	1,576	1,581	0	3	32	35

Table 2.A.4. MODERN METHOD OF FAMILY PLANNING
Current User (Ending)
Philippines, 2022

Area	IMPLANTS			Total	NFP-CCM			Total	NFP-BBT			Total
	Age group				Age group				Age group			
	10-14	15-19	20-49		10-14	15-19	20-49		10-14	15-19	20-49	
Camarines Norte	0	148	2,121	2,269	0	1	366	367	0	0	26	26
Camarines Sur	1	502	11,728	12,231	0	10	1,421	1,431	0	2	74	76
Catanduanes	0	76	2,756	2,832	0	10	1,397	1,407	0	2	12	14
Masbate	0	184	4,986	5,170	17	400	6,367	6,784	0	3	39	42
Sorsogon	0	130	3,489	3,619	0	44	4,752	4,796	0	0	0	0
Iriga City	0	34	914	948	0	1	204	205	0	1	126	127
Legaspi City	0	27	645	672	0	0	44	44	0	0	0	0
Naga City	0	12	735	747	0	0	69	69	0	0	41	41
**Region 6**	**29**	**1,949**	**30,707**	**32,685**	**0**	**79**	**4,498**	**4,577**	**60**	**15**	**191**	**266**
Aklan	2	52	1,598	1,652	0	1	63	64	0	0	11	11
Antique	1	76	2,133	2,210	0	75	4,224	4,299	0	0	4	4
Capiz	0	63	2,211	2,274	0	1	79	80	0	1	7	8
Guimaras	0	8	298	306	0	0	0	0	0	0	5	5
Iloilo	3	454	9,186	9,643	0	1	62	63	0	2	84	86
Negros Occidental	11	965	11,585	12,561	0	0	40	40	0	4	41	45
Bacolod City	12	228	1,496	1,736	0	0	29	29	60	8	34	102
Iloilo City	0	103	2,200	2,303	0	1	1	2	0	0	5	5
**Region 7**	**16**	**2,625**	**57,709**	**60,350**	**0**	**8**	**475**	**483**	**0**	**54**	**237**	**291**
Bohol	5	518	8,598	9,121	0	0	0	0	0	0	3	3
Cebu	7	1,368	35,619	36,994	0	1	24	25	0	44	181	225
Negros Oriental	3	178	3,257	3,438	0	7	451	458	0	7	37	44
Siquijor	0	18	366	384	0	0	0	0	0	0	0	0
Cebu City	1	404	4,353	4,758	0	0	0	0	0	3	16	19
Lapu-Lapu City	0	58	1,289	1,347	0	0	0	0	0	0	0	0
Mandaue City	0	81	4,227	4,308	0	0	0	0	0	0	0	0
**Region 8**	**8**	**1,162**	**29,854**	**31,024**	**0**	**14**	**1,999**	**2,013**	**0**	**7**	**407**	**414**
Biliran	0	92	1,060	1,152	0	0	132	132	0	1	5	6
Eastern Samar	0	86	2,506	2,592	0	0	257	257	0	0	24	24
Northern Leyte	5	309	11,181	11,495	0	5	123	128	0	6	175	181
Northern Samar	1	126	4,170	4,297	0	0	110	110	0	0	2	2
Southern Leyte	1	133	3,901	4,035	0	0	1,147	1,147	0	0	163	163
Western Samar	0	63	1,768	1,831	0	0	145	145	0	0	4	4
Calbayog City	0	13	472	485	0	0	0	0	0	0	0	0
Maasin City	0	6	383	389	0	0	83	83	0	0	33	33
Ormoc City	0	95	2,168	2,263	0	9	2	11	0	0	1	1
Tacloban City	1	239	2,245	2,485	0	0	0	0	0	0	0	0
**Region 9**	**13**	**2,896**	**42,199**	**45,108**	**0**	**2**	**75**	**77**	**0**	**0**	**13**	**13**
Zamboanga del Norte	4	646	10,445	11,095	0	0	64	64	0	0	13	13
Zamboanga del Sur	3	876	14,023	14,902	0	0	0	0	0	0	0	0
Zamboanga Sibugay	1	647	8,062	8,710	0	2	11	13	0	0	0	0
Dapitan City	0	38	261	299	0	0	0	0	0	0	0	0
Dipolog City	0	92	1,196	1,288	0	0	0	0	0	0	0	0
Isabela City	0	67	946	1,013	0	0	0	0	0	0	0	0
Pagadian City	0	55	1,229	1,284	0	0	0	0	0	0	0	0
Zamboanga City	5	475	6,037	6,517	0	0	0	0	0	0	0	0
**Region 10**	**52**	**2,935**	**35,159**	**38,146**	**0**	**240**	**4,094**	**4,334**	**1**	**112**	**881**	**994**
Bukidnon	7	1,449	12,841	14,297	0	52	707	759	0	4	381	385
Camiguin	0	10	153	163	0	0	0	0	0	0	18	18
Lanao del Norte	1	225	4,208	4,434	0	1	97	98	0	0	15	15
Misamis Occidental	0	17	485	502	0	22	44	66	0	45	48	93
Misamis Oriental	12	355	5,040	5,407	0	31	1,539	1,570	1	33	67	101

Table 2.A.4. MODERN METHOD OF FAMILY PLANNING
Current User (Ending)
Philippines, 2022

Area	IMPLANTS Age group			Total	NFP-CCM Age group			Total	NFP-BBT Age group			Total
	10-14	15-19	20-49		10-14	15-19	20-49		10-14	15-19	20-49	
Cagayan de Oro City	2	317	4,051	4,370	0	0	0	0	0	3	53	56
El Salvador City	2	71	684	757	0	0	0	0	0	0	0	0
Gingoog City	0	111	1,098	1,209	0	107	1,098	1,205	0	15	138	153
Iligan City	19	56	1,453	1,528	0	0	180	180	0	2	14	16
Malaybalay City	0	192	2,401	2,593	0	0	2	2	0	2	0	2
Oroquieta City	0	9	188	197	0	0	0	0	0	0	0	0
Ozamis City	0	85	1,525	1,610	0	2	257	259	0	2	10	12
Tangub City	0	0	25	25	0	0	0	0	0	0	0	0
Valencia City	9	38	1,007	1,054	0	25	170	195	0	6	137	143
**Region 11**	**100**	**3,687**	**41,943**	**45,730**	**1**	**84**	**2,116**	**2,201**	**-1**	**5**	**346**	**350**
Davao de Oro	11	585	5,664	6,260	0	11	472	483	0	0	34	34
Davao del Norte	30	670	7,832	8,532	0	30	151	181	0	0	100	100
Davao Oriental	14	414	4,159	4,587	0	19	302	321	-1	0	-11	-12
Davao del Sur	12	333	2,799	3,144	0	0	0	0	0	0	-3	-3
Davao Occidental	3	164	3,215	3,382	0	5	692	697	0	2	74	76
Davao City	30	1,521	18,274	19,825	1	19	499	519	0	3	152	155
**Region 12**	**70**	**4,052**	**41,951**	**46,073**	**0**	**4**	**668**	**672**	**6**	**29**	**111**	**146**
North Cotabato	0	750	13,059	13,809	0	1	309	310	0	2	61	63
Sarangani	16	552	5,587	6,155	0	0	193	193	2	5	15	22
South Cotabato	12	1,298	8,326	9,636	0	3	140	143	2	10	22	34
Sultan Kudarat	11	878	8,551	9,440	0	0	20	20	0	0	7	7
Gen. Santos City	31	574	6,428	7,033	0	0	6	6	2	12	6	20
**BARMM**	**22**	**1,763**	**24,156**	**25,941**	**0**	**0**	**57**	**57**	**0**	**4**	**3**	**7**
Basilan	5	254	1,763	2,022	0	0	3	3	0	0	0	0
Lanao del Sur	1	38	1,414	1,453	0	0	0	0	0	0	0	0
Maguindanao	6	655	6,444	7,105	0	0	6	6	0	4	3	7
Sulu	8	439	8,899	9,346	0	0	-8	-8	0	0	0	0
Tawi-Tawi	1	125	2,869	2,995	0	0	0	0	0	0	0	0
Lamitan City	1	107	645	753	0	0	0	0	0	0	0	0
Marawi City	0	3	316	319	0	0	0	0	0	0	0	0
Cotabato City	0	142	1,806	1,948	0	0	56	56	0	0	0	0
**CARAGA**	**24**	**2,230**	**26,512**	**28,766**	**0**	**29**	**1,540**	**1,569**	**0**	**21**	**147**	**168**
Agusan del Norte	4	372	3,563	3,939	0	0	37	37	0	0	2	2
Agusan del Sur	10	751	8,737	9,498	0	22	1,157	1,179	0	16	33	49
Surigao del Norte	3	257	3,269	3,529	0	4	148	152	0	2	72	74
Surigao del Sur	2	303	4,334	4,639	0	0	72	72	0	3	39	42
Province of Dinagat	3	117	1,887	2,007	0	0	0	0	0	0	1	1
Bislig City	0	17	574	591	0	3	107	110	0	0	0	0
Butuan City	2	319	3,033	3,354	0	0	19	19	0	0	0	0
Surigao City	0	94	1,115	1,209	0	0	0	0	0	0	0	0

Table 2.A.4. MODERN METHOD OF FAMILY PLANNING

Current User (Ending)

Philippines, 2022

Area	NFP-STM			Total	NFP-SDM			Total	NFP-LAM			Total
	Age group				Age group				Age group			
	10-14	15-19	20-49		10-14	15-19	20-49		10-14	15-19	20-49	
**PHILIPPINES**	17	156	3,384	3,557	35	3,552	115,059	118,646	1,551	68,880	601,164	671,595
**N C R**	0	7	90	97	1	18	1,864	1,883	177	10,126	85,696	95,999
Malabon	0	0	0	0	0	0	0	0	6	973	2,563	3,542
Navotas	0	0	0	0	0	0	0	0	1	262	1,041	1,304
Valenzuela City	0	0	0	0	0	0	11	11	3	176	2,758	2,937
Caloocan City	0	0	0	0	0	11	116	127	2	693	5,779	6,474
Marikina City	0	0	0	0	0	0	3	3	2	203	878	1,083
Pasig City	0	0	0	0	0	0	22	22	8	470	6,709	7,187
Pateros	0	0	0	0	0	0	0	0	0	14	186	200
Taguig	0	0	0	0	1	0	11	12	27	1,671	18,895	20,593
Quezon City	0	0	0	0	0	0	1,476	1,476	41	1,671	12,176	13,888
Makati City	0	0	0	0	0	0	0	0	0	93	3,635	3,728
Mandaluyong City	0	0	81	81	0	0	93	93	0	184	4,149	4,333
San Juan	0	0	0	0	0	0	10	10	0	27	360	387
Manila City	0	0	9	9	0	2	14	16	77	743	6,477	7,297
Las Piñas City	0	0	0	0	0	0	24	24	2	164	1,665	1,831
Muntinlupa City	0	0	0	0	0	0	0	0	1	1,484	7,938	9,423
Parañaque City	0	7	0	7	0	5	81	86	7	1,056	8,615	9,678
Pasay City	0	0	0	0	0	0	3	3	0	242	1,872	2,114
**C A R**	0	1	68	69	0	33	5,985	6,018	84	1,148	8,368	9,600
Abra	0	1	39	40	0	7	162	169	70	360	1,498	1,928
Apayao	0	0	0	0	0	0	8	8	4	133	997	1,134
Benguet	0	0	0	0	0	3	1,959	1,962	0	110	1,687	1,797
Ifugao	0	0	8	8	0	18	2,447	2,465	5	227	1,719	1,951
Kalinga	0	0	19	19	0	2	463	465	0	214	1,076	1,290
Mt. Province	0	0	2	2	0	3	857	860	5	82	927	1,014
Baguio City	0	0	0	0	0	0	89	89	0	22	464	486
**Region 1**	0	8	226	234	1	164	6,062	6,227	48	3,977	40,208	44,233
Ilocos Norte	0	0	64	64	0	34	1,152	1,186	1	194	3,571	3,766
Ilocos Sur	0	8	9	17	1	93	3,393	3,487	5	508	7,696	8,209
La Union	0	0	32	32	0	2	398	400	8	440	3,421	3,869
Pangasinan	0	0	1	1	0	32	591	623	31	2,387	19,794	22,212
Alaminos City	0	0	0	0	0	0	0	0	1	87	1,042	1,130
Candon City	0	0	31	31	0	0	27	27	0	0	572	572
Dagupan City	0	0	0	0	0	0	35	35	1	47	681	729
Laoag City	0	0	0	0	0	0	0	0	0	20	1,344	1,364
San Carlos City	0	0	0	0	0	0	0	0	0	31	753	784
San Fernando City	0	0	0	0	0	3	463	466	0	28	377	405
Urdaneta City	0	0	0	0	0	0	0	0	1	212	768	981
Vigan City	0	0	89	89	0	0	3	3	0	23	189	212
**Region 2**	0	2	79	81	0	3	447	450	49	2,319	16,466	18,834
Batanes	0	0	0	0	0	0	18	18	0	5	103	108
Cagayan	0	0	6	6	0	0	142	142	21	692	3,918	4,631
Isabela	0	0	2	2	0	2	54	56	13	546	4,421	4,980
Nueva Vizcaya	0	2	71	73	0	0	200	200	11	379	4,097	4,487
Quirino	0	0	0	0	0	0	15	15	1	198	827	1,026
Cauayan City	0	0	0	0	0	0	2	2	1	119	632	752
Ilagan City	0	0	0	0	0	0	0	0	1	83	838	922
Santiago City	0	0	0	0	0	0	0	0	1	278	1,313	1,592
Tuguegarao City	0	0	0	0	0	1	16	17	0	19	317	336

Table 2.A.4. MODERN METHOD OF FAMILY PLANNING
Current User (Ending)
Philippines, 2022

Area	NFP-STM Age group 10-14	NFP-STM Age group 15-19	NFP-STM Age group 20-49	Total	NFP-SDM Age group 10-14	NFP-SDM Age group 15-19	NFP-SDM Age group 20-49	Total	NFP-LAM Age group 10-14	NFP-LAM Age group 15-19	NFP-LAM Age group 20-49	Total
**Region 3**	**0**	**9**	**13**	**22**	**0**	**18**	**4,587**	**4,605**	**50**	**4,596**	**42,452**	**47,098**
Aurora	0	0	0	0	0	0	43	43	3	202	1,240	1,445
Bataan	0	0	0	0	0	1	116	117	12	496	3,767	4,275
Bulacan	0	0	0	0	0	12	2,926	2,938	1	560	7,106	7,667
Nueva Ecija	0	0	0	0	0	2	12	14	16	590	3,902	4,508
Pampanga	0	0	0	0	0	1	1,251	1,252	0	267	4,901	5,168
Tarlac	0	0	0	0	0	0	7	7	3	297	5,181	5,481
Zambales	0	0	5	5	0	0	45	45	8	353	2,458	2,819
Angeles City	0	0	3	3	0	2	116	118	1	147	1,365	1,513
Balanga City	0	0	0	0	0	0	15	15	1	44	217	262
Cabanatuan City	0	0	0	0	0	0	0	0	0	175	1,143	1,318
City of San Fernando	0	9	5	14	0	0	51	51	2	158	1,401	1,561
Gapan City	0	0	0	0	0	0	0	0	0	96	725	821
Mabalacat City	0	0	0	0	0	0	1	1	0	248	3,034	3,282
Malolos City	0	0	0	0	0	0	0	0	0	0	1	1
Meycauayan	0	0	0	0	0	0	0	0	0	153	1,093	1,246
Olongapo	0	0	0	0	0	0	4	4	1	69	1,091	1,161
Palayan City	0	0	0	0	0	0	0	0	0	11	93	104
San Jose City	0	0	0	0	0	0	0	0	2	158	763	923
San Jose del Monte City	0	0	0	0	0	0	0	0	0	0	0	0
Science City of Munoz	0	0	0	0	0	0	0	0	0	0	0	0
Tarlac City	0	0	0	0	0	0	0	0	0	572	2,971	3,543
**Region 4A**	**18**	**2**	**358**	**378**	**20**	**1,560**	**21,964**	**23,544**	**133**	**6,084**	**58,262**	**64,479**
Batangas	0	0	4	4	0	289	18,732	19,021	64	236	3,913	4,213
Cavite	0	0	0	0	0	4	350	354	13	245	4,715	4,973
Laguna	0	0	12	12	1	16	1,346	1,363	2	416	2,799	3,217
Quezon	0	0	16	16	10	1,133	920	2,063	0	504	5,027	5,531
Rizal	0	0	2	2	0	0	126	126	12	1,002	8,151	9,165
Antipolo City	0	0	321	321	0	0	62	62	0	0	5,646	5,646
Bacoor City	0	0	0	0	0	0	0	0	0	136	1,474	1,610
Batangas City	0	0	0	0	0	1	80	81	0	11	328	339
Biñan City	0	0	0	0	0	0	7	7	0	1,158	9,441	10,599
Cabuyao City	0	0	0	0	0	0	0	0	2	394	1,697	2,093
Calamba City	0	0	0	0	0	30	157	187	3	167	1,173	1,343
Cavite City	0	0	0	0	0	1	2	3	0	40	127	167
Dasmariñas City	0	2	0	2	0	84	26	110	0	173	2,582	2,755
General Trias City	0	0	0	0	0	0	0	0	4	46	556	606
Imus City	0	0	0	0	0	0	0	0	0	16	345	361
Lipa City	0	0	0	0	0	0	5	5	0	0	0	0
Lucena City	0	0	0	0	0	0	0	0	0	30	23	53
San Pablo City	0	0	0	0	0	0	61	61	5	156	889	1,050
San Pedro City	0	0	0	0	0	2	60	62	4	120	1,885	2,009
Santa Rosa City	18	0	0	18	9	0	11	20	21	1,150	6,227	7,398
Tagaytay City	0	0	1	1	0	0	0	0	1	27	441	469
Tanauan City	0	0	0	0	0	0	0	0	0	1	63	64
Tayabas City	0	0	2	2	0	0	12	12	2	55	709	766
Trece Martires City	0	0	0	0	0	0	7	7	0	1	51	52
**Region 4B**	**0**	**0**	**50**	**50**	**0**	**15**	**2,317**	**2,332**	**62**	**1,733**	**15,992**	**17,787**
Marinduque	0	0	0	0	0	0	44	44	4	74	1,326	1,404
Mindoro Occidental	0	0	10	10	0	4	454	458	16	607	3,808	4,431
Mindoro Oriental	0	0	13	13	0	0	62	62	9	202	4,186	4,397
Palawan	0	0	27	27	0	8	952	960	28	620	4,482	5,130
Romblon	0	0	0	0	0	3	668	671	1	141	1,693	1,835
Puerto Princesa City	0	0	0	0	0	0	137	137	4	89	497	590
**Region 5**	**0**	**49**	**1,698**	**1,747**	**1**	**634**	**33,427**	**34,062**	**52**	**4,028**	**40,815**	**44,895**
Albay	0	1	10	11	0	34	6,128	6,162	5	493	6,392	6,890

Table 2.A.4. MODERN METHOD OF FAMILY PLANNING
Current User (Ending)
Philippines, 2022

Area	NFP-STM			Total	NFP-SDM			Total	NFP-LAM			Total
	Age group				Age group				Age group			
	10-14	15-19	20-49		10-14	15-19	20-49		10-14	15-19	20-49	
Camarines Norte	0	37	876	913	0	16	1,426	1,442	12	455	4,604	5,071
Camarines Sur	0	10	496	506	0	44	7,315	7,359	8	935	8,573	9,516
Catanduanes	0	0	49	49	0	2	2,164	2,166	4	98	3,615	3,717
Masbate	0	1	11	12	1	473	7,872	8,346	13	1,253	7,502	8,768
Sorsogon	0	0	40	40	0	41	4,883	4,924	8	642	5,678	6,328
Iriga City	0	0	124	124	0	23	2,146	2,169	2	50	586	638
Legaspi City	0	0	54	54	0	0	82	82	0	71	756	827
Naga City	0	0	38	38	0	1	1,411	1,412	0	31	3,109	3,140
**Region 6**	**0**	**3**	**67**	**70**	**2**	**88**	**7,614**	**7,704**	**59**	**4,267**	**42,282**	**46,608**
Aklan	0	3	25	28	0	2	302	304	7	380	4,156	4,543
Antique	0	0	10	10	0	0	296	296	3	176	2,419	2,598
Capiz	0	0	0	0	0	0	2	2	1	188	2,586	2,775
Guimaras	0	0	0	0	0	1	33	34	0	132	1,033	1,165
Iloilo	0	0	32	32	2	12	3,216	3,230	24	1,109	12,516	13,649
Negros Occidental	0	0	0	0	0	73	3,545	3,618	21	2,196	14,890	17,107
Bacolod City	0	0	0	0	0	0	41	41	3	59	4,479	4,541
Iloilo City	0	0	0	0	0	0	179	179	0	27	203	230
**Region 7**	**0**	**2**	**17**	**19**	**0**	**12**	**1,793**	**1,805**	**100**	**5,874**	**48,093**	**54,067**
Bohol	0	0	0	0	0	0	642	642	3	281	2,620	2,904
Cebu	0	0	0	0	0	10	518	528	53	2,897	22,650	25,600
Negros Oriental	0	2	17	19	0	1	175	176	23	1,087	8,066	9,176
Siquijor	0	0	0	0	0	1	456	457	0	66	278	344
Cebu City	0	0	0	0	0	0	2	2	21	1,174	8,091	9,286
Lapu-Lapu City	0	0	0	0	0	0	0	0	0	367	5,825	6,192
Mandaue City	0	0	0	0	0	0	0	0	0	2	563	565
**Region 8**	**0**	**3**	**101**	**104**	**0**	**46**	**3,056**	**3,102**	**22**	**2,818**	**31,209**	**34,049**
Biliran	0	0	0	0	0	2	243	245	0	103	911	1,014
Eastern Samar	0	0	86	86	0	11	1,573	1,584	4	203	2,286	2,493
Northern Leyte	0	0	1	1	0	14	167	181	1	1,058	10,536	11,595
Northern Samar	0	0	4	4	0	9	394	403	1	400	8,852	9,253
Southern Leyte	0	3	9	12	0	0	0	0	14	375	2,300	2,689
Western Samar	0	0	1	1	0	8	139	147	0	245	1,984	2,229
Calbayog City	0	0	0	0	0	0	0	0	1	125	513	639
Maasin City	0	0	0	0	0	0	84	84	0	5	173	178
Ormoc City	0	0	0	0	0	2	456	458	0	168	1,215	1,383
Tacloban City	0	0	0	0	0	0	0	0	1	136	2,439	2,576
**Region 9**	**0**	**0**	**11**	**11**	**0**	**75**	**2,347**	**2,422**	**39**	**3,473**	**27,544**	**31,056**
Zamboanga del Norte	0	0	0	0	0	74	2,082	2,156	13	892	7,133	8,038
Zamboanga del Sur	0	0	0	0	0	0	0	0	5	284	1,907	2,196
Zamboanga Sibugay	0	0	0	0	0	1	208	209	1	460	2,568	3,029
Dapitan City	0	0	0	0	0	0	6	6	0	112	420	532
Dipolog City	0	0	11	11	0	0	17	17	7	102	836	945
Isabela City	0	0	0	0	0	0	34	34	0	328	1,344	1,672
Pagadian City	0	0	0	0	0	0	0	0	1	364	5,419	5,784
Zamboanga City	0	0	0	0	0	0	0	0	12	931	7,917	8,860
**Region 10**	**0**	**53**	**505**	**558**	**5**	**757**	**15,794**	**16,556**	**134**	**5,628**	**40,431**	**46,193**
Bukidnon	0	6	251	257	2	160	2,831	2,993	31	1,526	7,948	9,505
Camiguin	0	0	1	1	0	0	80	80	3	69	673	745
Lanao del Norte	0	2	7	9	0	2	306	308	11	368	5,274	5,653
Misamis Occidental	0	37	55	92	0	21	663	684	8	295	4,559	4,862
Misamis Oriental	0	0	35	35	0	11	1,896	1,907	14	760	4,811	5,585

Table 2.A.4. MODERN METHOD OF FAMILY PLANNING
Current User (Ending)
Philippines, 2022

Area	NFP-STM			Total	NFP-SDM			Total	NFP-LAM			Total
	Age group				Age group				Age group			
	10-14	15-19	20-49		10-14	15-19	20-49		10-14	15-19	20-49	
Cagayan de Oro City	0	0	0	0	0	0	61	61	0	525	5,544	6,069
El Salvador City	0	0	0	0	0	0	29	29	1	44	199	244
Gingoog City	0	8	137	145	3	75	2,752	2,830	6	566	2,792	3,364
Iligan City	0	0	2	2	0	42	775	817	14	421	2,344	2,779
Malaybalay City	0	0	0	0	0	19	2,144	2,163	3	162	678	843
Oroquieta City	0	0	0	0	0	0	264	264	0	93	605	698
Ozamis City	0	0	0	0	0	51	1,743	1,794	2	421	3,452	3,875
Tangub City	0	0	13	13	0	362	1,553	1,915	5	21	635	661
Valencia City	0	0	4	4	0	14	697	711	36	357	917	1,310
**Region 11**	**0**	**11**	**171**	**182**	**1**	**43**	**2,391**	**2,435**	**266**	**3,459**	**35,839**	**39,564**
Davao de Oro	0	0	3	3	0	2	190	192	19	306	914	1,239
Davao del Norte	0	1	42	43	0	4	238	242	68	727	3,265	4,060
Davao Oriental	0	0	16	16	0	2	147	149	19	161	9,506	9,686
Davao del Sur	0	0	-3	-3	0	17	252	269	2	209	487	698
Davao Occidental	0	3	0	3	0	12	1,318	1,330	21	616	3,144	3,781
Davao City	0	7	113	120	1	6	246	253	137	1,440	18,523	20,100
**Region 12**	**0**	**5**	**38**	**43**	**0**	**5**	**714**	**719**	**174**	**4,458**	**22,879**	**27,511**
North Cotabato	0	0	0	0	0	0	120	120	4	679	4,900	5,583
Sarangani	0	0	21	21	0	1	105	106	26	1,594	5,278	6,898
South Cotabato	0	0	1	1	0	4	346	350	4	829	4,163	4,996
Sultan Kudarat	0	0	0	0	0	0	138	138	52	803	4,106	4,961
Gen. Santos City	0	5	16	21	0	0	5	5	88	553	4,432	5,073
**BARMM**	**-1**	**0**	**-121**	**-122**	**0**	**7**	**334**	**341**	**52**	**2,550**	**27,070**	**29,672**
Basilan	-1	0	-124	-125	0	0	20	20	1	51	654	706
Lanao del Sur	0	0	1	1	0	1	87	88	0	399	6,497	6,896
Maguindanao	0	0	0	0	0	6	100	106	24	1,271	10,665	11,960
Sulu	0	0	2	2	0	0	15	15	20	408	4,767	5,195
Tawi-Tawi	0	0	0	0	0	0	34	34	-1	123	1,919	2,041
Lamitan City	0	0	0	0	0	0	2	2	8	122	259	389
Marawi City	0	0	0	0	0	0	11	11	0	43	1,008	1,051
Cotabato City	0	0	0	0	0	0	65	65	0	133	1,301	1,434
**CARAGA**	**0**	**1**	**13**	**14**	**4**	**74**	**4,363**	**4,441**	**50**	**2,342**	**17,558**	**19,950**
Agusan del Norte	0	0	0	0	0	1	138	139	3	236	1,851	2,090
Agusan del Sur	0	0	0	0	0	41	1,311	1,352	20	627	4,151	4,798
Surigao del Norte	0	0	9	9	0	9	1,590	1,599	2	186	1,743	1,931
Surigao del Sur	0	1	4	5	0	9	613	622	8	343	3,828	4,179
Province of Dinagat	0	0	0	0	4	2	266	272	0	109	719	828
Bislig City	0	0	0	0	0	10	217	227	8	321	905	1,234
Butuan City	0	0	0	0	0	2	110	112	9	405	3,220	3,634
Surigao City	0	0	0	0	0	0	118	118	0	115	1,141	1,256

Table 2.A.5. MODERN FAMILY PLANNING
No. and proportion of WRA 15-49 yrs old with unmet needs
Philippines, 2022

Area	Estimated No. of WRA (TP x GR Factor)	Total No. of WRA (15-49 yrs old) with Unmet Needs	%
**PHILIPPINES**	28,157,813	1,186,993	4.22
**N C R**	3,879,805	27,734	0.71
Malabon	**110,130**	*	**0.00**
Navotas	**75,163**	893	**1.19**
Valenzuela City	**186,929**	1,540	**0.82**
Caloocan City	**477,239**	*	**0.00**
Marikina City	**135,803**	646	**0.48**
Pasig City	**227,565**	5,411	**2.38**
Pateros	**19,234**	*	**0.00**
Taguig	**242,515**	*	**0.00**
Quezon City	**884,626**	8,000	**0.90**
Makati City	**175,529**	2,656	**1.51**
Mandaluyong City	**116,381**	2,845	**2.44**
San Juan	**36,814**	*	**0.00**
Manila City	**536,343**	*	**0.00**
Las Piñas City	**177,428**	287	**0.16**
Muntinlupa City	**152,005**	1,799	**1.18**
Parañaque City	**200,606**	1,712	**0.85**
Pasay City	**125,495**	1,945	**1.55**
**C A R**	482,129	52,667	10.92
Abra	**62,613**	45,684	**72.96**
Apayao	**31,056**	256	**0.82**
Benguet	**136,100**	0	**0.00**
Ifugao	**53,406**	17	**0.03**
Kalinga	**54,877**	145	**0.26**
Mt. Province	**38,751**	186	**0.48**
Baguio City	**105,326**	6,379	**6.06**
**Region 1**	1,329,788	5,432	0.41
Ilocos Norte	**129,307**	210	**0.16**
Ilocos Sur	**153,735**	0	**0.00**
La Union	**182,779**	2,041	**1.12**
Pangasinan	**618,362**	0	**0.00**
Alaminos City	**23,364**	0	**0.00**
Candon City	**16,202**	0	**0.00**
Dagupan City	**44,604**	0	**0.00**
Laoag City	**29,814**	0	**0.00**
San Carlos City	**49,110**	0	**0.00**
San Fernando City	**33,485**	3,181	**9.50**
Urdaneta City	**34625.00**	0.00	**0.00**
Vigan City	**14,401**	0	**0.00**

Table 2.A.5. MODERN FAMILY PLANNING
No. and proportion of WRA 15-49 yrs old with unmet needs
Philippines, 2022

Area	Estimated No. of WRA (TP x GR Factor)	Total No. of WRA (15-49 yrs old) with Unmet Needs	%
**Region 2**	**930,979**	**6,313**	**0.68**
Batanes	**4,084**	0	**0.00**
Cagayan	**282,852**	0	**0.00**
Isabela	**319,557**	3,952	**1.24**
Nueva Vizcaya	**123,112**	351	**0.29**
Quirino	**49,172**	23	**0.05**
Cauayan City	**34,971**	583	**1.67**
Ilagan City	**39,304**	125	**0.32**
Santiago City	**36,405**	0	**0.00**
Tuguegarao City	**41,522**	1,279	**3.08**
**Region 3**	**3,325,039**	**81,649**	**2.46**
Aurora	**57,929**	652	**1.13**
Bataan	**197,290**	12,849	**6.51**
Bulacan	**686,770**	0	**0.00**
Nueva Ecija	**429,222**	14,549	**3.39**
Pampanga	**501,185**	20,333	**4.06**
Tarlac	**285,019**	0	**0.00**
Zambales	**169,616**	10,156	**5.99**
Angeles City	**125,745**	224	**0.18**
Balanga City	**28,521**	719	**2.52**
Cabanatuan City	**87,850**	311	**0.35**
City of San Fernando	**93,677**	2,312	**2.47**
Gapan City	**32,061**	0	**0.00**
Mabalacat City	**76,615**	423	**0.55**
Malolos City	**76,708**	9,172	**11.96**
Meycauayan	**63,625**	41	**0.06**
Olongapo	**66,901**	0	**0.00**
Palayan City	**11,929**	1,946	**16.31**
San Jose City	**40,617**	6,207	**15.28**
San Jose del Monte City	**174,707**	1,513	**0.87**
Science City of Munoz	**23,685**	0	**0.00**
Tarlac City	**95,367**	242	**0.25**
**Region 4A**	**4,469,317**	**129,616**	**2.90**
Batangas	**579,946**	26,468	**4.56**
Cavite	**451,749**	6,242	**1.38**
Laguna	**310,498**	36,715	**11.82**
Quezon	**472,796**	24,503	**5.18**
Rizal	**655,080**	2,954	**0.45**
Antipolo City	**241,285**	330	**0.14**
Bacoor City	**197,922**	289	**0.15**
Batangas City	**102,926**	210	**0.20**
Biñan City	**104,118**	1,086	**1.04**

Table 2.A.5. MODERN FAMILY PLANNING
No. and proportion of WRA 15-49 yrs old with unmet needs
Philippines, 2022

Area	Estimated No. of WRA (TP x GR Factor)	Total No. of WRA (15-49 yrs old) with Unmet Needs	%
Cabuyao City	**96,524**	428	**0.44**
Calamba City	**142,086**	22,301	**15.70**
Cavite City	**33,880**	92	**0.27**
Dasmariñas City	**217,166**	1,817	**0.84**
General Trias City	**103,574**	1,446	**1.40**
Imus City	**133,060**	165	**0.12**
Lipa City	**103,711**	164	**0.16**
Lucena City	**71,650**	0	**0.00**
San Pablo City	**83,182**	362	**0.44**
San Pedro City	**101,857**	147	**0.14**
Santa Rosa City	**110,598**	126	**0.11**
Tagaytay City	**23,457**	152	**0.65**
Tanauan City	**54,092**	2,087	**3.86**
Tayabas City	**26,850**	*	**0.00**
Trece Martires City	**51,310**	1,532	**2.99**
**Region 4B**	780,430	**40,381**	**5.17**
Marinduque	**55,840**	122	**0.22**
Mindoro Occidental	**122,939**	15,207	**12.37**
Mindoro Oriental	**222,263**	18,294	**8.23**
Palawan	**238,299**	3,717	**1.56**
Romblon	**69,521**	3,041	**4.37**
Puerto Princesa City	**71,568**	0	**0.00**
**Region 5**	1,502,027	**9,819**	**0.65**
Albay	**298,304**	46	**0.02**
Camarines Norte	**153,828**	18	**0.01**
Camarines Sur	**424,771**	195	**0.05**
Catanduanes	**64,694**	3,329	**5.15**
Masbate	**224,351**	2,040	**0.91**
Sorsogon	**204,137**	1,088	**0.53**
Iriga City	**28,863**	22	**0.08**
Legaspi City	**52,459**	2,496	**4.76**
Naga City	**50,620**	585	**1.16**
**Region 6**	2,004,932	**132,307**	**6.60**
Aklan	**154,969**	22,572	**14.57**
Antique	**145,574**	4,574	**3.14**
Capiz	**209,959**	3,475	**1.66**
Guimaras	**47,306**	3,140	**6.64**
Iloilo	**528,604**	13,632	**2.58**
Negros Occidental	**649,987**	59,982	**9.23**
Bacolod City	**146,244**	15,912	**10.88**
Iloilo City	**122,289**	9,020	**7.38**

Table 2.A.5. MODERN FAMILY PLANNING
No. and proportion of WRA 15-49 yrs old with unmet needs
Philippines, 2022

Area	Estimated No. of WRA (TP x GR Factor)	Total No. of WRA (15-49 yrs old) with Unmet Needs	%
**Region 7**	**2,062,659**	**59,601**	**2.89**
Bohol	**325,382**	0	**0.00**
Cebu	**863,719**	57,850	**6.70**
Negros Oriental	**351,729**	1,560	**0.44**
Siquijor	**24,172**	0	**0.00**
Cebu City	**271,142**	191	**0.07**
Lapu-Lapu City	**119,937**	0	**0.00**
Mandaue City	**106,578**	0	**0.00**
**Region 8**	**639,619**	**25,899**	**4.05**
Biliran	**23,571**		**0.00**
Eastern Samar	**67,455**	22,355	**33.14**
Northern Leyte	**220,352**	10	**0.00**
Northern Samar	**93,304**		**0.00**
Southern Leyte	**44,746**	3,097	**6.92**
Western Samar	**85,673**	45	**0.05**
Calbayog City	**26,405**		**0.00**
Maasin City	**11,389**	0	**0.00**
Ormoc City	**31,381**		**0.00**
Tacloban City	**35,343**	392	**1.11**
**Region 9**	**972,456**	**2,960**	**0.30**
Zamboanga del Norte	**206,060**		**0.00**
Zamboanga del Sur	**218,096**	2,409	**1.10**
Zamboanga Sibugay	**172,938**		**0.00**
Dapitan City	**21,277**		**0.00**
Dipolog City	**33,759**		**0.00**
Isabela City	**35,241**	63	**0.18**
Pagadian City	**53,491**	0	**0.00**
Zamboanga City	**231,594**	488	**0.21**
**Region 10**	**1,300,778**	**10,851**	**0.83**
Bukidnon	**288,352**	628	**0.22**
Camiguin	**22,163**	0	**0.00**
Lanao del Norte	**192,400**	442	**0.23**
Misamis Occidental	**83,296**	495	**0.59**
Misamis Oriental	**203,269**	594	**0.29**
Cagayan de Oro City	**192,533**	289	**0.15**
El Salvador City	**14,301**	0	**0.00**

Table 2.A.5. MODERN FAMILY PLANNING
No. and proportion of WRA 15-49 yrs old with unmet needs
Philippines, 2022

Area	Estimated No. of WRA (TP x GR Factor)	Total No. of WRA (15-49 yrs old) with Unmet Needs	%
Gingoog City	35,507	1,347	3.79
Iligan City	97,461	84	0.09
Malaybalay City	48,068	3,984	8.29
Oroquieta City	18,050	0	0.00
Ozamis City	36,179	0	0.00
Tangub City	16,074	2,724	16.95
Valencia City	53,125	264	0.50
**Region 11**	1,375,458	554,265	40.30
Davao de Oro	194,199	141,300	72.76
Davao del Norte	270,148	178,021	65.90
Davao Oriental	145,336	62,757	43.18
Davao del Sur	189,658	33,669	17.75
Davao Occidental	86,515	88,689	102.51
Davao City	489,602	49,829	10.18
**Region 12**	1,213,620	8,013	0.66
North Cotabato	397,991	3,129	0.79
Sarangani	150,329	159	0.11
South Cotabato	266,955	1,106	0.41
Sultan Kudarat	224,964	3,187	1.42
Gen. Santos City	173,381	432	0.25
**BARMM**	1,207,699	6,880	0.57
Basilan	79,958	0	0.00
Lanao del Sur	253,423	943	0.37
Maguindanao	351,760	0	0.00
Sulu	245,800		0.00
Tawi-Tawi	108,569	2,225	2.05
Lamitan City	22,001	0	0.00
Marawi City	60,620	3,712	6.12
Cotabato City	85,568	0	0.00
**CARAGA**	681,078	32,606	4.79
Agusan del Norte	95,802	0	0.00
Agusan del Sur	180,600	0	0.00
Surigao del Norte	90,066	0	0.00
Surigao del Sur	126,339	2,048	1.62
Province of Dinagat	31,235	242	0.77
Bislig City	23,996	2,204	9.18
Butuan City	91,095	745	0.82
Surigao City	41,945	27,367	65.24

Legend: * - No Report   0 - No data

Table 2.B.1. Prenatal Care

Number and proportion of Women who gave birth with at least 4 or more Prenatal Check-ups
Philippines, 2022

Area	Total Deliveries	Women who gave birth with at least 4 prenatal check-ups							
		Age Group						Total	%
		10-14 yrs old		15-19 yrs old		20-49 yrs old			
		No.	%	No.	%	No.	%		
**PHILIPPINES**	**1,419,070**	**2,381**	**0.17**	**124,144**	**8.75**	**1,018,630**	**71.78**	**1,145,162**	**80.70**
**N C R**	**186,890**	**197**	**0.11**	**12,653**	**6.77**	**125,166**	**66.97**	**138,016**	**73.85**
Malabon	**3,327**	11	**0.33**	463	**13.92**	2,853	**85.75**	**3,327**	**100.00**
Navotas	**3,980**	8	**0.20**	448	**11.26**	2,916	**73.27**	**3,372**	**84.72**
Valenzuela City	**6,488**	8	**0.12**	436	**6.72**	5,096	**78.55**	**5,540**	**85.39**
Caloocan City	**12,459**	27	**0.22**	2,224	**17.85**	10,208	**81.93**	**12,459**	**100.00**
Marikina City	**7,167**	6	**0.08**	298	**4.16**	3,433	**47.90**	**3,737**	**52.14**
Pasig City	**11,385**	9	**0.08**	635	**5.58**	8,178	**71.83**	**8,822**	**77.49**
Pateros	**873**	4	**0.46**	48	**5.50**	410	**46.96**	**462**	**52.92**
Taguig	**11,981**	20	**0.17**	978	**8.16**	8,819	**73.61**	**9,817**	**81.94**
Quezon City	**43,348**	37	**0.09**	2,717	**6.27**	40,594	**93.65**	**43,348**	**100.00**
Makati City	**5,606**	5	**0.09**	153	**2.73**	2,926	**52.19**	**3,084**	**55.01**
Mandaluyong City	**6,814**	4	**0.06**	393	**5.77**	6,359	**93.32**	**6,756**	**99.15**
San Juan	**1,550**	0	**0.00**	69	**4.45**	760	**49.03**	**829**	**53.48**
Manila City	**44,644**	25	**0.06**	1,787	**4.00**	11,872	**26.59**	**13,684**	**30.65**
Las Piñas City	**6,428**	12	**0.19**	532	**8.28**	4,979	**77.46**	**5,523**	**85.92**
Muntinlupa City	**7,675**	15	**0.20**	687	**8.95**	6,973	**90.85**	**7,675**	**100.00**
Parañaque City	**7,879**	6	**0.08**	454	**5.76**	4,336	**55.03**	**4,796**	**60.87**
Pasay City	**5,286**	0	**0.00**	331	**6.26**	4,454	**84.26**	**4,785**	**90.52**
**C A R**	**24,090**	**18**	**0.07**	**1,553**	**6.45**	**16,127**	**66.94**	**17,698**	**73.47**
Abra	**2,872**	4	**0.14**	237	**8.25**	1,869	**65.08**	**2,110**	**73.47**
Apayao	**1,636**	5	**0.31**	155	**9.47**	1,061	**64.85**	**1,221**	**74.63**
Benguet	**4,048**	2	**0.05**	237	**5.85**	2,940	**72.63**	**3,179**	**78.53**
Ifugao	**2,745**	2	**0.07**	207	**7.54**	2,075	**75.59**	**2,284**	**83.21**
Kalinga	**2,646**	0	**0.00**	191	**7.22**	2,210	**83.52**	**2,401**	**90.74**
Mt. Province	**3,233**	1	**0.03**	117	**3.62**	1,808	**55.92**	**1,926**	**59.57**
Baguio City	**6,910**	4	**0.06**	409	**5.92**	4,164	**60.26**	**4,577**	**66.24**
**Region 1**	**49,615**	**57**	**0.11**	**4,393**	**8.85**	**48,998**	**98.76**	**53,448**	**107.73**
Ilocos Norte	**4,303**	9	**0.21**	364	**8.46**	3,800	**88.31**	**4,173**	**96.98**
Ilocos Sur	**4,687**	10	**0.21**	438	**9.34**	6,467	**137.98**	**6,915**	**147.54**
La Union	**4,346**	8	**0.18**	680	**15.65**	6,524	**150.12**	**7,212**	**165.95**
Pangasinan	**13,824**	18	**0.13**	1,932	**13.98**	21,824	**157.87**	**23,774**	**171.98**
Alaminos City	**1,320**	0	**0.00**	82	**6.21**	839	**63.56**	**921**	**69.77**
Candon City	**840**	0	**0.00**	2	**0.24**	838	**99.76**	**840**	**100.00**
Dagupan City	**8,504**	6	**0.07**	325	**3.82**	1,881	**22.12**	**2,212**	**26.01**
Laoag City	**1,963**	0	**0.00**	21	**1.07**	1,430	**72.85**	**1,451**	**73.92**
San Carlos City	**2,735**	1	**0.04**	178	**6.51**	2,167	**79.23**	**2,346**	**85.78**
San Fernando City	**3,574**	2	**0.06**	179	**5.01**	1,151	**32.20**	**1,332**	**37.27**
Urdaneta City	**1,843**	2	**0.11**	146	**7.92**	1,612	**87.47**	**1,760**	**95.50**
Vigan City	**1,676**	1	**0.06**	46	**2.74**	465	**27.74**	**512**	**30.55**
**Region 2**	**39,828**	**75**	**0.19**	**4,442**	**11.15**	**35,325**	**88.69**	**39,842**	**100.04**
Batanes	**254**	0	**0.00**	4	**1.57**	143	**56.30**	**147**	**57.87**
Cagayan	**8,786**	19	**0.22**	1,063	**12.10**	8,256	**93.97**	**9,338**	**106.28**

Table 2.B.1. Prenatal Care

Number and proportion of Women who gave birth with at least 4 or more Prenatal Check-ups
Philippines, 2022

Area	Total Deliveries	Women who gave birth with at least 4 prenatal check-ups							
		Age Group						Total	%
		10-14 yrs old		15-19 yrs old		20-49 yrs old			
		No.	%	No.	%	No.	%		
Isabela	**10,651**	38	**0.36**	1,627	**15.28**	12,630	**118.58**	**14,295**	**134.21**
Nueva Vizcaya	**7,453**	9	**0.12**	793	**10.64**	5,743	**77.06**	**6,545**	**87.82**
Quirino	**2,614**	2	**0.08**	173	**6.62**	2,439	**93.31**	**2,614**	**100.00**
Cauayan City	**1,880**	1	**0.05**	219	**11.65**	1,281	**68.14**	**1,501**	**79.84**
Ilagan City	**3,596**	2	**0.06**	207	**5.76**	1,514	**42.10**	**1,723**	**47.91**
Santiago City	**3,030**	4	**0.13**	276	**9.11**	2,642	**87.19**	**2,922**	**96.44**
Tuguegarao City	**1,564**	0	**0.00**	80	**5.12**	677	**43.29**	**757**	**48.40**
**Region 3**	**133,760**	**310**	**0.23**	**15,525**	**11.61**	**123,383**	**92.24**	**139,218**	**104.08**
Aurora	**3,023**	4	**0.13**	312	**10.32**	2,436	**80.58**	**2,752**	**91.04**
Bataan	**4,993**	22	**0.44**	1,042	**20.87**	6,881	**137.81**	**7,945**	**159.12**
Bulacan	**20,098**	55	**0.27**	2,876	**14.31**	22,647	**112.68**	**25,578**	**127.27**
Nueva Ecija	**12,191**	52	**0.43**	1,674	**13.73**	12,078	**99.07**	**13,804**	**113.23**
Pampanga	**17,975**	36	**0.20**	1,845	**10.26**	15,946	**88.71**	**17,827**	**99.18**
Tarlac	**10,541**	45	**0.43**	1,571	**14.90**	13,226	**125.47**	**14,842**	**140.80**
Zambales	**8,746**	8	**0.09**	464	**5.31**	3,788	**43.31**	**4,260**	**48.71**
Angeles City	**5,738**	17	**0.30**	866	**15.09**	7,475	**130.27**	**8,358**	**145.66**
Balanga City	**1,300**	3	**0.23**	96	**7.38**	793	**61.00**	**892**	**68.62**
Cabanatuan City	**6,167**	10	**0.16**	421	**6.83**	3,652	**59.22**	**4,083**	**66.21**
City of San Fernando	**4,691**	4	**0.09**	391	**8.34**	2,766	**58.96**	**3,161**	**67.38**
Gapan City	**834**	0	**0.00**	88	**10.55**	701	**84.05**	**789**	**94.60**
Mabalacat City	**4,196**	7	**0.17**	637	**15.18**	4,708	**112.20**	**5,352**	**127.55**
Malolos City	**4,724**	8	**0.17**	286	**6.05**	2,138	**45.26**	**2,432**	**51.48**
Meycauayan	**2,236**	1	**0.04**	242	**10.82**	2,578	**115.30**	**2,821**	**126.16**
Olongapo	**3,691**	2	**0.05**	204	**5.53**	2,018	**54.67**	**2,224**	**60.25**
Palayan City	**109**	0	**0.00**	29	**26.61**	185	**169.72**	**214**	**196.33**
San Jose City	**2,115**	5	**0.24**	246	**11.63**	1,544	**73.00**	**1,795**	**84.87**
San Jose del Monte City	**8,415**	23	**0.27**	1,141	**13.56**	11,346	**134.83**	**12,510**	**148.66**
Science City of Munoz	**129**	2	**1.55**	88	**68.22**	582	**451.16**	**672**	**520.93**
Tarlac City	**11,848**	6	**0.05**	1,006	**8.49**	5,895	**49.76**	**6,907**	**58.30**
**Region 4A**	**195,187**	**300**	**0.15**	**14,675**	**7.52**	**120,267**	**61.62**	**135,242**	**69.29**
Batangas	**24,328**	38	**0.16**	2,026	**8.33**	19,343	**79.51**	**21,407**	**87.99**
Cavite	**19,628**	29	**0.15**	1,172	**5.97**	9,824	**50.05**	**11,025**	**56.17**
Laguna	**12,194**	30	**0.25**	1,341	**11.00**	8,878	**72.81**	**10,249**	**84.05**
Quezon	**25,230**	81	**0.32**	1,785	**7.07**	13,737	**54.45**	**15,603**	**61.84**
Rizal	**30,278**	48	**0.16**	2,404	**7.94**	16,071	**53.08**	**18,523**	**61.18**
Antipolo City	**9,873**	8	**0.08**	1,037	**10.50**	6,885	**69.74**	**7,930**	**80.32**
Bacoor City	**7,255**	1	**0.01**	222	**3.06**	1,308	**18.03**	**1,531**	**21.10**
Batangas City	**3,345**	3	**0.09**	318	**9.51**	2,971	**88.82**	**3,292**	**98.42**
Biñan City	**7,204**	3	**0.04**	780	**10.83**	6,235	**86.55**	**7,018**	**97.42**
Cabuyao City	**3,348**	6	**0.18**	324	**9.68**	3,793	**113.29**	**4,123**	**123.15**
Calamba City	**6,653**	9	**0.14**	610	**9.17**	5,552	**83.45**	**6,171**	**92.76**
Cavite City	**1,321**	1	**0.08**	104	**7.87**	372	**28.16**	**477**	**36.11**
Dasmariñas City	**8,174**	5	**0.06**	480	**5.87**	4,555	**55.73**	**5,040**	**61.66**
General Trias City	**2,775**	4	**0.14**	197	**7.10**	1,640	**59.10**	**1,841**	**66.34**
Imus City	**4,653**	2	**0.04**	79	**1.70**	865	**18.59**	**946**	**20.33**
Lipa City	**4,649**	1	**0.02**	137	**2.95**	2,072	**44.57**	**2,210**	**47.54**
Lucena City	**3,206**	1	**0.03**	196	**6.11**	1,421	**44.32**	**1,618**	**50.47**
San Pablo City	**3,415**	10	**0.29**	376	**11.01**	2,783	**81.49**	**3,169**	**92.80**
San Pedro City	**2,970**	2	**0.07**	129	**4.34**	1,059	**35.66**	**1,190**	**40.07**

Table 2.B.1. Prenatal Care

Number and proportion of Women who gave birth with at least 4 or more Prenatal Check-ups
Philippines, 2022

Area	Total Deliveries	Women who gave birth with at least 4 prenatal check-ups							
		Age Group						Total	%
		10-14 yrs old		15-19 yrs old		20-49 yrs old			
		No.	%	No.	%	No.	%		
Santa Rosa City	**5,405**	3	**0.06**	513	**9.49**	4,685	**86.68**	**5,201**	**96.23**
Tagaytay City	**1,305**	1	**0.08**	75	**5.75**	1,223	**93.72**	**1,299**	**99.54**
Tanauan City	**3,459**	10	**0.29**	59	**1.71**	1,775	**51.32**	**1,844**	**53.31**
Tayabas City	**1,409**	1	**0.07**	142	**10.08**	1,250	**88.72**	**1,393**	**98.86**
Trece Martires City	**3,110**	3	**0.10**	169	**5.43**	1,970	**63.34**	**2,142**	**68.87**
**Region 4B**	**41,033**	**71**	**0.17**	**3,825**	**9.32**	**27,893**	**67.98**	**31,789**	**77.47**
Marinduque	**3,070**	5	**0.16**	232	**7.56**	2,306	**75.11**	**2,543**	**82.83**
Mindoro Occidental	**7,546**	4	**0.05**	834	**11.05**	5,339	**70.75**	**6,177**	**81.86**
Mindoro Oriental	**9,492**	30	**0.32**	830	**8.74**	7,711	**81.24**	**8,571**	**90.30**
Palawan	**12,965**	21	**0.16**	1,478	**11.40**	8,843	**68.21**	**10,342**	**79.77**
Romblon	**4,089**	2	**0.05**	213	**5.21**	2,244	**54.88**	**2,459**	**60.14**
Puerto Princesa City	**3,871**	9	**0.23**	238	**6.15**	1,450	**37.46**	**1,697**	**43.84**
**Region 5**	**90,156**	**40**	**0.04**	**5,527**	**6.13**	**54,948**	**60.95**	**60,515**	**67.12**
Albay	**12,749**	2	**0.02**	624	**4.89**	8,996	**70.56**	**9,622**	**75.47**
Camarines Norte	**10,484**	10	**0.10**	1,181	**11.26**	8,722	**83.19**	**9,913**	**94.55**
Camarines Sur	**17,803**	3	**0.02**	864	**4.85**	11,364	**63.83**	**12,231**	**68.70**
Catanduanes	**4,348**	3	**0.07**	226	**5.20**	2,610	**60.03**	**2,839**	**65.29**
Masbate	**14,257**	15	**0.11**	1,394	**9.78**	9,354	**65.61**	**10,763**	**75.49**
Sorsogon	**13,063**	4	**0.03**	853	**6.53**	8,497	**65.05**	**9,354**	**71.61**
Iriga City	**1,705**	1	**0.06**	97	**5.69**	1,439	**84.40**	**1,537**	**90.15**
Legaspi City	**3,426**	1	**0.03**	142	**4.14**	2,249	**65.65**	**2,392**	**69.82**
Naga City	**12,321**	1	**0.01**	146	**1.18**	1,717	**13.94**	**1,864**	**15.13**
**Region 6**	**91,080**	**126**	**0.14**	**7,244**	**7.95**	**63,349**	**69.55**	**70,719**	**77.64**
Aklan	**5,918**	4	**0.07**	445	**7.52**	5,128	**86.65**	**5,577**	**94.24**
Antique	**7,882**	8	**0.10**	396	**5.02**	4,724	**59.93**	**5,128**	**65.06**
Capiz	**7,735**	14	**0.18**	428	**5.53**	4,919	**63.59**	**5,361**	**69.31**
Guimaras	**2,248**	6	**0.27**	210	**9.34**	1,928	**85.77**	**2,144**	**95.37**
Iloilo	**22,304**	38	**0.17**	1,578	**7.07**	16,863	**75.61**	**18,479**	**82.85**
Negros Occidental	**33,405**	51	**0.15**	3,625	**10.85**	23,574	**70.57**	**27,250**	**81.57**
Bacolod City	**5,982**	1	**0.02**	177	**2.96**	1,864	**31.16**	**2,042**	**34.14**
Iloilo City	**5,606**	4	**0.07**	385	**6.87**	4,349	**77.58**	**4,738**	**84.52**
**Region 7**	**133,214**	**189**	**0.14**	**10,735**	**8.06**	**92,394**	**69.36**	**103,318**	**77.56**
Bohol	**18,027**	18	**0.10**	1,167	**6.47**	12,191	**67.63**	**13,376**	**74.20**
Cebu	**53,485**	70	**0.13**	4,306	**8.05**	33,035	**61.76**	**37,411**	**69.95**
Negros Oriental	**19,600**	28	**0.14**	1,642	**8.38**	11,757	**59.98**	**13,427**	**68.51**
Siquijor	**1,098**	1	**0.09**	77	**7.01**	743	**67.67**	**821**	**74.77**
Cebu City	**22,881**	23	**0.10**	1,438	**6.28**	12,126	**53.00**	**13,587**	**59.38**
Lapu-Lapu City	**8,517**	9	**0.11**	402	**4.72**	7,048	**82.75**	**7,459**	**87.58**
Mandaue City	**9,606**	40	**0.42**	1,703	**17.73**	15,494	**161.30**	**17,237**	**179.44**
**Region 8**	**59,532**	**80**	**0.13**	**4,574**	**7.68**	**38,689**	**64.99**	**43,343**	**72.81**
Biliran	**3,303**	5	**0.15**	290	**8.78**	1,931	**58.46**	**2,226**	**67.39**

Table 2.B.1. Prenatal Care
Number and proportion of Women who gave birth with at least 4 or more Prenatal Check-ups
Philippines, 2022

Area	Total Deliveries	Women who gave birth with at least 4 prenatal check-ups							
		Age Group						Total	%
		10-14 yrs old		15-19 yrs old		20-49 yrs old			
		No.	%	No.	%	No.	%		
Eastern Samar	**7,154**	13	**0.18**	642	**8.97**	5,720	**79.96**	**6,375**	**89.11**
Northern Leyte	**13,721**	25	**0.18**	1,242	**9.05**	11,099	**80.89**	**12,366**	**90.12**
Northern Samar	**10,410**	9	**0.09**	610	**5.86**	5,262	**50.55**	**5,881**	**56.49**
Southern Leyte	**2,990**	7	**0.23**	345	**11.54**	2,638	**88.23**	**2,990**	**100.00**
Western Samar	**8,510**	6	**0.07**	626	**7.36**	4,601	**54.07**	**5,233**	**61.49**
Calbayog City	**2,290**	5	**0.22**	196	**8.56**	1,408	**61.48**	**1,609**	**70.26**
Maasin City	**2,390**	0	**0.00**	52	**2.18**	711	**29.75**	**763**	**31.92**
Ormoc City	**5,850**	6	**0.10**	375	**6.41**	2,622	**44.82**	**3,003**	**51.33**
Tacloban City	**2,914**	4	**0.14**	196	**6.73**	2,697	**92.55**	**2,897**	**99.42**
**Region 9**	**49,894**	**64**	**0.13**	**4,612**	**9.24**	**32,638**	**65.41**	**37,321**	**74.80**
Zamboanga del Norte	**9,771**	7	**0.07**	868	**8.88**	5,338	**54.63**	**6,220**	**63.66**
Zamboanga del Sur	**6,479**	17	**0.26**	453	**6.99**	3,406	**52.57**	**3,872**	**59.76**
Zamboanga Sibugay	**8,998**	8	**0.09**	694	**7.71**	4,901	**54.47**	**5,601**	**62.25**
Dapitan City	**1,558**	2	**0.13**	163	**10.46**	1,354	**86.91**	**1,520**	**97.56**
Dipolog City	**2,220**	4	**0.18**	226	**10.18**	1,465	**65.99**	**1,695**	**76.35**
Isabela City	**1,693**	4	**0.24**	271	**16.01**	1,329	**78.50**	**1,603**	**94.68**
Pagadian City	**2,413**	3	**0.12**	224	**9.28**	1,282	**53.13**	**1,512**	**62.66**
Zamboanga City	**16,762**	19	**0.11**	1,713	**10.22**	13,563	**80.92**	**15,298**	**91.27**
**Region 10**	**76,762**	**185**	**0.24**	**10,260**	**13.37**	**60,615**	**78.96**	**71,060**	**92.57**
Bukidnon	**20,001**	71	**0.35**	3,176	**15.88**	13,872	**69.36**	**17,119**	**85.59**
Camiguin	**1,154**	0	**0.00**	63	**5.46**	508	**44.02**	**571**	**49.48**
Lanao del Norte	**8,532**	16	**0.19**	975	**11.43**	7,546	**88.44**	**8,537**	**100.06**
Misamis Occidental	**4,066**	10	**0.25**	275	**6.76**	3,203	**78.78**	**3,488**	**85.78**
Misamis Oriental	**8,271**	45	**0.54**	1,786	**21.59**	8,937	**108.05**	**10,768**	**130.19**
Cagayan de Oro City	**16,044**	29	**0.18**	1,658	**10.33**	10,190	**63.51**	**11,877**	**74.03**
El Salvador City	**125**	1	**0.80**	42	**33.60**	331	**264.80**	**374**	**299.20**
Gingoog City	**2,109**	3	**0.14**	324	**15.36**	1,780	**84.40**	**2,107**	**99.91**
Iligan City	**7,370**	4	**0.05**	876	**11.89**	6,668	**90.47**	**7,548**	**102.42**
Malaybalay City	**3,112**	3	**0.10**	364	**11.70**	2,321	**74.58**	**2,688**	**86.38**
Oroquieta City	**1,117**	0	**0.00**	55	**4.92**	495	**44.32**	**550**	**49.24**
Ozamis City	**1,955**	0	**0.00**	219	**11.20**	1,641	**83.94**	**1,860**	**95.14**
Tangub City	**953**	0	**0.00**	94	**9.86**	859	**90.14**	**953**	**100.00**
Valencia City	**1,953**	3	**0.15**	353	**18.07**	2,264	**115.92**	**2,620**	**134.15**
**Region 11**	**82,240**	**392**	**0.48**	**8,053**	**9.79**	**54,977**	**66.85**	**63,422**	**77.12**
Davao de Oro	**11,860**	115	**0.97**	1,723	**14.53**	8,703	**73.38**	**10,541**	**88.88**
Davao del Norte	**17,930**	73	**0.41**	1,791	**9.99**	11,934	**66.56**	**13,798**	**76.95**
Davao Oriental	**8,773**	38	**0.43**	988	**11.26**	5,793	**66.03**	**6,819**	**77.73**
Davao del Sur	**9,089**	43	**0.47**	784	**8.63**	4,535	**49.90**	**5,362**	**58.99**
Davao Occidental	**4,596**	24	**0.52**	387	**8.42**	1,071	**23.30**	**1,482**	**32.25**
Davao City	**29,992**	99	**0.33**	2,380	**7.94**	22,941	**76.49**	**25,420**	**84.76**
**Region 12**	**64,935**	**210**	**0.32**	**8,344**	**12.85**	**50,310**	**77.48**	**58,864**	**90.65**
North Cotabato	**19,637**	47	**0.24**	2,338	**11.91**	15,052	**76.65**	**17,437**	**88.80**
Sarangani	**10,342**	41	**0.40**	1,808	**17.48**	7,650	**73.97**	**9,499**	**91.85**

Table 2.B.1. Prenatal Care

Number and proportion of Women who gave birth with at least 4 or more Prenatal Check-ups
Philippines, 2022

Area	Total Deliveries	Women who gave birth with at least 4 prenatal check-ups							
		Age Group						Total	%
		10-14 yrs old		15-19 yrs old		20-49 yrs old			
		No.	%	No.	%	No.	%		
South Cotabato	**16,503**	61	**0.37**	2,212	**13.40**	13,032	**78.97**	**15,305**	**92.74**
Sultan Kudarat	**12,587**	55	**0.44**	1,584	**12.58**	9,696	**77.03**	**11,335**	**90.05**
Gen. Santos City	**5,866**	6	**0.10**	402	**6.85**	4,880	**83.19**	**5,288**	**90.15**
**BARMM**	**65,614**	**38**	**0.06**	**5,029**	**7.66**	**49,484**	**75.42**	**54,551**	**83.14**
Basilan	**3,804**	5	**0.13**	305	**8.02**	2,062	**54.21**	**2,372**	**62.36**
Lanao del Sur	**18,053**	5	**0.03**	760	**4.21**	15,578	**86.29**	**16,343**	**90.53**
Maguindanao	**19,085**	12	**0.06**	1,897	**9.94**	16,030	**83.99**	**17,939**	**94.00**
Sulu	**9,870**	4	**0.04**	625	**6.33**	7,094	**71.87**	**7,723**	**78.25**
Tawi-Tawi	**5,365**	9	**0.17**	269	**5.01**	4,120	**76.79**	**4,398**	**81.98**
Lamitan City	**1,366**	3	**0.22**	191	**13.98**	724	**53.00**	**918**	**67.20**
Marawi City	**4,890**	0	**0.00**	982	**20.08**	3,876	**79.26**	**4,858**	**99.35**
Cotabato City	**3,181**	1	**0.03**	262	**8.24**	2,608	**81.99**	**2,871**	**90.25**
**CARAGA**	**35,240**	**29**	**0.08**	**2,700**	**7.66**	**24,067**	**68.29**	**26,796**	**76.04**
Agusan del Norte	**3,646**	5	**0.14**	311	**8.53**	2,433	**66.73**	**2,749**	**75.40**
Agusan del Sur	**10,056**	10	**0.10**	814	**8.09**	7,242	**72.02**	**8,066**	**80.21**
Surigao del Norte	**3,060**	1	**0.03**	283	**9.25**	2,653	**86.70**	**2,937**	**95.98**
Surigao del Sur	**6,361**	4	**0.06**	455	**7.15**	3,736	**58.73**	**4,195**	**65.95**
Province of Dinagat	**913**	0	**0.00**	55	**6.02**	550	**60.24**	**605**	**66.27**
Bislig City	**1,482**	0	**0.00**	154	**10.39**	1,088	**73.41**	**1,242**	**83.81**
Butuan City	**5,989**	9	**0.15**	522	**8.72**	5,458	**91.13**	**5,989**	**100.00**
Surigao City	**3,733**	0	**0.00**	106	**2.84**	907	**24.30**	**1,013**	**27.14**

Table 2.B.1.2. Prenatal Care

Number and proportion of pregnant women according to their Nutritional Status
Philippines, 2022

Area	Eligible Pop.	Pregnant women seen during the 1st trimester according to their Body Mass Index (BMI)														
		Normal BMI					Low BMI					High BMI				
		Age Group			Total	%	Age Group			Total	%	Age Group			Total	%
		10-14	15-19	20-49			10-14	15-19	20-49			10-14	15-19	20-49		
**PHILIPPINES**	**2,131,496**	**2,713**	**107,825**	**752,429**	**862,967**	**40.49**	**476**	**12,249**	**39,955**	**52,680**	**2.47**	**237**	**7,968**	**99,247**	**107,452**	**5.04**
**N C R**	**236,901**	**154**	**6,588**	**68,562**	**75,304**	**31.79**	**42**	**1,622**	**4,209**	**5,873**	**2.48**	**21**	**1,071**	**15,145**	**16,237**	**6.85**
Malabon	**6,724**	10	289	1,711	**2,010**	**29.89**	5	132	288	**425**	**6.32**	3	70	889	**962**	**14.31**
Navotas	**4,591**	16	510	2,433	**2,959**	**64.45**	0	25	129	**154**	**3.35**	1	20	286	**307**	**6.69**
Valenzuela City	**11,418**	6	339	3,166	**3,511**	**30.75**	1	56	234	**291**	**2.55**	0	88	1,252	**1,340**	**11.74**
Caloocan City	**29,146**	9	891	6,417	**7,317**	**25.10**	5	180	391	**576**	**1.98**	1	96	1,053	**1,150**	**3.95**
Marikina City	**8,294**	1	59	480	**540**	**6.51**	1	15	79	**95**	**1.15**	0	35	515	**550**	**6.63**
Pasig City	**13,894**	11	262	2,115	**2,388**	**17.19**	2	72	295	**369**	**2.66**	5	75	1,220	**1,300**	**9.36**
Pateros	**1,176**	3	78	498	**579**	**49.23**	0	5	6	**11**	**0.94**	0	0	26	**26**	**2.21**
Taguig	**14,807**	11	368	3,069	**3,448**	**23.29**	7	154	392	**553**	**3.73**	3	163	1,941	**2,107**	**14.23**
Quezon City	**54,011**	27	1,077	25,869	**26,973**	**49.94**	9	379	680	**1,068**	**1.98**	0	87	1,202	**1,289**	**2.39**
Makati City	**10,718**	2	63	724	**789**	**7.36**	0	28	138	**166**	**1.55**	0	16	715	**731**	**6.82**
Mandaluyong City	**7,111**	4	237	2,991	**3,232**	**45.45**	0	15	72	**87**	**1.22**	1	17	299	**317**	**4.46**
San Juan	**2,246**	0	66	781	**847**	**37.71**	0	0	16	**16**	**0.71**	0	1	55	**56**	**2.49**
Manila City	**32,743**	26	1,091	6,359	**7,476**	**22.83**	5	228	572	**805**	**2.46**	2	171	1,970	**2,143**	**6.54**
Las Piñas City	**10,832**	7	261	2,537	**2,805**	**25.90**	4	84	198	**286**	**2.64**	4	68	1,203	**1,275**	**11.77**
Muntinlupa City	**9,281**	10	336	3,592	**3,938**	**42.43**	2	122	379	**503**	**5.42**	0	75	1,236	**1,311**	**14.13**
Parañaque City	**12,249**	11	411	3,400	**3,822**	**31.20**	1	38	126	**165**	**1.35**	1	36	418	**455**	**3.71**
Pasay City	**7,660**	0	250	2,420	**2,670**	**34.86**	0	89	214	**303**	**3.96**	0	53	865	**918**	**11.98**
**C A R**	**35,179**	**38**	**1,711**	**14,330**	**16,079**	**45.71**	**3**	**104**	**360**	**467**	**1.33**	**1**	**139**	**2,177**	**2,317**	**6.59**
Abra	**4,275**	6	291	1,600	**1,897**	**44.37**	0	22	70	**92**	**2.15**	0	8	169	**177**	**4.14**
Apayao	**2,502**	5	166	1,080	**1,251**	**50.00**	1	19	54	**74**	**2.96**	0	10	206	**216**	**8.63**
Benguet	**9,086**	5	446	4,799	**5,250**	**57.78**	0	9	76	**85**	**0.94**	0	19	555	**574**	**6.32**
Ifugao	**4,486**	11	260	2,220	**2,491**	**55.53**	0	18	60	**78**	**1.74**	0	21	238	**259**	**5.77**
Kalinga	**4,701**	1	217	1,905	**2,123**	**45.16**	1	10	30	**41**	**0.87**	1	15	302	**318**	**6.76**
Mt. Province	**3,103**	7	139	1,296	**1,442**	**46.47**	0	4	31	**35**	**1.13**	0	17	311	**328**	**10.57**
Baguio City	**7,026**	3	192	1,430	**1,625**	**23.13**	1	22	39	**62**	**0.88**	0	49	396	**445**	**6.33**
**Region 1**	**97,099**	**47**	**3,754**	**38,525**	**42,326**	**43.59**	**7**	**218**	**688**	**913**	**0.94**	**1**	**142**	**2,084**	**2,227**	**2.29**
Ilocos Norte	**7,854**	5	289	3,186	**3,480**	**44.31**	1	10	75	**86**	**1.09**	0	14	184	**198**	**2.52**
Ilocos Sur	**9,072**	8	367	5,189	**5,564**	**61.33**	0	25	53	**78**	**0.86**	0	8	138	**146**	**1.61**
La Union	**11,338**	6	524	4,625	**5,155**	**45.47**	3	43	84	**130**	**1.15**	0	28	441	**469**	**4.14**
Pangasinan	**50,710**	16	1,893	18,577	**20,486**	**40.40**	3	109	372	**484**	**0.95**	1	79	1,058	**1,138**	**2.24**
Alaminos City	**1,910**	0	96	1,007	**1,103**	**57.75**	0	0	0	**0**	**0.00**	0	0	0	**0**	**0.00**
Candon City	**954**	0	2	565	**567**	**59.43**	0	0	2	**2**	**0.21**	0	0	0	**0**	**0.00**
Dagupan City	**3,657**	6	168	838	**1,012**	**27.67**	0	2	15	**17**	**0.46**	0	0	2	**2**	**0.05**
Laoag City	**1,812**	0	21	1,430	**1,451**	**80.08**	0	0	0	**0**	**0.00**	0	0	0	**0**	**0.00**
San Carlos City	**4,028**	3	98	911	**1,012**	**25.12**	0	10	24	**34**	**0.84**	0	0	52	**52**	**1.29**
San Fernando City	**2,074**	1	106	773	**880**	**42.43**	0	3	3	**6**	**0.29**	0	0	3	**3**	**0.14**
Urdaneta City	**2,836**	0	161	1,186	**1,347**	**47.50**	0	14	45	**59**	**2.08**	0	11	145	**156**	**5.50**
Vigan City	**854**	2	29	238	**269**	**31.50**	0	2	15	**17**	**1.99**	0	2	61	**63**	**7.38**
**Region 2**	**68,960**	**98**	**3,855**	**27,313**	**31,266**	**45.34**	**15**	**335**	**985**	**1,335**	**1.94**	**7**	**247**	**3,052**	**3,306**	**4.79**
Batanes	**362**	0	4	91	**95**	**26.24**	0	2	6	**8**	**2.21**	0	2	69	**71**	**19.61**
Cagayan	**20,431**	25	925	6,792	**7,742**	**37.89**	2	104	341	**447**	**2.19**	4	38	471	**513**	**2.51**
Isabela	**23,449**	49	1,378	9,470	**10,897**	**46.47**	8	130	336	**474**	**2.02**	3	96	1,296	**1,395**	**5.95**
Nueva Vizcaya	**9,538**	7	622	3,909	**4,538**	**47.58**	2	43	141	**186**	**1.95**	0	59	514	**573**	**6.01**
Quirino	**4,051**	5	220	2,251	**2,476**	**61.12**	0	24	55	**79**	**1.95**	0	15	202	**217**	**5.36**
Cauayan City	**2,565**	4	200	996	**1,200**	**46.78**	0	9	17	**26**	**1.01**	0	8	41	**49**	**1.91**
Ilagan City	**2,889**	3	142	1,222	**1,367**	**47.32**	0	13	50	**63**	**2.18**	0	6	123	**129**	**4.47**
Santiago City	**2,671**	4	299	2,039	**2,342**	**87.68**	0	5	15	**20**	**0.75**	0	16	136	**152**	**5.69**
Tuguegarao City	**3,004**	1	65	543	**609**	**20.27**	3	5	24	**32**	**1.07**	0	7	200	**207**	**6.89**
**Region 3**	**220,155**	**393**	**12,944**	**86,211**	**99,548**	**45.22**	**38**	**904**	**2,624**	**3,566**	**1.62**	**22**	**660**	**7,925**	**8,607**	**3.91**
Aurora	**4,754**	5	345	2,222	**2,572**	**54.10**	0	34	58	**92**	**1.94**	0	13	117	**130**	**2.73**
Bataan	**13,789**	25	813	4,756	**5,594**	**40.57**	15	114	407	**536**	**3.89**	3	77	1,135	**1,215**	**8.81**
Bulacan	**43,760**	168	2,828	19,210	**22,206**	**50.74**	4	239	735	**978**	**2.23**	6	190	2,305	**2,501**	**5.72**
Nueva Ecija	**29,039**	47	1,420	9,425	**10,892**	**37.51**	1	128	460	**589**	**2.03**	1	65	765	**831**	**2.86**
Pampanga	**31,620**	37	1,762	11,698	**13,497**	**42.69**	6	112	259	**377**	**1.19**	4	96	1,114	**1,214**	**3.84**
Tarlac	**19,319**	21	1,132	8,065	**9,218**	**47.71**	0	48	131	**179**	**0.93**	1	44	368	**413**	**2.14**
Zambales	**12,476**	12	527	3,011	**3,550**	**28.45**	2	42	77	**121**	**0.97**	3	20	395	**418**	**3.35**
Angeles City	**7,932**	3	357	2,155	**2,515**	**31.71**	1	10	19	**30**	**0.38**	0	1	94	**95**	**1.20**
Balanga City	**1,992**	4	99	595	**698**	**35.04**	2	22	42	**66**	**3.31**	1	9	173	**183**	**9.19**
Cabanatuan City	**5,945**	7	210	1,485	**1,702**	**28.63**	0	1	15	**16**	**0.27**	0	10	153	**163**	**2.74**
City of San Fernando	**5,907**	5	408	2,299	**2,712**	**45.91**	0	7	25	**32**	**0.54**	0	2	58	**60**	**1.02**
Gapan City	**2,170**	0	92	525	**617**	**28.43**	1	2	5	**8**	**0.37**	1	1	11	**13**	**0.60**
Mabalacat City	**4,831**	9	378	2,914	**3,301**	**68.33**	2	39	60	**101**	**2.09**	0	30	278	**308**	**6.38**
Malolos City	**4,890**	7	246	1,911	**2,164**	**44.25**	0	20	85	**105**	**2.15**	0	31	379	**410**	**8.38**
Meycauayan	**4,055**	1	326	2,827	**3,154**	**77.78**	0	16	35	**51**	**1.26**	1	11	63	**75**	**1.85**
Olongapo	**4,922**	3	166	1,149	**1,318**	**26.78**	0	27	119	**146**	**2.97**	0	36	293	**329**	**6.68**
Palayan City	**808**	3	53	248	**304**	**37.62**	0	11	7	**18**	**2.23**	0	5	31	**36**	**4.46**
San Jose City	**2,748**	9	240	1,408	**1,657**	**60.30**	3	15	26	**44**	**1.60**	0	6	88	**94**	**3.42**
San Jose del Monte City	**11,133**	22	577	5,094	**5,693**	**51.14**	0	17	32	**49**	**0.44**	1	10	87	**98**	**0.88**
Science City of Munoz	**1,601**	0	76	388	**464**	**28.98**	1	0	3	**4**	**0.25**	0	3	6	**9**	**0.56**
Tarlac City	**6,464**	5	889	4,826	**5,720**	**88.49**	0	0	24	**24**	**0.37**	0	0	12	**12**	**0.19**

Table 2.B.1.2. Prenatal Care

Number and proportion of pregnant women according to their Nutritional Status
Philippines, 2022

Area	Eligible Pop.	Pregnant women seen during the 1st trimester according to their Body Mass Index (BMI)														
		Normal BMI					Low BMI					High BMI				
		Age Group			Total	%	Age Group			Total	%	Age Group			Total	%
		10-14	15-19	20-49			10-14	15-19	20-49			10-14	15-19	20-49		
**Region 4A**	**299,627**	**261**	**11,584**	**81,364**	**93,209**	**31.11**	**71**	**1,841**	**7,313**	**9,225**	**3.08**	**39**	**1,006**	**11,127**	**12,172**	**4.06**
Batangas	**39,255**	22	1,449	12,272	**13,743**	**35.01**	10	284	983	**1,277**	**3.25**	2	108	674	**784**	**2.00**
Cavite	**28,151**	20	906	6,900	**7,826**	**27.80**	8	132	538	**678**	**2.41**	3	82	1,015	**1,100**	**3.91**
Laguna	**19,696**	22	1,021	5,705	**6,748**	**34.26**	8	167	564	**739**	**3.75**	1	90	1,067	**1,158**	**5.88**
Quezon	**37,853**	68	1,741	11,379	**13,188**	**34.84**	11	279	1,143	**1,433**	**3.79**	10	133	1,660	**1,803**	**4.76**
Rizal	**44,798**	36	1,861	10,527	**12,424**	**27.73**	10	242	1,006	**1,258**	**2.81**	3	135	905	**1,043**	**2.33**
Antipolo City	**16,498**	2	682	3,999	**4,683**	**28.39**	2	127	838	**967**	**5.86**	0	78	579	**657**	**3.98**
Bacoor City	**12,341**	7	203	1,094	**1,304**	**10.57**	1	11	71	**83**	**0.67**	0	15	133	**148**	**1.20**
Batangas City	**6,965**	2	146	873	**1,021**	**14.66**	1	42	120	**163**	**2.34**	2	22	484	**508**	**7.29**
Biñan City	**6,597**	7	741	5,646	**6,394**	**96.92**	1	17	74	**92**	**1.39**	0	22	102	**124**	**1.88**
Cabuyao City	**6,119**	11	280	2,845	**3,136**	**51.25**	1	61	412	**474**	**7.75**	10	28	512	**550**	**8.99**
Calamba City	**9,005**	11	362	3,000	**3,373**	**37.46**	3	132	344	**479**	**5.32**	0	63	1,104	**1,167**	**12.96**
Cavite City	**2,105**	3	59	243	**305**	**14.49**	0	20	40	**60**	**2.85**	0	18	110	**128**	**6.08**
Dasmariñas City	**13,538**	4	358	3,030	**3,392**	**25.06**	1	33	205	**239**	**1.77**	0	29	454	**483**	**3.57**
General Trias City	**6,457**	2	130	825	**957**	**14.82**	1	10	29	**40**	**0.62**	0	4	96	**100**	**1.55**
Imus City	**8,292**	3	115	746	**864**	**10.42**	6	29	72	**107**	**1.29**	1	14	268	**283**	**3.41**
Lipa City	**7,018**	4	176	2,106	**2,286**	**32.57**	1	17	102	**120**	**1.71**	0	0	53	**53**	**0.76**
Lucena City	**5,738**	9	324	1,359	**1,692**	**29.49**	1	49	156	**206**	**3.59**	2	14	140	**156**	**2.72**
San Pablo City	**5,272**	6	156	889	**1,051**	**19.94**	2	37	67	**106**	**2.01**	1	29	310	**340**	**6.45**
San Pedro City	**6,455**	10	157	970	**1,137**	**17.61**	0	19	144	**163**	**2.53**	0	15	121	**136**	**2.11**
Santa Rosa City	**7,009**	6	324	2,662	**2,992**	**42.69**	2	84	213	**299**	**4.27**	1	68	789	**858**	**12.24**
Tagaytay City	**1,460**	1	65	1,076	**1,142**	**78.22**	0	0	0	**0**	**0.00**	0	0	2	**2**	**0.14**
Tanauan City	**3,661**	1	95	1,573	**1,669**	**45.59**	0	17	82	**99**	**2.70**	0	9	56	**65**	**1.78**
Tayabas City	**2,145**	1	134	1,143	**1,278**	**59.58**	0	9	53	**62**	**2.89**	0	3	93	**96**	**4.48**
Trece Martires City	**3,199**	3	99	502	**604**	**18.88**	1	23	57	**81**	**2.53**	3	27	400	**430**	**13.44**
**Region 4B**	**72,791**	**89**	**3,599**	**22,038**	**25,726**	**35.34**	**26**	**492**	**1,472**	**1,990**	**2.73**	**7**	**317**	**3,051**	**3,375**	**4.64**
Marinduque	**4,990**	5	196	1,493	**1,694**	**33.95**	2	21	106	**129**	**2.59**	1	11	229	**241**	**4.83**
Mindoro Occidental	**12,479**	3	526	3,356	**3,885**	**31.13**	1	50	124	**175**	**1.40**	1	31	419	**451**	**3.61**
Mindoro Oriental	**20,189**	26	828	6,146	**7,000**	**34.67**	2	104	354	**460**	**2.28**	0	26	288	**314**	**1.56**
Palawan	**21,963**	36	1,584	8,031	**9,651**	**43.94**	9	222	600	**831**	**3.78**	2	85	781	**868**	**3.95**
Romblon	**6,569**	13	214	1,854	**2,081**	**31.68**	9	31	132	**172**	**2.62**	0	22	195	**217**	**3.30**
Puerto Princesa City	**6,601**	6	251	1,158	**1,415**	**21.44**	3	64	156	**223**	**3.38**	3	142	1,139	**1,284**	**19.45**
**Region 5**	**138,457**	**80**	**5,410**	**46,410**	**51,900**	**37.48**	**14**	**845**	**3,280**	**4,139**	**2.99**	**14**	**343**	**6,271**	**6,628**	**4.79**
Albay	**24,081**	7	831	9,852	**10,690**	**44.39**	3	69	492	**564**	**2.34**	2	29	559	**590**	**2.45**
Camarines Norte	**14,384**	31	856	5,504	**6,391**	**44.43**	6	281	700	**987**	**6.86**	1	73	937	**1,011**	**7.03**
Camarines Sur	**39,486**	19	1,205	10,494	**11,718**	**29.68**	2	185	826	**1,013**	**2.57**	9	74	1,730	**1,813**	**4.59**
Catanduanes	**6,459**	2	206	1,973	**2,181**	**33.77**	0	63	180	**243**	**3.76**	2	24	446	**472**	**7.31**
Masbate	**22,600**	11	1,155	7,073	**8,239**	**36.46**	1	94	278	**373**	**1.65**	0	60	842	**902**	**3.99**
Sorsogon	**19,828**	4	766	7,119	**7,889**	**39.79**	1	123	660	**784**	**3.95**	0	67	1,497	**1,564**	**7.89**
Iriga City	**2,679**	2	105	974	**1,081**	**40.35**	0	9	46	**55**	**2.05**	0	10	144	**154**	**5.75**
Legaspi City	**4,233**	4	173	2,427	**2,604**	**61.52**	1	13	67	**81**	**1.91**	0	3	39	**42**	**0.99**
Naga City	**4,707**	0	113	994	**1,107**	**23.52**	0	8	31	**39**	**0.83**	0	3	77	**80**	**1.70**
**Region 6**	**146,449**	**134**	**6,302**	**47,325**	**53,761**	**36.71**	**21**	**712**	**2,417**	**3,150**	**2.15**	**8**	**315**	**4,975**	**5,298**	**3.62**
Aklan	**11,288**	3	349	3,606	**3,958**	**35.06**	1	47	216	**264**	**2.34**	0	13	575	**588**	**5.21**
Antique	**13,132**	11	524	4,516	**5,051**	**38.46**	4	63	220	**287**	**2.19**	2	14	538	**554**	**4.22**
Capiz	**13,975**	16	409	3,557	**3,982**	**28.49**	0	22	168	**190**	**1.36**	0	30	263	**293**	**2.10**
Guimaras	**3,084**	7	155	1,437	**1,599**	**51.85**	3	22	81	**106**	**3.44**	0	19	308	**327**	**10.60**
Iloilo	**36,267**	32	1,356	12,996	**14,384**	**39.66**	6	131	564	**701**	**1.93**	1	48	641	**690**	**1.90**
Negros Occidental	**49,230**	57	3,093	17,749	**20,899**	**42.45**	7	377	985	**1,369**	**2.78**	5	139	1,842	**1,986**	**4.03**
Bacolod City	**11,082**	4	182	1,196	**1,382**	**12.47**	0	30	101	**131**	**1.18**	0	35	557	**592**	**5.34**
Iloilo City	**8,391**	4	234	2,268	**2,506**	**29.87**	0	20	82	**102**	**1.22**	0	17	251	**268**	**3.19**
**Region 7**	**162,308**	**140**	**6,845**	**53,588**	**60,573**	**37.32**	**24**	**826**	**2,985**	**3,835**	**2.36**	**15**	**679**	**10,326**	**11,020**	**6.79**
Bohol	**27,992**	14	816	8,304	**9,134**	**32.63**	5	87	409	**501**	**1.79**	0	46	1,243	**1,289**	**4.60**
Cebu	**66,463**	64	2,819	19,668	**22,551**	**33.93**	2	349	1,338	**1,689**	**2.54**	8	296	3,580	**3,884**	**5.84**
Negros Oriental	**27,890**	34	1,394	7,780	**9,208**	**33.02**	5	185	430	**620**	**2.22**	2	99	1,721	**1,822**	**6.53**
Siquijor	**1,661**	1	44	440	**485**	**29.20**	2	14	63	**79**	**4.76**	0	12	171	**183**	**11.02**
Cebu City	**20,866**	18	991	6,797	**7,806**	**37.41**	8	138	388	**534**	**2.56**	3	164	2,742	**2,909**	**13.94**
Lapu-Lapu City	**9,232**	4	391	5,510	**5,905**	**63.96**	1	24	248	**273**	**2.96**	1	28	550	**579**	**6.27**
Mandaue City	**8,204**	5	390	5,089	**5,484**	**66.85**	1	29	109	**139**	**1.69**	1	34	319	**354**	**4.31**
**Region 8**	**105,471**	**108**	**4,666**	**33,147**	**37,921**	**35.95**	**30**	**488**	**2,538**	**3,056**	**2.90**	**4**	**169**	**2,689**	**2,862**	**2.71**
Biliran	**3,873**	10	262	1,574	**1,846**	**47.66**	2	37	115	**154**	**3.98**	0	14	312	**326**	**8.42**
Eastern Samar	**11,908**	18	590	4,139	**4,747**	**39.86**	1	11	88	**100**	**0.84**	0	13	225	**238**	**2.00**
Northern Leyte	**35,422**	30	1,721	12,251	**14,002**	**39.53**	14	135	937	**1,086**	**3.07**	1	46	546	**593**	**1.67**
Northern Samar	**15,921**	20	637	4,128	**4,785**	**30.05**	6	67	180	**253**	**1.59**	2	21	253	**276**	**1.73**
Southern Leyte	**6,662**	3	220	2,103	**2,326**	**34.91**	1	34	141	**176**	**2.64**	0	9	323	**332**	**4.98**
Western Samar	**14,732**	17	449	3,072	**3,538**	**24.02**	2	93	743	**838**	**5.69**	1	34	342	**377**	**2.56**
Calbayog City	**4,535**	3	195	1,089	**1,287**	**28.38**	3	62	184	**249**	**5.49**	0	3	112	**115**	**2.54**
Maasin City	**1,696**	0	56	399	**455**	**26.83**	0	5	25	**30**	**1.77**	0	4	132	**136**	**8.02**
Ormoc City	**5,044**	4	310	2,046	**2,360**	**46.79**	1	35	64	**100**	**1.98**	0	21	413	**434**	**8.60**
Tacloban City	**5,678**	3	226	2,346	**2,575**	**45.35**	0	9	61	**70**	**1.23**	0	4	31	**35**	**0.62**

Table 2.B.1.2. Prenatal Care

Number and proportion of pregnant women according to their Nutritional Status
Philippines, 2022

Area	Eligible Pop.	Pregnant women seen during the 1st trimester according to their Body Mass Index (BMI)														
		Normal BMI					Low BMI					High BMI				
		Age Group			Total	%	Age Group			Total	%	Age Group			Total	%
		10-14	15-19	20-49			10-14	15-19	20-49			10-14	15-19	20-49		
**Region 9**	**79,007**	**135**	**5,948**	**33,662**	**39,745**	**50.31**	**11**	**464**	**1,425**	**1,900**	**2.40**	**9**	**227**	**2,292**	**2,528**	**3.20**
Zamboanga del Norte	**16,854**	27	1,233	6,250	**7,510**	**44.56**	3	88	288	**379**	**2.25**	1	34	258	**293**	**1.74**
Zamboanga del Sur	**17,368**	25	995	5,338	**6,358**	**36.61**	1	86	254	**341**	**1.96**	3	36	502	**541**	**3.11**
Zamboanga Sibugay	**15,021**	17	944	5,430	**6,391**	**42.55**	2	77	249	**328**	**2.18**	1	38	444	**483**	**3.22**
Dapitan City	**1,737**	1	113	973	**1,087**	**62.58**	1	13	13	**27**	**1.55**	0	3	17	**20**	**1.15**
Dipolog City	**2,762**	10	327	1,971	**2,308**	**83.56**	1	27	58	**86**	**3.11**	0	11	164	**175**	**6.34**
Isabela City	**2,563**	3	314	1,298	**1,615**	**63.01**	0	30	76	**106**	**4.14**	0	6	34	**40**	**1.56**
Pagadian City	**4,257**	13	290	1,731	**2,034**	**47.78**	0	10	84	**94**	**2.21**	0	4	81	**85**	**2.00**
Zamboanga City	**18,445**	39	1,732	10,671	**12,442**	**67.45**	3	133	403	**539**	**2.92**	4	95	792	**891**	**4.83**
**Region 10**	**99,908**	**168**	**7,910**	**42,003**	**50,081**	**50.13**	**19**	**579**	**1,578**	**2,176**	**2.18**	**7**	**615**	**5,136**	**5,758**	**5.76**
Bukidnon	**22,900**	72	2,629	9,854	**12,555**	**54.83**	9	247	561	**817**	**3.57**	5	206	1,646	**1,857**	**8.11**
Camiguin	**1,854**	0	42	339	**381**	**20.55**	0	2	5	**7**	**0.38**	0	1	7	**8**	**0.43**
Lanao del Norte	**14,930**	14	915	7,515	**8,444**	**56.56**	1	52	269	**322**	**2.16**	0	15	128	**143**	**0.96**
Misamis Occidental	**6,420**	6	208	2,296	**2,510**	**39.10**	1	2	24	**27**	**0.42**	0	10	56	**66**	**1.03**
Misamis Oriental	**14,963**	32	1,151	5,972	**7,155**	**47.82**	3	54	121	**178**	**1.19**	0	37	323	**360**	**2.41**
Cagayan de Oro City	**14,172**	20	1,003	5,429	**6,452**	**45.53**	3	120	282	**405**	**2.86**	0	182	1,684	**1,866**	**13.17**
El Salvador City	**1,052**	3	50	314	**367**	**34.89**	0	11	27	**38**	**3.61**	0	6	81	**87**	**8.27**
Gingoog City	**2,608**	2	354	1,719	**2,075**	**79.56**	1	24	120	**145**	**5.56**	1	56	205	**262**	**10.05**
Iligan City	**7,565**	2	471	2,815	**3,288**	**43.46**	0	19	43	**62**	**0.82**	0	26	315	**341**	**4.51**
Malaybalay City	**3,817**	3	260	1,314	**1,577**	**41.32**	0	16	28	**44**	**1.15**	0	10	174	**184**	**4.82**
Oroquieta City	**1,389**	3	52	373	**428**	**30.81**	0	1	0	**1**	**0.07**	0	0	5	**5**	**0.36**
Ozamis City	**2,786**	0	213	1,544	**1,757**	**63.07**	0	3	14	**17**	**0.61**	0	3	83	**86**	**3.09**
Tangub City	**1,234**	0	45	569	**614**	**49.76**	0	4	21	**25**	**2.03**	0	0	9	**9**	**0.73**
Valencia City	**4,218**	11	517	1,950	**2,478**	**58.75**	1	24	63	**88**	**2.09**	1	63	420	**484**	**11.47**
**Region 11**	**108,407**	**498**	**8,824**	**44,492**	**53,814**	**49.64**	**90**	**1,181**	**3,050**	**4,321**	**3.99**	**62**	**1,076**	**12,833**	**13,971**	**12.89**
Davao de Oro	**15,490**	110	1,570	6,526	**8,206**	**52.98**	40	239	478	**757**	**4.89**	21	246	3,499	**3,766**	**24.31**
Davao del Norte	**21,017**	136	1,915	10,579	**12,630**	**60.09**	18	315	726	**1,059**	**5.04**	10	233	3,270	**3,513**	**16.72**
Davao Oriental	**13,229**	43	952	4,388	**5,383**	**40.69**	6	122	320	**448**	**3.39**	2	122	1,416	**1,540**	**11.64**
Davao del Sur	**14,564**	53	990	4,112	**5,155**	**35.40**	10	125	383	**518**	**3.56**	12	194	1,471	**1,677**	**11.51**
Davao Occidental	**6,510**	42	484	1,048	**1,574**	**24.18**	1	50	80	**131**	**2.01**	5	41	280	**326**	**5.01**
Davao City	**37,597**	114	2,913	17,839	**20,866**	**55.50**	15	330	1,063	**1,408**	**3.74**	12	240	2,897	**3,149**	**8.38**
**Region 12**	**98,859**	**230**	**8,905**	**42,654**	**51,789**	**52.39**	**41**	**834**	**2,277**	**3,152**	**3.19**	**17**	**580**	**6,235**	**6,832**	**6.91**
North Cotabato	**34,150**	45	2,473	12,294	**14,812**	**43.37**	19	219	799	**1,037**	**3.04**	11	118	1,136	**1,265**	**3.70**
Sarangani	**12,907**	56	1,780	6,786	**8,622**	**66.80**	6	134	322	**462**	**3.58**	1	138	1,277	**1,416**	**10.97**
South Cotabato	**20,917**	37	2,115	11,163	**13,315**	**63.66**	7	190	444	**641**	**3.06**	3	108	1,578	**1,689**	**8.07**
Sultan Kudarat	**17,299**	73	1,488	7,300	**8,861**	**51.22**	3	110	336	**449**	**2.60**	1	99	1,214	**1,314**	**7.60**
Gen. Santos City	**13,586**	19	1,049	5,111	**6,179**	**45.48**	6	181	376	**563**	**4.14**	1	117	1,030	**1,148**	**8.45**
**BARMM**	**101,343**	**52**	**4,627**	**43,639**	**48,318**	**47.68**	**6**	**490**	**1,778**	**2,274**	**2.24**	**1**	**228**	**1,806**	**2,035**	**2.01**
Basilan	**7,823**	6	301	1,690	**1,997**	**25.53**	0	47	86	**133**	**1.70**	0	12	68	**80**	**1.02**
Lanao del Sur	**21,639**	8	767	14,429	**15,204**	**70.26**	0	35	50	**85**	**0.39**	0	0	41	**41**	**0.19**
Maguindanao	**32,198**	13	1,527	12,884	**14,424**	**44.80**	1	101	416	**518**	**1.61**	0	90	818	**908**	**2.82**
Sulu	**17,165**	16	591	5,560	**6,167**	**35.93**	4	94	743	**841**	**4.90**	0	36	465	**501**	**2.92**
Tawi-Tawi	**9,369**	2	225	3,115	**3,342**	**35.67**	1	41	123	**165**	**1.76**	0	13	215	**228**	**2.43**
Lamitan City	**2,154**	1	114	355	**470**	**21.82**	0	2	15	**17**	**0.79**	1	4	43	**48**	**2.23**
Marawi City	**5,173**	0	781	3,501	**4,282**	**82.78**	0	158	321	**479**	**9.26**	0	61	54	**115**	**2.22**
Cotabato City	**5,822**	6	321	2,105	**2,432**	**41.77**	0	12	24	**36**	**0.62**	0	12	102	**114**	**1.96**
**CARAGA**	**60,575**	**88**	**4,353**	**27,166**	**31,607**	**52.18**	**18**	**314**	**976**	**1,308**	**2.16**	**2**	**154**	**2,123**	**2,279**	**3.76**
Agusan del Norte	**8,127**	8	553	3,263	**3,824**	**47.05**	2	26	47	**75**	**0.92**	1	9	168	**178**	**2.19**
Agusan del Sur	**17,709**	31	1,570	9,023	**10,624**	**59.99**	4	63	197	**264**	**1.49**	1	36	463	**500**	**2.82**
Surigao del Norte	**7,280**	12	525	3,860	**4,397**	**60.40**	3	28	89	**120**	**1.65**	0	17	223	**240**	**3.30**
Surigao del Sur	**11,553**	17	589	4,071	**4,677**	**40.48**	8	58	169	**235**	**2.03**	0	25	436	**461**	**3.99**
Province of Dinagat	**2,576**	1	169	1,067	**1,237**	**48.02**	1	11	39	**51**	**1.98**	0	4	118	**122**	**4.74**
Bislig City	**2,192**	2	180	1,025	**1,207**	**55.06**	0	16	38	**54**	**2.46**	0	20	255	**275**	**12.55**
Butuan City	**7,743**	11	541	3,272	**3,824**	**49.39**	0	69	256	**325**	**4.20**	0	10	164	**174**	**2.25**
Surigao City	**3,395**	6	226	1,585	**1,817**	**53.52**	0	43	141	**184**	**5.42**	0	33	296	**329**	**9.69**

Table 2.B.1.3. Prenatal Care

Number and Proportion of Pregnant Women for the 1st time given at least two (2) doses of Tetanus Diphtheria (Td) vaccination
Philippines, 2022

Area	Eligible Pop.	Women pregnant for the 1st time given at least 2 doses of Td Vaccine							
		Age Group						Total	%
		10-14 yrs old		15-19 yrs old		20-49 yrs old			
		No.	%	No.	%	No.	%		
**PHILIPPINES**	**2,131,496**	**2,244**	**0.11**	**95,373**	**4.47**	**404,529**	**18.98**	**502,146**	**23.56**
**N C R**	**236,901**	**155**	**0.07**	**7,789**	**3.29**	**48,233**	**20.36**	**56,177**	**23.71**
Malabon	**6,724**	6	**0.09**	241	**3.58**	1,056	**15.70**	**1,303**	**19.38**
Navotas	**4,591**	10	**0.22**	290	**6.32**	1,030	**22.44**	**1,330**	**28.97**
Valenzuela City	**11,418**	5	**0.04**	344	**3.01**	4,137	**36.23**	**4,486**	**39.29**
Caloocan City	**29,146**	8	**0.03**	1,183	**4.06**	8,172	**28.04**	**9,363**	**32.12**
Marikina City	**8,294**	7	**0.08**	215	**2.59**	1,222	**14.73**	**1,444**	**17.41**
Pasig City	**13,894**	7	**0.05**	315	**2.27**	2,630	**18.93**	**2,952**	**21.25**
Pateros	**1,176**	3	**0.26**	27	**2.30**	154	**13.10**	**184**	**15.65**
Taguig	**14,807**	13	**0.09**	586	**3.96**	2,906	**19.63**	**3,505**	**23.67**
Quezon City	**54,011**	55	**0.10**	1,931	**3.58**	11,381	**21.07**	**13,367**	**24.75**
Makati City	**10,718**	4	**0.04**	129	**1.20**	990	**9.24**	**1,123**	**10.48**
Mandaluyong City	**7,111**	3	**0.04**	170	**2.39**	2,049	**28.81**	**2,222**	**31.25**
San Juan	**2,246**	0	**0.00**	18	**0.80**	279	**12.42**	**297**	**13.22**
Manila City	**32,743**	12	**0.04**	1,090	**3.33**	5,069	**15.48**	**6,171**	**18.85**
Las Piñas City	**10,832**	5	**0.05**	265	**2.45**	2,056	**18.98**	**2,326**	**21.47**
Muntinlupa City	**9,281**	6	**0.06**	361	**3.89**	2,305	**24.84**	**2,672**	**28.79**
Parañaque City	**12,249**	11	**0.09**	332	**2.71**	1,679	**13.71**	**2,022**	**16.51**
Pasay City	**7,660**	0	**0.00**	292	**3.81**	1,118	**14.60**	**1,410**	**18.41**
**C A R**	**35,179**	**20**	**0.06**	**1,052**	**2.99**	**5,472**	**15.55**	**6,544**	**18.60**
Abra	**4,275**	3	**0.07**	154	**3.60**	672	**15.72**	**829**	**19.39**
Apayao	**2,502**	4	**0.16**	133	**5.32**	339	**13.55**	**476**	**19.02**
Benguet	**9,086**	1	**0.01**	198	**2.18**	1,523	**16.76**	**1,722**	**18.95**
Ifugao	**4,486**	5	**0.11**	143	**3.19**	809	**18.03**	**957**	**21.33**
Kalinga	**4,701**	0	**0.00**	194	**4.13**	797	**16.95**	**991**	**21.08**
Mt. Province	**3,103**	4	**0.13**	83	**2.67**	451	**14.53**	**538**	**17.34**
Baguio City	**7,026**	3	**0.04**	147	**2.09**	881	**12.54**	**1,031**	**14.67**
**Region 1**	**97,099**	**62**	**0.06**	**3,532**	**3.64**	**22,370**	**23.04**	**25,964**	**26.74**
Ilocos Norte	**7,854**	4	**0.05**	181	**2.30**	1,654	**21.06**	**1,839**	**23.41**
Ilocos Sur	**9,072**	7	**0.08**	268	**2.95**	2,490	**27.45**	**2,765**	**30.48**
La Union	**11,338**	5	**0.04**	442	**3.90**	2,774	**24.47**	**3,221**	**28.41**
Pangasinan	**50,710**	34	**0.07**	1,816	**3.58**	9,720	**19.17**	**11,570**	**22.82**
Alaminos City	**1,910**	0	**0.00**	56	**2.93**	411	**21.52**	**467**	**24.45**
Candon City	**954**	0	**0.00**	1	**0.10**	480	**50.31**	**481**	**50.42**
Dagupan City	**3,657**	3	**0.08**	178	**4.87**	554	**15.15**	**735**	**20.10**
Laoag City	**1,812**	0	**0.00**	39	**2.15**	1,180	**65.12**	**1,219**	**67.27**
San Carlos City	**4,028**	3	**0.07**	189	**4.69**	609	**15.12**	**801**	**19.89**
San Fernando City	**2,074**	3	**0.14**	166	**8.00**	1,589	**76.62**	**1,758**	**84.76**
Urdaneta City	**2,836**	2	**0.07**	162	**5.71**	749	**26.41**	**913**	**32.19**
Vigan City	**854**	1	**0.12**	34	**3.98**	160	**18.74**	**195**	**22.83**
**Region 2**	**68,960**	**77**	**0.11**	**3,237**	**4.69**	**12,473**	**18.09**	**15,787**	**22.89**
Batanes	**362**	0	**0.00**	6	**1.66**	53	**14.64**	**59**	**16.30**

Table 2.B.1.3. Prenatal Care

Number and Proportion of Pregnant Women for the 1st time given at least two (2) doses of Tetanus Diphtheria (Td) vaccination
Philippines, 2022

Area	Eligible Pop.	Women pregnant for the 1st time given at least 2 doses of Td Vaccine							
		Age Group						Total	%
		10-14 yrs old		15-19 yrs old		20-49 yrs old			
		No.	%	No.	%	No.	%		
Cagayan	**20,431**	16	**0.08**	893	**4.37**	3,019	**14.78**	**3,928**	**19.23**
Isabela	**23,449**	49	**0.21**	1,226	**5.23**	4,552	**19.41**	**5,827**	**24.85**
Nueva Vizcaya	**9,538**	4	**0.04**	445	**4.67**	1,781	**18.67**	**2,230**	**23.38**
Quirino	**4,051**	3	**0.07**	147	**3.63**	766	**18.91**	**916**	**22.61**
Cauayan City	**2,565**	3	**0.12**	154	**6.00**	482	**18.79**	**639**	**24.91**
Ilagan City	**2,889**	1	**0.03**	124	**4.29**	591	**20.46**	**716**	**24.78**
Santiago City	**2,671**	1	**0.04**	205	**7.68**	851	**31.86**	**1,057**	**39.57**
Tuguegarao City	**3,004**	0	**0.00**	37	**1.23**	378	**12.58**	**415**	**13.81**
			**0.00**		**0.00**		**0.00**		
**Region 3**	**220,155**	**198**	**0.09**	**9,406**	**4.27**	**42,082**	**19.11**	**51,686**	**23.48**
Aurora	**4,754**	6	**0.13**	239	**5.03**	857	**18.03**	**1,102**	**23.18**
Bataan	**13,789**	24	**0.17**	481	**3.49**	1,543	**11.19**	**2,048**	**14.85**
Bulacan	**43,760**	48	**0.11**	2,096	**4.79**	8,792	**20.09**	**10,936**	**24.99**
Nueva Ecija	**29,039**	24	**0.08**	998	**3.44**	3,699	**12.74**	**4,721**	**16.26**
Pampanga	**31,620**	21	**0.07**	1,191	**3.77**	6,072	**19.20**	**7,284**	**23.04**
Tarlac	**19,319**	11	**0.06**	1,009	**5.22**	4,596	**23.79**	**5,616**	**29.07**
Zambales	**12,476**	8	**0.06**	414	**3.32**	1,358	**10.88**	**1,780**	**14.27**
Angeles City	**7,932**	0	**0.00**	129	**1.63**	1,188	**14.98**	**1,317**	**16.60**
Balanga City	**1,992**	2	**0.10**	74	**3.71**	217	**10.89**	**293**	**14.71**
Cabanatuan City	**5,945**	6	**0.10**	232	**3.90**	982	**16.52**	**1,220**	**20.52**
City of San Fernando	**5,907**	5	**0.08**	192	**3.25**	911	**15.42**	**1,108**	**18.76**
Gapan City	**2,170**	0	**0.00**	48	**2.21**	208	**9.59**	**256**	**11.80**
Mabalacat City	**4,831**	2	**0.04**	329	**6.81**	1,343	**27.80**	**1,674**	**34.65**
Malolos City	**4,890**	8	**0.16**	105	**2.15**	689	**14.09**	**802**	**16.40**
Meycauayan	**4,055**	0	**0.00**	281	**6.93**	1,695	**41.80**	**1,976**	**48.73**
Olongapo	**4,922**	0	**0.00**	71	**1.44**	515	**10.46**	**586**	**11.91**
Palayan City	**808**	1	**0.12**	17	**2.10**	42	**5.20**	**60**	**7.43**
San Jose City	**2,748**	6	**0.22**	199	**7.24**	630	**22.93**	**835**	**30.39**
San Jose del Monte City	**11,133**	19	**0.17**	605	**5.43**	4,637	**41.65**	**5,261**	**47.26**
Science City of Munoz	**1,601**	1	**0.06**	65	**4.06**	186	**11.62**	**252**	**15.74**
Tarlac City	**6,464**	6	**0.09**	631	**9.76**	1,922	**29.73**	**2,559**	**39.59**
**Region 4A**	**299,627**	**192**	**0.06**	**10,377**	**3.46**	**50,567**	**16.88**	**61,136**	**20.40**
Batangas	**39,255**	26	**0.07**	1,393	**3.55**	8,447	**21.52**	**9,866**	**25.13**
Cavite	**28,151**	17	**0.06**	956	**3.40**	4,516	**16.04**	**5,489**	**19.50**
Laguna	**19,696**	23	**0.12**	964	**4.89**	3,789	**19.24**	**4,776**	**24.25**
Quezon	**37,853**	52	**0.14**	1,729	**4.57**	6,673	**17.63**	**8,454**	**22.33**
Rizal	**44,798**	17	**0.04**	1,151	**2.57**	4,331	**9.67**	**5,499**	**12.28**
Antipolo City	**16,498**	3	**0.02**	544	**3.30**	2,528	**15.32**	**3,075**	**18.64**
Bacoor City	**12,341**	3	**0.02**	180	**1.46**	881	**7.14**	**1,064**	**8.62**
Batangas City	**6,965**	4	**0.06**	248	**3.56**	1,406	**20.19**	**1,658**	**23.80**
Biñan City	**6,597**	3	**0.05**	667	**10.11**	3,977	**60.28**	**4,647**	**70.44**
Cabuyao City	**6,119**	7	**0.11**	271	**4.43**	1,370	**22.39**	**1,648**	**26.93**
Calamba City	**9,005**	8	**0.09**	365	**4.05**	1,521	**16.89**	**1,894**	**21.03**
Cavite City	**2,105**	1	**0.05**	92	**4.37**	260	**12.35**	**353**	**16.77**
Dasmariñas City	**13,538**	0	**0.00**	297	**2.19**	1,939	**14.32**	**2,236**	**16.52**
General Trias City	**6,457**	8	**0.12**	152	**2.35**	784	**12.14**	**944**	**14.62**
Imus City	**8,292**	1	**0.01**	103	**1.24**	642	**7.74**	**746**	**9.00**
Lipa City	**7,018**	1	**0.01**	178	**2.54**	1,257	**17.91**	**1,436**	**20.46**
Lucena City	**5,738**	1	**0.02**	206	**3.59**	1,143	**19.92**	**1,350**	**23.53**
San Pablo City	**5,272**	7	**0.13**	246	**4.67**	713	**13.52**	**966**	**18.32**

Table 2.B.1.3. Prenatal Care

Number and Proportion of Pregnant Women for the 1st time given at least two (2) doses of Tetanus Diphtheria (Td) vaccination
Philippines, 2022

Area	Eligible Pop.	Women pregnant for the 1st time given at least 2 doses of Td Vaccine							
		Age Group						Total	%
		10-14 yrs old		15-19 yrs old		20-49 yrs old			
		No.	%	No.	%	No.	%		
San Pedro City	**6,455**	1	**0.02**	58	**0.90**	392	**6.07**	**451**	**6.99**
Santa Rosa City	**7,009**	7	**0.10**	367	**5.24**	2,103	**30.00**	**2,477**	**35.34**
Tagaytay City	**1,460**	0	**0.00**	51	**3.49**	514	**35.21**	**565**	**38.70**
Tanauan City	**3,661**	1	**0.03**	26	**0.71**	895	**24.45**	**922**	**25.18**
Tayabas City	**2,145**	0	**0.00**	77	**3.59**	263	**12.26**	**340**	**15.85**
Trece Martires City	**3,199**	1	**0.03**	56	**1.75**	223	**6.97**	**280**	**8.75**
**Region 4B**	**72,791**	**95**	**0.13**	**3,569**	**4.90**	**12,463**	**17.12**	**16,127**	**22.16**
Marinduque	**4,990**	5	**0.10**	179	**3.59**	667	**13.37**	**851**	**17.05**
Mindoro Occidental	**12,479**	6	**0.05**	722	**5.79**	1,789	**14.34**	**2,517**	**20.17**
Mindoro Oriental	**20,189**	17	**0.08**	824	**4.08**	3,916	**19.40**	**4,757**	**23.56**
Palawan	**21,963**	29	**0.13**	1,039	**4.73**	3,568	**16.25**	**4,636**	**21.11**
Romblon	**6,569**	15	**0.23**	242	**3.68**	1,033	**15.73**	**1,290**	**19.64**
Puerto Princesa City	**6,601**	23	**0.35**	563	**8.53**	1,490	**22.57**	**2,076**	**31.45**
**Region 5**	**138,457**	**54**	**0.04**	**3,957**	**2.86**	**18,853**	**13.62**	**22,864**	**16.51**
Albay	**24,081**	3	**0.01**	520	**2.16**	3,629	**15.07**	**4,152**	**17.24**
Camarines Norte	**14,384**	4	**0.03**	421	**2.93**	1,728	**12.01**	**2,153**	**14.97**
Camarines Sur	**39,486**	15	**0.04**	845	**2.14**	4,545	**11.51**	**5,405**	**13.69**
Catanduanes	**6,459**	5	**0.08**	172	**2.66**	760	**11.77**	**937**	**14.51**
Masbate	**22,600**	15	**0.07**	1,223	**5.41**	4,021	**17.79**	**5,259**	**23.27**
Sorsogon	**19,828**	7	**0.04**	530	**2.67**	2,132	**10.75**	**2,669**	**13.46**
Iriga City	**2,679**	1	**0.04**	41	**1.53**	247	**9.22**	**289**	**10.79**
Legaspi City	**4,233**	3	**0.07**	96	**2.27**	1,079	**25.49**	**1,178**	**27.83**
Naga City	**4,707**	1	**0.02**	109	**2.32**	712	**15.13**	**822**	**17.46**
**Region 6**	**146,449**	**151**	**0.10**	**6,197**	**4.23**	**26,876**	**18.35**	**33,224**	**22.69**
Aklan	**11,288**	4	**0.04**	376	**3.33**	2,053	**18.19**	**2,433**	**21.55**
Antique	**13,132**	7	**0.05**	398	**3.03**	2,079	**15.83**	**2,484**	**18.92**
Capiz	**13,975**	17	**0.12**	374	**2.68**	1,897	**13.57**	**2,288**	**16.37**
Guimaras	**3,084**	2	**0.06**	120	**3.89**	645	**20.91**	**767**	**24.87**
Iloilo	**36,267**	60	**0.17**	1,271	**3.50**	7,494	**20.66**	**8,825**	**24.33**
Negros Occidental	**49,230**	53	**0.11**	3,039	**6.17**	9,364	**19.02**	**12,456**	**25.30**
Bacolod City	**11,082**	5	**0.05**	353	**3.19**	1,741	**15.71**	**2,099**	**18.94**
Iloilo City	**8,391**	3	**0.04**	266	**3.17**	1,603	**19.10**	**1,872**	**22.31**
**Region 7**	**162,308**	**168**	**0.10**	**8,461**	**5.21**	**35,327**	**21.77**	**43,956**	**27.08**
Bohol	**27,992**	16	**0.06**	1,041	**3.72**	4,705	**16.81**	**5,762**	**20.58**
Cebu	**66,463**	61	**0.09**	3,051	**4.59**	10,478	**15.77**	**13,590**	**20.45**
Negros Oriental	**27,890**	36	**0.13**	1,636	**5.87**	4,614	**16.54**	**6,286**	**22.54**
Siquijor	**1,661**	1	**0.06**	64	**3.85**	248	**14.93**	**313**	**18.84**
Cebu City	**20,866**	23	**0.11**	1,231	**5.90**	5,013	**24.02**	**6,267**	**30.03**
Lapu-Lapu City	**9,232**	5	**0.05**	378	**4.09**	2,920	**31.63**	**3,303**	**35.78**
Mandaue City	**8,204**	26	**0.32**	1,060	**12.92**	7,349	**89.58**	**8,435**	**102.82**
**Region 8**	**105,471**	**57**	**0.05**	**3,497**	**3.32**	**15,204**	**14.42**	**18,758**	**17.78**
Biliran	**3,873**	5	**0.13**	266	**6.87**	776	**20.04**	**1,047**	**27.03**

Table 2.B.1.3. Prenatal Care

Number and Proportion of Pregnant Women for the 1st time given at least two (2) doses of Tetanus Diphtheria (Td) vaccination
Philippines, 2022

Area	Eligible Pop.	Women pregnant for the 1st time given at least 2 doses of Td Vaccine							
		Age Group						Total	%
		10-14 yrs old		15-19 yrs old		20-49 yrs old			
		No.	%	No.	%	No.	%		
Eastern Samar	**11,908**	7	**0.06**	370	**3.11**	1,575	**13.23**	**1,952**	**16.39**
Northern Leyte	**35,422**	16	**0.05**	867	**2.45**	3,781	**10.67**	**4,664**	**13.17**
Northern Samar	**15,921**	15	**0.09**	609	**3.83**	2,626	**16.49**	**3,250**	**20.41**
Southern Leyte	**6,662**	4	**0.06**	225	**3.38**	1,469	**22.05**	**1,698**	**25.49**
Western Samar	**14,732**	3	**0.02**	431	**2.93**	1,388	**9.42**	**1,822**	**12.37**
Calbayog City	**4,535**	2	**0.04**	239	**5.27**	830	**18.30**	**1,071**	**23.62**
Maasin City	**1,696**	0	**0.00**	23	**1.36**	273	**16.10**	**296**	**17.45**
Ormoc City	**5,044**	5	**0.10**	309	**6.13**	883	**17.51**	**1,197**	**23.73**
Tacloban City	**5,678**	0	**0.00**	158	**2.78**	1,603	**28.23**	**1,761**	**31.01**
**Region 9**	**79,007**	**108**	**0.14**	**4,855**	**6.15**	**16,044**	**20.31**	**21,007**	**26.59**
Zamboanga del Norte	**16,854**	18	**0.11**	977	**5.80**	3,701	**21.96**	**4,696**	**27.86**
Zamboanga del Sur	**17,368**	20	**0.12**	967	**5.57**	2,897	**16.68**	**3,884**	**22.36**
Zamboanga Sibugay	**15,021**	21	**0.14**	843	**5.61**	2,486	**16.55**	**3,350**	**22.30**
Dapitan City	**1,737**	1	**0.06**	55	**3.17**	599	**34.48**	**655**	**37.71**
Dipolog City	**2,762**	6	**0.22**	245	**8.87**	1,096	**39.68**	**1,347**	**48.77**
Isabela City	**2,563**	5	**0.20**	183	**7.14**	816	**31.84**	**1,004**	**39.17**
Pagadian City	**4,257**	2	**0.05**	191	**4.49**	646	**15.18**	**839**	**19.71**
Zamboanga City	**18,445**	35	**0.19**	1,394	**7.56**	3,803	**20.62**	**5,232**	**28.37**
**Region 10**	**99,908**	**139**	**0.14**	**6,864**	**6.87**	**19,481**	**19.50**	**26,484**	**26.51**
Bukidnon	**22,900**	54	**0.24**	1,978	**8.64**	3,544	**15.48**	**5,576**	**24.35**
Camiguin	**1,854**	0	**0.00**	59	**3.18**	155	**8.36**	**214**	**11.54**
Lanao del Norte	**14,930**	18	**0.12**	766	**5.13**	4,722	**31.63**	**5,506**	**36.88**
Misamis Occidental	**6,420**	7	**0.11**	225	**3.50**	1,004	**15.64**	**1,236**	**19.25**
Misamis Oriental	**14,963**	24	**0.16**	1,081	**7.22**	2,689	**17.97**	**3,794**	**25.36**
Cagayan de Oro City	**14,172**	19	**0.13**	1,229	**8.67**	2,980	**21.03**	**4,228**	**29.83**
El Salvador City	**1,052**	6	**0.57**	80	**7.60**	182	**17.30**	**268**	**25.48**
Gingoog City	**2,608**	1	**0.04**	185	**7.09**	493	**18.90**	**679**	**26.04**
Iligan City	**7,565**	4	**0.05**	420	**5.55**	1,530	**20.22**	**1,954**	**25.83**
Malaybalay City	**3,817**	1	**0.03**	261	**6.84**	650	**17.03**	**912**	**23.89**
Oroquieta City	**1,389**	0	**0.00**	57	**4.10**	212	**15.26**	**269**	**19.37**
Ozamis City	**2,786**	0	**0.00**	181	**6.50**	425	**15.25**	**606**	**21.75**
Tangub City	**1,234**	0	**0.00**	90	**7.29**	318	**25.77**	**408**	**33.06**
Valencia City	**4,218**	5	**0.12**	252	**5.97**	577	**13.68**	**834**	**19.77**
**Region 11**	**108,407**	**492**	**0.45**	**8,682**	**8.01**	**23,833**	**21.98**	**33,007**	**30.45**
Davao de Oro	**15,490**	90	**0.58**	1,399	**9.03**	2,698	**17.42**	**4,187**	**27.03**
Davao del Norte	**21,017**	97	**0.46**	1,660	**7.90**	4,632	**22.04**	**6,389**	**30.40**
Davao Oriental	**13,229**	38	**0.29**	858	**6.49**	2,170	**16.40**	**3,066**	**23.18**
Davao del Sur	**14,564**	76	**0.52**	1,132	**7.77**	2,264	**15.55**	**3,472**	**23.84**
Davao Occidental	**6,510**	44	**0.68**	786	**12.07**	689	**10.58**	**1,519**	**23.33**
Davao City	**37,597**	147	**0.39**	2,847	**7.57**	11,380	**30.27**	**14,374**	**38.23**
**Region 12**	**98,859**	**172**	**0.17**	**7,032**	**7.11**	**23,322**	**23.59**	**30,526**	**30.88**
North Cotabato	**34,150**	31	**0.09**	1,943	**5.69**	7,729	**22.63**	**9,703**	**28.41**
Sarangani	**12,907**	42	**0.33**	1,351	**10.47**	3,374	**26.14**	**4,767**	**36.93**
South Cotabato	**20,917**	42	**0.20**	1,644	**7.86**	5,435	**25.98**	**7,121**	**34.04**

Table 2.B.1.3. Prenatal Care

Number and Proportion of Pregnant Women for the 1st time given at least two (2) doses of Tetanus Diphtheria (Td) vaccination
Philippines, 2022

Area	Eligible Pop.	Women pregnant for the 1st time given at least 2 doses of Td Vaccine							
		Age Group						Total	%
		10-14 yrs old		15-19 yrs old		20-49 yrs old			
		No.	%	No.	%	No.	%		
Sultan Kudarat	**17,299**	44	**0.25**	1,255	**7.25**	4,186	**24.20**	**5,485**	**31.71**
Gen. Santos City	**13,586**	13	**0.10**	839	**6.18**	2,598	**19.12**	**3,450**	**25.39**
**BARMM**	**101,343**	**51**	**0.05**	**4,271**	**4.21**	**23,356**	**23.05**	**27,678**	**27.31**
Basilan	**7,823**	5	**0.06**	220	**2.81**	1,062	**13.58**	**1,287**	**16.45**
Lanao del Sur	**21,639**	5	**0.02**	652	**3.01**	7,545	**34.87**	**8,202**	**37.90**
Maguindanao	**32,198**	12	**0.04**	1,452	**4.51**	4,372	**13.58**	**5,836**	**18.13**
Sulu	**17,165**	20	**0.12**	561	**3.27**	3,389	**19.74**	**3,970**	**23.13**
Tawi-Tawi	**9,369**	5	**0.05**	342	**3.65**	2,058	**21.97**	**2,405**	**25.67**
Lamitan City	**2,154**	3	**0.14**	127	**5.90**	258	**11.98**	**388**	**18.01**
Marawi City	**5,173**	0	**0.00**	728	**14.07**	3,003	**58.05**	**3,731**	**72.12**
Cotabato City	**5,822**	1	**0.02**	189	**3.25**	1,669	**28.67**	**1,859**	**31.93**
**CARAGA**	**60,575**	**53**	**0.09**	**2,595**	**4.28**	**8,573**	**14.15**	**11,221**	**18.52**
Agusan del Norte	**8,127**	11	**0.14**	303	**3.73**	1,153	**14.19**	**1,467**	**18.05**
Agusan del Sur	**17,709**	12	**0.07**	655	**3.70**	2,024	**11.43**	**2,691**	**15.20**
Surigao del Norte	**7,280**	6	**0.08**	298	**4.09**	1,301	**17.87**	**1,605**	**22.05**
Surigao del Sur	**11,553**	7	**0.06**	528	**4.57**	1,753	**15.17**	**2,288**	**19.80**
Province of Dinagat	**2,576**	0	**0.00**	117	**4.54**	395	**15.33**	**512**	**19.88**
Bislig City	**2,192**	1	**0.05**	130	**5.93**	288	**13.14**	**419**	**19.11**
Butuan City	**7,743**	11	**0.14**	381	**4.92**	1,074	**13.87**	**1,466**	**18.93**
Surigao City	**3,395**	5	**0.15**	183	**5.39**	585	**17.23**	**773**	**22.77**

Note: Put asterisk (*) for No Report and Zero (0) for No Case

Table 2.B.1.4 - Prenatal Care

Number and Proportion of Pregnant Women for the 2nd or more times given at least three (3) doses of Td vaccination (Td2 Plus)
Philippines, 2022

Area	Eligible Pop.	Number and Proportion of Pregnant Women for the 2nd or more times given at least three doses of Td2 Plus							
		Age Group						Total	%
		10-14 yrs old		15-19 yrs old		20-49 yrs old			
		No.	%	No.	%	No.	%		
**PHILIPPINES**	**2,131,496**	**1,071**	**0.05**	**57,926**	**2.72**	**739,363**	**34.69**	**798,360**	**37.46**
**N C R**	**236,901**	**50**	**0.02**	**4,446**	**1.88**	**96,201**	**40.61**	**100,697**	**42.51**
Malabon	**6,724**	3	**0.04**	112	**1.67**	1,797	**26.73**	**1,912**	**28.44**
Navotas	**4,591**	2	**0.04**	205	**4.47**	1,740	**37.90**	**1,947**	**42.41**
Valenzuela City	**11,418**	2	**0.02**	198	**1.73**	2,645	**23.17**	**2,845**	**24.92**
Caloocan City	**29,146**	3	**0.01**	500	**1.72**	10,051	**34.49**	**10,554**	**36.21**
Marikina City	**8,294**	0	**0.00**	86	**1.04**	2,053	**24.75**	**2,139**	**25.79**
Pasig City	**13,894**	6	**0.04**	258	**1.86**	4,643	**33.42**	**4,907**	**35.32**
Pateros	**1,176**	2	**0.17**	17	**1.45**	263	**22.36**	**282**	**23.98**
Taguig	**14,807**	9	**0.06**	651	**4.40**	7,047	**47.59**	**7,707**	**52.05**
Quezon City	**54,011**	2	**0.00**	410	**0.76**	35,663	**66.03**	**36,075**	**66.79**
Makati City	**10,718**	4	**0.04**	142	**1.32**	2,372	**22.13**	**2,518**	**23.49**
Mandaluyong City	**7,111**	3	**0.04**	186	**2.62**	6,598	**92.79**	**6,787**	**95.44**
San Juan	**2,246**	0	**0.00**	6	**0.27**	292	**13.00**	**298**	**13.27**
Manila City	**32,743**	4	**0.01**	787	**2.40**	8,593	**26.24**	**9,384**	**28.66**
Las Piñas City	**10,832**	4	**0.04**	266	**2.46**	3,458	**31.92**	**3,728**	**34.42**
Muntinlupa City	**9,281**	0	**0.00**	155	**1.67**	3,340	**35.99**	**3,495**	**37.66**
Parañaque City	**12,249**	6	**0.05**	270	**2.20**	3,286	**26.83**	**3,562**	**29.08**
Pasay City	**7,660**	0	**0.00**	197	**2.57**	2,360	**30.81**	**2,557**	**33.38**
**C A R**	**35,179**	**8**	**0.02**	**777**	**2.21**	**10,094**	**28.69**	**10,879**	**30.92**
Abra	**4,275**	3	**0.07**	169	**3.95**	1,297	**30.34**	**1,469**	**34.36**
Apayao	**2,502**	1	**0.04**	77	**3.08**	842	**33.65**	**920**	**36.77**
Benguet	**9,086**	0	**0.00**	97	**1.07**	2,343	**25.79**	**2,440**	**26.85**
Ifugao	**4,486**	3	**0.07**	126	**2.81**	1,361	**30.34**	**1,490**	**33.21**
Kalinga	**4,701**	0	**0.00**	188	**4.00**	2,141	**45.54**	**2,329**	**49.54**
Mt. Province	**3,103**	1	**0.03**	54	**1.74**	974	**31.39**	**1,029**	**33.16**
Baguio City	**7,026**	0	**0.00**	66	**0.94**	1,136	**16.17**	**1,202**	**17.11**
**Region 1**	**97,099**	**24**	**0.02**	**2,393**	**2.46**	**39,187**	**40.36**	**41,604**	**42.85**
Ilocos Norte	**7,854**	2	**0.03**	122	**1.55**	2,142	**27.27**	**2,266**	**28.85**
Ilocos Sur	**9,072**	1	**0.01**	112	**1.23**	4,186	**46.14**	**4,299**	**47.39**
La Union	**11,338**	3	**0.03**	277	**2.44**	4,063	**35.84**	**4,343**	**38.30**
Pangasinan	**50,710**	14	**0.03**	1,376	**2.71**	21,572	**42.54**	**22,962**	**45.28**
Alaminos City	**1,910**	0	**0.00**	51	**2.67**	796	**41.68**	**847**	**44.35**
Candon City	**954**	0	**0.00**	1	**0.10**	360	**37.74**	**361**	**37.84**
Dagupan City	**3,657**	1	**0.03**	116	**3.17**	1,231	**33.66**	**1,348**	**36.86**
Laoag City	**1,812**	0	**0.00**	35	**1.93**	940	**51.88**	**975**	**53.81**
San Carlos City	**4,028**	0	**0.00**	33	**0.82**	1,504	**37.34**	**1,537**	**38.16**
San Fernando City	**2,074**	2	**0.10**	134	**6.46**	1,022	**49.28**	**1,158**	**55.83**
Urdaneta City	**2,836**	0	**0.00**	105	**3.70**	1,064	**37.52**	**1,169**	**41.22**
Vigan City	**854**	1	**0.12**	31	**3.63**	307	**35.95**	**339**	**39.70**
**Region 2**	**68,960**	**26**	**0.04**	**1,991**	**2.89**	**25,081**	**36.37**	**27,098**	**39.30**
Batanes	**362**	1	**0.28**	1	**0.28**	46	**12.71**	**48**	**13.26**
Cagayan	**20,431**	2	**0.01**	334	**1.63**	6,119	**29.95**	**6,455**	**31.59**
Isabela	**23,449**	17	**0.07**	851	**3.63**	9,389	**40.04**	**10,257**	**43.74**
Nueva Vizcaya	**9,538**	2	**0.02**	223	**2.34**	3,276	**34.35**	**3,501**	**36.71**
Quirino	**4,051**	1	**0.02**	93	**2.30**	1,049	**25.89**	**1,143**	**28.22**

Table 2.B.1.4 - Prenatal Care

Number and Proportion of Pregnant Women for the 2nd or more times given at least three (3) doses of Td vaccination (Td2 Plus)
Philippines, 2022

Area	Eligible Pop.	Number and Proportion of Pregnant Women for the 2nd or more times given at least three doses of Td2 Plus							
		Age Group						Total	%
		10-14 yrs old		15-19 yrs old		20-49 yrs old			
		No.	%	No.	%	No.	%		
Cauayan City	**2,565**	1	**0.04**	103	**4.02**	836	**32.59**	**940**	**36.65**
Ilagan City	**2,889**	1	**0.03**	106	**3.67**	1,274	**44.10**	**1,381**	**47.80**
Santiago City	**2,671**	1	**0.04**	254	**9.51**	2,416	**90.45**	**2,671**	**100.00**
Tuguegarao City	**3,004**	0	**0.00**	26	**0.87**	676	**22.50**	**702**	**23.37**
**Region 3**	**220,155**	**287**	**0.13**	**7,111**	**3.23**	**80,033**	**36.35**	**87,431**	**39.71**
Aurora	**4,754**	0	**0.00**	71	**1.49**	1,659	**34.90**	**1,730**	**36.39**
Bataan	**13,789**	9	**0.07**	338	**2.45**	4,393	**31.86**	**4,740**	**34.38**
Bulacan	**43,760**	169	**0.39**	1,434	**3.28**	16,067	**36.72**	**17,670**	**40.38**
Nueva Ecija	**29,039**	15	**0.05**	738	**2.54**	8,482	**29.21**	**9,235**	**31.80**
Pampanga	**31,620**	16	**0.05**	956	**3.02**	11,015	**34.84**	**11,987**	**37.91**
Tarlac	**19,319**	6	**0.03**	802	**4.15**	8,038	**41.61**	**8,846**	**45.79**
Zambales	**12,476**	28	**0.22**	191	**1.53**	3,093	**24.79**	**3,312**	**26.55**
Angeles City	**7,932**	0	**0.00**	221	**2.79**	2,572	**32.43**	**2,793**	**35.21**
Balanga City	**1,992**	0	**0.00**	35	**1.76**	564	**28.31**	**599**	**30.07**
Cabanatuan City	**5,945**	9	**0.15**	108	**1.82**	1,903	**32.01**	**2,020**	**33.98**
City of San Fernando	**5,907**	2	**0.03**	145	**2.45**	1,358	**22.99**	**1,505**	**25.48**
Gapan City	**2,170**	0	**0.00**	35	**1.61**	455	**20.97**	**490**	**22.58**
Mabalacat City	**4,831**	2	**0.04**	401	**8.30**	2,687	**55.62**	**3,090**	**63.96**
Malolos City	**4,890**	11	**0.22**	158	**3.23**	1,587	**32.45**	**1,756**	**35.91**
Meycauayan	**4,055**	6	**0.15**	209	**5.15**	1,758	**43.35**	**1,973**	**48.66**
Olongapo	**4,922**	1	**0.02**	47	**0.95**	1,159	**23.55**	**1,207**	**24.52**
Palayan City	**808**	0	**0.00**	1	**0.12**	120	**14.85**	**121**	**14.98**
San Jose City	**2,748**	4	**0.15**	153	**5.57**	1,009	**36.72**	**1,166**	**42.43**
San Jose del Monte City	**11,133**	5	**0.04**	546	**4.90**	8,366	**75.15**	**8,917**	**80.10**
Science City of Munoz	**1,601**	1	**0.06**	82	**5.12**	605	**37.79**	**688**	**42.97**
Tarlac City	**6,464**	3	**0.05**	440	**6.81**	3,143	**48.62**	**3,586**	**55.48**
**Region 4A**	**299,627**	**87**	**0.03**	**7,333**	**2.45**	**87,542**	**29.22**	**94,962**	**31.69**
Batangas	**39,255**	5	**0.01**	825	**2.10**	13,702	**34.91**	**14,532**	**37.02**
Cavite	**28,151**	12	**0.04**	837	**2.97**	9,524	**33.83**	**10,373**	**36.85**
Laguna	**19,696**	9	**0.05**	629	**3.19**	5,936	**30.14**	**6,574**	**33.38**
Quezon	**37,853**	12	**0.03**	916	**2.42**	11,828	**31.25**	**12,756**	**33.70**
Rizal	**44,798**	12	**0.03**	1,088	**2.43**	9,139	**20.40**	**10,239**	**22.86**
Antipolo City	**16,498**	3	**0.02**	570	**3.45**	4,837	**29.32**	**5,410**	**32.79**
Bacoor City	**12,341**	1	**0.01**	128	**1.04**	994	**8.05**	**1,123**	**9.10**
Batangas City	**6,965**	0	**0.00**	61	**0.88**	1,670	**23.98**	**1,731**	**24.85**
Biñan City	**6,597**	0	**0.00**	583	**8.84**	4,508	**68.33**	**5,091**	**77.17**
Cabuyao City	**6,119**	5	**0.08**	228	**3.73**	3,097	**50.61**	**3,330**	**54.42**
Calamba City	**9,005**	0	**0.00**	95	**1.05**	3,424	**38.02**	**3,519**	**39.08**
Cavite City	**2,105**	0	**0.00**	84	**3.99**	513	**24.37**	**597**	**28.36**
Dasmariñas City	**13,538**	8	**0.06**	189	**1.40**	3,060	**22.60**	**3,257**	**24.06**
General Trias City	**6,457**	3	**0.05**	129	**2.00**	1,253	**19.41**	**1,385**	**21.45**
Imus City	**8,292**	0	**0.00**	57	**0.69**	997	**12.02**	**1,054**	**12.71**
Lipa City	**7,018**	5	**0.07**	147	**2.09**	2,869	**40.88**	**3,021**	**43.05**
Lucena City	**5,738**	1	**0.02**	137	**2.39**	1,522	**26.52**	**1,660**	**28.93**
San Pablo City	**5,272**	7	**0.13**	211	**4.00**	1,841	**34.92**	**2,059**	**39.06**
San Pedro City	**6,455**	1	**0.02**	83	**1.29**	786	**12.18**	**870**	**13.48**
Santa Rosa City	**7,009**	2	**0.03**	197	**2.81**	2,441	**34.83**	**2,640**	**37.67**
Tagaytay City	**1,460**	0	**0.00**	46	**3.15**	881	**60.34**	**927**	**63.49**
Tanauan City	**3,661**	1	**0.03**	29	**0.79**	1,420	**38.79**	**1,450**	**39.61**
Tayabas City	**2,145**	0	**0.00**	48	**2.24**	861	**40.14**	**909**	**42.38**
Trece Martires City	**3,199**	0	**0.00**	16	**0.50**	439	**13.72**	**455**	**14.22**

Table 2.B.1.4 - Prenatal Care

Number and Proportion of Pregnant Women for the 2nd or more times given at least three (3) doses of Td vaccination (Td2 Plus)
Philippines, 2022

Area	Eligible Pop.	Number and Proportion of Pregnant Women for the 2nd or more times given at least three doses of Td2 Plus							
		Age Group						Total	%
		10-14 yrs old		15-19 yrs old		20-49 yrs old			
		No.	%	No.	%	No.	%		
**Region 4B**	**72,791**	**30**	**0.04**	**1,748**	**2.40**	**22,962**	**31.55**	**24,740**	**33.99**
									**0.00**
Marinduque	**4,990**	0	**0.00**	48	**0.96**	1,382	**27.70**	**1,430**	**28.66**
Mindoro Occidental	**12,479**	0	**0.00**	307	**2.46**	4,252	**34.07**	**4,559**	**36.53**
Mindoro Oriental	**20,189**	10	**0.05**	610	**3.02**	7,606	**37.67**	**8,226**	**40.74**
Palawan	**21,963**	7	**0.03**	576	**2.62**	5,924	**26.97**	**6,507**	**29.63**
Romblon	**6,569**	11	**0.17**	117	**1.78**	2,693	**41.00**	**2,821**	**42.94**
Puerto Princesa City	**6,601**	2	**0.03**	90	**1.36**	1,105	**16.74**	**1,197**	**18.13**
**Region 5**	**138,457**	**15**	**0.01**	**1,918**	**1.39**	**34,456**	**24.89**	**36,389**	**26.28**
Albay	**24,081**	0	**0.00**	194	**0.81**	6,619	**27.49**	**6,813**	**28.29**
Camarines Norte	**14,384**	2	**0.01**	178	**1.24**	2,737	**19.03**	**2,917**	**20.28**
Camarines Sur	**39,486**	1	**0.00**	294	**0.74**	7,625	**19.31**	**7,920**	**20.06**
Catanduanes	**6,459**	1	**0.02**	79	**1.22**	1,142	**17.68**	**1,222**	**18.92**
Masbate	**22,600**	5	**0.02**	739	**3.27**	7,790	**34.47**	**8,534**	**37.76**
Sorsogon	**19,828**	4	**0.02**	291	**1.47**	5,557	**28.03**	**5,852**	**29.51**
Iriga City	**2,679**	0	**0.00**	19	**0.71**	540	**20.16**	**559**	**20.87**
Legaspi City	**4,233**	2	**0.05**	36	**0.85**	1,453	**34.33**	**1,491**	**35.22**
Naga City	**4,707**	0	**0.00**	88	**1.87**	993	**21.10**	**1,081**	**22.97**
**Region 6**	**146,449**	**64**	**0.04**	**3,973**	**2.71**	**50,769**	**34.67**	**54,806**	**37.42**
Aklan	**11,288**	2	**0.02**	190	**1.68**	3,730	**33.04**	**3,922**	**34.74**
Antique	**13,132**	5	**0.04**	323	**2.46**	4,244	**32.32**	**4,572**	**34.82**
Capiz	**13,975**	10	**0.07**	213	**1.52**	3,820	**27.33**	**4,043**	**28.93**
Guimaras	**3,084**	3	**0.10**	121	**3.92**	1,566	**50.78**	**1,690**	**54.80**
Iloilo	**36,267**	16	**0.04**	884	**2.44**	12,509	**34.49**	**13,409**	**36.97**
Negros Occidental	**49,230**	24	**0.05**	1,943	**3.95**	20,172	**40.98**	**22,139**	**44.97**
Bacolod City	**11,082**	4	**0.04**	193	**1.74**	2,598	**23.44**	**2,795**	**25.22**
Iloilo City	**8,391**	0	**0.00**	106	**1.26**	2,130	**25.38**	**2,236**	**26.65**
**Region 7**	**162,308**	**96**	**0.06**	**6,078**	**3.74**	**68,986**	**42.50**	**75,160**	**46.31**
Bohol	**27,992**	12	**0.04**	870	**3.11**	10,796	**38.57**	**11,678**	**41.72**
Cebu	**66,463**	30	**0.05**	1,897	**2.85**	21,550	**32.42**	**23,477**	**35.32**
Negros Oriental	**27,890**	23	**0.08**	945	**3.39**	9,162	**32.85**	**10,130**	**36.32**
Siquijor	**1,661**	1	**0.06**	66	**3.97**	753	**45.33**	**820**	**49.37**
Cebu City	**20,866**	19	**0.09**	1,150	**5.51**	12,700	**60.86**	**13,869**	**66.47**
Lapu-Lapu City	**9,232**	3	**0.03**	350	**3.79**	5,806	**62.89**	**6,159**	**66.71**
Mandaue City	**8,204**	8	**0.10**	800	**9.75**	8,219	**100.18**	**9,027**	**110.03**
**Region 8**	**105,471**	**16**	**0.02**	**1,613**	**1.53**	**28,453**	**26.98**	**30,082**	**28.52**
Biliran	**3,873**	2	**0.05**	112	**2.89**	1,770	**45.70**	**1,884**	**48.64**
Eastern Samar	**11,908**	1	**0.01**	162	**1.36**	3,117	**26.18**	**3,280**	**27.54**
Northern Leyte	**35,422**	0	**0.00**	660	**1.86**	10,125	**28.58**	**10,785**	**30.45**
Northern Samar	**15,921**	1	**0.01**	160	**1.00**	3,158	**19.84**	**3,319**	**20.85**
Southern Leyte	**6,662**	1	**0.02**	62	**0.93**	1,732	**26.00**	**1,795**	**26.94**
Western Samar	**14,732**	4	**0.03**	179	**1.22**	2,856	**19.39**	**3,039**	**20.63**
Calbayog City	**4,535**	6	**0.13**	141	**3.11**	1,079	**23.79**	**1,226**	**27.03**
Maasin City	**1,696**	0	**0.00**	4	**0.24**	245	**14.45**	**249**	**14.68**
Ormoc City	**5,044**	1	**0.02**	69	**1.37**	2,078	**41.20**	**2,148**	**42.59**

Table 2.B.1.4 - Prenatal Care

Number and Proportion of Pregnant Women for the 2nd or more times given at least three (3) doses of Td vaccination (Td2 Plus)
Philippines, 2022

Area	Eligible Pop.	Number and Proportion of Pregnant Women for the 2nd or more times given at least three doses of Td2 Plus							
		Age Group						Total	%
		10-14 yrs old		15-19 yrs old		20-49 yrs old			
		No.	%	No.	%	No.	%		
Tacloban City	5,678	0	0.00	64	1.13	2,293	40.38	2,357	41.51
**Region 9**	79,007	26	0.03	2,999	3.80	29,933	37.89	32,958	41.72
Zamboanga del Norte	16,854	2	0.01	426	2.53	5,692	33.77	6,120	36.31
Zamboanga del Sur	17,368	1	0.01	341	1.96	4,653	26.79	4,995	28.76
Zamboanga Sibugay	15,021	7	0.05	566	3.77	5,246	34.92	5,819	38.74
Dapitan City	1,737	0	0.00	49	2.82	473	27.23	522	30.05
Dipolog City	2,762	5	0.18	201	7.28	1,218	44.10	1,424	51.56
Isabela City	2,563	0	0.00	50	1.95	607	23.68	657	25.63
Pagadian City	4,257	0	0.00	138	3.24	1,682	39.51	1,820	42.75
Zamboanga City	18,445	11	0.06	1,228	6.66	10,362	56.18	11,601	62.90
**Region 10**	99,908	51	0.05	4,570	4.57	38,971	39.01	43,592	43.63
Bukidnon	22,900	21	0.09	1,396	6.10	8,556	37.36	9,973	43.55
Camiguin	1,854	0	0.00	25	1.35	524	28.26	549	29.61
Lanao del Norte	14,930	5	0.03	506	3.39	5,376	36.01	5,887	39.43
Misamis Occidental	6,420	1	0.02	103	1.60	1,939	30.20	2,043	31.82
Misamis Oriental	14,963	9	0.06	551	3.68	5,702	38.11	6,262	41.85
Cagayan de Oro City	14,172	7	0.05	886	6.25	6,264	44.20	7,157	50.50
El Salvador City	1,052	0	0.00	31	2.95	457	43.44	488	46.39
Gingoog City	2,608	2	0.08	167	6.40	1,238	47.47	1,407	53.95
Iligan City	7,565	3	0.04	282	3.73	3,105	41.04	3,390	44.81
Malaybalay City	3,817	0	0.00	111	2.91	1,472	38.56	1,583	41.47
Oroquieta City	1,389	0	0.00	24	1.73	497	35.78	521	37.51
Ozamis City	2,786	0	0.00	38	1.36	1,216	43.65	1,254	45.01
Tangub City	1,234	0	0.00	128	10.37	640	51.86	768	62.24
Valencia City	4,218	3	0.07	322	7.63	1,985	47.06	2,310	54.77
**Region 11**	108,407	136	0.13	2,956	2.73	41,428	38.22	44,520	41.07
Davao de Oro	15,490	45	0.29	391	2.52	6,047	39.04	6,483	41.85
Davao del Norte	21,017	31	0.15	628	2.99	9,079	43.20	9,738	46.33
Davao Oriental	13,229	2	0.02	315	2.38	4,973	37.59	5,290	39.99
Davao del Sur	14,564	17	0.12	433	2.97	5,337	36.65	5,787	39.73
Davao Occidental	6,510	5	0.08	222	3.41	2,355	36.18	2,582	39.66
Davao City	37,597	36	0.10	967	2.57	13,637	36.27	14,640	38.94
**Region 12**	98,859	108	0.11	4,842	4.90	36,855	37.28	41,805	42.29
North Cotabato	34,150	47	0.14	1,298	3.80	10,291	30.13	11,636	34.07
Sarangani	12,907	11	0.09	554	4.29	4,548	35.24	5,113	39.61
South Cotabato	20,917	17	0.08	1,356	6.48	9,433	45.10	10,806	51.66
Sultan Kudarat	17,299	29	0.17	918	5.31	7,584	43.84	8,531	49.31
Gen. Santos City	13,586	4	0.03	716	5.27	4,999	36.80	5,719	42.09
**BARMM**	101,343	22	0.02	1,909	1.88	29,392	29.00	31,323	30.91
Basilan	7,823	7	0.09	157	2.01	1,236	15.80	1,400	17.90
Lanao del Sur	21,639	2	0.01	230	1.06	8,614	39.81	8,846	40.88
Maguindanao	32,198	1	0.00	561	1.74	9,332	28.98	9,894	30.73
Sulu	17,165	9	0.05	381	2.22	4,864	28.34	5,254	30.61
Tawi-Tawi	9,369	0	0.00	169	1.80	2,940	31.38	3,109	33.18

Table 2.B.1.4 - Prenatal Care

Number and Proportion of Pregnant Women for the 2nd or more times given at least three (3) doses of Td vaccination (Td2 Plus)
Philippines, 2022

Area	Eligible Pop.	Number and Proportion of Pregnant Women for the 2nd or more times given at least three doses of Td2 Plus							
		Age Group						Total	%
		10-14 yrs old		15-19 yrs old		20-49 yrs old			
		No.	%	No.	%	No.	%		
Lamitan City	**2,154**	3	**0.14**	61	**2.83**	385	**17.87**	**449**	**20.84**
Marawi City	**5,173**	0	**0.00**	254	**4.91**	879	**16.99**	**1,133**	**21.90**
Cotabato City	**5,822**	0	**0.00**	96	**1.65**	1,142	**19.62**	**1,238**	**21.26**
**CARAGA**	**60,575**	**25**	**0.04**	**1,269**	**2.09**	**19,020**	**31.40**	**20,314**	**33.54**
Agusan del Norte	**8,127**	5	**0.06**	111	**1.37**	1,862	**22.91**	**1,978**	**24.34**
Agusan del Sur	**17,709**	15	**0.08**	374	**2.11**	5,491	**31.01**	**5,880**	**33.20**
Surigao del Norte	**7,280**	1	**0.01**	102	**1.40**	2,485	**34.13**	**2,588**	**35.55**
Surigao del Sur	**11,553**	4	**0.03**	333	**2.88**	3,481	**30.13**	**3,818**	**33.05**
Province of Dinagat	**2,576**	0	**0.00**	22	**0.85**	679	**26.36**	**701**	**27.21**
Bislig City	**2,192**	0	**0.00**	84	**3.83**	871	**39.74**	**955**	**43.57**
Butuan City	**7,743**	0	**0.00**	170	**2.20**	2,902	**37.48**	**3,072**	**39.67**
Surigao City	**3,395**	0	**0.00**	73	**2.15**	1,249	**36.79**	**1,322**	**38.94**

Table 2.B.1.5. Prenatal Care

No. of Pregnant women who Completed Iron with Folic Acid
Philippines, 2022

Area	Eligible Pop.	Iron with Folic Acid Supplementation							
		Age Group						Total	%
		10-14 yrs old		15-19 yrs old		20-49 yrs old			
		No.	%	No.	%	No.	%		
**PHILIPPINES**	**2,131,496**	**2,441**	**0.11**	**122,623**	**5.75**	**974,367**	**45.71**	**1,099,431**	**51.58**
**N C R**	**236,901**	**255**	**0.11**	**13,220**	**5.58**	**125,872**	**53.13**	**139,347**	**58.82**
Malabon	**6,724**	23	**0.34**	778	**11.57**	4,853	**72.17**	**5,654**	**84.09**
Navotas	**4,591**	11	**0.24**	517	**11.26**	2,681	**58.40**	**3,209**	**69.90**
Valenzuela City	**11,418**	6	**0.05**	448	**3.92**	5,066	**44.37**	**5,520**	**48.34**
Caloocan City	**29,146**	16	**0.05**	1,953	**6.70**	18,914	**64.89**	**20,883**	**71.65**
Marikina City	**8,294**	6	**0.07**	292	**3.52**	3,399	**40.98**	**3,697**	**44.57**
Pasig City	**13,894**	14	**0.10**	595	**4.28**	6,206	**44.67**	**6,815**	**49.05**
Pateros	**1,176**	4	**0.34**	110	**9.35**	768	**65.31**	**882**	**75.00**
Taguig	**14,807**	38	**0.26**	1,404	**9.48**	13,035	**88.03**	**14,477**	**97.77**
Quezon City	**54,011**	48	**0.09**	1,742	**3.23**	24,305	**45.00**	**26,095**	**48.31**
Makati City	**10,718**	2	**0.02**	180	**1.68**	3,134	**29.24**	**3,316**	**30.94**
Mandaluyong City	**7,111**	2	**0.03**	327	**4.60**	4,516	**63.51**	**4,845**	**68.13**
San Juan	**2,246**	0	**0.00**	40	**1.78**	716	**31.88**	**756**	**33.66**
Manila City	**32,743**	46	**0.14**	2,425	**7.41**	15,371	**46.94**	**17,842**	**54.49**
Las Piñas City	**10,832**	9	**0.08**	516	**4.76**	4,373	**40.37**	**4,898**	**45.22**
Muntinlupa City	**9,281**	9	**0.10**	763	**8.22**	8,173	**88.06**	**8,945**	**96.38**
Parañaque City	**12,249**	21	**0.17**	741	**6.05**	6,308	**51.50**	**7,070**	**57.72**
Pasay City	**7,660**	0	**0.00**	389	**5.08**	4,054	**52.92**	**4,443**	**58.00**
**C A R**	**35,179**	**20**	**0.06**	**1,438**	**4.09**	**16,614**	**47.23**	**18,072**	**51.37**
Abra	**4,275**	5	**0.12**	253	**5.92**	1,762	**41.22**	**2,020**	**47.25**
Apayao	**2,502**	3	**0.12**	136	**5.44**	1,052	**42.05**	**1,191**	**47.60**
Benguet	**9,086**	1	**0.01**	376	**4.14**	5,549	**61.07**	**5,926**	**65.22**
Ifugao	**4,486**	7	**0.16**	200	**4.46**	2,347	**52.32**	**2,554**	**56.93**
Kalinga	**4,701**	1	**0.02**	188	**4.00**	2,196	**46.71**	**2,385**	**50.73**
Mt. Province	**3,103**	2	**0.06**	108	**3.48**	1,536	**49.50**	**1,646**	**53.05**
Baguio City	**7,026**	1	**0.01**	177	**2.52**	2,172	**30.91**	**2,350**	**33.45**
**Region 1**	**97,099**	**50**	**0.05**	**5,060**	**5.21**	**53,958**	**55.57**	**59,068**	**60.83**
Ilocos Norte	**7,854**	4	**0.05**	260	**3.31**	3,707	**47.20**	**3,971**	**50.56**
Ilocos Sur	**9,072**	14	**0.15**	415	**4.57**	6,116	**67.42**	**6,545**	**72.15**
La Union	**11,338**	4	**0.04**	600	**5.29**	6,318	**55.72**	**6,922**	**61.05**
Pangasinan	**50,710**	17	**0.03**	2,757	**5.44**	27,847	**54.91**	**30,621**	**60.38**
Alaminos City	**1,910**	0	**0.00**	120	**6.28**	1,162	**60.84**	**1,282**	**67.12**
Candon City	**954**	0	**0.00**	2	**0.21**	838	**87.84**	**840**	**88.05**
Dagupan City	**3,657**	6	**0.16**	312	**8.53**	1,741	**47.61**	**2,059**	**56.30**
Laoag City	**1,812**	0	**0.00**	21	**1.16**	1,430	**78.92**	**1,451**	**80.08**
San Carlos City	**4,028**	1	**0.02**	140	**3.48**	1,648	**40.91**	**1,789**	**44.41**
San Fernando City	**2,074**	1	**0.05**	180	**8.68**	1,103	**53.18**	**1,284**	**61.91**
Urdaneta City	**2,836**	2	**0.07**	207	**7.30**	1,579	**55.68**	**1,788**	**63.05**
Vigan City	**854**	1	**0.12**	46	**5.39**	469	**54.92**	**516**	**60.42**
**Region 2**	**68,960**	**70**	**0.10**	**4,275**	**6.20**	**33,779**	**48.98**	**38,124**	**55.28**
Batanes	**362**	0	**0.00**	6	**1.66**	135	**37.29**	**141**	**38.95**
Cagayan	**20,431**	14	**0.07**	908	**4.44**	7,308	**35.77**	**8,230**	**40.28**
Isabela	**23,449**	39	**0.17**	1,639	**6.99**	12,268	**52.32**	**13,946**	**59.47**
Nueva Vizcaya	**9,538**	6	**0.06**	725	**7.60**	5,293	**55.49**	**6,024**	**63.16**
Quirino	**4,051**	5	**0.12**	277	**6.84**	2,593	**64.01**	**2,875**	**70.97**
Cauayan City	**2,565**	2	**0.08**	220	**8.58**	1,372	**53.49**	**1,594**	**62.14**
Ilagan City	**2,889**	0	**0.00**	175	**6.06**	1,501	**51.96**	**1,676**	**58.01**
Santiago City	**2,671**	3	**0.11**	264	**9.88**	2,404	**90.00**	**2,671**	**100.00**

Table 2.B.1.5. Prenatal Care
No. of Pregnant women who Completed Iron with Folic Acid
Philippines, 2022

Area	Eligible Pop.	Iron with Folic Acid Supplementation							
		Age Group						Total	%
		10-14 yrs old		15-19 yrs old		20-49 yrs old			
		No.	%	No.	%	No.	%		
Tuguegarao City	3,004	1	0.03	61	2.03	905	30.13	967	32.19
**Region 3**	220,155	282	0.13	14,893	6.76	109,266	49.63	124,441	56.52
Aurora	4,754	1	0.02	240	5.05	1,966	41.35	2,207	46.42
Bataan	13,789	16	0.12	927	6.72	6,497	47.12	7,440	53.96
Bulacan	43,760	72	0.16	3,165	7.23	22,647	51.75	25,884	59.15
Nueva Ecija	29,039	50	0.17	1,315	4.53	9,576	32.98	10,941	37.68
Pampanga	31,620	27	0.09	1,745	5.52	13,868	43.86	15,640	49.46
Tarlac	19,319	39	0.20	1,587	8.21	12,290	63.62	13,916	72.03
Zambales	12,476	11	0.09	417	3.34	3,142	25.18	3,570	28.61
Angeles City	7,932	4	0.05	402	5.07	2,403	30.30	2,809	35.41
Balanga City	1,992	4	0.20	132	6.63	994	49.90	1,130	56.73
Cabanatuan City	5,945	9	0.15	387	6.51	2,827	47.55	3,223	54.21
City of San Fernando	5,907	7	0.12	362	6.13	2,694	45.61	3,063	51.85
Gapan City	2,170	0	0.00	89	4.10	616	28.39	705	32.49
Mabalacat City	4,831	5	0.10	820	16.97	4,751	98.34	5,576	115.42
Malolos City	4,890	2	0.04	166	3.39	1,316	26.91	1,484	30.35
Meycauayan	4,055	1	0.02	363	8.95	2,825	69.67	3,189	78.64
Olongapo	4,922	2	0.04	239	4.86	2,020	41.04	2,261	45.94
Palayan City	808	1	0.12	57	7.05	259	32.05	317	39.23
San Jose City	2,748	6	0.22	273	9.93	1,553	56.51	1,832	66.67
San Jose del Monte City	11,133	18	0.16	1,170	10.51	11,351	101.96	12,539	112.63
Science City of Munoz	1,601	1	0.06	90	5.62	615	38.41	706	44.10
Tarlac City	6,464	6	0.09	947	14.65	5,056	78.22	6,009	92.96
**Region 4A**	299,627	200	0.07	13,497	4.50	112,049	37.40	125,746	41.97
Batangas	39,255	20	0.05	1,865	4.75	18,032	45.94	19,917	50.74
Cavite	28,151	8	0.03	788	2.80	7,627	27.09	8,423	29.92
Laguna	19,696	29	0.15	1,294	6.57	8,682	44.08	10,005	50.80
Quezon	37,853	36	0.10	1,586	4.19	12,028	31.78	13,650	36.06
Rizal	44,798	46	0.10	2,331	5.20	14,881	33.22	17,258	38.52
Antipolo City	16,498	8	0.05	898	5.44	7,282	44.14	8,188	49.63
Bacoor City	12,341	2	0.02	219	1.77	1,352	10.96	1,573	12.75
Batangas City	6,965	4	0.06	351	5.04	2,943	42.25	3,298	47.35
Biñan City	6,597	4	0.06	895	13.57	6,412	97.20	7,311	110.82
Cabuyao City	6,119	5	0.08	360	5.88	4,500	73.54	4,865	79.51
Calamba City	9,005	6	0.07	595	6.61	5,232	58.10	5,833	64.78
Cavite City	2,105	2	0.10	32	1.52	165	7.84	199	9.45
Dasmariñas City	13,538	4	0.03	333	2.46	3,351	24.75	3,688	27.24
General Trias City	6,457	0	0.00	104	1.61	750	11.62	854	13.23
Imus City	8,292	3	0.04	61	0.74	731	8.82	795	9.59
Lipa City	7,018	0	0.00	187	2.66	2,720	38.76	2,907	41.42
Lucena City	5,738	3	0.05	296	5.16	2,017	35.15	2,316	40.36
San Pablo City	5,272	8	0.15	312	5.92	2,203	41.79	2,523	47.86
San Pedro City	6,455	2	0.03	155	2.40	1,301	20.15	1,458	22.59
Santa Rosa City	7,009	5	0.07	519	7.40	4,915	70.12	5,439	77.60
Tagaytay City	1,460	0	0.00	64	4.38	1,232	84.38	1,296	88.77
Tanauan City	3,661	3	0.08	65	1.78	1,920	52.44	1,988	54.30
Tayabas City	2,145	1	0.05	128	5.97	1,221	56.92	1,350	62.94
Trece Martires City	3,199	1	0.03	59	1.84	552	17.26	612	19.13
**Region 4B**	72,791	82	0.11	4,097	5.63	29,065	39.93	33,244	45.67
Marinduque	4,990	6	0.12	189	3.79	2,279	45.67	2,474	49.58
Mindoro Occidental	12,479	10	0.08	873	7.00	5,610	44.96	6,493	52.03
Mindoro Oriental	20,189	8	0.04	820	4.06	7,992	39.59	8,820	43.69
Palawan	21,963	33	0.15	1,575	7.17	9,151	41.67	10,759	48.99
Romblon	6,569	13	0.20	222	3.38	2,314	35.23	2,549	38.80
Puerto Princesa City	6,601	12	0.18	418	6.33	1,719	26.04	2,149	32.56

Table 2.B.1.5. Prenatal Care

No. of Pregnant women who Completed Iron with Folic Acid
Philippines, 2022

Area	Eligible Pop.	Iron with Folic Acid Supplementation							
		Age Group						Total	%
		10-14 yrs old		15-19 yrs old		20-49 yrs old			
		No.	%	No.	%	No.	%		
**Region 5**	**138,457**	**40**	**0.03**	**5,172**	**3.74**	**49,807**	**35.97**	**55,019**	**39.74**
Albay	24,081	5	0.02	736	3.06	10,830	44.97	11,571	48.05
Camarines Norte	14,384	2	0.01	855	5.94	5,626	39.11	6,483	45.07
Camarines Sur	39,486	11	0.03	910	2.30	10,297	26.08	11,218	28.41
Catanduanes	6,459	0	0.00	247	3.82	2,249	34.82	2,496	38.64
Masbate	22,600	12	0.05	1,416	6.27	8,942	39.57	10,370	45.88
Sorsogon	19,828	7	0.04	706	3.56	7,937	40.03	8,650	43.63
Iriga City	2,679	1	0.04	65	2.43	718	26.80	784	29.26
Legaspi City	4,233	2	0.05	124	2.93	2,047	48.36	2,173	51.33
Naga City	4,707	0	0.00	113	2.40	1,161	24.67	1,274	27.07
**Region 6**	**146,449**	**134**	**0.09**	**6,726**	**4.59**	**58,479**	**39.93**	**65,339**	**44.62**
Aklan	11,288	5	0.04	463	4.10	5,137	45.51	5,605	49.65
Antique	13,132	5	0.04	334	2.54	4,108	31.28	4,447	33.86
Capiz	13,975	13	0.09	330	2.36	3,917	28.03	4,260	30.48
Guimaras	3,084	7	0.23	159	5.16	1,937	62.81	2,103	68.19
Iloilo	36,267	45	0.12	1,379	3.80	15,060	41.53	16,484	45.45
Negros Occidental	49,230	52	0.11	3,451	7.01	22,303	45.30	25,806	52.42
Bacolod City	11,082	4	0.04	217	1.96	1,853	16.72	2,074	18.72
Iloilo City	8,391	3	0.04	393	4.68	4,164	49.62	4,560	54.34
**Region 7**	**162,308**	**127**	**0.08**	**9,036**	**5.57**	**78,087**	**48.11**	**87,250**	**53.76**
Bohol	27,992	15	0.05	1,022	3.65	11,356	40.57	12,393	44.27
Cebu	66,463	47	0.07	3,757	5.65	28,258	42.52	32,062	48.24
Negros Oriental	27,890	21	0.08	1,637	5.87	11,435	41.00	13,093	46.95
Siquijor	1,661	2	0.12	65	3.91	738	44.43	805	48.46
Cebu City	20,866	24	0.12	1,476	7.07	12,704	60.88	14,204	68.07
Lapu-Lapu City	9,232	9	0.10	488	5.29	6,954	75.32	7,451	80.71
Mandaue City	8,204	9	0.11	591	7.20	6,642	80.96	7,242	88.27
**Region 8**	**105,471**	**70**	**0.07**	**4,246**	**4.03**	**37,072**	**35.15**	**41,388**	**39.24**
Biliran	3,873	8	0.21	234	6.04	1,880	48.54	2,122	54.79
Eastern Samar	11,908	6	0.05	546	4.59	4,686	39.35	5,238	43.99
Northern Leyte	35,422	26	0.07	1,448	4.09	13,119	37.04	14,593	41.20
Northern Samar	15,921	9	0.06	589	3.70	4,752	29.85	5,350	33.60
Southern Leyte	6,662	5	0.08	269	4.04	2,978	44.70	3,252	48.81
Western Samar	14,732	9	0.06	528	3.58	4,053	27.51	4,590	31.16
Calbayog City	4,535	3	0.07	102	2.25	591	13.03	696	15.35
Maasin City	1,696	0	0.00	46	2.71	697	41.10	743	43.81
Ormoc City	5,044	4	0.08	349	6.92	2,514	49.84	2,867	56.84
Tacloban City	5,678	0	0.00	135	2.38	1,802	31.74	1,937	34.11
**Region 9**	**79,007**	**86**	**0.11**	**5,356**	**6.78**	**34,737**	**43.97**	**40,179**	**50.85**
Zamboanga del Norte	16,854	18	0.11	1,117	6.63	7,261	43.08	8,396	49.82
Zamboanga del Sur	17,368	12	0.07	726	4.18	4,630	26.66	5,368	30.91
Zamboanga Sibugay	15,021	10	0.07	756	5.03	5,072	33.77	5,838	38.87
Dapitan City	1,737	3	0.17	182	10.48	1,375	79.16	1,560	89.81
Dipolog City	2,762	9	0.33	276	9.99	1,713	62.02	1,998	72.34
Isabela City	2,563	4	0.16	203	7.92	1,097	42.80	1,304	50.88
Pagadian City	4,257	0	0.00	223	5.24	1,435	33.71	1,658	38.95
Zamboanga City	18,445	30	0.16	1,873	10.15	12,154	65.89	14,057	76.21

Table 2.B.1.5. Prenatal Care

No. of Pregnant women who Completed Iron with Folic Acid

Philippines, 2022

Area	Eligible Pop.	Iron with Folic Acid Supplementation							
		Age Group						Total	%
		10-14 yrs old		15-19 yrs old		20-49 yrs old			
		No.	%	No.	%	No.	%		
**Region 10**	**99,908**	**176**	**0.18**	**9,657**	**9.67**	**56,506**	**56.56**	**66,339**	**66.40**
Bukidnon	**22,900**	66	**0.29**	3,078	**13.44**	13,287	**58.02**	**16,431**	**71.75**
Camiguin	**1,854**	1	**0.05**	84	**4.53**	751	**40.51**	**836**	**45.09**
Lanao del Norte	**14,930**	12	**0.08**	995	**6.66**	7,984	**53.48**	**8,991**	**60.22**
Misamis Occidental	**6,420**	11	**0.17**	251	**3.91**	2,950	**45.95**	**3,212**	**50.03**
Misamis Oriental	**14,963**	38	**0.25**	1,494	**9.98**	8,267	**55.25**	**9,799**	**65.49**
Cagayan de Oro City	**14,172**	29	**0.20**	1,447	**10.21**	8,526	**60.16**	**10,002**	**70.58**
El Salvador City	**1,052**	2	**0.19**	63	**5.99**	469	**44.58**	**534**	**50.76**
Gingoog City	**2,608**	2	**0.08**	376	**14.42**	1,938	**74.31**	**2,316**	**88.80**
Iligan City	**7,565**	5	**0.07**	682	**9.02**	4,718	**62.37**	**5,405**	**71.45**
Malaybalay City	**3,817**	3	**0.08**	364	**9.54**	2,321	**60.81**	**2,688**	**70.42**
Oroquieta City	**1,389**	0	**0.00**	47	**3.38**	527	**37.94**	**574**	**41.32**
Ozamis City	**2,786**	0	**0.00**	183	**6.57**	1,409	**50.57**	**1,592**	**57.14**
Tangub City	**1,234**	0	**0.00**	101	**8.18**	929	**75.28**	**1,030**	**83.47**
Valencia City	**4,218**	7	**0.17**	492	**11.66**	2,430	**57.61**	**2,929**	**69.44**
**Region 11**	**108,407**	**558**	**0.51**	**9,044**	**8.34**	**59,402**	**54.80**	**69,004**	**63.65**
Davao de Oro	**15,490**	137	**0.88**	1,812	**11.70**	9,074	**58.58**	**11,023**	**71.16**
Davao del Norte	**21,017**	129	**0.61**	2,031	**9.66**	12,643	**60.16**	**14,803**	**70.43**
Davao Oriental	**13,229**	70	**0.53**	1,142	**8.63**	6,433	**48.63**	**7,645**	**57.79**
Davao del Sur	**14,564**	112	**0.77**	997	**6.85**	5,455	**37.46**	**6,564**	**45.07**
Davao Occidental	**6,510**	18	**0.28**	326	**5.01**	971	**14.92**	**1,315**	**20.20**
Davao City	**37,597**	92	**0.24**	2,736	**7.28**	24,826	**66.03**	**27,654**	**73.55**
**Region 12**	**98,859**	**192**	**0.19**	**9,312**	**9.42**	**51,469**	**52.06**	**60,973**	**61.68**
North Cotabato	**34,150**	39	**0.11**	2,356	**6.90**	14,685	**43.00**	**17,080**	**50.01**
Sarangani	**12,907**	47	**0.36**	2,024	**15.68**	8,317	**64.44**	**10,388**	**80.48**
South Cotabato	**20,917**	44	**0.21**	2,255	**10.78**	12,723	**60.83**	**15,022**	**71.82**
Sultan Kudarat	**17,299**	54	**0.31**	1,647	**9.52**	9,977	**57.67**	**11,678**	**67.51**
Gen. Santos City	**13,586**	8	**0.06**	1,030	**7.58**	5,767	**42.45**	**6,805**	**50.09**
**BARMM**	**101,343**	**29**	**0.03**	**4,006**	**3.95**	**40,213**	**39.68**	**44,248**	**43.66**
Basilan	**7,823**	4	**0.05**	250	**3.20**	1,537	**19.65**	**1,791**	**22.89**
Lanao del Sur	**21,639**	4	**0.02**	544	**2.51**	11,195	**51.74**	**11,743**	**54.27**
Maguindanao	**32,198**	8	**0.02**	1,384	**4.30**	13,346	**41.45**	**14,738**	**45.77**
Sulu	**17,165**	10	**0.06**	557	**3.24**	5,557	**32.37**	**6,124**	**35.68**
Tawi-Tawi	**9,369**	0	**0.00**	220	**2.35**	2,943	**31.41**	**3,163**	**33.76**
Lamitan City	**2,154**	3	**0.14**	120	**5.57**	430	**19.96**	**553**	**25.67**
Marawi City	**5,173**	0	**0.00**	786	**15.19**	3,563	**68.88**	**4,349**	**84.07**
Cotabato City	**5,822**	0	**0.00**	145	**2.49**	1,642	**28.20**	**1,787**	**30.69**
**CARAGA**	**60,575**	**70**	**0.12**	**3,588**	**5.92**	**27,992**	**46.21**	**31,650**	**52.25**
Agusan del Norte	**8,127**	11	**0.14**	367	**4.52**	2,879	**35.43**	**3,257**	**40.08**
Agusan del Sur	**17,709**	25	**0.14**	1,324	**7.48**	9,507	**53.68**	**10,856**	**61.30**
Surigao del Norte	**7,280**	6	**0.08**	328	**4.51**	3,449	**47.38**	**3,783**	**51.96**
Surigao del Sur	**11,553**	13	**0.11**	412	**3.57**	3,499	**30.29**	**3,924**	**33.97**
Province of Dinagat	**2,576**	0	**0.00**	125	**4.85**	1,111	**43.13**	**1,236**	**47.98**
Bislig City	**2,192**	3	**0.14**	106	**4.84**	991	**45.21**	**1,100**	**50.18**
Butuan City	**7,743**	7	**0.09**	663	**8.56**	4,749	**61.33**	**5,419**	**69.99**
Surigao City	**3,395**	5	**0.15**	263	**7.75**	1,807	**53.23**	**2,075**	**61.12**

Table 2.B.1.6. Prenatal Care

No. of Pregnant women who Completed Calcium Carbonate supplementation
Philippines, 2022

Area	Eligible Pop.	Calcium Carbonate Supplementation							
		Age Group						Total	%
		10-14 yrs old		15-19 yrs old		20-49 yrs old			
		No.	%	No.	%	No.	%		
**PHILIPPINES**	**2,131,496**	**1,627**	**0.08**	**70,007**	**3.28**	**579,894**	**27.21**	**651,528**	**30.57**
**N C R**	**236,901**	**106**	**0.04**	**6,392**	**2.70**	**63,765**	**26.92**	**70,263**	**29.66**
Malabon	**6,724**	13	**0.19**	513	**7.63**	3,591	**53.41**	**4,117**	**61.23**
Navotas	**4,591**	4	**0.09**	264	**5.75**	1,554	**33.85**	**1,822**	**39.69**
Valenzuela City	**11,418**	7	**0.06**	208	**1.82**	2,190	**19.18**	**2,405**	**21.06**
Caloocan City	**29,146**	13	**0.04**	1,267	**4.35**	12,260	**42.06**	**13,540**	**46.46**
Marikina City	**8,294**	7	**0.08**	281	**3.39**	2,171	**26.18**	**2,459**	**29.65**
Pasig City	**13,894**	4	**0.03**	338	**2.43**	6,200	**44.62**	**6,542**	**47.09**
Pateros	**1,176**	0	**0.00**	28	**2.38**	51	**4.34**	**79**	**6.72**
Taguig	**14,807**	28	**0.19**	1,106	**7.47**	10,874	**73.44**	**12,008**	**81.10**
Quezon City	**54,011**	6	**0.01**	428	**0.79**	5,421	**10.04**	**5,855**	**10.84**
Makati City	**10,718**	5	**0.05**	224	**2.09**	3,443	**32.12**	**3,672**	**34.26**
Mandaluyong City	**7,111**	0	**0.00**	74	**1.04**	1,607	**22.60**	**1,681**	**23.64**
San Juan	**2,246**	0	**0.00**	45	**2.00**	684	**30.45**	**729**	**32.46**
Manila City	**32,743**	15	**0.05**	770	**2.35**	5,298	**16.18**	**6,083**	**18.58**
Las Piñas City	**10,832**	0	**0.00**	43	**0.40**	414	**3.82**	**457**	**4.22**
Muntinlupa City	**9,281**	0	**0.00**	85	**0.92**	438	**4.72**	**523**	**5.64**
Parañaque City	**12,249**	4	**0.03**	365	**2.98**	3,627	**29.61**	**3,996**	**32.62**
Pasay City	**7,660**	0	**0.00**	353	**4.61**	3,942	**51.46**	**4,295**	**56.07**
**C A R**	**35,179**	**9**	**0.03**	**1,052**	**2.99**	**12,064**	**34.29**	**13,125**	**37.31**
Abra	**4,275**	2	**0.05**	180	**4.21**	1,351	**31.60**	**1,533**	**35.86**
Apayao	**2,502**	3	**0.12**	125	**5.00**	959	**38.33**	**1,087**	**43.45**
Benguet	**9,086**	0	**0.00**	287	**3.16**	4,424	**48.69**	**4,711**	**51.85**
Ifugao	**4,486**	2	**0.04**	146	**3.25**	1,546	**34.46**	**1,694**	**37.76**
Kalinga	**4,701**	0	**0.00**	109	**2.32**	1,248	**26.55**	**1,357**	**28.87**
Mt. Province	**3,103**	1	**0.03**	43	**1.39**	1,014	**32.68**	**1,058**	**34.10**
Baguio City	**7,026**	1	**0.01**	162	**2.31**	1,522	**21.66**	**1,685**	**23.98**
**Region 1**	**97,099**	**50**	**0.05**	**4,338**	**4.47**	**48,120**	**49.56**	**52,508**	**54.08**
Ilocos Norte	**7,854**	4	**0.05**	153	**1.95**	2,530	**32.21**	**2,687**	**34.21**
Ilocos Sur	**9,072**	11	**0.12**	375	**4.13**	5,671	**62.51**	**6,057**	**66.77**
La Union	**11,338**	4	**0.04**	588	**5.19**	6,054	**53.40**	**6,646**	**58.62**
Pangasinan	**50,710**	21	**0.04**	2,233	**4.40**	24,538	**48.39**	**26,792**	**52.83**
Alaminos City	**1,910**	0	**0.00**	119	**6.23**	1,149	**60.16**	**1,268**	**66.39**
Candon City	**954**	0	**0.00**	2	**0.21**	838	**87.84**	**840**	**88.05**
Dagupan City	**3,657**	6	**0.16**	300	**8.20**	1,612	**44.08**	**1,918**	**52.45**
Laoag City	**1,812**	0	**0.00**	21	**1.16**	1,430	**78.92**	**1,451**	**80.08**
San Carlos City	**4,028**	1	**0.02**	120	**2.98**	1,298	**32.22**	**1,419**	**35.23**
San Fernando City	**2,074**	1	**0.05**	172	**8.29**	954	**46.00**	**1,127**	**54.34**
Urdaneta City	**2,836**	2	**0.07**	210	**7.40**	1,587	**55.96**	**1,799**	**63.43**
Vigan City	**854**	0	**0.00**	45	**5.27**	459	**53.75**	**504**	**59.02**
**Region 2**	**68,960**	**155**	**0.22**	**3,683**	**5.34**	**27,585**	**40.00**	**31,423**	**45.57**
Batanes	**362**	0	**0.00**	2	**0.55**	37	**10.22**	**39**	**10.77**
Cagayan	**20,431**	11	**0.05**	530	**2.59**	4,445	**21.76**	**4,986**	**24.40**
Isabela	**23,449**	39	**0.17**	1,436	**6.12**	10,613	**45.26**	**12,088**	**51.55**
Nueva Vizcaya	**9,538**	94	**0.99**	841	**8.82**	4,635	**48.60**	**5,570**	**58.40**
Quirino	**4,051**	4	**0.10**	250	**6.17**	2,353	**58.08**	**2,607**	**64.35**

Table 2.B.1.6. Prenatal Care
No. of Pregnant women who Completed Calcium Carbonate supplementation
Philippines, 2022

Area	Eligible Pop.	Calcium Carbonate Supplementation							
		Age Group						Total	%
		10-14 yrs old		15-19 yrs old		20-49 yrs old			
		No.	%	No.	%	No.	%		
Cauayan City	**2,565**	2	**0.08**	160	**6.24**	945	**36.84**	**1,107**	**43.16**
Ilagan City	**2,889**	1	**0.03**	141	**4.88**	1,297	**44.89**	**1,439**	**49.81**
Santiago City	**2,671**	4	**0.15**	256	**9.58**	2,406	**90.08**	**2,666**	**99.81**
Tuguegarao City	**3,004**	0	**0.00**	67	**2.23**	854	**28.43**	**921**	**30.66**
**Region 3**	**220,155**	**189**	**0.09**	**9,841**	**4.47**	**71,111**	**32.30**	**81,141**	**36.86**
Aurora	**4,754**	0	**0.00**	133	**2.80**	996	**20.95**	**1,129**	**23.75**
Bataan	**13,789**	10	**0.07**	490	**3.55**	3,360	**24.37**	**3,860**	**27.99**
Bulacan	**43,760**	36	**0.08**	2,396	**5.48**	17,526	**40.05**	**19,958**	**45.61**
Nueva Ecija	**29,039**	48	**0.17**	1,254	**4.32**	7,095	**24.43**	**8,397**	**28.92**
Pampanga	**31,620**	29	**0.09**	1,182	**3.74**	9,126	**28.86**	**10,337**	**32.69**
Tarlac	**19,319**	19	**0.10**	768	**3.98**	6,428	**33.27**	**7,215**	**37.35**
Zambales	**12,476**	0	**0.00**	61	**0.49**	606	**4.86**	**667**	**5.35**
Angeles City	**7,932**	0	**0.00**	213	**2.69**	1,267	**15.97**	**1,480**	**18.66**
Balanga City	**1,992**	3	**0.15**	87	**4.37**	517	**25.95**	**607**	**30.47**
Cabanatuan City	**5,945**	5	**0.08**	213	**3.58**	1,541	**25.92**	**1,759**	**29.59**
City of San Fernando	**5,907**	4	**0.07**	257	**4.35**	1,989	**33.67**	**2,250**	**38.09**
Gapan City	**2,170**	0	**0.00**	67	**3.09**	480	**22.12**	**547**	**25.21**
Mabalacat City	**4,831**	6	**0.12**	410	**8.49**	2,960	**61.27**	**3,376**	**69.88**
Malolos City	**4,890**	0	**0.00**	36	**0.74**	416	**8.51**	**452**	**9.24**
Meycauayan	**4,055**	1	**0.02**	260	**6.41**	2,002	**49.37**	**2,263**	**55.81**
Olongapo	**4,922**	1	**0.02**	120	**2.44**	918	**18.65**	**1,039**	**21.11**
Palayan City	**808**	0	**0.00**	48	**5.94**	204	**25.25**	**252**	**31.19**
San Jose City	**2,748**	3	**0.11**	199	**7.24**	1,115	**40.57**	**1,317**	**47.93**
San Jose del Monte City	**11,133**	20	**0.18**	936	**8.41**	8,771	**78.78**	**9,727**	**87.37**
Science City of Munoz	**1,601**	0	**0.00**	51	**3.19**	344	**21.49**	**395**	**24.67**
Tarlac City	**6,464**	4	**0.06**	660	**10.21**	3,450	**53.37**	**4,114**	**63.64**
**Region 4A**	**299,627**	**324**	**0.11**	**8,619**	**2.88**	**69,610**	**23.23**	**78,553**	**26.22**
Batangas	**39,255**	215	**0.55**	1,222	**3.11**	9,195	**23.42**	**10,632**	**27.08**
Cavite	**28,151**	4	**0.01**	542	**1.93**	4,675	**16.61**	**5,221**	**18.55**
Laguna	**19,696**	16	**0.08**	773	**3.92**	5,421	**27.52**	**6,210**	**31.53**
Quezon	**37,853**	14	**0.04**	632	**1.67**	4,677	**12.36**	**5,323**	**14.06**
Rizal	**44,798**	21	**0.05**	1,468	**3.28**	9,886	**22.07**	**11,375**	**25.39**
Antipolo City	**16,498**	8	**0.05**	647	**3.92**	4,611	**27.95**	**5,266**	**31.92**
Bacoor City	**12,341**	0	**0.00**	143	**1.16**	658	**5.33**	**801**	**6.49**
Batangas City	**6,965**	3	**0.04**	228	**3.27**	2,228	**31.99**	**2,459**	**35.31**
Biñan City	**6,597**	3	**0.05**	859	**13.02**	5,939	**90.03**	**6,801**	**103.09**
Cabuyao City	**6,119**	4	**0.07**	277	**4.53**	3,634	**59.39**	**3,915**	**63.98**
Calamba City	**9,005**	6	**0.07**	345	**3.83**	3,299	**36.64**	**3,650**	**40.53**
Cavite City	**2,105**	0	**0.00**	9	**0.43**	35	**1.66**	**44**	**2.09**
Dasmariñas City	**13,538**	3	**0.02**	188	**1.39**	1,770	**13.07**	**1,961**	**14.49**
General Trias City	**6,457**	1	**0.02**	70	**1.08**	539	**8.35**	**610**	**9.45**
Imus City	**8,292**	3	**0.04**	34	**0.41**	385	**4.64**	**422**	**5.09**
Lipa City	**7,018**	0	**0.00**	59	**0.84**	1,483	**21.13**	**1,542**	**21.97**
Lucena City	**5,738**	7	**0.12**	185	**3.22**	1,305	**22.74**	**1,497**	**26.09**
San Pablo City	**5,272**	5	**0.09**	191	**3.62**	1,281	**24.30**	**1,477**	**28.02**
San Pedro City	**6,455**	2	**0.03**	114	**1.77**	1,015	**15.72**	**1,131**	**17.52**
Santa Rosa City	**7,009**	2	**0.03**	398	**5.68**	4,077	**58.17**	**4,477**	**63.88**
Tagaytay City	**1,460**	0	**0.00**	61	**4.18**	1,218	**83.42**	**1,279**	**87.60**
Tanauan City	**3,661**	3	**0.08**	60	**1.64**	1,207	**32.97**	**1,270**	**34.69**
Tayabas City	**2,145**	0	**0.00**	64	**2.98**	629	**29.32**	**693**	**32.31**
Trece Martires City	**3,199**	4	**0.13**	50	**1.56**	443	**13.85**	**497**	**15.54**

Table 2.B.1.6. Prenatal Care

No. of Pregnant women who Completed Calcium Carbonate supplementation
Philippines, 2022

Area	Eligible Pop.	Calcium Carbonate Supplementation							
		Age Group						Total	%
		10-14 yrs old		15-19 yrs old		20-49 yrs old			
		No.	%	No.	%	No.	%		
**Region 4B**	**72,791**	**35**	**0.05**	**1,633**	**2.24**	**12,375**	**17.00**	**14,043**	**19.29**
Marinduque	4,990	4	0.08	155	3.11	1,449	29.04	1,608	32.22
Mindoro Occidental	12,479	5	0.04	414	3.32	2,662	21.33	3,081	24.69
Mindoro Oriental	20,189	5	0.02	441	2.18	4,208	20.84	4,654	23.05
Palawan	21,963	11	0.05	430	1.96	2,594	11.81	3,035	13.82
Romblon	6,569	10	0.15	133	2.02	1,234	18.79	1,377	20.96
Puerto Princesa City	6,601	0	0.00	60	0.91	228	3.45	288	4.36
**Region 5**	**138,457**	**18**	**0.01**	**2,898**	**2.09**	**30,833**	**22.27**	**33,749**	**24.38**
Albay	24,081	1	0.00	619	2.57	9,151	38.00	9,771	40.58
Camarines Norte	14,384	1	0.01	331	2.30	2,734	19.01	3,066	21.32
Camarines Sur	39,486	5	0.01	575	1.46	6,551	16.59	7,131	18.06
Catanduanes	6,459	0	0.00	122	1.89	1,243	19.24	1,365	21.13
Masbate	22,600	5	0.02	720	3.19	4,496	19.89	5,221	23.10
Sorsogon	19,828	3	0.02	376	1.90	4,307	21.72	4,686	23.63
Iriga City	2,679	1	0.04	19	0.71	194	7.24	214	7.99
Legaspi City	4,233	2	0.05	114	2.69	1,871	44.20	1,987	46.94
Naga City	4,707	0	0.00	22	0.47	286	6.08	308	6.54
**Region 6**	**146,449**	**74**	**0.05**	**3,568**	**2.44**	**34,425**	**23.51**	**38,067**	**25.99**
Aklan	11,288	3	0.03	231	2.05	3,214	28.47	3,448	30.55
Antique	13,132	5	0.04	145	1.10	1,691	12.88	1,841	14.02
Capiz	13,975	6	0.04	191	1.37	2,219	15.88	2,416	17.29
Guimaras	3,084	5	0.16	128	4.15	1,403	45.49	1,536	49.81
Iloilo	36,267	32	0.09	938	2.59	10,093	27.83	11,063	30.50
Negros Occidental	49,230	18	0.04	1,567	3.18	11,797	23.96	13,382	27.18
Bacolod City	11,082	2	0.02	60	0.54	630	5.68	692	6.24
Iloilo City	8,391	3	0.04	308	3.67	3,378	40.26	3,689	43.96
**Region 7**	**162,308**	**59**	**0.04**	**3,396**	**2.09**	**34,919**	**21.51**	**38,374**	**23.64**
Bohol	27,992	8	0.03	405	1.45	4,481	16.01	4,894	17.48
Cebu	66,463	8	0.01	594	0.89	5,723	8.61	6,325	9.52
Negros Oriental	27,890	1	0.00	123	0.44	775	2.78	899	3.22
Siquijor	1,661	0	0.00	20	1.20	243	14.63	263	15.83
Cebu City	20,866	23	0.11	1,367	6.55	11,937	57.21	13,327	63.87
Lapu-Lapu City	9,232	8	0.09	381	4.13	5,986	64.84	6,375	69.05
Mandaue City	8,204	11	0.13	506	6.17	5,774	70.38	6,291	76.68
**Region 8**	**105,471**	**39**	**0.04**	**1,981**	**1.88**	**17,558**	**16.65**	**19,578**	**18.56**
Biliran	3,873	4	0.10	131	3.38	1,206	31.14	1,341	34.62
Eastern Samar	11,908	5	0.04	329	2.76	2,900	24.35	3,234	27.16
Northern Leyte	35,422	11	0.03	661	1.87	5,680	16.04	6,352	17.93
Northern Samar	15,921	5	0.03	243	1.53	2,288	14.37	2,536	15.93
Southern Leyte	6,662	2	0.03	213	3.20	2,408	36.15	2,623	39.37
Western Samar	14,732	6	0.04	256	1.74	1,859	12.62	2,121	14.40
Calbayog City	4,535	5	0.11	43	0.95	188	4.15	236	5.20
Maasin City	1,696	0	0.00	29	1.71	421	24.82	450	26.53
Ormoc City	5,044	1	0.02	76	1.51	608	12.05	685	13.58
Tacloban City	5,678	0	0.00	0	0.00	0	0.00	0	0.00

Table 2.B.1.6. Prenatal Care

No. of Pregnant women who Completed Calcium Carbonate supplementation
Philippines, 2022

Area	Eligible Pop.	Calcium Carbonate Supplementation							
		Age Group						Total	%
		10-14 yrs old		15-19 yrs old		20-49 yrs old			
		No.	%	No.	%	No.	%		
**Region 9**	**79,007**	**25**	**0.03**	**2,061**	**2.61**	**13,352**	**16.90**	**15,438**	**19.54**
Zamboanga del Norte	**16,854**	4	**0.02**	444	**2.63**	2,968	**17.61**	**3,416**	**20.27**
Zamboanga del Sur	**17,368**	6	**0.03**	420	**2.42**	2,266	**13.05**	**2,692**	**15.50**
Zamboanga Sibugay	**15,021**	3	**0.02**	222	**1.48**	1,698	**11.30**	**1,923**	**12.80**
Dapitan City	**1,737**	3	**0.17**	183	**10.54**	1,332	**76.68**	**1,518**	**87.39**
Dipolog City	**2,762**	2	**0.07**	32	**1.16**	252	**9.12**	**286**	**10.35**
Isabela City	**2,563**	0	**0.00**	18	**0.70**	38	**1.48**	**56**	**2.18**
Pagadian City	**4,257**	0	**0.00**	66	**1.55**	457	**10.74**	**523**	**12.29**
Zamboanga City	**18,445**	7	**0.04**	676	**3.66**	4,341	**23.53**	**5,024**	**27.24**
**Region 10**	**99,908**	**113**	**0.11**	**6,090**	**6.10**	**36,724**	**36.76**	**42,927**	**42.97**
Bukidnon	**22,900**	42	**0.18**	1,975	**8.62**	9,199	**40.17**	**11,216**	**48.98**
Camiguin	**1,854**	0	**0.00**	36	**1.94**	360	**19.42**	**396**	**21.36**
Lanao del Norte	**14,930**	9	**0.06**	814	**5.45**	6,135	**41.09**	**6,958**	**46.60**
Misamis Occidental	**6,420**	4	**0.06**	186	**2.90**	2,044	**31.84**	**2,234**	**34.80**
Misamis Oriental	**14,963**	23	**0.15**	1,045	**6.98**	5,664	**37.85**	**6,732**	**44.99**
Cagayan de Oro City	**14,172**	22	**0.16**	874	**6.17**	5,324	**37.57**	**6,220**	**43.89**
El Salvador City	**1,052**	3	**0.29**	83	**7.89**	363	**34.51**	**449**	**42.68**
Gingoog City	**2,608**	3	**0.12**	151	**5.79**	818	**31.37**	**972**	**37.27**
Iligan City	**7,565**	5	**0.07**	532	**7.03**	3,669	**48.50**	**4,206**	**55.60**
Malaybalay City	**3,817**	1	**0.03**	90	**2.36**	660	**17.29**	**751**	**19.68**
Oroquieta City	**1,389**	0	**0.00**	25	**1.80**	334	**24.05**	**359**	**25.85**
Ozamis City	**2,786**	0	**0.00**	102	**3.66**	792	**28.43**	**894**	**32.09**
Tangub City	**1,234**	0	**0.00**	81	**6.56**	833	**67.50**	**914**	**74.07**
Valencia City	**4,218**	1	**0.02**	96	**2.28**	529	**12.54**	**626**	**14.84**
**Region 11**	**108,407**	**259**	**0.24**	**6,331**	**5.84**	**44,920**	**41.44**	**51,510**	**47.52**
Davao de Oro	**15,490**	94	**0.61**	1,477	**9.54**	7,467	**48.21**	**9,038**	**58.35**
Davao del Norte	**21,017**	55	**0.26**	1,625	**7.73**	10,551	**50.20**	**12,231**	**58.20**
Davao Oriental	**13,229**	18	**0.14**	671	**5.07**	4,073	**30.79**	**4,762**	**36.00**
Davao del Sur	**14,564**	28	**0.19**	563	**3.87**	3,366	**23.11**	**3,957**	**27.17**
Davao Occidental	**6,510**	2	**0.03**	55	**0.84**	197	**3.03**	**254**	**3.90**
Davao City	**37,597**	62	**0.16**	1,940	**5.16**	19,266	**51.24**	**21,268**	**56.57**
**Region 12**	**98,859**	**116**	**0.12**	**4,713**	**4.77**	**29,273**	**29.61**	**34,102**	**34.50**
North Cotabato	**34,150**	35	**0.10**	1,359	**3.98**	9,021	**26.42**	**10,415**	**30.50**
Sarangani	**12,907**	23	**0.18**	699	**5.42**	3,127	**24.23**	**3,849**	**29.82**
South Cotabato	**20,917**	21	**0.10**	831	**3.97**	5,906	**28.24**	**6,758**	**32.31**
Sultan Kudarat	**17,299**	32	**0.18**	1,202	**6.95**	7,667	**44.32**	**8,901**	**51.45**
Gen. Santos City	**13,586**	5	**0.04**	622	**4.58**	3,552	**26.14**	**4,179**	**30.76**
**BARMM**	**101,343**	**21**	**0.02**	**1,291**	**1.27**	**16,704**	**16.48**	**18,016**	**17.78**
Basilan	**7,823**	0	**0.00**	45	**0.58**	313	**4.00**	**358**	**4.58**
Lanao del Sur	**21,639**	2	**0.01**	416	**1.92**	8,388	**38.76**	**8,806**	**40.70**
Maguindanao	**32,198**	8	**0.02**	680	**2.11**	6,588	**20.46**	**7,276**	**22.60**
Sulu	**17,165**	2	**0.01**	56	**0.33**	371	**2.16**	**429**	**2.50**
Tawi-Tawi	**9,369**	9	**0.10**	28	**0.30**	205	**2.19**	**242**	**2.58**
Lamitan City	**2,154**	0	**0.00**	7	**0.32**	29	**1.35**	**36**	**1.67**
Marawi City	**5,173**	0	**0.00**	0	**0.00**	0	**0.00**	**0**	**0.00**
Cotabato City	**5,822**	0	**0.00**	59	**1.01**	810	**13.91**	**869**	**14.93**

Table 2.B.1.6. Prenatal Care

No. of Pregnant women who Completed Calcium Carbonate supplementation
Philippines, 2022

Area	Eligible Pop.	Calcium Carbonate Supplementation							
		Age Group						Total	%
		10-14 yrs old		15-19 yrs old		20-49 yrs old			
		No.	%	No.	%	No.	%		
**CARAGA**	**60,575**	**35**	**0.06**	**2,120**	**3.50**	**16,556**	**27.33**	**18,711**	**30.89**
Agusan del Norte	**8,127**	0	**0.00**	0	**0.00**	0	**0.00**	**0**	**0.00**
Agusan del Sur	**17,709**	19	**0.11**	895	**5.05**	6,475	**36.56**	**7,389**	**41.72**
Surigao del Norte	**7,280**	5	**0.07**	263	**3.61**	2,300	**31.59**	**2,568**	**35.27**
Surigao del Sur	**11,553**	2	**0.02**	255	**2.21**	1,873	**16.21**	**2,130**	**18.44**
Province of Dinagat	**2,576**	2	**0.08**	77	**2.99**	744	**28.88**	**823**	**31.95**
Bislig City	**2,192**	2	**0.09**	92	**4.20**	957	**43.66**	**1,051**	**47.95**
Butuan City	**7,743**	2	**0.03**	335	**4.33**	2,569	**33.18**	**2,906**	**37.53**
Surigao City	**3,395**	3	**0.09**	203	**5.98**	1,638	**48.25**	**1,844**	**54.32**

Table 2.B.1.6. Prenatal Care

No. of Pregnant women who Completed Iodine Supplementation
Philippines, 2022

Area	Eligible Pop.	Iodine Supplementation							
		Age Group						Total	%
		10-14 yrs old		15-19 yrs old		20-49 yrs old			
		No.	%	No.	%	No.	%		
**PHILIPPINES**	**2,131,496**	**192**	**0.01**	**5,617**	**0.26**	**37,926**	**1.78**	**43,735**	**2.05**
**N C R**	**236,901**	**1**	**0.00**	**101**	**0.04**	**467**	**0.20**	**569**	**0.24**
Malabon	**6,724**	0	**0.00**	0	**0.00**	11	**0.16**	**11**	**0.16**
Navotas	**4,591**	0	**0.00**	25	**0.54**	71	**1.55**	**96**	**2.09**
Valenzuela City	**11,418**	0	**0.00**	17	**0.15**	138	**1.21**	**155**	**1.36**
Caloocan City	**29,146**	0	**0.00**	0	**0.00**	0	**0.00**	**0**	**0.00**
Marikina City	**8,294**	0	**0.00**	0	**0.00**	0	**0.00**	**0**	**0.00**
Pasig City	**13,894**	0	**0.00**	0	**0.00**	0	**0.00**	**0**	**0.00**
Pateros	**1,176**	0	**0.00**	1	**0.09**	3	**0.26**	**4**	**0.34**
Taguig	**14,807**	0	**0.00**	23	**0.16**	144	**0.97**	**167**	**1.13**
Quezon City	**54,011**	0	**0.00**	0	**0.00**	0	**0.00**	**0**	**0.00**
Makati City	**10,718**	0	**0.00**	0	**0.00**	0	**0.00**	**0**	**0.00**
Mandaluyong City	**7,111**	0	**0.00**	2	**0.03**	11	**0.15**	**13**	**0.18**
San Juan	**2,246**	0	**0.00**	0	**0.00**	0	**0.00**	**0**	**0.00**
Manila City	**32,743**	1	**0.00**	31	**0.09**	76	**0.23**	**108**	**0.33**
Las Piñas City	**10,832**	0	**0.00**	2	**0.02**	13	**0.12**	**15**	**0.14**
Muntinlupa City	**9,281**	0	**0.00**	0	**0.00**	0	**0.00**	**0**	**0.00**
Parañaque City	**12,249**	0	**0.00**	0	**0.00**	0	**0.00**	**0**	**0.00**
Pasay City	**7,660**	0	**0.00**	0	**0.00**	0	**0.00**	**0**	**0.00**
**C A R**	**35,179**	**3**	**0.01**	**168**	**0.48**	**1,559**	**4.43**	**1,730**	**4.92**
Abra	**4,275**	1	**0.02**	54	**1.26**	300	**7.02**	**355**	**8.30**
Apayao	**2,502**	0	**0.00**	5	**0.20**	75	**3.00**	**80**	**3.20**
Benguet	**9,086**	2	**0.02**	76	**0.84**	888	**9.77**	**966**	**10.63**
Ifugao	**4,486**	0	**0.00**	10	**0.22**	56	**1.25**	**66**	**1.47**
Kalinga	**4,701**	0	**0.00**	13	**0.28**	93	**1.98**	**106**	**2.25**
Mt. Province	**3,103**	0	**0.00**	2	**0.06**	19	**0.61**	**21**	**0.68**
Baguio City	**7,026**	0	**0.00**	8	**0.11**	128	**1.82**	**136**	**1.94**
**Region 1**	**97,099**	**2**	**0.00**	**504**	**0.52**	**5,067**	**5.22**	**5,573**	**5.74**
Ilocos Norte	**7,854**	1	**0.01**	52	**0.66**	820	**10.44**	**873**	**11.12**
Ilocos Sur	**9,072**	0	**0.00**	23	**0.25**	221	**2.44**	**244**	**2.69**
La Union	**11,338**	0	**0.00**	30	**0.26**	237	**2.09**	**267**	**2.35**
Pangasinan	**50,710**	1	**0.00**	298	**0.59**	2,764	**5.45**	**3,063**	**6.04**
Alaminos City	**1,910**	0	**0.00**	0	**0.00**	0	**0.00**	**0**	**0.00**
Candon City	**954**	0	**0.00**	2	**0.21**	161	**16.88**	**163**	**17.09**
Dagupan City	**3,657**	0	**0.00**	39	**1.07**	160	**4.38**	**199**	**5.44**
Laoag City	**1,812**	0	**0.00**	17	**0.94**	470	**25.94**	**487**	**26.88**
San Carlos City	**4,028**	0	**0.00**	0	**0.00**	0	**0.00**	**0**	**0.00**
San Fernando City	**2,074**	0	**0.00**	33	**1.59**	176	**8.49**	**209**	**10.08**
Urdaneta City	**2,836**	0	**0.00**	10	**0.35**	58	**2.05**	**68**	**2.40**
Vigan City	**854**	0	**0.00**	0	**0.00**	0	**0.00**	**0**	**0.00**
**Region 2**	**68,960**	**1**	**0.00**	**87**	**0.13**	**577**	**0.84**	**665**	**0.96**
Batanes	**362**	0	**0.00**	0	**0.00**	0	**0.00**	**0**	**0.00**
Cagayan	**20,431**	0	**0.00**	14	**0.07**	60	**0.29**	**74**	**0.36**
Isabela	**23,449**	0	**0.00**	25	**0.11**	151	**0.64**	**176**	**0.75**
Nueva Vizcaya	**9,538**	0	**0.00**	30	**0.31**	192	**2.01**	**222**	**2.33**
Quirino	**4,051**	1	**0.02**	1	**0.02**	15	**0.37**	**17**	**0.42**
Cauayan City	**2,565**	0	**0.00**	17	**0.66**	159	**6.20**	**176**	**6.86**
Ilagan City	**2,889**	0	**0.00**	0	**0.00**	0	**0.00**	**0**	**0.00**

Table 2.B.1.6. Prenatal Care

No. of Pregnant women who Completed Iodine Supplementation
Philippines, 2022

Area	Eligible Pop.	Iodine Supplementation							
		Age Group						Total	%
		10-14 yrs old		15-19 yrs old		20-49 yrs old			
		No.	%	No.	%	No.	%		
Santiago City	2,671	0	0.00	0	0.00	0	0.00	0	0.00
Tuguegarao City	3,004	0	0.00	0	0.00	0	0.00	0	0.00
**Region 3**	**220,155**	**7**	**0.00**	**238**	**0.11**	**2,412**	**1.10**	**2,657**	**1.21**
Aurora	4,754	0	0.00	0	0.00	5	0.11	5	0.11
Bataan	13,789	1	0.01	4	0.03	51	0.37	56	0.41
Bulacan	43,760	2	0.00	123	0.28	1,365	3.12	1,490	3.40
Nueva Ecija	29,039	0	0.00	8	0.03	127	0.44	135	0.46
Pampanga	31,620	0	0.00	10	0.03	43	0.14	53	0.17
Tarlac	19,319	3	0.02	41	0.21	403	2.09	447	2.31
Zambales	12,476	0	0.00	3	0.02	18	0.14	21	0.17
Angeles City	7,932	0	0.00	2	0.03	2	0.03	4	0.05
Balanga City	1,992	0	0.00	0	0.00	0	0.00	0	0.00
Cabanatuan City	5,945	0	0.00	0	0.00	0	0.00	0	0.00
City of San Fernando	5,907	0	0.00	0	0.00	0	0.00	0	0.00
Gapan City	2,170	0	0.00	0	0.00	10	0.46	10	0.46
Mabalacat City	4,831	0	0.00	4	0.08	25	0.52	29	0.60
Malolos City	4,890	0	0.00	0	0.00	2	0.04	2	0.04
Meycauayan	4,055	0	0.00	26	0.64	220	5.43	246	6.07
Olongapo	4,922	0	0.00	0	0.00	0	0.00	0	0.00
Palayan City	808	0	0.00	0	0.00	0	0.00	0	0.00
San Jose City	2,748	0	0.00	11	0.40	55	2.00	66	2.40
San Jose del Monte City	11,133	0	0.00	0	0.00	9	0.08	9	0.08
Science City of Munoz	1,601	0	0.00	0	0.00	0	0.00	0	0.00
Tarlac City	6,464	1	0.02	6	0.09	77	1.19	84	1.30
**Region 4A**	**299,627**	**19**	**0.01**	**834**	**0.28**	**6,029**	**2.01**	**6,882**	**2.30**
Batangas	39,255	1	0.00	94	0.24	568	1.45	663	1.69
Cavite	28,151	8	0.03	36	0.13	190	0.67	234	0.83
Laguna	19,696	3	0.02	29	0.15	177	0.90	209	1.06
Quezon	37,853	0	0.00	41	0.11	258	0.68	299	0.79
Rizal	44,798	2	0.00	207	0.46	1,042	2.33	1,251	2.79
Antipolo City	16,498	0	0.00	29	0.18	356	2.16	385	2.33
Bacoor City	12,341	2	0.02	15	0.12	51	0.41	68	0.55
Batangas City	6,965	0	0.00	0	0.00	0	0.00	0	0.00
Biñan City	6,597	2	0.03	295	4.47	2,651	40.18	2,948	44.69
Cabuyao City	6,119	0	0.00	4	0.07	16	0.26	20	0.33
Calamba City	9,005	0	0.00	23	0.26	117	1.30	140	1.55
Cavite City	2,105	0	0.00	0	0.00	2	0.10	2	0.10
Dasmariñas City	13,538	0	0.00	23	0.17	194	1.43	217	1.60
General Trias City	6,457	0	0.00	0	0.00	9	0.14	9	0.14
Imus City	8,292	1	0.01	0	0.00	6	0.07	7	0.08
Lipa City	7,018	0	0.00	1	0.01	95	1.35	96	1.37
Lucena City	5,738	0	0.00	21	0.37	141	2.46	162	2.82
San Pablo City	5,272	0	0.00	0	0.00	2	0.04	2	0.04
San Pedro City	6,455	0	0.00	9	0.14	51	0.79	60	0.93
Santa Rosa City	7,009	0	0.00	7	0.10	68	0.97	75	1.07
Tagaytay City	1,460	0	0.00	0	0.00	0	0.00	0	0.00
Tanauan City	3,661	0	0.00	0	0.00	28	0.76	28	0.76
Tayabas City	2,145	0	0.00	0	0.00	7	0.33	7	0.33
Trece Martires City	3,199	0	0.00	0	0.00	0	0.00	0	0.00
**Region 4B**	**72,791**	**13**	**0.02**	**101**	**0.14**	**571**	**0.78**	**685**	**0.94**
Marinduque	4,990	0	0.00	2	0.04	11	0.22	13	0.26
Mindoro Occidental	12,479	0	0.00	5	0.04	45	0.36	50	0.40
Mindoro Oriental	20,189	2	0.01	6	0.03	67	0.33	75	0.37
Palawan	21,963	2	0.01	57	0.26	301	1.37	360	1.64
Romblon	6,569	9	0.14	16	0.24	52	0.79	77	1.17

Table 2.B.1.6. Prenatal Care

No. of Pregnant women who Completed Iodine Supplementation
Philippines, 2022

Area	Eligible Pop.	Iodine Supplementation							
		Age Group						Total	%
		10-14 yrs old		15-19 yrs old		20-49 yrs old			
		No.	%	No.	%	No.	%		
Puerto Princesa City	**6,601**	0	**0.00**	15	**0.23**	95	**1.44**	**110**	**1.67**
**Region 5**	**138,457**	**2**	**0.00**	**122**	**0.09**	**1,019**	**0.74**	**1,143**	**0.83**
Albay	**24,081**	0	**0.00**	8	**0.03**	175	**0.73**	**183**	**0.76**
Camarines Norte	**14,384**	2	**0.01**	93	**0.65**	542	**3.77**	**637**	**4.43**
Camarines Sur	**39,486**	0	**0.00**	6	**0.02**	142	**0.36**	**148**	**0.37**
Catanduanes	**6,459**	0	**0.00**	0	**0.00**	0	**0.00**	**0**	**0.00**
Masbate	**22,600**	0	**0.00**	13	**0.06**	91	**0.40**	**104**	**0.46**
Sorsogon	**19,828**	0	**0.00**	0	**0.00**	28	**0.14**	**28**	**0.14**
Iriga City	**2,679**	0	**0.00**	0	**0.00**	4	**0.15**	**4**	**0.15**
Legaspi City	**4,233**	0	**0.00**	2	**0.05**	37	**0.87**	**39**	**0.92**
Naga City	**4,707**	0	**0.00**	0	**0.00**	0	**0.00**	**0**	**0.00**
**Region 6**	**146,449**	**6**	**0.00**	**190**	**0.13**	**1,282**	**0.88**	**1,478**	**1.01**
Aklan	**11,288**	0	**0.00**	0	**0.00**	12	**0.11**	**12**	**0.11**
Antique	**13,132**	2	**0.02**	0	**0.00**	21	**0.16**	**23**	**0.18**
Capiz	**13,975**	0	**0.00**	11	**0.08**	106	**0.76**	**117**	**0.84**
Guimaras	**3,084**	0	**0.00**	0	**0.00**	0	**0.00**	**0**	**0.00**
Iloilo	**36,267**	1	**0.00**	44	**0.12**	446	**1.23**	**491**	**1.35**
Negros Occidental	**49,230**	2	**0.00**	107	**0.22**	472	**0.96**	**581**	**1.18**
Bacolod City	**11,082**	1	**0.01**	13	**0.12**	67	**0.60**	**81**	**0.73**
Iloilo City	**8,391**	0	**0.00**	15	**0.18**	158	**1.88**	**173**	**2.06**
**Region 7**	**162,308**	**5**	**0.00**	**82**	**0.05**	**921**	**0.57**	**1,008**	**0.62**
Bohol	**27,992**	0	**0.00**	3	**0.01**	39	**0.14**	**42**	**0.15**
Cebu	**66,463**	5	**0.01**	54	**0.08**	346	**0.52**	**405**	**0.61**
Negros Oriental	**27,890**	0	**0.00**	3	**0.01**	26	**0.09**	**29**	**0.10**
Siquijor	**1,661**	0	**0.00**	0	**0.00**	11	**0.66**	**11**	**0.66**
Cebu City	**20,866**	0	**0.00**	11	**0.05**	204	**0.98**	**215**	**1.03**
Lapu-Lapu City	**9,232**	0	**0.00**	0	**0.00**	71	**0.77**	**71**	**0.77**
Mandaue City	**8,204**	0	**0.00**	11	**0.13**	224	**2.73**	**235**	**2.86**
**Region 8**	**105,471**	**2**	**0.00**	**94**	**0.09**	**952**	**0.90**	**1,048**	**0.99**
Biliran	**3,873**	0	**0.00**	0	**0.00**	0	**0.00**	**0**	**0.00**
Eastern Samar	**11,908**	0	**0.00**	15	**0.13**	162	**1.36**	**177**	**1.49**
Northern Leyte	**35,422**	0	**0.00**	39	**0.11**	420	**1.19**	**459**	**1.30**
Northern Samar	**15,921**	0	**0.00**	14	**0.09**	211	**1.33**	**225**	**1.41**
Southern Leyte	**6,662**	0	**0.00**	4	**0.06**	24	**0.36**	**28**	**0.42**
Western Samar	**14,732**	2	**0.01**	22	**0.15**	127	**0.86**	**151**	**1.02**
Calbayog City	**4,535**	0	**0.00**	0	**0.00**	8	**0.18**	**8**	**0.18**
Maasin City	**1,696**	0	**0.00**	0	**0.00**	0	**0.00**	**0**	**0.00**
Ormoc City	**5,044**	0	**0.00**	0	**0.00**	0	**0.00**	**0**	**0.00**
Tacloban City	**5,678**	0	**0.00**	0	**0.00**	0	**0.00**	**0**	**0.00**
**Region 9**	**79,007**	**7**	**0.01**	**275**	**0.35**	**1,715**	**2.17**	**1,997**	**2.53**
Zamboanga del Norte	**16,854**	1	**0.01**	34	**0.20**	351	**2.08**	**386**	**2.29**
Zamboanga del Sur	**17,368**	0	**0.00**	3	**0.02**	8	**0.05**	**11**	**0.06**
Zamboanga Sibugay	**15,021**	0	**0.00**	0	**0.00**	0	**0.00**	**0**	**0.00**
Dapitan City	**1,737**	0	**0.00**	0	**0.00**	0	**0.00**	**0**	**0.00**
Dipolog City	**2,762**	4	**0.14**	184	**6.66**	966	**34.97**	**1,154**	**41.78**
Isabela City	**2,563**	0	**0.00**	5	**0.20**	6	**0.23**	**11**	**0.43**
Pagadian City	**4,257**	0	**0.00**	19	**0.45**	209	**4.91**	**228**	**5.36**

Table 2.B.1.6. Prenatal Care

No. of Pregnant women who Completed Iodine Supplementation
Philippines, 2022

Area	Eligible Pop.	Iodine Supplementation							
		Age Group						Total	%
		10-14 yrs old		15-19 yrs old		20-49 yrs old			
		No.	%	No.	%	No.	%		
Zamboanga City	**18,445**	2	**0.01**	30	**0.16**	175	**0.95**	**207**	**1.12**
**Region 10**	**99,908**	**26**	**0.03**	**840**	**0.84**	**4,736**	**4.74**	**5,602**	**5.61**
Bukidnon	**22,900**	14	**0.06**	297	**1.30**	1,450	**6.33**	**1,761**	**7.69**
Camiguin	**1,854**	0	**0.00**	0	**0.00**	0	**0.00**	**0**	**0.00**
Lanao del Norte	**14,930**	7	**0.05**	346	**2.32**	2,078	**13.92**	**2,431**	**16.28**
Misamis Occidental	**6,420**	1	**0.02**	20	**0.31**	215	**3.35**	**236**	**3.68**
Misamis Oriental	**14,963**	0	**0.00**	27	**0.18**	168	**1.12**	**195**	**1.30**
Cagayan de Oro City	**14,172**	0	**0.00**	26	**0.18**	226	**1.59**	**252**	**1.78**
El Salvador City	**1,052**	4	**0.38**	36	**3.42**	176	**16.73**	**216**	**20.53**
Gingoog City	**2,608**	0	**0.00**	7	**0.27**	38	**1.46**	**45**	**1.73**
Iligan City	**7,565**	0	**0.00**	14	**0.19**	67	**0.89**	**81**	**1.07**
Malaybalay City	**3,817**	0	**0.00**	0	**0.00**	1	**0.03**	**1**	**0.03**
Oroquieta City	**1,389**	0	**0.00**	0	**0.00**	0	**0.00**	**0**	**0.00**
Ozamis City	**2,786**	0	**0.00**	4	**0.14**	50	**1.79**	**54**	**1.94**
Tangub City	**1,234**	0	**0.00**	44	**3.57**	176	**14.26**	**220**	**17.83**
Valencia City	**4,218**	0	**0.00**	19	**0.45**	91	**2.16**	**110**	**2.61**
**Region 11**	**108,407**	**48**	**0.04**	**926**	**0.85**	**4,575**	**4.22**	**5,549**	**5.12**
Davao de Oro	**15,490**	5	**0.03**	170	**1.10**	837	**5.40**	**1,012**	**6.53**
Davao del Norte	**21,017**	6	**0.03**	177	**0.84**	1,012	**4.82**	**1,195**	**5.69**
Davao Oriental	**13,229**	0	**0.00**	5	**0.04**	116	**0.88**	**121**	**0.91**
Davao del Sur	**14,564**	2	**0.01**	26	**0.18**	109	**0.75**	**137**	**0.94**
Davao Occidental	**6,510**	7	**0.11**	155	**2.38**	300	**4.61**	**462**	**7.10**
Davao City	**37,597**	28	**0.07**	393	**1.05**	2,201	**5.85**	**2,622**	**6.97**
**Region 12**	**98,859**	**46**	**0.05**	**945**	**0.96**	**5,212**	**5.27**	**6,203**	**6.27**
North Cotabato	**34,150**	29	**0.08**	242	**0.71**	1,572	**4.60**	**1,843**	**5.40**
Sarangani	**12,907**	3	**0.02**	319	**2.47**	1,238	**9.59**	**1,560**	**12.09**
South Cotabato	**20,917**	0	**0.00**	64	**0.31**	462	**2.21**	**526**	**2.51**
Sultan Kudarat	**17,299**	10	**0.06**	247	**1.43**	1,484	**8.58**	**1,741**	**10.06**
Gen. Santos City	**13,586**	4	**0.03**	73	**0.54**	456	**3.36**	**533**	**3.92**
**BARMM**	**101,343**	**1**	**0.00**	**63**	**0.06**	**475**	**0.47**	**539**	**0.53**
Basilan	**7,823**	0	**0.00**	0	**0.00**	4	**0.05**	**4**	**0.05**
Lanao del Sur	**21,639**	0	**0.00**	0	**0.00**	18	**0.08**	**18**	**0.08**
Maguindanao	**32,198**	0	**0.00**	0	**0.00**	0	**0.00**	**0**	**0.00**
Sulu	**17,165**	0	**0.00**	13	**0.08**	12	**0.07**	**25**	**0.15**
Tawi-Tawi	**9,369**	0	**0.00**	0	**0.00**	9	**0.10**	**9**	**0.10**
Lamitan City	**2,154**	0	**0.00**	10	**0.46**	24	**1.11**	**34**	**1.58**
Marawi City	**5,173**	0	**0.00**	0	**0.00**	0	**0.00**	**0**	**0.00**
Cotabato City	**5,822**	1	**0.02**	40	**0.69**	408	**7.01**	**449**	**7.71**
**CARAGA**	**60,575**	**3**	**0.00**	**47**	**0.08**	**357**	**0.59**	**407**	**0.67**
Agusan del Norte	**8,127**	0	**0.00**	0	**0.00**	22	**0.27**	**22**	**0.27**
Agusan del Sur	**17,709**	2	**0.01**	35	**0.20**	212	**1.20**	**249**	**1.41**
Surigao del Norte	**7,280**	0	**0.00**	3	**0.04**	41	**0.56**	**44**	**0.60**
Surigao del Sur	**11,553**	1	**0.01**	4	**0.03**	50	**0.43**	**55**	**0.48**
Province of Dinagat	**2,576**	0	**0.00**	4	**0.16**	14	**0.54**	**18**	**0.70**
Bislig City	**2,192**	0	**0.00**	0	**0.00**	0	**0.00**	**0**	**0.00**
Butuan City	**7,743**	0	**0.00**	1	**0.01**	18	**0.23**	**19**	**0.25**
Surigao City	**3,395**	0	**0.00**	0	**0.00**	0	**0.00**	**0**	**0.00**

Table 2.B.1.8. Prenatal Care

Number and proportion of Pregnant Women given one (1) dose of deworming tablet
Philippines, 2022

Area	Eligible Pop.	Pregnant women given one (1) dose of Deworming Tablet							
		Age Group						Total	%
		10-14 yrs old		15-19 yrs old		20-49 yrs old			
		No.	%	No.	%	No.	%		
**PHILIPPINES**	**2,131,496**	**845**	**0.040**	**34,819**	**1.63**	**261,282**	**12.26**	**296,946**	**13.93**
**N C R**	**236,901**	**50**	**0.021**	**2,992**	**1.26**	**38,305**	**16.17**	**41,347**	**17.45**
Malabon	**6,724**	0	**0.000**	1	**0.01**	4	**0.06**	**5**	**0.07**
Navotas	**4,591**	0	**0.000**	9	**0.20**	36	**0.78**	**45**	**0.98**
Valenzuela City	**11,418**	0	**0.000**	8	**0.07**	36	**0.32**	**44**	**0.39**
Caloocan City	**29,146**	0	**0.000**	0	**0.00**	0	**0.00**	**0**	**0.00**
Marikina City	**8,294**	5	**0.060**	239	**2.88**	1,772	**21.36**	**2,016**	**24.31**
Pasig City	**13,894**	0	**0.000**	17	**0.12**	159	**1.14**	**176**	**1.27**
Pateros	**1,176**	1	**0.085**	24	**2.04**	156	**13.27**	**181**	**15.39**
Taguig	**14,807**	2	**0.014**	276	**1.86**	2,093	**14.14**	**2,371**	**16.01**
Quezon City	**54,011**	41	**0.076**	2,304	**4.27**	32,689	**60.52**	**35,034**	**64.86**
Makati City	**10,718**	0	**0.000**	14	**0.13**	140	**1.31**	**154**	**1.44**
Mandaluyong City	**7,111**	0	**0.000**	0	**0.00**	0	**0.00**	**0**	**0.00**
San Juan	**2,246**	0	**0.000**	8	**0.36**	86	**3.83**	**94**	**4.19**
Manila City	**32,743**	1	**0.003**	12	**0.04**	164	**0.50**	**177**	**0.54**
Las Piñas City	**10,832**	0	**0.000**	0	**0.00**	0	**0.00**	**0**	**0.00**
Muntinlupa City	**9,281**	0	**0.000**	0	**0.00**	0	**0.00**	**0**	**0.00**
Parañaque City	**12,249**	0	**0.000**	20	**0.16**	471	**3.85**	**491**	**4.01**
Pasay City	**7,660**	0	**0.000**	60	**0.78**	499	**6.51**	**559**	**7.30**
**C A R**	**35,179**	**10**	**0.028**	**706**	**2.01**	**7,490**	**21.29**	**8,206**	**23.33**
Abra	**4,275**	2	**0.047**	30	**0.70**	296	**6.92**	**328**	**7.67**
Apayao	**2,502**	3	**0.120**	174	**6.95**	1,160	**46.36**	**1,337**	**53.44**
Benguet	**9,086**	2	**0.022**	85	**0.94**	1,097	**12.07**	**1,184**	**13.03**
Ifugao	**4,486**	0	**0.000**	122	**2.72**	1,464	**32.63**	**1,586**	**35.35**
Kalinga	**4,701**	2	**0.043**	151	**3.21**	1,839	**39.12**	**1,992**	**42.37**
Mt. Province	**3,103**	0	**0.000**	9	**0.29**	91	**2.93**	**100**	**3.22**
Baguio City	**7,026**	1	**0.014**	135	**1.92**	1,543	**21.96**	**1,679**	**23.90**
**Region 1**	**97,099**	**43**	**0.044**	**1,180**	**1.22**	**11,557**	**11.90**	**12,780**	**13.16**
Ilocos Norte	**7,854**	0	**0.000**	92	**1.17**	1,442	**18.36**	**1,534**	**19.53**
Ilocos Sur	**9,072**	0	**0.000**	3	**0.03**	83	**0.91**	**86**	**0.95**
La Union	**11,338**	1	**0.009**	183	**1.61**	2,092	**18.45**	**2,276**	**20.07**
Pangasinan	**50,710**	4	**0.008**	495	**0.98**	5,848	**11.53**	**6,347**	**12.52**
Alaminos City	**1,910**	0	**0.000**	0	**0.00**	0	**0.00**	**0**	**0.00**
Candon City	**954**	0	**0.000**	0	**0.00**	267	**27.99**	**267**	**27.99**
Dagupan City	**3,657**	0	**0.000**	5	**0.14**	38	**1.04**	**43**	**1.18**
Laoag City	**1,812**	0	**0.000**	17	**0.94**	470	**25.94**	**487**	**26.88**
San Carlos City	**4,028**	0	**0.000**	25	**0.62**	228	**5.66**	**253**	**6.28**
San Fernando City	**2,074**	0	**0.000**	25	**1.21**	163	**7.86**	**188**	**9.06**
Urdaneta City	**2,836**	38	**1.340**	335	**11.81**	926	**32.65**	**1,299**	**45.80**
Vigan City	**854**	0	**0.000**	0	**0.00**	0	**0.00**	**0**	**0.00**
**Region 2**	**68,960**	**21**	**0.030**	**951**	**1.38**	**7,583**	**11.00**	**8,555**	**12.41**
Batanes	**362**	0	**0.000**	1	**0.28**	15	**4.14**	**16**	**4.42**

Table 2.B.1.8. Prenatal Care
Number and proportion of Pregnant Women given one (1) dose of deworming tablet
Philippines, 2022

Area	Eligible Pop.	Pregnant women given one (1) dose of Deworming Tablet							
		Age Group						Total	%
		10-14 yrs old		15-19 yrs old		20-49 yrs old			
		No.	%	No.	%	No.	%		
Cagayan	**20,431**	4	**0.020**	267	**1.31**	1,726	**8.45**	**1,997**	**9.77**
Isabela	**23,449**	12	**0.051**	302	**1.29**	2,013	**8.58**	**2,327**	**9.92**
Nueva Vizcaya	**9,538**	2	**0.021**	142	**1.49**	1,248	**13.08**	**1,392**	**14.59**
Quirino	**4,051**	0	**0.000**	2	**0.05**	20	**0.49**	**22**	**0.54**
Cauayan City	**2,565**	0	**0.000**	24	**0.94**	192	**7.49**	**216**	**8.42**
Ilagan City	**2,889**	0	**0.000**	52	**1.80**	545	**18.86**	**597**	**20.66**
Santiago City	**2,671**	1	**0.037**	113	**4.23**	1,182	**44.25**	**1,296**	**48.52**
Tuguegarao City	**3,004**	2	**0.067**	48	**1.60**	642	**21.37**	**692**	**23.04**
**Region 3**	**220,155**	**13**	**0.006**	**450**	**0.20**	**3,550**	**1.61**	**4,013**	**1.82**
Aurora	**4,754**	1	**0.021**	54	**1.14**	243	**5.11**	**298**	**6.27**
Bataan	**13,789**	0	**0.000**	4	**0.03**	26	**0.19**	**30**	**0.22**
Bulacan	**43,760**	0	**0.000**	21	**0.05**	168	**0.38**	**189**	**0.43**
Nueva Ecija	**29,039**	2	**0.007**	41	**0.14**	182	**0.63**	**225**	**0.77**
Pampanga	**31,620**	0	**0.000**	0	**0.00**	0	**0.00**	**0**	**0.00**
Tarlac	**19,319**	4	**0.021**	184	**0.95**	1,945	**10.07**	**2,133**	**11.04**
Zambales	**12,476**	0	**0.000**	45	**0.36**	220	**1.76**	**265**	**2.12**
Angeles City	**7,932**	5	**0.063**	5	**0.06**	6	**0.08**	**16**	**0.20**
Balanga City	**1,992**	0	**0.000**	0	**0.00**	0	**0.00**	**0**	**0.00**
Cabanatuan City	**5,945**	0	**0.000**	0	**0.00**	0	**0.00**	**0**	**0.00**
City of San Fernando	**5,907**	0	**0.000**	8	**0.14**	130	**2.20**	**138**	**2.34**
Gapan City	**2,170**	0	**0.000**	0	**0.00**	0	**0.00**	**0**	**0.00**
Mabalacat City	**4,831**	0	**0.000**	0	**0.00**	0	**0.00**	**0**	**0.00**
Malolos City	**4,890**	0	**0.000**	0	**0.00**	0	**0.00**	**0**	**0.00**
Meycauayan	**4,055**	0	**0.000**	4	**0.10**	19	**0.47**	**23**	**0.57**
Olongapo	**4,922**	0	**0.000**	62	**1.26**	497	**10.10**	**559**	**11.36**
Palayan City	**808**	1	**0.124**	4	**0.50**	10	**1.24**	**15**	**1.86**
San Jose City	**2,748**	0	**0.000**	0	**0.00**	0	**0.00**	**0**	**0.00**
San Jose del Monte City	**11,133**	0	**0.000**	0	**0.00**	0	**0.00**	**0**	**0.00**
Science City of Munoz	**1,601**	0	**0.000**	18	**1.12**	83	**5.18**	**101**	**6.31**
Tarlac City	**6,464**	0	**0.000**	0	**0.00**	21	**0.32**	**21**	**0.32**
**Region 4A**	**299,627**	**34**	**0.011**	**1,833**	**0.61**	**13,103**	**4.37**	**14,970**	**5.00**
Batangas	**39,255**	0	**0.000**	51	**0.13**	617	**1.57**	**668**	**1.70**
Cavite	**28,151**	10	**0.036**	57	**0.20**	407	**1.45**	**474**	**1.68**
Laguna	**19,696**	4	**0.020**	242	**1.23**	1,446	**7.34**	**1,692**	**8.59**
Quezon	**37,853**	7	**0.018**	218	**0.58**	1,352	**3.57**	**1,577**	**4.17**
Rizal	**44,798**	7	**0.016**	377	**0.84**	2,251	**5.02**	**2,635**	**5.88**
Antipolo City	**16,498**	0	**0.000**	23	**0.14**	103	**0.62**	**126**	**0.76**
Bacoor City	**12,341**	0	**0.000**	19	**0.15**	113	**0.92**	**132**	**1.07**
Batangas City	**6,965**	0	**0.000**	0	**0.00**	0	**0.00**	**0**	**0.00**
Biñan City	**6,597**	3	**0.045**	562	**8.52**	4,838	**73.34**	**5,403**	**81.90**
Cabuyao City	**6,119**	1	**0.016**	21	**0.34**	68	**1.11**	**90**	**1.47**
Calamba City	**9,005**	1	**0.011**	56	**0.62**	452	**5.02**	**509**	**5.65**
Cavite City	**2,105**	0	**0.000**	0	**0.00**	0	**0.00**	**0**	**0.00**
Dasmariñas City	**13,538**	0	**0.000**	32	**0.24**	442	**3.26**	**474**	**3.50**
General Trias City	**6,457**	0	**0.000**	21	**0.33**	146	**2.26**	**167**	**2.59**
Imus City	**8,292**	0	**0.000**	3	**0.04**	45	**0.54**	**48**	**0.58**
Lipa City	**7,018**	0	**0.000**	2	**0.03**	7	**0.10**	**9**	**0.13**
Lucena City	**5,738**	0	**0.000**	14	**0.24**	56	**0.98**	**70**	**1.22**
San Pablo City	**5,272**	0	**0.000**	0	**0.00**	0	**0.00**	**0**	**0.00**

Table 2.B.1.8. Prenatal Care

Number and proportion of Pregnant Women given one (1) dose of deworming tablet
Philippines, 2022

Area	Eligible Pop.	Pregnant women given one (1) dose of Deworming Tablet							
		Age Group						Total	%
		10-14 yrs old		15-19 yrs old		20-49 yrs old			
		No.	%	No.	%	No.	%		
San Pedro City	**6,455**	0	**0.000**	14	**0.22**	115	**1.78**	**129**	**2.00**
Santa Rosa City	**7,009**	0	**0.000**	64	**0.91**	315	**4.49**	**379**	**5.41**
Tagaytay City	**1,460**	0	**0.000**	0	**0.00**	7	**0.48**	**7**	**0.48**
Tanauan City	**3,661**	0	**0.000**	5	**0.14**	79	**2.16**	**84**	**2.29**
Tayabas City	**2,145**	0	**0.000**	0	**0.00**	0	**0.00**	**0**	**0.00**
Trece Martires City	**3,199**	1	**0.031**	52	**1.63**	244	**7.63**	**297**	**9.28**
**Region 4B**	**72,791**	**15**	**0.021**	**791**	**1.09**	**5,755**	**7.91**	**6,561**	**9.01**
Marinduque	**4,990**	1	**0.020**	91	**1.82**	693	**13.89**	**785**	**15.73**
Mindoro Occidental	**12,479**	1	**0.008**	130	**1.04**	720	**5.77**	**851**	**6.82**
Mindoro Oriental	**20,189**	1	**0.005**	244	**1.21**	1,918	**9.50**	**2,163**	**10.71**
Palawan	**21,963**	3	**0.014**	301	**1.37**	2,306	**10.50**	**2,610**	**11.88**
Romblon	**6,569**	9	**0.137**	25	**0.38**	99	**1.51**	**133**	**2.02**
Puerto Princesa City	**6,601**	0	**0.000**	0	**0.00**	19	**0.29**	**19**	**0.29**
**Region 5**	**138,457**	**34**	**0.025**	**3,302**	**2.38**	**27,123**	**19.59**	**30,459**	**22.00**
Albay	**24,081**	2	**0.008**	239	**0.99**	3,633	**15.09**	**3,874**	**16.09**
Camarines Norte	**14,384**	6	**0.042**	743	**5.17**	5,080	**35.32**	**5,829**	**40.52**
Camarines Sur	**39,486**	11	**0.028**	771	**1.95**	6,814	**17.26**	**7,596**	**19.24**
Catanduanes	**6,459**	5	**0.077**	187	**2.90**	1,498	**23.19**	**1,690**	**26.17**
Masbate	**22,600**	8	**0.035**	836	**3.70**	4,865	**21.53**	**5,709**	**25.26**
Sorsogon	**19,828**	1	**0.005**	394	**1.99**	3,898	**19.66**	**4,293**	**21.65**
Iriga City	**2,679**	0	**0.000**	74	**2.76**	701	**26.17**	**775**	**28.93**
Legaspi City	**4,233**	0	**0.000**	4	**0.09**	82	**1.94**	**86**	**2.03**
Naga City	**4,707**	1	**0.021**	54	**1.15**	552	**11.73**	**607**	**12.90**
**Region 6**	**146,449**	**25**	**0.017**	**1,333**	**0.91**	**12,617**	**8.62**	**13,975**	**9.54**
Aklan	**11,288**	2	**0.018**	120	**1.06**	1,289	**11.42**	**1,411**	**12.50**
Antique	**13,132**	4	**0.030**	72	**0.55**	758	**5.77**	**834**	**6.35**
Capiz	**13,975**	4	**0.029**	229	**1.64**	2,541	**18.18**	**2,774**	**19.85**
Guimaras	**3,084**	3	**0.097**	49	**1.59**	496	**16.08**	**548**	**17.77**
Iloilo	**36,267**	6	**0.017**	237	**0.65**	2,579	**7.11**	**2,822**	**7.78**
Negros Occidental	**49,230**	5	**0.010**	614	**1.25**	4,876	**9.90**	**5,495**	**11.16**
Bacolod City	**11,082**	1	**0.009**	7	**0.06**	33	**0.30**	**41**	**0.37**
Iloilo City	**8,391**	0	**0.000**	5	**0.06**	45	**0.54**	**50**	**0.60**
**Region 7**	**162,308**	**66**	**0.041**	**4,314**	**2.66**	**36,781**	**22.66**	**41,161**	**25.36**
Bohol	**27,992**	3	**0.011**	497	**1.78**	5,073	**18.12**	**5,573**	**19.91**
Cebu	**66,463**	27	**0.041**	1,556	**2.34**	11,912	**17.92**	**13,495**	**20.30**
Negros Oriental	**27,890**	5	**0.018**	402	**1.44**	2,362	**8.47**	**2,769**	**9.93**
Siquijor	**1,661**	0	**0.000**	44	**2.65**	408	**24.56**	**452**	**27.21**
Cebu City	**20,866**	23	**0.110**	1,224	**5.87**	10,215	**48.96**	**11,462**	**54.93**
Lapu-Lapu City	**9,232**	1	**0.011**	119	**1.29**	1,372	**14.86**	**1,492**	**16.16**
Mandaue City	**8,204**	7	**0.085**	472	**5.75**	5,439	**66.30**	**5,918**	**72.14**
**Region 8**	**105,471**	**23**	**0.022**	**1,416**	**1.34**	**11,638**	**11.03**	**13,077**	**12.40**
Biliran	**3,873**	3	**0.077**	255	**6.58**	1,991	**51.41**	**2,249**	**58.07**

Table 2.B.1.8. Prenatal Care

Number and proportion of Pregnant Women given one (1) dose of deworming tablet
Philippines, 2022

Area	Eligible Pop.	Pregnant women given one (1) dose of Deworming Tablet							
		Age Group						Total	%
		10-14 yrs old		15-19 yrs old		20-49 yrs old			
		No.	%	No.	%	No.	%		
Eastern Samar	**11,908**	6	**0.050**	241	**2.02**	2,299	**19.31**	**2,546**	**21.38**
Northern Leyte	**35,422**	3	**0.008**	62	**0.18**	694	**1.96**	**759**	**2.14**
Northern Samar	**15,921**	4	**0.025**	186	**1.17**	1,492	**9.37**	**1,682**	**10.56**
Southern Leyte	**6,662**	3	**0.045**	182	**2.73**	1,504	**22.58**	**1,689**	**25.35**
Western Samar	**14,732**	2	**0.014**	190	**1.29**	1,376	**9.34**	**1,568**	**10.64**
Calbayog City	**4,535**	0	**0.000**	43	**0.95**	328	**7.23**	**371**	**8.18**
Maasin City	**1,696**	0	**0.000**	9	**0.53**	327	**19.28**	**336**	**19.81**
Ormoc City	**5,044**	2	**0.040**	247	**4.90**	1,564	**31.01**	**1,813**	**35.94**
Tacloban City	**5,678**	0	**0.000**	1	**0.02**	63	**1.11**	**64**	**1.13**
**Region 9**	**79,007**	**26**	**0.033**	**1,752**	**2.22**	**10,829**	**13.71**	**12,607**	**15.96**
Zamboanga del Norte	**16,854**	3	**0.018**	352	**2.09**	1,904	**11.30**	**2,259**	**13.40**
Zamboanga del Sur	**17,368**	2	**0.012**	149	**0.86**	721	**4.15**	**872**	**5.02**
Zamboanga Sibugay	**15,021**	2	**0.013**	204	**1.36**	1,210	**8.06**	**1,416**	**9.43**
Dapitan City	**1,737**	0	**0.000**	80	**4.61**	890	**51.24**	**970**	**55.84**
Dipolog City	**2,762**	4	**0.145**	75	**2.72**	517	**18.72**	**596**	**21.58**
Isabela City	**2,563**	1	**0.039**	66	**2.58**	270	**10.53**	**337**	**13.15**
Pagadian City	**4,257**	0	**0.000**	1	**0.02**	45	**1.06**	**46**	**1.08**
Zamboanga City	**18,445**	14	**0.076**	825	**4.47**	5,272	**28.58**	**6,111**	**33.13**
**Region 10**	**99,908**	**53**	**0.053**	**2,470**	**2.47**	**12,101**	**12.11**	**14,624**	**14.64**
Bukidnon	**22,900**	34	**0.148**	1,374	**6.00**	4,620	**20.17**	**6,028**	**26.32**
Camiguin	**1,854**	0	**0.000**	7	**0.38**	102	**5.50**	**109**	**5.88**
Lanao del Norte	**14,930**	4	**0.027**	123	**0.82**	1,220	**8.17**	**1,347**	**9.02**
Misamis Occidental	**6,420**	2	**0.031**	40	**0.62**	416	**6.48**	**458**	**7.13**
Misamis Oriental	**14,963**	3	**0.020**	105	**0.70**	635	**4.24**	**743**	**4.97**
Cagayan de Oro City	**14,172**	0	**0.000**	0	**0.00**	1	**0.01**	**1**	**0.01**
El Salvador City	**1,052**	1	**0.095**	9	**0.86**	59	**5.61**	**69**	**6.56**
Gingoog City	**2,608**	0	**0.000**	4	**0.15**	46	**1.76**	**50**	**1.92**
Iligan City	**7,565**	7	**0.093**	490	**6.48**	3,248	**42.93**	**3,745**	**49.50**
Malaybalay City	**3,817**	0	**0.000**	21	**0.55**	145	**3.80**	**166**	**4.35**
Oroquieta City	**1,389**	0	**0.000**	0	**0.00**	0	**0.00**	**0**	**0.00**
Ozamis City	**2,786**	0	**0.000**	19	**0.68**	180	**6.46**	**199**	**7.14**
Tangub City	**1,234**	0	**0.000**	9	**0.73**	37	**3.00**	**46**	**3.73**
Valencia City	**4,218**	2	**0.047**	269	**6.38**	1,392	**33.00**	**1,663**	**39.43**
**Region 11**	**108,407**	**299**	**0.276**	**4,466**	**4.12**	**23,018**	**21.23**	**27,783**	**25.63**
Davao de Oro	**15,490**	110	**0.710**	1,195	**7.71**	5,601	**36.16**	**6,906**	**44.58**
Davao del Norte	**21,017**	82	**0.390**	1,197	**5.70**	6,324	**30.09**	**7,603**	**36.18**
Davao Oriental	**13,229**	26	**0.197**	634	**4.79**	3,459	**26.15**	**4,119**	**31.14**
Davao del Sur	**14,564**	20	**0.137**	177	**1.22**	909	**6.24**	**1,106**	**7.59**
Davao Occidental	**6,510**	29	**0.445**	621	**9.54**	1,643	**25.24**	**2,293**	**35.22**
Davao City	**37,597**	32	**0.085**	642	**1.71**	5,082	**13.52**	**5,756**	**15.31**
**Region 12**	**98,859**	**120**	**0.121**	**5,568**	**5.63**	**28,369**	**28.70**	**34,057**	**34.45**
North Cotabato	**34,150**	12	**0.035**	754	**2.21**	3,851	**11.28**	**4,617**	**13.52**
Sarangani	**12,907**	42	**0.325**	1,912	**14.81**	7,743	**59.99**	**9,697**	**75.13**
South Cotabato	**20,917**	16	**0.076**	1,104	**5.28**	5,670	**27.11**	**6,790**	**32.46**

Table 2.B.1.8. Prenatal Care

Number and proportion of Pregnant Women given one (1) dose of deworming tablet
Philippines, 2022

Area	Eligible Pop.	Pregnant women given one (1) dose of Deworming Tablet							
		Age Group						Total	%
		10-14 yrs old		15-19 yrs old		20-49 yrs old			
		No.	%	No.	%	No.	%		
Sultan Kudarat	**17,299**	43	**0.249**	1,463	**8.46**	9,422	**54.47**	**10,928**	**63.17**
Gen. Santos City	**13,586**	7	**0.052**	335	**2.47**	1,683	**12.39**	**2,025**	**14.91**
**BARMM**	**101,343**	**4**	**0.004**	**866**	**0.85**	**8,945**	**8.83**	**9,815**	**9.68**
Basilan	**7,823**	0	**0.000**	47	**0.60**	444	**5.68**	**491**	**6.28**
Lanao del Sur	**21,639**	0	**0.000**	151	**0.70**	3,142	**14.52**	**3,293**	**15.22**
Maguindanao	**32,198**	0	**0.000**	107	**0.33**	1,353	**4.20**	**1,460**	**4.53**
Sulu	**17,165**	2	**0.012**	301	**1.75**	1,900	**11.07**	**2,203**	**12.83**
Tawi-Tawi	**9,369**	1	**0.011**	134	**1.43**	1,108	**11.83**	**1,243**	**13.27**
Lamitan City	**2,154**	0	**0.000**	3	**0.14**	14	**0.65**	**17**	**0.79**
Marawi City	**5,173**	0	**0.000**	19	**0.37**	207	**4.00**	**226**	**4.37**
Cotabato City	**5,822**	1	**0.017**	104	**1.79**	777	**13.35**	882	**15.15**
**CARAGA**	**60,575**	**9**	**0.015**	**429**	**0.71**	**2,518**	**4.16**	**2,956**	**4.88**
Agusan del Norte	**8,127**	0	**0.000**	5	**0.06**	5	**0.06**	**10**	**0.12**
Agusan del Sur	**17,709**	0	**0.000**	92	**0.52**	621	**3.51**	**713**	**4.03**
Surigao del Norte	**7,280**	1	**0.014**	12	**0.16**	109	**1.50**	**122**	**1.68**
Surigao del Sur	**11,553**	6	**0.052**	205	**1.77**	1,156	**10.01**	**1,367**	**11.83**
Province of Dinagat	**2,576**	1	**0.039**	16	**0.62**	91	**3.53**	**108**	**4.19**
Bislig City	**2,192**	0	**0.000**	4	**0.18**	62	**2.83**	**66**	**3.01**
Butuan City	**7,743**	0	**0.000**	10	**0.13**	16	**0.21**	**26**	**0.34**
Surigao City	**3,395**	1	**0.029**	85	**2.50**	458	**13.49**	**544**	**16.02**

Table 2.B.1.9. Prenatal Care

Number and proportion of pregnant women screened for Syphilis
Philippines, 2022

Area	Eligible Pop.	Screened for Syphilis							
		Age Group						Total	%
		10-14 yrs old		15-19 yrs old		20-49 yrs old			
		No.	%	No.	%	No.	%		
**PHILIPPINES**	**2,131,496**	**1,708**	**0.08**	**66,494**	**3.12**	**504,462**	**23.67**	**572,664**	**26.87**
**N C R**	**236,901**	**268**	**0.11**	**12,050**	**5.09**	**110,302**	**46.56**	**122,620**	**51.76**
Malabon	**6,724**	13	**0.19**	385	**5.73**	2,268	**33.73**	2,666	**39.65**
Navotas	**4,591**	14	**0.30**	507	**11.04**	2,693	**58.66**	3,214	**70.01**
Valenzuela City	**11,418**	10	**0.09**	554	**4.85**	5,353	**46.88**	5,917	**51.82**
Caloocan City	**29,146**	17	**0.06**	1,102	**3.78**	9,638	**33.07**	10,757	**36.91**
								0	
Marikina City	**8,294**	5	**0.06**	181	**2.18**	1,632	**19.68**	1,818	**21.92**
Pasig City	**13,894**	15	**0.11**	830	**5.97**	11,562	**83.22**	12,407	**89.30**
Pateros	**1,176**	4	**0.34**	98	**8.33**	772	**65.65**	874	**74.32**
Taguig	**14,807**	34	**0.23**	1,024	**6.92**	8,045	**54.33**	9,103	**61.48**
Quezon City	**54,011**	54	**0.10**	2,627	**4.86**	28,993	**53.68**	31,674	**58.64**
Makati City	**10,718**	3	**0.03**	196	**1.83**	3,244	**30.27**	3,443	**32.12**
Mandaluyong City	**7,111**	4	**0.06**	389	**5.47**	6,287	**88.41**	6,680	**93.94**
San Juan	**2,246**	0	**0.00**	62	**2.76**	895	**39.85**	957	**42.61**
Manila City	**32,743**	62	**0.19**	2,491	**7.61**	14,941	**45.63**	17,494	**53.43**
Las Piñas City	**10,832**	7	**0.06**	274	**2.53**	2,478	**22.88**	2,759	**25.47**
Muntinlupa City	**9,281**	10	**0.11**	277	**2.98**	2,464	**26.55**	2,751	**29.64**
Parañaque City	**12,249**	16	**0.13**	627	**5.12**	5,429	**44.32**	6,072	**49.57**
Pasay City	**7,660**	0	**0.00**	426	**5.56**	3,608	**47.10**	4,034	**52.66**
**C A R**	**35,179**	**35**	**0.10**	**1,738**	**4.94**	**15,169**	**43.12**	**16,942**	**48.16**
Abra	**4,275**	2	**0.05**	182	**4.26**	860	**20.12**	1,044	**24.42**
Apayao	**2,502**	7	**0.28**	253	**10.11**	1,339	**53.52**	1,599	**63.91**
Benguet	**9,086**	13	**0.14**	638	**7.02**	6,311	**69.46**	6,962	**76.62**
Ifugao	**4,486**	3	**0.07**	180	**4.01**	1,648	**36.74**	1,831	**40.82**
Kalinga	**4,701**	2	**0.04**	86	**1.83**	968	**20.59**	1,056	**22.46**
Mt. Province	**3,103**	2	**0.06**	104	**3.35**	1,054	**33.97**	1,160	**37.38**
Baguio City	**7,026**	6	**0.09**	295	**4.20**	2,989	**42.54**	3,290	**46.83**
**Region 1**	**97,099**	**41**	**0.04**	**2,581**	**2.66**	**28,667**	**29.52**	**31,289**	**32.22**
Ilocos Norte	**7,854**	5	**0.06**	286	**3.64**	3,149	**40.09**	3,440	**43.80**
Ilocos Sur	**9,072**	7	**0.08**	379	**4.18**	4,903	**54.05**	5,289	**58.30**
La Union	**11,338**	11	**0.10**	474	**4.18**	4,185	**36.91**	4,670	**41.19**
Pangasinan	**50,710**	10	**0.02**	908	**1.79**	10,299	**20.31**	11,217	**22.12**
Alaminos City	**1,910**	0	**0.00**	119	**6.23**	1,153	**60.37**	1,272	**66.60**
Candon City	**954**	0	**0.00**	2	**0.21**	467	**48.95**	469	**49.16**
Dagupan City	**3,657**	4	**0.11**	54	**1.48**	396	**10.83**	454	**12.41**
Laoag City	**1,812**	0	**0.00**	21	**1.16**	1,430	**78.92**	1,451	**80.08**
San Carlos City	**4,028**	0	**0.00**	49	**1.22**	504	**12.51**	553	**13.73**
San Fernando City	**2,074**	1	**0.05**	74	**3.57**	620	**29.89**	695	**33.51**
Urdaneta City	**2,836**	2	**0.07**	199	**7.02**	1,421	**50.11**	1,622	**57.19**
Vigan City	**854**	1	**0.12**	16	**1.87**	140	**16.39**	157	**18.38**
**Region 2**	**68,960**	**52**	**0.08**	**1,908**	**2.77**	**13,336**	**19.34**	**15,296**	**22.18**
Batanes	**362**	1	**0.28**	3	**0.83**	165	**45.58**	169	**46.69**
Cagayan	**20,431**	6	**0.03**	193	**0.94**	1,128	**5.52**	1,327	**6.50**
Isabela	**23,449**	33	**0.14**	929	**3.96**	5,961	**25.42**	6,923	**29.52**
Nueva Vizcaya	**9,538**	2	**0.02**	281	**2.95**	2,236	**23.44**	2,519	**26.41**

Table 2.B.1.9. Prenatal Care

Number and proportion of pregnant women screened for Syphilis
Philippines, 2022

Area	Eligible Pop.	Screened for Syphilis							
		Age Group						Total	%
		10-14 yrs old		15-19 yrs old		20-49 yrs old			
		No.	%	No.	%	No.	%		
Quirino	4,051	4	0.10	172	4.25	1,349	33.30	1,525	37.65
Cauayan City	2,565	3	0.12	91	3.55	644	25.11	738	28.77
Ilagan City	2,889	1	0.03	155	5.37	1,205	41.71	1,361	47.11
Santiago City	2,671	2	0.07	73	2.73	428	16.02	503	18.83
Tuguegarao City	3,004	0	0.00	11	0.37	220	7.32	231	7.69
**Region 3**	**220,155**	**285**	**0.13**	**10,282**	**4.67**	**68,989**	**31.34**	**79,556**	**36.14**
Aurora	4,754	8	0.17	254	5.34	1,617	34.01	1,879	39.52
Bataan	13,789	26	0.19	958	6.95	5,631	40.84	6,615	47.97
Bulacan	43,760	58	0.13	1,841	4.21	13,002	29.71	14,901	34.05
Nueva Ecija	29,039	25	0.09	789	2.72	4,246	14.62	5,060	17.42
Pampanga	31,620	29	0.09	1,367	4.32	9,543	30.18	10,939	34.60
Tarlac	19,319	31	0.16	616	3.19	4,839	25.05	5,486	28.40
Zambales	12,476	14	0.11	543	4.35	3,294	26.40	3,851	30.87
Angeles City	7,932	2	0.03	174	2.19	995	12.54	1,171	14.76
Balanga City	1,992	7	0.35	168	8.43	1,027	51.56	1,202	60.34
Cabanatuan City	5,945	5	0.08	208	3.50	1,212	20.39	1,425	23.97
City of San Fernando	5,907	10	0.17	273	4.62	1,913	32.39	2,196	37.18
Gapan City	2,170	1	0.05	74	3.41	321	14.79	396	18.25
Mabalacat City	4,831	14	0.29	543	11.24	3,018	62.47	3,575	74.00
Malolos City	4,890	9	0.18	200	4.09	1,241	25.38	1,450	29.65
Meycauayan	4,055	0	0.00	251	6.19	1,510	37.24	1,761	43.43
Olongapo	4,922	3	0.06	173	3.51	1,076	21.86	1,252	25.44
Palayan City	808	0	0.00	13	1.61	100	12.38	113	13.99
San Jose City	2,748	3	0.11	85	3.09	381	13.86	469	17.07
San Jose del Monte City	11,133	33	0.30	1,200	10.78	11,128	99.96	12,361	111.03
Science City of Munoz	1,601	2	0.12	100	6.25	561	35.04	663	41.41
Tarlac City	6,464	5	0.08	452	6.99	2,334	36.11	2,791	43.18
**Region 4A**	**299,627**	**139**	**0.05**	**7,905**	**2.64**	**61,654**	**20.58**	**69,698**	**23.26**
Batangas	39,255	0	0.00	378	0.96	3,538	9.01	3,916	9.98
Cavite	28,151	19	0.07	681	2.42	6,367	22.62	7,067	25.10
Laguna	19,696	16	0.08	717	3.64	5,104	25.91	5,837	29.64
Quezon	37,853	34	0.09	843	2.23	5,864	15.49	6,741	17.81
Rizal	44,798	27	0.06	1,765	3.94	11,419	25.49	13,211	29.49
Antipolo City	16,498	0	0.00	303	1.84	1,811	10.98	2,114	12.81
Bacoor City	12,341	1	0.01	185	1.50	1,313	10.64	1,499	12.15
Batangas City	6,965	2	0.03	133	1.91	1,263	18.13	1,398	20.07
Biñan City	6,597	4	0.06	798	12.10	5,845	88.60	6,647	100.76
Cabuyao City	6,119	3	0.05	159	2.60	1,805	29.50	1,967	32.15
Calamba City	9,005	8	0.09	289	3.21	2,907	32.28	3,204	35.58
Cavite City	2,105	2	0.10	79	3.75	322	15.30	403	19.14
Dasmariñas City	13,538	4	0.03	346	2.56	3,103	22.92	3,453	25.51
General Trias City	6,457	2	0.03	53	0.82	586	9.08	641	9.93
Imus City	8,292	3	0.04	66	0.80	508	6.13	577	6.96
Lipa City	7,018	1	0.01	141	2.01	1,687	24.04	1,829	26.06
Lucena City	5,738	3	0.05	288	5.02	1,422	24.78	1,713	29.85
San Pablo City	5,272	1	0.02	21	0.40	196	3.72	218	4.14
San Pedro City	6,455	2	0.03	158	2.45	1,040	16.11	1,200	18.59
Santa Rosa City	7,009	2	0.03	278	3.97	2,376	33.90	2,656	37.89
Tagaytay City	1,460	0	0.00	61	4.18	1,165	79.79	1,226	83.97
Tanauan City	3,661	3	0.08	70	1.91	1,243	33.95	1,316	35.95
Tayabas City	2,145	0	0.00	7	0.33	124	5.78	131	6.11
Trece Martires City	3,199	2	0.06	86	2.69	646	20.19	734	22.94

Table 2.B.1.9. Prenatal Care

Number and proportion of pregnant women screened for Syphilis
Philippines, 2022

Area	Eligible Pop.	Screened for Syphilis							
		Age Group						Total	%
		10-14 yrs old		15-19 yrs old		20-49 yrs old			
		No.	%	No.	%	No.	%		
**Region 4B**	**72,791**	**23**	**0.03**	**1,394**	**1.92**	**10,909**	**14.99**	**12,326**	**16.93**
Marinduque	**4,990**	1	**0.02**	63	**1.26**	711	**14.25**	775	**15.53**
Mindoro Occidental	**12,479**	2	**0.02**	265	**2.12**	1,631	**13.07**	1,898	**15.21**
Mindoro Oriental	**20,189**	3	**0.01**	418	**2.07**	4,075	**20.18**	4,496	**22.27**
Palawan	**21,963**	8	**0.04**	424	**1.93**	2,654	**12.08**	3,086	**14.05**
Romblon	**6,569**	9	**0.14**	169	**2.57**	1,524	**23.20**	1,702	**25.91**
Puerto Princesa City	**6,601**	0	**0.00**	55	**0.83**	314	**4.76**	369	**5.59**
**Region 5**	**138,457**	**67**	**0.05**	**2,153**	**1.55**	**19,555**	**14.12**	**21,775**	**15.73**
Albay	**24,081**	43	**0.18**	324	**1.35**	4,920	**20.43**	5,287	**21.96**
Camarines Norte	**14,384**	5	**0.03**	294	**2.04**	2,060	**14.32**	2,359	**16.40**
Camarines Sur	**39,486**	0	**0.00**	201	**0.51**	2,359	**5.97**	2,560	**6.48**
Catanduanes	**6,459**	4	**0.06**	237	**3.67**	1,987	**30.76**	2,228	**34.49**
Masbate	**22,600**	9	**0.04**	666	**2.95**	3,707	**16.40**	4,382	**19.39**
Sorsogon	**19,828**	1	**0.01**	187	**0.94**	1,945	**9.81**	2,133	**10.76**
Iriga City	**2,679**	0	**0.00**	48	**1.79**	477	**17.81**	525	**19.60**
Legaspi City	**4,233**	4	**0.09**	90	**2.13**	957	**22.61**	1,051	**24.83**
Naga City	**4,707**	1	**0.02**	106	**2.25**	1,143	**24.28**	1,250	**26.56**
**Region 6**	**146,449**	**81**	**0.06**	**4,626**	**3.16**	**39,741**	**27.14**	**44,448**	**30.35**
Aklan	**11,288**	4	**0.04**	294	**2.60**	3,501	**31.02**	3,799	**33.66**
Antique	**13,132**	9	**0.07**	328	**2.50**	2,929	**22.30**	3,266	**24.87**
Capiz	**13,975**	8	**0.06**	246	**1.76**	2,763	**19.77**	3,017	**21.59**
Guimaras	**3,084**	7	**0.23**	194	**6.29**	1,809	**58.66**	2,010	**65.18**
Iloilo	**36,267**	35	**0.10**	1,497	**4.13**	13,985	**38.56**	15,517	**42.79**
Negros Occidental	**49,230**	7	**0.01**	1,122	**2.28**	7,235	**14.70**	8,364	**16.99**
Bacolod City	**11,082**	9	**0.08**	622	**5.61**	4,583	**41.36**	5,214	**47.05**
Iloilo City	**8,391**	2	**0.02**	323	**3.85**	2,936	**34.99**	3,261	**38.86**
**Region 7**	**162,308**	**314**	**0.19**	**7,973**	**4.91**	**49,469**	**30.48**	**57,756**	**35.58**
Bohol	**27,992**	5	**0.02**	742	**2.65**	7,240	**25.86**	7,987	**28.53**
Cebu	**66,463**	28	**0.04**	2,013	**3.03**	12,121	**18.24**	14,162	**21.31**
Negros Oriental	**27,890**	18	**0.06**	474	**1.70**	2,991	**10.72**	3,483	**12.49**
Siquijor	**1,661**	1	**0.06**	76	**4.58**	750	**45.15**	827	**49.79**
Cebu City	**20,866**	23	**0.11**	1,166	**5.59**	9,210	**44.14**	10,399	**49.84**
Lapu-Lapu City	**9,232**	6	**0.06**	284	**3.08**	4,166	**45.13**	4,456	**48.27**
Mandaue City	**8,204**	233	**2.84**	3,218	**39.22**	12,991	**158.35**	16,442	**200.41**
**Region 8**	**105,471**	**37**	**0.04**	**1,684**	**1.60**	**13,213**	**12.53**	**14,934**	**14.16**
Biliran	**3,873**	2	**0.05**	175	**4.52**	1,225	**31.63**	1,402	**36.20**
Eastern Samar	**11,908**	5	**0.04**	177	**1.49**	1,403	**11.78**	1,585	**13.31**
Northern Leyte	**35,422**	3	**0.01**	159	**0.45**	1,426	**4.03**	1,588	**4.48**
Northern Samar	**15,921**	14	**0.09**	604	**3.79**	3,961	**24.88**	4,579	**28.76**
Southern Leyte	**6,662**	3	**0.05**	35	**0.53**	415	**6.23**	453	**6.80**
Western Samar	**14,732**	0	**0.00**	79	**0.54**	528	**3.58**	607	**4.12**
Calbayog City	**4,535**	0	**0.00**	32	**0.71**	240	**5.29**	272	**6.00**
Maasin City	**1,696**	0	**0.00**	0	**0.00**	3	**0.18**	3	**0.18**
Ormoc City	**5,044**	5	**0.10**	153	**3.03**	1,101	**21.83**	1,259	**24.96**

Table 2.B.1.9. Prenatal Care

Number and proportion of pregnant women screened for Syphilis
Philippines, 2022

Area	Eligible Pop.	Screened for Syphilis							
		Age Group						Total	%
		10-14 yrs old		15-19 yrs old		20-49 yrs old			
		No.	%	No.	%	No.	%		
Tacloban City	**5,678**	5	**0.09**	270	**4.76**	2,911	**51.27**	3,186	**56.11**
**Region 9**	**79,007**	**17**	**0.02**	**897**	**1.14**	**5,314**	**6.73**	**6,228**	**7.88**
Zamboanga del Norte	**16,854**	0	**0.00**	39	**0.23**	209	**1.24**	248	**1.47**
Zamboanga del Sur	**17,368**	3	**0.02**	146	**0.84**	797	**4.59**	946	**5.45**
Zamboanga Sibugay	**15,021**	9	**0.06**	470	**3.13**	3,020	**20.11**	3,499	**23.29**
Dapitan City	**1,737**	2	**0.12**	44	**2.53**	189	**10.88**	235	**13.53**
Dipolog City	**2,762**	1	**0.04**	0	**0.00**	10	**0.36**	11	**0.40**
Isabela City	**2,563**	0	**0.00**	54	**2.11**	250	**9.75**	304	**11.86**
Pagadian City	**4,257**	0	**0.00**	57	**1.34**	281	**6.60**	338	**7.94**
Zamboanga City	**18,445**	2	**0.01**	87	**0.47**	558	**3.03**	647	**3.51**
**Region 10**	**99,908**	**43**	**0.04**	**2,449**	**2.45**	**13,940**	**13.95**	**16,432**	**16.45**
Bukidnon	**22,900**	4	**0.02**	378	**1.65**	1,845	**8.06**	2,227	**9.72**
Camiguin	**1,854**	1	**0.05**	47	**2.54**	390	**21.04**	438	**23.62**
Lanao del Norte	**14,930**	4	**0.03**	55	**0.37**	428	**2.87**	487	**3.26**
Misamis Occidental	**6,420**	1	**0.02**	15	**0.23**	202	**3.15**	218	**3.40**
Misamis Oriental	**14,963**	10	**0.07**	471	**3.15**	2,198	**14.69**	2,679	**17.90**
Cagayan de Oro City	**14,172**	14	**0.10**	827	**5.84**	4,955	**34.96**	5,796	**40.90**
El Salvador City	**1,052**	4	**0.38**	72	**6.84**	304	**28.90**	380	**36.12**
Gingoog City	**2,608**	1	**0.04**	58	**2.22**	244	**9.36**	303	**11.62**
Iligan City	**7,565**	0	**0.00**	91	**1.20**	835	**11.04**	926	**12.24**
Malaybalay City	**3,817**	2	**0.05**	129	**3.38**	739	**19.36**	870	**22.79**
Oroquieta City	**1,389**	0	**0.00**	4	**0.29**	11	**0.79**	15	**1.08**
Ozamis City	**2,786**	0	**0.00**	141	**5.06**	999	**35.86**	1,140	**40.92**
Tangub City	**1,234**	0	**0.00**	129	**10.45**	593	**48.06**	722	**58.51**
Valencia City	**4,218**	2	**0.05**	32	**0.76**	197	**4.67**	231	**5.48**
**Region 11**	**108,407**	**190**	**0.18**	**3,540**	**3.27**	**22,743**	**20.98**	**26,473**	**24.42**
Davao de Oro	**15,490**	2	**0.01**	31	**0.20**	286	**1.85**	319	**2.06**
Davao del Norte	**21,017**	49	**0.23**	898	**4.27**	5,764	**27.43**	6,711	**31.93**
Davao Oriental	**13,229**	7	**0.05**	51	**0.39**	302	**2.28**	360	**2.72**
Davao del Sur	**14,564**	11	**0.08**	217	**1.49**	1,030	**7.07**	1,258	**8.64**
Davao Occidental	**6,510**	2	**0.03**	57	**0.88**	136	**2.09**	195	**3.00**
Davao City	**37,597**	119	**0.32**	2,286	**6.08**	15,225	**40.50**	17,630	**46.89**
**Region 12**	**98,859**	**97**	**0.10**	**3,880**	**3.92**	**21,586**	**21.84**	**25,563**	**25.86**
North Cotabato	**34,150**	11	**0.03**	440	**1.29**	2,535	**7.42**	2,986	**8.74**
Sarangani	**12,907**	23	**0.18**	617	**4.78**	2,937	**22.76**	3,577	**27.71**
South Cotabato	**20,917**	20	**0.10**	1,164	**5.56**	6,544	**31.29**	7,728	**36.95**
Sultan Kudarat	**17,299**	29	**0.17**	1,103	**6.38**	6,558	**37.91**	7,690	**44.45**
Gen. Santos City	**13,586**	14	**0.10**	556	**4.09**	3,012	**22.17**	3,582	**26.37**
**BARMM**	**101,343**	**0**	**0.00**	**66**	**0.07**	**565**	**0.56**	**631**	**0.62**
Basilan	**7,823**	0	**0.00**	0	**0.00**	0	**0.00**	0	**0.00**
Lanao del Sur	**21,639**	0	**0.00**	2	**0.01**	75	**0.35**	77	**0.36**
Maguindanao	**32,198**	0	**0.00**	7	**0.02**	22	**0.07**	29	**0.09**
Sulu	**17,165**	0	**0.00**	28	**0.16**	97	**0.57**	125	**0.73**
Tawi-Tawi	**9,369**	0	**0.00**	0	**0.00**	0	**0.00**	0	**0.00**

Table 2.B.1.9. Prenatal Care

Number and proportion of pregnant women screened for Syphilis

Philippines, 2022

Area	Eligible Pop.	Screened for Syphilis							
		Age Group						Total	%
		10-14 yrs old		15-19 yrs old		20-49 yrs old			
		No.	%	No.	%	No.	%		
Lamitan City	**2,154**	0	**0.00**	3	**0.14**	11	**0.51**	14	**0.65**
Marawi City	**5,173**	0	**0.00**	0	**0.00**	0	**0.00**	0	**0.00**
Cotabato City	**5,822**	0	**0.00**	26	**0.45**	360	**6.18**	386	**6.63**
**CARAGA**	**60,575**	**19**	**0.03**	**1,368**	**2.26**	**9,310**	**15.37**	**10,697**	**17.66**
Agusan del Norte	**8,127**	1	**0.01**	145	**1.78**	1,005	**12.37**	1,151	**14.16**
Agusan del Sur	**17,709**	2	**0.01**	397	**2.24**	2,233	**12.61**	2,632	**14.86**
Surigao del Norte	**7,280**	0	**0.00**	48	**0.66**	560	**7.69**	608	**8.35**
Surigao del Sur	**11,553**	1	**0.01**	86	**0.74**	598	**5.18**	685	**5.93**
Province of Dinagat	**2,576**	2	**0.08**	112	**4.35**	733	**28.45**	847	**32.88**
Bislig City	**2,192**	0	**0.00**	4	**0.18**	51	**2.33**	55	**2.51**
Butuan City	**7,743**	8	**0.10**	371	**4.79**	3,025	**39.07**	3,404	**43.96**
Surigao City	**3,395**	5	**0.15**	205	**6.04**	1,105	**32.55**	1,315	**38.73**

Table 2.B.1.10. Prenatal Care

Number and proportion of pregnant women tested positive for Syphilis

Philippines, 2022

Area	Total No. of Screened for syphillis	Tested Positive for Syphilis							
		Age Group						Total	%
		10-14 yrs old		15-19 yrs old		20-49 yrs old			
		No.	%	No.	%	No.	%		
**PHILIPPINES**	**572,664**	**43**	**0.01**	**884**	**0.15**	**6,168**	**1.08**	**7,095**	**1.24**
**N C R**	**122,620**	**1**	**0.00**	**104**	**0.08**	**718**	**0.59**	**823**	**0.67**
Malabon	**2,666**	0	**0.00**	1	**0.04**	13	**0.49**	14	**0.53**
Navotas	**3,214**	0	**0.00**	1	**0.03**	75	**2.33**	76	**2.36**
Valenzuela City	**5,917**	0	**0.00**	8	**0.14**	113	**1.91**	121	**2.04**
Caloocan City	**10,757**	0	**0.00**	2	**0.02**	23	**0.21**	25	**0.23**
								0	
Marikina City	**1,818**	0	**0.00**	2	**0.11**	12	**0.66**	14	**0.77**
Pasig City	**12,407**	1	**0.01**	6	**0.05**	27	**0.22**	34	**0.27**
Pateros	**874**	0	**0.00**	0	**0.00**	2	**0.23**	2	**0.23**
Taguig	**9,103**	0	**0.00**	6	**0.07**	59	**0.65**	65	**0.71**
Quezon City	**31,674**	0	**0.00**	7	**0.02**	64	**0.20**	71	**0.22**
Makati City	**3,443**	0	**0.00**	0	**0.00**	19	**0.55**	19	**0.55**
Mandaluyong City	**6,680**	0	**0.00**	1	**0.01**	2	**0.03**	3	**0.04**
San Juan	**957**	0	**0.00**	0	**0.00**	2	**0.21**	2	**0.21**
Manila City	**17,494**	0	**0.00**	37	**0.21**	98	**0.56**	135	**0.77**
Las Piñas City	**2,759**	0	**0.00**	4	**0.14**	31	**1.12**	35	**1.27**
Muntinlupa City	**2,751**	0	**0.00**	6	**0.22**	15	**0.55**	21	**0.76**
Parañaque City	**6,072**	0	**0.00**	19	**0.31**	135	**2.22**	154	**2.54**
Pasay City	**4,034**	0	**0.00**	4	**0.10**	28	**0.69**	32	**0.79**
**C A R**	**16,942**	**0**	**0.00**	**11**	**0.06**	**44**	**0.26**	**55**	**0.32**
Abra	**1,044**	0	**0.00**	0	**0.00**	0	**0.00**	0	**0.00**
Apayao	**1,599**	0	**0.00**	4	**0.25**	3	**0.19**	7	**0.44**
Benguet	**6,962**	0	**0.00**	0	**0.00**	13	**0.19**	13	**0.19**
Ifugao	**1,831**	0	**0.00**	0	**0.00**	9	**0.49**	9	**0.49**
Kalinga	**1,056**	0	**0.00**	0	**0.00**	0	**0.00**	0	**0.00**
Mt. Province	**1,160**	0	**0.00**	0	**0.00**	0	**0.00**	0	**0.00**
Baguio City	**3,290**	0	**0.00**	7	**0.21**	19	**0.58**	26	**0.79**
**Region 1**	**31,289**	**0**	**0.00**	**11**	**0.04**	**80**	**0.26**	**91**	**0.29**
Ilocos Norte	**3,440**	0	**0.00**	0	**0.00**	5	**0.15**	5	**0.15**
Ilocos Sur	**5,289**	0	**0.00**	0	**0.00**	2	**0.04**	2	**0.04**
La Union	**4,670**	0	**0.00**	4	**0.09**	21	**0.45**	25	**0.54**
Pangasinan	**11,217**	0	**0.00**	7	**0.06**	46	**0.41**	53	**0.47**
Alaminos City	**1,272**	0	**0.00**	0	**0.00**	0	**0.00**	0	**0.00**
Candon City	**469**	0	**0.00**	0	**0.00**	1	**0.21**	1	**0.21**
Dagupan City	**454**	0	**0.00**	0	**0.00**	2	**0.44**	2	**0.44**
Laoag City	**1,451**	0	**0.00**	0	**0.00**	0	**0.00**	0	**0.00**
San Carlos City	**553**	0	**0.00**	0	**0.00**	3	**0.54**	3	**0.54**
San Fernando City	**695**	0	**0.00**	0	**0.00**	0	**0.00**	0	**0.00**
Urdaneta City	**1,622**	0	**0.00**	0	**0.00**	0	**0.00**	0	**0.00**
Vigan City	**157**	0	**0.00**	0	**0.00**	0	**0.00**	0	**0.00**
**Region 2**	**15,296**	**1**	**0.01**	**31**	**0.20**	**245**	**1.60**	**277**	**1.81**
Batanes	**169**	0	**0.00**	0	**0.00**	0	**0.00**	0	**0.00**
Cagayan	**1,327**	0	**0.00**	1	**0.08**	5	**0.38**	6	**0.45**
Isabela	**6,923**	1	**0.01**	0	**0.00**	41	**0.59**	42	**0.61**
Nueva Vizcaya	**2,519**	0	**0.00**	25	**0.99**	149	**5.92**	174	**6.91**
Quirino	**1,525**	0	**0.00**	4	**0.26**	27	**1.77**	31	**2.03**
Cauayan City	**738**	0	**0.00**	0	**0.00**	0	**0.00**	0	**0.00**
Ilagan City	**1,361**	0	**0.00**	0	**0.00**	0	**0.00**	0	**0.00**

Table 2.B.1.10. Prenatal Care
Number and proportion of pregnant women tested positive for Syphilis
Philippines, 2022

Area	Total No. of Screened for syphillis	Tested Positive for Syphilis							
		Age Group						Total	%
		10-14 yrs old		15-19 yrs old		20-49 yrs old			
		No.	%	No.	%	No.	%		
Santiago City	**503**	0	**0.00**	0	**0.00**	1	**0.20**	1	**0.20**
Tuguegarao City	**231**	0	**0.00**	1	**0.43**	22	**9.52**	23	**9.96**
**Region 3**	**79,556**	**6**	**0.01**	**78**	**0.10**	**552**	**0.69**	**636**	**0.80**
Aurora	**1,879**	0	**0.00**	0	**0.00**	0	**0.00**	0	**0.00**
Bataan	**6,615**	0	**0.00**	12	**0.18**	53	**0.80**	65	**0.98**
Bulacan	**14,901**	0	**0.00**	2	**0.01**	149	**1.00**	151	**1.01**
Nueva Ecija	**5,060**	2	**0.04**	9	**0.18**	83	**1.64**	94	**1.86**
Pampanga	**10,939**	3	**0.03**	27	**0.25**	156	**1.43**	186	**1.70**
Tarlac	**5,486**	1	**0.02**	0	**0.00**	8	**0.15**	9	**0.16**
Zambales	**3,851**	0	**0.00**	3	**0.08**	16	**0.42**	19	**0.49**
Angeles City	**1,171**	0	**0.00**	1	**0.09**	17	**1.45**	18	**1.54**
Balanga City	**1,202**	0	**0.00**	1	**0.08**	7	**0.58**	8	**0.67**
Cabanatuan City	**1,425**	0	**0.00**	0	**0.00**	0	**0.00**	0	**0.00**
City of San Fernando	**2,196**	0	**0.00**	0	**0.00**	4	**0.18**	4	**0.18**
Gapan City	**396**	0	**0.00**	0	**0.00**	1	**0.25**	1	**0.25**
Mabalacat City	**3,575**	0	**0.00**	12	**0.34**	7	**0.20**	19	**0.53**
Malolos City	**1,450**	0	**0.00**	1	**0.07**	3	**0.21**	4	**0.28**
Meycauayan	**1,761**	0	**0.00**	3	**0.17**	31	**1.76**	34	**1.93**
Olongapo	**1,252**	0	**0.00**	3	**0.24**	6	**0.48**	9	**0.72**
Palayan City	**113**	0	**0.00**	0	**0.00**	0	**0.00**	0	**0.00**
San Jose City	**469**	0	**0.00**	0	**0.00**	0	**0.00**	0	**0.00**
San Jose del Monte City	**12,361**	0	**0.00**	1	**0.01**	6	**0.05**	7	**0.06**
Science City of Munoz	**663**	0	**0.00**	0	**0.00**	0	**0.00**	0	**0.00**
Tarlac City	**2,791**	0	**0.00**	3	**0.11**	5	**0.18**	8	**0.29**
**Region 4A**	**69,698**	**7**	**0.01**	**189**	**0.27**	**1,481**	**2.12**	**1,677**	**2.41**
Batangas	**3,916**	2	**0.05**	5	**0.13**	52	**1.33**	59	**1.51**
Cavite	**7,067**	0	**0.00**	23	**0.33**	190	**2.69**	213	**3.01**
Laguna	**5,837**	0	**0.00**	17	**0.29**	109	**1.87**	126	**2.16**
Quezon	**6,741**	2	**0.03**	15	**0.22**	132	**1.96**	149	**2.21**
Rizal	**13,211**	0	**0.00**	33	**0.25**	155	**1.17**	188	**1.42**
Antipolo City	**2,114**	1	**0.05**	27	**1.28**	182	**8.61**	210	**9.93**
Bacoor City	**1,499**	0	**0.00**	3	**0.20**	15	**1.00**	18	**1.20**
Batangas City	**1,398**	0	**0.00**	0	**0.00**	12	**0.86**	12	**0.86**
Biñan City	**6,647**	0	**0.00**	10	**0.15**	57	**0.86**	67	**1.01**
Cabuyao City	**1,967**	2	**0.10**	0	**0.00**	12	**0.61**	14	**0.71**
Calamba City	**3,204**	0	**0.00**	7	**0.22**	101	**3.15**	108	**3.37**
Cavite City	**403**	0	**0.00**	0	**0.00**	9	**2.23**	9	**2.23**
Dasmariñas City	**3,453**	0	**0.00**	9	**0.26**	22	**0.64**	31	**0.90**
General Trias City	**641**	0	**0.00**	2	**0.31**	21	**3.28**	23	**3.59**
Imus City	**577**	0	**0.00**	1	**0.17**	17	**2.95**	18	**3.12**
Lipa City	**1,829**	0	**0.00**	15	**0.82**	112	**6.12**	127	**6.94**
Lucena City	**1,713**	0	**0.00**	9	**0.53**	39	**2.28**	48	**2.80**
San Pablo City	**218**	0	**0.00**	1	**0.46**	6	**2.75**	7	**3.21**
San Pedro City	**1,200**	0	**0.00**	3	**0.25**	39	**3.25**	42	**3.50**
Santa Rosa City	**2,656**	0	**0.00**	5	**0.19**	182	**6.85**	187	**7.04**
Tagaytay City	**1,226**	0	**0.00**	0	**0.00**	0	**0.00**	0	**0.00**
Tanauan City	**1,316**	0	**0.00**	0	**0.00**	7	**0.53**	7	**0.53**
Tayabas City	**131**	0	**0.00**	2	**1.53**	1	**0.76**	3	**2.29**
Trece Martires City	**734**	0	**0.00**	2	**0.27**	9	**1.23**	11	**1.50**
**Region 4B**	**12,326**	**16**	**0.13**	**28**	**0.23**	**171**	**1.39**	**215**	**1.74**
Marinduque	**775**	0	**0.00**	0	**0.00**	13	**1.68**	13	**1.68**
Mindoro Occidental	**1,898**	0	**0.00**	0	**0.00**	11	**0.58**	11	**0.58**
Mindoro Oriental	**4,496**	7	**0.16**	3	**0.07**	35	**0.78**	45	**1.00**
Palawan	**3,086**	0	**0.00**	8	**0.26**	75	**2.43**	83	**2.69**
Romblon	**1,702**	9	**0.53**	13	**0.76**	32	**1.88**	54	**3.17**

Table 2.B.1.10. Prenatal Care

Number and proportion of pregnant women tested positive for Syphilis
Philippines, 2022

Area	Total No. of Screened for syphillis	Tested Positive for Syphilis							
		Age Group						Total	%
		10-14 yrs old		15-19 yrs old		20-49 yrs old			
		No.	%	No.	%	No.	%		
Puerto Princesa City	**369**	0	**0.00**	4	**1.08**	5	**1.36**	9	**2.44**
**Region 5**	**21,775**	**0**	**0.00**	**22**	**0.10**	**299**	**1.37**	**321**	**1.47**
Albay	**5,287**	0	**0.00**	2	**0.04**	121	**2.29**	123	**2.33**
Camarines Norte	**2,359**	0	**0.00**	9	**0.38**	51	**2.16**	60	**2.54**
Camarines Sur	**2,560**	0	**0.00**	0	**0.00**	3	**0.12**	3	**0.12**
Catanduanes	**2,228**	0	**0.00**	1	**0.04**	6	**0.27**	7	**0.31**
Masbate	**4,382**	0	**0.00**	10	**0.23**	73	**1.67**	83	**1.89**
Sorsogon	**2,133**	0	**0.00**	0	**0.00**	30	**1.41**	30	**1.41**
Iriga City	**525**	0	**0.00**	0	**0.00**	6	**1.14**	6	**1.14**
Legaspi City	**1,051**	0	**0.00**	0	**0.00**	4	**0.38**	4	**0.38**
Naga City	**1,250**	0	**0.00**	0	**0.00**	5	**0.40**	5	**0.40**
**Region 6**	**44,448**	**3**	**0.01**	**93**	**0.21**	**587**	**1.32**	**683**	**1.54**
Aklan	**3,799**	0	**0.00**	12	**0.32**	41	**1.08**	53	**1.40**
Antique	**3,266**	2	**0.06**	6	**0.18**	42	**1.29**	50	**1.53**
Capiz	**3,017**	0	**0.00**	7	**0.23**	44	**1.46**	51	**1.69**
Guimaras	**2,010**	0	**0.00**	0	**0.00**	6	**0.30**	6	**0.30**
Iloilo	**15,517**	0	**0.00**	21	**0.14**	212	**1.37**	233	**1.50**
Negros Occidental	**8,364**	0	**0.00**	33	**0.39**	138	**1.65**	171	**2.04**
Bacolod City	**5,214**	1	**0.02**	9	**0.17**	86	**1.65**	96	**1.84**
Iloilo City	**3,261**	0	**0.00**	5	**0.15**	18	**0.55**	23	**0.71**
**Region 7**	**57,756**	**1**	**0.00**	**93**	**0.16**	**770**	**1.33**	**864**	**1.50**
Bohol	**7,987**	1	**0.01**	4	**0.05**	79	**0.99**	84	**1.05**
Cebu	**14,162**	0	**0.00**	60	**0.42**	466	**3.29**	526	**3.71**
Negros Oriental	**3,483**	0	**0.00**	0	**0.00**	7	**0.20**	7	**0.20**
Siquijor	**827**	0	**0.00**	0	**0.00**	4	**0.48**	4	**0.48**
Cebu City	**10,399**	0	**0.00**	27	**0.26**	213	**2.05**	240	**2.31**
Lapu-Lapu City	**4,456**	0	**0.00**	0	**0.00**	0	**0.00**	0	**0.00**
Mandaue City	**16,442**	0	**0.00**	2	**0.01**	1	**0.01**	3	**0.02**
**Region 8**	**14,934**	**0**	**0.00**	**55**	**0.37**	**147**	**0.98**	**202**	**1.35**
Biliran	**1,402**	0	**0.00**	1	**0.07**	7	**0.50**	8	**0.57**
Eastern Samar	**1,585**	0	**0.00**	4	**0.25**	24	**1.51**	28	**1.77**
Northern Leyte	**1,588**	0	**0.00**	46	**2.90**	50	**3.15**	96	**6.05**
Northern Samar	**4,579**	0	**0.00**	2	**0.04**	37	**0.81**	39	**0.85**
Southern Leyte	**453**	0	**0.00**	0	**0.00**	0	**0.00**	0	**0.00**
Western Samar	**607**	0	**0.00**	2	**0.33**	17	**2.80**	19	**3.13**
Calbayog City	**272**	0	**0.00**	0	**0.00**	0	**0.00**	0	**0.00**
Maasin City	**3**	0	**0.00**	0	**0.00**	0	**0.00**	0	**0.00**
Ormoc City	**1,259**	0	**0.00**	0	**0.00**	7	**0.56**	7	**0.56**
Tacloban City	**3,186**	0	**0.00**	0	**0.00**	5	**0.16**	5	**0.16**
**Region 9**	**6,228**	**0**	**0.00**	**4**	**0.06**	**70**	**1.12**	**74**	**1.19**
Zamboanga del Norte	**248**	0	**0.00**	0	**0.00**	0	**0.00**	0	**0.00**
Zamboanga del Sur	**946**	0	**0.00**	3	**0.32**	33	**3.49**	36	**3.81**
Zamboanga Sibugay	**3,499**	0	**0.00**	0	**0.00**	14	**0.40**	14	**0.40**
Dapitan City	**235**	0	**0.00**	0	**0.00**	0	**0.00**	0	**0.00**
Dipolog City	**11**	0	**0.00**	0	**0.00**	0	**0.00**	0	**0.00**
Isabela City	**304**	0	**0.00**	0	**0.00**	2	**0.66**	2	**0.66**
Pagadian City	**338**	0	**0.00**	0	**0.00**	13	**3.85**	13	**3.85**

Table 2.B.1.10. Prenatal Care

Number and proportion of pregnant women tested positive for Syphilis
Philippines, 2022

Area	Total No. of Screened for syphillis	Tested Positive for Syphilis							
		Age Group						Total	%
		10-14 yrs old		15-19 yrs old		20-49 yrs old			
		No.	%	No.	%	No.	%		
Zamboanga City	647	0	0.00	1	0.15	8	1.24	9	1.39
**Region 10**	**16,432**	**0**	**0.00**	**26**	**0.16**	**238**	**1.45**	**264**	**1.61**
Bukidnon	2,227	0	0.00	6	0.27	36	1.62	42	1.89
Camiguin	438	0	0.00	0	0.00	1	0.23	1	0.23
Lanao del Norte	487	0	0.00	0	0.00	0	0.00	0	0.00
Misamis Occidental	218	0	0.00	0	0.00	0	0.00	0	0.00
Misamis Oriental	2,679	0	0.00	0	0.00	2	0.07	2	0.07
Cagayan de Oro City	5,796	0	0.00	4	0.07	55	0.95	59	1.02
El Salvador City	380	0	0.00	0	0.00	0	0.00	0	0.00
Gingoog City	303	0	0.00	0	0.00	0	0.00	0	0.00
Iligan City	926	0	0.00	10	1.08	91	9.83	101	10.91
Malaybalay City	870	0	0.00	6	0.69	35	4.02	41	4.71
Oroquieta City	15	0	0.00	0	0.00	0	0.00	0	0.00
Ozamis City	1,140	0	0.00	0	0.00	16	1.40	16	1.40
Tangub City	722	0	0.00	0	0.00	0	0.00	0	0.00
Valencia City	231	0	0.00	0	0.00	2	0.87	2	0.87
**Region 11**	**26,473**	**0**	**0.00**	**12**	**0.05**	**91**	**0.34**	**103**	**0.39**
Davao de Oro	319	0	0.00	0	0.00	1	0.31	1	0.31
Davao del Norte	6,711	0	0.00	7	0.10	30	0.45	37	0.55
Davao Oriental	360	0	0.00	0	0.00	2	0.56	2	0.56
Davao del Sur	1,258	0	0.00	3	0.24	5	0.40	8	0.64
Davao Occidental	195	0	0.00	1	0.51	0	0.00	1	0.51
Davao City	17,630	0	0.00	1	0.01	53	0.30	54	0.31
**Region 12**	**25,563**	**7**	**0.03**	**118**	**0.46**	**560**	**2.19**	**685**	**2.68**
North Cotabato	2,986	3	0.10	17	0.57	135	4.52	155	5.19
Sarangani	3,577	4	0.11	53	1.48	237	6.63	294	8.22
South Cotabato	7,728	0	0.00	16	0.21	54	0.70	70	0.91
Sultan Kudarat	7,690	0	0.00	14	0.18	94	1.22	108	1.40
Gen. Santos City	3,582	0	0.00	18	0.50	40	1.12	58	1.62
**BARMM**	**631**	**1**	**0.16**	**1**	**0.16**	**15**	**2.38**	**17**	**2.69**
Basilan	0	0	0.00	0	0.00	0	0.00	0	0.00
Lanao del Sur	77	0	0.00	0	0.00	10	12.99	10	12.99
Maguindanao	29	0	0.00	0	0.00	0	0.00	0	0.00
Sulu	125	1	0.80	1	0.80	2	1.60	4	3.20
Tawi-Tawi	0	0	0.00	0	0.00	0	0.00	0	0.00
Lamitan City	14	0	0.00	0	0.00	0	0.00	0	0.00
Marawi City	0	0	0.00	0	0.00	0	0.00	0	0.00
Cotabato City	386	0	0.00	0	0.00	3	0.78	3	0.78
**CARAGA**	**10,697**	**0**	**0.00**	**8**	**0.07**	**100**	**0.93**	**108**	**1.01**
Agusan del Norte	1,151	0	0.00	0	0.00	2	0.17	2	0.17
Agusan del Sur	2,632	0	0.00	6	0.23	45	1.71	51	1.94
Surigao del Norte	608	0	0.00	0	0.00	10	1.64	10	1.64
Surigao del Sur	685	0	0.00	2	0.29	30	4.38	32	4.67
Province of Dinagat	847	0	0.00	0	0.00	6	0.71	6	0.71
Bislig City	55	0	0.00	0	0.00	0	0.00	0	0.00
Butuan City	3,404	0	0.00	0	0.00	3	0.09	3	0.09
Surigao City	1,315	0	0.00	0	0.00	4	0.30	4	0.30

Table 2.B.1.11. Prenatal Care

Number and proportion of pregnant women screened for Hepatitis B
Philippines, 2022

Area	Eligible Pop.	Screened for Hepatitis B							
		Age Group						Total	%
		10-14 yrs old		15-19 yrs old		20-49 yrs old			
		No.	%	No.	%	No.	%		
**PHILIPPINES**	**2,131,496**	**1,576**	**0.07**	**72,003**	**3.38**	**560,420**	**26.29**	**633,999**	**29.74**
**N C R**	**236,901**	**245**	**0.10**	**10,508**	**4.44**	**100,426**	**42.39**	**111,179**	**46.93**
Malabon	**6,724**	13	**0.19**	375	**5.58**	2,321	**34.52**	**2,709**	**40.29**
Navotas	**4,591**	13	**0.28**	513	**11.17**	2,617	**57.00**	**3,143**	**68.46**
Valenzuela City	**11,418**	13	**0.11**	504	**4.41**	5,105	**44.71**	**5,622**	**49.24**
Caloocan City	**29,146**	14	**0.05**	922	**3.16**	9,192	**31.54**	**10,128**	**34.75**
Marikina City	**8,294**	4	**0.05**	175	**2.11**	1,616	**19.48**	**1,795**	**21.64**
Pasig City	**13,894**	16	**0.12**	708	**5.10**	11,768	**84.70**	**12,492**	**89.91**
Pateros	**1,176**	4	**0.34**	97	**8.25**	775	**65.90**	**876**	**74.49**
Taguig	**14,807**	33	**0.22**	1,043	**7.04**	8,102	**54.72**	**9,178**	**61.98**
Quezon City	**54,011**	47	**0.09**	1,888	**3.50**	22,233	**41.16**	**24,168**	**44.75**
Makati City	**10,718**	3	**0.03**	189	**1.76**	3,451	**32.20**	**3,643**	**33.99**
Mandaluyong City	**7,111**	4	**0.06**	382	**5.37**	6,146	**86.43**	**6,532**	**91.86**
San Juan	**2,246**	0	**0.00**	62	**2.76**	865	**38.51**	**927**	**41.27**
Manila City	**32,743**	51	**0.16**	2,234	**6.82**	13,944	**42.59**	**16,229**	**49.56**
Las Piñas City	**10,832**	7	**0.06**	287	**2.65**	2,580	**23.82**	**2,874**	**26.53**
Muntinlupa City	**9,281**	8	**0.09**	221	**2.38**	2,008	**21.64**	**2,237**	**24.10**
Parañaque City	**12,249**	15	**0.12**	647	**5.28**	5,526	**45.11**	**6,188**	**50.52**
Pasay City	**7,660**	0	**0.00**	261	**3.41**	2,177	**28.42**	**2,438**	**31.83**
**C A R**	**35,179**	**33**	**0.09**	**1,727**	**4.91**	**14,965**	**42.54**	**16,725**	**47.54**
Abra	**4,275**	2	**0.05**	143	**3.35**	783	**18.32**	**928**	**21.71**
Apayao	**2,502**	7	**0.28**	248	**9.91**	1,305	**52.16**	**1,560**	**62.35**
Benguet	**9,086**	13	**0.14**	643	**7.08**	6,407	**70.52**	**7,063**	**77.73**
Ifugao	**4,486**	3	**0.07**	190	**4.24**	1,669	**37.20**	**1,862**	**41.51**
Kalinga	**4,701**	2	**0.04**	89	**1.89**	769	**16.36**	**860**	**18.29**
Mt. Province	**3,103**	0	**0.00**	104	**3.35**	1,035	**33.35**	**1,139**	**36.71**
Baguio City	**7,026**	6	**0.09**	310	**4.41**	2,997	**42.66**	**3,313**	**47.15**
**Region 1**	**97,099**	**42**	**0.04**	**2,747**	**2.83**	**30,471**	**31.38**	**33,260**	**34.25**
Ilocos Norte	**7,854**	6	**0.08**	292	**3.72**	3,247	**41.34**	**3,545**	**45.14**
Ilocos Sur	**9,072**	7	**0.08**	428	**4.72**	5,180	**57.10**	**5,615**	**61.89**
La Union	**11,338**	10	**0.09**	451	**3.98**	4,150	**36.60**	**4,611**	**40.67**
Pangasinan	**50,710**	10	**0.02**	1,001	**1.97**	11,549	**22.77**	**12,560**	**24.77**
Alaminos City	**1,910**	0	**0.00**	119	**6.23**	1,153	**60.37**	**1,272**	**66.60**
Candon City	**954**	0	**0.00**	2	**0.21**	426	**44.65**	**428**	**44.86**
Dagupan City	**3,657**	5	**0.14**	96	**2.63**	653	**17.86**	**754**	**20.62**
Laoag City	**1,812**	0	**0.00**	21	**1.16**	1,430	**78.92**	**1,451**	**80.08**
San Carlos City	**4,028**	0	**0.00**	48	**1.19**	469	**11.64**	**517**	**12.84**
San Fernando City	**2,074**	1	**0.05**	77	**3.71**	663	**31.97**	**741**	**35.73**
Urdaneta City	**2,836**	2	**0.07**	194	**6.84**	1,412	**49.79**	**1,608**	**56.70**
Vigan City	**854**	1	**0.12**	18	**2.11**	139	**16.28**	**158**	**18.50**
**Region 2**	**68,960**	**72**	**0.10**	**2,395**	**3.47**	**16,015**	**23.22**	**18,482**	**26.80**
Batanes	**362**	1	**0.28**	3	**0.83**	169	**46.69**	**173**	**47.79**
Cagayan	**20,431**	9	**0.04**	240	**1.17**	1,402	**6.86**	**1,651**	**8.08**
Isabela	**23,449**	37	**0.16**	1,093	**4.66**	6,949	**29.63**	**8,079**	**34.45**
Nueva Vizcaya	**9,538**	15	**0.16**	477	**5.00**	3,232	**33.89**	**3,724**	**39.04**
Quirino	**4,051**	4	**0.10**	196	**4.84**	1,533	**37.84**	**1,733**	**42.78**
Cauayan City	**2,565**	3	**0.12**	136	**5.30**	801	**31.23**	**940**	**36.65**
Ilagan City	**2,889**	1	**0.03**	153	**5.30**	1,220	**42.23**	**1,374**	**47.56**
Santiago City	**2,671**	2	**0.07**	95	**3.56**	608	**22.76**	**705**	**26.39**
Tuguegarao City	**3,004**	0	**0.00**	2	**0.07**	101	**3.36**	**103**	**3.43**

Table 2.B.1.11. Prenatal Care

Number and proportion of pregnant women screened for Hepatitis B
Philippines, 2022

Area	Eligible Pop.	Screened for Hepatitis B							
		Age Group						Total	%
		10-14 yrs old		15-19 yrs old		20-49 yrs old			
		No.	%	No.	%	No.	%		
**Region 3**	**220,155**	**220**	**0.10**	**8,939**	**4.06**	**62,569**	**28.42**	**71,728**	**32.58**
Aurora	4,754	8	0.17	235	4.94	1,468	30.88	1,711	35.99
Bataan	13,789	28	0.20	1,025	7.43	6,057	43.93	7,110	51.56
Bulacan	43,760	53	0.12	1,899	4.34	13,461	30.76	15,413	35.22
Nueva Ecija	29,039	23	0.08	761	2.62	4,350	14.98	5,134	17.68
Pampanga	31,620	18	0.06	1,073	3.39	7,631	24.13	8,722	27.58
Tarlac	19,319	17	0.09	422	2.18	3,062	15.85	3,501	18.12
Zambales	12,476	8	0.06	309	2.48	2,046	16.40	2,363	18.94
Angeles City	7,932	0	0.00	60	0.76	447	5.64	507	6.39
Balanga City	1,992	5	0.25	166	8.33	1,013	50.85	1,184	59.44
Cabanatuan City	5,945	3	0.05	112	1.88	794	13.36	909	15.29
City of San Fernando	5,907	11	0.19	301	5.10	2,068	35.01	2,380	40.29
Gapan City	2,170	1	0.05	88	4.06	371	17.10	460	21.20
Mabalacat City	4,831	2	0.04	276	5.71	1,868	38.67	2,146	44.42
Malolos City	4,890	2	0.04	50	1.02	491	10.04	543	11.10
Meycauayan	4,055	0	0.00	221	5.45	1,657	40.86	1,878	46.31
Olongapo	4,922	3	0.06	170	3.45	1,072	21.78	1,245	25.29
Palayan City	808	0	0.00	13	1.61	72	8.91	85	10.52
San Jose City	2,748	1	0.04	58	2.11	288	10.48	347	12.63
San Jose del Monte City	11,133	32	0.29	1,132	10.17	11,121	99.89	12,285	110.35
Science City of Munoz	1,601	1	0.06	37	2.31	193	12.05	231	14.43
Tarlac City	6,464	4	0.06	531	8.21	3,039	47.01	3,574	55.29
**Region 4A**	**299,627**	**194**	**0.06**	**10,769**	**3.59**	**80,794**	**26.96**	**91,757**	**30.62**
Batangas	39,255	1	0.00	755	1.92	7,706	19.63	8,462	21.56
Cavite	28,151	29	0.10	818	2.91	7,453	26.48	8,300	29.48
Laguna	19,696	22	0.11	955	4.85	6,253	31.75	7,230	36.71
Quezon	37,853	56	0.15	1,301	3.44	9,003	23.78	10,360	27.37
Rizal	44,798	29	0.06	2,044	4.56	13,037	29.10	15,110	33.73
Antipolo City	16,498	3	0.02	787	4.77	2,265	13.73	3,055	18.52
Bacoor City	12,341	1	0.01	231	1.87	1,552	12.58	1,784	14.46
Batangas City	6,965	2	0.03	134	1.92	1,308	18.78	1,444	20.73
Biñan City	6,597	3	0.05	826	12.52	6,049	91.69	6,878	104.26
Cabuyao City	6,119	8	0.13	311	5.08	3,404	55.63	3,723	60.84
Calamba City	9,005	11	0.12	376	4.18	3,601	39.99	3,988	44.29
Cavite City	2,105	2	0.10	81	3.85	283	13.44	366	17.39
Dasmariñas City	13,538	3	0.02	365	2.70	3,438	25.40	3,806	28.11
General Trias City	6,457	2	0.03	84	1.30	690	10.69	776	12.02
Imus City	8,292	3	0.04	113	1.36	824	9.94	940	11.34
Lipa City	7,018	1	0.01	218	3.11	2,448	34.88	2,667	38.00
Lucena City	5,738	4	0.07	311	5.42	1,565	27.27	1,880	32.76
San Pablo City	5,272	3	0.06	127	2.41	984	18.66	1,114	21.13
San Pedro City	6,455	2	0.03	165	2.56	1,150	17.82	1,317	20.40
Santa Rosa City	7,009	4	0.06	398	5.68	3,228	46.06	3,630	51.79
Tagaytay City	1,460	0	0.00	61	4.18	1,148	78.63	1,209	82.81
Tanauan City	3,661	3	0.08	72	1.97	1,466	40.04	1,541	42.09
Tayabas City	2,145	1	0.05	136	6.34	1,252	58.37	1,389	64.76
Trece Martires City	3,199	1	0.03	100	3.13	687	21.48	788	24.63
**Region 4B**	**72,791**	**33**	**0.05**	**1,752**	**2.41**	**13,492**	**18.54**	**15,277**	**20.99**
Marinduque	4,990	2	0.04	86	1.72	983	19.70	1,071	21.46
Mindoro Occidental	12,479	3	0.02	394	3.16	2,457	19.69	2,854	22.87
Mindoro Oriental	20,189	5	0.02	507	2.51	4,890	24.22	5,402	26.76
Palawan	21,963	9	0.04	392	1.78	2,526	11.50	2,927	13.33
Romblon	6,569	12	0.18	205	3.12	2,030	30.90	2,247	34.21
Puerto Princesa City	6,601	2	0.03	168	2.55	606	9.18	776	11.76
**Region 5**	**138,457**	**36**	**0.03**	**2,514**	**1.82**	**21,826**	**15.76**	**24,376**	**17.61**
Albay	24,081	9	0.04	363	1.51	5,379	22.34	5,751	23.88

Table 2.B.1.11. Prenatal Care

Number and proportion of pregnant women screened for Hepatitis B
Philippines, 2022

Area	Eligible Pop.	Screened for Hepatitis B							
		Age Group						Total	%
		10-14 yrs old		15-19 yrs old		20-49 yrs old			
		No.	%	No.	%	No.	%		
Camarines Norte	14,384	9	0.06	391	2.72	2,662	18.51	3,062	21.29
Camarines Sur	39,486	0	0.00	213	0.54	2,681	6.79	2,894	7.33
Catanduanes	6,459	3	0.05	306	4.74	1,909	29.56	2,218	34.34
Masbate	22,600	10	0.04	721	3.19	3,713	16.43	4,444	19.66
Sorsogon	19,828	1	0.01	359	1.81	3,573	18.02	3,933	19.84
Iriga City	2,679	0	0.00	36	1.34	520	19.41	556	20.75
Legaspi City	4,233	3	0.07	70	1.65	614	14.51	687	16.23
Naga City	4,707	1	0.02	55	1.17	775	16.46	831	17.65
**Region 6**	146,449	97	0.07	6,084	4.15	49,418	33.74	55,599	37.96
Aklan	11,288	4	0.04	333	2.95	3,796	33.63	4,133	36.61
Antique	13,132	5	0.04	359	2.73	3,189	24.28	3,553	27.06
Capiz	13,975	16	0.11	330	2.36	3,583	25.64	3,929	28.11
Guimaras	3,084	8	0.26	163	5.29	1,783	57.81	1,954	63.36
Iloilo	36,267	35	0.10	1,654	4.56	15,383	42.42	17,072	47.07
Negros Occidental	49,230	17	0.03	2,176	4.42	13,913	28.26	16,106	32.72
Bacolod City	11,082	9	0.08	752	6.79	4,724	42.63	5,485	49.49
Iloilo City	8,391	3	0.04	317	3.78	3,047	36.31	3,367	40.13
**Region 7**	162,308	139	0.09	5,685	3.50	51,942	32.00	57,766	35.59
Bohol	27,992	42	0.15	1,497	5.35	12,773	45.63	14,312	51.13
Cebu	66,463	8	0.01	401	0.60	5,569	8.38	5,978	8.99
Negros Oriental	27,890	25	0.09	915	3.28	6,263	22.46	7,203	25.83
Siquijor	1,661	2	0.12	63	3.79	747	44.97	812	48.89
Cebu City	20,866	23	0.11	1,234	5.91	9,647	46.23	10,904	52.26
Lapu-Lapu City	9,232	3	0.03	256	2.77	4,230	45.82	4,489	48.62
Mandaue City	8,204	36	0.44	1,319	16.08	12,713	154.96	14,068	171.48
**Region 8**	105,471	34	0.03	2,915	2.76	23,915	22.67	26,864	25.47
Biliran	3,873	3	0.08	250	6.45	1,692	43.69	1,945	50.22
Eastern Samar	11,908	4	0.03	251	2.11	2,209	18.55	2,464	20.69
Northern Leyte	35,422	11	0.03	794	2.24	6,722	18.98	7,527	21.25
Northern Samar	15,921	8	0.05	638	4.01	4,404	27.66	5,050	31.72
Southern Leyte	6,662	0	0.00	250	3.75	2,650	39.78	2,900	43.53
Western Samar	14,732	1	0.01	197	1.34	1,163	7.89	1,361	9.24
Calbayog City	4,535	0	0.00	34	0.75	269	5.93	303	6.68
Maasin City	1,696	0	0.00	22	1.30	393	23.17	415	24.47
Ormoc City	5,044	2	0.04	211	4.18	1,423	28.21	1,636	32.43
Tacloban City	5,678	5	0.09	268	4.72	2,990	52.66	3,263	57.47
**Region 9**	79,007	14	0.02	569	0.72	3,255	4.12	3,838	4.86
Zamboanga del Norte	16,854	1	0.01	77	0.46	457	2.71	535	3.17
Zamboanga del Sur	17,368	4	0.02	162	0.93	643	3.70	809	4.66
Zamboanga Sibugay	15,021	0	0.00	99	0.66	619	4.12	718	4.78
Dapitan City	1,737	2	0.12	47	2.71	247	14.22	296	17.04
Dipolog City	2,762	1	0.04	0	0.00	21	0.76	22	0.80
Isabela City	2,563	2	0.08	13	0.51	86	3.36	101	3.94
Pagadian City	4,257	0	0.00	10	0.23	126	2.96	136	3.19
Zamboanga City	18,445	4	0.02	161	0.87	1,056	5.73	1,221	6.62
**Region 10**	99,908	73	0.07	4,662	4.67	25,401	25.42	30,136	30.16
Bukidnon	22,900	13	0.06	845	3.69	3,202	13.98	4,060	17.73
Camiguin	1,854	1	0.05	64	3.45	549	29.61	614	33.12
Lanao del Norte	14,930	1	0.01	97	0.65	724	4.85	822	5.51
Misamis Occidental	6,420	1	0.02	55	0.86	457	7.12	513	7.99
Misamis Oriental	14,963	23	0.15	1,243	8.31	6,056	40.47	7,322	48.93

Table 2.B.1.11. Prenatal Care
Number and proportion of pregnant women screened for Hepatitis B
Philippines, 2022

Area	Eligible Pop.	Screened for Hepatitis B							
		Age Group						Total	%
		10-14 yrs old		15-19 yrs old		20-49 yrs old			
		No.	%	No.	%	No.	%		
Cagayan de Oro City	**14,172**	21	**0.15**	1,326	**9.36**	7,889	**55.67**	**9,236**	**65.17**
El Salvador City	**1,052**	5	**0.48**	106	**10.08**	474	**45.06**	**585**	**55.61**
Gingoog City	**2,608**	2	**0.08**	220	**8.44**	1,211	**46.43**	**1,433**	**54.95**
Iligan City	**7,565**	1	**0.01**	252	**3.33**	1,744	**23.05**	**1,997**	**26.40**
Malaybalay City	**3,817**	3	**0.08**	159	**4.17**	1,169	**30.63**	**1,331**	**34.87**
Oroquieta City	**1,389**	0	**0.00**	3	**0.22**	20	**1.44**	**23**	**1.66**
Ozamis City	**2,786**	0	**0.00**	139	**4.99**	985	**35.36**	**1,124**	**40.34**
Tangub City	**1,234**	0	**0.00**	104	**8.43**	659	**53.40**	**763**	**61.83**
Valencia City	**4,218**	2	**0.05**	49	**1.16**	262	**6.21**	**313**	**7.42**
**Region 11**	**108,407**	**205**	**0.19**	**3,838**	**3.54**	**24,416**	**22.52**	**28,459**	**26.25**
Davao de Oro	**15,490**	9	**0.06**	94	**0.61**	578	**3.73**	**681**	**4.40**
Davao del Norte	**21,017**	21	**0.10**	721	**3.43**	5,002	**23.80**	**5,744**	**27.33**
Davao Oriental	**13,229**	1	**0.01**	76	**0.57**	425	**3.21**	**502**	**3.79**
Davao del Sur	**14,564**	22	**0.15**	303	**2.08**	1,413	**9.70**	**1,738**	**11.93**
Davao Occidental	**6,510**	3	**0.05**	122	**1.87**	358	**5.50**	**483**	**7.42**
Davao City	**37,597**	149	**0.40**	2,522	**6.71**	16,640	**44.26**	**19,311**	**51.36**
**Region 12**	**98,859**	**101**	**0.10**	**4,957**	**5.01**	**26,936**	**27.25**	**31,994**	**32.36**
North Cotabato	**34,150**	22	**0.06**	1,071	**3.14**	6,209	**18.18**	**7,302**	**21.38**
Sarangani	**12,907**	26	**0.20**	984	**7.62**	4,385	**33.97**	**5,395**	**41.80**
South Cotabato	**20,917**	15	**0.07**	1,110	**5.31**	6,249	**29.88**	**7,374**	**35.25**
Sultan Kudarat	**17,299**	28	**0.16**	1,070	**6.19**	6,188	**35.77**	**7,286**	**42.12**
Gen. Santos City	**13,586**	10	**0.07**	722	**5.31**	3,905	**28.74**	**4,637**	**34.13**
**BARMM**	**101,343**	**1**	**0.00**	**161**	**0.16**	**1,722**	**1.70**	**1,884**	**1.86**
Basilan	**7,823**	0	**0.00**	0	**0.00**	0	**0.00**	**0**	**0.00**
Lanao del Sur	**21,639**	0	**0.00**	16	**0.07**	184	**0.85**	**200**	**0.92**
Maguindanao	**32,198**	0	**0.00**	6	**0.02**	57	**0.18**	**63**	**0.20**
Sulu	**17,165**	0	**0.00**	79	**0.46**	884	**5.15**	**963**	**5.61**
Tawi-Tawi	**9,369**	0	**0.00**	0	**0.00**	0	**0.00**	**0**	**0.00**
Lamitan City	**2,154**	0	**0.00**	1	**0.05**	1	**0.05**	**2**	**0.09**
Marawi City	**5,173**	0	**0.00**	0	**0.00**	0	**0.00**	**0**	**0.00**
Cotabato City	**5,822**	1	**0.02**	59	**1.01**	596	**10.24**	**656**	**11.27**
**CARAGA**	**60,575**	**37**	**0.06**	**1,781**	**2.94**	**12,857**	**21.22**	**14,675**	**24.23**
Agusan del Norte	**8,127**	4	**0.05**	235	**2.89**	1,695	**20.86**	**1,934**	**23.80**
Agusan del Sur	**17,709**	11	**0.06**	379	**2.14**	2,176	**12.29**	**2,566**	**14.49**
Surigao del Norte	**7,280**	3	**0.04**	188	**2.58**	1,931	**26.52**	**2,122**	**29.15**
Surigao del Sur	**11,553**	3	**0.03**	188	**1.63**	1,234	**10.68**	**1,425**	**12.33**
Province of Dinagat	**2,576**	2	**0.08**	123	**4.77**	773	**30.01**	**898**	**34.86**
Bislig City	**2,192**	0	**0.00**	0	**0.00**	15	**0.68**	**15**	**0.68**
Butuan City	**7,743**	8	**0.10**	449	**5.80**	3,534	**45.64**	**3,991**	**51.54**
Surigao City	**3,395**	6	**0.18**	219	**6.45**	1,499	**44.15**	**1,724**	**50.78**

Table 1.B.1.12 - Prenatal Care

Number and proportion of pregnant women tested positive for Hepatitis B
Philippines, 2022

Area	Total No. of Screened for Hepatitis B	Tested Positive for Hepatitis B							
		Age Group						Total	%
		10-14 yrs old		15-19 yrs old		20-49 yrs old			
		No.	%	No.	%	No.	%		
**PHILIPPINES**	**633,343**	**55**	**0.01**	**1,209**	**0.19**	**11,477**	**1.81**	**12,741**	**2.01**
**N C R**	**111,179**	**2**	**0.00**	**110**	**0.10**	**2,032**	**1.83**	**2,144**	**1.93**
Malabon	**2,709**	0	**0.00**	2	**0.07**	23	**0.85**	**25**	**0.92**
Navotas	**3,143**	0	**0.00**	5	**0.16**	87	**2.77**	**92**	**2.93**
Valenzuela City	**5,622**	0	**0.00**	23	**0.41**	157	**2.79**	**180**	**3.20**
Caloocan City	**10,128**	0	**0.00**	8	**0.08**	722	**7.13**	**730**	**7.21**
Marikina City	**1,795**	0	**0.00**	1	**0.06**	27	**1.50**	**28**	**1.56**
Pasig City	**12,492**	0	**0.00**	6	**0.05**	158	**1.26**	**164**	**1.31**
Pateros	**876**	0	**0.00**	0	**0.00**	2	**0.23**	**2**	**0.23**
Taguig	**9,178**	2	**0.02**	25	**0.27**	209	**2.28**	**236**	**2.57**
Quezon City	**24,168**	0	**0.00**	6	**0.02**	188	**0.78**	**194**	**0.80**
Makati City	**3,643**	0	**0.00**	0	**0.00**	51	**1.40**	**51**	**1.40**
Mandaluyong City	**6,532**	0	**0.00**	2	**0.03**	19	**0.29**	**21**	**0.32**
San Juan	**927**	0	**0.00**	0	**0.00**	4	**0.43**	**4**	**0.43**
Manila City	**16,229**	0	**0.00**	17	**0.10**	180	**1.11**	**197**	**1.21**
Las Piñas City	**2,874**	0	**0.00**	10	**0.35**	92	**3.20**	**102**	**3.55**
Muntinlupa City	**2,237**	0	**0.00**	0	**0.00**	28	**1.25**	**28**	**1.25**
Parañaque City	**6,188**	0	**0.00**	0	**0.00**	22	**0.36**	**22**	**0.36**
Pasay City	**2,438**	0	**0.00**	5	**0.21**	63	**2.58**	**68**	**2.79**
**C A R**	**16,725**	**0**	**0.00**	**27**	**0.16**	**211**	**1.26**	**238**	**1.42**
Abra	**928**	0	**0.00**	1	**0.11**	2	**0.22**	**3**	**0.32**
Apayao	**1,560**	0	**0.00**	6	**0.38**	23	**1.47**	**29**	**1.86**
Benguet	**7,063**	0	**0.00**	1	**0.01**	72	**1.02**	**73**	**1.03**
Ifugao	**1,862**	0	**0.00**	1	**0.05**	19	**1.02**	**20**	**1.07**
Kalinga	**860**	0	**0.00**	4	**0.47**	32	**3.72**	**36**	**4.19**
Mt. Province	**1,139**	0	**0.00**	0	**0.00**	1	**0.09**	**1**	**0.09**
Baguio City	**3,313**	0	**0.00**	14	**0.42**	62	**1.87**	**76**	**2.29**
**Region 1**	**33,260**	**0**	**0.00**	**35**	**0.11**	**220**	**0.66**	**255**	**0.77**
Ilocos Norte	**3,545**	0	**0.00**	2	**0.06**	9	**0.25**	**11**	**0.31**
Ilocos Sur	**5,615**	0	**0.00**	0	**0.00**	6	**0.11**	**6**	**0.11**
La Union	**4,611**	0	**0.00**	6	**0.13**	51	**1.11**	**57**	**1.24**
Pangasinan	**12,560**	0	**0.00**	23	**0.18**	128	**1.02**	**151**	**1.20**
Alaminos City	**1,272**	0	**0.00**	0	**0.00**	0	**0.00**	**0**	**0.00**
Candon City	**428**	0	**0.00**	0	**0.00**	1	**0.23**	**1**	**0.23**
Dagupan City	**754**	0	**0.00**	3	**0.40**	16	**2.12**	**19**	**2.52**
Laoag City	**1,451**	0	**0.00**	0	**0.00**	0	**0.00**	**0**	**0.00**
San Carlos City	**517**	0	**0.00**	0	**0.00**	3	**0.58**	**3**	**0.58**
San Fernando City	**741**	0	**0.00**	0	**0.00**	0	**0.00**	**0**	**0.00**
Urdaneta City	**1,608**	0	**0.00**	1	**0.06**	6	**0.37**	**7**	**0.44**
Vigan City	**158**	0	**0.00**	0	**0.00**	0	**0.00**	**0**	**0.00**
**Region 2**	**18,482**	**0**	**0.00**	**26**	**0.14**	**259**	**1.40**	**285**	**1.54**
Batanes	**173**	0	**0.00**	0	**0.00**	7	**4.05**	**7**	**4.05**
Cagayan	**1,651**	0	**0.00**	0	**0.00**	5	**0.30**	**5**	**0.30**
Isabela	**8,079**	0	**0.00**	6	**0.07**	107	**1.32**	**113**	**1.40**
Nueva Vizcaya	**3,724**	0	**0.00**	17	**0.46**	118	**3.17**	**135**	**3.63**
Quirino	**1,733**	0	**0.00**	2	**0.12**	14	**0.81**	**16**	**0.92**
Cauayan City	**940**	0	**0.00**	0	**0.00**	0	**0.00**	**0**	**0.00**

Table 1.B.1.12 - Prenatal Care
Number and proportion of pregnant women tested positive for Hepatitis B
Philippines, 2022

Area	Total No. of Screened for Hepatitis B	Tested Positive for Hepatitis B							
		Age Group						Total	%
		10-14 yrs old		15-19 yrs old		20-49 yrs old			
		No.	%	No.	%	No.	%		
Ilagan City	1,374	0	0.00	0	0.00	7	0.51	7	0.51
Santiago City	705	0	0.00	1	0.14	0	0.00	1	0.14
Tuguegarao City	103	0	0.00	0	0.00	1	0.97	1	0.97
**Region 3**	71,728	12	0.02	100	0.14	779	1.09	891	1.24
Aurora	1,711	0	0.00	1	0.06	33	1.93	34	1.99
Bataan	7,110	3	0.04	25	0.35	108	1.52	136	1.91
Bulacan	15,413	0	0.00	12	0.08	139	0.90	151	0.98
Nueva Ecija	5,134	0	0.00	12	0.23	128	2.49	140	2.73
Pampanga	8,722	0	0.00	19	0.22	149	1.71	168	1.93
Tarlac	3,501	0	0.00	1	0.03	18	0.51	19	0.54
Zambales	2,363	1	0.04	9	0.38	27	1.14	37	1.57
Angeles City	507	0	0.00	1	0.20	12	2.37	13	2.56
Balanga City	1,184	0	0.00	2	0.17	8	0.68	10	0.84
Cabanatuan City	909	0	0.00	0	0.00	3	0.33	3	0.33
City of San Fernando	2,380	0	0.00	2	0.08	41	1.72	43	1.81
Gapan City	460	0	0.00	0	0.00	2	0.43	2	0.43
Mabalacat City	2,146	0	0.00	1	0.05	23	1.07	24	1.12
Malolos City	543	0	0.00	0	0.00	0	0.00	0	0.00
Meycauayan	1,878	0	0.00	0	0.00	4	0.21	4	0.21
Olongapo	1,245	8	0.64	10	0.80	18	1.45	36	2.89
Palayan City	85	0	0.00	0	0.00	0	0.00	0	0.00
San Jose City	347	0	0.00	0	0.00	1	0.29	1	0.29
San Jose del Monte City	12,285	0	0.00	4	0.03	39	0.32	43	0.35
Science City of Munoz	231	0	0.00	0	0.00	3	1.30	3	1.30
Tarlac City	3,574	0	0.00	1	0.03	23	0.64	24	0.67
**Region 4A**	91,757	20	0.02	228	0.25	1,842	2.01	2,090	2.28
Batangas	8,462	0	0.00	7	0.08	72	0.85	79	0.93
Cavite	8,300	1	0.01	10	0.12	142	1.71	153	1.84
Laguna	7,230	1	0.01	21	0.29	135	1.87	157	2.17
Quezon	10,360	7	0.07	36	0.35	287	2.77	330	3.19
Rizal	15,110	9	0.06	42	0.28	306	2.03	357	2.36
Antipolo City	3,055	0	0.00	23	0.75	150	4.91	173	5.66
Bacoor City	1,784	0	0.00	4	0.22	32	1.79	36	2.02
Batangas City	1,444	0	0.00	4	0.28	24	1.66	28	1.94
Biñan City	6,878	0	0.00	1	0.01	7	0.10	8	0.12
Cabuyao City	3,723	0	0.00	15	0.40	88	2.36	103	2.77
Calamba City	3,988	0	0.00	7	0.18	69	1.73	76	1.91
Cavite City	366	0	0.00	1	0.27	5	1.37	6	1.64
Dasmariñas City	3,806	0	0.00	13	0.34	128	3.36	141	3.70
General Trias City	776	0	0.00	2	0.26	14	1.80	16	2.06
Imus City	940	0	0.00	4	0.43	17	1.81	21	2.23
Lipa City	2,667	0	0.00	4	0.15	50	1.87	54	2.02
Lucena City	1,880	0	0.00	10	0.53	48	2.55	58	3.09
San Pablo City	1,114	0	0.00	2	0.18	11	0.99	13	1.17
San Pedro City	1,317	0	0.00	2	0.15	39	2.96	41	3.11
Santa Rosa City	3,630	1	0.03	17	0.47	170	4.68	188	5.18
Tagaytay City	1,209	0	0.00	1	0.08	1	0.08	2	0.17
Tanauan City	1,541	0	0.00	2	0.13	15	0.97	17	1.10
Tayabas City	1,389	0	0.00	0	0.00	25	1.80	25	1.80
Trece Martires City	788	1	0.13	0	0.00	7	0.89	8	1.02
**Region 4B**	15,277	2	0.01	41	0.27	358	2.34	401	2.62
Marinduque	1,071	1	0.09	0	0.00	15	1.40	16	1.49
Mindoro Occidental	2,854	0	0.00	7	0.25	61	2.14	68	2.38
Mindoro Oriental	5,402	0	0.00	2	0.04	54	1.00	56	1.04
Palawan	2,927	0	0.00	14	0.48	111	3.79	125	4.27
Romblon	2,247	1	0.04	8	0.36	76	3.38	85	3.78

Table 1.B.1.12 - Prenatal Care

Number and proportion of pregnant women tested positive for Hepatitis B
Philippines, 2022

Area	Total No. of Screened for Hepatitis B	Tested Positive for Hepatitis B							
		Age Group						Total	%
		10-14 yrs old		15-19 yrs old		20-49 yrs old			
		No.	%	No.	%	No.	%		
Puerto Princesa City	**776**	0	**0.00**	10	**1.29**	41	**5.28**	**51**	**6.57**
**Region 5**	**24,376**	**1**	**0.00**	**56**	**0.23**	**824**	**3.38**	**881**	**3.61**
Albay	**5,751**	0	**0.00**	9	**0.16**	208	**3.62**	**217**	**3.77**
Camarines Norte	**3,062**	1	**0.03**	10	**0.33**	158	**5.16**	**169**	**5.52**
Camarines Sur	**2,894**	0	**0.00**	3	**0.10**	39	**1.35**	**42**	**1.45**
Catanduanes	**2,218**	0	**0.00**	4	**0.18**	65	**2.93**	**69**	**3.11**
Masbate	**4,444**	0	**0.00**	16	**0.36**	121	**2.72**	**137**	**3.08**
Sorsogon	**3,933**	0	**0.00**	13	**0.33**	206	**5.24**	**219**	**5.57**
Iriga City	**556**	0	**0.00**	0	**0.00**	11	**1.98**	**11**	**1.98**
Legaspi City	**687**	0	**0.00**	1	**0.15**	6	**0.87**	**7**	**1.02**
Naga City	**831**	0	**0.00**	0	**0.00**	10	**1.20**	**10**	**1.20**
**Region 6**	**55,599**	**1**	**0.00**	**110**	**0.20**	**1,008**	**1.81**	**1,119**	**2.01**
Aklan	**4,133**	0	**0.00**	5	**0.12**	53	**1.28**	**58**	**1.40**
Antique	**3,553**	0	**0.00**	9	**0.25**	99	**2.79**	**108**	**3.04**
Capiz	**3,929**	0	**0.00**	8	**0.20**	58	**1.48**	**66**	**1.68**
Guimaras	**1,954**	0	**0.00**	1	**0.05**	21	**1.07**	**22**	**1.13**
Iloilo	**17,072**	0	**0.00**	24	**0.14**	180	**1.05**	**204**	**1.19**
Negros Occidental	**16,106**	1	**0.01**	56	**0.35**	448	**2.78**	**505**	**3.14**
Bacolod City	**5,485**	0	**0.00**	6	**0.11**	103	**1.88**	**109**	**1.99**
Iloilo City	**3,367**	0	**0.00**	1	**0.03**	46	**1.37**	**47**	**1.40**
**Region 7**	**57,766**	**4**	**0.01**	**65**	**0.11**	**790**	**1.37**	**859**	**1.49**
Bohol	**14,312**	1	**0.01**	14	**0.10**	258	**1.80**	**273**	**1.91**
Cebu	**5,978**	1	**0.02**	4	**0.07**	99	**1.66**	**104**	**1.74**
Negros Oriental	**7,203**	2	**0.03**	18	**0.25**	163	**2.26**	**183**	**2.54**
Siquijor	**812**	0	**0.00**	7	**0.86**	8	**0.99**	**15**	**1.85**
Cebu City	**10,904**	0	**0.00**	16	**0.15**	188	**1.72**	**204**	**1.87**
Lapu-Lapu City	**4,489**	0	**0.00**	4	**0.09**	60	**1.34**	**64**	**1.43**
Mandaue City	**14,068**	0	**0.00**	2	**0.01**	14	**0.10**	**16**	**0.11**
**Region 8**	**26,864**	**0**	**0.00**	**108**	**0.40**	**980**	**3.65**	**1,088**	**4.05**
Biliran	**1,945**	0	**0.00**	4	**0.21**	41	**2.11**	**45**	**2.31**
Eastern Samar	**2,464**	0	**0.00**	6	**0.24**	51	**2.07**	**57**	**2.31**
Northern Leyte	**7,527**	0	**0.00**	66	**0.88**	610	**8.10**	**676**	**8.98**
Northern Samar	**5,050**	0	**0.00**	11	**0.22**	91	**1.80**	**102**	**2.02**
Southern Leyte	**2,900**	0	**0.00**	5	**0.17**	79	**2.72**	**84**	**2.90**
Western Samar	**1,361**	0	**0.00**	8	**0.59**	52	**3.82**	**60**	**4.41**
Calbayog City	**303**	0	**0.00**	0	**0.00**	3	**0.99**	**3**	**0.99**
Maasin City	**415**	0	**0.00**	2	**0.48**	25	**6.02**	**27**	**6.51**
Ormoc City	**1,636**	0	**0.00**	3	**0.18**	13	**0.79**	**16**	**0.98**
Tacloban City	**3,263**	0	**0.00**	3	**0.09**	15	**0.46**	**18**	**0.55**
**Region 9**	**3,838**	**0**	**0.00**	**18**	**0.47**	**108**	**2.81**	**126**	**3.28**
Zamboanga del Norte	**535**	0	**0.00**	0	**0.00**	7	**1.31**	**7**	**1.31**
Zamboanga del Sur	**809**	0	**0.00**	6	**0.74**	19	**2.35**	**25**	**3.09**
Zamboanga Sibugay	**718**	0	**0.00**	0	**0.00**	11	**1.53**	**11**	**1.53**
Dapitan City	**296**	0	**0.00**	1	**0.34**	6	**2.03**	**7**	**2.36**
Dipolog City	**22**	0	**0.00**	0	**0.00**	0	**0.00**	**0**	**0.00**
Isabela City	**101**	0	**0.00**	0	**0.00**	0	**0.00**	**0**	**0.00**
Pagadian City	**136**	0	**0.00**	0	**0.00**	6	**4.41**	**6**	**4.41**
Zamboanga City	**1,221**	0	**0.00**	11	**0.90**	59	**4.83**	**70**	**5.73**

Table 1.B.1.12 - Prenatal Care

Number and proportion of pregnant women tested positive for Hepatitis B

Philippines, 2022

Area	Total No. of Screened for Hepatitis B	Tested Positive for Hepatitis B							
		Age Group						Total	%
		10-14 yrs old		15-19 yrs old		20-49 yrs old			
		No.	%	No.	%	No.	%		
**Region 10**	**30,136**	**2**	**0.01**	**64**	**0.21**	**523**	**1.74**	**589**	**1.95**
Bukidnon	4,060	2	0.05	18	0.44	134	3.30	154	3.79
Camiguin	614	0	0.00	2	0.33	7	1.14	9	1.47
Lanao del Norte	822	0	0.00	0	0.00	4	0.49	4	0.49
Misamis Occidental	513	0	0.00	1	0.19	1	0.19	2	0.39
Misamis Oriental	7,322	0	0.00	13	0.18	82	1.12	95	1.30
Cagayan de Oro City	9,236	0	0.00	10	0.11	134	1.45	144	1.56
El Salvador City	585	0	0.00	4	0.68	19	3.25	23	3.93
Gingoog City	1,433	0	0.00	9	0.63	43	3.00	52	3.63
Iligan City	1,997	0	0.00	7	0.35	71	3.56	78	3.91
Malaybalay City	1,331	0	0.00	0	0.00	22	1.65	22	1.65
Oroquieta City	23	0	0.00	0	0.00	0	0.00	0	0.00
Ozamis City	1,124	0	0.00	0	0.00	2	0.18	2	0.18
Tangub City	763	0	0.00	0	0.00	2	0.26	2	0.26
Valencia City	313	0	0.00	0	0.00	2	0.64	2	0.64
**Region 11**	**28,459**	**3**	**0.01**	**33**	**0.12**	**223**	**0.78**	**259**	**0.91**
Davao de Oro	681	0	0.00	1	0.15	11	1.62	12	1.76
Davao del Norte	5,744	2	0.03	7	0.12	64	1.11	73	1.27
Davao Oriental	502	0	0.00	5	1.00	2	0.40	7	1.39
Davao del Sur	1,738	0	0.00	1	0.06	23	1.32	24	1.38
Davao Occidental	483	0	0.00	10	2.07	27	5.59	37	7.66
Davao City	19,311	1	0.01	9	0.05	96	0.50	106	0.55
**Region 12**	**31,994**	**8**	**0.03**	**137**	**0.43**	**953**	**2.98**	**1,098**	**3.43**
North Cotabato	7,302	8	0.11	41	0.56	294	4.03	343	4.70
Sarangani	5,395	0	0.00	59	1.09	251	4.65	310	5.75
South Cotabato	7,374	0	0.00	13	0.18	134	1.82	147	1.99
Sultan Kudarat	7,286	0	0.00	14	0.19	162	2.22	176	2.42
Gen. Santos City	4,637	0	0.00	10	0.22	112	2.42	122	2.63
**BARMM**	**1,228**	**0**	**0.00**	**0**	**0.00**	**30**	**2.44**	**30**	**2.44**
Basilan	0	0	0.00	0	0.00	0	0.00	0	0.00
Lanao del Sur	200	0	0.00	0	0.00	0	0.00	0	0.00
Maguindanao	63	0	0.00	0	0.00	0	0.00	0	0.00
Sulu	963	0	0.00	0	0.00	24	2.49	24	2.49
Tawi-Tawi	0	0	0.00	0	0.00	0	0.00	0	0.00
Lamitan City	2	0	0.00	0	0.00	0	0.00	0	0.00
Marawi City	0	0	0.00	0	0.00	0	0.00	0	0.00
Cotabato City	0	0	0.00	0	0.00	6	0.00	6	0.00
**CARAGA**	**14,675**	**0**	**0.00**	**51**	**0.35**	**337**	**2.30**	**388**	**2.64**
Agusan del Norte	1,934	0	0.00	1	0.05	13	0.67	14	0.72
Agusan del Sur	2,566	0	0.00	17	0.66	64	2.49	81	3.16
Surigao del Norte	2,122	0	0.00	9	0.42	71	3.35	80	3.77
Surigao del Sur	1,425	0	0.00	12	0.84	65	4.56	77	5.40
Province of Dinagat	898	0	0.00	2	0.22	50	5.57	52	5.79
Bislig City	15	0	0.00	0	0.00	0	0.00	0	0.00
Butuan City	3,991	0	0.00	9	0.23	54	1.35	63	1.58
Surigao City	1,724	0	0.00	1	0.06	20	1.16	21	1.22

Table 2.B.1.13. Prenatal Care

Number and proportion of pregnant women screened for HIV
Philippines, 2022

Area	Eligible Pop.	Screened for HIV							
		Age Group						Total	%
		10-14 yrs old		15-19 yrs old		20-49 yrs old			
		No.	%	No.	%	No.	%		
**PHILIPPINES**	**2,131,496**	**1,091**	**0.05**	**49,433**	**2.32**	**391,380**	**18.36**	**441,546**	**20.72**
**N C R**	**236,901**	**264**	**0.11**	**12,230**	**5.16**	**110,645**	**46.71**	**123,139**	**51.98**
Malabon	**6,724**	13	**0.19**	395	**5.87**	2,547	**37.88**	**2,955**	**43.95**
Navotas	**4,591**	15	**0.33**	514	**11.20**	2,544	**55.41**	**3,073**	**66.94**
Valenzuela City	**11,418**	9	**0.08**	615	**5.39**	6,079	**53.24**	**6,703**	**58.71**
Caloocan City	**29,146**	17	**0.06**	1,082	**3.71**	9,439	**32.39**	**10,538**	**36.16**
Marikina City	**8,294**	4	**0.05**	92	**1.11**	965	**11.63**	**1,061**	**12.79**
Pasig City	**13,894**	16	**0.12**	831	**5.98**	11,338	**81.60**	**12,185**	**87.70**
Pateros	**1,176**	1	**0.09**	62	**5.27**	480	**40.82**	**543**	**46.17**
Taguig	**14,807**	34	**0.23**	1,045	**7.06**	8,242	**55.66**	**9,321**	**62.95**
Quezon City	**54,011**	51	**0.09**	2,662	**4.93**	29,046	**53.78**	**31,759**	**58.80**
Makati City	**10,718**	5	**0.05**	227	**2.12**	3,479	**32.46**	**3,711**	**34.62**
Mandaluyong City	**7,111**	4	**0.06**	389	**5.47**	6,240	**87.75**	**6,633**	**93.28**
San Juan	**2,246**	0	**0.00**	61	**2.72**	895	**39.85**	**956**	**42.56**
Manila City	**32,743**	63	**0.19**	2,696	**8.23**	15,839	**48.37**	**18,598**	**56.80**
Las Piñas City	**10,832**	7	**0.06**	167	**1.54**	1,760	**16.25**	**1,934**	**17.85**
Muntinlupa City	**9,281**	10	**0.11**	330	**3.56**	2,929	**31.56**	**3,269**	**35.22**
Parañaque City	**12,249**	15	**0.12**	630	**5.14**	5,198	**42.44**	**5,843**	**47.70**
Pasay City	**7,660**	0	**0.00**	432	**5.64**	3,625	**47.32**	**4,057**	**52.96**
**C A R**	**35,179**	**18**	**0.05**	**988**	**2.81**	**8,878**	**25.24**	**9,884**	**28.10**
Abra	**4,275**	1	**0.02**	111	**2.60**	573	**13.40**	**685**	**16.02**
Apayao	**2,502**	0	**0.00**	50	**2.00**	227	**9.07**	**277**	**11.07**
Benguet	**9,086**	6	**0.07**	355	**3.91**	3,454	**38.01**	**3,815**	**41.99**
Ifugao	**4,486**	3	**0.07**	88	**1.96**	938	**20.91**	**1,029**	**22.94**
Kalinga	**4,701**	1	**0.02**	51	**1.08**	427	**9.08**	**479**	**10.19**
Mt. Province	**3,103**	0	**0.00**	25	**0.81**	397	**12.79**	**422**	**13.60**
Baguio City	**7,026**	7	**0.10**	308	**4.38**	2,862	**40.73**	**3,177**	**45.22**
**Region 1**	**97,099**	**26**	**0.03**	**1,563**	**1.61**	**16,922**	**17.43**	**18,511**	**19.06**
Ilocos Norte	**7,854**	2	**0.03**	146	**1.86**	1,689	**21.50**	**1,837**	**23.39**
Ilocos Sur	**9,072**	5	**0.06**	253	**2.79**	3,489	**38.46**	**3,747**	**41.30**
La Union	**11,338**	8	**0.07**	275	**2.43**	2,064	**18.20**	**2,347**	**20.70**
Pangasinan	**50,710**	7	**0.01**	413	**0.81**	4,350	**8.58**	**4,770**	**9.41**
Alaminos City	**1,910**	0	**0.00**	119	**6.23**	1,153	**60.37**	**1,272**	**66.60**
Candon City	**954**	0	**0.00**	0	**0.00**	176	**18.45**	**176**	**18.45**
Dagupan City	**3,657**	1	**0.03**	52	**1.42**	335	**9.16**	**388**	**10.61**
Laoag City	**1,812**	0	**0.00**	21	**1.16**	1,430	**78.92**	**1,451**	**80.08**
San Carlos City	**4,028**	0	**0.00**	22	**0.55**	246	**6.11**	**268**	**6.65**
San Fernando City	**2,074**	1	**0.05**	74	**3.57**	620	**29.89**	**695**	**33.51**
Urdaneta City	**2,836**	2	**0.07**	187	**6.59**	1,366	**48.17**	**1,555**	**54.83**
Vigan City	**854**	0	**0.00**	1	**0.12**	4	**0.47**	**5**	**0.59**
**Region 2**	**68,960**	**31**	**0.04**	**993**	**1.44**	**6,853**	**9.94**	**7,877**	**11.42**
Batanes	**362**	1	**0.28**	3	**0.83**	130	**35.91**	**134**	**37.02**
Cagayan	**20,431**	5	**0.02**	143	**0.70**	831	**4.07**	**979**	**4.79**
Isabela	**23,449**	13	**0.06**	366	**1.56**	2,397	**10.22**	**2,776**	**11.84**
Nueva Vizcaya	**9,538**	5	**0.05**	140	**1.47**	1,016	**10.65**	**1,161**	**12.17**
Quirino	**4,051**	3	**0.07**	157	**3.88**	1,317	**32.51**	**1,477**	**36.46**
Cauayan City	**2,565**	3	**0.12**	65	**2.53**	384	**14.97**	**452**	**17.62**
Ilagan City	**2,889**	0	**0.00**	20	**0.69**	134	**4.64**	**154**	**5.33**
Santiago City	**2,671**	1	**0.04**	98	**3.67**	600	**22.46**	**699**	**26.17**

Table 2.B.1.13. Prenatal Care

Number and proportion of pregnant women screened for HIV
Philippines, 2022

Area	Eligible Pop.	Screened for HIV							
		Age Group						Total	%
		10-14 yrs old		15-19 yrs old		20-49 yrs old			
		No.	%	No.	%	No.	%		
Tuguegarao City	**3,004**	0	**0.00**	1	**0.03**	44	**1.46**	**45**	**1.50**
**Region 3**	**220,155**	**241**	**0.11**	**8,656**	**3.93**	**58,778**	**26.70**	**67,675**	**30.74**
Aurora	**4,754**	8	**0.17**	272	**5.72**	1,695	**35.65**	**1,975**	**41.54**
Bataan	**13,789**	24	**0.17**	921	**6.68**	5,573	**40.42**	**6,518**	**47.27**
Bulacan	**43,760**	33	**0.08**	1,404	**3.21**	10,077	**23.03**	**11,514**	**26.31**
Nueva Ecija	**29,039**	20	**0.07**	615	**2.12**	3,174	**10.93**	**3,809**	**13.12**
Pampanga	**31,620**	26	**0.08**	1,147	**3.63**	8,360	**26.44**	**9,533**	**30.15**
Tarlac	**19,319**	29	**0.15**	478	**2.47**	3,220	**16.67**	**3,727**	**19.29**
Zambales	**12,476**	15	**0.12**	411	**3.29**	2,551	**20.45**	**2,977**	**23.86**
Angeles City	**7,932**	2	**0.03**	162	**2.04**	931	**11.74**	**1,095**	**13.80**
Balanga City	**1,992**	6	**0.30**	157	**7.88**	970	**48.69**	**1,133**	**56.88**
Cabanatuan City	**5,945**	3	**0.05**	196	**3.30**	1,146	**19.28**	**1,345**	**22.62**
City of San Fernando	**5,907**	10	**0.17**	272	**4.60**	1,909	**32.32**	**2,191**	**37.09**
Gapan City	**2,170**	2	**0.09**	72	**3.32**	313	**14.42**	**387**	**17.83**
Mabalacat City	**4,831**	14	**0.29**	438	**9.07**	2,281	**47.22**	**2,733**	**56.57**
Malolos City	**4,890**	6	**0.12**	168	**3.44**	1,061	**21.70**	**1,235**	**25.26**
Meycauayan	**4,055**	0	**0.00**	239	**5.89**	1,468	**36.20**	**1,707**	**42.10**
Olongapo	**4,922**	2	**0.04**	152	**3.09**	957	**19.44**	**1,111**	**22.57**
Palayan City	**808**	0	**0.00**	13	**1.61**	85	**10.52**	**98**	**12.13**
San Jose City	**2,748**	3	**0.11**	86	**3.13**	397	**14.45**	**486**	**17.69**
San Jose del Monte City	**11,133**	32	**0.29**	1,129	**10.14**	11,209	**100.68**	**12,370**	**111.11**
Science City of Munoz	**1,601**	2	**0.12**	97	**6.06**	553	**34.54**	**652**	**40.72**
Tarlac City	**6,464**	4	**0.06**	227	**3.51**	848	**13.12**	**1,079**	**16.69**
**Region 4A**	**299,627**	**132**	**0.04**	**7,059**	**2.36**	**53,288**	**17.78**	**60,479**	**20.18**
Batangas	**39,255**	0	**0.00**	316	**0.80**	3,259	**8.30**	**3,575**	**9.11**
Cavite	**28,151**	11	**0.04**	532	**1.89**	4,721	**16.77**	**5,264**	**18.70**
Laguna	**19,696**	20	**0.10**	583	**2.96**	4,268	**21.67**	**4,871**	**24.73**
Quezon	**37,853**	34	**0.09**	758	**2.00**	4,947	**13.07**	**5,739**	**15.16**
Rizal	**44,798**	38	**0.08**	1,893	**4.23**	11,975	**26.73**	**13,906**	**31.04**
Antipolo City	**16,498**	1	**0.01**	426	**2.58**	2,675	**16.21**	**3,102**	**18.80**
Bacoor City	**12,341**	1	**0.01**	213	**1.73**	1,438	**11.65**	**1,652**	**13.39**
Batangas City	**6,965**	0	**0.00**	18	**0.26**	205	**2.94**	**223**	**3.20**
Biñan City	**6,597**	4	**0.06**	769	**11.66**	5,695	**86.33**	**6,468**	**98.04**
Cabuyao City	**6,119**	0	**0.00**	87	**1.42**	827	**13.52**	**914**	**14.94**
Calamba City	**9,005**	4	**0.04**	89	**0.99**	918	**10.19**	**1,011**	**11.23**
Cavite City	**2,105**	2	**0.10**	79	**3.75**	335	**15.91**	**416**	**19.76**
Dasmariñas City	**13,538**	2	**0.01**	304	**2.25**	3,039	**22.45**	**3,345**	**24.71**
General Trias City	**6,457**	0	**0.00**	50	**0.77**	539	**8.35**	**589**	**9.12**
Imus City	**8,292**	3	**0.04**	72	**0.87**	655	**7.90**	**730**	**8.80**
Lipa City	**7,018**	1	**0.01**	112	**1.60**	1,314	**18.72**	**1,427**	**20.33**
Lucena City	**5,738**	0	**0.00**	156	**2.72**	749	**13.05**	**905**	**15.77**
San Pablo City	**5,272**	1	**0.02**	52	**0.99**	366	**6.94**	**419**	**7.95**
San Pedro City	**6,455**	1	**0.02**	98	**1.52**	801	**12.41**	**900**	**13.94**
Santa Rosa City	**7,009**	6	**0.09**	315	**4.49**	2,646	**37.75**	**2,967**	**42.33**
Tagaytay City	**1,460**	0	**0.00**	61	**4.18**	1,142	**78.22**	**1,203**	**82.40**
Tanauan City	**3,661**	3	**0.08**	35	**0.96**	463	**12.65**	**501**	**13.68**
Tayabas City	**2,145**	0	**0.00**	13	**0.61**	139	**6.48**	**152**	**7.09**
Trece Martires City	**3,199**	0	**0.00**	28	**0.88**	172	**5.38**	**200**	**6.25**
**Region 4B**	**72,791**	**10**	**0.01**	**1,062**	**1.46**	**8,233**	**11.31**	**9,305**	**12.78**
Marinduque	**4,990**	0	**0.00**	55	**1.10**	563	**11.28**	**618**	**12.38**
Mindoro Occidental	**12,479**	1	**0.01**	30	**0.24**	282	**2.26**	**313**	**2.51**
Mindoro Oriental	**20,189**	2	**0.01**	329	**1.63**	3,120	**15.45**	**3,451**	**17.09**
Palawan	**21,963**	6	**0.03**	390	**1.78**	2,665	**12.13**	**3,061**	**13.94**
Romblon	**6,569**	0	**0.00**	116	**1.77**	920	**14.01**	**1,036**	**15.77**
Puerto Princesa City	**6,601**	1	**0.02**	142	**2.15**	683	**10.35**	**826**	**12.51**

Table 2.B.1.13. Prenatal Care

Number and proportion of pregnant women screened for HIV
Philippines, 2022

Area	Eligible Pop.	Screened for HIV							
		Age Group						Total	%
		10-14 yrs old		15-19 yrs old		20-49 yrs old			
		No.	%	No.	%	No.	%		
**Region 5**	**138,457**	**18**	**0.01**	**1,278**	**0.92**	**13,282**	**9.59**	**14,578**	**10.53**
Albay	**24,081**	3	**0.01**	238	**0.99**	3,669	**15.24**	**3,910**	**16.24**
Camarines Norte	**14,384**	4	**0.03**	111	**0.77**	891	**6.19**	**1,006**	**6.99**
Camarines Sur	**39,486**	1	**0.00**	164	**0.42**	2,023	**5.12**	**2,188**	**5.54**
Catanduanes	**6,459**	3	**0.05**	72	**1.11**	893	**13.83**	**968**	**14.99**
Masbate	**22,600**	2	**0.01**	318	**1.41**	1,782	**7.88**	**2,102**	**9.30**
Sorsogon	**19,828**	0	**0.00**	90	**0.45**	858	**4.33**	**948**	**4.78**
Iriga City	**2,679**	0	**0.00**	55	**2.05**	601	**22.43**	**656**	**24.49**
Legaspi City	**4,233**	4	**0.09**	113	**2.67**	1,210	**28.58**	**1,327**	**31.35**
Naga City	**4,707**	1	**0.02**	117	**2.49**	1,355	**28.79**	**1,473**	**31.29**
**Region 6**	**146,449**	**39**	**0.03**	**2,328**	**1.59**	**20,268**	**13.84**	**22,635**	**15.46**
Aklan	**11,288**	2	**0.02**	183	**1.62**	2,022	**17.91**	**2,207**	**19.55**
Antique	**13,132**	2	**0.02**	157	**1.20**	1,659	**12.63**	**1,818**	**13.84**
Capiz	**13,975**	2	**0.01**	77	**0.55**	961	**6.88**	**1,040**	**7.44**
Guimaras	**3,084**	6	**0.19**	84	**2.72**	960	**31.13**	**1,050**	**34.05**
Iloilo	**36,267**	10	**0.03**	437	**1.20**	4,898	**13.51**	**5,345**	**14.74**
Negros Occidental	**49,230**	7	**0.01**	740	**1.50**	4,419	**8.98**	**5,166**	**10.49**
Bacolod City	**11,082**	8	**0.07**	378	**3.41**	2,844	**25.66**	**3,230**	**29.15**
Iloilo City	**8,391**	2	**0.02**	272	**3.24**	2,505	**29.85**	**2,779**	**33.12**
**Region 7**	**162,308**	**96**	**0.06**	**4,784**	**2.95**	**41,639**	**25.65**	**46,519**	**28.66**
Bohol	**27,992**	4	**0.01**	515	**1.84**	5,015	**17.92**	**5,534**	**19.77**
Cebu	**66,463**	29	**0.04**	1,176	**1.77**	7,632	**11.48**	**8,837**	**13.30**
Negros Oriental	**27,890**	1	**0.00**	140	**0.50**	905	**3.24**	**1,046**	**3.75**
Siquijor	**1,661**	1	**0.06**	60	**3.61**	748	**45.03**	**809**	**48.71**
Cebu City	**20,866**	22	**0.11**	1,195	**5.73**	9,448	**45.28**	**10,665**	**51.11**
Lapu-Lapu City	**9,232**	3	**0.03**	360	**3.90**	5,046	**54.66**	**5,409**	**58.59**
Mandaue City	**8,204**	36	**0.44**	1,338	**16.31**	12,845	**156.57**	**14,219**	**173.32**
**Region 8**	**105,471**	**37**	**0.04**	**1,654**	**1.57**	**11,886**	**11.27**	**13,577**	**12.87**
Biliran	**3,873**	2	**0.05**	183	**4.73**	1,299	**33.54**	**1,484**	**38.32**
Eastern Samar	**11,908**	8	**0.07**	164	**1.38**	1,290	**10.83**	**1,462**	**12.28**
Northern Leyte	**35,422**	10	**0.03**	152	**0.43**	1,112	**3.14**	**1,274**	**3.60**
Northern Samar	**15,921**	5	**0.03**	584	**3.67**	3,808	**23.92**	**4,397**	**27.62**
Southern Leyte	**6,662**	1	**0.02**	71	**1.07**	747	**11.21**	**819**	**12.29**
Western Samar	**14,732**	3	**0.02**	92	**0.62**	645	**4.38**	**740**	**5.02**
Calbayog City	**4,535**	0	**0.00**	0	**0.00**	24	**0.53**	**24**	**0.53**
Maasin City	**1,696**	0	**0.00**	0	**0.00**	0	**0.00**	**0**	**0.00**
Ormoc City	**5,044**	4	**0.08**	164	**3.25**	1,244	**24.66**	**1,412**	**27.99**
Tacloban City	**5,678**	4	**0.07**	244	**4.30**	1,717	**30.24**	**1,965**	**34.61**
**Region 9**	**79,007**	**7**	**0.01**	**322**	**0.41**	**1,866**	**2.36**	**2,195**	**2.78**
Zamboanga del Norte	**16,854**	0	**0.00**	38	**0.23**	205	**1.22**	**243**	**1.44**
Zamboanga del Sur	**17,368**	1	**0.01**	30	**0.17**	261	**1.50**	**292**	**1.68**
Zamboanga Sibugay	**15,021**	0	**0.00**	31	**0.21**	229	**1.52**	**260**	**1.73**
Dapitan City	**1,737**	3	**0.17**	55	**3.17**	285	**16.41**	**343**	**19.75**
Dipolog City	**2,762**	1	**0.04**	0	**0.00**	8	**0.29**	**9**	**0.33**
Isabela City	**2,563**	0	**0.00**	54	**2.11**	266	**10.38**	**320**	**12.49**
Pagadian City	**4,257**	0	**0.00**	7	**0.16**	60	**1.41**	**67**	**1.57**
Zamboanga City	**18,445**	2	**0.01**	107	**0.58**	552	**2.99**	**661**	**3.58**

Table 2.B.1.13. Prenatal Care

Number and proportion of pregnant women screened for HIV
Philippines, 2022

Area	Eligible Pop.	Screened for HIV							
		Age Group						Total	%
		10-14 yrs old		15-19 yrs old		20-49 yrs old			
		No.	%	No.	%	No.	%		
**Region 10**	**99,908**	**24**	**0.02**	**1,649**	**1.65**	**8,622**	**8.63**	**10,295**	**10.30**
Bukidnon	**22,900**	3	**0.01**	362	**1.58**	1,324	**5.78**	**1,689**	**7.38**
Camiguin	**1,854**	0	**0.00**	31	**1.67**	323	**17.42**	**354**	**19.09**
Lanao del Norte	**14,930**	0	**0.00**	44	**0.29**	438	**2.93**	**482**	**3.23**
Misamis Occidental	**6,420**	1	**0.02**	44	**0.69**	398	**6.20**	**443**	**6.90**
Misamis Oriental	**14,963**	7	**0.05**	308	**2.06**	1,275	**8.52**	**1,590**	**10.63**
Cagayan de Oro City	**14,172**	4	**0.03**	237	**1.67**	1,281	**9.04**	**1,522**	**10.74**
El Salvador City	**1,052**	4	**0.38**	61	**5.80**	274	**26.05**	**339**	**32.22**
Gingoog City	**2,608**	1	**0.04**	182	**6.98**	708	**27.15**	**891**	**34.16**
Iligan City	**7,565**	0	**0.00**	88	**1.16**	608	**8.04**	**696**	**9.20**
Malaybalay City	**3,817**	2	**0.05**	21	**0.55**	159	**4.17**	**182**	**4.77**
Oroquieta City	**1,389**	0	**0.00**	14	**1.01**	40	**2.88**	**54**	**3.89**
Ozamis City	**2,786**	0	**0.00**	141	**5.06**	1,010	**36.25**	**1,151**	**41.31**
Tangub City	**1,234**	0	**0.00**	88	**7.13**	590	**47.81**	**678**	**54.94**
Valencia City	**4,218**	2	**0.05**	28	**0.66**	194	**4.60**	**224**	**5.31**
**Region 11**	**108,407**	**86**	**0.08**	**1,753**	**1.62**	**11,759**	**10.85**	**13,598**	**12.54**
Davao de Oro	**15,490**	2	**0.01**	11	**0.07**	108	**0.70**	**121**	**0.78**
Davao del Norte	**21,017**	9	**0.04**	302	**1.44**	2,028	**9.65**	**2,339**	**11.13**
Davao Oriental	**13,229**	2	**0.02**	47	**0.36**	204	**1.54**	**253**	**1.91**
Davao del Sur	**14,564**	12	**0.08**	202	**1.39**	1,108	**7.61**	**1,322**	**9.08**
Davao Occidental	**6,510**	15	**0.23**	111	**1.71**	378	**5.81**	**504**	**7.74**
Davao City	**37,597**	46	**0.12**	1,080	**2.87**	7,933	**21.10**	**9,059**	**24.10**
**Region 12**	**98,859**	**37**	**0.04**	**1,825**	**1.85**	**10,377**	**10.50**	**12,239**	**12.38**
North Cotabato	**34,150**	8	**0.02**	211	**0.62**	1,127	**3.30**	**1,346**	**3.94**
Sarangani	**12,907**	4	**0.03**	188	**1.46**	973	**7.54**	**1,165**	**9.03**
South Cotabato	**20,917**	18	**0.09**	1,010	**4.83**	5,873	**28.08**	**6,901**	**32.99**
Sultan Kudarat	**17,299**	4	**0.02**	143	**0.83**	574	**3.32**	**721**	**4.17**
Gen. Santos City	**13,586**	3	**0.02**	273	**2.01**	1,830	**13.47**	**2,106**	**15.50**
**BARMM**	**101,343**	**1**	**0.00**	**239**	**0.24**	**672**	**0.66**	**554**	**0.55**
Basilan	**7,823**	0	**0.00**	0	**0.00**	2	**0.03**	**2**	**0.03**
Lanao del Sur	**21,639**	0	**0.00**	0	**0.00**	0	**0.00**	**0**	**0.00**
Maguindanao	**32,198**	0	**0.00**	0	**0.00**	2	**0.01**	**2**	**0.01**
Sulu	**17,165**	0	**0.00**	214	**1.25**	335	**1.95**	**549**	**3.20**
Tawi-Tawi	**9,369**	0	**0.00**	0	**0.00**	0	**0.00**	**0**	**0.00**
Lamitan City	**2,154**	0	**0.00**	0	**0.00**	1	**0.05**	**1**	**0.05**
Marawi City	**5,173**	0	**0.00**	0	**0.00**	0	**0.00**	**0**	**0.00**
Cotabato City	**5,822**	1	**0.02**	25	**0.43**	332	**5.70**		**0.00**
**CARAGA**	**60,575**	**24**	**0.04**	**1,050**	**1.73**	**7,412**	**12.24**	**8,486**	**14.01**
Agusan del Norte	**8,127**	1	**0.01**	73	**0.90**	750	**9.23**	**824**	**10.14**
Agusan del Sur	**17,709**	10	**0.06**	207	**1.17**	1,246	**7.04**	**1,463**	**8.26**
Surigao del Norte	**7,280**	0	**0.00**	28	**0.38**	372	**5.11**	**400**	**5.49**
Surigao del Sur	**11,553**	0	**0.00**	53	**0.46**	302	**2.61**	**355**	**3.07**
Province of Dinagat	**2,576**	1	**0.04**	113	**4.39**	702	**27.25**	**816**	**31.68**
Bislig City	**2,192**	0	**0.00**	3	**0.14**	31	**1.41**	**34**	**1.55**
Butuan City	**7,743**	8	**0.10**	358	**4.62**	2,821	**36.43**	**3,187**	**41.16**
Surigao City	**3,395**	4	**0.12**	215	**6.33**	1,188	**34.99**	**1,407**	**41.44**

Table 2.B.1.14. Prenatal Care

Number and proportion of pregnant women tested for CBC or Hemoglobin and Hematocrit Count
Philippines, 2022

Area	Eligible Pop.	Tested for CBC/Hgb and Hct							
		Age Group						Total	%
		10-14 yrs old		15-19 yrs old		20-49 yrs old			
		No.	%	No.	%	No.	%		
**PHILIPPINES**	**2,125,674**	**2,288**	**0.11**	**99,645**	**4.69**	**739,730**	**34.80**	**841,663**	**39.60**
**N C R**	**236,901**	**231**	**0.10**	**11,523**	**4.86**	**110,377**	**46.59**	**122,131**	**51.55**
Malabon	**6,724**	9	**0.13**	399	**5.93**	2,437	**36.24**	**2,845**	**42.31**
Navotas	**4,591**	13	**0.28**	516	**11.24**	2,547	**55.48**	**3,076**	**67.00**
Valenzuela City	**11,418**	10	**0.09**	602	**5.27**	6,065	**53.12**	**6,677**	**58.48**
Caloocan City	**29,146**	12	**0.04**	886	**3.04**	8,388	**28.78**	**9,286**	**31.86**
Marikina City	**8,294**	4	**0.05**	171	**2.06**	1,631	**19.66**	**1,806**	**21.77**
Pasig City	**13,894**	13	**0.09**	839	**6.04**	11,636	**83.75**	**12,488**	**89.88**
Pateros	**1,176**	4	**0.34**	102	**8.67**	785	**66.75**	**891**	**75.77**
Taguig	**14,807**	36	**0.24**	1,200	**8.10**	10,415	**70.34**	**11,651**	**78.69**
Quezon City	**54,011**	58	**0.11**	2,533	**4.69**	29,025	**53.74**	**31,616**	**58.54**
Makati City	**10,718**	3	**0.03**	217	**2.02**	3,707	**34.59**	**3,927**	**36.64**
Mandaluyong City	**7,111**	4	**0.06**	390	**5.48**	5,930	**83.39**	**6,324**	**88.93**
San Juan	**2,246**	0	**0.00**	62	**2.76**	900	**40.07**	**962**	**42.83**
Manila City	**32,743**	35	**0.11**	1,894	**5.78**	12,360	**37.75**	**14,289**	**43.64**
Las Piñas City	**10,832**	5	**0.05**	274	**2.53**	2,467	**22.78**	**2,746**	**25.35**
Muntinlupa City	**9,281**	8	**0.09**	308	**3.32**	2,412	**25.99**	**2,728**	**29.39**
Parañaque City	**12,249**	16	**0.13**	639	**5.22**	5,533	**45.17**	**6,188**	**50.52**
Pasay City	**7,660**	1	**0.01**	491	**6.41**	4,139	**54.03**	**4,631**	**60.46**
**C A R**	**35,179**	**37**	**0.11**	**1,904**	**5.41**	**16,188**	**46.02**	**18,129**	**51.53**
Abra	**4,275**	2	**0.05**	173	**4.05**	874	**20.44**	**1,049**	**24.54**
Apayao	**2,502**	9	**0.36**	329	**13.15**	1,703	**68.07**	**2,041**	**81.57**
Benguet	**9,086**	9	**0.10**	474	**5.22**	4,618	**50.83**	**5,101**	**56.14**
Ifugao	**4,486**	5	**0.11**	269	**6.00**	2,518	**56.13**	**2,792**	**62.24**
Kalinga	**4,701**	4	**0.09**	240	**5.11**	2,259	**48.05**	**2,503**	**53.24**
Mt. Province	**3,103**	1	**0.03**	124	**4.00**	1,293	**41.67**	**1,418**	**45.70**
Baguio City	**7,026**	7	**0.10**	295	**4.20**	2,923	**41.60**	**3,225**	**45.90**
**Region 1**	**97,099**	**52**	**0.05**	**3,471**	**3.57**	**37,929**	**39.06**	**41,452**	**42.69**
Ilocos Norte	**7,854**	5	**0.06**	288	**3.67**	3,500	**44.56**	**3,793**	**48.29**
Ilocos Sur	**9,072**	9	**0.10**	452	**4.98**	5,312	**58.55**	**5,773**	**63.64**
La Union	**11,338**	8	**0.07**	519	**4.58**	4,610	**40.66**	**5,137**	**45.31**
Pangasinan	**50,710**	22	**0.04**	1,640	**3.23**	18,010	**35.52**	**19,672**	**38.79**
Alaminos City	**1,910**	0	**0.00**	119	**6.23**	1,153	**60.37**	**1,272**	**66.60**
Candon City	**954**	0	**0.00**	3	**0.31**	421	**44.13**	**424**	**44.44**
Dagupan City	**3,657**	4	**0.11**	36	**0.98**	255	**6.97**	**295**	**8.07**
Laoag City	**1,812**	0	**0.00**	21	**1.16**	1,430	**78.92**	**1,451**	**80.08**
San Carlos City	**4,028**	0	**0.00**	53	**1.32**	649	**16.11**	**702**	**17.43**
San Fernando City	**2,074**	1	**0.05**	102	**4.92**	773	**37.27**	**876**	**42.24**
Urdaneta City	**2,836**	3	**0.11**	219	**7.72**	1,681	**59.27**	**1,903**	**67.10**
Vigan City	**854**	0	**0.00**	19	**2.22**	135	**15.81**	**154**	**18.03**
**Region 2**	**68,960**	**98**	**0.14**	**3,739**	**5.42**	**26,972**	**39.11**	**30,809**	**44.68**
Batanes	**362**	1	**0.28**	3	**0.83**	160	**44.20**	**164**	**45.30**
Cagayan	**20,431**	25	**0.12**	951	**4.65**	6,337	**31.02**	**7,313**	**35.79**
Isabela	**23,449**	52	**0.22**	1,412	**6.02**	9,636	**41.09**	**11,100**	**47.34**
Nueva Vizcaya	**9,538**	3	**0.03**	580	**6.08**	3,870	**40.57**	**4,453**	**46.69**
Quirino	**4,051**	4	**0.10**	267	**6.59**	2,989	**73.78**	**3,260**	**80.47**
Cauayan City	**2,565**	7	**0.27**	206	**8.03**	1,101	**42.92**	**1,314**	**51.23**
Ilagan City	**2,889**	1	**0.03**	144	**4.98**	1,204	**41.68**	**1,349**	**46.69**
Santiago City	**2,671**	2	**0.07**	110	**4.12**	752	**28.15**	**864**	**32.35**

Table 2.B.1.14. Prenatal Care

Number and proportion of pregnant women tested for CBC or Hemoglobin and Hematocrit Count

Philippines, 2022

Area	Eligible Pop.	Tested for CBC/Hgb and Hct							
		Age Group						Total	%
		10-14 yrs old		15-19 yrs old		20-49 yrs old			
		No.	%	No.	%	No.	%		
Tuguegarao City	3,004	3	0.10	66	2.20	923	30.73	992	33.02
**Region 3**	220,155	271	0.12	10,404	4.73	71,275	32.37	81,950	37.22
Aurora	4,754	7	0.15	193	4.06	1,263	26.57	1,463	30.77
Bataan	13,789	29	0.21	1,050	7.61	6,380	46.27	7,459	54.09
Bulacan	43,760	59	0.13	2,180	4.98	16,376	37.42	18,615	42.54
Nueva Ecija	29,039	32	0.11	1,037	3.57	6,067	20.89	7,136	24.57
Pampanga	31,620	19	0.06	851	2.69	5,833	18.45	6,703	21.20
Tarlac	19,319	27	0.14	793	4.10	5,049	26.13	5,869	30.38
Zambales	12,476	16	0.13	454	3.64	2,775	22.24	3,245	26.01
Angeles City	7,932	1	0.01	145	1.83	779	9.82	925	11.66
Balanga City	1,992	8	0.40	187	9.39	1,216	61.04	1,411	70.83
Cabanatuan City	5,945	5	0.08	186	3.13	1,125	18.92	1,316	22.14
City of San Fernando	5,907	11	0.19	330	5.59	2,499	42.31	2,840	48.08
Gapan City	2,170	2	0.09	108	4.98	514	23.69	624	28.76
Mabalacat City	4,831	0	0.00	184	3.81	1,567	32.44	1,751	36.25
Malolos City	4,890	6	0.12	198	4.05	1,166	23.84	1,370	28.02
Meycauayan	4,055	1	0.02	317	7.82	1,645	40.57	1,963	48.41
Olongapo	4,922	3	0.06	167	3.39	1,044	21.21	1,214	24.66
Palayan City	808	1	0.12	16	1.98	84	10.40	101	12.50
San Jose City	2,748	5	0.18	168	6.11	726	26.42	899	32.71
San Jose del Monte City	11,133	32	0.29	1,182	10.62	11,348	101.93	12,562	112.84
Science City of Munoz	1,601	3	0.19	97	6.06	543	33.92	643	40.16
Tarlac City	6,464	4	0.06	561	8.68	3,276	50.68	3,841	59.42
**Region 4A**	299,627	195	0.07	11,057	3.69	87,696	29.27	98,948	33.02
Batangas	39,255	6	0.02	886	2.26	8,945	22.79	9,837	25.06
Cavite	28,151	25	0.09	895	3.18	7,999	28.41	8,919	31.68
Laguna	19,696	20	0.10	961	4.88	6,653	33.78	7,634	38.76
Quezon	37,853	52	0.14	1,542	4.07	11,115	29.36	12,709	33.57
Rizal	44,798	28	0.06	1,989	4.44	12,987	28.99	15,004	33.49
Antipolo City	16,498	1	0.01	470	2.85	2,888	17.51	3,359	20.36
Bacoor City	12,341	2	0.02	235	1.90	1,569	12.71	1,806	14.63
Batangas City	6,965	2	0.03	149	2.14	1,395	20.03	1,546	22.20
Biñan City	6,597	4	0.06	838	12.70	6,067	91.97	6,909	104.73
Cabuyao City	6,119	8	0.13	310	5.07	3,356	54.85	3,674	60.04
Calamba City	9,005	10	0.11	444	4.93	4,251	47.21	4,705	52.25
Cavite City	2,105	3	0.14	83	3.94	333	15.82	419	19.90
Dasmariñas City	13,538	4	0.03	372	2.75	3,590	26.52	3,966	29.30
General Trias City	6,457	2	0.03	92	1.42	710	11.00	804	12.45
Imus City	8,292	2	0.02	87	1.05	883	10.65	972	11.72
Lipa City	7,018	1	0.01	263	3.75	2,896	41.27	3,160	45.03
Lucena City	5,738	4	0.07	334	5.82	1,711	29.82	2,049	35.71
San Pablo City	5,272	5	0.09	134	2.54	1,025	19.44	1,164	22.08
San Pedro City	6,455	3	0.05	182	2.82	1,219	18.88	1,404	21.75
Santa Rosa City	7,009	7	0.10	417	5.95	3,562	50.82	3,986	56.87
Tagaytay City	1,460	0	0.00	60	4.11	1,160	79.45	1,220	83.56
Tanauan City	3,661	3	0.08	72	1.97	1,475	40.29	1,550	42.34
Tayabas City	2,145	1	0.05	133	6.20	1,244	58.00	1,378	64.24
Trece Martires City	3,199	2	0.06	109	3.41	663	20.73	774	24.20
**Region 4B**	72,791	38	0.05	2,445	3.36	17,487	24.02	19,970	27.43
Marinduque	4,990	2	0.04	102	2.04	1,085	21.74	1,189	23.83
Mindoro Occidental	12,479	3	0.02	421	3.37	2,698	21.62	3,122	25.02
Mindoro Oriental	20,189	9	0.04	566	2.80	5,298	26.24	5,873	29.09
Palawan	21,963	19	0.09	767	3.49	4,572	20.82	5,358	24.40
Romblon	6,569	1	0.02	199	3.03	1,970	29.99	2,170	33.03
Puerto Princesa City	6,601	4	0.06	390	5.91	1,864	28.24	2,258	34.21

Table 2.B.1.14. Prenatal Care

Number and proportion of pregnant women tested for CBC or Hemoglobin and Hematocrit Count
Philippines, 2022

Area	Eligible Pop.	Tested for CBC/Hgb and Hct							
		Age Group						Total	%
		10-14 yrs old		15-19 yrs old		20-49 yrs old			
		No.	%	No.	%	No.	%		
**Region 5**	**138,457**	**42**	**0.03**	**3,428**	**2.48**	**32,428**	**23.42**	**35,898**	**25.93**
Albay	**24,081**	4	**0.02**	551	**2.29**	7,674	**31.87**	**8,229**	**34.17**
Camarines Norte	**14,384**	9	**0.06**	562	**3.91**	3,889	**27.04**	**4,460**	**31.01**
Camarines Sur	**39,486**	1	**0.00**	280	**0.71**	3,298	**8.35**	**3,579**	**9.06**
Catanduanes	**6,459**	3	**0.05**	317	**4.91**	2,413	**37.36**	**2,733**	**42.31**
Masbate	**22,600**	11	**0.05**	745	**3.30**	4,374	**19.35**	**5,130**	**22.70**
Sorsogon	**19,828**	4	**0.02**	786	**3.96**	7,792	**39.30**	**8,582**	**43.28**
Iriga City	**2,679**	1	**0.04**	16	**0.60**	155	**5.79**	**172**	**6.42**
Legaspi City	**4,233**	9	**0.21**	164	**3.87**	2,755	**65.08**	**2,928**	**69.17**
Naga City	**4,707**	0	**0.00**	7	**0.15**	78	**1.66**	**85**	**1.81**
**Region 6**	**146,449**	**109**	**0.07**	**6,893**	**4.71**	**54,297**	**37.08**	**61,299**	**41.86**
Aklan	**11,288**	3	**0.03**	378	**3.35**	4,111	**36.42**	**4,492**	**39.79**
Antique	**13,132**	10	**0.08**	420	**3.20**	3,581	**27.27**	**4,011**	**30.54**
Capiz	**13,975**	11	**0.08**	361	**2.58**	3,817	**27.31**	**4,189**	**29.97**
Guimaras	**3,084**	8	**0.26**	179	**5.80**	1,859	**60.28**	**2,046**	**66.34**
Iloilo	**36,267**	37	**0.10**	1,671	**4.61**	15,797	**43.56**	**17,505**	**48.27**
Negros Occidental	**49,230**	28	**0.06**	2,844	**5.78**	17,678	**35.91**	**20,550**	**41.74**
Bacolod City	**11,082**	9	**0.08**	694	**6.26**	4,329	**39.06**	**5,032**	**45.41**
Iloilo City	**8,391**	3	**0.04**	346	**4.12**	3,125	**37.24**	**3,474**	**41.40**
**Region 7**	**162,308**	**179**	**0.11**	**8,538**	**5.26**	**71,270**	**43.91**	**79,987**	**49.28**
Bohol	**27,992**	36	**0.13**	1,565	**5.59**	13,401	**47.87**	**15,002**	**53.59**
Cebu	**66,463**	52	**0.08**	2,725	**4.10**	16,884	**25.40**	**19,661**	**29.58**
Negros Oriental	**27,890**	24	**0.09**	1,283	**4.60**	8,444	**30.28**	**9,751**	**34.96**
Siquijor	**1,661**	2	**0.12**	50	**3.01**	610	**36.72**	**662**	**39.86**
Cebu City	**20,866**	22	**0.11**	1,201	**5.76**	9,576	**45.89**	**10,799**	**51.75**
Lapu-Lapu City	**9,232**	7	**0.08**	378	**4.09**	9,625	**104.26**	**10,010**	**108.43**
Mandaue City	**8,204**	36	**0.44**	1,336	**16.28**	12,730	**155.17**	**14,102**	**171.89**
**Region 8**	**105,471**	**50**	**0.05**	**3,570**	**3.38**	**29,824**	**28.28**	**33,444**	**31.71**
Biliran	**3,873**	4	**0.10**	245	**6.33**	1,797	**46.40**	**2,046**	**52.83**
Eastern Samar	**11,908**	6	**0.05**	298	**2.50**	2,562	**21.51**	**2,866**	**24.07**
Northern Leyte	**35,422**	16	**0.05**	1,143	**3.23**	9,598	**27.10**	**10,757**	**30.37**
Northern Samar	**15,921**	8	**0.05**	764	**4.80**	5,462	**34.31**	**6,234**	**39.16**
Southern Leyte	**6,662**	3	**0.05**	310	**4.65**	3,217	**48.29**	**3,530**	**52.99**
Western Samar	**14,732**	0	**0.00**	223	**1.51**	1,578	**10.71**	**1,801**	**12.23**
Calbayog City	**4,535**	0	**0.00**	31	**0.68**	313	**6.90**	**344**	**7.59**
Maasin City	**1,696**	3	**0.18**	40	**2.36**	558	**32.90**	**601**	**35.44**
Ormoc City	**5,044**	5	**0.10**	249	**4.94**	1,755	**34.79**	**2,009**	**39.83**
Tacloban City	**5,678**	5	**0.09**	267	**4.70**	2,984	**52.55**	**3,256**	**57.34**
**Region 9**	**79,007**	**42**	**0.05**	**1,941**	**2.46**	**12,707**	**16.08**	**14,690**	**18.59**
Zamboanga del Norte	**16,854**	5	**0.03**	406	**2.41**	3,007	**17.84**	**3,418**	**20.28**
Zamboanga del Sur	**17,368**	14	**0.08**	373	**2.15**	2,001	**11.52**	**2,388**	**13.75**
Zamboanga Sibugay	**15,021**	10	**0.07**	581	**3.87**	3,524	**23.46**	**4,115**	**27.39**
Dapitan City	**1,737**	2	**0.12**	47	**2.71**	201	**11.57**	**250**	**14.39**
Dipolog City	**2,762**	1	**0.04**	1	**0.04**	42	**1.52**	**44**	**1.59**
Isabela City	**2,563**	2	**0.08**	32	**1.25**	182	**7.10**	**216**	**8.43**
Pagadian City	**4,257**	1	**0.02**	194	**4.56**	1,632	**38.34**	**1,827**	**42.92**
Zamboanga City	**18,445**	7	**0.04**	307	**1.66**	2,118	**11.48**	**2,432**	**13.19**

Table 2.B.1.14. Prenatal Care

Number and proportion of pregnant women tested for CBC or Hemoglobin and Hematocrit Count
Philippines, 2022

Area	Eligible Pop.	Tested for CBC/Hgb and Hct							
		Age Group						Total	%
		10-14 yrs old		15-19 yrs old		20-49 yrs old			
		No.	%	No.	%	No.	%		
**Region 10**	**99,908**	**160**	**0.16**	**7,472**	**7.48**	**39,649**	**39.69**	**47,281**	**47.32**
Bukidnon	22,900	49	0.21	2,549	11.13	10,069	43.97	12,667	55.31
Camiguin	1,854	1	0.05	48	2.59	536	28.91	585	31.55
Lanao del Norte	14,930	1	0.01	133	0.89	1,015	6.80	1,149	7.70
Misamis Occidental	6,420	13	0.20	177	2.76	1,868	29.10	2,058	32.06
Misamis Oriental	14,963	39	0.26	1,454	9.72	7,552	50.47	9,045	60.45
Cagayan de Oro City	14,172	34	0.24	1,341	9.46	7,950	56.10	9,325	65.80
El Salvador City	1,052	7	0.67	117	11.12	597	56.75	721	68.54
Gingoog City	2,608	2	0.08	291	11.16	1,231	47.20	1,524	58.44
Iligan City	7,565	2	0.03	379	5.01	2,853	37.71	3,234	42.75
Malaybalay City	3,817	4	0.10	249	6.52	1,575	41.26	1,828	47.89
Oroquieta City	1,389	0	0.00	18	1.30	139	10.01	157	11.30
Ozamis City	2,786	0	0.00	186	6.68	1,380	49.53	1,566	56.21
Tangub City	1,234	2	0.16	111	9.00	1,121	90.84	1,234	100.00
Valencia City	4,218	6	0.14	419	9.93	1,763	41.80	2,188	51.87
**Region 11**	**108,407**	**544**	**0.50**	**10,862**	**10.02**	**60,236**	**55.56**	**71,642**	**66.09**
Davao de Oro	15,490	113	0.73	1,918	12.38	9,600	61.98	11,631	75.09
Davao del Norte	21,017	146	0.69	2,552	12.14	14,493	68.96	17,191	81.80
Davao Oriental	13,229	56	0.42	1,207	9.12	6,263	47.34	7,526	56.89
Davao del Sur	14,564	70	0.48	1,303	8.95	6,124	42.05	7,497	51.48
Davao Occidental	6,510	21	0.32	477	7.33	1,345	20.66	1,843	28.31
Davao City	37,597	138	0.37	3,405	9.06	22,411	59.61	25,954	69.03
**Region 12**	**98,859**	**159**	**0.16**	**7,522**	**7.61**	**40,087**	**40.55**	**47,768**	**48.32**
North Cotabato	34,150	36	0.11	2,005	5.87	11,341	33.21	13,382	39.19
Sarangani	12,907	42	0.33	1,642	12.72	6,887	53.36	8,571	66.41
South Cotabato	20,917	28	0.13	1,749	8.36	10,035	47.98	11,812	56.47
Sultan Kudarat	17,299	40	0.23	1,340	7.75	7,948	45.94	9,328	53.92
Gen. Santos City	13,586	13	0.10	786	5.79	3,876	28.53	4,675	34.41
**BARMM**	**95,521**	**5**	**0.01**	**669**	**0.70**	**4,437**	**4.65**	**5,111**	**5.35**
Basilan	7,823	0	0.00	6	0.08	18	0.23	24	0.31
Lanao del Sur	21,639	1	0.00	215	0.99	1,615	7.46	1,831	8.46
Maguindanao	32,198	1	0.00	172	0.53	1,090	3.39	1,263	3.92
Sulu	17,165	0	0.00	118	0.69	716	4.17	834	4.86
Tawi-Tawi	9,369	0	0.00	2	0.02	9	0.10	11	0.12
Lamitan City	2,154	0	0.00	48	2.23	136	6.31	184	8.54
Marawi City	5,173	0	0.00	0	0.00	0	0.00	0	0.00
Cotabato City	0	3	0.00	108	0.00	853	0.00	964	0.00
**CARAGA**	**60,575**	**76**	**0.13**	**4,207**	**6.95**	**26,871**	**44.36**	**31,154**	**51.43**
Agusan del Norte	8,127	7	0.09	440	5.41	2,803	34.49	3,250	39.99
Agusan del Sur	17,709	24	0.14	1,372	7.75	8,113	45.81	9,509	53.70
Surigao del Norte	7,280	5	0.07	361	4.96	3,294	45.25	3,660	50.27
Surigao del Sur	11,553	19	0.16	754	6.53	4,487	38.84	5,260	45.53
Province of Dinagat	2,576	1	0.04	144	5.59	1,005	39.01	1,150	44.64
Bislig City	2,192	4	0.18	257	11.72	1,429	65.19	1,690	77.10
Butuan City	7,743	11	0.14	556	7.18	3,982	51.43	4,549	58.75
Surigao City	3,395	5	0.15	323	9.51	1,758	51.78	2,086	61.44

Table 2.B.1.15. Prenatal Care

Number and proportion of pregnant women tested for Complete Blood Count or Hemoglobin and Hematocrit Count diagnosed with anemia
Philippines, 2022

Area	Total No. tested for CBC or Hgb or Hct count	Tested for CBC/Hgb and Hct diagnosed with anemia							
		Age Group						Total	%
		10-14 yrs old		15-19 yrs old		20-49 yrs old			
		No.	%	No.	%	No.	%		
**PHILIPPINES**	**841,663**	**436**	**0.05**	**14,600**	**1.73**	**78,073**	**9.28**	**93,109**	**11.06**
**N C R**	**122,131**	**34**	**0.03**	**1,507**	**1.23**	**10,358**	**8.48**	**11,899**	**9.74**
Malabon	**2,845**	1	**0.04**	37	**1.30**	236	**8.30**	**274**	**9.63**
Navotas	**3,076**	0	**0.00**	16	**0.52**	120	**3.90**	**136**	**4.42**
Valenzuela City	**6,677**	0	**0.00**	33	**0.49**	317	**4.75**	**350**	**5.24**
Caloocan City	**9,286**	3	**0.03**	185	**1.99**	1,201	**12.93**	**1,389**	**14.96**
Marikina City	**1,806**	1	**0.06**	14	**0.78**	105	**5.81**	**120**	**6.64**
Pasig City	**12,488**	1	**0.01**	118	**0.94**	799	**6.40**	**918**	**7.35**
Pateros	**891**	0	**0.00**	0	**0.00**	56	**6.29**	**56**	**6.29**
Taguig	**11,651**	4	**0.03**	149	**1.28**	1,112	**9.54**	**1,265**	**10.86**
Quezon City	**31,616**	13	**0.04**	397	**1.26**	2,500	**7.91**	**2,910**	**9.20**
Makati City	**3,927**	0	**0.00**	23	**0.59**	570	**14.51**	**593**	**15.10**
Mandaluyong City	**6,324**	1	**0.02**	28	**0.44**	234	**3.70**	**263**	**4.16**
San Juan	**962**	0	**0.00**	5	**0.52**	26	**2.70**	**31**	**3.22**
Manila City	**14,289**	4	**0.03**	201	**1.41**	1,188	**8.31**	**1,393**	**9.75**
Las Piñas City	**2,746**	0	**0.00**	47	**1.71**	354	**12.89**	**401**	**14.60**
Muntinlupa City	**2,728**	0	**0.00**	66	**2.42**	439	**16.09**	**505**	**18.51**
Parañaque City	**6,188**	6	**0.10**	78	**1.26**	510	**8.24**	**594**	**9.60**
Pasay City	**4,631**	0	**0.00**	110	**2.38**	591	**12.76**	**701**	**15.14**
**C A R**	**18,129**	**9**	**0.05**	**219**	**1.21**	**1,341**	**7.40**	**1,569**	**8.65**
Abra	**1,049**	0	**0.00**	9	**0.86**	33	**3.15**	**42**	**4.00**
Apayao	**2,041**	6	**0.29**	36	**1.76**	120	**5.88**	**162**	**7.94**
Benguet	**5,101**	0	**0.00**	14	**0.27**	136	**2.67**	**150**	**2.94**
Ifugao	**2,792**	2	**0.07**	38	**1.36**	225	**8.06**	**265**	**9.49**
Kalinga	**2,503**	1	**0.04**	103	**4.12**	732	**29.24**	**836**	**33.40**
Mt. Province	**1,418**	0	**0.00**	15	**1.06**	56	**3.95**	**71**	**5.01**
Baguio City	**3,225**	0	**0.00**	4	**0.12**	39	**1.21**	**43**	**1.33**
**Region 1**	**41,452**	**1**	**0.00**	**263**	**0.63**	**2,206**	**5.32**	**2,470**	**5.96**
Ilocos Norte	**3,793**	0	**0.00**	4	**0.11**	159	**4.19**	**163**	**4.30**
Ilocos Sur	**5,773**	0	**0.00**	8	**0.14**	152	**2.63**	**160**	**2.77**
La Union	**5,137**	0	**0.00**	57	**1.11**	280	**5.45**	**337**	**6.56**
Pangasinan	**19,672**	1	**0.01**	165	**0.84**	1,359	**6.91**	**1,525**	**7.75**
Alaminos City	**1,272**	0	**0.00**	1	**0.08**	0	**0.00**	**1**	**0.08**
Candon City	**424**	0	**0.00**	0	**0.00**	8	**1.89**	**8**	**1.89**
Dagupan City	**295**	0	**0.00**	20	**6.78**	164	**55.59**	**184**	**62.37**
Laoag City	**1,451**	0	**0.00**	0	**0.00**	0	**0.00**	**0**	**0.00**
San Carlos City	**702**	0	**0.00**	8	**1.14**	62	**8.83**	**70**	**9.97**
San Fernando City	**876**	0	**0.00**	0	**0.00**	10	**1.14**	**10**	**1.14**
Urdaneta City	**1,903**	0	**0.00**	0	**0.00**	7	**0.37**	**7**	**0.37**
Vigan City	**154**	0	**0.00**	0	**0.00**	5	**3.25**	**5**	**3.25**
**Region 2**	**30,809**	**12**	**0.04**	**386**	**1.25**	**2,167**	**7.03**	**2,565**	**8.33**
Batanes	**164**	0	**0.00**	1	**0.61**	13	**7.93**	**14**	**8.54**
Cagayan	**7,313**	3	**0.04**	79	**1.08**	456	**6.24**	**538**	**7.36**
Isabela	**11,100**	8	**0.07**	187	**1.68**	956	**8.61**	**1,151**	**10.37**
Nueva Vizcaya	**4,453**	1	**0.02**	56	**1.26**	338	**7.59**	**395**	**8.87**
Quirino	**3,260**	0	**0.00**	30	**0.92**	213	**6.53**	**243**	**7.45**
Cauayan City	**1,314**	0	**0.00**	5	**0.38**	23	**1.75**	**28**	**2.13**
Ilagan City	**1,349**	0	**0.00**	7	**0.52**	40	**2.97**	**47**	**3.48**
Santiago City	**864**	0	**0.00**	19	**2.20**	88	**10.19**	**107**	**12.38**

Table 2.B.1.15. Prenatal Care

Number and proportion of pregnant women tested for Complete Blood Count or Hemoglobin and Hematocrit Count diagnosed with anemia
Philippines, 2022

Area	Total No. tested for CBC or Hgb or Hct count	Tested for CBC/Hgb and Hct diagnosed with anemia							
		Age Group						Total	%
		10-14 yrs old		15-19 yrs old		20-49 yrs old			
		No.	%	No.	%	No.	%		
Tuguegarao City	992	0	0.00	2	0.20	40	4.03	42	4.23
**Region 3**	81,950	31	0.04	848	1.03	4,368	5.33	5,247	6.40
Aurora	1,463	0	0.00	6	0.41	46	3.14	52	3.55
Bataan	7,459	6	0.08	114	1.53	591	7.92	711	9.53
Bulacan	18,615	4	0.02	180	0.97	937	5.03	1,121	6.02
Nueva Ecija	7,136	3	0.04	109	1.53	759	10.64	871	12.21
Pampanga	6,703	2	0.03	36	0.54	221	3.30	259	3.86
Tarlac	5,869	0	0.00	15	0.26	61	1.04	76	1.29
Zambales	3,245	1	0.03	24	0.74	180	5.55	205	6.32
Angeles City	925	1	0.11	7	0.76	49	5.30	57	6.16
Balanga City	1,411	4	0.28	51	3.61	244	17.29	299	21.19
Cabanatuan City	1,316	0	0.00	0	0.00	4	0.30	4	0.30
City of San Fernando	2,840	6	0.21	68	2.39	317	11.16	391	13.77
Gapan City	624	0	0.00	0	0.00	5	0.80	5	0.80
Mabalacat City	1,751	0	0.00	1	0.06	12	0.69	13	0.74
Malolos City	1,370	0	0.00	5	0.36	32	2.34	37	2.70
Meycauayan	1,963	0	0.00	9	0.46	28	1.43	37	1.88
Olongapo	1,214	0	0.00	34	2.80	112	9.23	146	12.03
Palayan City	101	0	0.00	0	0.00	0	0.00	0	0.00
San Jose City	899	0	0.00	2	0.22	4	0.44	6	0.67
San Jose del Monte City	12,562	3	0.02	135	1.07	526	4.19	664	5.29
Science City of Munoz	643	0	0.00	24	3.73	92	14.31	116	18.04
Tarlac City	3,841	1	0.03	28	0.73	148	3.85	177	4.61
**Region 4A**	98,948	33	0.03	1,382	1.40	8,630	8.72	10,045	10.15
Batangas	9,837	0	0.00	64	0.65	418	4.25	482	4.90
Cavite	8,919	4	0.04	118	1.32	874	9.80	996	11.17
Laguna	7,634	7	0.09	149	1.95	768	10.06	924	12.10
Quezon	12,709	8	0.06	248	1.95	1,591	12.52	1,847	14.53
Rizal	15,004	2	0.01	177	1.18	1,023	6.82	1,202	8.01
Antipolo City	3,359	1	0.03	41	1.22	339	10.09	381	11.34
Bacoor City	1,806	0	0.00	19	1.05	132	7.31	151	8.36
Batangas City	1,546	0	0.00	26	1.68	162	10.48	188	12.16
Biñan City	6,909	0	0.00	30	0.43	105	1.52	135	1.95
Cabuyao City	3,674	1	0.03	70	1.91	420	11.43	491	13.36
Calamba City	4,705	3	0.06	101	2.15	747	15.88	851	18.09
Cavite City	419	0	0.00	5	1.19	24	5.73	29	6.92
Dasmariñas City	3,966	1	0.03	60	1.51	391	9.86	452	11.40
General Trias City	804	0	0.00	11	1.37	127	15.80	138	17.16
Imus City	972	1	0.10	9	0.93	85	8.74	95	9.77
Lipa City	3,160	1	0.03	37	1.17	220	6.96	258	8.16
Lucena City	2,049	1	0.05	74	3.61	301	14.69	376	18.35
San Pablo City	1,164	0	0.00	15	1.29	141	12.11	156	13.40
San Pedro City	1,404	2	0.14	30	2.14	123	8.76	155	11.04
Santa Rosa City	3,986	0	0.00	82	2.06	496	12.44	578	14.50
Tagaytay City	1,220	0	0.00	1	0.08	8	0.66	9	0.74
Tanauan City	1,550	0	0.00	4	0.26	31	2.00	35	2.26
Tayabas City	1,378	0	0.00	1	0.07	12	0.87	13	0.94
Trece Martires City	774	1	0.13	10	1.29	92	11.89	103	13.31
**Region 4B**	19,970	7	0.04	312	1.56	1,422	7.12	1,741	8.72
Marinduque	1,189	0	0.00	5	0.42	47	3.95	52	4.37
Mindoro Occidental	3,122	0	0.00	46	1.47	211	6.76	257	8.23
Mindoro Oriental	5,873	3	0.05	33	0.56	256	4.36	292	4.97
Palawan	5,358	4	0.07	122	2.28	500	9.33	626	11.68
Romblon	2,170	0	0.00	22	1.01	120	5.53	142	6.54
Puerto Princesa City	2,258	0	0.00	84	3.72	288	12.75	372	16.47

Table 2.B.1.15. Prenatal Care

Number and proportion of pregnant women tested for Complete Blood Count or Hemoglobin and Hematocrit Count diagnosed with anemia
Philippines, 2022

Area	Total No. tested for CBC or Hgb or Hct count	Tested for CBC/Hgb and Hct diagnosed with anemia							
		Age Group						Total	%
		10-14 yrs old		15-19 yrs old		20-49 yrs old			
		No.	%	No.	%	No.	%		
**Region 5**	**35,898**	**15**	**0.04**	**515**	**1.43**	**3,635**	**10.13**	**4,165**	**11.60**
Albay	**8,229**	1	**0.01**	54	**0.66**	577	**7.01**	**632**	**7.68**
Camarines Norte	**4,460**	2	**0.04**	152	**3.41**	953	**21.37**	**1,107**	**24.82**
Camarines Sur	**3,579**	0	**0.00**	11	**0.31**	98	**2.74**	**109**	**3.05**
Catanduanes	**2,733**	8	**0.29**	29	**1.06**	132	**4.83**	**169**	**6.18**
Masbate	**5,130**	3	**0.06**	127	**2.48**	527	**10.27**	**657**	**12.81**
Sorsogon	**8,582**	1	**0.01**	130	**1.51**	1,156	**13.47**	**1,287**	**15.00**
Iriga City	**172**	0	**0.00**	1	**0.58**	4	**2.33**	**5**	**2.91**
Legaspi City	**2,928**	0	**0.00**	9	**0.31**	180	**6.15**	**189**	**6.45**
Naga City	**85**	0	**0.00**	2	**2.35**	8	**9.41**	**10**	**11.76**
**Region 6**	**61,299**	**9**	**0.01**	**619**	**1.01**	**3,763**	**6.14**	**4,391**	**7.16**
Aklan	**4,492**	0	**0.00**	58	**1.29**	363	**8.08**	**421**	**9.37**
Antique	**4,011**	2	**0.05**	33	**0.82**	221	**5.51**	**256**	**6.38**
Capiz	**4,189**	0	**0.00**	38	**0.91**	243	**5.80**	**281**	**6.71**
Guimaras	**2,046**	0	**0.00**	32	**1.56**	196	**9.58**	**228**	**11.14**
Iloilo	**17,505**	3	**0.02**	82	**0.47**	718	**4.10**	**803**	**4.59**
Negros Occidental	**20,550**	2	**0.01**	301	**1.46**	1,406	**6.84**	**1,709**	**8.32**
Bacolod City	**5,032**	2	**0.04**	48	**0.95**	433	**8.60**	**483**	**9.60**
Iloilo City	**3,474**	0	**0.00**	27	**0.78**	183	**5.27**	**210**	**6.04**
**Region 7**	**79,987**	**22**	**0.03**	**799**	**1.00**	**5,137**	**6.42**	**5,958**	**7.45**
Bohol	**15,002**	4	**0.03**	89	**0.59**	775	**5.17**	**868**	**5.79**
Cebu	**19,661**	11	**0.06**	347	**1.76**	1,678	**8.53**	**2,036**	**10.36**
Negros Oriental	**9,751**	4	**0.04**	201	**2.06**	1,141	**11.70**	**1,346**	**13.80**
Siquijor	**662**	2	**0.30**	8	**1.21**	52	**7.85**	**62**	**9.37**
Cebu City	**10,799**	1	**0.01**	84	**0.78**	622	**5.76**	**707**	**6.55**
Lapu-Lapu City	**10,010**	0	**0.00**	44	**0.44**	574	**5.73**	**618**	**6.17**
Mandaue City	**14,102**	0	**0.00**	26	**0.18**	295	**2.09**	**321**	**2.28**
**Region 8**	**33,444**	**5**	**0.01**	**446**	**1.33**	**2,771**	**8.29**	**3,222**	**9.63**
Biliran	**2,046**	1	**0.05**	65	**3.18**	357	**17.45**	**423**	**20.67**
Eastern Samar	**2,866**	1	**0.03**	19	**0.66**	177	**6.18**	**197**	**6.87**
Northern Leyte	**10,757**	2	**0.02**	166	**1.54**	1,124	**10.45**	**1,292**	**12.01**
Northern Samar	**6,234**	1	**0.02**	69	**1.11**	410	**6.58**	**480**	**7.70**
Southern Leyte	**3,530**	0	**0.00**	26	**0.74**	185	**5.24**	**211**	**5.98**
Western Samar	**1,801**	0	**0.00**	46	**2.55**	236	**13.10**	**282**	**15.66**
Calbayog City	**344**	0	**0.00**	1	**0.29**	15	**4.36**	**16**	**4.65**
Maasin City	**601**	0	**0.00**	16	**2.66**	135	**22.46**	**151**	**25.12**
Ormoc City	**2,009**	0	**0.00**	35	**1.74**	119	**5.92**	**154**	**7.67**
Tacloban City	**3,256**	0	**0.00**	3	**0.09**	13	**0.40**	**16**	**0.49**
**Region 9**	**14,690**	**5**	**0.03**	**247**	**1.68**	**1,295**	**8.82**	**1,547**	**10.53**
Zamboanga del Norte	**3,418**	1	**0.03**	41	**1.20**	289	**8.46**	**331**	**9.68**
Zamboanga del Sur	**2,388**	3	**0.13**	110	**4.61**	449	**18.80**	**562**	**23.53**
Zamboanga Sibugay	**4,115**	0	**0.00**	39	**0.95**	196	**4.76**	**235**	**5.71**
Dapitan City	**250**	0	**0.00**	8	**3.20**	31	**12.40**	**39**	**15.60**
Dipolog City	**44**	0	**0.00**	0	**0.00**	1	**2.27**	**1**	**2.27**
Isabela City	**216**	0	**0.00**	10	**4.63**	32	**14.81**	**42**	**19.44**
Pagadian City	**1,827**	0	**0.00**	17	**0.93**	134	**7.33**	**151**	**8.26**
Zamboanga City	**2,432**	1	**0.04**	22	**0.90**	163	**6.70**	**186**	**7.65**

Table 2.B.1.15. Prenatal Care

Number and proportion of pregnant women tested for Complete Blood Count or Hemoglobin and Hematocrit Count diagnosed with anemia

Philippines, 2022

Area	Total No. tested for CBC or Hgb or Hct count	Tested for CBC/Hgb and Hct diagnosed with anemia							
		Age Group						Total	%
		10-14 yrs old		15-19 yrs old		20-49 yrs old			
		No.	%	No.	%	No.	%		
**Region 10**	**47,281**	**25**	**0.05**	**1,232**	**2.61**	**4,992**	**10.56**	**6,249**	**13.22**
Bukidnon	**12,667**	15	**0.12**	589	**4.65**	1,990	**15.71**	**2,594**	**20.48**
Camiguin	**585**	1	**0.17**	3	**0.51**	11	**1.88**	**15**	**2.56**
Lanao del Norte	**1,149**	0	**0.00**	13	**1.13**	253	**22.02**	**266**	**23.15**
Misamis Occidental	**2,058**	0	**0.00**	0	**0.00**	22	**1.07**	**22**	**1.07**
Misamis Oriental	**9,045**	4	**0.04**	160	**1.77**	635	**7.02**	**799**	**8.83**
Cagayan de Oro City	**9,325**	1	**0.01**	182	**1.95**	743	**7.97**	**926**	**9.93**
El Salvador City	**721**	2	**0.28**	26	**3.61**	77	**10.68**	**105**	**14.56**
Gingoog City	**1,524**	0	**0.00**	57	**3.74**	235	**15.42**	**292**	**19.16**
Iligan City	**3,234**	0	**0.00**	24	**0.74**	290	**8.97**	**314**	**9.71**
Malaybalay City	**1,828**	1	**0.05**	43	**2.35**	218	**11.93**	**262**	**14.33**
Oroquieta City	**157**	0	**0.00**	2	**1.27**	2	**1.27**	**4**	**2.55**
Ozamis City	**1,566**	0	**0.00**	14	**0.89**	100	**6.39**	**114**	**7.28**
Tangub City	**1,234**	0	**0.00**	12	**0.97**	105	**8.51**	**117**	**9.48**
Valencia City	**2,188**	1	**0.05**	107	**4.89**	311	**14.21**	**419**	**19.15**
**Region 11**	**71,642**	**165**	**0.23**	**3,715**	**5.19**	**16,182**	**22.59**	**20,062**	**28.00**
Davao de Oro	**11,631**	36	**0.31**	699	**6.01**	2,863	**24.62**	**3,598**	**30.93**
Davao del Norte	**17,191**	38	**0.22**	840	**4.89**	3,436	**19.99**	**4,314**	**25.09**
Davao Oriental	**7,526**	18	**0.24**	455	**6.05**	1,991	**26.45**	**2,464**	**32.74**
Davao del Sur	**7,497**	27	**0.36**	494	**6.59**	2,043	**27.25**	**2,564**	**34.20**
Davao Occidental	**1,843**	4	**0.22**	122	**6.62**	288	**15.63**	**414**	**22.46**
Davao City	**25,954**	42	**0.16**	1,105	**4.26**	5,561	**21.43**	**6,708**	**25.85**
**Region 12**	**47,768**	**47**	**0.10**	**1,081**	**2.26**	**4,942**	**10.35**	**6,070**	**12.71**
North Cotabato	**13,382**	25	**0.19**	368	**2.75**	2,042	**15.26**	**2,435**	**18.20**
Sarangani	**8,571**	9	**0.11**	317	**3.70**	1,175	**13.71**	**1,501**	**17.51**
South Cotabato	**11,812**	1	**0.01**	152	**1.29**	696	**5.89**	**849**	**7.19**
Sultan Kudarat	**9,328**	3	**0.03**	91	**0.98**	412	**4.42**	**506**	**5.42**
Gen. Santos City	**4,675**	9	**0.19**	153	**3.27**	617	**13.20**	**779**	**16.66**
**BARMM**	**5,111**	**2**	**0.04**	**78**	**1.53**	**329**	**6.44**	**409**	**8.00**
Basilan	**24**	0	**0.00**	0	**0.00**	1	**4.17**	**1**	**4.17**
Lanao del Sur	**1,831**	0	**0.00**	7	**0.38**	45	**2.46**	**52**	**2.84**
Maguindanao	**1,263**	2	**0.16**	31	**2.45**	182	**14.41**	**215**	**17.02**
Sulu	**834**	0	**0.00**	37	**4.44**	73	**8.75**	**110**	**13.19**
Tawi-Tawi	**11**	0	**0.00**	1	**9.09**	3	**27.27**	**4**	**36.36**
Lamitan City	**184**	0	**0.00**	0	**0.00**	4	**2.17**	**4**	**2.17**
Marawi City	**0**	0	**0.00**	0	**0.00**	0	**0.00**	**0**	**0.00**
Cotabato City	**964**	0	**0.00**	2	**0.21**	21	**2.18**	**23**	**2.39**
**CARAGA**	**31,154**	**14**	**0.04**	**951**	**3.05**	**4,535**	**14.56**	**5,500**	**17.65**
Agusan del Norte	**3,250**	1	**0.03**	47	**1.45**	171	**5.26**	**219**	**6.74**
Agusan del Sur	**9,509**	3	**0.03**	315	**3.31**	1,414	**14.87**	**1,732**	**18.21**
Surigao del Norte	**3,660**	1	**0.03**	59	**1.61**	526	**14.37**	**586**	**16.01**
Surigao del Sur	**5,260**	5	**0.10**	224	**4.26**	1,076	**20.46**	**1,305**	**24.81**
Province of Dinagat	**1,150**	0	**0.00**	17	**1.48**	126	**10.96**	**143**	**12.43**
Bislig City	**1,690**	2	**0.12**	128	**7.57**	552	**32.66**	**682**	**40.36**
Butuan City	**4,549**	2	**0.04**	83	**1.82**	442	**9.72**	**527**	**11.58**
Surigao City	**2,086**	0	**0.00**	78	**3.74**	228	**10.93**	**306**	**14.67**

Tabe 2.B.1.16. Prenatal Care

Number and proportion of pregnant women screened for Gestational Diabetes
Philippines, 2022

Area	Eligible Pop.	Screened for Gestational Diabetes							
		Age Group						Total	%
		10-14 yrs old		15-19 yrs old		20-49 yrs old			
		No.	%	No.	%	No.	%		
**PHILIPPINES**	**2,131,496**	**696**	**0.033**	**32,285**	**1.51**	**286,744**	**13.45**	**319,725**	**15.00**
**N C R**	**236,901**	**135**	**0.057**	**6,043**	**2.55**	**75,537**	**31.89**	**81,715**	**34.49**
Malabon	6,724	2	0.030	53	0.79	373	5.55	428	6.37
Navotas	4,591	7	0.152	179	3.90	1,114	24.26	1,300	28.32
Valenzuela City	11,418	4	0.035	141	1.23	983	8.61	1,128	9.88
Caloocan City	29,146	2	0.007	336	1.15	4,032	13.83	4,370	14.99
Marikina City	8,294	5	0.060	189	2.28	1,833	22.10	2,027	24.44
Pasig City	13,894	5	0.036	456	3.28	8,012	57.67	8,473	60.98
Pateros	1,176	3	0.255	40	3.40	509	43.28	552	46.94
Taguig	14,807	14	0.095	372	2.51	3,997	26.99	4,383	29.60
Quezon City	54,011	37	0.069	1,274	2.36	30,088	55.71	31,399	58.13
Makati City	10,718	2	0.019	91	0.85	1,988	18.55	2,081	19.42
Mandaluyong City	7,111	3	0.042	159	2.24	2,172	30.54	2,334	32.82
San Juan	2,246	0	0.000	69	3.07	873	38.87	942	41.94
Manila City	32,743	39	0.119	1,665	5.09	10,986	33.55	12,690	38.76
Las Piñas City	10,832	2	0.018	79	0.73	883	8.15	964	8.90
Muntinlupa City	9,281	2	0.022	109	1.17	885	9.54	996	10.73
Parañaque City	12,249	8	0.065	491	4.01	3,936	32.13	4,435	36.21
Pasay City	7,660	0	0.000	340	4.44	2,873	37.51	3,213	41.95
**C A R**	**35,179**	**23**	**0.065**	**758**	**2.15**	**7,039**	**20.01**	**7,820**	**22.23**
Abra	4,275	0	0.000	48	1.12	261	6.11	309	7.23
Apayao	2,502	5	0.200	88	3.52	478	19.10	571	22.82
Benguet	9,086	7	0.077	222	2.44	2,571	28.30	2,800	30.82
Ifugao	4,486	2	0.045	30	0.67	354	7.89	386	8.60
Kalinga	4,701	0	0.000	31	0.66	229	4.87	260	5.53
Mt. Province	3,103	0	0.000	21	0.68	417	13.44	438	14.12
Baguio City	7,026	9	0.128	318	4.53	2,729	38.84	3,056	43.50
**Region 1**	**97,099**	**26**	**0.027**	**1,538**	**1.58**	**17,497**	**18.02**	**19,061**	**19.63**
Ilocos Norte	7,854	1	0.013	115	1.46	1,692	21.54	1,808	23.02
Ilocos Sur	9,072	6	0.066	334	3.68	4,327	47.70	4,667	51.44
La Union	11,338	3	0.026	187	1.65	1,891	16.68	2,081	18.35
Pangasinan	50,710	12	0.024	540	1.06	5,805	11.45	6,357	12.54
Alaminos City	1,910	0	0.000	119	6.23	1,153	60.37	1,272	66.60
Candon City	954	0	0.000	2	0.21	316	33.12	318	33.33
Dagupan City	3,657	0	0.000	5	0.14	88	2.41	93	2.54
Laoag City	1,812	0	0.000	1	0.06	475	26.21	476	26.27
San Carlos City	4,028	0	0.000	23	0.57	267	6.63	290	7.20
San Fernando City	2,074	1	0.048	85	4.10	573	27.63	659	31.77
Urdaneta City	2,836	3	0.106	127	4.48	910	32.09	1,040	36.67
Vigan City	854	0	0.000	0	0.00	0	0.00	0	0.00
**Region 2**	**68,960**	**13**	**0.019**	**769**	**1.12**	**5,390**	**7.82**	**6,172**	**8.95**
Batanes	362	0	0.000	1	0.28	62	17.13	63	17.40
Cagayan	20,431	0	0.000	78	0.38	475	2.32	553	2.71
Isabela	23,449	6	0.026	299	1.28	2,116	9.02	2,421	10.32
Nueva Vizcaya	9,538	0	0.000	181	1.90	1,341	14.06	1,522	15.96
Quirino	4,051	2	0.049	61	1.51	445	10.98	508	12.54
Cauayan City	2,565	4	0.156	101	3.94	495	19.30	600	23.39
Ilagan City	2,889	1	0.035	19	0.66	141	4.88	161	5.57
Santiago City	2,671	0	0.000	29	1.09	300	11.23	329	12.32

Tabe 2.B.1.16. Prenatal Care
Number and proportion of pregnant women screened for Gestational Diabetes
Philippines, 2022

Area	Eligible Pop.	Screened for Gestational Diabetes							
		Age Group						Total	%
		10-14 yrs old		15-19 yrs old		20-49 yrs old			
		No.	%	No.	%	No.	%		
Tuguegarao City	3,004	0	0.000	0	0.00	15	0.50	15	0.50
**Region 3**	220,155	106	0.048	3,771	1.71	29,545	13.42	33,422	15.18
Aurora	4,754	0	0.000	47	0.99	297	6.25	344	7.24
Bataan	13,789	7	0.051	340	2.47	2,030	14.72	2,377	17.24
Bulacan	43,760	20	0.046	800	1.83	5,937	13.57	6,757	15.44
Nueva Ecija	29,039	11	0.038	404	1.39	2,592	8.93	3,007	10.36
Pampanga	31,620	9	0.028	340	1.08	3,226	10.20	3,575	11.31
Tarlac	19,319	15	0.078	196	1.01	1,288	6.67	1,499	7.76
Zambales	12,476	5	0.040	125	1.00	703	5.63	833	6.68
Angeles City	7,932	0	0.000	5	0.06	197	2.48	202	2.55
Balanga City	1,992	0	0.000	23	1.15	228	11.45	251	12.60
Cabanatuan City	5,945	1	0.017	46	0.77	290	4.88	337	5.67
City of San Fernando	5,907	11	0.186	290	4.91	2,089	35.36	2,390	40.46
Gapan City	2,170	0	0.000	20	0.92	116	5.35	136	6.27
Mabalacat City	4,831	0	0.000	5	0.10	283	5.86	288	5.96
Malolos City	4,890	0	0.000	4	0.08	51	1.04	55	1.12
Meycauayan	4,055	0	0.000	43	1.06	236	5.82	279	6.88
Olongapo	4,922	0	0.000	57	1.16	434	8.82	491	9.98
Palayan City	808	0	0.000	0	0.00	0	0.00	0	0.00
San Jose City	2,748	0	0.000	0	0.00	14	0.51	14	0.51
San Jose del Monte City	11,133	26	0.234	800	7.19	8,655	77.74	9,481	85.16
Science City of Munoz	1,601	0	0.000	0	0.00	0	0.00	0	0.00
Tarlac City	6,464	1	0.015	226	3.50	879	13.60	1,106	17.11
**Region 4A**	299,627	68	0.023	4,329	1.44	37,047	12.36	41,444	13.83
Batangas	39,255	0	0.000	361	0.92	3,827	9.75	4,188	10.67
Cavite	28,151	15	0.053	344	1.22	3,369	11.97	3,728	13.24
Laguna	19,696	11	0.056	217	1.10	1,987	10.09	2,215	11.25
Quezon	37,853	6	0.016	296	0.78	2,324	6.14	2,626	6.94
Rizal	44,798	16	0.036	1,185	2.65	8,210	18.33	9,411	21.01
Antipolo City	16,498	0	0.000	186	1.13	1,099	6.66	1,285	7.79
Bacoor City	12,341	0	0.000	123	1.00	818	6.63	941	7.62
Batangas City	6,965	1	0.014	66	0.95	560	8.04	627	9.00
Biñan City	6,597	2	0.030	645	9.78	4,918	74.55	5,565	84.36
Cabuyao City	6,119	3	0.049	115	1.88	1,698	27.75	1,816	29.68
Calamba City	9,005	2	0.022	143	1.59	1,857	20.62	2,002	22.23
Cavite City	2,105	0	0.000	0	0.00	20	0.95	20	0.95
Dasmariñas City	13,538	0	0.000	93	0.69	1,185	8.75	1,278	9.44
General Trias City	6,457	1	0.015	32	0.50	323	5.00	356	5.51
Imus City	8,292	2	0.024	14	0.17	295	3.56	311	3.75
Lipa City	7,018	1	0.014	37	0.53	350	4.99	388	5.53
Lucena City	5,738	1	0.017	117	2.04	570	9.93	688	11.99
San Pablo City	5,272	2	0.038	36	0.68	328	6.22	366	6.94
San Pedro City	6,455	1	0.015	42	0.65	359	5.56	402	6.23
Santa Rosa City	7,009	3	0.043	191	2.73	1,551	22.13	1,745	24.90
Tagaytay City	1,460	0	0.000	61	4.18	1,123	76.92	1,184	81.10
Tanauan City	3,661	1	0.027	10	0.27	186	5.08	197	5.38
Tayabas City	2,145	0	0.000	1	0.05	2	0.09	3	0.14
Trece Martires City	3,199	0	0.000	14	0.44	88	2.75	102	3.19
**Region 4B**	72,791	10	0.014	616	0.85	5,125	7.04	5,751	7.90
Marinduque	4,990	0	0.000	55	1.10	593	11.88	648	12.99
Mindoro Occidental	12,479	1	0.008	28	0.22	259	2.08	288	2.31
Mindoro Oriental	20,189	3	0.015	202	1.00	2,008	9.95	2,213	10.96
Palawan	21,963	6	0.027	233	1.06	1,425	6.49	1,664	7.58
Romblon	6,569	0	0.000	91	1.39	780	11.87	871	13.26
Puerto Princesa City	6,601	0	0.000	7	0.11	60	0.91	67	1.01

Tabe 2.B.1.16. Prenatal Care

Number and proportion of pregnant women screened for Gestational Diabetes
Philippines, 2022

Area	Eligible Pop.	Screened for Gestational Diabetes							
		Age Group						Total	%
		10-14 yrs old		15-19 yrs old		20-49 yrs old			
		No.	%	No.	%	No.	%		
**Region 5**	**138,457**	**11**	**0.008**	**933**	**0.67**	**9,435**	**6.81**	**10,379**	**7.50**
Albay	**24,081**	2	**0.008**	147	**0.61**	2,474	**10.27**	**2,623**	**10.89**
Camarines Norte	**14,384**	2	**0.014**	68	**0.47**	597	**4.15**	**667**	**4.64**
Camarines Sur	**39,486**	4	**0.010**	187	**0.47**	1,735	**4.39**	**1,926**	**4.88**
Catanduanes	**6,459**	1	**0.015**	37	**0.57**	368	**5.70**	**406**	**6.29**
Masbate	**22,600**	1	**0.004**	272	**1.20**	1,582	**7.00**	**1,855**	**8.21**
Sorsogon	**19,828**	1	**0.005**	167	**0.84**	1,686	**8.50**	**1,854**	**9.35**
Iriga City	**2,679**	0	**0.000**	3	**0.11**	35	**1.31**	**38**	**1.42**
Legaspi City	**4,233**	0	**0.000**	50	**1.18**	893	**21.10**	**943**	**22.28**
Naga City	**4,707**	0	**0.000**	2	**0.04**	65	**1.38**	**67**	**1.42**
**Region 6**	**146,449**	**63**	**0.043**	**3,906**	**2.67**	**33,701**	**23.01**	**37,670**	**25.72**
Aklan	**11,288**	4	**0.035**	232	**2.06**	2,581	**22.86**	**2,817**	**24.96**
Antique	**13,132**	4	**0.030**	107	**0.81**	1,226	**9.34**	**1,337**	**10.18**
Capiz	**13,975**	5	**0.036**	211	**1.51**	2,087	**14.93**	**2,303**	**16.48**
Guimaras	**3,084**	4	**0.130**	130	**4.22**	1,521	**49.32**	**1,655**	**53.66**
Iloilo	**36,267**	25	**0.069**	1,179	**3.25**	11,614	**32.02**	**12,818**	**35.34**
Negros Occidental	**49,230**	12	**0.024**	1,366	**2.77**	8,566	**17.40**	**9,944**	**20.20**
Bacolod City	**11,082**	8	**0.072**	442	**3.99**	3,865	**34.88**	**4,315**	**38.94**
Iloilo City	**8,391**	1	**0.012**	239	**2.85**	2,241	**26.71**	**2,481**	**29.57**
**Region 7**	**162,308**	**36**	**0.022**	**2,633**	**1.62**	**20,776**	**12.80**	**23,445**	**14.44**
Bohol	**27,992**	2	**0.007**	137	**0.49**	2,059	**7.36**	**2,198**	**7.85**
Cebu	**66,463**	14	**0.021**	1,436	**2.16**	7,334	**11.03**	**8,784**	**13.22**
Negros Oriental	**27,890**	3	**0.011**	142	**0.51**	1,069	**3.83**	**1,214**	**4.35**
Siquijor	**1,661**	0	**0.000**	11	**0.66**	115	**6.92**	**126**	**7.59**
Cebu City	**20,866**	8	**0.038**	457	**2.19**	4,042	**19.37**	**4,507**	**21.60**
Lapu-Lapu City	**9,232**	0	**0.000**	90	**0.97**	2,127	**23.04**	**2,217**	**24.01**
Mandaue City	**8,204**	9	**0.110**	360	**4.39**	4,030	**49.12**	**4,399**	**53.62**
**Region 8**	**105,471**	**13**	**0.012**	**767**	**0.73**	**5,817**	**5.52**	**6,597**	**6.25**
Biliran	**3,873**	2	**0.052**	50	**1.29**	303	**7.82**	**355**	**9.17**
Eastern Samar	**11,908**	1	**0.008**	69	**0.58**	767	**6.44**	**837**	**7.03**
Northern Leyte	**35,422**	5	**0.014**	106	**0.30**	856	**2.42**	**967**	**2.73**
Northern Samar	**15,921**	3	**0.019**	282	**1.77**	1,467	**9.21**	**1,752**	**11.00**
Southern Leyte	**6,662**	0	**0.000**	114	**1.71**	969	**14.55**	**1,083**	**16.26**
Western Samar	**14,732**	0	**0.000**	33	**0.22**	266	**1.81**	**299**	**2.03**
Calbayog City	**4,535**	0	**0.000**	10	**0.22**	99	**2.18**	**109**	**2.40**
Maasin City	**1,696**	0	**0.000**	1	**0.06**	5	**0.29**	**6**	**0.35**
Ormoc City	**5,044**	0	**0.000**	49	**0.97**	437	**8.66**	**486**	**9.64**
Tacloban City	**5,678**	2	**0.035**	53	**0.93**	648	**11.41**	**703**	**12.38**
**Region 9**	**79,007**	**11**	**0.014**	**285**	**0.36**	**1,467**	**1.86**	**1,763**	**2.23**
Zamboanga del Norte	**16,854**	1	**0.006**	85	**0.50**	492	**2.92**	**578**	**3.43**
Zamboanga del Sur	**17,368**	3	**0.017**	70	**0.40**	280	**1.61**	**353**	**2.03**
Zamboanga Sibugay	**15,021**	2	**0.013**	41	**0.27**	204	**1.36**	**247**	**1.64**
Dapitan City	**1,737**	0	**0.000**	8	**0.46**	37	**2.13**	**45**	**2.59**
Dipolog City	**2,762**	1	**0.036**	0	**0.00**	8	**0.29**	**9**	**0.33**
Isabela City	**2,563**	0	**0.000**	8	**0.31**	32	**1.25**	**40**	**1.56**
Pagadian City	**4,257**	0	**0.000**	2	**0.05**	18	**0.42**	**20**	**0.47**
Zamboanga City	**18,445**	4	**0.022**	71	**0.38**	396	**2.15**	**471**	**2.55**

Tabe 2.B.1.16. Prenatal Care

Number and proportion of pregnant women screened for Gestational Diabetes

Philippines, 2022

Area	Eligible Pop.	Screened for Gestational Diabetes							
		Age Group						Total	%
		10-14 yrs old		15-19 yrs old		20-49 yrs old			
		No.	%	No.	%	No.	%		
**Region 10**	**99,908**	**25**	**0.025**	**1,778**	**1.78**	**10,844**	**10.85**	**12,647**	**12.66**
Bukidnon	**22,900**	4	**0.017**	283	**1.24**	1,414	**6.17**	**1,701**	**7.43**
Camiguin	**1,854**	0	**0.000**	39	**2.10**	385	**20.77**	**424**	**22.87**
Lanao del Norte	**14,930**	0	**0.000**	11	**0.07**	345	**2.31**	**356**	**2.38**
Misamis Occidental	**6,420**	1	**0.016**	41	**0.64**	321	**5.00**	**363**	**5.65**
Misamis Oriental	**14,963**	9	**0.060**	493	**3.29**	2,406	**16.08**	**2,908**	**19.43**
Cagayan de Oro City	**14,172**	8	**0.056**	495	**3.49**	2,948	**20.80**	**3,451**	**24.35**
El Salvador City	**1,052**	1	**0.095**	21	**2.00**	147	**13.97**	**169**	**16.06**
Gingoog City	**2,608**	1	**0.038**	69	**2.65**	380	**14.57**	**450**	**17.25**
Iligan City	**7,565**	0	**0.000**	117	**1.55**	726	**9.60**	**843**	**11.14**
Malaybalay City	**3,817**	1	**0.026**	19	**0.50**	189	**4.95**	**209**	**5.48**
Oroquieta City	**1,389**	0	**0.000**	0	**0.00**	14	**1.01**	**14**	**1.01**
Ozamis City	**2,786**	0	**0.000**	134	**4.81**	1,015	**36.43**	**1,149**	**41.24**
Tangub City	**1,234**	0	**0.000**	55	**4.46**	535	**43.35**	**590**	**47.81**
Valencia City	**4,218**	0	**0.000**	1	**0.02**	19	**0.45**	**20**	**0.47**
**Region 11**	**108,407**	**92**	**0.085**	**1,501**	**1.38**	**11,158**	**10.29**	**12,751**	**11.76**
Davao de Oro	**15,490**	2	**0.013**	57	**0.37**	354	**2.29**	**413**	**2.67**
Davao del Norte	**21,017**	8	**0.038**	153	**0.73**	1,410	**6.71**	**1,571**	**7.47**
Davao Oriental	**13,229**	8	**0.060**	61	**0.46**	307	**2.32**	**376**	**2.84**
Davao del Sur	**14,564**	5	**0.034**	74	**0.51**	436	**2.99**	**515**	**3.54**
Davao Occidental	**6,510**	2	**0.031**	47	**0.72**	171	**2.63**	**220**	**3.38**
Davao City	**37,597**	67	**0.178**	1,109	**2.95**	8,480	**22.55**	**9,656**	**25.68**
**Region 12**	**98,859**	**54**	**0.055**	**1,903**	**1.92**	**10,342**	**10.46**	**12,299**	**12.44**
North Cotabato	**34,150**	18	**0.053**	462	**1.35**	2,606	**7.63**	**3,086**	**9.04**
Sarangani	**12,907**	20	**0.155**	553	**4.28**	2,526	**19.57**	**3,099**	**24.01**
South Cotabato	**20,917**	5	**0.024**	301	**1.44**	1,942	**9.28**	**2,248**	**10.75**
Sultan Kudarat	**17,299**	9	**0.052**	393	**2.27**	2,165	**12.52**	**2,567**	**14.84**
Gen. Santos City	**13,586**	2	**0.015**	194	**1.43**	1,103	**8.12**	**1,299**	**9.56**
**BARMM**	**101,343**	**1**	**0.001**	**217**	**0.21**	**2,098**	**2.07**	**2,316**	**2.29**
Basilan	**7,823**	0	**0.000**	0	**0.00**	0	**0.00**	**0**	**0.00**
Lanao del Sur	**21,639**	0	**0.000**	51	**0.24**	894	**4.13**	**945**	**4.37**
Maguindanao	**32,198**	1	**0.003**	89	**0.28**	639	**1.98**	**729**	**2.26**
Sulu	**17,165**	0	**0.000**	53	**0.31**	305	**1.78**	**358**	**2.09**
Tawi-Tawi	**9,369**	0	**0.000**	0	**0.00**	1	**0.01**	**1**	**0.01**
Lamitan City	**2,154**	0	**0.000**	1	**0.05**	6	**0.28**	**7**	**0.32**
Marawi City	**5,173**	0	**0.000**	0	**0.00**	0	**0.00**	**0**	**0.00**
Cotabato City	**5,822**	0	**0.000**	23	**0.40**	253	**4.35**	**276**	**4.74**
**CARAGA**	**60,575**	**9**	**0.015**	**538**	**0.89**	**3,926**	**6.48**	**4,473**	**7.38**
Agusan del Norte	**8,127**	0	**0.000**	31	**0.38**	375	**4.61**	**406**	**5.00**
Agusan del Sur	**17,709**	2	**0.011**	157	**0.89**	982	**5.55**	**1,141**	**6.44**
Surigao del Norte	**7,280**	2	**0.027**	45	**0.62**	396	**5.44**	**443**	**6.09**
Surigao del Sur	**11,553**	0	**0.000**	56	**0.48**	487	**4.22**	**543**	**4.70**
Province of Dinagat	**2,576**	1	**0.039**	23	**0.89**	220	**8.54**	**244**	**9.47**
Bislig City	**2,192**	0	**0.000**	24	**1.09**	108	**4.93**	**132**	**6.02**
Butuan City	**7,743**	2	**0.026**	123	**1.59**	906	**11.70**	**1,031**	**13.32**
Surigao City	**3,395**	2	**0.059**	79	**2.33**	452	**13.31**	**533**	**15.70**

Table 2.B.1.17. Prenatal Care

Number and proportion of pregnant women tested positive for Gestational Diabetes
Philippines, 2022

Area	Total No. of Screened for Gestational Diabetes	Tested positive for Gestational Diabetes							
		Age Group						Total	%
		10-14 yrs old		15-19 yrs old		20-49 yrs old			
		No.	%	No.	%	No.	%		
**PHILIPPINES**	**319,725**	**22**	**0.007**	**682**	**0.21**	**8,061**	**2.52**	**8,765**	**2.74**
**N C R**	**81,715**	**1**	**0.001**	**96**	**0.12**	**1,966**	**2.41**	**2,063**	**2.52**
Malabon	428	0	0.000	2	0.47	26	6.07	28	6.54
Navotas	1,300	0	0.000	24	1.85	39	3.00	63	4.85
Valenzuela City	1,128	0	0.000	3	0.27	78	6.91	81	7.18
Caloocan City	4,370	0	0.000	9	0.21	207	4.74	216	4.94
Marikina City	2,027	0	0.000	0	0.00	18	0.89	18	0.89
Pasig City	8,473	0	0.000	22	0.26	638	7.53	660	7.79
Pateros	552	0	0.000	0	0.00	1	0.18	1	0.18
Taguig	4,383	0	0.000	4	0.09	166	3.79	170	3.88
Quezon City	31,399	0	0.000	5	0.02	353	1.12	358	1.14
Makati City	2,081	0	0.000	0	0.00	106	5.09	106	5.09
Mandaluyong City	2,334	0	0.000	0	0.00	5	0.21	5	0.21
San Juan	942	0	0.000	0	0.00	4	0.42	4	0.42
Manila City	12,690	1	0.008	22	0.17	170	1.34	193	1.52
Las Piñas City	964	0	0.000	1	0.10	52	5.39	53	5.50
Muntinlupa City	996	0	0.000	3	0.30	37	3.71	40	4.02
Parañaque City	4,435	0	0.000	1	0.02	22	0.50	23	0.52
Pasay City	3,213	0	0.000	0	0.00	44	1.37	44	1.37
**C A R**	**7,820**	**0**	**0.000**	**8**	**0.10**	**73**	**0.93**	**81**	**1.04**
Abra	309	0	0.000	1	0.32	5	1.62	6	1.94
Apayao	571	0	0.000	2	0.35	21	3.68	23	4.03
Benguet	2,800	0	0.000	0	0.00	12	0.43	12	0.43
Ifugao	386	0	0.000	0	0.00	2	0.52	2	0.52
Kalinga	260	0	0.000	1	0.38	12	4.62	13	5.00
Mt. Province	438	0	0.000	0	0.00	12	2.74	12	2.74
Baguio City	3,056	0	0.000	4	0.13	9	0.29	13	0.43
**Region 1**	**19,061**	**0**	**0.000**	**7**	**0.04**	**133**	**0.70**	**140**	**0.73**
Ilocos Norte	1,808	0	0.000	0	0.00	16	0.88	16	0.88
Ilocos Sur	4,667	0	0.000	1	0.02	16	0.34	17	0.36
La Union	2,081	0	0.000	1	0.05	13	0.62	14	0.67
Pangasinan	6,357	0	0.000	5	0.08	58	0.91	63	0.99
Alaminos City	1,272	0	0.000	0	0.00	0	0.00	0	0.00
Candon City	318	0	0.000	0	0.00	2	0.63	2	0.63
Dagupan City	93	0	0.000	0	0.00	18	19.35	18	19.35
Laoag City	476	0	0.000	0	0.00	0	0.00	0	0.00
San Carlos City	290	0	0.000	0	0.00	10	3.45	10	3.45
San Fernando City	659	0	0.000	0	0.00	0	0.00	0	0.00
Urdaneta City	1,040	0	0.000	0	0.00	0	0.00	0	0.00
Vigan City	0	0	0.000	0	0.00	0	0.00	0	0.00
**Region 2**	**6,172**	**0**	**0.000**	**21**	**0.34**	**192**	**3.11**	**213**	**3.45**
Batanes	63	0	0.000	0	0.00	10	15.87	10	15.87
Cagayan	553	0	0.000	0	0.00	13	2.35	13	2.35
Isabela	2,421	0	0.000	6	0.25	78	3.22	84	3.47
Nueva Vizcaya	1,522	0	0.000	14	0.92	43	2.83	57	3.75
Quirino	508	0	0.000	0	0.00	10	1.97	10	1.97
Cauayan City	600	0	0.000	0	0.00	0	0.00	0	0.00
Ilagan City	161	0	0.000	0	0.00	6	3.73	6	3.73
Santiago City	329	0	0.000	1	0.30	32	9.73	33	10.03

Table 2.B.1.17. Prenatal Care

Number and proportion of pregnant women tested positive for Gestational Diabetes
Philippines, 2022

Area	Total No. of Screened for Gestational Diabetes	Tested positive for Gestational Diabetes							
		Age Group						Total	%
		10-14 yrs old		15-19 yrs old		20-49 yrs old			
		No.	%	No.	%	No.	%		
Tuguegarao City	15	0	0.000	0	0.00	0	0.00	0	0.00
**Region 3**	33,422	5	0.015	44	0.13	404	1.21	453	1.36
Aurora	344	0	0.000	0	0.00	2	0.58	2	0.58
Bataan	2,377	0	0.000	12	0.50	94	3.95	106	4.46
Bulacan	6,757	0	0.000	1	0.01	40	0.59	41	0.61
Nueva Ecija	3,007	0	0.000	3	0.10	38	1.26	41	1.36
Pampanga	3,575	0	0.000	1	0.03	45	1.26	46	1.29
Tarlac	1,499	0	0.000	0	0.00	3	0.20	3	0.20
Zambales	833	0	0.000	4	0.48	36	4.32	40	4.80
Angeles City	202	0	0.000	0	0.00	1	0.50	1	0.50
Balanga City	251	0	0.000	2	0.80	28	11.16	30	11.95
Cabanatuan City	337	4	1.187	3	0.89	3	0.89	10	2.97
City of San Fernando	2,390	1	0.042	17	0.71	89	3.72	107	4.48
Gapan City	136	0	0.000	0	0.00	0	0.00	0	0.00
Mabalacat City	288	0	0.000	0	0.00	0	0.00	0	0.00
Malolos City	55	0	0.000	0	0.00	1	1.82	1	1.82
Meycauayan	279	0	0.000	0	0.00	0	0.00	0	0.00
Olongapo	491	0	0.000	0	0.00	3	0.61	3	0.61
Palayan City	0	0	0.000	0	0.00	0	0.00	0	0.00
San Jose City	14	0	0.000	0	0.00	0	0.00	0	0.00
San Jose del Monte City	9,481	0	0.000	1	0.01	19	0.20	20	0.21
Science City of Munoz	0	0	0.000	0	0.00	0	0.00	0	0.00
Tarlac City	1,106	0	0.000	0	0.00	2	0.18	2	0.18
**Region 4A**	41,444	1	0.002	85	0.21	1,026	2.48	1,112	2.68
Batangas	4,188	0	0.000	11	0.26	66	1.58	77	1.84
Cavite	3,728	0	0.000	15	0.40	116	3.11	131	3.51
Laguna	2,215	1	0.045	7	0.32	71	3.21	79	3.57
Quezon	2,626	0	0.000	10	0.38	124	4.72	134	5.10
Rizal	9,411	0	0.000	16	0.17	118	1.25	134	1.42
Antipolo City	1,285	0	0.000	14	1.09	40	3.11	54	4.20
Bacoor City	941	0	0.000	0	0.00	16	1.70	16	1.70
Batangas City	627	0	0.000	1	0.16	24	3.83	25	3.99
Biñan City	5,565	0	0.000	2	0.04	5	0.09	7	0.13
Cabuyao City	1,816	0	0.000	1	0.06	20	1.10	21	1.16
Calamba City	2,002	0	0.000	1	0.05	107	5.34	108	5.39
Cavite City	20	0	0.000	0	0.00	0	0.00	0	0.00
Dasmariñas City	1,278	0	0.000	1	0.08	38	2.97	39	3.05
General Trias City	356	0	0.000	2	0.56	17	4.78	19	5.34
Imus City	311	0	0.000	0	0.00	7	2.25	7	2.25
Lipa City	388	0	0.000	0	0.00	11	2.84	11	2.84
Lucena City	688	0	0.000	1	0.15	8	1.16	9	1.31
San Pablo City	366	0	0.000	0	0.00	16	4.37	16	4.37
San Pedro City	402	0	0.000	0	0.00	4	1.00	4	1.00
Santa Rosa City	1,745	0	0.000	2	0.11	203	11.63	205	11.75
Tagaytay City	1,184	0	0.000	0	0.00	3	0.25	3	0.25
Tanauan City	197	0	0.000	0	0.00	10	5.08	10	5.08
Tayabas City	3	0	0.000	1	33.33	1	33.33	2	66.67
Trece Martires City	102	0	0.000	0	0.00	1	0.98	1	0.98
**Region 4B**	5,751	0	0.000	8	0.14	107	1.86	115	2.00
Marinduque	648	0	0.000	0	0.00	7	1.08	7	1.08
Mindoro Occidental	288	0	0.000	2	0.69	21	7.29	23	7.99
Mindoro Oriental	2,213	0	0.000	3	0.14	24	1.08	27	1.22
Palawan	1,664	0	0.000	3	0.18	33	1.98	36	2.16
Romblon	871	0	0.000	0	0.00	13	1.49	13	1.49
Puerto Princesa City	67	0	0.000	0	0.00	9	13.43	9	13.43

Table 2.B.1.17. Prenatal Care

Number and proportion of pregnant women tested positive for Gestational Diabetes
Philippines, 2022

Area	Total No. of Screened for Gestational Diabetes	Tested positive for Gestational Diabetes							
		Age Group						Total	%
		10-14 yrs old		15-19 yrs old		20-49 yrs old			
		No.	%	No.	%	No.	%		
**Region 5**	**10,379**	**1**	**0.010**	**13**	**0.13**	**273**	**2.63**	**287**	**2.77**
Albay	2,623	0	0.000	4	0.15	97	3.70	101	3.85
Camarines Norte	667	0	0.000	1	0.15	21	3.15	22	3.30
Camarines Sur	1,926	1	0.052	3	0.16	37	1.92	41	2.13
Catanduanes	406	0	0.000	1	0.25	7	1.72	8	1.97
Masbate	1,855	0	0.000	0	0.00	20	1.08	20	1.08
Sorsogon	1,854	0	0.000	4	0.22	52	2.80	56	3.02
Iriga City	38	0	0.000	0	0.00	0	0.00	0	0.00
Legaspi City	943	0	0.000	0	0.00	36	3.82	36	3.82
Naga City	67	0	0.000	0	0.00	3	4.48	3	4.48
**Region 6**	**37,670**	**0**	**0.000**	**65**	**0.17**	**852**	**2.26**	**917**	**2.43**
Aklan	2,817	0	0.000	1	0.04	34	1.21	35	1.24
Antique	1,337	0	0.000	8	0.60	107	8.00	115	8.60
Capiz	2,303	0	0.000	3	0.13	18	0.78	21	0.91
Guimaras	1,655	0	0.000	2	0.12	24	1.45	26	1.57
Iloilo	12,818	0	0.000	11	0.09	171	1.33	182	1.42
Negros Occidental	9,944	0	0.000	8	0.08	183	1.84	191	1.92
Bacolod City	4,315	0	0.000	31	0.72	264	6.12	295	6.84
Iloilo City	2,481	0	0.000	1	0.04	51	2.06	52	2.10
**Region 7**	**23,445**	**4**	**0.017**	**168**	**0.72**	**1,777**	**7.58**	**1,949**	**8.31**
Bohol	2,198	0	0.000	9	0.41	485	22.07	494	22.47
Cebu	8,784	2	0.023	41	0.47	197	2.24	240	2.73
Negros Oriental	1,214	0	0.000	6	0.49	51	4.20	57	4.70
Siquijor	126	1	0.794	0	0.00	21	16.67	22	17.46
Cebu City	4,507	1	0.022	110	2.44	964	21.39	1,075	23.85
Lapu-Lapu City	2,217	0	0.000	0	0.00	38	1.71	38	1.71
Mandaue City	4,399	0	0.000	2	0.05	21	0.48	23	0.52
**Region 8**	**6,597**	**0**	**0.000**	**13**	**0.20**	**133**	**2.02**	**146**	**2.21**
Biliran	355	0	0.000	1	0.28	5	1.41	6	1.69
Eastern Samar	837	0	0.000	0	0.00	10	1.19	10	1.19
Northern Leyte	967	0	0.000	7	0.72	54	5.58	61	6.31
Northern Samar	1,752	0	0.000	3	0.17	10	0.57	13	0.74
Southern Leyte	1,083	0	0.000	1	0.09	9	0.83	10	0.92
Western Samar	299	0	0.000	1	0.33	28	9.36	29	9.70
Calbayog City	109	0	0.000	0	0.00	8	7.34	8	7.34
Maasin City	6	0	0.000	0	0.00	0	0.00	0	0.00
Ormoc City	486	0	0.000	0	0.00	9	1.85	9	1.85
Tacloban City	703	0	0.000	0	0.00	0	0.00	0	0.00
**Region 9**	**1,763**	**1**	**0.057**	**11**	**0.62**	**63**	**3.57**	**75**	**4.25**
Zamboanga del Norte	578	0	0.000	2	0.35	11	1.90	13	2.25
Zamboanga del Sur	353	1	0.283	4	1.13	16	4.53	21	5.95
Zamboanga Sibugay	247	0	0.000	3	1.21	9	3.64	12	4.86
Dapitan City	45	0	0.000	0	0.00	0	0.00	0	0.00
Dipolog City	9	0	0.000	0	0.00	0	0.00	0	0.00
Isabela City	40	0	0.000	0	0.00	0	0.00	0	0.00
Pagadian City	20	0	0.000	0	0.00	2	10.00	2	10.00
Zamboanga City	471	0	0.000	2	0.42	25	5.31	27	5.73

Table 2.B.1.17. Prenatal Care

Number and proportion of pregnant women tested positive for Gestational Diabetes
Philippines, 2022

Area	Total No. of Screened for Gestational Diabetes	Tested positive for Gestational Diabetes							
		Age Group						Total	%
		10-14 yrs old		15-19 yrs old		20-49 yrs old			
		No.	%	No.	%	No.	%		
**Region 10**	**12,647**	**1**	**0.008**	**65**	**0.51**	**340**	**2.69**	**406**	**3.21**
Bukidnon	1,701	0	0.000	20	1.18	69	4.06	89	5.23
Camiguin	424	0	0.000	0	0.00	1	0.24	1	0.24
Lanao del Norte	356	0	0.000	0	0.00	1	0.28	1	0.28
Misamis Occidental	363	0	0.000	0	0.00	1	0.28	1	0.28
Misamis Oriental	2,908	0	0.000	5	0.17	53	1.82	58	1.99
Cagayan de Oro City	3,451	0	0.000	6	0.17	107	3.10	113	3.27
El Salvador City	169	0	0.000	1	0.59	9	5.33	10	5.92
Gingoog City	450	0	0.000	5	1.11	12	2.67	17	3.78
Iligan City	843	1	0.119	28	3.32	74	8.78	103	12.22
Malaybalay City	209	0	0.000	0	0.00	8	3.83	8	3.83
Oroquieta City	14	0	0.000	0	0.00	1	7.14	1	7.14
Ozamis City	1,149	0	0.000	0	0.00	3	0.26	3	0.26
Tangub City	590	0	0.000	0	0.00	0	0.00	0	0.00
Valencia City	20	0	0.000	0	0.00	1	5.00	1	5.00
**Region 11**	**12,751**	**0**	**0.000**	**7**	**0.05**	**234**	**1.84**	**241**	**1.89**
Davao de Oro	413	0	0.000	1	0.24	37	8.96	38	9.20
Davao del Norte	1,571	0	0.000	2	0.13	102	6.49	104	6.62
Davao Oriental	376	0	0.000	1	0.27	7	1.86	8	2.13
Davao del Sur	515	0	0.000	0	0.00	23	4.47	23	4.47
Davao Occidental	220	0	0.000	1	0.45	8	3.64	9	4.09
Davao City	9,656	0	0.000	2	0.02	57	0.59	59	0.61
**Region 12**	**12,299**	**6**	**0.049**	**56**	**0.46**	**301**	**2.45**	**363**	**2.95**
North Cotabato	3,086	3	0.097	7	0.23	92	2.98	102	3.31
Sarangani	3,099	1	0.032	32	1.03	108	3.48	141	4.55
South Cotabato	2,248	0	0.000	5	0.22	42	1.87	47	2.09
Sultan Kudarat	2,567	2	0.078	8	0.31	44	1.71	54	2.10
Gen. Santos City	1,299	0	0.000	4	0.31	15	1.15	19	1.46
**BARMM**	**2,316**	**2**	**0.086**	**3**	**0.13**	**23**	**0.99**	**28**	**1.21**
Basilan	0	0	0.000	0	0.00	0	0.00	0	0.00
Lanao del Sur	945	0	0.000	0	0.00	8	0.85	8	0.85
Maguindanao	729	0	0.000	0	0.00	5	0.69	5	0.69
Sulu	358	2	0.559	3	0.84	5	1.40	10	2.79
Tawi-Tawi	1	0	0.000	0	0.00	0	0.00	0	0.00
Lamitan City	7	0	0.000	0	0.00	0	0.00	0	0.00
Marawi City	0	0	0.000	0	0.00	0	0.00	0	0.00
Cotabato City	276	0	0.000	0	0.00	5	1.81	5	1.81
**CARAGA**	**4,473**	**0**	**0.000**	**12**	**0.27**	**164**	**3.67**	**176**	**3.93**
Agusan del Norte	406	0	0.000	0	0.00	6	1.48	6	1.48
Agusan del Sur	1,141	0	0.000	2	0.18	51	4.47	53	4.65
Surigao del Norte	443	0	0.000	1	0.23	23	5.19	24	5.42
Surigao del Sur	543	0	0.000	3	0.55	40	7.37	43	7.92
Province of Dinagat	244	0	0.000	2	0.82	21	8.61	23	9.43
Bislig City	132	0	0.000	2	1.52	10	7.58	12	9.09
Butuan City	1,031	0	0.000	2	0.19	9	0.87	11	1.07
Surigao City	533	0	0.000	0	0.00	4	0.75	4	0.75

Table 2.B.2.1. Intrapartum Care and Delivery Outcome

Total Number of women who delivered a live baby or stillbirth/fetal death, deliveries attended by skilled health professionals & deliveries in health facilities

Philippines, 2022

Area	Total Deliveries				Skilled Health Professional								Facility Based Delivery					
	Total Number women who delivered				Deliveries attended by skilled health professional								Type of Health Facility				Total	%
	Age Group (in Years)			Total	MD	%	Nurses	%	Midwives	%	Total	%	Public	%	Private	%		
	10-14	15-19	20-49															
**PHILIPPINES**	2,881	143,466	1,272,723	1,419,070	817,546	57.61	19,295	1.36	482,240	33.98	1,319,081	92.95	877,277	61.82	423,872	29.87	1,301,149	91.69
**N C R**	356	15,736	170,798	186,890	137,999	73.84	749	0.40	40,095	21.45	178,843	95.69	116,186	62.17	59,830	32.01	176,016	94.18
Malabon	7	391	2,929	**3,327**	1,892	**56.87**	1	**0.03**	1,298	**39.01**	**3,191**	**95.91**	1,885	**56.66**	1,280	**38.47**	**3,165**	**95.13**
Navotas	3	465	3,512	**3,980**	2,225	**55.90**	0	**0.00**	1,738	**43.67**	**3,963**	**99.57**	2,207	**55.45**	1,744	**43.82**	**3,951**	**99.27**
Valenzuela City	9	525	5,954	**6,488**	3,866	**59.59**	22	**0.34**	2,534	**39.06**	**6,422**	**98.98**	4,209	**64.87**	2,081	**32.07**	**6,290**	**96.95**
Caloocan City	12	745	11,702	**12,459**	5,565	**44.67**	303	**2.43**	5,883	**47.22**	**11,751**	**94.32**	11,144	**89.45**	0	**0.00**	**11,144**	**89.45**
Marikina City	8	544	6,615	**7,167**	4,865	**67.88**	102	**1.42**	2,013	**28.09**	**6,980**	**97.39**	2,727	**38.05**	4,193	**58.50**	**6,920**	**96.55**
Pasig City	13	782	10,590	**11,385**	9,700	**85.20**	1	**0.01**	1,271	**11.16**	**10,972**	**96.37**	6,714	**58.97**	4,067	**35.72**	**10,781**	**94.69**
Pateros	1	57	815	**873**	402	**46.05**	0	**0.00**	468	**53.61**	**870**	**99.66**	255	**29.21**	603	**69.07**	**858**	**98.28**
Taguig	25	1,085	10,871	**11,981**	6,937	**57.90**	14	**0.12**	4,234	**35.34**	**11,185**	**93.36**	7,500	**62.60**	3,681	**30.72**	**11,181**	**93.32**
Quezon City	80	3,387	39,881	**43,348**	32,223	**74.34**	90	**0.21**	7,711	**17.79**	**40,024**	**92.33**	22,469	**51.83**	17,000	**39.22**	**39,469**	**91.05**
Makati City	4	253	5,349	**5,606**	4,875	**86.96**	8	**0.14**	565	**10.08**	**5,448**	**97.18**	2,923	**52.14**	2,469	**44.04**	**5,392**	**96.18**
Mandaluyong City	4	395	6,415	**6,814**	5,769	**84.66**	0	**0.00**	1,038	**15.23**	**6,807**	**99.90**	5,524	**81.07**	1,272	**18.67**	**6,796**	**99.74**
San Juan	0	49	1,501	**1,550**	1,493	**96.32**	48	**3.10**	6	**0.39**	**1,547**	**99.81**	724	**46.71**	820	**52.90**	**1,544**	**99.61**
Manila City	123	4,481	40,040	**44,644**	42,086	**94.27**	0	**0.00**	2,558	**5.73**	**44,644**	**100.00**	34,084	**76.35**	9,876	**22.12**	**43,960**	**98.47**
Las Piñas City	9	603	5,816	**6,428**	2,083	**32.41**	44	**0.68**	3,906	**60.77**	**6,033**	**93.86**	3,662	**56.97**	2,313	**35.98**	**5,975**	**92.95**
Muntinlupa City	19	799	6,857	**7,675**	4,814	**62.72**	19	**0.25**	1,491	**19.43**	**6,324**	**82.40**	1,630	**21.24**	4,378	**57.04**	**6,008**	**78.28**
Parañaque City	38	851	6,990	**7,879**	4,722	**59.93**	96	**1.22**	2,634	**33.43**	**7,452**	**94.58**	4,417	**56.06**	2,945	**37.38**	**7,362**	**93.44**
Pasay City	1	324	4,961	**5,286**	4,482	**84.79**	1	**0.02**	747	**14.13**	**5,230**	**98.94**	4,112	**77.79**	1,108	**20.96**	**5,220**	**98.75**
**C A R**	38	2,110	21,942	24,090	20,734	86.07	418	1.74	2,309	9.58	23,461	97.39	19,275	80.01	3,759	15.60	23,034	95.62
Abra	4	350	2,518	**2,872**	2,474	**86.14**	71	**2.47**	206	**7.17**	**2,751**	**95.79**	2,551	**88.82**	134	**4.67**	**2,685**	**93.49**
Apayao	8	275	1,353	**1,636**	1,403	**85.76**	31	**1.89**	72	**4.40**	**1,506**	**92.05**	1,399	**85.51**	80	**4.89**	**1,479**	**90.40**
Benguet	4	285	3,759	**4,048**	3,689	**91.13**	32	**0.79**	136	**3.36**	**3,857**	**95.28**	2,968	**73.32**	826	**20.41**	**3,794**	**93.73**
Ifugao	6	268	2,471	**2,745**	1,922	**70.02**	65	**2.37**	672	**24.48**	**2,659**	**96.87**	2,271	**82.73**	314	**11.44**	**2,585**	**94.17**
Kalinga	0	204	2,442	**2,646**	1,593	**60.20**	77	**2.91**	960	**36.28**	**2,630**	**99.40**	1,831	**69.20**	751	**28.38**	**2,582**	**97.58**
Mt. Province	5	255	2,973	**3,233**	2,796	**86.48**	142	**4.39**	226	**6.99**	**3,164**	**97.87**	2,949	**91.22**	78	**2.41**	**3,027**	**93.63**
Baguio City	11	473	6,426	**6,910**	6,857	**99.23**	0	**0.00**	37	**0.54**	**6,894**	**99.77**	5,306	**76.79**	1,576	**22.81**	**6,882**	**99.59**
**Region 1**	70	3,861	45,684	49,615	39,526	79.67	124	0.25	9,851	19.85	49,501	99.77	37,609	75.80	11,439	23.06	49,048	98.86
Ilocos Norte	3	229	4,071	**4,303**	4,002	**93.00**	18	**0.42**	283	**6.58**	**4,303**	**100.00**	4,054	**94.21**	246	**5.72**	**4,300**	**99.93**
Ilocos Sur	7	307	4,373	**4,687**	4,512	**96.27**	4	**0.09**	162	**3.46**	**4,678**	**99.81**	3,057	**65.22**	1,615	**34.46**	**4,672**	**99.68**
La Union	7	402	3,937	**4,346**	3,552	**81.73**	6	**0.14**	763	**17.56**	**4,321**	**99.42**	4,030	**92.73**	286	**6.58**	**4,316**	**99.31**
Pangasinan	19	1,154	12,651	**13,824**	8,497	**61.47**	51	**0.37**	5,244	**37.93**	**13,792**	**99.77**	11,400	**82.47**	2,383	**17.24**	**13,783**	**99.70**
Alaminos City	0	74	1,246	**1,320**	1,319	**99.92**	0	**0.00**	1	**0.08**	**1,320**	**100.00**	1,234	**93.48**	86	**6.52**	**1,320**	**100.00**
Candon City	0	2	838	**840**	827	**98.45**	0	**0.00**	13	**1.55**	**840**	**100.00**	48	**5.71**	792	**94.29**	**840**	**100.00**
Dagupan City	19	735	7,750	**8,504**	6,302	**74.11**	13	**0.15**	2,163	**25.44**	**8,478**	**99.69**	4,641	**54.57**	3,816	**44.87**	**8,457**	**99.45**
Laoag City	0	47	1,916	**1,963**	1,963	**100.00**	0	**0.00**	0	**0.00**	**1,963**	**100.00**	1,488	**75.80**	68	**3.46**	**1,556**	**79.27**
San Carlos City	2	234	2,499	**2,735**	2,044	**74.73**	12	**0.44**	657	**24.02**	**2,713**	**99.20**	1,489	**54.44**	1,224	**44.75**	**2,713**	**99.20**
San Fernando City	5	296	3,273	**3,574**	3,525	**98.63**	17	**0.48**	32	**0.90**	**3,574**	**100.00**	3,169	**88.67**	405	**11.33**	**3,574**	**100.00**
Urdaneta City	3	226	1,614	**1,843**	1,307	**70.92**	3	**0.16**	533	**28.92**	**1,843**	**100.00**	1,337	**72.54**	504	**27.35**	**1,841**	**99.89**
Vigan City	5	155	1,516	**1,676**	1,676	**100.00**	0	**0.00**	0	**0.00**	**1,676**	**100.00**	1,662	**99.16**	14	**0.84**	**1,676**	**100.00**
**Region 2**	77	4,453	35,298	39,828	26,780	67.24	267	0.67	11,877	29.82	38,924	97.73	29,197	73.31	9,580	24.05	38,777	97.36
Batanes	0	13	241	**254**	246	**96.85**	2	**0.79**	1	**0.39**	**249**	**98.03**	252	**99.21**	0	**0.00**	**252**	**99.21**
Cagayan	13	1,053	7,720	**8,786**	5,280	**60.10**	124	**1.41**	3,163	**36.00**	**8,567**	**97.51**	7,553	**85.97**	907	**10.32**	**8,460**	**96.29**
Isabela	18	1,046	9,587	**10,651**	5,795	**54.41**	93	**0.87**	4,555	**42.77**	**10,443**	**98.05**	5,625	**52.81**	4,793	**45.00**	**10,418**	**97.81**
Nueva Vizcaya	29	935	6,489	**7,453**	6,404	**85.93**	36	**0.48**	710	**9.53**	**7,150**	**95.93**	6,868	**92.15**	282	**3.78**	**7,150**	**95.93**
Quirino	5	388	2,221	**2,614**	1,985	**75.94**	10	**0.38**	564	**21.58**	**2,559**	**97.90**	2,324	**88.91**	235	**8.99**	**2,559**	**97.90**
Cauayan City	1	244	1,635	**1,880**	1,182	**62.87**	2	**0.11**	668	**35.53**	**1,852**	**98.51**	1,073	**57.07**	766	**40.74**	**1,839**	**97.82**
Ilagan City	6	383	3,207	**3,596**	3,466	**96.38**	0	**0.00**	54	**1.50**	**3,520**	**97.89**	3,305	**91.91**	210	**5.84**	**3,515**	**97.75**
Santiago City	4	277	2,749	**3,030**	1,242	**40.99**	0	**0.00**	1,784	**58.88**	**3,026**	**99.87**	1,059	**34.95**	1,967	**64.92**	**3,026**	**99.87**
Tuguegarao City	1	114	1,449	**1,564**	1,180	**75.45**	0	**0.00**	378	**24.17**	**1,558**	**99.62**	1,138	**72.76**	420	**26.85**	**1,558**	**99.62**
**Region 3**	234	11,925	121,601	133,760	79,385	59.35	713	0.53	50,442	37.71	130,540	97.59	66,954	50.06	62,655	46.84	129,609	96.90
Aurora	2	358	2,663	**3,023**	2,095	**69.30**	5	**0.17**	719	**23.78**	**2,819**	**93.25**	2,436	**80.58**	364	**12.04**	**2,800**	**92.62**
Bataan	17	567	4,409	**4,993**	3,564	**71.38**	32	**0.64**	1,279	**25.62**	**4,875**	**97.64**	3,782	**75.75**	973	**19.49**	**4,755**	**95.23**
Bulacan	21	1,326	18,751	**20,098**	7,019	**34.92**	26	**0.13**	12,519	**62.29**	**19,564**	**97.34**	2,579	**12.83**	16,808	**83.63**	**19,387**	**96.46**
Nueva Ecija	20	775	11,396	**12,191**	6,241	**51.19**	20	**0.16**	5,168	**42.39**	**11,429**	**93.75**	4,321	**35.44**	6,930	**56.85**	**11,251**	**92.29**
Pampanga	40	1,773	16,162	**17,975**	11,021	**61.31**	151	**0.84**	6,464	**35.96**	**17,636**	**98.11**	11,899	**66.20**	5,737	**31.92**	**17,636**	**98.11**
Tarlac	14	842	9,685	**10,541**	5,786	**54.89**	60	**0.57**	4,572	**43.37**	**10,418**	**98.83**	7,250	**68.78**	3,127	**29.67**	**10,377**	**98.44**
Zambales	22	930	7,794	**8,746**	6,349	**72.59**	76	**0.87**	2,022	**23.12**	**8,447**	**96.58**	8,128	**92.93**	252	**2.88**	**8,380**	**95.82**
Angeles City	11	455	5,272	**5,738**	2,165	**37.73**	11	**0.19**	3,448	**60.09**	**5,624**	**98.01**	1,902	**33.15**	3,614	**62.98**	**5,516**	**96.13**
Balanga City	7	161	1,132	**1,300**	1,262	**97.08**	0	**0.00**	27	**2.08**	**1,289**	**99.15**	1,047	**80.54**	241	**18.54**	**1,288**	**99.08**
Cabanatuan City	12	516	5,639	**6,167**	5,375	**87.16**	3	**0.05**	686	**11.12**	**6,064**	**98.33**	4,304	**69.79**	1,728	**28.02**	**6,032**	**97.81**
City of San Fernando	7	497	4,187	**4,691**	3,599	**76.72**	3	**0.06**	895	**19.08**	**4,497**	**95.86**	3,353	**71.48**	1,059	**22.58**	**4,412**	**94.05**
Gapan City	0	92	742	**834**	471	**56.47**	5	**0.60**	358	**42.93**	**834**	**100.00**	417	**50.00**	417	**50.00**	**834**	**100.00**
Mabalacat City	6	358	3,832	**4,196**	2,468	**58.82**	41	**0.98**	1,650	**39.32**	**4,159**	**99.12**	1,290	**30.74**	2,858	**68.11**	**4,148**	**98.86**
Malolos City	1	112	4,611	**4,724**	4,068	**86.11**	22	**0.47**	581	**12.30**	**4,671**	**98.88**	39	**0.83**	4,628	**97.97**	**4,667**	**98.79**
Meycauayan	0	134	2,102	**2,236**	91	**4.07**	0	**0.00**	2,097	**93.78**	**2,188**	**97.85**	24	**1.07**	2,147	**96.02**	**2,171**	**97.09**
Olongapo	6	314	3,371	**3,691**	2,808	**76.08**	48	**1.30**	713	**19.32**	**3,569**	**96.69**	1,837	**49.77**	1,686	**45.68**	**3,523**	**95.45**
Palayan City	0	11	98	**109**	0	**0.00**	2	**1.83**	85	**77.98**	**87**	**79.82**	87	**79.82**	0	**0.00**	**87**	**79.82**
San Jose City	8	315	1,792	**2,115**	839	**39.67**	155	**7.33**	1,086	**51.35**	**2,080**	**98.35**	1,414	**66.86**	659	**31.16**	**2,073**	**98.01**
San Jose del Monte City	29	1,070	7,316	**8,415**	5,976	**71.02**	1	**0.01**	2,426	**28.83**	**8,403**	**99.86**	4,246	**50.46**	4,151	**49.33**	**8,397**	**99.79**
Science City of Munoz	1	17	111	**129**	4	**3.10**	0	**0.00**	98	**75.97**	**102**	**79.07**	3	**2.33**	96	**74.42**	**99**	**76.74**
Tarlac City	10	1,302	10,536	**11,848**	8,184	**69.07**	52	**0.44**	3,549	**29.95**	**11,785**	**99.47**	6,596	**55.67**	5,180	**43.72**	**11,776**	**99.39**
**Region 4A**	278	18,031	176,878	195,187	101,328	51.91	2,066	1.06	75,198	38.53	178,592	91.50	84,394	43.24	90,778	46.51	175,172	89.75
Batangas	35	2,217	22,076	**24,328**	16,723	**68.74**	60	**0.25**	5,559	**22.85**	**22,342**	**91.84**	7,125	**29.29**	14,241	**58.54**	**21,366**	**87.82**
Cavite	39	1,520	18,069	**19,628**	7,118	**36.26**	80	**0.41**	11,272	**57.43**	**18,470**	**94.10**	5,030	**25.63**	13,053	**66.50**	**18,083**	**92.13**
Laguna	33	1,393	10,768	**12,194**	7,560	**62.00**	28	**0.23**	3,721	**30.52**	**11,309**	**92.74**	8,034	**65.88**	3,096	**25.39**	**11,130**	**91.27**
Quezon	59	3,030	22,141	**25,230**	11,963	**47.42**	312	**1.24**	9,684	**38.38**	**21,959**	**87.04**	15,845	**62.80**	5,963	**23.63**	**21,808**	**86.44**
Rizal	43	3,078	27,157	**30,278**	16,921	**55.89**	189	**0.62**	9,755	**32.22**	**26,865**	**88.73**	17,226	**56.89**	9,334	**30.83**	**26,560**	**87.72**

Table 2.B.2.1. Intrapartum Care and Delivery Outcome

Total Number of women who delivered a live baby or stillbirth/fetal death, deliveries attended by skilled health professionals & deliveries in health facilities
Philippines, 2022

Area	Total Deliveries				Skilled Health Professional								Facility Based Delivery					
	Total Number women who delivered				Deliveries attended by skilled health professional								Type of Health Facility				Total	%
	Age Group (in Years)			Total	MD	%	Nurses	%	Midwives	%	Total	%	Public	%	Private	%		
	10-14	15-19	20-49															
Antipolo City	2	1,506	8,365	**9,873**	5,043	**51.08**	126	**1.28**	2,584	**26.17**	**7,753**	**78.53**	5,300	**53.68**	2,169	**21.97**	**7,469**	**75.65**
Bacoor City	4	518	6,733	**7,255**	2,987	**41.17**	23	**0.32**	3,551	**48.95**	**6,561**	**90.43**	1,791	**24.69**	4,684	**64.56**	**6,475**	**89.25**
Batangas City	6	344	2,995	**3,345**	2,773	**82.90**	0	**0.00**	300	**8.97**	**3,073**	**91.87**	1,577	**47.14**	1,494	**44.66**	**3,071**	**91.81**
Biñan City	3	657	6,544	**7,204**	2,228	**30.93**	134	**1.86**	4,644	**64.46**	**7,006**	**97.25**	1,883	**26.14**	4,917	**68.25**	**6,800**	**94.39**
Cabuyao City	0	174	3,174	**3,348**	1,189	**35.51**	0	**0.00**	2,118	**63.26**	**3,307**	**98.78**	847	**25.30**	2,393	**71.48**	**3,240**	**96.77**
Calamba City	7	428	6,218	**6,653**	3,684	**55.37**	277	**4.16**	2,508	**37.70**	**6,469**	**97.23**	3,328	**50.02**	3,161	**47.51**	**6,489**	**97.53**
Cavite City	1	102	1,218	**1,321**	809	**61.24**	37	**2.80**	475	**35.96**	**1,321**	**100.00**	271	**20.51**	1,042	**78.88**	**1,313**	**99.39**
Dasmariñas City	4	584	7,586	**8,174**	2,823	**34.54**	331	**4.05**	4,998	**61.15**	**8,152**	**99.73**	2,404	**29.41**	5,723	**70.01**	**8,127**	**99.43**
General Trias City	8	187	2,580	**2,775**	1,012	**36.47**	8	**0.29**	1,522	**54.85**	**2,542**	**91.60**	568	**20.47**	1,947	**70.16**	**2,515**	**90.63**
Imus City	6	169	4,478	**4,653**	2,541	**54.61**	57	**1.23**	1,897	**40.77**	**4,495**	**96.60**	1,152	**24.76**	3,226	**69.33**	**4,378**	**94.09**
Lipa City	5	297	4,347	**4,649**	3,028	**65.13**	23	**0.49**	1,074	**23.10**	**4,125**	**88.73**	2,235	**48.07**	1,630	**35.06**	**3,865**	**83.14**
Lucena City	4	371	2,831	**3,206**	1,626	**50.72**	13	**0.41**	1,470	**45.85**	**3,109**	**96.97**	1,988	**62.01**	1,108	**34.56**	**3,096**	**96.57**
San Pablo City	4	354	3,057	**3,415**	1,800	**52.71**	316	**9.25**	1,075	**31.48**	**3,191**	**93.44**	1,194	**34.96**	1,944	**56.93**	**3,138**	**91.89**
San Pedro City	2	214	2,754	**2,970**	1,586	**53.40**	35	**1.18**	1,189	**40.03**	**2,810**	**94.61**	1,360	**45.79**	1,338	**45.05**	**2,698**	**90.84**
Santa Rosa City	6	332	5,067	**5,405**	3,370	**62.35**	8	**0.15**	1,575	**29.14**	**4,953**	**91.64**	2,117	**39.17**	2,821	**52.19**	**4,938**	**91.36**
Tagaytay City	1	67	1,237	**1,305**	526	**40.31**	0	**0.00**	773	**59.23**	**1,299**	**99.54**	70	**5.36**	1,229	**94.18**	**1,299**	**99.54**
Tanauan City	3	120	3,336	**3,459**	2,105	**60.86**	6	**0.17**	1,033	**29.86**	**3,144**	**90.89**	1,003	**29.00**	2,005	**57.96**	**3,008**	**86.96**
Tayabas City	1	142	1,266	**1,409**	642	**45.56**	3	**0.21**	740	**52.52**	**1,385**	**98.30**	1,154	**81.90**	231	**16.39**	**1,385**	**98.30**
Trece Martires City	2	227	2,881	**3,110**	1,271	**40.87**	0	**0.00**	1,681	**54.05**	**2,952**	**94.92**	892	**28.68**	2,029	**65.24**	**2,921**	**93.92**
**Region 4B**	**68**	**4,980**	**35,985**	**41,033**	**21,965**	**53.53**	**698**	**1.70**	**10,691**	**26.05**	**33,354**	**81.29**	**27,595**	**67.25**	**5,288**	**12.89**	**32,883**	**80.14**
Marinduque	7	316	2,747	**3,070**	2,688	**87.56**	93	**3.03**	225	**7.33**	**3,006**	**97.92**	3,000	**97.72**	0	**0.00**	**3,000**	**97.72**
Mindoro Occidental	8	1,051	6,487	**7,546**	3,239	**42.92**	52	**0.69**	2,051	**27.18**	**5,342**	**70.79**	4,638	**61.46**	629	**8.34**	**5,267**	**69.80**
Mindoro Oriental	16	727	8,749	**9,492**	6,434	**67.78**	119	**1.25**	1,329	**14.00**	**7,882**	**83.04**	6,185	**65.16**	1,744	**18.37**	**7,929**	**83.53**
Palawan	22	1,980	10,963	**12,965**	5,108	**39.40**	377	**2.91**	3,997	**30.83**	**9,482**	**73.14**	8,121	**62.64**	969	**7.47**	**9,090**	**70.11**
Romblon	5	447	3,637	**4,089**	3,077	**75.25**	28	**0.68**	920	**22.50**	**4,025**	**98.43**	3,847	**94.08**	145	**3.55**	**3,992**	**97.63**
Puerto Princesa City	10	459	3,402	**3,871**	1,419	**36.66**	29	**0.75**	2,169	**56.03**	**3,617**	**93.44**	1,804	**46.60**	1,801	**46.53**	**3,605**	**93.13**
**Region 5**	**119**	**8,784**	**81,253**	**90,156**	**38,344**	**42.53**	**5,712**	**6.34**	**41,711**	**46.27**	**85,767**	**95.13**	**57,258**	**63.51**	**28,192**	**31.27**	**85,450**	**94.78**
Albay	10	908	11,831	**12,749**	5,383	**42.22**	233	**1.83**	6,842	**53.67**	**12,458**	**97.72**	6,965	**54.63**	5,483	**43.01**	**12,448**	**97.64**
Camarines Norte	11	1,228	9,245	**10,484**	2,307	**22.00**	3,470	**33.10**	4,346	**41.45**	**10,123**	**96.56**	7,297	**69.60**	2,822	**26.92**	**10,119**	**96.52**
Camarines Sur	10	1,412	16,381	**17,803**	2,991	**16.80**	966	**5.43**	11,663	**65.51**	**15,620**	**87.74**	5,321	**29.89**	10,121	**56.85**	**15,442**	**86.74**
Catanduanes	6	445	3,897	**4,348**	3,886	**89.37**	64	**1.47**	277	**6.37**	**4,227**	**97.22**	3,737	**85.95**	460	**10.58**	**4,197**	**96.53**
Masbate	24	2,009	12,224	**14,257**	2,078	**14.58**	435	**3.05**	10,657	**74.75**	**13,170**	**92.38**	12,285	**86.17**	846	**5.93**	**13,131**	**92.10**
Sorsogon	16	1,177	11,870	**13,063**	8,426	**64.50**	451	**3.45**	4,026	**30.82**	**12,903**	**98.78**	10,566	**80.88**	2,322	**17.78**	**12,888**	**98.66**
Iriga City	1	107	1,597	**1,705**	554	**32.49**	53	**3.11**	1,087	**63.75**	**1,694**	**99.35**	107	**6.28**	1,584	**92.90**	**1,691**	**99.18**
Legaspi City	4	227	3,195	**3,426**	1,967	**57.41**	40	**1.17**	1,403	**40.95**	**3,410**	**99.53**	1,737	**50.70**	1,673	**48.83**	**3,410**	**99.53**
Naga City	37	1,271	11,013	**12,321**	10,752	**87.27**	0	**0.00**	1,410	**11.44**	**12,162**	**98.71**	9,243	**75.02**	2,881	**23.38**	**12,124**	**98.40**
**Region 6**	**171**	**9,471**	**81,438**	**91,080**	**53,640**	**58.89**	**445**	**0.49**	**31,665**	**34.77**	**85,750**	**94.15**	**71,050**	**78.01**	**14,244**	**15.64**	**85,294**	**93.65**
Aklan	4	502	5,412	**5,918**	4,381	**74.03**	54	**0.91**	973	**16.44**	**5,408**	**91.38**	4,736	**80.03**	483	**8.16**	**5,219**	**88.19**
Antique	2	751	7,129	**7,882**	4,940	**62.67**	1	**0.01**	2,183	**27.70**	**7,124**	**90.38**	6,728	**85.36**	339	**4.30**	**7,067**	**89.66**
Capiz	18	702	7,015	**7,735**	3,878	**50.14**	16	**0.21**	3,379	**43.68**	**7,273**	**94.03**	3,768	**48.71**	3,481	**45.00**	**7,249**	**93.72**
Guimaras	6	226	2,016	**2,248**	2,101	**93.46**	0	**0.00**	130	**5.78**	**2,231**	**99.24**	2,140	**95.20**	69	**3.07**	**2,209**	**98.27**
Iloilo	48	2,054	20,202	**22,304**	17,984	**80.63**	166	**0.74**	3,625	**16.25**	**21,775**	**97.63**	20,490	**91.87**	1,199	**5.38**	**21,689**	**97.24**
Negros Occidental	77	4,205	29,123	**33,405**	11,654	**34.89**	180	**0.54**	18,778	**56.21**	**30,612**	**91.64**	25,662	**76.82**	4,904	**14.68**	**30,566**	**91.50**
Bacolod City	7	558	5,417	**5,982**	4,466	**74.66**	24	**0.40**	1,278	**21.36**	**5,768**	**96.42**	3,836	**64.13**	1,911	**31.95**	**5,747**	**96.07**
Iloilo City	9	473	5,124	**5,606**	4,236	**75.56**	4	**0.07**	1,319	**23.53**	**5,559**	**99.16**	3,690	**65.82**	1,858	**33.14**	**5,548**	**98.97**
**Region 7**	**181**	**9,580**	**123,453**	**133,214**	**75,846**	**56.94**	**502**	**0.38**	**51,816**	**38.90**	**128,164**	**96.21**	**88,102**	**66.14**	**38,672**	**29.03**	**126,774**	**95.17**
Bohol	30	1,630	16,367	**18,027**	11,558	**64.11**	69	**0.38**	6,324	**35.08**	**17,951**	**99.58**	14,132	**78.39**	3,739	**20.74**	**17,871**	**99.13**
Cebu	50	2,826	50,609	**53,485**	24,618	**46.03**	240	**0.45**	26,868	**50.23**	**51,726**	**96.71**	36,898	**68.99**	14,353	**26.84**	**51,251**	**95.82**
Negros Oriental	45	2,249	17,306	**19,600**	11,283	**57.57**	109	**0.56**	5,598	**28.56**	**16,990**	**86.68**	13,652	**69.65**	3,263	**16.65**	**16,915**	**86.30**
Siquijor	2	118	978	**1,098**	1,017	**92.62**	0	**0.00**	68	**6.19**	**1,085**	**98.82**	1,072	**97.63**	0	**0.00**	**1,072**	**97.63**
Cebu City	28	1,522	21,331	**22,881**	15,005	**65.58**	79	**0.35**	7,531	**32.91**	**22,615**	**98.84**	13,058	**57.07**	9,307	**40.68**	**22,365**	**97.74**
Lapu-Lapu City	9	483	8,025	**8,517**	4,266	**50.09**	5	**0.06**	4,183	**49.11**	**8,454**	**99.26**	3,980	**46.73**	4,173	**49.00**	**8,153**	**95.73**
Mandaue City	17	752	8,837	**9,606**	8,099	**84.31**	0	**0.00**	1,244	**12.95**	**9,343**	**97.26**	5,310	**55.28**	3,837	**39.94**	**9,147**	**95.22**
**Region 8**	**165**	**6,490**	**52,877**	**59,532**	**35,408**	**59.48**	**1,012**	**1.70**	**19,049**	**32.00**	**55,469**	**93.18**	**39,341**	**66.08**	**15,719**	**26.40**	**55,060**	**92.49**
Biliran	11	403	2,889	**3,303**	1,749	**52.95**	64	**1.94**	1,464	**44.32**	**3,277**	**99.21**	3,259	**98.67**	0	**0.00**	**3,259**	**98.67**
Eastern Samar	14	753	6,387	**7,154**	5,513	**77.06**	60	**0.84**	1,244	**17.39**	**6,817**	**95.29**	3,420	**47.81**	3,343	**46.73**	**6,763**	**94.53**
Northern Leyte	87	1,302	12,332	**13,721**	5,137	**37.44**	353	**2.57**	7,363	**53.66**	**12,853**	**93.67**	7,895	**57.54**	4,726	**34.44**	**12,621**	**91.98**
Northern Samar	21	1,194	9,195	**10,410**	6,484	**62.29**	50	**0.48**	2,356	**22.63**	**8,890**	**85.40**	8,483	**81.49**	381	**3.66**	**8,864**	**85.15**
Southern Leyte	7	345	2,638	**2,990**	2,707	**90.54**	1	**0.03**	276	**9.23**	**2,984**	**99.80**	2,428	**81.20**	510	**17.06**	**2,938**	**98.26**
Western Samar	6	1,142	7,362	**8,510**	4,984	**58.57**	341	**4.01**	2,577	**30.28**	**7,902**	**92.86**	6,960	**81.79**	920	**10.81**	**7,880**	**92.60**
Calbayog City	4	262	2,024	**2,290**	1,314	**57.38**	11	**0.48**	364	**15.90**	**1,689**	**73.76**	1,481	**64.67**	206	**9.00**	**1,687**	**73.67**
Maasin City	1	208	2,181	**2,390**	2,388	**99.92**	0	**0.00**	1	**0.04**	**2,389**	**99.96**	1,069	**44.73**	1,319	**55.19**	**2,388**	**99.92**
Ormoc City	10	679	5,161	**5,850**	2,972	**50.80**	113	**1.93**	2,669	**45.62**	**5,754**	**98.36**	2,034	**34.77**	3,713	**63.47**	**5,747**	**98.24**
Tacloban City	4	202	2,708	**2,914**	2,160	**74.12**	19	**0.65**	735	**25.22**	**2,914**	**100.00**	2,312	**79.34**	601	**20.62**	**2,913**	**99.97**
**Region 9**	**101**	**6,373**	**43,420**	**49,894**	**23,077**	**46.25**	**1,139**	**2.28**	**18,518**	**37.11**	**42,734**	**85.65**	**37,631**	**75.42**	**4,799**	**9.62**	**42,430**	**85.04**
Zamboanga del Norte	25	1,499	8,247	**9,771**	2,917	**29.85**	175	**1.79**	5,137	**52.57**	**8,229**	**84.22**	8,151	**83.42**	2	**0.02**	**8,153**	**83.44**
Zamboanga del Sur	19	830	5,630	**6,479**	846	**13.06**	125	**1.93**	4,093	**63.17**	**5,064**	**78.16**	3,943	**60.86**	1,015	**15.67**	**4,958**	**76.52**
Zamboanga Sibugay	11	1,195	7,792	**8,998**	2,337	**25.97**	329	**3.66**	5,242	**58.26**	**7,908**	**87.89**	7,358	**81.77**	539	**5.99**	**7,897**	**87.76**
Dapitan City	3	174	1,381	**1,558**	1,558	**100.00**	0	**0.00**	0	**0.00**	**1,558**	**100.00**	1,553	**99.68**	5	**0.32**	**1,558**	**100.00**
Dipolog City	7	275	1,938	**2,220**	2,155	**97.07**	0	**0.00**	23	**1.04**	**2,178**	**98.11**	2,152	**96.94**	24	**1.08**	**2,176**	**98.02**
Isabela City	4	205	1,484	**1,693**	946	**55.88**	19	**1.12**	66	**3.90**	**1,031**	**60.90**	984	**58.12**	47	**2.78**	**1,031**	**60.90**
Pagadian City	2	229	2,182	**2,413**	1,113	**46.13**	0	**0.00**	1,232	**51.06**	**2,345**	**97.18**	1,110	**46.00**	1,234	**51.14**	**2,344**	**97.14**
Zamboanga City	30	1,966	14,766	**16,762**	11,205	**66.85**	491	**2.93**	2,725	**16.26**	**14,421**	**86.03**	12,380	**73.86**	1,933	**11.53**	**14,313**	**85.39**
**Region 10**	**231**	**10,452**	**66,079**	**76,762**	**46,007**	**59.93**	**884**	**1.15**	**22,558**	**29.39**	**69,449**	**90.47**	**56,392**	**73.46**	**11,830**	**15.41**	**68,222**	**88.87**
Bukidnon	99	3,714	16,188	**20,001**	10,748	**53.74**	123	**0.61**	7,673	**38.36**	**18,544**	**92.72**	15,580	**77.90**	2,565	**12.82**	**18,145**	**90.72**
Camiguin	2	130	1,022	**1,154**	981	**85.01**	7	**0.61**	163	**14.12**	**1,151**	**99.74**	996	**86.31**	155	**13.43**	**1,151**	**99.74**
Lanao del Norte	20	910	7,602	**8,532**	3,698	**43.34**	175	**2.05**	2,725	**31.94**	**6,598**	**77.33**	5,804	**68.03**	320	**3.75**	**6,124**	**71.78**
Misamis Occidental	7	270	3,789	**4,066**	2,264	**55.68**	191	**4.70**	1,611	**39.62**	**4,066**	**100.00**	2,916	**71.72**	1,099	**27.03**	**4,015**	**98.75**
Misamis Oriental	29	1,166	7,076	**8,271**	5,076	**61.37**	112	**1.35**	2,695	**32.58**	**7,883**	**95.31**	6,643	**80.32**	1,198	**14.48**	**7,841**	**94.80**

Table 2.B.2.1. Intrapartum Care and Delivery Outcome

Total Number of women who delivered a live baby or stillbirth/fetal death, deliveries attended by skilled health professionals & deliveries in health facilities

Philippines, 2022

Area	Total Deliveries				Skilled Health Professional								Facility Based Delivery					
	Total Number women who delivered				Deliveries attended by skilled health professional								Type of Health Facility				Total	%
	Age Group (in Years)			Total	MD	%	Nurses	%	Midwives	%	Total	%	Public	%	Private	%		
	10-14	15-19	20-49															
Cagayan de Oro City	58	1,952	14,034	**16,044**	12,274	**76.50**	130	**0.81**	3,445	**21.47**	**15,849**	**98.78**	12,718	**79.27**	3,090	**19.26**	**15,808**	**98.53**
El Salvador City	0	4	121	**125**	123	**98.40**	0	**0.00**	1	**0.80**	**124**	**99.20**	123	**98.40**	0	**0.00**	**123**	**98.40**
Gingoog City	2	327	1,780	**2,109**	1,006	**47.70**	99	**4.69**	949	**45.00**	**2,054**	**97.39**	1,659	**78.66**	313	**14.84**	**1,972**	**93.50**
Iligan City	4	821	6,545	**7,370**	4,588	**62.25**	12	**0.16**	364	**4.94**	**4,964**	**67.35**	4,141	**56.19**	724	**9.82**	**4,865**	**66.01**
Malaybalay City	4	480	2,628	**3,112**	1,612	**51.80**	8	**0.26**	1,134	**36.44**	**2,754**	**88.50**	1,707	**54.85**	1,035	**33.26**	**2,742**	**88.11**
Oroquieta City	0	133	984	**1,117**	956	**85.59**	6	**0.54**	154	**13.79**	**1,116**	**99.91**	921	**82.45**	187	**16.74**	**1,108**	**99.19**
Ozamis City	1	242	1,712	**1,955**	1,604	**82.05**	0	**0.00**	351	**17.95**	**1,955**	**100.00**	1,774	**90.74**	181	**9.26**	**1,955**	**100.00**
Tangub City	0	73	880	**953**	694	**72.82**	19	**1.99**	142	**14.90**	**855**	**89.72**	830	**87.09**	25	**2.62**	**855**	**89.72**
Valencia City	5	230	1,718	**1,953**	383	**19.61**	2	**0.10**	1,151	**58.93**	**1,536**	**78.65**	580	**29.70**	938	**48.03**	**1,518**	**77.73**
**Region 11**	**472**	**11,523**	**70,245**	**82,240**	**50,882**	**61.87**	**375**	**0.46**	**25,812**	**31.39**	**77,069**	**93.71**	**47,789**	**58.11**	**28,695**	**34.89**	**76,484**	**93.00**
Davao de Oro	125	1,961	9,774	**11,860**	8,566	**72.23**	32	**0.27**	2,989	**25.20**	**11,587**	**97.70**	9,466	**79.81**	2,101	**17.72**	**11,567**	**97.53**
Davao del Norte	93	2,538	15,299	**17,930**	11,556	**64.45**	112	**0.62**	5,496	**30.65**	**17,164**	**95.73**	11,258	**62.79**	5,813	**32.42**	**17,071**	**95.21**
Davao Oriental	47	1,317	7,409	**8,773**	7,063	**80.51**	58	**0.66**	1,322	**15.07**	**8,443**	**96.24**	8,158	**92.99**	117	**1.33**	**8,275**	**94.32**
Davao del Sur	67	1,427	7,595	**9,089**	5,187	**57.07**	44	**0.48**	3,362	**36.99**	**8,593**	**94.54**	2,657	**29.23**	5,931	**65.25**	**8,588**	**94.49**
Davao Occidental	48	1,127	3,421	**4,596**	2,141	**46.58**	36	**0.78**	799	**17.38**	**2,976**	**64.75**	2,066	**44.95**	866	**18.84**	**2,932**	**63.79**
Davao City	92	3,153	26,747	**29,992**	16,369	**54.58**	93	**0.31**	11,844	**39.49**	**28,306**	**94.38**	14,184	**47.29**	13,867	**46.24**	**28,051**	**93.53**
**Region 12**	**215**	**9,288**	**55,432**	**64,935**	**31,928**	**49.17**	**540**	**0.83**	**26,649**	**41.04**	**59,117**	**91.04**	**40,929**	**63.03**	**17,917**	**27.59**	**58,846**	**90.62**
North Cotabato	41	2,653	16,943	**19,637**	10,991	**55.97**	189	**0.96**	5,345	**27.22**	**16,525**	**84.15**	11,002	**56.03**	5,372	**27.36**	**16,374**	**83.38**
Sarangani	38	1,947	8,357	**10,342**	3,738	**36.14**	58	**0.56**	5,791	**55.99**	**9,587**	**92.70**	6,649	**64.29**	2,900	**28.04**	**9,549**	**92.33**
South Cotabato	61	2,420	14,022	**16,503**	10,688	**64.76**	83	**0.50**	4,833	**29.29**	**15,604**	**94.55**	12,267	**74.33**	3,321	**20.12**	**15,588**	**94.46**
Sultan Kudarat	68	1,790	10,729	**12,587**	5,641	**44.82**	75	**0.60**	6,039	**47.98**	**11,755**	**93.39**	9,342	**74.22**	2,364	**18.78**	**11,706**	**93.00**
Gen. Santos City	7	478	5,381	**5,866**	870	**14.83**	135	**2.30**	4,641	**79.12**	**5,646**	**96.25**	1,669	**28.45**	3,960	**67.51**	**5,629**	**95.96**
**BARMM**	**49**	**6,240**	**59,325**	**65,614**	**13,878**	**21.15**	**3,402**	**5.18**	**31,709**	**48.33**	**48,989**	**74.66**	**35,450**	**54.03**	**9,392**	**14.31**	**44,842**	**68.34**
Basilan	14	583	3,207	**3,804**	135	**3.55**	870	**22.87**	1,181	**31.05**	**2,186**	**57.47**	1,627	**42.77**	1	**0.03**	**1,628**	**42.80**
Lanao del Sur	7	949	17,097	**18,053**	6,816	**37.76**	663	**3.67**	8,750	**48.47**	**16,229**	**89.90**	11,178	**61.92**	4,719	**26.14**	**15,897**	**88.06**
Maguindanao	13	1,782	17,290	**19,085**	810	**4.24**	1,123	**5.88**	11,490	**60.20**	**13,423**	**70.33**	7,914	**41.47**	2,648	**13.87**	**10,562**	**55.34**
Sulu	5	993	8,872	**9,870**	508	**5.15**	135	**1.37**	3,705	**37.54**	**4,348**	**44.05**	4,316	**43.73**	96	**0.97**	**4,412**	**44.70**
Tawi-Tawi	1	516	4,848	**5,365**	390	**7.27**	380	**7.08**	3,512	**65.46**	**4,282**	**79.81**	3,895	**72.60**	14	**0.26**	**3,909**	**72.86**
Lamitan City	8	238	1,120	**1,366**	206	**15.08**	26	**1.90**	1,100	**80.53**	**1,332**	**97.51**	1,236	**90.48**	19	**1.39**	**1,255**	**91.87**
Marawi City	0	899	3,991	**4,890**	3,106	**63.52**	204	**4.17**	958	**19.59**	**4,268**	**87.28**	3,288	**67.24**	980	**20.04**	**4,268**	**87.28**
Cotabato City	1	280	2,900	**3,181**	1,907	**59.95**	1	**0.03**	1,013	**31.85**	**2,921**	**91.83**	1,996	**62.75**	915	**28.76**	**2,911**	**91.51**
**CARAGA**	**56**	**4,169**	**31,015**	**35,240**	**20,819**	**59.08**	**249**	**0.71**	**12,290**	**34.88**	**33,358**	**94.66**	**22,125**	**62.78**	**11,083**	**31.45**	**33,208**	**94.23**
Agusan del Norte	5	453	3,188	**3,646**	1,991	**54.61**	9	**0.25**	1,344	**36.86**	**3,344**	**91.72**	1,301	**35.68**	2,039	**55.92**	**3,340**	**91.61**
Agusan del Sur	18	1,131	8,907	**10,056**	4,487	**44.62**	13	**0.13**	4,627	**46.01**	**9,127**	**90.76**	5,421	**53.91**	3,612	**35.92**	**9,033**	**89.83**
Surigao del Norte	8	320	2,732	**3,060**	1,683	**55.00**	196	**6.41**	1,026	**33.53**	**2,905**	**94.93**	2,884	**94.25**	3	**0.10**	**2,887**	**94.35**
Surigao del Sur	13	876	5,472	**6,361**	5,127	**80.60**	12	**0.19**	833	**13.10**	**5,972**	**93.88**	5,771	**90.72**	185	**2.91**	**5,956**	**93.63**
Province of Dinagat	0	115	798	**913**	641	**70.21**	18	**1.97**	208	**22.78**	**867**	**94.96**	856	**93.76**	0	**0.00**	**856**	**93.76**
Bislig City	3	199	1,280	**1,482**	1,468	**99.06**	0	**0.00**	2	**0.13**	**1,470**	**99.19**	1,176	**79.35**	294	**19.84**	**1,470**	**99.19**
Butuan City	9	522	5,458	**5,989**	2,149	**35.88**	1	**0.02**	3,798	**63.42**	**5,948**	**99.32**	1,494	**24.95**	4,452	**74.34**	**5,946**	**99.28**
Surigao City	0	553	3,180	**3,733**	3,273	**87.68**	0	**0.00**	452	**12.11**	**3,725**	**99.79**	3,222	**86.31**	498	**13.34**	**3,720**	**99.65**

Table 2.B.2.2. Intrapartum Care and Delivery Outcome

Number and proportion of delivey by type

Philippines, 2022

Area	Total number of Deliveries	Delivery by Type									
		Vaginal					Cesarean Section				
		Age Group in Year			Total	%	Age Group in Year			Total	%
		10-14	15-19	20-49			10-14	15-19	20-49		
**PHILIPPINES**	**1,419,070**	**2,364**	**126,071**	**1,071,036**	**1,199,471**	**84.53**	**237**	**10,396**	**152,330**	**162,963**	**11.48**
**N C R**	**186,890**	**188**	**11,503**	**118,776**	**130,467**	**69.81**	**10**	**1,048**	**13,849**	**14,907**	**7.98**
Malabon	**3,327**	6	368	2,617	**2,991**	**89.90**	1	23	312	**336**	**10.10**
Navotas	**3,980**	3	429	2,983	**3,415**	**85.80**	0	35	530	**565**	**14.20**
Valenzuela City	**6,488**	9	438	5,018	**5,465**	**84.23**	0	87	936	**1,023**	**15.77**
Caloocan City	**12,459**	16	1,356	10,063	**11,435**	**91.78**	1	88	935	**1,024**	**8.22**
Marikina City	**7,167**	0	53	573	**626**	**8.73**	1	4	161	**166**	**2.32**
Pasig City	**11,385**	8	631	9,921	**10,560**	**92.75**	0	89	736	**825**	**7.25**
Pateros	**873**	0	36	728	**764**	**87.51**	1	21	87	**109**	**12.49**
Taguig	**11,981**	23	992	9,897	**10,912**	**91.08**	2	60	1,007	**1,069**	**8.92**
Quezon City	**43,348**	79	3,241	36,979	**40,299**	**92.97**	1	146	2,902	**3,049**	**7.03**
Makati City	**5,606**	4	233	4,671	**4,908**	**87.55**	0	20	678	**698**	**12.45**
Mandaluyong City	**6,814**	3	332	5,606	**5,941**	**87.19**	1	63	809	**873**	**12.81**
San Juan	**1,550**	0	0	0	**0**	**0.00**	0	0	0	**0**	**0.00**
Manila City	**44,644**	22	1,774	14,416	**16,212**	**36.31**	0	209	2,307	**2,516**	**5.64**
Las Piñas City	**6,428**	8	544	5,200	**5,752**	**89.48**	1	59	616	**676**	**10.52**
Muntinlupa City	**7,675**	0	0	0	**0**	**0.00**	0	0	0	**0**	**0.00**
Parañaque City	**7,879**	6	814	5,806	**6,626**	**84.10**	1	87	1,165	**1,253**	**15.90**
Pasay City	**5,286**	1	262	4,298	**4,561**	**86.28**	0	57	668	**725**	**13.72**
**C A R**	**24,090**	**28**	**1,804**	**15,283**	**17,115**	**71.05**	**10**	**306**	**6,659**	**6,975**	**28.95**
Abra	**2,872**	2	288	1,728	**2,018**	**70.26**	2	62	790	**854**	**29.74**
Apayao	**1,636**	6	258	1,160	**1,424**	**87.04**	2	17	193	**212**	**12.96**
Benguet	**4,048**	3	241	2,750	**2,994**	**73.96**	1	44	1,009	**1,054**	**26.04**
Ifugao	**2,745**	5	230	2,024	**2,259**	**82.30**	1	38	447	**486**	**17.70**
Kalinga	**2,646**	0	189	2,047	**2,236**	**84.50**	0	15	395	**410**	**15.50**
Mt. Province	**3,233**	4	206	2,025	**2,235**	**69.13**	1	49	948	**998**	**30.87**
Baguio City	**6,910**	8	392	3,549	**3,949**	**57.15**	3	81	2,877	**2,961**	**42.85**
**Region 1**	**49,615**	**67**	**3,516**	**39,212**	**42,795**	**86.25**	**3**	**345**	**6,472**	**6,820**	**13.75**
Ilocos Norte	**4,303**	3	181	3,545	**3,729**	**86.66**	0	48	526	**574**	**13.34**
Ilocos Sur	**4,687**	5	257	3,233	**3,495**	**74.57**	2	50	1,140	**1,192**	**25.43**
La Union	**4,346**	7	381	3,756	**4,144**	**95.35**	0	21	181	**202**	**4.65**
Pangasinan	**13,824**	18	1,115	12,144	**13,277**	**96.04**	1	39	507	**547**	**3.96**
Alaminos City	**1,320**	0	64	1,080	**1,144**	**86.67**	0	10	166	**176**	**13.33**
Candon City	**840**	0	1	618	**619**	**73.69**	0	1	220	**221**	**26.31**
Dagupan City	**8,504**	19	634	5,275	**5,928**	**69.71**	0	101	2,475	**2,576**	**30.29**
Laoag City	**1,963**	0	47	1,865	**1,912**	**97.40**	0	0	51	**51**	**2.60**
San Carlos City	**2,735**	2	210	2,089	**2,301**	**84.13**	0	24	410	**434**	**15.87**
San Fernando City	**3,574**	5	272	2,907	**3,184**	**89.09**	0	24	366	**390**	**10.91**
Urdaneta City	**1,843**	3	207	1,431	**1,641**	**89.04**	0	19	183	**202**	**10.96**
Vigan City	**1,676**	5	147	1,269	**1,421**	**84.79**	0	8	247	**255**	**15.21**
**Region 2**	**39,828**	**76**	**3,961**	**27,762**	**31,799**	**79.84**	**5**	**654**	**7,370**	**8,029**	**20.16**
Batanes	**254**	0	12	188	**200**	**78.74**	0	1	53	**54**	**21.26**
Cagayan	**8,786**	15	1,031	7,083	**8,129**	**92.52**	0	60	597	**657**	**7.48**
Isabela	**10,651**	17	974	7,485	**8,476**	**79.58**	4	100	2,071	**2,175**	**20.42**

Table 2.B.2.2. Intrapartum Care and Delivery Outcome

Number and proportion of delivey by type

Philippines, 2022

Area	Total number of Deliveries	Delivery by Type									
		Vaginal					Cesarean Section				
		Age Group in Year			Total	%	Age Group in Year			Total	%
		10-14	15-19	20-49			10-14	15-19	20-49		
Nueva Vizcaya	**7,453**	29	814	4,375	**5,218**	**70.01**	0	273	1,962	**2,235**	**29.99**
Quirino	**2,614**	4	279	1,760	**2,043**	**78.16**	1	66	504	**571**	**21.84**
Cauayan City	**1,880**	1	189	1,145	**1,335**	**71.01**	0	56	489	**545**	**28.99**
Ilagan City	**3,596**	5	325	2,328	**2,658**	**73.92**	0	57	881	**938**	**26.08**
Santiago City	**3,030**	4	250	2,428	**2,682**	**88.51**	0	26	322	**348**	**11.49**
Tuguegarao City	**1,564**	1	87	970	**1,058**	**67.65**	0	15	491	**506**	**32.35**
**Region 3**	**133,760**	**190**	**9,791**	**87,645**	**97,626**	**72.99**	**36**	**1,785**	**29,248**	**31,069**	**23.23**
Aurora	**3,023**	3	345	2,286	**2,634**	**87.13**	0	12	377	**389**	**12.87**
Bataan	**4,993**	15	515	3,693	**4,223**	**84.58**	2	51	717	**770**	**15.42**
Bulacan	**20,098**	18	1,356	15,165	**16,539**	**82.29**	4	179	4,061	**4,244**	**21.12**
Nueva Ecija	**12,191**	15	643	5,986	**6,644**	**54.50**	4	83	5,460	**5,547**	**45.50**
Pampanga	**17,975**	30	1,463	11,924	**13,417**	**74.64**	10	315	4,238	**4,563**	**25.39**
Tarlac	**10,541**	13	794	7,964	**8,771**	**83.21**	1	124	1,645	**1,770**	**16.79**
Zambales	**8,746**	16	713	5,317	**6,046**	**69.13**	6	204	2,490	**2,700**	**30.87**
Angeles City	**5,738**	11	376	4,694	**5,081**	**88.55**	0	92	565	**657**	**11.45**
Balanga City	**1,300**	6	123	721	**850**	**65.38**	1	40	409	**450**	**34.62**
Cabanatuan City	**6,167**	11	450	4,832	**5,293**	**85.83**	1	66	807	**874**	**14.17**
City of San Fernando	**4,691**	0	51	760	**811**	**17.29**	0	2	18	**20**	**0.43**
Gapan City	**834**	0	50	492	**542**	**64.99**	0	47	245	**292**	**35.01**
Mabalacat City	**4,196**	2	187	2,058	**2,247**	**53.55**	0	2	52	**54**	**1.29**
Malolos City	**4,724**	0	71	1,241	**1,312**	**27.77**	2	63	3,347	**3,412**	**72.23**
Meycauayan	**2,236**	0	133	2,096	**2,229**	**99.69**	0	1	6	**7**	**0.31**
Olongapo	**3,691**	6	314	3,335	**3,655**	**99.02**	0	0	35	**35**	**0.95**
Palayan City	**109**	0	11	98	**109**	**100.00**	0	0	0	**0**	**0.00**
San Jose City	**2,115**	6	239	1,307	**1,552**	**73.38**	1	78	484	**563**	**26.62**
San Jose del Monte City	**8,415**	27	1,037	5,888	**6,952**	**82.61**	2	39	1,422	**1,463**	**17.39**
Science City of Munoz	**129**	2	22	105	**129**	**100.00**	0	0	0	**0**	**0.00**
Tarlac City	**11,848**	9	898	7,683	**8,590**	**72.50**	2	387	2,870	**3,259**	**27.51**
**Region 4A**	**195,187**	**224**	**14,727**	**144,072**	**159,023**	**81.47**	**24**	**1,838**	**30,596**	**32,458**	**16.63**
Batangas	**24,328**	31	1,707	15,701	**17,439**	**71.68**	4	411	6,271	**6,686**	**27.48**
Cavite	**19,628**	20	1,289	15,302	**16,611**	**84.63**	5	135	2,666	**2,806**	**14.30**
Laguna	**12,194**	27	1,215	8,269	**9,511**	**78.00**	3	136	2,474	**2,613**	**21.43**
Quezon	**25,230**	52	2,688	19,174	**21,914**	**86.86**	6	176	2,752	**2,934**	**11.63**
Rizal	**30,278**	29	2,516	22,252	**24,797**	**81.90**	4	275	4,136	**4,415**	**14.58**
Antipolo City	**9,873**	2	812	6,564	**7,378**	**74.73**	0	103	965	**1,068**	**10.82**
Bacoor City	**7,255**	4	465	5,725	**6,194**	**85.38**	0	38	1,003	**1,041**	**14.35**
Batangas City	**3,345**	6	281	2,145	**2,432**	**72.71**	0	59	854	**913**	**27.29**
Biñan City	**7,204**	1	518	5,948	**6,467**	**89.77**	2	126	597	**725**	**10.06**
Cabuyao City	**3,348**	0	143	2,782	**2,925**	**87.37**	0	16	368	**384**	**11.47**
Calamba City	**6,653**	7	372	4,534	**4,913**	**73.85**	0	56	1,684	**1,740**	**26.15**
Cavite City	**1,321**	1	83	1,117	**1,201**	**90.92**	0	9	111	**120**	**9.08**
Dasmariñas City	**8,174**	4	514	6,655	**7,173**	**87.75**	0	59	942	**1,001**	**12.25**
General Trias City	**2,775**	8	179	2,306	**2,493**	**89.84**	0	9	268	**277**	**9.98**
Imus City	**4,653**	6	150	3,486	**3,642**	**78.27**	0	19	962	**981**	**21.08**
Lipa City	**4,649**	3	244	2,807	**3,054**	**65.69**	0	61	1,471	**1,532**	**32.95**
Lucena City	**3,206**	4	332	2,477	**2,813**	**87.74**	0	40	352	**392**	**12.23**
San Pablo City	**3,415**	4	300	2,323	**2,627**	**76.93**	0	40	650	**690**	**20.20**
San Pedro City	**2,970**	2	161	2,532	**2,695**	**90.74**	0	6	248	**254**	**8.55**
Santa Rosa City	**5,405**	6	295	4,789	**5,090**	**94.17**	0	32	283	**315**	**5.83**

Table 2.B.2.2. Intrapartum Care and Delivery Outcome

Number and proportion of delivey by type
Philippines, 2022

Area	Total number of Deliveries	Delivery by Type									
		Vaginal					Cesarean Section				
		Age Group in Year			Total	%	Age Group in Year			Total	%
		10-14	15-19	20-49			10-14	15-19	20-49		
Tagaytay City	**1,305**	1	47	1,033	**1,081**	**82.84**	0	5	214	**219**	**16.78**
Tanauan City	**3,459**	3	83	2,561	**2,647**	**76.53**	0	9	764	**773**	**22.35**
Tayabas City	**1,409**	1	119	1,092	**1,212**	**86.02**	0	6	191	**197**	**13.98**
Trece Martires City	**3,110**	2	214	2,498	**2,714**	**87.27**	0	12	370	**382**	**12.28**
**Region 4B**	**41,033**	**63**	**4,795**	**33,753**	**38,611**	**94.10**	**3**	**130**	**2,198**	**2,331**	**5.68**
Marinduque	**3,070**	6	301	2,339	**2,646**	**86.19**	1	15	408	**424**	**13.81**
Mindoro Occidental	**7,546**	8	1,015	6,219	**7,242**	**95.97**	0	22	289	**311**	**4.12**
Mindoro Oriental	**9,492**	14	700	8,117	**8,831**	**93.04**	0	16	639	**655**	**6.90**
Palawan	**12,965**	21	1,907	10,538	**12,466**	**96.15**	1	49	352	**402**	**3.10**
Romblon	**4,089**	5	429	3,395	**3,829**	**93.64**	0	12	253	**265**	**6.48**
Puerto Princesa City	**3,871**	9	443	3,145	**3,597**	**92.92**	1	16	257	**274**	**7.08**
**Region 5**	**90,156**	**79**	**8,096**	**71,073**	**79,248**	**87.90**	**27**	**405**	**8,023**	**8,455**	**9.38**
Albay	**12,749**	10	929	11,586	**12,525**	**98.24**	0	0	224	**224**	**1.76**
Camarines Norte	**10,484**	8	1,124	7,968	**9,100**	**86.80**	3	96	1,285	**1,384**	**13.20**
Camarines Sur	**17,803**	10	1,409	16,281	**17,700**	**99.42**	0	3	100	**103**	**0.58**
Catanduanes	**4,348**	5	403	3,071	**3,479**	**80.01**	1	42	826	**869**	**19.99**
Masbate	**14,257**	25	1,994	12,153	**14,172**	**99.40**	0	7	78	**85**	**0.60**
Sorsogon	**13,063**	14	1,111	10,179	**11,304**	**86.53**	2	66	1,691	**1,759**	**13.47**
Iriga City	**1,705**	1	104	1,279	**1,384**	**81.17**	0	3	318	**321**	**18.83**
Legaspi City	**3,426**	3	215	2,859	**3,077**	**89.81**	1	11	337	**349**	**10.19**
Naga City	**12,321**	3	807	5,697	**6,507**	**52.81**	20	177	3,164	**3,361**	**27.28**
**Region 6**	**91,080**	**161**	**9,033**	**73,765**	**82,959**	**91.08**	**10**	**405**	**7,706**	**8,121**	**8.92**
Aklan	**5,918**	4	445	4,858	**5,307**	**89.68**	0	21	590	**611**	**10.32**
Antique	**7,882**	2	734	6,428	**7,164**	**90.89**	0	16	702	**718**	**9.11**
Capiz	**7,735**	17	665	6,322	**7,004**	**90.55**	1	37	693	**731**	**9.45**
Guimaras	**2,248**	6	210	1,764	**1,980**	**88.08**	0	16	252	**268**	**11.92**
Iloilo	**22,304**	42	1,946	18,562	**20,550**	**92.14**	6	108	1,640	**1,754**	**7.86**
Negros Occidental	**33,405**	74	4,053	26,636	**30,763**	**92.09**	3	160	2,479	**2,642**	**7.91**
Bacolod City	**5,982**	7	543	4,722	**5,272**	**88.13**	0	15	695	**710**	**11.87**
Iloilo City	**5,606**	9	437	4,473	**4,919**	**87.75**	0	32	655	**687**	**12.25**
**Region 7**	**133,214**	**157**	**9,020**	**115,063**	**124,240**	**93.26**	**5**	**409**	**8,559**	**8,973**	**6.74**
Bohol	**18,027**	26	1,590	14,829	**16,445**	**91.22**	0	47	1,535	**1,582**	**8.78**
Cebu	**53,485**	38	2,508	48,674	**51,220**	**95.77**	2	160	2,103	**2,265**	**4.23**
Negros Oriental	**19,600**	43	2,200	16,195	**18,438**	**94.07**	2	49	1,111	**1,162**	**5.93**
Siquijor	**1,098**	2	95	812	**909**	**82.79**	0	20	169	**189**	**17.21**
Cebu City	**22,881**	24	1,489	20,136	**21,649**	**94.62**	0	35	1,197	**1,232**	**5.38**
Lapu-Lapu City	**8,517**	8	438	7,367	**7,813**	**91.73**	0	45	659	**704**	**8.27**
Mandaue City	**9,606**	16	700	7,050	**7,766**	**80.85**	1	53	1,785	**1,839**	**19.14**
**Region 8**	**59,532**	**157**	**6,040**	**47,076**	**53,273**	**89.49**	**8**	**450**	**5,801**	**6,259**	**10.51**
Biliran	**3,303**	11	398	2,674	**3,083**	**93.34**	0	5	215	**220**	**6.66**
Eastern Samar	**7,154**	11	674	5,454	**6,139**	**85.81**	3	79	933	**1,015**	**14.19**
Northern Leyte	**13,721**	87	1,237	11,444	**12,768**	**93.05**	0	65	888	**953**	**6.95**

Table 2.B.2.2. Intrapartum Care and Delivery Outcome

Number and proportion of delivey by type

Philippines, 2022

Area	Total number of Deliveries	Delivery by Type									
		Vaginal					Cesarean Section				
		Age Group in Year			Total	%	Age Group in Year			Total	%
		10-14	15-19	20-49			10-14	15-19	20-49		
Northern Samar	**10,410**	17	1,112	8,405	**9,534**	**91.59**	4	82	790	**876**	**8.41**
Southern Leyte	**2,990**	7	333	2,359	**2,699**	**90.27**	0	12	279	**291**	**9.73**
Western Samar	**8,510**	6	1,057	6,669	**7,732**	**90.86**	0	85	693	**778**	**9.14**
Calbayog City	**2,290**	4	255	1,895	**2,154**	**94.06**	0	7	129	**136**	**5.94**
Maasin City	**2,390**	1	182	1,570	**1,753**	**73.35**	0	26	611	**637**	**26.65**
Ormoc City	**5,850**	10	606	4,300	**4,916**	**84.03**	0	73	861	**934**	**15.97**
Tacloban City	**2,914**	3	186	2,306	**2,495**	**85.62**	1	16	402	**419**	**14.38**
**Region 9**	**49,894**	**93**	**6,197**	**41,706**	**47,996**	**96.20**	**6**	**173**	**1,719**	**1,898**	**3.80**
Zamboanga del Norte	**9,771**	25	1,499	8,247	**9,771**	**100.00**	0	0	0	**0**	**0.00**
Zamboanga del Sur	**6,479**	18	822	5,598	**6,438**	**99.37**	0	3	38	**41**	**0.63**
Zamboanga Sibugay	**8,998**	11	1,166	7,320	**8,497**	**94.43**	0	29	472	**501**	**5.57**
Dapitan City	**1,558**	1	162	1,356	**1,519**	**97.50**	2	12	25	**39**	**2.50**
Dipolog City	**2,220**	6	261	1,712	**1,979**	**89.14**	1	14	226	**241**	**10.86**
Isabela City	**1,693**	4	195	1,393	**1,592**	**94.03**	0	10	91	**101**	**5.97**
Pagadian City	**2,413**	1	220	2,095	**2,316**	**95.98**	0	9	88	**97**	**4.02**
Zamboanga City	**16,762**	27	1,872	13,985	**15,884**	**94.76**	3	96	779	**878**	**5.24**
**Region 10**	**76,762**	**188**	**9,494**	**61,803**	**71,485**	**93.13**	**14**	**488**	**4,775**	**5,277**	**6.87**
Bukidnon	**20,001**	92	3,508	14,601	**18,201**	**91.00**	8	210	1,582	**1,800**	**9.00**
Camiguin	**1,154**	2	125	969	**1,096**	**94.97**	0	5	53	**58**	**5.03**
Lanao del Norte	**8,532**	17	789	7,421	**8,227**	**96.43**	5	51	249	**305**	**3.57**
Misamis Occidental	**4,066**	7	235	3,530	**3,772**	**92.77**	0	14	280	**294**	**7.23**
Misamis Oriental	**8,271**	29	1,069	6,992	**8,090**	**97.81**	0	16	165	**181**	**2.19**
Cagayan de Oro City	**16,044**	25	1,548	13,535	**15,108**	**94.17**	0	73	863	**936**	**5.83**
El Salvador City	**125**	1	32	92	**125**	**100.00**	0	0	0	**0**	**0.00**
Gingoog City	**2,109**	2	307	1,633	**1,942**	**92.08**	0	18	149	**167**	**7.92**
Iligan City	**7,370**	4	795	5,931	**6,730**	**91.32**	0	26	614	**640**	**8.68**
Malaybalay City	**3,112**	4	442	2,289	**2,735**	**87.89**	0	37	340	**377**	**12.11**
Oroquieta City	**1,117**	0	128	860	**988**	**88.45**	0	5	124	**129**	**11.55**
Ozamis City	**1,955**	0	215	1,488	**1,703**	**87.11**	1	27	224	**252**	**12.89**
Tangub City	**953**	0	75	865	**940**	**98.64**	0	1	12	**13**	**1.36**
Valencia City	**1,953**	5	226	1,597	**1,828**	**93.60**	0	5	120	**125**	**6.40**
**Region 11**	**82,240**	**397**	**9,891**	**57,338**	**67,626**	**82.23**	**56**	**907**	**10,176**	**11,139**	**13.54**
Davao de Oro	**11,860**	105	1,796	8,171	**10,072**	**84.92**	15	155	1,371	**1,541**	**12.99**
Davao del Norte	**17,930**	96	2,264	12,445	**14,805**	**82.57**	11	233	2,371	**2,615**	**14.58**
Davao Oriental	**8,773**	44	1,260	6,603	**7,907**	**90.13**	2	44	709	**755**	**8.61**
Davao del Sur	**9,089**	50	1,259	6,748	**8,057**	**88.65**	2	89	941	**1,032**	**11.35**
Davao Occidental	**4,596**	30	717	1,954	**2,701**	**58.77**	2	26	189	**217**	**4.72**
Davao City	**29,992**	72	2,595	21,417	**24,084**	**80.30**	24	360	4,595	**4,979**	**16.60**
**Region 12**	**64,935**	**197**	**8,745**	**49,887**	**58,829**	**90.60**	**18**	**551**	**5,550**	**6,119**	**9.42**
North Cotabato	**19,637**	38	2,469	15,158	**17,665**	**89.96**	3	184	1,785	**1,972**	**10.04**
Sarangani	**10,342**	36	1,886	7,883	**9,805**	**94.81**	2	69	479	**550**	**5.32**
South Cotabato	**16,503**	57	2,305	12,355	**14,717**	**89.18**	4	115	1,667	**1,786**	**10.82**
Sultan Kudarat	**12,587**	63	1,688	9,413	**11,164**	**88.69**	5	102	1,316	**1,423**	**11.31**

Table 2.B.2.2. Intrapartum Care and Delivery Outcome

Number and proportion of delivey by type
Philippines, 2022

Area	Total number of Deliveries	Delivery by Type									
		Vaginal					Cesarean Section				
		Age Group in Year			Total	%	Age Group in Year			Total	%
		10-14	15-19	20-49			10-14	15-19	20-49		
Gen. Santos City	**5,866**	3	397	5,078	**5,478**	**93.39**	4	81	303	**388**	**6.61**
**BARMM**	**65,614**	**45**	**5,425**	**57,207**	**62,677**	**95.52**	**0**	**373**	**2,222**	**2,595**	**3.95**
Basilan	**3,804**	14	561	3,137	**3,712**	**97.58**	0	17	75	**92**	**2.42**
Lanao del Sur	**18,053**	7	917	16,555	**17,479**	**96.82**	0	32	542	**574**	**3.18**
Maguindanao	**19,085**	13	1,745	16,887	**18,645**	**97.69**	0	38	402	**440**	**2.31**
Sulu	**9,870**	2	612	8,684	**9,298**	**94.20**	0	20	258	**278**	**2.82**
Tawi-Tawi	**5,365**	1	449	4,825	**5,275**	**98.32**	0	2	36	**38**	**0.71**
Lamitan City	**1,366**	7	225	1,100	**1,332**	**97.51**	0	4	30	**34**	**2.49**
Marawi City	**4,890**	0	655	3,511	**4,166**	**85.19**	0	248	480	**728**	**14.89**
Cotabato City	**3,181**	1	261	2,508	**2,770**	**87.08**	0	12	399	**411**	**12.92**
**CARAGA**	**35,240**	**54**	**4,033**	**29,615**	**33,702**	**95.64**	**2**	**129**	**1,407**	**1,538**	**4.36**
Agusan del Norte	**3,646**	5	453	3,183	**3,641**	**99.86**	0	0	5	**5**	**0.14**
Agusan del Sur	**10,056**	18	1,115	8,621	**9,754**	**97.00**	0	16	286	**302**	**3.00**
Surigao del Norte	**3,060**	8	320	2,732	**3,060**	**100.00**	0	0	0	**0**	**0.00**
Surigao del Sur	**6,361**	13	869	5,397	**6,279**	**98.71**	0	4	78	**82**	**1.29**
Province of Dinagat	**913**	0	115	798	**913**	**100.00**	0	0	0	**0**	**0.00**
Bislig City	**1,482**	2	185	1,159	**1,346**	**90.82**	1	11	124	**136**	**9.18**
Butuan City	**5,989**	8	490	4,638	**5,136**	**85.76**	1	32	820	**853**	**14.24**
Surigao City	**3,733**	0	486	3,087	**3,573**	**95.71**	0	66	94	**160**	**4.29**

Deliveries should be reported by place of occurrence.
Vaginal Delivery - deliveries by NSD, Vaccum and Forcep
CS Delivery - refers to deliveries by caesarian section

Table 2.B.2.3. Intrapartum Care and Delivery Outcome

Number and proportion of pregnancy outcome (Full term and Pre-term)
Philippines, 2022

Area	Total number of Deliveries	Full term (37-42 weeks AOG)					Pre-term (22-36 weeks AOG)				
		Age Group in Year			Total	%	Age Group in Year			Total	%
		10-14	15-19	20-49			10-14	15-19	20-49		
**PHILIPPINES**	**1,419,070**	**2,628**	**132,431**	**1,201,506**	**1,336,565**	**94.19**	**107**	**2,922**	**16,847**	**19,876**	**1.40**
**N C R**	**186,890**	**186**	**12,819**	**134,736**	**147,741**	**79.05**	**6**	**179**	**1,394**	**1,579**	**0.84**
Malabon	**3,327**	7	386	2,897	**3,290**	**98.89**	0	4	14	**18**	**0.54**
Navotas	**3,980**	3	461	3,481	**3,945**	**99.12**	0	0	28	**28**	**0.70**
Valenzuela City	**6,488**	9	512	5,850	**6,371**	**98.20**	1	10	59	**70**	**1.08**
Caloocan City	**12,459**	16	1,425	10,947	**12,388**	**99.43**	1	10	20	**31**	**0.25**
Marikina City	**7,167**	1	67	967	**1,035**	**14.44**	0	8	120	**128**	**1.79**
Pasig City	**11,385**	8	715	10,450	**11,173**	**98.14**	0	5	73	**78**	**0.69**
Pateros	**873**	1	55	798	**854**	**97.82**	0	2	11	**13**	**1.49**
Taguig	**11,981**	25	1,066	10,762	**11,853**	**98.93**	0	3	62	**65**	**0.54**
Quezon City	**43,348**	70	3,344	39,423	**42,837**	**98.82**	1	19	92	**112**	**0.26**
Makati City	**5,606**	4	234	5,265	**5,503**	**98.16**	0	6	55	**61**	**1.09**
Mandaluyong City	**6,814**	4	394	6,364	**6,762**	**99.24**	0	1	26	**27**	**0.40**
San Juan	**1,550**	0	43	1,427	**1,470**	**94.84**	0	6	58	**64**	**4.13**
Manila City	**44,644**	20	1,957	16,184	**18,161**	**40.68**	2	27	128	**157**	**0.35**
Las Piñas City	**6,428**	8	602	5,742	**6,352**	**98.82**	1	1	12	**14**	**0.22**
Muntinlupa City	**7,675**	2	386	2,543	**2,931**	**38.19**	0	60	397	**457**	**5.95**
Parañaque City	**7,879**	7	859	6,894	**7,760**	**98.49**	0	13	46	**59**	**0.75**
Pasay City	**5,286**	1	313	4,742	**5,056**	**95.65**	0	4	193	**197**	**3.73**
**C A R**	**24,090**	**38**	**1,963**	**21,057**	**23,058**	**95.72**	**0**	**138**	**650**	**788**	**3.27**
Abra	**2,872**	4	319	2,368	**2,691**	**93.70**	0	30	122	**152**	**5.29**
Apayao	**1,636**	8	227	1,316	**1,551**	**94.80**	0	48	27	**75**	**4.58**
Benguet	**4,048**	4	271	3,633	**3,908**	**96.54**	0	10	97	**107**	**2.64**
Ifugao	**2,745**	6	254	2,397	**2,657**	**96.79**	0	12	43	**55**	**2.00**
Kalinga	**2,646**	0	200	2,412	**2,612**	**98.72**	0	4	18	**22**	**0.83**
Mt. Province	**3,233**	5	248	2,890	**3,143**	**97.22**	0	6	54	**60**	**1.86**
Baguio City	**6,910**	11	444	6,041	**6,496**	**94.01**	0	28	289	**317**	**4.59**
**Region 1**	**49,615**	**68**	**3,663**	**42,959**	**46,690**	**94.10**	**2**	**200**	**2,723**	**2,925**	**5.90**
Ilocos Norte	**4,303**	3	211	3,869	**4,083**	**94.89**	0	18	202	**220**	**5.11**
Ilocos Sur	**4,687**	7	304	4,352	**4,663**	**99.49**	0	3	21	**24**	**0.51**
La Union	**4,346**	7	400	3,905	**4,312**	**99.22**	0	2	32	**34**	**0.78**
Pangasinan	**13,824**	19	1,114	12,564	**13,697**	**99.08**	0	40	87	**127**	**0.92**
Alaminos City	**1,320**	0	74	1,241	**1,315**	**99.62**	0	0	5	**5**	**0.38**
Candon City	**840**	0	1	806	**807**	**96.07**	0	1	32	**33**	**3.93**
Dagupan City	**8,504**	19	666	5,946	**6,631**	**77.98**	0	71	1,802	**1,873**	**22.02**
Laoag City	**1,963**	0	47	1,916	**1,963**	**100.00**	0	0	0	**0**	**0.00**
San Carlos City	**2,735**	2	228	2,469	**2,699**	**98.68**	0	6	30	**36**	**1.32**
San Fernando City	**3,574**	4	271	3,006	**3,281**	**91.80**	1	25	267	**293**	**8.20**
Urdaneta City	**1,843**	3	222	1,591	**1,816**	**98.53**	0	4	23	**27**	**1.47**
Vigan City	**1,676**	4	125	1,294	**1,423**	**84.90**	1	30	222	**253**	**15.10**
**Region 2**	**39,828**	**72**	**4,335**	**34,640**	**39,047**	**98.04**	**1**	**75**	**450**	**526**	**1.32**
Batanes	**254**	0	12	238	**250**	**98.43**	0	0	2	**2**	**0.79**
Cagayan	**8,786**	14	1,065	7,541	**8,620**	**98.11**	0	14	104	**118**	**1.34**
Isabela	**10,651**	18	1,019	9,394	**10,431**	**97.93**	0	20	141	**161**	**1.51**
Nueva Vizcaya	**7,453**	25	939	6,271	**7,235**	**97.08**	0	21	111	**132**	**1.77**

Table 2.B.2.3. Intrapartum Care and Delivery Outcome

Number and proportion of pregnancy outcome (Full term and Pre-term)
Philippines, 2022

Area	Total number of Deliveries	Full term (37-42 weeks AOG)					Pre-term (22-36 weeks AOG)				
		Age Group in Year			Total	%	Age Group in Year			Total	%
		10-14	15-19	20-49			10-14	15-19	20-49		
Quirino	**2,614**	4	307	2,241	**2,552**	**97.63**	1	11	35	**47**	**1.80**
Cauayan City	**1,880**	1	243	1,608	**1,852**	**98.51**	0	1	13	**14**	**0.74**
Ilagan City	**3,596**	6	377	3,173	**3,556**	**98.89**	0	4	12	**16**	**0.44**
Santiago City	**3,030**	4	274	2,743	**3,021**	**99.70**	0	2	7	**9**	**0.30**
Tuguegarao City	**1,564**	0	99	1,431	**1,530**	**97.83**	0	2	25	**27**	**1.73**
**Region 3**	**133,760**	**214**	**11,463**	**114,859**	**126,536**	**94.60**	**7**	**264**	**1,192**	**1,463**	**1.09**
Aurora	**3,023**	2	349	2,600	**2,951**	**97.62**	0	6	43	**49**	**1.62**
Bataan	**4,993**	15	542	4,338	**4,895**	**98.04**	2	16	58	**76**	**1.52**
Bulacan	**20,098**	22	1,482	19,011	**20,515**	**102.07**	0	28	114	**142**	**0.71**
Nueva Ecija	**12,191**	16	912	11,218	**12,146**	**99.63**	0	6	29	**35**	**0.29**
Pampanga	**17,975**	39	1,760	16,065	**17,864**	**99.38**	1	12	59	**72**	**0.40**
Tarlac	**10,541**	14	916	9,575	**10,505**	**99.66**	0	2	27	**29**	**0.28**
Zambales	**8,746**	21	891	7,538	**8,450**	**96.62**	2	38	201	**241**	**2.76**
Angeles City	**5,738**	11	416	5,078	**5,505**	**95.94**	0	39	192	**231**	**4.03**
Balanga City	**1,300**	6	153	1,098	**1,257**	**96.69**	0	10	22	**32**	**2.46**
Cabanatuan City	**6,167**	12	507	5,538	**6,057**	**98.22**	0	6	22	**28**	**0.45**
City of San Fernando	**4,691**	0	64	945	**1,009**	**21.51**	0	0	22	**22**	**0.47**
Gapan City	**834**	0	93	738	**831**	**99.64**	0	0	3	**3**	**0.36**
Mabalacat City	**4,196**	2	166	1,759	**1,927**	**45.92**	0	0	24	**24**	**0.57**
Malolos City	**4,724**	2	148	4,553	**4,703**	**99.56**	0	5	15	**20**	**0.42**
Meycauayan	**2,236**	0	134	2,100	**2,234**	**99.91**	0	0	0	**0**	**0.00**
Olongapo	**3,691**	6	312	3,310	**3,628**	**98.29**	0	0	3	**3**	**0.08**
Palayan City	**109**	0	11	98	**109**	**100.00**	0	0	0	**0**	**0.00**
San Jose City	**2,115**	8	308	1,771	**2,087**	**98.68**	0	4	10	**14**	**0.66**
San Jose del Monte City	**8,415**	29	1,065	7,296	**8,390**	**99.70**	0	8	1	**9**	**0.11**
Science City of Munoz	**129**	1	23	104	**128**	**99.22**	0	0	1	**1**	**0.78**
Tarlac City	**11,848**	8	1,211	10,126	**11,345**	**95.75**	2	84	346	**432**	**3.65**
**Region 4A**	**195,187**	**230**	**14,905**	**165,814**	**180,949**	**92.71**	**20**	**290**	**1,800**	**2,110**	**1.08**
Batangas	**24,328**	33	1,971	21,357	**23,361**	**96.03**	1	21	145	**167**	**0.69**
Cavite	**19,628**	19	1,365	17,705	**19,089**	**97.25**	2	23	166	**191**	**0.97**
Laguna	**12,194**	36	637	7,510	**8,183**	**67.11**	1	14	126	**141**	**1.16**
Quezon	**25,230**	53	2,761	21,431	**24,245**	**96.10**	1	43	213	**257**	**1.02**
Rizal	**30,278**	29	2,624	24,865	**27,518**	**90.88**	10	31	208	**249**	**0.82**
Antipolo City	**9,873**	1	707	6,118	**6,826**	**69.14**	0	28	105	**133**	**1.35**
Bacoor City	**7,255**	4	498	6,684	**7,186**	**99.05**	0	2	32	**34**	**0.47**
Batangas City	**3,345**	6	333	2,949	**3,288**	**98.30**	0	3	47	**50**	**1.49**
Biñan City	**7,204**	2	609	6,526	**7,137**	**99.07**	1	12	33	**46**	**0.64**
Cabuyao City	**3,348**	0	128	2,905	**3,033**	**90.59**	0	21	113	**134**	**4.00**
Calamba City	**6,653**	7	407	6,094	**6,508**	**97.82**	0	21	112	**133**	**2.00**
Cavite City	**1,321**	1	85	1,229	**1,315**	**99.55**	0	1	5	**6**	**0.45**
Dasmariñas City	**8,174**	4	563	7,515	**8,082**	**98.87**	0	14	78	**92**	**1.13**
General Trias City	**2,775**	7	173	2,576	**2,756**	**99.32**	1	2	11	**14**	**0.50**
Imus City	**4,653**	5	150	4,308	**4,463**	**95.92**	1	5	38	**44**	**0.95**
Lipa City	**4,649**	2	283	4,128	**4,413**	**94.92**	0	9	29	**38**	**0.82**
Lucena City	**3,206**	3	358	2,766	**3,127**	**97.54**	0	14	57	**71**	**2.21**
San Pablo City	**3,415**	4	323	2,877	**3,204**	**93.82**	0	7	34	**41**	**1.20**
San Pedro City	**2,970**	2	154	2,719	**2,875**	**96.80**	0	5	27	**32**	**1.08**
Santa Rosa City	**5,405**	6	323	4,896	**5,225**	**96.67**	0	5	161	**166**	**3.07**
Tagaytay City	**1,305**	0	49	1,252	**1,301**	**99.69**	1	0	3	**4**	**0.31**
Tanauan City	**3,459**	3	63	3,316	**3,382**	**97.77**	1	4	9	**14**	**0.40**

Table 2.B.2.3. Intrapartum Care and Delivery Outcome

Number and proportion of pregnancy outcome (Full term and Pre-term)
Philippines, 2022

Area	Total number of Deliveries	Full term (37-42 weeks AOG)					Pre-term (22-36 weeks AOG)				
		Age Group in Year			Total	%	Age Group in Year			Total	%
		10-14	15-19	20-49			10-14	15-19	20-49		
Tayabas City	**1,409**	1	121	1,277	**1,399**	**99.29**	0	1	8	**9**	**0.64**
Trece Martires City	**3,110**	2	220	2,811	**3,033**	**97.52**	0	4	40	**44**	**1.41**
**Region 4B**	**41,033**	**64**	**4,777**	**35,314**	**40,155**	**97.86**	**3**	**97**	**322**	**422**	**1.03**
Marinduque	**3,070**	7	305	2,689	**3,001**	**97.75**	0	6	33	**39**	**1.27**
Mindoro Occidental	**7,546**	8	1,012	6,388	**7,408**	**98.17**	0	15	65	**80**	**1.06**
Mindoro Oriental	**9,492**	13	702	8,693	**9,408**	**99.12**	0	6	31	**37**	**0.39**
Palawan	**12,965**	20	1,884	10,657	**12,561**	**96.88**	2	45	112	**159**	**1.23**
Romblon	**4,089**	6	428	3,559	**3,993**	**97.65**	1	14	55	**70**	**1.71**
Puerto Princesa City	**3,871**	10	446	3,328	**3,784**	**97.75**	0	11	26	**37**	**0.96**
**Region 5**	**90,156**	**114**	**8,319**	**78,832**	**87,265**	**96.79**	**5**	**326**	**1,719**	**2,050**	**2.27**
Albay	**12,749**	10	883	11,769	**12,662**	**99.32**	0	12	46	**58**	**0.45**
Camarines Norte	**10,484**	9	1,014	8,228	**9,251**	**88.24**	2	195	948	**1,145**	**10.92**
Camarines Sur	**17,803**	10	1,393	16,246	**17,649**	**99.13**	0	11	50	**61**	**0.34**
Catanduanes	**4,348**	5	437	3,849	**4,291**	**98.69**	0	4	5	**9**	**0.21**
Masbate	**14,257**	25	1,954	12,065	**14,044**	**98.51**	0	21	52	**73**	**0.51**
Sorsogon	**13,063**	13	1,083	11,229	**12,325**	**94.35**	3	73	479	**555**	**4.25**
Iriga City	**1,705**	1	105	1,564	**1,670**	**97.95**	0	2	17	**19**	**1.11**
Legaspi City	**3,426**	4	222	3,161	**3,387**	**98.86**	0	4	27	**31**	**0.90**
Naga City	**12,321**	37	1,228	10,721	**11,986**	**97.28**	0	4	95	**99**	**0.80**
**Region 6**	**91,080**	**164**	**9,176**	**79,847**	**89,187**	**97.92**	**7**	**221**	**1,150**	**1,378**	**1.51**
Aklan	**5,918**	5	457	5,268	**5,730**	**96.82**	0	9	65	**74**	**1.25**
Antique	**7,882**	2	727	6,913	**7,642**	**96.96**	0	23	154	**177**	**2.25**
Capiz	**7,735**	17	692	6,937	**7,646**	**98.85**	0	8	58	**66**	**0.85**
Guimaras	**2,248**	6	216	1,955	**2,177**	**96.84**	0	10	61	**71**	**3.16**
Iloilo	**22,304**	47	2,009	19,893	**21,949**	**98.41**	1	34	223	**258**	**1.16**
Negros Occidental	**33,405**	72	4,088	28,518	**32,678**	**97.82**	5	97	437	**539**	**1.61**
Bacolod City	**5,982**	6	529	5,290	**5,825**	**97.38**	1	28	89	**118**	**1.97**
Iloilo City	**5,606**	9	458	5,073	**5,540**	**98.82**	0	12	63	**75**	**1.34**
**Region 7**	**133,214**	**166**	**9,048**	**120,803**	**130,017**	**97.60**	**8**	**303**	**2,020**	**2,331**	**1.75**
Bohol	**18,027**	28	1,573	15,635	**17,236**	**95.61**	1	58	590	**649**	**3.60**
Cebu	**53,485**	46	2,510	49,972	**52,528**	**98.21**	2	123	591	**716**	**1.34**
Negros Oriental	**19,600**	42	2,169	16,858	**19,069**	**97.29**	4	68	316	**388**	**1.98**
Siquijor	**1,098**	2	113	953	**1,068**	**97.27**	0	0	15	**15**	**1.37**
Cebu City	**22,881**	24	1,507	21,011	**22,542**	**98.52**	0	9	167	**176**	**0.77**
Lapu-Lapu City	**8,517**	9	497	7,878	**8,384**	**98.44**	0	7	54	**61**	**0.72**
Mandaue City	**9,606**	15	679	8,496	**9,190**	**95.67**	1	38	287	**326**	**3.39**
**Region 8**	**59,532**	**147**	**6,110**	**51,447**	**57,704**	**96.93**	**11**	**290**	**1,084**	**1,385**	**2.33**
Biliran	**3,303**	9	392	2,805	**3,206**	**97.06**	2	6	46	**54**	**1.63**
Eastern Samar	**7,154**	12	717	6,256	**6,985**	**97.64**	0	25	66	**91**	**1.27**
Northern Leyte	**13,721**	84	1,215	11,995	**13,294**	**96.89**	4	60	309	**373**	**2.72**
Northern Samar	**10,410**	12	1,127	8,967	**10,106**	**97.08**	4	32	145	**181**	**1.74**
Southern Leyte	**2,990**	7	337	2,619	**2,963**	**99.10**	0	4	9	**13**	**0.43**
Western Samar	**8,510**	5	1,042	7,100	**8,147**	**95.73**	0	99	257	**356**	**4.18**

Table 2.B.2.3. Intrapartum Care and Delivery Outcome

Number and proportion of pregnancy outcome (Full term and Pre-term)
Philippines, 2022

Area	Total number of Deliveries	Full term (37-42 weeks AOG)					Pre-term (22-36 weeks AOG)				
		Age Group in Year			Total	%	Age Group in Year			Total	%
		10-14	15-19	20-49			10-14	15-19	20-49		
Calbayog City	**2,290**	4	254	1,992	**2,250**	**98.25**	0	3	17	**20**	**0.87**
Maasin City	**2,390**	1	188	2,115	**2,304**	**96.40**	0	18	27	**45**	**1.88**
Ormoc City	**5,850**	9	638	4,917	**5,564**	**95.11**	1	41	202	**244**	**4.17**
Tacloban City	**2,914**	4	200	2,681	**2,885**	**99.00**	0	2	6	**8**	**0.27**
**Region 9**	**49,894**	**94**	**6,252**	**42,975**	**49,321**	**98.85**	**5**	**71**	**220**	**296**	**0.59**
Zamboanga del Norte	**9,771**	23	1,470	8,166	**9,659**	**98.85**	1	11	29	**41**	**0.42**
Zamboanga del Sur	**6,479**	16	812	5,578	**6,406**	**98.87**	2	10	31	**43**	**0.66**
Zamboanga Sibugay	**8,998**	11	1,169	7,661	**8,841**	**98.26**	0	14	49	**63**	**0.70**
Dapitan City	**1,558**	2	168	1,373	**1,543**	**99.04**	1	4	8	**13**	**0.83**
Dipolog City	**2,220**	7	271	1,906	**2,184**	**98.38**	0	4	18	**22**	**0.99**
Isabela City	**1,693**	4	203	1,480	**1,687**	**99.65**	0	0	1	**1**	**0.06**
Pagadian City	**2,413**	2	226	2,165	**2,393**	**99.17**	0	3	16	**19**	**0.79**
Zamboanga City	**16,762**	29	1,933	14,646	**16,608**	**99.08**	1	25	68	**94**	**0.56**
**Region 10**	**76,762**	**291**	**9,842**	**65,555**	**75,688**	**98.60**	**4**	**114**	**496**	**614**	**0.80**
Bukidnon	**20,001**	97	3,638	15,900	**19,635**	**98.17**	0	48	186	**234**	**1.17**
Camiguin	**1,154**	2	130	1,006	**1,138**	**98.61**	0	0	0	**0**	**0.00**
Lanao del Norte	**8,532**	123	781	7,572	**8,476**	**99.34**	0	7	23	**30**	**0.35**
Misamis Occidental	**4,066**	7	261	3,786	**4,054**	**99.70**	0	0	6	**6**	**0.15**
Misamis Oriental	**8,271**	27	1,100	7,069	**8,196**	**99.09**	2	10	47	**59**	**0.71**
Cagayan de Oro City	**16,044**	19	1,636	14,138	**15,793**	**98.44**	1	22	101	**124**	**0.77**
El Salvador City	**125**	1	32	92	**125**	**100.00**	0	0	0	**0**	**0.00**
Gingoog City	**2,109**	2	324	1,741	**2,067**	**98.01**	0	3	20	**23**	**1.09**
Iligan City	**7,370**	4	810	6,428	**7,242**	**98.26**	0	0	60	**60**	**0.81**
Malaybalay City	**3,112**	4	458	2,593	**3,055**	**98.17**	0	19	21	**40**	**1.29**
Oroquieta City	**1,117**	0	132	964	**1,096**	**98.12**	0	1	11	**12**	**1.07**
Ozamis City	**1,955**	0	237	1,679	**1,916**	**98.01**	1	1	15	**17**	**0.87**
Tangub City	**953**	0	76	877	**953**	**100.00**	0	0	0	**0**	**0.00**
Valencia City	**1,953**	5	227	1,710	**1,942**	**99.44**	0	3	6	**9**	**0.46**
**Region 11**	**82,240**	**472**	**11,333**	**68,742**	**80,547**	**97.94**	**17**	**155**	**762**	**934**	**1.14**
Davao de Oro	**11,860**	127	1,926	9,556	**11,609**	**97.88**	2	27	121	**150**	**1.26**
Davao del Norte	**17,930**	107	2,470	14,739	**17,316**	**96.58**	5	39	208	**252**	**1.41**
Davao Oriental	**8,773**	51	1,302	7,251	**8,604**	**98.07**	1	12	55	**68**	**0.78**
Davao del Sur	**9,089**	52	1,404	7,383	**8,839**	**97.25**	6	34	110	**150**	**1.65**
Davao Occidental	**4,596**	46	1,113	3,261	**4,420**	**96.17**	1	13	149	**163**	**3.55**
Davao City	**29,992**	89	3,118	26,552	**29,759**	**99.22**	2	30	119	**151**	**0.50**
**Region 12**	**64,935**	**207**	**9,099**	**54,716**	**64,022**	**98.59**	**7**	**118**	**484**	**609**	**0.94**
North Cotabato	**19,637**	39	2,600	16,739	**19,378**	**98.68**	2	32	123	**157**	**0.80**
Sarangani	**10,342**	36	1,918	8,255	**10,209**	**98.71**	2	15	50	**67**	**0.65**
South Cotabato	**16,503**	60	2,368	13,807	**16,235**	**98.38**	1	37	153	**191**	**1.16**
Sultan Kudarat	**12,587**	65	1,765	10,562	**12,392**	**98.45**	2	22	120	**144**	**1.14**
Gen. Santos City	**5,866**	7	448	5,353	**5,808**	**99.01**	0	12	38	**50**	**0.85**

Table 2.B.2.3. Intrapartum Care and Delivery Outcome

Number and proportion of pregnancy outcome (Full term and Pre-term)
Philippines, 2022

Area	Total number of Deliveries	Full term (37-42 weeks AOG)					Pre-term (22-36 weeks AOG)				
		Age Group in Year			Total	%	Age Group in Year			Total	%
		10-14	15-19	20-49			10-14	15-19	20-49		
**BARMM**	**65,614**	**48**	**5,226**	**58,571**	**63,845**	**97.30**	**2**	**44**	**191**	**237**	**0.36**
Basilan	**3,804**	15	547	3,170	**3,732**	**98.11**	0	21	30	**51**	**1.34**
Lanao del Sur	**18,053**	7	946	17,053	**18,006**	**99.74**	0	2	35	**37**	**0.20**
Maguindanao	**19,085**	13	1,763	17,151	**18,927**	**99.17**	0	13	46	**59**	**0.31**
Sulu	**9,870**	2	616	8,935	**9,553**	**96.79**	0	0	34	**34**	**0.34**
Tawi-Tawi	**5,365**	1	443	4,891	**5,335**	**99.44**	0	2	22	**24**	**0.45**
Lamitan City	**1,366**	7	228	1,117	**1,352**	**98.98**	1	2	10	**13**	**0.95**
Marawi City	**4,890**	0	413	3,361	**3,774**	**77.18**	0	4	2	**6**	**0.12**
Cotabato City	**3,181**	3	270	2,893	**3,166**	**99.53**	1	0	12	**13**	**0.41**
**CARAGA**	**35,240**	**53**	**4,101**	**30,639**	**34,793**	**98.73**	**2**	**37**	**190**	**229**	**0.65**
Agusan del Norte	**3,646**	5	452	3,184	**3,641**	**99.86**	0	1	1	**2**	**0.05**
Agusan del Sur	**10,056**	18	1,114	8,835	**9,967**	**99.11**	0	5	27	**32**	**0.32**
Surigao del Norte	**3,060**	8	314	2,700	**3,022**	**98.76**	0	2	5	**7**	**0.23**
Surigao del Sur	**6,361**	12	857	5,386	**6,255**	**98.33**	1	10	34	**45**	**0.71**
Province of Dinagat	**913**	0	113	787	**900**	**98.58**	0	2	2	**4**	**0.44**
Bislig City	**1,482**	3	197	1,251	**1,451**	**97.91**	0	0	20	**20**	**1.35**
Butuan City	**5,989**	7	514	5,338	**5,859**	**97.83**	1	4	81	**86**	**1.44**
Surigao City	**3,733**	0	540	3,158	**3,698**	**99.06**	0	13	20	**33**	**0.88**

Table 2.B.2.4. Intrapartum Care and Delivery Outcome

Number and proportion of pregnancy outcome (Fetal Deaths and Abortion)
Philippines, 2022

Area	Total number of Deliveries	Fetal Death					Abortion (Counts Only)			
		Age Group			Total	%	Age Group			Total
		10-14	15-19	20-49			10-14	15-19	20-49	
**PHILIPPINES**	**1,419,070**	**34**	**878**	**7,594**	**8,506**	**0.60**	**56**	**840**	**7,733**	**8,629**
**N C R**	**186,890**	**1**	**73**	**1,432**	**1,506**	**0.81**	**1**	**31**	**331**	**363**
Malabon	**3,327**	0	1	18	**19**	**0.57**	0	4	20	**24**
Navotas	**3,980**	0	0	7	**7**	**0.18**	0	0	1	**1**
Valenzuela City	**6,488**	0	2	45	**47**	**0.72**	0	1	34	**35**
Caloocan City	**12,459**	0	22	18	**40**	**0.32**	0	0	2	**2**
Marikina City	**7,167**	0	1	71	**72**	**1.00**	0	0	0	**0**
Pasig City	**11,385**	0	9	125	**134**	**1.18**	0	0	4	**4**
Pateros	**873**	0	0	6	**6**	**0.69**	0	0	7	**7**
Taguig	**11,981**	0	3	60	**63**	**0.53**	1	12	103	**116**
Quezon City	**43,348**	1	24	374	**399**	**0.92**	0	8	64	**72**
Makati City	**5,606**	0	2	40	**42**	**0.75**	0	1	21	**22**
Mandaluyong City	**6,814**	0	1	24	**25**	**0.37**	0	0	10	**10**
San Juan	**1,550**	0	0	16	**16**	**1.03**	0	0	0	**0**
Manila City	**44,644**	0	2	408	**410**	**0.92**	0	3	54	**57**
Las Piñas City	**6,428**	0	0	62	**62**	**0.96**	0	0	0	**0**
Muntinlupa City	**7,675**	0	0	71	**71**	**0.93**	0	0	0	**0**
Parañaque City	**7,879**	0	0	60	**60**	**0.76**	0	1	4	**5**
Pasay City	**5,286**	0	6	27	**33**	**0.62**	0	1	7	**8**
**C A R**	**24,090**	**0**	**9**	**235**	**244**	**1.01**	**1**	**43**	**878**	**922**
Abra	**2,872**	0	1	28	**29**	**1.01**	0	2	29	**31**
Apayao	**1,636**	0	0	10	**10**	**0.61**	0	1	26	**27**
Benguet	**4,048**	0	4	29	**33**	**0.82**	0	13	189	**202**
Ifugao	**2,745**	0	2	31	**33**	**1.20**	0	4	49	**53**
Kalinga	**2,646**	0	0	12	**12**	**0.45**	0	1	31	**32**
Mt. Province	**3,233**	0	1	29	**30**	**0.93**	1	10	95	**106**
Baguio City	**6,910**	0	1	96	**97**	**1.40**	0	12	459	**471**
**Region 1**	**49,615**	**0**	**12**	**255**	**267**	**0.54**	**0**	**16**	**116**	**132**
Ilocos Norte	**4,303**	0	0	4	**4**	**0.09**	0	4	17	**21**
Ilocos Sur	**4,687**	0	1	4	**5**	**0.11**	0	2	18	**20**
La Union	**4,346**	0	0	19	**19**	**0.44**	0	4	36	**40**
Pangasinan	**13,824**	0	3	25	**28**	**0.20**	0	3	13	**16**
Alaminos City	**1,320**	0	0	0	**0**	**0.00**	0	2	2	**4**
Candon City	**840**	0	0	2	**2**	**0.24**	0	0	0	**0**
Dagupan City	**8,504**	0	3	105	**108**	**1.27**	0	1	0	**1**
Laoag City	**1,963**	0	0	0	**0**	**0.00**	0	0	0	**0**
San Carlos City	**2,735**	0	5	15	**20**	**0.73**	0	0	0	**0**
San Fernando City	**3,574**	0	0	71	**71**	**1.99**	0	0	9	**9**
Urdaneta City	**1,843**	0	0	10	**10**	**0.54**	0	0	21	**21**
Vigan City	**1,676**	0	0	0	**0**	**0.00**	0	0	0	**0**

Table 2.B.2.4. Intrapartum Care and Delivery Outcome

Number and proportion of pregnancy outcome (Fetal Deaths and Abortion)
Philippines, 2022

Area	Total number of Deliveries	Fetal Death					Abortion (Counts Only)			
		Age Group			Total	%	Age Group			Total
		10-14	15-19	20-49			10-14	15-19	20-49	
**Region 2**	**39,828**	**1**	**36**	**218**	**255**	**0.64**	**1**	**50**	**714**	**765**
Batanes	**254**	0	1	1	**2**	**0.79**	0	1	12	**13**
Cagayan	**8,786**	1	13	34	**48**	**0.55**	0	2	135	**137**
Isabela	**10,651**	0	6	53	**59**	**0.55**	0	8	107	**115**
Nueva Vizcaya	**7,453**	0	9	77	**86**	**1.15**	0	37	421	**458**
Quirino	**2,614**	0	1	14	**15**	**0.57**	0	2	20	**22**
Cauayan City	**1,880**	0	1	13	**14**	**0.74**	1	0	9	**10**
Ilagan City	**3,596**	0	5	19	**24**	**0.67**	0	0	2	**2**
Santiago City	**3,030**	0	0	0	**0**	**0.00**	0	0	0	**0**
Tuguegarao City	**1,564**	0	0	7	**7**	**0.45**	0	0	8	**8**
**Region 3**	**133,760**	**2**	**36**	**414**	**452**	**0.34**	**2**	**53**	**505**	**560**
Aurora	**3,023**	0	3	20	**23**	**0.76**	0	1	10	**11**
Bataan	**4,993**	0	1	21	**22**	**0.44**	2	15	152	**169**
Bulacan	**20,098**	0	6	55	**61**	**0.30**	0	16	136	**152**
Nueva Ecija	**12,191**	0	1	9	**10**	**0.08**	0	3	18	**21**
Pampanga	**17,975**	0	3	41	**44**	**0.24**	0	4	36	**40**
Tarlac	**10,541**	0	0	7	**7**	**0.07**	0	0	0	**0**
Zambales	**8,746**	0	6	49	**55**	**0.63**	0	7	101	**108**
Angeles City	**5,738**	0	0	1	**1**	**0.02**	0	0	0	**0**
Balanga City	**1,300**	1	0	10	**11**	**0.85**	0	4	32	**36**
Cabanatuan City	**6,167**	0	3	79	**82**	**1.33**	0	1	1	**2**
City of San Fernando	**4,691**	0	0	4	**4**	**0.09**	0	0	0	**0**
Gapan City	**834**	0	0	0	**0**	**0.00**	0	0	0	**0**
Mabalacat City	**4,196**	0	0	19	**19**	**0.45**	0	0	0	**0**
Malolos City	**4,724**	0	0	1	**1**	**0.02**	0	1	9	**10**
Meycauayan	**2,236**	0	0	2	**2**	**0.09**	0	0	0	**0**
Olongapo	**3,691**	0	0	3	**3**	**0.08**	0	0	0	**0**
Palayan City	**109**	0	0	0	**0**	**0.00**	0	0	0	**0**
San Jose City	**2,115**	0	3	11	**14**	**0.66**	0	0	5	**5**
San Jose del Monte City	**8,415**	1	0	15	**16**	**0.19**	0	1	3	**4**
Science City of Munoz	**129**	0	0	0	**0**	**0.00**	0	0	0	**0**
Tarlac City	**11,848**	0	10	67	**77**	**0.65**	0	0	2	**2**
**Region 4A**	**195,187**	**4**	**39**	**420**	**463**	**0.24**	**5**	**54**	**435**	**494**
Batangas	**24,328**	0	8	110	**118**	**0.49**	0	4	67	**71**
Cavite	**19,628**	0	7	49	**56**	**0.29**	1	12	64	**77**
Laguna	**12,194**	0	0	12	**12**	**0.10**	0	3	24	**27**
Quezon	**25,230**	2	18	127	**147**	**0.58**	0	18	134	**152**
Rizal	**30,278**	0	0	19	**19**	**0.06**	0	5	25	**30**
						**0.00**				
Antipolo City	**9,873**	0	0	0	**0**	**0.00**	0	0	2	**2**
Bacoor City	**7,255**	0	0	0	**0**	**0.00**	0	0	0	**0**
Batangas City	**3,345**	0	0	7	**7**	**0.21**	0	2	35	**37**
Biñan City	**7,204**	0	0	6	**6**	**0.08**	0	0	0	**0**
Cabuyao City	**3,348**	0	0	0	**0**	**0.00**	0	0	1	**1**

Table 2.B.2.4. Intrapartum Care and Delivery Outcome

Number and proportion of pregnancy outcome (Fetal Deaths and Abortion)
Philippines, 2022

Area	Total number of Deliveries	Fetal Death					Abortion (Counts Only)			
		Age Group			Total	%	Age Group			Total
		10-14	15-19	20-49			10-14	15-19	20-49	
Calamba City	**6,653**	0	0	8	**8**	**0.12**	0	1	20	**21**
Cavite City	**1,321**	0	0	0	**0**	**0.00**	0	0	0	**0**
Dasmariñas City	**8,174**	0	0	0	**0**	**0.00**	0	0	7	**7**
General Trias City	**2,775**	0	0	0	**0**	**0.00**	0	0	1	**1**
Imus City	**4,653**	0	0	3	**3**	**0.06**	0	0	2	**2**
Lipa City	**4,649**	1	3	30	**34**	**0.73**	2	3	24	**29**
Lucena City	**3,206**	1	0	5	**6**	**0.19**	0	0	2	**2**
San Pablo City	**3,415**	0	3	20	**23**	**0.67**	1	2	10	**13**
San Pedro City	**2,970**	0	0	0	**0**	**0.00**	0	0	1	**1**
Santa Rosa City	**5,405**	0	0	14	**14**	**0.26**	1	1	3	**5**
Tagaytay City	**1,305**	0	0	0	**0**	**0.00**	0	0	0	**0**
Tanauan City	**3,459**	0	0	5	**5**	**0.14**	0	2	2	**4**
Tayabas City	**1,409**	0	0	1	**1**	**0.07**	0	0	0	**0**
Trece Martires City	**3,110**	0	0	4	**4**	**0.13**	0	1	11	**12**
**Region 4B**	**41,033**	**1**	**43**	**296**	**340**	**0.83**	**3**	**46**	**239**	**288**
Marinduque	**3,070**	0	5	25	**30**	**0.98**	1	3	27	**31**
Mindoro Occidental	**7,546**	0	10	66	**76**	**1.01**	0	8	39	**47**
Mindoro Oriental	**9,492**	0	3	28	**31**	**0.33**	2	2	16	**20**
Palawan	**12,965**	1	21	103	**125**	**0.96**	0	26	113	**139**
Romblon	**4,089**	0	2	26	**28**	**0.68**	0	6	33	**39**
Puerto Princesa City	**3,871**	0	2	48	**50**	**1.29**	0	1	11	**12**
**Region 5**	**90,156**	**1**	**107**	**735**	**843**	**0.94**	**2**	**88**	**1,027**	**1,117**
Albay	**12,749**	0	2	27	**29**	**0.23**	0	4	56	**60**
Camarines Norte	**10,484**	0	9	79	**88**	**0.84**	0	1	58	**59**
Camarines Sur	**17,803**	0	8	85	**93**	**0.52**	0	11	108	**119**
Catanduanes	**4,348**	1	5	42	**48**	**1.10**	0	2	21	**23**
Masbate	**14,257**	0	24	118	**142**	**1.00**	0	19	87	**106**
Sorsogon	**13,063**	0	19	164	**183**	**1.40**	2	47	631	**680**
Iriga City	**1,705**	0	0	16	**16**	**0.94**	0	0	4	**4**
Legaspi City	**3,426**	0	1	7	**8**	**0.23**	0	2	49	**51**
Naga City	**12,321**	0	39	197	**236**	**1.92**	0	2	13	**15**
**Region 6**	**91,080**	**1**	**70**	**535**	**606**	**0.67**	**2**	**69**	**576**	**647**
Aklan	**5,918**	0	1	31	**32**	**0.54**	0	2	72	**74**
Antique	**7,882**	0	5	92	**97**	**1.23**	0	13	68	**81**
Capiz	**7,735**	1	2	25	**28**	**0.36**	0	5	44	**49**
Guimaras	**2,248**	0	1	18	**19**	**0.85**	0	4	45	**49**
Iloilo	**22,304**	0	11	86	**97**	**0.43**	0	7	87	**94**
Negros Occidental	**33,405**	0	48	228	**276**	**0.83**	2	35	228	**265**
Bacolod City	**5,982**	0	1	38	**39**	**0.65**	0	2	11	**13**
Iloilo City	**5,606**	0	1	17	**18**	**0.32**	0	1	21	**22**

Table 2.B.2.4. Intrapartum Care and Delivery Outcome

Number and proportion of pregnancy outcome (Fetal Deaths and Abortion)
Philippines, 2022

Area	Total number of Deliveries	Fetal Death					Abortion (Counts Only)			
		Age Group			Total	%	Age Group			Total
		10-14	15-19	20-49			10-14	15-19	20-49	
**Region 7**	**133,214**	**2**	**66**	**798**	**866**	**0.65**	**0**	**41**	**397**	**438**
Bohol	**18,027**	0	11	131	**142**	**0.79**	0	6	63	**69**
Cebu	**53,485**	1	18	222	**241**	**0.45**	0	17	223	**240**
Negros Oriental	**19,600**	1	20	122	**143**	**0.73**	0	8	59	**67**
Siquijor	**1,098**	0	2	13	**15**	**1.37**	0	1	17	**18**
Cebu City	**22,881**	0	0	163	**163**	**0.71**	0	0	1	**1**
Lapu-Lapu City	**8,517**	0	1	71	**72**	**0.85**	0	1	0	**1**
Mandaue City	**9,606**	0	14	76	**90**	**0.94**	0	8	34	**42**
**Region 8**	**59,532**	**7**	**90**	**346**	**443**	**0.74**	**2**	**48**	**394**	**444**
Biliran	**3,303**	0	5	38	**43**	**1.30**	0	13	119	**132**
Eastern Samar	**7,154**	2	11	65	**78**	**1.09**	0	14	59	**73**
Northern Leyte	**13,721**	0	27	27	**54**	**0.39**	0	13	140	**153**
Northern Samar	**10,410**	5	35	83	**123**	**1.18**	2	4	58	**64**
Southern Leyte	**2,990**	0	4	10	**14**	**0.47**	0	1	10	**11**
Western Samar	**8,510**	0	1	6	**7**	**0.08**	0	0	1	**1**
Calbayog City	**2,290**	0	5	15	**20**	**0.87**	0	0	5	**5**
Maasin City	**2,390**	0	2	39	**41**	**1.72**	0	2	2	**4**
Ormoc City	**5,850**	0	0	42	**42**	**0.72**	0	0	0	**0**
Tacloban City	**2,914**	0	0	21	**21**	**0.72**	0	1	0	**1**
**Region 9**	**49,894**	**1**	**49**	**224**	**274**	**0.55**	**5**	**38**	**246**	**289**
Zamboanga del Norte	**9,771**	1	18	52	**71**	**0.73**	0	20	73	**93**
Zamboanga del Sur	**6,479**	0	6	24	**30**	**0.46**	4	5	17	**26**
Zamboanga Sibugay	**8,998**	0	12	82	**94**	**1.04**	1	9	99	**109**
Dapitan City	**1,558**	0	1	1	**2**	**0.13**	0	0	0	**0**
Dipolog City	**2,220**	0	0	11	**11**	**0.50**	0	0	12	**12**
Isabela City	**1,693**	0	2	3	**5**	**0.30**	0	0	2	**2**
Pagadian City	**2,413**	0	0	1	**1**	**0.04**	0	0	0	**0**
Zamboanga City	**16,762**	0	10	50	**60**	**0.36**	0	4	43	**47**
**Region 10**	**76,762**	**2**	**57**	**401**	**460**	**0.60**	**2**	**49**	**295**	**346**
Bukidnon	**20,001**	1	19	112	**132**	**0.66**	1	31	165	**197**
Camiguin	**1,154**	0	0	16	**16**	**1.39**	0	0	1	**1**
Lanao del Norte	**8,532**	0	2	24	**26**	**0.30**	0	0	2	**2**
Misamis Occidental	**4,066**	0	0	6	**6**	**0.15**	0	0	3	**3**
Misamis Oriental	**8,271**	0	6	10	**16**	**0.19**	0	3	14	**17**
Cagayan de Oro City	**16,044**	1	6	120	**127**	**0.79**	1	4	55	**60**
El Salvador City	**125**	0	0	0	**0**	**0.00**	0	1	7	**8**
Gingoog City	**2,109**	0	5	14	**19**	**0.90**	0	4	9	**13**
Iligan City	**7,370**	0	11	57	**68**	**0.92**	0	2	18	**20**
Malaybalay City	**3,112**	0	3	14	**17**	**0.55**	0	2	12	**14**

Table 2.B.2.4. Intrapartum Care and Delivery Outcome

Number and proportion of pregnancy outcome (Fetal Deaths and Abortion)
Philippines, 2022

Area	Total number of Deliveries	Fetal Death					Abortion (Counts Only)			
		Age Group			Total	%	Age Group			Total
		10-14	15-19	20-49			10-14	15-19	20-49	
Oroquieta City	**1,117**	0	0	9	**9**	**0.81**	0	1	4	**5**
Ozamis City	**1,955**	0	4	18	**22**	**1.13**	0	1	5	**6**
Tangub City	**953**	0	0	0	**0**	**0.00**	0	0	0	**0**
Valencia City	**1,953**	0	1	1	**2**	**0.10**	0	0	0	**0**
**Region 11**	**82,240**	**9**	**77**	**607**	**693**	**0.84**	**25**	**136**	**867**	**1,028**
Davao de Oro	**11,860**	0	10	85	**95**	**0.80**	1	26	174	**201**
Davao del Norte	**17,930**	3	20	220	**243**	**1.36**	14	37	288	**339**
Davao Oriental	**8,773**	0	10	93	**103**	**1.17**	0	6	75	**81**
Davao del Sur	**9,089**	1	5	77	**83**	**0.91**	2	24	157	**183**
Davao Occidental	**4,596**	4	21	62	**87**	**1.89**	5	24	49	**78**
Davao City	**29,992**	1	11	70	**82**	**0.27**	3	19	124	**146**
**Region 12**	**64,935**	**1**	**66**	**237**	**304**	**0.47**	**3**	**52**	**397**	**452**
North Cotabato	**19,637**	0	18	84	**102**	**0.52**	0	18	129	**147**
Sarangani	**10,342**	0	14	52	**66**	**0.64**	0	11	54	**65**
South Cotabato	**16,503**	0	21	56	**77**	**0.47**	1	16	130	**147**
Sultan Kudarat	**12,587**	1	13	37	**51**	**0.41**	2	7	84	**93**
Gen. Santos City	**5,866**	0	0	8	**8**	**0.14**	0	0	0	**0**
**BARMM**	**65,614**	**0**	**17**	**255**	**272**	**0.41**	**1**	**19**	**188**	**208**
Basilan	**3,804**	0	4	23	**27**	**0.71**	0	6	76	**82**
Lanao del Sur	**18,053**	0	0	37	**37**	**0.20**	0	0	40	**40**
Maguindanao	**19,085**	0	6	92	**98**	**0.51**	1	4	24	**29**
Sulu	**9,870**	0	2	82	**84**	**0.85**	0	6	34	**40**
Tawi-Tawi	**5,365**	0	3	13	**16**	**0.30**	0	0	10	**10**
Lamitan City	**1,366**	0	1	0	**1**	**0.07**	0	0	2	**2**
Marawi City	**4,890**	0	1	1	**2**	**0.04**	0	3	2	**5**
Cotabato City	**3,181**	0	0	7	**7**	**0.22**	0	0	0	**0**
**CARAGA**	**35,240**	**1**	**31**	**186**	**218**	**0.62**	**1**	**7**	**128**	**136**
Agusan del Norte	**3,646**	0	0	3	**3**	**0.08**	0	0	1	**1**
Agusan del Sur	**10,056**	0	12	45	**57**	**0.57**	1	3	29	**33**
Surigao del Norte	**3,060**	0	4	27	**31**	**1.01**	0	1	19	**20**
Surigao del Sur	**6,361**	0	9	52	**61**	**0.96**	0	2	38	**40**
Province of Dinagat	**913**	0	0	9	**9**	**0.99**	0	1	10	**11**
Bislig City	**1,482**	0	2	9	**11**	**0.74**	0	0	24	**24**
Butuan City	**5,989**	1	4	39	**44**	**0.73**	0	0	7	**7**
Surigao City	**3,733**	0	0	2	**2**	**0.05**	0	0	0	**0**

Note: Put asterisk (*) for No Report and Zero (0) for No Case

Table 2.B.2.5. Intrapartum Care and Delivery Outcome

Number and proportion of livebirths by birth weight
Philippines, 2022

Area	Total No. of Livebirths	NBW (≥ 2500 grams)		LBW (< 2500)		UBW	
		No.	%	No.	%	No.	%
**PHILIPPINES**	**1,413,107**	**1,291,220**	**91.37**	**88,879**	**6.29**	**33,008**	**2.34**
**N C R**	**186,955**	**163,223**	**87.31**	**22,261**	**11.91**	**1,471**	**0.79**
Malabon	**3,325**	3,220	**96.84**	75	**2.26**	30	**0.90**
Navotas	**3,975**	3,768	**94.79**	128	**3.22**	79	**1.99**
Valenzuela City	**6,488**	6,072	**93.59**	337	**5.19**	79	**1.22**
Caloocan City	**12,459**	11,500	**92.30**	957	**7.68**	2	**0.02**
Marikina City	**7,095**	6,154	**86.74**	941	**13.26**	0	**0.00**
Pasig City	**11,325**	9,561	**84.42**	1,758	**15.52**	6	**0.05**
Pateros	**874**	853	**97.60**	21	**2.40**	0	**0.00**
Taguig	**11,962**	11,477	**95.95**	207	**1.73**	278	**2.32**
Quezon City	**42,950**	35,736	**83.20**	7,132	**16.61**	82	**0.19**
Makati City	**5,565**	4,828	**86.76**	614	**11.03**	123	**2.21**
Mandaluyong City	**6,817**	6,692	**98.17**	125	**1.83**	0	**0.00**
San Juan	**1,540**	1,377	**89.42**	162	**10.52**	1	**0.06**
Manila City	**45,321**	37,472	**82.68**	7,849	**17.32**	0	**0.00**
Las Piñas City	**6,425**	6,210	**96.65**	122	**1.90**	93	**1.45**
Muntinlupa City	**7,604**	6,630	**87.19**	974	**12.81**	0	**0.00**
Parañaque City	**7,939**	6,770	**85.28**	493	**6.21**	676	**8.51**
Pasay City	**5,291**	4,903	**92.67**	366	**6.92**	22	**0.42**
**C A R**	**23,980**	**21,853**	**91.13**	**2,066**	**8.62**	**61**	**0.25**
Abra	**2,816**	2,566	**91.12**	230	**8.17**	20	**0.71**
Apayao	**1,625**	1,501	**92.37**	124	**7.63**	0	**0.00**
Benguet	**4,029**	3,672	**91.14**	341	**8.46**	16	**0.40**
Ifugao	**2,737**	2,605	**95.18**	127	**4.64**	5	**0.18**
Kalinga	**2,637**	2,548	**96.62**	88	**3.34**	1	**0.04**
Mt. Province	**3,217**	2,990	**92.94**	226	**7.03**	1	**0.03**
Baguio City	**6,919**	5,971	**86.30**	930	**13.44**	18	**0.26**
**Region 1**	**49,509**	**46,021**	**92.95**	**3,297**	**6.66**	**191**	**0.39**
Ilocos Norte	**4,303**	3,974	**92.35**	329	**7.65**	0	**0.00**
Ilocos Sur	**4,694**	4,530	**96.51**	162	**3.45**	2	**0.04**
La Union	**4,327**	4,012	**92.72**	304	**7.03**	11	**0.25**
Pangasinan	**13,810**	13,488	**97.67**	322	**2.33**	0	**0.00**
Alaminos City	**1,324**	1,317	**99.47**	7	**0.53**	0	**0.00**
Candon City	**838**	828	**98.81**	10	**1.19**	0	**0.00**
Dagupan City	**8,447**	6,971	**82.53**	1,452	**17.19**	24	**0.28**
Laoag City	**1,963**	1,963	**100.00**	0	**0.00**	0	**0.00**
San Carlos City	**2,730**	2,675	**97.99**	55	**2.01**	0	**0.00**
San Fernando City	**3,552**	3,038	**85.53**	361	**10.16**	153	**4.31**
Urdaneta City	**1,842**	1,806	**98.05**	35	**1.90**	1	**0.05**
Vigan City	**1,679**	1,419	**84.51**	260	**15.49**	0	**0.00**
**Region 2**	**39,561**	**38,005**	**96.07**	**1,499**	**3.79**	**57**	**0.14**
Batanes	**254**	238	**93.70**	16	**6.30**	0	**0.00**
Cagayan	**8,759**	8,505	**97.10**	224	**2.56**	30	**0.34**
Isabela	**10,609**	10,272	**96.82**	326	**3.07**	11	**0.10**
Nueva Vizcaya	**7,377**	6,672	**90.44**	698	**9.46**	7	**0.09**

Table 2.B.2.5. Intrapartum Care and Delivery Outcome

Number and proportion of livebirths by birth weight

Philippines, 2022

Area	Total No. of Livebirths	NBW (≥ 2500 grams)		LBW (< 2500)		UBW	
		No.	%	No.	%	No.	%
Quirino	**2,609**	2,481	**95.09**	127	**4.87**	1	**0.04**
Cauayan City	**1,877**	1,849	**98.51**	27	**1.44**	1	**0.05**
Ilagan City	**3,578**	3,533	**98.74**	38	**1.06**	7	**0.20**
Santiago City	**2,935**	2,916	**99.35**	19	**0.65**	0	**0.00**
Tuguegarao City	**1,563**	1,539	**98.46**	24	**1.54**	0	**0.00**
**Region 3**	**133,782**	**127,230**	**95.10**	**5,717**	**4.27**	**835**	**0.62**
Aurora	**3,007**	2,742	**91.19**	230	**7.65**	35	**1.16**
Bataan	**4,985**	4,784	**95.97**	183	**3.67**	18	**0.36**
Bulacan	**20,016**	19,012	**94.98**	717	**3.58**	287	**1.43**
Nueva Ecija	**12,432**	12,108	**97.39**	301	**2.42**	23	**0.19**
Pampanga	**18,077**	17,362	**96.04**	452	**2.50**	263	**1.45**
Tarlac	**10,543**	10,513	**99.72**	24	**0.23**	6	**0.06**
Zambales	**8,718**	7,758	**88.99**	881	**10.11**	79	**0.91**
Angeles City	**5,733**	5,508	**96.08**	167	**2.91**	58	**1.01**
Balanga City	**1,300**	1,213	**93.31**	87	**6.69**	0	**0.00**
Cabanatuan City	**6,111**	5,931	**97.05**	167	**2.73**	13	**0.21**
City of San Fernando	**4,691**	4,158	**88.64**	533	**11.36**	0	**0.00**
Gapan City	**833**	827	**99.28**	6	**0.72**	0	**0.00**
Mabalacat City	**4,178**	3,777	**90.40**	401	**9.60**	0	**0.00**
Malolos City	**4,736**	4,678	**98.78**	56	**1.18**	2	**0.04**
Meycauayan	**2,234**	2,214	**99.10**	3	**0.13**	17	**0.76**
Olongapo	**3,687**	3,254	**88.26**	414	**11.23**	19	**0.52**
Palayan City	**109**	103	**94.50**	0	**0.00**	6	**5.50**
San Jose City	**2,108**	2,036	**96.58**	64	**3.04**	8	**0.38**
San Jose del Monte City	**8,400**	7,810	**92.98**	590	**7.02**	0	**0.00**
Science City of Munoz	**129**	127	**98.45**	2	**1.55**	0	**0.00**
Tarlac City	**11,755**	11,315	**96.26**	439	**3.73**	1	**0.01**
**Region 4A**	**191,030**	**175,615**	**91.93**	**9,916**	**5.19**	**5,499**	**2.88**
Batangas	**23,639**	22,772	**96.33**	404	**1.71**	463	**1.96**
Cavite	**19,509**	18,007	**92.30**	952	**4.88**	550	**2.82**
Laguna	**12,133**	11,027	**90.88**	1,040	**8.57**	66	**0.54**
Quezon	**24,660**	22,610	**91.69**	1,236	**5.01**	814	**3.30**
Rizal	**28,316**	25,927	**91.56**	1,093	**3.86**	1,296	**4.58**
Antipolo City	**9,153**	7,815	**85.38**	833	**9.10**	505	**5.52**
Bacoor City	**7,278**	6,422	**88.24**	327	**4.49**	529	**7.27**
Batangas City	**3,360**	3,061	**91.10**	291	**8.66**	8	**0.24**
Biñan City	**7,198**	7,037	**97.76**	135	**1.88**	26	**0.36**
Cabuyao City	**3,371**	3,196	**94.81**	156	**4.63**	19	**0.56**
Calamba City	**6,681**	6,034	**90.32**	552	**8.26**	95	**1.42**
Cavite City	**1,334**	1,265	**94.83**	68	**5.10**	1	**0.07**
Dasmariñas City	**8,217**	7,658	**93.20**	489	**5.95**	70	**0.85**
General Trias City	**2,772**	2,580	**93.07**	149	**5.38**	43	**1.55**
Imus City	**4,638**	4,304	**92.80**	155	**3.34**	179	**3.86**
Lipa City	**4,480**	4,250	**94.87**	76	**1.70**	154	**3.44**
Lucena City	**3,263**	2,950	**90.41**	247	**7.57**	66	**2.02**
San Pablo City	**3,312**	3,022	**91.24**	211	**6.37**	79	**2.39**
San Pedro City	**3,202**	2,643	**82.54**	537	**16.77**	22	**0.69**
Santa Rosa City	**5,392**	4,559	**84.55**	486	**9.01**	347	**6.44**
Tagaytay City	**1,308**	1,298	**99.24**	10	**0.76**	0	**0.00**
Tanauan City	**3,320**	3,055	**92.02**	186	**5.60**	79	**2.38**
Tayabas City	**1,428**	1,391	**97.41**	33	**2.31**	4	**0.28**
Trece Martires City	**3,066**	2,732	**89.11**	250	**8.15**	84	**2.74**

Table 2.B.2.5. Intrapartum Care and Delivery Outcome

Number and proportion of livebirths by birth weight

Philippines, 2022

Area	Total No. of Livebirths	NBW (≥ 2500 grams)		LBW (< 2500)		UBW	
		No.	%	No.	%	No.	%
**Region 4B**	**41,186**	**37,251**	**90.45**	**1,244**	**3.02**	**2,691**	**6.53**
Marinduque	**3,050**	2,832	**92.85**	212	**6.95**	6	**0.20**
Mindoro Occidental	**7,521**	6,861	**91.22**	251	**3.34**	409	**5.44**
Mindoro Oriental	**9,685**	8,640	**89.21**	135	**1.39**	910	**9.40**
Palawan	**12,929**	11,646	**90.08**	297	**2.30**	986	**7.63**
Romblon	**4,086**	3,678	**90.01**	220	**5.38**	188	**4.60**
Puerto Princesa City	**3,915**	3,594	**91.80**	129	**3.30**	192	**4.90**
**Region 5**	**89,935**	**79,040**	**87.89**	**8,545**	**9.50**	**2,350**	**2.61**
Albay	**12,789**	11,743	**91.82**	936	**7.32**	110	**0.86**
Camarines Norte	**10,393**	8,980	**86.40**	1,405	**13.52**	8	**0.08**
Camarines Sur	**17,741**	14,962	**84.34**	1,130	**6.37**	1,649	**9.29**
Catanduanes	**4,329**	3,825	**88.36**	498	**11.50**	6	**0.14**
Masbate	**14,162**	12,773	**90.19**	848	**5.99**	541	**3.82**
Sorsogon	**12,901**	11,648	**90.29**	1,239	**9.60**	14	**0.11**
Iriga City	**1,689**	1,507	**89.22**	176	**10.42**	6	**0.36**
Legaspi City	**3,429**	3,304	**96.35**	117	**3.41**	8	**0.23**
Naga City	**12,502**	10,298	**82.37**	2,196	**17.57**	8	**0.06**
**Region 6**	**90,983**	**83,126**	**91.36**	**6,683**	**7.35**	**1,174**	**1.29**
Aklan	**5,950**	5,359	**90.07**	415	**6.97**	176	**2.96**
Antique	**7,837**	6,363	**81.19**	1,329	**16.96**	145	**1.85**
Capiz	**7,755**	7,287	**93.97**	379	**4.89**	89	**1.15**
Guimaras	**2,248**	2,016	**89.68**	232	**10.32**	0	**0.00**
Iloilo	**22,289**	20,850	**93.54**	1,317	**5.91**	122	**0.55**
Negros Occidental	**33,282**	30,555	**91.81**	2,199	**6.61**	528	**1.59**
Bacolod City	**6,007**	5,344	**88.96**	555	**9.24**	108	**1.80**
Iloilo City	**5,615**	5,352	**95.32**	257	**4.58**	6	**0.11**
**Region 7**	**133,214**	**122,508**	**91.96**	**8,832**	**6.63**	**1,874**	**1.41**
Bohol	**18,027**	15,798	**87.64**	2,223	**12.33**	6	**0.03**
Cebu	**53,485**	50,394	**94.22**	2,643	**4.94**	448	**0.84**
Negros Oriental	**19,600**	16,826	**85.85**	1,365	**6.96**	1,409	**7.19**
Siquijor	**1,098**	940	**85.61**	155	**14.12**	3	**0.27**
Cebu City	**22,881**	22,123	**96.69**	758	**3.31**	0	**0.00**
Lapu-Lapu City	**8,517**	7,825	**91.88**	684	**8.03**	8	**0.09**
Mandaue City	**9,606**	8,602	**89.55**	1,004	**10.45**	0	**0.00**
**Region 8**	**59,604**	**53,197**	**89.25**	**4,585**	**7.69**	**1,822**	**3.06**
Biliran	**3,274**	2,914	**89.00**	360	**11.00**	0	**0.00**
Eastern Samar	**7,094**	6,715	**94.66**	228	**3.21**	151	**2.13**
Northern Leyte	**14,018**	12,705	**90.63**	833	**5.94**	480	**3.42**
Northern Samar	**10,288**	9,036	**87.83**	613	**5.96**	639	**6.21**
Southern Leyte	**2,984**	2,697	**90.38**	286	**9.58**	1	**0.03**
Western Samar	**8,500**	7,433	**87.45**	847	**9.96**	220	**2.59**
Calbayog City	**2,290**	1,879	**82.05**	80	**3.49**	331	**14.45**
Maasin City	**2,382**	2,040	**85.64**	342	**14.36**	0	**0.00**
Ormoc City	**5,866**	4,891	**83.38**	975	**16.62**	0	**0.00**
Tacloban City	**2,908**	2,887	**99.28**	21	**0.72**	0	**0.00**

Table 2.B.2.5. Intrapartum Care and Delivery Outcome

Number and proportion of livebirths by birth weight

Philippines, 2022

Area	Total No. of Livebirths	NBW (≥ 2500 grams)		LBW (< 2500)		UBW	
		No.	%	No.	%	No.	%
**Region 9**	**49,149**	**45,632**	**92.84**	**1,384**	**2.82**	**2,133**	**4.34**
Zamboanga del Norte	**9,740**	8,468	**86.94**	350	**3.59**	922	**9.47**
Zamboanga del Sur	**6,475**	5,473	**84.53**	217	**3.35**	785	**12.12**
Zamboanga Sibugay	**8,019**	7,391	**92.17**	404	**5.04**	224	**2.79**
Dapitan City	**1,557**	1,512	**97.11**	45	**2.89**	0	**0.00**
Dipolog City	**2,218**	2,122	**95.67**	60	**2.71**	36	**1.62**
Isabela City	**1,694**	1,586	**93.62**	1	**0.06**	107	**6.32**
Pagadian City	**2,708**	2,646	**97.71**	59	**2.18**	3	**0.11**
Zamboanga City	**16,738**	16,434	**98.18**	248	**1.48**	56	**0.33**
**Region 10**	**76,401**	**70,941**	**92.85**	**3,712**	**4.86**	**1,748**	**2.29**
Bukidnon	**19,904**	18,804	**94.47**	631	**3.17**	469	**2.36**
Camiguin	**1,143**	1,103	**96.50**	40	**3.50**	0	**0.00**
Lanao del Norte	**8,508**	7,091	**83.35**	344	**4.04**	1,073	**12.61**
Misamis Occidental	**4,064**	4,015	**98.79**	30	**0.74**	19	**0.47**
Misamis Oriental	**8,260**	7,854	**95.08**	288	**3.49**	118	**1.43**
Cagayan de Oro City	**15,917**	14,008	**88.01**	1,909	**11.99**	0	**0.00**
El Salvador City	**125**	123	**98.40**	1	**0.80**	1	**0.80**
Gingoog City	**2,084**	2,036	**97.70**	30	**1.44**	18	**0.86**
Iligan City	**7,317**	7,169	**97.98**	142	**1.94**	6	**0.08**
Malaybalay City	**3,114**	2,971	**95.41**	113	**3.63**	30	**0.96**
Oroquieta City	**1,116**	1,019	**91.31**	97	**8.69**	0	**0.00**
Ozamis City	**1,938**	1,913	**98.71**	25	**1.29**	0	**0.00**
Tangub City	**953**	929	**97.48**	24	**2.52**	0	**0.00**
Valencia City	**1,958**	1,906	**97.34**	38	**1.94**	14	**0.72**
**Region 11**	**82,907**	**74,432**	**89.78**	**4,839**	**5.84**	**3,636**	**4.39**
Davao de Oro	**11,820**	10,977	**92.87**	694	**5.87**	149	**1.26**
Davao del Norte	**18,059**	16,389	**90.75**	1,163	**6.44**	507	**2.81**
Davao Oriental	**8,835**	8,165	**92.42**	511	**5.78**	159	**1.80**
Davao del Sur	**9,217**	8,263	**89.65**	499	**5.41**	455	**4.94**
Davao Occidental	**4,576**	3,440	**75.17**	119	**2.60**	1,017	**22.22**
Davao City	**30,400**	27,198	**89.47**	1,853	**6.10**	1,349	**4.44**
**Region 12**	**64,775**	**60,305**	**93.10**	**1,994**	**3.08**	**2,476**	**3.82**
North Cotabato	**19,628**	17,295	**88.11**	622	**3.17**	1,711	**8.72**
Sarangani	**10,324**	9,924	**96.13**	236	**2.29**	164	**1.59**
South Cotabato	**16,426**	15,624	**95.12**	519	**3.16**	283	**1.72**
Sultan Kudarat	**12,536**	11,820	**94.29**	463	**3.69**	253	**2.02**
Gen. Santos City	**5,861**	5,642	**96.26**	154	**2.63**	65	**1.11**
**BARMM**	**65,177**	**60,425**	**92.71**	**814**	**1.25**	**3,938**	**6.04**
Basilan	**3,807**	2,873	**75.47**	188	**4.94**	746	**19.60**
Lanao del Sur	**18,045**	17,179	**95.20**	41	**0.23**	825	**4.57**
Maguindanao	**19,033**	17,515	**92.02**	356	**1.87**	1,162	**6.11**
Sulu	**9,614**	8,750	**91.01**	90	**0.94**	774	**8.05**
Tawi-Tawi	**5,208**	4,992	**95.85**	53	**1.02**	163	**3.13**
Lamitan City	**1,366**	1,331	**97.44**	20	**1.46**	15	**1.10**
Marawi City	**4,923**	4,735	**96.18**	30	**0.61**	158	**3.21**
Cotabato City	**3,181**	3,050	**95.88**	36	**1.13**	95	**2.99**

Table 2.B.2.5. Intrapartum Care and Delivery Outcome

Number and proportion of livebirths by birth weight

Philippines, 2022

Area	Total No. of Livebirths	NBW (≥ 2500 grams)		LBW (< 2500)		UBW	
		No.	%	No.	%	No.	%
**CARAGA**	**34,959**	**32,416**	**92.73**	**1,491**	**4.26**	**1,052**	**3.01**
Agusan del Norte	**3,505**	3,081	**87.90**	212	**6.05**	212	**6.05**
Agusan del Sur	**10,021**	9,498	**94.78**	202	**2.02**	321	**3.20**
Surigao del Norte	**3,039**	2,689	**88.48**	194	**6.38**	156	**5.13**
Surigao del Sur	**6,314**	5,687	**90.07**	318	**5.04**	309	**4.89**
Province of Dinagat	**912**	809	**88.71**	91	**9.98**	12	**1.32**
Bislig City	**1,477**	1,444	**97.77**	32	**2.17**	1	**0.07**
Butuan City	**5,958**	5,723	**96.06**	202	**3.39**	33	**0.55**
Surigao City	**3,733**	3,485	**93.36**	240	**6.43**	8	**0.21**

Live births should be reported by place of occurrence.

NBW - Normal Birth Weight

LBW - Low Birth Weight

UBW - Unknown Birth Weight

Table 2.B.3.1. Postpartum and Newborn Care

Number and proportion of Postpartum women together with their neeborn who completed at least 2 postpartum check-ups
Philippines, 2022

Area	Total Deliveries	Completed at least 2 postpartum check-ups							
		Age Group in Year						Total	%
		10 - 14 yrs old		15-19 yrs old		20-49 yrs old			
		No.	%	No.	%	No.	%		
**PHILIPPINES**	**1,419,070**	**2,613**	**0.18**	**131,433**	**9.26**	**1,124,573**	**79.25**	**1,258,619**	**88.69**
**N C R**	**186,890**	**179**	**0.10**	**12,236**	**6.55**	**136,061**	**72.80**	**148,476**	**79.45**
Malabon	**3,327**	6	**0.18**	389	**11.69**	2,926	**87.95**	**3,321**	**99.82**
Navotas	**3,980**	3	**0.08**	415	**10.43**	3,257	**81.83**	**3,675**	**92.34**
Valenzuela City	**6,488**	8	**0.12**	502	**7.74**	5,310	**81.84**	**5,820**	**89.70**
Caloocan City	**12,459**	15	**0.12**	1,692	**13.58**	10,752	**86.30**	**12,459**	**100.00**
Marikina City	**7,167**	1	**0.01**	148	**2.07**	2,427	**33.86**	**2,576**	**35.94**
Pasig City	**11,385**	11	**0.10**	739	**6.49**	8,936	**78.49**	**9,686**	**85.08**
Pateros	**873**	1	**0.11**	57	**6.53**	814	**93.24**	**872**	**99.89**
Taguig	**11,981**	25	**0.21**	1,080	**9.01**	10,835	**90.43**	**11,940**	**99.66**
Quezon City	**43,348**	47	**0.11**	2,417	**5.58**	40,883	**94.31**	**43,347**	**100.00**
Makati City	**5,606**	7	**0.12**	221	**3.94**	3,453	**61.59**	**3,681**	**65.66**
Mandaluyong City	**6,814**	4	**0.06**	384	**5.64**	6,339	**93.03**	**6,727**	**98.72**
San Juan	**1,550**	0	**0.00**	45	**2.90**	859	**55.42**	**904**	**58.32**
Manila City	**44,644**	19	**0.04**	1,864	**4.18**	15,579	**34.90**	**17,462**	**39.11**
Las Piñas City	**6,428**	8	**0.12**	575	**8.95**	5,626	**87.52**	**6,209**	**96.59**
Muntinlupa City	**7,675**	17	**0.22**	592	**7.71**	7,066	**92.07**	**7,675**	**100.00**
Parañaque City	**7,879**	6	**0.08**	763	**9.68**	6,103	**77.46**	**6,872**	**87.22**
Pasay City	**5,286**	1	**0.02**	353	**6.68**	4,896	**92.62**	**5,250**	**99.32**
**C A R**	**24,090**	**35**	**0.15**	**1,716**	**7.12**	**17,543**	**72.82**	**19,294**	**80.09**
Abra	**2,872**	3	**0.10**	297	**10.34**	2,163	**75.31**	**2,463**	**85.76**
Apayao	**1,636**	12	**0.73**	237	**14.49**	1,049	**64.12**	**1,298**	**79.34**
Benguet	**4,048**	3	**0.07**	222	**5.48**	3,418	**84.44**	**3,643**	**90.00**
Ifugao	**2,745**	6	**0.22**	221	**8.05**	2,131	**77.63**	**2,358**	**85.90**
Kalinga	**2,646**	0	**0.00**	217	**8.20**	2,410	**91.08**	**2,627**	**99.28**
Mt. Province	**3,233**	9	**0.28**	166	**5.13**	1,952	**60.38**	**2,127**	**65.79**
Baguio City	**6,910**	2	**0.03**	356	**5.15**	4,420	**63.97**	**4,778**	**69.15**
**Region 1**	**49,615**	**72**	**0.15**	**4,095**	**8.25**	**47,109**	**94.95**	**51,276**	**103.35**
Ilocos Norte	**4,303**	6	**0.14**	271	**6.30**	3,653	**84.89**	**3,930**	**91.33**
Ilocos Sur	**4,687**	12	**0.26**	436	**9.30**	6,159	**131.41**	**6,607**	**140.96**
La Union	**4,346**	18	**0.41**	691	**15.90**	6,618	**152.28**	**7,327**	**168.59**
Pangasinan	**13,824**	25	**0.18**	1,677	**12.13**	19,484	**140.94**	**21,186**	**153.26**
Alaminos City	**1,320**	0	**0.00**	64	**4.85**	904	**68.48**	**968**	**73.33**
Candon City	**840**	0	**0.00**	2	**0.24**	836	**99.52**	**838**	**99.76**
Dagupan City	**8,504**	2	**0.02**	245	**2.88**	1,986	**23.35**	**2,233**	**26.26**
Laoag City	**1,963**	0	**0.00**	12	**0.61**	1,932	**98.42**	**1,944**	**99.03**
San Carlos City	**2,735**	2	**0.07**	216	**7.90**	2,352	**86.00**	**2,570**	**93.97**
San Fernando City	**3,574**	3	**0.08**	296	**8.28**	1,451	**40.60**	**1,750**	**48.96**
Urdaneta City	**1,843**	3	**0.16**	150	**8.14**	1,278	**69.34**	**1,431**	**77.65**
Vigan City	**1,676**	1	**0.06**	35	**2.09**	456	**27.21**	**492**	**29.36**
**Region 2**	**39,828**	**128**	**0.32**	**4,804**	**12.06**	**37,167**	**93.32**	**42,099**	**105.70**
Batanes	**254**	0	**0.00**	12	**4.72**	239	**94.09**	**251**	**98.82**
Cagayan	**8,786**	68	**0.77**	1,366	**15.55**	9,541	**108.59**	**10,975**	**124.91**
Isabela	**10,651**	36	**0.34**	1,674	**15.72**	12,978	**121.85**	**14,688**	**137.90**
Nueva Vizcaya	**7,453**	13	**0.17**	779	**10.45**	5,095	**68.36**	**5,887**	**78.99**
Quirino	**2,614**	2	**0.08**	142	**5.43**	2,470	**94.49**	**2,614**	**100.00**

Table 2.B.3.1. Postpartum and Newborn Care

Number and proportion of Postpartum women together with their neeborn who completed at least 2 postpartum check-ups
Philippines, 2022

Area	Total Deliveries	Completed at least 2 postpartum check-ups							
		Age Group in Year						Total	%
		10 - 14 yrs old		15-19 yrs old		20-49 yrs old			
		No.	%	No.	%	No.	%		
Cauayan City	**1,880**	2	**0.11**	234	**12.45**	1,457	**77.50**	**1,693**	**90.05**
Ilagan City	**3,596**	2	**0.06**	210	**5.84**	1,333	**37.07**	**1,545**	**42.96**
Santiago City	**3,030**	4	**0.13**	276	**9.11**	2,642	**87.19**	**2,922**	**96.44**
Tuguegarao City	**1,564**	1	**0.06**	111	**7.10**	1,412	**90.28**	**1,524**	**97.44**
	**0**								
**Region 3**	**133,760**	**329**	**0.25**	**15,132**	**11.31**	**130,833**	**97.81**	**146,294**	**109.37**
Aurora	**3,023**	2	**0.07**	297	**9.82**	2,285	**75.59**	**2,584**	**85.48**
Bataan	**4,993**	24	**0.48**	907	**18.17**	6,901	**138.21**	**7,832**	**156.86**
Bulacan	**20,098**	54	**0.27**	3,011	**14.98**	26,424	**131.48**	**29,489**	**146.73**
Nueva Ecija	**12,191**	32	**0.26**	1,557	**12.77**	11,404	**93.54**	**12,993**	**106.58**
Pampanga	**17,975**	62	**0.34**	1,912	**10.64**	17,443	**97.04**	**19,417**	**108.02**
Tarlac	**10,541**	35	**0.33**	1,263	**11.98**	11,937	**113.24**	**13,235**	**125.56**
Zambales	**8,746**	14	**0.16**	555	**6.35**	4,934	**56.41**	**5,503**	**62.92**
Angeles City	**5,738**	13	**0.23**	695	**12.11**	7,735	**134.80**	**8,443**	**147.14**
Balanga City	**1,300**	6	**0.46**	167	**12.85**	1,102	**84.77**	**1,275**	**98.08**
Cabanatuan City	**6,167**	11	**0.18**	458	**7.43**	4,110	**66.65**	**4,579**	**74.25**
City of San Fernando	**4,691**	11	**0.23**	484	**10.32**	3,454	**73.63**	**3,949**	**84.18**
Gapan City	**834**	0	**0.00**	91	**10.91**	694	**83.21**	**785**	**94.12**
Mabalacat City	**4,196**	15	**0.36**	561	**13.37**	4,697	**111.94**	**5,273**	**125.67**
Malolos City	**4,724**	8	**0.17**	246	**5.21**	2,397	**50.74**	**2,651**	**56.12**
Meycauayan	**2,236**	0	**0.00**	276	**12.34**	2,667	**119.28**	**2,943**	**131.62**
Olongapo	**3,691**	6	**0.16**	159	**4.31**	2,785	**75.45**	**2,950**	**79.92**
Palayan City	**109**	0	**0.00**	24	**22.02**	171	**156.88**	**195**	**178.90**
San Jose City	**2,115**	8	**0.38**	304	**14.37**	1,715	**81.09**	**2,027**	**95.84**
San Jose del Monte City	**8,415**	18	**0.21**	1,107	**13.16**	11,921	**141.66**	**13,046**	**155.03**
Science City of Munoz	**129**	4	**3.10**	91	**70.54**	688	**533.33**	**783**	**606.98**
Tarlac City	**11,848**	6	**0.05**	967	**8.16**	5,369	**45.32**	**6,342**	**53.53**
**Region 4A**	**195,187**	**252**	**0.13**	**15,113**	**7.74**	**150,530**	**77.12**	**165,895**	**84.99**
Batangas	**24,328**	37	**0.15**	1,985	**8.16**	20,832	**85.63**	**22,854**	**93.94**
Cavite	**19,628**	27	**0.14**	1,217	**6.20**	15,384	**78.38**	**16,628**	**84.72**
Laguna	**12,194**	18	**0.15**	1,144	**9.38**	8,914	**73.10**	**10,076**	**82.63**
Quezon	**25,230**	56	**0.22**	2,442	**9.68**	18,802	**74.52**	**21,300**	**84.42**
Rizal	**30,278**	28	**0.09**	2,475	**8.17**	21,701	**71.67**	**24,204**	**79.94**
Antipolo City	**9,873**	14	**0.14**	666	**6.75**	6,797	**68.84**	**7,477**	**75.73**
Bacoor City	**7,255**	1	**0.01**	268	**3.69**	3,409	**46.99**	**3,678**	**50.70**
Batangas City	**3,345**	6	**0.18**	343	**10.25**	3,105	**92.83**	**3,454**	**103.26**
Biñan City	**7,204**	3	**0.04**	673	**9.34**	6,024	**83.62**	**6,700**	**93.00**
Cabuyao City	**3,348**	1	**0.03**	299	**8.93**	4,241	**126.67**	**4,541**	**135.63**
Calamba City	**6,653**	6	**0.09**	588	**8.84**	6,171	**92.76**	**6,765**	**101.68**
Cavite City	**1,321**	2	**0.15**	85	**6.43**	652	**49.36**	**739**	**55.94**
Dasmariñas City	**8,174**	5	**0.06**	545	**6.67**	6,703	**82.00**	**7,253**	**88.73**
General Trias City	**2,775**	12	**0.43**	198	**7.14**	2,045	**73.69**	**2,255**	**81.26**
Imus City	**4,653**	5	**0.11**	119	**2.56**	2,502	**53.77**	**2,626**	**56.44**
Lipa City	**4,649**	3	**0.06**	268	**5.76**	4,080	**87.76**	**4,351**	**93.59**
Lucena City	**3,206**	2	**0.06**	332	**10.36**	2,557	**79.76**	**2,891**	**90.17**
San Pablo City	**3,415**	11	**0.32**	373	**10.92**	2,767	**81.02**	**3,151**	**92.27**
San Pedro City	**2,970**	3	**0.10**	217	**7.31**	2,191	**73.77**	**2,411**	**81.18**
Santa Rosa City	**5,405**	4	**0.07**	428	**7.92**	4,865	**90.01**	**5,297**	**98.00**
Tagaytay City	**1,305**	1	**0.08**	65	**4.98**	1,233	**94.48**	**1,299**	**99.54**
Tanauan City	**3,459**	4	**0.12**	60	**1.73**	1,912	**55.28**	**1,976**	**57.13**
Tayabas City	**1,409**	1	**0.07**	144	**10.22**	1,224	**86.87**	**1,369**	**97.16**
Trece Martires City	**3,110**	2	**0.06**	179	**5.76**	2,419	**77.78**	**2,600**	**83.60**

Table 2.B.3.1. Postpartum and Newborn Care

Number and proportion of Postpartum women together with their neeborn who completed at least 2 postpartum check-ups
Philippines, 2022

Area	Total Deliveries	Completed at least 2 postpartum check-ups							
		Age Group in Year						Total	%
		10 - 14 yrs old		15-19 yrs old		20-49 yrs old			
		No.	%	No.	%	No.	%		
**Region 4B**	**41,033**	**58**	**0.14**	**4,750**	**11.58**	**34,129**	**83.17**	**38,937**	**94.89**
Marinduque	**3,070**	7	**0.23**	308	**10.03**	2,697	**87.85**	**3,012**	**98.11**
Mindoro Occidental	**7,546**	7	**0.09**	1,014	**13.44**	6,466	**85.69**	**7,487**	**99.22**
Mindoro Oriental	**9,492**	11	**0.12**	890	**9.38**	8,442	**88.94**	**9,343**	**98.43**
Palawan	**12,965**	16	**0.12**	1,806	**13.93**	10,313	**79.54**	**12,135**	**93.60**
Romblon	**4,089**	7	**0.17**	361	**8.83**	3,389	**82.88**	**3,757**	**91.88**
Puerto Princesa City	**3,871**	10	**0.26**	371	**9.58**	2,822	**72.90**	**3,203**	**82.74**
**Region 5**	**90,156**	**104**	**0.12**	**6,955**	**7.71**	**65,119**	**72.23**	**72,178**	**80.06**
Albay	**12,749**	12	**0.09**	676	**5.30**	9,671	**75.86**	**10,359**	**81.25**
Camarines Norte	**10,484**	11	**0.10**	914	**8.72**	7,044	**67.19**	**7,969**	**76.01**
Camarines Sur	**17,803**	26	**0.15**	1,525	**8.57**	16,466	**92.49**	**18,017**	**101.20**
Catanduanes	**4,348**	6	**0.14**	390	**8.97**	3,317	**76.29**	**3,713**	**85.40**
Masbate	**14,257**	22	**0.15**	1,892	**13.27**	11,753	**82.44**	**13,667**	**95.86**
Sorsogon	**13,063**	10	**0.08**	1,073	**8.21**	10,668	**81.67**	**11,751**	**89.96**
Iriga City	**1,705**	2	**0.12**	101	**5.92**	1,071	**62.82**	**1,174**	**68.86**
Legaspi City	**3,426**	2	**0.06**	124	**3.62**	2,181	**63.66**	**2,307**	**67.34**
Naga City	**12,321**	13	**0.11**	260	**2.11**	2,948	**23.93**	**3,221**	**26.14**
**Region 6**	**91,080**	**189**	**0.21**	**8,609**	**9.45**	**72,014**	**79.07**	**80,812**	**88.73**
Aklan	**5,918**	7	**0.12**	480	**8.11**	4,978	**84.12**	**5,465**	**92.35**
Antique	**7,882**	8	**0.10**	602	**7.64**	5,829	**73.95**	**6,439**	**81.69**
Capiz	**7,735**	13	**0.17**	506	**6.54**	5,541	**71.64**	**6,060**	**78.35**
Guimaras	**2,248**	5	**0.22**	221	**9.83**	1,943	**86.43**	**2,169**	**96.49**
Iloilo	**22,304**	42	**0.19**	1,708	**7.66**	17,782	**79.73**	**19,532**	**87.57**
Negros Occidental	**33,405**	74	**0.22**	3,924	**11.75**	26,968	**80.73**	**30,966**	**92.70**
Bacolod City	**5,982**	29	**0.48**	669	**11.18**	4,066	**67.97**	**4,764**	**79.64**
Iloilo City	**5,606**	11	**0.20**	499	**8.90**	4,907	**87.53**	**5,417**	**96.63**
**Region 7**	**133,214**	**147**	**0.11**	**9,566**	**7.18**	**92,422**	**69.38**	**102,135**	**76.67**
Bohol	**18,027**	14	**0.08**	1,260	**6.99**	13,494	**74.85**	**14,768**	**81.92**
Cebu	**53,485**	60	**0.11**	3,674	**6.87**	34,080	**63.72**	**37,814**	**70.70**
Negros Oriental	**19,600**	31	**0.16**	1,877	**9.58**	14,301	**72.96**	**16,209**	**82.70**
Siquijor	**1,098**	2	**0.18**	97	**8.83**	866	**78.87**	**965**	**87.89**
Cebu City	**22,881**	27	**0.12**	1,594	**6.97**	16,019	**70.01**	**17,640**	**77.09**
Lapu-Lapu City	**8,517**	5	**0.06**	478	**5.61**	6,876	**80.73**	**7,359**	**86.40**
Mandaue City	**9,606**	8	**0.08**	586	**6.10**	6,786	**70.64**	**7,380**	**76.83**
**Region 8**	**59,532**	**76**	**0.13**	**5,021**	**8.43**	**43,637**	**73.30**	**48,734**	**81.86**
Biliran	**3,303**	8	**0.24**	359	**10.87**	2,623	**79.41**	**2,990**	**90.52**
Eastern Samar	**7,154**	13	**0.18**	721	**10.08**	6,183	**86.43**	**6,917**	**96.69**
Northern Leyte	**13,721**	24	**0.17**	1,233	**8.99**	12,178	**88.75**	**13,435**	**97.92**
Northern Samar	**10,410**	7	**0.07**	753	**7.23**	6,699	**64.35**	**7,459**	**71.65**
Southern Leyte	**2,990**	7	**0.23**	345	**11.54**	2,638	**88.23**	**2,990**	**100.00**
Western Samar	**8,510**	3	**0.04**	693	**8.14**	5,324	**62.56**	**6,020**	**70.74**
Calbayog City	**2,290**	1	**0.04**	229	**10.00**	1,604	**70.04**	**1,834**	**80.09**
Maasin City	**2,390**	1	**0.04**	58	**2.43**	707	**29.58**	**766**	**32.05**

Table 2.B.3.1. Postpartum and Newborn Care

Number and proportion of Postpartum women together with their neeborn who completed at least 2 postpartum check-ups
Philippines, 2022

Area	Total Deliveries	Completed at least 2 postpartum check-ups							
		Age Group in Year						Total	%
		10 - 14 yrs old		15-19 yrs old		20-49 yrs old			
		No.	%	No.	%	No.	%		
Ormoc City	**5,850**	8	**0.14**	431	**7.37**	3,014	**51.52**	**3,453**	**59.03**
Tacloban City	**2,914**	4	**0.14**	199	**6.83**	2,667	**91.52**	**2,870**	**98.49**
**Region 9**	**49,894**	**90**	**0.18**	**5,645**	**11.31**	**39,461**	**79.09**	**45,196**	**90.58**
Zamboanga del Norte	**9,771**	17	**0.17**	1,222	**12.51**	7,272	**74.42**	**8,511**	**87.10**
Zamboanga del Sur	**6,479**	21	**0.32**	753	**11.62**	4,996	**77.11**	**5,770**	**89.06**
Zamboanga Sibugay	**8,998**	9	**0.10**	1,007	**11.19**	6,606	**73.42**	**7,622**	**84.71**
Dapitan City	**1,558**	1	**0.06**	153	**9.82**	1,351	**86.71**	**1,505**	**96.60**
Dipolog City	**2,220**	7	**0.32**	275	**12.39**	1,921	**86.53**	**2,203**	**99.23**
Isabela City	**1,693**	4	**0.24**	95	**5.61**	737	**43.53**	**836**	**49.38**
Pagadian City	**2,413**	2	**0.08**	229	**9.49**	2,177	**90.22**	**2,408**	**99.79**
Zamboanga City	**16,762**	29	**0.17**	1,911	**11.40**	14,401	**85.91**	**16,341**	**97.49**
**Region 10**	**76,762**	**204**	**0.27**	**9,647**	**12.57**	**60,633**	**78.99**	**70,484**	**91.82**
Bukidnon	**20,001**	77	**0.38**	3,063	**15.31**	13,796	**68.98**	**16,936**	**84.68**
Camiguin	**1,154**	2	**0.17**	129	**11.18**	990	**85.79**	**1,121**	**97.14**
Lanao del Norte	**8,532**	20	**0.23**	682	**7.99**	7,244	**84.90**	**7,946**	**93.13**
Misamis Occidental	**4,066**	13	**0.32**	254	**6.25**	3,590	**88.29**	**3,857**	**94.86**
Misamis Oriental	**8,271**	35	**0.42**	1,483	**17.93**	8,677	**104.91**	**10,195**	**123.26**
Cagayan de Oro City	**16,044**	21	**0.13**	1,527	**9.52**	9,421	**58.72**	**10,969**	**68.37**
El Salvador City	**125**	3	**2.40**	105	**84.00**	668	**534.40**	**776**	**620.80**
Gingoog City	**2,109**	0	**0.00**	343	**16.26**	1,730	**82.03**	**2,073**	**98.29**
Iligan City	**7,370**	4	**0.05**	782	**10.61**	6,286	**85.29**	**7,072**	**95.96**
Malaybalay City	**3,112**	4	**0.13**	476	**15.30**	2,615	**84.03**	**3,095**	**99.45**
Oroquieta City	**1,117**	0	**0.00**	133	**11.91**	984	**88.09**	**1,117**	**100.00**
Ozamis City	**1,955**	1	**0.05**	238	**12.17**	1,694	**86.65**	**1,933**	**98.87**
Tangub City	**953**	0	**0.00**	73	**7.66**	877	**92.03**	**950**	**99.69**
Valencia City	**1,953**	24	**1.23**	359	**18.38**	2,061	**105.53**	**2,444**	**125.14**
**Region 11**	**82,240**	**446**	**0.54**	**10,608**	**12.90**	**65,316**	**79.42**	**76,370**	**92.86**
Davao de Oro	**11,860**	122	**1.03**	1,916	**16.16**	9,482	**79.95**	**11,520**	**97.13**
Davao del Norte	**17,930**	121	**0.67**	2,411	**13.45**	14,527	**81.02**	**17,059**	**95.14**
Davao Oriental	**8,773**	47	**0.54**	1,288	**14.68**	7,141	**81.40**	**8,476**	**96.61**
Davao del Sur	**9,089**	54	**0.59**	1,320	**14.52**	6,990	**76.91**	**8,364**	**92.02**
Davao Occidental	**4,596**	36	**0.78**	806	**17.54**	2,267	**49.33**	**3,109**	**67.65**
Davao City	**29,992**	66	**0.22**	2,867	**9.56**	24,909	**83.05**	**27,842**	**92.83**
**Region 12**	**64,935**	**209**	**0.32**	**9,080**	**13.98**	**53,139**	**81.83**	**62,428**	**96.14**
North Cotabato	**19,637**	42	**0.21**	2,508	**12.77**	15,805	**80.49**	**18,355**	**93.47**
Sarangani	**10,342**	36	**0.35**	1,918	**18.55**	8,253	**79.80**	**10,207**	**98.69**
South Cotabato	**16,503**	59	**0.36**	2,371	**14.37**	13,771	**83.45**	**16,201**	**98.17**
Sultan Kudarat	**12,587**	66	**0.52**	1,816	**14.43**	10,437	**82.92**	**12,319**	**97.87**
Gen. Santos City	**5,866**	6	**0.10**	467	**7.96**	4,873	**83.07**	**5,346**	**91.14**
**BARMM**	**65,614**	**41**	**0.06**	**5,194**	**7.92**	**54,077**	**82.42**	**59,312**	**90.40**
Basilan	**3,804**	9	**0.24**	343	**9.02**	2,206	**57.99**	**2,558**	**67.25**
Lanao del Sur	**18,053**	7	**0.04**	874	**4.84**	16,278	**90.17**	**17,159**	**95.05**
Maguindanao	**19,085**	14	**0.07**	1,825	**9.56**	16,443	**86.16**	**18,282**	**95.79**
Sulu	**9,870**	3	**0.03**	574	**5.82**	7,327	**74.24**	**7,904**	**80.08**

Table 2.B.3.1. Postpartum and Newborn Care

Number and proportion of Postpartum women together with their neeborn who completed at least 2 postpartum check-ups
Philippines, 2022

Area	Total Deliveries	Completed at least 2 postpartum check-ups							
		Age Group in Year						Total	%
		10 - 14 yrs old		15-19 yrs old		20-49 yrs old			
		No.	%	No.	%	No.	%		
Tawi-Tawi	**5,365**	0	**0.00**	373	**6.95**	4,608	**85.89**	**4,981**	**92.84**
Lamitan City	**1,366**	7	**0.51**	232	**16.98**	1,134	**83.02**	**1,373**	**100.51**
Marawi City	**4,890**	0	**0.00**	736	**15.05**	3,486	**71.29**	**4,222**	**86.34**
Cotabato City	**3,181**	1	**0.03**	237	**7.45**	2,595	**81.58**	**2,833**	**89.06**
**CARAGA**	**35,240**	**54**	**0.15**	**3,262**	**9.26**	**25,383**	**72.03**	**28,699**	**81.44**
Agusan del Norte	**3,646**	4	**0.11**	315	**8.64**	2,430	**66.65**	**2,749**	**75.40**
Agusan del Sur	**10,056**	17	**0.17**	964	**9.59**	7,686	**76.43**	**8,667**	**86.19**
Surigao del Norte	**3,060**	8	**0.26**	298	**9.74**	2,656	**86.80**	**2,962**	**96.80**
Surigao del Sur	**6,361**	10	**0.16**	656	**10.31**	4,673	**73.46**	**5,339**	**83.93**
Province of Dinagat	**913**	0	**0.00**	90	**9.86**	665	**72.84**	**755**	**82.69**
Bislig City	**1,482**	3	**0.20**	193	**13.02**	1,269	**85.63**	**1,465**	**98.85**
Butuan City	**5,989**	8	**0.13**	539	**9.00**	4,925	**82.23**	**5,472**	**91.37**
Surigao City	**3,733**	4	**0.11**	207	**5.55**	1,079	**28.90**	**1,290**	**34.56**

Table 2.B.3.2. Postpartum and Newborn Care

Number and proportion of postpartum women who completed Iron with Folic Acid
Philippines, 2022

Area	Eligible Pop.	Iron with Folic Supplementation							
		Age Group in Year						Total	%
		10-14		15-19		20-49			
		No.	%	No.	%	No.	%		
**PHILIPPINES**	**2,131,496**	**2,319**	**0.11**	**117,327**	**5.50**	**1,019,405**	**47.83**	**1,139,051**	**53.44**
**N C R**	**236,901**	**170**	**0.07**	**10,995**	**4.64**	**125,647**	**53.04**	**136,812**	**57.75**
Malabon	**6,724**	12	**0.18**	490	**7.29**	3,560	**52.94**	**4,062**	**60.41**
Navotas	**4,591**	8	**0.17**	437	**9.52**	3,168	**69.00**	**3,613**	**78.70**
Valenzuela City	**11,418**	7	**0.06**	490	**4.29**	6,381	**55.89**	**6,878**	**60.24**
Caloocan City	**29,146**	13	**0.04**	1,549	**5.31**	17,557	**60.24**	**19,119**	**65.60**
Marikina City	**8,294**	1	**0.01**	197	**2.38**	3,183	**38.38**	**3,381**	**40.76**
Pasig City	**13,894**	11	**0.08**	622	**4.48**	5,535	**39.84**	**6,168**	**44.39**
Pateros	**1,176**	1	**0.09**	50	**4.25**	794	**67.52**	**845**	**71.85**
Taguig	**14,807**	27	**0.18**	1,120	**7.56**	11,226	**75.82**	**12,373**	**83.56**
Quezon City	**54,011**	39	**0.07**	1,497	**2.77**	25,476	**47.17**	**27,012**	**50.01**
Makati City	**10,718**	3	**0.03**	228	**2.13**	3,638	**33.94**	**3,869**	**36.10**
Mandaluyong City	**7,111**	4	**0.06**	346	**4.87**	5,912	**83.14**	**6,262**	**88.06**
San Juan	**2,246**	0	**0.00**	43	**1.91**	862	**38.38**	**905**	**40.29**
Manila City	**32,743**	18	**0.05**	1,836	**5.61**	14,936	**45.62**	**16,790**	**51.28**
Las Piñas City	**10,832**	4	**0.04**	567	**5.23**	5,335	**49.25**	**5,906**	**54.52**
Muntinlupa City	**9,281**	15	**0.16**	611	**6.58**	7,387	**79.59**	**8,013**	**86.34**
Parañaque City	**12,249**	6	**0.05**	558	**4.56**	5,806	**47.40**	**6,370**	**52.00**
Pasay City	**7,660**	1	**0.01**	354	**4.62**	4,891	**63.85**	**5,246**	**68.49**
**C A R**	**35,179**	**33**	**0.09**	**1,529**	**4.35**	**17,856**	**50.76**	**19,418**	**55.20**
Abra	**4,275**	10	**0.23**	278	**6.50**	1,946	**45.52**	**2,234**	**52.26**
Apayao	**2,502**	6	**0.24**	223	**8.91**	1,360	**54.36**	**1,589**	**63.51**
Benguet	**9,086**	5	**0.06**	324	**3.57**	4,926	**54.22**	**5,255**	**57.84**
Ifugao	**4,486**	3	**0.07**	126	**2.81**	1,896	**42.26**	**2,025**	**45.14**
Kalinga	**4,701**	1	**0.02**	244	**5.19**	2,547	**54.18**	**2,792**	**59.39**
Mt. Province	**3,103**	6	**0.19**	115	**3.71**	1,545	**49.79**	**1,666**	**53.69**
Baguio City	**7,026**	2	**0.03**	219	**3.12**	3,636	**51.75**	**3,857**	**54.90**
**Region 1**	**97,099**	**52**	**0.05**	**3,564**	**3.67**	**45,937**	**47.31**	**49,553**	**51.03**
Ilocos Norte	**7,854**	3	**0.04**	248	**3.16**	3,661	**46.61**	**3,912**	**49.81**
Ilocos Sur	**9,072**	10	**0.11**	394	**4.34**	6,037	**66.55**	**6,441**	**71.00**
La Union	**11,338**	14	**0.12**	578	**5.10**	5,780	**50.98**	**6,372**	**56.20**
Pangasinan	**50,710**	15	**0.03**	1,583	**3.12**	20,136	**39.71**	**21,734**	**42.86**
Alaminos City	**1,910**	0	**0.00**	56	**2.93**	865	**45.29**	**921**	**48.22**
Candon City	**954**	0	**0.00**	2	**0.21**	838	**87.84**	**840**	**88.05**
Dagupan City	**3,657**	4	**0.11**	234	**6.40**	2,110	**57.70**	**2,348**	**64.21**
Laoag City	**1,812**	0	**0.00**	12	**0.66**	1,932	**106.62**	**1,944**	**107.28**
San Carlos City	**4,028**	1	**0.02**	171	**4.25**	1,724	**42.80**	**1,896**	**47.07**
San Fernando City	**2,074**	1	**0.05**	89	**4.29**	1,260	**60.75**	**1,350**	**65.09**
Urdaneta City	**2,836**	3	**0.11**	152	**5.36**	1,126	**39.70**	**1,281**	**45.17**
Vigan City	**854**	1	**0.12**	45	**5.27**	468	**54.80**	**514**	**60.19**
**Region 2**	**68,960**	**98**	**0.14**	**4,350**	**6.31**	**34,288**	**49.72**	**38,736**	**56.17**
Batanes	**362**	0	**0.00**	7	**1.93**	207	**57.18**	**214**	**59.12**
Cagayan	**20,431**	53	**0.26**	1,101	**5.39**	8,428	**41.25**	**9,582**	**46.90**
Isabela	**23,449**	23	**0.10**	1,498	**6.39**	11,541	**49.22**	**13,062**	**55.70**
Nueva Vizcaya	**9,538**	13	**0.14**	682	**7.15**	5,184	**54.35**	**5,879**	**61.64**
Quirino	**4,051**	3	**0.07**	232	**5.73**	2,422	**59.79**	**2,657**	**65.59**
Cauayan City	**2,565**	1	**0.04**	262	**10.21**	1,375	**53.61**	**1,638**	**63.86**
Ilagan City	**2,889**	2	**0.07**	243	**8.41**	1,328	**45.97**	**1,573**	**54.45**
Santiago City	**2,671**	3	**0.11**	248	**9.28**	2,655	**99.40**	**2,906**	**108.80**
Tuguegarao City	**3,004**	0	**0.00**	77	**2.56**	1,148	**38.22**	**1,225**	**40.78**
**Region 3**	**220,155**	**218**	**0.10**	**13,930**	**6.33**	**121,978**	**55.41**	**136,126**	**61.83**
Aurora	**4,754**	2	**0.04**	292	**6.14**	2,083	**43.82**	**2,377**	**50.00**

Table 2.B.3.2. Postpartum and Newborn Care

Number and proportion of postpartum women who completed Iron with Folic Acid
Philippines, 2022

Area	Eligible Pop.	Iron with Folic Supplementation							
		Age Group in Year						Total	%
		10-14		15-19		20-49			
		No.	%	No.	%	No.	%		
Bataan	13,789	26	0.19	995	7.22	7,111	51.57	8,132	58.97
Bulacan	43,760	33	0.08	2,796	6.39	23,698	54.15	26,527	60.62
Nueva Ecija	29,039	22	0.08	1,286	4.43	9,743	33.55	11,051	38.06
Pampanga	31,620	33	0.10	1,662	5.26	16,260	51.42	17,955	56.78
Tarlac	19,319	23	0.12	1,186	6.14	11,832	61.25	13,041	67.50
Zambales	12,476	4	0.03	435	3.49	4,082	32.72	4,521	36.24
Angeles City	7,932	13	0.16	700	8.83	8,068	101.71	8,781	110.70
Balanga City	1,992	3	0.15	137	6.88	1,064	53.41	1,204	60.44
Cabanatuan City	5,945	5	0.08	358	6.02	3,075	51.72	3,438	57.83
City of San Fernando	5,907	12	0.20	464	7.86	3,380	57.22	3,856	65.28
Gapan City	2,170	0	0.00	79	3.64	643	29.63	722	33.27
Mabalacat City	4,831	6	0.12	565	11.70	4,709	97.47	5,280	109.29
Malolos City	4,890	2	0.04	172	3.52	1,395	28.53	1,569	32.09
Meycauayan	4,055	0	0.00	297	7.32	2,655	65.47	2,952	72.80
Olongapo	4,922	1	0.02	147	2.99	2,501	50.81	2,649	53.82
Palayan City	808	0	0.00	35	4.33	215	26.61	250	30.94
San Jose City	2,748	14	0.51	281	10.23	1,642	59.75	1,937	70.49
San Jose del Monte City	11,133	9	0.08	1,059	9.51	12,014	107.91	13,082	117.51
Science City of Munoz	1,601	4	0.25	72	4.50	744	46.47	820	51.22
Tarlac City	6,464	6	0.09	912	14.11	5,064	78.34	5,982	92.54
**Region 4A**	**299,627**	**216**	**0.07**	**13,008**	**4.34**	**130,701**	**43.62**	**143,925**	**48.03**
Batangas	39,255	23	0.06	1,748	4.45	19,520	49.73	21,291	54.24
Cavite	28,151	21	0.07	867	3.08	11,317	40.20	12,205	43.36
Laguna	19,696	10	0.05	1,008	5.12	8,230	41.79	9,248	46.95
Quezon	37,853	79	0.21	1,927	5.09	15,309	40.44	17,315	45.74
Rizal	44,798	24	0.05	2,361	5.27	19,387	43.28	21,772	48.60
Antipolo City	16,498	14	0.08	683	4.14	6,065	36.76	6,762	40.99
Bacoor City	12,341	0	0.00	236	1.91	3,078	24.94	3,314	26.85
Batangas City	6,965	4	0.06	310	4.45	3,088	44.34	3,402	48.84
Biñan City	6,597	2	0.03	659	9.99	5,928	89.86	6,589	99.88
Cabuyao City	6,119	1	0.02	293	4.79	4,165	68.07	4,459	72.87
Calamba City	9,005	4	0.04	543	6.03	5,807	64.49	6,354	70.56
Cavite City	2,105	1	0.05	31	1.47	282	13.40	314	14.92
Dasmariñas City	13,538	2	0.01	432	3.19	5,360	39.59	5,794	42.80
General Trias City	6,457	2	0.03	127	1.97	1,187	18.38	1,316	20.38
Imus City	8,292	2	0.02	84	1.01	1,722	20.77	1,808	21.80
Lipa City	7,018	2	0.03	176	2.51	3,507	49.97	3,685	52.51
Lucena City	5,738	1	0.02	278	4.84	2,142	37.33	2,421	42.19
San Pablo City	5,272	9	0.17	283	5.37	2,209	41.90	2,501	47.44
San Pedro City	6,455	5	0.08	203	3.14	2,129	32.98	2,337	36.20
Santa Rosa City	7,009	4	0.06	419	5.98	4,669	66.61	5,092	72.65
Tagaytay City	1,460	1	0.07	46	3.15	1,227	84.04	1,274	87.26
Tanauan City	3,661	2	0.05	40	1.09	1,852	50.59	1,894	51.73
Tayabas City	2,145	1	0.05	145	6.76	1,226	57.16	1,372	63.96
Trece Martires City	3,199	2	0.06	109	3.41	1,295	40.48	1,406	43.95
**Region 4B**	**72,791**	**65**	**0.09**	**4,247**	**5.83**	**31,525**	**43.31**	**35,837**	**49.23**
Marinduque	4,990	5	0.10	243	4.87	2,508	50.26	2,756	55.23
Mindoro Occidental	12,479	14	0.11	1,024	8.21	6,580	52.73	7,618	61.05
Mindoro Oriental	20,189	10	0.05	733	3.63	7,701	38.14	8,444	41.82
Palawan	21,963	25	0.11	1,635	7.44	9,906	45.10	11,566	52.66
Romblon	6,569	4	0.06	324	4.93	2,940	44.76	3,268	49.75
Puerto Princesa City	6,601	7	0.11	288	4.36	1,890	28.63	2,185	33.10
**Region 5**	**138,457**	**75**	**0.05**	**5,498**	**3.97**	**54,574**	**39.42**	**60,147**	**43.44**
Albay	24,081	8	0.03	664	2.76	10,556	43.84	11,228	46.63
Camarines Norte	14,384	7	0.05	785	5.46	6,082	42.28	6,874	47.79
Camarines Sur	39,486	14	0.04	1,092	2.77	11,782	29.84	12,888	32.64
Catanduanes	6,459	5	0.08	290	4.49	2,862	44.31	3,157	48.88
Masbate	22,600	17	0.08	1,460	6.46	9,587	42.42	11,064	48.96
Sorsogon	19,828	8	0.04	879	4.43	9,278	46.79	10,165	51.27

Table 2.B.3.2. Postpartum and Newborn Care

Number and proportion of postpartum women who completed Iron with Folic Acid
Philippines, 2022

Area	Eligible Pop.	Iron with Folic Supplementation							
		Age Group in Year						Total	%
		10-14		15-19		20-49			
		No.	%	No.	%	No.	%		
Iriga City	2,679	3	0.11	69	2.58	825	30.80	897	33.48
Legaspi City	4,233	2	0.05	103	2.43	1,878	44.37	1,983	46.85
Naga City	4,707	11	0.23	156	3.31	1,724	36.63	1,891	40.17
**Region 6**	**146,449**	**158**	**0.11**	**7,214**	**4.93**	**61,757**	**42.17**	**69,129**	**47.20**
Aklan	11,288	10	0.09	476	4.22	4,245	37.61	4,731	41.91
Antique	13,132	7	0.05	513	3.91	5,003	38.10	5,523	42.06
Capiz	13,975	8	0.06	368	2.63	4,434	31.73	4,810	34.42
Guimaras	3,084	3	0.10	154	4.99	1,830	59.34	1,987	64.43
Iloilo	36,267	40	0.11	1,397	3.85	15,936	43.94	17,373	47.90
Negros Occidental	49,230	63	0.13	3,407	6.92	23,601	47.94	27,071	54.99
Bacolod City	11,082	25	0.23	487	4.39	2,646	23.88	3,158	28.50
Iloilo City	8,391	2	0.02	412	4.91	4,062	48.41	4,476	53.34
**Region 7**	**162,308**	**114**	**0.07**	**8,374**	**5.16**	**82,175**	**50.63**	**90,663**	**55.86**
Bohol	27,992	10	0.04	1,034	3.69	12,147	43.39	13,191	47.12
Cebu	66,463	40	0.06	3,088	4.65	28,753	43.26	31,881	47.97
Negros Oriental	27,890	24	0.09	1,553	5.57	12,274	44.01	13,851	49.66
Siquijor	1,661	0	0.00	104	6.26	850	51.17	954	57.44
Cebu City	20,866	22	0.11	1,543	7.39	14,707	70.48	16,272	77.98
Lapu-Lapu City	9,232	8	0.09	449	4.86	6,667	72.22	7,124	77.17
Mandaue City	8,204	10	0.12	603	7.35	6,777	82.61	7,390	90.08
**Region 8**	**105,471**	**66**	**0.06**	**4,356**	**4.13**	**38,440**	**36.45**	**42,862**	**40.64**
Biliran	3,873	6	0.15	332	8.57	2,487	64.21	2,825	72.94
Eastern Samar	11,908	12	0.10	582	4.89	5,131	43.09	5,725	48.08
Northern Leyte	35,422	24	0.07	1,232	3.48	11,466	32.37	12,722	35.92
Northern Samar	15,921	10	0.06	630	3.96	6,114	38.40	6,754	42.42
Southern Leyte	6,662	4	0.06	428	6.42	3,420	51.34	3,852	57.82
Western Samar	14,732	1	0.01	437	2.97	3,805	25.83	4,243	28.80
Calbayog City	4,535	1	0.02	130	2.87	948	20.90	1,079	23.79
Maasin City	1,696	1	0.06	48	2.83	630	37.15	679	40.04
Ormoc City	5,044	6	0.12	423	8.39	2,842	56.34	3,271	64.85
Tacloban City	5,678	1	0.02	114	2.01	1,597	28.13	1,712	30.15
**Region 9**	**79,007**	**96**	**0.12**	**5,527**	**7.00**	**38,815**	**49.13**	**44,438**	**56.25**
Zamboanga del Norte	16,854	23	0.14	1,273	7.55	7,503	44.52	8,799	52.21
Zamboanga del Sur	17,368	31	0.18	848	4.88	5,916	34.06	6,795	39.12
Zamboanga Sibugay	15,021	4	0.03	810	5.39	5,119	34.08	5,933	39.50
Dapitan City	1,737	1	0.06	157	9.04	1,404	80.83	1,562	89.93
Dipolog City	2,762	7	0.25	248	8.98	1,837	66.51	2,092	75.74
Isabela City	2,563	4	0.16	195	7.61	1,423	55.52	1,622	63.29
Pagadian City	4,257	2	0.05	229	5.38	2,177	51.14	2,408	56.57
Zamboanga City	18,445	24	0.13	1,767	9.58	13,436	72.84	15,227	82.55
**Region 10**	**99,908**	**191**	**0.19**	**9,495**	**9.50**	**57,053**	**57.11**	**66,739**	**66.80**
Bukidnon	22,900	87	0.38	3,084	13.47	13,651	59.61	16,822	73.46
Camiguin	1,854	2	0.11	128	6.90	996	53.72	1,126	60.73
Lanao del Norte	14,930	13	0.09	652	4.37	6,413	42.95	7,078	47.41
Misamis Occidental	6,420	7	0.11	249	3.88	3,401	52.98	3,657	56.96
Misamis Oriental	14,963	33	0.22	1,444	9.65	8,677	57.99	10,154	67.86
Cagayan de Oro City	14,172	30	0.21	1,522	10.74	9,160	64.63	10,712	75.59
El Salvador City	1,052	4	0.38	102	9.70	660	62.74	766	72.81
Gingoog City	2,608	3	0.12	318	12.19	1,615	61.92	1,936	74.23
Iligan City	7,565	1	0.01	491	6.49	4,167	55.08	4,659	61.59
Malaybalay City	3,817	4	0.10	480	12.58	2,628	68.85	3,112	81.53
Oroquieta City	1,389	0	0.00	116	8.35	812	58.46	928	66.81
Ozamis City	2,786	1	0.04	219	7.86	1,573	56.46	1,793	64.36
Tangub City	1,234	0	0.00	174	14.10	944	76.50	1,118	90.60

Table 2.B.3.2. Postpartum and Newborn Care

Number and proportion of postpartum women who completed Iron with Folic Acid
Philippines, 2022

Area	Eligible Pop.	Iron with Folic Supplementation							
		Age Group in Year						Total	%
		10-14		15-19		20-49			
		No.	%	No.	%	No.	%		
Valencia City	4,218	6	0.14	516	12.23	2,356	55.86	2,878	68.23
**Region 11**	108,407	398	0.37	9,171	8.46	56,941	52.53	66,510	61.35
Davao de Oro	15,490	118	0.76	1,759	11.36	8,508	54.93	10,385	67.04
Davao del Norte	21,017	121	0.58	2,382	11.33	14,242	67.76	16,745	79.67
Davao Oriental	13,229	49	0.37	1,170	8.84	6,291	47.55	7,510	56.77
Davao del Sur	14,564	50	0.34	1,024	7.03	5,218	35.83	6,292	43.20
Davao Occidental	6,510	18	0.28	326	5.01	971	14.92	1,315	20.20
Davao City	37,597	42	0.11	2,510	6.68	21,711	57.75	24,263	64.53
**Region 12**	98,859	203	0.21	8,427	8.52	49,987	50.56	58,617	59.29
North Cotabato	34,150	37	0.11	2,097	6.14	13,935	40.81	16,069	47.05
Sarangani	12,907	32	0.25	1,803	13.97	7,964	61.70	9,799	75.92
South Cotabato	20,917	66	0.32	2,346	11.22	13,465	64.37	15,877	75.90
Sultan Kudarat	17,299	63	0.36	1,770	10.23	10,278	59.41	12,111	70.01
Gen. Santos City	13,586	5	0.04	411	3.03	4,345	31.98	4,761	35.04
**BARMM**	101,343	23	0.02	4,019	3.97	44,228	43.64	48,270	47.63
Basilan	7,823	5	0.06	280	3.58	1,755	22.43	2,040	26.08
Lanao del Sur	21,639	3	0.01	519	2.40	12,418	57.39	12,940	59.80
Maguindanao	32,198	8	0.02	1,465	4.55	14,498	45.03	15,971	49.60
Sulu	17,165	1	0.01	490	2.85	6,919	40.31	7,410	43.17
Tawi-Tawi	9,369	2	0.02	216	2.31	3,063	32.69	3,281	35.02
Lamitan City	2,154	4	0.19	214	9.94	1,053	48.89	1,271	59.01
Marawi City	5,173	0	0.00	676	13.07	2,683	51.87	3,359	64.93
Cotabato City	5,822	0	0.00	159	2.73	1,839	31.59	1,998	34.32
**CARAGA**	60,575	143	0.24	3,623	5.98	27,503	45.40	31,269	51.62
Agusan del Norte	8,127	83	1.02	410	5.04	2,564	31.55	3,057	37.62
Agusan del Sur	17,709	27	0.15	1,105	6.24	8,703	49.14	9,835	55.54
Surigao del Norte	7,280	2	0.03	464	6.37	3,966	54.48	4,432	60.88
Surigao del Sur	11,553	14	0.12	489	4.23	3,641	31.52	4,144	35.87
Province of Dinagat	2,576	1	0.04	116	4.50	922	35.79	1,039	40.33
Bislig City	2,192	3	0.14	137	6.25	1,046	47.72	1,186	54.11
Butuan City	7,743	10	0.13	639	8.25	5,047	65.18	5,696	73.56
Surigao City	3,395	3	0.09	263	7.75	1,614	47.54	1,880	55.38

Table 2.B.3.3. Postpartum and Newborn Care

Number and proportion of postpartum women who completed Vitamin A Suplementation
Philippines, 2022

Area	Eligible Pop.	Vitamin A Suplementation							
		Age Group in Year						Total	%
		10-14		15-19		20-49			
		No.	%	No.	%	No.	%		
**PHILIPPINES**	**2,131,496**	**2,544**	**0.12**	**121,922**	**5.72**	**1,058,038**	**49.64**	**1,182,504**	**55.48**
**N C R**	**236,901**	**199**	**0.08**	**12,191**	**5.15**	**151,632**	**64.01**	**164,022**	**69.24**
Malabon	**6,724**	12	**0.18**	466	**6.93**	3,353	**49.87**	**3,831**	**56.98**
Navotas	**4,591**	5	**0.11**	422	**9.19**	3,292	**71.71**	**3,719**	**81.01**
Valenzuela City	**11,418**	10	**0.09**	461	**4.04**	5,275	**46.20**	**5,746**	**50.32**
Caloocan City	**29,146**	15	**0.05**	1,535	**5.27**	21,189	**72.70**	**22,739**	**78.02**
Marikina City	**8,294**	1	**0.01**	181	**2.18**	2,597	**31.31**	**2,779**	**33.51**
Pasig City	**13,894**	12	**0.09**	715	**5.15**	5,995	**43.15**	**6,722**	**48.38**
Pateros	**1,176**	1	**0.09**	57	**4.85**	807	**68.62**	**865**	**73.55**
Taguig	**14,807**	25	**0.17**	1,094	**7.39**	11,158	**75.36**	**12,277**	**82.91**
Quezon City	**54,011**	54	**0.10**	2,481	**4.59**	47,079	**87.17**	**49,614**	**91.86**
Makati City	**10,718**	7	**0.07**	245	**2.29**	3,686	**34.39**	**3,938**	**36.74**
Mandaluyong City	**7,111**	4	**0.06**	362	**5.09**	6,111	**85.94**	**6,477**	**91.08**
San Juan	**2,246**	0	**0.00**	45	**2.00**	860	**38.29**	**905**	**40.29**
Manila City	**32,743**	20	**0.06**	1,832	**5.60**	15,299	**46.72**	**17,151**	**52.38**
Las Piñas City	**10,832**	9	**0.08**	584	**5.39**	5,574	**51.46**	**6,167**	**56.93**
Muntinlupa City	**9,281**	17	**0.18**	598	**6.44**	8,307	**89.51**	**8,922**	**96.13**
Parañaque City	**12,249**	6	**0.05**	775	**6.33**	6,257	**51.08**	**7,038**	**57.46**
Pasay City	**7,660**	1	**0.01**	338	**4.41**	4,793	**62.57**	**5,132**	**67.00**
**C A R**	**35,179**	**36**	**0.10**	**1,687**	**4.80**	**19,270**	**54.78**	**20,993**	**59.67**
Abra	**4,275**	3	**0.07**	287	**6.71**	2,038	**47.67**	**2,328**	**54.46**
Apayao	**2,502**	15	**0.60**	240	**9.59**	1,455	**58.15**	**1,710**	**68.35**
Benguet	**9,086**	4	**0.04**	347	**3.82**	5,105	**56.19**	**5,456**	**60.05**
Ifugao	**4,486**	6	**0.13**	223	**4.97**	2,328	**51.89**	**2,557**	**57.00**
Kalinga	**4,701**	1	**0.02**	225	**4.79**	2,780	**59.14**	**3,006**	**63.94**
Mt. Province	**3,103**	5	**0.16**	197	**6.35**	2,259	**72.80**	**2,461**	**79.31**
Baguio City	**7,026**	2	**0.03**	168	**2.39**	3,305	**47.04**	**3,475**	**49.46**
**Region 1**	**97,099**	**62**	**0.06**	**3,650**	**3.76**	**44,535**	**45.87**	**48,247**	**49.69**
Ilocos Norte	**7,854**	6	**0.08**	290	**3.69**	3,783	**48.17**	**4,079**	**51.94**
Ilocos Sur	**9,072**	11	**0.12**	408	**4.50**	6,136	**67.64**	**6,555**	**72.26**
La Union	**11,338**	16	**0.14**	626	**5.52**	5,847	**51.57**	**6,489**	**57.23**
Pangasinan	**50,710**	20	**0.04**	1,442	**2.84**	17,771	**35.04**	**19,233**	**37.93**
Alaminos City	**1,910**	0	**0.00**	64	**3.35**	904	**47.33**	**968**	**50.68**
Candon City	**954**	0	**0.00**	2	**0.21**	838	**87.84**	**840**	**88.05**
Dagupan City	**3,657**	2	**0.05**	245	**6.70**	1,986	**54.31**	**2,233**	**61.06**
Laoag City	**1,812**	0	**0.00**	12	**0.66**	1,932	**106.62**	**1,944**	**107.28**
San Carlos City	**4,028**	2	**0.05**	207	**5.14**	2,351	**58.37**	**2,560**	**63.56**
San Fernando City	**2,074**	2	**0.10**	126	**6.08**	944	**45.52**	**1,072**	**51.69**
Urdaneta City	**2,836**	2	**0.07**	186	**6.56**	1,589	**56.03**	**1,777**	**62.66**
Vigan City	**854**	1	**0.12**	42	**4.92**	454	**53.16**	**497**	**58.20**
**Region 2**	**68,960**	**114**	**0.17**	**4,669**	**6.77**	**35,764**	**51.86**	**40,547**	**58.80**
Batanes	**362**	0	**0.00**	12	**3.31**	204	**56.35**	**216**	**59.67**
Cagayan	**20,431**	60	**0.29**	1,262	**6.18**	9,122	**44.65**	**10,444**	**51.12**
Isabela	**23,449**	34	**0.14**	1,575	**6.72**	12,254	**52.26**	**13,863**	**59.12**
Nueva Vizcaya	**9,538**	7	**0.07**	683	**7.16**	5,110	**53.58**	**5,800**	**60.81**
Quirino	**4,051**	4	**0.10**	271	**6.69**	2,552	**63.00**	**2,827**	**69.79**
Cauayan City	**2,565**	3	**0.12**	228	**8.89**	1,430	**55.75**	**1,661**	**64.76**
Ilagan City	**2,889**	2	**0.07**	265	**9.17**	1,219	**42.19**	**1,486**	**51.44**
Santiago City	**2,671**	4	**0.15**	276	**10.33**	2,391	**89.52**	**2,671**	**100.00**
Tuguegarao City	**3,004**	0	**0.00**	97	**3.23**	1,482	**49.33**	**1,579**	**52.56**

Table 2.B.3.3. Postpartum and Newborn Care

Number and proportion of postpartum women who completed Vitamin A Suplementation
Philippines, 2022

Area	Eligible Pop.	Vitamin A Suplementation							
		Age Group in Year						Total	%
		10-14		15-19		20-49			
		No.	%	No.	%	No.	%		
**Region 3**	**220,155**	**264**	**0.12**	**12,929**	**5.87**	**112,487**	**51.09**	**125,680**	**57.09**
Aurora	4,754	3	0.06	277	5.83	2,091	43.98	2,371	49.87
Bataan	13,789	24	0.17	955	6.93	7,129	51.70	8,108	58.80
Bulacan	43,760	60	0.14	2,495	5.70	21,354	48.80	23,909	54.64
Nueva Ecija	29,039	19	0.07	1,048	3.61	7,318	25.20	8,385	28.87
Pampanga	31,620	35	0.11	1,692	5.35	16,101	50.92	17,828	56.38
Tarlac	19,319	31	0.16	1,115	5.77	10,693	55.35	11,839	61.28
Zambales	12,476	7	0.06	397	3.18	3,603	28.88	4,007	32.12
Angeles City	7,932	12	0.15	631	7.96	7,221	91.04	7,864	99.14
Balanga City	1,992	7	0.35	162	8.13	1,108	55.62	1,277	64.11
Cabanatuan City	5,945	9	0.15	448	7.54	3,886	65.37	4,343	73.05
City of San Fernando	5,907	11	0.19	482	8.16	3,513	59.47	4,006	67.82
Gapan City	2,170	0	0.00	22	1.01	174	8.02	196	9.03
Mabalacat City	4,831	9	0.19	459	9.50	3,778	78.20	4,246	87.89
Malolos City	4,890	2	0.04	190	3.89	1,862	38.08	2,054	42.00
Meycauayan	4,055	0	0.00	195	4.81	1,797	44.32	1,992	49.12
Olongapo	4,922	1	0.02	156	3.17	2,311	46.95	2,468	50.14
Palayan City	808	0	0.00	31	3.84	241	29.83	272	33.66
San Jose City	2,748	8	0.29	299	10.88	1,741	63.36	2,048	74.53
San Jose del Monte City	11,133	17	0.15	1,101	9.89	11,914	107.02	13,032	117.06
Science City of Munoz	1,601	3	0.19	70	4.37	551	34.42	624	38.98
Tarlac City	6,464	6	0.09	704	10.89	4,101	63.44	4,811	74.43
**Region 4A**	**299,627**	**196**	**0.07**	**13,792**	**4.60**	**136,945**	**45.71**	**150,933**	**50.37**
Batangas	39,255	28	0.07	1,331	3.39	14,235	36.26	15,594	39.72
Cavite	28,151	20	0.07	1,230	4.37	14,768	52.46	16,018	56.90
Laguna	19,696	22	0.11	1,092	5.54	8,548	43.40	9,662	49.06
Quezon	37,853	44	0.12	2,243	5.93	18,281	48.29	20,568	54.34
Rizal	44,798	22	0.05	2,325	5.19	19,410	43.33	21,757	48.57
Antipolo City	16,498	3	0.02	633	3.84	6,006	36.40	6,642	40.26
Bacoor City	12,341	2	0.02	267	2.16	3,345	27.10	3,614	29.28
Batangas City	6,965	6	0.09	355	5.10	3,157	45.33	3,518	50.51
Biñan City	6,597	2	0.03	602	9.13	5,784	87.68	6,388	96.83
Cabuyao City	6,119	1	0.02	298	4.87	4,042	66.06	4,341	70.94
Calamba City	9,005	7	0.08	556	6.17	6,075	67.46	6,638	73.71
Cavite City	2,105	1	0.05	91	4.32	693	32.92	785	37.29
Dasmariñas City	13,538	4	0.03	497	3.67	6,316	46.65	6,817	50.35
General Trias City	6,457	3	0.05	145	2.25	1,718	26.61	1,866	28.90
Imus City	8,292	3	0.04	113	1.36	2,153	25.96	2,269	27.36
Lipa City	7,018	2	0.03	257	3.66	3,709	52.85	3,968	56.54
Lucena City	5,738	4	0.07	327	5.70	2,510	43.74	2,841	49.51
San Pablo City	5,272	9	0.17	366	6.94	2,697	51.16	3,072	58.27
San Pedro City	6,455	3	0.05	227	3.52	2,306	35.72	2,536	39.29
Santa Rosa City	7,009	3	0.04	391	5.58	4,639	66.19	5,033	71.81
Tagaytay City	1,460	1	0.07	65	4.45	1,233	84.45	1,299	88.97
Tanauan City	3,661	3	0.08	59	1.61	1,809	49.41	1,871	51.11
Tayabas City	2,145	1	0.05	142	6.62	1,224	57.06	1,367	63.73
Trece Martires City	3,199	2	0.06	180	5.63	2,287	71.49	2,469	77.18
**Region 4B**	**72,791**	**73**	**0.10**	**3,718**	**5.11**	**27,748**	**38.12**	**31,539**	**43.33**
Marinduque	4,990	7	0.14	310	6.21	2,754	55.19	3,071	61.54
Mindoro Occidental	12,479	10	0.08	819	6.56	5,403	43.30	6,232	49.94
Mindoro Oriental	20,189	22	0.11	751	3.72	7,273	36.02	8,046	39.85
Palawan	21,963	24	0.11	1,428	6.50	8,506	38.73	9,958	45.34
Romblon	6,569	6	0.09	320	4.87	2,924	44.51	3,250	49.47
Puerto Princesa City	6,601	4	0.06	90	1.36	888	13.45	982	14.88

Table 2.B.3.3. Postpartum and Newborn Care

Number and proportion of postpartum women who completed Vitamin A Suplementation

Philippines, 2022

Area	Eligible Pop.	Vitamin A Suplementation							
		Age Group in Year						Total	%
		10-14		15-19		20-49			
		No.	%	No.	%	No.	%		
**Region 5**	**138,457**	**103**	**0.07**	**6,573**	**4.75**	**60,732**	**43.86**	**67,408**	**48.69**
Albay	24,081	10	0.04	686	2.85	10,322	42.86	11,018	45.75
Camarines Norte	14,384	9	0.06	812	5.65	6,843	47.57	7,664	53.28
Camarines Sur	39,486	26	0.07	1,169	2.96	12,695	32.15	13,890	35.18
Catanduanes	6,459	7	0.11	424	6.56	3,602	55.77	4,033	62.44
Masbate	22,600	32	0.14	1,943	8.60	11,689	51.72	13,664	60.46
Sorsogon	19,828	12	0.06	1,149	5.79	10,512	53.02	11,673	58.87
Iriga City	2,679	2	0.07	104	3.88	1,078	40.24	1,184	44.20
Legaspi City	4,233	2	0.05	140	3.31	2,216	52.35	2,358	55.71
Naga City	4,707	3	0.06	146	3.10	1,775	37.71	1,924	40.88
**Region 6**	**146,449**	**195**	**0.13**	**8,231**	**5.62**	**67,252**	**45.92**	**75,678**	**51.68**
Aklan	11,288	4	0.04	421	3.73	4,483	39.71	4,908	43.48
Antique	13,132	8	0.06	571	4.35	5,407	41.17	5,986	45.58
Capiz	13,975	12	0.09	465	3.33	5,186	37.11	5,663	40.52
Guimaras	3,084	4	0.13	220	7.13	1,963	63.65	2,187	70.91
Iloilo	36,267	52	0.14	1,711	4.72	17,050	47.01	18,813	51.87
Negros Occidental	49,230	79	0.16	3,786	7.69	25,693	52.19	29,558	60.04
Bacolod City	11,082	25	0.23	568	5.13	3,003	27.10	3,596	32.45
Iloilo City	8,391	11	0.13	489	5.83	4,467	53.24	4,967	59.19
**Region 7**	**162,308**	**101**	**0.06**	**7,294**	**4.49**	**72,535**	**44.69**	**79,930**	**49.25**
Bohol	27,992	17	0.06	1,300	4.64	13,358	47.72	14,675	52.43
Cebu	66,463	16	0.02	1,572	2.37	16,694	25.12	18,282	27.51
Negros Oriental	27,890	26	0.09	1,731	6.21	13,174	47.24	14,931	53.54
Siquijor	1,661	2	0.12	74	4.46	813	48.95	889	53.52
Cebu City	20,866	20	0.10	1,574	7.54	15,545	74.50	17,139	82.14
Lapu-Lapu City	9,232	10	0.11	461	4.99	6,245	67.65	6,716	72.75
Mandaue City	8,204	10	0.12	582	7.09	6,706	81.74	7,298	88.96
**Region 8**	**105,471**	**68**	**0.06**	**4,197**	**3.98**	**35,340**	**33.51**	**39,605**	**37.55**
Biliran	3,873	9	0.23	355	9.17	2,603	67.21	2,967	76.61
Eastern Samar	11,908	13	0.11	660	5.54	5,503	46.21	6,176	51.86
Northern Leyte	35,422	15	0.04	758	2.14	7,190	20.30	7,963	22.48
Northern Samar	15,921	12	0.08	739	4.64	6,459	40.57	7,210	45.29
Southern Leyte	6,662	7	0.11	467	7.01	3,590	53.89	4,064	61.00
Western Samar	14,732	1	0.01	467	3.17	3,799	25.79	4,267	28.96
Calbayog City	4,535	2	0.04	233	5.14	1,664	36.69	1,899	41.87
Maasin City	1,696	1	0.06	55	3.24	688	40.57	744	43.87
Ormoc City	5,044	8	0.16	434	8.60	3,030	60.07	3,472	68.83
Tacloban City	5,678	0	0.00	29	0.51	814	14.34	843	14.85
**Region 9**	**79,007**	**104**	**0.13**	**6,104**	**7.73**	**41,274**	**52.24**	**47,482**	**60.10**
Zamboanga del Norte	16,854	22	0.13	1,421	8.43	8,286	49.16	9,729	57.73
Zamboanga del Sur	17,368	21	0.12	1,023	5.89	6,420	36.96	7,464	42.98
Zamboanga Sibugay	15,021	7	0.05	893	5.95	5,615	37.38	6,515	43.37
Dapitan City	1,737	2	0.12	186	10.71	1,362	78.41	1,550	89.23
Dipolog City	2,762	7	0.25	275	9.96	1,983	71.80	2,265	82.01
Isabela City	2,563	3	0.12	211	8.23	1,407	54.90	1,621	63.25
Pagadian City	4,257	2	0.05	229	5.38	2,177	51.14	2,408	56.57
Zamboanga City	18,445	40	0.22	1,866	10.12	14,024	76.03	15,930	86.36
**Region 10**	**99,908**	**160**	**0.16**	**8,491**	**8.50**	**52,504**	**52.55**	**61,155**	**61.21**
Bukidnon	22,900	72	0.31	2,829	12.35	12,787	55.84	15,688	68.51

Table 2.B.3.3. Postpartum and Newborn Care

Number and proportion of postpartum women who completed Vitamin A Suplementation

Philippines, 2022

Area	Eligible Pop.	Vitamin A Suplementation							
		Age Group in Year						Total	%
		10-14		15-19		20-49			
		No.	%	No.	%	No.	%		
Camiguin	**1,854**	2	**0.11**	129	**6.96**	1,014	**54.69**	**1,145**	**61.76**
Lanao del Norte	**14,930**	16	**0.11**	660	**4.42**	6,478	**43.39**	**7,154**	**47.92**
Misamis Occidental	**6,420**	9	**0.14**	212	**3.30**	2,805	**43.69**	**3,026**	**47.13**
Misamis Oriental	**14,963**	18	**0.12**	881	**5.89**	5,012	**33.50**	**5,911**	**39.50**
Cagayan de Oro City	**14,172**	24	**0.17**	1,594	**11.25**	10,390	**73.31**	**12,008**	**84.73**
El Salvador City	**1,052**	4	**0.38**	102	**9.70**	656	**62.36**	**762**	**72.43**
Gingoog City	**2,608**	0	**0.00**	128	**4.91**	763	**29.26**	**891**	**34.16**
Iligan City	**7,565**	3	**0.04**	537	**7.10**	4,382	**57.92**	**4,922**	**65.06**
Malaybalay City	**3,817**	4	**0.10**	480	**12.58**	2,628	**68.85**	**3,112**	**81.53**
Oroquieta City	**1,389**	0	**0.00**	118	**8.50**	882	**63.50**	**1,000**	**71.99**
Ozamis City	**2,786**	0	**0.00**	217	**7.79**	1,618	**58.08**	**1,835**	**65.87**
Tangub City	**1,234**	0	**0.00**	49	**3.97**	767	**62.16**	**816**	**66.13**
Valencia City	**4,218**	8	**0.19**	555	**13.16**	2,322	**55.05**	**2,885**	**68.40**
**Region 11**	**108,407**	**487**	**0.45**	**10,535**	**9.72**	**66,579**	**61.42**	**77,601**	**71.58**
Davao de Oro	**15,490**	126	**0.81**	1,459	**9.42**	9,580	**61.85**	**11,165**	**72.08**
Davao del Norte	**21,017**	116	**0.55**	2,473	**11.77**	14,790	**70.37**	**17,379**	**82.69**
Davao Oriental	**13,229**	60	**0.45**	1,303	**9.85**	7,069	**53.44**	**8,432**	**63.74**
Davao del Sur	**14,564**	56	**0.38**	1,397	**9.59**	7,336	**50.37**	**8,789**	**60.35**
Davao Occidental	**6,510**	46	**0.71**	962	**14.78**	2,782	**42.73**	**3,790**	**58.22**
Davao City	**37,597**	83	**0.22**	2,941	**7.82**	25,022	**66.55**	**28,046**	**74.60**
**Region 12**	**98,859**	**214**	**0.22**	**8,573**	**8.67**	**49,187**	**49.75**	**57,974**	**58.64**
North Cotabato	**34,150**	43	**0.13**	2,135	**6.25**	12,995	**38.05**	**15,173**	**44.43**
Sarangani	**12,907**	38	**0.29**	1,933	**14.98**	8,284	**64.18**	**10,255**	**79.45**
South Cotabato	**20,917**	60	**0.29**	2,393	**11.44**	13,788	**65.92**	**16,241**	**77.64**
Sultan Kudarat	**17,299**	68	**0.39**	1,782	**10.30**	10,391	**60.07**	**12,241**	**70.76**
Gen. Santos City	**13,586**	5	**0.04**	330	**2.43**	3,729	**27.45**	**4,064**	**29.91**
**BARMM**	**101,343**	**42**	**0.04**	**5,182**	**5.11**	**55,940**	**55.20**	**61,164**	**60.35**
Basilan	**7,823**	7	**0.09**	360	**4.60**	2,386	**30.50**	**2,753**	**35.19**
Lanao del Sur	**21,639**	4	**0.02**	766	**3.54**	13,480	**62.29**	**14,250**	**65.85**
Maguindanao	**32,198**	20	**0.06**	1,928	**5.99**	19,625	**60.95**	**21,573**	**67.00**
Sulu	**17,165**	4	**0.02**	604	**3.52**	8,588	**50.03**	**9,196**	**53.57**
Tawi-Tawi	**9,369**	1	**0.01**	338	**3.61**	4,663	**49.77**	**5,002**	**53.39**
Lamitan City	**2,154**	6	**0.28**	239	**11.10**	1,153	**53.53**	**1,398**	**64.90**
Marawi City	**5,173**	0	**0.00**	726	**14.03**	3,486	**67.39**	**4,212**	**81.42**
Cotabato City	**5,822**	0	**0.00**	221	**3.80**	2,559	**43.95**	**2,780**	**47.75**
**CARAGA**	**60,575**	**126**	**0.21**	**4,106**	**6.78**	**28,314**	**46.74**	**32,546**	**53.73**
Agusan del Norte	**8,127**	72	**0.89**	436	**5.36**	2,517	**30.97**	**3,025**	**37.22**
Agusan del Sur	**17,709**	24	**0.14**	1,117	**6.31**	8,445	**47.69**	**9,586**	**54.13**
Surigao del Norte	**7,280**	3	**0.04**	183	**2.51**	2,199	**30.21**	**2,385**	**32.76**
Surigao del Sur	**11,553**	10	**0.09**	780	**6.75**	5,120	**44.32**	**5,910**	**51.16**
Province of Dinagat	**2,576**	1	**0.04**	131	**5.09**	974	**37.81**	**1,106**	**42.93**
Bislig City	**2,192**	2	**0.09**	188	**8.58**	1,279	**58.35**	**1,469**	**67.02**
Butuan City	**7,743**	11	**0.14**	675	**8.72**	5,203	**67.20**	**5,889**	**76.06**
Surigao City	**3,395**	3	**0.09**	596	**17.56**	2,577	**75.91**	**3,176**	**93.55**

Table 2.C.1.1. Immunization Services for Infants and Children

Number and Proportion of Children Protected at Birth (CPAB), Newborn / Infants Vaccinated with BCG and Hepatitis B antigen
Philippines, 2022

Area	Eligible Pop (0-11 mos. old)	Child Protected at Birth (CPAB)				BCG				Hepa B1 (w/in 24 Hrs.) after birth			
		Male	Female	Total	%	Male	Female	Total	%	Male	Female	Total	%
**PHILIPPINES**	**2,131,496**	**627,631**	**616,740**	**1,244,371**	**58.38**	**715,028**	**688,107**	**1,403,135**	**65.83**	**600,877**	**583,106**	**1,183,983**	**55.55**
**N C R**	**236,901**	**82,315**	**82,072**	**164,387**	**69.39**	**94,028**	**91,969**	**185,997**	**78.51**	**88,330**	**85,919**	**174,249**	**73.55**
Malabon	**6,724**	2460	2578	**5,038**	**74.93**	2,297	2,229	**4,526**	**67.31**	2,263	2,168	**4,431**	**65.90**
Navotas	**4,591**	1651	1615	**3,266**	**71.14**	2,111	2,025	**4,136**	**90.09**	1,918	1,812	**3,730**	**81.25**
Valenzuela City	**11,418**	2647	2706	**5,353**	**46.88**	3,226	3,249	**6,475**	**56.71**	2,885	2,932	**5,817**	**50.95**
Caloocan City	**29,146**	5649	5562	**11,211**	**38.46**	7,719	7,469	**15,188**	**52.11**	6,017	5,776	**11,793**	**40.46**
Marikina City	**8,294**	2050	2092	**4,142**	**49.94**	3,927	4,016	**7,943**	**95.77**	3,579	3,672	**7,251**	**87.42**
Pasig City	**13,894**	4951	5008	**9,959**	**71.68**	7,028	6,866	**13,894**	**100.00**	7,003	6,891	**13,894**	**100.00**
Pateros	**1,176**	442	451	**893**	**75.94**	467	475	**942**	**80.10**	451	461	**912**	**77.55**
Taguig	**14,807**	4202	4610	**8,812**	**59.51**	3,484	3,499	**6,983**	**47.16**	2,608	2,556	**5,164**	**34.88**
Quezon City	**54,011**	26675	27336	**54,011**	**100.00**	26,639	27,372	**54,011**	**100.00**	26,687	27,324	**54,011**	**100.00**
Makati City	**10,718**	3200	3255	**6,455**	**60.23**	4,313	3,972	**8,285**	**77.30**	4,254	3,785	**8,039**	**75.00**
Mandaluyong City	**7,111**	1810	1851	**3,661**	**51.48**	1,954	1,985	**3,939**	**55.39**	1,846	1,714	**3,560**	**50.06**
San Juan	**2,246**	330	362	**692**	**30.81**	581	526	**1,107**	**49.29**	510	454	**964**	**42.92**
Manila City	**32,743**	13178	12519	**25,697**	**78.48**	14,643	13,914	**28,557**	**87.22**	14,328	13,623	**27,951**	**85.36**
Las Piñas City	**10,832**	2730	2686	**5,416**	**50.00**	3,580	3,318	**6,898**	**63.68**	3,172	2,874	**6,046**	**55.82**
Muntinlupa City	**9,281**	3341	3057	**6,398**	**68.94**	3,608	3,338	**6,946**	**74.84**	3,043	2,873	**5,916**	**63.74**
Parañaque City	**12,249**	3441	3148	**6,589**	**53.79**	4,453	4,054	**8,507**	**69.45**	3,934	3,486	**7,420**	**60.58**
Pasay City	**7,660**	3558	3236	**6,794**	**88.69**	3,998	3,662	**7,660**	**100.00**	3,832	3,518	**7,350**	**95.95**
**C A R**	**35,179**	**9,254**	**9,019**	**18,273**	**51.94**	**12,283**	**11,527**	**23,810**	**67.68**	**11,867**	**11,135**	**23,002**	**65.39**
Abra	**4,275**	1,215	1,300	**2,515**	**58.83**	1,440	1,357	**2,797**	**65.43**	1,417	1,328	**2,745**	**64.21**
Apayao	**2,502**	809	705	**1,514**	**60.51**	604	557	**1,161**	**46.40**	504	488	**992**	**39.65**
Benguet	**9,086**	2,175	2,054	**4,229**	**46.54**	2,247	2,094	**4,341**	**47.78**	2,186	2,055	**4,241**	**46.68**
Ifugao	**4,486**	1,011	1,043	**2,054**	**45.79**	1,441	1,372	**2,813**	**62.71**	1,295	1,193	**2,488**	**55.46**
Kalinga	**4,701**	1,496	1,437	**2,933**	**62.39**	1,367	1,328	**2,695**	**57.33**	1,302	1,269	**2,571**	**54.69**
Mt. Province	**3,103**	1,103	1,024	**2,127**	**68.55**	1,634	1,510	**3,144**	**101.32**	1,624	1,515	**3,139**	**101.16**
Baguio City	**7,026**	1,445	1,456	**2,901**	**41.29**	3,550	3,309	**6,859**	**97.62**	3,539	3,287	**6,826**	**97.15**
**Region 1**	**97,099**	**28,924**	**28,503**	**57,427**	**59.14**	**25,967**	**25,129**	**51,096**	**52.62**	**24,408**	**23,605**	**48,013**	**49.45**
Ilocos Norte	**7,854**	1,886	2,649	**4,535**	**57.74**	1,965	2,727	**4,692**	**59.74**	1,931	2,698	**4,629**	**58.94**
Ilocos Sur	**9,072**	3,827	3,822	**7,649**	**84.31**	3,849	3,833	**7,682**	**84.68**	3,842	3,817	**7,659**	**84.42**
La Union	**11,338**	3,333	3,078	**6,411**	**56.54**	3,388	3,103	**6,491**	**57.25**	3,398	3,124	**6,522**	**57.52**
Pangasinan	**50,710**	13,852	12,973	**26,825**	**52.90**	10,002	8,811	**18,813**	**37.10**	8,614	7,444	**16,058**	**31.67**
Alaminos City	**1,910**	668	656	**1,324**	**69.32**	668	656	**1,324**	**69.32**	668	656	**1,324**	**69.32**
Candon City	**954**	424	414	**838**	**87.84**	424	414	**838**	**87.84**	424	414	**838**	**87.84**
Dagupan City	**3,657**	1,011	999	**2,010**	**54.96**	1,395	1,295	**2,690**	**73.56**	1,377	1,285	**2,662**	**72.79**
Laoag City	**1,812**	923	992	**1,915**	**105.68**	923	992	**1,915**	**105.68**	923	992	**1,915**	**105.68**
San Carlos City	**4,028**	1,192	1,153	**2,345**	**58.22**	1,612	1,545	**3,157**	**78.38**	1,487	1,442	**2,929**	**72.72**
San Fernando City	**2,074**	616	603	**1,219**	**58.78**	614	602	**1,216**	**58.63**	614	602	**1,216**	**58.63**
Urdaneta City	**2,836**	913	927	**1,840**	**64.88**	860	919	**1,779**	**62.73**	860	919	**1,779**	**62.73**
Vigan City	**854**	279	237	**516**	**60.42**	267	232	**499**	**58.43**	270	212	**482**	**56.44**
**Region 2**	**68,960**	**21,919**	**20,802**	**42,721**	**61.95**	**19,931**	**18,960**	**38,891**	**56.40**	**19,466**	**18,748**	**38,214**	**55.41**
Batanes	**362**	143	108	**251**	**69.34**	124	111	**235**	**64.92**	142	110	**252**	**69.61**
Cagayan	**20,431**	5,693	5,047	**10,740**	**52.57**	4,978	4,518	**9,496**	**46.48**	4,741	4,496	**9,237**	**45.21**
Isabela	**23,449**	7,359	7,103	**14,462**	**61.67**	7,334	7,299	**14,633**	**62.40**	7,249	7,158	**14,407**	**61.44**
Nueva Vizcaya	**9,538**	3,013	2,960	**5,973**	**62.62**	1,641	1,658	**3,299**	**34.59**	1,562	1,676	**3,238**	**33.95**
Quirino	**4,051**	1,566	1,432	**2,998**	**74.01**	1,618	1,442	**3,060**	**75.54**	1,603	1,435	**3,038**	**74.99**
Cauayan City	**2,565**	853	855	**1,708**	**66.59**	799	790	**1,589**	**61.95**	758	729	**1,487**	**57.97**
Ilagan City	**2,889**	1,429	1,456	**2,885**	**99.86**	1,429	1,447	**2,876**	**99.55**	1,429	1,456	**2,885**	**99.86**
Santiago City	**2,671**	1,425	1,246	**2,671**	**100.00**	1,425	1,246	**2,671**	**100.00**	1,425	1,246	**2,671**	**100.00**
Tuguegarao City	**3,004**	438	595	**1,033**	**34.39**	583	449	**1,032**	**34.35**	557	442	**999**	**33.26**
**Region 3**	**220,155**	**66,695**	**65,957**	**132,652**	**60.25**	**78,755**	**75,028**	**153,783**	**69.85**	**61,162**	**58,772**	**119,934**	**54.48**
Aurora	**4,754**	1,161	1,086	**2,247**	**47.27**	1,098	966	**2,064**	**43.42**	913	821	**1,734**	**36.47**
Bataan	**13,789**	3,719	3,673	**7,392**	**53.61**	4,596	3,968	**8,564**	**62.11**	4,087	3,554	**7,641**	**55.41**
Bulacan	**43,760**	11,730	11,613	**23,343**	**53.34**	19,271	18,077	**37,348**	**85.35**	14,246	13,586	**27,832**	**63.60**
Nueva Ecija	**29,039**	7,715	7,632	**15,347**	**52.85**	6,881	6,711	**13,592**	**46.81**	4,451	4,453	**8,904**	**30.66**
Pampanga	**31,620**	10,770	10,539	**21,309**	**67.39**	11,409	10,893	**22,302**	**70.53**	10,818	10,454	**21,272**	**67.27**
Tarlac	**19,319**	5,568	5,646	**11,214**	**58.05**	5,551	5,504	**11,055**	**57.22**	3,739	3,639	**7,378**	**38.19**
Zambales	**12,476**	2,656	2,775	**5,431**	**43.53**	3,278	3,221	**6,499**	**52.09**	1,753	1,917	**3,670**	**29.42**

Table 2.C.1.1. Immunization Services for Infants and Children

Number and Proportion of Children Protected at Birth (CPAB), Newborn / Infants Vaccinated with BCG and Hepatitis B antigen
Philippines, 2022

Area	Eligible Pop (0-11 mos. old)	Child Protected at Birth (CPAB)				BCG				Hepa B1 (w/in 24 Hrs.) after birth			
		Male	Female	Total	%	Male	Female	Total	%	Male	Female	Total	%
Angeles City	**7,932**	1,666	1,761	**3,427**	**43.20**	3,735	3,768	**7,503**	**94.59**	2,781	2,780	**5,561**	**70.11**
Balanga City	**1,992**	517	447	**964**	**48.39**	2,081	1,848	**3,929**	**197.24**	1,879	1,649	**3,528**	**177.11**
Cabanatuan City	**5,945**	2,597	2,354	**4,951**	**83.28**	2,706	2,485	**5,191**	**87.32**	2,286	2,075	**4,361**	**73.36**
City of San Fernando	**5,907**	1,625	1,595	**3,220**	**54.51**	1,130	1,136	**2,266**	**38.36**	968	948	**1,916**	**32.44**
Gapan City	**2,170**	489	474	**963**	**44.38**	715	633	**1,348**	**62.12**	426	377	**803**	**37.00**
Mabalacat City	**4,831**	2,376	2,423	**4,799**	**99.34**	1,539	1,594	**3,133**	**64.85**	1,478	1,524	**3,002**	**62.14**
Malolos City	**4,890**	1,021	999	**2,020**	**41.31**	3,202	2,853	**6,055**	**123.82**	2,171	1,911	**4,082**	**83.48**
Meycauayan	**4,055**	1,489	1,566	**3,055**	**75.34**	1,753	1,843	**3,596**	**88.68**	1,538	1,614	**3,152**	**77.73**
Olongapo	**4,922**	1,683	1,631	**3,314**	**67.33**	480	490	**970**	**19.71**	149	193	**342**	**6.95**
Palayan City	**808**	270	235	**505**	**62.50**	180	162	**342**	**42.33**	31	42	**73**	**9.03**
San Jose City	**2,748**	1,012	862	**1,874**	**68.20**	1,347	1,137	**2,484**	**90.39**	1,138	947	**2,085**	**75.87**
San Jose del Monte City	**11,133**	5,413	5,414	**10,827**	**97.25**	5,089	5,005	**10,094**	**90.67**	4,228	4,154	**8,382**	**75.29**
Science City of Munoz	**1,601**	573	500	**1,073**	**67.02**	393	353	**746**	**46.60**	124	115	**239**	**14.93**
Tarlac City	**6,464**	2,645	2,732	**5,377**	**83.18**	2,321	2,381	**4,702**	**72.74**	1,958	2,019	**3,977**	**61.53**
**Region 4A**	**299,627**	**79,744**	**77,772**	**157,516**	**52.57**	**101,443**	**96,656**	**198,099**	**66.12**	**88,533**	**84,885**	**173,418**	**57.88**
Batangas	**39,255**	11,399	10,985	**22,384**	**57.02**	12,327	11,704	**24,031**	**61.22**	11,358	11,031	**22,389**	**57.03**
Cavite	**28,151**	8,324	8,213	**16,537**	**58.74**	10,225	9,908	**20,133**	**71.52**	9,154	9,017	**18,171**	**64.55**
Laguna	**19,696**	3,694	3,705	**7,399**	**37.57**	4,124	3,975	**8,099**	**41.12**	2,680	2,627	**5,307**	**26.94**
Quezon	**37,853**	9,822	9,449	**19,271**	**50.91**	11,629	11,003	**22,632**	**59.79**	9,443	8,891	**18,334**	**48.43**
Rizal	**44,798**	11,531	11,115	**22,646**	**50.55**	15,496	14,547	**30,043**	**67.06**	11,699	11,171	**22,870**	**51.05**
Antipolo City	**16,498**	3,907	3,855	**7,762**	**47.05**	8,822	8,015	**16,837**	**102.05**	8,267	7,349	**15,616**	**94.65**
Bacoor City	**12,341**	2,638	2,505	**5,143**	**41.67**	3,590	3,247	**6,837**	**55.40**	2,961	2,533	**5,494**	**44.52**
Batangas City	**6,965**	1,702	1,575	**3,277**	**47.05**	2,330	2,122	**4,452**	**63.92**	2,230	2,041	**4,271**	**61.32**
Biñan City	**6,597**	3,061	3,261	**6,322**	**95.83**	3,216	3,425	**6,641**	**100.67**	3,084	3,280	**6,364**	**96.47**
Cabuyao City	**6,119**	2,116	2,203	**4,319**	**70.58**	2,428	2,502	**4,930**	**80.57**	2,172	2,179	**4,351**	**71.11**
Calamba City	**9,005**	2,539	2,393	**4,932**	**54.77**	2,675	2,498	**5,173**	**57.45**	3,273	3,128	**6,401**	**71.08**
Cavite City	**2,105**	344	370	**714**	**33.92**	535	522	**1,057**	**50.21**	431	425	**856**	**40.67**
Dasmariñas City	**13,538**	3,421	3,387	**6,808**	**50.29**	4,165	4,054	**8,219**	**60.71**	3,982	3,922	**7,904**	**58.38**
General Trias City	**6,457**	1,535	1,412	**2,947**	**45.64**	2,772	2,718	**5,490**	**85.02**	2,443	2,430	**4,873**	**75.47**
Imus City	**8,292**	1,516	1,424	**2,940**	**35.46**	2,244	2,099	**4,343**	**52.38**	1,999	1,881	**3,880**	**46.79**
Lipa City	**7,018**	2,175	2,135	**4,310**	**61.41**	2,597	2,445	**5,042**	**71.84**	2,046	1,933	**3,979**	**56.70**
Lucena City	**5,738**	1,314	1,405	**2,719**	**47.39**	1,708	1,711	**3,419**	**59.59**	1,626	1,673	**3,299**	**57.49**
San Pablo City	**5,272**	1,524	1,471	**2,995**	**56.81**	2,221	2,214	**4,435**	**84.12**	2,152	2,162	**4,314**	**81.83**
San Pedro City	**6,455**	1,172	1,085	**2,257**	**34.97**	1,850	1,813	**3,663**	**56.75**	1,631	1,627	**3,258**	**50.47**
Santa Rosa City	**7,009**	2,692	2,573	**5,265**	**75.12**	2,497	2,376	**4,873**	**69.52**	2,320	2,171	**4,491**	**64.07**
Tagaytay City	**1,460**	683	616	**1,299**	**88.97**	682	617	**1,299**	**88.97**	679	616	**1,295**	**88.70**
Tanauan City	**3,661**	905	899	**1,804**	**49.28**	1,008	883	**1,891**	**51.65**	711	641	**1,352**	**36.93**
Tayabas City	**2,145**	641	736	**1,377**	**64.20**	654	742	**1,396**	**65.08**	651	744	**1,395**	**65.03**
Trece Martires City	**3,199**	1,089	1,000	**2,089**	**65.30**	1,648	1,516	**3,164**	**98.91**	1,541	1,413	**2,954**	**92.34**
**Region 4B**	**72,791**	**17,625**	**16,923**	**34,548**	**47.46**	**21,873**	**20,556**	**42,429**	**58.29**	**15,499**	**14,572**	**30,071**	**41.31**
Marinduque	**4,990**	1,483	1,354	**2,837**	**56.85**	1,569	1,399	**2,968**	**59.48**	1,565	1,388	**2,953**	**59.18**
Mindoro Occidental	**12,479**	3,230	3,017	**6,247**	**50.06**	4,158	3,998	**8,156**	**65.36**	2,907	2,635	**5,542**	**44.41**
Mindoro Oriental	**20,189**	4,232	4,211	**8,443**	**41.82**	5,672	5,493	**11,165**	**55.30**	4,166	4,114	**8,280**	**41.01**
Palawan	**21,963**	5,189	5,135	**10,324**	**47.01**	6,900	6,369	**13,269**	**60.42**	3,964	3,804	**7,768**	**35.37**
Romblon	**6,569**	1,852	1,696	**3,548**	**54.01**	1,923	1,775	**3,698**	**56.29**	1,765	1,617	**3,382**	**51.48**
Puerto Princesa City	**6,601**	1,639	1,510	**3,149**	**47.70**	1,651	1,522	**3,173**	**48.07**	1,132	1,014	**2,146**	**32.51**
**Region 5**	**138,457**	**32,007**	**29,196**	**61,203**	**44.20**	**38,695**	**35,995**	**74,690**	**53.94**	**32,767**	**30,234**	**63,001**	**45.50**
Albay	**24,081**	5,684	5,162	**10,846**	**45.04**	5,618	5,161	**10,779**	**44.76**	5,302	4,822	**10,124**	**42.04**
Camarines Norte	**14,384**	2,728	2,861	**5,589**	**38.86**	3,156	3,101	**6,257**	**43.50**	2,811	2,658	**5,469**	**38.02**
Camarines Sur	**39,486**	5,701	5,486	**11,187**	**28.33**	9,325	8,689	**18,014**	**45.62**	6,611	6,213	**12,824**	**32.48**
Catanduanes	**6,459**	1,784	1,680	**3,464**	**53.63**	1,951	1,806	**3,757**	**58.17**	1,921	1,740	**3,661**	**56.68**
Masbate	**22,600**	7,866	6,487	**14,353**	**63.51**	8,118	7,691	**15,809**	**69.95**	5,806	5,394	**11,200**	**49.56**
Sorsogon	**19,828**	5,238	4,757	**9,995**	**50.41**	7,499	6,690	**14,189**	**71.56**	7,345	6,639	**13,984**	**70.53**
Iriga City	**2,679**	771	680	**1,451**	**54.16**	690	641	**1,331**	**49.68**	717	646	**1,363**	**50.88**
Legaspi City	**4,233**	1,269	1,149	**2,418**	**57.12**	1,211	1,112	**2,323**	**54.88**	1,170	1,076	**2,246**	**53.06**
Naga City	**4,707**	966	934	**1,900**	**40.37**	1,127	1,104	**2,231**	**47.40**	1,084	1,046	**2,130**	**45.25**
**Region 6**	**146,449**	**41,741**	**40,668**	**82,409**	**56.27**	**43,639**	**42,025**	**85,664**	**58.49**	**40,567**	**39,509**	**80,076**	**54.68**
Aklan	**11,288**	2,484	2,626	**5,110**	**45.27**	2,556	2,569	**5,125**	**45.40**	1,739	1,747	**3,486**	**30.88**
Antique	**13,132**	3,255	3,083	**6,338**	**48.26**	2,851	2,731	**5,582**	**42.51**	3,406	3,781	**7,187**	**54.73**
Capiz	**13,975**	3,107	2,982	**6,089**	**43.57**	3,977	3,791	**7,768**	**55.58**	3,777	3,597	**7,374**	**52.77**
Guimaras	**3,084**	1,117	1,049	**2,166**	**70.23**	1,557	1,502	**3,059**	**99.19**	1,353	1,319	**2,672**	**86.64**
Iloilo	**36,267**	10,247	9,952	**20,199**	**55.70**	9,657	9,080	**18,737**	**51.66**	9,185	8,666	**17,851**	**49.22**
Negros Occidental	**49,230**	15,799	15,395	**31,194**	**63.36**	18,543	17,751	**36,294**	**73.72**	16,906	16,063	**32,969**	**66.97**

Table 2.C.1.1. Immunization Services for Infants and Children

Number and Proportion of Children Protected at Birth (CPAB), Newborn / Infants Vaccinated with BCG and Hepatitis B antigen
Philippines, 2022

Area	Eligible Pop (0-11 mos. old)	Child Protected at Birth (CPAB)				BCG				Hepa B1 (w/in 24 Hrs.) after birth			
		Male	Female	Total	%	Male	Female	Total	%	Male	Female	Total	%
Bacolod City	**11,082**	3,100	3,030	**6,130**	**55.31**	2,181	2,486	**4,667**	**42.11**	2,080	2,359	**4,439**	**40.06**
Iloilo City	**8,391**	2,632	2,551	**5,183**	**61.77**	2,317	2,115	**4,432**	**52.82**	2,121	1,977	**4,098**	**48.84**
**Region 7**	**162,308**	**46,515**	**46,287**	**92,802**	**57.18**	**55,944**	**54,954**	**110,898**	**68.33**	**41,009**	**40,076**	**81,085**	**49.96**
Bohol	**27,992**	6,136	5,973	**12,109**	**43.26**	7,962	7,562	**15,524**	**55.46**	7,087	6,883	**13,970**	**49.91**
Cebu	**66,463**	17,669	17,212	**34,881**	**52.48**	20,128	19,502	**39,630**	**59.63**	14,395	13,909	**28,304**	**42.59**
Negros Oriental	**27,890**	7,444	7,889	**15,333**	**54.98**	10,192	10,742	**20,934**	**75.06**	6,109	6,296	**12,405**	**44.48**
Siquijor	**1,661**	490	512	**1,002**	**60.33**	542	512	**1,054**	**63.46**	507	464	**971**	**58.46**
Cebu City	**20,866**	8,832	8,560	**17,392**	**83.35**	8,567	8,142	**16,709**	**80.08**	8,282	8,034	**16,316**	**78.19**
Lapu-Lapu City	**9,232**	3,812	4,019	**7,831**	**84.82**	4,014	4,078	**8,092**	**87.65**	3,141	3,085	**6,226**	**67.44**
Mandaue City	**8,204**	2,132	2,122	**4,254**	**51.85**	4,539	4,416	**8,955**	**109.15**	1,488	1,405	**2,893**	**35.26**
**Region 8**	**105,471**	**25,441**	**24,840**	**50,281**	**47.67**	**31,386**	**30,751**	**62,137**	**58.91**	**25,014**	**24,654**	**49,668**	**47.09**
Biliran	**3,873**	1,558	1,527	**3,085**	**79.65**	1,591	1,596	**3,187**	**82.29**	1,616	1,600	**3,216**	**83.04**
Eastern Samar	**11,908**	3,377	3,245	**6,622**	**55.61**	3,512	3,519	**7,031**	**59.04**	2,200	2,293	**4,493**	**37.73**
Northern Leyte	**35,422**	8,364	7,966	**16,330**	**46.10**	9,595	9,087	**18,682**	**52.74**	8,077	7,838	**15,915**	**44.93**
Northern Samar	**15,921**	3,065	3,125	**6,190**	**38.88**	5,059	4,891	**9,950**	**62.50**	3,896	3,739	**7,635**	**47.96**
Southern Leyte	**6,662**	1,959	1,877	**3,836**	**57.58**	2,016	1,901	**3,917**	**58.80**	1,783	1,755	**3,538**	**53.11**
Western Samar	**14,732**	3,037	2,877	**5,914**	**40.14**	4,929	5,097	**10,026**	**68.06**	3,835	3,868	**7,703**	**52.29**
Calbayog City	**4,535**	715	786	**1,501**	**33.10**	1,209	1,139	**2,348**	**51.78**	358	259	**617**	**13.61**
Maasin City	**1,696**	296	325	**621**	**36.62**	359	368	**727**	**42.87**	185	204	**389**	**22.94**
Ormoc City	**5,044**	1,711	1,640	**3,351**	**66.44**	1,719	1,653	**3,372**	**66.85**	1,748	1,658	**3,406**	**67.53**
Tacloban City	**5,678**	1,359	1,472	**2,831**	**49.86**	1,397	1,500	**2,897**	**51.02**	1,316	1,440	**2,756**	**48.54**
**Region 9**	**79,007**	**23,922**	**24,382**	**48,304**	**61.14**	**21,050**	**21,087**	**42,137**	**53.33**	**15,892**	**16,501**	**32,393**	**41.00**
Zamboanga del Norte	**16,854**	4,854	4,782	**9,636**	**57.17**	5,121	4,972	**10,093**	**59.88**	3,897	3,918	**7,815**	**46.37**
Zamboanga del Sur	**17,368**	3,255	3,353	**6,608**	**38.05**	3,660	3,605	**7,265**	**41.83**	2,405	2,642	**5,047**	**29.06**
Zamboanga Sibugay	**15,021**	3,856	3,822	**7,678**	**51.12**	3,829	3,942	**7,771**	**51.73**	3,373	3,421	**6,794**	**45.23**
Dapitan City	**1,737**	672	883	**1,555**	**89.52**	672	883	**1,555**	**89.52**	671	882	**1,553**	**89.41**
Dipolog City	**2,762**	1,125	1,065	**2,190**	**79.29**	1,139	1,081	**2,220**	**80.38**	998	943	**1,941**	**70.28**
Isabela City	**2,563**	669	670	**1,339**	**52.24**	964	959	**1,923**	**75.03**	643	646	**1,289**	**50.29**
Pagadian City	**4,257**	1,214	1,243	**2,457**	**57.72**	1,206	1,205	**2,411**	**56.64**	1,206	1,143	**2,349**	**55.18**
Zamboanga City	**18,445**	8,277	8,564	**16,841**	**91.30**	4,459	4,440	**8,899**	**48.25**	2,699	2,906	**5,605**	**30.39**
**Region 10**	**99,908**	**32,815**	**32,896**	**65,711**	**65.77**	**35,579**	**34,560**	**70,139**	**70.20**	**28,576**	**28,599**	**57,175**	**57.23**
Bukidnon	**22,900**	7,847	8,101	**15,948**	**69.64**	9,122	8,738	**17,860**	**77.99**	6,802	6,941	**13,743**	**60.01**
Camiguin	**1,854**	522	578	**1,100**	**59.33**	500	535	**1,035**	**55.83**	536	584	**1,120**	**60.41**
Lanao del Norte	**14,930**	3,925	3,989	**7,914**	**53.01**	4,652	4,640	**9,292**	**62.24**	3,301	3,199	**6,500**	**43.54**
Misamis Occidental	**6,420**	1,852	2,060	**3,912**	**60.93**	1,872	1,958	**3,830**	**59.66**	1,590	1,736	**3,326**	**51.81**
Misamis Oriental	**14,963**	5,262	5,044	**10,306**	**68.88**	5,755	5,372	**11,127**	**74.36**	5,194	4,939	**10,133**	**67.72**
Cagayan de Oro City	**14,172**	6,306	6,003	**12,309**	**86.85**	4,389	4,183	**8,572**	**60.49**	3,815	3,716	**7,531**	**53.14**
El Salvador City	**1,052**	298	326	**624**	**59.32**	427	410	**837**	**79.56**	352	387	**739**	**70.25**
Gingoog City	**2,608**	894	968	**1,862**	**71.40**	1,044	1,105	**2,149**	**82.40**	943	1,067	**2,010**	**77.07**
Iligan City	**7,565**	1,684	1,679	**3,363**	**44.45**	2,380	2,218	**4,598**	**60.78**	2,028	1,980	**4,008**	**52.98**
Malaybalay City	**3,817**	1,411	1,332	**2,743**	**71.86**	2,248	2,158	**4,406**	**115.43**	1,348	1,343	**2,691**	**70.50**
Oroquieta City	**1,389**	444	457	**901**	**64.87**	208	258	**466**	**33.55**	206	250	**456**	**32.83**
Ozamis City	**2,786**	932	920	**1,852**	**66.48**	932	920	**1,852**	**66.48**	932	920	**1,852**	**66.48**
Tangub City	**1,234**	514	477	**991**	**80.31**	416	432	**848**	**68.72**	334	334	**668**	**54.13**
Valencia City	**4,218**	924	962	**1,886**	**44.71**	1,634	1,633	**3,267**	**77.45**	1,195	1,203	**2,398**	**56.85**
**Region 11**	**108,407**	**41,304**	**37,853**	**79,157**	**73.02**	**46,446**	**40,187**	**86,633**	**79.91**	**38,959**	**35,307**	**74,266**	**68.51**
Davao de Oro	**15,490**	5,987	5,497	**11,484**	**74.14**	6,809	5,911	**12,720**	**82.12**	5,853	5,399	**11,252**	**72.64**
Davao del Norte	**21,017**	8,939	8,204	**17,143**	**81.57**	9,504	8,711	**18,215**	**86.67**	8,863	7,939	**16,802**	**79.94**
Davao Oriental	**13,229**	4,669	3,989	**8,658**	**65.45**	5,622	4,064	**9,686**	**73.22**	4,427	3,707	**8,134**	**61.49**
Davao del Sur	**14,564**	4,645	4,228	**8,873**	**60.92**	4,660	4,254	**8,914**	**61.21**	4,293	3,968	**8,261**	**56.72**
Davao Occidental	**6,510**	1,866	1,776	**3,642**	**55.94**	2,330	2,169	**4,499**	**69.11**	1,209	1,189	**2,398**	**36.84**
Davao City	**37,597**	15,198	14,159	**29,357**	**78.08**	17,521	15,078	**32,599**	**86.71**	14,314	13,105	**27,419**	**72.93**
**Region 12**	**98,859**	**30,050**	**29,698**	**59,748**	**60.44**	**31,652**	**31,351**	**63,003**	**63.73**	**27,147**	**26,978**	**54,125**	**54.75**
North Cotabato	**34,150**	8,392	8,590	**16,982**	**49.73**	9,751	9,479	**19,230**	**56.31**	7,170	7,289	**14,459**	**42.34**
Sarangani	**12,907**	4,638	4,571	**9,209**	**71.35**	4,587	4,994	**9,581**	**74.23**	4,368	4,422	**8,790**	**68.10**
South Cotabato	**20,917**	8,112	7,888	**16,000**	**76.49**	8,266	8,038	**16,304**	**77.95**	7,624	7,385	**15,009**	**71.76**
Sultan Kudarat	**17,299**	6,107	5,840	**11,947**	**69.06**	6,680	6,304	**12,984**	**75.06**	5,866	5,639	**11,505**	**66.51**

Table 2.C.1.1. Immunization Services for Infants and Children

Number and Proportion of Children Protected at Birth (CPAB), Newborn / Infants Vaccinated with BCG and Hepatitis B antigen
Philippines, 2022

Area	Eligible Pop (0-11 mos. old)	Child Protected at Birth (CPAB)				BCG				Hepa B1 (w/in 24 Hrs.) after birth			
		Male	Female	Total	%	Male	Female	Total	%	Male	Female	Total	%
Gen. Santos City	**13,586**	2,801	2,809	**5,610**	**41.29**	2,368	2,536	**4,904**	**36.10**	2,119	2,243	**4,362**	**32.11**
**BARMM**	**101,343**	**30,478**	**32,556**	**63,034**	**62.20**	**35,356**	**37,103**	**72,459**	**71.50**	**25,285**	**27,151**	**52,436**	**51.74**
Basilan	**7,823**	989	1,120	**2,109**	**26.96**	1,781	1,843	**3,624**	**46.32**	835	870	**1,705**	**21.79**
Lanao del Sur	**21,639**	9,397	10,847	**20,244**	**93.55**	8,713	9,949	**18,662**	**86.24**	8,312	9,585	**17,897**	**82.71**
Maguindanao	**32,198**	9,308	9,429	**18,737**	**58.19**	12,066	12,396	**24,462**	**75.97**	6,451	6,520	**12,971**	**40.29**
Sulu	**17,165**	4,260	4,524	**8,784**	**51.17**	5,294	5,374	**10,668**	**62.15**	3,808	3,867	**7,675**	**44.71**
Tawi-Tawi	**9,369**	2,525	2,541	**5,066**	**54.07**	2,907	2,910	**5,817**	**62.09**	2,162	2,150	**4,312**	**46.02**
Lamitan City	**2,154**	442	449	**891**	**41.36**	679	678	**1,357**	**63.00**	628	583	**1,211**	**56.22**
Marawi City	**5,173**	2,165	2,232	**4,397**	**85.00**	2,210	2,289	**4,499**	**86.97**	1,993	2,505	**4,498**	**86.95**
Cotabato City	**5,822**	1,392	1,414	**2,806**	**48.20**	1,706	1,664	**3,370**	**57.88**	1,096	1,071	**2,167**	**37.22**
**CARAGA**	**60,575**	**16,882**	**17,316**	**34,198**	**56.46**	**21,001**	**20,269**	**41,270**	**68.13**	**16,396**	**16,461**	**32,857**	**54.24**
Agusan del Norte	**8,127**	2,471	2,338	**4,809**	**59.17**	2,523	2,518	**5,041**	**62.03**	1,727	1,669	**3,396**	**41.79**
Agusan del Sur	**17,709**	5,117	4,973	**10,090**	**56.98**	6,627	6,184	**12,811**	**72.34**	5,216	5,109	**10,325**	**58.30**
Surigao del Norte	**7,280**	1,722	1,907	**3,629**	**49.85**	2,197	2,174	**4,371**	**60.04**	1,259	1,273	**2,532**	**34.78**
Surigao del Sur	**11,553**	2,544	2,794	**5,338**	**46.20**	3,555	3,419	**6,974**	**60.37**	2,778	2,932	**5,710**	**49.42**
Province of Dinagat	**2,576**	524	499	**1,023**	**39.71**	708	695	**1,403**	**54.46**	436	399	**835**	**32.41**
Bislig City	**2,192**	724	726	**1,450**	**66.15**	728	679	**1,407**	**64.19**	727	727	**1,454**	**66.33**
Butuan City	**7,743**	2,808	3,052	**5,860**	**75.68**	2,813	2,739	**5,552**	**71.70**	2,386	2,547	**4,933**	**63.71**
Surigao City	**3,395**	972	1,027	**1,999**	**58.88**	1,850	1,861	**3,711**	**109.31**	1,867	1,805	**3,672**	**108.16**

Table 2.C.1.2. Immunization Services for Infants

Number and Proportion of Infants who completed 3 doses of HiB-HepB antigen
Philippines, 2022

Area	Eligible Pop (0-11 mos. old)	DPT-HiB-HepB 1				DPT-HiB-HepB 2				DPT-HiB-HepB 3			
		Male	Female	Total	%	Male	Female	Total	%	Male	Female	Total	%
**PHILIPPINES**	**2,131,496**	**846,809**	**803,150**	**1,649,959**	**77.41**	**831,513**	**796,328**	**1,627,841**	**76.37**	**812,142**	**778,830**	**1,590,972**	**74.64**
**N C R**	**236,901**	**100,711**	**97,708**	**198,419**	**83.76**	**98,878**	**100,295**	**199,173**	**84.07**	**98,553**	**96,201**	**194,754**	**82.21**
Malabon	**6,724**	2662	2489	**5,151**	**76.61**	2,553	2,481	**5,034**	**74.87**	2,547	2,393	**4,940**	**73.47**
Navotas	**4,591**	2356	2235	**4,591**	**100.00**	2,217	2,070	**4,287**	**93.38**	2,121	2,084	**4,205**	**91.59**
Valenzuela City	**11,418**	3956	3764	**7,720**	**67.61**	3,976	3,831	**7,807**	**68.37**	4,090	3,924	**8,014**	**70.19**
Caloocan City	**29,146**	10412	10037	**20,449**	**70.16**	10,218	13,792	**24,010**	**82.38**	10,111	9,534	**19,645**	**67.40**
Marikina City	**8,294**	3023	3075	**6,098**	**73.52**	2,906	2,857	**5,763**	**69.48**	2,884	2,806	**5,690**	**68.60**
Pasig City	**13,894**	6825	6439	**13,264**	**95.47**	6,777	6,545	**13,322**	**95.88**	6,828	6,595	**13,423**	**96.61**
Pateros	**1,176**	476	477	**953**	**81.04**	483	464	**947**	**80.53**	494	490	**984**	**83.67**
Taguig	**14,807**	5957	5943	**11,900**	**80.37**	6,022	5,934	**11,956**	**80.75**	6,020	5,937	**11,957**	**80.75**
Quezon City	**54,011**	26795	27216	**54,011**	**100.00**	26,755	27,256	**54,011**	**100.00**	26,911	27,100	**54,011**	**100.00**
Makati City	**10,718**	5035	4828	**9,863**	**92.02**	4,931	4,494	**9,425**	**87.94**	4,869	4,679	**9,548**	**89.08**
Mandaluyong City	**7,111**	2296	2393	**4,689**	**65.94**	2,397	2,469	**4,866**	**68.43**	2,449	2,629	**5,078**	**71.41**
San Juan	**2,246**	666	650	**1,316**	**58.59**	673	633	**1,306**	**58.15**	689	672	**1,361**	**60.60**
Manila City	**32,743**	14465	13697	**28,162**	**86.01**	13,545	13,078	**26,623**	**81.31**	13,194	13,022	**26,216**	**80.07**
Las Piñas City	**10,832**	4211	3916	**8,127**	**75.03**	4,182	3,913	**8,095**	**74.73**	4,186	4,021	**8,207**	**75.77**
Muntinlupa City	**9,281**	3824	3529	**7,353**	**79.23**	3,743	3,566	**7,309**	**78.75**	3,814	3,616	**7,430**	**80.06**
Parañaque City	**12,249**	4209	3842	**8,051**	**65.73**	4,078	3,694	**7,772**	**63.45**	4,052	3,575	**7,627**	**62.27**
Pasay City	**7,660**	3543	3178	**6,721**	**87.74**	3,422	3,218	**6,640**	**86.68**	3,294	3,124	**6,418**	**83.79**
**C A R**	**35,179**	**12,497**	**11,665**	**24,162**	**68.68**	**12,455**	**11,730**	**24,185**	**68.75**	**12,276**	**11,559**	**23,835**	**67.75**
Abra	**4,275**	1,397	1,314	**2,711**	**63.42**	1,374	1,331	**2,705**	**63.27**	1,384	1,333	**2,717**	**63.56**
Apayao	**2,502**	995	895	**1,890**	**75.54**	1,041	941	**1,982**	**79.22**	1,017	906	**1,923**	**76.86**
Benguet	**9,086**	3,037	2,798	**5,835**	**64.22**	3,042	2,846	**5,888**	**64.80**	2,935	2,825	**5,760**	**63.39**
Ifugao	**4,486**	1,617	1,478	**3,095**	**68.99**	1,623	1,528	**3,151**	**70.24**	1,612	1,501	**3,113**	**69.39**
Kalinga	**4,701**	1,997	1,817	**3,814**	**81.13**	1,996	1,798	**3,794**	**80.71**	2,003	1,803	**3,806**	**80.96**
Mt. Province	**3,103**	1,135	1,071	**2,206**	**71.09**	1,112	1,088	**2,200**	**70.90**	1,118	1,032	**2,150**	**69.29**
Baguio City	**7,026**	2,319	2,292	**4,611**	**65.63**	2,267	2,198	**4,465**	**63.55**	2,207	2,159	**4,366**	**62.14**
**Region 1**	**97,099**	**37,392**	**34,574**	**71,966**	**74.12**	**37,591**	**34,961**	**72,552**	**74.72**	**37,488**	**34,813**	**72,301**	**74.46**
Ilocos Norte	**7,854**	2,832	2,755	**5,587**	**71.14**	2,807	2,817	**5,624**	**71.61**	2,826	2,851	**5,677**	**72.28**
Ilocos Sur	**9,072**	3,554	3,474	**7,028**	**77.47**	3,692	3,525	**7,217**	**79.55**	3,701	3,438	**7,139**	**78.69**
La Union	**11,338**	3,895	3,753	**7,648**	**67.45**	3,965	3,775	**7,740**	**68.27**	3,996	3,744	**7,740**	**68.27**
Pangasinan	**50,710**	19,932	17,798	**37,730**	**74.40**	19,929	17,837	**37,766**	**74.47**	19,887	18,029	**37,916**	**74.77**
Alaminos City	**1,910**	740	696	**1,436**	**75.18**	742	912	**1,654**	**86.60**	748	674	**1,422**	**74.45**
Candon City	**954**	456	472	**928**	**97.27**	494	469	**963**	**100.94**	449	428	**877**	**91.93**
Dagupan City	**3,657**	1,198	1,058	**2,256**	**61.69**	1,184	1,064	**2,248**	**61.47**	1,187	1,057	**2,244**	**61.36**
Laoag City	**1,812**	931	986	**1,917**	**105.79**	923	996	**1,919**	**105.91**	930	987	**1,917**	**105.79**
San Carlos City	**4,028**	1,722	1,621	**3,343**	**82.99**	1,677	1,587	**3,264**	**81.03**	1,634	1,605	**3,239**	**80.41**
San Fernando City	**2,074**	659	643	**1,302**	**62.78**	689	677	**1,366**	**65.86**	714	711	**1,425**	**68.71**
Urdaneta City	**2,836**	1,139	1,020	**2,159**	**76.13**	1,120	1,049	**2,169**	**76.48**	1,089	1,030	**2,119**	**74.72**
Vigan City	**854**	334	298	**632**	**74.00**	369	253	**622**	**72.83**	327	259	**586**	**68.62**
**Region 2**	**68,960**	**26,123**	**24,239**	**50,362**	**73.03**	**26,607**	**24,822**	**51,429**	**74.58**	**26,087**	**24,018**	**50,105**	**72.66**
Batanes	**362**	137	119	**256**	**70.72**	134	135	**269**	**74.31**	116	137	**253**	**69.89**
Cagayan	**20,431**	7,202	6,510	**13,712**	**67.11**	7,149	6,600	**13,749**	**67.29**	7,040	6,561	**13,601**	**66.57**
Isabela	**23,449**	8,658	8,380	**17,038**	**72.66**	9,161	8,796	**17,957**	**76.58**	8,923	8,261	**17,184**	**73.28**
Nueva Vizcaya	**9,538**	3,491	3,208	**6,699**	**70.23**	3,416	3,202	**6,618**	**69.39**	3,343	2,751	**6,094**	**63.89**
Quirino	**4,051**	1,811	1,541	**3,352**	**82.75**	1,896	1,622	**3,518**	**86.84**	1,876	1,757	**3,633**	**89.68**
Cauayan City	**2,565**	1,062	1,012	**2,074**	**80.86**	1,025	995	**2,020**	**78.75**	1,007	984	**1,991**	**77.62**
Ilagan City	**2,889**	1,314	1,329	**2,643**	**91.48**	1,349	1,319	**2,668**	**92.35**	1,342	1,380	**2,722**	**94.22**
Santiago City	**2,671**	1,429	1,242	**2,671**	**100.00**	1,455	1,216	**2,671**	**100.00**	1,413	1,258	**2,671**	**100.00**
Tuguegarao City	**3,004**	1,019	898	**1,917**	**63.81**	1,022	937	**1,959**	**65.21**	1,027	929	**1,956**	**65.11**
**Region 3**	**220,155**	**98,299**	**92,433**	**190,732**	**86.64**	**97,626**	**92,626**	**190,252**	**86.42**	**96,604**	**92,197**	**188,801**	**85.76**
Aurora	**4,754**	1,947	1,716	**3,663**	**77.05**	2,012	1,821	**3,833**	**80.63**	2,055	1,802	**3,857**	**81.13**
Bataan	**13,789**	5,953	5,478	**11,431**	**82.90**	5,869	5,433	**11,302**	**81.96**	5,728	5,431	**11,159**	**80.93**
Bulacan	**43,760**	19,821	18,530	**38,351**	**87.64**	20,198	18,943	**39,141**	**89.44**	19,805	18,996	**38,801**	**88.67**
Nueva Ecija	**29,039**	11,738	11,016	**22,754**	**78.36**	11,405	10,852	**22,257**	**76.65**	10,925	10,506	**21,431**	**73.80**
Pampanga	**31,620**	14,599	13,913	**28,512**	**90.17**	14,472	13,988	**28,460**	**90.01**	14,592	13,843	**28,435**	**89.93**
Tarlac	**19,319**	9,219	8,900	**18,119**	**93.79**	9,128	8,989	**18,117**	**93.78**	9,222	9,136	**18,358**	**95.03**
Zambales	**12,476**	4,682	4,430	**9,112**	**73.04**	4,511	4,314	**8,825**	**70.74**	4,320	4,126	**8,446**	**67.70**
Angeles City	**7,932**	3,594	3,535	**7,129**	**89.88**	3,537	3,510	**7,047**	**88.84**	3,581	3,517	**7,098**	**89.49**
Balanga City	**1,992**	819	670	**1,489**	**74.75**	797	675	**1,472**	**73.90**	783	698	**1,481**	**74.35**
Cabanatuan City	**5,945**	2,676	2,419	**5,095**	**85.70**	2,628	2,447	**5,075**	**85.37**	2,667	2,400	**5,067**	**85.23**
City of San Fernando	**5,907**	2,614	2,385	**4,999**	**84.63**	2,602	2,376	**4,978**	**84.27**	2,625	2,439	**5,064**	**85.73**
Gapan City	**2,170**	1,037	874	**1,911**	**88.06**	1,035	912	**1,947**	**89.72**	1,038	891	**1,929**	**88.89**
Mabalacat City	**4,831**	2,429	2,379	**4,808**	**99.52**	2,395	2,374	**4,769**	**98.72**	2,369	2,379	**4,748**	**98.28**
Malolos City	**4,890**	1,759	1,539	**3,298**	**67.44**	1,735	1,591	**3,326**	**68.02**	1,725	1,598	**3,323**	**67.96**
Meycauayan	**4,055**	1,900	1,828	**3,728**	**91.94**	1,797	1,770	**3,567**	**87.97**	1,861	1,810	**3,671**	**90.53**
Olongapo	**4,922**	1,699	1,636	**3,335**	**67.76**	1,674	1,582	**3,256**	**66.15**	1,652	1,526	**3,178**	**64.57**
Palayan City	**808**	352	345	**697**	**86.26**	377	336	**713**	**88.24**	365	330	**695**	**86.01**

Table 2.C.1.2. Immunization Services for Infants

Number and Proportion of Infants who completed 3 doses of HiB-HepB antigen
Philippines, 2022

Area	Eligible Pop (0-11 mos. old)	DPT-HiB-HepB 1				DPT-HiB-HepB 2				DPT-HiB-HepB 3			
		Male	Female	Total	%	Male	Female	Total	%	Male	Female	Total	%
San Jose City	**2,748**	1,400	1,203	**2,603**	**94.72**	1,357	1,137	**2,494**	**90.76**	1,260	1,144	**2,404**	**87.48**
San Jose del Monte City	**11,133**	6,143	6,081	**12,224**	**109.80**	6,236	6,042	**12,278**	**110.28**	6,159	6,110	**12,269**	**110.20**
Science City of Munoz	**1,601**	682	570	**1,252**	**78.20**	671	586	**1,257**	**78.51**	659	595	**1,254**	**78.33**
Tarlac City	**6,464**	3,236	2,986	**6,222**	**96.26**	3,190	2,948	**6,138**	**94.96**	3,213	2,920	**6,133**	**94.88**
**Region 4A**	**299,627**	**121,813**	**114,280**	**236,093**	**78.80**	**119,437**	**113,093**	**232,530**	**77.61**	**116,474**	**110,816**	**227,290**	**75.86**
Batangas	**39,255**	15,023	13,675	**28,698**	**73.11**	14,938	14,106	**29,044**	**73.99**	14,775	14,154	**28,929**	**73.70**
Cavite	**28,151**	12,653	12,335	**24,988**	**88.76**	12,566	12,236	**24,802**	**88.10**	12,516	12,065	**24,581**	**87.32**
Laguna	**19,696**	7,666	7,004	**14,670**	**74.48**	7,459	6,902	**14,361**	**72.91**	7,326	6,768	**14,094**	**71.56**
Quezon	**37,853**	14,422	13,513	**27,935**	**73.80**	13,648	12,956	**26,604**	**70.28**	12,825	12,260	**25,085**	**66.27**
Rizal	**44,798**	17,948	16,775	**34,723**	**77.51**	17,462	16,369	**33,831**	**75.52**	16,836	15,971	**32,807**	**73.23**
Antipolo City	**16,498**	7,134	6,724	**13,858**	**84.00**	6,971	6,517	**13,488**	**81.76**	6,595	6,169	**12,764**	**77.37**
Bacoor City	**12,341**	4,201	4,000	**8,201**	**66.45**	4,044	3,767	**7,811**	**63.29**	4,016	3,728	**7,744**	**62.75**
Batangas City	**6,965**	2,410	2,186	**4,596**	**65.99**	2,359	2,086	**4,445**	**63.82**	2,232	2,046	**4,278**	**61.42**
Biñan City	**6,597**	3,662	3,647	**7,309**	**110.79**	3,491	3,605	**7,096**	**107.56**	3,499	3,586	**7,085**	**107.40**
Cabuyao City	**6,119**	3,000	3,020	**6,020**	**98.38**	2,944	2,884	**5,828**	**95.24**	2,946	2,929	**5,875**	**96.01**
Calamba City	**9,005**	4,241	3,912	**8,153**	**90.54**	4,224	3,846	**8,070**	**89.62**	4,101	3,898	**7,999**	**88.83**
Cavite City	**2,105**	603	579	**1,182**	**56.15**	588	582	**1,170**	**55.58**	532	568	**1,100**	**52.26**
Dasmariñas City	**13,538**	5,181	4,853	**10,034**	**74.12**	5,332	4,978	**10,310**	**76.16**	5,234	4,924	**10,158**	**75.03**
General Trias City	**6,457**	3,113	2,961	**6,074**	**94.07**	3,118	2,957	**6,075**	**94.08**	3,071	2,935	**6,006**	**93.02**
Imus City	**8,292**	2,698	2,430	**5,128**	**61.84**	2,722	2,497	**5,219**	**62.94**	2,750	2,550	**5,300**	**63.92**
Lipa City	**7,018**	3,200	2,859	**6,059**	**86.34**	3,090	2,836	**5,926**	**84.44**	3,018	2,833	**5,851**	**83.37**
Lucena City	**5,738**	2,518	2,359	**4,877**	**84.99**	2,531	2,380	**4,911**	**85.59**	2,466	2,280	**4,746**	**82.71**
San Pablo City	**5,272**	2,034	1,998	**4,032**	**76.48**	2,019	2,042	**4,061**	**77.03**	1,861	1,878	**3,739**	**70.92**
San Pedro City	**6,455**	2,072	1,974	**4,046**	**62.68**	1,988	1,955	**3,943**	**61.08**	1,951	1,884	**3,835**	**59.41**
Santa Rosa City	**7,009**	3,198	2,979	**6,177**	**88.13**	3,200	3,062	**6,262**	**89.34**	3,205	2,921	**6,126**	**87.40**
Tagaytay City	**1,460**	711	620	**1,331**	**91.16**	684	639	**1,323**	**90.62**	721	648	**1,369**	**93.77**
Tanauan City	**3,661**	1,493	1,416	**2,909**	**79.46**	1,495	1,501	**2,996**	**81.84**	1,539	1,468	**3,007**	**82.14**
Tayabas City	**2,145**	893	816	**1,709**	**79.67**	878	784	**1,662**	**77.48**	818	745	**1,563**	**72.87**
Trece Martires City	**3,199**	1,739	1,645	**3,384**	**105.78**	1,686	1,606	**3,292**	**102.91**	1,641	1,608	**3,249**	**101.56**
**Region 4B**	**72,791**	**25,816**	**24,076**	**49,892**	**68.54**	**24,957**	**23,756**	**48,713**	**66.92**	**24,227**	**22,992**	**47,219**	**64.87**
Marinduque	**4,990**	1,591	1,481	**3,072**	**61.56**	1,616	1,512	**3,128**	**62.69**	1,574	1,453	**3,027**	**60.66**
Mindoro Occidental	**12,479**	4,722	4,491	**9,213**	**73.83**	4,554	4,332	**8,886**	**71.21**	4,363	4,154	**8,517**	**68.25**
Mindoro Oriental	**20,189**	6,796	6,446	**13,242**	**65.59**	6,613	6,265	**12,878**	**63.79**	6,444	5,988	**12,432**	**61.58**
Palawan	**21,963**	7,947	7,276	**15,223**	**69.31**	7,555	7,188	**14,743**	**67.13**	7,279	7,005	**14,284**	**65.04**
Romblon	**6,569**	2,136	2,024	**4,160**	**63.33**	2,080	2,013	**4,093**	**62.31**	2,048	2,001	**4,049**	**61.64**
Puerto Princesa City	**6,601**	2,624	2,358	**4,982**	**75.47**	2,539	2,446	**4,985**	**75.52**	2,519	2,391	**4,910**	**74.38**
**Region 5**	**138,457**	**50,214**	**46,780**	**96,994**	**70.05**	**48,368**	**44,740**	**93,108**	**67.25**	**46,202**	**43,738**	**89,940**	**64.96**
Albay	**24,081**	8,646	8,015	**16,661**	**69.19**	8,610	8,093	**16,703**	**69.36**	8,459	8,065	**16,524**	**68.62**
Camarines Norte	**14,384**	5,503	5,203	**10,706**	**74.43**	5,419	5,159	**10,578**	**73.54**	5,312	5,070	**10,382**	**72.18**
Camarines Sur	**39,486**	14,287	13,172	**27,459**	**69.54**	13,410	12,642	**26,052**	**65.98**	12,566	11,844	**24,410**	**61.82**
Catanduanes	**6,459**	2,245	2,045	**4,290**	**66.42**	2,106	1,380	**3,486**	**53.97**	1,998	1,923	**3,921**	**60.71**
Masbate	**22,600**	8,440	8,025	**16,465**	**72.85**	8,024	7,288	**15,312**	**67.75**	7,442	6,955	**14,397**	**63.70**
Sorsogon	**19,828**	6,983	6,535	**13,518**	**68.18**	6,744	6,439	**13,183**	**66.49**	6,506	6,208	**12,714**	**64.12**
Iriga City	**2,679**	889	900	**1,789**	**66.78**	896	888	**1,784**	**66.59**	864	858	**1,722**	**64.28**
Legaspi City	**4,233**	1,640	1,379	**3,019**	**71.32**	1,546	1,325	**2,871**	**67.82**	1,447	1,319	**2,766**	**65.34**
Naga City	**4,707**	1,581	1,506	**3,087**	**65.58**	1,613	1,526	**3,139**	**66.69**	1,608	1,496	**3,104**	**65.94**
**Region 6**	**146,449**	**54,154**	**51,040**	**105,194**	**71.83**	**53,776**	**50,802**	**104,578**	**71.41**	**53,278**	**50,349**	**103,627**	**70.76**
Aklan	**11,288**	3,781	3,502	**7,283**	**64.52**	3,808	3,592	**7,400**	**65.56**	3,784	3,541	**7,325**	**64.89**
Antique	**13,132**	4,265	4,089	**8,354**	**63.62**	4,243	4,058	**8,301**	**63.21**	4,190	3,929	**8,119**	**61.83**
Capiz	**13,975**	4,912	4,504	**9,416**	**67.38**	4,869	4,633	**9,502**	**67.99**	4,996	4,703	**9,699**	**69.40**
Guimaras	**3,084**	1,213	1,159	**2,372**	**76.91**	1,199	1,153	**2,352**	**76.26**	1,209	1,191	**2,400**	**77.82**
Iloilo	**36,267**	12,687	11,861	**24,548**	**67.69**	12,778	11,943	**24,721**	**68.16**	12,596	11,811	**24,407**	**67.30**
Negros Occidental	**49,230**	20,023	19,146	**39,169**	**79.56**	19,752	18,805	**38,557**	**78.32**	19,476	18,587	**38,063**	**77.32**
Bacolod City	**11,082**	4,067	3,713	**7,780**	**70.20**	3,823	3,524	**7,347**	**66.30**	3,759	3,563	**7,322**	**66.07**
Iloilo City	**8,391**	3,206	3,066	**6,272**	**74.75**	3,304	3,094	**6,398**	**76.25**	3,268	3,024	**6,292**	**74.99**
**Region 7**	**162,308**	**63,559**	**61,431**	**124,990**	**77.01**	**63,016**	**60,497**	**123,513**	**76.10**	**61,122**	**59,605**	**120,727**	**74.38**
Bohol	**27,992**	9,158	8,401	**17,559**	**62.73**	9,007	8,323	**17,330**	**61.91**	8,724	8,170	**16,894**	**60.35**
Cebu	**66,463**	25,495	24,644	**50,139**	**75.44**	25,076	24,348	**49,424**	**74.36**	24,752	23,895	**48,647**	**73.19**
Negros Oriental	**27,890**	11,788	12,430	**24,218**	**86.83**	11,483	12,111	**23,594**	**84.60**	11,207	12,046	**23,253**	**83.37**
Siquijor	**1,661**	560	551	**1,111**	**66.89**	553	548	**1,101**	**66.29**	589	562	**1,151**	**69.30**
Cebu City	**20,866**	8,557	7,853	**16,410**	**78.64**	9,106	7,611	**16,717**	**80.12**	8,200	7,520	**15,720**	**75.34**
Lapu-Lapu City	**9,232**	4,642	4,289	**8,931**	**96.74**	4,391	4,194	**8,585**	**92.99**	4,277	4,123	**8,400**	**90.99**
Mandaue City	**8,204**	3,359	3,263	**6,622**	**80.72**	3,400	3,362	**6,762**	**82.42**	3,373	3,289	**6,662**	**81.20**
**Region 8**	**105,471**	**36,156**	**34,252**	**70,408**	**66.76**	**35,509**	**33,947**	**69,456**	**65.85**	**33,496**	**32,305**	**65,801**	**62.39**
Biliran	**3,873**	1,600	1,584	**3,184**	**82.21**	1,555	1,551	**3,106**	**80.20**	1,487	1,470	**2,957**	**76.35**
Eastern Samar	**11,908**	3,918	3,823	**7,741**	**65.01**	3,836	3,778	**7,614**	**63.94**	3,692	3,563	**7,255**	**60.93**
Northern Leyte	**35,422**	11,657	10,777	**22,434**	**63.33**	11,606	10,752	**22,358**	**63.12**	11,083	10,324	**21,407**	**60.43**
Northern Samar	**15,921**	5,538	5,173	**10,711**	**67.28**	5,429	5,138	**10,567**	**66.37**	5,001	4,766	**9,767**	**61.35**
Southern Leyte	**6,662**	2,218	2,106	**4,324**	**64.91**	2,266	2,082	**4,348**	**65.27**	2,191	2,018	**4,209**	**63.18**

Table 2.C.1.2. Immunization Services for Infants

Number and Proportion of Infants who completed 3 doses of HiB-HepB antigen
Philippines, 2022

Area	Eligible Pop (0-11 mos. old)	DPT-HiB-HepB 1				DPT-HiB-HepB 2				DPT-HiB-HepB 3			
		Male	Female	Total	%	Male	Female	Total	%	Male	Female	Total	%
Western Samar	14,732	5,411	5,090	10,501	71.28	5,002	4,917	9,919	67.33	4,539	4,602	9,141	62.05
Calbayog City	4,535	1,570	1,508	3,078	67.87	1,428	1,388	2,816	62.09	1,299	1,315	2,614	57.64
Maasin City	1,696	437	429	866	51.06	449	469	918	54.13	445	439	884	52.12
Ormoc City	5,044	1,895	1,807	3,702	73.39	1,942	1,823	3,765	74.64	1,849	1,777	3,626	71.89
Tacloban City	5,678	1,912	1,955	3,867	68.10	1,996	2,049	4,045	71.24	1,910	2,031	3,941	69.41
**Region 9**	**79,007**	**32,745**	**31,318**	**64,063**	**81.09**	**31,292**	**30,474**	**61,766**	**78.18**	**30,316**	**29,860**	**60,176**	**76.17**
Zamboanga del Norte	16,854	7,237	6,689	13,926	82.63	6,865	6,729	13,594	80.66	6,718	6,448	13,166	78.12
Zamboanga del Sur	17,368	6,557	6,221	12,778	73.57	6,321	6,100	12,421	71.52	5,983	5,833	11,816	68.03
Zamboanga Sibugay	15,021	5,291	5,182	10,473	69.72	5,133	4,938	10,071	67.05	4,956	4,780	9,736	64.82
Dapitan City	1,737	683	890	1,573	90.56	692	882	1,574	90.62	707	868	1,575	90.67
Dipolog City	2,762	1,206	1,184	2,390	86.53	1,175	1,109	2,284	82.69	1,163	1,141	2,304	83.42
Isabela City	2,563	1,145	1,102	2,247	87.67	1,192	1,086	2,278	88.88	1,132	1,067	2,199	85.80
Pagadian City	4,257	1,521	1,546	3,067	72.05	1,570	1,492	3,062	71.93	1,578	1,484	3,062	71.93
Zamboanga City	18,445	9,105	8,504	17,609	95.47	8,344	8,138	16,482	89.36	8,079	8,239	16,318	88.47
**Region 10**	**99,908**	**42,120**	**39,567**	**81,687**	**81.76**	**40,723**	**38,677**	**79,400**	**79.47**	**39,598**	**37,718**	**77,316**	**77.39**
Bukidnon	22,900	10,611	9,914	20,525	89.63	9,997	9,504	19,501	85.16	9,602	9,272	18,874	82.42
Camiguin	1,854	663	644	1,307	70.50	643	617	1,260	67.96	604	606	1,210	65.26
Lanao del Norte	14,930	5,679	5,391	11,070	74.15	5,449	5,217	10,666	71.44	5,204	5,094	10,298	68.98
Misamis Occidental	6,420	2,642	2,433	5,075	79.05	2,550	2,481	5,031	78.36	2,506	2,421	4,927	76.74
Misamis Oriental	14,963	5,184	4,941	10,125	67.67	5,254	4,879	10,133	67.72	5,186	4,792	9,978	66.68
Cagayan de Oro City	14,172	6,219	5,760	11,979	84.53	5,986	5,696	11,682	82.43	5,971	5,513	11,484	81.03
El Salvador City	1,052	450	432	882	83.84	432	423	855	81.27	407	445	852	80.99
Gingoog City	2,608	1,303	1,252	2,555	97.97	1,260	1,292	2,552	97.85	1,281	1,252	2,533	97.12
Iligan City	7,565	3,226	2,908	6,134	81.08	3,050	2,761	5,811	76.81	2,896	2,684	5,580	73.76
Malaybalay City	3,817	1,830	1,693	3,523	92.30	1,812	1,688	3,500	91.70	1,781	1,684	3,465	90.78
Oroquieta City	1,389	512	524	1,036	74.59	535	522	1,057	76.10	495	541	1,036	74.59
Ozamis City	2,786	1,094	1,040	2,134	76.60	1,046	969	2,015	72.33	1,021	903	1,924	69.06
Tangub City	1,234	578	573	1,151	93.27	668	640	1,308	106.00	680	606	1,286	104.21
Valencia City	4,218	2,129	2,062	4,191	99.36	2,041	1,988	4,029	95.52	1,964	1,905	3,869	91.73
**Region 11**	**108,407**	**45,267**	**42,077**	**87,344**	**80.57**	**44,403**	**40,482**	**84,885**	**78.30**	**42,906**	**39,975**	**82,881**	**76.45**
Davao de Oro	15,490	6,635	6,112	12,747	82.29	6,494	5,895	12,389	79.98	6,352	5,831	12,183	78.65
Davao del Norte	21,017	9,431	8,672	18,103	86.14	9,472	8,478	17,950	85.41	8,993	8,367	17,360	82.60
Davao Oriental	13,229	5,540	4,816	10,356	78.28	5,520	4,688	10,208	77.16	5,305	4,471	9,776	73.90
Davao del Sur	14,564	5,263	4,802	10,065	69.11	5,195	4,875	10,070	69.14	4,996	4,802	9,798	67.28
Davao Occidental	6,510	2,525	2,334	4,859	74.64	2,344	2,235	4,579	70.34	2,209	2,114	4,323	66.41
Davao City	37,597	15,873	15,341	31,214	83.02	15,378	14,311	29,689	78.97	15,051	14,390	29,441	78.31
**Region 12**	**98,859**	**38,585**	**36,621**	**75,206**	**76.07**	**37,214**	**35,681**	**72,895**	**73.74**	**35,495**	**34,348**	**69,843**	**70.65**
North Cotabato	34,150	11,715	11,030	22,745	66.60	11,078	10,610	21,688	63.51	10,463	10,157	20,620	60.38
Sarangani	12,907	5,940	5,679	11,619	90.02	5,744	5,406	11,150	86.39	5,427	5,200	10,627	82.34
South Cotabato	20,917	8,658	8,146	16,804	80.34	8,450	8,135	16,585	79.29	8,315	8,055	16,370	78.26
Sultan Kudarat	17,299	7,110	6,853	13,963	80.72	7,120	6,803	13,923	80.48	6,960	6,684	13,644	78.87
Gen. Santos City	13,586	5,162	4,913	10,075	74.16	4,822	4,727	9,549	70.29	4,330	4,252	8,582	63.17
**BARMM**	**101,343**	**36,669**	**37,718**	**74,387**	**73.40**	**35,925**	**37,177**	**73,102**	**72.13**	**35,086**	**36,366**	**71,452**	**70.51**
Basilan	7,823	2,005	2,008	4,013	51.30	1,736	1,820	3,556	45.46	1,643	1,602	3,245	41.48
Lanao del Sur	21,639	9,555	10,602	20,157	93.15	9,596	10,613	20,209	93.39	9,600	10,599	20,199	93.35
Maguindanao	32,198	12,426	12,397	24,823	77.09	12,134	12,171	24,305	75.49	11,782	12,039	23,821	73.98
Sulu	17,165	4,939	4,996	9,935	57.88	4,883	5,043	9,926	57.83	4,628	4,750	9,378	54.63
Tawi-Tawi	9,369	2,879	2,899	5,778	61.67	2,878	2,877	5,755	61.43	2,856	2,799	5,655	60.36
Lamitan City	2,154	705	700	1,405	65.23	650	633	1,283	59.56	592	600	1,192	55.34
Marawi City	5,173	2,202	2,249	4,451	86.04	2,179	2,248	4,427	85.58	2,221	2,248	4,469	86.39
Cotabato City	5,822	1,958	1,867	3,825	65.70	1,869	1,772	3,641	62.54	1,764	1,729	3,493	60.00
**CARAGA**	**60,575**	**24,689**	**23,371**	**48,060**	**79.34**	**23,736**	**22,568**	**46,304**	**76.44**	**22,934**	**21,970**	**44,904**	**74.13**
Agusan del Norte	8,127	3,137	3,054	6,191	76.18	3,007	2,897	5,904	72.65	2,879	2,764	5,643	69.44
Agusan del Sur	17,709	7,321	6,901	14,222	80.31	6,986	6,599	13,585	76.71	6,845	6,499	13,344	75.35
Surigao del Norte	7,280	3,108	2,959	6,067	83.34	3,006	2,952	5,958	81.84	2,973	2,793	5,766	79.20
Surigao del Sur	11,553	4,745	4,526	9,271	80.25	4,569	4,298	8,867	76.75	4,340	4,192	8,532	73.85
Province of Dinagat	2,576	887	788	1,675	65.02	811	776	1,587	61.61	781	754	1,535	59.59
Bislig City	2,192	841	768	1,609	73.40	793	728	1,521	69.39	733	687	1,420	64.78
Butuan City	7,743	3,214	3,009	6,223	80.37	3,131	2,957	6,088	78.63	3,010	2,938	5,948	76.82
Surigao City	3,395	1,436	1,366	2,802	82.53	1,433	1,361	2,794	82.30	1,373	1,343	2,716	80.00

Table 2.C.1.3. Immunization Services for Infants and Children

Number and Proportion of Infants who completed 3 doses of Oral Polio Vaccine (OPV)
Philippines, 2022

Area	Eligible Pop (0-11 mos. old)	OPV 1				OPV 2				OPV 3			
		Male	Female	Total	%	Male	Female	Total	%	Male	Female	Total	%
**PHILIPPINES**	**2,131,496**	**848,852**	**806,661**	**1,655,513**	**77.67**	**836,086**	**798,967**	**1,635,053**	**76.71**	**825,702**	**789,758**	**1,615,460**	**75.79**
**N C R**	**236,901**	**100,777**	**97,067**	**197,844**	**83.51**	**98,349**	**95,517**	**193,866**	**81.83**	**99,072**	**95,931**	**195,003**	**82.31**
Malabon	**6,724**	2644	2506	**5,150**	**76.59**	2,550	2,443	**4,993**	**74.26**	2,499	2,388	**4,887**	**72.68**
Navotas	**4,591**	2352	2239	**4,591**	**100.00**	2,216	2,087	**4,303**	**93.73**	2,111	2,066	**4,177**	**90.98**
Valenzuela City	**11,418**	3861	3698	**7,559**	**66.20**	3,957	3,736	**7,693**	**67.38**	4,112	3,868	**7,980**	**69.89**
Caloocan City	**29,146**	10490	9809	**20,299**	**69.65**	9,749	9,282	**19,031**	**65.30**	10,691	9,043	**19,734**	**67.71**
Marikina City	**8,294**	3051	3066	**6,117**	**73.75**	2,906	2,861	**5,767**	**69.53**	2,891	2,783	**5,674**	**68.41**
Pasig City	**13,894**	6796	6398	**13,194**	**94.96**	6,768	6,496	**13,264**	**95.47**	6,724	6,544	**13,268**	**95.49**
Pateros	**1,176**	476	477	**953**	**81.04**	483	464	**947**	**80.53**	494	490	**984**	**83.67**
Taguig	**14,807**	5983	5940	**11,923**	**80.52**	6,035	5,954	**11,989**	**80.97**	6,043	5,926	**11,969**	**80.83**
Quezon City	**54,011**	26998	27013	**54,011**	**100.00**	26,780	27,231	**54,011**	**100.00**	26,785	27,226	**54,011**	**100.00**
Makati City	**10,718**	5015	4826	**9,841**	**91.82**	4,929	4,503	**9,432**	**88.00**	4,845	4,689	**9,534**	**88.95**
Mandaluyong City	**7,111**	2280	2372	**4,652**	**65.42**	2,384	2,459	**4,843**	**68.11**	2,421	2,615	**5,036**	**70.82**
San Juan	**2,246**	650	653	**1,303**	**58.01**	667	636	**1,303**	**58.01**	657	636	**1,293**	**57.57**
Manila City	**32,743**	14476	13723	**28,199**	**86.12**	13,558	13,087	**26,645**	**81.38**	13,196	13,006	**26,202**	**80.02**
Las Piñas City	**10,832**	4190	3893	**8,083**	**74.62**	4,192	3,935	**8,127**	**75.03**	4,227	4,048	**8,275**	**76.39**
Muntinlupa City	**9,281**	3767	3465	**7,232**	**77.92**	3,716	3,530	**7,246**	**78.07**	3,766	3,595	**7,361**	**79.31**
Parañaque City	**12,249**	4212	3806	**8,018**	**65.46**	4,103	3,674	**7,777**	**63.49**	4,290	3,854	**8,144**	**66.49**
Pasay City	**7,660**	3536	3183	**6,719**	**87.72**	3,356	3,139	**6,495**	**84.79**	3,320	3,154	**6,474**	**84.52**
**C A R**	**35,179**	**12,538**	**11,724**	**24,262**	**68.97**	**12,501**	**11,737**	**24,238**	**68.90**	**12,457**	**11,658**	**24,115**	**68.55**
Abra	**4,275**	1,390	1,316	**2,706**	**63.30**	1,361	1,328	**2,689**	**62.90**	1,399	1,332	**2,731**	**63.88**
Apayao	**2,502**	1,038	913	**1,951**	**77.98**	1,058	981	**2,039**	**81.49**	1,088	977	**2,065**	**82.53**
Benguet	**9,086**	3,028	2,817	**5,845**	**64.33**	3,085	2,845	**5,930**	**65.27**	2,960	2,857	**5,817**	**64.02**
Ifugao	**4,486**	1,599	1,446	**3,045**	**67.88**	1,558	1,459	**3,017**	**67.25**	1,593	1,457	**3,050**	**67.99**
Kalinga	**4,701**	2,037	1,858	**3,895**	**82.85**	2,011	1,793	**3,804**	**80.92**	2,060	1,836	**3,896**	**82.88**
Mt. Province	**3,103**	1,132	1,084	**2,216**	**71.41**	1,113	1,090	**2,203**	**71.00**	1,128	1,069	**2,197**	**70.80**
Baguio City	**7,026**	2,314	2,290	**4,604**	**65.53**	2,315	2,241	**4,556**	**64.84**	2,229	2,130	**4,359**	**62.04**
**Region 1**	**97,099**	**37,342**	**34,421**	**71,763**	**73.91**	**37,751**	**35,297**	**73,048**	**75.23**	**37,860**	**35,208**	**73,068**	**75.25**
Ilocos Norte	**7,854**	2,833	2,753	**5,586**	**71.12**	2,809	2,806	**5,615**	**71.49**	2,828	2,862	**5,690**	**72.45**
Ilocos Sur	**9,072**	3,660	3,506	**7,166**	**78.99**	3,813	3,597	**7,410**	**81.68**	3,831	3,529	**7,360**	**81.13**
La Union	**11,338**	3,904	3,763	**7,667**	**67.62**	3,955	3,775	**7,730**	**68.18**	4,099	3,822	**7,921**	**69.86**
Pangasinan	**50,710**	19,833	17,730	**37,563**	**74.07**	20,009	17,892	**37,901**	**74.74**	20,044	18,261	**38,305**	**75.54**
Alaminos City	**1,910**	684	639	**1,323**	**69.27**	726	639	**1,365**	**71.47**	683	621	**1,304**	**68.27**
Candon City	**954**	456	472	**928**	**97.27**	494	469	**963**	**100.94**	449	430	**879**	**92.14**
Dagupan City	**3,657**	1,198	1,058	**2,256**	**61.69**	1,189	1,057	**2,246**	**61.42**	1,203	1,057	**2,260**	**61.80**
Laoag City	**1,812**	931	986	**1,917**	**105.79**	923	996	**1,919**	**105.91**	930	987	**1,917**	**105.79**
San Carlos City	**4,028**	1,714	1,609	**3,323**	**82.50**	1,684	2,092	**3,776**	**93.74**	1,656	1,624	**3,280**	**81.43**
San Fernando City	**2,074**	657	635	**1,292**	**62.30**	689	670	**1,359**	**65.53**	710	698	**1,408**	**67.89**
Urdaneta City	**2,836**	1,139	1,020	**2,159**	**76.13**	1,123	1,052	**2,175**	**76.69**	1,089	1,052	**2,141**	**75.49**
Vigan City	**854**	333	250	**583**	**68.27**	337	252	**589**	**68.97**	338	265	**603**	**70.61**
**Region 2**	**68,960**	**26,243**	**24,047**	**50,290**	**72.93**	**26,445**	**25,487**	**51,932**	**75.31**	**27,468**	**25,007**	**52,475**	**76.09**
Batanes	**362**	137	120	**257**	**70.99**	134	135	**269**	**74.31**	117	137	**254**	**70.17**
Cagayan	**20,431**	7,179	6,513	**13,692**	**67.02**	7,181	6,630	**13,811**	**67.60**	7,138	6,610	**13,748**	**67.29**
Isabela	**23,449**	8,663	8,013	**16,676**	**71.12**	8,887	9,129	**18,016**	**76.83**	8,877	8,485	**17,362**	**74.04**
Nueva Vizcaya	**9,538**	3,633	3,319	**6,952**	**72.89**	3,508	3,393	**6,901**	**72.35**	4,646	3,481	**8,127**	**85.21**
Quirino	**4,051**	1,782	1,581	**3,363**	**83.02**	1,867	1,699	**3,566**	**88.03**	1,885	1,782	**3,667**	**90.52**
Cauayan City	**2,565**	1,054	1,021	**2,075**	**80.90**	1,059	1,011	**2,070**	**80.70**	1,006	991	**1,997**	**77.86**
Ilagan City	**2,889**	1,340	1,344	**2,684**	**92.90**	1,331	1,333	**2,664**	**92.21**	1,344	1,351	**2,695**	**93.28**
Santiago City	**2,671**	1,431	1,240	**2,671**	**100.00**	1,448	1,214	**2,662**	**99.66**	1,402	1,239	**2,641**	**98.88**
Tuguegarao City	**3,004**	1,024	896	**1,920**	**63.91**	1,030	943	**1,973**	**65.68**	1,053	931	**1,984**	**66.05**
**Region 3**	**220,155**	**98,203**	**92,323**	**190,526**	**86.54**	**97,517**	**92,475**	**189,992**	**86.30**	**97,723**	**93,262**	**190,985**	**86.75**
Aurora	**4,754**	1,917	1,685	**3,602**	**75.77**	2,046	1,845	**3,891**	**81.85**	2,126	1,922	**4,048**	**85.15**
Bataan	**13,789**	5,916	5,446	**11,362**	**82.40**	5,887	5,460	**11,347**	**82.29**	5,911	5,567	**11,478**	**83.24**
Bulacan	**43,760**	20,115	18,860	**38,975**	**89.07**	20,068	18,777	**38,845**	**88.77**	20,017	19,078	**39,095**	**89.34**
Nueva Ecija	**29,039**	11,284	10,518	**21,802**	**75.08**	11,025	10,532	**21,557**	**74.23**	11,125	10,553	**21,678**	**74.65**
Pampanga	**31,620**	14,593	13,882	**28,475**	**90.05**	14,468	13,958	**28,426**	**89.90**	14,631	13,959	**28,590**	**90.42**
Tarlac	**19,319**	9,196	8,935	**18,131**	**93.85**	9,252	9,065	**18,317**	**94.81**	9,284	9,156	**18,440**	**95.45**
Zambales	**12,476**	4,665	4,410	**9,075**	**72.74**	4,507	4,284	**8,791**	**70.46**	4,330	4,295	**8,625**	**69.13**
Angeles City	**7,932**	3,644	3,582	**7,226**	**91.10**	3,642	3,614	**7,256**	**91.48**	3,687	3,602	**7,289**	**91.89**
Balanga City	**1,992**	816	673	**1,489**	**74.75**	798	674	**1,472**	**73.90**	783	698	**1,481**	**74.35**
Cabanatuan City	**5,945**	2,680	2,406	**5,086**	**85.55**	2,616	2,434	**5,050**	**84.95**	2,690	2,425	**5,115**	**86.04**
City of San Fernando	**5,907**	2,609	2,395	**5,004**	**84.71**	2,643	2,411	**5,054**	**85.56**	2,653	2,461	**5,114**	**86.58**
Gapan City	**2,170**	1,075	912	**1,987**	**91.57**	1,036	920	**1,956**	**90.14**	1,028	897	**1,925**	**88.71**
Mabalacat City	**4,831**	2,426	2,389	**4,815**	**99.67**	2,415	2,379	**4,794**	**99.23**	2,374	2,412	**4,786**	**99.07**
Malolos City	**4,890**	1,771	1,538	**3,309**	**67.67**	1,733	1,588	**3,321**	**67.91**	1,729	1,606	**3,335**	**68.20**
Meycauayan	**4,055**	1,912	1,824	**3,736**	**92.13**	1,801	1,800	**3,601**	**88.80**	1,875	1,812	**3,687**	**90.92**
Olongapo	**4,922**	1,706	1,649	**3,355**	**68.16**	1,710	1,583	**3,293**	**66.90**	1,673	1,543	**3,216**	**65.34**
Palayan City	**808**	351	351	**702**	**86.88**	373	330	**703**	**87.00**	370	376	**746**	**92.33**
San Jose City	**2,748**	1,399	1,190	**2,589**	**94.21**	1,377	1,185	**2,562**	**93.23**	1,286	1,171	**2,457**	**89.41**

Table 2.C.1.3. Immunization Services for Infants and Children

Number and Proportion of Infants who completed 3 doses of Oral Polio Vaccine (OPV)
Philippines, 2022

Area	Eligible Pop (0-11 mos. old)	OPV 1				OPV 2				OPV 3			
		Male	Female	Total	%	Male	Female	Total	%	Male	Female	Total	%
San Jose del Monte City	**11,133**	6,164	6,099	**12,263**	**110.15**	6,240	6,083	**12,323**	**110.69**	6,286	6,227	**12,513**	**112.40**
Science City of Munoz	**1,601**	682	575	**1,257**	**78.51**	671	591	**1,262**	**78.83**	660	597	**1,257**	**78.51**
Tarlac City	**6,464**	3,282	3,004	**6,286**	**97.25**	3,209	2,962	**6,171**	**95.47**	3,205	2,905	**6,110**	**94.52**
**Region 4A**	**299,627**	**121,744**	**114,090**	**235,834**	**78.71**	**120,441**	**114,027**	**234,468**	**78.25**	**118,018**	**111,945**	**229,963**	**76.75**
Batangas	**39,255**	14,944	13,670	**28,614**	**72.89**	15,043	14,125	**29,168**	**74.30**	14,959	14,234	**29,193**	**74.37**
Cavite	**28,151**	12,600	12,207	**24,807**	**88.12**	12,645	12,249	**24,894**	**88.43**	12,549	12,117	**24,666**	**87.62**
Laguna	**19,696**	7,640	6,981	**14,621**	**74.23**	7,463	6,984	**14,447**	**73.35**	7,354	6,798	**14,152**	**71.85**
Quezon	**37,853**	14,628	13,539	**28,167**	**74.41**	14,084	13,304	**27,388**	**72.35**	13,485	12,847	**26,332**	**69.56**
Rizal	**44,798**	18,014	16,905	**34,919**	**77.95**	17,659	16,548	**34,207**	**76.36**	16,941	16,150	**33,091**	**73.87**
Antipolo City	**16,498**	7,169	6,754	**13,923**	**84.39**	6,981	6,522	**13,503**	**81.85**	6,612	6,169	**12,781**	**77.47**
Bacoor City	**12,341**	4,201	3,853	**8,054**	**65.26**	4,093	3,742	**7,835**	**63.49**	4,035	3,755	**7,790**	**63.12**
Batangas City	**6,965**	2,415	2,171	**4,586**	**65.84**	2,355	2,082	**4,437**	**63.70**	2,228	2,047	**4,275**	**61.38**
Biñan City	**6,597**	3,524	3,635	**7,159**	**108.52**	3,484	3,623	**7,107**	**107.73**	3,495	3,604	**7,099**	**107.61**
Cabuyao City	**6,119**	2,999	3,031	**6,030**	**98.55**	3,003	2,927	**5,930**	**96.91**	2,993	2,960	**5,953**	**97.29**
Calamba City	**9,005**	4,237	3,904	**8,141**	**90.41**	4,201	3,944	**8,145**	**90.45**	4,093	3,854	**7,947**	**88.25**
Cavite City	**2,105**	597	584	**1,181**	**56.10**	597	584	**1,181**	**56.10**	538	579	**1,117**	**53.06**
Dasmariñas City	**13,538**	5,144	4,852	**9,996**	**73.84**	5,313	5,023	**10,336**	**76.35**	5,308	4,994	**10,302**	**76.10**
General Trias City	**6,457**	3,124	2,995	**6,119**	**94.77**	3,136	3,167	**6,303**	**97.61**	3,100	2,915	**6,015**	**93.15**
Imus City	**8,292**	2,699	2,415	**5,114**	**61.67**	2,697	2,501	**5,198**	**62.69**	2,769	2,556	**5,325**	**64.22**
Lipa City	**7,018**	3,180	2,820	**6,000**	**85.49**	3,173	2,801	**5,974**	**85.12**	3,004	2,825	**5,829**	**83.06**
Lucena City	**5,738**	2,515	2,344	**4,859**	**84.68**	2,544	2,353	**4,897**	**85.34**	2,623	2,271	**4,894**	**85.29**
San Pablo City	**5,272**	2,024	1,995	**4,019**	**76.23**	2,025	2,066	**4,091**	**77.60**	2,010	2,010	**4,020**	**76.25**
San Pedro City	**6,455**	2,056	1,967	**4,023**	**62.32**	1,973	1,943	**3,916**	**60.67**	1,951	1,896	**3,847**	**59.60**
Santa Rosa City	**7,009**	3,201	2,975	**6,176**	**88.12**	3,203	3,059	**6,262**	**89.34**	3,217	2,932	**6,149**	**87.73**
Tagaytay City	**1,460**	710	619	**1,329**	**91.03**	684	641	**1,325**	**90.75**	718	650	**1,368**	**93.70**
Tanauan City	**3,661**	1,465	1,400	**2,865**	**78.26**	1,524	1,450	**2,974**	**81.23**	1,558	1,432	**2,990**	**81.67**
Tayabas City	**2,145**	915	812	**1,727**	**80.51**	887	789	**1,676**	**78.14**	830	757	**1,587**	**73.99**
Trece Martires City	**3,199**	1,743	1,662	**3,405**	**106.44**	1,674	1,600	**3,274**	**102.34**	1,648	1,593	**3,241**	**101.31**
**Region 4B**	**72,791**	**25,810**	**23,967**	**49,777**	**68.38**	**25,015**	**23,657**	**48,672**	**66.87**	**24,552**	**23,315**	**47,867**	**65.76**
Marinduque	**4,990**	1,596	1,485	**3,081**	**61.74**	1,610	1,506	**3,116**	**62.44**	1,601	1,484	**3,085**	**61.82**
Mindoro Occidental	**12,479**	4,752	4,430	**9,182**	**73.58**	4,634	4,397	**9,031**	**72.37**	4,487	4,259	**8,746**	**70.09**
Mindoro Oriental	**20,189**	6,691	6,291	**12,982**	**64.30**	6,522	6,137	**12,659**	**62.70**	6,432	6,044	**12,476**	**61.80**
Palawan	**21,963**	7,962	7,326	**15,288**	**69.61**	7,540	7,116	**14,656**	**66.73**	7,332	7,026	**14,358**	**65.37**
Romblon	**6,569**	2,131	2,021	**4,152**	**63.21**	2,081	1,998	**4,079**	**62.09**	2,080	2,035	**4,115**	**62.64**
Puerto Princesa City	**6,601**	2,678	2,414	**5,092**	**77.14**	2,628	2,503	**5,131**	**77.73**	2,620	2,467	**5,087**	**77.06**
**Region 5**	**138,457**	**50,521**	**46,757**	**97,278**	**70.26**	**50,350**	**47,294**	**97,644**	**70.52**	**49,482**	**46,811**	**96,293**	**69.55**
Albay	**24,081**	8,661	7,919	**16,580**	**68.85**	8,752	8,201	**16,953**	**70.40**	8,601	8,098	**16,699**	**69.35**
Camarines Norte	**14,384**	5,612	5,363	**10,975**	**76.30**	5,710	5,488	**11,198**	**77.85**	5,598	5,330	**10,928**	**75.97**
Camarines Sur	**39,486**	14,186	13,153	**27,339**	**69.24**	14,027	13,162	**27,189**	**68.86**	13,606	12,898	**26,504**	**67.12**
Catanduanes	**6,459**	2,161	1,968	**4,129**	**63.93**	2,069	1,995	**4,064**	**62.92**	2,063	2,025	**4,088**	**63.29**
Masbate	**22,600**	8,748	8,051	**16,799**	**74.33**	8,745	8,106	**16,851**	**74.56**	8,798	8,188	**16,986**	**75.16**
Sorsogon	**19,828**	7,051	6,530	**13,581**	**68.49**	6,924	6,553	**13,477**	**67.97**	6,803	6,461	**13,264**	**66.90**
Iriga City	**2,679**	889	897	**1,786**	**66.67**	920	900	**1,820**	**67.94**	886	867	**1,753**	**65.43**
Legaspi City	**4,233**	1,644	1,373	**3,017**	**71.27**	1,593	1,360	**2,953**	**69.76**	1,515	1,422	**2,937**	**69.38**
Naga City	**4,707**	1,569	1,503	**3,072**	**65.26**	1,610	1,529	**3,139**	**66.69**	1,612	1,522	**3,134**	**66.58**
**Region 6**	**146,449**	**54,197**	**50,975**	**105,172**	**71.81**	**53,991**	**50,834**	**104,825**	**71.58**	**53,470**	**50,454**	**103,924**	**70.96**
Aklan	**11,288**	3,810	3,553	**7,363**	**65.23**	3,823	3,611	**7,434**	**65.86**	3,805	3,533	**7,338**	**65.01**
Antique	**13,132**	4,256	4,044	**8,300**	**63.20**	4,252	4,093	**8,345**	**63.55**	4,261	4,004	**8,265**	**62.94**
Capiz	**13,975**	4,926	4,529	**9,455**	**67.66**	4,888	4,610	**9,498**	**67.96**	5,007	4,711	**9,718**	**69.54**
Guimaras	**3,084**	1,212	1,161	**2,373**	**76.95**	1,198	1,141	**2,339**	**75.84**	1,216	1,192	**2,408**	**78.08**
Iloilo	**36,267**	12,845	11,957	**24,802**	**68.39**	12,912	12,059	**24,971**	**68.85**	12,602	11,897	**24,499**	**67.55**
Negros Occidental	**49,230**	20,035	19,012	**39,047**	**79.32**	19,752	18,665	**38,417**	**78.04**	19,511	18,504	**38,015**	**77.22**
Bacolod City	**11,082**	3,914	3,649	**7,563**	**68.25**	3,859	3,573	**7,432**	**67.06**	3,804	3,589	**7,393**	**66.71**
Iloilo City	**8,391**	3,199	3,070	**6,269**	**74.71**	3,307	3,082	**6,389**	**76.14**	3,264	3,024	**6,288**	**74.94**
**Region 7**	**162,308**	**63,531**	**62,415**	**125,946**	**77.60**	**62,168**	**60,690**	**122,858**	**75.69**	**61,547**	**60,054**	**121,601**	**74.92**
Bohol	**27,992**	9,146	8,383	**17,529**	**62.62**	9,022	8,370	**17,392**	**62.13**	8,828	8,319	**17,147**	**61.26**
Cebu	**66,463**	25,475	25,618	**51,093**	**76.87**	24,937	24,199	**49,136**	**73.93**	24,808	23,969	**48,777**	**73.39**
Negros Oriental	**27,890**	11,882	12,494	**24,376**	**87.40**	11,716	12,450	**24,166**	**86.65**	11,328	12,405	**23,733**	**85.10**
Siquijor	**1,661**	567	557	**1,124**	**67.67**	558	550	**1,108**	**66.71**	603	569	**1,172**	**70.56**
Cebu City	**20,866**	8,569	7,851	**16,420**	**78.69**	8,244	7,597	**15,841**	**75.92**	8,259	7,448	**15,707**	**75.28**
Lapu-Lapu City	**9,232**	4,606	4,269	**8,875**	**96.13**	4,327	4,169	**8,496**	**92.03**	4,347	4,092	**8,439**	**91.41**
Mandaue City	**8,204**	3,286	3,243	**6,529**	**79.58**	3,364	3,355	**6,719**	**81.90**	3,374	3,252	**6,626**	**80.77**
**Region 8**	**105,471**	**36,179**	**34,449**	**70,628**	**66.96**	**35,607**	**34,156**	**69,763**	**66.14**	**34,640**	**33,259**	**67,899**	**64.38**
Biliran	**3,873**	1,604	1,573	**3,177**	**82.03**	1,539	1,538	**3,077**	**79.45**	1,500	1,449	**2,949**	**76.14**
Eastern Samar	**11,908**	4,035	3,879	**7,914**	**66.46**	3,871	3,894	**7,765**	**65.21**	3,887	3,709	**7,596**	**63.79**
Northern Leyte	**35,422**	11,463	10,666	**22,129**	**62.47**	11,646	10,667	**22,313**	**62.99**	11,387	10,580	**21,967**	**62.02**
Northern Samar	**15,921**	5,504	5,148	**10,652**	**66.91**	5,339	5,108	**10,447**	**65.62**	5,046	4,945	**9,991**	**62.75**
Southern Leyte	**6,662**	2,229	2,095	**4,324**	**64.91**	2,235	2,059	**4,294**	**64.46**	2,250	2,018	**4,268**	**64.06**
Western Samar	**14,732**	5,501	5,384	**10,885**	**73.89**	5,173	5,105	**10,278**	**69.77**	4,913	4,897	**9,810**	**66.59**

Table 2.C.1.3. Immunization Services for Infants and Children

Number and Proportion of Infants who completed 3 doses of Oral Polio Vaccine (OPV)
Philippines, 2022

Area	Eligible Pop (0-11 mos. old)	OPV 1				OPV 2				OPV 3			
		Male	Female	Total	%	Male	Female	Total	%	Male	Female	Total	%
Calbayog City	4,535	1,559	1,499	3,058	67.43	1,422	1,418	2,840	62.62	1,364	1,347	2,711	59.78
Maasin City	1,696	448	445	893	52.65	457	485	942	55.54	458	448	906	53.42
Ormoc City	5,044	1,909	1,799	3,708	73.51	1,923	1,833	3,756	74.46	1,899	1,834	3,733	74.01
Tacloban City	5,678	1,927	1,961	3,888	68.47	2,002	2,049	4,051	71.35	1,936	2,032	3,968	69.88
**Region 9**	79,007	32,691	31,409	64,100	81.13	31,754	30,570	62,324	78.88	30,678	29,834	60,512	76.59
Zamboanga del Norte	16,854	7,348	6,814	14,162	84.03	6,915	6,665	13,580	80.57	6,794	6,454	13,248	78.60
Zamboanga del Sur	17,368	6,648	6,228	12,876	74.14	6,485	6,230	12,715	73.21	6,192	6,076	12,268	70.64
Zamboanga Sibugay	15,021	5,278	5,123	10,401	69.24	5,174	4,925	10,099	67.23	4,924	4,788	9,712	64.66
Dapitan City	1,737	682	885	1,567	90.21	688	875	1,563	89.98	683	859	1,542	88.77
Dipolog City	2,762	1,207	1,161	2,368	85.73	1,191	1,113	2,304	83.42	1,184	1,131	2,315	83.82
Isabela City	2,563	1,147	1,110	2,257	88.06	1,188	1,093	2,281	89.00	1,149	1,064	2,213	86.34
Pagadian City	4,257	1,529	1,550	3,079	72.33	1,569	1,489	3,058	71.83	1,607	1,474	3,081	72.37
Zamboanga City	18,445	8,852	8,538	17,390	94.28	8,544	8,180	16,724	90.67	8,145	7,988	16,133	87.47
**Region 10**	99,908	44,097	44,101	88,198	88.28	42,823	40,738	83,561	83.64	41,509	39,694	81,203	81.28
Bukidnon	22,900	11,225	10,490	21,715	94.83	10,712	10,124	20,836	90.99	10,222	9,788	20,010	87.38
Camiguin	1,854	654	647	1,301	70.17	639	624	1,263	68.12	600	605	1,205	64.99
Lanao del Norte	14,930	5,598	5,431	11,029	73.87	5,424	5,189	10,613	71.09	5,354	5,259	10,613	71.09
Misamis Occidental	6,420	2,406	4,875	7,281	113.41	2,340	2,350	4,690	73.05	2,226	2,211	4,437	69.11
Misamis Oriental	14,963	6,447	6,155	12,602	84.22	6,460	6,117	12,577	84.05	6,389	5,964	12,353	82.56
Cagayan de Oro City	14,172	6,705	6,215	12,920	91.17	6,418	6,103	12,521	88.35	6,399	6,016	12,415	87.60
El Salvador City	1,052	443	426	869	82.60	433	429	862	81.94	419	445	864	82.13
Gingoog City	2,608	1,308	1,245	2,553	97.89	1,296	1,257	2,553	97.89	1,293	1,247	2,540	97.39
Iligan City	7,565	3,262	2,860	6,122	80.93	3,066	2,814	5,880	77.73	2,891	2,672	5,563	73.54
Malaybalay City	3,817	1,841	1,703	3,544	92.85	1,819	1,678	3,497	91.62	1,793	1,714	3,507	91.88
Oroquieta City	1,389	496	504	1,000	71.99	498	487	985	70.91	408	456	864	62.20
Ozamis City	2,786	1,105	1,018	2,123	76.20	1,055	995	2,050	73.58	1,000	922	1,922	68.99
Tangub City	1,234	489	488	977	79.17	593	561	1,154	93.52	534	504	1,038	84.12
Valencia City	4,218	2,118	2,044	4,162	98.67	2,070	2,010	4,080	96.73	1,981	1,891	3,872	91.80
**Region 11**	108,407	45,075	41,278	86,353	79.66	44,139	40,401	84,540	77.98	42,944	39,784	82,728	76.31
Davao de Oro	15,490	6,705	6,198	12,903	83.30	6,627	6,096	12,723	82.14	6,520	6,003	12,523	80.85
Davao del Norte	21,017	9,365	8,548	17,913	85.23	9,260	8,486	17,746	84.44	8,946	8,228	17,174	81.71
Davao Oriental	13,229	5,416	4,698	10,114	76.45	5,466	4,602	10,068	76.11	5,353	4,565	9,918	74.97
Davao del Sur	14,564	5,211	4,756	9,967	68.44	5,152	4,713	9,865	67.74	4,933	4,783	9,716	66.71
Davao Occidental	6,510	2,539	2,286	4,825	74.12	2,393	2,327	4,720	72.50	2,266	2,158	4,424	67.96
Davao City	37,597	15,839	14,792	30,631	81.47	15,241	14,177	29,418	78.25	14,926	14,047	28,973	77.06
**Region 12**	98,859	37,924	36,149	74,073	74.93	36,929	35,648	72,577	73.41	36,228	35,016	71,244	72.07
North Cotabato	34,150	11,546	10,835	22,381	65.54	10,668	10,360	21,028	61.58	10,319	10,154	20,473	59.95
Sarangani	12,907	5,618	5,300	10,918	84.59	5,511	5,187	10,698	82.89	5,379	5,110	10,489	81.27
South Cotabato	20,917	8,521	8,067	16,588	79.30	8,497	8,145	16,642	79.56	8,365	8,139	16,504	78.90
Sultan Kudarat	17,299	7,054	6,790	13,844	80.03	7,001	6,769	13,770	79.60	6,968	6,689	13,657	78.95
Gen. Santos City	13,586	5,185	5,157	10,342	76.12	5,252	5,187	10,439	76.84	5,197	4,924	10,121	74.50
**BARMM**	101,343	37,094	38,197	75,291	74.29	36,193	37,447	73,640	72.66	35,051	36,354	71,405	70.46
Basilan	7,823	1,915	1,953	3,868	49.44	1,717	1,743	3,460	44.23	1,544	1,563	3,107	39.72
Lanao del Sur	21,639	9,765	10,847	20,612	95.25	9,704	10,743	20,447	94.49	9,583	10,595	20,178	93.25
Maguindanao	32,198	12,621	12,495	25,116	78.00	12,327	12,364	24,691	76.68	11,969	12,080	24,049	74.69
Sulu	17,165	4,960	5,137	10,097	58.82	4,747	4,985	9,732	56.70	4,424	4,686	9,110	53.07
Tawi-Tawi	9,369	2,939	2,936	5,875	62.71	2,925	2,924	5,849	62.43	2,889	2,852	5,741	61.28
Lamitan City	2,154	700	717	1,417	65.78	648	641	1,289	59.84	635	612	1,247	57.89
Marawi City	5,173	2,232	2,241	4,473	86.47	2,225	2,273	4,498	86.95	2,231	2,230	4,461	86.24
Cotabato City	5,822	1,962	1,871	3,833	65.84	1,900	1,774	3,674	63.11	1,776	1,736	3,512	60.32
**CARAGA**	60,575	24,886	23,292	48,178	79.53	24,113	22,992	47,105	77.76	23,003	22,172	45,175	74.58
Agusan del Norte	8,127	3,165	2,979	6,144	75.60	3,021	2,883	5,904	72.65	2,849	2,749	5,598	68.88
Agusan del Sur	17,709	7,297	6,794	14,091	79.57	7,058	6,676	13,734	77.55	6,852	6,481	13,333	75.29
Surigao del Norte	7,280	3,171	2,948	6,119	84.05	3,020	2,983	6,003	82.46	2,869	2,817	5,686	78.10
Surigao del Sur	11,553	4,843	4,603	9,446	81.76	4,714	4,555	9,269	80.23	4,443	4,297	8,740	75.65
Province of Dinagat	2,576	856	782	1,638	63.59	826	755	1,581	61.37	733	720	1,453	56.41
Bislig City	2,192	838	762	1,600	72.99	788	735	1,523	69.48	741	703	1,444	65.88
Butuan City	7,743	3,261	3,077	6,338	81.85	3,274	3,050	6,324	81.67	3,150	3,084	6,234	80.51
Surigao City	3,395	1,455	1,347	2,802	82.53	1,412	1,355	2,767	81.50	1,366	1,321	2,687	79.15

Table 2.C.1.3. Immunization Services for Infants and Children

Number and Proportion of Infants who completed 2 doses of Inactivated Polio Vaccine (IPV)
Philippines, 2022

Area	Eligible Pop (0-11mos. old)	IPV 1				IPV 2 (Routine)				Eligible Pop (0-23mos. old)	IPV 2 (Catch-up)			
		Male	Female	Total	%	Male	Female	Total	%		Male	Female	Total	%
**PHILIPPINES**	**2,131,496**	**806,671**	**771,692**	**1,578,363**	**74.05**	**499,731**	**477,690**	**977,421**	**45.86**	**4,278,249**	**73,260**	**69,435**	**142,695**	**3.34**
**N C R**	**236,901**	**96,826**	**94,089**	**190,915**	**80.59**	**86,436**	**84,342**	**170,778**	**72.09**	**471,146**	**11,120**	**10,508**	**21,628**	**4.59**
Malabon	**6,724**	2,497	2,418	**4,915**	**73.10**	1,791	1,711	**3,502**	**52.08**	**13,377**	544	480	**1,024**	**7.65**
Navotas	**4,591**	1,873	1,722	**3,595**	**78.31**	2,285	2,177	**4,462**	**97.19**	**9,127**	318	253	**571**	**6.26**
Valenzuela City	**11,418**	4,017	3,712	**7,729**	**67.69**	3,569	3,396	**6,965**	**61.00**	**22,701**	0	0	**0**	**0.00**
Caloocan City	**29,146**	9,544	8,901	**18,445**	**63.28**	8,510	7,996	**16,506**	**56.63**	**57,953**	0	0	**0**	**0.00**
Marikina City	**8,294**	2,719	2,625	**5,344**	**64.43**	2,486	2,241	**4,727**	**56.99**	**16,492**	0	0	**0**	**0.00**
Pasig City	**13,894**	6,592	6,288	**12,880**	**92.70**	4,915	5,655	**10,570**	**76.08**	**27,634**	2,008	1,895	**3,903**	**14.12**
Pateros	**1,176**	495	490	**985**	**83.76**	509	475	**984**	**83.67**	**2,336**	0	0	**0**	**0.00**
Taguig	**14,807**	6,013	5,765	**11,778**	**79.54**	5,812	5,605	**11,417**	**77.11**	**29,450**	0	0	**0**	**0.00**
Quezon City	**54,011**	25,893	26,205	**52,098**	**96.46**	22,331	23,181	**45,512**	**84.26**	**107,432**	0	0	**0**	**0.00**
Makati City	**10,718**	4,706	4,443	**9,149**	**85.36**	3,569	3,448	**7,017**	**65.47**	**21,316**	211	211	**422**	**1.98**
Mandaluyong City	**7,111**	2,837	3,123	**5,960**	**83.81**	1,518	1,581	**3,099**	**43.58**	**14,134**	0	0	**0**	**0.00**
San Juan	**2,246**	680	689	**1,369**	**60.95**	801	825	**1,626**	**72.40**	**4,469**	0	0	**0**	**0.00**
Manila City	**32,743**	13,586	13,367	**26,953**	**82.32**	12,878	12,521	**25,399**	**77.57**	**65,131**	4,581	4,382	**8,963**	**13.76**
Las Piñas City	**10,832**	4,197	4,037	**8,234**	**76.02**	3,939	3,847	**7,786**	**71.88**	**21,544**	0	0	**0**	**0.00**
Muntinlupa City	**9,281**	3,705	3,494	**7,199**	**77.57**	3,546	3,390	**6,936**	**74.73**	**18,459**	947	906	**1,853**	**10.04**
Parañaque City	**12,249**	4,008	3,614	**7,622**	**62.23**	4,219	2,860	**7,079**	**57.79**	**24,360**	1,083	1,007	**2,090**	**8.58**
Pasay City	**7,660**	3,464	3,196	**6,660**	**86.95**	3,758	3,433	**7,191**	**93.88**	**15,231**	1,428	1,374	**2,802**	**18.40**
**C A R**	**35,179**	**12,140**	**11,415**	**23,555**	**66.96**	**7,686**	**7,456**	**15,142**	**43.04**	**71,132**	**771**	**736**	**1,507**	**2.12**
Abra	**4,275**	1,302	1,227	**2,529**	**59.16**	1,140	1,093	**2,233**	**52.23**	**8,668**	0	0	**0**	**0.00**
Apayao	**2,502**	1,079	992	**2,071**	**82.77**	836	732	**1,568**	**62.67**	**5,047**	0	0	**0**	**0.00**
Benguet	**9,086**	2,902	2,823	**5,725**	**63.01**	1,943	1,831	**3,774**	**41.54**	**18,290**	636	622	**1,258**	**6.88**
Ifugao	**4,486**	1,475	1,304	**2,779**	**61.95**	231	260	**491**	**10.95**	**9,266**	0	0	**0**	**0.00**
Kalinga	**4,701**	2,020	1,775	**3,795**	**80.73**	995	990	**1,985**	**42.23**	**9,502**	131	107	**238**	**2.50**
Mt. Province	**3,103**	1,101	1,040	**2,141**	**69.00**	653	620	**1,273**	**41.02**	**6,199**	4	7	**11**	**0.18**
Baguio City	**7,026**	2,261	2,254	**4,515**	**64.26**	1,888	1,930	**3,818**	**54.34**	**14,160**	0	0	**0**	**0.00**
**Region 1**	**97,099**	**38,381**	**36,867**	**75,248**	**77.50**	**16,364**	**14,659**	**31,023**	**31.95**	**194,342**	**266**	**243**	**509**	**0.26**
Ilocos Norte	**7,854**	2,872	2,851	**5,723**	**72.87**	1,091	1,082	**2,173**	**27.67**	**15,438**	0	0	**0**	**0.00**
Ilocos Sur	**9,072**	3,881	3,613	**7,494**	**82.61**	3,117	2,956	**6,073**	**66.94**	**18,422**	0	0	**0**	**0.00**
La Union	**11,338**	4,329	4,048	**8,377**	**73.88**	1,975	1,908	**3,883**	**34.25**	**22,633**	0	0	**0**	**0.00**
Pangasinan	**50,710**	20,241	19,618	**39,859**	**78.60**	7,249	6,006	**13,255**	**26.14**	**101,567**	0	0	**0**	**0.00**
Alaminos City	**1,910**	712	644	**1,356**	**70.99**	597	552	**1,149**	**60.16**	**3,840**	0	0	**0**	**0.00**
Candon City	**954**	449	430	**879**	**92.14**	220	232	**452**	**47.38**	**1,937**	0	0	**0**	**0.00**
Dagupan City	**3,657**	1,203	1,057	**2,260**	**61.80**	770	736	**1,506**	**41.18**	**7,323**	266	243	**509**	**6.95**
Laoag City	**1,812**	940	997	**1,937**	**106.90**	168	180	**348**	**19.21**	**3,559**	0	0	**0**	**0.00**
San Carlos City	**4,028**	1,638	1,630	**3,268**	**81.13**	409	391	**800**	**19.86**	**8,068**	0	0	**0**	**0.00**
San Fernando City	**2,074**	688	673	**1,361**	**65.62**	99	68	**167**	**8.05**	**4,141**	0	0	**0**	**0.00**
Urdaneta City	**2,836**	1,082	1,046	**2,128**	**75.04**	648	535	**1,183**	**41.71**	**5,685**	0	0	**0**	**0.00**
Vigan City	**854**	346	260	**606**	**70.96**	21	13	**34**	**3.98**	**1,729**	0	0	**0**	**0.00**
**Region 2**	**68,960**	**26,541**	**24,542**	**51,083**	**74.08**	**10,200**	**9,435**	**19,635**	**28.47**	**138,670**	**973**	**811**	**1,784**	**1.29**
Batanes	**362**	117	136	**253**	**69.89**	93	107	**200**	**55.25**	**729**	0	0	**0**	**0.00**
Cagayan	**20,431**	7,223	6,528	**13,751**	**67.30**	1,941	1,725	**3,666**	**17.94**	**41,280**	510	527	**1,037**	**2.51**
Isabela	**23,449**	9,061	8,394	**17,455**	**74.44**	5,575	5,146	**10,721**	**45.72**	**46,823**	463	284	**747**	**1.60**
Nueva Vizcaya	**9,538**	3,429	3,294	**6,723**	**70.49**	0	0	**0**	**0.00**	**19,245**	0	0	**0**	**0.00**
Quirino	**4,051**	1,896	1,702	**3,598**	**88.82**	986	843	**1,829**	**45.15**	**8,315**	0	0	**0**	**0.00**
Cauayan City	**2,565**	1,029	970	**1,999**	**77.93**	75	69	**144**	**5.61**	**5,118**	0	0	**0**	**0.00**
Ilagan City	**2,889**	1,336	1,354	**2,690**	**93.11**	587	609	**1,196**	**41.40**	**5,762**	0	0	**0**	**0.00**
Santiago City	**2,671**	1,401	1,239	**2,640**	**98.84**	855	838	**1,693**	**63.38**	**5,333**	0	0	**0**	**0.00**
Tuguegarao City	**3,004**	1,049	925	**1,974**	**65.71**	88	98	**186**	**6.19**	**6,065**	0	0	**0**	**0.00**
**Region 3**	**220,155**	**99,223**	**94,084**	**193,307**	**87.80**	**83,854**	**79,633**	**163,487**	**74.26**	**438,467**	**16,060**	**15,400**	**31,460**	**7.17**
Aurora	**4,754**	2,126	1,905	**4,031**	**84.79**	1,816	1,652	**3,468**	**72.95**	**9,695**	0	0	**0**	**0.00**
Bataan	**13,789**	5,932	5,554	**11,486**	**83.30**	4,884	4,422	**9,306**	**67.49**	**27,103**	2,656	2,434	**5,090**	**18.78**
Bulacan	**43,760**	20,446	19,305	**39,751**	**90.84**	16,799	16,457	**33,256**	**76.00**	**87,057**	4,842	4,758	**9,600**	**11.03**
Nueva Ecija	**29,039**	12,711	11,979	**24,690**	**85.02**	6,460	6,001	**12,461**	**42.91**	**58,193**	1,698	1,751	**3,449**	**5.93**
Pampanga	**31,620**	14,483	13,818	**28,301**	**89.50**	13,907	12,638	**26,545**	**83.95**	**62,829**	2,146	1,935	**4,081**	**6.50**
Tarlac	**19,319**	9,361	9,229	**18,590**	**96.23**	10,709	10,395	**21,104**	**109.24**	**38,414**	1,615	1,536	**3,151**	**8.20**
Zambales	**12,476**	4,209	4,018	**8,227**	**65.94**	3,061	3,009	**6,070**	**48.65**	**24,929**	37	44	**81**	**0.32**
Angeles City	**7,932**	3,612	3,580	**7,192**	**90.67**	3,233	3,175	**6,408**	**80.79**	**15,763**	0	0	**0**	**0.00**
Balanga City	**1,992**	785	698	**1,483**	**74.45**	762	684	**1,446**	**72.59**	**3,916**	430	389	**819**	**20.91**
Cabanatuan City	**5,945**	2,655	2,376	**5,031**	**84.63**	2,672	2,577	**5,249**	**88.29**	**11,917**	467	452	**919**	**7.71**
City of San Fernando	**5,907**	2,636	2,422	**5,058**	**85.63**	2,203	1,957	**4,160**	**70.42**	**11,747**	0	0	**0**	**0.00**
Gapan City	**2,170**	1,036	991	**2,027**	**93.41**	651	726	**1,377**	**63.46**	**4,347**	221	159	**380**	**8.74**
Mabalacat City	**4,831**	2,351	2,306	**4,657**	**96.40**	2,132	1,992	**4,124**	**85.37**	**9,604**	0	0	**0**	**0.00**
Malolos City	**4,890**	1,732	1,612	**3,344**	**68.38**	1,650	1,583	**3,233**	**66.11**	**9,721**	1	2	**3**	**0.03**
Meycauayan	**4,055**	1,927	1,838	**3,765**	**92.85**	1,131	1,124	**2,255**	**55.61**	**8,067**	132	143	**275**	**3.41**
Olongapo	**4,922**	1,594	1,429	**3,023**	**61.42**	1,303	1,133	**2,436**	**49.49**	**9,837**	0	0	**0**	**0.00**
Palayan City	**808**	353	341	**694**	**85.89**	213	200	**413**	**51.11**	**1,614**	414	397	**811**	**50.25**
San Jose City	**2,748**	1,280	1,167	**2,447**	**89.05**	862	787	**1,649**	**60.01**	**5,507**	536	544	**1,080**	**19.61**

Table 2.C.1.3. Immunization Services for Infants and Children

Number and Proportion of Infants who completed 2 doses of Inactivated Polio Vaccine (IPV)
Philippines, 2022

Area	Eligible Pop (0-11mos. old)	IPV 1				IPV 2 (Routine)				Eligible Pop (0-23mos. old)	IPV 2 (Catch-up)			
		Male	Female	Total	%	Male	Female	Total	%		Male	Female	Total	%
San Jose del Monte City	11,133	6,279	6,136	12,415	111.52	6,065	6,002	12,067	108.39	22,140	748	738	1,486	6.71
Science City of Munoz	1,601	673	610	1,283	80.14	638	579	1,217	76.01	3,211	0	0	0	0.00
Tarlac City	6,464	3,042	2,770	5,812	89.91	2,703	2,540	5,243	81.11	12,856	117	118	235	1.83
**Region 4A**	299,627	116,259	110,744	227,003	75.76	67,964	64,497	132,461	44.21	597,437	15,288	14,475	29,763	4.98
Batangas	39,255	15,029	14,194	29,223	74.44	10,410	9,796	20,206	51.47	78,750	4,237	3,913	8,150	10.35
Cavite	28,151	12,663	12,154	24,817	88.16	7,792	7,477	15,269	54.24	55,842	1,239	1,171	2,410	4.32
Laguna	19,696	7,270	6,735	14,005	71.11	3,368	3,307	6,675	33.89	39,127	999	854	1,853	4.74
Quezon	37,853	12,403	12,016	24,419	64.51	5,828	5,535	11,363	30.02	76,326	1,196	1,169	2,365	3.10
Rizal	44,798	16,566	15,858	32,424	72.38	10,164	9,663	19,827	44.26	88,903	582	586	1,168	1.31
Antipolo City	16,498	6,500	6,120	12,620	76.49	1,870	1,771	3,641	22.07	32,750	1,589	1,645	3,234	9.87
Bacoor City	12,341	4,052	3,756	7,808	63.27	2,613	2,395	5,008	40.58	24,462	379	353	732	2.99
Batangas City	6,965	2,241	2,038	4,279	61.44	1,412	1,333	2,745	39.41	13,978	342	286	628	4.49
Biñan City	6,597	3,589	3,678	7,267	110.16	2,680	2,772	5,452	82.64	13,124	194	199	393	2.99
Cabuyao City	6,119	2,896	2,901	5,797	94.74	2,411	2,216	4,627	75.62	12,161	199	173	372	3.06
Calamba City	9,005	4,162	3,900	8,062	89.53	2,521	2,410	4,931	54.76	17,904	2,190	2,199	4,389	24.51
Cavite City	2,105	593	628	1,221	58.00	256	257	513	24.37	4,194	8	6	14	0.33
Dasmariñas City	13,538	5,589	5,310	10,899	80.51	2,158	1,973	4,131	30.51	26,832	270	234	504	1.88
General Trias City	6,457	3,142	3,127	6,269	97.09	2,007	1,864	3,871	59.95	12,802	24	19	43	0.34
Imus City	8,292	2,671	2,496	5,167	62.31	1,932	1,721	3,653	44.05	16,435	253	197	450	2.74
Lipa City	7,018	2,822	2,522	5,344	76.15	953	926	1,879	26.77	14,083	246	204	450	3.20
Lucena City	5,738	2,564	2,331	4,895	85.31	1,681	1,609	3,290	57.34	11,570	64	52	116	1.00
San Pablo City	5,272	1,948	1,908	3,856	73.14	1,497	1,472	2,969	56.32	10,485	138	134	272	2.59
San Pedro City	6,455	1,951	1,896	3,847	59.60	1,192	1,196	2,388	36.99	12,834	3	7	10	0.08
Santa Rosa City	7,009	2,909	2,699	5,608	80.01	1,821	1,590	3,411	48.67	13,939	0	0	0	0.00
Tagaytay City	1,460	706	661	1,367	93.63	640	572	1,212	83.01	2,901	192	174	366	12.62
Tanauan City	3,661	1,508	1,452	2,960	80.85	908	849	1,757	47.99	7,352	339	301	640	8.71
Tayabas City	2,145	808	733	1,541	71.84	631	646	1,277	59.53	4,343	206	265	471	10.85
Trece Martires City	3,199	1,677	1,631	3,308	103.41	1,219	1,147	2,366	73.96	6,340	399	334	733	11.56
**Region 4B**	72,791	23,378	22,126	45,504	62.51	7,779	7,518	15,297	21.01	145,581	1,071	1,029	2,100	1.44
Marinduque	4,990	1,589	1,493	3,082	61.76	1,088	1,067	2,155	43.19	10,041	147	147	294	2.93
Mindoro Occidental	12,479	4,150	4,023	8,173	65.49	1,052	997	2,049	16.42	25,047	272	265	537	2.14
Mindoro Oriental	20,189	6,308	5,874	12,182	60.34	1,849	1,764	3,613	17.90	40,162	205	187	392	0.98
Palawan	21,963	6,753	6,348	13,101	59.65	1,419	1,331	2,750	12.52	43,897	371	366	737	1.68
Romblon	6,569	2,019	1,960	3,979	60.57	1,086	1,091	2,177	33.14	13,258	28	27	55	0.41
Puerto Princesa City	6,601	2,559	2,428	4,987	75.55	1,285	1,268	2,553	38.68	13,176	48	37	85	0.65
**Region 5**	138,457	48,295	45,417	93,712	67.68	26,233	24,951	51,184	36.97	279,883	10,297	9,531	19,828	7.08
Albay	24,081	8,877	8,385	17,262	71.68	6,167	6,052	12,219	50.74	48,776	70	84	154	0.32
Camarines Norte	14,384	5,603	5,325	10,928	75.97	2,267	2,034	4,301	29.90	28,421	1,030	989	2,019	7.10
Camarines Sur	39,486	12,886	11,956	24,842	62.91	6,976	6,515	13,491	34.17	79,682	5,395	4,839	10,234	12.84
Catanduanes	6,459	2,027	1,927	3,954	61.22	1,517	1,507	3,024	46.82	13,096	516	456	972	7.42
Masbate	22,600	8,304	7,633	15,937	70.52	2,574	2,455	5,029	22.25	46,418	839	765	1,604	3.46
Sorsogon	19,828	6,881	6,680	13,561	68.39	4,191	3,924	8,115	40.93	40,010	2,009	1,903	3,912	9.78
Iriga City	2,679	863	837	1,700	63.46	655	678	1,333	49.76	5,409	141	168	309	5.71
Legaspi City	4,233	1,287	1,176	2,463	58.19	473	424	897	21.19	8,577	121	126	247	2.88
Naga City	4,707	1,567	1,498	3,065	65.12	1,413	1,362	2,775	58.95	9,494	176	201	377	3.97
**Region 6**	146,449	52,879	49,853	102,732	70.15	33,435	31,192	64,627	44.13	294,166	7,520	7,142	14,662	4.98
Aklan	11,288	3,857	3,551	7,408	65.63	1,420	1,162	2,582	22.87	22,860	0	0	0	0.00
Antique	13,132	4,201	4,000	8,201	62.45	1,459	1,328	2,787	21.22	26,309	271	241	512	1.95
Capiz	13,975	4,952	4,646	9,598	68.68	2,150	1,937	4,087	29.25	28,242	841	751	1,592	5.64
Guimaras	3,084	1,198	1,170	2,368	76.78	454	426	880	28.53	6,438	0	0	0	0.00
Iloilo	36,267	12,491	11,640	24,131	66.54	8,123	7,813	15,936	43.94	72,479	2,852	2,756	5,608	7.74
Negros Occidental	49,230	19,219	18,323	37,542	76.26	13,700	12,872	26,572	53.98	98,851	3,094	2,963	6,057	6.13
Bacolod City	11,082	3,869	3,682	7,551	68.14	3,879	3,566	7,445	67.18	22,228	291	251	542	2.44
Iloilo City	8,391	3,092	2,841	5,933	70.71	2,250	2,088	4,338	51.70	16,759	171	180	351	2.09
**Region 7**	162,308	59,969	58,599	118,568	73.05	39,252	38,612	77,864	47.97	321,087	0	0	0	0.00
Bohol	27,992	8,919	8,388	17,307	61.83	7,843	7,316	15,159	54.15	55,999	0	0	0	0.00
Cebu	66,463	24,205	23,529	47,734	71.82	18,559	18,021	36,580	55.04	130,689	0	0	0	0.00
Negros Oriental	27,890	11,019	11,850	22,869	82.00	6,406	7,113	13,519	48.47	55,654	0	0	0	0.00
Siquijor	1,661	602	577	1,179	70.98	597	582	1,179	70.98	3,446	0	0	0	0.00
Cebu City	20,866	8,161	7,419	15,580	74.67	0	0	0	0.00	41,026	0	0	0	0.00
Lapu-Lapu City	9,232	3,977	3,814	7,791	84.39	3,055	2,807	5,862	63.50	18,149	0	0	0	0.00
Mandaue City	8,204	3,086	3,022	6,108	74.45	2,792	2,773	5,565	67.83	16,124	0	0	0	0.00

Table 2.C.1.3. Immunization Services for Infants and Children

Number and Proportion of Infants who completed 2 doses of Inactivated Polio Vaccine (IPV)
Philippines, 2022

Area	Eligible Pop (0-11mos. old)	IPV 1				IPV 2 (Routine)				Eligible Pop (0-23mos. old)	IPV 2 (Catch-up)			
		Male	Female	Total	%	Male	Female	Total	%		Male	Female	Total	%
**Region 8**	105,471	31,109	30,003	61,112	57.94	8,871	8,655	17,526	16.62	210,801	1,162	968	2,130	1.01
Biliran	**3,873**	1,479	1,461	**2,940**	**75.91**	833	782	**1,615**	**41.70**	**7,946**	528	433	**961**	**12.09**
Eastern Samar	**11,908**	3,780	3,624	**7,404**	**62.18**	1,345	1,181	**2,526**	**21.21**	**23,437**	42	44	**86**	**0.37**
Northern Leyte	**35,422**	8,569	8,061	**16,630**	**46.95**	786	756	**1,542**	**4.35**	**70,168**	0	0	**0**	**0.00**
Northern Samar	**15,921**	4,793	4,663	**9,456**	**59.39**	1,129	1,124	**2,253**	**14.15**	**32,213**	0	0	**0**	**0.00**
Southern Leyte	**6,662**	2,289	2,035	**4,324**	**64.91**	868	778	**1,646**	**24.71**	**13,696**	0	0	**0**	**0.00**
Western Samar	**14,732**	4,351	4,332	**8,683**	**58.94**	971	1,034	**2,005**	**13.61**	**29,527**	0	0	**0**	**0.00**
Calbayog City	**4,535**	1,346	1,325	**2,671**	**58.90**	0	0	**0**	**0.00**	**9,099**	0	0	**0**	**0.00**
Maasin City	**1,696**	446	433	**879**	**51.83**	213	256	**469**	**27.65**	**3,486**	0	0	**0**	**0.00**
Ormoc City	**5,044**	2,126	2,025	**4,151**	**82.30**	1,357	1,333	**2,690**	**53.33**	**9,991**	592	491	**1,083**	**10.84**
Tacloban City	**5,678**	1,930	2,044	**3,974**	**69.99**	1,369	1,411	**2,780**	**48.96**	**11,238**	0	0	**0**	**0.00**
**Region 9**	79,007	30,881	29,766	60,647	76.76	22,996	22,140	45,136	57.13	158,303	4,796	4,750	9,546	6.03
Zamboanga del Norte	**16,854**	6,780	6,387	**13,167**	**78.12**	4,466	4,067	**8,533**	**50.63**	**33,979**	940	904	**1,844**	**5.43**
Zamboanga del Sur	**17,368**	6,336	5,968	**12,304**	**70.84**	4,169	3,961	**8,130**	**46.81**	**34,373**	878	784	**1,662**	**4.84**
Zamboanga Sibugay	**15,021**	4,936	4,746	**9,682**	**64.46**	3,299	3,291	**6,590**	**43.87**	**30,508**	317	285	**602**	**1.97**
Dapitan City	**1,737**	695	850	**1,545**	**88.95**	578	728	**1,306**	**75.19**	**3,512**	31	44	**75**	**2.14**
Dipolog City	**2,762**	1,147	1,088	**2,235**	**80.92**	1,266	1,227	**2,493**	**90.26**	**5,565**	239	177	**416**	**7.48**
Isabela City	**2,563**	1,139	1,034	**2,173**	**84.78**	1,061	989	**2,050**	**79.98**	**5,439**	120	98	**218**	**4.01**
Pagadian City	**4,257**	1,616	1,485	**3,101**	**72.84**	1,390	1,423	**2,813**	**66.08**	**8,434**	154	198	**352**	**4.17**
Zamboanga City	**18,445**	8,232	8,208	**16,440**	**89.13**	6,767	6,454	**13,221**	**71.68**	**36,493**	2,117	2,260	**4,377**	**11.99**
**Region 10**	99,908	37,979	35,798	73,777	73.84	9,916	10,551	20,467	20.49	202,551	566	579	1,145	0.57
Bukidnon	**22,900**	8,906	8,427	**17,333**	**75.69**	1,122	1,128	**2,250**	**9.83**	**46,425**	273	324	**597**	**1.29**
Camiguin	**1,854**	647	625	**1,272**	**68.61**	403	341	**744**	**40.13**	**3,725**	0	0	**0**	**0.00**
Lanao del Norte	**14,930**	4,503	4,319	**8,822**	**59.09**	963	898	**1,861**	**12.46**	**30,651**	6	4	**10**	**0.03**
Misamis Occidental	**6,420**	2,033	1,955	**3,988**	**62.12**	72	70	**142**	**2.21**	**12,996**	79	77	**156**	**1.20**
Misamis Oriental	**14,963**	5,994	5,663	**11,657**	**77.91**	355	348	**703**	**4.70**	**30,092**	0	0	**0**	**0.00**
Cagayan de Oro City	**14,172**	6,255	5,718	**11,973**	**84.48**	3,589	3,620	**7,209**	**50.87**	**28,500**	0	0	**0**	**0.00**
El Salvador City	**1,052**	404	414	**818**	**77.76**	174	172	**346**	**32.89**	**2,116**	0	0	**0**	**0.00**
Gingoog City	**2,608**	1,245	1,360	**2,605**	**99.88**	224	242	**466**	**17.87**	**5,251**	0	0	**0**	**0.00**
Iligan City	**7,565**	2,495	2,287	**4,782**	**63.21**	848	766	**1,614**	**21.34**	**15,525**	208	174	**382**	**2.46**
Malaybalay City	**3,817**	1,766	1,646	**3,412**	**89.39**	1,255	2,077	**3,332**	**87.29**	**7,742**	0	0	**0**	**0.00**
Oroquieta City	**1,389**	454	446	**900**	**64.79**	768	746	**1,514**	**109.00**	**2,819**	0	0	**0**	**0.00**
Ozamis City	**2,786**	1,054	934	**1,988**	**71.36**	0	0	**0**	**0.00**	**5,643**	0	0	**0**	**0.00**
Tangub City	**1,234**	371	317	**688**	**55.75**	38	23	**61**	**4.94**	**2,511**	0	0	**0**	**0.00**
Valencia City	**4,218**	1,852	1,687	**3,539**	**83.90**	105	120	**225**	**5.33**	**8,555**	0	0	**0**	**0.00**
**Region 11**	108,407	42,732	39,772	82,504	76.11	33,067	30,272	63,339	58.43	217,671	0	0	0	0.00
Davao de Oro	**15,490**	6,461	5,954	**12,415**	**80.15**	5,702	4,992	**10,694**	**69.04**	**31,609**	0	0	**0**	**0.00**
Davao del Norte	**21,017**	8,766	8,001	**16,767**	**79.78**	7,333	6,775	**14,108**	**67.13**	**42,041**	0	0	**0**	**0.00**
Davao Oriental	**13,229**	5,243	4,443	**9,686**	**73.22**	4,019	3,510	**7,529**	**56.91**	**26,688**	0	0	**0**	**0.00**
Davao del Sur	**14,564**	5,014	4,760	**9,774**	**67.11**	5,058	4,691	**9,749**	**66.94**	**28,992**	0	0	**0**	**0.00**
Davao Occidental	**6,510**	2,136	2,045	**4,181**	**64.22**	1,392	1,415	**2,807**	**43.12**	**13,502**	0	0	**0**	**0.00**
Davao City	**37,597**	15,112	14,569	**29,681**	**78.95**	9,563	8,889	**18,452**	**49.08**	**74,839**	0	0	**0**	**0.00**
**Region 12**	98,859	35,683	34,166	69,849	70.66	21,725	20,529	42,254	42.74	197,872	2,848	2,772	5,620	2.84
North Cotabato	**34,150**	9,833	9,571	**19,404**	**56.82**	4,116	3,823	**7,939**	**23.25**	**68,063**	657	640	**1,297**	**1.91**
Sarangani	**12,907**	5,266	4,987	**10,253**	**79.44**	2,893	2,730	**5,623**	**43.57**	**26,207**	730	626	**1,356**	**5.17**
South Cotabato	**20,917**	8,463	8,079	**16,542**	**79.08**	6,142	5,887	**12,029**	**57.51**	**41,559**	1,207	1,290	**2,497**	**6.01**
Sultan Kudarat	**17,299**	6,918	6,571	**13,489**	**77.98**	5,517	5,291	**10,808**	**62.48**	**35,051**	195	162	**357**	**1.02**
Gen. Santos City	**13,586**	5,203	4,958	**10,161**	**74.79**	3,057	2,798	**5,855**	**43.10**	**26,992**	59	54	**113**	**0.42**
**BARMM**	101,343	32,253	33,268	65,521	64.65	12,630	12,416	25,046	24.71	216,396	522	491	1,013	0.47
Basilan	**7,823**	1,152	1,125	**2,277**	**29.11**	83	86	**169**	**2.16**	**15,979**	0	0	**0**	**0.00**
Lanao del Sur	**21,639**	8,664	9,773	**18,437**	**85.20**	2,982	3,021	**6,003**	**27.74**	**46,996**	0	0	**0**	**0.00**
Maguindanao	**32,198**	11,439	11,339	**22,778**	**70.74**	7,797	7,644	**15,441**	**47.96**	**67,544**	0	0	**0**	**0.00**
Sulu	**17,165**	3,742	3,891	**7,633**	**44.47**	151	129	**280**	**1.63**	**39,037**	0	0	**0**	**0.00**
Tawi-Tawi	**9,369**	2,737	2,702	**5,439**	**58.05**	0	0	**0**	**0.00**	**19,223**	0	0	**0**	**0.00**
Lamitan City	**2,154**	619	599	**1,218**	**56.55**	0	0	**0**	**0.00**	**4,395**	0	0	**0**	**0.00**
Marawi City	**5,173**	2,128	2,107	**4,235**	**81.87**	19	22	**41**	**0.79**	**11,237**	0	0	**0**	**0.00**
Cotabato City	**5,822**	1,772	1,732	**3,504**	**60.19**	1,598	1,514	**3,112**	**53.45**	**11,985**	522	491	**1,013**	**8.45**
**CARAGA**	60,575	22,143	21,183	43,326	71.52	11,323	10,832	22,155	36.57	122,744	0	0	0	0.00
Agusan del Norte	**8,127**	2,762	2,622	**5,384**	**66.25**	1,900	1,828	**3,728**	**45.87**	**16,353**	0	0	**0**	**0.00**
Agusan del Sur	**17,709**	6,792	6,456	**13,248**	**74.81**	1,616	1,547	**3,163**	**17.86**	**36,252**	0	0	**0**	**0.00**
Surigao del Norte	**7,280**	2,744	2,730	**5,474**	**75.19**	1,379	1,342	**2,721**	**37.38**	**14,992**	0	0	**0**	**0.00**
Surigao del Sur	**11,553**	4,117	3,962	**8,079**	**69.93**	1,967	1,831	**3,798**	**32.87**	**22,992**	0	0	**0**	**0.00**
Province of Dinagat	**2,576**	786	735	**1,521**	**59.05**	446	400	**846**	**32.84**	**5,246**	0	0	**0**	**0.00**
Bislig City	**2,192**	662	624	**1,286**	**58.67**	511	501	**1,012**	**46.17**	**4,366**	0	0	**0**	**0.00**
Butuan City	**7,743**	3,002	2,909	**5,911**	**76.34**	2,390	2,288	**4,678**	**60.42**	**15,557**	0	0	**0**	**0.00**
Surigao City	**3,395**	1,278	1,145	**2,423**	**71.37**	1,114	1,095	**2,209**	**65.07**	**6,986**	0	0	**0**	**0.00**

Table 2.C.1.4. Immunization Services for Infants and Children

Number and Proportion of Infants who completed 3 doses of Pneumococcal Conjugate Vaccine (PCV)
Philippines, 2022

Area	Eligible Pop (0-11 mos. old)	PCV 1				PCV 2				PCV 3			
		Male	Female	Total	%	Male	Female	Total	%	Male	Female	Total	%
**PHILIPPINES**	**2,131,496**	**848,156**	**801,927**	**1,650,083**	**77.41**	**833,999**	**797,454**	**1,631,453**	**76.54**	**808,196**	**776,028**	**1,584,224**	**74.32**
**N C R**	**236,901**	**101,785**	**97,898**	**199,683**	**84.29**	**99,759**	**97,112**	**196,871**	**83.10**	**99,370**	**97,000**	**196,370**	**82.89**
Malabon	**6,724**	2739	2577	**5,316**	**79.06**	2,612	2,581	**5,193**	**77.23**	2,550	2,589	**5,139**	**76.43**
Navotas	**4,591**	2366	2225	**4,591**	**100.00**	2,181	2,056	**4,237**	**92.29**	2,063	2,024	**4,087**	**89.02**
Valenzuela City	**11,418**	4070	3827	**7,897**	**69.16**	4,184	3,962	**8,146**	**71.34**	4,192	3,949	**8,141**	**71.30**
Caloocan City	**29,146**	11497	10851	**22,348**	**76.68**	10,787	10,299	**21,086**	**72.35**	10,507	9,727	**20,234**	**69.42**
Marikina City	**8,294**	2869	2879	**5,748**	**69.30**	2,765	2,707	**5,472**	**65.98**	2,729	2,664	**5,393**	**65.02**
Pasig City	**13,894**	6918	6594	**13,512**	**97.25**	7,010	6,701	**13,711**	**98.68**	7,034	6,860	**13,894**	**100.00**
Pateros	**1,176**	481	471	**952**	**80.95**	489	478	**967**	**82.23**	495	500	**995**	**84.61**
Taguig	**14,807**	6169	6075	**12,244**	**82.69**	6,160	6,054	**12,214**	**82.49**	6,249	6,106	**12,355**	**83.44**
Quezon City	**54,011**	26970	27041	**54,011**	**100.00**	26,877	27,134	**54,011**	**100.00**	27,028	26,983	**54,011**	**100.00**
Makati City	**10,718**	4194	3984	**8,178**	**76.30**	4,153	3,914	**8,067**	**75.27**	3,673	3,605	**7,278**	**67.90**
Mandaluyong City	**7,111**	2505	2415	**4,920**	**69.19**	2,502	2,581	**5,083**	**71.48**	2,584	2,739	**5,323**	**74.86**
San Juan	**2,246**	724	680	**1,404**	**62.51**	695	669	**1,364**	**60.73**	712	703	**1,415**	**63.00**
Manila City	**32,743**	14639	13922	**28,561**	**87.23**	13,847	13,333	**27,180**	**83.01**	13,643	13,268	**26,911**	**82.19**
Las Piñas City	**10,832**	4275	3978	**8,253**	**76.19**	4,216	3,985	**8,201**	**75.71**	4,242	4,137	**8,379**	**77.35**
Muntinlupa City	**9,281**	3815	3541	**7,356**	**79.26**	3,908	3,820	**7,728**	**83.27**	3,991	3,895	**7,886**	**84.97**
Parañaque City	**12,249**	4245	3878	**8,123**	**66.32**	4,183	3,893	**8,076**	**65.93**	4,754	4,356	**9,110**	**74.37**
Pasay City	**7,660**	3309	2960	**6,269**	**81.84**	3,190	2,945	**6,135**	**80.09**	2,924	2,895	**5,819**	**75.97**
**C A R**	**35,179**	**12,466**	**11,639**	**24,105**	**68.52**	**12,387**	**11,631**	**24,018**	**68.27**	**12,350**	**11,459**	**23,809**	**67.68**
Abra	**4,275**	1,425	1,328	**2,753**	**64.40**	1,391	1,344	**2,735**	**63.98**	1,417	1,331	**2,748**	**64.28**
Apayao	**2,502**	1,016	907	**1,923**	**76.86**	1,043	920	**1,963**	**78.46**	1,002	900	**1,902**	**76.02**
Benguet	**9,086**	3,012	2,742	**5,754**	**63.33**	2,957	2,830	**5,787**	**63.69**	3,006	2,804	**5,810**	**63.94**
Ifugao	**4,486**	1,605	1,460	**3,065**	**68.32**	1,655	1,504	**3,159**	**70.42**	1,610	1,478	**3,088**	**68.84**
Kalinga	**4,701**	1,994	1,841	**3,835**	**81.58**	1,997	1,785	**3,782**	**80.45**	1,995	1,785	**3,780**	**80.41**
Mt. Province	**3,103**	1,135	1,083	**2,218**	**71.48**	1,126	1,075	**2,201**	**70.93**	1,120	1,016	**2,136**	**68.84**
Baguio City	**7,026**	2,279	2,278	**4,557**	**64.86**	2,218	2,173	**4,391**	**62.50**	2,200	2,145	**4,345**	**61.84**
**Region 1**	**97,099**	**37,690**	**34,786**	**72,476**	**74.64**	**38,040**	**35,991**	**74,031**	**76.24**	**37,528**	**34,878**	**72,406**	**74.57**
Ilocos Norte	**7,854**	2,838	2,765	**5,603**	**71.34**	2,810	2,804	**5,614**	**71.48**	2,811	2,832	**5,643**	**71.85**
Ilocos Sur	**9,072**	3,653	3,534	**7,187**	**79.22**	3,763	3,583	**7,346**	**80.97**	3,742	3,525	**7,267**	**80.10**
La Union	**11,338**	3,913	3,776	**7,689**	**67.82**	3,969	3,784	**7,753**	**68.38**	3,921	3,751	**7,672**	**67.67**
Pangasinan	**50,710**	20,089	17,999	**38,088**	**75.11**	20,251	18,879	**39,130**	**77.16**	19,811	17,842	**37,653**	**74.25**
Alaminos City	**1,910**	754	690	**1,444**	**75.60**	749	705	**1,454**	**76.13**	769	695	**1,464**	**76.65**
Candon City	**954**	456	472	**928**	**97.27**	494	469	**963**	**100.94**	449	430	**879**	**92.14**
Dagupan City	**3,657**	1,192	1,051	**2,243**	**61.33**	1,209	1,093	**2,302**	**62.95**	1,266	1,150	**2,416**	**66.07**
Laoag City	**1,812**	957	971	**1,928**	**106.40**	914	973	**1,887**	**104.14**	918	986	**1,904**	**105.08**
San Carlos City	**4,028**	1,710	1,627	**3,337**	**82.85**	1,711	1,640	**3,351**	**83.19**	1,687	1,643	**3,330**	**82.67**
San Fernando City	**2,074**	652	631	**1,283**	**61.86**	620	642	**1,262**	**60.85**	668	641	**1,309**	**63.11**
Urdaneta City	**2,836**	1,135	1,017	**2,152**	**75.88**	1,129	1,062	**2,191**	**77.26**	1,090	1,050	**2,140**	**75.46**
Vigan City	**854**	341	253	**594**	**69.56**	421	357	**778**	**91.10**	396	333	**729**	**85.36**
**Region 2**	**68,960**	**26,110**	**24,193**	**50,303**	**72.95**	**26,726**	**24,233**	**50,959**	**73.90**	**25,553**	**23,986**	**49,539**	**71.84**
Batanes	**362**	137	120	**257**	**70.99**	135	134	**269**	**74.31**	117	136	**253**	**69.89**
Cagayan	**20,431**	7,125	6,605	**13,730**	**67.20**	7,192	6,625	**13,817**	**67.63**	7,056	6,435	**13,491**	**66.03**
Isabela	**23,449**	8,773	8,076	**16,849**	**71.85**	9,346	8,353	**17,699**	**75.48**	8,604	8,004	**16,608**	**70.83**
Nueva Vizcaya	**9,538**	3,425	3,202	**6,627**	**69.48**	3,299	3,087	**6,386**	**66.95**	3,171	3,126	**6,297**	**66.02**
Quirino	**4,051**	1,801	1,628	**3,429**	**84.65**	1,853	1,694	**3,547**	**87.56**	1,805	1,610	**3,415**	**84.30**
Cauayan City	**2,565**	1,073	1,043	**2,116**	**82.50**	1,093	1,055	**2,148**	**83.74**	1,043	1,022	**2,065**	**80.51**
Ilagan City	**2,889**	1,342	1,367	**2,709**	**93.77**	1,323	1,145	**2,468**	**85.43**	1,319	1,321	**2,640**	**91.38**
Santiago City	**2,671**	1,410	1,255	**2,665**	**99.78**	1,444	1,199	**2,643**	**98.95**	1,433	1,238	**2,671**	**100.00**
Tuguegarao City	**3,004**	1,024	897	**1,921**	**63.95**	1,041	941	**1,982**	**65.98**	1,005	1,094	**2,099**	**69.87**
**Region 3**	**220,155**	**99,380**	**93,315**	**192,695**	**87.53**	**99,595**	**94,579**	**194,174**	**88.20**	**98,799**	**94,178**	**192,977**	**87.66**
Aurora	**4,754**	1,911	1,692	**3,603**	**75.79**	2,012	1,831	**3,843**	**80.84**	2,084	1,808	**3,892**	**81.87**
Bataan	**13,789**	5,802	5,465	**11,267**	**81.71**	5,855	5,472	**11,327**	**82.15**	5,781	5,456	**11,237**	**81.49**
Bulacan	**43,760**	20,525	19,093	**39,618**	**90.53**	20,594	19,660	**40,254**	**91.99**	20,166	19,205	**39,371**	**89.97**
Nueva Ecija	**29,039**	11,902	10,990	**22,892**	**78.83**	12,198	11,512	**23,710**	**81.65**	12,305	11,827	**24,132**	**83.10**
Pampanga	**31,620**	14,638	13,954	**28,592**	**90.42**	14,510	13,923	**28,433**	**89.92**	14,676	13,956	**28,632**	**90.55**
Tarlac	**19,319**	9,300	8,991	**18,291**	**94.68**	9,253	9,053	**18,306**	**94.76**	9,352	9,209	**18,561**	**96.08**
Zambales	**12,476**	4,575	4,420	**8,995**	**72.10**	4,472	4,234	**8,706**	**69.78**	4,199	4,046	**8,245**	**66.09**
Angeles City	**7,932**	3,822	3,708	**7,530**	**94.93**	3,735	3,762	**7,497**	**94.52**	3,733	3,669	**7,402**	**93.32**
Balanga City	**1,992**	828	668	**1,496**	**75.10**	801	676	**1,477**	**74.15**	787	704	**1,491**	**74.85**

Table 2.C.1.4. Immunization Services for Infants and Children

Number and Proportion of Infants who completed 3 doses of Pneumococcal Conjugate Vaccine (PCV)
Philippines, 2022

Area	Eligible Pop (0-11 mos. old)	PCV 1				PCV 2				PCV 3			
		Male	Female	Total	%	Male	Female	Total	%	Male	Female	Total	%
Cabanatuan City	**5,945**	2,664	2,419	**5,083**	**85.50**	2,609	2,395	**5,004**	**84.17**	2,612	2,379	**4,991**	**83.95**
City of San Fernando	**5,907**	2,650	2,427	**5,077**	**85.95**	2,677	2,451	**5,128**	**86.81**	2,735	2,507	**5,242**	**88.74**
Gapan City	**2,170**	1,200	971	**2,171**	**100.05**	1,185	964	**2,149**	**99.03**	1,160	927	**2,087**	**96.18**
Mabalacat City	**4,831**	2,407	2,428	**4,835**	**100.08**	2,570	2,405	**4,975**	**102.98**	2,304	2,385	**4,689**	**97.06**
Malolos City	**4,890**	1,764	1,542	**3,306**	**67.61**	1,741	1,619	**3,360**	**68.71**	1,742	1,620	**3,362**	**68.75**
Meycauayan	**4,055**	1,951	1,840	**3,791**	**93.49**	1,920	1,931	**3,851**	**94.97**	1,967	1,903	**3,870**	**95.44**
Olongapo	**4,922**	1,737	1,677	**3,414**	**69.36**	1,739	1,626	**3,365**	**68.37**	1,721	1,612	**3,333**	**67.72**
Palayan City	**808**	357	350	**707**	**87.50**	381	332	**713**	**88.24**	380	337	**717**	**88.74**
San Jose City	**2,748**	1,394	1,180	**2,574**	**93.67**	1,374	1,220	**2,594**	**94.40**	1,299	1,174	**2,473**	**89.99**
San Jose del Monte City	**11,133**	6,167	6,057	**12,224**	**109.80**	6,217	6,070	**12,287**	**110.37**	6,051	6,011	**12,062**	**108.34**
Science City of Munoz	**1,601**	680	573	**1,253**	**78.26**	676	589	**1,265**	**79.01**	658	593	**1,251**	**78.14**
Tarlac City	**6,464**	3,106	2,870	**5,976**	**92.45**	3,076	2,854	**5,930**	**91.74**	3,087	2,850	**5,937**	**91.85**
**Region 4A**	**299,627**	**123,542**	**116,366**	**239,908**	**80.07**	**121,858**	**115,942**	**237,800**	**79.37**	**115,979**	**111,582**	**227,561**	**75.95**
Batangas	**39,255**	15,184	13,939	**29,123**	**74.19**	15,481	14,617	**30,098**	**76.67**	15,362	14,671	**30,033**	**76.51**
Cavite	**28,151**	12,882	12,515	**25,397**	**90.22**	13,167	12,777	**25,944**	**92.16**	13,200	12,755	**25,955**	**92.20**
Laguna	**19,696**	7,763	6,976	**14,739**	**74.83**	7,573	7,019	**14,592**	**74.09**	7,151	6,827	**13,978**	**70.97**
Quezon	**37,853**	14,864	13,740	**28,604**	**75.57**	14,054	13,440	**27,494**	**72.63**	12,504	12,154	**24,658**	**65.14**
Rizal	**44,798**	17,669	17,151	**34,820**	**77.73**	17,527	16,889	**34,416**	**76.82**	16,784	16,166	**32,950**	**73.55**
Antipolo City	**16,498**	7,286	6,781	**14,067**	**85.26**	7,024	6,541	**13,565**	**82.22**	6,325	5,987	**12,312**	**74.63**
Bacoor City	**12,341**	4,415	4,010	**8,425**	**68.27**	4,262	3,921	**8,183**	**66.31**	3,889	3,559	**7,448**	**60.35**
Batangas City	**6,965**	2,394	2,187	**4,581**	**65.77**	2,179	2,073	**4,252**	**61.05**	1,977	1,854	**3,831**	**55.00**
Biñan City	**6,597**	3,469	3,920	**7,389**	**112.01**	3,413	3,501	**6,914**	**104.81**	3,392	3,438	**6,830**	**103.53**
Cabuyao City	**6,119**	3,040	3,031	**6,071**	**99.22**	2,956	2,943	**5,899**	**96.40**	2,948	2,925	**5,873**	**95.98**
Calamba City	**9,005**	4,364	4,010	**8,374**	**92.99**	4,369	4,001	**8,370**	**92.95**	3,919	3,793	**7,712**	**85.64**
Cavite City	**2,105**	586	585	**1,171**	**55.63**	591	560	**1,151**	**54.68**	535	588	**1,123**	**53.35**
Dasmariñas City	**13,538**	5,217	4,879	**10,096**	**74.58**	5,297	4,891	**10,188**	**75.25**	4,889	4,697	**9,586**	**70.81**
General Trias City	**6,457**	3,286	3,064	**6,350**	**98.34**	3,271	3,134	**6,405**	**99.19**	3,215	3,144	**6,359**	**98.48**
Imus City	**8,292**	2,797	2,470	**5,267**	**63.52**	2,832	2,579	**5,411**	**65.26**	2,798	2,595	**5,393**	**65.04**
Lipa City	**7,018**	3,146	2,823	**5,969**	**85.05**	3,092	2,858	**5,950**	**84.78**	3,038	2,908	**5,946**	**84.72**
Lucena City	**5,738**	2,525	2,497	**5,022**	**87.52**	2,535	2,328	**4,863**	**84.75**	2,306	2,168	**4,474**	**77.97**
San Pablo City	**5,272**	2,368	2,332	**4,700**	**89.15**	2,303	2,321	**4,624**	**87.71**	2,151	2,156	**4,307**	**81.70**
San Pedro City	**6,455**	2,158	1,985	**4,143**	**64.18**	1,973	1,972	**3,945**	**61.12**	1,861	1,892	**3,753**	**58.14**
Santa Rosa City	**7,009**	3,239	2,991	**6,230**	**88.89**	3,217	3,041	**6,258**	**89.29**	3,173	2,828	**6,001**	**85.62**
Tagaytay City	**1,460**	709	618	**1,327**	**90.89**	678	638	**1,316**	**90.14**	705	666	**1,371**	**93.90**
Tanauan City	**3,661**	1,426	1,359	**2,785**	**76.07**	1,457	1,439	**2,896**	**79.10**	1,452	1,401	**2,853**	**77.93**
Tayabas City	**2,145**	985	839	**1,824**	**85.03**	902	823	**1,725**	**80.42**	767	772	**1,539**	**71.75**
Trece Martires City	**3,199**	1,770	1,664	**3,434**	**107.35**	1,705	1,636	**3,341**	**104.44**	1,638	1,638	**3,276**	**102.41**
**Region 4B**	**72,791**	**25,058**	**23,414**	**48,472**	**66.59**	**24,092**	**22,922**	**47,014**	**64.59**	**22,633**	**21,563**	**44,196**	**60.72**
Marinduque	**4,990**	1,603	1,496	**3,099**	**62.10**	1,600	1,502	**3,102**	**62.16**	1,580	1,473	**3,053**	**61.18**
Mindoro Occidental	**12,479**	4,368	4,193	**8,561**	**68.60**	4,049	3,947	**7,996**	**64.08**	3,710	3,593	**7,303**	**58.52**
Mindoro Oriental	**20,189**	6,644	6,155	**12,799**	**63.40**	6,383	6,034	**12,417**	**61.50**	5,917	5,644	**11,561**	**57.26**
Palawan	**21,963**	7,553	7,004	**14,557**	**66.28**	7,240	6,821	**14,061**	**64.02**	6,851	6,463	**13,314**	**60.62**
Romblon	**6,569**	2,270	2,099	**4,369**	**66.51**	2,186	2,074	**4,260**	**64.85**	2,084	2,009	**4,093**	**62.31**
Puerto Princesa City	**6,601**	2,620	2,467	**5,087**	**77.06**	2,634	2,544	**5,178**	**78.44**	2,491	2,381	**4,872**	**73.81**
**Region 5**	**138,457**	**49,363**	**45,887**	**95,250**	**68.79**	**48,053**	**45,581**	**93,634**	**67.63**	**45,181**	**42,759**	**87,940**	**63.51**
Albay	**24,081**	8,605	7,899	**16,504**	**68.54**	8,621	8,106	**16,727**	**69.46**	8,301	7,826	**16,127**	**66.97**
Camarines Norte	**14,384**	5,495	5,065	**10,560**	**73.41**	5,500	5,109	**10,609**	**73.76**	5,286	4,972	**10,258**	**71.32**
Camarines Sur	**39,486**	14,135	13,072	**27,207**	**68.90**	13,584	12,801	**26,385**	**66.82**	12,862	12,018	**24,880**	**63.01**
Catanduanes	**6,459**	2,141	2,000	**4,141**	**64.11**	2,054	2,017	**4,071**	**63.03**	2,001	1,947	**3,948**	**61.12**
Masbate	**22,600**	8,025	7,593	**15,618**	**69.11**	7,369	7,344	**14,713**	**65.10**	6,607	6,078	**12,685**	**56.13**
Sorsogon	**19,828**	6,936	6,470	**13,406**	**67.61**	6,827	6,449	**13,276**	**66.96**	6,358	6,340	**12,698**	**64.04**
Iriga City	**2,679**	891	893	**1,784**	**66.59**	927	897	**1,824**	**68.09**	800	819	**1,619**	**60.43**
Legaspi City	**4,233**	1,603	1,398	**3,001**	**70.90**	1,573	1,337	**2,910**	**68.75**	1,448	1,296	**2,744**	**64.82**
Naga City	**4,707**	1,532	1,497	**3,029**	**64.35**	1,598	1,521	**3,119**	**66.26**	1,518	1,463	**2,981**	**63.33**
**Region 6**	**146,449**	**54,147**	**50,840**	**104,987**	**71.69**	**53,855**	**50,707**	**104,562**	**71.40**	**53,074**	**50,069**	**103,143**	**70.43**
Aklan	**11,288**	3,769	3,508	**7,277**	**64.47**	3,858	3,598	**7,456**	**66.05**	3,808	3,557	**7,365**	**65.25**
Antique	**13,132**	4,249	4,034	**8,283**	**63.07**	4,210	4,021	**8,231**	**62.68**	4,209	3,926	**8,135**	**61.95**
Capiz	**13,975**	4,930	4,509	**9,439**	**67.54**	4,864	4,565	**9,429**	**67.47**	4,992	4,681	**9,673**	**69.22**
Guimaras	**3,084**	1,211	1,159	**2,370**	**76.85**	1,196	1,190	**2,386**	**77.37**	1,214	1,185	**2,399**	**77.79**
Iloilo	**36,267**	12,559	11,729	**24,288**	**66.97**	12,589	11,781	**24,370**	**67.20**	12,336	11,469	**23,805**	**65.64**
Negros Occidental	**49,230**	20,254	19,201	**39,455**	**80.14**	19,801	18,719	**38,520**	**78.24**	19,142	18,362	**37,504**	**76.18**
Bacolod City	**11,082**	3,965	3,638	**7,603**	**68.61**	4,031	3,722	**7,753**	**69.96**	4,098	3,862	**7,960**	**71.83**
Iloilo City	**8,391**	3,210	3,062	**6,272**	**74.75**	3,306	3,111	**6,417**	**76.47**	3,275	3,027	**6,302**	**75.10**

Table 2.C.1.4. Immunization Services for Infants and Children

Number and Proportion of Infants who completed 3 doses of Pneumococcal Conjugate Vaccine (PCV)
Philippines, 2022

Area	Eligible Pop (0-11 mos. old)	PCV 1				PCV 2				PCV 3			
		Male	Female	Total	%	Male	Female	Total	%	Male	Female	Total	%
**Region 7**	**162,308**	**63,151**	**61,269**	**124,420**	**76.66**	**61,451**	**59,494**	**120,945**	**74.52**	**61,100**	**58,488**	**119,588**	**73.68**
Bohol	**27,992**	9,179	8,438	**17,617**	**62.94**	8,922	8,305	**17,227**	**61.54**	8,294	7,898	**16,192**	**57.85**
Cebu	**66,463**	25,347	24,474	**49,821**	**74.96**	25,421	24,443	**49,864**	**75.03**	25,845	24,172	**50,017**	**75.26**
Negros Oriental	**27,890**	11,899	12,578	**24,477**	**87.76**	11,796	12,209	**24,005**	**86.07**	10,971	11,581	**22,552**	**80.86**
Siquijor	**1,661**	489	490	**979**	**58.94**	473	483	**956**	**57.56**	498	497	**995**	**59.90**
Cebu City	**20,866**	8,494	7,860	**16,354**	**78.38**	7,362	6,782	**14,144**	**67.78**	7,953	7,317	**15,270**	**73.18**
Lapu-Lapu City	**9,232**	4,567	4,245	**8,812**	**95.45**	4,254	4,118	**8,372**	**90.68**	4,355	3,961	**8,316**	**90.08**
Mandaue City	**8,204**	3,176	3,184	**6,360**	**77.52**	3,223	3,154	**6,377**	**77.73**	3,184	3,062	**6,246**	**76.13**
**Region 8**	**105,471**	**36,255**	**34,326**	**70,581**	**66.92**	**35,494**	**33,953**	**69,447**	**65.84**	**33,239**	**32,036**	**65,275**	**61.89**
Biliran	**3,873**	1,602	1,580	**3,182**	**82.16**	1,531	1,558	**3,089**	**79.76**	1,509	1,463	**2,972**	**76.74**
Eastern Samar	**11,908**	3,962	3,869	**7,831**	**65.76**	3,858	3,805	**7,663**	**64.35**	3,772	3,571	**7,343**	**61.66**
Northern Leyte	**35,422**	11,780	10,862	**22,642**	**63.92**	11,715	10,657	**22,372**	**63.16**	11,041	10,309	**21,350**	**60.27**
Northern Samar	**15,921**	5,421	5,070	**10,491**	**65.89**	5,251	5,133	**10,384**	**65.22**	4,948	4,765	**9,713**	**61.01**
Southern Leyte	**6,662**	2,195	2,057	**4,252**	**63.82**	2,240	2,091	**4,331**	**65.01**	2,190	1,965	**4,155**	**62.37**
Western Samar	**14,732**	5,377	5,098	**10,475**	**71.10**	5,068	4,939	**10,007**	**67.93**	4,446	4,466	**8,912**	**60.49**
Calbayog City	**4,535**	1,543	1,498	**3,041**	**67.06**	1,433	1,397	**2,830**	**62.40**	1,099	1,138	**2,237**	**49.33**
Maasin City	**1,696**	446	445	**891**	**52.54**	446	438	**884**	**52.12**	444	454	**898**	**52.95**
Ormoc City	**5,044**	2,007	1,905	**3,912**	**77.56**	2,028	1,972	**4,000**	**79.30**	1,858	1,867	**3,725**	**73.85**
Tacloban City	**5,678**	1,922	1,942	**3,864**	**68.05**	1,924	1,963	**3,887**	**68.46**	1,932	2,038	**3,970**	**69.92**
**Region 9**	**79,007**	**32,254**	**30,705**	**62,959**	**79.69**	**30,958**	**30,102**	**61,060**	**77.28**	**28,968**	**28,101**	**57,069**	**72.23**
Zamboanga del Norte	**16,854**	7,205	6,567	**13,772**	**81.71**	6,828	6,650	**13,478**	**79.97**	6,414	6,087	**12,501**	**74.17**
Zamboanga del Sur	**17,368**	6,349	6,068	**12,417**	**71.49**	6,141	5,876	**12,017**	**69.19**	5,578	5,452	**11,030**	**63.51**
Zamboanga Sibugay	**15,021**	5,293	5,087	**10,380**	**69.10**	5,055	4,920	**9,975**	**66.41**	4,731	4,595	**9,326**	**62.09**
Dapitan City	**1,737**	678	871	**1,549**	**89.18**	672	870	**1,542**	**88.77**	680	851	**1,531**	**88.14**
Dipolog City	**2,762**	1,196	1,160	**2,356**	**85.30**	1,168	1,089	**2,257**	**81.72**	1,157	1,132	**2,289**	**82.87**
Isabela City	**2,563**	1,114	1,069	**2,183**	**85.17**	1,121	1,075	**2,196**	**85.68**	1,050	1,002	**2,052**	**80.06**
Pagadian City	**4,257**	1,539	1,505	**3,044**	**71.51**	1,547	1,604	**3,151**	**74.02**	1,567	1,485	**3,052**	**71.69**
Zamboanga City	**18,445**	8,880	8,378	**17,258**	**93.56**	8,426	8,018	**16,444**	**89.15**	7,791	7,497	**15,288**	**82.88**
**Region 10**	**99,908**	**45,136**	**41,784**	**86,920**	**87.00**	**43,613**	**41,821**	**85,434**	**85.51**	**42,467**	**40,976**	**83,443**	**83.52**
Bukidnon	**22,900**	11,198	10,428	**21,626**	**94.44**	10,756	10,311	**21,067**	**92.00**	10,544	10,188	**20,732**	**90.53**
Camiguin	**1,854**	655	645	**1,300**	**70.12**	639	615	**1,254**	**67.64**	599	613	**1,212**	**65.37**
Lanao del Norte	**14,930**	5,403	5,271	**10,674**	**71.49**	5,403	5,281	**10,684**	**71.56**	5,255	5,183	**10,438**	**69.91**
Misamis Occidental	**6,420**	2,736	2,571	**5,307**	**82.66**	2,435	2,412	**4,847**	**75.50**	2,473	2,492	**4,965**	**77.34**
Misamis Oriental	**14,963**	6,484	6,006	**12,490**	**83.47**	6,565	6,249	**12,814**	**85.64**	6,672	6,135	**12,807**	**85.59**
Cagayan de Oro City	**14,172**	6,650	6,211	**12,861**	**90.75**	6,451	6,259	**12,710**	**89.68**	6,452	6,094	**12,546**	**88.53**
El Salvador City	**1,052**	448	433	**881**	**83.75**	439	432	**871**	**82.79**	428	450	**878**	**83.46**
Gingoog City	**2,608**	1,312	1,245	**2,557**	**98.04**	1,303	1,254	**2,557**	**98.04**	1,060	1,253	**2,313**	**88.69**
Iligan City	**7,565**	3,437	3,046	**6,483**	**85.70**	3,395	3,125	**6,520**	**86.19**	3,086	2,867	**5,953**	**78.69**
Malaybalay City	**3,817**	2,421	1,757	**4,178**	**109.46**	1,875	1,730	**3,605**	**94.45**	1,863	1,779	**3,642**	**95.42**
Oroquieta City	**1,389**	538	553	**1,091**	**78.55**	605	604	**1,209**	**87.04**	604	695	**1,299**	**93.52**
Ozamis City	**2,786**	1,107	1,016	**2,123**	**76.20**	1,070	967	**2,037**	**73.12**	966	873	**1,839**	**66.01**
Tangub City	**1,234**	578	490	**1,068**	**86.55**	559	503	**1,062**	**86.06**	400	415	**815**	**66.05**
Valencia City	**4,218**	2,169	2,112	**4,281**	**101.49**	2,118	2,079	**4,197**	**99.50**	2,065	1,939	**4,004**	**94.93**
**Region 11**	**108,407**	**45,700**	**41,906**	**87,606**	**80.81**	**45,100**	**41,428**	**86,528**	**79.82**	**43,552**	**40,718**	**84,270**	**77.73**
Davao de Oro	**15,490**	6,680	6,231	**12,911**	**83.35**	6,664	6,138	**12,802**	**82.65**	6,487	6,041	**12,528**	**80.88**
Davao del Norte	**21,017**	9,296	8,465	**17,761**	**84.51**	9,253	8,438	**17,691**	**84.17**	8,842	8,230	**17,072**	**81.23**
Davao Oriental	**13,229**	5,436	4,643	**10,079**	**76.19**	5,472	4,596	**10,068**	**76.11**	5,300	4,516	**9,816**	**74.20**
Davao del Sur	**14,564**	5,290	4,799	**10,089**	**69.27**	5,102	4,818	**9,920**	**68.11**	5,071	4,820	**9,891**	**67.91**
Davao Occidental	**6,510**	3,420	3,129	**6,549**	**100.60**	3,288	3,113	**6,401**	**98.33**	3,016	3,013	**6,029**	**92.61**
Davao City	**37,597**	15,578	14,639	**30,217**	**80.37**	15,321	14,325	**29,646**	**78.85**	14,836	14,098	**28,934**	**76.96**

Table 2.C.1.4. Immunization Services for Infants and Children

Number and Proportion of Infants who completed 3 doses of Pneumococcal Conjugate Vaccine (PCV)
Philippines, 2022

Area	Eligible Pop (0-11 mos. old)	PCV 1				PCV 2				PCV 3			
		Male	Female	Total	%	Male	Female	Total	%	Male	Female	Total	%
**Region 12**	**98,859**	**37,729**	**35,646**	**73,375**	**74.22**	**36,657**	**35,297**	**71,954**	**72.78**	**34,792**	**33,862**	**68,654**	**69.45**
North Cotabato	**34,150**	11,507	10,688	**22,195**	**64.99**	10,735	10,359	**21,094**	**61.77**	10,050	9,807	**19,857**	**58.15**
Sarangani	**12,907**	5,500	5,115	**10,615**	**82.24**	5,294	4,960	**10,254**	**79.45**	4,939	4,746	**9,685**	**75.04**
South Cotabato	**20,917**	8,500	8,053	**16,553**	**79.14**	8,460	8,080	**16,540**	**79.07**	8,102	8,023	**16,125**	**77.09**
Sultan Kudarat	**17,299**	7,064	6,741	**13,805**	**79.80**	7,043	6,847	**13,890**	**80.29**	6,873	6,581	**13,454**	**77.77**
Gen. Santos City	**13,586**	5,158	5,049	**10,207**	**75.13**	5,125	5,051	**10,176**	**74.90**	4,828	4,705	**9,533**	**70.17**
**BARMM**	**101,343**	**33,528**	**34,698**	**68,226**	**67.32**	**32,486**	**33,774**	**66,260**	**65.38**	**30,972**	**32,498**	**63,470**	**62.63**
Basilan	**7,823**	1,674	1,688	**3,362**	**42.98**	1,429	1,469	**2,898**	**37.04**	1,222	1,299	**2,521**	**32.23**
Lanao del Sur	**21,639**	9,541	10,569	**20,110**	**92.93**	9,515	10,529	**20,044**	**92.63**	9,481	10,503	**19,984**	**92.35**
Maguindanao	**32,198**	9,856	9,859	**19,715**	**61.23**	9,373	9,465	**18,838**	**58.51**	8,761	8,922	**17,683**	**54.92**
Sulu	**17,165**	4,786	4,922	**9,708**	**56.56**	4,571	4,798	**9,369**	**54.58**	4,225	4,473	**8,698**	**50.67**
Tawi-Tawi	**9,369**	2,802	2,806	**5,608**	**59.86**	2,785	2,747	**5,532**	**59.05**	2,729	2,686	**5,415**	**57.80**
Lamitan City	**2,154**	673	642	**1,315**	**61.05**	628	631	**1,259**	**58.45**	574	587	**1,161**	**53.90**
Marawi City	**5,173**	2,293	2,353	**4,646**	**89.81**	2,275	2,338	**4,613**	**89.17**	2,259	2,319	**4,578**	**88.50**
Cotabato City	**5,822**	1,903	1,859	**3,762**	**64.62**	1,910	1,797	**3,707**	**63.67**	1,721	1,709	**3,430**	**58.91**
**CARAGA**	**60,575**	**24,862**	**23,255**	**48,117**	**79.43**	**23,875**	**22,887**	**46,762**	**77.20**	**22,639**	**21,875**	**44,514**	**73.49**
Agusan del Norte	**8,127**	3,165	3,034	**6,199**	**76.28**	3,015	2,949	**5,964**	**73.39**	2,885	2,807	**5,692**	**70.04**
Agusan del Sur	**17,709**	7,323	6,840	**14,163**	**79.98**	6,952	6,614	**13,566**	**76.61**	6,679	6,339	**13,018**	**73.51**
Surigao del Norte	**7,280**	3,130	2,918	**6,048**	**83.08**	3,067	2,953	**6,020**	**82.69**	2,843	2,801	**5,644**	**77.53**
Surigao del Sur	**11,553**	4,774	4,542	**9,316**	**80.64**	4,549	4,380	**8,929**	**77.29**	4,285	4,191	**8,476**	**73.37**
Province of Dinagat	**2,576**	906	757	**1,663**	**64.56**	826	785	**1,611**	**62.54**	730	763	**1,493**	**57.96**
Bislig City	**2,192**	845	748	**1,593**	**72.67**	775	747	**1,522**	**69.43**	750	706	**1,456**	**66.42**
Butuan City	**7,743**	3,245	3,075	**6,320**	**81.62**	3,215	3,068	**6,283**	**81.14**	3,021	2,887	**5,908**	**76.30**
Surigao City	**3,395**	1,474	1,341	**2,815**	**82.92**	1,476	1,391	**2,867**	**84.45**	1,446	1,381	**2,827**	**83.27**

Table 2.C.1.5. Immunization Services for Infants and Children

Number and Proportion of Children vaccinated with 2 doses of Measles Containing Vaccine (MCV)
Philippines, 2022

Area	Eligible Pop (0-11 mos. old)	MCV 1				Eligible Pop (0-12 mos. old)	MCV 2			
		Male	Female	Total	%		Male	Female	Total	%
**PHILIPPINES**	2,131,496	791,395	745,828	1,537,223	72.12	2,310,426	774,607	736,842	1,511,449	65.42
**N C R**	236,901	99,201	95,440	194,641	82.16	256,420	100,966	97,768	198,734	77.50
Malabon	6,724	2,256	2,077	4,333	64.44	7,280	1,830	1,615	3,445	47.32
Navotas	4,591	2,145	2,076	4,221	91.94	4,969	2,274	2,086	4,360	87.74
Valenzuela City	11,418	3,761	3,512	7,273	63.70	12,358	3,667	3,479	7,146	57.82
Caloocan City	29,146	10,266	9,414	19,680	67.52	31,541	9,369	8,559	17,928	56.84
Marikina City	8,294	2,757	2,495	5,252	63.32	8,977	2,358	2,144	4,502	50.15
Pasig City	13,894	7,145	6,675	13,820	99.47	15,041	7,333	6,770	14,103	93.76
Pateros	1,176	509	475	984	83.67	1,271	481	460	941	74.04
Taguig	14,807	5,875	5,769	11,644	78.64	16,028	5,952	5,691	11,643	72.64
Quezon City	54,011	26,752	27,259	54,011	100.00	58,456	28,012	28,690	56,702	97.00
Makati City	10,718	4,559	4,180	8,739	81.54	11,600	4,888	4,359	9,247	79.72
Mandaluyong City	7,111	2,820	2,924	5,744	80.78	7,690	3,777	3,836	7,613	99.00
San Juan	2,246	694	672	1,366	60.82	2,434	821	804	1,625	66.76
Manila City	32,743	13,419	12,786	26,205	80.03	35,447	13,581	13,217	26,798	75.60
Las Piñas City	10,832	4,111	3,856	7,967	73.55	11,726	4,278	4,223	8,501	72.50
Muntinlupa City	9,281	3,978	3,806	7,784	83.87	10,045	4,231	4,119	8,350	83.13
Parañaque City	12,249	4,594	4,202	8,796	71.81	13,256	4,131	3,869	8,000	60.35
Pasay City	7,660	3,560	3,262	6,822	89.06	8,301	3,983	3,847	7,830	94.33
**C A R**	35,179	11,625	10,810	22,435	63.77	38,179	11,508	10,778	22,286	58.37
Abra	4,275	1,296	1,181	2,477	57.94	4,649	1,270	1,202	2,472	53.17
Apayao	2,502	924	892	1,816	72.58	2,715	904	828	1,732	63.79
Benguet	9,086	2,851	2,627	5,478	60.29	9,860	2,682	2,589	5,271	53.46
Ifugao	4,486	1,528	1,437	2,965	66.09	4,869	1,516	1,385	2,901	59.58
Kalinga	4,701	1,896	1,678	3,574	76.03	5,105	1,926	1,679	3,605	70.62
Mt. Province	3,103	1,022	962	1,984	63.94	3,357	930	877	1,807	53.83
Baguio City	7,026	2,108	2,033	4,141	58.94	7,624	2,280	2,218	4,498	59.00
**Region 1**	97,099	36,978	33,406	70,384	72.49	105,233	36,789	34,366	71,155	67.62
Ilocos Norte	7,854	2,775	2,507	5,282	67.25	8,499	2,723	2,521	5,244	61.70
Ilocos Sur	9,072	3,747	3,489	7,236	79.76	9,875	3,835	3,562	7,397	74.91
La Union	11,338	4,008	3,682	7,690	67.83	12,268	3,982	3,781	7,763	63.28
Pangasinan	50,710	19,486	17,240	36,726	72.42	54,942	19,336	18,116	37,452	68.17
Alaminos City	1,910	699	607	1,306	68.38	2,078	668	606	1,274	61.31
Candon City	954	386	368	754	79.04	1,042	525	479	1,004	96.35
Dagupan City	3,657	1,028	972	2,000	54.69	3,963	1,030	954	1,984	50.06
Laoag City	1,812	934	981	1,915	105.68	1,953	875	873	1,748	89.50
San Carlos City	4,028	1,779	1,663	3,442	85.45	4,362	1,729	1,607	3,336	76.48
San Fernando City	2,074	688	594	1,282	61.81	2,249	683	559	1,242	55.22
Urdaneta City	2,836	1,106	1,038	2,144	75.60	3,078	1,073	1,015	2,088	67.84
Vigan City	854	342	265	607	71.08	924	330	293	623	67.42
**Region 2**	68,960	25,440	23,526	48,966	71.01	74,767	25,170	23,730	48,900	65.40
Batanes	362	137	122	259	71.55	395	121	117	238	60.25
Cagayan	20,431	6,804	6,113	12,917	63.22	22,189	6,435	6,105	12,540	56.51
Isabela	23,449	8,622	7,978	16,600	70.79	25,388	8,566	8,035	16,601	65.39
Nueva Vizcaya	9,538	3,446	3,262	6,708	70.33	10,349	3,394	3,225	6,619	63.96
Quirino	4,051	1,762	1,610	3,372	83.24	4,404	1,854	1,689	3,543	80.45
Cauayan City	2,565	984	948	1,932	75.32	2,775	942	882	1,824	65.73
Ilagan City	2,889	1,372	1,311	2,683	92.87	3,118	1,448	1,427	2,875	92.21
Santiago City	2,671	1,349	1,302	2,651	99.25	2,891	1,509	1,382	2,891	100.00
Tuguegarao City	3,004	964	880	1,844	61.38	3,258	901	868	1,769	54.30
**Region 3**	220,155	95,422	89,749	185,171	84.11	238,292	91,226	87,203	178,429	74.88
Aurora	4,754	1,993	1,808	3,801	79.95	5,169	2,129	2,012	4,141	80.11
Bataan	13,789	5,622	5,157	10,779	78.17	14,894	5,043	4,459	9,502	63.80

Table 2.C.1.5. Immunization Services for Infants and Children

Number and Proportion of Children vaccinated with 2 doses of Measles Containing Vaccine (MCV)
Philippines, 2022

Area	Eligible Pop (0-11 mos. old)	MCV 1				Eligible Pop (0-12 mos. old)	MCV 2			
		Male	Female	Total	%		Male	Female	Total	%
Bulacan	43,760	19,827	18,587	38,414	87.78	47,363	20,090	19,153	39,243	82.86
Nueva Ecija	29,039	10,437	9,710	20,147	69.38	31,452	9,435	9,072	18,507	58.84
Pampanga	31,620	14,166	13,405	27,571	87.19	34,194	13,063	12,820	25,883	75.69
Tarlac	19,319	9,306	8,877	18,183	94.12	20,918	9,396	9,231	18,627	89.05
Zambales	12,476	4,160	3,884	8,044	64.48	13,516	3,591	3,390	6,981	51.65
Angeles City	7,932	3,634	3,490	7,124	89.81	8,586	3,504	3,315	6,819	79.42
Balanga City	1,992	787	691	1,478	74.20	2,152	666	619	1,285	59.71
Cabanatuan City	5,945	2,702	2,546	5,248	88.28	6,443	2,489	2,459	4,948	76.80
City of San Fernando	5,907	2,617	2,359	4,976	84.24	6,395	2,420	2,146	4,566	71.40
Gapan City	2,170	984	850	1,834	84.52	2,349	845	760	1,605	68.33
Mabalacat City	4,831	2,426	2,295	4,721	97.72	5,226	2,581	2,491	5,072	97.05
Malolos City	4,890	1,602	1,521	3,123	63.87	5,290	1,396	1,338	2,734	51.68
Meycauayan	4,055	1,977	2,021	3,998	98.59	4,390	1,839	1,865	3,704	84.37
Olongapo	4,922	1,629	1,521	3,150	64.00	5,334	1,452	1,372	2,824	52.94
Palayan City	808	336	296	632	78.22	874	296	316	612	70.02
San Jose City	2,748	1,332	1,209	2,541	92.47	2,978	1,280	1,118	2,398	80.52
San Jose del Monte City	11,133	5,977	5,893	11,870	106.62	12,043	5,828	5,631	11,459	95.15
Science City of Munoz	1,601	640	604	1,244	77.70	1,737	635	575	1,210	69.66
Tarlac City	6,464	3,268	3,025	6,293	97.35	6,989	3,248	3,061	6,309	90.27
**Region 4A**	299,627	110,138	103,235	213,373	71.21	324,408	104,364	98,536	202,900	62.54
Batangas	39,255	14,450	13,387	27,837	70.91	42,544	14,074	13,125	27,199	63.93
Cavite	28,151	11,502	11,045	22,547	80.09	30,459	11,571	11,017	22,588	74.16
Laguna	19,696	6,675	6,248	12,923	65.61	21,291	6,116	5,694	11,810	55.47
Quezon	37,853	11,680	10,979	22,659	59.86	41,028	10,536	10,072	20,608	50.23
Rizal	44,798	15,739	14,800	30,539	68.17	48,473	13,902	13,275	27,177	56.07
Antipolo City	16,498	6,193	5,684	11,877	71.99	17,853	5,372	5,021	10,393	58.21
Bacoor City	12,341	3,758	3,321	7,079	57.36	13,347	3,490	3,248	6,738	50.48
Batangas City	6,965	1,993	1,882	3,875	55.64	7,553	1,851	1,786	3,637	48.15
Biñan City	6,597	3,335	3,376	6,711	101.73	7,143	3,378	3,303	6,681	93.53
Cabuyao City	6,119	2,906	2,785	5,691	93.01	6,621	2,897	2,847	5,744	86.75
Calamba City	9,005	4,513	4,356	8,869	98.49	9,751	4,520	4,196	8,716	89.39
Cavite City	2,105	497	475	972	46.18	2,289	449	394	843	36.83
Dasmariñas City	13,538	5,018	4,757	9,775	72.20	14,647	5,071	4,759	9,830	67.11
General Trias City	6,457	2,936	2,718	5,654	87.56	6,985	2,637	2,452	5,089	72.86
Imus City	8,292	2,706	2,360	5,066	61.10	8,972	2,459	2,300	4,759	53.04
Lipa City	7,018	2,741	2,587	5,328	75.92	7,604	2,572	2,431	5,003	65.79
Lucena City	5,738	2,330	2,159	4,489	78.23	6,223	2,113	2,174	4,287	68.89
San Pablo City	5,272	1,942	1,878	3,820	72.46	5,702	1,946	1,749	3,695	64.80
San Pedro City	6,455	1,763	1,726	3,489	54.05	6,989	1,622	1,530	3,152	45.10
Santa Rosa City	7,009	2,961	2,571	5,532	78.93	7,587	3,026	2,660	5,686	74.94
Tagaytay City	1,460	714	641	1,355	92.81	1,584	775	708	1,483	93.62
Tanauan City	3,661	1,369	1,261	2,630	71.84	3,966	1,798	1,722	3,520	88.75
Tayabas City	2,145	784	718	1,502	70.02	2,335	725	654	1,379	59.06
Trece Martires City	3,199	1,633	1,521	3,154	98.59	3,462	1,464	1,419	2,883	83.28
**Region 4B**	72,791	23,624	22,331	45,955	63.13	78,852	23,275	21,671	44,946	57.00
Marinduque	4,990	1,478	1,431	2,909	58.30	5,400	1,452	1,372	2,824	52.30
Mindoro Occidental	12,479	4,246	3,969	8,215	65.83	13,530	4,003	3,619	7,622	56.33
Mindoro Oriental	20,189	6,209	5,685	11,894	58.91	21,869	6,802	6,222	13,024	59.55
Palawan	21,963	7,214	7,017	14,231	64.80	23,795	6,732	6,388	13,120	55.14
Romblon	6,569	1,989	1,904	3,893	59.26	7,110	1,893	1,849	3,742	52.63
Puerto Princesa City	6,601	2,488	2,325	4,813	72.91	7,148	2,393	2,221	4,614	64.55
**Region 5**	138,457	45,214	41,730	86,944	62.79	150,220	42,863	38,585	81,448	54.22
Albay	24,081	8,150	7,615	15,765	65.47	26,117	8,001	6,076	14,077	53.90
Camarines Norte	14,384	4,923	4,561	9,484	65.93	15,550	4,735	4,434	9,169	58.96
Camarines Sur	39,486	12,562	11,419	23,981	60.73	42,828	11,306	10,413	21,719	50.71
Catanduanes	6,459	2,007	1,902	3,909	60.52	7,032	1,905	1,864	3,769	53.60
Masbate	22,600	7,273	6,654	13,927	61.62	24,582	6,679	6,253	12,932	52.61
Sorsogon	19,828	6,522	6,017	12,539	63.24	21,498	6,351	5,929	12,280	57.12
Iriga City	2,679	769	767	1,536	57.33	2,914	726	756	1,482	50.86

Table 2.C.1.5. Immunization Services for Infants and Children

Number and Proportion of Children vaccinated with 2 doses of Measles Containing Vaccine (MCV)
Philippines, 2022

Area	Eligible Pop (0-11 mos. old)	MCV 1				Eligible Pop (0-12 mos. old)	MCV 2			
		Male	Female	Total	%		Male	Female	Total	%
Legaspi City	**4,233**	1,427	1,287	**2,714**	**64.12**	**4,593**	1,592	1,393	**2,985**	**64.99**
Naga City	**4,707**	1,581	1,508	**3,089**	**65.63**	**5,106**	1,568	1,467	**3,035**	**59.44**
**Region 6**	**146,449**	**52,045**	**48,255**	**100,300**	**68.49**	**158,796**	**53,646**	**49,652**	**103,298**	**65.05**
Aklan	**11,288**	3,783	3,506	**7,289**	**64.57**	**12,266**	3,781	3,558	**7,339**	**59.83**
Antique	**13,132**	4,196	3,839	**8,035**	**61.19**	**14,222**	4,193	3,849	**8,042**	**56.55**
Capiz	**13,975**	4,858	4,495	**9,353**	**66.93**	**15,161**	4,713	4,427	**9,140**	**60.29**
Guimaras	**3,084**	1,269	1,221	**2,490**	**80.74**	**3,371**	1,272	1,241	**2,513**	**74.55**
Iloilo	**36,267**	12,214	11,138	**23,352**	**64.39**	**39,296**	13,091	11,909	**25,000**	**63.62**
Negros Occidental	**49,230**	18,895	17,741	**36,636**	**74.42**	**53,380**	19,433	18,182	**37,615**	**70.47**
Bacolod City	**11,082**	3,644	3,388	**7,032**	**63.45**	**12,007**	3,680	3,327	**7,007**	**58.36**
Iloilo City	**8,391**	3,186	2,927	**6,113**	**72.85**	**9,093**	3,483	3,159	**6,642**	**73.05**
**Region 7**	**162,308**	**59,442**	**56,951**	**116,393**	**71.71**	**175,584**	**58,864**	**56,673**	**115,537**	**65.80**
Bohol	**27,992**	8,755	8,100	**16,855**	**60.21**	**30,336**	8,318	7,829	**16,147**	**53.23**
Cebu	**66,463**	24,178	22,993	**47,171**	**70.97**	**71,834**	23,728	22,535	**46,263**	**64.40**
Negros Oriental	**27,890**	10,756	11,090	**21,846**	**78.33**	**30,202**	10,056	10,474	**20,530**	**67.98**
Siquijor	**1,661**	470	494	**964**	**58.04**	**1,827**	445	458	**903**	**49.43**
Cebu City	**20,866**	7,509	6,984	**14,493**	**69.46**	**22,546**	7,939	7,388	**15,327**	**67.98**
Lapu-Lapu City	**9,232**	4,204	3,937	**8,141**	**88.18**	**9,978**	4,506	4,349	**8,855**	**88.75**
Mandaue City	**8,204**	3,570	3,353	**6,923**	**84.39**	**8,861**	3,872	3,640	**7,512**	**84.78**
**Region 8**	**105,471**	**31,759**	**29,756**	**61,515**	**58.32**	**114,239**	**29,909**	**28,560**	**58,469**	**51.18**
Biliran	**3,873**	1,332	1,261	**2,593**	**66.95**	**4,199**	1,223	1,186	**2,409**	**57.37**
Eastern Samar	**11,908**	3,608	3,282	**6,890**	**57.86**	**12,870**	3,373	3,205	**6,578**	**51.11**
Northern Leyte	**35,422**	10,544	9,692	**20,236**	**57.13**	**38,298**	9,856	9,099	**18,955**	**49.49**
Northern Samar	**15,921**	4,705	4,333	**9,038**	**56.77**	**17,281**	4,369	4,105	**8,474**	**49.04**
Southern Leyte	**6,662**	2,103	1,895	**3,998**	**60.01**	**7,254**	2,027	1,898	**3,925**	**54.11**
Western Samar	**14,732**	4,236	4,183	**8,419**	**57.15**	**15,975**	3,901	3,831	**7,732**	**48.40**
Calbayog City	**4,535**	1,170	1,069	**2,239**	**49.37**	**4,922**	1,035	1,031	**2,066**	**41.97**
Maasin City	**1,696**	425	427	**852**	**50.24**	**1,846**	434	473	**907**	**49.13**
Ormoc City	**5,044**	1,737	1,728	**3,465**	**68.70**	**5,446**	1,731	1,743	**3,474**	**63.79**
Tacloban City	**5,678**	1,899	1,886	**3,785**	**66.66**	**6,148**	1,960	1,989	**3,949**	**64.23**
**Region 9**	**79,007**	**28,969**	**27,834**	**56,803**	**71.90**	**85,598**	**28,683**	**27,576**	**56,259**	**65.72**
Zamboanga del Norte	**16,854**	6,235	5,892	**12,127**	**71.95**	**18,295**	6,153	5,884	**12,037**	**65.79**
Zamboanga del Sur	**17,368**	5,608	5,244	**10,852**	**62.48**	**18,780**	5,535	5,266	**10,801**	**57.51**
Zamboanga Sibugay	**15,021**	4,572	4,371	**8,943**	**59.54**	**16,286**	4,437	4,271	**8,708**	**53.47**
Dapitan City	**1,737**	698	816	**1,514**	**87.16**	**1,886**	748	853	**1,601**	**84.89**
Dipolog City	**2,762**	1,173	1,061	**2,234**	**80.88**	**2,994**	1,071	964	**2,035**	**67.97**
Isabela City	**2,563**	1,096	1,034	**2,130**	**83.11**	**2,803**	1,289	1,088	**2,377**	**84.80**
Pagadian City	**4,257**	1,404	1,448	**2,852**	**67.00**	**4,609**	1,272	1,337	**2,609**	**56.61**
Zamboanga City	**18,445**	8,183	7,968	**16,151**	**87.56**	**19,945**	8,178	7,913	**16,091**	**80.68**
**Region 10**	**99,908**	**40,159**	**37,888**	**78,047**	**78.12**	**108,469**	**39,338**	**37,757**	**77,095**	**71.08**
Bukidnon	**22,900**	9,716	9,207	**18,923**	**82.63**	**24,850**	9,171	8,809	**17,980**	**72.35**
Camiguin	**1,854**	567	545	**1,112**	**59.98**	**2,014**	547	457	**1,004**	**49.85**
Lanao del Norte	**14,930**	4,755	4,662	**9,417**	**63.07**	**16,254**	4,774	4,867	**9,641**	**59.31**
Misamis Occidental	**6,420**	2,251	2,322	**4,573**	**71.23**	**6,958**	2,205	2,209	**4,414**	**63.44**
Misamis Oriental	**14,963**	6,117	5,554	**11,671**	**78.00**	**16,228**	5,951	5,525	**11,476**	**70.72**
Cagayan de Oro City	**14,172**	6,506	6,254	**12,760**	**90.04**	**15,363**	6,874	6,622	**13,496**	**87.85**
El Salvador City	**1,052**	432	401	**833**	**79.18**	**1,143**	453	395	**848**	**74.19**
Gingoog City	**2,608**	1,303	1,250	**2,553**	**97.89**	**2,832**	1,141	1,334	**2,475**	**87.39**
Iligan City	**7,565**	2,788	2,501	**5,289**	**69.91**	**8,226**	2,766	2,593	**5,359**	**65.15**
Malaybalay City	**3,817**	1,796	1,546	**3,342**	**87.56**	**4,143**	1,742	1,448	**3,190**	**77.00**
Oroquieta City	**1,389**	447	496	**943**	**67.89**	**1,513**	411	440	**851**	**56.25**
Ozamis City	**2,786**	1,018	924	**1,942**	**69.71**	**3,021**	934	839	**1,773**	**58.69**
Tangub City	**1,234**	543	453	**996**	**80.71**	**1,342**	487	417	**904**	**67.36**
Valencia City	**4,218**	1,920	1,773	**3,693**	**87.55**	**4,582**	1,882	1,802	**3,684**	**80.40**

Table 2.C.1.5. Immunization Services for Infants and Children

Number and Proportion of Children vaccinated with 2 doses of Measles Containing Vaccine (MCV)
Philippines, 2022

Area	Eligible Pop (0-11 mos. old)	MCV 1				Eligible Pop (0-12 mos. old)	MCV 2			
		Male	Female	Total	%		Male	Female	Total	%
**Region 11**	**108,407**	**42,569**	**39,026**	**81,595**	**75.27**	**117,503**	**41,125**	**38,472**	**79,597**	**67.74**
Davao de Oro	**15,490**	6,542	5,848	**12,390**	**79.99**	**16,846**	6,302	5,678	**11,980**	**71.11**
Davao del Norte	**21,017**	9,087	8,425	**17,512**	**83.32**	**22,765**	9,129	8,556	**17,685**	**77.69**
Davao Oriental	**13,229**	5,203	4,524	**9,727**	**73.53**	**14,342**	4,891	4,397	**9,288**	**64.76**
Davao del Sur	**14,564**	4,862	4,500	**9,362**	**64.28**	**15,771**	4,616	4,375	**8,991**	**57.01**
Davao Occidental	**6,510**	2,171	2,031	**4,202**	**64.55**	**7,091**	2,025	1,859	**3,884**	**54.77**
Davao City	**37,597**	14,704	13,698	**28,402**	**75.54**	**40,688**	14,162	13,607	**27,769**	**68.25**
**Region 12**	**98,859**	**33,869**	**31,831**	**65,700**	**66.46**	**107,130**	**32,630**	**31,163**	**63,793**	**59.55**
North Cotabato	**34,150**	9,743	9,149	**18,892**	**55.32**	**36,967**	8,950	8,656	**17,606**	**47.63**
Sarangani	**12,907**	5,018	4,713	**9,731**	**75.39**	**14,021**	4,993	4,719	**9,712**	**69.27**
South Cotabato	**20,917**	8,176	7,744	**15,920**	**76.11**	**22,633**	8,345	7,975	**16,320**	**72.11**
Sultan Kudarat	**17,299**	6,555	6,135	**12,690**	**73.36**	**18,806**	6,467	6,149	**12,616**	**67.08**
Gen. Santos City	**13,586**	4,377	4,090	**8,467**	**62.32**	**14,703**	3,875	3,664	**7,539**	**51.28**
**BARMM**	**101,343**	**34,575**	**34,967**	**69,542**	**68.62**	**110,934**	**32,888**	**33,811**	**66,699**	**60.12**
Basilan	**7,823**	1,404	1,392	**2,796**	**35.74**	**8,503**	1,141	1,194	**2,335**	**27.46**
Lanao del Sur	**21,639**	9,788	10,395	**20,183**	**93.27**	**23,729**	9,766	10,180	**19,946**	**84.06**
Maguindanao	**32,198**	12,009	11,717	**23,726**	**73.69**	**35,160**	11,340	11,384	**22,724**	**64.63**
Sulu	**17,165**	4,209	4,358	**8,567**	**49.91**	**18,995**	3,921	4,218	**8,139**	**42.85**
Tawi-Tawi	**9,369**	2,650	2,700	**5,350**	**57.10**	**10,190**	2,470	2,530	**5,000**	**49.07**
Lamitan City	**2,154**	537	499	**1,036**	**48.10**	**2,340**	453	458	**911**	**38.93**
Marawi City	**5,173**	2,255	2,249	**4,504**	**87.07**	**5,681**	2,055	2,143	**4,198**	**73.90**
Cotabato City	**5,822**	1,723	1,657	**3,380**	**58.06**	**6,336**	1,742	1,704	**3,446**	**54.39**
**CARAGA**	**60,575**	**20,366**	**19,093**	**39,459**	**65.14**	**65,802**	**21,363**	**20,541**	**41,904**	**63.68**
Agusan del Norte	**8,127**	2,884	2,667	**5,551**	**68.30**	**8,821**	3,091	2,892	**5,983**	**67.83**
Agusan del Sur	**17,709**	4,637	4,370	**9,007**	**50.86**	**19,262**	4,658	4,651	**9,309**	**48.33**
Surigao del Norte	**7,280**	2,786	2,610	**5,396**	**74.12**	**7,951**	2,869	2,731	**5,600**	**70.43**
Surigao del Sur	**11,553**	4,182	3,855	**8,037**	**69.57**	**12,510**	4,134	3,837	**7,971**	**63.72**
Province of Dinagat	**2,576**	716	704	**1,420**	**55.12**	**2,794**	754	730	**1,484**	**53.11**
Bislig City	**2,192**	686	610	**1,296**	**59.12**	**2,377**	631	594	**1,225**	**51.54**
Butuan City	**7,743**	3,231	3,089	**6,320**	**81.62**	**8,383**	3,782	3,754	**7,536**	**89.90**
Surigao City	**3,395**	1,244	1,188	**2,432**	**71.63**	**3,704**	1,444	1,352	**2,796**	**75.49**

Table 2.C.1.6. Immunization Services for Infants and Children

Number and Proportion of Fully Immunized Children & Completely Immunized Children
Philippines, 2022

Area	Eligible Pop (0-12 mos. old)	FIC				Eligible Pop (13-23 mos. old)	CIC			
		Male	Female	Total	%		Male	Female	Total	%
**PHILIPPINES**	2,310,426	708,410	676,062	1,384,472	59.92	1,967,820	129,958	122,741	252,699	12.84
**N C R**	256,420	93,837	91,132	184,969	72.14	214,752	31,627	30,751	62,378	29.05
Malabon	**7,280**	2,258	2,014	**4,272**	**58.68**	**6,096**	1,851	1,690	**3,541**	**58.09**
Navotas	**4,969**	1,914	1,779	**3,693**	**74.32**	**4,160**	313	270	**583**	**14.01**
Valenzuela City	**12,358**	3,144	3,052	**6,196**	**50.14**	**10,346**	960	842	**1,802**	**17.42**
Caloocan City	**31,541**	6,424	6,108	**12,532**	**39.73**	**26,420**	4,082	3,845	**7,927**	**30.00**
							0			
Marikina City	**8,977**	2,151	1,965	**4,116**	**45.85**	**7,518**	406	370	**776**	**10.32**
Pasig City	**15,041**	6,721	5,876	**12,597**	**83.75**	**12,593**	2,195	2,239	**4,434**	**35.21**
Pateros	**1,271**	479	454	**933**	**73.41**	**1,065**	44	43	**87**	**8.17**
Taguig	**16,028**	4,911	4,869	**9,780**	**61.02**	**13,423**	2,296	2,140	**4,436**	**33.05**
Quezon City	**58,456**	27,938	28,739	**56,677**	**96.96**	**48,959**	5,544	5,652	**11,196**	**22.87**
Makati City	**11,600**	4,892	4,378	**9,270**	**79.91**	**9,715**	994	958	**1,952**	**20.09**
Mandaluyong City	**7,690**	3,603	3,698	**7,301**	**94.94**	**6,442**	815	769	**1,584**	**24.59**
San Juan	**2,434**	527	553	**1,080**	**44.37**	**2,039**	294	251	**545**	**26.73**
Manila City	**35,447**	13,598	13,181	**26,779**	**75.55**	**29,687**	6,466	6,426	**12,892**	**43.43**
Las Piñas City	**11,726**	3,585	3,447	**7,032**	**59.97**	**9,820**	1,979	1,988	**3,967**	**40.40**
Muntinlupa City	**10,045**	3,112	2,965	**6,077**	**60.50**	**8,413**	956	952	**1,908**	**22.68**
Parañaque City	**13,256**	5,894	5,521	**11,415**	**86.11**	**11,103**	1,121	995	**2,116**	**19.06**
Pasay City	**8,301**	2,686	2,533	**5,219**	**62.87**	**6,953**	1,311	1,321	**2,632**	**37.85**
**C A R**	38,179	10,986	10,307	21,293	55.77	32,929	433	380	813	2.47
Abra	**4,649**	1,238	1,185	**2,423**	**52.12**	**4,023**	36	21	**57**	**1.42**
Apayao	**2,715**	874	797	**1,671**	**61.55**	**2,317**	37	25	**62**	**2.68**
Benguet	**9,860**	2,541	2,465	**5,006**	**50.77**	**8,436**	132	108	**240**	**2.84**
Ifugao	**4,869**	1,416	1,331	**2,747**	**56.42**	**4,379**	81	71	**152**	**3.47**
Kalinga	**5,105**	1,925	1,677	**3,602**	**70.56**	**4,403**	0	3	**3**	**0.07**
Mt. Province	**3,357**	893	825	**1,718**	**51.18**	**2,842**	40	43	**83**	**2.92**
Baguio City	**7,624**	2,099	2,027	**4,126**	**54.12**	**6,529**	107	109	**216**	**3.31**
**Region 1**	105,233	35,656	33,181	68,837	65.41	89,113	1,133	1,186	2,319	2.60
Ilocos Norte	**8,499**	2,722	2,521	**5,243**	**61.69**	**6,959**	1	1	**2**	**0.03**
Ilocos Sur	**9,875**	3,826	3,546	**7,372**	**74.65**	**8,550**	9	16	**25**	**0.29**
La Union	**12,268**	3,856	3,658	**7,514**	**61.25**	**10,363**	126	123	**249**	**2.40**
Pangasinan	**54,942**	18,742	17,509	**36,251**	**65.98**	**46,596**	594	607	**1,201**	**2.58**
Alaminos City	**2,078**	658	591	**1,249**	**60.11**	**1,770**	10	15	**25**	**1.41**
Candon City	**1,042**	525	479	**1,004**	**96.35**	**901**	0	0	**0**	**0.00**
Dagupan City	**3,963**	679	577	**1,256**	**31.69**	**3,364**	351	377	**728**	**21.64**
Laoag City	**1,953**	875	873	**1,748**	**89.50**	**1,596**	0	0	**0**	**0.00**
San Carlos City	**4,362**	1,708	1,584	**3,292**	**75.47**	**3,700**	21	23	**44**	**1.19**
San Fernando City	**2,249**	682	559	**1,241**	**55.18**	**1,908**	1	0	**1**	**0.05**
Urdaneta City	**3,078**	1,057	1,002	**2,059**	**66.89**	**2,606**	16	13	**29**	**1.11**
Vigan City	**924**	326	282	**608**	**65.80**	**800**	4	11	**15**	**1.88**
**Region 2**	74,767	24,319	22,788	47,107	63.01	63,897	1,262	1,235	2,497	3.91
Batanes	**395**	121	116	**237**	**60.00**	**333**	0	0	**0**	**0.00**
Cagayan	**22,189**	6,109	5,724	**11,833**	**53.33**	**19,099**	397	368	**765**	**4.01**
Isabela	**25,388**	8,193	7,612	**15,805**	**62.25**	**21,431**	390	421	**811**	**3.78**
Nueva Vizcaya	**10,349**	3,357	3,214	**6,571**	**63.49**	**8,892**	124	114	**238**	**2.68**
Quirino	**4,404**	1,853	1,688	**3,541**	**80.40**	**3,917**	54	67	**121**	**3.09**
Cauayan City	**2,775**	890	835	**1,725**	**62.16**	**2,345**	156	145	**301**	**12.84**
Ilagan City	**3,118**	1,448	1,437	**2,885**	**92.53**	**2,634**	20	22	**42**	**1.59**
Santiago City	**2,891**	1,509	1,382	**2,891**	**100.00**	**2,444**	3	4	**7**	**0.29**
Tuguegarao City	**3,258**	839	780	**1,619**	**49.69**	**2,802**	118	94	**212**	**7.57**

Table 2.C.1.6. Immunization Services for Infants and Children

Number and Proportion of Fully Immunized Children & Completely Immunized Children
Philippines, 2022

Area	Eligible Pop (0-12 mos. old)	FIC				Eligible Pop (13-23 mos. old)	CIC			
		Male	Female	Total	%		Male	Female	Total	%
**Region 3**	**238,292**	**84,088**	**80,244**	**164,332**	**68.96**	**200,102**	**15,682**	**14,666**	**30,348**	**15.17**
Aurora	**5,169**	2,129	2,010	**4,139**	**80.07**	**4,519**	88	82	**170**	**3.76**
Bataan	**14,894**	4,808	4,238	**9,046**	**60.74**	**12,196**	838	729	**1,567**	**12.85**
Bulacan	**47,363**	16,332	15,637	**31,969**	**67.50**	**39,669**	4,732	4,351	**9,083**	**22.90**
Nueva Ecija	**31,452**	8,828	8,469	**17,297**	**54.99**	**26,733**	1,686	1,610	**3,296**	**12.33**
Pampanga	**34,194**	12,947	12,670	**25,617**	**74.92**	**28,603**	1,625	1,465	**3,090**	**10.80**
Tarlac	**20,918**	8,935	8,696	**17,631**	**84.29**	**17,523**	711	675	**1,386**	**7.91**
Zambales	**13,516**	3,564	3,334	**6,898**	**51.04**	**11,413**	726	689	**1,415**	**12.40**
Angeles City	**8,586**	3,253	3,082	**6,335**	**73.78**	**7,182**	566	589	**1,155**	**16.08**
Balanga City	**2,152**	671	609	**1,280**	**59.48**	**1,763**	43	45	**88**	**4.99**
Cabanatuan City	**6,443**	2,021	1,964	**3,985**	**61.85**	**5,474**	462	494	**956**	**17.46**
City of San Fernando	**6,395**	2,156	1,968	**4,124**	**64.49**	**5,349**	676	561	**1,237**	**23.13**
Gapan City	**2,349**	844	759	**1,603**	**68.24**	**1,996**	128	153	**281**	**14.08**
Mabalacat City	**5,226**	2,391	2,292	**4,683**	**89.61**	**4,372**	222	179	**401**	**9.17**
Malolos City	**5,290**	1,367	1,281	**2,648**	**50.06**	**4,434**	325	322	**647**	**14.59**
Meycauayan	**4,390**	1,842	1,818	**3,660**	**83.37**	**3,673**	585	621	**1,206**	**32.83**
Olongapo	**5,334**	1,063	1,020	**2,083**	**39.05**	**4,503**	432	373	**805**	**17.88**
Palayan City	**874**	240	266	**506**	**57.89**	**742**	71	52	**123**	**16.58**
San Jose City	**2,978**	1,158	1,024	**2,182**	**73.27**	**2,530**	206	155	**361**	**14.27**
San Jose del Monte City	**12,043**	5,828	5,631	**11,459**	**95.15**	**10,093**	1,100	1,096	**2,196**	**21.76**
Science City of Munoz	**1,737**	584	528	**1,112**	**64.02**	**1,474**	48	47	**95**	**6.45**
Tarlac City	**6,989**	3,127	2,948	**6,075**	**86.92**	**5,861**	412	378	**790**	**13.48**
**Region 4A**	**324,408**	**89,511**	**85,185**	**174,696**	**53.85**	**273,030**	**23,614**	**21,900**	**45,514**	**16.67**
Batangas	**42,544**	11,720	11,011	**22,731**	**53.43**	**36,225**	3,704	3,413	**7,117**	**19.65**
Cavite	**30,459**	10,540	10,119	**20,659**	**67.83**	**25,359**	1,605	1,532	**3,137**	**12.37**
Laguna	**21,291**	4,837	4,527	**9,364**	**43.98**	**17,828**	1,446	1,341	**2,787**	**15.63**
Quezon	**41,028**	8,336	8,056	**16,392**	**39.95**	**35,307**	2,219	2,008	**4,227**	**11.97**
Rizal	**48,473**	12,053	11,402	**23,455**	**48.39**	**40,444**	3,835	3,591	**7,426**	**18.36**
Antipolo City	**17,853**	4,942	4,609	**9,551**	**53.50**	**14,897**	1,166	1,082	**2,248**	**15.09**
Bacoor City	**13,347**	2,664	2,567	**5,231**	**39.19**	**11,111**	876	808	**1,684**	**15.16**
Batangas City	**7,553**	1,521	1,535	**3,056**	**40.46**	**6,428**	334	267	**601**	**9.35**
Biñan City	**7,143**	3,343	3,241	**6,584**	**92.17**	**5,977**	671	695	**1,366**	**22.85**
Cabuyao City	**6,621**	2,617	2,596	**5,213**	**78.73**	**5,542**	780	739	**1,519**	**27.41**
Calamba City	**9,751**	4,052	3,749	**7,801**	**80.00**	**8,160**	466	447	**913**	**11.19**
Cavite City	**2,289**	389	333	**722**	**31.54**	**1,906**	10	9	**19**	**1.00**
Dasmariñas City	**14,647**	4,859	4,557	**9,416**	**64.29**	**12,194**	1,281	1,170	**2,451**	**20.10**
General Trias City	**6,985**	2,010	1,896	**3,906**	**55.92**	**5,815**	654	617	**1,271**	**21.86**
Imus City	**8,972**	1,976	1,835	**3,811**	**42.48**	**7,470**	493	465	**958**	**12.82**
Lipa City	**7,604**	2,150	2,086	**4,236**	**55.71**	**6,471**	745	685	**1,430**	**22.10**
Lucena City	**6,223**	1,705	1,780	**3,485**	**56.00**	**5,351**	732	679	**1,411**	**26.37**
San Pablo City	**5,702**	1,534	1,441	**2,975**	**52.17**	**4,775**	394	322	**716**	**14.99**
San Pedro City	**6,989**	1,224	1,307	**2,531**	**36.21**	**5,842**	428	339	**767**	**13.13**
Santa Rosa City	**7,587**	2,949	2,637	**5,586**	**73.63**	**6,352**	608	586	**1,194**	**18.80**
Tagaytay City	**1,584**	771	703	**1,474**	**93.06**	**1,317**	3	9	**12**	**0.91**
Tanauan City	**3,966**	1,611	1,593	**3,204**	**80.79**	**3,375**	621	547	**1,168**	**34.61**
Tayabas City	**2,335**	659	588	**1,247**	**53.40**	**2,005**	111	110	**221**	**11.02**
Trece Martires City	**3,462**	1,049	1,017	**2,066**	**59.68**	**2,879**	432	439	**871**	**30.25**
**Region 4B**	**78,852**	**20,911**	**19,585**	**40,496**	**51.36**	**66,715**	**3,274**	**3,100**	**6,374**	**9.55**
Marinduque	**5,400**	1,326	1,257	**2,583**	**47.83**	**4,629**	104	117	**221**	**4.77**
Mindoro Occidental	**13,530**	3,197	2,886	**6,083**	**44.96**	**11,512**	676	629	**1,305**	**11.34**
Mindoro Oriental	**21,869**	6,540	6,036	**12,576**	**57.51**	**18,296**	816	742	**1,558**	**8.52**
Palawan	**23,795**	6,085	5,763	**11,848**	**49.79**	**20,092**	975	981	**1,956**	**9.74**
Romblon	**7,110**	1,778	1,769	**3,547**	**49.89**	**6,148**	104	77	**181**	**2.94**
Puerto Princesa City	**7,148**	1,985	1,874	**3,859**	**53.99**	**6,038**	599	554	**1,153**	**19.10**
**Region 5**	**150,220**	**36,523**	**34,430**	**70,953**	**47.23**	**129,669**	**8,036**	**7,254**	**15,290**	**11.79**
Albay	**26,117**	6,855	6,545	**13,400**	**51.31**	**22,658**	1,406	1,294	**2,700**	**11.92**
Camarines Norte	**15,550**	4,138	3,817	**7,955**	**51.16**	**12,859**	789	809	**1,598**	**12.43**
Camarines Sur	**42,828**	9,112	8,523	**17,635**	**41.18**	**36,843**	2,719	2,283	**5,002**	**13.58**
Catanduanes	**7,032**	1,732	1,764	**3,496**	**49.72**	**6,073**	215	184	**399**	**6.57**
Masbate	**24,582**	5,596	5,282	**10,878**	**44.25**	**21,859**	1,357	1,273	**2,630**	**12.03**
Sorsogon	**21,498**	5,550	5,223	**10,773**	**50.11**	**18,495**	880	802	**1,682**	**9.09**

Table 2.C.1.6. Immunization Services for Infants and Children

Number and Proportion of Fully Immunized Children & Completely Immunized Children
Philippines, 2022

Area	Eligible Pop (0-12 mos. old)	FIC				Eligible Pop (13-23 mos. old)	CIC			
		Male	Female	Total	%		Male	Female	Total	%
Iriga City	**2,914**	658	643	**1,301**	**44.65**	**2,502**	103	120	**223**	**8.91**
Legaspi City	**4,593**	1,375	1,224	**2,599**	**56.59**	**3,987**	329	259	**588**	**14.75**
Naga City	**5,106**	1,507	1,409	**2,916**	**57.11**	**4,393**	238	230	**468**	**10.65**
**Region 6**	**158,796**	**48,763**	**45,748**	**94,511**	**59.52**	**135,387**	**5,485**	**4,850**	**10,335**	**7.63**
Aklan	**12,266**	3,487	3,316	**6,803**	**55.46**	**10,600**	595	554	**1,149**	**10.84**
Antique	**14,222**	3,939	3,651	**7,590**	**53.37**	**12,076**	190	185	**375**	**3.11**
Capiz	**15,161**	4,038	3,910	**7,948**	**52.42**	**13,069**	774	642	**1,416**	**10.83**
Guimaras	**3,371**	1,199	1,170	**2,369**	**70.28**	**3,070**	72	62	**134**	**4.36**
Iloilo	**39,296**	12,960	11,941	**24,901**	**63.37**	**33,203**	132	146	**278**	**0.84**
Negros Occidental	**53,380**	17,154	16,263	**33,417**	**62.60**	**45,461**	2,412	2,096	**4,508**	**9.92**
Bacolod City	**12,007**	2,607	2,429	**5,036**	**41.94**	**10,224**	1,206	1,074	**2,280**	**22.30**
Iloilo City	**9,093**	3,379	3,068	**6,447**	**70.90**	**7,684**	104	91	**195**	**2.54**
**Region 7**	**175,584**	**50,530**	**48,633**	**99,163**	**56.48**	**145,510**	**8,903**	**8,595**	**17,498**	**12.03**
Bohol	**30,336**	6,605	6,278	**12,883**	**42.47**	**25,662**	1,712	1,546	**3,258**	**12.70**
Cebu	**71,834**	20,355	19,431	**39,786**	**55.39**	**58,863**	3,582	3,357	**6,939**	**11.79**
Negros Oriental	**30,202**	8,016	8,187	**16,203**	**53.65**	**25,460**	2,383	2,537	**4,920**	**19.32**
Siquijor	**1,827**	417	423	**840**	**45.98**	**1,627**	28	35	**63**	**3.87**
Cebu City	**22,546**	7,171	6,744	**13,915**	**61.72**	**18,469**	787	700	**1,487**	**8.05**
Lapu-Lapu City	**9,978**	4,317	4,185	**8,502**	**85.21**	**8,168**	188	165	**353**	**4.32**
Mandaue City	**8,861**	3,649	3,385	**7,034**	**79.38**	**7,261**	223	255	**478**	**6.58**
**Region 8**	**114,239**	**26,981**	**25,794**	**52,775**	**46.20**	**96,565**	**4,384**	**4,118**	**8,502**	**8.80**
Biliran	**4,199**	1,101	1,076	**2,177**	**51.85**	**3,740**	129	139	**268**	**7.17**
Eastern Samar	**12,870**	3,158	2,833	**5,991**	**46.55**	**10,563**	651	585	**1,236**	**11.70**
Northern Leyte	**38,298**	9,330	8,807	**18,137**	**47.36**	**31,834**	1,121	1,017	**2,138**	**6.72**
Northern Samar	**17,281**	3,433	3,227	**6,660**	**38.54**	**14,926**	1,007	919	**1,926**	**12.90**
Southern Leyte	**7,254**	1,854	1,756	**3,610**	**49.77**	**6,444**	173	142	**315**	**4.89**
Western Samar	**15,975**	3,364	3,371	**6,735**	**42.16**	**13,591**	778	759	**1,537**	**11.31**
Calbayog City	**4,922**	863	849	**1,712**	**34.78**	**4,195**	168	171	**339**	**8.08**
Maasin City	**1,846**	413	445	**858**	**46.48**	**1,640**	31	38	**69**	**4.21**
Ormoc City	**5,446**	1,515	1,489	**3,004**	**55.16**	**4,527**	235	272	**507**	**11.20**
Tacloban City	**6,148**	1,950	1,941	**3,891**	**63.29**	**5,105**	91	76	**167**	**3.27**
**Region 9**	**85,598**	**24,364**	**23,201**	**47,565**	**55.57**	**72,699**	**4,931**	**4,832**	**9,763**	**13.43**
Zamboanga del Norte	**18,295**	5,481	5,105	**10,586**	**57.86**	**15,682**	753	771	**1,524**	**9.72**
Zamboanga del Sur	**18,780**	4,260	4,088	**8,348**	**44.45**	**15,593**	1,119	1,050	**2,169**	**13.91**
Zamboanga Sibugay	**16,286**	3,708	3,549	**7,257**	**44.56**	**14,216**	668	647	**1,315**	**9.25**
Dapitan City	**1,886**	743	847	**1,590**	**84.31**	**1,618**	26	26	**52**	**3.21**
Dipolog City	**2,994**	758	678	**1,436**	**47.96**	**2,569**	266	240	**506**	**19.70**
Isabela City	**2,803**	1,090	975	**2,065**	**73.67**	**2,644**	398	276	**674**	**25.49**
Pagadian City	**4,609**	1,130	1,133	**2,263**	**49.10**	**3,823**	195	211	**406**	**10.62**
Zamboanga City	**19,945**	7,194	6,826	**14,020**	**70.29**	**16,554**	1,506	1,611	**3,117**	**18.83**
**Region 10**	**108,469**	**37,935**	**36,291**	**74,226**	**68.43**	**94,121**	**5,118**	**4,945**	**10,063**	**10.69**
Bukidnon	**24,850**	9,310	9,005	**18,315**	**73.70**	**21,589**	1,239	1,149	**2,388**	**11.06**
Camiguin	**2,014**	492	416	**908**	**45.08**	**1,711**	55	42	**97**	**5.67**
Lanao del Norte	**16,254**	4,489	4,511	**9,000**	**55.37**	**14,414**	580	560	**1,140**	**7.91**
Misamis Occidental	**6,958**	2,148	2,151	**4,299**	**61.78**	**6,033**	803	872	**1,675**	**27.76**
Misamis Oriental	**16,228**	5,481	5,105	**10,586**	**65.23**	**13,877**	537	497	**1,034**	**7.45**
Cagayan de Oro City	**15,363**	6,986	6,665	**13,651**	**88.86**	**13,140**	587	629	**1,216**	**9.25**
El Salvador City	**1,143**	394	349	**743**	**65.00**	**977**	59	46	**105**	**10.75**
Gingoog City	**2,832**	1,101	1,208	**2,309**	**81.53**	**2,424**	79	87	**166**	**6.85**
Iligan City	**8,226**	2,599	2,418	**5,017**	**60.99**	**7,294**	473	422	**895**	**12.27**
Malaybalay City	**4,143**	1,455	1,212	**2,667**	**64.37**	**3,599**	340	287	**627**	**17.42**
Oroquieta City	**1,513**	397	416	**813**	**53.73**	**1,306**	9	11	**20**	**1.53**
Ozamis City	**3,021**	930	813	**1,743**	**57.70**	**2,618**	22	12	**34**	**1.30**
Tangub City	**1,342**	484	422	**906**	**67.51**	**1,166**	119	107	**226**	**19.38**
Valencia City	**4,582**	1,669	1,600	**3,269**	**71.34**	**3,973**	216	224	**440**	**11.07**

Table 2.C.1.6. Immunization Services for Infants and Children

Number and Proportion of Fully Immunized Children & Completely Immunized Children
Philippines, 2022

Area	Eligible Pop (0-12 mos. old)	FIC				Eligible Pop (13-23 mos. old)	CIC			
		Male	Female	Total	%		Male	Female	Total	%
**Region 11**	**117,503**	**39,437**	**36,627**	**76,064**	**64.73**	**100,126**	**6,468**	**5,622**	**12,090**	**12.07**
Davao de Oro	**16,846**	6,165	5,674	**11,839**	**70.28**	**14,770**	310	245	**555**	**3.76**
Davao del Norte	**22,765**	8,859	8,266	**17,125**	**75.23**	**19,272**	462	398	**860**	**4.46**
Davao Oriental	**14,342**	4,981	4,439	**9,420**	**65.68**	**12,330**	869	699	**1,568**	**12.72**
Davao del Sur	**15,771**	4,616	4,375	**8,991**	**57.01**	**13,212**	888	720	**1,608**	**12.17**
Davao Occidental	**7,091**	1,736	1,646	**3,382**	**47.69**	**6,404**	429	326	**755**	**11.79**
Davao City	**40,688**	13,080	12,227	**25,307**	**62.20**	**34,138**	3,510	3,234	**6,744**	**19.76**
**Region 12**	**107,130**	**30,302**	**29,011**	**59,313**	**55.37**	**90,752**	**3,517**	**3,296**	**6,813**	**7.51**
North Cotabato	**36,967**	7,896	7,667	**15,563**	**42.10**	**31,064**	1,669	1,579	**3,248**	**10.46**
Sarangani	**14,021**	4,762	4,520	**9,282**	**66.20**	**12,200**	277	265	**542**	**4.44**
South Cotabato	**22,633**	7,926	7,580	**15,506**	**68.51**	**18,924**	592	570	**1,162**	**6.14**
Sultan Kudarat	**18,806**	6,227	5,932	**12,159**	**64.65**	**16,275**	311	272	**583**	**3.58**
Gen. Santos City	**14,703**	3,491	3,312	**6,803**	**46.27**	**12,289**	668	610	**1,278**	**10.40**
**BARMM**	**110,934**	**31,582**	**32,074**	**63,656**	**57.38**	**105,470**	**4,694**	**4,746**	**9,440**	**8.95**
Basilan	**8,503**	1,036	1,032	**2,068**	**24.32**	**7,485**	367	381	**748**	**9.99**
Lanao del Sur	**23,729**	9,761	10,176	**19,937**	**84.02**	**23,216**	284	330	**614**	**2.64**
Maguindanao	**35,160**	10,316	10,196	**20,512**	**58.34**	**32,422**	1,822	1,746	**3,568**	**11.00**
Sulu	**18,995**	3,533	3,739	**7,272**	**38.28**	**20,046**	928	920	**1,848**	**9.22**
Tawi-Tawi	**10,190**	2,578	2,570	**5,148**	**50.52**	**9,035**	154	178	**332**	**3.67**
Lamitan City	**2,340**	432	436	**868**	**37.09**	**2,059**	54	56	**110**	**5.34**
Marawi City	**5,681**	2,237	2,278	**4,515**	**79.48**	**5,555**	1,021	1,075	**2,096**	**37.73**
Cotabato City	**6,336**	1,689	1,647	**3,336**	**52.65**	**5,652**	64	60	**124**	**2.19**
**CARAGA**	**65,802**	**22,685**	**21,831**	**44,516**	**67.65**	**56,983**	**1,397**	**1,265**	**2,662**	**4.67**
Agusan del Norte	**8,821**	2,918	2,718	**5,636**	**63.89**	**7,539**	213	185	**398**	**5.28**
Agusan del Sur	**19,262**	7,037	6,884	**13,921**	**72.27**	**16,996**	133	154	**287**	**1.69**
Surigao del Norte	**7,951**	2,744	2,599	**5,343**	**67.20**	**7,062**	143	143	**286**	**4.05**
Surigao del Sur	**12,510**	3,687	3,469	**7,156**	**57.20**	**10,497**	538	466	**1,004**	**9.56**
Province of Dinagat	**2,794**	681	691	**1,372**	**49.11**	**2,434**	93	62	**155**	**6.37**
Bislig City	**2,377**	563	531	**1,094**	**46.02**	**1,995**	94	79	**173**	**8.67**
Butuan City	**8,383**	3,709	3,685	**7,394**	**88.20**	**7,171**	73	69	**142**	**1.98**
Surigao City	**3,704**	1,346	1,254	**2,600**	**70.19**	**3,289**	110	107	**217**	**6.60**

Table 2.C.2.1. Nutrition Services for Infants and Children

Numbers and Proportion of newborns initiated on breastfeeding immediately after birth lasting for at least 90 minutes, infants exclusively breastfed until 6th month, and infants who continued breastfeeding and were introduced to complementary feeding beginning at 6 months of age

Philippines, 2022

Area	Live births	Newborns initiated on breastfeeding immediately after birth				E.Pop (Under 1y.o.)	Infants Exclusively Breastfed until 5th month and 29 days				E.Pop (6-11 Mos.)	Infants who continued breastfeeding and were introduced to complementary feeding			
		Male	Female	Total	%		Male	Female	Total	%		Male	Female	Total	%
**PHILIPPINES**	**1,413,107**	**576,072**	**582,385**	**1,158,457**	**81.98**	**2,131,496**	**555,077**	**551,814**	**1,106,891**	**51.93**	**1,065,764**	**505,898**	**493,432**	**999,330**	**93.77**
**N C R**	**186,955**	**65,240**	**67,270**	**132,510**	**70.88**	**236,901**	**68,793**	**68,838**	**137,631**	**58.10**	**118,428**	**49,420**	**49,005**	**98,425**	**83.11**
Malabon	3,325	1,440	1,360	**2,800**	**84.21**	6,724	1,783	1,660	**3,443**	**51.20**	**3,361**	1,589	1,546	**3,135**	**93.28**
Navotas	3,975	1,679	1,924	**3,603**	**90.64**	4,591	1,603	1,510	**3,113**	**67.81**	**2,292**	1,228	1,064	**2,292**	**100.00**
Valenzuela City	6,488	1,790	1,381	**3,171**	**48.87**	11,418	2,901	2,585	**5,486**	**48.05**	**5,705**	1,766	1,474	**3,240**	**56.79**
Caloocan City	12,459	5,860	6,599	**12,459**	**100.00**	29,146	6,210	6,383	**12,593**	**43.21**	**14,561**	4,976	5,102	**10,078**	**69.21**
Marikina City	7,095	1,142	1,301	**2,443**	**34.43**	8,294	1,469	1,459	**2,928**	**35.30**	**4,143**	1,333	1,337	**2,670**	**64.45**
Pasig City	11,325	3,034	3,134	**6,168**	**54.46**	13,894	1,619	1,665	**3,284**	**23.64**	**6,951**	1,683	1,691	**3,374**	**48.54**
Pateros	874	430	428	**858**	**98.17**	1,176	419	398	**817**	**69.47**	**585**	292	293	**585**	**100.00**
Taguig	11,962	4,534	4,737	**9,271**	**77.50**	14,807	6,392	6,420	**12,812**	**86.53**	**7,405**	3,756	3,649	**7,405**	**100.00**
Quezon City	42,950	21,137	21,813	**42,950**	**100.00**	54,011	22,534	23,603	**46,137**	**85.42**	**27,001**	13,109	13,891	**27,000**	**100.00**
Makati City	5,565	1,454	1,503	**2,957**	**53.14**	10,718	1,239	1,181	**2,420**	**22.58**	**5,364**	1,281	1,158	**2,439**	**45.47**
Mandaluyong City	6,817	2,674	2,732	**5,406**	**79.30**	7,111	1,694	1,774	**3,468**	**48.77**	**3,546**	1,422	1,457	**2,879**	**81.19**
San Juan	1,540	380	453	**833**	**54.09**	2,246	285	363	**648**	**28.85**	**1,122**	256	298	**554**	**49.38**
Manila City	45,321	9,285	9,462	**18,747**	**41.36**	32,743	10,232	9,726	**19,958**	**60.95**	**16,375**	8,401	7,974	**16,375**	**100.00**
Las Piñas City	6,425	3,013	2,934	**5,947**	**92.56**	10,832	3,267	3,195	**6,462**	**59.66**	**5,415**	2,731	2,684	**5,415**	**100.00**
Muntinlupa City	7,604	1,574	1,598	**3,172**	**41.71**	9,281	2,117	2,017	**4,134**	**44.54**	**4,639**	799	788	**1,587**	**34.21**
Parañaque City	7,939	3,224	3,230	**6,454**	**81.29**	12,249	2,979	2,929	**5,908**	**48.23**	**6,123**	2,812	2,745	**5,557**	**90.76**
Pasay City	5,291	2,590	2,681	**5,271**	**99.62**	7,660	2,050	1,970	**4,020**	**52.48**	**3,840**	1,986	1,854	**3,840**	**100.00**
**C A R**	**23,980**	**11,832**	**11,358**	**23,190**	**96.71**	**35,179**	**9,163**	**9,104**	**18,267**	**51.93**	**17,595**	**8,597**	**8,519**	**17,116**	**97.28**
Abra	2,816	1,430	1,336	**2,766**	**98.22**	4,275	907	1,068	**1,975**	**46.20**	**2,134**	880	1,029	**1,909**	**89.46**
Apayao	1,625	727	804	**1,531**	**94.22**	2,502	851	809	**1,660**	**66.35**	**1,253**	777	736	**1,513**	**120.75**
Benguet	4,029	2,021	1,953	**3,974**	**98.63**	9,086	2,397	2,395	**4,792**	**52.74**	**4,538**	2,292	2,274	**4,566**	**100.62**
Ifugao	2,737	1,217	1,215	**2,432**	**88.86**	4,486	935	927	**1,862**	**41.51**	**2,240**	835	822	**1,657**	**73.97**
Kalinga	2,637	1,270	1,207	**2,477**	**93.93**	4,701	1,450	1,294	**2,744**	**58.37**	**2,353**	1,431	1,280	**2,711**	**115.21**
Mt. Province	3,217	1,591	1,507	**3,098**	**96.30**	3,103	950	941	**1,891**	**60.94**	**1,550**	877	899	**1,776**	**114.58**
Baguio City	6,919	3,576	3,336	**6,912**	**99.90**	7,026	1,673	1,670	**3,343**	**47.58**	**3,527**	1,505	1,479	**2,984**	**84.60**
**Region 1**	**49,509**	**24,751**	**23,892**	**48,643**	**98.25**	**97,099**	**32,544**	**31,012**	**63,556**	**65.45**	**48,526**	**30,123**	**28,842**	**58,965**	**121.51**
Ilocos Norte	4,303	2,118	2,185	**4,303**	**100.00**	7,854	2,144	2,171	**4,315**	**54.94**	**3,928**	2,280	2,238	**4,518**	**115.02**
Ilocos Sur	4,694	2,142	2,220	**4,362**	**92.93**	9,072	3,601	3,512	**7,113**	**78.41**	**4,541**	3,519	3,414	**6,933**	**152.68**
La Union	4,327	2,202	2,121	**4,323**	**99.91**	11,338	3,542	3,403	**6,945**	**61.25**	**5,663**	3,445	3,163	**6,608**	**116.69**
Pangasinan	13,810	6,992	6,619	**13,611**	**98.56**	50,710	16,781	15,726	**32,507**	**64.10**	**25,326**	14,984	14,297	**29,281**	**115.62**
Alaminos City	1,324	668	656	**1,324**	**100.00**	1,910	796	747	**1,543**	**80.79**	**961**	796	747	**1,543**	**160.56**
Candon City	838	428	406	**834**	**99.52**	954	482	432	**914**	**95.81**	**477**	398	400	**798**	**167.30**
Dagupan City	8,447	4,441	4,006	**8,447**	**100.00**	3,657	1,087	1,068	**2,155**	**58.93**	**1,828**	833	814	**1,647**	**90.10**
Laoag City	1,963	967	994	**1,961**	**99.90**	1,812	700	725	**1,425**	**78.64**	**901**	700	725	**1,425**	**158.16**
San Carlos City	2,730	1,358	1,339	**2,697**	**98.79**	4,028	1,039	967	**2,006**	**49.80**	**2,011**	1,029	957	**1,986**	**98.76**
San Fernando City	3,552	1,796	1,756	**3,552**	**100.00**	2,074	743	768	**1,511**	**72.85**	**1,038**	542	624	**1,166**	**112.33**
Urdaneta City	1,842	858	867	**1,725**	**93.65**	2,836	924	913	**1,837**	**64.77**	**1,424**	898	885	**1,783**	**125.21**
Vigan City	1,679	781	723	**1,504**	**89.58**	854	705	580	**1,285**	**150.47**	**428**	699	578	**1,277**	**298.36**
**Region 2**	**39,561**	**18,234**	**17,450**	**35,684**	**90.20**	**68,960**	**20,275**	**19,627**	**39,902**	**57.86**	**34,463**	**16,782**	**16,806**	**33,588**	**97.46**
Batanes	254	142	110	**252**	**99.21**	362	93	101	**194**	**53.59**	**185**	79	95	**174**	**94.05**
Cagayan	8,759	4,231	3,989	**8,220**	**93.85**	20,431	5,586	4,875	**10,461**	**51.20**	**10,202**	4,957	4,533	**9,490**	**93.02**
Isabela	10,609	4,994	4,935	**9,929**	**93.59**	23,449	6,298	6,356	**12,654**	**53.96**	**11,728**	5,905	5,773	**11,678**	**99.57**
Nueva Vizcaya	7,377	2,654	2,710	**5,364**	**72.71**	9,538	3,085	2,985	**6,070**	**63.64**	**4,762**	2,054	2,606	**4,660**	**97.86**
Quirino	2,609	1,286	1,172	**2,458**	**94.21**	4,051	1,332	1,366	**2,698**	**66.60**	**2,023**	1,012	1,011	**2,023**	**100.00**
															**0.00**
Cauayan City	1,877	955	876	**1,831**	**97.55**	2,565	762	735	**1,497**	**58.36**	**1,282**	635	647	**1,282**	**100.00**
Ilagan City	3,578	1,641	1,540	**3,181**	**88.90**	2,889	914	1,087	**2,001**	**69.26**	**1,440**	663	777	**1,440**	**100.00**
Santiago City	2,935	1,557	1,378	**2,935**	**100.00**	2,671	1,347	1,324	**2,671**	**100.00**	**1,336**	703	633	**1,336**	**100.00**
Tuguegarao City	1,563	774	740	**1,514**	**96.87**	3,004	858	798	**1,656**	**55.13**	**1,505**	774	731	**1,505**	**100.00**
**Region 3**	**133,782**	**52,203**	**51,607**	**103,810**	**77.60**	**220,155**	**53,315**	**51,673**	**104,988**	**47.69**	**110,043**	**50,299**	**48,715**	**99,014**	**89.98**
Aurora	3,007	1,019	981	**2,000**	**66.51**	4,754	1,273	1,150	**2,423**	**50.97**	**2,380**	1,438	1,243	**2,681**	**112.65**
Bataan	4,985	3,205	3,108	**6,313**	**126.64**	13,789	4,465	4,003	**8,468**	**61.41**	**6,896**	3,479	3,527	**7,006**	**101.60**
Bulacan	20,016	9,240	9,516	**18,756**	**93.71**	43,760	7,821	7,790	**15,611**	**35.67**	**21,863**	8,813	8,462	**17,275**	**79.01**
Nueva Ecija	12,432	5,112	3,694	**8,806**	**70.83**	29,039	6,449	6,079	**12,528**	**43.14**	**14,499**	5,030	4,721	**9,751**	**67.25**
Pampanga	18,077	7,002	7,066	**14,068**	**77.82**	31,620	6,419	6,285	**12,704**	**40.18**	**15,802**	6,697	6,417	**13,114**	**82.99**
Tarlac	10,543	4,335	4,366	**8,701**	**82.53**	19,319	6,716	6,558	**13,274**	**68.71**	**9,655**	5,222	5,240	**10,462**	**108.36**
Zambales	8,718	4,155	4,179	**8,334**	**95.60**	12,476	2,584	2,552	**5,136**	**41.17**	**6,251**	2,466	2,416	**4,882**	**78.10**
Angeles City	5,733	171	177	**348**	**6.07**	7,932	1,200	1,244	**2,444**	**30.81**	**3,965**	1,292	1,446	**2,738**	**69.05**
Balanga City	1,300	669	593	**1,262**	**97.08**	1,992	616	544	**1,160**	**58.23**	**997**	605	527	**1,132**	**113.54**
Cabanatuan City	6,111	2,934	2,807	**5,741**	**93.95**	5,945	1,354	1,250	**2,604**	**43.80**	**2,971**	1,532	1,433	**2,965**	**99.80**
City of San Fernando	4,691	635	700	**1,335**	**28.46**	5,907	1,684	1,772	**3,456**	**58.51**	**2,953**	1,768	1,834	**3,602**	**121.98**
Gapan City	833	379	400	**779**	**93.52**	2,170	564	551	**1,115**	**51.38**	**1,084**	546	543	**1,089**	**100.46**
Mabalacat City	4,178	1,757	1,713	**3,470**	**83.05**	4,831	1,529	1,424	**2,953**	**61.13**	**2,414**	1,362	1,291	**2,653**	**109.90**
Malolos City	4,736	1,063	1,112	**2,175**	**45.92**	4,890	548	543	**1,091**	**22.31**	**2,441**	528	525	**1,053**	**43.14**
Meycauayan	2,234	1,288	1,439	**2,727**	**122.07**	4,055	1,395	1,487	**2,882**	**71.07**	**2,027**	1,418	1,470	**2,888**	**142.48**
Olongapo	3,687	1,326	1,269	**2,595**	**70.38**	4,922	692	709	**1,401**	**28.46**	**2,463**	294	307	**601**	**24.40**
Palayan City	109	45	56	**101**	**92.66**	808	342	323	**665**	**82.30**	**404**	283	240	**523**	**129.46**
San Jose City	2,108	1,084	950	**2,034**	**96.49**	2,748	813	771	**1,584**	**57.64**	**1,373**	520	518	**1,038**	**75.60**
San Jose del Monte City	8,400	3,966	4,134	**8,100**	**96.43**	11,133	4,459	4,377	**8,836**	**79.37**	**5,566**	3,921	3,712	**7,633**	**137.14**
Science City of Munoz	129	90	75	**165**	**127.91**	1,601	688	624	**1,312**	**81.95**	**801**	688	624	**1,312**	**163.80**
Tarlac City	11,755	2,728	3,272	**6,000**	**51.04**	6,464	1,704	1,637	**3,341**	**51.69**	**3,238**	2,397	2,219	**4,616**	**142.56**

Table 2.C.2.1. Nutrition Services for Infants and Children

Numbers and Proportion of newborns initiated on breastfeeding immediately after birth lasting for at least 90 minutes, infants exclusively breastfed until 6th month, and infants who continued breastfeeding and were introduced to complementary feeding beginning at 6 months of age

Philippines, 2022

Area	Live births	Newborns initiated on breastfeeding immediately after birth				E.Pop (Under 1y.o.)	Infants Exclusively Breastfed until 5th month and 29 days				E.Pop (6-11 Mos.)	Infants who continued breastfeeding and were introduced to complementary feeding			
		Male	Female	Total	%		Male	Female	Total	%		Male	Female	Total	%
**Region 4A**	**191,030**	**77,114**	**79,322**	**156,436**	**81.89**	**299,627**	**58,168**	**58,568**	**116,736**	**38.96**	**149,838**	**56,938**	**56,768**	**113,706**	**75.89**
Batangas	23,639	10,002	10,122	**20,124**	**85.13**	39,255	7,522	7,332	**14,854**	**37.84**	**19,634**	7,834	7,664	**15,498**	**78.93**
Cavite	19,509	8,070	8,421	**16,491**	**84.53**	28,151	6,224	6,472	**12,696**	**45.10**	**14,086**	5,676	5,822	**11,498**	**81.63**
Laguna	12,133	3,698	3,872	**7,570**	**62.39**	19,696	3,919	4,011	**7,930**	**40.26**	**9,851**	3,901	3,929	**7,830**	**79.48**
Quezon	24,660	9,053	9,066	**18,119**	**73.48**	37,853	7,307	7,186	**14,493**	**38.29**	**18,915**	6,838	6,743	**13,581**	**71.80**
Rizal	28,316	12,184	12,322	**24,506**	**86.54**	44,798	8,694	8,788	**17,482**	**39.02**	**22,406**	7,968	7,945	**15,913**	**71.02**
Antipolo City	9,153	2,379	2,731	**5,110**	**55.83**	16,498	2,207	2,232	**4,439**	**26.91**	**8,249**	2,034	2,114	**4,148**	**50.28**
Bacoor City	7,278	2,323	2,174	**4,497**	**61.79**	12,341	1,622	1,611	**3,233**	**26.20**	**6,167**	1,436	1,363	**2,799**	**45.39**
Batangas City	3,360	1,569	1,487	**3,056**	**90.95**	6,965	509	518	**1,027**	**14.75**	**3,485**	942	880	**1,822**	**52.28**
Biñan City	7,198	3,440	3,687	**7,127**	**99.01**	6,597	2,952	3,150	**6,102**	**92.50**	**3,297**	2,822	2,854	**5,676**	**172.16**
Cabuyao City	3,371	2,172	2,536	**4,708**	**139.66**	6,119	1,697	1,853	**3,550**	**58.02**	**3,060**	1,691	2,015	**3,706**	**121.11**
Calamba City	6,681	3,388	3,263	**6,651**	**99.55**	9,005	3,062	3,028	**6,090**	**67.63**	**4,502**	3,193	3,031	**6,224**	**138.25**
Cavite City	1,334	178	202	**380**	**28.49**	2,105	122	174	**296**	**14.06**	**1,056**	148	171	**319**	**30.21**
Dasmariñas City	8,217	3,804	3,795	**7,599**	**92.48**	13,538	2,854	2,824	**5,678**	**41.94**	**6,767**	2,963	2,911	**5,874**	**86.80**
General Trias City	2,772	1,266	1,297	**2,563**	**92.46**	6,457	668	682	**1,350**	**20.91**	**3,227**	647	627	**1,274**	**39.48**
Imus City	4,638	1,728	1,757	**3,485**	**75.14**	8,292	1,072	1,043	**2,115**	**25.51**	**4,147**	873	898	**1,771**	**42.71**
Lipa City	4,480	2,054	2,075	**4,129**	**92.17**	7,018	596	602	**1,198**	**17.07**	**3,513**	1,198	1,118	**2,316**	**65.93**
Lucena City	3,263	1,410	1,437	**2,847**	**87.25**	5,738	910	873	**1,783**	**31.07**	**2,868**	947	908	**1,855**	**64.68**
San Pablo City	3,312	1,445	1,440	**2,885**	**87.11**	5,272	1,036	1,040	**2,076**	**39.38**	**2,639**	749	734	**1,483**	**56.20**
San Pedro City	3,202	1,303	1,308	**2,611**	**81.54**	6,455	932	932	**1,864**	**28.88**	**3,230**	774	785	**1,559**	**48.27**
Santa Rosa City	5,392	2,077	2,746	**4,823**	**89.45**	7,009	1,880	1,917	**3,797**	**54.17**	**3,507**	1,681	1,660	**3,341**	**95.27**
Tagaytay City	1,308	680	614	**1,294**	**98.93**	1,460	659	647	**1,306**	**89.45**	**731**	639	614	**1,253**	**171.41**
Tanauan City	3,320	994	1,038	**2,032**	**61.20**	3,661	295	283	**578**	**15.79**	**1,833**	632	635	**1,267**	**69.12**
Tayabas City	1,428	726	727	**1,453**	**101.75**	2,145	805	764	**1,569**	**73.15**	**1,068**	794	755	**1,549**	**145.04**
Trece Martires City	3,066	1,171	1,205	**2,376**	**77.50**	3,199	624	606	**1,230**	**38.45**	**1,600**	558	592	**1,150**	**71.88**
**Region 4B**	**41,186**	**18,902**	**18,858**	**37,760**	**91.68**	**72,791**	**16,588**	**16,680**	**33,268**	**45.70**	**36,417**	**15,978**	**15,979**	**31,957**	**87.75**
Marinduque	3,050	1,566	1,387	**2,953**	**96.82**	4,990	1,157	1,152	**2,309**	**46.27**	**2,496**	1,261	1,086	**2,347**	**94.03**
Mindoro Occidental	7,521	3,930	3,783	**7,713**	**102.55**	12,479	3,803	3,719	**7,522**	**60.28**	**6,251**	3,768	3,625	**7,393**	**118.27**
Mindoro Oriental	9,685	4,018	4,271	**8,289**	**85.59**	20,189	3,603	3,946	**7,549**	**37.39**	**10,098**	3,247	3,582	**6,829**	**67.63**
Palawan	12,929	5,786	5,982	**11,768**	**91.02**	21,963	5,905	5,952	**11,857**	**53.99**	**10,993**	5,816	5,886	**11,702**	**106.45**
Romblon	4,086	1,931	1,817	**3,748**	**91.73**	6,569	1,603	1,470	**3,073**	**46.78**	**3,280**	1,409	1,370	**2,779**	**84.73**
Puerto Princesa City	3,915	1,671	1,618	**3,289**	**84.01**	6,601	517	441	**958**	**14.51**	**3,299**	477	430	**907**	**27.49**
**Region 5**	**89,935**	**36,162**	**35,335**	**71,497**	**79.50**	**138,457**	**36,922**	**34,942**	**71,864**	**51.90**	**69,222**	**29,617**	**26,964**	**56,581**	**81.74**
Albay	12,789	5,132	5,186	**10,318**	**80.68**	24,081	6,963	6,766	**13,729**	**57.01**	**12,031**	5,362	4,891	**10,253**	**85.22**
Camarines Norte	10,393	4,919	5,004	**9,923**	**95.48**	14,384	3,797	3,641	**7,438**	**51.71**	**7,180**	3,416	3,160	**6,576**	**91.59**
Camarines Sur	17,741	7,819	7,528	**15,347**	**86.51**	39,486	9,948	9,021	**18,969**	**48.04**	**19,757**	9,433	8,351	**17,784**	**90.01**
Catanduanes	4,329	2,195	2,126	**4,321**	**99.82**	6,459	1,700	1,604	**3,304**	**51.15**	**3,229**	1,558	1,506	**3,064**	**94.89**
Masbate	14,162	6,506	6,376	**12,882**	**90.96**	22,600	5,588	5,267	**10,855**	**48.03**	**11,291**	6,440	5,950	**12,390**	**109.73**
Sorsogon	12,901	6,418	6,137	**12,555**	**97.32**	19,828	5,637	5,452	**11,089**	**55.93**	**9,925**	1,000	844	**1,844**	**18.58**
Iriga City	1,689	774	697	**1,471**	**87.09**	2,679	232	237	**469**	**17.51**	**1,343**	368	377	**745**	**55.47**
Legaspi City	3,429	1,268	1,216	**2,484**	**72.44**	4,233	1,858	1,792	**3,650**	**86.23**	**2,115**	1,071	1,041	**2,112**	**99.86**
Naga City	12,502	1,131	1,065	**2,196**	**17.57**	4,707	1,199	1,162	**2,361**	**50.16**	**2,351**	969	844	**1,813**	**77.12**
**Region 6**	**90,983**	**39,667**	**40,044**	**79,711**	**87.61**	**146,449**	**29,957**	**29,959**	**59,916**	**40.91**	**73,254**	**28,357**	**28,262**	**56,619**	**77.29**
Aklan	5,950	2,371	2,815	**5,186**	**87.16**	11,288	2,813	2,913	**5,726**	**50.73**	**5,637**	2,626	2,674	**5,300**	**94.02**
Antique	7,837	3,572	4,041	**7,613**	**97.14**	13,132	2,551	2,506	**5,057**	**38.51**	**6,576**	2,451	2,403	**4,854**	**73.81**
Capiz	7,755	3,835	3,713	**7,548**	**97.33**	13,975	3,138	3,097	**6,235**	**44.62**	**6,987**	2,961	2,825	**5,786**	**82.81**
Guimaras	2,248	1,136	1,059	**2,195**	**97.64**	3,084	995	1,027	**2,022**	**65.56**	**1,549**	984	1,017	**2,001**	**129.18**
Iloilo	22,289	9,537	9,727	**19,264**	**86.43**	36,267	8,604	8,491	**17,095**	**47.14**	**18,145**	7,739	7,696	**15,435**	**85.06**
Negros Occidental	33,282	14,910	14,861	**29,771**	**89.45**	49,230	9,011	9,170	**18,181**	**36.93**	**24,624**	8,667	8,864	**17,531**	**71.19**
Bacolod City	6,007	1,745	1,320	**3,065**	**51.02**	11,082	1,027	1,023	**2,050**	**18.50**	**5,538**	1,231	1,192	**2,423**	**43.75**
Iloilo City	5,615	2,561	2,508	**5,069**	**90.28**	8,391	1,818	1,732	**3,550**	**42.31**	**4,198**	1,698	1,591	**3,289**	**78.35**
**Region 7**	**133,214**	**48,091**	**48,496**	**96,587**	**72.50**	**162,308**	**47,666**	**49,070**	**96,736**	**59.60**	**81,144**	**45,687**	**47,186**	**92,873**	**114.45**
Bohol	18,027	5,329	5,289	**10,618**	**58.90**	27,992	6,388	6,203	**12,591**	**44.98**	**13,994**	6,353	6,264	**12,617**	**90.16**
Cebu	53,485	17,588	17,776	**35,364**	**66.12**	66,463	20,329	21,334	**41,663**	**62.69**	**33,237**	18,511	19,480	**37,991**	**114.30**
Negros Oriental	19,600	8,666	9,074	**17,740**	**90.51**	27,890	8,648	9,649	**18,297**	**65.60**	**13,946**	9,338	10,261	**19,599**	**140.53**
Siquijor	1,098	483	457	**940**	**85.61**	1,661	475	503	**978**	**58.88**	**821**	464	490	**954**	**116.20**
Cebu City	22,881	8,376	8,306	**16,682**	**72.91**	20,866	6,170	5,710	**11,880**	**56.93**	**10,429**	5,803	5,415	**11,218**	**107.57**
Lapu-Lapu City	8,517	2,960	3,118	**6,078**	**71.36**	9,232	2,981	2,971	**5,952**	**64.47**	**4,615**	2,792	2,822	**5,614**	**121.65**
Mandaue City	9,606	4,689	4,476	**9,165**	**95.40**	8,204	2,675	2,700	**5,375**	**65.52**	**4,102**	2,426	2,454	**4,880**	**118.97**
**Region 8**	**59,604**	**22,371**	**23,505**	**45,876**	**76.97**	**105,471**	**22,044**	**21,822**	**43,866**	**41.59**	**52,765**	**18,486**	**19,016**	**37,502**	**71.07**
Biliran	3,274	1,530	1,542	**3,072**	**93.83**	3,873	1,139	1,140	**2,279**	**58.84**	**1,925**	951	974	**1,925**	**100.00**
Eastern Samar	7,094	3,208	3,403	**6,611**	**93.19**	11,908	2,465	2,571	**5,036**	**42.29**	**5,980**	1,974	2,112	**4,086**	**68.33**
Northern Leyte	14,018	5,823	6,049	**11,872**	**84.69**	35,422	8,100	7,870	**15,970**	**45.08**	**17,713**	5,773	5,806	**11,579**	**65.37**
Northern Samar	10,288	3,076	3,566	**6,642**	**64.56**	15,921	3,411	3,351	**6,762**	**42.47**	**7,964**	2,965	3,189	**6,154**	**77.27**
Southern Leyte	2,984	1,170	1,276	**2,446**	**81.97**	6,662	1,772	1,724	**3,496**	**52.48**	**3,335**	1,402	1,429	**2,831**	**84.89**
Western Samar	8,500	3,196	3,128	**6,324**	**74.40**	14,732	2,581	2,551	**5,132**	**34.84**	**7,359**	2,444	2,493	**4,937**	**67.09**
	0	0	0				0	0				0	0		
Calbayog City	2,290	742	757	**1,499**	**65.46**	4,535	634	695	**1,329**	**29.31**	**2,270**	611	658	**1,269**	**55.90**
Maasin City	2,382	594	564	**1,158**	**48.61**	1,696	303	286	**589**	**34.73**	**847**	240	258	**498**	**58.80**
Ormoc City	5,866	1,727	1,659	**3,386**	**57.72**	5,044	1,426	1,387	**2,813**	**55.77**	**2,524**	1,261	1,257	**2,518**	**99.76**
Tacloban City	2,908	1,305	1,561	**2,866**	**98.56**	5,678	213	247	**460**	**8.10**	**2,848**	865	840	**1,705**	**59.87**

Table 2.C.2.1. Nutrition Services for Infants and Children

Numbers and Proportion of newborns initiated on breastfeeding immediately after birth lasting for at least 90 minutes, infants exclusively breastfed until 6th month, and infants who continued breastfeeding and were introduced to complementary feeding beginning at 6 months of age

Philippines, 2022

Area	Live births	Newborns initiated on breastfeeding immediately after birth				E.Pop (Under 1y.o.)	Infants Exclusively Breastfed until 5th month and 29 days				E.Pop (6-11 Mos.)	Infants who continued breastfeeding and were introduced to complementary feeding			
		Male	Female	Total	%		Male	Female	Total	%		Male	Female	Total	%
Region 9	49,149	20,943	23,186	44,129	89.79	79,007	20,791	21,319	42,110	53.30	39,497	18,496	18,688	37,184	94.14
Zamboanga del Norte	9,740	4,093	4,500	8,593	88.22	16,854	4,505	4,463	8,968	53.21	8,438	4,063	4,080	8,143	96.50
Zamboanga del Sur	6,475	2,496	2,944	5,440	84.02	17,368	4,078	4,189	8,267	47.60	8,678	3,605	3,730	7,335	84.52
Zamboanga Sibugay	8,019	3,429	3,512	6,941	86.56	15,021	2,917	3,089	6,006	39.98	7,506	2,574	2,701	5,275	70.28
Dapitan City	1,557	665	878	1,543	99.10	1,737	61	76	137	7.89	872	61	72	133	15.25
Dipolog City	2,218	1,125	1,095	2,220	100.09	2,762	1,162	1,078	2,240	81.10	1,381	1,115	1,036	2,151	155.76
Isabela City	1,694	563	545	1,108	65.41	2,563	545	603	1,148	44.79	1,277	514	502	1,016	79.56
Pagadian City	2,708	953	1,448	2,401	88.66	4,257	1,223	1,141	2,364	55.53	2,128	1,091	1,065	2,156	101.32
Zamboanga City	16,738	7,619	8,264	15,883	94.89	18,445	6,300	6,680	12,980	70.37	9,217	5,473	5,502	10,975	119.07
Region 10	76,401	29,209	31,885	61,094	79.96	99,908	34,209	33,562	67,771	67.83	49,952	38,047	29,979	68,026	136.18
Bukidnon	19,904	7,078	8,020	15,098	75.85	22,900	8,022	8,511	16,533	72.20	11,449	6,861	7,286	14,147	123.57
Camiguin	1,143	516	551	1,067	93.35	1,854	493	447	940	50.70	929	574	541	1,115	120.02
Lanao del Norte	8,508	4,071	3,590	7,661	90.04	14,930	5,408	4,010	9,418	63.08	7,475	11,540	3,364	14,904	199.38
Misamis Occidental	4,064	1,439	1,721	3,160	77.76	6,420	1,854	1,991	3,845	59.89	3,196	1,694	1,795	3,489	109.17
Misamis Oriental	8,260	2,911	3,035	5,946	71.99	14,963	4,245	4,253	8,498	56.79	7,478	3,579	3,530	7,109	95.07
Cagayan de Oro City	15,917	5,622	6,417	12,039	75.64	14,172	6,230	5,999	12,229	86.29	7,076	5,911	5,686	11,597	163.89
El Salvador City	125	152	150	302	241.60	1,052	282	274	556	52.85	527	286	270	556	105.50
Gingoog City	2,084	516	1,137	1,653	79.32	2,608	580	1,089	1,669	64.00	1,314	771	913	1,684	128.16
Iligan City	7,317	2,355	2,496	4,851	66.30	7,565	2,509	2,266	4,775	63.12	3,784	2,427	2,154	4,581	121.06
Malaybalay City	3,114	1,574	1,503	3,077	98.81	3,817	1,530	1,516	3,046	79.80	1,908	1,569	1,540	3,109	162.95
Oroquieta City	1,116	739	795	1,534	137.46	1,389	550	613	1,163	83.73	693	544	588	1,132	163.35
Ozamis City	1,938	906	903	1,809	93.34	2,786	767	790	1,557	55.89	1,393	681	711	1,392	99.93
Tangub City	953	374	566	940	98.64	1,234	511	481	992	80.39	621	430	431	861	138.65
Valencia City	1,958	956	1,001	1,957	99.95	4,218	1,228	1,322	2,550	60.46	2,109	1,180	1,170	2,350	111.43
Region 11	82,907	40,147	36,744	76,891	92.74	108,407	34,497	32,202	66,699	61.53	54,210	35,750	33,055	68,805	126.92
Davao de Oro	11,820	6,007	5,513	11,520	97.46	15,490	5,541	4,967	10,508	67.84	7,750	5,724	5,119	10,843	139.91
Davao del Norte	18,059	9,097	8,321	17,418	96.45	21,017	7,086	6,798	13,884	66.06	10,511	7,348	6,905	14,253	135.60
Davao Oriental	8,835	4,750	4,026	8,776	99.33	13,229	5,004	4,309	9,313	70.40	6,614	5,180	4,430	9,610	145.30
Davao del Sur	9,217	4,341	3,953	8,294	89.99	14,564	3,967	3,700	7,667	52.64	7,283	3,780	3,490	7,270	99.82
Davao Occidental	4,576	1,516	1,430	2,946	64.38	6,510	1,881	1,803	3,684	56.59	3,253	1,834	1,798	3,632	111.65
Davao City	30,400	14,436	13,501	27,937	91.90	37,597	11,018	10,625	21,643	57.57	18,799	11,884	11,313	23,197	123.39
Region 12	64,775	30,256	30,973	61,229	94.53	98,859	30,196	30,919	61,115	61.82	49,447	28,076	28,519	56,595	114.46
North Cotabato	19,628	8,783	9,508	18,291	93.19	34,150	9,257	9,814	19,071	55.84	17,074	8,456	8,777	17,233	100.93
Sarangani	10,324	4,971	5,082	10,053	97.38	12,907	5,773	5,579	11,352	87.95	6,453	4,850	4,556	9,406	145.76
South Cotabato	16,426	8,096	7,920	16,016	97.50	20,917	7,130	7,589	14,719	70.37	10,458	6,120	6,516	12,636	120.83
Sultan Kudarat	12,536	6,189	5,962	12,151	96.93	17,299	5,913	5,631	11,544	66.73	8,668	6,714	6,681	13,395	154.53
Gen. Santos City	5,861	2,217	2,501	4,718	80.50	13,586	2,123	2,306	4,429	32.60	6,794	1,936	1,989	3,925	57.77
BARMM	65,177	24,657	25,568	50,225	77.06	101,343	21,438	22,968	44,406	43.82	50,645	19,261	20,581	39,842	78.67
Basilan	3,807	1,075	1,158	2,233	58.66	7,823	569	617	1,186	15.16	3,915	661	709	1,370	34.99
Lanao del Sur	18,045	7,602	8,161	15,763	87.35	21,639	7,289	7,811	15,100	69.78	10,799	6,270	6,904	13,174	121.99
Maguindanao	19,033	6,608	6,293	12,901	67.78	32,198	7,326	7,880	15,206	47.23	16,086	6,546	6,962	13,508	83.97
Sulu	9,614	3,793	3,328	7,121	74.07	17,165	2,199	2,386	4,585	26.71	8,590	2,137	2,245	4,382	51.01
Tawi-Tawi	5,208	2,051	2,229	4,280	82.18	9,369	1,877	2,012	3,889	41.51	4,683	1,365	1,513	2,878	61.46
Lamitan City	1,366	537	564	1,101	80.60	2,154	425	443	868	40.30	1,076	238	242	480	44.61
Marawi City	4,923	1,489	2,297	3,786	76.90	5,173	91	120	211	4.08	2,584	225	200	425	16.45
Cotabato City	3,181	1,502	1,538	3,040	95.57	5,822	1,662	1,699	3,361	57.73	2,912	1,819	1,806	3,625	124.48
CARAGA	34,959	16,293	16,892	33,185	94.93	60,575	18,511	19,549	38,060	62.83	30,318	15,984	16,548	32,532	107.30
Agusan del Norte	3,505	1,652	1,725	3,377	96.35	8,127	2,271	2,347	4,618	56.82	4,072	1,707	1,749	3,456	84.87
Agusan del Sur	10,021	4,863	4,847	9,710	96.90	17,709	6,378	7,097	13,475	76.09	8,857	5,725	6,068	11,793	133.15
Surigao del Norte	3,039	1,398	1,471	2,869	94.41	7,280	1,901	1,974	3,875	53.23	3,655	1,519	1,599	3,118	85.31
Surigao del Sur	6,314	3,079	3,050	6,129	97.07	11,553	3,096	3,157	6,253	54.12	5,778	3,153	3,106	6,259	108.32
Province of Dinagat	912	416	416	832	91.23	2,576	555	569	1,124	43.63	1,292	422	432	854	66.10
Bislig City	1,477	742	735	1,477	100.00	2,192	677	635	1,312	59.85	1,096	642	609	1,251	114.14
Butuan City	5,958	2,335	2,768	5,103	85.65	7,743	2,684	2,814	5,498	71.01	3,867	1,951	2,076	4,027	104.14
Surigao City	3,733	1,808	1,880	3,688	98.79	3,395	949	956	1,905	56.11	1,701	865	909	1,774	104.29

Table 2.C.2.2. Nutrition Services for Infants and Children

Number of Infants born pre-term or with low birth weight given iron supplements and infants 6-11 mos. old and children 12-59 mos. old given Vitamin A supplementation
Philippines, 2022

Area	Total No. of live births with low birth weight	Infants pre-term or with LBW (< 2500 grams) given Iron				Eligible Pop (6-11 months)	Infants 6-11 mos. old Given Vitamin A				Eligible Pop (12-59 months)	Children 12-59 mos. Old Given Vitamin A			
		Male	Female	Total	%		Male	Female	Total	%		Male	Female	Total	%
**PHILIPPINES**	**88,879**	**9,039**	**9,932**	**18,971**	**21.34**	**1,065,764**	**674,178**	**654,624**	**1,328,802**	**124.68**	**8,976,673**	**2,785,698**	**2,689,923**	**5,475,621**	**61.00**
**NCR**	**22,261**	**1,346**	**1,560**	**2,906**	**13.05**	**118,428**	**103,995**	**105,083**	**209,078**	**176.54**	**951,556**	**347,539**	**340,842**	**688,381**	**72.34**
Malabon City	75	37	38	**75**	**100.00**	**3,361**	2,891	2,689	**5,580**	**166.02**	**27,010**	10,455	10,028	**20,483**	**75.83**
Navotas City	128	53	42	**95**	**74.22**	**2,292**	2,999	2,857	**5,856**	**255.50**	**18,431**	7,395	7,351	**14,746**	**80.01**
Valenzuela City	337	40	297	**337**	**100.00**	**5,705**	3,397	5,384	**8,781**	**153.92**	**45,846**	13,209	10,725	**23,934**	**52.21**
Caloocan City	957	0	1	**1**	**0.10**	**14,561**	11,933	11,485	**23,418**	**160.83**	**117,048**	59,950	57,098	**117,048**	**100.00**
Marikina City	941	171	168	**339**	**36.03**	**4,143**	2,507	2,386	**4,893**	**118.10**	**33,305**	5,615	5,926	**11,541**	**34.65**
Pasig City	1,758	47	29	**76**	**4.32**	**6,951**	4,576	4,222	**8,798**	**126.57**	**55,813**	24,378	23,590	**47,968**	**85.94**
Pateros City	21	7	6	**13**	**61.90**	**585**	469	451	**920**	**157.26**	**4,718**	1,099	1,045	**2,144**	**45.44**
Taguig City	207	93	97	**190**	**91.79**	**7,405**	7,118	7,268	**14,386**	**194.27**	**59,478**	30,033	29,445	**59,478**	**100.00**
Quezon City	7,132	146	113	**259**	**3.63**	**27,001**	33,514	34,592	**68,106**	**252.24**	**216,968**	72,045	74,101	**146,146**	**67.36**
Makati City	614	68	70	**138**	**22.48**	**5,364**	2,386	2,204	**4,590**	**85.57**	**43,053**	20,574	19,142	**39,716**	**92.25**
Mandaluyong City	125	26	15	**41**	**32.80**	**3,546**	1,870	2,052	**3,922**	**110.60**	**28,541**	9,049	9,096	**18,145**	**63.58**
San Juan City	162	6	8	**14**	**8.64**	**1,122**	566	599	**1,165**	**103.83**	**9,028**	4,745	3,396	**8,141**	**90.18**
Manila City	7,849	293	321	**614**	**7.82**	**16,375**	16,820	16,390	**33,210**	**202.81**	**131,547**	26,934	26,485	**53,419**	**40.61**
Las Piñas City	122	47	49	**96**	**78.69**	**5,415**	3,893	3,559	**7,452**	**137.62**	**43,514**	8,141	8,046	**16,187**	**37.20**
Muntinlupa City	974	57	48	**105**	**10.78**	**4,639**	2,400	2,542	**4,942**	**106.53**	**37,280**	17,931	19,573	**37,504**	**100.60**
Parañaque City	493	94	118	**212**	**43.00**	**6,123**	3,463	3,309	**6,772**	**110.60**	**49,198**	21,133	20,983	**42,116**	**85.61**
Pasay City	366	161	140	**301**	**82.24**	**3,840**	3,193	3,094	**6,287**	**163.72**	**30,778**	14,853	14,812	**29,665**	**96.38**
**C A R**	**2,066**	**205**	**205**	**410**	**19.85**	**17,595**	**10,124**	**9,925**	**20,049**	**113.95**	**147,190**	**50,095**	**47,881**	**97,976**	**66.56**
Abra	230	49	57	**106**	**46.09**	**2,134**	1,296	1,181	**2,477**	**116.07**	**18,948**	7,365	7,167	**14,532**	**76.69**
Apayao	124	23	15	**38**	**30.65**	**1,253**	966	898	**1,864**	**148.76**	**11,111**	3,602	3,253	**6,855**	**61.70**
Benguet	341	41	35	**76**	**22.29**	**4,538**	2,448	2,699	**5,147**	**113.42**	**36,006**	11,982	11,353	**23,335**	**64.81**
Ifugao	127	7	3	**10**	**7.87**	**2,240**	1,089	1,098	**2,187**	**97.63**	**19,435**	7,877	7,263	**15,140**	**77.90**
Kalinga	88	18	16	**34**	**38.64**	**2,353**	1,223	1,111	**2,334**	**99.19**	**20,071**	5,775	5,242	**11,017**	**54.89**
Mt. Province	226	13	22	**35**	**15.49**	**1,550**	1,302	1,238	**2,540**	**163.87**	**13,756**	4,623	4,167	**8,790**	**63.90**
Baguio City	930	54	57	**111**	**11.94**	**3,527**	1,800	1,700	**3,500**	**99.23**	**27,863**	8,871	9,436	**18,307**	**65.70**
**Region 1**	**3,297**	**352**	**355**	**707**	**21.44**	**48,526**	**21,433**	**19,452**	**40,885**	**84.25**	**406,852**	**137,390**	**130,191**	**267,581**	**65.77**
Ilocos Norte	329	110	92	**202**	**61.40**	**3,928**	1,645	1,575	**3,220**	**81.98**	**31,951**	15,019	13,887	**28,906**	**90.47**
Ilocos Sur	162	56	66	**122**	**75.31**	**4,541**	2,338	2,187	**4,525**	**99.65**	**39,653**	28,930	28,421	**57,351**	**144.63**
La Union	304	45	50	**95**	**31.25**	**5,663**	1,971	1,778	**3,749**	**66.20**	**47,886**	27,218	25,678	**52,896**	**110.46**
Pangasinan	322	13	25	**38**	**11.80**	**25,326**	10,729	9,358	**20,087**	**79.31**	**211,444**	45,297	42,433	**87,730**	**41.49**
Alaminos City	7	0	2	**2**	**28.57**	**961**	809	734	**1,543**	**160.56**	**7,986**	3,928	3,825	**7,753**	**97.08**
Candon City	10	6	4	**10**	**100.00**	**477**	497	395	**892**	**187.00**	**4,182**	5,577	5,166	**10,743**	**256.89**
Dagupan City	1,452	49	42	**91**	**6.27**	**1,828**	834	840	**1,674**	**91.58**	**15,255**	5,236	5,206	**10,442**	**68.45**
Laoag City	0	0	0	**0**	**0.00**	**901**	533	518	**1,051**	**116.65**	**7,365**	2,474	2,059	**4,533**	**61.55**
San Carlos City	55	20	24	**44**	**80.00**	**2,011**	502	467	**969**	**48.18**	**16,796**	585	621	**1,206**	**7.18**
San Fernando City	361	8	10	**18**	**4.99**	**1,038**	542	686	**1,228**	**118.30**	**8,776**	632	632	**1,264**	**14.40**
Urdaneta City	35	10	11	**21**	**60.00**	**1,424**	586	565	**1,151**	**80.83**	**11,842**	666	654	**1,320**	**11.15**
Vigan City	260	35	29	**64**	**24.62**	**428**	447	349	**796**	**185.98**	**3,716**	1,828	1,609	**3,437**	**92.49**
**Region 2**	**1,499**	**344**	**369**	**713**	**47.57**	**34,463**	**17,781**	**16,322**	**34,103**	**98.96**	**289,361**	**114,460**	**104,942**	**219,402**	**75.82**
Batanes	16	1	0	**1**	**6.25**	**185**	24	24	**48**	**25.95**	**1,627**	99	150	**249**	**15.30**
Cagayan	224	76	81	**157**	**70.09**	**10,202**	5,498	4,597	**10,095**	**98.95**	**85,819**	28,682	26,032	**54,714**	**63.76**
Isabela	326	100	118	**218**	**66.87**	**11,728**	6,082	5,646	**11,728**	**100.00**	**97,953**	42,639	39,564	**82,203**	**83.92**
Nueva Vizcaya	698	88	106	**194**	**27.79**	**4,762**	2,367	2,338	**4,705**	**98.80**	**40,154**	17,953	16,702	**34,655**	**86.31**
Quirino	127	47	44	**91**	**71.65**	**2,023**	1,010	1,013	**2,023**	**100.00**	**17,287**	5,154	5,090	**10,244**	**59.26**
Cauayan City	27	13	8	**21**	**77.78**	**1,282**	677	605	**1,282**	**100.00**	**10,715**	2,203	1,923	**4,126**	**38.51**
Ilagan City	38	13	11	**24**	**63.16**	**1,440**	645	736	**1,381**	**95.90**	**12,047**	4,714	4,738	**9,452**	**78.46**
Santiago City	19	3	0	**3**	**15.79**	**1,336**	704	632	**1,336**	**100.00**	**11,159**	5,816	5,343	**11,159**	**100.00**
Tuguegarao City	24	3	1	**4**	**16.67**	**1,505**	774	731	**1,505**	**100.00**	**12,600**	7,200	5,400	**12,600**	**100.00**
**Region 3**	**5,717**	**777**	**900**	**1,677**	**29.33**	**110,043**	**84,866**	**82,367**	**167,233**	**151.97**	**913,147**	**338,458**	**324,786**	**663,244**	**72.63**
Aurora	230	6	10	**16**	**6.96**	**2,380**	2,251	2,126	**4,377**	**183.91**	**20,431**	8,366	8,324	**16,690**	**81.69**
Bataan	183	40	52	**92**	**50.27**	**6,896**	6,410	6,186	**12,596**	**182.66**	**54,674**	41,023	39,002	**80,025**	**146.37**
Bulacan	717	264	267	**531**	**74.06**	**21,863**	16,174	15,799	**31,973**	**146.24**	**181,432**	67,374	65,186	**132,560**	**73.06**
Nueva Ecija	301	62	162	**224**	**74.42**	**14,499**	9,476	9,507	**18,983**	**130.93**	**122,537**	45,941	45,942	**91,883**	**74.98**
Pampanga	452	133	160	**293**	**64.82**	**15,802**	12,742	11,972	**24,714**	**156.40**	**131,844**	49,749	43,219	**92,968**	**70.51**
Tarlac	24	9	13	**22**	**91.67**	**9,655**	4,779	5,245	**10,024**	**103.82**	**79,847**	13,477	15,576	**29,053**	**36.39**
Zambales	881	123	111	**234**	**26.56**	**6,251**	3,264	3,187	**6,451**	**103.20**	**50,671**	17,511	16,620	**34,131**	**67.36**
Angeles City	167	0	2	**2**	**1.20**	**3,965**	2,899	2,812	**5,711**	**144.04**	**33,080**	4,039	3,621	**7,660**	**23.16**
Balanga City	87	11	4	**15**	**17.24**	**997**	546	513	**1,059**	**106.22**	**7,905**	4,155	3,958	**8,113**	**102.63**
Cabanatuan City	167	71	60	**131**	**78.44**	**2,971**	2,644	2,575	**5,219**	**175.66**	**25,080**	3,251	3,250	**6,501**	**25.92**
City of San Fernando	533	0	0	**0**	**0.00**	**2,953**	4,198	3,824	**8,022**	**271.66**	**24,640**	15,731	14,069	**29,800**	**120.94**
Gapan City	6	0	0	**0**	**0.00**	**1,084**	682	691	**1,373**	**126.66**	**9,152**	5,393	5,392	**10,785**	**117.84**
Mabalacat City	401	0	1	**1**	**0.25**	**2,414**	2,630	2,433	**5,063**	**209.73**	**20,155**	3,782	3,728	**7,510**	**37.26**
Malolos City	56	17	15	**32**	**57.14**	**2,441**	1,609	1,574	**3,183**	**130.40**	**20,270**	13,478	13,568	**27,046**	**133.43**
Meycauayan	3	4	3	**7**	**233.33**	**2,027**	1,621	1,690	**3,311**	**163.34**	**16,812**	2,528	2,757	**5,285**	**31.44**
Olongapo	414	4	3	**7**	**1.69**	**2,463**	1,827	1,850	**3,677**	**149.29**	**19,984**	4,611	4,238	**8,849**	**44.28**
Palayan City	0	0	0	**0**	**0.00**	**404**	282	271	**553**	**136.88**	**3,405**	1,678	1,657	**3,335**	**97.94**
San Jose City	64	25	21	**46**	**71.88**	**1,373**	1,059	902	**1,961**	**142.83**	**11,599**	7,837	6,908	**14,745**	**127.12**
San Jose del Monte City	590	7	13	**20**	**3.39**	**5,566**	6,878	6,451	**13,329**	**239.47**	**46,153**	21,700	20,951	**42,651**	**92.41**
Science City of Munoz	2	0	2	**2**	**100.00**	**801**	0	0	**0**	**0.00**	**6,759**	0	0	**0**	**0.00**
Tarlac City	439	1	1	**2**	**0.46**	**3,238**	2,895	2,759	**5,654**	**174.61**	**26,717**	6,834	6,820	**13,654**	**51.11**
**Region 4A**	**9,916**	**968**	**1,101**	**2,069**	**20.87**	**149,838**	**97,503**	**93,235**	**190,738**	**127.30**	**1,238,585**	**294,007**	**282,154**	**576,161**	**46.52**
Batangas	404	97	110	**207**	**51.24**	**19,634**	14,548	13,631	**28,179**	**143.52**	**165,380**	59,561	56,074	**115,635**	**69.92**
Cavite	952	68	67	**135**	**14.18**	**14,086**	10,706	10,713	**21,419**	**152.06**	**115,721**	36,817	35,945	**72,762**	**62.88**
Laguna	1,040	50	45	**95**	**9.13**	**9,851**	5,930	5,625	**11,555**	**117.30**	**80,424**	22,480	20,446	**42,926**	**53.37**
Quezon	1,236	155	148	**303**	**24.51**	**18,915**	9,806	9,436	**19,242**	**101.73**	**160,430**	36,208	34,736	**70,944**	**44.22**
Rizal	1,093	191	277	**468**	**42.82**	**22,406**	12,571	12,316	**24,887**	**111.07**	**181,775**	22,179	21,975	**44,154**	**24.29**

Table 2.C.2.2. Nutrition Services for Infants and Children

Number of Infants born pre-term or with low birth weight given iron supplements and infants 6-11 mos. old and children 12-59 mos. old given Vitamin A supplementation
Philippines, 2022

Area	Total No. of live births with low birth weight	Infants pre-term or with LBW (< 2500 grams) given Iron				Eligible Pop (6-11 months)	Infants 6-11 mos. old Given Vitamin A				Eligible Pop (12-59 months)	Children 12-59 mos. Old Given Vitamin A			
		Male	Female	Total	%		Male	Female	Total	%		Male	Female	Total	%
Antipolo City	833	14	42	**56**	**6.72**	**8,249**	4,797	4,696	**9,493**	**115.08**	**66,955**	8,729	8,219	**16,948**	**25.31**
Bacoor City	327	26	27	**53**	**16.21**	**6,167**	3,378	3,137	**6,515**	**105.64**	**50,703**	4,756	4,631	**9,387**	**18.51**
Batangas City	291	18	23	**41**	**14.09**	**3,485**	2,115	1,954	**4,069**	**116.76**	**29,351**	4,157	3,866	**8,023**	**27.33**
Biñan City	135	0	3	**3**	**2.22**	**3,297**	3,281	3,234	**6,515**	**197.60**	**26,965**	10,521	10,127	**20,648**	**76.57**
Cabuyao City	156	12	13	**25**	**16.03**	**3,060**	2,588	2,552	**5,140**	**167.97**	**24,996**	4,656	4,616	**9,272**	**37.09**
Calamba City	552	25	22	**47**	**8.51**	**4,502**	4,064	3,861	**7,925**	**176.03**	**36,801**	15,805	14,787	**30,592**	**83.13**
Cavite City	68	9	4	**13**	**19.12**	**1,056**	374	399	**773**	**73.20**	**8,682**	2,182	2,040	**4,222**	**48.63**
Dasmariñas City	489	68	73	**141**	**28.83**	**6,767**	4,311	4,074	**8,385**	**123.91**	**55,633**	14,045	14,044	**28,089**	**50.49**
General Trias City	149	2	6	**8**	**5.37**	**3,227**	1,641	1,572	**3,213**	**99.57**	**26,535**	1,577	1,470	**3,047**	**11.48**
Imus City	155	15	10	**25**	**16.13**	**4,147**	2,479	2,258	**4,737**	**114.23**	**34,083**	5,954	5,746	**11,700**	**34.33**
Lipa City	76	4	10	**14**	**18.42**	**3,513**	882	841	**1,723**	**49.05**	**29,578**	1,474	1,344	**2,818**	**9.53**
Lucena City	247	54	50	**104**	**42.11**	**2,868**	2,650	2,547	**5,197**	**181.21**	**24,313**	7,792	7,467	**15,259**	**62.76**
San Pablo City	211	6	3	**9**	**4.27**	**2,639**	2,330	2,089	**4,419**	**167.45**	**21,543**	6,878	6,866	**13,744**	**63.80**
San Pedro City	537	133	143	**276**	**51.40**	**3,230**	1,426	1,458	**2,884**	**89.29**	**26,381**	2,130	2,021	**4,151**	**15.73**
Santa Rosa City	486	4	1	**5**	**1.03**	**3,507**	3,253	2,766	**6,019**	**171.63**	**28,642**	6,708	6,433	**13,141**	**45.88**
Tagaytay City	10	3	1	**4**	**40.00**	**731**	668	646	**1,314**	**179.75**	**6,012**	1,099	1,065	**2,164**	**35.99**
Tanauan City	186	2	4	**6**	**3.23**	**1,833**	1,197	1,110	**2,307**	**125.86**	**15,428**	3,475	3,354	**6,829**	**44.26**
Tayabas City	33	8	10	**18**	**54.55**	**1,068**	1,083	931	**2,014**	**188.58**	**9,107**	10,907	10,800	**21,707**	**238.36**
Trece Martires City	250	4	9	**13**	**5.20**	**1,600**	1,425	1,389	**2,814**	**175.88**	**13,147**	3,917	4,082	**7,999**	**60.84**
**Region 4B**	**1,244**	**466**	**500**	**966**	**77.65**	**36,417**	**19,912**	**19,370**	**39,282**	**107.87**	**304,748**	**111,216**	**108,092**	**219,308**	**71.96**
Marinduque	212	53	42	**95**	**44.81**	**2,496**	1,743	1,785	**3,528**	**141.35**	**22,343**	8,501	8,131	**16,632**	**74.44**
Mindoro Occidental	251	49	51	**100**	**39.84**	**6,251**	2,771	2,760	**5,531**	**88.48**	**50,640**	22,678	21,476	**44,154**	**87.19**
Mindoro Oriental	135	200	227	**427**	**316.30**	**10,098**	5,594	5,482	**11,076**	**109.69**	**84,729**	35,200	32,832	**68,032**	**80.29**
Palawan	297	116	117	**233**	**78.45**	**10,993**	6,959	6,697	**13,656**	**124.22**	**90,740**	33,270	34,083	**67,353**	**74.23**
Romblon	220	28	38	**66**	**30.00**	**3,280**	2,018	1,861	**3,879**	**118.26**	**29,043**	11,378	11,360	**22,738**	**78.29**
Puerto Princesa City	129	20	25	**45**	**34.88**	**3,299**	827	785	**1,612**	**48.86**	**27,253**	189	210	**399**	**1.46**
**Region 5**	**8,545**	**715**	**798**	**1,513**	**17.71**	**69,222**	**36,991**	**35,200**	**72,191**	**104.29**	**595,917**	**176,774**	**165,824**	**342,598**	**57.49**
Albay	936	190	227	**417**	**44.55**	**12,031**	6,193	5,661	**11,854**	**98.53**	**104,616**	49,263	45,829	**95,092**	**90.90**
Camarines Norte	1,405	76	82	**158**	**11.25**	**7,180**	3,589	3,319	**6,908**	**96.21**	**60,281**	23,928	22,853	**46,781**	**77.60**
Camarines Sur	1,130	105	95	**200**	**17.70**	**19,757**	9,220	8,941	**18,161**	**91.92**	**169,027**	36,299	34,315	**70,614**	**41.78**
Catanduanes	498	119	115	**234**	**46.99**	**3,229**	2,030	2,121	**4,151**	**128.55**	**27,331**	8,794	8,149	**16,943**	**61.99**
Masbate	848	79	105	**184**	**21.70**	**11,291**	4,797	4,521	**9,318**	**82.53**	**99,694**	21,640	19,588	**41,228**	**41.35**
Sorsogon	1,239	106	96	**202**	**16.30**	**9,925**	8,010	7,482	**15,492**	**156.09**	**84,950**	26,386	25,058	**51,444**	**60.56**
Iriga City	176	11	26	**37**	**21.02**	**1,343**	606	607	**1,213**	**90.32**	**11,482**	3,346	3,244	**6,590**	**57.39**
Legaspi City	117	19	25	**44**	**37.61**	**2,115**	1,032	1,017	**2,049**	**96.88**	**18,395**	1,090	1,093	**2,183**	**11.87**
Naga City	2,196	10	27	**37**	**1.68**	**2,351**	1,514	1,531	**3,045**	**129.52**	**20,141**	6,028	5,695	**11,723**	**58.20**
**Region 6**	**6,683**	**663**	**719**	**1,382**	**20.68**	**73,254**	**38,269**	**36,390**	**74,659**	**101.92**	**623,453**	**164,553**	**155,771**	**320,324**	**51.38**
Aklan	415	67	77	**144**	**34.70**	**5,637**	3,713	3,535	**7,248**	**128.58**	**48,370**	10,269	9,749	**20,018**	**41.39**
Antique	1,329	36	49	**85**	**6.40**	**6,576**	3,021	2,935	**5,956**	**90.57**	**55,203**	7,985	7,444	**15,429**	**27.95**
Capiz	379	24	43	**67**	**17.68**	**6,987**	4,326	4,164	**8,490**	**121.51**	**60,007**	16,250	15,296	**31,546**	**52.57**
Guimaras	232	35	55	**90**	**38.79**	**1,549**	914	932	**1,846**	**119.17**	**14,498**	5,062	4,601	**9,663**	**66.65**
Iloilo	1,317	169	188	**357**	**27.11**	**18,145**	7,771	7,376	**15,147**	**83.48**	**152,501**	68,191	64,381	**132,572**	**86.93**
Negros Occidental	2,199	203	210	**413**	**18.78**	**24,624**	13,134	12,541	**25,675**	**104.27**	**210,270**	48,109	46,377	**94,486**	**44.94**
Bacolod City	555	113	77	**190**	**34.23**	**5,538**	2,617	2,321	**4,938**	**89.17**	**47,314**	2,828	2,667	**5,495**	**11.61**
Iloilo City	257	16	20	**36**	**14.01**	**4,198**	2,773	2,586	**5,359**	**127.66**	**35,290**	5,859	5,256	**11,115**	**31.50**
**Region 7**	**8,832**	**535**	**515**	**1,050**	**11.89**	**81,144**	**34,884**	**32,797**	**67,681**	**83.41**	**648,479**	**121,181**	**115,810**	**236,991**	**36.55**
Bohol	2,223	92	94	**186**	**8.37**	**13,994**	5,806	5,490	**11,296**	**80.72**	**117,825**	31,685	30,006	**61,691**	**52.36**
Cebu	2,643	175	159	**334**	**12.64**	**33,237**	10,836	10,176	**21,012**	**63.22**	**259,039**	35,753	34,273	**70,026**	**27.03**
Negros Oriental	1,365	26	27	**53**	**3.88**	**13,946**	2,144	1,944	**4,088**	**29.31**	**114,713**	9,065	9,141	**18,206**	**15.87**
Siquijor	155	0	0	**0**	**0.00**	**821**	145	143	**288**	**35.08**	**7,654**	535	533	**1,068**	**13.95**
Cebu City	758	134	103	**237**	**31.27**	**10,429**	8,654	8,050	**16,704**	**160.17**	**81,316**	33,956	32,008	**65,964**	**81.12**
Lapu-Lapu City	684	90	111	**201**	**29.39**	**4,615**	4,168	4,020	**8,188**	**177.42**	**35,968**	5,689	5,647	**11,336**	**31.52**
Mandaue City	1,004	18	21	**39**	**3.88**	**4,102**	3,131	2,974	**6,105**	**148.83**	**31,964**	4,498	4,202	**8,700**	**27.22**
**Region 8**	**4,585**	**502**	**635**	**1,137**	**24.80**	**52,765**	**26,035**	**25,718**	**51,753**	**98.08**	**445,167**	**110,855**	**108,175**	**219,030**	**49.20**
Biliran	360	42	75	**117**	**32.50**	**1,925**	968	955	**1,923**	**99.90**	**16,955**	5,765	5,092	**10,857**	**64.03**
Eastern Samar	228	22	32	**54**	**23.68**	**5,980**	2,925	3,055	**5,980**	**100.00**	**48,401**	14,479	14,770	**29,249**	**60.43**
Northern Leyte	833	191	246	**437**	**52.46**	**17,713**	8,934	8,777	**17,711**	**99.99**	**146,531**	36,013	34,786	**70,799**	**48.32**
Northern Samar	613	71	72	**143**	**23.33**	**7,964**	4,022	3,942	**7,964**	**100.00**	**69,862**	18,470	18,978	**37,448**	**53.60**
Southern Leyte	286	29	42	**71**	**24.83**	**3,335**	1,751	1,584	**3,335**	**100.00**	**29,766**	11,614	10,891	**22,505**	**75.61**
Western Samar	847	37	35	**72**	**8.50**	**7,359**	3,693	3,666	**7,359**	**100.00**	**62,466**	12,416	11,823	**24,239**	**38.80**
Calbayog City	80	7	20	**27**	**33.75**	**2,270**	999	907	**1,906**	**83.96**	**19,250**	2,124	1,927	**4,051**	**21.04**
Maasin City	342	19	18	**37**	**10.82**	**847**	325	334	**659**	**77.80**	**7,575**	1,278	1,144	**2,422**	**31.97**
Ormoc City	975	80	93	**173**	**17.74**	**2,524**	1,223	1,260	**2,483**	**98.38**	**20,865**	5,048	4,673	**9,721**	**46.59**
Tacloban City	21	4	2	**6**	**28.57**	**2,848**	1,195	1,238	**2,433**	**85.43**	**23,496**	3,648	4,091	**7,739**	**32.94**
**Region 9**	**1,384**	**241**	**271**	**512**	**36.99**	**39,497**	**23,506**	**23,302**	**46,808**	**118.51**	**335,069**	**66,421**	**66,513**	**132,934**	**39.67**
Zamboanga del Norte	350	74	91	**165**	**47.14**	**8,438**	5,517	5,420	**10,937**	**129.62**	**73,193**	16,465	17,096	**33,561**	**45.85**
Zamboanga del Sur	217	54	60	**114**	**52.53**	**8,678**	5,150	5,129	**10,279**	**118.45**	**70,988**	17,046	16,567	**33,613**	**47.35**
Zamboanga Sibugay	404	39	29	**68**	**16.83**	**7,506**	3,382	3,320	**6,702**	**89.29**	**66,344**	14,259	13,680	**27,939**	**42.11**
Dapitan City	45	16	11	**27**	**60.00**	**872**	366	450	**816**	**93.58**	**7,557**	2,798	3,390	**6,188**	**81.88**
Dipolog City	60	14	11	**25**	**41.67**	**1,381**	1,211	1,196	**2,407**	**174.29**	**11,987**	4,102	4,038	**8,140**	**67.91**
Isabela City	1	0	1	**1**	**100.00**	**1,277**	567	527	**1,094**	**85.67**	**12,229**	342	295	**637**	**5.21**
Pagadian City	59	14	25	**39**	**66.10**	**2,128**	1,547	1,558	**3,105**	**145.91**	**17,408**	1,470	1,463	**2,933**	**16.85**
Zamboanga City	248	30	43	**73**	**29.44**	**9,217**	5,766	5,702	**11,468**	**124.42**	**75,363**	9,939	9,984	**19,923**	**26.44**
**Region 10**	**3,712**	**365**	**351**	**716**	**19.29**	**49,952**	**30,389**	**29,489**	**59,878**	**119.87**	**428,356**	**127,999**	**128,306**	**256,305**	**59.83**
Bukidnon	631	89	98	**187**	**29.64**	**11,449**	7,493	7,084	**14,577**	**127.32**	**97,101**	41,085	39,545	**80,630**	**83.04**
Camiguin	40	22	18	**40**	**100.00**	**929**	222	212	**434**	**46.72**	**7,996**	1,817	1,694	**3,511**	**43.91**
Lanao del Norte	344	72	44	**116**	**33.72**	**7,475**	6,274	6,321	**12,595**	**168.49**	**67,342**	25,506	25,829	**51,335**	**76.23**
Misamis Occidental	30	9	7	**16**	**53.33**	**3,196**	3,058	3,209	**6,267**	**196.09**	**27,481**	19,921	22,534	**42,455**	**154.49**

Table 2.C.2.2. Nutrition Services for Infants and Children

Number of Infants born pre-term or with low birth weight given iron supplements and infants 6-11 mos. old and children 12-59 mos. old given Vitamin A supplementation
Philippines, 2022

Area	Total No. of live births with low birth weight	Infants pre-term or with LBW (< 2500 grams) given Iron				Eligible Pop (6-11 months)	Infants 6-11 mos. old Given Vitamin A				Eligible Pop (12-59 months)	Children 12-59 mos. Old Given Vitamin A			
		Male	Female	Total	%		Male	Female	Total	%		Male	Female	Total	%
Misamis Oriental	288	27	30	**57**	**19.79**	**7,478**	1,706	1,546	**3,252**	**43.49**	**62,525**	8,756	8,768	**17,524**	**28.03**
Cagayan de Oro City	1,909	52	53	**105**	**5.50**	**7,076**	4,244	4,159	**8,403**	**118.75**	**59,215**	8,346	7,895	**16,241**	**27.43**
El Salvador City	1	12	5	**17**	**1700.00**	**527**	130	171	**301**	**57.12**	**4,397**	419	634	**1,053**	**23.95**
Gingoog City	30	10	15	**25**	**83.33**	**1,314**	934	974	**1,908**	**145.21**	**10,919**	3,982	3,564	**7,546**	**69.11**
Iligan City	142	33	30	**63**	**44.37**	**3,784**	3,813	3,393	**7,206**	**190.43**	**34,120**	2,877	2,906	**5,783**	**16.95**
Malaybalay City	113	8	8	**16**	**14.16**	**1,908**	308	295	**603**	**31.60**	**16,184**	2,109	2,143	**4,252**	**26.27**
Oroquieta City	97	6	14	**20**	**20.62**	**693**	603	574	**1,177**	**169.84**	**5,954**	4,051	3,894	**7,945**	**133.44**
Ozamis City	25	6	7	**13**	**52.00**	**1,393**	725	693	**1,418**	**101.79**	**11,936**	4,967	4,717	**9,684**	**81.13**
Tangub City	24	2	3	**5**	**20.83**	**621**	66	67	**133**	**21.42**	**5,300**	325	285	**610**	**11.51**
Valencia City	38	17	19	**36**	**94.74**	**2,109**	813	791	**1,604**	**76.06**	**17,886**	3,838	3,898	**7,736**	**43.25**
**Region 11**	**4,839**	**621**	**699**	**1,320**	**27.28**	**54,210**	**39,354**	**36,558**	**75,912**	**140.03**	**452,922**	**263,765**	**251,876**	**515,641**	**113.85**
Davao de Oro	694	97	149	**246**	**35.45**	**7,750**	6,587	5,937	**12,524**	**161.60**	**67,865**	45,077	41,985	**87,062**	**128.29**
Davao del Norte	1,163	184	181	**365**	**31.38**	**10,511**	8,314	8,080	**16,394**	**155.97**	**86,391**	68,319	65,016	**133,335**	**154.34**
Davao Oriental	511	63	56	**119**	**23.29**	**6,614**	6,187	5,294	**11,481**	**173.59**	**57,129**	26,374	24,960	**51,334**	**89.86**
Davao del Sur	499	68	75	**143**	**28.66**	**7,283**	1,627	1,356	**2,983**	**40.96**	**59,042**	26,911	25,078	**51,989**	**88.05**
Davao Occidental	119	11	17	**28**	**23.53**	**3,253**	1,849	1,804	**3,653**	**112.30**	**30,091**	12,798	12,807	**25,605**	**85.09**
Davao City	1,853	198	221	**419**	**22.61**	**18,799**	14,790	14,087	**28,877**	**153.61**	**152,404**	84,286	82,030	**166,316**	**109.13**
**Region 12**	**1,994**	**500**	**519**	**1,019**	**51.10**	**49,447**	**38,420**	**37,414**	**75,834**	**153.36**	**409,593**	**167,215**	**161,082**	**328,297**	**80.15**
North Cotabato	622	97	114	**211**	**33.92**	**17,074**	8,608	8,351	**16,959**	**99.33**	**141,044**	52,709	50,170	**102,879**	**72.94**
Sarangani	236	64	50	**114**	**48.31**	**6,453**	7,968	7,703	**15,671**	**242.85**	**54,628**	28,441	28,162	**56,603**	**103.62**
South Cotabato	519	129	168	**297**	**57.23**	**10,458**	8,857	8,606	**17,463**	**166.98**	**84,448**	42,214	39,804	**82,018**	**97.12**
Sultan Kudarat	463	132	117	**249**	**53.78**	**8,668**	8,199	8,129	**16,328**	**188.37**	**74,631**	32,342	32,252	**64,594**	**86.55**
Gen. Santos City	154	78	70	**148**	**96.10**	**6,794**	4,788	4,625	**9,413**	**138.55**	**54,842**	11,509	10,694	**22,203**	**40.49**
**BARMM**	**814**	**196**	**173**	**369**	**45.33**	**50,645**	**29,271**	**30,809**	**60,080**	**118.63**	**525,340**	**87,094**	**93,641**	**180,735**	**34.40**
Basilan	188	65	2	**67**	**35.64**	**3,915**	1,420	1,235	**2,655**	**67.82**	**36,827**	4,258	4,028	**8,286**	**22.50**
Lanao del Sur	41	1	5	**6**	**14.63**	**10,799**	8,078	8,822	**16,900**	**156.50**	**112,850**	25,596	29,004	**54,600**	**48.38**
Maguindanao	356	20	23	**43**	**12.08**	**16,086**	9,370	9,858	**19,228**	**119.53**	**154,029**	7,761	7,999	**15,760**	**10.23**
Sulu	90	81	114	**195**	**216.67**	**8,590**	4,867	5,596	**10,463**	**121.80**	**110,769**	16,649	19,554	**36,203**	**32.68**
Tawi-Tawi	53	9	12	**21**	**39.62**	**4,683**	2,525	2,560	**5,085**	**108.58**	**45,443**	5,304	5,550	**10,854**	**23.88**
Lamitan City	20	2	2	**4**	**20.00**	**1,076**	203	177	**380**	**35.32**	**10,136**	328	318	**646**	**6.37**
Marawi City	30	5	3	**8**	**26.67**	**2,584**	1,246	1,325	**2,571**	**99.50**	**26,991**	13,431	13,066	**26,497**	**98.17**
Cotabato City	36	13	12	**25**	**69.44**	**2,912**	1,562	1,236	**2,798**	**96.09**	**28,295**	13,767	14,122	**27,889**	**98.57**
**CARAGA**	**1,491**	**243**	**262**	**505**	**33.87**	**30,318**	**21,445**	**21,193**	**42,638**	**140.64**	**260,938**	**106,676**	**104,037**	**210,713**	**80.75**
Agusan del Norte	212	70	73	**143**	**67.45**	**4,072**	2,323	2,292	**4,615**	**113.33**	**34,222**	10,509	10,068	**20,577**	**60.13**
Agusan del Sur	202	26	27	**53**	**26.24**	**8,857**	6,050	6,188	**12,238**	**138.17**	**77,316**	32,584	32,891	**65,475**	**84.68**
Surigao del Norte	194	22	26	**48**	**24.74**	**3,655**	2,311	2,323	**4,634**	**126.79**	**32,414**	14,954	14,775	**29,729**	**91.72**
Surigao del Sur	318	41	49	**90**	**28.30**	**5,778**	3,989	3,909	**7,898**	**136.69**	**48,819**	21,293	20,689	**41,982**	**86.00**
Province of Dinagat	91	9	11	**20**	**21.98**	**1,292**	587	538	**1,125**	**87.07**	**11,259**	3,853	3,671	**7,524**	**66.83**
Bislig City	32	13	12	**25**	**78.13**	**1,096**	578	518	**1,096**	**100.00**	**9,270**	3,691	3,571	**7,262**	**78.34**
Butuan City	202	9	11	**20**	**9.90**	**3,867**	4,504	4,364	**8,868**	**229.33**	**32,536**	16,539	15,415	**31,954**	**98.21**
Surigao City	240	53	53	**106**	**44.17**	**1,701**	1,103	1,061	**2,164**	**127.22**	**15,102**	3,253	2,957	**6,210**	**41.12**

Table 2.C.2.3. Nutrition Services for Infants and Children

Number and Proportion of infants 6-11 mos. old and children 12-23 mos. old who completed Micronutrient Powder supplementation
Philippines, 2022

Area	Eligible Pop (6-11 mos.)	Infants 6-11 mos. old who completed MNP supplementation				Eligible Pop (12-23 mos.)	Children 12-23 mos. old who completed MNP supplementation			
		Male	Female	Total	%		Male	Female	Total	%
PHILIPPINES	1,065,764	85,978	83,449	169,427	15.90	2,146,851	138,928	141,279	280,207	13.05
N C R	118,428	18,771	18,465	37,236	31.44	234,258	29,344	33,598	62,942	26.87
Malabon City	3,361	1794	1423	3,217	95.72	6,648	2,416	2,413	4,829	72.64
Navotas City	2,292	301	320	621	27.09	4,540	240	300	540	11.89
Valenzuela City	5,705	531	569	1,100	19.28	11,286	136	4,490	4,626	40.99
Caloocan City	14,561	426	763	1,189	8.17	28,815	810	727	1,537	5.33
Marikina City	4,143	402	396	798	19.26	8,202	370	341	711	8.67
Pasig City	6,951	556	539	1,095	15.75	13,743	673	741	1,414	10.29
Pateros City	585	224	197	421	71.97	1,162	179	178	357	30.72
Taguig City	7,405	3630	3775	7,405	100.00	14,642	7,180	6,920	14,100	96.30
Quezon City	27,001	815	829	1,644	6.09	53,416	1,099	1,083	2,182	4.08
Makati City	5,364	1512	1433	2,945	54.90	10,598	1,089	958	2,047	19.31
Mandaluyong City	3,546	341	397	738	20.81	7,024	432	738	1,170	16.66
San Juan City	1,122	355	210	565	50.36	2,224	313	397	710	31.92
Manila City	16,375	3012	3050	6,062	37.02	32,387	2,217	2,223	4,440	13.71
Las Piñas City	5,415	95	78	173	3.19	10,714	134	167	301	2.81
Muntinlupa City	4,639	2398	2241	4,639	100.00	9,180	4,505	4,675	9,180	100.00
Parañaque City	6,123	389	395	784	12.80	12,112	628	615	1,243	10.26
Pasay City	3,840	1990	1850	3,840	100.00	7,565	6,923	6,632	13,555	179.18
C A R	17,595	637	659	1,296	7.37	35,961	337	470	807	2.24
Abra	2,134	58	76	134	6.28	4,394	74	77	151	3.44
Apayao	1,253	14	19	33	2.63	2,543	5	15	20	0.79
Benguet	4,538	487	478	965	21.26	9,214	169	296	465	5.05
Ifugao	2,240	10	10	20	0.89	4,779	31	30	61	1.28
Kalinga	2,353	40	46	86	3.65	4,796	55	48	103	2.15
Mt. Province	1,550	23	19	42	2.71	3,106	1	1	2	0.06
Baguio City	3,527	5	11	16	0.45	7,129	2	3	5	0.07
Region 1	48,526	6,688	6,172	12,860	26.50	97,313	15,854	15,026	30,880	31.73
Ilocos Norte	3,928	505	487	992	25.25	7,574	812	832	1,644	21.71
Ilocos Sur	4,541	883	814	1697	37.37	9,348	1778	1813	3,591	38.41
La Union	5,663	410	432	842	14.87	11,341	824	716	1,540	13.58
Pangasinan	25,326	3235	3010	6245	24.66	50,878	3304	3221	6,525	12.82
Alaminos City	961	484	401	885	92.09	1,920	480	432	912	47.50
Candon City	477	525	420	945	198.11	990	1099	1040	2,139	216.06
Dagupan City	1,828	330	301	631	34.52	3,670	605	589	1,194	32.53
Laoag City	901	0	0	0	0.00	1,747	0	0	0	0.00
San Carlos City	2,011	0	0	0	0.00	4,044	0	0	0	0.00
San Fernando City	1,038	0	0	0	0.00	2,072	0	0	0	0.00
Urdaneta City	1,424	156	151	307	21.56	2,848	6534	6008	12,542	440.38
Vigan City	428	160	156	316	73.83	881	418	375	793	90.01
Region 2	34,463	3,988	3,706	7,694	22.33	69,717	6,776	6,469	13,245	19.00
Batanes	185	2	0	2	1.08	364	0	0	0	0.00
Cagayan	10,202	769	637	1406	13.78	20,842	644	580	1,224	5.87
Isabela	11,728	1644	1570	3214	27.40	23,384	2720	2691	5,411	23.14
Nueva Vizcaya	4,762	428	378	806	16.93	9,702	1161	1104	2,265	23.35

Table 2.C.2.3. Nutrition Services for Infants and Children

Number and Proportion of infants 6-11 mos. old and children 12-23 mos. old who completed Micronutrient Powder supplementation
Philippines, 2022

Area	Eligible Pop (6-11 mos.)	Infants 6-11 mos. old who completed MNP supplementation				Eligible Pop (12-23 mos.)	Children 12-23 mos. old who completed MNP supplementation			
		Male	Female	Total	%		Male	Female	Total	%
Quirino	2,023	27	32	59	2.92	4,268	106	119	225	5.27
Cauayan City	1,282	338	292	630	49.14	2,558	389	371	760	29.71
Ilagan City	1,440	2	4	6	0.42	2,881	112	90	202	7.01
Santiago City	1,336	646	628	1274	95.36	2,662	1097	1047	2,144	80.54
Tuguegarao City	1,505	132	165	297	19.73	3,056	547	467	1,014	33.18
**Region 3**	**110,043**	**6,258**	**5,837**	**12,095**	**10.99**	**218,354**	**10,576**	**10,738**	**21,314**	**9.76**
Aurora	2,380	10	9	19	0.80	4,936	181	184	365	7.39
Bataan	6,896	729	733	1,462	21.20	13,317	1,009	1,001	2,010	15.09
Bulacan	21,863	1,132	982	2,114	9.67	43,284	4,292	4,510	8,802	20.34
Nueva Ecija	14,499	727	695	1,422	9.81	29,187	1,504	1,509	3,013	10.32
Pampanga	15,802	799	646	1,445	9.14	31,200	922	944	1,866	5.98
Tarlac	9,655	181	193	374	3.87	19,109	196	196	392	2.05
Zambales	6,251	188	167	355	5.68	12,457	451	462	913	7.33
Angeles City	3,965	388	434	822	20.73	7,830	371	380	751	9.59
Balanga City	997	1	3	4	0.40	1,926	2	3	5	0.26
Cabanatuan City	2,971	34	28	62	2.09	5,973	49	43	92	1.54
City of San Fernando	2,953	133	119	252	8.53	5,834	99	79	178	3.05
Gapan City	1,084	4	4	8	0.74	2,180	2	0	2	0.09
Mabalacat City	2,414	0	0	0	0.00	4,777	0	0	0	0.00
Malolos City	2,441	0	0	0	0.00	4,839	204	178	382	7.89
Meycauayan	2,027	231	252	483	23.83	4,012	252	233	485	12.09
Olongapo	2,463	97	103	200	8.12	4,914	239	187	426	8.67
Palayan City	404	1	1	2	0.50	810	9	5	14	1.73
San Jose City	1,373	5	4	9	0.66	2,763	2	1	3	0.11
San Jose del Monte City	5,566	1,312	1,186	2,498	44.88	11,010	428	424	852	7.74
Science City of Munoz	801	0	0	0	0.00	1,611	0	0	0	0.00
Tarlac City	3,238	286	278	564	17.42	6,385	364	399	763	11.95
**Region 4A**	**149,838**	**16,815**	**16,845**	**33,660**	**22.46**	**297,796**	**19,237**	**19,039**	**38,276**	**12.85**
Batangas	19,634	685	616	1,301	6.63	39,506	1,184	1,055	2,239	5.67
Cavite	14,086	1,636	1,664	3,300	23.43	27,663	879	819	1,698	6.14
Laguna	9,851	695	608	1,303	13.23	19,448	340	441	781	4.02
Quezon	18,915	1,203	1,144	2,347	12.41	38,492	2,069	1,979	4,048	10.52
Rizal	22,406	4,881	5,354	10,235	45.68	44,115	1,520	1,704	3,224	7.31
Antipolo City	8,249	295	337	632	7.66	16,248	147	216	363	2.23
Bacoor City	6,167	593	522	1,115	18.08	12,127	530	511	1,041	8.58
Batangas City	3,485	53	28	81	2.32	7,006	23	28	51	0.73
Biñan City	3,297	3,520	3,570	7,090	215.04	6,523	9,783	9,690	19,473	298.53
Cabuyao City	3,060	41	40	81	2.65	6,044	44	41	85	1.41
Calamba City	4,502	257	262	519	11.53	8,899	237	228	465	5.23
Cavite City	1,056	5	7	12	1.14	2,074	0	0	0	0.00
Dasmariñas City	6,767	977	930	1,907	28.18	13,297	657	623	1,280	9.63
General Trias City	3,227	2	3	5	0.15	6,344	1	1	2	0.03
Imus City	4,147	87	87	174	4.20	8,145	40	31	71	0.87
Lipa City	3,513	313	215	528	15.03	7,064	39	1	40	0.57
Lucena City	2,868	0	0	0	0.00	5,836	0	0	0	0.00
San Pablo City	2,639	7	4	11	0.42	5,208	0	2	2	0.04
San Pedro City	3,230	567	543	1,110	34.37	6,376	565	565	1,130	17.72
Santa Rosa City	3,507	381	328	709	20.22	6,927	371	329	700	10.11
Tagaytay City	731	609	580	1,189	162.65	1,437	808	775	1,583	110.16
Tanauan City	1,833	0	0	0	0.00	3,684	0	0	0	0.00
Tayabas City	1,068	0	0	0	0.00	2,190	0	0	0	0.00

Table 2.C.2.3. Nutrition Services for Infants and Children

Number and Proportion of infants 6-11 mos. old and children 12-23 mos. old who completed Micronutrient Powder supplementation
Philippines, 2022

Area	Eligible Pop (6-11 mos.)	Infants 6-11 mos. old who completed MNP supplementation				Eligible Pop (12-23 mos.)	Children 12-23 mos. old who completed MNP supplementation			
		Male	Female	Total	%		Male	Female	Total	%
Trece Martires City	1,600	8	3	11	0.69	3,143	0	0	0	0.00
**Region 4B**	**36,417**	**2,012**	**1,796**	**3,808**	**10.46**	**72,783**	**2,994**	**2,855**	**5,849**	**8.04**
Marinduque	2,496	56	49	105	4.21	5,050	75	64	139	2.75
Mindoro Occidental	6,251	604	548	1,152	18.43	12,570	326	265	591	4.70
Mindoro Oriental	10,098	216	145	361	3.57	19,961	222	208	430	2.15
Palawan	10,993	1,111	1,026	2,137	19.44	21,910	2,311	2,248	4,559	20.81
Romblon	3,280	9	12	21	0.64	6,710	15	14	29	0.43
Puerto Princesa City	3,299	16	16	32	0.97	6,582	45	56	101	1.53
**Region 5**	**69,222**	**3,435**	**3,420**	**6,855**	**9.90**	**141,458**	**5,779**	**5,989**	**11,768**	**8.32**
Albay	12,031	822	847	1,669	13.87	24,724	1,473	1,503	2,976	12.04
Camarines Norte	7,180	154	160	314	4.37	14,031	401	431	832	5.93
Camarines Sur	19,757	539	542	1,081	5.47	40,179	475	593	1,068	2.66
Catanduanes	3,229	456	407	863	26.73	6,647	1,208	1,252	2,460	37.01
Masbate	11,291	619	652	1,271	11.26	23,839	984	1,015	1,999	8.39
Sorsogon	9,925	620	584	1,204	12.13	20,171	998	911	1,909	9.46
Iriga City	1,343	36	36	72	5.36	2,728	79	76	155	5.68
Legaspi City	2,115	105	110	215	10.17	4,350	125	160	285	6.55
Naga City	2,351	84	82	166	7.06	4,789	36	48	84	1.75
**Region 6**	**73,254**	**6,098**	**5,945**	**12,043**	**16.44**	**147,721**	**8,615**	**8,365**	**16,980**	**11.49**
Aklan	5,637	374	349	723	12.83	11,587	542	543	1,085	9.36
Antique	6,576	147	132	279	4.24	13,176	243	249	492	3.73
Capiz	6,987	419	389	808	11.56	14,254	341	361	702	4.92
Guimaras	1,549	294	354	648	41.83	3,339	587	549	1,136	34.02
Iloilo	18,145	1,221	1,137	2,358	13.00	36,235	1,618	1,587	3,205	8.85
Negros Occidental	24,624	2,457	2,475	4,932	20.03	49,584	2,905	2,670	5,575	11.24
Bacolod City	5,538	191	174	365	6.59	11,160	223	223	446	4.00
Iloilo City	4,198	995	935	1,930	45.97	8,386	2,156	2,183	4,339	51.74
**Region 7**	**81,144**	**5,133**	**4,798**	**9,931**	**12.24**	**158,778**	**6,049**	**6,012**	**12,061**	**7.60**
Bohol	13,994	469	455	924	6.60	28,019	877	784	1,661	5.93
Cebu	33,237	802	749	1,551	4.67	64,208	995	981	1,976	3.08
Negros Oriental	13,946	327	301	628	4.50	27,762	272	341	613	2.21
Siquijor	821	0	0	0	0.00	1,785	0	0	0	0.00
Cebu City	10,429	1,261	1,056	2,317	22.22	20,163	2,014	2,021	4,035	20.01
Lapu-Lapu City	4,615	1,282	1,234	2,516	54.52	8,916	1,137	1,140	2,277	25.54
Mandaue City	4,102	992	1,003	1,995	48.63	7,925	754	745	1,499	18.91
**Region 8**	**52,765**	**5,075**	**4,984**	**10,059**	**19.06**	**105,345**	**8,993**	**8,979**	**17,972**	**17.06**
Biliran	1,925	272	270	542	28.16	4,097	908	942	1,850	45.15
Eastern Samar	5,980	311	308	619	10.35	11,519	607	588	1,195	10.37
Northern Leyte	17,713	1,848	1,782	3,630	20.49	34,704	2,840	2,855	5,695	16.41
Northern Samar	7,964	1,113	1,199	2,312	29.03	16,279	2,135	2,359	4,494	27.61
Southern Leyte	3,335	351	339	690	20.69	7,048	1,009	858	1,867	26.49
Western Samar	7,359	948	872	1,820	24.73	14,829	1,065	994	2,059	13.88
Calbayog City	2,270	19	33	52	2.29	4,562	118	111	229	5.02

Table 2.C.2.3. Nutrition Services for Infants and Children

Number and Proportion of infants 6-11 mos. old and children 12-23 mos. old who completed Micronutrient Powder supplementation
Philippines, 2022

Area	Eligible Pop (6-11 mos.)	Infants 6-11 mos. old who completed MNP supplementation				Eligible Pop (12-23 mos.)	Children 12-23 mos. old who completed MNP supplementation			
		Male	Female	Total	%		Male	Female	Total	%
Maasin City	847	135	124	259	30.58	1,791	229	202	431	24.06
Ormoc City	2,524	78	57	135	5.35	4,947	82	70	152	3.07
Tacloban City	2,848	0	0	0	0.00	5,569	0	0	0	0.00
**Region 9**	39,497	572	563	1,135	2.87	79,262	382	406	788	0.99
Zamboanga del Norte	8,438	85	80	165	1.96	17,109	82	80	162	0.95
Zamboanga del Sur	8,678	41	47	88	1.01	16,995	37	49	86	0.51
Zamboanga Sibugay	7,506	82	82	164	2.18	15,488	82	83	165	1.07
Dapitan City	872	0	0	0	0.00	1,764	0	0	0	0.00
Dipolog City	1,381	14	15	29	2.10	2,804	38	41	79	2.82
Isabela City	1,277	0	0	0	0.00	2,880	0	0	0	0.00
Pagadian City	2,128	4	6	10	0.47	4,173	12	9	21	0.50
Zamboanga City	9,217	346	333	679	7.37	18,049	131	144	275	1.52
**Region 10**	49,952	3,085	2,971	6,056	12.12	102,627	3,711	3,583	7,294	7.11
Bukidnon	11,449	727	795	1,522	13.29	23,542	996	981	1,977	8.40
Camiguin	929	17	15	32	3.44	1,870	74	68	142	7.59
Lanao del Norte	7,475	144	220	364	4.87	15,705	100	86	186	1.18
Misamis Occidental	3,196	497	494	991	31.01	6,571	605	694	1,299	19.77
Misamis Oriental	7,478	46	64	110	1.47	15,136	58	82	140	0.92
Cagayan de Oro City	7,076	682	490	1,172	16.56	14,335	80	74	154	1.07
El Salvador City	527	0	0	0	0.00	1,066	0	0	0	0.00
Gingoog City	1,314	353	308	661	50.30	2,642	659	566	1,225	46.37
Iligan City	3,784	227	218	445	11.76	7,958	332	296	628	7.89
Malaybalay City	1,908	14	15	29	1.52	3,920	109	102	211	5.38
Oroquieta City	693	105	122	227	32.76	1,424	181	212	393	27.60
Ozamis City	1,393	60	49	109	7.82	2,856	112	106	218	7.63
Tangub City	621	1	2	3	0.48	1,267	1	2	3	0.24
Valencia City	2,109	212	179	391	18.54	4,335	404	314	718	16.56
**Region 11**	54,210	1,269	1,181	2,450	4.52	109,260	5,754	5,699	11,453	10.48
Davao de Oro	7,750	75	72	147	1.90	16,118	254	212	466	2.89
Davao del Norte	10,511	115	111	226	2.15	21,038	493	519	1,012	4.81
Davao Oriental	6,614	120	75	195	2.95	13,465	1,485	1,403	2,888	21.45
Davao del Sur	7,283	95	81	176	2.42	14,420	178	147	325	2.25
Davao Occidental	3,253	5	5	10	0.31	6,983	17	14	31	0.44
Davao City	18,799	859	837	1,696	9.02	37,236	3,327	3,404	6,731	18.08
**Region 12**	49,447	1,135	1,160	2,295	4.64	98,991	1,801	1,901	3,702	3.74
North Cotabato	17,074	229	226	455	2.66	33,912	317	313	630	1.86
Sarangani	6,453	186	191	377	5.84	13,292	168	244	412	3.10
South Cotabato	10,458	366	411	777	7.43	20,640	862	933	1,795	8.70
Sultan Kudarat	8,668	235	225	460	5.31	17,741	403	374	777	4.38
Gen. Santos City	6,794	119	107	226	3.33	13,406	51	37	88	0.66
**BARMM**	50,645	1,690	1,671	3,361	6.64	115,067	2,540	2,484	5,024	4.37
Basilan	3,915	64	78	142	3.63	8,158	95	85	180	2.21
Lanao del Sur	10,799	933	806	1,739	16.10	25,346	1,552	1,606	3,158	12.46
Maguindanao	16,086	199	203	402	2.50	35,358	149	150	299	0.85

Table 2.C.2.3. Nutrition Services for Infants and Children

Number and Proportion of infants 6-11 mos. old and children 12-23 mos. old who completed Micronutrient Powder supplementation
Philippines, 2022

Area	Eligible Pop (6-11 mos.)	Infants 6-11 mos. old who completed MNP supplementation				Eligible Pop (12-23 mos.)	Children 12-23 mos. old who completed MNP supplementation			
		Male	Female	Total	%		Male	Female	Total	%
Sulu	8,590	7	8	15	0.17	21,868	7	7	14	0.06
Tawi-Tawi	4,683	0	0	0	0.00	9,852	0	0	0	0.00
Lamitan City	1,076	0	0	0	0.00	2,252	0	0	0	0.00
Marawi City	2,584	480	565	1,045	40.44	6,062	723	626	1,349	22.25
Cotabato City	2,912	7	11	18	0.62	6,171	14	10	24	0.39
CARAGA	30,318	3,317	3,276	6,593	21.75	62,160	10,186	9,666	19,852	31.94
Agusan del Norte	4,072	34	38	72	1.77	8,226	28	27	55	0.67
Agusan del Sur	8,857	719	694	1,413	15.95	18,532	4,564	4,124	8,688	46.88
Surigao del Norte	3,655	333	324	657	17.98	7,694	1,544	1,535	3,079	40.02
Surigao del Sur	5,778	1,128	1,093	2,221	38.44	11,452	1,829	1,848	3,677	32.11
Province of Dinagat	1,292	16	18	34	2.63	2,662	30	20	50	1.88
Bislig City	1,096	132	134	266	24.27	2,176	311	273	584	26.84
Butuan City	3,867	380	415	795	20.56	7,826	935	907	1,842	23.54
Surigao City	1,701	575	560	1,135	66.73	3,592	945	932	1,877	52.26

Table 2.C.2.4. Nutrition Services for Infants and Children

Number and Proportion of children 0-59 mos. old whose nutritional status are normal, stunted and overweight/obese

Philippines, 2022

Area	Eligible Pop (0-59 mos)	Normal				Stunted				Overweight/obese			
		Male	Female	Total	%	Male	Female	Total	%	Male	Female	Total	%
**PHILIPPINES**	**11,108,117**	**3,167,620**	**3,170,922**	**6,338,542**	**57.06**	**196,308**	**175,518**	**371,826**	**3.35**	**82,410**	**71,221**	**153,631**	**1.38**
**N C R**	**1,188,449**	**261,031**	**248,473**	**509,504**	**42.87**	**7,456**	**6,098**	**13,554**	**1.14**	**3,122**	**2,791**	**5,913**	**0.50**
Malabon City	**33,737**	14,090	13,365	**27,455**	**81.38**	377	340	**717**	**2.13**	105	82	**187**	**0.55**
Navotas City	**23,024**	7,252	7,222	**14,474**	**62.86**	75	67	**142**	**0.62**	76	66	**142**	**0.62**
Valenzuela City	**57,258**	24,762	23,765	**48,527**	**84.75**	356	235	**591**	**1.03**	174	150	**324**	**0.57**
Caloocan City	**146,178**	59,328	56,180	**115,508**	**79.02**	745	529	**1,274**	**0.87**	696	636	**1,332**	**0.91**
Marikina City	**41,598**	*	*	**0**	**0.00**	*	*	**0**	**0.00**	*	*	**0**	**0.00**
Pasig City	**69,710**	*	*	**0**	**0.00**	*	*	**0**	**0.00**	*	*	**0**	**0.00**
Pateros	**5,893**	2,160	1,995	**4,155**	**70.51**	60	42	**102**	**1.73**	52	35	**87**	**1.48**
Taguig City	**74,287**	*	*	**0**	**0.00**	*	*	**0**	**0.00**	*	*	**0**	**0.00**
Quezon City	**270,977**	*	*	**0**	**0.00**	*	*	**0**	**0.00**	*	*	**0**	**0.00**
Makati City	**53,769**	19,871	18,307	**38,178**	**71.00**	108	115	**223**	**0.41**	133	109	**242**	**0.45**
Mandaluyong City	**35,649**	16,294	15,866	**32,160**	**90.21**	42	40	**82**	**0.23**	11	5	**16**	**0.04**
San Juan City	**11,276**	4,533	4,284	**8,817**	**78.19**	44	38	**82**	**0.73**	38	51	**89**	**0.79**
Manila City	**164,290**	27,235	25,921	**53,156**	**32.35**	4,137	3,491	**7,628**	**4.64**	1,333	1,244	**2,577**	**1.57**
Las Piñas City	**54,353**	23,136	22,167	**45,303**	**83.35**	393	301	**694**	**1.28**	114	117	**231**	**0.42**
Muntinlupa City	**46,560**	22,288	21,561	**43,849**	**94.18**	93	83	**176**	**0.38**	23	18	**41**	**0.09**
Parañaque City	**61,447**	24,537	23,056	**47,593**	**77.45**	708	548	**1,256**	**2.04**	223	183	**406**	**0.66**
Pasay City	**38,443**	15,545	14,784	**30,329**	**78.89**	318	269	**587**	**1.53**	144	95	**239**	**0.62**
**C A R**	**182,376**	**55,506**	**51,729**	**107,235**	**58.80**	**5,937**	**5,087**	**11,024**	**6.04**	**1,944**	**1,497**	**3,441**	**1.89**
Abra	**23,221**	11,015	10,492	**21,507**	**92.62**	1,672	1,496	**3,168**	**13.64**	647	557	**1,204**	**5.18**
Apayao	**13,618**	3,584	3,547	**7,131**	**52.36**	631	567	**1,198**	**8.80**	175	129	**304**	**2.23**
Benguet	**45,087**	13,352	12,204	**25,556**	**56.68**	1,063	873	**1,936**	**4.29**	269	228	**497**	**1.10**
Ifugao	**23,917**	8,797	8,124	**16,921**	**70.75**	411	357	**768**	**3.21**	223	135	**358**	**1.50**
Kalinga	**24,778**	9,875	9,164	**19,039**	**76.84**	1,124	839	**1,963**	**7.92**	304	211	**515**	**2.08**
Mt. Province	**16,861**	5,674	5,238	**10,912**	**64.72**	825	751	**1,576**	**9.35**	207	166	**373**	**2.21**
Baguio City	**34,894**	3,209	2,960	**6,169**	**17.68**	211	204	**415**	**1.19**	119	71	**190**	**0.54**
**Region 1**	**503,968**	**202,052**	**189,899**	**391,951**	**77.77**	**7,443**	**6,107**	**13,550**	**2.69**	**3,331**	**2,421**	**5,752**	**1.14**
Ilocos Norte	**39,803**	13,944	13,065	**27,009**	**67.86**	859	697	**1,556**	**3.91**	414	288	**702**	**1.76**
Ilocos Sur	**48,732**	18,516	17,278	**35,794**	**73.45**	1,087	878	**1,965**	**4.03**	508	281	**789**	**1.62**
La Union	**59,231**	22,703	21,055	**43,758**	**73.88**	891	817	**1,708**	**2.88**	336	220	**556**	**0.94**
Pangasinan	**262,152**	113,137	107,676	**220,813**	**84.23**	2,868	2,266	**5,134**	**1.96**	1,054	848	**1,902**	**0.73**
Alaminos City	**9,900**	3,761	3,509	**7,270**	**73.43**	84	84	**168**	**1.70**	104	64	**168**	**1.70**
Candon City	**5,144**	2,060	1,924	**3,984**	**77.45**	60	61	**121**	**2.35**	39	31	**70**	**1.36**
Dagupan City	**18,915**	4,920	4,628	**9,548**	**50.48**	783	647	**1,430**	**7.56**	273	214	**487**	**2.57**
Laoag City	**9,182**	3,496	3,090	**6,586**	**71.73**	59	44	**103**	**1.12**	56	26	**82**	**0.89**
San Carlos City	**20,817**	7,474	6,978	**14,452**	**69.42**	418	337	**755**	**3.63**	334	274	**608**	**2.92**
San Fernando City	**10,849**	3,820	3,587	**7,407**	**68.27**	205	154	**359**	**3.31**	141	112	**253**	**2.33**
Urdaneta City	**14,679**	6,296	5,789	**12,085**	**82.33**	101	100	**201**	**1.37**	69	60	**129**	**0.88**
Vigan City	**4,564**	1,925	1,320	**3,245**	**71.10**	28	22	**50**	**1.10**	3	3	**6**	**0.13**
**Region 2**	**358,323**	**125,482**	**119,437**	**244,919**	**68.35**	**8,024**	**7,034**	**15,058**	**4.20**	**6,112**	**5,242**	**11,354**	**3.17**
Batanes	**1,992**	344	280	**624**	**31.33**	8	14	**22**	**1.10**	20	29	**49**	**2.46**
Cagayan	**106,266**	34,154	32,495	**66,649**	**62.72**	2,316	1,917	**4,233**	**3.98**	2,698	2,477	**5,175**	**4.87**
Isabela	**121,394**	37,600	36,712	**74,312**	**61.22**	4,279	3,805	**8,084**	**6.66**	2,733	2,278	**5,011**	**4.13**
Nueva Vizcaya	**49,695**	18,636	17,597	**36,233**	**72.91**	800	697	**1,497**	**3.01**	175	136	**311**	**0.63**
Quirino	**21,331**	9,075	8,491	**17,566**	**82.35**	284	309	**593**	**2.78**	273	176	**449**	**2.10**
Cauayan City	**13,286**	5,766	5,268	**11,034**	**83.05**	119	123	**242**	**1.82**	90	76	**166**	**1.25**
Ilagan City	**14,927**	6,174	5,823	**11,997**	**80.37**	193	147	**340**	**2.28**	93	56	**149**	**1.00**
Santiago City	**13,826**	7,060	6,766	**13,826**	**100.00**	19	20	**39**	**0.28**	11	7	**18**	**0.13**
Tuguegarao City	**15,606**	6,673	6,005	**12,678**	**81.24**	6	2	**8**	**0.05**	19	7	**26**	**0.17**
**Region 3**	**1,133,260**	**486,498**	**552,953**	**1,039,451**	**91.72**	**27,016**	**30,546**	**57,562**	**5.08**	**16,889**	**18,519**	**35,408**	**3.12**
Aurora	**25,190**	8,059	7,605	**15,664**	**62.18**	1,289	1,099	**2,388**	**9.48**	421	292	**713**	**2.83**
Bataan	**68,478**	31,058	29,281	**60,339**	**88.11**	1,031	938	**1,969**	**2.88**	955	724	**1,679**	**2.45**
Bulacan	**225,205**	111,105	104,526	**215,631**	**95.75**	3,071	2,517	**5,588**	**2.48**	2,497	2,057	**4,554**	**2.02**
Nueva Ecija	**151,544**	56,539	54,228	**110,767**	**73.09**	4,788	4,068	**8,856**	**5.84**	3,787	3,161	**6,948**	**4.58**
Pampanga	**163,443**	67,401	64,553	**131,954**	**80.73**	4,011	3,305	**7,316**	**4.48**	2,554	2,120	**4,674**	**2.86**
Tarlac	**99,162**	4,083	89,004	**93,087**	**93.87**	13	8,096	**8,109**	**8.18**	59	4,356	**4,415**	**4.45**
Zambales	**63,158**	31,150	29,181	**60,331**	**95.52**	3,641	3,096	**6,737**	**10.67**	1,156	935	**2,091**	**3.31**

Table 2.C.2.4. Nutrition Services for Infants and Children

Number and Proportion of children 0-59 mos. old whose nutritional status are normal, stunted and overweight/obese
Philippines, 2022

Area	Eligible Pop (0-59 mos)	Normal				Stunted				Overweight/obese			
		Male	Female	Total	%	Male	Female	Total	%	Male	Female	Total	%
Angeles City	**41,010**	15,392	14,421	**29,813**	**72.70**	1,196	833	**2,029**	**4.95**	519	423	**942**	**2.30**
Balanga City	**9,894**	4,575	4,559	**9,134**	**92.32**	148	142	**290**	**2.93**	176	114	**290**	**2.93**
Cabanatuan City	**31,015**	18,738	24,332	**43,070**	**138.87**	110	107	**217**	**0.70**	120	110	**230**	**0.74**
City of San Fernando	**30,545**	22,422	20,824	**43,246**	**141.58**	670	490	**1,160**	**3.80**	1,074	1,188	**2,262**	**7.41**
Gapan City	**11,319**	5,022	4,879	**9,901**	**87.47**	252	224	**476**	**4.21**	174	157	**331**	**2.92**
Mabalacat City	**24,985**	14,427	14,294	**28,721**	**114.95**	94	77	**171**	**0.68**	92	67	**159**	**0.64**
Malolos City	**25,157**	7,493	7,127	**14,620**	**58.12**	682	606	**1,288**	**5.12**	715	648	**1,363**	**5.42**
Meycauayan	**20,863**	10,508	9,917	**20,425**	**97.90**	524	422	**946**	**4.53**	159	107	**266**	**1.27**
Olongapo	**24,909**	4,739	4,685	**9,424**	**37.83**	45	31	**76**	**0.31**	24	22	**46**	**0.18**
Palayan City	**4,211**	1,432	1,335	**2,767**	**65.71**	205	196	**401**	**9.52**	125	102	**227**	**5.39**
San Jose City	**14,339**	15,744	14,953	**30,697**	**214.08**	478	432	**910**	**6.35**	306	252	**558**	**3.89**
San Jose del Monte City	**57,281**	26,190	24,821	**51,011**	**89.05**	3,935	3,084	**7,019**	**12.25**	1,659	1,421	**3,080**	**5.38**
Science City of Munoz	**8,365**	3,023	2,820	**5,843**	**69.85**	315	277	**592**	**7.08**	76	59	**135**	**1.61**
Tarlac City	**33,187**	27,398	25,608	**53,006**	**159.72**	518	506	**1,024**	**3.09**	241	204	**445**	**1.34**
**Region 4A**	**1,538,257**	**422,798**	**404,748**	**827,546**	**53.80**	**26,797**	**22,672**	**49,469**	**3.22**	**13,297**	**11,183**	**24,480**	**1.59**
Batangas	**204,669**	57,750	52,430	**110,180**	**53.83**	3,387	2,950	**6,337**	**3.10**	2,497	1,974	**4,471**	**2.18**
Cavite	**143,888**	40,062	38,242	**78,304**	**54.42**	3,935	3,243	**7,178**	**4.99**	3,366	2,858	**6,224**	**4.33**
Laguna	**100,090**	49,028	46,431	**95,459**	**95.37**	2,471	1,891	**4,362**	**4.36**	1,305	1,114	**2,419**	**2.42**
Quezon	**198,276**	31,264	29,840	**61,104**	**30.82**	5,833	4,874	**10,707**	**5.40**	1,340	1,097	**2,437**	**1.23**
Rizal	**226,578**	6,169	5,702	**11,871**	**5.24**	1,236	1,178	**2,414**	**1.07**	413	280	**693**	**0.31**
Antipolo City	**83,454**	*	*	**0**	**0.00**	*	*	**0**	**0.00**	*	*	**0**	**0.00**
Bacoor City	**63,043**	27,400	25,878	**53,278**	**84.51**	811	715	**1,526**	**2.42**	732	644	**1,376**	**2.18**
Batangas City	**36,328**	7,763	7,068	**14,831**	**40.83**	256	202	**458**	**1.26**	227	139	**366**	**1.01**
Biñan City	**33,565**	46,320	49,053	**95,373**	**284.14**	*	*	**0**	**0.00**	*	*	**0**	**0.00**
Cabuyao City	**31,118**	1,023	945	**1,968**	**6.32**	20	11	**31**	**0.10**	8	8	**16**	**0.05**
Calamba City	**45,802**	76,768	72,582	**149,350**	**326.08**	4,644	4,039	**8,683**	**18.96**	1,412	1,323	**2,735**	**5.97**
Cavite City	**10,792**	*	*	**0**	**0.00**	12	17	**29**	**0.27**	*	*	**0**	**0.00**
Dasmariñas City	**69,171**	26,064	25,712	**51,776**	**74.85**	947	853	**1,800**	**2.60**	669	577	**1,246**	**1.80**
General Trias City	**32,987**	12,336	11,880	**24,216**	**73.41**	619	518	**1,137**	**3.45**	641	627	**1,268**	**3.84**
Imus City	**42,383**	2,524	2,425	**4,949**	**11.68**	244	184	**428**	**1.01**	126	95	**221**	**0.52**
Lipa City	**36,596**	*	*	**0**	**0.00**	1	*	**1**	**0.00**	*	*	**0**	**0.00**
Lucena City	**30,046**	48	44	**92**	**0.31**	1,113	960	**2,073**	**6.90**	*	1	**1**	**0.00**
San Pablo City	**26,811**	4,260	4,230	**8,490**	**31.67**	199	157	**356**	**1.33**	121	91	**212**	**0.79**
San Pedro City	**32,836**	2,927	2,768	**5,695**	**17.34**	122	96	**218**	**0.66**	76	89	**165**	**0.50**
Santa Rosa City	**35,649**	21,323	20,513	**41,836**	**117.36**	375	314	**689**	**1.93**	8	6	**14**	**0.04**
Tagaytay City	**7,475**	3,316	3,083	**6,399**	**85.61**	105	96	**201**	**2.69**	145	72	**217**	**2.90**
Tanauan City	**19,088**	5,957	5,532	**11,489**	**60.19**	409	326	**735**	**3.85**	197	164	**361**	**1.89**
Tayabas City	**11,266**	*	*	**0**	**0.00**	*	*	**0**	**0.00**	*	*	**0**	**0.00**
Trece Martires City	**16,346**	496	390	**886**	**5.42**	58	48	**106**	**0.65**	14	24	**38**	**0.23**
**Region 4B**	**377,603**	**140,407**	**135,566**	**275,973**	**73.09**	**14,300**	**12,946**	**27,246**	**7.22**	**3,137**	**2,700**	**5,837**	**1.55**
Marinduque	**27,335**	6,599	6,039	**12,638**	**46.23**	610	549	**1,159**	**4.24**	148	104	**252**	**0.92**
Mindoro Occidental	**63,123**	21,826	21,071	**42,897**	**67.96**	4,121	3,346	**7,467**	**11.83**	561	416	**977**	**1.55**
Mindoro Oriental	**104,948**	32,174	31,460	**63,634**	**60.63**	2,320	2,042	**4,362**	**4.16**	889	726	**1,615**	**1.54**
Palawan	**112,731**	41,727	39,927	**81,654**	**72.43**	4,799	4,571	**9,370**	**8.31**	858	753	**1,611**	**1.43**
Romblon	**35,614**	37,182	36,172	**73,354**	**205.97**	2,257	2,272	**4,529**	**12.72**	681	701	**1,382**	**3.88**
Puerto Princesa City	**33,852**	899	897	**1,796**	**5.31**	193	166	**359**	**1.06**	0	0	**0**	**0.00**
**Region 5**	**734,359**	**258,357**	**240,971**	**499,328**	**68.00**	**23,110**	**19,517**	**42,627**	**5.80**	**7,833**	**6,191**	**14,024**	**1.91**
Albay	**128,685**	52,417	50,220	**102,637**	**79.76**	3,198	2,569	**5,767**	**4.48**	1,635	1,354	**2,989**	**2.32**
Camarines Norte	**74,678**	30,327	28,607	**58,934**	**78.92**	3,348	2,655	**6,003**	**8.04**	778	636	**1,414**	**1.89**
Camarines Sur	**208,510**	48,960	46,446	**95,406**	**45.76**	4,179	3,726	**7,905**	**3.79**	1,646	1,266	**2,912**	**1.40**
Catanduanes	**33,791**	24,919	27,538	**52,457**	**155.24**	3,571	3,126	**6,697**	**19.82**	767	583	**1,350**	**4.00**
Masbate	**122,281**	47,056	40,689	**87,745**	**71.76**	4,888	4,150	**9,038**	**7.39**	1,271	995	**2,266**	**1.85**
Sorsogon	**104,768**	42,089	35,798	**77,887**	**74.34**	3,546	3,013	**6,559**	**6.26**	1,549	1,221	**2,770**	**2.64**
Iriga City	**14,168**	2,116	2,086	**4,202**	**29.66**	159	113	**272**	**1.92**	80	40	**120**	**0.85**
Legaspi City	**22,629**	9,701	8,706	**18,407**	**81.34**	204	146	**350**	**1.55**	105	94	**199**	**0.88**
Naga City	**24,849**	772	881	**1,653**	**6.65**	17	19	**36**	**0.14**	2	2	**4**	**0.02**
**Region 6**	**769,881**	**271,629**	**255,385**	**527,014**	**68.45**	**12,403**	**10,491**	**22,894**	**2.97**	**7,210**	**4,417**	**11,627**	**1.51**
Aklan	**59,656**	14,094	11,900	**25,994**	**43.57**	869	518	**1,387**	**2.32**	2,088	231	**2,319**	**3.89**
Antique	**68,339**	24,882	23,467	**48,349**	**70.75**	2,451	2,148	**4,599**	**6.73**	679	569	**1,248**	**1.83**
Capiz	**73,990**	28,981	27,575	**56,556**	**76.44**	714	569	**1,283**	**1.73**	614	573	**1,187**	**1.60**
Guimaras	**17,582**	5,406	6,294	**11,700**	**66.55**	427	398	**825**	**4.69**	269	224	**493**	**2.80**
Iloilo	**188,737**	79,348	74,300	**153,648**	**81.41**	3,510	3,015	**6,525**	**3.46**	2,302	1,782	**4,084**	**2.16**
Negros Occidental	**259,519**	79,347	75,246	**154,593**	**59.57**	3,778	3,321	**7,099**	**2.74**	1,022	878	**1,900**	**0.73**

Table 2.C.2.4. Nutrition Services for Infants and Children

Number and Proportion of children 0-59 mos. old whose nutritional status are normal, stunted and overweight/obese

Philippines, 2022

Area	Eligible Pop (0-59 mos)	Normal				Stunted				Overweight/obese			
		Male	Female	Total	%	Male	Female	Total	%	Male	Female	Total	%
Bacolod City	**58,394**	26,640	24,360	**51,000**	**87.34**	547	433	**980**	**1.68**	104	73	**177**	**0.30**
Iloilo City	**43,664**	12,931	12,243	**25,174**	**57.65**	107	89	**196**	**0.45**	132	87	**219**	**0.50**
**Region 7**	**810,776**	**62,800**	**60,795**	**123,595**	**15.24**	**4,078**	**3,769**	**7,847**	**0.97**	**900**	**664**	**1,564**	**0.19**
Bohol	**145,820**	2,323	2,163	**4,486**	**3.08**	77	48	**125**	**0.09**	47	23	**70**	**0.05**
Cebu	**325,498**	60,477	58,632	**119,109**	**36.59**	4,001	3,721	**7,722**	**2.37**	853	641	**1,494**	**0.46**
Negros Oriental	**142,596**	0	0	**0**	**0.00**	0	0	**0**	**0.00**	0	0	**0**	**0.00**
Siquijor	**9,320**	0	0	**0**	**0.00**	0	0	**0**	**0.00**	0	0	**0**	**0.00**
Cebu City	**102,179**	0	0	**0**	**0.00**	0	0	**0**	**0.00**	0	0	**0**	**0.00**
Lapu-Lapu City	**45,198**	0	0	**0**	**0.00**	0	0	**0**	**0.00**	0	0	**0**	**0.00**
Mandaue City	**40,165**	0	0	**0**	**0.00**	0	0	**0**	**0.00**	0	0	**0**	**0.00**
**Region 8**	**550,559**	**62,180**	**74,191**	**136,371**	**24.77**	**10,108**	**9,950**	**20,058**	**3.64**	**3,324**	**3,176**	**6,500**	**1.18**
Biliran	**20,815**	6,957	6,645	**13,602**	**65.35**	1,125	905	**2,030**	**9.75**	363	262	**625**	**3.00**
Eastern Samar	**60,293**	11,955	12,835	**24,790**	**41.12**	1,899	1,696	**3,595**	**5.96**	658	597	**1,255**	**2.08**
Northern Leyte	**181,930**	2,786	2,546	**5,332**	**2.93**	96	90	**186**	**0.10**	56	52	**108**	**0.06**
Northern Samar	**85,776**	21,804	20,949	**42,753**	**49.84**	3,934	3,601	**7,535**	**8.78**	1,224	1,018	**2,242**	**2.61**
Southern Leyte	**36,430**	3,070	2,878	**5,948**	**16.33**	537	470	**1,007**	**2.76**	201	130	**331**	**0.91**
Western Samar	**77,165**	2,759	2,521	**5,280**	**6.84**	550	440	**990**	**1.28**	124	143	**267**	**0.35**
Calbayog City	**23,791**	0	0	**0**	**0.00**	0	0	**0**	**0.00**	0	0	**0**	**0.00**
Maasin City	**9,269**	2,427	2,246	**4,673**	**50.42**	140	126	**266**	**2.87**	85	50	**135**	**1.46**
Ormoc City	**25,915**	7,825	7,687	**15,512**	**59.86**	1,564	1,220	**2,784**	**10.74**	494	438	**932**	**3.60**
Tacloban City	**29,175**	2,597	15,884	**18,481**	**63.35**	263	1,402	**1,665**	**5.71**	119	486	**605**	**2.07**
**Region 9**	**414,018**	**73,925**	**73,026**	**146,951**	**35.49**	**3,782**	**3,332**	**7,114**	**1.72**	**695**	**597**	**1,292**	**0.31**
Zamboanga del Norte	**90,053**	8,148	7,956	**16,104**	**17.88**	210	179	**389**	**0.43**	62	59	**121**	**0.13**
Zamboanga del Sur	**88,339**	6,212	6,052	**12,264**	**13.88**	712	638	**1,350**	**1.53**	210	209	**419**	**0.47**
Zamboanga Sibugay	**81,330**	21,557	21,178	**42,735**	**52.55**	2,013	1,749	**3,762**	**4.63**	262	184	**446**	**0.55**
Dapitan City	**9,293**	3,915	3,668	**7,583**	**81.60**	280	207	**487**	**5.24**	36	36	**72**	**0.77**
Dipolog City	**14,750**	6,725	6,449	**13,174**	**89.32**	16	17	**33**	**0.22**	2	4	**6**	**0.04**
Isabela City	**14,783**	1,855	1,706	**3,561**	**24.09**	163	158	**321**	**2.17**	9	10	**19**	**0.13**
Pagadian City	**21,668**	6	10	**16**	**0.07**	0	0	**0**	**0.00**	0	0	**0**	**0.00**
Zamboanga City	**93,802**	25,507	26,007	**51,514**	**54.92**	388	384	**772**	**0.82**	114	95	**209**	**0.22**
**Region 10**	**528,284**	**147,133**	**192,983**	**340,116**	**64.38**	**10,641**	**8,511**	**19,152**	**3.63**	**2,560**	**2,057**	**4,617**	**0.87**
Bukidnon	**120,012**	5,539	56,463	**62,002**	**51.66**	3,357	2,729	**6,086**	**5.07**	1,038	819	**1,857**	**1.55**
Camiguin	**9,858**	3,299	5,248	**8,547**	**86.70**	334	271	**605**	**6.14**	87	66	**153**	**1.55**
Lanao del Norte	**82,295**	35,144	35,233	**70,377**	**85.52**	3,327	2,631	**5,958**	**7.24**	450	430	**880**	**1.07**
Misamis Occidental	**33,888**	3,414	4,588	**8,002**	**23.61**	251	240	**491**	**1.45**		29	**29**	**0.09**
Misamis Oriental	**77,473**	34,218	31,696	**65,914**	**85.08**	1,183	857	**2,040**	**2.63**	399	298	**697**	**0.90**
Cagayan de Oro City	**73,382**	35,128	32,252	**67,380**	**91.82**	159	144	**303**	**0.41**	52	37	**89**	**0.12**
El Salvador City	**5,453**	2,812	2,620	**5,432**	**99.61**	9	6	**15**	**0.28**	6	0	**6**	**0.11**
Gingoog City	**13,532**	7,065	5,621	**12,686**	**93.75**	682	570	**1,252**	**9.25**	56	45	**101**	**0.75**
Iligan City	**41,681**	0	0	**0**	**0.00**	377	371	**748**	**1.79**	103	59	**162**	**0.39**
Malaybalay City	**20,001**	8,606	7,744	**16,350**	**81.75**	540	440	**980**	**4.90**	145	106	**251**	**1.25**
Oroquieta City	**7,343**	5,111	5,173	**10,284**	**140.05**	392	217	**609**	**8.29**	181	130	**311**	**4.24**
Ozamis City	**14,725**	1,759	1,558	**3,317**	**22.53**	5	2	**7**	**0.05**	2	1	**3**	**0.02**
Tangub City	**6,538**	2,818	2,612	**5,430**	**83.05**	11	14	**25**	**0.38**	38	33	**71**	**1.09**
Valencia City	**22,103**	2,220	2,175	**4,395**	**19.88**	14	19	**33**	**0.15**	3	4	**7**	**0.03**
**Region 11**	**561,320**	**236,495**	**220,375**	**456,870**	**81.39**	**15,123**	**12,418**	**27,541**	**4.91**	**4,424**	**3,270**	**7,694**	**1.37**
Davao de Oro	**83,372**	33,061	30,786	**63,847**	**76.58**	2,325	1,939	**4,264**	**5.11**	717	499	**1,216**	**1.46**
Davao del Norte	**107,397**	49,504	42,918	**92,422**	**86.06**	2,025	1,753	**3,778**	**3.52**	821	541	**1,362**	**1.27**
Davao Oriental	**70,353**	26,311	24,740	**51,051**	**72.56**	2,026	1,601	**3,627**	**5.16**	416	303	**719**	**1.02**
Davao del Sur	**73,592**	26,671	25,217	**51,888**	**70.51**	2,043	1,630	**3,673**	**4.99**	745	588	**1,333**	**1.81**
Davao Occidental	**36,611**	11,734	11,344	**23,078**	**63.04**	2,717	2,091	**4,808**	**13.13**	825	655	**1,480**	**4.04**
Davao City	**189,995**	89,214	85,370	**174,584**	**91.89**	3,987	3,404	**7,391**	**3.89**	900	684	**1,584**	**0.83**
**Region 12**	**508,469**	**200,254**	**191,849**	**392,103**	**77.11**	**8,606**	**7,091**	**15,697**	**3.09**	**3,572**	**2,980**	**6,552**	**1.29**
North Cotabato	**175,185**	63,790	60,559	**124,349**	**70.98**	2,963	2,435	**5,398**	**3.08**	873	744	**1,617**	**0.92**
Sarangani	**67,550**	31,874	30,371	**62,245**	**92.15**	1,094	894	**1,988**	**2.94**	285	235	**520**	**0.77**

Table 2.C.2.4. Nutrition Services for Infants and Children

Number and Proportion of children 0-59 mos. old whose nutritional status are normal, stunted and overweight/obese

Philippines, 2022

Area	Eligible Pop (0-59 mos)	Normal				Stunted				Overweight/obese			
		Male	Female	Total	%	Male	Female	Total	%	Male	Female	Total	%
South Cotabato	**105,361**	46,403	43,136	**89,539**	**84.98**	1,192	1,079	**2,271**	**2.16**	518	431	**949**	**0.90**
Sultan Kudarat	**91,944**	31,996	30,368	**62,364**	**67.83**	2,184	1,846	**4,030**	**4.38**	832	713	**1,545**	**1.68**
Gen. Santos City	**68,429**	26,191	27,415	**53,606**	**78.34**	1,173	837	**2,010**	**2.94**	1,064	857	**1,921**	**2.81**
**BARMM**	**626,669**	**62,525**	**63,945**	**126,470**	**20.18**	**3,462**	**3,116**	**6,578**	**1.05**	**977**	**928**	**1,905**	**0.30**
Basilan	**44,651**	0	0	**0**	**0.00**	0	0	**0**	**0.00**	0	0	**0**	**0.00**
Lanao del Sur	**134,457**	37,704	38,355	**76,059**	**56.57**	1,671	1,508	**3,179**	**2.36**	220	200	**420**	**0.31**
Maguindanao	**186,234**	0	0	**0**	**0.00**	3	3	**6**	**0.00**	0	0	**0**	**0.00**
Sulu	**127,946**	0	0	**0**	**0.00**	0	0	**0**	**0.00**	0	0	**0**	**0.00**
Tawi-Tawi	**54,806**	13,546	14,523	**28,069**	**51.22**	1,291	1,182	**2,473**	**4.51**	503	490	**993**	**1.81**
Lamitan City	**12,292**	0	0	**0**	**0.00**	0	0	**0**	**0.00**	0	0	**0**	**0.00**
Marawi City	**32,165**	5,605	5,978	**11,583**	**36.01**	272	236	**508**	**1.58**	183	169	**352**	**1.09**
Cotabato City	**34,118**	5,670	5,089	**10,759**	**31.53**	225	187	**412**	**1.21**	71	69	**140**	**0.41**
**CARAGA**	**321,546**	**98,548**	**94,597**	**193,145**	**60.07**	**8,022**	**6,833**	**14,855**	**4.62**	**3,083**	**2,588**	**5,671**	**1.76**
Agusan del Norte	**42,357**	15,710	15,321	**31,031**	**73.26**	495	505	**1,000**	**2.36**	177	143	**320**	**0.76**
Agusan del Sur	**95,042**	16,324	15,804	**32,128**	**33.80**	914	843	**1,757**	**1.85**	237	200	**437**	**0.46**
Surigao del Norte	**39,725**	13,478	12,970	**26,448**	**66.58**	2,587	2,148	**4,735**	**11.92**	891	760	**1,651**	**4.16**
Surigao del Sur	**60,346**	23,088	22,429	**45,517**	**75.43**	2,267	1,962	**4,229**	**7.01**	978	845	**1,823**	**3.02**
Province of Dinagat	**13,838**	2,741	2,510	**5,251**	**37.95**	225	174	**399**	**2.88**	108	131	**239**	**1.73**
Bislig City	**11,466**	4,507	4,371	**8,878**	**77.43**	597	475	**1,072**	**9.35**	289	211	**500**	**4.36**
Butuan City	**40,269**	17,592	16,366	**33,958**	**84.33**	230	173	**403**	**1.00**	119	102	**221**	**0.55**
Surigao City	**18,503**	5,108	4,826	**9,934**	**53.69**	707	553	**1,260**	**6.81**	284	196	**480**	**2.59**

Table 2.C.2.5 - Nutrition Services for Infants and Children

Number and Proportion of children 0-59 mos. old whose nutritional status are Wasted-MAM, Wasted-SAM and Wasted
Philippines, 2022

Area	Eligible Pop (0-59 mos)	Wasted-MAM				Wasted-SAM				Wasted			
		Male	Female	Total	%	Male	Female	Total	%	Male	Female	Total	%
**PHILIPPINES**	**11,108,117**	**41,894**	**38,811**	**80,705**	**0.73**	**15,636**	**14,091**	**29,727**	**0.27**	**57,530**	**52,902**	**110,432**	**0.99**
**N C R**	**1,188,449**	**0**	**0**	**0**	**0.00**	**0**	**0**	**0**	**0.00**	**0**	**0**	**0**	**0.00**
Malabon City	**33,737**	*	*	**0**	**0.00**	*	*	**0**	**0.00**	0	0	**0**	**0.00**
Navotas City	**23,024**	*	*	**0**	**0.00**	*	*	**0**	**0.00**	0	0	**0**	**0.00**
Valenzuela City	**57,258**	*	*	**0**	**0.00**	*	*	**0**	**0.00**	0	0	**0**	**0.00**
Caloocan City	**146,178**	*	*	**0**	**0.00**	*	*	**0**	**0.00**	0	0	**0**	**0.00**
Marikina City	**41,598**	*	*	**0**	**0.00**	*	*	**0**	**0.00**	0	0	**0**	**0.00**
Pasig City	**69,710**	*	*	**0**	**0.00**	*	*	**0**	**0.00**	0	0	**0**	**0.00**
Pateros	**5,893**	*	*	**0**	**0.00**	*	*	**0**	**0.00**	0	0	**0**	**0.00**
Taguig City	**74,287**	*	*	**0**	**0.00**	*	*	**0**	**0.00**	0	0	**0**	**0.00**
Quezon City	**270,977**	*	*	**0**	**0.00**	*	*	**0**	**0.00**	0	0	**0**	**0.00**
										0	0		
Makati City	**53,769**	*	*	**0**	**0.00**	*	*	**0**	**0.00**	0	0	**0**	**0.00**
Mandaluyong City	**35,649**	*	*	**0**	**0.00**	*	*	**0**	**0.00**	0	0	**0**	**0.00**
San Juan City	**11,276**	*	*	**0**	**0.00**	*	*	**0**	**0.00**	0	0	**0**	**0.00**
Manila City	**164,290**	*	*	**0**	**0.00**	*	*	**0**	**0.00**	0	0	**0**	**0.00**
Las Piñas City	**54,353**	*	*	**0**	**0.00**	*	*	**0**	**0.00**	0	0	**0**	**0.00**
Muntinlupa City	**46,560**	*	*	**0**	**0.00**	*	*	**0**	**0.00**	0	0	**0**	**0.00**
Parañaque City	**61,447**	*	*	**0**	**0.00**	*	*	**0**	**0.00**	0	0	**0**	**0.00**
Pasay City	**38,443**	*	*	**0**	**0.00**	*	*	**0**	**0.00**	0	0	**0**	**0.00**
**C A R**	**182,376**	**996**	**704**	**1,700**	**0.93**	**448**	**266**	**714**	**0.39**	**1,444**	**970**	**2,414**	**1.32**
Abra	**23,221**	499	392	**891**	**3.84**	196	151	**347**	**1.49**	695	543	**1,238**	**5.33**
Apayao	**13,618**	90	94	**184**	**1.35**	27	29	**56**	**0.41**	117	123	**240**	**1.76**
Benguet	**45,087**	46	29	**75**	**0.17**	17	10	**27**	**0.06**	63	39	**102**	**0.23**
Ifugao	**23,917**	51	51	**102**	**0.43**	16	8	**24**	**0.10**	67	59	**126**	**0.53**
Kalinga	**24,778**	205	51	**256**	**1.03**	139	39	**178**	**0.72**	344	90	**434**	**1.75**
Mt. Province	**16,861**	80	72	**152**	**0.90**	45	27	**72**	**0.43**	125	99	**224**	**1.33**
Baguio City	**34,894**	25	15	**40**	**0.11**	8	2	**10**	**0.03**	33	17	**50**	**0.14**
**Region 1**	**503,968**	**3,662**	**3,264**	**6,926**	**1.37**	**798**	**744**	**1,542**	**0.31**	**4,460**	**4,008**	**8,468**	**1.68**
Ilocos Norte	**39,803**	439	347	**786**	**1.97**	76	65	**141**	**0.35**	515	412	**927**	**2.33**
Ilocos Sur	**48,732**	470	437	**907**	**1.86**	62	53	**115**	**0.24**	532	490	**1,022**	**2.10**
La Union	**59,231**	533	524	**1,057**	**1.78**	68	62	**130**	**0.22**	601	586	**1,187**	**2.00**
Pangasinan	**262,152**	1,392	1,226	**2,618**	**1.00**	260	259	**519**	**0.20**	1,652	1,485	**3,137**	**1.20**
Alaminos City	**9,900**	55	47	**102**	**1.03**	6	15	**21**	**0.21**	61	62	**123**	**1.24**
Candon City	**5,144**	30	28	**58**	**1.13**	5	4	**9**	**0.17**	35	32	**67**	**1.30**
Dagupan City	**18,915**	274	232	**506**	**2.68**	155	143	**298**	**1.58**	429	375	**804**	**4.25**
Laoag City	**9,182**	30	21	**51**	**0.56**	2	5	**7**	**0.08**	32	26	**58**	**0.63**
San Carlos City	**20,817**	276	225	**501**	**2.41**	95	83	**178**	**0.86**	371	308	**679**	**3.26**
San Fernando City	**10,849**	86	75	**161**	**1.48**	40	35	**75**	**0.69**	126	110	**236**	**2.18**
Urdaneta City	**14,679**	67	92	**159**	**1.08**	22	18	**40**	**0.27**	89	110	**199**	**1.36**
Vigan City	**4,564**	10	10	**20**	**0.44**	7	2	**9**	**0.20**	17	12	**29**	**0.64**
**Region 2**	**358,323**	**1,888**	**1,744**	**3,632**	**1.01**	**756**	**712**	**1,468**	**0.41**	**2,644**	**2,456**	**5,100**	**1.42**
Batanes	**1,992**	33	10	**43**	**2.16**	1	1	**2**	**0.10**	34	11	**45**	**2.26**
Cagayan	**106,266**	608	505	**1,113**	**1.05**	229	170	**399**	**0.38**	837	675	**1,512**	**1.42**
Isabela	**121,394**	1,190	1,161	**2,351**	**1.94**	490	511	**1,001**	**0.82**	1,680	1,672	**3,352**	**2.76**
Nueva Vizcaya	**49,695**	0	0	**0**	**0.00**	0	0	**0**	**0.00**	0	0	**0**	**0.00**
Quirino	**21,331**	6	5	**11**	**0.05**	4	4	**8**	**0.04**	10	9	**19**	**0.09**
Cauayan City	**13,286**	20	36	**56**	**0.42**	26	20	**46**	**0.35**	46	56	**102**	**0.77**
Ilagan City	**14,927**	27	21	**48**	**0.32**	6	6	**12**	**0.08**	33	27	**60**	**0.40**
Santiago City	**13,826**	4	6	**10**	**0.07**	0	0	**0**	**0.00**	4	6	**10**	**0.07**
Tuguegarao City	**15,606**	0	0	**0**	**0.00**	0	0	**0**	**0.00**	0	0	**0**	**0.00**
**Region 3**	**1,133,260**	**10,193**	**11,054**	**21,247**	**1.87**	**5,056**	**5,353**	**10,409**	**0.92**	**15,249**	**16,407**	**31,656**	**2.79**
Aurora	**25,190**	202	233	**435**	**1.73**	60	74	**134**	**0.53**	262	307	**569**	**2.26**
Bataan	**68,478**	535	481	**1,016**	**1.48**	168	140	**308**	**0.45**	703	621	**1,324**	**1.93**
Bulacan	**225,205**	1,052	829	**1,881**	**0.84**	574	457	**1,031**	**0.46**	1,626	1,286	**2,912**	**1.29**
Nueva Ecija	**151,544**	1,117	1,036	**2,153**	**1.42**	396	328	**724**	**0.48**	1,513	1,364	**2,877**	**1.90**
Pampanga	**163,443**	2,230	1,899	**4,129**	**2.53**	1,293	987	**2,280**	**1.39**	3,523	2,886	**6,409**	**3.92**

Table 2.C.2.5 - Nutrition Services for Infants and Children

Number and Proportion of children 0-59 mos. old whose nutritional status are Wasted-MAM, Wasted-SAM and Wasted

Philippines, 2022

Area	Eligible Pop (0-59 mos)	Wasted-MAM				Wasted-SAM				Wasted			
		Male	Female	Total	%	Male	Female	Total	%	Male	Female	Total	%
Tarlac	**99,162**	0	2,558	**2,558**	**2.58**	0	1,499	**1,499**	**1.51**	0	4,057	**4,057**	**4.09**
Zambales	**63,158**	1,831	1,333	**3,164**	**5.01**	812	672	**1,484**	**2.35**	2,643	2,005	**4,648**	**7.36**
Angeles City	**41,010**	234	282	**516**	**1.26**	340	101	**441**	**1.08**	574	383	**957**	**2.33**
Balanga City	**9,894**	58	57	**115**	**1.16**	29	23	**52**	**0.53**	87	80	**167**	**1.69**
Cabanatuan City	**31,015**	93	96	**189**	**0.61**	0	0	**0**	**0.00**	93	96	**189**	**0.61**
City of San Fernando	**30,545**	191	156	**347**	**1.14**	11	6	**17**	**0.06**	202	162	**364**	**1.19**
Gapan City	**11,319**	91	75	**166**	**1.47**	58	59	**117**	**1.03**	149	134	**283**	**2.50**
Mabalacat City	**24,985**	60	56	**116**	**0.46**	0	0	**0**	**0.00**	60	56	**116**	**0.46**
Malolos City	**25,157**	1,026	762	**1,788**	**7.11**	504	426	**930**	**3.70**	1,530	1,188	**2,718**	**10.80**
Meycauayan	**20,863**	139	138	**277**	**1.33**	53	49	**102**	**0.49**	192	187	**379**	**1.82**
Olongapo	**24,909**	0	0	**0**	**0.00**	0	0	**0**	**0.00**	0	0	**0**	**0.00**
Palayan City	**4,211**	0	0	**0**	**0.00**	0	0	**0**	**0.00**	0	0	**0**	**0.00**
San Jose City	**14,339**	290	252	**542**	**3.78**	78	69	**147**	**1.03**	368	321	**689**	**4.81**
San Jose del Monte City	**57,281**	839	626	**1,465**	**2.56**	569	405	**974**	**1.70**	1,408	1,031	**2,439**	**4.26**
Science City of Munoz	**8,365**	0	0	**0**	**0.00**	0	0	**0**	**0.00**	0	0	**0**	**0.00**
Tarlac City	**33,187**	205	185	**390**	**1.18**	111	58	**169**	**0.51**	316	243	**559**	**1.68**
**Region 4A**	**1,538,257**	**3,463**	**3,330**	**6,793**	**0.44**	**1,316**	**993**	**2,309**	**0.15**	**4,779**	**4,323**	**9,102**	**0.59**
Batangas	**204,669**	151	114	**265**	**0.13**	6	3	**9**	**0.00**	157	117	**274**	**0.13**
Cavite	**143,888**	1,005	876	**1,881**	**1.31**	399	298	**697**	**0.48**	1,404	1,174	**2,578**	**1.79**
Laguna	**100,090**	356	280	**636**	**0.64**	153	139	**292**	**0.29**	509	419	**928**	**0.93**
Quezon	**198,276**	848	691	**1,539**	**0.78**	240	174	**414**	**0.21**	1,088	865	**1,953**	**0.98**
Rizal	**226,578**	53	60	**113**	**0.05**	1	2	**3**	**0.00**	54	62	**116**	**0.05**
Antipolo City	**83,454**	*	*	**0**	**0.00**	*	*	**0**	**0.00**	0	0	**0**	**0.00**
Bacoor City	**63,043**	278	236	**514**	**0.82**	207	180	**387**	**0.61**	485	416	**901**	**1.43**
Batangas City	**36,328**	6	2	**8**	**0.02**	3	*	**3**	**0.01**	9	2	**11**	**0.03**
Biñan City	**33,565**	*	*	**0**	**0.00**	*	*	**0**	**0.00**	0	0	**0**	**0.00**
Cabuyao City	**31,118**	7	4	**11**	**0.04**	*	*	**0**	**0.00**	7	4	**11**	**0.04**
Calamba City	**45,802**	241	156	**397**	**0.87**	43	25	**68**	**0.15**	284	181	**465**	**1.02**
Cavite City	**10,792**	2	486	**488**	**4.52**	*	*	**0**	**0.00**	2	486	**488**	**4.52**
Dasmariñas City	**69,171**	*	*	**0**	**0.00**	*	*	**0**	**0.00**	0	0	**0**	**0.00**
General Trias City	**32,987**	*	*	**0**	**0.00**	*	*	**0**	**0.00**	0	0	**0**	**0.00**
Imus City	**42,383**	21	17	**38**	**0.09**	1	1	**2**	**0.00**	22	18	**40**	**0.09**
Lipa City	**36,596**	*	*	**0**	**0.00**	*	*	**0**	**0.00**	0	0	**0**	**0.00**
Lucena City	**30,046**	365	288	**653**	**2.17**	262	170	**432**	**1.44**	627	458	**1,085**	**3.61**
San Pablo City	**26,811**	2	*	**2**	**0.01**	*	*	**0**	**0.00**	2	0	**2**	**0.01**
San Pedro City	**32,836**	54	48	**102**	**0.31**	*	*	**0**	**0.00**	54	48	**102**	**0.31**
Santa Rosa City	**35,649**	65	70	**135**	**0.38**	*	*	**0**	**0.00**	65	70	**135**	**0.38**
Tagaytay City	**7,475**	9	2	**11**	**0.15**	1	1	**2**	**0.03**	10	3	**13**	**0.17**
Tanauan City	**19,088**	*	*	**0**	**0.00**	*	*	**0**	**0.00**	0	0	**0**	**0.00**
Tayabas City	**11,266**	*	*	**0**	**0.00**	*	*	**0**	**0.00**	0	0	**0**	**0.00**
Trece Martires City	**16,346**	*	*	**0**	**0.00**	*	*	**0**	**0.00**	0	0	**0**	**0.00**
**Region 4B**	**377,603**	**1,730**	**1,444**	**3,174**	**0.84**	**647**	**523**	**1,170**	**0.31**	**2,377**	**1,967**	**4,344**	**1.15**
Marinduque	**27,335**	80	62	**142**	**0.52**	11	7	**18**	**0.07**	91	69	**160**	**0.59**
Mindoro Occidental	**63,123**	796	603	**1,399**	**2.22**	292	231	**523**	**0.83**	1,088	834	**1,922**	**3.04**
Mindoro Oriental	**104,948**	132	101	**233**	**0.22**	21	19	**40**	**0.04**	153	120	**273**	**0.26**
Palawan	**112,731**	573	549	**1,122**	**1.00**	279	236	**515**	**0.46**	852	785	**1,637**	**1.45**
Romblon	**35,614**	149	129	**278**	**0.78**	44	30	**74**	**0.21**	193	159	**352**	**0.99**
Puerto Princesa City	**33,852**	0	0	**0**	**0.00**	0	0	**0**	**0.00**	0	0	**0**	**0.00**
**Region 5**	**734,359**	**5,170**	**4,909**	**10,079**	**1.37**	**1,325**	**1,201**	**2,526**	**0.34**	**6,495**	**6,110**	**12,605**	**1.72**
Albay	**128,685**	366	300	**666**	**0.52**	128	187	**315**	**0.24**	494	487	**981**	**0.76**
Camarines Norte	**74,678**	420	272	**692**	**0.93**	0	0	**0**	**0.00**	420	272	**692**	**0.93**
Camarines Sur	**208,510**	2,096	1,813	**3,909**	**1.87**	642	529	**1,171**	**0.56**	2,738	2,342	**5,080**	**2.44**
Catanduanes	**33,791**	605	1,040	**1,645**	**4.87**	23	17	**40**	**0.12**	628	1,057	**1,685**	**4.99**
Masbate	**122,281**	1,095	935	**2,030**	**1.66**	343	268	**611**	**0.50**	1,438	1,203	**2,641**	**2.16**
Sorsogon	**104,768**	547	507	**1,054**	**1.01**	148	151	**299**	**0.29**	695	658	**1,353**	**1.29**
Iriga City	**14,168**	5	2	**7**	**0.05**	3	2	**5**	**0.04**	8	4	**12**	**0.08**
Legaspi City	**22,629**	36	40	**76**	**0.34**	38	47	**85**	**0.38**	74	87	**161**	**0.71**
Naga City	**24,849**	0	0	**0**	**0.00**	0	0	**0**	**0.00**	0	0	**0**	**0.00**

Table 2.C.2.5 - Nutrition Services for Infants and Children

Number and Proportion of children 0-59 mos. old whose nutritional status are Wasted-MAM, Wasted-SAM and Wasted
Philippines, 2022

Area	Eligible Pop (0-59 mos)	Wasted-MAM				Wasted-SAM				Wasted			
		Male	Female	Total	%	Male	Female	Total	%	Male	Female	Total	%
**Region 6**	**769,881**	**2,904**	**2,495**	**5,399**	**0.70**	**825**	**688**	**1,513**	**0.20**	**3,729**	**3,183**	**6,912**	**0.90**
Aklan	**59,656**	106	119	**225**	**0.38**	41	40	**81**	**0.14**	147	159	**306**	**0.51**
Antique	**68,339**	347	228	**575**	**0.84**	104	67	**171**	**0.25**	451	295	**746**	**1.09**
Capiz	**73,990**	146	129	**275**	**0.37**	81	50	**131**	**0.18**	227	179	**406**	**0.55**
Guimaras	**17,582**	60	47	**107**	**0.61**	19	14	**33**	**0.19**	79	61	**140**	**0.80**
Iloilo	**188,737**	812	677	**1,489**	**0.79**	284	251	**535**	**0.28**	1,096	928	**2,024**	**1.07**
Negros Occidental	**259,519**	1,290	1,177	**2,467**	**0.95**	268	247	**515**	**0.20**	1,558	1,424	**2,982**	**1.15**
Bacolod City	**58,394**	118	98	**216**	**0.37**	28	18	**46**	**0.08**	146	116	**262**	**0.45**
Iloilo City	**43,664**	25	20	**45**	**0.10**	0	1	**1**	**0.00**	25	21	**46**	**0.11**
**Region 7**	**810,776**	**661**	**444**	**1,105**	**0.14**	**159**	**74**	**233**	**0.03**	**820**	**518**	**1,338**	**0.17**
Bohol	**145,820**	18	15	**33**	**0.02**	5	8	**13**	**0.01**	23	23	**46**	**0.03**
Cebu	**325,498**	643	429	**1,072**	**0.33**	154	66	**220**	**0.07**	797	495	**1,292**	**0.40**
Negros Oriental	**142,596**	0	0	**0**	**0.00**	0	0	**0**	**0.00**	0	0	**0**	**0.00**
Siquijor	**9,320**	0	0	**0**	**0.00**	0	0	**0**	**0.00**	0	0	**0**	**0.00**
Cebu City	**102,179**	0	0	**0**	**0.00**	0	0	**0**	**0.00**	0	0	**0**	**0.00**
Lapu-Lapu City	**45,198**	0	0	**0**	**0.00**	0	0	**0**	**0.00**	0	0	**0**	**0.00**
Mandaue City	**40,165**	0	0	**0**	**0.00**	0	0	**0**	**0.00**	0	0	**0**	**0.00**
**Region 8**	**550,559**	**1,262**	**1,290**	**2,552**	**0.46**	**781**	**762**	**1,543**	**0.28**	**2,043**	**2,052**	**4,095**	**0.74**
Biliran	**20,815**	267	201	**468**	**2.25**	151	84	**235**	**1.13**	418	285	**703**	**3.38**
Eastern Samar	**60,293**	142	159	**301**	**0.50**	123	63	**186**	**0.31**	265	222	**487**	**0.81**
Northern Leyte	**181,930**	0	0	**0**	**0.00**	7	4	**11**	**0.01**	7	4	**11**	**0.01**
Northern Samar	**85,776**	323	233	**556**	**0.65**	296	316	**612**	**0.71**	619	549	**1,168**	**1.36**
Southern Leyte	**36,430**	77	62	**139**	**0.38**	21	13	**34**	**0.09**	98	75	**173**	**0.47**
Western Samar	**77,165**	18	33	**51**	**0.07**	1	4	**5**	**0.01**	19	37	**56**	**0.07**
Calbayog City	**23,791**	0	0	**0**	**0.00**	0	0	**0**	**0.00**	0	0	**0**	**0.00**
Maasin City	**9,269**	14	8	**22**	**0.24**	0	1	**1**	**0.01**	14	9	**23**	**0.25**
Ormoc City	**25,915**	316	246	**562**	**2.17**	135	106	**241**	**0.93**	451	352	**803**	**3.10**
Tacloban City	**29,175**	105	348	**453**	**1.55**	47	171	**218**	**0.75**	152	519	**671**	**2.30**
**Region 9**	**414,018**	**835**	**696**	**1,531**	**0.37**	**237**	**201**	**438**	**0.11**	**1,072**	**897**	**1,969**	**0.48**
Zamboanga del Norte	**90,053**	70	56	**126**	**0.14**	15	20	**35**	**0.04**	85	76	**161**	**0.18**
Zamboanga del Sur	**88,339**	257	201	**458**	**0.52**	96	64	**160**	**0.18**	353	265	**618**	**0.70**
Zamboanga Sibugay	**81,330**	142	130	**272**	**0.33**	22	24	**46**	**0.06**	164	154	**318**	**0.39**
Dapitan City	**9,293**	2	3	**5**	**0.05**	2	1	**3**	**0.03**	4	4	**8**	**0.09**
Dipolog City	**14,750**	17	10	**27**	**0.18**	2	4	**6**	**0.04**	19	14	**33**	**0.22**
Isabela City	**14,783**	115	43	**158**	**1.07**	23	14	**37**	**0.25**	138	57	**195**	**1.32**
Pagadian City	**21,668**	0	0	**0**	**0.00**	0	0	**0**	**0.00**	0	0	**0**	**0.00**
Zamboanga City	**93,802**	232	253	**485**	**0.52**	77	74	**151**	**0.16**	309	327	**636**	**0.68**
**Region 10**	**528,284**	**1,758**	**1,442**	**3,200**	**0.61**	**545**	**500**	**1,045**	**0.20**	**2,303**	**1,942**	**4,245**	**0.80**
Bukidnon	**120,012**	764	614	**1,378**	**1.15**	221	213	**434**	**0.36**	985	827	**1,812**	**1.51**
Camiguin	**9,858**	0	0	**0**	**0.00**	0	0	**0**	**0.00**	0	0	**0**	**0.00**
Lanao del Norte	**82,295**	565	467	**1,032**	**1.25**	208	185	**393**	**0.48**	773	652	**1,425**	**1.73**
Misamis Occidental	**33,888**	16	27	**43**	**0.13**	0	0	**0**	**0.00**	16	27	**43**	**0.13**
Misamis Oriental	**77,473**	227	151	**378**	**0.49**	64	54	**118**	**0.15**	291	205	**496**	**0.64**
Cagayan de Oro City	**73,382**	18	12	**30**	**0.04**	10	15	**25**	**0.03**	28	27	**55**	**0.07**
El Salvador City	**5,453**	0	0	**0**	**0.00**	0	0	**0**	**0.00**	0	0	**0**	**0.00**
Gingoog City	**13,532**	0	0	**0**	**0.00**	0	0	**0**	**0.00**	0	0	**0**	**0.00**
Iligan City	**41,681**	27	22	**49**	**0.12**	5	6	**11**	**0.03**	32	28	**60**	**0.14**
Malaybalay City	**20,001**	111	86	**197**	**0.98**	37	27	**64**	**0.32**	148	113	**261**	**1.30**
Oroquieta City	**7,343**	4	5	**9**	**0.12**	0	0	**0**	**0.00**	4	5	**9**	**0.12**
Ozamis City	**14,725**	3	7	**10**	**0.07**	0	0	**0**	**0.00**	3	7	**10**	**0.07**
Tangub City	**6,538**	23	50	**73**	**1.12**	0	0	**0**	**0.00**	23	50	**73**	**1.12**
Valencia City	**22,103**	0	1	**1**	**0.00**	0	0	**0**	**0.00**	0	1	**1**	**0.00**

Table 2.C.2.5 - Nutrition Services for Infants and Children

Number and Proportion of children 0-59 mos. old whose nutritional status are Wasted-MAM, Wasted-SAM and Wasted

Philippines, 2022

Area	Eligible Pop (0-59 mos)	Wasted-MAM				Wasted-SAM				Wasted			
		Male	Female	Total	%	Male	Female	Total	%	Male	Female	Total	%
**Region 11**	**561,320**	**2,175**	**1,871**	**4,046**	**0.72**	**707**	**549**	**1,256**	**0.22**	**2,882**	**2,420**	**5,302**	**0.94**
Davao de Oro	**83,372**	208	169	**377**	**0.45**	94	65	**159**	**0.19**	302	234	**536**	**0.64**
Davao del Norte	**107,397**	295	289	**584**	**0.54**	45	39	**84**	**0.08**	340	328	**668**	**0.62**
Davao Oriental	**70,353**	463	373	**836**	**1.19**	221	177	**398**	**0.57**	684	550	**1,234**	**1.75**
Davao del Sur	**73,592**	457	364	**821**	**1.12**	87	61	**148**	**0.20**	544	425	**969**	**1.32**
Davao Occidental	**36,611**	517	444	**961**	**2.62**	198	165	**363**	**0.99**	715	609	**1,324**	**3.62**
Davao City	**189,995**	235	232	**467**	**0.25**	62	42	**104**	**0.05**	297	274	**571**	**0.30**
**Region 12**	**508,469**	**3,019**	**2,219**	**5,238**	**1.03**	**1,064**	**734**	**1,798**	**0.35**	**4,083**	**2,953**	**7,036**	**1.38**
North Cotabato	**175,185**	728	576	**1,304**	**0.74**	256	158	**414**	**0.24**	984	734	**1,718**	**0.98**
Sarangani	**67,550**	211	149	**360**	**0.53**	59	51	**110**	**0.16**	270	200	**470**	**0.70**
South Cotabato	**105,361**	227	185	**412**	**0.39**	50	42	**92**	**0.09**	277	227	**504**	**0.48**
Sultan Kudarat	**91,944**	896	679	**1,575**	**1.71**	304	236	**540**	**0.59**	1,200	915	**2,115**	**2.30**
Gen. Santos City	**68,429**	957	630	**1,587**	**2.32**	395	247	**642**	**0.94**	1,352	877	**2,229**	**3.26**
**BARMM**	**626,669**	**901**	**816**	**1,717**	**0.27**	**502**	**429**	**931**	**0.15**	**1,403**	**1,245**	**2,648**	**0.42**
Basilan	**44,651**	0	0	**0**	**0.00**	0	0	**0**	**0.00**	0	0	**0**	**0.00**
Lanao del Sur	**134,457**	175	174	**349**	**0.26**	57	34	**91**	**0.07**	232	208	**440**	**0.33**
Maguindanao	**186,234**	17	21	**38**	**0.02**	62	78	**140**	**0.08**	79	99	**178**	**0.10**
Sulu	**127,946**	0	0	**0**	**0.00**	0	0	**0**	**0.00**	0	0	**0**	**0.00**
Tawi-Tawi	**54,806**	545	493	**1,038**	**1.89**	305	271	**576**	**1.05**	850	764	**1,614**	**2.94**
Lamitan City	**12,292**	0	0	**0**	**0.00**	0	0	**0**	**0.00**	0	0	**0**	**0.00**
Marawi City	**32,165**	131	98	**229**	**0.71**	70	41	**111**	**0.35**	201	139	**340**	**1.06**
Cotabato City	**34,118**	33	30	**63**	**0.18**	8	5	**13**	**0.04**	41	35	**76**	**0.22**
**CARAGA**	**321,546**	**1,277**	**1,089**	**2,366**	**0.74**	**470**	**362**	**832**	**0.26**	**1,747**	**1,451**	**3,198**	**0.99**
Agusan del Norte	**42,357**	79	73	**152**	**0.36**	19	22	**41**	**0.10**	98	95	**193**	**0.46**
Agusan del Sur	**95,042**	119	130	**249**	**0.26**	40	27	**67**	**0.07**	159	157	**316**	**0.33**
Surigao del Norte	**39,725**	403	322	**725**	**1.83**	163	115	**278**	**0.70**	566	437	**1,003**	**2.52**
Surigao del Sur	**60,346**	433	305	**738**	**1.22**	152	122	**274**	**0.45**	585	427	**1,012**	**1.68**
Province of Dinagat	**13,838**	31	41	**72**	**0.52**	5	3	**8**	**0.06**	36	44	**80**	**0.58**
Bislig City	**11,466**	2	0	**2**	**0.02**	2	0	**2**	**0.02**	4	0	**4**	**0.03**
Butuan City	**40,269**	46	50	**96**	**0.24**	13	10	**23**	**0.06**	59	60	**119**	**0.30**
Surigao City	**18,503**	164	168	**332**	**1.79**	76	63	**139**	**0.75**	240	231	**471**	**2.55**

Table 2.C.3.1 -Deworming Services for Children and Adolescents

Number and Proportion of Children/Adolescents who completed 2 doses of deworming tablet
Philippines, 2022

Area	Eligible Pop (1-4 years old)	No. of children 1-4 y/o given 2 doses of deworming drugs				Eligible Pop (5-9 years old)	No. of children 5-9 y/o given 2 doses of deworming drugs				Eligible Pop (10-19 years old)	No. of children 10-19 y/o given 2 doses of deworming drugs			
		Male	Female	Total	%		Male	Female	Total	%		Male	Female	Total	%
**PHILIPPINES**	**8,976,673**	**2,772,787**	**2,630,782**	**5,403,569**	**60.20**	**11,132,656**	**2,602,254**	**2,444,532**	**5,046,786**	**45.33**	**21,215,201**	**3,880,661**	**3,697,117**	**7,577,778**	**35.72**
**N C R**	**951,556**	**270,396**	**269,261**	**539,657**	**56.71**	**1,234,875**	**171,445**	**151,802**	**323,247**	**26.18**	**2,440,227**	**145,763**	**130,994**	**276,757**	**11.34**
Malabon City	**27,010**	4571	4579	**9,150**	**33.88**	**35,052**	3035	2882	**5,917**	**16.88**	**69,262**	2806	2833	**5,639**	**8.14**
Navotas City	**18,431**	1964	2098	**4,062**	**22.04**	**23,921**	1316	1505	**2,821**	**11.79**	**47,273**	976	909	**1,885**	**3.99**
Valenzuela City	**45,846**	9127	9335	**18,462**	**40.27**	**59,496**	5856	6635	**12,491**	**20.99**	**117,570**	5870	3920	**9,790**	**8.33**
Caloocan City	**117,048**	59861	57187	**117,048**	**100.00**	**151,892**	37202	36894	**74,096**	**48.78**	**300,158**	24729	24626	**49,355**	**16.44**
Marikina City	**33,305**	4526	4979	**9,505**	**28.54**	**43,224**	1410	1330	**2,740**	**6.34**	**85,416**	896	930	**1,826**	**2.14**
Pasig City	**55,813**	5826	5454	**11,280**	**20.21**	**72,427**	3578	3265	**6,843**	**9.45**	**143,127**	3222	3007	**6,229**	**4.35**
Pateros City	**4,718**	876	822	**1,698**	**35.99**	**6,121**	470	476	**946**	**15.45**	**12,099**	433	416	**849**	**7.02**
Taguig City	**59,478**	29267	30211	**59,478**	**100.00**	**77,193**	35533	35234	**70,767**	**91.68**	**152,530**	48390	44720	**93,110**	**61.04**
Quezon City	**216,968**	61252	63859	**125,111**	**57.66**	**281,564**	33019	22313	**55,332**	**19.65**	**556,389**	22652	15146	**37,798**	**6.79**
Makati City	**43,053**	11581	10745	**22,326**	**51.86**	**55,870**	5256	4884	**10,140**	**18.15**	**110,404**	2160	2104	**4,264**	**3.86**
Mandaluyong City	**28,541**	3848	3996	**7,844**	**27.48**	**37,044**	623	740	**1,363**	**3.68**	**73,200**	264	308	**572**	**0.78**
San Juan City	**9,028**	577	521	**1,098**	**12.16**	**11,714**	475	505	**980**	**8.37**	**23,154**	785	837	**1,622**	**7.01**
Manila City	**131,547**	25433	24834	**50,267**	**38.21**	**170,708**	16072	15733	**31,805**	**18.63**	**337,336**	15158	15306	**30,464**	**9.03**
Las Piñas City	**43,514**	1968	1723	**3,691**	**8.48**	**56,474**	660	626	**1,286**	**2.28**	**111,596**	294	283	**577**	**0.52**
Muntinlupa City	**37,280**	18162	18447	**36,609**	**98.20**	**48,380**	18444	10826	**29,270**	**60.50**	**95,603**	12488	12000	**24,488**	**25.61**
Parañaque City	**49,198**	16025	15225	**31,250**	**63.52**	**63,849**	4808	4083	**8,891**	**13.93**	**126,174**	2298	1337	**3,635**	**2.88**
Pasay City	**30,778**	15532	15246	**30,778**	**100.00**	**39,946**	3688	3871	**7,559**	**18.92**	**78,936**	2342	2312	**4,654**	**5.90**
**C A R**	**147,190**	**50,278**	**47,892**	**98,170**	**66.70**	**180,107**	**32,300**	**30,055**	**62,355**	**34.62**	**346,319**	**50,017**	**46,611**	**96,628**	**27.90**
Abra	**18,948**	7,406	7,104	**14,510**	**76.58**	**23,334**	9,466	8,522	**17,988**	**77.09**	**46,682**	14,694	12,660	**27,354**	**58.60**
Apayao	**11,111**	3,792	3,537	**7,329**	**65.96**	**13,346**	1,201	1,260	**2,461**	**18.44**	**24,870**	1,614	1,487	**3,101**	**12.47**
Benguet	**36,006**	11,759	11,221	**22,980**	**63.82**	**44,205**	3,785	3,363	**7,148**	**16.17**	**87,502**	5,975	5,548	**11,523**	**13.17**
Ifugao	**19,435**	7,148	6,888	**14,036**	**72.22**	**23,529**	8,730	8,384	**17,114**	**72.74**	**43,068**	15,091	14,391	**29,482**	**68.45**
Kalinga	**20,071**	5,350	4,828	**10,178**	**50.71**	**24,443**	4,296	3,561	**7,857**	**32.14**	**44,965**	7,426	6,965	**14,391**	**32.00**
Mt. Province	**13,756**	4,358	4,023	**8,381**	**60.93**	**17,025**	1,665	1,602	**3,267**	**19.19**	**31,507**	2,460	2,250	**4,710**	**14.95**
Baguio City	**27,863**	10,465	10,291	**20,756**	**74.49**	**34,225**	3,157	3,363	**6,520**	**19.05**	**67,725**	2,757	3,310	**6,067**	**8.96**
**Region 1**	**406,852**	**113,866**	**115,547**	**229,413**	**56.39**	**519,612**	**100,272**	**98,235**	**198,507**	**38.20**	**1,002,199**	**167,933**	**169,332**	**337,265**	**33.65**
Ilocos Norte	**31,951**	8,236	7,726	**15,962**	**49.96**	**40,888**	1,424	1,297	**2,721**	**6.65**	**85,884**	1,730	1,822	**3,552**	**4.14**
Ilocos Sur	**39,653**	16,429	16,217	**32,646**	**82.33**	**51,586**	20,487	20,080	**40,567**	**78.64**	**105,601**	34,997	35,529	**70,526**	**66.79**
La Union	**47,886**	18,340	17,128	**35,468**	**74.07**	**61,561**	21,843	20,488	**42,331**	**68.76**	**125,987**	42,265	39,476	**81,741**	**64.88**
Pangasinan	**211,444**	56,769	60,727	**117,496**	**55.57**	**268,691**	42,482	43,374	**85,856**	**31.95**	**498,531**	64,939	69,954	**134,893**	**27.06**
Alaminos City	**7,986**	3,644	4,070	**7,714**	**96.59**	**10,153**	4,816	4,342	**9,158**	**90.20**	**18,841**	8,898	8,467	**17,365**	**92.17**
Candon City	**4,182**	2,320	1,861	**4,181**	**99.98**	**5,431**	628	523	**1,151**	**21.19**	**11,126**	775	620	**1,395**	**12.54**
Dagupan City	**15,255**	2,549	2,450	**4,999**	**32.77**	**19,383**	2,901	2,783	**5,684**	**29.32**	**35,957**	4,101	3,884	**7,985**	**22.21**
Laoag City	**7,365**	0	0	**0**	**0.00**	**9,425**	0	0	**0**	**0.00**	**19,796**	0	0	**0**	**0.00**
San Carlos City	**16,796**	0	0	**0**	**0.00**	**21,342**	0	0	**0**	**0.00**	**39,591**	0	0	**0**	**0.00**
San Fernando City	**8,776**	3,510	3,431	**6,941**	**79.09**	**11,278**	3,555	3,292	**6,847**	**60.71**	**23,080**	6,699	6,071	**12,770**	**55.33**
Urdaneta City	**11,842**	398	357	**755**	**6.38**	**15,045**	564	590	**1,154**	**7.67**	**27,914**	1,180	1,098	**2,278**	**8.16**
Vigan City	**3,716**	1,671	1,580	**3,251**	**87.49**	**4,829**	1,572	1,466	**3,038**	**62.91**	**9,891**	2,349	2,411	**4,760**	**48.12**
**Region 2**	**289,361**	**115,851**	**109,735**	**225,586**	**77.96**	**365,905**	**62,149**	**59,998**	**122,147**	**33.38**	**688,024**	**86,149**	**82,886**	**169,035**	**24.57**
Batanes	**1,627**	238	271	**509**	**31.28**	**1,970**	1	3	**4**	**0.20**	**3,424**	12	15	**27**	**0.79**
Cagayan	**85,819**	34,949	33,046	**67,995**	**79.23**	**108,623**	13,446	12,570	**26,016**	**23.95**	**206,463**	18,034	16,929	**34,963**	**16.93**
Isabela	**97,953**	47,520	45,214	**92,734**	**94.67**	**124,800**	29,060	28,641	**57,701**	**46.23**	**234,701**	42,516	40,998	**83,514**	**35.58**
Nueva Vizcaya	**40,154**	10,341	9,741	**20,082**	**50.01**	**49,688**	2,369	2,459	**4,828**	**9.72**	**93,592**	1,901	2,166	**4,067**	**4.35**
Quirino	**17,287**	4,644	4,365	**9,009**	**52.11**	**21,668**	1,786	1,679	**3,465**	**15.99**	**38,258**	1,087	929	**2,016**	**5.27**
Cauayan City	**10,715**	634	603	**1,237**	**11.54**	**13,657**	293	296	**589**	**4.31**	**25,680**	195	168	**363**	**1.41**
Ilagan City	**12,047**	6,244	5,682	**11,926**	**99.00**	**15,344**	7,754	7,416	**15,170**	**98.87**	**28,865**	10,762	10,502	**21,264**	**73.67**
Santiago City	**11,159**	5,725	5,434	**11,159**	**100.00**	**14,212**	6,355	5,890	**12,245**	**86.16**	**26,736**	11,170	10,701	**21,871**	**81.80**
Tuguegarao City	**12,600**	5,556	5,379	**10,935**	**86.79**	**15,943**	1,085	1,044	**2,129**	**13.35**	**30,305**	472	478	**950**	**3.13**
**Region 3**	**913,147**	**446,061**	**373,409**	**819,470**	**89.74**	**1,160,787**	**460,725**	**376,639**	**837,364**	**72.14**	**2,330,062**	**684,726**	**576,785**	**1,261,511**	**54.14**
Aurora	**20,431**	8,612	8,482	**17,094**	**83.67**	**24,576**	9,979	9,686	**19,665**	**80.02**	**45,578**	17,102	16,713	**33,815**	**74.19**
Bataan	**54,674**	22,596	21,985	**44,581**	**81.54**	**69,465**	15,501	13,295	**28,796**	**41.45**	**137,149**	14,122	13,332	**27,454**	**20.02**
Bulacan	**181,432**	90,746	90,182	**180,928**	**99.72**	**232,852**	116,770	112,081	**228,851**	**98.28**	**472,290**	186,828	195,194	**382,022**	**80.89**
Nueva Ecija	**122,537**	39,120	37,090	**76,210**	**62.19**	**156,072**	22,397	21,212	**43,609**	**27.94**	**306,967**	28,329	27,429	**55,758**	**18.16**
Pampanga	**131,844**	60,964	60,974	**121,938**	**92.49**	**167,978**	67,697	67,706	**135,403**	**80.61**	**344,023**	105,352	105,359	**210,711**	**61.25**
Tarlac	**79,847**	71,669	8,483	**80,152**	**100.38**	**101,338**	76,812	7,860	**84,672**	**83.55**	**206,110**	117,936	13,012	**130,948**	**63.53**
Zambales	**50,671**	19,036	17,941	**36,977**	**72.97**	**62,546**	21,091	19,567	**40,658**	**65.00**	**121,213**	31,896	30,373	**62,269**	**51.37**
Angeles City	**33,080**	3,579	3,228	**6,807**	**20.58**	**42,137**	2,708	2,468	**5,176**	**12.28**	**86,316**	1,597	1,554	**3,151**	**3.65**
Balanga City	**7,905**	3,404	3,290	**6,694**	**84.68**	**10,039**	3,875	3,467	**7,342**	**73.13**	**19,820**	5,106	4,110	**9,216**	**46.50**
Cabanatuan City	**25,080**	16,054	16,108	**32,162**	**128.24**	**31,953**	14,594	13,313	**27,907**	**87.34**	**62,823**	20,389	18,374	**38,763**	**61.70**
City of San Fernando	**24,640**	26,103	23,641	**49,744**	**201.88**	**31,391**	23,058	20,895	**43,953**	**140.02**	**64,303**	26,310	24,161	**50,471**	**78.49**
Gapan City	**9,152**	4,197	3,997	**8,194**	**89.53**	**11,662**	2,032	1,945	**3,977**	**34.10**	**22,931**	787	708	**1,495**	**6.52**
Mabalacat City	**20,155**	20,519	18,867	**39,386**	**195.42**	**25,675**	22,459	23,283	**45,742**	**178.16**	**52,590**	37,324	35,117	**72,441**	**137.75**
Malolos City	**20,270**	6,939	6,654	**13,593**	**67.06**	**26,007**	8,767	8,336	**17,103**	**65.76**	**52,753**	13,984	13,244	**27,228**	**51.61**
Meycauayan	**16,812**	1,073	1,006	**2,079**	**12.37**	**21,570**	1,309	1,361	**2,670**	**12.38**	**43,757**	1,746	2,100	**3,846**	**8.79**
Olongapo	**19,984**	5,168	4,818	**9,986**	**49.97**	**24,668**	4,189	4,055	**8,244**	**33.42**	**47,810**	4,310	4,313	**8,623**	**18.04**
Palayan City	**3,405**	1,550	1,529	**3,079**	**90.43**	**4,338**	1,784	1,727	**3,511**	**80.94**	**8,532**	1,962	1,989	**3,951**	**46.31**
San Jose City	**11,599**	4,337	4,042	**8,379**	**72.24**	**14,771**	5,726	5,206	**10,932**	**74.01**	**29,049**	8,801	8,179	**16,980**	**58.45**
San Jose del Monte City	**46,153**	18,089	18,364	**36,453**	**78.98**	**59,227**	14,947	14,655	**29,602**	**49.98**	**120,139**	17,966	17,094	**35,060**	**29.18**
Science City of Munoz	**6,759**	2,790	2,643	**5,433**	**80.38**	**8,615**	629	592	**1,221**	**14.17**	**16,939**	980	971	**1,951**	**11.52**
Tarlac City	**26,717**	19,516	20,085	**39,601**	**148.22**	**33,907**	24,401	23,929	**48,330**	**142.54**	**68,970**	41,899	43,459	**85,358**	**123.76**
**Region 4A**	**1,238,585**	**304,194**	**287,314**	**591,508**	**47.76**	**1,557,324**	**318,035**	**304,281**	**622,316**	**39.96**	**3,088,213**	**431,743**	**425,059**	**856,802**	**27.74**
Batangas	**165,380**	69,069	62,933	**132,002**	**79.82**	**204,337**	85,082	78,919	**164,001**	**80.26**	**406,963**	124,011	121,098	**245,109**	**60.23**
Cavite	**115,721**	46,457	41,774	**88,231**	**76.24**	**147,524**	45,943	42,687	**88,630**	**60.08**	**299,232**	55,968	54,197	**110,165**	**36.82**
Laguna	**80,424**	20,296	19,497	**39,793**	**49.48**	**101,782**	19,149	19,147	**38,296**	**37.63**	**201,518**	25,610	24,641	**50,251**	**24.94**
Quezon	**160,430**	35,571	32,905	**68,476**	**42.68**	**199,889**	36,306	35,021	**71,327**	**35.68**	**380,590**	60,761	59,799	**120,560**	**31.68**
Rizal	**181,775**	18,043	18,441	**36,484**	**20.07**	**228,559**	16,779	17,743	**34,522**	**15.10**	**452,884**	15,454	15,272	**30,726**	**6.78**
Antipolo City	**66,955**	873	998	**1,871**	**2.79**	**84,187**	86	89	**175**	**0.21**	**166,808**	140	52	**192**	**0.12**
Bacoor City	**50,703**	3,356	3,373	**6,729**	**13.27**	**64,626**	1,519	1,423	**2,942**	**4.55**	**131,095**	804	786	**1,590**	**1.21**
Batangas City	**29,351**	1,537	1,418	**2,955**	**10.07**	**36,266**	1,162	1,090	**2,252**	**6.21**	**72,227**	1,588	1,624	**3,212**	**4.45**
Biñan City	**26,965**	19,006	18,738	**37,744**	**139.97**	**34,128**	22,406	21,385	**43,791**	**128.31**	**67,575**	35,049	36,491	**71,540**	**105.87**
Cabuyao City	**24,996**	3,940	3,913	**7,853**	**31.42**	**31,640**	3,077	2,951	**6,028**	**19.05**	**62,650**	7,441	7,152	**14,593**	**23.29**
Calamba City	**36,801**	8,650	8,039	**16,689**	**45.35**	**46,570**	11,103	10,361	**21,464**	**46.09**	**92,218**	10,332	10,692	**21,024**	**22.80**
Cavite City	**8,682**	679	682	**1,361**	**15.68**	**11,068**	892	844	**1,736**	**15.68**	**22,434**	988	1,101	**2,089**	**9.31**
Dasmariñas City	**55,633**	15,759	15,437	**31,196**	**56.07**	**70,910**	10,417	10,218	**20,635**	**29.10**	**143,849**	10,806	10,634	**21,440**	**14.90**
General Trias City	**26,535**	10,701	10,670	**21,371**	**80.54**	**33,821**	13,325	13,342	**26,667**	**78.85**	**68,606**	15,724	15,741	**31,465**	**45.86**
Imus City	**34,083**	7,423	7,081	**14,504**	**42.55**	**43,453**	6,303	5,978	**12,281**	**28.26**	**88,135**	7,200	7,794	**14,994**	**17.01**
Lipa City	**29,578**	445	497	**942**	**3.18**	**36,537**	11	9	**20**	**0.05**	**72,772**	0	0	**0**	**0.00**
Lucena City	**24,313**	7,197	6,952	**14,149**	**58.20**	**30,295**	6,052	6,191	**12,243**	**40.41**	**57,681**	8,521	8,826	**17,347**	**30.07**
San Pablo City	**21,543**	3,010	2,768	**5,778**	**26.82**	**27,260**	2,809	2,748	**5,557**	**20.39**	**53,987**	2,842	2,704	**5,546**	**10.27**
San Pedro City	**26,381**	1,795	1,944	**3,739**	**14.17**	**33,387**	1,041	961	**2,002**	**6.00**	**66,110**	848	849	**1,697**	**2.57**
Santa Rosa City	**28,642**	15,085	14,985	**30,070**	**104.99**	**36,253**	15,667	14,976	**30,643**	**84.53**	**71,784**	16,911	15,871	**32,782**	**45.67**
Tagaytay City	**6,012**	2,990	2,767	**5,757**	**95.76**	**7,663**	3,466	3,365	**6,831**	**89.14**	**15,536**	7,064	7,169	**14,233**	**91.61**
Tanauan City	**15,428**	1,947	1,964	**3,911**	**25.35**	**19,060**	1,527	1,541	**3,068**	**16.10**	**37,952**	1,395	1,370	**2,765**	**7.29**
Tayabas City	**9,107**	7,654	7,186	**14,840**	**162.95**	**11,353**	9,982	9,378	**19,360**	**170.53**	**21,617**	15,927	15,424	**31,351**	**145.03**
Trece Martires City	**13,147**	2,711	2,352	**5,063**	**38.51**	**16,756**	3,931	3,914	**7,845**	**46.82**	**33,990**	6,359	5,772	**12,131**	**35.69**
**Region 4B**	**304,748**	**116,800**	**110,542**	**227,342**	**74.60**	**375,540**	**127,043**	**128,303**	**255,346**	**67.99**	**669,431**	**213,634**	**204,475**	**418,109**	**62.46**
Marinduque	**22,343**	9,866	8,815	**18,681**	**83.61**	**27,473**	9,886	9,032	**18,918**	**68.86**	**48,572**	17,354	16,810	**34,164**	**70.34**

Table 2.C.3.1 -Deworming Services for Children and Adolescents

Number and Proportion of Children/Adolescents who completed 2 doses of deworming tablet
Philippines, 2022

Area	Eligible Pop (1-4 years old)	No. of children 1-4 y/o given 2 doses of deworming drugs				Eligible Pop (5-9 years old)	No. of children 5-9 y/o given 2 doses of deworming drugs				Eligible Pop (10-19 years old)	No. of children 10-19 y/o given 2 doses of deworming drugs			
		Male	Female	Total	%		Male	Female	Total	%		Male	Female	Total	%
Mindoro Occidental	**50,640**	20,482	19,651	**40,133**	**79.25**	**62,712**	27,803	25,992	**53,795**	**85.78**	**110,861**	45,866	45,217	**91,083**	**82.16**
Mindoro Oriental	**84,729**	35,676	33,203	**68,879**	**81.29**	**105,481**	40,018	37,780	**77,798**	**73.76**	**187,248**	73,075	69,628	**142,703**	**76.21**
Palawan	**90,740**	33,681	32,572	**66,253**	**73.01**	**111,233**	33,663	31,897	**65,560**	**58.94**	**200,456**	54,948	51,180	**106,128**	**52.94**
Romblon	**29,043**	9,655	8,992	**18,647**	**64.20**	**35,224**	5,860	5,503	**11,363**	**32.26**	**62,087**	7,765	7,466	**15,231**	**24.53**
Puerto Princesa City	**27,253**	7,440	7,309	**14,749**	**54.12**	**33,417**	9,813	18,099	**27,912**	**83.53**	**60,207**	14,626	14,174	**28,800**	**47.83**
**Region 5**	595,917	169,888	160,873	330,761	55.50	706,890	250,770	229,818	480,588	67.99	1,291,879	379,035	359,842	738,877	57.19
Albay	**104,616**	24,039	23,027	**47,066**	**44.99**	**127,856**	35,018	31,631	**66,649**	**52.13**	**241,395**	57,114	54,796	**111,910**	**46.36**
Camarines Norte	**60,281**	18,210	17,370	**35,580**	**59.02**	**70,639**	27,598	25,787	**53,385**	**75.57**	**128,624**	44,981	43,498	**88,479**	**68.79**
Camarines Sur	**169,027**	50,921	48,804	**99,725**	**59.00**	**201,633**	82,701	78,846	**161,547**	**80.12**	**367,442**	134,806	127,956	**262,762**	**71.51**
Catanduanes	**27,331**	7,543	7,477	**15,020**	**54.96**	**32,117**	12,849	11,482	**24,331**	**75.76**	**57,004**	20,404	19,082	**39,486**	**69.27**
Masbate	**99,694**	26,185	25,101	**51,286**	**51.44**	**114,354**	47,961	39,384	**87,345**	**76.38**	**207,060**	47,689	44,433	**92,122**	**44.49**
Sorsogon	**84,950**	27,396	24,881	**52,277**	**61.54**	**100,075**	25,592	23,955	**49,547**	**49.51**	**179,147**	44,114	41,617	**85,731**	**47.86**
Iriga City	**11,482**	3,933	3,394	**7,327**	**63.81**	**13,702**	5,346	5,112	**10,458**	**76.32**	**24,970**	9,965	9,663	**19,628**	**78.61**
Legaspi City	**18,395**	7,624	7,014	**14,638**	**79.58**	**22,488**	9,051	9,053	**18,104**	**80.51**	**42,448**	14,259	13,288	**27,547**	**64.90**
Naga City	**20,141**	4,037	3,805	**7,842**	**38.94**	**24,026**	4,654	4,568	**9,222**	**38.38**	**43,789**	5,703	5,509	**11,212**	**25.60**
**Region 6**	623,453	205,260	198,309	403,569	64.73	775,969	194,670	188,970	383,640	49.44	1,496,586	335,090	324,573	659,663	44.08
Aklan	**48,370**	17,942	17,260	**35,202**	**72.78**	**60,505**	23,187	23,145	**46,332**	**76.58**	**113,962**	42,934	41,655	**84,589**	**74.23**
Antique	**55,203**	19,677	18,709	**38,386**	**69.54**	**68,857**	23,492	22,470	**45,962**	**66.75**	**122,111**	40,944	39,731	**80,675**	**66.07**
Capiz	**60,007**	23,094	22,918	**46,012**	**76.68**	**73,668**	26,050	27,173	**53,223**	**72.25**	**147,929**	43,690	43,347	**87,037**	**58.84**
Guimaras	**14,498**	5,265	5,017	**10,282**	**70.92**	**17,617**	7,534	7,180	**14,714**	**83.52**	**34,038**	14,669	14,228	**28,897**	**84.90**
Iloilo	**152,501**	65,793	63,222	**129,015**	**84.60**	**191,695**	80,929	77,699	**158,628**	**82.75**	**379,498**	145,931	141,400	**287,331**	**75.71**
Negros Occidental	**210,270**	47,584	47,589	**95,173**	**45.26**	**260,638**	21,834	20,703	**42,537**	**16.32**	**498,985**	28,921	28,119	**57,040**	**11.43**
Bacolod City	**47,314**	12,737	11,323	**24,060**	**50.85**	**58,643**	11,644	10,600	**22,244**	**37.93**	**112,269**	18,001	16,093	**34,094**	**30.37**
Iloilo City	**35,290**	13,168	12,271	**25,439**	**72.09**	**44,346**	0	0	**0**	**0.00**	**87,794**	0	0	**0**	**0.00**
**Region 7**	648,479	152,737	143,802	296,539	45.73	812,744	73,553	68,943	142,496	17.53	1,542,671	94,219	89,318	183,537	11.90
Bohol	**117,825**	38,887	37,030	**75,917**	**64.43**	**146,548**	12,020	11,182	**23,202**	**15.83**	**271,202**	12,656	11,473	**24,129**	**8.90**
Cebu	**259,039**	57,991	53,913	**111,904**	**43.20**	**326,959**	29,216	27,456	**56,672**	**17.33**	**623,368**	42,836	41,642	**84,478**	**13.55**
Negros Oriental	**114,713**	9,727	9,008	**18,735**	**16.33**	**141,100**	3,674	3,660	**7,334**	**5.20**	**270,562**	5,824	2,679	**8,503**	**3.14**
Siquijor	**7,654**	3,523	3,607	**7,130**	**93.15**	**9,756**	4,424	4,324	**8,748**	**89.67**	**18,358**	10,075	10,443	**20,518**	**111.77**
Cebu City	**81,316**	30,500	28,559	**59,059**	**72.63**	**102,635**	8,172	7,655	**15,827**	**15.42**	**195,693**	3,935	4,065	**8,000**	**4.09**
Lapu-Lapu City	**35,968**	3,460	3,244	**6,704**	**18.64**	**45,399**	1,309	1,249	**2,558**	**5.63**	**86,564**	682	794	**1,476**	**1.71**
Mandaue City	**31,964**	8,649	8,441	**17,090**	**53.47**	**40,347**	14,738	13,417	**28,155**	**69.78**	**76,924**	18,211	18,222	**36,433**	**47.36**
**Region 8**	445,167	82,188	82,940	165,128	37.09	531,452	52,186	51,978	104,164	19.60	980,557	74,376	74,302	148,678	15.16
Biliran	**16,955**	6,020	5,408	**11,428**	**67.40**	**19,880**	5,767	5,004	**10,771**	**54.18**	**35,845**	8,952	9,249	**18,201**	**50.78**
Eastern Samar	**48,401**	11,004	11,131	**22,135**	**45.73**	**58,185**	2,425	2,381	**4,806**	**8.26**	**105,352**	1,643	1,690	**3,333**	**3.16**
Northern Leyte	**146,531**	22,438	22,306	**44,744**	**30.54**	**176,953**	8,947	9,084	**18,031**	**10.19**	**332,231**	12,409	12,364	**24,773**	**7.46**
Northern Samar	**69,862**	15,813	15,403	**31,216**	**44.68**	**81,192**	15,379	15,885	**31,264**	**38.51**	**145,644**	28,352	27,710	**56,062**	**38.49**
Southern Leyte	**29,766**	9,674	9,057	**18,731**	**62.93**	**36,786**	6,864	6,176	**13,040**	**35.45**	**68,278**	8,936	8,596	**17,532**	**25.68**
Western Samar	**62,466**	4,569	7,102	**11,671**	**18.68**	**73,014**	1,622	2,038	**3,660**	**5.01**	**133,960**	2,519	2,414	**4,933**	**3.68**
Calbayog City	**19,250**	7,600	7,700	**15,300**	**79.48**	**22,514**	7,591	7,655	**15,246**	**67.72**	**41,280**	8,612	8,909	**17,521**	**42.44**
Maasin City	**7,575**	1,197	1,107	**2,304**	**30.42**	**9,362**	1,053	935	**1,988**	**21.23**	**17,375**	1,910	2,171	**4,081**	**23.49**
Ormoc City	**20,865**	2,439	2,201	**4,640**	**22.24**	**25,196**	1,163	1,275	**2,438**	**9.68**	**47,315**	227	301	**528**	**1.12**
Tacloban City	**23,496**	1,434	1,525	**2,959**	**12.59**	**28,370**	1,375	1,545	**2,920**	**10.29**	**53,277**	816	898	**1,714**	**3.22**
**Region 9**	335,069	144,330	146,209	290,539	86.71	406,544	195,303	195,101	390,404	96.03	771,448	343,082	343,480	686,562	89.00
Zamboanga del Norte	**73,193**	33,416	33,660	**67,076**	**91.64**	**87,946**	44,000	43,465	**87,465**	**99.45**	**166,998**	80,701	82,352	**163,053**	**97.64**
Zamboanga del Sur	**70,988**	29,775	29,325	**59,100**	**83.25**	**87,332**	38,913	39,725	**78,638**	**90.04**	**168,775**	73,832	75,473	**149,305**	**88.46**
Zamboanga Sibugay	**66,344**	24,517	23,540	**48,057**	**72.44**	**79,178**	33,556	33,809	**67,365**	**85.08**	**143,298**	63,596	60,733	**124,329**	**86.76**
Dapitan City	**7,557**	3,516	3,966	**7,482**	**99.01**	**9,082**	4,203	4,707	**8,910**	**98.11**	**17,244**	7,899	8,713	**16,612**	**96.33**
Dipolog City	**11,987**	5,361	7,839	**13,200**	**110.12**	**14,406**	6,801	9,945	**16,746**	**116.24**	**27,362**	12,234	17,912	**30,146**	**110.17**
Isabela City	**12,229**	6,267	7,385	**13,652**	**111.64**	**14,461**	8,378	8,610	**16,988**	**117.47**	**27,173**	10,693	13,069	**23,762**	**87.45**
Pagadian City	**17,408**	18,219	15,751	**33,970**	**195.14**	**21,417**	24,437	20,050	**44,487**	**207.72**	**41,391**	40,661	27,584	**68,245**	**164.88**
Zamboanga City	**75,363**	23,259	24,743	**48,002**	**63.69**	**92,722**	35,015	34,790	**69,805**	**75.28**	**179,207**	53,466	57,644	**111,110**	**62.00**
**Region 10**	428,356	125,290	124,550	249,840	58.33	521,123	173,993	170,535	344,528	66.11	994,909	296,247	287,919	584,166	58.72
Bukidnon	**97,101**	26,102	27,673	**53,775**	**55.38**	**117,189**	36,719	36,673	**73,392**	**62.63**	**223,834**	67,189	63,886	**131,075**	**58.56**
Camiguin	**7,996**	2,869	2,794	**5,663**	**70.82**	**9,551**	4,212	4,051	**8,263**	**86.51**	**17,941**	8,150	8,268	**16,418**	**91.51**
Lanao del Norte	**67,342**	9,122	9,208	**18,330**	**27.22**	**80,919**	11,379	10,957	**22,336**	**27.60**	**153,243**	18,598	19,705	**38,303**	**24.99**
Misamis Occidental	**27,481**	10,049	10,226	**20,275**	**73.78**	**34,162**	15,692	15,771	**31,463**	**92.10**	**65,668**	27,114	28,250	**55,364**	**84.31**
Misamis Oriental	**62,525**	18,859	17,139	**35,998**	**57.57**	**76,800**	25,934	23,407	**49,341**	**64.25**	**147,169**	41,459	40,440	**81,899**	**55.65**
Cagayan de Oro City	**59,215**	14,247	16,103	**30,350**	**51.25**	**72,747**	18,867	21,068	**39,935**	**54.90**	**139,390**	18,348	20,067	**38,415**	**27.56**
El Salvador City	**4,397**	2,485	1,658	**4,143**	**94.22**	**5,404**	4,079	2,719	**6,798**	**125.80**	**10,355**	6,147	4,098	**10,245**	**98.94**
Gingoog City	**10,919**	6,710	6,313	**13,023**	**119.27**	**13,423**	10,316	10,874	**21,190**	**157.86**	**25,703**	21,142	19,375	**40,517**	**157.64**
Iligan City	**34,120**	13,571	12,678	**26,249**	**76.93**	**40,984**	14,310	14,445	**28,755**	**70.16**	**77,623**	21,448	21,056	**42,504**	**54.76**
Malaybalay City	**16,184**	6,595	6,231	**12,826**	**79.25**	**19,533**	11,367	10,584	**21,951**	**112.38**	**37,310**	19,575	17,917	**37,492**	**100.49**
Oroquieta City	**5,954**	3,886	3,642	**7,528**	**126.44**	**7,401**	5,456	5,103	**10,559**	**142.67**	**14,233**	19,669	18,315	**37,984**	**266.87**
Ozamis City	**11,936**	5,761	5,928	**11,689**	**97.93**	**14,834**	7,497	7,494	**14,991**	**101.06**	**28,528**	14,697	14,382	**29,079**	**101.93**
Tangub City	**5,300**	2,378	2,197	**4,575**	**86.32**	**6,590**	4,783	4,347	**9,130**	**138.54**	**12,673**	7,271	7,006	**14,277**	**112.66**
Valencia City	**17,886**	2,656	2,760	**5,416**	**30.28**	**21,586**	3,382	3,042	**6,424**	**29.76**	**41,239**	5,440	5,154	**10,594**	**25.69**
**Region 11**	452,922	200,482	191,347	391,829	86.51	563,918	96,430	89,108	185,538	32.90	1,051,315	104,546	97,867	202,413	19.25
Davao de Oro	**67,865**	29,816	27,970	**57,786**	**85.15**	**82,518**	10,057	9,780	**19,837**	**24.04**	**151,192**	15,762	14,719	**30,481**	**20.16**
Davao del Norte	**86,391**	57,405	55,478	**112,883**	**130.67**	**108,621**	17,594	16,562	**34,156**	**31.45**	**203,223**	17,018	16,313	**33,331**	**16.40**
Davao Oriental	**57,129**	16,460	14,625	**31,085**	**54.41**	**68,687**	5,620	3,895	**9,515**	**13.85**	**125,328**	3,792	3,431	**7,223**	**5.76**
Davao del Sur	**59,042**	12,901	12,134	**25,035**	**42.40**	**74,330**	4,620	4,499	**9,119**	**12.27**	**140,515**	8,467	7,946	**16,413**	**11.68**
Davao Occidental	**30,091**	5,841	5,989	**11,830**	**39.31**	**37,881**	6,035	5,487	**11,522**	**30.42**	**68,338**	11,080	10,877	**21,957**	**32.13**
Davao City	**152,404**	78,059	75,151	**153,210**	**100.53**	**191,881**	52,504	48,885	**101,389**	**52.84**	**362,719**	48,427	44,581	**93,008**	**25.64**
**Region 12**	409,593	124,919	120,061	244,980	59.81	505,576	121,590	118,013	239,603	47.39	942,855	190,680	187,462	378,142	40.11
North Cotabato	**141,044**	43,980	42,352	**86,332**	**61.21**	**173,779**	34,002	32,934	**66,936**	**38.52**	**319,178**	46,264	45,660	**91,924**	**28.80**
Sarangani	**54,628**	15,892	14,765	**30,657**	**56.12**	**66,433**	15,527	14,964	**30,491**	**45.90**	**124,927**	30,328	28,838	**59,166**	**47.36**
South Cotabato	**84,448**	19,071	18,981	**38,052**	**45.06**	**104,409**	18,652	18,133	**36,785**	**35.23**	**197,535**	26,483	25,746	**52,229**	**26.44**
Sultan Kudarat	**74,631**	25,992	25,388	**51,380**	**68.85**	**93,146**	31,704	31,846	**63,550**	**68.23**	**172,914**	60,280	60,705	**120,985**	**69.97**
Gen. Santos City	**54,842**	19,984	18,575	**38,559**	**70.31**	**67,809**	21,705	20,136	**41,841**	**61.70**	**128,301**	27,325	26,513	**53,838**	**41.96**
**B.A.R.M.M.**	525,340	61,632	63,023	124,655	23.73	605,438	62,757	76,917	139,674	23.07	1,020,727	97,261	117,840	215,101	21.07
Basilan	**36,827**	1,117	1,183	**2,300**	**6.25**	**42,144**	1,216	1,449	**2,665**	**6.32**	**70,689**	2,007	2,503	**4,510**	**6.38**
Lanao del Sur	**112,850**	0	0	**0**	**0.00**	**128,261**	0	0	**0**	**0.00**	**209,923**	0	0	**0**	**0.00**
Maguindanao	**154,029**	4,416	4,043	**8,459**	**5.49**	**179,697**	5,000	4,141	**9,141**	**5.09**	**305,831**	6,464	5,882	**12,346**	**4.04**
Sulu	**110,769**	19,215	22,014	**41,229**	**37.22**	**125,008**	21,516	28,009	**49,525**	**39.62**	**207,875**	34,824	40,404	**75,228**	**36.19**
Tawi-Tawi	**45,443**	15,307	15,166	**30,473**	**67.06**	**53,100**	14,587	20,684	**35,271**	**66.42**	**92,418**	22,331	27,535	**49,866**	**53.96**
Lamitan City	**10,136**	9	8	**17**	**0.17**	**11,601**	22	25	**47**	**0.41**	**19,449**	16	15	**31**	**0.16**
Marawi City	**26,991**	10,369	10,620	**20,989**	**77.76**	**30,680**	10,497	10,723	**21,220**	**69.17**	**50,191**	14,995	24,545	**39,540**	**78.78**
Cotabato City	**28,295**	11,199	9,989	**21,188**	**74.88**	**34,947**	9,919	11,886	**21,805**	**62.39**	**64,351**	16,624	16,956	**33,580**	**52.18**

Table 2.C.3.1 -Deworming Services for Children and Adolescents

Number and Proportion of Children/Adolescents who completed 2 doses of deworming tablet
Philippines, 2022

Area	Eligible Pop (1-4 years old)	No. of children 1-4 y/o given 2 doses of deworming drugs				Eligible Pop (5-9 years old)	No. of children 5-9 y/o given 2 doses of deworming drugs				Eligible Pop (10-19 years old)	No. of children 10-19 y/o given 2 doses of deworming drugs			
		Male	Female	Total	%		Male	Female	Total	%		Male	Female	Total	%
**CARAGA**	**260,938**	**88,615**	**85,968**	**174,583**	**66.91**	**308,852**	**109,033**	**105,836**	**214,869**	**69.57**	**557,779**	**186,160**	**178,372**	**364,532**	**65.35**
Agusan del Norte	**34,222**	16,600	15,810	**32,410**	**94.71**	**40,653**	19,238	18,592	**37,830**	**93.06**	**75,499**	33,806	31,528	**65,334**	**86.54**
Agusan del Sur	**77,316**	21,148	21,389	**42,537**	**55.02**	**87,642**	21,202	20,976	**42,178**	**48.13**	**150,989**	33,320	31,737	**65,057**	**43.09**
Surigao del Norte	**32,414**	8,240	8,043	**16,283**	**50.23**	**39,889**	12,750	12,136	**24,886**	**62.39**	**74,105**	23,778	22,704	**46,482**	**62.72**
Surigao del Sur	**48,819**	19,804	18,800	**38,604**	**79.08**	**58,177**	27,802	26,637	**54,439**	**93.57**	**105,260**	47,188	45,051	**92,239**	**87.63**
Province of Dinagat	**11,259**	2,951	2,783	**5,734**	**50.93**	**14,214**	4,296	4,151	**8,447**	**59.43**	**25,630**	7,640	6,989	**14,629**	**57.08**
Bislig City	**9,270**	2,311	2,175	**4,486**	**48.39**	**11,049**	1,188	1,124	**2,312**	**20.92**	**19,992**	2,341	2,115	**4,456**	**22.29**
Butuan City	**32,536**	13,261	13,018	**26,279**	**80.77**	**38,652**	16,807	16,836	**33,643**	**87.04**	**71,789**	26,149	26,786	**52,935**	**73.74**
Surigao City	**15,102**	4,300	3,950	**8,250**	**54.63**	**18,576**	5,750	5,384	**11,134**	**59.94**	**34,515**	11,938	11,462	**23,400**	**67.80**

Table 2.C.4.1. Management of Sick Infants and Children

Number of high risk infants and children with measles and/or persistent diarrhea who received Vitamin A capsule aside from routine supplementation
Philippines, 2022

Area	Sick Infants aged 6-11 mos.							Sick Children aged 12-59 mos.						
	Seen			given vitamin A 100,000 IU				Seen			given vitamin A 200,000 IU			
	Male	Female	Total	Male	Female	Total	%	Male	Female	Total	Male	Female	Total	%
**PHILIPPINES**	**70,427**	**66,725**	**137,152**	**35,996**	**34,030**	**70,026**	**51.06**	**146,406**	**136,041**	**282,447**	**63,376**	**60,733**	**124,109**	**43.94**
**N C R**	**2,057**	**1,978**	**4,035**	**1,459**	**1,441**	**2,900**	**71.87**	**4,364**	**4,236**	**8,600**	**2,518**	**2,454**	**4,972**	**57.81**
Malabon	545	486	**1,031**	222	203	**425**	**41.22**	1,773	1,795	**3,568**	591	527	**1,118**	**31.33**
Navotas	294	304	**598**	226	230	**456**	**76.25**	636	686	**1,322**	476	530	**1,006**	**76.10**
Valenzuela City	39	10	**49**	8	2	**10**	**20.41**	61	25	**86**	8	15	**23**	**26.74**
Caloocan City	123	230	**353**	123	190	**313**	**88.67**	131	149	**280**	131	149	**280**	**100.00**
Marikina City	10	6	**16**	10	6	**16**	**100.00**	19	18	**37**	13	15	**28**	**75.68**
Pasig City	0	0	**0**	0	0	**0**	**0.00**	0	1	**1**	0	1	**1**	**100.00**
Pateros	5	7	**12**	5	7	**12**	**100.00**	12	7	**19**	12	7	**19**	**100.00**
Taguig	253	216	**469**	164	143	**307**	**65.46**	535	514	**1,049**	386	384	**770**	**73.40**
Quezon City	431	399	**830**	410	393	**803**	**96.75**	385	338	**723**	357	326	**683**	**94.47**
							**0.00**							**0.00**
Makati City	53	61	**114**	53	61	**114**	**100.00**	108	103	**211**	108	103	**211**	**100.00**
Mandaluyong City	5	3	**8**	5	3	**8**	**100.00**	2	1	**3**	2	1	**3**	**100.00**
San Juan	2	4	**6**	2	4	**6**	**100.00**	9	12	**21**	9	12	**21**	**100.00**
Manila City	86	72	**158**	86	72	**158**	**100.00**	191	183	**374**	191	183	**374**	**100.00**
							**0.00**							**0.00**
Las Piñas City	0	0	**0**	0	0	**0**	**0.00**	3	1	**4**	3	1	**4**	**100.00**
Muntinlupa City	73	63	**136**	73	63	**136**	**100.00**	100	93	**193**	100	93	**193**	**100.00**
Parañaque City	112	85	**197**	59	43	**102**	**51.78**	348	248	**596**	99	70	**169**	**28.36**
Pasay City	26	32	**58**	13	21	**34**	**58.62**	51	62	**113**	32	37	**69**	**61.06**
**C A R**	**592**	**479**	**1,071**	**372**	**300**	**672**	**62.75**	**1,481**	**1,388**	**2,869**	**737**	**643**	**1,380**	**48.10**
Abra	279	238	**517**	156	136	**292**	**56.48**	762	731	**1,493**	355	317	**672**	**45.01**
Apayao	83	69	**152**	24	18	**42**	**27.63**	153	175	**328**	53	54	**107**	**32.62**
Benguet	26	25	**51**	26	25	**51**	**100.00**	18	17	**35**	18	17	**35**	**100.00**
Ifugao	103	68	**171**	78	52	**130**	**76.02**	184	140	**324**	106	70	**176**	**54.32**
Kalinga	98	72	**170**	87	64	**151**	**88.82**	323	284	**607**	174	150	**324**	**53.38**
Mt. Province	2	2	**4**	0	0	**0**	**0.00**	15	12	**27**	5	6	**11**	**40.74**
Baguio City	1	5	**6**	1	5	**6**	**100.00**	26	29	**55**	26	29	**55**	**100.00**
**Region 1**	**2,843**	**2,509**	**5,352**	**2,245**	**1,990**	**4,235**	**79.13**	**4,942**	**4,465**	**9,407**	**3,431**	**3,118**	**6,549**	**69.62**
Ilocos Norte	89	105	**194**	69	76	**145**	**74.74**	220	189	**409**	183	160	**343**	**83.86**
Ilocos Sur	234	207	**441**	134	112	**246**	**55.78**	546	496	**1,042**	302	241	**543**	**52.11**
La Union	151	108	**259**	44	29	**73**	**28.19**	442	339	**781**	90	58	**148**	**18.95**
Pangasinan	1,934	1,744	**3,678**	1,563	1,429	**2,992**	**81.35**	3,179	2,943	**6,122**	2,415	2,273	**4,688**	**76.58**
Alaminos City	318	239	**557**	318	239	**557**	**100.00**	283	238	**521**	283	238	**521**	**100.00**
Candon City	40	29	**69**	40	28	**68**	**98.55**	72	54	**126**	72	54	**126**	**100.00**
Dagupan City	0	0	**0**	0	0	**0**	**0.00**	0	0	**0**	0	0	**0**	**0.00**
Laoag City	0	0	**0**	0	0	**0**	**0.00**	0	0	**0**	0	0	**0**	**0.00**
San Carlos City	69	72	**141**	69	72	**141**	**100.00**	74	83	**157**	74	83	**157**	**100.00**
San Fernando City	0	0	**0**	0	0	**0**	**0.00**	121	117	**238**	7	5	**12**	**5.04**
Urdaneta City	8	5	**13**	8	5	**13**	**100.00**	5	6	**11**	5	6	**11**	**100.00**
Vigan City	0	0	**0**	0	0	**0**	**0.00**	0	0	**0**	0	0	**0**	**0.00**
**Region 2**	**4,730**	**4,156**	**8,886**	**1,988**	**1,854**	**3,842**	**43.24**	**8,236**	**7,589**	**15,825**	**3,571**	**3,496**	**7,067**	**44.66**
Batanes	0	0	**0**	0	0	**0**	**0.00**	0	3	**3**	0	3	**3**	**100.00**
Cagayan	942	815	**1,757**	337	290	**627**	**35.69**	1,085	841	**1,926**	435	342	**777**	**40.34**
Isabela	2,843	2,464	**5,307**	811	791	**1,602**	**30.19**	5,074	4,688	**9,762**	1,624	1,697	**3,321**	**34.02**
Nueva Vizcaya	148	156	**304**	128	133	**261**	**85.86**	374	348	**722**	197	193	**390**	**54.02**
Quirino	120	130	**250**	110	116	**226**	**90.40**	248	285	**533**	192	214	**406**	**76.17**
Cauayan City	501	432	**933**	496	428	**924**	**99.04**	925	840	**1,765**	859	785	**1,644**	**93.14**
Ilagan City	174	155	**329**	105	95	**200**	**60.79**	529	582	**1,111**	263	260	**523**	**47.07**
Santiago City	0	0	**0**	0	0	**0**	**0.00**	0	0	**0**	0	0	**0**	**0.00**
Tuguegarao City	2	4	**6**	1	1	**2**	**33.33**	1	2	**3**	1	2	**3**	**100.00**
**Region 3**	**9,783**	**9,143**	**18,926**	**4,855**	**4,604**	**9,459**	**49.98**	**22,950**	**21,005**	**43,955**	**8,390**	**8,018**	**16,408**	**37.33**
Aurora	55	59	**114**	40	45	**85**	**74.56**	149	138	**287**	113	104	**217**	**75.61**
Bataan	757	662	**1,419**	124	121	**245**	**17.27**	1,736	1,551	**3,287**	274	235	**509**	**15.49**
Bulacan	2,371	2,212	**4,583**	1,367	1,301	**2,668**	**58.22**	6,293	6,039	**12,332**	3,103	3,014	**6,117**	**49.60**
Nueva Ecija	1,206	1,075	**2,281**	318	257	**575**	**25.21**	2,844	2,461	**5,305**	650	645	**1,295**	**24.41**
Pampanga	288	321	**609**	246	271	**517**	**84.89**	444	453	**897**	337	356	**693**	**77.26**
Tarlac	1,595	1,580	**3,175**	666	627	**1,293**	**40.72**	5,066	4,369	**9,435**	873	750	**1,623**	**17.20**
Zambales	435	448	**883**	159	169	**328**	**37.15**	1,218	1,195	**2,413**	387	375	**762**	**31.58**
Angeles City	494	426	**920**	384	316	**700**	**76.09**	738	679	**1,417**	499	442	**941**	**66.41**
Balanga City	0	1	**1**	0	0	**0**	**0.00**	0	0	**0**	0	0	**0**	**0.00**
Cabanatuan City	5	6	**11**	5	6	**11**	**100.00**	3	4	**7**	3	4	**7**	**100.00**
City of San Fernando	512	491	**1,003**	509	478	**987**	**98.40**	705	669	**1,374**	696	659	**1,355**	**98.62**
Gapan City	47	41	**88**	11	10	**21**	**23.86**	22	23	**45**	9	8	**17**	**37.78**

Table 2.C.4.1. Management of Sick Infants and Children

Number of high risk infants and children with measles and/or persistent diarrhea who received Vitamin A capsule aside from routine supplementation
Philippines, 2022

Area	Sick Infants aged 6-11 mos.							Sick Children aged 12-59 mos.						
	Seen			given vitamin A 100,000 IU				Seen			given vitamin A 200,000 IU			
	Male	Female	Total	Male	Female	Total	%	Male	Female	Total	Male	Female	Total	%
Mabalacat City	104	90	**194**	90	82	**172**	**88.66**	231	231	**462**	187	184	**371**	**80.30**
Malolos City	3	5	**8**	0	0	**0**	**0.00**	9	8	**17**	0	0	**0**	**0.00**
Meycauayan	1,462	1,251	**2,713**	582	547	**1,129**	**41.61**	2,643	2,404	**5,047**	696	710	**1,406**	**27.86**
Olongapo	52	62	**114**	11	14	**25**	**21.93**	169	136	**305**	27	14	**41**	**13.44**
Palayan City	0	0	**0**	0	0	**0**	**0.00**	0	0	**0**	0	0	**0**	**0.00**
San Jose City	3	2	**5**	3	2	**5**	**100.00**	21	27	**48**	19	25	**44**	**91.67**
San Jose del Monte City	71	71	**142**	71	71	**142**	**100.00**	129	142	**271**	129	142	**271**	**100.00**
Science City of Munoz	5	4	**9**	0	0	**0**	**0.00**	36	32	**68**	0	0	**0**	**0.00**
Tarlac City	318	336	**654**	269	287	**556**	**85.02**	494	444	**938**	388	351	**739**	**78.78**
**Region 4A**	**10,465**	**10,117**	**20,582**	**3,197**	**3,152**	**6,349**	**30.85**	**23,080**	**22,043**	**45,123**	**5,323**	**5,203**	**10,526**	**23.33**
Batangas	214	169	**383**	9	11	**20**	**5.22**	523	512	**1,035**	14	19	**33**	**3.19**
Cavite	2,491	2,593	**5,084**	601	615	**1,216**	**23.92**	4,722	4,540	**9,262**	792	759	**1,551**	**16.75**
Laguna	375	385	**760**	24	35	**59**	**7.76**	1,123	971	**2,094**	66	50	**116**	**5.54**
Quezon	1,925	1,774	**3,699**	467	426	**893**	**24.14**	4,106	3,731	**7,837**	723	651	**1,374**	**17.53**
Rizal	1,275	1,242	**2,517**	583	590	**1,173**	**46.60**	2,848	2,995	**5,843**	730	799	**1,529**	**26.17**
Antipolo City	169	235	**404**	115	178	**293**	**72.52**	432	456	**888**	192	193	**385**	**43.36**
Bacoor City	1,513	1,422	**2,935**	488	445	**933**	**31.79**	3,804	3,598	**7,402**	1,006	957	**1,963**	**26.52**
Batangas City	17	15	**32**	4	0	**4**	**12.50**	14	7	**21**	1	1	**2**	**9.52**
Biñan City	90	105	**195**	84	97	**181**	**92.82**	99	112	**211**	68	84	**152**	**72.04**
Cabuyao City	71	54	**125**	30	17	**47**	**37.60**	118	103	**221**	23	16	**39**	**17.65**
Calamba City	15	19	**34**	13	16	**29**	**85.29**	34	37	**71**	11	18	**29**	**40.85**
Cavite City	108	72	**180**	0	0	**0**	**0.00**	140	125	**265**	0	1	**1**	**0.38**
Dasmariñas City	564	494	**1,058**	266	248	**514**	**48.58**	879	861	**1,740**	414	409	**823**	**47.30**
General Trias City	151	169	**320**	19	15	**34**	**10.63**	364	342	**706**	23	25	**48**	**6.80**
Imus City	842	743	**1,585**	161	147	**308**	**19.43**	2,447	2,322	**4,769**	744	737	**1,481**	**31.05**
Lipa City	3	1	**4**	1	1	**2**	**50.00**	3	4	**7**	3	4	**7**	**100.00**
Lucena City	219	209	**428**	217	206	**423**	**98.83**	302	300	**602**	300	296	**596**	**99.00**
San Pablo City	68	59	**127**	8	11	**19**	**14.96**	79	63	**142**	11	15	**26**	**18.31**
San Pedro City	4	5	**9**	2	3	**5**	**55.56**	3	3	**6**	0	0	**0**	**0.00**
Santa Rosa City	195	206	**401**	66	56	**122**	**30.42**	309	329	**638**	104	97	**201**	**31.50**
Tagaytay City	94	95	**189**	12	13	**25**	**13.23**	240	294	**534**	19	18	**37**	**6.93**
Tanauan City	62	50	**112**	27	22	**49**	**43.75**	490	337	**827**	79	54	**133**	**16.08**
Tayabas City	0	0	**0**	0	0	**0**	**0.00**	1	1	**2**	0	0	**0**	**0.00**
Trece Martires City	0	1	**1**	0	0	**0**	**0.00**	0	0	**0**	0	0	**0**	**0.00**
**Region 4B**	**1,365**	**1,295**	**2,660**	**565**	**507**	**1,072**	**40.30**	**3,817**	**3,615**	**7,432**	**1,349**	**1,315**	**2,664**	**35.84**
Marinduque	72	85	**157**	49	61	**110**	**70.06**	168	138	**306**	127	86	**213**	**69.61**
Mindoro Occidental	575	540	**1,115**	194	177	**371**	**33.27**	1,275	1,157	**2,432**	219	215	**434**	**17.85**
Mindoro Oriental	270	260	**530**	118	103	**221**	**41.70**	707	712	**1,419**	197	208	**405**	**28.54**
Palawan	293	257	**550**	193	154	**347**	**63.09**	1,031	1,039	**2,070**	780	776	**1,556**	**75.17**
Romblon	139	135	**274**	11	7	**18**	**6.57**	575	508	**1,083**	15	20	**35**	**3.23**
Puerto Princesa City	16	18	**34**	0	5	**5**	**14.71**	61	61	**122**	11	10	**21**	**17.21**
**Region 5**	**4,117**	**3,962**	**8,079**	**1,914**	**1,724**	**3,638**	**45.03**	**9,328**	**8,983**	**18,311**	**4,103**	**4,096**	**8,199**	**44.78**
Albay	1,222	1,170	**2,392**	445	389	**834**	**34.87**	3,213	3,152	**6,365**	1,037	1,022	**2,059**	**32.35**
Camarines Norte	414	400	**814**	336	313	**649**	**79.73**	770	767	**1,537**	607	599	**1,206**	**78.46**
Camarines Sur	76	66	**142**	22	13	**35**	**24.65**	217	247	**464**	72	85	**157**	**33.84**
Catanduanes	43	25	**68**	37	23	**60**	**88.24**	400	368	**768**	17	20	**37**	**4.82**
Masbate	999	1,016	**2,015**	578	538	**1,116**	**55.38**	2,325	2,174	**4,499**	1,677	1,591	**3,268**	**72.64**
Sorsogon	817	821	**1,638**	228	226	**454**	**27.72**	1,420	1,415	**2,835**	332	344	**676**	**23.84**
Iriga City	55	50	**105**	54	45	**99**	**94.29**	73	84	**157**	63	79	**142**	**90.45**
Legaspi City	454	373	**827**	182	144	**326**	**39.42**	803	665	**1,468**	201	265	**466**	**31.74**
Naga City	37	41	**78**	32	33	**65**	**83.33**	107	111	**218**	97	91	**188**	**86.24**
**Region 6**	**4,569**	**4,325**	**8,894**	**2,020**	**2,049**	**4,069**	**45.75**	**10,973**	**10,470**	**21,443**	**3,930**	**3,773**	**7,703**	**35.92**
Aklan	510	466	**976**	352	338	**690**	**70.70**	1,351	1,323	**2,674**	1,113	1,081	**2,194**	**82.05**
Antique	509	531	**1,040**	131	133	**264**	**25.38**	1,008	1,006	**2,014**	217	220	**437**	**21.70**
Capiz	57	76	**133**	9	16	**25**	**18.80**	117	124	**241**	22	20	**42**	**17.43**
Guimaras	49	39	**88**	39	23	**62**	**70.45**	107	109	**216**	97	97	**194**	**89.81**
Iloilo	1,639	1,492	**3,131**	688	693	**1,381**	**44.11**	4,175	3,747	**7,922**	1,330	1,286	**2,616**	**33.02**
Negros Occidental	1,577	1,511	**3,088**	707	753	**1,460**	**47.28**	3,716	3,705	**7,421**	990	939	**1,929**	**25.99**
Bacolod City	103	105	**208**	67	69	**136**	**65.38**	135	133	**268**	103	86	**189**	**70.52**
Iloilo City	125	105	**230**	27	24	**51**	**22.17**	364	323	**687**	58	44	**102**	**14.85**
**Region 7**	**1,645**	**1,446**	**3,091**	**864**	**908**	**1,772**	**57.33**	**3,257**	**2,978**	**6,235**	**1,432**	**1,526**	**2,958**	**47.44**
Bohol	139	108	**247**	6	16	**22**	**8.91**	478	402	**880**	26	22	**48**	**5.45**
Cebu	566	546	**1,112**	282	347	**629**	**56.56**	1,150	1,118	**2,268**	535	689	**1,224**	**53.97**
Negros Oriental	17	23	**40**	20	29	**49**	**122.50**	15	29	**44**	4	12	**16**	**36.36**
Siquijor	64	61	**125**	1	0	**1**	**0.80**	313	264	**577**	2	0	**2**	**0.35**

Table 2.C.4.1. Management of Sick Infants and Children

Number of high risk infants and children with measles and/or persistent diarrhea who received Vitamin A capsule aside from routine supplementation
Philippines, 2022

Area	Sick Infants aged 6-11 mos.							Sick Children aged 12-59 mos.						
	Seen			given vitamin A 100,000 IU				Seen			given vitamin A 200,000 IU			
	Male	Female	Total	Male	Female	Total	%	Male	Female	Total	Male	Female	Total	%
Cebu City	613	464	**1,077**	346	298	**644**	**59.80**	963	817	**1,780**	611	522	**1,133**	**63.65**
Lapu-Lapu City	184	181	**365**	184	184	**368**	**100.82**	255	263	**518**	241	259	**500**	**96.53**
Mandaue City	62	63	**125**	25	34	**59**	**47.20**	83	85	**168**	13	22	**35**	**20.83**
**Region 8**	**6,080**	**5,632**	**11,712**	**2,647**	**2,415**	**5,062**	**43.22**	**10,791**	**10,240**	**21,031**	**3,520**	**3,247**	**6,767**	**32.18**
Biliran	241	213	**454**	46	49	**95**	**20.93**	560	610	**1,170**	80	92	**172**	**14.70**
Eastern Samar	1,371	1,255	**2,626**	838	718	**1,556**	**59.25**	1,670	1,587	**3,257**	211	185	**396**	**12.16**
Northern Leyte	760	750	**1,510**	417	399	**816**	**54.04**	1,401	1,403	**2,804**	601	559	**1,160**	**41.37**
Northern Samar	1,628	1,499	**3,127**	483	433	**916**	**29.29**	3,574	3,302	**6,876**	988	903	**1,891**	**27.50**
Southern Leyte	138	101	**239**	55	41	**96**	**40.17**	330	303	**633**	146	118	**264**	**41.71**
Western Samar	1,401	1,278	**2,679**	515	453	**968**	**36.13**	2,053	1,836	**3,889**	1,067	951	**2,018**	**51.89**
Calbayog City	84	73	**157**	2	7	**9**	**5.73**	223	211	**434**	4	5	**9**	**2.07**
Maasin City	8	13	**21**	4	4	**8**	**38.10**	22	24	**46**	19	21	**40**	**86.96**
Ormoc City	11	13	**24**	11	13	**24**	**100.00**	16	14	**30**	16	14	**30**	**100.00**
Tacloban City	438	437	**875**	276	298	**574**	**65.60**	942	950	**1,892**	388	399	**787**	**41.60**
**Region 9**	**3,476**	**3,305**	**6,781**	**2,372**	**2,271**	**4,643**	**68.47**	**6,420**	**6,092**	**12,512**	**4,185**	**3,909**	**8,094**	**64.69**
Zamboanga del Norte	1,316	1,269	**2,585**	818	816	**1,634**	**63.21**	2,148	2,129	**4,277**	1,303	1,269	**2,572**	**60.14**
Zamboanga del Sur	875	850	**1,725**	731	676	**1,407**	**81.57**	1,732	1,644	**3,376**	1,458	1,307	**2,765**	**81.90**
Zamboanga Sibugay	529	475	**1,004**	238	223	**461**	**45.92**	1,023	943	**1,966**	367	334	**701**	**35.66**
Dapitan City	24	23	**47**	23	23	**46**	**97.87**	33	36	**69**	33	36	**69**	**100.00**
Dipolog City	45	33	**78**	23	12	**35**	**44.87**	64	55	**119**	37	27	**64**	**53.78**
Isabela City	85	74	**159**	77	65	**142**	**89.31**	167	131	**298**	136	106	**242**	**81.21**
Pagadian City	0	0	**0**	0	0	**0**	**0.00**	0	0	**0**	0	0	**0**	**0.00**
Zamboanga City	602	581	**1,183**	462	456	**918**	**77.60**	1,253	1,154	**2,407**	851	830	**1,681**	**69.84**
**Region 10**	**6,247**	**6,446**	**12,693**	**3,510**	**3,173**	**6,683**	**52.65**	**14,262**	**11,231**	**25,493**	**6,385**	**6,010**	**12,395**	**48.62**
Bukidnon	1,478	1,622	**3,100**	621	659	**1,280**	**41.29**	2,561	2,658	**5,219**	1,065	1,025	**2,090**	**40.05**
Camiguin	8	8	**16**	1	2	**3**	**18.75**	73	36	**109**	14	6	**20**	**18.35**
Lanao del Norte	1,141	1,175	**2,316**	821	842	**1,663**	**71.80**	1,879	1,848	**3,727**	1,201	1,183	**2,384**	**63.97**
Misamis Occidental	803	572	**1,375**	701	496	**1,197**	**87.05**	1,853	1,529	**3,382**	1,568	1,645	**3,213**	**95.00**
Misamis Oriental	1,016	1,235	**2,251**	216	228	**444**	**19.72**	2,149	2,247	**4,396**	510	400	**910**	**20.70**
Cagayan de Oro City	309	323	**632**	80	96	**176**	**27.85**	329	295	**624**	191	170	**361**	**57.85**
El Salvador City	0	0	**0**	0	0	**0**	**0.00**	0	0	**0**	0	0	**0**	**0.00**
Gingoog City	265	331	**596**	218	41	**259**	**43.46**	3,141	538	**3,679**	273	181	**454**	**12.34**
Iligan City	850	779	**1,629**	736	662	**1,398**	**85.82**	1,504	1,310	**2,814**	1,303	1,136	**2,439**	**86.67**
Malaybalay City	219	215	**434**	25	27	**52**	**11.98**	424	383	**807**	44	24	**68**	**8.43**
Oroquieta City	0	0	**0**	0	0	**0**	**0.00**	0	0	**0**	0	0	**0**	**0.00**
Ozamis City	3	4	**7**	0	1	**1**	**14.29**	27	32	**59**	2	0	**2**	**3.39**
Tangub City	58	71	**129**	58	71	**129**	**100.00**	93	102	**195**	93	102	**195**	**100.00**
Valencia City	97	111	**208**	33	48	**81**	**38.94**	229	253	**482**	121	138	**259**	**53.73**
**Region 11**	**1,882**	**1,534**	**3,416**	**1,752**	**1,400**	**3,152**	**92.27**	**3,098**	**2,778**	**5,876**	**2,896**	**2,629**	**5,525**	**94.03**
Davao de Oro	43	29	**72**	36	21	**57**	**79.17**	86	69	**155**	70	58	**128**	**82.58**
Davao del Norte	302	259	**561**	285	241	**526**	**93.76**	730	649	**1,379**	689	625	**1,314**	**95.29**
Davao Oriental	740	597	**1,337**	727	583	**1,310**	**97.98**	1,055	1,015	**2,070**	1,022	990	**2,012**	**97.20**
Davao del Sur	76	61	**137**	63	48	**111**	**81.02**	127	110	**237**	111	97	**208**	**87.76**
Davao Occidental	53	53	**106**	51	45	**96**	**90.57**	53	47	**100**	47	44	**91**	**91.00**
Davao City	668	535	**1,203**	590	462	**1,052**	**87.45**	1,047	888	**1,935**	957	815	**1,772**	**91.58**
**Region 12**	**4,634**	**4,343**	**8,977**	**2,693**	**2,627**	**5,320**	**59.26**	**7,803**	**7,635**	**15,438**	**4,893**	**4,805**	**9,698**	**62.82**
North Cotabato	2,268	2,202	**4,470**	832	900	**1,732**	**38.75**	3,566	3,531	**7,097**	1,447	1,451	**2,898**	**40.83**
Sarangani	1,828	1,697	**3,525**	1,418	1,348	**2,766**	**78.47**	2,926	2,930	**5,856**	2,365	2,377	**4,742**	**80.98**
South Cotabato	274	218	**492**	201	163	**364**	**73.98**	800	775	**1,575**	659	629	**1,288**	**81.78**
Sultan Kudarat	30	36	**66**	26	32	**58**	**87.88**	28	36	**64**	25	36	**61**	**95.31**
Gen. Santos City	234	190	**424**	216	184	**400**	**94.34**	483	363	**846**	397	312	**709**	**83.81**
**BARMM**	**3,945**	**4,089**	**8,034**	**2,805**	**2,863**	**5,668**	**70.55**	**7,433**	**7,248**	**14,681**	**4,857**	**4,748**	**9,605**	**65.42**
Basilan	898	875	**1,773**	615	636	**1,251**	**70.56**	1,598	1,512	**3,110**	1,110	1,076	**2,186**	**70.29**
Lanao del Sur	759	758	**1,517**	481	462	**943**	**62.16**	1,512	1,491	**3,003**	1,056	1,088	**2,144**	**71.40**
Maguindanao	0	1	**1**	0	0	**0**	**0.00**	1	0	**1**	0	0	**0**	**0.00**
Sulu	1,486	1,580	**3,066**	1,057	1,049	**2,106**	**68.69**	2,803	2,925	**5,728**	1,924	1,959	**3,883**	**67.79**
Tawi-Tawi	176	187	**363**	55	64	**119**	**32.78**	745	679	**1,424**	101	100	**201**	**14.12**
Lamitan City	8	8	**16**	1	2	**3**	**18.75**	8	10	**18**	0	4	**4**	**22.22**
Marawi City	573	628	**1,201**	573	628	**1,201**	**100.00**	635	502	**1,137**	579	437	**1,016**	**89.36**
Cotabato City	45	52	**97**	23	22	**45**	**46.39**	131	129	**260**	87	84	**171**	**65.77**

Table 2.C.4.1. Management of Sick Infants and Children

Number of high risk infants and children with measles and/or persistent diarrhea who received Vitamin A capsule aside from routine supplementation
Philippines, 2022

Area	Sick Infants aged 6-11 mos.							Sick Children aged 12-59 mos.							
	Seen			given vitamin A 100,000 IU				Seen			given vitamin A 200,000 IU				
	Male	Female	Total	Male	Female	Total	%	Male	Female	Total	Male	Female	Total	%
**CARAGA**	**1,997**	**1,966**	**3,963**	**738**	**752**	**1,490**	**37.60**	**4,171**	**4,045**	**8,216**	**1,856**	**1,743**	**3,599**	**43.80**
Agusan del Norte	252	274	**526**	78	84	**162**	**30.80**	474	424	**898**	233	211	**444**	**49.44**
Agusan del Sur	832	782	**1,614**	339	364	**703**	**43.56**	1,618	1,639	**3,257**	716	743	**1,459**	**44.80**
Surigao del Norte	621	608	**1,229**	141	106	**247**	**20.10**	1,328	1,352	**2,680**	352	358	**710**	**26.49**
Surigao del Sur	52	61	**113**	50	58	**108**	**95.58**	101	93	**194**	101	90	**191**	**98.45**
Province of Dinagat	33	58	**91**	18	36	**54**	**59.34**	247	168	**415**	216	145	**361**	**86.99**
Bislig City	14	10	**24**	11	9	**20**	**83.33**	51	36	**87**	51	36	**87**	**100.00**
Butuan City	47	38	**85**	29	25	**54**	**63.53**	47	35	**82**	43	28	**71**	**86.59**
Surigao City	146	135	**281**	72	70	**142**	**50.53**	305	298	**603**	144	132	**276**	**45.77**

Table 2.C.4.2. Management of Sick Infants and Children

Number and proportion of diarrhea cases 0-59 mos. old who received Oral Rehydration Salt (ORS) solution and ORS with Zinc drops/syrup and Pneumonia cases among children 0-59 mos. old who received treatment

Philippines, 2022

Area	Children 0-59 mos. old with Diarrhea											Children 0-59 mos. old with Pneumonia						
	Seen			Given ORS				Given ORS w/ zinc				Seen			Received treatment			
	Male	Female	Total	Male	Female	Total	%	Male	Female	Total	%	Male	Female	Total	Male	Female	Total	%
PHILIPPINES	46,239	42,578	88,817	29,031	27,065	56,096	63.16	26,798	24,783	51,581	58.08	34,779	31,398	66,177	33,320	30,186	63,506	95.96
N C R	4,669	4,153	8,822	1,933	1,676	3,609	40.91	2,721	2,466	5,187	58.80	3,117	2,698	5,815	3,058	2,652	5,710	98.19
Malabon City	351	224	575	136	77	213	37.04	215	147	362	62.96	320	285	605	291	265	556	91.90
Navotas City	453	442	895	147	156	303	33.85	302	283	585	65.36	49	47	96	48	47	95	98.96
Valenzuela City	305	261	566	235	198	433	76.50	67	62	129	22.79	399	364	763	396	359	755	98.95
Caloocan City	4	128	132	1	122	123	93.18	3	6	9	6.82	69	251	320	69	251	320	100.00
Marikina City	15	10	25	7	4	11	44.00	8	6	14	56.00	11	6	17	10	6	16	94.12
Pasig City	188	188	376	97	101	198	52.66	91	87	178	47.34	179	165	344	179	165	344	100.00
Pateros City	30	9	39	29	9	38	97.44	1	0	1	2.56	24	9	33	24	9	33	100.00
Taguig City	982	885	1,867	217	168	385	20.62	757	710	1,467	78.58	143	132	275	120	114	234	85.09
Quezon City	516	488	1,004	206	173	379	37.75	310	315	625	62.25	752	539	1,291	752	538	1,290	99.92
Makati City	94	68	162	0	0	0	0.00	94	68	162	100.00	72	49	121	72	49	121	100.00
Mandaluyong City	74	60	134	54	43	97	72.39	20	17	37	27.61	60	46	106	60	46	106	100.00
San Juan City	39	29	68	0	0	0	0.00	39	29	68	100.00	42	28	70	42	28	70	100.00
Manila City	1,036	848	1,884	587	454	1,041	55.25	449	394	843	44.75	661	552	1,213	661	552	1,213	100.00
Las Piñas City	268	274	542	115	107	222	40.96	153	167	320	59.04	38	26	64	38	26	64	100.00
Muntinlupa City	193	144	337	50	28	78	23.15	143	116	259	76.85	155	90	245	155	90	245	100.00
Parañaque City	64	39	103	45	23	68	66.02	19	16	35	33.98	49	38	87	48	36	84	96.55
Pasay City	57	56	113	7	13	20	17.70	50	43	93	82.30	94	71	165	93	71	164	99.39
C A R	2,299	1,926	4,225	735	617	1,352	32.00	1,536	1,277	2,813	66.58	1,675	1,377	3,052	1,648	1,358	3,006	98.49
Abra	203	185	388	43	34	77	19.85	153	144	297	76.55	164	155	319	161	152	313	98.12
Apayao	255	285	540	54	78	132	24.44	188	193	381	70.56	162	134	296	160	131	291	98.31
Benguet	317	230	547	105	56	161	29.43	212	174	386	70.57	321	247	568	315	242	557	98.06
Ifugao	607	469	1,076	252	229	481	44.70	354	240	594	55.20	488	359	847	483	358	841	99.29
Kalinga	583	485	1,068	171	137	308	28.84	409	344	753	70.51	98	75	173	91	73	164	94.80
Mt. Province	235	188	423	110	82	192	45.39	125	103	228	53.90	156	140	296	154	137	291	98.31
Baguio City	99	84	183	0	1	1	0.55	95	79	174	95.08	286	267	553	284	265	549	99.28
Region 1	2,255	1,895	4,150	1,322	1,129	2,451	59.06	890	721	1,611	38.82	1,425	1,276	2,701	1,409	1,266	2,675	99.04
Ilocos Norte	110	80	190	43	24	67	35.26	72	58	130	68.42	31	34	65	30	34	64	98.46
Ilocos Sur	267	268	535	39	51	90	16.82	228	217	445	83.18	243	228	471	240	228	468	99.36
La Union	133	90	223	13	17	30	13.45	114	69	183	82.06	225	183	408	215	179	394	96.57
Pangasinan	1,164	920	2,084	855	703	1,558	74.76	309	217	526	25.24	560	493	1,053	558	490	1,048	99.53
Alaminos City	255	209	464	159	127	286	61.64	96	82	178	38.36	227	218	445	227	218	445	100.00
Candon City	45	51	96	10	17	27	28.13	0	0	0	0.00	9	12	21	9	12	21	100.00
Dagupan City	0	0	0	0	0	0	0.00	0	0	0	0.00	0	0	0	0	0	0	0.00
Laoag City	0	0	0	0	0	0	0.00	0	0	0	0.00	0	0	0	0	0	0	0.00
San Carlos City	222	213	435	188	171	359	82.53	34	42	76	17.47	62	52	114	62	52	114	100.00
San Fernando City	26	31	57	7	16	23	40.35	12	6	18	31.58	6	11	17	6	8	14	82.35
Urdaneta City	6	7	13	0	0	0	0.00	6	7	13	100.00	1	0	1	1	0	1	100.00
Vigan City	27	26	53	8	3	11	20.75	19	23	42	79.25	61	45	106	61	45	106	100.00
Region 2	2,481	2,107	4,588	1,903	1,608	3,511	76.53	2,006	1,687	3,693	80.49	1,787	1,479	3,266	1,720	1,411	3,131	95.87
Batanes	1	0	1	1	0	1	100.00	0	0	0	0.00	10	13	23	10	13	23	100.00
Cagayan	345	278	623	331	265	596	95.67	272	224	496	79.61	216	151	367	210	144	354	96.46
Isabela	1,515	1,325	2,840	1,305	1,099	2,404	84.65	1,260	1,071	2,331	82.08	1,469	1,240	2,709	1,414	1,190	2,604	96.12
Nueva Vizcaya	224	180	404	135	132	267	66.09	122	104	226	55.94	25	20	45	25	19	44	97.78
Quirino	42	46	88	38	36	74	84.09	24	26	50	56.82	8	5	13	8	5	13	100.00
Cauayan City	253	189	442	8	2	10	2.26	245	187	432	97.74	5	3	8	4	1	5	62.50
Ilagan City	79	68	147	79	68	147	100.00	79	68	147	100.00	42	34	76	42	34	76	100.00
Santiago City	0	0	0	0	0	0	0.00	0	0	0	0.00	0	0	0	0	0	0	0.00
Tuguegarao City	22	21	43	6	6	12	27.91	4	7	11	25.58	12	13	25	7	5	12	48.00
Region 3	7,806	7,508	15,314	4,639	4,538	9,177	59.93	4,290	4,134	8,424	55.01	1,509	1,427	2,936	1,424	1,354	2,778	94.62
Aurora	66	43	109	60	39	99	90.83	22	11	33	30.28	37	36	73	36	35	71	97.26
Bataan	685	596	1,281	299	257	556	43.40	419	337	756	59.02	129	124	253	126	118	244	96.44
Bulacan	2,304	2,235	4,539	1,703	1,704	3,407	75.06	1,334	1,344	2,678	59.00	466	461	927	461	451	912	98.38
Nueva Ecija	549	569	1,118	406	410	816	72.99	299	296	595	53.22	298	268	566	281	254	535	94.52
Pampanga	289	278	567	265	250	515	90.83	143	134	277	48.85	69	68	137	70	65	135	98.54
Tarlac	1,131	1,060	2,191	773	758	1,531	69.88	312	261	573	26.15	147	120	267	147	120	267	100.00
Zambales	224	194	418	161	137	298	71.29	91	93	184	44.02	91	95	186	48	61	109	58.60
Angeles City	185	183	368	119	129	248	67.39	115	98	213	57.88	7	13	20	6	13	19	95.00
Balanga City	57	47	104	2	2	4	3.85	55	45	100	96.15	2	1	3	2	1	3	100.00
Cabanatuan City	223	207	430	169	166	335	77.91	48	42	90	20.93	9	13	22	9	13	22	100.00
City of San Fernando	269	280	549	221	238	459	83.61	46	55	101	18.40	17	18	35	14	16	30	85.71
Gapan City	87	86	173	16	16	32	18.50	75	75	150	86.71	1	0	1	1	0	1	100.00

Table 2.C.4.2. Management of Sick Infants and Children

Number and proportion of diarrhea cases 0-59 mos. old who received Oral Rehydration Salt (ORS) solution and ORS with Zinc drops/syrup and Pneumonia cases among children 0-59 mos. old who received treatment

Philippines, 2022

Area	Children 0-59 mos. old with Diarrhea											Children 0-59 mos. old with Pneumonia						
	Seen			Given ORS				Given ORS w/ zinc				Seen			Received treatment			
	Male	Female	Total	Male	Female	Total	%	Male	Female	Total	%	Male	Female	Total	Male	Female	Total	%
Mabalacat City	47	36	**83**	6	6	**12**	**14.46**	41	30	**71**	**85.54**	142	146	**288**	142	146	**288**	**100.00**
Malolos City	30	32	**62**	29	29	**58**	**93.55**	21	24	**45**	**72.58**	1	0	**1**	1	0	**1**	**100.00**
Meycauayan	593	573	**1,166**	247	226	**473**	**40.57**	345	347	**692**	**59.35**	16	5	**21**	16	5	**21**	**100.00**
Olongapo	25	26	**51**	4	8	**12**	**23.53**	24	23	**47**	**92.16**	19	20	**39**	12	17	**29**	**74.36**
Palayan City	16	6	**22**	2	1	**3**	**13.64**	16	6	**22**	**100.00**	1	0	**1**	0	1	**1**	**100.00**
San Jose City	10	9	**19**	4	5	**9**	**47.37**	6	4	**10**	**52.63**	0	0	**0**	0	0	**0**	**0.00**
San Jose del Monte City	535	553	**1,088**	118	118	**236**	**21.69**	417	435	**852**	**78.31**	9	6	**15**	9	6	**15**	**100.00**
Science City of Munoz	97	85	**182**	8	7	**15**	**8.24**	89	78	**167**	**91.76**	42	30	**72**	37	29	**66**	**91.67**
Tarlac City	384	410	**794**	27	32	**59**	**7.43**	372	396	**768**	**96.73**	6	3	**9**	6	3	**9**	**100.00**
**Region 4A**	**4,100**	**3,722**	**7,822**	**2,913**	**2,775**	**5,688**	**72.72**	**2,140**	**1,991**	**4,131**	**52.81**	**1,011**	**945**	**1,956**	**929**	**878**	**1,807**	**92.38**
Batangas	101	113	**214**	52	59	**111**	**51.87**	73	88	**161**	**75.23**	60	67	**127**	60	67	**127**	**100.00**
Cavite	376	392	**768**	288	283	**571**	**74.35**	139	143	**282**	**36.72**	44	43	**87**	29	27	**56**	**64.37**
Laguna	193	178	**371**	171	146	**317**	**85.44**	110	108	**218**	**58.76**	29	21	**50**	24	18	**42**	**84.00**
Quezon	630	556	**1,186**	577	500	**1,077**	**90.81**	389	334	**723**	**60.96**	475	432	**907**	453	426	**879**	**96.91**
Rizal	703	682	**1,385**	685	656	**1,341**	**96.82**	473	439	**912**	**65.85**	162	164	**326**	153	150	**303**	**92.94**
Antipolo City	33	67	**100**	28	48	**76**	**76.00**	22	42	**64**	**64.00**	24	37	**61**	24	37	**61**	**100.00**
Bacoor City	253	236	**489**	217	214	**431**	**88.14**	233	221	**454**	**92.84**	8	10	**18**	7	8	**15**	**83.33**
Batangas City	34	29	**63**	19	19	**38**	**60.32**	28	25	**53**	**84.13**	3	6	**9**	2	6	**8**	**88.89**
Biñan City	187	201	**388**	182	197	**379**	**97.68**	97	100	**197**	**50.77**	0	0	**0**	0	0	**0**	**0.00**
Cabuyao City	123	142	**265**	106	122	**228**	**86.04**	80	105	**185**	**69.81**	6	7	**13**	2	1	**3**	**23.08**
Calamba City	107	97	**204**	103	96	**199**	**97.55**	32	30	**62**	**30.39**	1	3	**4**	1	1	**2**	**50.00**
Cavite City	10	8	**18**	2	4	**6**	**33.33**	0	0	**0**	**0.00**	1	1	**2**	1	1	**2**	**100.00**
Dasmariñas City	137	108	**245**	111	95	**206**	**84.08**	82	59	**141**	**57.55**	105	66	**171**	91	58	**149**	**87.13**
General Trias City	20	15	**35**	17	11	**28**	**80.00**	7	3	**10**	**28.57**	34	29	**63**	34	29	**63**	**100.00**
Imus City	104	89	**193**	83	68	**151**	**78.24**	9	8	**17**	**8.81**	12	16	**28**	1	6	**7**	**25.00**
Lipa City	0	0	**0**	0	0	**0**	**0.00**	0	0	**0**	**0.00**	0	0	**0**	0	0	**0**	**0.00**
Lucena City	196	192	**388**	174	156	**330**	**85.05**	173	156	**329**	**84.79**	31	34	**65**	31	34	**65**	**100.00**
San Pablo City	24	21	**45**	20	18	**38**	**84.44**	8	3	**11**	**24.44**	1	0	**1**	1	0	**1**	**100.00**
San Pedro City	2	0	**2**	2	0	**2**	**100.00**	2	0	**2**	**100.00**	0	0	**0**	0	0	**0**	**0.00**
Santa Rosa City	74	74	**148**	65	67	**132**	**89.19**	5	5	**10**	**6.76**	14	8	**22**	14	8	**22**	**100.00**
Tagaytay City	0	5	**5**	0	5	**5**	**100.00**	0	2	**2**	**40.00**	0	0	**0**	0	0	**0**	**0.00**
Tanauan City	790	514	**1,304**	8	9	**17**	**1.30**	177	119	**296**	**22.70**	0	0	**0**	0	0	**0**	**0.00**
Tayabas City	1	1	**2**	1	0	**1**	**50.00**	1	1	**2**	**100.00**	1	1	**2**	1	1	**2**	**100.00**
Trece Martires City	2	2	**4**	2	2	**4**	**100.00**	0	0	**0**	**0.00**	0	0	**0**	0	0	**0**	**0.00**
**Region 4B**	**1,381**	**1,236**	**2,617**	**1,229**	**1,106**	**2,335**	**89.22**	**692**	**624**	**1,316**	**50.29**	**358**	**272**	**630**	**343**	**260**	**603**	**95.71**
Marinduque	39	34	**73**	35	31	**66**	**90.41**	22	16	**38**	**52.05**	22	17	**39**	22	17	**39**	**100.00**
Mindoro Occidental	384	335	**719**	309	277	**586**	**81.50**	91	70	**161**	**22.39**	15	18	**33**	13	16	**29**	**87.88**
Mindoro Oriental	212	180	**392**	181	151	**332**	**84.69**	147	129	**276**	**70.41**	133	93	**226**	123	84	**207**	**91.59**
Palawan	645	578	**1,223**	637	572	**1,209**	**98.86**	427	400	**827**	**67.62**	143	109	**252**	142	107	**249**	**98.81**
Romblon	39	46	**85**	23	31	**54**	**63.53**	2	3	**5**	**5.88**	44	33	**77**	42	34	**76**	**98.70**
Puerto Princesa City	62	63	**125**	44	44	**88**	**70.40**	3	6	**9**	**7.20**	1	2	**3**	1	2	**3**	**100.00**
**Region 5**	**1,856**	**1,616**	**3,472**	**1,355**	**1,197**	**2,552**	**73.50**	**936**	**819**	**1,755**	**50.55**	**1,118**	**996**	**2,114**	**1,054**	**936**	**1,990**	**94.13**
Albay	398	328	**726**	323	291	**614**	**84.57**	186	168	**354**	**48.76**	168	147	**315**	153	136	**289**	**91.75**
Camarines Norte	317	296	**613**	282	260	**542**	**88.42**	182	162	**344**	**56.12**	159	151	**310**	154	149	**303**	**97.74**
Camarines Sur	402	366	**768**	194	180	**374**	**48.70**	205	176	**381**	**49.61**	223	198	**421**	208	191	**399**	**94.77**
Catanduanes	121	82	**203**	75	56	**131**	**64.53**	46	26	**72**	**35.47**	68	62	**130**	67	62	**129**	**99.23**
Masbate	357	330	**687**	297	259	**556**	**80.93**	234	229	**463**	**67.39**	317	260	**577**	296	224	**520**	**90.12**
Sorsogon	200	152	**352**	136	98	**234**	**66.48**	71	51	**122**	**34.66**	161	162	**323**	160	162	**322**	**99.69**
Iriga City	12	19	**31**	12	17	**29**	**93.55**	0	2	**2**	**6.45**	6	3	**9**	4	3	**7**	**77.78**
Legaspi City	47	36	**83**	34	32	**66**	**79.52**	12	4	**16**	**19.28**	12	9	**21**	9	7	**16**	**76.19**
Naga City	2	7	**9**	2	4	**6**	**66.67**	0	1	**1**	**11.11**	4	4	**8**	3	2	**5**	**62.50**
**Region 6**	**2,154**	**1,885**	**4,039**	**1,666**	**1,463**	**3,129**	**77.47**	**1,409**	**1,266**	**2,675**	**66.23**	**4,002**	**3,691**	**7,693**	**3,836**	**3,551**	**7,387**	**96.02**
Aklan	192	168	**360**	183	159	**342**	**95.00**	93	97	**190**	**52.78**	803	683	**1,486**	802	682	**1,484**	**99.87**
Antique	114	105	**219**	96	88	**184**	**84.02**	53	57	**110**	**50.23**	722	723	**1,445**	701	700	**1,401**	**96.96**
Capiz	44	78	**122**	24	35	**59**	**48.36**	20	43	**63**	**51.64**	164	200	**364**	164	200	**364**	**100.00**
Guimaras	6	6	**12**	6	6	**12**	**100.00**	4	6	**10**	**83.33**	18	26	**44**	18	26	**44**	**100.00**
Iloilo	388	306	**694**	366	278	**644**	**92.80**	243	203	**446**	**64.27**	902	778	**1,680**	776	677	**1,453**	**86.49**
Negros Occidental	1,142	1,007	**2,149**	798	727	**1,525**	**70.96**	849	736	**1,585**	**73.76**	1,095	1,039	**2,134**	1,085	1,032	**2,117**	**99.20**
Bacolod City	82	84	**166**	77	81	**158**	**95.18**	45	57	**102**	**61.45**	52	43	**95**	44	35	**79**	**83.16**
Iloilo City	186	131	**317**	116	89	**205**	**64.67**	102	67	**169**	**53.31**	246	199	**445**	246	199	**445**	**100.00**
**Region 7**	**1,665**	**1,633**	**3,298**	**1,207**	**1,180**	**2,387**	**72.38**	**873**	**858**	**1,731**	**52.49**	**2,579**	**2,440**	**5,019**	**2,295**	**2,190**	**4,485**	**89.36**
Bohol	850	829	**1,679**	828	803	**1,631**	**97.14**	278	256	**534**	**31.80**	276	224	**500**	261	212	**473**	**94.60**
Cebu	297	254	**551**	226	229	**455**	**82.58**	133	120	**253**	**45.92**	1,040	956	**1,996**	848	779	**1,627**	**81.51**
Negros Oriental	218	260	**478**	0	0	**0**	**0.00**	214	247	**461**	**96.44**	617	697	**1,314**	612	692	**1,304**	**99.24**
Siquijor	8	10	**18**	3	1	**4**	**22.22**	5	7	**12**	**66.67**	57	57	**114**	54	51	**105**	**92.11**

Table 2.C.4.2. Management of Sick Infants and Children

Number and proportion of diarrhea cases 0-59 mos. old who received Oral Rehydration Salt (ORS) solution and ORS with Zinc drops/syrup and Pneumonia cases among children 0-59 mos. old who received treatment

Philippines, 2022

Area	Children 0-59 mos. old with Diarrhea											Children 0-59 mos. old with Pneumonia						
	Seen			Given ORS				Given ORS w/ zinc				Seen			Received treatment			
	Male	Female	Total	Male	Female	Total	%	Male	Female	Total	%	Male	Female	Total	Male	Female	Total	%
Cebu City	105	97	202	62	61	123	60.89	81	72	153	75.74	412	342	754	349	298	647	85.81
Lapu-Lapu City	152	152	304	59	59	118	38.82	132	128	260	85.53	139	124	263	136	124	260	98.86
Mandaue City	35	31	66	29	27	56	84.85	30	28	58	87.88	38	40	78	35	34	69	88.46
**Region 8**	**1,927**	**1,685**	**3,612**	**1,409**	**1,223**	**2,632**	**72.87**	**1,246**	**1,080**	**2,326**	**64.40**	**2,873**	**2,441**	**5,314**	**2,652**	**2,291**	**4,943**	**93.02**
Biliran	103	98	201	43	48	91	45.27	82	71	153	76.12	131	120	251	130	119	249	99.20
Eastern Samar	182	165	347	166	152	318	91.64	100	86	186	53.60	608	525	1,133	504	434	938	82.79
Northern Leyte	383	370	753	214	199	413	54.85	144	144	288	38.25	543	436	979	543	430	973	99.39
Northern Samar	509	476	985	441	430	871	88.43	395	362	757	76.85	344	264	608	328	247	575	94.57
Southern Leyte	69	60	129	24	17	41	31.78	44	41	85	65.89	87	62	149	87	62	149	100.00
Western Samar	513	375	888	403	282	685	77.14	358	268	626	70.50	747	655	1,402	657	627	1,284	91.58
Calbayog City	9	6	15	9	6	15	100.00	0	0	0	0.00	1	3	4	0	2	2	50.00
Maasin City	35	23	58	33	22	55	94.83	22	15	37	63.79	4	1	5	1	0	1	20.00
Ormoc City	58	56	114	13	13	26	22.81	45	43	88	77.19	248	215	463	248	215	463	100.00
Tacloban City	66	56	122	63	54	117	95.90	56	50	106	86.89	160	160	320	154	155	309	96.56
**Region 9**	**1,989**	**1,910**	**3,899**	**391**	**368**	**759**	**19.47**	**1,541**	**1,470**	**3,011**	**77.22**	**1,835**	**1,708**	**3,543**	**1,800**	**1,679**	**3,479**	**98.19**
Zamboanga del Norte	748	770	1,518	198	199	397	26.15	536	547	1,083	71.34	1,043	1,051	2,094	1,034	1,038	2,072	98.95
Zamboanga del Sur	401	349	750	51	45	96	12.80	344	296	640	85.33	547	450	997	544	448	992	99.50
Zamboanga Sibugay	187	159	346	68	54	122	35.26	113	93	206	59.54	122	115	237	111	111	222	93.67
Dapitan City	9	10	19	0	0	0	0.00	9	10	19	100.00	2	2	4	2	2	4	100.00
Dipolog City	21	22	43	4	6	10	23.26	12	12	24	55.81	20	13	33	13	10	23	69.70
Isabela City	215	198	413	15	9	24	5.81	200	189	389	94.19	25	25	50	25	24	49	98.00
Pagadian City	1	0	1	0	0	0	0.00	1	0	1	100.00	0	0	0	0	0	0	0.00
Zamboanga City	407	402	809	55	55	110	13.60	326	323	649	80.22	76	52	128	71	46	117	91.41
**Region 10**	**2,234**	**2,279**	**4,513**	**1,858**	**1,898**	**3,756**	**83.23**	**1,431**	**1,453**	**2,884**	**63.90**	**3,090**	**2,966**	**6,056**	**3,001**	**2,886**	**5,887**	**97.21**
Bukidnon	507	513	1,020	495	506	1,001	98.14	311	325	636	62.35	656	668	1,324	620	636	1,256	94.86
Camiguin	1	0	1	1	0	1	100.00	0	0	0	0.00	18	7	25	18	7	25	100.00
Lanao del Norte	271	256	527	259	250	509	96.58	214	205	419	79.51	652	611	1,263	647	602	1,249	98.89
Misamis Occidental	508	517	1,025	394	400	794	77.46	363	401	764	74.54	543	552	1,095	540	551	1,091	99.63
Misamis Oriental	203	205	408	135	128	263	64.46	146	145	291	71.32	310	278	588	305	272	577	98.13
Cagayan de Oro City	163	146	309	134	106	240	77.67	107	90	197	63.75	202	173	375	177	148	325	86.67
El Salvador City	17	23	40	17	23	40	100.00	0	0	0	0.00	63	65	128	63	65	128	100.00
Gingoog City	44	107	151	44	100	144	95.36	14	13	27	17.88	46	38	84	37	38	75	89.29
Iligan City	149	140	289	124	119	243	84.08	95	97	192	66.44	102	78	180	96	72	168	93.33
Malaybalay City	86	93	179	37	45	82	45.81	83	89	172	96.09	73	58	131	73	58	131	100.00
Oroquieta City	0	0	0	0	0	0	0.00	0	0	0	0.00	0	0	0	0	0	0	0.00
Ozamis City	229	244	473	166	186	352	74.42	85	81	166	35.10	296	296	592	296	295	591	99.83
Tangub City	0	0	0	0	0	0	0.00	0	0	0	0.00	0	0	0	0	0	0	0.00
Valencia City	56	35	91	52	35	87	95.60	13	7	20	21.98	129	142	271	129	142	271	100.00
**Region 11**	**1,403**	**1,097**	**2,500**	**527**	**367**	**894**	**35.76**	**911**	**753**	**1,664**	**66.56**	**2,365**	**1,925**	**4,290**	**2,301**	**1,862**	**4,163**	**97.04**
Davao de Oro	67	49	116	42	23	65	56.03	25	23	48	41.38	181	118	299	172	114	286	95.65
Davao del Norte	303	225	528	90	57	147	27.84	207	162	369	69.89	501	401	902	486	381	867	96.12
Davao Oriental	312	248	560	64	42	106	18.93	260	217	477	85.18	842	680	1,522	836	674	1,510	99.21
Davao del Sur	104	66	170	31	14	45	26.47	65	51	116	68.24	196	163	359	188	155	343	95.54
Davao Occidental	54	65	119	37	41	78	65.55	18	24	42	35.29	41	58	99	40	44	84	84.85
Davao City	563	444	1,007	263	190	453	44.99	336	276	612	60.77	604	505	1,109	579	494	1,073	96.75
**Region 12**	**1,671**	**1,628**	**3,299**	**1,424**	**1,381**	**2,805**	**85.03**	**513**	**505**	**1,018**	**30.86**	**2,327**	**2,203**	**4,530**	**2,283**	**2,164**	**4,447**	**98.17**
North Cotabato	506	538	1,044	452	475	927	88.79	138	155	293	28.07	852	840	1,692	840	819	1,659	98.05
Sarangani	407	423	830	396	412	808	97.35	0	0	0	0.00	264	233	497	252	221	473	95.17
South Cotabato	393	339	732	293	239	532	72.68	242	225	467	63.80	875	817	1,692	864	813	1,677	99.11
Sultan Kudarat	250	212	462	181	151	332	71.86	69	61	130	28.14	277	260	537	273	260	533	99.26
Gen. Santos City	115	116	231	102	104	206	89.18	64	64	128	55.41	59	53	112	54	51	105	93.75
**B.A.R.M.M.**	**5,740**	**5,722**	**11,462**	**4,106**	**4,137**	**8,243**	**71.92**	**3,437**	**3,471**	**6,908**	**60.27**	**2,987**	**2,853**	**5,840**	**2,852**	**2,752**	**5,604**	**95.96**
Basilan	674	616	1,290	565	572	1,137	88.14	399	389	788	61.09	466	510	976	381	440	821	84.12
Lanao del Sur	1,135	1,111	2,246	972	956	1,928	85.84	724	714	1,438	64.02	351	357	708	341	346	687	97.03
Maguindanao	2,009	2,063	4,072	770	781	1,551	38.09	1,237	1,284	2,521	61.91	1,368	1,334	2,702	1,350	1,316	2,666	98.67
Sulu	1,521	1,548	3,069	1,408	1,453	2,861	93.22	1,064	1,071	2,135	69.57	600	493	1,093	578	493	1,071	97.99
Tawi-Tawi	0	0	0	0	0	0	0.00	0	0	0	0.00	0	0	0	0	0	0	0.00
Lamitan City	13	7	20	7	5	12	60.00	6	2	8	40.00	13	5	18	12	5	17	94.44
Marawi City	362	352	714	360	345	705	98.74	0	0	0	0.00	96	87	183	96	87	183	100.00
Cotabato City	26	25	51	24	25	49	96.08	7	11	18	35.29	93	67	160	94	65	159	99.38

Table 2.C.4.2. Management of Sick Infants and Children

Number and proportion of diarrhea cases 0-59 mos. old who received Oral Rehydration Salt (ORS) solution and ORS with Zinc drops/syrup and Pneumonia cases among children 0-59 mos. old who received treatment

Philippines, 2022

Area	Children 0-59 mos. old with Diarrhea											Children 0-59 mos. old with Pneumonia						
	Seen			Given ORS				Given ORS w/ zinc				Seen			Received treatment			
	Male	Female	Total	Male	Female	Total	%	Male	Female	Total	%	Male	Female	Total	Male	Female	Total	%
**CARAGA**	**609**	**576**	**1,185**	**414**	**402**	**816**	**68.86**	**226**	**208**	**434**	**36.62**	**721**	**701**	**1,422**	**715**	**696**	**1,411**	**99.23**
Agusan del Norte	21	41	**62**	15	37	**52**	**83.87**	6	14	**20**	**32.26**	13	6	**19**	13	6	**19**	**100.00**
Agusan del Sur	146	151	**297**	99	102	**201**	**67.68**	44	46	**90**	**30.30**	224	264	**488**	224	264	**488**	**100.00**
Surigao del Norte	200	166	**366**	150	131	**281**	**76.78**	82	69	**151**	**41.26**	203	207	**410**	199	203	**402**	**98.05**
Surigao del Sur	114	114	**228**	47	54	**101**	**44.30**	66	54	**120**	**52.63**	233	191	**424**	233	190	**423**	**99.76**
Province of Dinagat	40	29	**69**	23	14	**37**	**53.62**	20	15	**35**	**50.72**	10	6	**16**	10	6	**16**	**100.00**
Bislig City	3	5	**8**	2	3	**5**	**62.50**	1	2	**3**	**37.50**	1	1	**2**	1	1	**2**	**100.00**
Butuan City	5	8	**13**	4	7	**11**	**84.62**	1	1	**2**	**15.38**	6	2	**8**	4	2	**6**	**75.00**
Surigao City	80	62	**142**	74	54	**128**	**90.14**	6	7	**13**	**9.15**	31	24	**55**	31	24	**55**	**100.00**

Table 2.D.1. Oral Health Care

Number and proportion of children 12-59 months old who are orally fit upon oral examination or after oral rehabilitation

Philippines, 2022

Area	Elig Pop x (Service Target 20%)	Orally fit		Total	%
		Male	Female		
**PHILIPPINES**	**1,787,969**	**200,402**	**217,220**	**417,622**	**23.36**
**NCR**	**190,314**	**57,027**	**60,343**	**117,370**	**61.67**
Malabon	**5,402**	96	147	**243**	**4.50**
Navotas	**3,686**	875	967	**1,842**	**49.97**
Valenzuela City	**9,169**	1,637	1,652	**3,289**	**35.87**
Caloocan City	**23,410**	6,821	7,449	**14,270**	**60.96**
Marikina City	**6,661**	1,438	1,425	**2,863**	**42.98**
Pasig City	**11,163**	3,204	3,457	**6,661**	**59.67**
Pateros	**944**	495	449	**944**	**100.00**
Taguig	**11,896**	656	638	**1,294**	**10.88**
Quezon City	**43,394**	19,230	19,882	**39,112**	**90.13**
Makati City	**8,611**	3,194	3,462	**6,656**	**77.30**
Mandaluyong City	**5,708**	1,833	1,804	**3,637**	**63.72**
San Juan	**1,806**	349	372	**721**	**39.92**
Manila City	**26,309**	12,527	13,782	**26,309**	**100.00**
Las Piñas City	**8,703**	1,099	1,089	**2,188**	**25.14**
Muntinlupa City	**7,456**	1,415	1,529	**2,944**	**39.48**
Parañaque City	**9,840**	1,839	1,891	**3,730**	**37.91**
Pasay City	**6,156**	319	348	**667**	**10.83**
**CAR**	**29,438**	**3,114**	**3,150**	**6,264**	**21.28**
Abra	**3,790**	146	136	**282**	**7.44**
Apayao	**2,222**	272	280	**552**	**24.84**
Benguet	**7,201**	1,353	1,270	**2,623**	**36.43**
Ifugao	**3,887**	154	223	**377**	**9.70**
Kalinga	**4,014**	342	398	**740**	**18.44**
Mt. Province	**2,751**	183	173	**356**	**12.94**
Baguio City	**5,573**	664	670	**1,334**	**23.94**
**Region 1**	**81,369**	**10,055**	**11,366**	**21,421**	**26.33**
Ilocos Norte	**6,390**	661	740	**1,401**	**21.92**
Ilocos Sur	**7,931**	1,576	1,711	**3,287**	**41.44**
La Union	**9,577**	3,042	3,624	**6,666**	**69.60**
Pangasinan	**42,289**	2,933	3,395	**6,328**	**14.96**
Alaminos City	**1,597**	265	285	**550**	**34.44**
Candon City	**836**	243	246	**489**	**58.49**
Dagupan City	**3,051**	138	155	**293**	**9.60**
Laoag City	**1,473**	395	287	**682**	**46.30**
San Carlos City	**3,359**	249	225	**474**	**14.11**
San Fernando City	**1,755**	107	125	**232**	**13.22**
Urdaneta City	**2,368**	277	413	**690**	**29.14**
Vigan City	**743**	169	160	**329**	**44.28**

Table 2.D.1. Oral Health Care

Number and proportion of children 12-59 months old who are orally fit upon oral examination or after oral rehabilitation

Philippines, 2022

Area	Elig Pop x (Service Target 20%)	Orally fit		Total	%
		Male	Female		
**Region 2**	**57,872**	**6,937**	**8,100**	**15,037**	**25.98**
Batanes	**325**	119	131	**250**	**76.92**
Cagayan	**17,164**	986	1,613	**2,599**	**15.14**
Isabela	**19,591**	2,352	2,770	**5,122**	**26.14**
Nueva Vizcaya	**8,031**	786	909	**1,695**	**21.11**
Quirino	**3,457**	669	771	**1,440**	**41.65**
Cauayan City	**2,143**	480	556	**1,036**	**48.34**
Ilagan City	**2,409**	1,299	1,108	**2,407**	**99.92**
Santiago City	**2,232**	246	242	**488**	**21.86**
Tuguegarao City	**2,520**	0	0	**0**	**0.00**
**Region 3**	**182,628**	**20,784**	**22,603**	**43,387**	**23.76**
Aurora	**4,086**	455	451	**906**	**22.17**
Bataan	**10,935**	879	932	**1,811**	**16.56**
Bulacan	**36,286**	2,247	2,319	**4,566**	**12.58**
Nueva Ecija	**24,507**	3,843	4,138	**7,981**	**32.57**
Pampanga	**26,369**	2,973	3,125	**6,098**	**23.13**
Tarlac	**15,969**	2,352	2,637	**4,989**	**31.24**
Zambales	**10,134**	2,019	2,569	**4,588**	**45.27**
Angeles City	**6,616**	6	5	**11**	**0.17**
Balanga City	**1,581**	246	341	**587**	**37.13**
Cabanatuan City	**5,016**	245	296	**541**	**10.79**
City of San Fernando	**4,928**	372	429	**801**	**16.25**
Gapan City	**1,830**	93	86	**179**	**9.78**
Mabalacat City	**4,031**	1,985	2,050	**4,035**	**100.10**
Malolos City	**4,054**	324	345	**669**	**16.50**
Meycauayan	**3,362**	364	405	**769**	**22.87**
Olongapo	**3,997**	129	161	**290**	**7.26**
Palayan City	**681**	14	20	**34**	**4.99**
San Jose City	**2,320**	673	735	**1,408**	**60.69**
San Jose del Monte City	**9,231**	286	238	**524**	**5.68**
Science City of Munoz	**1,352**	272	336	**608**	**44.97**
Tarlac City	**5,343**	1,007	985	**1,992**	**37.28**
**Region 4A**	**247,717**	**13,092**	**14,010**	**27,102**	**10.94**
Batangas	**33,076**	1,998	2,011	**4,009**	**12.12**
Cavite	**23,144**	1,079	1,135	**2,214**	**9.57**
Laguna	**16,085**	1,231	1,252	**2,483**	**15.44**
Quezon	**32,086**	1,948	2,148	**4,096**	**12.77**
Rizal	**36,355**	1,501	1,866	**3,367**	**9.26**
Antipolo City	**13,391**	1,335	1,427	**2,762**	**20.63**
Bacoor City	**10,141**	16	23	**39**	**0.38**

Table 2.D.1. Oral Health Care

Number and proportion of children 12-59 months old who are orally fit upon oral examination or after oral rehabilitation

Philippines, 2022

Area	Elig Pop x (Service Target 20%)	Orally fit		Total	%
		Male	Female		
Batangas City	**5,870**	394	472	**866**	**14.75**
Biñan City	**5,393**	371	480	**851**	**15.78**
Cabuyao City	**4,999**	70	89	**159**	**3.18**
Calamba City	**7,360**	50	45	**95**	**1.29**
Cavite City	**1,736**	0	0	**0**	**0.00**
Dasmariñas City	**11,127**	0	0	**0**	**0.00**
General Trias City	**5,307**	3	2	**5**	**0.09**
Imus City	**6,817**	342	306	**648**	**9.51**
Lipa City	**5,916**	201	260	**461**	**7.79**
Lucena City	**4,863**	203	238	**441**	**9.07**
San Pablo City	**4,309**	2	7	**9**	**0.21**
San Pedro City	**5,276**	90	112	**202**	**3.83**
Santa Rosa City	**5,728**	1,182	1,111	**2,293**	**40.03**
Tagaytay City	**1,202**	41	60	**101**	**8.40**
Tanauan City	**3,086**	217	243	**460**	**14.91**
Tayabas City	**1,821**	617	560	**1,177**	**64.63**
Trece Martires City	**2,629**	201	163	**364**	**13.85**
**Region 4B**	**60,951**	**1,165**	**1,033**	**2,198**	**3.61**
Marinduque	**4,469**	197	188	**385**	**8.61**
Mindoro Occidental	**10,128**	270	52	**322**	**3.18**
Mindoro Oriental	**16,946**	321	308	**629**	**3.71**
Palawan	**18,148**	249	309	**558**	**3.07**
Romblon	**5,809**	98	122	**220**	**3.79**
Puerto Princesa City	**5,451**	30	54	**84**	**1.54**
**Region 5**	**119,182**	**21,871**	**23,155**	**45,026**	**37.78**
Albay	**20,923**	3,752	3,832	**7,584**	**36.25**
Camarines Norte	**12,056**	2,965	3,116	**6,081**	**50.44**
Camarines Sur	**33,805**	2,785	2,944	**5,729**	**16.95**
Catanduanes	**5,466**	1,721	1,795	**3,516**	**64.32**
Masbate	**19,939**	2,579	2,615	**5,194**	**26.05**
Sorsogon	**16,990**	6,535	7,131	**13,666**	**80.44**
Iriga City	**2,296**	288	352	**640**	**27.87**
Legaspi City	**3,679**	1,087	1,162	**2,249**	**61.13**
Naga City	**4,028**	159	208	**367**	**9.11**
**Region 6**	**124,691**	**16,317**	**17,292**	**33,609**	**26.95**
Aklan	**9,674**	376	401	**777**	**8.03**
Antique	**11,041**	366	360	**726**	**6.58**
Capiz	**12,001**	675	786	**1,461**	**12.17**
Guimaras	**2,900**	273	319	**592**	**20.41**
Iloilo	**30,500**	11,152	11,952	**23,104**	**75.75**
Negros Occidental	**42,054**	556	790	**1,346**	**3.20**
Bacolod City	**9,463**	822	1,032	**1,854**	**19.59**
Iloilo City	**7,058**	2,097	1,652	**3,749**	**53.12**

Table 2.D.1. Oral Health Care

Number and proportion of children 12-59 months old who are orally fit upon oral examination or after oral rehabilitation

Philippines, 2022

Area	Elig Pop x (Service Target 20%)	Orally fit		Total	%
		Male	Female		
**Region 7**	**129,697**	**19,893**	**22,953**	**42,846**	**33.04**
Bohol	**23,565**	1,183	1,354	**2,537**	**10.77**
Cebu	**51,808**	4,475	4,706	**9,181**	**17.72**
Negros Oriental	**22,943**	959	1,030	**1,989**	**8.67**
Siquijor	**1,531**	63	60	**123**	**8.03**
Cebu City	**16,263**	6,419	8,248	**14,667**	**90.19**
Lapu-Lapu City	**7,194**	5,207	5,954	**11,161**	**155.14**
Mandaue City	**6,393**	1,587	1,601	**3,188**	**49.87**
**Region 8**	**89,032**	**2,105**	**1,927**	**4,032**	**4.53**
Biliran	**3,391**	204	164	**368**	**10.85**
Eastern Samar	**9,680**	202	288	**490**	**5.06**
Northern Leyte	**29,306**	509	201	**710**	**2.42**
Northern Samar	**13,972**	247	269	**516**	**3.69**
Southern Leyte	**5,953**	0	0	**0**	**0.00**
Western Samar	**12,493**	63	100	**163**	**1.30**
Calbayog City	**3,850**	67	94	**161**	**4.18**
Maasin City	**1,515**	207	200	**407**	**26.86**
Ormoc City	**4,173**	368	360	**728**	**17.45**
Tacloban City	**4,699**	238	251	**489**	**10.41**
**Region 9**	**67,015**	**4,000**	**4,393**	**8,393**	**12.52**
Zamboanga Del Norte	**14,639**	402	350	**752**	**5.14**
Zamboanga Del Sur	**14,198**	2,226	2,326	**4,552**	**32.06**
Zamboanga Sibugay	**13,269**	528	794	**1,322**	**9.96**
Dapitan City	**1,511**	219	249	**468**	**30.97**
Dipolog City	**2,397**	256	285	**541**	**22.57**
Isabela City	**2,446**	39	64	**103**	**4.21**
Pagadian City	**3,482**	329	325	**654**	**18.78**
Zamboanga City	**15,073**	1	0	**1**	**0.01**
**Region 10**	**85,670**	**3,733**	**4,568**	**8,301**	**9.69**
Bukidnon	**19,420**	660	898	**1,558**	**8.02**
Camiguin	**1,599**	80	53	**133**	**8.32**
Lanao Del Norte	**13,468**	692	665	**1,357**	**10.08**
Misamis Occidental	**5,496**	442	590	**1,032**	**18.78**
Misamis Oriental	**12,505**	439	501	**940**	**7.52**
Cagayan de Oro City	**11,843**	348	551	**899**	**7.59**
El Salvador City	**879**	55	51	**106**	**12.06**
Gingoog City	**2,184**	1	1	**2**	**0.09**
Iligan City	**6,824**	337	316	**653**	**9.57**
Malaybalay City	**3,237**	316	274	**590**	**18.23**
Oroquieta City	**1,191**	0	0	**0**	**0.00**

Table 2.D.1. Oral Health Care

Number and proportion of children 12-59 months old who are orally fit upon oral examination or after oral rehabilitation

Philippines, 2022

Area	Elig Pop x (Service Target 20%)	Orally fit		Total	%
		Male	Female		
Ozamis City	**2,387**	165	423	**588**	**24.63**
Tangub City	**1,060**	129	127	**256**	**24.15**
Valencia City	**3,577**	69	118	**187**	**5.23**
**Region 11**	**90,584**	**2,288**	**2,517**	**4,805**	**5.30**
Davao de Oro	**13,573**	408	447	**855**	**6.30**
Davao del Norte	**17,278**	568	560	**1,128**	**6.53**
Davao Oriental	**11,426**	399	437	**836**	**7.32**
Davao del Sur	**11,808**	204	218	**422**	**3.57**
Davao Occidental	**6,018**	137	166	**303**	**5.03**
Davao City	**30,481**	572	689	**1,261**	**4.14**
**Region 12**	**81,919**	**6,372**	**6,778**	**13,150**	**16.05**
North Cotabato	**28,209**	1,157	1,297	**2,454**	**8.70**
Sarangani	**10,926**	899	971	**1,870**	**17.12**
South Cotabato	**16,890**	1,850	1,783	**3,633**	**21.51**
Sultan Kudarat	**14,926**	2,137	2,334	**4,471**	**29.95**
Gen. Santos City	**10,968**	329	393	**722**	**6.58**
**BARMM**	**97,703**	**7,615**	**8,532**	**16,147**	**16.53**
Basilan	**7,365**	43	36	**79**	**1.07**
Lanao Del Sur	**22,570**	1,545	1,766	**3,311**	**14.67**
Maguindanao	**30,806**	3,384	3,707	**7,091**	**23.02**
Sulu	**22,154**	1,955	2,215	**4,170**	**18.82**
Tawi-Tawi	**9,089**	445	494	**939**	**10.33**
Lamitan City	**2,027**	18	14	**32**	**1.58**
Marawi City	**5,398**	126	164	**290**	**5.37**
Cotabato City	**5,659**	99	136	**235**	**4.15**
**CARAGA**	**52,187**	**4,034**	**4,500**	**8,534**	**16.35**
Agusan del Norte	**6,844**	442	667	**1,109**	**16.20**
Agusan del Sur	**15,463**	676	672	**1,348**	**8.72**
Surigao del Norte	**6,483**	233	269	**502**	**7.74**
Surigao del Sur	**9,764**	768	828	**1,596**	**16.35**
Province of Dinagat	**2,252**	603	611	**1,214**	**53.91**
Bislig City	**1,854**	105	97	**202**	**10.90**
Butuan City	**6,507**	1,086	1,232	**2,318**	**35.62**
Surigao City	**3,020**	121	124	**245**	**8.11**

Table 2.D.1. Oral Health Care

Number and Proportion of children 5 years old and above with cases of decayed, missing, filled teeth (DMFT)
Philippines, 2022

Area	Total Examined	Cases of 5 y/o and above with DMFT		Total with DMFT	%
		Male	Female		
**PHILIPPINES**	**3,643,478**	**1,211,469**	**1,791,180**	**3,002,649**	**82.41**
**NCR**	**864,004**	**327,192**	**536,812**	**864,004**	**100.00**
Malabon	3,217	962	2,255	**3,217**	**100.00**
Navotas	21,076	8,568	12,508	**21,076**	**100.00**
Valenzuela City	34,842	12,422	22,420	**34,842**	**100.00**
Caloocan City	76,149	29,711	46,438	**76,149**	**100.00**
Marikina City	9,926	3,596	6,330	**9,926**	**100.00**
Pasig City	54,776	21,725	33,051	**54,776**	**100.00**
Pateros	2,582	1,113	1,469	**2,582**	**100.00**
Taguig	12,111	4,287	7,824	**12,111**	**100.00**
Quezon City	153,232	42,939	110,293	**153,232**	**100.00**
Makati City	36,033	14,061	21,972	**36,033**	**100.00**
Mandaluyong City	17,228	5,842	11,386	**17,228**	**100.00**
San Juan	2,850	1,108	1,742	**2,850**	**100.00**
Manila City	254,260	112,563	141,697	**254,260**	**100.00**
Las Piñas City	4,540	2,275	2,265	**4,540**	**100.00**
Muntinlupa City	45,045	18,115	26,930	**45,045**	**100.00**
Parañaque City	13,197	4,763	8,434	**13,197**	**100.00**
Pasay City	122,940	43,142	79,798	**122,940**	**100.00**
**CAR**	**55,399**	**11,392**	**20,899**	**32,291**	**58.29**
Abra	2,871	916	1,402	**2,318**	**80.74**
Apayao	2,614	827	1,152	**1,979**	**75.71**
Benguet	18,155	3,206	5,512	**8,718**	**48.02**
Ifugao	4,135	938	2,003	**2,941**	**71.12**
Kalinga	6,054	670	1,132	**1,802**	**29.77**
Mt. Province	7,212	1,527	2,167	**3,694**	**51.22**
Baguio City	14,358	3,308	7,531	**10,839**	**75.49**
**Region 1**	**309,314**	**35,420**	**51,824**	**87,244**	**28.21**
Ilocos Norte	25,987	1,637	2,069	**3,706**	**14.26**
Ilocos Sur	57,715	1,818	2,890	**4,708**	**8.16**
La Union	42,719	8,091	12,769	**20,860**	**48.83**
Pangasinan	132,344	20,989	29,083	**50,072**	**37.83**
Alaminos City	21,726	810	1,505	**2,315**	**10.66**
Candon City	3,839	65	50	**115**	**3.00**
Dagupan City	2,577	296	1,325	**1,621**	**62.90**
Laoag City	2,883	81	75	**156**	**5.41**
San Carlos City	2,747	50	64	**114**	**4.15**
San Fernando City	5,456	534	749	**1,283**	**23.52**
Urdaneta City	6,787	897	953	**1,850**	**27.26**
Vigan City	4,534	152	292	**444**	**9.79**

Table 2.D.1. Oral Health Care

Number and Proportion of children 5 years old and above with cases of decayed, missing, filled teeth (DMFT)
Philippines, 2022

Area	Total Examined	Cases of 5 y/o and above with DMFT		Total with DMFT	%
		Male	Female		
**Region 2**	**116,680**	**46,555**	**70,125**	**116,680**	**100.00**
Batanes	0	0	0	**0**	**0.00**
Cagayan	9,839	3,955	5,884	**9,839**	**100.00**
Isabela	42,143	16,545	25,598	**42,143**	**100.00**
Nueva Vizcaya	44,708	17,323	27,385	**44,708**	**100.00**
Quirino	7,830	4,042	3,788	**7,830**	**100.00**
Cauayan City	1,256	543	713	**1,256**	**100.00**
Ilagan City	4,941	2,380	2,561	**4,941**	**100.00**
Santiago City	3,354	1,156	2,198	**3,354**	**100.00**
Tuguegarao City	2,609	611	1,998	**2,609**	**100.00**
**Region 3**	**204,257**	**69,421**	**93,859**	**163,280**	**79.94**
Aurora	1,854	697	958	**1,655**	**89.27**
Bataan	4,135	1,606	2,529	**4,135**	**100.00**
Bulacan	31,445	11,318	18,201	**29,519**	**93.88**
Nueva Ecija	42,071	11,902	12,446	**24,348**	**57.87**
Pampanga	8,007	2,712	4,244	**6,956**	**86.87**
Tarlac	33,832	8,709	15,479	**24,188**	**71.49**
Zambales	7,374	2,924	3,972	**6,896**	**93.52**
Angeles City	0	0	0	**0**	**0.00**
Balanga City	2,976	943	1,954	**2,897**	**97.35**
Cabanatuan City	3,168	1,344	1,801	**3,145**	**99.27**
City of San Fernando	1,581	572	1,009	**1,581**	**100.00**
Gapan City	1,477	545	552	**1,097**	**74.27**
Mabalacat City	49,196	19,427	21,866	**41,293**	**83.94**
Malolos City	378	110	268	**378**	**100.00**
Meycauayan	663	348	315	**663**	**100.00**
Olongapo	517	252	265	**517**	**100.00**
Palayan City	27	7	20	**27**	**100.00**
San Jose City	1,681	730	951	**1,681**	**100.00**
San Jose del Monte City	1,004	84	82	**166**	**16.53**
Science City of Munoz	4,211	1,436	2,618	**4,054**	**96.27**
Tarlac City	8,660	3,755	4,329	**8,084**	**93.35**
**Region 4A**	**963,409**	**382,634**	**530,614**	**913,248**	**94.79**
Batangas	52,597	21,790	23,307	**45,097**	**85.74**
Cavite	6,271	1,817	2,934	**4,751**	**75.76**
Laguna	238,752	97,963	132,959	**230,922**	**96.72**
Quezon	11,945	2,653	4,573	**7,226**	**60.49**
Rizal	9,092	3,200	5,299	**8,499**	**93.48**
Antipolo City	6,936	1,634	3,499	**5,133**	**74.01**
Bacoor City	4,427	614	1,098	**1,712**	**38.67**
Batangas City	8,667	3,151	5,481	**8,632**	**99.60**
Biñan City	223,966	103,629	119,804	**223,433**	**99.76**
Cabuyao City	16,459	6,657	9,802	**16,459**	**100.00**

Table 2.D.1. Oral Health Care

Number and Proportion of children 5 years old and above with cases of decayed, missing, filled teeth (DMFT)
Philippines, 2022

Area	Total Examined	Cases of 5 y/o and above with DMFT		Total with DMFT	%
		Male	Female		
Calamba City	219,927	88,089	131,795	**219,884**	**99.98**
Cavite City	0	0	0	**0**	**0.00**
Dasmariñas City	984	245	729	**974**	**98.98**
General Trias City	22	11	11	**22**	**100.00**
Imus City	4,561	62	155	**217**	**4.76**
Lipa City	971	341	369	**710**	**73.12**
Lucena City	1,931	722	1,142	**1,864**	**96.53**
San Pablo City	13,612	581	3,016	**3,597**	**26.43**
San Pedro City	88,239	26,472	54,100	**80,572**	**91.31**
Santa Rosa City	45,637	19,792	25,845	**45,637**	**100.00**
Tagaytay City	936	242	622	**864**	**92.31**
Tanauan City	4,898	1,790	2,704	**4,494**	**91.75**
Tayabas City	26	0	0	**0**	**0.00**
Trece Martires City	2,553	1,179	1,370	**2,549**	**99.84**
**Region 4B**	**58,156**	**17,635**	**38,302**	**55,937**	**96.18**
Marinduque	1,070	291	600	**891**	**83.27**
Mindoro Occidental	42,217	12,480	29,737	**42,217**	**100.00**
Mindoro Oriental	6,968	2,499	3,204	**5,703**	**81.85**
Palawan	4,263	1,076	2,502	**3,578**	**83.93**
Romblon	1,614	571	965	**1,536**	**95.17**
Puerto Princesa City	2,024	718	1,294	**2,012**	**99.41**
**Region 5**	**155,040**	**39,874**	**63,010**	**102,884**	**66.36**
Albay	12,098	4,737	6,250	**10,987**	**90.82**
Camarines Norte	42,525	11,652	16,486	**28,138**	**66.17**
Camarines Sur	31,906	5,110	10,598	**15,708**	**49.23**
Catanduanes	5,938	1,702	2,214	**3,916**	**65.95**
Masbate	13,455	703	721	**1,424**	**10.58**
Sorsogon	31,302	10,111	18,354	**28,465**	**90.94**
Iriga City	3,944	1,397	2,302	**3,699**	**93.79**
Legaspi City	9,371	4,006	5,365	**9,371**	**100.00**
Naga City	4,501	456	720	**1,176**	**26.13**
**Region 6**	**234,632**	**58,896**	**92,362**	**151,258**	**64.47**
Aklan	14,233	4,330	7,664	**11,994**	**84.27**
Antique	8,470	2,183	3,603	**5,786**	**68.31**
Capiz	25,587	5,753	7,419	**13,172**	**51.48**
Guimaras	1,719	294	561	**855**	**49.74**
Iloilo	104,409	29,931	44,430	**74,361**	**71.22**
Negros Occidental	42,626	7,776	11,174	**18,950**	**44.46**
Bacolod City	13,901	648	4,877	**5,525**	**39.75**
Iloilo City	23,687	7,981	12,634	**20,615**	**87.03**

Table 2.D.1. Oral Health Care

Number and Proportion of children 5 years old and above with cases of decayed, missing, filled teeth (DMFT)
Philippines, 2022

Area	Total Examined	Cases of 5 y/o and above with DMFT		Total with DMFT	%
		Male	Female		
**Region 7**	**277,130**	**123,150**	**154,053**	**277,203**	**100.03**
Bohol	16,526	6,838	9,688	**16,526**	**100.00**
Cebu	24,944	10,041	14,903	**24,944**	**100.00**
Negros Oriental	78	18	60	**78**	**100.00**
Siquijor	0	0	0	**0**	**0.00**
Cebu City	43,899	13,210	30,689	**43,899**	**100.00**
Lapu-Lapu City	187,552	90,851	96,701	**187,552**	**100.00**
Mandaue City	4,131	2,192	2,012	**4,204**	**101.77**
**Region 8**	**26,124**	**4,622**	**6,081**	**10,703**	**40.97**
Biliran	17,964	1,639	1,897	**3,536**	**19.68**
Eastern Samar	196	63	94	**157**	**80.10**
Northern Leyte	3,458	1,152	2,306	**3,458**	**100.00**
Northern Samar	454	266	188	**454**	**100.00**
Southern Leyte	0	0	0	**0**	**0.00**
Western Samar	1,751	804	947	**1,751**	**100.00**
Calbayog City	694	330	364	**694**	**100.00**
Maasin City	907	17	40	**57**	**6.28**
Ormoc City	504	286	210	**496**	**98.41**
Tacloban City	196	65	35	**100**	**51.02**
**Region 9**	**40,361**	**16,649**	**21,660**	**38,309**	**94.92**
Zamboanga Del Norte	1,022	214	266	**480**	**46.97**
Zamboanga Del Sur	13,830	6,445	7,385	**13,830**	**100.00**
Zamboanga Sibugay	9,397	3,826	5,571	**9,397**	**100.00**
Dapitan City	903	74	88	**162**	**17.94**
Dipolog City	6,034	2,472	2,793	**5,265**	**87.26**
Isabela City	9,171	3,617	5,554	**9,171**	**100.00**
Pagadian City	0	0	0	**0**	**0.00**
Zamboanga City	4	1	3	**4**	**100.00**
**Region 10**	**185,474**	**16,454**	**22,771**	**39,225**	**21.15**
Bukidnon	39,661	2,574	3,941	**6,515**	**16.43**
Camiguin	3,005	72	50	**122**	**4.06**
Lanao Del Norte	29,600	2,558	4,033	**6,591**	**22.27**
Misamis Occidental	17,900	6,562	7,509	**14,071**	**78.61**
Misamis Oriental	27,410	1,210	2,016	**3,226**	**11.77**
Cagayan de Oro City	21,824	0	0	**0**	**0.00**
El Salvador City	1,635	37	47	**84**	**5.14**
Gingoog City	4,533	289	407	**696**	**15.35**
Iligan City	12,554	312	389	**701**	**5.58**
Malaybalay City	5,862	7	2	**9**	**0.15**
Oroquieta City	2,220	0	0	**0**	**0.00**
Ozamis City	4,990	302	587	**889**	**17.82**

Table 2.D.1. Oral Health Care

Number and Proportion of children 5 years old and above with cases of decayed, missing, filled teeth (DMFT)
Philippines, 2022

Area	Total Examined	Cases of 5 y/o and above with DMFT		Total with DMFT	%
		Male	Female		
Tangub City	2,331	232	256	**488**	**20.94**
Valencia City	11,949	2,299	3,534	**5,833**	**48.82**
**Region 11**	**6,808**	**2,918**	**3,501**	**6,419**	**94.29**
Davao de Oro	1,456	681	668	**1,349**	**92.65**
Davao del Norte	1,023	422	530	**952**	**93.06**
Davao Oriental	947	351	553	**904**	**95.46**
Davao del Sur	781	344	393	**737**	**94.37**
Davao Occidental	659	240	369	**609**	**92.41**
Davao City	1,942	880	988	**1,868**	**96.19**
**Region 12**	**55,904**	**21,783**	**38,413**	**60,196**	**107.68**
North Cotabato	18,820	6,599	13,059	**19,658**	**104.45**
Sarangani	8,965	2,019	6,946	**8,965**	**100.00**
South Cotabato	5,225	3,483	5,632	**9,115**	**174.45**
Sultan Kudarat	20,397	8,890	11,071	**19,961**	**97.86**
Gen. Santos City	2,497	792	1,705	**2,497**	**100.00**
**BARMM**	**53,167**	**24,441**	**27,413**	**51,854**	**97.53**
Basilan	1,032	398	634	**1,032**	**100.00**
Lanao Del Sur	16,705	7,758	8,947	**16,705**	**100.00**
Maguindanao	21,292	9,935	11,357	**21,292**	**100.00**
Sulu	7,826	3,830	3,996	**7,826**	**100.00**
Tawi-Tawi	0	0	0	**0**	**0.00**
Lamitan City	996	381	615	**996**	**100.00**
Marawi City	253	112	141	**253**	**100.00**
Cotabato City	5,063	2,027	1,723	**3,750**	**74.07**
**CARAGA**	**37,619**	**12,433**	**19,481**	**31,914**	**84.83**
Agusan del Norte	12,005	2,624	3,676	**6,300**	**52.48**
Agusan del Sur	4,513	1,558	2,955	**4,513**	**100.00**
Surigao del Norte	3,016	1,120	1,896	**3,016**	**100.00**
Surigao del Sur	12,394	4,533	7,861	**12,394**	**100.00**
Province of Dinagat	937	455	482	**937**	**100.00**
Bislig City	612	297	315	**612**	**100.00**
Butuan City	3,052	1,367	1,685	**3,052**	**100.00**
Surigao City	1,090	479	611	**1,090**	**100.00**

Table 2.D.1. Oral Health Care

(BOHC)

Philippines, 2022

Area	Infants 0-11 months old who received BOHC				
	Eligible Population	Male	Female	Total	%
**Philippines**	**639,457**	**133,955**	**137,690**	**271,645**	**42.48**
**NCR**	**71,069**	**31,858**	**31,892**	**63,750**	**89.70**
Malabon	**2,017**	157	203	**360**	**17.85**
Navotas	**1,377**	608	769	**1,377**	**100.00**
Valenzuela City	**3,425**	1,845	1,580	**3,425**	**100.00**
Caloocan City	**8,744**	4,470	4,274	**8,744**	**100.00**
Marikina City	**2,488**	1,244	1,244	**2,488**	**100.00**
Pasig City	**4,168**	1,987	2,180	**4,167**	**99.98**
Pateros	**353**	156	197	**353**	**100.00**
Taguig	**4,442**	1,835	1,887	**3,722**	**83.79**
Quezon City	**16,203**	8,007	8,196	**16,203**	**100.00**
Makati City	**3,215**	1,289	1,123	**2,412**	**75.02**
Mandaluyong City	**2,133**	1,042	1,091	**2,133**	**100.00**
San Juan	**674**	345	329	**674**	**100.00**
Manila City	**9,823**	4,911	4,912	**9,823**	**100.00**
Las Piñas City	**3,250**	1,533	1,574	**3,107**	**95.60**
Muntinlupa City	**2,784**	1,392	1,392	**2,784**	**100.00**
Parañaque City	**3,675**	635	565	**1,200**	**32.65**
Pasay City	**2,298**	402	376	**778**	**33.86**
**CAR**	**10,555**	**2,365**	**2,394**	**4,759**	**45.09**
Abra	**1,283**	237	230	**467**	**36.40**
Apayao	**751**	117	122	**239**	**31.82**
Benguet	**2,726**	841	873	**1,714**	**62.88**
Ifugao	**1,346**	16	22	**38**	**2.82**
Kalinga	**1,410**	623	580	**1,203**	**85.32**
Mt. Province	**931**	123	141	**264**	**28.36**
Baguio City	**2,108**	408	426	**834**	**39.56**
**Region 1**	**29,129**	**5,880**	**6,326**	**12,206**	**41.90**
Ilocos Norte	**2,356**	431	480	**911**	**38.67**
Ilocos Sur	**2,722**	1,242	1,167	**2,409**	**88.50**
La Union	**3,401**	1,152	1,198	**2,350**	**69.10**
Pangasinan	**15,213**	2,227	2,533	**4,760**	**31.29**
Alaminos City	**573**	205	275	**480**	**83.77**
Candon City	**286**	148	138	**286**	**100.00**
Dagupan City	**1,097**	217	245	**462**	**42.11**
Laoag City	**544**	25	30	**55**	**10.11**
San Carlos City	**1,208**	95	140	**235**	**19.45**
San Fernando City	**622**	0	0	**0**	**0.00**
Urdaneta City	**851**	2	0	**2**	**0.24**
Vigan City	**256**	136	120	**256**	**100.00**

Table 2.D.1. Oral Health Care
(BOHC)
Philippines, 2022

Area	Infants 0-11 months old who received BOHC				
	Eligible Population	Male	Female	Total	%
**Region 2**	**20,688**	**6,318**	**6,420**	**12,738**	**61.57**
Batanes	**109**	0	0	**0**	**0.00**
Cagayan	**6,129**	361	318	**679**	**11.08**
Isabela	**7,035**	3,395	3,640	**7,035**	**100.00**
Nueva Vizcaya	**2,861**	1,222	1,039	**2,261**	**79.03**
Quirino	**1,215**	158	168	**326**	**26.83**
Cauayan City	**770**	351	419	**770**	**100.00**
Ilagan City	**867**	414	452	**866**	**99.88**
Santiago City	**801**	417	384	**801**	**100.00**
Tuguegarao City	**901**	0	0	**0**	**0.00**
**Region 3**	**66,048**	**16,551**	**17,022**	**33,573**	**50.83**
Aurora	**1,426**	310	327	**637**	**44.67**
Bataan	**4,137**	166	168	**334**	**8.07**
Bulacan	**13,128**	4,394	4,200	**8,594**	**65.46**
Nueva Ecija	**8,712**	2,748	2,993	**5,741**	**65.90**
Pampanga	**9,486**	1,362	1,467	**2,829**	**29.82**
Tarlac	**5,796**	1,025	1,103	**2,128**	**36.71**
Zambales	**3,743**	998	963	**1,961**	**52.39**
Angeles City	**2,380**	0	0	**0**	**0.00**
Balanga City	**598**	180	188	**368**	**61.54**
Cabanatuan City	**1,784**	40	52	**92**	**5.16**
City of San Fernando	**1,772**	355	376	**731**	**41.25**
Gapan City	**651**	132	172	**304**	**46.70**
Mabalacat City	**1,449**	1,349	1,267	**2,616**	**180.54**
Malolos City	**1,467**	337	378	**715**	**48.74**
Meycauayan	**1,217**	67	66	**133**	**10.93**
Olongapo	**1,477**	203	215	**418**	**28.30**
Palayan City	**242**	2	4	**6**	**2.48**
San Jose City	**824**	902	865	**1,767**	**214.44**
San Jose del Monte City	**3,340**	861	970	**1,831**	**54.82**
Science City of Munoz	**480**	55	33	**88**	**18.33**
Tarlac City	**1,939**	1,065	1,215	**2,280**	**117.59**
**Region 4A**	**89,890**	**14,901**	**14,965**	**29,866**	**33.23**
Batangas	**11,777**	1,584	1,470	**3,054**	**25.93**
Cavite	**8,445**	315	290	**605**	**7.16**
Laguna	**5,909**	802	835	**1,637**	**27.70**
Quezon	**11,356**	1,510	1,457	**2,967**	**26.13**
Rizal	**13,439**	3,360	3,334	**6,694**	**49.81**
Antipolo City	**4,949**	2,734	2,886	**5,620**	**113.56**
Bacoor City	**3,702**	318	306	**624**	**16.86**
Batangas City	**2,090**	161	180	**341**	**16.32**
Biñan City	**1,979**	1,938	2,209	**4,147**	**209.55**
Cabuyao City	**1,836**	0	0	**0**	**0.00**
Calamba City	**2,702**	0	0	**0**	**0.00**
Cavite City	**632**	0	0	**0**	**0.00**

Table 2.D.1. Oral Health Care

(BOHC)

Philippines, 2022

Area	Infants 0-11 months old who received BOHC				
	Eligible Population	Male	Female	Total	%
Dasmariñas City	**4,061**	0	1	**1**	**0.02**
General Trias City	**1,937**	53	37	**90**	**4.65**
Imus City	**2,488**	695	652	**1,347**	**54.14**
Lipa City	**2,105**	162	183	**345**	**16.39**
Lucena City	**1,721**	98	79	**177**	**10.28**
San Pablo City	**1,582**	0	4	**4**	**0.25**
San Pedro City	**1,937**	0	0	**0**	**0.00**
Santa Rosa City	**2,103**	749	691	**1,440**	**68.47**
Tagaytay City	**438**	2	2	**4**	**0.91**
Tanauan City	**1,098**	3	6	**9**	**0.82**
Tayabas City	**644**	417	343	**760**	**118.01**
Trece Martires City	**960**	0	0	**0**	**0.00**
**Region 4B**	**21,838**	**1,254**	**1,096**	**2,350**	**10.76**
Marinduque	**1,497**	99	95	**194**	**12.96**
Mindoro Occidental	**3,744**	567	498	**1,065**	**28.45**
Mindoro Oriental	**6,057**	288	256	**544**	**8.98**
Palawan	**6,589**	163	136	**299**	**4.54**
Romblon	**1,971**	132	108	**240**	**12.18**
Puerto Princesa City	**1,980**	5	3	**8**	**0.40**
**Region 5**	**41,537**	**6,076**	**5,995**	**12,071**	**29.06**
Albay	**7,224**	599	674	**1,273**	**17.62**
Camarines Norte	**4,315**	1,056	791	**1,847**	**42.80**
Camarines Sur	**11,846**	1,180	968	**2,148**	**18.13**
Catanduanes	**1,938**	314	327	**641**	**33.08**
Masbate	**6,780**	703	721	**1,424**	**21.00**
Sorsogon	**5,948**	1,750	1,985	**3,735**	**62.79**
Iriga City	**804**	338	398	**736**	**91.54**
Legaspi City	**1,270**	110	112	**222**	**17.48**
Naga City	**1,412**	26	19	**45**	**3.19**
**Region 6**	**43,935**	**9,025**	**9,534**	**18,559**	**42.24**
Aklan	**3,386**	467	398	**865**	**25.55**
Antique	**3,940**	221	251	**472**	**11.98**
Capiz	**4,193**	908	1,056	**1,964**	**46.84**
Guimaras	**925**	29	32	**61**	**6.59**
Iloilo	**10,880**	4,048	4,521	**8,569**	**78.76**
Negros Occidental	**14,769**	408	384	**792**	**5.36**
Bacolod City	**3,325**	651	569	**1,220**	**36.69**
Iloilo City	**2,517**	2,293	2,323	**4,616**	**183.39**
**Region 7**	**48,693**	**12,614**	**12,770**	**25,384**	**52.13**
Bohol	**8,398**	309	327	**636**	**7.57**
Cebu	**19,939**	4,055	4,265	**8,320**	**41.73**
Negros Oriental	**8,367**	752	774	**1,526**	**18.24**

Table 2.D.1. Oral Health Care
(BOHC)
Philippines, 2022

Area	Infants 0-11 months old who received BOHC				
	Eligible Population	Male	Female	Total	%
Siquijor	**498**	0	0	**0**	**0.00**
Cebu City	**6,260**	4,478	4,418	**8,896**	**142.11**
Lapu-Lapu City	**2,770**	1,487	1,531	**3,018**	**108.95**
Mandaue City	**2,461**	1,533	1,455	**2,988**	**121.41**
**Region 8**	**31,642**	**1,905**	**2,112**	**4,017**	**12.70**
Biliran	**1,162**	73	70	**143**	**12.31**
Eastern Samar	**3,572**	176	275	**451**	**12.63**
Northern Leyte	**10,627**	525	578	**1,103**	**10.38**
Northern Samar	**4,776**	11	13	**24**	**0.50**
Southern Leyte	**1,999**	0	0	**0**	**0.00**
Western Samar	**4,420**	52	65	**117**	**2.65**
Calbayog City	**1,361**	57	63	**120**	**8.82**
Maasin City	**509**	4	8	**12**	**2.36**
Ormoc City	**1,513**	574	585	**1,159**	**76.60**
Tacloban City	**1,703**	433	455	**888**	**52.14**
**Region 9**	**23,702**	**1,691**	**1,693**	**3,384**	**14.28**
Zamboanga Del Norte	**5,056**	286	286	**572**	**11.31**
Zamboanga Del Sur	**5,210**	1,046	1,046	**2,092**	**40.15**
Zamboanga Sibugay	**4,506**	146	146	**292**	**6.48**
Dapitan City	**521**	1	5	**6**	**1.15**
Dipolog City	**829**	13	10	**23**	**2.77**
Isabela City	**769**	0	0	**0**	**0.00**
Pagadian City	**1,277**	105	105	**210**	**16.44**
Zamboanga City	**5,534**	94	95	**189**	**3.42**
**Region 10**	**29,973**	**2,851**	**3,784**	**6,635**	**22.14**
Bukidnon	**6,870**	298	362	**660**	**9.61**
Camiguin	**556**	144	123	**267**	**48.02**
Lanao Del Norte	**4,479**	119	124	**243**	**5.43**
Misamis Occidental	**1,926**	743	925	**1,668**	**86.60**
Misamis Oriental	**4,489**	258	270	**528**	**11.76**
Cagayan de Oro City	**4,252**	247	641	**888**	**20.88**
El Salvador City	**316**	6	13	**19**	**6.01**
Gingoog City	**782**	0	0	**0**	**0.00**
Iligan City	**2,270**	209	195	**404**	**17.80**
Malaybalay City	**1,145**	373	351	**724**	**63.23**
Oroquieta City	**417**	0	0	**0**	**0.00**
Ozamis City	**836**	242	508	**750**	**89.71**
Tangub City	**370**	126	194	**320**	**86.49**
Valencia City	**1,265**	86	78	**164**	**12.96**
**Region 11**	**32,522**	**1,731**	**2,097**	**3,828**	**11.77**
Davao de Oro	**4,647**	255	323	**578**	**12.44**

Table 2.D.1. Oral Health Care

(BOHC)

Philippines, 2022

Area	Infants 0-11 months old who received BOHC				
	Eligible Population	Male	Female	Total	%
Davao del Norte	**6,305**	328	433	**761**	**12.07**
Davao Oriental	**3,969**	263	284	**547**	**13.78**
Davao del Sur	**4,369**	130	171	**301**	**6.89**
Davao Occidental	**1,953**	102	129	**231**	**11.83**
Davao City	**11,279**	653	757	**1,410**	**12.50**
**Region 12**	**29,658**	**9,429**	**9,609**	**19,038**	**64.19**
North Cotabato	**10,245**	3,545	3,702	**7,247**	**70.74**
Sarangani	**3,872**	580	513	**1,093**	**28.23**
South Cotabato	**6,275**	1,998	1,848	**3,846**	**61.29**
Sultan Kudarat	**5,190**	820	986	**1,806**	**34.80**
Gen. Santos City	**4,076**	2,486	2,560	**5,046**	**123.80**
**BARMM**	**30,404**	**5,068**	**5,598**	**10,666**	**35.08**
Basilan	**2,347**	37	38	**75**	**3.20**
Lanao Del Sur	**6,492**	1,690	1,881	**3,571**	**55.01**
Maguindanao	**9,659**	1,829	2,158	**3,987**	**41.28**
Sulu	**5,150**	1,112	1,092	**2,204**	**42.80**
Tawi-Tawi	**2,811**	0	0	**0**	**0.00**
Lamitan City	**646**	9	14	**23**	**3.56**
Marawi City	**1,552**	134	153	**287**	**18.49**
Cotabato City	**1,747**	257	262	**519**	**29.71**
**CARAGA**	**18,174**	**4,438**	**4,383**	**8,821**	**48.54**
Agusan del Norte	**2,438**	1,044	900	**1,944**	**79.74**
Agusan del Sur	**5,313**	166	212	**378**	**7.11**
Surigao del Norte	**2,184**	614	585	**1,199**	**54.90**
Surigao del Sur	**3,466**	483	606	**1,089**	**31.42**
Province of Dinagat	**773**	428	320	**748**	**96.77**
Bislig City	**658**	89	95	**184**	**27.96**
Butuan City	**2,323**	1,112	1,171	**2,283**	**98.28**
Surigao City	**1,019**	502	494	**996**	**97.74**

Table 2.D.2. Oral Health Care

Number and proportion of children 1-4 years old who received Basic Oral Health Care (BOHC)
Philippines, 2022

Area	Infants 1-4 years old who received BOHC				
	Eligible Population	Male	Female	Total	%
**Philippines**	**2,693,010**	**436,136**	**463,051**	**899,187**	**33.39**
**NCR**	**285,464**	**98,654**	**102,893**	**201,547**	**70.60**
Malabon	**8,103**	932	1,158	**2,090**	**25.79**
Navotas	**5,529**	2,743	2,786	**5,529**	**100.00**
Valenzuela City	**13,754**	3,550	3,699	**7,249**	**52.70**
Caloocan City	**35,114**	12,805	13,590	**26,395**	**75.17**
Marikina City	**9,992**	2,079	2,105	**4,184**	**41.87**
Pasig City	**16,744**	8,904	7,840	**16,744**	**100.00**
Pateros	**1,415**	752	663	**1,415**	**100.00**
Taguig	**17,843**	1,319	1,332	**2,651**	**14.86**
Quezon City	**65,090**	28,603	29,681	**58,284**	**89.54**
Makati City	**12,916**	4,281	4,268	**8,549**	**66.19**
Mandaluyong City	**8,562**	2,390	2,525	**4,915**	**57.40**
San Juan	**2,708**	792	775	**1,567**	**57.87**
Manila City	**39,464**	18,686	20,778	**39,464**	**100.00**
Las Piñas City	**13,054**	2,113	2,048	**4,161**	**31.88**
Muntinlupa City	**11,184**	5,286	5,898	**11,184**	**100.00**
Parañaque City	**14,759**	2,264	2,405	**4,669**	**31.63**
Pasay City	**9,233**	1,155	1,342	**2,497**	**27.04**
**CAR**	**44,157**	**6,939**	**7,488**	**14,427**	**32.67**
Abra	**5,684**	519	539	**1,058**	**18.61**
Apayao	**3,333**	1,344	1,384	**2,728**	**81.85**
Benguet	**10,802**	1,815	2,057	**3,872**	**35.85**
Ifugao	**5,831**	670	673	**1,343**	**23.03**
Kalinga	**6,021**	714	818	**1,532**	**25.44**
Mt. Province	**4,127**	419	418	**837**	**20.28**
Baguio City	**8,359**	1,458	1,599	**3,057**	**36.57**
**Region 1**	**122,058**	**24,969**	**28,435**	**53,404**	**43.75**
Ilocos Norte	**9,585**	2,313	2,618	**4,931**	**51.44**
Ilocos Sur	**11,896**	4,075	4,367	**8,442**	**70.97**
La Union	**14,366**	5,566	5,996	**11,562**	**80.48**
Pangasinan	**63,433**	8,467	9,399	**17,866**	**28.17**
Alaminos City	**2,396**	741	1,017	**1,758**	**73.37**
Candon City	**1,255**	545	533	**1,078**	**85.90**
Dagupan City	**4,577**	258	273	**531**	**11.60**
Laoag City	**2,210**	116	1,043	**1,159**	**52.44**
San Carlos City	**5,039**	1,146	1,230	**2,376**	**47.15**
San Fernando City	**2,633**	789	872	**1,661**	**63.08**
Urdaneta City	**3,553**	388	478	**866**	**24.37**
Vigan City	**1,115**	565	609	**1,174**	**105.29**

Table 2.D.2. Oral Health Care

Number and proportion of children 1-4 years old who received Basic Oral Health Care (BOHC)
Philippines, 2022

Area	Infants 1-4 years old who received BOHC				
	Eligible Population	Male	Female	Total	%
**Region 2**	**86,809**	**18,740**	**20,910**	**39,650**	**45.67**
Batanes	**488**	147	162	**309**	**63.32**
Cagayan	**25,746**	2,049	2,159	**4,208**	**16.34**
Isabela	**29,386**	6,953	7,322	**14,275**	**48.58**
Nueva Vizcaya	**12,046**	2,375	3,275	**5,650**	**46.90**
Quirino	**5,186**	2,438	2,676	**5,114**	**98.61**
Cauayan City	**3,215**	1,310	1,905	**3,215**	**100.00**
Ilagan City	**3,614**	1,703	1,810	**3,513**	**97.21**
Santiago City	**3,348**	1,756	1,592	**3,348**	**100.00**
Tuguegarao City	**3,780**	9	9	**18**	**0.48**
**Region 3**	**273,946**	**48,517**	**51,490**	**100,007**	**36.51**
Aurora	**6,129**	842	916	**1,758**	**28.68**
Bataan	**16,402**	1,615	1,600	**3,215**	**19.60**
Bulacan	**54,430**	5,631	5,944	**11,575**	**21.27**
Nueva Ecija	**36,761**	6,340	6,940	**13,280**	**36.13**
Pampanga	**39,553**	9,155	9,940	**19,095**	**48.28**
Tarlac	**23,954**	5,911	5,841	**11,752**	**49.06**
Zambales	**15,201**	2,179	2,232	**4,411**	**29.02**
Angeles City	**9,924**	478	489	**967**	**9.74**
Balanga City	**2,372**	878	998	**1,876**	**79.09**
Cabanatuan City	**7,524**	858	1,113	**1,971**	**26.20**
City of San Fernando	**7,392**	773	847	**1,620**	**21.92**
Gapan City	**2,746**	147	150	**297**	**10.82**
Mabalacat City	**6,047**	5,567	5,772	**11,339**	**187.51**
Malolos City	**6,081**	1,102	1,178	**2,280**	**37.49**
Meycauayan	**5,044**	398	401	**799**	**15.84**
Olongapo	**5,995**	463	494	**957**	**15.96**
Palayan City	**1,022**	59	58	**117**	**11.45**
San Jose City	**3,480**	2,160	2,161	**4,321**	**124.17**
San Jose del Monte Cit	**13,846**	1,237	1,359	**2,596**	**18.75**
Science City of Munoz	**2,028**	910	1,122	**2,032**	**100.20**
Tarlac City	**8,015**	1,814	1,935	**3,749**	**46.77**
**Region 4A**	**371,577**	**45,367**	**49,129**	**94,496**	**25.43**
Batangas	**49,614**	9,252	9,189	**18,441**	**37.17**
Cavite	**34,716**	2,497	2,740	**5,237**	**15.09**
Laguna	**24,127**	4,484	5,868	**10,352**	**42.91**
Quezon	**48,129**	4,885	5,273	**10,158**	**21.11**
Rizal	**54,533**	5,668	5,783	**11,451**	**21.00**
Antipolo City	**20,087**	2,316	2,523	**4,839**	**24.09**
Bacoor City	**15,211**	112	147	**259**	**1.70**
Batangas City	**8,805**	2,145	2,231	**4,376**	**49.70**
Biñan City	**8,090**	2,628	3,009	**5,637**	**69.68**
Cabuyao City	**7,499**	3,777	4,004	**7,781**	**103.76**
Calamba City	**11,040**	258	328	**586**	**5.31**
Cavite City	**2,605**	0	0	**0**	**0.00**

Table 2.D.2. Oral Health Care

Number and proportion of children 1-4 years old who received Basic Oral Health Care (BOHC)
Philippines, 2022

Area	Infants 1-4 years old who received BOHC				
	Eligible Population	Male	Female	Total	%
Dasmariñas City	**16,690**	9	5	**14**	**0.08**
General Trias City	**7,961**	33	32	**65**	**0.82**
Imus City	**10,225**	194	187	**381**	**3.73**
Lipa City	**8,873**	837	926	**1,763**	**19.87**
Lucena City	**7,294**	750	830	**1,580**	**21.66**
San Pablo City	**6,463**	1,234	1,521	**2,755**	**42.63**
San Pedro City	**7,914**	30	4	**34**	**0.43**
Santa Rosa City	**8,593**	2,236	2,327	**4,563**	**53.10**
Tagaytay City	**1,804**	238	257	**495**	**27.44**
Tanauan City	**4,628**	557	617	**1,174**	**25.37**
Tayabas City	**2,732**	367	336	**703**	**25.73**
Trece Martires City	**3,944**	860	992	**1,852**	**46.96**
**Region 4B**	**91,425**	**4,734**	**4,963**	**9,697**	**10.61**
Marinduque	**6,703**	404	467	**871**	**12.99**
Mindoro Occidental	**15,192**	1,200	1,218	**2,418**	**15.92**
Oriental Mindoro	**25,419**	1,626	1,744	**3,370**	**13.26**
Palawan	**27,222**	1,099	1,134	**2,233**	**8.20**
Romblon	**8,713**	348	346	**694**	**7.97**
Puerto Princesa City	**8,176**	57	54	**111**	**1.36**
**Region 5**	**178,775**	**49,638**	**48,567**	**98,205**	**54.93**
Albay	**31,385**	6,833	6,326	**13,159**	**41.93**
Camarines Norte	**18,084**	6,379	6,187	**12,566**	**69.49**
Camarines Sur	**50,708**	9,515	9,167	**18,682**	**36.84**
Catanduanes	**8,199**	5,301	5,270	**10,571**	**128.93**
Masbate	**29,908**	4,714	4,669	**9,383**	**31.37**
Sorsogon	**25,485**	12,297	12,078	**24,375**	**95.64**
Iriga City	**3,445**	1,023	1,081	**2,104**	**61.07**
Legaspi City	**5,519**	2,280	2,308	**4,588**	**83.13**
Naga City	**6,042**	1,296	1,481	**2,777**	**45.96**
**Region 6**	**187,035**	**28,520**	**30,312**	**58,832**	**31.46**
Aklan	**14,511**	1,295	1,354	**2,649**	**18.26**
Antique	**16,561**	842	970	**1,812**	**10.94**
Capiz	**18,002**	2,019	2,234	**4,253**	**23.63**
Guimaras	**4,349**	753	797	**1,550**	**35.64**
Iloilo	**45,750**	17,314	18,043	**35,357**	**77.28**
Negros Occidental	**63,081**	2,654	3,619	**6,273**	**9.94**
Bacolod City	**14,194**	829	902	**1,731**	**12.20**
Iloilo City	**10,587**	2,814	2,393	**5,207**	**49.18**
**Region 7**	**194,544**	**32,927**	**34,050**	**66,977**	**34.43**
Bohol	**35,348**	2,928	3,075	**6,003**	**16.98**
Cebu	**77,712**	8,829	9,697	**18,526**	**23.84**
Negros Oriental	**34,414**	2,115	2,507	**4,622**	**13.43**

Table 2.D.2. Oral Health Care

Number and proportion of children 1-4 years old who received Basic Oral Health Care (BOHC)
Philippines, 2022

Area	Infants 1-4 years old who received BOHC				
	Eligible Population	Male	Female	Total	%
Siquijor	**2,296**	356	346	**702**	**30.57**
Cebu City	**24,395**	9,251	10,043	**19,294**	**79.09**
Lapu-Lapu City	**10,790**	7,468	6,396	**13,864**	**128.49**
Mandaue City	**9,589**	1,980	1,986	**3,966**	**41.36**
**Region 8**	**133,552**	**7,169**	**7,661**	**14,830**	**11.10**
Biliran	**5,087**	538	581	**1,119**	**22.00**
Eastern Samar	**14,520**	1,011	1,227	**2,238**	**15.41**
Northern Leyte	**43,959**	1,266	1,339	**2,605**	**5.93**
Northern Samar	**20,959**	754	799	**1,553**	**7.41**
Southern Leyte	**8,930**	0	0	**0**	**0.00**
Western Samar	**18,740**	628	564	**1,192**	**6.36**
Calbayog City	**5,775**	894	996	**1,890**	**32.73**
Maasin City	**2,273**	695	696	**1,391**	**61.20**
Ormoc City	**6,260**	890	900	**1,790**	**28.59**
Tacloban City	**7,049**	493	559	**1,052**	**14.92**
**Region 9**	**100,520**	**6,484**	**7,119**	**13,603**	**13.53**
Zamboanga Del Norte	**21,958**	693	696	**1,389**	**6.33**
Zamboanga Del Sur	**21,296**	3,439	3,951	**7,390**	**34.70**
Zamboanga Sibugay	**19,903**	666	715	**1,381**	**6.94**
Dapitan City	**2,267**	221	247	**468**	**20.64**
Dipolog City	**3,596**	915	927	**1,842**	**51.22**
Isabela City	**3,669**	279	270	**549**	**14.96**
Pagadian City	**5,222**	197	221	**418**	**8.00**
Zamboanga City	**22,609**	74	92	**166**	**0.73**
**Region 10**	**128,508**	**14,519**	**17,268**	**31,787**	**24.74**
Bukidnon	**29,130**	2,456	2,740	**5,196**	**17.84**
Camiguin	**2,399**	147	164	**311**	**12.96**
Lanao Del Norte	**20,203**	1,145	1,039	**2,184**	**10.81**
Misamis Occidental	**8,244**	2,483	3,245	**5,728**	**69.48**
Misamis Oriental	**18,758**	2,226	2,373	**4,599**	**24.52**
Cagayan de Oro City	**17,765**	1,305	1,489	**2,794**	**15.73**
El Salvador City	**1,319**	157	175	**332**	**25.17**
Gingoog City	**3,276**	725	785	**1,510**	**46.09**
Iligan City	**10,236**	1,716	1,827	**3,543**	**34.61**
Malaybalay City	**4,855**	113	127	**240**	**4.94**
Oroquieta City	**1,786**	134	115	**249**	**13.94**
Ozamis City	**3,581**	1,467	2,655	**4,122**	**115.11**
Tangub City	**1,590**	304	315	**619**	**38.93**
Valencia City	**5,366**	141	219	**360**	**6.71**
**Region 11**	**135,877**	**4,103**	**4,601**	**8,704**	**6.41**
Davao de Oro	**20,360**	745	688	**1,433**	**7.04**

Table 2.D.2. Oral Health Care

Number and proportion of children 1-4 years old who received Basic Oral Health Care (BOHC)
Philippines, 2022

Area	Infants 1-4 years old who received BOHC				
	Eligible Population	Male	Female	Total	%
Davao del Norte	**25,917**	889	1,025	**1,914**	**7.39**
Davao Oriental	**17,139**	625	679	**1,304**	**7.61**
Davao del Sur	**17,713**	266	389	**655**	**3.70**
Davao Occidental	**9,027**	255	327	**582**	**6.45**
Davao City	**45,721**	1,323	1,493	**2,816**	**6.16**
**Region 12**	**122,877**	**17,493**	**18,337**	**35,830**	**29.16**
North Cotabato	**42,313**	3,595	3,860	**7,455**	**17.62**
Sarangani	**16,388**	2,924	3,006	**5,930**	**36.19**
South Cotabato	**25,334**	4,670	4,761	**9,431**	**37.23**
Sultan Kudarat	**22,389**	2,518	2,627	**5,145**	**22.98**
Gen. Santos City	**16,453**	3,786	4,083	**7,869**	**47.83**
**BARMM**	**157,603**	**15,464**	**16,701**	**32,165**	**20.41**
Basilan	**11,048**	63	77	**140**	**1.27**
Lanao Del Sur	**33,855**	3,954	4,444	**8,398**	**24.81**
Maguindanao	**46,209**	4,769	5,712	**10,481**	**22.68**
Sulu	**33,231**	3,831	4,078	**7,909**	**23.80**
Tawi-Tawi	**13,633**	1,895	1,433	**3,328**	**24.41**
Lamitan City	**3,041**	16	14	**30**	**0.99**
Marawi City	**8,097**	651	718	**1,369**	**16.91**
Cotabato City	**8,489**	285	225	**510**	**6.01**
**CARAGA**	**78,283**	**11,899**	**13,127**	**25,026**	**31.97**
Agusan del Norte	**10,267**	1,796	2,044	**3,840**	**37.40**
Agusan del Sur	**23,195**	1,546	1,836	**3,382**	**14.58**
Surigao del Norte	**9,724**	1,479	1,543	**3,022**	**31.08**
Surigao del Sur	**14,646**	1,892	1,999	**3,891**	**26.57**
Province of Dinagat	**3,378**	1,485	1,622	**3,107**	**91.98**
Bislig City	**2,781**	607	741	**1,348**	**48.47**
Butuan City	**9,761**	2,470	2,725	**5,195**	**53.22**
Surigao City	**4,531**	624	617	**1,241**	**27.39**

Table 2.D.3. Oral Health Care

Number and proportion of children 5-9 years old who received Basic Oral Health Care (BOHC)
Philippines, 2022

Area	Children 5-9 years old who received BOHC				
	Eligible Population	Male	Female	Total	%
**Philippines**	**3,339,797**	**387,942**	**413,635**	**801,577**	**24.00**
**NCR**	**370,463**	**73,119**	**76,471**	**149,590**	**40.38**
Malabon	**10,516**	407	2,016	**2,423**	**23.04**
Navotas	**7,176**	3,229	3,947	**7,176**	**100.00**
Valenzuela City	**17,849**	2,597	3,126	**5,723**	**32.06**
Caloocan City	**45,568**	5,483	5,771	**11,254**	**24.70**
Marikina City	**12,967**	1,945	2,005	**3,950**	**30.46**
Pasig City	**21,728**	9,359	10,126	**19,485**	**89.68**
Pateros	**1,836**	889	947	**1,836**	**99.98**
Taguig	**23,158**	1,095	1,171	**2,266**	**9.78**
Quezon City	**84,469**	4,416	4,693	**9,109**	**10.78**
Makati City	**16,761**	6,851	6,890	**13,741**	**81.98**
Mandaluyong City	**11,113**	1,156	1,249	**2,405**	**21.64**
San Juan	**3,514**	752	749	**1,501**	**42.71**
Manila City	**51,212**	26,406	24,806	**51,212**	**100.00**
Las Piñas City	**16,942**	2,745	2,764	**5,509**	**32.52**
Muntinlupa City	**14,514**	3,723	3,833	**7,556**	**52.06**
Parañaque City	**19,155**	1,128	1,251	**2,379**	**12.42**
Pasay City	**11,984**	938	1,127	**2,065**	**17.23**
**CAR**	**54,032**	**12,472**	**13,875**	**26,347**	**48.76**
Abra	**7,000**	317	334	**651**	**9.30**
Apayao	**4,004**	932	954	**1,886**	**47.11**
Benguet	**13,262**	2,635	2,624	**5,259**	**39.66**
Ifugao	**7,059**	739	788	**1,527**	**21.63**
Kalinga	**7,333**	1,289	1,438	**2,727**	**37.19**
Mt. Province	**5,108**	987	1,010	**1,997**	**39.10**
Baguio City	**10,268**	5,573	6,727	**12,300**	**119.80**
**Region 1**	**155,884**	**24,026**	**25,835**	**49,861**	**31.99**
Ilocos Norte	**12,266**	2,210	2,316	**4,526**	**36.90**
Ilocos Sur	**15,476**	4,241	4,516	**8,757**	**56.59**
La Union	**18,468**	4,474	4,502	**8,976**	**48.60**
Pangasinan	**80,607**	9,609	10,898	**20,507**	**25.44**
Alaminos City	**3,046**	1,137	1,210	**2,347**	**77.05**
Candon City	**1,629**	344	334	**678**	**41.61**
Dagupan City	**5,815**	88	98	**186**	**3.20**
Laoag City	**2,828**	250	185	**435**	**15.38**
San Carlos City	**6,403**	324	331	**655**	**10.23**
San Fernando City	**3,383**	88	71	**159**	**4.70**
Urdaneta City	**4,514**	897	953	**1,850**	**40.99**
Vigan City	**1,449**	364	421	**785**	**54.19**

Table 2.D.3. Oral Health Care

Number and proportion of children 5-9 years old who received Basic Oral Health Care (BOHC)
Philippines, 2022

Area	Children 5-9 years old who received BOHC				
	Eligible Population	Male	Female	Total	%
**Region 2**	**109,772**	**22,498**	**24,412**	**46,910**	**42.73**
Batanes	**591**	134	135	**269**	**45.52**
Cagayan	**32,587**	1,792	1,855	**3,647**	**11.19**
Isabela	**37,440**	5,010	5,482	**10,492**	**28.02**
Nueva Vizcaya	**14,906**	6,743	7,507	**14,250**	**95.60**
Quirino	**6,500**	3,170	3,330	**6,500**	**99.99**
Cauayan City	**4,097**	1,866	2,192	**4,058**	**99.05**
Ilagan City	**4,603**	2,117	2,209	**4,326**	**93.98**
Santiago City	**4,264**	1,624	1,650	**3,274**	**76.79**
Tuguegarao City	**4,783**	42	52	**94**	**1.97**
**Region 3**	**348,236**	**31,495**	**37,430**	**68,925**	**19.79**
Aurora	**7,373**	832	906	**1,738**	**23.57**
Bataan	**20,840**	532	502	**1,034**	**4.96**
Bulacan	**69,856**	3,880	4,558	**8,438**	**12.08**
Nueva Ecija	**46,822**	6,028	11,014	**17,042**	**36.40**
Pampanga	**50,393**	2,858	3,025	**5,883**	**11.67**
Tarlac	**30,401**	3,789	3,859	**7,648**	**25.16**
Zambales	**18,764**	2,192	2,204	**4,396**	**23.43**
Angeles City	**12,641**	0	0	**0**	**0.00**
Balanga City	**3,012**	425	404	**829**	**27.53**
Cabanatuan City	**9,586**	413	459	**872**	**9.10**
City of San Fernando	**9,417**	115	104	**219**	**2.33**
Gapan City	**3,499**	1,149	1,155	**2,304**	**65.85**
Mabalacat City	**7,703**	4,874	4,723	**9,597**	**124.60**
Malolos City	**7,802**	457	481	**938**	**12.02**
Meycauayan	**6,471**	416	437	**853**	**13.18**
Olongapo	**7,400**	469	492	**961**	**12.99**
Palayan City	**1,301**	13	8	**21**	**1.61**
San Jose City	**4,431**	683	707	**1,390**	**31.37**
San Jose del Monte City	**17,768**	874	912	**1,786**	**10.05**
Science City of Munoz	**2,585**	305	348	**653**	**25.27**
Tarlac City	**10,172**	1,191	1,132	**2,323**	**22.84**
**Region 4A**	**467,197**	**47,073**	**48,830**	**95,903**	**20.53**
Batangas	**61,301**	11,991	11,810	**23,801**	**38.83**
Cavite	**44,257**	1,787	1,829	**3,616**	**8.17**
Laguna	**30,535**	12,047	13,094	**25,141**	**82.34**
Quezon	**59,967**	3,492	3,583	**7,075**	**11.80**
Rizal	**68,568**	2,187	2,340	**4,527**	**6.60**
Antipolo City	**25,256**	387	403	**790**	**3.13**
Bacoor City	**19,388**	141	179	**320**	**1.65**
Batangas City	**10,880**	419	414	**833**	**7.66**
Biñan City	**10,238**	3,029	3,086	**6,115**	**59.73**
Cabuyao City	**9,492**	242	241	**483**	**5.09**
Calamba City	**13,971**	1,385	1,594	**2,979**	**21.32**
Cavite City	**3,320**	0	0	**0**	**0.00**

Table 2.D.3. Oral Health Care

Number and proportion of children 5-9 years old who received Basic Oral Health Care (BOHC)
Philippines, 2022

Area	Children 5-9 years old who received BOHC				
	Eligible Population	Male	Female	Total	%
Dasmariñas City	**21,273**	22	31	**53**	**0.25**
General Trias City	**10,146**	88	125	**213**	**2.10**
Imus City	**13,036**	818	948	**1,766**	**13.55**
Lipa City	**10,961**	543	586	**1,129**	**10.30**
Lucena City	**9,089**	241	287	**528**	**5.81**
San Pablo City	**8,178**	2,451	2,169	**4,620**	**56.49**
San Pedro City	**10,016**	2,374	2,303	**4,677**	**46.69**
Santa Rosa City	**10,876**	1,446	1,486	**2,932**	**26.96**
Tagaytay City	**2,299**	74	98	**172**	**7.48**
Tanauan City	**5,718**	620	679	**1,299**	**22.72**
Tayabas City	**3,406**	120	127	**247**	**7.25**
Trece Martires City	**5,027**	1,169	1,418	**2,587**	**51.46**
**Region 4B**	**112,662**	**4,497**	**4,751**	**9,248**	**8.21**
Marinduque	**8,242**	411	356	**767**	**9.31**
Mindoro Occidental	**18,814**	330	382	**712**	**3.78**
Mindoro Oriental	**31,644**	2,057	2,090	**4,147**	**13.11**
Palawan	**33,370**	956	1,119	**2,075**	**6.22**
Romblon	**10,567**	597	665	**1,262**	**11.94**
Puerto Princesa City	**10,025**	146	139	**285**	**2.84**
**Region 5**	**212,067**	**29,550**	**29,493**	**59,043**	**27.84**
Albay	**38,357**	5,785	5,456	**11,241**	**29.31**
Camarines Norte	**21,192**	5,359	5,386	**10,745**	**50.70**
Camarines Sur	**60,490**	3,895	4,297	**8,192**	**13.54**
Catanduanes	**9,635**	3,095	2,380	**5,475**	**56.82**
Masbate	**34,306**	3,370	3,425	**6,795**	**19.81**
Sorsogon	**30,023**	5,237	5,666	**10,903**	**36.32**
Iriga City	**4,111**	422	500	**922**	**22.43**
Legaspi City	**6,746**	1,828	1,805	**3,633**	**53.85**
Naga City	**7,208**	559	578	**1,137**	**15.77**
**Region 6**	**232,791**	**27,552**	**29,403**	**56,955**	**24.47**
Aklan	**18,152**	1,228	1,294	**2,522**	**13.89**
Antique	**20,657**	1,030	1,149	**2,179**	**10.55**
Capiz	**22,100**	3,028	3,441	**6,469**	**29.27**
Guimaras	**5,285**	253	246	**499**	**9.44**
Iloilo	**57,509**	12,733	13,324	**26,057**	**45.31**
Negros Occidental	**78,191**	4,911	5,442	**10,353**	**13.24**
Bacolod City	**17,593**	807	810	**1,617**	**9.19**
Iloilo City	**13,304**	3,562	3,697	**7,259**	**54.56**
**Region 7**	**243,823**	**34,695**	**34,840**	**69,535**	**28.52**
Bohol	**43,964**	5,268	5,417	**10,685**	**24.30**
Cebu	**98,088**	9,337	10,051	**19,388**	**19.77**
Negros Oriental	**42,330**	1,388	1,569	**2,957**	**6.99**

Table 2.D.3. Oral Health Care

Number and proportion of children 5-9 years old who received Basic Oral Health Care (BOHC)
Philippines, 2022

Area	Children 5-9 years old who received BOHC				
	Eligible Population	Male	Female	Total	%
Siquijor	**2,927**	464	436	**900**	**30.75**
Cebu City	**30,791**	3,533	2,849	**6,382**	**20.73**
Lapu-Lapu City	**13,620**	10,250	10,295	**20,545**	**150.85**
Mandaue City	**12,104**	4,455	4,223	**8,678**	**71.69**
**Region 8**	**159,436**	**7,015**	**7,320**	**14,335**	**8.99**
Biliran	**5,964**	1,750	1,768	**3,518**	**58.99**
Eastern Samar	**17,456**	681	917	**1,598**	**9.15**
Northern Leyte	**53,086**	1,534	1,586	**3,120**	**5.88**
Northern Samar	**24,358**	855	923	**1,778**	**7.30**
Southern Leyte	**11,036**	0	0	**0**	**0.00**
Western Samar	**21,904**	841	881	**1,722**	**7.86**
Calbayog City	**6,754**	321	344	**665**	**9.85**
Maasin City	**2,809**	143	149	**292**	**10.40**
Ormoc City	**7,559**	286	210	**496**	**6.56**
Tacloban City	**8,511**	604	542	**1,146**	**13.46**
**Region 9**	**121,963**	**7,073**	**7,672**	**14,745**	**12.09**
Zamboanga Del Norte	**26,384**	468	493	**961**	**3.64**
Zamboanga Del Sur	**26,200**	3,962	4,556	**8,518**	**32.51**
Zamboanga Sibugay	**23,753**	1,085	1,055	**2,140**	**9.01**
Dapitan City	**2,725**	73	84	**157**	**5.76**
Dipolog City	**4,322**	571	402	**973**	**22.51**
Isabela City	**4,338**	160	146	**306**	**7.05**
Pagadian City	**6,425**	708	887	**1,595**	**24.82**
Zamboanga City	**27,817**	46	49	**95**	**0.34**
**Region 10**	**156,337**	**14,954**	**17,091**	**32,045**	**20.50**
Bukidnon	**35,157**	2,566	2,708	**5,274**	**15.00**
Camiguin	**2,865**	1,361	1,355	**2,716**	**94.79**
Lanao Del Norte	**24,276**	1,702	1,574	**3,276**	**13.49**
Misamis Occidental	**10,249**	3,261	3,914	**7,175**	**70.01**
Misamis Oriental	**23,040**	1,608	1,787	**3,395**	**14.74**
Cagayan de Oro City	**21,824**	221	260	**481**	**2.20**
El Salvador City	**1,621**	50	56	**106**	**6.54**
Gingoog City	**4,027**	285	271	**556**	**13.81**
Iligan City	**12,295**	1,789	1,711	**3,500**	**28.47**
Malaybalay City	**5,860**	152	151	**303**	**5.17**
Oroquieta City	**2,220**	99	94	**193**	**8.69**
Ozamis City	**4,450**	1,129	2,272	**3,401**	**76.42**
Tangub City	**1,977**	425	446	**871**	**44.06**
Valencia City	**6,476**	306	492	**798**	**12.32**
**Region 11**	**169,175**	**2,590**	**3,263**	**5,853**	**3.46**
Davao de Oro	**24,755**	402	498	**900**	**3.64**

Table 2.D.3. Oral Health Care

Number and proportion of children 5-9 years old who received Basic Oral Health Care (BOHC)
Philippines, 2022

Area	Children 5-9 years old who received BOHC				
	Eligible Population	Male	Female	Total	%
Davao del Norte	**32,586**	526	701	**1,227**	**3.77**
Davao Oriental	**20,606**	448	553	**1,001**	**4.86**
Davao del Sur	**22,299**	198	280	**478**	**2.14**
Davao Occidental	**11,364**	140	160	**300**	**2.64**
Davao City	**57,564**	876	1,071	**1,947**	**3.38**
**Region 12**	**151,673**	**14,799**	**16,235**	**31,034**	**20.46**
North Cotabato	**52,134**	4,183	4,671	**8,854**	**16.98**
Sarangani	**19,930**	2,800	3,018	**5,818**	**29.19**
South Cotabato	**31,323**	3,431	3,685	**7,116**	**22.72**
Sultan Kudarat	**27,944**	3,015	3,506	**6,521**	**23.34**
Gen. Santos City	**20,343**	1,370	1,355	**2,725**	**13.40**
**BARMM**	**181,631**	**20,416**	**20,793**	**41,209**	**22.69**
Basilan	**12,643**	96	108	**204**	**1.61**
Lanao Del Sur	**38,478**	7,079	7,232	**14,311**	**37.19**
Maguindanao	**53,909**	5,698	6,112	**11,810**	**21.91**
Sulu	**37,502**	5,553	5,599	**11,152**	**29.74**
Tawi-Tawi	**15,930**	0	0	**0**	**0.00**
Lamitan City	**3,480**	52	51	**103**	**2.96**
Marawi City	**9,204**	1,315	1,185	**2,500**	**27.16**
Cotabato City	**10,484**	623	506	**1,129**	**10.77**
**CARAGA**	**92,656**	**14,118**	**15,921**	**30,039**	**32.42**
Agusan del Norte	**12,196**	1,550	1,497	**3,047**	**24.98**
Agusan del Sur	**26,293**	2,582	3,380	**5,962**	**22.68**
Surigao del Norte	**11,967**	2,679	3,433	**6,112**	**51.08**
Surigao del Sur	**17,453**	2,482	2,522	**5,004**	**28.67**
Province of Dinagat	**4,264**	1,908	2,095	**4,003**	**93.87**
Bislig City	**3,315**	365	329	**694**	**20.94**
Butuan City	**11,596**	2,083	2,223	**4,306**	**37.13**
Surigao City	**5,573**	469	442	**911**	**16.35**

Table 2.D.4. Oral Health Care

Number and proportion of adolescents 10-14 years who received Basic Oral Health Care (BOHC)
Philippines, 2022

Area	Adolescents 10-14 years old who received BOHC				
	Eligible Population	Male	Female	Total	%
**Philippines**	**3,260,685**	**231,937**	**262,655**	**494,592**	**15.17**
**NCR**	**380,579**	**61,364**	**64,887**	**126,251**	**33.17**
Malabon	**10,804**	274	357	**631**	**5.84**
Navotas	**7,373**	2,035	2,235	**4,270**	**57.91**
Valenzuela City	**18,336**	1,842	1,999	**3,841**	**20.95**
Caloocan City	**46,812**	3,228	3,912	**7,140**	**15.25**
Marikina City	**13,321**	458	600	**1,058**	**7.94**
Pasig City	**22,322**	8,136	8,881	**17,017**	**76.23**
Pateros	**1,887**	563	598	**1,161**	**61.53**
Taguig	**23,788**	1,322	1,325	**2,647**	**11.13**
Quezon City	**86,776**	5,138	5,602	**10,740**	**12.38**
Makati City	**17,219**	6,416	6,809	**13,225**	**76.80**
Mandaluyong City	**11,417**	791	841	**1,632**	**14.29**
San Juan	**3,611**	401	380	**781**	**21.63**
Manila City	**52,611**	26,182	26,429	**52,611**	**100.00**
Las Piñas City	**17,404**	179	168	**347**	**1.99**
Muntinlupa City	**14,910**	3,017	3,087	**6,104**	**40.94**
Parañaque City	**19,677**	401	463	**864**	**4.39**
Pasay City	**12,311**	981	1,201	**2,182**	**17.72**
**CAR**	**52,205**	**7,419**	**9,319**	**16,738**	**32.06**
Abra	**6,950**	283	316	**599**	**8.62**
Apayao	**3,839**	484	561	**1,045**	**27.22**
Benguet	**12,834**	1,643	2,044	**3,687**	**28.73**
Ifugao	**6,711**	341	573	**914**	**13.62**
Kalinga	**7,004**	895	1,025	**1,920**	**27.41**
Mt. Province	**4,931**	638	752	**1,390**	**28.19**
Baguio City	**9,936**	3,135	4,048	**7,183**	**72.29**
**Region 1**	**156,010**	**13,087**	**14,346**	**27,433**	**17.58**
Ilocos Norte	**12,494**	1,027	1,108	**2,135**	**17.09**
Ilocos Sur	**15,824**	2,537	2,930	**5,467**	**34.55**
La Union	**18,653**	1,724	1,953	**3,677**	**19.71**
Pangasinan	**79,970**	5,419	6,232	**11,651**	**14.57**
Alaminos City	**3,021**	200	160	**360**	**11.92**
Candon City	**1,668**	372	180	**552**	**33.09**
Dagupan City	**5,771**	60	76	**136**	**2.36**
Laoag City	**2,881**	260	212	**472**	**16.38**
San Carlos City	**6,351**	102	137	**239**	**3.76**
San Fernando City	**3,417**	100	83	**183**	**5.36**
Urdaneta City	**4,478**	903	896	**1,799**	**40.17**
Vigan City	**1,482**	383	379	**762**	**51.42**

Table 2.D.4. Oral Health Care

Number and proportion of adolescents 10-14 years who received Basic Oral Health Care (BOHC)

Philippines, 2022

Area	Adolescents 10-14 years old who received BOHC				
	Eligible Population	Male	Female	Total	%
**Region 2**	**108,092**	**11,951**	**15,449**	**27,400**	**25.35**
Batanes	**592**	131	180	**311**	**52.53**
Cagayan	**32,000**	1,063	1,363	**2,426**	**7.58**
Isabela	**36,990**	3,154	3,855	**7,009**	**18.95**
Nueva Vizcaya	**14,666**	3,107	5,074	**8,181**	**55.78**
Quirino	**6,339**	807	916	**1,723**	**27.18**
Cauayan City	**4,048**	395	515	**910**	**22.48**
Ilagan City	**4,550**	1,844	1,923	**3,767**	**82.79**
Santiago City	**4,212**	1,331	1,379	**2,710**	**64.34**
Tuguegarao City	**4,695**	119	244	**363**	**7.73**
**Region 3**	**347,595**	**16,236**	**17,765**	**34,001**	**9.78**
Aurora	**6,924**	567	651	**1,218**	**17.59**
Bataan	**20,712**	279	298	**577**	**2.79**
Bulacan	**69,828**	1,841	2,071	**3,912**	**5.60**
Nueva Ecija	**46,726**	4,598	5,123	**9,721**	**20.80**
Pampanga	**51,041**	1,111	1,322	**2,433**	**4.77**
Tarlac	**30,292**	390	416	**806**	**2.66**
Zambales	**18,225**	1,443	1,638	**3,081**	**16.91**
Angeles City	**12,805**	23	17	**40**	**0.31**
Balanga City	**2,993**	157	171	**328**	**10.96**
Cabanatuan City	**9,563**	189	170	**359**	**3.75**
City of San Fernando	**9,539**	69	69	**138**	**1.45**
Gapan City	**3,490**	144	114	**258**	**7.39**
Mabalacat City	**7,802**	2,016	1,997	**4,013**	**51.44**
Malolos City	**7,800**	180	234	**414**	**5.31**
Meycauayan	**6,468**	449	488	**937**	**14.49**
Olongapo	**7,189**	521	577	**1,098**	**15.27**
Palayan City	**1,299**	8	10	**18**	**1.39**
San Jose City	**4,422**	446	398	**844**	**19.09**
San Jose del Monte City	**17,763**	579	744	**1,323**	**7.45**
Science City of Munoz	**2,578**	160	236	**396**	**15.36**
Tarlac City	**10,136**	1,066	1,021	**2,087**	**20.59**
**Region 4A**	**464,777**	**23,897**	**27,364**	**51,261**	**11.03**
Batangas	**60,748**	10,521	10,658	**21,179**	**34.86**
Cavite	**44,608**	970	1,338	**2,308**	**5.17**
Laguna	**30,596**	1,211	1,720	**2,931**	**9.58**
Quezon	**58,465**	1,938	2,093	**4,031**	**6.89**
Rizal	**67,874**	3,352	3,756	**7,108**	**10.47**
Antipolo City	**25,001**	521	808	**1,329**	**5.32**
Bacoor City	**19,545**	162	214	**376**	**1.92**
Batangas City	**10,783**	343	436	**779**	**7.22**
Biñan City	**10,259**	1,147	1,485	**2,632**	**25.66**
Cabuyao City	**9,512**	1,021	1,557	**2,578**	**27.10**
Calamba City	**14,000**	393	487	**880**	**6.29**
Cavite City	**3,347**	0	0	**0**	**0.00**

Table 2.D.4. Oral Health Care

Number and proportion of adolescents 10-14 years who received Basic Oral Health Care (BOHC)

Philippines, 2022

Area	Adolescents 10-14 years old who received BOHC				
	Eligible Population	Male	Female	Total	%
Dasmariñas City	**21,446**	21	33	**54**	**0.25**
General Trias City	**10,228**	159	288	**447**	**4.37**
Imus City	**13,141**	931	975	**1,906**	**14.50**
Lipa City	**10,864**	467	488	**955**	**8.79**
Lucena City	**8,862**	121	146	**267**	**3.01**
San Pablo City	**8,195**	99	109	**208**	**2.54**
San Pedro City	**10,036**	0	0	**0**	**0.00**
Santa Rosa City	**10,898**	81	160	**241**	**2.21**
Tagaytay City	**2,317**	20	18	**38**	**1.64**
Tanauan City	**5,666**	112	213	**325**	**5.74**
Tayabas City	**3,319**	85	124	**209**	**6.30**
Trece Martires City	**5,067**	222	258	**480**	**9.47**
**Region 4B**	**107,294**	**2,184**	**2,597**	**4,781**	**4.46**
Marinduque	**7,853**	195	230	**425**	**5.41**
Mindoro Occidental	**18,051**	0	0	**0**	**0.00**
Mindoro Oriental	**30,270**	823	1,005	**1,828**	**6.04**
Palawan	**31,538**	583	795	**1,378**	**4.37**
Romblon	**10,109**	313	384	**697**	**6.89**
Puerto Princesa City	**9,473**	270	183	**453**	**4.78**
**Region 5**	**200,487**	**9,260**	**10,727**	**19,987**	**9.97**
Albay	**37,003**	1,732	1,880	**3,612**	**9.76**
Camarines Norte	**19,831**	2,156	2,846	**5,002**	**25.22**
Camarines Sur	**57,413**	795	934	**1,729**	**3.01**
Catanduanes	**9,062**	486	474	**960**	**10.59**
Masbate	**31,445**	1,578	1,634	**3,212**	**10.21**
Sorsogon	**28,480**	1,733	1,986	**3,719**	**13.06**
Iriga City	**3,902**	90	151	**241**	**6.18**
Legaspi City	**6,509**	404	509	**913**	**14.03**
Naga City	**6,842**	286	313	**599**	**8.75**
**Region 6**	**226,360**	**18,582**	**21,090**	**39,672**	**17.53**
Aklan	**17,435**	1,151	1,451	**2,602**	**14.92**
Antique	**20,117**	636	716	**1,352**	**6.72**
Capiz	**21,543**	1,891	2,170	**4,061**	**18.85**
Guimaras	**5,090**	67	105	**172**	**3.38**
Iloilo	**56,535**	7,395	8,153	**15,548**	**27.50**
Negros Occidental	**75,561**	3,738	4,277	**8,015**	**10.61**
Bacolod City	**17,001**	1,205	1,394	**2,599**	**15.29**
Iloilo City	**13,078**	2,499	2,824	**5,323**	**40.70**
**Region 7**	**238,017**	**18,847**	**20,761**	**39,608**	**16.64**
Bohol	**43,339**	2,048	2,521	**4,569**	**10.54**
Cebu	**95,723**	3,824	4,808	**8,632**	**9.02**

Table 2.D.4. Oral Health Care

Number and proportion of adolescents 10-14 years who received Basic Oral Health Care (BOHC)

Philippines, 2022

Area	Adolescents 10-14 years old who received BOHC				
	Eligible Population	Male	Female	Total	%
Negros Oriental	**40,869**	1,003	1,269	**2,272**	**5.56**
Siquijor	**2,929**	172	293	**465**	**15.88**
Cebu City	**30,052**	2,064	2,490	**4,554**	**15.15**
Lapu-Lapu City	**13,293**	7,677	6,747	**14,424**	**108.51**
Mandaue City	**11,812**	2,059	2,633	**4,692**	**39.72**
**Region 8**	**150,335**	**3,276**	**4,635**	**7,911**	**5.26**
Biliran	**5,477**	129	122	**251**	**4.58**
Eastern Samar	**16,412**	92	129	**221**	**1.35**
Northern Leyte	**50,215**	1,628	2,552	**4,180**	**8.32**
Northern Samar	**22,628**	584	884	**1,468**	**6.49**
Southern Leyte	**10,662**	0	0	**0**	**0.00**
Western Samar	**20,655**	411	531	**942**	**4.56**
Calbayog City	**6,368**	108	113	**221**	**3.47**
Maasin City	**2,713**	37	42	**79**	**2.91**
Ormoc City	**7,154**	0	0	**0**	**0.00**
Tacloban City	**8,051**	287	262	**549**	**6.82**
**Region 9**	**116,216**	**3,556**	**4,369**	**7,925**	**6.82**
Zamboanga Del Norte	**25,052**	248	311	**559**	**2.23**
Zamboanga Del Sur	**25,158**	1,836	2,209	**4,045**	**16.08**
Zamboanga Sibugay	**22,262**	414	329	**743**	**3.34**
Dapitan City	**2,588**	84	82	**166**	**6.41**
Dipolog City	**4,104**	245	288	**533**	**12.99**
Isabela City	**4,173**	204	229	**433**	**10.38**
Pagadian City	**6,168**	511	896	**1,407**	**22.81**
Zamboanga City	**26,711**	14	25	**39**	**0.15**
**Region 10**	**149,903**	**10,122**	**12,943**	**23,065**	**15.39**
Bukidnon	**33,174**	1,589	1,858	**3,447**	**10.39**
Camiguin	**2,768**	335	311	**646**	**23.34**
Lanao Del Norte	**23,076**	967	1,062	**2,029**	**8.79**
Misamis Occidental	**10,102**	3,774	4,379	**8,153**	**80.71**
Misamis Oriental	**22,321**	574	748	**1,322**	**5.92**
Cagayan de Oro City	**21,141**	225	346	**571**	**2.70**
El Salvador City	**1,570**	42	57	**99**	**6.31**
Gingoog City	**3,900**	140	148	**288**	**7.38**
Iligan City	**11,688**	968	1,195	**2,163**	**18.51**
Malaybalay City	**5,531**	63	128	**191**	**3.45**
Oroquieta City	**2,187**	100	125	**225**	**10.29**
Ozamis City	**4,384**	403	1,462	**1,865**	**42.54**
Tangub City	**1,949**	288	291	**579**	**29.71**
Valencia City	**6,112**	654	833	**1,487**	**24.33**

Table 2.D.4. Oral Health Care

Number and proportion of adolescents 10-14 years who received Basic Oral Health Care (BOHC)

Philippines, 2022

Area	Adolescents 10-14 years old who received BOHC				
	Eligible Population	Male	Female	Total	%
**Region 11**	**164,500**	**2,966**	**3,593**	**6,559**	**3.99**
Davao de Oro	**23,649**	520	561	**1,081**	**4.57**
Davao del Norte	**31,700**	618	710	**1,328**	**4.19**
Davao Oriental	**19,580**	414	579	**993**	**5.07**
Davao del Sur	**21,952**	244	321	**565**	**2.57**
Davao Occidental	**10,944**	161	228	**389**	**3.55**
Davao City	**56,675**	1,009	1,194	**2,203**	**3.89**
**Region 12**	**145,576**	**7,339**	**9,401**	**16,740**	**11.50**
North Cotabato	**49,541**	1,927	2,187	**4,114**	**8.30**
Sarangani	**19,029**	769	864	**1,633**	**8.58**
South Cotabato	**30,382**	1,932	2,317	**4,249**	**13.99**
Sultan Kudarat	**26,891**	2,267	3,539	**5,806**	**21.59**
Gen. Santos City	**19,733**	444	494	**938**	**4.75**
**BARMM**	**165,509**	**14,950**	**15,899**	**30,849**	**18.64**
Basilan	**11,352**	80	92	**172**	**1.52**
Lanao Del Sur	**33,954**	3,737	4,131	**7,868**	**23.17**
Maguindanao	**48,877**	3,947	4,182	**8,129**	**16.63**
Sulu	**34,852**	5,360	5,352	**10,712**	**30.74**
Tawi-Tawi	**14,916**	0	0	**0**	**0.00**
Lamitan City	**3,122**	53	71	**124**	**3.97**
Marawi City	**8,122**	1,380	1,682	**3,062**	**37.70**
Cotabato City	**10,314**	393	389	**782**	**7.58**
**CARAGA**	**87,230**	**6,901**	**7,510**	**14,411**	**16.52**
Agusan del Norte	**11,480**	1,041	824	**1,865**	**16.25**
Agusan del Sur	**23,877**	747	966	**1,713**	**7.17**
Surigao del Norte	**11,779**	435	585	**1,020**	**8.66**
Surigao del Sur	**16,401**	1,726	1,863	**3,589**	**21.88**
Province of Dinagat	**4,175**	1,140	1,201	**2,341**	**56.07**
Bislig City	**3,116**	114	118	**232**	**7.45**
Butuan City	**10,917**	1,307	1,581	**2,888**	**26.45**
Surigao City	**5,485**	391	372	**763**	**13.91**

Table 2.D.5. Oral Health Care

Number and proportion of adolescents 15- 19 years old who received Basic Oral Health Care (BOHC)
Philippines, 2022

Area	Adolescents 15-19 years old who received BOHC				
	Eligible Population	Male	Female	Total	%
**Philippines**	**3,103,896**	**253,860**	**311,485**	**565,345**	**18.21**
**NCR**	**351,488**	**46,335**	**54,055**	**100,390**	**28.56**
Malabon	**9,978**	467	415	**882**	**8.84**
Navotas	**6,809**	1,024	1,295	**2,319**	**34.06**
Valenzuela City	**16,934**	1,938	2,652	**4,590**	**27.10**
Caloocan City	**43,236**	5,055	6,275	**11,330**	**26.20**
Marikina City	**12,302**	197	208	**405**	**3.29**
Pasig City	**20,616**	7,698	9,217	**16,915**	**82.05**
Pateros	**1,742**	181	259	**440**	**25.26**
Taguig	**21,971**	1,387	1,623	**3,010**	**13.70**
Quezon City	**80,142**	4,492	5,857	**10,349**	**12.91**
Makati City	**15,902**	4,201	4,559	**8,760**	**55.09**
Mandaluyong City	**10,544**	554	679	**1,233**	**11.69**
San Juan	**3,334**	61	90	**151**	**4.53**
Manila City	**48,590**	14,220	15,404	**29,624**	**60.97**
Las Piñas City	**16,074**	146	161	**307**	**1.91**
Muntinlupa City	**13,770**	2,830	3,123	**5,953**	**43.23**
Parañaque City	**18,174**	453	517	**970**	**5.34**
Pasay City	**11,369**	1,431	1,721	**3,152**	**27.72**
**CAR**	**51,692**	**4,682**	**7,343**	**12,025**	**23.26**
Abra	**7,064**	381	482	**863**	**12.22**
Apayao	**3,623**	234	395	**629**	**17.36**
Benguet	**13,412**	2,058	3,035	**5,093**	**37.97**
Ifugao	**6,208**	263	528	**791**	**12.74**
Kalinga	**6,485**	559	953	**1,512**	**23.32**
Mt. Province	**4,519**	405	661	**1,066**	**23.59**
Baguio City	**10,382**	782	1,289	**2,071**	**19.95**
**Region 1**	**144,640**	**18,807**	**22,672**	**41,479**	**28.68**
Ilocos Norte	**13,266**	1,389	1,551	**2,940**	**22.16**
Ilocos Sur	**15,846**	2,917	3,789	**6,706**	**42.32**
La Union	**19,142**	1,684	2,157	**3,841**	**20.07**
Pangasinan	**69,588**	9,430	11,424	**20,854**	**29.97**
Alaminos City	**2,630**	2,040	2,221	**4,261**	**161.99**
Candon City	**1,670**	98	161	**259**	**15.51**
Dagupan City	**5,021**	49	83	**132**	**2.63**
Laoag City	**3,059**	182	170	**352**	**11.51**
San Carlos City	**5,528**	130	168	**298**	**5.39**
San Fernando City	**3,508**	153	163	**316**	**9.01**
Urdaneta City	**3,896**	431	446	**877**	**22.51**
Vigan City	**1,485**	304	339	**643**	**43.30**

Table 2.D.5. Oral Health Care

Number and proportion of adolescents 15- 19 years old who received Basic Oral Health Care (BOHC)
Philippines, 2022

Area	Adolescents 15-19 years old who received BOHC				
	Eligible Population	Male	Female	Total	%
**Region 2**	**98,321**	**10,707**	**15,290**	**25,997**	**26.44**
Batanes	**433**	118	177	**295**	**68.10**
Cagayan	**29,946**	1,153	1,589	**2,742**	**9.16**
Isabela	**33,425**	4,306	4,923	**9,229**	**27.61**
Nueva Vizcaya	**13,406**	1,628	3,217	**4,845**	**36.14**
Quirino	**5,140**	200	375	**575**	**11.19**
Cauayan City	**3,657**	324	528	**852**	**23.30**
Ilagan City	**4,111**	1,597	1,966	**3,563**	**86.68**
Santiago City	**3,807**	1,158	1,227	**2,385**	**62.65**
Tuguegarao City	**4,397**	223	1,288	**1,511**	**34.37**
**Region 3**	**351,401**	**19,872**	**22,704**	**42,576**	**12.12**
Aurora	**6,751**	241	246	**487**	**7.21**
Bataan	**20,429**	173	202	**375**	**1.84**
Bulacan	**71,858**	1,882	2,528	**4,410**	**6.14**
Nueva Ecija	**45,359**	4,786	5,291	**10,077**	**22.22**
Pampanga	**52,165**	1,475	1,742	**3,217**	**6.17**
Tarlac	**31,536**	2,593	2,794	**5,387**	**17.08**
Zambales	**18,137**	1,512	1,986	**3,498**	**19.29**
Angeles City	**13,088**	26	26	**52**	**0.40**
Balanga City	**2,954**	134	169	**303**	**10.26**
Cabanatuan City	**9,284**	206	205	**411**	**4.43**
City of San Fernando	**9,751**	67	76	**143**	**1.47**
Gapan City	**3,388**	0	0	**0**	**0.00**
Mabalacat City	**7,975**	2,232	2,234	**4,466**	**56.00**
Malolos City	**8,027**	189	259	**448**	**5.58**
Meycauayan	**6,657**	460	486	**946**	**14.21**
Olongapo	**7,154**	715	765	**1,480**	**20.69**
Palayan City	**1,261**	3	21	**24**	**1.90**
San Jose City	**4,293**	840	915	**1,755**	**40.88**
San Jose del Monte City	**18,279**	785	1,114	**1,899**	**10.39**
Science City of Munoz	**2,504**	134	236	**370**	**14.78**
Tarlac City	**10,552**	1,419	1,409	**2,828**	**26.80**
**Region 4A**	**461,707**	**68,365**	**82,396**	**150,761**	**32.65**
Batangas	**61,352**	11,519	12,091	**23,610**	**38.48**
Cavite	**45,158**	1,364	2,057	**3,421**	**7.58**
Laguna	**29,864**	20,883	24,625	**45,508**	**152.39**
Quezon	**55,708**	2,348	2,925	**5,273**	**9.47**
Rizal	**67,987**	4,126	4,965	**9,091**	**13.37**
Antipolo City	**25,041**	126	244	**370**	**1.48**
Bacoor City	**19,787**	210	307	**517**	**2.61**
Batangas City	**10,886**	436	665	**1,101**	**10.11**
Biñan City	**10,013**	8,348	10,432	**18,780**	**187.56**
Cabuyao City	**9,284**	1,229	1,592	**2,821**	**30.39**
Calamba City	**13,666**	6,191	8,572	**14,763**	**108.03**
Cavite City	**3,389**	0	0	**0**	**0.00**

Table 2.D.5. Oral Health Care

Number and proportion of adolescents 15- 19 years old who received Basic Oral Health Care (BOHC)
Philippines, 2022

Area	Adolescents 15-19 years old who received BOHC				
	Eligible Population	Male	Female	Total	%
Dasmariñas City	**21,710**	33	57	**90**	**0.41**
General Trias City	**10,354**	473	1,051	**1,524**	**14.72**
Imus City	**13,301**	350	578	**928**	**6.98**
Lipa City	**10,970**	479	496	**975**	**8.89**
Lucena City	**8,443**	107	159	**266**	**3.15**
San Pablo City	**7,998**	1,263	1,558	**2,821**	**35.27**
San Pedro City	**9,797**	6,833	7,288	**14,121**	**144.14**
Santa Rosa City	**10,638**	1,326	1,735	**3,061**	**28.78**
Tagaytay City	**2,346**	33	59	**92**	**3.92**
Tanauan City	**5,723**	174	258	**432**	**7.55**
Tayabas City	**3,165**	249	379	**628**	**19.84**
Trece Martires City	**5,130**	265	303	**568**	**11.07**
**Region 4B**	**93,542**	**1,869**	**3,044**	**4,913**	**5.25**
Marinduque	**6,721**	236	301	**537**	**7.99**
Mindoro Occidental	**15,212**	0	0	**0**	**0.00**
Mindoro Oriental	**25,911**	511	763	**1,274**	**4.92**
Palawan	**28,589**	520	785	**1,305**	**4.56**
Romblon	**8,520**	314	458	**772**	**9.06**
Puerto Princesa City	**8,590**	288	737	**1,025**	**11.93**
**Region 5**	**187,077**	**8,912**	**13,116**	**22,028**	**11.77**
Albay	**35,418**	1,372	1,714	**3,086**	**8.71**
Camarines Norte	**18,761**	1,737	2,471	**4,208**	**22.43**
Camarines Sur	**52,818**	672	1,130	**1,802**	**3.41**
Catanduanes	**8,033**	476	485	**961**	**11.96**
Masbate	**30,671**	1,179	1,497	**2,676**	**8.72**
Sorsogon	**25,265**	2,162	4,073	**6,235**	**24.68**
Iriga City	**3,590**	238	327	**565**	**15.74**
Legaspi City	**6,227**	755	1,050	**1,805**	**28.99**
Naga City	**6,294**	321	369	**690**	**10.96**
**Region 6**	**222,611**	**13,171**	**16,708**	**29,879**	**13.42**
Aklan	**16,750**	391	563	**954**	**5.70**
Antique	**16,523**	636	716	**1,352**	**8.18**
Capiz	**22,834**	1,418	1,558	**2,976**	**13.03**
Guimaras	**5,124**	61	93	**154**	**3.01**
Iloilo	**57,305**	5,185	6,286	**11,471**	**20.02**
Negros Occidental	**74,134**	2,518	3,670	**6,188**	**8.35**
Bacolod City	**16,681**	954	1,303	**2,257**	**13.53**
Iloilo City	**13,261**	2,008	2,519	**4,527**	**34.14**
**Region 7**	**224,784**	**16,885**	**20,283**	**37,168**	**16.53**
Bohol	**38,018**	541	760	**1,301**	**3.42**
Cebu	**91,289**	3,169	4,261	**7,430**	**8.14**
Negros Oriental	**40,298**	1,349	2,314	**3,663**	**9.09**

Table 2.D.5. Oral Health Care

Number and proportion of adolescents 15- 19 years old who received Basic Oral Health Care (BOHC)
Philippines, 2022

Area	Adolescents 15-19 years old who received BOHC				
	Eligible Population	Male	Female	Total	%
Siquijor	**2,578**	77	84	**161**	**6.24**
Cebu City	**28,660**	1,371	2,484	**3,855**	**13.45**
Lapu-Lapu City	**12,677**	8,958	8,503	**17,461**	**137.74**
Mandaue City	**11,263**	1,420	1,877	**3,297**	**29.27**
**Region 8**	**143,828**	**2,980**	**4,231**	**7,211**	**5.01**
Biliran	**5,278**	350	370	**720**	**13.64**
Eastern Samar	**15,198**	63	107	**170**	**1.12**
Northern Leyte	**49,447**	115	140	**255**	**0.52**
Northern Samar	**21,060**	945	1,770	**2,715**	**12.89**
Southern Leyte	**9,820**	0	0	**0**	**0.00**
Western Samar	**19,535**	438	530	**968**	**4.96**
Calbayog City	**6,018**	220	285	**505**	**8.39**
Maasin City	**2,501**	31	54	**85**	**3.40**
Ormoc City	**7,041**	538	692	**1,230**	**17.47**
Tacloban City	**7,930**	280	283	**563**	**7.10**
**Region 9**	**115,220**	**3,506**	**4,194**	**7,700**	**6.68**
Zamboanga Del Norte	**25,054**	165	279	**444**	**1.77**
Zamboanga Del Sur	**25,473**	1,714	2,028	**3,742**	**14.69**
Zamboanga Sibugay	**20,723**	233	325	**558**	**2.69**
Dapitan City	**2,586**	84	82	**166**	**6.42**
Dipolog City	**4,103**	174	191	**365**	**8.90**
Isabela City	**3,981**	363	478	**841**	**21.12**
Pagadian City	**6,249**	754	761	**1,515**	**24.24**
Zamboanga City	**27,050**	19	50	**69**	**0.26**
**Region 10**	**148,596**	**9,178**	**12,082**	**21,260**	**14.31**
Bukidnon	**33,982**	1,015	1,481	**2,496**	**7.35**
Camiguin	**2,617**	396	341	**737**	**28.17**
Lanao Del Norte	**22,904**	584	691	**1,275**	**5.57**
Misamis Occidental	**9,604**	4,264	4,709	**8,973**	**93.43**
Misamis Oriental	**21,831**	781	930	**1,711**	**7.84**
Cagayan de Oro City	**20,678**	216	529	**745**	**3.60**
El Salvador City	**1,536**	32	39	**71**	**4.62**
Gingoog City	**3,813**	135	120	**255**	**6.69**
Iligan City	**11,600**	260	485	**745**	**6.42**
Malaybalay City	**5,665**	144	183	**327**	**5.77**
Oroquieta City	**2,081**	7	6	**13**	**0.62**
Ozamis City	**4,173**	373	1,374	**1,747**	**41.87**
Tangub City	**1,854**	310	320	**630**	**33.98**
Valencia City	**6,259**	661	874	**1,535**	**24.52**
**Region 11**	**150,897**	**2,822**	**3,308**	**6,130**	**4.06**
Davao de Oro	**21,711**	425	461	**886**	**4.08**

Table 2.D.5. Oral Health Care

Number and proportion of adolescents 15- 19 years old who received Basic Oral Health Care (BOHC)
Philippines, 2022

Area	Adolescents 15-19 years old who received BOHC				
	Eligible Population	Male	Female	Total	%
Davao del Norte	**29,262**	580	679	**1,259**	**4.30**
Davao Oriental	**18,019**	389	467	**856**	**4.75**
Davao del Sur	**20,200**	278	351	**629**	**3.11**
Davao Occidental	**9,557**	184	236	**420**	**4.39**
Davao City	**52,149**	966	1,114	**2,080**	**3.99**
**Region 12**	**137,294**	**6,519**	**8,868**	**15,387**	**11.21**
North Cotabato	**46,220**	1,299	1,928	**3,227**	**6.98**
Sarangani	**18,449**	810	1,127	**1,937**	**10.50**
South Cotabato	**28,882**	1,593	2,211	**3,804**	**13.17**
Sultan Kudarat	**24,987**	2,635	3,127	**5,762**	**23.06**
Gen. Santos City	**18,756**	182	475	**657**	**3.50**
**BARMM**	**140,706**	**13,151**	**14,195**	**27,346**	**19.43**
Basilan	**9,857**	47	99	**146**	**1.48**
Lanao Del Sur	**29,015**	2,546	2,955	**5,501**	**18.96**
Maguindanao	**42,869**	3,670	4,068	**7,738**	**18.05**
Sulu	**27,513**	5,850	5,857	**11,707**	**42.55**
Tawi-Tawi	**12,810**	0	0	**0**	**0.00**
Lamitan City	**2,712**	35	77	**112**	**4.13**
Marawi City	**6,940**	732	856	**1,588**	**22.88**
Cotabato City	**8,991**	271	283	**554**	**6.16**
**CARAGA**	**80,094**	**6,099**	**6,996**	**13,095**	**16.35**
Agusan del Norte	**11,167**	602	634	**1,236**	**11.07**
Agusan del Sur	**21,418**	638	892	**1,530**	**7.14**
Surigao del Norte	**10,454**	458	621	**1,079**	**10.32**
Surigao del Sur	**15,173**	1,212	1,289	**2,501**	**16.48**
Province of Dinagat	**3,513**	1,361	1,308	**2,669**	**75.97**
Bislig City	**2,882**	117	183	**300**	**10.41**
Butuan City	**10,619**	1,173	1,505	**2,678**	**25.22**
Surigao City	**4,868**	538	564	**1,102**	**22.64**

Table 2.D.6. Oral Health Care

Number and proportion of adolescents 20 -59 years old who received Basic Oral Health Care (BOHC)
Philippines, 2022

Area	Adults 20-59 years old who received BOHC				
	Eligible Population	Male	Female	Total	%
**Philippines**	**17,356,856**	**620,613**	**966,690**	**1,587,303**	**9.15**
**NCR**	**2,369,150**	**99,251**	**168,690**	**267,941**	**11.31**
Malabon	**67,250**	440	931	**1,371**	**2.04**
Navotas	**45,896**	3,548	5,397	**8,945**	**19.49**
Valenzuela City	**114,144**	6,382	12,902	**19,284**	**16.89**
Caloocan City	**291,419**	12,456	19,039	**31,495**	**10.81**
Marikina City	**82,927**	950	1,261	**2,211**	**2.67**
Pasig City	**138,960**	16,382	34,605	**50,987**	**36.69**
Pateros	**11,745**	462	1,176	**1,638**	**13.95**
Taguig	**148,087**	3,619	6,996	**10,615**	**7.17**
Quezon City	**540,185**	8,545	16,329	**24,874**	**4.60**
Makati City	**107,186**	6,406	11,355	**17,761**	**16.57**
Mandaluyong City	**71,066**	1,722	2,475	**4,197**	**5.91**
San Juan	**22,479**	270	643	**913**	**4.06**
Manila City	**327,510**	23,703	36,564	**60,267**	**18.40**
Las Piñas City	**108,345**	311	850	**1,161**	**1.07**
Muntinlupa City	**92,819**	9,945	12,507	**22,452**	**24.19**
Parañaque City	**122,498**	774	960	**1,734**	**1.42**
Pasay City	**76,634**	3,336	4,700	**8,036**	**10.49**
**CAR**	**290,738**	**12,099**	**26,107**	**38,206**	**13.14**
Abra	**38,701**	1,207	2,288	**3,495**	**9.03**
Apayao	**18,963**	1,025	2,416	**3,441**	**18.15**
Benguet	**81,428**	5,081	8,765	**13,846**	**17.00**
Ifugao	**31,952**	979	2,427	**3,406**	**10.66**
Kalinga	**33,172**	966	2,825	**3,791**	**11.43**
Mt. Province	**23,499**	914	2,031	**2,945**	**12.53**
Baguio City	**63,023**	1,927	5,355	**7,282**	**11.55**
**Region 1**	**821,270**	**57,194**	**78,095**	**135,289**	**16.47**
Ilocos Norte	**81,800**	2,956	3,955	**6,911**	**8.45**
Ilocos Sur	**96,700**	10,939	15,122	**26,061**	**26.95**
La Union	**114,185**	6,065	10,064	**16,129**	**14.13**
Pangasinan	**377,045**	28,064	36,724	**64,788**	**17.18**
Alaminos City	**14,248**	6,032	6,411	**12,443**	**87.33**
Candon City	**10,194**	424	1,195	**1,619**	**15.88**
Dagupan City	**27,199**	166	310	**476**	**1.75**
Laoag City	**18,862**	302	295	**597**	**3.17**
San Carlos City	**29,946**	422	748	**1,170**	**3.91**
San Fernando City	**20,922**	638	923	**1,561**	**7.46**
Urdaneta City	**21,112**	690	1,358	**2,048**	**9.70**
Vigan City	**9,059**	496	990	**1,486**	**16.40**

Table 2.D.6. Oral Health Care

Number and proportion of adolescents 20 -59 years old who received Basic Oral Health Care (BOHC)
Philippines, 2022

Area	Adults 20-59 years old who received BOHC				
	Eligible Population	Male	Female	Total	%
**Region 2**	**580,589**	**28,806**	**49,099**	**77,905**	**13.42**
Batanes	**2,616**	419	713	**1,132**	**43.27**
Cagayan	**176,429**	3,538	6,267	**9,805**	**5.56**
Isabela	**200,108**	11,481	14,082	**25,563**	**12.77**
Nueva Vizcaya	**75,706**	3,745	10,380	**14,125**	**18.66**
Quirino	**30,535**	1,289	3,185	**4,474**	**14.65**
Cauayan City	**21,898**	753	2,156	**2,909**	**13.28**
Ilagan City	**24,609**	4,479	8,077	**12,556**	**51.02**
Santiago City	**22,792**	2,977	3,774	**6,751**	**29.62**
Tuguegarao City	**25,896**	125	465	**590**	**2.28**
**Region 3**	**2,049,217**	**49,874**	**70,691**	**120,565**	**5.88**
Aurora	**35,048**	777	938	**1,715**	**4.89**
Bataan	**121,360**	603	1,160	**1,763**	**1.45**
Bulacan	**421,077**	5,848	9,931	**15,779**	**3.75**
Nueva Ecija	**267,319**	6,156	7,258	**13,414**	**5.02**
Pampanga	**306,325**	5,423	8,083	**13,506**	**4.41**
Tarlac	**179,183**	3,677	5,175	**8,852**	**4.94**
Zambales	**104,100**	3,309	5,202	**8,511**	**8.18**
Angeles City	**76,854**	106	138	**244**	**0.32**
Balanga City	**17,541**	754	2,165	**2,919**	**16.64**
Cabanatuan City	**54,712**	478	734	**1,212**	**2.22**
City of San Fernando	**57,256**	103	195	**298**	**0.52**
Gapan City	**19,968**	6	14	**20**	**0.10**
Mabalacat City	**46,827**	9,075	9,252	**18,327**	**39.14**
Malolos City	**47,030**	670	1,164	**1,834**	**3.90**
Meycauayan	**39,010**	796	1,143	**1,939**	**4.97**
Olongapo	**41,058**	2,183	2,149	**4,332**	**10.55**
Palayan City	**7,430**	44	57	**101**	**1.36**
San Jose City	**25,297**	2,014	2,594	**4,608**	**18.22**
San Jose del Monte City	**107,111**	4,135	8,352	**12,487**	**11.66**
Science City of Munoz	**14,752**	674	1,783	**2,457**	**16.66**
Tarlac City	**59,959**	3,043	3,204	**6,247**	**10.42**
**Region 4A**	**2,691,816**	**149,296**	**223,801**	**373,097**	**13.86**
Batangas	**350,891**	40,699	45,630	**86,329**	**24.60**
Cavite	**270,989**	5,439	11,005	**16,444**	**6.07**
Laguna	**187,162**	29,771	38,035	**67,806**	**36.23**
Quezon	**286,460**	5,253	9,478	**14,731**	**5.14**
Rizal	**393,165**	8,955	13,388	**22,343**	**5.68**
Antipolo City	**144,817**	1,140	2,994	**4,134**	**2.85**
Bacoor City	**118,724**	830	1,835	**2,665**	**2.24**
Batangas City	**62,273**	2,376	6,211	**8,587**	**13.79**
Biñan City	**62,758**	15,238	31,018	**46,256**	**73.71**
Cabuyao City	**58,181**	1,252	1,460	**2,712**	**4.66**

Table 2.D.6. Oral Health Care

Number and proportion of adolescents 20 -59 years old who received Basic Oral Health Care (BOHC)
Philippines, 2022

Area	Adults 20-59 years old who received BOHC				
	Eligible Population	Male	Female	Total	%
Calamba City	**85,645**	19,934	28,385	**48,319**	**56.42**
Cavite City	**20,322**	0	0	**0**	**0.00**
Dasmariñas City	**130,271**	149	585	**734**	**0.56**
General Trias City	**62,129**	1,016	2,077	**3,093**	**4.98**
Imus City	**79,817**	1,264	2,245	**3,509**	**4.40**
Lipa City	**62,749**	990	1,238	**2,228**	**3.55**
Lucena City	**43,413**	304	661	**965**	**2.22**
San Pablo City	**50,139**	1,944	7,772	**9,716**	**19.38**
San Pedro City	**61,397**	7,009	10,974	**17,983**	**29.29**
Santa Rosa City	**66,666**	2,703	4,239	**6,942**	**10.41**
Tagaytay City	**14,070**	95	203	**298**	**2.12**
Tanauan City	**32,728**	1,450	2,181	**3,631**	**11.09**
Tayabas City	**16,270**	1,021	1,648	**2,669**	**16.40**
Trece Martires City	**30,781**	464	539	**1,003**	**3.26**
**Region 4B**	**464,516**	**6,072**	**11,535**	**17,607**	**3.79**
Marinduque	**33,995**	567	906	**1,473**	**4.33**
Mindoro Occidental	**73,823**	804	1,573	**2,377**	**3.22**
Mindoro Oriental	**132,604**	1,468	2,514	**3,982**	**3.00**
Palawan	**139,938**	1,228	2,637	**3,865**	**2.76**
Romblon	**42,129**	806	1,503	**2,309**	**5.48**
Puerto Princesa City	**42,029**	1,199	2,402	**3,601**	**8.57**
**Region 5**	**889,920**	**24,646**	**43,099**	**67,745**	**7.61**
Albay	**178,579**	5,033	6,420	**11,453**	**6.41**
Camarines Norte	**92,090**	5,568	8,264	**13,832**	**15.02**
Camarines Sur	**250,927**	1,560	6,849	**8,409**	**3.35**
Catanduanes	**38,798**	937	1,370	**2,307**	**5.95**
Masbate	**129,903**	2,996	4,091	**7,087**	**5.46**
Sorsogon	**121,267**	5,484	12,134	**17,618**	**14.53**
Iriga City	**17,050**	620	1,119	**1,739**	**10.20**
Legaspi City	**31,405**	1,634	2,159	**3,793**	**12.08**
Naga City	**29,902**	814	693	**1,507**	**5.04**
**Region 6**	**1,237,730**	**31,108**	**53,994**	**85,102**	**6.88**
Aklan	**95,170**	1,939	3,289	**5,228**	**5.49**
Antique	**90,455**	1,833	2,671	**4,504**	**4.98**
Capiz	**129,714**	2,634	4,252	**6,886**	**5.31**
Guimaras	**29,701**	143	386	**529**	**1.78**
Iloilo	**326,747**	11,538	19,933	**31,471**	**9.63**
Negros Occidental	**400,287**	7,713	13,950	**21,663**	**5.41**
Bacolod City	**90,064**	2,442	3,446	**5,888**	**6.54**
Iloilo City	**75,592**	2,866	6,067	**8,933**	**11.82**
**Region 7**	**1,255,265**	**79,028**	**106,610**	**185,638**	**14.79**
Bohol	**200,702**	1,355	3,450	**4,805**	**2.39**

Table 2.D.6. Oral Health Care

Number and proportion of adolescents 20 -59 years old who received Basic Oral Health Care (BOHC)
Philippines, 2022

Area	Adults 20-59 years old who received BOHC				
	Eligible Population	Male	Female	Total	%
Cebu	**522,225**	7,957	14,331	**22,288**	**4.27**
Negros Oriental	**216,015**	2,672	4,636	**7,308**	**3.38**
Siquijor	**15,430**	119	204	**323**	**2.09**
Cebu City	**163,937**	6,428	18,419	**24,847**	**15.16**
Lapu-Lapu City	**72,517**	56,715	59,816	**116,531**	**160.70**
Mandaue City	**64,439**	3,782	5,754	**9,536**	**14.80**
**Region 8**	**703,230**	**6,110**	**12,684**	**18,794**	**2.67**
Biliran	**26,362**	244	485	**729**	**2.77**
Eastern Samar	**73,909**	552	1,329	**1,881**	**2.55**
Northern Leyte	**247,818**	2,641	6,362	**9,003**	**3.63**
Northern Samar	**94,693**	223	357	**580**	**0.61**
Southern Leyte	**53,603**	0	0	**0**	**0.00**
Western Samar	**90,332**	901	1,271	**2,172**	**2.40**
Calbayog City	**27,836**	412	478	**890**	**3.20**
Maasin City	**13,642**	83	187	**270**	**1.98**
Ormoc City	**35,295**	828	1,617	**2,445**	**6.93**
Tacloban City	**39,741**	226	598	**824**	**2.07**
**Region 9**	**576,370**	**9,468**	**13,932**	**23,400**	**4.06**
Zamboanga Del Norte	**124,573**	259	628	**887**	**0.71**
Zamboanga Del Sur	**128,560**	3,491	4,557	**8,048**	**6.26**
Zamboanga Sibugay	**102,020**	941	997	**1,938**	**1.90**
Dapitan City	**12,863**	95	112	**207**	**1.61**
Dipolog City	**20,407**	528	1,018	**1,546**	**7.58**
Isabela City	**19,916**	1,729	2,503	**4,232**	**21.25**
Pagadian City	**31,529**	2,407	3,998	**6,405**	**20.31**
Zamboanga City	**136,503**	18	119	**137**	**0.10**
**Region 10**	**789,758**	**22,336**	**34,281**	**56,617**	**7.17**
Bukidnon	**176,927**	2,350	3,815	**6,165**	**3.48**
Camiguin	**13,783**	771	869	**1,640**	**11.90**
Lanao Del Norte	**111,375**	885	1,344	**2,229**	**2.00**
Misamis Occidental	**51,979**	11,764	13,228	**24,992**	**48.08**
Misamis Oriental	**124,680**	1,786	3,002	**4,788**	**3.84**
Cagayan de Oro City	**118,094**	440	1,633	**2,073**	**1.76**
El Salvador City	**8,772**	81	168	**249**	**2.84**
Gingoog City	**21,776**	155	151	**306**	**1.41**
Iligan City	**56,415**	958	3,705	**4,663**	**8.27**
Malaybalay City	**29,491**	281	600	**881**	**2.99**
Oroquieta City	**11,264**	142	285	**427**	**3.79**
Ozamis City	**22,577**	611	2,400	**3,011**	**13.34**
Tangub City	**10,030**	979	893	**1,872**	**18.66**
Valencia City	**32,594**	1,133	2,188	**3,321**	**10.19**

Table 2.D.6. Oral Health Care

Number and proportion of adolescents 20 -59 years old who received Basic Oral Health Care (BOHC)
Philippines, 2022

Area	Adults 20-59 years old who received BOHC				
	Eligible Population	Male	Female	Total	%
**Region 11**	**838,779**	**2,665**	**3,498**	**6,163**	**0.73**
Davao de Oro	**121,504**	366	511	**877**	**0.72**
Davao del Norte	**166,565**	476	758	**1,234**	**0.74**
Davao Oriental	**89,031**	430	558	**988**	**1.11**
Davao del Sur	**114,308**	242	309	**551**	**0.48**
Davao Occidental	**52,286**	178	206	**384**	**0.73**
Davao City	**295,085**	973	1,156	**2,129**	**0.72**
**Region 12**	**725,255**	**16,543**	**32,122**	**48,665**	**6.71**
North Cotabato	**236,471**	3,652	7,002	**10,654**	**4.51**
Sarangani	**89,207**	1,408	2,461	**3,869**	**4.34**
South Cotabato	**160,672**	6,534	10,715	**17,249**	**10.74**
Sultan Kudarat	**134,557**	4,099	6,720	**10,819**	**8.04**
Gen. Santos City	**104,349**	850	5,224	**6,074**	**5.82**
**BARMM**	**659,689**	**15,545**	**18,621**	**34,166**	**5.18**
Basilan	**43,350**	216	401	**617**	**1.42**
Lanao Del Sur	**134,129**	2,853	3,544	**6,397**	**4.77**
Maguindanao	**196,325**	4,728	5,880	**10,608**	**5.40**
Sulu	**133,785**	6,398	6,304	**12,702**	**9.49**
Tawi-Tawi	**60,176**	0	0	**0**	**0.00**
Lamitan City	**11,927**	208	387	**595**	**4.99**
Marawi City	**32,084**	646	1,630	**2,276**	**7.09**
Cotabato City	**47,912**	496	475	**971**	**2.03**
**CARAGA**	**413,565**	**10,572**	**19,831**	**30,403**	**7.35**
Agusan del Norte	**58,094**	1,818	5,130	**6,948**	**11.96**
Agusan del Sur	**107,950**	1,556	2,547	**4,103**	**3.80**
Surigao del Norte	**55,211**	2,357	3,446	**5,803**	**10.51**
Surigao del Sur	**77,315**	1,958	2,853	**4,811**	**6.22**
Province of Dinagat	**19,358**	326	474	**800**	**4.13**
Bislig City	**14,684**	477	1,064	**1,541**	**10.49**
Butuan City	**55,237**	1,929	4,102	**6,031**	**10.92**
Surigao City	**25,715**	151	215	**366**	**1.42**

Table 2.D.7. Oral Health Care

Number and proportion of senior citizens 60 years old and above who received Basic Oral Health Care (BOHC)

Philippines, 2022

Area	Senior citizens 60 years old and above who received BOHC				
	Eligible Population	Male	Female	Total	%
**Philippines**	**3,078,111**	**193,325**	**237,774**	**431,099**	**14.01**
**NCR**	**407,382**	**36,647**	**46,721**	**83,368**	**20.46**
Malabon	**11,564**	219	339	**558**	**4.83**
Navotas	**7,892**	1,417	1,591	**3,008**	**38.11**
Valenzuela City	**19,628**	2,106	2,594	**4,700**	**23.95**
Caloocan City	**50,110**	3,618	4,374	**7,992**	**15.95**
Marikina City	**14,260**	849	1,243	**2,092**	**14.67**
Pasig City	**23,895**	7,412	9,599	**17,011**	**71.19**
Pateros	**2,020**	152	235	**387**	**19.16**
Taguig	**25,465**	554	746	**1,300**	**5.11**
Quezon City	**92,885**	4,656	6,210	**10,866**	**11.70**
Makati City	**18,431**	812	1,289	**2,101**	**11.40**
Mandaluyong City	**12,220**	439	584	**1,023**	**8.37**
San Juan	**3,865**	186	274	**460**	**11.90**
Manila City	**56,317**	9,591	11,801	**21,392**	**37.98**
Las Piñas City	**18,631**	942	1,431	**2,373**	**12.74**
Muntinlupa City	**15,961**	2,545	3,091	**5,636**	**35.31**
Parañaque City	**21,064**	395	437	**832**	**3.95**
Pasay City	**13,174**	754	883	**1,637**	**12.43**
**CAR**	**47,679**	**3,957**	**5,817**	**9,774**	**20.50**
Abra	**8,169**	390	656	**1,046**	**12.80**
Apayao	**3,138**	325	472	**797**	**25.40**
Benguet	**12,297**	1,775	2,342	**4,117**	**33.48**
Ifugao	**4,909**	343	563	**906**	**18.46**
Kalinga	**5,421**	435	803	**1,238**	**22.84**
Mt. Province	**4,229**	312	376	**688**	**16.27**
Baguio City	**9,516**	377	605	**982**	**10.32**
**Region 1**	**176,969**	**14,822**	**18,472**	**33,294**	**18.81**
Ilocos Norte	**19,390**	1,180	1,351	**2,531**	**13.05**
Ilocos Sur	**22,772**	3,090	3,715	**6,805**	**29.88**
La Union	**25,289**	1,791	2,611	**4,402**	**17.41**
Pangasinan	**76,997**	6,729	7,859	**14,588**	**18.95**
Alaminos City	**2,909**	1,033	1,282	**2,315**	**79.58**
Candon City	**2,398**	136	381	**517**	**21.56**
Dagupan City	**5,553**	43	46	**89**	**1.60**
Laoag City	**4,469**	108	95	**203**	**4.54**
San Carlos City	**6,117**	157	228	**385**	**6.29**
San Fernando City	**4,633**	361	624	**985**	**21.26**
Urdaneta City	**4,310**	94	119	**213**	**4.94**
Vigan City	**2,132**	100	161	**261**	**12.24**

Table 2.D.7. Oral Health Care

Number and proportion of senior citizens 60 years old and above who received Basic Oral Health Care (BOHC)

Philippines, 2022

Area	Senior citizens 60 years old and above who received BOHC				
	Eligible Population	Male	Female	Total	%
**Region 2**	**114,007**	**11,973**	**16,221**	**28,194**	**24.73**
Batanes	**614**	93	165	**258**	**42.02**
Cagayan	**36,800**	1,246	1,557	**2,803**	**7.62**
Isabela	**38,195**	3,881	5,339	**9,220**	**24.14**
Nueva Vizcaya	**14,391**	1,606	2,289	**3,895**	**27.07**
Quirino	**5,376**	424	769	**1,193**	**22.19**
Cauayan City	**4,180**	603	927	**1,530**	**36.60**
Ilagan City	**4,698**	2,154	2,694	**4,848**	**103.19**
Santiago City	**4,351**	1,911	2,440	**4,351**	**100.00**
Tuguegarao City	**5,402**	55	41	**96**	**1.78**
**Region 3**	**375,547**	**15,979**	**17,818**	**33,797**	**9.00**
Aurora	**5,960**	350	392	**742**	**12.45**
Bataan	**22,124**	130	119	**249**	**1.13**
Bulacan	**74,667**	1,589	2,046	**3,635**	**4.87**
Nueva Ecija	**51,510**	2,209	2,506	**4,715**	**9.15**
Pampanga	**55,046**	1,018	1,132	**2,150**	**3.91**
Tarlac	**34,222**	1,752	1,831	**3,583**	**10.47**
Zambales	**19,410**	1,130	1,326	**2,456**	**12.65**
Angeles City	**13,810**	18	13	**31**	**0.22**
Balanga City	**3,198**	162	191	**353**	**11.04**
Cabanatuan City	**10,544**	88	84	**172**	**1.63**
City of San Fernando	**10,287**	127	133	**260**	**2.53**
Gapan City	**3,849**	0	0	**0**	**0.00**
Mabalacat City	**8,413**	2,741	2,706	**5,447**	**64.75**
Malolos City	**8,341**	152	171	**323**	**3.87**
Meycauayan	**6,917**	380	419	**799**	**11.55**
Olongapo	**7,654**	655	849	**1,504**	**19.65**
Palayan City	**1,432**	16	9	**25**	**1.75**
San Jose City	**4,875**	492	482	**974**	**19.98**
San Jose del Monte City	**18,994**	1,269	1,506	**2,775**	**14.61**
Science City of Munoz	**2,842**	211	404	**615**	**21.64**
Tarlac City	**11,452**	1,490	1,499	**2,989**	**26.10**
**Region 4A**	**449,558**	**44,322**	**49,404**	**93,726**	**20.85**
Batangas	**63,263**	9,147	9,507	**18,654**	**29.49**
Cavite	**43,313**	903	1,050	**1,953**	**4.51**
Laguna	**30,362**	9,663	9,235	**18,898**	**62.24**
Quezon	**52,601**	1,294	1,653	**2,947**	**5.60**
Rizal	**62,337**	2,417	2,944	**5,361**	**8.60**
Antipolo City	**22,960**	99	147	**246**	**1.07**
Bacoor City	**18,978**	228	301	**529**	**2.79**
Batangas City	**11,225**	466	584	**1,050**	**9.35**
Biñan City	**10,181**	6,468	7,086	**13,554**	**133.13**
Cabuyao City	**9,438**	3,185	2,806	**5,991**	**63.48**
Calamba City	**13,894**	3,914	3,909	**7,823**	**56.30**
Cavite City	**3,248**	0	0	**0**	**0.00**

Table 2.D.7. Oral Health Care

Number and proportion of senior citizens 60 years old and above who received Basic Oral Health Care (BOHC)

Philippines, 2022

Area	Senior citizens 60 years old and above who received BOHC				
	Eligible Population	Male	Female	Total	%
Dasmariñas City	**20,822**	26	60	**86**	**0.41**
General Trias City	**9,932**	153	175	**328**	**3.30**
Imus City	**12,759**	402	540	**942**	**7.38**
Lipa City	**11,311**	448	512	**960**	**8.49**
Lucena City	**7,971**	49	53	**102**	**1.28**
San Pablo City	**8,131**	964	3,717	**4,681**	**57.57**
San Pedro City	**9,959**	3,370	3,679	**7,049**	**70.78**
Santa Rosa City	**10,815**	549	753	**1,302**	**12.04**
Tagaytay City	**2,249**	21	14	**35**	**1.56**
Tanauan City	**5,901**	176	213	**389**	**6.59**
Tayabas City	**2,988**	141	190	**331**	**11.08**
Trece Martires City	**4,920**	239	276	**515**	**10.47**
**Region 4B**	**83,042**	**1,295**	**1,677**	**2,972**	**3.58**
Marinduque	**8,145**	365	404	**769**	**9.44**
Mindoro Occidental	**12,550**	123	170	**293**	**2.33**
Mindoro Oriental	**24,347**	262	362	**624**	**2.56**
Palawan	**22,072**	196	249	**445**	**2.02**
Romblon	**9,298**	180	275	**455**	**4.89**
Puerto Princesa City	**6,630**	169	217	**386**	**5.82**
**Region 5**	**164,785**	**5,525**	**7,607**	**13,132**	**7.97**
Albay	**35,272**	1,464	1,699	**3,163**	**8.97**
Camarines Norte	**16,501**	1,053	1,625	**2,678**	**16.23**
Camarines Sur	**45,481**	296	627	**923**	**2.03**
Catanduanes	**7,432**	231	306	**537**	**7.23**
Masbate	**22,707**	1,185	1,386	**2,571**	**11.32**
Sorsogon	**22,679**	885	1,401	**2,286**	**10.08**
Iriga City	**3,090**	89	171	**260**	**8.41**
Legaspi City	**6,201**	178	211	**389**	**6.27**
Naga City	**5,422**	144	181	**325**	**5.99**
**Region 6**	**259,467**	**10,604**	**14,860**	**25,464**	**9.81**
Aklan	**19,385**	448	681	**1,129**	**5.82**
Antique	**18,609**	459	572	**1,031**	**5.54**
Capiz	**27,897**	1,006	1,499	**2,505**	**8.98**
Guimaras	**6,152**	45	61	**106**	**1.72**
Iloilo	**71,747**	5,620	7,515	**13,135**	**18.31**
Negros Occidental	**80,881**	2,004	2,750	**4,754**	**5.88**
Bacolod City	**18,198**	387	663	**1,050**	**5.77**
Iloilo City	**16,598**	635	1,119	**1,754**	**10.57**

Table 2.D.7. Oral Health Care

Number and proportion of senior citizens 60 years old and above who received Basic Oral Health Care (BOHC)

Philippines, 2022

Area	Senior citizens 60 years old and above who received BOHC				
	Eligible Population	Male	Female	Total	%
**Region 7**	**238,981**	**18,123**	**22,054**	**40,177**	**16.81**
Bohol	**44,995**	382	629	**1,011**	**2.25**
Cebu	**92,161**	2,804	3,563	**6,367**	**6.91**
Negros Oriental	**44,794**	504	621	**1,125**	**2.51**
Siquijor	**3,922**	17	21	**38**	**0.97**
Cebu City	**28,934**	1,308	1,837	**3,145**	**10.87**
Lapu-Lapu City	**12,800**	8,179	9,049	**17,228**	**134.59**
Mandaue City	**11,375**	4,929	6,334	**11,263**	**99.02**
**Region 8**	**137,524**	**1,306**	**1,703**	**3,009**	**2.19**
Biliran	**5,279**	48	75	**123**	**2.33**
Eastern Samar	**14,690**	228	364	**592**	**4.03**
Northern Leyte	**49,675**	512	660	**1,172**	**2.36**
Northern Samar	**15,917**	11	14	**25**	**0.16**
Southern Leyte	**12,530**	0	0	**0**	**0.00**
Western Samar	**16,205**	189	180	**369**	**2.28**
Calbayog City	**4,997**	67	85	**152**	**3.04**
Maasin City	**3,189**	13	35	**48**	**1.51**
Ormoc City	**7,077**	150	157	**307**	**4.34**
Tacloban City	**7,965**	88	133	**221**	**2.77**
**Region 9**	**96,481**	**1,900**	**2,325**	**4,225**	**4.38**
Zamboanga Del Norte	**22,765**	78	113	**191**	**0.84**
Zamboanga Del Sur	**21,322**	1,297	1,503	**2,800**	**13.13**
Zamboanga Sibugay	**15,422**	131	141	**272**	**1.76**
Dapitan City	**2,349**	44	41	**85**	**3.62**
Dipolog City	**3,729**	40	86	**126**	**3.38**
Isabela City	**3,031**	89	106	**195**	**6.43**
Pagadian City	**5,230**	220	327	**547**	**10.46**
Zamboanga City	**22,633**	1	8	**9**	**0.04**
**Region 10**	**135,640**	**7,233**	**9,627**	**16,860**	**12.43**
Bukidnon	**27,324**	622	963	**1,585**	**5.80**
Camiguin	**3,141**	304	360	**664**	**21.14**
Lanao Del Norte	**17,007**	194	257	**451**	**2.65**
Misamis Occidental	**11,305**	2,111	2,597	**4,708**	**41.65**
Misamis Oriental	**22,405**	345	462	**807**	**3.60**
Cagayan de Oro City	**21,225**	101	107	**208**	**0.98**
El Salvador City	**1,577**	57	116	**173**	**10.97**
Gingoog City	**3,915**	602	850	**1,452**	**37.09**
Iligan City	**8,613**	143	205	**348**	**4.04**
Malaybalay City	**4,554**	1,894	2,228	**4,122**	**90.51**
Oroquieta City	**2,451**	79	134	**213**	**8.69**
Ozamis City	**4,910**	254	772	**1,026**	**20.90**

Table 2.D.7. Oral Health Care

Number and proportion of senior citizens 60 years old and above who received Basic Oral Health Care (BOHC)

Philippines, 2022

Area	Senior citizens 60 years old and above who received BOHC				
	Eligible Population	Male	Female	Total	%
Tangub City	**2,181**	151	133	**284**	**13.02**
Valencia City	**5,032**	376	443	**819**	**16.28**
**Region 11**	**143,689**	**1,090**	**1,352**	**2,442**	**1.70**
Davao de Oro	**19,481**	141	179	**320**	**1.64**
Davao del Norte	**28,658**	238	302	**540**	**1.88**
Davao Oriental	**16,101**	162	197	**359**	**2.23**
Davao del Sur	**19,713**	103	131	**234**	**1.19**
Davao Occidental	**8,843**	62	86	**148**	**1.67**
Davao City	**50,893**	384	457	**841**	**1.65**
**Region 12**	**108,290**	**4,224**	**5,872**	**10,096**	**9.32**
North Cotabato	**36,377**	1,203	1,638	**2,841**	**7.81**
Sarangani	**12,320**	524	714	**1,238**	**10.05**
South Cotabato	**24,601**	1,230	1,715	**2,945**	**11.97**
Sultan Kudarat	**19,014**	1,150	1,478	**2,628**	**13.82**
Gen. Santos City	**15,978**	117	327	**444**	**2.78**
**BARMM**	**65,957**	**10,967**	**11,700**	**22,667**	**34.37**
Basilan	**4,564**	52	75	**127**	**2.78**
Lanao Del Sur	**12,266**	892	1,069	**1,961**	**15.99**
Maguindanao	**19,036**	2,134	2,635	**4,769**	**25.05**
Sulu	**13,783**	6,442	6,548	**12,990**	**94.25**
Tawi-Tawi	**6,181**	800	832	**1,632**	**26.40**
Lamitan City	**1,256**	21	38	**59**	**4.70**
Marawi City	**2,932**	68	136	**204**	**6.96**
Cotabato City	**5,939**	558	367	**925**	**15.58**
**CARAGA**	**73,113**	**3,358**	**4,544**	**7,902**	**10.81**
Agusan del Norte	**10,439**	534	975	**1,509**	**14.46**
Agusan del Sur	**15,986**	371	525	**896**	**5.60**
Surigao del Norte	**10,585**	361	451	**812**	**7.67**
Surigao del Sur	**14,417**	737	792	**1,529**	**10.61**
Province of Dinagat	**4,091**	309	368	**677**	**16.55**
Bislig City	**2,737**	119	143	**262**	**9.57**
Butuan City	**9,927**	860	1,196	**2,056**	**20.71**
Surigao City	**4,931**	67	94	**161**	**3.27**

Table 2.D.8. Oral Health Care

Number and proportion of pregnant women 10-14, 15-19, 20-49 years old who received
Basic Oral Health Care (BOHC)
Philippines, 2022

Area	Eligible Pop.	Pregnant Women 10-14 y/o received BOHC		Pregnant Women 15-19 y/o received BOHC		Pregnant Women 20-49 y/o received BOHC	
		No.	%	No.	%	No.	%
**Philippines**	**2,131,496**	**4,363**	**0.20**	**47,208**	**2.21**	**339,554**	**15.93**
**NCR**	**236,901**	**450**	**0.19**	**13,165**	**5.56**	**79,248**	**33.45**
Malabon	**6,724**	6	**0.09**	133	**1.98**	780	**11.60**
Navotas	**4,591**	68	**1.48**	723	**15.75**	2,779	**60.53**
Valenzuela City	**11,418**	2	**0.02**	719	**6.30**	2,516	**22.04**
Caloocan City	**29,146**	50	**0.17**	2,245	**7.70**	7,978	**27.37**
Marikina City	**8,294**	3	**0.04**	242	**2.92**	1,803	**21.74**
Pasig City	**13,894**	30	**0.22**	1,165	**8.38**	7,673	**55.23**
Pateros	**1,176**	1	**0.09**	89	**7.57**	505	**42.94**
Taguig	**14,807**	30	**0.20**	437	**2.95**	2,475	**16.72**
Quezon City	**54,011**	48	**0.09**	1,605	**2.97**	17,137	**31.73**
Makati City	**10,718**	9	**0.08**	399	**3.72**	4,326	**40.36**
Mandaluyong City	**7,111**	50	**0.70**	722	**10.15**	3,733	**52.50**
San Juan	**2,246**	0	**0.00**	37	**1.65**	423	**18.83**
Manila City	**32,743**	75	**0.23**	2,436	**7.44**	13,752	**42.00**
Las Piñas City	**10,832**	33	**0.30**	373	**3.44**	3,073	**28.37**
Muntinlupa City	**9,281**	5	**0.05**	773	**8.33**	5,158	**55.58**
Parañaque City	**12,249**	17	**0.14**	282	**2.30**	1,342	**10.96**
Pasay City	**7,660**	23	**0.30**	785	**10.25**	3,795	**49.54**
**CAR**	**35,179**	**50**	**0.14**	**902**	**2.56**	**6,414**	**18.23**
Abra	**4,275**	28	**0.65**	164	**3.84**	946	**22.13**
Apayao	**2,502**	3	**0.12**	67	**2.68**	512	**20.46**
Benguet	**9,086**	4	**0.04**	193	**2.12**	1,576	**17.35**
Ifugao	**4,486**	2	**0.04**	65	**1.45**	432	**9.63**
Kalinga	**4,701**	0	**0.00**	142	**3.02**	1,106	**23.53**
Mt. Province	**3,103**	8	**0.26**	81	**2.61**	376	**12.12**
Baguio City	**7,026**	5	**0.07**	190	**2.70**	1,466	**20.87**
**Region 1**	**97,099**	**132**	**0.14**	**2,664**	**2.74**	**21,216**	**21.85**
Ilocos Norte	**7,854**	1	**0.01**	177	**2.25**	1,329	**16.92**
Ilocos Sur	**9,072**	2	**0.02**	336	**3.70**	3,581	**39.47**
La Union	**11,338**	47	**0.41**	740	**6.53**	2,508	**22.12**
Pangasinan	**50,710**	67	**0.13**	794	**1.57**	8,285	**16.34**
Alaminos City	**1,910**	0	**0.00**	20	**1.05**	1,415	**74.08**
Candon City	**954**	1	**0.10**	34	**3.56**	179	**18.76**
Dagupan City	**3,657**	9	**0.25**	171	**4.68**	719	**19.66**
Laoag City	**1,812**	0	**0.00**	0	**0.00**	0	**0.00**
San Carlos City	**4,028**	0	**0.00**	25	**0.62**	533	**13.23**
San Fernando City	**2,074**	2	**0.10**	73	**3.52**	566	**27.29**
Urdaneta City	**2,836**	3	**0.11**	195	**6.88**	1,603	**56.52**
Vigan City	**854**	0	**0.00**	99	**11.59**	498	**58.31**
**Region 2**	**68,960**	**116**	**0.17**	**3,407**	**4.94**	**14,356**	**20.82**
Batanes	**362**	0	**0.00**	1	**0.28**	78	**21.55**
Cagayan	**20,431**	9	**0.04**	363	**1.78**	1,914	**9.37**
Isabela	**23,449**	53	**0.23**	855	**3.65**	4,520	**19.28**
Nueva Vizcaya	**9,538**	26	**0.27**	1,205	**12.63**	2,593	**27.19**

Table 2.D.8. Oral Health Care

Number and proportion of pregnant women 10-14, 15-19, 20-49 years old who received
Basic Oral Health Care (BOHC)
Philippines, 2022

Area	Eligible Pop.	Pregnant Women 10-14 y/o received BOHC		Pregnant Women 15-19 y/o received BOHC		Pregnant Women 20-49 y/o received BOHC	
		No.	%	No.	%	No.	%
Quirino	**4,051**	6	**0.15**	123	**3.04**	713	**17.60**
Cauayan City	**2,565**	7	**0.27**	206	**8.03**	684	**26.67**
Ilagan City	**2,889**	8	**0.28**	228	**7.89**	1,131	**39.15**
Santiago City	**2,671**	6	**0.22**	367	**13.74**	2,298	**86.04**
Tuguegarao City	**3,004**	1	**0.03**	59	**1.96**	425	**14.15**
**Region 3**	**220,155**	**1,056**	**0.48**	**8,524**	**3.87**	**35,486**	**16.12**
Aurora	**4,754**	0	**0.00**	174	**3.66**	773	**16.26**
Bataan	**13,789**	338	**2.45**	164	**1.19**	759	**5.50**
Bulacan	**43,760**	91	**0.21**	1,633	**3.73**	8,538	**19.51**
Nueva Ecija	**29,039**	46	**0.16**	855	**2.94**	3,000	**10.33**
Pampanga	**31,620**	10	**0.03**	406	**1.28**	2,647	**8.37**
Tarlac	**19,319**	493	**2.55**	1,788	**9.26**	5,208	**26.96**
Zambales	**12,476**	11	**0.09**	349	**2.80**	2,181	**17.48**
Angeles City	**7,932**	0	**0.00**	82	**1.03**	337	**4.25**
Balanga City	**1,992**	4	**0.20**	129	**6.48**	806	**40.46**
Cabanatuan City	**5,945**	0	**0.00**	70	**1.18**	102	**1.72**
City of San Fernando	**5,907**	19	**0.32**	90	**1.52**	337	**5.71**
Gapan City	**2,170**	0	**0.00**	7	**0.32**	29	**1.34**
Mabalacat City	**4,831**	3	**0.06**	1,423	**29.46**	2,315	**47.92**
Malolos City	**4,890**	10	**0.20**	95	**1.94**	544	**11.12**
Meycauayan	**4,055**	2	**0.05**	124	**3.06**	393	**9.69**
Olongapo	**4,922**	3	**0.06**	206	**4.19**	1,397	**28.38**
Palayan City	**808**	0	**0.00**	1	**0.12**	1	**0.12**
San Jose City	**2,748**	20	**0.73**	287	**10.44**	636	**23.14**
San Jose del Monte City	**11,133**	4	**0.04**	454	**4.08**	3,921	**35.22**
Science City of Munoz	**1,601**	2	**0.12**	21	**1.31**	121	**7.56**
Tarlac City	**6,464**	0	**0.00**	166	**2.57**	1,441	**22.29**
**Region 4A**	**299,627**	**882**	**0.29**	**5,554**	**1.85**	**43,101**	**14.38**
Batangas	**39,255**	618	**1.57**	1,457	**3.71**	9,163	**23.34**
Cavite	**28,151**	8	**0.03**	124	**0.44**	852	**3.03**
Laguna	**19,696**	0	**0.00**	92	**0.47**	734	**3.73**
Quezon	**37,853**	62	**0.16**	1,642	**4.34**	7,818	**20.65**
Rizal	**44,798**	61	**0.14**	867	**1.94**	4,458	**9.95**
Antipolo City	**16,498**	5	**0.03**	246	**1.49**	1,668	**10.11**
Bacoor City	**12,341**	2	**0.02**	59	**0.48**	326	**2.64**
Batangas City	**6,965**	2	**0.03**	68	**0.98**	404	**5.80**
Biñan City	**6,597**	3	**0.05**	253	**3.84**	4,278	**64.85**
Cabuyao City	**6,119**	0	**0.00**	0	**0.00**	480	**7.84**
Calamba City	**9,005**	12	**0.13**	8	**0.09**	6,997	**77.70**
Cavite City	**2,105**	0	**0.00**	0	**0.00**	0	**0.00**
Dasmariñas City	**13,538**	0	**0.00**	0	**0.00**	0	**0.00**
General Trias City	**6,457**	2	**0.03**	108	**1.67**	593	**9.18**
Imus City	**8,292**	2	**0.02**	40	**0.48**	293	**3.53**
Lipa City	**7,018**	10	**0.14**	218	**3.11**	1,011	**14.41**
Lucena City	**5,738**	1	**0.02**	6	**0.10**	130	**2.27**
San Pablo City	**5,272**	0	**0.00**	3	**0.06**	17	**0.32**
San Pedro City	**6,455**	0	**0.00**	0	**0.00**	0	**0.00**
Santa Rosa City	**7,009**	32	**0.46**	199	**2.84**	962	**13.73**
Tagaytay City	**1,460**	2	**0.14**	35	**2.40**	370	**25.34**
Tanauan City	**3,661**	1	**0.03**	6	**0.16**	328	**8.96**
Tayabas City	**2,145**	0	**0.00**	0	**0.00**	1,778	**82.89**
Trece Martires City	**3,199**	59	**1.84**	123	**3.84**	441	**13.79**

Table 2.D.8. Oral Health Care

Number and proportion of pregnant women 10-14, 15-19, 20-49 years old who received Basic Oral Health Care (BOHC)
Philippines, 2022

Area	Eligible Pop.	Pregnant Women 10-14 y/o received BOHC		Pregnant Women 15-19 y/o received BOHC		Pregnant Women 20-49 y/o received BOHC	
		No.	%	No.	%	No.	%
**Region 4B**	**72,791**	**58**	**0.08**	**522**	**0.72**	**3,561**	**4.89**
Marinduque	**4,990**	12	**0.24**	42	**0.84**	546	**10.94**
Mindoro Occidental	**12,479**	0	**0.00**	0	**0.00**	0	**0.00**
Mindoro Oriental	**20,189**	38	**0.19**	156	**0.77**	908	**4.50**
Palawan	**21,963**	3	**0.01**	169	**0.77**	994	**4.53**
Romblon	**6,569**	1	**0.02**	46	**0.70**	577	**8.78**
Puerto Princesa City	**6,601**	4	**0.06**	109	**1.65**	536	**8.12**
**Region 5**	**138,457**	**124**	**0.09**	**3,041**	**2.20**	**16,351**	**11.81**
Albay	**24,081**	18	**0.07**	155	**0.64**	1,177	**4.89**
Camarines Norte	**14,384**	52	**0.36**	808	**5.62**	3,428	**23.83**
Camarines Sur	**39,486**	11	**0.03**	198	**0.50**	1,392	**3.53**
Catanduanes	**6,459**	1	**0.02**	231	**3.58**	1,472	**22.79**
Masbate	**22,600**	33	**0.15**	411	**1.82**	1,734	**7.67**
Sorsogon	**19,828**	6	**0.03**	1,017	**5.13**	5,797	**29.24**
Iriga City	**2,679**	2	**0.07**	43	**1.61**	383	**14.30**
Legaspi City	**4,233**	0	**0.00**	133	**3.14**	567	**13.39**
Naga City	**4,707**	1	**0.02**	45	**0.96**	401	**8.52**
**Region 6**	**146,449**	**233**	**0.16**	**3,673**	**2.51**	**22,441**	**15.32**
Aklan	**11,288**	69	**0.61**	387	**3.43**	1,339	**11.86**
Antique	**13,132**	0	**0.00**	58	**0.44**	1,286	**9.79**
Capiz	**13,975**	12	**0.09**	591	**4.23**	2,087	**14.93**
Guimaras	**3,084**	0	**0.00**	1	**0.03**	136	**4.41**
Iloilo	**36,267**	7	**0.02**	760	**2.10**	5,925	**16.34**
Negros Occidental	**49,230**	21	**0.04**	891	**1.81**	5,364	**10.90**
Bacolod City	**11,082**	38	**0.34**	548	**4.94**	3,269	**29.50**
Iloilo City	**8,391**	86	**1.02**	437	**5.21**	3,035	**36.17**
**Region 7**	**162,308**	**226**	**0.14**	**3,171**	**1.95**	**26,027**	**16.04**
Bohol	**27,992**	0	**0.00**	269	**0.96**	1,406	**5.02**
Cebu	**66,463**	154	**0.23**	948	**1.43**	7,575	**11.40**
Negros Oriental	**27,890**	15	**0.05**	586	**2.10**	1,850	**6.63**
Siquijor	**1,661**	0	**0.00**	0	**0.00**	42	**2.53**
Cebu City	**20,866**	0	**0.00**	0	**0.00**	7,231	**34.65**
Lapu-Lapu City	**9,232**	6	**0.06**	240	**2.60**	346	**3.75**
Mandaue City	**8,204**	51	**0.62**	1,128	**13.75**	7,577	**92.36**
**Region 8**	**105,471**	**18**	**0.02**	**1,395**	**1.32**	**8,903**	**8.44**
Biliran	**3,873**	0	**0.00**	96	**2.48**	532	**13.74**
Eastern Samar	**11,908**	17	**0.14**	347	**2.91**	966	**8.11**
Northern Leyte	**35,422**	1	**0.00**	661	**1.87**	2,549	**7.20**
Northern Samar	**15,921**	0	**0.00**	0	**0.00**	1,017	**6.39**
Southern Leyte	**6,662**	0	**0.00**	0	**0.00**	0	**0.00**
Western Samar	**14,732**	0	**0.00**	10	**0.07**	188	**1.28**
Calbayog City	**4,535**	0	**0.00**	14	**0.31**	165	**3.64**
Maasin City	**1,696**	0	**0.00**	17	**1.00**	116	**6.84**
Ormoc City	**5,044**	0	**0.00**	0	**0.00**	2,418	**47.94**

Table 2.D.8. Oral Health Care

Number and proportion of pregnant women 10-14, 15-19, 20-49 years old who received
Basic Oral Health Care (BOHC)
Philippines, 2022

Area	Eligible Pop.	Pregnant Women 10-14 y/o received BOHC		Pregnant Women 15-19 y/o received BOHC		Pregnant Women 20-49 y/o received BOHC	
		No.	%	No.	%	No.	%
Tacloban City	**5,678**	0	**0.00**	250	**4.40**	952	**16.77**
**Region 9**	**79,007**	**11**	**0.01**	**788**	**1.00**	**4,854**	**6.14**
Zamboanga Del Norte	**16,854**	1	**0.01**	285	**1.69**	1,440	**8.54**
Zamboanga Del Sur	**17,368**	5	**0.03**	264	**1.52**	2,267	**13.05**
Zamboanga Sibugay	**15,021**	4	**0.03**	109	**0.73**	578	**3.85**
Dapitan City	**1,737**	0	**0.00**	2	**0.12**	10	**0.58**
Dipolog City	**2,762**	0	**0.00**	0	**0.00**	1	**0.04**
Isabela City	**2,563**	0	**0.00**	5	**0.20**	16	**0.62**
Pagadian City	**4,257**	0	**0.00**	68	**1.60**	260	**6.11**
Zamboanga City	**18,445**	1	**0.01**	55	**0.30**	282	**1.53**
**Region 10**	**99,908**	**208**	**0.21**	**3,586**	**3.59**	**12,141**	**12.15**
Bukidnon	**22,900**	5	**0.02**	418	**1.83**	1,969	**8.60**
Camiguin	**1,854**	0	**0.00**	28	**1.51**	188	**10.14**
Lanao Del Norte	**14,930**	1	**0.01**	39	**0.26**	193	**1.29**
Misamis Occidental	**6,420**	43	**0.67**	1,001	**15.59**	2,673	**41.64**
Misamis Oriental	**14,963**	33	**0.22**	386	**2.58**	1,541	**10.30**
Cagayan de Oro City	**14,172**	64	**0.45**	290	**2.05**	1,258	**8.88**
El Salvador City	**1,052**	3	**0.29**	12	**1.14**	96	**9.13**
Gingoog City	**2,608**	0	**0.00**	400	**15.34**	565	**21.66**
Iligan City	**7,565**	1	**0.01**	82	**1.08**	468	**6.19**
Malaybalay City	**3,817**	10	**0.26**	105	**2.75**	545	**14.28**
Oroquieta City	**1,389**	30	**2.16**	120	**8.64**	128	**9.22**
Ozamis City	**2,786**	0	**0.00**	103	**3.70**	976	**35.03**
Tangub City	**1,234**	3	**0.24**	54	**4.38**	226	**18.31**
Valencia City	**4,218**	15	**0.36**	548	**12.99**	1,315	**31.18**
**Region 11**	**108,407**	**1**	**0.00**	**397**	**0.37**	**3,338**	**3.08**
Davao de Oro	**15,490**	0	**0.00**	63	**0.41**	461	**2.98**
Davao del Norte	**21,017**	0	**0.00**	79	**0.38**	658	**3.13**
Davao Oriental	**13,229**	1	**0.01**	52	**0.39**	449	**3.39**
Davao del Sur	**14,564**	0	**0.00**	36	**0.25**	359	**2.46**
Davao Occidental	**6,510**	0	**0.00**	24	**0.37**	181	**2.78**
Davao City	**37,597**	0	**0.00**	143	**0.38**	1,230	**3.27**
**Region 12**	**98,859**	**405**	**0.41**	**4,229**	**4.28**	**22,829**	**23.09**
North Cotabato	**34,150**	83	**0.24**	913	**2.67**	3,943	**11.55**
Sarangani	**12,907**	244	**1.89**	411	**3.18**	2,619	**20.29**
South Cotabato	**20,917**	32	**0.15**	1,248	**5.97**	6,819	**32.60**
Sultan Kudarat	**17,299**	19	**0.11**	798	**4.61**	4,757	**27.50**
Gen. Santos City	**13,586**	27	**0.20**	859	**6.32**	4,691	**34.53**
**BARMM**	**101,343**	**170**	**0.17**	**3,056**	**3.02**	**7,460**	**7.36**
Basilan	**7,823**	1	**0.01**	30	**0.38**	96	**1.23**
Lanao Del Sur	**21,639**	0	**0.00**	1,244	**5.75**	2,158	**9.97**
Maguindanao	**32,198**	147	**0.46**	1,401	**4.35**	4,090	**12.70**
Sulu	**17,165**	7	**0.04**	163	**0.95**	425	**2.48**
Tawi-Tawi	**9,369**	0	**0.00**	89	**0.95**	474	**5.06**

Table 2.D.8. Oral Health Care

Number and proportion of pregnant women 10-14, 15-19, 20-49 years old who received
Basic Oral Health Care (BOHC)
Philippines, 2022

Area	Eligible Pop.	Pregnant Women 10-14 y/o received BOHC		Pregnant Women 15-19 y/o received BOHC		Pregnant Women 20-49 y/o received BOHC	
		No.	%	No.	%	No.	%
Lamitan City	**2,154**	1	**0.05**	22	**1.02**	45	**2.09**
Marawi City	**5,173**	14	**0.27**	107	**2.07**	172	**3.32**
Cotabato City	**5,822**	0	**0.00**	0	**0.00**	0	**0.00**
**CARAGA**	**60,575**	**223**	**0.37**	**2,299**	**3.80**	**11,828**	**19.53**
Agusan del Norte	**8,127**	154	**1.89**	212	**2.61**	2,179	**26.81**
Agusan del Sur	**17,709**	14	**0.08**	319	**1.80**	1,443	**8.15**
Surigao del Norte	**7,280**	11	**0.15**	369	**5.07**	1,395	**19.16**
Surigao del Sur	**11,553**	19	**0.16**	641	**5.55**	2,226	**19.27**
Province of Dinagat	**2,576**	0	**0.00**	127	**4.93**	791	**30.71**
Bislig City	**2,192**	11	**0.50**	277	**12.64**	1,367	**62.36**
Butuan City	**7,743**	4	**0.05**	229	**2.96**	1,674	**21.62**
Surigao City	**3,395**	10	**0.29**	125	**3.68**	753	**22.18**

Table 2.E.1.1. Filariasis Prevention and Control

Case Detection Rate (CDR) and Clinical Rate of Lymphatic Filariasis

Philippines, 2022

Area	Total Examined for lymphatic filariasis	Number positive cases found				Lymphatic filariasis cases examined with disabilities/deformities			
		Male	Female	Total	Case Det. Rate	Male	Female	Total	Clinical Rate
**PHILIPPINES**	**11,460**	**81**	**57**	**138**	**1.20**	**0**	**0**	**0**	**0.00**
**N C R**	**0**	**0**	**0**	**0**	**0.00**	**0**	**0**	**0**	**0.00**
Malabon City	0	0	0	0	**0.00**	0	0	0	**0.00**
Navotas City	0	0	0	0	**0.00**	0	0	0	**0.00**
Valenzuela City	0	0	0	0	**0.00**	0	0	0	**0.00**
Caloocan City	0	0	0	0	**0.00**	0	0	0	**0.00**
Marikina City	0	0	0	0	**0.00**	0	0	0	**0.00**
Pasig City	0	0	0	0	**0.00**	0	0	0	**0.00**
Pateros	0	0	0	0	**0.00**	0	0	0	**0.00**
Taguig City	0	0	0	0	**0.00**	0	0	0	**0.00**
Quezon City	0	0	0	0	**0.00**	0	0	0	**0.00**
Makati City	0	0	0	0	**0.00**	0	0	0	**0.00**
Mandaluyong City	0	0	0	0	**0.00**	0	0	0	**0.00**
San Juan City	0	0	0	0	**0.00**	0	0	0	**0.00**
Manila City	0	0	0	0	**0.00**	0	0	0	**0.00**
Las Piñas City	0	0	0	0	**0.00**	0	0	0	**0.00**
Muntinlupa City	0	0	0	0	**0.00**	0	0	0	**0.00**
Parañaque City	0	0	0	0	**0.00**	0	0	0	**0.00**
Pasay City	0	0	0	0	**0.00**	0	0	0	**0.00**
**C A R**	**0**	**0**	**0**	**0**	**0.00**	**0**	**0**	**0**	**0.00**
Abra	0	0	0	0	**0.00**	0	0	0	**0.00**
Apayao	0	0	0	0	**0.00**	0	0	0	**0.00**
Benguet	0	0	0	0	**0.00**	0	0	0	**0.00**
Ifugao	0	0	0	0	**0.00**	0	0	0	**0.00**
Kalinga	0	0	0	0	**0.00**	0	0	0	**0.00**
Mt. Province	0	0	0	0	**0.00**	0	0	0	**0.00**
Baguio City	0	0	0	0	**0.00**	0	0	0	**0.00**
**Region 1**	**0**	**0**	**0**	**0**	**0.00**	**0**	**0**	**0**	**0.00**
Ilocos Norte	0	0	0	0	**0.00**	0	0	0	**0.00**
Ilocos Sur	0	0	0	0	**0.00**	0	0	0	**0.00**
La Union	0	0	0	0	**0.00**	0	0	0	**0.00**
Pangasinan	0	0	0	0	**0.00**	0	0	0	**0.00**
Alaminos City	0	0	0	0	**0.00**	0	0	0	**0.00**
Candon City	0	0	0	0	**0.00**	0	0	0	**0.00**
Dagupan City	0	0	0	0	**0.00**	0	0	0	**0.00**
Laoag City	0	0	0	0	**0.00**	0	0	0	**0.00**
San Carlos City	0	0	0	0	**0.00**	0	0	0	**0.00**
San Fernando City	0	0	0	0	**0.00**	0	0	0	**0.00**
Urdaneta City	0	0	0	0	**0.00**	0	0	0	**0.00**
Vigan City	0	0	0	0	**0.00**	0	0	0	**0.00**

Table 2.E.1.1. Filariasis Prevention and Control

Case Detection Rate (CDR) and Clinical Rate of Lymphatic Filariasis

Philippines, 2022

Area	Total Examined for lymphatic filariasis	Number positive cases found				Lymphatic filariasis cases examined with disabilities/deformities			
		Male	Female	Total	Case Det. Rate	Male	Female	Total	Clinical Rate
**Region 2**	**0**	**0**	**0**	**0**	**0.00**	**0**	**0**	**0**	**0.00**
Batanes	0	0	0	0	**0.00**	0	0	0	**0.00**
Cagayan	0	0	0	0	**0.00**	0	0	0	**0.00**
Isabela	0	0	0	0	**0.00**	0	0	0	**0.00**
Nueva Vizcaya	0	0	0	0	**0.00**	0	0	0	**0.00**
Quirino	0	0	0	0	**0.00**	0	0	0	**0.00**
Cauayan City	0	0	0	0	**0.00**	0	0	0	**0.00**
Ilagan City	0	0	0	0	**0.00**	0	0	0	**0.00**
Santiago City	0	0	0	0	**0.00**	0	0	0	**0.00**
Tuguegarao City	0	0	0	0	**0.00**	0	0	0	**0.00**
**Region 3**	**0**	**0**	**0**	**0**	**0.00**	**0**	**0**	**0**	**0.00**
Aurora	0	0	0	0	**0.00**	0	0	0	**0.00**
Bataan	0	0	0	0	**0.00**	0	0	0	**0.00**
Bulacan	0	0	0	0	**0.00**	0	0	0	**0.00**
Nueva Ecija	0	0	0	0	**0.00**	0	0	0	**0.00**
Pampanga	0	0	0	0	**0.00**	0	0	0	**0.00**
Tarlac	0	0	0	0	**0.00**	0	0	0	**0.00**
Zambales	0	0	0	0	**0.00**	0	0	0	**0.00**
Angeles City	0	0	0	0	**0.00**	0	0	0	**0.00**
Balanga City	0	0	0	0	**0.00**	0	0	0	**0.00**
Cabanatuan City	0	0	0	0	**0.00**	0	0	0	**0.00**
City of San Fernando	0	0	0	0	**0.00**	0	0	0	**0.00**
Gapan City	0	0	0	0	**0.00**	0	0	0	**0.00**
Mabalacat City	0	0	0	0	**0.00**	0	0	0	**0.00**
Malolos City	0	0	0	0	**0.00**	0	0	0	**0.00**
Meycauayan City	0	0	0	0	**0.00**	0	0	0	**0.00**
Olongapo City	0	0	0	0	**0.00**	0	0	0	**0.00**
Palayan City	0	0	0	0	**0.00**	0	0	0	**0.00**
San Jose City	0	0	0	0	**0.00**	0	0	0	**0.00**
San Jose del Monte City	0	0	0	0	**0.00**	0	0	0	**0.00**
Science City of Munoz	0	0	0	0	**0.00**	0	0	0	**0.00**
Tarlac City	0	0	0	0	**0.00**	0	0	0	**0.00**
**Region 4A**	**3**	**0**	**0**	**0**	**0.00**	**0**	**0**	**0**	**0.00**
Batangas	0	0	0	0	**0.00**	0	0	0	**0.00**
Cavite	0	0	0	0	**0.00**	0	0	0	**0.00**
Laguna	0	0	0	0	**0.00**	0	0	0	**0.00**
Quezon	3	0	0	0	**0.00**	0	0	0	**0.00**
Rizal	0	0	0	0	**0.00**	0	0	0	**0.00**
Antipolo City	0	0	0	0	**0.00**	0	0	0	**0.00**
Bacoor City	0	0	0	0	**0.00**	0	0	0	**0.00**
Batangas City	0	0	0	0	**0.00**	0	0	0	**0.00**
Biñan City	0	0	0	0	**0.00**	0	0	0	**0.00**
Cabuyao City	0	0	0	0	**0.00**	0	0	0	**0.00**
Calamba City	0	0	0	0	**0.00**	0	0	0	**0.00**
Cavite City	0	0	0	0	**0.00**	0	0	0	**0.00**

Table 2.E.1.1. Filariasis Prevention and Control

Case Detection Rate (CDR) and Clinical Rate of Lymphatic Filariasis
Philippines, 2022

Area	Total Examined for lymphatic filariasis	Number positive cases found				Lymphatic filariasis cases examined with disabilities/deformities			
		Male	Female	Total	Case Det. Rate	Male	Female	Total	Clinical Rate
Dasmariñas City	0	0	0	0	**0.00**	0	0	0	**0.00**
General Trias City	0	0	0	0	**0.00**	0	0	0	**0.00**
Imus City	0	0	0	0	**0.00**	0	0	0	**0.00**
Lipa City	0	0	0	0	**0.00**	0	0	0	**0.00**
Lucena City	0	0	0	0	**0.00**	0	0	0	**0.00**
San Pablo City	0	0	0	0	**0.00**	0	0	0	**0.00**
San Pedro City	0	0	0	0	**0.00**	0	0	0	**0.00**
Santa Rosa City	0	0	0	0	**0.00**	0	0	0	**0.00**
Tagaytay City	0	0	0	0	**0.00**	0	0	0	**0.00**
Tanauan City	0	0	0	0	**0.00**	0	0	0	**0.00**
Tayabas City	0	0	0	0	**0.00**	0	0	0	**0.00**
Trece Martires City	0	0	0	0	**0.00**	0	0	0	**0.00**
**Region 4B**	**1,679**	**68**	**53**	**121**	**7.21**	**0**	**0**	**0**	**0.00**
Marinduque	1	0	0	0	**0.00**	0	0	0	**0.00**
Mindoro Occidental	0	0	0	0	**0.00**	0	0	0	**0.00**
Mindoro Oriental	1675	68	53	121	**7.22**	0	0	0	**0.00**
Palawan	3	0	0	0	**0.00**	0	0	0	**0.00**
Romblon	0	0	0	0	**0.00**	0	0	0	**0.00**
Puerto Princesa City	0	0	0	0	**0.00**	0	0	0	**0.00**
**Region 5**	**0**	**0**	**0**	**0**	**0.00**	**0**	**0**	**0**	**0.00**
Albay	0	0	0	0	**0.00**	0	0	0	**0.00**
Camarines Norte	0	0	0	0	**0.00**	0	0	0	**0.00**
Camarines Sur	0	0	0	0	**0.00**	0	0	0	**0.00**
Catanduanes	0	0	0	0	**0.00**	0	0	0	**0.00**
Masbate	0	0	0	0	**0.00**	0	0	0	**0.00**
Sorsogon	0	0	0	0	**0.00**	0	0	0	**0.00**
Iriga City	0	0	0	0	**0.00**	0	0	0	**0.00**
Legaspi City	0	0	0	0	**0.00**	0	0	0	**0.00**
Naga City	0	0	0	0	**0.00**	0	0	0	**0.00**
**Region 6**	**359**	**1**	**1**	**2**	**0.56**	**0**	**0**	**0**	**0.00**
Aklan	0	0	0	0	**0.00**	0	0	0	**0.00**
Antique	18	0	0	0	**0.00**	0	0	0	**0.00**
Capiz	0	0	0	0	**0.00**	0	0	0	**0.00**
Guimaras	0	0	0	0	**0.00**	0	0	0	**0.00**
Iloilo	341	1	1	2	**0.59**	0	0	0	**0.00**
Negros Occidental	0	0	0	0	**0.00**	0	0	0	**0.00**
Bacolod City	0	0	0	0	**0.00**	0	0	0	**0.00**
Iloilo City	0	0	0	0	**0.00**	0	0	0	**0.00**
**Region 7**	**0**	**0**	**0**	**0**	**0.00**	**0**	**0**	**0**	**0.00**
Bohol	0	0	0	0	**0.00**	0	0	0	**0.00**
Cebu	0	0	0	0	**0.00**	0	0	0	**0.00**
Negros Oriental	0	0	0	0	**0.00**	0	0	0	**0.00**

Table 2.E.1.1. Filariasis Prevention and Control

Case Detection Rate (CDR) and Clinical Rate of Lymphatic Filariasis

Philippines, 2022

Area	Total Examined for lymphatic filariasis	Number positive cases found				Lymphatic filariasis cases examined with disabilities/deformities			
		Male	Female	Total	Case Det. Rate	Male	Female	Total	Clinical Rate
Siquijor	0	0	0	0	**0.00**	0	0	0	**0.00**
Cebu City	0	0	0	0	**0.00**	0	0	0	**0.00**
Lapu-Lapu City	0	0	0	0	**0.00**	0	0	0	**0.00**
Mandaue City	0	0	0	0	**0.00**	0	0	0	**0.00**
**Region 8**	**0**	**0**	**0**	**0**	**0.00**	**0**	**0**	**0**	**0.00**
Biliran	0	0	0	0	**0.00**	0	0	0	**0.00**
Eastern Samar	0	0	0	0	**0.00**	0	0	0	**0.00**
Northern Leyte	0	0	0	0	**0.00**	0	0	0	**0.00**
Northern Samar	0	0	0	0	**0.00**	0	0	0	**0.00**
Southern Leyte	0	0	0	0	**0.00**	0	0	0	**0.00**
Western Samar	0	0	0	0	**0.00**	0	0	0	**0.00**
Calbayog City	0	0	0	0	**0.00**	0	0	0	**0.00**
Maasin City	0	0	0	0	**0.00**	0	0	0	**0.00**
Ormoc City	0	0	0	0	**0.00**	0	0	0	**0.00**
Tacloban City	0	0	0	0	**0.00**	0	0	0	**0.00**
**Region 9**	**0**	**0**	**0**	**0**	**0.00**	**0**	**0**	**0**	**0.00**
Zamboanga del Norte	0	0	0	0	**0.00**	0	0	0	**0.00**
Zamboanga del Sur	0	0	0	0	**0.00**	0	0	0	**0.00**
Zamboanga Sibugay	0	0	0	0	**0.00**	0	0	0	**0.00**
Dapitan City	0	0	0	0	**0.00**	0	0	0	**0.00**
Dipolog City	0	0	0	0	**0.00**	0	0	0	**0.00**
Isabela City	0	0	0	0	**0.00**	0	0	0	**0.00**
Pagadian City	0	0	0	0	**0.00**	0	0	0	**0.00**
Zamboanga City	0	0	0	0	**0.00**	0	0	0	**0.00**
**Region 10**	**0**	**0**	**0**	**0**	**0.00**	**0**	**0**	**0**	**0.00**
Bukidnon	0	0	0	0	**0.00**	0	0	0	**0.00**
Camiguin	0	0	0	0	**0.00**	0	0	0	**0.00**
Lanao del Norte	0	0	0	0	**0.00**	0	0	0	**0.00**
Misamis Occidental	0	0	0	0	**0.00**	0	0	0	**0.00**
Misamis Oriental	0	0	0	0	**0.00**	0	0	0	**0.00**
Cagayan de Oro City	0	0	0	0	**0.00**	0	0	0	**0.00**
El Salvador City	0	0	0	0	**0.00**	0	0	0	**0.00**
Gingoog City	0	0	0	0	**0.00**	0	0	0	**0.00**
Iligan City	0	0	0	0	**0.00**	0	0	0	**0.00**
Malaybalay City	0	0	0	0	**0.00**	0	0	0	**0.00**
Oroquieta City	0	0	0	0	**0.00**	0	0	0	**0.00**
Ozamis City	0	0	0	0	**0.00**	0	0	0	**0.00**
Tangub City	0	0	0	0	**0.00**	0	0	0	**0.00**
Valencia City	0	0	0	0	**0.00**	0	0	0	**0.00**
**Region 11**	**650**	**2**	**0**	**2**	**0.31**	**0**	**0**	**0**	**0.00**
Davao de Oro	0	0	0	0	**0.00**	0	0	0	**0.00**

Table 2.E.1.1. Filariasis Prevention and Control

Case Detection Rate (CDR) and Clinical Rate of Lymphatic Filariasis
Philippines, 2022

Area	Total Examined for lymphatic filariasis	Number positive cases found				Lymphatic filariasis cases examined with disabilities/deformities			
		Male	Female	Total	Case Det. Rate	Male	Female	Total	Clinical Rate
Davao del Norte	0	0	0	0	**0.00**	0	0	0	**0.00**
Davao del Sur	0	0	0	0	**0.00**	0	0	0	**0.00**
Davao Oriental	0	0	0	0	**0.00**	0	0	0	**0.00**
Davao Occidental	0	0	0	0	**0.00**	0	0	0	**0.00**
Davao City	650	2	0	2	**0.31**	0	0	0	**0.00**
**Region 12**	**2,297**	**9**	**2**	**11**	**0.48**	**0**	**0**	**0**	**0.00**
North Cotabato	0	0	0	0	**0.00**	0	0	0	**0.00**
Sarangani	1,801	4	1	5	**0.28**	0	0	0	**0.00**
South Cotabato	0	0	0	0	**0.00**	0	0	0	**0.00**
Sultan Kudarat	496	5	1	6	**1.21**	0	0	0	**0.00**
Gen. Santos City	0	0	0	0	**0.00**	0	0	0	**0.00**
**BARMM**	**0**	**0**	**0**	**0**	**0.00**	**0**	**0**	**0**	**0.00**
Basilan	0	0	0	0	**0.00**	0	0	0	**0.00**
Lanao del Sur	0	0	0	0	**0.00**	0	0	0	**0.00**
Maguindanao	0	0	0	0	**0.00**	0	0	0	**0.00**
Sulu	0	0	0	0	**0.00**	0	0	0	**0.00**
Tawi-Tawi	0	0	0	0	**0.00**	0	0	0	**0.00**
Cotabato City	0	0	0	0	**0.00**	0	0	0	**0.00**
Lamitan City	0	0	0	0	**0.00**	0	0	0	**0.00**
Marawi City	0	0	0	0	**0.00**	0	0	0	**0.00**
**CARAGA**	**6,472**	**1**	**1**	**2**	**0.03**	**0**	**0**	**0**	**0.00**
Agusan del Norte	824	1	1	2	**0.24**	0	0	0	**0.00**
Agusan del Sur	620	0	0	0	**0.00**	0	0	0	**0.00**
Surigao del Norte	1726	0	0	0	**0.00**	0	0	0	**0.00**
Surigao del Sur	2785	0	0	0	**0.00**	0	0	0	**0.00**
Province of Dinagat	360	0	0	0	**0.00**	0	0	0	**0.00**
Bislig City	0	0	0	0	**0.00**	0	0	0	**0.00**
Butuan City	114	0	0	0	**0.00**	0	0	0	**0.00**
Surigao City	43	0	0	0	**0.00**	0	0	0	**0.00**

Table 2.E.2.1. Schistosomiasis Prevention and Control

No. and Proportion of Suspected Schistosomiasis cases seen, Acute clinically diagnosed cases seen and Acute Confirmed Cases by Stool Examination

Philippines, 2022

Area	Total Patients seen	Number of Suspected Cases				No. of Acute Clinically Diagnosed Cases				No. of Acute Cases Confirmed Positive by Stool Examination			
		Male	Female	Total	%	Male	Female	Total	%	Male	Female	Total	%
**PHILIPPINES**	**59,083**	**12,012**	**10,609**	**22,621**	**38.29**	**754**	**506**	**1,260**	**5.57**	**255**	**168**	**423**	**33.57**
**N C R**	**0**	**0**	**0**	**0**	**0.00**	**0**	**0**	**0**	**0.00**	**0**	**0**	**0**	**0.00**
Malabon City	**0**	0	0	**0**	**0.00**	0	0	**0**	**0.00**	0	0	**0**	**0.00**
Navotas City	**0**	0	0	**0**	**0.00**	0	0	**0**	**0.00**	0	0	**0**	**0.00**
Valenzuela City	**0**	0	0	**0**	**0.00**	0	0	**0**	**0.00**	0	0	**0**	**0.00**
Caloocan City	**0**	0	0	**0**	**0.00**	0	0	**0**	**0.00**	0	0	**0**	**0.00**
Marikina City	**0**	0	0	**0**	**0.00**	0	0	**0**	**0.00**	0	0	**0**	**0.00**
Pasig City	**0**	0	0	**0**	**0.00**	0	0	**0**	**0.00**	0	0	**0**	**0.00**
Pateros	**0**	0	0	**0**	**0.00**	0	0	**0**	**0.00**	0	0	**0**	**0.00**
Taguig City	**0**	0	0	**0**	**0.00**	0	0	**0**	**0.00**	0	0		**0.00**
Quezon City	**0**	0	0	**0**	**0.00**	0	0	**0**	**0.00**	0	0	**0**	**0.00**
Makati City	**0**	0	0	**0**	**0.00**	0	0	**0**	**0.00**	0	0	**0**	**0.00**
Mandaluyong City	**0**	0	0	**0**	**0.00**	0	0	**0**	**0.00**	0	0	**0**	**0.00**
San Juan City	**0**	0	0	**0**	**0.00**	0	0	**0**	**0.00**	0	0	**0**	**0.00**
Manila City	**0**	0	0	**0**	**0.00**	0	0	**0**	**0.00**	0	0	**0**	**0.00**
Las Piñas City	**0**	0	0	**0**	**0.00**	0	0	**0**	**0.00**	0	0	**0**	**0.00**
Muntinlupa City	**0**	0	0	**0**	**0.00**	0	0	**0**	**0.00**	0	0	**0**	**0.00**
Parañaque City	**0**	0	0	**0**	**0.00**	0	0	**0**	**0.00**	0	0	**0**	**0.00**
Pasay City	**0**	0	0	**0**	**0.00**	0	0	**0**	**0.00**	0	0	**0**	**0.00**
**C A R**	**0**	**0**	**0**	**0**	**0.00**	**0**	**0**	**0**	**0.00**	**0**	**0**	**0**	**0.00**
Abra	**0**	0	0	**0**	**0.00**	0	0	**0**	**0.00**	0	0	**0**	**0.00**
Apayao	**0**	0	0	**0**	**0.00**	0	0	**0**	**0.00**	0	0	**0**	**0.00**
Benguet	**0**	0	0	**0**	**0.00**	0	0	**0**	**0.00**	0	0	**0**	**0.00**
Ifugao	**0**	0	0	**0**	**0.00**	0	0	**0**	**0.00**	0	0	**0**	**0.00**
Kalinga	**0**	0	0	**0**	**0.00**	0	0	**0**	**0.00**	0	0	**0**	**0.00**
Mt. Province	**0**	0	0	**0**	**0.00**	0	0	**0**	**0.00**	0	0	**0**	**0.00**
Baguio City	**0**	0	0	**0**	**0.00**	0	0	**0**	**0.00**	0	0	**0**	**0.00**
**Region 1**	**0**	**0**	**0**	**0**	**0.00**	**0**	**0**	**0**	**0.00**	**0**	**0**	**0**	**0.00**
Ilocos Norte	**0**	0	0	**0**	**0.00**	0	0	**0**	**0.00**	0	0	**0**	**0.00**
Ilocos Sur	**0**	0	0	**0**	**0.00**	0	0	**0**	**0.00**	0	0	**0**	**0.00**
La Union	**0**	0	0	**0**	**0.00**	0	0	**0**	**0.00**	0	0	**0**	**0.00**
Pangasinan	**0**	0	0	**0**	**0.00**	0	0	**0**	**0.00**	0	0	**0**	**0.00**
Alaminos City	**0**	0	0	**0**	**0.00**	0	0	**0**	**0.00**	0	0	**0**	**0.00**
Candon City	**0**	0	0	**0**	**0.00**	0	0	**0**	**0.00**	0	0	**0**	**0.00**
Dagupan City	**0**	0	0	**0**	**0.00**	0	0	**0**	**0.00**	0	0	**0**	**0.00**
Laoag City	**0**	0	0	**0**	**0.00**	0	0	**0**	**0.00**	0	0	**0**	**0.00**
San Carlos City	**0**	0	0	**0**	**0.00**	0	0	**0**	**0.00**	0	0	**0**	**0.00**

Table 2.E.2.1. Schistosomiasis Prevention and Control

No. and Proportion of Suspected Schistosomiasis cases seen, Acute clinically diagnosed cases seen and Acute Confirmed Cases by Stool Examination

Philippines, 2022

Area	Total Patients seen	Number of Suspected Cases				No. of Acute Clinically Diagnosed Cases				No. of Acute Cases Confirmed Positive by Stool Examination			
		Male	Female	Total	%	Male	Female	Total	%	Male	Female	Total	%
San Fernando City	**0**	0	0	**0**	**0.00**	0	0	**0**	**0.00**	0	0	**0**	**0.00**
Urdaneta City	**0**	0	0	**0**	**0.00**	0	0	**0**	**0.00**	0	0	**0**	**0.00**
Vigan City	**0**	0	0	**0**	**0.00**	0	0	**0**	**0.00**	0	0	**0**	**0.00**
**Region 2**	**0**	**0**	**0**	**0**	**0.00**	**0**	**0**	**0**	**0.00**	**0**	**0**	**0**	**0.00**
Batanes	**0**	0	0	**0**	**0.00**	0	0	**0**	**0.00**	0	0	**0**	**0.00**
Cagayan	**0**	0	0	**0**	**0.00**	0	0	**0**	**0.00**	0	0	**0**	**0.00**
Isabela	**0**	0	0	**0**	**0.00**	0	0	**0**	**0.00**	0	0	**0**	**0.00**
Nueva Vizcaya	**0**	0	0	**0**	**0.00**	0	0	**0**	**0.00**	0	0	**0**	**0.00**
Quirino	**0**	0	0	**0**	**0.00**	0	0	**0**	**0.00**	0	0	**0**	**0.00**
Cauayan City	**0**	0	0	**0**	**0.00**	0	0	**0**	**0.00**	0	0	**0**	**0.00**
Ilagan City	**0**	0	0	**0**	**0.00**	0	0	**0**	**0.00**	0	0	**0**	**0.00**
Santiago City	**0**	0	0	**0**	**0.00**	0	0	**0**	**0.00**	0	0	**0**	**0.00**
Tuguegarao City	**0**	0	0	**0**	**0.00**	0	0	**0**	**0.00**	0	0	**0**	**0.00**
**Region 3**	**0**	**0**	**0**	**0**	**0.00**	**0**	**0**	**0**	**0.00**	**0**	**0**	**0**	**0.00**
Aurora	**0**	0	0	**0**	**0.00**	0	0	**0**	**0.00**	0	0	**0**	**0.00**
Bataan	**0**	0	0	**0**	**0.00**	0	0	**0**	**0.00**	0	0	**0**	**0.00**
Bulacan	**0**	0	0	**0**	**0.00**	0	0	**0**	**0.00**	0	0	**0**	**0.00**
Nueva Ecija	**0**	0	0	**0**	**0.00**	0	0	**0**	**0.00**	0	0	**0**	**0.00**
Pampanga	**0**	0	0	**0**	**0.00**	0	0	**0**	**0.00**	0	0	**0**	**0.00**
Tarlac	**0**	0	0	**0**	**0.00**	0	0	**0**	**0.00**	0	0	**0**	**0.00**
Zambales	**0**	0	0	**0**	**0.00**	0	0	**0**	**0.00**	0	0	**0**	**0.00**
Angeles City	**0**	0	0	**0**	**0.00**	0	0	**0**	**0.00**	0	0	**0**	**0.00**
Balanga City	**0**	0	0	**0**	**0.00**	0	0	**0**	**0.00**	0	0	**0**	**0.00**
Cabanatuan City	**0**	0	0	**0**	**0.00**	0	0	**0**	**0.00**	0	0	**0**	**0.00**
City of San Fernando	**0**	0	0	**0**	**0.00**	0	0	**0**	**0.00**	0	0	**0**	**0.00**
Gapan City	**0**	0	0	**0**	**0.00**	0	0	**0**	**0.00**	0	0	**0**	**0.00**
Mabalacat City	**0**	0	0	**0**	**0.00**	0	0	**0**	**0.00**	0	0	**0**	**0.00**
Malolos City	**0**	0	0	**0**	**0.00**	0	0	**0**	**0.00**	0	0	**0**	**0.00**
Meycauayan City	**0**	0	0	**0**	**0.00**	0	0	**0**	**0.00**	0	0	**0**	**0.00**
Olongapo City	**0**	0	0	**0**	**0.00**	0	0	**0**	**0.00**	0	0	**0**	**0.00**
Palayan City	**0**	0	0	**0**	**0.00**	0	0	**0**	**0.00**	0	0	**0**	**0.00**
San Jose City	**0**	0	0	**0**	**0.00**	0	0	**0**	**0.00**	0	0	**0**	**0.00**
San Jose del Monte City	**0**	0	0	**0**	**0.00**	0	0	**0**	**0.00**	0	0	**0**	**0.00**
Science City of Munoz	**0**	0	0	**0**	**0.00**	0	0	**0**	**0.00**	0	0	**0**	**0.00**
Tarlac City	**0**	0	0	**0**	**0.00**	0	0	**0**	**0.00**	0	0	**0**	**0.00**
**Region 4A**	**110**	**32**	**3**	**35**	**31.82**	**10**	**2**	**12**	**34.29**	**1**	**2**	**3**	**25.00**
Batangas	**4**	0	0	**0**	**0.00**	0	0	**0**	**0.00**	0	0	**0**	**0.00**
Cavite	**18**	5	0	**5**	**27.78**	5	0	**5**	**100.00**	0	0	**0**	**0.00**
Laguna	**3**	3	0	**3**	**100.00**	3	0	**3**	**100.00**	0	0	**0**	**0.00**
Quezon	**10**	2	0	**2**	**20.00**	1	0	**1**	**50.00**	0	0	**0**	**0.00**

Table 2.E.2.1. Schistosomiasis Prevention and Control

No. and Proportion of Suspected Schistosomiasis cases seen, Acute clinically diagnosed cases seen and Acute Confirmed Cases by Stool Examination

Philippines, 2022

Area	Total Patients seen	Number of Suspected Cases				No. of Acute Clinically Diagnosed Cases				No. of Acute Cases Confirmed Positive by Stool Examination			
		Male	Female	Total	%	Male	Female	Total	%	Male	Female	Total	%
Rizal	**11**	0	1	**1**	**9.09**	0	1	**1**	**100.00**	0	1	**1**	**100.00**
Antipolo City	**5**	0	0	**0**	**0.00**	0	0	**0**	**0.00**	0	0	**0**	**0.00**
Bacoor City	**0**	0	0	**0**	**0.00**	0	0	**0**	**0.00**	0	0	**0**	**0.00**
Batangas City	**2**	1	1	**2**	**100.00**	0	1	**1**	**50.00**	0	1	**1**	**100.00**
Biñan City	**0**	0	0	**0**	**0.00**	0	0	**0**	**0.00**	0	0	**0**	**0.00**
Cabuyao City	**3**	2	1	**3**	**100.00**	1	0	**1**	**33.33**	1	0	**1**	**100.00**
Calamba City	**3**	3	0	**3**	**100.00**	0	0	**0**	**0.00**	0	0	**0**	**0.00**
Cavite City	**0**	0	0	**0**	**0.00**	0	0	**0**	**0.00**	0	0	**0**	**0.00**
Dasmariñas City	**0**	11	0	**11**	**0.00**	0	0	**0**	**0.00**	0	0	**0**	**0.00**
General Trias City	**0**	0	0	**0**	**0.00**	0	0	**0**	**0.00**	0	0	**0**	**0.00**
Imus City	**3**	0	0	**0**	**0.00**	0	0	**0**	**0.00**	0	0	**0**	**0.00**
Lipa City	**0**	0	0	**0**	**0.00**	0	0	**0**	**0.00**	0	0	**0**	**0.00**
Lucena City	**2**	0	0	**0**	**0.00**	0	0	**0**	**0.00**	0	0	**0**	**0.00**
San Pablo City	**3**	1	0	**1**	**33.33**	0	0	**0**	**0.00**	0	0	**0**	**0.00**
San Pedro City	**0**	0	0	**0**	**0.00**	0	0	**0**	**0.00**	0	0	**0**	**0.00**
Santa Rosa City	**0**	0	0	**0**	**0.00**	0	0	**0**	**0.00**	0	0	**0**	**0.00**
Tagaytay City	**40**	1	0	**1**	**2.50**	0	0	**0**	**0.00**	0	0	**0**	**0.00**
Tanauan City	**0**	0	0	**0**	**0.00**	0	0	**0**	**0.00**	0	0	**0**	**0.00**
Tayabas City	**0**	0	0	**0**	**0.00**	0	0	**0**	**0.00**	0	0	**0**	**0.00**
Trece Martires City	**3**	3	0	**3**	**100.00**	0	0	**0**	**0.00**	0	0	**0**	**0.00**
**Region 4B**	**471**	**53**	**26**	**79**	**16.77**	**2**	**0**	**2**	**2.53**	**0**	**0**	**0**	**0.00**
Marinduque	**4**	0	2	**2**	**50.00**	0	0	**0**	**0.00**	0	0	**0**	**0.00**
Mindoro Occidental	**0**	0	0	**0**	**0.00**	0	0	**0**	**0.00**	0	0	**0**	**0.00**
Mindoro Oriental	**448**	51	24	**75**	**16.74**	0	0	**0**	**0.00**	0	0	**0**	**0.00**
Palawan	**18**	2	0	**2**	**11.11**	2	0	**2**	**100.00**	0	0	**0**	**0.00**
Romblon	**1**	0	0	**0**	**0.00**	0	0	**0**	**0.00**	0	0	**0**	**0.00**
Puerto Princesa City	**0**	0	0	**0**	**0.00**	0	0	**0**	**0.00**	0	0	**0**	**0.00**
**Region 5**	**67**	**42**	**15**	**57**	**85.07**	**14**	**7**	**21**	**36.84**	**0**	**1**	**1**	**4.76**
Albay	**11**	0	1	**1**	**9.09**	0	1	**1**	**100.00**	0	0	**0**	**0.00**
Camarines Norte	**0**	0	0	**0**	**0.00**	0	0	**0**	**0.00**	0	0	**0**	**0.00**
Camarines Sur	**0**	0	0	**0**	**0.00**	0	0	**0**	**0.00**	0	0	**0**	**0.00**
Catanduanes	**0**	0	0	**0**	**0.00**	0	0	**0**	**0.00**	0	0	**0**	**0.00**
Masbate	**0**	0	0	**0**	**0.00**	0	0	**0**	**0.00**	0	0	**0**	**0.00**
Sorsogon	**56**	42	14	**56**	**100.00**	14	6	**20**	**35.71**	0	1	**1**	**5.00**
Iriga City	**0**	0	0	**0**	**0.00**	0	0	**0**	**0.00**	0	0	**0**	**0.00**
Legaspi City	**0**	0	0	**0**	**0.00**	0	0	**0**	**0.00**	0	0	**0**	**0.00**
Naga City	**0**	0	0	**0**	**0.00**	0	0	**0**	**0.00**	0	0	**0**	**0.00**
**Region 6**	**0**	**0**	**0**	**0**	**0.00**	**0**	**0**	**0**	**0.00**	**0**	**0**	**0**	**0.00**
Aklan	**0**	0	0	**0**	**0.00**	0	0	**0**	**0.00**	0	0	**0**	**0.00**

Table 2.E.2.1. Schistosomiasis Prevention and Control

No. and Proportion of Suspected Schistosomiasis cases seen, Acute clinically diagnosed cases seen and Acute Confirmed Cases by Stool Examination

Philippines, 2022

Area	Total Patients seen	Number of Suspected Cases				No. of Acute Clinically Diagnosed Cases				No. of Acute Cases Confirmed Positive by Stool Examination			
		Male	Female	Total	%	Male	Female	Total	%	Male	Female	Total	%
Antique	**0**	0	0	**0**	**0.00**	0	0	**0**	**0.00**	0	0	**0**	**0.00**
Capiz	**0**	0	0	**0**	**0.00**	0	0	**0**	**0.00**	0	0	**0**	**0.00**
Guimaras	**0**	0	0	**0**	**0.00**	0	0	**0**	**0.00**	0	0	**0**	**0.00**
Iloilo	**0**	0	0	**0**	**0.00**	0	0	**0**	**0.00**	0	0	**0**	**0.00**
Negros Occidental	**0**	0	0	**0**	**0.00**	0	0	**0**	**0.00**	0	0	**0**	**0.00**
Bacolod City	**0**	0	0	**0**	**0.00**	0	0	**0**	**0.00**	0	0	**0**	**0.00**
Bago City	**0**	0	0	**0**	**0.00**	0	0	**0**	**0.00**	0	0	**0**	**0.00**
**Region 7**	**0**	**0**	**0**	**0**	**0.00**	**0**	**0**	**0**	**0.00**	**0**	**0**	**0**	**0.00**
Bohol	**0**	0	0	**0**	**0.00**	0	0	**0**	**0.00**	0	0	**0**	**0.00**
Cebu	**0**	0	0	**0**	**0.00**	0	0	**0**	**0.00**	0	0	**0**	**0.00**
Negros Oriental	**0**	0	0	**0**	**0.00**	0	0	**0**	**0.00**	0	0	**0**	**0.00**
Siquijor	**0**	0	0	**0**	**0.00**	0	0	**0**	**0.00**	0	0	**0**	**0.00**
Cebu City	**0**	0	0	**0**	**0.00**	0	0	**0**	**0.00**	0	0	**0**	**0.00**
Lapu-Lapu City	**0**	0	0	**0**	**0.00**	0	0	**0**	**0.00**	0	0	**0**	**0.00**
Mandaue City	**0**	0	0	**0**	**0.00**	0	0	**0**	**0.00**	0	0	**0**	**0.00**
**Region 8**	**39,982**	**6,187**	**5,873**	**12,060**	**30.16**	**57**	**31**	**88**	**0.73**	**41**	**29**	**70**	**79.55**
Biliran	**0**	0	0	**0**	**0.00**	0	0	**0**	**0.00**	0	0	**0**	**0.00**
Eastern Samar	**15,755**	77	79	**156**	**0.99**	8	14	**22**	**14.10**	7	14	**21**	**95.45**
Northern Leyte	**10,032**	8	3	**11**	**0.11**	2	1	**3**	**27.27**	2	1	**3**	**100.00**
Northern Samar	**3,217**	1,391	1,588	**2,979**	**92.60**	41	14	**55**	**1.85**	24	12	**36**	**65.45**
Southern Leyte	**0**	0	0	**0**	**0.00**	0	0	**0**	**0.00**	0	0	**0**	**0.00**
Western Samar	**10,973**	4,708	4,201	**8,909**	**81.19**	6	2	**8**	**0.09**	6	2	**8**	**100.00**
Calbayog City	**5**	3	2	**5**	**100.00**	0	0	**0**	**0.00**	2	0	**2**	**0.00**
Maasin City	**0**	0	0	**0**	**0.00**	0	0	**0**	**0.00**	0	0	**0**	**0.00**
Ormoc City	**0**	0	0	**0**	**0.00**	0	0	**0**	**0.00**	0	0	**0**	**0.00**
Tacloban City	**0**	0	0	**0**	**0.00**	0	0	**0**	**0.00**	0	0	**0**	**0.00**
**Region 9**	**70**	**50**	**20**	**70**	**100.00**	**50**	**20**	**70**	**100.00**	**9**	**4**	**13**	**18.57**
Zamboanga del Norte	**2**	1	1	**2**	**100.00**	1	1	**2**	**100.00**	1	1	**2**	**100.00**
Zamboanga del Sur	**67**	48	19	**67**	**100.00**	48	19	**67**	**100.00**	7	3	**10**	**14.93**
Zamboanga Sibugay	**1**	1	0	**1**	**100.00**	1	0	**1**	**100.00**	1	0	**1**	**100.00**
Dapitan City	**0**	0	0	**0**	**0.00**	0	0	**0**	**0.00**	0	0	**0**	**0.00**
Dipolog City	**0**	0	0	**0**	**0.00**	0	0	**0**	**0.00**	0	0	**0**	**0.00**
Isabela City	**0**	0	0	**0**	**0.00**	0	0	**0**	**0.00**	0	0	**0**	**0.00**
Pagadian City	**0**	0	0	**0**	**0.00**	0	0	**0**	**0.00**	0	0	**0**	**0.00**
Zamboanga City	**0**	0	0	**0**	**0.00**	0	0	**0**	**0.00**	0	0	**0**	**0.00**

Table 2.E.2.1. Schistosomiasis Prevention and Control

No. and Proportion of Suspected Schistosomiasis cases seen, Acute clinically diagnosed cases seen and Acute Confirmed Cases by Stool Examination

Philippines, 2022

Area	Total Patients seen	Number of Suspected Cases				No. of Acute Clinically Diagnosed Cases				No. of Acute Cases Confirmed Positive by Stool Examination			
		Male	Female	Total	%	Male	Female	Total	%	Male	Female	Total	%
**Region 10**	**1,842**	**615**	**512**	**1,127**	**61.18**	**209**	**116**	**325**	**28.84**	**59**	**33**	**92**	**28.31**
Bukidnon	**52**	35	17	**52**	**100.00**	27	11	**38**	**73.08**	11	10	**21**	**55.26**
Camiguin	**0**	0	0	**0**	**0.00**	0	0	**0**	**0.00**	0	0	**0**	**0.00**
Lanao del Norte	**670**	306	341	**647**	**96.57**	27	26	**53**	**8.19**	8	5	**13**	**24.53**
Misamis Occidental	**1**	0	1	**1**	**100.00**	0	1	**1**	**100.00**	0	1	**1**	**100.00**
Misamis Oriental	**0**	0	0	**0**	**0.00**	0	0	**0**	**0.00**	0	0	**0**	**0.00**
Cagayan de Oro City	**0**	0	0	**0**	**0.00**	0	0	**0**	**0.00**	0	0	**0**	**0.00**
El Salvador City	**0**	0	0	**0**	**0.00**	0	0	**0**	**0.00**	0	0	**0**	**0.00**
Gingoog City	**0**	0	0	**0**	**0.00**	0	0	**0**	**0.00**	0	0	**0**	**0.00**
Iligan City	**0**	0	0	**0**	**0.00**	0	0	**0**	**0.00**	0	0	**0**	**0.00**
Malaybalay City	**53**	33	20	**53**	**100.00**	33	20	**53**	**100.00**	4	3	**7**	**13.21**
Oroquieta City	**486**	0	0	**0**	**0.00**	0	0	**0**	**0.00**	0	0	**0**	**0.00**
Ozamis City	**34**	28	6	**34**	**100.00**	20	3	**23**	**67.65**	17	5	**22**	**95.65**
Tangub City	**0**	0	0	**0**	**0.00**	0	0	**0**	**0.00**	0	0	**0**	**0.00**
Valencia City	**546**	213	127	**340**	**62.27**	102	55	**157**	**46.18**	19	9	**28**	**17.83**
**Region 11**	**3,785**	**221**	**165**	**386**	**10.20**	**37**	**25**	**62**	**16.06**	**3**	**2**	**5**	**8.06**
Davao de Oro	**5**	0	0	**0**	**0.00**	0	0	**0**	**0.00**	2	2	**4**	**0.00**
Davao del Norte	**596**	41	35	**76**	**12.75**	36	25	**61**	**80.26**	0	0	**0**	**0.00**
Davao del Sur	**2,872**	2	0	**2**	**0.07**	1	0	**1**	**50.00**	0	0	**0**	**0.00**
Davao Oriental	**308**	178	130	**308**	**100.00**	0	0	**0**	**0.00**	1	0	**1**	**0.00**
Davao Occidental	**0**	0	0	**0**	**0.00**	0	0	**0**	**0.00**	0	0	**0**	**0.00**
Davao City	**4**	0	0	**0**	**0.00**	0	0	**0**	**0.00**	0	0	**0**	**0.00**
**Region 12**	**37**	**14**	**15**	**29**	**78.38**	**6**	**8**	**14**	**48.28**	**5**	**7**	**12**	**85.71**
North Cotabato	**30**	9	13	**22**	**73.33**	3	8	**11**	**50.00**	3	7	**10**	**90.91**
Sarangani	**0**	0	0	**0**	**0.00**	0	0	**0**	**0.00**	0	0	**0**	**0.00**
South Cotabato	**6**	4	2	**6**	**100.00**	2	0	**2**	**33.33**	2	0	**2**	**100.00**
Sultan Kudarat	**0**	0	0	**0**	**0.00**	0	0	**0**	**0.00**	0	0	**0**	**0.00**
Gen. Santos City	**1**	1	0	**1**	**100.00**	1	0	**1**	**100.00**	0	0	**0**	**0.00**
**BARMM**	**2,416**	**1,380**	**1,034**	**2,414**	**99.92**	**30**	**18**	**48**	**1.99**	**16**	**12**	**28**	**58.33**
Basilan	**0**	0	0	**0**	**0.00**	0	0	**0**	**0.00**	0	0	**0**	**0.00**
Lanao del Sur	**2,390**	1,362	1,026	**2,388**	**99.92**	14	11	**25**	**1.05**	14	11	**25**	**100.00**
Maguindanao	**26**	18	8	**26**	**100.00**	16	7	**23**	**88.46**	2	1	**3**	**13.04**
Sulu	**0**	0	0	**0**	**0.00**	0	0	**0**	**0.00**	0	0	**0**	**0.00**
Tawi-Tawi	**0**	0	0	**0**	**0.00**	0	0	**0**	**0.00**	0	0	**0**	**0.00**
Cotabato City	**0**	0	0	**0**	**0.00**	0	0	**0**	**0.00**	0	0	**0**	**0.00**
Lamitan City	**0**	0	0	**0**	**0.00**	0	0	**0**	**0.00**	0	0	**0**	**0.00**

Table 2.E.2.1. Schistosomiasis Prevention and Control

No. and Proportion of Suspected Schistosomiasis cases seen, Acute clinically diagnosed cases seen and Acute Confirmed Cases by Stool Examination

Philippines, 2022

Area	Total Patients seen	Number of Suspected Cases				No. of Acute Clinically Diagnosed Cases				No. of Acute Cases Confirmed Positive by Stool Examination			
		Male	Female	Total	%	Male	Female	Total	%	Male	Female	Total	%
Marawi City	**0**	0	0	**0**	**0.00**	0	0	**0**	**0.00**	0	0	**0**	**0.00**
**CARAGA**	**10,303**	**3,418**	**2,946**	**6,364**	**61.77**	**339**	**279**	**618**	**9.71**	**121**	**78**	**199**	**32.20**
Agusan del Norte	**509**	100	43	**143**	**28.09**	2	1	**3**	**2.10**	0	1	**1**	**33.33**
Agusan del Sur	**8,632**	3,000	2,606	**5,606**	**64.94**	107	75	**182**	**3.25**	73	58	**131**	**71.98**
Surigao del Norte	**821**	315	293	**608**	**74.06**	229	199	**428**	**70.39**	47	18	**65**	**15.19**
Surigao del Sur	**0**	0	0	**0**	**0.00**	0	0	**0**	**0.00**	0	0	**0**	**0.00**
Province of Dinagat	**2**	0	1	**1**	**50.00**	0	1	**1**	**100.00**	0	0	**0**	**0.00**
Bislig City	**0**	0	0	**0**	**0.00**	0	0	**0**	**0.00**	0	0	**0**	**0.00**
Butuan City	**339**	3	3	**6**	**1.77**	1	3	**4**	**66.67**	1	1	**2**	**50.00**
Surigao City	**0**	0	0	**0**	**0.00**	0	0	**0**	**0.00**	0	0	**0**	**0.00**

Legend:

0 = No Case

** = No Report*

Table 2.E.2.2. Schistosomiasis Prevention and Control

No. and Proportion of Chronic Clinically Diagnosed Cases and Chronic Cllinically diagnosed cases confirmed by stool examination

Philippines, 2022

Area	No. of Clinically Diagnosed Chronic Cases				No. of chronic clinically diagnoses cases confirmed by stool examination (KKT) or rectal biopsy			
	Male	Female	Total	%	Male	Female	Total	%
**PHILIPPINES**	**276**	**148**	**424**	**1.87**	**107**	**53**	**160**	**37.74**
**N C R**	0	0	**0**	**0.00**	0	0	**0**	**0.00**
Malabon City	0	0	**0**	**0.00**	0	0	**0**	**0.00**
Navotas City	0	0	**0**	**0.00**	0	0	**0**	**0.00**
Valenzuela City	0	0	**0**	**0.00**	0	0	**0**	**0.00**
Caloocan City	0	0	**0**	**0.00**	0	0	**0**	**0.00**
Marikina City	0	0	**0**	**0.00**	0	0	**0**	**0.00**
Pasig City	0	0	**0**	**0.00**	0	0	**0**	**0.00**
Pateros	0	0	**0**	**0.00**	0	0	**0**	**0.00**
Taguig City	0	0	**0**	**0.00**	0	0	**0**	**0.00**
Quezon City	0	0	**0**	**0.00**	0	0	**0**	**0.00**
Makati City	0	0	**0**	**0.00**	0	0	**0**	**0.00**
Mandaluyong City	0	0	**0**	**0.00**	0	0	**0**	**0.00**
San Juan City	0	0	**0**	**0.00**	0	0	**0**	**0.00**
Manila City	0	0	**0**	**0.00**	0	0	**0**	**0.00**
Las Piñas City	0	0	**0**	**0.00**	0	0	**0**	**0.00**
Muntinlupa City	0	0	**0**	**0.00**	0	0	**0**	**0.00**
Parañaque City	0	0	**0**	**0.00**	0	0	**0**	**0.00**
Pasay City	0	0	**0**	**0.00**	0	0	**0**	**0.00**
**C A R**	0	0	**0**	**0.00**	0	0	**0**	**0.00**
Abra	0	0	**0**	**0.00**	0	0	**0**	**0.00**
Apayao	0	0	**0**	**0.00**	0	0	**0**	**0.00**
Benguet	0	0	**0**	**0.00**	0	0	**0**	**0.00**
Ifugao	0	0	**0**	**0.00**	0	0	**0**	**0.00**
Kalinga	0	0	**0**	**0.00**	0	0	**0**	**0.00**
Mt. Province	0	0	**0**	**0.00**	0	0	**0**	**0.00**
Baguio City	0	0	**0**	**0.00**	0	0	**0**	**0.00**
**Region 1**	0	0	**0**	**0.00**	0	0	**0**	**0.00**
Ilocos Norte	0	0	**0**	**0.00**	0	0	**0**	**0.00**
Ilocos Sur	0	0	**0**	**0.00**	0	0	**0**	**0.00**
La Union	0	0	**0**	**0.00**	0	0	**0**	**0.00**
Pangasinan	0	0	**0**	**0.00**	0	0	**0**	**0.00**

Table 2.E.2.2. Schistosomiasis Prevention and Control

No. and Proportion of Chronic Clinically Diagnosed Cases and Chronic Cllinically diagnosed cases confirmed by stool examination

Philippines, 2022

Area	No. of Clinically Diagnosed Chronic Cases				No. of chronic clinically diagnoses cases confirmed by stool examination (KKT) or rectal biopsy			
	Male	Female	Total	%	Male	Female	Total	%
Alaminos City	0	0	**0**	**0.00**	0	0	**0**	**0.00**
Candon City	0	0	**0**	**0.00**	0	0	**0**	**0.00**
Dagupan City	0	0	**0**	**0.00**	0	0	**0**	**0.00**
Laoag City	0	0	**0**	**0.00**	0	0	**0**	**0.00**
San Carlos City	0	0	**0**	**0.00**	0	0	**0**	**0.00**
San Fernando City	0	0	**0**	**0.00**	0	0	**0**	**0.00**
Urdaneta City	0	0	**0**	**0.00**	0	0	**0**	**0.00**
Vigan City	0	0	**0**	**0.00**	0	0	**0**	**0.00**
**Region 2**	0	0	**0**	**0.00**	0	0	**0**	**0.00**
							**0**	**0.00**
Batanes	0	0	**0**	**0.00**	0	0	**0**	**0.00**
Cagayan	0	0	**0**	**0.00**	0	0	**0**	**0.00**
Isabela	0	0	**0**	**0.00**	0	0	**0**	**0.00**
Nueva Vizcaya	0	0	**0**	**0.00**	0	0	**0**	**0.00**
Quirino	0	0	**0**	**0.00**	0	0	**0**	**0.00**
Cauayan City	0	0	**0**	**0.00**	0	0	**0**	**0.00**
Ilagan City	0	0	**0**	**0.00**	0	0	**0**	**0.00**
Santiago City	0	0	**0**	**0.00**	0	0	**0**	**0.00**
Tuguegarao City	0	0	**0**	**0.00**	0	0	**0**	**0.00**
**Region 3**	0	0	**0**	**0.00**	0	0	**0**	**0.00**
Aurora	0	0	**0**	**0.00**	0	0	**0**	**0.00**
Bataan	0	0	**0**	**0.00**	0	0	**0**	**0.00**
Bulacan	0	0	**0**	**0.00**	0	0	**0**	**0.00**
Nueva Ecija	0	0	**0**	**0.00**	0	0	**0**	**0.00**
Pampanga	0	0	**0**	**0.00**	0	0	**0**	**0.00**
Tarlac	0	0	**0**	**0.00**	0	0	**0**	**0.00**
Zambales	0	0	**0**	**0.00**	0	0	**0**	**0.00**
Angeles City	0	0	**0**	**0.00**	0	0	**0**	**0.00**
Balanga City	0	0	**0**	**0.00**	0	0	**0**	**0.00**
Cabanatuan City	0	0	**0**	**0.00**	0	0	**0**	**0.00**
City of San Fernando	0	0	**0**	**0.00**	0	0	**0**	**0.00**
Gapan City	0	0	**0**	**0.00**	0	0	**0**	**0.00**
Mabalacat City	0	0	**0**	**0.00**	0	0	**0**	**0.00**
Malolos City	0	0	**0**	**0.00**	0	0	**0**	**0.00**
Meycauayan City	0	0	**0**	**0.00**	0	0	**0**	**0.00**
Olongapo City	0	0	**0**	**0.00**	0	0	**0**	**0.00**
Palayan City	0	0	**0**	**0.00**	0	0	**0**	**0.00**

Table 2.E.2.2. Schistosomiasis Prevention and Control

No. and Proportion of Chronic Clinically Diagnosed Cases and Chronic Cllinically diagnosed cases confirmed by stool examination

Philippines, 2022

Area	No. of Clinically Diagnosed Chronic Cases				No. of chronic clinically diagnoses cases confirmed by stool examination (KKT) or rectal biopsy			
	Male	Female	Total	%	Male	Female	Total	%
San Jose City	0	0	0	0.00	0	0	0	0.00
San Jose del Monte City	0	0	0	0.00	0	0	0	0.00
Science City of Munoz	0	0	0	0.00	0	0	0	0.00
Tarlac City	0	0	0	0.00	0	0	0	0.00
**Region 4A**	5	0	5	14.29	5	0	5	100.00
Batangas	0	0	0	0.00	0	0	0	0.00
Cavite	0	0	0	0.00	0	0	0	0.00
Laguna	0	0	0	0.00	0	0	0	0.00
Quezon	2	0	2	100.00	2	0	2	100.00
Rizal	0	0	0	0.00	0	0	0	0.00
Antipolo City	0	0	0	0.00	0	0	0	0.00
Bacoor City	0	0	0	0.00	0	0	0	0.00
Batangas City	1	0	1	50.00	1	0	1	100.00
Biñan City	0	0	0	0.00	0	0	0	0.00
Cabuyao City	0	0	0	0.00	0	0	0	0.00
Calamba City	0	0	0	0.00	0	0	0	0.00
Cavite City	0	0	0	0.00	0	0	0	0.00
Dasmariñas City	0	0	0	0.00	0	0	0	0.00
General Trias City	0	0	0	0.00	0	0	0	0.00
Imus City	0	0	0	0.00	0	0	0	0.00
Lipa City	0	0	0	0.00	0	0	0	0.00
Lucena City	0	0	0	0.00	0	0	0	0.00
San Pablo City	1	0	1	100.00	1	0	1	100.00
San Pedro City	0	0	0	0.00	0	0	0	0.00
Santa Rosa City	0	0	0	0.00	0	0	0	0.00
Tagaytay City	1	0	1	100.00	1	0	1	100.00
Tanauan City	0	0	0	0.00	0	0	0	0.00
Tayabas City	0	0	0	0.00	0	0	0	0.00
Trece Martires City	0	0	0	0.00	0	0	0	0.00
**Region 4B**	4	5	9	11.39	4	5	9	100.00
Marinduque	0	0	0	0.00	0	0	0	0.00
Mindoro Occidental	0	0	0	0.00	0	0	0	0.00
Mindoro Oriental	4	5	9	12.00	4	5	9	100.00
Palawan	0	0	0	0.00	0	0	0	0.00
Romblon	0	0	0	0.00	0	0	0	0.00
Puerto Princesa City	0	0	0	0.00	0	0	0	0.00

Table 2.E.2.2. Schistosomiasis Prevention and Control

No. and Proportion of Chronic Clinically Diagnosed Cases and Chronic Cllinically diagnosed cases confirmed by stool examination

Philippines, 2022

Area	No. of Clinically Diagnosed Chronic Cases				No. of chronic clinically diagnoses cases confirmed by stool examination (KKT) or rectal biopsy			
	Male	Female	Total	%	Male	Female	Total	%
**Region 5**	10	2	**12**	**21.05**	10	2	**12**	**100.00**
Albay	0	1	**1**	**100.00**	0	1	**1**	**100.00**
Camarines Norte	0	0	**0**	**0.00**	0	0	**0**	**0.00**
Camarines Sur	0	0	**0**	**0.00**	0	0	**0**	**0.00**
Catanduanes	0	0	**0**	**0.00**	0	0	**0**	**0.00**
Masbate	0	0	**0**	**0.00**	0	0	**0**	**0.00**
Sorsogon	10	1	**11**	**19.64**	10	1	**11**	**100.00**
Iriga City	0	0	**0**	**0.00**	0	0	**0**	**0.00**
Legaspi City	0	0	**0**	**0.00**	0	0	**0**	**0.00**
Naga City	0	0	**0**	**0.00**	0	0	**0**	**0.00**
**Region 6**	0	0	**0**	**0.00**	0	0	**0**	**0.00**
Aklan	0	0	**0**	**0.00**	0	0	**0**	**0.00**
Antique	0	0	**0**	**0.00**	0	0	**0**	**0.00**
Capiz	0	0	**0**	**0.00**	0	0	**0**	**0.00**
Guimaras	0	0	**0**	**0.00**	0	0	**0**	**0.00**
Iloilo	0	0	**0**	**0.00**	0	0	**0**	**0.00**
Negros Occidental	0	0	**0**	**0.00**	0	0	**0**	**0.00**
Bacolod City	0	0	**0**	**0.00**	0	0	**0**	**0.00**
Bago City	0	0	**0**	**0.00**	0	0	**0**	**0.00**
**Region 7**	0	0	**0**	**0.00**	0	0	**0**	**0.00**
Bohol	0	0	**0**	**0.00**	0	0	**0**	**0.00**
Cebu	0	0	**0**	**0.00**	0	0	**0**	**0.00**
Negros Oriental	0	0	**0**	**0.00**	0	0	**0**	**0.00**
Siquijor	0	0	**0**	**0.00**	0	0	**0**	**0.00**
				**0.00**				
Cebu City	0	0	**0**	**0.00**	0	0	**0**	**0.00**
Lapu-Lapu City	0	0	**0**	**0.00**	0	0	**0**	**0.00**
Mandaue City	0	0	**0**	**0.00**	0	0	**0**	**0.00**
**Region 8**	20	20	**40**	**0.33**	13	19	**32**	**80.00**
Biliran	0	0	**0**	**0.00**	0	0	**0**	**0.00**
Eastern Samar	7	14	**21**	**13.46**	7	14	**21**	**100.00**
Northern Leyte	0	1	**1**	**9.09**	0	1	**1**	**100.00**
Northern Samar	10	3	**13**	**0.44**	3	2	**5**	**38.46**
Southern Leyte	0	0	**0**	**0.00**	0	0	**0**	**0.00**

Table 2.E.2.2. Schistosomiasis Prevention and Control

No. and Proportion of Chronic Clinically Diagnosed Cases and Chronic Cllinically diagnosed cases confirmed by stool examination

Philippines, 2022

Area	No. of Clinically Diagnosed Chronic Cases				No. of chronic clinically diagnoses cases confirmed by stool examination (KKT) or rectal biopsy			
	Male	Female	Total	%	Male	Female	Total	%
Western Samar	3	2	**5**	**0.06**	3	2	**5**	**100.00**
Calbayog City	0	0	**0**	**0.00**	0	0	**0**	**0.00**
Maasin City	0	0	**0**	**0.00**	0	0	**0**	**0.00**
Ormoc City	0	0	**0**	**0.00**	0	0	**0**	**0.00**
Tacloban City	0	0	**0**	**0.00**	0	0	**0**	**0.00**
**Region 9**	0	0	**0**	**0.00**	0	0	**0**	**0.00**
Zamboanga del Norte	0	0	**0**	**0.00**	0	0	**0**	**0.00**
Zamboanga del Sur	0	0	**0**	**0.00**	0	0	**0**	**0.00**
Zamboanga Sibugay	0	0	**0**	**0.00**	0	0	**0**	**0.00**
Dapitan City	0	0	**0**	**0.00**	0	0	**0**	**0.00**
Dipolog City	0	0	**0**	**0.00**	0	0	**0**	**0.00**
Isabela City	0	0	**0**	**0.00**	0	0	**0**	**0.00**
Pagadian City	0	0	**0**	**0.00**	0	0	**0**	**0.00**
Zamboanga City	0	0	**0**	**0.00**	0	0	**0**	**0.00**
**Region 10**	188	96	**284**	**25.20**	63	16	**79**	**27.82**
Bukidnon	13	7	**20**	**38.46**	8	4	**12**	**60.00**
Camiguin	0	0	**0**	**0.00**	0	0	**0**	**0.00**
Lanao del Norte	5	0	**5**	**0.77**	0	0	**0**	**0.00**
Misamis Occidental	0	0	**0**	**0.00**	0	0	**0**	**0.00**
Misamis Oriental	0	0	**0**	**0.00**	0	0	**0**	**0.00**
Cagayan de Oro City	0	0	**0**	**0.00**	0	0	**0**	**0.00**
El Salvador City	0	0	**0**	**0.00**	0	0	**0**	**0.00**
Gingoog City	0	0	**0**	**0.00**	0	0	**0**	**0.00**
Iligan City	0	0	**0**	**0.00**	0	0	**0**	**0.00**
Malaybalay City	0	0	**0**	**0.00**	0	0	**0**	**0.00**
Oroquieta City	0	0	**0**	**0.00**	0	0	**0**	**0.00**
Ozamis City	7	1	**8**	**23.53**	7	1	**8**	**100.00**
Tangub City	0	0	**0**	**0.00**	0	0	**0**	**0.00**
Valencia City	163	88	**251**	**73.82**	48	11	**59**	**23.51**
**Region 11**	12	7	**19**	**4.92**	9	7	**16**	**84.21**
Davao de Oro	0	0	**0**	**0.00**	0	0	**0**	**0.00**
Davao del Norte	10	5	**15**	**19.74**	7	5	**12**	**80.00**
Davao del Sur	0	0	**0**	**0.00**	0	0	**0**	**0.00**
Davao Oriental	0	0	**0**	**0.00**	0	0	**0**	**0.00**

Table 2.E.2.2. Schistosomiasis Prevention and Control

No. and Proportion of Chronic Clinically Diagnosed Cases and Chronic Cllinically diagnosed cases confirmed by stool examination

Philippines, 2022

Area	No. of Clinically Diagnosed Chronic Cases				No. of chronic clinically diagnoses cases confirmed by stool examination (KKT) or rectal biopsy			
	Male	Female	Total	%	Male	Female	Total	%
Davao Occidental	0	0	**0**	**0.00**	0	0	**0**	**0.00**
							**0**	**0.00**
Davao City	2	2	**4**	**0.00**	2	2	**4**	**100.00**
**Region 12**	1	2	3	**10.34**	0	1	1	**33.33**
North Cotabato	0	2	**2**	**9.09**	0	1	**1**	**50.00**
Sarangani	0	0	**0**	**0.00**	0	0	**0**	**0.00**
South Cotabato	0	0	**0**	**0.00**	0	0	**0**	**0.00**
Sultan Kudarat	0	0	**0**	**0.00**	0	0	**0**	**0.00**
Gen. Santos City	1	0	**1**	**100.00**	0	0	**0**	**0.00**
**BARMM**	0	0	**0**	**0.00**	0	0	**0**	**0.00**
Basilan	0	0	**0**	**0.00**	0	0	**0**	**0.00**
Lanao del Sur	0	0	**0**	**0.00**	0	0	**0**	**0.00**
Maguindanao	0	0	**0**	**0.00**	0	0	**0**	**0.00**
Sulu	0	0	**0**	**0.00**	0	0	**0**	**0.00**
Tawi-Tawi	0	0	**0**	**0.00**	0	0	**0**	**0.00**
Cotabato City	0	0	**0**	**0.00**	0	0	**0**	**0.00**
Lamitan City	0	0	**0**	**0.00**	0	0	**0**	**0.00**
Marawi City	0	0	**0**	**0.00**	0	0	**0**	**0.00**
**CARAGA**	36	16	**52**	**0.82**	3	3	**6**	**11.54**
Agusan del Norte	0	0	**0**	**0.0**	0	0	**0**	**0.00**
Agusan del Sur	8	9	**17**	**0.30**	1	1	**2**	**11.76**
Surigao del Norte	27	7	**34**	**5.59**	2	2	**4**	**11.76**
Surigao del Sur	0	0	**0**	**0.00**	0	0	**0**	**0.00**
Province of Dinagat	0	0	**0**	**0.00**	0	0	**0**	**0.00**
Bislig City	0	0	**0**	**0.00**	0	0	**0**	**0.00**
Butuan City	1	0	**1**	**16.67**	0	0	**0**	**0.00**
Surigao City	0	0	**0**	**0.00**	0	0	**0**	**0.00**

Legend:

0 = No Case

** = No Report*

Table 2.E.2.3. Schistosomiasis Prevention and Control

No. and Proportion of Chronic Clinically Diagnosed cases treated in the Health Facility and Confirmed Chronic Cases referred to a Hospital Facility
Philippines, 2022

Area	No. of Chronic Clinically Diagnosed Cases treated in the health facility				No. of Confirmed Chronic Cases referred to Hospital Facility			
	Male	Female	Total	%	Male	Female	Total	%
**PHILIPPINES**	**169**	**69**	**238**	**40.82**	**3**	**18**	**21**	**13.13**
**N C R**	**0**	**0**	**0**	**0.00**	**0**	**0**	**0**	**0.00**
Malabon City	0	0	**0**	**0.00**	0	0	**0**	**0.00**
Navotas City	0	0	**0**	**0.00**	0	0	**0**	**0.00**
Valenzuela City	0	0	**0**	**0.00**	0	0	**0**	**0.00**
Caloocan City	0	0	**0**	**0.00**	0	0	**0**	**0.00**
Marikina City	0	0	**0**	**0.00**	0	0	**0**	**0.00**
Pasig City	0	0	**0**	**0.00**	0	0	**0**	**0.00**
Pateros	0	0	**0**	**0.00**	0	0	**0**	**0.00**
Taguig City	0	0	**0**	**0.00**	0	0	**0**	**0.00**
Quezon City	0	0	**0**	**0.00**	0	0	**0**	**0.00**
Makati City	0	0	**0**	**0.00**	0	0	**0**	**0.00**
Mandaluyong City	0	0	**0**	**0.00**	0	0	**0**	**0.00**
San Juan City	0	0	**0**	**0.00**	0	0	**0**	**0.00**
Manila City	0	0	**0**	**0.00**	0	0	**0**	**0.00**
Las Piñas City	0	0	**0**	**0.00**	0	0	**0**	**0.00**
Muntinlupa City	0	0	**0**	**0.00**	0	0	**0**	**0.00**
Parañaque City	0	0	**0**	**0.00**	0	0	**0**	**0.00**
Pasay City	0	0	**0**	**0.00**	0	0	**0**	**0.00**
**C A R**	**0**	**0**	**0**	**0.00**	**0**	**0**	**0**	**0.00**
Abra	0	0	**0**	**0.00**	0	0	**0**	**0.00**
Apayao	0	0	**0**	**0.00**	0	0	**0**	**0.00**
Benguet	0	0	**0**	**0.00**	0	0	**0**	**0.00**
Ifugao	0	0	**0**	**0.00**	0	0	**0**	**0.00**
Kalinga	0	0	**0**	**0.00**	0	0	**0**	**0.00**
Mt. Province	0	0	**0**	**0.00**	0	0	**0**	**0.00**
Baguio City	0	0	**0**	**0.00**	0	0	**0**	**0.00**
**Region 1**	**0**	**0**	**0**	**0.00**	**0**	**0**	**0**	**0.00**
Ilocos Norte	0	0	**0**	**0.00**	0	0	**0**	**0.00**
Ilocos Sur	0	0	**0**	**0.00**	0	0	**0**	**0.00**
La Union	0	0	**0**	**0.00**	0	0	**0**	**0.00**
Pangasinan	0	0	**0**	**0.00**	0	0	**0**	**0.00**
Alaminos City	0	0	**0**	**0.00**	0	0	**0**	**0.00**
Candon City	0	0	**0**	**0.00**	0	0	**0**	**0.00**
Dagupan City	0	0	**0**	**0.00**	0	0	**0**	**0.00**
Laoag City	0	0	**0**	**0.00**	0	0	**0**	**0.00**
San Carlos City	0	0	**0**	**0.00**	0	0	**0**	**0.00**
San Fernando City	0	0	**0**	**0.00**	0	0	**0**	**0.00**
Urdaneta City	0	0	**0**	**0.00**	0	0	**0**	**0.00**
Vigan City	0	0	**0**	**0.00**	0	0	**0**	**0.00**
**Region 2**	**0**	**0**	**0**	**0.00**	**0**	**0**	**0**	**0.00**
Batanes	0	0	**0**	**0.00**	0	0	**0**	**0.00**

Table 2.E.2.3. Schistosomiasis Prevention and Control

No. and Proportion of Chronic Clinically Diagnosed cases treated in the Health Facility and Confirmed Chronic Cases referred to a Hospital Facility
Philippines, 2022

Area	No. of Chronic Clinically Diagnosed Cases treated in the health facility				No. of Confirmed Chronic Cases referred to Hospital Facility			
	Male	Female	Total	%	Male	Female	Total	%
Cagayan	0	0	**0**	**0.00**	0	0	**0**	**0.00**
Isabela	0	0	**0**	**0.00**	0	0	**0**	**0.00**
Nueva Vizcaya	0	0	**0**	**0.00**	0	0	**0**	**0.00**
Quirino	0	0	**0**	**0.00**	0	0	**0**	**0.00**
Cauayan City	0	0	**0**	**0.00**	0	0	**0**	**0.00**
Ilagan City	0	0	**0**	**0.00**	0	0	**0**	**0.00**
Santiago City	0	0	**0**	**0.00**	0	0	**0**	**0.00**
Tuguegarao City	0	0	**0**	**0.00**	0	0	**0**	**0.00**
**Region 3**	**0**	**0**	**0**	**0.00**	**0**	**0**	**0**	**0.00**
Aurora	0	0	**0**	**0.00**	0	0	**0**	**0.00**
Bataan	0	0	**0**	**0.00**	0	0	**0**	**0.00**
Bulacan	0	0	**0**	**0.00**	0	0	**0**	**0.00**
Nueva Ecija	0	0	**0**	**0.00**	0	0	**0**	**0.00**
Pampanga	0	0	**0**	**0.00**	0	0	**0**	**0.00**
Tarlac	0	0	**0**	**0.00**	0	0	**0**	**0.00**
Zambales	0	0	**0**	**0.00**	0	0	**0**	**0.00**
Angeles City	0	0	**0**	**0.00**	0	0	**0**	**0.00**
Balanga City	0	0	**0**	**0.00**	0	0	**0**	**0.00**
Cabanatuan City	0	0	**0**	**0.00**	0	0	**0**	**0.00**
City of San Fernando	0	0	**0**	**0.00**	0	0	**0**	**0.00**
Gapan City	0	0	**0**	**0.00**	0	0	**0**	**0.00**
Mabalacat City	0	0	**0**	**0.00**	0	0	**0**	**0.00**
Malolos City	0	0	**0**	**0.00**	0	0	**0**	**0.00**
Meycauayan City	0	0	**0**	**0.00**	0	0	**0**	**0.00**
Olongapo City	0	0	**0**	**0.00**	0	0	**0**	**0.00**
Palayan City	0	0	**0**	**0.00**	0	0	**0**	**0.00**
San Jose City	0	0	**0**	**0.00**	0	0	**0**	**0.00**
San Jose del Monte City	0	0	**0**	**0.00**	0	0	**0**	**0.00**
Science City of Munoz	0	0	**0**	**0.00**	0	0	**0**	**0.00**
Tarlac City	0	0	**0**	**0.00**	0	0	**0**	**0.00**
**Region 4A**	**3**	**0**	**3**	**37.50**	**0**	**0**	**0**	**0.00**
Batangas	0	0	**0**	**0.00**	0	0	**0**	**0.00**
Cavite	0	0	**0**	**0.00**	0	0	**0**	**0.00**
Laguna	0	0	**0**	**0.00**	0	0	**0**	**0.00**
Quezon	2	0	**2**	**100.00**	0	0	**0**	**0.00**
Rizal	0	0	**0**	**0.00**	0	0	**0**	**0.00**
Antipolo City	0	0	**0**	**0.00**	0	0	**0**	**0.00**
Bacoor City	0	0	**0**	**0.00**	0	0	**0**	**0.00**
Batangas City	1	0	**1**	**50.00**	0	0	**0**	**0.00**
Biñan City	0	0	**0**	**0.00**	0	0	**0**	**0.00**
Cabuyao City	0	0	**0**	**0.00**	0	0	**0**	**0.00**
Calamba City	0	0	**0**	**0.00**	0	0	**0**	**0.00**
Cavite City	0	0	**0**	**0.00**	0	0	**0**	**0.00**
Dasmariñas City	0	0	**0**	**0.00**	0	0	**0**	**0.00**
General Trias City	0	0	**0**	**0.00**	0	0	**0**	**0.00**
Imus City	0	0	**0**	**0.00**	0	0	**0**	**0.00**
Lipa City	0	0	**0**	**0.00**	0	0	**0**	**0.00**
Lucena City	0	0	**0**	**0.00**	0	0	**0**	**0.00**

Table 2.E.2.3. Schistosomiasis Prevention and Control

No. and Proportion of Chronic Clinically Diagnosed cases treated in the Health Facility and Confirmed Chronic Cases referred to a Hospital Facility

Philippines, 2022

Area	No. of Chronic Clinically Diagnosed Cases treated in the health facility				No. of Confirmed Chronic Cases referred to Hospital Facility			
	Male	Female	Total	%	Male	Female	Total	%
San Pablo City	0	0	**0**	**0.00**	0	0	**0**	**0.00**
San Pedro City	0	0	**0**	**0.00**	0	0	**0**	**0.00**
Santa Rosa City	0	0	**0**	**0.00**	0	0	**0**	**0.00**
Tagaytay City	0	0	**0**	**0.00**	0	0	**0**	**0.00**
Tanauan City	0	0	**0**	**0.00**	0	0	**0**	**0.00**
Tayabas City	0	0	**0**	**0.00**	0	0	**0**	**0.00**
Trece Martires City	0	0	**0**	**0.00**	0	0	**0**	**0.00**
**Region 4B**	**4**	**5**	**9**	**100.00**	**0**	**0**	**0**	**0.00**
Marinduque	0	0	**0**	**0.00**	0	0	**0**	**0.00**
Mindoro Occidental	0	0	**0**	**0.00**	0	0	**0**	**0.00**
Mindoro Oriental	4	5	**9**	**100.00**	0	0	**0**	**0.00**
Palawan	0	0	**0**	**0.00**	0	0	**0**	**0.00**
Romblon	0	0	**0**	**0.00**	0	0	**0**	**0.00**
Puerto Princesa City	0	0	**0**	**0.00**	0	0	**0**	**0.00**
**Region 5**	**10**	**3**	**13**	**100.00**	**0**	**0**	**0**	**0.00**
Albay	0	1	**1**	**100.00**	0	0	**0**	**0.00**
Camarines Norte	0	0	**0**	**0.00**	0	0	**0**	**0.00**
Camarines Sur	0	0	**0**	**0.00**	0	0	**0**	**0.00**
Catanduanes	0	0	**0**	**0.00**	0	0	**0**	**0.00**
Masbate	0	0	**0**	**0.00**	0	0	**0**	**0.00**
Sorsogon	10	2	**12**	**100.00**	0	0	**0**	**0.00**
Iriga City	0	0	**0**	**0.00**	0	0	**0**	**0.00**
Legaspi City	0	0	**0**	**0.00**	0	0	**0**	**0.00**
Naga City	0	0	**0**	**0.00**	0	0	**0**	**0.00**
**Region 6**	**0**	**0**	**0**	**0.00**	**0**	**0**	**0**	**0.00**
Aklan	0	0	**0**	**0.00**	0	0	**0**	**0.00**
Antique	0	0	**0**	**0.00**	0	0	**0**	**0.00**
Capiz	0	0	**0**	**0.00**	0	0	**0**	**0.00**
Guimaras	0	0	**0**	**0.00**	0	0	**0**	**0.00**
Iloilo	0	0	**0**	**0.00**	0	0	**0**	**0.00**
Negros Occidental	0	0	**0**	**0.00**	0	0	**0**	**0.00**
Bacolod City	0	0	**0**	**0.00**	0	0	**0**	**0.00**
Bago City	0	0	**0**	**0.00**	0	0	**0**	**0.00**
**Region 7**	**0**	**0**	**0**	**0.00**	**0**	**2**	**2**	**0.00**
Bohol	0	0	**0**	**0.00**	0	0	**0**	**0.00**
Cebu	0	0	**0**	**0.00**	0	2	**2**	**0.00**
Negros Oriental	0	0	**0**	**0.00**	0	0	**0**	**0.00**
Siquijor	0	0	**0**	**0.00**	0	0	**0**	**0.00**
Cebu City	0	0	**0**	**0.00**	0	0	**0**	**0.00**
Lapu-Lapu City	0	0	**0**	**0.00**	0	0	**0**	**0.00**
Mandaue City	0	0	**0**	**0.00**	0	0	**0**	**0.00**

Table 2.E.2.3. Schistosomiasis Prevention and Control

No. and Proportion of Chronic Clinically Diagnosed cases treated in the Health Facility and Confirmed Chronic Cases referred to a Hospital Facility

Philippines, 2022

Area	No. of Chronic Clinically Diagnosed Cases treated in the health facility				No. of Confirmed Chronic Cases referred to Hospital Facility			
	Male	Female	Total	%	Male	Female	Total	%
**Region 8**	**16**	**10**	**26**	**25.49**	**3**	**12**	**15**	**46.88**
Biliran	0	0	**0**	**0.00**	0	0	**0**	**0.00**
Eastern Samar	2	4	**6**	**14.29**	2	10	**12**	**57.14**
Northern Leyte	1	1	**2**	**50.00**	0	1	**1**	**100.00**
Northern Samar	10	3	**13**	**31.71**	1	1	**2**	**40.00**
Southern Leyte	0	0	**0**	**0.00**	0	0	**0**	**0.00**
Western Samar	3	2	**5**	**38.46**	0	0	**0**	**0.00**
Calbayog City	0	0	**0**	**0.00**	0	0	**0**	**0.00**
Maasin City	0	0	**0**	**0.00**	0	0	**0**	**0.00**
Ormoc City	0	0	**0**	**0.00**	0	0	**0**	**0.00**
Tacloban City	0	0	**0**	**0.00**	0	0	**0**	**0.00**
**Region 9**	**0**	**0**	**0**	**0.00**	**0**	**0**	**0**	**0.00**
Zamboanga del Norte	0	0	**0**	**0.00**	0	0	**0**	**0.00**
Zamboanga del Sur	0	0	**0**	**0.00**	0	0	**0**	**0.00**
Zamboanga Sibugay	0	0	**0**	**0.00**	0	0	**0**	**0.00**
Dapitan City	0	0	**0**	**0.00**	0	0	**0**	**0.00**
Dipolog City	0	0	**0**	**0.00**	0	0	**0**	**0.00**
Isabela City	0	0	**0**	**0.00**	0	0	**0**	**0.00**
Pagadian City	0	0	**0**	**0.00**	0	0	**0**	**0.00**
Zamboanga City	0	0	**0**	**0.00**	0	0	**0**	**0.00**
**Region 10**	**89**	**36**	**125**	**73.10**	**0**	**0**	**0**	**0.00**
Bukidnon	13	9	**22**	**66.67**	0	0	**0**	**0.00**
Camiguin	0	0	**0**	**0.00**	0	0	**0**	**0.00**
Lanao del Norte	5	0	**5**	**38.46**	0	0	**0**	**0.00**
Misamis Occidental	0	1	**1**	**100.00**	0	0	**0**	**0.00**
Misamis Oriental	0	0	**0**	**0.00**	0	0	**0**	**0.00**
Cagayan de Oro City	0	0	**0**	**0.00**	0	0	**0**	**0.00**
El Salvador City	0	0	**0**	**0.00**	0	0	**0**	**0.00**
Gingoog City	0	2	**2**	**0.00**	0	0	**0**	**0.00**
Iligan City	0	0	**0**	**0.00**	0	0	**0**	**0.00**
Malaybalay City	2	0	**2**	**28.57**	0	0	**0**	**0.00**
Oroquieta City	4	3	**7**	**0.00**	0	0	**0**	**0.00**
Ozamis City	7	1	**8**	**26.67**	0	0	**0**	**0.00**
Tangub City	27	13	**40**	**0.00**	0	0	**0**	**0.00**
Valencia City	31	7	**38**	**43.68**	0	0	**0**	**0.00**
**Region 11**	**8**	**4**	**12**	**57.14**	**0**	**1**	**1**	**6.25**
Davao de Oro	0	0	**0**	**0.00**	0	0	**0**	**0.00**
Davao del Norte	6	2	**8**	**66.67**	0	1	**1**	**8.33**
Davao del Sur	0	0	**0**	**0.00**	0	0	**0**	**0.00**
Davao Oriental	0	0	**0**	**0.00**	0	0	**0**	**0.00**
Davao Occidental	0	0	**0**	**0.00**	0	0	**0**	**0.00**
Davao City	2	2	**4**	**100.00**	0	0	**0**	**0.00**

Table 2.E.2.3. Schistosomiasis Prevention and Control

No. and Proportion of Chronic Clinically Diagnosed cases treated in the Health Facility and Confirmed Chronic Cases referred to a Hospital Facility

Philippines, 2022

Area	No. of Chronic Clinically Diagnosed Cases treated in the health facility				No. of Confirmed Chronic Cases referred to Hospital Facility			
	Male	Female	Total	%	Male	Female	Total	%
**Region 12**	**0**	**1**	**1**	**7.69**	**0**	**1**	**1**	**100.00**
North Cotabato	0	1	**1**	**9.09**	0	1	**1**	**100.00**
Sarangani	0	0	**0**	**0.00**	0	0	**0**	**0.00**
South Cotabato	0	0	**0**	**0.00**	0	0	**0**	**0.00**
Sultan Kudarat	0	0	**0**	**0.00**	0	0	**0**	**0.00**
Gen. Santos City	0	0	**0**	**0.00**	0	0	**0**	**0.00**
**BARMM**	**0**	**0**	**0**	**0.00**	**0**	**0**	**0**	**0.00**
Basilan	0	0	**0**	**0.00**	0	0	**0**	**0.00**
Lanao del Sur	0	0	**0**	**0.00**	0	0	**0**	**0.00**
Maguindanao	0	0	**0**	**0.00**	0	0	**0**	**0.00**
Sulu	0	0	**0**	**0.00**	0	0	**0**	**0.00**
Tawi-Tawi	0	0	**0**	**0.00**	0	0	**0**	**0.00**
Cotabato City	0	0	**0**	**0.00**	0	0	**0**	**0.00**
Lamitan City	0	0	**0**	**0.00**	0	0	**0**	**0.00**
Marawi City	0	0	**0**	**0.00**	0	0	**0**	**0.00**
**CARAGA**	**39**	**10**	**49**	**23.90**	**0**	**2**	**2**	**33.33**
Agusan del Norte	0	0	**0**	**0.00**	0	0	**0**	**0.00**
Agusan del Sur	15	2	**17**	**12.78**	0	0	**0**	**0.00**
Surigao del Norte	23	7	**30**	**43.48**	0	1	**1**	**25.00**
Surigao del Sur	0	0	**0**	**0.00**	0	0	**0**	**0.00**
Province of Dinagat	0	0	**0**	**0.00**	0	0	**0**	**0.00**
Bislig City	0	0	**0**	**0.00**	0	0	**0**	**0.00**
Butuan City	1	1	**2**	**100.00**	0	1	**1**	**0.00**
Surigao City	0	0	**0**	**0.00**	0	0	**0**	**0.00**

Legend:
0 = No Case
** = No Report*

Table 2.E.3.1. Soil Transmitted Helminthiasis Prevention and Control

No. and Proportion of PSAC [1](1-4) and SAC [2](5-9) years old who completed 2 doses of deworming tablets
Philippines, 2022

Area	Elig. Pop	Number PSAC [1] (1-4 yrs. Old) who completed 2 doses of deworming tablets				Elig. Pop	Number SAC [2] (5-9 yrs. Old) who completed 2 doses of deworming tablets			
		Male	Female	Total	%		Male	Female	Total	%
**PHILIPPINES**	**8,976,673**	**2,765,096**	**2,622,616**	**5,387,712**	**60.02**	**11,132,656**	**2,596,884**	**2,433,504**	**5,030,388**	**45.19**
**NCR**	**951,556**	**270,396**	**269,261**	**539,657**	**56.71**	**1,234,875**	**171,445**	**151,802**	**323,247**	**26.18**
Malabon City	**27,010**	4,571	4,579	**9,150**	**33.88**	35,052	3,035	2,882	**5,917**	**16.88**
Navotas City	**18,431**	1,964	2,098	**4,062**	**22.04**	23,921	1,316	1,505	**2,821**	**11.79**
Valenzuela City	**45,846**	9,127	9,335	**18,462**	**40.27**	59,496	5,856	6,635	**12,491**	**20.99**
Caloocan City	**117,048**	59,861	57,187	**117,048**	**100.00**	151,892	37,202	36,894	**74,096**	**48.78**
Marikina City	**33,305**	4,526	4,979	**9,505**	**28.54**	43,224	1,410	1,330	**2,740**	**6.34**
Pasig City	**55,813**	5,826	5,454	**11,280**	**20.21**	72,427	3,578	3,265	**6,843**	**9.45**
Pateros	**4,718**	876	822	**1,698**	**35.99**	6,121	470	476	**946**	**15.45**
Taguig City	**59,478**	29,267	30,211	**59,478**	**100.00**	77,193	35,533	35,234	**70,767**	**91.68**
Quezon City	**216,968**	61,252	63,859	**125,111**	**57.66**	281,564	33,019	22,313	**55,332**	**19.65**
Makati City	**43,053**	11,581	10,745	**22,326**	**51.86**	55,870	5,256	4,884	**10,140**	**18.15**
Mandaluyong City	**28,541**	3,848	3,996	**7,844**	**27.48**	37,044	623	740	**1,363**	**3.68**
San Juan City	**9,028**	577	521	**1,098**	**12.16**	11,714	475	505	**980**	**8.37**
Manila City	**131,547**	25,433	24,834	**50,267**	**38.21**	170,708	16,072	15,733	**31,805**	**18.63**
Las Piñas City	**43,514**	1,968	1,723	**3,691**	**8.48**	56,474	660	626	**1,286**	**2.28**
Muntinlupa City	**37,280**	18,162	18,447	**36,609**	**98.20**	48,380	18,444	10,826	**29,270**	**60.50**
Parañaque City	**49,198**	16,025	15,225	**31,250**	**63.52**	63,849	4,808	4,083	**8,891**	**13.93**
Pasay City	**30,778**	15,532	15,246	**30,778**	**100.00**	39,946	3,688	3,871	**7,559**	**18.92**
**CAR**	**147,190**	**50,278**	**47,892**	**98,170**	**66.70**	**180,107**	**32,300**	**32,300**	**64,600**	**35.87**
Abra	**18,948**	7,406	7,104	**14,510**	**76.58**	23,334	9,466	9,466	**18,932**	**81.13**
Apayao	**11,111**	3,792	3,537	**7,329**	**65.96**	13,346	1,201	1,201	**2,402**	**18.00**
Benguet	**36,006**	11,759	11,221	**22,980**	**63.82**	44,205	3,785	3,785	**7,570**	**17.12**
Ifugao	**19,435**	7,148	6,888	**14,036**	**72.22**	23,529	8,730	8,730	**17,460**	**74.21**
Kalinga	**20,071**	5,350	4,828	**10,178**	**50.71**	24,443	4,296	4,296	**8,592**	**35.15**
Mt. Province	**13,756**	4,358	4,023	**8,381**	**60.93**	17,025	1,665	1,665	**3,330**	**19.56**
Baguio City	**27,863**	10,465	10,291	**20,756**	**74.49**	34,225	3,157	3,157	**6,314**	**18.45**
**Region 1**	**406,852**	**113,866**	**115,547**	**229,413**	**56.39**	**519,612**	**100,272**	**98,235**	**198,507**	**38.20**
Ilocos Norte	**31,951**	8,236	7,726	**15,962**	**49.96**	40,888	1,424	1,297	**2,721**	**6.65**
Ilocos Sur	**39,653**	16,429	16,217	**32,646**	**82.33**	51,586	20,487	20,080	**40,567**	**78.64**
La Union	**47,886**	18,340	17,128	**35,468**	**74.07**	61,561	21,843	20,488	**42,331**	**68.76**
Pangasinan	**211,444**	56,769	60,727	**117,496**	**55.57**	268,691	42,482	43,374	**85,856**	**31.95**
Alaminos City	**7,986**	3,644	4,070	**7,714**	**96.59**	10,153	4,816	4,342	**9,158**	**90.20**
Candon City	**4,182**	2,320	1,861	**4,181**	**99.98**	5,431	628	523	**1,151**	**21.19**
Dagupan City	**15,255**	2,549	2,450	**4,999**	**32.77**	19,383	2,901	2,783	**5,684**	**29.32**
Laoag City	**7,365**	0	0	**0**	**0.00**	9,425	0	0	**0**	**0.00**
San Carlos City	**16,796**	0	0	**0**	**0.00**	21,342	0	0	**0**	**0.00**
San Fernando City	**8,776**	3,510	3,431	**6,941**	**79.09**	11,278	3,555	3,292	**6,847**	**60.71**
Urdaneta City	**11,842**	398	357	**755**	**6.38**	15,045	564	590	**1,154**	**7.67**
Vigan City	**3,716**	1,671	1,580	**3,251**	**87.49**	4,829	1,572	1,466	**3,038**	**62.91**
**Region 2**	**289,361**	**115,851**	**109,735**	**225,586**	**77.96**	**365,905**	**62,149**	**59,998**	**122,147**	**33.38**
Batanes	**1,627**	238	271	**509**	**31.28**	1,970	1	3	**4**	**0.20**

Table 2.E.3.1. Soil Transmitted Helminthiasis Prevention and Control

No. and Proportion of PSAC [1](1-4) and SAC [2](5-9) years old who completed 2 doses of deworming tablets
Philippines, 2022

Area	Elig. Pop	Number PSAC [1] (1-4 yrs. Old) who completed 2 doses of deworming tablets				Elig. Pop	Number SAC [2] (5-9 yrs. Old) who completed 2 doses of deworming tablets			
		Male	Female	Total	%		Male	Female	Total	%
Cagayan	**85,819**	34,949	33,046	**67,995**	**79.23**	108,623	13,446	12,570	**26,016**	**23.95**
Isabela	**97,953**	47,520	45,214	**92,734**	**94.67**	124,800	29,060	28,641	**57,701**	**46.23**
Nueva Vizcaya	**40,154**	10,341	9,741	**20,082**	**50.01**	49,688	2,369	2,459	**4,828**	**9.72**
Quirino	**17,287**	4,644	4,365	**9,009**	**52.11**	21,668	1,786	1,679	**3,465**	**15.99**
Cauayan City	**10,715**	634	603	**1,237**	**11.54**	13,657	293	296	**589**	**4.31**
Ilagan City	**12,047**	6,244	5,682	**11,926**	**99.00**	15,344	7,754	7,416	**15,170**	**98.87**
Santiago City	**11,159**	5,725	5,434	**11,159**	**100.00**	14,212	6,355	5,890	**12,245**	**86.16**
Tuguegarao City	**12,600**	5,556	5,379	**10,935**	**86.79**	15,943	1,085	1,044	**2,129**	**13.35**
**Region 3**	**913,147**	**446,061**	**373,409**	**819,470**	**89.74**	**1,160,787**	**460,725**	**376,639**	**837,364**	**72.14**
Aurora	**20,431**	8,612	8,482	**17,094**	**83.67**	24,576	9,979	9,686	**19,665**	**80.02**
Bataan	**54,674**	22,596	21,985	**44,581**	**81.54**	69,465	15,501	13,295	**28,796**	**41.45**
Bulacan	**181,432**	90,746	90,182	**180,928**	**99.72**	232,852	116,770	112,081	**228,851**	**98.28**
Nueva Ecija	**122,537**	39,120	37,090	**76,210**	**62.19**	156,072	22,397	21,212	**43,609**	**27.94**
Pampanga	**131,844**	60,964	60,974	**121,938**	**92.49**	167,978	67,697	67,706	**135,403**	**80.61**
Tarlac	**79,847**	71,669	8,483	**80,152**	**100.38**	101,338	76,812	7,860	**84,672**	**83.55**
Zambales	**50,671**	19,036	17,941	**36,977**	**72.97**	62,546	21,091	19,567	**40,658**	**65.00**
Angeles City	**33,080**	3,579	3,228	**6,807**	**20.58**	42,137	2,708	2,468	**5,176**	**12.28**
Balanga City	**7,905**	3,404	3,290	**6,694**	**84.68**	10,039	3,875	3,467	**7,342**	**73.13**
Cabanatuan City	**25,080**	16,054	16,108	**32,162**	**128.24**	31,953	14,594	13,313	**27,907**	**87.34**
City of San Fernando	**24,640**	26,103	23,641	**49,744**	**201.88**	31,391	23,058	20,895	**43,953**	**140.02**
Gapan City	**9,152**	4,197	3,997	**8,194**	**89.53**	11,662	2,032	1,945	**3,977**	**34.10**
Mabalacat City	**20,155**	20,519	18,867	**39,386**	**195.42**	25,675	22,459	23,283	**45,742**	**178.16**
Malolos City	**20,270**	6,939	6,654	**13,593**	**67.06**	26,007	8,767	8,336	**17,103**	**65.76**
Meycauayan City	**16,812**	1,073	1,006	**2,079**	**12.37**	21,570	1,309	1,361	**2,670**	**12.38**
Olongapo City	**19,984**	5,168	4,818	**9,986**	**49.97**	24,668	4,189	4,055	**8,244**	**33.42**
Palayan City	**3,405**	1,550	1,529	**3,079**	**90.43**	4,338	1,784	1,727	**3,511**	**80.94**
San Jose City	**11,599**	4,337	4,042	**8,379**	**72.24**	14,771	5,726	5,206	**10,932**	**74.01**
San Jose del Monte City	**46,153**	18,089	18,364	**36,453**	**78.98**	59,227	14,947	14,655	**29,602**	**49.98**
Science City of Munoz	**6,759**	2,790	2,643	**5,433**	**80.38**	8,615	629	592	**1,221**	**14.17**
Tarlac City	**26,717**	19,516	20,085	**39,601**	**148.22**	33,907	24,401	23,929	**48,330**	**142.54**
**Region 4A**	**1,238,585**	**304,194**	**287,314**	**591,508**	**47.76**	**1,557,324**	**318,035**	**304,281**	**622,316**	**39.96**
Batangas	**165,380**	69,069	62,933	**132,002**	**79.82**	204,337	85,082	78,919	**164,001**	**80.26**
Cavite	**115,721**	46,457	41,774	**88,231**	**76.24**	147,524	45,943	42,687	**88,630**	**60.08**
Laguna	**80,424**	20,296	19,497	**39,793**	**49.48**	101,782	19,149	19,147	**38,296**	**37.63**
Quezon	**160,430**	35,571	32,905	**68,476**	**42.68**	199,889	36,306	35,021	**71,327**	**35.68**
Rizal	**181,775**	18,043	18,441	**36,484**	**20.07**	228,559	16,779	17,743	**34,522**	**15.10**
Antipolo City	**66,955**	873	998	**1,871**	**2.79**	84,187	86	89	**175**	**0.21**
Bacoor City	**50,703**	3,356	3,373	**6,729**	**13.27**	64,626	1,519	1,423	**2,942**	**4.55**
Batangas City	**29,351**	1,537	1,418	**2,955**	**10.07**	36,266	1,162	1,090	**2,252**	**6.21**
Biñan City	**26,965**	19,006	18,738	**37,744**	**139.97**	34,128	22,406	21,385	**43,791**	**128.31**
Cabuyao City	**24,996**	3,940	3,913	**7,853**	**31.42**	31,640	3,077	2,951	**6,028**	**19.05**
Calamba City	**36,801**	8,650	8,039	**16,689**	**45.35**	46,570	11,103	10,361	**21,464**	**46.09**
Cavite City	**8,682**	679	682	**1,361**	**15.68**	11,068	892	844	**1,736**	**15.68**
Dasmariñas City	**55,633**	15,759	15,437	**31,196**	**56.07**	70,910	10,417	10,218	**20,635**	**29.10**
General Trias City	**26,535**	10,701	10,670	**21,371**	**80.54**	33,821	13,325	13,342	**26,667**	**78.85**
Imus City	**34,083**	7,423	7,081	**14,504**	**42.55**	43,453	6,303	5,978	**12,281**	**28.26**
Lipa City	**29,578**	445	497	**942**	**3.18**	36,537	11	9	**20**	**0.05**
Lucena City	**24,313**	7,197	6,952	**14,149**	**58.20**	30,295	6,052	6,191	**12,243**	**40.41**
San Pablo City	**21,543**	3,010	2,768	**5,778**	**26.82**	27,260	2,809	2,748	**5,557**	**20.39**
San Pedro City	**26,381**	1,795	1,944	**3,739**	**14.17**	33,387	1,041	961	**2,002**	**6.00**

Table 2.E.3.1. Soil Transmitted Helminthiasis Prevention and Control

No. and Proportion of PSAC [1](1-4) and SAC [2](5-9) years old who completed 2 doses of deworming tablets
Philippines, 2022

Area	Elig. Pop	Number PSAC [1] (1-4 yrs. Old) who completed 2 doses of deworming tablets				Elig. Pop	Number SAC [2] (5-9 yrs. Old) who completed 2 doses of deworming tablets			
		Male	Female	Total	%		Male	Female	Total	%
Santa Rosa City	**28,642**	15,085	14,985	**30,070**	**104.99**	36,253	15,667	14,976	**30,643**	**84.53**
Tagaytay City	**6,012**	2,990	2,767	**5,757**	**95.76**	7,663	3,466	3,365	**6,831**	**89.14**
Tanauan City	**15,428**	1,947	1,964	**3,911**	**25.35**	19,060	1,527	1,541	**3,068**	**16.10**
Tayabas City	**9,107**	7,654	7,186	**14,840**	**162.95**	11,353	9,982	9,378	**19,360**	**170.53**
Trece Martires City	**13,147**	2,711	2,352	**5,063**	**38.51**	16,756	3,931	3,914	**7,845**	**46.82**
**Region 4B**	**304,748**	**130,679**	**123,991**	**254,670**	**83.57**	**375,540**	**145,534**	**145,735**	**291,269**	**77.56**
Marinduque	**22,343**	9,866	8,815	**18,681**	**83.61**	27,473	9,886	9,032	**18,918**	**68.86**
Mindoro Occidental	**50,640**	34,361	33,100	**67,461**	**133.22**	62,712	46,294	43,424	**89,718**	**143.06**
Mindoro Oriental	**84,729**	35,676	33,203	**68,879**	**81.29**	105,481	40,018	37,780	**77,798**	**73.76**
Palawan	**90,740**	33,681	32,572	**66,253**	**73.01**	111,233	33,663	31,897	**65,560**	**58.94**
Romblon	**29,043**	9,655	8,992	**18,647**	**64.20**	35,224	5,860	5,503	**11,363**	**32.26**
Puerto Princesa City	**27,253**	7,440	7,309	**14,749**	**54.12**	33,417	9,813	18,099	**27,912**	**83.53**
**Region 5**	**595,917**	**169,888**	**160,873**	**330,761**	**55.50**	**706,890**	**250,770**	**229,818**	**480,588**	**67.99**
Albay	**104,616**	24,039	23,027	**47,066**	**44.99**	127,856	35,018	31,631	**66,649**	**52.13**
Camarines Norte	**60,281**	18,210	17,370	**35,580**	**59.02**	70,639	27,598	25,787	**53,385**	**75.57**
Camarines Sur	**169,027**	50,921	48,804	**99,725**	**59.00**	201,633	82,701	78,846	**161,547**	**80.12**
Catanduanes	**27,331**	7,543	7,477	**15,020**	**54.96**	32,117	12,849	11,482	**24,331**	**75.76**
Masbate	**99,694**	26,185	25,101	**51,286**	**51.44**	114,354	47,961	39,384	**87,345**	**76.38**
Sorsogon	**84,950**	27,396	24,881	**52,277**	**61.54**	100,075	25,592	23,955	**49,547**	**49.51**
Iriga City	**11,482**	3,933	3,394	**7,327**	**63.81**	13,702	5,346	5,112	**10,458**	**76.32**
Legaspi City	**18,395**	7,624	7,014	**14,638**	**79.58**	22,488	9,051	9,053	**18,104**	**80.51**
Naga City	**20,141**	4,037	3,805	**7,842**	**38.94**	24,026	4,654	4,568	**9,222**	**38.38**
**Region 6**	**623,453**	**205,260**	**198,309**	**403,569**	**64.73**	**775,969**	**194,670**	**188,970**	**383,640**	**49.44**
Aklan	**48,370**	17,942	17,260	**35,202**	**72.78**	60,505	23,187	23,145	**46,332**	**76.58**
Antique	**55,203**	19,677	18,709	**38,386**	**69.54**	68,857	23,492	22,470	**45,962**	**66.75**
Capiz	**60,007**	23,094	22,918	**46,012**	**76.68**	73,668	26,050	27,173	**53,223**	**72.25**
Guimaras	**14,498**	5,265	5,017	**10,282**	**70.92**	17,617	7,534	7,180	**14,714**	**83.52**
Iloilo	**152,501**	65,793	63,222	**129,015**	**84.60**	191,695	80,929	77,699	**158,628**	**82.75**
Negros Occidental	**210,270**	47,584	47,589	**95,173**	**45.26**	260,638	21,834	20,703	**42,537**	**16.32**
Bacolod City	**47,314**	12,737	11,323	**24,060**	**50.85**	58,643	11,644	10,600	**22,244**	**37.93**
Iloilo City	**35,290**	13,168	12,271	**25,439**	**72.09**	44,346	0	0	**0**	**0.00**
**Region 7**	**648,479**	**152,737**	**143,802**	**296,539**	**45.73**	**812,744**	**73,553**	**68,943**	**142,496**	**17.53**
Bohol	**117,825**	38,887	37,030	**75,917**	**64.43**	146,548	12,020	11,182	**23,202**	**15.83**
Cebu	**259,039**	57,991	53,913	**111,904**	**43.20**	326,959	29,216	27,456	**56,672**	**17.33**
Negros Oriental	**114,713**	9,727	9,008	**18,735**	**16.33**	141,100	3,674	3,660	**7,334**	**5.20**
Siquijor	**7,654**	3,523	3,607	**7,130**	**93.15**	9,756	4,424	4,324	**8,748**	**89.67**
Cebu City	**81,316**	30,500	28,559	**59,059**	**72.63**	102,635	8,172	7,655	**15,827**	**15.42**
Lapu-Lapu City	**35,968**	3,460	3,244	**6,704**	**18.64**	45,399	1,309	1,249	**2,558**	**5.63**
Mandaue City	**31,964**	8,649	8,441	**17,090**	**53.47**	40,347	14,738	13,417	**28,155**	**69.78**
**Region 8**	**445,167**	**82,188**	**82,940**	**165,128**	**37.09**	**531,452**	**52,186**	**51,978**	**104,164**	**19.60**
Biliran	**16,955**	6,020	5,408	**11,428**	**67.40**	19,880	5,767	5,004	**10,771**	**54.18**
Eastern Samar	**48,401**	11,004	11,131	**22,135**	**45.73**	58,185	2,425	2,381	**4,806**	**8.26**
Northern Leyte	**146,531**	22,438	22,306	**44,744**	**30.54**	176,953	8,947	9,084	**18,031**	**10.19**

Table 2.E.3.1. Soil Transmitted Helminthiasis Prevention and Control

No. and Proportion of PSAC [1](1-4) and SAC [2](5-9) years old who completed 2 doses of deworming tablets
Philippines, 2022

Area	Elig. Pop	Number PSAC [1] (1-4 yrs. Old) who completed 2 doses of deworming tablets				Elig. Pop	Number SAC [2] (5-9 yrs. Old) who completed 2 doses of deworming tablets			
		Male	Female	Total	%		Male	Female	Total	%
Northern Samar	**69,862**	15,813	15,403	**31,216**	**44.68**	81,192	15,379	15,885	**31,264**	**38.51**
Southern Leyte	**29,766**	9,674	9,057	**18,731**	**62.93**	36,786	6,864	6,176	**13,040**	**35.45**
Western Samar	**62,466**	4,569	7,102	**11,671**	**18.68**	73,014	1,622	2,038	**3,660**	**5.01**
Calbayog City	**19,250**	7,600	7,700	**15,300**	**79.48**	22,514	7,591	7,655	**15,246**	**67.72**
Maasin City	**7,575**	1,197	1,107	**2,304**	**30.42**	9,362	1,053	935	**1,988**	**21.23**
Ormoc City	**20,865**	2,439	2,201	**4,640**	**22.24**	25,196	1,163	1,275	**2,438**	**9.68**
Tacloban City	**23,496**	1,434	1,525	**2,959**	**12.59**	28,370	1,375	1,545	**2,920**	**10.29**
**Region 9**	**335,069**	**120,703**	**122,780**	**243,483**	**72.67**	**406,544**	**171,094**	**169,939**	**341,033**	**83.89**
Zamboanga del Norte	**73,193**	30,556	30,828	**61,384**	**83.87**	87,946	40,333	39,913	**80,246**	**91.24**
Zamboanga del Sur	**70,988**	12,419	12,197	**24,616**	**34.68**	87,332	15,558	15,749	**31,307**	**35.85**
Zamboanga Sibugay	**66,344**	22,446	21,789	**44,235**	**66.68**	79,178	33,556	33,809	**67,365**	**85.08**
Dapitan City	**7,557**	0	0	**0**	**0.00**	9,082	0	0	**0**	**0.00**
Dipolog City	**11,987**	5,361	7,839	**13,200**	**110.12**	14,406	6,801	9,945	**16,746**	**116.24**
Isabela City	**12,229**	6,267	7,385	**13,652**	**111.64**	14,461	8,378	8,610	**16,988**	**117.47**
Pagadian City	**17,408**	18,219	15,751	**33,970**	**195.14**	21,417	24,437	20,050	**44,487**	**207.72**
Zamboanga City	**75,363**	25,435	26,991	**52,426**	**69.56**	92,722	42,031	41,863	**83,894**	**90.48**
**Region 10**	**428,356**	**125,290**	**124,550**	**249,840**	**58.33**	**521,123**	**173,993**	**170,535**	**344,528**	**66.11**
Bukidnon	**97,101**	26,102	27,673	**53,775**	**55.38**	117,189	36,719	36,673	**73,392**	**62.63**
Camiguin	**7,996**	2,869	2,794	**5,663**	**70.82**	9,551	4,212	4,051	**8,263**	**86.51**
Lanao del Norte	**67,342**	9,122	9,208	**18,330**	**27.22**	80,919	11,379	10,957	**22,336**	**27.60**
Misamis Occidental	**27,481**	10,049	10,226	**20,275**	**73.78**	34,162	15,692	15,771	**31,463**	**92.10**
Misamis Oriental	**62,525**	18,859	17,139	**35,998**	**57.57**	76,800	25,934	23,407	**49,341**	**64.25**
Cagayan de Oro City	**59,215**	14,247	16,103	**30,350**	**51.25**	72,747	18,867	21,068	**39,935**	**54.90**
El Salvador City	**4,397**	2,485	1,658	**4,143**	**94.22**	5,404	4,079	2,719	**6,798**	**125.80**
Gingoog City	**10,919**	6,710	6,313	**13,023**	**119.27**	13,423	10,316	10,874	**21,190**	**157.86**
Iligan City	**34,120**	13,571	12,678	**26,249**	**76.93**	40,984	14,310	14,445	**28,755**	**70.16**
Malaybalay City	**16,184**	6,595	6,231	**12,826**	**79.25**	19,533	11,367	10,584	**21,951**	**112.38**
Oroquieta City	**5,954**	3,886	3,642	**7,528**	**126.44**	7,401	5,456	5,103	**10,559**	**142.67**
Ozamis City	**11,936**	5,761	5,928	**11,689**	**97.93**	14,834	7,497	7,494	**14,991**	**101.06**
Tangub City	**5,300**	2,378	2,197	**4,575**	**86.32**	6,590	4,783	4,347	**9,130**	**138.54**
Valencia City	**17,886**	2,656	2,760	**5,416**	**30.28**	21,586	3,382	3,042	**6,424**	**29.76**
**Region 11**	**452,922**	**202,539**	**193,161**	**395,700**	**87.37**	**563,918**	**95,839**	**83,113**	**178,952**	**31.73**
Davao de Oro	**67,865**	29,631	27,754	**57,385**	**84.56**	82,518	9,674	9,355	**19,029**	**23.06**
Davao del Norte	**86,391**	57,405	55,478	**112,883**	**130.67**	108,621	17,602	16,562	**34,164**	**31.45**
Davao del Sur	**57,129**	12,901	12,134	**25,035**	**43.82**	68,687	4,620	4,499	**9,119**	**13.28**
Davao Oriental	**59,042**	16,460	14,625	**31,085**	**52.65**	74,330	5,620	3,895	**9,515**	**12.80**
Davao Occidental	**30,091**	8,083	8,019	**16,102**	**53.51**	37,881	6,434	5,826	**12,260**	**32.36**
Davao City	**152,404**	78,059	75,151	**153,210**	**100.53**	191,881	51,889	42,976	**94,865**	**49.44**
**Region 12**	**409,593**	**124,919**	**120,061**	**244,980**	**59.81**	**505,576**	**133,026**	**129,188**	**262,214**	**51.86**
North Cotabato	**141,044**	43,980	42,352	**86,332**	**61.21**	173,779	34,002	32,934	**66,936**	**38.52**
Sarangani	**54,628**	15,892	14,765	**30,657**	**56.12**	66,433	26,963	26,139	**53,102**	**79.93**
South Cotabato	**84,448**	19,071	18,981	**38,052**	**45.06**	104,409	18,652	18,133	**36,785**	**35.23**
Sultan Kudarat	**74,631**	25,992	25,388	**51,380**	**68.85**	93,146	31,704	31,846	**63,550**	**68.23**
Gen. Santos City	**54,842**	19,984	18,575	**38,559**	**70.31**	67,809	21,705	20,136	**41,841**	**61.70**

Table 2.E.3.1. Soil Transmitted Helminthiasis Prevention and Control

No. and Proportion of PSAC [1](1-4) and SAC [2](5-9) years old who completed 2 doses of deworming tablets
Philippines, 2022

Area	Elig. Pop	Number PSAC [1] (1-4 yrs. Old) who completed 2 doses of deworming tablets				Elig. Pop	Number SAC [2] (5-9 yrs. Old) who completed 2 doses of deworming tablets			
		Male	Female	Total	%		Male	Female	Total	%
**BARMM**	**525,340**	**61,632**	**63,023**	**124,655**	**23.73**	**605,438**	**52,260**	**66,194**	**118,454**	**19.57**
Basilan	**36,827**	1,117	1,183	**2,300**	**6.25**	42,144	1,216	1,449	**2,665**	**6.32**
Lanao del Sur	**112,850**	0	0	**0**	**0.00**	128,261	0	0	**0**	**0.00**
Maguindanao	**154,029**	4,416	4,043	**8,459**	**5.49**	179,697	5,000	4,141	**9,141**	**5.09**
Sulu	**110,769**	19,215	22,014	**41,229**	**37.22**	125,008	21,516	28,009	**49,525**	**39.62**
Tawi-Tawi	**45,443**	15,307	15,166	**30,473**	**67.06**	53,100	14,587	20,684	**35,271**	**66.42**
Cotabato City	**28,295**	11,199	9,989	**21,188**	**74.88**	34,947	9,919	11,886	**21,805**	**62.39**
Lamitan City	**10,136**	9	8	**17**	**0.17**	11,601	22	25	**47**	**0.41**
Marawi City	**26,991**	10,369	10,620	**20,989**	**77.76**	30,680	10,497	10,723	**21,220**	**69.17**
**CARAGA**	**260,938**	**88,615**	**85,968**	**174,583**	**66.91**	**308,852**	**109,033**	**105,836**	**214,869**	**69.57**
Agusan del Norte	**34,222**	16,600	15,810	**32,410**	**94.71**	40,653	19,238	18,592	**37,830**	**93.06**
Agusan del Sur	**77,316**	21,148	21,389	**42,537**	**55.02**	87,642	21,202	20,976	**42,178**	**48.13**
Surigao del Norte	**32,414**	8,240	8,043	**16,283**	**50.23**	39,889	12,750	12,136	**24,886**	**62.39**
Surigao del Sur	**48,819**	19,804	18,800	**38,604**	**79.08**	58,177	27,802	26,637	**54,439**	**93.57**
Province of Dinagat	**11,259**	2,951	2,783	**5,734**	**50.93**	14,214	4,296	4,151	**8,447**	**59.43**
Bislig City	**9,270**	2,311	2,175	**4,486**	**48.39**	11,049	1,188	1,124	**2,312**	**20.92**
Butuan City	**32,536**	13,261	13,018	**26,279**	**80.77**	38,652	16,807	16,836	**33,643**	**87.04**
Surigao City	**15,102**	4,300	3,950	**8,250**	**54.63**	18,576	5,750	5,384	**11,134**	**59.94**

Legend:
0 = No Case
** = No Report*

PSAC [1] - Pre School Age Children
SAC [2] - School Age Children

Table 2.E.3.2. Soil Transmitted Helminthiasis Prevention and Control

No. and Proportion of 10-19 and Women 20-49 years old who completed 2 doses of deworming tablets

Philippines, 2022

Area	Elig. Pop.	No. of Adolecents (10-19 yrs. Old) who completed 2 doses of deworming tablets				Elig. Pop.	No of WRA (20 - 49 yrs. Old) who completed 2 doses of deworming tablets	%
		Male	Female	Total	%			
**PHILIPPINES**	**21,215,204**	**3,909,886**	**3,742,010**	**7,651,896**	**36.07**	**23,093,019**	**381,342**	**1.65**
**N C R**	**2,440,231**	**145,763**	**130,994**	**276,757**	**11.34**	**3,296,083**	**36,112**	**1.10**
Malabon City	**69,266**	2,806	2,833	**5,639**	**8.14**	93,559	**0**	**0.00**
Navotas City	**47,271**	976	909	**1,885**	**3.99**	63,853	**4**	**0.01**
Valenzuela City	**117,572**	5,870	3,920	**9,790**	**8.33**	158,802	**8**	**0.01**
Caloocan City	**300,161**	24,729	24,626	**49,355**	**16.44**	405,437	**17,546**	**4.33**
Marikina City	**85,416**	896	930	**1,826**	**2.14**	115,371	**1,371**	**1.19**
Pasig City	**143,132**	3,222	3,007	**6,229**	**4.35**	193,328	**325**	**0.17**
Pateros	**12,097**	433	416	**849**	**7.02**	16,340	**46**	**0.28**
Taguig City	**152,532**	48,390	44,720	**93,110**	**61.04**	206,029	**975**	**0.47**
Quezon City	**556,391**	22,652	15,146	**37,798**	**6.79**	751,537	**7,219**	**0.96**
Makati City	**110,402**	2,160	2,104	**4,264**	**3.86**	149,126	**1,303**	**0.87**
Mandaluyong City	**73,197**	264	308	**572**	**0.78**	98,871	**0**	**0.00**
San Juan City	**23,152**	785	837	**1,622**	**7.01**	31,272	**63**	**0.20**
Manila City	**337,335**	15,158	15,306	**30,464**	**9.03**	455,649	**6,218**	**1.36**
Las Piñas City	**111,594**	294	283	**577**	**0.52**	150,733	**0**	**0.00**
Muntinlupa City	**95,605**	12,488	12,000	**24,488**	**25.61**	129,135	**0**	**0.00**
Parañaque City	**126,173**	2,298	1,337	**3,635**	**2.88**	170,425	**1,034**	**0.61**
Pasay City	**78,935**	2,342	2,312	**4,654**	**5.90**	106,616	**0**	**0.00**
**C A R**	**346,319**	**50,017**	**46,611**	**96,628**	**27.90**	**397,511**	**0**	**0.00**
Abra	**46,682**	14,694	12,660	**27,354**	**58.60**	51,244	**0**	**0.00**
Apayao	**24,870**	1,614	1,487	**3,101**	**12.47**	25,203	**0**	**0.00**
Benguet	**87,502**	5,975	5,548	**11,523**	**13.17**	113,710	**0**	**0.00**
Ifugao	**43,068**	15,091	14,391	**29,482**	**68.45**	43,326	**0**	**0.00**
Kalinga	**44,965**	7,426	6,965	**14,391**	**32.00**	44,568	**0**	**0.00**
Mt. Province	**31,507**	2,460	2,250	**4,710**	**14.95**	31,447	**0**	**0.00**
Baguio City	**67,725**	2,757	3,310	**6,067**	**8.96**	88,013	**0**	**0.00**
**Region 1**	**1,002,199**	**167,933**	**169,332**	**337,265**	**33.65**	**1,097,113**	**13,098**	**1.19**
Ilocos Norte	**85,884**	1,730	1,822	**3,552**	**4.14**	108,065	**252**	**0.23**
Ilocos Sur	**105,601**	34,997	35,529	**70,526**	**66.79**	128,224	**1,907**	**1.49**
La Union	**125,987**	42,265	39,476	**81,741**	**64.88**	151,872	**10,939**	**7.20**
Pangasinan	**498,531**	64,939	69,954	**134,893**	**27.06**	506,433	**0**	**0.00**
Alaminos City	**18,841**	8,898	8,467	**17,365**	**92.17**	19,135	**0**	**0.00**
Candon City	**11,126**	775	620	**1,395**	**12.54**	13,516	**0**	**0.00**
Dagupan City	**35,957**	4,101	3,884	**7,985**	**22.21**	36,538	**0**	**0.00**
Laoag City	**19,796**	0	0	**0**	**0.00**	24,913	**0**	**0.00**
San Carlos City	**39,591**	0	0	**0**	**0.00**	40,222	**0**	**0.00**

Table 2.E.3.2. Soil Transmitted Helminthiasis Prevention and Control

No. and Proportion of 10-19 and Women 20-49 years old who completed 2 doses of deworming tablets

Philippines, 2022

Area	Elig. Pop.	No. of Adolecents (10-19 yrs. Old) who completed 2 doses of deworming tablets				Elig. Pop.	No of WRA (20 - 49 yrs. Old) who completed 2 doses of deworming tablets	%
		Male	Female	Total	%			
San Fernando City	**23,080**	6,699	6,071	**12,770**	**55.33**	27,823	**0**	**0.00**
Urdaneta City	**27,914**	1,180	1,098	**2,278**	**8.16**	28,357	**0**	**0.00**
Vigan City	**9,891**	2,349	2,411	**4,760**	**48.12**	12,015	**0**	**0.00**
**Region 2**	**688,024**	**86,149**	**82,886**	**169,035**	**24.57**	**772,000**	**1,418**	**0.18**
Batanes	**3,424**	12	15	**27**	**0.79**	3,371	**0**	**0.00**
Cagayan	**206,463**	18,034	16,929	**34,963**	**16.93**	234,612	**703**	**0.30**
Isabela	**234,701**	42,516	40,998	**83,514**	**35.58**	265,644	**0**	**0.00**
Nueva Vizcaya	**93,592**	1,901	2,166	**4,067**	**4.35**	101,030	**0**	**0.00**
Quirino	**38,258**	1,087	929	**2,016**	**5.27**	40,898	**0**	**0.00**
Cauayan City	**25,680**	195	168	**363**	**1.41**	29,075	**21**	**0.07**
Ilagan City	**28,865**	10,762	10,502	**21,264**	**73.67**	32,671	**0**	**0.00**
Santiago City	**26,736**	11,170	10,701	**21,871**	**81.80**	30,262	**694**	**2.29**
Tuguegarao City	**30,305**	472	478	**950**	**3.13**	34,437	**0**	**0.00**
**Region 3**	**2,330,062**	**684,726**	**576,785**	**1,261,511**	**54.14**	**2,754,870**	**41,943**	**1.52**
Aurora	**45,578**	17,102	16,713	**33,815**	**74.19**	47,031	**6**	**0.01**
Bataan	**137,149**	14,122	13,332	**27,454**	**20.02**	164,282	**6**	**0.00**
Bulacan	**472,290**	186,828	195,194	**382,022**	**80.89**	569,815	**30,011**	**5.27**
Nueva Ecija	**306,967**	28,329	27,429	**55,758**	**18.16**	355,856	**0**	**0.00**
Pampanga	**344,023**	105,352	105,359	**210,711**	**61.25**	416,314	**0**	**0.00**
Tarlac	**206,110**	117,936	13,012	**130,948**	**63.53**	234,068	**0**	**0.00**
Zambales	**121,213**	31,896	30,373	**62,269**	**51.37**	140,226	**202**	**0.14**
Angeles City	**86,316**	1,597	1,554	**3,151**	**3.65**	104,450	**37**	**0.04**
Balanga City	**19,820**	5,106	4,110	**9,216**	**46.50**	23,746	**0**	**0.00**
Cabanatuan City	**62,823**	20,389	18,374	**38,763**	**61.70**	72,836	**15**	**0.02**
City of San Fernando	**64,303**	26,310	24,161	**50,471**	**78.49**	77,813	**64**	**0.08**
Gapan City	**22,931**	787	708	**1,495**	**6.52**	26,582	**0**	**0.00**
Mabalacat City	**52,590**	37,324	35,117	**72,441**	**137.75**	63,640	**5,237**	**8.23**
Malolos City	**52,753**	13,984	13,244	**27,228**	**51.61**	63,644	**0**	**0.00**
Meycauayan City	**43,757**	1,746	2,100	**3,846**	**8.79**	52,789	**143**	**0.27**
Olongapo City	**47,810**	4,310	4,313	**8,623**	**18.04**	55,308	**3,604**	**6.52**
Palayan City	**8,532**	1,962	1,989	**3,951**	**46.31**	9,891	**2,596**	**26.25**
San Jose City	**29,049**	8,801	8,179	**16,980**	**58.45**	33,678	**0**	**0.00**
San Jose del Monte City	**120,139**	17,966	17,094	**35,060**	**29.18**	144,945	**0**	**0.00**
Science City of Munoz	**16,939**	980	971	**1,951**	**11.52**	19,634	**0**	**0.00**
Tarlac City	**68,970**	41,899	43,459	**85,358**	**123.76**	78,322	**22**	**0.03**
**Region 4A**	**3,088,212**	**431,743**	**425,059**	**856,802**	**27.74**	**3,716,041**	**13,644**	**0.37**
Batangas	**406,963**	124,011	121,098	**245,109**	**60.23**	480,114	**3,105**	**0.65**
Cavite	**299,232**	55,968	54,197	**110,165**	**36.82**	377,839	**5**	**0.00**
Laguna	**201,518**	25,610	24,641	**50,251**	**24.94**	261,618	**419**	**0.16**
Quezon	**380,590**	60,761	59,799	**120,560**	**31.68**	382,787	**535**	**0.14**

Table 2.E.3.2. Soil Transmitted Helminthiasis Prevention and Control

No. and Proportion of 10-19 and Women 20-49 years old who completed 2 doses of deworming tablets
Philippines, 2022

Area	Elig. Pop.	No. of Adolecents (10-19 yrs. Old) who completed 2 doses of deworming tablets				Elig. Pop.	No of WRA (20 - 49 yrs. Old) who completed 2 doses of deworming tablets	%
		Male	Female	Total	%			
Rizal	**452,883**	15,454	15,272	**30,726**	**6.78**	543,904	**1,009**	**0.19**
Antipolo City	**166,808**	140	52	**192**	**0.12**	200,337	**0**	**0.00**
Bacoor City	**131,095**	804	786	**1,590**	**1.21**	165,540	**1**	**0.00**
Batangas City	**72,227**	1,588	1,624	**3,212**	**4.45**	85,209	**0**	**0.00**
Biñan City	**67,575**	35,049	36,491	**71,540**	**105.87**	87,727	**446**	**0.51**
Cabuyao City	**62,650**	7,441	7,152	**14,593**	**23.29**	81,328	**8**	**0.01**
Calamba City	**92,218**	10,332	10,692	**21,024**	**22.80**	119,721	**153**	**0.13**
Cavite City	**22,434**	988	1,101	**2,089**	**9.31**	28,334	**2**	**0.01**
Dasmariñas City	**143,849**	10,806	10,634	**21,440**	**14.90**	181,640	**4**	**0.00**
General Trias City	**68,606**	15,724	15,741	**31,465**	**45.86**	86,629	**81**	**0.09**
Imus City	**88,135**	7,200	7,794	**14,994**	**17.01**	111,286	**2,996**	**2.69**
Lipa City	**72,772**	0	0	**0**	**0.00**	85,860	**0**	**0.00**
Lucena City	**57,681**	8,521	8,826	**17,347**	**30.07**	58,012	**4,534**	**7.82**
San Pablo City	**53,987**	2,842	2,704	**5,546**	**10.27**	70,084	**0**	**0.00**
San Pedro City	**66,110**	848	849	**1,697**	**2.57**	85,824	**0**	**0.00**
Santa Rosa City	**71,784**	16,911	15,871	**32,782**	**45.67**	93,190	**346**	**0.37**
Tagaytay City	**15,536**	7,064	7,169	**14,233**	**91.61**	19,620	**0**	**0.00**
Tanauan City	**37,952**	1,395	1,370	**2,765**	**7.29**	44,780	**0**	**0.00**
Tayabas City	**21,617**	15,927	15,424	**31,351**	**145.03**	21,742	**0**	**0.00**
Trece Martires City	**33,990**	6,359	5,772	**12,131**	**35.69**	42,916	**0**	**0.00**
**Region 4B**	**669,431**	**213,634**	**204,475**	**418,109**	**62.46**	**628,629**	**38,520**	**6.13**
Marinduque	**48,572**	17,354	16,810	**34,164**	**70.34**	45,047	**0**	**0.00**
Mindoro Occidental	**110,861**	45,866	45,217	**91,083**	**82.16**	98,846	**0**	**0.00**
Mindoro Oriental	**187,248**	73,075	69,628	**142,703**	**76.21**	180,072	**6,988**	**3.88**
Palawan	**200,456**	54,948	51,180	**106,128**	**52.94**	191,398	**4,160**	**2.17**
Romblon	**62,087**	7,765	7,466	**15,231**	**24.53**	55,781	**27,372**	**49.07**
Puerto Princesa City	**60,207**	14,626	14,174	**28,800**	**47.83**	57,485	**0**	**0.00**
**Region 5**	**1,291,879**	**379,035**	**359,842**	**738,877**	**57.19**	**1,199,805**	**21,891**	**1.82**
Albay	**241,395**	57,114	54,796	**111,910**	**46.36**	241,323	**1,329**	**0.55**
Camarines Norte	**128,624**	44,981	43,498	**88,479**	**68.79**	123,012	**4,101**	**3.33**
Camarines Sur	**367,442**	134,806	127,956	**262,762**	**71.51**	339,702	**5,401**	**1.59**
Catanduanes	**57,004**	20,404	19,082	**39,486**	**69.27**	51,849	**324**	**0.62**
Masbate	**207,060**	47,689	44,433	**92,122**	**44.49**	175,052	**0**	**0.00**
Sorsogon	**179,147**	44,114	41,617	**85,731**	**47.86**	162,866	**4,886**	**3.00**
Iriga City	**24,970**	9,965	9,663	**19,628**	**78.61**	23,082	**5,692**	**24.66**
Legaspi City	**42,448**	14,259	13,288	**27,547**	**64.90**	42,436	**158**	**0.37**
Naga City	**43,789**	5,703	5,509	**11,212**	**25.60**	40,483	**0**	**0.00**
**Region 6**	**1,496,586**	**335,090**	**324,573**	**659,663**	**44.08**	**1,644,018**	**27,029**	**1.64**
Aklan	**113,962**	42,934	41,655	**84,589**	**74.23**	127,468	**17,051**	**13.38**

Table 2.E.3.2. Soil Transmitted Helminthiasis Prevention and Control

No. and Proportion of 10-19 and Women 20-49 years old who completed 2 doses of deworming tablets
Philippines, 2022

Area	Elig. Pop.	No. of Adolecents (10-19 yrs. Old) who completed 2 doses of deworming tablets				Elig. Pop.	No of WRA (20 - 49 yrs. Old) who completed 2 doses of deworming tablets	%
		Male	Female	Total	%			
Antique	**122,111**	40,944	39,731	**80,675**	**66.07**	119,111	**1,541**	**1.29**
Capiz	**147,929**	43,690	43,347	**87,037**	**58.84**	172,342	**2,921**	**1.69**
Guimaras	**34,038**	14,669	14,228	**28,897**	**84.90**	39,027	**382**	**0.98**
Iloilo	**379,498**	145,931	141,400	**287,331**	**75.71**	435,752	**1,949**	**0.45**
Negros Occidental	**498,985**	28,921	28,119	**57,040**	**11.43**	530,220	**1,501**	**0.28**
Bacolod City	**112,269**	18,001	16,093	**34,094**	**30.37**	119,295	**1,684**	**1.41**
Iloilo City	**87,794**	0	0	**0**	**0.00**	100,803	**0**	**0.00**
**Region 7**	**1,542,671**	**94,219**	**89,318**	**183,537**	**11.90**	**1,697,318**	**390**	**0.02**
Bohol	**271,202**	12,656	11,473	**24,129**	**8.90**	264,737	**0**	**0.00**
Cebu	**623,368**	42,836	41,642	**84,478**	**13.55**	714,987	**0**	**0.00**
Negros Oriental	**270,562**	5,824	2,679	**8,503**	**3.14**	285,584	**0**	**0.00**
Siquijor	**18,358**	10,075	10,443	**20,518**	**111.77**	20,045	**0**	**0.00**
Cebu City	**195,693**	3,935	4,065	**8,000**	**4.09**	224,450	**0**	**0.00**
Lapu-Lapu City	**86,564**	682	794	**1,476**	**1.71**	99,288	**390**	**0.39**
Mandaue City	**76,924**	18,211	18,222	**36,433**	**47.36**	88,227	**0**	**0.00**
**Region 8**	**980,557**	**74,376**	**74,302**	**148,678**	**15.16**	**407,913**	**4,477**	**1.10**
Biliran	**35,845**	8,952	9,249	**18,201**	**50.78**	15,289	**1,991**	**13.02**
Eastern Samar	**105,352**	1,643	1,690	**3,333**	**3.16**	43,133	**300**	**0.70**
Northern Leyte	**332,231**	12,409	12,364	**24,773**	**7.46**	140,302	**395**	**0.28**
Northern Samar	**145,644**	28,352	27,710	**56,062**	**38.49**	59,368	**1,266**	**2.13**
Southern Leyte	**133,960**	8,936	8,596	**17,532**	**13.09**	54,290	**68**	**0.13**
Western Samar	**68,278**	2,519	2,414	**4,933**	**7.22**	28,943	**0**	**0.00**
Calbayog City	**41,280**	8,612	8,909	**17,521**	**42.44**	16,733	**0**	**0.00**
Maasin City	**17,375**	1,910	2,171	**4,081**	**23.49**	7,364	**92**	**1.25**
Ormoc City	**47,315**	227	301	**528**	**1.12**	19,984	**296**	**1.48**
Tacloban City	**53,277**	816	898	**1,714**	**3.22**	22,507	**69**	**0.31**
**Region 9**	**771,448**	**381,594**	**394,541**	**776,135**	**100.61**	**783,502**	**7,229**	**0.92**
Zamboanga del Norte	**166,998**	109,290	118,944	**228,234**	**136.67**	165,395	**854**	**0.52**
Zamboanga del Sur	**168,775**	73,832	75,473	**149,305**	**88.46**	176,126	**15**	**0.01**
Zamboanga Sibugay	**143,298**	45,378	43,824	**89,202**	**62.25**	139,019	**365**	**0.26**
Dapitan City	**17,244**	16,598	17,389	**33,987**	**197.09**	17,074	**0**	**0.00**
Dipolog City	**27,362**	25,063	33,591	**58,654**	**214.36**	27,094	**36**	**0.13**
Isabela City	**27,173**	10,693	13,069	**23,762**	**87.45**	28,567	**1,030**	**3.61**
Pagadian City	**41,391**	40,661	27,584	**68,245**	**164.88**	43,199	**0**	**0.00**
Zamboanga City	**179,207**	60,079	64,667	**124,746**	**69.61**	187,028	**4,929**	**2.64**

Table 2.E.3.2. Soil Transmitted Helminthiasis Prevention and Control

No. and Proportion of 10-19 and Women 20-49 years old who completed 2 doses of deworming tablets
Philippines, 2022

Area	Elig. Pop.	No. of Adolecents (10-19 yrs. Old) who completed 2 doses of deworming tablets				Elig. Pop.	No of WRA (20 - 49 yrs. Old) who completed 2 doses of deworming tablets	%
		Male	Female	Total	%			
**Region 10**	**994,909**	**296,247**	**287,919**	**584,166**	**58.72**	**1,057,276**	**33,475**	**3.17**
Bukidnon	**223,834**	67,189	63,886	**131,075**	**58.56**	233,127	**242**	**0.10**
Camiguin	**17,941**	8,150	8,268	**16,418**	**91.51**	17,926	**35**	**0.20**
Lanao del Norte	**153,243**	18,598	19,705	**38,303**	**24.99**	154,278	**222**	**0.14**
Misamis Occidental	**65,668**	27,114	28,250	**55,364**	**84.31**	67,695	**8,111**	**11.98**
Misamis Oriental	**147,169**	41,459	40,440	**81,899**	**55.65**	167,488	**0**	**0.00**
Cagayan de Oro City	**139,390**	18,348	20,067	**38,415**	**27.56**	158,639	**0**	**0.00**
El Salvador City	**10,355**	6,147	4,098	**10,245**	**98.94**	11,781	**0**	**0.00**
Gingoog City	**25,703**	21,142	19,375	**40,517**	**157.64**	29,252	**0**	**0.00**
Iligan City	**77,623**	21,448	21,056	**42,504**	**54.76**	78,149	**22,373**	**28.63**
Malaybalay City	**37,310**	19,575	17,917	**37,492**	**100.49**	38,858	**232**	**0.60**
Oroquieta City	**14,233**	19,669	18,315	**37,984**	**266.87**	14,669	**0**	**0.00**
Ozamis City	**28,528**	14,697	14,382	**29,079**	**101.93**	29,405	**172**	**0.58**
Tangub City	**12,673**	7,271	7,006	**14,277**	**112.66**	13,063	**2,088**	**15.98**
Valencia City	**41,239**	5,440	5,154	**10,594**	**25.69**	42,946	**0**	**0.00**
**Region 11**	**1,051,315**	**105,530**	**98,607**	**204,137**	**19.42**	**1,128,101**	**17,628**	**1.56**
Davao de Oro	**151,192**	15,243	13,954	**29,197**	**19.31**	159,674	**1,858**	**1.16**
Davao del Norte	**203,223**	17,018	16,313	**33,331**	**16.40**	223,241	**4,698**	**2.10**
Davao Oriental	**125,328**	3,792	3,431	**7,223**	**5.76**	116,705	**2,155**	**1.85**
Davao del Sur	**140,515**	8,467	7,946	**16,413**	**11.68**	155,762	**820**	**0.53**
Davao Occidental	**68,338**	12,583	12,382	**24,965**	**36.53**	70,631	**501**	**0.71**
Davao City	**362,719**	48,427	44,581	**93,008**	**25.64**	402,088	**7,596**	**1.89**
**Region 12**	**942,855**	**190,680**	**187,462**	**378,142**	**40.11**	**988,637**	**111,922**	**11.32**
North Cotabato	**319,178**	46,264	45,660	**91,924**	**28.80**	321,590	**0**	**0.00**
Sarangani	**124,927**	30,328	28,838	**59,166**	**47.36**	120,465	**2,514**	**2.09**
South Cotabato	**197,535**	26,483	25,746	**52,229**	**26.44**	219,691	**2,542**	**1.16**
Sultan Kudarat	**172,914**	60,280	60,705	**120,985**	**69.97**	184,212	**106,574**	**57.85**
Gen. Santos City	**128,301**	27,325	26,513	**53,838**	**41.96**	142,679	**292**	**0.20**
**BARMM**	**1,020,727**	**86,990**	**110,932**	**197,922**	**19.39**	**973,312**	**277**	**0.03**
Basilan	**70,689**	2,007	2,503	**4,510**	**6.38**	63,785	**0**	**0.00**
Lanao del Sur	**209,923**	0	0	**0**	**0.00**	204,025	**0**	**0.00**
Maguindanao	**305,831**	6,464	5,882	**12,346**	**4.04**	282,420	**187**	**0.07**
Sulu	**207,875**	34,824	40,404	**75,228**	**36.19**	199,204	**0**	**0.00**
Tawi-Tawi	**92,418**	12,060	18,708	**30,768**	**33.29**	87,256	**0**	**0.00**
Cotabato City	**64,351**	16,624	16,956	**33,580**	**52.18**	70,262	**90**	**0.13**
Lamitan City	**19,449**	16	15	**31**	**0.16**	17,552	**0**	**0.00**
Marawi City	**50,191**	14,995	26,464	**41,459**	**82.60**	48,808	**0**	**0.00**

Table 2.E.3.2. Soil Transmitted Helminthiasis Prevention and Control

No. and Proportion of 10-19 and Women 20-49 years old who completed 2 doses of deworming tablets
Philippines, 2022

Area	Elig. Pop.	No. of Adolecents (10-19 yrs. Old) who completed 2 doses of deworming tablets				Elig. Pop.	No of WRA (20 - 49 yrs. Old) who completed 2 doses of deworming tablets	%
		Male	Female	Total	%			
**CARAGA**	**557,779**	**186,160**	**178,372**	**364,532**	**65.35**	**550,890**	**12,289**	**2.23**
Agusan del Norte	**75,499**	33,806	31,528	**65,334**	**86.54**	77,426	**1**	**0.00**
Agusan del Sur	**150,989**	33,320	31,737	**65,057**	**43.09**	145,433	**111**	**0.08**
Surigao del Norte	**25,630**	23,778	22,704	**46,482**	**181.36**	25,603	**2,223**	**8.68**
Surigao del Sur	**74,105**	47,188	45,051	**92,239**	**124.47**	73,367	**0**	**0.00**
Province of Dinagat	**105,260**	7,640	6,989	**14,629**	**13.90**	101,910	**30**	**0.03**
								**0.00**
Bislig City	**19,992**	2,341	2,115	**4,456**	**22.29**	19,357	**0**	**0.00**
Butuan City	**71,789**	26,149	26,786	**52,935**	**73.74**	73,624	**48**	**0.07**
Surigao City	**34,515**	11,938	11,462	**23,400**	**67.80**	34,170	**9,876**	**28.90**

Table 2.E.3.3. Soil Transmitted Helminthiasis Prevention and Control
No. and Proportion of Pregnant Women who completed 1 dose of deworming tablet
Philippines, 2022

Area	Elig. Pop	Pregnant Women who completed 1 dose of deworming tablet	
		No.	%
**PHILIPPINES**	**2,131,496**	**295,824**	**13.88**
**N C R**	**236,901**	**41,347**	**17.45**
Malabon City	**6,724**	5	**0.07**
Navotas City	**4,591**	45	**0.98**
Valenzuela City	**11,418**	44	**0.39**
Caloocan City	**29,146**	0	**0.00**
Marikina City	**8,294**	2,016	**24.31**
Pasig City	**13,894**	176	**1.27**
Pateros	**1,176**	181	**15.39**
Taguig City	**14,807**	2,371	**16.01**
Quezon City	**54,011**	35,034	**64.86**
Makati City	**10,718**	154	**1.44**
Mandaluyong City	**7,111**	0	**0.00**
San Juan City	**2,246**	94	**4.19**
Manila City	**32,743**	177	**0.54**
Las Piñas City	**10,832**	0	**0.00**
Muntinlupa City	**9,281**	0	**0.00**
Parañaque City	**12,249**	491	**4.01**
Pasay City	**7,660**	559	**7.30**
**C A R**	**35,179**	**8,206**	**23.33**
Abra	**4,275**	328	**7.67**
Apayao	**2,502**	1,337	**53.44**
Benguet	**9,086**	1,184	**13.03**
Ifugao	**4,486**	1,586	**35.35**
Kalinga	**4,701**	1,992	**42.37**
Mt. Province	**3,103**	100	**3.22**
Baguio City	**7,026**	1,679	**23.90**
**Region 1**	**97,099**	**12,452**	**12.82**
Ilocos Norte	**7,854**	1,534	**19.53**
Ilocos Sur	**9,072**	86	**0.95**
La Union	**11,338**	2,276	**20.07**
Pangasinan	**50,710**	6,347	**12.52**
Alaminos City	**1,910**	0	**0.00**
Candon City	**954**	267	**27.99**
Dagupan City	**3,657**	0	**0.00**
Laoag City	**1,812**	487	**26.88**
San Carlos City	**4,028**	253	**6.28**
San Fernando City	**2,074**	188	**9.06**
Urdaneta City	**2,836**	1,014	**35.75**
Vigan City	**854**	0	**0.00**
**Region 2**	**68,960**	**8,555**	**12.41**
Batanes	**362**	16	**4.42**
Cagayan	**20,431**	1,997	**9.77**
Isabela	**23,449**	2,327	**9.92**
Nueva Vizcaya	**9,538**	1,392	**14.59**
Quirino	**4,051**	22	**5.33**
Cauayan City	**2,565**	216	**8.42**
Ilagan City	**2,889**	597	**20.66**
Santiago City	**2,671**	1,296	**48.52**
Tuguegarao City	**3,004**	692	**23.04**
**Region 3**	**220,155**	**4,256**	**1.93**
Aurora	**4,754**	298	**6.27**
Bataan	**13,789**	30	**0.22**
Bulacan	**43,760**	189	**0.43**
Nueva Ecija	**29,039**	225	**0.77**
Pampanga	**31,620**	0	**0.00**
Tarlac	**19,319**	2,133	**11.04**

Table 2.E.3.3. Soil Transmitted Helminthiasis Prevention and Control

No. and Proportion of Pregnant Women who completed 1 dose of deworming tablet
Philippines, 2022

Area	Elig. Pop	Pregnant Women who completed 1 dose of deworming tablet	
		No.	%
Zambales	**12,476**	265	**2.12**
Angeles City	**7,932**	16	**0.20**
Balanga City	**1,992**	0	**0.00**
Cabanatuan City	**5,945**	0	**0.00**
City of San Fernando	**5,907**	138	**2.34**
Gapan City	**2,170**	0	**0.00**
Mabalacat City	**4,831**	0	**0.00**
Malolos City	**4,890**	0	**0.00**
Meycauayan City	**4,055**	23	**0.57**
Olongapo City	**4,922**	559	**11.36**
Palayan City	**808**	15	**1.86**
San Jose City	**2,748**	0	**0.00**
San Jose del Monte City	**11,133**	0	**0.00**
Science City of Munoz	**1,601**	344	**21.49**
Tarlac City	**6,464**	21	**0.32**
**Region 4A**	**299,627**	**14,970**	**5.00**
Batangas	**39,255**	668	**1.70**
Cavite	**28,151**	474	**1.68**
Laguna	**19,696**	1,692	**8.59**
Quezon	**37,853**	1,577	**4.17**
Rizal	**44,798**	2,635	**5.88**
Antipolo City	**16,498**	126	**0.76**
Bacoor City	**12,341**	132	**1.07**
Batangas City	**6,965**	0	**0.00**
Biñan City	**6,597**	5,403	**81.90**
Cabuyao City	**6,119**	90	**1.47**
Calamba City	**9,005**	509	**5.65**
Cavite City	**2,105**	0	**0.00**
Dasmariñas City	**13,538**	474	**3.50**
General Trias City	**6,457**	167	**2.59**
Imus City	**8,292**	48	**0.58**
Lipa City	**7,018**	9	**0.13**
Lucena City	**5,738**	70	**1.22**
San Pablo City	**5,272**	0	**0.00**
San Pedro City	**6,455**	129	**2.00**
Santa Rosa City	**7,009**	379	**5.41**
Tagaytay City	**1,460**	7	**0.48**
Tanauan City	**3,661**	84	**2.29**
Tayabas City	**2,145**	0	**0.00**
Trece Martires City	**3,199**	297	**9.28**
**Region 4B**	**72,791**	**6,561**	**9.01**
Marinduque	**4,990**	785	**15.73**
Mindoro Occidental	**12,479**	851	**6.82**
Mindoro Oriental	**20,189**	2,163	**10.71**
Palawan	**21,963**	2,610	**11.88**
Romblon	**6,569**	133	**2.02**
Puerto Princesa City	**6,601**	19	**0.29**
**Region 5**	**138,457**	**30,459**	**22.00**
Albay	**24,081**	3,874	**16.09**
Camarines Norte	**14,384**	5,829	**40.52**
Camarines Sur	**39,486**	7,596	**19.24**
Catanduanes	**6,459**	1,690	**26.17**
Masbate	**22,600**	5,709	**25.26**
Sorsogon	**19,828**	4,293	**21.65**
Iriga City	**2,679**	775	**28.93**
Legaspi City	**4,233**	86	**2.03**
Naga City	**4,707**	607	**12.90**
**Region 6**	**146,449**	**13,975**	**9.54**
Aklan	**11,288**	1,411	**12.50**
Antique	**13,132**	834	**6.35**

Table 2.E.3.3. Soil Transmitted Helminthiasis Prevention and Control

No. and Proportion of Pregnant Women who completed 1 dose of deworming tablet

Philippines, 2022

Area	Elig. Pop	Pregnant Women who completed 1 dose of deworming tablet	
		No.	%
Capiz	13,975	2,774	19.85
Guimaras	3,084	548	17.77
Iloilo	36,267	2,822	7.78
Negros Occidental	49,230	5,495	11.16
Bacolod City	11,082	41	0.37
Iloilo City	8,391	50	0.60
**Region 7**	**162,308**	**41,161**	**25.36**
Bohol	27,992	5,573	19.91
Cebu	66,463	13,495	20.30
Negros Oriental	27,890	2,769	9.93
Siquijor	1,661	452	27.21
Cebu City	20,866	11,462	54.93
Lapu-Lapu City	9,232	1,492	16.16
Mandaue City	8,204	5,918	72.14
**Region 8**	**105,471**	**13,077**	**12.40**
Biliran	3,873	2,249	58.07
Eastern Samar	11,908	2,546	21.38
Northern Leyte	35,422	759	2.14
Northern Samar	15,921	1,682	10.56
Southern Leyte	6,662	1,689	25.35
Western Samar	14,732	1,568	10.64
Calbayog City	4,535	371	8.18
Maasin City	1,696	336	19.81
Ormoc City	5,044	1,813	35.94
Tacloban City	5,678	64	1.13
**Region 9**	**79,007**	**11,427**	**14.46**
Zamboanga del Norte	16,854	1,502	8.91
Zamboanga del Sur	17,368	872	5.02
Zamboanga Sibugay	15,021	1,411	9.39
Dapitan City	1,737	760	43.75
Dipolog City	2,762	408	14.77
Isabela City	2,563	337	13.15
Pagadian City	4,257	26	0.61
Zamboanga City	18,445	6,111	33.13
**Region 10**	**99,908**	**14,723**	**14.74**
Bukidnon	22,900	6,028	26.32
Camiguin	1,854	109	5.88
Lanao del Norte	14,930	1,347	9.02
Misamis Occidental	6,420	557	8.68
Misamis Oriental	14,963	743	4.97
Cagayan de Oro City	14,172	1	0.01
El Salvador City	1,052	69	6.56
Gingoog City	2,608	50	1.92
Iligan City	7,565	3,745	49.50
Malaybalay City	3,817	166	4.35
Oroquieta City	1,389	0	0.00
Ozamis City	2,786	199	7.14
Tangub City	1,234	46	3.73
Valencia City	4,218	1,663	39.43
**Region 11**	**108,407**	**27,827**	**25.67**
Davao De Oro	15,490	5,014	32.37
Davao del Norte	21,017	7,606	36.19
Davao del Sur	13,229	1,106	8.36
Davao Occidental	14,564	4,119	28.28
Davao Oriental	6,510	4,226	64.92
Davao City	37,597	5,756	15.31

Table 2.E.3.3. Soil Transmitted Helminthiasis Prevention and Control
No. and Proportion of Pregnant Women who completed 1 dose of deworming tablet
Philippines, 2022

Area	Elig. Pop	Pregnant Women who completed 1 dose of deworming tablet	
		No.	%
**Region 12**	**98,859**	**34,057**	**34.45**
North Cotabato	**34,150**	4,617	**13.52**
Sarangani	**12,907**	9,697	**75.13**
South Cotabato	**20,917**	6,790	**32.46**
Sultan Kudarat	**17,299**	10,928	**63.17**
Gen. Santos City	**13,586**	2,025	**14.91**
**BARMM**	**101,343**	**9,815**	**9.68**
Basilan	**7,823**	491	**6.28**
Lanao del Sur	**21,639**	3,293	**15.22**
Maguindanao	**32,198**	1,460	**4.53**
Sulu	**17,165**	2,203	**12.83**
Tawi-Tawi	**9,369**	1,243	**13.27**
Cotabato City	**5,822**	882	**15.15**
Lamitan City	**2,154**	17	**0.79**
Marawi City	**5,173**	226	**4.37**
**CARAGA**	**60,575**	**2,956**	**4.88**
Agusan del Norte	**8,127**	10	**0.12**
Agusan del Sur	**17,709**	713	**4.03**
Surigao del Norte	**7,280**	122	**1.68**
Surigao del Sur	**11,553**	1,367	**11.83**
Province of Dinagat	**2,576**	108	**4.19**
Bislig City	**2,192**	66	**3.01**
Butuan City	**7,743**	26	**0.34**
Surigao City	**3,395**	544	**16.02**

Table 2.E.4.1. HIV-AIDS/STI Prevention and Control Services

Proportion of Pregnant Women Screened for HIV, Screened and Positive for Syphilis
Philippines, 2022

Area	Eligible Pop.	Pregnant Women					
		Screened for Syphilis	%	Positive for Sphilis	%	Screened for HIV	%
**PHILIPPINES**	**2,131,496**	**570,716**	**26.78**	**6,845**	**1.20**	**439,888**	**20.64**
**NCR**	**236,901**	**122,620**	**51.76**	**905**	**0.74**	**123,139**	**51.98**
Malabon City	6,724	2,666	39.65	16	0.60	2,955	43.95
Navotas City	4,591	3,214	70.01	117	3.64	3,073	66.94
Valenzuela City	11,418	5,917	51.82	116	1.96	6,703	58.71
Caloocan City	29,146	10,757	36.91	29	0.27	10,538	36.16
Marikina City	8,294	1,818	21.92	8	0.44	1,061	12.79
Pasig City	13,894	12,407	89.30	31	0.25	12,185	87.70
Pateros	1,176	874	74.32	2	0.23	543	46.17
Taguig City	14,807	9,103	61.48	100	1.10	9,321	62.95
Quezon City	54,011	31,674	58.64	68	0.21	31,759	58.80
Makati City	10,718	3,443	32.12	16	0.46	3,711	34.62
Mandaluyong City	7,111	6,680	93.94	5	0.07	6,633	93.28
San Juan City	2,246	957	42.61	1	0.10	956	42.56
Manila City	32,743	17,494	53.43	142	0.81	18,598	56.80
Las Piñas City	10,832	2,759	25.47	34	1.23	1,934	17.85
Muntinlupa City	9,281	2,751	29.64	34	1.24	3,269	35.22
Parañaque City	12,249	6,072	49.57	141	2.32	5,843	47.70
Pasay City	7,660	4,034	52.66	45	1.12	4,057	52.96
**CAR**	**35,179**	**16,942**	**48.16**	**57**	**0.34**	**9,884**	**28.10**
Abra	4,275	1,044	24.42	0	0.00	685	16.02
Apayao	2,502	1,599	63.91	7	0.44	277	11.07
Benguet	9,086	6,962	76.62	15	0.22	3,815	41.99
Ifugao	4,486	1,831	40.82	7	0.38	1,029	22.94
Kalinga	4,701	1,056	22.46	0	0.00	479	10.19
Mt. Province	3,103	1,160	37.38	0	0.00	422	13.60
				0			
Baguio City	7,026	3,290	46.83	28	0.85	3,177	45.22
**Region 1**	**97,099**	**31,289**	**32.22**	**107**	**0.34**	**18,511**	**19.06**
Ilocos Norte	7,854	3,440	43.80	10	0.29	1,837	23.39
Ilocos Sur	9,072	5,289	58.30	2	0.04	3,747	41.30
La Union	11,338	4,670	41.19	24	0.51	2,347	20.70
Pangasinan	50,710	11,217	22.12	54	0.48	4,770	9.41
Alaminos City	1,910	1,272	66.60	0	0.00	1,272	66.60
Candon City	954	469	49.16	1	0.21	176	18.45
Dagupan City	3,657	454	12.41	1	0.22	388	10.61
Laoag City	1,812	1,451	80.08	0	0.00	1,451	80.08
San Carlos City	4,028	553	13.73	4	0.72	268	6.65
San Fernando City	2,074	695	33.51	0	0.00	695	33.51
Urdaneta City	2,836	1,622	57.19	11	0.68	1,555	54.83
Vigan City	854	157	18.38	0	0.00	5	0.59
**Region 2**	**68,960**	**15,296**	**22.18**	**222**	**1.45**	**7,877**	**11.42**
Batanes	362	169	46.69	1	0.59	134	37.02
Cagayan	20,431	1,327	6.50	8	0.60	979	4.79
Isabela	23,449	6,923	29.52	49	0.71	2,776	11.84
Nueva Vizcaya	9,538	2,519	26.41	109	4.33	1,161	12.17

Table 2.E.4.1. HIV-AIDS/STI Prevention and Control Services

Proportion of Pregnant Women Screened for HIV, Screened and Positive for Syphilis

Philippines, 2022

Area	Eligible Pop.	Pregnant Women					
		Screened for Syphilis	%	Positive for Sphilis	%	Screened for HIV	%
Quirino	**4,051**	1,525	**37.65**	32	**2.10**	1,477	**36.46**
Cauayan City	**2,565**	738	**28.77**	0	**0.00**	452	**17.62**
Ilagan City	**2,889**	1,361	**47.11**	2	**0.15**	154	**5.33**
Santiago City	**2,671**	503	**18.83**	2	**0.40**	699	**26.17**
Tuguegarao City	**3,004**	231	**7.69**	19	**8.23**	45	**1.50**
**Region 3**	**220,155**	**79,556**	**36.14**	**576**	**0.72**	**67,675**	**30.74**
Aurora	**4,754**	1,879	**39.52**	0	**0.00**	1,975	**41.54**
Bataan	**13,789**	6,615	**47.97**	59	**0.89**	6,518	**47.27**
Bulacan	**43,760**	14,901	**34.05**	85	**0.57**	11,514	**26.31**
Nueva Ecija	**29,039**	5,060	**17.42**	104	**2.06**	3,809	**13.12**
Pampanga	**31,620**	10,939	**34.60**	186	**1.70**	9,533	**30.15**
Tarlac	**19,319**	5,486	**28.40**	23	**0.42**	3,727	**19.29**
Zambales	**12,476**	3,851	**30.87**	17	**0.44**	2,977	**23.86**
Angeles City	**7,932**	1,171	**14.76**	15	**1.28**	1,095	**13.80**
Balanga City	**1,992**	1,202	**60.34**	7	**0.58**	1,133	**56.88**
Cabanatuan City	**5,945**	1,425	**23.97**	0	**0.00**	1,345	**22.62**
City of San Fernando	**5,907**	2,196	**37.18**	4	**0.18**	2,191	**37.09**
Gapan City	**2,170**	396	**18.25**	1	**0.25**	387	**17.83**
Mabalacat City	**4,831**	3,575	**74.00**	22	**0.62**	2,733	**56.57**
Malolos City	**4,890**	1,450	**29.65**	4	**0.28**	1,235	**25.26**
Meycauayan City	**4,055**	1,761	**43.43**	26	**1.48**	1,707	**42.10**
Olongapo City	**4,922**	1,252	**25.44**	6	**0.48**	1,111	**22.57**
Palayan City	**808**	113	**13.99**	0	**0.00**	98	**12.13**
San Jose City	**2,748**	469	**17.07**	2	**0.43**	486	**17.69**
San Jose del Monte City	**11,133**	12,361	**111.03**	8	**0.06**	12,370	**111.11**
Science City of Munoz	**1,601**	663	**41.41**	0	**0.00**	652	**40.72**
Tarlac City	**6,464**	2,791	**43.18**	7	**0.25**	1,079	**16.69**
**Region 4A**	**299,627**	**69,698**	**23.26**	**1,603**	**2.30**	**60,479**	**20.18**
Batangas	**39,255**	3,916	**9.98**	64	**1.63**	3,575	**9.11**
Cavite	**28,151**	7,067	**25.10**	183	**2.59**	5,264	**18.70**
Laguna	**19,696**	5,837	**29.64**	144	**2.47**	4,871	**24.73**
Quezon	**37,853**	6,741	**17.81**	158	**2.34**	5,739	**15.16**
Rizal	**44,798**	13,211	**29.49**	155	**1.17**	13,906	**31.04**
Antipolo City	**16,498**	2,114	**12.81**	300	**14.19**	3,102	**18.80**
Bacoor City	**12,341**	1,499	**12.15**	22	**1.47**	1,652	**13.39**
Batangas City	**6,965**	1,398	**20.07**	7	**0.50**	223	**3.20**
Biñan City	**6,597**	6,647	**100.76**	62	**0.93**	6,468	**98.04**
Cabuyao City	**6,119**	1,967	**32.15**	9	**0.46**	914	**14.94**
Calamba City	**9,005**	3,204	**35.58**	108	**3.37**	1,011	**11.23**
Cavite City	**2,105**	403	**19.14**	7	**1.74**	416	**19.76**
Dasmariñas City	**13,538**	3,453	**25.51**	41	**1.19**	3,345	**24.71**
General Trias City	**6,457**	641	**9.93**	14	**2.18**	589	**9.12**
Imus City	**8,292**	577	**6.96**	20	**3.47**	730	**8.80**
Lipa City	**7,018**	1,829	**26.06**	89	**4.87**	1,427	**20.33**
Lucena City	**5,738**	1,713	**29.85**	33	**1.93**	905	**15.77**
San Pablo City	**5,272**	218	**4.14**	6	**2.75**	419	**7.95**
San Pedro City	**6,455**	1,200	**18.59**	25	**2.08**	900	**13.94**
Santa Rosa City	**7,009**	2,656	**37.89**	137	**5.16**	2,967	**42.33**
Tagaytay City	**1,460**	1,226	**83.97**	0	**0.00**	1,203	**82.40**
Tanauan City	**3,661**	1,316	**35.95**	9	**0.68**	501	**13.68**
Tayabas City	**2,145**	131	**6.11**	2	**1.53**	152	**7.09**

Table 2.E.4.1. HIV-AIDS/STI Prevention and Control Services

Proportion of Pregnant Women Screened for HIV, Screened and Positive for Syphilis
Philippines, 2022

Area	Eligible Pop.	Pregnant Women					
		Screened for Syphilis	%	Positive for Sphilis	%	Screened for HIV	%
Trece Martires City	**3,199**	734	**22.94**	8	**1.09**	200	**6.25**
**Region 4B**	**72,791**	**12,326**	**16.93**	**238**	**1.93**	**9,305**	**12.78**
Marinduque	**4,990**	775	**15.53**	14	**1.81**	618	**12.38**
Mindoro Occidental	**12,479**	1,889	**15.14**	11	**0.58**	313	**2.51**
Mindoro Oriental	**20,189**	4,496	**22.27**	55	**1.22**	3,451	**17.09**
Palawan	**21,963**	3,086	**14.05**	76	**2.46**	3,061	**13.94**
Romblon	**6,569**	1,711	**26.05**	62	**3.62**	1,036	**15.77**
Puerto Princesa City	**6,601**	369	**5.59**	20	**5.42**	826	**12.51**
**Region 5**	**138,457**	**21,775**	**15.73**	**300**	**1.38**	**14,578**	**10.53**
Albay	**24,081**	5,287	**21.96**	124	**2.35**	3,910	**16.24**
Camarines Norte	**14,384**	2,359	**16.40**	52	**2.20**	1,006	**6.99**
Camarines Sur	**39,486**	2,560	**6.48**	5	**0.20**	2,188	**5.54**
Catanduanes	**6,459**	2,228	**34.49**	6	**0.27**	968	**14.99**
Masbate	**22,600**	4,382	**19.39**	76	**1.73**	2,102	**9.30**
Sorsogon	**19,828**	2,133	**10.76**	23	**1.08**	948	**4.78**
Iriga City	**2,679**	525	**19.60**	3	**0.57**	656	**24.49**
Legaspi City	**4,233**	1,051	**24.83**	2	**0.19**	1,327	**31.35**
Naga City	**4,707**	1,250	**26.56**	9	**0.72**	1,473	**31.29**
**Region 6**	**146,449**	**44,448**	**30.35**	**636**	**1.43**	**22,635**	**15.46**
Aklan	**11,288**	3,799	**33.66**	49	**1.29**	2,207	**19.55**
Antique	**13,132**	3,266	**24.87**	57	**1.75**	1,818	**13.84**
Capiz	**13,975**	3,017	**21.59**	38	**1.26**	1,040	**7.44**
Guimaras	**3,084**	2,010	**65.18**	4	**0.20**	1,050	**34.05**
Iloilo	**36,267**	15,517	**42.79**	228	**1.47**	5,345	**14.74**
Negros Occidental	**49,230**	8,364	**16.99**	155	**1.85**	5,166	**10.49**
Bacolod City	**11,082**	5,214	**47.05**	87	**1.67**	3,230	**29.15**
Iloilo City	**8,391**	3,261	**38.86**	18	**0.55**	2,779	**33.12**
**Region 7**	**162,308**	**57,756**	**35.58**	**743**	**1.29**	**46,519**	**28.66**
Bohol	**27,992**	7,987	**28.53**	76	**0.95**	5,534	**19.77**
Cebu	**66,463**	14,162	**21.31**	442	**3.12**	8,837	**13.30**
Negros Oriental	**27,890**	3,483	**12.49**	9	**0.26**	1,046	**3.75**
Siquijor	**1,661**	827	**49.79**	5	**0.60**	809	**48.71**
Cebu City	**20,866**	10,399	**49.84**	200	**1.92**	10,665	**51.11**
Lapu-Lapu City	**9,232**	4,456	**48.27**	0	**0.00**	5,409	**58.59**
Mandaue City	**8,204**	16,442	**200.41**	11	**0.07**	14,219	**173.32**
**Region 8**	**105,471**	**14,934**	**14.16**	**304**	**2.04**	**13,577**	**12.87**
Biliran	**3,873**	1,402	**36.20**	5	**0.36**	1,484	**38.32**
Eastern Samar	**11,908**	1,585	**13.31**	27	**1.70**	1,462	**12.28**
Northern Leyte	**35,422**	1,588	**4.48**	190	**11.96**	1,274	**3.60**
Northern Samar	**15,921**	4,579	**28.76**	40	**0.87**	4,397	**27.62**
Southern Leyte	**6,662**	453	**6.80**	0	**0.00**	819	**12.29**
Western Samar	**14,732**	607	**4.12**	32	**5.27**	740	**5.02**
Calbayog City	**4,535**	272	**6.00**	0	**0.00**	24	**0.53**

Table 2.E.4.1. HIV-AIDS/STI Prevention and Control Services

Proportion of Pregnant Women Screened for HIV, Screened and Positive for Syphilis
Philippines, 2022

Area	Eligible Pop.	Pregnant Women					
		Screened for Syphilis	%	Positive for Sphilis	%	Screened for HIV	%
Maasin City	**1,696**	3	**0.18**	0	**0.00**	0	**0.00**
Ormoc City	**5,044**	1,259	**24.96**	5	**0.40**	1,412	**27.99**
Tacloban City	**5,678**	3,186	**56.11**	5	**0.16**	1,965	**34.61**
**Region 9**	**79,007**	**5,234**	**6.62**	**25**	**0.48**	**2,008**	**2.54**
Zamboanga del Norte	**16,854**	247	**1.47**	0	**0.00**	235	**1.39**
Zamboanga del Sur	**17,368**	553	**3.18**	4	**0.72**	194	**1.12**
Zamboanga Sibugay	**15,021**	3,120	**20.77**	11	**0.35**	257	**1.71**
Dapitan City	**1,737**	235	**13.53**	0	**0.00**	342	**19.69**
Dipolog City	**2,762**	11	**0.40**	0	**0.00**	9	**0.33**
Isabela City	**2,563**	304	**11.86**	1	**0.33**	239	**9.33**
Pagadian City	**4,257**	117	**2.75**	1	**0.85**	71	**1.67**
Zamboanga City	**18,445**	647	**3.51**	8	**1.24**	661	**3.58**
**Region 10**	**99,908**	**14,936**	**14.95**	**214**	**1.43**	**8,902**	**8.91**
Bukidnon	**22,900**	2,226	**9.72**	48	**2.16**	1,658	**7.24**
Camiguin	**1,854**	438	**23.62**	1	**0.23**	354	**19.09**
Lanao del Norte	**14,930**	188	**1.26**	1	**0.53**	216	**1.45**
Misamis Occidental	**6,420**	0	**0.00**	0	**0.00**	121	**1.88**
Misamis Oriental	**14,963**	2,400	**16.04**	3	**0.13**	1,522	**10.17**
Cagayan de Oro City	**14,172**	5,796	**40.90**	34	**0.59**	1,522	**10.74**
El Salvador City	**1,052**	380	**36.12**	0	**0.00**	339	**32.22**
Gingoog City	**2,608**	241	**9.24**	12	**4.98**	847	**32.48**
Iligan City	**7,565**	950	**12.56**	70	**7.37**	696	**9.20**
Malaybalay City	**3,817**	919	**24.08**	35	**3.81**	189	**4.95**
Oroquieta City	**1,389**	10	**0.72**	0	**0.00**	63	**4.54**
Ozamis City	**2,786**	1,140	**40.92**	9	**0.79**	1,151	**41.31**
Tangub City	**1,234**	17	**1.38**	0	**0.00**	0	**0.00**
Valencia City	**4,218**	231	**5.48**	1	**0.43**	224	**5.31**
**Region 11**	**108,407**	**27,073**	**24.97**	**106**	**0.39**	**13,706**	**12.64**
Davao de Oro	**15,490**	329	**2.12**	4	**1.22**	121	**0.78**
Davao del Norte	**21,017**	6,742	**32.08**	37	**0.55**	2,339	**11.13**
Davao Oriental	**13,229**	1,288	**9.74**	7	**0.54**	1,322	**9.99**
Davao del Sur	**14,564**	198	**1.36**	6	**3.03**	504	**3.46**
Davao Occidental	**6,510**	364	**5.59**	1	**0.27**	252	**3.87**
Davao City	**37,597**	18,152	**48.28**	51	**0.28**	9,168	**24.38**
**Region 12**	**98,859**	**25,617**	**25.91**	**669**	**2.61**	**12,239**	**12.38**
North Cotabato	**34,150**	2,986	**8.74**	164	**5.49**	1,346	**3.94**
Sarangani	**12,907**	3,577	**27.71**	264	**7.38**	1,165	**9.03**
South Cotabato	**20,917**	7,782	**37.20**	48	**0.62**	6,901	**32.99**
Sultan Kudarat	**17,299**	7,690	**44.45**	110	**1.43**	721	**4.17**
Gen. Santos City	**13,586**	3,582	**26.37**	83	**2.32**	2,106	**15.50**
**BARMM**	**101,343**	**519**	**0.51**	**22**	**4.24**	**374**	**0.37**
Basilan	**7,823**	0	**0.00**	0	**0.00**	0	**0.00**
Lanao del Sur	**21,639**	67	**0.31**	10	**14.93**	0	**0.00**
Maguindanao	**32,198**	28	**0.09**	0	**0.00**	0	**0.00**

Table 2.E.4.1. HIV-AIDS/STI Prevention and Control Services

Proportion of Pregnant Women Screened for HIV, Screened and Positive for Syphilis
Philippines, 2022

Area	Eligible Pop.	Pregnant Women					
		Screened for Syphilis	%	Positive for Sphilis	%	Screened for HIV	%
Sulu	**17,165**	73	**0.43**	4	**5.48**	0	**0.00**
Tawi-Tawi	**9,369**	0	**0.00**	0	**0.00**	77	**0.82**
Cotabato City	**5,822**	337	**5.79**	8	**2.37**	296	**5.08**
Lamitan City	**2,154**	14	**0.65**	0	**0.00**	1	**0.05**
Marawi City	**5,173**	0	**0.00**	0	**0.00**	0	**0.00**
**CARAGA**	**60,575**	**10,697**	**17.66**	**118**	**1.10**	**8,480**	**14.00**
Agusan del Norte	**8,127**	1,151	**14.16**	2	**0.17**	824	**10.14**
Agusan del Sur	**17,709**	2,632	**14.86**	52	**1.98**	1,463	**8.26**
Surigao del Norte	**7,280**	608	**8.35**	10	**1.64**	400	**5.49**
Surigao del Sur	**11,553**	685	**5.93**	20	**2.92**	349	**3.02**
Province of Dinagat	**2,576**	847	**32.88**	27	**3.19**	816	**31.68**
Bislig City	**2,192**	55	**2.51**	0	**0.00**	34	**1.55**
Butuan City	**7,743**	3,404	**43.96**	2	**0.06**	3,187	**41.16**
Surigao City	**3,395**	1,315	**38.73**	5	**0.38**	1,407	**41.44**

Legend:
0 = No Case
** = No Report*

Table 2.E.5.1. Leprosy

Prevalence and Detection Rate
Philippines, 2022

Area	Population	Leprosy Cases undergoing Treatment				Total Newly Detected Case			
		Male	Female	Total	Prev. Rate	Male	Female	Total	Case Detection Rate
**PHILIPPINES**	**111,572,254**	**1,100**	**608**	**1,708**	**0.15**	**329**	**173**	**502**	**0.45**
**N C R**	**14,118,673**	**5**	**0**	**5**	**0.004**	**2**	**0**	**2**	**0.014**
Malabon City	**400,763**	0	0	**0**	**0.00**	0	0	**0**	**0.00**
Navotas City	**273,513**	0	0	**0**	**0.00**	0	0	**0**	**0.00**
Valenzuela City	**680,233**	0	0	**0**	**0.00**	0	0	**0**	**0.00**
Caloocan City	**1,736,680**	0	0	**0**	**0.00**	0	0	**0**	**0.00**
Marikina City	**494,193**	1	0	**1**	**0.02**	0	0	**0**	**0.00**
Pasig City	**828,114**	0	0	**0**	**0.00**	0	0	**0**	**0.00**
Pateros	**69,994**	0	0	**0**	**0.00**	0	0	**0**	**0.00**
Taguig City	**882,512**	4	0	**4**	**0.05**	2	0	**2**	**0.23**
Quezon City	**3,219,173**	0	0	**0**	**0.00**	0	0	**0**	**0.00**
Makati City	**638,764**	0	0	**0**	**0.00**	0	0	**0**	**0.00**
Mandaluyong City	**423,514**	0	0	**0**	**0.00**	0	0	**0**	**0.00**
San Juan City	**133,959**	0	0	**0**	**0.00**	0	0	**0**	**0.00**
Manila City	**1,951,758**	0	0	**0**	**0.00**	0	0	**0**	**0.00**
Las Piñas City	**645,666**	0	0	**0**	**0.00**	0	0	**0**	**0.00**
Muntinlupa City	**553,146**	0	0	**0**	**0.00**	0	0	**0**	**0.00**
Parañaque City	**730,009**	0	0	**0**	**0.00**	0	0	**0**	**0.00**
Pasay City	**456,682**	0	0	**0**	**0.00**	0	0	**0**	**0.00**
**C A R**	**1,836,857**	**28**	**24**	**52**	**0.28**	**7**	**5**	**12**	**0.65**
Abra	**249,467**	10	12	**22**	**0.88**	2	3	**5**	**2.00**
Apayao	**125,511**	0	0	**0**	**0.00**	0	0	**0**	**0.00**
Benguet	**489,214**	4	4	**8**	**0.16**	0	0	**0**	**0.00**
Ifugao	**213,380**	6	0	**6**	**0.28**	1	0	**1**	**0.47**
Kalinga	**222,826**	0	0	**0**	**0.00**	0	0	**0**	**0.00**
Mt. Province	**157,820**	0	1	**1**	**0.06**	0	0	**0**	**0.00**
Baguio City	**378,639**	8	7	**15**	**0.40**	4	2	**6**	**1.58**
**Region 1**	**5,353,139**	**70**	**38**	**108**	**0.20**	**11**	**9**	**20**	**0.37**
Ilocos Norte	**503,876**	5	5	**10**	**0.20**	2	4	**6**	**1.19**
Ilocos Sur	**604,123**	11	2	**13**	**0.22**	0	0	**0**	**0.00**
La Union	**711,657**	6	2	**8**	**0.11**	2	0	**2**	**0.28**
Pangasinan	**2,542,805**	37	24	**61**	**0.24**	7	5	**12**	**0.47**
Alaminos City	**96,076**	2	1	**3**	**0.31**	0	0	**0**	**0.00**
Candon City	**63,678**	4	0	**4**	**0.63**	0	0	**0**	**0.00**
Dagupan City	**183,431**	0	0	**0**	**0.00**	0	0	**0**	**0.00**
Laoag City	**116,176**	1	0	**1**	**0.09**	0	0	**0**	**0.00**
San Carlos City	**201,955**	2	0	**2**	**0.10**	0	0	**0**	**0.00**
San Fernando City	**130,390**	1	4	**5**	**0.38**	0	0	**0**	**0.00**
Urdaneta City	**142,378**	0	0	**0**	**0.00**	0	0	**0**	**0.00**
Vigan City	**56,594**	1	0	**1**	**0.18**	0	0	**0**	**0.00**
**Region 2**	**3,727,548**	**36**	**30**	**66**	**0.18**	**10**	**12**	**22**	**0.59**
Batanes	**18,145**	0	0	**0**	**0.00**	0	0	**0**	**0.00**
Cagayan	**1,132,144**	5	2	**7**	**0.06**	0	2	**2**	**0.18**
Isabela	**1,275,221**	16	18	**34**	**0.27**	5	6	**11**	**0.86**
Nueva Vizcaya	**493,293**	6	4	**10**	**0.20**	2	2	**4**	**0.81**
Quirino	**200,940**	0	1	**1**	**0.05**	1	1	**2**	**1.00**
Cauayan City	**139,546**	5	0	**5**	**0.36**	1	0	**1**	**0.72**
Ilagan City	**156,829**	0	0	**0**	**0.00**	0	0	**0**	**0.00**

Table 2.E.5.1. Leprosy
Prevalence and Detection Rate
Philippines, 2022

Area	Population	Leprosy Cases undergoing Treatment				Total Newly Detected Case			
		Male	Female	Total	Prev. Rate	Male	Female	Total	Case Detection Rate
Santiago City	145,258	4	5	9	0.62	1	1	2	1.38
Tuguegarao City	166,172	0	0	0	0.00	0	0	0	0.00
**Region 3**	**12,706,460**	**84**	**86**	**170**	**0.13**	**11**	**10**	**21**	**0.17**
Aurora	232,022	0	0	0	0.00	0	0	0	0.00
Bataan	753,343	0	0	0	0.00	0	0	0	0.00
Bulacan	2,582,769	9	0	9	0.03	0	0	0	0.00
Nueva Ecija	1,677,367	9	3	12	0.07	2	1	3	0.18
Pampanga	1,879,959	15	5	20	0.11	6	1	7	0.37
Tarlac	1,117,955	0	35	35	0.31	0	6	6	0.54
Zambales	658,590	1	0	1	0.02	1	0	1	0.15
						0	0		
Angeles City	471,675	7	3	10	0.21	1	0	1	0.21
Balanga City	108,890	0	0	0	0.00	0	0	0	0.00
Cabanatuan City	343,309	7	9	16	0.47	0	1	1	0.29
City of San Fernando	351,388	0	0	0	0.00	0	0	0	0.00
Gapan City	125,295	0	0	0	0.00	0	0	0	0.00
Mabalacat City	287,381	0	0	0	0.00	0	0	0	0.00
Malolos City	288,480	0	0	0	0.00	0	0	0	0.00
Meycauayan City	239,282	0	0	0	0.00	0	0	0	0.00
Olongapo City	259,761	0	0	0	0.00	0	0	0	0.00
Palayan City	46,619	0	0	0	0.00	0	0	0	0.00
San Jose City	158,730	7	0	7	0.44	1	0	1	0.63
San Jose del Monte City	657,003	0	0	0	0.00	0	0	0	0.00
Science City of Munoz	92,559	2	2	4	0.43	0	0	0	0.00
Tarlac City	374,083	27	29	56	1.50	0	1	1	0.27
**Region 4A**	**16,654,975**	**58**	**21**	**79**	**0.05**	**23**	**10**	**33**	**0.20**
Batangas	2,196,463	1	0	1	0.00	3	1	4	0.18
Cavite	1,638,293	16	12	28	0.17	4	0	4	0.24
Laguna	1,128,465	1	1	2	0.02	1	1	2	0.18
Quezon	1,908,946	22	3	25	0.13	6	6	12	0.63
Rizal	2,426,363	1	0	1	0.00	0	0	0	0.00
Antipolo City	893,708	0	0	0	0.00	0	0	0	0.00
Bacoor City	717,766	0	0	0	0.00	0	0	0	0.00
Batangas City	389,811	1	0	1	0.03	2	0	2	0.51
Biñan City	378,391	0	1	1	0.03	0	0	0	0.00
Cabuyao City	350,801	0	0	0	0.00	0	0	0	0.00
Calamba City	516,395	5	0	5	0.10	0	0	0	0.00
Cavite City	122,859	0	0	0	0.00	0	0	0	0.00
Dasmariñas City	787,570	3	0	3	0.04	3	0	3	0.38
General Trias City	375,612	1	2	3	0.08	0	0	0	0.00
Imus City	482,549	0	0	0	0.00	0	0	0	0.00
Lipa City	392,788	4	0	4	0.10	1	0	1	0.25
Lucena City	289,304	3	2	5	0.17	3	2	5	1.73
San Pablo City	302,307	0	0	0	0.00	0	0	0	0.00
San Pedro City	370,189	0	0	0	0.00	0	0	0	0.00
Santa Rosa City	401,955	0	0	0	0.00	0	0	0	0.00
Tagaytay City	85,067	0	0	0	0.00	0	0	0	0.00
Tanauan City	204,868	0	0	0	0.00	0	0	0	0.00
Tayabas City	108,418	0	0	0	0.00	0	0	0	0.00
Trece Martires City	186,087	0	0	0	0.00	0	0	0	0.00
**Region 4B**	**3,247,680**	**29**	**17**	**46**	**0.14**	**14**	**5**	**19**	**0.59**
Marinduque	243,827	3	0	3	0.12	3	1	4	1.64
Mindoro Occidental	524,583	3	0	3	0.06	0	1	1	0.19
Mindoro Oriental	920,817	14	7	21	0.23	5	1	6	0.65
Palawan	964,459	8	8	16	0.17	5	2	7	0.73
Romblon	304,342	0	2	2	0.07	0	0	0	0.00

Table 2.E.5.1. Leprosy
Prevalence and Detection Rate
Philippines, 2022

Area	Population	Leprosy Cases undergoing Treatment				Total Newly Detected Case			
		Male	Female	Total	Prev. Rate	Male	Female	Total	Case Detection Rate
Puerto Princesa City	289,652	1	0	1	0.03	1	0	1	0.35
**Region 5**	6,248,843	51	12	63	0.10	4	3	7	0.11
Albay	1,210,784	12	4	16	0.13	0	2	2	0.17
Camarines Norte	635,921	4	0	4	0.06	0	0	0	0.00
Camarines Sur	1,765,618	0	0	0	0.00	0	0	0	0.00
Catanduanes	277,033	0	0	0	0.00	0	0	0	0.00
Masbate	952,388	19	3	22	0.23	2	0	2	0.21
Sorsogon	863,803	16	5	21	0.24	2	1	3	0.35
Iriga City	119,968	0	0	0	0.00	0	0	0	0.00
Legaspi City	212,924	0	0	0	0.00	0	0	0	0.00
Naga City	210,404	0	0	0	0.00	0	0	0	0.00
**Region 6**	8,033,041	55	46	101	0.13	11	12	23	0.29
Aklan	616,000	1	2	3	0.05	0	1	1	0.16
Antique	622,828	5	2	7	0.11	3	2	5	0.80
Capiz	820,940	2	8	10	0.12	1	2	3	0.37
Guimaras	188,747	0	0	0	0.00	0	0	0	0.00
Iloilo	2,088,223	5	9	14	0.07	2	2	4	0.19
Negros Occidental	2,623,026	32	16	48	0.18	5	2	7	0.27
Bacolod City	590,174	10	9	19	0.32	0	3	3	0.51
Iloilo City	483,103	0	0	0	0.00	0	0	0	0.00
**Region 7**	8,147,078	32	2	34	0.04	12	6	18	0.22
Bohol	1,382,606	20	0	20	0.14	6	0	6	0.43
Cebu	3,323,807	0	0	0	0.00	0	0	0	0.00
Negros Oriental	1,423,606	10	1	11	0.08	0	0	0	0.00
Siquijor	101,951	0	0	0	0.00	0	0	0	0.00
Cebu City	1,043,417	1	0	1	0.01	1	1	2	0.19
Lapu-Lapu City	461,551	1	1	2	0.04	5	5	10	2.17
Mandaue City	410,140	0	0	0	0.00	0	0	0	0.00
**Region 8**	4,865,157	161	75	236	0.49	54	15	69	1.42
Biliran	182,002	2	1	3	0.16	1	2	3	1.65
Eastern Samar	519,219	5	6	11	0.21	2	0	2	0.39
Northern Leyte	1,682,729	28	10	38	0.23	17	2	19	1.13
Northern Samar	681,299	25	12	37	0.54	3	0	3	0.44
Southern Leyte	361,920	11	3	14	0.39	5	1	6	1.66
Western Samar	639,344	40	5	45	0.70	8	0	8	1.25
Calbayog City	197,014	12	22	34	1.73	2	2	4	2.03
Maasin City	92,110	19	2	21	2.28	5	0	5	5.43
Ormoc City	239,675	8	8	16	0.67	4	2	6	2.50
Tacloban City	269,845	11	6	17	0.63	7	6	13	4.82
**Region 9**	3,834,801	74	47	121	0.32	27	8	35	0.91
Zamboanga del Norte	836,122	17	11	28	0.33	11	2	13	1.55
Zamboanga del Sur	844,012	5	1	6	0.07	6	1	7	0.83
Zamboanga Sibugay	695,261	1	0	1	0.01	5	0	5	0.72
Dapitan City	86,329	1	2	3	0.35	1	2	3	3.48
Dipolog City	136,968	0	0	0	0.00	0	0	0	0.00
Isabela City	132,907	5	2	7	0.53	1	1	2	1.50

Table 2.E.5.1. Leprosy

Prevalence and Detection Rate
Philippines, 2022

Area	Population	Leprosy Cases undergoing Treatment				Total Newly Detected Case			
		Male	Female	Total	Prev. Rate	Male	Female	Total	Case Detection Rate
Pagadian City	207,002	24	14	38	1.84	0	0	0	0.00
Zamboanga City	896,200	21	17	38	0.42	3	2	5	0.56
**Region 10**	5,128,964	247	76	323	0.63	70	29	99	1.93
Bukidnon	1,141,852	41	20	61	0.53	11	5	16	1.40
Camiguin	93,769	1	0	1	0.11	0	2	2	2.13
Lanao del Norte	744,374	10	1	11	0.15	3	0	3	0.40
Misamis Occidental	344,680	0	0	0	0.00	0	0	0	0.00
Misamis Oriental	791,744	57	29	86	1.09	16	7	23	2.90
Cagayan de Oro City	749,911	28	8	36	0.48	7	0	7	0.93
El Salvador City	55,698	4	1	5	0.90	3	1	4	7.18
Gingoog City	138,285	16	0	16	1.16	2	0	2	1.45
Iligan City	377,050	32	5	37	0.98	15	9	24	6.37
Malaybalay City	190,337	9	1	10	0.53	4	1	5	2.63
Oroquieta City	74,688	5	2	7	0.94	0	1	1	1.34
Ozamis City	149,710	17	1	18	1.20	4	1	5	3.34
Tangub City	66,511	2	2	4	0.60	1	1	2	3.01
Valencia City	210,355	25	6	31	1.47	4	1	5	2.38
**Region 11**	5,451,448	63	81	144	0.26	15	10	25	0.46
Davao de Oro	787,004	3	3	6	0.08	2	0	2	0.25
Davao del Norte	1,069,992	13	20	33	0.31	3	4	7	0.65
Davao del Sur	614,795	14	32	46	0.75	2	2	4	0.65
Davao Oriental	735,194	8	9	17	0.23	1	1	2	0.27
Davao Occidental	346,602	15	14	29	0.84	3	3	6	1.73
Davao City	1,897,861	10	3	13	0.07	4	0	4	0.21
**Region 12**	4,735,406	64	43	107	0.23	36	23	59	1.25
North Cotabato	1,577,659	8	16	24	0.15	4	3	7	0.44
Sarangani	597,339	20	8	28	0.47	5	0	5	0.84
South Cotabato	1,024,887	22	10	32	0.31	6	14	20	1.95
Sultan Kudarat	869,896	5	6	11	0.13	12	4	16	1.84
Gen. Santos City	665,625	9	3	12	0.18	9	2	11	1.65
**BARMM**	4,671,758	10	4	14	0.03	14	16	30	0.64
Basilan	317,215	4	1	5	0.16	4	2	6	1.89
Lanao del Sur	960,666	0	2	2	0.02	0	2	2	0.21
Maguindanao	1,389,643	5	0	5	0.04	4	0	4	0.29
Sulu	952,737	0	0	0	0.00	4	9	13	1.36
Tawi-Tawi	421,522	0	0	0	0.00	0	0	0	0.00
Cotabato City	312,921	1	1	2	0.06	2	3	5	1.60
Lamitan City	87,277	0	0	0	0.00	0	0	0	0.00
Marawi City	229,777	0	0	0	0.00	0	0	0	0.00
**CARAGA**	2,810,426	33	6	39	0.14	8	0	8	0.28
Agusan del Norte	386,955	1	0	1	0.03	0	0	0	0.00
Agusan del Sur	746,771	4	1	5	0.07	8	0	8	1.07
Surigao del Norte	373,053	10	2	12	0.32	0	0	0	0.00
Surigao del Sur	529,552	0	2	2	0.04	0	0	0	0.00
Province of Dinagat	131,841	1	0	1	0.08	0	0	0	0.00
Bislig City	100,580	9	0	9	0.89	0	0	0	0.00
Butuan City	367,928	0	0	0	0.00	0	0	0	0.00
Surigao City	173,746	8	1	9	0.52	0	0	0	0.00

Table 2.E.6.1. Rabies

Number and Proportion of Deaths due to Rabies
Philippines, 2022

Area	Animal Bites			No. of Deaths due to rabies			Rate
	Male	Female	Total	Male	Female	Total	
**PHILIPPINES**	**505,467**	**544,815**	**1,050,282**	**196**	**88**	**284**	**0.03**
**N C R**	**100,808**	**118,122**	**218,930**	**8**	**3**	**11**	**0.01**
Malabon City	4,484	4,245	**8,729**	0	0	**0**	**0.00**
Navotas City	2,048	2,400	**4,448**	0	0	**0**	**0.00**
Valenzuela City	3,334	4,338	**7,672**	1	0	**1**	**0.01**
Caloocan City	5,731	6,171	**11,902**	1	0	**1**	**0.01**
Marikina City	4,427	5,009	**9,436**	0	0	**0**	**0.00**
Pasig City	12,419	15,092	**27,511**	1	1	**2**	**0.01**
Pateros	1,070	1,314	**2,384**	0	0	**0**	**0.00**
Taguig City	18,540	22,063	**40,603**	1	0	**1**	**0.00**
Quezon City	22,009	26,052	**48,061**	3	1	**4**	**0.01**
Makati City	1,900	2,440	**4,340**	0	0	**0**	**0.00**
Mandaluyong City	3,939	4,717	**8,656**	0	0	**0**	**0.00**
San Juan City	1,548	1,960	**3,508**	0	0	**0**	**0.00**
Manila City	4,075	4,732	**8,807**	0	1	**1**	**0.01**
Las Piñas City	3,243	3,592	**6,835**	0	0	**0**	**0.00**
Muntinlupa City	4,944	5,734	**10,678**	1	0	**1**	**0.01**
Parañaque City	4,564	5,287	**9,851**	0	0	**0**	**0.00**
Pasay City	2,533	2,976	**5,509**	0	0	**0**	**0.00**
**C A R**	**16,907**	**15,246**	**32,153**	**2**	**0**	**2**	**0.01**
Abra	1,761	1,667	**3,428**	0	0	**0**	**0.00**
Apayao	798	820	**1,618**	2	0	**2**	**0.12**
Benguet	3,688	3,273	**6,961**	0	0	**0**	**0.00**
Ifugao	1,431	1,244	**2,675**	0	0	**0**	**0.00**
Kalinga	1,384	1,267	**2,651**	0	0	**0**	**0.00**
Mt. Province	1,469	1,151	**2,620**	0	0	**0**	**0.00**
Baguio City	6,376	5,824	**12,200**	0	0	**0**	**0.00**
**Region 1**	**33,165**	**34,351**	**67,516**	**14**	**0**	**14**	**0.02**
Ilocos Norte	1,327	1,442	**2,769**	0	0	**0**	**0.00**
Ilocos Sur	3,877	3,878	**7,755**	6	0	**6**	**0.08**
La Union	9,888	9,369	**19,257**	1	0	**1**	**0.01**
Pangasinan	7,108	7,767	**14,875**	6	0	**6**	**0.04**
Alaminos City	206	242	**448**	1	0	**1**	**0.22**
Candon City	644	340	**984**	0	0	**0**	**0.00**
Dagupan City	3,417	3,841	**7,258**	0	0	**0**	**0.00**
Laoag City	431	479	**910**	0	0	**0**	**0.00**
San Carlos City	1,391	1,536	**2,927**	0	0	**0**	**0.00**
San Fernando City	2,524	2,588	**5,112**	0	0	**0**	**0.00**
Urdaneta City	920	1,068	**1,988**	0	0	**0**	**0.00**
Vigan City	1,432	1,801	**3,233**	0	0	**0**	**0.00**
**Region 2**	**32,805**	**32,773**	**65,578**	**10**	**1**	**11**	**0.02**
Batanes	5	5	**10**	0	0	**0**	**0.00**
Cagayan	3,037	2,945	**5,982**	0	1	**1**	**0.02**

Table 2.E.6.1. Rabies

Number and Proportion of Deaths due to Rabies
Philippines, 2022

Area	Animal Bites			No. of Deaths due to rabies			Rate
	Male	Female	Total	Male	Female	Total	
Isabela	16,952	17,640	**34,592**	8	0	**8**	**0.02**
Nueva Vizcaya	7,033	6,012	**13,045**	0	0	**0**	**0.00**
Quirino	1,556	1,649	**3,205**	1	0	**1**	**0.03**
Cauayan City	1,157	1,320	**2,477**	0	0	**0**	**0.00**
Ilagan City	1,539	1,558	**3,097**	1	0	**1**	**0.03**
Santiago City	1,526	1,644	**3,170**	0	0	**0**	**0.00**
Tuguegarao City	0	0	**0**	0	0	**0**	**0.00**
**Region 3**	**52,419**	**58,143**	**110,562**	**33**	**21**	**54**	**0.05**
Aurora	3,188	3,332	**6,520**	0	0	**0**	**0.00**
Bataan	3,581	3,960	**7,541**	1	1	**2**	**0.03**
Bulacan	9,882	10,444	**20,326**	4	1	**5**	**0.02**
Nueva Ecija	8,518	9,701	**18,219**	11	1	**12**	**0.07**
Pampanga	1,681	1,802	**3,483**	1	0	**1**	**0.03**
Tarlac	3,519	3,904	**7,423**	1	4	**5**	**0.07**
Zambales	3,026	3,455	**6,481**	5	5	**10**	**0.15**
Angeles City	1,000	1,060	**2,060**	6	6	**12**	**0.58**
Balanga City	640	738	**1,378**	0	0	**0**	**0.00**
Cabanatuan City	2,423	2,764	**5,187**	1	0	**1**	**0.02**
City of San Fernando	1,512	1,727	**3,239**	0	0	**0**	**0.00**
Gapan City	4	3	**7**	0	0	**0**	**0.00**
Mabalacat City	1	0	**1**	0	0	**0**	**0.00**
Malolos City	2	4	**6**	0	1	**1**	**16.67**
Meycauayan City	1,296	1,408	**2,704**	1	0	**1**	**0.04**
Olongapo City	861	887	**1,748**	0	0	**0**	**0.00**
Palayan City	40	28	**68**	0	0	**0**	**0.00**
San Jose City	2,046	2,388	**4,434**	0	0	**0**	**0.00**
San Jose del Monte City	3,673	4,063	**7,736**	2	2	**4**	**0.05**
Science City of Munoz	704	853	**1,557**	0	0	**0**	**0.00**
Tarlac City	4,822	5,622	**10,444**	0	0	**0**	**0.00**
**Region 4A**	**78,363**	**87,246**	**165,609**	**14**	**11**	**25**	**0.02**
Batangas	3,930	4,368	**8,298**	1	0	**1**	**0.01**
Cavite	10,380	11,540	**21,920**	0	2	**2**	**0.01**
Laguna	6,445	7,537	**13,982**	0	1	**1**	**0.01**
Quezon	4,112	5,016	**9,128**	4	3	**7**	**0.08**
Rizal	15,732	17,829	**33,561**	3	3	**6**	**0.02**
Antipolo City	14,238	15,302	**29,540**	0	0	**0**	**0.00**
Bacoor City	1,023	1,256	**2,279**	0	2	**2**	**0.09**
Batangas City	140	162	**302**	0	0	**0**	**0.00**
Biñan City	1,278	1,392	**2,670**	0	0	**0**	**0.00**
Cabuyao City	142	164	**306**	1	0	**1**	**0.33**
Calamba City	3,926	4,381	**8,307**	0	0	**0**	**0.00**
Cavite City	1,147	1,301	**2,448**	0	0	**0**	**0.00**
Dasmariñas City	244	291	**535**	2	0	**2**	**0.37**
General Trias City	749	763	**1,512**	0	0	**0**	**0.00**
Imus City	867	988	**1,855**	0	0	**0**	**0.00**
Lipa City	1,273	1,217	**2,490**	0	0	**0**	**0.00**
Lucena City	2,985	2,944	**5,929**	2	0	**2**	**0.03**
San Pablo City	975	1,005	**1,980**	0	0	**0**	**0.00**
San Pedro City	2,589	2,914	**5,503**	0	0	**0**	**0.00**

Table 2.E.6.1. Rabies

Number and Proportion of Deaths due to Rabies

Philippines, 2022

Area	Animal Bites			No. of Deaths due to rabies			Rate
	Male	Female	Total	Male	Female	Total	
Santa Rosa City	2,282	2,577	**4,859**	1	0	**1**	**0.02**
Tagaytay City	869	881	**1,750**	0	0	**0**	**0.00**
Tanauan City	2,074	2,289	**4,363**	0	0	**0**	**0.00**
Tayabas City	0	0	**0**	0	0	**0**	**0.00**
Trece Martires City	963	1,129	**2,092**	0	0	**0**	**0.00**
**Region 4B**	**12,133**	**12,579**	**24,712**	**8**	**9**	**17**	**0.07**
Marinduque	1,423	1,738	**3,161**	0	0	**0**	**0.00**
Mindoro Occidental	4,464	4,624	**9,088**	0	0	**0**	**0.00**
Mindoro Oriental	1,030	1,122	**2,152**	2	2	**4**	**0.19**
Palawan	3,081	2,964	**6,045**	6	7	**13**	**0.22**
Romblon	373	379	**752**	0	0	**0**	**0.00**
Puerto Princesa City	1,762	1,752	**3,514**	0	0	**0**	**0.00**
**Region 5**	**17,864**	**18,934**	**36,798**	**4**	**0**	**4**	**0.01**
Albay	2,425	2,558	**4,983**	1	0	**1**	**0.02**
Camarines Norte	416	469	**885**	0	0	**0**	**0.00**
Camarines Sur	5,439	5,733	**11,172**	0	0	**0**	**0.00**
Catanduanes	1,838	1,533	**3,371**	0	0	**0**	**0.00**
Masbate	167	144	**311**	1	0	**1**	**0.32**
Sorsogon	4,562	5,104	**9,666**	1	0	**1**	**0.01**
Iriga City	1,143	1,194	**2,337**	0	0	**0**	**0.00**
Legaspi City	0	0	**0**	0	0	**0**	**0.00**
Naga City	1,874	2,199	**4,073**	1	0	**1**	**0.02**
**Region 6**	**70,530**	**77,006**	**147,536**	**19**	**10**	**29**	**0.02**
Aklan	4,956	5,581	**10,537**	4	1	**5**	**0.05**
Antique	4,101	4,334	**8,435**	1	0	**1**	**0.01**
Capiz	9,545	10,511	**20,056**	5	1	**6**	**0.03**
Guimaras	1,638	1,723	**3,361**	0	0	**0**	**0.00**
Iloilo	21,699	24,473	**46,172**	2	3	**5**	**0.01**
Negros Occidental	13,757	13,957	**27,714**	6	4	**10**	**0.04**
Bacolod City	5,381	5,727	**11,108**	0	1	**1**	**0.01**
Bago City	9,453	10,700	**20,153**	1	0	**1**	**0.00**
**Region 7**	**6,191**	**6,761**	**12,952**	**1**	**1**	**2**	**0.02**
Bohol	592	662	**1,254**	0	1	**1**	**0.08**
Cebu	0	0	**0**	0	0	**0**	**0.00**
Negros Oriental	3,613	4,006	**7,619**	0	0	**0**	**0.00**
Siquijor	0	0	**0**	0	0	**0**	**0.00**
Cebu City	0	0	**0**	0	0	**0**	**0.00**
Lapu-Lapu City	1,986	2,093	**4,079**	1	0	**1**	**0.02**
Mandaue City	0	0	**0**	0	0	**0**	**0.00**
**Region 8**	**8,610**	**8,193**	**16,803**	**5**	**3**	**8**	**0.05**
Biliran	1,007	1,027	**2,034**	0	0	**0**	**0.00**
Eastern Samar	226	245	**471**	0	0	**0**	**0.00**
Northern Leyte	749	777	**1,526**	1	1	**2**	**0.13**

Table 2.E.6.1. Rabies

Number and Proportion of Deaths due to Rabies
Philippines, 2022

Area	Animal Bites			No. of Deaths due to rabies			Rate
	Male	Female	Total	Male	Female	Total	
Northern Samar	73	61	**134**	1	0	**1**	**0.75**
Southern Leyte	1,470	1,647	**3,117**	0	0	**0**	**0.00**
Western Samar	136	123	**259**	0	0	**0**	**0.00**
Calbayog City	642	616	**1,258**	1	0	**1**	**0.08**
Maasin City	393	421	**814**	1	0	**1**	**0.12**
Ormoc City	1,905	1,930	**3,835**	0	0	**0**	**0.00**
Tacloban City	2,009	1,346	**3,355**	1	2	**3**	**0.09**
**Region 9**	**6,380**	**6,605**	**12,985**	**4**	**0**	**4**	**0.03**
							**0.00**
Zamboanga del Norte	878	861	**1,739**	2	0	**2**	**0.12**
Zamboanga del Sur	7	8	**15**	0	0	**0**	**0.00**
Zamboanga Sibugay	3212	3335	**6,547**	2	0	**2**	**0.03**
Dapitan City	0	0	**0**	0	0	**0**	**0.00**
Dipolog City	424	479	**903**	0	0	**0**	**0.00**
Isabela City	367	372	**739**	0	0	**0**	**0.00**
Pagadian City	1,380	1,440	**2,820**	0	0	**0**	**0.00**
Zamboanga City	112	110	**222**	0	0	**0**	**0.00**
**Region 10**	**24,835**	**25,076**	**49,911**	**9**	**5**	**14**	**0.03**
Bukidnon	513	419	**932**	2	2	**4**	**0.43**
Camiguin	621	616	**1,237**	0	0	**0**	**0.00**
Lanao del Norte	1,360	1,576	**2,936**	2	1	**3**	**0.10**
Misamis Occidental	42	53	**95**	0	0	**0**	**0.00**
Misamis Oriental	7,209	7,065	**14,274**	2	0	**2**	**0.01**
Cagayan de Oro City	5,052	5,556	**10,608**	1	1	**2**	**0.02**
El Salvador City	383	342	**725**	0	0	**0**	**0.00**
Gingoog City	990	929	**1,919**	0	1	**1**	**0.05**
Iligan City	3,318	3,333	**6,651**	0	0	**0**	**0.00**
Malaybalay City	1,742	1,713	**3,455**	0	0	**0**	**0.00**
Oroquieta City	1,153	1,085	**2,238**	1	0	**1**	**0.04**
Ozamis City	627	644	**1,271**	1	0	**1**	**0.08**
Tangub City	37	28	**65**	0	0	**0**	**0.00**
Valencia City	1,788	1,717	**3,505**	0	0	**0**	**0.00**
**Region 11**	**9,483**	**9,845**	**19,328**	**29**	**13**	**42**	**0.22**
Davao de Oro	1,039	1,095	**2,134**	2	1	**3**	**0.14**
Davao del Norte	4,731	4,889	**9,620**	4	2	**6**	**0.06**
Davao del Sur	2,618	2,616	**5,234**	8	4	**12**	**0.23**
Davao Occidental	199	258	**457**	4	1	**5**	**1.09**
Davao Oriental	96	83	**179**	1	2	**3**	**1.68**
Davao City	800	904	**1,704**	10	3	**13**	**0.76**
**Region 12**	**18,212**	**17,991**	**36,203**	**34**	**11**	**45**	**0.12**
North Cotabato	3,692	3,787	**7,479**	11	4	**15**	**0.20**
Sarangani	1,417	1,395	**2,812**	6	2	**8**	**0.28**
South Cotabato	5,020	4,895	**9,915**	10	2	**12**	**0.12**
Sultan Kudarat	6,784	6,545	**13,329**	4	3	**7**	**0.05**

Table 2.E.6.1. Rabies

Number and Proportion of Deaths due to Rabies
Philippines, 2022

Area	Animal Bites			No. of Deaths due to rabies			Rate
	Male	Female	Total	Male	Female	Total	
Gen. Santos City	1,299	1,369	**2,668**	3	0	**3**	**0.11**
**BARMM**	**2,446**	**2,326**	**4,772**	**2**	**0**	**2**	**0.04**
Basilan	128	90	**218**	0	0	**0**	**0.00**
Lanao del Sur	458	418	**876**	0	0	**0**	**0.00**
Maguindanao	293	265	**558**	2	0	**2**	**0.36**
Sulu	298	306	**604**	0	0	**0**	**0.00**
Tawi-Tawi	325	254	**579**	0	0	**0**	**0.00**
Cotabato City	751	831	**1,582**	0	0	**0**	**0.00**
Lamitan City	133	119	**252**	0	0	**0**	**0.00**
Marawi City	60	43	**103**	0	0	**0**	**0.00**
**CARAGA**	**14,316**	**13,618**	**27,934**	**0**	**0**	**0**	**0.00**
Agusan del Norte	2,202	1,769	**3,971**	0	0	**0**	**0.00**
Agusan del Sur	3,774	3,590	**7,364**	0	0	**0**	**0.00**
Surigao del Norte	998	1,110	**2,108**	0	0	**0**	**0.00**
Surigao del Sur	4,294	4,200	**8,494**	0	0	**0**	**0.00**
Province of Dinagat	435	436	**871**	0	0	**0**	**0.00**
Bislig City	723	741	**1,464**	0	0	**0**	**0.00**
Butuan City	39	36	**75**	0	0	**0**	**0.00**
Surigao City	1,851	1,736	**3,587**	0	0	**0**	**0.00**

Table 2.E.7.1. Tuberculosis

Case Notification Rate (All forms)

Philippines, 2022

Area	Total Population	Notified TB cases all forms			CNR (All Forms)
		Male	Female	Total	
**PHILIPPINES**	**111,572,254**	**324,564**	**179,200**	**503,764**	**451.51**
**N C R**	**14,118,673**	**61,361**	**38,105**	**99,466**	**704.50**
Malabon City	**400,763**	2,398	1,313	**3,711**	**925.98**
Navotas City	**273,513**	1573	995	**2568**	**938.90**
Valenzuela City	**680,233**	3471	2003	**5474**	**804.72**
Caloocan City	**1,736,680**	6,604	4,438	**11,042**	**635.81**
Marikina City	**494,193**	2,246	1,367	**3,613**	**731.09**
Pasig City	**828,114**	3641	2375	**6016**	**726.47**
Pateros	**69,994**	155	81	**236**	**337.17**
Taguig City	**882,512**	3554	2458	**6012**	**681.24**
Quezon City	**3,219,173**	13612	8482	**22094**	**686.33**
Makati City	**638,764**	2,209	1,775	**3,984**	**623.70**
Mandaluyong City	**423,514**	2,113	954	**3,067**	**724.18**
San Juan City	**133,959**	636	342	**978**	**730.07**
Manila City	**1,951,758**	10495	6723	**17218**	**882.18**
Las Piñas City	**645,666**	2,204	1,310	**3,514**	**544.24**
Muntinlupa City	**553,146**	2398	920	**3318**	**599.84**
Parañaque City	**730,009**	2255	1270	**3525**	**482.87**
Pasay City	**456,682**	1797	1299	**3096**	**677.93**
**C A R**	**1,836,857**	**2,760**	**1,362**	**4,122**	**224.41**
Abra	**249,467**	600	252	**852**	**341.53**
Apayao	**125,511**	228	113	**341**	**271.69**
Benguet	**489,214**	294	135	**429**	**87.69**
Ifugao	**213,380**	228	122	**350**	**164.03**
Kalinga	**222,826**	486	225	**711**	**319.08**
Mt. Province	**157,820**	199	114	**313**	**198.33**
Baguio City	**378,639**	725	401	**1,126**	**297.38**
**Region 1**	**5,353,139**	**11,997**	**6,443**	**18,440**	**344.47**
Ilocos Norte	**503,876**	673	269	**942**	**186.95**
Ilocos Sur	**604,123**	974	577	**1,551**	**256.74**
La Union	**711,657**	1,347	754	**2,101**	**295.23**
Pangasinan	**2,542,805**	5,427	2,974	**8,401**	**330.38**
Alaminos City	**96,076**	376	191	**567**	**590.16**
Candon City	**63,678**	147	78	**225**	**353.34**
Dagupan City	**183,431**	994	526	**1,520**	**828.65**
Laoag City	**116,176**	108	54	**162**	**139.44**
San Carlos City	**201,955**	565	330	**895**	**443.17**
San Fernando City	**130,390**	633	317	**950**	**728.58**
Urdaneta City	**142,378**	616	299	**915**	**642.66**
Vigan City	**56,594**	137	74	**211**	**372.83**

Table 2.E.7.1. Tuberculosis

Case Notification Rate (All forms)

Philippines, 2022

Area	Total Population	Notified TB cases all forms			CNR (All Forms)
		Male	Female	Total	
**Region 2**	**3,727,548**	**9,253**	**5,329**	**14,582**	**391.20**
Batanes	**18,145**	54	36	**90**	**496.00**
Cagayan	**1,132,144**	2,114	1,236	**3,350**	**295.90**
Isabela	**1,275,221**	2,938	1,757	**4,695**	**368.17**
Nueva Vizcaya	**493,293**	804	414	**1,218**	**246.91**
Quirino	**200,940**	372	207	**579**	**288.15**
Cauayan City	**139,546**	635	311	**946**	**677.91**
Ilagan City	**156,829**	850	540	**1,390**	**886.32**
Santiago City	**145,258**	812	481	**1,293**	**890.14**
Tuguegarao City	**166,172**	674	347	**1,021**	**614.42**
**Region 3**	**12,706,460**	**37,954**	**21,578**	**59,532**	**468.52**
Aurora	**232,022**	685	341	**1,026**	**442.20**
Bataan	**753,343**	2,403	1,310	**3,713**	**492.87**
Bulacan	**2,582,769**	8,259	4,751	**13,010**	**503.72**
Nueva Ecija	**1,677,367**	3,016	1,551	**4,567**	**272.27**
Pampanga	**1,879,959**	4782	2581	**7,363**	**391.66**
Tarlac	**1,117,955**	2,457	1,214	**3,671**	**328.37**
Zambales	**658,590**	2560	1684	**4,244**	**644.41**
Angeles City	**471,675**	1421	772	**2,193**	**464.94**
Balanga City	**108,890**	571	342	**913**	**838.46**
Cabanatuan City	**343,309**	1358	801	**2,159**	**628.88**
City of San Fernando	**351,388**	1362	726	**2,088**	**594.21**
Gapan City	**125,295**	388	246	**634**	**506.01**
Mabalacat City	**287,381**	822	540	**1,362**	**473.94**
Malolos City	**288,480**	849	443	**1,292**	**447.86**
Meycauayan City	**239,282**	881	570	**1,451**	**606.40**
Olongapo City	**259,761**	1261	766	**2,027**	**780.33**
Palayan City	**46,619**	126	60	**186**	**398.98**
San Jose City	**158,730**	501	265	**766**	**482.58**
San Jose del Monte City	**657,003**	2675	1757	**4,432**	**674.58**
Science City of Munoz	**92,559**	205	127	**332**	**358.69**
Tarlac City	**374,083**	1372	731	**2,103**	**562.17**
**Region 4A**	**16,654,975**	**57,034**	**33,426**	**90,460**	**543.14**
Batangas	**2,196,463**	7,275	4,477	**11,752**	**535.04**
Cavite	**1,638,293**	5,204	2,939	**8,143**	**497.04**
Laguna	**1,128,465**	4,465	2,706	**7,171**	**635.46**
Quezon	**1,908,946**	5,319	2,930	**8,249**	**432.12**
Rizal	**2,426,363**	8,213	4,880	**13,093**	**539.61**
Antipolo City	**893,708**	2656	1548	**4,204**	**470.40**
Bacoor City	**717,766**	1536	813	**2,349**	**327.27**
Batangas City	**389,811**	1936	982	**2,918**	**748.57**
Biñan City	**378,391**	1581	937	**2,518**	**665.45**
Cabuyao City	**350,801**	1322	897	**2,219**	**632.55**
Calamba City	**516,395**	2096	1249	**3,345**	**647.76**

Table 2.E.7.1. Tuberculosis

Case Notification Rate (All forms)

Philippines, 2022

Area	Total Population	Notified TB cases all forms			CNR (All Forms)
		Male	Female	Total	
Cavite City	122,859	335	227	562	457.43
Dasmariñas City	787,570	2395	1379	3,774	479.20
General Trias City	375,612	1182	646	1,828	486.67
Imus City	482,549	1521	928	2,449	507.51
Lipa City	392,788	1525	927	2,452	624.26
Lucena City	289,304	1268	741	2,009	694.43
San Pablo City	302,307	1724	1123	2,847	941.76
San Pedro City	370,189	1730	995	2,725	736.11
Santa Rosa City	401,955	1385	784	2,169	539.61
Tagaytay City	85,067	268	116	384	451.41
Tanauan City	204,868	698	389	1,087	530.59
Tayabas City	108,418	262	159	421	388.31
Trece Martires City	186,087	1138	654	1,792	962.99
**Region 4B**	**3,247,680**	**9,652**	**5,303**	**14,955**	**460.48**
Marinduque	243,827	845	510	1,355	555.72
Mindoro Occidental	524,583	1,452	756	2,208	420.91
Mindoro Oriental	920,817	3,161	1,822	4,983	541.15
Palawan	964,459	2,101	1,135	3,236	335.52
Romblon	304,342	841	451	1,292	424.52
				0	
Puerto Princesa City	289,652	1252	629	1,881	649.40
**Region 5**	**6,248,843**	**20,264**	**11,025**	**31,289**	**500.72**
Albay	1,210,784	3,859	2,102	5,961	492.33
Camarines Norte	635,921	1964	773	2,737	430.40
Camarines Sur	1,765,618	4,711	2,596	7,307	413.85
Catanduanes	277,033	734	386	1,120	404.28
Masbate	952,388	2567	1440	4,007	420.73
Sorsogon	863,803	2421	1298	3,719	430.54
				0	
Iriga City	119,968	610	331	941	784.38
Legaspi City	212,924	1801	981	2,782	1306.57
Naga City	210,404	1597	1118	2,715	1290.37
**Region 6**	**8,033,041**	**26,680**	**13,081**	**39,761**	**494.97**
Aklan	616,000	1720	870	2,590	420.45
Antique	622,828	1912	1050	2,962	475.57
Capiz	820,940	2069	1150	3,219	392.11
Guimaras	188,747	425	212	637	337.49
Iloilo	2,088,223	5264	2332	7,596	363.75
Negros Occidental	2,623,026	9214	4269	13,483	514.02
Bacolod City	590,174	3115	1685	4,800	813.32
Iloilo City	483,103	2961	1513	4,474	926.10

Table 2.E.7.1. Tuberculosis
Case Notification Rate (All forms)
Philippines, 2022

Area	Total Population	Notified TB cases all forms			CNR (All Forms)
		Male	Female	Total	
**Region 7**	**8,147,078**	**19,379**	**9,620**	**28,999**	**355.94**
Bohol	**1,382,606**	2395	1261	**3,656**	**264.43**
Cebu	**3,323,807**	6538	3127	**9,665**	**290.78**
Negros Oriental	**1,423,606**	3399	1755	**5,154**	**362.04**
Siquijor	**101,951**	226	77	**303**	**297.20**
Cebu City	**1,043,417**	3792	1893	**5,685**	**544.84**
Lapu-Lapu City	**461,551**	1634	895	**2,529**	**547.94**
Mandaue City	**410,140**	1395	612	**2,007**	**489.35**
**Region 8**	**4,865,157**	**10,026**	**5,482**	**15,508**	**318.76**
Biliran	**182,002**	469	218	**687**	**377.47**
Eastern Samar	**519,219**	928	513	**1,441**	**277.53**
Northern Leyte	**1,682,729**	3,367	1,643	**5,010**	**297.73**
Northern Samar	**681,299**	1347	797	**2,144**	**314.69**
Southern Leyte	**361,920**	522	265	**787**	**217.45**
Western Samar	**639,344**	1,027	601	**1,628**	**254.64**
Calbayog City	**197,014**	483	267	**750**	**380.68**
Maasin City	**92,110**	180	119	**299**	**324.61**
Ormoc City	**239,675**	517	260	**777**	**324.19**
Tacloban City	**269,845**	1186	799	**1,985**	**735.61**
**Region 9**	**3,834,801**	**8,908**	**4,072**	**12,980**	**338.48**
Zamboanga del Norte	**836,122**	1,290	558	**1,848**	**221.02**
Zamboanga del Sur	**844,012**	1,578	646	**2,224**	**263.50**
Zamboanga Sibugay	**695,261**	1418	582	**2,000**	**287.66**
Dapitan City	**86,329**	186	71	**257**	**297.70**
Dipolog City	**136,968**	392	226	**618**	**451.20**
Isabela City	**132,907**	224	135	**359**	**270.11**
Pagadian City	**207,002**	610	288	**898**	**433.81**
Zamboanga City	**896,200**	3210	1566	**4,776**	**532.92**
**Region 10**	**5,128,964**	**10,632**	**5,140**	**15,772**	**307.51**
Bukidnon	**1,141,852**	1,103	528	**1,631**	**142.84**
Camiguin	**93,769**	137	51	**188**	**200.49**
Lanao del Norte	**744,374**	1275	662	**1,937**	**260.22**
Misamis Occidental	**344,680**	487	211	**698**	**202.51**
Misamis Oriental	**791,744**	1,411	566	**1,977**	**249.70**
Cagayan de Oro City	**749,911**	2411	1295	**3,706**	**494.19**
El Salvador City	**55,698**	158	55	**213**	**382.42**
Gingoog City	**138,285**	434	229	**663**	**479.44**
Iligan City	**377,050**	1784	888	**2,672**	**708.66**
Malaybalay City	**190,337**	342	121	**463**	**243.25**

Table 2.E.7.1. Tuberculosis
Case Notification Rate (All forms)
Philippines, 2022

Area	Total Population	Notified TB cases all forms			CNR (All Forms)
		Male	Female	Total	
Oroquieta City	74,688	132	80	212	283.85
Ozamis City	149,710	442	235	677	452.21
Tangub City	66,511	75	19	94	141.33
Valencia City	210,355	441	200	641	304.72
**Region 11**	**5,451,448**	**12,536**	**5,636**	**18,172**	**333.34**
Compostela Valley	787,004	1440	472	1,912	242.95
Davao del Norte	1,069,992	3123	1236	4,359	407.39
Davao del Sur	614,795	1218	558	1,776	288.88
Davao Oriental	735,194	1231	547	1,778	241.84
Davao Occidental	346,602	405	191	596	171.96
Davao City	1,897,861	5119	2632	7,751	408.41
**Region 12**	**4,735,406**	**12,710**	**5,965**	**18,675**	**394.37**
North Cotabato	1,577,659	3261	1592	4,853	307.61
Sarangani	597,339	963	490	1,453	243.25
South Cotabato	1,024,887	3534	1495	5,029	490.69
Sultan Kudarat	869,896	2341	1206	3,547	407.75
Gen. Santos City	665,625	2611	1182	3,793	569.84
**BARMM**	**4,671,758**	**5,650**	**3,682**	**9,332**	**199.75**
Basilan	317,215	158	109	267	84.17
Lanao del Sur	960,666	709	676	1,385	144.17
Maguindanao	1,389,643	1,562	775	2,337	168.17
Sulu	952,737	723	418	1,141	119.76
Tawi-Tawi	421,522	420	254	674	159.90
Cotabato City	312,921	1560	1016	2,576	823.21
Lamitan City	87,277	158	103	261	299.05
Marawi City	229,777	360	331	691	300.73
**CARAGA**	**2,810,426**	**7,768**	**3,951**	**11,719**	**417**
Agusan del Norte	386,955	665	295	960	248.09
Agusan del Sur	746,771	1815	800	2,615	350.17
Surigao del Norte	373,053	863	439	1,302	349.01
Surigao del Sur	529,552	1,284	676	1,960	370.12
Province of Dinagat	131,841	197	97	294	223.00
Bislig City	100,580	416	277	693	689.00
Butuan City	367,928	1495	810	2,305	626.48
Surigao City	173,746	1033	557	1,590	915.13

Table 2.E.7.2. Tuberculosis
Case Notification Rate (DRTB)
Philippines, 2022

Area	Registered Bacteriologically Confirmed Drug Resistant			Estimated MDR among New & Retreatment			CNR (DRTB)
	Male	Female	Total	Male	Female	Total	
**PHILIPPINES**	**5,392**	**2,312**	**7,704**	**0**	**0**	**22,493**	**34.3**
**N C R**	**1,277**	**562**	**1,839**	**0**	**0**	**5,023**	**36.6**
Malabon City	66	26	**92**			**268**	**34.3**
Navotas City	51	11	**62**			**128**	**48.3**
Valenzuela City	50	31	**81**			**288**	**28.2**
Caloocan City	143	63	**206**			**542**	**38.0**
Marikina City	32	20	**52**			**204**	**25.5**
Pasig City	51	28	**79**			**272**	**29.0**
Pateros	0	0	**0**			**0**	**0.0**
Taguig City	106	24	**130**			**311**	**41.8**
Quezon City	243	155	**398**			**1,016**	**39.2**
Makati City	42	7	**49**			**156**	**31.5**
Mandaluyong City	30	14	**44**			**149**	**29.6**
San Juan City	10	8	**18**			**32**	**56.2**
Manila City	258	124	**382**			**1,001**	**38.2**
Las Piñas City	36	20	**56**			**122**	**45.8**
Muntinlupa City	88	7	**95**			**188**	**50.5**
Parañaque City	38	11	**49**			**166**	**29.5**
Pasay City	33	13	**46**			**180**	**25.5**
**C A R**	**35**	**6**	**41**	**0**	**0**	**135**	**30.4**
Abra	11	2	**13**			**35**	**37.2**
Apayao	6	2	**8**			**9**	**85.4**
Benguet	0	0	**0**			**9**	**0.0**
Ifugao	3	1	**4**			**10**	**40.1**
Kalinga	4	1	**5**			**24**	**20.9**
Mt. Province	1	0	**1**			**12**	**8.1**
Baguio City	10	0	**10**			**35**	**28.5**
**Region 1**	**240**	**99**	**339**	**0**	**0**	**736**	**46.1**
Ilocos Norte	8	5	**13**			**36**	**36.0**
Ilocos Sur	13	4	**17**			**59**	**28.6**
La Union	13	3	**16**			**66**	**24.4**
Pangasinan	54	24	**78**			**351**	**22.2**
Alaminos City	14	4	**18**			**22**	**83.6**
Candon City	3	1	**4**			**7**	**56.3**
Dagupan City	59	21	**80**			**71**	**111.9**
Laoag City	0	0	**0**			**0**	**0.0**

Table 2.E.7.2. Tuberculosis
Case Notification Rate (DRTB)
Philippines, 2022

Area	Registered Bacteriologically Confirmed Drug Resistant			Estimated MDR among New & Retreatment			CNR (DRTB)
	Male	Female	Total	Male	Female	Total	
San Carlos City	25	15	**40**			**33**	**119.5**
San Fernando City	23	8	**31**			**40**	**76.7**
Urdaneta City	23	8	**31**			**41**	**75.7**
Vigan City	5	6	**11**			**9**	**127.9**
**Region 2**	**92**	**43**	**135**	**0**	**0**	**240**	**56.3**
Batanes	0	0	**0**			**0**	**0.0**
Cagayan	16	6	**22**			**101**	**21.7**
Isabela	0	0	**0**			**0**	**0.0**
Nueva Vizcaya	11	3	**14**			**53**	**26.6**
Quirino	5	3	**8**			**19**	**42.5**
Cauayan City	0	0	**0**			**0**	**0.0**
Ilagan City	0	0	**0**			**0**	**0.0**
Santiago City	22	19	**41**			**31**	**134.4**
Tuguegarao City	38	12	**50**			**37**	**136.1**
**Region 3**	**746**	**321**	**1,067**	**0**	**0**	**2,617**	**40.8**
Aurora	17	4	**21**			**63**	**33.1**
Bataan	14	9	**23**			**190**	**12.1**
Bulacan	137	55	**192**			**585**	
Nueva Ecija	39	22	**61**			**190**	**32.0**
Pampanga	67	31	**98**			**368**	**26.6**
Tarlac	34	15	**49**			**163**	**30.1**
Zambales	44	15	**59**			**235**	**25.1**
Angeles City	32	11	**43**			**93**	**46.4**
Balanga City	40	17	**57**			**49**	**116.3**
Cabanatuan City	33	23	**56**			**66**	**84.4**
City of San Fernando	58	33	**91**			**92**	**99.3**
Gapan City	0	0	**0**			**0**	**0.0**
Mabalacat City	28	6	**34**			**69**	**49.1**
Malolos City	69	24	**93**			**66**	**140.2**
Meycauayan City	0	0	**0**			**0**	**0.0**
Olongapo City	24	16	**40**			**108**	**37.2**
Palayan City	0	0	**0**			**0**	**0.0**
San Jose City	23	2	**25**			**30**	**84.6**
San Jose del Monte City	60	24	**84**			**158**	**53.1**
Science City of Munoz	0	0	**0**			**0**	**0.0**
Tarlac City	27	14	**41**			**91**	**44.9**
**Region 4A**	**924**	**455**	**1,379**	**0**	**0**	**3,815**	**36.1**
Batangas	53	19	**72**			**542**	**13.3**

Table 2.E.7.2. Tuberculosis
Case Notification Rate (DRTB)
Philippines, 2022

Area	Registered Bacteriologically Confirmed Drug Resistant			Estimated MDR among New & Retreatment			CNR (DRTB)
	Male	Female	Total	Male	Female	Total	
Cavite	16	11	**27**			**335**	**8.1**
Laguna	73	33	**106**			**409**	**25.9**
Quezon	75	39	**114**			**433**	**26.3**
Rizal	162	83	**245**			**696**	**35.2**
Antipolo City	67	45	**112**			**213**	**52.5**
Bacoor City	28	13	**41**			**120**	**34.3**
Batangas City	37	14	**51**			**93**	**54.7**
Biñan City	0	0	**0**			**0**	**0.0**
Cabuyao City	0	0	**0**			**0**	**0.0**
Calamba City	70	29	**99**			**186**	**53.3**
Cavite City	0	0	**0**			**0**	**0.0**
Dasmariñas City	51	25	**76**			**184**	**41.3**
General Trias City	0	0	**0**			**0**	**0.0**
Imus City	38	25	**63**			**92**	**68.5**
Lipa City	34	18	**52**			**102**	**51.0**
Lucena City	66	25	**91**			**146**	**62.3**
San Pablo City	0	1	**1**			**98**	**1.0**
San Pedro City	0	0	**0**			**0**	**0.0**
Santa Rosa City	69	37	**106**			**89**	**119.3**
Tagaytay City	17	8	**25**			**18**	**142.9**
Tanauan City	0	0	**0**			**0**	**0.0**
Tayabas City	0	0	**0**			**0**	**0.0**
Trece Martires City	68	30	**98**			**61**	**159.4**
**Region 4B**	**207**	**85**	**292**	**0**	**0**	**892**	**32.7**
Marinduque	16	2	**18**			**87**	**20.7**
Mindoro Occidental	46	22	**68**			**127**	**53.4**
Mindoro Oriental	60	24	**84**			**299**	**28.1**
Palawan	34	16	**50**			**215**	**23.3**
Romblon	12	8	**20**			**74**	**27.2**
Puerto Princesa City	39	13	**52**			**91**	**57.4**
**Region 5**	**319**	**168**	**487**	**0**	**0**	**1,634**	**29.8**
Albay	35	14	**49**			**361**	**13.6**
Camarines Norte	55	12	**67**			**133**	**50.3**
Camarines Sur	24	10	**34**			**440**	**7.7**
Catanduanes	10	14	**24**			**73**	**33.1**
Masbate	30	16	**46**			**208**	**22.1**
Sorsogon	44	13	**57**			**174**	**32.7**
Iriga City	0	0	**0**			**0**	**0.0**
Legaspi City	55	41	**96**			**153**	**62.9**

Table 2.E.7.2. Tuberculosis
Case Notification Rate (DRTB)
Philippines, 2022

Area	Registered Bacteriologically Confirmed Drug Resistant			Estimated MDR among New & Retreatment			CNR (DRTB)
	Male	Female	Total	Male	Female	Total	
Naga City	66	48	**114**			**92**	**123.9**
**Region 6**	**400**	**138**	**538**	**0**	**0**	**2,317**	**23.2**
Aklan	28	12	**40**			**125**	**31.9**
Antique	23	13	**36**			**163**	**22.0**
Capiz	39	17	**56**			**121**	**46.4**
Guimaras	0	1	**1**			**21**	**4.8**
Iloilo	45	10	**55**			**420**	**13.1**
Negros Occidental	153	49	**202**			**968**	**20.9**
Bacolod City	57	17	**74**			**280**	**26.4**
Iloilo City	55	19	**74**			**218**	**33.9**
**Region 7**	**276**	**101**	**377**	**0**	**0**	**1,258**	**30.0**
Bohol	53	12	**65**			**167**	**39.0**
Cebu	0	0	**0**			**412**	**0.0**
Negros Oriental	35	12	**47**			**272**	**17.3**
Siquijor	0	0	**0**				**0.0**
Cebu City	118	41	**159**			**315**	**50.4**
Lapu-Lapu City	0	0	**0**			**0**	**0.0**
Mandaue City	70	36	**106**			**92**	**114.8**
**Region 8**	**183**	**57**	**240**	**0**	**0**	**447**	**53.6**
Biliran	0	0	**0**			**0**	**0.0**
Eastern Samar	29	7	**36**			**73**	**49.0**
Northern Leyte	83	32	**115**			**218**	**52.9**
Northern Samar	21	3	**24**			**102**	**23.5**
Southern Leyte	0	0	**0**			**0**	**0.0**
Western Samar	0	0	**0**			**0**	**0.0**
Calbayog City	22	7	**29**			**32**	**91.5**
Maasin City	28	8	**36**			**23**	**159.1**
Ormoc City	0	0	**0**			**0**	**0.0**
Tacloban City	0	0	**0**			**0**	**0.0**
**Region 9**	**129**	**46**	**175**	**0**	**0**	**405**	**43.2**
Zamboanga del Norte	0	0	**0**			**0**	**0.0**
Zamboanga del Sur	31	7	**38**			**103**	**36.9**
Zamboanga Sibugay	20	8	**28**			**96**	**29.2**
Dapitan City	40	12	**52**			**18**	**293.1**

Table 2.E.7.2. Tuberculosis
Case Notification Rate (DRTB)
Philippines, 2022

Area	Registered Bacteriologically Confirmed Drug Resistant			Estimated MDR among New & Retreatment			CNR (DRTB)
	Male	Female	Total	Male	Female	Total	
Dipolog City	0	0	**0**			**0**	**0.0**
Isabela City	2	1	**3**			**12**	**24.3**
Pagadian City	0	0	**0**			**0**	**0.0**
Zamboanga City	36	18	**54**			**176**	**30.7**
**Region 10**	**104**	**43**	**147**	**0**	**0**	**594**	**24.8**
Bukidnon	3	0	**3**			**66**	**4.5**
Camiguin	1	0	**1**			**6**	**15.8**
Lanao del Norte	19	9	**28**			**102**	**27.5**
Misamis Occidental	0	0	**0**			**0**	**0.0**
Misamis Oriental	3	1	**4**			**95**	**4.2**
Cagayan de Oro City	25	8	**33**			**126**	**26.2**
El Salvador City	0	0	**0**			**14**	**0.0**
Gingoog City	6	1	**7**			**0**	**0.0**
Iligan City	19	12	**31**			**118**	**26.2**
Malaybalay City	13	4	**17**			**24**	**71.8**
Oroquieta City	9	5	**14**			**12**	**114.5**
Ozamis City	6	3	**9**			**29**	**30.6**
Tangub City	0	0	**0**			**0**	**0.0**
Valencia City	0	0	**0**			**0**	**0.0**
**Region 11**	**136**	**47**	**183**	**0**	**0**	**663**	**27.6**
Compostela Valley	0	0	**0**			**0**	**0.0**
Davao del Norte	51	9	**60**			**177**	**34.0**
Davao del Sur	14	3	**17**			**59**	**29.0**
Davao Oriental	9	5	**14**			**104**	**13.4**
Davao Occidental	1	1	**2**			**20**	**9.8**
Davao City	61	29	**90**			**303**	**29.7**
**Region 12**	**119**	**46**	**165**	**0**	**0**	**865**	**19.1**
North Cotabato	29	13	**42**			**219**	**19.1**
Sarangani	4	1	**5**			**79**	**6.3**
South Cotabato	24	13	**37**			**175**	**21.2**
Sultan Kudarat	26	9	**35**			**145**	**24.1**
Gen. Santos City	36	10	**46**			**246**	**18.7**
**BARMM**	**90**	**57**	**147**	**0**	**0**	**404**	**36.4**
Basilan	0	0	**0**			**0**	**0.0**
Lanao del Sur	3	8	**11**			**55**	**19.8**
Maguindanao	26	15	**41**			**111**	**37.0**

Table 2.E.7.2. Tuberculosis
Case Notification Rate (DRTB)
Philippines, 2022

Area	Registered Bacteriologically Confirmed Drug Resistant			Estimated MDR among New & Retreatment			CNR (DRTB)
	Male	Female	Total	Male	Female	Total	
Sulu	12	5	**17**			**34**	**50.1**
Tawi-Tawi	3	3	**6**			**26**	**23.5**
Cotabato City	24	9	**33**			**120**	**27.5**
Lamitan City	2	2	**4**			**14**	**29.4**
Marawi City	20	15	**35**			**45**	**78.4**
**CARAGA**	**115**	**38**	**153**	**0**	**0**	**448**	**34.1**
Agusan del Norte	0	0	**0**			**0**	**0.0**
Agusan del Sur	12	8	**20**			**131**	**15.2**
Surigao del Norte	9	1	**10**			**73**	**13.7**
Surigao del Sur	31	9	**40**			**91**	**43.9**
Province of Dinagat	3	1	**4**			**9**	**42.9**
Bislig City	19	9	**28**			**30**	**93.3**
Butuan City	27	4	**31**			**62**	**49.9**
Surigao City	14	6	**20**			**52**	**38.7**

Table 2.E.7.3. Tuberculosis

Treatment Success Rate DSTB - All Forms

Philippines,2022

Area	TB all forms cured and Completely Treated			Registered TB all forms Registered during Specified Period			Treatment Success Rate
	Male	Female	Total	Male	Female	Total	
**PHILIPPINES**	**152,287**	**73,954**	**226,241**	**163,846**	**83,587**	**247,433**	**91.44**
**N C R**	**22,833**	**11,433**	**34,266**	**25,047**	**12,408**	**37,455**	**91.49**
Malabon City	614	378	**992**	684	418	**1,102**	**90.02**
Navotas City	554	271	**825**	615	294	**909**	**90.76**
Valenzuela City	1,491	813	**2,304**	1,638	892	**2,530**	**91.07**
Caloocan City	2,374	1,158	**3,532**	2,565	1,252	**3,817**	**92.53**
Marikina City	973	498	**1,471**	1,119	547	**1,666**	**88.30**
Pasig City	1,160	702	**1,862**	1,390	822	**2,212**	**84.18**
Pateros	75	22	**97**	79	24	**103**	**94.17**
Taguig City	3,933	2,141	**6,074**	2,169	1,139	**3,308**	**183.62**
Quezon City	2,072	1,094	**3,166**	4,205	2,267	**6,472**	**48.92**
Makati City	841	399	**1,240**	935	452	**1,387**	**89.40**
Mandaluyong City	955	388	**1,343**	1,060	429	**1,489**	**90.19**
San Juan City	167	77	**244**	177	82	**259**	**94.21**
Manila City	4,275	2,061	**6,336**	4,650	2,231	**6,881**	**92.08**
Las Piñas City	741	396	**1,137**	816	432	**1,248**	**91.11**
Muntinlupa City	1,071	370	**1,441**	1,244	418	**1,662**	**86.70**
Parañaque City	838	383	**1,221**	903	406	**1,309**	**93.28**
Pasay City	699	282	**981**	798	303	**1,101**	**89.10**
**C A R**	**1,389**	**633**	**2,022**	**1,533**	**699**	**2,232**	**90.59**
Abra	247	103	**350**	269	119	**388**	**90.21**
Apayao	156	106	**262**	174	111	**285**	**91.93**
Benguet	173	53	**226**	195	60	**255**	**88.63**
Ifugao	154	72	**226**	165	75	**240**	**94.17**
Kalinga	260	131	**391**	284	135	**419**	**93.32**
Mt. Province	104	36	**140**	112	39	**151**	**92.72**
Baguio City	295	132	**427**	334	160	**494**	**86.44**
**Region 1**	**6,407**	**3,153**	**9,560**	**6,835**	**3,341**	**10,176**	**93.95**
Ilocos Norte	289	159	**448**	334	177	**511**	**87.67**
Ilocos Sur	404	161	**565**	455	182	**637**	**88.70**
La Union	755	418	**1,173**	791	443	**1,234**	**95.06**
Pangasinan	3,456	1,754	**5,210**	3,573	1,799	**5,372**	**96.98**
Alaminos City	122	64	**186**	127	67	**194**	**95.88**
Candon City	46	15	**61**	47	17	**64**	**73.44**
Dagupan City	394	151	**545**	433	165	**598**	**91.14**
Laoag City	48	25	**73**	58	28	**86**	**84.88**
San Carlos City	292	132	**424**	312	146	**458**	**92.58**
San Fernando City	284	120	**404**	335	140	**475**	**85.05**
Urdaneta City	283	135	**418**	309	145	**454**	**92.07**
Vigan City	34	19	**53**	61	32	**93**	**56.99**
**Region 2**	**3,497**	**1,801**	**5,298**	**3,793**	**1,904**	**5,697**	**93.00**
Batanes	16	14	**30**	**20**	**14**	**34**	**88.24**
Cagayan	1,010	481	**1,491**	1,066	501	**1,567**	**95.15**
Isabela	1,214	604	**1,818**	1,316	644	**1,960**	**92.76**
Nueva Vizcaya	353	183	**536**	**407**	**204**	**611**	**87.73**
Quirino	206	135	**341**	**213**	**138**	**351**	**97.15**
Cauayan City	89	50	**139**	112	57	**169**	**82.25**
Ilagan City	225	142	**367**	250	150	**400**	**91.75**

Table 2.E.7.3. Tuberculosis
Treatment Success Rate DSTB - All Forms
Philippines,2022

Area	TB all forms cured and Completely Treated			Registered TB all forms Registered during Specified Period			Treatment Success Rate
	Male	Female	Total	Male	Female	Total	
Santiago City	199	124	**323**	215	128	**343**	**94.17**
Tuguegarao City	185	68	**253**	194	68	**262**	**96.56**
**Region 3**	**16,277**	**8,064**	**19,123**	**17,389**	**8,580**	**20,368**	**93.89**
Aurora	434	203	**637**	452	214	**666**	**95.65**
Bataan	1,433	715	**2,148**	1,512	750	**2,262**	**94.96**
Bulacan	3,077	1,543	**4,620**	3,356	1,672	**5,028**	**91.89**
Nueva Ecija	1,510	751	**2,261**	1,627	795	**2,422**	**93.35**
Pampanga	1,993	918	**2,911**	2,121	968	**3,089**	**94.24**
Tarlac	1,380	657	**2,037**	1,414	682	**2,096**	**97.19**
Zambales	1,323	720	**2,043**	1,434	769	**2,203**	**92.74**
Angeles City	491	255	**746**	515	266	**781**	**95.52**
Balanga City	225	96	**321**	255	111	**366**	**87.70**
Cabanatuan City	405	179	**584**	420	188	**608**	**96.05**
City of San Fernando	381	183	**564**	391	191	**582**	**96.91**
Gapan City	164	87	**251**	171	94	**265**	**94.72**
Mabalacat City	348	199	**547**	391	217	**608**	**89.97**
Malolos City	395	143	**538**	440	164	**604**	**89.07**
Meycauayan City	347	172	**519**	387	186	**573**	**90.58**
Olongapo City	425	208	**633**	470	246	**716**	**88.41**
Palayan City	54	22	**76**	59	23	**82**	**92.68**
San Jose City	174	105	**279**	193	111	**304**	**91.78**
San Jose del Monte City	969	554	**1,523**	986	559	**1,545**	**98.58**
Science City of Munoz	99	58	**157**	112	64	**176**	**89.20**
Tarlac City	650	296	**946**	683	310	**993**	**95.27**
**Region 4A**	**23,293**	**12,075**	**35,368**	**25,257**	**12,912**	**38,169**	**92.66**
Batangas	2,865	1,428	**4,293**	3,098	1,494	**4,592**	**93.49**
Cavite	2,385	1,312	**3,697**	2,589	1,391	**3,980**	**92.89**
Laguna	1,570	790	**2,360**	1,697	834	**2,531**	**93.24**
Quezon	2,377	1,197	**3,574**	2,672	1,340	**4,012**	**89.08**
Rizal	3,689	2,045	**5,734**	3,975	2,182	**6,157**	**93.13**
Antipolo City	1,429	825	**2,254**	1,531	862	**2,393**	**94.19**
Bacoor City	731	404	**1,135**	824	444	**1,268**	**89.51**
Batangas City	405	175	**580**	482	195	**677**	**85.67**
Biñan City	872	436	**1,308**	880	439	**1,319**	**99.17**
Cabuyao City	451	187	**638**	470	194	**664**	**96.08**
Calamba City	1,154	446	**1,600**	1,174	453	**1,627**	**98.34**
Cavite City	230	113	**343**	245	127	**372**	**92.20**
Dasmariñas City	1,070	566	**1,636**	1,191	636	**1,827**	**89.55**
General Trias City	552	317	**869**	595	337	**932**	**93.24**
Imus City	577	314	**891**	638	341	**979**	**91.01**
Lipa City	431	191	**622**	451	201	**652**	**95.40**
Lucena City	449	255	**704**	504	287	**791**	**89.00**
San Pablo City	296	150	**446**	340	166	**506**	**88.14**
San Pedro City	280	115	**395**	299	122	**421**	**93.82**
Santa Rosa City	550	251	**801**	585	263	**848**	**94.46**
Tagaytay City	64	25	**89**	69	26	**95**	**93.68**
Tanauan City	314	221	**535**	349	242	**591**	**90.52**
Tayabas City	194	109	**303**	220	124	**344**	**88.08**
Trece Martires City	358	203	**561**	379	212	**591**	**94.92**
**Region 4B**	**5,218**	**2,743**	**7,961**	**5,710**	**3,036**	**8,746**	**91.02**
Marinduque	444	202	**646**	521	230	**751**	**86.02**
Mindoro Occidental	1,057	510	**1,567**	1,148	561	**1,709**	**91.69**
Mindoro Oriental	1,780	1,016	**2,796**	1,867	1,084	**2,951**	**94.75**
Palawan	1,072	609	**1,681**	1,225	710	**1,935**	**86.87**

Table 2.E.7.3. Tuberculosis
Treatment Success Rate DSTB - All Forms
Philippines,2022

Area	TB all forms cured and Completely Treated			Registered TB all forms Registered during Specified Period			Treatment Success Rate
	Male	Female	Total	Male	Female	Total	
Romblon	364	161	**525**	431	199	**630**	**83.33**
Puerto Princesa City	501	245	**746**	518	252	**770**	**96.88**
**Region 5**	**11,393**	**5,434**	**16,827**	**12,236**	**5,825**	**18,061**	**93.17**
Albay	2,235	1,044	**3,279**	2,392	1,114	**3,506**	**93.53**
Camarines Norte	1,219	402	**1,621**	**1,336**	**455**	**1,791**	**90.51**
Camarines Sur	3,482	1,795	**5,277**	3,714	1,915	**5,629**	**93.75**
Catanduanes	442	243	**685**	**475**	**255**	**730**	**93.84**
Masbate	1,482	760	**2,242**	**1,616**	**808**	**2,424**	**92.49**
Sorsogon	1,350	647	**1,997**	**1,427**	**700**	**2,127**	**93.89**
							**0.00**
Iriga City	271	134	**405**	291	142	**433**	**93.53**
Legaspi City	390	194	**584**	432	211	**643**	**90.82**
Naga City	522	215	**737**	553	225	**778**	**94.73**
**Region 6**	**15,975**	**7,042**	**23,017**	**17,196**	**7,515**	**24,711**	**93.14**
Aklan	850	348	**1,198**	933	376	**1,309**	**91.52**
Antique	1,026	529	**1,555**	1,107	579	**1,686**	**92.23**
Capiz	1,335	584	**1,919**	1,449	629	**2,078**	**92.35**
Guimaras	346	154	**500**	357	157	**514**	**97.28**
Iloilo	3,296	1,371	**4,667**	3,453	1,432	**4,885**	**95.54**
Negros Occidental	6,016	2,744	**8,760**	6,620	2,960	**9,580**	**91.44**
Bacolod City	1,803	758	**2,561**	1,892	788	**2,680**	**95.56**
Iloilo City	1,303	554	**1,857**	1,385	594	**1,979**	**93.84**
**Region 7**	**10,050**	**4,562**	**14,612**	**11,099**	**4,980**	**16,079**	**90.88**
Bohol	1,311	608	**1,919**	1,424	660	**2,084**	**92.08**
Cebu	3,899	1,710	**5,609**	4,300	1,862	**6,162**	**91.03**
Negros Oriental	1,771	758	**2,529**	1,929	816	**2,745**	**92.13**
Siquijor	118	47	**165**	129	48	**177**	**93.22**
Cebu City	1,704	802	**2,506**	1,968	920	**2,888**	**86.77**
Lapu-Lapu City	744	356	**1,100**	795	379	**1,174**	**93.70**
Mandaue City	503	281	**784**	554	295	**849**	**92.34**
**Region 8**	**5,214**	**2,649**	**7,863**	**5,906**	**2,981**	**8,887**	**88.48**
Biliran	243	115	**358**	264	125	**389**	**92.03**
Eastern Samar	428	217	**645**	468	236	**704**	**91.62**
Northern Leyte	1,683	741	**2,424**	1,906	824	**2,730**	**88.79**
Northern Samar	723	449	**1,172**	813	505	**1,318**	**88.92**
Southern Leyte	311	144	**455**	369	167	**536**	**84.89**
Western Samar	681	336	**1,017**	771	380	**1,151**	**88.36**
Calbayog City	232	124	**356**	267	152	**419**	**84.96**
Maasin City	86	39	**125**	94	43	**137**	**91.24**
Ormoc City	261	164	**425**	325	203	**528**	**80.49**
Tacloban City	566	320	**886**	629	346	**975**	**90.87**
**Region 9**	**4,626**	**2,036**	**6,662**	**5,017**	**2,183**	**7,200**	**92.53**
Zamboanga del Norte	728	287	**1,015**	767	301	**1,068**	**95.04**
Zamboanga del Sur	960	419	**1,379**	1,065	460	**1,525**	**90.43**
Zamboanga Sibugay	612	290	**902**	705	326	**1,031**	**87.49**
Dapitan City	109	38	**147**	111	40	**151**	**97.35**
Dipolog City	175	59	**234**	188	62	**250**	**93.60**

Table 2.E.7.3. Tuberculosis

Treatment Success Rate DSTB - All Forms

Philippines,2022

Area	TB all forms cured and Completely Treated			Registered TB all forms Registered during Specified Period			Treatment Success Rate
	Male	Female	Total	Male	Female	Total	
Isabela City	58	40	**98**	72	45	**117**	**83.76**
Pagadian City	341	159	**500**	388	168	**556**	**89.93**
Zamboanga City	1,643	744	**2,387**	1,721	781	**2,502**	**95.40**
**Region 10**	**5,530**	**2,563**	**8,093**	**6,041**	**2,786**	**8,827**	**91.68**
Bukidnon	660	258	**918**	714	278	**992**	**92.54**
Camiguin	83	43	**126**	**91**	**49**	**140**	**90.00**
Lanao del Norte	808	408	**1,216**	**845**	**434**	**1,279**	**95.07**
Misamis Occidental	289	97	**386**	312	110	**422**	**91.47**
Misamis Oriental	846	351	**1,197**	896	370	**1,266**	**94.55**
			**0**				
Cagayan de Oro City	986	490	**1,476**	**1,186**	**578**	**1,764**	**83.67**
El Salvador City	83	32	**115**	89	33	**122**	**94.26**
Gingoog City	181	89	**270**	198	95	**293**	**92.15**
Iligan City	898	483	**1,381**	**970**	**508**	**1,478**	**93.44**
Malaybalay City	159	59	**218**	175	66	**241**	**90.46**
Oroquieta City	99	39	**138**	106	41	**147**	**93.88**
Ozamis City	204	110	**314**	213	115	**328**	**95.73**
Tangub City	12	4	**16**	12	5	**17**	**94.12**
Valencia City	222	100	**322**	234	104	**338**	**95.27**
**Region 11**	**6,344**	**2,612**	**8,956**	**7,053**	**2,930**	**9,983**	**89.71**
Davao de Oro	823	275	**1,098**	894	302	**1,196**	**91.81**
Davao del Norte	1,708	646	**2,354**	1,890	716	**2,606**	**90.33**
Davao del Sur	743	285	**1,028**	773	300	**1,073**	**95.81**
Davao Occidental	293	128	**421**	310	138	**448**	**93.97**
Davao Oriental	686	282	**968**	726	301	**1,027**	**94.26**
Davao City	2,091	996	**3,087**	2,460	1,173	**3,633**	**84.97**
**Region 12**	**7,009**	**3,109**	**10,118**	**7,656**	**3,358**	**11,014**	**91.86**
North Cotabato	1,772	755	**2,527**	1,968	835	**2,803**	**90.15**
Sarangani	752	337	**1,089**	835	378	**1,213**	**89.78**
South Cotabato	1,736	798	**2,534**	1,872	836	**2,708**	**93.57**
Sultan Kudarat	1,441	664	**2,105**	1,519	691	**2,210**	**95.25**
Gen. Santos City	1,308	555	**1,863**	1,462	618	**2,080**	**89.57**
**BARMM**	**2,782**	**1,863**	**4,645**	**3,155**	**2,031**	**5,186**	**89.57**
Basilan	305	227	**532**	320	236	**556**	**95.68**
Lanao del Sur	581	448	**1,029**	598	456	**1,054**	**97.63**
Maguindanao	662	360	**1,022**	689	365	**1,054**	**96.96**
Sulu	172	147	**319**	288	210	**498**	**64.06**
Tawi-Tawi	172	145	**317**	240	178	**418**	**75.84**
Cotabato City	572	323	**895**	680	362	**1,042**	**85.89**
Lamitan City	125	63	**188**	133	71	**204**	**92.16**
Marawi City	193	150	**343**	207	153	**360**	**95.28**
**CARAGA**	**4,450**	**2,182**	**6,632**	**4,812**	**2,325**	**7,137**	**92.92**
Agusan del Norte	500	211	**711**	544	223	**767**	**92.70**
Agusan del Sur	921	367	**1,288**	958	380	**1,338**	**96.26**
Surigao del Norte	645	373	**1,018**	706	406	**1,112**	**91.55**
Surigao del Sur	914	445	**1,359**	1,003	469	**1,472**	**92.32**
Province of Dinagat	138	55	**193**	141	59	**200**	**96.50**
			**0**				
Bislig City	336	247	**583**	354	254	**608**	**95.89**

Table 2.E.7.3. Tuberculosis
Treatment Success Rate DSTB - All Forms
Philippines,2022

Area	TB all forms cured and Completely Treated			Registered TB all forms Registered during Specified Period			Treatment Success Rate
	Male	Female	Total	Male	Female	Total	
Butuan City	614	279	**893**	679	307	**986**	**90.57**
Surigao City	382	205	**587**	427	227	**654**	**89.76**

Legend:
0 = No Case
** = No Report*

Table 2.E.7.4. Tuberculosis

Treatment Success Rate - MDRTB

Philippines, 2022

Area	Registered Bacteriology Confirmed Drug Resistant			Registered Bacteriologically Confirmed Drug Resistant Specified Period			Treatment Success Rate
	Male	Female	Total	Male	Female	Total	
**PHILIPPINES**	**2,757**	**1,122**	**3,879**	**3,417**	**1,479**	**4,896**	**79.23**
**N C R**	477	208	**685**	626	302	**928**	**73.81**
Malabon City	23	9	**32**	24	14	**38**	**84.21**
Navotas City	24	10	**34**	30	13	**43**	**79.07**
Valenzuela City	22	11	**33**	32	16	**48**	**68.75**
Caloocan City	41	21	**62**	55	26	**81**	**76.54**
Marikina City	35	10	**45**	41	21	**62**	**72.58**
Pasig City	19	16	**35**	30	22	**52**	**67.31**
Pateros			**0**			**0**	**0.00**
Taguig City	43	14	**57**	54	20	**74**	**77.03**
Quezon City	119	60	**179**	158	79	**237**	**75.53**
Makati City	23	5	**28**	25	13	**38**	**73.68**
Mandaluyong City	16	4	**20**	22	8	**30**	**66.67**
San Juan City	8	1	**9**	8	1	**9**	**100.00**
Manila City	42	21	**63**	66	38	**104**	**60.58**
Las Piñas City	10	6	**16**	14	7	**21**	**76.19**
Muntinlupa City	25	4	**29**	33	4	**37**	**78.38**
Parañaque City	11	10	**21**	15	13	**28**	**75.00**
Pasay City	16	6	**22**	19	7	**26**	**84.62**
**C A R**	17	9	**26**	23	9	**32**	**81.25**
Abra	4	0	**4**	4	0	**4**	**100.00**
Apayao	3	2	**5**	3	2	**5**	**100.00**
Benguet	0	0	**0**	0	0	**0**	**0.00**
Ifugao	0	1	**1**	1	1	**2**	**50.00**
Kalinga	2	2	**4**	2	2	**4**	**100.00**
Mt. Province	0	1	**1**	0	1	**1**	**100.00**
						**0**	
Baguio City	8	3	**11**	13	3	**16**	**68.75**
**Region 1**	143	57	**200**	172	80	**252**	**79.37**
Ilocos Norte	4	0	**4**	4	1	**5**	**80.00**
Ilocos Sur	7	3	**10**	8	3	**11**	**90.91**
La Union	8	2	**10**	12	3	**15**	**66.67**
Pangasinan	0	0	**0**	0	0	**0**	**0.00**
Alaminos City	12	4	**16**	12	5	**17**	**94.12**
Candon City	0	0	**0**	0	0	**0**	**0.00**
Dagupan City	33	9	**42**	37	15	**52**	**80.77**
Laoag City	0	0	**0**	0	0	**0**	**0.00**
San Carlos City	36	21	**57**	46	31	**77**	**74.03**
San Fernando City	14	6	**20**	18	6	**24**	**83.33**
Urdaneta City	26	12	**38**	30	16	**46**	**82.61**
Vigan City	3	0	**3**	5	0	**5**	**60.00**

Table 2.E.7.4. Tuberculosis

Treatment Success Rate - MDRTB

Philippines, 2022

Area	Registered Bacteriology Confirmed Drug Resistant			Registered Bacteriologically Confirmed Drug Resistant Specified Period			Treatment Success Rate
	Male	Female	Total	Male	Female	Total	
**Region 2**	55	23	**78**	71	28	**99**	**78.79**
Batanes	0	0	**0**	0	0	**0**	**0.00**
Cagayan	8	3	**11**	8	3	**11**	**100.00**
Isabela	0	0	**0**	0	0	**0**	**0.00**
Nueva Vizcaya	6	4	**10**	7	4	**11**	**90.91**
Quirino	3	2	**5**	3	2	**5**	**100.00**
Cauayan City	0	0	**0**	0	0	**0**	**0.00**
Ilagan City	0	0	**0**	0	0	**0**	**0.00**
Santiago City	21	3	**24**	28	5	**33**	**72.73**
Tuguegarao City	17	11	**28**	25	14	**39**	**71.79**
**Region 3**	383	174	**557**	478	221	**699**	**79.69**
Aurora	8	1	**9**	10	1	**11**	**90.91**
Bataan	8	4	**12**	11	5	**16**	**68.75**
Bulacan	76	37	**113**	90	44	**134**	**84.33**
Nueva Ecija	16	7	**23**	19	10	**29**	**65.52**
Pampanga	33	13	**46**	42	19	**61**	**68.85**
Tarlac	28	14	**42**	35	16	**51**	**78.00**
Zambales	22	5	**27**	30	6	**36**	**83.33**
						**0**	
Angeles City	13	13	**26**	17	14	**31**	**54.84**
Balanga City	14	8	**22**	20	10	**30**	**73.33**
Cabanatuan City	23	13	**36**	27	15	**42**	**85.71**
City of San Fernando	18	10	**28**	22	15	**37**	**75.68**
Gapan City			**0**			**0**	**0.00**
Mabalacat City	23	5	**28**	24	8	**32**	**87.50**
Malolos City	31	12	**43**	41	15	**56**	**76.79**
Meycauayan City			**0**			**0**	**0.00**
Olongapo City	11	5	**16**	12	7	**19**	**84.21**
Palayan City	0	0	**0**	0	0	**0**	**0.00**
San Jose City	4	3	**7**	10	4	**14**	**50.00**
San Jose del Monte City	32	18	**50**	40	23	**63**	**79.37**
Science City of Munoz	0	0	**0**	0	0	**0**	**0.00**
Tarlac City	23	6	**29**	28	9	**37**	**78.38**
**Region 4A**	470	196	**666**	576	263	**839**	**79.38**
Batangas	33	7	**40**	36	11	**47**	**85.11**
Cavite	10	6	**16**	10	7	**17**	**94.12**
Laguna	29	15	**44**	38	16	**54**	**81.48**
Quezon	37	12	**49**	44	16	**60**	**81.67**
Rizal	84	39	**123**	113	57	**170**	**72.35**
Antipolo City	37	16	**53**	46	22	**68**	**77.94**
Bacoor City	12	6	**18**	16	7	**23**	**78.26**
Batangas City	10	6	**16**	13	8	**21**	**76.19**
Biñan City	0	0	**0**	0	0	**0**	**0.00**
Cabuyao City	0	0	**0**	0	0	**0**	**0.00**
Calamba City	30	5	**35**	35	6	**41**	**85.37**
Cavite City			**0**			**0**	**0.00**
Dasmariñas City	36	9	**45**	40	10	**50**	**90.00**

Table 2.E.7.4. Tuberculosis

Treatment Success Rate - MDRTB

Philippines, 2022

Area	Registered Bacteriology Confirmed Drug Resistant			Registered Bacteriologically Confirmed Drug Resistant Specified Period			Treatment Success Rate
	Male	Female	Total	Male	Female	Total	
General Trias City	0	0	**0**		0	**0**	**0.00**
Imus City	11	10	**21**	15	12	**27**	**77.78**
Lipa City	24	4	**28**	32	8	**40**	**70.00**
Lucena City	24	12	**42**	26	15	**41**	**102.44**
San Pablo City	0	0	**0**	0	0	**0**	**0.00**
San Pedro City	0	0	**0**	0	0	**0**	**0.00**
Santa Rosa City	51	18	**69**	60	31	**91**	**75.82**
Tagaytay City	9	5	**14**	11	6	**17**	**82.35**
Tanauan City	0	0	**0**	0	0	**0**	**0.00**
Tayabas City	0	0	**0**	0	0	**0**	**0.00**
Trece Martires City	33	26	**59**	41	31	**72**	**81.94**
**Region 4B**	83	41	**124**	107	48	**155**	**80.00**
Marinduque	10	3	**13**	13	4	**17**	**76.47**
Mindoro Occidental	15	8	**23**	22	8	**30**	**76.67**
Mindoro Oriental	33	11	**44**	41	15	**56**	**78.57**
Palawan	9	9	**18**	12	9	**21**	**85.71**
Romblon	8	2	**10**	11	3	**14**	**71.43**
Puerto Princesa City	8	8	**16**	8	9	**17**	**94.12**
**Region 5**	246	91	**337**	282	108	**390**	**86.41**
Albay	22	5	**27**	23	6	**29**	**93.10**
Camarines Norte	45	12	**57**	50	12	**62**	**91.94**
Camarines Sur	15	4	**19**	17	5	**22**	**86.36**
Catanduanes	13	4	**17**	15	4	**19**	**89.47**
Masbate	22	10	**32**	29	13	**42**	**76.19**
Sorsogon	33	9	**42**	36	10	**46**	**91.30**
Iriga City	0	0	**0**	0	0	**0**	**0.00**
Legaspi City	39	12	**51**	45	15	**60**	**85.00**
Naga City	57	35	**0**	67	43	**110**	**0.00**
**Region 6**	245	71	**316**	291	90	**381**	**82.94**
Aklan	15	6	**21**	20	10	**30**	**70.00**
Antique	19	8	**27**	21	8	**29**	**93.10**
Capiz	19	2	**21**	22	2	**24**	**87.50**
Guimaras	0	0	**0**	0	0	**0**	**0.00**
Iloilo	27	6	**33**	32	9	**41**	**80.49**
Negros Occidental	102	30	**132**	121	36	**157**	**84.08**
			**0**			**0**	
Bacolod City	19	8	**27**	25	10	**35**	**77.14**
Iloilo City	44	11	**55**	50	15	**65**	**84.62**
**Region 7**	154	53	**207**	199	73	**272**	**76.10**
Bohol	24	15	**39**	31	20	**51**	**76.47**
Cebu	0	0	**0**	0	0	**0**	**0.00**
Negros Oriental	31	4	**35**	35	5	**40**	**87.50**
Siquijor			**0**			**0**	**0.00**

Table 2.E.7.4. Tuberculosis

Treatment Success Rate - MDRTB
Philippines, 2022

Area	Registered Bacteriology Confirmed Drug Resistant			Registered Bacteriologically Confirmed Drug Resistant Specified Period			Treatment Success Rate
	Male	Female	Total	Male	Female	Total	
Cebu City	47	21	**68**	65	25	**90**	**75.56**
Lapu-Lapu City	0	0	**0**	0	0	**0**	**0.00**
Mandaue City	52	13	**65**	68	23	**91**	**71.43**
**Region 8**	69	35	**104**	78	38	**116**	**89.66**
Biliran	0	0	**0**	0	0	**0**	**0.00**
Eastern Samar	9	5	**14**	9	6	**15**	**93.33**
Northern Leyte	29	12	**41**	33	14	**47**	**87.23**
Northern Samar	7	5	**12**	8	5	**13**	**92.31**
Southern Leyte	0	0	**0**	0	0	**0**	**0.00**
Western Samar	0	0	**0**	0	0	**0**	**0.00**
Calbayog City	13	4	**17**	13	4	**17**	**100.00**
Maasin City	11	9	**20**	15	9	**24**	**83.33**
Ormoc City	0	0	**0**	0	0	**0**	**0.00**
Tacloban City	0	0	**0**	0	0	**0**	**0.00**
**Region 9**	54	23	**77**	64	29	**93**	**82.80**
Zamboanga del Norte	0	0	**0**	0	0	**0**	**0.00**
Zamboanga del Sur	10	4	**14**	15	6	**21**	**66.67**
Zamboanga Sibugay	11	7	**18**	12	8	**20**	**90.00**
						**0**	
Dapitan City	9	4	**13**	11	5	**16**	**81.25**
Dipolog City	0	0	**0**	0	0	**0**	**0.00**
Isabela City	2	1	**3**	2	1	**3**	**100.00**
Pagadian City	0	0	**0**	0	0	**0**	**0.00**
Zamboanga City	22	7	**29**	24	9	**33**	**87.88**
**Region 10**	75	34	**109**	93	43	**136**	**80.15**
Bukidnon	0	0	**0**	0	1	**1**	**0.00**
Camiguin	0	0	**0**	0	0	**0**	**0.00**
Lanao del Norte	15	5	**20**	17	8	**25**	**80.00**
Misamis Occidental	0	0	**0**	0	0	**0**	**0.00**
Misamis Oriental	0	0	**0**	0	0	**0**	**0.00**
			**0**				
Cagayan de Oro City	24	11	**35**	28	12	**40**	**87.50**
El Salvador City	0	0	**0**	0	0	**0**	**0.00**
Gingoog City	5	2	**7**	6	2	**8**	**87.50**
Iligan City	12	7	**19**	18	8	**26**	**73.08**
Malaybalay City	11	3	**14**	13	3	**16**	**87.50**
Oroquieta City	4	4	**8**	5	6	**11**	**72.73**
Ozamis City	4	2	**6**	6	3	**9**	**66.67**
Tangub City	0	0	**0**	0	0	**0**	**0.00**
Valencia City	0	0	**0**	0	0	**0**	**0.00**
**Region 11**	105	35	**140**	134	49	**183**	**76.50**
Compostela Valley	0	0	**0**	0	0	**0**	**0.00**
Davao del Norte	54	9	**63**	61	14	**75**	**84.00**
Davao del Sur	7	3	**10**	14	3	**17**	**58.82**
Davao Occidental	1	0	**1**	3	0	**3**	**33.33**

Table 2.E.7.4. Tuberculosis
Treatment Success Rate - MDRTB
Philippines, 2022

Area	Registered Bacteriology Confirmed Drug Resistant			Registered Bacteriologically Confirmed Drug Resistant Specified Period			Treatment Success Rate
	Male	Female	Total	Male	Female	Total	
Davao Oriental	6	9	**15**	8	9	**17**	**88.24**
Davao City	37	14	**51**	48	23	**71**	**71.83**
**Region 12**	91	22	**113**	115	28	**143**	**79.02**
North Cotabato	28	4	**32**	38	7	**45**	**71.11**
Sarangani	2	1	**3**	3	1	**4**	**75.00**
South Cotabato	14	10	**24**	21	11	**32**	**75.00**
Sultan Kudarat	19	4	**23**	21	5	**26**	**88.46**
Gen. Santos City	28	3	**31**	32	4	**36**	**86.11**
**BARMM**	33	18	**51**	38	31	**69**	**73.91**
Basilan	0	0	**0**	0	0	**0**	**0.00**
Lanao del Sur	0	0	**0**	0	0	**0**	**0.00**
Maguindanao	8	1	**9**	10	2	**12**	**75.00**
Sulu	3	2	**5**	4	3	**7**	**71.43**
Tawi-Tawi	0	0	**0**	0	0	**0**	**0.00**
Cotabato City	14	8	**22**	16	12	**28**	**78.57**
Lamitan City	0	0	**0**	0	0	**0**	**0.00**
Marawi City	8	7	**15**	8	14	**22**	**68.18**
**CARAGA**	57	32	**89**	70	39	**109**	**81.65**
Agusan del Norte	0	0	**0**	0	0	**0**	**0.00**
Agusan del Sur	10	8	**18**	16	11	**27**	**66.67**
Surigao del Norte	2	1	**3**	3	3	**6**	**50.00**
Surigao del Sur	10	5	**15**	14	7	**21**	**71.43**
Province of Dinagat	2	2	**4**	2	2	**4**	**100.00**
Bislig City	8	6	**14**	9	6	**15**	**93.33**
Butuan City	13	6	**19**	13	6	**19**	**100.00**
Surigao City	12	4	**16**	13	4	**17**	**94.12**

Table 2.F.1.1. Lifestyle Related Diseases (Risk Assessment)

Number and proportion of adults age 20 yrs old and above who were risk-assessed using the PhilPEN
Philippines, 2022

Area	Eligible Pop. (20 y/o & above)	Adults who were risk-assessed			
		Male	Female	Total	%
**Philippines**	**68,116,194**	**3,417,054**	**3,783,001**	**7,200,055**	**10.57**
**NCR**	**9,255,105**	**210,226**	**364,674**	**574,900**	**6.21**
Malabon	**262,707**	9,244	14,738	**23,982**	**9.13**
Navotas	**179,295**	8,351	15,161	**23,512**	**13.11**
Valenzuela City	**445,903**	16,529	25,329	**41,858**	**9.39**
Caloocan City	**1,138,437**	9,395	26,780	**36,175**	**3.18**
Marikina City	**323,954**	1,929	3,169	**5,098**	**1.57**
Pasig City	**542,850**	19,830	34,184	**54,014**	**9.95**
Pateros	**45,883**	948	1,587	**2,535**	**5.52**
Taguig	**578,508**	30,664	58,323	**88,987**	**15.38**
Quezon City	**2,110,240**	38,519	57,778	**96,297**	**4.56**
Makati City	**418,722**	7,379	17,010	**24,389**	**5.82**
Mandaluyong City	**277,629**	9,873	15,556	**25,429**	**9.16**
San Juan	**87,811**	2,375	4,671	**7,046**	**8.02**
Manila City	**1,279,423**	26,470	45,552	**72,022**	**5.63**
Las Piñas City	**423,249**	1,963	2,714	**4,677**	**1.11**
Muntinlupa City	**362,602**	6,825	11,109	**17,934**	**4.95**
Parañaque City	**478,536**	7,313	13,945	**21,258**	**4.44**
Pasay City	**299,356**	12,619	17,068	**29,687**	**9.92**
**CAR**	**1,128,030**	**74,860**	**104,204**	**179,064**	**15.87**
Abra	**156,220**	23,006	25,918	**48,924**	**31.32**
Apayao	**73,675**	12,723	19,133	**31,856**	**43.24**
Benguet	**312,406**	11,130	16,587	**27,717**	**8.87**
Ifugao	**122,865**	5,102	8,928	**14,030**	**11.42**
Kalinga	**128,638**	10,407	12,725	**23,132**	**17.98**
Mt. Province	**92,433**	9,422	13,293	**22,715**	**24.57**
Baguio City	**241,793**	3,070	7,620	**10,690**	**4.42**
**Region 1**	**3,327,393**	**239,312**	**263,834**	**503,146**	**15.12**
Ilocos Norte	**337,295**	4,128	4,825	**8,953**	**2.65**
Ilocos Sur	**398,222**	30,174	36,131	**66,305**	**16.65**
La Union	**464,902**	44,830	46,896	**91,726**	**19.73**
Pangasinan	**1,513,447**	98,723	92,811	**191,534**	**12.66**
Alaminos City	**57,184**	2,631	2,351	**4,982**	**8.71**
Candon City	**41,974**	305	275	**580**	**1.38**
Dagupan City	**109,173**	5,328	5,394	**10,722**	**9.82**
Laoag City	**77,767**	655	587	**1,242**	**1.60**
San Carlos City	**120,198**	2,545	2,482	**5,027**	**4.18**
San Fernando City	**85,182**	10,179	13,705	**23,884**	**28.04**
Urdaneta City	**84,741**	38,476	56,794	**95,270**	**112.42**
Vigan City	**37,308**	1,338	1,583	**2,921**	**7.83**

Table 2.F.1.1. Lifestyle Related Diseases (Risk Assessment)

Number and proportion of adults age 20 yrs old and above who were risk-assessed using the PhilPEN
Philippines, 2022

Area	Eligible Pop. (20 y/o & above)	Adults who were risk-assessed			
		Male	Female	Total	%
**Region 2**	**2,315,282**	**324,259**	**395,472**	**719,731**	**31.09**
Batanes	**10,762**	1,109	2,045	**3,154**	**29.31**
Cagayan	**710,775**	52,844	52,879	**105,723**	**14.87**
Isabela	**794,323**	114,699	151,725	**266,424**	**33.54**
Nueva Vizcaya	**300,303**	29,118	39,369	**68,487**	**22.81**
Quirino	**119,703**	48,529	55,818	**104,347**	**87.17**
Cauayan City	**86,922**	2,780	4,944	**7,724**	**8.89**
Ilagan City	**97,682**	30,058	41,556	**71,614**	**73.31**
Santiago City	**90,480**	44,329	46,151	**90,480**	**100.00**
Tuguegarao City	**104,332**	793	985	**1,778**	**1.70**
**Region 3**	**8,082,454**	**578,078**	**651,594**	**1,229,672**	**15.21**
Aurora	**136,678**	42,427	50,179	**92,606**	**67.75**
Bataan	**478,275**	20,890	32,930	**53,820**	**11.25**
Bulacan	**1,652,464**	115,286	121,898	**237,184**	**14.35**
Nueva Ecija	**1,062,780**	38,143	40,112	**78,255**	**7.36**
Pampanga	**1,204,521**	21,896	27,611	**49,507**	**4.11**
Tarlac	**711,338**	43,086	46,967	**90,053**	**12.66**
Zambales	**411,689**	39,960	49,916	**89,876**	**21.83**
Angeles City	**302,221**	1,870	2,259	**4,129**	**1.37**
Balanga City	**69,134**	1,010	1,280	**2,290**	**3.31**
Cabanatuan City	**217,520**	12,076	16,609	**28,685**	**13.19**
City of San Fernando	**225,146**	4,735	6,247	**10,982**	**4.88**
Gapan City	**79,388**	80	70	**150**	**0.19**
Mabalacat City	**184,135**	25,844	38,845	**64,689**	**35.13**
Malolos City	**184,576**	3,795	4,965	**8,760**	**4.75**
Meycauayan	**153,092**	794	811	**1,605**	**1.05**
Olongapo	**162,374**	1,218	2,626	**3,844**	**2.37**
Palayan City	**29,538**	119	98	**217**	**0.73**
San Jose City	**100,568**	10,022	6,873	**16,895**	**16.80**
San Jose del Monte Cit	**420,348**	182,583	189,305	**371,888**	**88.47**
Science City of Munoz	**58,643**	10,956	10,627	**21,583**	**36.80**
Tarlac City	**238,026**	1,288	1,366	**2,654**	**1.12**
**Region 4A**	**10,471,181**	**175,201**	**179,812**	**355,013**	**3.39**
Batangas	**1,380,486**	36,102	32,291	**68,393**	**4.95**
Cavite	**1,047,657**	35,520	38,567	**74,087**	**7.07**
Laguna	**725,078**	6,970	6,481	**13,451**	**1.86**
Quezon	**1,130,173**	22,332	21,457	**43,789**	**3.87**
Rizal	**1,518,351**	9,308	8,924	**18,232**	**1.20**
Antipolo City	**559,259**	0	0	**0**	**0.00**
Bacoor City	**459,001**	328	357	**685**	**0.15**
Batangas City	**244,997**	758	950	**1,708**	**0.70**
Biñan City	**243,128**	2,338	2,749	**5,087**	**2.09**
Cabuyao City	**225,399**	480	395	**875**	**0.39**

Table 2.F.1.1. Lifestyle Related Diseases (Risk Assessment)

Number and proportion of adults age 20 yrs old and above who were risk-assessed using the PhilPEN
Philippines, 2022

Area	Eligible Pop. (20 y/o & above)	Adults who were risk-assessed			
		Male	Female	Total	%
Calamba City	**331,801**	2,810	3,113	**5,923**	**1.79**
Cavite City	**78,563**	228	233	**461**	**0.59**
Dasmariñas City	**503,637**	21,498	28,212	**49,710**	**9.87**
General Trias City	**240,198**	3,603	5,047	**8,650**	**3.60**
Imus City	**308,590**	1,343	1,787	**3,130**	**1.01**
Lipa City	**246,866**	5,879	1,548	**7,427**	**3.01**
Lucena City	**171,283**	636	846	**1,482**	**0.87**
San Pablo City	**194,243**	492	732	**1,224**	**0.63**
San Pedro City	**237,856**	19	23	**42**	**0.02**
Santa Rosa City	**258,264**	5,440	6,818	**12,258**	**4.75**
Tagaytay City	**54,398**	7,988	8,068	**16,056**	**29.52**
Tanauan City	**128,760**	4,162	2,334	**6,496**	**5.05**
Tayabas City	**64,192**	4,135	5,507	**9,642**	**15.02**
Trece Martires City	**119,001**	2,832	3,373	**6,205**	**5.21**
**Region 4B**	**1,825,131**	**136,106**	**85,733**	**221,839**	**12.15**
Marinduque	**140,451**	4,032	2,795	**6,827**	**4.86**
Mindoro Occidental	**287,885**	9,345	10,899	**20,244**	**7.03**
Mindoro Oriental	**523,141**	30,978	11,075	**42,053**	**8.04**
Palawan	**540,053**	87,183	56,389	**143,572**	**26.58**
Romblon	**171,414**	3,649	3,225	**6,874**	**4.01**
Puerto Princesa City	**162,187**	919	1,350	**2,269**	**1.40**
**Region 5**	**3,515,705**	**220,275**	**260,358**	**480,633**	**13.67**
Albay	**712,843**	19,864	21,901	**41,765**	**5.86**
Camarines Norte	**361,986**	20,030	25,649	**45,679**	**12.62**
Camarines Sur	**988,031**	85,601	106,600	**192,201**	**19.45**
Catanduanes	**154,126**	11,013	12,902	**23,915**	**15.52**
Masbate	**508,674**	20,482	11,731	**32,213**	**6.33**
Sorsogon	**479,811**	50,797	64,417	**115,214**	**24.01**
Iriga City	**67,134**	6,547	9,779	**16,326**	**24.32**
Legaspi City	**125,358**	2,893	3,415	**6,308**	**5.03**
Naga City	**117,742**	3,048	3,964	**7,012**	**5.96**
**Region 6**	**4,990,616**	**274,702**	**310,235**	**584,937**	**11.72**
Aklan	**381,865**	30,586	44,398	**74,984**	**19.64**
Antique	**363,531**	20,029	29,848	**49,877**	**13.72**
Capiz	**525,351**	18,597	25,866	**44,463**	**8.46**
Guimaras	**119,494**	16,635	20,590	**37,225**	**31.15**
Iloilo	**1,328,314**	86,093	69,630	**155,723**	**11.72**
Negros Occidental	**1,603,902**	88,289	95,892	**184,181**	**11.48**
Bacolod City	**360,867**	2,867	6,685	**9,552**	**2.65**
Iloilo City	**307,292**	11,606	17,326	**28,932**	**9.42**

Table 2.F.1.1. Lifestyle Related Diseases (Risk Assessment)

Number and proportion of adults age 20 yrs old and above who were risk-assessed using the PhilPEN
Philippines, 2022

Area	Eligible Pop. (20 y/o & above)	Adults who were risk-assessed			
		Male	Female	Total	%
**Region 7**	**4,980,831**	**187,771**	**278,121**	**465,892**	**9.35**
Bohol	**819,036**	25,951	43,312	**69,263**	**8.46**
Cebu	**2,047,936**	110,566	168,935	**279,501**	**13.65**
Negros Oriental	**869,370**	10,230	11,058	**21,288**	**2.45**
Siquijor	**64,498**	5,016	7,064	**12,080**	**18.73**
Cebu City	**642,902**	3,428	5,414	**8,842**	**1.38**
Lapu-Lapu City	**284,384**	3,061	3,680	**6,741**	**2.37**
Mandaue City	**252,705**	29,519	38,658	**68,177**	**26.98**
					**0.00**
**Region 8**	**2,802,545**	**187,826**	**203,477**	**391,303**	**13.96**
Biliran	**105,448**	2,499	3,757	**6,256**	**5.93**
Eastern Samar	**295,358**	30,032	35,297	**65,329**	**22.12**
Northern Leyte	**991,606**	26,705	33,845	**60,550**	**6.11**
Northern Samar	**368,719**	21,954	15,840	**37,794**	**10.25**
Southern Leyte	**220,448**	32,805	27,765	**60,570**	**27.48**
Western Samar	**355,167**	45,372	49,128	**94,500**	**26.61**
Calbayog City	**109,441**	360	721	**1,081**	**0.99**
Maasin City	**56,096**	1,050	1,018	**2,068**	**3.69**
Ormoc City	**141,246**	3,328	4,436	**7,764**	**5.50**
Tacloban City	**159,016**	23,721	31,670	**55,391**	**34.83**
**Region 9**	**2,242,795**	**102,381**	**114,016**	**216,397**	**9.65**
Zamboanga Del Norte	**491,126**	20,850	21,859	**42,709**	**8.70**
Zamboanga Del Sur	**499,579**	20,407	21,496	**41,903**	**8.39**
Zamboanga Sibugay	**391,467**	48,781	53,789	**102,570**	**26.20**
Dapitan City	**50,704**	1,530	1,889	**3,419**	**6.74**
Dipolog City	**80,454**	1,361	1,461	**2,822**	**3.51**
Isabela City	**76,485**	953	441	**1,394**	**1.82**
Pagadian City	**122,528**	149	660	**809**	**0.66**
Zamboanga City	**530,452**	8,350	12,421	**20,771**	**3.92**
**Region 10**	**3,084,632**	**152,384**	**61,822**	**214,206**	**6.94**
Bukidnon	**680,822**	72,572	13,143	**85,715**	**12.59**
Camiguin	**56,410**	84	133	**217**	**0.38**
Lanao Del Norte	**427,934**	19,515	11,624	**31,139**	**7.28**
Misamis Occidental	**210,949**	12,136	9,808	**21,944**	**10.40**
Misamis Oriental	**490,301**	126	128	**254**	**0.05**
Cagayan de Oro City	**464,385**	15,939	5,280	**21,219**	**4.57**
El Salvador City	**34,493**	102	520	**622**	**1.80**
Gingoog City	**85,632**	58	74	**132**	**0.15**
Iligan City	**216,758**	3,438	2,063	**5,501**	**2.54**
Malaybalay City	**113,486**	5,558	641	**6,199**	**5.46**
Oroquieta City	**45,710**	9,816	1,517	**11,333**	**24.79**
Ozamis City	**91,623**	7,021	9,071	**16,092**	**17.56**

Table 2.F.1.1. Lifestyle Related Diseases (Risk Assessment)

Number and proportion of adults age 20 yrs old and above who were risk-assessed using the PhilPEN
Philippines, 2022

Area	Eligible Pop. (20 y/o & above)	Adults who were risk-assessed			
		Male	Female	Total	%
Tangub City	**40,708**	227	171	**398**	**0.98**
Valencia City	**125,421**	5,792	7,649	**13,441**	**10.72**
**Region 11**	**3,274,878**	**263,794**	**242,215**	**506,009**	**15.45**
Davao de Oro	**469,935**	62,604	52,098	**114,702**	**24.41**
Davao del Norte	**650,744**	73,927	65,507	**139,434**	**21.43**
Davao Oriental	**350,420**	30,457	31,995	**62,452**	**17.82**
Davao del Sur	**446,748**	50,093	41,989	**92,082**	**20.61**
Davao Occidental	**203,774**	7,915	7,503	**15,418**	**7.57**
Davao City	**1,153,257**	38,798	43,123	**81,921**	**7.10**
**Region 12**	**2,778,484**	**181,333**	**186,408**	**367,741**	**13.24**
North Cotabato	**909,490**	41,714	46,120	**87,834**	**9.66**
Sarangani	**338,425**	45,168	30,052	**75,220**	**22.23**
South Cotabato	**617,577**	26,419	28,069	**54,488**	**8.82**
Sultan Kudarat	**511,900**	66,151	79,709	**145,860**	**28.49**
Gen. Santos City	**401,092**	1,881	2,458	**4,339**	**1.08**
**BARMM**	**2,418,859**	**32,996**	**33,473**	**66,469**	**2.75**
Basilan	**159,715**	4,258	1,044	**5,302**	**3.32**
Lanao Del Sur	**487,992**	9,125	10,485	**19,610**	**4.02**
Maguindanao	**717,889**	7,108	6,434	**13,542**	**1.89**
Sulu	**491,900**	191	85	**276**	**0.06**
Tawi-Tawi	**221,191**	1,954	1,080	**3,034**	**1.37**
Lamitan City	**43,940**	1,064	328	**1,392**	**3.17**
Marawi City	**116,733**	4,515	7,224	**11,739**	**10.06**
Cotabato City	**179,499**	4,781	6,793	**11,574**	**6.45**
**CARAGA**	**1,622,273**	**75,550**	**47,553**	**123,103**	**7.59**
Agusan del Norte	**228,449**	11,423	6,465	**17,888**	**7.83**
Agusan del Sur	**413,122**	30,963	9,015	**39,978**	**9.68**
Surigao del Norte	**219,323**	5,673	1,653	**7,326**	**3.34**
Surigao del Sur	**305,771**	5,058	5,335	**10,393**	**3.40**
Province of Dinagat	**78,175**	750	659	**1,409**	**1.80**
Bislig City	**58,072**	8,914	10,423	**19,337**	**33.30**
Butuan City	**217,214**	11,253	12,882	**24,135**	**11.11**
Surigao City	**102,147**	1,516	1,121	**2,637**	**2.58**

Table 2.F.1.2. Lifestyle Related Diseases (Tobacco Use, Harmful Use of Alcohol, Unhealthy Diet)

Number and proportion of adults ages 20 yrs. old and above who are current smokers, binge drinkers and overweight or obese

Philippines, 2022

Area	Total No. of clients ≥ 20 y/o risk-assesed[1]	Current Smokers				Binge Drinkers				Overweight or obese			
		Male	Female	Total	%	Male	Female	Total	%	Male	Female	Total	%
**PHILIPPINES**	**7,190,509**	**1,301,629**	**325,411**	**1,627,040**	**22.63**	**984,594**	**339,920**	**1,324,514**	**18.42**	**390,302**	**530,954**	**921,256**	**12.81**
**N C R**	**574,900**	**34,386**	**22,239**	**56,625**	**9.85**	**44,553**	**37,896**	**82,449**	**14.34**	**80,563**	**141,832**	**222,395**	**38.68**
Malabon	**23,982**	1,481	988	**2,469**	**10.30**	1,576	1,363	**2,939**	**12.26**	3,125	5,558	**8,683**	**36.21**
Navotas	**23,512**	1,658	870	**2,528**	**10.75**	2,098	1,677	**3,775**	**16.06**	3,446	6,519	**9,965**	**42.38**
Valenzuela City	**41,858**	2,392	1,047	**3,439**	**8.22**	3,607	2,186	**5,793**	**13.84**	6,433	9,787	**16,220**	**38.75**
Caloocan City	**36,175**	1,603	1,448	**3,051**	**8.43**	2,141	2,809	**4,950**	**13.68**	3,189	10,526	**13,715**	**37.91**
Marikina City	**5,098**	577	242	**819**	**16.07**	530	221	**751**	**14.73**	910	1,695	**2,605**	**51.10**
Pasig City	**54,014**	2,861	1,644	**4,505**	**8.34**	3,528	2,781	**6,309**	**11.68**	6,588	12,047	**18,635**	**34.50**
Pateros	**2,535**	230	107	**337**	**13.29**	218	126	**344**	**13.57**	494	928	**1,422**	**56.09**
Taguig	**88,987**	6,525	3,405	**9,930**	**11.16**	11,455	9,237	**20,692**	**23.25**	14,993	28,701	**43,694**	**49.10**
Quezon City	**96,297**	3,242	4,863	**8,105**	**8.42**	5,621	8,430	**14,051**	**14.59**	11,438	17,157	**28,595**	**29.69**
Makati City	**24,389**	1,186	735	**1,921**	**7.88**	1,463	983	**2,446**	**10.03**	3,297	5,509	**8,806**	**36.11**
Mandaluyong City	**25,429**	1,856	961	**2,817**	**11.08**	1,190	806	**1,996**	**7.85**	4,468	7,277	**11,745**	**46.19**
San Juan	**7,046**	445	321	**766**	**10.87**	607	374	**981**	**13.92**	1,578	2,965	**4,543**	**64.48**
Manila City	**72,022**	4,919	3,192	**8,111**	**11.26**	5,467	3,965	**9,432**	**13.10**	8,964	16,311	**25,275**	**35.09**
Las Piñas City	**4,677**	261	142	**403**	**8.62**	344	196	**540**	**11.55**	413	697	**1,110**	**23.73**
Muntinlupa City	**17,934**	1,336	506	**1,842**	**10.27**	1,070	644	**1,714**	**9.56**	3,284	5,391	**8,675**	**48.37**
Parañaque City	**21,258**	1,218	522	**1,740**	**8.19**	1,444	770	**2,214**	**10.41**	1,755	3,124	**4,879**	**22.95**
Pasay City	**29,687**	2,596	1,246	**3,842**	**12.94**	2,194	1,328	**3,522**	**11.86**	6,188	7,640	**13,828**	**46.58**
**C A R**	**179,064**	**16,720**	**5,022**	**21,742**	**12.14**	**19,056**	**6,404**	**25,460**	**14.22**	**13,042**	**21,807**	**34,849**	**19.46**
Abra	**48,924**	6,530	2,161	**8,691**	**17.76**	6,631	2,383	**9,014**	**18.42**	2,320	3,167	**5,487**	**11.22**
Apayao	**31,856**	3,129	1,037	**4,166**	**13.08**	4,075	1,323	**5,398**	**16.95**	1,842	2,876	**4,718**	**14.81**
Benguet	**27,717**	1,799	426	**2,225**	**8.03**	2,111	585	**2,696**	**9.73**	3,153	4,932	**8,085**	**29.17**
Ifugao	**14,030**	309	120	**429**	**3.06**	582	302	**884**	**6.30**	543	1,029	**1,572**	**11.20**
Kalinga	**23,132**	2,860	738	**3,598**	**15.55**	2,986	561	**3,547**	**15.33**	459	690	**1,149**	**4.97**
Mt. Province	**22,715**	1,384	106	**1,490**	**6.56**	1,686	342	**2,028**	**8.93**	3,250	5,178	**8,428**	**37.10**
Baguio City	**10,690**	709	434	**1,143**	**10.69**	985	908	**1,893**	**17.71**	1,475	3,935	**5,410**	**50.61**
**Region 1**	**503,146**	**52,891**	**17,592**	**70,483**	**14.01**	**50,462**	**16,373**	**66,835**	**13.28**	**21,304**	**23,410**	**44,714**	**8.89**
Ilocos Norte	**8,953**	532	134	**666**	**7.44**	553	138	**691**	**7.72**	296	460	**756**	**8.44**
Ilocos Sur	**66,305**	7,160	1,668	**8,828**	**13.31**	5,145	1,347	**6,492**	**9.79**	4,034	4,918	**8,952**	**13.50**
La Union	**91,726**	6,495	1,689	**8,184**	**8.92**	7,061	1,909	**8,970**	**9.78**	4,999	6,335	**11,334**	**12.36**
Pangasinan	**191,534**	33,497	12,138	**45,635**	**23.83**	32,808	10,943	**43,751**	**22.84**	9,267	8,869	**18,136**	**9.47**
Alaminos City	**4,982**	1,113	543	**1,656**	**33.24**	813	303	**1,116**	**22.40**	341	228	**569**	**11.42**
Candon City	**580**	90	19	**109**	**18.79**	24	5	**29**	**5.00**	89	70	**159**	**27.41**
Dagupan City	**10,722**	545	266	**811**	**7.56**	526	261	**787**	**7.34**	465	267	**732**	**6.83**
Laoag City	**1,242**	87	37	**124**	**9.98**	84	28	**112**	**9.02**	19	17	**36**	**2.90**
San Carlos City	**5,027**	1,078	513	**1,591**	**31.65**	958	464	**1,422**	**28.29**	762	946	**1,708**	**33.98**
San Fernando City	**23,884**	426	127	**553**	**2.32**	665	178	**843**	**3.53**	29	30	**59**	**0.25**
Urdaneta City	**95,270**	1,798	442	**2,240**	**2.35**	1,791	788	**2,579**	**2.71**	857	1,060	**1,917**	**2.01**
Vigan City	**2,921**	70	16	**86**	**2.94**	34	9	**43**	**1.47**	146	210	**356**	**12.19**
**Region 2**	**719,731**	**73,084**	**16,647**	**89,731**	**12.47**	**68,745**	**16,961**	**85,706**	**11.91**	**20,550**	**27,335**	**47,885**	**6.65**
Batanes	**3,154**	198	72	**270**	**8.56**	484	301	**785**	**24.89**	280	506	**786**	**24.92**
Cagayan	**105,723**	12,398	2,470	**14,868**	**14.06**	10,529	2,127	**12,656**	**11.97**	2,908	4,210	**7,118**	**6.73**
Isabela	**266,424**	35,488	6,201	**41,689**	**15.65**	25,416	4,653	**30,069**	**11.29**	8,331	12,055	**20,386**	**7.65**
Nueva Vizcaya	**68,487**	3,783	1,572	**5,355**	**7.82**	7,196	2,410	**9,606**	**14.03**	2,002	3,127	**5,129**	**7.49**
Quirino	**104,347**	5,830	2,238	**8,068**	**7.73**	6,760	2,013	**8,773**	**8.41**	2,265	2,625	**4,890**	**4.69**
Cauayan City	**7,724**	86	18	**104**	**1.35**	93	22	**115**	**1.49**	4	1	**5**	**0.06**
Ilagan City	**71,614**	4,268	1,296	**5,564**	**7.77**	4,877	1,236	**6,113**	**8.54**	543	715	**1,258**	**1.76**
Santiago City	**90,480**	10,880	2,745	**13,625**	**15.06**	13,218	4,164	**17,382**	**19.21**	4,056	3,855	**7,911**	**8.74**
Tuguegarao City	**1,778**	153	35	**188**	**10.57**	172	35	**207**	**11.64**	161	241	**402**	**22.61**
**Region 3**	**1,229,672**	**110,597**	**50,447**	**161,044**	**13.10**	**108,424**	**52,660**	**161,084**	**13.10**	**38,961**	**53,583**	**92,544**	**7.53**
Aurora	**92,606**	5,718	1,872	**7,590**	**8.20**	7,394	3,368	**10,762**	**11.62**	2,915	4,005	**6,920**	**7.47**
Bataan	**53,820**	3,659	921	**4,580**	**8.51**	2,581	721	**3,302**	**6.14**	1,991	3,065	**5,056**	**9.39**
Bulacan	**237,184**	21,260	9,758	**31,018**	**13.08**	20,497	10,450	**30,947**	**13.05**	11,206	17,910	**29,116**	**12.28**
Nueva Ecija	**78,255**	11,546	3,019	**14,565**	**18.61**	11,577	2,842	**14,419**	**18.43**	4,884	5,607	**10,491**	**13.41**
Pampanga	**49,507**	5,723	1,356	**7,079**	**14.30**	4,515	1,124	**5,639**	**11.39**	2,675	3,537	**6,212**	**12.55**
Tarlac	**90,053**	6,278	4,238	**10,516**	**11.68**	4,562	3,074	**7,636**	**8.48**	3,638	3,636	**7,274**	**8.08**
Zambales	**89,876**	7,144	2,595	**9,739**	**10.84**	6,878	2,387	**9,265**	**10.31**	2,291	2,765	**5,056**	**5.63**
Angeles City	**4,129**	644	315	**959**	**23.23**	299	133	**432**	**10.46**	60	152	**212**	**5.13**
Balanga City	**2,290**	345	48	**393**	**17.16**	376	70	**446**	**19.48**	268	380	**648**	**28.30**
Cabanatuan City	**28,685**	4,596	1,451	**6,047**	**21.08**	5,739	2,276	**8,015**	**27.94**	292	468	**760**	**2.65**
City of San Fernando	**10,982**	1,219	499	**1,718**	**15.64**	664	298	**962**	**8.76**	610	989	**1,599**	**14.56**
Gapan City	**150**	43	24	**67**	**44.67**	28	13	**41**	**27.33**	23	27	**50**	**33.33**
Mabalacat City	**64,689**	8,291	3,153	**11,444**	**17.69**	9,380	4,143	**13,523**	**20.90**	4,832	6,793	**11,625**	**17.97**
Malolos City	**8,760**	785	390	**1,175**	**13.41**	707	420	**1,127**	**12.87**	113	191	**304**	**3.47**
Meycauayan	**1,605**	157	61	**218**	**13.58**	151	41	**192**	**11.96**	34	39	**73**	**4.55**
Olongapo	**3,844**	299	146	**445**	**11.58**	213	162	**375**	**9.76**	54	89	**143**	**3.72**
Palayan City	**217**	65	40	**105**	**48.39**	16	0	**16**	**7.37**	0	5	**5**	**2.30**
San Jose City	**16,895**	3,901	1,164	**5,065**	**29.98**	3,951	1,561	**5,512**	**32.63**	339	550	**889**	**5.26**
San Jose del Monte City	**371,888**	26,340	18,965	**45,305**	**12.18**	26,613	19,057	**45,670**	**12.28**	2,550	3,051	**5,601**	**1.51**

Table 2.F.1.2. Lifestyle Related Diseases (Tobacco Use, Harmful Use of Alcohol, Unhealthy Diet)

Number and proportion of adults ages 20 yrs. old and above who are current smokers, binge drinkers and overweight or obese

Philippines, 2022

Area	Total No. of clients ≥ 20 y/o risk-assesed[1]	Current Smokers				Binge Drinkers				Overweight or obese			
		Male	Female	Total	%	Male	Female	Total	%	Male	Female	Total	%
Science City of Munoz	21,583	2,163	332	2,495	11.56	2,056	481	2,537	11.75	37	44	81	0.38
Tarlac City	2,654	421	100	521	19.63	227	39	266	10.02	149	280	429	16.16
**Region 4A**	355,013	59,476	18,313	77,789	21.91	48,916	16,702	65,618	18.48	19,451	25,194	44,645	12.58
Batangas	68,393	15,379	1,168	16,547	24.19	14,936	1,825	16,761	24.51	837	769	1,606	2.35
Cavite	74,087	10,370	3,614	13,984	18.88	8,212	2,860	11,072	14.94	7,921	8,855	16,776	22.64
Laguna	13,451	1,936	343	2,279	16.94	1,904	509	2,413	17.94	509	600	1,109	8.24
Quezon	43,789	14,320	5,908	20,228	46.19	8,073	4,827	12,900	29.46	2,387	3,786	6,173	14.10
Rizal	18,232	3,202	1,935	5,137	28.18	2,137	1,234	3,371	18.49	770	1,036	1,806	9.91
Antipolo City	0	0	0	0	0.00	0	0	0	0.00	0	0	0	0.00
Bacoor City	685	79	21	100	14.60	70	23	93	13.58	41	53	94	13.72
Batangas City	1,708	232	373	605	35.42	156	48	204	11.94	71	109	180	10.54
Biñan City	5,087	196	53	249	4.89	128	48	176	3.46	1	24	25	0.49
Cabuyao City	875	306	116	422	48.23	258	87	345	39.43	62	84	146	16.69
Calamba City	5,923	1,061	448	1,509	25.48	1,002	469	1,471	24.84	335	508	843	14.23
Cavite City	461	58	44	102	22.13	46	26	72	15.62	21	25	46	9.98
Dasmariñas City	49,710	5,715	2,754	8,469	17.04	4,836	2,638	7,474	15.04	2,949	4,179	7,128	14.34
General Trias City	8,650	589	251	840	9.71	461	190	651	7.53	1,993	2,991	4,984	57.62
Imus City	3,130	460	287	747	23.87	442	238	680	21.73	61	64	125	3.99
Lipa City	7,427	2,180	176	2,356	31.72	2,394	241	2,635	35.48	165	265	430	5.79
Lucena City	1,482	86	17	103	6.95	50	26	76	5.13	79	134	213	14.37
San Pablo City	1,224	72	12	84	6.86	92	11	103	8.42	63	94	157	12.83
San Pedro City	42	7	5	12	28.57	8	2	10	23.81	1	3	4	9.52
Santa Rosa City	12,258	577	134	711	5.80	538	170	708	5.78	151	257	408	3.33
Tagaytay City	16,056	524	166	690	4.30	473	213	686	4.27	40	37	77	0.48
Tanauan City	6,496	937	231	1,168	17.98	985	172	1,157	17.81	321	344	665	10.24
Tayabas City	9,642	292	73	365	3.79	714	610	1,324	13.73	609	866	1,475	15.30
Trece Martires City	6,205	898	184	1,082	17.44	1,001	235	1,236	19.92	64	111	175	2.82
**Region 4B**	221,839	91,932	10,708	102,640	46.27	76,095	14,376	90,471	40.78	5,999	8,747	14,746	6.65
Marinduque	6,827	2,016	147	2,163	31.68	494	105	599	8.77	278	603	881	12.90
Mindoro Occidental	20,244	3,168	852	4,020	19.86	2,866	822	3,688	18.22	118	259	377	1.86
Mindoro Oriental	42,053	21,349	1,989	23,338	55.50	29,019	7,619	36,638	87.12	2,014	2,765	4,779	11.36
Palawan	143,572	63,760	7,267	71,027	49.47	41,641	4,808	46,449	32.35	3,199	4,612	7,811	5.44
Romblon	6,874	1,443	360	1,803	26.23	2,045	1,015	3,060	44.52	388	506	894	13.01
Puerto Princesa City	2,269	196	93	289	12.74	30	7	37	1.63	2	2	4	0.18
**Region 5**	480,633	68,825	14,752	83,577	17.39	66,328	20,561	86,889	18.08	21,184	33,031	54,215	11.28
Albay	41,765	5,274	1,139	6,413	15.35	4,605	1,409	6,014	14.40	1,354	1,701	3,055	7.31
Camarines Norte	45,679	5,687	1,429	7,116	15.58	3,284	1,360	4,644	10.17	2,585	3,968	6,553	14.35
Camarines Sur	192,201	24,378	4,146	28,524	14.84	22,437	8,201	30,638	15.94	7,024	12,329	19,353	10.07
Catanduanes	23,915	4,558	627	5,185	21.68	5,395	1,372	6,767	28.30	2,225	3,662	5,887	24.62
Masbate	32,213	11,575	3,490	15,065	46.77	11,147	1,941	13,088	40.63	525	756	1,281	3.98
Sorsogon	115,214	14,625	3,369	17,994	15.62	16,642	5,386	22,028	19.12	5,500	7,532	13,032	11.31
Iriga City	16,326	1,222	194	1,416	8.67	1,298	338	1,636	10.02	1,482	2,423	3,905	23.92
Legaspi City	6,308	667	162	829	13.14	541	126	667	10.57	87	127	214	3.39
Naga City	7,012	839	196	1,035	14.76	979	428	1,407	20.07	402	533	935	13.33
**Region 6**	584,937	97,433	20,285	117,718	20.12	88,344	26,110	114,454	19.57	26,908	38,382	65,290	11.16
Aklan	74,984	3,706	462	4,168	5.56	4,734	1,208	5,942	7.92	1,169	1,663	2,832	3.78
Antique	49,877	5,458	2,087	7,545	15.13	4,566	880	5,446	10.92	721	1,289	2,010	4.03
Capiz	44,463	4,502	617	5,119	11.51	5,415	1,241	6,656	14.97	2,320	3,602	5,922	13.32
Guimaras	37,225	4,847	357	5,204	13.98	5,109	695	5,804	15.59	2,753	3,530	6,283	16.88
Iloilo	155,723	38,276	8,217	46,493	29.86	25,680	5,879	31,559	20.27	5,080	5,291	10,371	6.66
Negros Occidental	184,181	38,212	7,518	45,730	24.83	40,545	15,264	55,809	30.30	13,641	20,359	34,000	18.46
Bacolod City	9,552	806	516	1,322	13.84	933	502	1,435	15.02	612	1,460	2,072	21.69
Iloilo City	28,932	1,626	511	2,137	7.39	1,362	441	1,803	6.23	612	1,188	1,800	6.22
**Region 7**	465,892	43,625	15,221	58,846	12.63	44,045	21,419	65,464	14.05	35,623	35,191	70,814	15.20
Bohol	69,263	5,378	2,317	7,695	11.11	5,838	2,919	8,757	12.64	5,382	4,653	10,035	14.49
Cebu	279,501	26,764	9,373	36,137	12.93	27,985	14,839	42,824	15.32	25,904	23,181	49,085	17.56
Negros Oriental	21,288	4,781	952	5,733	26.93	4,817	1,128	5,945	27.93	1,181	2,973	4,154	19.51
Siquijor	12,080	2,368	386	2,754	22.80	1,896	719	2,615	21.65	1,181	1,633	2,814	23.29
Cebu City	8,842	1,478	631	2,109	23.85	1,355	916	2,271	25.68	897	1,503	2,400	27.14
Lapu-Lapu City	6,741	2,094	1,420	3,514	52.13	1,603	717	2,320	34.42	865	1,005	1,870	27.74
Mandaue City	68,177	762	142	904	1.33	551	181	732	1.07	213	243	456	0.67
**Region 8**	391,303	66,835	15,668	82,503	21.08	74,163	40,483	114,646	29.30	15,053	17,686	32,739	8.37
Biliran	6,256	724	119	843	13.48	824	607	1,431	22.87	635	1,041	1,676	26.79
Eastern Samar	65,329	16,484	3,718	20,202	30.92	25,961	15,542	41,503	63.53	3,074	3,319	6,393	9.79
Northern Leyte	60,550	8,883	2,031	10,914	18.02	7,513	3,738	11,251	18.58	1,911	2,457	4,368	7.21
Northern Samar	37,794	14,322	3,232	17,554	46.45	16,300	6,608	22,908	60.61	4,185	4,164	8,349	22.09
Southern Leyte	60,570	12,621	2,756	15,377	25.39	11,444	4,584	16,028	26.46	3,476	4,368	7,844	12.95
Western Samar	94,500	9,156	2,394	11,550	12.22	7,735	6,216	13,951	14.76	425	619	1,044	1.10
Calbayog City	1,081	115	28	143	13.23	79	44	123	11.38	39	83	122	11.29
Maasin City	2,068	615	56	671	32.45	396	115	511	24.71	146	215	361	17.46

Table 2.F.1.2. Lifestyle Related Diseases (Tobacco Use, Harmful Use of Alcohol, Unhealthy Diet)

Number and proportion of adults ages 20 yrs. old and above who are current smokers, binge drinkers and overweight or obese

Philippines, 2022

Area	Total No. of clients ≥ 20 y/o risk-assesed[1]	Current Smokers				Binge Drinkers				Overweight or obese			
		Male	Female	Total	%	Male	Female	Total	%	Male	Female	Total	%
Ormoc City	**7,764**	427	414	**841**	**10.83**	588	624	**1,212**	**15.61**	487	673	**1,160**	**14.94**
Tacloban City	**55,391**	3,488	920	**4,408**	**7.96**	3,323	2,405	**5,728**	**10.34**	675	747	**1,422**	**2.57**
**Region 9**	**215,477**	**28,534**	**6,891**	**35,425**	**16.44**	**25,494**	**7,660**	**33,154**	**15.39**	**7,403**	**9,548**	**16,951**	**7.87**
Zamboanga del Norte	**42,709**	5,835	1,778	**7,613**	**17.83**	4,599	1,607	**6,206**	**14.53**	1,302	1,877	**3,179**	**7.44**
Zamboanga del Sur	**41,903**	6,442	1,820	**8,262**	**19.72**	5,266	1,821	**7,087**	**16.91**	2,577	2,654	**5,231**	**12.48**
Zamboanga Sibugay	**102,570**	11,862	2,082	**13,944**	**13.59**	12,120	3,204	**15,324**	**14.94**	2,064	2,617	**4,681**	**4.56**
Dapitan City	**3,419**	28	9	**37**	**1.08**	3	3	**6**	**0.18**	26	31	**57**	**1.67**
Dipolog City	**1,900**	717	113	**830**	**43.68**	876	458	**1,334**	**70.21**	477	610	**1,087**	**57.21**
Isabela City	**1,394**	762	67	**829**	**59.47**	194	22	**216**	**15.49**	31	45	**76**	**5.45**
Pagadian City	**809**	26	264	**290**	**35.85**	0	36	**36**	**4.45**	10	146	**156**	**19.28**
Zamboanga City	**20,773**	2,862	758	**3,620**	**17.43**	2,436	509	**2,945**	**14.18**	916	1,568	**2,484**	**11.96**
**Region 10**	**214,206**	**115,891**	**17,295**	**133,186**	**62.18**	**80,251**	**16,146**	**96,397**	**45.00**	**11,785**	**11,219**	**23,004**	**10.74**
Bukidnon	**85,715**	52,567	5,347	**57,914**	**67.57**	44,286	5,490	**49,776**	**58.07**	5,536	4,857	**10,393**	**12.13**
Camiguin	**217**	0	0	**0**	**0.00**	0	0	**0**	**0.00**	0	0	**0**	**0.00**
Lanao del Norte	**31,139**	10,161	3,216	**13,377**	**42.96**	4,379	1,937	**6,316**	**20.28**	1,274	1,365	**2,639**	**8.47**
Misamis Occidental	**21,944**	14,151	2,157	**16,308**	**74.32**	2,899	1,111	**4,010**	**18.27**	785	791	**1,576**	**7.18**
Misamis Oriental	**254**	8	4	**12**	**4.72**	0	0	**0**	**0.00**	0	0	**0**	**0.00**
Cagayan de Oro City	**21,219**	7,409	2,201	**9,610**	**45.29**	7,185	2,323	**9,508**	**44.81**	843	609	**1,452**	**6.84**
El Salvador City	**622**	43	1	**44**	**7.07**	30	3	**33**	**5.31**	8	18	**26**	**4.18**
Gingoog City	**132**	19	7	**26**	**19.70**	16	8	**24**	**18.18**	21	15	**36**	**27.27**
Iligan City	**5,501**	2,340	852	**3,192**	**58.03**	1,967	857	**2,824**	**51.34**	238	238	**476**	**8.65**
Malaybalay City	**6,199**	3,994	381	**4,375**	**70.58**	1,509	177	**1,686**	**27.20**	49	72	**121**	**1.95**
Oroquieta City	**11,333**	22,567	2,624	**25,191**	**222.28**	14,982	3,550	**18,532**	**163.52**	993	1,211	**2,204**	**19.45**
Ozamis City	**16,092**	1,155	236	**1,391**	**8.64**	1,236	329	**1,565**	**9.73**	370	421	**791**	**4.92**
Tangub City	**398**	162	131	**293**	**73.62**	215	145	**360**	**90.45**	3	3	**6**	**1.51**
Valencia City	**13,441**	1,315	138	**1,453**	**10.81**	1,547	216	**1,763**	**13.12**	1,665	1,619	**3,284**	**24.43**
**Region 11**	**506,009**	**313,538**	**71,236**	**384,774**	**76.04**	**116,739**	**29,782**	**146,521**	**28.96**	**48,459**	**53,039**	**101,498**	**20.06**
Compostela Valley	**114,702**	59,914	13,640	**73,554**	**64.13**	22,105	4,093	**26,198**	**22.84**	14,428	14,235	**28,663**	**24.99**
Davao del Norte	**139,434**	106,172	13,727	**119,899**	**85.99**	36,350	7,115	**43,465**	**31.17**	12,973	13,709	**26,682**	**19.14**
Davao Oriental	**62,452**	29,742	7,166	**36,908**	**59.10**	18,637	7,746	**26,383**	**42.25**	9,133	11,631	**20,764**	**33.25**
Davao del Sur	**92,082**	66,387	12,170	**78,557**	**85.31**	19,493	4,026	**23,519**	**25.54**	2,862	2,233	**5,095**	**5.53**
Davao Occidental	**15,418**	44,045	21,482	**65,527**	**425.00**	13,849	3,738	**17,587**	**114.07**	1,781	1,594	**3,375**	**21.89**
Davao City	**81,921**	7,278	3,051	**10,329**	**12.61**	6,305	3,064	**9,369**	**11.44**	7,282	9,637	**16,919**	**20.65**
**Region 12**	**359,115**	**59,970**	**9,493**	**69,463**	**19.34**	**45,599**	**8,616**	**54,215**	**15.10**	**16,806**	**20,995**	**37,801**	**10.53**
North Cotabato	**87,834**	18,885	3,159	**22,044**	**25.10**	16,550	2,739	**19,289**	**21.96**	4,232	5,851	**10,083**	**11.48**
Sarangani	**66,594**	18,263	2,718	**20,981**	**31.51**	12,606	2,981	**15,587**	**23.41**	3,290	4,553	**7,843**	**11.78**
South Cotabato	**54,488**	7,091	1,302	**8,393**	**15.40**	4,885	1,235	**6,120**	**11.23**	1,658	2,547	**4,205**	**7.72**
Sultan Kudarat	**145,860**	15,314	2,274	**17,588**	**12.06**	11,240	1,613	**12,853**	**8.81**	7,334	7,749	**15,083**	**10.34**
Gen. Santos City	**4,339**	417	40	**457**	**10.53**	318	48	**366**	**8.44**	292	295	**587**	**13.53**
**BARMM**	**66,469**	**33,935**	**8,124**	**42,059**	**63.28**	**3,185**	**454**	**3,639**	**5.47**	**2,748**	**3,610**	**6,358**	**9.57**
Basilan	**5,302**	5,212	1,031	**6,243**	**117.75**	211	51	**262**	**4.94**	34	62	**96**	**1.81**
Lanao del Sur	**19,610**	9,731	3,699	**13,430**	**68.49**	750	84	**834**	**4.25**	612	787	**1,399**	**7.13**
Maguindanao	**13,542**	4,706	1,249	**5,955**	**43.97**	691	106	**797**	**5.89**	457	655	**1,112**	**8.21**
Sulu	**276**	659	319	**978**	**354.35**	5	0	**5**	**1.81**	10	7	**17**	**6.16**
Tawi-Tawi	**3,034**	10,123	372	**10,495**	**345.91**	995	155	**1,150**	**37.90**	364	412	**776**	**25.58**
Lamitan City	**1,392**	690	189	**879**	**63.15**	83	7	**90**	**6.47**	12	14	**26**	**1.87**
Marawi City	**11,739**	1,998	1,167	**3,165**	**26.96**	213	27	**240**	**2.04**	768	936	**1,704**	**14.52**
Cotabato City	**11,574**	816	98	**914**	**7.90**	237	24	**261**	**2.26**	491	737	**1,228**	**10.61**
**CARAGA**	**123,103**	**33,957**	**5,478**	**39,435**	**32.03**	**24,195**	**7,317**	**31,512**	**25.60**	**4,463**	**6,345**	**10,808**	**8.78**
Agusan del Norte	**17,888**	5,768	1,119	**6,887**	**38.50**	4,254	2,323	**6,577**	**36.77**	226	244	**470**	**2.63**
Agusan del Sur	**39,978**	11,951	1,327	**13,278**	**33.21**	9,943	1,821	**11,764**	**29.43**	201	326	**527**	**1.32**
Surigao del Norte	**7,326**	4,461	697	**5,158**	**70.41**	2,746	1,061	**3,807**	**51.97**	392	445	**837**	**11.43**
Surigao del Sur	**10,393**	2,666	587	**3,253**	**31.30**	1,549	577	**2,126**	**20.46**	1,140	1,534	**2,674**	**25.73**
Province of Dinagat	**1,409**	433	27	**460**	**32.65**	517	132	**649**	**46.06**	69	101	**170**	**12.07**
Bislig City	**19,337**	5,123	597	**5,720**	**29.58**	2,830	346	**3,176**	**16.42**	241	1,242	**1,483**	**7.67**
Butuan City	**24,135**	3,228	973	**4,201**	**17.41**	1,941	828	**2,769**	**11.47**	1,974	2,191	**4,165**	**17.26**
Surigao City	**2,637**	327	151	**478**	**18.13**	415	229	**644**	**24.42**	220	262	**482**	**18.28**

Table 2.F.2.1. Cancer Prevention and Control (Cervical and Breast Cancer Screening)

Number and proportion of women ages 20 yrs old and above screened for Cervical and Breast Cancer

Philippines, 2022

Area	Eligible Pop. (20 y/o and above female)	Women screened for Cervical Cancer		Women found positive or suspected for Cervical Cancer		Women screened for breast mass		Women with suspicious breast mass	
		Total	%	Total	%	Total	%	Total	%
**Philippines**	**34,111,514**	**132,238**	**0.39**	**5,365**	**4.06**	**410,336**	**1.20**	**6,909**	**1.68**
**NCR**	**4,773,695**	**31,107**	**0.65**	**617**	**1.98**	**62,461**	**1.31**	**663**	**1.06**
Malabon	**135,502**	0	**0.00**	0	**0.00**	0	**0.00**	0	**0.00**
Navotas	**92,479**	772	**0.83**	0	**0.00**	772	**0.83**	2	**0.26**
Valenzuela City	**229,990**	707	**0.31**	21	**2.97**	477	**0.21**	12	**2.52**
Caloocan City	**587,195**	3,958	**0.67**	9	**0.23**	4,372	**0.74**	0	**0.00**
Marikina City	**167,093**	132	**0.08**	0	**0.00**	131	**0.08**	0	**0.00**
Pasig City	**279,998**	304	**0.11**	34	**11.18**	919	**0.33**	24	**2.61**
Pateros	**23,666**	306	**1.29**	6	**1.96**	440	**1.86**	0	**0.00**
Taguig	**298,389**	7,525	**2.52**	142	**1.89**	11,113	**3.72**	365	**3.28**
Quezon City	**1,088,441**	3,378	**0.31**	130	**3.85**	3,420	**0.31**	0	**0.00**
Makati City	**215,974**	3,204	**1.48**	51	**1.59**	4,471	**2.07**	107	**2.39**
Mandaluyong City	**143,199**	760	**0.53**	40	**5.26**	1,351	**0.94**	3	**0.22**
San Juan	**45,291**	121	**0.27**	15	**12.40**	126	**0.28**	1	**0.79**
Manila City	**659,914**	7,903	**1.20**	142	**1.80**	23,466	**3.56**	130	**0.55**
Las Piñas City	**218,310**	17	**0.01**	1	**5.88**	569	**0.26**	4	**0.70**
Muntinlupa City	**187,026**	244	**0.13**	5	**2.05**	596	**0.32**	15	**2.52**
Parañaque City	**246,824**	1,505	**0.61**	21	**1.40**	6,229	**2.52**	0	**0.00**
Pasay City	**154,404**	271	**0.18**	0	**0.00**	4,009	**2.60**	0	**0.00**
**CAR**	**556,626**	**2,277**	**0.41**	**80**	**3.51**	**22,727**	**4.08**	**276**	**1.21**
Abra	**76,934**	1	**0.00**	1	**100.00**	1,520	**1.98**	4	**0.26**
Apayao	**35,702**	0	**0.00**	0	**0.00**	3,718	**10.41**	21	**0.56**
Benguet	**156,157**	440	**0.28**	12	**2.73**	5,228	**3.35**	164	**3.14**
Ifugao	**59,543**	265	**0.45**	13	**4.91**	431	**0.72**	14	**3.25**
Kalinga	**62,564**	36	**0.06**	0	**0.00**	729	**1.17**	19	**2.61**
Mt. Province	**44,863**	276	**0.62**	0	**0.00**	3,147	**7.01**	15	**0.48**
Baguio City	**120,863**	1,259	**1.04**	54	**4.29**	7,954	**6.58**	39	**0.49**
**Region 1**	**1,672,668**	**1,365**	**0.08**	**79**	**5.79**	**10,858**	**0.65**	**92**	**0.85**
Ilocos Norte	**170,305**	86	**0.05**	37	**43.02**	178	**0.10**	3	**1.69**
Ilocos Sur	**200,905**	580	**0.29**	5	**0.86**	5,185	**2.58**	29	**0.56**
La Union	**233,732**	44	**0.02**	0	**0.00**	3,520	**1.51**	8	**0.23**
Pangasinan	**759,339**	640	**0.08**	33	**5.16**	1,698	**0.22**	41	**2.41**
Alaminos City	**28,691**	0	**0.00**	0	**0.00**	0	**0.00**	0	**0.00**
Candon City	**21,178**	10	**0.05**	0	**0.00**	42	**0.20**	6	**14.29**
Dagupan City	**54,775**	5	**0.01**	4	**80.00**	5	**0.01**	5	**100.00**
Laoag City	**39,267**	0	**0.00**	0	**0.00**	0	**0.00**	0	**0.00**
San Carlos City	**60,308**	0	**0.00**	0	**0.00**	230	**0.38**	0	**0.00**
San Fernando City	**42,827**	0	**0.00**	0	**0.00**	0	**0.00**	0	**0.00**
Urdaneta City	**42,517**	0	**0.00**	0	**0.00**	0	**0.00**	0	**0.00**
Vigan City	**18,824**	0	**0.00**	0	**0.00**	0	**0.00**	0	**0.00**
**Region 2**	**1,148,264**	**1,064**	**0.09**	**83**	**7.80**	**28,127**	**2.45**	**426**	**1.51**
Batanes	**5,356**	0	**0.00**	0	**0.00**	0	**0.00**	0	**0.00**
Cagayan	**354,159**	163	**0.05**	5	**3.07**	1,962	**0.55**	143	**7.29**
Isabela	**393,410**	145	**0.04**	9	**6.21**	662	**0.17**	27	**4.08**
Nueva Vizcaya	**148,305**	471	**0.32**	66	**14.01**	7,254	**4.89**	25	**0.34**
Quirino	**58,805**	7	**0.01**	3	**42.86**	1,058	**1.80**	5	**0.47**
Cauayan City	**43,050**	3	**0.01**	0	**0.00**	136	**0.32**	0	**0.00**
Ilagan City	**48,379**	212	**0.44**	0	**0.00**	8,036	**16.61**	31	**0.39**
Santiago City	**44,815**	63	**0.14**	0	**0.00**	9,019	**20.12**	195	**2.16**
Tuguegarao City	**51,985**	0	**0.00**	0	**0.00**	0	**0.00**	0	**0.00**
**Region 3**	**4,045,036**	**7,771**	**0.19**	**61**	**0.78**	**42,510**	**1.05**	**434**	**1.02**
Aurora	**66,828**	0	**0.00**	0	**0.00**	2,472	**3.70**	7	**0.28**
Bataan	**240,166**	324	**0.13**	9	**2.78**	2,448	**1.02**	67	**2.74**
Bulacan	**833,404**	3,113	**0.37**	7	**0.22**	12,701	**1.52**	72	**0.57**

Table 2.F.2.1. Cancer Prevention and Control (Cervical and Breast Cancer Screening)

Number and proportion of women ages 20 yrs old and above screened for Cervical and Breast Cancer

Philippines, 2022

Area	Eligible Pop. (20 y/o and above female)	Women screened for Cervical Cancer		Women found positive or suspected for Cervical Cancer		Women screened for breast mass		Women with suspicious breast mass	
		Total	%	Total	%	Total	%	Total	%
Nueva Ecija	**529,718**	98	**0.02**	4	**4.08**	136	**0.03**	24	**17.65**
Pampanga	**604,280**	127	**0.02**	23	**18.11**	1,284	**0.21**	8	**0.62**
Tarlac	**351,801**	138	**0.04**	3	**2.17**	346	**0.10**	26	**7.51**
Zambales	**204,464**	137	**0.07**	0	**0.00**	1,096	**0.54**	23	**2.10**
Angeles City	**151,618**	270	**0.18**	1	**0.37**	644	**0.42**	10	**1.55**
Balanga City	**34,716**	105	**0.30**	4	**3.81**	946	**2.72**	8	**0.85**
Cabanatuan City	**108,417**	0	**0.00**	0	**0.00**	1	**0.00**	0	**0.00**
City of San Fernando	**112,950**	1	**0.00**	1	**100.00**	15	**0.01**	3	**20.00**
Gapan City	**39,569**	0	**0.00**	0	**0.00**	56	**0.14**	0	**0.00**
Mabalacat City	**92,376**	325	**0.35**	0	**0.00**	12,136	**13.14**	18	**0.15**
Malolos City	**93,089**	8	**0.01**	0	**0.00**	648	**0.70**	3	**0.46**
Meycauayan	**77,212**	31	**0.04**	0	**0.00**	51	**0.07**	0	**0.00**
Olongapo	**80,643**	32	**0.04**	0	**0.00**	71	**0.09**	2	**2.82**
Palayan City	**14,721**	0	**0.00**	0	**0.00**	0	**0.00**	0	**0.00**
San Jose City	**50,124**	233	**0.46**	0	**0.00**	0	**0.00**	0	**0.00**
San Jose del Monte Cit	**211,997**	2,827	**1.33**	7	**0.25**	7,035	**3.32**	154	**2.19**
Science City of Munoz	**29,227**	2	**0.01**	2	**100.00**	408	**1.40**	7	**1.72**
Tarlac City	**117,716**	0	**0.00**	0	**0.00**	16	**0.01**	2	**12.50**
**Region 4A**	**5,322,343**	**9,249**	**0.17**	**456**	**4.93**	**11,883**	**0.22**	**326**	**2.74**
Batangas	**698,066**	513	**0.07**	6	**1.17**	127	**0.02**	1	**0.79**
Cavite	**538,064**	3,090	**0.57**	123	**3.98**	2,289	**0.43**	5	**0.22**
Laguna	**371,378**	1,026	**0.28**	10	**0.97**	1,302	**0.35**	22	**1.69**
Quezon	**560,316**	1,462	**0.26**	131	**8.96**	4,720	**0.84**	173	**3.67**
Rizal	**770,839**	654	**0.08**	29	**4.43**	1,386	**0.18**	17	**1.23**
Antipolo City	**283,925**	0	**0.00**	0	**0.00**	0	**0.00**	0	**0.00**
Bacoor City	**235,740**	74	**0.03**	1	**1.35**	7	**0.00**	5	**71.43**
Batangas City	**123,888**	320	**0.26**	36	**11.25**	473	**0.38**	26	**5.50**
Biñan City	**124,528**	31	**0.02**	11	**35.48**	4	**0.00**	1	**25.00**
Cabuyao City	**115,447**	54	**0.05**	3	**5.56**	38	**0.03**	1	**2.63**
Calamba City	**169,943**	0	**0.00**	0	**0.00**	6	**0.00**	2	**33.33**
Cavite City	**40,350**	0	**0.00**	0	**0.00**	1	**0.00**	1	**100.00**
Dasmariñas City	**258,666**	476	**0.18**	11	**2.31**	450	**0.17**	24	**5.33**
General Trias City	**123,365**	356	**0.29**	4	**1.12**	2	**0.00**	0	**0.00**
Imus City	**158,491**	126	**0.08**	51	**40.48**	48	**0.03**	1	**2.08**
Lipa City	**124,830**	0	**0.00**	0	**0.00**	0	**0.00**	0	**0.00**
Lucena City	**84,920**	100	**0.12**	0	**0.00**	10	**0.01**	6	**60.00**
San Pablo City	**99,488**	31	**0.03**	0	**0.00**	100	**0.10**	4	**4.00**
San Pedro City	**121,827**	0	**0.00**	0	**0.00**	1	**0.00**	1	**100.00**
Santa Rosa City	**132,280**	94	**0.07**	7	**7.45**	350	**0.26**	1	**0.29**
Tagaytay City	**27,939**	248	**0.89**	4	**1.61**	248	**0.89**	16	**6.45**
Tanauan City	**65,109**	308	**0.47**	2	**0.65**	45	**0.07**	11	**24.44**
Tayabas City	**31,826**	286	**0.90**	27	**9.44**	276	**0.87**	8	**2.90**
Trece Martires City	**61,118**	0	**0.00**	0	**0.00**	0	**0.00**	0	**0.00**
**Region 4B**	**895,715**	**1,059**	**0.12**	**62**	**5.85**	**1,841**	**0.21**	**178**	**9.67**
Marinduque	**70,314**	300	**0.43**	4	**1.33**	6	**0.01**	0	**0.00**
Mindoro Occidental	**139,734**	25	**0.02**	2	**8.00**	28	**0.02**	28	**100.00**
Mindoro Oriental	**260,131**	234	**0.09**	16	**6.84**	101	**0.04**	28	**27.72**
Palawan	**261,696**	278	**0.11**	22	**7.91**	1,521	**0.58**	84	**5.52**
Romblon	**85,245**	18	**0.02**	16	**88.89**	21	**0.02**	18	**85.71**
Puerto Princesa City	**78,595**	204	**0.26**	2	**0.98**	164	**0.21**	20	**12.20**
**Region 5**	**1,740,830**	**1,329**	**0.08**	**105**	**7.90**	**15,582**	**0.90**	**266**	**1.71**
Albay	**356,093**	91	**0.03**	8	**8.79**	8,153	**2.29**	75	**0.92**
Camarines Norte	**177,844**	0	**0.00**	0	**0.00**	81	**0.05**	13	**16.05**
Camarines Sur	**489,138**	740	**0.15**	67	**9.05**	3,383	**0.69**	63	**1.86**
Catanduanes	**75,717**	51	**0.07**	9	**17.65**	114	**0.15**	4	**3.51**
Masbate	**250,378**	0	**0.00**	0	**0.00**	156	**0.06**	27	**17.31**
Sorsogon	**237,515**	81	**0.03**	16	**19.75**	1,378	**0.58**	61	**4.43**
Iriga City	**33,236**	88	**0.26**	2	**2.27**	928	**2.79**	7	**0.75**
Legaspi City	**62,621**	34	**0.05**	0	**0.00**	44	**0.07**	12	**27.27**
Naga City	**58,288**	244	**0.42**	3	**1.23**	1,345	**2.31**	4	**0.30**

Table 2.F.2.1. Cancer Prevention and Control (Cervical and Breast Cancer Screening)

Number and proportion of women ages 20 yrs old and above screened for Cervical and Breast Cancer
Philippines, 2022

Area	Eligible Pop. (20 y/o and above female)	Women screened for Cervical Cancer		Women found positive or suspected for Cervical Cancer		Women screened for breast mass		Women with suspicious breast mass	
		Total	%	Total	%	Total	%	Total	%
**Region 6**	**2,480,318**	**2,125**	**0.09**	**203**	**9.55**	**44,828**	**1.81**	**575**	**1.28**
Aklan	**189,518**	33	**0.02**	4	**12.12**	185	**0.10**	28	**15.14**
Antique	**177,991**	135	**0.08**	9	**6.67**	4,142	**2.33**	57	**1.38**
Capiz	**261,776**	95	**0.04**	19	**20.00**	8,121	**3.10**	51	**0.63**
Guimaras	**58,404**	28	**0.05**	0	**0.00**	9,211	**15.77**	21	**0.23**
Iloilo	**665,798**	459	**0.07**	96	**20.92**	8,136	**1.22**	112	**1.38**
Negros Occidental	**794,133**	1,151	**0.14**	69	**5.99**	5,737	**0.72**	243	**4.24**
Bacolod City	**178,674**	9	**0.01**	3	**33.33**	418	**0.23**	25	**5.98**
Iloilo City	**154,024**	215	**0.14**	3	**1.40**	8,878	**5.76**	38	**0.43**
**Region 7**	**2,483,319**	**7,548**	**0.30**	**205**	**2.72**	**40,736**	**1.64**	**1,004**	**2.46**
Bohol	**405,574**	0	**0.00**	0	**0.00**	46	**0.01**	16	**34.78**
Cebu	**1,026,179**	5,717	**0.56**	70	**1.22**	31,246	**3.04**	901	**2.88**
Negros Oriental	**428,275**	29	**0.01**	1	**3.45**	541	**0.13**	6	**1.11**
Siquijor	**32,016**	16	**0.05**	2	**12.50**	150	**0.47**	0	**0.00**
Cebu City	**322,151**	479	**0.15**	132	**27.56**	7,695	**2.39**	79	**1.03**
Lapu-Lapu City	**142,500**	0	**0.00**	0	**0.00**	157	**0.11**	2	**1.27**
Mandaue City	**126,624**	1,307	**1.03**	0	**0.00**	901	**0.71**	0	**0.00**
**Region 8**	**1,367,588**	**729**	**0.05**	**25**	**3.43**	**1,752**	**0.13**	**93**	**5.31**
Biliran	**51,959**	163	**0.31**	0	**0.00**	320	**0.62**	20	**6.25**
Eastern Samar	**142,645**	3	**0.00**	0	**0.00**	44	**0.03**	7	**15.91**
Northern Leyte	**484,353**	218	**0.05**	2	**0.92**	44	**0.01**	13	**29.55**
Northern Samar	**180,755**	250	**0.14**	22	**8.80**	95	**0.05**	29	**30.53**
Southern Leyte	**108,842**	92	**0.08**	1	**1.09**	1,241	**1.14**	20	**1.61**
Western Samar	**171,747**	1	**0.00**	0	**0.00**	5	**0.00**	1	**20.00**
Calbayog City	**52,930**	0	**0.00**	0	**0.00**	0	**0.00**	0	**0.00**
Maasin City	**27,695**	2	**0.01**	0	**0.00**	3	**0.01**	3	**100.00**
Ormoc City	**68,994**	0	**0.00**	0	**0.00**	0	**0.00**	0	**0.00**
Tacloban City	**77,668**	0	**0.00**	0	**0.00**	0	**0.00**	0	**0.00**
**Region 9**	**1,108,842**	**263**	**0.02**	**4**	**1.52**	**3,630**	**0.33**	**146**	**4.02**
Zamboanga Del Norte	**240,008**	7	**0.00**	0	**0.00**	906	**0.38**	36	**3.97**
Zamboanga Del Sur	**248,901**	4	**0.00**	2	**50.00**	118	**0.05**	11	**9.32**
Zamboanga Sibugay	**191,257**	101	**0.05**	0	**0.00**	685	**0.36**	14	**2.04**
Dapitan City	**24,779**	0	**0.00**	0	**0.00**	0	**0.00**	0	**0.00**
Dipolog City	**39,315**	4	**0.01**	0	**0.00**	10	**0.03**	3	**30.00**
Isabela City	**39,260**	1	**0.00**	0	**0.00**	4	**0.01**	3	**75.00**
Pagadian City	**61,044**	0	**0.00**	0	**0.00**	0	**0.00**	0	**0.00**
Zamboanga City	**264,278**	146	**0.06**	2	**1.37**	1,907	**0.72**	79	**4.14**
**Region 10**	**1,515,153**	**484**	**0.03**	**45**	**9.30**	**3,217**	**0.21**	**189**	**5.88**
Bukidnon	**325,386**	240	**0.07**	14	**5.83**	806	**0.25**	15	**1.86**
Camiguin	**27,772**	2	**0.01**	0	**0.00**	8	**0.03**	1	**12.50**
Lanao Del Norte	**214,287**	1	**0.00**	0	**0.00**	731	**0.34**	7	**0.96**
Misamis Occidental	**104,397**	22	**0.02**	7	**31.82**	38	**0.04**	0	**0.00**
Misamis Oriental	**242,903**	71	**0.03**	2	**2.82**	281	**0.12**	33	**11.74**
Cagayan de Oro City	**230,063**	60	**0.03**	11	**18.33**	211	**0.09**	20	**9.48**
El Salvador City	**17,090**	2	**0.01**	2	**100.00**	715	**4.18**	67	**9.37**
Gingoog City	**42,422**	10	**0.02**	6	**60.00**	14	**0.03**	13	**92.86**
Iligan City	**108,536**	14	**0.01**	0	**0.00**	62	**0.06**	7	**11.29**
Malaybalay City	**54,239**	27	**0.05**	0	**0.00**	11	**0.02**	1	**9.09**
Oroquieta City	**22,623**	0	**0.00**	0	**0.00**	0	**0.00**	0	**0.00**
Ozamis City	**45,348**	35	**0.08**	3	**8.57**	340	**0.75**	25	**7.35**
Tangub City	**20,145**	0	**0.00**	0	**0.00**	0	**0.00**	0	**0.00**
Valencia City	**59,942**	0	**0.00**	0	**0.00**	0	**0.00**	0	**0.00**
**Region 11**	**1,606,611**	**59,329**	**3.69**	**3,182**	**5.36**	**56,793**	**3.53**	**1,028**	**1.81**
Davao de Oro	**224,221**	10,297	**4.59**	1,852	**17.99**	11,222	**5.00**	32	**0.29**
Davao del Norte	**318,257**	16,711	**5.25**	589	**3.52**	9,547	**3.00**	195	**2.04**

Table 2.F.2.1. Cancer Prevention and Control (Cervical and Breast Cancer Screening)

Number and proportion of women ages 20 yrs old and above screened for Cervical and Breast Cancer

Philippines, 2022

Area	Eligible Pop. (20 y/o and above female)	Women screened for Cervical Cancer		Women found positive or suspected for Cervical Cancer		Women screened for breast mass		Women with suspicious breast mass	
		Total	%	Total	%	Total	%	Total	%
Davao Oriental	**169,198**	4,448	**2.63**	34	**0.76**	2,885	**1.71**	295	**10.23**
Davao del Sur	**221,923**	20,898	**9.42**	635	**3.04**	6,134	**2.76**	99	**1.61**
Davao Occidental	**100,136**	189	**0.19**	3	**1.32**	436	**0.44**	10	**2.29**
Davao City	**572,876**	6,786	**1.18**	69	**1.02**	26,569	**4.64**	397	**1.49**
**Region 12**	**1,366,343**	**4,414**	**0.32**	**64**	**1.45**	**49,772**	**3.64**	**1,070**	**2.15**
North Cotabato	**446,496**	211	**0.05**	12	**5.69**	2,680	**0.60**	159	**5.93**
Sarangani	**163,318**	682	**0.42**	24	**3.52**	664	**0.41**	193	**29.07**
South Cotabato	**306,127**	270	**0.09**	4	**1.48**	4,421	**1.44**	28	**0.63**
Sultan Kudarat	**251,585**	3,048	**1.21**	22	**0.72**	38,584	**15.34**	673	**1.74**
Gen. Santos City	**198,817**	203	**0.10**	2	**0.99**	3,423	**1.72**	17	**0.50**
**BARMM**	**1,238,154**	**855**	**0.07**	**18**	**2.11**	**11,284**	**0.91**	**78**	**0.69**
Basilan	**81,472**	0	**0.00**	0	**0.00**	1	**0.00**	1	**100.00**
Lanao Del Sur	**254,382**	33	**0.01**	0	**0.00**	5,322	**2.09**	17	**0.32**
Maguindanao	**358,678**	764	**0.21**	4	**0.52**	5,715	**1.59**	42	**0.73**
Sulu	**255,075**	0	**0.00**	0	**0.00**	0	**0.00**	0	**0.00**
Tawi-Tawi	**111,813**	0	**0.00**	11	**0.00**	0	**0.00**	0	**0.00**
Lamitan City	**22,413**	0	**0.00**	0	**0.00**	0	**0.00**	0	**0.00**
Marawi City	**60,847**	15	**0.02**	0	**0.00**	25	**0.04**	0	**0.00**
Cotabato City	**93,474**	43	**0.05**	3	**6.98**	221	**0.24**	18	**8.14**
**CARAGA**	**790,009**	**1,270**	**0.16**	**76**	**5.98**	**2,335**	**0.30**	**65**	**2.78**
Agusan del Norte	**111,689**	235	**0.21**	0	**0.00**	638	**0.57**	5	**0.78**
Agusan del Sur	**198,437**	53	**0.03**	0	**0.00**	36	**0.02**	11	**30.56**
Surigao del Norte	**108,116**	638	**0.59**	21	**3.29**	3	**0.00**	1	**33.33**
Surigao del Sur	**148,306**	179	**0.12**	50	**27.93**	452	**0.30**	23	**5.09**
Province of Dinagat	**38,743**	38	**0.10**	0	**0.00**	24	**0.06**	10	**41.67**
Bislig City	**28,167**	103	**0.37**	5	**4.85**	261	**0.93**	4	**1.53**
Butuan City	**106,198**	22	**0.02**	0	**0.00**	300	**0.28**	1	**0.33**
Surigao City	**50,353**	2	**0.00**	0	**0.00**	621	**1.23**	10	**1.61**

Table 2.F.3.1. Cardiovascular Disease and Diabetes Mellitus Prevention and Control

Number and proportion of newly-identified 20 yrs old and above adults with Hypertension and Type 2 Diabetes Mellitus
Philippines, 2022

Area	Total No. of clients ≥ 20 y/o risk-assesed[1]	Newly identified Hypertensive Adults				Newly identified with Type 2 Diabetes Mellitus			
		Male	Female	Total	%	Male	Female	Total	%
**Philippines**	**7,200,055**	**273,104**	**346,615**	**619,719**	**8.61**	**69,997**	**89,908**	**159,905**	**2.22**
**NCR**	**574,900**	**41,420**	**59,801**	**101,221**	**17.61**	**10,585**	**15,410**	**25,995**	**4.52**
Malabon	23,982	2,278	2,805	**5,083**	**21.20**	499	590	**1,089**	**4.54**
Navotas	23,512	793	1,085	**1,878**	**7.99**	164	209	**373**	**1.59**
Valenzuela City	41,858	2,259	3,267	**5,526**	**13.20**	614	890	**1,504**	**3.59**
Caloocan City	36,175	1,373	2,244	**3,617**	**10.00**	338	378	**716**	**1.98**
Marikina City	5,098	650	902	**1,552**	**30.44**	196	245	**441**	**8.65**
Pasig City	54,014	4,197	6,252	**10,449**	**19.34**	1,723	2,762	**4,485**	**8.30**
Pateros	2,535	340	486	**826**	**32.58**	116	177	**293**	**11.56**
Taguig	88,987	3,969	5,982	**9,951**	**11.18**	1,357	1,975	**3,332**	**3.74**
Quezon City	96,297	14,237	21,354	**35,591**	**36.96**	2,856	4,283	**7,139**	**7.41**
Makati City	24,389	1,068	1,431	**2,499**	**10.25**	603	796	**1,399**	**5.74**
Mandaluyong City	25,429	1,808	2,183	**3,991**	**15.69**	253	306	**559**	**2.20**
San Juan	7,046	45	59	**104**	**1.48**	5	14	**19**	**0.27**
Manila City	72,022	4,114	5,951	**10,065**	**13.97**	648	1,074	**1,722**	**2.39**
Las Piñas City	4,677	863	1,095	**1,958**	**41.86**	239	354	**593**	**12.68**
Muntinlupa City	17,934	891	1,008	**1,899**	**10.59**	174	253	**427**	**2.38**
Parañaque City	21,258	1,566	2,570	**4,136**	**19.46**	521	744	**1,265**	**5.95**
Pasay City	29,687	969	1,127	**2,096**	**7.06**	279	360	**639**	**2.15**
**CAR**	**179,064**	**3,385**	**4,630**	**8,015**	**4.48**	**446**	**724**	**1,170**	**0.65**
Abra	48,924	508	531	**1,039**	**2.12**	52	66	**118**	**0.24**
Apayao	31,856	127	166	**293**	**0.92**	27	32	**59**	**0.19**
Benguet	27,717	1,256	1,903	**3,159**	**11.40**	172	322	**494**	**1.78**
Ifugao	14,030	165	160	**325**	**2.32**	12	26	**38**	**0.27**
Kalinga	23,132	550	691	**1,241**	**5.36**	115	156	**271**	**1.17**
Mt. Province	22,715	263	325	**588**	**2.59**	28	58	**86**	**0.38**
Baguio City	10,690	516	854	**1,370**	**12.82**	40	64	**104**	**0.97**
**Region 1**	**503,146**	**13,501**	**15,116**	**28,617**	**5.69**	**3,204**	**3,970**	**7,174**	**1.43**
Ilocos Norte	8,953	750	792	**1,542**	**17.22**	135	149	**284**	**3.17**
Ilocos Sur	66,305	1,839	2,122	**3,961**	**5.97**	288	528	**816**	**1.23**
La Union	91,726	2,700	3,157	**5,857**	**6.39**	515	583	**1,098**	**1.20**
Pangasinan	191,534	6,252	6,983	**13,235**	**6.91**	1,509	1,914	**3,423**	**1.79**
Alaminos City	4,982	166	138	**304**	**6.10**	60	60	**120**	**2.41**
Candon City	580	100	74	**174**	**30.00**	34	33	**67**	**11.55**
Dagupan City	10,722	592	421	**1,013**	**9.45**	336	257	**593**	**5.53**
Laoag City	1,242	156	128	**284**	**22.87**	23	16	**39**	**3.14**
San Carlos City	5,027	355	391	**746**	**14.84**	132	137	**269**	**5.35**
San Fernando City	23,884	371	588	**959**	**4.02**	120	207	**327**	**1.37**
Urdaneta City	95,270	125	252	**377**	**0.40**	41	78	**119**	**0.12**
Vigan City	2,921	95	70	**165**	**5.65**	11	8	**19**	**0.65**
**Region 2**	**719,731**	**9,991**	**12,495**	**22,486**	**3.12**	**1,340**	**1,967**	**3,307**	**0.46**
Batanes	3,154	11	6	**17**	**0.54**	5	16	**21**	**0.67**
Cagayan	105,723	1,001	1,187	**2,188**	**2.07**	188	266	**454**	**0.43**
Isabela	266,424	6,053	6,694	**12,747**	**4.78**	525	813	**1,338**	**0.50**
Nueva Vizcaya	68,487	578	529	**1,107**	**1.62**	95	159	**254**	**0.37**
Quirino	104,347	954	1,138	**2,092**	**2.00**	248	286	**534**	**0.51**
Cauayan City	7,724	446	576	**1,022**	**13.23**	45	63	**108**	**1.40**
Ilagan City	71,614	557	1,771	**2,328**	**3.25**	112	138	**250**	**0.35**
Santiago City	90,480	296	471	**767**	**0.85**	110	210	**320**	**0.35**
Tuguegarao City	1,778	95	123	**218**	**12.26**	12	16	**28**	**1.57**

Table 2.F.3.1. Cardiovascular Disease and Diabetes Mellitus Prevention and Control

Number and proportion of newly-identified 20 yrs old and above adults with Hypertension and Type 2 Diabetes Mellitus
Philippines, 2022

Area	Total No. of clients ≥ 20 y/o risk-assesed[1]	Newly identified Hypertensive Adults				Newly identified with Type 2 Diabetes Mellitus			
		Male	Female	Total	%	Male	Female	Total	%
**Region 3**	**1,229,672**	**42,572**	**51,733**	**94,305**	**7.67**	**12,650**	**15,627**	**28,277**	**2.30**
Aurora	92,606	1,626	2,228	**3,854**	**4.16**	132	190	**322**	**0.35**
Bataan	53,820	1,593	2,324	**3,917**	**7.28**	337	503	**840**	**1.56**
Bulacan	237,184	11,386	12,743	**24,129**	**10.17**	4,423	5,231	**9,654**	**4.07**
Nueva Ecija	78,255	4,989	5,386	**10,375**	**13.26**	1,225	1,336	**2,561**	**3.27**
Pampanga	49,507	4,476	6,006	**10,482**	**21.17**	1,701	2,043	**3,744**	**7.56**
Tarlac	90,053	4,939	6,620	**11,559**	**12.84**	945	1,080	**2,025**	**2.25**
Zambales	89,876	1,589	2,342	**3,931**	**4.37**	297	486	**783**	**0.87**
Angeles City	4,129	395	527	**922**	**22.33**	126	193	**319**	**7.73**
Balanga City	2,290	324	333	**657**	**28.69**	87	119	**206**	**9.00**
Cabanatuan City	28,685	1,229	1,568	**2,797**	**9.75**	304	379	**683**	**2.38**
City of San Fernando	10,982	628	784	**1,412**	**12.86**	208	300	**508**	**4.63**
Gapan City	150	51	55	**106**	**70.67**	7	18	**25**	**16.67**
Mabalacat City	64,689	491	662	**1,153**	**1.78**	161	263	**424**	**0.66**
Malolos City	8,760	432	473	**905**	**10.33**	146	185	**331**	**3.78**
Meycauayan	1,605	460	510	**970**	**60.44**	134	127	**261**	**16.26**
Olongapo	3,844	270	357	**627**	**16.31**	43	69	**112**	**2.91**
Palayan City	217	39	38	**77**	**35.48**	8	14	**22**	**10.14**
San Jose City	16,895	72	86	**158**	**0.94**	17	15	**32**	**0.19**
San Jose del Monte Cit	371,888	5,065	5,557	**10,622**	**2.86**	1,641	2,155	**3,796**	**1.02**
Science City of Munoz	21,583	1,926	2,487	**4,413**	**20.45**	475	625	**1,100**	**5.10**
Tarlac City	2,654	592	647	**1,239**	**46.68**	233	296	**529**	**19.93**
**Region 4A**	**355,013**	**29,929**	**40,280**	**70,209**	**19.78**	**11,208**	**10,825**	**22,033**	**6.21**
Batangas	68,393	3,398	4,331	**7,729**	**11.30**	1,016	1,219	**2,235**	**3.27**
Cavite	74,087	6,734	12,421	**19,155**	**25.85**	1,637	2,086	**3,723**	**5.03**
Laguna	13,451	2,515	3,300	**5,815**	**43.23**	400	574	**974**	**7.24**
Quezon	43,789	2,640	4,002	**6,642**	**15.17**	460	708	**1,168**	**2.67**
Rizal	18,232	3,402	3,606	**7,008**	**38.44**	1,556	1,600	**3,156**	**17.31**
Antipolo City	0	0	0	**0**	**0.00**	0	0	**0**	**0.00**
Bacoor City	685	159	209	**368**	**53.72**	50	71	**121**	**17.66**
Batangas City	1,708	278	373	**651**	**38.11**	106	127	**233**	**13.64**
Biñan City	5,087	307	275	**582**	**11.44**	127	141	**268**	**5.27**
Cabuyao City	875	127	144	**271**	**30.97**	46	70	**116**	**13.26**
Calamba City	5,923	719	1,037	**1,756**	**29.65**	177	268	**445**	**7.51**
Cavite City	461	151	145	**296**	**64.21**	33	29	**62**	**13.45**
Dasmariñas City	49,710	2,367	1,894	**4,261**	**8.57**	1,282	661	**1,943**	**3.91**
General Trias City	8,650	441	572	**1,013**	**11.71**	664	995	**1,659**	**19.18**
Imus City	3,130	440	522	**962**	**30.73**	156	139	**295**	**9.42**
Lipa City	7,427	552	398	**950**	**12.79**	576	480	**1,056**	**14.22**
Lucena City	1,482	128	174	**302**	**20.38**	12	38	**50**	**3.37**
San Pablo City	1,224	344	463	**807**	**65.93**	90	117	**207**	**16.91**
San Pedro City	42	7	15	**22**	**52.38**	2	2	**4**	**9.52**
Santa Rosa City	12,258	1,569	2,294	**3,863**	**31.51**	348	586	**934**	**7.62**
Tagaytay City	16,056	2,218	2,214	**4,432**	**27.60**	513	504	**1,017**	**6.33**
Tanauan City	6,496	979	1,171	**2,150**	**33.10**	1,877	283	**2,160**	**33.25**
Tayabas City	9,642	267	385	**652**	**6.76**	21	25	**46**	**0.48**
Trece Martires City	6,205	187	335	**522**	**8.41**	59	102	**161**	**2.59**
**Region 4B**	**221,839**	**8,037**	**10,877**	**18,914**	**8.53**	**1,823**	**2,544**	**4,367**	**1.97**
Marinduque	6,827	485	681	**1,166**	**17.08**	217	279	**496**	**7.27**
Mindoro Occidental	20,244	1,067	1,248	**2,315**	**11.44**	134	141	**275**	**1.36**
Mindoro Oriental	42,053	2,496	3,593	**6,089**	**14.48**	732	1,068	**1,800**	**4.28**
Palawan	143,572	2,969	3,815	**6,784**	**4.73**	476	673	**1,149**	**0.80**
Romblon	6,874	283	363	**646**	**9.40**	67	107	**174**	**2.53**
Puerto Princesa City	2,269	737	1,177	**1,914**	**84.35**	197	276	**473**	**20.85**
**Region 5**	**480,633**	**9,269**	**12,773**	**22,042**	**4.59**	**1,160**	**1,767**	**2,927**	**0.61**
Albay	41,765	1,781	2,398	**4,179**	**10.01**	208	254	**462**	**1.11**
Camarines Norte	45,679	771	969	**1,740**	**3.81**	107	186	**293**	**0.64**

Table 2.F.3.1. Cardiovascular Disease and Diabetes Mellitus Prevention and Control

Number and proportion of newly-identified 20 yrs old and above adults with Hypertension and Type 2 Diabetes Mellitus

Philippines, 2022

Area	Total No. of clients ≥ 20 y/o risk-assesed[1]	Newly identified Hypertensive Adults				Newly identified with Type 2 Diabetes Mellitus			
		Male	Female	Total	%	Male	Female	Total	%
Camarines Sur	192,201	2,432	3,680	**6,112**	**3.18**	236	471	**707**	**0.37**
Catanduanes	23,915	468	535	**1,003**	**4.19**	56	77	**133**	**0.56**
Masbate	32,213	1,153	1,687	**2,840**	**8.82**	208	322	**530**	**1.65**
Sorsogon	115,214	1,443	1,914	**3,357**	**2.91**	224	264	**488**	**0.42**
Iriga City	16,326	223	348	**571**	**3.50**	20	35	**55**	**0.34**
Legaspi City	6,308	457	538	**995**	**15.77**	38	66	**104**	**1.65**
Naga City	7,012	541	704	**1,245**	**17.76**	63	92	**155**	**2.21**
**Region 6**	**584,937**	**29,913**	**37,468**	**67,381**	**11.52**	**6,533**	**8,605**	**15,138**	**2.59**
Aklan	74,984	4,211	5,587	**9,798**	**13.07**	606	818	**1,424**	**1.90**
Antique	49,877	7,398	9,534	**16,932**	**33.95**	1,807	2,623	**4,430**	**8.88**
Capiz	44,463	3,870	5,310	**9,180**	**20.65**	864	1,180	**2,044**	**4.60**
Guimaras	37,225	1,434	1,600	**3,034**	**8.15**	196	248	**444**	**1.19**
Iloilo	155,723	6,405	6,490	**12,895**	**8.28**	1,707	1,924	**3,631**	**2.33**
Negros Occidental	184,181	4,471	6,066	**10,537**	**5.72**	714	946	**1,660**	**0.90**
Bacolod City	9,552	652	1,092	**1,744**	**18.26**	98	147	**245**	**2.56**
Iloilo City	28,932	1,472	1,789	**3,261**	**11.27**	541	719	**1,260**	**4.36**
**Region 7**	**465,892**	**11,054**	**14,882**	**25,936**	**5.57**	**2,256**	**3,100**	**5,356**	**1.15**
Bohol	69,263	616	953	**1,569**	**2.27**	117	199	**316**	**0.46**
Cebu	279,501	6,560	8,619	**15,179**	**5.43**	1,266	1,678	**2,944**	**1.05**
Negros Oriental	21,288	713	1,103	**1,816**	**8.53**	247	401	**648**	**3.04**
Siquijor	12,080	230	384	**614**	**5.08**	41	80	**121**	**1.00**
Cebu City	8,842	1,216	1,667	**2,883**	**32.61**	280	345	**625**	**7.07**
Lapu-Lapu City	6,741	692	931	**1,623**	**24.08**	148	208	**356**	**5.28**
Mandaue City	68,177	1,027	1,225	**2,252**	**3.30**	157	189	**346**	**0.51**
**Region 8**	**391,303**	**8,593**	**11,646**	**20,239**	**5.17**	**1,706**	**2,316**	**4,022**	**1.03**
Biliran	6,256	144	196	**340**	**5.43**	24	38	**62**	**0.99**
Eastern Samar	65,329	500	665	**1,165**	**1.78**	73	129	**202**	**0.31**
Northern Leyte	60,550	2,158	3,049	**5,207**	**8.60**	582	710	**1,292**	**2.13**
Northern Samar	37,794	861	1,141	**2,002**	**5.30**	109	128	**237**	**0.63**
Southern Leyte	60,570	461	507	**968**	**1.60**	94	143	**237**	**0.39**
Western Samar	94,500	1,185	1,581	**2,766**	**2.93**	252	389	**641**	**0.68**
Calbayog City	1,081	215	324	**539**	**49.86**	46	48	**94**	**8.70**
Maasin City	2,068	61	58	**119**	**5.75**	19	17	**36**	**1.74**
Ormoc City	7,764	2,611	3,506	**6,117**	**78.79**	438	611	**1,049**	**13.51**
Tacloban City	55,391	397	619	**1,016**	**1.83**	69	103	**172**	**0.31**
**Region 9**	**216,397**	**5,020**	**5,973**	**10,993**	**5.08**	**1,021**	**1,470**	**2,491**	**1.15**
Zamboanga Del Norte	42,709	1,161	1,357	**2,518**	**5.90**	295	471	**766**	**1.79**
Zamboanga Del Sur	41,903	1,167	1,289	**2,456**	**5.86**	193	311	**504**	**1.20**
Zamboanga Sibugay	102,570	882	933	**1,815**	**1.77**	133	178	**311**	**0.30**
Dapitan City	3,419	24	24	**48**	**1.40**	12	7	**19**	**0.56**
Dipolog City	2,822	423	534	**957**	**33.91**	33	40	**73**	**2.59**
Isabela City	1,394	178	215	**393**	**28.19**	90	138	**228**	**16.36**
Pagadian City	809	14	57	**71**	**8.78**	24	41	**65**	**8.03**
Zamboanga City	20,771	1,171	1,564	**2,735**	**13.17**	241	284	**525**	**2.53**
**Region 10**	**214,206**	**8,425**	**8,829**	**17,254**	**8.05**	**1,942**	**2,368**	**4,310**	**2.01**
Bukidnon	85,715	691	605	**1,296**	**1.51**	73	98	**171**	**0.20**
Camiguin	217	79	115	**194**	**89.40**	5	18	**23**	**10.60**
Lanao Del Norte	31,139	815	932	**1,747**	**5.61**	168	220	**388**	**1.25**
Misamis Occidental	21,944	2,268	1,615	**3,883**	**17.70**	478	421	**899**	**4.10**
Misamis Oriental	254	0	0	**0**	**0.00**	0	0	**0**	**0.00**

Table 2.F.3.1. Cardiovascular Disease and Diabetes Mellitus Prevention and Control

Number and proportion of newly-identified 20 yrs old and above adults with Hypertension and Type 2 Diabetes Mellitus
Philippines, 2022

Area	Total No. of clients ≥ 20 y/o risk-assesed[1]	Newly identified Hypertensive Adults				Newly identified with Type 2 Diabetes Mellitus			
		Male	Female	Total	%	Male	Female	Total	%
Cagayan de Oro City	21,219	471	441	**912**	**4.30**	121	103	**224**	**1.06**
El Salvador City	622	46	81	**127**	**20.42**	6	38	**44**	**7.07**
Gingoog City	132	17	13	**30**	**22.73**	8	1	**9**	**6.82**
Iligan City	5,501	1,398	1,553	**2,951**	**53.64**	319	338	**657**	**11.94**
Malaybalay City	6,199	19	30	**49**	**0.79**	13	14	**27**	**0.44**
Oroquieta City	11,333	1,792	2,557	**4,349**	**38.37**	606	958	**1,564**	**13.80**
Ozamis City	16,092	630	639	**1,269**	**7.89**	104	118	**222**	**1.38**
Tangub City	398	11	15	**26**	**6.53**	4	2	**6**	**1.51**
Valencia City	13,441	188	233	**421**	**3.13**	37	39	**76**	**0.57**
**Region 11**	**506,009**	**31,109**	**35,454**	**66,563**	**13.15**	**8,657**	**11,972**	**20,629**	**4.08**
Davao de Oro	114,702	3,198	3,945	**7,143**	**6.23**	418	586	**1,004**	**0.88**
Davao del Norte	139,434	9,791	8,630	**18,421**	**13.21**	1,938	3,620	**5,558**	**3.99**
Davao Oriental	62,452	3,934	5,351	**9,285**	**14.87**	1,454	2,184	**3,638**	**5.83**
Davao del Sur	92,082	6,823	8,777	**15,600**	**16.94**	2,519	2,749	**5,268**	**5.72**
Davao Occidental	15,418	1,138	1,448	**2,586**	**16.77**	227	344	**571**	**3.70**
Davao City	81,921	6,225	7,303	**13,528**	**16.51**	2,101	2,489	**4,590**	**5.60**
**Region 12**	**367,741**	**9,252**	**11,171**	**20,423**	**5.55**	**2,800**	**3,615**	**6,415**	**1.74**
North Cotabato	87,834	2,359	2,666	**5,025**	**5.72**	464	651	**1,115**	**1.27**
Sarangani	75,220	1,506	1,931	**3,437**	**4.57**	287	435	**722**	**0.96**
South Cotabato	54,488	1,642	2,054	**3,696**	**6.78**	352	442	**794**	**1.46**
Sultan Kudarat	145,860	3,045	3,591	**6,636**	**4.55**	1,408	1,687	**3,095**	**2.12**
Gen. Santos City	4,339	700	929	**1,629**	**37.54**	289	400	**689**	**15.88**
**BARMM**	**66,469**	**4,514**	**6,119**	**10,633**	**16.00**	**1,060**	**1,552**	**2,612**	**3.93**
Basilan	5,302	193	278	**471**	**8.88**	31	35	**66**	**1.24**
Lanao Del Sur	19,610	1,582	2,166	**3,748**	**19.11**	302	522	**824**	**4.20**
Maguindanao	13,542	1,346	1,815	**3,161**	**23.34**	281	390	**671**	**4.95**
Sulu	276	480	704	**1,184**	**428.99**	124	169	**293**	**106.16**
Tawi-Tawi	3,034	317	393	**710**	**23.40**	63	69	**132**	**4.35**
Lamitan City	1,392	24	34	**58**	**4.17**	5	7	**12**	**0.86**
Marawi City	11,739	311	409	**720**	**6.13**	196	256	**452**	**3.85**
Cotabato City	11,574	261	320	**581**	**5.02**	58	104	**162**	**1.40**
**CARAGA**	**123,103**	**7,120**	**7,368**	**14,488**	**11.77**	**1,606**	**2,076**	**3,682**	**2.99**
Agusan del Norte	17,888	2,399	2,110	**4,509**	**25.21**	627	634	**1,261**	**7.05**
Agusan del Sur	39,978	1,168	1,454	**2,622**	**6.56**	230	259	**489**	**1.22**
Surigao del Norte	7,326	505	494	**999**	**13.64**	131	247	**378**	**5.16**
Surigao del Sur	10,393	764	942	**1,706**	**16.41**	119	216	**335**	**3.22**
Province of Dinagat	1,409	109	132	**241**	**17.10**	17	41	**58**	**4.12**
Bislig City	19,337	1,137	1,012	**2,149**	**11.11**	269	368	**637**	**3.29**
Butuan City	24,135	768	895	**1,663**	**6.89**	124	203	**327**	**1.35**
Surigao City	2,637	270	329	**599**	**22.72**	89	108	**197**	**7.47**

Table 2.F.4.1. Blindness Prevention Program

Number and proportion of senior citizens 60 yrs. old and above screened visual acuity and diagnosed with eye diseases
Philippines, 2022

Area	Eligible Pop.(60 y/o and above)	Screened for Visual Acuity				Diagnosed with eye diseases			
		Male	Female	Total	%	Male	Female	Total	%
**PHILIPPINES**	**10,260,325**	**101,417**	**128,072**	**229,489**	**2.24**	**12,761**	**17,703**	**30,464**	**13.27**
**NCR**	**1,357,932**	**14,120**	**21,301**	**35,421**	**2.61**	**2,370**	**4,488**	**6,858**	**19.36**
Malabon	38,545	149	309	**458**	**1.19**	11	27	**38**	**8.30**
Navotas	26,307	3	5	**8**	**0.03**	0	5	**5**	**62.50**
Valenzuela City	65,426	133	186	**319**	**0.49**	21	46	**67**	**21.00**
Caloocan City	167,033	1,761	2,353	**4,114**	**2.46**	131	585	**716**	**17.40**
Marikina City	47,534	112	218	**330**	**0.69**	0	0	**0**	**0.00**
Pasig City	79,649	5,148	8,230	**13,378**	**16.80**	1,478	2,766	**4,244**	**31.72**
Pateros	6,732	602	833	**1,435**	**21.32**	102	247	**349**	**24.32**
Taguig	84,882	1,154	2,003	**3,157**	**3.72**	106	162	**268**	**8.49**
Quezon City	309,616	757	1,082	**1,839**	**0.59**	38	53	**91**	**4.95**
Makati City	61,438	96	134	**230**	**0.37**	88	126	**214**	**93.04**
Mandaluyong City	40,733	39	46	**85**	**0.21**	2	4	**6**	**7.06**
San Juan	12,883	0	0	**0**	**0.00**	0	0	**0**	**0.00**
Manila City	187,723	3,997	5,678	**9,675**	**5.15**	270	313	**583**	**6.03**
Las Piñas City	62,104	94	97	**191**	**0.31**	94	97	**191**	**100.00**
Muntinlupa City	53,202	16	15	**31**	**0.06**	10	10	**20**	**64.52**
Parañaque City	70,212	54	101	**155**	**0.22**	15	36	**51**	**32.90**
Pasay City	43,913	5	11	**16**	**0.04**	4	11	**15**	**93.75**
**CAR**	**158,933**	**1,329**	**2,358**	**3,687**	**2.32**	**114**	**247**	**361**	**9.79**
Abra	27,231	245	319	**564**	**2.07**	9	8	**17**	**3.01**
Apayao	10,461	193	304	**497**	**4.75**	10	16	**26**	**5.23**
Benguet	40,989	267	479	**746**	**1.82**	65	153	**218**	**29.22**
Ifugao	16,362	40	41	**81**	**0.50**	3	8	**11**	**13.58**
Kalinga	18,071	46	81	**127**	**0.70**	11	30	**41**	**32.28**
Mountain Province	14,098	354	706	**1,060**	**7.52**	11	26	**37**	**3.49**
Baguio City	31,721	184	428	**612**	**1.93**	5	6	**11**	**1.80**
**Region 1**	**589,897**	**1,510**	**2,124**	**3,634**	**0.62**	**221**	**361**	**582**	**16.02**
Ilocos Norte	64,634	203	337	**540**	**0.84**	47	96	**143**	**26.48**
Ilocos Sur	75,905	937	1,331	**2,268**	**2.99**	92	154	**246**	**10.85**
La Union	84,296	28	49	**77**	**0.09**	8	13	**21**	**27.27**
Pangasinan	256,657	331	391	**722**	**0.28**	72	87	**159**	**22.02**
Alaminos City	9,695	0	0	**0**	**0.00**	0	0	**0**	**0.00**
Candon City	7,993	10	14	**24**	**0.30**	2	2	**4**	**16.67**
Dagupan City	18,510	0	0	**0**	**0.00**	0	0	**0**	**0.00**
Laoag City	14,898	0	0	**0**	**0.00**	0	0	**0**	**0.00**
San Carlos City	20,389	0	0	**0**	**0.00**	0	7	**7**	**0.00**
San Fernando City	15,444	0	0	**0**	**0.00**	0	0	**0**	**0.00**
Urdaneta City	14,368	1	2	**3**	**0.02**	0	2	**2**	**66.67**
Vigan City	7,108	0	0	**0**	**0.00**	0	0	**0**	**0.00**
**Region 2**	**380,020**	**6,952**	**9,667**	**16,619**	**4.37**	**442**	**622**	**1,064**	**6.40**
Batanes	2,048	0	0	**0**	**0.00**	0	0	**0**	**0.00**
Cagayan	122,665	811	919	**1,730**	**1.41**	25	33	**58**	**3.35**
Isabela	127,318	835	1,262	**2,097**	**1.65**	119	174	**293**	**13.97**
Nueva Vizcaya	47,970	131	244	**375**	**0.78**	31	47	**78**	**20.80**
Quirino	17,919	68	98	**166**	**0.93**	14	43	**57**	**34.34**
Cauayan City	13,932	1	4	**5**	**0.04**	1	4	**5**	**100.00**
Ilagan City	15,659	1,671	2,530	**4,201**	**26.83**	167	208	**375**	**8.93**
Santiago City	14,502	3,435	4,610	**8,045**	**55.48**	85	113	**198**	**2.46**
Tuguegarao City	18,007	0	0	**0**	**0.00**	0	0	**0**	**0.00**

Table 2.F.4.1. Blindness Prevention Program

Number and proportion of senior citizens 60 yrs. old and above screened visual acuity and diagnosed with eye diseases
Philippines, 2022

Area	Eligible Pop.(60 y/o and above)	Screened for Visual Acuity				Diagnosed with eye diseases			
		Male	Female	Total	%	Male	Female	Total	%
**Region 3**	**1,251,818**	**11,027**	**13,903**	**24,930**	**1.99**	**476**	**577**	**1,053**	**4.22**
Aurora	19,865	10	31	**41**	**0.21**	0	9	**9**	**21.95**
Bataan	73,747	65	85	**150**	**0.20**	2	8	**10**	**6.67**
Bulacan	248,890	6,936	9,376	**16,312**	**6.55**	130	131	**261**	**1.60**
Nueva Ecija	171,700	497	673	**1,170**	**0.68**	153	177	**330**	**28.21**
Pampanga	183,485	243	246	**489**	**0.27**	2	2	**4**	**0.82**
Tarlac	114,073	297	365	**662**	**0.58**	82	98	**180**	**27.19**
Zambales	64,701	0	1	**1**	**0.00**	0	1	**1**	**100.00**
Angeles City	46,032	0	0	**0**	**0.00**	0	0	**0**	**0.00**
Balanga City	10,661	0	0	**0**	**0.00**	0	0	**0**	**0.00**
Cabanatuan City	35,147	0	4	**4**	**0.01**	0	0	**0**	**0.00**
City of San Fernando	34,291	0	0	**0**	**0.00**	0	0	**0**	**0.00**
Gapan City	12,830	0	0	**0**	**0.00**	0	0	**0**	**0.00**
Mabalacat City	28,044	588	682	**1,270**	**4.53**	82	115	**197**	**15.51**
Malolos City	27,804	19	33	**52**	**0.19**	0	1	**1**	**1.92**
Meycauayan	23,055	0	0	**0**	**0.00**	0	0	**0**	**0.00**
Olongapo	25,513	0	0	**0**	**0.00**	0	0	**0**	**0.00**
Palayan City	4,772	0	0	**0**	**0.00**	0	0	**0**	**0.00**
San Jose City	16,250	0	0	**0**	**0.00**	0	0	**0**	**0.00**
San Jose del Monte City	63,314	2,335	2,351	**4,686**	**7.40**	17	25	**42**	**0.90**
Science City of Munoz	9,472	33	49	**82**	**0.87**	4	7	**11**	**13.41**
Tarlac City	38,172	4	7	**11**	**0.03**	4	3	**7**	**63.64**
**Region 4A**	**1,498,518**	**2,822**	**4,933**	**7,755**	**0.52**	**691**	**1,100**	**1,791**	**23.09**
Batangas	210,875	162	214	**376**	**0.18**	122	127	**249**	**66.22**
Cavite	144,377	742	1,344	**2,086**	**1.44**	33	50	**83**	**3.98**
Laguna	101,206	14	30	**44**	**0.04**	7	11	**18**	**40.91**
Quezon	175,335	223	336	**559**	**0.32**	53	79	**132**	**23.61**
Rizal	207,791	31	57	**88**	**0.04**	28	57	**85**	**96.59**
Antipolo City	76,533	0	0	**0**	**0.00**	0	0	**0**	**0.00**
Bacoor City	63,259	503	844	**1,347**	**2.13**	75	141	**216**	**16.04**
Batangas City	37,418	0	0	**0**	**0.00**	0	0	**0**	**0.00**
Biñan City	33,935	7	6	**13**	**0.04**	0	2	**2**	**15.38**
Cabuyao City	31,459	53	49	**102**	**0.32**	7	5	**12**	**11.76**
Calamba City	46,312	241	359	**600**	**1.30**	207	307	**514**	**85.67**
Cavite City	10,828	0	0	**0**	**0.00**	0	0	**0**	**0.00**
Dasmariñas City	69,408	60	95	**155**	**0.22**	31	30	**61**	**39.35**
General Trias City	33,105	0	0	**0**	**0.00**	0	0	**0**	**0.00**
Imus City	42,530	0	0	**0**	**0.00**	0	0	**0**	**0.00**
Lipa City	37,703	4	7	**11**	**0.03**	1	6	**7**	**63.64**
Lucena City	26,569	1	2	**3**	**0.01**	0	0	**0**	**0.00**
San Pablo City	27,103	8	34	**42**	**0.15**	3	17	**20**	**47.62**
San Pedro City	33,197	0	0	**0**	**0.00**	0	0	**0**	**0.00**
Santa Rosa City	36,049	565	1,020	**1,585**	**4.40**	14	32	**46**	**2.90**
Tagaytay City	7,495	27	123	**150**	**2.00**	0	10	**10**	**6.67**
Tanauan City	19,669	30	21	**51**	**0.26**	5	4	**9**	**17.65**
Tayabas City	9,961	151	392	**543**	**5.45**	105	222	**327**	**60.22**
Trece Martires City	16,401	0	0	**0**	**0.00**	0	0	**0**	**0.00**
**Region 4B**	**276,804**	**200**	**256**	**456**	**0.16**	**95**	**113**	**208**	**45.61**
Marinduque	27,151	0	0	**0**	**0.00**	0	0	**0**	**0.00**
Mindoro Occidental	41,833	42	58	**100**	**0.24**	12	13	**25**	**25.00**
Mindoro Occidental	81,156	42	58	**100**	**0.12**	12	13	**25**	**25.00**
Palawan	73,572	68	86	**154**	**0.21**	56	65	**121**	**78.57**
Romblon	30,992	17	23	**40**	**0.13**	14	21	**35**	**87.50**
Puerto Princesa City	22,100	31	31	**62**	**0.28**	1	1	**2**	**3.23**

Table 2.F.4.1. Blindness Prevention Program

Number and proportion of senior citizens 60 yrs. old and above screened visual acuity and diagnosed with eye diseases
Philippines, 2022

Area	Eligible Pop.(60 y/o and above)	Screened for Visual Acuity				Diagnosed with eye diseases			
		Male	Female	Total	%	Male	Female	Total	%
**Region 5**	**549,286**	**2,109**	**3,311**	**5,420**	**0.99**	**372**	**693**	**1,065**	**19.65**
Albay	117,574	421	588	**1,009**	**0.86**	69	106	**175**	**17.34**
Camarines Norte	55,004	38	49	**87**	**0.16**	4	10	**14**	**16.09**
Camarines Sur	151,603	805	1,286	**2,091**	**1.38**	121	214	**335**	**16.02**
Catanduanes	24,774	94	156	**250**	**1.01**	40	55	**95**	**38.00**
Masbate	75,689	37	53	**90**	**0.12**	14	18	**32**	**35.56**
Sorsogon	75,597	299	541	**840**	**1.11**	54	151	**205**	**24.40**
Iriga City	10,301	134	224	**358**	**3.48**	29	49	**78**	**21.79**
Legaspi City	20,671	197	255	**452**	**2.19**	39	88	**127**	**28.10**
Naga City	18,073	84	159	**243**	**1.34**	2	2	**4**	**1.65**
**Region 6**	**864,891**	**2,575**	**3,578**	**6,153**	**0.71**	**493**	**878**	**1,371**	**22.28**
Aklan	64,618	129	129	**258**	**0.40**	17	19	**36**	**13.95**
Antique	62,029	207	420	**627**	**1.01**	160	318	**478**	**76.24**
Capiz	92,991	336	649	**985**	**1.06**	48	152	**200**	**20.30**
Guimaras	20,507	254	364	**618**	**3.01**	6	8	**14**	**2.27**
Iloilo	239,155	435	480	**915**	**0.38**	46	74	**120**	**13.11**
Negros Occidental	269,602	812	1,169	**1,981**	**0.73**	160	217	**377**	**19.03**
Bacolod City	60,661	300	165	**465**	**0.77**	47	74	**121**	**26.02**
Iloilo City	55,328	102	202	**304**	**0.55**	9	16	**25**	**8.22**
**Region 7**	**796,596**	**512**	**782**	**1,294**	**0.16**	**529**	**576**	**1,105**	**85.39**
Bohol	149,983	0	5	**5**	**0.00**	0	1	**1**	**20.00**
Cebu	307,202	263	507	**770**	**0.25**	140	197	**337**	**43.77**
Negros Oriental	149,312	179	207	**386**	**0.26**	98	127	**225**	**58.29**
Siquijor	13,072	21	11	**32**	**0.24**	0	0	**0**	**0.00**
Cebu City	96,447	27	36	**63**	**0.07**	12	22	**34**	**53.97**
Lapu-Lapu City	42,665	22	16	**38**	**0.09**	4	7	**11**	**28.95**
Mandaue City	37,915	0	0	**0**	**0.00**	275	222	**497**	**0.00**
**Region 8**	**458,406**	**747**	**1,153**	**1,900**	**0.41**	**169**	**239**	**408**	**21.47**
Biliran	17,595	16	24	**40**	**0.23**	16	24	**40**	**100.00**
Eastern Samar	48,965	148	385	**533**	**1.09**	3	2	**5**	**0.94**
Northern Leyte	165,582	249	327	**576**	**0.35**	40	77	**117**	**20.31**
Northern Samar	53,055	116	147	**263**	**0.50**	75	69	**144**	**54.75**
Southern Leyte	41,767	105	126	**231**	**0.55**	18	43	**61**	**26.41**
Western Samar	54,015	113	144	**257**	**0.48**	17	24	**41**	**15.95**
Calbayog City	16,655	0	0	**0**	**0.00**	0	0	**0**	**0.00**
Maasin City	10,631	0	0	**0**	**0.00**	0	0	**0**	**0.00**
Ormoc City	23,590	0	0	**0**	**0.00**	0	0	**0**	**0.00**
Tacloban City	26,551	0	0	**0**	**0.00**	0	0	**0**	**0.00**
**Region 9**	**321,602**	**371**	**426**	**797**	**0.25**	**74**	**104**	**178**	**22.33**
Zamboanga Del Norte	75,882	198	216	**414**	**0.55**	21	27	**48**	**11.59**
Zamboanga Del Sur	71,073	74	71	**145**	**0.20**	19	20	**39**	**26.90**
Zamboanga Sibugay	51,407	27	42	**69**	**0.13**	1	2	**3**	**4.35**
Dapitan City	7,831	0	0	**0**	**0.00**	0	0	**0**	**0.00**
Dipolog City	12,429	33	38	**71**	**0.57**	11	20	**31**	**43.66**
Isabela City	10,103	3	4	**7**	**0.07**	3	4	**7**	**100.00**
Pagadian City	17,433	0	0	**0**	**0.00**	0	0	**0**	**0.00**
Zamboanga City	75,444	36	55	**91**	**0.12**	19	31	**50**	**54.95**
**Region 10**	**452,130**	**3,155**	**3,785**	**6,940**	**1.53**	**883**	**1,105**	**1,988**	**28.65**
Bukidnon	91,081	88	95	**183**	**0.20**	9	16	**25**	**13.66**

Table 2.F.4.1. Blindness Prevention Program

Number and proportion of senior citizens 60 yrs. old and above screened visual acuity and diagnosed with eye diseases
Philippines, 2022

Area	Eligible Pop.(60 y/o and above)	Screened for Visual Acuity				Diagnosed with eye diseases			
		Male	Female	Total	%	Male	Female	Total	%
Camiguin	10,469	0	0	**0**	**0.00**	0	0	**0**	**0.00**
Lanao Del Norte	56,689	743	493	**1,236**	**2.18**	72	77	**149**	**12.06**
Misamis Occidental	37,683	46	54	**100**	**0.27**	20	16	**36**	**36.00**
Misamis Oriental	74,683	17	26	**43**	**0.06**	5	6	**11**	**25.58**
Cagayan de Oro City	70,750	30	42	**72**	**0.10**	23	14	**37**	**51.39**
El Salvador City	5,255	2	10	**12**	**0.23**	0	0	**0**	**0.00**
Gingoog City	13,049	15	10	**25**	**0.19**	10	9	**19**	**76.00**
Iligan City	28,710	38	34	**72**	**0.25**	16	21	**37**	**51.39**
Malaybalay City	15,180	1	5	**6**	**0.04**	1	4	**5**	**83.33**
Oroquieta City	8,169	0	0	**0**	**0.00**	0	0	**0**	**0.00**
Ozamis City	16,368	2,047	2,895	**4,942**	**30.19**	607	829	**1,436**	**29.06**
Tangub City	7,270	126	119	**245**	**3.37**	118	113	**231**	**94.29**
Valencia City	16,774	2	2	**4**	**0.02**	2	0	**2**	**50.00**
**Region 11**	**478,960**	**47,763**	**51,848**	**99,611**	**20.80**	**4,322**	**4,539**	**8,861**	**8.90**
Davao de Oro	64,938	6,283	6,611	**12,894**	**19.86**	231	290	**521**	**4.04**
Davao del Norte	95,525	11,829	12,462	**24,291**	**25.43**	792	854	**1,646**	**6.78**
Davao Oriental	53,669	7,611	8,508	**16,119**	**30.03**	360	488	**848**	**5.26**
Davao del Sur	65,711	9,417	10,578	**19,995**	**30.43**	36	57	**93**	**0.47**
Davao Occidental	29,475	1,003	1,153	**2,156**	**7.31**	143	130	**273**	**12.66**
Davao City	169,642	11,620	12,536	**24,156**	**14.24**	2,760	2,720	**5,480**	**22.69**
**Region 12**	**360,969**	**5,208**	**7,097**	**12,305**	**3.41**	**1,126**	**1,467**	**2,593**	**21.07**
North Cotabato	121,258	429	538	**967**	**0.80**	164	212	**376**	**38.88**
Sarangani	41,067	1,820	2,746	**4,566**	**11.12**	369	498	**867**	**18.99**
South Cotabato	82,003	256	352	**608**	**0.74**	20	23	**43**	**7.07**
Sultan Kudarat	63,380	2,648	3,283	**5,931**	**9.36**	566	723	**1,289**	**21.73**
Gen. Santos City	53,261	55	178	**233**	**0.44**	7	11	**18**	**7.73**
**BARMM**	**219,854**	**368**	**471**	**839**	**0.38**	**99**	**127**	**226**	**26.94**
Basilan	15,213	1	7	**8**	**0.05**	0	0	**0**	**0.00**
Lanao Del Sur	40,887	13	9	**22**	**0.05**	3	36	**39**	**177.27**
Maguindanao	63,452	253	343	**596**	**0.94**	80	78	**158**	**26.51**
Sulu	45,942	0	0	**0**	**0.00**	0	0	**0**	**0.00**
Tawi-Tawi	20,602	0	0	**0**	**0.00**	0	0	**0**	**0.00**
Lamitan City	4,186	0	0	**0**	**0.00**	0	0	**0**	**0.00**
Marawi City	9,774	0	0	**0**	**0.00**	0	0	**0**	**0.00**
Cotabato City	19,798	101	112	**213**	**1.08**	16	13	**29**	**13.62**
**CARAGA**	**243,709**	**649**	**1,079**	**1,728**	**0.71**	**285**	**467**	**752**	**43.52**
Agusan del Norte	34,797	32	38	**70**	**0.20**	10	9	**19**	**27.14**
Agusan del Sur	53,286	11	22	**33**	**0.06**	4	10	**14**	**42.42**
Surigao del Norte	35,283	89	168	**257**	**0.73**	12	37	**49**	**19.07**
Surigao del Sur	48,057	77	86	**163**	**0.34**	45	55	**100**	**61.35**
Province of Dinagat	13,636	31	60	**91**	**0.67**	5	9	**14**	**15.38**
Bislig City	9,124	205	290	**495**	**5.43**	151	228	**379**	**76.57**
Butuan City	33,089	1	0	**1**	**0.00**	1	0	**1**	**100.00**
Surigao City	16,437	203	415	**618**	**3.76**	57	119	**176**	**28.48**

Table 2.F.5.1. Immunization for Senior Citizen

Number and proportion of senior citizen 60 yrs old and above who received one (1) dose of Pneumococcal Polysaccharide Vaccine (PPV) and one (1) dose of Influenza Vaccine

Philippines, 2022

Area	Eligible Pop. (60 y/o & above)	Received one (1) dose of PPV				Received one (1) dose of Influenza Vaccine			
		Male	Female	Total	%	Male	Female	Total	%
**Philippines**	**10,260,325**	**75,151**	**107,449**	**182,600**	**1.78**	**356,943**	**546,868**	**903,811**	**8.81**
**NCR**	**1,357,932**	**14,869**	**21,327**	**36,196**	**2.67**	**74,399**	**113,532**	**187,931**	**13.84**
Malabon	**38,545**	809	1,077	**1,886**	**4.89**	2,530	4,040	**6,570**	**17.05**
Navotas	**26,307**	128	223	**351**	**1.33**	2,357	3,084	**5,441**	**20.68**
Valenzuela City	**65,426**	277	435	**712**	**1.09**	1,635	2,989	**4,624**	**7.07**
Caloocan City	**167,033**	4,238	5,367	**9,605**	**5.75**	12,542	20,012	**32,554**	**19.49**
Marikina City	**47,534**	478	800	**1,278**	**2.69**	2,380	4,136	**6,516**	**13.71**
Pasig City	**79,649**	887	1,243	**2,130**	**2.67**	3,553	4,515	**8,068**	**10.13**
Pateros	**6,732**	46	78	**124**	**1.84**	786	736	**1,522**	**22.61**
Taguig	**84,882**	99	187	**286**	**0.34**	2,459	4,697	**7,156**	**8.43**
Quezon City	**309,616**	4,823	7,384	**12,207**	**3.94**	18,588	25,812	**44,400**	**14.34**
Makati City	**61,438**	542	995	**1,537**	**2.50**	3,726	6,512	**10,238**	**16.66**
Mandaluyong City	**40,733**	273	341	**614**	**1.51**	1,180	2,127	**3,307**	**8.12**
San Juan	**12,883**	109	182	**291**	**2.26**	383	490	**873**	**6.78**
Manila City	**187,723**	565	776	**1,341**	**0.71**	13,612	20,665	**34,277**	**18.26**
Las Piñas City	**62,104**	428	560	**988**	**1.59**	2,551	4,632	**7,183**	**11.57**
Muntinlupa City	**53,202**	48	52	**100**	**0.19**	994	1,653	**2,647**	**4.98**
Parañaque City	**70,212**	1,084	1,577	**2,661**	**3.79**	3,970	5,603	**9,573**	**13.63**
Pasay City	**43,913**	35	50	**85**	**0.19**	1,153	1,829	**2,982**	**6.79**
**CAR**	**158,933**	**959**	**1,737**	**2,696**	**1.70**	**4,402**	**7,486**	**11,888**	**7.48**
Abra	**27,231**	52	73	**125**	**0.46**	717	1,105	**1,822**	**6.69**
Apayao	**10,461**	53	98	**151**	**1.44**	308	550	**858**	**8.20**
Benguet	**40,989**	285	546	**831**	**2.03**	1,124	1,981	**3,105**	**7.58**
Ifugao	**16,362**	42	89	**131**	**0.80**	341	702	**1,043**	**6.37**
Kalinga	**18,071**	14	24	**38**	**0.21**	440	765	**1,205**	**6.67**
Mt. Province	**14,098**	119	286	**405**	**2.87**	157	504	**661**	**4.69**
Baguio City	**31,721**	394	621	**1,015**	**3.20**	1,315	1,879	**3,194**	**10.07**
**Region 1**	**589,897**	**6,942**	**9,933**	**16,875**	**2.86**	**20,205**	**34,830**	**55,035**	**9.33**
Ilocos Norte	**64,634**	1,070	1,396	**2,466**	**3.82**	1,610	3,146	**4,756**	**7.36**
Ilocos Sur	**75,905**	1,126	1,891	**3,017**	**3.97**	2,022	3,665	**5,687**	**7.49**
La Union	**84,296**	381	455	**836**	**0.99**	2,010	3,724	**5,734**	**6.80**
Pangasinan	**256,657**	3,361	4,926	**8,287**	**3.23**	12,074	21,234	**33,308**	**12.98**
Alaminos City	**9,695**	45	101	**146**	**1.51**	0	0	**0**	**0.00**
Candon City	**7,993**	337	453	**790**	**9.88**	0	0	**0**	**0.00**
Dagupan City	**18,510**	0	0	**0**	**0.00**	0	0	**0**	**0.00**
Laoag City	**14,898**	0	0	**0**	**0.00**	0	0	**0**	**0.00**
San Carlos City	**20,389**	193	319	**512**	**2.51**	544	913	**1,457**	**7.15**
San Fernando City	**15,444**	235	135	**370**	**2.40**	192	331	**523**	**3.39**
Urdaneta City	**14,368**	178	205	**383**	**2.67**	1,753	1,817	**3,570**	**24.85**
Vigan City	**7,108**	16	52	**68**	**0.96**	0	0	**0**	**0.00**
**Region 2**	**380,020**	**3,615**	**5,649**	**9,264**	**2.44**	**14,548**	**23,165**	**37,713**	**9.92**
Batanes	**2,048**	18	28	**46**	**2.25**	68	103	**171**	**8.35**
Cagayan	**122,665**	1,767	2,862	**4,629**	**3.77**	5,048	8,153	**13,201**	**10.76**
Isabela	**127,318**	1,132	1,690	**2,822**	**2.22**	4,550	7,790	**12,340**	**9.69**
Nueva Vizcaya	**47,970**	80	129	**209**	**0.44**	704	977	**1,681**	**3.50**
Quirino	**17,919**	144	176	**320**	**1.79**	392	424	**816**	**4.55**
Cauayan City	**13,932**	136	160	**296**	**2.12**	1,405	1,753	**3,158**	**22.67**
Ilagan City	**15,659**	74	144	**218**	**1.39**	896	1,297	**2,193**	**14.00**
Santiago City	**14,502**	150	266	**416**	**2.87**	726	1,298	**2,024**	**13.96**
Tuguegarao City	**18,007**	114	194	**308**	**1.71**	759	1,370	**2,129**	**11.82**
**Region 3**	**1,251,818**	**12,440**	**17,243**	**29,683**	**2.37**	**86,103**	**117,714**	**203,817**	**16.28**
Aurora	**19,865**	58	71	**129**	**0.65**	1,224	1,841	**3,065**	**15.43**
Bataan	**73,747**	478	742	**1,220**	**1.65**	4,522	6,596	**11,118**	**15.08**
Bulacan	**248,890**	3,315	5,326	**8,641**	**3.47**	17,283	24,755	**42,038**	**16.89**
Nueva Ecija	**171,700**	1,864	2,160	**4,024**	**2.34**	9,957	13,296	**23,253**	**13.54**
Pampanga	**183,485**	3,067	3,718	**6,785**	**3.70**	15,132	18,015	**33,147**	**18.07**

Table 2.F.5.1. Immunization for Senior Citizen

Number and proportion of senior citizen 60 yrs old and above who received one (1) dose of Pneumococcal Polysaccharide Vaccine (PPV) and one (1) dose of Influenza Vaccine

Philippines, 2022

Area	Eligible Pop. (60 y/o & above)	Received one (1) dose of PPV				Received one (1) dose of Influenza Vaccine			
		Male	Female	Total	%	Male	Female	Total	%
Tarlac	**114,073**	392	485	**877**	**0.77**	4,123	4,949	**9,072**	**7.95**
Zambales	**64,701**	343	677	**1,020**	**1.58**	4,082	7,235	**11,317**	**17.49**
Angeles City	**46,032**	394	538	**932**	**2.02**	2,745	3,434	**6,179**	**13.42**
Balanga City	**10,661**	79	113	**192**	**1.80**	879	1,417	**2,296**	**21.54**
Cabanatuan City	**35,147**	396	498	**894**	**2.54**	2,298	3,182	**5,480**	**15.59**
City of San Fernando	**34,291**	237	378	**615**	**1.79**	2,835	3,975	**6,810**	**19.86**
Gapan City	**12,830**	51	49	**100**	**0.78**	20	43	**63**	**0.49**
Mabalacat City	**28,044**	0	0	**0**	**0.00**	2,154	3,287	**5,441**	**19.40**
Malolos City	**27,804**	0	0	**0**	**0.00**	2,096	3,548	**5,644**	**20.30**
Meycauayan	**23,055**	278	504	**782**	**3.39**	1,554	2,364	**3,918**	**16.99**
Olongapo	**25,513**	209	260	**469**	**1.84**	827	1,129	**1,956**	**7.67**
Palayan City	**4,772**	90	152	**242**	**5.07**	770	856	**1,626**	**34.07**
San Jose City	**16,250**	0	0	**0**	**0.00**	639	1,028	**1,667**	**10.26**
San Jose del Monte City	**63,314**	447	584	**1,031**	**1.63**	7,595	10,372	**17,967**	**28.38**
Science City of Munoz	**9,472**	69	131	**200**	**2.11**	863	1,037	**1,900**	**20.06**
Tarlac City	**38,172**	673	857	**1,530**	**4.01**	4,505	5,355	**9,860**	**25.83**
**Region 4A**	**1,498,518**	**8,572**	**12,560**	**21,132**	**1.41**	**44,452**	**74,398**	**118,850**	**7.93**
Batangas	**210,875**	699	1,522	**2,221**	**1.05**	5,334	9,002	**14,336**	**6.80**
Cavite	**144,377**	4,805	6,160	**10,965**	**7.59**	14,807	23,694	**38,501**	**26.67**
Laguna	**101,206**	522	1,102	**1,624**	**1.60**	1,216	2,577	**3,793**	**3.75**
Quezon	**175,335**	236	416	**652**	**0.37**	1,282	4,110	**5,392**	**3.08**
Rizal	**207,791**	233	259	**492**	**0.24**	2,301	4,050	**6,351**	**3.06**
Antipolo City	**76,533**	0	0	**0**	**0.00**	0	0	**0**	**0.00**
Bacoor City	**63,259**	243	380	**623**	**0.98**	1,724	2,794	**4,518**	**7.14**
Batangas City	**37,418**	17	6	**23**	**0.06**	351	748	**1,099**	**2.94**
Biñan City	**33,935**	70	50	**120**	**0.35**	1,582	1,519	**3,101**	**9.14**
Cabuyao City	**31,459**	0	0	**0**	**0.00**	952	896	**1,848**	**5.87**
Calamba City	**46,312**	274	504	**778**	**1.68**	1,330	2,529	**3,859**	**8.33**
Cavite City	**10,828**	5	5	**10**	**0.09**	0	0	**0**	**0.00**
Dasmariñas City	**69,408**	189	273	**462**	**0.67**	3,327	5,691	**9,018**	**12.99**
General Trias City	**33,105**	55	73	**128**	**0.39**	1,129	2,027	**3,156**	**9.53**
Imus City	**42,530**	158	216	**374**	**0.88**	1,695	2,793	**4,488**	**10.55**
Lipa City	**37,703**	48	84	**132**	**0.35**	0	0	**0**	**0.00**
Lucena City	**26,569**	0	0	**0**	**0.00**	212	132	**344**	**1.29**
San Pablo City	**27,103**	347	575	**922**	**3.40**	1,622	2,862	**4,484**	**16.54**
San Pedro City	**33,197**	1	0	**1**	**0.00**	47	68	**115**	**0.35**
Santa Rosa City	**36,049**	516	632	**1,148**	**3.18**	2,832	4,291	**7,123**	**19.76**
Tagaytay City	**7,495**	0	0	**0**	**0.00**	415	605	**1,020**	**13.61**
Tanauan City	**19,669**	139	268	**407**	**2.07**	1,405	2,492	**3,897**	**19.81**
Tayabas City	**9,961**	1	3	**4**	**0.04**	596	891	**1,487**	**14.93**
Trece Martires City	**16,401**	14	32	**46**	**0.28**	293	627	**920**	**5.61**
**Region 4B**	**276,804**	**487**	**752**	**1,239**	**0.45**	**5,507**	**8,480**	**13,987**	**5.05**
Marinduque	**27,151**	102	242	**344**	**1.27**	1,065	1,757	**2,822**	**10.39**
Mindoro Occidental	**41,833**	84	82	**166**	**0.40**	1,179	1,380	**2,559**	**6.12**
Mindoro Oriental	**81,156**	53	57	**110**	**0.14**	1,306	2,105	**3,411**	**4.20**
Palawan	**73,572**	183	239	**422**	**0.57**	945	1,552	**2,497**	**3.39**
Romblon	**30,992**	60	123	**183**	**0.59**	402	791	**1,193**	**3.85**
Puerto Princesa City	**22,100**	5	9	**14**	**0.06**	610	895	**1,505**	**6.81**
**Region 5**	**549,286**	**2,856**	**4,828**	**7,684**	**1.40**	**12,317**	**24,415**	**36,732**	**6.69**
Albay	**117,574**	753	1,201	**1,954**	**1.66**	3,500	6,727	**10,227**	**8.70**
Camarines Norte	**55,004**	203	445	**648**	**1.18**	1,257	2,387	**3,644**	**6.62**
Camarines Sur	**151,603**	97	232	**329**	**0.22**	2,878	6,090	**8,968**	**5.92**
Catanduanes	**24,774**	74	118	**192**	**0.78**	1,468	2,256	**3,724**	**15.03**
Masbate	**75,689**	801	1,232	**2,033**	**2.69**	472	817	**1,289**	**1.70**
Sorsogon	**75,597**	335	533	**868**	**1.15**	1,831	4,052	**5,883**	**7.78**
Iriga City	**10,301**	7	24	**31**	**0.30**	158	424	**582**	**5.65**
Legaspi City	**20,671**	270	513	**783**	**3.79**	164	338	**502**	**2.43**
Naga City	**18,073**	316	530	**846**	**4.68**	589	1,324	**1,913**	**10.58**
**Region 6**	**864,891**	**6,934**	**10,439**	**17,373**	**2.01**	**30,107**	**44,178**	**74,285**	**8.59**
Aklan	**64,618**	647	1,120	**1,767**	**2.73**	972	1,778	**2,750**	**4.26**
Antique	**62,029**	309	550	**859**	**1.38**	1,163	1,877	**3,040**	**4.90**
Capiz	**92,991**	1,133	1,407	**2,540**	**2.73**	8,248	8,504	**16,752**	**18.01**

Table 2.F.5.1. Immunization for Senior Citizen

Number and proportion of senior citizen 60 yrs old and above who received one (1) dose of Pneumococcal Polysaccharide Vaccine (PPV) and one (1) dose of Influenza Vaccine

Philippines, 2022

Area	Eligible Pop. (60 y/o & above)	Received one (1) dose of PPV				Received one (1) dose of Influenza Vaccine			
		Male	Female	Total	%	Male	Female	Total	%
Guimaras	**20,507**	760	1,402	**2,162**	**10.54**	379	616	**995**	**4.85**
Iloilo	**239,155**	2,105	2,741	**4,846**	**2.03**	8,065	12,220	**20,285**	**8.48**
Negros Occidental	**269,602**	753	1,308	**2,061**	**0.76**	8,382	14,230	**22,612**	**8.39**
Bacolod City	**60,661**	516	972	**1,488**	**2.45**	1,006	1,657	**2,663**	**4.39**
Iloilo City	**55,328**	711	939	**1,650**	**2.98**	1,892	3,296	**5,188**	**9.38**
**Region 7**	**796,596**	**3,071**	**4,608**	**7,679**	**0.96**	**7,000**	**12,840**	**19,840**	**2.49**
Bohol	**149,983**	257	478	**735**	**0.49**	1,214	2,312	**3,526**	**2.35**
Cebu	**307,202**	510	938	**1,448**	**0.47**	2,180	4,039	**6,219**	**2.02**
Negros Oriental	**149,312**	865	1,319	**2,184**	**1.46**	1,851	3,552	**5,403**	**3.62**
Siquijor	**13,072**	51	73	**124**	**0.95**	142	288	**430**	**3.29**
Cebu City	**96,447**	615	1,006	**1,621**	**1.68**	1,252	2,109	**3,361**	**3.48**
Lapu-Lapu City	**42,665**	252	435	**687**	**1.61**	203	311	**514**	**1.20**
Mandaue City	**37,915**	521	359	**880**	**2.32**	158	229	**387**	**1.02**
**Region 8**	**458,406**	**1,176**	**1,740**	**2,916**	**0.64**	**6,670**	**12,140**	**18,810**	**4.10**
Biliran	**17,595**	49	97	**146**	**0.83**	480	927	**1,407**	**8.00**
Eastern Samar	**48,965**	93	147	**240**	**0.49**	118	183	**301**	**0.61**
Northern Leyte	**165,582**	93	250	**343**	**0.21**	1,869	4,398	**6,267**	**3.78**
Northern Samar	**53,055**	82	164	**246**	**0.46**	858	1,313	**2,171**	**4.09**
Southern Leyte	**41,767**	212	268	**480**	**1.15**	1,244	2,102	**3,346**	**8.01**
Western Samar	**54,015**	40	59	**99**	**0.18**	102	172	**274**	**0.51**
Calbayog City	**16,655**	10	18	**28**	**0.17**	141	249	**390**	**2.34**
Maasin City	**10,631**	39	102	**141**	**1.33**	409	951	**1,360**	**12.79**
Ormoc City	**23,590**	343	476	**819**	**3.47**	652	890	**1,542**	**6.54**
Tacloban City	**26,551**	215	159	**374**	**1.41**	797	955	**1,752**	**6.60**
**Region 9**	**321,602**	**1,506**	**2,113**	**3,619**	**1.13**	**6,487**	**10,531**	**17,018**	**5.29**
Zamboanga Del Norte	**75,882**	311	442	**753**	**0.99**	1,646	2,532	**4,178**	**5.51**
Zamboanga Del Sur	**71,073**	87	146	**233**	**0.33**	288	584	**872**	**1.23**
Zamboanga Sibugay	**51,407**	62	88	**150**	**0.29**	253	335	**588**	**1.14**
Dapitan City	**7,831**	96	89	**185**	**2.36**	490	1,090	**1,580**	**20.18**
Dipolog City	**12,429**	56	106	**162**	**1.30**	514	926	**1,440**	**11.59**
Isabela City	**10,103**	132	140	**272**	**2.69**	426	445	**871**	**8.62**
Pagadian City	**17,433**	0	0	**0**	**0.00**	66	97	**163**	**0.94**
Zamboanga City	**75,444**	762	1,102	**1,864**	**2.47**	2,804	4,522	**7,326**	**9.71**
**Region 10**	**452,130**	**3,481**	**4,334**	**7,815**	**1.73**	**11,745**	**15,743**	**27,488**	**6.08**
Bukidnon	**91,081**	293	417	**710**	**0.78**	499	629	**1,128**	**1.24**
Camiguin	**10,469**	103	178	**281**	**2.68**	428	601	**1,029**	**9.83**
Lanao Del Norte	**56,689**	1,306	1,378	**2,684**	**4.73**	1,213	1,301	**2,514**	**4.43**
Misamis Occidental	**37,683**	135	164	**299**	**0.79**	927	997	**1,924**	**5.11**
Misamis Oriental	**74,683**	102	167	**269**	**0.36**	2,331	3,516	**5,847**	**7.83**
Cagayan de Oro City	**70,750**	395	631	**1,026**	**1.45**	517	794	**1,311**	**1.85**
El Salvador City	**5,255**	12	23	**35**	**0.67**	261	539	**800**	**15.22**
Gingoog City	**13,049**	412	323	**735**	**5.63**	947	871	**1,818**	**13.93**
Iligan City	**28,710**	62	82	**144**	**0.50**	2,209	2,928	**5,137**	**17.89**
Malaybalay City	**15,180**	101	189	**290**	**1.91**	290	491	**781**	**5.14**
Oroquieta City	**8,169**	120	80	**200**	**2.45**	608	607	**1,215**	**14.87**
Ozamis City	**16,368**	123	148	**271**	**1.66**	433	780	**1,213**	**7.41**
Tangub City	**7,270**	0	0	**0**	**0.00**	2	2	**4**	**0.06**
Valencia City	**16,774**	317	554	**871**	**5.19**	1,080	1,687	**2,767**	**16.50**
**Region 11**	**478,960**	**4,424**	**5,007**	**9,431**	**1.97**	**12,738**	**17,824**	**30,562**	**6.38**
Davao de Oro	**64,938**	352	441	**793**	**1.22**	1,597	2,020	**3,617**	**5.57**
Davao del Norte	**95,525**	723	974	**1,697**	**1.78**	4,183	5,469	**9,652**	**10.10**
Davao Oriental	**53,669**	621	789	**1,410**	**2.63**	1,268	1,846	**3,114**	**5.80**
Davao del Sur	**65,711**	347	425	**772**	**1.17**	1,032	1,353	**2,385**	**3.63**
Davao Occidental	**29,475**	392	541	**933**	**3.17**	673	744	**1,417**	**4.81**
Davao City	**169,642**	1,989	1,837	**3,826**	**2.26**	3,985	6,392	**10,377**	**6.12**

Table 2.F.5.1. Immunization for Senior Citizen

Number and proportion of senior citizen 60 yrs old and above who received one (1) dose of Pneumococcal Polysaccharide Vaccine (PPV) and one (1) dose of Influenza Vaccine

Philippines, 2022

Area	Eligible Pop. (60 y/o & above)	Received one (1) dose of PPV				Received one (1) dose of Influenza Vaccine			
		Male	Female	Total	%	Male	Female	Total	%
**Region 12**	**360,969**	**2,603**	**3,433**	**6,036**	**1.67**	**12,958**	**18,595**	**31,553**	**8.74**
North Cotabato	**121,258**	510	835	**1,345**	**1.11**	2,545	4,033	**6,578**	**5.42**
Sarangani	**41,067**	521	701	**1,222**	**2.98**	2,963	4,051	**7,014**	**17.08**
South Cotabato	**82,003**	307	443	**750**	**0.91**	3,339	4,697	**8,036**	**9.80**
Sultan Kudarat	**63,380**	1,159	1,261	**2,420**	**3.82**	3,205	4,369	**7,574**	**11.95**
Gen. Santos City	**53,261**	106	193	**299**	**0.56**	906	1,445	**2,351**	**4.41**
**BARMM**	**219,854**	**1,725**	**2,352**	**4,077**	**1.85**	**2,846**	**3,200**	**6,046**	**2.75**
Basilan	**15,213**	1	6	**7**	**0.05**	0	0	**0**	**0.00**
Lanao Del Sur	**40,887**	34	86	**120**	**0.29**	24	58	**82**	**0.20**
Maguindanao	**63,452**	1,159	1,536	**2,695**	**4.25**	2,543	2,865	**5,408**	**8.52**
Sulu	**45,942**	0	0	**0**	**0.00**	0	0	**0**	**0.00**
Tawi-Tawi	**20,602**	138	90	**228**	**1.11**	220	172	**392**	**1.90**
Lamitan City	**4,186**	0	0	**0**	**0.00**	0	0	**0**	**0.00**
Marawi City	**9,774**	290	530	**820**	**8.39**	0	0	**0**	**0.00**
Cotabato City	**19,798**	103	104	**207**	**1.05**	59	105	**164**	**0.83**
**CARAGA**	**243,709**	**1,216**	**1,746**	**2,962**	**1.22**	**7,305**	**10,997**	**18,302**	**7.51**
Agusan del Norte	**34,797**	61	90	**151**	**0.43**	481	649	**1,130**	**3.25**
Agusan del Sur	**53,286**	524	872	**1,396**	**2.62**	444	722	**1,166**	**2.19**
Surigao del Norte	**35,283**	91	200	**291**	**0.82**	563	1,106	**1,669**	**4.73**
Surigao del Sur	**48,057**	327	373	**700**	**1.46**	2,013	3,183	**5,196**	**10.81**
Province of Dinagat	**13,636**	5	5	**10**	**0.07**	114	199	**313**	**2.30**
Bislig City	**9,124**	180	167	**347**	**3.80**	2,341	3,287	**5,628**	**61.68**
Butuan City	**33,089**	22	26	**48**	**0.15**	1,094	1,558	**2,652**	**8.01**
Surigao City	**16,437**	6	13	**19**	**0.12**	255	293	**548**	**3.33**

Table 2.G.1. Environmental Health and Sanitation Services

Proportion and Total No. of Households (HHs) with access to basic safe water supply and manage drinking water
Philippines, 2022

Area	Projected No. of HHs	HHs with Access to Basic Safe Water Supply								HHs using Safely Managed Drinking Water Services	
		Level 1		Level 2		Level 3		Total	%		
		No.	%	No.	%	No.	%			No.	%
**PHILIPPINES**	**25,481,993**	**5,252,600**	**23.23**	**2,997,478**	**13.26**	**14,362,750**	**63.52**	**22,612,828**	**88.74**	**16,516,197**	**64.82**
**NCR**	**3,416,247**	**3,613**	**0.11**	**126,255**	**3.86**	**3,144,322**	**96.03**	**3,274,190**	**95.84**	**3,169,273**	**92.77**
Malabon City	94,825	0	0.00	0	0.00	94,825	100.00	94,825	100.00	94,825	100.00
Navotas City	66,853	0	0.00	0	0.00	65,984	100.00	65,984	98.70	65,984	98.70
Valenzuela City	168,093	0	0.00	0	0.00	166,278	100.00	166,278	98.92	166,278	98.92
Caloocan City	404,100	0	0.00	0	0.00	350,802	100.00	350,802	86.81	350,802	86.81
Marikina City	108,152	0	0.00	0	0.00	108,152	100.00	108,152	100.00	108,152	100.00
Pasig City	198,622	0	0.00	0	0.00	198,622	100.00	198,622	100.00	198,622	100.00
Pateros	15,604	0	0.00	953	5.76	15,604	94.24	16,557	106.11	14,358	92.01
Taguig City	220,634	0	0.00	0	0.00	186,458	100.00	186,458	84.51	186,458	84.51
Quezon City	748,646	0	0.00	24	0.00	701,215	100.00	701,239	93.67	700,639	93.59
		0									
Makati City	169,874	0	0.00	1,928	1.13	167,946	98.87	169,874	100.00	169,874	100.00
Mandaluyong City	112,485	0	0.00	291	0.26	112,194	99.74	112,485	100.00	112,485	100.00
San Juan City	31,765	0	0.00	0	0.00	31,765	100.00	31,765	100.00	31,765	100.00
Manila City	481,750	0	0.00	102,110	21.20	379,648	78.80	481,758	100.00	379,648	78.81
Las Piñas City	155,928	0	0.00	1,273	0.82	154,655	99.18	155,928	100.00	155,928	100.00
Muntinlupa City	140,406	3,613	2.59	1,794	1.29	133,972	96.12	139,379	99.27	139,384	99.27
Parañaque City	179,359	0	0.00	17,882	9.97	161,490	90.03	179,372	100.01	179,359	100.00
Pasay City	119,151	0	0.00	0	0.00	114,712	100.00	114,712	96.27	114,712	96.27
**CAR**	**425,062**	**97,793**	**23.15**	**78,320**	**18.54**	**246,351**	**58.31**	**422,464**	**99.39**	**261,553**	**61.53**
Abra	55,106	13,943	24.59	16,406	28.94	26,345	46.47	56,694	102.88	21,394	38.82
Apayao	27,036	17,341	66.61	4,496	17.27	4,197	16.12	26,034	96.29	2,398	8.87
Benguet	117,566	22,158	21.01	12,943	12.27	70,344	66.71	105,445	89.69	74,549	63.41
Ifugao	45,597	19,178	39.08	16,790	34.21	13,106	26.71	49,074	107.63	23,206	50.89
Kalinga	44,270	13,937	27.85	14,352	28.68	21,749	43.46	50,038	113.03	38,006	85.85
Mt. Province	35,919	5,682	14.83	5,299	13.83	27,337	71.34	38,318	106.68	10,930	30.43
Baguio City	99,568	5,554	5.73	8,034	8.29	83,273	85.97	96,861	97.28	91,070	91.47
**Region 1**	**1,229,862**	**619,617**	**50.77**	**60,420**	**4.95**	**540,318**	**44.28**	**1,220,355**	**99.23**	**727,041**	**59.12**
Ilocos Norte	119,513	49,873	43.12	12,283	10.62	53,504	46.26	115,660	96.78	76,800	64.26
Ilocos Sur	138,487	59,109	41.92	10,434	7.40	71,458	50.68	141,001	101.82	60,540	43.72
La Union	163,492	110,897	65.54	18,152	10.73	40,166	23.74	169,215	103.50	57,626	35.25
Pangasinan	581,803	315,315	55.11	19,339	3.38	237,480	41.51	572,134	98.34	358,985	61.70
Alaminos City	23,157	0	0.00	0	0.00	23,138	100.00	23,138	99.92	23,138	99.92
Candon City	14,562	9,214	62.80	0	0.00	5,457	37.20	14,671	100.75	9,271	63.67
Dagupan City	41,145	1,489	3.62	0	0.00	39,656	96.38	41,145	100.00	39,656	96.38
Laoag City	26,612	3,206	13.78	0	0.00	20,066	86.22	23,272	87.45	22,006	82.69
San Carlos City	45,106	33,710	74.74	93	0.21	11,303	25.06	45,106	100.00	37,412	82.94
San Fernando City	30,657	18,888	62.54	119	0.39	11,193	37.06	30,200	98.51	7,377	24.06
Urdaneta City	32,668	17,621	54.99	0	0.00	14,425	45.01	32,046	98.10	21,758	66.60
Vigan City	12,660	295	2.31	0	0.00	12,472	97.69	12,767	100.85	12,472	98.52
**Region 2**	**870,916**	**483,846**	**58.34**	**68,123**	**8.21**	**277,394**	**33.45**	**829,363**	**95.23**	**326,401**	**37.48**
Batanes	5,049	0	0.00	0	0.00	5,049	100.00	5,049	100.00	0	0.00
Cagayan	258,783	140,732	58.66	30,012	12.51	69,171	28.83	239,915	92.71	75,355	29.12
Isabela	298,933	198,976	69.26	5,245	1.83	83,058	28.91	287,279	96.10	100,928	33.76
Nueva Vizcaya	121,760	66,988	56.51	20,054	16.92	31,500	26.57	118,542	97.36	35,332	29.02
Quirino	46,324	27,378	59.10	11,397	24.60	7,549	16.30	46,324	100.00	10,231	22.09
Cauayan City	33,115	12,117	39.58	504	1.65	17,993	58.77	30,614	92.45	30,614	92.45
Ilagan City	37,439	23,665	63.21	911	2.43	12,863	34.36	37,439	100.00	23,587	63.00
Santiago City	32,953	11,458	34.77	0	0.00	21,495	65.23	32,953	100.00	21,638	65.66
Tuguegarao City	36,560	2,532	8.10	0	0.00	28,716	91.90	31,248	85.47	28,716	78.54
**Region 3**	**2,913,980**	**595,037**	**21.86**	**64,914**	**2.38**	**2,062,226**	**75.76**	**2,722,177**	**93.42**	**2,557,165**	**87.76**
Aurora	53,645	27,574	47.19	9,661	16.53	21,197	36.28	58,432	108.92	56,008	104.40
Bataan	173,197	29,216	17.65	7,658	4.63	128,681	77.73	165,555	95.59	149,176	86.13
Bulacan	600,553	54,430	9.30	959	0.16	529,830	90.54	585,219	97.45	526,456	87.66
Nueva Ecija	389,283	141,426	41.46	16,495	4.84	183,173	53.70	341,094	87.62	288,968	74.23
Pampanga	406,365	77,346	20.26	9,847	2.58	294,507	77.16	381,700	93.93	349,129	85.92

Table 2.G.1. Environmental Health and Sanitation Services

Proportion and Total No. of Households (HHs) with access to basic safe water supply and manage drinking water
Philippines, 2022

Area	Projected No. of HHs	HHs with Access to Basic Safe Water Supply								HHs using Safely Managed Drinking Water Services	
		Level 1		Level 2		Level 3		Total	%		
		No.	%	No.	%	No.	%			No.	%
Tarlac	258,975	128,767	50.08	638	0.25	127,717	49.67	257,122	99.28	247,739	95.66
Zambales	158,172	54,773	33.89	18,436	11.41	88,389	54.70	161,598	102.17	152,251	96.26
Angeles City	110,996	0	0.00	0	0.00	110,390	100.00	110,390	99.45	111,164	100.15
Balanga City	24,579	1,130	4.92	312	1.36	21,545	93.73	22,987	93.52	22,401	91.14
Cabanatuan City	77,925	23,011	32.02	0	0.00	48,848	67.98	71,859	92.22	52,181	66.96
City of San Fernando	78,861	44	1.24	52	1.47	3,444	97.29	3,540	4.49	76,783	97.36
Gapan City	28,302	6,207	22.90	0	0.00	20,895	77.10	27,102	95.76	27,102	95.76
Mabalacat City	64,948	1,721	2.72	0	0.00	61,512	97.28	63,233	97.36	60,493	93.14
Malolos City	66,845	1,846	2.90	0	0.00	61,842	97.10	63,688	95.28	63,688	95.28
Meycauayan City	60,173	4,073	6.95	0	0.00	54,547	93.05	58,620	97.42	54,547	90.65
Olongapo City	63,291	0	0.00	0	0.00	61,549	100.00	61,549	97.25	61,549	97.25
Palayan City	10,751	1,443	13.32	0	0.00	9,388	86.68	10,831	100.74	9,388	87.32
San Jose City	37,384	10,897	33.23	0	0.00	21,900	66.77	32,797	87.73	32,797	87.73
San Jose del Monte City	144,995	1,276	0.89	0	0.00	142,267	99.11	143,543	99.00	142,734	98.44
Science City of Munoz	21,863	1,216	5.59	856	3.93	19,686	90.48	21,758	99.52	17,913	81.93
Tarlac City	82,877	28,641	36.00	0	0.00	50,919	64.00	79,560	96.00	54,698	66.00
**Region 4A**	**3,937,696**	**395,320**	**10.62**	**216,694**	**5.82**	**3,109,694**	**83.56**	**3,721,708**	**94.51**	**3,246,157**	**82.44**
Batangas	502,937	75,085	15.93	24,395	5.17	372,010	78.90	471,490	93.75	446,715	88.82
Cavite	391,310	23,929	6.18	11,569	2.99	351,924	90.84	387,422	99.01	349,060	89.20
Laguna	271,300	47,104	18.65	19,839	7.86	185,600	73.49	252,543	93.09	156,113	57.54
Quezon	438,052	154,750	40.42	81,665	21.33	146,482	38.26	382,897	87.41	129,089	29.47
Rizal	556,257	22,309	4.11	34,477	6.36	485,542	89.53	542,328	97.50	528,368	94.99
Antipolo City	196,712	10,637	5.71	28,961	15.54	146,717	78.75	186,315	94.71	178,875	90.93
Bacoor City	178,634	1,608	1.29	247	0.20	123,235	98.52	125,090	70.03	146,245	81.87
Batangas City	92,793	727	0.86	272	0.32	83,746	98.82	84,745	91.33	86,893	93.64
Biñan City	98,821	2,274	2.35	14	0.01	94,446	97.63	96,734	97.89	92,822	93.93
Cabuyao City	92,909	20,747	24.81	0	0.00	62,884	75.19	83,631	90.01	82,986	89.32
Calamba City	141,258	18,270	13.22	5,980	4.33	113,969	82.46	138,219	97.85	138,648	98.15
Cavite City	31,155	0	0.00	0	0.00	31,148	100.00	31,148	99.98	31,053	99.67
Dasmariñas City	177,028	298	0.17	0	0.00	175,570	99.83	175,868	99.34	162,497	91.79
General Trias City	96,312	393	0.42	0	0.00	93,943	99.58	94,336	97.95	94,336	97.95
Imus City	116,694	0	0.00	0	0.00	138,248	100.00	138,248	118.47	135,004	115.69
Lipa City	87,524	0	0.00	0	0.00	86,534	100.00	86,534	98.87	86,347	98.66
Lucena City	65,323	78	0.12	4,668	7.15	60,573	92.73	65,319	99.99	60,873	93.19
San Pablo City	71,096	1,963	3.06	0	0.00	62,250	96.94	64,213	90.32	43,518	61.21
San Pedro City	83,120	3,053	4.06	0	0.00	72,120	95.94	75,173	90.44	72,120	86.77
Santa Rosa City	115,252	9,813	8.91	0	0.00	100,285	91.09	110,098	95.53	100,285	87.01
Tagaytay City	19,670	122	0.62	0	0.00	19,548	99.38	19,670	100.00	19,670	100.00
Tanauan City	45,285	397	0.96	0	0.00	41,036	99.04	41,433	91.49	42,756	94.42
Tayabas City	25,927	1,763	6.80	4,607	17.77	19,557	75.43	25,927	100.00	19,557	75.43
Trece Martires City	42,327	0	0.00	0	0.00	42,327	100.00	42,327	100.00	42,327	100.00
**Region 4B**	**752,631**	**291,145**	**41.54**	**125,111**	**17.85**	**284,673**	**40.61**	**700,929**	**93.13**	**485,017**	**64.44**
Marinduque	56,786	13,976	25.22	12,774	23.05	28,673	51.73	55,423	97.60	38,337	67.51
Mindoro Occidental	121,218	60,902	57.83	13,369	12.70	31,034	29.47	105,305	86.87	82,078	67.71
Mindoro Oriental	208,718	110,411	54.97	36,549	18.20	53,907	26.84	200,867	96.24	107,450	51.48
Palawan	222,467	80,259	47.88	31,008	18.50	56,371	33.63	167,638	75.35	113,605	51.07
Romblon	71,371	21,758	29.84	16,458	22.57	34,706	47.59	72,922	102.17	60,436	84.68
Puerto Princesa City	72,071	3,839	3.89	14,953	15.14	79,982	80.97	98,774	137.05	83,111	115.32
**Region 5**	**1,315,025**	**318,563**	**29.02**	**316,679**	**28.85**	**462,553**	**42.13**	**1,097,795**	**83.48**	**694,133**	**52.78**
Albay	255,270	76,608	33.52	65,449	28.64	86,473	37.84	228,530	89.52	219,355	85.93
Camarines Norte	142,092	26,838	19.89	48,432	35.90	59,653	44.21	134,923	94.95	129,232	90.95
Camarines Sur	361,980	65,892	31.56	70,881	33.95	72,012	34.49	208,785	57.68	26,318	7.27
Catanduanes	57,589	11,849	21.25	11,780	21.13	32,122	57.62	55,751	96.81	26,745	46.44
Masbate	201,151	77,340	49.34	46,028	29.37	33,366	21.29	156,734	77.92	111,367	55.36
Sorsogon	182,373	42,074	21.87	59,249	30.80	91,016	47.32	192,339	105.46	99,523	54.57
Iriga City	23,556	2,568	10.93	5,167	21.99	15,764	67.08	23,499	99.76	13,139	55.78
Legaspi City	45,378	15,196	29.45	8,566	16.60	27,836	53.95	51,598	113.71	23,016	50.72
Naga City	45,636	198	0.43	1,127	2.47	44,311	97.10	45,636	100.00	45,438	99.57
**Region 6**	**1,825,050**	**771,786**	**43.48**	**270,923**	**15.26**	**732,141**	**41.25**	**1,774,850**	**97.25**	**957,001**	**52.44**
Aklan	143,827	52,297	39.38	22,954	17.28	57,548	43.33	132,799	92.33	90,904	63.20
Antique	139,142	42,379	28.03	56,385	37.30	52,422	34.67	151,186	108.66	87,473	62.87

Table 2.G.1. Environmental Health and Sanitation Services

Proportion and Total No. of Households (HHs) with access to basic safe water supply and manage drinking water
Philippines, 2022

Area	Projected No. of HHs	HHs with Access to Basic Safe Water Supply								HHs using Safely Managed Drinking Water Services	
		Level 1		Level 2		Level 3		Total	%		
		No.	%	No.	%	No.	%			No.	%
Capiz	194,450	107,214	55.59	17,248	8.94	68,420	35.47	192,882	99.19	97,659	50.22
Guimaras	43,939	22,834	46.87	3,772	7.74	22,113	45.39	48,719	110.88	27,940	63.59
Iloilo	471,075	281,681	60.37	60,801	13.03	124,137	26.60	466,619	99.05	149,733	31.79
Negros Occidental	592,946	224,780	40.32	105,885	18.99	226,802	40.68	557,467	94.02	336,088	56.68
Bacolod City	136,312	10,905	8.75	3,878	3.11	109,798	88.13	124,581	91.39	96,277	70.63
Iloilo City	103,359	29,696	29.52	0	0.00	70,901	70.48	100,597	97.33	70,927	68.62
**Region 7**	**1,882,116**	**181,464**	**20.55**	**161,525**	**18.29**	**540,239**	**61.17**	**883,228**	**46.93**	**589,202**	**31.31**
Bohol	304,765	57,108	20.51	26,662	9.58	194,656	69.91	278,426	91.36	118,401	38.85
Cebu	759,825	31,944	21.15	44,091	29.19	74,992	49.65	151,027	19.88	146,706	19.31
Negros Oriental	327,390	42,323	13.90	77,321	25.39	184,899	60.71	304,543	93.02	183,483	56.04
Siquijor	24,312	1,747	60.85	62	2.16	1,062	36.99	2,871	11.81	5,324	21.90
Cebu City	244,943	468	3.91	1,986	16.58	9,526	79.52	11,980	4.89	928	0.38
Lapu-Lapu City	113,099	46,275	41.09	10,513	9.33	55,832	49.58	112,620	99.58	112,594	99.55
Mandaue City	107,782	1,599	7.35	890	4.09	19,272	88.56	21,761	20.19	21,766	20.19
**Region 8**	**1,085,539**	**262,701**	**25.94**	**355,696**	**35.12**	**394,298**	**38.94**	**1,012,695**	**93.29**	**564,164**	**51.97**
Biliran	41,059	3,085	6.98	11,164	25.26	29,943	67.76	44,192	107.63	31,547	76.83
Eastern Samar	118,610	31,813	29.64	57,067	53.18	18,439	17.18	107,319	90.48	4	0.00
Northern Leyte	398,319	91,211	24.83	119,331	32.48	156,832	42.69	367,374	92.23	177,377	44.53
Northern Samar	136,774	64,730	56.92	40,843	35.91	8,154	7.17	113,727	83.15	49,335	36.07
Southern Leyte	80,084	5,973	8.07	11,625	15.71	56,383	76.21	73,981	92.38	61,879	77.27
Western Samar	136,204	43,212	32.79	54,956	41.71	33,598	25.50	131,766	96.74	92,473	67.89
Calbayog City	41,325	9,527	23.03	14,785	35.74	17,055	41.23	41,367	100.10	26,532	64.20
Maasin City	20,197	2,953	13.63	4,157	19.19	14,551	67.18	21,661	107.25	17,652	87.40
Ormoc City	56,258	4,140	7.36	17,893	31.81	34,212	60.83	56,245	99.98	53,777	95.59
Tacloban City	56,709	6,057	11.00	23,875	43.36	25,131	45.64	55,063	97.10	53,588	94.50
**Region 9**	**847,632**	**146,632**	**20.38**	**220,444**	**30.64**	**352,448**	**48.98**	**719,524**	**84.89**	**459,097**	**54.16**
		146,632	20.38	220,444	30.64	352,448	48.98				
Zamboanga del Norte	185,543	57,540	34.32	58,604	34.95	51,533	30.73	167,677	90.37	114,280	61.59
Zamboanga del Sur	182,760	25,207	17.63	57,433	40.16	60,364	42.21	143,004	78.25	83,039	45.44
Zamboanga Sibugay	154,850	31,606	26.77	44,325	37.55	42,125	35.68	118,056	76.24	54,319	35.08
Dapitan City	19,068	1,290	7.09	4,656	25.60	12,241	67.31	18,187	95.38	16,674	87.44
Dipolog City	31,700	8,150	25.78	1,235	3.91	22,232	70.32	31,617	99.74	31,617	99.74
Isabela City	27,165	3,407	13.00	12,710	48.48	10,098	38.52	26,215	96.50	24,508	90.22
Pagadian City	43,329	896	3.62	12,710	51.40	11,122	44.98	24,728	57.07	11,298	26.07
Zamboanga City	203,217	18,536	9.75	28,771	15.14	142,733	75.11	190,040	93.52	123,362	60.70
**Region 10**	**1,147,397**	**162,215**	**15.37**	**225,465**	**21.37**	**667,417**	**63.26**	**1,055,097**	**91.96**	**590,634**	**51.48**
Bukidnon	246,975	23,299	12.89	49,477	27.36	108,028	59.75	180,804	73.21	99,602	40.33
Camiguin	21,326	88	0.50	1,104	6.31	16,297	93.18	17,489	82.01	12,103	56.75
Lanao del Norte	154,854	45,550	40.09	28,272	24.88	39,809	35.03	113,631	73.38	35,890	23.18
Misamis Occidental	79349	16,561	19.16	22,761	26.33	47,109	54.50	86,431	108.93	59,335	74.78
Misamis Oriental	179,047	31,125	17.10	47,756	26.23	103,179	56.67	182,060	101.68	68,351	38.17
Cagayan de Oro City	182,549	2,118	1.16	16,099	8.84	163,800	89.99	182,017	99.71	140,400	76.91
El Salvador City	13,274	14	0.11	935	7.04	12,325	92.85	13,274	100.00	10,213	76.94
Gingoog City	29,517	2,868	9.49	11,358	37.58	16,000	52.93	30,226	102.40	16,000	54.21
Iligan City	85,177	9,470	11.25	11,947	14.19	62,776	74.56	84,193	98.84	52,580	61.73
Malaybalay City	43,125	5,219	11.55	13,921	30.80	26,061	57.66	45,201	104.81	25,998	60.29
Oroquieta City	17,634	1,027	5.47	3,168	16.87	14,579	77.66	18,774	106.46	10,514	59.62
Ozamis City	32,257	12,608	37.99	3,097	9.33	17,484	52.68	33,189	102.89	19,837	61.50
Tangub City	13,812	2,983	18.22	6,794	41.49	6,599	40.30	16,376	118.56	9,156	66.29
Valencia City	48,501	9,285	18.05	8,776	17.06	33,371	64.88	51,432	106.04	30,655	63.20
**Region 11**	**1,321,012**	**240,118**	**20.00**	**239,965**	**19.98**	**720,691**	**60.02**	**1,200,774**	**90.90**	**892,507**	**67.56**
Davao de Oro	181,621	70,051	38.73	56,111	31.02	54,706	30.25	180,868	99.59	145,855	80.31
Davao del Norte	247,830	53,479	28.39	23,925	12.70	110,988	58.91	188,392	76.02	181,104	73.08
Davao Oriental	141,913	48,795	33.43	40,419	27.69	56,749	38.88	145,963	102.85	57,009	40.17
Davao del Sur	185,113	42,433	26.71	44,871	28.24	71,570	45.05	158,874	85.83	54,293	29.33
Davao Occidental	84,993	22,787	30.79	43,060	58.19	8,158	11.02	74,005	87.07	35,726	42.03
Davao City	479,542	2,573	0.57	31,579	6.98	418,520	92.46	452,672	94.40	418,520	87.27

Table 2.G.1. Environmental Health and Sanitation Services
Proportion and Total No. of Households (HHs) with access to basic safe water supply and manage drinking water
Philippines, 2022

Area	Projected No. of HHs	HHs with Access to Basic Safe Water Supply								HHs using Safely Managed Drinking Water Services	
		Level 1		Level 2		Level 3		Total	%		
		No.	%	No.	%	No.	%			No.	%
**Region 12**	**1,109,718**	**351,373**	**35.16**	**185,693**	**18.58**	**462,325**	**46.26**	**999,391**	**90.06**	**552,987**	**49.83**
North Cotabato	368,078	108,966	33.34	68,974	21.11	148,866	45.55	326,806	88.79	174,963	47.53
Sarangani	136,651	53,508	45.85	38,939	33.37	24,252	20.78	116,699	85.40	88,972	65.11
South Cotabato	244,678	52,151	24.18	34,590	16.04	128,928	59.78	215,669	88.14	196,901	80.47
Sultan Kudarat	198,263	115,246	63.16	35,347	19.37	31,864	17.46	182,457	92.03	22,966	11.58
Gen. Santos City	162,048	21,502	13.63	7,843	4.97	128,415	81.40	157,760	97.35	69,185	42.69
**BARMM**	**778,286**	**216,294**	**49.16**	**131,552**	**29.90**	**92,127**	**20.94**	**439,973**	**56.53**	**141,656**	**18.20**
Basilan	52,314	14,688	47.32	14,017	45.16	2,335	7.52	31,040	59.33	**0**	0.00
Lanao del Sur	148,786	4,656	44.27	5,645	53.68	216	2.05	10,517	7.07	2,323	1.56
Maguindanao	229,173	100,502	53.90	60,361	32.37	25,593	13.73	186,456	81.36	16,938	7.39
Sulu	160,918	59,927	60.29	25,724	25.88	13,741	13.83	99,392	61.77	41,375	25.71
Tawi-Tawi	73,049	18,503	81.59	3,648	16.09	526	2.32	22,677	31.04	8,647	11.84
Cotabato City	61,767	3,948	9.43	6,944	16.59	30,956	73.97	41,848	67.75	34,904	56.51
Lamitan City	17,602	5,605	32.92	4,959	29.13	6,460	37.95	17,024	96.72	9,264	52.63
Marawi City	34,677	8,465	27.29	10,254	33.06	12,300	39.65	31,019	89.45	28,205	81.34
**Caraga**	**623,824**	**115,083**	**21.38**	**149,699**	**27.81**	**273,533**	**50.81**	**538,315**	**86.29**	**302,209**	**48.44**
Agusan del Norte	84,957	21,325	25.50	20,917	25.01	41,389	49.49	83,631	98.44	63,008	74.16
Agusan del Sur	164,223	57,848	38.37	43,339	28.75	49,572	32.88	150,759	91.80	50,110	30.51
Surigao del Norte	82,635	7,975	10.83	22,622	30.72	43,048	58.45	73,645	89.12	51,282	62.06
Surigao del Sur	115,311	19,448	16.79	34,130	29.46	62,284	53.76	115,862	100.48	70,490	61.13
Province of Dinagat	29,664	3,733	12.70	11,150	37.93	14,511	49.37	29,394	99.09	777	2.62
Bislig City	22,813	878	4.38	3,813	19.03	15,343	76.58	20,034	87.82	19,156	83.97
Butuan City	83,613	2,205	9.48	1,066	4.58	19,994	85.94	23,265	27.82	19,994	23.91
Surigao City	40,608	1,671	4.00	12,662	30.35	27,392	65.65	41,725	102.75	27,392	67.45

Legend:
0 = No Case
** = No Report*

Table 2.G.2. Environmental Health and Sanitation Services Proportion and Total No. of Households (HHs) with access to basic sanitation facility
Philippines, 2022

Area	Projected No. of HHs	HH with Basic Sanitation Facility								HHs using Safely Managed Sanitation Service	
		Total	%	Pour/Flush Toilet							
				Septic Tank		Community sewer/sewerage system		Ventillated improved Pit Latrine (VIP)			
				No.	%	No.	%	No.	%	No.	%
**PHILIPPINES**	**25,481,993**	**21,312,775**	**83.64**	**19,496,666**	**91.48**	**620,468**	**2.91**	**1,195,641**	**5.61**	**16,842,599**	**66.10**
**NCR**	**3,416,247**	**3,199,166**	**93.65**	**2,737,295**	**85.56**	**459,062**	**14.35**	**2,809**	**0.09**	**3,158,351**	**92.45**
Malabon City	94,825	94,826	100.00	87,293	92.06	4,724	4.98	2,809	2.96	94,825	100.00
Navotas City	66,853	61,438	91.90	61,438	100.00	0	0.00	0	0.00	61,438	91.90
Valenzuela City	168,093	166,131	98.83	166,131	100.00	0	0.00	0	0.00	166,218	98.88
Caloocan City	404,100	350,782	86.81	350,782	100.00	0	0.00	0	0.00	350,802	86.81
Marikina City	108,152	108,152	100.00	108,152	100.00	0	0.00	0	0.00	108,152	100.00
Pasig City	198,622	180,103	90.68	130,519	72.47	49,584	27.53	0	0.00	179,355	90.30
Pateros	15,604	15,580	99.85	15,580	100.00	0	0.00	0	0.00	14,559	93.30
Taguig City	220,634	184,570	83.65	184,570	100.00	0	0.00	0	0.00	184,570	83.65
Quezon City	748,646	697,863	93.22	697,863	100.00	0	0.00	0	0.00	700,378	93.55
Makati City	169,874	167,233	98.45	153,368	91.71	13,865	8.29	0	0.00	137,678	81.05
Mandaluyong City	112,485	112,485	100.00	112,485	100.00	0	0.00	0	0.00	112,485	100.00
San Juan City	31,765	31,765	100.00	31,417	98.90	348	1.10	0	0.00	31,765	100.00
Manila City	481,750	438,398	91.00	76,896	17.54	361,502	82.46	0	0.00	438,398	91.00
Las Piñas City	155,928	155,928	100.00	155,507	99.73	421	0.27	0	0.00	155,507	99.73
Muntinlupa City	140,406	139,423	99.30	126,337	90.61	13,086	9.39	0	0.00	139,423	99.30
Parañaque City	179,359	179,359	100.00	163,827	91.34	15,532	8.66	0	0.00	167,668	93.48
Pasay City	119,151	115,130	96.63	115,130	100.00	0	0.00	0	0.00	115,130	96.63
**CAR**	**425,062**	**400,604**	**94.25**	**356,447**	**88.98**	**11,677**	**2.91**	**32,480**	**8.11**	**389,999**	**91.75**
Abra	55,106	54,516	98.93	50,293	92.25	0	0.00	4,223	7.75	49,623	90.05
Apayao	27,036	25,704	95.07	18,909	73.56	0	0.00	6,795	26.44	25,704	95.07
Benguet	117,566	102,877	87.51	98,315	95.57	0	0.00	4,562	4.43	100,553	85.53
Ifugao	45,597	42,855	93.99	35,462	82.75	0	0.00	7,393	17.25	42,792	93.85
Kalinga	44,270	42,232	95.40	34,675	82.11	0	0.00	7,557	17.89	38,954	87.99
Mt. Province	35,919	35,613	99.15	33,698	94.62	0	0.00	1,915	5.38	35,613	99.15
Baguio City	99,568	96,807	97.23	85,095	87.90	11,677	12.06	35	0.04	96,760	97.18
**Region 1**	**1,229,862**	**1,193,688**	**97.06**	**1,169,509**	**97.97**	**11,106**	**0.93**	**13,073**	**1.10**	**418,720**	**34.05**
Ilocos Norte	119,513	114,739	96.01	114,632	99.91	0	0.00	107	0.09	62,233	52.07
Ilocos Sur	138,487	134,340	97.01	134,340	100.00	0	0.00	0	0.00	27,977	20.20
La Union	163,492	165,809	101.42	153,943	92.84	1,675	1.01	10,191	6.15	11,866	7.26
Pangasinan	581,803	558,934	96.07	556,561	99.58	202	0.04	2,171	0.39	209,226	35.96
						0		0			
Alaminos City	23,157	21,556	93.09	21,556	100.00	0	0.00	0	0.00	10,137	43.78
Candon City	14,562	14,353	98.56	14,353	100.00	0	0.00	0	0.00	7,769	53.35
Dagupan C	41,145	38,588	93.79	38,588	100.00	0	0.00	0	0.00	6,303	15.32
Laoag City	26,612	23,272	87.45	23,272	100.00	0	0.00	0	0.00	23,272	87.45
San Carlos City	45,106	41,658	92.36	41,658	100.00	0	0.00	0	0.00	31,108	68.97
San Fernando City	30,657	35,860	116.97	26,027	72.58	9,229	25.74	604	1.68	9,833	32.07
Urdaneta City	32,668	32,009	97.98	32,009	100.00	0	0.00	0	0.00	13,288	40.68
Vigan City	12,660	12,570	99.29	12,570	100.00	0	0.00	0	0.00	5,708	45.09
**Region 2**	**870,916**	**783,128**	**89.92**	**738,028**	**94.24**	**0**	**0.00**	**45,100**	**5.76**	**734,234**	**84.31**
Batanes	5,049	5,049	100.00	5,049	100.00	0	0.00	0	0.00	5,049	100.00
Cagayan	258,783	224,556	86.77	188,941	84.14	0	0.00	35,615	15.86	203,524	78.65
Isabela	298,933	270,217	90.39	266,083	98.47	0	0.00	4,134	1.53	248,940	83.28
Nueva Vizcaya	121,760	107,233	88.07	104,718	97.65	0	0.00	2,515	2.35	106,750	87.67
Quirino	46,324	45,188	97.55	43,308	95.84	0	0.00	1,880	4.16	44,194	95.40
Cauayan City	33,115	31,035	93.72	31,035	100.00	0	0.00	0	0.00	30,614	92.45
Ilagan City	37,439	37,439	100.00	36,483	97.45	0	0.00	956	2.55	37,439	100.00
Santiago City	32,953	32,953	100.00	32,953	100.00	0	0.00	0	0.00	29,008	88.03
Tuguegarao City	36,560	29,458	80.57	29,458	100.00	0	0.00	0	0.00	28,716	78.54
**Region 3**	**2,913,980**	**2,685,518**	**92.16**	**2,600,212**	**96.82**	**46,283**	**1.72**	**39,023**	**1.45**	**2,582,833**	**88.64**
Aurora	53,645	53,835	100.35	51,863	96.34	49	0.09	1,923	3.57	51,481	95.97
Bataan	173,197	167,045	96.45	165,572	99.12	1,421	0.85	52	0.03	163,705	94.52
Bulacan	600,553	554,485	92.33	540,971	97.56	12,962	2.34	552	0.10	503,795	83.89

Table 2.G.2. Environmental Health and Sanitation Services Proportion and Total No. of Households (HHs) with access to basic sanitation facility
Philippines, 2022

Area	Projected No. of HHs	HH with Basic Sanitation Facility								HHs using Safely Managed Sanitation Service	
		Total	%	Pour/Flush Toilet							
				Septic Tank		Community sewer/sewerage system		Ventillated improved Pit Latrine (VIP)			
				No.	%	No.	%	No.	%	No.	%
Nueva Ecija	389,283	331,104	85.05	326,224	98.53	0	0.00	4,880	1.47	312,132	80.18
Pampanga	406,365	351,489	86.50	348,093	99.03	77	0.02	3,319	0.94	349,276	85.95
Tarlac	258,975	248,477	95.95	224,288	90.27	23,766	9.56	423	0.17	246,481	95.18
Zambales	158,172	150,019	94.85	118,678	79.11	7,998	5.33	23,343	15.56	147,293	93.12
Angeles City	110,996	109,225	98.40	109,225	100.00	0	0.00	0	0.00	109,225	98.40
Balanga City	24,579	22,523	91.64	22,523	100.00	0	0.00	0	0.00	22,280	90.65
Cabanatuan City	77,925	57,586	73.90	56,323	97.81	0	0.00	1,263	2.19	56,323	72.28
City of San Fernando	78,861	78,861	100.00	76,732	97.30	0	0.00	2,129	2.70	76,732	97.30
Gapan City	28,302	26,379	93.21	26,379	100.00	0	0.00	0	0.00	27,102	95.76
Mabalacat City	64,948	62,818	96.72	61,727	98.26	10	0.02	1,081	1.72	59,795	92.07
Malolos City	66,845	63,307	94.71	63,307	100.00	0	0.00	0	0.00	48,998	73.30
Meycauayan City	60,173	58,217	96.75	58,210	99.99	0	0.00	7	0.01	58,217	96.75
Olongapo City	63,291	62,796	99.22	62,796	100.00	0	0.00	0	0.00	62,796	99.22
Palayan City	10,751	10,079	93.75	10,079	100.00	0	0.00	0	0.00	10,079	93.75
San Jose City	37,384	32,352	86.54	32,352	100.00	0	0.00	0	0.00	32,253	86.27
San Jose del Monte City	144,995	143,543	99.00	143,543	100.00	0	0.00	0	0.00	143,543	99.00
Science City of Munoz	21,863	20,987	95.99	20,936	99.76	0	0.00	51	0.24	20,936	95.76
Tarlac City	82,877	80,391	97.00	80,391	100.00	0	0.00	0	0.00	80,391	97.00
**Region 4A**	**3,937,696**	**3,606,031**	**91.58**	**3,418,484**	**94.80**	**18,532**	**0.51**	**169,015**	**4.69**	**3,240,746**	**82.30**
Batangas	502,937	445868	88.65	407,476	91.39	16,945	3.80	21,447	4.81	402,604	80.05
Cavite	391,310	365088	93.30	364,684	99.89	390	0.11	14	0.00	361,210	92.31
Laguna	271,300	245690	90.56	243,663	99.17	0	0.00	2,027	0.83	91,325	33.66
Quezon	438,052	352515	80.47	319,306	90.58	0	0.00	33,209	9.42	281,576	64.28
Rizal	556,257	523599	94.13	508,472	97.11	601	0.11	14,526	2.77	496,294	89.22
Antipolo City	196,712	183310	93.19	183,310	100.00	0	0.00	0	0.00	171,315	87.09
Bacoor City	178,634	124676	69.79	124,676	100.00	0	0.00	0	0.00	124,675	69.79
Batangas City	92,793	86877	93.62	86,405	99.46	0	0.00	472	0.54	86,811	93.55
Biñan City	98,821	96699	97.85	96,699	100.00	0	0.00	0	0.00	90,315	91.39
Cabuyao City	92,909	84919	91.40	84,919	100.00	0	0.00	0	0.00	81,695	87.93
Calamba City	141,258	139433	98.71	139,433	100.00	0	0.00	0	0.00	132,115	93.53
Cavite City	31,155	26668	85.60	26,668	100.00	0	0.00	0	0.00	26,668	85.60
Dasmariñas City	177,028	174449	98.54	174,449	100.00	0	0.00	0	0.00	162,431	91.75
General Trias City	96,312	94067	97.67	94,067	100.00	0	0.00	0	0.00	94,067	97.67
Imus City	116,694	137669	117.97	137,669	100.00	0	0.00	0	0.00	134,451	115.22
Lipa City	87,524	86535	98.87	0	0.00	0	0.00	86,535	100.00	86,534	98.87
Lucena City	65,323	62355	95.46	55,060	88.30	0	0.00	7,295	11.70	54,589	83.57
San Pablo City	71,096	63243	88.95	63,243	100.00	0	0.00	0	0.00	62,192	87.48
San Pedro City	83,120	72071	86.71	72,071	100.00	0	0.00	0	0.00	72,071	86.71
Santa Rosa City	115,252	111566	96.80	111,566	100.00	0	0.00	0	0.00	102,581	89.01
Tagaytay City	19,670	19670	100.00	19,074	96.97	596	3.03	0	0.00	19,670	100.00
Tanauan City	45,285	41654	91.98	41,654	100.00	0	0.00	0	0.00	41,653	91.98
Tayabas City	25,927	25083	96.74	21,593	86.09	0	0.00	3,490	13.91	21,577	83.22
Trece Martires City	42,327	42327	100.00	42,327	100.00	0	0.00	0	0.00	42,327	100.00
**Region 4B**	**752,631**	**619,029**	**82.25**	**595,021**	**96.12**	**723**	**0.12**	**23,285**	**3.76**	**469,340**	**62.36**
Marinduque	56,786	53060	93.44	53,060	100.00	0	0.00	0	0.00	53,060	93.44
Mindoro Occidental	121,218	92540	76.34	91,037	98.38	0	0.00	1,503	1.62	60,994	50.32
Mindoro Oriental	208,718	189973	91.02	187,500	98.70	114	0.06	2,359	1.24	152,011	72.83
Palawan	222,467	157021	70.58	139,964	89.14	609	0.39	16,448	10.48	90,399	40.63
Romblon	71,371	68941	96.60	68,077	98.75	0	0.00	864	1.25	54,998	77.06
Puerto Princesa City	72,071	57494	79.77	55,383	96.33	0	0.00	2,111	3.67	57,878	80.31
**Region 5**	**1,315,025**	**983,585**	**74.80**	**918,350**	**93.37**	**23,039**	**2.34**	**42,196**	**4.29**	**679,933**	**51.70**
Albay	255,270	214,979	84.22	191,713	89.18	333	0.15	22,933	10.67	146,659	57.45
Camarines Norte	142,092	139,851	98.42	126,437	90.41	11,576	8.28	1,838	1.31	130,049	91.52
Camarines Sur	361,980	167,016	46.14	158,565	94.94	4,433	2.65	4,018	2.41	1	0.00
[truncated: 2,113,497 more chars]
