# Supplementary material for: Neglected Tropical Diseases Elimination in the Philippines: Challenges and Gaps
Source: Trop Med Infect Dis. 2026 Apr 17;11(4):106. doi: 10.3390/tropicalmed11040106 (PMC13120366; doi:10.3390/tropicalmed11040106)
Supplement: Supplementary file 1 [file tropicalmed-11-00106-s001.zip › Supplementary File S5. FHSIS 2023.pdf]

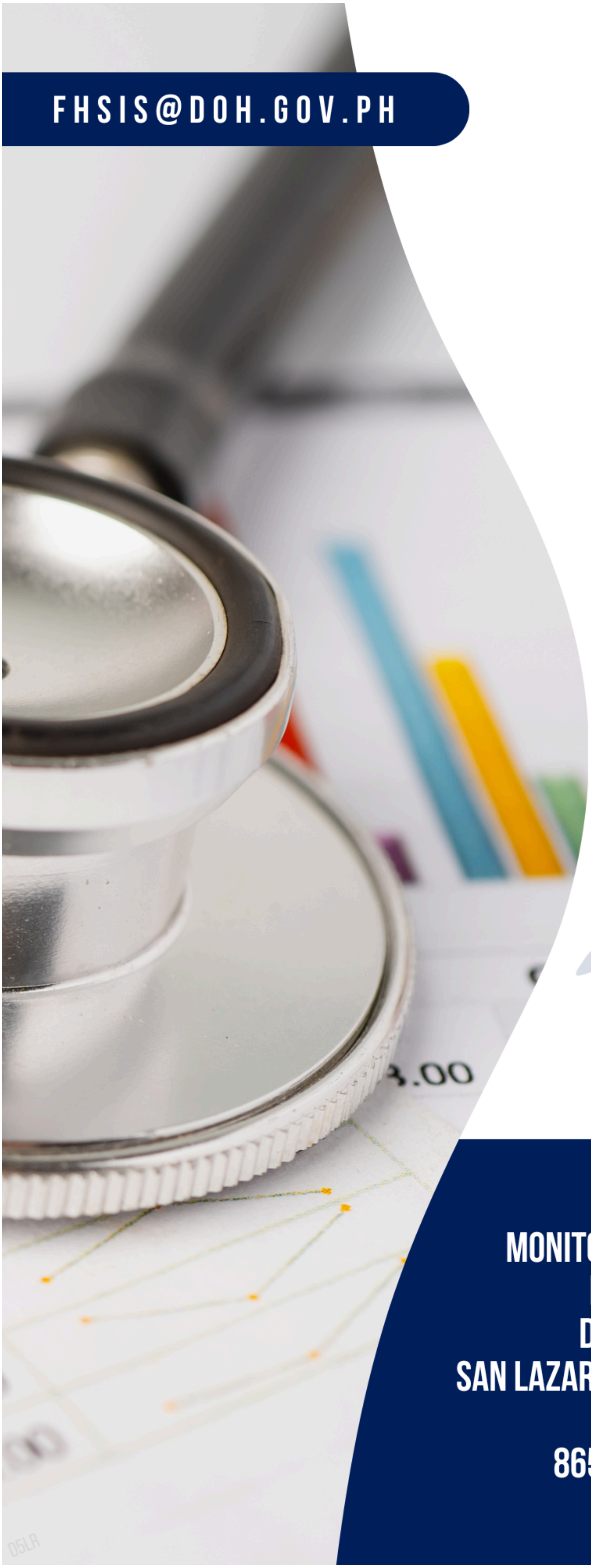

FHSIS@DOH.GOV.PH

2023 ANNUAL REPORT

FIELD HEALTH SERVICES INFORMATION SYSTEM

MONITORING & EVALUATION DIVISION
EPIDEMIOLOGY BUREAU
DEPARTMENT OF HEALTH
SAN LAZARO COMPOUND, STA. CRUZ, MANILA

8651-7800 LOC. 2928/2953

Republic of the Philippines
Department of Health
OFFICE OF THE SECRETARY

MESSAGE

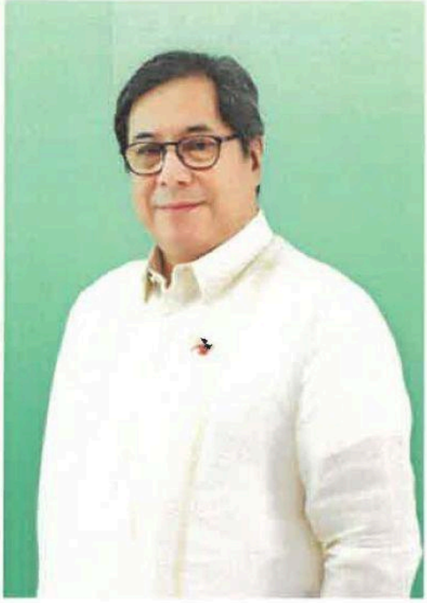

As one Department of Health (DOH), it is with great pride that we present the **CY 2023 Field Health Services Information System (FHSIS) Annual Report**. Mandated by Executive Order No. 352 s. 1996, the FHSIS stands as our cornerstone statistical system, furnishing crucial data on public health programs, morbidity, and demographics for a more evidence-informed decision-making and action in alignment with the **DOH's 8-Point Action Agenda** under the banner of ***"Sa Bagong Pilipinas, Bawat Buhay Mahalaga"***, and the Universal Health Care Act.

Throughout the years, FHSIS reports have not only been invaluable to the Department but also to a diverse array of stakeholders including the Local Government Units (LGUs), academic institutions, non-government organizations, and legislative bodies. For the health workers and program managers at all levels of our healthcare system, these reports are instrumental in crafting pertinent health policies and guidelines, allocating resources, and monitoring and evaluation of healthcare services and interventions. For the LGUs, it provides an opportunity to strategize and recalibrate public health programs, projects, and activities at the grassroots. For policy- and decision-makers, it advances the crafting of responsive and evidence-informed legislation and plans. And for the academicians, researchers, and media practitioners, it serves as an avenue to further the studies on and awareness of health-related problems and as such, prompts quality and timely response.

We extend our heartfelt appreciation to the diligent health workers and data managers at every level of the health system - barangay, municipal, city, provincial, regional, and national, and to our partners from the LGUs, Centers for Health Development, Ministry of Health - Bangsamoro Autonomous Region in Muslim Mindanao, Disease Prevention and Control Bureau, and Health Promotion Bureau for their tireless efforts in producing and disseminating this report. Special recognition goes to the Epidemiology Bureau for spearheading the validation process, ensuring the integrity and quality of the data. We urge all stakeholders to utilize and champion this report as we center our healthcare reforms around every Filipino, fostering community engagement and recognizing the indispensable role of healthcare workers.

Together, let us continue our collaborative efforts to enhance health outcomes, fortify health systems, and ensure equitable access to healthcare for all Filipinos, *dahil sa Bagong Pilipinas, bawat buhay mahalaga!*

TEODORO J. HERBOSA, MD
Secretary of Health

Republic of the Philippines
DEPARTMENT OF HEALTH

GLENN MATHEW G. BAGGAO, MD, MHA, MSN, FPSMS, FPCHA
Undersecretary of Health, Public Health Services Cluster

ALETHEA R. DE GUZMAN, MD, MCHM, PHSAE
Director IV, Epidemiology Bureau

ALFONSO MIGUEL R. REGALA, MD, MBA
Medical Officer V, Monitoring and Evaluation Division

HEALTH STATISTICS SECTION

MARIEL A. DEJESA, MD, MPM
Medical Officer IV

JOSE M. HERNAEZ
Information Systems Analyst III

ROMCY A. SAGUCIO
Senior Health Program Officer

DEXTER JAY B. FLORES
Health Program Officer II

JOEL V. CANTERO
Computer Programmer II

HERNAN T. TAYER
Computer Programmer II

JULIUS T. CASTRO
Computer Programmer II

THERESA D. TIMBANG
Supervising Health Program Officer

RHENCY M. LEGASPI
Supervising Health Program Officer

FRANCES MAE L. RIVERA
Supervising Health Program Officer

KARLA MAE C. RAMIREZ
Senior Health Program Officer

ALLAN P. IGNACIO
Statistician II

TABLE OF CONTENTS

FOREWORD .... **i**
AUTHORS AND PUBLISHER .... **ii**
TABLE OF CONTENTS .... **iii**

CHAPTER I **1-67**
- **1.A. FAMILY PLANNING SERVICES** **2-9**
- **1.B. MATERNAL CARE AND SERVICES** **10-18**
  - 1.B.1. Prenatal Care 11-12
  - 1.B.2. Intrapartum Care and Delivery Outcome 13-14
  - 1.B.3. Postnatal Care 15-16
  - 1.B.4. Maternal Mortality 17-18
- **1.C. CHILDCARE AND SERVICES** **19-32**
  - 1.C.1. Immunization Services for Infants and Children 20-22
  - 1.C.2. Nutrition Services for Infants and Children 23-25
  - 1.C.3. Management of Sick Infants and Children 26-28
  - 1.C.4. Child Mortality 29-32
- **1.D. ORAL HEALTH CARE SERVICES** **33-37**
- **1.E. NON-COMMUNICABLE DISEASES PREVENTION AND CONTROL SERVICES** **38-43**
- **1.F. ENVIRONMENTAL HEALTH AND SANITATION SERVICES** **44-48**
- **1.G. MORBIDITY** **49-50**
- **1.H. DEMOGRAPHIC DATA** **51-55**
- **1.I. INFECTIOUS DISEASE PREVENTION AND CONTROL SERVICES** **56-67**
  - 1.I.1. Filariasis 57-58
  - 1.I.2. Leprosy 59-60
  - 1.I.3 Rabies 61-62
  - 1.I.4. Schistosomiasis 63-64
  - 1.I.5. Soil Transmitted Helminthiasis 65
  - 1.I.6. Tuberculosis 66-67

CHAPTER II **68-1381**
- **2.A. FAMILY PLANNING SERVICES** **69-218**
  - **2.A.1. New Acceptors** **69-103**
  - **2.A.2. Other Acceptors** **104-138**
  - **2.A.3. Drop-outs** **139-173**
  - **2.A.4. Current Users** **174-208**
  - **2.A.5. Demand Satisfied** **209-213**
  - **2.A.6. Unmet Needs** **214-218**
- **2.B. MATERNAL CARE AND SERVICES** **211-353**
  - **2.B.1. Prenatal Care** **219-308**
    - 2.B.1.1. Women who gave birth with at least 4 or more Prenatal Check-ups 219-223
    - 2.B.1.2. Pregnant women according to their nutritional status 224-233
    - 2.B.1.3. Pregnant women given Tetanus Diphtheria (Td) Vaccination 234-243
    - 2.B.1.4. Pregnant women who Completed Iron with Folic Acid 244-248
    - 2.B.1.5. Pregnant women who Completed Calcium Carbonate 249-253
    - 2.B.1.6. Pregnant women who Completed Iodine Supplementation 254-263
    - 2.B.1.7. Pregnant women screened for Syphilis 264-268
    - 2.B.1.8. Pregnant women tested positive for Syphilis 269-273
    - 2.B.1.9. Pregnant women screened for Hepatitis B 274-278

2.B.1.10. Pregnant women tested positive for Hepatitis B.......... 279-283
2.B.1.11. Pregnant women screened for HIV.......... 284-288
2.B.1.12. Pregnant women tested for CBC or Hemoglobin and Hematocrit Count.......... 289-293
2.B.1.13. Pregnant women diagnosed with anemia.......... 294-298
2.B.1.14. Pregnant women screened for Gestational Diabetes.......... 299-303
2.B.1.15. Pregnant women tested positive for Gestational Diabetes.......... 304-308
2.B.2. Intrapartum Care and Delivery Outcome.......... 309-338
2.B.2.1. Skilled Health Professionals & Facility Based Delivery 309-317
2.B.2.2. Delivery by type.......... 318-323
2.B.2.3. Pregnancy outcome (Full term and Preterm).......... 324-333
2.B.2.4. Pregnancy outcome (Fetal Deaths and Abortion).......... 334-338
2.B.3. Postpartum and Newborn Care.......... 339-353
2.B.3.1. 2 Postpartum women who completed postpartum check-ups.......... 339-343
2.B.3.2. Postpartum women who completed Iron with Folic Acid.......... 344-348
2.B.3.3. Postpartum women who completed Vitamin A Supplementation.......... 349-353
2.C. CHILDCARE AND SERVICES.......... 354-553
2.C.1. Immunization Services for Infants and Children.......... 354-438
2.C.1.1. Child Protected at Birth (CPAB), Newborn / Infants Vaccinated with Bacillus Calmette-Guérin (BCG), Newborn Vaccinated with Hepatitis B antigen.......... 354-368
2.C.1.2. Infants who completed 3 doses of DPT-HiB-HepB antigen.......... 369-383
2.C.1.3. Infants who completed 3 doses of Oral Polio Vaccine (OPV) and Inactivated Polio Vaccine (IPV).......... 384-408
2.C.1.4. Infants who completed 3 doses of Pneumococcal Conjugate Vaccine (PCV).......... 409-418
2.C.1.5. Children vaccinated with 2 doses of Measles Containing Vaccine (MCV).......... 419-428
2.C.1.6. Fully Immunized Children (FIC) and Completely Immunized Children (CIC).......... 429-438
2.C.2. Nutrition Services for Infants and Children.......... 439-498
2.C.2.1. Newborns initiated on breastfeeding immediately after birth lasting for at least 90 minutes, Infants Exclusively Breastfed until 5th months and 29 days, Infant who continued Breastfeeding and were introduced to complementary feeding beginning at 6 months of age.......... 439-453
2.C.2.2. Infants born preterm or with low birth weight given iron supplements Infants/Children 6-11 and 12-59 months old who completed Vitamin A Supplementation.......... 454-468
2.C.2.3. Infants 6-11 months old and Children 12-23 months old who completed Micronutrient Powder (MNP) Supplementation.......... 469-472
2.C.2.4. Children 0-59 months old whose Nutritional Status are Normal, Stunted and Overweight/Obese Children 0-59 months old whose Nutritional Status are Wasted-MAM, Wasted-SAM and Wasted.......... 473-498
2.C.3. Deworming and Services for Children and Adolescents.......... 499-513
2.C.4. Management of Sick Infants and Children.......... 514-553
2.C.4.1. High Risk Infants and Children with Measles and/or Persistent Diarrhea who received Vitamin A capsule aside from routine supplementation.......... 514-523
2.C.4.2. Diarrhea cases seen (0-59 months old) who received Oral Rehydration Salt Solution (ORS) and ORS with Zinc drops or syrup.......... 524-528
Pneumonia cases seen (0-59 months old) and received treatment.......... 529-533
2.C.4.3. Moderately Wasted Children enrolled and cured in Supplementary Feeding Program (SFP).......... 534-543
2.C.4.4. Severe Acute Malnutrition Children enrolled and cured in Supplementary Feeding Program (SFP).......... 534-553
2.D. ORAL HEALTH CARE SERVICES.......... 554-603
2.D.1. Children 12-59 months old orally fit.......... 554-558

2.D.2. 5 years old and above with cases of decayed, missing, filled teeth (DMFT)........................ 559-563
2.D.3. Basic Oral Health Care (BOHC).. 564-603
2.D.3.1. Infants 0-11 months old.. 564-568
2.D.3.2. Children 1-4 years old.. 569-573
2.D.1.3. Children 5-9 years old.. 574-578
2.D.1.4. Adolescents 10-14 years old.. 579-583
2.D.1.5. Adolescents 15- 19 years old.. 584-588
2.D.1.6. Adults 20 -59 years old.. 589-593
2.D.1.7. Senior citizens 60 years old and above.. 594-598
2.D.1.8. Pregnant women 10-14, 15-19, 20-49 years old.. 599-603
2.E. INFECTIOUS DISEASE PREVENTION AND CONTROL SERVICES.. 604-681
2.E.1. Filariasis Prevention and Control.. 604-608
2.E.2. HIV-AIDS/STI Prevention and Control Services.. 609-613
2.E.3. Leprosy.. 614-615
2.E.4. Rabies.. 619-623
2.E.5. Schistosomiasis Prevention and Control.. 624-640
2.E.5.1. Acute clinically diagnosed cases seen & Acute Confirmed Cases by Stool Examination...... 624-629
2.E.5.2. Chronic Clinically Diagnosed Cases & Chronic Clinically diagnosed cases
confirmed by stool examination.. 630-635
2.E.5.3. Chronic Clinically Diagnosed cases treated in the Health Facility
and Confirmed Chronic Cases referred to a Hospital Facility.. 636-640
2.E.6 Soil Transmitted Helminthiasis Prevention and Control.. 641-661
2.E.6.1. Children 1-4 who completed 2 doses of deworming tablets.. 641-645
Children 5-9 years old who completed 2 doses of deworming tablets.................................. 646-650
2.E.6.2. Adolescents 10-19 and Women 20-49 years old who completed
Two (2) doses of deworming tablets.. 651-656
2.E.6.3 Pregnant Women who completed 1 dose of deworming tablets.. 657-661
2.E.7. Tuberculosis Prevention and Control.. 662-681
2.E.7.1. Treatment Success Rate DSTB - All Forms.. 662-666
2.E.7.2. Treatment Success Rate - MDRTB.. 667-671
2.E.7.3. Case Notification Rate (All forms).. 672-676
2.E.7.4. Case Notification Rate (DRTB).. 677-681
2.F. NON-COMMUNICABLE DISEASES PREVENTION AND CONTROL SERVICES.......................... 682-693
2.F.1. Lifestyle Related Diseases (Risk Assessment using PhilPEN protocol).............................. 682-696
2.F.1.1 Adults aged 20 yrs old and above who were risk-assessed
using the PhilPEN protocol.. 682-686
2.F.1.2 Adults aged 20 yrs old and above who are current smokers,
binge drinkers and overweight or obese.. 687-696
2.F.2. Cancer Prevention and Control.. 670-678
2.F.2.1 Women aged 20 yrs old and above screened for Cervical Cancer... 670-673
Women aged 20 yrs old and above screened for Breast Cancer.. 674-678
2.F.3. Cardiovascular Disease and Diabetes Mellitus Prevention and Control.............................. 679-683
2.F.3.1 Identified 20 yrs old and above adults with Hypertension (HPN)
and Type 2 Diabetes Mellitus (DM).. 679-683
2.F.4. Blindness Prevention Program.. 684-688
2.F.4.1 Senior citizens 60 yrs old and above screened for visual acuity
and diagnosed with eye diseases.. 684-688
2.F.5. Immunization for Senior Citizens.. 689-693
2.F.6.1 Pneumococcal Polysaccharide Vaccine (PPV) and one (1) dose of Influenza Vaccine........... 689-693
2.G. ENVIRONMENTAL HEALTH AND SANITATION SERVICES.. 694-721
2.G.1 Households (HHs) with access to basic safe water supply and manage drinking water..... 694-703

2.G.2 Households (HHs) with access to basic sanitation facility.... 704-715
2.G.3 Municipalities, Cities and Chartered Cities Certified as ZOD....716-721
2.H. MORBIDITY....722-1316
2.H.1. Top Ten Leading Causes of Morbidity.... 722
2.H.2. Morbidity Rate (by Type of Diseases, Sex and Age Group)....723-1316
2.H.2.1. Acute Bloody Diarrhea....724-738
2.H.2.2. Acute Febrile Illness.... 739-753
2.H.2.3. Acute Flaccid Paralysis....754-768
2.H.2.4. Acute Hemorrhagic Fever....769-783
2.H.2.5. Acute Lower Respiratory Tract Infection....784-796
2.H.2.6. Acute Respiratory Infection (Less than 5 years old)....797-801
2.H.2.7. Acute Respiratory Infection (More than 5 years old).... 802-816
2.H.2.8. Acute Watery Diarrhea 817-828
2.H.2.9. Animal Bites....829-837
2.H.2.10. Bronchitis....838-849
2.H.2.11. Cholera.... 850-864
2.H.2.12. Chronic Obstructive Pulmonary Disease....865-879
2.H.2.13. Diphtheria.... 880-894
2.H.2.14. Diseases of the Heart.... 895-909
2.H.2.15. Filariasis....910-924
2.H.2.16. Fever of Unknown Origin....925-939
2.H.2.17. Genital Ulcer....940-954
2.H.2.18. Gonorrhea....955-969
2.H.2.19. Hypertension....967-981
2.H.2.20. Influenza-Like Illness.... 982-996
2.H.2.21. Influenza.... 997-1011
2.H.2.22. Leprosy....1012-1026
2.H.2.23. Leptospirosis....1027-1041
2.H.2.24. Malaria....1042-1056
2.H.2.25. Measles.... 1057-1071
2.H.2.26. Meningococcal Infection.... 1072-1086
2.H.2.27. Neonatal Tetanus....1087-1091
2.H.2.28. Non-neonatal Tetanus....1092-1106
2.H.2.29. Pneumonia.... 1107-1121
2.H.2.30. Rabies (Human).... 1122-1136
2.H.2.31. Paralytic Shellfish Poisoning.... 1137-1151
2.H.2.32. Schistosomiasis....1152-1166
2.H.2.33. Skin Diseases....1167-1181
2.H.2.34. Syphilis.... 1182-1196
2.H.2.35. Tuberculosis (All Forms).... 1197-1211
2.H.2.36. Typhoid and Paratyphoid Fever....1212-1226
2.H.2.37. Urethral Discharge....1227-1241
2.H.2.38. Urinary Tract Infection....1242-1256
2.H.2.39. Viral Encephalitis.... 1257-1271
2.H.2.40. Viral Hepatitis....1272-1286
2.H.2.41. Viral Meningitis.... 1287-1301
2.H.2.42. Whooping Cough....1302-1316
2.I. MORTALITY....1317-1341
2.I.1 Maternal Mortality Ratio (MMR) and Under Five Mortality Ratio (UFMR)....1317-1331
2.I.2 Infant Mortality Rate (IMR), Neonatal Mortality and Perinatal Mortality Rate.... 1332-1341
2.J. NATALITY.... 1342-1346

2.J.1 Live Births, Crude Birth Rate.. 1342-1346
2.K. DEMOGRAPHIC DATA...1347-1381
2.K.1 Number and Ratio by Barangays, Health Centers, and Barangay Health Stations..........1347-1356
2.K.2 Number and Ration by Health Professional and Active BHW..1357-1381

CHAPTER I

Main Report Analysis

1.A Family Planning Services

2023 KEY FINDINGS

MODERN METHODS OF FAMILY PLANNING

8,683,596
women aged 10-49 years old were using modern methods of family planning

67.01%
of women have their family planning needs met by modern family planning methods

Combined-Oral-Contraceptive (COC) Pills
are the most common method used by 34.48% of the total current modern family planning users

24.11% increase
in the users of implants

13.9% increase
in the users of Postpartum-Intrauterine device (IUD)

851,531
women of reproductive age were reported to have unmet need for modern family planning

ADOLESCENT BIRTH

25.20 births per 1,000 women
Adolescent Birth Rate (ABR)

The Philippines commits to full and effective implementation of the country's Universal Health Care (UHC) Law, along with the Reproductive Health (RH) Law, that will guarantee all Filipinos equitable and inclusive access to quality and affordable health care, goods, and services, including family planning (FP).

At present, the Philippines is in the stage of transitioning from a low to a high percentage of modern contraceptive use and has the potential to substantially expand modern contraceptive use among the eligible population[1]. During this particular stage, it is important that there are no barriers to accessing family planning by ensuring contraceptive availability, high-quality services, and continued demand generation.

This chapter hereby presents the service statistics for family planning provided by public health facilities.

Modern methods of FP have been utilized by an expanding number of women aged 10 to 49 years in the country. Modern FP methods include bilateral tubal ligation (BTL), no-scalpel vasectomy (NSV), condoms, intrauterine devices (IUD, interval, and postpartum), oral pills (combined-oral-contraceptives [COC] and progestin-only pills [POP]), injectables, implants, and natural family planning methods (NFP), namely cervical mucus method (CCM), basal body temperature (BBT), symptothermal method (STM), standard days method (SDM), and lactational amenorrhea (LAM).

In 2023, 8,683,596 women aged 10 to 49 years were using modern FP methods, which is 4% higher than the number of modern FP method users in 2022 and corresponds to an increase of 311,037 women *(Figure 1.A.1)*.

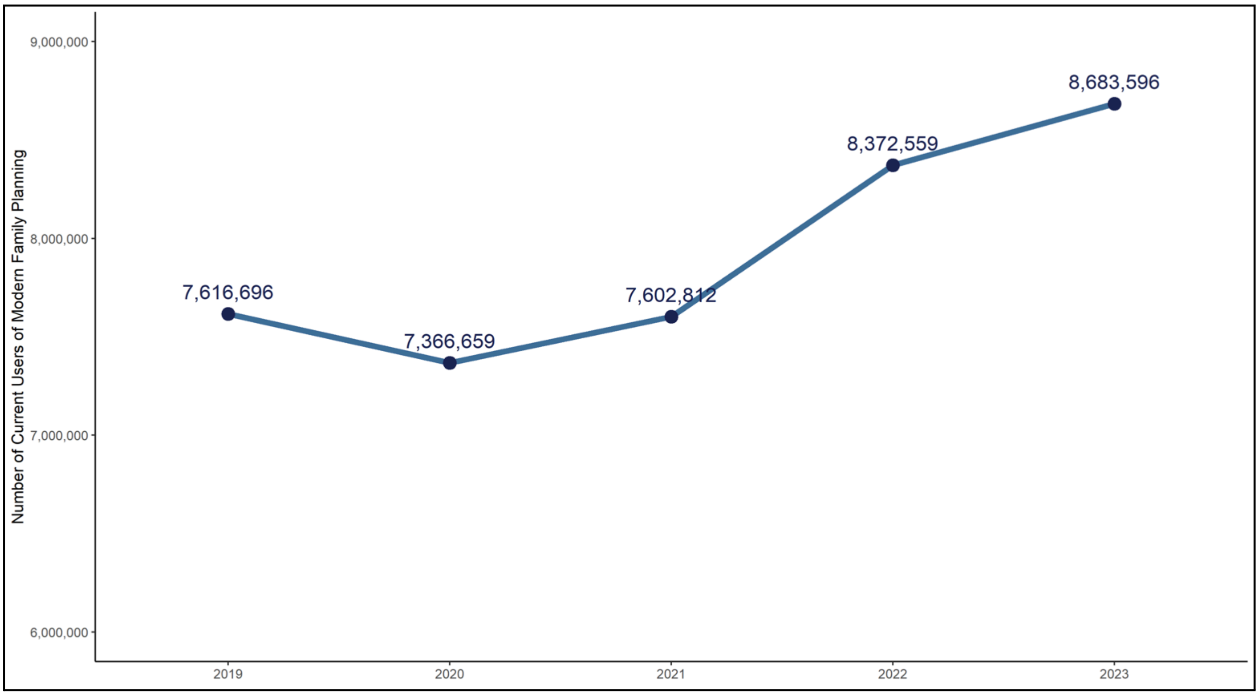

Figure 1.A.1: Current Users of Modern Family Planning, Philippines, 2019 to 2023

In the Philippines, oral pills, specifically combined-oral-contraceptives or COCs, continue to be the most commonly used modern FP method, accounting for 34.48% (2,994,278) of the total current users in 2023. Injectables, which accounted for 20.66% (1,794,406) of the total current users, were the next most commonly used in the country, followed by female sterilization/bilateral tubal ligation, which accounted for 9.48% (822,843) of the total modern FP users *(Table 1.A.1)*.

[1] Exploring Opportunities for mCPR Growth in Philippines, Track20, December 2022

Among modern FP methods, implants had the highest increase in the number of users from 2022 to 2023. The users of implants have grown by 24.11% (636,689) in 2023, making implants the fourth most common modern FP method. The observed increase in the number of implant users is a positive finding, as it supports the efficacy of these long-acting reversible FP methods. The World Health Organization (WHO) also highlighted implants in preventing pregnancies, stating that although oral pills, which are commonly used by women, may lead to 7 pregnancies per 100 women, using implants will only result in 0.1 pregnancies per 100 women.[2]

In contrast, the number of users of the majority of natural FP methods (LAM, CCM, BBT, STM), IUD-Interval, and male sterilization/no-scalpel vasectomy decreased in 2023, despite a 13.9% increase in postpartum IUD users compared to 2022 *(Table 1.A.1)*.

Method	2023		2022	% change 2022 vs. 2023
	Number of Users	Percent	Number of Users	
PILLS-COC	2,994,278	34.48%	2,929,378	2.22% (↑)
INJECTABLES	1,794,406	20.66%	1,702,295	5.41% (↑)
FSTR/BTL	822,843	9.48%	819,597	0.40% (↑)
IMPLANTS	790,220	9.10%	636,689	24.11% (↑)
NFP-LAM	638,588	7.35%	671,595	-4.91% (↓)
PILLS-POP	457,336	5.27%	423,170	8.07% (↑)
CONDOM	426,324	4.91%	412,406	3.37% (↑)
IUD-INTERVAL	415,634	4.79%	445,713	-6.75% (↓)
IUD-POSTPARTUM	166,849	1.92%	146,484	13.90% (↑)
NFP-SDM	120,665	1.39%	118,646	1.70% (↑)
NFP-CCM	43,540	0.50%	49,437	-11.93% (↓)
MSTR/NSV	7,499	0.09%	9,393	-20.16% (↓)
NFP-BBT	3,214	0.04%	4,199	-23.46% (↓)
NFP-STM	2,200	0.03%	3,557	-38.15% (↓)
Total Users	8,683,596	100.00%	8,372,559	3.71% (↑)

Table 1.A.1: Modern Contraception Use by Method, Philippines, 2023

New acceptors of modern FP methods are defined as clients who use a contraceptive method for the first time or have never accepted any modern FP method. In 2023, 1,078,501 women were reported as new acceptors of modern FP methods, representing an 8.92% increase from 2022 and the highest number of new acceptors in the last five years *(Figure 1.A.2)*.

[2] Family planning/contraception methods Fact Sheet, World Health Organization, 2023

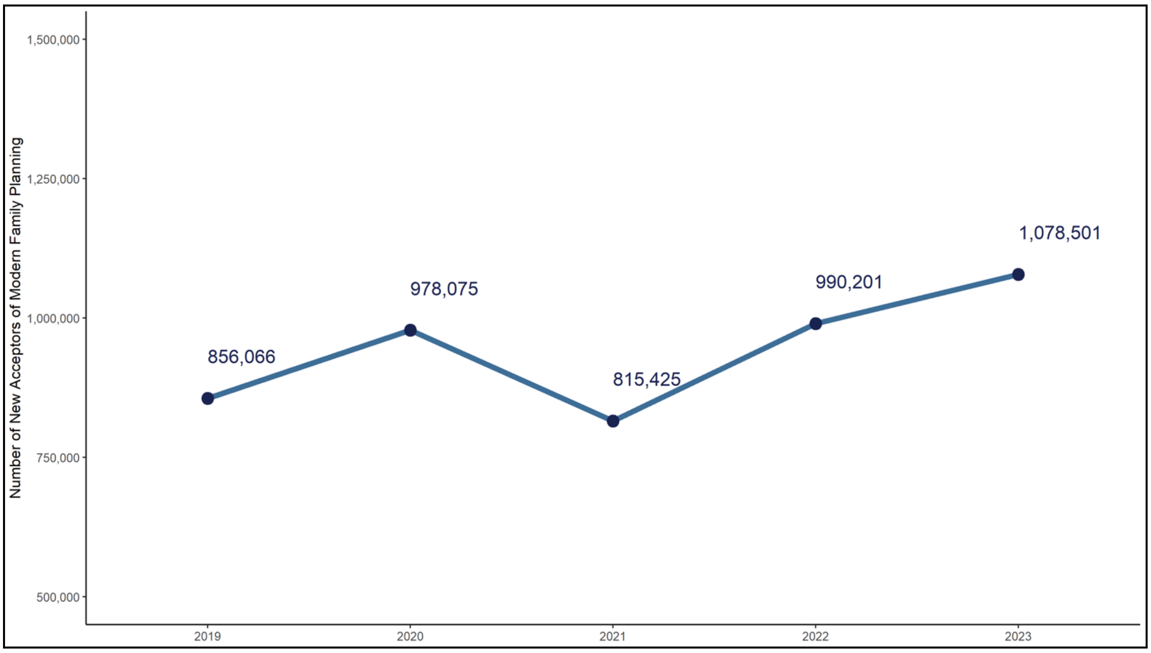

Figure 1.A.2: New Acceptors of Modern Family Planning, Philippines, 2019 to 2023

Similar to the previous year, there was a decrease in the number of new acceptors of all natural FP methods (except LAM). Consequently, injectables and NFP-LAM comprise over 50% of the total number of new acceptors of modern FP, with 204,866 and 410,749 new acceptors, respectively *(Table 1.A.2)*. NFP-LAM or lactational amenorrhea is the temporary postnatal infertility that occurs when a woman is not menstruating and is fully breastfeeding. It is therefore important to keep track of women using LAM, provide them with information about additional postpartum contraceptive options, and effectively assist them in transitioning to alternative contemporary techniques once their temporary infertility has passed.

On the other hand, an increase of 33.81% was observed in the new acceptors of female sterilization or bilateral tubal ligation in 2023. The number of new implant acceptors is continuing to increase, albeit at a reduced pace (23.28% increase in 2023 vs. 88.41% increase in 2022) *(Table 1.A.2)*.

Method	2023		2022	% change 2022 vs. 2023
	Number	Percent	Number	
NFP-LAM	410,749	38.09%	408,032	0.67% (↑)
INJECTABLES	204,866	19.00%	175,664	16.62% (↑)
PILLS-COC	143,393	13.30%	131,606	8.96% (↑)
IMPLANTS	104,684	9.71%	84,913	23.28% (↑)
PILLS-POP	90,084	8.35%	87,609	2.83% (↑)
CONDOM	62,711	5.81%	48,188	30.14% (↑)
FSTR/BTL	24,294	2.25%	18,094	34.27% (↑)
IUD-POSTPARTUM	18,251	1.69%	16,701	9.28% (↑)
IUD-INTERVAL	12,766	1.18%	11,057	15.46% (↑)
NFP-SDM	3,844	0.36%	4,549	-15.50% (↓)
NFP-CCM	1,381	0.13%	1,908	-27.62% (↓)
NFP-BBT	816	0.08%	979	-16.65% (↓)
MSTR/NSV	458	0.04%	408	12.25% (↑)
NFP-STM	204	0.02%	493	-58.62% (↓)

Method	2023		2022	% change 2022 vs. 2023
	Number	Percent	Number	
Total New Acceptors	1,078,501	100.00%	990,201	8.92% (↑)

Table 1.A.2: New Acceptors of Modern Family Planning by Method, Philippines, 2023

Aside from tracking the provision of family planning commodities, it is also vital to look at women who wish to delay or prevent pregnancies but are not using modern FP methods. This group refers to women with unmet need for modern FP and those that are currently using traditional FP methods (e.g., withdrawal) but wish to transition to modern FP methods. Given that the intention to delay or limit childbearing is already present, it is important to engage with this group in order to determine and address the rationale behind their non-use of modern FP methods. After all, reproductive health entails that people have the capability and the freedom to decide if and when to have children. Furthermore, studies have linked unintended pregnancies to poor health outcomes for both the mother and the child, as well as social and economic repercussions.

Along with the increase in the number of current users of modern family planning methods, the number of women with unmet need for modern family planning has decreased by 23.26% from 1,186,993 women in 2022 to 851,531 in 2023. Relative to the estimated population of women of reproductive age (WRA) in the country, this is the lowest percentage with unmet need for modern contraception since the pandemic, albeit still lower than pre-pandemic in 2019 (*Table 1.A.3).*

Unmet Need for Modern Family Planning	2019	2020	2021	2022	2023
No. of women with unmet need for modern family planning	504,219	844,752	930,909	1,186,993	851,531
% among estimated women of reproductive age (15-49 y.o)	1.80	3.02	3.28	4.22	2.98

Table 1.A.3. Unmet Need for Modern Family Planning, Philippines, 2019 to 2023

Similar to 2022, the region contributing the most in the total number of WRA (15-49 year old) with unmet need for modern FP is Region 11, followed by Regions 6 and 4A. Cumulatively, these three (3) regions comprise more than 50% of the total WRA with unmet need for modern contraceptives in the whole country. Accounting for the regions' respective populations of WRA, Region 11, CAR, and Region 6 recorded the highest percentage of WRA with unmet need for modern FP *(Figure 1.A.3)*.

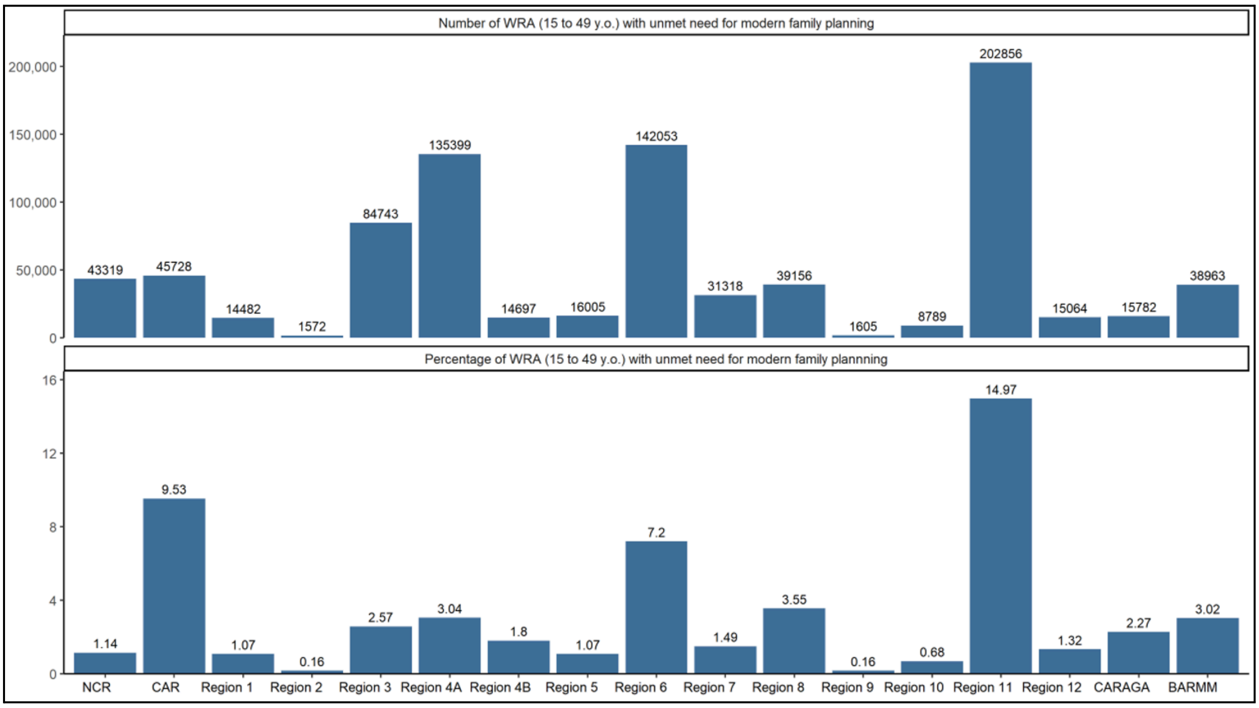

Figure 1.A.3. Unmet Need for Modern Family Planning, by Region, 2023

From 2023 onward, the indicator “Proportion of the Demand for Family Planning Satisfied by Modern Methods” was included in the FHSIS. This is a more specific indicator than the modern contraceptive prevalence rate as it considers whether or not a woman has a need for modern family planning in the first place. Moreover, this indicator requires an estimate of the total demand for family planning, which was constructed by getting 11.6% of the total WRA population, as stipulated in the Department Memorandum 2022-0417 entitled “Guideline on the New Indicator Demand Satisfied with Modern Family Planning Methods for the Program Expenditure Classification (PREXC) for Family Planning".

Nationally, 8,677,668 women of reproductive age (15-49 years old) were reported to have their need for family planning satisfied by any modern method, corresponding to 67.01% of the total demand for FP. Regionally, the proportion of demand satisfied by modern FP methods ranged from 46.17% to 91.02%. Region 12 had the highest demand satisfied, while 11 other regions had percent demand satisfied by modern FP higher than the national rate. Meanwhile, CARAGA, Region 4A, and Region 7 had the least proportion of demand satisfied by any modern method (Table 1.A.4).

Areas	Estimated Demand for Family Planning (Total WRA Population x 11.6%)	Current Users of Modern FP (WRA only)	%Demand Satisfied by Modern FP
**PHILIPPINES**	**12,950,068**	**8,677,668**	**67.01**
NCR	1,601,070	1,040,250	64.97
CAR	214,794	165,546	77.07
Region 1	625,265	498,112	79.66
Region 2	437,033	343,128	78.51
Region 3	1,473,863	984,838	66.82
Region 4A	1,923,189	887,882	46.17
Region 4B	383,240	313,342	81.76
Region 5	726,847	490,831	67.53
Region 6	933,549	667,254	71.47
Region 7	963,385	483,253	50.16
Region 8	539,553	340,540	63.12
Region 9	461,042	354,575	76.91
Region 10	602,303	511,350	84.90
Region 11	624,165	535,603	85.81
Region 12	522,884	475,905	91.02
CARAGA	333,262	292,364	87.73
BARMM	584,624	292,895	50.10

Note: This only considers women of reproductive age (15 to 49 years old).

Table 1.A.4. Demand for Family Planning Satisfied by Modern Methods, by Region, 2023

Sustainable Development Goal (SDG) Target 3.7 aims to “ensure universal access to sexual and reproductive health-care services, including family planning, information and education, and the integration of reproductive health into national strategies and programmes”. In line with this, the Philippine adolescent (aged 15 to 19 years) birth rate (ABR) per 1,000 women is aimed to be lowered to 30.3 by 2030. The Philippine Statistics Authority's (PSA’s) National Demographic and Health Surveys (NDHS) are the official sources for monitoring the said SDG target. However, the FHSIS also provides data on live births in

the 15 to 19 age group that may be useful for local government unit (LGU) performance monitoring.

Early pregnancies among adolescent women come with increased health risks for mothers and newborns. Babies born to mothers under 20 years of age face higher risks of low birth weight (LBW), preterm delivery and severe neonatal conditions, while young mothers have higher risks of eclampsia, puerperal endometritis and systemic infections. In addition to health risks, adolescent pregnancies may also come with social and economic consequences. Early pregnancies among adolescent women are preventable by ensuring information and services on reproductive health, including family planning, are made accessible to the population.

Although still lower than the SDG target, the ABR has increased since 2019 (14.67%), such that the ABR in the Philippines was 24.36% in 2022 and 25.50% in 2023. Moreover, when comparing the ABRs from 2020 to 2023, it is evident that the trend is observing a slower rate of increase, with an average percentage increase of 14.61%. On the other hand, comparing the ABR in 2023 (25.30%) to the ABR during the pre-pandemic in 2019, there is a substantially higher percentage change in the trend, with an average percentage increase of 73.82% *(Figure 1.A.4)*.

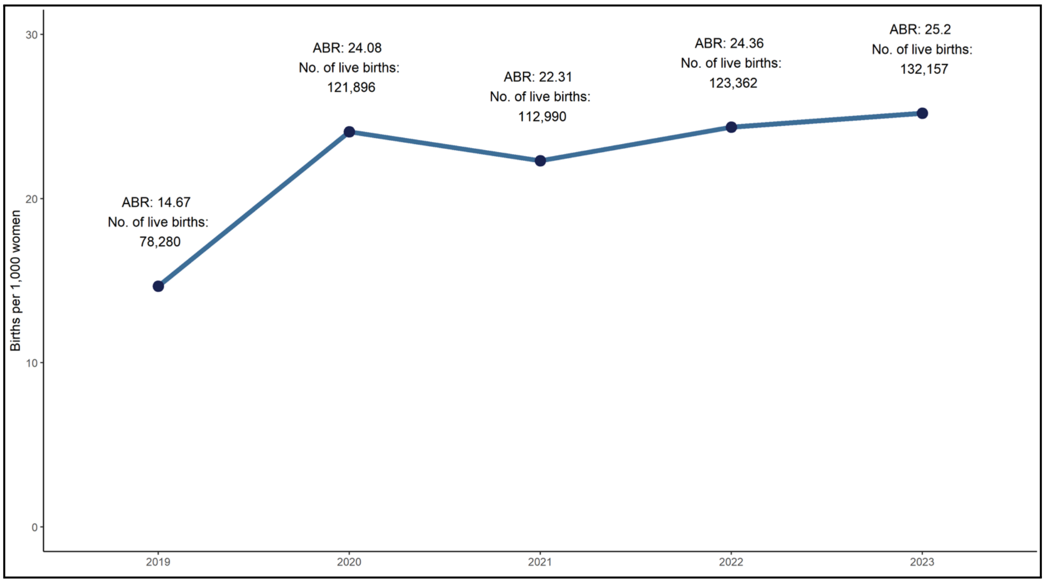

Figure 1.A.4. Adolescent Birth Rate, Philippines, 2019 to 2023

In terms of absolute counts, NCR, Region 4A, Region 3, and Region 10 had the most recorded live births among adolescent women in 2022. Cumulatively, the four (4) aforementioned regions comprised about 40% of the total adolescent live births in the country *(Table 1.A.4).*

Based on the estimated number of adolescent women in the population, Region 10 had the highest ABR of 41.66 births per 1,000 women. This was followed by Region 12 and Region 11 with an ABR of 41.15 and 38.56, respectively. Meanwhile, Region 7 had the lowest ABR, with 11.99 births per 1,000 women *(Table 1.A.5)*.

Region	Live births among women aged 15 to 19 y.o.	ABR per 1,000 women
**PHILIPPINES**	**132,157**	**25.2**
NCR	11,955	21.22
CAR	2,022	22.4
Region 1	3,683	14.93

Region	Live births among women aged 15 to 19 y.o.	ABR per 1,000 women
Region 2	3,724	21.7
Region 3	13,798	24.06
Region 4A	12,786	17.4
Region 4B	5,348	31.52
Region 5	9,272	27.71
Region 6	6,862	18.29
Region 7	11,286	28.8
Region 8	6,330	26.55
Region 9	5,953	29.76
Region 10	9,304	36.49
Region 11	11,091	44.27
Region 12	8,935	39.66
CARAGA	4,841	33.5
BARMM	4,967	17.74

Table 1.A.5. Adolescent Birth Rate, by Region, 2023

1.B Maternal Health Care and Services

2023 KEY FINDINGS

ANTENATAL CARE SERVICES

76.68%

of women who gave birth had at least four antenatal care (4ANC) visits

1st Pregnancy

19.85%

of women who were pregnant for the first time completed Tetanus-Diphtheria vaccination

2 or more Pregnancies

32.16%

of women with repeat pregnancies completed Tetanus-Diphtheria (Td2 Plus) vaccination

INTRAPARTUM AND POSTNATAL CARE SERVICES

92.96%

of deliveries occurred in health facilities

93.97%

of deliveries were attended by Skilled Health Professionals

90.58%

postpartum women had at least two (2) postnatal check-ups

MATERNAL MORTALITY

63.22

Deaths per 100,000 live births

Maternal Mortality Ratio (MMR)

1.B.1 Antenatal Care

Formula:

Women who gave birth and had at least 4ANC check-ups

Numerator:	Number of pregnant women who gave birth with at least 4 or more antenatal check-ups
Denominator:	Total number of deliveries

Women pregnant for the first time given Tetanus Diphtheria (Td) Vaccination

Numerator:	Number of pregnant women for the first time given at least 2 doses of Td vaccine
Denominator:	Eligible population under 1

Pregnant women with repeat pregnancy given Tetanus Diphtheria (Td) Vaccination

Numerator:	Number of pregnant women for the 2nd or more times given at least 3 doses of Td vaccine (Td2 Plus)
Denominator:	Eligible population under 1

Maternal Health Services are essential for ensuring that women have access to high-quality healthcare, especially during their pregnancy and delivery. Most maternal deaths are preventable, which underscores the necessity of services that are designed to promote the health and well-being of mothers in Filipino families.

By 2030, the global maternal mortality ratio (MMR) will be reduced to 70 deaths per 100,000 live births in accordance with SDG Target 3.1, which will subsequently result in a decrease in newborn deaths. Under the guidance of Universal Health Care, the Philippines is committed to providing women with full access to health services to ensure the safety of their pregnancy and childbirth. The goal is to reach target outcomes for antenatal care, facility-based delivery, and postnatal care at 95% or higher.

This chapter hereby presents data on prenatal care nationwide including antenatal care visits and Tetanus-Diphtheria vaccination, intrapartum care and delivery outcomes, and postnatal care.

Getting timely and quality care before childbirth is vital to ensuring the safety and health of both the mother and the child. To detect and manage the underlying conditions of mothers, as well as to provide them with essential supplements and vaccines, prenatal care is essential. In the Philippines, the proportion of women who had at least four (4) antenatal care (ANC) check-ups among those who have given birth has decreased compared to previous years. In 2023, 76.68% of the women who had given birth had at least 4 ANC visits, which was lower by 4.98% compared to 2022. This indicator increased in 2021, albeit still lower than the pre-pandemic year 2019, but started to decrease in 2022. This is the first time in five years that the 4ANC coverage reported in the FHSIS has gone below 80% (Figure 1.B.1.1).

A similar decrease in the coverage of Tetanus-Diphtheria (Td) vaccine was observed both in first time pregnant women and in women with repeat pregnancies. Td vaccination coverage in women who were pregnant for the first time was 19.85% (compared to 23.56% in 2022), while the coverage in women with repeat pregnancies was 32.16% (compared to 37.46% in 2022). Nonetheless, both groups have exhibited a trend decline in recent years (Figure 1.B.1.1). Therefore, it is important to broaden the coverage of Td among pregnant women, as the antibodies developed through vaccination not only protect the mother but also provide protection for the child.

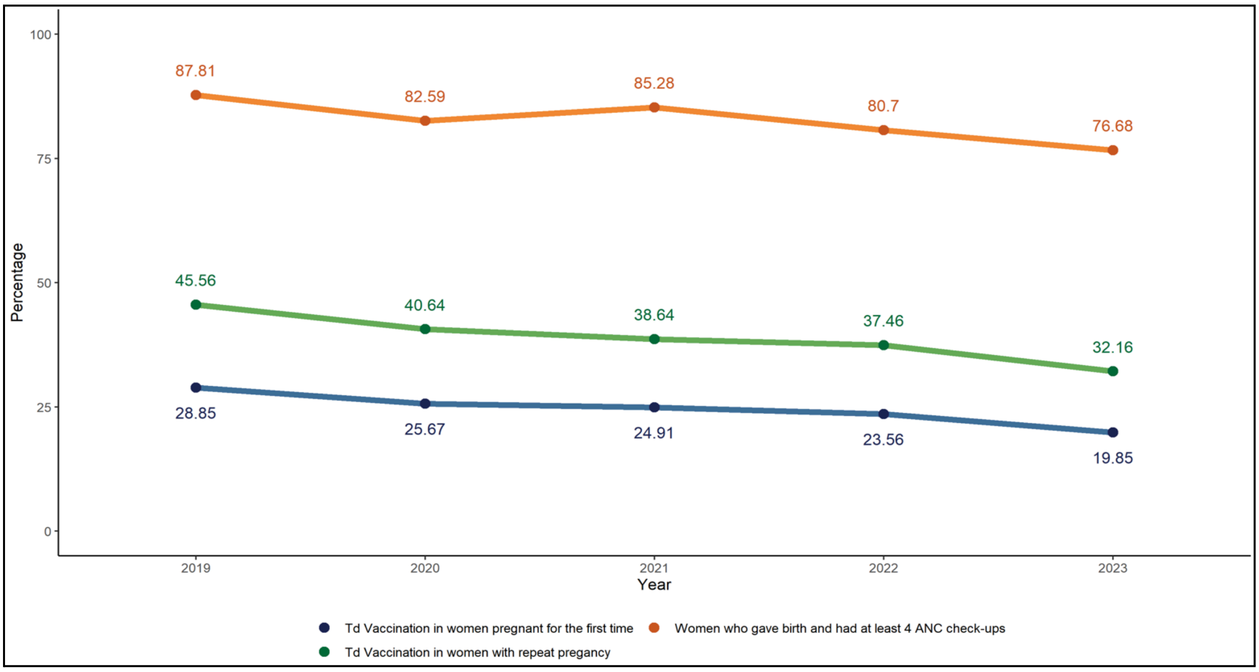

Figure 1.B.1.1: Antenatal Care Indicators, Philippines, 2019 to 2023

Looking at the regions, Region 3 and Region 1 reached the 95% target for ANC check-ups in 2023. Region 2 had the third highest ANC coverage at 88%, followed by Region 12 with 87.72%, and NCR with 87.14%. The target accomplishments of the remaining regions ranged from 28.57% to 84.11%. Regions 7 and 4B had the lowest ANC coverage of 28.57% and 46.02%, respectively (Figure 1.B.1.2).

Overall, the coverage of Td vaccination is consistently higher in women with repeat pregnancies across all regions, but it is less than 50% in both groups, which is still below the nationwide target of 95%. Similar to 2022, Td vaccination coverage in women with repeat pregnancies was highest in Region 7, while Region 12 had the highest coverage for first time pregnancies. In both groups, BARMM had the lowest Td vaccination coverage (Figure 1.B.1.2).

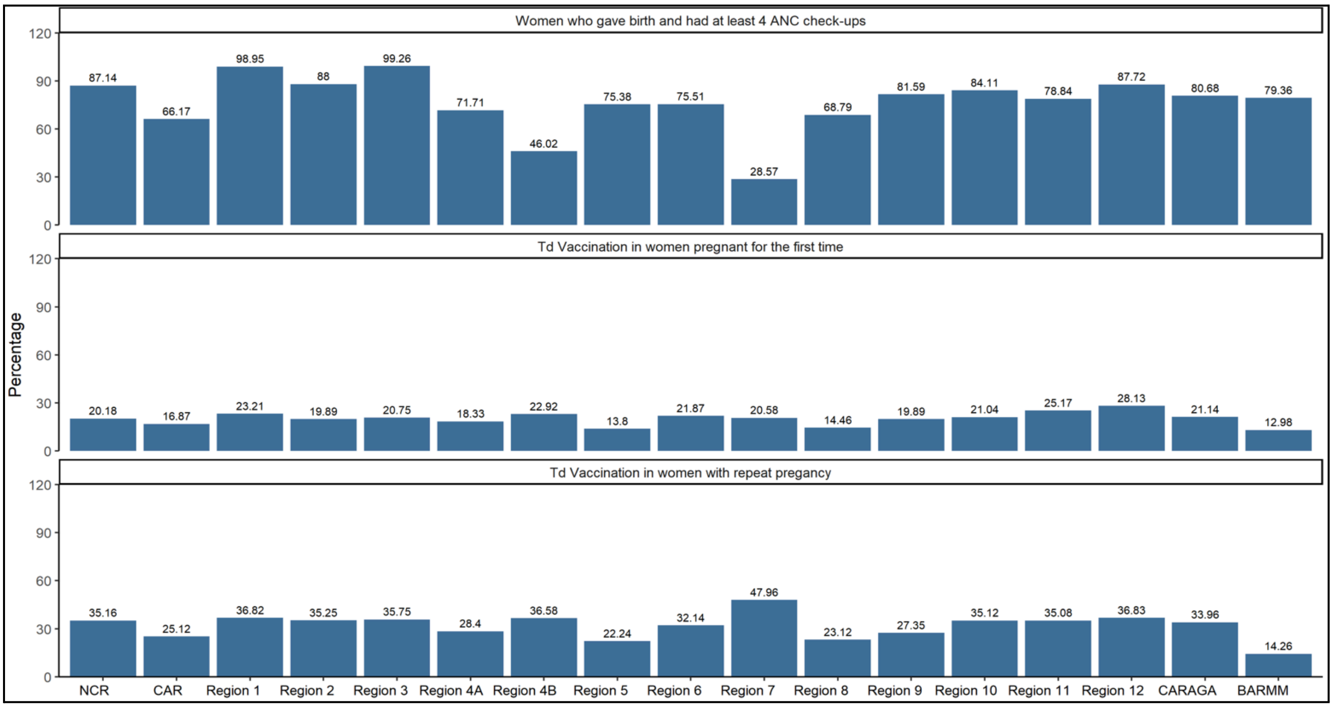

Figure 1.B.1.2: Antenatal Care Indicators, by Region, 2023

1.B.2 Intrapartum Care and Delivery Outcomes

Formula:

Facility-based Deliveries (FBD)

Numerator: Number of deliveries in health facilities (clinics, BHS, RHUs, lying-ins, hospitals, etc.)

Denominator: Total number of deliveries

Births attended by Skilled Health Professionals (Skilled Birth Attendance or SBA)

Numerator: Number of deliveries attended by skilled health professionals (licensed midwives, doctors, or nurses)

Denominator: Total number of deliveries

A positive childbirth experience is a critical component of maternal health care. Globally, over a third of maternal deaths occur during labor and childbirth. Complications that may arise during labor and childbirth can be prevented and/or managed by ensuring that births are conducted by skilled professionals in facilities that are equipped with the necessary tools.

This chapter hereby presents data on facility-based deliveries (FBD), skilled birth attendance (SBA), and live births with low birth weights (LBW) across the country.

The FBD and SBA nationwide have only marginally changed over the past five years. The decrease in both indicators' figures in 2020 may have been attributed by the COVID-19 pandemic onset, which impeded access to health services and mobility. However, these indicators have been rising since 2021 and have surpassed their 2019 coverages, but were still below the 95% FBD/SBA targets (Figure 1.B.2.1).

In 2023, 92.96% (1,320,227) of the total deliveries reported to the FHSIS occurred in health facilities such as clinics, barangay health stations (BHS), rural health units (RHUs), lying-ins, birthing clinics, hospitals, and DOH-licensed ambulances. Meanwhile, 93.97% (1,334,479) of total deliveries were attended by skilled health professionals. Particularly, 61.77% were attended by doctors, 31% were attended by midwives, and 1.20% were attended by nurses (Figure 1.B.2.1).

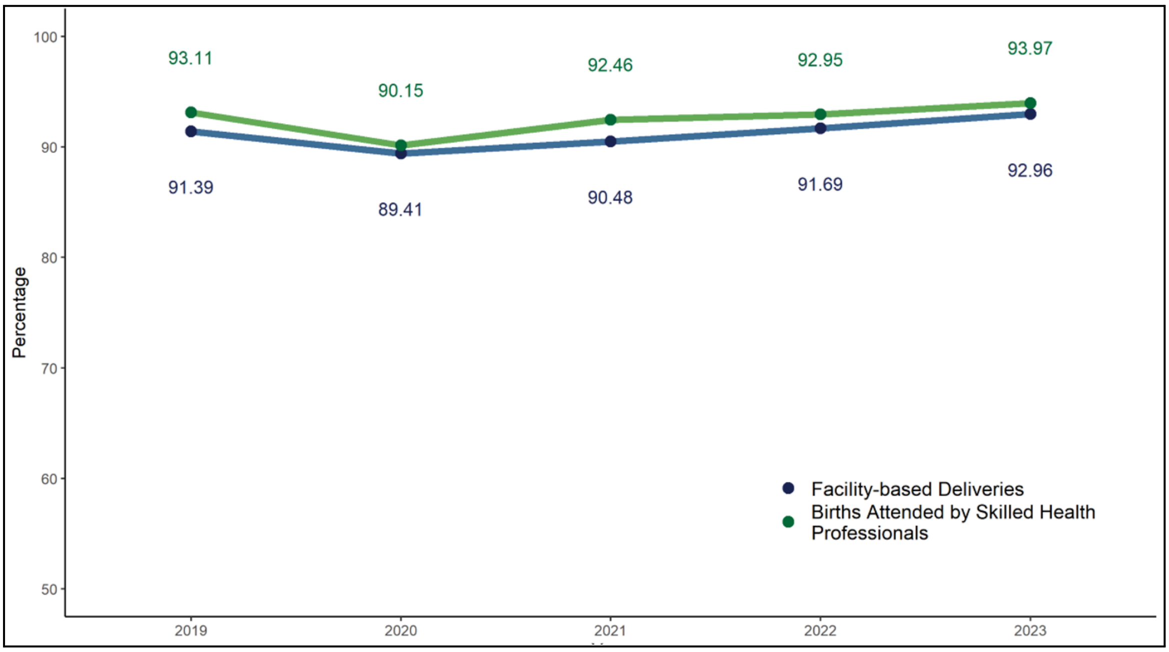

Figure 1.B.2.1: Facility-based Deliveries and Skilled Birth Attendance, Philippines, 2019 to 2023

As shown in *Table 1.B.2.1*, Regions 4A, 4B, 8, 9, 10, 11, 12, and BARMM are almost at the 95% target, but they were still a little short in terms of FBD and SBA. As it was the previous year, BARMM appeared to be trailing in both indicators, with only 70.13% of its births are attended by skilled health professionals, and 61.49% of them were done in health facilities. On the other hand, Region 1, akin to 2022, had the highest FBD and SBA of 99.6% and 99.83%, respectively *(Table 1.B.2.1)*.

Region	Total Deliveries	Skilled Birth Attendance	Facility-based Deliveries
NCR	184,090	97.83%	97.39%
CAR	24,874	98.42%	96.88%
Region 1	50,550	99.83%	99.60%
Region 2	36,823	98.42%	97.92%
Region 3	148,973	97.61%	96.99%
Region 4A	202,438	92.8%	91.52%
Region 4B	47,523	85.12%	84.41%
Region 5	84,229	96.05%	95.46%
Region 6	96,790	95.54%	95.28%
Region 7	112,305	96.32%	95.91%
Region 8	59,834	94.99%	94.27%
Region 9	47,886	86.40%	86.25%
Region 10	71,891	94.02%	92.23%
Region 11	81,389	94.48%	94.43%
Region 12	64,112	93.12%	92.97%
CARAGA	41,335	96.37%	96.06%
BARMM	65,140	70.13%	61.49%

Table 1.B.2.1: Facility-based Deliveries and Skilled Birth Attendance, by Region, 2023

Additionally, among the recorded 1,426,704 live births in 2023, 5.98% (85,337) of them had LBW (weighed less than 2500 grams). This figure had generally decreased compared to 2019, and was roughly 5% lower compared to 2022 data.

Regionally, the percentage of live births with LBW was highest in Region 5 (10.52%) and lowest in BARMM (2.58%). In terms of absolute counts, the highest number of newborns with LBW were observed in Region 4A (10,276), Region 3 (10,259), and Region 7 (9,640) [Figure 1.B.2.3].

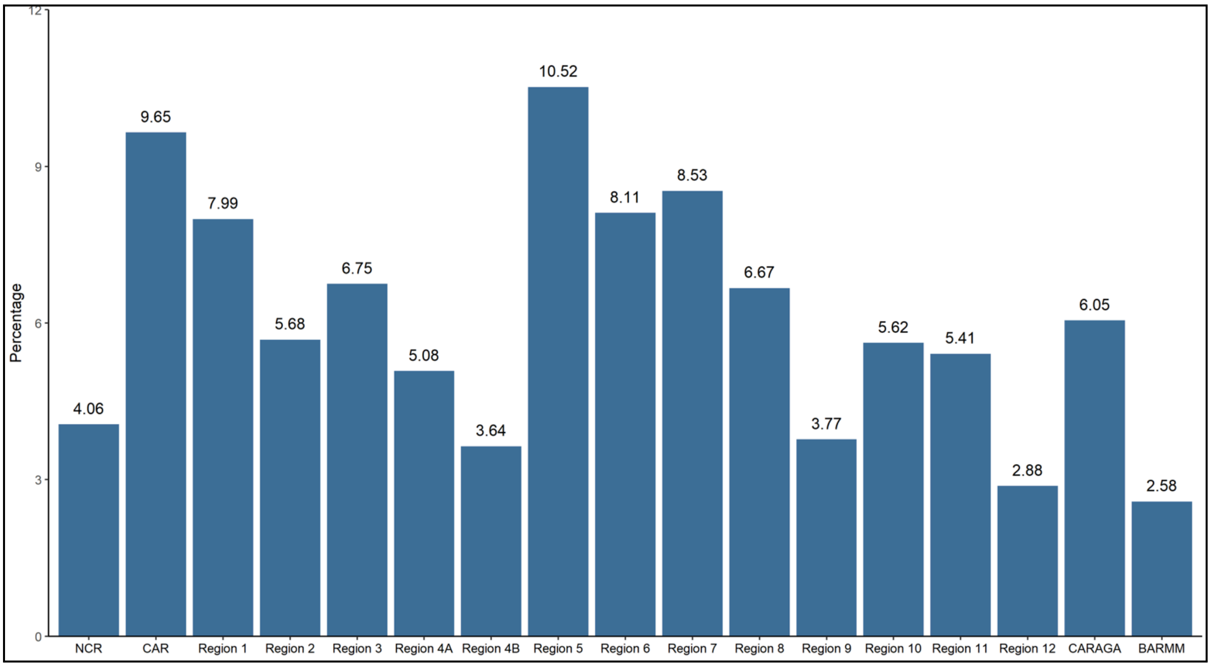

Figure 1.B.2.3: Live Births with Low Birth Weight (<2500 grams), by Region, 2023

1.B.3 Postnatal Care

Formula:

Women together with their newborn who completed at least 2 postpartum check-ups

Numerator:	Number of postpartum women and their newborn who completed at least 2 postnatal check-ups
Denominator:	Total number of deliveries

Postpartum women who completed Iron with Folic Acid supplementation

Numerator:	Number of postpartum women who completed iron with folic acid supplementation
Denominator:	Eligible population under 1

Postpartum women who completed Vitamin A supplementation

Numerator:	Number of postpartum women who completed Vitamin A supplementation
Denominator:	Eligible population under 1

Most maternal and infant deaths occur in the first six (6) weeks after delivery were often due to preventable causes. Therefore, it is important to provide utmost care and monitoring during the postpartum phase to prevent and manage potential complications, ensure health of the mother and the child, and promote birth planning. The first check-up shall be administered within 24 hours of delivery, followed by a subsequent second check-up within seven (7) days. After delivery, women who experienced stillbirths are required to undergo a minimum of two (2) check-ups.

This chapter hereby presents the percentage of postpartum women who completed at least two (2) postnatal check-ups, together with the percentages of vitamin A and/or iron supplementation among postpartum women.

Of the total number of women who gave birth, 90.58% had at least two (2) postnatal check-ups, which was about 2% increase from the previous year (2022). However, the said figure was still three (3) percentage points below from pre-pandemic's figure (93.54%) in 2019 (Figure 1.B.3.1).

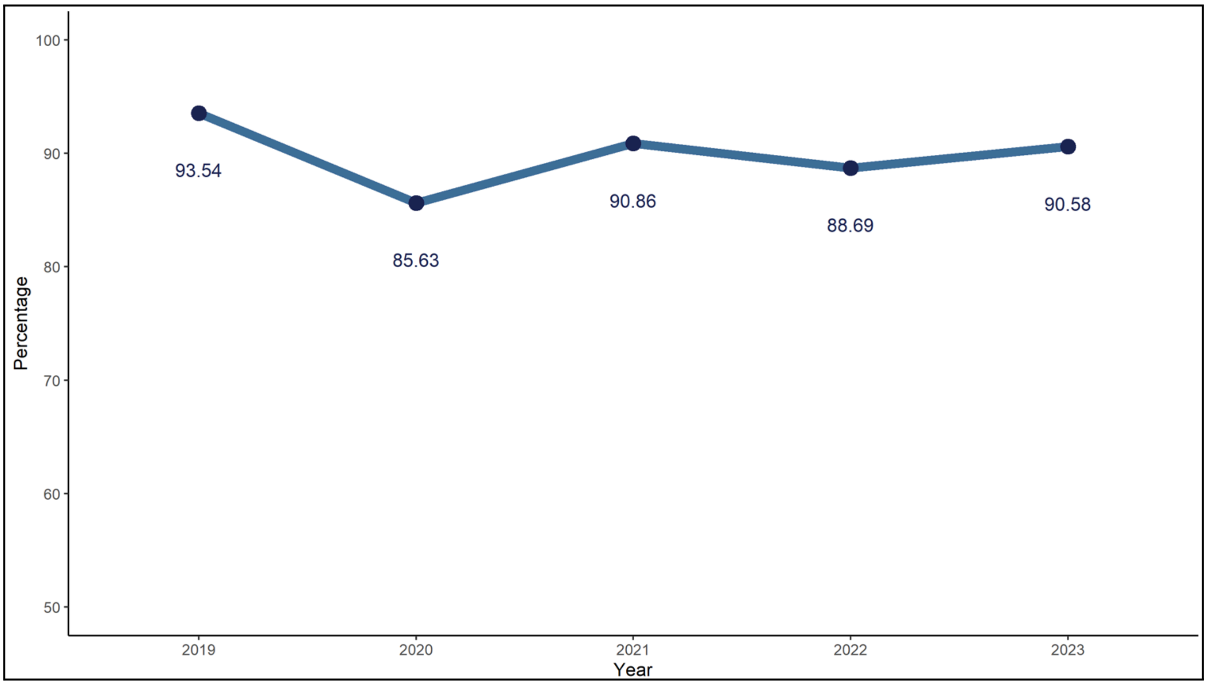

Figure 1.B.3.1: Women with at least 2 Postnatal Check-Ups, Philippines, 2019 to 2023

Looking at the regional disaggregation, NCR, Regions 1, 3, 9, and 12 achieved the target of 95% of postpartum women completing at least two (2) postnatal check-ups. Notably, Regions 1, 2, and 3 exhibited the highest percentage of women who had undergone at least four (4) ANC check-ups, as well as the highest percentages of FBD and SBA (Figure 1.B.3.2).

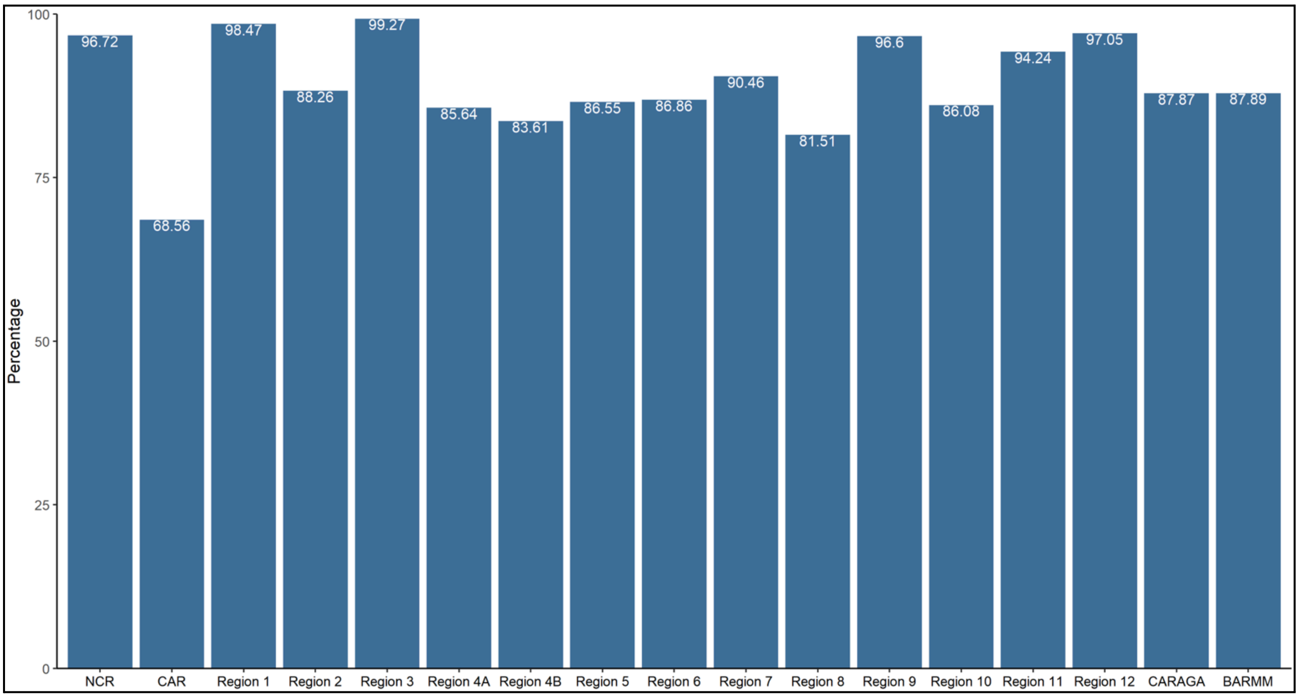

Figure 1.B.3.2: Women with at least 2 Postnatal Check-Ups, by Region, 2023

Moreover, there has been a gradual increase in the percentage of postpartum women who have completed vitamin A supplementation and iron with folic acid supplementation since 2021. In 2023, 57.43% of postpartum women completed vitamin A supplementation, while 56.42% completed iron with folic acid supplementation. Although we are still in the process of approaching the target of 85%, the highest percentage we reached was in 2019 during the pre-pandemic. In that particular year, 75.22% of postpartum women completed Vitamin A supplementation, while 75.68% of them completed iron with folic acid supplementation *(Figure 1.B.3.3)*.

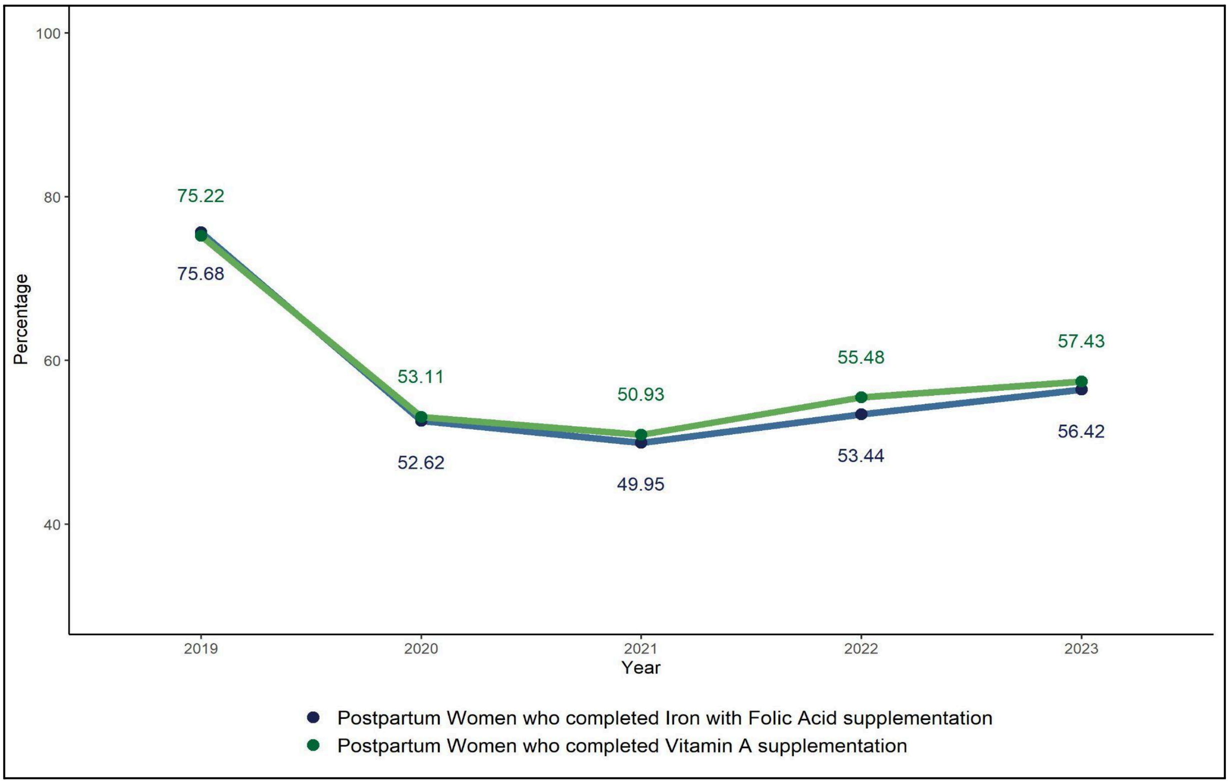

Figure 1.B.3.3:Postpartum Women who completed Iron with Folic Acid and Vitamin A supplementation

1.B.4 Maternal Mortality

Formula:
Maternal Mortality Ratio per 100,000 live births
Numerator: Number of maternal deaths
Denominator: Total number of live births
Multiplier 100,000

Maternal health services aim to prevent and manage complications during pregnancy, labor, and postpartum in an effort to reduce maternal and child mortality. The maternal mortality ratio (MMR) is a widely used indicator for evaluating the effectiveness of maternal health programs. Maternal death, however, is defined as the death of a woman while pregnant or within 42 days of termination of pregnancy, irrespective of the duration and the site of the pregnancy, from any cause related to or aggravated by the pregnancy or its management, but not from accidental or incidental causes.

Based on the SDG 3, the goal is to reduce the global MMR to less than 70 per 100,000 live births. In 2023, the FHSIS recorded 902 maternal deaths, which was approximately 1.31% decrease from the maternal deaths in 2022. As a result, the MMR per 100,000 live births in 2023 lowered to 63.22, which was the lowest ratio since 2020 *(Figure 1.B.4.1).* Note that the deaths reported in the FHSIS come from death registries or logbooks in health facilities and from local civil registries, and therefore may differ from the official death statistics released by the PSA.

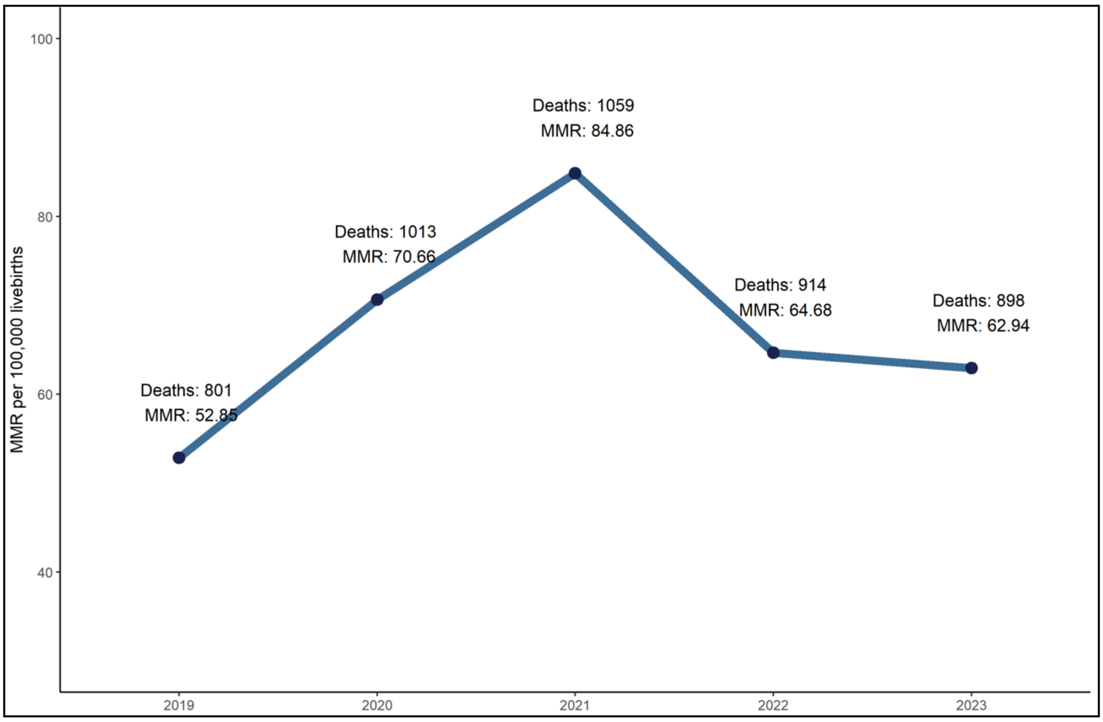

Figure 1.B.4.1: Maternal Mortality Ratio, Philippines, 2019 to 2023

Similar to 2022, the biggest contributor in terms of the number of maternal deaths in the country was NCR, possibly due to its larger total population. Region 4B has the highest MMR (91.96) among all regions, with Region 11 (89.90) and NCR (85.16) following closely behind. Region 9 had the lowest MMR of 10.49 deaths per 100,000 live births, with a total of 5 reported maternal deaths *(Table 1.B.4.1).*

Conversely, the trend of maternal mortality in the country differs across different regions.. CARAGA showed the highest decrease in MMR, from an MMR of 77.23 in 2022 to 43.87 in 2023. Eight (8) other regions also showed a trend decrease in both the maternal death counts and MMR. Among regions with an observed increased MMR, the increase was most prominent in Region 10, from an MMR of 49.74 in 2022 to 81.11 in 2023 *(Table 1.B.4.1)*.

While this report does not include data on the causes of death wherein the pregnancy, delivery, or postpartum phase occurred, further empowering of maternal programs is essential to prevent deaths, particularly given their significant impact on child health.

Region	2023		2022		%change	
	Maternal Deaths	MMR per 100,000 live births	Maternal Deaths	MMR per 100,000 live births	Maternal Deaths	MMR per 100,000 live births
NCR	155	85.16	178	95.21	-12.36%	-10.56% (↓)
CAR	14	56.42	16	66.72	-12.50%	-15.44% (↓)
Region 1	26	51.47	38	76.75	-31.58%	-32.94% (↓)
Region 2	22	59.97	31	78.36	-29.03%	-23.47% (↓)
Region 3	84	55.24	60	44.85	40.00%	23.17% (↑)
Region 4A	85	42.06	71	37.17	19.72%	13.16% (↑)
Region 4B	44	91.96	34	82.55	29.41%	11.40% (↑)
Region 5	71	79.35	90	100.07	-21.11%	-20.71% (↓)
Region 6	73	75.56	68	74.74	7.35%	1.10% (↑)
Region 7	60	53.09	67	50.30	-10.45%	5.55% (↑)
Region 8	34	56.76	46	77.18	-26.09%	-26.46% (↓)
Region 9	5	10.46	8	16.28	-37.50%	-35.75% (↓)
Region 10	58	81.11	38	49.74	52.63%	63.07% (↑)
Region 11	73	89.90	80	96.49	-8.75%	-6.83% (↓)
Region 12	35	54.70	30	46.31	16.67%	18.12% (↑)
CARAGA	18	43.87	27	77.23	-33.33%	-43.20% (↓)
BARMM	41	63.11	32	49.10	28.13%	28.53% (↑)

Table 1.B.4.1: Maternal Mortality Ratio, by Region, 2023 vs. 2022

1.C Child Care and Services

2023 KEY FINDINGS

INFANTS & CHILDREN IMMUNIZATION

62.34%
of children aged 0-12 months were fully immunized

66.95%
Bacillus Calmette Guérin vaccine (BCG)

66.86%
Oral Polio Vaccine (OPV 3)

76.81%
Diphtheria, Pertussis, Tetanus, Haemophilus influenzae type B, Hepatitis B vaccine (DPT-HiB-HepB 3)

68.47%
Measles-Containing Vaccine (MCV 2)

INFANT & CHILDREN NUTRITION

84.85%
of newborns have been immediately initiated on breastfeeding after birth

52.82%
of infants have been exclusively breastfed

44.25%
children aged 0-59 months old with diarrhea were given Oral Rehydration Solution (ORS)

43.20%
sick infants aged 12-59 months were given Vitamin A 100,000 IU

48.69%
sick infants aged 6-11 months were given Vitamin A 100,000 IU

CHILD MORTALITY

14.50
deaths per 1,000 live births
Under Five Mortality Rate (UFMR)

10.64
deaths per 1,000 live births
Infant Mortality Rate (IMR)

6.66
deaths per 1,000 live births
Neonatal Mortality Rate (NMR)

Health is a critical and fundamental element in everyone's welfare, especially children. Child health is important due to the heightened vulnerability throughout the early years of life, which increases the risk of illnesses and complications in younger children. Ensuring the protection of children from diseases is of utmost importance, as it grants them the opportunity to grow into healthy adults and actively contribute to the societal development. Access to basic lifesaving interventions such as immunization, breastfeeding, adequate nutrition, and treatment for common childhood diseases, among others, are some key interventions to achieve this vision.

This chapter hereby presents data on childhood immunization, nutrition services, management of sick children, and mortality.

1.C.1 Immunization Services for Infants and Children

Formula:

Bacillus Calmette–Guérin (BCG)
Numerator: Number of newborn / infants vaccinated with BCG antigen
Denominator: Eligible population 0-11 months

Children Protected at Birth (CPAB)
Numerator: Number of newborns whose mothers pregnant are for the first time who have received at least 2 doses of TT/Td vaccination at least a month prior to delivery PLUS number of newborns whose mothers are pregnant for the 2nd or more times and received at least 3 doses of TT/Td
Denominator: Eligible population aged 0-11 months

Oral Polio Vaccine (OPV)
Numerator: Number of infants vaccinated with OPV 1, 2, 3
Denominator: Eligible population aged 0-11 months

Pneumococcal Conjugate Vaccine (PCV)
Numerator: Number of infants vaccinated with PCV 1, 2, 3
Denominator: Eligible population aged 0-11 months

Hepatitis B within 24 hours after birth
Numerator: Number of newborn vaccinated with birth dose of HepB antigen within 24hrs
Denominator: Eligible population aged 0-11 months

DPT-HiB-HepB
Numerator: Number of infants vaccinated with DPT-HiB-HepB 1, 2, 3
Denominator: Eligible population aged 0-11 months

Inactivated Polio Vaccine (IPV) 1
Numerator: Number of infants vaccinated with IPV1
Denominator: Eligible population 0-11 months

Measles Containing Vaccine (MCV) 2
Numerator: Number of children vaccinated with MCV2
Denominator: Eligible Population aged 0-12 months

Fully-Immunized Children (FIC)
Numerator: Number of FIC
Denominator: Eligible population aged 0-12 months

Completely Immunized Children (CIC)
Numerator: Number of CIC
Denominator: Eligible population aged 13-23 months

Immunization protects us, including children, from life-threatening diseases. Routine vaccines given to children in the Philippines include: BCG for Tuberculosis, Pentavalent for Diphtheria, Pertussis (whooping cough), Tetanus, Haemophilus Influenzae type B and Hepatitis B (DPT-HiB-HepB), Oral Polio and Inactivated Polio, PCV, and MCV. Moreover, doses against neonatal tetanus are also given to pregnant mothers to provide additional protection to children.

For vaccines to be effective, timely immunization is of importance. A child is considered fully immunized if they received 1 dose of BCG, 3 doses of OPV, 3 doses of DPT-HiB-HepB vaccines and 2 doses of MCV by 12 months (MCV1 at 9 months and MCV2 at 12 months). Those who missed any of the routine vaccines could still complete the prescribed doses before they reach two (2) years old and are tagged as completely immunized children (CIC).

From 2019 to 2022, the proportion of fully immunized children (FIC) has decreased in the Philippines. Despite the COVID-19 pandemic becoming more manageable in 2022, the FIC still declined, dropping below 60%. In 2023, there was an improvement in the said trend as the FIC coverage increased to 62.34%, representing a total of 1,482,484 children. Additionally, 213,979 children were tagged as CIC in the FHSIS, indicating that they had missed doses but were able to complete the routine immunization before reaching two years of age.

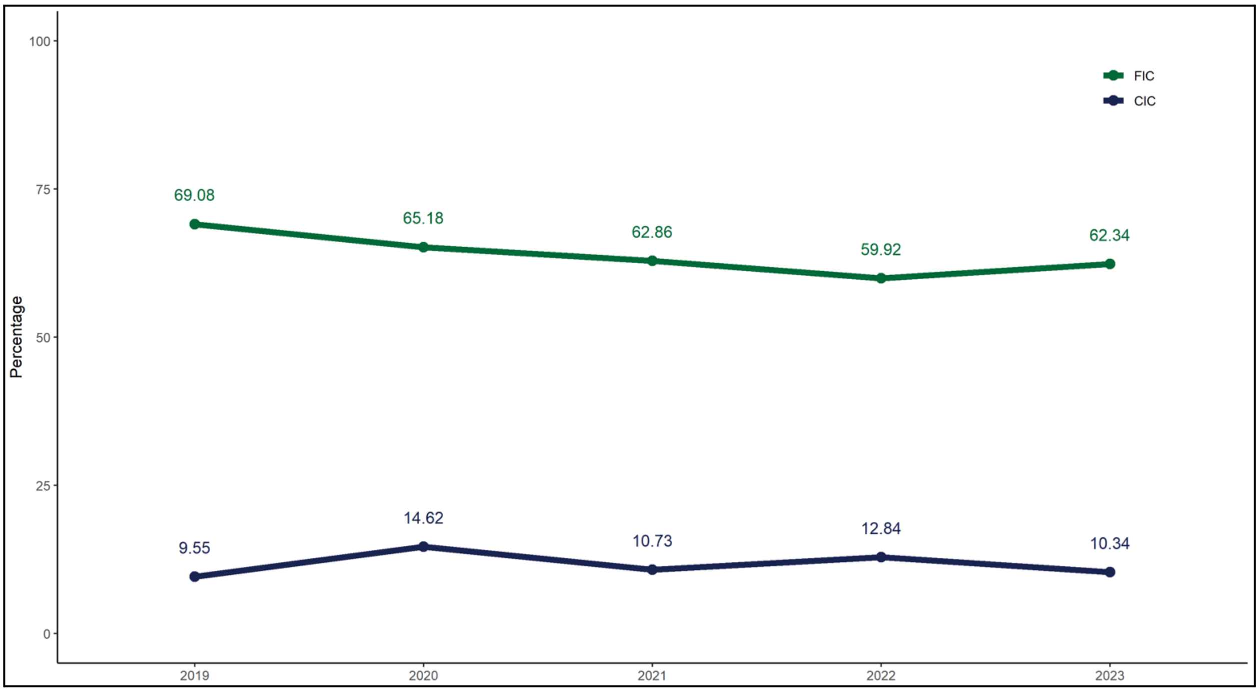

Figure 1.C.1. FIC and CIC, Philippines, 2019 to 2023

Regionally, the FIC coverage ranged from 43.78% in BARMM to 79.34% in CARAGA. The other leading regions in terms of coverage were NCR (78.57%), Region 1 (71.28%), and Region 3 (70.98%). In terms of absolute count, NCR, Region 3, and Region 4A vaccinated the most children. These three (3) regions were also the top regions with the most number of CIC *(Table 1.C.1)*. Moreover, 9 out of the 17 regions in the country had FIC coverage less than the national coverage of 62.34%. Achieving the 95% FIC target on a national and regional scale is essential in order to protect children from vaccine-preventable diseases, improve their chances of survival, and help them in developing into healthy adults.

Region	FIC (0-12 months)		CIC (13-23 months)	
	Count	%	Count	%
NCR	222,827	78.57%	32,836	13.44%
CAR	22,116	65.03%	1,236	4.16%
Region 1	74,391	71.28%	1,749	1.95%
Region 2	48,081	69.00%	2,090	3.46%
Region 3	180,339	70.98%	22,389	10.01%
Region 4A	175,677	52.46%	47,349	16.15%
Region 4B	41,912	58.40%	6,695	10.89%

Region	FIC (0-12 months)		CIC (13-23 months)	
	Count	%	Count	%
Region 5	75,696	51.35%	13,942	10.92%
Region 6	98,795	61.36%	9,003	6.36%
Region 7	101,260	56.31%	20,200	13.20%
Region 8	58,971	57.97%	7,169	8.17%
Region 9	50,183	54.43%	11,561	14.70%
Region 10	83,473	70.40%	9,026	8.89%
Region 11	73,480	63.22%	9,083	9.04%
Region 12	64,111	61.96%	6,959	7.81%
CARAGA	47,510	79.34%	2,283	4.40%
BARMM	63,662	43.78%	10,409	7.69%

Table 1.C.1.1. FIC and CIC, by Region, 2023

The analysis of the FHSIS five-year trend on immunization on children revealed varying coverage rates for different vaccines. Across the different antigens, coverage for DPT-HiB-HepB and IPV were generally higher than others from in the recent five years. Compared to 2019, DPT-HiB-HepB, IPV, and PCV3 coverages increased in 2023 while all other coverage declined. The increase in the coverage was particularly noticeable in PCV3, which jumped to 75.39% from 52.96% in 2019. Moreover, with the exception of MCV2 and PCV3, all antigens reached their lowest levels in the trend during 2021, coinciding with the peak of the COVID-19 pandemic, during which healthcare services were disrupted and population mobility was restricted. None of the antigens in the routine immunization reached the 95% target and OPV3 and BCG had the lowest coverages. Increasing the FIC coverage entails improvements on each of the antigens it comprises.

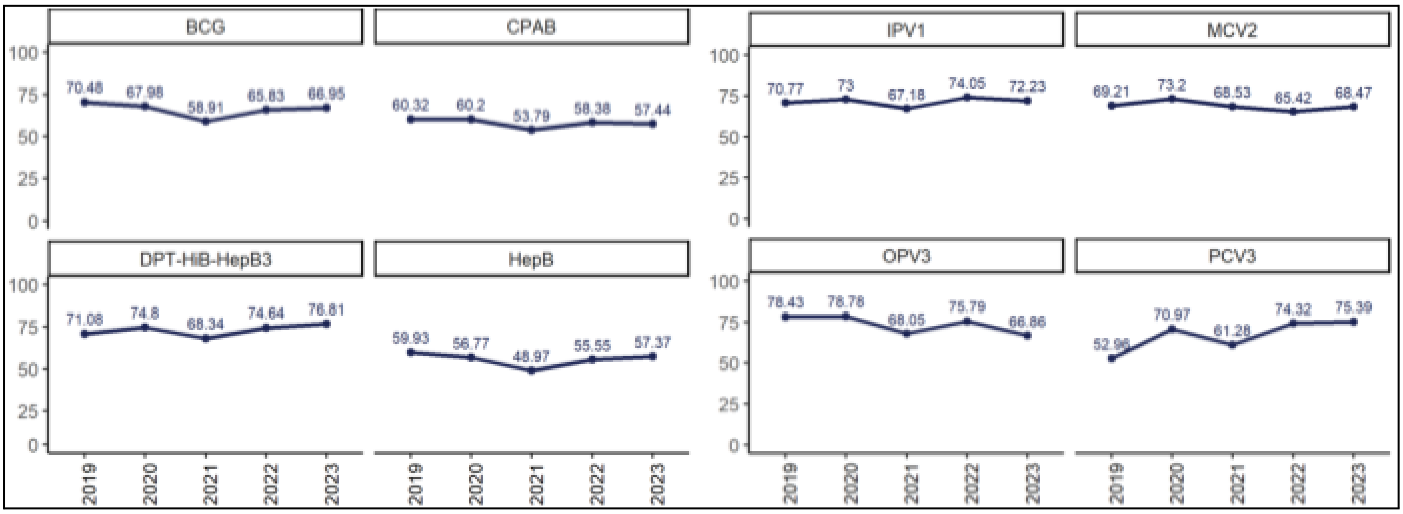

Figure 1.C.1.2. Immunization in Children, Philippines, 2019 to 2023

As shown in Table 1.C.1.2, CARAGA had the highest coverages for DPT-HiB-HepB3 (84.96%), IPV1 (80.13%), MCV2 (83.21%), and PCV3 (86.09%). Meanwhile, NCR had the highest vaccine coverage for the following antigens: BCG (81.89%), HepB (78.68%), and OPV3 (76.62%). Consequently, these two (2) regions also had the highest FIC coverage. Conversely, BARMM, Region 5, and Region 8 were consistently in the lower end of coverages across all antigens. BARMM had the lowest coverages for DPT-HiB-HepB3, OPV3, IPV1, MCV2, PCV3, as well as for CPAB (neonatal tetanus).

Overall, the data emphasizes the importance of addressing regional disparities in National Immunization Program (NIP) coverage to ensure all children in the Philippines have equitable access to vaccines. In regions with lower coverage rates, it is imperative to improve public health strategies, raise awareness, and strengthen immunization programs.

Region	BCG	HepB	DPT-HiB-HepB3	OPV3	IPV1	MCV2	PCV3	CPAB
NCR	81.89	78.68	78.59	76.62	67.53	80.18	73.09	70.04
CAR	78.15	75.86	78.15	58.17	71.56	68.85	79.41	54.18
Region 1	52.48	50.44	80.09	71.37	77.60	72.96	80.57	62.30
Region 2	70.29	69.37	79.85	65.59	74.52	72.79	80.47	71.63
Region 3	69.30	58.27	83.14	70.86	80.12	74.20	82.95	61.08
Region 4A	63.83	57.93	75.55	72.62	72.95	63.68	74.41	53.32
Region 4B	66.79	47.74	78.20	63.70	68.65	66.53	76.35	52.19
Region 5	57.13	49.61	72.29	55.00	67.92	58.46	70.08	43.72
Region 6	63.45	60.31	74.23	62.86	70.86	66.32	74.94	56.16
Region 7	69.21	54.22	75.92	59.56	71.82	66.98	75.91	54.59
Region 8	60.62	49.15	72.40	57.38	65.45	63.69	73.22	47.04
Region 9	46.90	35.55	79.74	75.60	77.21	67.55	77.59	54.42
Region 10	65.61	44.01	80.09	73.31	75.18	74.05	78.27	60.61
Region 11	77.39	68.89	78.48	75.20	76.56	68.28	77.22	69.97
Region 12	65.90	57.40	76.54	66.95	69.71	67.00	73.57	60.33
CARAGA	77.16	59.33	84.96	68.58	80.13	83.21	86.09	64.04
BARMM	64.63	41.41	62.82	42.26	63.50	50.01	59.68	39.57

Table 1.C.1.2: Immunization in Children, by Region, 2023

1.C.2 Nutrition Services for Infants and Children

Formula:

Initiated Breastfeeding

Numerator:	Number of newborns who were initiated on breastfeeding immediately after birth lasting for at least 90 minutes
Denominator:	Total number of live births

Exclusively Breastfed

Numerator:	Total number of infants age 5 months and 29 days old who have been exclusively breastfed from birth until 5th month and 1 day before the child turns 6 months
Denominator:	Eligible population under 1

Vitamin A Supplementation 6-11 months

Numerator:	Number of Infant 6-11 months old who completed 1 dose of Vitamin A
Denominator:	Eligible population 6-11 months

Vitamin A Supplementation 12-59 months

Numerator:	Number of children with ages 12-59 months old who completed 2 doses Vitamin A
Denominator:	Eligible population 12-59 months

Iron Supplementation

Numerator:	Number of preterm infants and/or infants with low birth weight (less than 2500 grams) given iron supplement starting 1 month until 3 months
Denominator:	Total number of live births with low birth weight

Breastfeeding is one key and impactful practice to ensure child health and survival. Breast milk contains all nutrients that infants need in their first months of life, promoting optimal child growth and development. Moreover, breast milk contains antibodies that can help protect infants from childhood diseases. The World Health Organization (WHO) and the United Nations International Children's Emergency Fund (UNICEF) recommend that children initiate breastfeeding within the first hour of birth and be exclusively breastfed for the first 6 months of life.

In the Philippines, the percentage of newborns initiated on breastfeeding immediately after birth varies across regions. Similar to the previous year (2022), the highest percentage was observed in Region 1 with 96.85% of newborns being breastfed immediately after birth. This was followed by CARAGA with 96.21%, and Region 12 with 93.89%. The lowest percentages were seen in Region 3 with 74.80%, BARMM with 75.74%, and Region 2 with 76.35% *(Figure 1.C.2.1)*. It is worth noting that a quarter of the total deliveries in Region 3 were Cesarean sections, which usually pose challenges in immediate breastfeeding due to the effects of anesthesia, recovery from surgery, and mothers needing assistance in holding their babies safely[3]. Trained birth attendants or health workers are necessary to overcome these challenges. In CAR, although 30% of the total deliveries were Cesarean sections, the immediate breastfeeding rate remained high at 90.35%.

For children who were exclusively breastfed up to 5th month and 29 days, Figure 1.C.2.1 shows that Region 10 has the highest percentage at 68.60% coverage, followed by CARAGA with 66.54%, and Region 11 with 65.93%. On the other hand, BARMM had the lowest percentage with 34.79%, followed by Region 4A with 40.58%, and Region 3 with 46.26%.

[3] *Selim, Leah. "Breastfeeding from the First Hour of Birth: What Works and What Hurts." UNICEF, July 31, 2018.*

These analyses revealed regional variations in both the initiation of breastfeeding and exclusive breastfeeding practices. Barriers to the implementation of both practices shall be identified to properly come up with targeted interventions that will improve breastfeeding rates, provide appropriate support, and raise awareness on the benefits of breastfeeding for newborns and infants. In addition, the data collected for initial breastfeeding immediately after birth is based on observation in facilities, whereas the data for exclusively breastfeeding is solely based on the responses of mothers during their visits to primary care facilities.

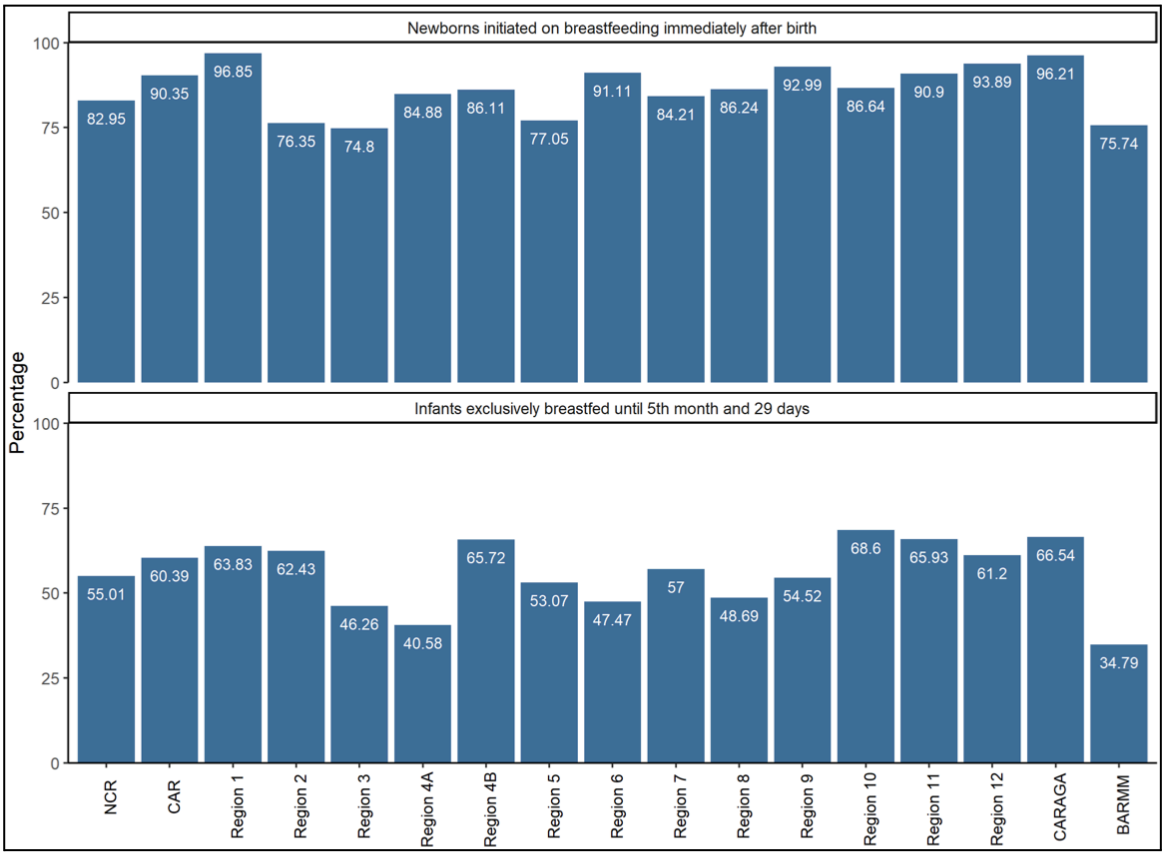

Figure 1.C.2.1. Initial Breastfeeding and Exclusive Breastfeeding, by Region, 2023

The Vitamin A deficiency is a major public health problem affecting an estimated 190 million preschool-age children, mostly from the WHO regions of Africa and South-East Asia. Infants and children have increased Vitamin A requirements to promote rapid growth and to help combat infections. Inadequate intakes of Vitamin A could lead to Vitamin A deficiency which may cause visual impairment (night blindness) or increased risk of illness and mortality from childhood infections such as measles and those causing diarrhea[4]. Meanwhile, iron, a mineral necessary to carry oxygen in hemoglobin, is an essential nutrient for development and cell growth in the immune and neural systems and in muscle metabolism. Iron deficiency in children under-two years of age can pose significant and irreversible effects on brain development, which consequently leads to negative consequences on learning and school performance later in life.

[4] *WHO Guidelines: Vitamin A supplementation in infants and children 6–59 months of age Geneva, World Health Organization, 2011*

Iron supplementation in newborns with LBW is generally low in the country. Iron supplementation was given to 23.37% of the total newborns with LBW in 2023, which was already a 9.51% increase from previous year's coverage. Regionally, the highest coverage was in Region 12 with only 58.91% of LBW infants provided with iron, while BARMM had the lowest coverage of 10.01%. The data shows that Vitamin A supplementation for infants aged 6 to 11 months was high and had a coverage of over 100% in almost all regions, except for Region 1. It is important to note that the denominator used to calculate coverage is an estimate and may not precisely reflect the actual number of eligible children. On the other hand, Vitamin A supplementation in children aged 12 to 59 months was lower, only ranging from 45.25% in Region 9 to 101.8% in Region 11 *(Figure 1.C.2.2)*.

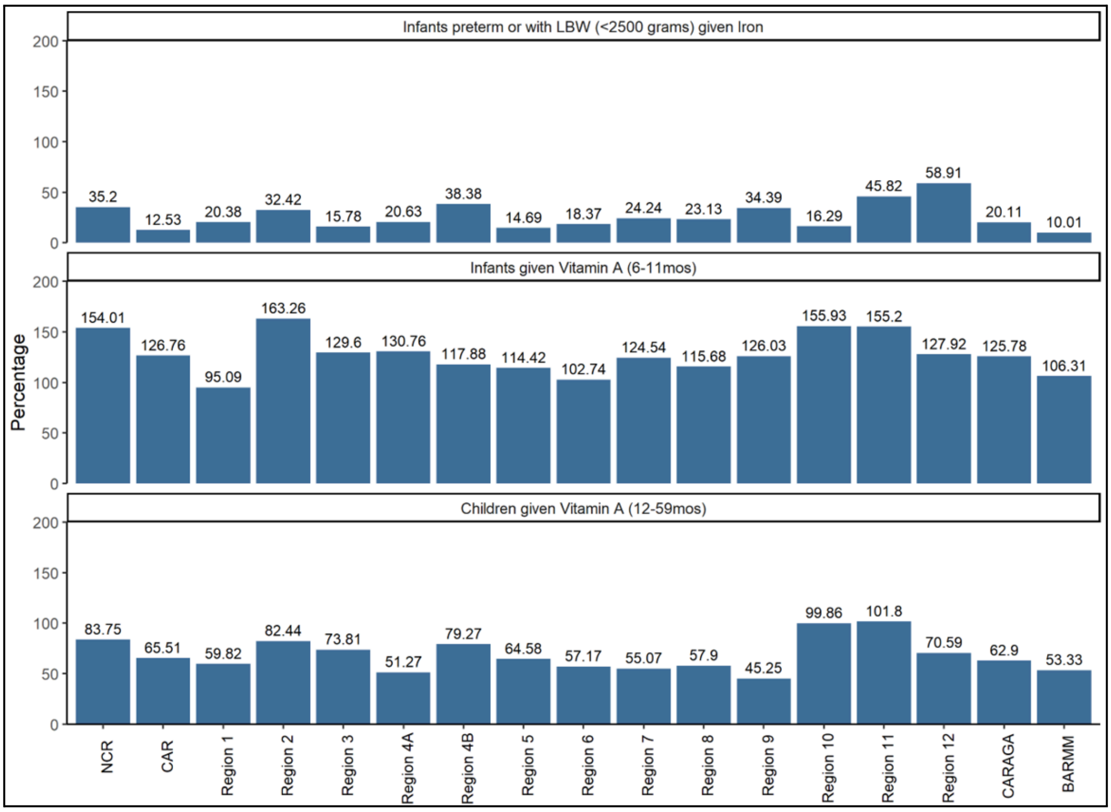

Figure 1.C.2.2. Supplementation in Children, by Region, 2023

1.C.3 Management of Sick Infants and Children

Formula:

Diarrhea Cases who received Oral Rehydration Salt Solution (ORS) and ORS with Zinc

Numerator: Number of diarrhea cases 0-59 months old who received ORS or ORS with Zinc

Denominator: Number of acute diarrhea cases 0-59 months old seen

Pneumonia cases and received treatment

Numerator: Number of pneumonia cases 0-59 months old who received treatment

Denominator: Number of pneumonia cases 0-59 months old seen

Children with Measles and/or Persistent Diarrhea who received Vitamin A capsule

Numerator: Number of sick infants who received Vitamin A capsule aside from routine supplementation (6-11 and 12-59 months)

Denominator: Total number of sick infants seen (6-11 and 12-59 months)

Globally, over 80% of the under five deaths are due to neonatal conditions and infectious diseases like pneumonia, diarrhea, malaria, measles and meningitis, often compounded by malnutrition. Most childhood deaths can be prevented with effective interventions that are feasible for implementation, even in resource constrained settings[5]. Management of sick children is a cost-effective health intervention. In that regard, the WHO and UNICEF designed the Integrated Management of Childhood Illness (IMCI), which aims to strengthen prevention and management of common childhood illnesses and support children's healthy growth and development. The DOH Administrative Order No. 119 s. 2003 dated 2 December 2003, "Updated Guidelines on Micronutrient Supplementation (Vitamin A, Iron, and Iodine)", provides for the supplementation of Vitamin A to high risk children to help reestablish body reserves drained by chronic or repeated infectious diseases and protects the children against the severity of subsequent infection.

The percentage of sick infants aged 6 to 11 months given 100,000 IU of Vitamin A has further decreased to 48.69% in 2023, from 51.06% in 2022. Similarly, the proportion of sick infants aged 12 to 59 months given 200,000 IU of Vitamin A marginally decreased from 43.94% in 2022 to 43.2% in 2023. The percentage of children with diarrhea aged 0-59 months old that were given ORS or ORS with Zinc also decreased, but the decrease in the proportion of children with diarrhea given ORS was steep from 63.16% in 2022 to only 44.25% in 2023. The coverage for pneumonia treatment was consistently high at 97.30%*(Figure 1.C.3.1)*.

[5] *"Integrated Management of Childhood Illness." World Health Organization, n.d.*

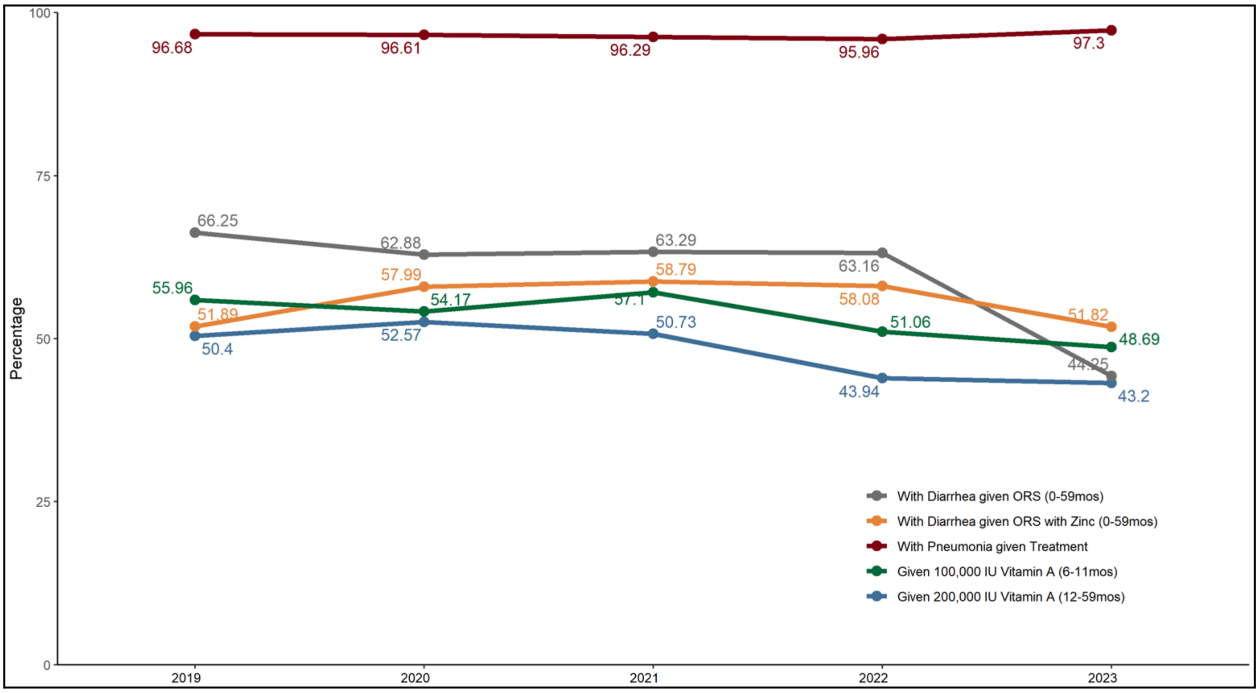

Figure 1.C.3.1. Management of Sick Children, Philippines, 2019 to 2023

There are noticeable differences in how essential healthcare services for children are provided in different regions. For Vitamin A (both 1000IU and 2000IU), Region 4A and Region 4B had the lowest coverages in the country ranging from 21% to 35%, while Region 1 and Region 7 had the highest coverages at 68% to 81%. For the provision of ORS and ORS with Zinc to children with diarrhea, Region 12 recorded the lowest coverage of 12.93% and 5.86%, respectively *(Figure 1.C.3.2)*. These variations implicate the presence of gaps in access to healthcare, available resources, and the quality of infrastructure across regions. Meanwhile, the provision of pneumonia treatment was more consistent across regions, from 92% in Region 4B to 99% in NCR *(Figure 1.C.3.2)*.

These findings highlight the need for sustained efforts to improve provision of Vitamin A and ORS to sick infants and to continue the successful service provision of treatment to children with pneumonia. Moreover, understanding the reasons behind these regional variations in service delivery would help in designing interventions. Factors like availability and access to healthcare resources, limitations in health infrastructure, and variations in regional healthcare strategies could all be contributing factors. It is crucial to conduct further assessments to identify the specific reasons behind these variations and to implement the best practices from well-performing regions.

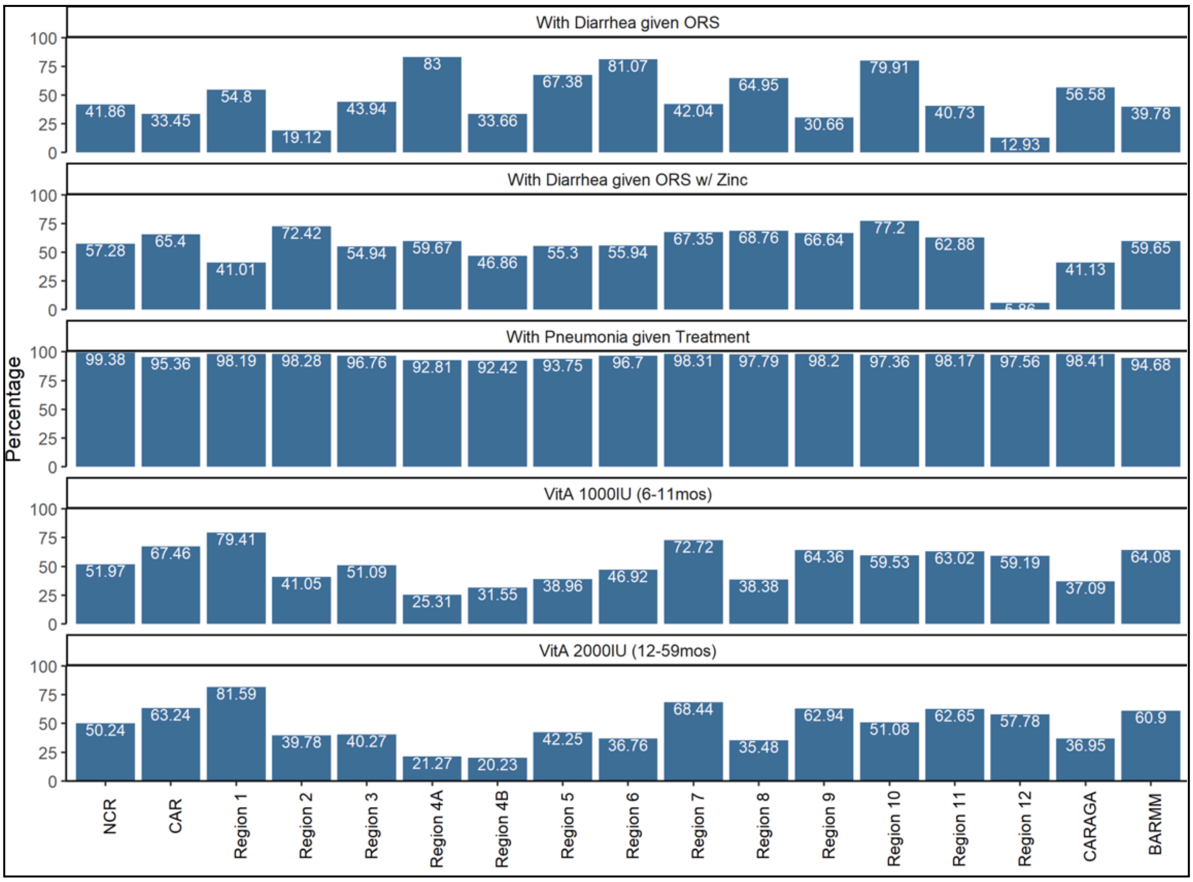

Figure 1.C.3.2: Management of Sick Children, by Region, 2023

1.C.4 Child Mortality

Article 24 of the United Nations Convention on the Rights of the Child recognizes that no child shall be deprived of his or her right of access to health care services, and that all States shall take appropriate measures to diminish infant and child mortality[6]. Consequently, the SDGs aim to end preventable deaths in children under-five years of age globally by 2030. Specifically, the under-five mortality rate is targeted to be lowered to 25 deaths per 1,000 live births, while neonatal mortality is aimed to be as low as 12 deaths per 1,000 live births.

Monitoring mortality in children is essential in assessing the effectiveness of the existing child care programs. As the health of the child is heavily dependent on the mother's health, indicators of child mortality may also be linked to maternal health care programs. This chapter presents data on under-five mortality, as well as infant (under 1 year old), neonatal (0 to 28 days), and perinatal mortality. Note, however, that the official deaths statistics shall come from the PSA and numbers in this chapter were from reports submitted in the FHSIS.

[6]How the Convention on the Rights of the Child works, UNICEF

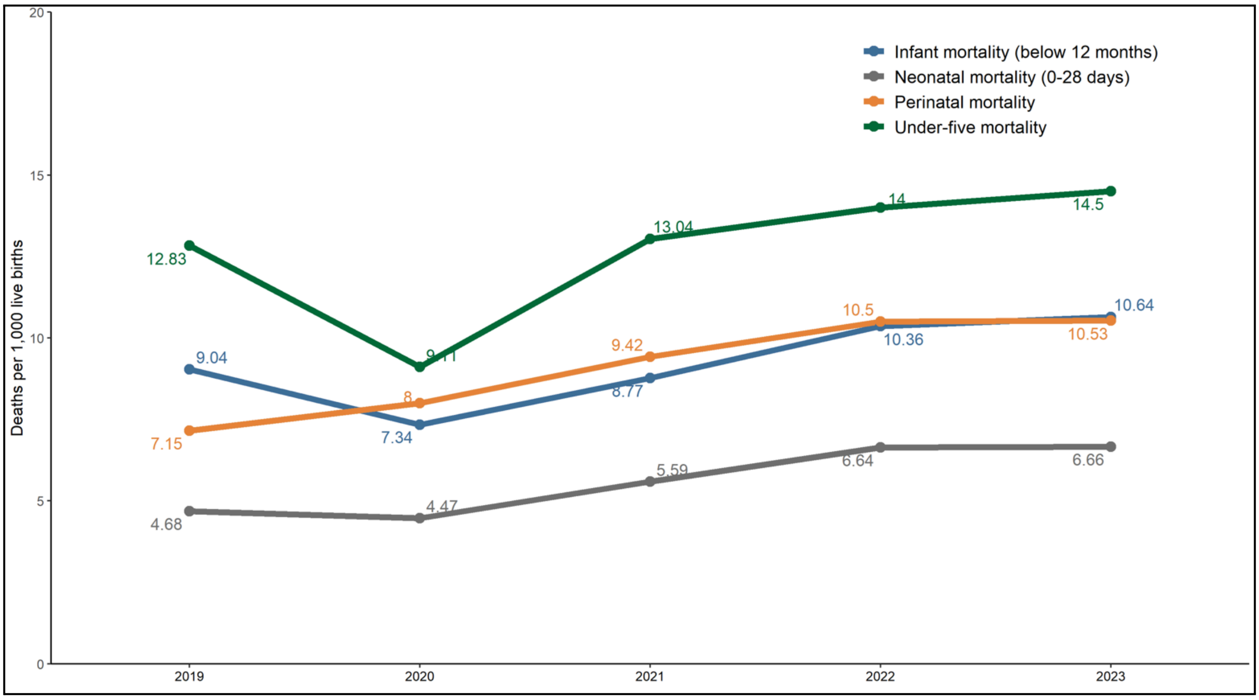

Figure 1.C.4.1. Child Mortality, Philippines, 2019 to 2023

Nationally, the child mortality rates computed from deaths and live births reported in the FHSIS have been increasing since 2021. Perinatal mortality, which includes stillbirths and early neonatal deaths, has been steadily increasing since 2019, while neonatal mortality, infant mortality, and under-five mortality, all have the same trend where a drop was observed in 2020, but has increased thereafter.

In 2023, there were 20,689 deaths among the under-five population, which was 905 deaths higher than reported in 2022. This translates to an under-five mortality rate of 14.50 deaths per 1,000 live births, from 14.00 in the previous year *(Figure 1.C.4.1)*. While this rate is already below the SDG target, it is important to note that this is based on mortality data collected by the municipal and city health offices. Thus, this information may be different from the reports of the Philippine Statistics Authority (PSA) which is the government agency mandated to release official data on vital statistics. Nonetheless, monitoring this in the FHSIS is beneficial in the assessment of the programs being implemented by the government, as well as for planning and resource allocation.

Furthermore, 15,186 deaths of the total under-five deaths were among infants aged below 1 year old, which corresponds to an infant mortality ratio of 10.64 deaths per 1,000 live births *(Table 1.C.4.1, Figure 1.C.4.1)*. Further disaggregation shows that 9,496 of these deaths occurred in the neonatal period or the first 28 days of life. In summary, the majority or approximately 7 out of 10 under-five deaths happen before the child reaches 1 year old, while roughly 4 out of 10 under-five deaths occur in the neonatal period *(Table 1.C.4.1)*.

Regional data showed that under-five mortality ratios range from as low as 7.43 deaths to 25.70 deaths for every 1,000 live births. Region 1 with 1,298 under-five deaths recorded the highest mortality rate. Meanwhile, Region 2 had the lowest number of under-five deaths (277 deaths) and BARMM had the lowest under-five mortality rate (7.43 deaths per 1,000 live births) *(Figure 1.C.4.2)*. Of note was Region 9 which registered an under-five mortality rate of 8.01 deaths per 1,000 live births which was roughly five times its reported mortality rate of 1.57 in 2022. CARAGA, despite the minute change in its mortality rate, reported twice its reported under-five deaths in 2022 (634 deaths in 2023 vs. 309 deaths in 2022).

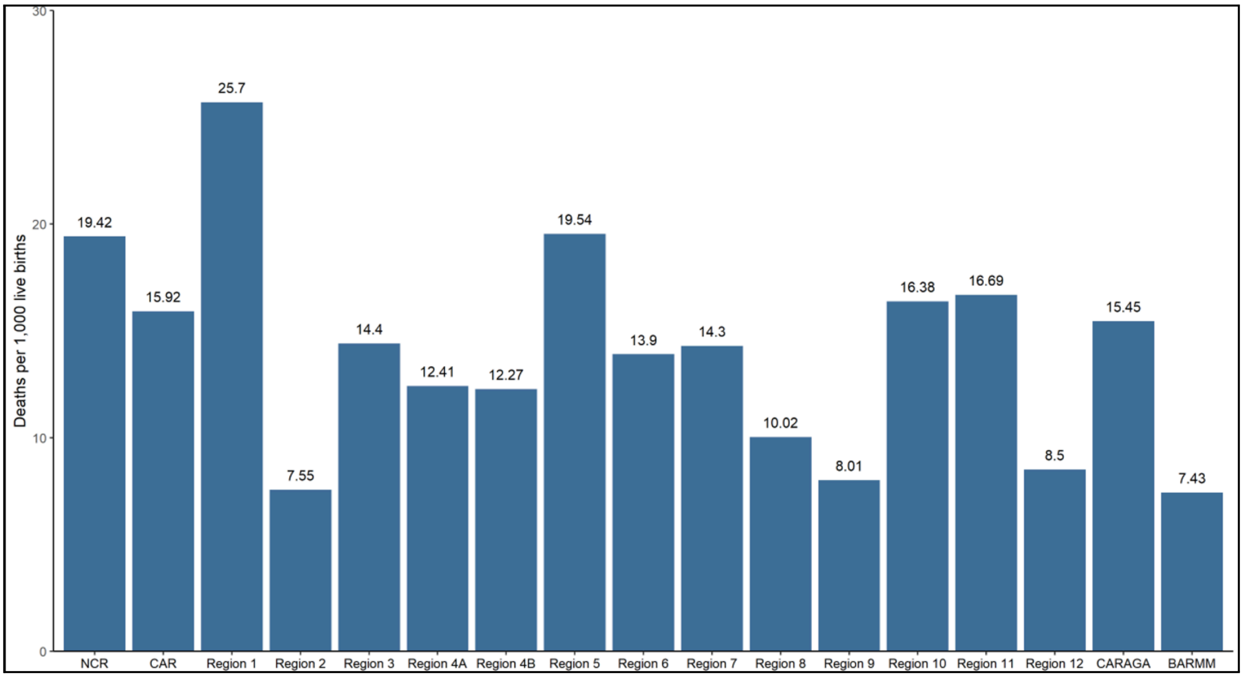

Table 1.C.4.2. Under-five Mortality Rate, by Region, 2023

Evidently, at least half of the under-five deaths in all regions were infant deaths or deaths of children less than 1 year old. In CAR, Region 1, and Region 11, approximately 8 out of 10 under-five deaths were among the under 1 age group. Furthermore, in CAR and Region 1, 6 out of 10 under-five deaths were among neonates or infants aged 0 to 28 days. Although this report does not include data on the causes of deaths, previous data from the PSA suggest that most infant deaths were due to conditions originating in the perinatal period, infectious and parasitic diseases, and diseases of the respiratory system. These conditions are often preventable, thus the need to strengthen relevant maternal and child care services, including nutrition and immunization. Furthermore, literature shows that in most economies, a child has a high chance of reaching their fifth birthday if he or she survives the first year of life[7].

These findings highlight the need for continued attention and efforts to address child mortality in the Philippines. It is crucial to understand the underlying factors contributing to these deaths, implement targeted interventions to reduce child mortality and improve the well-being of children in the country. Further analysis and consideration of other factors, such as causes of deaths, access to health care, and socio-economic conditions, would provide a more comprehensive understanding of the situation and guide effective strategies to end preventable deaths in children.

[7] *Key Indicators for Asia and the Pacific 2009, Asian Development Bank, 2009*

Region	Under-five (U5) deaths	Infant deaths		Neonatal deaths	
		Count	% of U5 deaths	Count	% of U5 deaths
PHILIPPINES	20,689	15,186	73.40%	9,496	45.90%
NCR	3,557	2,737	76.95%	1,626	45.71%
CAR	395	325	82.28%	237	60.00%
Region 1	1,298	1,072	82.59%	820	63.17%
Region 2	277	137	49.46%	88	31.77%
Region 3	2,190	1,647	75.21%	1,036	47.31%
Region 4A	2,508	1,738	69.30%	1,014	40.43%
Region 4B	587	421	71.72%	277	47.19%
Region 5	1,748	1,238	70.82%	795	45.48%
Region 6	1,343	974	72.52%	631	46.98%
Region 7	1,616	1,221	75.56%	695	43.01%
Region 8	600	429	71.50%	218	36.33%
Region 9	383	212	55.35%	129	33.68%
Region 10	1,171	862	73.61%	510	43.55%
Region 11	1,355	1,092	80.59%	758	55.94%
Region 12	544	388	71.32%	249	45.77%
CARAGA	634	391	61.67%	245	38.64%
BARMM	483	302	62.53%	168	34.78%

Table 1.C.4.1. Under-five, Infant, and Neonatal Deaths, by Region, 2023

1.D Oral Health Care and Services

2023 KEY FINDINGS

Basic Oral Health Care (BOHC) Coverage Among Specified Age Groups

Age Group	BOHC Coverage
Infants 0-11 months old	64.07%
Children 1-4 years old	50.74%
School-Aged Children 5-9 years old	33.18%
Adolescents 10-19 years old	23.09%
Adults 20-59 years old	11.00%
Senior Citizens 60 years old and above	18.95%

BOHC Coverage Among Pregnant Women

Age Group	BOHC Coverage
Pregnant Women 10-14 years old	0.31%
Pregnant Women 15-19 years old	3.41%
Pregnant Women 20-49 years old	19.30%

Formula:

BOHC proportion specified age groups

Numerator: Total number of clients per specified age groups who received BOHC

Denominator: Eligible population of the specified age group x 30% (service target)

BOHC proportion among pregnant women

Numerator: Total number of pregnant women in specified age groups who received BOHC

Denominator: Eligible population of under 1 year old

Provision of affordable, quality, and comprehensive health services to encourage Filipinos to obtain oral health services was one of the goals of the enactment of Republic Act 11223, or the Universal Health Care (UHC) Act. Good oral health is fundamental to the overall health and well-being of Filipinos. In 2007, the Department of Health issued Administrative Order 2007-0007 providing for Guidelines in the Implementation of Oral Health Program for Public Health Services. The program provides preventive, curative, and promotive dental health care to Filipinos through a lifecycle approach. A package of essential basic oral health care (BOHC) was established to provide a continuum of quality care. FHSIS monitors and tracks the provision of BOHC at the RHU/MHC levels which are targeted at 100% among the target population of the specified age groups and 50% of the target population among pregnant women. Preventive oral health habits, such as BOHC, when developed early in life can lead to better overall oral health throughout a person's life.

This chapter presents data on the proportion of clients provided with BOHC across different lifecycle stages including during pregnancy. The indicators on Oral Health Care in the FHSIS aim to track routine BOHC services provided in the public Primary Care Facilities (PCF), which do not include dental missions and other similar activities to measure the outcome of total health care for planning and evaluation. Routine BOHC services in private facilities are also excluded in this report.

Nationally, across different lifecycle stages, the trend of BOHC coverage was generally increasing from 2019 to 2023. The BOHC coverage among infants remained the highest from 2019 to 2023. Between 2019 and 2023, BOHC coverage among infants increased from 20.86% to 64.07%. However, rates of progress for all lifecycle stages of BOHC coverage would need to accelerate to achieve the targets of 100% among the target population (30% of the estimated age group population) and 50% of the pregnant women target population.

In 2023, the highest BOHC coverage was among 0-11 months old infants (64.07% of the service target) and lowest among adults 20-59 years old (11.00% of the target). The low BOHC coverage in this age group may be explained by the fact that this is composed of the workforce population, and thus has less time to avail of the services. A strategy to possibly increase the coverage among this age group is to collaborate with labor agencies in the government and private sectors to advocate BOHC among the workforce, highlighting that BOHC services are provided free of charge in public facilities. Additionally, the BOHC coverage among adolescents (10-19 years old) was 23.05% and among senior citizens (60 years old and above) was 18.95% of the target population *(Figure 1.D.1)*. Oral health problems among the senior citizens are imminent with the low coverage of BOHC among this group. There is a need to develop effective strategies to encourage elderly Filipinos to avail the BOHC services since there is an increased risk of some oral health problems among the elderly as teeth become weaker with age.

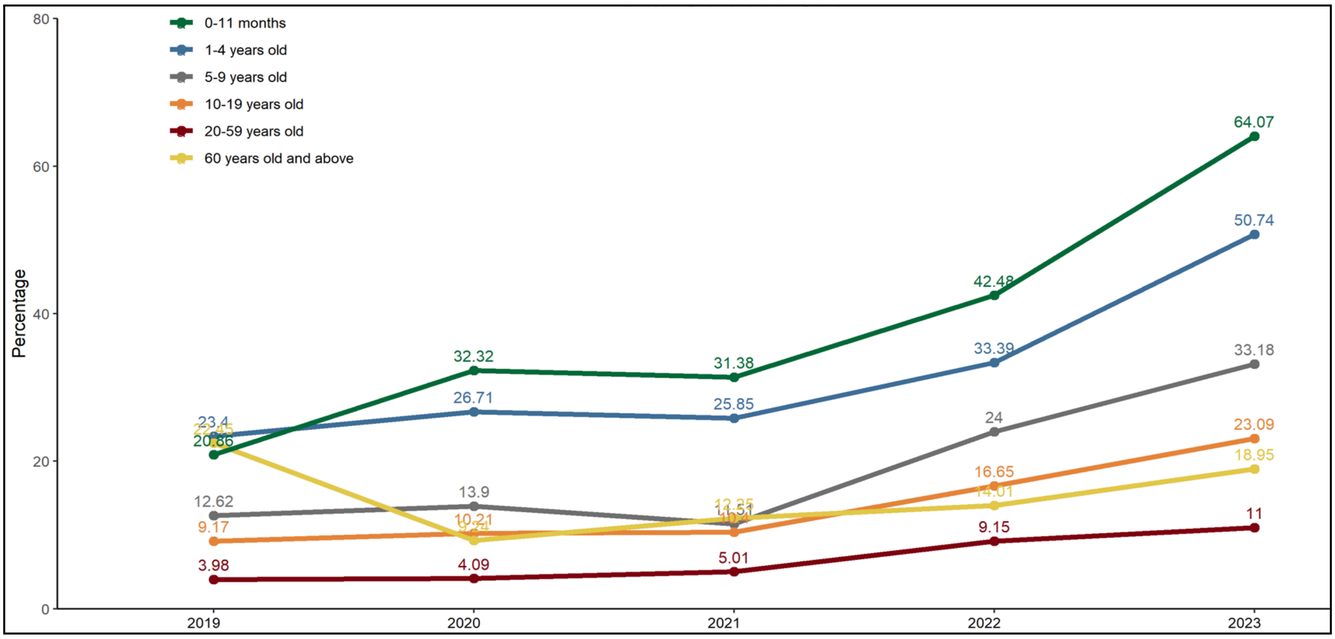

Figure 1.D.1: BOHC Coverage by Age Group, Philippines, 2019-2023

Likewise, the 23.02% BOHC coverage among pregnant women aged 10-49 years old was only almost half of the 50% target coverage *(Figure 1.D.2)*. The reported coverage may pose a public health concern as poor oral health during pregnancy can lead to poor health outcomes for mothers and their babies since pregnancy may make women more prone to oral diseases. Advocacy campaigns should be strengthened to encourage Filipinos to practice good oral health care and avail of the BOHC services for every lifecycle stage, starting from infancy to old age, including during pregnancy.

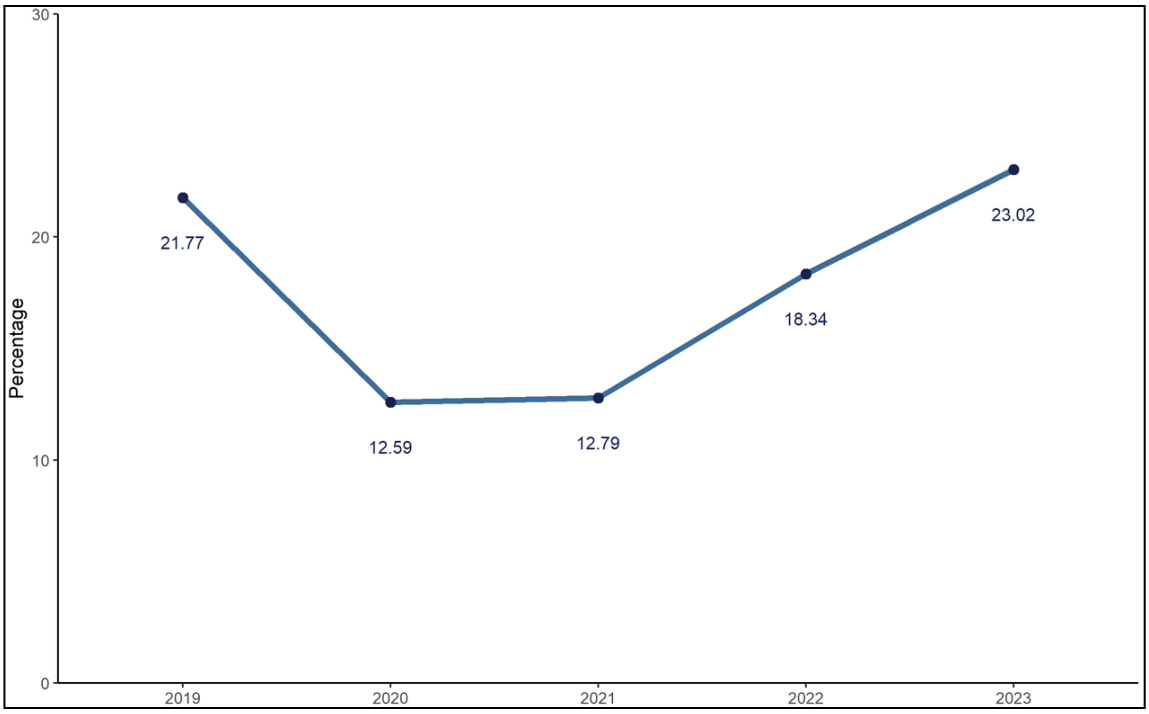

Figure 1.D.2: BOHC Coverage among Pregnant Women (10-49yo), Philippines, 2019-2023

In 2023, regional data showed that BOHC coverage among infants is highest in NCR (164.23%) and lowest in Region 4B (12.32%) *[Figure 1.D.3]*. Among the 17 regions, 11 regions (NCR, CAR, 1, 2, 4A, 6, 7, 10, 12, CARAGA, and BARMM) had at least one province/city with 100% BOHC coverage, these have reached and gone beyond the set service target of 30% of their total estimated population. However, there were also provinces/cities with less than 50% coverage and these areas have to exert more efforts to advocate for BOHC, especially among infants as this would impact the overall oral health throughout their life.

For children aged 1-4 years old, NCR also had the highest BOHC coverage of 85.81% and lowest in Region 4B with 21.50% coverage. In school-aged children (5-9 years old), BOHC coverage was highest in CAR (81.39%) and lowest again in Region 9 (13.51%) *[Figure 1.D.3]*. Among adolescents (10-19 years old), BOHC coverage was highest in NCR (62.71%) and lowest in Region 9 (6.36%) *[Figure 1.D.4]*.

Among adults aged 20-59 years old, BOHC was availed by 24.17% of the service target population in CAR (highest) and only 4.17% in Region 11 (lowest) *[Figure 1.D.4]*. BOHC coverage among the elderly (60 years old and above) was highest in BARMM (50.96%) and lowest in Region 11 (3.56%) *[Figure 1.D.5]*. Lastly, the vulnerable group of pregnant women (10-49 years old) had the highest BOHC coverage in NCR at 49.30% and lowest in Region 9 (6.12%).

Overall, regional data in 2023 showed that all regions still had a lot to do to achieve the 100% BOHC target coverage particularly Region 4B with the lowest coverage reported among infants and children aged 1-4 years old, Region 9 among adolescents, adults, and pregnant women, and Region 11 among the elderly.

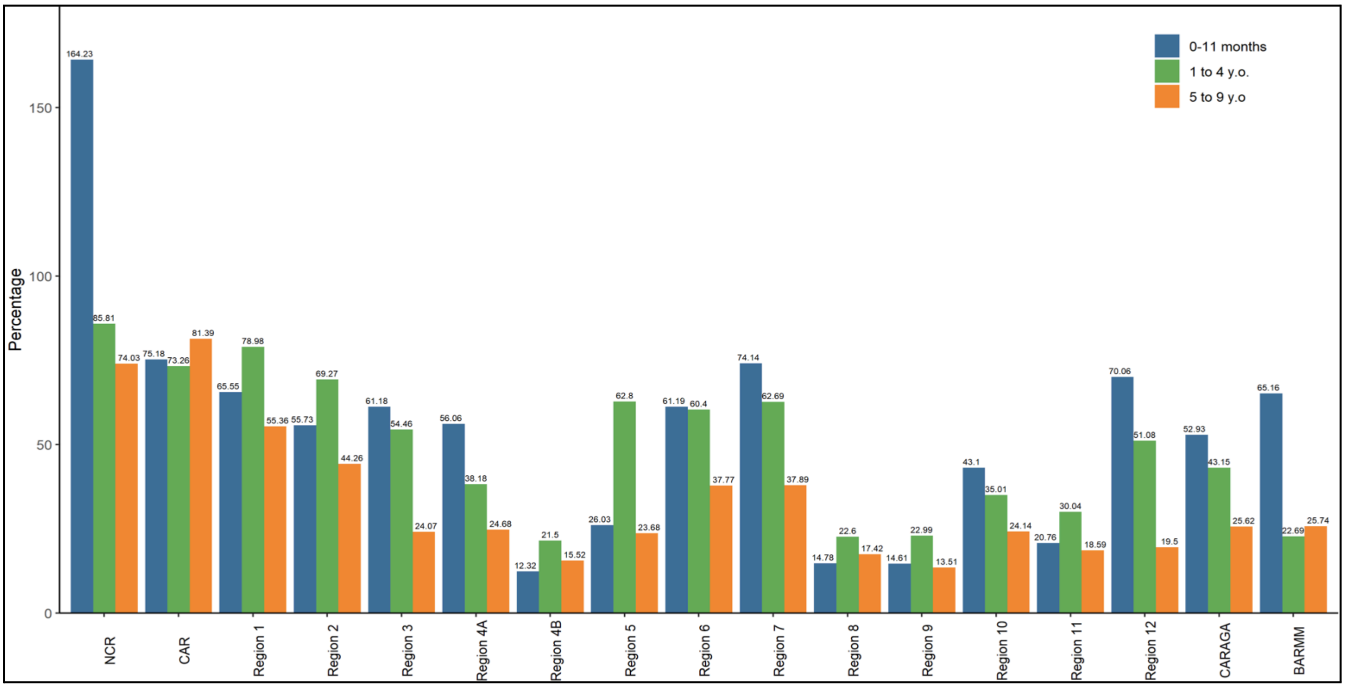

Figure 1.D.3: BOHC Coverage among Infants and Children, by Region, 2023

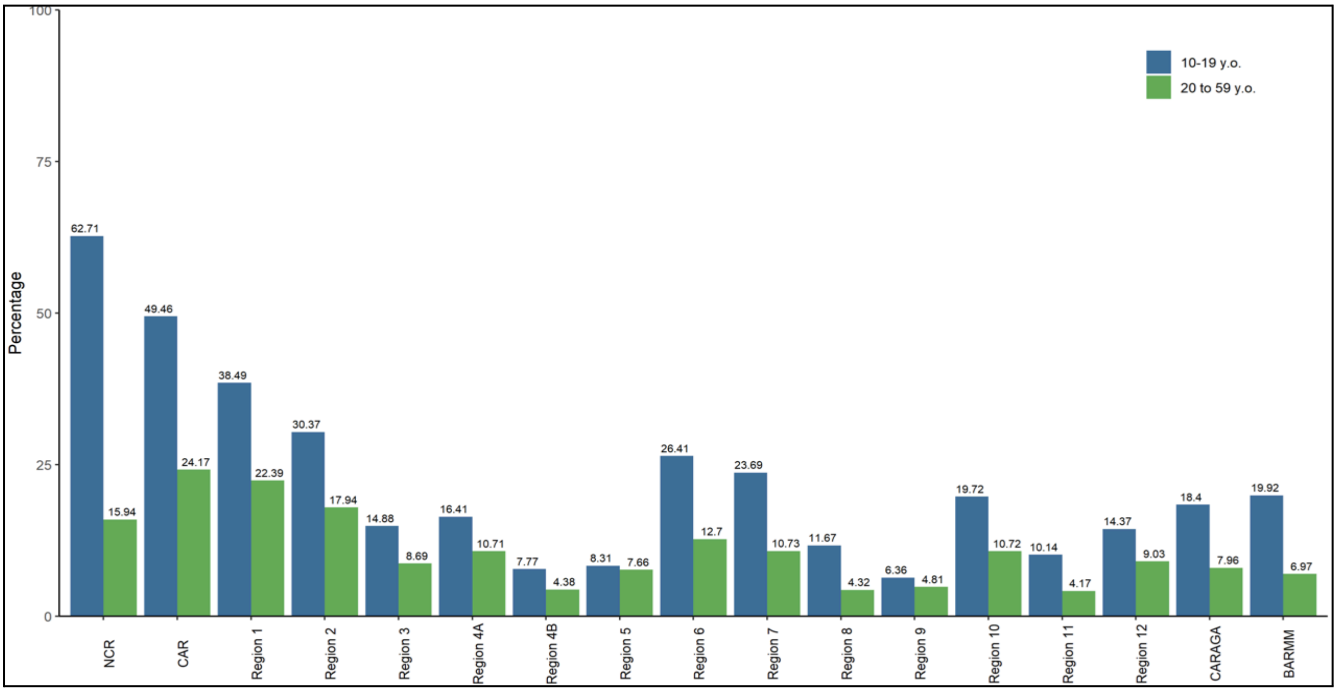

Figure 1.D.4: BOHC Coverage among Adolescents and Adults, by Region, 2023

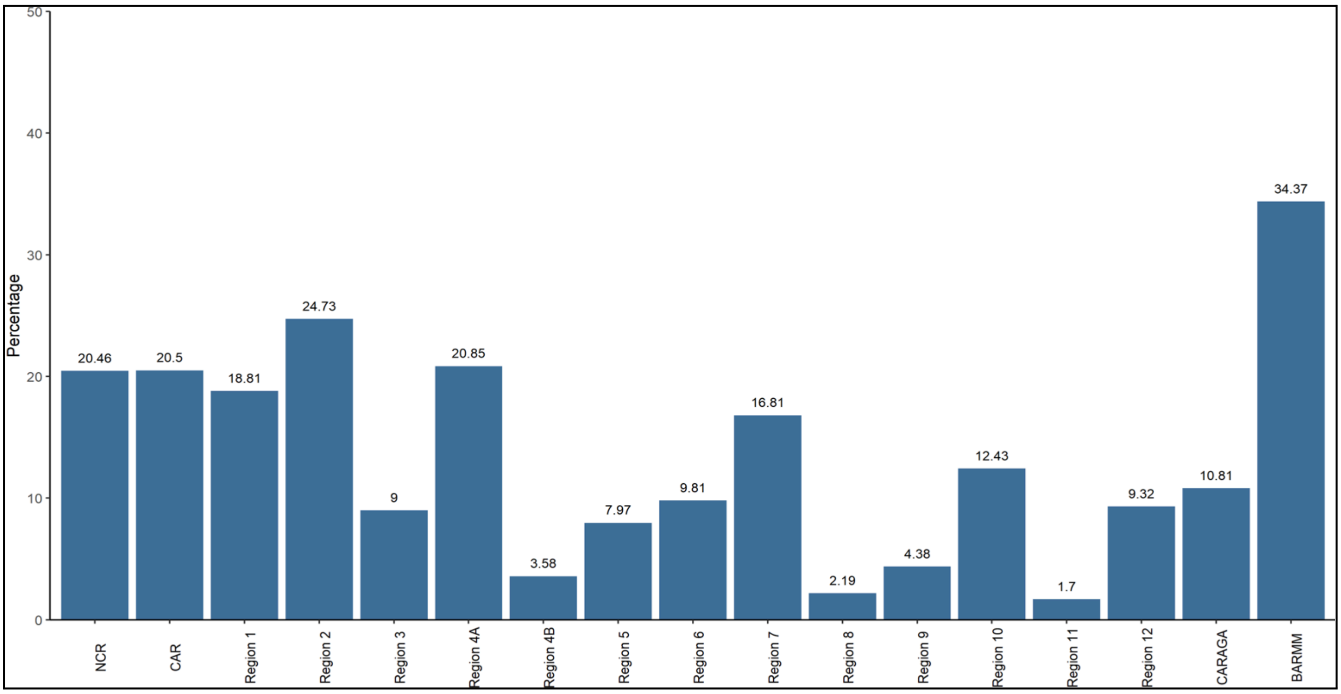

Figure 1.D.5: BOHC Coverage among Senior 60 years old and above , by Region, 2023

1.E Non Communicable Disease Prevention And Control Services

2023 KEY FINDINGS

Hypertension & Diabetes Mellitus Type 2

20.90%
20 years old and above adults were assessed using PhilPEN protocol

13.44%
identified hypertensives among risk-assessed

2.92%
identified with Diabetes Mellitus Type 2 among risk-assessed

Vaccination in Senior Citizens

15.05%
of elderlies received one dose of Influenza Vaccine

6.99%
of elderlies received one dose of Pneumococcal Polysaccharide Vaccine (PPV)

Cancer Prevention and Control

0.60%
of women aged 20 years and above were screened for Cervical Cancer

2.50%
of women aged 20 years and above were screened for Breast Mass

2.31%
of women who were screened were found positive for or suspected with Cervical Cancer

1.48%
of women who were screened were found with suspicious breast mass

Noncommunicable diseases (NCDs), also known as chronic diseases, tend to be of long duration and are the result of a combination of genetic, physiological, environmental, and behavioral factors. The rise of NCDs, including Cardiovascular Diseases (CVDs), is largely attributed to the high prevalence of risk factors – namely smoking, unhealthy diet, physical inactivity, alcohol use, and obesity. Detection, screening, and treatment of NCDs, as well as palliative care, are key components of the response to NCDs[8]. The Department of Health (DOH) Administrative Order (AO) No. 2012-0029 dated 04 December 2012 prescribes the implementing guidelines on the institutionalization of the Philippine Package of Essential NCD Interventions (PhilPEN). PhilPEN is a protocol for the Integrated Management of Hypertension and Diabetes in primary healthcare settings adapted from the WHOPEN. Specifically, the AO mandates the Local Government Units (LGUs) to adopt and implement the PhilPEN and provide services and products in primary healthcare facilities and hospitals in their localities.

Likewise, the most cost-effective long-term strategy for the control of cancer is prevention. Among women, cervical and breast cancers are the most preventable types of cancer. Regular screening can help prevent cervical and breast cancers or identify the conditions early when they are most treatable.

On the other hand, the elderly population is especially vulnerable to developing serious complications from diseases that are vaccine preventable such as influenza and pneumococcal infections due to age-related impairment of the immune system which is worsened by decreased physical activity and poor nutrition. In recognition of this, the Republic Act 9994 or the “Expanded Senior Citizens Act,” was signed,mandating the DOH to provide free influenza and pneumococcal immunization for indigent senior citizens.

The FHSIS monitors indicators on the NCD Program including the adoption of PhilPEN which reflects the ability of the local health system in integrating risk assessment in the routine delivery of health care and properly classifying those who have found to have risk. Early identification of risk factors among the 20 years old and above population could lead to responsive or appropriate preventive measures[9]. Further, for the elderly, FHSIS tracks the provision of Pneumococcal Polysaccharide Vaccine (PPV) and Influenza vaccine.

NCD indicators were included in the FHSIS monitoring only in 2019 and data from 2019 to 2023 showed a steady but slow incremental increase in the percentage of those risk-assessed among 20 years old and above Filipinos. In 2023, the proportion of those risk-assessed using the PhilPEN protocol among the target population (20.9%) was still much lower than the 70% target. However, it has doubled from the previous year’s coverage of 10.57%. Among those risk-assessed, 13.44% were identified as hypertensives and 2.92% were classified with Diabetes Mellitus Type 2.

[8] Noncommunicable diseases Fact Sheet, - World Health Organization (WHO), 2022
[9] Field Health Services Information System MOP version 2018

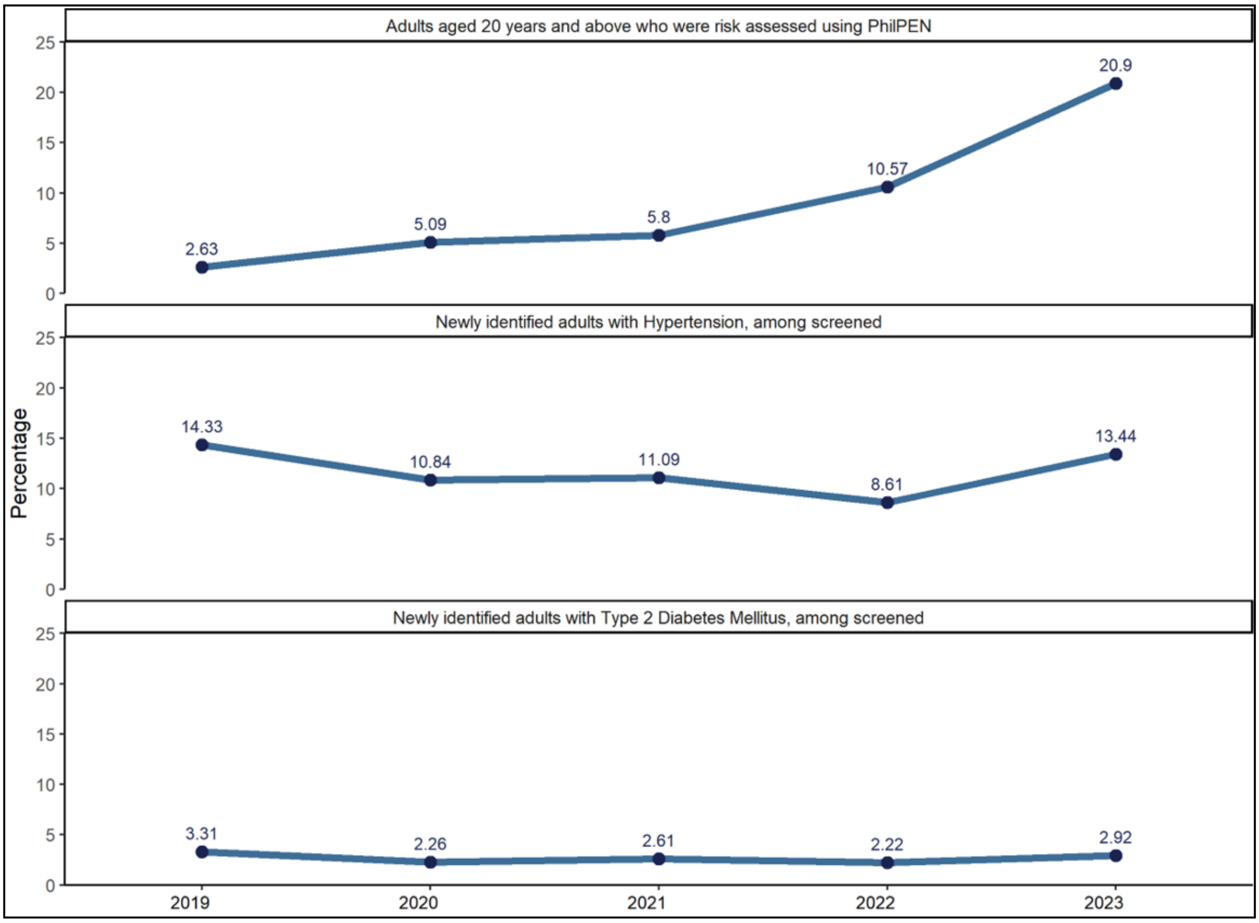

Figure 1.E.1. Screening for Hypertension and Diabetes Mellitus 2 using the PhilPEN Protocol, Philippines, 2019 to 2023

For the Cancer Prevention and Control Program, screening for cervical cancer using Visual Inspection with Acetic Acid (VIA) or Pap Smear among 20 years old and above women was at 0.71% in 2019 and dropped to 0.12% in 2020 then steadily increased in 2021, 2022 and 2023 at 0.22%, 0.39%, and 0.60% respectively *(Figure 1.E.2)*. While the screening increased by 53.85% from 2022, it remained extremely low and did not meet the 70% target. Among those screened, 2.31% were found positive or suspected for cervical cancer, which was a 43.10% decrease from 2022.

Similarly, breast cancer screening in the same target population was at 0.91% in 2019, and further decreased to 0.57% in 2020 and 0.54% in 2021. In 2022 it was registered at 1.21% and doubled at 2.5% in 2023 which is still below the 70% target. Among those screened in 2023, there were 1.48% detected with a suspicious breast mass which was a 11.90% decrease from 2022. Health promotion activities on the importance and availability of screening for cervical and breast cancer need to be continuously strengthened to break possible barriers such as lack of knowledge, fear, and poor health-seeking behavior among others that could be preventing the target population from having the screening.

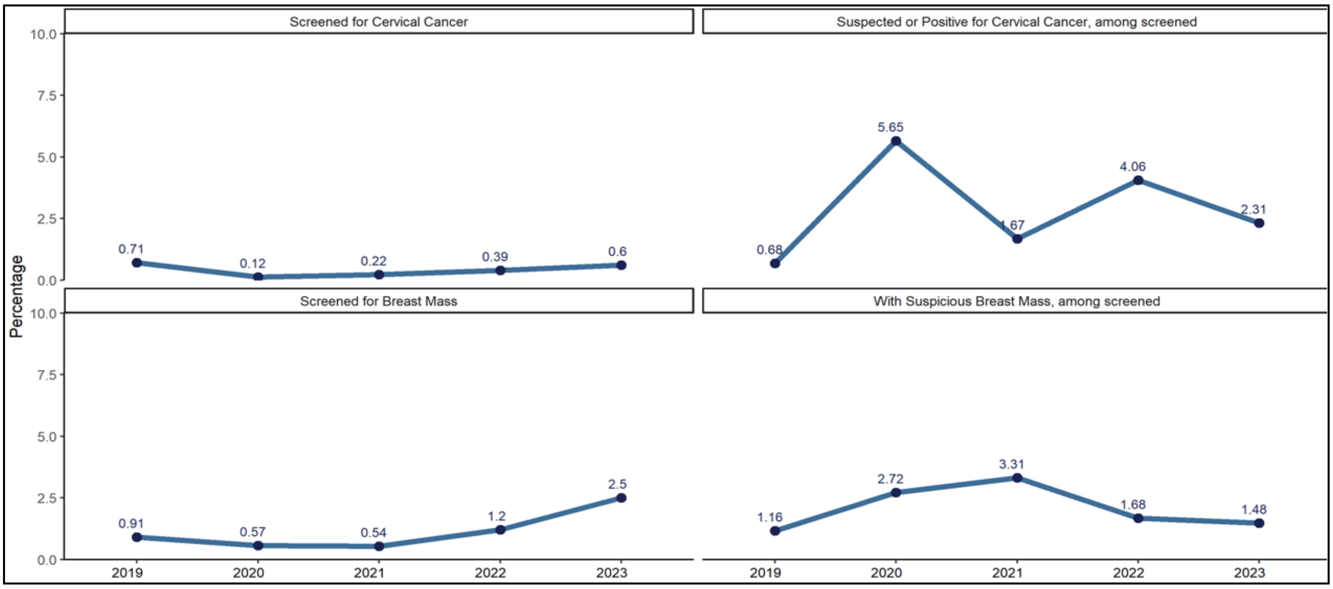

Figure 1.E.2. Screening for Cervical Cancer and Breast Mass, Philippines, 2019 to 2023

In 2019, RA No. 11215 or the National Integrated Cancer Control Act was enacted. The Act backs the establishment of the National Integrated Cancer Control Program aiming to decrease the overall mortality and impact of all adult and child cancer and lessen the incidence of preventable cancer in adults and children by scaling up essential programs and increasing investments for the prevention of cancer, better screening, prompt and accurate diagnosis, timely and optimal treatment among others.

The Immunization Program for senior citizens (60 years old and above) for PPV showed an unstable trend from 2019 to 2022, but leaped to 6.99% in 2023 from 1.78% in 2022. Further, for Influenza vaccine a gradual increase was noted from 2019 at 2.64% to 8.81% in 2022, but almost doubled in 2023 at 15.05%.

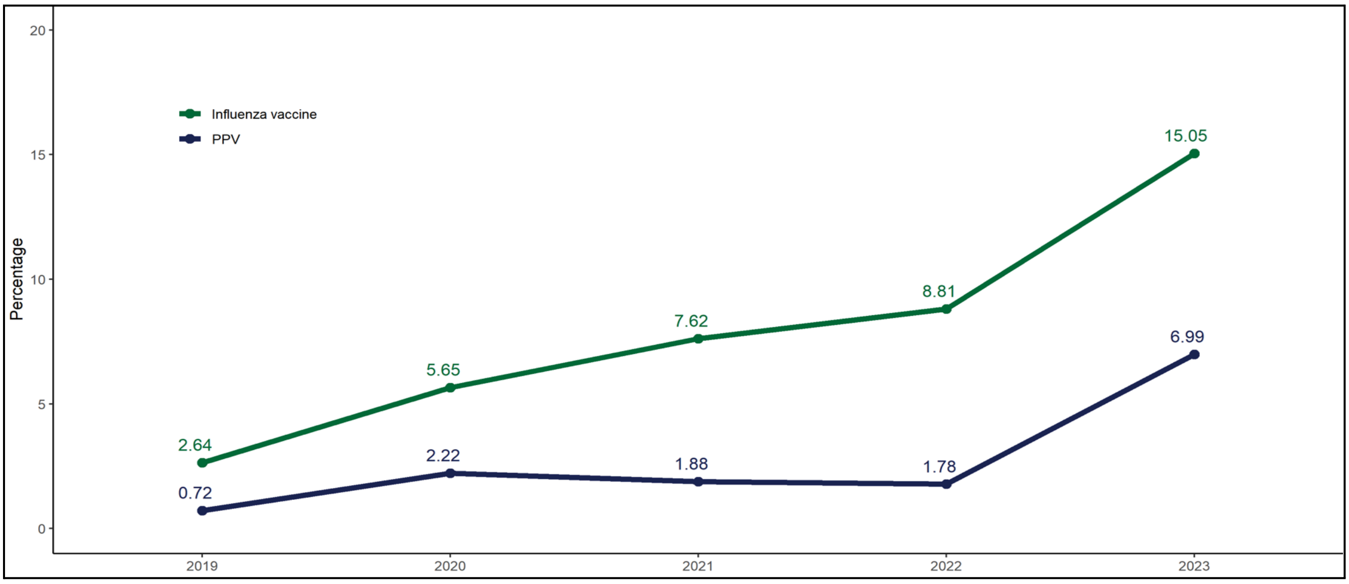

Figure 1.E.3. PPV and Influenza Vaccination in Senior Citizens, Philippines, 2019 to 2023

For risk assessment of adults aged 20 years and above using the PhilPEN protocol, FHSIS reports showed that none of the 17 regions met the 70% target in 2023. However, Region 5 nearly reached the target with 69.78% which was the highest registered coverage, while Region 4A had the lowest coverage at 6.76%.

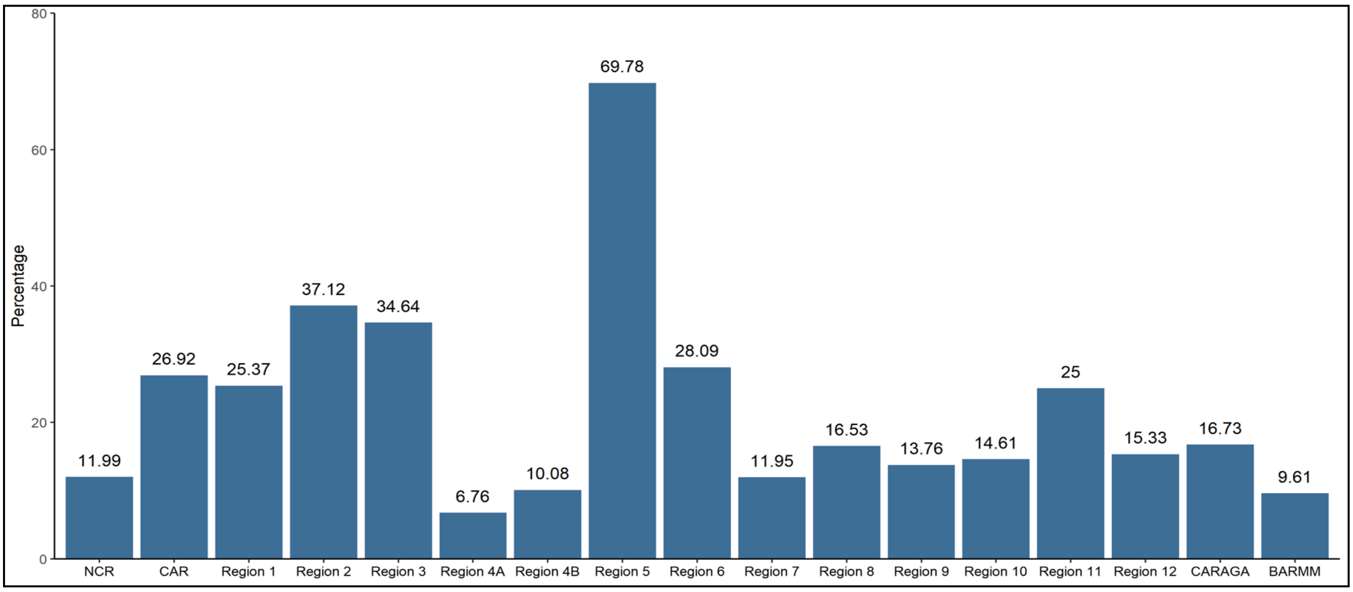

Figure 1.E.4. Risk Assessment using the PhilPEN Protocol, by Region, 2023

FHSIS reports for 2023 showed 43 identified hypertensives per 100 individuals who were risk assessed in BARMM (highest) and only 6 out of 100 in Region 9 (lowest). Similarly, identified Type 2 Diabetes Mellitus among those who were risk assessed was highest in NCR (5.99%) and lowest in Region 5 with 0.46%

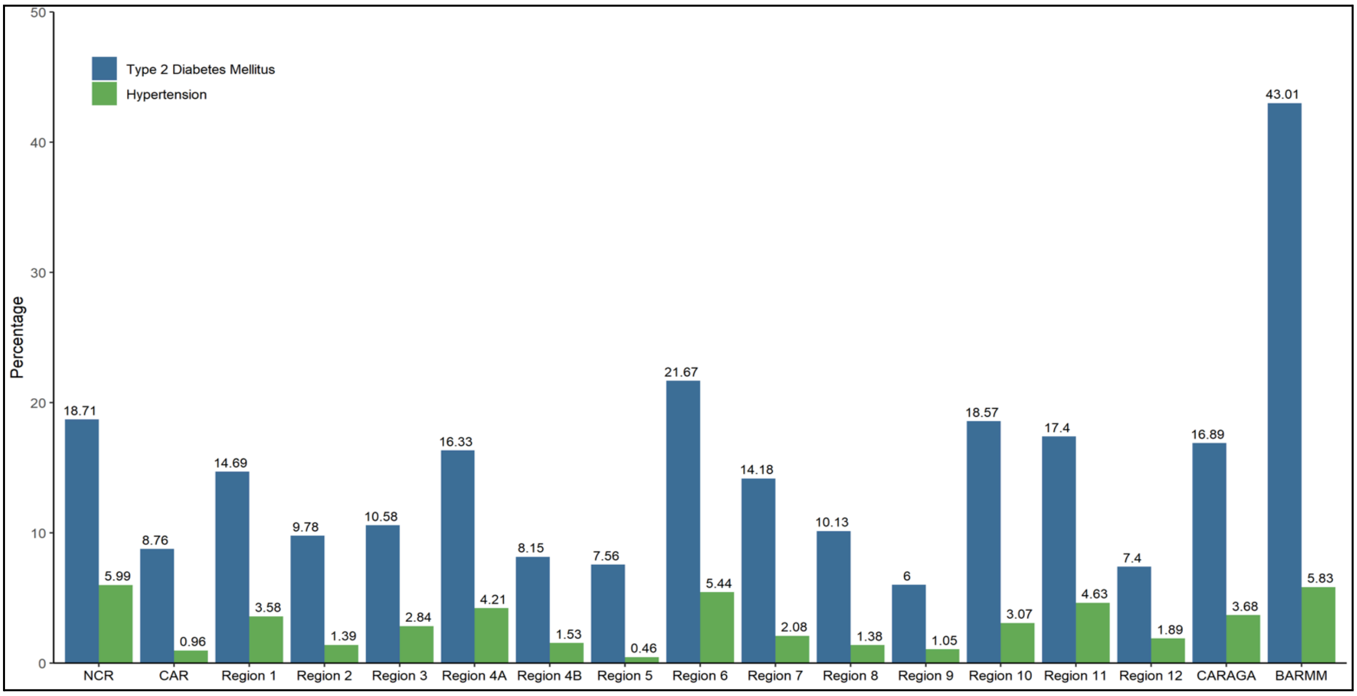

Figure 1.E.5. Adults 20 years old and above identified as Hypertensive or with Type 2 Diabetes Mellitus using the PhilPEN Protocol, by Region, 2023

For cervical cancer screening using VIA or Pap Smear, all regions had less than 1.0% of their eligible population screened except for NCR and Region 11 at 2.11% and 1.46%, respectively. All regions were far below the target of 70% of the eligible population being screened for cervical cancer. Among those screened, Region 4B had the highest (16.2%) detected positive or suspected for cervical cancer. Screening for breast mass among women aged 20 years old and above also seemed to be unpopular with all regions registering less than 5% women screened among their eligible population except Regions CAR and 11 with 5.52% (highest) and 5.06%, respectively. The lowest screening coverage was in Region 8 at 0.28%. Among those screened for breast mass, the region with the highest percentage of detected suspicious breast mass was Region 8 at 9.68% and the lowest in Region 1 at 0.43%. Treatment of cancer at an early stage increases the chance of survival, thus early detection through screening is very vital in reducing both cancer incidence and mortality.

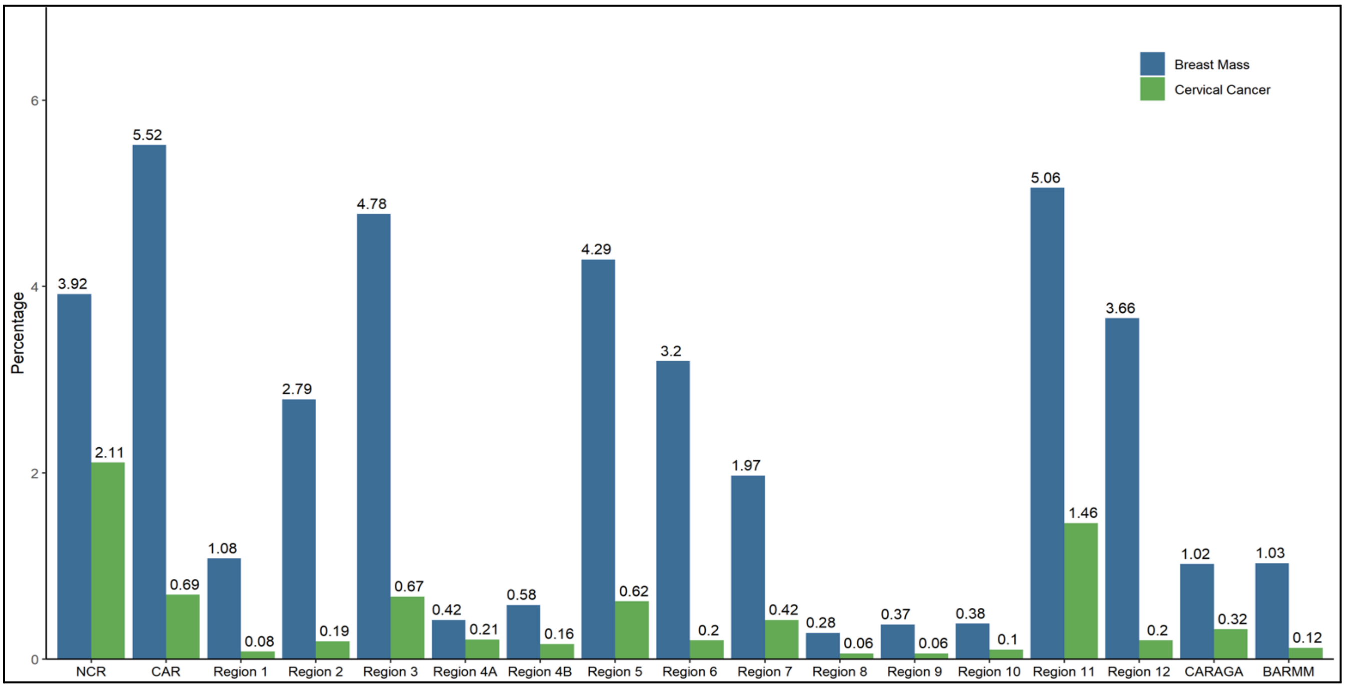

Figure 1.E.6. Screening for Cervical Cancer and Breast Mass, by Region, 2023

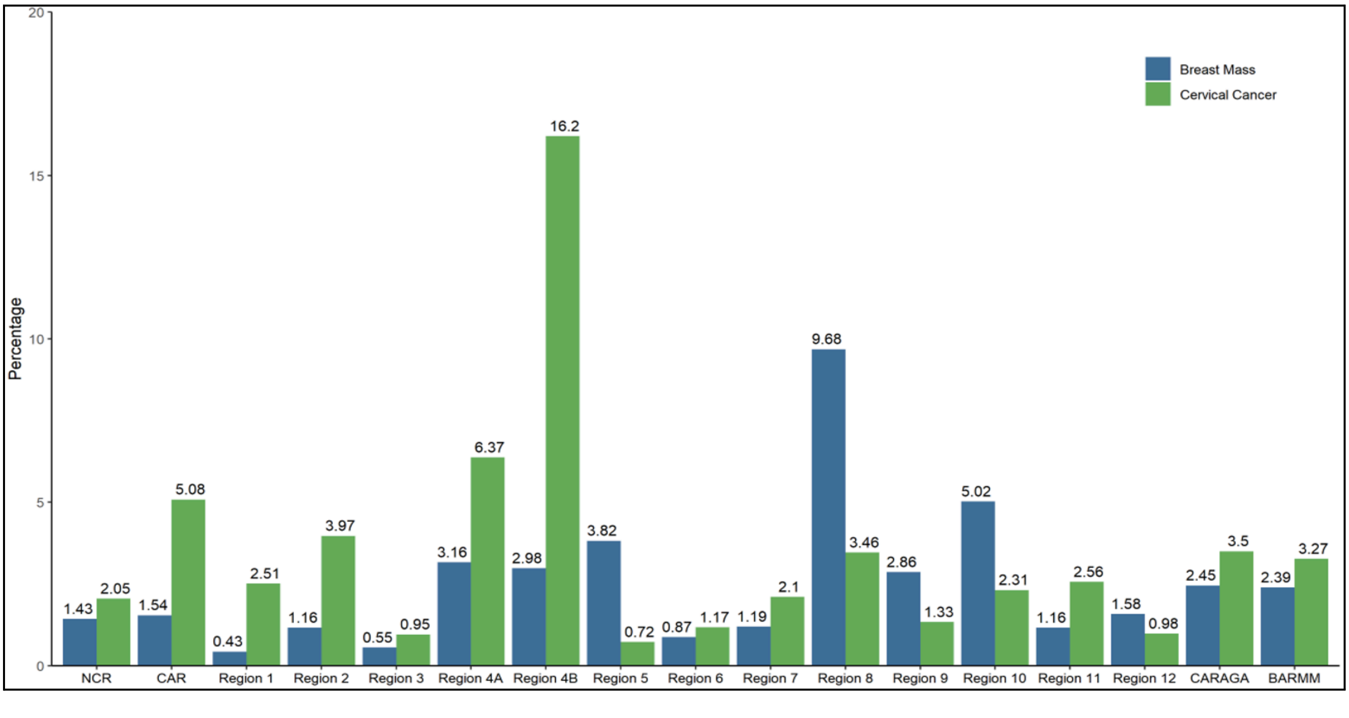

Figure 1.E.7. Suspicious Cervical Cancer and Breast Mass among Screened, by Region, 2023

Immunization coverage among seniors for PPV and Influenza vaccines in 2023 remained low for all regions. The highest coverage for PPV was reported in NCR at 10.48%, while it was Region 3 for Influenza with a coverage of 24.92%. Region 7 had the lowest coverages for both PPV and Influenza for senior citizens, at 1.88% and 4.12%, respectively. Older people have higher chances of deterioration due to the natural wear and tear of bodies, therefore ensuring that they receive additional protection for preventable diseases is crucial for healthy aging.

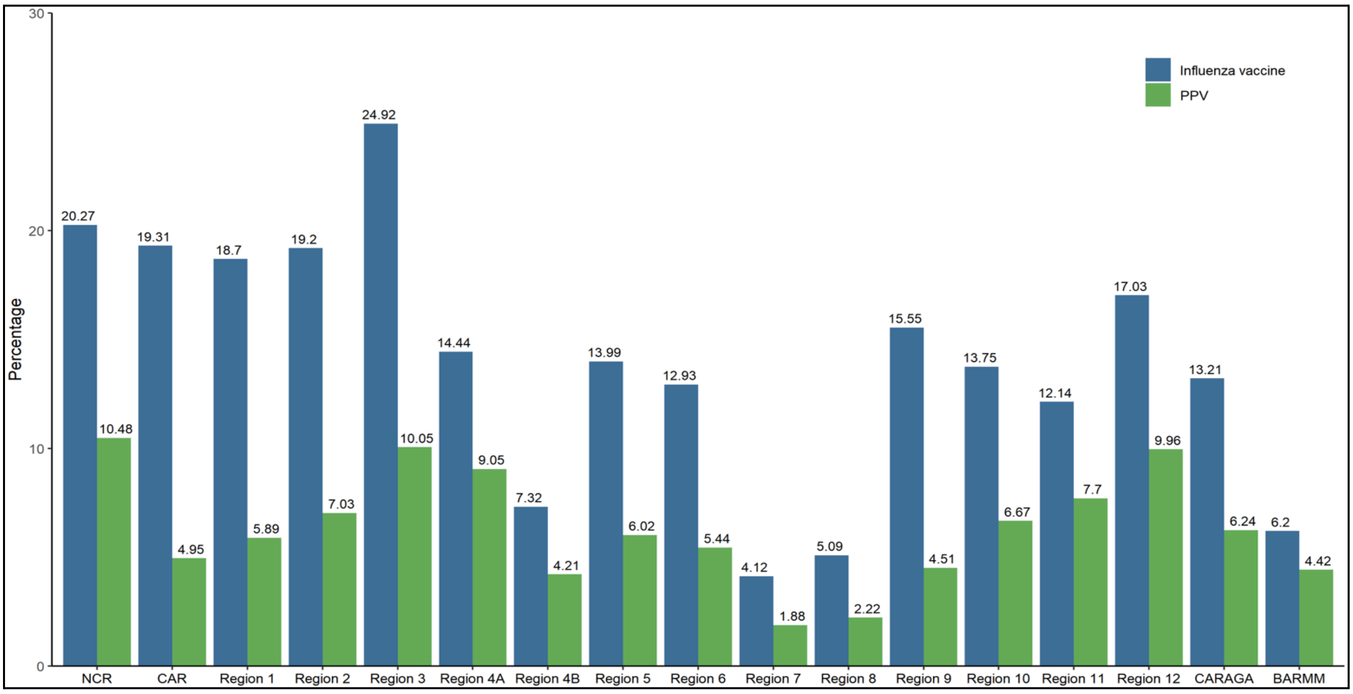

Figure 1.E.8. PPV and Influenza Vaccination in Senior Citizens, by Region, 2023

1.F Environmental Health and Sanitation Services

2023 KEY FINDINGS

ACCESS AND USE OF SAFE WATER SUPPLY AND SANITATION FACILITIES

27,025,049
Estimated Number of Households (HH)

91.98%
HHs with access to Basic Safe Water Supply (BSWS)

85.01%
HHs with Basic Sanitation Facility (BSF)

67.38%
HHs using Safely Managed Drinking Water Services (SMDWS)

63.68%
HHs using Safely Managed Sanitation Services (SMSS)

ZERO OPEN DEFECATION (ZOD)

34.88%
Municipalities/Cities were certified as ZOD

Formula:

Proportion of Households with Basic Safe Water Supply (BSWS)
Numerator: Total number of households (HH) with access to BSWS
Denominator: Estimated total number of HHs

Proportion of Households using Safely Managed Drinking Water Services (SMDWS)
Numerator: Total number of HHs using SMDWS
Denominator: Estimated total number of HHs

Proportion of Households with Basic Sanitation Facility (BSF)
Numerator: Total number of HHs with BSF
Denominator: Estimated total number of HHs

Proportion of Households using Safely Managed Sanitation Services (SMSS)
Numerator: Total number of HHs using SMSS
Denominator: Estimated total number of HHs

Proportion of Municipalities/Cities Certified as Zero Open Defecation (ZOD) Area
Numerator: Total number of municipalities/cities certified as ZOD areas
Denominator: Total number of municipalities/cities

The environment is inevitably linked to health, thus, unhealthy environmental conditions contribute to the exposure pathways of both communicable and noncommunicable diseases. In 2010, the United Nations General Assembly explicitly recognized the human right to water and sanitation. Everyone has the right to sufficient, continuous, safe, acceptable, physically accessible and affordable water for personal and domestic use. The Department of Health, through Administrative Order No. 2010-0021 issued on 25 June 2010, declared sustainable sanitation as a national policy and program priority. Further, Administrative Order (AO) No. 2019-0054 or the “Guidelines on the Implementation of the Philippine Approach to Sustainable Sanitation (PhATSS)” was issued in 2019. The AO aims to contribute to the reduction of risks and diseases related to environmental sanitation through the provision of the implementing guidelines to operationalize the national policy on sustainable sanitation and achieve the Sustainable Development Goal 6 (SDG 6) on sanitation. Specifically, SDG 6 calls for ensuring universal access to safe and affordable drinking water, sanitation, and hygiene (WASH), and ending open defecation.

This chapter presents data on environmental and health sanitation services, particularly indicators on SDG 6 targets.

In 2023, there were 27,025,049 estimated total number of households. Of these, 24,857,831 or 91.98% were reported to have access to Basic Safe Water Supply (BSWS). BSWS refers to different types of improved drinking-water supply including point sources, communal faucet system, stand posts, waterworks system, and individual house connection. A BSWS is considered a SMDWS if it is (1) located inside the household or within its premises, (2) available at least 12 hours per day, and (3) the water supplied should be free of fecal contamination.

The proportion of households with access to BSWS has steadily increased over the years (Figure 1.F.1). However, there was a substantial gap between the proportion of households with access to BSWS and those using safely managed drinking water services (SMDWS). Only 67.38% of the total estimated households were using SMDWS. Further investigation of this finding is crucial, especially for BSWS, which does not meet the third criterion for SMDWS since contamination may lead to outbreaks of water-borne diseases.

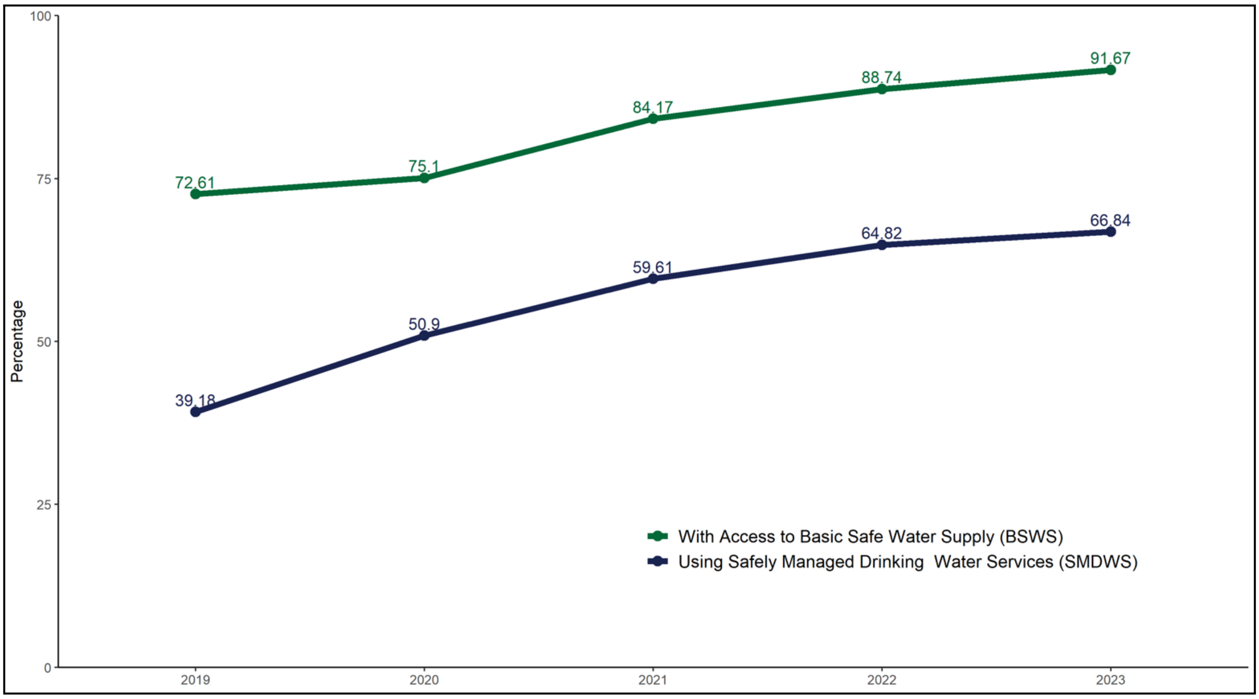

Figure 1.F.1. Access to Basic Safe Water Supply and Safely Managed Drinking Water Services, Philippines, 2019 to 2023

In 12 out of the 17 regions in the country, more than 90% of the total estimated households had access to basic safe water supply. Region 1 had the highest coverage with 98.83% of its households having access to BSWS, followed by Region 3 (97.57%), and NCR (96.82%). On the other hand, BARMM had the lowest coverage with only 67.86% of its households having access to BSWS, followed by Region 9 (74.95%), and Region 5 (81.66%). In terms of SMSS, BARMM still had the lowest coverage at 18.97%, and NCR had the highest at 93.56% (Table 1.F.1).

Region	Estimated Number of Households	Basic Safe Water Supply		Safely Managed Drinking Water Services	
		Number of HHs	%	Number of HHs	%
NCR	3,582,003	3,468,273	96.82	3,351,424	93.56
CAR	452,303	436,773	96.57	282,867	62.54
Region 1	1,328,091	1,312,515	98.83	829,295	62.44
Region 2	927,620	880,356	94.90	550,672	59.36
Region 3	3,109,563	3,034,031	97.57	2,803,798	90.17
Region 4A	4,159,056	3,927,616	94.44	3,440,872	82.73
Region 4B	811,290	748,565	92.27	400,661	49.39
Region 5	1,406,129	1,148,209	81.66	665,646	47.34
Region 6	1,962,744	1,867,577	95.15	1,078,261	54.94
Region 7	2,021,190	1,758,990	87.03	1,225,213	60.62
Region 8	1,106,829	1,032,146	93.25	538,878	48.69
Region 9	918,762	688,624	74.95	435,026	47.35
Region 10	1,238,184	1,135,687	91.72	633,954	51.20
Region 11	1,372,765	1,177,083	85.75	885,308	64.49
Region 12	1,101,285	1,031,395	93.65	556,097	50.50
CARAGA	677,878	633,595	93.47	370,057	54.59
BARMM	849,357	576,396	67.86	161,125	18.97

Table 1.F.1. Access to Basic Safe Water Supply and Safely Managed Drinking Water Services, by Region, 2023

Sanitation services, along with safe drinking water services, reduce the risk of diarrhea, the spread of intestinal worms, schistosomiasis/trachoma, malnutrition, etc. Similar to safe drinking water services, there was a gap between the number of households with access to BSF and those households with safely managed sanitation services (SMSS). A BSF is considered an SMSS when it is not shared with other households and its sewage/excreta is properly stored, disposed of, or treated (the actual criteria are listed in the FHSIS MOP 2018).

In 2023, 22,972,827 households (85.01% of the total estimated households) were reported to have access to basic sanitation facilities (BSF), approximately 7.79% higher than the number of households with access to BSF in 2022. A total of 17,208,867 households were identified as having SMSS, representing 63.68% of the total estimated households in the country. In terms of absolute counts, this figure was equivalent to a 2.17% increase from the 2022 data, but its proportion relative to the estimated number of households has decreased from 66.1% in 2022 to 63.68% in 2023 [Figure 1.F.2].

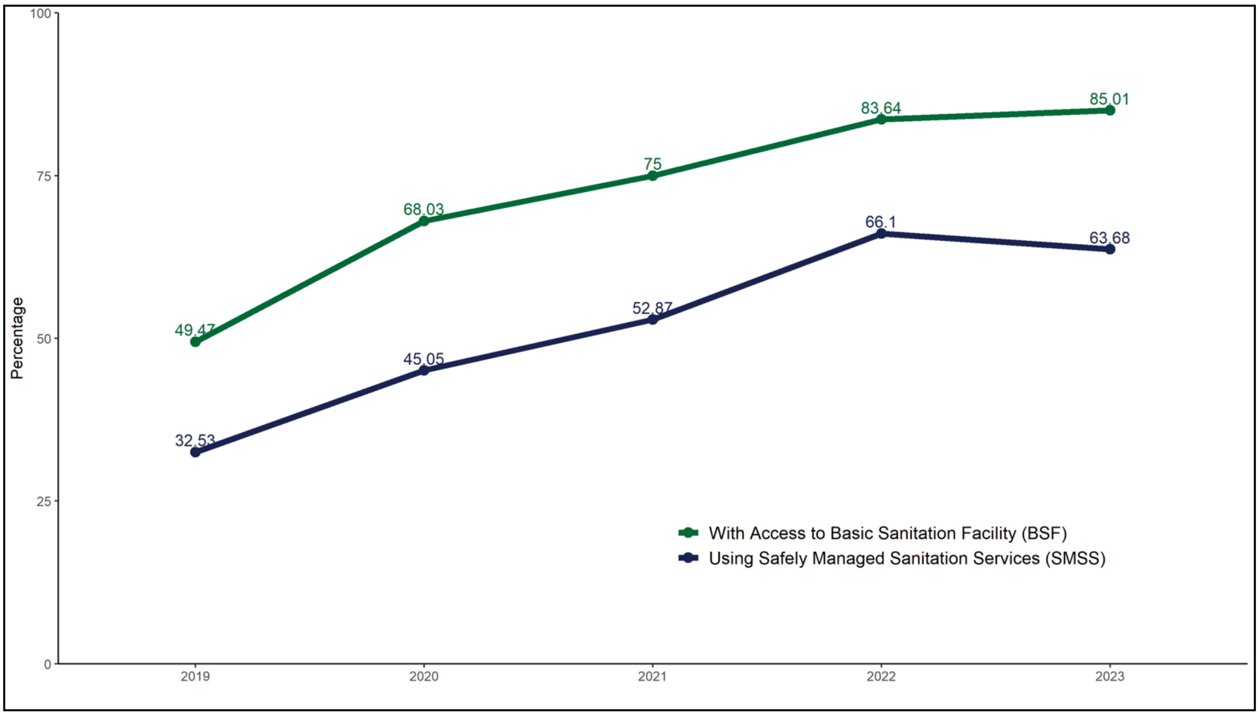

Figure 1.F.2. Access to Basic Sanitation Facility and Safely Managed Sanitation Services, Philippines, 2019 to 2023

The percentage of households with access to basic sanitation facilities has been consistently lower than those with access to basic safe water supply across all regions. For sanitation services, the highest coverage was observed in Region 1 and Region 3 (94.53%), followed by CAR (93.70%). Similar to BSWS, access to BSF was lowest in BARMM (47.66%), Region 9 (57.48%), and Region 4B (68.49%). BARMM had low SMSS coverage of 9.25%, followed by Region 10 with 17.83%, and Region 8 with 21.97%. Notably, CARAGA indicated that no households utilized SMSS [Table 1.F.2].

Region	Estimated Number of Households	Basic Sanitation Facility		Safely Managed Sanitation Services	
		Number of HHs	%	Number of HHs	%
NCR	3,582,003	3,222,685	89.97	2,513,362	70.17
CAR	452,303	423,820	93.70	408,127	90.23
Region 1	1,328,091	1,255,448	94.53	460,044	34.64
Region 2	927,620	850,652	91.70	794,606	85.66
Region 3	3,109,563	2,939,464	94.53	2,796,882	89.94
Region 4A	4,159,056	3,833,979	92.18	3,420,133	82.23
Region 4B	811,290	555,651	68.49	341,449	42.09
Region 5	1,406,129	1,014,492	72.15	806,070	57.33

Region 6	1,962,744	1,789,256	91.16	1,369,605	69.78
Region 7	2,021,190	1,650,396	81.65	1,488,299	73.63
Region 8	1,106,829	917,061	82.85	243,161	21.97
Region 9	918,762	528,063	57.48	433,711	47.21
Region 10	1,238,184	976,725	78.88	220,720	17.83
Region 11	1,372,765	1,134,414	82.64	1,058,367	77.10
Region 12	1,101,285	897,080	81.46	775,784	70.44
CARAGA	677,878	578,863	85.39	0	0.00
BARMM	849,357	404,778	47.66	78,547	9.25

Table 1.F.2. Access to Basic Sanitation Facility and Safely Managed Sanitation Services, by Region, 2023

Starting in 2023, the units of analysis used for Zero Open Defecation (ZOD) are municipalities or cities instead of barangays as per Department Memorandum 2022-0511 entitled "Redefining the Zero Open Defecation (ZOD) Grade 1 Level under the Philippine Approach to Sustainable Sanitation (PhATSS) and the reporting of ZOD data in the DOH-Field Health Service Information System (FHSIS)". A municipality/city is considered a ZOD area if at least 95% of its total households have BSF or if it has undergone the usual ZOD certification process.

Following this new reporting mechanism, a total of 569 municipalities/cities in the Philippines were certified to be ZOD areas in 2023. The ZOD coverage in the country ranged from 3.79% to 84%. The lowest coverage was in Region 7, where only the provinces of Bohol and Cebu had cities and municipalities reported as ZOD. Region 1, the region with the highest ZOD coverage in 2022 when the considered reporting units were barangays, still had the highest coverage in 2023 [Table 1.F.3].

Region	Number of ZOD Municipalities/Cities	% of Total Municipalities/Cities
**PHILIPPINES**	**570**	**34.88**
NCR	13	76.47
CAR	45	58.44
Region 1	105	84.00
Region 2	16	17.20
Region 3	83	63.85
Region 4A	62	43.66
Region 4B	18	25.00
Region 5	7	6.14
Region 6	75	56.39
Region 7	5	3.79
Region 8	47	32.87
Region 9	4	5.56
Region 10	20	21.51
Region 11	6	12.24
Region 12	11	22.45
CARAGA	18	24.66
BARMM	35	29.17

Table 1.F.3. ZOD Municipalities/Cities, by Region, 2023

1.G Morbidity

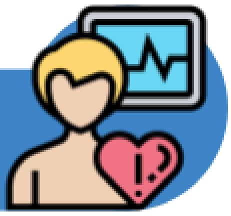

The FHSIS collects morbidity data using a pre-specified list of 42 diseases from public primary care facilities. These are submitted to the DOH-EB on a monthly basis and are consolidated for the annual report. While this chapter does not provide a full picture of the country's morbidity statistics, the DOH-EB is currently exploring mechanisms to expand coverage and include all diseases and related health problems. Nonetheless, this chapter presents data on the top 10 leading causes of morbidity based on the pre-selected 42 diseases, as well as the diseases with the most increase in cases compared to the previous year. This chapter will be helpful in tracking the trends in cases, especially for priority diseases, which aids in public health decision-making and strategic planning.

Rank				2023 Top 10 Causes of Morbidity	2023		
2019	2020	2021	2022		Count	Rate per 100k population	%change vs. 2022
1	1	1	1	1. Acute Respiratory Infection (ARI)	1,897,902	1,700.04	-38.39%
2	2	2	2	2. Hypertension	861,049	771.28	28.73%
8	4	3	3	3. Animal Bites	607,031	543.75	20.64%
3	3	4	4	4. Urinary Tract Infection (UTI)	392,413	351.5	12.81%
6	7	6	7	5. Pneumonia	304,912	273.12	51.10%
7	5	5	6	6. Skin Diseases	256,897	230.11	21.34%
4	6	7	5	7. Acute Lower Respiratory Tract Infection	243,236	217.88	-13.77%
>10	10	8	9	8. Tuberculosis (all forms)	145,412	130.25	21.62%
9	9	>10	>10	9. Bronchitis	90,165	80.77	96.62%
>10	>10	9	10	10. Fever of Unknown Origin	86,503	77.48	-9.19%

Table 1.G.1. Leading Causes of Morbidity, Philippines, 2019 to 2023

The table above consistently showed ARI and Hypertension as the Top 1 and 2 causes of morbidity, respectively, in the last five (5) years. Despite ARI being the top leading cause of morbidity, the number of cases in 2023 were actually 38% lower than in 2022 (3,080,648). Hypertension, Animal Bites, and UTI retained the same rankings since 2021, but increased in cases in 2023. Bronchitis reverted to its rank in 2019 and 2020 as the ninth leading cause of morbidity, from being the top 15 in 2021 and 2022. Furthermore, bronchitis cases in 2023 were almost twice as high as the cases reported in 2022 (45,858).

Lastly, shown in Table 1.G.2 are the diseases among the 42 pre-selected morbidities which had the most increase in cases compared to the previous year. These diseases may often be neglected as some have relatively less in number of cases than others, however, tracking these will help decision-makers, program managers and program implementers at all levels in controlling and preventing further increase/reoccurrence. There were more than thrice the cases of Paralytic Shellfish Poisoning, Malaria, Filariasis, and Viral encephalitis seen in 2023 compared to 2022. Of note are Pneumonia and Bronchitis which were both included in the list of top diseases based on absolute counts and based on increase in cases from last year. Furthermore, attention shall be paid to Malaria, Filariasis, and Schistosomiasis - all seeing 150% to 265% increase in cases, since these are part of the Multi-Disease Elimination Plan (MDEP) which aims to achieve zero or reduced new infections of priority diseases by 2030.

Diseases	Number of Cases (2022)	Number of Cases (2023)	% change
1. Paralytic Shellfish Poisoning	44	169	284.09%
2. Malaria	401	1,467	265.84%
3. Filariasis	33	104	215.15%
4. Viral encephalitis	92	285	209.78%
5. Diphtheria	90	250	177.78%
6. Schistosomiasis	926	2,348	153.56%
7. Bronchitis	45,858	90,165	96.62%
8. Viral hepatitis	746	1,373	84.05%
9. Typhoid and Paratyphoid Fever	2,815	4,313	53.21%
10. Pneumonia	201,798	304,912	51.10%

Table 1.G.2. Top 10 Diseases based on Increase in Cases, Philippines

1.H Demographics

2023 KEY FINDINGS

PUBLIC HEALTH PROFESSIONALS/WORKERS TO POPULATION RATIO

1 : 25,280
Physician/Doctor to Population Ratio

1 : 59,099
Dentist to Population Ratio

1 : 4,985
Midwife to Population Ratio

1 : 157,905
Nutritionist to Population Ratio

1 : 5,863
Public Health Nurse to Population Ratio

1 : 33,255
Medical Technologist to Population Ratio

1 : 37,908
Sanitary Inspector to Population Ratio

1 : 3,721,285
Sanitary Engineer to Household Ratio

HEALTH FACILITY TO POPULATION RATIO
Health Center (HC) and Barangay Health Station (BHS)

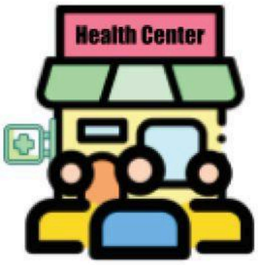

1 : 42,497
HC to Population Ratio

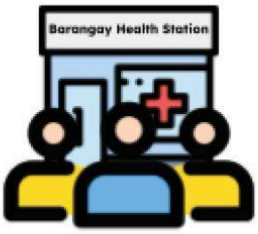

1 : 4,589
BHS to Population Ratio

Formula:

Barangay to Population Ratio
Numerator: Total number of barangays
Denominator: Estimated total population

BHS to Population Ratio
Numerator: Total number of BHS
Denominator: Estimated total population

Health Center (HC) to Population Ratio
Numerator: Total number of health centers
Denominator: Estimated total population

Active Barangay Health Worker (BHW) to HH Ratio
Numerator: Total number of active BHW
Denominator: Estimated total number of households

Public Health Worker to Population Ratio
Numerator: Total number of public health workers (per group)
Denominator: Estimated total population

The FHSIS continues to track and monitor community-based or population-based data, specifically demographic data that are only reported on an annual basis. Demographic data includes information about the area in terms of its total catchment population, households, barangays, number of existing health facilities (HCand BHS), and healthcare worker (HCW). The provision of quality healthcare services is anchored on the equitable distribution of health facilities and health care workers.

At the Local Government Unit (LGU) level, the DOH sets the ideal distribution ratios of health professionals in a primary care facility (PCF) with its catchment population. These ratios are used as a norm by both the DOH and the LGUs in identifying staffing needs and in measuring accomplishment of target healthcare workers at the PCFs. Additionally, the DOH and the Human Resources for Health Network Philippines developed the National Human Resources for Health Master Plan (NHRHMP) 2020-2040 to guide the management and development of the human resources for health (HRH) of the Philippines according to the goals of Universal Health Care.[10] Below are the recommended HCW to population ratios:

Physician/Doctor	1:20,000	Nutritionist	1:20,000
Dentist	1:50,000	Medical Technologist	1:50,000
Public Health Nurse	1:10,000	Sanitary Inspector	1:20,000
Midwife	1:5,000	Sanitary Engineer	-

This chapter presents select demographic data of the country in 2023, specifically data on the ratio of health facilities and HCW to the estimated population. The numbers presented here are submitted by the Centers for Health Development (CHD) and the Ministry of Health - Bangsamoro Autonomous Region in Muslim Mindanao.

Over the past five years, the growth in the country's population has slowed down. From 2019 to 2023, the population has increased by 3.35%. A decreasing trend in the number of barangays reporting to the FHSIS, from 42,134 in 2020 to 41,539 in 2023, was also observed.

[10] *https://hhrdb.doh.gov.ph/hrh-network/*

However, note that the official statistics on the geographic areas in the Philippines shall come from the PSA.

Meanwhile, the number of BHS has generally increased over the years *(Figure 1.H.1)*. In 2023, a total of 24,327 BHS were reporting to the FHSIS, resulting in a BHS to population ratio of 1:4,589. This essentially means that on the average, one (1) BHS caters to approximately 4,589 individuals. In addition, there were 2,627 HC in 2023, which translated to an HC to population ratio of 1:42,497.

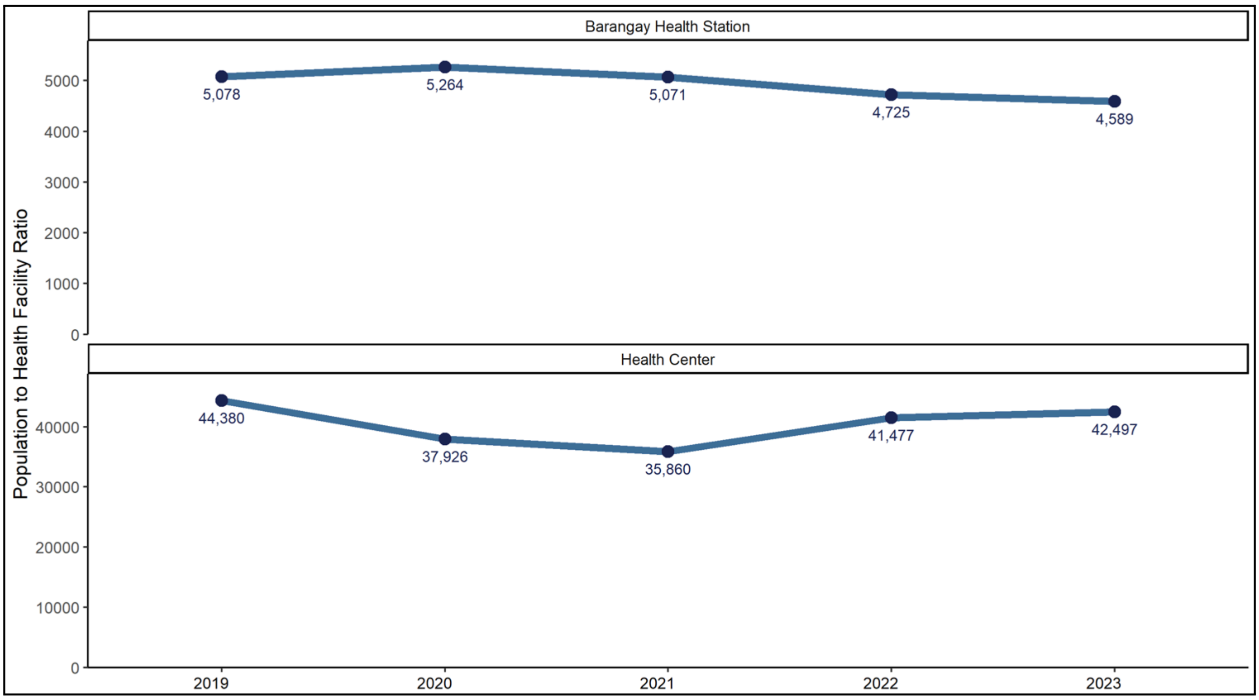

Figure 1.H.1: Health Facility to Population Ratio, Philippines, 2019 to 2023

In terms of HCW, the ratio of HCW to population was decreasing for doctors, medical technologists, sanitary inspectors, midwives, and nutritionists. On the contrary, it was increasing for dentists, public health nurses (PHNs), and sanitary engineers. The increase was more prominent for PHNs, in which the ratio grew to 5,863 from 4,911 in 2022. Despite this, it was still within the recommended PHN to population ratio of 1:10,000. Conversely, the ratio for doctors, sanitary inspectors, and nutritionists were still below the recommended level despite the decrease. Note that this only covers personnel hired by either the DOH or the LGUs. Nonetheless, this means that the growth in the number of several HCWs in PCFs hired by the government is not proportional to the growth in the population. The increase in the population has slowed down in recent years which may be an opportunity to hone and grow the workforce, especially in the health sector. Ensuring that every Filipino has access to quality health care does not only entail improvement in terms of facilities, but also includes the people who will man these facilities.

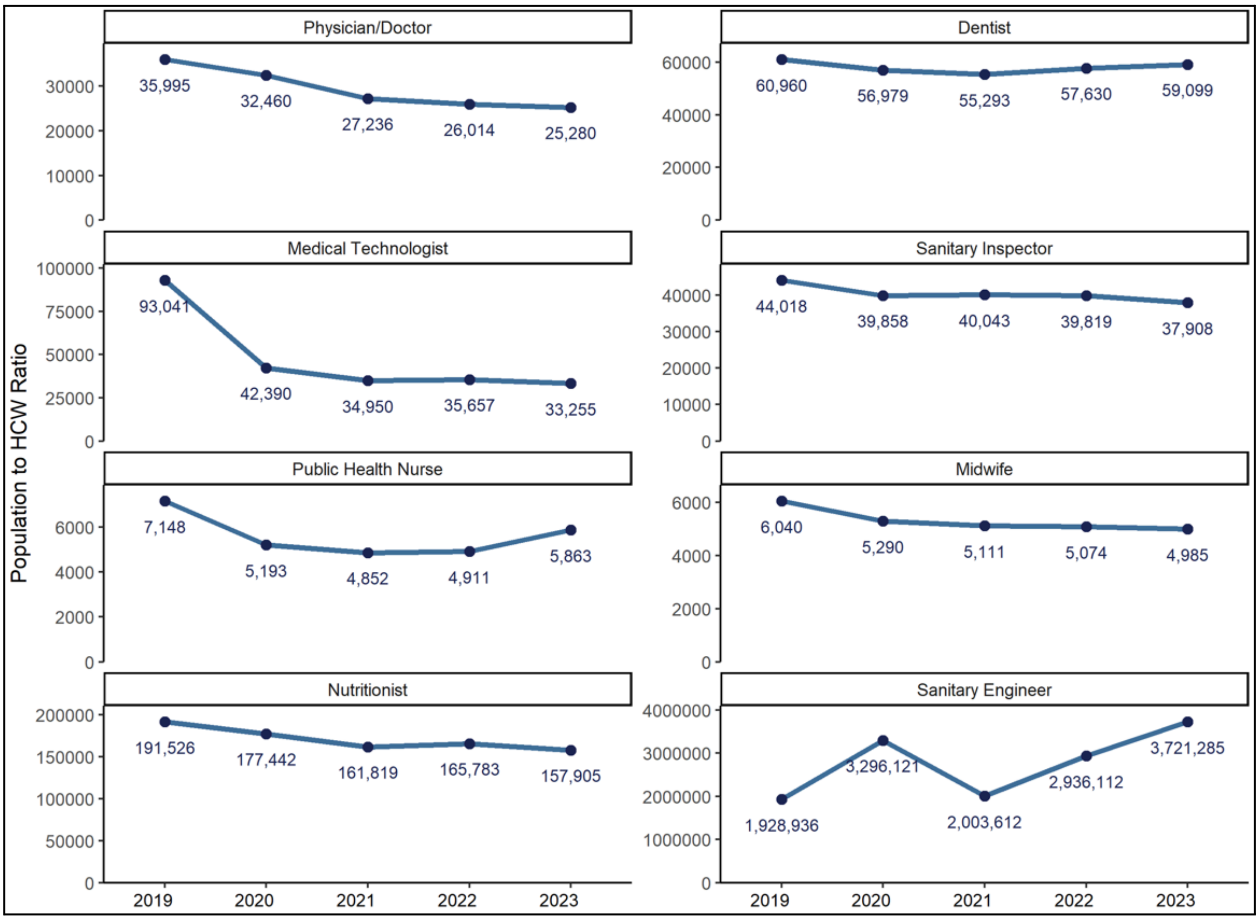

Figure 1.H.2: Health Care Worker to Population Ratio, Philippines, 2019 to 2023

Looking at the regional statistics, Region 4A was the most populous region in the country with an estimated population of 16,579,218, while CAR had the least population of 1,851,672. Consequently, Region 4A had the highest number of households (4,159,056) and CAR had the lowest (452,303). Meanwhile, the total number of barangays was highest in Region 8 (4,390) and lowest in CARAGA (1,311).

The number of HCs was highest in NCR (467) with a 1:29,555 HC to population ratio and lowest in Region 11 (52 HC) with 1:103,476 HC to population ratio. As to the number of BHS, Region 4A had the most with 2,935 BHS or a 1:5,649 BHS to population ratio, while NCR had the least with only 31 BHS resulting in a 1:445,237 BHS to population ratio *(Table 1.H.1)*.

Region	No. of BHS	BHS to Population Ratio	No. of Health Centers	HC to Population Ratio
NCR	31	445,237	467	29,555
CAR	1,129	1,640	98	18,895
Region 1	1,622	3,323	165	32,668
Region 2	1,623	2,321	98	38,444
Region 3	2,212	5,744	307	41,387
Region 4A	2,935	5,649	303	54,717
Region 4B	1,178	2,805	80	41,297
Region 5	2,533	2,474	140	44,757
Region 6	2,166	3,716	144	55,888

Region	No. of BHS	BHS to Population Ratio	No. of Health Centers	HC to Population Ratio
Region 7	2,248	3,694	154	53,929
Region 8	867	5,365	159	29,254
Region 9	801	4,962	114	34,864
Region 10	1,156	4,492	93	55,831
Region 11	917	5,868	52	103,476
Region 12	1,092	4,128	65	69,348
CARAGA	1,075	2,673	85	33,799
BARMM	742	6,792	103	48,931

Table 1.H.1: Health Facility to Population Ratio, by Region, 2023

For the public healthcare workforce in PCFs, only CAR met the recommended HCW to population ratio across the following professions: doctor, dentist, PHN, midwife, medical technologist, and sanitary inspector. The NCR met the recommended ratios for almost the same professions as CAR, with the exception of the ratio for midwives and sanity inspectors *(Table 1.H.2)*. Meanwhile, all regions met the recommended 1 PHN per 10,000 population.. Notably, several regions still fell short of the recommended ratios for midwives and medical technologists despite the national ratios being within the recommendations. In the NCR, for example, 1 midwife had a catchment population of 10,954 or double the recommended 5,000. A complete table found in Table 1.H.2 would show that all regions had very few nutritionists, sanitary engineers, and sanity inspectors. Further, Region 2, Region 9, CARAGA, and BARMM reported zero sanitary engineers.

Region	HCW to Population Ratio						
	Doctor	Dentist	Public Health Nurse	Midwife	Medical Technologist	Sanitary Inspector	Sanitary Engineer
**RECOMMENDED RATIO**	**20,000**	**50,000**	**10,000**	**5,000**	**50,000**	**20,000**	**20,000**
NCR	14,215	24,429	7,901	10,954	30,070	48,092	94,537
CAR	16,101	33,667	2,547	2,088	19,288	16,386	71,218
Region 1	24,280	59,233	5,802	3,730	27,362	27,929	256,677
Region 2	20,701	62,792	5,084	3,334	24,465	32,479	221,620
Region 3	31,764	65,493	7,936	6,939	40,593	52,073	239,731
Region 4A	37,007	80,482	7,963	8,559	59,211	56,584	312,815
Region 4B	18,772	54,161	4,761	3,371	22,785	25,220	173,884
Region 5	32,133	80,332	4,327	4,498	17,852	37,746	122,861
Region 6	20,583	62,386	6,312	3,716	26,473	32,715	335,326
Region 7	26,619	79,856	6,448	4,862	37,923	26,035	110,734
Region 8	22,913	84,569	4,275	3,366	26,732	22,255	357,794
Region 9	39,745	101,910	4,506	4,297	46,215	46,215	68,526
Region 10	30,906	110,474	6,218	5,007	45,150	36,310	148,351
Region 11	40,155	70,799	6,059	6,375	43,393	61,847	168,148
Region 12	24,498	100,169	5,424	3,271	25,467	42,525	81,957
CARAGA	25,424	58,632	2,896	3,508	27,361	26,118	261,177
BARMM	49,410	143,996	5,070	3,962	86,894	52,499	0

Table 1.H.2: Health Care Worker to Population Ratio, by Region, 2023

1.1 Infectious Diseases

2023 KEY FINDINGS

FILARIASIS PREVENTION AND CONTROL

2.56
Number positive cases found
Case Detection Rate (CDR)

4.84
Lymphatic filariasis cases examined with disabilities/deformities
Clinical Rate (CR)

HIV-AIDS/STI PREVENTION AND CONTROL SERVICES

29.73%
650,225 Pregnant women screened for Syphilis

26.10%
570,963 Pregnant women screened for HIV

LEPROSY PREVENTION AND CONTROL

0.08
924 Leprosy cases undergoing treatment per 1,000 population

0.97
1,086 Newly detected cases per 100,000 population

SCHISTOSOMIASIS PREVENTION AND CONTROL

4.19%
Acute clinically diagnosed cases

2.21%
Chronic clinically diagnosed cases

SOIL TRANSMITTED HELMINTHIASIS PREVENTION AND CONTROL

45.60%
1-4 yrs. old who completed 2 doses of deworming tablets

23.57%
5-9 yrs. old who completed 2 doses of deworming tablets

17.01%
10-19 yrs. old who completed 2 doses of deworming tablets

23.57%
20-49 yrs. old who completed 2 doses of deworming tablets

11%
Pregnant Women who completed 1 dose of deworming tablets

1.I.1. Filariasis

Lymphatic filariasis is a vector borne disease in which people are infected via certain species of mosquitoes namely *Wuchereria bancrofti* and *Brugia malayi* which is endemic in the southern half of the Philippines. Symptoms include lymphedema of the limbs, genital disease and elephantiasis causing temporary and permanent disability.

As of 2023, the number of declared filaria-free provinces is 44 out of 46 endemic provinces. The FHSIS monitors the program accomplishments of the Filariasis Prevention and Control services through case detection, diagnosis and management and treatment.

The Case Detection Rate of lymphatic filariasis refers to the number of blood slides found positive for lymphatic filariasis among the total number of individuals examined either from Nocturnal Blood Examination (NBE), Immunochromatographic Test (ICT) or Filariasis Test Strips (FTS) within a given period of time while Clinical Rate refers to the proportion of lymphatic filariasis cases examined with disabilities/deformities such as lymphedema, hydrocoele and elephantiasis.

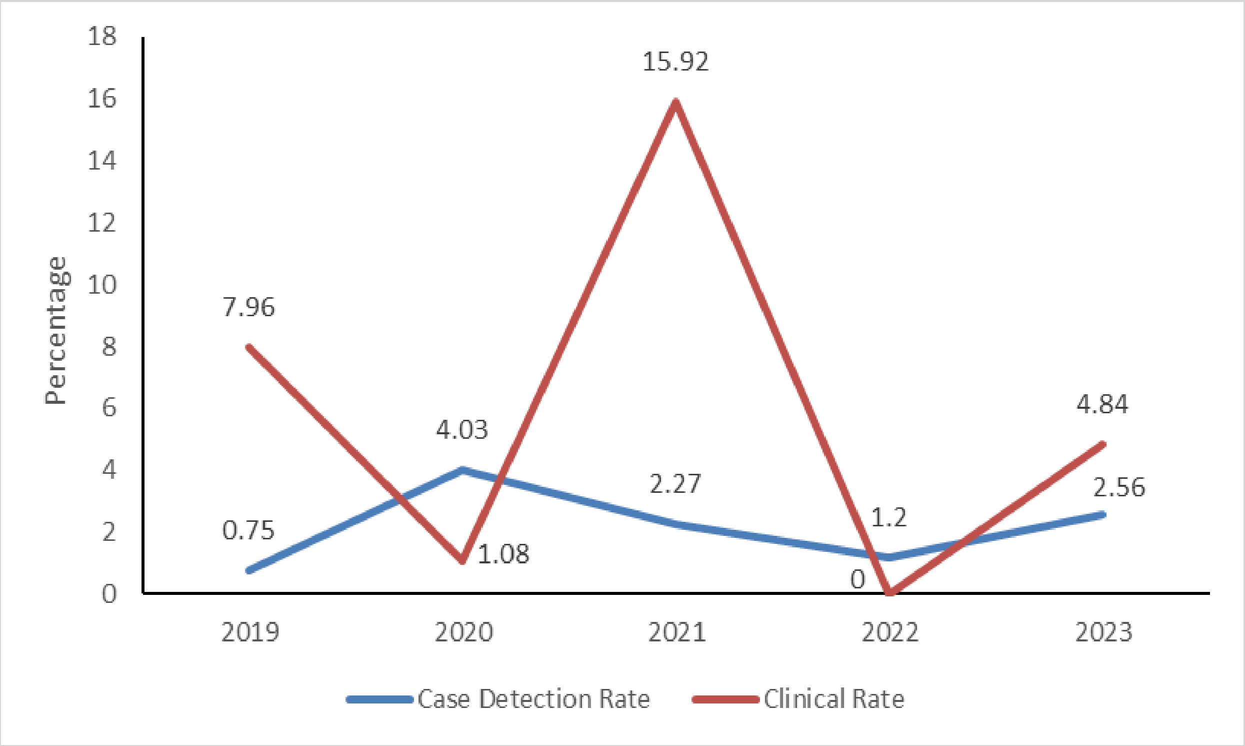

Figure 1.I 1 Case Detection Rate (CDR) and Clinical Rate of Lymphatic Filariasis Philippines, 2019 to 2023

The Case Detection Rate increased in 2020 and 2023 at 4.03% and 2.56% respectively. A decreasing trend was observed from 2020 at 4.03% to 1.2% in 2022 and the target of less than 1% has not been achieved since 2020 up to 2023. The Clinical Rate had the sharpest increase from 2020 to 2021 at 15.92% (Figure 1.I 1).

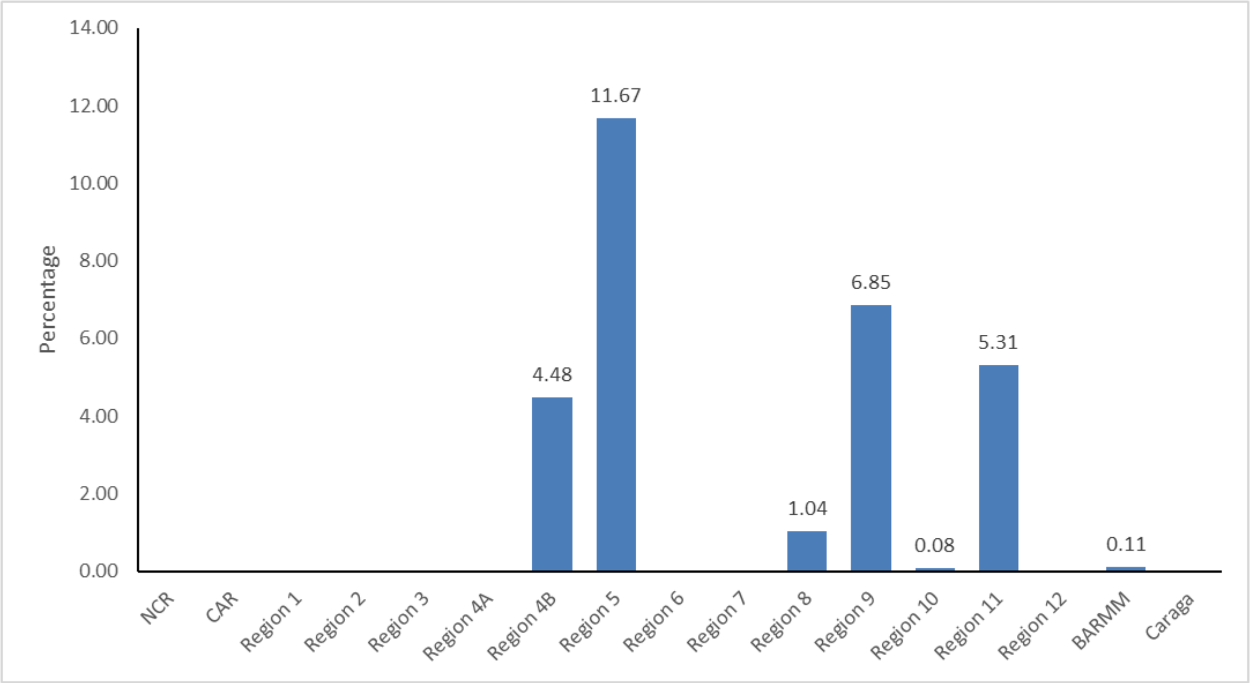

Figure 1.I 2 Case Detection Rate (CDR) of Lymphatic Filariasis by Region, 2023

For 2023, the top 3 regions with the highest reported cases are: Region 5 with the highest case detection rate at 11.67%, Region 9 is the second highest reported cases at 6.85% and Region 11 with the third highest rate at 5.31%(Figure 1.I 2).

HIV-AIDS/STI Prevention and Control Services

Sexually transmitted infections (STIs) during pregnancy are associated with adverse birth outcomes, including preterm birth, LBW, perinatal death, and congenital infections such as increased mother-to-child HIV transmission.

FHSIS Indicators for HIV-AIDS/STI Prevention and Control Services are used to provide information on how pregnant women are provided with appropriate screening, management and treatment on HIV-AIDS/STI to prevent maternal complications and help service providers and health managers prevent further maternal and newborn complications.

In 2023, there were 26.10% pregnant women screened for HIV and 29.73% pregnant women screened for Syphilis. Out of the pregnant women screened for syphilis, 1% tested positive.

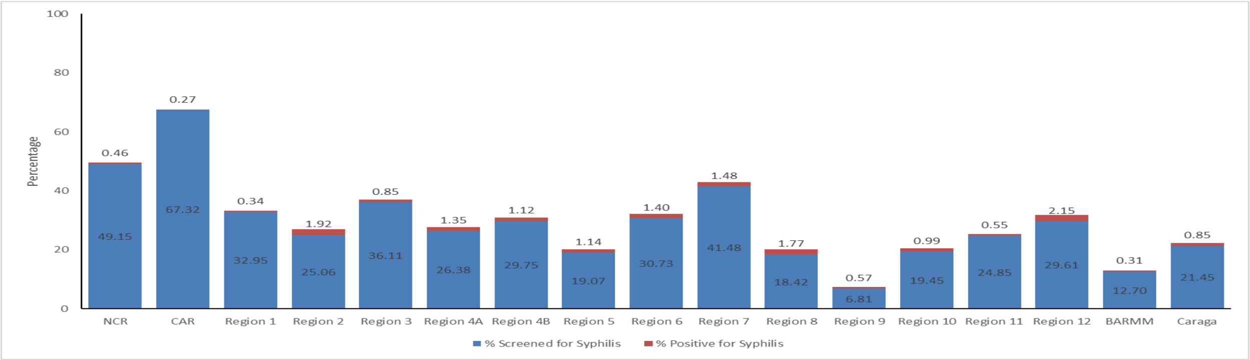

Figure 1.I 3 Proportion of Pregnant Women Screened and Positive for Syphilis by Region, 2023

The highest proportion of pregnant women screened for syphilis was reported in Cordillera Administrative Region (CAR) at 67.32%, followed by National Capital Region (NCR) at 49.15%. Positivity rate for syphilis is highest for Region 12 and Region 2 at 2.15% and 1.92%, respectively and lowest for CAR and BARMM at 0.27% and 0.31% respectively (Figure 1.I 3).

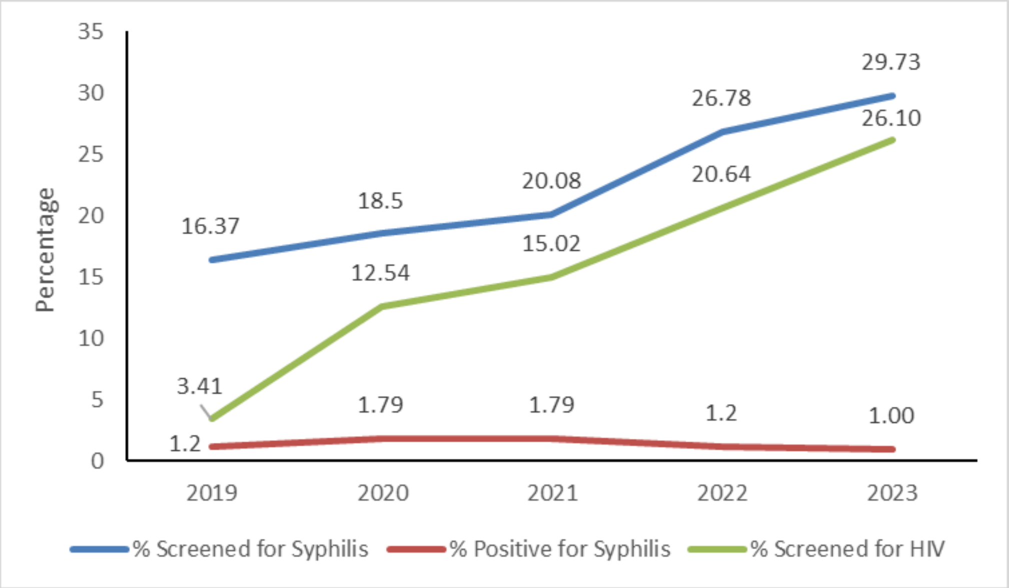

Figure 1.I 4 Proportion of Pregnant Women Screened and Positive for Syphilis and Screened for HIV Philippines, 2019 to 2023

The proportion of pregnant women screened for Syphilis and HIV had an increasing trend from 2019 to 2023 while the percentage for those positive for syphilis had an unstable trend from 2019 to 2023.

1.I.2. Leprosy

Leprosy is a neglected tropical disease which still occurs in more than 120 countries, with more than 200,000 new cases reported every year. It is reported from all the six (6) WHO regions with the majority of annual new case detections are from South-East Asia with the inclusion of the Philippines.

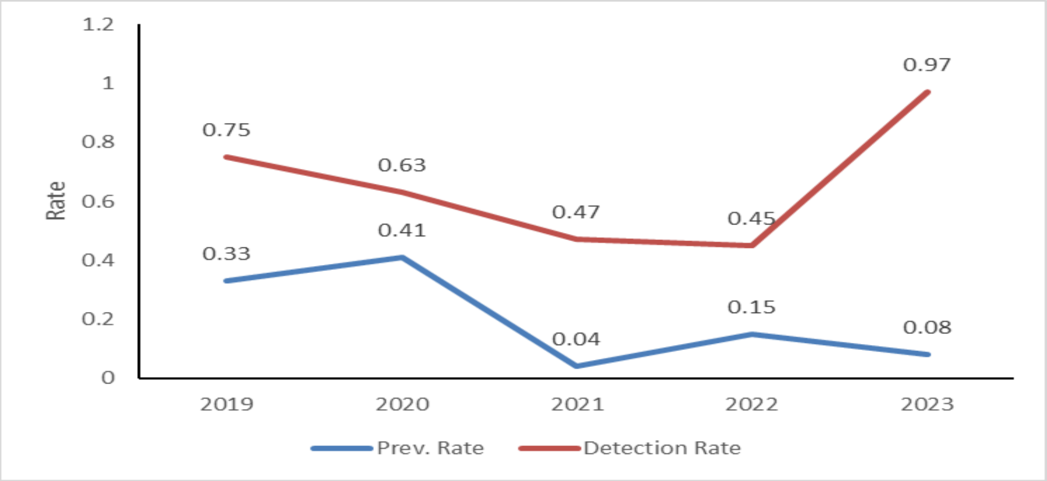

Figure 1.l 5 Prevalence and Detection Rate for Leprosy Philippines, 2019 to 2023

The prevalence rate for leprosy had a fluctuating trend from 2019 to 2023 with a slight increase in 2019 to 2020 and 2022 to 2023 and large decrease from 2020 to 2021 from 0.41 per 10,000 persons in 2020 to 0.04 per 10,000 persons in 2021 while the detection rate had a sharp decline from 2019 to 2022 from 0.75 cases per 100,000 persons in 2019 to 0.45 cases per 100,000 persons in 2022. A sharp increase in the cases detected was seen in 2023 at 0.97 cases per 100,000 persons. The prevalence rate has continually reached its target since 2019 at less than 1 case per 10,000 persons, for the case detection rate, the sharp increase from 2022 and 2023 data would cause not reaching the target in 2023 of less than 5% from the 2022 data which is 0.43 per 100,000 persons.

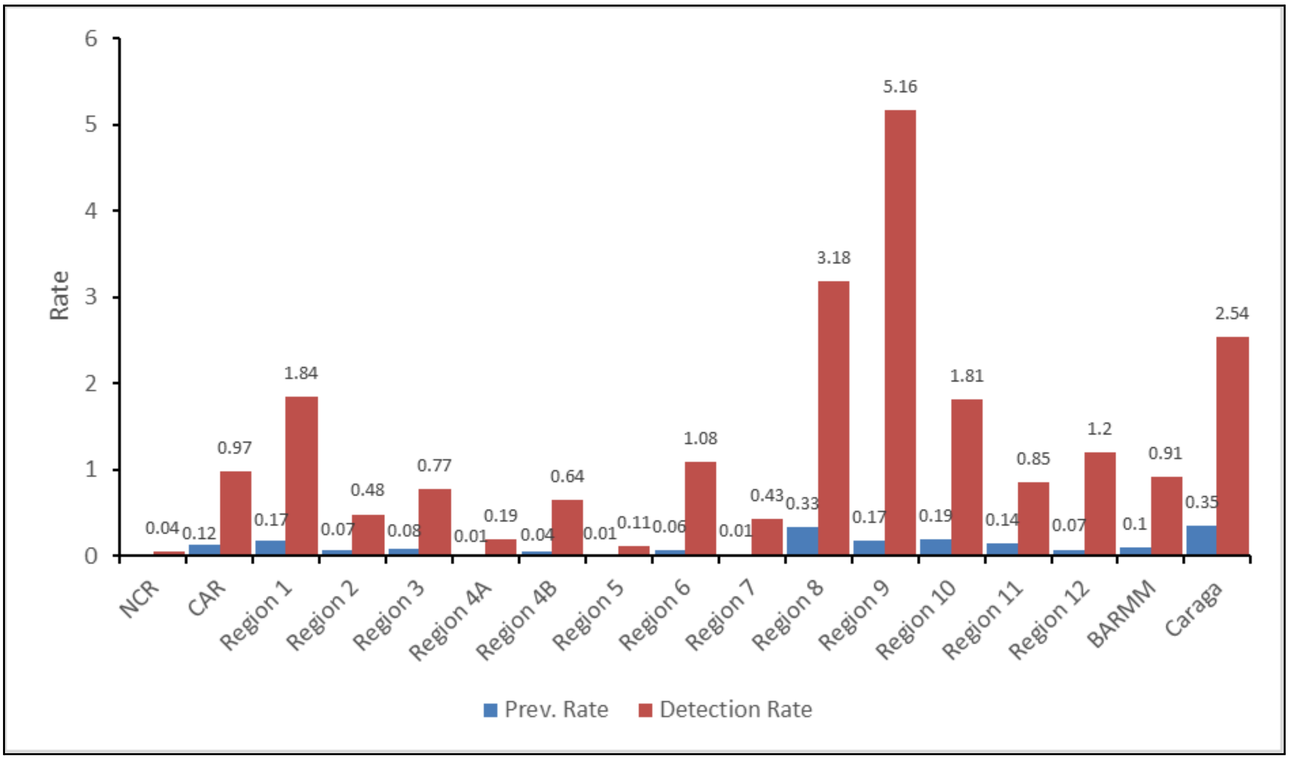

Figure 1.l 6 Prevalence and Detection Rate for Leprosy by Region, 2023

The regions with the highest prevalence rate in 2023 are CARAGA with 0.35 cases per 10,000 persons and Region 8 with 0.33 cases per 10,000 persons while the regions with the lowest prevalence rate are NCR with no reported cases, and Regions 4A, 5 and 7 with 0.01 cases per 10,000 persons.

For the detection rate, Region 9 had the highest detection rate at 5.16 cases per 100,000 persons followed by Region 8 with 3.18 cases per 100,000 persons and CARAGA with 2.54 cases per 100,000 persons while the regions with the lowest detection rate are NCR with 0.04 cases per 100,000 persons, Region 5 with 0.11 cases per 100,000 persons and Region 4A with 0.19 cases per 100,000 persons.

1.I.3 Rabies

Rabies is a viral zoonotic disease that causes progressive and fatal inflammation of the brain and spinal cord. This fatal disease is usually seen in developing countries where animal immunization and control of dogs are inadequate.

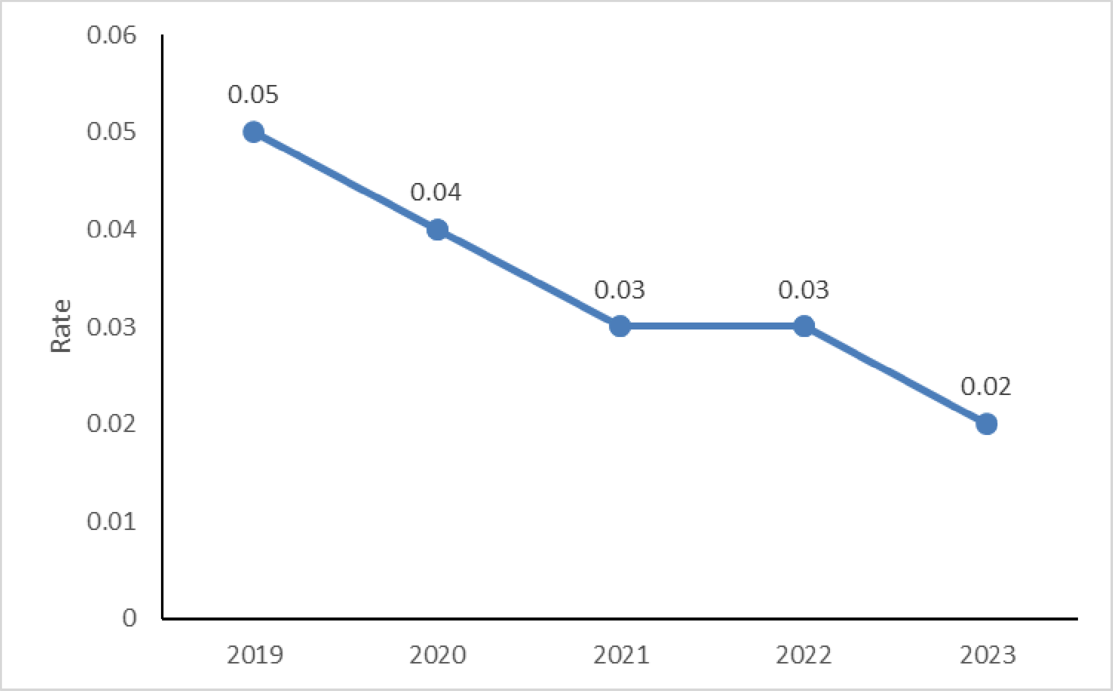

Figure 1.I 7 Proportion of Deaths due to Rabies Philippines, 2019 to 2023

The proportion of deaths due to rabies had a steady decline from 2019 to 2023 with 0.05 cases per 1,000,000 persons in 2019 to 0.02 cases per 1,000,000 persons in 2023 (Figure 1.I 7).

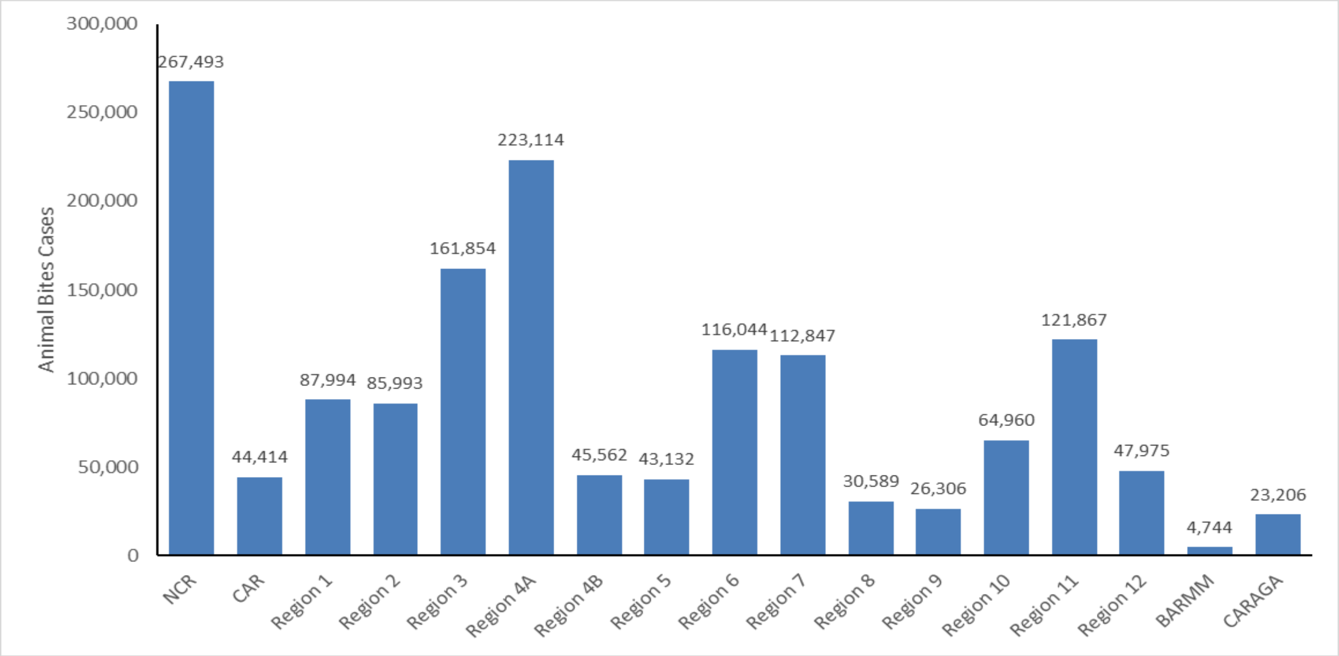

Figure 1.I 8 Animal Bite Cases by Region, 2023

In 2023, the regions with the highest number of reported animal bite cases were NCR with 267,493 cases, Region 4A with 223,114 cases and Region 3 with 161,854 cases while the regions with the least cases were BARMM with 4,744 cases, followed by CARAGA with 23,206 cases and Region 9 with 26,306 cases(Figure 1.I 8). For the proportion of deaths due to rabies, BARMM had the highest proportion of deaths due to rabies among the regions with 0.08 deaths per 1,000,000 persons, followed by Regions 5 and 12 with 0.06 deaths per 1,000,000 persons and Region 9 with 0.05 deaths per 1,000,000 persons (Figure 1.I 9).

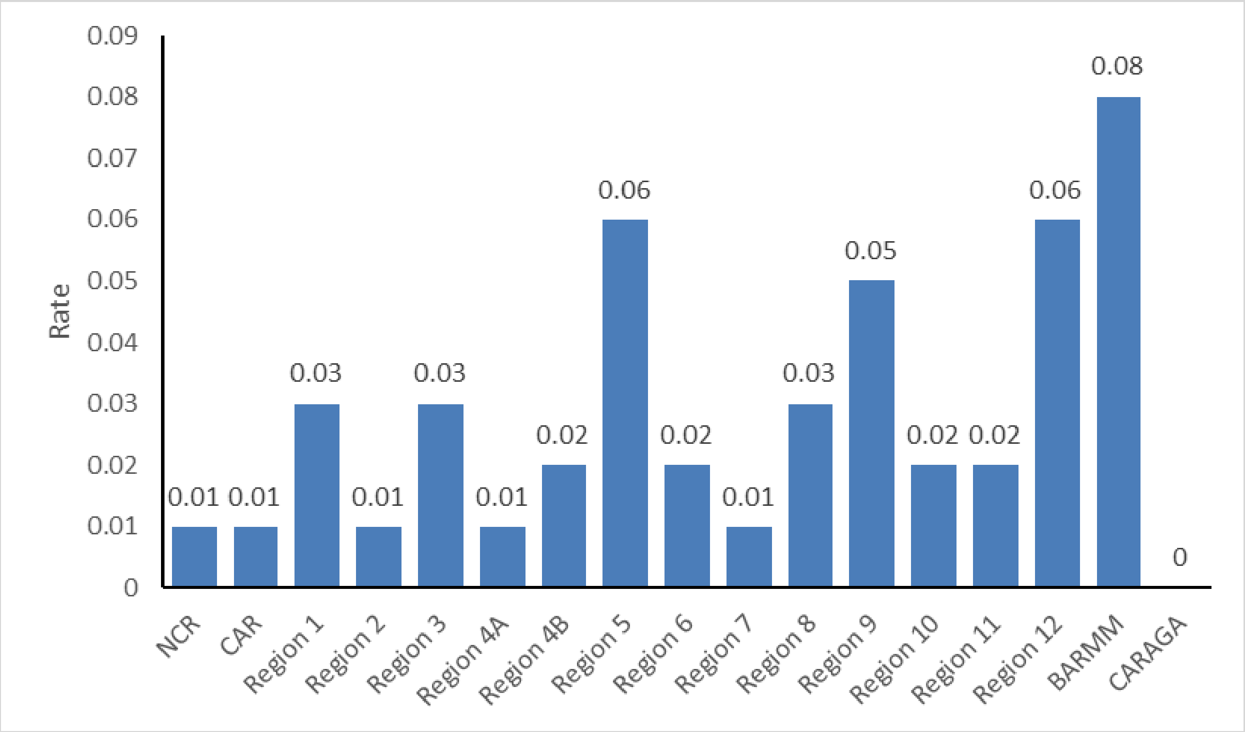

Figure 1.I 9 Proportion of Deaths due to Rabies by Region, 2023

1.I.4. Schistosomiasis

Schistosomiasis is an acute and chronic disease caused by parasitic worms. Key factors that lead to vulnerability in infection include lack of hygiene, swimming and fishing in infested water and during routine agricultural, domestic and occupational and recreational activities which expose people to infested water.

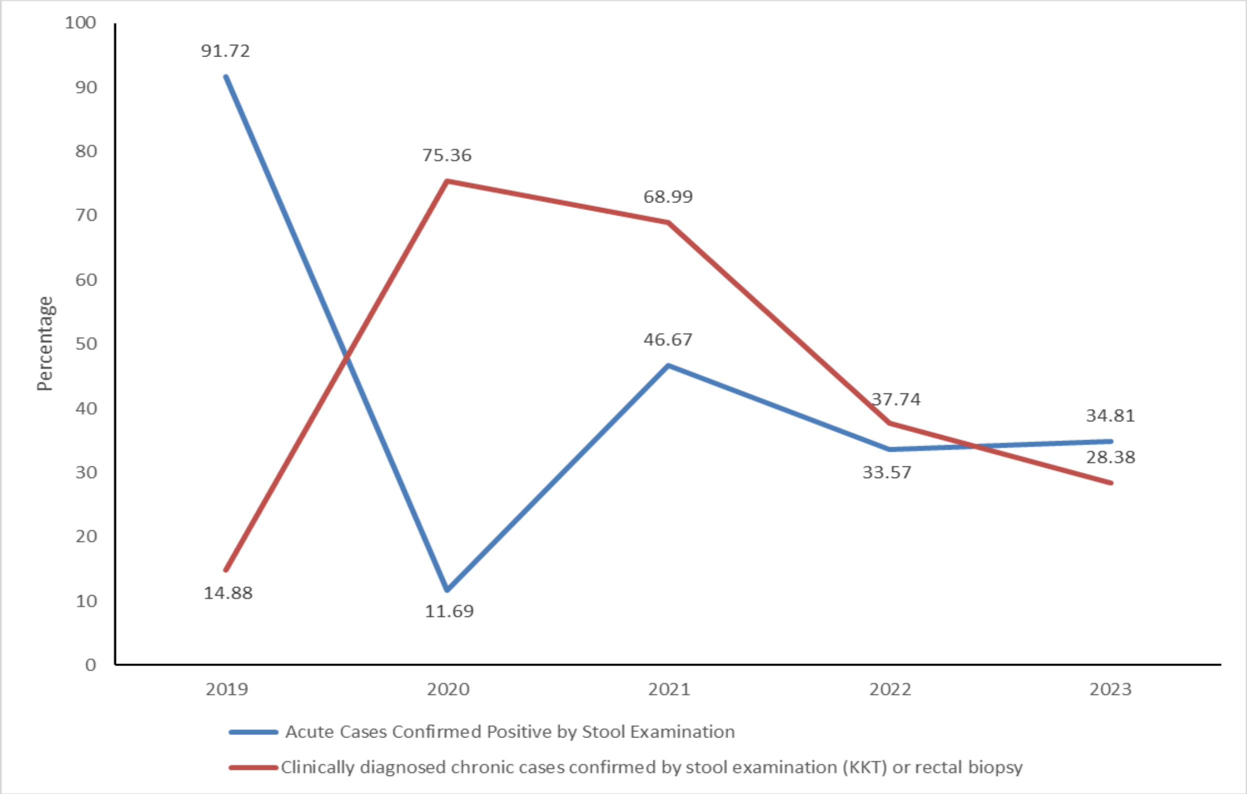

Figure 1.I 10 Proportion Acute and Clinically Diagnosed Chronic Cases Confirmed by Stool Examination Philippines, 2019 to 2023

In the acute cases confirmed positive by stool examination, a sharp decline was seen in 2019 at 91.72% to 11.69% in 2020 and in 2021 at 46.67% to 2022 at 33.57% while a notable increase was seen from 2020 to 2021 at 11.69% and 46.67%, respectively and a slight increase from 2022 at 33.57% to 34.81% in 2023. For the proportion of clinically diagnosed chronic cases confirmed by stool examination of rectal biopsy, a considerable increase was noted from 2019 to 2020 with 14.88% and 75.36% respectively while a gradual decrease from 2020 to 2023 was observed from 75.36% in 2020 to 28.38% in 2023.

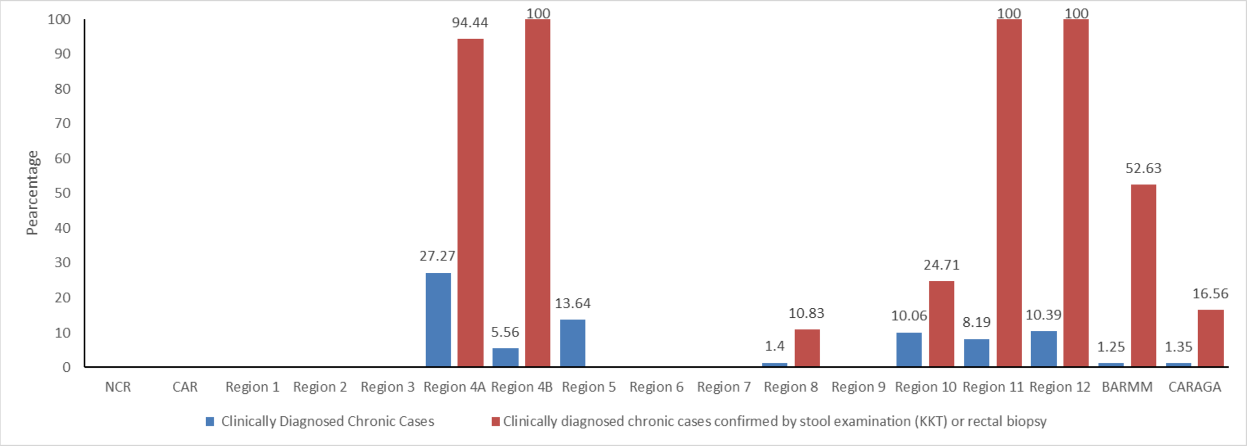

Figure 1.I 11 Proportion of Clinically Diagnosed Chronic Cases and Clinically Diagnosed Chronic Cases Confirmed by Stool Examination (KKT) or Rectal Biopsy by Region, 2023

The regions with the highest proportion of clinically diagnosed chronic cases are Region 4A with 27.27%, Region 5 with 13.64% and Regions 12 and 10 with 10.39% and 10.06% respectively. Among the clinically diagnosed cases confirmed by stool examination (KKT) or rectal biopsy, the regions with 100% confirmation are Regions 4B, 11 and 12, followed by Region 4A at 94.44% and BARMM at 52.63%.

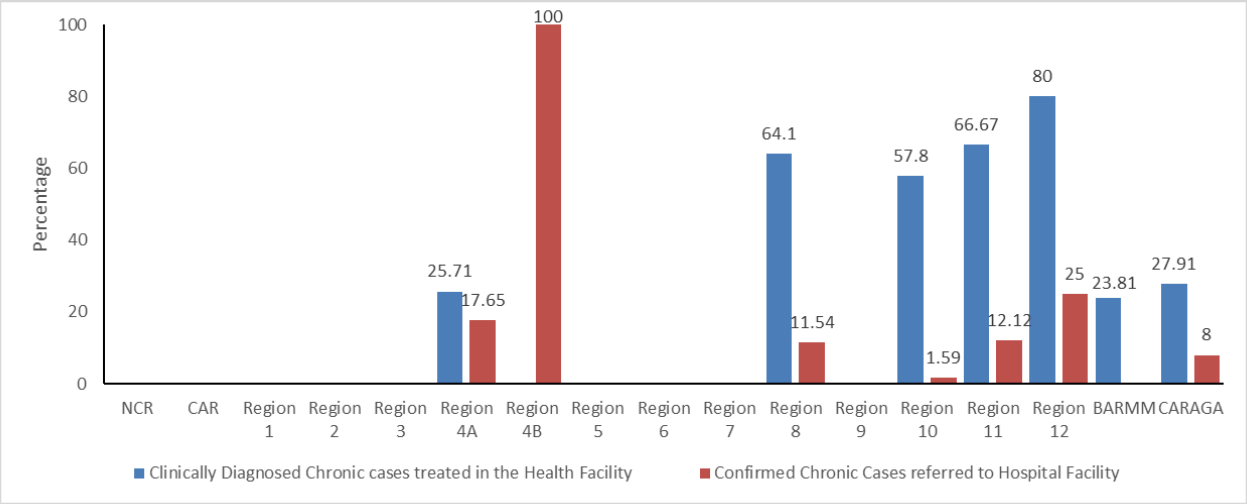

Figure 1.I 12 Proportion of Clinically Diagnosed Chronic Cases Treated in the Health Facility and Confirmed Chronic Cases Referred to a Hospital Facility by Region, 2023

In the proportion of clinically diagnosed chronic cases treated in the health facility, the regions with the highest proportion of cases are Region 12 with 80% of the clinically diagnosed chronic cases being treated in the health facility followed by Region 11 at 67.77% and Region 8 with 64.1%. Even with the high proportion of chronic clinically diagnosed cases treated in the health facility, the regional data did not reach the target of 100%. For the confirmed chronic cases referred to a hospital facility, the regions with the highest proportion of confirmed chronic cases referred to a hospital facility are Region 4B with 100% of their confirmed chronic cases being referred to a hospital facility followed by Region 12 at 25% and Region 4A at 17.65%.

1.I.5. Soil Transmitted Helminthiasis

The soil transmitted helminthiasis infections have been an important public health concern in the Philippines and FHSIS tracks the soil transmitted helminthiasis mass drug administration (MDA) program among the most vulnerable groups specifically children in the 1-4 years old age group, 5-9 years old, 10-19 years old, women of reproductive age 20-49 years old and pregnant women.

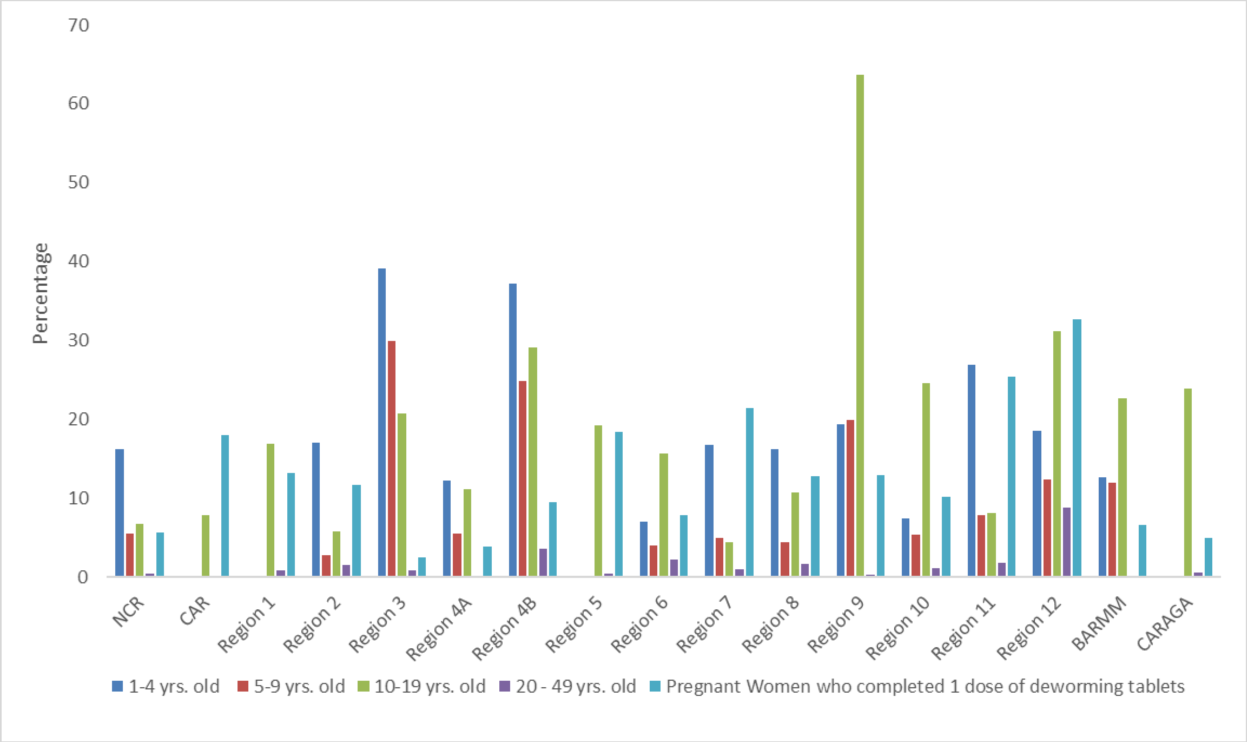

Figure 1.I 12 Proportion of 1 to 4 Years Old, 5-9 Years Old, 10-19 Years Old, 20-49 Years Old and Pregnant Women Who Completed Their Doses of Deworming Tablets by Region, 2023

In the soil transmitted helminthiasis mass drug administration program, the regions with the highest proportion of administered STH drugs are Regions 9, 4B and 12.

The target in the mass drug administration of STH drugs is at 85% and among the age groups, the 1-4 years old had the highest proportion of STH drugs administered at 45.6% but still did not meet the national target.

1.I.6. Tuberculosis

Tuberculosis is an infectious disease that most often affects the lungs and can be prevented and cured. Ending the TB epidemic by 2030 is among the health targets of the United Nations' SDGs.

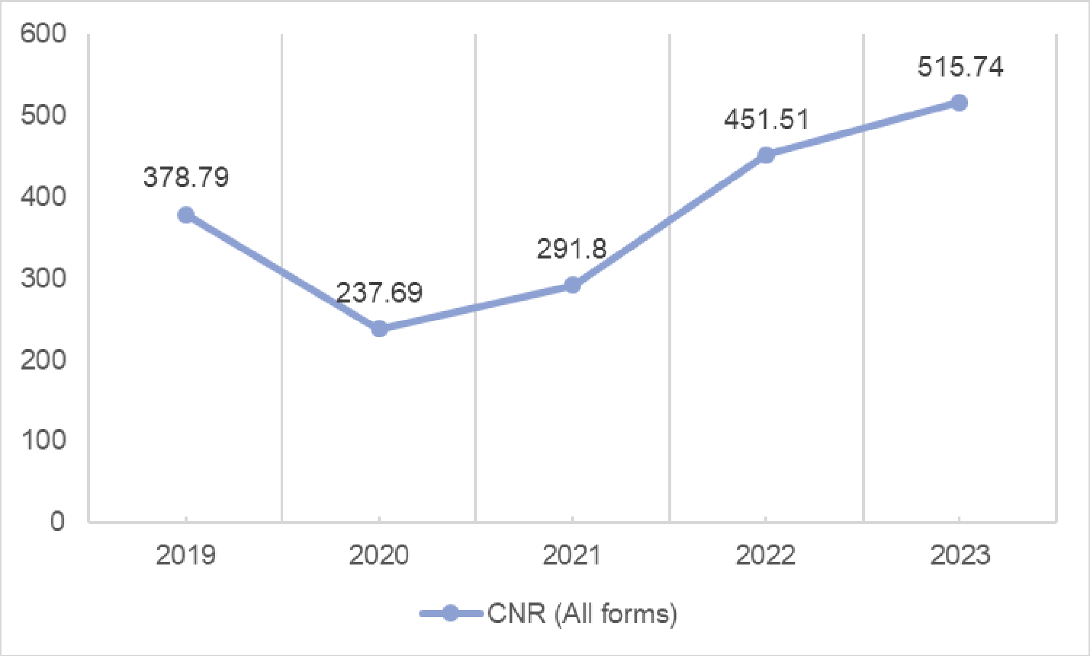

Figure 1.I 13 Case Notification Rate (All forms)
Philippines, 2019 to 2023

For the case notification rate of all forms of TB, a decrease was noted from 2019 to 2020 from 378.79 cases per 100,000 persons in 2019 to 237.49 cases per 100,000 persons in 2020 while a steady and gradual increase was noted from 2020 to 515.74 cases per 100,000 persons in 2023.

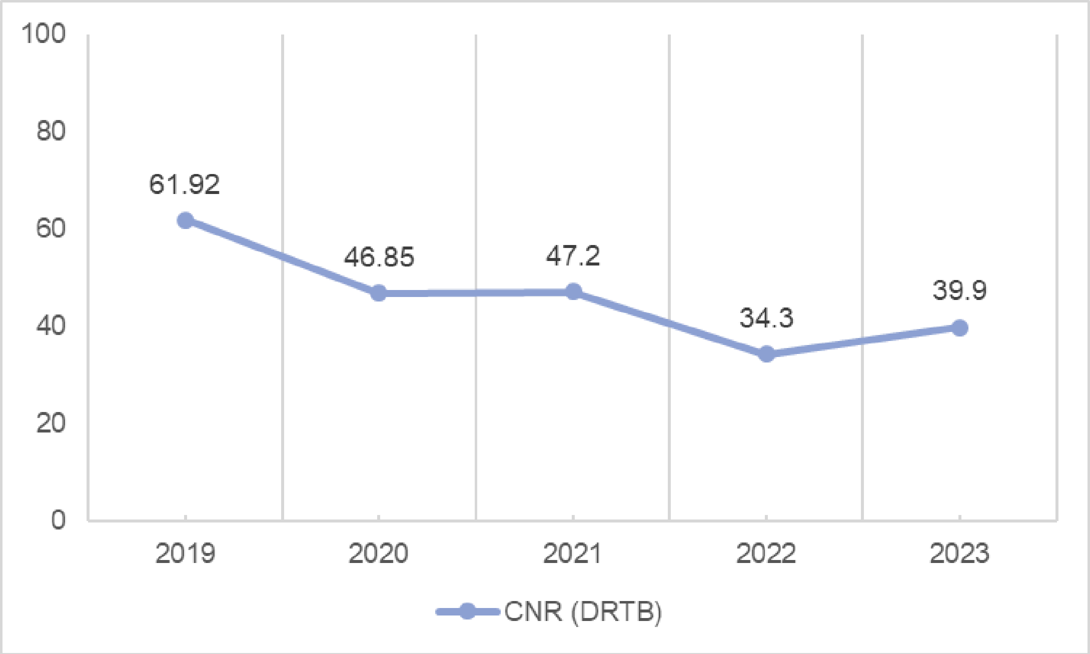

Figure 1.I 14 Case Notification Rate (Drug Resistant TB) Philippines, 2019 to 2023

In the case notification rate of drug resistant TB, a gradual decline was noted from 2019 with 61.92% to 34.3% in 2022 and a notable increase from 2022 to 39.9% in 2023. This further moves away from the target of a 90% case notification rate for drug resistant tuberculosis.

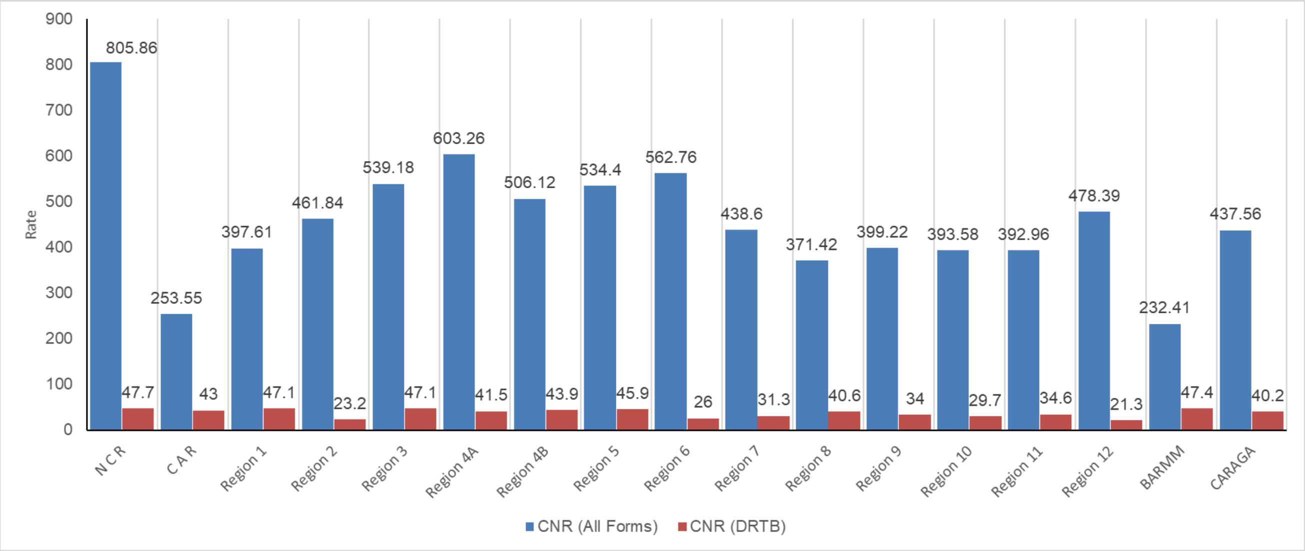

Figure 1.I 15 Case Notification Rate, All Forms and Drug Resistant Tuberculosis by Region, 2023

The regions with the highest case notification rate of all forms of TB are NCR with 805.86 cases per 100,000 persons, Region 4A with 603.26 cases per 100,000 persons and Region 6 with 562.76 cases per 100,000 persons while the regions with the lowest case notification rate of all forms of TB are BARMM with 232.41 cases per 100,000 persons, CAR with 253.55 cases per 100,000 persons and Region 8 with 371.42 cases per 100,000 persons.

As for the regions with highest case notification rate of drug resistant TB, NCR has the highest CNR-DRTB at 47.7%, followed by BARMM at 47.4% and Region 1 at 47.1% while the regions with the lowest CNR-DRTB are Region 12 at 21.3%, followed by Region 2 at 23.2% and Region 6 at 26%.

CHAPTER II

Summary Tables

Table 2.A.1 - MODERN METHOD OF FAMILY PLANNING

New Acceptors

Philippines, 2023

Area	Total Current Users	FSTR/BTL			Total	MSTR/NSV			Total
		Age group				Age group			
		10-14	15-19	20-49		10-14	15-19	20-49	
**Philippines**	**8,353,912**	**3**	**79**	**24,212**	**24,294**	**0**	**11**	**447**	**458**
**N C R**	**859,210**	**0**	**2**	**3,624**	**3,626**	**0**	**5**	**102**	**107**
Malabon City	33,641	0	1	17	18	0	0	0	0
Navotas City	25,067	0	0	46	46	0	0	2	2
Valenzuela City	37,966	0	0	73	73	0	0	4	4
Caloocan City	61,903	0	0	85	85	0	0	1	1
Marikina City	20,095	0	0	10	10	0	0	1	1
Pasig City	55,229	0	1	269	270	0	0	13	13
Pateros	3,813	0	0	18	18	0	0	1	1
Taguig City	71,609	0	0	0	0	0	5	12	17
Quezon City	306,956	0	0	494	494	0	0	0	0
Makati City	20,752	0	0	143	143	0	0	8	8
Mandaluyong City	23,665	0	0	170	170	0	0	5	5
San Juan City	5,890	0	0	0	0	0	0	0	0
Manila City	50,873	0	0	1,849	1,849	0	0	51	51
Las Piñas City	20,551	0	0	82	82	0	0	0	0
Muntinlupa City	41,934	0	0	186	186	0	0	4	4
Parañaque City	42,631	0	0	164	164	0	0	0	0
Pasay City	36,635	0	0	18	18	0	0	0	0
**C A R**	**161,545**	**0**	**0**	**288**	**288**	**0**	**0**	**37**	**37**
Abra	19,903	0	0	118	118	0	0	0	0
Apayao	24,278	0	0	0	0	0	0	1	1
Benguet	34,934	0	0	7	7	0	0	1	1
Ifugao	21,809	0	0	0	0	0	0	0	0
Kalinga	18,022	0	0	133	133	0	0	0	0
Mountain Province	13,937	0	0	14	14	0	0	0	0
City of Baguio	28,662	0	0	16	16	0	0	35	35
**Region 1**	**481,911**	**0**	**2**	**912**	**914**	**0**	**0**	**8**	**8**
Ilocos Norte	51,402	0	0	314	314	0	0	2	2
Ilocos Sur	76,206	0	2	160	162	0	0	0	0
La Union	69,182	0	0	82	82	0	0	0	0

Table 2.A.1 - MODERN METHOD OF FAMILY PLANNING

New Acceptors

Philippines, 2023

Area	Total Current Users	FSTR/BTL			Total	MSTR/NSV			Total
		Age group				Age group			
		10-14	15-19	20-49		10-14	15-19	20-49	
Pangasinan	278,036	0	0	356	356	0	0	6	6
City of Dagupan	7,085	0	0	0	0	0	0	0	0
**Region 2**	**320,396**	**0**	**3**	**603**	**606**	**0**	**0**	**1**	**1**
Batanes	945	0	0	0	0	0	0	0	0
Cagayan	106,997	0	1	78	79	0	0	0	0
Isabela	130,614	0	2	87	89	0	0	1	1
Nueva Vizcaya	49,739	0	0	432	432	0	0	0	0
Quirino	19,516	0	0	6	6	0	0	0	0
City of Santiago	12,585	0	0	0	0	0	0	0	0
**Region 3**	**884,246**	**3**	**3**	**9,462**	**9,468**	**0**	**1**	**15**	**16**
Aurora	22,956	2	0	5	7	0	0	0	0
Bataan	63,188	0	0	41	41	0	0	0	0
Bulacan	245,933	0	0	6,325	6,325	0	1	9	10
Nueva Ecija	186,491	0	1	774	775	0	0	0	0
Pampanga	159,224	1	2	1,226	1,229	0	0	5	5
Tarlac	115,785	0	0	321	321	0	0	1	1
Zambales	48,549	0	0	230	230	0	0	0	0
City of Angeles	29,054	0	0	315	315	0	0	0	0
City of Olongapo	13,066	0	0	225	225	0	0	0	0
**Region 4A**	**910,368**	**0**	**1**	**1,928**	**1,929**	**0**	**0**	**23**	**23**
Batangas	162,745	0	0	431	431	0	0	2	2
Cavite	164,389	0	0	382	382	0	0	5	5
Laguna	320,343	0	0	632	632	0	0	8	8
Quezon	105,489	0	0	124	124	0	0	2	2
Rizal	152,400	0	1	333	334	0	0	6	6
City of Lucena	5,002	0	0	26	26	0	0	0	0
**Region 4B**	**266,333**	**0**	**1**	**534**	**535**	**0**	**4**	**17**	**21**
Marinduque	15,205	0	0	56	56	0	0	1	1
Occidental Mindoro	40,816	0	0	194	194	0	0	0	0
Oriental Mindoro	63,083	0	0	161	161	0	0	2	2

Table 2.A.1 - MODERN METHOD OF FAMILY PLANNING

New Acceptors

Philippines, 2023

Area	Total Current Users	FSTR/BTL Age group 10-14	FSTR/BTL Age group 15-19	FSTR/BTL Age group 20-49	Total	MSTR/NSV Age group 10-14	MSTR/NSV Age group 15-19	MSTR/NSV Age group 20-49	Total
Palawan	93,326	0	0	47	47	0	4	12	16
Romblon	22,228	0	0	60	60	0	0	1	1
City of Puerto Princesa	31,675	0	1	16	17	0	0	1	1
**Region 5**	**484,529**	**0**	**5**	**300**	**305**	**0**	**0**	**2**	**2**
Albay	120,028	0	3	146	149	0	0	1	1
Camarines Norte	46,841	0	0	19	19	0	0	0	0
Camarines Sur	107,402	0	0	4	4	0	0	0	0
Catanduanes	23,049	0	2	10	12	0	0	0	0
Masbate	91,907	0	0	38	38	0	0	0	0
Sorsogon	65,058	0	0	25	25	0	0	0	0
City of Naga	30,244	0	0	58	58	0	0	1	1
**Region 6**	**639,939**	**0**	**4**	**989**	**993**	**0**	**1**	**39**	**40**
Aklan	46,162	0	0	56	56	0	0	0	0
Antique	47,022	0	1	266	267	0	1	0	1
Capiz	75,514	0	0	83	83	0	0	3	3
Guimaras	16,676	0	0	15	15	0	0	0	0
Iloilo	169,693	0	3	210	213	0	0	0	0
Negros Occidental	199,968	0	0	297	297	0	0	36	36
City of Bacolod	28,243	0	0	41	41	0	0	0	0
City of Iloilo	56,661	0	0	21	21	0	0	0	0
**Region 7**	**597,007**	**0**	**1**	**683**	**684**	**0**	**0**	**31**	**31**
Bohol	86,129	0	0	302	302	0	0	22	22
Cebu	309,013	0	1	170	171	0	0	3	3
Negros Oriental	90,628	0	0	48	48	0	0	0	0
Siquijor	9,078	0	0	0	0	0	0	0	0
City of Cebu	39,810	0	0	63	63	0	0	6	6
City of Lapu-Lapu	35,757	0	0	2	2	0	0	0	0
City of Mandaue	26,592	0	0	98	98	0	0	0	0
**Region 8**	**332,967**	**0**	**35**	**1,582**	**1,617**	**0**	**0**	**54**	**54**
Biliran	11,240	0	0	0	0	0	0	0	0

Table 2.A.1 - MODERN METHOD OF FAMILY PLANNING

New Acceptors

Philippines, 2023

Area	Total Current Users	FSTR/BTL			Total	MSTR/NSV			Total
		Age group				Age group			
		10-14	15-19	20-49		10-14	15-19	20-49	
Eastern Samar	35,729	0	34	1,055	1,089	0	0	51	51
Leyte	122,333	0	1	333	334	0	0	2	2
Northern Samar	49,058	0	0	15	15	0	0	0	0
Southern Leyte	27,698	0	0	12	12	0	0	0	0
Samar	41,329	0	0	112	112	0	0	1	1
Ormoc City	19,622	0	0	3	3	0	0	0	0
City of Tacloban	25,958	0	0	52	52	0	0	0	0
**Region 9**	**339,891**	**0**	**0**	**356**	**356**	**0**	**0**	**3**	**3**
Zamboanga del Norte	124,603	0	0	0	0	0	0	0	0
Zamboanga del Sur	94,708	0	0	12	12	0	0	1	1
Zamboanga Sibugay	44,556	0	0	0	0	0	0	0	0
City of Isabela	9,211	0	0	16	16	0	0	0	0
City of Zamboanga	66,813	0	0	328	328	0	0	2	2
**Region 10**	**514,846**	**0**	**1**	**766**	**767**	**0**	**0**	**19**	**19**
Bukidnon	158,694	0	1	65	66	0	0	2	2
Camiguin	6,535	0	0	6	6	0	0	0	0
Lanao del Norte	67,645	0	0	51	51	0	0	10	10
Misamis Occidental	69,918	0	0	67	67	0	0	1	1
Misamis Oriental	127,215	0	0	161	161	0	0	4	4
City of Cagayan De Oro	54,303	0	0	89	89	0	0	1	1
City of Iligan	30,536	0	0	327	327	0	0	1	1
**Region 11**	**532,707**	**0**	**14**	**1,142**	**1,156**	**0**	**0**	**71**	**71**
Davao de Oro	83,298	0	0	68	68	0	0	3	3
Davao del Norte	108,564	0	9	308	317	0	0	6	6
Davao Oriental	54,593	0	0	79	79	0	0	0	0
Davao del Sur	67,257	0	5	154	159	0	0	0	0
Davao Occidental	31,707	0	0	12	12	0	0	18	18
City of Davao	187,288	0	0	521	521	0	0	44	44
**Region 12**	**453,032**	**0**	**5**	**471**	**476**	**0**	**0**	**19**	**19**
Cotabato	134,232	0	0	236	236	0	0	14	14

Table 2.A.1 - MODERN METHOD OF FAMILY PLANNING

New Acceptors

Philippines, 2023

Area	Total Current Users	FSTR/BTL			Total	MSTR/NSV			Total
		Age group				Age group			
		10-14	15-19	20-49		10-14	15-19	20-49	
Sarangani	72,262	0	5	25	30	0	0	0	0
South Cotabato	96,119	0	0	95	95	0	0	3	3
Sultan Kudarat	101,026	0	0	82	82	0	0	1	1
					0				0
City of General Santos	49,393	0	0	33	33	0	0	1	1
**BARMM**	**290,634**	**0**	**0**	**178**	**178**	**0**	**0**	**5**	**5**
Basilan	15,552	0	0	5	5	0	0	0	0
Lanao del Sur	37,406	0	0	82	82	0	0	5	5
Maguindanao	92,272	0	0	29	29	0	0	0	0
Sulu	82,939	0	0	20	20	0	0	0	0
Tawi-Tawi	18,014	0	0	6	6	0	0	0	0
SGU	8,193	0	0	1	1	0	0	0	0
City of Cotabato	36,258	0	0	35	35	0	0	0	0
**Caraga**	**284,351**	**0**	**2**	**394**	**396**	**0**	**0**	**1**	**1**
Agusan del Norte	40,460	0	0	118	118	0	0	0	0
Agusan del Sur	86,938	0	0	10	10	0	0	0	0
Surigao del Norte	50,319	0	2	217	219	0	0	0	0
Surigao del Sur	56,051	0	0	8	8	0	0	0	0
Dinagat Islands	10,768	0	0	0	0	0	0	0	0
City of Butuan	39,815	0	0	41	41	0	0	1	1

Table 2.A.1 - MODERN METHOD OF FAMILY PLANNING

New Acceptors

Philippines, 2023

Area	Total Current Users	CONDOM			Total	IUD-INTERVAL			Total
		Age group				Age group			
		10-14	15-19	20-49		10-14	15-19	20-49	
**Philippines**	**8,353,912**	**79**	**4,653**	**57,979**	**62,711**	**18**	**860**	**11,888**	**12,766**
**N C R**	**859,210**	**25**	**1,724**	**27,853**	**29,602**	**2**	**133**	**1,744**	**1,879**
Malabon City	33,641	2	71	599	672	0	1	8	9
Navotas City	25,067	2	261	439	702	0	21	44	65
Valenzuela City	37,966	0	1	276	277	0	0	91	91
Caloocan City	61,903	1	59	674	734	0	10	18	28
Marikina City	20,095	0	5	71	76	0	5	4	9
Pasig City	55,229	0	10	733	743	0	13	179	192
Pateros	3,813	0	1	61	62	0	0	12	12
Taguig City	71,609	6	182	3,361	3,549	0	0	23	23
Quezon City	306,956	0	392	3,305	3,697	0	44	323	367
Makati City	20,752	1	30	1,341	1,372	1	1	121	123
Mandaluyong City	23,665	0	33	555	588	1	4	117	122
San Juan City	5,890	0	0	69	69	0	3	46	49
Manila City	50,873	7	325	12,279	12,611	0	28	260	288
Las Piñas City	20,551	4	67	2,107	2,178	0	2	29	31
Muntinlupa City	41,934	0	4	150	154	0	0	31	31
Parañaque City	42,631	2	72	939	1,013	0	0	17	17
Pasay City	36,635	0	211	894	1,105	0	1	421	422
**C A R**	**161,545**	**0**	**27**	**305**	**332**	**0**	**13**	**37**	**50**
Abra	19,903	0	5	98	103	0	1	0	1
Apayao	24,278	0	10	17	27	0	0	0	0
Benguet	34,934	0	4	72	76	0	2	11	13
Ifugao	21,809	0	0	0	0	0	0	1	1
Kalinga	18,022	0	1	34	35	0	0	5	5
Mountain Province	13,937	0	2	5	7	0	0	2	2
City of Baguio	28,662	0	5	79	84	0	10	18	28
**Region 1**	**481,911**	**1**	**93**	**1,955**	**2,049**	**1**	**30**	**300**	**331**
Ilocos Norte	51,402	0	1	14	15	0	1	78	79
Ilocos Sur	76,206	0	8	104	112	0	0	8	8
La Union	69,182	1	26	82	109	0	10	38	48

Table 2.A.1 - MODERN METHOD OF FAMILY PLANNING

New Acceptors

Philippines, 2023

Area	Total Current Users	CONDOM			Total	IUD-INTERVAL			Total
		Age group				Age group			
		10-14	15-19	20-49		10-14	15-19	20-49	
Pangasinan	278,036	0	58	1,755	1,813	1	17	173	191
City of Dagupan	7,085	0	0	0	0	0	2	3	5
**Region 2**	**320,396**	**1**	**12**	**165**	**178**	**1**	**29**	**431**	**461**
Batanes	945	0	0	0	0	0	0	0	0
Cagayan	106,997	0	1	54	55	0	9	68	77
Isabela	130,614	1	11	74	86	1	7	22	30
Nueva Vizcaya	49,739	0	0	35	35	0	13	340	353
Quirino	19,516	0	0	2	2	0	0	1	1
City of Santiago	12,585	0	0	0	0	0	0	0	0
**Region 3**	**884,246**	**3**	**318**	**5,321**	**5,642**	**6**	**97**	**2,850**	**2,953**
Aurora	22,956	0	1	10	11	0	0	1	1
Bataan	63,188	0	24	366	390	0	8	37	45
Bulacan	245,933	0	140	1,917	2,057	6	68	2,628	2,702
Nueva Ecija	186,491	0	46	473	519	0	7	90	97
Pampanga	159,224	2	62	1,260	1,324	0	7	41	48
Tarlac	115,785	1	31	645	677	0	3	24	27
Zambales	48,549	0	9	378	387	0	0	4	4
City of Angeles	29,054	0	0	26	26	0	0	4	4
City of Olongapo	13,066	0	5	246	251	0	4	21	25
**Region 4A**	**910,368**	**5**	**1,073**	**6,740**	**7,818**	**3**	**67**	**657**	**727**
Batangas	162,745	1	13	438	452	1	3	98	102
Cavite	164,389	2	41	459	502	1	17	166	184
Laguna	320,343	1	969	5,271	6,241	0	18	146	164
Quezon	105,489	1	19	152	172	1	17	86	104
Rizal	152,400	0	28	401	429	0	12	159	171
City of Lucena	5,002	0	3	19	22	0	0	2	2
**Region 4B**	**266,333**	**0**	**25**	**328**	**353**	**0**	**5**	**207**	**212**
Marinduque	15,205	0	0	10	10	0	0	2	2
Occidental Mindoro	40,816	0	2	119	121	0	0	135	135
Oriental Mindoro	63,083	0	4	70	74	0	2	47	49

Table 2.A.1 - MODERN METHOD OF FAMILY PLANNING

New Acceptors

Philippines, 2023

Area	Total Current Users	CONDOM			Total	IUD-INTERVAL			Total
		Age group				Age group			
		10-14	15-19	20-49		10-14	15-19	20-49	
Palawan	93,326	0	13	55	68	0	0	12	12
Romblon	22,228	0	4	31	35	0	0	10	10
City of Puerto Princesa	31,675	0	2	43	45	0	3	1	4
**Region 5**	**484,529**	**14**	**60**	**1,962**	**2,036**	**0**	**16**	**84**	**100**
Albay	120,028	0	27	591	618	0	1	38	39
Camarines Norte	46,841	0	9	92	101	0	0	4	4
Camarines Sur	107,402	0	13	682	695	0	3	4	7
Catanduanes	23,049	14	1	23	38	0	0	4	4
Masbate	91,907	0	7	99	106	0	3	18	21
Sorsogon	65,058	0	1	10	11	0	0	0	0
City of Naga	30,244	0	2	465	467	0	9	16	25
**Region 6**	**639,939**	**0**	**218**	**2,382**	**2,600**	**1**	**62**	**580**	**643**
Aklan	46,162	0	89	671	760	0	0	15	15
Antique	47,022	0	4	135	139	0	0	13	13
Capiz	75,514	0	2	280	282	0	0	65	65
Guimaras	16,676	0	2	15	17	0	1	8	9
Iloilo	169,693	0	26	513	539	1	18	144	163
Negros Occidental	199,968	0	84	626	710	0	32	257	289
City of Bacolod	28,243	0	8	93	101	0	4	32	36
City of Iloilo	56,661	0	3	49	52	0	7	46	53
**Region 7**	**597,007**	**0**	**161**	**1,927**	**2,088**	**0**	**103**	**1,354**	**1,457**
Bohol	86,129	0	32	315	347	0	22	361	383
Cebu	309,013	0	48	822	870	0	45	508	553
Negros Oriental	90,628	0	14	204	218	0	11	129	140
Siquijor	9,078	0	0	16	16	0	0	2	2
City of Cebu	39,810	0	6	220	226	0	17	267	284
City of Lapu-Lapu	35,757	0	46	215	261	0	3	31	34
City of Mandaue	26,592	0	15	135	150	0	5	56	61
**Region 8**	**332,967**	**2**	**82**	**671**	**755**	**0**	**35**	**324**	**359**
Biliran	11,240	0	4	18	22	0	2	9	11

Table 2.A.1 - MODERN METHOD OF FAMILY PLANNING

New Acceptors

Philippines, 2023

Area	Total Current Users	CONDOM			Total	IUD-INTERVAL			Total
		Age group				Age group			
		10-14	15-19	20-49		10-14	15-19	20-49	
Eastern Samar	35,729	1	32	252	285	0	8	47	55
Leyte	122,333	1	21	140	162	0	9	138	147
Northern Samar	49,058	0	4	59	63	0	0	15	15
Southern Leyte	27,698	0	2	39	41	0	1	34	35
Samar	41,329	0	6	110	116	0	13	28	41
Ormoc City	19,622	0	2	9	11	0	0	0	0
City of Tacloban	25,958	0	11	44	55	0	2	53	55
**Region 9**	**339,891**	**0**	**113**	**535**	**648**	**0**	**31**	**559**	**590**
Zamboanga del Norte	124,603	0	50	77	127	0	4	417	421
Zamboanga del Sur	94,708	0	9	85	94	0	10	63	73
Zamboanga Sibugay	44,556	0	7	74	81	0	14	40	54
City of Isabela	9,211	0	33	176	209	0	0	0	0
City of Zamboanga	66,813	0	14	123	137	0	3	39	42
**Region 10**	**514,846**	**12**	**174**	**1,914**	**2,100**	**2**	**120**	**1,237**	**1,359**
Bukidnon	158,694	3	23	186	212	2	32	142	176
Camiguin	6,535	0	0	1	1	0	0	1	1
Lanao del Norte	67,645	0	29	761	790	0	4	78	82
Misamis Occidental	69,918	0	12	346	358	0	0	28	28
Misamis Oriental	127,215	9	48	147	204	0	26	271	297
City of Cagayan De Oro	54,303	0	31	259	290	0	46	664	710
City of Iligan	30,536	0	31	214	245	0	12	53	65
**Region 11**	**532,707**	**13**	**314**	**2,385**	**2,712**	**0**	**57**	**864**	**921**
Davao de Oro	83,298	0	14	128	142	0	10	72	82
Davao del Norte	108,564	0	17	293	310	0	5	198	203
Davao Oriental	54,593	0	3	31	34	0	1	19	20
Davao del Sur	67,257	2	12	125	139	0	11	108	119
Davao Occidental	31,707	1	3	26	30	0	0	54	54
City of Davao	187,288	10	265	1,782	2,057	0	30	413	443
**Region 12**	**453,032**	**2**	**126**	**1,195**	**1,323**	**1**	**30**	**259**	**290**
Cotabato	134,232	0	42	365	407	0	13	123	136

Table 2.A.1 - MODERN METHOD OF FAMILY PLANNING

New Acceptors

Philippines, 2023

Area	Total Current Users	CONDOM			Total	IUD-INTERVAL			Total
		Age group				Age group			
		10-14	15-19	20-49		10-14	15-19	20-49	
Sarangani	72,262	0	4	186	190	0	0	9	9
South Cotabato	96,119	2	33	195	230	1	6	49	56
Sultan Kudarat	101,026	0	14	79	93	0	1	22	23
					0				0
City of General Santos	49,393	0	33	370	403	0	10	56	66
**BARMM**	**290,634**	**1**	**67**	**1,621**	**1,689**	**0**	**4**	**80**	**84**
Basilan	15,552	0	6	26	32	0	1	12	13
Lanao del Sur	37,406	0	15	664	679	0	0	26	26
Maguindanao	92,272	1	34	426	461	0	0	3	3
Sulu	82,939	0	3	137	140	0	0	6	6
Tawi-Tawi	18,014	0	1	16	17	0	0	0	0
SGU	8,193	0	3	52	55	0	0	1	1
City of Cotabato	36,258	0	5	300	305	0	3	32	35
**Caraga**	**284,351**	**0**	**66**	**720**	**786**	**1**	**28**	**321**	**350**
Agusan del Norte	40,460	0	14	86	100	1	5	41	47
Agusan del Sur	86,938	0	17	197	214	0	15	93	108
Surigao del Norte	50,319	0	12	206	218	0	2	126	128
Surigao del Sur	56,051	0	11	105	116	0	4	31	35
Dinagat Islands	10,768	0	7	57	64	0	2	22	24
City of Butuan	39,815	0	5	69	74	0	0	8	8

Table 2.A.1 - MODERN METHOD OF FAMILY PLANNING

New Acceptors

Philippines, 2023

Area	Total Current Users	IUD-POSTPARTUM Age group			Total	PILLS-POP Age group			Total
		10-14	15-19	20-49		10-14	15-19	20-49	
**Philippines**	**8,353,912**	**140**	**3,450**	**14,661**	**18,251**	**197**	**11,672**	**78,215**	**90,084**
**N C R**	**859,210**	**43**	**970**	**5,603**	**6,616**	**30**	**1,762**	**21,767**	**23,559**
Malabon City	33,641	0	2	4	6	2	81	508	591
Navotas City	25,067	0	0	0	0	4	300	477	781
Valenzuela City	37,966	0	1	219	220	0	9	921	930
Caloocan City	61,903	13	319	686	1,018	1	165	1,001	1,167
Marikina City	20,095	7	152	1,077	1,236	0	11	202	213
Pasig City	55,229	6	105	582	693	0	32	826	858
Pateros	3,813	0	1	0	1	0	0	27	27
Taguig City	71,609	1	9	146	156	1	103	1,467	1,571
Quezon City	306,956	9	227	896	1,132	1	291	2,983	3,275
Makati City	20,752	0	29	413	442	0	15	930	945
Mandaluyong City	23,665	0	8	85	93	0	23	400	423
San Juan City	5,890	0	0	0	0	0	2	95	97
Manila City	50,873	7	110	1,091	1,208	20	508	8,758	9,286
Las Piñas City	20,551	0	2	12	14	0	82	1,548	1,630
Muntinlupa City	41,934	0	0	0	0	0	30	259	289
Parañaque City	42,631	0	3	12	15	1	66	601	668
Pasay City	36,635	0	2	380	382	0	44	764	808
**C A R**	**161,545**	**0**	**1**	**21**	**22**	**1**	**99**	**808**	**908**
Abra	19,903	0	0	0	0	0	10	65	75
Apayao	24,278	0	0	4	4	0	13	44	57
Benguet	34,934	0	1	4	5	1	35	218	254
Ifugao	21,809	0	0	0	0	0	0	19	19
Kalinga	18,022	0	0	2	2	0	7	50	57
Mountain Province	13,937	0	0	0	0	0	11	29	40
City of Baguio	28,662	0	0	11	11	0	23	383	406
**Region 1**	**481,911**	**1**	**36**	**76**	**113**	**0**	**79**	**801**	**880**
Ilocos Norte	51,402	0	2	16	18	0	21	184	205
Ilocos Sur	76,206	0	1	15	16	0	20	497	517
La Union	69,182	1	27	30	58	0	32	95	127

Table 2.A.1 - MODERN METHOD OF FAMILY PLANNING

New Acceptors

Philippines, 2023

Area	Total Current Users	IUD-POSTPARTUM			Total	PILLS-POP			Total
		Age group				Age group			
		10-14	15-19	20-49		10-14	15-19	20-49	
Pangasinan	278,036	0	0	0	0	0	0	0	0
City of Dagupan	7,085	0	6	15	21	0	6	25	31
**Region 2**	**320,396**	**1**	**39**	**159**	**199**	**4**	**277**	**1,287**	**1,568**
Batanes	945	0	0	0	0	0	0	2	2
Cagayan	106,997	0	9	47	56	1	98	577	676
Isabela	130,614	0	12	15	27	3	152	627	782
Nueva Vizcaya	49,739	1	18	96	115	0	22	55	77
Quirino	19,516	0	0	1	1	0	5	26	31
City of Santiago	12,585	0	0	0	0	0	0	0	0
**Region 3**	**884,246**	**16**	**172**	**875**	**1,063**	**43**	**1,310**	**8,169**	**9,522**
Aurora	22,956	1	2	5	8	0	0	21	21
Bataan	63,188	0	1	4	5	0	280	841	1,121
Bulacan	245,933	0	27	196	223	1	308	2,570	2,879
Nueva Ecija	186,491	14	123	375	512	37	347	1,609	1,993
Pampanga	159,224	1	17	191	209	3	186	1,324	1,513
Tarlac	115,785	0	1	75	76	1	94	923	1,018
Zambales	48,549	0	0	11	11	1	39	298	338
City of Angeles	29,054	0	0	6	6	0	22	156	178
City of Olongapo	13,066	0	1	12	13	0	34	427	461
**Region 4A**	**910,368**	**4**	**67**	**442**	**513**	**14**	**1,598**	**10,247**	**11,859**
Batangas	162,745	0	9	77	86	5	96	929	1,030
Cavite	164,389	1	16	96	113	3	151	1,217	1,371
Laguna	320,343	3	11	120	134	1	961	6,010	6,972
Quezon	105,489	0	13	35	48	3	127	611	741
Rizal	152,400	0	17	113	130	2	229	1,336	1,567
City of Lucena	5,002	0	1	1	2	0	34	144	178
**Region 4B**	**266,333**	**12**	**54**	**184**	**250**	**9**	**282**	**1,315**	**1,606**
Marinduque	15,205	0	1	1	2	0	15	92	107
Occidental Mindoro	40,816	0	0	1	1	0	14	429	443
Oriental Mindoro	63,083	3	48	150	201	1	27	231	259

Table 2.A.1 - MODERN METHOD OF FAMILY PLANNING

New Acceptors

Philippines, 2023

Area	Total Current Users	IUD-POSTPARTUM			Total	PILLS-POP			Total
		Age group				Age group			
		10-14	15-19	20-49		10-14	15-19	20-49	
Palawan	93,326	9	4	18	31	0	167	379	546
Romblon	22,228	0	0	9	9	5	13	64	82
City of Puerto Princesa	31,675	0	1	5	6	3	46	120	169
**Region 5**	**484,529**	**0**	**14**	**75**	**89**	**1**	**246**	**2,046**	**2,293**
Albay	120,028	0	6	39	45	1	62	822	885
Camarines Norte	46,841	0	7	7	14	0	33	93	126
Camarines Sur	107,402	0	1	11	12	0	97	782	879
Catanduanes	23,049	0	0	1	1	0	6	77	83
Masbate	91,907	0	0	2	2	0	34	160	194
Sorsogon	65,058	0	0	1	1	0	6	40	46
City of Naga	30,244	0	0	14	14	0	8	72	80
**Region 6**	**639,939**	**10**	**252**	**1,075**	**1,337**	**8**	**978**	**4,633**	**5,619**
Aklan	46,162	0	5	104	109	0	92	544	636
Antique	47,022	0	0	6	6	0	54	319	373
Capiz	75,514	0	0	10	10	0	55	472	527
Guimaras	16,676	0	1	5	6	1	10	40	51
Iloilo	169,693	1	115	400	516	0	116	769	885
Negros Occidental	199,968	5	48	201	254	5	618	2,248	2,871
City of Bacolod	28,243	0	1	34	35	2	30	229	261
City of Iloilo	56,661	4	82	315	401	0	3	12	15
**Region 7**	**597,007**	**6**	**332**	**1,309**	**1,647**	**7**	**835**	**5,589**	**6,431**
Bohol	86,129	2	145	553	700	1	143	991	1,135
Cebu	309,013	4	164	592	760	3	297	1,845	2,145
Negros Oriental	90,628	0	4	21	25	2	150	686	838
Siquijor	9,078	0	0	0	0	0	5	65	70
City of Cebu	39,810	0	8	48	56	1	113	1,007	1,121
City of Lapu-Lapu	35,757	0	0	5	5	0	66	426	492
City of Mandaue	26,592	0	11	90	101	0	61	569	630
**Region 8**	**332,967**	**0**	**38**	**176**	**214**	**4**	**191**	**1,278**	**1,473**
Biliran	11,240	0	1	3	4	2	20	73	95

Table 2.A.1 - MODERN METHOD OF FAMILY PLANNING

New Acceptors

Philippines, 2023

Area	Total Current Users	IUD-POSTPARTUM			Total	PILLS-POP			Total
		Age group				Age group			
		10-14	15-19	20-49		10-14	15-19	20-49	
Eastern Samar	35,729	0	13	109	122	0	32	324	356
Leyte	122,333	0	8	15	23	1	40	324	365
Northern Samar	49,058	0	3	1	4	1	26	126	153
Southern Leyte	27,698	0	6	18	24	0	8	78	86
Samar	41,329	0	0	19	19	0	38	243	281
Ormoc City	19,622	0	1	0	1	0	14	25	39
City of Tacloban	25,958	0	6	11	17	0	13	85	98
**Region 9**	**339,891**	**17**	**538**	**1,304**	**1,859**	**8**	**350**	**1,287**	**1,645**
Zamboanga del Norte	124,603	0	4	13	17	0	21	155	176
Zamboanga del Sur	94,708	12	470	807	1,289	6	87	266	359
Zamboanga Sibugay	44,556	1	7	19	27	0	44	203	247
City of Isabela	9,211	0	0	0	0	0	77	203	280
City of Zamboanga	66,813	4	57	465	526	2	121	460	583
**Region 10**	**514,846**	**27**	**582**	**1,345**	**1,954**	**9**	**694**	**3,003**	**3,706**
Bukidnon	158,694	0	28	71	99	2	290	529	821
Camiguin	6,535	0	0	12	12	0	1	2	3
Lanao del Norte	67,645	0	3	56	59	0	63	710	773
Misamis Occidental	69,918	0	1	16	17	6	40	427	473
Misamis Oriental	127,215	15	302	633	950	0	99	389	488
City of Cagayan De Oro	54,303	10	161	252	423	0	117	550	667
City of Iligan	30,536	2	87	305	394	1	84	396	481
**Region 11**	**532,707**	**2**	**91**	**771**	**864**	**29**	**1,321**	**7,075**	**8,425**
Davao de Oro	83,298	0	8	19	27	4	286	794	1,084
Davao del Norte	108,564	1	14	108	123	11	173	975	1,159
Davao Oriental	54,593	0	1	16	17	0	82	456	538
Davao del Sur	67,257	0	6	23	29	5	223	769	997
Davao Occidental	31,707	0	3	9	12	3	68	126	197
City of Davao	187,288	1	59	596	656	6	489	3,955	4,450
**Region 12**	**453,032**	**0**	**39**	**201**	**240**	**16**	**660**	**3,270**	**3,946**
Cotabato	134,232	0	11	76	87	7	271	1,388	1,666

Table 2.A.1 - MODERN METHOD OF FAMILY PLANNING

New Acceptors

Philippines, 2023

Area	Total Current Users	IUD-POSTPARTUM			Total	PILLS-POP			Total
		Age group				Age group			
		10-14	15-19	20-49		10-14	15-19	20-49	
Sarangani	72,262	0	0	3	3	0	87	216	303
South Cotabato	96,119	0	11	56	67	4	114	786	904
Sultan Kudarat	101,026	0	15	20	35	2	113	450	565
					0				0
City of General Santos	49,393	0	2	46	48	3	75	430	508
**BARMM**	**290,634**	**0**	**45**	**430**	**475**	**6**	**407**	**3,019**	**3,432**
Basilan	15,552	0	6	23	29	1	49	210	260
Lanao del Sur	37,406	0	0	54	54	2	51	604	657
Maguindanao	92,272	0	13	166	179	0	177	971	1,148
Sulu	82,939	0	1	0	1	1	71	543	615
Tawi-Tawi	18,014	0	0	0	0	0	13	112	125
SGU	8,193	0	0	0	0	0	32	275	307
City of Cotabato	36,258	0	25	187	212	2	14	304	320
**Caraga**	**284,351**	**1**	**180**	**615**	**796**	**8**	**583**	**2,621**	**3,212**
Agusan del Norte	40,460	0	6	50	56	2	186	614	802
Agusan del Sur	86,938	0	40	79	119	3	143	591	737
Surigao del Norte	50,319	1	124	376	501	0	83	682	765
Surigao del Sur	56,051	0	5	83	88	2	84	346	432
Dinagat Islands	10,768	0	0	3	3	1	18	83	102
City of Butuan	39,815	0	5	24	29	0	69	305	374

Table 2.A.1 - MODERN METHOD OF FAMILY PLANNING

New Acceptors

Philippines, 2023

Area	Total Current Users	PILLS-COC			Total	INJECTABLES			Total
		Age group				Age group			
		10-14	15-19	20-49		10-14	15-19	20-49	
**Philippines**	**8,353,912**	**118**	**10,026**	**133,249**	**143,393**	**296**	**21,358**	**183,212**	**204,866**
**N C R**	**859,210**	**16**	**1,319**	**30,544**	**31,879**	**71**	**3,665**	**49,762**	**53,498**
Malabon City	33,641	1	35	377	413	2	143	835	980
Navotas City	25,067	3	316	609	928	7	433	1,207	1,647
Valenzuela City	37,966	1	24	1,600	1,625	1	179	3,083	3,263
Caloocan City	61,903	0	129	945	1,074	2	505	2,931	3,438
Marikina City	20,095	0	12	146	158	0	105	1,138	1,243
Pasig City	55,229	0	47	1,752	1,799	1	140	2,251	2,392
Pateros	3,813	0	2	102	104	0	5	143	148
Taguig City	71,609	0	62	1,365	1,427	5	230	3,561	3,796
Quezon City	306,956	1	159	3,306	3,466	3	414	3,739	4,156
Makati City	20,752	0	18	1,047	1,065	0	49	1,381	1,430
Mandaluyong City	23,665	0	22	667	689	0	38	888	926
San Juan City	5,890	0	3	115	118	1	14	212	227
Manila City	50,873	10	269	15,203	15,482	43	729	21,619	22,391
Las Piñas City	20,551	0	51	1,768	1,819	4	316	2,986	3,306
Muntinlupa City	41,934	0	23	277	300	1	170	1,566	1,737
Parañaque City	42,631	0	30	530	560	1	160	1,282	1,443
Pasay City	36,635	0	117	735	852	0	35	940	975
**C A R**	**161,545**	**0**	**48**	**587**	**635**	**1**	**157**	**1,161**	**1,319**
Abra	19,903	0	6	113	119	0	16	77	93
Apayao	24,278	0	11	56	67	1	16	47	64
Benguet	34,934	0	24	209	233	0	35	294	329
Ifugao	21,809	0	0	5	5	0	0	14	14
Kalinga	18,022	0	1	40	41	0	2	46	48
Mountain Province	13,937	0	3	18	21	0	6	27	33
City of Baguio	28,662	0	3	146	149	0	82	656	738
**Region 1**	**481,911**	**9**	**370**	**9,161**	**9,540**	**5**	**416**	**5,728**	**6,149**
Ilocos Norte	51,402	1	28	432	461	0	19	218	237
Ilocos Sur	76,206	0	37	1,042	1,079	1	15	593	609
La Union	69,182	0	39	245	284	0	87	483	570

Table 2.A.1 - MODERN METHOD OF FAMILY PLANNING

New Acceptors

Philippines, 2023

Area	Total Current Users	PILLS-COC			Total	INJECTABLES			Total
		Age group				Age group			
		10-14	15-19	20-49		10-14	15-19	20-49	
Pangasinan	278,036	8	264	7,425	7,697	4	283	4,293	4,580
City of Dagupan	7,085	0	2	17	19	0	12	141	153
**Region 2**	**320,396**	**1**	**166**	**1,631**	**1,798**	**4**	**330**	**1,450**	**1,784**
Batanes	945	0	1	0	1	0	0	1	1
Cagayan	106,997	1	60	895	956	1	67	491	559
Isabela	130,614	0	75	473	548	3	216	722	941
Nueva Vizcaya	49,739	0	17	187	204	0	33	187	220
Quirino	19,516	0	13	75	88	0	14	49	63
City of Santiago	12,585	0	0	1	1	0	0	0	0
**Region 3**	**884,246**	**8**	**1,709**	**21,498**	**23,215**	**40**	**3,350**	**29,163**	**32,553**
Aurora	22,956	0	0	74	74	0	3	65	68
Bataan	63,188	1	82	1,066	1,149	3	275	1,856	2,134
Bulacan	245,933	1	597	7,261	7,859	8	1,047	11,082	12,137
Nueva Ecija	186,491	4	343	4,286	4,633	11	650	4,123	4,784
Pampanga	159,224	0	258	2,999	3,257	8	576	4,725	5,309
Tarlac	115,785	1	176	2,905	3,082	4	227	2,750	2,981
Zambales	48,549	0	64	880	944	2	242	1,450	1,694
City of Angeles	29,054	1	26	512	539	1	96	1,307	1,404
City of Olongapo	13,066	0	163	1,515	1,678	3	234	1,805	2,042
**Region 4A**	**910,368**	**10**	**1,196**	**12,841**	**14,047**	**37**	**3,058**	**26,412**	**29,507**
Batangas	162,745	4	59	1,495	1,558	6	195	2,490	2,691
Cavite	164,389	2	155	1,981	2,138	6	623	5,554	6,183
Laguna	320,343	1	794	7,005	7,800	6	997	9,469	10,472
Quezon	105,489	2	76	753	831	10	451	2,405	2,866
Rizal	152,400	1	106	1,470	1,577	9	731	6,125	6,865
City of Lucena	5,002	0	6	137	143	0	61	369	430
**Region 4B**	**266,333**	**5**	**184**	**1,161**	**1,350**	**21**	**782**	**2,635**	**3,438**
Marinduque	15,205	0	7	109	116	1	22	213	236
Occidental Mindoro	40,816	0	27	90	117	0	25	102	127
Oriental Mindoro	63,083	0	29	386	415	2	85	543	630

Table 2.A.1 - MODERN METHOD OF FAMILY PLANNING

New Acceptors

Philippines, 2023

Area	Total Current Users	PILLS-COC			Total	INJECTABLES			Total
		Age group				Age group			
		10-14	15-19	20-49		10-14	15-19	20-49	
Palawan	93,326	3	106	343	452	14	467	1,215	1,696
Romblon	22,228	2	4	144	150	1	21	164	186
City of Puerto Princesa	31,675	0	11	89	100	3	162	398	563
**Region 5**	**484,529**	**2**	**221**	**4,677**	**4,900**	**4**	**575**	**6,317**	**6,896**
Albay	120,028	2	59	1,422	1,483	1	158	2,087	2,246
Camarines Norte	46,841	0	25	79	104	1	85	474	560
Camarines Sur	107,402	0	52	1,544	1,596	2	172	2,354	2,528
Catanduanes	23,049	0	4	71	75	0	20	103	123
Masbate	91,907	0	47	465	512	0	70	638	708
Sorsogon	65,058	0	18	119	137	0	44	209	253
City of Naga	30,244	0	16	977	993	0	26	452	478
**Region 6**	**639,939**	**2**	**765**	**8,104**	**8,871**	**8**	**1,332**	**9,471**	**10,811**
Aklan	46,162	0	62	633	695	1	157	988	1,146
Antique	47,022	1	14	311	326	0	144	955	1,099
Capiz	75,514	0	60	818	878	0	106	1,001	1,107
Guimaras	16,676	0	19	264	283	0	9	74	83
Iloilo	169,693	0	206	2,307	2,513	1	299	2,469	2,769
Negros Occidental	199,968	1	363	3,169	3,533	6	499	3,180	3,685
City of Bacolod	28,243	0	23	275	298	0	91	613	704
City of Iloilo	56,661	0	18	327	345	0	27	191	218
**Region 7**	**597,007**	**3**	**424**	**7,388**	**7,815**	**15**	**1,596**	**11,562**	**13,173**
Bohol	86,129	0	95	844	939	1	146	1,071	1,218
Cebu	309,013	1	125	3,435	3,561	3	451	4,071	4,525
Negros Oriental	90,628	1	88	946	1,035	4	411	1,939	2,354
Siquijor	9,078	0	1	28	29	0	10	79	89
City of Cebu	39,810	1	53	1,429	1,483	4	388	2,534	2,926
City of Lapu-Lapu	35,757	0	56	444	500	1	121	1,105	1,227
City of Mandaue	26,592	0	6	262	268	2	69	763	834
**Region 8**	**332,967**	**1**	**575**	**5,908**	**6,484**	**6**	**601**	**4,613**	**5,220**
Biliran	11,240	0	17	70	87	4	23	107	134

Table 2.A.1 - MODERN METHOD OF FAMILY PLANNING

New Acceptors

Philippines, 2023

Area	Total Current Users	PILLS-COC Age group			Total	INJECTABLES Age group			Total
		10-14	15-19	20-49		10-14	15-19	20-49	
Eastern Samar	35,729	0	145	3,002	3,147	1	160	1,506	1,667
Leyte	122,333	0	223	943	1,166	0	100	882	982
Northern Samar	49,058	0	35	446	481	0	93	646	739
Southern Leyte	27,698	0	8	171	179	0	32	180	212
Samar	41,329	1	120	1,017	1,138	1	152	1,034	1,187
Ormoc City	19,622	0	6	33	39	0	21	43	64
City of Tacloban	25,958	0	21	226	247	0	20	215	235
**Region 9**	**339,891**	**3**	**291**	**2,079**	**2,373**	**6**	**501**	**2,851**	**3,358**
Zamboanga del Norte	124,603	1	42	291	334	2	76	393	471
Zamboanga del Sur	94,708	1	38	372	411	0	72	408	480
Zamboanga Sibugay	44,556	0	68	388	456	2	94	393	489
City of Isabela	9,211	0	13	197	210	0	31	243	274
City of Zamboanga	66,813	1	130	831	962	2	228	1,414	1,644
**Region 10**	**514,846**	**7**	**622**	**6,578**	**7,207**	**10**	**754**	**5,226**	**5,990**
Bukidnon	158,694	4	209	1,334	1,547	6	253	781	1,040
Camiguin	6,535	0	0	4	4	0	0	6	6
Lanao del Norte	67,645	2	97	1,178	1,277	1	88	1,122	1,211
Misamis Occidental	69,918	0	49	979	1,028	0	41	631	672
Misamis Oriental	127,215	0	78	501	579	3	122	551	676
City of Cagayan De Oro	54,303	1	124	2,095	2,220	0	174	1,555	1,729
City of Iligan	30,536	0	65	487	552	0	76	580	656
**Region 11**	**532,707**	**13**	**867**	**7,610**	**8,490**	**37**	**1,417**	**7,304**	**8,758**
Davao de Oro	83,298	2	103	1,220	1,325	1	193	687	881
Davao del Norte	108,564	1	94	1,410	1,505	8	263	1,254	1,525
Davao Oriental	54,593	0	64	463	527	1	84	377	462
Davao del Sur	67,257	4	108	748	860	11	213	854	1,078
Davao Occidental	31,707	5	99	381	485	10	197	426	633
City of Davao	187,288	1	399	3,388	3,788	6	467	3,706	4,179
**Region 12**	**453,032**	**30**	**573**	**5,384**	**5,987**	**12**	**1,219**	**6,351**	**7,582**
Cotabato	134,232	27	161	1,921	2,109	3	338	1,942	2,283

Table 2.A.1 - MODERN METHOD OF FAMILY PLANNING

New Acceptors

Philippines, 2023

Area	Total Current Users	PILLS-COC			Total	INJECTABLES			Total
		Age group				Age group			
		10-14	15-19	20-49		10-14	15-19	20-49	
Sarangani	72,262	0	116	660	776	0	141	711	852
South Cotabato	96,119	0	84	1,082	1,166	2	222	1,119	1,343
Sultan Kudarat	101,026	1	107	970	1,078	7	266	1,179	1,452
					0				0
City of General Santos	49,393	2	105	751	858	0	252	1,400	1,652
**BARMM**	**290,634**	**3**	**368**	**5,292**	**5,663**	**8**	**1,232**	**10,444**	**11,684**
Basilan	15,552	0	62	397	459	5	172	935	1,112
Lanao del Sur	37,406	1	16	488	505	1	62	891	954
Maguindanao	92,272	1	134	1,551	1,686	2	554	3,840	4,396
Sulu	82,939	0	52	967	1,019	0	168	1,731	1,899
Tawi-Tawi	18,014	0	52	547	599	0	103	814	917
SGU	8,193	0	11	100	111	0	36	363	399
City of Cotabato	36,258	1	41	1,242	1,284	0	137	1,870	2,007
**Caraga**	**284,351**	**5**	**328**	**2,806**	**3,139**	**11**	**373**	**2,762**	**3,146**
Agusan del Norte	40,460	0	55	348	403	4	80	376	460
Agusan del Sur	86,938	4	128	691	823	2	91	531	624
Surigao del Norte	50,319	0	61	779	840	0	85	1,007	1,092
Surigao del Sur	56,051	1	36	413	450	0	43	192	235
Dinagat Islands	10,768	0	13	153	166	0	16	192	208
City of Butuan	39,815	0	35	422	457	5	58	464	527

Table 2.A.1 - MODERN METHOD OF FAMILY PLANNING

New Acceptors

Philippines, 2023

Area	Total Current Users	IMPLANTS Age group 10-14	IMPLANTS Age group 15-19	IMPLANTS Age group 20-49	Total	NFP-CCM Age group 10-14	NFP-CCM Age group 15-19	NFP-CCM Age group 20-49	Total
**Philippines**	**8,353,912**	**374**	**16,681**	**87,629**	**104,684**	**134**	**65**	**1,182**	**1,381**
**N C R**	**859,210**	**84**	**2,957**	**21,139**	**24,180**	**0**	**19**	**213**	**232**
Malabon City	33,641	3	101	274	378	0	0	0	0
Navotas City	25,067	1	194	509	704	0	0	0	0
Valenzuela City	37,966	0	58	905	963	0	0	6	6
Caloocan City	61,903	8	250	1,379	1,637	0	0	0	0
Marikina City	20,095	1	75	584	660	0	0	0	0
Pasig City	55,229	10	203	1,687	1,900	0	0	0	0
Pateros	3,813	0	0	25	25	0	0	0	0
Taguig City	71,609	0	56	841	897	0	0	0	0
Quezon City	306,956	7	433	2,365	2,805	0	19	174	193
Makati City	20,752	0	17	601	618	0	0	0	0
Mandaluyong City	23,665	1	43	686	730	0	0	23	23
San Juan City	5,890	0	10	98	108	0	0	0	0
Manila City	50,873	45	1,219	9,224	10,488	0	0	1	1
Las Piñas City	20,551	7	237	686	930	0	0	0	0
Muntinlupa City	41,934	0	6	231	237	0	0	0	0
Parañaque City	42,631	1	39	487	527	0	0	0	0
Pasay City	36,635	0	16	557	573	0	0	9	9
**C A R**	**161,545**	**1**	**119**	**661**	**781**	**0**	**0**	**1**	**1**
Abra	19,903	0	12	64	76	0	0	1	1
Apayao	24,278	0	7	40	47	0	0	0	0
Benguet	34,934	0	6	102	108	0	0	0	0
Ifugao	21,809	0	2	8	10	0	0	0	0
Kalinga	18,022	0	14	54	68	0	0	0	0
Mountain Province	13,937	1	3	47	51	0	0	0	0
City of Baguio	28,662	0	75	346	421	0	0	0	0
**Region 1**	**481,911**	**2**	**151**	**1,543**	**1,696**	**0**	**1**	**43**	**44**
Ilocos Norte	51,402	2	9	140	151	0	0	5	5
Ilocos Sur	76,206	0	14	231	245	0	1	18	19
La Union	69,182	0	39	119	158	0	0	18	18

Table 2.A.1 - MODERN METHOD OF FAMILY PLANNING

New Acceptors

Philippines, 2023

Area	Total Current Users	IMPLANTS			Total	NFP-CCM			Total
		Age group				Age group			
		10-14	15-19	20-49		10-14	15-19	20-49	
Pangasinan	278,036	0	88	1,039	1,127	0	0	2	2
City of Dagupan	7,085	0	1	14	15	0	0	0	0
**Region 2**	**320,396**	**2**	**158**	**631**	**791**	**0**	**0**	**0**	**0**
Batanes	945	0	0	1	1	0	0	0	0
Cagayan	106,997	0	57	236	293	0	0	0	0
Isabela	130,614	2	88	287	377	0	0	0	0
Nueva Vizcaya	49,739	0	9	69	78	0	0	0	0
Quirino	19,516	0	4	38	42	0	0	0	0
City of Santiago	12,585	0	0	0	0	0	0	0	0
**Region 3**	**884,246**	**41**	**1,660**	**9,512**	**11,213**	**123**	**2**	**83**	**208**
Aurora	22,956	0	8	10	18	0	0	0	0
Bataan	63,188	8	305	524	837	0	0	0	0
Bulacan	245,933	8	255	3,883	4,146	0	0	2	2
Nueva Ecija	186,491	19	513	1,505	2,037	0	2	8	10
Pampanga	159,224	3	253	1,364	1,620	0	0	73	73
Tarlac	115,785	0	42	582	624	0	0	0	0
Zambales	48,549	1	119	505	625	0	0	0	0
City of Angeles	29,054	0	27	373	400	0	0	0	0
City of Olongapo	13,066	2	138	766	906	123	0	0	123
**Region 4A**	**910,368**	**22**	**1,047**	**7,169**	**8,238**	**0**	**0**	**11**	**11**
Batangas	162,745	3	58	713	774	0	0	5	5
Cavite	164,389	10	260	1,698	1,968	0	0	6	6
Laguna	320,343	5	376	2,458	2,839	0	0	0	0
Quezon	105,489	2	82	628	712	0	0	0	0
Rizal	152,400	2	264	1,594	1,860	0	0	0	0
City of Lucena	5,002	0	7	78	85	0	0	0	0
**Region 4B**	**266,333**	**6**	**366**	**1,591**	**1,963**	**0**	**12**	**66**	**78**
Marinduque	15,205	1	49	251	301	0	0	0	0
Occidental Mindoro	40,816	0	30	166	196	0	2	14	16
Oriental Mindoro	63,083	0	46	286	332	0	0	1	1

Table 2.A.1 - MODERN METHOD OF FAMILY PLANNING

New Acceptors

Philippines, 2023

Area	Total Current Users	IMPLANTS Age group 10-14	IMPLANTS Age group 15-19	IMPLANTS Age group 20-49	Total	NFP-CCM Age group 10-14	NFP-CCM Age group 15-19	NFP-CCM Age group 20-49	Total
Palawan	93,326	2	171	547	720	0	5	18	23
Romblon	22,228	1	38	260	299	0	5	33	38
City of Puerto Princesa	31,675	2	32	81	115	0	0	0	0
**Region 5**	**484,529**	**7**	**408**	**3,636**	**4,051**	**4**	**15**	**252**	**271**
Albay	120,028	3	83	1,117	1,203	0	0	20	20
Camarines Norte	46,841	0	24	124	148	0	0	4	4
Camarines Sur	107,402	0	189	1,713	1,902	0	5	18	23
Catanduanes	23,049	3	39	153	195	0	0	15	15
Masbate	91,907	1	32	270	303	4	9	187	200
Sorsogon	65,058	0	18	79	97	0	1	6	7
City of Naga	30,244	0	23	180	203	0	0	2	2
**Region 6**	**639,939**	**9**	**505**	**3,334**	**3,848**	**0**	**3**	**189**	**192**
Aklan	46,162	0	16	89	105	0	0	0	0
Antique	47,022	1	19	136	156	0	0	172	172
Capiz	75,514	1	24	307	332	0	0	0	0
Guimaras	16,676	0	2	23	25	0	0	2	2
Iloilo	169,693	1	148	1,000	1,149	0	1	12	13
Negros Occidental	199,968	6	176	1,139	1,321	0	2	3	5
City of Bacolod	28,243	0	108	564	672	0	0	0	0
City of Iloilo	56,661	0	12	76	88	0	0	0	0
**Region 7**	**597,007**	**24**	**992**	**6,043**	**7,059**	**0**	**2**	**96**	**98**
Bohol	86,129	8	320	1,312	1,640	0	0	0	0
Cebu	309,013	12	338	2,306	2,656	0	0	0	0
Negros Oriental	90,628	3	75	413	491	0	2	72	74
Siquijor	9,078	0	6	21	27	0	0	0	0
City of Cebu	39,810	1	155	1,193	1,349	0	0	24	24
City of Lapu-Lapu	35,757	0	29	254	283	0	0	0	0
City of Mandaue	26,592	0	69	544	613	0	0	0	0
**Region 8**	**332,967**	**1**	**418**	**2,363**	**2,782**	**0**	**2**	**72**	**74**
Biliran	11,240	0	47	114	161	0	0	1	1

Table 2.A.1 - MODERN METHOD OF FAMILY PLANNING

New Acceptors

Philippines, 2023

Area	Total Current Users	IMPLANTS			Total	NFP-CCM			Total
		Age group				Age group			
		10-14	15-19	20-49		10-14	15-19	20-49	
Eastern Samar	35,729	0	60	600	660	0	0	35	35
Leyte	122,333	0	116	576	692	0	2	32	34
Northern Samar	49,058	0	37	270	307	0	0	0	0
Southern Leyte	27,698	0	17	146	163	0	0	1	1
Samar	41,329	1	86	370	457	0	0	3	3
Ormoc City	19,622	0	5	19	24	0	0	0	0
City of Tacloban	25,958	0	50	268	318	0	0	0	0
**Region 9**	**339,891**	**34**	**1,951**	**5,165**	**7,150**	**0**	**0**	**0**	**0**
Zamboanga del Norte	124,603	4	325	876	1,205	0	0	0	0
Zamboanga del Sur	94,708	8	631	915	1,554	0	0	0	0
Zamboanga Sibugay	44,556	2	332	794	1,128	0	0	0	0
City of Isabela	9,211	6	104	298	408	0	0	0	0
City of Zamboanga	66,813	14	559	2,282	2,855	0	0	0	0
**Region 10**	**514,846**	**16**	**1,079**	**5,273**	**6,368**	**7**	**0**	**23**	**30**
Bukidnon	158,694	8	483	1,318	1,809	0	0	19	19
Camiguin	6,535	0	0	9	9	0	0	0	0
Lanao del Norte	67,645	2	67	606	675	0	0	0	0
Misamis Occidental	69,918	0	36	1,030	1,066	7	0	0	7
Misamis Oriental	127,215	3	169	654	826	0	0	4	4
City of Cagayan De Oro	54,303	2	263	1,257	1,522	0	0	0	0
City of Iligan	30,536	1	61	399	461	0	0	0	0
**Region 11**	**532,707**	**56**	**1,645**	**7,036**	**8,737**	**0**	**7**	**108**	**115**
Davao de Oro	83,298	6	154	476	636	0	0	30	30
Davao del Norte	108,564	12	347	1,432	1,791	0	0	22	22
Davao Oriental	54,593	2	81	383	466	0	7	14	21
Davao del Sur	67,257	8	193	589	790	0	0	6	6
Davao Occidental	31,707	2	161	321	484	0	0	0	0
City of Davao	187,288	26	709	3,835	4,570	0	0	36	36
**Region 12**	**453,032**	**24**	**1,433**	**4,865**	**6,322**	**0**	**2**	**23**	**25**
Cotabato	134,232	5	359	1,624	1,988	0	0	4	4

Table 2.A.1 - MODERN METHOD OF FAMILY PLANNING

New Acceptors

Philippines, 2023

Area	Total Current Users	IMPLANTS			Total	NFP-CCM			Total
		Age group				Age group			
		10-14	15-19	20-49		10-14	15-19	20-49	
Sarangani	72,262	0	143	500	643	0	1	1	2
South Cotabato	96,119	6	359	1,072	1,437	0	1	18	19
Sultan Kudarat	101,026	12	332	663	1,007	0	0	0	0
					0				0
City of General Santos	49,393	1	240	1,006	1,247	0	0	0	0
**BARMM**	**290,634**	**14**	**753**	**4,340**	**5,107**	**0**	**0**	**0**	**0**
Basilan	15,552	2	113	492	607	0	0	0	0
Lanao del Sur	37,406	2	30	307	339	0	0	0	0
Maguindanao	92,272	5	312	1,176	1,493	0	0	0	0
Sulu	82,939	1	122	849	972	0	0	0	0
Tawi-Tawi	18,014	2	134	1,056	1,192	0	0	0	0
SGU	8,193	0	0	8	8	0	0	0	0
City of Cotabato	36,258	2	42	452	496	0	0	0	0
**Caraga**	**284,351**	**31**	**1,039**	**3,328**	**4,398**	**0**	**0**	**2**	**2**
Agusan del Norte	40,460	8	162	349	519	0	0	0	0
Agusan del Sur	86,938	12	387	904	1,303	0	0	1	1
Surigao del Norte	50,319	4	248	1,072	1,324	0	0	1	1
Surigao del Sur	56,051	6	126	566	698	0	0	0	0
Dinagat Islands	10,768	1	41	169	211	0	0	0	0
City of Butuan	39,815	0	75	268	343	0	0	0	0

Table 2.A.1 - MODERN METHOD OF FAMILY PLANNING

New Acceptors

Philippines, 2023

Area	Total Current Users	NFP-BBT Age group 10-14	NFP-BBT Age group 15-19	NFP-BBT Age group 20-49	Total	NFP-STM Age group 10-14	NFP-STM Age group 15-19	NFP-STM Age group 20-49	Total
**Philippines**	**8,353,912**	**9**	**105**	**702**	**816**	**0**	**4**	**200**	**204**
**N C R**	**859,210**	**0**	**30**	**197**	**227**	**0**	**0**	**61**	**61**
Malabon City	33,641	0	0	0	0	0	0	0	0
Navotas City	25,067	0	14	8	22	0	0	0	0
Valenzuela City	37,966	0	9	82	91	0	0	1	1
Caloocan City	61,903	0	0	0	0	0	0	0	0
Marikina City	20,095	0	0	30	30	0	0	0	0
Pasig City	55,229	0	0	0	0	0	0	0	0
Pateros	3,813	0	0	0	0	0	0	0	0
Taguig City	71,609	0	0	0	0	0	0	0	0
Quezon City	306,956	0	6	42	48	0	0	3	3
Makati City	20,752	0	0	2	2	0	0	0	0
Mandaluyong City	23,665	0	0	0	0	0	0	57	57
San Juan City	5,890	0	0	0	0	0	0	0	0
Manila City	50,873	0	1	33	34	0	0	0	0
Las Piñas City	20,551	0	0	0	0	0	0	0	0
Muntinlupa City	41,934	0	0	0	0	0	0	0	0
Parañaque City	42,631	0	0	0	0	0	0	0	0
Pasay City	36,635	0	0	0	0	0	0	0	0
**C A R**	**161,545**	**0**	**2**	**19**	**21**	**0**	**0**	**0**	**0**
Abra	19,903	0	0	0	0	0	0	0	0
Apayao	24,278	0	0	0	0	0	0	0	0
Benguet	34,934	0	2	14	16	0	0	0	0
Ifugao	21,809	0	0	0	0	0	0	0	0
Kalinga	18,022	0	0	0	0	0	0	0	0
Mountain Province	13,937	0	0	2	2	0	0	0	0
City of Baguio	28,662	0	0	3	3	0	0	0	0
**Region 1**	**481,911**	**1**	**1**	**14**	**16**	**0**	**1**	**3**	**4**
Ilocos Norte	51,402	0	0	0	0	0	0	0	0
Ilocos Sur	76,206	0	0	2	2	0	1	3	4
La Union	69,182	0	0	4	4	0	0	0	0

Table 2.A.1 - MODERN METHOD OF FAMILY PLANNING

New Acceptors

Philippines, 2023

Area	Total Current Users	NFP-BBT			Total	NFP-STM			Total
		Age group				Age group			
		10-14	15-19	20-49		10-14	15-19	20-49	
Pangasinan	278,036	1	1	8	10	0	0	0	0
City of Dagupan	7,085	0	0	0	0	0	0	0	0
**Region 2**	**320,396**	**0**	**2**	**17**	**19**	**0**	**0**	**3**	**3**
Batanes	945	0	0	0	0	0	0	0	0
Cagayan	106,997	0	0	0	0	0	0	0	0
Isabela	130,614	0	2	11	13	0	0	3	3
Nueva Vizcaya	49,739	0	0	0	0	0	0	0	0
Quirino	19,516	0	0	6	6	0	0	0	0
City of Santiago	12,585	0	0	0	0	0	0	0	0
**Region 3**	**884,246**	**0**	**0**	**138**	**138**	**0**	**0**	**0**	**0**
Aurora	22,956	0	0	1	1	0	0	0	0
Bataan	63,188	0	0	5	5	0	0	0	0
Bulacan	245,933	0	0	0	0	0	0	0	0
Nueva Ecija	186,491	0	0	0	0	0	0	0	0
Pampanga	159,224	0	0	0	0	0	0	0	0
Tarlac	115,785	0	0	1	1	0	0	0	0
Zambales	48,549	0	0	0	0	0	0	0	0
City of Angeles	29,054	0	0	7	7	0	0	0	0
City of Olongapo	13,066	0	0	124	124	0	0	0	0
**Region 4A**	**910,368**	**0**	**6**	**23**	**29**	**0**	**0**	**14**	**14**
Batangas	162,745	0	0	18	18	0	0	0	0
Cavite	164,389	0	0	3	3	0	0	0	0
Laguna	320,343	0	0	1	1	0	0	0	0
Quezon	105,489	0	4	1	5	0	0	9	9
Rizal	152,400	0	2	0	2	0	0	5	5
City of Lucena	5,002	0	0	0	0	0	0	0	0
**Region 4B**	**266,333**	**0**	**5**	**13**	**18**	**0**	**0**	**1**	**1**
Marinduque	15,205	0	0	0	0	0	0	0	0
Occidental Mindoro	40,816	0	0	0	0	0	0	0	0
Oriental Mindoro	63,083	0	2	10	12	0	0	1	1

Table 2.A.1 - MODERN METHOD OF FAMILY PLANNING

New Acceptors

Philippines, 2023

Area	Total Current Users	NFP-BBT			Total	NFP-STM			Total
		Age group				Age group			
		10-14	15-19	20-49		10-14	15-19	20-49	
Palawan	93,326	0	1	3	4	0	0	0	0
Romblon	22,228	0	2	0	2	0	0	0	0
City of Puerto Princesa	31,675	0	0	0	0	0	0	0	0
**Region 5**	**484,529**	**1**	**14**	**76**	**91**	**0**	**1**	**49**	**50**
Albay	120,028	0	1	18	19	0	0	4	4
Camarines Norte	46,841	0	1	1	2	0	0	4	4
Camarines Sur	107,402	0	4	14	18	0	1	38	39
Catanduanes	23,049	1	8	29	38	0	0	0	0
Masbate	91,907	0	0	4	4	0	0	2	2
Sorsogon	65,058	0	0	0	0	0	0	0	0
City of Naga	30,244	0	0	10	10	0	0	1	1
**Region 6**	**639,939**	**0**	**1**	**7**	**8**	**0**	**0**	**0**	**0**
Aklan	46,162	0	0	0	0	0	0	0	0
Antique	47,022	0	0	0	0	0	0	0	0
Capiz	75,514	0	0	3	3	0	0	0	0
Guimaras	16,676	0	0	0	0	0	0	0	0
Iloilo	169,693	0	0	2	2	0	0	0	0
Negros Occidental	199,968	0	1	2	3	0	0	0	0
City of Bacolod	28,243	0	0	0	0	0	0	0	0
City of Iloilo	56,661	0	0	0	0	0	0	0	0
**Region 7**	**597,007**	**0**	**1**	**5**	**6**	**0**	**0**	**2**	**2**
Bohol	86,129	0	0	0	0	0	0	0	0
Cebu	309,013	0	0	0	0	0	0	0	0
Negros Oriental	90,628	0	1	3	4	0	0	2	2
Siquijor	9,078	0	0	0	0	0	0	0	0
City of Cebu	39,810	0	0	2	2	0	0	0	0
City of Lapu-Lapu	35,757	0	0	0	0	0	0	0	0
City of Mandaue	26,592	0	0	0	0	0	0	0	0
**Region 8**	**332,967**	**0**	**4**	**68**	**72**	**0**	**0**	**1**	**1**
Biliran	11,240	0	0	0	0	0	0	0	0

Table 2.A.1 - MODERN METHOD OF FAMILY PLANNING

New Acceptors

Philippines, 2023

Area	Total Current Users	NFP-BBT			Total	NFP-STM			Total
		Age group				Age group			
		10-14	15-19	20-49		10-14	15-19	20-49	
Eastern Samar	35,729	0	0	16	16	0	0	1	1
Leyte	122,333	0	2	33	35	0	0	0	0
Northern Samar	49,058	0	1	12	13	0	0	0	0
Southern Leyte	27,698	0	0	5	5	0	0	0	0
Samar	41,329	0	1	1	2	0	0	0	0
Ormoc City	19,622	0	0	1	1	0	0	0	0
City of Tacloban	25,958	0	0	0	0	0	0	0	0
**Region 9**	**339,891**	**0**	**0**	**7**	**7**	**0**	**0**	**0**	**0**
Zamboanga del Norte	124,603	0	0	0	0	0	0	0	0
Zamboanga del Sur	94,708	0	0	7	7	0	0	0	0
Zamboanga Sibugay	44,556	0	0	0	0	0	0	0	0
City of Isabela	9,211	0	0	0	0	0	0	0	0
City of Zamboanga	66,813	0	0	0	0	0	0	0	0
**Region 10**	**514,846**	**6**	**17**	**65**	**88**	**0**	**2**	**23**	**25**
Bukidnon	158,694	1	8	34	43	0	0	0	0
Camiguin	6,535	0	0	0	0	0	0	0	0
Lanao del Norte	67,645	5	1	5	11	0	2	12	14
Misamis Occidental	69,918	0	4	1	5	0	0	10	10
Misamis Oriental	127,215	0	4	9	13	0	0	1	1
City of Cagayan De Oro	54,303	0	0	3	3	0	0	0	0
City of Iligan	30,536	0	0	13	13	0	0	0	0
**Region 11**	**532,707**	**0**	**3**	**22**	**25**	**0**	**0**	**33**	**33**
Davao de Oro	83,298	0	2	3	5	0	0	0	0
Davao del Norte	108,564	0	1	10	11	0	0	20	20
Davao Oriental	54,593	0	0	0	0	0	0	0	0
Davao del Sur	67,257	0	0	0	0	0	0	11	11
Davao Occidental	31,707	0	0	1	1	0	0	0	0
City of Davao	187,288	0	0	8	8	0	0	2	2
**Region 12**	**453,032**	**1**	**9**	**9**	**19**	**0**	**0**	**10**	**10**
Cotabato	134,232	0	0	6	6	0	0	0	0

Table 2.A.1 - MODERN METHOD OF FAMILY PLANNING

New Acceptors

Philippines, 2023

Area	Total Current Users	NFP-BBT			Total	NFP-STM			Total
		Age group				Age group			
		10-14	15-19	20-49		10-14	15-19	20-49	
Sarangani	72,262	0	0	0	0	0	0	10	10
South Cotabato	96,119	1	9	0	10	0	0	0	0
Sultan Kudarat	101,026	0	0	3	3	0	0	0	0
					0				0
City of General Santos	49,393	0	0	0	0	0	0	0	0
**BARMM**	**290,634**	**0**	**4**	**15**	**19**	**0**	**0**	**0**	**0**
Basilan	15,552	0	0	0	0	0	0	0	0
Lanao del Sur	37,406	0	0	2	2	0	0	0	0
Maguindanao	92,272	0	0	1	1	0	0	0	0
Sulu	82,939	0	0	0	0	0	0	0	0
Tawi-Tawi	18,014	0	0	0	0	0	0	0	0
SGU	8,193	0	0	0	0	0	0	0	0
City of Cotabato	36,258	0	4	12	16	0	0	0	0
**Caraga**	**284,351**	**0**	**6**	**7**	**13**	**0**	**0**	**0**	**0**
Agusan del Norte	40,460	0	0	0	0	0	0	0	0
Agusan del Sur	86,938	0	0	0	0	0	0	0	0
Surigao del Norte	50,319	0	2	2	4	0	0	0	0
Surigao del Sur	56,051	0	1	3	4	0	0	0	0
Dinagat Islands	10,768	0	0	0	0	0	0	0	0
City of Butuan	39,815	0	3	2	5	0	0	0	0

Table 2.A.1 - MODERN METHOD OF FAMILY PLANNING

New Acceptors

Philippines, 2023

Area	Total Current Users	NFP-SDM			Total	NFP-LAM			Total	Total New Acceptors
		Age group				Age group				
		10-14	15-19	20-49		10-14	15-19	20-49		
**Philippines**	**8,353,912**	**58**	**360**	**3,426**	**3,844**	**1,126**	**59,047**	**350,576**	**410,749**	**1,078,501**
**N C R**	**859,210**	**0**	**21**	**179**	**200**	**130**	**6,903**	**74,788**	**81,821**	**257,487**
Malabon City	33,641	0	0	0	0	4	191	828	1,023	4,090
Navotas City	25,067	0	0	0	0	7	345	2,075	2,427	7,324
Valenzuela City	37,966	0	1	2	3	0	89	1,984	2,073	9,620
Caloocan City	61,903	0	1	12	13	2	755	3,877	4,634	13,829
Marikina City	20,095	0	0	0	0	2	104	1,252	1,358	4,994
Pasig City	55,229	0	0	14	14	10	256	2,920	3,186	12,060
Pateros	3,813	0	0	0	0	0	1	738	739	1,137
Taguig City	71,609	0	0	0	0	25	1,428	14,322	15,775	27,211
Quezon City	306,956	0	18	141	159	12	1,292	10,684	11,988	31,783
Makati City	20,752	0	0	0	0	0	78	2,590	2,668	8,816
Mandaluyong City	23,665	0	0	4	4	9	254	4,796	5,059	8,889
San Juan City	5,890	0	0	0	0	0	51	756	807	1,475
Manila City	50,873	0	1	3	4	43	824	12,374	13,241	86,934
Las Piñas City	20,551	0	0	0	0	6	318	4,465	4,789	14,779
Muntinlupa City	41,934	0	0	0	0	9	265	2,939	3,213	6,151
Parañaque City	42,631	0	0	0	0	0	445	6,337	6,782	11,189
Pasay City	36,635	0	0	3	3	1	207	1,851	2,059	7,206
**C A R**	**161,545**	**0**	**1**	**14**	**15**	**16**	**1,045**	**3,774**	**4,835**	**9,244**
Abra	19,903	0	0	2	2	4	256	714	974	1,562
Apayao	24,278	0	0	0	0	2	114	352	468	735
Benguet	34,934	0	0	3	3	2	149	819	970	2,015
Ifugao	21,809	0	0	1	1	2	177	445	624	674
Kalinga	18,022	0	1	7	8	3	187	836	1,026	1,423
Mountain Province	13,937	0	0	1	1	3	141	358	502	673
City of Baguio	28,662	0	0	0	0	0	21	250	271	2,162
**Region 1**	**481,911**	**0**	**15**	**203**	**218**	**43**	**2,269**	**18,386**	**20,698**	**42,660**
Ilocos Norte	51,402	0	0	3	3	0	137	3,009	3,146	4,636
Ilocos Sur	76,206	0	5	167	172	6	287	4,027	4,320	7,265
La Union	69,182	0	7	18	25	9	377	2,106	2,492	3,975

Table 2.A.1 - MODERN METHOD OF FAMILY PLANNING

New Acceptors

Philippines, 2023

Area	Total Current Users	NFP-SDM			Total	NFP-LAM			Total	Total New Acceptors
		Age group				Age group				
		10-14	15-19	20-49		10-14	15-19	20-49		
Pangasinan	278,036	0	3	15	18	25	1,358	8,633	10,016	25,816
City of Dagupan	7,085	0	0	0	0	3	110	611	724	968
**Region 2**	**320,396**	**0**	**0**	**4**	**4**	**97**	**1,806**	**6,911**	**8,814**	**16,226**
Batanes	945	0	0	0	0	1	16	57	74	79
Cagayan	106,997	0	0	1	1	6	349	1,704	2,059	4,811
Isabela	130,614	0	0	1	1	25	755	2,564	3,344	6,242
Nueva Vizcaya	49,739	0	0	2	2	11	325	1,843	2,179	3,695
Quirino	19,516	0	0	0	0	0	73	195	268	508
City of Santiago	12,585	0	0	0	0	54	288	548	890	891
**Region 3**	**884,246**	**6**	**1**	**132**	**139**	**79**	**4,141**	**31,092**	**35,312**	**131,442**
Aurora	22,956	0	0	0	0	9	228	497	734	943
Bataan	63,188	0	0	0	0	9	533	3,211	3,753	9,480
Bulacan	245,933	0	0	44	44	4	815	7,521	8,340	46,724
Nueva Ecija	186,491	0	0	3	3	26	1,143	5,173	6,342	21,705
Pampanga	159,224	0	0	75	75	4	592	6,711	7,307	21,969
Tarlac	115,785	0	1	4	5	2	380	4,322	4,704	13,517
Zambales	48,549	0	0	2	2	11	281	1,413	1,705	5,940
City of Angeles	29,054	6	0	0	6	13	92	1,374	1,479	4,364
City of Olongapo	13,066	0	0	4	4	1	77	870	948	6,800
**Region 4A**	**910,368**	**0**	**14**	**338**	**352**	**100**	**4,514**	**37,513**	**42,127**	**117,194**
Batangas	162,745	0	0	64	64	34	306	4,049	4,389	11,602
Cavite	164,389	0	0	6	6	11	850	10,257	11,118	23,979
Laguna	320,343	0	13	203	216	23	1,453	12,234	13,710	49,189
Quezon	105,489	0	0	59	59	14	608	2,446	3,068	8,741
Rizal	152,400	0	1	6	7	18	1,295	8,517	9,830	22,783
City of Lucena	5,002	0	0	0	0	0	2	10	12	900
**Region 4B**	**266,333**	**0**	**0**	**52**	**52**	**51**	**2,783**	**9,140**	**11,974**	**21,851**
Marinduque	15,205	0	0	0	0	1	32	351	384	1,215
Occidental Mindoro	40,816	0	0	0	0	16	911	1,716	2,643	3,993
Oriental Mindoro	63,083	0	0	4	4	8	430	1,840	2,278	4,419

Table 2.A.1 - MODERN METHOD OF FAMILY PLANNING

New Acceptors

Philippines, 2023

Area	Total Current Users	NFP-SDM			Total	NFP-LAM			Total	Total New Acceptors
		Age group				Age group				
		10-14	15-19	20-49		10-14	15-19	20-49		
Palawan	93,326	0	0	1	1	17	1,028	2,939	3,984	7,600
Romblon	22,228	0	0	44	44	7	284	2,074	2,365	3,281
City of Puerto Princesa	31,675	0	0	3	3	2	98	220	320	1,343
**Region 5**	**484,529**	**51**	**130**	**884**	**1,065**	**70**	**4,720**	**20,070**	**24,860**	**47,009**
Albay	120,028	0	1	224	225	14	385	4,378	4,777	11,714
Camarines Norte	46,841	0	0	4	4	9	535	1,839	2,383	3,473
Camarines Sur	107,402	0	2	170	172	12	1,348	5,644	7,004	14,879
Catanduanes	23,049	0	0	176	176	8	313	902	1,223	1,983
Masbate	91,907	51	122	238	411	14	1,312	3,521	4,847	7,348
Sorsogon	65,058	0	4	13	17	13	799	2,689	3,501	4,095
City of Naga	30,244	0	1	59	60	0	28	1,097	1,125	3,517
**Region 6**	**639,939**	**0**	**2**	**183**	**185**	**70**	**4,147**	**24,298**	**28,515**	**63,662**
Aklan	46,162	0	0	6	6	3	305	2,202	2,510	6,038
Antique	47,022	0	0	17	17	4	202	2,030	2,236	4,805
Capiz	75,514	0	0	0	0	1	128	2,099	2,228	5,518
Guimaras	16,676	0	0	0	0	4	166	627	797	1,288
Iloilo	169,693	0	0	64	64	20	893	6,389	7,302	16,128
Negros Occidental	199,968	0	2	96	98	38	2,321	9,830	12,189	25,291
City of Bacolod	28,243	0	0	0	0	0	131	1,104	1,235	3,383
City of Iloilo	56,661	0	0	0	0	0	1	17	18	1,211
**Region 7**	**597,007**	**0**	**0**	**61**	**61**	**80**	**4,630**	**28,400**	**33,110**	**73,662**
Bohol	86,129	0	0	44	44	7	351	1,924	2,282	9,012
Cebu	309,013	0	0	1	1	22	1,641	10,503	12,166	27,411
Negros Oriental	90,628	0	0	1	1	37	1,252	5,064	6,353	11,583
Siquijor	9,078	0	0	15	15	0	31	148	179	427
City of Cebu	39,810	0	0	0	0	12	1,030	8,246	9,288	16,828
City of Lapu-Lapu	35,757	0	0	0	0	2	318	2,346	2,666	5,470
City of Mandaue	26,592	0	0	0	0	0	7	169	176	2,931

Table 2.A.1 - MODERN METHOD OF FAMILY PLANNING

New Acceptors

Philippines, 2023

Area	Total Current Users	NFP-SDM			Total	NFP-LAM			Total	Total New Acceptors
		Age group				Age group				
		10-14	15-19	20-49		10-14	15-19	20-49		
**Region 8**	**332,967**	**0**	**134**	**568**	**702**	**24**	**2,106**	**11,295**	**13,425**	**33,232**
Biliran	11,240	0	0	0	0	2	82	505	589	1,104
Eastern Samar	35,729	0	129	394	523	7	386	1,892	2,285	10,292
Leyte	122,333	0	0	162	162	2	526	3,623	4,151	8,255
Northern Samar	49,058	0	0	1	1	1	298	1,520	1,819	3,610
Southern Leyte	27,698	0	0	6	6	0	179	868	1,047	1,811
Samar	41,329	0	5	5	10	10	260	1,610	1,880	5,247
Ormoc City	19,622	0	0	0	0	0	194	481	675	857
City of Tacloban	25,958	0	0	0	0	2	181	796	979	2,056
**Region 9**	**339,891**	**0**	**0**	**6**	**6**	**55**	**3,591**	**9,383**	**13,029**	**31,024**
Zamboanga del Norte	124,603	0	0	1	1	26	1,230	3,054	4,310	7,062
Zamboanga del Sur	94,708	0	0	2	2	7	492	1,245	1,744	6,026
Zamboanga Sibugay	44,556	0	0	3	3	4	399	894	1,297	3,782
City of Isabela	9,211	0	0	0	0	0	120	591	711	2,108
City of Zamboanga	66,813	0	0	0	0	18	1,350	3,599	4,967	12,046
**Region 10**	**514,846**	**0**	**11**	**317**	**328**	**121**	**5,752**	**22,060**	**27,933**	**57,874**
Bukidnon	158,694	0	6	108	114	55	2,248	4,389	6,692	12,640
Camiguin	6,535	0	0	0	0	0	99	212	311	353
Lanao del Norte	67,645	0	0	31	31	7	538	3,572	4,117	9,101
Misamis Occidental	69,918	0	3	135	138	4	354	2,293	2,651	6,521
Misamis Oriental	127,215	0	1	24	25	25	954	3,198	4,177	8,405
City of Cagayan De Oro	54,303	0	0	0	0	26	1,194	6,484	7,704	15,358
City of Iligan	30,536	0	1	19	20	4	365	1,912	2,281	5,496
**Region 11**	**532,707**	**1**	**15**	**199**	**215**	**84**	**2,447**	**12,974**	**15,505**	**56,027**
Davao de Oro	83,298	1	0	1	2	5	109	411	525	4,810
Davao del Norte	108,564	0	0	91	91	12	420	1,885	2,317	9,400
Davao Oriental	54,593	0	4	18	22	32	679	1,745	2,456	4,642
Davao del Sur	67,257	0	1	5	6	11	182	835	1,028	5,222
Davao Occidental	31,707	0	1	5	6	12	218	306	536	2,468
City of Davao	187,288	0	9	79	88	12	839	7,792	8,643	29,485

Table 2.A.1 - MODERN METHOD OF FAMILY PLANNING

New Acceptors

Philippines, 2023

Area	Total Current Users	NFP-SDM			Total	NFP-LAM			Total	Total New Acceptors
		Age group				Age group				
		10-14	15-19	20-49		10-14	15-19	20-49		
**Region 12**	**453,032**	**0**	**5**	**34**	**39**	**55**	**3,663**	**13,920**	**17,638**	**43,916**
Cotabato	134,232	0	0	14	14	15	716	3,464	4,195	13,145
Sarangani	72,262	0	0	4	4	0	1,133	2,416	3,549	6,371
South Cotabato	96,119	0	2	14	16	8	754	2,869	3,631	8,977
Sultan Kudarat	101,026	0	3	0	3	30	774	2,641	3,445	7,787
					0				0	
City of General Santos	49,393	0	0	2	2	2	286	2,530	2,818	7,636
**BARMM**	**290,634**	**0**	**0**	**22**	**22**	**26**	**2,634**	**18,911**	**21,571**	**49,929**
Basilan	15,552	0	0	0	0	6	153	629	788	3,305
Lanao del Sur	37,406	0	0	3	3	5	463	6,099	6,567	9,873
Maguindanao	92,272	0	0	0	0	3	1,172	5,895	7,070	16,466
Sulu	82,939	0	0	0	0	2	158	929	1,089	5,761
Tawi-Tawi	18,014	0	0	19	19	1	248	1,363	1,612	4,487
SGU	8,193	0	0	0	0	0	83	500	583	1,465
City of Cotabato	36,258	0	0	0	0	9	357	3,496	3,862	8,572
**Caraga**	**284,351**	**0**	**11**	**230**	**241**	**25**	**1,896**	**7,661**	**9,582**	**26,062**
Agusan del Norte	40,460	0	0	6	6	2	229	667	898	3,409
Agusan del Sur	86,938	0	0	53	53	10	471	1,206	1,687	5,679
Surigao del Norte	50,319	0	9	149	158	1	198	1,763	1,962	7,212
Surigao del Sur	56,051	0	0	3	3	6	479	2,524	3,009	5,078
Dinagat Islands	10,768	0	2	14	16	0	63	257	320	1,114
City of Butuan	39,815	0	0	5	5	6	456	1,244	1,706	3,570

Table 2.A.2 - MODERN METHOD OF FAMILY PLANNING

Other Acceptors

Philippines, 2023

Area	Total Current Users	FSTR/BTL			Total	MSTR/NSV			Total
		Age group				Age group			
		10-14	15-19	20-49		10-14	15-19	20-49	
**Philippines**	**8,353,912**	**29**	**181**	**76,237**	**76,447**	**0**	**14**	**703**	**717**
**N C R**	**859,210**	**0**	**3**	**9,488**	**9,491**	**0**	**0**	**108**	**108**
Malabon City	33,641	0	0	564	564	0	0	0	0
Navotas City	25,067	0	0	51	51	0	0	3	3
Valenzuela City	37,966	0	0	1,029	1,029	0	0	22	22
Caloocan City	61,903	0	0	1,195	1,195	0	0	7	7
Marikina City	20,095	0	0	76	76	0	0	0	0
Pasig City	55,229	0	0	468	468	0	0	8	8
Pateros	3,813	0	0	130	130	0	0	0	0
Taguig City	71,609	0	1	728	729	0	0	7	7
Quezon City	306,956	0	1	3,202	3,203	0	0	25	25
Makati City	20,752	0	0	49	49	0	0	7	7
Mandaluyong City	23,665	0	0	59	59	0	0	2	2
San Juan City	5,890	0	0	0	0	0	0	0	0
Manila City	50,873	0	1	800	801	0	0	20	20
Las Piñas City	20,551	0	0	603	603	0	0	4	4
Muntinlupa City	41,934	0	0	60	60	0	0	1	1
Parañaque City	42,631	0	0	95	95	0	0	0	0
Pasay City	36,635	0	0	379	379	0	0	2	2
**C A R**	**161,545**	**0**	**2**	**2,558**	**2,560**	**0**	**0**	**33**	**33**
Abra	19,903	0	0	27	27	0	0	1	1
Apayao	24,278	0	0	8	8	0	0	0	0
Benguet	34,934	0	2	452	454	0	0	5	5
Ifugao	21,809	0	0	429	429	0	0	19	19
Kalinga	18,022	0	0	87	87	0	0	0	0
Mountain Province	13,937	0	0	521	521	0	0	0	0
City of Baguio	28,662	0	0	1,034	1,034	0	0	8	8
**Region 1**	**481,911**	**0**	**2**	**2,647**	**2,649**	**0**	**0**	**3**	**3**
Ilocos Norte	51,402	0	2	1,150	1,152	0	0	0	0
Ilocos Sur	76,206	0	0	188	188	0	0	0	0
La Union	69,182	0	0	426	426	0	0	2	2

Table 2.A.2 - MODERN METHOD OF FAMILY PLANNING

Other Acceptors

Philippines, 2023

Area	Total Current Users	FSTR/BTL			Total	MSTR/NSV			Total
		Age group				Age group			
		10-14	15-19	20-49		10-14	15-19	20-49	
Pangasinan	278,036	0	0	880	880	0	0	1	1
City of Dagupan	7,085	0	0	3	3	0	0	0	0
**Region 2**	**320,396**	**0**	**9**	**4,446**	**4,455**	**0**	**0**	**18**	**18**
Batanes	945	0	0	15	15	0	0	0	0
Cagayan	106,997	0	1	316	317	0	0	0	0
Isabela	130,614	0	7	2,371	2,378	0	0	15	15
Nueva Vizcaya	49,739	0	1	768	769	0	0	3	3
Quirino	19,516	0	0	894	894	0	0	0	0
City of Santiago	12,585	0	0	82	82	0	0	0	0
**Region 3**	**884,246**	**25**	**17**	**22,888**	**22,930**	**0**	**0**	**30**	**30**
Aurora	22,956	7	0	331	338	0	0	2	2
Bataan	63,188	0	0	451	451	0	0	0	0
Bulacan	245,933	0	3	11,003	11,006	0	0	11	11
Nueva Ecija	186,491	17	4	4,480	4,501	0	0	3	3
Pampanga	159,224	1	8	2,613	2,622	0	0	0	0
Tarlac	115,785	0	2	3,516	3,518	0	0	0	0
Zambales	48,549	0	0	192	192	0	0	5	5
City of Angeles	29,054	0	0	250	250	0	0	7	7
City of Olongapo	13,066	0	0	52	52	0	0	2	2
**Region 4A**	**910,368**	**4**	**37**	**7,412**	**7,453**	**0**	**7**	**19**	**26**
Batangas	162,745	0	0	1,018	1,018	0	0	0	0
Cavite	164,389	0	16	2,458	2,474	0	0	10	10
Laguna	320,343	0	5	1,350	1,355	0	0	5	5
Quezon	105,489	4	14	1,101	1,119	0	6	2	8
Rizal	152,400	0	2	1,409	1,411	0	1	2	3
City of Lucena	5,002	0	0	76	76	0	0	0	0
**Region 4B**	**266,333**	**0**	**31**	**3,886**	**3,917**	**0**	**4**	**61**	**65**
Marinduque	15,205	0	0	81	81	0	0	1	1
Occidental Mindoro	40,816	0	0	237	237	0	0	0	0
Oriental Mindoro	63,083	0	26	2,913	2,939	0	0	34	34

Table 2.A.2 - MODERN METHOD OF FAMILY PLANNING

Other Acceptors

Philippines, 2023

Area	Total Current Users	FSTR/BTL Age group 10-14	FSTR/BTL Age group 15-19	FSTR/BTL Age group 20-49	Total	MSTR/NSV Age group 10-14	MSTR/NSV Age group 15-19	MSTR/NSV Age group 20-49	Total
Palawan	93,326	0	4	371	375	0	4	24	28
Romblon	22,228	0	0	60	60	0	0	0	0
City of Puerto Princesa	31,675	0	1	224	225	0	0	2	2
**Region 5**	**484,529**	**0**	**3**	**1,497**	**1,500**	**0**	**0**	**12**	**12**
Albay	120,028	0	0	332	332	0	0	5	5
Camarines Norte	46,841	0	0	45	45	0	0	0	0
Camarines Sur	107,402	0	0	24	24	0	0	0	0
Catanduanes	23,049	0	3	254	257	0	0	1	1
Masbate	91,907	0	0	240	240	0	0	6	6
Sorsogon	65,058	0	0	556	556	0	0	0	0
City of Naga	30,244	0	0	46	46	0	0	0	0
**Region 6**	**639,939**	**0**	**8**	**3,884**	**3,892**	**0**	**0**	**74**	**74**
Aklan	46,162	0	0	80	80	0	0	0	0
Antique	47,022	0	0	267	267	0	0	2	2
Capiz	75,514	0	0	545	545	0	0	3	3
Guimaras	16,676	0	0	128	128	0	0	0	0
Iloilo	169,693	0	0	337	337	0	0	0	0
Negros Occidental	199,968	0	6	1,669	1,675	0	0	68	68
City of Bacolod	28,243	0	1	374	375	0	0	1	1
City of Iloilo	56,661	0	1	484	485	0	0	0	0
**Region 7**	**597,007**	**0**	**0**	**3,624**	**3,624**	**0**	**0**	**71**	**71**
Bohol	86,129	0	0	2,166	2,166	0	0	4	4
Cebu	309,013	0	0	1,017	1,017	0	0	19	19
Negros Oriental	90,628	0	0	228	228	0	0	12	12
Siquijor	9,078	0	0	1	1	0	0	0	0
City of Cebu	39,810	0	0	211	211	0	0	36	36
City of Lapu-Lapu	35,757	0	0	0	0	0	0	0	0
City of Mandaue	26,592	0	0	1	1	0	0	0	0
**Region 8**	**332,967**	**0**	**0**	**1,103**	**1,103**	**0**	**0**	**10**	**10**
Biliran	11,240	0	0	358	358	0	0	1	1

Table 2.A.2 - MODERN METHOD OF FAMILY PLANNING

Other Acceptors

Philippines, 2023

Area	Total Current Users	FSTR/BTL			Total	MSTR/NSV			Total
		Age group				Age group			
		10-14	15-19	20-49		10-14	15-19	20-49	
Eastern Samar	35,729	0	0	59	59	0	0	1	1
Leyte	122,333	0	0	215	215	0	0	2	2
Northern Samar	49,058	0	0	90	90	0	0	4	4
Southern Leyte	27,698	0	0	119	119	0	0	0	0
Samar	41,329	0	0	142	142	0	0	0	0
Ormoc City	19,622	0	0	61	61	0	0	2	2
City of Tacloban	25,958	0	0	59	59	0	0	0	0
**Region 9**	**339,891**	**0**	**2**	**647**	**649**	**0**	**0**	**2**	**2**
Zamboanga del Norte	124,603	0	0	189	189	0	0	0	0
Zamboanga del Sur	94,708	0	0	329	329	0	0	2	2
Zamboanga Sibugay	44,556	0	0	21	21	0	0	0	0
City of Isabela	9,211	0	0	1	1	0	0	0	0
City of Zamboanga	66,813	0	2	107	109	0	0	0	0
**Region 10**	**514,846**	**0**	**14**	**4,794**	**4,808**	**0**	**0**	**79**	**79**
Bukidnon	158,694	0	3	1,391	1,394	0	0	18	18
Camiguin	6,535	0	0	26	26	0	0	0	0
Lanao del Norte	67,645	0	0	72	72	0	0	12	12
Misamis Occidental	69,918	0	9	1,333	1,342	0	0	25	25
Misamis Oriental	127,215	0	2	1,158	1,160	0	0	15	15
City of Cagayan De Oro	54,303	0	0	625	625	0	0	7	7
City of Iligan	30,536	0	0	189	189	0	0	2	2
**Region 11**	**532,707**	**0**	**7**	**2,619**	**2,626**	**0**	**1**	**41**	**42**
Davao de Oro	83,298	0	1	438	439	0	0	3	3
Davao del Norte	108,564	0	0	776	776	0	0	10	10
Davao Oriental	54,593	0	3	444	447	0	0	2	2
Davao del Sur	67,257	0	3	164	167	0	0	0	0
Davao Occidental	31,707	0	0	269	269	0	0	18	18
City of Davao	187,288	0	0	528	528	0	1	8	9
**Region 12**	**453,032**	**0**	**44**	**2,591**	**2,635**	**0**	**2**	**111**	**113**
Cotabato	134,232	0	0	979	979	0	0	26	26

Table 2.A.2 - MODERN METHOD OF FAMILY PLANNING

Other Acceptors

Philippines, 2023

Area	Total Current Users	FSTR/BTL			Total	MSTR/NSV			Total
		Age group				Age group			
		10-14	15-19	20-49		10-14	15-19	20-49	
Sarangani	72,262	0	41	324	365	0	2	75	77
South Cotabato	96,119	0	0	429	429	0	0	5	5
Sultan Kudarat	101,026	0	3	496	499	0	0	0	0
					0				0
City of General Santos	49,393	0	0	363	363	0	0	5	5
**BARMM**	**290,634**	**0**	**0**	**471**	**471**	**0**	**0**	**11**	**11**
Basilan	15,552	0	0	13	13	0	0	0	0
Lanao del Sur	37,406	0	0	165	165	0	0	9	9
Maguindanao	92,272	0	0	117	117	0	0	0	0
Sulu	82,939	0	0	57	57	0	0	2	2
Tawi-Tawi	18,014	0	0	50	50	0	0	0	0
SGU	8,193	0	0	1	1	0	0	0	0
City of Cotabato	36,258	0	0	68	68	0	0	0	0
**Caraga**	**284,351**	**0**	**2**	**1,682**	**1,684**	**0**	**0**	**20**	**20**
Agusan del Norte	40,460	0	0	159	159	0	0	0	0
Agusan del Sur	86,938	0	0	369	369	0	0	1	1
Surigao del Norte	50,319	0	2	388	390	0	0	5	5
Surigao del Sur	56,051	0	0	563	563	0	0	14	14
Dinagat Islands	10,768	0	0	69	69	0	0	0	0
City of Butuan	39,815	0	0	134	134	0	0	0	0

Table 2.A.2 - MODERN METHOD OF FAMILY PLANNING

Other Acceptors

Philippines, 2023

Area	Total Current Users	CONDOM			Total	IUD-INTERVAL			Total
		Age group				Age group			
		10-14	15-19	20-49		10-14	15-19	20-49	
**Philippines**	**8,353,912**	**146**	**5,029**	**73,595**	**78,770**	**32**	**1,651**	**38,369**	**40,052**
**N C R**	**859,210**	**56**	**2,126**	**25,962**	**28,144**	**7**	**337**	**4,689**	**5,033**
Malabon City	33,641	4	29	907	940	0	8	210	218
Navotas City	25,067	6	301	684	991	1	35	98	134
Valenzuela City	37,966	5	3	418	426	0	1	169	170
Caloocan City	61,903	0	20	657	677	2	114	850	966
Marikina City	20,095	0	1	308	309	0	5	67	72
Pasig City	55,229	0	3	262	265	0	7	170	177
Pateros	3,813	0	2	78	80	0	2	61	63
Taguig City	71,609	0	95	670	765	0	17	314	331
Quezon City	306,956	1	543	16,399	16,943	1	68	1,604	1,673
Makati City	20,752	0	7	974	981	1	0	40	41
Mandaluyong City	23,665	12	1	156	169	0	1	35	36
San Juan City	5,890	0	0	1	1	0	0	0	0
Manila City	50,873	28	335	2,629	2,992	1	39	246	286
Las Piñas City	20,551	0	8	244	252	1	6	147	154
Muntinlupa City	41,934	0	0	27	27	0	0	14	14
Parañaque City	42,631	0	14	248	262	0	0	22	22
Pasay City	36,635	0	764	1,300	2,064	0	34	642	676
**C A R**	**161,545**	**2**	**29**	**1,784**	**1,815**	**1**	**17**	**542**	**560**
Abra	19,903	0	6	191	197	0	1	3	4
Apayao	24,278	0	3	55	58	0	1	18	19
Benguet	34,934	0	5	615	620	1	6	235	242
Ifugao	21,809	0	1	180	181	0	2	42	44
Kalinga	18,022	1	3	160	164	0	2	123	125
Mountain Province	13,937	0	5	113	118	0	3	57	60
City of Baguio	28,662	1	6	470	477	0	2	64	66
**Region 1**	**481,911**	**1**	**59**	**1,326**	**1,386**	**0**	**61**	**936**	**997**
Ilocos Norte	51,402	0	1	74	75	0	0	4	4
Ilocos Sur	76,206	0	4	107	111	0	2	14	16
La Union	69,182	0	27	114	141	0	28	208	236
Pangasinan	278,036	1	27	1,011	1,039	0	31	708	739

Table 2.A.2 - MODERN METHOD OF FAMILY PLANNING

Other Acceptors

Philippines, 2023

Area	Total Current Users	CONDOM			Total	IUD-INTERVAL			Total
		Age group				Age group			
		10-14	15-19	20-49		10-14	15-19	20-49	
City of Dagupan	7,085	0	0	20	20	0	0	2	2
**Region 2**	**320,396**	**0**	**64**	**1,158**	**1,222**	**2**	**99**	**1,609**	**1,710**
Batanes	945	0	0	9	9	0	0	0	0
Cagayan	106,997	0	32	107	139	0	18	420	438
Isabela	130,614	0	18	648	666	1	38	911	950
Nueva Vizcaya	49,739	0	10	281	291	0	23	182	205
Quirino	19,516	0	2	72	74	0	16	60	76
City of Santiago	12,585	0	2	41	43	1	4	36	41
**Region 3**	**884,246**	**8**	**327**	**7,077**	**7,412**	**8**	**36**	**3,570**	**3,614**
Aurora	22,956	0	6	606	612	0	2	44	46
Bataan	63,188	0	12	152	164	0	0	11	11
Bulacan	245,933	0	146	2,874	3,020	0	7	2,758	2,765
Nueva Ecija	186,491	0	25	914	939	8	14	520	542
Pampanga	159,224	0	20	781	801	0	12	109	121
Tarlac	115,785	2	102	1,213	1,317	0	1	61	62
Zambales	48,549	6	11	185	202	0	0	14	14
City of Angeles	29,054	0	1	14	15	0	0	53	53
City of Olongapo	13,066	0	4	338	342	0	0	0	0
**Region 4A**	**910,368**	**2**	**256**	**4,769**	**5,027**	**3**	**128**	**3,120**	**3,251**
Batangas	162,745	1	2	647	650	0	11	242	253
Cavite	164,389	0	49	1,429	1,478	1	30	898	929
Laguna	320,343	0	99	1,405	1,504	0	28	508	536
Quezon	105,489	1	25	665	691	2	36	911	949
Rizal	152,400	0	79	582	661	0	23	545	568
City of Lucena	5,002	0	2	41	43	0	0	16	16
**Region 4B**	**266,333**	**2**	**47**	**1,297**	**1,346**	**1**	**24**	**637**	**662**
Marinduque	15,205	0	0	24	24	0	0	14	14
Occidental Mindoro	40,816	0	2	194	196	0	0	89	89
Oriental Mindoro	63,083	2	9	403	414	1	14	395	410
Palawan	93,326	0	15	365	380	0	7	77	84

Table 2.A.2 - MODERN METHOD OF FAMILY PLANNING

Other Acceptors

Philippines, 2023

Area	Total Current Users	CONDOM			Total	IUD-INTERVAL			Total
		Age group				Age group			
		10-14	15-19	20-49		10-14	15-19	20-49	
Romblon	22,228	0	0	36	36	0	2	18	20
City of Puerto Princesa	31,675	0	21	275	296	0	1	44	45
**Region 5**	**484,529**	**0**	**137**	**5,074**	**5,211**	**0**	**59**	**500**	**559**
Albay	120,028	0	13	601	614	0	2	133	135
Camarines Norte	46,841	0	39	279	318	0	47	38	85
Camarines Sur	107,402	0	5	1,111	1,116	0	1	44	45
Catanduanes	23,049	0	7	364	371	0	1	16	17
Masbate	91,907	0	60	2,125	2,185	0	7	203	210
Sorsogon	65,058	0	13	472	485	0	1	54	55
City of Naga	30,244	0	0	122	122	0	0	12	12
**Region 6**	**639,939**	**0**	**145**	**3,755**	**3,900**	**0**	**76**	**1,868**	**1,944**
Aklan	46,162	0	66	380	446	0	0	13	13
Antique	47,022	0	0	224	224	0	0	45	45
Capiz	75,514	0	0	242	242	0	1	145	146
Guimaras	16,676	0	4	309	313	0	1	35	36
Iloilo	169,693	0	30	823	853	0	18	340	358
Negros Occidental	199,968	0	31	1,286	1,317	0	25	719	744
City of Bacolod	28,243	0	8	120	128	0	8	210	218
City of Iloilo	56,661	0	6	371	377	0	23	361	384
**Region 7**	**597,007**	**1**	**99**	**2,765**	**2,865**	**0**	**110**	**6,441**	**6,551**
Bohol	86,129	0	7	406	413	0	23	1,920	1,943
Cebu	309,013	0	48	1,274	1,322	0	41	1,772	1,813
Negros Oriental	90,628	0	26	710	736	0	18	2,343	2,361
Siquijor	9,078	0	0	41	41	0	1	23	24
City of Cebu	39,810	0	2	141	143	0	26	342	368
City of Lapu-Lapu	35,757	1	16	175	192	0	1	34	35
City of Mandaue	26,592	0	0	18	18	0	0	7	7
**Region 8**	**332,967**	**3**	**96**	**1,364**	**1,463**	**0**	**12**	**721**	**733**
Biliran	11,240	0	4	90	94	0	0	139	139
Eastern Samar	35,729	0	13	112	125	0	0	44	44

Table 2.A.2 - MODERN METHOD OF FAMILY PLANNING

Other Acceptors

Philippines, 2023

Area	Total Current Users	CONDOM			Total	IUD-INTERVAL			Total
		Age group				Age group			
		10-14	15-19	20-49		10-14	15-19	20-49	
Leyte	122,333	0	15	180	195	0	7	136	143
Northern Samar	49,058	3	36	282	321	0	1	122	123
Southern Leyte	27,698	0	2	212	214	0	1	139	140
Samar	41,329	0	8	207	215	0	1	40	41
Ormoc City	19,622	0	10	216	226	0	2	88	90
City of Tacloban	25,958	0	8	65	73	0	0	13	13
**Region 9**	**339,891**	**1**	**85**	**1,705**	**1,791**	**0**	**89**	**1,762**	**1,851**
Zamboanga del Norte	124,603	1	18	565	584	0	13	355	368
Zamboanga del Sur	94,708	0	26	548	574	0	45	949	994
Zamboanga Sibugay	44,556	0	36	290	326	0	19	397	416
City of Isabela	9,211	0	2	172	174	0	0	1	1
City of Zamboanga	66,813	0	3	130	133	0	12	60	72
**Region 10**	**514,846**	**21**	**930**	**7,039**	**7,990**	**9**	**279**	**4,899**	**5,187**
Bukidnon	158,694	2	95	1,508	1,605	2	115	2,301	2,418
Camiguin	6,535	0	4	33	37	0	0	12	12
Lanao del Norte	67,645	0	17	991	1,008	0	3	67	70
Misamis Occidental	69,918	19	621	2,317	2,957	7	12	122	141
Misamis Oriental	127,215	0	107	603	710	0	26	976	1,002
City of Cagayan De Oro	54,303	0	78	1,368	1,446	0	117	1,209	1,326
City of Iligan	30,536	0	8	219	227	0	6	212	218
**Region 11**	**532,707**	**48**	**250**	**2,162**	**2,460**	**0**	**111**	**1,926**	**2,037**
Davao de Oro	83,298	0	6	372	378	0	5	301	306
Davao del Norte	108,564	3	36	600	639	0	16	620	636
Davao Oriental	54,593	0	6	211	217	0	8	264	272
Davao del Sur	67,257	0	11	162	173	0	11	217	228
Davao Occidental	31,707	0	4	59	63	0	7	152	159
City of Davao	187,288	45	187	758	990	0	64	372	436
**Region 12**	**453,032**	**0**	**147**	**2,428**	**2,575**	**0**	**65**	**3,121**	**3,186**
Cotabato	134,232	0	47	933	980	0	29	1,068	1,097
Sarangani	72,262	0	23	300	323	0	7	149	156

Table 2.A.2 - MODERN METHOD OF FAMILY PLANNING

Other Acceptors

Philippines, 2023

Area	Total Current Users	CONDOM			Total	IUD-INTERVAL			Total
		Age group				Age group			
		10-14	15-19	20-49		10-14	15-19	20-49	
South Cotabato	96,119	0	24	481	505	0	4	190	194
Sultan Kudarat	101,026	0	36	311	347	0	10	289	299
					0				0
City of General Santos	49,393	0	17	403	420	0	15	1,425	1,440
**BARMM**	**290,634**	**0**	**79**	**1,718**	**1,797**	**1**	**7**	**177**	**185**
Basilan	15,552	0	1	36	37	0	0	38	38
Lanao del Sur	37,406	0	37	1,124	1,161	0	2	16	18
Maguindanao	92,272	0	38	372	410	1	3	98	102
Sulu	82,939	0	3	114	117	0	2	9	11
Tawi-Tawi	18,014	0	0	20	20	0	0	2	2
SGU	8,193	0	0	9	9	0	0	0	0
City of Cotabato	36,258	0	0	43	43	0	0	14	14
**Caraga**	**284,351**	**1**	**153**	**2,212**	**2,366**	**0**	**141**	**1,851**	**1,992**
Agusan del Norte	40,460	1	23	374	398	0	35	264	299
Agusan del Sur	86,938	0	85	458	543	0	40	601	641
Surigao del Norte	50,319	0	14	618	632	0	37	394	431
Surigao del Sur	56,051	0	26	458	484	0	28	481	509
Dinagat Islands	10,768	0	3	142	145	0	1	74	75
City of Butuan	39,815	0	2	162	164	0	0	37	37

Table 2.A.2 - MODERN METHOD OF FAMILY PLANNING

Other Acceptors

Philippines, 2023

Area	Total Current Users	IUD-POSTPARTUM			Total	PILLS-POP			Total
		Age group				Age group			
		10-14	15-19	20-49		10-14	15-19	20-49	
**Philippines**	**8,353,912**	**55**	**1,749**	**27,285**	**29,089**	**172**	**31,378**	**111,152**	**142,702**
**N C R**	**859,210**	**31**	**413**	**7,736**	**8,180**	**26**	**1,391**	**19,376**	**20,793**
Malabon City	33,641	0	32	258	290	0	23	907	930
Navotas City	25,067	0	1	8	9	15	446	931	1,392
Valenzuela City	37,966	24	1	220	245	2	12	844	858
Caloocan City	61,903	1	72	1,173	1,246	0	64	1,030	1,094
Marikina City	20,095	0	0	113	113	0	2	87	89
Pasig City	55,229	0	20	356	376	0	10	226	236
Pateros	3,813	0	0	4	4	0	0	12	12
Taguig City	71,609	1	13	191	205	3	32	755	790
Quezon City	306,956	5	147	3,778	3,930	0	408	9,152	9,560
Makati City	20,752	0	8	45	53	0	5	498	503
Mandaluyong City	23,665	0	13	106	119	0	2	84	86
San Juan City	5,890	0	0	0	0	0	0	31	31
Manila City	50,873	0	2	141	143	6	182	2,498	2,686
Las Piñas City	20,551	0	0	23	23	0	15	307	322
Muntinlupa City	41,934	0	0	0	0	0	7	62	69
Parañaque City	42,631	0	1	9	10	0	16	207	223
Pasay City	36,635	0	103	1,311	1,414	0	167	1,745	1,912
**C A R**	**161,545**	**0**	**6**	**209**	**215**	**8**	**337**	**3,676**	**4,021**
Abra	19,903	0	0	12	12	0	48	486	534
Apayao	24,278	0	1	18	19	0	73	460	533
Benguet	34,934	0	2	60	62	1	38	708	747
Ifugao	21,809	0	1	51	52	3	29	499	531
Kalinga	18,022	0	2	4	6	1	39	465	505
Mountain Province	13,937	0	0	31	31	0	57	365	422
City of Baguio	28,662	0	0	33	33	3	53	693	749
**Region 1**	**481,911**	**1**	**38**	**112**	**151**	**0**	**80**	**994**	**1,074**
Ilocos Norte	51,402	0	1	12	13	0	14	315	329
Ilocos Sur	76,206	0	0	4	4	0	21	345	366
La Union	69,182	1	35	94	130	0	44	323	367
Pangasinan	278,036	0	0	0	0	0	0	0	0

Table 2.A.2 - MODERN METHOD OF FAMILY PLANNING

Other Acceptors

Philippines, 2023

Area	Total Current Users	IUD-POSTPARTUM			Total	PILLS-POP			Total
		Age group				Age group			
		10-14	15-19	20-49		10-14	15-19	20-49	
City of Dagupan	7,085	0	2	2	4	0	1	11	12
**Region 2**	**320,396**	**4**	**118**	**666**	**788**	**12**	**468**	**6,858**	**7,338**
Batanes	945	0	0	0	0	0	2	21	23
Cagayan	106,997	0	23	144	167	2	100	997	1,099
Isabela	130,614	2	47	271	320	8	253	4,714	4,975
Nueva Vizcaya	49,739	1	45	231	277	0	77	779	856
Quirino	19,516	1	3	20	24	2	36	347	385
City of Santiago	12,585	0	0	0	0	0	0	0	0
**Region 3**	**884,246**	**1**	**81**	**751**	**833**	**14**	**1,093**	**7,845**	**8,952**
Aurora	22,956	0	0	20	20	1	25	256	282
Bataan	63,188	0	1	4	5	0	29	1,067	1,096
Bulacan	245,933	1	11	247	259	0	240	2,191	2,431
Nueva Ecija	186,491	0	17	264	281	3	210	1,652	1,865
Pampanga	159,224	0	13	94	107	8	441	919	1,368
Tarlac	115,785	0	36	87	123	2	113	1,165	1,280
Zambales	48,549	0	3	33	36	0	21	333	354
City of Angeles	29,054	0	0	2	2	0	8	102	110
City of Olongapo	13,066	0	0	0	0	0	6	160	166
**Region 4A**	**910,368**	**5**	**77**	**1,245**	**1,327**	**9**	**599**	**8,327**	**8,935**
Batangas	162,745	0	12	226	238	1	24	632	657
Cavite	164,389	1	11	163	175	1	89	1,727	1,817
Laguna	320,343	1	14	137	152	0	145	1,741	1,886
Quezon	105,489	0	12	90	102	4	100	1,387	1,491
Rizal	152,400	3	28	623	654	3	193	2,401	2,597
City of Lucena	5,002	0	0	6	6	0	48	439	487
**Region 4B**	**266,333**	**0**	**23**	**1,955**	**1,978**	**35**	**22,504**	**5,926**	**28,465**
Marinduque	15,205	0	0	2	2	0	4	331	335
Occidental Mindoro	40,816	0	2	6	8	1	110	1,160	1,271
Oriental Mindoro	63,083	0	16	1,865	1,881	2	78	982	1,062
Palawan	93,326	0	3	35	38	31	22,195	2,192	24,418

Table 2.A.2 - MODERN METHOD OF FAMILY PLANNING

Other Acceptors

Philippines, 2023

Area	Total Current Users	IUD-POSTPARTUM			Total	PILLS-POP			Total
		Age group				Age group			
		10-14	15-19	20-49		10-14	15-19	20-49	
Romblon	22,228	0	1	15	16	0	9	254	263
City of Puerto Princesa	31,675	0	1	32	33	1	108	1,007	1,116
**Region 5**	**484,529**	**0**	**13**	**125**	**138**	**0**	**371**	**10,115**	**10,486**
Albay	120,028	0	4	31	35	0	34	2,701	2,735
Camarines Norte	46,841	0	2	12	14	0	46	2,203	2,249
Camarines Sur	107,402	0	3	30	33	0	69	1,769	1,838
Catanduanes	23,049	0	0	3	3	0	21	288	309
Masbate	91,907	0	1	10	11	0	123	1,821	1,944
Sorsogon	65,058	0	1	31	32	0	77	1,318	1,395
City of Naga	30,244	0	2	8	10	0	1	15	16
**Region 6**	**639,939**	**0**	**54**	**838**	**892**	**3**	**370**	**4,419**	**4,792**
Aklan	46,162	0	0	3	3	0	39	273	312
Antique	47,022	0	0	2	2	0	16	495	511
Capiz	75,514	0	0	23	23	0	14	331	345
Guimaras	16,676	0	0	19	19	0	25	336	361
Iloilo	169,693	0	4	144	148	1	60	627	688
Negros Occidental	199,968	0	31	496	527	2	192	2,047	2,241
City of Bacolod	28,243	0	4	41	45	0	14	159	173
City of Iloilo	56,661	0	15	110	125	0	10	151	161
**Region 7**	**597,007**	**0**	**132**	**1,728**	**1,860**	**4**	**408**	**5,468**	**5,880**
Bohol	86,129	0	59	761	820	0	12	682	694
Cebu	309,013	0	67	630	697	1	163	2,298	2,462
Negros Oriental	90,628	0	1	241	242	2	133	1,357	1,492
Siquijor	9,078	0	0	2	2	0	4	103	107
City of Cebu	39,810	0	5	93	98	0	60	643	703
City of Lapu-Lapu	35,757	0	0	0	0	0	33	288	321
City of Mandaue	26,592	0	0	1	1	1	3	97	101
**Region 8**	**332,967**	**0**	**28**	**432**	**460**	**4**	**197**	**2,246**	**2,447**
Biliran	11,240	0	4	25	29	0	9	240	249
Eastern Samar	35,729	0	2	32	34	0	32	290	322

Table 2.A.2 - MODERN METHOD OF FAMILY PLANNING

Other Acceptors

Philippines, 2023

Area	Total Current Users	IUD-POSTPARTUM			Total	PILLS-POP			Total
		Age group				Age group			
		10-14	15-19	20-49		10-14	15-19	20-49	
Leyte	122,333	0	2	33	35	0	24	360	384
Northern Samar	49,058	0	8	141	149	0	61	329	390
Southern Leyte	27,698	0	0	164	164	0	8	354	362
Samar	41,329	0	9	12	21	4	27	306	337
Ormoc City	19,622	0	3	16	19	0	36	331	367
City of Tacloban	25,958	0	0	9	9	0	0	36	36
**Region 9**	**339,891**	**3**	**136**	**2,163**	**2,302**	**4**	**341**	**4,358**	**4,703**
Zamboanga del Norte	124,603	0	6	285	291	2	99	940	1,041
Zamboanga del Sur	94,708	3	126	1,774	1,903	1	89	1,893	1,983
Zamboanga Sibugay	44,556	0	3	64	67	1	108	756	865
City of Isabela	9,211	0	0	0	0	0	15	229	244
City of Zamboanga	66,813	0	1	40	41	0	30	540	570
**Region 10**	**514,846**	**10**	**394**	**3,653**	**4,057**	**18**	**965**	**7,358**	**8,341**
Bukidnon	158,694	1	47	195	243	2	506	2,509	3,017
Camiguin	6,535	0	0	6	6	0	6	30	36
Lanao del Norte	67,645	0	5	40	45	0	21	719	740
Misamis Occidental	69,918	0	25	512	537	4	86	785	875
Misamis Oriental	127,215	9	176	1,824	2,009	12	168	1,355	1,535
City of Cagayan De Oro	54,303	0	127	1,007	1,134	0	161	1,727	1,888
City of Iligan	30,536	0	14	69	83	0	17	233	250
**Region 11**	**532,707**	**0**	**45**	**897**	**942**	**18**	**684**	**7,647**	**8,349**
Davao de Oro	83,298	0	3	44	47	0	83	1,257	1,340
Davao del Norte	108,564	0	12	238	250	3	148	1,499	1,650
Davao Oriental	54,593	0	1	38	39	0	112	822	934
Davao del Sur	67,257	0	2	129	131	5	43	535	583
Davao Occidental	31,707	0	0	25	25	2	48	268	318
City of Davao	187,288	0	27	423	450	8	250	3,266	3,524
**Region 12**	**453,032**	**0**	**66**	**746**	**812**	**6**	**637**	**6,177**	**6,820**
Cotabato	134,232	0	11	281	292	0	167	2,673	2,840
Sarangani	72,262	0	11	15	26	0	211	1,082	1,293

Table 2.A.2 - MODERN METHOD OF FAMILY PLANNING

Other Acceptors

Philippines, 2023

Area	Total Current Users	IUD-POSTPARTUM Age group			Total	PILLS-POP Age group			Total
		10-14	15-19	20-49		10-14	15-19	20-49	
South Cotabato	96,119	0	6	161	167	0	102	987	1,089
Sultan Kudarat	101,026	0	15	186	201	5	72	942	1,019
					0				0
City of General Santos	49,393	0	23	103	126	1	85	493	579
**BARMM**	**290,634**	**0**	**22**	**186**	**208**	**7**	**400**	**4,301**	**4,708**
Basilan	15,552	0	0	27	27	0	12	192	204
Lanao del Sur	37,406	0	1	14	15	1	72	1,425	1,498
Maguindanao	92,272	0	8	79	87	1	111	1,437	1,549
Sulu	82,939	0	0	3	3	5	186	690	881
Tawi-Tawi	18,014	0	0	0	0	0	4	391	395
SGU	8,193	0	5	5	10	0	11	78	89
City of Cotabato	36,258	0	8	58	66	0	4	88	92
**Caraga**	**284,351**	**0**	**103**	**3,843**	**3,946**	**4**	**533**	**6,061**	**6,598**
Agusan del Norte	40,460	0	40	45	85	0	81	1,045	1,126
Agusan del Sur	86,938	0	27	232	259	0	167	1,352	1,519
Surigao del Norte	50,319	0	24	799	823	0	81	1,137	1,218
Surigao del Sur	56,051	0	7	2,710	2,717	4	154	1,746	1,904
Dinagat Islands	10,768	0	2	15	17	0	8	130	138
City of Butuan	39,815	0	3	42	45	0	42	651	693

Table 2.A.2 - MODERN METHOD OF FAMILY PLANNING

Other Acceptors

Philippines, 2023

Area	Total Current Users	PILLS-COC			Total	INJECTABLES			Total
		Age group				Age group			
		10-14	15-19	20-49		10-14	15-19	20-49	
**Philippines**	**8,353,912**	**231**	**16,881**	**386,970**	**404,082**	**411**	**18,481**	**300,148**	**319,040**
**N C R**	**859,210**	**33**	**1,653**	**52,206**	**53,892**	**94**	**3,098**	**50,826**	**54,018**
Malabon City	33,641	1	46	1,714	1,761	3	439	2,188	2,630
Navotas City	25,067	6	583	1,045	1,634	11	667	2,152	2,830
Valenzuela City	37,966	16	44	3,843	3,903	15	168	3,715	3,898
Caloocan City	61,903	0	113	2,611	2,724	1	270	6,351	6,622
Marikina City	20,095	0	2	357	359	0	33	741	774
Pasig City	55,229	0	35	988	1,023	0	35	1,326	1,361
Pateros	3,813	0	2	184	186	0	4	103	107
Taguig City	71,609	0	34	2,052	2,086	1	39	1,582	1,622
Quezon City	306,956	0	416	29,112	29,528	1	596	16,559	17,156
Makati City	20,752	0	6	867	873	0	25	950	975
Mandaluyong City	23,665	0	1	220	221	0	2	173	175
San Juan City	5,890	0	1	68	69	0	6	106	112
Manila City	50,873	10	206	4,965	5,181	62	600	9,121	9,783
Las Piñas City	20,551	0	17	823	840	0	71	1,606	1,677
Muntinlupa City	41,934	0	13	633	646	0	32	928	960
Parañaque City	42,631	0	21	445	466	0	49	1,018	1,067
Pasay City	36,635	0	113	2,279	2,392	0	62	2,207	2,269
**C A R**	**161,545**	**5**	**300**	**12,112**	**12,417**	**8**	**474**	**7,884**	**8,366**
Abra	19,903	1	46	1,079	1,126	0	41	691	732
Apayao	24,278	0	66	1,425	1,491	2	80	755	837
Benguet	34,934	1	43	2,023	2,067	1	54	1,420	1,475
Ifugao	21,809	3	41	1,537	1,581	3	46	1,087	1,136
Kalinga	18,022	0	48	4,019	4,067	0	73	1,146	1,219
Mountain Province	13,937	0	26	719	745	1	71	711	783
City of Baguio	28,662	0	30	1,310	1,340	1	109	2,074	2,184
**Region 1**	**481,911**	**1**	**417**	**9,787**	**10,205**	**5**	**420**	**7,215**	**7,640**
Ilocos Norte	51,402	0	13	632	645	0	10	295	305
Ilocos Sur	76,206	0	35	823	858	0	20	444	464
La Union	69,182	0	102	1,204	1,306	2	159	1,407	1,568
Pangasinan	278,036	1	267	7,088	7,356	3	231	4,994	5,228

Table 2.A.2 - MODERN METHOD OF FAMILY PLANNING

Other Acceptors

Philippines, 2023

Area	Total Current Users	PILLS-COC			Total	INJECTABLES			Total
		Age group				Age group			
		10-14	15-19	20-49		10-14	15-19	20-49	
City of Dagupan	7,085	0	0	40	40	0	0	75	75
**Region 2**	**320,396**	**22**	**778**	**31,016**	**31,816**	**32**	**747**	**10,616**	**11,395**
Batanes	945	0	2	60	62	1	8	138	147
Cagayan	106,997	10	149	3,626	3,785	16	156	1,941	2,113
Isabela	130,614	5	439	22,270	22,714	13	354	5,859	6,226
Nueva Vizcaya	49,739	6	110	2,967	3,083	1	97	1,451	1,549
Quirino	19,516	1	40	769	810	0	65	521	586
City of Santiago	12,585	0	38	1,324	1,362	1	67	706	774
**Region 3**	**884,246**	**40**	**1,869**	**44,970**	**46,879**	**62**	**2,057**	**34,350**	**36,469**
Aurora	22,956	0	54	1,885	1,939	0	75	1,139	1,214
Bataan	63,188	0	61	1,484	1,545	3	163	3,001	3,167
Bulacan	245,933	0	770	18,558	19,328	3	751	13,675	14,429
Nueva Ecija	186,491	32	358	10,451	10,841	40	350	5,752	6,142
Pampanga	159,224	1	270	2,767	3,038	3	323	2,395	2,721
Tarlac	115,785	7	200	5,301	5,508	12	205	4,257	4,474
Zambales	48,549	0	94	2,372	2,466	1	134	2,411	2,546
City of Angeles	29,054	0	48	555	603	0	25	509	534
City of Olongapo	13,066	0	14	1,597	1,611	0	31	1,211	1,242
**Region 4A**	**910,368**	**10**	**1,322**	**32,114**	**33,446**	**34**	**2,078**	**39,565**	**41,677**
Batangas	162,745	0	94	4,831	4,925	4	46	2,554	2,604
Cavite	164,389	4	233	10,889	11,126	9	385	11,176	11,570
Laguna	320,343	0	222	5,104	5,326	0	279	4,722	5,001
Quezon	105,489	2	177	6,695	6,874	9	339	8,660	9,008
Rizal	152,400	4	542	4,241	4,787	10	908	11,180	12,098
City of Lucena	5,002	0	54	354	408	2	121	1,273	1,396
**Region 4B**	**266,333**	**26**	**736**	**15,120**	**15,882**	**63**	**1,213**	**15,435**	**16,711**
Marinduque	15,205	0	0	578	578	0	3	181	184
Occidental Mindoro	40,816	0	125	3,740	3,865	1	124	2,365	2,490
Oriental Mindoro	63,083	1	116	3,602	3,719	5	161	2,391	2,557
Palawan	93,326	25	384	4,922	5,331	51	584	7,040	7,675

Table 2.A.2 - MODERN METHOD OF FAMILY PLANNING

Other Acceptors

Philippines, 2023

Area	Total Current Users	PILLS-COC			Total	INJECTABLES			Total
		Age group				Age group			
		10-14	15-19	20-49		10-14	15-19	20-49	
Romblon	22,228	0	8	519	527	1	30	475	506
City of Puerto Princesa	31,675	0	103	1,759	1,862	5	311	2,983	3,299
**Region 5**	**484,529**	**5**	**515**	**17,722**	**18,242**	**7**	**776**	**18,325**	**19,108**
Albay	120,028	0	57	2,783	2,840	1	54	2,295	2,350
Camarines Norte	46,841	0	41	1,298	1,339	0	147	3,035	3,182
Camarines Sur	107,402	1	87	4,369	4,457	1	178	5,172	5,351
Catanduanes	23,049	0	12	843	855	2	92	1,569	1,663
Masbate	91,907	2	216	4,928	5,146	1	94	2,217	2,312
Sorsogon	65,058	2	101	3,241	3,344	2	207	3,870	4,079
City of Naga	30,244	0	1	260	261	0	4	167	171
**Region 6**	**639,939**	**13**	**866**	**23,635**	**24,514**	**4**	**713**	**14,169**	**14,886**
Aklan	46,162	0	48	838	886	0	47	824	871
Antique	47,022	0	31	1,335	1,366	1	62	1,990	2,053
Capiz	75,514	0	41	1,560	1,601	0	27	704	731
Guimaras	16,676	0	33	1,025	1,058	0	18	383	401
Iloilo	169,693	0	132	4,160	4,292	0	99	2,236	2,335
Negros Occidental	199,968	13	480	11,572	12,065	3	363	6,615	6,981
City of Bacolod	28,243	0	39	1,277	1,316	0	46	690	736
City of Iloilo	56,661	0	62	1,868	1,930	0	51	727	778
**Region 7**	**597,007**	**2**	**503**	**18,855**	**19,360**	**10**	**823**	**17,386**	**18,219**
Bohol	86,129	0	32	1,843	1,875	0	68	1,611	1,679
Cebu	309,013	0	202	7,703	7,905	3	232	6,259	6,494
Negros Oriental	90,628	2	179	6,124	6,305	7	318	5,930	6,255
Siquijor	9,078	0	9	429	438	0	23	371	394
City of Cebu	39,810	0	56	2,242	2,298	0	143	1,944	2,087
City of Lapu-Lapu	35,757	0	23	373	396	0	27	864	891
City of Mandaue	26,592	0	2	141	143	0	12	407	419
**Region 8**	**332,967**	**21**	**514**	**13,019**	**13,554**	**29**	**520**	**10,741**	**11,290**
Biliran	11,240	0	35	595	630	0	25	711	736
Eastern Samar	35,729	0	45	1,124	1,169	0	60	1,042	1,102

Table 2.A.2 - MODERN METHOD OF FAMILY PLANNING

Other Acceptors

Philippines, 2023

Area	Total Current Users	PILLS-COC			Total	INJECTABLES			Total
		Age group				Age group			
		10-14	15-19	20-49		10-14	15-19	20-49	
Leyte	122,333	0	62	1,295	1,357	0	53	1,028	1,081
Northern Samar	49,058	0	166	3,242	3,408	0	121	3,522	3,643
Southern Leyte	27,698	0	20	1,167	1,187	0	30	903	933
Samar	41,329	21	95	3,741	3,857	29	137	2,115	2,281
Ormoc City	19,622	0	77	1,649	1,726	0	93	1,349	1,442
City of Tacloban	25,958	0	14	206	220	0	1	71	72
**Region 9**	**339,891**	**3**	**800**	**15,534**	**16,337**	**0**	**634**	**11,581**	**12,215**
Zamboanga del Norte	124,603	2	289	4,376	4,667	0	199	3,276	3,475
Zamboanga del Sur	94,708	1	245	5,189	5,435	0	163	3,662	3,825
Zamboanga Sibugay	44,556	0	170	3,762	3,932	0	142	2,293	2,435
City of Isabela	9,211	0	18	363	381	0	18	465	483
City of Zamboanga	66,813	0	78	1,844	1,922	0	112	1,885	1,997
**Region 10**	**514,846**	**16**	**2,687**	**32,286**	**34,989**	**7**	**1,470**	**15,407**	**16,884**
Bukidnon	158,694	3	988	15,178	16,169	5	751	6,346	7,102
Camiguin	6,535	0	7	241	248	0	11	87	98
Lanao del Norte	67,645	1	134	2,289	2,424	0	87	1,380	1,467
Misamis Occidental	69,918	3	1,013	2,517	3,533	0	162	1,850	2,012
Misamis Oriental	127,215	2	188	4,045	4,235	2	198	2,393	2,593
City of Cagayan De Oro	54,303	7	333	7,352	7,692	0	228	2,700	2,928
City of Iligan	30,536	0	24	664	688	0	33	651	684
**Region 11**	**532,707**	**17**	**1,217**	**22,623**	**23,857**	**28**	**984**	**11,327**	**12,339**
Davao de Oro	83,298	1	138	4,285	4,424	2	126	1,841	1,969
Davao del Norte	108,564	1	247	5,743	5,991	4	158	2,680	2,842
Davao Oriental	54,593	0	179	4,488	4,667	0	125	1,559	1,684
Davao del Sur	67,257	1	132	1,764	1,897	0	100	1,226	1,326
Davao Occidental	31,707	2	255	2,600	2,857	1	210	1,441	1,652
City of Davao	187,288	12	266	3,743	4,021	21	265	2,580	2,866
**Region 12**	**453,032**	**9**	**1,441**	**22,754**	**24,204**	**16**	**1,119**	**15,024**	**16,159**
Cotabato	134,232	1	446	8,115	8,562	4	231	4,331	4,566
Sarangani	72,262	0	319	3,182	3,501	0	316	2,773	3,089

Table 2.A.2 - MODERN METHOD OF FAMILY PLANNING

Other Acceptors

Philippines, 2023

Area	Total Current Users	PILLS-COC			Total	INJECTABLES			Total
		Age group				Age group			
		10-14	15-19	20-49		10-14	15-19	20-49	
South Cotabato	96,119	5	231	5,080	5,316	1	201	2,623	2,825
Sultan Kudarat	101,026	3	322	4,886	5,211	7	192	2,992	3,191
					0				0
City of General Santos	49,393	0	123	1,491	1,614	4	179	2,305	2,488
**BARMM**	**290,634**	**0**	**401**	**7,593**	**7,994**	**7**	**839**	**11,919**	**12,765**
Basilan	15,552	0	46	666	712	2	84	1,065	1,151
Lanao del Sur	37,406	0	63	1,175	1,238	2	67	1,755	1,824
Maguindanao	92,272	0	168	3,099	3,267	2	519	5,574	6,095
Sulu	82,939	0	21	1,261	1,282	0	84	2,028	2,112
Tawi-Tawi	18,014	0	40	677	717	1	22	882	905
SGU	8,193	0	10	100	110	0	22	186	208
City of Cotabato	36,258	0	53	615	668	0	41	429	470
**Caraga**	**284,351**	**8**	**862**	**15,624**	**16,494**	**5**	**516**	**8,378**	**8,899**
Agusan del Norte	40,460	0	131	1,949	2,080	3	96	1,286	1,385
Agusan del Sur	86,938	7	343	3,486	3,836	0	119	1,913	2,032
Surigao del Norte	50,319	0	190	3,657	3,847	0	66	1,969	2,035
Surigao del Sur	56,051	0	141	3,575	3,716	2	170	1,772	1,944
Dinagat Islands	10,768	0	6	826	832	0	20	452	472
City of Butuan	39,815	1	51	2,131	2,183	0	45	986	1,031

Table 2.A.2 - MODERN METHOD OF FAMILY PLANNING

Other Acceptors

Philippines, 2023

Area	Total Current Users	IMPLANTS			Total	NFP-CCM			Total
		Age group				Age group			
		10-14	15-19	20-49		10-14	15-19	20-49	
**Philippines**	**8,353,912**	**355**	**14,277**	**173,850**	**188,482**	**10**	**297**	**9,109**	**9,416**
**N C R**	**859,210**	**85**	**2,177**	**24,887**	**27,149**	**0**	**1**	**83**	**84**
Malabon City	33,641	56	324	1,297	1,677	0	0	0	0
Navotas City	25,067	2	377	1,071	1,450	0	0	0	0
Valenzuela City	37,966	10	47	1,530	1,587	0	0	4	4
Caloocan City	61,903	4	224	3,337	3,565	0	0	0	0
Marikina City	20,095	0	16	477	493	0	0	13	13
Pasig City	55,229	0	36	1,057	1,093	0	0	0	0
Pateros	3,813	0	2	67	69	0	0	0	0
Taguig City	71,609	0	29	664	693	0	1	0	1
Quezon City	306,956	8	498	8,244	8,750	0	0	0	0
Makati City	20,752	0	4	263	267	0	0	0	0
Mandaluyong City	23,665	1	4	140	145	0	0	30	30
San Juan City	5,890	0	0	0	0	0	0	0	0
Manila City	50,873	2	380	4,058	4,440	0	0	0	0
Las Piñas City	20,551	2	108	1,395	1,505	0	0	0	0
Muntinlupa City	41,934	0	1	58	59	0	0	0	0
Parañaque City	42,631	0	11	359	370	0	0	0	0
Pasay City	36,635	0	116	870	986	0	0	36	36
**C A R**	**161,545**	**11**	**277**	**4,249**	**4,537**	**0**	**36**	**917**	**953**
Abra	19,903	0	35	332	367	0	1	3	4
Apayao	24,278	2	56	552	610	0	0	1	1
Benguet	34,934	0	44	693	737	0	0	4	4
Ifugao	21,809	6	40	506	552	0	9	166	175
Kalinga	18,022	0	27	622	649	0	26	605	631
Mountain Province	13,937	1	49	498	548	0	0	137	137
City of Baguio	28,662	2	26	1,046	1,074	0	0	1	1
**Region 1**	**481,911**	**3**	**201**	**3,129**	**3,333**	**0**	**27**	**378**	**405**
Ilocos Norte	51,402	0	18	162	180	0	10	125	135
Ilocos Sur	76,206	0	11	169	180	0	17	216	233
La Union	69,182	2	77	470	549	0	0	37	37
Pangasinan	278,036	1	95	2,310	2,406	0	0	0	0

Table 2.A.2 - MODERN METHOD OF FAMILY PLANNING

Other Acceptors

Philippines, 2023

Area	Total Current Users	IMPLANTS			Total	NFP-CCM			Total
		Age group				Age group			
		10-14	15-19	20-49		10-14	15-19	20-49	
City of Dagupan	7,085	0	0	18	18	0	0	0	0
**Region 2**	**320,396**	**17**	**347**	**4,950**	**5,314**	**0**	**1**	**99**	**100**
Batanes	945	0	2	39	41	0	0	14	14
Cagayan	106,997	5	61	888	954	0	0	0	0
Isabela	130,614	11	194	2,784	2,989	0	1	3	4
Nueva Vizcaya	49,739	1	48	724	773	0	0	61	61
Quirino	19,516	0	27	274	301	0	0	21	21
City of Santiago	12,585	0	15	241	256	0	0	0	0
**Region 3**	**884,246**	**25**	**1,136**	**12,421**	**13,582**	**0**	**0**	**56**	**56**
Aurora	22,956	1	60	588	649	0	0	2	2
Bataan	63,188	0	63	1,197	1,260	0	0	0	0
Bulacan	245,933	1	220	4,397	4,618	0	0	8	8
Nueva Ecija	186,491	8	228	2,529	2,765	0	0	40	40
Pampanga	159,224	12	447	1,437	1,896	0	0	1	1
Tarlac	115,785	3	46	1,119	1,168	0	0	0	0
Zambales	48,549	0	46	765	811	0	0	5	5
City of Angeles	29,054	0	14	239	253	0	0	0	0
City of Olongapo	13,066	0	12	150	162	0	0	0	0
**Region 4A**	**910,368**	**25**	**921**	**12,943**	**13,889**	**0**	**2**	**413**	**415**
Batangas	162,745	3	30	945	978	0	0	358	358
Cavite	164,389	0	180	3,311	3,491	0	1	13	14
Laguna	320,343	16	165	2,350	2,531	0	0	33	33
Quezon	105,489	0	214	2,195	2,409	0	1	9	10
Rizal	152,400	6	315	3,969	4,290	0	0	0	0
City of Lucena	5,002	0	17	173	190	0	0	0	0
**Region 4B**	**266,333**	**74**	**727**	**7,955**	**8,756**	**0**	**59**	**997**	**1,056**
Marinduque	15,205	2	8	182	192	0	0	0	0
Occidental Mindoro	40,816	0	174	2,367	2,541	0	58	865	923
Oriental Mindoro	63,083	4	95	1,151	1,250	0	0	87	87
Palawan	93,326	66	334	2,965	3,365	0	1	16	17

Table 2.A.2 - MODERN METHOD OF FAMILY PLANNING

Other Acceptors

Philippines, 2023

Area	Total Current Users	IMPLANTS			Total	NFP-CCM			Total
		Age group				Age group			
		10-14	15-19	20-49		10-14	15-19	20-49	
Romblon	22,228	0	35	328	363	0	0	29	29
City of Puerto Princesa	31,675	2	81	962	1,045	0	0	0	0
**Region 5**	**484,529**	**8**	**642**	**10,666**	**11,316**	**4**	**88**	**3,618**	**3,710**
Albay	120,028	2	27	1,291	1,320	0	2	171	173
Camarines Norte	46,841	0	64	1,024	1,088	0	0	14	14
Camarines Sur	107,402	4	162	3,464	3,630	0	1	27	28
Catanduanes	23,049	2	69	839	910	0	28	743	771
Masbate	91,907	0	204	1,598	1,802	4	26	1,924	1,954
Sorsogon	65,058	0	110	2,298	2,408	0	31	739	770
City of Naga	30,244	0	6	152	158	0	0	0	0
**Region 6**	**639,939**	**4**	**305**	**6,137**	**6,446**	**0**	**16**	**683**	**699**
Aklan	46,162	0	11	211	222	0	0	0	0
Antique	47,022	0	24	695	719	0	16	671	687
Capiz	75,514	0	22	486	508	0	0	0	0
Guimaras	16,676	0	2	71	73	0	0	0	0
Iloilo	169,693	0	67	1,146	1,213	0	0	0	0
Negros Occidental	199,968	0	122	2,207	2,329	0	0	12	12
City of Bacolod	28,243	4	34	983	1,021	0	0	0	0
City of Iloilo	56,661	0	23	338	361	0	0	0	0
**Region 7**	**597,007**	**6**	**669**	**11,014**	**11,689**	**0**	**0**	**105**	**105**
Bohol	86,129	2	224	3,118	3,344	0	0	33	33
Cebu	309,013	3	225	4,246	4,474	0	0	0	0
Negros Oriental	90,628	0	79	1,389	1,468	0	0	70	70
Siquijor	9,078	0	12	147	159	0	0	0	0
City of Cebu	39,810	0	101	1,161	1,262	0	0	2	2
City of Lapu-Lapu	35,757	1	12	396	409	0	0	0	0
City of Mandaue	26,592	0	16	557	573	0	0	0	0
**Region 8**	**332,967**	**3**	**347**	**5,689**	**6,039**	**0**	**9**	**31**	**40**
Biliran	11,240	1	43	678	722	0	0	3	3
Eastern Samar	35,729	0	58	538	596	0	0	2	2

Table 2.A.2 - MODERN METHOD OF FAMILY PLANNING

Other Acceptors

Philippines, 2023

Area	Total Current Users	IMPLANTS			Total	NFP-CCM			Total
		Age group				Age group			
		10-14	15-19	20-49		10-14	15-19	20-49	
Leyte	122,333	0	31	575	606	0	0	0	0
Northern Samar	49,058	0	50	1,298	1,348	0	0	1	1
Southern Leyte	27,698	0	25	478	503	0	0	9	9
Samar	41,329	2	51	1,161	1,214	0	9	16	25
Ormoc City	19,622	0	77	765	842	0	0	0	0
City of Tacloban	25,958	0	12	196	208	0	0	0	0
**Region 9**	**339,891**	**12**	**1,167**	**15,854**	**17,033**	**0**	**0**	**0**	**0**
Zamboanga del Norte	124,603	5	448	4,651	5,104	0	0	0	0
Zamboanga del Sur	94,708	1	405	5,704	6,110	0	0	0	0
Zamboanga Sibugay	44,556	2	176	2,794	2,972	0	0	0	0
City of Isabela	9,211	2	20	432	454	0	0	0	0
City of Zamboanga	66,813	2	118	2,273	2,393	0	0	0	0
**Region 10**	**514,846**	**20**	**1,987**	**15,009**	**17,016**	**6**	**37**	**669**	**712**
Bukidnon	158,694	13	1,331	8,778	10,122	0	12	374	386
Camiguin	6,535	0	5	61	66	0	0	0	0
Lanao del Norte	67,645	0	59	784	843	0	0	0	0
Misamis Occidental	69,918	0	120	893	1,013	0	6	36	42
Misamis Oriental	127,215	4	223	2,308	2,535	6	19	178	203
City of Cagayan De Oro	54,303	3	222	1,628	1,853	0	0	0	0
City of Iligan	30,536	0	27	557	584	0	0	81	81
**Region 11**	**532,707**	**19**	**860**	**9,850**	**10,729**	**0**	**2**	**119**	**121**
Davao de Oro	83,298	1	83	1,346	1,430	0	0	8	8
Davao del Norte	108,564	1	287	2,666	2,954	0	0	6	6
Davao Oriental	54,593	1	116	1,480	1,597	0	0	54	54
Davao del Sur	67,257	3	73	946	1,022	0	1	22	23
Davao Occidental	31,707	2	124	1,080	1,206	0	0	0	0
City of Davao	187,288	11	177	2,332	2,520	0	1	29	30
**Region 12**	**453,032**	**25**	**1,299**	**13,743**	**15,067**	**0**	**3**	**593**	**596**
Cotabato	134,232	4	298	4,230	4,532	0	1	509	510
Sarangani	72,262	0	203	2,237	2,440	0	1	6	7

Table 2.A.2 - MODERN METHOD OF FAMILY PLANNING

Other Acceptors

Philippines, 2023

Area	Total Current Users	IMPLANTS			Total	NFP-CCM			Total
		Age group				Age group			
		10-14	15-19	20-49		10-14	15-19	20-49	
South Cotabato	96,119	7	317	2,501	2,825	0	1	72	73
Sultan Kudarat	101,026	10	242	2,200	2,452	0	0	6	6
					0				0
City of General Santos	49,393	4	239	2,575	2,818	0	0	0	0
**BARMM**	**290,634**	**9**	**435**	**4,948**	**5,392**	**0**	**0**	**8**	**8**
Basilan	15,552	1	82	664	747	0	0	0	0
Lanao del Sur	37,406	0	17	517	534	0	0	0	0
Maguindanao	92,272	8	246	1,777	2,031	0	0	0	0
Sulu	82,939	0	43	852	895	0	0	8	8
Tawi-Tawi	18,014	0	36	949	985	0	0	0	0
SGU	8,193	0	1	30	31	0	0	0	0
City of Cotabato	36,258	0	10	159	169	0	0	0	0
**Caraga**	**284,351**	**9**	**780**	**10,406**	**11,195**	**0**	**16**	**340**	**356**
Agusan del Norte	40,460	2	88	1,189	1,279	0	0	3	3
Agusan del Sur	86,938	4	304	3,047	3,355	0	15	234	249
Surigao del Norte	50,319	1	102	1,912	2,015	0	0	24	24
Surigao del Sur	56,051	2	211	2,862	3,075	0	1	79	80
Dinagat Islands	10,768	0	22	542	564	0	0	0	0
City of Butuan	39,815	0	53	854	907	0	0	0	0

Table 2.A.2 - MODERN METHOD OF FAMILY PLANNING

Other Acceptors

Philippines, 2023

Area	Total Current Users	NFP-BBT			Total	NFP-STM			Total
		Age group				Age group			
		10-14	15-19	20-49		10-14	15-19	20-49	
**Philippines**	**8,353,912**	**6**	**102**	**1,015**	**1,123**	**4**	**8**	**724**	**736**
**N C R**	**859,210**	**0**	**29**	**158**	**187**	**0**	**0**	**9**	**9**
Malabon City	33,641	0	0	0	0	0	0	0	0
Navotas City	25,067	0	0	0	0	0	0	0	0
Valenzuela City	37,966	0	0	3	3	0	0	0	0
Caloocan City	61,903	0	0	0	0	0	0	0	0
Marikina City	20,095	0	0	0	0	0	0	0	0
Pasig City	55,229	0	0	0	0	0	0	0	0
Pateros	3,813	0	0	0	0	0	0	0	0
Taguig City	71,609	0	0	0	0	0	0	0	0
Quezon City	306,956	0	0	0	0	0	0	3	3
Makati City	20,752	0	0	0	0	0	0	0	0
Mandaluyong City	23,665	0	0	0	0	0	0	0	0
San Juan City	5,890	0	0	0	0	0	0	0	0
Manila City	50,873	0	29	26	55	0	0	0	0
Las Piñas City	20,551	0	0	0	0	0	0	0	0
Muntinlupa City	41,934	0	0	0	0	0	0	0	0
Parañaque City	42,631	0	0	0	0	0	0	0	0
Pasay City	36,635	0	0	129	129	0	0	6	6
**C A R**	**161,545**	**0**	**0**	**20**	**20**	**0**	**0**	**8**	**8**
Abra	19,903	0	0	1	1	0	0	2	2
Apayao	24,278	0	0	0	0	0	0	0	0
Benguet	34,934	0	0	12	12	0	0	0	0
Ifugao	21,809	0	0	0	0	0	0	4	4
Kalinga	18,022	0	0	3	3	0	0	0	0
Mountain Province	13,937	0	0	2	2	0	0	0	0
City of Baguio	28,662	0	0	2	2	0	0	2	2
**Region 1**	**481,911**	**0**	**0**	**116**	**116**	**0**	**0**	**68**	**68**
Ilocos Norte	51,402	0	0	0	0	0	0	0	0
Ilocos Sur	76,206	0	0	115	115	0	0	64	64
La Union	69,182	0	0	0	0	0	0	0	0
Pangasinan	278,036	0	0	1	1	0	0	4	4

Table 2.A.2 - MODERN METHOD OF FAMILY PLANNING

Other Acceptors

Philippines, 2023

Area	Total Current Users	NFP-BBT			Total	NFP-STM			Total
		Age group				Age group			
		10-14	15-19	20-49		10-14	15-19	20-49	
City of Dagupan	7,085	0	0	0	0	0	0	0	0
**Region 2**	**320,396**	**0**	**8**	**21**	**29**	**1**	**2**	**11**	**14**
Batanes	945	0	0	0	0	0	0	0	0
Cagayan	106,997	0	2	0	2	0	0	0	0
Isabela	130,614	0	6	19	25	0	0	0	0
Nueva Vizcaya	49,739	0	0	0	0	1	2	11	14
Quirino	19,516	0	0	2	2	0	0	0	0
City of Santiago	12,585	0	0	0	0	0	0	0	0
**Region 3**	**884,246**	**0**	**1**	**11**	**12**	**0**	**0**	**1**	**1**
Aurora	22,956	0	0	6	6	0	0	0	0
Bataan	63,188	0	0	0	0	0	0	0	0
Bulacan	245,933	0	0	0	0	0	0	0	0
Nueva Ecija	186,491	0	0	1	1	0	0	0	0
Pampanga	159,224	0	0	0	0	0	0	0	0
Tarlac	115,785	0	1	4	5	0	0	1	1
Zambales	48,549	0	0	0	0	0	0	0	0
City of Angeles	29,054	0	0	0	0	0	0	0	0
City of Olongapo	13,066	0	0	0	0	0	0	0	0
**Region 4A**	**910,368**	**3**	**2**	**29**	**34**	**0**	**0**	**13**	**13**
Batangas	162,745	3	0	3	6	0	0	0	0
Cavite	164,389	0	0	0	0	0	0	0	0
Laguna	320,343	0	0	7	7	0	0	1	1
Quezon	105,489	0	2	18	20	0	0	12	12
Rizal	152,400	0	0	1	1	0	0	0	0
City of Lucena	5,002	0	0	0	0	0	0	0	0
**Region 4B**	**266,333**	**0**	**2**	**47**	**49**	**0**	**0**	**37**	**37**
Marinduque	15,205	0	0	1	1	0	0	0	0
Occidental Mindoro	40,816	0	0	0	0	0	0	7	7
Oriental Mindoro	63,083	0	1	30	31	0	0	25	25
Palawan	93,326	0	0	8	8	0	0	2	2

Table 2.A.2 - MODERN METHOD OF FAMILY PLANNING

Other Acceptors

Philippines, 2023

Area	Total Current Users	NFP-BBT Age group 10-14	NFP-BBT Age group 15-19	NFP-BBT Age group 20-49	Total	NFP-STM Age group 10-14	NFP-STM Age group 15-19	NFP-STM Age group 20-49	Total
Romblon	22,228	0	1	2	3	0	0	2	2
City of Puerto Princesa	31,675	0	0	6	6	0	0	1	1
**Region 5**	**484,529**	**0**	**4**	**123**	**127**	**0**	**1**	**78**	**79**
Albay	120,028	0	1	38	39	0	0	20	20
Camarines Norte	46,841	0	0	2	2	0	0	3	3
Camarines Sur	107,402	0	1	13	14	0	0	8	8
Catanduanes	23,049	0	2	59	61	0	0	6	6
Masbate	91,907	0	0	10	10	0	1	17	18
Sorsogon	65,058	0	0	1	1	0	0	24	24
City of Naga	30,244	0	0	0	0	0	0	0	0
**Region 6**	**639,939**	**0**	**0**	**16**	**16**	**0**	**0**	**7**	**7**
Aklan	46,162	0	0	0	0	0	0	0	0
Antique	47,022	0	0	1	1	0	0	1	1
Capiz	75,514	0	0	0	0	0	0	0	0
Guimaras	16,676	0	0	2	2	0	0	0	0
Iloilo	169,693	0	0	0	0	0	0	5	5
Negros Occidental	199,968	0	0	11	11	0	0	1	1
City of Bacolod	28,243	0	0	0	0	0	0	0	0
City of Iloilo	56,661	0	0	2	2	0	0	0	0
**Region 7**	**597,007**	**0**	**0**	**6**	**6**	**0**	**0**	**4**	**4**
Bohol	86,129	0	0	0	0	0	0	0	0
Cebu	309,013	0	0	0	0	0	0	0	0
Negros Oriental	90,628	0	0	6	6	0	0	4	4
Siquijor	9,078	0	0	0	0	0	0	0	0
City of Cebu	39,810	0	0	0	0	0	0	0	0
City of Lapu-Lapu	35,757	0	0	0	0	0	0	0	0
City of Mandaue	26,592	0	0	0	0	0	0	0	0
**Region 8**	**332,967**	**0**	**1**	**180**	**181**	**0**	**0**	**39**	**39**
Biliran	11,240	0	0	0	0	0	0	0	0
Eastern Samar	35,729	0	0	0	0	0	0	0	0

Table 2.A.2 - MODERN METHOD OF FAMILY PLANNING

Other Acceptors

Philippines, 2023

Area	Total Current Users	NFP-BBT			Total	NFP-STM			Total
		Age group				Age group			
		10-14	15-19	20-49		10-14	15-19	20-49	
Leyte	122,333	0	0	5	5	0	0	39	39
Northern Samar	49,058	0	0	172	172	0	0	0	0
Southern Leyte	27,698	0	0	3	3	0	0	0	0
Samar	41,329	0	1	0	1	0	0	0	0
Ormoc City	19,622	0	0	0	0	0	0	0	0
City of Tacloban	25,958	0	0	0	0	0	0	0	0
**Region 9**	**339,891**	**0**	**0**	**0**	**0**	**0**	**0**	**19**	**19**
Zamboanga del Norte	124,603	0	0	0	0	0	0	0	0
Zamboanga del Sur	94,708	0	0	0	0	0	0	3	3
Zamboanga Sibugay	44,556	0	0	0	0	0	0	16	16
City of Isabela	9,211	0	0	0	0	0	0	0	0
City of Zamboanga	66,813	0	0	0	0	0	0	0	0
**Region 10**	**514,846**	**3**	**12**	**160**	**175**	**2**	**4**	**242**	**248**
Bukidnon	158,694	0	2	25	27	0	0	113	113
Camiguin	6,535	0	0	0	0	0	0	0	0
Lanao del Norte	67,645	0	0	12	12	0	0	46	46
Misamis Occidental	69,918	0	4	28	32	0	0	32	32
Misamis Oriental	127,215	3	6	67	76	2	4	51	57
City of Cagayan De Oro	54,303	0	0	28	28	0	0	0	0
City of Iligan	30,536	0	0	0	0	0	0	0	0
**Region 11**	**532,707**	**0**	**18**	**43**	**61**	**0**	**0**	**16**	**16**
Davao de Oro	83,298	0	1	20	21	0	0	0	0
Davao del Norte	108,564	0	7	0	7	0	0	3	3
Davao Oriental	54,593	0	0	0	0	0	0	1	1
Davao del Sur	67,257	0	0	0	0	0	0	0	0
Davao Occidental	31,707	0	10	0	10	0	0	0	0
City of Davao	187,288	0	0	23	23	0	0	12	12
**Region 12**	**453,032**	**0**	**22**	**62**	**84**	**0**	**0**	**27**	**27**
Cotabato	134,232	0	0	17	17	0	0	0	0
Sarangani	72,262	0	0	0	0	0	0	0	0

Table 2.A.2 - MODERN METHOD OF FAMILY PLANNING

Other Acceptors

Philippines, 2023

Area	Total Current Users	NFP-BBT			Total	NFP-STM			Total
		Age group				Age group			
		10-14	15-19	20-49		10-14	15-19	20-49	
South Cotabato	96,119	0	0	21	21	0	0	27	27
Sultan Kudarat	101,026	0	0	9	9	0	0	0	0
					0				0
City of General Santos	49,393	0	22	15	37	0	0	0	0
**BARMM**	**290,634**	**0**	**0**	**1**	**1**	**1**	**0**	**124**	**125**
Basilan	15,552	0	0	0	0	1	0	124	125
Lanao del Sur	37,406	0	0	1	1	0	0	0	0
Maguindanao	92,272	0	0	0	0	0	0	0	0
Sulu	82,939	0	0	0	0	0	0	0	0
Tawi-Tawi	18,014	0	0	0	0	0	0	0	0
SGU	8,193	0	0	0	0	0	0	0	0
City of Cotabato	36,258	0	0	0	0	0	0	0	0
**Caraga**	**284,351**	**0**	**3**	**22**	**25**	**0**	**1**	**21**	**22**
Agusan del Norte	40,460	0	0	1	1	0	0	0	0
Agusan del Sur	86,938	0	0	4	4	0	0	1	1
Surigao del Norte	50,319	0	3	17	20	0	1	15	16
Surigao del Sur	56,051	0	0	0	0	0	0	5	5
Dinagat Islands	10,768	0	0	0	0	0	0	0	0
City of Butuan	39,815	0	0	0	0	0	0	0	0

Table 2.A.2 - MODERN METHOD OF FAMILY PLANNING

Other Acceptors

Philippines, 2023

Area	Total Current Users	NFP-SDM			Total	NFP-LAM			Total	Total Other Acceptors
		Age group				Age group				
		10-14	15-19	20-49		10-14	15-19	20-49		
**Philippines**	**8,353,912**	**96**	**921**	**23,625**	**24,642**	**465**	**18,327**	**358,611**	**377,403**	**1,692,701**
**N C R**	**859,210**	**92**	**0**	**46**	**138**	**102**	**1,934**	**39,369**	**41,405**	**248,631**
Malabon City	33,641	0	0	1	1	0	44	1,222	1,266	10,277
Navotas City	25,067	0	0	0	0	6	192	1,853	2,051	10,545
Valenzuela City	37,966	92	0	2	94	60	46	1,530	1,636	13,875
Caloocan City	61,903	0	0	4	4	0	211	4,029	4,240	22,340
Marikina City	20,095	0	0	1	1	0	1	87	88	2,387
Pasig City	55,229	0	0	2	2	0	6	306	312	5,321
Pateros	3,813	0	0	0	0	0	3	39	42	693
Taguig City	71,609	0	0	0	0	0	23	326	349	7,578
Quezon City	306,956	0	0	35	35	1	604	25,116	25,721	116,527
Makati City	20,752	0	0	0	0	0	9	80	89	3,838
Mandaluyong City	23,665	0	0	0	0	0	8	101	109	1,151
San Juan City	5,890	0	0	0	0	0	0	8	8	221
Manila City	50,873	0	0	0	0	27	59	563	649	27,036
Las Piñas City	20,551	0	0	0	0	0	3	83	86	5,466
Muntinlupa City	41,934	0	0	0	0	0	0	48	48	1,884
Parañaque City	42,631	0	0	0	0	0	62	646	708	3,223
Pasay City	36,635	0	0	1	1	8	663	3,332	4,003	16,269
**C A R**	**161,545**	**2**	**23**	**2,195**	**2,220**	**9**	**300**	**10,011**	**10,320**	**48,045**
Abra	19,903	0	3	183	186	0	62	1,574	1,636	4,829
Apayao	24,278	0	0	1	1	1	47	856	904	4,481
Benguet	34,934	0	1	1,033	1,034	2	63	2,238	2,303	9,762
Ifugao	21,809	2	15	625	642	4	47	1,741	1,792	7,138
Kalinga	18,022	0	0	41	41	0	35	2,033	2,068	9,565
Mountain Province	13,937	0	4	295	299	2	44	1,255	1,301	4,967
City of Baguio	28,662	0	0	17	17	0	2	314	316	7,303
**Region 1**	**481,911**	**0**	**26**	**823**	**849**	**5**	**710**	**19,306**	**20,021**	**48,897**
Ilocos Norte	51,402	0	0	191	191	0	42	1,242	1,284	4,313
Ilocos Sur	76,206	0	7	294	301	0	59	1,356	1,415	4,315
La Union	69,182	0	12	189	201	3	187	4,195	4,385	9,348
Pangasinan	278,036	0	7	149	156	2	416	12,494	12,912	30,722

Table 2.A.2 - MODERN METHOD OF FAMILY PLANNING

Other Acceptors

Philippines, 2023

Area	Total Current Users	NFP-SDM			Total	NFP-LAM			Total	Total Other Acceptors
		Age group				Age group				
		10-14	15-19	20-49		10-14	15-19	20-49		
City of Dagupan	7,085	0	0	0	0	0	6	19	25	199
**Region 2**	**320,396**	**0**	**2**	**364**	**366**	**18**	**817**	**13,996**	**14,831**	**79,396**
Batanes	945	0	0	2	2	0	3	127	130	443
Cagayan	106,997	0	0	0	0	0	95	2,160	2,255	11,269
Isabela	130,614	0	0	166	166	11	356	5,677	6,044	47,472
Nueva Vizcaya	49,739	0	2	162	164	6	278	3,250	3,534	11,579
Quirino	19,516	0	0	8	8	1	47	705	753	3,934
City of Santiago	12,585	0	0	26	26	0	38	2,077	2,115	4,699
**Region 3**	**884,246**	**0**	**4**	**426**	**430**	**10**	**1,092**	**19,194**	**20,296**	**161,496**
Aurora	22,956	0	0	9	9	1	85	1,924	2,010	7,129
Bataan	63,188	0	0	0	0	1	105	1,509	1,615	9,314
Bulacan	245,933	0	1	325	326	1	211	5,007	5,219	63,420
Nueva Ecija	186,491	0	0	46	46	3	300	4,793	5,096	33,062
Pampanga	159,224	0	0	35	35	2	117	1,168	1,287	13,997
Tarlac	115,785	0	1	4	5	2	176	2,595	2,773	20,234
Zambales	48,549	0	2	4	6	0	76	1,905	1,981	8,618
City of Angeles	29,054	0	0	0	0	0	16	62	78	1,905
City of Olongapo	13,066	0	0	3	3	0	6	231	237	3,817
**Region 4A**	**910,368**	**0**	**12**	**518**	**530**	**47**	**1,585**	**29,971**	**31,603**	**147,626**
Batangas	162,745	0	0	17	17	2	147	5,505	5,654	17,358
Cavite	164,389	0	0	47	47	5	257	6,821	7,083	40,214
Laguna	320,343	0	11	368	379	3	315	4,736	5,054	23,770
Quezon	105,489	0	1	82	83	28	272	5,137	5,437	28,213
Rizal	152,400	0	0	4	4	9	592	7,752	8,353	35,427
City of Lucena	5,002	0	0	0	0	0	2	20	22	2,644
**Region 4B**	**266,333**	**0**	**9**	**792**	**801**	**62**	**1,107**	**20,127**	**21,296**	**101,021**
Marinduque	15,205	0	0	0	0	0	2	281	283	1,695
Occidental Mindoro	40,816	0	6	506	512	0	213	6,213	6,426	18,565
Oriental Mindoro	63,083	0	0	32	32	3	283	4,291	4,577	19,018
Palawan	93,326	0	3	83	86	43	460	7,125	7,628	49,435

Table 2.A.2 - MODERN METHOD OF FAMILY PLANNING

Other Acceptors

Philippines, 2023

Area	Total Current Users	NFP-SDM			Total	NFP-LAM			Total	Total Other Acceptors
		Age group				Age group				
		10-14	15-19	20-49		10-14	15-19	20-49		
Romblon	22,228	0	0	111	111	14	58	1,186	1,258	3,194
City of Puerto Princesa	31,675	0	0	60	60	2	91	1,031	1,124	9,114
**Region 5**	**484,529**	**0**	**599**	**8,878**	**9,477**	**9**	**1,386**	**37,200**	**38,595**	**118,560**
Albay	120,028	0	7	1,119	1,126	0	101	3,589	3,690	15,414
Camarines Norte	46,841	0	42	655	697	3	206	3,424	3,633	12,669
Camarines Sur	107,402	0	4	879	883	0	301	10,425	10,726	28,153
Catanduanes	23,049	0	6	1,202	1,208	1	151	2,258	2,410	8,842
Masbate	91,907	0	513	3,545	4,058	2	406	8,899	9,307	29,203
Sorsogon	65,058	0	26	1,454	1,480	3	220	8,426	8,649	23,278
City of Naga	30,244	0	1	24	25	0	1	179	180	1,001
**Region 6**	**639,939**	**0**	**53**	**2,290**	**2,343**	**8**	**814**	**20,469**	**21,291**	**85,696**
Aklan	46,162	0	0	11	11	0	54	1,393	1,447	4,291
Antique	47,022	0	0	131	131	0	29	1,382	1,411	7,420
Capiz	75,514	0	0	1	1	0	13	803	816	4,961
Guimaras	16,676	0	0	16	16	0	50	1,318	1,368	3,775
Iloilo	169,693	0	25	1,271	1,296	0	131	3,906	4,037	15,562
Negros Occidental	199,968	0	27	849	876	8	473	10,935	11,416	40,263
City of Bacolod	28,243	0	0	0	0	0	56	610	666	4,679
City of Iloilo	56,661	0	1	11	12	0	8	122	130	4,745
**Region 7**	**597,007**	**0**	**1**	**207**	**208**	**8**	**831**	**23,488**	**24,327**	**94,769**
Bohol	86,129	0	0	17	17	0	93	2,826	2,919	15,907
Cebu	309,013	0	0	45	45	4	314	9,037	9,355	35,603
Negros Oriental	90,628	0	0	116	116	3	235	6,960	7,198	26,493
Siquijor	9,078	0	1	29	30	0	9	254	263	1,459
City of Cebu	39,810	0	0	0	0	1	117	2,485	2,603	9,811
City of Lapu-Lapu	35,757	0	0	0	0	0	62	1,841	1,903	4,147
City of Mandaue	26,592	0	0	0	0	0	1	85	86	1,349
**Region 8**	**332,967**	**0**	**7**	**417**	**424**	**41**	**672**	**10,700**	**11,413**	**49,196**
Biliran	11,240	0	0	9	9	0	34	730	764	3,734

Table 2.A.2 - MODERN METHOD OF FAMILY PLANNING

Other Acceptors

Philippines, 2023

Area	Total Current Users	NFP-SDM			Total	NFP-LAM			Total	Total Other Acceptors
		Age group				Age group				
		10-14	15-19	20-49		10-14	15-19	20-49		
Eastern Samar	35,729	0	4	287	291	0	83	1,181	1,264	5,009
Leyte	122,333	0	2	25	27	3	110	2,023	2,136	6,225
Northern Samar	49,058	0	0	26	26	0	97	2,341	2,438	12,113
Southern Leyte	27,698	0	0	33	33	0	65	1,470	1,535	5,202
Samar	41,329	0	0	0	0	38	186	1,137	1,361	9,495
Ormoc City	19,622	0	1	37	38	0	96	1,694	1,790	6,603
City of Tacloban	25,958	0	0	0	0	0	1	124	125	815
**Region 9**	**339,891**	**0**	**19**	**482**	**501**	**12**	**1,087**	**20,462**	**21,561**	**78,964**
Zamboanga del Norte	124,603	0	19	326	345	8	414	7,130	7,552	23,616
Zamboanga del Sur	94,708	0	0	4	4	3	195	3,980	4,178	25,340
Zamboanga Sibugay	44,556	0	0	152	152	0	87	2,443	2,530	13,732
City of Isabela	9,211	0	0	0	0	1	36	546	583	2,321
City of Zamboanga	66,813	0	0	0	0	0	355	6,363	6,718	13,955
**Region 10**	**514,846**	**1**	**144**	**3,746**	**3,891**	**40**	**2,282**	**29,173**	**31,495**	**135,872**
Bukidnon	158,694	0	71	1,926	1,997	14	1,100	12,616	13,730	58,341
Camiguin	6,535	0	0	5	5	0	26	771	797	1,331
Lanao del Norte	67,645	0	0	20	20	1	211	2,808	3,020	9,779
Misamis Occidental	69,918	0	4	465	469	6	140	3,104	3,250	16,260
Misamis Oriental	127,215	1	63	1,223	1,287	14	482	5,868	6,364	23,781
City of Cagayan De Oro	54,303	0	0	2	2	5	255	2,008	2,268	21,197
City of Iligan	30,536	0	6	105	111	0	68	1,998	2,066	5,183
**Region 11**	**532,707**	**0**	**8**	**712**	**720**	**48**	**901**	**11,192**	**12,141**	**76,440**
Davao de Oro	83,298	0	0	8	8	0	22	402	424	10,797
Davao del Norte	108,564	0	3	95	98	3	106	2,094	2,203	18,065
Davao Oriental	54,593	0	2	370	372	6	238	3,904	4,148	14,434
Davao del Sur	67,257	0	0	14	14	0	36	232	268	5,832
Davao Occidental	31,707	0	3	142	145	0	68	515	583	7,305
City of Davao	187,288	0	0	83	83	39	431	4,045	4,515	20,007
**Region 12**	**453,032**	**0**	**1**	**210**	**211**	**21**	**1,222**	**16,829**	**18,072**	**90,561**

Table 2.A.2 - MODERN METHOD OF FAMILY PLANNING

Other Acceptors

Philippines, 2023

Area	Total Current Users	NFP-SDM			Total	NFP-LAM			Total	Total Other Acceptors
		Age group				Age group				
		10-14	15-19	20-49		10-14	15-19	20-49		
Cotabato	134,232	0	0	140	140	1	155	2,495	2,651	27,192
Sarangani	72,262	0	1	27	28	0	322	4,762	5,084	16,389
South Cotabato	96,119	0	0	38	38	4	271	3,520	3,795	17,309
Sultan Kudarat	101,026	0	0	4	4	16	325	4,298	4,639	17,877
					0				0	
City of General Santos	49,393	0	0	1	1	0	149	1,754	1,903	11,794
**BARMM**	**290,634**	**0**	**0**	**38**	**38**	**7**	**699**	**22,234**	**22,940**	**56,643**
Basilan	15,552	0	0	0	0	2	44	732	778	3,832
Lanao del Sur	37,406	0	0	26	26	1	140	8,492	8,633	15,122
Maguindanao	92,272	0	0	0	0	1	345	7,996	8,342	22,000
Sulu	82,939	0	0	3	3	2	54	2,694	2,750	8,121
Tawi-Tawi	18,014	0	0	9	9	1	67	1,939	2,007	5,090
SGU	8,193	0	0	0	0	0	17	86	103	561
City of Cotabato	36,258	0	0	0	0	0	32	295	327	1,917
**Caraga**	**284,351**	**1**	**13**	**1,481**	**1,495**	**18**	**888**	**14,890**	**15,796**	**70,888**
Agusan del Norte	40,460	0	2	83	85	2	75	1,378	1,455	8,355
Agusan del Sur	86,938	0	2	285	287	3	207	3,847	4,057	17,153
Surigao del Norte	50,319	1	8	673	682	0	61	1,088	1,149	13,287
Surigao del Sur	56,051	0	1	288	289	10	407	5,075	5,492	20,792
Dinagat Islands	10,768	0	0	144	144	0	6	399	405	2,861
City of Butuan	39,815	0	0	8	8	3	132	3,103	3,238	8,440

Table 2.A.3 - MODERN METHOD OF FAMILY PLANNING

Drop Outs

Philippines, 2023

Area	Total Current Users	FSTR/BTL			Total	MSTR/NSV			Total
		Age group				Age group			
		10-14	15-19	20-49		10-14	15-19	20-49	
**Philippines**	**8,353,912**	**38**	**297**	**101,089**	**101,424**	**0**	**18**	**2,670**	**2,688**
**N C R**	**859,210**	**0**	**4**	**10,107**	**10,111**	**0**	**4**	**76**	**80**
Malabon City	33,641	0	1	133	134	0	0	1	1
Navotas City	25,067	0	0	178	178	0	4	5	9
Valenzuela City	37,966	0	0	885	885	0	0	31	31
Caloocan City	61,903	0	1	295	296	0	0	6	6
Marikina City	20,095	0	0	0	0	0	0	0	0
Pasig City	55,229	0	1	113	114	0	0	1	1
Pateros	3,813	0	0	105	105	0	0	0	0
Taguig City	71,609	0	0	38	38	0	0	0	0
Quezon City	306,956	0	1	6,095	6,096	0	0	27	27
Makati City	20,752	0	0	3	3	0	0	0	0
Mandaluyong City	23,665	0	0	27	27	0	0	1	1
San Juan City	5,890	0	0	0	0	0	0	0	0
Manila City	50,873	0	0	75	75	0	0	4	4
Las Piñas City	20,551	0	0	277	277	0	0	0	0
Muntinlupa City	41,934	0	0	0	0	0	0	0	0
Parañaque City	42,631	0	0	38	38	0	0	0	0
Pasay City	36,635	0	0	1,845	1,845	0	0	0	0
**C A R**	**161,545**	**0**	**0**	**2,399**	**2,399**	**0**	**0**	**53**	**53**
Abra	19,903	0	0	743	743	0	0	2	2
Apayao	24,278	0	0	41	41	0	0	0	0
Benguet	34,934	0	0	528	528	0	0	9	9
Ifugao	21,809	0	0	505	505	0	0	40	40
Kalinga	18,022	0	0	185	185	0	0	0	0
Mountain Province	13,937	0	0	277	277	0	0	0	0
City of Baguio	28,662	0	0	120	120	0	0	2	2
**Region 1**	**481,911**	**0**	**10**	**4,609**	**4,619**	**0**	**0**	**29**	**29**
Ilocos Norte	51,402	0	0	741	741	0	0	0	0
Ilocos Sur	76,206	0	10	616	626	0	0	0	0
La Union	69,182	0	0	1,357	1,357	0	0	19	19

Table 2.A.3 - MODERN METHOD OF FAMILY PLANNING

Drop Outs

Philippines, 2023

Area	Total Current Users	FSTR/BTL			Total	MSTR/NSV			Total
		Age group				Age group			
		10-14	15-19	20-49		10-14	15-19	20-49	
Pangasinan	278,036	0	0	1,871	1,871	0	0	10	10
City of Dagupan	7,085	0	0	24	24	0	0	0	0
**Region 2**	**320,396**	**0**	**14**	**4,622**	**4,636**	**0**	**0**	**31**	**31**
Batanes	945	0	0	18	18	0	0	0	0
Cagayan	106,997	0	0	763	763	0	0	4	4
Isabela	130,614	0	14	2,925	2,939	0	0	20	20
Nueva Vizcaya	49,739	0	0	696	696	0	0	6	6
Quirino	19,516	0	0	118	118	0	0	1	1
City of Santiago	12,585	0	0	102	102	0	0	0	0
**Region 3**	**884,246**	**28**	**28**	**13,395**	**13,451**	**0**	**0**	**34**	**34**
Aurora	22,956	9	0	143	152	0	0	0	0
Bataan	63,188	0	0	599	599	0	0	0	0
Bulacan	245,933	0	3	3,953	3,956	0	0	17	17
Nueva Ecija	186,491	17	4	3,325	3,346	0	0	0	0
Pampanga	159,224	2	14	2,931	2,947	0	0	10	10
Tarlac	115,785	0	3	1,595	1,598	0	0	3	3
Zambales	48,549	0	2	604	606	0	0	0	0
City of Angeles	29,054	0	0	230	230	0	0	2	2
City of Olongapo	13,066	0	2	15	17	0	0	2	2
**Region 4A**	**910,368**	**4**	**14**	**9,062**	**9,080**	**0**	**1**	**29**	**30**
Batangas	162,745	0	0	1,735	1,735	0	0	1	1
Cavite	164,389	0	7	2,945	2,952	0	0	14	14
Laguna	320,343	0	0	1,482	1,482	0	0	5	5
Quezon	105,489	4	7	1,217	1,228	0	0	3	3
Rizal	152,400	0	0	1,591	1,591	0	1	6	7
City of Lucena	5,002	0	0	92	92	0	0	0	0
**Region 4B**	**266,333**	**0**	**20**	**2,301**	**2,321**	**0**	**0**	**23**	**23**
Marinduque	15,205	0	0	60	60	0	0	0	0
Occidental Mindoro	40,816	0	0	222	222	0	0	0	0
Oriental Mindoro	63,083	0	14	871	885	0	0	11	11

Table 2.A.3 - MODERN METHOD OF FAMILY PLANNING

Drop Outs

Philippines, 2023

Area	Total Current Users	FSTR/BTL			Total	MSTR/NSV			Total
		Age group				Age group			
		10-14	15-19	20-49		10-14	15-19	20-49	
Palawan	93,326	0	6	629	635	0	0	10	10
Romblon	22,228	0	0	279	279	0	0	0	0
City of Puerto Princesa	31,675	0	0	240	240	0	0	2	2
**Region 5**	**484,529**	**0**	**14**	**3,269**	**3,283**	**0**	**0**	**40**	**40**
Albay	120,028	0	4	940	944	0	0	8	8
Camarines Norte	46,841	0	7	302	309	0	0	2	2
Camarines Sur	107,402	0	0	625	625	0	0	5	5
Catanduanes	23,049	0	0	354	354	0	0	4	4
Masbate	91,907	0	0	214	214	0	0	9	9
Sorsogon	65,058	0	3	786	789	0	0	7	7
City of Naga	30,244	0	0	48	48	0	0	5	5
**Region 6**	**639,939**	**0**	**11**	**5,917**	**5,928**	**0**	**0**	**201**	**201**
Aklan	46,162	0	0	201	201	0	0	1	1
Antique	47,022	0	0	607	607	0	0	9	9
Capiz	75,514	0	0	243	243	0	0	23	23
Guimaras	16,676	0	0	106	106	0	0	0	0
Iloilo	169,693	0	2	593	595	0	0	21	21
Negros Occidental	199,968	0	8	1,493	1,501	0	0	133	133
City of Bacolod	28,243	0	0	110	110	0	0	14	14
City of Iloilo	56,661	0	1	2,564	2,565	0	0	0	0
**Region 7**	**597,007**	**0**	**7**	**19,035**	**19,042**	**0**	**0**	**799**	**799**
Bohol	86,129	0	5	3,615	3,620	0	0	20	20
Cebu	309,013	0	0	14,322	14,322	0	0	682	682
Negros Oriental	90,628	0	2	430	432	0	0	27	27
Siquijor	9,078	0	0	33	33	0	0	0	0
City of Cebu	39,810	0	0	180	180	0	0	63	63
City of Lapu-Lapu	35,757	0	0	451	451	0	0	7	7
City of Mandaue	26,592	0	0	4	4	0	0	0	0
**Region 8**	**332,967**	**0**	**5**	**3,795**	**3,800**	**0**	**1**	**88**	**89**
Biliran	11,240	0	0	92	92	0	0	1	1

Table 2.A.3 - MODERN METHOD OF FAMILY PLANNING

Drop Outs

Philippines, 2023

Area	Total Current Users	FSTR/BTL			Total	MSTR/NSV			Total
		Age group				Age group			
		10-14	15-19	20-49		10-14	15-19	20-49	
Eastern Samar	35,729	0	0	1,010	1,010	0	0	44	44
Leyte	122,333	0	1	370	371	0	0	7	7
Northern Samar	49,058	0	4	239	243	0	1	11	12
Southern Leyte	27,698	0	0	508	508	0	0	11	11
Samar	41,329	0	0	1,044	1,044	0	0	6	6
Ormoc City	19,622	0	0	211	211	0	0	8	8
City of Tacloban	25,958	0	0	321	321	0	0	0	0
**Region 9**	**339,891**	**0**	**4**	**1,053**	**1,057**	**0**	**0**	**8**	**8**
Zamboanga del Norte	124,603	0	0	364	364	0	0	0	0
Zamboanga del Sur	94,708	0	3	212	215	0	0	2	2
Zamboanga Sibugay	44,556	0	0	172	172	0	0	6	6
City of Isabela	9,211	0	1	259	260	0	0	0	0
City of Zamboanga	66,813	0	0	46	46	0	0	0	0
**Region 10**	**514,846**	**0**	**18**	**8,056**	**8,074**	**0**	**9**	**673**	**682**
Bukidnon	158,694	0	5	1,823	1,828	0	1	58	59
Camiguin	6,535	0	0	59	59	0	0	3	3
Lanao del Norte	67,645	0	0	80	80	0	0	11	11
Misamis Occidental	69,918	0	9	1,506	1,515	0	0	36	36
Misamis Oriental	127,215	0	1	3,033	3,034	0	2	85	87
City of Cagayan De Oro	54,303	0	0	1,434	1,434	0	6	480	486
City of Iligan	30,536	0	3	121	124	0	0	0	0
**Region 11**	**532,707**	**6**	**94**	**6,214**	**6,314**	**0**	**1**	**365**	**366**
Davao de Oro	83,298	0	14	914	928	0	0	46	46
Davao del Norte	108,564	0	17	1,126	1,143	0	1	48	49
Davao Oriental	54,593	0	1	701	702	0	0	24	24
Davao del Sur	67,257	0	14	903	917	0	0	1	1
Davao Occidental	31,707	0	0	492	492	0	0	33	33
City of Davao	187,288	6	48	2,078	2,132	0	0	213	213
**Region 12**	**453,032**	**0**	**19**	**3,423**	**3,442**	**0**	**1**	**124**	**125**

Table 2.A.3 - MODERN METHOD OF FAMILY PLANNING

Drop Outs

Philippines, 2023

Area	Total Current Users	FSTR/BTL			Total	MSTR/NSV			Total
		Age group				Age group			
		10-14	15-19	20-49		10-14	15-19	20-49	
Cotabato	134,232	0	1	1,226	1,227	0	0	64	64
Sarangani	72,262	0	15	494	509	0	1	16	17
South Cotabato	96,119	0	0	500	500	0	0	26	26
Sultan Kudarat	101,026	0	3	781	784	0	0	7	7
					0				0
City of General Santos	49,393	0	0	422	422	0	0	11	11
**BARMM**	**290,634**	**0**	**31**	**1,218**	**1,249**	**0**	**0**	**20**	**20**
Basilan	15,552	0	0	19	19	0	0	0	0
Lanao del Sur	37,406	0	0	126	126	0	0	5	5
Maguindanao	92,272	0	4	870	874	0	0	15	15
Sulu	82,939	0	27	41	68	0	0	0	0
Tawi-Tawi	18,014	0	0	23	23	0	0	0	0
SGU	8,193	0	0	37	37	0	0	0	0
					0				0
City of Cotabato	36,258	0	0	102	102	0	0	0	0
**Caraga**	**284,351**	**0**	**4**	**2,614**	**2,618**	**0**	**1**	**77**	**78**
Agusan del Norte	40,460	0	0	409	409	0	0	6	6
Agusan del Sur	86,938	0	2	687	689	0	0	14	14
Surigao del Norte	50,319	0	0	451	451	0	1	29	30
Surigao del Sur	56,051	0	0	839	839	0	0	27	27
Dinagat Islands	10,768	0	2	69	71	0	0	1	1
City of Butuan	39,815	0	0	159	159	0	0	0	0

Table 2.A.3 - MODERN METHOD OF FAMILY PLANNING

Drop Outs

Philippines, 2023

Area	Total Current Users	CONDOM			Total	IUD-INTERVAL			Total
		Age group				Age group			
		10-14	15-19	20-49		10-14	15-19	20-49	
**Philippines**	**8,353,912**	**147**	**10,404**	**115,743**	**126,294**	**111**	**2,892**	**82,496**	**85,499**
**N C R**	**859,210**	**35**	**3,025**	**27,359**	**30,419**	**0**	**259**	**7,153**	**7,412**
Malabon City	33,641	6	907	2,129	3,042	0	72	260	332
Navotas City	25,067	11	627	2,022	2,660	0	38	404	442
Valenzuela City	37,966	0	8	729	737	0	1	364	365
Caloocan City	61,903	1	48	793	842	0	16	298	314
Marikina City	20,095	0	0	132	132	0	3	7	10
Pasig City	55,229	0	13	595	608	0	9	303	312
Pateros	3,813	0	3	82	85	0	0	33	33
Taguig City	71,609	0	24	569	593	0	4	1	5
Quezon City	306,956	0	629	13,815	14,444	0	78	3,207	3,285
Makati City	20,752	0	17	379	396	0	1	11	12
Mandaluyong City	23,665	12	106	326	444	0	0	8	8
San Juan City	5,890	0	1	35	36	0	0	27	27
Manila City	50,873	4	143	2,622	2,769	0	14	132	146
Las Piñas City	20,551	1	34	831	866	0	3	102	105
Muntinlupa City	41,934	0	5	41	46	0	0	214	214
Parañaque City	42,631	0	28	239	267	0	14	41	55
Pasay City	36,635	0	432	2,020	2,452	0	6	1,741	1,747
**C A R**	**161,545**	**0**	**46**	**1,724**	**1,770**	**2**	**23**	**740**	**765**
Abra	19,903	0	13	134	147	0	0	30	30
Apayao	24,278	0	3	75	78	0	0	45	45
Benguet	34,934	0	13	554	567	1	11	276	288
Ifugao	21,809	0	6	195	201	0	2	53	55
Kalinga	18,022	0	3	210	213	0	1	154	155
Mountain Province	13,937	0	6	166	172	1	7	94	102
City of Baguio	28,662	0	2	390	392	0	2	88	90
**Region 1**	**481,911**	**0**	**242**	**1,829**	**2,071**	**2**	**191**	**1,048**	**1,241**
Ilocos Norte	51,402	0	5	191	196	0	3	41	44
Ilocos Sur	76,206	0	11	185	196	0	0	77	77
La Union	69,182	0	24	336	360	0	19	208	227
Pangasinan	278,036	0	202	1,104	1,306	2	169	722	893

Table 2.A.3 - MODERN METHOD OF FAMILY PLANNING

Drop Outs

Philippines, 2023

Area	Total Current Users	CONDOM			Total	IUD-INTERVAL			Total
		Age group				Age group			
		10-14	15-19	20-49		10-14	15-19	20-49	
City of Dagupan	7,085	0	0	13	13	0	0	0	0
**Region 2**	**320,396**	**3**	**133**	**1,165**	**1,301**	**0**	**159**	**2,721**	**2,880**
Batanes	945	0	0	14	14	0	0	3	3
Cagayan	106,997	0	7	113	120	0	44	728	772
Isabela	130,614	3	112	527	642	0	92	1,354	1,446
Nueva Vizcaya	49,739	0	8	438	446	0	18	563	581
Quirino	19,516	0	4	34	38	0	0	40	40
City of Santiago	12,585	0	2	39	41	0	5	33	38
**Region 3**	**884,246**	**10**	**645**	**8,302**	**8,957**	**61**	**91**	**1,847**	**1,999**
Aurora	22,956	0	29	423	452	0	4	44	48
Bataan	63,188	0	53	458	511	0	1	91	92
Bulacan	245,933	0	250	3,182	3,432	0	8	557	565
Nueva Ecija	186,491	0	63	778	841	7	16	494	517
Pampanga	159,224	3	52	1,250	1,305	54	8	189	251
Tarlac	115,785	2	170	1,627	1,799	0	50	380	430
Zambales	48,549	5	21	384	410	0	4	44	48
City of Angeles	29,054	0	3	95	98	0	0	38	38
City of Olongapo	13,066	0	4	105	109	0	0	10	10
**Region 4A**	**910,368**	**7**	**3,328**	**18,742**	**22,077**	**1**	**157**	**4,336**	**4,494**
Batangas	162,745	1	26	1,381	1,408	0	13	433	446
Cavite	164,389	1	62	1,294	1,357	1	33	860	894
Laguna	320,343	0	3,149	13,595	16,744	0	45	1,272	1,317
Quezon	105,489	1	41	939	981	0	32	1,102	1,134
Rizal	152,400	3	45	1,465	1,513	0	34	657	691
City of Lucena	5,002	1	5	68	74	0	0	12	12
**Region 4B**	**266,333**	**0**	**30**	**1,484**	**1,514**	**1**	**18**	**953**	**972**
Marinduque	15,205	0	0	19	19	0	0	23	23
Occidental Mindoro	40,816	0	0	158	158	0	3	84	87
Oriental Mindoro	63,083	0	6	609	615	1	9	456	466
Palawan	93,326	0	14	377	391	0	3	181	184

Table 2.A.3 - MODERN METHOD OF FAMILY PLANNING

Drop Outs

Philippines, 2023

Area	Total Current Users	CONDOM			Total	IUD-INTERVAL			Total
		Age group				Age group			
		10-14	15-19	20-49		10-14	15-19	20-49	
Romblon	22,228	0	1	122	123	0	3	55	58
City of Puerto Princesa	31,675	0	9	199	208	0	0	154	154
**Region 5**	**484,529**	**12**	**296**	**6,744**	**7,052**	**0**	**89**	**991**	**1,080**
Albay	120,028	0	168	1,940	2,108	0	7	174	181
Camarines Norte	46,841	9	22	764	795	0	71	291	362
Camarines Sur	107,402	0	26	1,420	1,446	0	1	211	212
Catanduanes	23,049	2	9	208	219	0	0	13	13
Masbate	91,907	1	32	1,697	1,730	0	6	223	229
Sorsogon	65,058	0	34	529	563	0	4	74	78
City of Naga	30,244	0	5	186	191	0	0	5	5
**Region 6**	**639,939**	**1**	**164**	**4,122**	**4,287**	**0**	**120**	**3,151**	**3,271**
Aklan	46,162	0	27	373	400	0	0	60	60
Antique	47,022	1	1	260	262	0	0	61	61
Capiz	75,514	0	4	343	347	0	4	486	490
Guimaras	16,676	0	5	146	151	0	1	29	30
Iloilo	169,693	0	26	657	683	0	26	425	451
Negros Occidental	199,968	0	75	1,710	1,785	0	51	1,612	1,663
City of Bacolod	28,243	0	10	241	251	0	2	143	145
City of Iloilo	56,661	0	16	392	408	0	36	335	371
**Region 7**	**597,007**	**1**	**277**	**17,216**	**17,494**	**3**	**615**	**24,695**	**25,313**
Bohol	86,129	0	33	1,071	1,104	1	40	2,859	2,900
Cebu	309,013	0	171	14,256	14,427	2	464	19,935	20,401
Negros Oriental	90,628	1	23	917	941	0	10	849	859
Siquijor	9,078	0	0	46	46	0	0	53	53
City of Cebu	39,810	0	28	500	528	0	101	820	921
City of Lapu-Lapu	35,757	0	21	358	379	0	0	142	142
City of Mandaue	26,592	0	1	68	69	0	0	37	37
**Region 8**	**332,967**	**1**	**128**	**2,239**	**2,368**	**2**	**97**	**2,734**	**2,833**
Biliran	11,240	0	2	80	82	0	3	49	52
Eastern Samar	35,729	0	40	496	536	0	12	243	255

Table 2.A.3 - MODERN METHOD OF FAMILY PLANNING

Drop Outs

Philippines, 2023

Area	Total Current Users	CONDOM			Total	IUD-INTERVAL			Total
		Age group				Age group			
		10-14	15-19	20-49		10-14	15-19	20-49	
Leyte	122,333	0	16	234	250	0	15	578	593
Northern Samar	49,058	0	16	416	432	0	7	364	371
Southern Leyte	27,698	0	4	254	258	0	1	457	458
Samar	41,329	1	13	313	327	2	22	155	179
Ormoc City	19,622	0	11	173	184	0	2	199	201
City of Tacloban	25,958	0	26	273	299	0	35	689	724
**Region 9**	**339,891**	**16**	**158**	**2,581**	**2,755**	**0**	**107**	**2,682**	**2,789**
Zamboanga del Norte	124,603	1	53	874	928	0	12	1,007	1,019
Zamboanga del Sur	94,708	15	19	583	617	0	45	907	952
Zamboanga Sibugay	44,556	0	37	355	392	0	45	460	505
City of Isabela	9,211	0	35	355	390	0	0	36	36
City of Zamboanga	66,813	0	14	414	428	0	5	272	277
**Region 10**	**514,846**	**35**	**1,022**	**7,385**	**8,442**	**28**	**495**	**13,114**	**13,637**
Bukidnon	158,694	2	120	1,661	1,783	4	167	3,453	3,624
Camiguin	6,535	0	3	24	27	0	0	31	31
Lanao del Norte	67,645	0	44	466	510	0	3	305	308
Misamis Occidental	69,918	10	627	1,884	2,521	7	15	838	860
Misamis Oriental	127,215	23	121	1,819	1,963	17	168	5,328	5,513
City of Cagayan De Oro	54,303	0	89	1,402	1,491	0	137	3,005	3,142
City of Iligan	30,536	0	18	129	147	0	5	154	159
**Region 11**	**532,707**	**19**	**302**	**5,263**	**5,584**	**7**	**132**	**6,018**	**6,157**
Davao de Oro	83,298	0	8	578	586	0	10	963	973
Davao del Norte	108,564	4	45	667	716	0	56	1,016	1,072
Davao Oriental	54,593	0	11	270	281	0	2	411	413
Davao del Sur	67,257	0	8	237	245	2	16	1,059	1,077
Davao Occidental	31,707	0	9	84	93	0	17	298	315
City of Davao	187,288	15	221	3,427	3,663	5	31	2,271	2,307
**Region 12**	**453,032**	**0**	**203**	**2,598**	**2,801**	**2**	**129**	**4,299**	**4,430**
Cotabato	134,232	0	70	1,005	1,075	1	57	2,023	2,081

Table 2.A.3 - MODERN METHOD OF FAMILY PLANNING

Drop Outs

Philippines, 2023

Area	Total Current Users	CONDOM			Total	IUD-INTERVAL			Total
		Age group				Age group			
		10-14	15-19	20-49		10-14	15-19	20-49	
Sarangani	72,262	0	28	341	369	0	25	492	517
South Cotabato	96,119	0	39	590	629	1	15	537	553
Sultan Kudarat	101,026	0	36	411	447	0	22	655	677
					0				0
City of General Santos	49,393	0	30	251	281	0	10	592	602
**BARMM**	**290,634**	**4**	**138**	**3,620**	**3,762**	**1**	**29**	**855**	**885**
Basilan	15,552	0	7	77	84	0	17	46	63
Lanao del Sur	37,406	1	36	1,693	1,730	0	2	93	95
Maguindanao	92,272	3	70	1,085	1,158	1	7	665	673
Sulu	82,939	0	5	187	192	0	0	12	12
Tawi-Tawi	18,014	0	0	75	75	0	0	2	2
SGU	8,193	0	5	108	113	0	0	11	11
					0				0
City of Cotabato	36,258	0	15	395	410	0	3	26	29
**Caraga**	**284,351**	**3**	**267**	**3,370**	**3,640**	**1**	**181**	**5,159**	**5,341**
Agusan del Norte	40,460	2	50	511	563	0	28	680	708
Agusan del Sur	86,938	0	50	895	945	0	72	1,495	1,567
Surigao del Norte	50,319	0	91	632	723	1	25	1,698	1,724
Surigao del Sur	56,051	1	41	761	803	0	43	867	910
Dinagat Islands	10,768	0	11	382	393	0	3	303	306
City of Butuan	39,815	0	24	189	213	0	10	116	126

Table 2.A.3 - MODERN METHOD OF FAMILY PLANNING

Drop Outs

Philippines, 2023

Area	Total Current Users	IUD-POSTPARTUM Age group			Total	PILLS-POP Age group			Total
		10-14	15-19	20-49		10-14	15-19	20-49	
**Philippines**	**8,353,912**	**97**	**4,301**	**32,488**	**36,886**	**281**	**20,208**	**153,356**	**173,845**
**N C R**	**859,210**	**17**	**841**	**6,149**	**7,007**	**42**	**2,937**	**23,534**	**26,513**
Malabon City	33,641	0	98	240	338	2	991	2,672	3,665
Navotas City	25,067	0	2	46	48	19	335	1,337	1,691
Valenzuela City	37,966	0	3	161	164	1	32	1,472	1,505
Caloocan City	61,903	13	285	1,295	1,593	0	218	1,281	1,499
Marikina City	20,095	0	0	57	57	0	13	167	180
Pasig City	55,229	0	96	606	702	0	20	1,080	1,100
Pateros	3,813	0	1	7	8	0	0	14	14
Taguig City	71,609	0	1	11	12	0	25	763	788
Quezon City	306,956	0	215	1,451	1,666	1	634	7,785	8,420
Makati City	20,752	0	14	1	15	0	11	344	355
Mandaluyong City	23,665	0	3	7	10	1	35	198	234
San Juan City	5,890	0	0	0	0	0	2	112	114
Manila City	50,873	4	55	477	536	18	260	2,837	3,115
Las Piñas City	20,551	0	49	268	317	0	85	1,284	1,369
Muntinlupa City	41,934	0	0	20	20	0	9	138	147
Parañaque City	42,631	0	1	20	21	0	112	420	532
Pasay City	36,635	0	18	1,482	1,500	0	155	1,630	1,785
**C A R**	**161,545**	**0**	**16**	**170**	**186**	**6**	**361**	**7,358**	**7,725**
Abra	19,903	0	5	23	28	2	42	259	303
Apayao	24,278	0	1	10	11	0	75	516	591
Benguet	34,934	0	3	41	44	2	65	585	652
Ifugao	21,809	0	0	17	17	1	55	1,593	1,649
Kalinga	18,022	0	0	12	12	1	38	3,235	3,274
Mountain Province	13,937	0	3	17	20	0	52	359	411
City of Baguio	28,662	0	4	50	54	0	34	811	845
**Region 1**	**481,911**	**1**	**9**	**125**	**135**	**0**	**126**	**1,379**	**1,505**
Ilocos Norte	51,402	0	0	10	10	0	31	394	425
Ilocos Sur	76,206	1	0	13	14	0	47	487	534
La Union	69,182	0	9	99	108	0	48	494	542
Pangasinan	278,036	0	0	0	0	0	0	0	0

Table 2.A.3 - MODERN METHOD OF FAMILY PLANNING

Drop Outs

Philippines, 2023

Area	Total Current Users	IUD-POSTPARTUM			Total	PILLS-POP			Total
		Age group				Age group			
		10-14	15-19	20-49		10-14	15-19	20-49	
City of Dagupan	7,085	0	0	3	3	0	0	4	4
**Region 2**	**320,396**	**4**	**134**	**645**	**783**	**8**	**1,630**	**4,259**	**5,897**
Batanes	945	0	0	0	0	0	1	10	11
Cagayan	106,997	0	30	106	136	0	127	851	978
Isabela	130,614	2	80	425	507	8	1,336	2,416	3,760
Nueva Vizcaya	49,739	2	22	101	125	0	124	797	921
Quirino	19,516	0	2	13	15	0	42	185	227
City of Santiago	12,585	0	0	0	0	0	0	0	0
**Region 3**	**884,246**	**1**	**53**	**717**	**771**	**18**	**2,100**	**12,226**	**14,344**
Aurora	22,956	0	1	23	24	0	35	507	542
Bataan	63,188	0	2	2	4	3	439	1,764	2,206
Bulacan	245,933	0	2	152	154	0	454	2,785	3,239
Nueva Ecija	186,491	1	17	250	268	6	496	3,320	3,822
Pampanga	159,224	0	15	112	127	4	289	1,489	1,782
Tarlac	115,785	0	15	92	107	3	305	1,550	1,858
Zambales	48,549	0	1	24	25	2	31	403	436
City of Angeles	29,054	0	0	62	62	0	40	201	241
City of Olongapo	13,066	0	0	0	0	0	11	207	218
**Region 4A**	**910,368**	**4**	**182**	**1,338**	**1,524**	**23**	**2,923**	**22,992**	**25,938**
Batangas	162,745	1	63	287	351	1	151	3,046	3,198
Cavite	164,389	0	19	263	282	2	131	1,857	1,990
Laguna	320,343	1	22	191	214	3	2,171	13,562	15,736
Quezon	105,489	0	22	104	126	16	215	1,632	1,863
Rizal	152,400	2	55	489	546	1	206	2,632	2,839
City of Lucena	5,002	0	1	4	5	0	49	263	312
**Region 4B**	**266,333**	**0**	**20**	**359**	**379**	**17**	**528**	**4,850**	**5,395**
Marinduque	15,205	0	0	0	0	0	7	455	462
Occidental Mindoro	40,816	0	0	4	4	1	53	680	734
Oriental Mindoro	63,083	0	10	114	124	5	70	1,165	1,240
Palawan	93,326	0	8	16	24	10	256	1,764	2,030

Table 2.A.3 - MODERN METHOD OF FAMILY PLANNING

Drop Outs

Philippines, 2023

Area	Total Current Users	IUD-POSTPARTUM Age group 10-14	15-19	20-49	Total	PILLS-POP Age group 10-14	15-19	20-49	Total
Romblon	22,228	0	0	131	131	0	14	185	199
City of Puerto Princesa	31,675	0	2	94	96	1	128	601	730
**Region 5**	**484,529**	**0**	**24**	**430**	**454**	**3**	**571**	**6,730**	**7,304**
Albay	120,028	0	10	146	156	1	84	1,350	1,435
Camarines Norte	46,841	0	0	23	23	0	88	861	949
Camarines Sur	107,402	0	13	72	85	1	156	1,937	2,094
Catanduanes	23,049	0	0	9	9	0	3	130	133
Masbate	91,907	0	0	151	151	1	169	1,634	1,804
Sorsogon	65,058	0	0	29	29	0	68	802	870
City of Naga	30,244	0	1	0	1	0	3	16	19
**Region 6**	**639,939**	**22**	**348**	**1,923**	**2,293**	**8**	**933**	**5,493**	**6,434**
Aklan	46,162	0	0	2	2	0	55	330	385
Antique	47,022	0	0	14	14	0	49	510	559
Capiz	75,514	0	0	30	30	0	37	473	510
Guimaras	16,676	0	1	2	3	1	17	112	130
Iloilo	169,693	1	6	167	174	2	90	741	833
Negros Occidental	199,968	4	115	724	843	5	640	3,036	3,681
City of Bacolod	28,243	0	5	93	98	0	16	178	194
City of Iloilo	56,661	17	221	891	1,129	0	29	113	142
**Region 7**	**597,007**	**12**	**594**	**7,476**	**8,082**	**9**	**1,471**	**16,588**	**18,068**
Bohol	86,129	2	198	1,361	1,561	2	196	2,120	2,318
Cebu	309,013	10	301	5,842	6,153	4	717	10,697	11,418
Negros Oriental	90,628	0	19	79	98	2	214	1,195	1,411
Siquijor	9,078	0	0	2	2	0	13	266	279
City of Cebu	39,810	0	73	168	241	1	275	1,738	2,014
City of Lapu-Lapu	35,757	0	0	12	12	0	43	397	440
City of Mandaue	26,592	0	3	12	15	0	13	175	188
**Region 8**	**332,967**	**2**	**90**	**1,104**	**1,196**	**7**	**312**	**5,079**	**5,398**
Biliran	11,240	0	0	9	9	2	23	137	162
Eastern Samar	35,729	0	7	81	88	0	61	1,035	1,096

Table 2.A.3 - MODERN METHOD OF FAMILY PLANNING

Drop Outs

Philippines, 2023

Area	Total Current Users	IUD-POSTPARTUM Age group			Total	PILLS-POP Age group			Total
		10-14	15-19	20-49		10-14	15-19	20-49	
Leyte	122,333	1	2	86	89	0	39	563	602
Northern Samar	49,058	0	5	78	83	2	46	1,397	1,445
Southern Leyte	27,698	0	4	56	60	0	20	196	216
Samar	41,329	1	0	155	156	3	43	973	1,019
Ormoc City	19,622	0	1	4	5	0	54	380	434
City of Tacloban	25,958	0	71	635	706	0	26	398	424
**Region 9**	**339,891**	**7**	**979**	**3,501**	**4,487**	**2**	**560**	**5,768**	**6,330**
Zamboanga del Norte	124,603	0	14	129	143	1	91	2,656	2,748
Zamboanga del Sur	94,708	7	919	2,934	3,860	0	151	1,005	1,156
Zamboanga Sibugay	44,556	0	19	88	107	1	96	691	788
City of Isabela	9,211	0	0	2	2	0	94	465	559
City of Zamboanga	66,813	0	27	348	375	0	128	951	1,079
**Region 10**	**514,846**	**19**	**701**	**3,976**	**4,696**	**21**	**1,405**	**7,596**	**9,022**
Bukidnon	158,694	3	94	488	585	2	672	2,260	2,934
Camiguin	6,535	0	0	9	9	0	1	19	20
Lanao del Norte	67,645	0	6	64	70	0	101	1,094	1,195
Misamis Occidental	69,918	0	4	219	223	12	95	1,213	1,320
Misamis Oriental	127,215	12	238	1,391	1,641	5	392	2,140	2,537
City of Cagayan De Oro	54,303	3	331	1,698	2,032	1	98	539	638
City of Iligan	30,536	1	28	107	136	1	46	331	378
**Region 11**	**532,707**	**5**	**69**	**1,784**	**1,858**	**79**	**1,507**	**10,655**	**12,241**
Davao de Oro	83,298	0	11	84	95	5	217	1,572	1,794
Davao del Norte	108,564	2	24	260	286	8	342	2,373	2,723
Davao Oriental	54,593	0	4	164	168	2	168	818	988
Davao del Sur	67,257	0	4	85	89	15	132	811	958
Davao Occidental	31,707	0	3	65	68	2	50	208	260
City of Davao	187,288	3	23	1,126	1,152	47	598	4,873	5,518
**Region 12**	**453,032**	**1**	**92**	**1,097**	**1,190**	**16**	**1,158**	**6,753**	**7,927**
Cotabato	134,232	0	22	497	519	6	516	3,440	3,962

Table 2.A.3 - MODERN METHOD OF FAMILY PLANNING

Drop Outs

Philippines, 2023

Area	Total Current Users	IUD-POSTPARTUM			Total	PILLS-POP			Total
		Age group				Age group			
		10-14	15-19	20-49		10-14	15-19	20-49	
Sarangani	72,262	0	13	24	37	0	263	660	923
South Cotabato	96,119	0	17	231	248	2	127	1,037	1,166
Sultan Kudarat	101,026	1	29	298	328	5	128	828	961
					0				0
City of General Santos	49,393	0	11	47	58	3	124	788	915
**BARMM**	**290,634**	**1**	**30**	**349**	**380**	**8**	**613**	**5,229**	**5,850**
Basilan	15,552	0	2	35	37	1	82	436	519
Lanao del Sur	37,406	0	2	7	9	4	72	1,475	1,551
Maguindanao	92,272	0	20	271	291	3	314	1,666	1,983
Sulu	82,939	0	0	14	14	0	71	688	759
Tawi-Tawi	18,014	0	0	0	0	0	1	86	87
SGU	8,193	0	0	8	8	0	18	364	382
					0				0
City of Cotabato	36,258	1	6	14	21	0	55	514	569
**Caraga**	**284,351**	**1**	**119**	**1,345**	**1,465**	**14**	**1,073**	**6,867**	**7,954**
Agusan del Norte	40,460	0	27	516	543	7	258	1,591	1,856
Agusan del Sur	86,938	0	29	256	285	2	258	1,341	1,601
Surigao del Norte	50,319	0	29	275	304	1	189	1,447	1,637
Surigao del Sur	56,051	0	32	222	254	1	182	1,585	1,768
Dinagat Islands	10,768	0	1	54	55	3	59	310	372
City of Butuan	39,815	1	1	22	24	0	127	593	720

Table 2.A.3 - MODERN METHOD OF FAMILY PLANNING

Drop Outs

Philippines, 2023

Area	Total Current Users	PILLS-COC			Total	INJECTABLES			Total
		Age group				Age group			
		10-14	15-19	20-49		10-14	15-19	20-49	
**Philippines**	**8,353,912**	**342**	**29,996**	**462,841**	**493,179**	**606**	**33,980**	**390,741**	**425,327**
**N C R**	**859,210**	**23**	**3,008**	**49,295**	**52,326**	**165**	**6,272**	**50,908**	**57,345**
Malabon City	33,641	3	1,038	2,767	3,808	7	1,601	3,258	4,866
Navotas City	25,067	2	522	2,604	3,128	7	1,479	3,932	5,418
Valenzuela City	37,966	7	132	5,303	5,442	15	315	5,672	6,002
Caloocan City	61,903	0	253	2,761	3,014	10	549	6,236	6,795
Marikina City	20,095	0	12	505	517	0	80	913	993
Pasig City	55,229	0	31	2,355	2,386	1	109	3,837	3,947
Pateros	3,813	0	4	237	241	0	2	123	125
Taguig City	71,609	0	27	769	796	1	57	1,082	1,140
Quezon City	306,956	1	503	24,639	25,143	9	1,192	14,314	15,515
Makati City	20,752	0	7	304	311	0	19	894	913
Mandaluyong City	23,665	0	20	371	391	0	76	452	528
San Juan City	5,890	0	2	132	134	0	12	194	206
Manila City	50,873	10	89	1,387	1,486	111	259	2,388	2,758
Las Piñas City	20,551	0	76	1,905	1,981	2	289	3,269	3,560
Muntinlupa City	41,934	0	7	283	290	1	54	2,427	2,482
Parañaque City	42,631	0	137	547	684	1	145	865	1,011
Pasay City	36,635	0	148	2,426	2,574	0	34	1,052	1,086
**C A R**	**161,545**	**2**	**583**	**9,053**	**9,638**	**7**	**512**	**9,204**	**9,723**
Abra	19,903	0	286	834	1,120	0	106	547	653
Apayao	24,278	0	48	1,355	1,403	3	72	848	923
Benguet	34,934	1	46	1,674	1,721	1	58	1,547	1,606
Ifugao	21,809	1	35	1,401	1,437	3	66	1,116	1,185
Kalinga	18,022	0	101	1,406	1,507	0	72	1,351	1,423
Mountain Province	13,937	0	31	939	970	0	69	899	968
City of Baguio	28,662	0	36	1,444	1,480	0	69	2,896	2,965
**Region 1**	**481,911**	**12**	**1,387**	**11,210**	**12,609**	**4**	**637**	**6,890**	**7,531**
Ilocos Norte	51,402	0	42	1,080	1,122	0	20	454	474
Ilocos Sur	76,206	3	108	1,126	1,237	2	60	714	776
La Union	69,182	0	78	2,081	2,159	0	114	1,880	1,994
Pangasinan	278,036	9	1,144	6,826	7,979	2	412	3,774	4,188

Table 2.A.3 - MODERN METHOD OF FAMILY PLANNING

Drop Outs

Philippines, 2023

Area	Total Current Users	PILLS-COC			Total	INJECTABLES			Total
		Age group				Age group			
		10-14	15-19	20-49		10-14	15-19	20-49	
City of Dagupan	7,085	0	15	97	112	0	31	68	99
**Region 2**	**320,396**	**8**	**1,917**	**16,210**	**18,135**	**19**	**1,343**	**12,345**	**13,707**
Batanes	945	0	0	84	84	0	10	181	191
Cagayan	106,997	0	125	3,701	3,826	2	115	2,279	2,396
Isabela	130,614	6	1,554	8,348	9,908	13	996	7,250	8,259
Nueva Vizcaya	49,739	2	170	2,692	2,864	2	113	1,334	1,449
Quirino	19,516	0	42	790	832	0	60	431	491
City of Santiago	12,585	0	26	595	621	2	49	870	921
**Region 3**	**884,246**	**48**	**3,348**	**40,563**	**43,959**	**69**	**3,857**	**40,524**	**44,450**
Aurora	22,956	0	107	1,376	1,483	0	69	793	862
Bataan	63,188	0	146	2,355	2,501	6	379	4,398	4,783
Bulacan	245,933	0	1,010	13,337	14,347	2	1,142	11,773	12,917
Nueva Ecija	186,491	37	1,004	7,406	8,447	45	938	7,758	8,741
Pampanga	159,224	1	442	4,658	5,101	9	466	5,141	5,616
Tarlac	115,785	9	503	8,216	8,728	7	551	6,596	7,154
Zambales	48,549	0	50	1,412	1,462	0	173	1,859	2,032
City of Angeles	29,054	1	54	1,146	1,201	0	64	1,242	1,306
City of Olongapo	13,066	0	32	657	689	0	75	964	1,039
**Region 4A**	**910,368**	**72**	**4,179**	**58,463**	**62,714**	**105**	**4,897**	**63,036**	**68,038**
Batangas	162,745	1	167	6,224	6,392	5	237	4,633	4,875
Cavite	164,389	0	312	10,475	10,787	7	637	13,647	14,291
Laguna	320,343	1	2,826	23,150	25,977	10	2,205	18,093	20,308
Quezon	105,489	60	409	8,771	9,240	71	571	10,295	10,937
Rizal	152,400	10	375	8,981	9,366	11	1,081	15,057	16,149
City of Lucena	5,002	0	90	862	952	1	166	1,311	1,478
**Region 4B**	**266,333**	**21**	**685**	**15,197**	**15,903**	**15**	**1,343**	**13,189**	**14,547**
Marinduque	15,205	0	0	266	266	0	5	115	120
Occidental Mindoro	40,816	6	143	3,218	3,367	0	83	1,569	1,652
Oriental Mindoro	63,083	2	74	4,187	4,263	7	146	2,574	2,727
Palawan	93,326	12	379	4,812	5,203	7	800	6,049	6,856

Table 2.A.3 - MODERN METHOD OF FAMILY PLANNING

Drop Outs

Philippines, 2023

Area	Total Current Users	PILLS-COC			Total	INJECTABLES			Total
		Age group				Age group			
		10-14	15-19	20-49		10-14	15-19	20-49	
Romblon	22,228	0	5	648	653	0	27	751	778
City of Puerto Princesa	31,675	1	84	2,066	2,151	1	282	2,131	2,414
**Region 5**	**484,529**	**5**	**1,003**	**31,766**	**32,774**	**10**	**1,125**	**18,851**	**19,986**
Albay	120,028	1	265	10,207	10,473	1	219	3,727	3,947
Camarines Norte	46,841	1	129	3,778	3,908	2	176	2,300	2,478
Camarines Sur	107,402	0	196	7,621	7,817	2	237	5,093	5,332
Catanduanes	23,049	0	20	1,156	1,176	0	99	1,560	1,659
Masbate	91,907	2	225	4,994	5,221	3	185	2,693	2,881
Sorsogon	65,058	1	150	3,544	3,695	2	192	3,294	3,488
City of Naga	30,244	0	18	466	484	0	17	184	201
**Region 6**	**639,939**	**15**	**1,837**	**21,612**	**23,464**	**3**	**1,546**	**18,261**	**19,810**
Aklan	46,162	0	99	1,129	1,228	0	165	1,484	1,649
Antique	47,022	1	61	1,726	1,788	0	136	2,278	2,414
Capiz	75,514	1	60	1,492	1,553	0	74	1,510	1,584
Guimaras	16,676	0	49	771	820	0	20	350	370
Iloilo	169,693	0	256	2,984	3,240	1	260	1,833	2,094
Negros Occidental	199,968	13	1,030	10,444	11,487	2	674	8,796	9,472
City of Bacolod	28,243	0	113	949	1,062	0	126	1,369	1,495
City of Iloilo	56,661	0	169	2,117	2,286	0	91	641	732
**Region 7**	**597,007**	**2**	**1,318**	**44,150**	**45,470**	**17**	**2,250**	**42,992**	**45,259**
Bohol	86,129	0	108	3,722	3,830	1	216	2,954	3,171
Cebu	309,013	0	557	30,359	30,916	2	630	28,076	28,708
Negros Oriental	90,628	2	328	5,247	5,577	10	574	5,746	6,330
Siquijor	9,078	0	6	321	327	0	14	337	351
City of Cebu	39,810	0	282	3,907	4,189	4	764	4,728	5,496
City of Lapu-Lapu	35,757	0	37	483	520	0	47	1,003	1,050
City of Mandaue	26,592	0	0	111	111	0	5	148	153
**Region 8**	**332,967**	**18**	**698**	**14,571**	**15,287**	**45**	**986**	**12,179**	**13,210**
Biliran	11,240	0	43	725	768	3	42	436	481
Eastern Samar	35,729	1	134	1,879	2,014	4	140	1,619	1,763

Table 2.A.3 - MODERN METHOD OF FAMILY PLANNING

Drop Outs

Philippines, 2023

Area	Total Current Users	PILLS-COC			Total	INJECTABLES			Total
		Age group				Age group			
		10-14	15-19	20-49		10-14	15-19	20-49	
Leyte	122,333	0	55	1,796	1,851	0	71	1,182	1,253
Northern Samar	49,058	0	129	2,968	3,097	0	89	3,548	3,637
Southern Leyte	27,698	0	40	1,513	1,553	0	45	1,023	1,068
Samar	41,329	17	153	3,531	3,701	38	426	2,281	2,745
Ormoc City	19,622	0	99	1,628	1,727	0	105	1,125	1,230
City of Tacloban	25,958	0	45	531	576	0	68	965	1,033
**Region 9**	**339,891**	**2**	**882**	**16,824**	**17,708**	**6**	**922**	**13,613**	**14,541**
Zamboanga del Norte	124,603	2	263	3,948	4,213	1	220	3,413	3,634
Zamboanga del Sur	94,708	0	171	4,590	4,761	1	165	3,375	3,541
Zamboanga Sibugay	44,556	0	181	3,061	3,242	0	210	2,511	2,721
City of Isabela	9,211	0	46	1,249	1,295	0	106	1,400	1,506
City of Zamboanga	66,813	0	221	3,976	4,197	4	221	2,914	3,139
**Region 10**	**514,846**	**33**	**2,902**	**41,436**	**44,371**	**36**	**1,858**	**20,278**	**22,172**
Bukidnon	158,694	23	1,208	15,273	16,504	16	752	6,181	6,949
Camiguin	6,535	2	1	140	143	0	4	86	90
Lanao del Norte	67,645	0	229	1,864	2,093	1	114	1,245	1,360
Misamis Occidental	69,918	5	751	7,681	8,437	4	208	3,180	3,392
Misamis Oriental	127,215	2	391	9,155	9,548	10	392	4,951	5,353
City of Cagayan De Oro	54,303	1	289	6,523	6,813	2	347	4,272	4,621
City of Iligan	30,536	0	33	800	833	3	41	363	407
**Region 11**	**532,707**	**46**	**1,999**	**33,011**	**35,056**	**42**	**1,803**	**19,106**	**20,951**
Davao de Oro	83,298	6	227	4,735	4,968	2	230	2,856	3,088
Davao del Norte	108,564	28	505	5,793	6,326	7	345	3,985	4,337
Davao Oriental	54,593	0	216	2,906	3,122	1	132	1,602	1,735
Davao del Sur	67,257	3	145	2,151	2,299	1	193	1,497	1,691
Davao Occidental	31,707	3	201	2,339	2,543	13	391	2,021	2,425
City of Davao	187,288	6	705	15,087	15,798	18	512	7,145	7,675
**Region 12**	**453,032**	**14**	**2,065**	**23,191**	**25,270**	**37**	**2,199**	**17,953**	**20,189**
Cotabato	134,232	7	774	9,406	10,187	19	703	7,277	7,999

Table 2.A.3 - MODERN METHOD OF FAMILY PLANNING

Drop Outs

Philippines, 2023

Area	Total Current Users	PILLS-COC			Total	INJECTABLES			Total
		Age group				Age group			
		10-14	15-19	20-49		10-14	15-19	20-49	
Sarangani	72,262	0	391	3,506	3,897	0	502	2,086	2,588
South Cotabato	96,119	3	292	3,613	3,908	2	363	3,435	3,800
Sultan Kudarat	101,026	4	447	4,934	5,385	12	371	3,275	3,658
					0				0
City of General Santos	49,393	0	161	1,732	1,893	4	260	1,880	2,144
**BARMM**	**290,634**	**7**	**923**	**17,889**	**18,819**	**11**	**1,628**	**20,595**	**22,234**
Basilan	15,552	1	145	1,141	1,287	2	266	1,252	1,520
Lanao del Sur	37,406	1	77	2,980	3,058	0	104	3,437	3,541
Maguindanao	92,272	1	478	7,590	8,069	7	824	8,638	9,469
Sulu	82,939	4	129	3,152	3,285	2	275	4,310	4,587
Tawi-Tawi	18,014	0	18	1,665	1,683	0	42	1,501	1,543
SGU	8,193	0	38	507	545	0	35	629	664
					0				0
City of Cotabato	36,258	0	38	854	892	0	82	828	910
**Caraga**	**284,351**	**14**	**1,262**	**18,400**	**19,676**	**15**	**802**	**10,817**	**11,634**
Agusan del Norte	40,460	0	148	2,058	2,206	10	146	1,781	1,937
Agusan del Sur	86,938	7	463	5,109	5,579	0	182	3,127	3,309
Surigao del Norte	50,319	0	270	4,120	4,390	5	199	2,464	2,668
Surigao del Sur	56,051	6	214	4,753	4,973	0	141	1,963	2,104
Dinagat Islands	10,768	0	52	1,101	1,153	0	44	802	846
City of Butuan	39,815	1	115	1,259	1,375	0	90	680	770

Table 2.A.3 - MODERN METHOD OF FAMILY PLANNING

Drop Outs

Philippines, 2023

Area	Total Current Users	IMPLANTS			Total	NFP-CCM			Total
		Age group				Age group			
		10-14	15-19	20-49		10-14	15-19	20-49	
**Philippines**	**8,353,912**	**358**	**13,576**	**131,206**	**145,140**	**17**	**661**	**15,201**	**15,879**
**N C R**	**859,210**	**104**	**2,619**	**16,381**	**19,104**	**0**	**20**	**222**	**242**
Malabon City	33,641	55	552	456	1,063	0	0	0	0
Navotas City	25,067	4	305	1,376	1,685	0	0	0	0
Valenzuela City	37,966	13	106	1,707	1,826	0	0	3	3
Caloocan City	61,903	1	151	1,065	1,217	0	0	21	21
Marikina City	20,095	0	3	133	136	0	0	0	0
Pasig City	55,229	0	51	1,584	1,635	0	0	0	0
Pateros	3,813	0	0	59	59	0	0	0	0
Taguig City	71,609	0	2	72	74	0	1	0	1
Quezon City	306,956	10	477	3,871	4,358	0	19	174	193
Makati City	20,752	0	11	112	123	0	0	0	0
Mandaluyong City	23,665	0	8	176	184	0	0	24	24
San Juan City	5,890	0	0	13	13	0	0	0	0
Manila City	50,873	18	861	4,199	5,078	0	0	0	0
Las Piñas City	20,551	3	74	642	719	0	0	0	0
Muntinlupa City	41,934	0	0	113	113	0	0	0	0
Parañaque City	42,631	0	10	307	317	0	0	0	0
Pasay City	36,635	0	8	496	504	0	0	0	0
**C A R**	**161,545**	**4**	**249**	**2,816**	**3,069**	**0**	**19**	**879**	**898**
Abra	19,903	1	26	222	249	0	0	114	114
Apayao	24,278	0	32	265	297	0	0	1	1
Benguet	34,934	0	11	318	329	0	0	0	0
Ifugao	21,809	3	21	401	425	0	2	170	172
Kalinga	18,022	0	19	297	316	0	17	502	519
Mountain Province	13,937	0	22	409	431	0	0	92	92
City of Baguio	28,662	0	118	904	1,022	0	0	0	0
**Region 1**	**481,911**	**3**	**324**	**2,594**	**2,921**	**0**	**8**	**191**	**199**
Ilocos Norte	51,402	0	9	106	115	0	7	92	99
Ilocos Sur	76,206	0	3	90	93	0	1	47	48
La Union	69,182	2	44	598	644	0	0	48	48
Pangasinan	278,036	1	256	1,756	2,013	0	0	4	4

Table 2.A.3 - MODERN METHOD OF FAMILY PLANNING

Drop Outs

Philippines, 2023

Area	Total Current Users	IMPLANTS			Total	NFP-CCM			Total
		Age group				Age group			
		10-14	15-19	20-49		10-14	15-19	20-49	
City of Dagupan	7,085	0	12	44	56	0	0	0	0
**Region 2**	**320,396**	**7**	**252**	**2,294**	**2,553**	**0**	**0**	**89**	**89**
Batanes	945	0	0	0	0	0	0	21	21
Cagayan	106,997	0	51	521	572	0	0	4	4
Isabela	130,614	6	155	1,156	1,317	0	0	6	6
Nueva Vizcaya	49,739	1	35	366	402	0	0	38	38
Quirino	19,516	0	5	84	89	0	0	20	20
City of Santiago	12,585	0	6	167	173	0	0	0	0
**Region 3**	**884,246**	**41**	**979**	**6,706**	**7,726**	**0**	**7**	**207**	**214**
Aurora	22,956	2	28	197	227	0	0	88	88
Bataan	63,188	9	112	736	857	0	0	4	4
Bulacan	245,933	0	246	2,008	2,254	0	0	5	5
Nueva Ecija	186,491	16	334	1,571	1,921	0	3	50	53
Pampanga	159,224	9	176	807	992	0	4	49	53
Tarlac	115,785	3	54	716	773	0	0	0	0
Zambales	48,549	2	16	407	425	0	0	6	6
City of Angeles	29,054	0	11	202	213	0	0	5	5
City of Olongapo	13,066	0	2	62	64	0	0	0	0
**Region 4A**	**910,368**	**9**	**632**	**8,162**	**8,803**	**0**	**83**	**4,570**	**4,653**
Batangas	162,745	1	36	358	395	0	81	4,197	4,278
Cavite	164,389	1	210	2,928	3,139	0	0	56	56
Laguna	320,343	3	171	1,808	1,982	0	1	58	59
Quezon	105,489	4	45	603	652	0	1	259	260
Rizal	152,400	0	150	2,347	2,497	0	0	0	0
City of Lucena	5,002	0	20	118	138	0	0	0	0
**Region 4B**	**266,333**	**3**	**408**	**4,497**	**4,908**	**1**	**28**	**478**	**507**
Marinduque	15,205	0	7	60	67	0	0	11	11
Occidental Mindoro	40,816	0	53	734	787	0	10	158	168
Oriental Mindoro	63,083	3	36	412	451	0	0	166	166
Palawan	93,326	0	263	2,766	3,029	0	15	70	85

Table 2.A.3 - MODERN METHOD OF FAMILY PLANNING

Drop Outs

Philippines, 2023

Area	Total Current Users	IMPLANTS			Total	NFP-CCM			Total
		Age group				Age group			
		10-14	15-19	20-49		10-14	15-19	20-49	
Romblon	22,228	0	26	195	221	1	3	73	77
City of Puerto Princesa	31,675	0	23	330	353	0	0	0	0
**Region 5**	**484,529**	**3**	**510**	**7,181**	**7,694**	**2**	**285**	**4,367**	**4,654**
Albay	120,028	0	106	1,345	1,451	0	2	389	391
Camarines Norte	46,841	0	15	206	221	0	0	58	58
Camarines Sur	107,402	2	208	2,552	2,762	0	1	648	649
Catanduanes	23,049	0	50	533	583	0	23	677	700
Masbate	91,907	1	59	1,186	1,246	2	225	1,740	1,967
Sorsogon	65,058	0	72	1,315	1,387	0	34	840	874
City of Naga	30,244	0	0	44	44	0	0	15	15
**Region 6**	**639,939**	**13**	**552**	**5,895**	**6,460**	**0**	**8**	**342**	**350**
Aklan	46,162	0	10	240	250	0	0	3	3
Antique	47,022	2	49	604	655	0	7	283	290
Capiz	75,514	0	12	555	567	0	0	18	18
Guimaras	16,676	0	7	34	41	0	0	0	0
Iloilo	169,693	2	125	859	986	0	0	10	10
Negros Occidental	199,968	6	274	2,675	2,955	0	0	28	28
City of Bacolod	28,243	3	30	697	730	0	0	0	0
City of Iloilo	56,661	0	45	231	276	0	1	0	1
**Region 7**	**597,007**	**9**	**1,691**	**27,840**	**29,540**	**0**	**5**	**162**	**167**
Bohol	86,129	5	325	3,147	3,477	0	0	14	14
Cebu	309,013	2	994	22,316	23,312	0	1	3	4
Negros Oriental	90,628	0	42	660	702	0	4	143	147
Siquijor	9,078	0	5	89	94	0	0	0	0
City of Cebu	39,810	2	320	1,102	1,424	0	0	2	2
City of Lapu-Lapu	35,757	0	4	297	301	0	0	0	0
City of Mandaue	26,592	0	1	229	230	0	0	0	0
**Region 8**	**332,967**	**3**	**274**	**3,858**	**4,135**	**0**	**8**	**212**	**220**
Biliran	11,240	1	34	215	250	0	0	31	31
Eastern Samar	35,729	0	45	603	648	0	0	55	55

Table 2.A.3 - MODERN METHOD OF FAMILY PLANNING

Drop Outs

Philippines, 2023

Area	Total Current Users	IMPLANTS Age group			Total	NFP-CCM Age group			Total
		10-14	15-19	20-49		10-14	15-19	20-49	
Leyte	122,333	0	34	556	590	0	0	6	6
Northern Samar	49,058	1	54	906	961	0	0	1	1
Southern Leyte	27,698	0	22	415	437	0	0	28	28
Samar	41,329	1	32	431	464	0	8	91	99
Ormoc City	19,622	0	36	428	464	0	0	0	0
City of Tacloban	25,958	0	17	304	321	0	0	0	0
**Region 9**	**339,891**	**10**	**748**	**7,043**	**7,801**	**0**	**0**	**4**	**4**
Zamboanga del Norte	124,603	1	210	1,730	1,941	0	0	4	4
Zamboanga del Sur	94,708	4	320	2,371	2,695	0	0	0	0
Zamboanga Sibugay	44,556	1	114	1,208	1,323	0	0	0	0
City of Isabela	9,211	1	37	471	509	0	0	0	0
City of Zamboanga	66,813	3	67	1,263	1,333	0	0	0	0
**Region 10**	**514,846**	**55**	**1,177**	**9,864**	**11,096**	**13**	**174**	**2,070**	**2,257**
Bukidnon	158,694	16	692	4,180	4,888	0	50	621	671
Camiguin	6,535	0	5	43	48	0	0	0	0
Lanao del Norte	67,645	0	54	467	521	0	1	69	70
Misamis Occidental	69,918	0	114	1,714	1,828	7	9	166	182
Misamis Oriental	127,215	14	175	1,625	1,814	6	114	1,184	1,304
City of Cagayan De Oro	54,303	6	131	1,514	1,651	0	0	0	0
City of Iligan	30,536	19	6	321	346	0	0	30	30
**Region 11**	**532,707**	**49**	**930**	**7,672**	**8,651**	**1**	**7**	**616**	**624**
Davao de Oro	83,298	2	129	1,109	1,240	0	1	106	107
Davao del Norte	108,564	15	253	1,275	1,543	0	0	84	84
Davao Oriental	54,593	1	42	706	749	0	0	210	210
Davao del Sur	67,257	3	55	620	678	0	1	46	47
Davao Occidental	31,707	5	120	552	677	0	0	1	1
City of Davao	187,288	23	331	3,410	3,764	1	5	169	175
**Region 12**	**453,032**	**16**	**1,067**	**7,766**	**8,849**	**0**	**2**	**300**	**302**
Cotabato	134,232	2	360	2,808	3,170	0	0	238	238

Table 2.A.3 - MODERN METHOD OF FAMILY PLANNING

Drop Outs

Philippines, 2023

Area	Total Current Users	IMPLANTS			Total	NFP-CCM			Total
		Age group				Age group			
		10-14	15-19	20-49		10-14	15-19	20-49	
Sarangani	72,262	0	137	1,116	1,253	0	1	21	22
South Cotabato	96,119	5	218	1,477	1,700	0	1	30	31
Sultan Kudarat	101,026	9	247	1,375	1,631	0	0	11	11
					0				0
City of General Santos	49,393	0	105	990	1,095	0	0	0	0
**BARMM**	**290,634**	**14**	**449**	**4,760**	**5,223**	**0**	**0**	**6**	**6**
Basilan	15,552	1	68	548	617	0	0	0	0
Lanao del Sur	37,406	0	12	422	434	0	0	0	0
Maguindanao	92,272	11	251	1,868	2,130	0	0	6	6
Sulu	82,939	1	75	1,331	1,407	0	0	0	0
Tawi-Tawi	18,014	1	28	417	446	0	0	0	0
SGU	8,193	0	2	55	57	0	0	0	0
					0				0
City of Cotabato	36,258	0	13	119	132	0	0	0	0
**Caraga**	**284,351**	**15**	**715**	**5,877**	**6,607**	**0**	**7**	**486**	**493**
Agusan del Norte	40,460	1	120	722	843	0	0	9	9
Agusan del Sur	86,938	3	233	1,796	2,032	0	0	247	247
Surigao del Norte	50,319	4	115	1,062	1,181	0	4	143	147
Surigao del Sur	56,051	3	153	1,312	1,468	0	3	82	85
Dinagat Islands	10,768	4	71	579	654	0	0	0	0
City of Butuan	39,815	0	23	406	429	0	0	5	5

Table 2.A.3 - MODERN METHOD OF FAMILY PLANNING

Drop Outs

Philippines, 2023

Area	Total Current Users	NFP-BBT			Total	NFP-STM			Total
		Age group				Age group			
		10-14	15-19	20-49		10-14	15-19	20-49	
**Philippines**	**8,353,912**	**6**	**253**	**2,393**	**2,652**	**2**	**73**	**2,060**	**2,135**
**N C R**	**859,210**	**0**	**52**	**117**	**169**	**0**	**0**	**6**	**6**
Malabon City	33,641	0	0	0	0	0	0	0	0
Navotas City	25,067	0	14	8	22	0	0	0	0
Valenzuela City	37,966	0	0	6	6	0	0	0	0
Caloocan City	61,903	0	0	0	0	0	0	0	0
Marikina City	20,095	0	0	0	0	0	0	0	0
Pasig City	55,229	0	0	1	1	0	0	0	0
Pateros	3,813	0	0	0	0	0	0	0	0
Taguig City	71,609	0	0	0	0	0	0	0	0
Quezon City	306,956	0	6	42	48	0	0	6	6
Makati City	20,752	0	0	0	0	0	0	0	0
Mandaluyong City	23,665	0	1	0	1	0	0	0	0
San Juan City	5,890	0	0	0	0	0	0	0	0
Manila City	50,873	0	31	60	91	0	0	0	0
Las Piñas City	20,551	0	0	0	0	0	0	0	0
Muntinlupa City	41,934	0	0	0	0	0	0	0	0
Parañaque City	42,631	0	0	0	0	0	0	0	0
Pasay City	36,635	0	0	0	0	0	0	0	0
**C A R**	**161,545**	**0**	**2**	**115**	**117**	**0**	**0**	**7**	**7**
Abra	19,903	0	0	73	73	0	0	2	2
Apayao	24,278	0	0	0	0	0	0	0	0
Benguet	34,934	0	2	36	38	0	0	0	0
Ifugao	21,809	0	0	0	0	0	0	3	3
Kalinga	18,022	0	0	1	1	0	0	0	0
Mountain Province	13,937	0	0	4	4	0	0	2	2
City of Baguio	28,662	0	0	1	1	0	0	0	0
**Region 1**	**481,911**	**0**	**7**	**219**	**226**	**0**	**0**	**124**	**124**
Ilocos Norte	51,402	0	0	0	0	0	0	0	0
Ilocos Sur	76,206	0	0	106	106	0	0	96	96
La Union	69,182	0	0	0	0	0	0	28	28
Pangasinan	278,036	0	7	111	118	0	0	0	0

Table 2.A.3 - MODERN METHOD OF FAMILY PLANNING

Drop Outs

Philippines, 2023

Area	Total Current Users	NFP-BBT			Total	NFP-STM			Total
		Age group				Age group			
		10-14	15-19	20-49		10-14	15-19	20-49	
City of Dagupan	7,085	0	0	2	2	0	0	0	0
**Region 2**	**320,396**	**0**	**6**	**24**	**30**	**0**	**0**	**16**	**16**
Batanes	945	0	0	0	0	0	0	0	0
Cagayan	106,997	0	0	0	0	0	0	0	0
Isabela	130,614	0	5	24	29	0	0	2	2
Nueva Vizcaya	49,739	0	1	0	1	0	0	14	14
Quirino	19,516	0	0	0	0	0	0	0	0
City of Santiago	12,585	0	0	0	0	0	0	0	0
**Region 3**	**884,246**	**0**	**4**	**34**	**38**	**0**	**0**	**3**	**3**
Aurora	22,956	0	0	6	6	0	0	0	0
Bataan	63,188	0	0	0	0	0	0	0	0
Bulacan	245,933	0	0	0	0	0	0	0	0
Nueva Ecija	186,491	0	0	1	1	0	0	0	0
Pampanga	159,224	0	3	7	10	0	0	0	0
Tarlac	115,785	0	1	5	6	0	0	0	0
Zambales	48,549	0	0	0	0	0	0	0	0
City of Angeles	29,054	0	0	15	15	0	0	3	3
City of Olongapo	13,066	0	0	0	0	0	0	0	0
**Region 4A**	**910,368**	**0**	**4**	**71**	**75**	**0**	**0**	**25**	**25**
Batangas	162,745	0	0	10	10	0	0	0	0
Cavite	164,389	0	0	3	3	0	0	0	0
Laguna	320,343	0	1	3	4	0	0	0	0
Quezon	105,489	0	1	44	45	0	0	25	25
Rizal	152,400	0	2	11	13	0	0	0	0
City of Lucena	5,002	0	0	0	0	0	0	0	0
**Region 4B**	**266,333**	**0**	**6**	**126**	**132**	**0**	**0**	**57**	**57**
Marinduque	15,205	0	0	0	0	0	0	0	0
Occidental Mindoro	40,816	0	1	76	77	0	0	5	5
Oriental Mindoro	63,083	0	0	24	24	0	0	32	32
Palawan	93,326	0	2	17	19	0	0	18	18

Table 2.A.3 - MODERN METHOD OF FAMILY PLANNING

Drop Outs

Philippines, 2023

Area	Total Current Users	NFP-BBT Age group 10-14	NFP-BBT Age group 15-19	NFP-BBT Age group 20-49	Total	NFP-STM Age group 10-14	NFP-STM Age group 15-19	NFP-STM Age group 20-49	Total
Romblon	22,228	0	3	3	6	0	0	1	1
City of Puerto Princesa	31,675	0	0	6	6	0	0	1	1
**Region 5**	**484,529**	**0**	**9**	**177**	**186**	**0**	**16**	**1,251**	**1,267**
Albay	120,028	0	1	16	17	0	0	43	43
Camarines Norte	46,841	0	0	8	8	0	8	770	778
Camarines Sur	107,402	0	8	57	65	0	8	385	393
Catanduanes	23,049	0	0	47	47	0	0	5	5
Masbate	91,907	0	0	36	36	0	0	8	8
Sorsogon	65,058	0	0	0	0	0	0	29	29
City of Naga	30,244	0	0	13	13	0	0	11	11
**Region 6**	**639,939**	**0**	**0**	**59**	**59**	**0**	**0**	**10**	**10**
Aklan	46,162	0	0	2	2	0	0	0	0
Antique	47,022	0	0	0	0	0	0	5	5
Capiz	75,514	0	0	0	0	0	0	0	0
Guimaras	16,676	0	0	0	0	0	0	0	0
Iloilo	169,693	0	0	57	57	0	0	5	5
Negros Occidental	199,968	0	0	0	0	0	0	0	0
City of Bacolod	28,243	0	0	0	0	0	0	0	0
City of Iloilo	56,661	0	0	0	0	0	0	0	0
**Region 7**	**597,007**	**0**	**10**	**59**	**69**	**0**	**0**	**2**	**2**
Bohol	86,129	0	0	3	3	0	0	0	0
Cebu	309,013	0	0	4	4	0	0	0	0
Negros Oriental	90,628	0	7	36	43	0	0	2	2
Siquijor	9,078	0	0	0	0	0	0	0	0
City of Cebu	39,810	0	3	16	19	0	0	0	0
City of Lapu-Lapu	35,757	0	0	0	0	0	0	0	0
City of Mandaue	26,592	0	0	0	0	0	0	0	0
**Region 8**	**332,967**	**0**	**6**	**500**	**506**	**0**	**3**	**82**	**85**
Biliran	11,240	0	0	0	0	0	0	0	0
Eastern Samar	35,729	0	0	17	17	0	0	73	73

Table 2.A.3 - MODERN METHOD OF FAMILY PLANNING

Drop Outs

Philippines, 2023

Area	Total Current Users	NFP-BBT Age group 10-14	NFP-BBT Age group 15-19	NFP-BBT Age group 20-49	Total	NFP-STM Age group 10-14	NFP-STM Age group 15-19	NFP-STM Age group 20-49	Total
Leyte	122,333	0	6	122	128	0	0	0	0
Northern Samar	49,058	0	0	175	175	0	0	0	0
Southern Leyte	27,698	0	0	21	21	0	0	0	0
Samar	41,329	0	0	164	164	0	3	9	12
Ormoc City	19,622	0	0	1	1	0	0	0	0
City of Tacloban	25,958	0	0	0	0	0	0	0	0
**Region 9**	**339,891**	**0**	**0**	**12**	**12**	**0**	**0**	**6**	**6**
Zamboanga del Norte	124,603	0	0	5	5	0	0	5	5
Zamboanga del Sur	94,708	0	0	7	7	0	0	1	1
Zamboanga Sibugay	44,556	0	0	0	0	0	0	0	0
City of Isabela	9,211	0	0	0	0	0	0	0	0
City of Zamboanga	66,813	0	0	0	0	0	0	0	0
**Region 10**	**514,846**	**5**	**104**	**524**	**633**	**2**	**51**	**361**	**414**
Bukidnon	158,694	1	11	221	233	0	6	174	180
Camiguin	6,535	0	0	8	8	0	0	0	0
Lanao del Norte	67,645	0	0	4	4	0	0	8	8
Misamis Occidental	69,918	0	52	67	119	0	37	81	118
Misamis Oriental	127,215	4	37	140	181	2	8	98	108
City of Cagayan De Oro	54,303	0	3	74	77	0	0	0	0
City of Iligan	30,536	0	1	10	11	0	0	0	0
**Region 11**	**532,707**	**0**	**4**	**242**	**246**	**0**	**3**	**86**	**89**
Davao de Oro	83,298	0	0	32	32	0	0	0	0
Davao del Norte	108,564	0	0	1	1	0	0	1	1
Davao Oriental	54,593	0	2	14	16	0	2	1	3
Davao del Sur	67,257	0	2	91	93	0	0	0	0
Davao Occidental	31,707	0	0	1	1	0	0	0	0
City of Davao	187,288	0	0	103	103	0	1	84	85
**Region 12**	**453,032**	**1**	**15**	**26**	**42**	**0**	**0**	**0**	**0**
Cotabato	134,232	0	0	17	17	0	0	0	0

Table 2.A.3 - MODERN METHOD OF FAMILY PLANNING

Drop Outs

Philippines, 2023

Area	Total Current Users	NFP-BBT			Total	NFP-STM			Total
		Age group				Age group			
		10-14	15-19	20-49		10-14	15-19	20-49	
Sarangani	72,262	0	0	0	0	0	0	0	0
South Cotabato	96,119	1	11	3	15	0	0	0	0
Sultan Kudarat	101,026	0	0	4	4	0	0	0	0
					0				0
City of General Santos	49,393	0	4	2	6	0	0	0	0
**BARMM**	**290,634**	**0**	**4**	**6**	**10**	**0**	**0**	**0**	**0**
Basilan	15,552	0	0	0	0	0	0	0	0
Lanao del Sur	37,406	0	0	2	2	0	0	0	0
Maguindanao	92,272	0	4	4	8	0	0	0	0
Sulu	82,939	0	0	0	0	0	0	0	0
Tawi-Tawi	18,014	0	0	0	0	0	0	0	0
SGU	8,193	0	0	0	0	0	0	0	0
					0				0
City of Cotabato	36,258	0	0	0	0	0	0	0	0
**Caraga**	**284,351**	**0**	**20**	**82**	**102**	**0**	**0**	**24**	**24**
Agusan del Norte	40,460	0	0	0	0	0	0	0	0
Agusan del Sur	86,938	0	16	37	53	0	0	1	1
Surigao del Norte	50,319	0	4	42	46	0	0	19	19
Surigao del Sur	56,051	0	0	3	3	0	0	4	4
Dinagat Islands	10,768	0	0	0	0	0	0	0	0
City of Butuan	39,815	0	0	0	0	0	0	0	0

Table 2.A.3 - MODERN METHOD OF FAMILY PLANNING

Drop Outs

Philippines, 2023

Area	Total Current Users	NFP-SDM			Total	NFP-LAM			Total	Total Drop-outs
		Age group				Age group				
		10-14	15-19	20-49		10-14	15-19	20-49		
**Philippines**	**8,353,912**	**15**	**841**	**25,188**	**26,044**	**1,330**	**77,692**	**725,504**	**804,526**	**2,441,518**
**N C R**	**859,210**	**6**	**6**	**191**	**203**	**105**	**8,931**	**103,824**	**112,860**	**323,797**
Malabon City	33,641	0	0	0	0	10	1,063	3,422	4,495	21,744
Navotas City	25,067	0	0	0	0	9	689	3,141	3,839	19,120
Valenzuela City	37,966	6	0	13	19	4	165	3,853	4,022	21,007
Caloocan City	61,903	0	5	56	61	3	861	7,874	8,738	24,396
Marikina City	20,095	0	0	1	1	0	62	541	603	2,629
Pasig City	55,229	0	0	0	0	0	94	2,773	2,867	13,673
Pateros	3,813	0	0	0	0	0	17	752	769	1,439
Taguig City	71,609	0	0	1	1	24	892	9,981	10,897	14,345
Quezon City	306,956	0	0	94	94	30	2,070	35,194	37,294	116,589
Makati City	20,752	0	0	0	0	0	72	2,634	2,706	4,834
Mandaluyong City	23,665	0	0	1	1	4	289	4,363	4,656	6,509
San Juan City	5,890	0	0	10	10	0	62	845	907	1,447
Manila City	50,873	0	1	2	3	12	722	9,946	10,680	26,741
Las Piñas City	20,551	0	0	5	5	3	358	4,256	4,617	13,816
Muntinlupa City	41,934	0	0	0	0	0	181	2,290	2,471	5,783
Parañaque City	42,631	0	0	8	8	5	673	6,332	7,010	9,943
Pasay City	36,635	0	0	0	0	1	661	5,627	6,289	19,782
**C A R**	**161,545**	**0**	**21**	**1,326**	**1,347**	**82**	**1,418**	**13,974**	**15,474**	**53,171**
Abra	19,903	0	7	54	61	66	445	2,503	3,014	6,539
Apayao	24,278	0	0	1	1	2	154	1,195	1,351	4,742
Benguet	34,934	0	1	539	540	3	172	2,961	3,136	9,458
Ifugao	21,809	0	7	422	429	4	221	2,158	2,383	8,501
Kalinga	18,022	0	1	61	62	1	221	2,854	3,076	10,743
Mountain Province	13,937	0	5	197	202	6	201	1,762	1,969	5,620
City of Baguio	28,662	0	0	52	52	0	4	541	545	7,568
**Region 1**	**481,911**	**1**	**44**	**567**	**612**	**43**	**2,924**	**38,475**	**41,442**	**75,264**
Ilocos Norte	51,402	0	0	118	118	0	168	4,015	4,183	7,527
Ilocos Sur	76,206	1	40	190	231	4	321	5,302	5,627	9,661
La Union	69,182	0	4	97	101	14	621	6,499	7,134	14,721
Pangasinan	278,036	0	0	158	158	22	1,727	21,932	23,681	42,221

Table 2.A.3 - MODERN METHOD OF FAMILY PLANNING

Drop Outs

Philippines, 2023

Area	Total Current Users	NFP-SDM			Total	NFP-LAM			Total	Total Drop-outs
		Age group				Age group				
		10-14	15-19	20-49		10-14	15-19	20-49		
City of Dagupan	7,085	0	0	4	4	3	87	727	817	1,134
**Region 2**	**320,396**	**0**	**2**	**128**	**130**	**48**	**2,419**	**19,985**	**22,452**	**72,640**
Batanes	945	0	0	5	5	1	12	152	165	512
Cagayan	106,997	0	0	1	1	6	414	4,155	4,575	14,147
Isabela	130,614	0	2	8	10	26	1,159	8,167	9,352	38,197
Nueva Vizcaya	49,739	0	0	91	91	14	510	4,760	5,284	12,918
Quirino	19,516	0	0	23	23	0	101	723	824	2,718
City of Santiago	12,585	0	0	0	0	1	223	2,028	2,252	4,148
**Region 3**	**884,246**	**0**	**2**	**956**	**958**	**76**	**5,586**	**49,293**	**54,955**	**191,859**
Aurora	22,956	0	0	39	39	11	295	2,237	2,543	6,466
Bataan	63,188	0	0	8	8	10	571	4,120	4,701	16,266
Bulacan	245,933	0	1	158	159	3	1,047	11,078	12,128	53,173
Nueva Ecija	186,491	0	0	23	23	34	1,501	10,846	12,381	40,361
Pampanga	159,224	0	1	685	686	3	778	8,235	9,016	27,896
Tarlac	115,785	0	0	7	7	5	890	7,734	8,629	31,092
Zambales	48,549	0	0	3	3	9	343	2,976	3,328	8,781
City of Angeles	29,054	0	0	33	33	1	102	1,428	1,531	4,978
City of Olongapo	13,066	0	0	0	0	0	59	639	698	2,846
**Region 4A**	**910,368**	**0**	**88**	**761**	**849**	**75**	**6,247**	**71,874**	**78,196**	**286,496**
Batangas	162,745	0	1	110	111	13	485	9,438	9,936	33,136
Cavite	164,389	0	66	14	80	8	921	16,476	17,405	53,250
Laguna	320,343	0	9	238	247	14	2,375	20,822	23,211	107,286
Quezon	105,489	0	11	279	290	28	780	8,078	8,886	35,670
Rizal	152,400	0	1	120	121	12	1,683	17,028	18,723	54,056
City of Lucena	5,002	0	0	0	0	0	3	32	35	3,098
**Region 4B**	**266,333**	**0**	**13**	**1,018**	**1,031**	**87**	**2,779**	**24,985**	**27,851**	**75,540**
Marinduque	15,205	0	0	4	4	4	28	615	647	1,679
Occidental Mindoro	40,816	0	5	385	390	17	862	5,942	6,821	14,472
Oriental Mindoro	63,083	0	0	40	40	13	450	6,051	6,514	17,558
Palawan	93,326	0	7	440	447	34	1,071	8,151	9,256	28,187

Table 2.A.3 - MODERN METHOD OF FAMILY PLANNING

Drop Outs

Philippines, 2023

Area	Total Current Users	NFP-SDM Age group			Total	NFP-LAM Age group			Total	Total Drop-outs
		10-14	15-19	20-49		10-14	15-19	20-49		
Romblon	22,228	0	1	121	122	16	251	3,321	3,588	6,236
City of Puerto Princesa	31,675	0	0	28	28	3	117	905	1,025	7,408
**Region 5**	**484,529**	**0**	**252**	**8,151**	**8,403**	**72**	**5,940**	**58,903**	**64,915**	**159,092**
Albay	120,028	0	19	1,391	1,410	5	598	8,122	8,725	31,289
Camarines Norte	46,841	0	2	172	174	11	587	5,481	6,079	16,144
Camarines Sur	107,402	0	27	2,906	2,933	18	1,640	16,894	18,552	42,970
Catanduanes	23,049	0	3	504	507	8	328	3,803	4,139	9,548
Masbate	91,907	0	166	1,591	1,757	13	1,737	12,783	14,533	31,786
Sorsogon	65,058	0	35	1,499	1,534	17	1,036	11,079	12,132	25,475
City of Naga	30,244	0	0	88	88	0	14	741	755	1,880
**Region 6**	**639,939**	**0**	**36**	**909**	**945**	**65**	**4,941**	**43,144**	**48,150**	**121,662**
Aklan	46,162	0	0	22	22	2	323	3,410	3,735	7,938
Antique	47,022	0	0	55	55	5	204	3,003	3,212	9,931
Capiz	75,514	0	0	0	0	1	108	2,577	2,686	8,051
Guimaras	16,676	0	1	8	9	3	237	1,896	2,136	3,796
Iloilo	169,693	0	7	137	144	13	860	9,087	9,960	19,253
Negros Occidental	199,968	0	28	663	691	40	3,051	21,303	24,394	58,633
City of Bacolod	28,243	0	0	0	0	1	132	1,652	1,785	5,884
City of Iloilo	56,661	0	0	24	24	0	26	216	242	8,176
**Region 7**	**597,007**	**0**	**11**	**512**	**523**	**107**	**6,611**	**65,454**	**72,172**	**282,000**
Bohol	86,129	0	0	167	167	6	455	5,078	5,539	27,724
Cebu	309,013	0	9	164	173	42	2,222	27,993	30,257	180,777
Negros Oriental	90,628	0	0	130	130	28	1,665	12,098	13,791	30,490
Siquijor	9,078	0	2	51	53	0	46	455	501	1,739
City of Cebu	39,810	0	0	0	0	29	1,856	14,590	16,475	31,552
City of Lapu-Lapu	35,757	0	0	0	0	2	357	4,919	5,278	8,580
City of Mandaue	26,592	0	0	0	0	0	10	321	331	1,138
**Region 8**	**332,967**	**0**	**20**	**639**	**659**	**50**	**2,391**	**22,555**	**24,996**	**74,782**
Biliran	11,240	0	2	21	23	1	133	1,279	1,413	3,364

Table 2.A.3 - MODERN METHOD OF FAMILY PLANNING

Drop Outs

Philippines, 2023

Area	Total Current Users	NFP-SDM Age group 10-14	NFP-SDM Age group 15-19	NFP-SDM Age group 20-49	Total	NFP-LAM Age group 10-14	NFP-LAM Age group 15-19	NFP-LAM Age group 20-49	Total	Total Drop-outs
Eastern Samar	35,729	0	9	370	379	6	346	2,718	3,070	11,048
Leyte	122,333	0	2	12	14	6	542	4,880	5,428	11,182
Northern Samar	49,058	0	0	45	45	1	357	5,224	5,582	16,084
Southern Leyte	27,698	0	0	45	45	0	244	2,553	2,797	7,460
Samar	41,329	0	5	5	10	35	393	2,760	3,188	13,114
Ormoc City	19,622	0	2	141	143	0	272	2,031	2,303	6,911
City of Tacloban	25,958	0	0	0	0	1	104	1,110	1,215	5,619
**Region 9**	**339,891**	**0**	**34**	**475**	**509**	**64**	**4,661**	**32,389**	**37,114**	**95,121**
Zamboanga del Norte	124,603	0	33	381	414	34	1,670	12,078	13,782	29,200
Zamboanga del Sur	94,708	0	0	0	0	10	619	5,009	5,638	23,445
Zamboanga Sibugay	44,556	0	1	89	90	3	552	3,530	4,085	13,431
City of Isabela	9,211	0	0	5	5	0	309	1,602	1,911	6,473
City of Zamboanga	66,813	0	0	0	0	17	1,511	10,170	11,698	22,572
**Region 10**	**514,846**	**3**	**242**	**6,366**	**6,611**	**176**	**8,277**	**56,331**	**64,784**	**196,891**
Bukidnon	158,694	0	73	2,310	2,383	94	3,391	16,558	20,043	62,664
Camiguin	6,535	0	0	11	11	3	83	800	886	1,335
Lanao del Norte	67,645	0	2	134	136	2	484	5,775	6,261	12,627
Misamis Occidental	69,918	0	46	1,495	1,541	10	707	9,003	9,720	31,812
Misamis Oriental	127,215	3	92	2,274	2,369	43	1,783	11,112	12,938	48,390
City of Cagayan De Oro	54,303	0	0	0	0	13	1,352	9,309	10,674	33,059
City of Iligan	30,536	0	29	142	171	11	477	3,774	4,262	7,004
**Region 11**	**532,707**	**1**	**28**	**833**	**862**	**108**	**3,168**	**26,662**	**29,938**	**128,937**
Davao de Oro	83,298	0	1	59	60	6	139	972	1,117	15,034
Davao del Norte	108,564	0	0	113	113	17	492	4,212	4,721	23,115
Davao Oriental	54,593	0	13	245	258	23	759	5,576	6,358	15,027
Davao del Sur	67,257	0	1	12	13	3	148	959	1,110	9,218
Davao Occidental	31,707	0	2	203	205	6	294	848	1,148	8,261
City of Davao	187,288	1	11	201	213	53	1,336	14,095	15,484	58,282

Table 2.A.3 - MODERN METHOD OF FAMILY PLANNING

Drop Outs

Philippines, 2023

Area	Total Current Users	NFP-SDM			Total	NFP-LAM			Total	Total Drop-outs
		Age group				Age group				
		10-14	15-19	20-49		10-14	15-19	20-49		
**Region 12**	**453,032**	**0**	**3**	**314**	**317**	**77**	**5,221**	**31,108**	**36,406**	**111,290**
Cotabato	134,232	0	0	79	79	12	896	6,535	7,443	38,061
Sarangani	72,262	0	0	52	52	0	1,670	6,778	8,448	18,632
South Cotabato	96,119	0	0	130	130	24	1,092	6,318	7,434	20,140
Sultan Kudarat	101,026	0	3	53	56	35	1,079	7,109	8,223	22,172
					0				0	
City of General Santos	49,393	0	0	0	0	6	484	4,368	4,858	12,285
**BARMM**	**290,634**	**0**	**6**	**171**	**177**	**46**	**3,196**	**42,329**	**45,571**	**104,186**
Basilan	15,552	0	0	2	2	3	218	1,517	1,738	5,886
Lanao del Sur	37,406	0	0	50	50	4	563	14,872	15,439	26,040
Maguindanao	92,272	0	6	100	106	25	1,745	16,346	18,116	42,898
Sulu	82,939	0	0	0	0	10	184	4,328	4,522	14,846
Tawi-Tawi	18,014	0	0	19	19	0	251	3,395	3,646	7,524
SGU	8,193	0	0	0	0	0	64	521	585	2,402
					0				0	
City of Cotabato	36,258	0	0	0	0	4	171	1,350	1,525	4,590
**Caraga**	**284,351**	**4**	**33**	**1,871**	**1,908**	**49**	**2,982**	**24,219**	**27,250**	**88,790**
Agusan del Norte	40,460	0	0	32	32	4	323	2,637	2,964	12,076
Agusan del Sur	86,938	0	20	657	677	17	747	5,286	6,050	23,049
Surigao del Norte	50,319	0	3	745	748	3	303	3,285	3,591	17,659
Surigao del Sur	56,051	0	10	308	318	13	883	7,171	8,067	21,623
Dinagat Islands	10,768	4	0	123	127	0	122	1,037	1,159	5,137
City of Butuan	39,815	0	0	6	6	12	604	4,803	5,419	9,246

Table 2.A.4 - MODERN METHOD OF FAMILY PLANNING

Current User (Ending)

Philippines, 2023

Area	Total Current Users	FSTR/BTL			Total	MSTR/NSV			Total
		Age group				Age group			
		10-14	15-19	20-49		10-14	15-19	20-49	
**Philippines**	**8,683,596**	**0**	**756**	**822,087**	**822,843**	**0**	**35**	**7,464**	**7,499**
**N C R**	**1,041,531**	**0**	**83**	**95,453**	**95,536**	**0**	**8**	**904**	**912**
Malabon City	26,264	0	0	3,342	3,342	0	0	3	3
Navotas City	23,816	0	1	563	564	0	0	5	5
Valenzuela City	40,454	0	0	6,436	6,436	0	0	88	88
Caloocan City	73,676	0	0	9,591	9,591	0	0	12	12
Marikina City	24,847	0	0	2,347	2,347	0	0	200	200
Pasig City	58,937	0	1	5,100	5,101	0	0	48	48
Pateros	4,204	0	0	409	409	0	0	7	7
Taguig City	92,053	0	1	4,401	4,402	0	6	49	55
Quezon City	338,677	0	68	34,694	34,762	0	0	229	229
Makati City	28,572	0	0	1,857	1,857	0	0	39	39
Mandaluyong City	27,196	0	9	2,341	2,350	0	0	39	39
San Juan City	6,139	0	0	233	233	0	0	9	9
Manila City	138,102	0	2	8,652	8,654	0	0	110	110
Las Piñas City	26,980	0	0	3,837	3,837	0	0	15	15
Muntinlupa City	44,186	0	1	3,737	3,738	0	1	19	20
Parañaque City	47,100	0	0	3,190	3,190	0	1	30	31
Pasay City	40,328	0	0	4,723	4,723	0	0	2	2
**C A R**	**165,663**	**0**	**2**	**32,842**	**32,844**	**0**	**0**	**208**	**208**
Abra	19,755	0	0	3,297	3,297	0	0	2	2
Apayao	24,752	0	0	1,028	1,028	0	0	2	2
Benguet	37,253	0	2	8,061	8,063	0	0	32	32
Ifugao	21,120	0	0	2,879	2,879	0	0	66	66
Kalinga	18,267	0	0	3,146	3,146	0	0	3	3
Mountain Province	13,957	0	0	5,481	5,481	0	0	4	4
City of Baguio	30,559	0	0	8,950	8,950	0	0	99	99
**Region 1**	**498,204**	**0**	**31**	**67,213**	**67,244**	**0**	**0**	**84**	**84**
Ilocos Norte	52,824	0	11	10,257	10,268	0	0	33	33
Ilocos Sur	78,125	0	2	10,345	10,347	0	0	4	4
La Union	67,784	0	3	11,679	11,682	0	0	11	11

Table 2.A.4 - MODERN METHOD OF FAMILY PLANNING
Current User (Ending)
Philippines, 2023

Area	Total Current Users	FSTR/BTL Age group 10-14	FSTR/BTL Age group 15-19	FSTR/BTL Age group 20-49	Total	MSTR/NSV Age group 10-14	MSTR/NSV Age group 15-19	MSTR/NSV Age group 20-49	Total
Pangasinan	292,353	0	15	34,099	34,114	0	0	34	34
City of Dagupan	7,118	0	0	833	833	0	0	2	2
**Region 2**	**343,378**	**0**	**78**	**44,940**	**45,018**	**0**	**0**	**92**	**92**
Batanes	955	0	0	167	167	0	0	0	0
Cagayan	108,930	0	66	13,214	13,280	0	0	36	36
Isabela	146,131	0	10	18,355	18,365	0	0	17	17
Nueva Vizcaya	52,095	0	1	8,627	8,628	0	0	33	33
Quirino	21,240	0	1	3,272	3,273	0	0	6	6
City of Santiago	14,027	0	0	1,305	1,305	0	0	0	0
**Region 3**	**985,325**	**0**	**4**	**176,788**	**176,792**	**0**	**1**	**193**	**194**
Aurora	24,562	0	0	2,312	2,312	0	0	4	4
Bataan	65,716	0	0	8,217	8,217	0	0	20	20
Bulacan	302,904	0	0	50,145	50,145	0	1	53	54
Nueva Ecija	200,897	0	2	37,618	37,620	0	0	5	5
Pampanga	167,294	0	2	43,778	43,780	0	0	10	10
Tarlac	118,444	0	0	18,648	18,648	0	0	5	5
Zambales	54,326	0	0	7,417	7,417	0	0	14	14
City of Angeles	30,345	0	0	6,212	6,212	0	0	79	79
City of Olongapo	20,837	0	0	2,441	2,441	0	0	3	3
**Region 4A**	**888,692**	**0**	**56**	**97,591**	**97,647**	**0**	**6**	**261**	**267**
Batangas	158,569	0	8	20,983	20,991	0	0	25	25
Cavite	175,332	0	17	23,788	23,805	0	0	69	69
Laguna	286,016	0	13	28,801	28,814	0	0	93	93
Quezon	106,773	0	10	10,185	10,195	0	6	22	28
Rizal	156,554	0	8	13,647	13,655	0	0	49	49
City of Lucena	5,448	0	0	187	187	0	0	3	3
**Region 4B**	**313,665**	**0**	**62**	**24,349**	**24,411**	**0**	**8**	**283**	**291**
Marinduque	16,436	0	0	2,296	2,296	0	0	18	18
Occidental Mindoro	48,902	0	0	2,486	2,486	0	0	6	6
Oriental Mindoro	68,962	0	21	7,870	7,891	0	0	90	90

Table 2.A.4 - MODERN METHOD OF FAMILY PLANNING
Current User (Ending)
Philippines, 2023

Area	Total Current Users	FSTR/BTL			Total	MSTR/NSV			Total
		Age group				Age group			
		10-14	15-19	20-49		10-14	15-19	20-49	
Palawan	122,174	0	4	5,897	5,901	0	8	112	120
Romblon	22,467	0	0	3,344	3,344	0	0	41	41
City of Puerto Princesa	34,724	0	37	2,456	2,493	0	0	16	16
**Region 5**	**491,006**	**0**	**9**	**28,215**	**28,224**	**0**	**0**	**284**	**284**
Albay	115,867	0	2	7,491	7,493	0	0	66	66
Camarines Norte	46,839	0	0	1,459	1,459	0	0	5	5
Camarines Sur	107,464	0	0	4,570	4,570	0	0	17	17
Catanduanes	24,326	0	6	3,204	3,210	0	0	72	72
Masbate	96,672	0	1	2,914	2,915	0	0	32	32
Sorsogon	66,956	0	0	6,267	6,267	0	0	39	39
City of Naga	32,882	0	0	2,310	2,310	0	0	53	53
**Region 6**	**667,635**	**0**	**13**	**52,424**	**52,437**	**0**	**6**	**1,879**	**1,885**
Aklan	48,553	0	2	3,519	3,521	0	0	2	2
Antique	49,316	0	3	6,607	6,610	0	1	75	76
Capiz	77,942	0	0	4,559	4,559	0	0	328	328
Guimaras	17,943	0	0	1,286	1,286	0	0	6	6
Iloilo	182,130	0	1	12,690	12,691	0	0	263	263
Negros Occidental	206,889	0	5	14,860	14,865	0	5	1,122	1,127
City of Bacolod	30,421	0	1	3,752	3,753	0	0	70	70
City of Iloilo	54,441	0	1	5,151	5,152	0	0	13	13
**Region 7**	**483,438**	**0**	**25**	**23,716**	**23,741**	**0**	**1**	**745**	**746**
Bohol	83,324	0	0	8,819	8,819	0	0	216	216
Cebu	191,250	0	3	7,408	7,411	0	0	2	2
Negros Oriental	98,214	0	20	3,048	3,068	0	0	196	196
Siquijor	9,225	0	0	437	437	0	0	1	1
City of Cebu	34,897	0	2	1,928	1,930	0	1	283	284
City of Lapu-Lapu	36,794	0	0	1,727	1,727	0	0	47	47
City of Mandaue	29,734	0	0	349	349	0	0	0	0
**Region 8**	**340,613**	**0**	**41**	**19,294**	**19,335**	**0**	**0**	**253**	**253**
Biliran	12,714	0	0	1,410	1,410	0	0	35	35

Table 2.A.4 - MODERN METHOD OF FAMILY PLANNING
Current User (Ending)
Philippines, 2023

Area	Total Current Users	FSTR/BTL Age group 10-14	15-19	20-49	Total	MSTR/NSV Age group 10-14	15-19	20-49	Total
Eastern Samar	39,982	0	40	2,246	2,286	0	0	49	49
Leyte	125,631	0	0	9,881	9,881	0	0	88	88
Northern Samar	48,697	0	1	1,170	1,171	0	0	40	40
Southern Leyte	27,251	0	0	545	545	0	0	13	13
Samar	42,957	0	0	1,685	1,685	0	0	11	11
Ormoc City	20,171	0	0	1,246	1,246	0	0	11	11
City of Tacloban	23,210	0	0	1,111	1,111	0	0	6	6
**Region 9**	**354,758**	**0**	**6**	**14,735**	**14,741**	**0**	**0**	**25**	**25**
Zamboanga del Norte	126,081	0	0	5,359	5,359	0	0	3	3
Zamboanga del Sur	102,629	0	2	4,001	4,003	0	0	10	10
Zamboanga Sibugay	48,639	0	0	1,788	1,788	0	0	7	7
City of Isabela	7,167	0	1	577	578	0	0	2	2
City of Zamboanga	70,242	0	3	3,010	3,013	0	0	3	3
**Region 10**	**511,701**	**0**	**6**	**31,991**	**31,997**	**0**	**0**	**220**	**220**
Bukidnon	167,011	0	5	10,329	10,334	0	0	103	103
Camiguin	6,884	0	0	1,055	1,055	0	0	5	5
Lanao del Norte	73,898	0	0	1,205	1,205	0	0	19	19
Misamis Occidental	60,887	0	0	3,453	3,453	0	0	9	9
Misamis Oriental	111,011	0	1	9,906	9,907	0	0	58	58
City of Cagayan De Oro	57,799	0	0	3,865	3,865	0	0	7	7
City of Iligan	34,211	0	0	2,178	2,178	0	0	19	19
**Region 11**	**536,237**	**0**	**93**	**43,133**	**43,226**	**0**	**1**	**1,148**	**1,149**
Davao de Oro	83,871	0	3	6,947	6,950	0	0	110	110
Davao del Norte	112,914	0	9	11,507	11,516	0	0	143	143
Davao Oriental	58,642	0	22	5,419	5,441	0	0	109	109
Davao del Sur	69,093	0	59	4,313	4,372	0	0	92	92
Davao Occidental	33,219	0	0	3,659	3,659	0	0	62	62
City of Davao	178,498	0	0	11,288	11,288	0	1	632	633
**Region 12**	**476,219**	**0**	**222**	**41,087**	**41,309**	**0**	**4**	**742**	**746**
Cotabato	136,508	0	11	9,824	9,835	0	0	99	99

Table 2.A.4 - MODERN METHOD OF FAMILY PLANNING

Current User (Ending)

Philippines, 2023

Area	Total Current Users	FSTR/BTL			Total	MSTR/NSV			Total
		Age group				Age group			
		10-14	15-19	20-49		10-14	15-19	20-49	
Sarangani	76,390	0	202	4,378	4,580	0	4	151	155
South Cotabato	102,265	0	0	9,595	9,595	0	0	357	357
Sultan Kudarat	104,518	0	4	8,955	8,959	0	0	83	83
City of General Santos	56,538	0	5	8,335	8,340	0	0	52	52
**BARMM**	**293,020**	**0**	**2**	**8,410**	**8,412**	**0**	**0**	**19**	**19**
Basilan	16,803	0	0	517	517	0	0	0	0
Lanao del Sur	36,361	0	0	1,744	1,744	0	0	18	18
Maguindanao	87,840	0	2	1,978	1,980	0	0	1	1
Sulu	81,975	0	0	1,310	1,310	0	0	0	0
Tawi-Tawi	20,067	0	0	334	334	0	0	0	0
SGU	7,817	0	0	141	141	0	0	0	0
City of Cotabato	42,157	0	0	2,386	2,386	0	0	0	0
**Caraga**	**292,511**	**0**	**23**	**19,906**	**19,929**	**0**	**0**	**124**	**124**
Agusan del Norte	40,148	0	3	3,270	3,273	0	0	1	1
Agusan del Sur	86,721	0	1	6,516	6,517	0	0	35	35
Surigao del Norte	53,159	0	6	3,044	3,050	0	0	28	28
Surigao del Sur	60,298	0	1	3,975	3,976	0	0	43	43
Dinagat Islands	9,606	0	11	383	394	0	0	1	1
City of Butuan	42,579	0	1	2,718	2,719	0	0	16	16

Table 2.A.4. MODERN METHOD OF FAMILY PLANNING
Current User (Ending)
Philippines, 2023

Area	Total Current Users	CONDOM Age group 10-14	CONDOM Age group 15-19	CONDOM Age group 20-49	Total	IUD-INTERVAL Age group 10-14	IUD-INTERVAL Age group 15-19	IUD-INTERVAL Age group 20-49	Total
**Philippines**	**8,683,596**	**242**	**15,939**	**410,143**	**426,324**	**80**	**7,597**	**407,957**	**415,634**
**N C R**	**1,041,531**	**75**	**5,107**	**107,805**	**112,987**	**12**	**1,258**	**41,565**	**42,835**
Malabon City	26,264	1	119	1,574	1,694	0	47	1,305	1,352
Navotas City	23,816	0	330	1,100	1,430	1	25	101	127
Valenzuela City	40,454	5	4	806	815	0	0	268	268
Caloocan City	73,676	0	140	3,215	3,355	3	254	4,173	4,430
Marikina City	24,847	0	14	881	895	0	52	1,226	1,278
Pasig City	58,937	9	120	2,825	2,954	0	55	2,455	2,510
Pateros	4,204	0	1	283	284	0	2	482	484
Taguig City	92,053	15	593	8,588	9,196	1	82	2,501	2,584
Quezon City	338,677	6	1,775	56,467	58,248	1	431	17,804	18,236
Makati City	28,572	1	42	3,438	3,481	2	15	866	883
Mandaluyong City	27,196	0	49	2,085	2,134	1	16	663	680
San Juan City	6,139	0	0	152	152	0	3	1,347	1,350
Manila City	138,102	33	681	15,828	16,542	1	120	1,293	1,414
Las Piñas City	26,980	3	68	2,528	2,599	1	15	503	519
Muntinlupa City	44,186	0	64	1,220	1,284	1	68	2,738	2,807
Parañaque City	47,100	2	312	4,497	4,811	0	44	1,137	1,181
Pasay City	40,328	0	795	2,318	3,113	0	29	2,703	2,732
**C A R**	**165,663**	**3**	**209**	**7,040**	**7,252**	**0**	**71**	**5,631**	**5,702**
Abra	19,755	0	103	1,386	1,489	0	2	13	15
Apayao	24,752	0	13	168	181	0	1	254	255
Benguet	37,253	0	13	2,868	2,881	0	16	2,470	2,486
Ifugao	21,120	0	5	528	533	0	3	960	963
Kalinga	18,267	1	16	514	531	0	7	659	666
Mountain Province	13,957	0	4	468	472	0	2	407	409
City of Baguio	30,559	2	55	1,108	1,165	0	40	868	908
**Region 1**	**498,204**	**3**	**375**	**21,099**	**21,477**	**7**	**246**	**10,484**	**10,737**
Ilocos Norte	52,824	0	17	1,568	1,585	0	17	630	647
Ilocos Sur	78,125	0	58	2,628	2,686	0	4	492	496
La Union	67,784	1	52	1,063	1,116	0	55	1,559	1,614
Pangasinan	292,353	2	247	15,652	15,901	7	167	7,725	7,899

Table 2.A.4. MODERN METHOD OF FAMILY PLANNING
Current User (Ending)
Philippines, 2023

Area	Total Current Users	CONDOM			Total	IUD-INTERVAL			Total
		Age group				Age group			
		10-14	15-19	20-49		10-14	15-19	20-49	
City of Dagupan	7,118	0	1	188	189	0	3	78	81
**Region 2**	**343,378**	**2**	**89**	**3,500**	**3,591**	**7**	**361**	**16,578**	**16,946**
Batanes	955	0	0	16	16	0	0	8	8
Cagayan	108,930	1	41	458	500	4	219	10,766	10,989
Isabela	146,131	1	21	1,690	1,712	2	86	3,719	3,807
Nueva Vizcaya	52,095	0	14	996	1,010	0	31	1,086	1,117
Quirino	21,240	0	12	299	311	0	16	725	741
City of Santiago	14,027	0	1	41	42	1	9	274	284
**Region 3**	**985,325**	**21**	**1,334**	**42,446**	**43,801**	**7**	**255**	**16,522**	**16,784**
Aurora	24,562	0	19	1,175	1,194	0	3	267	270
Bataan	65,716	10	103	2,765	2,878	0	41	475	516
Bulacan	302,904	0	370	16,274	16,644	6	78	9,917	10,001
Nueva Ecija	200,897	0	219	4,194	4,413	1	66	2,921	2,988
Pampanga	167,294	4	343	9,374	9,721	0	37	1,037	1,074
Tarlac	118,444	1	229	4,950	5,180	0	18	949	967
Zambales	54,326	6	32	1,894	1,932	0	2	575	577
City of Angeles	30,345	0	13	621	634	0	3	294	297
City of Olongapo	20,837	0	6	1,199	1,205	0	7	87	94
**Region 4A**	**888,692**	**42**	**3,274**	**46,295**	**49,611**	**21**	**488**	**29,257**	**29,766**
Batangas	158,569	11	309	9,675	9,995	1	27	3,505	3,533
Cavite	175,332	3	220	6,683	6,906	6	113	5,431	5,550
Laguna	286,016	20	2,474	21,452	23,946	1	189	8,096	8,286
Quezon	106,773	6	59	2,453	2,518	12	70	8,146	8,228
Rizal	156,554	2	211	5,958	6,171	1	89	4,058	4,148
City of Lucena	5,448	0	1	74	75	0	0	21	21
**Region 4B**	**313,665**	**3**	**129**	**7,044**	**7,176**	**0**	**59**	**6,583**	**6,642**
Marinduque	16,436	0	0	335	335	0	0	387	387
Occidental Mindoro	48,902	0	11	1,138	1,149	0	7	1,273	1,280
Oriental Mindoro	68,962	2	15	1,592	1,609	0	31	2,846	2,877
Palawan	122,174	0	54	2,282	2,336	0	11	1,004	1,015

Table 2.A.4. MODERN METHOD OF FAMILY PLANNING
Current User (Ending)
Philippines, 2023

Area	Total Current Users	CONDOM			Total	IUD-INTERVAL			Total
		Age group				Age group			
		10-14	15-19	20-49		10-14	15-19	20-49	
Romblon	22,467	0	4	549	553	0	2	742	744
City of Puerto Princesa	34,724	1	45	1,148	1,194	0	8	331	339
**Region 5**	**491,006**	**13**	**357**	**28,997**	**29,367**	**0**	**116**	**6,048**	**6,164**
Albay	115,867	0	109	9,756	9,865	0	9	1,125	1,134
Camarines Norte	46,839	1	84	2,072	2,157	0	64	1,155	1,219
Camarines Sur	107,464	0	56	5,117	5,173	0	12	1,295	1,307
Catanduanes	24,326	12	5	653	670	0	1	57	58
Masbate	96,672	0	81	2,733	2,814	0	11	1,795	1,806
Sorsogon	66,956	0	14	2,388	2,402	0	0	213	213
City of Naga	32,882	0	8	6,278	6,286	0	19	408	427
**Region 6**	**667,635**	**1**	**774**	**32,880**	**33,655**	**10**	**580**	**35,802**	**36,392**
Aklan	48,553	0	181	3,659	3,840	0	2	634	636
Antique	49,316	0	17	1,440	1,457	0	1	624	625
Capiz	77,942	0	29	3,804	3,833	0	23	6,684	6,707
Guimaras	17,943	0	4	1,172	1,176	0	1	311	312
Iloilo	182,130	1	187	9,594	9,782	7	151	8,846	9,004
Negros Occidental	206,889	0	287	7,036	7,323	0	265	13,407	13,672
City of Bacolod	30,421	0	12	1,442	1,454	3	70	3,139	3,212
City of Iloilo	54,441	0	57	4,733	4,790	0	67	2,157	2,224
**Region 7**	**483,438**	**2**	**324**	**14,196**	**14,522**	**1**	**808**	**57,456**	**58,265**
Bohol	83,324	0	41	4,872	4,913	0	200	11,852	12,052
Cebu	191,250	1	0	485	486	1	355	25,710	26,066
Negros Oriental	98,214	0	121	3,155	3,276	0	134	9,386	9,520
Siquijor	9,225	0	3	420	423	0	4	450	454
City of Cebu	34,897	0	5	848	853	0	69	3,466	3,535
City of Lapu-Lapu	36,794	1	136	2,152	2,289	0	39	4,059	4,098
City of Mandaue	29,734	0	18	2,264	2,282	0	7	2,533	2,540
**Region 8**	**340,613**	**6**	**379**	**12,151**	**12,536**	**0**	**196**	**21,999**	**22,195**
Biliran	12,714	0	17	345	362	0	15	684	699
Eastern Samar	39,982	1	50	2,590	2,641	0	8	527	535

Table 2.A.4. MODERN METHOD OF FAMILY PLANNING
Current User (Ending)
Philippines, 2023

Area	Total Current Users	CONDOM			Total	IUD-INTERVAL			Total
		Age group				Age group			
		10-14	15-19	20-49		10-14	15-19	20-49	
Leyte	125,631	2	128	3,982	4,112	0	77	11,722	11,799
Northern Samar	48,697	3	47	1,313	1,363	0	20	1,764	1,784
Southern Leyte	27,251	0	14	970	984	0	28	4,114	4,142
Samar	42,957	0	15	739	754	0	24	785	809
Ormoc City	20,171	0	12	967	979	0	3	1,180	1,183
City of Tacloban	23,210	0	96	1,245	1,341	0	21	1,223	1,244
**Region 9**	**354,758**	**0**	**242**	**10,772**	**11,014**	**0**	**374**	**25,994**	**26,368**
Zamboanga del Norte	126,081	0	86	5,392	5,478	0	78	10,179	10,257
Zamboanga del Sur	102,629	0	42	2,812	2,854	0	173	9,377	9,550
Zamboanga Sibugay	48,639	0	75	1,671	1,746	0	82	4,006	4,088
City of Isabela	7,167	0	17	219	236	0	2	80	82
City of Zamboanga	70,242	0	22	678	700	0	39	2,352	2,391
**Region 10**	**511,701**	**27**	**869**	**22,429**	**23,325**	**4**	**1,117**	**46,075**	**47,196**
Bukidnon	167,011	4	144	3,938	4,086	2	432	14,590	15,024
Camiguin	6,884	0	6	242	248	0	3	716	719
Lanao del Norte	73,898	0	174	6,665	6,839	0	33	3,031	3,064
Misamis Occidental	60,887	20	233	4,463	4,716	0	27	2,796	2,823
Misamis Oriental	111,011	3	139	3,194	3,336	2	258	15,612	15,872
City of Cagayan De Oro	57,799	0	80	1,899	1,979	0	250	6,227	6,477
City of Iligan	34,211	0	93	2,028	2,121	0	114	3,103	3,217
**Region 11**	**536,237**	**39**	**1,262**	**19,931**	**21,232**	**3**	**609**	**37,029**	**37,641**
Davao de Oro	83,871	0	61	2,941	3,002	0	68	5,969	6,037
Davao del Norte	112,914	0	139	4,402	4,541	0	95	7,709	7,804
Davao Oriental	58,642	0	21	1,343	1,364	0	10	2,972	2,982
Davao del Sur	69,093	2	155	1,836	1,993	1	130	7,663	7,794
Davao Occidental	33,219	1	20	260	281	0	39	1,858	1,897
City of Davao	178,498	36	866	9,149	10,051	2	267	10,858	11,127
**Region 12**	**476,219**	**4**	**612**	**12,879**	**13,495**	**5**	**512**	**25,876**	**26,393**
Cotabato	136,508	0	112	3,712	3,824	1	148	11,257	11,406
Sarangani	76,390	0	131	2,347	2,478	0	48	2,231	2,279

Table 2.A.4. MODERN METHOD OF FAMILY PLANNING

Current User (Ending)

Philippines, 2023

Area	Total Current Users	CONDOM			Total	IUD-INTERVAL			Total
		Age group				Age group			
		10-14	15-19	20-49		10-14	15-19	20-49	
South Cotabato	102,265	4	116	2,962	3,082	0	137	5,197	5,334
Sultan Kudarat	104,518	0	173	2,362	2,535	0	105	3,843	3,948
City of General Santos	56,538	0	80	1,496	1,576	4	74	3,348	3,426
**BARMM**	**293,020**	**1**	**310**	**10,036**	**10,347**	**1**	**97**	**3,067**	**3,165**
Basilan	16,803	0	12	306	318	1	35	288	324
Lanao del Sur	36,361	0	92	4,031	4,123	0	23	723	746
Maguindanao	87,840	1	107	2,420	2,528	0	12	846	858
Sulu	81,975	0	32	486	518	0	0	312	312
Tawi-Tawi	20,067	0	1	423	424	0	0	1	1
SGU	7,817	0	13	251	264	0	0	12	12
City of Cotabato	42,157	0	53	2,119	2,172	0	27	885	912
**Caraga**	**292,511**	**0**	**293**	**10,643**	**10,936**	**2**	**450**	**21,991**	**22,443**
Agusan del Norte	40,148	0	42	1,495	1,537	1	185	4,079	4,265
Agusan del Sur	86,721	0	108	3,143	3,251	1	123	8,044	8,168
Surigao del Norte	53,159	0	39	1,691	1,730	0	56	2,864	2,920
Surigao del Sur	60,298	0	54	2,208	2,262	0	61	4,648	4,709
Dinagat Islands	9,606	0	13	539	552	0	11	680	691
City of Butuan	42,579	0	37	1,567	1,604	0	14	1,676	1,690

Table 2.A.4 - MODERN METHOD OF FAMILY PLANNING
Current User (Ending)
Philippines, 2023

Area	Total Current Users	IUD-POSTPARTUM Age group 10-14	15-19	20-49	Total	PILLS-POP Age group 10-14	15-19	20-49	Total
**Philippines**	**8,683,596**	**401**	**14,009**	**152,439**	**166,849**	**679**	**59,389**	**397,268**	**457,336**
**N C R**	**1,041,531**	**165**	**3,926**	**35,399**	**39,490**	**125**	**6,242**	**63,868**	**70,235**
Malabon City	26,264	0	34	256	290	0	201	852	1,053
Navotas City	23,816	0	2	8	10	6	555	1,311	1,872
Valenzuela City	40,454	24	28	807	859	1	22	737	760
Caloocan City	73,676	6	184	1,280	1,470	1	322	3,300	3,623
Marikina City	24,847	30	662	3,541	4,233	0	63	879	942
Pasig City	58,937	20	505	2,174	2,699	3	243	2,149	2,395
Pateros	4,204	0	0	1	1	0	0	76	76
Taguig City	92,053	4	51	685	740	22	1,138	5,773	6,933
Quezon City	338,677	67	1,405	20,166	21,638	64	1,960	23,449	25,473
Makati City	28,572	0	98	1,400	1,498	0	65	2,880	2,945
Mandaluyong City	27,196	7	216	1,070	1,293	0	151	1,842	1,993
San Juan City	6,139	0	0	0	0	0	4	166	170
Manila City	138,102	6	146	1,704	1,856	20	683	11,181	11,884
Las Piñas City	26,980	0	2	127	129	0	97	1,731	1,828
Muntinlupa City	44,186	0	7	37	44	7	285	1,263	1,555
Parañaque City	47,100	1	499	708	1,208	1	390	3,498	3,889
Pasay City	40,328	0	87	1,435	1,522	0	63	2,781	2,844
**C A R**	**165,663**	**0**	**27**	**6,583**	**6,610**	**14**	**732**	**7,939**	**8,685**
Abra	19,755	0	1	31	32	0	126	1,220	1,346
Apayao	24,752	0	9	292	301	3	139	966	1,108
Benguet	37,253	0	4	542	546	0	98	1,416	1,514
Ifugao	21,120	0	2	87	89	4	41	1,817	1,862
Kalinga	18,267	0	6	36	42	0	87	488	575
Mountain Province	13,957	0	5	77	82	0	51	448	499
City of Baguio	30,559	0	0	5,518	5,518	7	190	1,584	1,781
**Region 1**	**498,204**	**2**	**106**	**703**	**811**	**2**	**341**	**6,290**	**6,633**
Ilocos Norte	52,824	0	9	143	152	1	67	1,802	1,870
Ilocos Sur	78,125	0	3	245	248	0	148	3,569	3,717
La Union	67,784	2	81	232	315	1	114	831	946
Pangasinan	292,353	0	0	0	0	0	0	0	0

Table 2.A.4 - MODERN METHOD OF FAMILY PLANNING
Current User (Ending)
Philippines, 2023

Area	Total Current Users	IUD-POSTPARTUM			Total	PILLS-POP			Total
		Age group				Age group			
		10-14	15-19	20-49		10-14	15-19	20-49	
City of Dagupan	7,118	0	13	83	96	0	12	88	100
**Region 2**	**343,378**	**2**	**232**	**1,614**	**1,848**	**16**	**1,429**	**13,912**	**15,357**
Batanes	955	0	0	0	0	0	1	16	17
Cagayan	108,930	0	116	754	870	4	562	4,590	5,156
Isabela	146,131	1	61	455	517	9	666	6,508	7,183
Nueva Vizcaya	52,095	0	54	378	432	1	150	2,258	2,409
Quirino	21,240	1	1	27	29	2	50	540	592
City of Santiago	14,027	0	0	0	0	0	0	0	0
**Region 3**	**985,325**	**46**	**1,383**	**11,272**	**12,701**	**51**	**3,379**	**30,152**	**33,582**
Aurora	24,562	1	3	85	89	1	37	806	844
Bataan	65,716	1	19	515	535	1	194	2,133	2,328
Bulacan	302,904	1	65	1,924	1,990	1	813	8,579	9,393
Nueva Ecija	200,897	42	1,192	3,546	4,780	36	844	6,140	7,020
Pampanga	167,294	1	55	4,275	4,331	10	783	5,178	5,971
Tarlac	118,444	0	27	389	416	2	433	4,587	5,022
Zambales	54,326	0	7	236	243	0	139	1,426	1,565
City of Angeles	30,345	0	2	175	177	0	60	506	566
City of Olongapo	20,837	0	13	127	140	0	76	797	873
**Region 4A**	**888,692**	**29**	**422**	**12,040**	**12,491**	**78**	**4,637**	**70,837**	**75,552**
Batangas	158,569	0	167	3,935	4,102	29	664	11,768	12,461
Cavite	175,332	2	47	893	942	11	412	5,334	5,757
Laguna	286,016	22	93	5,236	5,351	33	2,502	31,110	33,645
Quezon	106,773	2	15	393	410	0	369	5,467	5,836
Rizal	156,554	3	100	1,578	1,681	5	622	16,736	17,363
City of Lucena	5,448	0	0	5	5	0	68	422	490
**Region 4B**	**313,665**	**12**	**82**	**3,425**	**3,519**	**45**	**23,267**	**14,976**	**38,288**
Marinduque	16,436	0	1	4	5	0	48	2,429	2,477
Occidental Mindoro	48,902	0	2	22	24	0	168	2,257	2,425
Oriental Mindoro	68,962	3	67	2,466	2,536	2	141	2,026	2,169
Palawan	122,174	9	7	95	111	31	22,582	5,285	27,898

Table 2.A.4 - MODERN METHOD OF FAMILY PLANNING
Current User (Ending)
Philippines, 2023

Area	Total Current Users	IUD-POSTPARTUM Age group			Total	PILLS-POP Age group			Total
		10-14	15-19	20-49		10-14	15-19	20-49	
Romblon	22,467	0	3	781	784	5	35	748	788
City of Puerto Princesa	34,724	0	2	57	59	7	293	2,231	2,531
**Region 5**	**491,006**	**0**	**87**	**948**	**1,035**	**0**	**1,070**	**21,490**	**22,560**
Albay	115,867	0	13	410	423	0	204	5,538	5,742
Camarines Norte	46,839	0	50	90	140	0	195	3,487	3,682
Camarines Sur	107,464	0	10	212	222	0	243	4,789	5,032
Catanduanes	24,326	0	0	46	46	0	24	362	386
Masbate	96,672	0	1	64	65	0	265	4,291	4,556
Sorsogon	66,956	0	7	61	68	0	97	2,192	2,289
City of Naga	32,882	0	6	65	71	0	42	831	873
**Region 6**	**667,635**	**12**	**755**	**12,036**	**12,803**	**139**	**2,337**	**19,612**	**22,088**
Aklan	48,553	0	5	156	161	3	161	2,838	3,002
Antique	49,316	0	0	130	130	0	98	1,448	1,546
Capiz	77,942	0	1	67	68	0	112	1,558	1,670
Guimaras	17,943	0	1	35	36	0	36	592	628
Iloilo	182,130	4	341	4,526	4,871	1	591	5,162	5,754
Negros Occidental	206,889	5	331	4,233	4,569	4	1,224	6,931	8,159
City of Bacolod	30,421	3	49	1,941	1,993	131	72	687	890
City of Iloilo	54,441	0	27	948	975	0	43	396	439
**Region 7**	**483,438**	**13**	**1,170**	**8,997**	**10,180**	**18**	**1,759**	**15,888**	**17,665**
Bohol	83,324	1	403	4,298	4,702	0	135	1,885	2,020
Cebu	191,250	12	710	2,941	3,663	9	696	2,609	3,314
Negros Oriental	98,214	0	10	497	507	6	371	2,853	3,230
Siquijor	9,225	0	1	110	111	0	26	304	330
City of Cebu	34,897	0	13	706	719	1	116	1,860	1,977
City of Lapu-Lapu	36,794	0	22	267	289	0	316	5,179	5,495
City of Mandaue	29,734	0	11	178	189	2	99	1,198	1,299
**Region 8**	**340,613**	**0**	**251**	**2,889**	**3,140**	**4**	**875**	**12,043**	**12,922**
Biliran	12,714	0	5	90	95	0	32	476	508
Eastern Samar	39,982	0	11	102	113	0	71	725	796

Table 2.A.4 - MODERN METHOD OF FAMILY PLANNING
Current User (Ending)
Philippines, 2023

Area	Total Current Users	IUD-POSTPARTUM Age group 10-14	IUD-POSTPARTUM Age group 15-19	IUD-POSTPARTUM Age group 20-49	Total	PILLS-POP Age group 10-14	PILLS-POP Age group 15-19	PILLS-POP Age group 20-49	Total
Leyte	125,631	0	91	1,251	1,342	2	304	6,264	6,570
Northern Samar	48,697	0	21	448	469	1	110	1,017	1,128
Southern Leyte	27,251	0	19	424	443	0	30	791	821
Samar	42,957	0	50	451	501	1	169	1,047	1,217
Ormoc City	20,171	0	4	47	51	0	77	644	721
City of Tacloban	23,210	0	50	76	126	0	82	1,079	1,161
**Region 9**	**354,758**	**33**	**1,788**	**15,979**	**17,800**	**21**	**880**	**11,230**	**12,131**
Zamboanga del Norte	126,081	0	82	3,615	3,697	11	235	4,602	4,848
Zamboanga del Sur	102,629	20	1,121	7,667	8,808	8	188	3,240	3,436
Zamboanga Sibugay	48,639	1	48	400	449	0	203	1,748	1,951
City of Isabela	7,167	0	0	2	2	0	79	382	461
City of Zamboanga	70,242	12	537	4,295	4,844	2	175	1,258	1,435
**Region 10**	**511,701**	**53**	**2,239**	**16,464**	**18,756**	**19**	**2,645**	**28,291**	**30,955**
Bukidnon	167,011	0	65	872	937	4	957	4,974	5,935
Camiguin	6,884	0	2	48	50	0	8	50	58
Lanao del Norte	73,898	0	31	622	653	0	376	11,171	11,547
Misamis Occidental	60,887	0	32	469	501	0	338	4,377	4,715
Misamis Oriental	111,011	37	1,490	7,493	9,020	9	450	3,349	3,808
City of Cagayan De Oro	57,799	11	421	6,185	6,617	0	262	2,685	2,947
City of Iligan	34,211	5	198	775	978	6	254	1,685	1,945
**Region 11**	**536,237**	**14**	**290**	**4,897**	**5,201**	**89**	**3,773**	**30,014**	**33,876**
Davao de Oro	83,871	0	21	547	568	8	591	3,819	4,418
Davao del Norte	112,914	1	63	1,082	1,146	14	502	5,244	5,760
Davao Oriental	58,642	0	1	197	198	4	203	2,215	2,422
Davao del Sur	69,093	1	36	688	725	19	636	3,370	4,025
Davao Occidental	33,219	1	6	94	101	8	146	559	713
City of Davao	178,498	11	163	2,289	2,463	36	1,695	14,807	16,538
**Region 12**	**476,219**	**7**	**412**	**9,025**	**9,444**	**25**	**2,820**	**19,740**	**22,585**
Cotabato	136,508	0	72	1,400	1,472	4	661	6,930	7,595
Sarangani	76,390	0	9	150	159	0	884	3,662	4,546

Table 2.A.4 - MODERN METHOD OF FAMILY PLANNING
Current User (Ending)
Philippines, 2023

Area	Total Current Users	IUD-POSTPARTUM			Total	PILLS-POP			Total
		Age group				Age group			
		10-14	15-19	20-49		10-14	15-19	20-49	
South Cotabato	102,265	0	109	1,972	2,081	10	541	4,221	4,772
Sultan Kudarat	104,518	0	106	3,947	4,053	5	369	2,618	2,992
City of General Santos	56,538	7	116	1,556	1,679	6	365	2,309	2,680
**BARMM**	**293,020**	**4**	**300**	**2,779**	**3,083**	**20**	**1,438**	**13,402**	**14,860**
Basilan	16,803	0	92	432	524	0	72	471	543
Lanao del Sur	36,361	0	4	283	287	3	194	3,263	3,460
Maguindanao	87,840	0	27	441	468	0	347	3,404	3,751
Sulu	81,975	3	1	117	121	15	639	3,492	4,146
Tawi-Tawi	20,067	0	0	14	14	0	18	528	546
SGU	7,817	0	5	7	12	0	99	1,067	1,166
City of Cotabato	42,157	1	171	1,485	1,657	2	69	1,177	1,248
**Caraga**	**292,511**	**9**	**539**	**7,389**	**7,937**	**13**	**1,765**	**17,584**	**19,362**
Agusan del Norte	40,148	2	86	883	971	0	307	2,573	2,880
Agusan del Sur	86,721	1	73	1,213	1,287	7	540	4,086	4,633
Surigao del Norte	53,159	1	171	1,227	1,399	0	188	3,985	4,173
Surigao del Sur	60,298	5	43	3,252	3,300	6	410	3,943	4,359
Dinagat Islands	9,606	0	2	25	27	0	19	287	306
City of Butuan	42,579	0	164	789	953	0	301	2,710	3,011

Table 2.A.4 - MODERN METHOD OF FAMILY PLANNING
Current User (Ending)
Philippines, 2023

Area	Total Current Users	PILLS-COC			Total	INJECTABLES			Total
		Age group				Age group			
		10-14	15-19	20-49		10-14	15-19	20-49	
**Philippines**	**8,683,596**	**510**	**76,131**	**2,917,637**	**2,994,278**	**848**	**92,866**	**1,700,692**	**1,794,406**
**N C R**	**1,041,531**	**82**	**7,497**	**210,261**	**217,840**	**145**	**14,568**	**217,236**	**231,949**
Malabon City	26,264	0	409	6,285	6,694	3	644	6,275	6,922
Navotas City	23,816	7	1,081	5,547	6,635	12	1,382	7,806	9,200
Valenzuela City	40,454	10	303	8,192	8,505	2	592	8,164	8,758
Caloocan City	73,676	3	370	9,160	9,533	5	1,372	21,350	22,727
Marikina City	24,847	0	35	2,235	2,270	6	560	5,704	6,270
Pasig City	58,937	4	413	10,650	11,067	10	1,106	15,959	17,075
Pateros	4,204	0	4	1,387	1,391	0	10	1,051	1,061
Taguig City	92,053	16	868	15,144	16,028	9	956	20,162	21,127
Quezon City	338,677	11	1,790	85,799	87,600	12	2,519	43,790	46,321
Makati City	28,572	0	61	4,413	4,474	0	188	7,022	7,210
Mandaluyong City	27,196	0	190	4,381	4,571	0	223	5,337	5,560
San Juan City	6,139	0	5	1,769	1,774	1	20	1,871	1,892
Manila City	138,102	21	618	23,094	23,733	76	1,670	36,650	38,396
Las Piñas City	26,980	0	104	4,507	4,611	3	342	5,565	5,910
Muntinlupa City	44,186	10	685	12,277	12,972	5	1,571	7,926	9,502
Parañaque City	47,100	0	475	7,202	7,677	1	1,350	10,509	11,860
Pasay City	40,328	0	86	8,219	8,305	0	63	12,095	12,158
**C A R**	**165,663**	**10**	**878**	**48,950**	**49,838**	**38**	**1,404**	**21,422**	**22,864**
Abra	19,755	6	213	7,495	7,714	15	174	2,285	2,474
Apayao	24,752	0	268	16,943	17,211	2	198	2,125	2,325
Benguet	37,253	0	105	9,639	9,744	1	162	5,423	5,586
Ifugao	21,120	4	81	4,428	4,513	2	119	3,123	3,244
Kalinga	18,267	0	72	5,636	5,708	2	138	2,783	2,923
Mountain Province	13,957	0	61	2,438	2,499	2	96	1,459	1,557
City of Baguio	30,559	0	78	2,371	2,449	14	517	4,224	4,755
**Region 1**	**498,204**	**7**	**3,075**	**208,476**	**211,558**	**13**	**3,251**	**103,067**	**106,331**
Ilocos Norte	52,824	2	164	21,642	21,808	0	125	8,151	8,276
Ilocos Sur	78,125	0	446	30,326	30,772	1	301	13,911	14,213
La Union	67,784	0	350	27,510	27,860	3	515	15,481	15,999

Table 2.A.4 - MODERN METHOD OF FAMILY PLANNING

Current User (Ending)

Philippines, 2023

Area	Total Current Users	PILLS-COC			Total	INJECTABLES			Total
		Age group				Age group			
		10-14	15-19	20-49		10-14	15-19	20-49	
Pangasinan	292,353	5	2,092	126,534	128,631	9	2,243	63,468	65,720
City of Dagupan	7,118	0	23	2,464	2,487	0	67	2,056	2,123
**Region 2**	**343,378**	**22**	**2,655**	**168,900**	**171,577**	**65**	**2,319**	**49,943**	**52,327**
Batanes	955	0	5	172	177	1	14	278	293
Cagayan	108,930	14	712	54,689	55,415	21	547	13,288	13,856
Isabela	146,131	2	1,089	76,753	77,844	13	1,086	21,302	22,401
Nueva Vizcaya	52,095	4	389	22,465	22,858	3	317	6,978	7,298
Quirino	21,240	2	352	9,264	9,618	27	165	4,503	4,695
City of Santiago	14,027	0	108	5,557	5,665	0	190	3,594	3,784
**Region 3**	**985,325**	**28**	**10,272**	**318,588**	**328,888**	**69**	**14,305**	**243,228**	**257,602**
Aurora	24,562	0	128	11,921	12,049	0	163	3,967	4,130
Bataan	65,716	7	609	14,945	15,561	8	1,742	21,868	23,618
Bulacan	302,904	2	2,939	98,027	100,968	13	3,982	76,486	80,481
Nueva Ecija	200,897	4	2,649	78,596	81,249	11	2,568	40,885	43,464
Pampanga	167,294	1	1,292	41,776	43,069	8	2,302	38,183	40,493
Tarlac	118,444	7	1,608	41,345	42,960	15	1,578	31,850	33,443
Zambales	54,326	6	459	17,361	17,826	6	1,114	16,086	17,206
City of Angeles	30,345	1	294	9,169	9,464	3	399	8,512	8,914
City of Olongapo	20,837	0	294	5,448	5,742	5	457	5,391	5,853
**Region 4A**	**888,692**	**121**	**8,043**	**248,780**	**256,944**	**88**	**11,154**	**207,728**	**218,970**
Batangas	158,569	48	714	52,254	53,016	10	932	26,009	26,951
Cavite	175,332	25	1,039	53,136	54,200	32	1,909	45,321	47,262
Laguna	286,016	35	4,520	77,385	81,940	21	4,145	51,376	55,542
Quezon	106,773	0	561	35,503	36,064	3	1,264	28,465	29,732
Rizal	156,554	13	1,186	29,225	30,424	20	2,749	53,568	56,337
City of Lucena	5,448	0	23	1,277	1,300	2	155	2,989	3,146
**Region 4B**	**313,665**	**27**	**2,067**	**99,262**	**101,356**	**69**	**3,460**	**64,799**	**68,328**
Marinduque	16,436	0	13	4,836	4,849	1	68	2,842	2,911
Occidental Mindoro	48,902	1	344	16,397	16,742	1	340	8,961	9,302
Oriental Mindoro	68,962	1	336	30,666	31,003	0	367	11,266	11,633

Table 2.A.4 - MODERN METHOD OF FAMILY PLANNING
Current User (Ending)
Philippines, 2023

Area	Total Current Users	PILLS-COC			Total	INJECTABLES			Total
		Age group				Age group			
		10-14	15-19	20-49		10-14	15-19	20-49	
Palawan	122,174	23	1,128	30,578	31,729	58	1,668	26,241	27,967
Romblon	22,467	2	22	6,172	6,196	2	87	3,477	3,566
City of Puerto Princesa	34,724	0	224	10,613	10,837	7	930	12,012	12,949
**Region 5**	**491,006**	**5**	**2,562**	**179,669**	**182,236**	**6**	**2,625**	**80,466**	**83,097**
Albay	115,867	1	589	43,129	43,719	2	551	24,473	25,026
Camarines Norte	46,839	0	426	18,554	18,980	1	542	7,902	8,445
Camarines Sur	107,464	1	447	37,085	37,533	1	582	18,603	19,186
Catanduanes	24,326	1	18	4,084	4,103	2	131	4,431	4,564
Masbate	96,672	1	851	40,500	41,352	0	428	10,447	10,875
Sorsogon	66,956	1	193	23,585	23,779	0	330	10,864	11,194
City of Naga	32,882	0	38	12,732	12,770	0	61	3,746	3,807
**Region 6**	**667,635**	**21**	**4,672**	**275,717**	**280,410**	**35**	**5,018**	**123,426**	**128,479**
Aklan	48,553	3	303	16,698	17,004	5	664	12,827	13,496
Antique	49,316	0	202	15,035	15,237	1	357	12,546	12,904
Capiz	77,942	2	414	35,975	36,391	0	572	18,058	18,630
Guimaras	17,943	0	73	10,616	10,689	0	42	2,161	2,203
Iloilo	182,130	13	735	73,286	74,034	8	1,103	33,998	35,109
Negros Occidental	206,889	2	2,562	86,848	89,412	10	1,930	32,258	34,198
City of Bacolod	30,421	1	147	6,540	6,688	11	183	4,639	4,833
City of Iloilo	54,441	0	236	30,719	30,955	0	167	6,939	7,106
**Region 7**	**483,438**	**6**	**2,482**	**156,137**	**158,625**	**27**	**4,883**	**103,567**	**108,477**
Bohol	83,324	0	221	23,320	23,541	1	493	12,818	13,312
Cebu	191,250	2	866	72,045	72,913	8	1,970	37,098	39,076
Negros Oriental	98,214	3	747	35,827	36,577	12	1,318	26,227	27,557
Siquijor	9,225	0	28	3,477	3,505	0	93	2,661	2,754
City of Cebu	34,897	1	369	7,191	7,561	3	435	6,925	7,363
City of Lapu-Lapu	36,794	0	212	5,530	5,742	1	412	9,473	9,886
City of Mandaue	29,734	0	39	8,747	8,786	2	162	8,365	8,529
**Region 8**	**340,613**	**11**	**2,551**	**130,983**	**133,545**	**7**	**2,454**	**58,924**	**61,385**
Biliran	12,714	0	56	4,826	4,882	1	59	1,582	1,642

Table 2.A.4 - MODERN METHOD OF FAMILY PLANNING

Current User (Ending)

Philippines, 2023

Area	Total Current Users	PILLS-COC			Total	INJECTABLES			Total
		Age group				Age group			
		10-14	15-19	20-49		10-14	15-19	20-49	
Eastern Samar	39,982	0	319	16,094	16,413	1	346	8,335	8,682
Leyte	125,631	0	991	45,518	46,509	2	788	19,238	20,028
Northern Samar	48,697	0	304	18,394	18,698	2	306	10,301	10,609
Southern Leyte	27,251	0	53	11,332	11,385	0	179	3,638	3,817
Samar	42,957	11	369	18,874	19,254	1	370	8,169	8,540
Ormoc City	20,171	0	142	9,081	9,223	0	131	2,051	2,182
City of Tacloban	23,210	0	317	6,864	7,181	0	275	5,610	5,885
**Region 9**	**354,758**	**11**	**2,529**	**117,260**	**119,800**	**17**	**2,677**	**58,538**	**61,232**
Zamboanga del Norte	126,081	3	895	45,300	46,198	4	882	20,342	21,228
Zamboanga del Sur	102,629	2	522	34,369	34,893	2	441	12,939	13,382
Zamboanga Sibugay	48,639	5	541	14,629	15,175	4	480	8,274	8,758
City of Isabela	7,167	0	62	1,317	1,379	0	63	1,398	1,461
City of Zamboanga	70,242	1	509	21,645	22,155	7	811	15,585	16,403
**Region 10**	**511,701**	**28**	**6,458**	**174,119**	**180,605**	**18**	**3,846**	**65,101**	**68,965**
Bukidnon	167,011	7	2,279	70,218	72,504	9	1,185	12,677	13,871
Camiguin	6,884	0	51	2,754	2,805	0	9	693	702
Lanao del Norte	73,898	10	799	19,594	20,403	1	659	17,202	17,862
Misamis Occidental	60,887	0	1,354	22,386	23,740	0	434	7,667	8,101
Misamis Oriental	111,011	2	1,030	33,788	34,820	5	673	12,077	12,755
City of Cagayan De Oro	57,799	7	510	15,592	16,109	0	424	7,840	8,264
City of Iligan	34,211	2	435	9,787	10,224	3	462	6,945	7,410
**Region 11**	**536,237**	**52**	**7,561**	**214,476**	**222,089**	**169**	**6,695**	**76,446**	**83,310**
Davao de Oro	83,871	14	1,064	42,143	43,221	6	669	10,349	11,024
Davao del Norte	112,914	5	977	51,014	51,996	8	838	13,523	14,369
Davao Oriental	58,642	0	505	28,397	28,902	0	348	6,250	6,598
Davao del Sur	69,093	5	1,324	29,206	30,535	106	1,595	10,895	12,596
Davao Occidental	33,219	4	970	13,116	14,090	10	867	6,441	7,318
City of Davao	178,498	24	2,721	50,600	53,345	39	2,378	28,988	31,405
**Region 12**	**476,219**	**62**	**6,790**	**178,474**	**185,326**	**41**	**7,206**	**85,135**	**92,382**
Cotabato	136,508	38	1,325	56,391	57,754	4	1,158	22,606	23,768

Table 2.A.4 - MODERN METHOD OF FAMILY PLANNING
Current User (Ending)
Philippines, 2023

Area	Total Current Users	PILLS-COC			Total	INJECTABLES			Total
		Age group				Age group			
		10-14	15-19	20-49		10-14	15-19	20-49	
Sarangani	76,390	0	1,704	30,412	32,116	0	1,356	13,973	15,329
South Cotabato	102,265	13	1,387	40,691	42,091	14	1,307	15,876	17,197
Sultan Kudarat	104,518	5	1,765	40,970	42,740	13	2,414	20,464	22,891
City of General Santos	56,538	6	609	10,010	10,625	10	971	12,216	13,197
**BARMM**	**293,020**	**4**	**3,436**	**77,247**	**80,687**	**25**	**5,131**	**106,375**	**111,531**
Basilan	16,803	1	281	3,778	4,060	14	463	5,582	6,059
Lanao del Sur	36,361	0	162	7,570	7,732	3	290	7,959	8,252
Maguindanao	87,840	1	961	22,092	23,054	6	2,262	35,177	37,445
Sulu	81,975	1	1,337	23,086	24,424	0	1,083	35,723	36,806
Tawi-Tawi	20,067	0	224	5,622	5,846	2	223	5,894	6,119
SGU	7,817	0	107	1,863	1,970	0	157	3,228	3,385
City of Cotabato	42,157	1	364	13,236	13,601	0	653	12,812	13,465
**Caraga**	**292,511**	**13**	**2,603**	**110,338**	**112,954**	**16**	**1,870**	**35,291**	**37,177**
Agusan del Norte	40,148	0	358	14,602	14,960	4	305	5,347	5,656
Agusan del Sur	86,721	8	768	33,544	34,320	3	530	9,164	9,697
Surigao del Norte	53,159	0	399	19,611	20,010	0	219	8,130	8,349
Surigao del Sur	60,298	5	476	22,086	22,567	4	335	4,312	4,651
Dinagat Islands	9,606	0	48	3,531	3,579	0	49	1,179	1,228
City of Butuan	42,579	0	554	16,964	17,518	5	432	7,159	7,596

Table 2.A.4 - MODERN METHOD OF FAMILY PLANNING

Current User (Ending)

Philippines, 2023

Area	Total Current Users	IMPLANTS			Total	NFP-CCM			Total
		Age group				Age group			
		10-14	15-19	20-49		10-14	15-19	20-49	
**Philippines**	**8,683,596**	**1,012**	**60,660**	**728,548**	**790,220**	**173**	**876**	**42,491**	**43,540**
**N C R**	**1,041,531**	**286**	**12,442**	**107,545**	**120,273**	**0**	**2**	**632**	**634**
Malabon City	26,264	5	447	3,125	3,577	0	0	0	0
Navotas City	23,816	2	485	1,543	2,030	0	0	0	0
Valenzuela City	40,454	5	1,498	9,652	11,155	0	0	8	8
Caloocan City	73,676	13	1,098	11,131	12,242	0	0	0	0
Marikina City	24,847	11	443	3,983	4,437	0	2	14	16
Pasig City	58,937	16	568	6,637	7,221	0	0	11	11
Pateros	4,204	0	3	276	279	0	0	0	0
Taguig City	92,053	4	163	4,990	5,157	0	0	0	0
Quezon City	338,677	79	2,840	27,359	30,278	0	0	13	13
Makati City	28,572	0	70	2,334	2,404	0	0	0	0
Mandaluyong City	27,196	4	155	2,949	3,108	0	0	388	388
San Juan City	6,139	0	14	250	264	0	0	0	0
Manila City	138,102	129	3,313	21,495	24,937	0	0	1	1
Las Piñas City	26,980	14	732	4,678	5,424	0	0	0	0
Muntinlupa City	44,186	1	119	1,931	2,051	0	0	0	0
Parañaque City	47,100	3	370	2,634	3,007	0	0	0	0
Pasay City	40,328	0	124	2,578	2,702	0	0	197	197
**C A R**	**165,663**	**23**	**759**	**12,311**	**13,093**	**0**	**90**	**2,113**	**2,203**
Abra	19,755	2	96	1,277	1,375	0	1	114	115
Apayao	24,752	4	85	1,088	1,177	0	0	1	1
Benguet	37,253	0	79	1,923	2,002	0	0	6	6
Ifugao	21,120	3	72	1,441	1,516	0	8	775	783
Kalinga	18,267	0	88	1,600	1,688	0	81	1,070	1,151
Mountain Province	13,957	3	78	917	998	0	0	145	145
City of Baguio	30,559	11	261	4,065	4,337	0	0	2	2
**Region 1**	**498,204**	**4**	**1,023**	**19,464**	**20,491**	**0**	**27**	**2,255**	**2,282**
Ilocos Norte	52,824	3	51	1,000	1,054	0	8	434	442
Ilocos Sur	78,125	0	61	975	1,036	0	18	1,457	1,475
La Union	67,784	0	243	2,627	2,870	0	0	345	345
Pangasinan	292,353	1	651	14,366	15,018	0	1	19	20

Table 2.A.4 - MODERN METHOD OF FAMILY PLANNING
Current User (Ending)
Philippines, 2023

Area	Total Current Users	IMPLANTS			Total	NFP-CCM			Total
		Age group				Age group			
		10-14	15-19	20-49		10-14	15-19	20-49	
City of Dagupan	7,118	0	17	496	513	0	0	0	0
**Region 2**	**343,378**	**19**	**835**	**14,604**	**15,458**	**0**	**1**	**287**	**288**
Batanes	955	0	2	42	44	0	0	71	71
Cagayan	108,930	9	219	3,702	3,930	0	0	8	8
Isabela	146,131	9	445	6,867	7,321	0	1	5	6
Nueva Vizcaya	52,095	1	106	2,754	2,861	0	0	185	185
Quirino	21,240	0	35	691	726	0	0	18	18
City of Santiago	14,027	0	28	548	576	0	0	0	0
**Region 3**	**985,325**	**73**	**5,044**	**57,242**	**62,359**	**123**	**28**	**321**	**472**
Aurora	24,562	0	98	1,777	1,875	0	0	131	131
Bataan	65,716	13	939	5,710	6,662	0	0	35	35
Bulacan	302,904	9	769	18,940	19,718	0	0	12	12
Nueva Ecija	200,897	33	1,626	10,928	12,587	0	0	0	0
Pampanga	167,294	7	706	7,643	8,356	0	28	120	148
Tarlac	118,444	1	117	3,802	3,920	0	0	0	0
Zambales	54,326	2	304	3,985	4,291	0	0	23	23
City of Angeles	30,345	1	110	2,261	2,372	0	0	0	0
City of Olongapo	20,837	7	375	2,196	2,578	123	0	0	123
**Region 4A**	**888,692**	**101**	**3,507**	**57,847**	**61,455**	**27**	**28**	**1,598**	**1,653**
Batangas	158,569	9	160	3,454	3,623	0	0	0	0
Cavite	175,332	15	882	17,538	18,435	0	1	157	158
Laguna	286,016	63	1,259	21,053	22,375	27	27	530	584
Quezon	106,773	1	354	5,102	5,457	0	0	312	312
Rizal	156,554	13	848	10,535	11,396	0	0	599	599
City of Lucena	5,448	0	4	165	169	0	0	0	0
**Region 4B**	**313,665**	**79**	**1,886**	**30,136**	**32,101**	**0**	**113**	**5,966**	**6,079**
Marinduque	16,436	3	93	1,592	1,688	0	0	4	4
Occidental Mindoro	48,902	2	315	4,764	5,081	0	98	3,038	3,136
Oriental Mindoro	68,962	1	246	3,813	4,060	0	0	240	240
Palawan	122,174	68	960	15,274	16,302	0	6	667	673

Table 2.A.4 - MODERN METHOD OF FAMILY PLANNING
Current User (Ending)
Philippines, 2023

Area	Total Current Users	IMPLANTS			Total	NFP-CCM			Total
		Age group				Age group			
		10-14	15-19	20-49		10-14	15-19	20-49	
Romblon	22,467	1	88	1,762	1,851	0	9	2,011	2,020
City of Puerto Princesa	34,724	4	184	2,931	3,119	0	0	6	6
**Region 5**	**491,006**	**16**	**1,892**	**39,536**	**41,444**	**23**	**302**	**15,957**	**16,282**
Albay	115,867	7	274	6,925	7,206	0	5	1,422	1,427
Camarines Norte	46,839	0	221	3,063	3,284	0	1	326	327
Camarines Sur	107,464	4	679	15,267	15,950	0	16	1,022	1,038
Catanduanes	24,326	5	134	3,215	3,354	0	15	1,478	1,493
Masbate	96,672	0	357	5,492	5,849	23	223	6,996	7,242
Sorsogon	66,956	0	186	4,551	4,737	0	42	4,657	4,699
City of Naga	32,882	0	41	1,023	1,064	0	0	56	56
**Region 6**	**667,635**	**29**	**2,207**	**34,283**	**36,519**	**0**	**90**	**5,028**	**5,118**
Aklan	48,553	2	69	1,658	1,729	0	1	60	61
Antique	49,316	0	70	2,360	2,430	0	84	4,784	4,868
Capiz	77,942	1	97	2,449	2,547	0	1	61	62
Guimaras	17,943	0	5	358	363	0	0	2	2
Iloilo	182,130	2	544	10,473	11,019	0	2	64	66
Negros Occidental	206,889	11	989	12,256	13,256	0	2	27	29
City of Bacolod	30,421	13	340	2,346	2,699	0	0	29	29
City of Iloilo	54,441	0	93	2,383	2,476	0	0	1	1
**Region 7**	**483,438**	**37**	**2,595**	**46,926**	**49,558**	**0**	**5**	**514**	**519**
Bohol	83,324	10	737	9,881	10,628	0	0	19	19
Cebu	191,250	20	937	19,855	20,812	0	0	21	21
Negros Oriental	98,214	6	290	4,399	4,695	0	5	450	455
Siquijor	9,225	0	31	445	476	0	0	0	0
City of Cebu	34,897	0	340	5,605	5,945	0	0	24	24
City of Lapu-Lapu	36,794	1	95	1,642	1,738	0	0	0	0
City of Mandaue	29,734	0	165	5,099	5,264	0	0	0	0
**Region 8**	**340,613**	**9**	**1,654**	**34,049**	**35,712**	**0**	**17**	**1,890**	**1,907**
Biliran	12,714	0	148	1,637	1,785	0	0	105	105
Eastern Samar	39,982	0	159	3,041	3,200	0	0	239	239

Table 2.A.4 - MODERN METHOD OF FAMILY PLANNING
Current User (Ending)
Philippines, 2023

Area	Total Current Users	IMPLANTS			Total	NFP-CCM			Total
		Age group				Age group			
		10-14	15-19	20-49		10-14	15-19	20-49	
Leyte	125,631	5	422	11,776	12,203	0	7	149	156
Northern Samar	48,697	0	160	4,833	4,993	0	0	110	110
Southern Leyte	27,251	0	89	2,360	2,449	0	0	210	210
Samar	42,957	3	251	5,473	5,727	0	1	1,075	1,076
Ormoc City	20,171	0	141	2,524	2,665	0	9	2	11
City of Tacloban	23,210	1	284	2,405	2,690	0	0	0	0
**Region 9**	**354,758**	**61**	**5,631**	**58,543**	**64,235**	**0**	**0**	**67**	**67**
Zamboanga del Norte	126,081	12	1,351	16,677	18,040	0	0	60	60
Zamboanga del Sur	102,629	12	1,884	20,340	22,236	0	0	0	0
Zamboanga Sibugay	48,639	4	1,059	10,563	11,626	0	0	7	7
City of Isabela	7,167	15	252	1,615	1,882	0	0	0	0
City of Zamboanga	70,242	18	1,085	9,348	10,451	0	0	0	0
**Region 10**	**511,701**	**53**	**4,840**	**47,532**	**52,425**	**0**	**103**	**2,522**	**2,625**
Bukidnon	167,011	40	2,817	24,081	26,938	0	39	457	496
Camiguin	6,884	0	10	180	190	0	0	0	0
Lanao del Norte	73,898	3	297	5,131	5,431	0	0	28	28
Misamis Occidental	60,887	0	153	2,432	2,585	0	21	171	192
Misamis Oriental	111,011	8	754	8,198	8,960	0	43	1,635	1,678
City of Cagayan De Oro	57,799	1	671	5,422	6,094	0	0	0	0
City of Iligan	34,211	1	138	2,088	2,227	0	0	231	231
**Region 11**	**536,237**	**80**	**4,861**	**52,342**	**57,283**	**0**	**26**	**1,115**	**1,141**
Davao de Oro	83,871	11	480	6,798	7,289	0	3	117	120
Davao del Norte	112,914	6	989	11,215	12,210	0	1	64	65
Davao Oriental	58,642	5	273	4,421	4,699	0	7	379	386
Davao del Sur	69,093	11	554	5,124	5,689	0	0	160	160
Davao Occidental	33,219	3	489	3,754	4,246	0	0	0	0
City of Davao	178,498	44	2,076	21,030	23,150	0	15	395	410
**Region 12**	**476,219**	**62**	**5,639**	**52,900**	**58,601**	**0**	**6**	**771**	**777**
Cotabato	136,508	8	992	15,778	16,778	0	1	563	564
Sarangani	76,390	0	622	7,200	7,822	0	1	37	38

Table 2.A.4 - MODERN METHOD OF FAMILY PLANNING
Current User (Ending)
Philippines, 2023

Area	Total Current Users	IMPLANTS			Total	NFP-CCM			Total
		Age group				Age group			
		10-14	15-19	20-49		10-14	15-19	20-49	
South Cotabato	102,265	20	1,799	10,501	12,320	0	4	150	154
Sultan Kudarat	104,518	25	1,278	10,404	11,707	0	0	15	15
City of General Santos	56,538	9	948	9,017	9,974	0	0	6	6
**BARMM**	**293,020**	**31**	**2,511**	**28,919**	**31,461**	**0**	**0**	**59**	**59**
Basilan	16,803	8	488	3,016	3,512	0	0	3	3
Lanao del Sur	36,361	3	76	2,132	2,211	0	0	0	0
Maguindanao	87,840	8	962	7,529	8,499	0	0	0	0
Sulu	81,975	8	529	9,269	9,806	0	0	0	0
Tawi-Tawi	20,067	2	267	4,457	4,726	0	0	0	0
SGU	7,817	0	8	218	226	0	0	0	0
City of Cotabato	42,157	2	181	2,298	2,481	0	0	56	56
**Caraga**	**292,511**	**49**	**3,334**	**34,369**	**37,752**	**0**	**38**	**1,396**	**1,434**
Agusan del Norte	40,148	13	502	4,379	4,894	0	0	31	31
Agusan del Sur	86,721	23	1,209	10,892	12,124	0	37	1,145	1,182
Surigao del Norte	53,159	4	586	6,306	6,896	0	0	30	30
Surigao del Sur	60,298	7	504	7,024	7,535	0	1	176	177
Dinagat Islands	9,606	0	109	2,019	2,128	0	0	0	0
City of Butuan	42,579	2	424	3,749	4,175	0	0	14	14

Table 2.A.4. MODERN METHOD OF FAMILY PLANNING

Current User (Ending)

Philippines, 2023

Area	Total Current Users	NFP-BBT			Total	NFP-STM			Total
		Age group				Age group			
		10-14	15-19	20-49		10-14	15-19	20-49	
**Philippines**	**8,683,596**	**129**	**277**	**2,808**	**3,214**	**19**	**88**	**2,093**	**2,200**
**N C R**	**1,041,531**	**0**	**39**	**256**	**295**	**0**	**7**	**154**	**161**
Malabon City	26,264	0	0	0	0	0	0	0	0
Navotas City	23,816	0	0	0	0	0	0	0	0
Valenzuela City	40,454	0	9	79	88	0	0	1	1
Caloocan City	73,676	0	0	0	0	0	0	0	0
Marikina City	24,847	0	0	30	30	0	0	0	0
Pasig City	58,937	0	0	0	0	0	0	0	0
Pateros	4,204	0	0	0	0	0	0	0	0
Taguig City	92,053	0	0	0	0	0	0	0	0
Quezon City	338,677	0	0	0	0	0	0	0	0
Makati City	28,572	0	0	2	2	0	0	0	0
Mandaluyong City	27,196	0	0	1	1	0	0	138	138
San Juan City	6,139	0	0	0	0	0	0	0	0
Manila City	138,102	0	30	12	42	0	0	9	9
Las Piñas City	26,980	0	0	0	0	0	0	0	0
Muntinlupa City	44,186	0	0	0	0	0	0	0	0
Parañaque City	47,100	0	0	3	3	0	7	0	7
Pasay City	40,328	0	0	129	129	0	0	6	6
**C A R**	**165,663**	**0**	**2**	**105**	**107**	**0**	**1**	**69**	**70**
Abra	19,755	0	0	36	36	0	1	39	40
Apayao	24,752	0	0	0	0	0	0	0	0
Benguet	37,253	0	0	0	0	0	0	0	0
Ifugao	21,120	0	0	0	0	0	0	9	9
Kalinga	18,267	0	2	53	55	0	0	19	19
Mountain Province	13,957	0	0	5	5	0	0	0	0
City of Baguio	30,559	0	0	11	11	0	0	2	2
**Region 1**	**498,204**	**1**	**4**	**214**	**219**	**0**	**9**	**173**	**182**
Ilocos Norte	52,824	0	1	22	23	0	0	64	64
Ilocos Sur	78,125	0	2	160	162	0	9	100	109
La Union	67,784	0	0	14	14	0	0	4	4
Pangasinan	292,353	1	1	16	18	0	0	5	5

Table 2.A.4. MODERN METHOD OF FAMILY PLANNING
Current User (Ending)
Philippines, 2023

Area	Total Current Users	NFP-BBT			Total	NFP-STM			Total
		Age group				Age group			
		10-14	15-19	20-49		10-14	15-19	20-49	
City of Dagupan	7,118	0	0	2	2	0	0	0	0
**Region 2**	**343,378**	**0**	**12**	**65**	**77**	**1**	**4**	**77**	**82**
Batanes	955	0	0	0	0	0	0	0	0
Cagayan	108,930	0	3	16	19	0	0	6	6
Isabela	146,131	0	9	41	50	0	0	3	3
Nueva Vizcaya	52,095	0	0	0	0	1	4	68	73
Quirino	21,240	0	0	8	8	0	0	0	0
City of Santiago	14,027	0	0	0	0	0	0	0	0
**Region 3**	**985,325**	**0**	**2**	**165**	**167**	**0**	**9**	**11**	**20**
Aurora	24,562	0	0	5	5	0	0	0	0
Bataan	65,716	0	2	19	21	0	0	0	0
Bulacan	302,904	0	0	4	4	0	0	0	0
Nueva Ecija	200,897	0	0	0	0	0	0	0	0
Pampanga	167,294	0	0	10	10	0	9	5	14
Tarlac	118,444	0	0	0	0	0	0	1	1
Zambales	54,326	0	0	0	0	0	0	5	5
City of Angeles	30,345	0	0	0	0	0	0	0	0
City of Olongapo	20,837	0	0	127	127	0	0	0	0
**Region 4A**	**888,692**	**60**	**14**	**292**	**366**	**18**	**2**	**360**	**380**
Batangas	158,569	33	0	35	68	0	0	4	4
Cavite	175,332	0	1	8	9	0	2	1	3
Laguna	286,016	27	5	67	99	18	0	13	31
Quezon	106,773	0	5	131	136	0	0	14	14
Rizal	156,554	0	3	51	54	0	0	328	328
City of Lucena	5,448	0	0	0	0	0	0	0	0
**Region 4B**	**313,665**	**0**	**3**	**80**	**83**	**0**	**0**	**31**	**31**
Marinduque	16,436	0	0	2	2	0	0	0	0
Occidental Mindoro	48,902	0	0	0	0	0	0	12	12
Oriental Mindoro	68,962	0	3	48	51	0	0	7	7
Palawan	122,174	0	0	25	25	0	0	11	11

Table 2.A.4. MODERN METHOD OF FAMILY PLANNING
Current User (Ending)
Philippines, 2023

Area	Total Current Users	NFP-BBT			Total	NFP-STM			Total
		Age group				Age group			
		10-14	15-19	20-49		10-14	15-19	20-49	
Romblon	22,467	0	0	5	5	0	0	1	1
City of Puerto Princesa	34,724	0	0	0	0	0	0	0	0
**Region 5**	**491,006**	**1**	**20**	**382**	**403**	**0**	**35**	**574**	**609**
Albay	115,867	0	4	72	76	0	1	45	46
Camarines Norte	46,839	0	1	21	22	0	29	113	142
Camarines Sur	107,464	0	0	170	170	0	3	281	284
Catanduanes	24,326	1	12	53	66	0	0	50	50
Masbate	96,672	0	3	27	30	0	2	22	24
Sorsogon	66,956	0	0	1	1	0	0	35	35
City of Naga	32,882	0	0	38	38	0	0	28	28
**Region 6**	**667,635**	**60**	**16**	**155**	**231**	**0**	**3**	**64**	**67**
Aklan	48,553	0	0	9	9	0	3	25	28
Antique	49,316	0	0	5	5	0	0	6	6
Capiz	77,942	0	1	10	11	0	0	0	0
Guimaras	17,943	0	0	7	7	0	0	0	0
Iloilo	182,130	0	2	29	31	0	0	32	32
Negros Occidental	206,889	0	5	54	59	0	0	1	1
City of Bacolod	30,421	60	8	34	102	0	0	0	0
City of Iloilo	54,441	0	0	7	7	0	0	0	0
**Region 7**	**483,438**	**0**	**45**	**189**	**234**	**0**	**2**	**21**	**23**
Bohol	83,324	0	0	0	0	0	0	0	0
Cebu	191,250	0	44	177	221	0	0	0	0
Negros Oriental	98,214	0	1	10	11	0	2	21	23
Siquijor	9,225	0	0	0	0	0	0	0	0
City of Cebu	34,897	0	0	2	2	0	0	0	0
City of Lapu-Lapu	36,794	0	0	0	0	0	0	0	0
City of Mandaue	29,734	0	0	0	0	0	0	0	0
**Region 8**	**340,613**	**0**	**6**	**155**	**161**	**0**	**0**	**59**	**59**
Biliran	12,714	0	1	5	6	0	0	0	0
Eastern Samar	39,982	0	0	23	23	0	0	14	14

Table 2.A.4. MODERN METHOD OF FAMILY PLANNING

Current User (Ending)

Philippines, 2023

Area	Total Current Users	NFP-BBT			Total	NFP-STM			Total
		Age group				Age group			
		10-14	15-19	20-49		10-14	15-19	20-49	
Leyte	125,631	0	2	91	93	0	0	40	40
Northern Samar	48,697	0	1	11	12	0	0	4	4
Southern Leyte	27,251	0	0	24	24	0	0	1	1
Samar	42,957	0	2	0	2	0	0	0	0
Ormoc City	20,171	0	0	1	1	0	0	0	0
City of Tacloban	23,210	0	0	0	0	0	0	0	0
**Region 9**	**354,758**	**0**	**0**	**8**	**8**	**0**	**0**	**30**	**30**
Zamboanga del Norte	126,081	0	0	8	8	0	0	12	12
Zamboanga del Sur	102,629	0	0	0	0	0	0	2	2
Zamboanga Sibugay	48,639	0	0	0	0	0	0	16	16
City of Isabela	7,167	0	0	0	0	0	0	0	0
City of Zamboanga	70,242	0	0	0	0	0	0	0	0
**Region 10**	**511,701**	**5**	**36**	**291**	**332**	**0**	**8**	**305**	**313**
Bukidnon	167,011	0	10	65	75	0	0	90	90
Camiguin	6,884	0	0	10	10	0	0	1	1
Lanao del Norte	73,898	5	1	28	34	0	4	57	61
Misamis Occidental	60,887	0	3	20	23	0	0	29	29
Misamis Oriental	111,011	0	21	141	162	0	4	126	130
City of Cagayan De Oro	57,799	0	0	10	10	0	0	0	0
City of Iligan	34,211	0	1	17	18	0	0	2	2
**Region 11**	**536,237**	**0**	**28**	**235**	**263**	**0**	**1**	**99**	**100**
Davao de Oro	83,871	0	3	16	19	0	0	0	0
Davao del Norte	112,914	0	8	10	18	0	0	22	22
Davao Oriental	58,642	0	0	29	29	0	0	43	43
Davao del Sur	69,093	0	4	100	104	0	0	16	16
Davao Occidental	33,219	0	10	0	10	0	0	0	0
City of Davao	178,498	0	3	80	83	0	1	18	19
**Region 12**	**476,219**	**2**	**36**	**109**	**147**	**0**	**5**	**53**	**58**
Cotabato	136,508	0	0	34	34	0	0	0	0
Sarangani	76,390	0	5	14	19	0	0	10	10

Table 2.A.4. MODERN METHOD OF FAMILY PLANNING
Current User (Ending)
Philippines, 2023

Area	Total Current Users	NFP-BBT			Total	NFP-STM			Total
		Age group				Age group			
		10-14	15-19	20-49		10-14	15-19	20-49	
South Cotabato	102,265	2	1	29	32	0	0	27	27
Sultan Kudarat	104,518	0	0	13	13	0	0	0	0
City of General Santos	56,538	0	30	19	49	0	5	16	21
**BARMM**	**293,020**	**0**	**4**	**13**	**17**	**0**	**0**	**3**	**3**
Basilan	16,803	0	0	0	0	0	0	0	0
Lanao del Sur	36,361	0	0	1	1	0	0	1	1
Maguindanao	87,840	0	0	0	0	0	0	0	0
Sulu	81,975	0	0	0	0	0	0	2	2
Tawi-Tawi	20,067	0	0	0	0	0	0	0	0
SGU	7,817	0	0	0	0	0	0	0	0
City of Cotabato	42,157	0	4	12	16	0	0	0	0
**Caraga**	**292,511**	**0**	**10**	**94**	**104**	**0**	**2**	**10**	**12**
Agusan del Norte	40,148	0	0	3	3	0	0	0	0
Agusan del Sur	86,721	0	0	0	0	0	0	0	0
Surigao del Norte	53,159	0	3	49	52	0	1	5	6
Surigao del Sur	60,298	0	4	39	43	0	1	5	6
Dinagat Islands	9,606	0	0	1	1	0	0	0	0
City of Butuan	42,579	0	3	2	5	0	0	0	0

Table 2.A.4 - MODERN METHOD OF FAMILY PLANNING
Current User (Ending)
Philippines, 2023

Area	Total Current Users	NFP-SDM			Total	NFP-LAM			Total
		Age group				Age group			
		10-14	15-19	20-49		10-14	15-19	20-49	
**Philippines**	**8,683,596**	**172**	**3,891**	**116,602**	**120,665**	**1,663**	**66,851**	**570,074**	**638,588**
**N C R**	**1,041,531**	**87**	**33**	**1,898**	**2,018**	**304**	**10,032**	**96,030**	**106,366**
Malabon City	26,264	0	0	1	1	0	145	1,191	1,336
Navotas City	23,816	0	0	0	0	5	110	1,828	1,943
Valenzuela City	40,454	86	1	2	89	59	146	2,419	2,624
Caloocan City	73,676	0	7	76	83	1	798	5,811	6,610
Marikina City	24,847	0	0	3	3	4	246	1,676	1,926
Pasig City	58,937	0	0	38	38	18	638	7,162	7,818
Pateros	4,204	0	0	0	0	0	1	211	212
Taguig City	92,053	1	0	10	11	28	2,230	23,562	25,820
Quezon City	338,677	0	18	1,558	1,576	24	1,497	12,782	14,303
Makati City	28,572	0	0	0	0	0	108	3,671	3,779
Mandaluyong City	27,196	0	0	96	96	5	157	4,683	4,845
San Juan City	6,139	0	0	0	0	0	16	279	295
Manila City	138,102	0	2	15	17	135	904	9,468	10,507
Las Piñas City	26,980	0	0	19	19	5	127	1,957	2,089
Muntinlupa City	44,186	0	0	0	0	10	1,568	8,635	10,213
Parañaque City	47,100	0	5	73	78	2	890	9,266	10,158
Pasay City	40,328	0	0	7	7	8	451	1,429	1,888
**C A R**	**165,663**	**2**	**36**	**6,868**	**6,906**	**27**	**1,075**	**8,179**	**9,281**
Abra	19,755	0	3	293	296	8	233	1,283	1,524
Apayao	24,752	0	0	8	8	5	140	1,010	1,155
Benguet	37,253	0	3	2,456	2,459	1	150	1,783	1,934
Ifugao	21,120	2	26	2,651	2,679	7	230	1,747	1,984
Kalinga	18,267	0	2	450	452	2	215	1,091	1,308
Mountain Province	13,957	0	2	956	958	4	66	778	848
City of Baguio	30,559	0	0	54	54	0	41	487	528
**Region 1**	**498,204**	**0**	**124**	**6,521**	**6,645**	**53**	**4,030**	**39,427**	**43,510**
Ilocos Norte	52,824	0	0	1,225	1,225	1	225	5,151	5,377
Ilocos Sur	78,125	0	65	3,694	3,759	7	556	8,538	9,101
La Union	67,784	0	17	974	991	6	409	3,602	4,017
Pangasinan	292,353	0	42	597	639	38	2,764	21,552	24,354

Table 2.A.4 - MODERN METHOD OF FAMILY PLANNING

Current User (Ending)

Philippines, 2023

Area	Total Current Users	NFP-SDM			Total	NFP-LAM			Total
		Age group				Age group			
		10-14	15-19	20-49		10-14	15-19	20-49	
City of Dagupan	7,118	0	0	31	31	1	76	584	661
**Region 2**	**343,378**	**0**	**3**	**687**	**690**	**116**	**2,523**	**17,388**	**20,027**
Batanes	955	0	0	15	15	0	12	135	147
Cagayan	108,930	0	1	158	159	21	741	3,944	4,706
Isabela	146,131	0	0	215	215	25	700	5,965	6,690
Nueva Vizcaya	52,095	0	2	273	275	14	472	4,430	4,916
Quirino	21,240	0	0	0	0	2	217	1,004	1,223
City of Santiago	14,027	0	0	26	26	54	381	1,910	2,345
**Region 3**	**985,325**	**6**	**21**	**4,186**	**4,213**	**63**	**4,242**	**43,445**	**47,750**
Aurora	24,562	0	0	13	13	2	220	1,424	1,646
Bataan	65,716	0	1	120	121	13	607	4,584	5,204
Bulacan	302,904	0	12	3,137	3,149	3	692	9,650	10,345
Nueva Ecija	200,897	0	2	38	40	13	972	5,746	6,731
Pampanga	167,294	0	0	728	728	5	604	8,980	9,589
Tarlac	118,444	0	2	8	10	2	535	7,335	7,872
Zambales	54,326	0	2	48	50	10	367	2,800	3,177
City of Angeles	30,345	6	2	83	91	13	153	1,373	1,539
City of Olongapo	20,837	0	0	11	11	2	92	1,553	1,647
**Region 4A**	**888,692**	**20**	**1,498**	**22,059**	**23,577**	**205**	**5,936**	**53,872**	**60,013**
Batangas	158,569	0	289	18,788	19,077	87	216	4,420	4,723
Cavite	175,332	0	23	424	447	26	870	10,893	11,789
Laguna	286,016	10	63	1,975	2,048	49	2,954	20,259	23,262
Quezon	106,773	10	1,123	794	1,927	16	659	5,241	5,916
Rizal	156,554	0	0	78	78	27	1,206	13,038	14,271
City of Lucena	5,448	0	0	0	0	0	31	21	52
**Region 4B**	**313,665**	**0**	**11**	**2,143**	**2,154**	**88**	**2,844**	**20,274**	**23,206**
Marinduque	16,436	0	0	40	40	1	80	1,343	1,424
Occidental Mindoro	48,902	0	5	575	580	15	869	5,795	6,679
Oriental Mindoro	68,962	0	0	58	58	7	465	4,266	4,738
Palawan	122,174	0	4	596	600	54	1,037	6,395	7,486

Table 2.A.4 - MODERN METHOD OF FAMILY PLANNING

Current User (Ending)

Philippines, 2023

Area	Total Current Users	NFP-SDM			Total	NFP-LAM			Total
		Age group				Age group			
		10-14	15-19	20-49		10-14	15-19	20-49	
Romblon	22,467	0	2	702	704	6	232	1,632	1,870
City of Puerto Princesa	34,724	0	0	172	172	5	161	843	1,009
**Region 5**	**491,006**	**52**	**1,109**	**34,864**	**36,025**	**59**	**4,175**	**39,042**	**43,276**
Albay	115,867	0	23	6,162	6,185	14	452	6,993	7,459
Camarines Norte	46,839	0	56	1,913	1,969	13	609	4,386	5,008
Camarines Sur	107,464	0	46	7,604	7,650	4	994	8,334	9,332
Catanduanes	24,326	0	5	3,038	3,043	5	234	2,972	3,211
Masbate	96,672	52	940	9,890	10,882	16	1,215	6,999	8,230
Sorsogon	66,956	0	36	4,851	4,887	7	625	5,714	6,346
City of Naga	32,882	0	3	1,406	1,409	0	46	3,644	3,690
**Region 6**	**667,635**	**2**	**107**	**9,178**	**9,287**	**72**	**4,287**	**43,905**	**48,264**
Aklan	48,553	0	2	297	299	8	416	4,341	4,765
Antique	49,316	0	0	389	389	2	203	2,828	3,033
Capiz	77,942	0	0	3	3	1	221	2,911	3,133
Guimaras	17,943	0	0	41	41	1	111	1,082	1,194
Iloilo	182,130	2	30	4,414	4,446	31	1,273	13,724	15,028
Negros Occidental	206,889	0	74	3,827	3,901	27	1,939	14,352	16,318
City of Bacolod	30,421	0	0	41	41	2	114	4,541	4,657
City of Iloilo	54,441	0	1	166	167	0	10	126	136
**Region 7**	**483,438**	**0**	**2**	**1,549**	**1,551**	**81**	**4,724**	**34,527**	**39,332**
Bohol	83,324	0	0	536	536	4	270	2,292	2,566
Cebu	191,250	0	1	400	401	37	2,630	14,197	16,864
Negros Oriental	98,214	0	1	162	163	35	909	7,992	8,936
Siquijor	9,225	0	0	449	449	0	60	225	285
City of Cebu	34,897	0	0	2	2	5	465	4,232	4,702
City of Lapu-Lapu	36,794	0	0	0	0	0	390	5,093	5,483
City of Mandaue	29,734	0	0	0	0	0	0	496	496
**Region 8**	**340,613**	**0**	**167**	**3,402**	**3,569**	**36**	**3,201**	**30,657**	**33,894**
Biliran	12,714	0	0	231	231	1	86	867	954
Eastern Samar	39,982	0	135	1,884	2,019	5	326	2,641	2,972

Table 2.A.4 - MODERN METHOD OF FAMILY PLANNING
Current User (Ending)
Philippines, 2023

Area	Total Current Users	NFP-SDM			Total	NFP-LAM			Total
		Age group				Age group			
		10-14	15-19	20-49		10-14	15-19	20-49	
Leyte	125,631	0	14	342	356	0	1,152	11,302	12,454
Northern Samar	48,697	0	9	376	385	0	434	7,497	7,931
Southern Leyte	27,251	0	8	217	225	0	250	1,942	2,192
Samar	42,957	0	0	0	0	28	553	2,800	3,381
Ormoc City	20,171	0	1	352	353	0	186	1,359	1,545
City of Tacloban	23,210	0	0	0	0	2	214	2,249	2,465
**Region 9**	**354,758**	**0**	**60**	**2,354**	**2,414**	**40**	**3,146**	**21,707**	**24,893**
Zamboanga del Norte	126,081	0	60	2,051	2,111	20	1,067	7,695	8,782
Zamboanga del Sur	102,629	0	0	6	6	4	386	3,059	3,449
Zamboanga Sibugay	48,639	0	0	268	268	2	393	2,365	2,760
City of Isabela	7,167	0	0	29	29	1	175	879	1,055
City of Zamboanga	70,242	0	0	0	0	13	1,125	7,709	8,847
**Region 10**	**511,701**	**1**	**590**	**13,477**	**14,068**	**143**	**5,290**	**34,486**	**39,919**
Bukidnon	167,011	0	117	5,382	5,499	69	1,907	9,143	11,119
Camiguin	6,884	0	0	74	74	0	111	856	967
Lanao del Norte	73,898	0	0	223	223	17	633	5,879	6,529
Misamis Occidental	60,887	0	395	3,328	3,723	15	617	5,645	6,277
Misamis Oriental	111,011	1	58	3,650	3,709	17	1,023	5,756	6,796
City of Cagayan De Oro	57,799	0	0	63	63	18	622	4,727	5,367
City of Iligan	34,211	0	20	757	777	7	377	2,480	2,864
**Region 11**	**536,237**	**1**	**57**	**2,372**	**2,430**	**187**	**2,696**	**24,413**	**27,296**
Davao de Oro	83,871	1	0	41	42	3	79	989	1,071
Davao del Norte	112,914	0	4	254	258	7	373	2,686	3,066
Davao Oriental	58,642	0	9	1,563	1,572	22	547	3,328	3,897
Davao del Sur	69,093	0	32	82	114	13	126	739	878
Davao Occidental	33,219	0	8	225	233	8	191	410	609
City of Davao	178,498	0	4	207	211	134	1,380	16,261	17,775
**Region 12**	**476,219**	**0**	**7**	**618**	**625**	**106**	**3,770**	**20,455**	**24,331**
Cotabato	136,508	0	0	101	101	8	391	2,879	3,278
Sarangani	76,390	0	1	137	138	0	1,254	5,467	6,721

Table 2.A.4 - MODERN METHOD OF FAMILY PLANNING

Current User (Ending)

Philippines, 2023

Area	Total Current Users	NFP-SDM			Total	NFP-LAM			Total
		Age group				Age group			
		10-14	15-19	20-49		10-14	15-19	20-49	
South Cotabato	102,265	0	6	278	284	15	758	4,166	4,939
Sultan Kudarat	104,518	0	0	94	94	30	863	3,595	4,488
City of General Santos	56,538	0	0	8	8	53	504	4,348	4,905
**BARMM**	**293,020**	**0**	**1**	**223**	**224**	**39**	**2,736**	**26,377**	**29,152**
Basilan	16,803	0	0	20	20	14	152	757	923
Lanao del Sur	36,361	0	1	77	78	2	482	7,224	7,708
Maguindanao	87,840	0	0	0	0	3	1,043	8,210	9,256
Sulu	81,975	0	0	18	18	14	436	4,062	4,512
Tawi-Tawi	20,067	0	0	43	43	1	187	1,826	2,014
SGU	7,817	0	0	0	0	0	85	556	641
City of Cotabato	42,157	0	0	65	65	5	351	3,742	4,098
**Caraga**	**292,511**	**1**	**65**	**4,203**	**4,269**	**44**	**2,144**	**15,890**	**18,078**
Agusan del Norte	40,148	0	3	195	198	3	217	1,259	1,479
Agusan del Sur	86,721	0	23	992	1,015	16	558	3,918	4,492
Surigao del Norte	53,159	1	23	1,785	1,809	0	257	2,450	2,707
Surigao del Sur	60,298	0	10	813	823	19	667	5,161	5,847
Dinagat Islands	9,606	0	4	301	305	0	56	338	394
City of Butuan	42,579	0	2	117	119	6	389	2,764	3,159

Table 2.A.5 - MODERN FAMILY PLANNING
WRA 15-49 years old who have demand for FP and currently using, or whose partner is currently using any modern FP methods
Philippines, 2023

Area	Elig. Pop (TPx11.6%)	FP Current User Ending (15-49 y.o.)	%
**Philippines**	**12,950,068**	**8,677,668**	**67.01**
**N C R**	**1,601,070**	**1,040,250**	**64.97**
Malabon City	**45,219**	26,255	**58.06**
Navotas City	**29,554**	23,783	**80.47**
Valenzuela City	**84,954**	40,262	**47.39**
Caloocan City	**198,172**	73,644	**37.16**
Marikina City	**53,936**	24,796	**45.97**
Pasig City	**95,744**	58,857	**61.47**
Pateros	**7,688**	4,204	**54.68**
Taguig City	**106,403**	91,953	**86.42**
Quezon City	**351,155**	338,413	**96.37**
Makati City	**73,993**	28,569	**38.61**
Mandaluyong City	**50,457**	27,179	**53.87**
San Juan City	**14,784**	6,138	**41.52**
Manila City	**219,163**	137,681	**62.82**
Las Piñas City	**71,788**	26,954	**37.55**
Muntinlupa City	**64,225**	44,152	**68.75**
Parañaque City	**81,769**	47,090	**57.59**
Pasay City	**52,066**	40,320	**77.44**
**C A R**	**214,794**	**165,546**	**77.07**
Abra	**29,413**	19,724	**67.06**
Apayao	**14,867**	24,738	**166.40**
Benguet	**54,982**	37,251	**67.75**
Ifugao	**25,108**	21,098	**84.03**
Kalinga	**27,720**	18,262	**65.88**
Mountain Province	**18,905**	13,948	**73.78**
City of Baguio	**43,799**	30,525	**69.69**
**Region 1**	**625,265**	**498,112**	**79.66**
Ilocos Norte	**71,552**	52,817	**73.82**
Ilocos Sur	**82,757**	78,117	**94.39**

Table 2.A.5 - MODERN FAMILY PLANNING
WRA 15-49 years old who have demand for FP and currently using, or whose partner is currently using any modern FP methods
Philippines, 2023

Area	Elig. Pop (TPx11.6%)	FP Current User Ending (15-49 y.o.)	%
La Union	**96,917**	67,771	**69.93**
Pangasinan	**353,315**	292,290	**82.73**
City of Dagupan	**20,724**	7,117	**34.34**
**Region 2**	**437,033**	**343,128**	**78.51**
Batanes	**2,216**	954	**43.05**
Cagayan	**149,609**	108,856	**72.76**
Isabela	**183,281**	146,069	**79.70**
Nueva Vizcaya	**59,648**	52,071	**87.30**
Quirino	**24,493**	21,206	**86.58**
City of Santiago	**17,786**	13,972	**78.56**
**Region 3**	**1,473,863**	**984,838**	**66.82**
Aurora	**28,208**	24,558	**87.06**
Bataan	**101,617**	65,663	**64.62**
Bulacan	**439,510**	302,869	**68.91**
Nueva Ecija	**272,967**	200,757	**73.55**
Pampanga	**290,991**	167,258	**57.48**
Tarlac	**177,719**	118,416	**66.63**
Zambales	**77,123**	54,296	**70.40**
City of Angeles	**55,074**	30,321	**55.06**
City of Olongapo	**30,654**	20,700	**67.53**
**Region 4A**	**1,923,189**	**887,882**	**46.17**
Batangas	**344,414**	158,341	**45.97**
Cavite	**516,321**	175,212	**33.93**
Laguna	**401,219**	285,690	**71.21**
Quezon	**231,536**	106,723	**46.09**
Rizal	**396,522**	156,470	**39.46**
City of Lucena	**33,177**	5,446	**16.41**
**Region 4B**	**383,240**	**313,342**	**81.76**
Marinduque	**28,124**	16,431	**58.42**

Table 2.A.5 - MODERN FAMILY PLANNING
WRA 15-49 years old who have demand for FP and currently using, or whose partner is currently using any modern FP methods
Philippines, 2023

Area	Elig. Pop (TPx11.6%)	FP Current User Ending (15-49 y.o.)	%
Occidental Mindoro	**62,586**	48,883	**78.11**
Oriental Mindoro	**107,492**	68,946	**64.14**
Palawan	**112,023**	121,931	**108.84**
Romblon	**36,491**	22,451	**61.52**
City of Puerto Princesa	**36,524**	34,700	**95.01**
**Region 5**	**726,847**	**490,831**	**67.53**
Albay	**164,227**	115,843	**70.54**
Camarines Norte	**75,824**	46,824	**61.75**
Camarines Sur	**222,271**	107,454	**48.34**
Catanduanes	**32,439**	24,300	**74.91**
Masbate	**108,138**	96,580	**89.31**
Sorsogon	**98,924**	66,948	**67.68**
City of Naga	**25,024**	32,882	**131.40**
**Region 6**	**933,549**	**667,254**	**71.47**
Aklan	**72,691**	48,532	**66.76**
Antique	**71,674**	49,313	**68.80**
Capiz	**94,868**	77,938	**82.15**
Guimaras	**22,205**	17,942	**80.80**
Iloilo	**241,402**	182,061	**75.42**
Negros Occidental	**307,719**	206,830	**67.21**
City of Bacolod	**69,422**	30,197	**43.50**
City of Iloilo	**53,568**	54,441	**101.63**
**Region 7**	**963,385**	**483,253**	**50.16**
Bohol	**164,892**	83,308	**50.52**
Cebu	**396,733**	191,160	**48.18**
Negros Oriental	**170,653**	98,152	**57.52**
Siquijor	**12,072**	9,225	**76.42**
City of Cebu	**115,189**	34,887	**30.29**
City of Lapu-Lapu	**60,063**	36,791	**61.25**
City of Mandaue	**43,783**	29,730	**67.90**

Table 2.A.5 - MODERN FAMILY PLANNING
WRA 15-49 years old who have demand for FP and currently using, or whose partner is currently using any modern FP methods
Philippines, 2023

Area	Elig. Pop (TPx11.6%)	FP Current User Ending (15-49 y.o.)	%
**Region 8**	**539,553**	**340,540**	**63.12**
Biliran	**21,316**	12,712	**59.64**
Eastern Samar	**56,655**	39,975	**70.56**
Leyte	**183,575**	125,620	**68.43**
Northern Samar	**75,665**	48,691	**64.35**
Southern Leyte	**50,294**	27,251	**54.18**
Samar	**94,388**	42,913	**45.46**
Ormoc City	**27,614**	20,171	**73.05**
City of Tacloban	**30,046**	23,207	**77.24**
**Region 9**	**461,042**	**354,575**	**76.91**
Zamboanga del Norte	**124,574**	126,031	**101.17**
Zamboanga del Sur	**125,309**	102,581	**81.86**
Zamboanga Sibugay	**79,643**	48,623	**61.05**
City of Isabela	**15,294**	7,151	**46.76**
City of Zamboanga	**116,222**	70,189	**60.39**
**Region 10**	**602,303**	**511,350**	**84.90**
Bukidnon	**186,311**	166,876	**89.57**
Camiguin	**10,956**	6,884	**62.83**
Lanao del Norte	**86,249**	73,862	**85.64**
Misamis Occidental	**73,411**	60,852	**82.89**
Misamis Oriental	**114,543**	110,927	**96.84**
City of Cagayan De Oro	**87,685**	57,762	**65.87**
City of Iligan	**43,148**	34,187	**79.23**
**Region 11**	**624,165**	**535,603**	**85.81**
Davao de Oro	**91,901**	83,828	**91.22**
Davao del Norte	**134,083**	112,873	**84.18**
Davao Oriental	**68,613**	58,611	**85.42**
Davao del Sur	**80,919**	68,935	**85.19**
Davao Occidental	**36,978**	33,184	**89.74**
City of Davao	**211,671**	178,172	**84.17**

Table 2.A.5 - MODERN FAMILY PLANNING
WRA 15-49 years old who have demand for FP and currently using, or whose partner is currently using any modern FP methods
Philippines, 2023

Area	Elig. Pop (TPx11.6%)	FP Current User Ending (15-49 y.o.)	%
**Region 12**	**522,884**	**475,905**	**91.02**
Cotabato	**152,838**	136,445	**89.27**
Sarangani	**67,264**	76,390	**113.57**
South Cotabato	**116,696**	102,187	**87.57**
Sultan Kudarat	**102,201**	104,440	**102.19**
City of General Santos	**83,885**	56,443	**67.29**
**BARMM**	**584,624**	**292,895**	**50.10**
Basilan	**50,016**	16,765	**33.52**
Lanao del Sur	**140,400**	36,350	**25.89**
Maguindanao	**161,361**	87,821	**54.43**
Sulu	**116,713**	81,934	**70.20**
Tawi-Tawi	**51,361**	20,062	**39.06**
SGU	**25,935**	7,817	**30.14**
City of Cotabato	**38,838**	42,146	**108.52**
**Caraga**	**333,262**	**292,364**	**87.73**
Agusan del Norte	**45,945**	40,125	**87.33**
Agusan del Sur	**88,094**	86,662	**98.37**
Surigao del Norte	**63,300**	53,153	**83.97**
Surigao del Sur	**76,699**	60,252	**78.56**
Dinagat Islands	**14,979**	9,606	**64.13**
City of Butuan	**44,245**	42,566	**96.21**

Table 2.A.6 - MODERN FAMILY PLANNING

WRA 15-49 yrs old with unmet needs
Philippines, 2023

Area	Estimated No. of WRA	Total No. of WRA (15-49 yrs old) with Unmet Needs	%
**Philippines**	**28,578,271**	**851,531**	**2.98**
**N C R**	**3,815,363**	**43,319**	**1.14**
Malabon City	**104,657**	2,019	**1.93**
Navotas City	**66,273**	1,563	**2.36**
Valenzuela City	**202,096**	1,715	**0.85**
Caloocan City	**460,510**	384	**0.08**
Marikina City	**124,737**	643	**0.52**
Pasig City	**229,465**	5,294	**2.31**
Pateros	**18,029**	0	**0.00**
Taguig City	**258,807**	6,251	**2.42**
Quezon City	**829,993**	7,431	**0.90**
Makati City	**184,254**	3,393	**1.84**
Mandaluyong City	**126,003**	2,096	**1.66**
San Juan City	**35,847**	62	**0.17**
Manila City	**520,580**	1,901	**0.37**
Las Piñas City	**172,892**	1,454	**0.84**
Muntinlupa City	**149,680**	-	**0.00**
Parañaque City	**196,455**	6,870	**3.50**
Pasay City	**135,085**	2,243	**1.66**
**C A R**	**480,029**	**45,728**	**9.53**
Abra	**62,404**	38,194	**61.20**
Apayao	**32,017**	80	**0.25**
Benguet	**122,502**	0	**0.00**
Ifugao	**53,447**	0	**0.00**
Kalinga	**60,028**	88	**0.15**
Mountain Province	**39,695**	20	**0.05**
City of Baguio	**109,936**	7,346	**6.68**
**Region 1**	**1,348,654**	**14,482**	**1.07**
Ilocos Norte	**156,771**	4,587	**2.93**
Ilocos Sur	**179,151**	8,158	**4.55**
La Union	**212,455**	1,737	**0.82**

Table 2.A.6 - MODERN FAMILY PLANNING

WRA 15-49 yrs old with unmet needs
Philippines, 2023

Area	Estimated No. of WRA	Total No. of WRA (15-49 yrs old) with Unmet Needs	%
Pangasinan	**754,466**	0	**0.00**
City of Dagupan	**45,811**	0	**0.00**
**Region 2**	**953,159**	**1,572**	**0.16**
Batanes	**4,685**	0	**0.00**
Cagayan	**326,251**	0	**0.00**
Isabela	**401,596**	1,548	**0.39**
Nueva Vizcaya	**127,721**	0	**0.00**
Quirino	**52,798**	24	**0.05**
City of Santiago	**40,108**	0	**0.00**
**Region 3**	**3,296,753**	**84,743**	**2.57**
Aurora	**60,340**	637	**1.06**
Bataan	**225,586**	10,849	**4.81**
Bulacan	**999,936**	30,152	**3.02**
Nueva Ecija	**597,132**	19,403	**3.25**
Pampanga	**659,415**	12,357	**1.87**
Tarlac	**382,292**	361	**0.09**
Zambales	**166,587**	10,809	**6.49**
City of Angeles	**132,844**	0	**0.00**
City of Olongapo	**72,621**	175	**0.24**
**Region 4A**	**4,448,928**	**135,399**	**3.04**
Batangas	**792,382**	26,807	**3.38**
Cavite	**1,227,026**	22,004	**1.79**
Laguna	**954,575**	48,866	**5.12**
Quezon	**484,670**	32,204	**6.64**
Rizal	**916,502**	5,413	**0.59**
City of Lucena	**73,773**	105	**0.14**
**Region 4B**	**815,800**	**14,697**	**1.80**
Marinduque	**55,684**	2	**0.00**
Mindoro Occidental	**129,977**	10,536	**8.11**

Table 2.A.6 - MODERN FAMILY PLANNING

WRA 15-49 yrs old with unmet needs

Philippines, 2023

Area	Estimated No. of WRA	Total No. of WRA (15-49 yrs old) with Unmet Needs	%
Mindoro Oriental	**231,105**	14	**0.01**
Palawan	**236,362**	3,838	**1.62**
Romblon	**74,707**	307	**0.41**
Puerto Princesa City	**87,965**	0	**0.00**
**Region 5**	**1,492,754**	**16,005**	**1.07**
Albay	**349,123**	6,261	**1.79**
Camarines Norte	**154,244**	79	**0.05**
Camarines Sur	**451,557**	39	**0.01**
Catanduanes	**64,816**	3,329	**5.14**
Masbate	**212,202**	2,040	**0.96**
Sorsogon	**202,509**	3,873	**1.91**
City of Naga	**58,303**	384	**0.66**
**Region 6**	**1,971,624**	**142,053**	**7.20**
Aklan	**155,029**	27,495	**17.74**
Antique	**147,073**	22,260	**15.14**
Capiz	**202,201**	7,707	**3.81**
Guimaras	**46,900**	2,702	**5.76**
Iloilo	**507,069**	18,497	**3.65**
Negros Occidental	**636,012**	53,095	**8.35**
City of Bacolod	**157,401**	5,982	**3.80**
City of Iloilo	**119,939**	4,315	**3.60**
**Region 7**	**2,105,782**	**31,318**	**1.49**
Bohol	**340,607**	167	**0.05**
Cebu	**853,047**	22,267	**2.61**
Negros Oriental	**357,168**	4,718	**1.32**
Siquijor	**25,376**	968	**3.81**
City of Cebu	**272,834**	2,520	**0.92**
City of Lapu-Lapu	**148,043**	660	**0.45**
City of Mandaue	**108,707**	18	**0.02**

Table 2.A.6 - MODERN FAMILY PLANNING

WRA 15-49 yrs old with unmet needs
Philippines, 2023

Area	Estimated No. of WRA	Total No. of WRA (15-49 yrs old) with Unmet Needs	%
**Region 8**	**1,102,370**	**39,156**	**3.55**
Biliran	**42,436**	518	**1.22**
Eastern Samar	**114,982**	11,179	**9.72**
Leyte	**369,240**	*	**0.00**
Northern Samar	**153,222**	27,185	**17.74**
Southern Leyte	**103,156**	0	**0.00**
Samar	**193,065**	0	**0.00**
Ormoc City	**59,441**	274	**0.46**
City of Tacloban	**66,828**	0	**0.00**
**Region 9**	**975,564**	**1,605**	**0.16**
Zamboanga del Norte	**253,737**	616	**0.24**
Zamboanga del Sur	**260,488**	447	**0.17**
Zamboanga Sibugay	**166,370**	-	**0.00**
City of Isabela	**33,313**	13	**0.04**
City of Zamboanga	**261,656**	529	**0.20**
**Region 10**	**1,288,120**	**8,789**	**0.68**
Bukidnon	**389,233**	1,083	**0.28**
Camiguin	**21,988**	0	**0.00**
Lanao del Norte	**186,472**	2,368	**1.27**
Misamis Occidental	**153,067**	4,919	**3.21**
Misamis Oriental	**238,154**	177	**0.07**
City of Cagayan De Oro	**203,323**	0	**0.00**
City of Iligan	**95,883**	242	**0.25**
**Region 11**	**1,355,476**	**202,856**	**14.97**
Davao de Oro	**191,617**	23,143	**12.08**
Davao del Norte	**291,100**	52,562	**18.06**
Davao Oriental	**139,819**	30,712	**21.97**
Davao del Sur	**171,842**	60,791	**35.38**
Davao Occidental	**73,856**	17,868	**24.19**
City of Davao	**487,242**	17,780	**3.65**

Table 2.A.6 - MODERN FAMILY PLANNING

WRA 15-49 yrs old with unmet needs
Philippines, 2023

Area	Estimated No. of WRA	Total No. of WRA (15-49 yrs old) with Unmet Needs	%
**Region 12**	**1,140,616**	**15,064**	**1.32**
Cotabato	**326,985**	12,148	**3.72**
Sarangani	**142,160**	418	**0.29**
South Cotabato	**254,294**	1,006	**0.40**
Sultan Kudarat	**223,495**	790	**0.35**
City of General Santos	**193,682**	702	**0.36**
**BARMM**	**1,291,984**	**38,963**	**3.02**
Basilan	**105,914**	15,548	**14.68**
Lanao del Sur	**314,416**	4,290	**1.36**
Maguindanao	**338,097**	1,388	**0.41**
Sulu	**268,384**	3,049	**1.14**
Tawi-Tawi	**112,878**	2,578	**2.28**
SGA	**57,236**	12,110	**21.16**
City of Cotabato	**95,059**	0	**0.00**
**Caraga**	**695,295**	**15,782**	**2.27**
Agusan del Norte	**95,399**	31	**0.03**
Agusan del Sur	**178,689**	3,247	**1.82**
Surigao del Norte	**136,352**	1,409	**1.03**
Surigao del Sur	**157,007**	5,176	**3.30**
Dinagat Islands	**30,296**	348	**1.15**
City of Butuan	**97,552**	5,571	**5.71**

Table 2.B.1.1 - Prenatal Care
Women who gave birth with at least 4 or more Prenatal Check-ups
Philippines, 2023

Area	Total Deliveries	Women who gave birth delivered with at least 4 prenatal check-ups							
		Age Group						Total	%
		10-14 yrs old		15-19 yrs old		20-49 yrs old			
		No.	%	No.	%	No.	%		
**Philippines**	**1,420,182**	**2,048**	**0.14**	**107,856**	**7.59**	**979,113**	**68.94**	**1,089,017**	**76.68**
**N C R**	**184,090**	**182**	**0.10**	**10,337**	**5.62**	**149,896**	**81.43**	**160,415**	**87.14**
Malabon City	**1,782**	6	**0.34**	163	**9.15**	1,613	**90.52**	**1,782**	**100.00**
Navotas City	**4,102**	12	**0.29**	328	**8.00**	3,531	**86.08**	**3,871**	**94.37**
Valenzuela City	**5,585**	11	**0.20**	393	**7.04**	4,962	**88.85**	**5,366**	**96.08**
Caloocan City	**22,186**	25	**0.11**	1,670	**7.53**	20,491	**92.36**	**22,186**	**100.00**
Marikina City	**2,526**	7	**0.28**	210	**8.31**	2,301	**91.09**	**2,518**	**99.68**
Pasig City	**11,370**	10	**0.09**	536	**4.71**	7,708	**67.79**	**8,254**	**72.59**
Pateros	**791**	0	**0.00**	12	**1.52**	478	**60.43**	**490**	**61.95**
Taguig City	**12,357**	20	**0.16**	830	**6.72**	8,876	**71.83**	**9,726**	**78.71**
Quezon City	**57,484**	34	**0.06**	2,308	**4.02**	51,629	**89.81**	**53,971**	**93.89**
Makati City	**3,534**	4	**0.11**	163	**4.61**	2,356	**66.67**	**2,523**	**71.39**
Mandaluyong City	**7,639**	9	**0.12**	294	**3.85**	7,244	**94.83**	**7,547**	**98.80**
San Juan City	**745**	0	**0.00**	40	**5.37**	526	**70.60**	**566**	**75.97**
Manila City	**23,640**	19	**0.08**	1,695	**7.17**	14,480	**61.25**	**16,194**	**68.50**
Las Piñas City	**6,851**	5	**0.07**	419	**6.12**	5,396	**78.76**	**5,820**	**84.95**
Muntinlupa City	**8,297**	10	**0.12**	550	**6.63**	7,360	**88.71**	**7,920**	**95.46**
Parañaque City	**8,296**	2	**0.02**	447	**5.39**	4,714	**56.82**	**5,163**	**62.23**
Pasay City	**6,905**	8	**0.12**	279	**4.04**	6,231	**90.24**	**6,518**	**94.40**
**C A R**	**24,874**	**18**	**0.07**	**1,303**	**5.24**	**15,139**	**60.86**	**16,460**	**66.17**
Abra	**2,730**	4	**0.15**	280	**10.26**	2,185	**80.04**	**2,469**	**90.44**
Apayao	**2,733**	3	**0.11**	124	**4.54**	925	**33.85**	**1,052**	**38.49**
Benguet	**4,082**	2	**0.05**	178	**4.36**	2,898	**70.99**	**3,078**	**75.40**
Ifugao	**2,414**	1	**0.04**	166	**6.88**	1,611	**66.74**	**1,778**	**73.65**
Kalinga	**2,387**	2	**0.08**	133	**5.57**	2,161	**90.53**	**2,296**	**96.19**
Mountain Province	**3,390**	4	**0.12**	217	**6.40**	2,302	**67.91**	**2,523**	**74.42**
City of Baguio	**7,138**	2	**0.03**	205	**2.87**	3,057	**42.83**	**3,264**	**45.73**
**Region 1**	**50,550**	**77**	**0.15**	**4,005**	**7.92**	**45,938**	**90.88**	**50,020**	**98.95**
Ilocos Norte	**6,049**	1	**0.02**	374	**6.18**	5,556	**91.85**	**5,931**	**98.05**
Ilocos Sur	**7,661**	11	**0.14**	391	**5.10**	7,068	**92.26**	**7,470**	**97.51**
La Union	**10,615**	11	**0.10**	840	**7.91**	9,632	**90.74**	**10,483**	**98.76**

Table 2.B.1.1 - Prenatal Care
Women who gave birth with at least 4 or more Prenatal Check-ups
Philippines, 2023

Area	Total Deliveries	Women who gave birth delivered with at least 4 prenatal check-ups							
		Age Group						Total	%
		10-14 yrs old		15-19 yrs old		20-49 yrs old			
		No.	%	No.	%	No.	%		
Pangasinan	**17,409**	48	**0.28**	1,678	**9.64**	15,647	**89.88**	**17,373**	**99.79**
City of Dagupan	**8,816**	6	**0.07**	722	**8.19**	8,035	**91.14**	**8,763**	**99.40**
**Region 2**	**36,823**	**76**	**0.21**	**3,547**	**9.63**	**28,783**	**78.17**	**32,406**	**88.00**
Batanes	**216**	0	**0.00**	5	**2.31**	130	**60.19**	**135**	**62.50**
Cagayan	**8,424**	10	**0.12**	728	**8.64**	6,558	**77.85**	**7,296**	**86.61**
Isabela	**15,263**	36	**0.24**	1,444	**9.46**	11,815	**77.41**	**13,295**	**87.11**
Nueva Vizcaya	**7,319**	15	**0.20**	725	**9.91**	5,340	**72.96**	**6,080**	**83.07**
Quirino	**2,776**	12	**0.43**	351	**12.64**	2,412	**86.89**	**2,775**	**99.96**
City of Santiago	**2,825**	3	**0.11**	294	**10.41**	2,528	**89.49**	**2,825**	**100.00**
**Region 3**	**148,973**	**317**	**0.21**	**15,469**	**10.38**	**132,081**	**88.66**	**147,867**	**99.26**
Aurora	**3,200**	7	**0.22**	340	**10.63**	2,732	**85.38**	**3,079**	**96.22**
Bataan	**11,009**	28	**0.25**	1,231	**11.18**	8,066	**73.27**	**9,325**	**84.70**
Bulacan	**40,824**	79	**0.19**	4,406	**10.79**	44,058	**107.92**	**48,543**	**118.91**
Nueva Ecija	**28,779**	65	**0.23**	2,639	**9.17**	20,266	**70.42**	**22,970**	**79.82**
Pampanga	**25,991**	51	**0.20**	2,847	**10.95**	23,534	**90.55**	**26,432**	**101.70**
Tarlac	**20,870**	44	**0.21**	2,212	**10.60**	18,822	**90.19**	**21,078**	**101.00**
Zambales	**8,677**	13	**0.15**	576	**6.64**	4,407	**50.79**	**4,996**	**57.58**
City of Angeles	**5,487**	14	**0.26**	761	**13.87**	7,781	**141.81**	**8,556**	**155.93**
City of Olongapo	**4,136**	16	**0.39**	457	**11.05**	2,415	**58.39**	**2,888**	**69.83**
**Region 4A**	**202,438**	**311**	**0.15**	**13,931**	**6.88**	**130,925**	**64.67**	**145,167**	**71.71**
Batangas	**38,479**	40	**0.10**	2,292	**5.96**	26,394	**68.59**	**28,726**	**74.65**
Cavite	**48,372**	55	**0.11**	2,541	**5.25**	27,498	**56.85**	**30,094**	**62.21**
Laguna	**45,570**	80	**0.18**	3,474	**7.62**	32,862	**72.11**	**36,416**	**79.91**
Quezon	**24,586**	57	**0.23**	1,971	**8.02**	15,725	**63.96**	**17,753**	**72.21**
Rizal	**42,344**	72	**0.17**	3,315	**7.83**	26,168	**61.80**	**29,555**	**69.80**
City of Lucena	**3,087**	7	**0.23**	338	**10.95**	2,278	**73.79**	**2,623**	**84.97**
**Region 4B**	**47,523**	**53**	**0.11**	**2,527**	**5.32**	**19,292**	**40.60**	**21,872**	**46.02**
Marinduque	**2,791**	4	**0.14**	216	**7.74**	2,078	**74.45**	**2,298**	**82.34**
Occidental Mindoro	**8,490**	25	**0.29**	850	**10.01**	6,140	**72.32**	**7,015**	**82.63**

Table 2.B.1.1 - Prenatal Care
Women who gave birth with at least 4 or more Prenatal Check-ups
Philippines, 2023

Area	Total Deliveries	Women who gave birth delivered with at least 4 prenatal check-ups							
		Age Group						Total	%
		10-14 yrs old		15-19 yrs old		20-49 yrs old			
		No.	%	No.	%	No.	%		
Oriental Mindoro	**14,579**	6	**0.04**	319	**2.19**	3,508	**24.06**	**3,833**	**26.29**
Palawan	**13,176**	16	**0.12**	827	**6.28**	4,733	**35.92**	**5,576**	**42.32**
Romblon	**4,369**	0	**0.00**	153	**3.50**	1,585	**36.28**	**1,738**	**39.78**
City of Puerto Princesa	**4,118**	2	**0.05**	162	**3.93**	1,248	**30.31**	**1,412**	**34.29**
**Region 5**	**84,229**	**53**	**0.06**	**5,831**	**6.92**	**57,609**	**68.40**	**63,493**	**75.38**
Albay	**16,099**	6	**0.04**	803	**4.99**	12,268	**76.20**	**13,077**	**81.23**
Camarines Norte	**10,605**	5	**0.05**	1,347	**12.70**	8,752	**82.53**	**10,104**	**95.28**
Camarines Sur	**17,655**	3	**0.02**	850	**4.81**	11,297	**63.99**	**12,150**	**68.82**
Catanduanes	**4,341**	5	**0.12**	278	**6.40**	2,706	**62.34**	**2,989**	**68.86**
Masbate	**14,258**	13	**0.09**	1,355	**9.50**	9,656	**67.72**	**11,024**	**77.32**
Sorsogon	**13,726**	11	**0.08**	1,073	**7.82**	11,466	**83.53**	**12,550**	**91.43**
City of Naga	**7,545**	10	**0.13**	125	**1.66**	1,464	**19.40**	**1,599**	**21.19**
**Region 6**	**96,790**	**107**	**0.11**	**7,276**	**7.52**	**65,700**	**67.88**	**73,083**	**75.51**
Aklan	**6,213**	4	**0.06**	432	**6.95**	5,033	**81.01**	**5,469**	**88.03**
Antique	**8,282**	5	**0.06**	378	**4.56**	5,207	**62.87**	**5,590**	**67.50**
Capiz	**10,750**	8	**0.07**	566	**5.27**	5,938	**55.24**	**6,512**	**60.58**
Guimaras	**2,370**	4	**0.17**	224	**9.45**	2,047	**86.37**	**2,275**	**95.99**
Iloilo	**22,667**	27	**0.12**	1,772	**7.82**	17,051	**75.22**	**18,850**	**83.16**
Negros Occidental	**34,588**	42	**0.12**	3,261	**9.43**	23,016	**66.54**	**26,319**	**76.09**
City of Bacolod	**6,150**	1	**0.02**	174	**2.83**	2,496	**40.59**	**2,671**	**43.43**
City of Iloilo	**5,770**	16	**0.28**	469	**8.13**	4,912	**85.13**	**5,397**	**93.54**
**Region 7**	**112,305**	**31**	**0.03**	**2,856**	**2.54**	**29,198**	**26.00**	**32,085**	**28.57**
Bohol	**18,420**	0	**0.00**	336	**1.82**	4,186	**22.73**	**4,522**	**24.55**
Cebu	**40,979**	12	**0.03**	1,102	**2.69**	11,721	**28.60**	**12,835**	**31.32**
Negros Oriental	**19,030**	0	**0.00**	0	**0.00**	0	**0.00**	**0**	**0.00**
Siquijor	**1,014**	2	**0.20**	83	**8.19**	904	**89.15**	**989**	**97.53**
City of Cebu	**15,467**	15	**0.10**	1,134	**7.33**	10,122	**65.44**	**11,271**	**72.87**
City of Lapu-Lapu	**7,557**	0	**0.00**	83	**1.10**	1,090	**14.42**	**1,173**	**15.52**
City of Mandaue	**9,838**	2	**0.02**	118	**1.20**	1,175	**11.94**	**1,295**	**13.16**
**Region 8**	**59,834**	**65**	**0.11**	**4,138**	**6.92**	**36,958**	**61.77**	**41,161**	**68.79**

Table 2.B.1.1 - Prenatal Care
Women who gave birth with at least 4 or more Prenatal Check-ups
Philippines, 2023

Area	Total Deliveries	Women who gave birth delivered with at least 4 prenatal check-ups							
		Age Group						Total	%
		10-14 yrs old		15-19 yrs old		20-49 yrs old			
		No.	%	No.	%	No.	%		
Biliran	**3,084**	5	**0.16**	205	**6.65**	1,937	**62.81**	**2,147**	**69.62**
Eastern Samar	**7,014**	6	**0.09**	793	**11.31**	5,672	**80.87**	**6,471**	**92.26**
Leyte	**12,068**	25	**0.21**	841	**6.97**	7,884	**65.33**	**8,750**	**72.51**
Northern Samar	**10,442**	3	**0.03**	394	**3.77**	4,235	**40.56**	**4,632**	**44.36**
Southern Leyte	**5,094**	1	**0.02**	348	**6.83**	3,473	**68.18**	**3,822**	**75.03**
Samar	**10,338**	14	**0.14**	822	**7.95**	5,895	**57.02**	**6,731**	**65.11**
Ormoc City	**5,965**	5	**0.08**	417	**6.99**	2,768	**46.40**	**3,190**	**53.48**
City of Tacloban	**5,829**	6	**0.10**	318	**5.46**	5,094	**87.39**	**5,418**	**92.95**
**Region 9**	**47,886**	**55**	**0.11**	**4,611**	**9.63**	**34,406**	**71.85**	**39,072**	**81.59**
Zamboanga del Norte	**13,451**	14	**0.10**	1,279	**9.51**	9,183	**68.27**	**10,476**	**77.88**
Zamboanga del Sur	**8,271**	15	**0.18**	588	**7.11**	4,615	**55.80**	**5,218**	**63.09**
Zamboanga Sibugay	**7,406**	5	**0.07**	620	**8.37**	4,683	**63.23**	**5,308**	**71.67**
City of Isabela	**1,797**	1	**0.06**	201	**11.19**	1,532	**85.25**	**1,734**	**96.49**
City of Zamboanga	**16,961**	20	**0.12**	1,923	**11.34**	14,393	**84.86**	**16,336**	**96.32**
**Region 10**	**71,891**	**182**	**0.25**	**8,561**	**11.91**	**51,721**	**71.94**	**60,464**	**84.11**
Bukidnon	**23,309**	75	**0.32**	3,325	**14.26**	16,832	**72.21**	**20,232**	**86.80**
Camiguin	**1,355**	0	**0.00**	69	**5.09**	605	**44.65**	**674**	**49.74**
Lanao del Norte	**6,720**	4	**0.06**	520	**7.74**	5,893	**87.69**	**6,417**	**95.49**
Misamis Occidental	**6,284**	24	**0.38**	676	**10.76**	5,238	**83.35**	**5,938**	**94.49**
Misamis Oriental	**9,358**	44	**0.47**	1,648	**17.61**	6,948	**74.25**	**8,640**	**92.33**
City of Cagayan De Oro	**17,552**	30	**0.17**	1,586	**9.04**	9,667	**55.08**	**11,283**	**64.28**
City of Iligan	**7,313**	5	**0.07**	737	**10.08**	6,538	**89.40**	**7,280**	**99.55**
**Region 11**	**81,389**	**257**	**0.32**	**8,046**	**9.89**	**55,863**	**68.64**	**64,166**	**78.84**
Davao de Oro	**11,522**	65	**0.56**	1,532	**13.30**	8,554	**74.24**	**10,151**	**88.10**
Davao del Norte	**17,062**	52	**0.30**	1,766	**10.35**	11,415	**66.90**	**13,233**	**77.56**
Davao Oriental	**8,590**	42	**0.49**	991	**11.54**	5,931	**69.05**	**6,964**	**81.07**
Davao del Sur	**9,438**	38	**0.40**	886	**9.39**	5,147	**54.53**	**6,071**	**64.33**
Davao Occidental	**4,886**	10	**0.20**	457	**9.35**	1,315	**26.91**	**1,782**	**36.47**
City of Davao	**29,891**	50	**0.17**	2,414	**8.08**	23,501	**78.62**	**25,965**	**86.87**

Table 2.B.1.1 - Prenatal Care
Women who gave birth with at least 4 or more Prenatal Check-ups
Philippines, 2023

Area	Total Deliveries	Women who gave birth delivered with at least 4 prenatal check-ups							
		Age Group						Total	%
		10-14 yrs old		15-19 yrs old		20-49 yrs old			
		No.	%	No.	%	No.	%		
**Region 12**	**64,112**	**160**	**0.25**	**7,616**	**11.88**	**48,465**	**75.59**	**56,241**	**87.72**
Cotabato	**17,294**	46	**0.27**	1,956	**11.31**	13,044	**75.43**	**15,046**	**87.00**
Sarangani	**9,947**	27	**0.27**	1,666	**16.75**	7,352	**73.91**	**9,045**	**90.93**
South Cotabato	**16,339**	37	**0.23**	1,980	**12.12**	12,639	**77.35**	**14,656**	**89.70**
Sultan Kudarat	**12,614**	45	**0.36**	1,441	**11.42**	9,430	**74.76**	**10,916**	**86.54**
City of General Santos	**7,918**	5	**0.06**	573	**7.24**	6,000	**75.78**	**6,578**	**83.08**
**BARMM**	**65,140**	**41**	**0.06**	**4,067**	**6.24**	**47,587**	**73.05**	**51,695**	**79.36**
Basilan	**4,718**	10	**0.21**	444	**9.41**	2,548	**54.01**	**3,002**	**63.63**
Lanao del Sur	**17,820**	7	**0.04**	804	**4.51**	15,235	**85.49**	**16,046**	**90.04**
Maguindanao	**19,942**	5	**0.03**	1,330	**6.67**	13,901	**69.71**	**15,236**	**76.40**
Sulu	**11,670**	10	**0.09**	737	**6.32**	8,400	**71.98**	**9,147**	**78.38**
Tawi-Tawi	**6,010**	0	**0.00**	360	**5.99**	4,191	**69.73**	**4,551**	**75.72**
SGA	**1,432**	2	**0.14**	87	**6.08**	773	**53.98**	**862**	**60.20**
City of Cotabato	**3,548**	7	**0.20**	305	**8.60**	2,539	**71.56**	**2,851**	**80.36**
**Caraga**	**41,335**	**63**	**0.15**	**3,735**	**9.04**	**29,552**	**71.49**	**33,350**	**80.68**
Agusan del Norte	**5,881**	12	**0.20**	763	**12.97**	4,530	**77.03**	**5,305**	**90.21**
Agusan del Sur	**10,972**	24	**0.22**	1,263	**11.51**	9,062	**82.59**	**10,349**	**94.32**
Surigao del Norte	**7,756**	2	**0.03**	332	**4.28**	4,147	**53.47**	**4,481**	**57.77**
Surigao del Sur	**9,826**	12	**0.12**	716	**7.29**	6,083	**61.91**	**6,811**	**69.32**
Dinagat Islands	**1,034**	2	**0.19**	57	**5.51**	547	**52.90**	**606**	**58.61**
City of Butuan	**5,866**	11	**0.19**	604	**10.30**	5,183	**88.36**	**5,798**	**98.84**

Table 2.B.1.2 - Prenatal Care
Pregnant women according to their nutritional status
Philippines, 2023

Area	Elig. Pop.	Pregnant women seen during the 1st trimester according to their Body Mass Index (BMI)									
		Normal BMI					Low BMI				
		Age Group			Total	%	Age Group			Total	%
		10-14	15-19	20-49			10-14	15-19	20-49		
**Philippines**	**2,187,333**	**2,791**	**100,242**	**734,040**	**837,073**	**38.27**	**499**	**11,793**	**39,354**	**51,646**	**2.36**
**N C R**	**261,228**	**149**	**6,159**	**77,564**	**83,872**	**32.11**	**60**	**1,619**	**4,679**	**6,358**	**2.43**
Malabon City	**7,385**	10	259	1,516	**1,785**	**24.17**	4	105	299	**408**	**5.52**
Navotas City	**5,210**	12	338	2,224	**2,574**	**49.40**	0	1	137	**138**	**2.65**
Valenzuela City	**13,477**	7	289	2,581	**2,877**	**21.35**	5	62	266	**333**	**2.47**
Caloocan City	**33,168**	13	725	5,811	**6,549**	**19.74**	5	128	360	**493**	**1.49**
Marikina City	**8,342**	0	47	406	**453**	**5.43**	1	21	67	**89**	**1.07**
Pasig City	**17,682**	10	205	1,733	**1,948**	**11.02**	1	65	202	**268**	**1.52**
Pateros	**1,116**	2	43	586	**631**	**56.54**	0	2	3	**5**	**0.45**
Taguig City	**18,992**	3	415	3,139	**3,557**	**18.73**	9	146	407	**562**	**2.96**
Quezon City	**59,136**	17	781	30,832	**31,630**	**53.49**	20	565	671	**1,256**	**2.12**
Makati City	**10,944**	2	54	632	**688**	**6.29**	1	21	108	**130**	**1.19**
Mandaluyong City	**7,922**	13	224	4,537	**4,774**	**60.26**	0	24	84	**108**	**1.36**
San Juan City	**2,168**	1	44	468	**513**	**23.66**	0	2	28	**30**	**1.38**
Manila City	**34,128**	25	1,261	8,828	**10,114**	**29.64**	5	208	929	**1,142**	**3.35**
Las Piñas City	**11,263**	2	226	2,367	**2,595**	**23.04**	3	65	236	**304**	**2.70**
Muntinlupa City	**9,881**	14	632	5,316	**5,962**	**60.34**	2	85	312	**399**	**4.04**
Parañaque City	**13,280**	12	312	3,170	**3,494**	**26.31**	2	55	295	**352**	**2.65**
Pasay City	**7,134**	6	304	3,418	**3,728**	**52.26**	2	64	275	**341**	**4.78**
**C A R**	**31,319**	**41**	**1,577**	**13,847**	**15,465**	**49.38**	**7**	**104**	**389**	**500**	**1.60**
Abra	**3,660**	4	253	1,590	**1,847**	**50.46**	2	31	87	**120**	**3.28**
Apayao	**2,238**	2	166	854	**1,022**	**45.67**	1	22	44	**67**	**2.99**
Benguet	**8,806**	7	409	4,738	**5,154**	**58.53**	2	15	87	**104**	**1.18**
Ifugao	**4,098**	8	202	1,941	**2,151**	**52.49**	0	6	45	**51**	**1.24**
Kalinga	**4,549**	3	207	1,796	**2,006**	**44.10**	1	16	47	**64**	**1.41**
Mountain Province	**2,619**	10	191	1,476	**1,677**	**64.03**	1	10	49	**60**	**2.29**
City of Baguio	**5,349**	7	149	1,452	**1,608**	**30.06**	0	4	30	**34**	**0.64**
**Region 1**	**96,016**	**67**	**3,455**	**37,294**	**40,816**	**42.51**	**1**	**250**	**1,027**	**1,278**	**1.33**
Ilocos Norte	**8,912**	7	353	5,262	**5,622**	**63.08**	0	17	35	**52**	**0.58**
Ilocos Sur	**9,914**	7	423	6,299	**6,729**	**67.87**	0	15	48	**63**	**0.64**

Table 2.B.1.2 - Prenatal Care
Pregnant women according to their nutritional status
Philippines, 2023

Area	Elig. Pop.	Pregnant women seen during the 1st trimester according to their Body Mass Index (BMI)									
		Normal BMI					Low BMI				
		Age Group			Total	%	Age Group			Total	%
		10-14	15-19	20-49			10-14	15-19	20-49		
La Union	**13,507**	12	604	5,155	**5,771**	**42.73**	0	45	194	**239**	**1.77**
Pangasinan	**60,360**	36	1,920	19,770	**21,726**	**35.99**	1	171	728	**900**	**1.49**
City of Dagupan	**3,323**	5	155	808	**968**	**29.13**	0	2	22	**24**	**0.72**
**Region 2**	**63,967**	**125**	**3,598**	**25,464**	**29,187**	**45.63**	**20**	**288**	**1,009**	**1,317**	**2.06**
Batanes	**240**	0	5	75	**80**	**33.33**	0	1	3	**4**	**1.67**
Cagayan	**20,786**	31	955	6,946	**7,932**	**38.16**	5	93	392	**490**	**2.36**
Isabela	**27,379**	63	1,531	10,974	**12,568**	**45.90**	6	96	289	**391**	**1.43**
Nueva Vizcaya	**8,798**	14	533	3,461	**4,008**	**45.56**	6	61	273	**340**	**3.86**
Quirino	**3,839**	12	280	1,921	**2,213**	**57.65**	2	29	43	**74**	**1.93**
City of Santiago	**2,925**	5	294	2,087	**2,386**	**81.57**	1	8	9	**18**	**0.62**
**Region 3**	**233,544**	**374**	**12,425**	**87,847**	**100,646**	**43.10**	**56**	**967**	**2,482**	**3,505**	**1.50**
Aurora	**4,537**	12	317	1,890	**2,219**	**48.91**	2	24	67	**93**	**2.05**
Bataan	**17,380**	39	827	5,228	**6,094**	**35.06**	3	130	325	**458**	**2.64**
Bulacan	**67,590**	135	4,458	32,386	**36,979**	**54.71**	30	392	914	**1,336**	**1.98**
Nueva Ecija	**43,425**	73	2,096	12,723	**14,892**	**34.29**	8	134	379	**521**	**1.20**
Pampanga	**45,651**	59	1,987	15,017	**17,063**	**37.38**	5	154	316	**475**	**1.04**
Tarlac	**29,503**	32	1,497	11,927	**13,456**	**45.61**	4	60	250	**314**	**1.06**
Zambales	**11,723**	13	631	3,751	**4,395**	**37.49**	3	34	92	**129**	**1.10**
City of Angeles	**9,146**	5	430	3,599	**4,034**	**44.11**	0	6	19	**25**	**0.27**
City of Olongapo	**4,589**	6	182	1,326	**1,514**	**32.99**	1	33	120	**154**	**3.36**
**Region 4A**	**307,982**	**287**	**10,331**	**81,575**	**92,193**	**29.93**	**67**	**1,635**	**5,698**	**7,400**	**2.40**
Batangas	**53,251**	32	1,682	17,215	**18,929**	**35.55**	11	306	1,083	**1,400**	**2.63**
Cavite	**77,964**	54	1,940	15,992	**17,986**	**23.07**	7	279	1,037	**1,323**	**1.70**
Laguna	**61,525**	72	2,495	21,284	**23,851**	**38.77**	17	507	1,576	**2,100**	**3.41**
Quezon	**40,810**	64	1,733	11,288	**13,085**	**32.06**	12	287	973	**1,272**	**3.12**
Rizal	**68,235**	58	2,168	14,229	**16,455**	**24.12**	17	214	951	**1,182**	**1.73**
City of Lucena	**6,197**	7	313	1,567	**1,887**	**30.45**	3	42	78	**123**	**1.98**
**Region 4B**	**66,121**	**80**	**3,495**	**23,779**	**27,354**	**41.37**	**38**	**538**	**1,730**	**2,306**	**3.49**
Marinduque	**4,144**	1	153	1,288	**1,442**	**34.80**	1	25	89	**115**	**2.78**

Table 2.B.1.2 - Prenatal Care
Pregnant women according to their nutritional status
Philippines, 2023

Area	Elig. Pop.	Pregnant women seen during the 1st trimester according to their Body Mass Index (BMI)									
		Normal BMI					Low BMI				
		Age Group			Total	%	Age Group			Total	%
		10-14	15-19	20-49			10-14	15-19	20-49		
Mindoro Occidental	**11,640**	8	639	3,832	**4,479**	**38.48**	3	76	150	**229**	**1.97**
Mindoro Oriental	**18,943**	18	762	8,500	**9,280**	**48.99**	12	104	401	**517**	**2.73**
Palawan	**20,317**	47	1,606	7,842	**9,495**	**46.73**	11	236	832	**1,079**	**5.31**
Romblon	**5,489**	4	205	1,718	**1,927**	**35.11**	10	48	171	**229**	**4.17**
Puerto Princesa City	**5,588**	2	130	599	**731**	**13.08**	1	49	87	**137**	**2.45**
**Region 5**	**135,480**	**72**	**5,242**	**41,133**	**46,447**	**34.28**	**26**	**844**	**2,822**	**3,692**	**2.73**
Albay	**27,440**	9	871	11,068	**11,948**	**43.54**	3	91	390	**484**	**1.76**
Camarines Norte	**14,695**	16	703	4,419	**5,138**	**34.96**	9	194	573	**776**	**5.28**
Camarines Sur	**43,452**	15	1,196	9,535	**10,746**	**24.73**	3	202	744	**949**	**2.18**
Catanduanes	**5,414**	2	216	1,671	**1,889**	**34.89**	2	55	136	**193**	**3.56**
Masbate	**22,365**	12	1,164	7,719	**8,895**	**39.77**	2	108	300	**410**	**1.83**
Sorsogon	**17,929**	13	763	5,941	**6,717**	**37.46**	7	179	631	**817**	**4.56**
City of Naga	**4,185**	5	329	780	**1,114**	**26.62**	0	15	48	**63**	**1.51**
**Region 6**	**148,020**	**98**	**5,749**	**45,480**	**51,327**	**34.68**	**23**	**706**	**2,649**	**3,378**	**2.28**
Aklan	**11,283**	2	338	3,508	**3,848**	**34.10**	0	52	215	**267**	**2.37**
Antique	**11,930**	7	448	4,140	**4,595**	**38.52**	0	75	271	**346**	**2.90**
Capiz	**13,931**	7	416	3,738	**4,161**	**29.87**	0	30	146	**176**	**1.26**
Guimaras	**3,351**	3	148	1,370	**1,521**	**45.39**	1	24	87	**112**	**3.34**
Iloilo	**36,140**	27	1,368	12,813	**14,208**	**39.31**	2	75	346	**423**	**1.17**
Negros Occidental	**52,270**	39	2,627	16,020	**18,686**	**35.75**	18	350	975	**1,343**	**2.57**
City of Bacolod	**10,373**	3	162	1,646	**1,811**	**17.46**	1	70	535	**606**	**5.84**
City of Iloilo	**8,742**	10	242	2,245	**2,497**	**28.56**	1	30	74	**105**	**1.20**
**Region 7**	**165,673**	**157**	**7,090**	**52,943**	**60,190**	**36.33**	**23**	**814**	**2,637**	**3,474**	**2.10**
Bohol	**26,235**	14	750	7,503	**8,267**	**31.51**	1	101	309	**411**	**1.57**
Cebu	**72,201**	68	3,354	22,598	**26,020**	**36.04**	4	351	1,086	**1,441**	**2.00**
Negros Oriental	**28,722**	35	1,354	7,724	**9,113**	**31.73**	10	166	579	**755**	**2.63**
Siquijor	**1,680**	2	44	390	**436**	**25.95**	0	9	36	**45**	**2.68**
City of Cebu	**19,267**	23	925	5,900	**6,848**	**35.54**	6	137	371	**514**	**2.67**
City of Lapu-Lapu	**10,679**	3	377	5,094	**5,474**	**51.26**	1	19	154	**174**	**1.63**
City of Mandaue	**6,889**	12	286	3,734	**4,032**	**58.53**	1	31	102	**134**	**1.95**

Table 2.B.1.2 - Prenatal Care
Pregnant women according to their nutritional status
Philippines, 2023

Area	Elig. Pop.	Pregnant women seen during the 1st trimester according to their Body Mass Index (BMI)									
		Normal BMI					Low BMI				
		Age Group			Total	%	Age Group			Total	%
		10-14	15-19	20-49			10-14	15-19	20-49		
**Region 8**	**93,813**	**88**	**4,028**	**28,776**	**32,892**	**35.06**	**12**	**339**	**1,313**	**1,664**	**1.77**
Biliran	**3,373**	3	232	1,250	**1,485**	**44.03**	1	36	96	**133**	**3.94**
Eastern Samar	**9,624**	11	625	3,811	**4,447**	**46.21**	0	37	89	**126**	**1.31**
Leyte	**31,559**	35	1,320	10,268	**11,623**	**36.83**	3	58	360	**421**	**1.33**
Northern Samar	**14,056**	8	535	3,566	**4,109**	**29.23**	1	47	207	**255**	**1.81**
Southern Leyte	**7,406**	0	206	2,516	**2,722**	**36.75**	0	21	125	**146**	**1.97**
Samar	**17,011**	9	510	3,286	**3,805**	**22.37**	2	63	240	**305**	**1.79**
Ormoc City	**5,292**	21	345	2,017	**2,383**	**45.03**	3	62	121	**186**	**3.51**
City of Tacloban	**5,492**	1	255	2,062	**2,318**	**42.21**	2	15	75	**92**	**1.68**
**Region 9**	**84,815**	**131**	**5,436**	**30,417**	**35,984**	**42.43**	**15**	**441**	**1,185**	**1,641**	**1.93**
Zamboanga del Norte	**24,841**	37	1,426	8,338	**9,801**	**39.45**	8	98	213	**319**	**1.28**
Zamboanga del Sur	**23,757**	27	1,191	6,244	**7,462**	**31.41**	3	117	386	**506**	**2.13**
Zamboanga Sibugay	**13,841**	22	869	4,554	**5,445**	**39.34**	2	65	152	**219**	**1.58**
City of Isabela	**3,201**	3	249	1,511	**1,763**	**55.08**	0	9	22	**31**	**0.97**
City of Zamboanga	**19,175**	42	1,701	9,770	**11,513**	**60.04**	2	152	412	**566**	**2.95**
**Region 10**	**109,169**	**331**	**7,857**	**43,508**	**51,696**	**47.35**	**25**	**534**	**1,163**	**1,722**	**1.58**
Bukidnon	**33,403**	72	2,908	11,999	**14,979**	**44.84**	13	257	510	**780**	**2.34**
Camiguin	**1,727**	2	60	362	**424**	**24.55**	1	3	9	**13**	**0.75**
Lanao del Norte	**17,760**	18	915	7,523	**8,456**	**47.61**	1	21	78	**100**	**0.56**
Misamis Occidental	**12,328**	166	843	6,415	**7,424**	**60.22**	3	13	33	**49**	**0.40**
Misamis Oriental	**21,975**	44	1,633	8,993	**10,670**	**48.56**	3	67	148	**218**	**0.99**
City of Cagayan De Oro	**14,308**	20	1,072	5,344	**6,436**	**44.98**	2	135	265	**402**	**2.81**
City of Iligan	**7,668**	9	426	2,872	**3,307**	**43.13**	2	38	120	**160**	**2.09**
**Region 11**	**107,001**	**383**	**7,966**	**39,521**	**47,870**	**44.74**	**75**	**1,127**	**2,700**	**3,902**	**3.65**
Davao de Oro	**15,253**	71	1,574	5,929	**7,574**	**49.66**	10	245	458	**713**	**4.67**
Davao del Norte	**23,453**	63	1,596	8,447	**10,106**	**43.09**	15	276	621	**912**	**3.89**
Davao Oriental	**11,935**	58	1,003	4,316	**5,377**	**45.05**	8	138	318	**464**	**3.89**
Davao del Sur	**13,083**	32	924	4,181	**5,137**	**39.26**	12	151	314	**477**	**3.65**
Davao Occidental	**6,629**	26	468	1,106	**1,600**	**24.14**	3	59	80	**142**	**2.14**
City of Davao	**36,648**	133	2,401	15,542	**18,076**	**49.32**	27	258	909	**1,194**	**3.26**

Table 2.B.1.2 - Prenatal Care
Pregnant women according to their nutritional status
Philippines, 2023

Area	Elig. Pop.	Pregnant women seen during the 1st trimester according to their Body Mass Index (BMI)									
		Normal BMI					Low BMI				
		Age Group			Total	%	Age Group			Total	%
		10-14	15-19	20-49			10-14	15-19	20-49		
**Region 12**	**95,279**	**259**	**7,906**	**37,686**	**45,851**	**48.12**	**34**	**814**	**1,776**	**2,624**	**2.75**
Cotabato	**26,521**	50	1,920	8,905	**10,875**	**41.01**	7	145	333	**485**	**1.83**
Sarangani	**13,322**	60	1,710	6,330	**8,100**	**60.80**	5	160	253	**418**	**3.14**
South Cotabato	**21,595**	54	1,982	10,447	**12,483**	**57.81**	10	211	506	**727**	**3.37**
Sultan Kudarat	**19,391**	79	1,336	6,147	**7,562**	**39.00**	12	108	276	**396**	**2.04**
City of General Santos	**14,450**	16	958	5,857	**6,831**	**47.27**	0	190	408	**598**	**4.14**
**BARMM**	**132,841**	**63**	**4,164**	**43,277**	**47,504**	**35.76**	**6**	**504**	**5,358**	**5,868**	**4.42**
Basilan	**11,636**	8	413	1,791	**2,212**	**19.01**	1	56	81	**138**	**1.19**
Lanao del Sur	**33,374**	12	946	16,767	**17,725**	**53.11**	3	249	4,489	**4,741**	**14.21**
Maguindanao	**41,650**	15	1,440	12,106	**13,561**	**32.56**	1	72	273	**346**	**0.83**
Sulu	**21,378**	7	601	6,347	**6,955**	**32.53**	0	58	255	**313**	**1.46**
Tawi-Tawi	**12,609**	3	290	3,303	**3,596**	**28.52**	0	46	158	**204**	**1.62**
SGU	**5,103**	8	175	875	**1,058**	**20.73**	1	16	59	**76**	**1.49**
City of Cotabato	**7,091**	10	299	2,088	**2,397**	**33.80**	0	7	43	**50**	**0.71**
**Caraga**	**55,065**	**86**	**3,764**	**23,929**	**27,779**	**50.45**	**11**	**269**	**737**	**1,017**	**1.85**
Agusan del Norte	**7,286**	11	585	3,031	**3,627**	**49.78**	2	35	70	**107**	**1.47**
Agusan del Sur	**16,038**	33	1,230	7,468	**8,731**	**54.44**	5	58	158	**221**	**1.38**
Surigao del Norte	**10,186**	14	613	4,363	**4,990**	**48.99**	1	70	180	**251**	**2.46**
Surigao del Sur	**12,736**	18	704	4,518	**5,240**	**41.14**	1	62	175	**238**	**1.87**
Dinagat Islands	**2,134**	1	129	908	**1,038**	**48.64**	0	17	38	**55**	**2.58**
City of Butuan	**6,685**	9	503	3,641	**4,153**	**62.12**	2	27	116	**145**	**2.17**

Table 2.B.1.2 - Prenatal Care
Pregnant women according to their nutritional status
Philippines, 2023

Area	Elig. Pop.	Pregnant women seen during the 1st trimester according to their Body Mass Index (BMI)				
		High BMI				
		Age Group			Total	%
		10-14	15-19	20-49		
**Philippines**	**2,187,333**	**225**	**8,473**	**122,498**	**131,196**	**6.00**
**N C R**	**261,228**	**30**	**1,019**	**19,182**	**20,231**	**7.74**
Malabon City	**7,385**	1	67	1,012	**1,080**	**14.62**
Navotas City	**5,210**	0	4	321	**325**	**6.24**
Valenzuela City	**13,477**	1	59	1,369	**1,429**	**10.60**
Caloocan City	**33,168**	4	74	1,060	**1,138**	**3.43**
Marikina City	**8,342**	0	23	458	**481**	**5.77**
Pasig City	**17,682**	1	69	1,328	**1,398**	**7.91**
Pateros	**1,116**	0	1	31	**32**	**2.87**
Taguig City	**18,992**	5	104	2,289	**2,398**	**12.63**
Quezon City	**59,136**	1	76	1,727	**1,804**	**3.05**
Makati City	**10,944**	0	33	957	**990**	**9.05**
Mandaluyong City	**7,922**	0	22	528	**550**	**6.94**
San Juan City	**2,168**	0	5	199	**204**	**9.41**
Manila City	**34,128**	5	194	2,628	**2,827**	**8.28**
Las Piñas City	**11,263**	8	85	1,456	**1,549**	**13.75**
Muntinlupa City	**9,881**	3	119	2,121	**2,243**	**22.70**
Parañaque City	**13,280**	0	33	590	**623**	**4.69**
Pasay City	**7,134**	1	51	1,108	**1,160**	**16.26**
**C A R**	**31,319**	**1**	**143**	**2,954**	**3,098**	**9.89**
Abra	**3,660**	0	16	232	**248**	**6.78**
Apayao	**2,238**	0	14	219	**233**	**10.41**
Benguet	**8,806**	0	27	678	**705**	**8.01**
Ifugao	**4,098**	0	12	242	**254**	**6.20**
Kalinga	**4,549**	0	6	407	**413**	**9.08**
Mountain Province	**2,619**	1	46	696	**743**	**28.37**
City of Baguio	**5,349**	0	22	480	**502**	**9.38**
**Region 1**	**96,016**	**1**	**144**	**2,040**	**2,185**	**2.28**
Ilocos Norte	**8,912**	0	17	340	**357**	**4.01**
Ilocos Sur	**9,914**	0	7	141	**148**	**1.49**

Table 2.B.1.2 - Prenatal Care
Pregnant women according to their nutritional status
Philippines, 2023

Area	Elig. Pop.	Pregnant women seen during the 1st trimester according to their Body Mass Index (BMI)				
		High BMI				
		Age Group			Total	%
		10-14	15-19	20-49		
La Union	**13,507**	1	30	343	**374**	**2.77**
Pangasinan	**60,360**	0	90	1,201	**1,291**	**2.14**
City of Dagupan	**3,323**	0	0	15	**15**	**0.45**
**Region 2**	**63,967**	**7**	**247**	**3,180**	**3,434**	**5.37**
Batanes	**240**	0	0	67	**67**	**27.92**
Cagayan	**20,786**	1	51	761	**813**	**3.91**
Isabela	**27,379**	3	116	1,462	**1,581**	**5.77**
Nueva Vizcaya	**8,798**	2	56	546	**604**	**6.87**
Quirino	**3,839**	0	13	175	**188**	**4.90**
City of Santiago	**2,925**	1	11	169	**181**	**6.19**
**Region 3**	**233,544**	**22**	**798**	**10,326**	**11,146**	**4.77**
Aurora	**4,537**	0	5	123	**128**	**2.82**
Bataan	**17,380**	2	89	1,540	**1,631**	**9.38**
Bulacan	**67,590**	8	322	4,125	**4,455**	**6.59**
Nueva Ecija	**43,425**	5	131	1,496	**1,632**	**3.76**
Pampanga	**45,651**	6	113	1,589	**1,708**	**3.74**
Tarlac	**29,503**	0	62	504	**566**	**1.92**
Zambales	**11,723**	0	39	455	**494**	**4.21**
City of Angeles	**9,146**	1	6	193	**200**	**2.19**
City of Olongapo	**4,589**	0	31	301	**332**	**7.23**
**Region 4A**	**307,982**	**15**	**1,051**	**13,711**	**14,777**	**4.80**
Batangas	**53,251**	4	204	1,935	**2,143**	**4.02**
Cavite	**77,964**	1	186	3,171	**3,358**	**4.31**
Laguna	**61,525**	5	316	4,422	**4,743**	**7.71**
Quezon	**40,810**	3	163	2,007	**2,173**	**5.32**
Rizal	**68,235**	2	167	2,006	**2,175**	**3.19**
City of Lucena	**6,197**	0	15	170	**185**	**2.99**
**Region 4B**	**66,121**	**6**	**235**	**2,940**	**3,181**	**4.81**
Marinduque	**4,144**	0	8	259	**267**	**6.44**

Table 2.B.1.2 - Prenatal Care
Pregnant women according to their nutritional status
Philippines, 2023

Area	Elig. Pop.	Pregnant women seen during the 1st trimester according to their Body Mass Index (BMI)				
		High BMI				
		Age Group			Total	%
		10-14	15-19	20-49		
Mindoro Occidental	**11,640**	1	30	488	**519**	**4.46**
Mindoro Oriental	**18,943**	1	41	461	**503**	**2.66**
Palawan	**20,317**	1	89	889	**979**	**4.82**
Romblon	**5,489**	2	9	212	**223**	**4.06**
Puerto Princesa City	**5,588**	1	58	631	**690**	**12.35**
**Region 5**	**135,480**	**10**	**418**	**8,307**	**8,735**	**6.45**
Albay	**27,440**	2	46	1,151	**1,199**	**4.37**
Camarines Norte	**14,695**	1	65	1,242	**1,308**	**8.90**
Camarines Sur	**43,452**	1	102	2,130	**2,233**	**5.14**
Catanduanes	**5,414**	1	25	414	**440**	**8.13**
Masbate	**22,365**	1	99	1,170	**1,270**	**5.68**
Sorsogon	**17,929**	4	72	2,029	**2,105**	**11.74**
City of Naga	**4,185**	0	9	171	**180**	**4.30**
**Region 6**	**148,020**	**9**	**353**	**6,506**	**6,868**	**4.64**
Aklan	**11,283**	0	32	831	**863**	**7.65**
Antique	**11,930**	0	20	632	**652**	**5.47**
Capiz	**13,931**	0	29	313	**342**	**2.45**
Guimaras	**3,351**	0	11	446	**457**	**13.64**
Iloilo	**36,140**	1	51	774	**826**	**2.29**
Negros Occidental	**52,270**	7	164	2,448	**2,619**	**5.01**
City of Bacolod	**10,373**	1	20	720	**741**	**7.14**
City of Iloilo	**8,742**	0	26	342	**368**	**4.21**
**Region 7**	**165,673**	**16**	**762**	**11,820**	**12,598**	**7.60**
Bohol	**26,235**	1	34	1,398	**1,433**	**5.46**
Cebu	**72,201**	6	289	4,368	**4,663**	**6.46**
Negros Oriental	**28,722**	5	169	2,098	**2,272**	**7.91**
Siquijor	**1,680**	0	7	292	**299**	**17.80**
City of Cebu	**19,267**	3	195	2,653	**2,851**	**14.80**
City of Lapu-Lapu	**10,679**	1	21	579	**601**	**5.63**
City of Mandaue	**6,889**	0	47	432	**479**	**6.95**

Table 2.B.1.2 - Prenatal Care
Pregnant women according to their nutritional status
Philippines, 2023

Area	Elig. Pop.	Pregnant women seen during the 1st trimester according to their Body Mass Index (BMI)				
		High BMI				
		Age Group			Total	%
		10-14	15-19	20-49		
**Region 8**	**93,813**	**5**	**218**	**2,837**	**3,060**	**3.26**
Biliran	**3,373**	0	23	300	**323**	**9.58**
Eastern Samar	**9,624**	0	23	231	**254**	**2.64**
Leyte	**31,559**	2	28	508	**538**	**1.70**
Northern Samar	**14,056**	1	33	371	**405**	**2.88**
Southern Leyte	**7,406**	1	18	422	**441**	**5.95**
Samar	**17,011**	0	41	426	**467**	**2.75**
Ormoc City	**5,292**	1	37	477	**515**	**9.73**
City of Tacloban	**5,492**	0	15	102	**117**	**2.13**
**Region 9**	**84,815**	**10**	**256**	**2,360**	**2,626**	**3.10**
Zamboanga del Norte	**24,841**	0	36	425	**461**	**1.86**
Zamboanga del Sur	**23,757**	0	49	540	**589**	**2.48**
Zamboanga Sibugay	**13,841**	2	46	489	**537**	**3.88**
City of Isabela	**3,201**	4	2	36	**42**	**1.31**
City of Zamboanga	**19,175**	4	123	870	**997**	**5.20**
**Region 10**	**109,169**	**18**	**491**	**5,514**	**6,023**	**5.52**
Bukidnon	**33,403**	11	241	2,425	**2,677**	**8.01**
Camiguin	**1,727**	0	0	21	**21**	**1.22**
Lanao del Norte	**17,760**	0	3	97	**100**	**0.56**
Misamis Occidental	**12,328**	0	12	115	**127**	**1.03**
Misamis Oriental	**21,975**	1	47	559	**607**	**2.76**
City of Cagayan De Oro	**14,308**	5	151	1,956	**2,112**	**14.76**
City of Iligan	**7,668**	1	37	341	**379**	**4.94**
**Region 11**	**107,001**	**50**	**1,225**	**15,354**	**16,629**	**15.54**
Davao de Oro	**15,253**	16	323	3,757	**4,096**	**26.85**
Davao del Norte	**23,453**	13	241	3,533	**3,787**	**16.15**
Davao Oriental	**11,935**	2	162	1,849	**2,013**	**16.87**
Davao del Sur	**13,083**	8	143	1,664	**1,815**	**13.87**
Davao Occidental	**6,629**	3	47	372	**422**	**6.37**
City of Davao	**36,648**	8	309	4,179	**4,496**	**12.27**

Table 2.B.1.2 - Prenatal Care
Pregnant women according to their nutritional status
Philippines, 2023

Area	Elig. Pop.	Pregnant women seen during the 1st trimester according to their Body Mass Index (BMI)				
		High BMI				
		Age Group			Total	%
		10-14	15-19	20-49		
**Region 12**	**95,279**	**17**	**607**	**6,412**	**7,036**	**7.38**
Cotabato	**26,521**	5	106	1,195	**1,306**	**4.92**
Sarangani	**13,322**	0	143	1,346	**1,489**	**11.18**
South Cotabato	**21,595**	3	134	1,694	**1,831**	**8.48**
Sultan Kudarat	**19,391**	9	125	1,255	**1,389**	**7.16**
City of General Santos	**14,450**	0	99	922	**1,021**	**7.07**
**BARMM**	**132,841**	**4**	**337**	**6,412**	**6,753**	**5.08**
Basilan	**11,636**	0	13	99	**112**	**0.96**
Lanao del Sur	**33,374**	3	207	4,409	**4,619**	**13.84**
Maguindanao	**41,650**	1	63	878	**942**	**2.26**
Sulu	**21,378**	0	34	526	**560**	**2.62**
Tawi-Tawi	**12,609**	0	12	282	**294**	**2.33**
SGU	**5,103**	0	4	48	**52**	**1.02**
City of Cotabato	**7,091**	0	4	170	**174**	**2.45**
**Caraga**	**55,065**	**4**	**169**	**2,643**	**2,816**	**5.11**
Agusan del Norte	**7,286**	1	20	263	**284**	**3.90**
Agusan del Sur	**16,038**	2	44	579	**625**	**3.90**
Surigao del Norte	**10,186**	0	25	597	**622**	**6.11**
Surigao del Sur	**12,736**	1	57	807	**865**	**6.79**
Dinagat Islands	**2,134**	0	8	139	**147**	**6.89**
City of Butuan	**6,685**	0	15	258	**273**	**4.08**

Table 2.B.1.3 - Prenatal Care
Pregnant women given Tetanus Diphtheria (Td) Vaccination
Philippines, 2023

Area	Eligible Pop.	Pregnant women for the 1st time given at least two (2) doses of Td Vaccine							
		Age Group						Total	%
		10-14 yrs old		15-19 yrs old		20-49 yrs old			
		No.	%	No.	%	No.	%		
**Philippines**	**2,187,333**	**1,903**	**0.09**	**80,116**	**3.66**	**352,248**	**16.10**	**434,267**	**19.85**
**N C R**	**261,228**	**155**	**0.06**	**6,763**	**2.59**	**45,800**	**17.53**	**52,718**	**20.18**
Malabon City	**7,385**	4	**0.05**	162	**2.19**	774	**10.48**	**940**	**12.73**
Navotas City	**5,210**	4	**0.08**	122	**2.34**	711	**13.65**	**837**	**16.07**
Valenzuela City	**13,477**	10	**0.07**	334	**2.48**	3,473	**25.77**	**3,817**	**28.32**
Caloocan City	**33,168**	16	**0.05**	931	**2.81**	5,223	**15.75**	**6,170**	**18.60**
Marikina City	**8,342**	6	**0.07**	310	**3.72**	977	**11.71**	**1,293**	**15.50**
Pasig City	**17,682**	6	**0.03**	220	**1.24**	2,446	**13.83**	**2,672**	**15.11**
Pateros	**1,116**	0	**0.00**	10	**0.90**	141	**12.63**	**151**	**13.53**
Taguig City	**18,992**	13	**0.07**	545	**2.87**	2,988	**15.73**	**3,546**	**18.67**
Quezon City	**59,136**	33	**0.06**	1,613	**2.73**	10,558	**17.85**	**12,204**	**20.64**
Makati City	**10,944**	22	**0.20**	105	**0.96**	920	**8.41**	**1,047**	**9.57**
Mandaluyong City	**7,922**	4	**0.05**	139	**1.75**	2,367	**29.88**	**2,510**	**31.68**
San Juan City	**2,168**	0	**0.00**	19	**0.88**	199	**9.18**	**218**	**10.06**
Manila City	**34,128**	18	**0.05**	1,188	**3.48**	6,308	**18.48**	**7,514**	**22.02**
Las Piñas City	**11,263**	7	**0.06**	270	**2.40**	2,516	**22.34**	**2,793**	**24.80**
Muntinlupa City	**9,881**	9	**0.09**	377	**3.82**	3,619	**36.63**	**4,005**	**40.53**
Parañaque City	**13,280**	0	**0.00**	258	**1.94**	1,500	**11.30**	**1,758**	**13.24**
Pasay City	**7,134**	3	**0.04**	160	**2.24**	1,080	**15.14**	**1,243**	**17.42**
**C A R**	**31,319**	**18**	**0.06**	**770**	**2.46**	**4,496**	**14.36**	**5,284**	**16.87**
Abra	**3,660**	0	**0.00**	114	**3.11**	550	**15.03**	**664**	**18.14**
Apayao	**2,238**	4	**0.18**	76	**3.40**	223	**9.96**	**303**	**13.54**
Benguet	**8,806**	1	**0.01**	124	**1.41**	1,063	**12.07**	**1,188**	**13.49**
Ifugao	**4,098**	6	**0.15**	144	**3.51**	758	**18.50**	**908**	**22.16**
Kalinga	**4,549**	1	**0.02**	177	**3.89**	782	**17.19**	**960**	**21.10**
Mountain Province	**2,619**	6	**0.23**	101	**3.86**	662	**25.28**	**769**	**29.36**
City of Baguio	**5,349**	0	**0.00**	34	**0.64**	458	**8.56**	**492**	**9.20**
**Region 1**	**96,016**	**51**	**0.05**	**3,047**	**3.17**	**19,190**	**19.99**	**22,288**	**23.21**
Ilocos Norte	**8,912**	5	**0.06**	269	**3.02**	2,638	**29.60**	**2,912**	**32.68**
Ilocos Sur	**9,914**	9	**0.09**	295	**2.98**	3,284	**33.12**	**3,588**	**36.19**
La Union	**13,507**	13	**0.10**	464	**3.44**	2,861	**21.18**	**3,338**	**24.71**
Pangasinan	**60,360**	19	**0.03**	1,854	**3.07**	10,021	**16.60**	**11,894**	**19.71**

Table 2.B.1.3 - Prenatal Care
Pregnant women given Tetanus Diphtheria (Td) Vaccination
Philippines, 2023

Area	Eligible Pop.	Pregnant women for the 1st time given at least two (2) doses of Td Vaccine							
		Age Group						Total	%
		10-14 yrs old		15-19 yrs old		20-49 yrs old			
		No.	%	No.	%	No.	%		
City of Dagupan	**3,323**	5	**0.15**	165	**4.97**	386	**11.62**	**556**	**16.73**
**Region 2**	**63,967**	**72**	**0.11**	**2,531**	**3.96**	**10,120**	**15.82**	**12,723**	**19.89**
Batanes	**240**	2	**0.83**	12	**5.00**	47	**19.58**	**61**	**25.42**
Cagayan	**20,786**	9	**0.04**	684	**3.29**	2,453	**11.80**	**3,146**	**15.14**
Isabela	**27,379**	48	**0.18**	1,144	**4.18**	4,878	**17.82**	**6,070**	**22.17**
Nueva Vizcaya	**8,798**	7	**0.08**	371	**4.22**	1,453	**16.52**	**1,831**	**20.81**
Quirino	**3,839**	6	**0.16**	189	**4.92**	797	**20.76**	**992**	**25.84**
City of Santiago	**2,925**	0	**0.00**	131	**4.48**	492	**16.82**	**623**	**21.30**
			**0.00**		**0.00**		**0.00**		
**Region 3**	**233,544**	**200**	**0.09**	**8,212**	**3.52**	**40,046**	**17.15**	**48,458**	**20.75**
Aurora	**4,537**	5	**0.11**	197	**4.34**	749	**16.51**	**951**	**20.96**
Bataan	**17,380**	18	**0.10**	694	**3.99**	2,233	**12.85**	**2,945**	**16.94**
Bulacan	**67,590**	62	**0.09**	2,900	**4.29**	16,324	**24.15**	**19,286**	**28.53**
Nueva Ecija	**43,425**	55	**0.13**	1,712	**3.94**	5,890	**13.56**	**7,657**	**17.63**
Pampanga	**45,651**	22	**0.05**	1,125	**2.46**	7,445	**16.31**	**8,592**	**18.82**
Tarlac	**29,503**	18	**0.06**	990	**3.36**	4,723	**16.01**	**5,731**	**19.43**
Zambales	**11,723**	18	**0.15**	390	**3.33**	1,123	**9.58**	**1,531**	**13.06**
City of Angeles	**9,146**	1	**0.01**	88	**0.96**	790	**8.64**	**879**	**9.61**
City of Olongapo	**4,589**	1	**0.02**	116	**2.53**	769	**16.76**	**886**	**19.31**
**Region 4A**	**307,982**	**193**	**0.06**	**8,761**	**2.84**	**47,498**	**15.42**	**56,452**	**18.33**
Batangas	**53,251**	24	**0.05**	1,651	**3.10**	11,615	**21.81**	**13,290**	**24.96**
Cavite	**77,964**	40	**0.05**	1,722	**2.21**	9,896	**12.69**	**11,658**	**14.95**
Laguna	**61,525**	61	**0.10**	2,324	**3.78**	14,084	**22.89**	**16,469**	**26.77**
Quezon	**40,810**	33	**0.08**	1,415	**3.47**	5,039	**12.35**	**6,487**	**15.90**
Rizal	**68,235**	30	**0.04**	1,416	**2.08**	5,952	**8.72**	**7,398**	**10.84**
City of Lucena	**6,197**	5	**0.08**	233	**3.76**	912	**14.72**	**1,150**	**18.56**
**Region 4B**	**66,121**	**60**	**0.09**	**3,135**	**4.74**	**11,958**	**18.09**	**15,153**	**22.92**
Marinduque	**4,144**	3	**0.07**	169	**4.08**	579	**13.97**	**751**	**18.12**
Mindoro Occidental	**11,640**	8	**0.07**	589	**5.06**	1,495	**12.84**	**2,092**	**17.97**
Mindoro Oriental	**18,943**	8	**0.04**	686	**3.62**	4,579	**24.17**	**5,273**	**27.84**
Palawan	**20,317**	27	**0.13**	1,069	**5.26**	2,995	**14.74**	**4,091**	**20.14**
Romblon	**5,489**	6	**0.11**	232	**4.23**	985	**17.94**	**1,223**	**22.28**

Table 2.B.1.3 - Prenatal Care
Pregnant women given Tetanus Diphtheria (Td) Vaccination
Philippines, 2023

Area	Eligible Pop.	Pregnant women for the 1st time given at least two (2) doses of Td Vaccine							
		Age Group						Total	%
		10-14 yrs old		15-19 yrs old		20-49 yrs old			
		No.	%	No.	%	No.	%		
Puerto Princesa City	**5,588**	8	**0.14**	390	**6.98**	1,325	**23.71**	**1,723**	**30.83**
**Region 5**	**135,480**	**39**	**0.03**	**3,439**	**2.54**	**15,217**	**11.23**	**18,695**	**13.80**
Albay	**27,440**	4	**0.01**	557	**2.03**	4,278	**15.59**	**4,839**	**17.63**
Camarines Norte	**14,695**	9	**0.06**	486	**3.31**	1,729	**11.77**	**2,224**	**15.13**
Camarines Sur	**43,452**	4	**0.01**	743	**1.71**	3,577	**8.23**	**4,324**	**9.95**
Catanduanes	**5,414**	7	**0.13**	247	**4.56**	746	**13.78**	**1,000**	**18.47**
Masbate	**22,365**	9	**0.04**	716	**3.20**	2,043	**9.13**	**2,768**	**12.38**
Sorsogon	**17,929**	4	**0.02**	581	**3.24**	2,296	**12.81**	**2,881**	**16.07**
City of Naga	**4,185**	2	**0.05**	109	**2.60**	548	**13.09**	**659**	**15.75**
**Region 6**	**148,020**	**100**	**0.07**	**5,161**	**3.49**	**27,112**	**18.32**	**32,373**	**21.87**
Aklan	**11,283**	5	**0.04**	355	**3.15**	2,265	**20.07**	**2,625**	**23.27**
Antique	**11,930**	2	**0.02**	379	**3.18**	2,167	**18.16**	**2,548**	**21.36**
Capiz	**13,931**	15	**0.11**	395	**2.84**	1,946	**13.97**	**2,356**	**16.91**
Guimaras	**3,351**	3	**0.09**	149	**4.45**	893	**26.65**	**1,045**	**31.18**
Iloilo	**36,140**	32	**0.09**	1,147	**3.17**	6,492	**17.96**	**7,671**	**21.23**
Negros Occidental	**52,270**	34	**0.07**	2,234	**4.27**	7,181	**13.74**	**9,449**	**18.08**
City of Bacolod	**10,373**	6	**0.06**	261	**2.52**	4,624	**44.58**	**4,891**	**47.15**
City of Iloilo	**8,742**	3	**0.03**	241	**2.76**	1,544	**17.66**	**1,788**	**20.45**
**Region 7**	**165,673**	**172**	**0.10**	**7,245**	**4.37**	**26,673**	**16.10**	**34,090**	**20.58**
Bohol	**26,235**	14	**0.05**	938	**3.58**	4,569	**17.42**	**5,521**	**21.04**
Cebu	**72,201**	90	**0.12**	3,158	**4.37**	9,870	**13.67**	**13,118**	**18.17**
Negros Oriental	**28,722**	27	**0.09**	1,276	**4.44**	3,996	**13.91**	**5,299**	**18.45**
Siquijor	**1,680**	1	**0.06**	66	**3.93**	306	**18.21**	**373**	**22.20**
City of Cebu	**19,267**	24	**0.12**	1,006	**5.22**	3,547	**18.41**	**4,577**	**23.76**
City of Lapu-Lapu	**10,679**	5	**0.05**	343	**3.21**	2,305	**21.58**	**2,653**	**24.84**
City of Mandaue	**6,889**	11	**0.16**	458	**6.65**	2,080	**30.19**	**2,549**	**37.00**
**Region 8**	**93,813**	**40**	**0.04**	**2,503**	**2.67**	**11,027**	**11.75**	**13,570**	**14.46**
Biliran	**3,373**	2	**0.06**	198	**5.87**	681	**20.19**	**881**	**26.12**
Eastern Samar	**9,624**	3	**0.03**	234	**2.43**	826	**8.58**	**1,063**	**11.05**
Leyte	**31,559**	15	**0.05**	673	**2.13**	3,257	**10.32**	**3,945**	**12.50**
Northern Samar	**14,056**	4	**0.03**	290	**2.06**	1,220	**8.68**	**1,514**	**10.77**

Table 2.B.1.3 - Prenatal Care
Pregnant women given Tetanus Diphtheria (Td) Vaccination
Philippines, 2023

Area	Eligible Pop.	Pregnant women for the 1st time given at least two (2) doses of Td Vaccine							
		Age Group						Total	%
		10-14 yrs old		15-19 yrs old		20-49 yrs old			
		No.	%	No.	%	No.	%		
Southern Leyte	**7,406**	2	**0.03**	197	**2.66**	1,389	**18.76**	**1,588**	**21.44**
Samar	**17,011**	8	**0.05**	363	**2.13**	1,597	**9.39**	**1,968**	**11.57**
Ormoc City	**5,292**	2	**0.04**	215	**4.06**	715	**13.51**	**932**	**17.61**
City of Tacloban	**5,492**	4	**0.07**	333	**6.06**	1,342	**24.44**	**1,679**	**30.57**
**Region 9**	**84,815**	**81**	**0.10**	**3,916**	**4.62**	**12,876**	**15.18**	**16,873**	**19.89**
Zamboanga del Norte	**24,841**	34	**0.14**	1,232	**4.96**	4,743	**19.09**	**6,009**	**24.19**
Zamboanga del Sur	**23,757**	20	**0.08**	951	**4.00**	3,067	**12.91**	**4,038**	**17.00**
Zamboanga Sibugay	**13,841**	15	**0.11**	810	**5.85**	2,167	**15.66**	**2,992**	**21.62**
City of Isabela	**3,201**	2	**0.06**	150	**4.69**	708	**22.12**	**860**	**26.87**
City of Zamboanga	**19,175**	10	**0.05**	773	**4.03**	2,191	**11.43**	**2,974**	**15.51**
**Region 10**	**109,169**	**157**	**0.14**	**6,137**	**5.62**	**16,676**	**15.28**	**22,970**	**21.04**
Bukidnon	**33,403**	71	**0.21**	2,296	**6.87**	4,582	**13.72**	**6,949**	**20.80**
Camiguin	**1,727**	1	**0.06**	83	**4.81**	192	**11.12**	**276**	**15.98**
Lanao del Norte	**17,760**	13	**0.07**	573	**3.23**	2,891	**16.28**	**3,477**	**19.58**
Misamis Occidental	**12,328**	24	**0.19**	605	**4.91**	2,101	**17.04**	**2,730**	**22.14**
Misamis Oriental	**21,975**	23	**0.10**	1,045	**4.76**	2,614	**11.90**	**3,682**	**16.76**
City of Cagayan De Oro	**14,308**	19	**0.13**	1,128	**7.88**	2,781	**19.44**	**3,928**	**27.45**
City of Iligan	**7,668**	6	**0.08**	407	**5.31**	1,515	**19.76**	**1,928**	**25.14**
**Region 11**	**107,001**	**323**	**0.30**	**7,203**	**6.73**	**19,410**	**18.14**	**26,936**	**25.17**
Davao de Oro	**15,253**	53	**0.35**	1,177	**7.72**	2,286	**14.99**	**3,516**	**23.05**
Davao del Norte	**23,453**	78	**0.33**	1,524	**6.50**	3,764	**16.05**	**5,366**	**22.88**
Davao Oriental	**11,935**	42	**0.35**	830	**6.95**	1,785	**14.96**	**2,657**	**22.26**
Davao del Sur	**13,083**	44	**0.34**	1,041	**7.96**	1,911	**14.61**	**2,996**	**22.90**
Davao Occidental	**6,629**	34	**0.51**	645	**9.73**	601	**9.07**	**1,280**	**19.31**
City of Davao	**36,648**	72	**0.20**	1,986	**5.42**	9,063	**24.73**	**11,121**	**30.35**
**Region 12**	**95,279**	**162**	**0.17**	**6,258**	**6.57**	**20,383**	**21.39**	**26,803**	**28.13**
Cotabato	**26,521**	44	**0.17**	1,719	**6.48**	6,223	**23.46**	**7,986**	**30.11**
Sarangani	**13,322**	22	**0.17**	1,085	**8.14**	2,691	**20.20**	**3,798**	**28.51**
South Cotabato	**21,595**	25	**0.12**	1,525	**7.06**	5,259	**24.35**	**6,809**	**31.53**
Sultan Kudarat	**19,391**	54	**0.28**	1,156	**5.96**	3,249	**16.76**	**4,459**	**23.00**

Table 2.B.1.3 - Prenatal Care
Pregnant women given Tetanus Diphtheria (Td) Vaccination
Philippines, 2023

Area	Eligible Pop.	Pregnant women for the 1st time given at least two (2) doses of Td Vaccine							
		Age Group						Total	%
		10-14 yrs old		15-19 yrs old		20-49 yrs old			
		No.	%	No.	%	No.	%		
City of General Santos	**14,450**	17	**0.12**	773	**5.35**	2,961	**20.49**	**3,751**	**25.96**
**BARMM**	**132,841**	**30**	**0.02**	**2,268**	**1.71**	**14,942**	**11.25**	**17,240**	**12.98**
Basilan	**11,636**	3	**0.03**	206	**1.77**	629	**5.41**	**838**	**7.20**
Lanao del Sur	**33,374**	3	**0.01**	332	**0.99**	5,347	**16.02**	**5,682**	**17.03**
Maguindanao	**41,650**	9	**0.02**	866	**2.08**	3,409	**8.18**	**4,284**	**10.29**
Sulu	**21,378**	1	**0.00**	258	**1.21**	2,058	**9.63**	**2,317**	**10.84**
Tawi-Tawi	**12,609**	4	**0.03**	315	**2.50**	1,551	**12.30**	**1,870**	**14.83**
SGU	**5,103**	3	**0.06**	72	**1.41**	349	**6.84**	**424**	**8.31**
City of Cotabato	**7,091**	7	**0.10**	219	**3.09**	1,599	**22.55**	**1,825**	**25.74**
**Caraga**	**55,065**	**50**	**0.09**	**2,767**	**5.02**	**8,824**	**16.02**	**11,641**	**21.14**
Agusan del Norte	**7,286**	4	**0.05**	365	**5.01**	968	**13.29**	**1,337**	**18.35**
Agusan del Sur	**16,038**	10	**0.06**	778	**4.85**	2,371	**14.78**	**3,159**	**19.70**
Surigao del Norte	**10,186**	6	**0.06**	435	**4.27**	1,702	**16.71**	**2,143**	**21.04**
Surigao del Sur	**12,736**	18	**0.14**	660	**5.18**	2,081	**16.34**	**2,759**	**21.66**
Dinagat Islands	**2,134**	1	**0.05**	89	**4.17**	364	**17.06**	**454**	**21.27**
City of Butuan	**6,685**	11	**0.16**	440	**6.58**	1,338	**20.01**	**1,789**	**26.76**

Table 1.B.1.4 - Prenatal Care
Pregnant women given Tetanus Diphtheria (Td) Vaccination
Philippines, 2023

Area	Eligible Pop.	Women pregnant for the 2nd or more times given at least three (3) doses of Td Vaccine							
		Age Group						Total	%
		10-14 yrs old		15-19 yrs old		20-49 yrs old			
		No.	%	No.	%	No.	%		
**Philippines**	2,187,333	832	0.04	45,849	2.10	656,737	30.02	703,418	32.16
**N C R**	261,228	33	0.01	2,752	1.05	89,054	34.09	91,839	35.16
Malabon City	7,385	0	0.00	59	0.80	1,226	16.60	1,285	17.40
Navotas City	5,210	1	0.02	91	1.75	1,239	23.78	1,331	25.55
Valenzuela City	13,477	5	0.04	184	1.37	2,719	20.18	2,908	21.58
Caloocan City	33,168	0	0.00	270	0.81	6,855	20.67	7,125	21.48
Marikina City	8,342	0	0.00	131	1.57	1,852	22.20	1,983	23.77
Pasig City	17,682	0	0.00	33	0.19	2,780	15.72	2,813	15.91
Pateros	1,116	1	0.09	5	0.45	188	16.85	194	17.38
Taguig City	18,992	8	0.04	434	2.29	5,995	31.57	6,437	33.89
Quezon City	59,136	8	0.01	343	0.58	37,379	63.21	37,730	63.80
Makati City	10,944	0	0.00	25	0.23	1,368	12.50	1,393	12.73
Mandaluyong City	7,922	4	0.05	158	1.99	5,001	63.13	5,163	65.17
San Juan City	2,168	0	0.00	5	0.23	182	8.39	187	8.63
Manila City	34,128	2	0.01	464	1.36	10,276	30.11	10,742	31.48
Las Piñas City	11,263	4	0.04	198	1.76	3,908	34.70	4,110	36.49
Muntinlupa City	9,881	0	0.00	104	1.05	3,404	34.45	3,508	35.50
Parañaque City	13,280	0	0.00	189	1.42	2,584	19.46	2,773	20.88
Pasay City	7,134	0	0.00	59	0.83	2,098	29.41	2,157	30.24
**C A R**	31,319	8	0.03	534	1.71	7,324	23.39	7,866	25.12
Abra	3,660	0	0.00	120	3.28	1,008	27.54	1,128	30.82
Apayao	2,238	0	0.00	32	1.43	497	22.21	529	23.64
Benguet	8,806	0	0.00	57	0.65	1,542	17.51	1,599	18.16
Ifugao	4,098	6	0.15	102	2.49	1,200	29.28	1,308	31.92
Kalinga	4,549	1	0.02	161	3.54	1,840	40.45	2,002	44.01
Mountain Province	2,619	0	0.00	57	2.18	756	28.87	813	31.04
City of Baguio	5,349	1	0.02	5	0.09	481	8.99	487	9.10
**Region 1**	96,016	28	0.03	1,858	1.94	33,468	34.86	35,354	36.82
Ilocos Norte	8,912	2	0.02	157	1.76	3,509	39.37	3,668	41.16
Ilocos Sur	9,914	5	0.05	134	1.35	4,721	47.62	4,860	49.02
La Union	13,507	7	0.05	329	2.44	4,468	33.08	4,804	35.57
Pangasinan	60,360	14	0.02	1,200	1.99	19,776	32.76	20,990	34.77

Table 1.B.1.4 - Prenatal Care
Pregnant women given Tetanus Diphtheria (Td) Vaccination
Philippines, 2023

Area	Eligible Pop.	Women pregnant for the 2nd or more times given at least three (3) doses of Td Vaccine							
		Age Group						Total	%
		10-14 yrs old		15-19 yrs old		20-49 yrs old			
		No.	%	No.	%	No.	%		
City of Dagupan	**3,323**	0	**0.00**	38	**1.14**	994	**29.91**	**1,032**	**31.06**
**Region 2**	**63,967**	**28**	**0.04**	**1,495**	**2.34**	**21,028**	**32.87**	**22,551**	**35.25**
Batanes	**240**	0	**0.00**	2	**0.83**	58	**24.17**	**60**	**25.00**
Cagayan	**20,786**	3	**0.01**	267	**1.28**	5,188	**24.96**	**5,458**	**26.26**
Isabela	**27,379**	21	**0.08**	741	**2.71**	10,654	**38.91**	**11,416**	**41.70**
Nueva Vizcaya	**8,798**	1	**0.01**	257	**2.92**	2,406	**27.35**	**2,664**	**30.28**
Quirino	**3,839**	3	**0.08**	69	**1.80**	1,167	**30.40**	**1,239**	**32.27**
City of Santiago	**2,925**	0	**0.00**	159	**5.44**	1,555	**53.16**	**1,714**	**58.60**
**Region 3**	**233,544**	**126**	**0.05**	**5,942**	**2.54**	**77,413**	**33.15**	**83,481**	**35.75**
Aurora	**4,537**	1	**0.02**	54	**1.19**	1,334	**29.40**	**1,389**	**30.61**
Bataan	**17,380**	6	**0.03**	348	**2.00**	5,303	**30.51**	**5,657**	**32.55**
Bulacan	**67,590**	45	**0.07**	2,066	**3.06**	28,774	**42.57**	**30,885**	**45.69**
Nueva Ecija	**43,425**	22	**0.05**	1,084	**2.50**	12,825	**29.53**	**13,931**	**32.08**
Pampanga	**45,651**	13	**0.03**	1,186	**2.60**	14,922	**32.69**	**16,121**	**35.31**
Tarlac	**29,503**	33	**0.11**	838	**2.84**	8,821	**29.90**	**9,692**	**32.85**
Zambales	**11,723**	4	**0.03**	146	**1.25**	2,674	**22.81**	**2,824**	**24.09**
City of Angeles	**9,146**	2	**0.02**	133	**1.45**	1,500	**16.40**	**1,635**	**17.88**
City of Olongapo	**4,589**	0	**0.00**	87	**1.90**	1,260	**27.46**	**1,347**	**29.35**
**Region 4A**	**307,982**	**121**	**0.04**	**5,352**	**1.74**	**82,009**	**26.63**	**87,482**	**28.40**
Batangas	**53,251**	13	**0.02**	870	**1.63**	18,600	**34.93**	**19,483**	**36.59**
Cavite	**77,964**	21	**0.03**	1,275	**1.64**	17,841	**22.88**	**19,137**	**24.55**
Laguna	**61,525**	18	**0.03**	1,279	**2.08**	21,268	**34.57**	**22,565**	**36.68**
Quezon	**40,810**	14	**0.03**	607	**1.49**	9,954	**24.39**	**10,575**	**25.91**
Rizal	**68,235**	51	**0.07**	1,170	**1.71**	12,901	**18.91**	**14,122**	**20.70**
City of Lucena	**6,197**	4	**0.06**	151	**2.44**	1,445	**23.32**	**1,600**	**25.82**
**Region 4B**	**66,121**	**20**	**0.03**	**1,427**	**2.16**	**22,740**	**34.39**	**24,187**	**36.58**
Marinduque	**4,144**	0	**0.00**	31	**0.75**	1,188	**28.67**	**1,219**	**29.42**
Mindoro Occidental	**11,640**	0	**0.00**	231	**1.98**	3,580	**30.76**	**3,811**	**32.74**
Mindoro Oriental	**18,943**	13	**0.07**	541	**2.86**	8,954	**47.27**	**9,508**	**50.19**
Palawan	**20,317**	5	**0.02**	432	**2.13**	5,325	**26.21**	**5,762**	**28.36**
Romblon	**5,489**	2	**0.04**	131	**2.39**	2,367	**43.12**	**2,500**	**45.55**

Table 1.B.1.4 - Prenatal Care

Pregnant women given Tetanus Diphtheria (Td) Vaccination

Philippines, 2023

Area	Eligible Pop.	Women pregnant for the 2nd or more times given at least three (3) doses of Td Vaccine							
		Age Group						Total	%
		10-14 yrs old		15-19 yrs old		20-49 yrs old			
		No.	%	No.	%	No.	%		
Puerto Princesa City	**5,588**	0	**0.00**	61	**1.09**	1,326	**23.73**	**1,387**	**24.82**
**Region 5**	**135,480**	**17**	**0.01**	**1,627**	**1.20**	**28,486**	**21.03**	**30,130**	**22.24**
Albay	**27,440**	1	**0.00**	179	**0.65**	7,042	**25.66**	**7,222**	**26.32**
Camarines Norte	**14,695**	3	**0.02**	239	**1.63**	3,765	**25.62**	**4,007**	**27.27**
Camarines Sur	**43,452**	3	**0.01**	235	**0.54**	5,434	**12.51**	**5,672**	**13.05**
Catanduanes	**5,414**	1	**0.02**	140	**2.59**	1,400	**25.86**	**1,541**	**28.46**
Masbate	**22,365**	4	**0.02**	494	**2.21**	4,842	**21.65**	**5,340**	**23.88**
Sorsogon	**17,929**	5	**0.03**	317	**1.77**	5,178	**28.88**	**5,500**	**30.68**
City of Naga	**4,185**	0	**0.00**	23	**0.55**	825	**19.71**	**848**	**20.26**
**Region 6**	**148,020**	**39**	**0.03**	**3,089**	**2.09**	**44,449**	**30.03**	**47,577**	**32.14**
Aklan	**11,283**	1	**0.01**	161	**1.43**	3,299	**29.24**	**3,461**	**30.67**
Antique	**11,930**	2	**0.02**	215	**1.80**	3,839	**32.18**	**4,056**	**34.00**
Capiz	**13,931**	5	**0.04**	248	**1.78**	3,897	**27.97**	**4,150**	**29.79**
Guimaras	**3,351**	3	**0.09**	141	**4.21**	1,779	**53.09**	**1,923**	**57.39**
Iloilo	**36,140**	14	**0.04**	788	**2.18**	11,722	**32.43**	**12,524**	**34.65**
Negros Occidental	**52,270**	13	**0.02**	1,307	**2.50**	15,474	**29.60**	**16,794**	**32.13**
City of Bacolod	**10,373**	1	**0.01**	133	**1.28**	2,240	**21.59**	**2,374**	**22.89**
City of Iloilo	**8,742**	0	**0.00**	96	**1.10**	2,199	**25.15**	**2,295**	**26.25**
**Region 7**	**165,673**	**123**	**0.07**	**6,963**	**4.20**	**72,377**	**43.69**	**79,463**	**47.96**
Bohol	**26,235**	10	**0.04**	752	**2.87**	9,307	**35.48**	**10,069**	**38.38**
Cebu	**72,201**	29	**0.04**	2,061	**2.85**	23,154	**32.07**	**25,244**	**34.96**
Negros Oriental	**28,722**	8	**0.03**	732	**2.55**	8,649	**30.11**	**9,389**	**32.69**
Siquijor	**1,680**	1	**0.06**	74	**4.40**	763	**45.42**	**838**	**49.88**
City of Cebu	**19,267**	20	**0.10**	1,095	**5.68**	9,900	**51.38**	**11,015**	**57.17**
City of Lapu-Lapu	**10,679**	0	**0.00**	159	**1.49**	4,298	**40.25**	**4,457**	**41.74**
City of Mandaue	**6,889**	55	**0.80**	2,090	**30.34**	16,306	**236.70**	**18,451**	**267.83**
**Region 8**	**93,813**	**14**	**0.01**	**1,061**	**1.13**	**20,611**	**21.97**	**21,686**	**23.12**
Biliran	**3,373**	1	**0.03**	100	**2.96**	1,396	**41.39**	**1,497**	**44.38**
Eastern Samar	**9,624**	0	**0.00**	119	**1.24**	1,870	**19.43**	**1,989**	**20.67**
Leyte	**31,559**	8	**0.03**	413	**1.31**	7,704	**24.41**	**8,125**	**25.75**
Northern Samar	**14,056**	3	**0.02**	78	**0.55**	1,909	**13.58**	**1,990**	**14.16**

Table 1.B.1.4 - Prenatal Care
Pregnant women given Tetanus Diphtheria (Td) Vaccination
Philippines, 2023

Area	Eligible Pop.	Women pregnant for the 2nd or more times given at least three (3) doses of Td Vaccine							
		Age Group						Total	%
		10-14 yrs old		15-19 yrs old		20-49 yrs old			
		No.	%	No.	%	No.	%		
Southern Leyte	**7,406**	0	**0.00**	69	**0.93**	1,694	**22.87**	**1,763**	**23.81**
Samar	**17,011**	0	**0.00**	122	**0.72**	2,515	**14.78**	**2,637**	**15.50**
Ormoc City	**5,292**	2	**0.04**	57	**1.08**	1,355	**25.60**	**1,414**	**26.72**
City of Tacloban	**5,492**	0	**0.00**	103	**1.88**	2,168	**39.48**	**2,271**	**41.35**
**Region 9**	**84,815**	**27**	**0.03**	**1,896**	**2.24**	**21,273**	**25.08**	**23,196**	**27.35**
Zamboanga del Norte	**24,841**	10	**0.04**	565	**2.27**	6,814	**27.43**	**7,389**	**29.75**
Zamboanga del Sur	**23,757**	3	**0.01**	337	**1.42**	4,666	**19.64**	**5,006**	**21.07**
Zamboanga Sibugay	**13,841**	6	**0.04**	365	**2.64**	3,919	**28.31**	**4,290**	**30.99**
City of Isabela	**3,201**	2	**0.06**	42	**1.31**	632	**19.74**	**676**	**21.12**
City of Zamboanga	**19,175**	6	**0.03**	587	**3.06**	5,242	**27.34**	**5,835**	**30.43**
**Region 10**	**109,169**	**59**	**0.05**	**3,721**	**3.41**	**34,560**	**31.66**	**38,340**	**35.12**
Bukidnon	**33,403**	22	**0.07**	1,352	**4.05**	10,937	**32.74**	**12,311**	**36.86**
Camiguin	**1,727**	1	**0.06**	51	**2.95**	529	**30.63**	**581**	**33.64**
Lanao del Norte	**17,760**	1	**0.01**	389	**2.19**	3,752	**21.13**	**4,142**	**23.32**
Misamis Occidental	**12,328**	3	**0.02**	279	**2.26**	5,391	**43.73**	**5,673**	**46.02**
Misamis Oriental	**21,975**	22	**0.10**	738	**3.36**	6,220	**28.30**	**6,980**	**31.76**
City of Cagayan De Oro	**14,308**	3	**0.02**	690	**4.82**	5,285	**36.94**	**5,978**	**41.78**
City of Iligan	**7,668**	7	**0.09**	222	**2.90**	2,446	**31.90**	**2,675**	**34.89**
**Region 11**	**107,001**	**53**	**0.05**	**2,114**	**1.98**	**35,369**	**33.05**	**37,536**	**35.08**
Davao de Oro	**15,253**	7	**0.05**	327	**2.14**	5,754	**37.72**	**6,088**	**39.91**
Davao del Norte	**23,453**	12	**0.05**	414	**1.77**	7,890	**33.64**	**8,316**	**35.46**
Davao Oriental	**11,935**	13	**0.11**	276	**2.31**	4,175	**34.98**	**4,464**	**37.40**
Davao del Sur	**13,083**	4	**0.03**	283	**2.16**	4,765	**36.42**	**5,052**	**38.61**
Davao Occidental	**6,629**	1	**0.02**	182	**2.75**	1,906	**28.75**	**2,089**	**31.51**
City of Davao	**36,648**	16	**0.04**	632	**1.72**	10,879	**29.69**	**11,527**	**31.45**
**Region 12**	**95,279**	**115**	**0.12**	**3,708**	**3.89**	**31,265**	**32.81**	**35,088**	**36.83**
Cotabato	**26,521**	19	**0.07**	1,082	**4.08**	8,451	**31.87**	**9,552**	**36.02**
Sarangani	**13,322**	4	**0.03**	361	**2.71**	3,340	**25.07**	**3,705**	**27.81**
South Cotabato	**21,595**	28	**0.13**	967	**4.48**	8,778	**40.65**	**9,773**	**45.26**
Sultan Kudarat	**19,391**	57	**0.29**	724	**3.73**	5,956	**30.72**	**6,737**	**34.74**

Table 1.B.1.4 - Prenatal Care
Pregnant women given Tetanus Diphtheria (Td) Vaccination
Philippines, 2023

Area	Eligible Pop.	Women pregnant for the 2nd or more times given at least three (3) doses of Td Vaccine							
		Age Group						Total	%
		10-14 yrs old		15-19 yrs old		20-49 yrs old			
		No.	%	No.	%	No.	%		
City of General Santos	14,450	7	0.05	574	3.97	4,740	32.80	5,321	36.82
**BARMM**	132,841	7	0.01	1,140	0.86	17,793	13.39	18,940	14.26
Basilan	11,636	1	0.01	79	0.68	727	6.25	807	6.94
Lanao del Sur	33,374	1	0.00	372	1.11	6,143	18.41	6,516	19.52
Maguindanao	41,650	1	0.00	300	0.72	5,128	12.31	5,429	13.03
Sulu	21,378	0	0.00	129	0.60	2,466	11.54	2,595	12.14
Tawi-Tawi	12,609	1	0.01	151	1.20	2,142	16.99	2,294	18.19
SGU	5,103	2	0.04	46	0.90	289	5.66	337	6.60
City of Cotabato	7,091	1	0.01	63	0.89	898	12.66	962	13.57
**Caraga**	55,065	14	0.03	1,170	2.12	17,518	31.81	18,702	33.96
Agusan del Norte	7,286	2	0.03	99	1.36	1,867	25.62	1,968	27.01
Agusan del Sur	16,038	0	0.00	333	2.08	4,856	30.28	5,189	32.35
Surigao del Norte	10,186	0	0.00	153	1.50	3,122	30.65	3,275	32.15
Surigao del Sur	12,736	9	0.07	447	3.51	4,414	34.66	4,870	38.24
Dinagat Islands	2,134	1	0.05	25	1.17	579	27.13	605	28.35
City of Butuan	6,685	2	0.03	113	1.69	2,680	40.09	2,795	41.81

Table 2.B.1.5 - Prenatal Care

Pregnant women who Completed Iron with Folic Acid

Philippines, 2023

Area	Eligible Pop.	Iron with Folic Acid Supplementation							
		Age Group						Total	%
		10-14 yrs old		15-19 yrs old		20-49 yrs old			
		No.	%	No.	%	No.	%		
**Philippines**	**2,187,333**	**2,762**	**0.13**	**119,639**	**5.47**	**1,047,572**	**47.89**	**1,169,973**	**53.49**
**N C R**	**261,228**	**297**	**0.11**	**12,464**	**4.77**	**140,842**	**53.92**	**153,603**	**58.80**
Malabon City	**7,385**	26	**0.35**	639	**8.65**	4,280	**57.96**	**4,945**	**66.96**
Navotas City	**5,210**	12	**0.23**	369	**7.08**	3,486	**66.91**	**3,867**	**74.22**
Valenzuela City	**13,477**	5	**0.04**	415	**3.08**	5,174	**38.39**	**5,594**	**41.51**
Caloocan City	**33,168**	26	**0.08**	1,907	**5.75**	19,735	**59.50**	**21,668**	**65.33**
Marikina City	**8,342**	7	**0.08**	368	**4.41**	3,537	**42.40**	**3,912**	**46.90**
Pasig City	**17,682**	6	**0.03**	589	**3.33**	8,617	**48.73**	**9,212**	**52.10**
Pateros	**1,116**	0	**0.00**	27	**2.42**	678	**60.75**	**705**	**63.17**
Taguig City	**18,992**	21	**0.11**	1,075	**5.66**	10,314	**54.31**	**11,410**	**60.08**
Quezon City	**59,136**	44	**0.07**	1,995	**3.37**	32,488	**54.94**	**34,527**	**58.39**
Makati City	**10,944**	4	**0.04**	190	**1.74**	2,537	**23.18**	**2,731**	**24.95**
Mandaluyong City	**7,922**	9	**0.11**	291	**3.67**	7,171	**90.52**	**7,471**	**94.31**
San Juan City	**2,168**	0	**0.00**	25	**1.15**	625	**28.83**	**650**	**29.98**
Manila City	**34,128**	52	**0.15**	2,077	**6.09**	15,919	**46.64**	**18,048**	**52.88**
Las Piñas City	**11,263**	6	**0.05**	519	**4.61**	5,760	**51.14**	**6,285**	**55.80**
Muntinlupa City	**9,881**	56	**0.57**	788	**7.97**	8,749	**88.54**	**9,593**	**97.09**
Parañaque City	**13,280**	15	**0.11**	767	**5.78**	6,164	**46.42**	**6,946**	**52.30**
Pasay City	**7,134**	8	**0.11**	423	**5.93**	5,608	**78.61**	**6,039**	**84.65**
**C A R**	**31,319**	**25**	**0.08**	**1,594**	**5.09**	**18,952**	**60.51**	**20,571**	**65.68**
Abra	**3,660**	5	**0.14**	282	**7.70**	2,396	**65.46**	**2,683**	**73.31**
Apayao	**2,238**	1	**0.04**	126	**5.63**	1,038	**46.38**	**1,165**	**52.06**
Benguet	**8,806**	1	**0.01**	353	**4.01**	5,313	**60.33**	**5,667**	**64.35**
Ifugao	**4,098**	8	**0.20**	193	**4.71**	2,180	**53.20**	**2,381**	**58.10**
Kalinga	**4,549**	1	**0.02**	243	**5.34**	2,660	**58.47**	**2,904**	**63.84**
Mountain Province	**2,619**	6	**0.23**	261	**9.97**	2,893	**110.46**	**3,160**	**120.66**
City of Baguio	**5,349**	3	**0.06**	136	**2.54**	2,472	**46.21**	**2,611**	**48.81**
**Region 1**	**96,016**	**67**	**0.07**	**4,423**	**4.61**	**52,677**	**54.86**	**57,167**	**59.54**
Ilocos Norte	**8,912**	0	**0.00**	352	**3.95**	5,414	**60.75**	**5,766**	**64.70**
Ilocos Sur	**9,914**	11	**0.11**	362	**3.65**	8,107	**81.77**	**8,480**	**85.54**
La Union	**13,507**	12	**0.09**	673	**4.98**	7,332	**54.28**	**8,017**	**59.35**
Pangasinan	**60,360**	38	**0.06**	2,793	**4.63**	30,188	**50.01**	**33,019**	**54.70**

Table 2.B.1.5 - Prenatal Care

Pregnant women who Completed Iron with Folic Acid

Philippines, 2023

Area	Eligible Pop.	Iron with Folic Acid Supplementation							
		Age Group						Total	%
		10-14 yrs old		15-19 yrs old		20-49 yrs old			
		No.	%	No.	%	No.	%		
City of Dagupan	**3,323**	6	**0.18**	243	**7.31**	1,636	**49.23**	**1,885**	**56.73**
**Region 2**	**63,967**	**100**	**0.16**	**4,063**	**6.35**	**34,736**	**54.30**	**38,899**	**60.81**
Batanes	**240**	0	**0.00**	3	**1.25**	104	**43.33**	**107**	**44.58**
Cagayan	**20,786**	16	**0.08**	908	**4.37**	8,436	**40.59**	**9,360**	**45.03**
Isabela	**27,379**	59	**0.22**	1,856	**6.78**	15,959	**58.29**	**17,874**	**65.28**
Nueva Vizcaya	**8,798**	16	**0.18**	683	**7.76**	4,950	**56.26**	**5,649**	**64.21**
Quirino	**3,839**	6	**0.16**	319	**8.31**	2,759	**71.87**	**3,084**	**80.33**
City of Santiago	**2,925**	3	**0.10**	294	**10.05**	2,528	**86.43**	**2,825**	**96.58**
**Region 3**	**233,544**	**285**	**0.12**	**14,449**	**6.19**	**124,386**	**53.26**	**139,120**	**59.57**
Aurora	**4,537**	8	**0.18**	302	**6.66**	2,366	**52.15**	**2,676**	**58.98**
Bataan	**17,380**	24	**0.14**	1,030	**5.93**	8,127	**46.76**	**9,181**	**52.83**
Bulacan	**67,590**	96	**0.14**	4,750	**7.03**	44,102	**65.25**	**48,948**	**72.42**
Nueva Ecija	**43,425**	53	**0.12**	2,523	**5.81**	18,540	**42.69**	**21,116**	**48.63**
Pampanga	**45,651**	39	**0.09**	2,509	**5.50**	21,907	**47.99**	**24,455**	**53.57**
Tarlac	**29,503**	41	**0.14**	2,057	**6.97**	17,246	**58.46**	**19,344**	**65.57**
Zambales	**11,723**	16	**0.14**	549	**4.68**	4,131	**35.24**	**4,696**	**40.06**
City of Angeles	**9,146**	4	**0.04**	484	**5.29**	5,623	**61.48**	**6,111**	**66.82**
City of Olongapo	**4,589**	4	**0.09**	245	**5.34**	2,344	**51.08**	**2,593**	**56.50**
**Region 4A**	**307,982**	**240**	**0.08**	**12,979**	**4.21**	**126,037**	**40.92**	**139,256**	**45.22**
Batangas	**53,251**	33	**0.06**	2,158	**4.05**	25,948	**48.73**	**28,139**	**52.84**
Cavite	**77,964**	35	**0.04**	2,030	**2.60**	22,030	**28.26**	**24,095**	**30.91**
Laguna	**61,525**	69	**0.11**	3,436	**5.58**	33,761	**54.87**	**37,266**	**60.57**
Quezon	**40,810**	39	**0.10**	1,840	**4.51**	16,654	**40.81**	**18,533**	**45.41**
Rizal	**68,235**	60	**0.09**	3,170	**4.65**	25,405	**37.23**	**28,635**	**41.97**
City of Lucena	**6,197**	4	**0.06**	345	**5.57**	2,239	**36.13**	**2,588**	**41.76**
**Region 4B**	**66,121**	**96**	**0.15**	**4,020**	**6.08**	**33,778**	**51.09**	**37,894**	**57.31**
Marinduque	**4,144**	3	**0.07**	210	**5.07**	2,145	**51.76**	**2,358**	**56.90**
Occidental Mindoro	**11,640**	14	**0.12**	901	**7.74**	6,227	**53.50**	**7,142**	**61.36**
Oriental Mindoro	**18,943**	17	**0.09**	844	**4.46**	11,996	**63.33**	**12,857**	**67.87**
Palawan	**20,317**	36	**0.18**	1,572	**7.74**	9,212	**45.34**	**10,820**	**53.26**
Romblon	**5,489**	10	**0.18**	265	**4.83**	2,793	**50.88**	**3,068**	**55.89**

Table 2.B.1.5 - Prenatal Care

Pregnant women who Completed Iron with Folic Acid

Philippines, 2023

Area	Eligible Pop.	Iron with Folic Acid Supplementation							
		Age Group						Total	%
		10-14 yrs old		15-19 yrs old		20-49 yrs old			
		No.	%	No.	%	No.	%		
City of Puerto Princesa	**5,588**	16	**0.29**	228	**4.08**	1,405	**25.14**	**1,649**	**29.51**
**Region 5**	**135,480**	**41**	**0.03**	**5,056**	**3.73**	**53,208**	**39.27**	**58,305**	**43.04**
Albay	**27,440**	8	**0.03**	724	**2.64**	13,195	**48.09**	**13,927**	**50.75**
Camarines Norte	**14,695**	5	**0.03**	717	**4.88**	6,034	**41.06**	**6,756**	**45.97**
Camarines Sur	**43,452**	7	**0.02**	1,024	**2.36**	11,476	**26.41**	**12,507**	**28.78**
Catanduanes	**5,414**	6	**0.11**	324	**5.98**	2,605	**48.12**	**2,935**	**54.21**
Masbate	**22,365**	6	**0.03**	1,333	**5.96**	9,709	**43.41**	**11,048**	**49.40**
Sorsogon	**17,929**	8	**0.04**	753	**4.20**	8,631	**48.14**	**9,392**	**52.38**
City of Naga	**4,185**	1	**0.02**	181	**4.32**	1,558	**37.23**	**1,740**	**41.58**
**Region 6**	**148,020**	**103**	**0.07**	**6,775**	**4.58**	**62,161**	**42.00**	**69,039**	**46.64**
Aklan	**11,283**	5	**0.04**	442	**3.92**	5,390	**47.77**	**5,837**	**51.73**
Antique	**11,930**	2	**0.02**	328	**2.75**	4,948	**41.48**	**5,278**	**44.24**
Capiz	**13,931**	6	**0.04**	413	**2.96**	4,936	**35.43**	**5,355**	**38.44**
Guimaras	**3,351**	3	**0.09**	164	**4.89**	2,028	**60.52**	**2,195**	**65.50**
Iloilo	**36,140**	34	**0.09**	1,359	**3.76**	14,744	**40.80**	**16,137**	**44.65**
Negros Occidental	**52,270**	35	**0.07**	3,281	**6.28**	22,572	**43.18**	**25,888**	**49.53**
City of Bacolod	**10,373**	4	**0.04**	341	**3.29**	2,901	**27.97**	**3,246**	**31.29**
City of Iloilo	**8,742**	14	**0.16**	447	**5.11**	4,642	**53.10**	**5,103**	**58.37**
**Region 7**	**165,673**	**185**	**0.11**	**9,233**	**5.57**	**80,372**	**48.51**	**89,790**	**54.20**
Bohol	**26,235**	16	**0.06**	1,033	**3.94**	12,052	**45.94**	**13,101**	**49.94**
Cebu	**72,201**	84	**0.12**	3,988	**5.52**	30,956	**42.87**	**35,028**	**48.51**
Negros Oriental	**28,722**	33	**0.11**	1,810	**6.30**	12,423	**43.25**	**14,266**	**49.67**
Siquijor	**1,680**	0	**0.00**	77	**4.58**	788	**46.90**	**865**	**51.49**
City of Cebu	**19,267**	26	**0.13**	1,284	**6.66**	10,920	**56.68**	**12,230**	**63.48**
City of Lapu-Lapu	**10,679**	14	**0.13**	476	**4.46**	6,985	**65.41**	**7,475**	**70.00**
City of Mandaue	**6,889**	12	**0.17**	565	**8.20**	6,248	**90.70**	**6,825**	**99.07**
**Region 8**	**93,813**	**71**	**0.08**	**4,422**	**4.71**	**38,785**	**41.34**	**43,278**	**46.13**
Biliran	**3,373**	6	**0.18**	176	**5.22**	1,838	**54.49**	**2,020**	**59.89**
Eastern Samar	**9,624**	4	**0.04**	623	**6.47**	4,672	**48.55**	**5,299**	**55.06**
Leyte	**31,559**	39	**0.12**	1,576	**4.99**	14,181	**44.93**	**15,796**	**50.05**
Northern Samar	**14,056**	6	**0.04**	474	**3.37**	4,470	**31.80**	**4,950**	**35.22**

Table 2.B.1.5 - Prenatal Care

Pregnant women who Completed Iron with Folic Acid

Philippines, 2023

Area	Eligible Pop.	Iron with Folic Acid Supplementation							
		Age Group						Total	%
		10-14 yrs old		15-19 yrs old		20-49 yrs old			
		No.	%	No.	%	No.	%		
Southern Leyte	7,406	3	0.04	314	4.24	3,705	50.03	4,022	54.31
Samar	17,011	8	0.05	672	3.95	5,246	30.84	5,926	34.84
Ormoc City	5,292	1	0.02	340	6.42	2,588	48.90	2,929	55.35
City of Tacloban	5,492	4	0.07	247	4.50	2,085	37.96	2,336	42.53
**Region 9**	**84,815**	**80**	**0.09**	**5,207**	**6.14**	**34,583**	**40.77**	**39,870**	**47.01**
Zamboanga del Norte	24,841	39	0.16	1,447	5.83	9,580	38.57	11,066	44.55
Zamboanga del Sur	23,757	13	0.05	1,184	4.98	7,073	29.77	8,270	34.81
Zamboanga Sibugay	13,841	7	0.05	741	5.35	4,890	35.33	5,638	40.73
City of Isabela	3,201	1	0.03	187	5.84	1,310	40.92	1,498	46.80
City of Zamboanga	19,175	20	0.10	1,648	8.59	11,730	61.17	13,398	69.87
**Region 10**	**109,169**	**218**	**0.20**	**9,725**	**8.91**	**61,735**	**56.55**	**71,678**	**65.66**
Bukidnon	33,403	86	0.26	3,374	10.10	17,465	52.29	20,925	62.64
Camiguin	1,727	1	0.06	125	7.24	883	51.13	1,009	58.43
Lanao del Norte	17,760	11	0.06	1,226	6.90	9,755	54.93	10,992	61.89
Misamis Occidental	12,328	32	0.26	1,008	8.18	7,778	63.09	8,818	71.53
Misamis Oriental	21,975	54	0.25	1,827	8.31	11,827	53.82	13,708	62.38
City of Cagayan De Oro	14,308	24	0.17	1,603	11.20	9,437	65.96	11,064	77.33
City of Iligan	7,668	10	0.13	562	7.33	4,590	59.86	5,162	67.32
**Region 11**	**107,001**	**489**	**0.46**	**9,141**	**8.54**	**61,242**	**57.23**	**70,872**	**66.23**
Davao de Oro	15,253	70	0.46	1,606	10.53	8,938	58.60	10,614	69.59
Davao del Norte	23,453	111	0.47	1,983	8.46	12,309	52.48	14,403	61.41
Davao Oriental	11,935	61	0.51	1,166	9.77	6,709	56.21	7,936	66.49
Davao del Sur	13,083	67	0.51	1,059	8.09	5,919	45.24	7,045	53.85
Davao Occidental	6,629	26	0.39	621	9.37	1,874	28.27	2,521	38.03
City of Davao	36,648	154	0.42	2,706	7.38	25,493	69.56	28,353	77.37
**Region 12**	**95,279**	**375**	**0.39**	**8,768**	**9.20**	**51,143**	**53.68**	**60,286**	**63.27**
Cotabato	26,521	37	0.14	2,115	7.97	13,074	49.30	15,226	57.41
Sarangani	13,322	36	0.27	1,846	13.86	7,914	59.41	9,796	73.53
South Cotabato	21,595	33	0.15	2,105	9.75	12,713	58.87	14,851	68.77
Sultan Kudarat	19,391	258	1.33	1,589	8.19	9,229	47.59	11,076	57.12

Table 2.B.1.5 - Prenatal Care

Pregnant women who Completed Iron with Folic Acid

Philippines, 2023

Area	Eligible Pop.	Iron with Folic Acid Supplementation							
		Age Group						Total	%
		10-14 yrs old		15-19 yrs old		20-49 yrs old			
		No.	%	No.	%	No.	%		
City of General Santos	**14,450**	11	**0.08**	1,113	**7.70**	8,213	**56.84**	**9,337**	**64.62**
**BARMM**	**132,841**	**36**	**0.03**	**3,797**	**2.86**	**44,817**	**33.74**	**48,650**	**36.62**
Basilan	**11,636**	8	**0.07**	377	**3.24**	2,091	**17.97**	**2,476**	**21.28**
Lanao del Sur	**33,374**	9	**0.03**	847	**2.54**	16,410	**49.17**	**17,266**	**51.73**
Maguindanao	**41,650**	5	**0.01**	1,430	**3.43**	13,631	**32.73**	**15,066**	**36.17**
Sulu	**21,378**	2	**0.01**	469	**2.19**	6,102	**28.54**	**6,573**	**30.75**
Tawi-Tawi	**12,609**	1	**0.01**	292	**2.32**	3,533	**28.02**	**3,826**	**30.34**
SGU	**5,103**	4	**0.08**	130	**2.55**	768	**15.05**	**902**	**17.68**
City of Cotabato	**7,091**	7	**0.10**	252	**3.55**	2,282	**32.18**	**2,541**	**35.83**
**Caraga**	**55,065**	**54**	**0.10**	**3,523**	**6.40**	**28,118**	**51.06**	**31,695**	**57.56**
Agusan del Norte	**7,286**	6	**0.08**	466	**6.40**	3,044	**41.78**	**3,516**	**48.26**
Agusan del Sur	**16,038**	13	**0.08**	1,143	**7.13**	8,350	**52.06**	**9,506**	**59.27**
Surigao del Norte	**10,186**	3	**0.03**	451	**4.43**	4,925	**48.35**	**5,379**	**52.81**
Surigao del Sur	**12,736**	18	**0.14**	662	**5.20**	5,507	**43.24**	**6,187**	**48.58**
Dinagat Islands	**2,134**	2	**0.09**	112	**5.25**	985	**46.16**	**1,099**	**51.50**
City of Butuan	**6,685**	12	**0.18**	689	**10.31**	5,307	**79.39**	**6,008**	**89.87**

Table 2.B.1.6 - Prenatal Care

Pregnant women who Completed Calcium Carbonate
Philippines, 2023

Area	Eligible Pop.	Calcium Carbonate							
		Age Group						Total	%
		10-14 yrs old		15-19 yrs old		20-49 yrs old			
		No.	%	No.	%	No.	%		
**Philippines**	**2,187,333**	**1,458**	**0.07**	**75,991**	**3.47**	**693,607**	**31.71**	**771,056**	**35.25**
**N C R**	**261,228**	**125**	**0.05**	**6,979**	**2.67**	**82,144**	**31.45**	**89,248**	**34.16**
Malabon City	**7,385**	4	**0.05**	223	**3.02**	1,814	**24.56**	**2,041**	**27.64**
Navotas City	**5,210**	9	**0.17**	297	**5.70**	3,515	**67.47**	**3,821**	**73.34**
Valenzuela City	**13,477**	7	**0.05**	272	**2.02**	3,041	**22.56**	**3,320**	**24.63**
Caloocan City	**33,168**	20	**0.06**	1,295	**3.90**	15,087	**45.49**	**16,402**	**49.45**
Marikina City	**8,342**	9	**0.11**	383	**4.59**	3,439	**41.23**	**3,831**	**45.92**
Pasig City	**17,682**	3	**0.02**	381	**2.15**	6,096	**34.48**	**6,480**	**36.65**
Pateros	**1,116**	0	**0.00**	14	**1.25**	351	**31.45**	**365**	**32.71**
Taguig City	**18,992**	13	**0.07**	950	**5.00**	9,976	**52.53**	**10,939**	**57.60**
Quezon City	**59,136**	5	**0.01**	307	**0.52**	6,791	**11.48**	**7,103**	**12.01**
Makati City	**10,944**	2	**0.02**	171	**1.56**	3,416	**31.21**	**3,589**	**32.79**
Mandaluyong City	**7,922**	3	**0.04**	151	**1.91**	5,033	**63.53**	**5,187**	**65.48**
San Juan City	**2,168**	0	**0.00**	29	**1.34**	600	**27.68**	**629**	**29.01**
Manila City	**34,128**	35	**0.10**	1,301	**3.81**	10,534	**30.87**	**11,870**	**34.78**
Las Piñas City	**11,263**	7	**0.06**	325	**2.89**	3,233	**28.70**	**3,565**	**31.65**
Muntinlupa City	**9,881**	1	**0.01**	52	**0.53**	265	**2.68**	**318**	**3.22**
Parañaque City	**13,280**	3	**0.02**	448	**3.37**	3,741	**28.17**	**4,192**	**31.57**
Pasay City	**7,134**	4	**0.06**	380	**5.33**	5,212	**73.06**	**5,596**	**78.44**
**C A R**	**31,319**	**9**	**0.03**	**1,315**	**4.20**	**15,700**	**50.13**	**17,024**	**54.36**
Abra	**3,660**	3	**0.08**	150	**4.10**	1,343	**36.69**	**1,496**	**40.87**
Apayao	**2,238**	1	**0.04**	154	**6.88**	1,045	**46.69**	**1,200**	**53.62**
Benguet	**8,806**	0	**0.00**	285	**3.24**	4,802	**54.53**	**5,087**	**57.77**
Ifugao	**4,098**	2	**0.05**	173	**4.22**	1,771	**43.22**	**1,946**	**47.49**
Kalinga	**4,549**	0	**0.00**	239	**5.25**	2,497	**54.89**	**2,736**	**60.15**
Mountain Province	**2,619**	3	**0.11**	194	**7.41**	2,255	**86.10**	**2,452**	**93.62**
City of Baguio	**5,349**	0	**0.00**	120	**2.24**	1,987	**37.15**	**2,107**	**39.39**
**Region 1**	**96,016**	**67**	**0.07**	**4,146**	**4.32**	**48,017**	**50.01**	**52,230**	**54.40**
Ilocos Norte	**8,912**	1	**0.01**	324	**3.64**	5,229	**58.67**	**5,554**	**62.32**
Ilocos Sur	**9,914**	11	**0.11**	350	**3.53**	7,039	**71.00**	**7,400**	**74.64**
La Union	**13,507**	13	**0.10**	638	**4.72**	7,159	**53.00**	**7,810**	**57.82**
Pangasinan	**60,360**	36	**0.06**	2,592	**4.29**	27,001	**44.73**	**29,629**	**49.09**

Table 2.B.1.6 - Prenatal Care

Pregnant women who Completed Calcium Carbonate

Philippines, 2023

Area	Eligible Pop.	Calcium Carbonate							
		Age Group						Total	%
		10-14 yrs old		15-19 yrs old		20-49 yrs old			
		No.	%	No.	%	No.	%		
City of Dagupan	**3,323**	6	**0.18**	242	**7.28**	1,589	**47.82**	**1,837**	**55.28**
**Region 2**	**63,967**	**85**	**0.13**	**3,362**	**5.26**	**29,350**	**45.88**	**32,797**	**51.27**
Batanes	**240**	0	**0.00**	2	**0.83**	38	**15.83**	**40**	**16.67**
Cagayan	**20,786**	7	**0.03**	589	**2.83**	5,310	**25.55**	**5,906**	**28.41**
Isabela	**27,379**	54	**0.20**	1,688	**6.17**	14,213	**51.91**	**15,955**	**58.27**
Nueva Vizcaya	**8,798**	15	**0.17**	487	**5.54**	4,687	**53.27**	**5,189**	**58.98**
Quirino	**3,839**	6	**0.16**	306	**7.97**	2,651	**69.05**	**2,963**	**77.18**
City of Santiago	**2,925**	3	**0.10**	290	**9.91**	2,451	**83.79**	**2,744**	**93.81**
**Region 3**	**233,544**	**215**	**0.09**	**10,439**	**4.47**	**94,001**	**40.25**	**104,655**	**44.81**
Aurora	**4,537**	6	**0.13**	203	**4.47**	1,604	**35.35**	**1,813**	**39.96**
Bataan	**17,380**	14	**0.08**	681	**3.92**	5,129	**29.51**	**5,824**	**33.51**
Bulacan	**67,590**	84	**0.12**	3,907	**5.78**	37,066	**54.84**	**41,057**	**60.74**
Nueva Ecija	**43,425**	49	**0.11**	1,950	**4.49**	14,806	**34.10**	**16,805**	**38.70**
Pampanga	**45,651**	31	**0.07**	1,902	**4.17**	17,962	**39.35**	**19,895**	**43.58**
Tarlac	**29,503**	24	**0.08**	1,342	**4.55**	10,776	**36.53**	**12,142**	**41.16**
Zambales	**11,723**	2	**0.02**	125	**1.07**	1,228	**10.48**	**1,355**	**11.56**
City of Angeles	**9,146**	1	**0.01**	175	**1.91**	4,045	**44.23**	**4,221**	**46.15**
City of Olongapo	**4,589**	4	**0.09**	154	**3.36**	1,385	**30.18**	**1,543**	**33.62**
**Region 4A**	**307,982**	**154**	**0.05**	**8,541**	**2.77**	**83,176**	**27.01**	**91,871**	**29.83**
Batangas	**53,251**	17	**0.03**	985	**1.85**	11,715	**22.00**	**12,717**	**23.88**
Cavite	**77,964**	21	**0.03**	1,359	**1.74**	15,234	**19.54**	**16,614**	**21.31**
Laguna	**61,525**	45	**0.07**	2,621	**4.26**	27,133	**44.10**	**29,799**	**48.43**
Quezon	**40,810**	24	**0.06**	1,124	**2.75**	9,520	**23.33**	**10,668**	**26.14**
Rizal	**68,235**	45	**0.07**	2,243	**3.29**	18,036	**26.43**	**20,324**	**29.79**
City of Lucena	**6,197**	2	**0.03**	209	**3.37**	1,538	**24.82**	**1,749**	**28.22**
**Region 4B**	**66,121**	**29**	**0.04**	**2,084**	**3.15**	**20,343**	**30.77**	**22,456**	**33.96**
Marinduque	**4,144**	3	**0.07**	180	**4.34**	1,633	**39.41**	**1,816**	**43.82**
Occidental Mindoro	**11,640**	5	**0.04**	649	**5.58**	4,021	**34.54**	**4,675**	**40.16**
Oriental Mindoro	**18,943**	11	**0.06**	613	**3.24**	9,854	**52.02**	**10,478**	**55.31**
Palawan	**20,317**	10	**0.05**	432	**2.13**	2,829	**13.92**	**3,271**	**16.10**
Romblon	**5,489**	0	**0.00**	156	**2.84**	1,670	**30.42**	**1,826**	**33.27**

Table 2.B.1.6 - Prenatal Care

Pregnant women who Completed Calcium Carbonate
Philippines, 2023

Area	Eligible Pop.	Calcium Carbonate							
		Age Group						Total	%
		10-14 yrs old		15-19 yrs old		20-49 yrs old			
		No.	%	No.	%	No.	%		
City of Puerto Princesa	**5,588**	0	**0.00**	54	**0.97**	336	**6.01**	**390**	**6.98**
**Region 5**	**135,480**	**32**	**0.02**	**3,502**	**2.58**	**37,690**	**27.82**	**41,224**	**30.43**
Albay	**27,440**	10	**0.04**	680	**2.48**	11,408	**41.57**	**12,098**	**44.09**
Camarines Norte	**14,695**	4	**0.03**	494	**3.36**	4,055	**27.59**	**4,553**	**30.98**
Camarines Sur	**43,452**	4	**0.01**	649	**1.49**	7,780	**17.90**	**8,433**	**19.41**
Catanduanes	**5,414**	6	**0.11**	249	**4.60**	2,125	**39.25**	**2,380**	**43.96**
Masbate	**22,365**	3	**0.01**	902	**4.03**	6,084	**27.20**	**6,989**	**31.25**
Sorsogon	**17,929**	5	**0.03**	459	**2.56**	5,538	**30.89**	**6,002**	**33.48**
City of Naga	**4,185**	0	**0.00**	69	**1.65**	700	**16.73**	**769**	**18.38**
**Region 6**	**148,020**	**62**	**0.04**	**4,329**	**2.92**	**41,335**	**27.93**	**45,726**	**30.89**
Aklan	**11,283**	4	**0.04**	306	**2.71**	4,039	**35.80**	**4,349**	**38.54**
Antique	**11,930**	0	**0.00**	159	**1.33**	2,397	**20.09**	**2,556**	**21.42**
Capiz	**13,931**	3	**0.02**	258	**1.85**	3,211	**23.05**	**3,472**	**24.92**
Guimaras	**3,351**	3	**0.09**	138	**4.12**	1,646	**49.12**	**1,787**	**53.33**
Iloilo	**36,140**	20	**0.06**	919	**2.54**	10,579	**29.27**	**11,518**	**31.87**
Negros Occidental	**52,270**	22	**0.04**	2,014	**3.85**	13,797	**26.40**	**15,833**	**30.29**
City of Bacolod	**10,373**	0	**0.00**	116	**1.12**	1,040	**10.03**	**1,156**	**11.14**
City of Iloilo	**8,742**	10	**0.11**	419	**4.79**	4,626	**52.92**	**5,055**	**57.82**
**Region 7**	**165,673**	**108**	**0.07**	**5,114**	**3.09**	**48,853**	**29.49**	**54,075**	**32.64**
Bohol	**26,235**	6	**0.02**	465	**1.77**	5,636	**21.48**	**6,107**	**23.28**
Cebu	**72,201**	44	**0.06**	2,180	**3.02**	17,407	**24.11**	**19,631**	**27.19**
Negros Oriental	**28,722**	8	**0.03**	294	**1.02**	2,688	**9.36**	**2,990**	**10.41**
Siquijor	**1,680**	0	**0.00**	17	**1.01**	279	**16.61**	**296**	**17.62**
City of Cebu	**19,267**	28	**0.15**	1,203	**6.24**	10,533	**54.67**	**11,764**	**61.06**
City of Lapu-Lapu	**10,679**	10	**0.09**	431	**4.04**	6,169	**57.77**	**6,610**	**61.90**
City of Mandaue	**6,889**	12	**0.17**	524	**7.61**	6,141	**89.14**	**6,677**	**96.92**
**Region 8**	**93,813**	**38**	**0.04**	**2,459**	**2.62**	**21,928**	**23.37**	**24,425**	**26.04**
Biliran	**3,373**	4	**0.12**	121	**3.59**	1,244	**36.88**	**1,369**	**40.59**
Eastern Samar	**9,624**	4	**0.04**	584	**6.07**	3,837	**39.87**	**4,425**	**45.98**
Leyte	**31,559**	16	**0.05**	593	**1.88**	6,681	**21.17**	**7,290**	**23.10**
Northern Samar	**14,056**	2	**0.01**	224	**1.59**	2,070	**14.73**	**2,296**	**16.33**

Table 2.B.1.6 - Prenatal Care

Pregnant women who Completed Calcium Carbonate

Philippines, 2023

Area	Eligible Pop.	Calcium Carbonate							
		Age Group						Total	%
		10-14 yrs old		15-19 yrs old		20-49 yrs old			
		No.	%	No.	%	No.	%		
Southern Leyte	**7,406**	2	**0.03**	279	**3.77**	3,244	**43.80**	**3,525**	**47.60**
Samar	**17,011**	7	**0.04**	383	**2.25**	2,705	**15.90**	**3,095**	**18.19**
Ormoc City	**5,292**	3	**0.06**	138	**2.61**	1,042	**19.69**	**1,183**	**22.35**
City of Tacloban	**5,492**	0	**0.00**	137	**2.49**	1,105	**20.12**	**1,242**	**22.61**
**Region 9**	**84,815**	**32**	**0.04**	**1,947**	**2.30**	**12,549**	**14.80**	**14,528**	**17.13**
Zamboanga del Norte	**24,841**	12	**0.05**	615	**2.48**	3,615	**14.55**	**4,242**	**17.08**
Zamboanga del Sur	**23,757**	7	**0.03**	526	**2.21**	2,932	**12.34**	**3,465**	**14.59**
Zamboanga Sibugay	**13,841**	2	**0.01**	203	**1.47**	1,638	**11.83**	**1,843**	**13.32**
City of Isabela	**3,201**	3	**0.09**	1	**0.03**	38	**1.19**	**42**	**1.31**
City of Zamboanga	**19,175**	8	**0.04**	602	**3.14**	4,326	**22.56**	**4,936**	**25.74**
**Region 10**	**109,169**	**112**	**0.10**	**6,713**	**6.15**	**41,552**	**38.06**	**48,377**	**44.31**
Bukidnon	**33,403**	39	**0.12**	2,030	**6.08**	10,469	**31.34**	**12,538**	**37.54**
Camiguin	**1,727**	0	**0.00**	63	**3.65**	397	**22.99**	**460**	**26.64**
Lanao del Norte	**17,760**	7	**0.04**	744	**4.19**	5,784	**32.57**	**6,535**	**36.80**
Misamis Occidental	**12,328**	15	**0.12**	739	**5.99**	5,256	**42.63**	**6,010**	**48.75**
Misamis Oriental	**21,975**	30	**0.14**	1,355	**6.17**	8,115	**36.93**	**9,500**	**43.23**
City of Cagayan De Oro	**14,308**	13	**0.09**	1,310	**9.16**	7,669	**53.60**	**8,992**	**62.85**
City of Iligan	**7,668**	8	**0.10**	472	**6.16**	3,862	**50.37**	**4,342**	**56.62**
**Region 11**	**107,001**	**225**	**0.21**	**6,914**	**6.46**	**49,536**	**46.29**	**56,675**	**52.97**
Davao de Oro	**15,253**	61	**0.40**	1,447	**9.49**	7,682	**50.36**	**9,190**	**60.25**
Davao del Norte	**23,453**	43	**0.18**	1,568	**6.69**	10,519	**44.85**	**12,130**	**51.72**
Davao Oriental	**11,935**	28	**0.23**	809	**6.78**	4,857	**40.70**	**5,694**	**47.71**
Davao del Sur	**13,083**	37	**0.28**	739	**5.65**	4,260	**32.56**	**5,036**	**38.49**
Davao Occidental	**6,629**	5	**0.08**	159	**2.40**	465	**7.01**	**629**	**9.49**
City of Davao	**36,648**	51	**0.14**	2,192	**5.98**	21,753	**59.36**	**23,996**	**65.48**
**Region 12**	**95,279**	**112**	**0.12**	**4,469**	**4.69**	**28,455**	**29.86**	**33,036**	**34.67**
Cotabato	**26,521**	29	**0.11**	1,262	**4.76**	7,815	**29.47**	**9,106**	**34.34**
Sarangani	**13,322**	12	**0.09**	674	**5.06**	2,892	**21.71**	**3,578**	**26.86**
South Cotabato	**21,595**	9	**0.04**	873	**4.04**	6,273	**29.05**	**7,155**	**33.13**
Sultan Kudarat	**19,391**	59	**0.30**	1,071	**5.52**	6,706	**34.58**	**7,836**	**40.41**

Table 2.B.1.6 - Prenatal Care

Pregnant women who Completed Calcium Carbonate
Philippines, 2023

Area	Eligible Pop.	Calcium Carbonate							
		Age Group						Total	%
		10-14 yrs old		15-19 yrs old		20-49 yrs old			
		No.	%	No.	%	No.	%		
City of General Santos	**14,450**	3	**0.02**	589	**4.08**	4,769	**33.00**	**5,361**	**37.10**
**BARMM**	**132,841**	**21**	**0.02**	**1,522**	**1.15**	**20,911**	**15.74**	**22,454**	**16.90**
Basilan	**11,636**	4	**0.03**	67	**0.58**	534	**4.59**	**605**	**5.20**
Lanao del Sur	**33,374**	8	**0.02**	383	**1.15**	10,052	**30.12**	**10,443**	**31.29**
Maguindanao	**41,650**	4	**0.01**	685	**1.64**	6,571	**15.78**	**7,260**	**17.43**
Sulu	**21,378**	0	**0.00**	95	**0.44**	1,151	**5.38**	**1,246**	**5.83**
Tawi-Tawi	**12,609**	0	**0.00**	52	**0.41**	715	**5.67**	**767**	**6.08**
SGU	**5,103**	1	**0.02**	91	**1.78**	602	**11.80**	**694**	**13.60**
City of Cotabato	**7,091**	4	**0.06**	149	**2.10**	1,286	**18.14**	**1,439**	**20.29**
**Caraga**	**55,065**	**32**	**0.06**	**2,156**	**3.92**	**18,067**	**32.81**	**20,255**	**36.78**
Agusan del Norte	**7,286**	4	**0.05**	268	**3.68**	1,973	**27.08**	**2,245**	**30.81**
Agusan del Sur	**16,038**	5	**0.03**	773	**4.82**	5,679	**35.41**	**6,457**	**40.26**
Surigao del Norte	**10,186**	3	**0.03**	347	**3.41**	3,618	**35.52**	**3,968**	**38.96**
Surigao del Sur	**12,736**	15	**0.12**	396	**3.11**	3,108	**24.40**	**3,519**	**27.63**
Dinagat Islands	**2,134**	0	**0.00**	89	**4.17**	793	**37.16**	**882**	**41.33**
City of Butuan	**6,685**	5	**0.07**	283	**4.23**	2,896	**43.32**	**3,184**	**47.63**

Table 2.B.1.7 - Prenatal Care

Pregnant women who Completed Iodine Supplementation
Philippines, 2023

Area	Eligible Pop.	Iodine Supplementation							
		Age Group						Total	%
		10-14 yrs old		15-19 yrs old		20-49 yrs old			
		No.	%	No.	%	No.	%		
**Philippines**	**2,187,333**	**109**	**0.00**	**2,999**	**0.14**	**26,269**	**1.20**	**29,377**	**1.34**
**N C R**	**261,228**	**7**	**0.00**	**391**	**0.15**	**4,544**	**1.74**	**4,942**	**1.89**
Malabon City	**7,385**	0	**0.00**	1	**0.01**	11	**0.15**	**12**	**0.16**
Navotas City	**5,210**	0	**0.00**	0	**0.00**	0	**0.00**	**0**	**0.00**
Valenzuela City	**13,477**	1	**0.01**	47	**0.35**	222	**1.65**	**270**	**2.00**
Caloocan City	**33,168**	6	**0.02**	304	**0.92**	3,853	**11.62**	**4,163**	**12.55**
Marikina City	**8,342**	0	**0.00**	0	**0.00**	0	**0.00**	**0**	**0.00**
Pasig City	**17,682**	0	**0.00**	0	**0.00**	0	**0.00**	**0**	**0.00**
Pateros	**1,116**	0	**0.00**	0	**0.00**	0	**0.00**	**0**	**0.00**
Taguig City	**18,992**	0	**0.00**	0	**0.00**	0	**0.00**	**0**	**0.00**
Quezon City	**59,136**	0	**0.00**	0	**0.00**	0	**0.00**	**0**	**0.00**
Makati City	**10,944**	0	**0.00**	0	**0.00**	0	**0.00**	**0**	**0.00**
Mandaluyong City	**7,922**	0	**0.00**	0	**0.00**	0	**0.00**	**0**	**0.00**
San Juan City	**2,168**	0	**0.00**	0	**0.00**	0	**0.00**	**0**	**0.00**
Manila City	**34,128**	0	**0.00**	0	**0.00**	0	**0.00**	**0**	**0.00**
Las Piñas City	**11,263**	0	**0.00**	1	**0.01**	85	**0.75**	**86**	**0.76**
Muntinlupa City	**9,881**	0	**0.00**	0	**0.00**	0	**0.00**	**0**	**0.00**
Parañaque City	**13,280**	0	**0.00**	0	**0.00**	0	**0.00**	**0**	**0.00**
Pasay City	**7,134**	0	**0.00**	38	**0.53**	373	**5.23**	**411**	**5.76**
**C A R**	**31,319**	**0**	**0.00**	**92**	**0.29**	**670**	**2.14**	**762**	**2.43**
Abra	**3,660**	0	**0.00**	24	**0.66**	182	**4.97**	**206**	**5.63**
Apayao	**2,238**	0	**0.00**	12	**0.54**	73	**3.26**	**85**	**3.80**
Benguet	**8,806**	0	**0.00**	14	**0.16**	255	**2.90**	**269**	**3.05**
Ifugao	**4,098**	0	**0.00**	1	**0.02**	50	**1.22**	**51**	**1.24**
Kalinga	**4,549**	0	**0.00**	10	**0.22**	100	**2.20**	**110**	**2.42**
Mountain Province	**2,619**	0	**0.00**	31	**1.18**	10	**0.38**	**41**	**1.57**
City of Baguio	**5,349**	0	**0.00**	0	**0.00**	0	**0.00**	**0**	**0.00**
**Region 1**	**96,016**	**4**	**0.00**	**280**	**0.29**	**2,930**	**3.05**	**3,214**	**3.35**
Ilocos Norte	**8,912**	0	**0.00**	101	**1.13**	1,101	**12.35**	**1,202**	**13.49**
Ilocos Sur	**9,914**	0	**0.00**	8	**0.08**	131	**1.32**	**139**	**1.40**
La Union	**13,507**	0	**0.00**	5	**0.04**	254	**1.88**	**259**	**1.92**
Pangasinan	**60,360**	4	**0.01**	145	**0.24**	1,311	**2.17**	**1,460**	**2.42**

Table 2.B.1.7 - Prenatal Care

Pregnant women who Completed Iodine Supplementation
Philippines, 2023

Area	Eligible Pop.	Iodine Supplementation							
		Age Group						Total	%
		10-14 yrs old		15-19 yrs old		20-49 yrs old			
		No.	%	No.	%	No.	%		
City of Dagupan	**3,323**	0	**0.00**	21	**0.63**	133	**4.00**	**154**	**4.63**
**Region 2**	**63,967**	**0**	**0.00**	**123**	**0.19**	**1,049**	**1.64**	**1,172**	**1.83**
Batanes	**240**	0	**0.00**	0	**0.00**	3	**1.25**	**3**	**1.25**
Cagayan	**20,786**	0	**0.00**	24	**0.12**	122	**0.59**	**146**	**0.70**
Isabela	**27,379**	0	**0.00**	20	**0.07**	262	**0.96**	**282**	**1.03**
Nueva Vizcaya	**8,798**	0	**0.00**	2	**0.02**	25	**0.28**	**27**	**0.31**
Quirino	**3,839**	0	**0.00**	15	**0.39**	113	**2.94**	**128**	**3.33**
City of Santiago	**2,925**	0	**0.00**	62	**2.12**	524	**17.91**	**586**	**20.03**
**Region 3**	**233,544**	**13**	**0.01**	**520**	**0.22**	**4,875**	**2.09**	**5,408**	**2.32**
Aurora	**4,537**	0	**0.00**	0	**0.00**	13	**0.29**	**13**	**0.29**
Bataan	**17,380**	0	**0.00**	1	**0.01**	6	**0.03**	**7**	**0.04**
Bulacan	**67,590**	11	**0.02**	370	**0.55**	3,418	**5.06**	**3,799**	**5.62**
Nueva Ecija	**43,425**	2	**0.00**	87	**0.20**	997	**2.30**	**1,086**	**2.50**
Pampanga	**45,651**	0	**0.00**	4	**0.01**	39	**0.09**	**43**	**0.09**
Tarlac	**29,503**	0	**0.00**	46	**0.16**	347	**1.18**	**393**	**1.33**
Zambales	**11,723**	0	**0.00**	3	**0.03**	13	**0.11**	**16**	**0.14**
City of Angeles	**9,146**	0	**0.00**	9	**0.10**	38	**0.42**	**47**	**0.51**
City of Olongapo	**4,589**	0	**0.00**	0	**0.00**	4	**0.09**	**4**	**0.09**
**Region 4A**	**307,982**	**26**	**0.01**	**280**	**0.09**	**2,374**	**0.77**	**2,680**	**0.87**
Batangas	**53,251**	8	**0.02**	13	**0.02**	198	**0.37**	**219**	**0.41**
Cavite	**77,964**	12	**0.02**	29	**0.04**	236	**0.30**	**277**	**0.36**
Laguna	**61,525**	0	**0.00**	111	**0.18**	850	**1.38**	**961**	**1.56**
Quezon	**40,810**	5	**0.01**	9	**0.02**	91	**0.22**	**105**	**0.26**
Rizal	**68,235**	1	**0.00**	70	**0.10**	833	**1.22**	**904**	**1.32**
City of Lucena	**6,197**	0	**0.00**	48	**0.77**	166	**2.68**	**214**	**3.45**
**Region 4B**	**66,121**	**10**	**0.02**	**60**	**0.09**	**402**	**0.61**	**472**	**0.71**
Marinduque	**4,144**	0	**0.00**	2	**0.05**	37	**0.89**	**39**	**0.94**
Mindoro Occidental	**11,640**	0	**0.00**	4	**0.03**	37	**0.32**	**41**	**0.35**
Mindoro Oriental	**18,943**	1	**0.01**	18	**0.10**	136	**0.72**	**155**	**0.82**
Palawan	**20,317**	9	**0.04**	31	**0.15**	117	**0.58**	**157**	**0.77**
Romblon	**5,489**	0	**0.00**	5	**0.09**	75	**1.37**	**80**	**1.46**

Table 2.B.1.7 - Prenatal Care

Pregnant women who Completed Iodine Supplementation
Philippines, 2023

Area	Eligible Pop.	Iodine Supplementation							
		Age Group						Total	%
		10-14 yrs old		15-19 yrs old		20-49 yrs old			
		No.	%	No.	%	No.	%		
Puerto Princesa City	**5,588**	0	**0.00**	0	**0.00**	0	**0.00**	**0**	**0.00**
**Region 5**	**135,480**	**2**	**0.00**	**56**	**0.04**	**570**	**0.42**	**628**	**0.46**
Albay	**27,440**	0	**0.00**	8	**0.03**	141	**0.51**	**149**	**0.54**
Camarines Norte	**14,695**	0	**0.00**	9	**0.06**	71	**0.48**	**80**	**0.54**
Camarines Sur	**43,452**	0	**0.00**	13	**0.03**	195	**0.45**	**208**	**0.48**
Catanduanes	**5,414**	0	**0.00**	0	**0.00**	0	**0.00**	**0**	**0.00**
Masbate	**22,365**	2	**0.01**	26	**0.12**	136	**0.61**	**164**	**0.73**
Sorsogon	**17,929**	0	**0.00**	0	**0.00**	27	**0.15**	**27**	**0.15**
City of Naga	**4,185**	0	**0.00**	0	**0.00**	0	**0.00**	**0**	**0.00**
**Region 6**	**148,020**	**1**	**0.00**	**74**	**0.05**	**695**	**0.47**	**770**	**0.52**
Aklan	**11,283**	0	**0.00**	1	**0.01**	14	**0.12**	**15**	**0.13**
Antique	**11,930**	0	**0.00**	1	**0.01**	15	**0.13**	**16**	**0.13**
Capiz	**13,931**	0	**0.00**	10	**0.07**	144	**1.03**	**154**	**1.11**
Guimaras	**3,351**	0	**0.00**	0	**0.00**	0	**0.00**	**0**	**0.00**
Iloilo	**36,140**	1	**0.00**	29	**0.08**	283	**0.78**	**313**	**0.87**
Negros Occidental	**52,270**	0	**0.00**	33	**0.06**	229	**0.44**	**262**	**0.50**
City of Bacolod	**10,373**	0	**0.00**	0	**0.00**	10	**0.10**	**10**	**0.10**
City of Iloilo	**8,742**	0	**0.00**	0	**0.00**	0	**0.00**	**0**	**0.00**
**Region 7**	**165,673**	**0**	**0.00**	**61**	**0.04**	**578**	**0.35**	**639**	**0.39**
Bohol	**26,235**	0	**0.00**	2	**0.01**	62	**0.24**	**64**	**0.24**
Cebu	**72,201**	0	**0.00**	35	**0.05**	221	**0.31**	**256**	**0.35**
Negros Oriental	**28,722**	0	**0.00**	5	**0.02**	112	**0.39**	**117**	**0.41**
Siquijor	**1,680**	0	**0.00**	0	**0.00**	0	**0.00**	**0**	**0.00**
City of Cebu	**19,267**	0	**0.00**	10	**0.05**	60	**0.31**	**70**	**0.36**
City of Lapu-Lapu	**10,679**	0	**0.00**	7	**0.07**	92	**0.86**	**99**	**0.93**
City of Mandaue	**6,889**	0	**0.00**	2	**0.03**	31	**0.45**	**33**	**0.48**
**Region 8**	**93,813**	**3**	**0.00**	**34**	**0.04**	**429**	**0.46**	**466**	**0.50**
Biliran	**3,373**	0	**0.00**	0	**0.00**	0	**0.00**	**0**	**0.00**
Eastern Samar	**9,624**	0	**0.00**	8	**0.08**	34	**0.35**	**42**	**0.44**
Leyte	**31,559**	3	**0.01**	13	**0.04**	223	**0.71**	**239**	**0.76**
Northern Samar	**14,056**	0	**0.00**	11	**0.08**	141	**1.00**	**152**	**1.08**

Table 2.B.1.7 - Prenatal Care

Pregnant women who Completed Iodine Supplementation
Philippines, 2023

Area	Eligible Pop.	Iodine Supplementation							
		Age Group						Total	%
		10-14 yrs old		15-19 yrs old		20-49 yrs old			
		No.	%	No.	%	No.	%		
Southern Leyte	**7,406**	0	**0.00**	2	**0.03**	31	**0.42**	**33**	**0.45**
Samar	**17,011**	0	**0.00**	0	**0.00**	0	**0.00**	**0**	**0.00**
Ormoc City	**5,292**	0	**0.00**	0	**0.00**	0	**0.00**	**0**	**0.00**
City of Tacloban	**5,492**	0	**0.00**	0	**0.00**	0	**0.00**	**0**	**0.00**
**Region 9**	**84,815**	**2**	**0.00**	**58**	**0.07**	**763**	**0.90**	**823**	**0.97**
Zamboanga del Norte	**24,841**	2	**0.01**	29	**0.12**	170	**0.68**	**201**	**0.81**
Zamboanga del Sur	**23,757**	0	**0.00**	4	**0.02**	33	**0.14**	**37**	**0.16**
Zamboanga Sibugay	**13,841**	0	**0.00**	0	**0.00**	2	**0.01**	**2**	**0.01**
City of Isabela	**3,201**	0	**0.00**	0	**0.00**	0	**0.00**	**0**	**0.00**
City of Zamboanga	**19,175**	0	**0.00**	25	**0.13**	558	**2.91**	**583**	**3.04**
**Region 10**	**109,169**	**5**	**0.00**	**257**	**0.24**	**1,298**	**1.19**	**1,560**	**1.43**
Bukidnon	**33,403**	0	**0.00**	70	**0.21**	404	**1.21**	**474**	**1.42**
Camiguin	**1,727**	0	**0.00**	1	**0.06**	1	**0.06**	**2**	**0.12**
Lanao del Norte	**17,760**	1	**0.01**	102	**0.57**	352	**1.98**	**455**	**2.56**
Misamis Occidental	**12,328**	0	**0.00**	25	**0.20**	162	**1.31**	**187**	**1.52**
Misamis Oriental	**21,975**	2	**0.01**	22	**0.10**	151	**0.69**	**175**	**0.80**
City of Cagayan De Oro	**14,308**	0	**0.00**	10	**0.07**	36	**0.25**	**46**	**0.32**
City of Iligan	**7,668**	2	**0.03**	27	**0.35**	192	**2.50**	**221**	**2.88**
**Region 11**	**107,001**	**16**	**0.01**	**302**	**0.28**	**2,008**	**1.88**	**2,326**	**2.17**
Davao de Oro	**15,253**	3	**0.02**	11	**0.07**	47	**0.31**	**61**	**0.40**
Davao del Norte	**23,453**	1	**0.00**	57	**0.24**	219	**0.93**	**277**	**1.18**
Davao Oriental	**11,935**	1	**0.01**	7	**0.06**	131	**1.10**	**139**	**1.16**
Davao del Sur	**13,083**	1	**0.01**	11	**0.08**	50	**0.38**	**62**	**0.47**
Davao Occidental	**6,629**	0	**0.00**	3	**0.05**	24	**0.36**	**27**	**0.41**
City of Davao	**36,648**	10	**0.03**	213	**0.58**	1,537	**4.19**	**1,760**	**4.80**
**Region 12**	**95,279**	**14**	**0.01**	**337**	**0.35**	**2,509**	**2.63**	**2,860**	**3.00**
Cotabato	**26,521**	11	**0.04**	102	**0.38**	656	**2.47**	**769**	**2.90**
Sarangani	**13,322**	1	**0.01**	89	**0.67**	294	**2.21**	**384**	**2.88**
South Cotabato	**21,595**	1	**0.00**	40	**0.19**	331	**1.53**	**372**	**1.72**
Sultan Kudarat	**19,391**	0	**0.00**	12	**0.06**	45	**0.23**	**57**	**0.29**

Table 2.B.1.7 - Prenatal Care

Pregnant women who Completed Iodine Supplementation
Philippines, 2023

Area	Eligible Pop.	Iodine Supplementation							
		Age Group						Total	%
		10-14 yrs old		15-19 yrs old		20-49 yrs old			
		No.	%	No.	%	No.	%		
City of General Santos	**14,450**	1	**0.01**	94	**0.65**	1,183	**8.19**	**1,278**	**8.84**
**BARMM**	**132,841**	**6**	**0.00**	**30**	**0.02**	**289**	**0.22**	**325**	**0.24**
Basilan	**11,636**	0	**0.00**	4	**0.03**	13	**0.11**	**17**	**0.15**
Lanao del Sur	**33,374**	0	**0.00**	2	**0.01**	132	**0.40**	**134**	**0.40**
Maguindanao	**41,650**	0	**0.00**	1	**0.00**	0	**0.00**	**1**	**0.00**
Sulu	**21,378**	0	**0.00**	0	**0.00**	1	**0.00**	**1**	**0.00**
Tawi-Tawi	**12,609**	0	**0.00**	4	**0.03**	11	**0.09**	**15**	**0.12**
SGU	**5,103**	2	**0.04**	6	**0.12**	31	**0.61**	**39**	**0.76**
City of Cotabato	**7,091**	4	**0.06**	13	**0.18**	101	**1.42**	**118**	**1.66**
**Caraga**	**55,065**	**0**	**0.00**	**44**	**0.08**	**286**	**0.52**	**330**	**0.60**
Agusan del Norte	**7,286**	0	**0.00**	1	**0.01**	23	**0.32**	**24**	**0.33**
Agusan del Sur	**16,038**	0	**0.00**	29	**0.18**	137	**0.85**	**166**	**1.04**
Surigao del Norte	**10,186**	0	**0.00**	0	**0.00**	36	**0.35**	**36**	**0.35**
Surigao del Sur	**12,736**	0	**0.00**	11	**0.09**	25	**0.20**	**36**	**0.28**
Dinagat Islands	**2,134**	0	**0.00**	0	**0.00**	14	**0.66**	**14**	**0.66**
City of Butuan	**6,685**	0	**0.00**	3	**0.04**	51	**0.76**	**54**	**0.81**

Table 2.B.1.8 - Prenatal Care

Pregnant Women given one (1) dose of deworming tablet

Philippines, 2023

Area	Eligible Pop.	Pregnant women given one (1) dose of Deworming Tablet							
		Age Group						Total	%
		10-14 yrs old		15-19 yrs old		20-49 yrs old			
		No.	%	No.	%	No.	%		
**Philippines**	**2,187,333**	**1,413**	**0.06**	**29,236**	**1.34**	**211,760**	**9.68**	**242,409**	**11.08**
**N C R**	**261,228**	**18**	**0.01**	**1,164**	**0.45**	**13,584**	**5.20**	**14,766**	**5.65**
Malabon City	**7,385**	0	**0.00**	1	**0.01**	12	**0.16**	**13**	**0.18**
Navotas City	**5,210**	0	**0.00**	0	**0.00**	7	**0.13**	**7**	**0.13**
Valenzuela City	**13,477**	0	**0.00**	21	**0.16**	82	**0.61**	**103**	**0.76**
Caloocan City	**33,168**	0	**0.00**	0	**0.00**	0	**0.00**	**0**	**0.00**
Marikina City	**8,342**	2	**0.02**	139	**1.67**	1,321	**15.84**	**1,462**	**17.53**
Pasig City	**17,682**	0	**0.00**	2	**0.01**	10	**0.06**	**12**	**0.07**
Pateros	**1,116**	0	**0.00**	5	**0.45**	77	**6.90**	**82**	**7.35**
Taguig City	**18,992**	7	**0.04**	489	**2.57**	4,487	**23.63**	**4,983**	**26.24**
Quezon City	**59,136**	7	**0.01**	371	**0.63**	5,753	**9.73**	**6,131**	**10.37**
Makati City	**10,944**	0	**0.00**	8	**0.07**	137	**1.25**	**145**	**1.32**
Mandaluyong City	**7,922**	0	**0.00**	0	**0.00**	0	**0.00**	**0**	**0.00**
San Juan City	**2,168**	0	**0.00**	1	**0.05**	17	**0.78**	**18**	**0.83**
Manila City	**34,128**	1	**0.00**	0	**0.00**	6	**0.02**	**7**	**0.02**
Las Piñas City	**11,263**	0	**0.00**	24	**0.21**	385	**3.42**	**409**	**3.63**
Muntinlupa City	**9,881**	0	**0.00**	0	**0.00**	0	**0.00**	**0**	**0.00**
Parañaque City	**13,280**	1	**0.01**	83	**0.63**	857	**6.45**	**941**	**7.09**
Pasay City	**7,134**	0	**0.00**	20	**0.28**	433	**6.07**	**453**	**6.35**
**C A R**	**31,319**	**12**	**0.04**	**468**	**1.49**	**5,155**	**16.46**	**5,635**	**17.99**
Abra	**3,660**	1	**0.03**	41	**1.12**	342	**9.34**	**384**	**10.49**
Apayao	**2,238**	1	**0.04**	115	**5.14**	812	**36.28**	**928**	**41.47**
Benguet	**8,806**	0	**0.00**	11	**0.12**	202	**2.29**	**213**	**2.42**
Ifugao	**4,098**	2	**0.05**	90	**2.20**	1,335	**32.58**	**1,427**	**34.82**
Kalinga	**4,549**	1	**0.02**	117	**2.57**	1,328	**29.19**	**1,446**	**31.79**
Mountain Province	**2,619**	0	**0.00**	17	**0.65**	85	**3.25**	**102**	**3.89**
City of Baguio	**5,349**	7	**0.13**	77	**1.44**	1,051	**19.65**	**1,135**	**21.22**
**Region 1**	**96,016**	**18**	**0.02**	**1,022**	**1.06**	**11,752**	**12.24**	**12,792**	**13.32**
Ilocos Norte	**8,912**	2	**0.02**	171	**1.92**	2,106	**23.63**	**2,279**	**25.57**
Ilocos Sur	**9,914**	0	**0.00**	18	**0.18**	651	**6.57**	**669**	**6.75**
La Union	**13,507**	8	**0.06**	209	**1.55**	2,113	**15.64**	**2,330**	**17.25**
Pangasinan	**60,360**	8	**0.01**	624	**1.03**	6,882	**11.40**	**7,514**	**12.45**

Table 2.B.1.8 - Prenatal Care

Pregnant Women given one (1) dose of deworming tablet

Philippines, 2023

Area	Eligible Pop.	Pregnant women given one (1) dose of Deworming Tablet							
		Age Group						Total	%
		10-14 yrs old		15-19 yrs old		20-49 yrs old			
		No.	%	No.	%	No.	%		
City of Dagupan	**3,323**	0	**0.00**	0	**0.00**	0	**0.00**	**0**	**0.00**
**Region 2**	**63,967**	**19**	**0.03**	**807**	**1.26**	**6,688**	**10.46**	**7,514**	**11.75**
Batanes	**240**	0	**0.00**	0	**0.00**	37	**15.42**	**37**	**15.42**
Cagayan	**20,786**	1	**0.00**	190	**0.91**	1,506	**7.25**	**1,697**	**8.16**
Isabela	**27,379**	13	**0.05**	386	**1.41**	3,292	**12.02**	**3,691**	**13.48**
Nueva Vizcaya	**8,798**	3	**0.03**	151	**1.72**	1,128	**12.82**	**1,282**	**14.57**
Quirino	**3,839**	0	**0.00**	0	**0.00**	7	**0.18**	**7**	**0.18**
City of Santiago	**2,925**	2	**0.07**	80	**2.74**	718	**24.55**	**800**	**27.35**
**Region 3**	**233,544**	**649**	**0.28**	**1,409**	**0.60**	**3,534**	**1.51**	**5,592**	**2.39**
Aurora	**4,537**	0	**0.00**	3	**0.07**	8	**0.18**	**11**	**0.24**
Bataan	**17,380**	0	**0.00**	0	**0.00**	0	**0.00**	**0**	**0.00**
Bulacan	**67,590**	0	**0.00**	10	**0.01**	108	**0.16**	**118**	**0.17**
Nueva Ecija	**43,425**	0	**0.00**	10	**0.02**	129	**0.30**	**139**	**0.32**
Pampanga	**45,651**	0	**0.00**	0	**0.00**	9	**0.02**	**9**	**0.02**
Tarlac	**29,503**	647	**2.19**	1,353	**4.59**	3,052	**10.34**	**5,052**	**17.12**
Zambales	**11,723**	2	**0.02**	33	**0.28**	226	**1.93**	**261**	**2.23**
City of Angeles	**9,146**	0	**0.00**	0	**0.00**	2	**0.02**	**2**	**0.02**
City of Olongapo	**4,589**	0	**0.00**	0	**0.00**	0	**0.00**	**0**	**0.00**
**Region 4A**	**307,982**	**28**	**0.01**	**1,125**	**0.37**	**10,877**	**3.53**	**12,030**	**3.91**
Batangas	**53,251**	0	**0.00**	16	**0.03**	291	**0.55**	**307**	**0.58**
Cavite	**77,964**	1	**0.00**	173	**0.22**	1,448	**1.86**	**1,622**	**2.08**
Laguna	**61,525**	17	**0.03**	596	**0.97**	6,731	**10.94**	**7,344**	**11.94**
Quezon	**40,810**	5	**0.01**	162	**0.40**	1,059	**2.59**	**1,226**	**3.00**
Rizal	**68,235**	5	**0.01**	177	**0.26**	1,299	**1.90**	**1,481**	**2.17**
City of Lucena	**6,197**	0	**0.00**	1	**0.02**	49	**0.79**	**50**	**0.81**
**Region 4B**	**66,121**	**15**	**0.02**	**787**	**1.19**	**5,429**	**8.21**	**6,231**	**9.42**
Marinduque	**4,144**	0	**0.00**	53	**1.28**	484	**11.68**	**537**	**12.96**
Mindoro Occidental	**11,640**	0	**0.00**	59	**0.51**	358	**3.08**	**417**	**3.58**
Mindoro Oriental	**18,943**	4	**0.02**	227	**1.20**	1,746	**9.22**	**1,977**	**10.44**
Palawan	**20,317**	11	**0.05**	408	**2.01**	2,374	**11.68**	**2,793**	**13.75**
Romblon	**5,489**	0	**0.00**	40	**0.73**	467	**8.51**	**507**	**9.24**

Table 2.B.1.8 - Prenatal Care

Pregnant Women given one (1) dose of deworming tablet

Philippines, 2023

Area	Eligible Pop.	Pregnant women given one (1) dose of Deworming Tablet							
		Age Group						Total	%
		10-14 yrs old		15-19 yrs old		20-49 yrs old			
		No.	%	No.	%	No.	%		
Puerto Princesa City	5,588	0	0.00	0	0.00	0	0.00	0	0.00
**Region 5**	135,480	40	0.03	2,616	1.93	22,360	16.50	25,016	18.46
Albay	27,440	3	0.01	152	0.55	2,496	9.10	2,651	9.66
Camarines Norte	14,695	7	0.05	574	3.91	4,068	27.68	4,649	31.64
Camarines Sur	43,452	11	0.03	714	1.64	6,237	14.35	6,962	16.02
Catanduanes	5,414	4	0.07	178	3.29	1,366	25.23	1,548	28.59
Masbate	22,365	8	0.04	601	2.69	3,921	17.53	4,530	20.25
Sorsogon	17,929	6	0.03	346	1.93	3,761	20.98	4,113	22.94
City of Naga	4,185	1	0.02	51	1.22	511	12.21	563	13.45
**Region 6**	148,020	101	0.07	1,410	0.95	12,033	8.13	13,544	9.15
Aklan	11,283	0	0.00	101	0.90	1,120	9.93	1,221	10.82
Antique	11,930	74	0.62	204	1.71	1,044	8.75	1,322	11.08
Capiz	13,931	9	0.06	263	1.89	2,513	18.04	2,785	19.99
Guimaras	3,351	1	0.03	56	1.67	671	20.02	728	21.72
Iloilo	36,140	3	0.01	288	0.80	2,984	8.26	3,275	9.06
Negros Occidental	52,270	14	0.03	445	0.85	3,043	5.82	3,502	6.70
City of Bacolod	10,373	0	0.00	51	0.49	645	6.22	696	6.71
City of Iloilo	8,742	0	0.00	2	0.02	13	0.15	15	0.17
**Region 7**	165,673	76	0.05	3,603	2.17	31,827	19.21	35,506	21.43
Bohol	26,235	6	0.02	405	1.54	4,419	16.84	4,830	18.41
Cebu	72,201	30	0.04	1,447	2.00	11,671	16.16	13,148	18.21
Negros Oriental	28,722	7	0.02	259	0.90	1,748	6.09	2,014	7.01
Siquijor	1,680	0	0.00	54	3.21	569	33.87	623	37.08
City of Cebu	19,267	18	0.09	952	4.94	7,939	41.21	8,909	46.24
City of Lapu-Lapu	10,679	0	0.00	89	0.83	942	8.82	1,031	9.65
City of Mandaue	6,889	15	0.22	397	5.76	4,539	65.89	4,951	71.87
**Region 8**	93,813	22	0.02	1,250	1.33	10,672	11.38	11,944	12.73
Biliran	3,373	3	0.09	167	4.95	1,326	39.31	1,496	44.35
Eastern Samar	9,624	1	0.01	222	2.31	1,769	18.38	1,992	20.70
Leyte	31,559	2	0.01	110	0.35	1,044	3.31	1,156	3.66
Northern Samar	14,056	5	0.04	136	0.97	1,180	8.39	1,321	9.40

Table 2.B.1.8 - Prenatal Care

Pregnant Women given one (1) dose of deworming tablet

Philippines, 2023

Area	Eligible Pop.	Pregnant women given one (1) dose of Deworming Tablet							
		Age Group						Total	%
		10-14 yrs old		15-19 yrs old		20-49 yrs old			
		No.	%	No.	%	No.	%		
Southern Leyte	**7,406**	2	**0.03**	179	**2.42**	1,653	**22.32**	**1,834**	**24.76**
Samar	**17,011**	0	**0.00**	168	**0.99**	1,644	**9.66**	**1,812**	**10.65**
Ormoc City	**5,292**	9	**0.17**	243	**4.59**	1,665	**31.46**	**1,917**	**36.22**
City of Tacloban	**5,492**	0	**0.00**	25	**0.46**	391	**7.12**	**416**	**7.57**
**Region 9**	**84,815**	**21**	**0.02**	**1,549**	**1.83**	**9,379**	**11.06**	**10,949**	**12.91**
Zamboanga del Norte	**24,841**	7	**0.03**	389	**1.57**	2,708	**10.90**	**3,104**	**12.50**
Zamboanga del Sur	**23,757**	3	**0.01**	194	**0.82**	865	**3.64**	**1,062**	**4.47**
Zamboanga Sibugay	**13,841**	5	**0.04**	171	**1.24**	1,041	**7.52**	**1,217**	**8.79**
City of Isabela	**3,201**	1	**0.03**	88	**2.75**	608	**18.99**	**697**	**21.77**
City of Zamboanga	**19,175**	5	**0.03**	707	**3.69**	4,157	**21.68**	**4,869**	**25.39**
**Region 10**	**109,169**	**30**	**0.03**	**1,602**	**1.47**	**9,431**	**8.64**	**11,063**	**10.13**
Bukidnon	**33,403**	21	**0.06**	820	**2.45**	3,618	**10.83**	**4,459**	**13.35**
Camiguin	**1,727**	0	**0.00**	32	**1.85**	229	**13.26**	**261**	**15.11**
Lanao del Norte	**17,760**	0	**0.00**	193	**1.09**	1,363	**7.67**	**1,556**	**8.76**
Misamis Occidental	**12,328**	0	**0.00**	64	**0.52**	554	**4.49**	**618**	**5.01**
Misamis Oriental	**21,975**	4	**0.02**	116	**0.53**	597	**2.72**	**717**	**3.26**
City of Cagayan De Oro	**14,308**	0	**0.00**	0	**0.00**	0	**0.00**	**0**	**0.00**
City of Iligan	**7,668**	5	**0.07**	377	**4.92**	3,070	**40.04**	**3,452**	**45.02**
**Region 11**	**107,001**	**194**	**0.18**	**4,364**	**4.08**	**22,700**	**21.21**	**27,258**	**25.47**
Davao de Oro	**15,253**	44	**0.29**	1,011	**6.63**	5,135	**33.67**	**6,190**	**40.58**
Davao del Norte	**23,453**	43	**0.18**	905	**3.86**	5,188	**22.12**	**6,136**	**26.16**
Davao Oriental	**11,935**	23	**0.19**	704	**5.90**	3,530	**29.58**	**4,257**	**35.67**
Davao del Sur	**13,083**	16	**0.12**	258	**1.97**	1,166	**8.91**	**1,440**	**11.01**
Davao Occidental	**6,629**	30	**0.45**	677	**10.21**	1,860	**28.06**	**2,567**	**38.72**
City of Davao	**36,648**	38	**0.10**	809	**2.21**	5,821	**15.88**	**6,668**	**18.19**
**Region 12**	**95,279**	**159**	**0.17**	**5,109**	**5.36**	**25,910**	**27.19**	**31,178**	**32.72**
Cotabato	**26,521**	19	**0.07**	686	**2.59**	3,300	**12.44**	**4,005**	**15.10**
Sarangani	**13,322**	31	**0.23**	1,755	**13.17**	6,875	**51.61**	**8,661**	**65.01**
South Cotabato	**21,595**	23	**0.11**	942	**4.36**	5,795	**26.83**	**6,760**	**31.30**
Sultan Kudarat	**19,391**	77	**0.40**	1,521	**7.84**	8,427	**43.46**	**10,025**	**51.70**

Table 2.B.1.8 - Prenatal Care

Pregnant Women given one (1) dose of deworming tablet

Philippines, 2023

Area	Eligible Pop.	Pregnant women given one (1) dose of Deworming Tablet							
		Age Group						Total	%
		10-14 yrs old		15-19 yrs old		20-49 yrs old			
		No.	%	No.	%	No.	%		
City of General Santos	**14,450**	9	**0.06**	205	**1.42**	1,513	**10.47**	**1,727**	**11.95**
**BARMM**	**132,841**	**6**	**0.00**	**650**	**0.49**	**8,036**	**6.05**	**8,692**	**6.54**
Basilan	**11,636**	1	**0.01**	40	**0.34**	205	**1.76**	**246**	**2.11**
Lanao del Sur	**33,374**	2	**0.01**	147	**0.44**	3,326	**9.97**	**3,475**	**10.41**
Maguindanao	**41,650**	0	**0.00**	106	**0.25**	1,537	**3.69**	**1,643**	**3.94**
Sulu	**21,378**	0	**0.00**	152	**0.71**	1,466	**6.86**	**1,618**	**7.57**
Tawi-Tawi	**12,609**	0	**0.00**	115	**0.91**	878	**6.96**	**993**	**7.88**
SGU	**5,103**	0	**0.00**	5	**0.10**	52	**1.02**	**57**	**1.12**
City of Cotabato	**7,091**	3	**0.04**	85	**1.20**	572	**8.07**	**660**	**9.31**
**Caraga**	**55,065**	**5**	**0.01**	**301**	**0.55**	**2,393**	**4.35**	**2,699**	**4.90**
Agusan del Norte	**7,286**	0	**0.00**	0	**0.00**	1	**0.01**	**1**	**0.01**
Agusan del Sur	**16,038**	0	**0.00**	59	**0.37**	345	**2.15**	**404**	**2.52**
Surigao del Norte	**10,186**	0	**0.00**	85	**0.83**	840	**8.25**	**925**	**9.08**
Surigao del Sur	**12,736**	5	**0.04**	151	**1.19**	1,075	**8.44**	**1,231**	**9.67**
Dinagat Islands	**2,134**	0	**0.00**	4	**0.19**	121	**5.67**	**125**	**5.86**
City of Butuan	**6,685**	0	**0.00**	2	**0.03**	11	**0.16**	**13**	**0.19**

Table 2.B.1.9 - Prenatal Care

Pregnant women screened for Syphilis

Philippines, 2023

Area	Eligible Pop.	Screened for Syphilis							
		Age Group						Total	%
		10-14 yrs old		15-19 yrs old		20-49 yrs old			
		No.	%	No.	%	No.	%		
**PHILIPPINES**	**2,187,333**	**1,610**	**0.07**	**68,803**	**3.15**	**579,812**	**26.51**	**650,225**	**29.73**
**N C R**	**261,228**	**242**	**0.09**	**10,673**	**4.09**	**117,473**	**44.97**	**128,388**	**49.15**
Malabon City	**7,385**	5	**0.07**	194	**2.63**	1,606	**21.75**	1,805	**24.44**
Navotas City	**5,210**	17	**0.33**	376	**7.22**	2,742	**52.63**	3,135	**60.17**
Valenzuela City	**13,477**	10	**0.07**	369	**2.74**	4,288	**31.82**	4,667	**34.63**
Caloocan City	**33,168**	17	**0.05**	1,039	**3.13**	10,607	**31.98**	11,663	**35.16**
								0	
Marikina City	**8,342**	4	**0.05**	338	**4.05**	3,013	**36.12**	3,355	**40.22**
Pasig City	**17,682**	16	**0.09**	700	**3.96**	11,716	**66.26**	12,432	**70.31**
Pateros	**1,116**	0	**0.00**	24	**2.15**	262	**23.48**	286	**25.63**
Taguig City	**18,992**	16	**0.08**	1,039	**5.47**	8,488	**44.69**	9,543	**50.25**
Quezon City	**59,136**	48	**0.08**	2,179	**3.68**	31,497	**53.26**	33,724	**57.03**
Makati City	**10,944**	7	**0.06**	173	**1.58**	2,536	**23.17**	2,716	**24.82**
Mandaluyong City	**7,922**	12	**0.15**	271	**3.42**	6,708	**84.68**	6,991	**88.25**
San Juan City	**2,168**	1	**0.05**	59	**2.72**	815	**37.59**	875	**40.36**
Manila City	**34,128**	43	**0.13**	2,239	**6.56**	16,232	**47.56**	18,514	**54.25**
Las Piñas City	**11,263**	5	**0.04**	265	**2.35**	3,278	**29.10**	3,548	**31.50**
Muntinlupa City	**9,881**	22	**0.22**	341	**3.45**	3,146	**31.84**	3,509	**35.51**
Parañaque City	**13,280**	9	**0.07**	603	**4.54**	5,597	**42.15**	6,209	**46.75**
Pasay City	**7,134**	10	**0.14**	464	**6.50**	4,942	**69.27**	5,416	**75.92**
**C A R**	**31,319**	**45**	**0.14**	**2,266**	**7.24**	**18,772**	**59.94**	**21,083**	**67.32**
Abra	**3,660**	5	**0.14**	214	**5.85**	1,182	**32.30**	1,401	**38.28**
Apayao	**2,238**	14	**0.63**	371	**16.58**	2,250	**100.54**	2,635	**117.74**
Benguet	**8,806**	10	**0.11**	725	**8.23**	6,406	**72.75**	7,141	**81.09**
Ifugao	**4,098**	4	**0.10**	190	**4.64**	1,672	**40.80**	1,866	**45.53**
Kalinga	**4,549**	2	**0.04**	198	**4.35**	1,461	**32.12**	1,661	**36.51**
Mountain Province	**2,619**	5	**0.19**	310	**11.84**	3,013	**115.04**	3,328	**127.07**
City of Baguio	**5,349**	5	**0.09**	258	**4.82**	2,788	**52.12**	3,051	**57.04**
**Region 1**	**96,016**	**44**	**0.05**	**2,627**	**2.74**	**28,967**	**30.17**	**31,638**	**32.95**
Ilocos Norte	**8,912**	5	**0.06**	332	**3.73**	4,734	**53.12**	5,071	**56.90**
Ilocos Sur	**9,914**	4	**0.04**	386	**3.89**	5,676	**57.25**	6,066	**61.19**
La Union	**13,507**	15	**0.11**	620	**4.59**	5,531	**40.95**	6,166	**45.65**
Pangasinan	**60,360**	18	**0.03**	1,245	**2.06**	12,814	**21.23**	14,077	**23.32**

Table 2.B.1.9 - Prenatal Care

Pregnant women screened for Syphilis

Philippines, 2023

Area	Eligible Pop.	Screened for Syphilis							
		Age Group						Total	%
		10-14 yrs old		15-19 yrs old		20-49 yrs old			
		No.	%	No.	%	No.	%		
City of Dagupan	**3,323**	2	**0.06**	44	**1.32**	212	**6.38**	258	**7.76**
**Region 2**	**63,967**	**56**	**0.09**	**1,675**	**2.62**	**14,302**	**22.36**	**16,033**	**25.06**
Batanes	**240**	0	**0.00**	12	**5.00**	112	**46.67**	124	**51.67**
Cagayan	**20,786**	10	**0.05**	191	**0.92**	1,388	**6.68**	1,589	**7.64**
Isabela	**27,379**	31	**0.11**	894	**3.27**	8,981	**32.80**	9,906	**36.18**
Nueva Vizcaya	**8,798**	5	**0.06**	297	**3.38**	2,265	**25.74**	2,567	**29.18**
Quirino	**3,839**	9	**0.23**	250	**6.51**	1,338	**34.85**	1,597	**41.60**
City of Santiago	**2,925**	1	**0.03**	31	**1.06**	218	**7.45**	250	**8.55**
**Region 3**	**233,544**	**225**	**0.10**	**10,114**	**4.33**	**73,999**	**31.69**	**84,338**	**36.11**
Aurora	**4,537**	10	**0.22**	270	**5.95**	1,802	**39.72**	2,082	**45.89**
Bataan	**17,380**	22	**0.13**	1,116	**6.42**	7,698	**44.29**	8,836	**50.84**
Bulacan	**67,590**	72	**0.11**	3,650	**5.40**	31,238	**46.22**	34,960	**51.72**
Nueva Ecija	**43,425**	43	**0.10**	1,344	**3.09**	7,623	**17.55**	9,010	**20.75**
Pampanga	**45,651**	58	**0.13**	1,916	**4.20**	13,733	**30.08**	15,707	**34.41**
Tarlac	**29,503**	9	**0.03**	965	**3.27**	6,357	**21.55**	7,331	**24.85**
Zambales	**11,723**	4	**0.03**	469	**4.00**	2,828	**24.12**	3,301	**28.16**
City of Angeles	**9,146**	3	**0.03**	194	**2.12**	1,386	**15.15**	1,583	**17.31**
City of Olongapo	**4,589**	4	**0.09**	190	**4.14**	1,334	**29.07**	1,528	**33.30**
**Region 4A**	**307,982**	**181**	**0.06**	**8,195**	**2.66**	**72,877**	**23.66**	**81,253**	**26.38**
Batangas	**53,251**	21	**0.04**	800	**1.50**	9,245	**17.36**	10,066	**18.90**
Cavite	**77,964**	33	**0.04**	1,804	**2.31**	17,418	**22.34**	19,255	**24.70**
Laguna	**61,525**	35	**0.06**	1,979	**3.22**	20,197	**32.83**	22,211	**36.10**
Quezon	**40,810**	25	**0.06**	926	**2.27**	6,580	**16.12**	7,531	**18.45**
Rizal	**68,235**	61	**0.09**	2,337	**3.42**	17,640	**25.85**	20,038	**29.37**
City of Lucena	**6,197**	6	**0.10**	349	**5.63**	1,797	**29.00**	2,152	**34.73**
**Region 4B**	**66,121**	**58**	**0.09**	**2,104**	**3.18**	**17,511**	**26.48**	**19,673**	**29.75**
Marinduque	**4,144**	11	**0.27**	119	**2.87**	1,131	**27.29**	1,261	**30.43**
Mindoro Occidental	**11,640**	4	**0.03**	332	**2.85**	2,328	**20.00**	2,664	**22.89**
Mindoro Oriental	**18,943**	9	**0.05**	443	**2.34**	6,418	**33.88**	6,870	**36.27**
Palawan	**20,317**	23	**0.11**	682	**3.36**	3,807	**18.74**	4,512	**22.21**
Romblon	**5,489**	2	**0.04**	180	**3.28**	1,767	**32.19**	1,949	**35.51**

Table 2.B.1.9 - Prenatal Care

Pregnant women screened for Syphilis

Philippines, 2023

Area	Eligible Pop.	Screened for Syphilis							
		Age Group						Total	%
		10-14 yrs old		15-19 yrs old		20-49 yrs old			
		No.	%	No.	%	No.	%		
Puerto Princesa City	**5,588**	9	**0.16**	348	**6.23**	2,060	**36.86**	2,417	**43.25**
**Region 5**	**135,480**	**40**	**0.03**	**2,511**	**1.85**	**23,290**	**17.19**	**25,841**	**19.07**
Albay	**27,440**	7	**0.03**	470	**1.71**	7,415	**27.02**	7,892	**28.76**
Camarines Norte	**14,695**	7	**0.05**	350	**2.38**	2,616	**17.80**	2,973	**20.23**
Camarines Sur	**43,452**	3	**0.01**	314	**0.72**	3,424	**7.88**	3,741	**8.61**
Catanduanes	**5,414**	10	**0.18**	353	**6.52**	2,081	**38.44**	2,444	**45.14**
Masbate	**22,365**	8	**0.04**	711	**3.18**	4,284	**19.15**	5,003	**22.37**
Sorsogon	**17,929**	4	**0.02**	229	**1.28**	2,589	**14.44**	2,822	**15.74**
City of Naga	**4,185**	1	**0.02**	84	**2.01**	881	**21.05**	966	**23.08**
**Region 6**	**148,020**	**153**	**0.10**	**4,611**	**3.12**	**40,725**	**27.51**	**45,489**	**30.73**
Aklan	**11,283**	4	**0.04**	284	**2.52**	3,629	**32.16**	3,917	**34.72**
Antique	**11,930**	78	**0.65**	389	**3.26**	3,089	**25.89**	3,556	**29.81**
Capiz	**13,931**	12	**0.09**	312	**2.24**	2,977	**21.37**	3,301	**23.70**
Guimaras	**3,351**	3	**0.09**	188	**5.61**	1,926	**57.48**	2,117	**63.18**
Iloilo	**36,140**	25	**0.07**	1,225	**3.39**	13,131	**36.33**	14,381	**39.79**
Negros Occidental	**52,270**	17	**0.03**	1,385	**2.65**	8,991	**17.20**	10,393	**19.88**
City of Bacolod	**10,373**	2	**0.02**	538	**5.19**	4,184	**40.34**	4,724	**45.54**
City of Iloilo	**8,742**	12	**0.14**	290	**3.32**	2,798	**32.01**	3,100	**35.46**
**Region 7**	**165,673**	**144**	**0.09**	**7,874**	**4.75**	**60,706**	**36.64**	**68,724**	**41.48**
Bohol	**26,235**	23	**0.09**	1,287	**4.91**	12,000	**45.74**	13,310	**50.73**
Cebu	**72,201**	28	**0.04**	2,406	**3.33**	13,922	**19.28**	16,356	**22.65**
Negros Oriental	**28,722**	6	**0.02**	574	**2.00**	3,847	**13.39**	4,427	**15.41**
Siquijor	**1,680**	2	**0.12**	78	**4.64**	810	**48.21**	890	**52.98**
City of Cebu	**19,267**	20	**0.10**	995	**5.16**	7,948	**41.25**	8,963	**46.52**
City of Lapu-Lapu	**10,679**	5	**0.05**	337	**3.16**	5,081	**47.58**	5,423	**50.78**
City of Mandaue	**6,889**	60	**0.87**	2,197	**31.89**	17,098	**248.19**	19,355	**280.96**
**Region 8**	**93,813**	**25**	**0.03**	**2,048**	**2.18**	**15,206**	**16.21**	**17,279**	**18.42**
Biliran	**3,373**	3	**0.09**	200	**5.93**	1,268	**37.59**	1,471	**43.61**
Eastern Samar	**9,624**	4	**0.04**	217	**2.25**	1,787	**18.57**	2,008	**20.86**
Leyte	**31,559**	3	**0.01**	308	**0.98**	2,798	**8.87**	3,109	**9.85**
Northern Samar	**14,056**	8	**0.06**	674	**4.80**	3,948	**28.09**	4,630	**32.94**

Table 2.B.1.9 - Prenatal Care

Pregnant women screened for Syphilis

Philippines, 2023

Area	Eligible Pop.	Screened for Syphilis							
		Age Group						Total	%
		10-14 yrs old		15-19 yrs old		20-49 yrs old			
		No.	%	No.	%	No.	%		
Southern Leyte	**7,406**	0	**0.00**	35	**0.47**	444	**6.00**	479	**6.47**
Samar	**17,011**	0	**0.00**	122	**0.72**	1,340	**7.88**	1,462	**8.59**
Ormoc City	**5,292**	4	**0.08**	220	**4.16**	1,369	**25.87**	1,593	**30.10**
City of Tacloban	**5,492**	3	**0.05**	272	**4.95**	2,252	**41.01**	2,527	**46.01**
**Region 9**	**84,815**	**16**	**0.02**	**927**	**1.09**	**4,829**	**5.69**	**5,772**	**6.81**
Zamboanga del Norte	**24,841**	0	**0.00**	103	**0.41**	475	**1.91**	578	**2.33**
Zamboanga del Sur	**23,757**	3	**0.01**	177	**0.75**	908	**3.82**	1,088	**4.58**
Zamboanga Sibugay	**13,841**	9	**0.07**	488	**3.53**	2,581	**18.65**	3,078	**22.24**
City of Isabela	**3,201**	2	**0.06**	29	**0.91**	195	**6.09**	226	**7.06**
City of Zamboanga	**19,175**	2	**0.01**	130	**0.68**	670	**3.49**	802	**4.18**
**Region 10**	**109,169**	**71**	**0.07**	**3,011**	**2.76**	**18,155**	**16.63**	**21,237**	**19.45**
Bukidnon	**33,403**	15	**0.04**	619	**1.85**	3,396	**10.17**	4,030	**12.06**
Camiguin	**1,727**	0	**0.00**	56	**3.24**	412	**23.86**	468	**27.10**
Lanao del Norte	**17,760**	4	**0.02**	126	**0.71**	941	**5.30**	1,071	**6.03**
Misamis Occidental	**12,328**	2	**0.02**	198	**1.61**	1,623	**13.17**	1,823	**14.79**
Misamis Oriental	**21,975**	34	**0.15**	932	**4.24**	5,071	**23.08**	6,037	**27.47**
City of Cagayan De Oro	**14,308**	14	**0.10**	921	**6.44**	5,767	**40.31**	6,702	**46.84**
City of Iligan	**7,668**	2	**0.03**	159	**2.07**	945	**12.32**	1,106	**14.42**
**Region 11**	**107,001**	**111**	**0.10**	**3,253**	**3.04**	**23,222**	**21.70**	**26,586**	**24.85**
Davao de Oro	**15,253**	3	**0.02**	26	**0.17**	327	**2.14**	356	**2.33**
Davao del Norte	**23,453**	19	**0.08**	677	**2.89**	5,057	**21.56**	5,753	**24.53**
Davao Oriental	**11,935**	8	**0.07**	51	**0.43**	284	**2.38**	343	**2.87**
Davao del Sur	**13,083**	9	**0.07**	238	**1.82**	1,339	**10.23**	1,586	**12.12**
Davao Occidental	**6,629**	1	**0.02**	53	**0.80**	136	**2.05**	190	**2.87**
City of Davao	**36,648**	71	**0.19**	2,208	**6.02**	16,079	**43.87**	18,358	**50.09**
**Region 12**	**95,279**	**155**	**0.16**	**4,321**	**4.54**	**23,739**	**24.92**	**28,215**	**29.61**
Cotabato	**26,521**	17	**0.06**	633	**2.39**	3,413	**12.87**	4,063	**15.32**
Sarangani	**13,322**	33	**0.25**	1,050	**7.88**	4,538	**34.06**	5,621	**42.19**
South Cotabato	**21,595**	23	**0.11**	1,014	**4.70**	6,646	**30.78**	7,683	**35.58**
Sultan Kudarat	**19,391**	79	**0.41**	1,272	**6.56**	6,476	**33.40**	7,827	**40.36**

Table 2.B.1.9 - Prenatal Care

Pregnant women screened for Syphilis

Philippines, 2023

Area	Eligible Pop.	Screened for Syphilis							
		Age Group						Total	%
		10-14 yrs old		15-19 yrs old		20-49 yrs old			
		No.	%	No.	%	No.	%		
City of General Santos	**14,450**	3	**0.02**	352	**2.44**	2,666	**18.45**	3,021	**20.91**
**BARMM**	**132,841**	**21**	**0.02**	**1,151**	**0.87**	**15,694**	**11.81**	**16,866**	**12.70**
Basilan	**11,636**	0	**0.00**	27	**0.23**	74	**0.64**	101	**0.87**
Lanao del Sur	**33,374**	14	**0.04**	829	**2.48**	13,597	**40.74**	14,440	**43.27**
Maguindanao	**41,650**	0	**0.00**	32	**0.08**	240	**0.58**	272	**0.65**
Sulu	**21,378**	2	**0.01**	44	**0.21**	205	**0.96**	251	**1.17**
Tawi-Tawi	**12,609**	0	**0.00**	7	**0.06**	58	**0.46**	65	**0.52**
SGU	**5,103**	0	**0.00**	0	**0.00**	0	**0.00**	0	**0.00**
City of Cotabato	**7,091**	5	**0.07**	212	**2.99**	1,520	**21.44**	1,737	**24.50**
**CARAGA**	**55,065**	**23**	**0.04**	**1,442**	**2.62**	**10,345**	**18.79**	**11,810**	**21.45**
Agusan del Norte	**7,286**	2	**0.03**	217	**2.98**	1,359	**18.65**	1,578	**21.66**
Agusan del Sur	**16,038**	4	**0.02**	285	**1.78**	1,982	**12.36**	2,271	**14.16**
Surigao del Norte	**10,186**	4	**0.04**	249	**2.44**	1,963	**19.27**	2,216	**21.76**
Surigao del Sur	**12,736**	1	**0.01**	142	**1.11**	727	**5.71**	870	**6.83**
Dinagat Islands	**2,134**	2	**0.09**	91	**4.26**	815	**38.19**	908	**42.55**
City of Butuan	**6,685**	10	**0.15**	458	**6.85**	3,499	**52.34**	3,967	**59.34**

Table 2.B.1.10 - Prenatal Care

Pregnant women tested positive for Syphilis

Philippines, 2023

Area	Total No. of Screened for syphilis	Tested Positive for Syphilis							
		Age Group						Total	%
		10-14 yrs old		15-19 yrs old		20-49 yrs old			
		No.	%	No.	%	No.	%		
**Philippines**	**648,488**	**36**	**0.01**	**839**	**0.13**	**5,627**	**0.87**	**6,502**	**1.00**
**N C R**	**128,388**	**0**	**0.00**	**77**	**0.06**	**512**	**0.40**	**589**	**0.46**
Malabon City	**1,805**	0	**0.00**	3	**0.17**	11	**0.61**	14	**0.78**
Navotas City	**3,135**	0	**0.00**	0	**0.00**	10	**0.32**	10	**0.32**
Valenzuela City	**4,667**	0	**0.00**	16	**0.34**	87	**1.86**	103	**2.21**
Caloocan City	**11,663**	0	**0.00**	9	**0.08**	41	**0.35**	50	**0.43**
								0	
Marikina City	**3,355**	0	**0.00**	1	**0.03**	6	**0.18**	7	**0.21**
Pasig City	**12,432**	0	**0.00**	2	**0.02**	20	**0.16**	22	**0.18**
Pateros	**286**	0	**0.00**	0	**0.00**	0	**0.00**	0	**0.00**
Taguig City	**9,543**	0	**0.00**	18	**0.19**	111	**1.16**	129	**1.35**
Quezon City	**33,724**	0	**0.00**	2	**0.01**	42	**0.12**	44	**0.13**
Makati City	**2,716**	0	**0.00**	2	**0.07**	6	**0.22**	8	**0.29**
Mandaluyong City	**6,991**	0	**0.00**	0	**0.00**	5	**0.07**	5	**0.07**
San Juan City	**875**	0	**0.00**	0	**0.00**	2	**0.23**	2	**0.23**
Manila City	**18,514**	0	**0.00**	10	**0.05**	60	**0.32**	70	**0.38**
Las Piñas City	**3,548**	0	**0.00**	3	**0.08**	12	**0.34**	15	**0.42**
Muntinlupa City	**3,509**	0	**0.00**	1	**0.03**	14	**0.40**	15	**0.43**
Parañaque City	**6,209**	0	**0.00**	2	**0.03**	11	**0.18**	13	**0.21**
Pasay City	**5,416**	0	**0.00**	8	**0.15**	74	**1.37**	82	**1.51**
**C A R**	**21,083**	**0**	**0.00**	**3**	**0.01**	**53**	**0.25**	**56**	**0.27**
Abra	**1,401**	0	**0.00**	1	**0.07**	5	**0.36**	6	**0.43**
Apayao	**2,635**	0	**0.00**	1	**0.04**	23	**0.87**	24	**0.91**
Benguet	**7,141**	0	**0.00**	0	**0.00**	1	**0.01**	1	**0.01**
Ifugao	**1,866**	0	**0.00**	0	**0.00**	1	**0.05**	1	**0.05**
Kalinga	**1,661**	0	**0.00**	0	**0.00**	1	**0.06**	1	**0.06**
Mountain Province	**3,328**	0	**0.00**	1	**0.03**	4	**0.12**	5	**0.15**
City of Baguio	**3,051**	0	**0.00**	0	**0.00**	18	**0.59**	18	**0.59**
**Region 1**	**31,638**	**0**	**0.00**	**8**	**0.03**	**99**	**0.31**	**107**	**0.34**
Ilocos Norte	**5,071**	0	**0.00**	0	**0.00**	7	**0.14**	7	**0.14**
Ilocos Sur	**6,066**	0	**0.00**	0	**0.00**	2	**0.03**	2	**0.03**
La Union	**6,166**	0	**0.00**	2	**0.03**	19	**0.31**	21	**0.34**
Pangasinan	**14,077**	0	**0.00**	6	**0.04**	69	**0.49**	75	**0.53**

Table 2.B.1.10 - Prenatal Care

Pregnant women tested positive for Syphilis

Philippines, 2023

Area	Total No. of Screened for syphilis	Tested Positive for Syphilis							
		Age Group						Total	%
		10-14 yrs old		15-19 yrs old		20-49 yrs old			
		No.	%	No.	%	No.	%		
City of Dagupan	**258**	0	**0.00**	0	**0.00**	2	**0.78**	2	**0.78**
**Region 2**	**16,033**	**4**	**0.02**	**50**	**0.31**	**254**	**1.58**	**308**	**1.92**
Batanes	**124**	0	**0.00**	0	**0.00**	1	**0.81**	1	**0.81**
Cagayan	**1,589**	0	**0.00**	13	**0.82**	24	**1.51**	37	**2.33**
Isabela	**9,906**	4	**0.04**	19	**0.19**	204	**2.06**	227	**2.29**
Nueva Vizcaya	**2,567**	0	**0.00**	15	**0.58**	16	**0.62**	31	**1.21**
Quirino	**1,597**	0	**0.00**	1	**0.06**	6	**0.38**	7	**0.44**
City of Santiago	**250**	0	**0.00**	2	**0.80**	3	**1.20**	5	**2.00**
**Region 3**	**84,338**	**3**	**0.00**	**112**	**0.13**	**602**	**0.71**	**717**	**0.85**
Aurora	**2,082**	0	**0.00**	1	**0.05**	10	**0.48**	11	**0.53**
Bataan	**8,836**	0	**0.00**	12	**0.14**	35	**0.40**	47	**0.53**
Bulacan	**34,960**	1	**0.00**	4	**0.01**	58	**0.17**	63	**0.18**
Nueva Ecija	**9,010**	1	**0.01**	56	**0.62**	243	**2.70**	300	**3.33**
Pampanga	**15,707**	1	**0.01**	32	**0.20**	186	**1.18**	219	**1.39**
Tarlac	**7,331**	0	**0.00**	3	**0.04**	41	**0.56**	44	**0.60**
Zambales	**3,301**	0	**0.00**	1	**0.03**	17	**0.51**	18	**0.55**
City of Angeles	**1,583**	0	**0.00**	2	**0.13**	2	**0.13**	4	**0.25**
City of Olongapo	**1,528**	0	**0.00**	1	**0.07**	10	**0.65**	11	**0.72**
**Region 4A**	**81,253**	**10**	**0.01**	**123**	**0.15**	**966**	**1.19**	**1,099**	**1.35**
Batangas	**10,066**	1	**0.01**	10	**0.10**	64	**0.64**	75	**0.75**
Cavite	**19,255**	0	**0.00**	24	**0.12**	211	**1.10**	235	**1.22**
Laguna	**22,211**	0	**0.00**	42	**0.19**	311	**1.40**	353	**1.59**
Quezon	**7,531**	1	**0.01**	18	**0.24**	122	**1.62**	141	**1.87**
Rizal	**20,038**	8	**0.04**	20	**0.10**	218	**1.09**	246	**1.23**
City of Lucena	**2,152**	0	**0.00**	9	**0.42**	40	**1.86**	49	**2.28**
**Region 4B**	**19,673**	**3**	**0.02**	**33**	**0.17**	**185**	**0.94**	**221**	**1.12**
Marinduque	**1,261**	0	**0.00**	1	**0.08**	6	**0.48**	7	**0.56**
Mindoro Occidental	**2,664**	0	**0.00**	6	**0.23**	42	**1.58**	48	**1.80**
Mindoro Oriental	**6,870**	3	**0.04**	1	**0.01**	28	**0.41**	32	**0.47**
Palawan	**4,512**	0	**0.00**	11	**0.24**	54	**1.20**	65	**1.44**
Romblon	**1,949**	0	**0.00**	2	**0.10**	26	**1.33**	28	**1.44**

Table 2.B.1.10 - Prenatal Care

Pregnant women tested positive for Syphilis

Philippines, 2023

Area	Total No. of Screened for syphilis	Tested Positive for Syphilis							
		Age Group						Total	%
		10-14 yrs old		15-19 yrs old		20-49 yrs old			
		No.	%	No.	%	No.	%		
Puerto Princesa City	**2,417**	0	**0.00**	12	**0.50**	29	**1.20**	41	**1.70**
**Region 5**	**25,841**	**1**	**0.00**	**40**	**0.15**	**254**	**0.98**	**295**	**1.14**
Albay	**7,892**	0	**0.00**	9	**0.11**	54	**0.68**	63	**0.80**
Camarines Norte	**2,973**	1	**0.03**	10	**0.34**	63	**2.12**	74	**2.49**
Camarines Sur	**3,741**	0	**0.00**	2	**0.05**	30	**0.80**	32	**0.86**
Catanduanes	**2,444**	0	**0.00**	0	**0.00**	9	**0.37**	9	**0.37**
Masbate	**5,003**	0	**0.00**	15	**0.30**	64	**1.28**	79	**1.58**
Sorsogon	**2,822**	0	**0.00**	3	**0.11**	27	**0.96**	30	**1.06**
City of Naga	**966**	0	**0.00**	1	**0.10**	7	**0.72**	8	**0.83**
**Region 6**	**45,489**	**4**	**0.01**	**82**	**0.18**	**552**	**1.21**	**638**	**1.40**
Aklan	**3,917**	1	**0.03**	7	**0.18**	15	**0.38**	23	**0.59**
Antique	**3,556**	3	**0.08**	7	**0.20**	53	**1.49**	63	**1.77**
Capiz	**3,301**	0	**0.00**	7	**0.21**	33	**1.00**	40	**1.21**
Guimaras	**2,117**	0	**0.00**	0	**0.00**	10	**0.47**	10	**0.47**
Iloilo	**14,381**	0	**0.00**	13	**0.09**	116	**0.81**	129	**0.90**
Negros Occidental	**10,393**	0	**0.00**	13	**0.13**	90	**0.87**	103	**0.99**
City of Bacolod	**4,724**	0	**0.00**	34	**0.72**	229	**4.85**	263	**5.57**
City of Iloilo	**3,100**	0	**0.00**	1	**0.03**	6	**0.19**	7	**0.23**
**Region 7**	**68,724**	**1**	**0.00**	**107**	**0.16**	**911**	**1.33**	**1,019**	**1.48**
Bohol	**13,310**	0	**0.00**	10	**0.08**	127	**0.95**	137	**1.03**
Cebu	**16,356**	1	**0.01**	71	**0.43**	467	**2.86**	539	**3.30**
Negros Oriental	**4,427**	0	**0.00**	4	**0.09**	45	**1.02**	49	**1.11**
Siquijor	**890**	0	**0.00**	0	**0.00**	5	**0.56**	5	**0.56**
City of Cebu	**8,963**	0	**0.00**	17	**0.19**	141	**1.57**	158	**1.76**
City of Lapu-Lapu	**5,423**	0	**0.00**	5	**0.09**	111	**2.05**	116	**2.14**
City of Mandaue	**19,355**	0	**0.00**	0	**0.00**	15	**0.08**	15	**0.08**
**Region 8**	**17,279**	**3**	**0.02**	**37**	**0.21**	**265**	**1.53**	**305**	**1.77**
Biliran	**1,471**	0	**0.00**	0	**0.00**	5	**0.34**	5	**0.34**
Eastern Samar	**2,008**	0	**0.00**	3	**0.15**	17	**0.85**	20	**1.00**
Leyte	**3,109**	0	**0.00**	21	**0.68**	126	**4.05**	147	**4.73**
Northern Samar	**4,630**	0	**0.00**	3	**0.06**	43	**0.93**	46	**0.99**

Table 2.B.1.10 - Prenatal Care

Pregnant women tested positive for Syphilis

Philippines, 2023

Area	Total No. of Screened for syphilis	Tested Positive for Syphilis							
		Age Group						Total	%
		10-14 yrs old		15-19 yrs old		20-49 yrs old			
		No.	%	No.	%	No.	%		
Southern Leyte	**479**	0	**0.00**	0	**0.00**	1	**0.21**	1	**0.21**
Samar	**1,462**	3	**0.21**	10	**0.68**	58	**3.97**	71	**4.86**
Ormoc City	**1,593**	0	**0.00**	0	**0.00**	9	**0.56**	9	**0.56**
City of Tacloban	**2,527**	0	**0.00**	0	**0.00**	6	**0.24**	6	**0.24**
**Region 9**	**5,772**	**1**	**0.02**	**3**	**0.05**	**29**	**0.50**	**33**	**0.57**
Zamboanga del Norte	**578**	0	**0.00**	0	**0.00**	2	**0.35**	2	**0.35**
Zamboanga del Sur	**1,088**	1	**0.09**	2	**0.18**	10	**0.92**	13	**1.19**
Zamboanga Sibugay	**3,078**	0	**0.00**	0	**0.00**	15	**0.49**	15	**0.49**
City of Isabela	**226**	0	**0.00**	0	**0.00**	0	**0.00**	0	**0.00**
City of Zamboanga	**802**	0	**0.00**	1	**0.12**	2	**0.25**	3	**0.37**
**Region 10**	**21,237**	**0**	**0.00**	**34**	**0.16**	**176**	**0.83**	**210**	**0.99**
Bukidnon	**4,030**	0	**0.00**	8	**0.20**	42	**1.04**	50	**1.24**
Camiguin	**468**	0	**0.00**	0	**0.00**	0	**0.00**	0	**0.00**
Lanao del Norte	**1,071**	0	**0.00**	0	**0.00**	0	**0.00**	0	**0.00**
Misamis Occidental	**1,823**	0	**0.00**	0	**0.00**	0	**0.00**	0	**0.00**
Misamis Oriental	**6,037**	0	**0.00**	7	**0.12**	29	**0.48**	36	**0.60**
City of Cagayan De Oro	**6,702**	0	**0.00**	19	**0.28**	105	**1.57**	124	**1.85**
City of Iligan	**1,106**	0	**0.00**	0	**0.00**	0	**0.00**	0	**0.00**
**Region 11**	**26,586**	**3**	**0.01**	**24**	**0.09**	**119**	**0.45**	**146**	**0.55**
Davao de Oro	**356**	0	**0.00**	0	**0.00**	7	**1.97**	7	**1.97**
Davao del Norte	**5,753**	1	**0.02**	8	**0.14**	23	**0.40**	32	**0.56**
Davao Oriental	**343**	0	**0.00**	0	**0.00**	5	**1.46**	5	**1.46**
Davao del Sur	**1,586**	0	**0.00**	2	**0.13**	8	**0.50**	10	**0.63**
Davao Occidental	**190**	0	**0.00**	0	**0.00**	2	**1.05**	2	**1.05**
City of Davao	**18,358**	2	**0.01**	14	**0.08**	74	**0.40**	90	**0.49**
**Region 12**	**28,215**	**2**	**0.01**	**86**	**0.30**	**519**	**1.84**	**607**	**2.15**
Cotabato	**4,063**	0	**0.00**	25	**0.62**	133	**3.27**	158	**3.89**
Sarangani	**5,621**	0	**0.00**	29	**0.52**	148	**2.63**	177	**3.15**
South Cotabato	**7,683**	0	**0.00**	10	**0.13**	116	**1.51**	126	**1.64**
Sultan Kudarat	**7,827**	2	**0.03**	9	**0.11**	58	**0.74**	69	**0.88**

Table 2.B.1.10 - Prenatal Care

Pregnant women tested positive for Syphilis

Philippines, 2023

Area	Total No. of Screened for syphilis	Tested Positive for Syphilis							
		Age Group						Total	%
		10-14 yrs old		15-19 yrs old		20-49 yrs old			
		No.	%	No.	%	No.	%		
City of General Santos	**3,021**	0	**0.00**	13	**0.43**	64	**2.12**	77	**2.55**
**BARMM**	**15,129**	**1**	**0.01**	**5**	**0.03**	**46**	**0.30**	**52**	**0.34**
Basilan	**101**	0	**0.00**	0	**0.00**	0	**0.00**	0	**0.00**
Lanao del Sur	**14,440**	0	**0.00**	0	**0.00**	0	**0.00**	0	**0.00**
Maguindanao	**272**	0	**0.00**	1	**0.37**	15	**5.51**	16	**5.88**
Sulu	**251**	1	**0.40**	3	**1.20**	5	**1.99**	9	**3.59**
Tawi-Tawi	**65**	0	**0.00**	0	**0.00**	1	**1.54**	1	**1.54**
SGU	**0**	0	**0.00**	0	**0.00**	0	**0.00**	0	**0.00**
City of Cotabato		0	**0.00**	1	**0.00**	25	**0.00**	26	**0.00**
**Caraga**	**11,810**	**0**	**0.00**	**15**	**0.13**	**85**	**0.72**	**100**	**0.85**
Agusan del Norte	**1,578**	0	**0.00**	1	**0.06**	22	**1.39**	23	**1.46**
Agusan del Sur	**2,271**	0	**0.00**	9	**0.40**	37	**1.63**	46	**2.03**
Surigao del Norte	**2,216**	0	**0.00**	3	**0.14**	13	**0.59**	16	**0.72**
Surigao del Sur	**870**	0	**0.00**	0	**0.00**	3	**0.34**	3	**0.34**
Dinagat Islands	**908**	0	**0.00**	0	**0.00**	9	**0.99**	9	**0.99**
City of Butuan	**3,967**	0	**0.00**	2	**0.05**	1	**0.03**	3	**0.08**

Table 2.B.1.11 - Prenatal Care

Pregnant women screened for Hepatitis B

Philippines, 2023

Area	Eligible Pop.	Screened for Hepatitis B							
		Age Group						Total	%
		10-14 yrs old		15-19 yrs old		20-49 yrs old			
		No.	%	No.	%	No.	%		
**Philippines**	**2,187,333**	**1,774**	**0.08**	**77,422**	**3.54**	**636,424**	**29.10**	**715,620**	**32.72**
**N C R**	**261,228**	**213**	**0.08**	**10,059**	**3.85**	**109,687**	**41.99**	**119,959**	**45.92**
Malabon City	**7,385**	7	**0.09**	229	**3.10**	1,746	**23.64**	**1,982**	**26.84**
Navotas City	**5,210**	16	**0.31**	376	**7.22**	2,728	**52.36**	**3,120**	**59.88**
Valenzuela City	**13,477**	14	**0.10**	439	**3.26**	4,978	**36.94**	**5,431**	**40.30**
Caloocan City	**33,168**	17	**0.05**	1,024	**3.09**	10,764	**32.45**	**11,805**	**35.59**
Marikina City	**8,342**	5	**0.06**	321	**3.85**	3,006	**36.03**	**3,332**	**39.94**
Pasig City	**17,682**	9	**0.05**	716	**4.05**	11,825	**66.88**	**12,550**	**70.98**
Pateros	**1,116**	0	**0.00**	23	**2.06**	263	**23.57**	**286**	**25.63**
Taguig City	**18,992**	15	**0.08**	1,041	**5.48**	8,522	**44.87**	**9,578**	**50.43**
Quezon City	**59,136**	28	**0.05**	1,737	**2.94**	24,555	**41.52**	**26,320**	**44.51**
Makati City	**10,944**	7	**0.06**	167	**1.53**	2,578	**23.56**	**2,752**	**25.15**
Mandaluyong City	**7,922**	10	**0.13**	261	**3.29**	6,544	**82.61**	**6,815**	**86.03**
San Juan City	**2,168**	1	**0.05**	59	**2.72**	815	**37.59**	**875**	**40.36**
Manila City	**34,128**	44	**0.13**	2,233	**6.54**	16,309	**47.79**	**18,586**	**54.46**
Las Piñas City	**11,263**	6	**0.05**	236	**2.10**	3,100	**27.52**	**3,342**	**29.67**
Muntinlupa City	**9,881**	19	**0.19**	291	**2.95**	2,780	**28.13**	**3,090**	**31.27**
Parañaque City	**13,280**	7	**0.05**	604	**4.55**	5,523	**41.59**	**6,134**	**46.19**
Pasay City	**7,134**	8	**0.11**	302	**4.23**	3,651	**51.18**	**3,961**	**55.52**
**C A R**	**31,319**	**47**	**0.15**	**2,197**	**7.01**	**18,604**	**59.40**	**20,848**	**66.57**
Abra	**3,660**	4	**0.11**	156	**4.26**	935	**25.55**	**1,095**	**29.92**
Apayao	**2,238**	16	**0.71**	366	**16.35**	2,231	**99.69**	**2,613**	**116.76**
Benguet	**8,806**	11	**0.12**	712	**8.09**	6,668	**75.72**	**7,391**	**83.93**
Ifugao	**4,098**	6	**0.15**	248	**6.05**	1,962	**47.88**	**2,216**	**54.08**
Kalinga	**4,549**	0	**0.00**	125	**2.75**	1,024	**22.51**	**1,149**	**25.26**
Mountain Province	**2,619**	5	**0.19**	327	**12.49**	3,057	**116.72**	**3,389**	**129.40**
City of Baguio	**5,349**	5	**0.09**	263	**4.92**	2,727	**50.98**	**2,995**	**55.99**
**Region 1**	**96,016**	**51**	**0.05**	**2,782**	**2.90**	**31,343**	**32.64**	**34,176**	**35.59**
Ilocos Norte	**8,912**	8	**0.09**	358	**4.02**	4,885	**54.81**	**5,251**	**58.92**
Ilocos Sur	**9,914**	5	**0.05**	368	**3.71**	6,469	**65.25**	**6,842**	**69.01**
La Union	**13,507**	16	**0.12**	622	**4.61**	5,497	**40.70**	**6,135**	**45.42**
Pangasinan	**60,360**	20	**0.03**	1,361	**2.25**	14,062	**23.30**	**15,443**	**25.58**

Table 2.B.1.11 - Prenatal Care

Pregnant women screened for Hepatitis B

Philippines, 2023

Area	Eligible Pop.	Screened for Hepatitis B							
		Age Group						Total	%
		10-14 yrs old		15-19 yrs old		20-49 yrs old			
		No.	%	No.	%	No.	%		
City of Dagupan	**3,323**	2	**0.06**	73	**2.20**	430	**12.94**	**505**	**15.20**
**Region 2**	**63,967**	**68**	**0.11**	**2,158**	**3.37**	**15,933**	**24.91**	**18,159**	**28.39**
Batanes	**240**	0	**0.00**	12	**5.00**	108	**45.00**	**120**	**50.00**
Cagayan	**20,786**	9	**0.04**	208	**1.00**	1,643	**7.90**	**1,860**	**8.95**
Isabela	**27,379**	43	**0.16**	1,253	**4.58**	9,520	**34.77**	**10,816**	**39.50**
Nueva Vizcaya	**8,798**	6	**0.07**	369	**4.19**	2,809	**31.93**	**3,184**	**36.19**
Quirino	**3,839**	10	**0.26**	279	**7.27**	1,532	**39.91**	**1,821**	**47.43**
City of Santiago	**2,925**	0	**0.00**	37	**1.26**	321	**10.97**	**358**	**12.24**
**Region 3**	**233,544**	**276**	**0.12**	**10,762**	**4.61**	**81,140**	**34.74**	**92,178**	**39.47**
Aurora	**4,537**	10	**0.22**	266	**5.86**	1,766	**38.92**	**2,042**	**45.01**
Bataan	**17,380**	28	**0.16**	1,175	**6.76**	8,083	**46.51**	**9,286**	**53.43**
Bulacan	**67,590**	107	**0.16**	4,092	**6.05**	36,261	**53.65**	**40,460**	**59.86**
Nueva Ecija	**43,425**	53	**0.12**	1,715	**3.95**	10,392	**23.93**	**12,160**	**28.00**
Pampanga	**45,651**	46	**0.10**	1,657	**3.63**	11,794	**25.84**	**13,497**	**29.57**
Tarlac	**29,503**	17	**0.06**	1,135	**3.85**	7,843	**26.58**	**8,995**	**30.49**
Zambales	**11,723**	9	**0.08**	497	**4.24**	3,194	**27.25**	**3,700**	**31.56**
City of Angeles	**9,146**	2	**0.02**	52	**0.57**	564	**6.17**	**618**	**6.76**
City of Olongapo	**4,589**	4	**0.09**	173	**3.77**	1,243	**27.09**	**1,420**	**30.94**
**Region 4A**	**307,982**	**206**	**0.07**	**9,738**	**3.16**	**88,730**	**28.81**	**98,674**	**32.04**
Batangas	**53,251**	22	**0.04**	1,056	**1.98**	13,228	**24.84**	**14,306**	**26.87**
Cavite	**77,964**	40	**0.05**	1,988	**2.55**	19,237	**24.67**	**21,265**	**27.28**
Laguna	**61,525**	45	**0.07**	2,483	**4.04**	24,951	**40.55**	**27,479**	**44.66**
Quezon	**40,810**	37	**0.09**	1,312	**3.21**	10,032	**24.58**	**11,381**	**27.89**
Rizal	**68,235**	55	**0.08**	2,546	**3.73**	19,418	**28.46**	**22,019**	**32.27**
City of Lucena	**6,197**	7	**0.11**	353	**5.70**	1,864	**30.08**	**2,224**	**35.89**
**Region 4B**	**66,121**	**51**	**0.08**	**2,286**	**3.46**	**19,185**	**29.01**	**21,522**	**32.55**
Marinduque	**4,144**	5	**0.12**	116	**2.80**	1,181	**28.50**	**1,302**	**31.42**
Mindoro Occidental	**11,640**	12	**0.10**	522	**4.48**	3,553	**30.52**	**4,087**	**35.11**
Mindoro Oriental	**18,943**	5	**0.03**	518	**2.73**	7,171	**37.86**	**7,694**	**40.62**
Palawan	**20,317**	20	**0.10**	714	**3.51**	3,980	**19.59**	**4,714**	**23.20**
Romblon	**5,489**	3	**0.05**	183	**3.33**	1,875	**34.16**	**2,061**	**37.55**

Table 2.B.1.11 - Prenatal Care

Pregnant women screened for Hepatitis B

Philippines, 2023

Area	Eligible Pop.	Screened for Hepatitis B							
		Age Group						Total	%
		10-14 yrs old		15-19 yrs old		20-49 yrs old			
		No.	%	No.	%	No.	%		
Puerto Princesa City	**5,588**	6	**0.11**	233	**4.17**	1,425	**25.50**	**1,664**	**29.78**
**Region 5**	**135,480**	**41**	**0.03**	**2,667**	**1.97**	**25,074**	**18.51**	**27,782**	**20.51**
Albay	**27,440**	8	**0.03**	490	**1.79**	7,768	**28.31**	**8,266**	**30.12**
Camarines Norte	**14,695**	7	**0.05**	343	**2.33**	2,805	**19.09**	**3,155**	**21.47**
Camarines Sur	**43,452**	3	**0.01**	323	**0.74**	3,636	**8.37**	**3,962**	**9.12**
Catanduanes	**5,414**	8	**0.15**	357	**6.59**	2,105	**38.88**	**2,470**	**45.62**
Masbate	**22,365**	8	**0.04**	714	**3.19**	4,342	**19.41**	**5,064**	**22.64**
Sorsogon	**17,929**	6	**0.03**	362	**2.02**	3,663	**20.43**	**4,031**	**22.48**
City of Naga	**4,185**	1	**0.02**	78	**1.86**	755	**18.04**	**834**	**19.93**
**Region 6**	**148,020**	**152**	**0.10**	**5,890**	**3.98**	**50,349**	**34.01**	**56,391**	**38.10**
Aklan	**11,283**	5	**0.04**	314	**2.78**	3,896	**34.53**	**4,215**	**37.36**
Antique	**11,930**	71	**0.60**	394	**3.30**	3,415	**28.63**	**3,880**	**32.52**
Capiz	**13,931**	10	**0.07**	420	**3.01**	3,784	**27.16**	**4,214**	**30.25**
Guimaras	**3,351**	3	**0.09**	189	**5.64**	1,926	**57.48**	**2,118**	**63.21**
Iloilo	**36,140**	28	**0.08**	1,472	**4.07**	14,924	**41.29**	**16,424**	**45.45**
Negros Occidental	**52,270**	23	**0.04**	2,266	**4.34**	15,214	**29.11**	**17,503**	**33.49**
City of Bacolod	**10,373**	1	**0.01**	541	**5.22**	4,390	**42.32**	**4,932**	**47.55**
City of Iloilo	**8,742**	11	**0.13**	294	**3.36**	2,800	**32.03**	**3,105**	**35.52**
**Region 7**	**165,673**	**176**	**0.11**	**9,783**	**5.91**	**72,019**	**43.47**	**81,978**	**49.48**
Bohol	**26,235**	25	**0.10**	1,490	**5.68**	14,307	**54.53**	**15,822**	**60.31**
Cebu	**72,201**	48	**0.07**	3,670	**5.08**	19,824	**27.46**	**23,542**	**32.61**
Negros Oriental	**28,722**	19	**0.07**	1,112	**3.87**	7,690	**26.77**	**8,821**	**30.71**
Siquijor	**1,680**	1	**0.06**	65	**3.87**	739	**43.99**	**805**	**47.92**
City of Cebu	**19,267**	20	**0.10**	1,028	**5.34**	8,179	**42.45**	**9,227**	**47.89**
City of Lapu-Lapu	**10,679**	5	**0.05**	375	**3.51**	5,479	**51.31**	**5,859**	**54.86**
City of Mandaue	**6,889**	58	**0.84**	2,043	**29.66**	15,801	**229.37**	**17,902**	**259.86**
**Region 8**	**93,813**	**45**	**0.05**	**2,902**	**3.09**	**23,992**	**25.57**	**26,939**	**28.72**
Biliran	**3,373**	3	**0.09**	239	**7.09**	1,597	**47.35**	**1,839**	**54.52**
Eastern Samar	**9,624**	4	**0.04**	304	**3.16**	2,409	**25.03**	**2,717**	**28.23**
Leyte	**31,559**	24	**0.08**	756	**2.40**	7,274	**23.05**	**8,054**	**25.52**
Northern Samar	**14,056**	6	**0.04**	624	**4.44**	3,942	**28.04**	**4,572**	**32.53**

Table 2.B.1.11 - Prenatal Care

Pregnant women screened for Hepatitis B

Philippines, 2023

Area	Eligible Pop.	Screened for Hepatitis B							
		Age Group						Total	%
		10-14 yrs old		15-19 yrs old		20-49 yrs old			
		No.	%	No.	%	No.	%		
Southern Leyte	**7,406**	1	**0.01**	231	**3.12**	2,646	**35.73**	**2,878**	**38.86**
Samar	**17,011**	2	**0.01**	199	**1.17**	2,021	**11.88**	**2,222**	**13.06**
Ormoc City	**5,292**	2	**0.04**	261	**4.93**	1,610	**30.42**	**1,873**	**35.39**
City of Tacloban	**5,492**	3	**0.05**	288	**5.24**	2,493	**45.39**	**2,784**	**50.69**
**Region 9**	**84,815**	**17**	**0.02**	**842**	**0.99**	**4,517**	**5.33**	**5,376**	**6.34**
Zamboanga del Norte	**24,841**	0	**0.00**	166	**0.67**	966	**3.89**	**1,132**	**4.56**
Zamboanga del Sur	**23,757**	7	**0.03**	278	**1.17**	1,170	**4.92**	**1,455**	**6.12**
Zamboanga Sibugay	**13,841**	5	**0.04**	208	**1.50**	1,284	**9.28**	**1,497**	**10.82**
City of Isabela	**3,201**	4	**0.12**	31	**0.97**	186	**5.81**	**221**	**6.90**
City of Zamboanga	**19,175**	1	**0.01**	159	**0.83**	911	**4.75**	**1,071**	**5.59**
**Region 10**	**109,169**	**112**	**0.10**	**4,708**	**4.31**	**28,522**	**26.13**	**33,342**	**30.54**
Bukidnon	**33,403**	34	**0.10**	978	**2.93**	5,077	**15.20**	**6,089**	**18.23**
Camiguin	**1,727**	1	**0.06**	75	**4.34**	526	**30.46**	**602**	**34.86**
Lanao del Norte	**17,760**	3	**0.02**	158	**0.89**	1,328	**7.48**	**1,489**	**8.38**
Misamis Occidental	**12,328**	3	**0.02**	250	**2.03**	2,153	**17.46**	**2,406**	**19.52**
Misamis Oriental	**21,975**	48	**0.22**	1,588	**7.23**	9,315	**42.39**	**10,951**	**49.83**
City of Cagayan De Oro	**14,308**	22	**0.15**	1,365	**9.54**	8,190	**57.24**	**9,577**	**66.93**
City of Iligan	**7,668**	1	**0.01**	294	**3.83**	1,933	**25.21**	**2,228**	**29.06**
**Region 11**	**107,001**	**121**	**0.11**	**3,314**	**3.10**	**22,838**	**21.34**	**26,273**	**24.55**
Davao de Oro	**15,253**	4	**0.03**	78	**0.51**	629	**4.12**	**711**	**4.66**
Davao del Norte	**23,453**	14	**0.06**	434	**1.85**	3,145	**13.41**	**3,593**	**15.32**
Davao Oriental	**11,935**	5	**0.04**	38	**0.32**	303	**2.54**	**346**	**2.90**
Davao del Sur	**13,083**	15	**0.11**	306	**2.34**	1,810	**13.83**	**2,131**	**16.29**
Davao Occidental	**6,629**	6	**0.09**	155	**2.34**	400	**6.03**	**561**	**8.46**
City of Davao	**36,648**	77	**0.21**	2,303	**6.28**	16,551	**45.16**	**18,931**	**51.66**
**Region 12**	**95,279**	**167**	**0.18**	**5,193**	**5.45**	**28,839**	**30.27**	**34,199**	**35.89**
Cotabato	**26,521**	24	**0.09**	1,153	**4.35**	6,347	**23.93**	**7,524**	**28.37**
Sarangani	**13,322**	39	**0.29**	1,258	**9.44**	5,564	**41.77**	**6,861**	**51.50**
South Cotabato	**21,595**	23	**0.11**	1,017	**4.71**	6,388	**29.58**	**7,428**	**34.40**
Sultan Kudarat	**19,391**	77	**0.40**	1,221	**6.30**	6,163	**31.78**	**7,461**	**38.48**

Table 2.B.1.11 - Prenatal Care

Pregnant women screened for Hepatitis B

Philippines, 2023

Area	Eligible Pop.	Screened for Hepatitis B							
		Age Group						Total	%
		10-14 yrs old		15-19 yrs old		20-49 yrs old			
		No.	%	No.	%	No.	%		
City of General Santos	**14,450**	4	**0.03**	544	**3.76**	4,377	**30.29**	**4,925**	**34.08**
**BARMM**	**132,841**	**2**	**0.00**	**281**	**0.21**	**2,414**	**1.82**	**2,697**	**2.03**
Basilan	**11,636**	0	**0.00**	36	**0.31**	76	**0.65**	**112**	**0.96**
Lanao del Sur	**33,374**	0	**0.00**	27	**0.08**	611	**1.83**	**638**	**1.91**
Maguindanao	**41,650**	0	**0.00**	27	**0.06**	235	**0.56**	**262**	**0.63**
Sulu	**21,378**	2	**0.01**	139	**0.65**	866	**4.05**	**1,007**	**4.71**
Tawi-Tawi	**12,609**	0	**0.00**	5	**0.04**	58	**0.46**	**63**	**0.50**
SGU	**5,103**	0	**0.00**	0	**0.00**	0	**0.00**	**0**	**0.00**
City of Cotabato	**7,091**	0	**0.00**	47	**0.66**	568	**8.01**	**615**	**8.67**
**Caraga**	**55,065**	**29**	**0.05**	**1,860**	**3.38**	**13,238**	**24.04**	**15,127**	**27.47**
Agusan del Norte	**7,286**	3	**0.04**	281	**3.86**	1,884	**25.86**	**2,168**	**29.76**
Agusan del Sur	**16,038**	3	**0.02**	299	**1.86**	2,034	**12.68**	**2,336**	**14.57**
Surigao del Norte	**10,186**	6	**0.06**	403	**3.96**	3,333	**32.72**	**3,742**	**36.74**
Surigao del Sur	**12,736**	4	**0.03**	237	**1.86**	1,205	**9.46**	**1,446**	**11.35**
Dinagat Islands	**2,134**	3	**0.14**	103	**4.83**	907	**42.50**	**1,013**	**47.47**
City of Butuan	**6,685**	10	**0.15**	537	**8.03**	3,875	**57.97**	**4,422**	**66.15**

Table 2.B.1.12 - Prenatal Care

Pregnant women tested positive for Hepatitis B

Philippines, 2023

Area	Total No. of Screened for Hepatitis B	Tested Positive for Hepatitis B							
		Age Group						Total	%
		10-14 yrs old		15-19 yrs old		20-49 yrs old			
		No.	%	No.	%	No.	%		
**Philippines**	**715,005**	**136**	**0.02**	**1,125**	**0.16**	**10,724**	**1.50**	**11,985**	**1.68**
**N C R**	**119,959**	**0**	**0.00**	**73**	**0.06**	**1,300**	**1.08**	**1,373**	**1.14**
Malabon City	**1,982**	0	**0.00**	1	**0.05**	27	**1.36**	**28**	**1.41**
Navotas City	**3,120**	0	**0.00**	0	**0.00**	19	**0.61**	**19**	**0.61**
Valenzuela City	**5,431**	0	**0.00**	12	**0.22**	149	**2.74**	**161**	**2.96**
Caloocan City	**11,805**	0	**0.00**	8	**0.07**	190	**1.61**	**198**	**1.68**
Marikina City	**3,332**	0	**0.00**	4	**0.12**	11	**0.33**	**15**	**0.45**
Pasig City	**12,550**	0	**0.00**	2	**0.02**	74	**0.59**	**76**	**0.61**
Pateros	**286**	0	**0.00**	0	**0.00**	1	**0.35**	**1**	**0.35**
Taguig City	**9,578**	0	**0.00**	14	**0.15**	209	**2.18**	**223**	**2.33**
Quezon City	**26,320**	0	**0.00**	6	**0.02**	175	**0.66**	**181**	**0.69**
Makati City	**2,752**	0	**0.00**	0	**0.00**	72	**2.62**	**72**	**2.62**
Mandaluyong City	**6,815**	0	**0.00**	0	**0.00**	18	**0.26**	**18**	**0.26**
San Juan City	**875**	0	**0.00**	1	**0.11**	2	**0.23**	**3**	**0.34**
Manila City	**18,586**	0	**0.00**	9	**0.05**	116	**0.62**	**125**	**0.67**
Las Piñas City	**3,342**	0	**0.00**	3	**0.09**	49	**1.47**	**52**	**1.56**
Muntinlupa City	**3,090**	0	**0.00**	2	**0.06**	26	**0.84**	**28**	**0.91**
Parañaque City	**6,134**	0	**0.00**	4	**0.07**	53	**0.86**	**57**	**0.93**
Pasay City	**3,961**	0	**0.00**	7	**0.18**	109	**2.75**	**116**	**2.93**
**C A R**	**20,848**	**0**	**0.00**	**11**	**0.05**	**150**	**0.72**	**161**	**0.77**
Abra	**1,095**	0	**0.00**	2	**0.18**	9	**0.82**	**11**	**1.00**
Apayao	**2,613**	0	**0.00**	4	**0.15**	51	**1.95**	**55**	**2.10**
Benguet	**7,391**	0	**0.00**	0	**0.00**	4	**0.05**	**4**	**0.05**
Ifugao	**2,216**	0	**0.00**	1	**0.05**	28	**1.26**	**29**	**1.31**
Kalinga	**1,149**	0	**0.00**	1	**0.09**	4	**0.35**	**5**	**0.44**
Mountain Province	**3,389**	0	**0.00**	1	**0.03**	6	**0.18**	**7**	**0.21**
City of Baguio	**2,995**	0	**0.00**	2	**0.07**	48	**1.60**	**50**	**1.67**
**Region 1**	**34,176**	**1**	**0.00**	**22**	**0.06**	**195**	**0.57**	**218**	**0.64**
Ilocos Norte	**5,251**	0	**0.00**	1	**0.02**	8	**0.15**	**9**	**0.17**
Ilocos Sur	**6,842**	0	**0.00**	2	**0.03**	7	**0.10**	**9**	**0.13**
La Union	**6,135**	1	**0.02**	1	**0.02**	33	**0.54**	**35**	**0.57**
Pangasinan	**15,443**	0	**0.00**	18	**0.12**	139	**0.90**	**157**	**1.02**

Table 2.B.1.12 - Prenatal Care

Pregnant women tested positive for Hepatitis B

Philippines, 2023

Area	Total No. of Screened for Hepatitis B	Tested Positive for Hepatitis B							
		Age Group						Total	%
		10-14 yrs old		15-19 yrs old		20-49 yrs old			
		No.	%	No.	%	No.	%		
City of Dagupan	**505**	0	**0.00**	0	**0.00**	8	**1.58**	**8**	**1.58**
**Region 2**	**18,159**	**1**	**0.01**	**22**	**0.12**	**254**	**1.40**	**277**	**1.53**
Batanes	**120**	0	**0.00**	0	**0.00**	1	**0.83**	**1**	**0.83**
Cagayan	**1,860**	0	**0.00**	1	**0.05**	7	**0.38**	**8**	**0.43**
Isabela	**10,816**	0	**0.00**	8	**0.07**	135	**1.25**	**143**	**1.32**
Nueva Vizcaya	**3,184**	1	**0.03**	12	**0.38**	99	**3.11**	**112**	**3.52**
Quirino	**1,821**	0	**0.00**	1	**0.05**	12	**0.66**	**13**	**0.71**
City of Santiago	**358**	0	**0.00**	0	**0.00**	0	**0.00**	**0**	**0.00**
**Region 3**	**92,178**	**0**	**0.00**	**83**	**0.09**	**911**	**0.99**	**994**	**1.08**
Aurora	**2,042**	0	**0.00**	3	**0.15**	21	**1.03**	**24**	**1.18**
Bataan	**9,286**	0	**0.00**	6	**0.06**	76	**0.82**	**82**	**0.88**
Bulacan	**40,460**	0	**0.00**	24	**0.06**	241	**0.60**	**265**	**0.65**
Nueva Ecija	**12,160**	0	**0.00**	10	**0.08**	102	**0.84**	**112**	**0.92**
Pampanga	**13,497**	0	**0.00**	25	**0.19**	234	**1.73**	**259**	**1.92**
Tarlac	**8,995**	0	**0.00**	4	**0.04**	116	**1.29**	**120**	**1.33**
Zambales	**3,700**	0	**0.00**	5	**0.14**	53	**1.43**	**58**	**1.57**
City of Angeles	**618**	0	**0.00**	0	**0.00**	8	**1.29**	**8**	**1.29**
City of Olongapo	**1,420**	0	**0.00**	6	**0.42**	60	**4.23**	**66**	**4.65**
**Region 4A**	**98,674**	**9**	**0.01**	**176**	**0.18**	**1,702**	**1.72**	**1,887**	**1.91**
Batangas	**14,306**	2	**0.01**	10	**0.07**	132	**0.92**	**144**	**1.01**
Cavite	**21,265**	0	**0.00**	34	**0.16**	329	**1.55**	**363**	**1.71**
Laguna	**27,479**	1	**0.00**	51	**0.19**	467	**1.70**	**519**	**1.89**
Quezon	**11,381**	4	**0.04**	24	**0.21**	257	**2.26**	**285**	**2.50**
Rizal	**22,019**	2	**0.01**	49	**0.22**	467	**2.12**	**518**	**2.35**
City of Lucena	**2,224**	0	**0.00**	8	**0.36**	50	**2.25**	**58**	**2.61**
**Region 4B**	**21,522**	**18**	**0.08**	**45**	**0.21**	**367**	**1.71**	**430**	**2.00**
Marinduque	**1,302**	0	**0.00**	2	**0.15**	18	**1.38**	**20**	**1.54**
Mindoro Occidental	**4,087**	12	**0.29**	12	**0.29**	81	**1.98**	**105**	**2.57**
Mindoro Oriental	**7,694**	6	**0.08**	1	**0.01**	37	**0.48**	**44**	**0.57**
Palawan	**4,714**	0	**0.00**	21	**0.45**	159	**3.37**	**180**	**3.82**
Romblon	**2,061**	0	**0.00**	2	**0.10**	41	**1.99**	**43**	**2.09**

Table 2.B.1.12 - Prenatal Care

Pregnant women tested positive for Hepatitis B

Philippines, 2023

Area	Total No. of Screened for Hepatitis B	Tested Positive for Hepatitis B							
		Age Group						Total	%
		10-14 yrs old		15-19 yrs old		20-49 yrs old			
		No.	%	No.	%	No.	%		
Puerto Princesa City	1,664	0	0.00	7	0.42	31	1.86	38	2.28
**Region 5**	**27,782**	**5**	**0.02**	**60**	**0.22**	**612**	**2.20**	**677**	**2.44**
Albay	8,266	0	0.00	6	0.07	119	1.44	125	1.51
Camarines Norte	3,155	0	0.00	10	0.32	72	2.28	82	2.60
Camarines Sur	3,962	0	0.00	4	0.10	61	1.54	65	1.64
Catanduanes	2,470	0	0.00	6	0.24	82	3.32	88	3.56
Masbate	5,064	5	0.10	30	0.59	172	3.40	207	4.09
Sorsogon	4,031	0	0.00	4	0.10	101	2.51	105	2.60
City of Naga	834	0	0.00	0	0.00	5	0.60	5	0.60
**Region 6**	**56,391**	**0**	**0.00**	**105**	**0.19**	**1,045**	**1.85**	**1,150**	**2.04**
Aklan	4,215	0	0.00	5	0.12	0	0.00	5	0.12
Antique	3,880	0	0.00	4	0.10	0	0.00	4	0.10
Capiz	4,214	0	0.00	12	0.28	0	0.00	12	0.28
Guimaras	2,118	0	0.00	4	0.19	0	0.00	4	0.19
Iloilo	16,424	0	0.00	30	0.18	896	5.46	926	5.64
Negros Occidental	17,503	0	0.00	37	0.21	0	0.00	37	0.21
City of Bacolod	4,932	0	0.00	13	0.26	76	1.54	89	1.80
City of Iloilo	3,105	0	0.00	0	0.00	73	2.35	73	2.35
**Region 7**	**81,978**	**4**	**0.00**	**126**	**0.15**	**1,195**	**1.46**	**1,325**	**1.62**
Bohol	15,822	0	0.00	14	0.09	282	1.78	296	1.87
Cebu	23,542	2	0.01	46	0.20	416	1.77	464	1.97
Negros Oriental	8,821	2	0.02	26	0.29	220	2.49	248	2.81
Siquijor	805	0	0.00	1	0.12	9	1.12	10	1.24
City of Cebu	9,227	0	0.00	24	0.26	156	1.69	180	1.95
City of Lapu-Lapu	5,859	0	0.00	15	0.26	108	1.84	123	2.10
City of Mandaue	17,902	0	0.00	0	0.00	4	0.02	4	0.02
**Region 8**	**26,939**	**89**	**0.33**	**53**	**0.20**	**513**	**1.90**	**655**	**2.43**
Biliran	1,839	0	0.00	0	0.00	18	0.98	18	0.98
Eastern Samar	2,717	0	0.00	7	0.26	43	1.58	50	1.84
Leyte	8,054	88	1.09	11	0.14	224	2.78	323	4.01
Northern Samar	4,572	1	0.02	20	0.44	118	2.58	139	3.04

Table 2.B.1.12 - Prenatal Care

Pregnant women tested positive for Hepatitis B

Philippines, 2023

Area	Total No. of Screened for Hepatitis B	Tested Positive for Hepatitis B							
		Age Group						Total	%
		10-14 yrs old		15-19 yrs old		20-49 yrs old			
		No.	%	No.	%	No.	%		
Southern Leyte	2,878	0	0.00	5	0.17	47	1.63	52	1.81
Samar	2,222	0	0.00	2	0.09	34	1.53	36	1.62
Ormoc City	1,873	0	0.00	6	0.32	20	1.07	26	1.39
City of Tacloban	2,784	0	0.00	2	0.07	9	0.32	11	0.40
**Region 9**	**5,376**	**0**	**0.00**	**12**	**0.22**	**106**	**1.97**	**118**	**2.19**
Zamboanga del Norte	1,132	0	0.00	4	0.35	19	1.68	23	2.03
Zamboanga del Sur	1,455	0	0.00	2	0.14	39	2.68	41	2.82
Zamboanga Sibugay	1,497	0	0.00	4	0.27	23	1.54	27	1.80
City of Isabela	221	0	0.00	0	0.00	0	0.00	0	0.00
City of Zamboanga	1,071	0	0.00	2	0.19	25	2.33	27	2.52
**Region 10**	**33,342**	**1**	**0.00**	**75**	**0.22**	**604**	**1.81**	**680**	**2.04**
Bukidnon	6,089	1	0.02	26	0.43	203	3.33	230	3.78
Camiguin	602	0	0.00	0	0.00	1	0.17	1	0.17
Lanao del Norte	1,489	0	0.00	7	0.47	43	2.89	50	3.36
Misamis Occidental	2,406	0	0.00	0	0.00	3	0.12	3	0.12
Misamis Oriental	10,951	0	0.00	14	0.13	127	1.16	141	1.29
City of Cagayan De Oro	9,577	0	0.00	27	0.28	211	2.20	238	2.49
City of Iligan	2,228	0	0.00	1	0.04	16	0.72	17	0.76
**Region 11**	**26,273**	**2**	**0.01**	**58**	**0.22**	**302**	**1.15**	**362**	**1.38**
Davao de Oro	711	0	0.00	0	0.00	14	1.97	14	1.97
Davao del Norte	3,593	0	0.00	8	0.22	34	0.95	42	1.17
Davao Oriental	346	0	0.00	0	0.00	17	4.91	17	4.91
Davao del Sur	2,131	1	0.05	2	0.09	28	1.31	31	1.45
Davao Occidental	561	0	0.00	10	1.78	40	7.13	50	8.91
City of Davao	18,931	1	0.01	38	0.20	169	0.89	208	1.10
**Region 12**	**34,199**	**5**	**0.01**	**162**	**0.47**	**1,046**	**3.06**	**1,213**	**3.55**
Cotabato	7,524	3	0.04	33	0.44	179	2.38	215	2.86
Sarangani	6,861	1	0.01	65	0.95	257	3.75	323	4.71
South Cotabato	7,428	0	0.00	15	0.20	116	1.56	131	1.76
Sultan Kudarat	7,461	1	0.01	17	0.23	99	1.33	117	1.57

Table 2.B.1.12 - Prenatal Care

Pregnant women tested positive for Hepatitis B

Philippines, 2023

Area	Total No. of Screened for Hepatitis B	Tested Positive for Hepatitis B							
		Age Group						Total	%
		10-14 yrs old		15-19 yrs old		20-49 yrs old			
		No.	%	No.	%	No.	%		
City of General Santos	**4,925**	0	**0.00**	32	**0.65**	395	**8.02**	**427**	**8.67**
**BARMM**	**2,082**	**0**	**0.00**	**5**	**0.24**	**56**	**2.69**	**61**	**2.93**
Basilan	**112**	0	**0.00**	0	**0.00**	1	**0.89**	**1**	**0.89**
Lanao del Sur	**638**	0	**0.00**	1	**0.16**	6	**0.94**	**7**	**1.10**
Maguindanao	**262**	0	**0.00**	1	**0.38**	21	**8.02**	**22**	**8.40**
Sulu	**1,007**	0	**0.00**	2	**0.20**	22	**2.18**	**24**	**2.38**
Tawi-Tawi	**63**	0	**0.00**	0	**0.00**	4	**6.35**	**4**	**6.35**
SGU	**0**	0	**0.00**	0	**0.00**	0	**0.00**	**0**	**0.00**
City of Cotabato		0	**0.00**	1	**0.00**	2	**0.00**	**3**	**0.00**
**Caraga**	**15,127**	**1**	**0.01**	**37**	**0.24**	**366**	**2.42**	**404**	**2.67**
Agusan del Norte	**2,168**	0	**0.00**	3	**0.14**	46	**2.12**	**49**	**2.26**
Agusan del Sur	**2,336**	0	**0.00**	9	**0.39**	54	**2.31**	**63**	**2.70**
Surigao del Norte	**3,742**	0	**0.00**	10	**0.27**	134	**3.58**	**144**	**3.85**
Surigao del Sur	**1,446**	0	**0.00**	8	**0.55**	56	**3.87**	**64**	**4.43**
Dinagat Islands	**1,013**	0	**0.00**	5	**0.49**	44	**4.34**	**49**	**4.84**
City of Butuan	**4,422**	1	**0.02**	2	**0.05**	32	**0.72**	**35**	**0.79**

Table 2.B.1.13 - Prenatal Care

Pregnant women screened for HIV

Philippines, 2023

Area	Eligible Pop.	Screened for HIV							
		Age Group						Total	%
		10-14 yrs old		15-19 yrs old		20-49 yrs old			
		No.	%	No.	%	No.	%		
**Philippines**	**2,187,333**	**1,383**	**0.06**	**60,216**	**2.75**	**509,364**	**23.29**	**570,963**	**26.10**
**N C R**	**261,228**	**248**	**0.09**	**11,220**	**4.30**	**120,713**	**46.21**	**132,181**	**50.60**
Malabon City	**7,385**	11	**0.15**	263	**3.56**	2,007	**27.18**	**2,281**	**30.89**
Navotas City	**5,210**	16	**0.31**	365	**7.01**	2,613	**50.15**	**2,994**	**57.47**
Valenzuela City	**13,477**	18	**0.13**	529	**3.93**	5,589	**41.47**	**6,136**	**45.53**
Caloocan City	**33,168**	19	**0.06**	1,030	**3.11**	10,509	**31.68**	**11,558**	**34.85**
Marikina City	**8,342**	3	**0.04**	259	**3.10**	2,266	**27.16**	**2,528**	**30.30**
Pasig City	**17,682**	7	**0.04**	694	**3.92**	11,722	**66.29**	**12,423**	**70.26**
Pateros	**1,116**	0	**0.00**	27	**2.42**	334	**29.93**	**361**	**32.35**
Taguig City	**18,992**	15	**0.08**	1,022	**5.38**	8,516	**44.84**	**9,553**	**50.30**
Quezon City	**59,136**	46	**0.08**	2,335	**3.95**	31,907	**53.96**	**34,288**	**57.98**
Makati City	**10,944**	9	**0.08**	215	**1.96**	2,970	**27.14**	**3,194**	**29.18**
Mandaluyong City	**7,922**	11	**0.14**	262	**3.31**	6,637	**83.78**	**6,910**	**87.23**
San Juan City	**2,168**	1	**0.05**	59	**2.72**	816	**37.64**	**876**	**40.41**
Manila City	**34,128**	45	**0.13**	2,309	**6.77**	16,628	**48.72**	**18,982**	**55.62**
Las Piñas City	**11,263**	11	**0.10**	358	**3.18**	4,017	**35.67**	**4,386**	**38.94**
Muntinlupa City	**9,881**	14	**0.14**	401	**4.06**	3,574	**36.17**	**3,989**	**40.37**
Parañaque City	**13,280**	12	**0.09**	622	**4.68**	5,314	**40.02**	**5,948**	**44.79**
Pasay City	**7,134**	10	**0.14**	470	**6.59**	5,294	**74.21**	**5,774**	**80.94**
**C A R**	**31,319**	**31**	**0.10**	**1,401**	**4.47**	**12,490**	**39.88**	**13,922**	**44.45**
Abra	**3,660**	5	**0.14**	199	**5.44**	1,115	**30.46**	**1,319**	**36.04**
Apayao	**2,238**	8	**0.36**	125	**5.59**	734	**32.80**	**867**	**38.74**
Benguet	**8,806**	4	**0.05**	345	**3.92**	3,602	**40.90**	**3,951**	**44.87**
Ifugao	**4,098**	4	**0.10**	130	**3.17**	1,150	**28.06**	**1,284**	**31.33**
Kalinga	**4,549**	3	**0.07**	141	**3.10**	1,346	**29.59**	**1,490**	**32.75**
Mountain Province	**2,619**	2	**0.08**	205	**7.83**	1,820	**69.49**	**2,027**	**77.40**
City of Baguio	**5,349**	5	**0.09**	256	**4.79**	2,723	**50.91**	**2,984**	**55.79**
**Region 1**	**96,016**	**37**	**0.04**	**1,727**	**1.80**	**19,334**	**20.14**	**21,098**	**21.97**
Ilocos Norte	**8,912**	5	**0.06**	247	**2.77**	3,902	**43.78**	**4,154**	**46.61**
Ilocos Sur	**9,914**	2	**0.02**	191	**1.93**	3,946	**39.80**	**4,139**	**41.75**
La Union	**13,507**	19	**0.14**	500	**3.70**	4,164	**30.83**	**4,683**	**34.67**
Pangasinan	**60,360**	9	**0.01**	716	**1.19**	6,946	**11.51**	**7,671**	**12.71**

Table 2.B.1.13 - Prenatal Care

Pregnant women screened for HIV

Philippines, 2023

Area	Eligible Pop.	Screened for HIV							
		Age Group						Total	%
		10-14 yrs old		15-19 yrs old		20-49 yrs old			
		No.	%	No.	%	No.	%		
City of Dagupan	3,323	2	0.06	73	2.20	376	11.32	451	13.57
**Region 2**	63,967	48	0.08	1,211	1.89	8,919	13.94	10,178	15.91
Batanes	240	0	0.00	10	4.17	95	39.58	105	43.75
Cagayan	20,786	7	0.03	149	0.72	1,075	5.17	1,231	5.92
Isabela	27,379	17	0.06	527	1.92	4,792	17.50	5,336	19.49
Nueva Vizcaya	8,798	16	0.18	227	2.58	1,275	14.49	1,518	17.25
Quirino	3,839	7	0.18	261	6.80	1,369	35.66	1,637	42.64
City of Santiago	2,925	1	0.03	37	1.26	313	10.70	351	12.00
**Region 3**	233,544	237	0.10	9,680	4.14	70,882	30.35	80,799	34.60
Aurora	4,537	10	0.22	333	7.34	2,093	46.13	2,436	53.69
Bataan	17,380	29	0.17	1,152	6.63	7,878	45.33	9,059	52.12
Bulacan	67,590	77	0.11	3,483	5.15	29,961	44.33	33,521	49.59
Nueva Ecija	43,425	42	0.10	1,259	2.90	7,014	16.15	8,315	19.15
Pampanga	45,651	47	0.10	1,776	3.89	12,808	28.06	14,631	32.05
Tarlac	29,503	14	0.05	738	2.50	5,091	17.26	5,843	19.80
Zambales	11,723	15	0.13	580	4.95	3,413	29.11	4,008	34.19
City of Angeles	9,146	3	0.03	180	1.97	1,335	14.60	1,518	16.60
City of Olongapo	4,589	0	0.00	179	3.90	1,289	28.09	1,468	31.99
**Region 4A**	307,982	164	0.05	7,411	2.41	65,727	21.34	73,302	23.80
Batangas	53,251	11	0.02	508	0.95	5,979	11.23	6,498	12.20
Cavite	77,964	35	0.04	1,629	2.09	15,980	20.50	17,644	22.63
Laguna	61,525	37	0.06	1,566	2.55	16,688	27.12	18,291	29.73
Quezon	40,810	22	0.05	908	2.22	6,393	15.67	7,323	17.94
Rizal	68,235	55	0.08	2,553	3.74	19,483	28.55	22,091	32.37
City of Lucena	6,197	4	0.06	247	3.99	1,204	19.43	1,455	23.48
**Region 4B**	66,121	42	0.06	1,890	2.86	15,296	23.13	17,228	26.06
Marinduque	4,144	3	0.07	101	2.44	969	23.38	1,073	25.89
Mindoro Occidental	11,640	0	0.00	50	0.43	325	2.79	375	3.22
Mindoro Oriental	18,943	3	0.02	392	2.07	5,655	29.85	6,050	31.94
Palawan	20,317	22	0.11	847	4.17	4,899	24.11	5,768	28.39
Romblon	5,489	5	0.09	123	2.24	1,174	21.39	1,302	23.72

Table 2.B.1.13 - Prenatal Care

Pregnant women screened for HIV

Philippines, 2023

Area	Eligible Pop.	Screened for HIV							
		Age Group						Total	%
		10-14 yrs old		15-19 yrs old		20-49 yrs old			
		No.	%	No.	%	No.	%		
Puerto Princesa City	**5,588**	9	**0.16**	377	**6.75**	2,274	**40.69**	**2,660**	**47.60**
**Region 5**	**135,480**	**49**	**0.04**	**1,841**	**1.36**	**18,007**	**13.29**	**19,897**	**14.69**
Albay	**27,440**	7	**0.03**	401	**1.46**	6,360	**23.18**	**6,768**	**24.66**
Camarines Norte	**14,695**	11	**0.07**	76	**0.52**	667	**4.54**	**754**	**5.13**
Camarines Sur	**43,452**	3	**0.01**	352	**0.81**	3,727	**8.58**	**4,082**	**9.39**
Catanduanes	**5,414**	4	**0.07**	323	**5.97**	1,988	**36.72**	**2,315**	**42.76**
Masbate	**22,365**	20	**0.09**	432	**1.93**	2,849	**12.74**	**3,301**	**14.76**
Sorsogon	**17,929**	2	**0.01**	110	**0.61**	1,115	**6.22**	**1,227**	**6.84**
City of Naga	**4,185**	2	**0.05**	147	**3.51**	1,301	**31.09**	**1,450**	**34.65**
**Region 6**	**148,020**	**44**	**0.03**	**2,918**	**1.97**	**26,339**	**17.79**	**29,301**	**19.80**
Aklan	**11,283**	2	**0.02**	159	**1.41**	2,142	**18.98**	**2,303**	**20.41**
Antique	**11,930**	2	**0.02**	178	**1.49**	1,847	**15.48**	**2,027**	**16.99**
Capiz	**13,931**	5	**0.04**	194	**1.39**	1,349	**9.68**	**1,548**	**11.11**
Guimaras	**3,351**	4	**0.12**	180	**5.37**	1,718	**51.27**	**1,902**	**56.76**
Iloilo	**36,140**	5	**0.01**	409	**1.13**	5,786	**16.01**	**6,200**	**17.16**
Negros Occidental	**52,270**	16	**0.03**	1,160	**2.22**	7,883	**15.08**	**9,059**	**17.33**
City of Bacolod	**10,373**	1	**0.01**	400	**3.86**	3,259	**31.42**	**3,660**	**35.28**
City of Iloilo	**8,742**	9	**0.10**	238	**2.72**	2,355	**26.94**	**2,602**	**29.76**
**Region 7**	**165,673**	**143**	**0.09**	**7,812**	**4.72**	**60,283**	**36.39**	**68,238**	**41.19**
Bohol	**26,235**	17	**0.06**	1,062	**4.05**	9,383	**35.77**	**10,462**	**39.88**
Cebu	**72,201**	36	**0.05**	2,861	**3.96**	17,547	**24.30**	**20,444**	**28.32**
Negros Oriental	**28,722**	6	**0.02**	302	**1.05**	2,181	**7.59**	**2,489**	**8.67**
Siquijor	**1,680**	2	**0.12**	67	**3.99**	801	**47.68**	**870**	**51.79**
City of Cebu	**19,267**	18	**0.09**	988	**5.13**	7,875	**40.87**	**8,881**	**46.09**
City of Lapu-Lapu	**10,679**	5	**0.05**	343	**3.21**	5,461	**51.14**	**5,809**	**54.40**
City of Mandaue	**6,889**	59	**0.86**	2,189	**31.78**	17,035	**247.28**	**19,283**	**279.91**
**Region 8**	**93,813**	**34**	**0.04**	**2,398**	**2.56**	**17,651**	**18.82**	**20,083**	**21.41**
Biliran	**3,373**	3	**0.09**	250	**7.41**	1,591	**47.17**	**1,844**	**54.67**
Eastern Samar	**9,624**	4	**0.04**	271	**2.82**	2,045	**21.25**	**2,320**	**24.11**
Leyte	**31,559**	5	**0.02**	353	**1.12**	3,114	**9.87**	**3,472**	**11.00**
Northern Samar	**14,056**	8	**0.06**	665	**4.73**	4,056	**28.86**	**4,729**	**33.64**

Table 2.B.1.13 - Prenatal Care

Pregnant women screened for HIV

Philippines, 2023

Area	Eligible Pop.	Screened for HIV							
		Age Group						Total	%
		10-14 yrs old		15-19 yrs old		20-49 yrs old			
		No.	%	No.	%	No.	%		
Southern Leyte	**7,406**	1	**0.01**	122	**1.65**	1,183	**15.97**	**1,306**	**17.63**
Samar	**17,011**	2	**0.01**	194	**1.14**	1,765	**10.38**	**1,961**	**11.53**
Ormoc City	**5,292**	2	**0.04**	242	**4.57**	1,509	**28.51**	**1,753**	**33.13**
City of Tacloban	**5,492**	9	**0.16**	301	**5.48**	2,388	**43.48**	**2,698**	**49.13**
**Region 9**	**84,815**	**8**	**0.01**	**435**	**0.51**	**1,984**	**2.34**	**2,427**	**2.86**
Zamboanga del Norte	**24,841**	1	**0.00**	124	**0.50**	579	**2.33**	**704**	**2.83**
Zamboanga del Sur	**23,757**	1	**0.00**	64	**0.27**	161	**0.68**	**226**	**0.95**
Zamboanga Sibugay	**13,841**	2	**0.01**	97	**0.70**	488	**3.53**	**587**	**4.24**
City of Isabela	**3,201**	2	**0.06**	29	**0.91**	195	**6.09**	**226**	**7.06**
City of Zamboanga	**19,175**	2	**0.01**	121	**0.63**	561	**2.93**	**684**	**3.57**
**Region 10**	**109,169**	**60**	**0.05**	**2,526**	**2.31**	**15,184**	**13.91**	**17,770**	**16.28**
Bukidnon	**33,403**	19	**0.06**	462	**1.38**	2,351	**7.04**	**2,832**	**8.48**
Camiguin	**1,727**	0	**0.00**	36	**2.08**	184	**10.65**	**220**	**12.74**
Lanao del Norte	**17,760**	4	**0.02**	176	**0.99**	1,509	**8.50**	**1,689**	**9.51**
Misamis Occidental	**12,328**	3	**0.02**	356	**2.89**	3,060	**24.82**	**3,419**	**27.73**
Misamis Oriental	**21,975**	29	**0.13**	1,020	**4.64**	5,006	**22.78**	**6,055**	**27.55**
City of Cagayan De Oro	**14,308**	1	**0.01**	243	**1.70**	1,538	**10.75**	**1,782**	**12.45**
City of Iligan	**7,668**	4	**0.05**	233	**3.04**	1,536	**20.03**	**1,773**	**23.12**
**Region 11**	**107,001**	**119**	**0.11**	**2,909**	**2.72**	**18,006**	**16.83**	**21,034**	**19.66**
Davao de Oro	**15,253**	9	**0.06**	124	**0.81**	820	**5.38**	**953**	**6.25**
Davao del Norte	**23,453**	19	**0.08**	588	**2.51**	3,398	**14.49**	**4,005**	**17.08**
Davao Oriental	**11,935**	5	**0.04**	91	**0.76**	363	**3.04**	**459**	**3.85**
Davao del Sur	**13,083**	16	**0.12**	387	**2.96**	2,031	**15.52**	**2,434**	**18.60**
Davao Occidental	**6,629**	13	**0.20**	377	**5.69**	1,089	**16.43**	**1,479**	**22.31**
City of Davao	**36,648**	57	**0.16**	1,342	**3.66**	10,305	**28.12**	**11,704**	**31.94**
**Region 12**	**95,279**	**76**	**0.08**	**2,372**	**2.49**	**13,425**	**14.09**	**15,873**	**16.66**
Cotabato	**26,521**	15	**0.06**	494	**1.86**	2,534	**9.55**	**3,043**	**11.47**
Sarangani	**13,322**	9	**0.07**	358	**2.69**	1,611	**12.09**	**1,978**	**14.85**
South Cotabato	**21,595**	25	**0.12**	975	**4.51**	6,034	**27.94**	**7,034**	**32.57**
Sultan Kudarat	**19,391**	25	**0.13**	333	**1.72**	1,369	**7.06**	**1,727**	**8.91**

Table 2.B.1.13 - Prenatal Care
Pregnant women screened for HIV
Philippines, 2023

Area	Eligible Pop.	Screened for HIV							
		Age Group						Total	%
		10-14 yrs old		15-19 yrs old		20-49 yrs old			
		No.	%	No.	%	No.	%		
City of General Santos	**14,450**	2	**0.01**	212	**1.47**	1,877	**12.99**	**2,091**	**14.47**
**BARMM**	**132,841**	**26**	**0.02**	**1,191**	**0.90**	**16,059**	**12.09**	**17,276**	**13.01**
Basilan	**11,636**	0	**0.00**	13	**0.11**	21	**0.18**	**34**	**0.29**
Lanao del Sur	**33,374**	11	**0.03**	747	**2.24**	12,855	**38.52**	**13,613**	**40.79**
Maguindanao	**41,650**	1	**0.00**	78	**0.19**	520	**1.25**	**599**	**1.44**
Sulu	**21,378**	2	**0.01**	42	**0.20**	208	**0.97**	**252**	**1.18**
Tawi-Tawi	**12,609**	4	**0.03**	12	**0.10**	376	**2.98**	**392**	**3.11**
SGU	**5,103**	0	**0.00**	0	**0.00**	0	**0.00**	**0**	**0.00**
City of Cotabato	**7,091**	8	**0.11**	299	**4.22**	2,079	**29.32**	**2,386**	**33.65**
**Caraga**	**55,065**	**17**	**0.03**	**1,274**	**2.31**	**9,065**	**16.46**	**10,356**	**18.81**
Agusan del Norte	**7,286**	2	**0.03**	171	**2.35**	1,245	**17.09**	**1,418**	**19.46**
Agusan del Sur	**16,038**	1	**0.01**	157	**0.98**	1,011	**6.30**	**1,169**	**7.29**
Surigao del Norte	**10,186**	3	**0.03**	255	**2.50**	1,887	**18.53**	**2,145**	**21.06**
Surigao del Sur	**12,736**	0	**0.00**	124	**0.97**	543	**4.26**	**667**	**5.24**
Dinagat Islands	**2,134**	3	**0.14**	100	**4.69**	835	**39.13**	**938**	**43.96**
City of Butuan	**6,685**	8	**0.12**	467	**6.99**	3,544	**53.01**	**4,019**	**60.12**

Table 2.B.1.14 - Prenatal Care

Pregnant women tested for CBC or Hemoglobin and Hematocrit Count

Philippines, 2023

Area	Eligible Pop.	Tested for CBC/Hgb and Hct							
		Age Group						Total	%
		10-14 yrs old		15-19 yrs old		20-49 yrs old			
		No.	%	No.	%	No.	%		
**Philippines**	**2,187,333**	**2,630**	**0.12**	**100,203**	**4.58**	**779,103**	**35.62**	**881,936**	**40.32**
**N C R**	**261,228**	**241**	**0.09**	**10,694**	**4.09**	**118,239**	**45.26**	**129,174**	**49.45**
Malabon City	**7,385**	13	**0.18**	269	**3.64**	2,029	**27.47**	**2,311**	**31.29**
Navotas City	**5,210**	16	**0.31**	368	**7.06**	2,718	**52.17**	**3,102**	**59.54**
Valenzuela City	**13,477**	19	**0.14**	523	**3.88**	5,472	**40.60**	**6,014**	**44.62**
Caloocan City	**33,168**	14	**0.04**	791	**2.38**	9,209	**27.76**	**10,014**	**30.19**
Marikina City	**8,342**	7	**0.08**	332	**3.98**	2,883	**34.56**	**3,222**	**38.62**
Pasig City	**17,682**	10	**0.06**	713	**4.03**	12,080	**68.32**	**12,803**	**72.41**
Pateros	**1,116**	0	**0.00**	22	**1.97**	265	**23.75**	**287**	**25.72**
Taguig City	**18,992**	18	**0.09**	1,079	**5.68**	9,586	**50.47**	**10,683**	**56.25**
Quezon City	**59,136**	46	**0.08**	2,290	**3.87**	31,144	**52.67**	**33,480**	**56.62**
Makati City	**10,944**	8	**0.07**	173	**1.58**	2,749	**25.12**	**2,930**	**26.77**
Mandaluyong City	**7,922**	11	**0.14**	248	**3.13**	6,416	**80.99**	**6,675**	**84.26**
San Juan City	**2,168**	1	**0.05**	58	**2.68**	776	**35.79**	**835**	**38.51**
Manila City	**34,128**	40	**0.12**	2,094	**6.14**	15,782	**46.24**	**17,916**	**52.50**
Las Piñas City	**11,263**	6	**0.05**	207	**1.84**	2,699	**23.96**	**2,912**	**25.85**
Muntinlupa City	**9,881**	14	**0.14**	340	**3.44**	3,166	**32.04**	**3,520**	**35.62**
Parañaque City	**13,280**	7	**0.05**	632	**4.76**	5,504	**41.45**	**6,143**	**46.26**
Pasay City	**7,134**	11	**0.15**	555	**7.78**	5,761	**80.75**	**6,327**	**88.69**
**C A R**	**31,319**	**40**	**0.13**	**2,268**	**7.24**	**19,381**	**61.88**	**21,689**	**69.25**
Abra	**3,660**	2	**0.05**	149	**4.07**	826	**22.57**	**977**	**26.69**
Apayao	**2,238**	11	**0.49**	454	**20.29**	2,564	**114.57**	**3,029**	**135.34**
Benguet	**8,806**	5	**0.06**	450	**5.11**	4,752	**53.96**	**5,207**	**59.13**
Ifugao	**4,098**	7	**0.17**	324	**7.91**	2,754	**67.20**	**3,085**	**75.28**
Kalinga	**4,549**	5	**0.11**	282	**6.20**	2,228	**48.98**	**2,515**	**55.29**
Mountain Province	**2,619**	5	**0.19**	345	**13.17**	3,459	**132.07**	**3,809**	**145.44**
City of Baguio	**5,349**	5	**0.09**	264	**4.94**	2,798	**52.31**	**3,067**	**57.34**
**Region 1**	**96,016**	**58**	**0.06**	**3,399**	**3.54**	**38,041**	**39.62**	**41,498**	**43.22**
Ilocos Norte	**8,912**	8	**0.09**	372	**4.17**	4,672	**52.42**	**5,052**	**56.69**
Ilocos Sur	**9,914**	5	**0.05**	373	**3.76**	6,883	**69.43**	**7,261**	**73.24**
La Union	**13,507**	15	**0.11**	602	**4.46**	5,401	**39.99**	**6,018**	**44.55**
Pangasinan	**60,360**	25	**0.04**	1,910	**3.16**	20,297	**33.63**	**22,232**	**36.83**

Table 2.B.1.14 - Prenatal Care

Pregnant women tested for CBC or Hemoglobin and Hematocrit Count

Philippines, 2023

Area	Eligible Pop.	Tested for CBC/Hgb and Hct							
		Age Group						Total	%
		10-14 yrs old		15-19 yrs old		20-49 yrs old			
		No.	%	No.	%	No.	%		
City of Dagupan	**3,323**	5	**0.15**	142	**4.27**	788	**23.71**	**935**	**28.14**
**Region 2**	**63,967**	**90**	**0.14**	**3,217**	**5.03**	**24,169**	**37.78**	**27,476**	**42.95**
Batanes	**240**	0	**0.00**	11	**4.58**	103	**42.92**	**114**	**47.50**
Cagayan	**20,786**	18	**0.09**	716	**3.44**	6,453	**31.04**	**7,187**	**34.58**
Isabela	**27,379**	50	**0.18**	1,579	**5.77**	11,673	**42.63**	**13,302**	**48.58**
Nueva Vizcaya	**8,798**	8	**0.09**	460	**5.23**	3,180	**36.14**	**3,648**	**41.46**
Quirino	**3,839**	13	**0.34**	402	**10.47**	2,350	**61.21**	**2,765**	**72.02**
City of Santiago	**2,925**	1	**0.03**	49	**1.68**	410	**14.02**	**460**	**15.73**
**Region 3**	**233,544**	**307**	**0.13**	**11,051**	**4.73**	**82,883**	**35.49**	**94,241**	**40.35**
Aurora	**4,537**	8	**0.18**	241	**5.31**	1,572	**34.65**	**1,821**	**40.14**
Bataan	**17,380**	23	**0.13**	1,170	**6.73**	7,806	**44.91**	**8,999**	**51.78**
Bulacan	**67,590**	128	**0.19**	4,478	**6.63**	38,127	**56.41**	**42,733**	**63.22**
Nueva Ecija	**43,425**	60	**0.14**	1,922	**4.43**	11,247	**25.90**	**13,229**	**30.46**
Pampanga	**45,651**	42	**0.09**	1,141	**2.50**	9,249	**20.26**	**10,432**	**22.85**
Tarlac	**29,503**	30	**0.10**	1,427	**4.84**	10,126	**34.32**	**11,583**	**39.26**
Zambales	**11,723**	11	**0.09**	440	**3.75**	2,842	**24.24**	**3,293**	**28.09**
City of Angeles	**9,146**	0	**0.00**	74	**0.81**	763	**8.34**	**837**	**9.15**
City of Olongapo	**4,589**	5	**0.11**	158	**3.44**	1,151	**25.08**	**1,314**	**28.63**
**Region 4A**	**307,982**	**227**	**0.07**	**10,431**	**3.39**	**93,983**	**30.52**	**104,641**	**33.98**
Batangas	**53,251**	20	**0.04**	1,226	**2.30**	15,709	**29.50**	**16,955**	**31.84**
Cavite	**77,964**	40	**0.05**	1,945	**2.49**	19,091	**24.49**	**21,076**	**27.03**
Laguna	**61,525**	58	**0.09**	2,616	**4.25**	25,703	**41.78**	**28,377**	**46.12**
Quezon	**40,810**	54	**0.13**	1,646	**4.03**	12,180	**29.85**	**13,880**	**34.01**
Rizal	**68,235**	47	**0.07**	2,586	**3.79**	19,329	**28.33**	**21,962**	**32.19**
City of Lucena	**6,197**	8	**0.13**	412	**6.65**	1,971	**31.81**	**2,391**	**38.58**
**Region 4B**	**66,121**	**63**	**0.10**	**2,970**	**4.49**	**23,152**	**35.01**	**26,185**	**39.60**
Marinduque	**4,144**	5	**0.12**	154	**3.72**	1,406	**33.93**	**1,565**	**37.77**
Mindoro Occidental	**11,640**	13	**0.11**	552	**4.74**	3,718	**31.94**	**4,283**	**36.80**
Mindoro Oriental	**18,943**	6	**0.03**	565	**2.98**	7,593	**40.08**	**8,164**	**43.10**
Palawan	**20,317**	26	**0.13**	986	**4.85**	5,389	**26.52**	**6,401**	**31.51**
Romblon	**5,489**	4	**0.07**	191	**3.48**	1,967	**35.84**	**2,162**	**39.39**

Table 2.B.1.14 - Prenatal Care

Pregnant women tested for CBC or Hemoglobin and Hematocrit Count

Philippines, 2023

Area	Eligible Pop.	Tested for CBC/Hgb and Hct							
		Age Group						Total	%
		10-14 yrs old		15-19 yrs old		20-49 yrs old			
		No.	%	No.	%	No.	%		
Puerto Princesa City	5,588	9	0.16	522	9.34	3,079	55.10	3,610	64.60
**Region 5**	135,480	203	0.15	3,524	2.60	33,445	24.69	37,172	27.44
Albay	27,440	10	0.04	643	2.34	10,478	38.19	11,131	40.56
Camarines Norte	14,695	12	0.08	653	4.44	4,496	30.60	5,161	35.12
Camarines Sur	43,452	3	0.01	303	0.70	3,523	8.11	3,829	8.81
Catanduanes	5,414	9	0.17	440	8.13	2,783	51.40	3,232	59.70
Masbate	22,365	152	0.68	734	3.28	4,693	20.98	5,579	24.95
Sorsogon	17,929	17	0.09	743	4.14	7,363	41.07	8,123	45.31
City of Naga	4,185	0	0.00	8	0.19	109	2.60	117	2.80
**Region 6**	148,020	195	0.13	6,666	4.50	54,050	36.52	60,911	41.15
Aklan	11,283	7	0.06	342	3.03	4,056	35.95	4,405	39.04
Antique	11,930	76	0.64	432	3.62	3,622	30.36	4,130	34.62
Capiz	13,931	15	0.11	427	3.07	4,048	29.06	4,490	32.23
Guimaras	3,351	4	0.12	187	5.58	2,009	59.95	2,200	65.65
Iloilo	36,140	49	0.14	1,642	4.54	15,268	42.25	16,959	46.93
Negros Occidental	52,270	32	0.06	2,808	5.37	17,671	33.81	20,511	39.24
City of Bacolod	10,373	0	0.00	521	5.02	4,435	42.76	4,956	47.78
City of Iloilo	8,742	12	0.14	307	3.51	2,941	33.64	3,260	37.29
**Region 7**	165,673	176	0.11	10,766	6.50	78,572	47.43	89,514	54.03
Bohol	26,235	26	0.10	1,638	6.24	15,239	58.09	16,903	64.43
Cebu	72,201	43	0.06	4,114	5.70	22,996	31.85	27,153	37.61
Negros Oriental	28,722	23	0.08	1,353	4.71	9,135	31.80	10,511	36.60
Siquijor	1,680	1	0.06	55	3.27	624	37.14	680	40.48
City of Cebu	19,267	19	0.10	1,009	5.24	8,067	41.87	9,095	47.21
City of Lapu-Lapu	10,679	6	0.06	417	3.90	5,630	52.72	6,053	56.68
City of Mandaue	6,889	58	0.84	2,180	31.64	16,881	245.04	19,119	277.53
**Region 8**	93,813	54	0.06	3,531	3.76	28,915	30.82	32,500	34.64
Biliran	3,373	1	0.03	239	7.09	1,683	49.90	1,923	57.01
Eastern Samar	9,624	5	0.05	331	3.44	2,616	27.18	2,952	30.67
Leyte	31,559	27	0.09	1,071	3.39	9,289	29.43	10,387	32.91
Northern Samar	14,056	8	0.06	749	5.33	4,962	35.30	5,719	40.69

Table 2.B.1.14 - Prenatal Care

Pregnant women tested for CBC or Hemoglobin and Hematocrit Count

Philippines, 2023

Area	Eligible Pop.	Tested for CBC/Hgb and Hct							
		Age Group						Total	%
		10-14 yrs old		15-19 yrs old		20-49 yrs old			
		No.	%	No.	%	No.	%		
Southern Leyte	**7,406**	2	**0.03**	289	**3.90**	3,238	**43.72**	**3,529**	**47.65**
Samar	**17,011**	0	**0.00**	243	**1.43**	2,638	**15.51**	**2,881**	**16.94**
Ormoc City	**5,292**	2	**0.04**	269	**5.08**	1,798	**33.98**	**2,069**	**39.10**
City of Tacloban	**5,492**	9	**0.16**	340	**6.19**	2,691	**49.00**	**3,040**	**55.35**
**Region 9**	**84,815**	**66**	**0.08**	**2,576**	**3.04**	**13,814**	**16.29**	**16,456**	**19.40**
Zamboanga del Norte	**24,841**	19	**0.08**	702	**2.83**	3,893	**15.67**	**4,614**	**18.57**
Zamboanga del Sur	**23,757**	12	**0.05**	717	**3.02**	3,936	**16.57**	**4,665**	**19.64**
Zamboanga Sibugay	**13,841**	13	**0.09**	646	**4.67**	3,423	**24.73**	**4,082**	**29.49**
City of Isabela	**3,201**	12	**0.37**	67	**2.09**	429	**13.40**	**508**	**15.87**
City of Zamboanga	**19,175**	10	**0.05**	444	**2.32**	2,133	**11.12**	**2,587**	**13.49**
**Region 10**	**109,169**	**179**	**0.16**	**7,373**	**6.75**	**41,860**	**38.34**	**49,412**	**45.26**
Bukidnon	**33,403**	75	**0.22**	2,823	**8.45**	12,792	**38.30**	**15,690**	**46.97**
Camiguin	**1,727**	1	**0.06**	72	**4.17**	553	**32.02**	**626**	**36.25**
Lanao del Norte	**17,760**	2	**0.01**	167	**0.94**	1,069	**6.02**	**1,238**	**6.97**
Misamis Occidental	**12,328**	23	**0.19**	735	**5.96**	5,730	**46.48**	**6,488**	**52.63**
Misamis Oriental	**21,975**	52	**0.24**	1,754	**7.98**	10,487	**47.72**	**12,293**	**55.94**
City of Cagayan De Oro	**14,308**	22	**0.15**	1,416	**9.90**	8,384	**58.60**	**9,822**	**68.65**
City of Iligan	**7,668**	4	**0.05**	406	**5.29**	2,845	**37.10**	**3,255**	**42.45**
**Region 11**	**107,001**	**444**	**0.41**	**9,930**	**9.28**	**56,680**	**52.97**	**67,054**	**62.67**
Davao de Oro	**15,253**	73	**0.48**	1,718	**11.26**	8,683	**56.93**	**10,474**	**68.67**
Davao del Norte	**23,453**	88	**0.38**	2,127	**9.07**	12,534	**53.44**	**14,749**	**62.89**
Davao Oriental	**11,935**	53	**0.44**	1,187	**9.95**	6,108	**51.18**	**7,348**	**61.57**
Davao del Sur	**13,083**	78	**0.60**	1,269	**9.70**	6,340	**48.46**	**7,687**	**58.76**
Davao Occidental	**6,629**	32	**0.48**	720	**10.86**	1,955	**29.49**	**2,707**	**40.84**
City of Davao	**36,648**	120	**0.33**	2,909	**7.94**	21,060	**57.47**	**24,089**	**65.73**
**Region 12**	**95,279**	**199**	**0.21**	**7,055**	**7.40**	**38,006**	**39.89**	**45,260**	**47.50**
Cotabato	**26,521**	41	**0.15**	1,758	**6.63**	9,620	**36.27**	**11,419**	**43.06**
Sarangani	**13,322**	39	**0.29**	1,618	**12.15**	6,964	**52.27**	**8,621**	**64.71**
South Cotabato	**21,595**	36	**0.17**	1,571	**7.27**	9,493	**43.96**	**11,100**	**51.40**
Sultan Kudarat	**19,391**	74	**0.38**	1,363	**7.03**	7,119	**36.71**	**8,556**	**44.12**

Table 2.B.1.14 - Prenatal Care

Pregnant women tested for CBC or Hemoglobin and Hematocrit Count

Philippines, 2023

Area	Eligible Pop.	Tested for CBC/Hgb and Hct							
		Age Group						Total	%
		10-14 yrs old		15-19 yrs old		20-49 yrs old			
		No.	%	No.	%	No.	%		
City of General Santos	**14,450**	9	**0.06**	745	**5.16**	4,810	**33.29**	**5,564**	**38.51**
**BARMM**	**132,841**	**17**	**0.01**	**825**	**0.62**	**7,479**	**5.63**	**8,321**	**6.26**
Basilan	**11,636**	2	**0.02**	70	**0.60**	283	**2.43**	**355**	**3.05**
Lanao del Sur	**33,374**	3	**0.01**	262	**0.79**	3,009	**9.02**	**3,274**	**9.81**
Maguindanao	**41,650**	6	**0.01**	198	**0.48**	2,000	**4.80**	**2,204**	**5.29**
Sulu	**21,378**	3	**0.01**	175	**0.82**	1,077	**5.04**	**1,255**	**5.87**
Tawi-Tawi	**12,609**	1	**0.01**	20	**0.16**	168	**1.33**	**189**	**1.50**
SGU	**5,103**	0	**0.00**	0	**0.00**	0	**0.00**	**0**	**0.00**
City of Cotabato	**7,091**	2	**0.03**	100	**1.41**	942	**13.28**	**1,044**	**14.72**
**Caraga**	**55,065**	**71**	**0.13**	**3,927**	**7.13**	**26,434**	**48.01**	**30,432**	**55.27**
Agusan del Norte	**7,286**	5	**0.07**	416	**5.71**	2,807	**38.53**	**3,228**	**44.30**
Agusan del Sur	**16,038**	36	**0.22**	1,286	**8.02**	8,352	**52.08**	**9,674**	**60.32**
Surigao del Norte	**10,186**	6	**0.06**	545	**5.35**	4,176	**41.00**	**4,727**	**46.41**
Surigao del Sur	**12,736**	14	**0.11**	976	**7.66**	6,072	**47.68**	**7,062**	**55.45**
Dinagat Islands	**2,134**	3	**0.14**	112	**5.25**	936	**43.86**	**1,051**	**49.25**
City of Butuan	**6,685**	7	**0.10**	592	**8.86**	4,091	**61.20**	**4,690**	**70.16**

Table 2.B.1.15 - Prenatal Care

Pregnant women tested for Complete Blood Count or Hemoglobin and Hematocrit Count diagnosed with anemia

Philippines, 2023

Area	Total No. tested for CBC or Hgb or Hct count	Tested for CBC/Hgb and Hct diagnosed with anemia							
		Age Group						Total	%
		10-14 yrs old		15-19 yrs old		20-49 yrs old			
		No.	%	No.	%	No.	%		
**Philippines**	**881,936**	**456**	**0.05**	**13,882**	**1.57**	**73,452**	**8.33**	**87,790**	**9.95**
**N C R**	**129,174**	**29**	**0.02**	**1,312**	**1.02**	**9,526**	**7.37**	**10,867**	**8.41**
Malabon City	**2,311**	0	**0.00**	24	**1.04**	128	**5.54**	**152**	**6.58**
Navotas City	**3,102**	1	**0.03**	36	**1.16**	400	**12.89**	**437**	**14.09**
Valenzuela City	**6,014**	1	**0.02**	43	**0.71**	321	**5.34**	**365**	**6.07**
Caloocan City	**10,014**	3	**0.03**	171	**1.71**	1,205	**12.03**	**1,379**	**13.77**
Marikina City	**3,222**	2	**0.06**	14	**0.43**	123	**3.82**	**139**	**4.31**
Pasig City	**12,803**	3	**0.02**	90	**0.70**	727	**5.68**	**820**	**6.40**
Pateros	**287**	0	**0.00**	1	**0.35**	25	**8.71**	**26**	**9.06**
Taguig City	**10,683**	3	**0.03**	192	**1.80**	1,195	**11.19**	**1,390**	**13.01**
Quezon City	**33,480**	7	**0.02**	300	**0.90**	2,295	**6.85**	**2,602**	**7.77**
Makati City	**2,930**	0	**0.00**	32	**1.09**	395	**13.48**	**427**	**14.57**
Mandaluyong City	**6,675**	1	**0.01**	24	**0.36**	193	**2.89**	**218**	**3.27**
San Juan City	**835**	0	**0.00**	1	**0.12**	15	**1.80**	**16**	**1.92**
Manila City	**17,916**	2	**0.01**	106	**0.59**	899	**5.02**	**1,007**	**5.62**
Las Piñas City	**2,912**	0	**0.00**	35	**1.20**	359	**12.33**	**394**	**13.53**
Muntinlupa City	**3,520**	3	**0.09**	73	**2.07**	258	**7.33**	**334**	**9.49**
Parañaque City	**6,143**	1	**0.02**	49	**0.80**	296	**4.82**	**346**	**5.63**
Pasay City	**6,327**	2	**0.03**	121	**1.91**	692	**10.94**	**815**	**12.88**
**C A R**	**21,689**	**5**	**0.02**	**217**	**1.00**	**1,161**	**5.35**	**1,383**	**6.38**
Abra	**977**	0	**0.00**	11	**1.13**	37	**3.79**	**48**	**4.91**
Apayao	**3,029**	2	**0.07**	60	**1.98**	176	**5.81**	**238**	**7.86**
Benguet	**5,207**	0	**0.00**	11	**0.21**	121	**2.32**	**132**	**2.54**
Ifugao	**3,085**	1	**0.03**	54	**1.75**	318	**10.31**	**373**	**12.09**
Kalinga	**2,515**	2	**0.08**	68	**2.70**	456	**18.13**	**526**	**20.91**
Mountain Province	**3,809**	0	**0.00**	8	**0.21**	40	**1.05**	**48**	**1.26**
City of Baguio	**3,067**	0	**0.00**	5	**0.16**	13	**0.42**	**18**	**0.59**
**Region 1**	**41,498**	**5**	**0.01**	**279**	**0.67**	**1,849**	**4.46**	**2,133**	**5.14**
Ilocos Norte	**5,052**	0	**0.00**	9	**0.18**	40	**0.79**	**49**	**0.97**
Ilocos Sur	**7,261**	0	**0.00**	16	**0.22**	115	**1.58**	**131**	**1.80**
La Union	**6,018**	2	**0.03**	57	**0.95**	441	**7.33**	**500**	**8.31**
Pangasinan	**22,232**	1	**0.00**	139	**0.63**	933	**4.20**	**1,073**	**4.83**

Table 2.B.1.15 - Prenatal Care

Pregnant women tested for Complete Blood Count or Hemoglobin and Hematocrit Count diagnosed with anemia

Philippines, 2023

Area	Total No. tested for CBC or Hgb or Hct count	Tested for CBC/Hgb and Hct diagnosed with anemia							
		Age Group						Total	%
		10-14 yrs old		15-19 yrs old		20-49 yrs old			
		No.	%	No.	%	No.	%		
City of Dagupan	**935**	2	**0.21**	58	**6.20**	320	**34.22**	**380**	**40.64**
**Region 2**	**27,476**	**16**	**0.06**	**382**	**1.39**	**1,762**	**6.41**	**2,160**	**7.86**
Batanes	**114**	0	**0.00**	0	**0.00**	0	**0.00**	**0**	**0.00**
Cagayan	**7,187**	2	**0.03**	106	**1.47**	515	**7.17**	**623**	**8.67**
Isabela	**13,302**	11	**0.08**	187	**1.41**	906	**6.81**	**1,104**	**8.30**
Nueva Vizcaya	**3,648**	0	**0.00**	47	**1.29**	190	**5.21**	**237**	**6.50**
Quirino	**2,765**	3	**0.11**	35	**1.27**	129	**4.67**	**167**	**6.04**
City of Santiago	**460**	0	**0.00**	7	**1.52**	22	**4.78**	**29**	**6.30**
**Region 3**	**94,241**	**30**	**0.03**	**756**	**0.80**	**4,027**	**4.27**	**4,813**	**5.11**
Aurora	**1,821**	0	**0.00**	4	**0.22**	17	**0.93**	**21**	**1.15**
Bataan	**8,999**	4	**0.04**	127	**1.41**	751	**8.35**	**882**	**9.80**
Bulacan	**42,733**	9	**0.02**	313	**0.73**	1,541	**3.61**	**1,863**	**4.36**
Nueva Ecija	**13,229**	5	**0.04**	74	**0.56**	397	**3.00**	**476**	**3.60**
Pampanga	**10,432**	3	**0.03**	85	**0.81**	500	**4.79**	**588**	**5.64**
Tarlac	**11,583**	8	**0.07**	110	**0.95**	588	**5.08**	**706**	**6.10**
Zambales	**3,293**	1	**0.03**	19	**0.58**	80	**2.43**	**100**	**3.04**
City of Angeles	**837**	0	**0.00**	1	**0.12**	10	**1.19**	**11**	**1.31**
City of Olongapo	**1,314**	0	**0.00**	23	**1.75**	143	**10.88**	**166**	**12.63**
**Region 4A**	**104,641**	**64**	**0.06**	**1,516**	**1.45**	**9,003**	**8.60**	**10,583**	**10.11**
Batangas	**16,955**	4	**0.02**	101	**0.60**	762	**4.49**	**867**	**5.11**
Cavite	**21,076**	11	**0.05**	232	**1.10**	1,746	**8.28**	**1,989**	**9.44**
Laguna	**28,377**	12	**0.04**	511	**1.80**	2,949	**10.39**	**3,472**	**12.24**
Quezon	**13,880**	13	**0.09**	285	**2.05**	1,553	**11.19**	**1,851**	**13.34**
Rizal	**21,962**	22	**0.10**	314	**1.43**	1,745	**7.95**	**2,081**	**9.48**
City of Lucena	**2,391**	2	**0.08**	73	**3.05**	248	**10.37**	**323**	**13.51**
**Region 4B**	**26,185**	**12**	**0.05**	**442**	**1.69**	**2,176**	**8.31**	**2,630**	**10.04**
Marinduque	**1,565**	1	**0.06**	22	**1.41**	113	**7.22**	**136**	**8.69**
Mindoro Occidental	**4,283**	0	**0.00**	37	**0.86**	258	**6.02**	**295**	**6.89**
Mindoro Oriental	**8,164**	0	**0.00**	28	**0.34**	211	**2.58**	**239**	**2.93**
Palawan	**6,401**	8	**0.12**	171	**2.67**	701	**10.95**	**880**	**13.75**
Romblon	**2,162**	0	**0.00**	18	**0.83**	100	**4.63**	**118**	**5.46**

Table 2.B.1.15 - Prenatal Care

Pregnant women tested for Complete Blood Count or Hemoglobin and Hematocrit Count diagnosed with anemia

Philippines, 2023

Area	Total No. tested for CBC or Hgb or Hct count	Tested for CBC/Hgb and Hct diagnosed with anemia							
		Age Group						Total	%
		10-14 yrs old		15-19 yrs old		20-49 yrs old			
		No.	%	No.	%	No.	%		
Puerto Princesa City	**3,610**	3	**0.08**	166	**4.60**	793	**21.97**	**962**	**26.65**
**Region 5**	**37,172**	**7**	**0.02**	**432**	**1.16**	**3,379**	**9.09**	**3,818**	**10.27**
Albay	**11,131**	1	**0.01**	50	**0.45**	789	**7.09**	**840**	**7.55**
Camarines Norte	**5,161**	2	**0.04**	121	**2.34**	790	**15.31**	**913**	**17.69**
Camarines Sur	**3,829**	1	**0.03**	11	**0.29**	110	**2.87**	**122**	**3.19**
Catanduanes	**3,232**	0	**0.00**	30	**0.93**	176	**5.45**	**206**	**6.37**
Masbate	**5,579**	2	**0.04**	117	**2.10**	530	**9.50**	**649**	**11.63**
Sorsogon	**8,123**	1	**0.01**	103	**1.27**	983	**12.10**	**1,087**	**13.38**
City of Naga	**117**	0	**0.00**	0	**0.00**	1	**0.85**	**1**	**0.85**
**Region 6**	**60,911**	**10**	**0.02**	**625**	**1.03**	**3,553**	**5.83**	**4,188**	**6.88**
Aklan	**4,405**	0	**0.00**	52	**1.18**	446	**10.12**	**498**	**11.31**
Antique	**4,130**	4	**0.10**	52	**1.26**	366	**8.86**	**422**	**10.22**
Capiz	**4,490**	3	**0.07**	25	**0.56**	208	**4.63**	**236**	**5.26**
Guimaras	**2,200**	0	**0.00**	18	**0.82**	153	**6.95**	**171**	**7.77**
Iloilo	**16,959**	0	**0.00**	80	**0.47**	593	**3.50**	**673**	**3.97**
Negros Occidental	**20,511**	3	**0.01**	259	**1.26**	1,223	**5.96**	**1,485**	**7.24**
City of Bacolod	**4,956**	0	**0.00**	108	**2.18**	401	**8.09**	**509**	**10.27**
City of Iloilo	**3,260**	0	**0.00**	31	**0.95**	163	**5.00**	**194**	**5.95**
**Region 7**	**89,514**	**16**	**0.02**	**1,053**	**1.18**	**5,455**	**6.09**	**6,524**	**7.29**
Bohol	**16,903**	1	**0.01**	94	**0.56**	785	**4.64**	**880**	**5.21**
Cebu	**27,153**	11	**0.04**	567	**2.09**	2,407	**8.86**	**2,985**	**10.99**
Negros Oriental	**10,511**	4	**0.04**	226	**2.15**	1,114	**10.60**	**1,344**	**12.79**
Siquijor	**680**	0	**0.00**	14	**2.06**	71	**10.44**	**85**	**12.50**
City of Cebu	**9,095**	0	**0.00**	76	**0.84**	490	**5.39**	**566**	**6.22**
City of Lapu-Lapu	**6,053**	0	**0.00**	76	**1.26**	520	**8.59**	**596**	**9.85**
City of Mandaue	**19,119**	0	**0.00**	0	**0.00**	68	**0.36**	**68**	**0.36**
**Region 8**	**32,500**	**12**	**0.04**	**342**	**1.05**	**2,226**	**6.85**	**2,580**	**7.94**
Biliran	**1,923**	2	**0.10**	38	**1.98**	253	**13.16**	**293**	**15.24**
Eastern Samar	**2,952**	0	**0.00**	25	**0.85**	97	**3.29**	**122**	**4.13**
Leyte	**10,387**	1	**0.01**	82	**0.79**	769	**7.40**	**852**	**8.20**
Northern Samar	**5,719**	0	**0.00**	83	**1.45**	428	**7.48**	**511**	**8.94**

Table 2.B.1.15 - Prenatal Care

Pregnant women tested for Complete Blood Count or Hemoglobin and Hematocrit Count diagnosed with anemia

Philippines, 2023

Area	Total No. tested for CBC or Hgb or Hct count	Tested for CBC/Hgb and Hct diagnosed with anemia							
		Age Group						Total	%
		10-14 yrs old		15-19 yrs old		20-49 yrs old			
		No.	%	No.	%	No.	%		
Southern Leyte	**3,529**	1	**0.03**	33	**0.94**	248	**7.03**	**282**	**7.99**
Samar	**2,881**	7	**0.24**	33	**1.15**	274	**9.51**	**314**	**10.90**
Ormoc City	**2,069**	0	**0.00**	39	**1.88**	149	**7.20**	**188**	**9.09**
City of Tacloban	**3,040**	1	**0.03**	9	**0.30**	8	**0.26**	**18**	**0.59**
**Region 9**	**16,456**	**4**	**0.02**	**332**	**2.02**	**1,486**	**9.03**	**1,822**	**11.07**
Zamboanga del Norte	**4,614**	2	**0.04**	87	**1.89**	480	**10.40**	**569**	**12.33**
Zamboanga del Sur	**4,665**	2	**0.04**	153	**3.28**	543	**11.64**	**698**	**14.96**
Zamboanga Sibugay	**4,082**	0	**0.00**	63	**1.54**	313	**7.67**	**376**	**9.21**
City of Isabela	**508**	0	**0.00**	7	**1.38**	33	**6.50**	**40**	**7.87**
City of Zamboanga	**2,587**	0	**0.00**	22	**0.85**	117	**4.52**	**139**	**5.37**
**Region 10**	**49,412**	**32**	**0.06**	**964**	**1.95**	**3,881**	**7.85**	**4,877**	**9.87**
Bukidnon	**15,690**	21	**0.13**	574	**3.66**	2,040	**13.00**	**2,635**	**16.79**
Camiguin	**626**	0	**0.00**	2	**0.32**	10	**1.60**	**12**	**1.92**
Lanao del Norte	**1,238**	0	**0.00**	21	**1.70**	103	**8.32**	**124**	**10.02**
Misamis Occidental	**6,488**	2	**0.03**	15	**0.23**	123	**1.90**	**140**	**2.16**
Misamis Oriental	**12,293**	5	**0.04**	178	**1.45**	636	**5.17**	**819**	**6.66**
City of Cagayan De Oro	**9,822**	4	**0.04**	152	**1.55**	681	**6.93**	**837**	**8.52**
City of Iligan	**3,255**	0	**0.00**	22	**0.68**	288	**8.85**	**310**	**9.52**
**Region 11**	**67,054**	**166**	**0.25**	**3,329**	**4.96**	**14,992**	**22.36**	**18,487**	**27.57**
Davao de Oro	**10,474**	23	**0.22**	621	**5.93**	2,691	**25.69**	**3,335**	**31.84**
Davao del Norte	**14,749**	36	**0.24**	629	**4.26**	2,632	**17.85**	**3,297**	**22.35**
Davao Oriental	**7,348**	20	**0.27**	382	**5.20**	1,749	**23.80**	**2,151**	**29.27**
Davao del Sur	**7,687**	35	**0.46**	530	**6.89**	2,373	**30.87**	**2,938**	**38.22**
Davao Occidental	**2,707**	5	**0.18**	200	**7.39**	507	**18.73**	**712**	**26.30**
City of Davao	**24,089**	47	**0.20**	967	**4.01**	5,040	**20.92**	**6,054**	**25.13**
**Region 12**	**45,260**	**20**	**0.04**	**793**	**1.75**	**3,457**	**7.64**	**4,270**	**9.43**
Cotabato	**11,419**	4	**0.04**	195	**1.71**	864	**7.57**	**1,063**	**9.31**
Sarangani	**8,621**	9	**0.10**	214	**2.48**	793	**9.20**	**1,016**	**11.79**
South Cotabato	**11,100**	5	**0.05**	115	**1.04**	697	**6.28**	**817**	**7.36**
Sultan Kudarat	**8,556**	1	**0.01**	86	**1.01**	352	**4.11**	**439**	**5.13**

Table 2.B.1.15 - Prenatal Care

Pregnant women tested for Complete Blood Count or Hemoglobin and Hematocrit Count diagnosed with anemia

Philippines, 2023

Area	Total No. tested for CBC or Hgb or Hct count	Tested for CBC/Hgb and Hct diagnosed with anemia							
		Age Group						Total	%
		10-14 yrs old		15-19 yrs old		20-49 yrs old			
		No.	%	No.	%	No.	%		
City of General Santos	**5,564**	1	**0.02**	183	**3.29**	751	**13.50**	**935**	**16.80**
**BARMM**	**8,321**	**1**	**0.01**	**78**	**0.94**	**467**	**5.61**	**546**	**6.56**
Basilan	**355**	0	**0.00**	13	**3.66**	21	**5.92**	**34**	**9.58**
Lanao del Sur	**3,274**	0	**0.00**	1	**0.03**	22	**0.67**	**23**	**0.70**
Maguindanao	**2,204**	0	**0.00**	30	**1.36**	242	**10.98**	**272**	**12.34**
Sulu	**1,255**	1	**0.08**	25	**1.99**	136	**10.84**	**162**	**12.91**
Tawi-Tawi	**189**	0	**0.00**	0	**0.00**	3	**1.59**	**3**	**1.59**
SGU	**0**	0	**0.00**	0	**0.00**	0	**0.00**	**0**	**0.00**
City of Cotabato	**1,044**	0	**0.00**	9	**0.86**	43	**4.12**	**52**	**4.98**
**Caraga**	**30,432**	**27**	**0.09**	**1,030**	**3.38**	**5,052**	**16.60**	**6,109**	**20.07**
Agusan del Norte	**3,228**	1	**0.03**	79	**2.45**	405	**12.55**	**485**	**15.02**
Agusan del Sur	**9,674**	14	**0.14**	387	**4.00**	1,930	**19.95**	**2,331**	**24.10**
Surigao del Norte	**4,727**	2	**0.04**	101	**2.14**	611	**12.93**	**714**	**15.10**
Surigao del Sur	**7,062**	9	**0.13**	338	**4.79**	1,570	**22.23**	**1,917**	**27.15**
Dinagat Islands	**1,051**	0	**0.00**	15	**1.43**	130	**12.37**	**145**	**13.80**
City of Butuan	**4,690**	1	**0.02**	110	**2.35**	406	**8.66**	**517**	**11.02**

Tabe 2.B.1.16 - Prenatal Care

Pregnant women screened for Gestational Diabetes

Philippines, 2023

Area	Eligible Pop.	Screened for Gestational Diabetes							
		Age Group						Total	%
		10-14 yrs old		15-19 yrs old		20-49 yrs old			
		No.	%	No.	%	No.	%		
**Philippines**	**2,187,333**	**804**	**0.04**	**34,996**	**1.60**	**334,978**	**15.31**	**370,778**	**16.95**
**N C R**	**261,228**	**128**	**0.05**	**5,990**	**2.29**	**83,266**	**31.87**	**89,384**	**34.22**
Malabon City	**7,385**	0	**0.00**	49	**0.66**	426	**5.77**	**475**	**6.43**
Navotas City	**5,210**	10	**0.19**	217	**4.17**	1,626	**31.21**	**1,853**	**35.57**
Valenzuela City	**13,477**	7	**0.05**	192	**1.42**	2,312	**17.16**	**2,511**	**18.63**
Caloocan City	**33,168**	2	**0.01**	222	**0.67**	4,575	**13.79**	**4,799**	**14.47**
Marikina City	**8,342**	6	**0.07**	187	**2.24**	1,962	**23.52**	**2,155**	**25.83**
Pasig City	**17,682**	1	**0.01**	468	**2.65**	9,955	**56.30**	**10,424**	**58.95**
Pateros	**1,116**	1	**0.09**	41	**3.67**	609	**54.57**	**651**	**58.33**
Taguig City	**18,992**	10	**0.05**	492	**2.59**	4,930	**25.96**	**5,432**	**28.60**
Quezon City	**59,136**	15	**0.03**	738	**1.25**	23,317	**39.43**	**24,070**	**40.70**
Makati City	**10,944**	8	**0.07**	140	**1.28**	2,086	**19.06**	**2,234**	**20.41**
Mandaluyong City	**7,922**	8	**0.10**	198	**2.50**	5,419	**68.40**	**5,625**	**71.00**
San Juan City	**2,168**	1	**0.05**	44	**2.03**	645	**29.75**	**690**	**31.83**
Manila City	**34,128**	40	**0.12**	2,073	**6.07**	15,597	**45.70**	**17,710**	**51.89**
Las Piñas City	**11,263**	0	**0.00**	62	**0.55**	1,050	**9.32**	**1,112**	**9.87**
Muntinlupa City	**9,881**	8	**0.08**	211	**2.14**	1,989	**20.13**	**2,208**	**22.35**
Parañaque City	**13,280**	5	**0.04**	375	**2.82**	3,441	**25.91**	**3,821**	**28.77**
Pasay City	**7,134**	6	**0.08**	281	**3.94**	3,327	**46.64**	**3,614**	**50.66**
**C A R**	**31,319**	**14**	**0.04**	**803**	**2.56**	**7,961**	**25.42**	**8,778**	**28.03**
Abra	**3,660**	1	**0.03**	51	**1.39**	261	**7.13**	**313**	**8.55**
Apayao	**2,238**	3	**0.13**	105	**4.69**	710	**31.72**	**818**	**36.55**
Benguet	**8,806**	4	**0.05**	212	**2.41**	2,405	**27.31**	**2,621**	**29.76**
Ifugao	**4,098**	1	**0.02**	50	**1.22**	585	**14.28**	**636**	**15.52**
Kalinga	**4,549**	0	**0.00**	33	**0.73**	332	**7.30**	**365**	**8.02**
Mountain Province	**2,619**	0	**0.00**	90	**3.44**	979	**37.38**	**1,069**	**40.82**
City of Baguio	**5,349**	5	**0.09**	262	**4.90**	2,689	**50.27**	**2,956**	**55.26**
**Region 1**	**96,016**	**24**	**0.02**	**873**	**0.91**	**13,240**	**13.79**	**14,137**	**14.72**
Ilocos Norte	**8,912**	0	**0.00**	111	**1.25**	1,466	**16.45**	**1,577**	**17.70**
Ilocos Sur	**9,914**	4	**0.04**	221	**2.23**	4,337	**43.75**	**4,562**	**46.02**
La Union	**13,507**	15	**0.11**	319	**2.36**	3,084	**22.83**	**3,418**	**25.31**
Pangasinan	**60,360**	4	**0.01**	221	**0.37**	4,337	**7.19**	**4,562**	**7.56**

Tabe 2.B.1.16 - Prenatal Care

Pregnant women screened for Gestational Diabetes

Philippines, 2023

Area	Eligible Pop.	Screened for Gestational Diabetes							
		Age Group						Total	%
		10-14 yrs old		15-19 yrs old		20-49 yrs old			
		No.	%	No.	%	No.	%		
City of Dagupan	3,323	1	0.03	1	0.03	16	0.48	18	0.54
**Region 2**	**63,967**	**20**	**0.03**	**761**	**1.19**	**5,746**	**8.98**	**6,527**	**10.20**
Batanes	240	0	0.00	5	2.08	43	17.92	48	20.00
Cagayan	20,786	2	0.01	77	0.37	539	2.59	618	2.97
Isabela	27,379	11	0.04	381	1.39	3,012	11.00	3,404	12.43
Nueva Vizcaya	8,798	3	0.03	196	2.23	1,499	17.04	1,698	19.30
Quirino	3,839	4	0.10	82	2.14	493	12.84	579	15.08
City of Santiago	2,925	0	0.00	20	0.68	160	5.47	180	6.15
**Region 3**	**233,544**	**112**	**0.05**	**4,633**	**1.98**	**40,361**	**17.28**	**45,106**	**19.31**
Aurora	4,537	2	0.04	57	1.26	277	6.11	336	7.41
Bataan	17,380	8	0.05	400	2.30	3,134	18.03	3,542	20.38
Bulacan	67,590	57	0.08	2,382	3.52	23,403	34.62	25,842	38.23
Nueva Ecija	43,425	19	0.04	724	1.67	4,560	10.50	5,303	12.21
Pampanga	45,651	18	0.04	498	1.09	4,788	10.49	5,304	11.62
Tarlac	29,503	3	0.01	370	1.25	2,689	9.11	3,062	10.38
Zambales	11,723	3	0.03	92	0.78	618	5.27	713	6.08
City of Angeles	9,146	0	0.00	16	0.17	246	2.69	262	2.86
City of Olongapo	4,589	2	0.04	94	2.05	646	14.08	742	16.17
**Region 4A**	**307,982**	**113**	**0.04**	**4,692**	**1.52**	**46,620**	**15.14**	**51,425**	**16.70**
Batangas	53,251	9	0.02	445	0.84	5,968	11.21	6,422	12.06
Cavite	77,964	21	0.03	1,005	1.29	10,672	13.69	11,698	15.00
Laguna	61,525	36	0.06	998	1.62	12,964	21.07	13,998	22.75
Quezon	40,810	10	0.02	378	0.93	2,964	7.26	3,352	8.21
Rizal	68,235	35	0.05	1,683	2.47	13,107	19.21	14,825	21.73
City of Lucena	6,197	2	0.03	183	2.95	945	15.25	1,130	18.23
**Region 4B**	**66,121**	**29**	**0.04**	**848**	**1.28**	**9,005**	**13.62**	**9,882**	**14.95**
Marinduque	4,144	3	0.07	66	1.59	779	18.80	848	20.46
Mindoro Occidental	11,640	1	0.01	78	0.67	536	4.60	615	5.28
Mindoro Oriental	18,943	7	0.04	210	1.11	4,273	22.56	4,490	23.70
Palawan	20,317	8	0.04	375	1.85	2,377	11.70	2,760	13.58
Romblon	5,489	0	0.00	94	1.71	907	16.52	1,001	18.24

Tabe 2.B.1.16 - Prenatal Care

Pregnant women screened for Gestational Diabetes

Philippines, 2023

Area	Eligible Pop.	Screened for Gestational Diabetes							
		Age Group						Total	%
		10-14 yrs old		15-19 yrs old		20-49 yrs old			
		No.	%	No.	%	No.	%		
Puerto Princesa City	**5,588**	10	**0.18**	25	**0.45**	133	**2.38**	**168**	**3.01**
**Region 5**	**135,480**	**12**	**0.01**	**1,017**	**0.75**	**10,302**	**7.60**	**11,331**	**8.36**
Albay	**27,440**	5	**0.02**	193	**0.70**	3,556	**12.96**	**3,754**	**13.68**
Camarines Norte	**14,695**	3	**0.02**	119	**0.81**	700	**4.76**	**822**	**5.59**
Camarines Sur	**43,452**	0	**0.00**	214	**0.49**	2,018	**4.64**	**2,232**	**5.14**
Catanduanes	**5,414**	0	**0.00**	56	**1.03**	460	**8.50**	**516**	**9.53**
Masbate	**22,365**	2	**0.01**	291	**1.30**	1,883	**8.42**	**2,176**	**9.73**
Sorsogon	**17,929**	2	**0.01**	140	**0.78**	1,577	**8.80**	**1,719**	**9.59**
City of Naga	**4,185**	0	**0.00**	4	**0.10**	108	**2.58**	**112**	**2.68**
**Region 6**	**148,020**	**67**	**0.05**	**3,986**	**2.69**	**35,218**	**23.79**	**39,271**	**26.53**
Aklan	**11,283**	3	**0.03**	185	**1.64**	2,568	**22.76**	**2,756**	**24.43**
Antique	**11,930**	8	**0.07**	144	**1.21**	1,300	**10.90**	**1,452**	**12.17**
Capiz	**13,931**	6	**0.04**	245	**1.76**	2,434	**17.47**	**2,685**	**19.27**
Guimaras	**3,351**	3	**0.09**	139	**4.15**	1,629	**48.61**	**1,771**	**52.85**
Iloilo	**36,140**	21	**0.06**	1,155	**3.20**	11,482	**31.77**	**12,658**	**35.02**
Negros Occidental	**52,270**	16	**0.03**	1,458	**2.79**	9,910	**18.96**	**11,384**	**21.78**
City of Bacolod	**10,373**	1	**0.01**	444	**4.28**	3,703	**35.70**	**4,148**	**39.99**
City of Iloilo	**8,742**	9	**0.10**	216	**2.47**	2,192	**25.07**	**2,417**	**27.65**
**Region 7**	**165,673**	**68**	**0.04**	**3,464**	**2.09**	**26,652**	**16.09**	**30,184**	**18.22**
Bohol	**26,235**	3	**0.01**	228	**0.87**	2,626	**10.01**	**2,857**	**10.89**
Cebu	**72,201**	31	**0.04**	1,779	**2.46**	9,276	**12.85**	**11,086**	**15.35**
Negros Oriental	**28,722**	2	**0.01**	181	**0.63**	1,632	**5.68**	**1,815**	**6.32**
Siquijor	**1,680**	0	**0.00**	9	**0.54**	160	**9.52**	**169**	**10.06**
City of Cebu	**19,267**	11	**0.06**	404	**2.10**	3,936	**20.43**	**4,351**	**22.58**
City of Lapu-Lapu	**10,679**	3	**0.03**	147	**1.38**	2,348	**21.99**	**2,498**	**23.39**
City of Mandaue	**6,889**	18	**0.26**	716	**10.39**	6,674	**96.88**	**7,408**	**107.53**
**Region 8**	**93,813**	**18**	**0.02**	**927**	**0.99**	**7,871**	**8.39**	**8,816**	**9.40**
Biliran	**3,373**	0	**0.00**	35	**1.04**	215	**6.37**	**250**	**7.41**
Eastern Samar	**9,624**	1	**0.01**	87	**0.90**	819	**8.51**	**907**	**9.42**
Leyte	**31,559**	10	**0.03**	184	**0.58**	1,518	**4.81**	**1,712**	**5.42**
Northern Samar	**14,056**	3	**0.02**	280	**1.99**	1,773	**12.61**	**2,056**	**14.63**

Tabe 2.B.1.16 - Prenatal Care

Pregnant women screened for Gestational Diabetes

Philippines, 2023

Area	Eligible Pop.	Screened for Gestational Diabetes							
		Age Group						Total	%
		10-14 yrs old		15-19 yrs old		20-49 yrs old			
		No.	%	No.	%	No.	%		
Southern Leyte	**7,406**	2	**0.03**	65	**0.88**	763	**10.30**	**830**	**11.21**
Samar	**17,011**	1	**0.01**	81	**0.48**	786	**4.62**	**868**	**5.10**
Ormoc City	**5,292**	0	**0.00**	73	**1.38**	564	**10.66**	**637**	**12.04**
City of Tacloban	**5,492**	1	**0.02**	122	**2.22**	1,433	**26.09**	**1,556**	**28.33**
**Region 9**	**84,815**	**18**	**0.02**	**305**	**0.36**	**1,917**	**2.26**	**2,240**	**2.64**
Zamboanga del Norte	**24,841**	1	**0.00**	63	**0.25**	507	**2.04**	**571**	**2.30**
Zamboanga del Sur	**23,757**	4	**0.02**	84	**0.35**	496	**2.09**	**584**	**2.46**
Zamboanga Sibugay	**13,841**	0	**0.00**	26	**0.19**	158	**1.14**	**184**	**1.33**
City of Isabela	**3,201**	11	**0.34**	28	**0.87**	201	**6.28**	**240**	**7.50**
City of Zamboanga	**19,175**	2	**0.01**	104	**0.54**	555	**2.89**	**661**	**3.45**
**Region 10**	**109,169**	**49**	**0.04**	**2,062**	**1.89**	**13,788**	**12.63**	**15,899**	**14.56**
Bukidnon	**33,403**	8	**0.02**	365	**1.09**	2,306	**6.90**	**2,679**	**8.02**
Camiguin	**1,727**	0	**0.00**	43	**2.49**	325	**18.82**	**368**	**21.31**
Lanao del Norte	**17,760**	1	**0.01**	5	**0.03**	166	**0.93**	**172**	**0.97**
Misamis Occidental	**12,328**	3	**0.02**	222	**1.80**	1,863	**15.11**	**2,088**	**16.94**
Misamis Oriental	**21,975**	26	**0.12**	830	**3.78**	4,740	**21.57**	**5,596**	**25.47**
City of Cagayan De Oro	**14,308**	10	**0.07**	469	**3.28**	3,395	**23.73**	**3,874**	**27.08**
City of Iligan	**7,668**	1	**0.01**	128	**1.67**	993	**12.95**	**1,122**	**14.63**
**Region 11**	**107,001**	**76**	**0.07**	**1,756**	**1.64**	**13,822**	**12.92**	**15,654**	**14.63**
Davao de Oro	**15,253**	3	**0.02**	41	**0.27**	445	**2.92**	**489**	**3.21**
Davao del Norte	**23,453**	4	**0.02**	169	**0.72**	1,825	**7.78**	**1,998**	**8.52**
Davao Oriental	**11,935**	3	**0.03**	55	**0.46**	330	**2.76**	**388**	**3.25**
Davao del Sur	**13,083**	8	**0.06**	76	**0.58**	643	**4.91**	**727**	**5.56**
Davao Occidental	**6,629**	7	**0.11**	220	**3.32**	699	**10.54**	**926**	**13.97**
City of Davao	**36,648**	51	**0.14**	1,195	**3.26**	9,880	**26.96**	**11,126**	**30.36**
**Region 12**	**95,279**	**40**	**0.04**	**1,812**	**1.90**	**10,333**	**10.84**	**12,185**	**12.79**
Cotabato	**26,521**	6	**0.02**	467	**1.76**	2,434	**9.18**	**2,907**	**10.96**
Sarangani	**13,322**	19	**0.14**	563	**4.23**	2,653	**19.91**	**3,235**	**24.28**
South Cotabato	**21,595**	3	**0.01**	281	**1.30**	2,261	**10.47**	**2,545**	**11.79**
Sultan Kudarat	**19,391**	10	**0.05**	329	**1.70**	1,912	**9.86**	**2,251**	**11.61**

Tabe 2.B.1.16 - Prenatal Care

Pregnant women screened for Gestational Diabetes

Philippines, 2023

Area	Eligible Pop.	Screened for Gestational Diabetes							
		Age Group						Total	%
		10-14 yrs old		15-19 yrs old		20-49 yrs old			
		No.	%	No.	%	No.	%		
City of General Santos	**14,450**	2	**0.01**	172	**1.19**	1,073	**7.43**	**1,247**	**8.63**
**BARMM**	**132,841**	**6**	**0.00**	**367**	**0.28**	**3,566**	**2.68**	**3,939**	**2.97**
Basilan	**11,636**	0	**0.00**	15	**0.13**	39	**0.34**	**54**	**0.46**
Lanao del Sur	**33,374**	0	**0.00**	43	**0.13**	1,048	**3.14**	**1,091**	**3.27**
Maguindanao	**41,650**	3	**0.01**	101	**0.24**	1,011	**2.43**	**1,115**	**2.68**
Sulu	**21,378**	2	**0.01**	96	**0.45**	665	**3.11**	**763**	**3.57**
Tawi-Tawi	**12,609**	0	**0.00**	2	**0.02**	7	**0.06**	**9**	**0.07**
SGU	**5,103**	0	**0.00**	8	**0.16**	47	**0.92**	**55**	**1.08**
City of Cotabato	**7,091**	1	**0.01**	102	**1.44**	749	**10.56**	**852**	**12.02**
**Caraga**	**55,065**	**10**	**0.02**	**700**	**1.27**	**5,310**	**9.64**	**6,020**	**10.93**
Agusan del Norte	**7,286**	0	**0.00**	74	**1.02**	436	**5.98**	**510**	**7.00**
Agusan del Sur	**16,038**	3	**0.02**	205	**1.28**	1,408	**8.78**	**1,616**	**10.08**
Surigao del Norte	**10,186**	2	**0.02**	136	**1.34**	1,168	**11.47**	**1,306**	**12.82**
Surigao del Sur	**12,736**	3	**0.02**	122	**0.96**	910	**7.15**	**1,035**	**8.13**
Dinagat Islands	**2,134**	1	**0.05**	30	**1.41**	381	**17.85**	**412**	**19.31**
City of Butuan	**6,685**	1	**0.01**	133	**1.99**	1,007	**15.06**	**1,141**	**17.07**

Table 2.B.1.17 - Prenatal Care

Pregnant women tested positive for Gestational Diabetes

Philippines, 2023

Area	Total No. of Screened for Gestational Diabetes	Tested positive for Gestational Diabetes							
		Age Group						Total	%
		10-14 yrs old		15-19 yrs old		20-49 yrs old			
		No.	%	No.	%	No.	%		
**Philippines**	**370,778**	**14**	**0.004**	**646**	**0.17**	**9,153**	**2.47**	**9,813**	**2.65**
**N C R**	**89,384**	**1**	**0.001**	**103**	**0.12**	**2,562**	**2.87**	**2,666**	**2.98**
Malabon City	**475**	0	**0.000**	1	**0.21**	30	**6.32**	**31**	**6.53**
Navotas City	**1,853**	0	**0.000**	20	**1.08**	186	**10.04**	**206**	**11.12**
Valenzuela City	**2,511**	0	**0.000**	5	**0.20**	125	**4.98**	**130**	**5.18**
Caloocan City	**4,799**	0	**0.000**	31	**0.65**	803	**16.73**	**834**	**17.38**
Marikina City	**2,155**	0	**0.000**	0	**0.00**	41	**1.90**	**41**	**1.90**
Pasig City	**10,424**	0	**0.000**	7	**0.07**	395	**3.79**	**402**	**3.86**
Pateros	**651**	0	**0.000**	0	**0.00**	12	**1.84**	**12**	**1.84**
Taguig City	**5,432**	1	**0.018**	17	**0.31**	209	**3.85**	**227**	**4.18**
Quezon City	**24,070**	0	**0.000**	4	**0.02**	297	**1.23**	**301**	**1.25**
Makati City	**2,234**	0	**0.000**	2	**0.09**	114	**5.10**	**116**	**5.19**
Mandaluyong City	**5,625**	0	**0.000**	2	**0.04**	16	**0.28**	**18**	**0.32**
San Juan City	**690**	0	**0.000**	1	**0.14**	12	**1.74**	**13**	**1.88**
Manila City	**17,710**	0	**0.000**	6	**0.03**	189	**1.07**	**195**	**1.10**
Las Piñas City	**1,112**	0	**0.000**	0	**0.00**	49	**4.41**	**49**	**4.41**
Muntinlupa City	**2,208**	0	**0.000**	6	**0.27**	31	**1.40**	**37**	**1.68**
Parañaque City	**3,821**	0	**0.000**	1	**0.03**	26	**0.68**	**27**	**0.71**
Pasay City	**3,614**	0	**0.000**	0	**0.00**	27	**0.75**	**27**	**0.75**
**C A R**	**8,778**	**0**	**0.000**	**6**	**0.07**	**121**	**1.38**	**127**	**1.45**
Abra	**313**	0	**0.000**	0	**0.00**	2	**0.64**	**2**	**0.64**
Apayao	**818**	0	**0.000**	0	**0.00**	14	**1.71**	**14**	**1.71**
Benguet	**2,621**	0	**0.000**	2	**0.08**	36	**1.37**	**38**	**1.45**
Ifugao	**636**	0	**0.000**	0	**0.00**	11	**1.73**	**11**	**1.73**
Kalinga	**365**	0	**0.000**	2	**0.55**	2	**0.55**	**4**	**1.10**
Mountain Province	**1,069**	0	**0.000**	0	**0.00**	20	**1.87**	**20**	**1.87**
City of Baguio	**2,956**	0	**0.000**	2	**0.07**	36	**1.22**	**38**	**1.29**
**Region 1**	**14,137**	**0**	**0.000**	**7**	**0.05**	**92**	**0.65**	**99**	**0.70**
Ilocos Norte	**1,577**	0	**0.000**	0	**0.00**	9	**0.57**	**9**	**0.57**
Ilocos Sur	**4,562**	0	**0.000**	0	**0.00**	2	**0.04**	**2**	**0.04**
La Union	**3,418**	0	**0.000**	2	**0.06**	21	**0.61**	**23**	**0.67**
Pangasinan	**4,562**	0	**0.000**	4	**0.09**	58	**1.27**	**62**	**1.36**

Table 2.B.1.17 - Prenatal Care

Pregnant women tested positive for Gestational Diabetes

Philippines, 2023

Area	Total No. of Screened for Gestational Diabetes	Tested positive for Gestational Diabetes							
		Age Group						Total	%
		10-14 yrs old		15-19 yrs old		20-49 yrs old			
		No.	%	No.	%	No.	%		
City of Dagupan	18	0	0.000	1	5.56	2	11.11	3	16.67
**Region 2**	**6,527**	**0**	**0.000**	**10**	**0.15**	**141**	**2.16**	**151**	**2.31**
Batanes	48	0	0.000	0	0.00	10	20.83	10	20.83
Cagayan	618	0	0.000	7	1.13	40	6.47	47	7.61
Isabela	3,404	0	0.000	2	0.06	68	2.00	70	2.06
Nueva Vizcaya	1,698	0	0.000	0	0.00	10	0.59	10	0.59
Quirino	579	0	0.000	1	0.17	8	1.38	9	1.55
City of Santiago	180	0	0.000	0	0.00	5	2.78	5	2.78
**Region 3**	**45,106**	**2**	**0.004**	**79**	**0.18**	**1,007**	**2.23**	**1,088**	**2.41**
Aurora	336	0	0.000	1	0.30	2	0.60	3	0.89
Bataan	3,542	0	0.000	6	0.17	79	2.23	85	2.40
Bulacan	25,842	1	0.004	44	0.17	713	2.76	758	2.93
Nueva Ecija	5,303	0	0.000	6	0.11	34	0.64	40	0.75
Pampanga	5,304	1	0.019	13	0.25	117	2.21	131	2.47
Tarlac	3,062	0	0.000	4	0.13	47	1.53	51	1.67
Zambales	713	0	0.000	0	0.00	4	0.56	4	0.56
City of Angeles	262	0	0.000	2	0.76	0	0.00	2	0.76
City of Olongapo	742	0	0.000	3	0.40	11	1.48	14	1.89
**Region 4A**	**51,425**	**1**	**0.002**	**80**	**0.16**	**1,371**	**2.67**	**1,452**	**2.82**
Batangas	6,422	0	0.000	11	0.17	158	2.46	169	2.63
Cavite	11,698	0	0.000	11	0.09	211	1.80	222	1.90
Laguna	13,998	1	0.007	39	0.28	555	3.96	595	4.25
Quezon	3,352	0	0.000	7	0.21	171	5.10	178	5.31
Rizal	14,825	0	0.000	10	0.07	244	1.65	254	1.71
City of Lucena	1,130	0	0.000	2	0.18	32	2.83	34	3.01
**Region 4B**	**9,882**	**1**	**0.010**	**9**	**0.09**	**186**	**1.88**	**196**	**1.98**
Marinduque	848	0	0.000	0	0.00	8	0.94	8	0.94
Mindoro Occidental	615	0	0.000	4	0.65	52	8.46	56	9.11
Mindoro Oriental	4,490	1	0.022	1	0.02	39	0.87	41	0.91
Palawan	2,760	0	0.000	2	0.07	62	2.25	64	2.32
Romblon	1,001	0	0.000	2	0.20	20	2.00	22	2.20

Table 2.B.1.17 - Prenatal Care

Pregnant women tested positive for Gestational Diabetes

Philippines, 2023

Area	Total No. of Screened for Gestational Diabetes	Tested positive for Gestational Diabetes							
		Age Group						Total	%
		10-14 yrs old		15-19 yrs old		20-49 yrs old			
		No.	%	No.	%	No.	%		
Puerto Princesa City	**168**	0	**0.000**	0	**0.00**	5	**2.98**	**5**	**2.98**
**Region 5**	**11,331**	**0**	**0.000**	**14**	**0.12**	**259**	**2.29**	**273**	**2.41**
Albay	**3,754**	0	**0.000**	5	**0.13**	98	**2.61**	**103**	**2.74**
Camarines Norte	**822**	0	**0.000**	2	**0.24**	17	**2.07**	**19**	**2.31**
Camarines Sur	**2,232**	0	**0.000**	0	**0.00**	28	**1.25**	**28**	**1.25**
Catanduanes	**516**	0	**0.000**	0	**0.00**	8	**1.55**	**8**	**1.55**
Masbate	**2,176**	0	**0.000**	4	**0.18**	47	**2.16**	**51**	**2.34**
Sorsogon	**1,719**	0	**0.000**	3	**0.17**	56	**3.26**	**59**	**3.43**
City of Naga	**112**	0	**0.000**	0	**0.00**	5	**4.46**	**5**	**4.46**
**Region 6**	**39,271**	**0**	**0.000**	**69**	**0.18**	**815**	**2.08**	**884**	**2.25**
Aklan	**2,756**	0	**0.000**	3	**0.11**	61	**2.21**	**64**	**2.32**
Antique	**1,452**	0	**0.000**	1	**0.07**	62	**4.27**	**63**	**4.34**
Capiz	**2,685**	0	**0.000**	2	**0.07**	34	**1.27**	**36**	**1.34**
Guimaras	**1,771**	0	**0.000**	1	**0.06**	19	**1.07**	**20**	**1.13**
Iloilo	**12,658**	0	**0.000**	6	**0.05**	122	**0.96**	**128**	**1.01**
Negros Occidental	**11,384**	0	**0.000**	16	**0.14**	165	**1.45**	**181**	**1.59**
City of Bacolod	**4,148**	0	**0.000**	38	**0.92**	315	**7.59**	**353**	**8.51**
City of Iloilo	**2,417**	0	**0.000**	2	**0.08**	37	**1.53**	**39**	**1.61**
**Region 7**	**30,184**	**1**	**0.003**	**51**	**0.17**	**967**	**3.20**	**1,019**	**3.38**
Bohol	**2,857**	0	**0.000**	14	**0.49**	573	**20.06**	**587**	**20.55**
Cebu	**11,086**	1	**0.009**	19	**0.17**	248	**2.24**	**268**	**2.42**
Negros Oriental	**1,815**	0	**0.000**	0	**0.00**	39	**2.15**	**39**	**2.15**
Siquijor	**169**	0	**0.000**	0	**0.00**	4	**2.37**	**4**	**2.37**
City of Cebu	**4,351**	0	**0.000**	11	**0.25**	74	**1.70**	**85**	**1.95**
City of Lapu-Lapu	**2,498**	0	**0.000**	7	**0.28**	29	**1.16**	**36**	**1.44**
City of Mandaue	**7,408**	0	**0.000**	0	**0.00**	0	**0.00**	**0**	**0.00**
**Region 8**	**8,816**	**3**	**0.034**	**13**	**0.15**	**109**	**1.24**	**125**	**1.42**
Biliran	**250**	0	**0.000**	0	**0.00**	6	**2.40**	**6**	**2.40**
Eastern Samar	**907**	0	**0.000**	0	**0.00**	6	**0.66**	**6**	**0.66**
Leyte	**1,712**	0	**0.000**	7	**0.41**	40	**2.34**	**47**	**2.75**
Northern Samar	**2,056**	0	**0.000**	1	**0.05**	12	**0.58**	**13**	**0.63**

Table 2.B.1.17 - Prenatal Care

Pregnant women tested positive for Gestational Diabetes

Philippines, 2023

Area	Total No. of Screened for Gestational Diabetes	Tested positive for Gestational Diabetes							
		Age Group						Total	%
		10-14 yrs old		15-19 yrs old		20-49 yrs old			
		No.	%	No.	%	No.	%		
Southern Leyte	**830**	0	**0.000**	0	**0.00**	12	**1.45**	**12**	**1.45**
Samar	**868**	3	**0.346**	3	**0.35**	19	**2.19**	**25**	**2.88**
Ormoc City	**637**	0	**0.000**	1	**0.16**	8	**1.26**	**9**	**1.41**
City of Tacloban	**1,556**	0	**0.000**	1	**0.06**	6	**0.39**	**7**	**0.45**
**Region 9**	**2,240**	**0**	**0.000**	**7**	**0.31**	**60**	**2.68**	**67**	**2.99**
Zamboanga del Norte	**571**	0	**0.000**	6	**1.05**	34	**5.95**	**40**	**7.01**
Zamboanga del Sur	**584**	0	**0.000**	1	**0.17**	7	**1.20**	**8**	**1.37**
Zamboanga Sibugay	**184**	0	**0.000**	0	**0.00**	4	**2.17**	**4**	**2.17**
City of Isabela	**240**	0	**0.000**	0	**0.00**	2	**0.83**	**2**	**0.83**
City of Zamboanga	**661**	0	**0.000**	0	**0.00**	13	**1.97**	**13**	**1.97**
**Region 10**	**15,899**	**1**	**0.006**	**60**	**0.38**	**441**	**2.77**	**502**	**3.16**
Bukidnon	**2,679**	1	**0.037**	23	**0.86**	148	**5.52**	**172**	**6.42**
Camiguin	**368**	0	**0.000**	0	**0.00**	0	**0.00**	**0**	**0.00**
Lanao del Norte	**172**	0	**0.000**	0	**0.00**	3	**1.74**	**3**	**1.74**
Misamis Occidental	**2,088**	0	**0.000**	0	**0.00**	4	**0.19**	**4**	**0.19**
Misamis Oriental	**5,596**	0	**0.000**	9	**0.16**	54	**0.96**	**63**	**1.13**
City of Cagayan De Oro	**3,874**	0	**0.000**	28	**0.72**	219	**5.65**	**247**	**6.38**
City of Iligan	**1,122**	0	**0.000**	0	**0.00**	13	**1.16**	**13**	**1.16**
**Region 11**	**15,654**	**1**	**0.006**	**47**	**0.30**	**350**	**2.24**	**398**	**2.54**
Davao de Oro	**489**	0	**0.000**	3	**0.61**	31	**6.34**	**34**	**6.95**
Davao del Norte	**1,998**	0	**0.000**	3	**0.15**	68	**3.40**	**71**	**3.55**
Davao Oriental	**388**	0	**0.000**	1	**0.26**	22	**5.67**	**23**	**5.93**
Davao del Sur	**727**	0	**0.000**	2	**0.28**	29	**3.99**	**31**	**4.26**
Davao Occidental	**926**	0	**0.000**	6	**0.65**	23	**2.48**	**29**	**3.13**
City of Davao	**11,126**	1	**0.009**	32	**0.29**	177	**1.59**	**210**	**1.89**
**Region 12**	**12,185**	**3**	**0.025**	**52**	**0.43**	**374**	**3.07**	**429**	**3.52**
Cotabato	**2,907**	0	**0.000**	16	**0.55**	117	**4.02**	**133**	**4.58**
Sarangani	**3,235**	0	**0.000**	12	**0.37**	73	**2.26**	**85**	**2.63**
South Cotabato	**2,545**	0	**0.000**	3	**0.12**	55	**2.16**	**58**	**2.28**
Sultan Kudarat	**2,251**	3	**0.133**	12	**0.53**	50	**2.22**	**65**	**2.89**

Table 2.B.1.17 - Prenatal Care

Pregnant women tested positive for Gestational Diabetes

Philippines, 2023

Area	Total No. of Screened for Gestational Diabetes	Tested positive for Gestational Diabetes							
		Age Group						Total	%
		10-14 yrs old		15-19 yrs old		20-49 yrs old			
		No.	%	No.	%	No.	%		
City of General Santos	**1,247**	0	**0.000**	9	**0.72**	79	**6.34**	**88**	**7.06**
**BARMM**	**3,939**	**0**	**0.000**	**6**	**0.15**	**29**	**0.74**	**35**	**0.89**
Basilan	**54**	0	**0.000**	2	**3.70**	2	**3.70**	**4**	**7.41**
Lanao del Sur	**1,091**	0	**0.000**	0	**0.00**	2	**0.18**	**2**	**0.18**
Maguindanao	**1,115**	0	**0.000**	0	**0.00**	11	**0.99**	**11**	**0.99**
Sulu	**763**	0	**0.000**	1	**0.13**	8	**1.05**	**9**	**1.18**
Tawi-Tawi	**9**	0	**0.000**	0	**0.00**	1	**11.11**	**1**	**11.11**
SGU	**55**	0	**0.000**	0	**0.00**	0	**0.00**	**0**	**0.00**
City of Cotabato	**852**	0	**0.000**	3	**0.35**	5	**0.59**	**8**	**0.94**
**Caraga**	**6,020**	**0**	**0.000**	**33**	**0.55**	**269**	**4.47**	**302**	**5.02**
Agusan del Norte	**510**	0	**0.000**	0	**0.00**	8	**1.57**	**8**	**1.57**
Agusan del Sur	**1,616**	0	**0.000**	9	**0.56**	71	**4.39**	**80**	**4.95**
Surigao del Norte	**1,306**	0	**0.000**	9	**0.69**	58	**4.44**	**67**	**5.13**
Surigao del Sur	**1,035**	0	**0.000**	12	**1.16**	112	**10.82**	**124**	**11.98**
Dinagat Islands	**412**	0	**0.000**	3	**0.73**	17	**4.13**	**20**	**4.85**
City of Butuan	**1,141**	0	**0.000**	0	**0.00**	3	**0.26**	**3**	**0.26**

Table 2.B.2.1 - Intrapartum Care and Delivery Outcome

Total Number of women who delivered a live baby or stillbirth/fetal death, deliveries attended by skilled health professionals
Philippines, 2023

Area	Total Number women who delivered	Skilled Health Professional							
		Deliveries attended by skilled health professional							
		MD	%	Nurses	%	Midwives	%	Total	%
**Philippines**	**1,420,182**	**877,253**	**61.77**	**16,980**	**1.20**	**440,246**	**31.00**	**1,334,479**	**93.97**
**N C R**	**184,090**	**135,796**	**73.77**	**322**	**0.17**	**43,982**	**23.89**	**180,100**	**97.83**
Malabon City	**1,782**	1,117	**62.68**	6	**0.34**	567	**31.82**	**1,690**	**94.84**
Navotas City	**4,102**	2,409	**58.73**	1	**0.02**	1,688	**41.15**	**4,098**	**99.90**
Valenzuela City	**5,585**	3,843	**68.81**	56	**1.00**	1,643	**29.42**	**5,542**	**99.23**
Caloocan City	**22,186**	11,154	**50.27**	99	**0.45**	10,790	**48.63**	**22,043**	**99.36**
Marikina City	**2,526**	1,459	**57.76**	1	**0.04**	1,047	**41.45**	**2,507**	**99.25**
Pasig City	**11,370**	10,589	**93.13**	16	**0.14**	654	**5.75**	**11,259**	**99.02**
Pateros	**791**	458	**57.90**	1	**0.13**	330	**41.72**	**789**	**99.75**
Taguig City	**12,357**	8,209	**66.43**	54	**0.44**	3,511	**28.41**	**11,774**	**95.28**
Quezon City	**57,484**	45,734	**79.56**	9	**0.02**	10,884	**18.93**	**56,627**	**98.51**
Makati City	**3,534**	3,177	**89.90**	1	**0.03**	345	**9.76**	**3,523**	**99.69**
Mandaluyong City	**7,639**	6,826	**89.36**	0	**0.00**	811	**10.62**	**7,637**	**99.97**
San Juan City	**745**	722	**96.91**	1	**0.13**	20	**2.68**	**743**	**99.73**
Manila City	**23,640**	21,816	**92.28**	2	**0.01**	1,520	**6.43**	**23,338**	**98.72**
Las Piñas City	**6,851**	2,652	**38.71**	31	**0.45**	3,909	**57.06**	**6,592**	**96.22**
Muntinlupa City	**8,297**	4,019	**48.44**	5	**0.06**	3,072	**37.03**	**7,096**	**85.52**
Parañaque City	**8,296**	5,944	**71.65**	20	**0.24**	1,999	**24.10**	**7,963**	**95.99**
Pasay City	**6,905**	5,668	**82.09**	19	**0.28**	1,192	**17.26**	**6,879**	**99.62**
**C A R**	**24,874**	**22,154**	**89.06**	**373**	**1.50**	**1,955**	**7.86**	**24,482**	**98.42**
Abra	**2,730**	2,442	**89.45**	64	**2.34**	153	**5.60**	**2,659**	**97.40**
Apayao	**2,733**	2,579	**94.37**	26	**0.95**	68	**2.49**	**2,673**	**97.80**
Benguet	**4,082**	3,814	**93.43**	22	**0.54**	112	**2.74**	**3,948**	**96.72**
Ifugao	**2,414**	1,696	**70.26**	44	**1.82**	627	**25.97**	**2,367**	**98.05**
Kalinga	**2,387**	1,534	**64.26**	51	**2.14**	796	**33.35**	**2,381**	**99.75**
Mountain Province	**3,390**	3,019	**89.06**	166	**4.90**	163	**4.81**	**3,348**	**98.76**
City of Baguio	**7,138**	7,070	**99.05**	0	**0.00**	36	**0.50**	**7,106**	**99.55**
**Region 1**	**50,550**	**42,956**	**84.98**	**58**	**0.11**	**7,452**	**14.74**	**50,466**	**99.83**
Ilocos Norte	**6,049**	5,883	**97.26**	4	**0.07**	160	**2.65**	**6,047**	**99.97**
Ilocos Sur	**7,661**	7,555	**98.62**	1	**0.01**	104	**1.36**	**7,660**	**99.99**
La Union	**10,615**	10,017	**94.37**	4	**0.04**	561	**5.28**	**10,582**	**99.69**
Pangasinan	**17,409**	12,539	**72.03**	49	**0.28**	4,788	**27.50**	**17,376**	**99.81**

Table 2.B.2.1 - Intrapartum Care and Delivery Outcome

Total Number of women who delivered a live baby or stillbirth/fetal death, deliveries attended by skilled health professionals
Philippines, 2023

Area	Total Number women who delivered	Skilled Health Professional							
		Deliveries attended by skilled health professional							
		MD	%	Nurses	%	Midwives	%	Total	%
City of Dagupan	**8,816**	6,962	**78.97**	0	**0.00**	1,839	**20.86**	**8,801**	**99.83**
**Region 2**	**36,823**	**26,351**	**71.56**	**220**	**0.60**	**9,670**	**26.26**	**36,241**	**98.42**
Batanes	**216**	212	**98.15**	2	**0.93**	2	**0.93**	**216**	**100.00**
Cagayan	**8,424**	5,488	**65.15**	25	**0.30**	2,729	**32.40**	**8,242**	**97.84**
Isabela	**15,263**	10,459	**68.53**	141	**0.92**	4,460	**29.22**	**15,060**	**98.67**
Nueva Vizcaya	**7,319**	6,670	**91.13**	50	**0.68**	431	**5.89**	**7,151**	**97.70**
Quirino	**2,776**	2,295	**82.67**	2	**0.07**	454	**16.35**	**2,751**	**99.10**
City of Santiago	**2,825**	1,227	**43.43**	0	**0.00**	1,594	**56.42**	**2,821**	**99.86**
**Region 3**	**148,973**	**102,270**	**68.65**	**662**	**0.44**	**42,478**	**28.51**	**145,410**	**97.61**
Aurora	**3,200**	2,491	**77.84**	1	**0.03**	561	**17.53**	**3,053**	**95.41**
Bataan	**11,009**	9,787	**88.90**	15	**0.14**	1,093	**9.93**	**10,895**	**98.96**
Bulacan	**40,824**	24,319	**59.57**	92	**0.23**	15,136	**37.08**	**39,547**	**96.87**
Nueva Ecija	**28,779**	22,455	**78.03**	145	**0.50**	5,622	**19.54**	**28,222**	**98.06**
Pampanga	**25,991**	17,649	**67.90**	286	**1.10**	7,269	**27.97**	**25,204**	**96.97**
Tarlac	**20,870**	12,733	**61.01**	30	**0.14**	7,913	**37.92**	**20,676**	**99.07**
Zambales	**8,677**	6,820	**78.60**	56	**0.65**	1,445	**16.65**	**8,321**	**95.90**
City of Angeles	**5,487**	2,589	**47.18**	35	**0.64**	2,828	**51.54**	**5,452**	**99.36**
City of Olongapo	**4,136**	3,427	**82.86**	2	**0.05**	611	**14.77**	**4,040**	**97.68**
**Region 4A**	**202,438**	**113,977**	**56.30**	**1,156**	**0.57**	**72,728**	**35.93**	**187,861**	**92.80**
Batangas	**38,479**	28,267	**73.46**	59	**0.15**	7,581	**19.70**	**35,907**	**93.32**
Cavite	**48,372**	22,437	**46.38**	349	**0.72**	23,475	**48.53**	**46,261**	**95.64**
Laguna	**45,570**	23,692	**51.99**	360	**0.79**	19,698	**43.23**	**43,750**	**96.01**
Quezon	**24,586**	12,729	**51.77**	123	**0.50**	8,810	**35.83**	**21,662**	**88.11**
Rizal	**42,344**	24,951	**58.92**	237	**0.56**	12,065	**28.49**	**37,253**	**87.98**
City of Lucena	**3,087**	1,901	**61.58**	28	**0.91**	1,099	**35.60**	**3,028**	**98.09**
**Region 4B**	**47,523**	**29,789**	**62.68**	**927**	**1.95**	**9,735**	**20.48**	**40,451**	**85.12**
Marinduque	**2,791**	2,446	**87.64**	85	**3.05**	222	**7.95**	**2,753**	**98.64**
Mindoro Occidental	**8,490**	4,634	**54.58**	48	**0.57**	1,953	**23.00**	**6,635**	**78.15**
Mindoro Oriental	**14,579**	12,080	**82.86**	164	**1.12**	863	**5.92**	**13,107**	**89.90**
Palawan	**13,176**	5,165	**39.20**	493	**3.74**	4,009	**30.43**	**9,667**	**73.37**
Romblon	**4,369**	3,508	**80.29**	78	**1.79**	725	**16.59**	**4,311**	**98.67**

Table 2.B.2.1 - Intrapartum Care and Delivery Outcome

Total Number of women who delivered a live baby or stillbirth/fetal death, deliveries attended by skilled health professionals
Philippines, 2023

Area	Total Number women who delivered	Skilled Health Professional							
		Deliveries attended by skilled health professional							
		MD	%	Nurses	%	Midwives	%	Total	%
Puerto Princesa City	**4,118**	1,956	**47.50**	59	**1.43**	1,963	**47.67**	**3,978**	**96.60**
**Region 5**	**84,229**	**35,925**	**42.65**	**5,235**	**6.22**	**39,742**	**47.18**	**80,902**	**96.05**
Albay	**16,099**	7,612	**47.28**	423	**2.63**	7,883	**48.97**	**15,918**	**98.88**
Camarines Norte	**10,605**	1,989	**18.76**	2,672	**25.20**	5,699	**53.74**	**10,360**	**97.69**
Camarines Sur	**17,655**	3,632	**20.57**	1,274	**7.22**	11,022	**62.43**	**15,928**	**90.22**
Catanduanes	**4,341**	3,930	**90.53**	76	**1.75**	219	**5.04**	**4,225**	**97.33**
Masbate	**14,258**	2,441	**17.12**	424	**2.97**	10,552	**74.01**	**13,417**	**94.10**
Sorsogon	**13,726**	9,761	**71.11**	360	**2.62**	3,479	**25.35**	**13,600**	**99.08**
City of Naga	**7,545**	6,560	**86.94**	6	**0.08**	888	**11.77**	**7,454**	**98.79**
**Region 6**	**96,790**	**64,823**	**66.97**	**290**	**0.30**	**27,361**	**28.27**	**92,474**	**95.54**
Aklan	**6,213**	4,983	**80.20**	22	**0.35**	740	**11.91**	**5,745**	**92.47**
Antique	**8,282**	5,663	**68.38**	17	**0.21**	2,041	**24.64**	**7,721**	**93.23**
Capiz	**10,750**	6,942	**64.58**	29	**0.27**	3,409	**31.71**	**10,380**	**96.56**
Guimaras	**2,370**	2,256	**95.19**	1	**0.04**	109	**4.60**	**2,366**	**99.83**
Iloilo	**22,667**	19,619	**86.55**	82	**0.36**	2,586	**11.41**	**22,287**	**98.32**
Negros Occidental	**34,588**	15,295	**44.22**	124	**0.36**	16,780	**48.51**	**32,199**	**93.09**
City of Bacolod	**6,150**	5,129	**83.40**	12	**0.20**	883	**14.36**	**6,024**	**97.95**
City of Iloilo	**5,770**	4,936	**85.55**	3	**0.05**	813	**14.09**	**5,752**	**99.69**
**Region 7**	**112,305**	**67,426**	**60.04**	**867**	**0.77**	**39,882**	**35.51**	**108,175**	**96.32**
Bohol	**18,420**	12,095	**65.66**	56	**0.30**	6,164	**33.46**	**18,315**	**99.43**
Cebu	**40,979**	19,699	**48.07**	517	**1.26**	19,393	**47.32**	**39,609**	**96.66**
Negros Oriental	**19,030**	12,142	**63.80**	78	**0.41**	4,565	**23.99**	**16,785**	**88.20**
Siquijor	**1,014**	955	**94.18**	0	**0.00**	54	**5.33**	**1,009**	**99.51**
City of Cebu	**15,467**	10,703	**69.20**	132	**0.85**	4,434	**28.67**	**15,269**	**98.72**
City of Lapu-Lapu	**7,557**	4,143	**54.82**	84	**1.11**	3,281	**43.42**	**7,508**	**99.35**
City of Mandaue	**9,838**	7,689	**78.16**	0	**0.00**	1,991	**20.24**	**9,680**	**98.39**
**Region 8**	**59,834**	**39,448**	**65.93**	**625**	**1.04**	**16,763**	**28.02**	**56,836**	**94.99**
Biliran	**3,084**	1,926	**62.45**	33	**1.07**	1,104	**35.80**	**3,063**	**99.32**
Eastern Samar	**7,014**	5,577	**79.51**	48	**0.68**	1,082	**15.43**	**6,707**	**95.62**
Leyte	**12,068**	4,921	**40.78**	82	**0.68**	6,697	**55.49**	**11,700**	**96.95**
Northern Samar	**10,442**	7,199	**68.94**	32	**0.31**	1,901	**18.21**	**9,132**	**87.45**
Southern Leyte	**5,094**	4,595	**90.20**	10	**0.20**	481	**9.44**	**5,086**	**99.84**

Table 2.B.2.1 - Intrapartum Care and Delivery Outcome

Total Number of women who delivered a live baby or stillbirth/fetal death, deliveries attended by skilled health professionals
Philippines, 2023

Area	Total Number women who delivered	Skilled Health Professional							
		Deliveries attended by skilled health professional							
		MD	%	Nurses	%	Midwives	%	Total	%
Samar	**10,338**	6,417	**62.07**	227	**2.20**	2,741	**26.51**	**9,385**	**90.78**
Ormoc City	**5,965**	3,433	**57.55**	174	**2.92**	2,327	**39.01**	**5,934**	**99.48**
City of Tacloban	**5,829**	5,380	**92.30**	19	**0.33**	430	**7.38**	**5,829**	**100.00**
**Region 9**	**47,886**	**24,211**	**50.56**	**847**	**1.77**	**16,317**	**34.07**	**41,375**	**86.40**
Zamboanga del Norte	**13,451**	7,095	**52.75**	128	**0.95**	4,688	**34.85**	**11,911**	**88.55**
Zamboanga del Sur	**8,271**	1,929	**23.32**	123	**1.49**	4,916	**59.44**	**6,968**	**84.25**
Zamboanga Sibugay	**7,406**	2,131	**28.77**	125	**1.69**	4,260	**57.52**	**6,516**	**87.98**
City of Isabela	**1,797**	1,041	**57.93**	19	**1.06**	24	**1.34**	**1,084**	**60.32**
City of Zamboanga	**16,961**	12,015	**70.84**	452	**2.66**	2,429	**14.32**	**14,896**	**87.83**
**Region 10**	**71,891**	**48,170**	**67.00**	**654**	**0.91**	**18,769**	**26.11**	**67,593**	**94.02**
Bukidnon	**23,309**	11,846	**50.82**	70	**0.30**	9,217	**39.54**	**21,133**	**90.66**
Camiguin	**1,355**	1,255	**92.62**	0	**0.00**	97	**7.16**	**1,352**	**99.78**
Lanao del Norte	**6,720**	2,373	**35.31**	104	**1.55**	2,803	**41.71**	**5,280**	**78.57**
Misamis Occidental	**6,284**	5,276	**83.96**	130	**2.07**	878	**13.97**	**6,284**	**100.00**
Misamis Oriental	**9,358**	5,950	**63.58**	212	**2.27**	2,835	**30.29**	**8,997**	**96.14**
City of Cagayan De Oro	**17,552**	14,714	**83.83**	136	**0.77**	2,518	**14.35**	**17,368**	**98.95**
City of Iligan	**7,313**	6,756	**92.38**	2	**0.03**	421	**5.76**	**7,179**	**98.17**
**Region 11**	**81,389**	**53,245**	**65.42**	**581**	**0.71**	**23,068**	**28.34**	**76,894**	**94.48**
Davao de Oro	**11,522**	8,419	**73.07**	284	**2.46**	2,611	**22.66**	**11,314**	**98.19**
Davao del Norte	**17,062**	12,058	**70.67**	110	**0.64**	4,207	**24.66**	**16,375**	**95.97**
Davao Oriental	**8,590**	7,188	**83.68**	54	**0.63**	1,108	**12.90**	**8,350**	**97.21**
Davao del Sur	**9,438**	5,694	**60.33**	13	**0.14**	3,345	**35.44**	**9,052**	**95.91**
Davao Occidental	**4,886**	2,499	**51.15**	55	**1.13**	898	**18.38**	**3,452**	**70.65**
City of Davao	**29,891**	17,387	**58.17**	65	**0.22**	10,899	**36.46**	**28,351**	**94.85**
**Region 12**	**64,112**	**34,985**	**54.57**	**413**	**0.64**	**24,304**	**37.91**	**59,702**	**93.12**
Cotabato	**17,294**	11,198	**64.75**	127	**0.73**	4,126	**23.86**	**15,451**	**89.34**
Sarangani	**9,947**	3,728	**37.48**	32	**0.32**	5,453	**54.82**	**9,213**	**92.62**
South Cotabato	**16,339**	11,402	**69.78**	142	**0.87**	3,983	**24.38**	**15,527**	**95.03**
Sultan Kudarat	**12,614**	6,781	**53.76**	41	**0.33**	4,955	**39.28**	**11,777**	**93.36**
City of General Santos	**7,918**	1,876	**23.69**	71	**0.90**	5,787	**73.09**	**7,734**	**97.68**

Table 2.B.2.1 - Intrapartum Care and Delivery Outcome

Total Number of women who delivered a live baby or stillbirth/fetal death, deliveries attended by skilled health professionals
Philippines, 2023

Area	Total Number women who delivered	Skilled Health Professional							
		Deliveries attended by skilled health professional							
		MD	%	Nurses	%	Midwives	%	Total	%
**BARMM**	**65,140**	**9,225**	**14.16**	**3,107**	**4.77**	**33,349**	**51.20**	**45,681**	**70.13**
Basilan	**4,718**	154	**3.26**	754	**15.98**	2,023	**42.88**	**2,931**	**62.12**
Lanao del Sur	**17,820**	4,441	**24.92**	1,160	**6.51**	9,208	**51.67**	**14,809**	**83.10**
Maguindanao	**19,942**	1,425	**7.15**	601	**3.01**	12,153	**60.94**	**14,179**	**71.10**
Sulu	**11,670**	519	**4.45**	273	**2.34**	4,862	**41.66**	**5,654**	**48.45**
Tawi-Tawi	**6,010**	483	**8.04**	317	**5.27**	3,953	**65.77**	**4,753**	**79.08**
SGA	**1,432**	0	**0.00**	0	**0.00**	257	**17.95**	**257**	**17.95**
City of Cotabato	**3,548**	2,203	**62.09**	2	**0.06**	893	**25.17**	**3,098**	**87.32**
**Caraga**	**41,335**	**26,502**	**64.12**	**643**	**1.56**	**12,691**	**30.70**	**39,836**	**96.37**
Agusan del Norte	**5,881**	4,259	**72.42**	6	**0.10**	1,451	**24.67**	**5,716**	**97.19**
Agusan del Sur	**10,972**	6,222	**56.71**	39	**0.36**	3,921	**35.74**	**10,182**	**92.80**
Surigao del Norte	**7,756**	5,966	**76.92**	288	**3.71**	1,314	**16.94**	**7,568**	**97.58**
Surigao del Sur	**9,826**	6,778	**68.98**	287	**2.92**	2,468	**25.12**	**9,533**	**97.02**
Dinagat Islands	**1,034**	845	**81.72**	23	**2.22**	134	**12.96**	**1,002**	**96.91**
City of Butuan	**5,866**	2,432	**41.46**	0	**0.00**	3,403	**58.01**	**5,835**	**99.47**

Table 2.B.2.1 - Intrapartum Care and Delivery Outcome

Total Number of women who delivered a live baby or stillbirth/fetal death, deliveries in health facilities
Philippines, 2023

Area	Total Number women who delivered	Facility Based Delivery					
		Type of Health Facility				Total	%
		Public	%	Private	%		
**Philippines**	**1,420,182**	**929,713**	**65.46**	**390,514**	**27.50**	**1,320,227**	**92.96**
**N C R**	**184,090**	**120,929**	**65.69**	**58,349**	**31.70**	**179,278**	**97.39**
Malabon City	**1,782**	1,131	**63.47**	553	**31.03**	**1,684**	**94.50**
Navotas City	**4,102**	2,472	**60.26**	1,604	**39.10**	**4,076**	**99.37**
Valenzuela City	**5,585**	3,921	**70.21**	1,513	**27.09**	**5,434**	**97.30**
Caloocan City	**22,186**	9,436	**42.53**	12,539	**56.52**	**21,975**	**99.05**
Marikina City	**2,526**	1,264	**50.04**	1,203	**47.62**	**2,467**	**97.66**
Pasig City	**11,370**	7,860	**69.13**	3,371	**29.65**	**11,231**	**98.78**
Pateros	**791**	289	**36.54**	481	**60.81**	**770**	**97.35**
Taguig City	**12,357**	8,276	**66.97**	3,488	**28.23**	**11,764**	**95.20**
Quezon City	**57,484**	36,634	**63.73**	19,708	**34.28**	**56,342**	**98.01**
Makati City	**3,534**	3,031	**85.77**	478	**13.53**	**3,509**	**99.29**
Mandaluyong City	**7,639**	6,681	**87.46**	956	**12.51**	**7,637**	**99.97**
San Juan City	**745**	664	**89.13**	75	**10.07**	**739**	**99.19**
Manila City	**23,640**	20,248	**85.65**	3,008	**12.72**	**23,256**	**98.38**
Las Piñas City	**6,851**	4,011	**58.55**	2,486	**36.29**	**6,497**	**94.83**
Muntinlupa City	**8,297**	4,379	**52.78**	2,705	**32.60**	**7,084**	**85.38**
Parañaque City	**8,296**	5,436	**65.53**	2,500	**30.14**	**7,936**	**95.66**
Pasay City	**6,905**	5,196	**75.25**	1,681	**24.34**	**6,877**	**99.59**
**C A R**	**24,874**	**20,087**	**80.76**	**4,012**	**16.13**	**24,099**	**96.88**
Abra	**2,730**	1,992	**72.97**	613	**22.45**	**2,605**	**95.42**
Apayao	**2,733**	2,559	**93.63**	42	**1.54**	**2,601**	**95.17**
Benguet	**4,082**	3,178	**77.85**	678	**16.61**	**3,856**	**94.46**
Ifugao	**2,414**	1,991	**82.48**	327	**13.55**	**2,318**	**96.02**
Kalinga	**2,387**	1,765	**73.94**	605	**25.35**	**2,370**	**99.29**
Mountain Province	**3,390**	3,208	**94.63**	40	**1.18**	**3,248**	**95.81**
City of Baguio	**7,138**	5,394	**75.57**	1,707	**23.91**	**7,101**	**99.48**
**Region 1**	**50,550**	**38,695**	**76.55**	**11,653**	**23.05**	**50,348**	**99.60**
Ilocos Norte	**6,049**	5,603	**92.63**	444	**7.34**	**6,047**	**99.97**
Ilocos Sur	**7,661**	4,536	**59.21**	3,122	**40.75**	**7,658**	**99.96**
La Union	**10,615**	9,678	**91.17**	899	**8.47**	**10,577**	**99.64**

Table 2.B.2.1 - Intrapartum Care and Delivery Outcome

Total Number of women who delivered a live baby or stillbirth/fetal death, deliveries in health facilities

Philippines, 2023

Area	Total Number women who delivered	Facility Based Delivery					
		Type of Health Facility				Total	%
		Public	%	Private	%		
Pangasinan	**17,409**	14,042	**80.66**	3,241	**18.62**	**17,283**	**99.28**
City of Dagupan	**8,816**	4,836	**54.85**	3,947	**44.77**	**8,783**	**99.63**
**Region 2**	**36,823**	**28,705**	**77.95**	**7,353**	**19.97**	**36,058**	**97.92**
Batanes	**216**	213	**98.61**	0	**0.00**	**213**	**98.61**
Cagayan	**8,424**	7,264	**86.23**	916	**10.87**	**8,180**	**97.10**
Isabela	**15,263**	10,616	**69.55**	4,397	**28.81**	**15,013**	**98.36**
Nueva Vizcaya	**7,319**	6,948	**94.93**	132	**1.80**	**7,080**	**96.73**
Quirino	**2,776**	2,492	**89.77**	259	**9.33**	**2,751**	**99.10**
City of Santiago	**2,825**	1,172	**41.49**	1,649	**58.37**	**2,821**	**99.86**
**Region 3**	**148,973**	**82,711**	**55.52**	**61,777**	**41.47**	**144,488**	**96.99**
Aurora	**3,200**	2,730	**85.31**	308	**9.63**	**3,038**	**94.94**
Bataan	**11,009**	8,893	**80.78**	1,901	**17.27**	**10,794**	**98.05**
Bulacan	**40,824**	11,673	**28.59**	27,748	**67.97**	**39,421**	**96.56**
Nueva Ecija	**28,779**	16,830	**58.48**	11,130	**38.67**	**27,960**	**97.15**
Pampanga	**25,991**	17,378	**66.86**	7,582	**29.17**	**24,960**	**96.03**
Tarlac	**20,870**	12,920	**61.91**	7,740	**37.09**	**20,660**	**98.99**
Zambales	**8,677**	8,108	**93.44**	147	**1.69**	**8,255**	**95.14**
City of Angeles	**5,487**	1,532	**27.92**	3,870	**70.53**	**5,402**	**98.45**
City of Olongapo	**4,136**	2,647	**64.00**	1,351	**32.66**	**3,998**	**96.66**
**Region 4A**	**202,438**	**98,143**	**48.48**	**87,132**	**43.04**	**185,275**	**91.52**
Batangas	**38,479**	15,688	**40.77**	19,424	**50.48**	**35,112**	**91.25**
Cavite	**48,372**	15,549	**32.14**	30,324	**62.69**	**45,873**	**94.83**
Laguna	**45,570**	24,361	**53.46**	18,914	**41.51**	**43,275**	**94.96**
Quezon	**24,586**	15,833	**64.40**	5,645	**22.96**	**21,478**	**87.36**
Rizal	**42,344**	24,502	**57.86**	12,018	**28.38**	**36,520**	**86.25**
City of Lucena	**3,087**	2,210	**71.59**	807	**26.14**	**3,017**	**97.73**
**Region 4B**	**47,523**	**35,311**	**74.30**	**4,805**	**10.11**	**40,116**	**84.41**
Marinduque	**2,791**	2,751	**98.57**	0	**0.00**	**2,751**	**98.57**
Mindoro Occidental	**8,490**	6,034	**71.07**	576	**6.78**	**6,610**	**77.86**
Mindoro Oriental	**14,579**	11,537	**79.13**	1,532	**10.51**	**13,069**	**89.64**
Palawan	**13,176**	8,454	**64.16**	968	**7.35**	**9,422**	**71.51**

Table 2.B.2.1 - Intrapartum Care and Delivery Outcome

Total Number of women who delivered a live baby or stillbirth/fetal death, deliveries in health facilities

Philippines, 2023

Area	Total Number women who delivered	Facility Based Delivery					
		Type of Health Facility				Total	%
		Public	%	Private	%		
Romblon	**4,369**	4,112	**94.12**	194	**4.44**	**4,306**	**98.56**
Puerto Princesa City	**4,118**	2,423	**58.84**	1,535	**37.28**	**3,958**	**96.11**
**Region 5**	**84,229**	**57,356**	**68.10**	**23,049**	**27.36**	**80,405**	**95.46**
Albay	**16,099**	10,070	**62.55**	5,829	**36.21**	**15,899**	**98.76**
Camarines Norte	**10,605**	8,059	**75.99**	2,288	**21.57**	**10,347**	**97.57**
Camarines Sur	**17,655**	5,188	**29.39**	10,635	**60.24**	**15,823**	**89.62**
Catanduanes	**4,341**	3,931	**90.56**	293	**6.75**	**4,224**	**97.30**
Masbate	**14,258**	12,488	**87.59**	594	**4.17**	**13,082**	**91.75**
Sorsogon	**13,726**	11,738	**85.52**	1,852	**13.49**	**13,590**	**99.01**
City of Naga	**7,545**	5,882	**77.96**	1,558	**20.65**	**7,440**	**98.61**
**Region 6**	**96,790**	**77,658**	**80.23**	**14,565**	**15.05**	**92,223**	**95.28**
Aklan	**6,213**	5,185	**83.45**	469	**7.55**	**5,654**	**91.00**
Antique	**8,282**	7,200	**86.94**	487	**5.88**	**7,687**	**92.82**
Capiz	**10,750**	6,086	**56.61**	4,277	**39.79**	**10,363**	**96.40**
Guimaras	**2,370**	2,242	**94.60**	109	**4.60**	**2,351**	**99.20**
Iloilo	**22,667**	21,046	**92.85**	1,195	**5.27**	**22,241**	**98.12**
Negros Occidental	**34,588**	27,674	**80.01**	4,480	**12.95**	**32,154**	**92.96**
City of Bacolod	**6,150**	3,963	**64.44**	2,059	**33.48**	**6,022**	**97.92**
City of Iloilo	**5,770**	4,262	**73.86**	1,489	**25.81**	**5,751**	**99.67**
**Region 7**	**112,305**	**79,154**	**70.48**	**28,556**	**25.43**	**107,710**	**95.91**
Bohol	**18,420**	15,403	**83.62**	2,859	**15.52**	**18,262**	**99.14**
Cebu	**40,979**	31,344	**76.49**	8,243	**20.12**	**39,587**	**96.60**
Negros Oriental	**19,030**	13,813	**72.59**	2,889	**15.18**	**16,702**	**87.77**
Siquijor	**1,014**	1,005	**99.11**	0	**0.00**	**1,005**	**99.11**
City of Cebu	**15,467**	9,027	**58.36**	6,022	**38.93**	**15,049**	**97.30**
City of Lapu-Lapu	**7,557**	3,159	**41.80**	4,345	**57.50**	**7,504**	**99.30**
City of Mandaue	**9,838**	5,403	**54.92**	4,198	**42.67**	**9,601**	**97.59**
**Region 8**	**59,834**	**40,934**	**68.41**	**15,474**	**25.86**	**56,408**	**94.27**
Biliran	**3,084**	3,051	**98.93**	0	**0.00**	**3,051**	**98.93**
Eastern Samar	**7,014**	3,401	**48.49**	3,251	**46.35**	**6,652**	**94.84**
Leyte	**12,068**	7,503	**62.17**	4,084	**33.84**	**11,587**	**96.01**

Table 2.B.2.1 - Intrapartum Care and Delivery Outcome

Total Number of women who delivered a live baby or stillbirth/fetal death, deliveries in health facilities

Philippines, 2023

Area	Total Number women who delivered	Facility Based Delivery					
		Type of Health Facility				Total	%
		Public	%	Private	%		
Northern Samar	**10,442**	8,725	**83.56**	380	**3.64**	**9,105**	**87.20**
Southern Leyte	**5,094**	3,826	**75.11**	1,224	**24.03**	**5,050**	**99.14**
Samar	**10,338**	8,081	**78.17**	1,157	**11.19**	**9,238**	**89.36**
Ormoc City	**5,965**	2,385	**39.98**	3,512	**58.88**	**5,897**	**98.86**
City of Tacloban	**5,829**	3,962	**67.97**	1,866	**32.01**	**5,828**	**99.98**
**Region 9**	**47,886**	**36,947**	**77.16**	**4,353**	**9.09**	**41,300**	**86.25**
Zamboanga del Norte	**13,451**	11,881	**88.33**	28	**0.21**	**11,909**	**88.54**
Zamboanga del Sur	**8,271**	4,768	**57.65**	2,159	**26.10**	**6,927**	**83.75**
Zamboanga Sibugay	**7,406**	6,237	**84.22**	281	**3.79**	**6,518**	**88.01**
City of Isabela	**1,797**	992	**55.20**	88	**4.90**	**1,080**	**60.10**
City of Zamboanga	**16,961**	13,069	**77.05**	1,797	**10.59**	**14,866**	**87.65**
**Region 10**	**71,891**	**56,194**	**78.17**	**10,108**	**14.06**	**66,302**	**92.23**
Bukidnon	**23,309**	17,235	**73.94**	3,747	**16.08**	**20,982**	**90.02**
Camiguin	**1,355**	1,269	**93.65**	82	**6.05**	**1,351**	**99.70**
Lanao del Norte	**6,720**	3,976	**59.17**	252	**3.75**	**4,228**	**62.92**
Misamis Occidental	**6,284**	5,746	**91.44**	538	**8.56**	**6,284**	**100.00**
Misamis Oriental	**9,358**	7,771	**83.04**	1,196	**12.78**	**8,967**	**95.82**
City of Cagayan De Oro	**17,552**	14,082	**80.23**	3,286	**18.72**	**17,368**	**98.95**
City of Iligan	**7,313**	6,115	**83.62**	1,007	**13.77**	**7,122**	**97.39**
**Region 11**	**81,389**	**51,080**	**62.76**	**25,773**	**31.67**	**76,853**	**94.43**
Davao de Oro	**11,522**	9,674	**83.96**	1,741	**15.11**	**11,415**	**99.07**
Davao del Norte	**17,062**	11,848	**69.44**	4,508	**26.42**	**16,356**	**95.86**
Davao Oriental	**8,590**	8,209	**95.56**	111	**1.29**	**8,320**	**96.86**
Davao del Sur	**9,438**	3,313	**35.10**	5,727	**60.68**	**9,040**	**95.78**
Davao Occidental	**4,886**	2,655	**54.34**	784	**16.05**	**3,439**	**70.38**
City of Davao	**29,891**	15,381	**51.46**	12,902	**43.16**	**28,283**	**94.62**
**Region 12**	**64,112**	**43,367**	**67.64**	**16,237**	**25.33**	**59,604**	**92.97**
Cotabato	**17,294**	10,442	**60.38**	4,986	**28.83**	**15,428**	**89.21**
Sarangani	**9,947**	8,387	**84.32**	797	**8.01**	**9,184**	**92.33**
South Cotabato	**16,339**	12,471	**76.33**	3,040	**18.61**	**15,511**	**94.93**
Sultan Kudarat	**12,614**	9,523	**75.50**	2,254	**17.87**	**11,777**	**93.36**

Table 2.B.2.1 - Intrapartum Care and Delivery Outcome

Total Number of women who delivered a live baby or stillbirth/fetal death, deliveries in health facilities
Philippines, 2023

Area	Total Number women who delivered	Facility Based Delivery					
		Type of Health Facility				Total	%
		Public	%	Private	%		
City of General Santos	**7,918**	2,544	**32.13**	5,160	**65.17**	**7,704**	**97.30**
**BARMM**	**65,140**	**32,641**	**50.11**	**7,413**	**11.38**	**40,054**	**61.49**
Basilan	**4,718**	2,287	**48.47**	13	**0.28**	**2,300**	**48.75**
Lanao del Sur	**17,820**	10,521	**59.04**	2,892	**16.23**	**13,413**	**75.27**
Maguindanao	**19,942**	7,777	**39.00**	3,490	**17.50**	**11,267**	**56.50**
Sulu	**11,670**	5,413	**46.38**	71	**0.61**	**5,484**	**46.99**
Tawi-Tawi	**6,010**	4,269	**71.03**	16	**0.27**	**4,285**	**71.30**
SGA	**1,432**	0	**0.00**	209	**14.59**	**209**	**14.59**
City of Cotabato	**3,548**	2,374	**66.91**	722	**20.35**	**3,096**	**87.26**
**Caraga**	**41,335**	**29,801**	**72.10**	**9,905**	**23.96**	**39,706**	**96.06**
Agusan del Norte	**5,881**	3,984	**67.74**	1,727	**29.37**	**5,711**	**97.11**
Agusan del Sur	**10,972**	7,125	**64.94**	3,035	**27.66**	**10,160**	**92.60**
Surigao del Norte	**7,756**	6,872	**88.60**	677	**8.73**	**7,549**	**97.33**
Surigao del Sur	**9,826**	8,732	**88.87**	725	**7.38**	**9,457**	**96.24**
Dinagat Islands	**1,034**	994	**96.13**	0	**0.00**	**994**	**96.13**
City of Butuan	**5,866**	2,094	**35.70**	3,741	**63.77**	**5,835**	**99.47**

Table 2.B.2.2 - Intrapartum Care and Delivery Outcome
Delivery by type
Philippines, 2023

Area	Total number of Deliveries	Delivery by Type									
		Vaginal					Cesarean Section				
		Age Group in Year			Total	%	Age Group in Year			Total	%
		10-14	15-19	20-49			10-14	15-19	20-49		
**Philippines**	**1,420,182**	**2,702**	**127,459**	**1,099,448**	**1,229,609**	**86.58**	**319**	**11,096**	**179,066**	**190,481**	**13.41**
**N C R**	**184,090**	**223**	**11,010**	**152,878**	**164,111**	**89.15**	**34**	**1,070**	**18,875**	**19,979**	**10.85**
Malabon City	**1,782**	6	156	1,475	**1,637**	**91.86**	0	7	138	**145**	**8.14**
Navotas City	**4,102**	8	335	3,161	**3,504**	**85.42**	0	2	596	**598**	**14.58**
Valenzuela City	**5,585**	10	346	4,262	**4,618**	**82.69**	1	64	902	**967**	**17.31**
Caloocan City	**22,186**	21	1,570	18,960	**20,551**	**92.63**	4	145	1,486	**1,635**	**7.37**
Marikina City	**2,526**	6	176	1,879	**2,061**	**81.59**	1	33	431	**465**	**18.41**
Pasig City	**11,370**	34	733	9,626	**10,393**	**91.41**	0	37	940	**977**	**8.59**
Pateros	**791**	1	30	679	**710**	**89.76**	0	1	80	**81**	**10.24**
Taguig City	**12,357**	16	1,001	9,877	**10,894**	**88.16**	0	71	1,392	**1,463**	**11.84**
Quezon City	**57,484**	49	2,561	50,894	**53,504**	**93.08**	2	137	3,841	**3,980**	**6.92**
Makati City	**3,534**	5	222	2,416	**2,643**	**74.79**	2	27	862	**891**	**25.21**
Mandaluyong City	**7,639**	9	259	6,568	**6,836**	**89.49**	0	40	763	**803**	**10.51**
San Juan City	**745**	0	42	533	**575**	**77.18**	0	8	162	**170**	**22.82**
Manila City	**23,640**	18	1,829	18,260	**20,107**	**85.05**	1	319	3,213	**3,533**	**14.95**
Las Piñas City	**6,851**	13	477	5,561	**6,051**	**88.32**	1	42	757	**800**	**11.68**
Muntinlupa City	**8,297**	16	517	6,920	**7,453**	**89.83**	0	47	797	**844**	**10.17**
Parañaque City	**8,296**	5	532	6,151	**6,688**	**80.62**	20	47	1,541	**1,608**	**19.38**
Pasay City	**6,905**	6	224	5,656	**5,886**	**85.24**	2	43	974	**1,019**	**14.76**
**C A R**	**24,874**	**33**	**1,674**	**15,252**	**16,959**	**68.18**	**7**	**361**	**7,547**	**7,915**	**31.82**
Abra	**2,730**	3	284	1,575	**1,862**	**68.21**	2	55	811	**868**	**31.79**
Apayao	**2,733**	8	326	1,823	**2,157**	**78.92**	2	34	540	**576**	**21.08**
Benguet	**4,082**	6	183	2,783	**2,972**	**72.81**	0	45	1,065	**1,110**	**27.19**
Ifugao	**2,414**	3	215	1,831	**2,049**	**84.88**	0	25	340	**365**	**15.12**
Kalinga	**2,387**	2	128	1,853	**1,983**	**83.07**	0	19	385	**404**	**16.93**
Mountain Province	**3,390**	6	257	2,105	**2,368**	**69.85**	1	49	972	**1,022**	**30.15**
City of Baguio	**7,138**	5	281	3,282	**3,568**	**49.99**	2	134	3,434	**3,570**	**50.01**
**Region 1**	**50,550**	**71**	**3,229**	**39,895**	**43,195**	**85.45**	**7**	**452**	**6,896**	**7,355**	**14.55**
Ilocos Norte	**6,049**	7	242	4,775	**5,024**	**83.06**	0	92	933	**1,025**	**16.94**
Ilocos Sur	**7,661**	6	308	5,363	**5,677**	**74.10**	3	87	1,894	**1,984**	**25.90**

Table 2.B.2.2 - Intrapartum Care and Delivery Outcome
Delivery by type
Philippines, 2023

Area	Total number of Deliveries	Delivery by Type									
		Vaginal					Cesarean Section				
		Age Group in Year			Total	%	Age Group in Year			Total	%
		10-14	15-19	20-49			10-14	15-19	20-49		
La Union	**10,615**	26	735	8,631	**9,392**	**88.48**	2	107	1,114	**1,223**	**11.52**
Pangasinan	**17,409**	13	1,233	14,668	**15,914**	**91.41**	2	145	1,348	**1,495**	**8.59**
City of Dagupan	**8,816**	19	711	6,458	**7,188**	**81.53**	0	21	1,607	**1,628**	**18.47**
**Region 2**	**36,823**	**58**	**3,214**	**25,596**	**28,868**	**78.40**	**18**	**559**	**7,378**	**7,955**	**21.60**
Batanes	**216**	2	14	140	**156**	**72.22**	1	2	57	**60**	**27.78**
Cagayan	**8,424**	12	835	6,617	**7,464**	**88.60**	1	42	917	**960**	**11.40**
Isabela	**15,263**	15	1,236	10,848	**12,099**	**79.27**	5	237	2,922	**3,164**	**20.73**
Nueva Vizcaya	**7,319**	19	600	4,229	**4,848**	**66.24**	6	162	2,303	**2,471**	**33.76**
Quirino	**2,776**	7	277	1,760	**2,044**	**73.63**	5	74	653	**732**	**26.37**
City of Santiago	**2,825**	3	252	2,002	**2,257**	**79.89**	0	42	526	**568**	**20.11**
**Region 3**	**148,973**	**287**	**10,759**	**99,409**	**110,455**	**74.14**	**70**	**2,157**	**36,199**	**38,426**	**25.79**
Aurora	**3,200**	11	366	2,353	**2,730**	**85.31**	1	21	448	**470**	**14.69**
Bataan	**11,009**	40	1,002	6,539	**7,581**	**68.86**	7	203	3,218	**3,428**	**31.14**
Bulacan	**40,824**	63	2,424	26,514	**29,001**	**71.04**	6	377	11,348	**11,731**	**28.74**
Nueva Ecija	**28,779**	37	1,796	18,127	**19,960**	**69.36**	36	634	8,149	**8,819**	**30.64**
Pampanga	**25,991**	58	2,367	18,460	**20,885**	**80.35**	7	329	4,770	**5,106**	**19.65**
Tarlac	**20,870**	31	1,357	14,951	**16,339**	**78.29**	5	366	4,160	**4,531**	**21.71**
Zambales	**8,677**	25	839	5,243	**6,107**	**70.38**	8	186	2,376	**2,570**	**29.62**
City of Angeles	**5,487**	11	283	3,460	**3,754**	**68.42**	0	41	1,692	**1,733**	**31.58**
City of Olongapo	**4,136**	11	325	3,762	**4,098**	**99.08**	0	0	38	**38**	**0.92**
**Region 4A**	**202,438**	**319**	**16,087**	**150,173**	**166,579**	**82.29**	**36**	**1,660**	**34,163**	**35,859**	**17.71**
Batangas	**38,479**	45	2,532	23,902	**26,479**	**68.81**	11	523	11,466	**12,000**	**31.19**
Cavite	**48,372**	45	3,040	37,518	**40,603**	**83.94**	6	278	7,485	**7,769**	**16.06**
Laguna	**45,570**	75	3,646	35,224	**38,945**	**85.46**	6	349	6,270	**6,625**	**14.54**
Quezon	**24,586**	75	2,815	19,336	**22,226**	**90.40**	5	90	2,265	**2,360**	**9.60**
Rizal	**42,344**	71	3,694	31,817	**35,582**	**84.03**	8	406	6,348	**6,762**	**15.97**
City of Lucena	**3,087**	8	360	2,376	**2,744**	**88.89**	0	14	329	**343**	**11.11**
**Region 4B**	**47,523**	**107**	**5,132**	**38,018**	**43,257**	**91.02**	**4**	**248**	**4,014**	**4,266**	**8.98**
Marinduque	**2,791**	5	255	2,111	**2,371**	**84.95**	0	28	392	**420**	**15.05**

Table 2.B.2.2 - Intrapartum Care and Delivery Outcome
Delivery by type
Philippines, 2023

Area	Total number of Deliveries	Delivery by Type									
		Vaginal					Cesarean Section				
		Age Group in Year			Total	%	Age Group in Year			Total	%
		10-14	15-19	20-49			10-14	15-19	20-49		
Mindoro Occidental	**8,490**	15	1,041	6,800	**7,856**	**92.53**	1	45	588	**634**	**7.47**
Mindoro Oriental	**14,579**	19	941	11,448	**12,408**	**85.11**	2	113	2,056	**2,171**	**14.89**
Palawan	**13,176**	51	2,054	10,671	**12,776**	**96.96**	1	43	356	**400**	**3.04**
Romblon	**4,369**	6	407	3,630	**4,043**	**92.54**	0	8	318	**326**	**7.46**
Puerto Princesa City	**4,118**	11	434	3,358	**3,803**	**92.35**	0	11	304	**315**	**7.65**
**Region 5**	**84,229**	**96**	**8,033**	**70,080**	**78,209**	**92.85**	**4**	**322**	**5,694**	**6,020**	**7.15**
Albay	**16,099**	17	1,103	14,543	**15,663**	**97.29**	1	4	431	**436**	**2.71**
Camarines Norte	**10,605**	9	1,279	7,832	**9,120**	**86.00**	0	124	1,361	**1,485**	**14.00**
Camarines Sur	**17,655**	5	1,411	15,828	**17,244**	**97.67**	0	4	407	**411**	**2.33**
Catanduanes	**4,341**	11	447	3,053	**3,511**	**80.88**	0	38	792	**830**	**19.12**
Masbate	**14,258**	19	1,907	12,225	**14,151**	**99.25**	0	10	97	**107**	**0.75**
Sorsogon	**13,726**	15	1,173	10,653	**11,841**	**86.27**	0	69	1,816	**1,885**	**13.73**
City of Naga	**7,545**	20	713	5,946	**6,679**	**88.52**	3	73	790	**866**	**11.48**
**Region 6**	**96,790**	**156**	**9,540**	**76,056**	**85,752**	**88.60**	**7**	**504**	**10,527**	**11,038**	**11.40**
Aklan	**6,213**	6	503	4,995	**5,504**	**88.59**	0	25	684	**709**	**11.41**
Antique	**8,282**	8	790	6,622	**7,420**	**89.59**	0	36	826	**862**	**10.41**
Capiz	**10,750**	10	824	7,934	**8,768**	**81.56**	1	69	1,912	**1,982**	**18.44**
Guimaras	**2,370**	4	215	1,802	**2,021**	**85.27**	0	21	328	**349**	**14.73**
Iloilo	**22,667**	38	2,083	18,663	**20,784**	**91.69**	1	86	1,796	**1,883**	**8.31**
Negros Occidental	**34,588**	71	4,178	26,821	**31,070**	**89.83**	3	204	3,311	**3,518**	**10.17**
City of Bacolod	**6,150**	4	487	4,786	**5,277**	**85.80**	0	7	866	**873**	**14.20**
City of Iloilo	**5,770**	15	460	4,433	**4,908**	**85.06**	2	56	804	**862**	**14.94**
**Region 7**	**112,305**	**178**	**10,652**	**89,360**	**100,190**	**89.21**	**10**	**544**	**11,561**	**12,115**	**10.79**
Bohol	**18,420**	26	1,527	14,274	**15,827**	**85.92**	3	87	2,503	**2,593**	**14.08**
Cebu	**40,979**	52	4,257	33,146	**37,455**	**91.40**	4	249	3,271	**3,524**	**8.60**
Negros Oriental	**19,030**	45	2,125	15,736	**17,906**	**94.09**	0	41	1,083	**1,124**	**5.91**
Siquijor	**1,014**	1	69	789	**859**	**84.71**	0	14	141	**155**	**15.29**
City of Cebu	**15,467**	21	1,428	12,428	**13,877**	**89.72**	1	79	1,510	**1,590**	**10.28**
City of Lapu-Lapu	**7,557**	15	529	6,087	**6,631**	**87.75**	0	36	890	**926**	**12.25**
City of Mandaue	**9,838**	18	717	6,900	**7,635**	**77.61**	2	38	2,163	**2,203**	**22.39**

Table 2.B.2.2 - Intrapartum Care and Delivery Outcome
Delivery by type
Philippines, 2023

Area	Total number of Deliveries	Delivery by Type									
		Vaginal					Cesarean Section				
		Age Group in Year			Total	%	Age Group in Year			Total	%
		10-14	15-19	20-49			10-14	15-19	20-49		
**Region 8**	**59,834**	**95**	**5,838**	**47,257**	**53,190**	**88.90**	**7**	**520**	**6,117**	**6,644**	**11.10**
Biliran	**3,084**	5	321	2,546	**2,872**	**93.13**	0	8	204	**212**	**6.87**
Eastern Samar	**7,014**	7	804	5,104	**5,915**	**84.33**	2	97	1,000	**1,099**	**15.67**
Leyte	**12,068**	24	1,116	10,241	**11,381**	**94.31**	1	84	602	**687**	**5.69**
Northern Samar	**10,442**	9	1,192	8,144	**9,345**	**89.49**	3	77	1,017	**1,097**	**10.51**
Southern Leyte	**5,094**	13	410	3,738	**4,161**	**81.68**	0	38	895	**933**	**18.32**
Samar	**10,338**	18	1,158	8,191	**9,367**	**90.61**	0	63	908	**971**	**9.39**
Ormoc City	**5,965**	13	535	4,312	**4,860**	**81.48**	1	134	970	**1,105**	**18.52**
City of Tacloban	**5,829**	6	302	4,981	**5,289**	**90.74**	0	19	521	**540**	**9.26**
**Region 9**	**47,886**	**90**	**5,840**	**40,251**	**46,181**	**96.44**	**3**	**139**	**1,563**	**1,705**	**3.56**
Zamboanga del Norte	**13,451**	37	1,832	11,296	**13,165**	**97.87**	2	11	273	**286**	**2.13**
Zamboanga del Sur	**8,271**	13	994	7,085	**8,092**	**97.84**	1	13	165	**179**	**2.16**
Zamboanga Sibugay	**7,406**	9	920	6,213	**7,142**	**96.44**	0	13	251	**264**	**3.56**
City of Isabela	**1,797**	2	202	1,498	**1,702**	**94.71**	0	7	88	**95**	**5.29**
City of Zamboanga	**16,961**	29	1,892	14,159	**16,080**	**94.81**	0	95	786	**881**	**5.19**
**Region 10**	**71,891**	**197**	**8,387**	**58,144**	**66,728**	**92.82**	**15**	**409**	**4,739**	**5,163**	**7.18**
Bukidnon	**23,309**	92	3,709	17,425	**21,226**	**91.06**	10	227	1,846	**2,083**	**8.94**
Camiguin	**1,355**	1	151	1,029	**1,181**	**87.16**	0	9	165	**174**	**12.84**
Lanao del Norte	**6,720**	4	494	6,099	**6,597**	**98.17**	0	13	110	**123**	**1.83**
Misamis Occidental	**6,284**	25	665	4,905	**5,595**	**89.04**	0	44	645	**689**	**10.96**
Misamis Oriental	**9,358**	28	1,101	7,837	**8,966**	**95.81**	1	24	367	**392**	**4.19**
City of Cagayan De Oro	**17,552**	41	1,549	14,941	**16,531**	**94.18**	2	61	958	**1,021**	**5.82**
City of Iligan	**7,313**	6	718	5,908	**6,632**	**90.69**	2	31	648	**681**	**9.31**
**Region 11**	**81,389**	**456**	**9,902**	**58,064**	**68,422**	**84.07**	**70**	**1,137**	**11,760**	**12,967**	**15.93**
Davao de Oro	**11,522**	188	1,597	7,910	**9,695**	**84.14**	30	252	1,545	**1,827**	**15.86**
Davao del Norte	**17,062**	70	2,263	11,968	**14,301**	**83.82**	12	228	2,521	**2,761**	**16.18**
Davao Oriental	**8,590**	54	1,230	6,467	**7,751**	**90.23**	5	68	766	**839**	**9.77**
Davao del Sur	**9,438**	55	1,359	6,756	**8,170**	**86.56**	5	103	1,160	**1,268**	**13.44**

Table 2.B.2.2 - Intrapartum Care and Delivery Outcome
Delivery by type
Philippines, 2023

Area	Total number of Deliveries	Delivery by Type									
		Vaginal					Cesarean Section				
		Age Group in Year			Total	%	Age Group in Year			Total	%
		10-14	15-19	20-49			10-14	15-19	20-49		
Davao Occidental	**4,886**	32	1,008	3,553	**4,593**	**94.00**	2	27	264	**293**	**6.00**
City of Davao	**29,891**	57	2,445	21,410	**23,912**	**80.00**	16	459	5,504	**5,979**	**20.00**
**Region 12**	**64,112**	**200**	**8,399**	**48,574**	**57,173**	**89.18**	**19**	**477**	**6,443**	**6,939**	**10.82**
Cotabato	**17,294**	48	2,175	13,046	**15,269**	**88.29**	5	147	1,873	**2,025**	**11.71**
Sarangani	**9,947**	41	1,750	7,535	**9,326**	**93.76**	2	79	540	**621**	**6.24**
South Cotabato	**16,339**	44	2,208	12,069	**14,321**	**87.65**	6	109	1,903	**2,018**	**12.35**
Sultan Kudarat	**12,614**	62	1,595	9,442	**11,099**	**87.99**	5	104	1,406	**1,515**	**12.01**
City of General Santos	**7,918**	5	671	6,482	**7,158**	**90.40**	1	38	721	**760**	**9.60**
**BARMM**	**65,140**	**39**	**5,014**	**57,902**	**62,955**	**96.65**	**5**	**290**	**1,890**	**2,185**	**3.35**
Basilan	**4,718**	10	691	3,977	**4,678**	**99.15**	0	7	33	**40**	**0.85**
Lanao del Sur	**17,820**	7	682	16,649	**17,338**	**97.30**	0	167	315	**482**	**2.70**
Maguindanao	**19,942**	6	1,744	17,639	**19,389**	**97.23**	1	51	501	**553**	**2.77**
Sulu	**11,670**	8	960	10,289	**11,257**	**96.46**	4	22	387	**413**	**3.54**
Tawi-Tawi	**6,010**	1	500	5,462	**5,963**	**99.22**	0	4	43	**47**	**0.78**
SGU	**1,432**	0	120	1,312	**1,432**	**100.00**	0	0	0	**0**	**0.00**
City of Cotabato	**3,548**	7	317	2,574	**2,898**	**81.68**	0	39	611	**650**	**18.32**
**Caraga**	**41,335**	**97**	**4,749**	**32,539**	**37,385**	**90.44**	**3**	**247**	**3,700**	**3,950**	**9.56**
Agusan del Norte	**5,881**	22	786	4,107	**4,915**	**83.57**	2	93	871	**966**	**16.43**
Agusan del Sur	**10,972**	25	1,340	8,589	**9,954**	**90.72**	1	60	957	**1,018**	**9.28**
Surigao del Norte	**7,756**	12	784	6,793	**7,589**	**97.85**	0	16	151	**167**	**2.15**
Surigao del Sur	**9,826**	23	1,150	7,819	**8,992**	**91.51**	0	44	790	**834**	**8.49**
Dinagat Islands	**1,034**	4	117	913	**1,034**	**100.00**	0	0	0	**0**	**0.00**
City of Butuan	**5,866**	11	572	4,318	**4,901**	**83.55**	0	34	931	**965**	**16.45**

Table 2.B.2.3 - Intrapartum Care and Delivery Outcome

Pregnancy outcome (Full term and Preterm)

Philippines, 2023

Area	Total number of Deliveries	Full term (37-42 weeks AOG)					Pre-term (22-36 weeks AOG)				
		Age Group in Year			Total	%	Age Group in Year			Total	%
		10-14	15-19	20-49			10-14	15-19	20-49		
**Philippines**	**1,420,182**	**2,838**	**133,835**	**1,247,044**	**1,383,717**	**97.43**	**238**	**3,452**	**18,321**	**22,011**	**1.55**
**N C R**	**184,090**	**245**	**11,867**	**169,824**	**181,936**	**98.83**	**9**	**113**	**904**	**1,026**	**0.56**
Malabon City	1,782	6	162	1,605	**1,773**	**99.49**	0	1	6	**7**	**0.39**
Navotas City	4,102	6	315	3,723	**4,044**	**98.59**	0	0	59	**59**	**1.44**
Valenzuela City	5,585	11	391	5,061	**5,463**	**97.82**	0	16	72	**88**	**1.58**
Caloocan City	22,186	24	1,703	20,397	**22,124**	**99.72**	1	4	42	**47**	**0.21**
Marikina City	2,526	7	208	2,288	**2,503**	**99.09**	0	2	13	**15**	**0.59**
Pasig City	11,370	32	750	10,354	**11,136**	**97.94**	1	15	112	**128**	**1.13**
Pateros	791	1	31	747	**779**	**98.48**	0	0	8	**8**	**1.01**
Taguig City	12,357	16	1,078	11,173	**12,267**	**99.27**	0	6	69	**75**	**0.61**
Quezon City	57,484	47	2,675	54,534	**57,256**	**99.60**	3	15	118	**136**	**0.24**
Makati City	3,534	7	244	3,182	**3,433**	**97.14**	0	5	89	**94**	**2.66**
Mandaluyong City	7,639	9	294	7,322	**7,625**	**99.82**	0	5	25	**30**	**0.39**
San Juan City	745	0	46	683	**729**	**97.85**	0	4	11	**15**	**2.01**
Manila City	23,640	18	2,133	21,366	**23,517**	**99.48**	1	19	133	**153**	**0.65**
Las Piñas City	6,851	13	511	6,272	**6,796**	**99.20**	1	4	26	**31**	**0.45**
Muntinlupa City	8,297	16	497	6,885	**7,398**	**89.16**	0	2	25	**27**	**0.33**
Parañaque City	8,296	25	573	7,669	**8,267**	**99.65**	0	6	23	**29**	**0.35**
Pasay City	6,905	7	256	6,563	**6,826**	**98.86**	2	9	73	**84**	**1.22**
**C A R**	**24,874**	**38**	**1,885**	**21,772**	**23,695**	**95.26**	**2**	**139**	**811**	**952**	**3.83**
Abra	2,730	5	287	2,223	**2,515**	**92.12**	0	48	139	**187**	**6.85**
Apayao	2,733	9	348	2,287	**2,644**	**96.74**	1	10	51	**62**	**2.27**
Benguet	4,082	6	208	3,715	**3,929**	**96.25**	0	20	115	**135**	**3.31**
Ifugao	2,414	3	230	2,131	**2,364**	**97.93**	0	10	21	**31**	**1.28**
Kalinga	2,387	2	143	2,190	**2,335**	**97.82**	0	4	30	**34**	**1.42**
Mountain Province	3,390	6	289	2,967	**3,262**	**96.22**	1	13	73	**87**	**2.57**
City of Baguio	7,138	7	380	6,259	**6,646**	**93.11**	0	34	382	**416**	**5.83**
**Region 1**	**50,550**	**75**	**3,528**	**44,414**	**48,017**	**94.99**	**3**	**153**	**2,377**	**2,533**	**5.01**
Ilocos Norte	6,049	6	294	5,313	**5,613**	**92.79**	1	40	395	**436**	**7.21**
Ilocos Sur	7,661	9	380	7,190	**7,579**	**98.93**	0	15	67	**82**	**1.07**

Table 2.B.2.3 - Intrapartum Care and Delivery Outcome

Pregnancy outcome (Full term and Preterm)

Philippines, 2023

Area	Total number of Deliveries	Full term (37-42 weeks AOG)					Pre-term (22-36 weeks AOG)				
		Age Group in Year			Total	%	Age Group in Year			Total	%
		10-14	15-19	20-49			10-14	15-19	20-49		
La Union	10,615	27	808	9,110	**9,945**	**93.69**	1	34	635	**670**	**6.31**
Pangasinan	17,409	15	1,354	15,939	**17,308**	**99.42**	0	24	77	**101**	**0.58**
City of Dagupan	8,816	18	692	6,862	**7,572**	**85.89**	1	40	1,203	**1,244**	**14.11**
**Region 2**	**36,823**	**75**	**3,674**	**31,951**	**35,700**	**96.95**	**7**	**156**	**705**	**868**	**2.36**
Batanes	216	3	16	191	**210**	**97.22**	0	0	5	**5**	**2.31**
Cagayan	8,424	16	829	7,240	**8,085**	**95.98**	3	55	241	**299**	**3.55**
Isabela	15,263	18	1,410	13,457	**14,885**	**97.52**	1	67	221	**289**	**1.89**
Nueva Vizcaya	7,319	25	823	6,193	**7,041**	**96.20**	1	19	185	**205**	**2.80**
Quirino	2,776	10	337	2,358	**2,705**	**97.44**	2	11	37	**50**	**1.80**
City of Santiago	2,825	3	259	2,512	**2,774**	**98.19**	0	4	16	**20**	**0.71**
**Region 3**	**148,973**	**320**	**11,652**	**126,623**	**138,595**	**93.03**	**32**	**473**	**2,679**	**3,184**	**2.14**
Aurora	3,200	10	337	2,717	**3,064**	**95.75**	1	18	77	**96**	**3.00**
Bataan	11,009	42	1,135	9,309	**10,486**	**95.25**	6	61	369	**436**	**3.96**
Bulacan	40,824	50	2,584	36,901	**39,535**	**96.84**	14	162	759	**935**	**2.29**
Nueva Ecija	28,779	71	2,385	25,535	**27,991**	**97.26**	0	24	667	**691**	**2.40**
Pampanga	25,991	46	2,007	17,383	**19,436**	**74.78**	4	31	92	**127**	**0.49**
Tarlac	20,870	51	1,651	18,659	**20,361**	**97.56**	2	58	330	**390**	**1.87**
Zambales	8,677	29	957	7,376	**8,362**	**96.37**	4	69	200	**273**	**3.15**
City of Angeles	5,487	10	271	4,945	**5,226**	**95.24**	1	50	185	**236**	**4.30**
City of Olongapo	4,136	11	325	3,798	**4,134**	**99.95**	0	0	0	**0**	**0.00**
**Region 4A**	**202,438**	**343**	**17,451**	**182,001**	**199,795**	**98.69**	**10**	**252**	**1,818**	**2,080**	**1.03**
Batangas	38,479	55	2,992	34,879	**37,926**	**98.56**	1	52	306	**359**	**0.93**
Cavite	48,372	48	3,268	44,574	**47,890**	**99.00**	3	47	384	**434**	**0.90**
Laguna	45,570	81	3,931	40,968	**44,980**	**98.71**	0	54	414	**468**	**1.03**
Quezon	24,586	75	2,854	21,222	**24,151**	**98.23**	3	39	241	**283**	**1.15**
Rizal	42,344	78	4,039	37,704	**41,821**	**98.76**	1	53	434	**488**	**1.15**
City of Lucena	3,087	6	367	2,654	**3,027**	**98.06**	2	7	39	**48**	**1.55**
**Region 4B**	**47,523**	**106**	**5,169**	**41,199**	**46,474**	**97.79**	**4**	**131**	**431**	**566**	**1.19**
Marinduque	2,791	4	276	2,470	**2,750**	**98.53**	1	3	13	**17**	**0.61**

Table 2.B.2.3 - Intrapartum Care and Delivery Outcome

Pregnancy outcome (Full term and Preterm)

Philippines, 2023

Area	Total number of Deliveries	Full term (37-42 weeks AOG)					Pre-term (22-36 weeks AOG)				
		Age Group in Year			Total	%	Age Group in Year			Total	%
		10-14	15-19	20-49			10-14	15-19	20-49		
Mindoro Occidental	8,490	16	1,042	7,220	**8,278**	**97.50**	0	25	74	**99**	**1.17**
Mindoro Oriental	14,579	21	1,019	13,251	**14,291**	**98.02**	0	33	134	**167**	**1.15**
Palawan	13,176	51	2,005	10,822	**12,878**	**97.74**	1	50	108	**159**	**1.21**
Romblon	4,369	4	394	3,850	**4,248**	**97.23**	2	14	77	**93**	**2.13**
Puerto Princesa City	4,118	10	433	3,586	**4,029**	**97.84**	0	6	25	**31**	**0.75**
**Region 5**	**84,229**	**91**	**7,857**	**73,672**	**81,620**	**96.90**	**7**	**394**	**1,510**	**1,911**	**2.27**
Albay	16,099	17	1,058	14,834	**15,909**	**98.82**	1	34	110	**145**	**0.90**
Camarines Norte	10,605	7	1,134	8,330	**9,471**	**89.31**	2	244	783	**1,029**	**9.70**
Camarines Sur	17,655	10	1,401	16,105	**17,516**	**99.21**	0	8	54	**62**	**0.35**
Catanduanes	4,341	11	480	3,781	**4,272**	**98.41**	0	0	10	**10**	**0.23**
Masbate	14,258	21	1,878	12,144	**14,043**	**98.49**	0	24	63	**87**	**0.61**
Sorsogon	13,726	12	1,131	11,859	**13,002**	**94.73**	3	81	477	**561**	**4.09**
City of Naga	7,545	13	775	6,619	**7,407**	**98.17**	1	3	13	**17**	**0.23**
**Region 6**	**96,790**	**155**	**9,699**	**84,478**	**94,332**	**97.46**	**8**	**409**	**1,570**	**1,987**	**2.05**
Aklan	6,213	6	510	5,555	**6,071**	**97.71**	0	15	83	**98**	**1.58**
Antique	8,282	6	781	7,212	**7,999**	**96.58**	2	42	186	**230**	**2.78**
Capiz	10,750	11	866	9,627	**10,504**	**97.71**	0	23	215	**238**	**2.21**
Guimaras	2,370	4	226	2,069	**2,299**	**97.00**	0	10	62	**72**	**3.04**
Iloilo	22,667	37	2,105	20,025	**22,167**	**97.79**	2	153	248	**403**	**1.78**
Negros Occidental	34,588	71	4,254	29,432	**33,757**	**97.60**	3	92	461	**556**	**1.61**
City of Bacolod	6,150	3	464	5,380	**5,847**	**95.07**	1	51	249	**301**	**4.89**
City of Iloilo	5,770	17	493	5,178	**5,688**	**98.58**	0	23	66	**89**	**1.54**
**Region 7**	**112,305**	**176**	**10,699**	**98,270**	**109,145**	**97.19**	**10**	**417**	**2,009**	**2,436**	**2.17**
Bohol	18,420	26	1,537	16,247	**17,810**	**96.69**	3	68	387	**458**	**2.49**
Cebu	40,979	51	4,305	35,569	**39,925**	**97.43**	5	167	635	**807**	**1.97**
Negros Oriental	19,030	44	2,071	16,319	**18,434**	**96.87**	0	80	372	**452**	**2.38**
Siquijor	1,014	1	77	916	**994**	**98.03**	0	6	11	**17**	**1.68**
City of Cebu	15,467	21	1,451	13,695	**15,167**	**98.06**	1	52	214	**267**	**1.73**
City of Lapu-Lapu	7,557	15	561	6,926	**7,502**	**99.27**	0	0	2	**2**	**0.03**
City of Mandaue	9,838	18	697	8,598	**9,313**	**94.66**	1	44	388	**433**	**4.40**

Table 2.B.2.3 - Intrapartum Care and Delivery Outcome

Pregnancy outcome (Full term and Preterm)

Philippines, 2023

Area	Total number of Deliveries	Full term (37-42 weeks AOG)					Pre-term (22-36 weeks AOG)				
		Age Group in Year			Total	%	Age Group in Year			Total	%
		10-14	15-19	20-49			10-14	15-19	20-49		
**Region 8**	**59,834**	**98**	**6,084**	**52,205**	**58,387**	**97.58**	**5**	**193**	**834**	**1,032**	**1.72**
Biliran	3,084	5	320	2,678	**3,003**	**97.37**	0	6	34	**40**	**1.30**
Eastern Samar	7,014	5	875	5,959	**6,839**	**97.50**	3	18	72	**93**	**1.33**
Leyte	12,068	26	1,143	10,777	**11,946**	**98.99**	0	23	60	**83**	**0.69**
Northern Samar	10,442	11	1,179	8,691	**9,881**	**94.63**	1	70	370	**441**	**4.22**
Southern Leyte	5,094	13	441	4,579	**5,033**	**98.80**	0	3	23	**26**	**0.51**
Samar	10,338	16	1,155	8,863	**10,034**	**97.06**	1	64	198	**263**	**2.54**
Ormoc City	5,965	16	652	5,187	**5,855**	**98.16**	0	7	72	**79**	**1.32**
City of Tacloban	5,829	6	319	5,471	**5,796**	**99.43**	0	2	5	**7**	**0.12**
**Region 9**	**47,886**	**91**	**5,898**	**41,422**	**47,411**	**99.01**	**1**	**88**	**171**	**260**	**0.54**
Zamboanga del Norte	13,451	39	1,829	11,436	**13,304**	**98.91**	0	28	40	**68**	**0.51**
Zamboanga del Sur	8,271	12	986	7,184	**8,182**	**98.92**	1	20	33	**54**	**0.65**
Zamboanga Sibugay	7,406	9	914	6,381	**7,304**	**98.62**	0	12	34	**46**	**0.62**
City of Isabela	1,797	2	208	1,579	**1,789**	**99.55**	0	0	2	**2**	**0.11**
City of Zamboanga	16,961	29	1,961	14,842	**16,832**	**99.24**	0	28	62	**90**	**0.53**
**Region 10**	**71,891**	**204**	**8,635**	**62,071**	**70,910**	**98.64**	**5**	**91**	**430**	**526**	**0.73**
Bukidnon	23,309	98	3,867	18,962	**22,927**	**98.36**	3	45	165	**213**	**0.91**
Camiguin	1,355	1	157	1,165	**1,323**	**97.64**	0	0	13	**13**	**0.96**
Lanao del Norte	6,720	4	494	6,180	**6,678**	**99.38**	0	6	34	**40**	**0.60**
Misamis Occidental	6,284	23	683	5,435	**6,141**	**97.72**	1	16	83	**100**	**1.59**
Misamis Oriental	9,358	29	1,116	8,146	**9,291**	**99.28**	0	8	24	**32**	**0.34**
City of Cagayan De Oro	17,552	41	1,570	15,726	**17,337**	**98.78**	1	15	71	**87**	**0.50**
City of Iligan	7,313	8	748	6,457	**7,213**	**98.63**	0	1	40	**41**	**0.56**
**Region 11**	**81,389**	**479**	**10,941**	**68,234**	**79,654**	**97.87**	**123**	**163**	**932**	**1,218**	**1.50**
Davao de Oro	11,522	182	1,731	9,200	**11,113**	**96.45**	113	33	344	**490**	**4.25**
Davao del Norte	17,062	74	2,433	14,130	**16,637**	**97.51**	4	40	227	**271**	**1.59**
Davao Oriental	8,590	57	1,277	7,031	**8,365**	**97.38**	1	10	93	**104**	**1.21**
Davao del Sur	9,438	55	1,432	7,729	**9,216**	**97.65**	4	30	91	**125**	**1.32**
Davao Occidental	4,886	40	1,174	3,467	**4,681**	**95.80**	0	19	39	**58**	**1.19**

Table 2.B.2.3 - Intrapartum Care and Delivery Outcome

Pregnancy outcome (Full term and Preterm)

Philippines, 2023

Area	Total number of Deliveries	Full term (37-42 weeks AOG)					Pre-term (22-36 weeks AOG)				
		Age Group in Year			Total	%	Age Group in Year			Total	%
		10-14	15-19	20-49			10-14	15-19	20-49		
City of Davao	29,891	71	2,894	26,677	**29,642**	**99.17**	1	31	138	**170**	**0.57**
**Region 12**	**64,112**	**208**	**8,715**	**54,080**	**63,003**	**98.27**	**5**	**98**	**539**	**642**	**1.00**
Cotabato	17,294	48	2,278	14,660	**16,986**	**98.22**	2	30	159	**191**	**1.10**
Sarangani	9,947	41	1,804	7,979	**9,824**	**98.76**	2	12	59	**73**	**0.73**
South Cotabato	16,339	48	2,260	13,667	**15,975**	**97.77**	1	37	178	**216**	**1.32**
Sultan Kudarat	12,614	65	1,669	10,608	**12,342**	**97.84**	0	14	116	**130**	**1.03**
City of General Santos	7,918	6	704	7,166	**7,876**	**99.47**	0	5	27	**32**	**0.40**
**BARMM**	**65,140**	**44**	**5,253**	**59,294**	**64,591**	**99.16**	**0**	**57**	**203**	**260**	**0.40**
Basilan	4,718	10	695	3,956	**4,661**	**98.79**	0	7	22	**29**	**0.61**
Lanao del Sur	17,820	7	871	16,893	**17,771**	**99.73**	0	6	24	**30**	**0.17**
Maguindanao	19,942	5	1,767	18,022	**19,794**	**99.26**	0	19	58	**77**	**0.39**
Sulu	11,670	14	955	10,507	**11,476**	**98.34**	0	13	64	**77**	**0.66**
Tawi-Tawi	6,010	1	489	5,456	**5,946**	**98.94**	0	10	19	**29**	**0.48**
SGA	1,432	0	120	1,312	**1,432**	**100.00**	0	0	0	**0**	**0.00**
City of Cotabato	3,548	7	356	3,148	**3,511**	**98.96**	0	2	16	**18**	**0.51**
**Caraga**	**41,335**	**90**	**4,828**	**35,534**	**40,452**	**97.86**	**7**	**125**	**398**	**530**	**1.28**
Agusan del Norte	5,881	15	844	4,898	**5,757**	**97.89**	7	25	34	**66**	**1.12**
Agusan del Sur	10,972	26	1,387	9,441	**10,854**	**98.92**	0	6	30	**36**	**0.33**
Surigao del Norte	7,756	11	793	6,911	**7,715**	**99.47**	0	1	10	**11**	**0.14**
Surigao del Sur	9,826	23	1,094	8,225	**9,342**	**95.07**	0	85	275	**360**	**3.66**
Dinagat Islands	1,034	4	114	907	**1,025**	**99.13**	0	3	1	**4**	**0.39**
City of Butuan	5,866	11	596	5,152	**5,759**	**98.18**	0	5	48	**53**	**0.90**

Table 2.B.2.3 - Intrapartum Care and Delivery Outcome

Pregnancy outcome (Fetal Deaths and Abortion)

Philippines, 2023

Area	Total number of Deliveries	Fetal Death					Abortion (Counts Only)			
		Age Group			Total	%	Age Group			Total
		10-14	15-19	20-49			10-14	15-19	20-49	
**Philippines**	**1,420,182**	**32**	**813**	**6,847**	**7,692**	**0.54**	**63**	**857**	**8,056**	**8,976**
**N C R**	**184,090**	**2**	**26**	**332**	**360**	**0.20**	**1**	**31**	**467**	**499**
Malabon City	**1,782**	0	0	2	**2**	**0.11**	0	7	37	**44**
Navotas City	**4,102**	0	0	1	**1**	**0.02**	0	0	2	**2**
Valenzuela City	**5,585**	0	4	21	**25**	**0.45**	0	4	35	**39**
Caloocan City	**22,186**	0	1	11	**12**	**0.05**	1	1	19	**21**
Marikina City	**2,526**	0	0	8	**8**	**0.32**	0	0	8	**8**
Pasig City	**11,370**	1	5	100	**106**	**0.93**	0	0	8	**8**
Pateros	**791**	0	0	6	**6**	**0.76**	0	0	8	**8**
Taguig City	**12,357**	0	1	14	**15**	**0.12**	0	5	52	**57**
Quezon City	**57,484**	1	8	83	**92**	**0.16**	0	7	92	**99**
Makati City	**3,534**	0	1	25	**26**	**0.74**	0	2	53	**55**
Mandaluyong City	**7,639**	0	0	11	**11**	**0.14**	0	1	4	**5**
San Juan City	**745**	0	0	1	**1**	**0.13**	0	1	4	**5**
Manila City	**23,640**	0	2	29	**31**	**0.13**	0	2	106	**108**
Las Piñas City	**6,851**	0	4	20	**24**	**0.35**	0	0	7	**7**
Muntinlupa City	**8,297**	0	0	0	**0**	**0.00**	0	1	15	**16**
Parañaque City	**8,296**	0	0	0	**0**	**0.00**	0	0	7	**7**
Pasay City	**6,905**	0	0	0	**0**	**0.00**	0	0	10	**10**
**C A R**	**24,874**	**0**	**11**	**216**	**227**	**0.91**	**1**	**46**	**1,018**	**1,065**
Abra	**2,730**	0	4	24	**28**	**1.03**	0	7	108	**115**
Apayao	**2,733**	0	2	25	**27**	**0.99**	1	16	107	**124**
Benguet	**4,082**	0	0	18	**18**	**0.44**	0	7	188	**195**
Ifugao	**2,414**	0	0	19	**19**	**0.79**	0	2	54	**56**
Kalinga	**2,387**	0	0	18	**18**	**0.75**	0	0	26	**26**
Mountain Province	**3,390**	0	4	37	**41**	**1.21**	0	10	186	**196**
City of Baguio	**7,138**	0	1	75	**76**	**1.06**	0	4	349	**353**
**Region 1**	**50,550**	**1**	**14**	**225**	**240**	**0.47**	**0**	**5**	**93**	**98**
Ilocos Norte	**6,049**	0	1	4	**5**	**0.08**	0	1	5	**6**
Ilocos Sur	**7,661**	0	2	10	**12**	**0.16**	0	1	13	**14**

Table 2.B.2.3 - Intrapartum Care and Delivery Outcome

Pregnancy outcome (Fetal Deaths and Abortion)

Philippines, 2023

Area	Total number of Deliveries	Fetal Death					Abortion (Counts Only)			
		Age Group			Total	%	Age Group			Total
		10-14	15-19	20-49			10-14	15-19	20-49	
La Union	**10,615**	0	4	60	**64**	**0.60**	0	3	37	**40**
Pangasinan	**17,409**	0	3	47	**50**	**0.29**	0	0	38	**38**
City of Dagupan	**8,816**	1	4	104	**109**	**1.24**	0	0	0	**0**
**Region 2**	**36,823**	**2**	**15**	**209**	**226**	**0.61**	**4**	**60**	**720**	**784**
Batanes	**216**	0	0	3	**3**	**1.39**	0	0	6	**6**
Cagayan	**8,424**	1	1	38	**40**	**0.47**	0	15	145	**160**
Isabela	**15,263**	1	6	82	**89**	**0.58**	1	8	140	**149**
Nueva Vizcaya	**7,319**	0	6	67	**73**	**1.00**	3	23	342	**368**
Quirino	**2,776**	0	2	19	**21**	**0.76**	0	14	87	**101**
City of Santiago	**2,825**	0	0	0	**0**	**0.00**	0	0	0	**0**
**Region 3**	**148,973**	**5**	**56**	**722**	**783**	**0.53**	**5**	**102**	**1,081**	**1,188**
Aurora	**3,200**	0	4	36	**40**	**1.25**	0	3	2	**5**
Bataan	**11,009**	2	11	74	**87**	**0.79**	1	30	562	**593**
Bulacan	**40,824**	2	20	232	**254**	**0.62**	2	37	216	**255**
Nueva Ecija	**28,779**	1	7	132	**140**	**0.49**	0	2	40	**42**
Pampanga	**25,991**	0	5	62	**67**	**0.26**	2	19	164	**185**
Tarlac	**20,870**	0	6	113	**119**	**0.57**	0	2	3	**5**
Zambales	**8,677**	0	1	48	**49**	**0.56**	0	9	93	**102**
City of Angeles	**5,487**	0	2	23	**25**	**0.46**	0	0	0	**0**
City of Olongapo	**4,136**	0	0	2	**2**	**0.05**	0	0	1	**1**
**Region 4A**	**202,438**	**2**	**44**	**517**	**563**	**0.28**	**4**	**64**	**623**	**691**
Batangas	**38,479**	0	11	183	**194**	**0.50**	3	18	153	**174**
Cavite	**48,372**	0	3	45	**48**	**0.10**	0	5	94	**99**
Laguna	**45,570**	0	10	112	**122**	**0.27**	0	16	152	**168**
Quezon	**24,586**	2	12	138	**152**	**0.62**	1	21	164	**186**
Rizal	**42,344**	0	8	27	**35**	**0.08**	0	4	55	**59**
						**0.00**				
City of Lucena	**3,087**	0	0	12	**12**	**0.39**	0	0	5	**5**
**Region 4B**	**47,523**	**1**	**68**	**360**	**429**	**0.90**	**3**	**32**	**269**	**304**
Marinduque	**2,791**	0	4	20	**24**	**0.86**	0	0	18	**18**

Table 2.B.2.3 - Intrapartum Care and Delivery Outcome

Pregnancy outcome (Fetal Deaths and Abortion)

Philippines, 2023

Area	Total number of Deliveries	Fetal Death					Abortion (Counts Only)			
		Age Group			Total	%	Age Group			Total
		10-14	15-19	20-49			10-14	15-19	20-49	
Occidental Mindoro	**8,490**	0	16	84	**100**	**1.18**	0	4	32	**36**
Oriental Mindoro	**14,579**	0	13	80	**93**	**0.64**	1	3	41	**45**
Palawan	**13,176**	0	26	100	**126**	**0.96**	1	20	137	**158**
Romblon	**4,369**	0	5	23	**28**	**0.64**	0	2	25	**27**
City of Puerto Princesa	**4,118**	1	4	53	**58**	**1.41**	1	3	16	**20**
**Region 5**	**84,229**	**0**	**82**	**610**	**692**	**0.82**	**9**	**101**	**1,048**	**1,158**
Albay	**16,099**	0	5	40	**45**	**0.28**	0	2	37	**39**
Camarines Norte	**10,605**	0	13	92	**105**	**0.99**	0	0	43	**43**
Camarines Sur	**17,655**	0	7	70	**77**	**0.44**	0	14	89	**103**
Catanduanes	**4,341**	0	5	54	**59**	**1.36**	1	4	30	**35**
Masbate	**14,258**	0	16	112	**128**	**0.90**	2	16	109	**127**
Sorsogon	**13,726**	0	27	139	**166**	**1.21**	6	65	726	**797**
City of Naga	**7,545**	0	9	103	**112**	**1.48**	0	0	14	**14**
**Region 6**	**96,790**	**0**	**66**	**538**	**604**	**0.62**	**3**	**70**	**683**	**756**
Aklan	**6,213**	0	5	25	**30**	**0.48**	1	7	72	**80**
Antique	**8,282**	0	7	94	**101**	**1.22**	0	6	78	**84**
Capiz	**10,750**	0	6	63	**69**	**0.64**	2	5	53	**60**
Guimaras	**2,370**	0	2	16	**18**	**0.76**	0	2	57	**59**
Iloilo	**22,667**	0	8	54	**62**	**0.27**	0	11	101	**112**
Negros Occidental	**34,588**	0	34	242	**276**	**0.80**	0	37	284	**321**
City of Bacolod	**6,150**	0	2	29	**31**	**0.50**	0	0	15	**15**
City of Iloilo	**5,770**	0	2	15	**17**	**0.29**	0	2	23	**25**
**Region 7**	**112,305**	**2**	**80**	**642**	**724**	**0.64**	**0**	**0**	**0**	**0**
Bohol	**18,420**	0	9	143	**152**	**0.83**	0	0	0	**0**
Cebu	**40,979**	0	34	213	**247**	**0.60**	0	0	0	**0**
Negros Oriental	**19,030**	1	15	128	**144**	**0.76**	0	0	0	**0**
Siquijor	**1,014**	0	0	3	**3**	**0.30**	0	0	0	**0**
City of Cebu	**15,467**	0	4	29	**33**	**0.21**	0	0	0	**0**
City of Lapu-Lapu	**7,557**	0	4	49	**53**	**0.70**	0	0	0	**0**
City of Mandaue	**9,838**	1	14	77	**92**	**0.94**	0	0	0	**0**

Table 2.B.2.3 - Intrapartum Care and Delivery Outcome

Pregnancy outcome (Fetal Deaths and Abortion)

Philippines, 2023

Area	Total number of Deliveries	Fetal Death					Abortion (Counts Only)			
		Age Group			Total	%	Age Group			Total
		10-14	15-19	20-49			10-14	15-19	20-49	
**Region 8**	**59,834**	**1**	**57**	**357**	**415**	**0.69**	**2**	**42**	**328**	**372**
Biliran	**3,084**	0	4	37	**41**	**1.33**	0	2	43	**45**
Eastern Samar	**7,014**	1	10	71	**82**	**1.17**	2	14	58	**74**
Leyte	**12,068**	0	3	36	**39**	**0.32**	0	16	132	**148**
Northern Samar	**10,442**	0	15	105	**120**	**1.15**	0	2	48	**50**
Southern Leyte	**5,094**	0	4	31	**35**	**0.69**	0	0	6	**6**
Samar	**10,338**	0	9	32	**41**	**0.40**	0	7	37	**44**
Ormoc City	**5,965**	0	12	19	**31**	**0.52**	0	0	0	**0**
City of Tacloban	**5,829**	0	0	26	**26**	**0.45**	0	1	4	**5**
**Region 9**	**47,886**	**1**	**29**	**182**	**212**	**0.44**	**0**	**0**	**0**	**0**
Zamboanga del Norte	**13,451**	0	8	71	**79**	**0.59**	0	0	0	**0**
Zamboanga del Sur	**8,271**	1	9	25	**35**	**0.42**	0	0	0	**0**
Zamboanga Sibugay	**7,406**	0	9	44	**53**	**0.72**	0	0	0	**0**
City of Isabela	**1,797**	0	1	5	**6**	**0.33**	0	0	0	**0**
City of Zamboanga	**16,961**	0	2	37	**39**	**0.23**	0	0	0	**0**
**Region 10**	**71,891**	**1**	**41**	**413**	**455**	**0.63**	**10**	**60**	**380**	**450**
Bukidnon	**23,309**	1	24	144	**169**	**0.73**	8	40	169	**217**
Camiguin	**1,355**	0	2	17	**19**	**1.40**	0	0	1	**1**
Lanao del Norte	**6,720**	0	0	2	**2**	**0.03**	0	0	1	**1**
Misamis Occidental	**6,284**	0	1	42	**43**	**0.68**	0	4	76	**80**
Misamis Oriental	**9,358**	0	2	33	**35**	**0.37**	1	4	13	**18**
City of Cagayan De Oro	**17,552**	0	8	120	**128**	**0.73**	1	11	100	**112**
City of Iligan	**7,313**	0	4	55	**59**	**0.81**	0	1	20	**21**
**Region 11**	**81,389**	**5**	**89**	**559**	**653**	**0.80**	**18**	**168**	**849**	**1,035**
Davao de Oro	**11,522**	3	13	85	**101**	**0.88**	5	29	175	**209**
Davao del Norte	**17,062**	1	17	136	**154**	**0.90**	0	55	234	**289**
Davao Oriental	**8,590**	1	11	109	**121**	**1.41**	4	16	74	**94**
Davao del Sur	**9,438**	0	13	84	**97**	**1.03**	4	26	129	**159**
Davao Occidental	**4,886**	0	26	75	**101**	**2.07**	4	17	74	**95**
City of Davao	**29,891**	0	9	70	**79**	**0.26**	1	25	163	**189**

Table 2.B.2.3 - Intrapartum Care and Delivery Outcome

Pregnancy outcome (Fetal Deaths and Abortion)

Philippines, 2023

Area	Total number of Deliveries	Fetal Death					Abortion (Counts Only)			
		Age Group			Total	%	Age Group			Total
		10-14	15-19	20-49			10-14	15-19	20-49	
**Region 12**	**64,112**	**4**	**63**	**400**	**467**	**0.73**	**2**	**50**	**321**	**373**
Cotabato	**17,294**	3	14	100	**117**	**0.68**	1	24	118	**143**
Sarangani	**9,947**	0	13	37	**50**	**0.50**	0	8	45	**53**
South Cotabato	**16,339**	1	20	127	**148**	**0.91**	1	14	98	**113**
Sultan Kudarat	**12,614**	0	16	126	**142**	**1.13**	0	4	60	**64**
City of General Santos	**7,918**	0	0	10	**10**	**0.13**	0	0	0	**0**
**BARMM**	**65,140**	**2**	**29**	**258**	**289**	**0.44**	**1**	**26**	**148**	**175**
Basilan	**4,718**	0	4	24	**28**	**0.59**	0	10	34	**44**
Lanao del Sur	**17,820**	0	3	16	**19**	**0.11**	0	3	18	**21**
Maguindanao	**19,942**	2	9	60	**71**	**0.36**	1	3	27	**31**
Sulu	**11,670**	0	8	109	**117**	**1.00**	0	5	35	**40**
Tawi-Tawi	**6,010**	0	4	31	**35**	**0.58**	0	4	25	**29**
SGA	**1,432**	0	0	0	**0**	**0.00**	0	0	0	**0**
City of Cotabato	**3,548**	0	1	18	**19**	**0.54**	0	1	9	**10**
**Caraga**	**41,335**	**3**	**43**	**307**	**353**	**0.85**	**0**	**0**	**28**	**28**
Agusan del Norte	**5,881**	2	10	46	**58**	**0.99**	0	0	0	**0**
Agusan del Sur	**10,972**	0	7	75	**82**	**0.75**	0	0	6	**6**
Surigao del Norte	**7,756**	1	6	23	**30**	**0.39**	0	0	3	**3**
Surigao del Sur	**9,826**	0	15	109	**124**	**1.26**	0	0	16	**16**
Dinagat Islands	**1,034**	0	0	5	**5**	**0.48**	0	0	2	**2**
City of Butuan	**5,866**	0	5	49	**54**	**0.92**	0	0	1	**1**

Table 2.B.2.4 - Intrapartum Care and Delivery Outcome
Live Births by birth weight
Philippines, 2023

Area	Total No. of Live Births	NBW (≥ 2500 grams)		LBW (< 2500)		UBW	
		No.	%	No.	%	No.	%
**Philippines**	**1,426,704**	**1,310,387**	**91.85**	**85,337**	**5.98**	**30,980**	**2.17**
**N C R**	**183,183**	**174,652**	**95.34**	**7,441**	**4.06**	**1,090**	**0.60**
Malabon City	**1,781**	1,742	**97.81**	28	**1.57**	11	**0.62**
Navotas City	**4,103**	3,882	**94.61**	143	**3.49**	78	**1.90**
Valenzuela City	**5,569**	5,098	**91.54**	417	**7.49**	54	**0.97**
Caloocan City	**22,175**	21,776	**98.20**	340	**1.53**	59	**0.27**
Marikina City	**2,518**	2,395	**95.12**	123	**4.88**	0	**0.00**
Pasig City	**11,340**	9,417	**83.04**	1,917	**16.90**	6	**0.05**
Pateros	**787**	768	**97.59**	19	**2.41**	0	**0.00**
Taguig City	**12,346**	11,933	**96.65**	242	**1.96**	171	**1.39**
Quezon City	**57,393**	55,669	**97.00**	1,664	**2.90**	60	**0.10**
Makati City	**3,527**	3,266	**92.60**	259	**7.34**	2	**0.06**
Mandaluyong City	**7,655**	7,517	**98.20**	138	**1.80**	0	**0.00**
San Juan City	**751**	707	**94.14**	44	**5.86**	0	**0.00**
Manila City	**23,674**	22,656	**95.70**	967	**4.08**	51	**0.22**
Las Piñas City	**6,827**	6,486	**95.01**	200	**2.93**	141	**2.07**
Muntinlupa City	**7,504**	7,263	**96.79**	170	**2.27**	71	**0.95**
Parañaque City	**8,323**	7,504	**90.16**	441	**5.30**	378	**4.54**
Pasay City	**6,910**	6,573	**95.12**	329	**4.76**	8	**0.12**
**C A R**	**24,815**	**22,373**	**90.16**	**2,394**	**9.65**	**48**	**0.19**
Abra	**2,716**	2,411	**88.77**	302	**11.12**	3	**0.11**
Apayao	**2,782**	2,539	**91.27**	242	**8.70**	1	**0.04**
Benguet	**4,086**	3,720	**91.04**	343	**8.39**	23	**0.56**
Ifugao	**2,401**	2,319	**96.58**	78	**3.25**	4	**0.17**
Kalinga	**2,376**	2,284	**96.13**	92	**3.87**	0	**0.00**
Mountain Province	**3,361**	3,082	**91.70**	278	**8.27**	1	**0.03**
City of Baguio	**7,093**	6,018	**84.84**	1,059	**14.93**	16	**0.23**
**Region 1**	**50,514**	**46,473**	**92.00**	**4,038**	**7.99**	**3**	**0.01**
Ilocos Norte	**6,048**	5,441	**89.96**	606	**10.02**	1	**0.02**
Ilocos Sur	**7,661**	7,274	**94.95**	387	**5.05**	0	**0.00**
La Union	**10,613**	9,467	**89.20**	1,146	**10.80**	0	**0.00**
Pangasinan	**17,403**	17,019	**97.79**	384	**2.21**	0	**0.00**
City of Dagupan	**8,789**	7,272	**82.74**	1,515	**17.24**	2	**0.02**

Table 2.B.2.4 - Intrapartum Care and Delivery Outcome
Live Births by birth weight
Philippines, 2023

Area	Total No. of Live Births	NBW (≥ 2500 grams)		LBW (< 2500)		UBW	
		No.	%	No.	%	No.	%
**Region 2**	**36,685**	**34,531**	**94.13**	**2,082**	**5.68**	**72**	**0.20**
Batanes	**216**	188	**87.04**	28	**12.96**	0	**0.00**
Cagayan	**8,404**	8,102	**96.41**	279	**3.32**	23	**0.27**
Isabela	**15,203**	14,453	**95.07**	710	**4.67**	40	**0.26**
Nueva Vizcaya	**7,254**	6,403	**88.27**	842	**11.61**	9	**0.12**
Quirino	**2,774**	2,596	**93.58**	178	**6.42**	0	**0.00**
City of Santiago	**2,834**	2,789	**98.41**	45	**1.59**	0	**0.00**
**Region 3**	**152,056**	**140,991**	**92.72**	**10,259**	**6.75**	**806**	**0.53**
Aurora	**3,171**	2,828	**89.18**	306	**9.65**	37	**1.17**
Bataan	**11,007**	9,750	**88.58**	1,233	**11.20**	24	**0.22**
Bulacan	**41,925**	38,351	**91.48**	3,477	**8.29**	97	**0.23**
Nueva Ecija	**28,807**	27,355	**94.96**	1,336	**4.64**	116	**0.40**
Pampanga	**25,935**	23,941	**92.31**	1,594	**6.15**	400	**1.54**
Tarlac	**20,924**	20,491	**97.93**	408	**1.95**	25	**0.12**
Zambales	**8,646**	7,582	**87.69**	978	**11.31**	86	**0.99**
City of Angeles	**7,506**	6,931	**92.34**	567	**7.55**	8	**0.11**
City of Olongapo	**4,135**	3,762	**90.98**	360	**8.71**	13	**0.31**
**Region 4A**	**202,104**	**185,980**	**92.02**	**10,276**	**5.08**	**5,848**	**2.89**
Batangas	**38,151**	35,992	**94.34**	1,576	**4.13**	583	**1.53**
Cavite	**50,543**	46,909	**92.81**	2,558	**5.06**	1,076	**2.13**
Laguna	**44,295**	41,486	**93.66**	1,980	**4.47**	829	**1.87**
Quezon	**24,211**	22,285	**92.04**	1,274	**5.26**	652	**2.69**
Rizal	**41,811**	36,520	**87.35**	2,621	**6.27**	2,670	**6.39**
City of Lucena	**3,093**	2,788	**90.14**	267	**8.63**	38	**1.23**
**Region 4B**	**47,849**	**43,093**	**90.06**	**1,743**	**3.64**	**3,013**	**6.30**
Marinduque	**2,772**	2,533	**91.38**	233	**8.41**	6	**0.22**
Mindoro Occidental	**8,414**	7,507	**89.22**	384	**4.56**	523	**6.22**
Mindoro Oriental	**15,155**	13,482	**88.96**	236	**1.56**	1,437	**9.48**
Palawan	**13,077**	11,910	**91.08**	459	**3.51**	708	**5.41**
Romblon	**4,355**	3,900	**89.55**	278	**6.38**	177	**4.06**
Puerto Princesa City	**4,076**	3,761	**92.27**	153	**3.75**	162	**3.97**
**Region 5**	**89,477**	**78,208**	**87.41**	**9,417**	**10.52**	**1,852**	**2.07**
Albay	**16,144**	14,575	**90.28**	1,437	**8.90**	132	**0.82**
Camarines Norte	**10,499**	9,125	**86.91**	1,364	**12.99**	10	**0.10**

Table 2.B.2.4 - Intrapartum Care and Delivery Outcome
Live Births by birth weight
Philippines, 2023

Area	Total No. of Live Births	NBW (≥ 2500 grams)		LBW (< 2500)		UBW	
		No.	%	No.	%	No.	%
Camarines Sur	**17,601**	15,059	**85.56**	1,295	**7.36**	1,247	**7.08**
Catanduanes	**4,304**	3,793	**88.13**	510	**11.85**	1	**0.02**
Masbate	**14,187**	12,803	**90.24**	927	**6.53**	457	**3.22**
Sorsogon	**13,580**	12,192	**89.78**	1,384	**10.19**	4	**0.03**
City of Naga	**13,162**	10,661	**81.00**	2,500	**18.99**	1	**0.01**
**Region 6**	**96,610**	**87,856**	**90.94**	**7,839**	**8.11**	**915**	**0.95**
Aklan	**6,225**	5,694	**91.47**	391	**6.28**	140	**2.25**
Antique	**8,240**	6,580	**79.85**	1,528	**18.54**	132	**1.60**
Capiz	**10,744**	9,957	**92.67**	759	**7.06**	28	**0.26**
Guimaras	**2,371**	2,103	**88.70**	267	**11.26**	1	**0.04**
Iloilo	**22,616**	21,134	**93.45**	1,410	**6.23**	72	**0.32**
Negros Occidental	**34,516**	31,445	**91.10**	2,552	**7.39**	519	**1.50**
City of Bacolod	**6,121**	5,507	**89.97**	594	**9.70**	20	**0.33**
City of Iloilo	**5,777**	5,436	**94.10**	338	**5.85**	3	**0.05**
**Region 7**	**113,020**	**101,871**	**90.14**	**9,640**	**8.53**	**1,509**	**1.34**
Bohol	**18,473**	16,570	**89.70**	1,891	**10.24**	12	**0.06**
Cebu	**41,522**	37,493	**90.30**	3,845	**9.26**	184	**0.44**
Negros Oriental	**19,266**	16,970	**88.08**	997	**5.17**	1,299	**6.74**
Siquijor	**1,014**	897	**88.46**	117	**11.54**	0	**0.00**
City of Cebu	**15,464**	14,559	**94.15**	891	**5.76**	14	**0.09**
City of Lapu-Lapu	**7,508**	6,779	**90.29**	729	**9.71**	0	**0.00**
City of Mandaue	**9,773**	8,603	**88.03**	1,170	**11.97**	0	**0.00**
**Region 8**	**59,902**	**54,479**	**90.95**	**3,995**	**6.67**	**1,428**	**2.38**
Biliran	**3,066**	2,796	**91.19**	270	**8.81**	0	**0.00**
Eastern Samar	**6,971**	6,575	**94.32**	238	**3.41**	158	**2.27**
Leyte	**12,199**	11,753	**96.34**	405	**3.32**	41	**0.34**
Northern Samar	**10,341**	8,612	**83.28**	998	**9.65**	731	**7.07**
Southern Leyte	**5,061**	4,565	**90.20**	496	**9.80**	0	**0.00**
Samar	**10,416**	9,135	**87.70**	783	**7.52**	498	**4.78**
Ormoc City	**6,003**	5,230	**87.12**	773	**12.88**	0	**0.00**
City of Tacloban	**5,845**	5,813	**99.45**	32	**0.55**	0	**0.00**
**Region 9**	**47,802**	**43,658**	**91.33**	**1,803**	**3.77**	**2,341**	**4.90**
Zamboanga del Norte	**13,406**	11,642	**86.84**	687	**5.12**	1,077	**8.03**
Zamboanga del Sur	**8,244**	7,109	**86.23**	370	**4.49**	765	**9.28**
Zamboanga Sibugay	**7,392**	6,657	**90.06**	540	**7.31**	195	**2.64**

Table 2.B.2.4 - Intrapartum Care and Delivery Outcome
Live Births by birth weight
Philippines, 2023

Area	Total No. of Live Births	NBW (≥ 2500 grams)		LBW (< 2500)		UBW	
		No.	%	No.	%	No.	%
City of Isabela	**1,793**	1,689	**94.20**	4	**0.22**	100	**5.58**
City of Zamboanga	**16,967**	16,561	**97.61**	202	**1.19**	204	**1.20**
**Region 10**	**71,506**	**65,672**	**91.84**	**4,020**	**5.62**	**1,814**	**2.54**
Bukidnon	**23,203**	21,712	**93.57**	947	**4.08**	544	**2.34**
Camiguin	**1,345**	1,279	**95.09**	66	**4.91**	0	**0.00**
Lanao del Norte	**6,718**	5,659	**84.24**	58	**0.86**	1,001	**14.90**
Misamis Occidental	**6,248**	6,033	**96.56**	203	**3.25**	12	**0.19**
Misamis Oriental	**9,329**	8,976	**96.22**	214	**2.29**	139	**1.49**
City of Cagayan De Oro	**17,424**	15,044	**86.34**	2,354	**13.51**	26	**0.15**
City of Iligan	**7,239**	6,969	**96.27**	178	**2.46**	92	**1.27**
**Region 11**	**81,198**	**74,087**	**91.24**	**4,391**	**5.41**	**2,720**	**3.35**
Davao de Oro	**11,482**	10,632	**92.60**	726	**6.32**	124	**1.08**
Davao del Norte	**17,050**	15,647	**91.77**	998	**5.85**	405	**2.38**
Davao Oriental	**8,540**	8,065	**94.44**	420	**4.92**	55	**0.64**
Davao del Sur	**9,423**	8,700	**92.33**	465	**4.93**	258	**2.74**
Davao Occidental	**4,769**	3,658	**76.70**	236	**4.95**	875	**18.35**
City of Davao	**29,934**	27,385	**91.48**	1,546	**5.16**	1,003	**3.35**
**Region 12**	**63,981**	**60,293**	**94.24**	**1,840**	**2.88**	**1,848**	**2.89**
Cotabato	**17,313**	15,766	**91.06**	621	**3.59**	926	**5.35**
Sarangani	**9,930**	9,584	**96.52**	104	**1.05**	242	**2.44**
South Cotabato	**16,287**	15,564	**95.56**	530	**3.25**	193	**1.18**
Sultan Kudarat	**12,543**	11,701	**93.29**	430	**3.43**	412	**3.28**
City of General Santos	**7,908**	7,678	**97.09**	155	**1.96**	75	**0.95**
**BARMM**	**64,968**	**58,600**	**90.20**	**1,678**	**2.58**	**4,690**	**7.22**
Basilan	**4,764**	4,016	**84.30**	101	**2.12**	647	**13.58**
Lanao del Sur	**17,803**	15,855	**89.06**	651	**3.66**	1,297	**7.29**
Maguindanao	**19,910**	18,498	**92.91**	443	**2.23**	969	**4.87**
Sulu	**11,518**	10,187	**88.44**	350	**3.04**	981	**8.52**
Tawi-Tawi	**5,994**	5,705	**95.18**	61	**1.02**	228	**3.80**
SGU	**1,431**	962	**67.23**	2	**0.14**	467	**32.63**
City of Cotabato	**3,548**	3,377	**95.18**	70	**1.97**	101	**2.85**

Table 2.B.2.4 - Intrapartum Care and Delivery Outcome
Live Births by birth weight
Philippines, 2023

Area	Total No. of Live Births	NBW (≥ 2500 grams)		LBW (< 2500)		UBW	
		No.	%	No.	%	No.	%
**Caraga**	**41,034**	**37,570**	**91.56**	**2,481**	**6.05**	**983**	**2.40**
Agusan del Norte	**5,748**	5,387	**93.72**	210	**3.65**	151	**2.63**
Agusan del Sur	**10,949**	10,263	**93.73**	336	**3.07**	350	**3.20**
Surigao del Norte	**7,722**	6,726	**87.10**	791	**10.24**	205	**2.65**
Surigao del Sur	**9,741**	8,752	**89.85**	735	**7.55**	254	**2.61**
Dinagat Islands	**1,031**	885	**85.84**	134	**13.00**	12	**1.16**
City of Butuan	**5,843**	5,557	**95.11**	275	**4.71**	11	**0.19**

Table 2.B.3.1 - Postpartum and Newborn Care
Postpartum women together with their newborn who completed at least 2 postpartum check-ups
Philippines, 2023

Area	Total Deliveries	Completed at least 2 postpartum check-ups							
		Age Group in Year						Total	%
		10 - 14		15-19		20-49			
		No.	%	No.	%	No.	%		
**Philippines**	**1,420,182**	**2,879**	**0.20**	**126,809**	**8.93**	**1,156,662**	**81.44**	**1,286,350**	**90.58**
**N C R**	**184,090**	**262**	**0.14**	**11,334**	**6.16**	**166,463**	**90.42**	**178,059**	**96.72**
Malabon City	**1,782**	6	**0.34**	162	**9.09**	1,600	**89.79**	**1,768**	**99.21**
Navotas City	**4,102**	8	**0.20**	334	**8.14**	3,760	**91.66**	**4,102**	**100.00**
Valenzuela City	**5,585**	8	**0.14**	407	**7.29**	5,027	**90.01**	**5,442**	**97.44**
Caloocan City	**22,186**	24	**0.11**	1,712	**7.72**	20,081	**90.51**	**21,817**	**98.34**
Marikina City	**2,526**	7	**0.28**	210	**8.31**	2,301	**91.09**	**2,518**	**99.68**
Pasig City	**11,370**	13	**0.11**	792	**6.97**	8,696	**76.48**	**9,501**	**83.56**
Pateros	**791**	1	**0.13**	30	**3.79**	755	**95.45**	**786**	**99.37**
Taguig City	**12,357**	33	**0.27**	1,159	**9.38**	11,123	**90.01**	**12,315**	**99.66**
Quezon City	**57,484**	48	**0.08**	1,963	**3.41**	53,474	**93.02**	**55,485**	**96.52**
Makati City	**3,534**	7	**0.20**	246	**6.96**	3,247	**91.88**	**3,500**	**99.04**
Mandaluyong City	**7,639**	9	**0.12**	299	**3.91**	7,308	**95.67**	**7,616**	**99.70**
San Juan City	**745**	0	**0.00**	50	**6.71**	695	**93.29**	**745**	**100.00**
Manila City	**23,640**	19	**0.08**	2,129	**9.01**	21,282	**90.03**	**23,430**	**99.11**
Las Piñas City	**6,851**	11	**0.16**	508	**7.41**	6,059	**88.44**	**6,578**	**96.02**
Muntinlupa City	**8,297**	55	**0.66**	570	**6.87**	7,657	**92.29**	**8,282**	**99.82**
Parañaque City	**8,296**	5	**0.06**	492	**5.93**	6,801	**81.98**	**7,298**	**87.97**
Pasay City	**6,905**	8	**0.12**	271	**3.92**	6,597	**95.54**	**6,876**	**99.58**
**C A R**	**24,874**	**9**	**0.04**	**1,334**	**5.36**	**15,711**	**63.16**	**17,054**	**68.56**
Abra	**2,730**	3	**0.11**	315	**11.54**	2,343	**85.82**	**2,661**	**97.47**
Apayao	**2,733**	1	**0.04**	109	**3.99**	793	**29.02**	**903**	**33.04**
Benguet	**4,082**	0	**0.00**	185	**4.53**	3,219	**78.86**	**3,404**	**83.39**
Ifugao	**2,414**	1	**0.04**	216	**8.95**	2,012	**83.35**	**2,229**	**92.34**
Kalinga	**2,387**	2	**0.08**	145	**6.07**	2,209	**92.54**	**2,356**	**98.70**
Mountain Province	**3,390**	2	**0.06**	185	**5.46**	1,818	**53.63**	**2,005**	**59.14**
City of Baguio	**7,138**	0	**0.00**	179	**2.51**	3,317	**46.47**	**3,496**	**48.98**
**Region 1**	**50,550**	**103**	**0.20**	**4,066**	**8.04**	**45,607**	**90.22**	**49,776**	**98.47**
Ilocos Norte	**6,049**	5	**0.08**	331	**5.47**	5,222	**86.33**	**5,558**	**91.88**
Ilocos Sur	**7,661**	12	**0.16**	358	**4.67**	6,474	**84.51**	**6,844**	**89.34**

Table 2.B.3.1 - Postpartum and Newborn Care
Postpartum women together with their newborn who completed at least 2 postpartum check-ups
Philippines, 2023

Area	Total Deliveries	Completed at least 2 postpartum check-ups							
		Age Group in Year						Total	%
		10 - 14		15-19		20-49			
		No.	%	No.	%	No.	%		
La Union	**10,615**	39	**0.37**	864	**8.14**	7,417	**69.87**	**8,320**	**78.38**
Pangasinan	**17,409**	41	**0.24**	2,239	**12.86**	24,382	**140.05**	**26,662**	**153.15**
City of Dagupan	**8,816**	6	**0.07**	274	**3.11**	2,112	**23.96**	**2,392**	**27.13**
**Region 2**	**36,823**	**93**	**0.25**	**3,594**	**9.76**	**28,813**	**78.25**	**32,500**	**88.26**
Batanes	**216**	3	**1.39**	14	**6.48**	195	**90.28**	**212**	**98.15**
Cagayan	**8,424**	15	**0.18**	832	**9.88**	6,645	**78.88**	**7,492**	**88.94**
Isabela	**15,263**	44	**0.29**	1,539	**10.08**	12,468	**81.69**	**14,051**	**92.06**
Nueva Vizcaya	**7,319**	18	**0.25**	543	**7.42**	5,228	**71.43**	**5,789**	**79.10**
Quirino	**2,776**	10	**0.36**	372	**13.40**	1,749	**63.00**	**2,131**	**76.77**
City of Santiago	**2,825**	3	**0.11**	294	**10.41**	2,528	**89.49**	**2,825**	**100.00**
	**0**								
**Region 3**	**148,973**	**306**	**0.21**	**13,674**	**9.18**	**133,899**	**89.88**	**147,879**	**99.27**
Aurora	**3,200**	15	**0.47**	363	**11.34**	2,803	**87.59**	**3,181**	**99.41**
Bataan	**11,009**	35	**0.32**	1,125	**10.22**	9,150	**83.11**	**10,310**	**93.65**
Bulacan	**40,824**	70	**0.17**	4,188	**10.26**	45,179	**110.67**	**49,437**	**121.10**
Nueva Ecija	**28,779**	60	**0.21**	2,476	**8.60**	20,009	**69.53**	**22,545**	**78.34**
Pampanga	**25,991**	68	**0.26**	2,306	**8.87**	21,930	**84.38**	**24,304**	**93.51**
Tarlac	**20,870**	33	**0.16**	1,846	**8.85**	19,090	**91.47**	**20,969**	**100.47**
Zambales	**8,677**	11	**0.13**	590	**6.80**	5,220	**60.16**	**5,821**	**67.09**
City of Angeles	**5,487**	9	**0.16**	620	**11.30**	7,555	**137.69**	**8,184**	**149.15**
City of Olongapo	**4,136**	5	**0.12**	160	**3.87**	2,963	**71.64**	**3,128**	**75.63**
**Region 4A**	**202,438**	**329**	**0.16**	**15,047**	**7.43**	**157,996**	**78.05**	**173,372**	**85.64**
Batangas	**38,479**	51	**0.13**	2,692	**7.00**	31,238	**81.18**	**33,981**	**88.31**
Cavite	**48,372**	54	**0.11**	2,624	**5.42**	35,222	**72.81**	**37,900**	**78.35**
Laguna	**45,570**	65	**0.14**	3,308	**7.26**	35,858	**78.69**	**39,231**	**86.09**
Quezon	**24,586**	70	**0.28**	2,593	**10.55**	20,145	**81.94**	**22,808**	**92.77**
Rizal	**42,344**	81	**0.19**	3,460	**8.17**	32,854	**77.59**	**36,395**	**85.95**
City of Lucena	**3,087**	8	**0.26**	370	**11.99**	2,679	**86.78**	**3,057**	**99.03**
**Region 4B**	**47,523**	**92**	**0.19**	**4,356**	**9.17**	**35,288**	**74.25**	**39,736**	**83.61**
Marinduque	**2,791**	4	**0.14**	191	**6.84**	1,898	**68.00**	**2,093**	**74.99**

Table 2.B.3.1 - Postpartum and Newborn Care
Postpartum women together with their newborn who completed at least 2 postpartum check-ups
Philippines, 2023

Area	Total Deliveries	Completed at least 2 postpartum check-ups							
		Age Group in Year						Total	%
		10 - 14		15-19		20-49			
		No.	%	No.	%	No.	%		
Mindoro Occidental	**8,490**	11	**0.13**	903	**10.64**	6,385	**75.21**	**7,299**	**85.97**
Mindoro Oriental	**14,579**	16	**0.11**	701	**4.81**	10,789	**74.00**	**11,506**	**78.92**
Palawan	**13,176**	48	**0.36**	1,889	**14.34**	10,200	**77.41**	**12,137**	**92.11**
Romblon	**4,369**	4	**0.09**	306	**7.00**	3,063	**70.11**	**3,373**	**77.20**
Puerto Princesa City	**4,118**	9	**0.22**	366	**8.89**	2,953	**71.71**	**3,328**	**80.82**
**Region 5**	**84,229**	**85**	**0.10**	**7,045**	**8.36**	**65,767**	**78.08**	**72,897**	**86.55**
Albay	**16,099**	13	**0.08**	966	**6.00**	12,958	**80.49**	**13,937**	**86.57**
Camarines Norte	**10,605**	8	**0.08**	841	**7.93**	6,569	**61.94**	**7,418**	**69.95**
Camarines Sur	**17,655**	19	**0.11**	1,755	**9.94**	17,677	**100.12**	**19,451**	**110.17**
Catanduanes	**4,341**	8	**0.18**	413	**9.51**	3,414	**78.65**	**3,835**	**88.34**
Masbate	**14,258**	19	**0.13**	1,811	**12.70**	11,841	**83.05**	**13,671**	**95.88**
Sorsogon	**13,726**	14	**0.10**	1,075	**7.83**	11,308	**82.38**	**12,397**	**90.32**
City of Naga	**7,545**	4	**0.05**	184	**2.44**	2,000	**26.51**	**2,188**	**29.00**
**Region 6**	**96,790**	**168**	**0.17**	**8,580**	**8.86**	**75,328**	**77.83**	**84,076**	**86.86**
Aklan	**6,213**	6	**0.10**	484	**7.79**	5,316	**85.56**	**5,806**	**93.45**
Antique	**8,282**	11	**0.13**	589	**7.11**	6,290	**75.95**	**6,890**	**83.19**
Capiz	**10,750**	11	**0.10**	587	**5.46**	6,360	**59.16**	**6,958**	**64.73**
Guimaras	**2,370**	4	**0.17**	227	**9.58**	2,086	**88.02**	**2,317**	**97.76**
Iloilo	**22,667**	35	**0.15**	1,792	**7.91**	18,383	**81.10**	**20,210**	**89.16**
Negros Occidental	**34,588**	82	**0.24**	4,079	**11.79**	27,927	**80.74**	**32,088**	**92.77**
City of Bacolod	**6,150**	2	**0.03**	311	**5.06**	3,788	**61.59**	**4,101**	**66.68**
City of Iloilo	**5,770**	17	**0.29**	511	**8.86**	5,178	**89.74**	**5,706**	**98.89**
**Region 7**	**112,305**	**208**	**0.19**	**10,048**	**8.95**	**91,332**	**81.32**	**101,588**	**90.46**
Bohol	**18,420**	20	**0.11**	1,283	**6.97**	13,558	**73.60**	**14,861**	**80.68**
Cebu	**40,979**	99	**0.24**	4,263	**10.40**	35,880	**87.56**	**40,242**	**98.20**
Negros Oriental	**19,030**	52	**0.27**	1,951	**10.25**	14,666	**77.07**	**16,669**	**87.59**
Siquijor	**1,014**	0	**0.00**	68	**6.71**	698	**68.84**	**766**	**75.54**
City of Cebu	**15,467**	20	**0.13**	1,499	**9.69**	13,585	**87.83**	**15,104**	**97.65**
City of Lapu-Lapu	**7,557**	6	**0.08**	413	**5.47**	6,519	**86.26**	**6,938**	**91.81**
City of Mandaue	**9,838**	11	**0.11**	571	**5.80**	6,426	**65.32**	**7,008**	**71.23**

Table 2.B.3.1 - Postpartum and Newborn Care
Postpartum women together with their newborn who completed at least 2 postpartum check-ups
Philippines, 2023

Area	Total Deliveries	Completed at least 2 postpartum check-ups							
		Age Group in Year						Total	%
		10 - 14		15-19		20-49			
		No.	%	No.	%	No.	%		
**Region 8**	**59,834**	**66**	**0.11**	**4,813**	**8.04**	**43,892**	**73.36**	**48,771**	**81.51**
Biliran	**3,084**	3	**0.10**	201	**6.52**	2,298	**74.51**	**2,502**	**81.13**
Eastern Samar	**7,014**	8	**0.11**	879	**12.53**	5,978	**85.23**	**6,865**	**97.88**
Leyte	**12,068**	17	**0.14**	1,114	**9.23**	10,449	**86.58**	**11,580**	**95.96**
Northern Samar	**10,442**	9	**0.09**	650	**6.22**	5,906	**56.56**	**6,565**	**62.87**
Southern Leyte	**5,094**	1	**0.02**	377	**7.40**	3,724	**73.11**	**4,102**	**80.53**
Samar	**10,338**	16	**0.15**	811	**7.84**	7,343	**71.03**	**8,170**	**79.03**
Ormoc City	**5,965**	6	**0.10**	463	**7.76**	2,912	**48.82**	**3,381**	**56.68**
City of Tacloban	**5,829**	6	**0.10**	318	**5.46**	5,282	**90.62**	**5,606**	**96.17**
**Region 9**	**47,886**	**98**	**0.20**	**5,742**	**11.99**	**40,418**	**84.40**	**46,258**	**96.60**
Zamboanga del Norte	**13,451**	31	**0.23**	1,446	**10.75**	10,066	**74.83**	**11,543**	**85.82**
Zamboanga del Sur	**8,271**	15	**0.18**	931	**11.26**	6,768	**81.83**	**7,714**	**93.27**
Zamboanga Sibugay	**7,406**	11	**0.15**	1,170	**15.80**	7,398	**99.89**	**8,579**	**115.84**
City of Isabela	**1,797**	2	**0.11**	206	**11.46**	1,571	**87.42**	**1,779**	**99.00**
City of Zamboanga	**16,961**	39	**0.23**	1,989	**11.73**	14,615	**86.17**	**16,643**	**98.13**
**Region 10**	**71,891**	**233**	**0.32**	**9,269**	**12.89**	**52,384**	**72.87**	**61,886**	**86.08**
Bukidnon	**23,309**	111	**0.48**	4,136	**17.74**	17,865	**76.64**	**22,112**	**94.86**
Camiguin	**1,355**	1	**0.07**	154	**11.37**	1,133	**83.62**	**1,288**	**95.06**
Lanao del Norte	**6,720**	5	**0.07**	492	**7.32**	5,511	**82.01**	**6,008**	**89.40**
Misamis Occidental	**6,284**	24	**0.38**	647	**10.30**	4,893	**77.86**	**5,564**	**88.54**
Misamis Oriental	**9,358**	42	**0.45**	1,509	**16.13**	6,845	**73.15**	**8,396**	**89.72**
City of Cagayan De Oro	**17,552**	43	**0.24**	1,563	**8.90**	9,782	**55.73**	**11,388**	**64.88**
City of Iligan	**7,313**	7	**0.10**	768	**10.50**	6,355	**86.90**	**7,130**	**97.50**
**Region 11**	**81,389**	**498**	**0.61**	**10,405**	**12.78**	**65,801**	**80.85**	**76,704**	**94.24**
Davao de Oro	**11,522**	196	**1.70**	1,757	**15.25**	9,246	**80.25**	**11,199**	**97.20**
Davao del Norte	**17,062**	76	**0.45**	2,342	**13.73**	13,879	**81.34**	**16,297**	**95.52**
Davao Oriental	**8,590**	56	**0.65**	1,274	**14.83**	6,989	**81.36**	**8,319**	**96.85**
Davao del Sur	**9,438**	62	**0.66**	1,361	**14.42**	7,276	**77.09**	**8,699**	**92.17**
Davao Occidental	**4,886**	29	**0.59**	917	**18.77**	2,581	**52.82**	**3,527**	**72.19**
City of Davao	**29,891**	79	**0.26**	2,754	**9.21**	25,830	**86.41**	**28,663**	**95.89**

Table 2.B.3.1 - Postpartum and Newborn Care
Postpartum women together with their newborn who completed at least 2 postpartum check-ups
Philippines, 2023

Area	Total Deliveries	Completed at least 2 postpartum check-ups							
		Age Group in Year						Total	%
		10 - 14		15-19		20-49			
		No.	%	No.	%	No.	%		
**Region 12**	**64,112**	**205**	**0.32**	**8,614**	**13.44**	**53,402**	**83.29**	**62,221**	**97.05**
Cotabato	**17,294**	49	**0.28**	2,218	**12.83**	14,369	**83.09**	**16,636**	**96.20**
Sarangani	**9,947**	42	**0.42**	1,784	**17.94**	7,895	**79.37**	**9,721**	**97.73**
South Cotabato	**16,339**	48	**0.29**	2,267	**13.87**	13,656	**83.58**	**15,971**	**97.75**
Sultan Kudarat	**12,614**	60	**0.48**	1,671	**13.25**	10,658	**84.49**	**12,389**	**98.22**
City of General Santos	**7,918**	6	**0.08**	674	**8.51**	6,824	**86.18**	**7,504**	**94.77**
**BARMM**	**65,140**	**36**	**0.06**	**4,603**	**7.07**	**52,615**	**80.77**	**57,254**	**87.89**
Basilan	**4,718**	10	**0.21**	577	**12.23**	3,341	**70.81**	**3,928**	**83.26**
Lanao del Sur	**17,820**	6	**0.03**	825	**4.63**	16,095	**90.32**	**16,926**	**94.98**
Maguindanao	**19,942**	7	**0.04**	1,440	**7.22**	14,915	**74.79**	**16,362**	**82.05**
Sulu	**11,670**	4	**0.03**	850	**7.28**	9,701	**83.13**	**10,555**	**90.45**
Tawi-Tawi	**6,010**	0	**0.00**	482	**8.02**	5,144	**85.59**	**5,626**	**93.61**
SGU	**1,432**	0	**0.00**	99	**6.91**	872	**60.89**	**971**	**67.81**
City of Cotabato	**3,548**	9	**0.25**	330	**9.30**	2,547	**71.79**	**2,886**	**81.34**
**Caraga**	**41,335**	**88**	**0.21**	**4,285**	**10.37**	**31,946**	**77.29**	**36,319**	**87.87**
Agusan del Norte	**5,881**	24	**0.41**	838	**14.25**	4,840	**82.30**	**5,702**	**96.96**
Agusan del Sur	**10,972**	26	**0.24**	1,312	**11.96**	9,337	**85.10**	**10,675**	**97.29**
Surigao del Norte	**7,756**	3	**0.04**	613	**7.90**	4,816	**62.09**	**5,432**	**70.04**
Surigao del Sur	**9,826**	20	**0.20**	893	**9.09**	7,022	**71.46**	**7,935**	**80.76**
Dinagat Islands	**1,034**	4	**0.39**	102	**9.86**	842	**81.43**	**948**	**91.68**
City of Butuan	**5,866**	11	**0.19**	527	**8.98**	5,089	**86.75**	**5,627**	**95.93**

Table 2.B.3.2 - Postpartum and Newborn Care
Postpartum women who completed Iron with Folic Acid
Philippines, 2023

Area	Eligible Pop.	Iron with Folic Supplementation							
		Age Group in Year						Total	%
		10-14		15-19		20-49			
		No.	%	No.	%	No.	%		
**Philippines**	**2,187,333**	**2,574**	**0.12**	**118,725**	**5.43**	**1,109,634**	**50.73**	**1,230,933**	**56.28**
**N C R**	**261,228**	**234**	**0.09**	**10,764**	**4.12**	**140,694**	**53.86**	**151,692**	**58.07**
Malabon City	**7,385**	14	**0.19**	416	**5.63**	3,394	**45.96**	**3,824**	**51.78**
Navotas City	**5,210**	8	**0.15**	334	**6.41**	3,760	**72.17**	**4,102**	**78.73**
Valenzuela City	**13,477**	8	**0.06**	386	**2.86**	5,199	**38.58**	**5,593**	**41.50**
Caloocan City	**33,168**	15	**0.05**	1,744	**5.26**	19,780	**59.64**	**21,539**	**64.94**
Marikina City	**8,342**	7	**0.08**	210	**2.52**	2,301	**27.58**	**2,518**	**30.18**
Pasig City	**17,682**	8	**0.05**	623	**3.52**	7,766	**43.92**	**8,397**	**47.49**
Pateros	**1,116**	1	**0.09**	29	**2.60**	696	**62.37**	**726**	**65.05**
Taguig City	**18,992**	28	**0.15**	1,206	**6.35**	11,158	**58.75**	**12,392**	**65.25**
Quezon City	**59,136**	28	**0.05**	1,489	**2.52**	27,766	**46.95**	**29,283**	**49.52**
Makati City	**10,944**	6	**0.05**	219	**2.00**	3,479	**31.79**	**3,704**	**33.85**
Mandaluyong City	**7,922**	15	**0.19**	288	**3.64**	7,350	**92.78**	**7,653**	**96.60**
San Juan City	**2,168**	0	**0.00**	44	**2.03**	679	**31.32**	**723**	**33.35**
Manila City	**34,128**	21	**0.06**	1,953	**5.72**	19,941	**58.43**	**21,915**	**64.21**
Las Piñas City	**11,263**	7	**0.06**	477	**4.24**	6,098	**54.14**	**6,582**	**58.44**
Muntinlupa City	**9,881**	55	**0.56**	570	**5.77**	8,007	**81.03**	**8,632**	**87.36**
Parañaque City	**13,280**	5	**0.04**	503	**3.79**	6,717	**50.58**	**7,225**	**54.41**
Pasay City	**7,134**	8	**0.11**	273	**3.83**	6,603	**92.56**	**6,884**	**96.50**
**C A R**	**31,319**	**38**	**0.12**	**1,513**	**4.83**	**18,141**	**57.92**	**19,692**	**62.88**
Abra	**3,660**	21	**0.57**	323	**8.83**	2,323	**63.47**	**2,667**	**72.87**
Apayao	**2,238**	1	**0.04**	157	**7.02**	1,394	**62.29**	**1,552**	**69.35**
Benguet	**8,806**	3	**0.03**	325	**3.69**	4,955	**56.27**	**5,283**	**59.99**
Ifugao	**4,098**	1	**0.02**	199	**4.86**	1,965	**47.95**	**2,165**	**52.83**
Kalinga	**4,549**	5	**0.11**	193	**4.24**	2,651	**58.28**	**2,849**	**62.63**
Mountain Province	**2,619**	7	**0.27**	137	**5.23**	1,507	**57.54**	**1,651**	**63.04**
City of Baguio	**5,349**	0	**0.00**	179	**3.35**	3,346	**62.55**	**3,525**	**65.90**
**Region 1**	**96,016**	**74**	**0.08**	**4,010**	**4.18**	**48,987**	**51.02**	**53,071**	**55.27**
Ilocos Norte	**8,912**	4	**0.04**	360	**4.04**	6,267	**70.32**	**6,631**	**74.41**
Ilocos Sur	**9,914**	15	**0.15**	373	**3.76**	7,589	**76.55**	**7,977**	**80.46**
La Union	**13,507**	17	**0.13**	685	**5.07**	7,404	**54.82**	**8,106**	**60.01**
Pangasinan	**60,360**	32	**0.05**	2,318	**3.84**	25,532	**42.30**	**27,882**	**46.19**

Table 2.B.3.2 - Postpartum and Newborn Care
Postpartum women who completed Iron with Folic Acid

Philippines, 2023

Area	Eligible Pop.	Iron with Folic Supplementation							
		Age Group in Year						Total	%
		10-14		15-19		20-49			
		No.	%	No.	%	No.	%		
City of Dagupan	**3,323**	6	**0.18**	274	**8.25**	2,195	**66.05**	**2,475**	**74.48**
**Region 2**	**63,967**	**93**	**0.15**	**3,924**	**6.13**	**35,772**	**55.92**	**39,789**	**62.20**
Batanes	**240**	2	**0.83**	15	**6.25**	166	**69.17**	**183**	**76.25**
Cagayan	**20,786**	21	**0.10**	1,122	**5.40**	9,897	**47.61**	**11,040**	**53.11**
Isabela	**27,379**	49	**0.18**	1,739	**6.35**	15,831	**57.82**	**17,619**	**64.35**
Nueva Vizcaya	**8,798**	10	**0.11**	432	**4.91**	4,704	**53.47**	**5,146**	**58.49**
Quirino	**3,839**	9	**0.23**	350	**9.12**	2,805	**73.07**	**3,164**	**82.42**
City of Santiago	**2,925**	2	**0.07**	266	**9.09**	2,369	**80.99**	**2,637**	**90.15**
**Region 3**	**233,544**	**286**	**0.12**	**13,144**	**5.63**	**132,294**	**56.65**	**145,724**	**62.40**
Aurora	**4,537**	10	**0.22**	349	**7.69**	2,697	**59.44**	**3,056**	**67.36**
Bataan	**17,380**	39	**0.22**	1,074	**6.18**	9,032	**51.97**	**10,145**	**58.37**
Bulacan	**67,590**	82	**0.12**	3,964	**5.86**	44,999	**66.58**	**49,045**	**72.56**
Nueva Ecija	**43,425**	61	**0.14**	2,312	**5.32**	19,046	**43.86**	**21,419**	**49.32**
Pampanga	**45,651**	39	**0.09**	2,323	**5.09**	22,398	**49.06**	**24,760**	**54.24**
Tarlac	**29,503**	31	**0.11**	1,795	**6.08**	18,274	**61.94**	**20,100**	**68.13**
Zambales	**11,723**	11	**0.09**	520	**4.44**	4,968	**42.38**	**5,499**	**46.91**
City of Angeles	**9,146**	9	**0.10**	643	**7.03**	7,852	**85.85**	**8,504**	**92.98**
City of Olongapo	**4,589**	4	**0.09**	164	**3.57**	3,028	**65.98**	**3,196**	**69.64**
**Region 4A**	**307,982**	**258**	**0.08**	**13,276**	**4.31**	**143,872**	**46.71**	**157,406**	**51.11**
Batangas	**53,251**	36	**0.07**	2,347	**4.41**	29,203	**54.84**	**31,586**	**59.32**
Cavite	**77,964**	36	**0.05**	2,306	**2.96**	30,217	**38.76**	**32,559**	**41.76**
Laguna	**61,525**	64	**0.10**	3,081	**5.01**	33,700	**54.77**	**36,845**	**59.89**
Quezon	**40,810**	48	**0.12**	1,973	**4.83**	16,634	**40.76**	**18,655**	**45.71**
Rizal	**68,235**	67	**0.10**	3,215	**4.71**	31,570	**46.27**	**34,852**	**51.08**
City of Lucena	**6,197**	7	**0.11**	354	**5.71**	2,548	**41.12**	**2,909**	**46.94**
**Region 4B**	**66,121**	**78**	**0.12**	**4,311**	**6.52**	**37,093**	**56.10**	**41,482**	**62.74**
Marinduque	**4,144**	6	**0.14**	202	**4.87**	2,627	**63.39**	**2,835**	**68.41**
Mindoro Occidental	**11,640**	13	**0.11**	1,048	**9.00**	7,474	**64.21**	**8,535**	**73.32**
Mindoro Oriental	**18,943**	14	**0.07**	659	**3.48**	11,249	**59.38**	**11,922**	**62.94**
Palawan	**20,317**	33	**0.16**	1,789	**8.81**	10,377	**51.08**	**12,199**	**60.04**
Romblon	**5,489**	4	**0.07**	362	**6.60**	3,404	**62.01**	**3,770**	**68.68**
Puerto Princesa City	**5,588**	8	**0.14**	251	**4.49**	1,962	**35.11**	**2,221**	**39.75**

Table 2.B.3.2 - Postpartum and Newborn Care
Postpartum women who completed Iron with Folic Acid

Philippines, 2023

Area	Eligible Pop.	Iron with Folic Supplementation							
		Age Group in Year						Total	%
		10-14		15-19		20-49			
		No.	%	No.	%	No.	%		
**Region 5**	**135,480**	**74**	**0.05**	**5,730**	**4.23**	**59,648**	**44.03**	**65,452**	**48.31**
Albay	**27,440**	14	**0.05**	783	**2.85**	13,229	**48.21**	**14,026**	**51.12**
Camarines Norte	**14,695**	5	**0.03**	761	**5.18**	6,283	**42.76**	**7,049**	**47.97**
Camarines Sur	**43,452**	14	**0.03**	1,242	**2.86**	14,269	**32.84**	**15,525**	**35.73**
Catanduanes	**5,414**	13	**0.24**	327	**6.04**	3,171	**58.57**	**3,511**	**64.85**
Masbate	**22,365**	12	**0.05**	1,542	**6.89**	10,471	**46.82**	**12,025**	**53.77**
Sorsogon	**17,929**	10	**0.06**	908	**5.06**	10,431	**58.18**	**11,349**	**63.30**
City of Naga	**4,185**	6	**0.14**	167	**3.99**	1,794	**42.87**	**1,967**	**47.00**
**Region 6**	**148,020**	**132**	**0.09**	**7,797**	**5.27**	**71,004**	**47.97**	**78,933**	**53.33**
Aklan	**11,283**	4	**0.04**	404	**3.58**	5,076	**44.99**	**5,484**	**48.60**
Antique	**11,930**	8	**0.07**	516	**4.33**	5,860	**49.12**	**6,384**	**53.51**
Capiz	**13,931**	8	**0.06**	480	**3.45**	5,629	**40.41**	**6,117**	**43.91**
Guimaras	**3,351**	3	**0.09**	170	**5.07**	1,979	**59.06**	**2,152**	**64.22**
Iloilo	**36,140**	31	**0.09**	1,516	**4.19**	16,475	**45.59**	**18,022**	**49.87**
Negros Occidental	**52,270**	59	**0.11**	3,626	**6.94**	25,599	**48.97**	**29,284**	**56.02**
City of Bacolod	**10,373**	5	**0.05**	625	**6.03**	5,515	**53.17**	**6,145**	**59.24**
City of Iloilo	**8,742**	14	**0.16**	460	**5.26**	4,871	**55.72**	**5,345**	**61.14**
**Region 7**	**165,673**	**142**	**0.09**	**8,739**	**5.27**	**87,321**	**52.71**	**96,202**	**58.07**
Bohol	**26,235**	8	**0.03**	1,040	**3.96**	12,718	**48.48**	**13,766**	**52.47**
Cebu	**72,201**	62	**0.09**	3,478	**4.82**	32,964	**45.66**	**36,504**	**50.56**
Negros Oriental	**28,722**	31	**0.11**	1,710	**5.95**	14,032	**48.85**	**15,773**	**54.92**
Siquijor	**1,680**	0	**0.00**	51	**3.04**	711	**42.32**	**762**	**45.36**
City of Cebu	**19,267**	24	**0.12**	1,414	**7.34**	13,472	**69.92**	**14,910**	**77.39**
City of Lapu-Lapu	**10,679**	6	**0.06**	453	**4.24**	6,787	**63.55**	**7,246**	**67.85**
City of Mandaue	**6,889**	11	**0.16**	593	**8.61**	6,637	**96.34**	**7,241**	**105.11**
**Region 8**	**93,813**	**69**	**0.07**	**4,595**	**4.90**	**41,676**	**44.42**	**46,340**	**49.40**
Biliran	**3,373**	1	**0.03**	228	**6.76**	2,171	**64.36**	**2,400**	**71.15**
Eastern Samar	**9,624**	7	**0.07**	761	**7.91**	5,399	**56.10**	**6,167**	**64.08**
Leyte	**31,559**	24	**0.08**	1,233	**3.91**	11,270	**35.71**	**12,527**	**39.69**
Northern Samar	**14,056**	8	**0.06**	560	**3.98**	5,606	**39.88**	**6,174**	**43.92**
Southern Leyte	**7,406**	4	**0.05**	336	**4.54**	3,482	**47.02**	**3,822**	**51.61**
Samar	**17,011**	15	**0.09**	793	**4.66**	6,232	**36.64**	**7,040**	**41.38**
Ormoc City	**5,292**	5	**0.09**	374	**7.07**	2,886	**54.54**	**3,265**	**61.70**
City of Tacloban	**5,492**	5	**0.09**	310	**5.64**	4,630	**84.30**	**4,945**	**90.04**

Table 2.B.3.2 - Postpartum and Newborn Care
Postpartum women who completed Iron with Folic Acid

Philippines, 2023

Area	Eligible Pop.	Iron with Folic Supplementation							
		Age Group in Year						Total	%
		10-14		15-19		20-49			
		No.	%	No.	%	No.	%		
**Region 9**	**84,815**	**110**	**0.13**	**5,800**	**6.84**	**41,949**	**49.46**	**47,859**	**56.43**
Zamboanga del Norte	**24,841**	27	**0.11**	1,501	**6.04**	11,342	**45.66**	**12,870**	**51.81**
Zamboanga del Sur	**23,757**	34	**0.14**	1,323	**5.57**	8,765	**36.89**	**10,122**	**42.61**
Zamboanga Sibugay	**13,841**	9	**0.07**	949	**6.86**	6,405	**46.28**	**7,363**	**53.20**
City of Isabela	**3,201**	2	**0.06**	173	**5.40**	1,426	**44.55**	**1,601**	**50.02**
City of Zamboanga	**19,175**	38	**0.20**	1,854	**9.67**	14,011	**73.07**	**15,903**	**82.94**
**Region 10**	**109,169**	**259**	**0.24**	**9,767**	**8.95**	**61,410**	**56.25**	**71,436**	**65.44**
Bukidnon	**33,403**	134	**0.40**	4,000	**11.97**	19,747	**59.12**	**23,881**	**71.49**
Camiguin	**1,727**	1	**0.06**	158	**9.15**	1,179	**68.27**	**1,338**	**77.48**
Lanao del Norte	**17,760**	8	**0.05**	805	**4.53**	7,958	**44.81**	**8,771**	**49.39**
Misamis Occidental	**12,328**	17	**0.14**	823	**6.68**	6,995	**56.74**	**7,835**	**63.55**
Misamis Oriental	**21,975**	49	**0.22**	1,909	**8.69**	11,450	**52.10**	**13,408**	**61.01**
City of Cagayan De Oro	**14,308**	46	**0.32**	1,580	**11.04**	9,756	**68.19**	**11,382**	**79.55**
City of Iligan	**7,668**	4	**0.05**	492	**6.42**	4,325	**56.40**	**4,821**	**62.87**
**Region 11**	**107,001**	**504**	**0.47**	**9,651**	**9.02**	**61,407**	**57.39**	**71,562**	**66.88**
Davao de Oro	**15,253**	205	**1.34**	1,751	**11.48**	9,329	**61.16**	**11,285**	**73.99**
Davao del Norte	**23,453**	70	**0.30**	2,269	**9.67**	13,942	**59.45**	**16,281**	**69.42**
Davao Oriental	**11,935**	47	**0.39**	1,178	**9.87**	6,765	**56.68**	**7,990**	**66.95**
Davao del Sur	**13,083**	63	**0.48**	1,112	**8.50**	5,974	**45.66**	**7,149**	**54.64**
Davao Occidental	**6,629**	36	**0.54**	779	**11.75**	2,178	**32.86**	**2,993**	**45.15**
City of Davao	**36,648**	83	**0.23**	2,562	**6.99**	23,219	**63.36**	**25,864**	**70.57**
**Region 12**	**95,279**	**143**	**0.15**	**8,089**	**8.49**	**50,319**	**52.81**	**58,551**	**61.45**
Cotabato	**26,521**	27	**0.10**	2,040	**7.69**	13,378	**50.44**	**15,445**	**58.24**
Sarangani	**13,322**	29	**0.22**	1,693	**12.71**	7,735	**58.06**	**9,457**	**70.99**
South Cotabato	**21,595**	29	**0.13**	2,181	**10.10**	13,195	**61.10**	**15,405**	**71.34**
Sultan Kudarat	**19,391**	52	**0.27**	1,577	**8.13**	9,917	**51.14**	**11,546**	**59.54**
City of General Santos	**14,450**	6	**0.04**	598	**4.14**	6,094	**42.17**	**6,698**	**46.35**
**BARMM**	**132,841**	**30**	**0.02**	**4,014**	**3.02**	**48,135**	**36.24**	**52,179**	**39.28**
Basilan	**11,636**	11	**0.09**	467	**4.01**	2,882	**24.77**	**3,360**	**28.88**
Lanao del Sur	**33,374**	2	**0.01**	740	**2.22**	14,694	**44.03**	**15,436**	**46.25**
Maguindanao	**41,650**	7	**0.02**	1,633	**3.92**	16,365	**39.29**	**18,005**	**43.23**

Table 2.B.3.2 - Postpartum and Newborn Care
Postpartum women who completed Iron with Folic Acid

Philippines, 2023

Area	Eligible Pop.	Iron with Folic Supplementation							
		Age Group in Year						Total	%
		10-14		15-19		20-49			
		No.	%	No.	%	No.	%		
Sulu	**21,378**	3	**0.01**	528	**2.47**	6,728	**31.47**	**7,259**	**33.96**
Tawi-Tawi	**12,609**	1	**0.01**	319	**2.53**	4,331	**34.35**	**4,651**	**36.89**
SGU	**5,103**	0	**0.00**	75	**1.47**	781	**15.30**	**856**	**16.77**
City of Cotabato	**7,091**	6	**0.08**	252	**3.55**	2,354	**33.20**	**2,612**	**36.84**
**Caraga**	**55,065**	**50**	**0.09**	**3,601**	**6.54**	**29,912**	**54.32**	**33,563**	**60.95**
Agusan del Norte	**7,286**	5	**0.07**	413	**5.67**	2,856	**39.20**	**3,274**	**44.94**
Agusan del Sur	**16,038**	10	**0.06**	1,090	**6.80**	8,493	**52.96**	**9,593**	**59.81**
Surigao del Norte	**10,186**	3	**0.03**	568	**5.58**	5,803	**56.97**	**6,374**	**62.58**
Surigao del Sur	**12,736**	17	**0.13**	760	**5.97**	6,294	**49.42**	**7,071**	**55.52**
Dinagat Islands	**2,134**	4	**0.19**	129	**6.04**	1,042	**48.83**	**1,175**	**55.06**
City of Butuan	**6,685**	11	**0.16**	641	**9.59**	5,424	**81.14**	**6,076**	**90.89**

Table 2.B.3.3 - Postpartum and Newborn Care
Postpartum women who completed Vitamin A Supplementation
Philippines, 2023

Area	Eligible Pop.	Vitamin A Supplementation							
		Age Group in Year						Total	%
		10-14		15-19		20-49			
		No.	%	No.	%	No.	%		
**Philippines**	**2,187,333**	**2,884**	**0.13**	**123,429**	**5.64**	**1,126,467**	**51.50**	**1,252,780**	**57.27**
**N C R**	**261,228**	**241**	**0.09**	**11,766**	**4.50**	**168,650**	**64.56**	**180,657**	**69.16**
Malabon City	**7,385**	14	**0.19**	416	**5.63**	3,375	**45.70**	**3,805**	**51.52**
Navotas City	**5,210**	8	**0.15**	334	**6.41**	3,760	**72.17**	**4,102**	**78.73**
Valenzuela City	**13,477**	7	**0.05**	418	**3.10**	5,083	**37.72**	**5,508**	**40.87**
Caloocan City	**33,168**	22	**0.07**	1,596	**4.81**	19,674	**59.32**	**21,292**	**64.19**
Marikina City	**8,342**	7	**0.08**	210	**2.52**	2,301	**27.58**	**2,518**	**30.18**
Pasig City	**17,682**	9	**0.05**	556	**3.14**	6,726	**38.04**	**7,291**	**41.23**
Pateros	**1,116**	1	**0.09**	27	**2.42**	757	**67.83**	**785**	**70.34**
Taguig City	**18,992**	15	**0.08**	1,053	**5.54**	11,000	**57.92**	**12,068**	**63.54**
Quezon City	**59,136**	45	**0.08**	2,585	**4.37**	56,341	**95.27**	**58,971**	**99.72**
Makati City	**10,944**	7	**0.06**	238	**2.17**	3,159	**28.87**	**3,404**	**31.10**
Mandaluyong City	**7,922**	9	**0.11**	280	**3.53**	7,027	**88.70**	**7,316**	**92.35**
San Juan City	**2,168**	0	**0.00**	50	**2.31**	710	**32.75**	**760**	**35.06**
Manila City	**34,128**	19	**0.06**	2,139	**6.27**	21,266	**62.31**	**23,424**	**68.64**
Las Piñas City	**11,263**	12	**0.11**	504	**4.47**	6,091	**54.08**	**6,607**	**58.66**
Muntinlupa City	**9,881**	50	**0.51**	570	**5.77**	8,003	**80.99**	**8,623**	**87.27**
Parañaque City	**13,280**	9	**0.07**	526	**3.96**	6,852	**51.60**	**7,387**	**55.63**
Pasay City	**7,134**	7	**0.10**	264	**3.70**	6,525	**91.46**	**6,796**	**95.26**
**C A R**	**31,319**	**17**	**0.05**	**1,600**	**5.11**	**18,090**	**57.76**	**19,707**	**62.92**
Abra	**3,660**	3	**0.08**	306	**8.36**	2,293	**62.65**	**2,602**	**71.09**
Apayao	**2,238**	4	**0.18**	170	**7.60**	1,383	**61.80**	**1,557**	**69.57**
Benguet	**8,806**	3	**0.03**	296	**3.36**	3,712	**42.15**	**4,011**	**45.55**
Ifugao	**4,098**	1	**0.02**	236	**5.76**	2,289	**55.86**	**2,526**	**61.64**
Kalinga	**4,549**	3	**0.07**	159	**3.50**	2,533	**55.68**	**2,695**	**59.24**
Mountain Province	**2,619**	3	**0.11**	255	**9.74**	2,538	**96.91**	**2,796**	**106.76**
City of Baguio	**5,349**	0	**0.00**	178	**3.33**	3,342	**62.48**	**3,520**	**65.81**
**Region 1**	**96,016**	**87**	**0.09**	**3,849**	**4.01**	**44,712**	**46.57**	**48,648**	**50.67**
Ilocos Norte	**8,912**	6	**0.07**	377	**4.23**	6,265	**70.30**	**6,648**	**74.60**
Ilocos Sur	**9,914**	19	**0.19**	412	**4.16**	7,060	**71.21**	**7,491**	**75.56**
La Union	**13,507**	23	**0.17**	743	**5.50**	7,467	**55.28**	**8,233**	**60.95**
Pangasinan	**60,360**	33	**0.05**	2,042	**3.38**	21,808	**36.13**	**23,883**	**39.57**
City of Dagupan	**3,323**	6	**0.18**	275	**8.28**	2,112	**63.56**	**2,393**	**72.01**

Table 2.B.3.3 - Postpartum and Newborn Care
Postpartum women who completed Vitamin A Supplementation
Philippines, 2023

Area	Eligible Pop.	Vitamin A Supplementation							
		Age Group in Year						Total	%
		10-14		15-19		20-49			
		No.	%	No.	%	No.	%		
**Region 2**	**63,967**	**137**	**0.21**	**4,362**	**6.82**	**35,780**	**55.94**	**40,279**	**62.97**
Batanes	**240**	3	**1.25**	13	**5.42**	195	**81.25**	**211**	**87.92**
Cagayan	**20,786**	20	**0.10**	1,229	**5.91**	9,888	**47.57**	**11,137**	**53.58**
Isabela	**27,379**	70	**0.26**	1,853	**6.77**	15,460	**56.47**	**17,383**	**63.49**
Nueva Vizcaya	**8,798**	31	**0.35**	646	**7.34**	5,033	**57.21**	**5,710**	**64.90**
Quirino	**3,839**	10	**0.26**	327	**8.52**	2,614	**68.09**	**2,951**	**76.87**
City of Santiago	**2,925**	3	**0.10**	294	**10.05**	2,590	**88.55**	**2,887**	**98.70**
**Region 3**	**233,544**	**240**	**0.10**	**11,548**	**4.94**	**113,475**	**48.59**	**125,263**	**53.64**
Aurora	**4,537**	9	**0.20**	299	**6.59**	2,424	**53.43**	**2,732**	**60.22**
Bataan	**17,380**	32	**0.18**	1,125	**6.47**	8,976	**51.65**	**10,133**	**58.30**
Bulacan	**67,590**	59	**0.09**	3,585	**5.30**	40,146	**59.40**	**43,790**	**64.79**
Nueva Ecija	**43,425**	53	**0.12**	1,918	**4.42**	14,705	**33.86**	**16,676**	**38.40**
Pampanga	**45,651**	43	**0.09**	2,196	**4.81**	21,542	**47.19**	**23,781**	**52.09**
Tarlac	**29,503**	23	**0.08**	1,492	**5.06**	15,457	**52.39**	**16,972**	**57.53**
Zambales	**11,723**	10	**0.09**	465	**3.97**	4,359	**37.18**	**4,834**	**41.24**
City of Angeles	**9,146**	7	**0.08**	312	**3.41**	3,374	**36.89**	**3,693**	**40.38**
City of Olongapo	**4,589**	4	**0.09**	156	**3.40**	2,492	**54.30**	**2,652**	**57.79**
**Region 4A**	**307,982**	**338**	**0.11**	**14,090**	**4.57**	**148,090**	**48.08**	**162,518**	**52.77**
Batangas	**53,251**	59	**0.11**	2,223	**4.17**	26,464	**49.70**	**28,746**	**53.98**
Cavite	**77,964**	61	**0.08**	2,661	**3.41**	34,683	**44.49**	**37,405**	**47.98**
Laguna	**61,525**	66	**0.11**	3,262	**5.30**	34,835	**56.62**	**38,163**	**62.03**
Quezon	**40,810**	60	**0.15**	2,389	**5.85**	18,800	**46.07**	**21,249**	**52.07**
Rizal	**68,235**	84	**0.12**	3,189	**4.67**	30,638	**44.90**	**33,911**	**49.70**
City of Lucena	**6,197**	8	**0.13**	366	**5.91**	2,670	**43.09**	**3,044**	**49.12**
**Region 4B**	**66,121**	**75**	**0.11**	**4,071**	**6.16**	**33,792**	**51.11**	**37,938**	**57.38**
Marinduque	**4,144**	5	**0.12**	312	**7.53**	2,685	**64.79**	**3,002**	**72.44**
Mindoro Occidental	**11,640**	11	**0.09**	972	**8.35**	6,435	**55.28**	**7,418**	**63.73**
Mindoro Oriental	**18,943**	14	**0.07**	741	**3.91**	11,239	**59.33**	**11,994**	**63.32**
Palawan	**20,317**	31	**0.15**	1,447	**7.12**	7,919	**38.98**	**9,397**	**46.25**
Romblon	**5,489**	4	**0.07**	338	**6.16**	3,186	**58.04**	**3,528**	**64.27**
Puerto Princesa City	**5,588**	10	**0.18**	261	**4.67**	2,328	**41.66**	**2,599**	**46.51**
**Region 5**	**135,480**	**112**	**0.08**	**6,510**	**4.81**	**62,204**	**45.91**	**68,826**	**50.80**
Albay	**27,440**	21	**0.08**	821	**2.99**	12,975	**47.28**	**13,817**	**50.35**

Table 2.B.3.3 - Postpartum and Newborn Care
Postpartum women who completed Vitamin A Supplementation
Philippines, 2023

Area	Eligible Pop.	Vitamin A Supplementation							
		Age Group in Year						Total	%
		10-14		15-19		20-49			
		No.	%	No.	%	No.	%		
Camarines Norte	**14,695**	11	**0.07**	838	**5.70**	6,623	**45.07**	**7,472**	**50.85**
Camarines Sur	**43,452**	34	**0.08**	1,441	**3.32**	14,799	**34.06**	**16,274**	**37.45**
Catanduanes	**5,414**	7	**0.13**	406	**7.50**	3,283	**60.64**	**3,696**	**68.27**
Masbate	**22,365**	19	**0.08**	1,740	**7.78**	11,499	**51.42**	**13,258**	**59.28**
Sorsogon	**17,929**	16	**0.09**	1,103	**6.15**	11,315	**63.11**	**12,434**	**69.35**
City of Naga	**4,185**	4	**0.10**	161	**3.85**	1,710	**40.86**	**1,875**	**44.80**
**Region 6**	**148,020**	**162**	**0.11**	**8,649**	**5.84**	**74,921**	**50.62**	**83,732**	**56.57**
Aklan	**11,283**	6	**0.05**	465	**4.12**	5,183	**45.94**	**5,654**	**50.11**
Antique	**11,930**	10	**0.08**	581	**4.87**	6,177	**51.78**	**6,768**	**56.73**
Capiz	**13,931**	8	**0.06**	563	**4.04**	6,262	**44.95**	**6,833**	**49.05**
Guimaras	**3,351**	4	**0.12**	232	**6.92**	2,117	**63.18**	**2,353**	**70.22**
Iloilo	**36,140**	37	**0.10**	1,767	**4.89**	17,537	**48.53**	**19,341**	**53.52**
Negros Occidental	**52,270**	75	**0.14**	3,906	**7.47**	27,029	**51.71**	**31,010**	**59.33**
City of Bacolod	**10,373**	6	**0.06**	632	**6.09**	5,605	**54.03**	**6,243**	**60.19**
City of Iloilo	**8,742**	16	**0.18**	503	**5.75**	5,011	**57.32**	**5,530**	**63.26**
**Region 7**	**165,673**	**163**	**0.10**	**8,649**	**5.22**	**81,826**	**49.39**	**90,638**	**54.71**
Bohol	**26,235**	22	**0.08**	1,188	**4.53**	13,395	**51.06**	**14,605**	**55.67**
Cebu	**72,201**	59	**0.08**	3,234	**4.48**	28,519	**39.50**	**31,812**	**44.06**
Negros Oriental	**28,722**	43	**0.15**	1,676	**5.84**	13,286	**46.26**	**15,005**	**52.24**
Siquijor	**1,680**	0	**0.00**	68	**4.05**	697	**41.49**	**765**	**45.54**
City of Cebu	**19,267**	20	**0.10**	1,476	**7.66**	13,637	**70.78**	**15,133**	**78.54**
City of Lapu-Lapu	**10,679**	5	**0.05**	458	**4.29**	5,973	**55.93**	**6,436**	**60.27**
City of Mandaue	**6,889**	14	**0.20**	549	**7.97**	6,319	**91.73**	**6,882**	**99.90**
**Region 8**	**93,813**	**74**	**0.08**	**4,862**	**5.18**	**42,739**	**45.56**	**47,675**	**50.82**
Biliran	**3,373**	3	**0.09**	211	**6.26**	2,064	**61.19**	**2,278**	**67.54**
Eastern Samar	**9,624**	7	**0.07**	770	**8.00**	5,317	**55.25**	**6,094**	**63.32**
Leyte	**31,559**	24	**0.08**	1,255	**3.98**	11,147	**35.32**	**12,426**	**39.37**
Northern Samar	**14,056**	8	**0.06**	704	**5.01**	6,195	**44.07**	**6,907**	**49.14**
Southern Leyte	**7,406**	3	**0.04**	325	**4.39**	3,272	**44.18**	**3,600**	**48.61**
Samar	**17,011**	20	**0.12**	994	**5.84**	8,136	**47.83**	**9,150**	**53.79**
Ormoc City	**5,292**	4	**0.08**	460	**8.69**	2,896	**54.72**	**3,360**	**63.49**
City of Tacloban	**5,492**	5	**0.09**	143	**2.60**	3,712	**67.59**	**3,860**	**70.28**
**Region 9**	**84,815**	**114**	**0.13**	**6,389**	**7.53**	**42,755**	**50.41**	**49,258**	**58.08**
Zamboanga del Norte	**24,841**	38	**0.15**	1,836	**7.39**	11,498	**46.29**	**13,372**	**53.83**

Table 2.B.3.3 - Postpartum and Newborn Care
Postpartum women who completed Vitamin A Supplementation
Philippines, 2023

Area	Eligible Pop.	Vitamin A Supplementation							
		Age Group in Year						Total	%
		10-14		15-19		20-49			
		No.	%	No.	%	No.	%		
Zamboanga del Sur	23,757	32	0.13	1,293	5.44	8,543	35.96	9,868	41.54
Zamboanga Sibugay	13,841	9	0.07	1,044	7.54	6,715	48.52	7,768	56.12
City of Isabela	3,201	0	0.00	185	5.78	1,409	44.02	1,594	49.80
City of Zamboanga	19,175	35	0.18	2,031	10.59	14,590	76.09	16,656	86.86
**Region 10**	109,169	224	0.21	8,913	8.16	56,409	51.67	65,546	60.04
Bukidnon	33,403	116	0.35	3,907	11.70	19,325	57.85	23,348	69.90
Camiguin	1,727	1	0.06	159	9.21	1,190	68.91	1,350	78.17
Lanao del Norte	17,760	7	0.04	675	3.80	7,223	40.67	7,905	44.51
Misamis Occidental	12,328	17	0.14	839	6.81	6,945	56.34	7,801	63.28
Misamis Oriental	21,975	37	0.17	1,225	5.57	7,562	34.41	8,824	40.15
City of Cagayan De Oro	14,308	42	0.29	1,582	11.06	9,782	68.37	11,406	79.72
City of Iligan	7,668	4	0.05	526	6.86	4,382	57.15	4,912	64.06
**Region 11**	107,001	580	0.54	10,841	10.13	67,363	62.96	78,784	73.63
Davao de Oro	15,253	290	1.90	1,745	11.44	9,244	60.60	11,279	73.95
Davao del Norte	23,453	78	0.33	2,440	10.40	14,118	60.20	16,636	70.93
Davao Oriental	11,935	55	0.46	1,287	10.78	7,083	59.35	8,425	70.59
Davao del Sur	13,083	58	0.44	1,462	11.17	7,652	58.49	9,172	70.11
Davao Occidental	6,629	35	0.53	1,092	16.47	3,104	46.82	4,231	63.83
City of Davao	36,648	64	0.17	2,815	7.68	26,162	71.39	29,041	79.24
**Region 12**	95,279	201	0.21	7,959	8.35	47,690	50.05	55,850	58.62
Cotabato	26,521	50	0.19	1,970	7.43	11,911	44.91	13,931	52.53
Sarangani	13,322	36	0.27	1,667	12.51	7,462	56.01	9,165	68.80
South Cotabato	21,595	52	0.24	2,255	10.44	13,585	62.91	15,892	73.59
Sultan Kudarat	19,391	58	0.30	1,643	8.47	10,396	53.61	12,097	62.38
City of General Santos	14,450	5	0.03	424	2.93	4,336	30.01	4,765	32.98
**BARMM**	132,841	43	0.03	5,355	4.03	57,639	43.39	63,037	47.45
Basilan	11,636	9	0.08	483	4.15	2,626	22.57	3,118	26.80
Lanao del Sur	33,374	6	0.02	1,018	3.05	17,206	51.56	18,230	54.62
Maguindanao	41,650	15	0.04	2,208	5.30	20,315	48.78	22,538	54.11
Sulu	21,378	6	0.03	890	4.16	10,000	46.78	10,896	50.97
Tawi-Tawi	12,609	2	0.02	450	3.57	4,826	38.27	5,278	41.86
SGU	5,103	1	0.02	71	1.39	611	11.97	683	13.38
City of Cotabato	7,091	4	0.06	235	3.31	2,055	28.98	2,294	32.35

Table 2.B.3.3 - Postpartum and Newborn Care
Postpartum women who completed Vitamin A Supplementation
Philippines, 2023

Area	Eligible Pop.	Vitamin A Supplementation							
		Age Group in Year						Total	%
		10-14		15-19		20-49			
		No.	%	No.	%	No.	%		
**Caraga**	**55,065**	**76**	**0.14**	**4,016**	**7.29**	**30,332**	**55.08**	**34,424**	**62.52**
Agusan del Norte	**7,286**	5	**0.07**	438	**6.01**	3,026	**41.53**	**3,469**	**47.61**
Agusan del Sur	**16,038**	23	**0.14**	1,448	**9.03**	9,628	**60.03**	**11,099**	**69.20**
Surigao del Norte	**10,186**	4	**0.04**	444	**4.36**	4,118	**40.43**	**4,566**	**44.83**
Surigao del Sur	**12,736**	23	**0.18**	877	**6.89**	6,931	**54.42**	**7,831**	**61.49**
Dinagat Islands	**2,134**	6	**0.28**	129	**6.04**	1,014	**47.52**	**1,149**	**53.84**
City of Butuan	**6,685**	15	**0.22**	680	**10.17**	5,615	**83.99**	**6,310**	**94.39**

Table 2.C.1.1 - Immunization Services for Infants and Children
Child Protected at Birth (CPAB)
Philippines, 2023

Area	Eligible Pop (0-11 mos. old)	Child Protected at Birth (CPAB)			
		Male	Female	Total	%
**Philippines**	**2,187,333**	**636,018**	**620,383**	**1,256,401**	**57.44**
**NCR**	**261,228**	**92,405**	**90,563**	**182,968**	**70.04**
Malabon City	7,385	2,349	2,248	4,597	62.25
Navotas City	5,210	1,786	1,827	3,613	69.35
Valenzuela City	13,477	3,306	3,232	6,538	48.51
Caloocan City	33,168	7,087	6,874	13,961	42.09
Marikina City	8,342	2,080	1,933	4,013	48.11
Pasig City	17,682	3,417	3,279	6,696	37.87
Pateros	1,116	399	372	771	69.09
Taguig City	18,992	5,737	5,512	11,249	59.23
Quezon City	59,136	33,785	34,093	67,878	114.78
Makati City	10,944	3,453	3,485	6,938	63.40
Mandaluyong City	7,922	3,023	2,892	5,915	74.67
San Juan City	2,168	294	313	607	28.00
Manila City	34,128	13,097	12,224	25,321	74.19
Las Piñas City	11,263	2,708	2,783	5,491	48.75
Muntinlupa City	9,881	2,932	2,871	5,803	58.73
Parañaque City	13,280	3,065	2,880	5,945	44.77
Pasay City	7,134	3,887	3,745	7,632	106.98
**CAR**	**31,319**	**8,642**	**8,327**	**16,969**	**54.18**
Abra	3,660	1,297	1,193	2,490	68.03
Apayao	2,238	696	803	1,499	66.98
Benguet	8,806	2,057	1,994	4,051	46.00
Ifugao	4,098	851	787	1,638	39.97
Kalinga	4,549	1,286	1,226	2,512	55.22
Mountain Province	2,619	943	832	1,775	67.77
City of Baguio	5,349	1,512	1,492	3,004	56.16
**Region 1**	**96,016**	**30,420**	**29,400**	**59,820**	**62.30**
Ilocos Norte	8,912	3,631	3,859	7,490	84.04
Ilocos Sur	9,914	4,732	4,473	9,205	92.85
La Union	13,507	4,721	4,376	9,097	67.35
Pangasinan	60,360	16,334	15,753	32,087	53.16
City of Dagupan	3,323	1,002	939	1,941	58.41

Table 2.C.1.1 - Immunization Services for Infants and Children
Child Protected at Birth (CPAB)
Philippines, 2023

Area	Eligible Pop (0-11 mos. old)	Child Protected at Birth (CPAB)			
		Male	Female	Total	%
**Region 2**	**63,967**	**22,992**	**22,830**	**45,822**	**71.63**
Batanes	240	125	90	215	89.58
Cagayan	20,786	5,817	5,224	11,041	53.12
Isabela	27,379	11,217	10,727	21,944	80.15
Nueva Vizcaya	8,798	2,865	3,889	6,754	76.77
Quirino	3,839	1,530	1,504	3,034	79.03
City of Santiago	2,925	1,438	1,396	2,834	96.89
**Region 3**	**233,544**	**71,936**	**70,719**	**142,655**	**61.08**
Aurora	4,537	1,539	1,523	3,062	67.49
Bataan	17,380	4,864	4,414	9,278	53.38
Bulacan	67,590	20,973	20,842	41,815	61.87
Nueva Ecija	43,425	13,050	12,704	25,754	59.31
Pampanga	45,651	13,731	13,764	27,495	60.23
Tarlac	29,503	8,102	8,211	16,313	55.29
Zambales	11,723	2,997	2,913	5,910	50.41
City of Angeles	9,146	4,942	4,724	9,666	105.69
City of Olongapo	4,589	1,738	1,624	3,362	73.26
**Region 4A**	**307,982**	**83,377**	**80,849**	**164,226**	**53.32**
Batangas	53,251	16,852	16,324	33,176	62.30
Cavite	77,964	20,937	20,331	41,268	52.93
Laguna	61,525	17,743	17,598	35,341	57.44
Quezon	40,810	10,490	9,908	20,398	49.98
Rizal	68,235	15,877	15,239	31,116	45.60
City of Lucena	6,197	1,478	1,449	2,927	47.23
**Region 4B**	**66,121**	**17,768**	**16,739**	**34,507**	**52.19**
Marinduque	4,144	1,384	1,149	2,533	61.12
Occidental Mindoro	11,640	3,126	2,949	6,075	52.19
Oriental Mindoro	18,943	4,217	4,062	8,279	43.70
Palawan	20,317	5,176	4,900	10,076	49.59
Romblon	5,489	2,003	1,901	3,904	71.12
City of Puerto Princesa	5,588	1,862	1,778	3,640	65.14

Table 2.C.1.1 - Immunization Services for Infants and Children

Child Protected at Birth (CPAB)

Philippines, 2023

Area	Eligible Pop (0-11 mos. old)	Child Protected at Birth (CPAB)			
		Male	Female	Total	%
**Region 5**	**135,480**	**30,109**	**29,129**	**59,238**	**43.72**
Albay	27,440	6,816	6,584	13,400	48.83
Camarines Norte	14,695	3,154	3,224	6,378	43.40
Camarines Sur	43,452	5,667	5,371	11,038	25.40
Catanduanes	5,414	2,037	1,918	3,955	73.05
Masbate	22,365	5,904	5,889	11,793	52.73
Sorsogon	17,929	5,645	5,328	10,973	61.20
City of Naga	4,185	886	815	1,701	40.65
**Region 6**	**148,020**	**42,302**	**40,832**	**83,134**	**56.16**
Aklan	11,283	2,694	2,648	5,342	47.35
Antique	11,930	3,506	3,337	6,843	57.36
Capiz	13,931	3,655	3,257	6,912	49.62
Guimaras	3,351	1,161	1,178	2,339	69.80
Iloilo	36,140	10,558	9,919	20,477	56.66
Negros Occidental	52,270	15,268	15,165	30,433	58.22
City of Bacolod	10,373	2,705	2,613	5,318	51.27
City of Iloilo	8,742	2,755	2,715	5,470	62.57
**Region 7**	**165,673**	**45,895**	**44,543**	**90,438**	**54.59**
Bohol	26,235	6,311	6,271	12,582	47.96
Cebu	72,201	17,300	16,700	34,000	47.09
Negros Oriental	28,722	7,009	6,946	13,955	48.59
Siquijor	1,680	457	419	876	52.14
City of Cebu	19,267	8,062	7,519	15,581	80.87
City of Lapu-Lapu	10,679	4,356	4,305	8,661	81.10
City of Mandaue	6,889	2,400	2,383	4,783	69.43
**Region 8**	**93,813**	**22,236**	**21,898**	**44,134**	**47.04**
Biliran	3,373	1,472	1,381	2,853	84.58
Eastern Samar	9,624	3,112	3,073	6,185	64.27
Leyte	31,559	7,086	6,949	14,035	44.47
Northern Samar	14,056	2,388	2,539	4,927	35.05
Southern Leyte	7,406	2,057	1,980	4,037	54.51
Samar	17,011	3,246	3,199	6,445	37.89
Ormoc City	5,292	1,633	1,519	3,152	59.56
City of Tacloban	5,492	1,242	1,258	2,500	45.52

Table 2.C.1.1 - Immunization Services for Infants and Children
Child Protected at Birth (CPAB)
Philippines, 2023

Area	Eligible Pop (0-11 mos. old)	Child Protected at Birth (CPAB)			
		Male	Female	Total	%
**Region 9**	**84,815**	**23,069**	**23,084**	**46,153**	**54.42**
Zamboanga del Norte	24,841	6,339	6,449	12,788	51.48
Zamboanga del Sur	23,757	4,591	4,628	9,219	38.81
Zamboanga Sibugay	13,841	3,891	3,686	7,577	54.74
City of Isabela	3,201	685	618	1,303	40.71
City of Zamboanga	19,175	7,563	7,703	15,266	79.61
**Region 10**	**109,169**	**33,444**	**32,720**	**66,164**	**60.61**
Bukidnon	33,403	10,453	10,234	20,687	61.93
Camiguin	1,727	670	661	1,331	77.07
Lanao del Norte	17,760	3,881	4,081	7,962	44.83
Misamis Occidental	12,328	3,961	3,793	7,754	62.90
Misamis Oriental	21,975	6,758	6,799	13,557	61.69
City of Cagayan De Oro	14,308	5,570	5,065	10,635	74.33
City of Iligan	7,668	2,151	2,087	4,238	55.27
**Region 11**	**107,001**	**38,616**	**36,250**	**74,866**	**69.97**
Davao de Oro	15,253	5,822	5,299	11,121	72.91
Davao del Norte	23,453	8,221	7,686	15,907	67.83
Davao Oriental	11,935	3,896	3,712	7,608	63.75
Davao del Sur	13,083	4,461	4,101	8,562	65.44
Davao Occidental	6,629	1,829	1,782	3,611	54.47
City of Davao	36,648	14,387	13,670	28,057	76.56
**Region 12**	**95,279**	**29,308**	**28,171**	**57,479**	**60.33**
Cotabato	26,521	7,668	7,203	14,871	56.07
Sarangani	13,322	4,502	4,288	8,790	65.98
South Cotabato	21,595	7,869	7,725	15,594	72.21
Sultan Kudarat	19,391	5,994	5,729	11,723	60.46
City of General Santos	14,450	3,275	3,226	6,501	44.99
**BARMM**	**132,841**	**25,739**	**26,824**	**52,563**	**39.57**
Basilan	11,636	1,410	1,383	2,793	24.00
Lanao del Sur	33,374	10,174	10,673	20,847	62.46
Maguindanao	41,650	6,475	6,840	13,315	31.97

Table 2.C.1.1 - Immunization Services for Infants and Children
Child Protected at Birth (CPAB)
Philippines, 2023

Area	Eligible Pop (0-11 mos. old)	Child Protected at Birth (CPAB)			
		Male	Female	Total	%
Sulu	21,378	3,911	4,226	8,137	38.06
Tawi-Tawi	12,609	2,130	2,064	4,194	33.26
SGU	5,103	276	200	476	9.33
City of Cotabato	7,091	1,363	1,438	2,801	39.50
**CARAGA**	**55,065**	**17,760**	**17,505**	**35,265**	**64.04**
Agusan del Norte	7,286	2,379	2,233	4,612	63.30
Agusan del Sur	16,038	4,692	4,844	9,536	59.46
Surigao del Norte	10,186	3,052	2,989	6,041	59.31
Surigao del Sur	12,736	4,220	4,052	8,272	64.95
Dinagat Islands	2,134	446	417	863	40.44
City of Butuan	6,685	2,971	2,970	5,941	88.87

Table 2.C.1.1 - Immunization Services for Infants and Children

Children Given Bacille Calmette-Guérin (BCG)

Philippines, 2023

Area	Eligible Pop (0-11 mos. old)	BCG			
		Male	Female	Total	%
**Philippines**	**2,187,333**	**747,817**	**716,698**	**1,464,515**	**66.95**
**NCR**	**261,228**	**108,807**	**105,103**	**213,910**	**81.89**
Malabon City	7,385	2,232	2,178	4,410	59.72
Navotas City	5,210	2,155	2,204	4,359	83.67
Valenzuela City	13,477	5,050	4,457	9,507	70.54
Caloocan City	33,168	9,555	9,003	18,558	55.95
Marikina City	8,342	4,673	4,515	9,188	110.14
Pasig City	17,682	8,194	7,847	16,041	90.72
Pateros	1,116	394	385	779	69.80
Taguig City	18,992	3,926	3,511	7,437	39.16
Quezon City	59,136	34,946	35,176	70,122	118.58
Makati City	10,944	4,265	4,258	8,523	77.88
Mandaluyong City	7,922	3,116	2,970	6,086	76.82
San Juan City	2,168	523	454	977	45.06
Manila City	34,128	14,711	13,708	28,419	83.27
Las Piñas City	11,263	3,867	3,731	7,598	67.46
Muntinlupa City	9,881	2,998	2,955	5,953	60.25
Parañaque City	13,280	3,853	3,612	7,465	56.21
Pasay City	7,134	4,349	4,139	8,488	118.98
**CAR**	**31,319**	**12,639**	**11,838**	**24,477**	**78.15**
Abra	3,660	1,394	1,343	2,737	74.78
Apayao	2,238	1,100	1,165	2,265	101.21
Benguet	8,806	2,191	2,135	4,326	49.13
Ifugao	4,098	1,249	1,097	2,346	57.25
Kalinga	4,549	1,242	1,179	2,421	53.22
Mountain Province	2,619	1,802	1,532	3,334	127.30
City of Baguio	5,349	3,661	3,387	7,048	131.76
**Region 1**	**96,016**	**25,770**	**24,620**	**50,390**	**52.48**
Ilocos Norte	8,912	3,549	3,679	7,228	81.10
Ilocos Sur	9,914	4,809	4,531	9,340	94.21
La Union	13,507	4,702	4,352	9,054	67.03
Pangasinan	60,360	11,554	10,966	22,520	37.31
City of Dagupan	3,323	1,156	1,092	2,248	67.65

Table 2.C.1.1 - Immunization Services for Infants and Children
Children Given Bacille Calmette-Guérin (BCG)
Philippines, 2023

Area	Eligible Pop (0-11 mos. old)	BCG			
		Male	Female	Total	%
**Region 2**	**63,967**	**23,202**	**21,761**	**44,963**	**70.29**
Batanes	240	123	93	216	90.00
Cagayan	20,786	5,656	5,082	10,738	51.66
Isabela	27,379	11,417	10,939	22,356	81.65
Nueva Vizcaya	8,798	3,010	2,745	5,755	65.41
Quirino	3,839	1,558	1,506	3,064	79.81
City of Santiago	2,925	1,438	1,396	2,834	96.89
**Region 3**	**233,544**	**82,688**	**79,157**	**161,845**	**69.30**
Aurora	4,537	1,735	1,632	3,367	74.21
Bataan	17,380	5,299	4,944	10,243	58.94
Bulacan	67,590	29,222	27,580	56,802	84.04
Nueva Ecija	43,425	14,645	14,138	28,783	66.28
Pampanga	45,651	13,146	12,651	25,797	56.51
Tarlac	29,503	9,482	9,660	19,142	64.88
Zambales	11,723	3,147	2,970	6,117	52.18
City of Angeles	9,146	4,223	4,004	8,227	89.95
City of Olongapo	4,589	1,789	1,578	3,367	73.37
**Region 4A**	**307,982**	**100,348**	**96,224**	**196,572**	**63.83**
Batangas	53,251	18,736	17,983	36,719	68.95
Cavite	77,964	26,348	25,654	52,002	66.70
Laguna	61,525	18,518	18,040	36,558	59.42
Quezon	40,810	12,193	11,587	23,780	58.27
Rizal	68,235	23,020	21,467	44,487	65.20
City of Lucena	6,197	1,533	1,493	3,026	48.83
**Region 4B**	**66,121**	**22,773**	**21,389**	**44,162**	**66.79**
Marinduque	4,144	1,421	1,200	2,621	63.25
Occidental Mindoro	11,640	4,365	4,078	8,443	72.53
Oriental Mindoro	18,943	6,038	5,657	11,695	61.74
Palawan	20,317	7,209	6,853	14,062	69.21
Romblon	5,489	2,077	1,963	4,040	73.60
City of Puerto Princesa	5,588	1,663	1,638	3,301	59.07

Table 2.C.1.1 - Immunization Services for Infants and Children
Children Given Bacille Calmette-Guérin (BCG)
Philippines, 2023

Area	Eligible Pop (0-11 mos. old)	BCG			
		Male	Female	Total	%
**Region 5**	**135,480**	**39,550**	**37,845**	**77,395**	**57.13**
Albay	27,440	6,600	6,423	13,023	47.46
Camarines Norte	14,695	6,275	6,007	12,282	83.58
Camarines Sur	43,452	8,800	8,509	17,309	39.83
Catanduanes	5,414	2,032	1,837	3,869	71.46
Masbate	22,365	8,538	8,189	16,727	74.79
Sorsogon	17,929	6,306	5,946	12,252	68.34
City of Naga	4,185	999	934	1,933	46.19
**Region 6**	**148,020**	**48,494**	**45,429**	**93,923**	**63.45**
Aklan	11,283	3,136	2,931	6,067	53.77
Antique	11,930	2,864	2,645	5,509	46.18
Capiz	13,931	5,127	4,554	9,681	69.49
Guimaras	3,351	1,150	1,128	2,278	67.98
Iloilo	36,140	9,952	9,398	19,350	53.54
Negros Occidental	52,270	18,229	17,181	35,410	67.74
City of Bacolod	10,373	3,202	2,994	6,196	59.73
City of Iloilo	8,742	4,834	4,598	9,432	107.89
**Region 7**	**165,673**	**58,821**	**55,844**	**114,665**	**69.21**
Bohol	26,235	9,285	8,985	18,270	69.64
Cebu	72,201	22,754	21,484	44,238	61.27
Negros Oriental	28,722	9,824	9,327	19,151	66.68
Siquijor	1,680	516	493	1,009	60.06
City of Cebu	19,267	8,078	7,327	15,405	79.96
City of Lapu-Lapu	10,679	3,846	3,839	7,685	71.96
City of Mandaue	6,889	4,518	4,389	8,907	129.29
**Region 8**	**93,813**	**29,046**	**27,825**	**56,871**	**60.62**
Biliran	3,373	1,587	1,474	3,061	90.75
Eastern Samar	9,624	3,349	3,227	6,576	68.33
Leyte	31,559	8,424	8,121	16,545	52.43
Northern Samar	14,056	5,284	4,965	10,249	72.92
Southern Leyte	7,406	2,144	2,018	4,162	56.20
Samar	17,011	5,031	4,860	9,891	58.14
Ormoc City	5,292	1,770	1,685	3,455	65.29
City of Tacloban	5,492	1,457	1,475	2,932	53.39

Table 2.C.1.1 - Immunization Services for Infants and Children
Children Given Bacille Calmette-Guérin (BCG)
Philippines, 2023

Area	Eligible Pop (0-11 mos. old)	BCG			
		Male	Female	Total	%
**Region 9**	**84,815**	**19,976**	**19,804**	**39,780**	**46.90**
Zamboanga del Norte	24,841	7,118	7,149	14,267	57.43
Zamboanga del Sur	23,757	4,836	4,748	9,584	40.34
Zamboanga Sibugay	13,841	3,763	3,663	7,426	53.65
City of Isabela	3,201	1,202	1,108	2,310	72.16
City of Zamboanga	19,175	3,057	3,136	6,193	32.30
**Region 10**	**109,169**	**36,310**	**35,319**	**71,629**	**65.61**
Bukidnon	33,403	11,116	10,676	21,792	65.24
Camiguin	1,727	679	658	1,337	77.42
Lanao del Norte	17,760	5,676	5,646	11,322	63.75
Misamis Occidental	12,328	4,045	3,752	7,797	63.25
Misamis Oriental	21,975	7,370	7,609	14,979	68.16
City of Cagayan De Oro	14,308	5,125	4,748	9,873	69.00
City of Iligan	7,668	2,299	2,230	4,529	59.06
**Region 11**	**107,001**	**42,674**	**40,130**	**82,804**	**77.39**
Davao de Oro	15,253	6,238	5,837	12,075	79.16
Davao del Norte	23,453	8,828	8,308	17,136	73.07
Davao Oriental	11,935	4,494	4,243	8,737	73.20
Davao del Sur	13,083	4,790	4,429	9,219	70.47
Davao Occidental	6,629	2,510	2,399	4,909	74.05
City of Davao	36,648	15,814	14,914	30,728	83.85
**Region 12**	**95,279**	**32,082**	**30,710**	**62,792**	**65.90**
Cotabato	26,521	8,975	8,553	17,528	66.09
Sarangani	13,322	4,928	4,637	9,565	71.80
South Cotabato	21,595	8,370	7,950	16,320	75.57
Sultan Kudarat	19,391	6,676	6,350	13,026	67.18
City of General Santos	14,450	3,133	3,220	6,353	43.97
**BARMM**	**132,841**	**42,700**	**43,150**	**85,850**	**64.63**
Basilan	11,636	3,004	3,032	6,036	51.87
Lanao del Sur	33,374	12,861	13,454	26,315	78.85
Maguindanao	41,650	14,256	14,337	28,593	68.65

Table 2.C.1.1 - Immunization Services for Infants and Children

Children Given Bacille Calmette-Guérin (BCG)

Philippines, 2023

Area	Eligible Pop (0-11 mos. old)	BCG			
		Male	Female	Total	%
Sulu	21,378	6,182	6,258	12,440	58.19
Tawi-Tawi	12,609	3,284	3,123	6,407	50.81
SGU	5,103	1,104	1,019	2,123	41.60
City of Cotabato	7,091	2,009	1,927	3,936	55.51
**CARAGA**	**55,065**	**21,937**	**20,550**	**42,487**	**77.16**
Agusan del Norte	7,286	3,449	3,115	6,564	90.09
Agusan del Sur	16,038	5,909	5,744	11,653	72.66
Surigao del Norte	10,186	3,941	3,730	7,671	75.31
Surigao del Sur	12,736	5,260	4,874	10,134	79.57
Dinagat Islands	2,134	546	493	1,039	48.69
City of Butuan	6,685	2,832	2,594	5,426	81.17

Table 2.C.1.1 - Immunization Services for Infants and Children
Children Given Hepatitis B Vaccines
Philippines, 2023

Area	Eligible Pop (0-11 mos. old)	Hepa B (w/in 24 Hrs.) after birth			
		Male	Female	Total	%
**Philippines**	**2,187,333**	**639,865**	**615,003**	**1,254,868**	**57.37**
**NCR**	**261,228**	**104,275**	**101,268**	**205,543**	**78.68**
Malabon City	7,385	2,246	2,198	4,444	60.18
Navotas City	5,210	2,035	2,062	4,097	78.64
Valenzuela City	13,477	4,825	4,784	9,609	71.30
Caloocan City	33,168	8,158	7,677	15,835	47.74
Marikina City	8,342	4,562	4,343	8,905	106.75
Pasig City	17,682	7,602	7,308	14,910	84.32
Pateros	1,116	395	376	771	69.09
Taguig City	18,992	3,232	2,906	6,138	32.32
Quezon City	59,136	34,290	34,603	68,893	116.50
Makati City	10,944	4,032	3,991	8,023	73.31
Mandaluyong City	7,922	3,202	2,916	6,118	77.23
San Juan City	2,168	487	424	911	42.02
Manila City	34,128	14,589	13,579	28,168	82.54
Las Piñas City	11,263	4,579	4,417	8,996	79.87
Muntinlupa City	9,881	2,406	2,365	4,771	48.28
Parañaque City	13,280	3,389	3,194	6,583	49.57
Pasay City	7,134	4,246	4,125	8,371	117.34
**CAR**	**31,319**	**12,352**	**11,407**	**23,759**	**75.86**
Abra	3,660	1,371	1,307	2,678	73.17
Apayao	2,238	1,022	1,070	2,092	93.48
Benguet	8,806	2,149	1,950	4,099	46.55
Ifugao	4,098	1,118	1,003	2,121	51.76
Kalinga	4,549	1,214	1,147	2,361	51.90
Mountain Province	2,619	1,813	1,542	3,355	128.10
City of Baguio	5,349	3,665	3,388	7,053	131.86
**Region 1**	**96,016**	**24,779**	**23,655**	**48,434**	**50.44**
Ilocos Norte	8,912	3,489	3,618	7,107	79.75
Ilocos Sur	9,914	4,782	4,496	9,278	93.58
La Union	13,507	4,705	4,349	9,054	67.03
Pangasinan	60,360	10,683	10,101	20,784	34.43
City of Dagupan	3,323	1,120	1,091	2,211	66.54

Table 2.C.1.1 - Immunization Services for Infants and Children
Children Given Hepatitis B Vaccines
Philippines, 2023

Area	Eligible Pop (0-11 mos. old)	Hepa B (w/in 24 Hrs.) after birth			
		Male	Female	Total	%
**Region 2**	**63,967**	**22,544**	**21,831**	**44,375**	**69.37**
Batanes	240	125	91	216	90.00
Cagayan	20,786	5,124	4,629	9,753	46.92
Isabela	27,379	11,301	11,437	22,738	83.05
Nueva Vizcaya	8,798	2,951	2,681	5,632	64.01
Quirino	3,839	1,605	1,597	3,202	83.41
City of Santiago	2,925	1,438	1,396	2,834	96.89
**Region 3**	**233,544**	**69,507**	**66,573**	**136,080**	**58.27**
Aurora	4,537	1,525	1,445	2,970	65.46
Bataan	17,380	4,950	4,625	9,575	55.09
Bulacan	67,590	22,935	21,465	44,400	65.69
Nueva Ecija	43,425	11,998	11,704	23,702	54.58
Pampanga	45,651	12,416	11,878	24,294	53.22
Tarlac	29,503	7,910	8,235	16,145	54.72
Zambales	11,723	2,560	2,403	4,963	42.34
City of Angeles	9,146	3,596	3,388	6,984	76.36
City of Olongapo	4,589	1,617	1,430	3,047	66.40
**Region 4A**	**307,982**	**90,811**	**87,592**	**178,403**	**57.93**
Batangas	53,251	17,498	16,913	34,411	64.62
Cavite	77,964	24,596	24,141	48,737	62.51
Laguna	61,525	16,724	16,292	33,016	53.66
Quezon	40,810	10,987	10,421	21,408	52.46
Rizal	68,235	19,487	18,342	37,829	55.44
City of Lucena	6,197	1,519	1,483	3,002	48.44
**Region 4B**	**66,121**	**16,305**	**15,264**	**31,569**	**47.74**
Marinduque	4,144	1,494	1,234	2,728	65.83
Occidental Mindoro	11,640	3,073	2,867	5,940	51.03
Oriental Mindoro	18,943	4,547	4,341	8,888	46.92
Palawan	20,317	4,057	3,776	7,833	38.55
Romblon	5,489	1,924	1,855	3,779	68.85
City of Puerto Princesa	5,588	1,210	1,191	2,401	42.97

Table 2.C.1.1 - Immunization Services for Infants and Children
Children Given Hepatitis B Vaccines
Philippines, 2023

Area	Eligible Pop (0-11 mos. old)	Hepa B (w/in 24 Hrs.) after birth			
		Male	Female	Total	%
**Region 5**	**135,480**	**34,330**	**32,887**	**67,217**	**49.61**
Albay	27,440	6,261	6,199	12,460	45.41
Camarines Norte	14,695	5,933	5,727	11,660	79.35
Camarines Sur	43,452	6,744	6,307	13,051	30.04
Catanduanes	5,414	2,014	1,841	3,855	71.20
Masbate	22,365	6,098	5,932	12,030	53.79
Sorsogon	17,929	6,288	5,943	12,231	68.22
City of Naga	4,185	992	938	1,930	46.12
**Region 6**	**148,020**	**46,156**	**43,122**	**89,278**	**60.31**
Aklan	11,283	2,279	2,180	4,459	39.52
Antique	11,930	3,544	3,397	6,941	58.18
Capiz	13,931	5,231	4,570	9,801	70.35
Guimaras	3,351	703	732	1,435	42.82
Iloilo	36,140	9,653	9,253	18,906	52.31
Negros Occidental	52,270	17,144	15,916	33,060	63.25
City of Bacolod	10,373	3,148	2,846	5,994	57.78
City of Iloilo	8,742	4,454	4,228	8,682	99.31
**Region 7**	**165,673**	**45,976**	**43,853**	**89,829**	**54.22**
Bohol	26,235	9,188	8,879	18,067	68.87
Cebu	72,201	18,543	17,547	36,090	49.99
Negros Oriental	28,722	6,476	6,128	12,604	43.88
Siquijor	1,680	314	310	624	37.14
City of Cebu	19,267	7,565	7,137	14,702	76.31
City of Lapu-Lapu	10,679	727	720	1,447	13.55
City of Mandaue	6,889	3,163	3,132	6,295	91.38
**Region 8**	**93,813**	**23,479**	**22,629**	**46,108**	**49.15**
Biliran	3,373	1,581	1,471	3,052	90.48
Eastern Samar	9,624	2,350	2,292	4,642	48.23
Leyte	31,559	7,365	7,172	14,537	46.06
Northern Samar	14,056	4,241	4,078	8,319	59.18
Southern Leyte	7,406	1,887	1,837	3,724	50.28
Samar	17,011	2,903	2,648	5,551	32.63
Ormoc City	5,292	1,747	1,697	3,444	65.08
City of Tacloban	5,492	1,405	1,434	2,839	51.69

Table 2.C.1.1 - Immunization Services for Infants and Children
Children Given Hepatitis B Vaccines
Philippines, 2023

Area	Eligible Pop (0-11 mos. old)	Hepa B (w/in 24 Hrs.) after birth			
		Male	Female	Total	%
**Region 9**	**84,815**	**15,146**	**15,004**	**30,150**	**35.55**
Zamboanga del Norte	24,841	5,798	5,898	11,696	47.08
Zamboanga del Sur	23,757	3,487	3,560	7,047	29.66
Zamboanga Sibugay	13,841	3,438	3,239	6,677	48.24
City of Isabela	3,201	761	679	1,440	44.99
City of Zamboanga	19,175	1,662	1,628	3,290	17.16
**Region 10**	**109,169**	**24,372**	**23,670**	**48,042**	**44.01**
Bukidnon	33,403	7,376	7,446	14,822	44.37
Camiguin	1,727	498	504	1,002	58.02
Lanao del Norte	17,760	1,628	1,688	3,316	18.67
Misamis Occidental	12,328	3,922	3,679	7,601	61.66
Misamis Oriental	21,975	3,761	3,663	7,424	33.78
City of Cagayan De Oro	14,308	5,399	4,876	10,275	71.81
City of Iligan	7,668	1,788	1,814	3,602	46.97
**Region 11**	**107,001**	**37,945**	**35,770**	**73,715**	**68.89**
Davao de Oro	15,253	5,700	5,371	11,071	72.58
Davao del Norte	23,453	8,258	7,774	16,032	68.36
Davao Oriental	11,935	4,036	3,856	7,892	66.12
Davao del Sur	13,083	4,525	4,187	8,712	66.59
Davao Occidental	6,629	1,620	1,581	3,201	48.29
City of Davao	36,648	13,806	13,001	26,807	73.15
**Region 12**	**95,279**	**27,967**	**26,720**	**54,687**	**57.40**
Cotabato	26,521	7,191	6,705	13,896	52.40
Sarangani	13,322	4,453	4,275	8,728	65.52
South Cotabato	21,595	7,604	7,302	14,906	69.03
Sultan Kudarat	19,391	5,942	5,585	11,527	59.45
City of General Santos	14,450	2,777	2,853	5,630	38.96
**BARMM**	**132,841**	**27,594**	**27,414**	**55,008**	**41.41**
Basilan	11,636	1,214	1,126	2,340	20.11
Lanao del Sur	33,374	11,644	11,425	23,069	69.12
Maguindanao	41,650	7,014	7,004	14,018	33.66

Table 2.C.1.1 - Immunization Services for Infants and Children
Children Given Hepatitis B Vaccines
Philippines, 2023

Area	Eligible Pop (0-11 mos. old)	Hepa B (w/in 24 Hrs.) after birth			
		Male	Female	Total	%
Sulu	21,378	4,192	4,303	8,495	39.74
Tawi-Tawi	12,609	2,414	2,421	4,835	38.35
SGU	5,103	132	168	300	5.88
City of Cotabato	7,091	984	967	1,951	27.51
**CARAGA**	**55,065**	**16,327**	**16,344**	**32,671**	**59.33**
Agusan del Norte	7,286	2,875	2,540	5,415	74.32
Agusan del Sur	16,038	3,317	3,587	6,904	43.05
Surigao del Norte	10,186	2,716	2,616	5,332	52.35
Surigao del Sur	12,736	4,668	4,773	9,441	74.13
Dinagat Islands	2,134	460	444	904	42.36
City of Butuan	6,685	2,291	2,384	4,675	69.93

Table 2.C.1.2 - Immunization Services for Infants
Infants who completed 3 doses of HiB-HepB antigen
Philippines, 2023

Area	Eligible Pop (0-11 mos. old)	DPT-HiB-HepB 1			
		Male	Female	Total	%
**Philippines**	**2,187,333**	**876,160**	**830,383**	**1,706,543**	**78.02**
**NCR**	**261,228**	**107,865**	**103,646**	**211,511**	**80.97**
Malabon City	7,385	2,658	2,528	5,186	70.22
Navotas City	5,210	2,403	2,406	4,809	92.30
Valenzuela City	13,477	5,071	4,829	9,900	73.46
Caloocan City	33,168	10,838	10,001	20,839	62.83
Marikina City	8,342	2,970	2,794	5,764	69.10
Pasig City	17,682	6,147	5,952	12,099	68.43
Pateros	1,116	398	399	797	71.42
Taguig City	18,992	6,127	5,471	11,598	61.07
Quezon City	59,136	33,286	33,390	66,676	112.75
Makati City	10,944	4,491	4,357	8,848	80.85
Mandaluyong City	7,922	3,151	3,081	6,232	78.67
San Juan City	2,168	548	546	1,094	50.46
Manila City	34,128	14,559	13,628	28,187	82.59
Las Piñas City	11,263	4,327	4,082	8,409	74.66
Muntinlupa City	9,881	3,794	3,558	7,352	74.41
Parañaque City	13,280	3,914	3,542	7,456	56.14
Pasay City	7,134	3,183	3,082	6,265	87.82
**CAR**	**31,319**	**12,741**	**11,769**	**24,510**	**78.26**
Abra	3,660	1,610	1,536	3,146	85.96
Apayao	2,238	993	934	1,927	86.10
Benguet	8,806	3,031	2,902	5,933	67.37
Ifugao	4,098	1,723	1,472	3,195	77.96
Kalinga	4,549	1,930	1,762	3,692	81.16
Mountain Province	2,619	1,183	1,035	2,218	84.69
City of Baguio	5,349	2,271	2,128	4,399	82.24
**Region 1**	**96,016**	**38,538**	**36,300**	**74,838**	**77.94**
Ilocos Norte	8,912	3,863	3,861	7,724	86.67
Ilocos Sur	9,914	4,647	4,305	8,952	90.30
La Union	13,507	5,077	4,511	9,588	70.99
Pangasinan	60,360	23,760	22,515	46,275	76.67
City of Dagupan	3,323	1,191	1,108	2,299	69.18

Table 2.C.1.2 - Immunization Services for Infants
Infants who completed 3 doses of HiB-HepB antigen
Philippines, 2023

Area	Eligible Pop (0-11 mos. old)	DPT-HiB-HepB 1			
		Male	Female	Total	%
**Region 2**	**63,967**	**26,123**	**24,982**	**51,105**	**79.89**
Batanes	240	123	90	213	88.75
Cagayan	20,786	8,164	8,161	16,325	78.54
Isabela	27,379	11,717	11,183	22,900	83.64
Nueva Vizcaya	8,798	3,102	2,766	5,868	66.70
Quirino	3,839	1,745	1,590	3,335	86.87
City of Santiago	2,925	1,272	1,192	2,464	84.24
**Region 3**	**233,544**	**98,641**	**93,560**	**192,201**	**82.30**
Aurora	4,537	2,020	1,884	3,904	86.05
Bataan	17,380	6,789	6,319	13,108	75.42
Bulacan	67,590	31,354	29,062	60,416	89.39
Nueva Ecija	43,425	16,843	16,150	32,993	75.98
Pampanga	45,651	19,176	18,706	37,882	82.98
Tarlac	29,503	12,450	12,136	24,586	83.33
Zambales	11,723	4,850	4,453	9,303	79.36
City of Angeles	9,146	3,419	3,284	6,703	73.29
City of Olongapo	4,589	1,740	1,566	3,306	72.04
**Region 4A**	**307,982**	**121,431**	**114,843**	**236,274**	**76.72**
Batangas	53,251	21,651	20,644	42,295	79.43
Cavite	77,964	31,434	29,837	61,271	78.59
Laguna	61,525	25,094	23,967	49,061	79.74
Quezon	40,810	15,430	14,536	29,966	73.43
Rizal	68,235	25,529	23,671	49,200	72.10
City of Lucena	6,197	2,293	2,188	4,481	72.31
**Region 4B**	**66,121**	**27,968**	**26,062**	**54,030**	**81.71**
Marinduque	4,144	1,703	1,448	3,151	76.04
Occidental Mindoro	11,640	5,124	4,824	9,948	85.46
Oriental Mindoro	18,943	7,180	6,669	13,849	73.11
Palawan	20,317	8,912	8,284	17,196	84.64
Romblon	5,489	2,348	2,204	4,552	82.93
City of Puerto Princesa	5,588	2,701	2,633	5,334	95.45
**Region 5**	**135,480**	**51,431**	**48,347**	**99,778**	**73.65**

Table 2.C.1.2 - Immunization Services for Infants
Infants who completed 3 doses of HiB-HepB antigen
Philippines, 2023

Area	Eligible Pop (0-11 mos. old)	DPT-HiB-HepB 1			
		Male	Female	Total	%
Albay	27,440	9,970	9,478	19,448	70.87
Camarines Norte	14,695	5,728	5,301	11,029	75.05
Camarines Sur	43,452	15,321	14,441	29,762	68.49
Catanduanes	5,414	2,177	2,099	4,276	78.98
Masbate	22,365	9,304	8,810	18,114	80.99
Sorsogon	17,929	7,358	6,787	14,145	78.89
City of Naga	4,185	1,573	1,431	3,004	71.78
**Region 6**	**148,020**	**56,747**	**53,349**	**110,096**	**74.38**
Aklan	11,283	3,820	3,586	7,406	65.64
Antique	11,930	4,549	4,256	8,805	73.81
Capiz	13,931	5,063	4,616	9,679	69.48
Guimaras	3,351	1,230	1,225	2,455	73.26
Iloilo	36,140	13,799	12,833	26,632	73.69
Negros Occidental	52,270	20,856	19,860	40,716	77.90
City of Bacolod	10,373	4,168	3,823	7,991	77.04
City of Iloilo	8,742	3,262	3,150	6,412	73.35
**Region 7**	**165,673**	**65,015**	**61,382**	**126,397**	**76.29**
Bohol	26,235	9,529	9,047	18,576	70.81
Cebu	72,201	26,493	24,966	51,459	71.27
Negros Oriental	28,722	11,762	11,112	22,874	79.64
Siquijor	1,680	588	548	1,136	67.62
City of Cebu	19,267	8,254	7,564	15,818	82.10
City of Lapu-Lapu	10,679	5,128	5,010	10,138	94.93
City of Mandaue	6,889	3,261	3,135	6,396	92.84
**Region 8**	**93,813**	**35,899**	**33,740**	**69,639**	**74.23**
Biliran	3,373	1,601	1,520	3,121	92.53
Eastern Samar	9,624	3,770	3,625	7,395	76.84
Leyte	31,559	11,469	10,703	22,172	70.26
Northern Samar	14,056	5,545	5,082	10,627	75.60
Southern Leyte	7,406	2,542	2,344	4,886	65.97
Samar	17,011	7,103	6,767	13,870	81.54
Ormoc City	5,292	1,947	1,810	3,757	70.99
City of Tacloban	5,492	1,922	1,889	3,811	69.39

Table 2.C.1.2 - Immunization Services for Infants
Infants who completed 3 doses of HiB-HepB antigen
Philippines, 2023

Area	Eligible Pop (0-11 mos. old)	DPT-HiB-HepB 1			
		Male	Female	Total	%
**Region 9**	**84,815**	**35,104**	**33,731**	**68,835**	**81.16**
Zamboanga del Norte	24,841	9,613	9,265	18,878	76.00
Zamboanga del Sur	23,757	8,689	8,370	17,059	71.81
Zamboanga Sibugay	13,841	5,556	5,421	10,977	79.31
City of Isabela	3,201	1,402	1,318	2,720	84.97
City of Zamboanga	19,175	9,844	9,357	19,201	100.14
**Region 10**	**109,169**	**46,237**	**43,735**	**89,972**	**82.42**
Bukidnon	33,403	15,319	14,260	29,579	88.55
Camiguin	1,727	669	712	1,381	79.97
Lanao del Norte	17,760	6,750	6,523	13,273	74.74
Misamis Occidental	12,328	5,403	4,901	10,304	83.58
Misamis Oriental	21,975	8,474	8,455	16,929	77.04
City of Cagayan De Oro	14,308	6,551	6,057	12,608	88.12
City of Iligan	7,668	3,071	2,827	5,898	76.92
**Region 11**	**107,001**	**44,865**	**41,716**	**86,581**	**80.92**
Davao de Oro	15,253	6,647	6,133	12,780	83.79
Davao del Norte	23,453	9,309	8,572	17,881	76.24
Davao Oriental	11,935	5,065	4,750	9,815	82.24
Davao del Sur	13,083	5,409	5,059	10,468	80.01
Davao Occidental	6,629	2,718	2,572	5,290	79.80
City of Davao	36,648	15,717	14,630	30,347	82.81
**Region 12**	**95,279**	**38,332**	**35,894**	**74,226**	**77.90**
Cotabato	26,521	11,045	10,235	21,280	80.24
Sarangani	13,322	5,680	5,141	10,821	81.23
South Cotabato	21,595	8,645	8,162	16,807	77.83
Sultan Kudarat	19,391	7,190	6,950	14,140	72.92
City of General Santos	14,450	5,772	5,406	11,178	77.36
**BARMM**	**132,841**	**44,556**	**44,451**	**89,007**	**67.00**
Basilan	11,636	3,333	3,199	6,532	56.14
Lanao del Sur	33,374	13,431	13,977	27,408	82.12
Maguindanao	41,650	14,846	14,543	29,389	70.56
Sulu	21,378	6,099	6,181	12,280	57.44

Table 2.C.1.2 - Immunization Services for Infants
Infants who completed 3 doses of HiB-HepB antigen
Philippines, 2023

Area	Eligible Pop (0-11 mos. old)	DPT-HiB-HepB 1			
		Male	Female	Total	%
Tawi-Tawi	12,609	3,262	3,270	6,532	51.80
SGA	5,103	1,370	1,217	2,587	50.70
City of Cotabato	7,091	2,215	2,064	4,279	60.34
**CARAGA**	**55,065**	**24,667**	**22,876**	**47,543**	**86.34**
Agusan del Norte	7,286	3,226	2,938	6,164	84.60
Agusan del Sur	16,038	6,841	6,661	13,502	84.19
Surigao del Norte	10,186	4,799	4,184	8,983	88.19
Surigao del Sur	12,736	5,704	5,193	10,897	85.56
Dinagat Islands	2,134	869	846	1,715	80.37
City of Butuan	6,685	3,228	3,054	6,282	93.97

Table 2.C.1.2 - Immunization Services for Infants
Infants who completed 3 doses of HiB-HepB antigen
Philippines, 2023

Area	Eligible Pop (0-11 mos. old)	DPT-HiB-HepB 2			
		Male	Female	Total	%
**Philippines**	**2,187,333**	**868,633**	**828,180**	**1,696,813**	**77.57**
**NCR**	**261,228**	**104,660**	**101,442**	**206,102**	**78.90**
Malabon City	7,385	2,598	2,466	5,064	68.57
Navotas City	5,210	2,300	2,332	4,632	88.91
Valenzuela City	13,477	5,117	4,899	10,016	74.32
Caloocan City	33,168	10,207	9,815	20,022	60.37
Marikina City	8,342	2,843	2,739	5,582	66.91
Pasig City	17,682	6,067	5,850	11,917	67.40
Pateros	1,116	395	372	767	68.73
Taguig City	18,992	6,145	5,624	11,769	61.97
Quezon City	59,136	32,174	32,368	64,542	109.14
Makati City	10,944	4,496	4,211	8,707	79.56
Mandaluyong City	7,922	3,104	3,151	6,255	78.96
San Juan City	2,168	527	524	1,051	48.48
Manila City	34,128	13,947	13,227	27,174	79.62
Las Piñas City	11,263	4,233	4,017	8,250	73.25
Muntinlupa City	9,881	3,732	3,437	7,169	72.55
Parañaque City	13,280	3,766	3,460	7,226	54.41
Pasay City	7,134	3,009	2,950	5,959	83.53
**CAR**	**31,319**	**12,636**	**11,833**	**24,469**	**78.13**
Abra	3,660	1,628	1,522	3,150	86.07
Apayao	2,238	964	912	1,876	83.82
Benguet	8,806	3,046	2,926	5,972	67.82
Ifugao	4,098	1,649	1,519	3,168	77.31
Kalinga	4,549	1,906	1,801	3,707	81.49
Mountain Province	2,619	1,210	1,060	2,270	86.67
City of Baguio	5,349	2,233	2,093	4,326	80.87
**Region 1**	**96,016**	**38,964**	**37,110**	**76,074**	**79.23**
Ilocos Norte	8,912	3,782	3,877	7,659	85.94
Ilocos Sur	9,914	4,705	4,378	9,083	91.62
La Union	13,507	5,307	4,816	10,123	74.95
Pangasinan	60,360	24,027	22,955	46,982	77.84
City of Dagupan	3,323	1,143	1,084	2,227	67.02

Table 2.C.1.2 - Immunization Services for Infants
Infants who completed 3 doses of HiB-HepB antigen
Philippines, 2023

Area	Eligible Pop (0-11 mos. old)	DPT-HiB-HepB 2			
		Male	Female	Total	%
**Region 2**	**63,967**	**26,588**	**25,157**	**51,745**	**80.89**
Batanes	240	134	91	225	93.75
Cagayan	20,786	8,253	7,831	16,084	77.38
Isabela	27,379	11,598	11,543	23,141	84.52
Nueva Vizcaya	8,798	3,628	2,900	6,528	74.20
Quirino	3,839	1,688	1,550	3,238	84.34
City of Santiago	2,925	1,287	1,242	2,529	86.46
**Region 3**	**233,544**	**98,926**	**93,421**	**192,347**	**82.36**
Aurora	4,537	2,063	1,905	3,968	87.46
Bataan	17,380	6,835	6,323	13,158	75.71
Bulacan	67,590	31,382	29,054	60,436	89.42
Nueva Ecija	43,425	17,028	16,403	33,431	76.99
Pampanga	45,651	19,205	18,456	37,661	82.50
Tarlac	29,503	12,435	12,125	24,560	83.25
Zambales	11,723	4,838	4,386	9,224	78.68
City of Angeles	9,146	3,467	3,214	6,681	73.05
City of Olongapo	4,589	1,673	1,555	3,228	70.34
**Region 4A**	**307,982**	**120,167**	**114,418**	**234,585**	**76.17**
Batangas	53,251	21,686	20,810	42,496	79.80
Cavite	77,964	31,191	29,635	60,826	78.02
Laguna	61,525	24,861	23,622	48,483	78.80
Quezon	40,810	15,261	14,486	29,747	72.89
Rizal	68,235	24,922	23,684	48,606	71.23
City of Lucena	6,197	2,246	2,181	4,427	71.44
**Region 4B**	**66,121**	**27,352**	**25,938**	**53,290**	**80.59**
Marinduque	4,144	1,740	1,487	3,227	77.87
Occidental Mindoro	11,640	5,011	4,816	9,827	84.42
Oriental Mindoro	18,943	6,987	6,782	13,769	72.69
Palawan	20,317	8,615	8,031	16,646	81.93
Romblon	5,489	2,353	2,276	4,629	84.33
City of Puerto Princesa	5,588	2,646	2,546	5,192	92.91
**Region 5**	**135,480**	**51,282**	**48,065**	**99,347**	**73.33**

Table 2.C.1.2 - Immunization Services for Infants
Infants who completed 3 doses of HiB-HepB antigen
Philippines, 2023

Area	Eligible Pop (0-11 mos. old)	DPT-HiB-HepB 2			
		Male	Female	Total	%
Albay	27,440	9,956	9,485	19,441	70.85
Camarines Norte	14,695	5,750	5,229	10,979	74.71
Camarines Sur	43,452	15,069	14,054	29,123	67.02
Catanduanes	5,414	2,257	2,145	4,402	81.31
Masbate	22,365	9,384	8,985	18,369	82.13
Sorsogon	17,929	7,340	6,749	14,089	78.58
City of Naga	4,185	1,526	1,418	2,944	70.35
**Region 6**	**148,020**	**56,947**	**53,940**	**110,887**	**74.91**
Aklan	11,283	3,813	3,743	7,556	66.97
Antique	11,930	4,532	4,373	8,905	74.64
Capiz	13,931	5,175	4,814	9,989	71.70
Guimaras	3,351	1,292	1,245	2,537	75.71
Iloilo	36,140	13,801	12,778	26,579	73.54
Negros Occidental	52,270	20,859	19,945	40,804	78.06
City of Bacolod	10,373	4,138	3,832	7,970	76.83
City of Iloilo	8,742	3,337	3,210	6,547	74.89
**Region 7**	**165,673**	**65,016**	**61,698**	**126,714**	**76.48**
Bohol	26,235	9,572	9,165	18,737	71.42
Cebu	72,201	26,816	25,207	52,023	72.05
Negros Oriental	28,722	11,677	11,112	22,789	79.34
Siquijor	1,680	580	566	1,146	68.21
City of Cebu	19,267	7,971	7,550	15,521	80.56
City of Lapu-Lapu	10,679	5,079	4,858	9,937	93.05
City of Mandaue	6,889	3,321	3,240	6,561	95.24
**Region 8**	**93,813**	**36,495**	**34,014**	**70,509**	**75.16**
Biliran	3,373	1,630	1,499	3,129	92.77
Eastern Samar	9,624	3,828	3,719	7,547	78.42
Leyte	31,559	12,267	10,872	23,139	73.32
Northern Samar	14,056	5,426	5,305	10,731	76.34
Southern Leyte	7,406	2,584	2,461	5,045	68.12
Samar	17,011	6,960	6,488	13,448	79.05
Ormoc City	5,292	1,922	1,836	3,758	71.01
City of Tacloban	5,492	1,878	1,834	3,712	67.59
**Region 9**	**84,815**	**34,873**	**33,503**	**68,376**	**80.62**

Table 2.C.1.2 - Immunization Services for Infants
Infants who completed 3 doses of HiB-HepB antigen
Philippines, 2023

Area	Eligible Pop (0-11 mos. old)	DPT-HiB-HepB 2			
		Male	Female	Total	%
Zamboanga del Norte	24,841	9,524	9,336	18,860	75.92
Zamboanga del Sur	23,757	8,783	8,420	17,203	72.41
Zamboanga Sibugay	13,841	5,646	5,403	11,049	79.83
City of Isabela	3,201	1,498	1,346	2,844	88.85
City of Zamboanga	19,175	9,422	8,998	18,420	96.06
**Region 10**	**109,169**	**45,313**	**43,950**	**89,263**	**81.77**
Bukidnon	33,403	14,656	14,002	28,658	85.79
Camiguin	1,727	677	687	1,364	78.98
Lanao del Norte	17,760	6,734	6,675	13,409	75.50
Misamis Occidental	12,328	5,042	5,165	10,207	82.80
Misamis Oriental	21,975	8,642	8,491	17,133	77.97
City of Cagayan De Oro	14,308	6,589	6,126	12,715	88.87
City of Iligan	7,668	2,973	2,804	5,777	75.34
**Region 11**	**107,001**	**44,110**	**41,329**	**85,439**	**79.85**
Davao de Oro	15,253	6,479	6,137	12,616	82.71
Davao del Norte	23,453	9,190	8,542	17,732	75.61
Davao Oriental	11,935	5,051	4,779	9,830	82.36
Davao del Sur	13,083	5,412	5,098	10,510	80.33
Davao Occidental	6,629	2,638	2,535	5,173	78.04
City of Davao	36,648	15,340	14,238	29,578	80.71
**Region 12**	**95,279**	**37,958**	**35,946**	**73,904**	**77.57**
Cotabato	26,521	10,656	9,986	20,642	77.83
Sarangani	13,322	5,676	5,310	10,986	82.47
South Cotabato	21,595	8,503	8,043	16,546	76.62
Sultan Kudarat	19,391	7,285	7,068	14,353	74.02
City of General Santos	14,450	5,838	5,539	11,377	78.73
**BARMM**	**132,841**	**42,949**	**43,249**	**86,198**	**64.89**
Basilan	11,636	2,964	3,106	6,070	52.17
Lanao del Sur	33,374	13,544	14,205	27,749	83.15
Maguindanao	41,650	14,435	14,056	28,491	68.41
Sulu	21,378	5,786	5,738	11,524	53.91
Tawi-Tawi	12,609	3,225	3,178	6,403	50.78

Table 2.C.1.2 - Immunization Services for Infants

Infants who completed 3 doses of HiB-HepB antigen

Philippines, 2023

Area	Eligible Pop (0-11 mos. old)	DPT-HiB-HepB 2			
		Male	Female	Total	%
SGA	5,103	950	942	1,892	37.08
City of Cotabato	7,091	2,045	2,024	4,069	57.38
**CARAGA**	**55,065**	**24,397**	**23,167**	**47,564**	**86.38**
Agusan del Norte	7,286	3,149	2,910	6,059	83.16
Agusan del Sur	16,038	6,739	6,653	13,392	83.50
Surigao del Norte	10,186	4,756	4,570	9,326	91.56
Surigao del Sur	12,736	5,633	5,164	10,797	84.78
Dinagat Islands	2,134	912	869	1,781	83.46
City of Butuan	6,685	3,208	3,001	6,209	92.88

Table 2.C.1.2 - Immunization Services for Infants
Infants who completed 3 doses of HiB-HepB antigen
Philippines, 2023

Area	Eligible Pop (0-11 mos. old)	DPT-HiB-HepB 3			
		Male	Female	Total	%
**Philippines**	**2,187,333**	**860,496**	**819,536**	**1,680,032**	**76.81**
**NCR**	**261,228**	**104,071**	**101,216**	**205,287**	**78.59**
Malabon City	7,385	2,549	2,422	4,971	67.31
Navotas City	5,210	2,357	2,318	4,675	89.73
Valenzuela City	13,477	4,979	4,941	9,920	73.61
Caloocan City	33,168	9,861	9,665	19,526	58.87
Marikina City	8,342	2,870	2,669	5,539	66.40
Pasig City	17,682	6,037	5,819	11,856	67.05
Pateros	1,116	418	376	794	71.15
Taguig City	18,992	6,143	5,585	11,728	61.75
Quezon City	59,136	31,888	31,770	63,658	107.65
Makati City	10,944	4,553	4,420	8,973	81.99
Mandaluyong City	7,922	3,250	3,276	6,526	82.38
San Juan City	2,168	558	535	1,093	50.42
Manila City	34,128	14,015	13,637	27,652	81.02
Las Piñas City	11,263	4,205	4,014	8,219	72.97
Muntinlupa City	9,881	3,744	3,526	7,270	73.58
Parañaque City	13,280	3,705	3,380	7,085	53.35
Pasay City	7,134	2,939	2,863	5,802	81.33
**CAR**	**31,319**	**12,581**	**11,894**	**24,475**	**78.15**
Abra	3,660	1,608	1,579	3,187	87.08
Apayao	2,238	957	945	1,902	84.99
Benguet	8,806	3,069	2,937	6,006	68.20
Ifugao	4,098	1,656	1,509	3,165	77.23
Kalinga	4,549	1,890	1,828	3,718	81.73
Mountain Province	2,619	1,229	1,010	2,239	85.49
City of Baguio	5,349	2,172	2,086	4,258	79.60
**Region 1**	**96,016**	**39,489**	**37,414**	**76,903**	**80.09**
Ilocos Norte	8,912	3,806	3,867	7,673	86.10
Ilocos Sur	9,914	4,733	4,411	9,144	92.23
La Union	13,507	5,297	4,908	10,205	75.55
Pangasinan	60,360	24,525	23,185	47,710	79.04
City of Dagupan	3,323	1,128	1,043	2,171	65.33

Table 2.C.1.2 - Immunization Services for Infants
Infants who completed 3 doses of HiB-HepB antigen
Philippines, 2023

Area	Eligible Pop (0-11 mos. old)	DPT-HiB-HepB 3			
		Male	Female	Total	%
**Region 2**	**63,967**	**26,467**	**24,613**	**51,080**	**79.85**
Batanes	240	134	94	228	95.00
Cagayan	20,786	8,078	7,696	15,774	75.89
Isabela	27,379	11,930	11,045	22,975	83.91
Nueva Vizcaya	8,798	3,317	2,791	6,108	69.42
Quirino	3,839	1,676	1,722	3,398	88.51
City of Santiago	2,925	1,332	1,265	2,597	88.79
**Region 3**	**233,544**	**99,597**	**94,579**	**194,176**	**83.14**
Aurora	4,537	2,075	1,925	4,000	88.16
Bataan	17,380	6,851	6,339	13,190	75.89
Bulacan	67,590	31,896	29,609	61,505	91.00
Nueva Ecija	43,425	17,186	16,426	33,612	77.40
Pampanga	45,651	19,184	18,649	37,833	82.87
Tarlac	29,503	12,715	12,429	25,144	85.23
Zambales	11,723	4,649	4,351	9,000	76.77
City of Angeles	9,146	3,356	3,219	6,575	71.89
City of Olongapo	4,589	1,685	1,632	3,317	72.28
**Region 4A**	**307,982**	**119,425**	**113,269**	**232,694**	**75.55**
Batangas	53,251	21,720	20,864	42,584	79.97
Cavite	77,964	30,809	29,513	60,322	77.37
Laguna	61,525	25,201	23,448	48,649	79.07
Quezon	40,810	15,022	14,154	29,176	71.49
Rizal	68,235	24,427	23,068	47,495	69.61
City of Lucena	6,197	2,246	2,222	4,468	72.10
**Region 4B**	**66,121**	**26,520**	**25,187**	**51,707**	**78.20**
Marinduque	4,144	1,703	1,454	3,157	76.18
Occidental Mindoro	11,640	4,911	4,746	9,657	82.96
Oriental Mindoro	18,943	6,901	6,640	13,541	71.48
Palawan	20,317	8,156	7,747	15,903	78.27
Romblon	5,489	2,279	2,138	4,417	80.47
City of Puerto Princesa	5,588	2,570	2,462	5,032	90.05
**Region 5**	**135,480**	**50,638**	**47,299**	**97,937**	**72.29**

Table 2.C.1.2 - Immunization Services for Infants
Infants who completed 3 doses of HiB-HepB antigen
Philippines, 2023

Area	Eligible Pop (0-11 mos. old)	DPT-HiB-HepB 3			
		Male	Female	Total	%
Albay	27,440	9,948	9,455	19,403	70.71
Camarines Norte	14,695	5,524	5,123	10,647	72.45
Camarines Sur	43,452	14,653	13,580	28,233	64.98
Catanduanes	5,414	2,202	2,053	4,255	78.59
Masbate	22,365	9,535	8,917	18,452	82.50
Sorsogon	17,929	7,261	6,700	13,961	77.87
City of Naga	4,185	1,515	1,471	2,986	71.35
**Region 6**	**148,020**	**56,896**	**52,975**	**109,871**	**74.23**
Aklan	11,283	3,732	3,677	7,409	65.67
Antique	11,930	4,445	4,331	8,776	73.56
Capiz	13,931	5,072	4,809	9,881	70.93
Guimaras	3,351	1,324	1,213	2,537	75.71
Iloilo	36,140	13,981	12,858	26,839	74.26
Negros Occidental	52,270	20,929	19,865	40,794	78.04
City of Bacolod	10,373	4,079	2,980	7,059	68.05
City of Iloilo	8,742	3,334	3,242	6,576	75.22
**Region 7**	**165,673**	**64,696**	**61,087**	**125,783**	**75.92**
Bohol	26,235	9,489	9,057	18,546	70.69
Cebu	72,201	26,632	25,134	51,766	71.70
Negros Oriental	28,722	11,539	10,859	22,398	77.98
Siquijor	1,680	567	567	1,134	67.50
City of Cebu	19,267	7,838	7,509	15,347	79.65
City of Lapu-Lapu	10,679	5,151	4,590	9,741	91.22
City of Mandaue	6,889	3,480	3,371	6,851	99.45
**Region 8**	**93,813**	**34,858**	**33,060**	**67,918**	**72.40**
Biliran	3,373	1,565	1,536	3,101	91.94
Eastern Samar	9,624	3,810	3,605	7,415	77.05
Leyte	31,559	11,192	10,597	21,789	69.04
Northern Samar	14,056	5,350	5,056	10,406	74.03
Southern Leyte	7,406	2,593	2,482	5,075	68.53
Samar	17,011	6,544	6,246	12,790	75.19
Ormoc City	5,292	1,935	1,790	3,725	70.39
City of Tacloban	5,492	1,869	1,748	3,617	65.86
**Region 9**	**84,815**	**34,405**	**33,229**	**67,634**	**79.74**

Table 2.C.1.2 - Immunization Services for Infants
Infants who completed 3 doses of HiB-HepB antigen
Philippines, 2023

Area	Eligible Pop (0-11 mos. old)	DPT-HiB-HepB 3			
		Male	Female	Total	%
Zamboanga del Norte	24,841	9,452	9,305	18,757	75.51
Zamboanga del Sur	23,757	8,670	8,298	16,968	71.42
Zamboanga Sibugay	13,841	5,525	5,452	10,977	79.31
City of Isabela	3,201	1,425	1,335	2,760	86.22
City of Zamboanga	19,175	9,333	8,839	18,172	94.77
**Region 10**	**109,169**	**44,811**	**42,624**	**87,435**	**80.09**
Bukidnon	33,403	14,444	13,627	28,071	84.04
Camiguin	1,727	664	687	1,351	78.23
Lanao del Norte	17,760	6,663	6,379	13,042	73.43
Misamis Occidental	12,328	4,893	4,782	9,675	78.48
Misamis Oriental	21,975	8,798	8,465	17,263	78.56
City of Cagayan De Oro	14,308	6,410	6,028	12,438	86.93
City of Iligan	7,668	2,939	2,656	5,595	72.97
**Region 11**	**107,001**	**43,117**	**40,861**	**83,978**	**78.48**
Davao de Oro	15,253	6,244	6,068	12,312	80.72
Davao del Norte	23,453	9,031	8,538	17,569	74.91
Davao Oriental	11,935	4,909	4,655	9,564	80.13
Davao del Sur	13,083	5,219	4,879	10,098	77.18
Davao Occidental	6,629	2,544	2,495	5,039	76.01
City of Davao	36,648	15,170	14,226	29,396	80.21
**Region 12**	**95,279**	**37,314**	**35,614**	**72,928**	**76.54**
Cotabato	26,521	10,378	9,593	19,971	75.30
Sarangani	13,322	5,516	5,252	10,768	80.83
South Cotabato	21,595	8,430	8,061	16,491	76.36
Sultan Kudarat	19,391	7,278	7,134	14,412	74.32
City of General Santos	14,450	5,712	5,574	11,286	78.10
**BARMM**	**132,841**	**41,584**	**41,861**	**83,445**	**62.82**
Basilan	11,636	2,732	2,735	5,467	46.98
Lanao del Sur	33,374	13,716	14,225	27,941	83.72
Maguindanao	41,650	13,988	13,770	27,758	66.65
Sulu	21,378	5,293	5,461	10,754	50.30
Tawi-Tawi	12,609	3,093	3,068	6,161	48.86

Table 2.C.1.2 - Immunization Services for Infants

Infants who completed 3 doses of HiB-HepB antigen

Philippines, 2023

Area	Eligible Pop (0-11 mos. old)	DPT-HiB-HepB 3			
		Male	Female	Total	%
SGA	5,103	818	725	1,543	30.24
City of Cotabato	7,091	1,944	1,877	3,821	53.89
**CARAGA**	**55,065**	**24,027**	**22,754**	**46,781**	**84.96**
Agusan del Norte	7,286	3,138	2,868	6,006	82.43
Agusan del Sur	16,038	6,558	6,462	13,020	81.18
Surigao del Norte	10,186	4,858	4,380	9,238	90.69
Surigao del Sur	12,736	5,466	5,134	10,600	83.23
Dinagat Islands	2,134	932	881	1,813	84.96
City of Butuan	6,685	3,075	3,029	6,104	91.31

Table 2.C.1.3 - Immunization Services for Infants and Children

Infants who completed 3 doses of Oral Polio Vaccine (OPV)

Philippines, 2023

Area	Eligible Pop (0-11 mos. old)	OPV 1			
		Male	Female	Total	%
**Philippines**	**2,187,333**	**840,676**	**802,871**	**1,643,547**	**75.14**
**NCR**	**261,228**	**106,948**	**103,078**	**210,026**	**80.40**
Malabon City	7,385	2,680	2,574	5,254	71.14
Navotas City	5,210	2,408	2,398	4,806	92.25
Valenzuela City	13,477	4,801	4,560	9,361	69.46
Caloocan City	33,168	9,878	9,141	19,019	57.34
Marikina City	8,342	2,989	2,798	5,787	69.37
Pasig City	17,682	6,077	5,905	11,982	67.76
Pateros	1,116	399	399	798	71.51
Taguig City	18,992	6,137	5,507	11,644	61.31
Quezon City	59,136	34,084	34,091	68,175	115.29
Makati City	10,944	4,391	4,260	8,651	79.05
Mandaluyong City	7,922	2,961	2,897	5,858	73.95
San Juan City	2,168	573	539	1,112	51.29
Manila City	34,128	14,458	13,729	28,187	82.59
Las Piñas City	11,263	4,211	4,055	8,266	73.39
Muntinlupa City	9,881	3,806	3,562	7,368	74.57
Parañaque City	13,280	3,894	3,562	7,456	56.14
Pasay City	7,134	3,201	3,101	6,302	88.34
**CAR**	**31,319**	**11,513**	**10,741**	**22,254**	**71.06**
Abra	3,660	1,388	1,329	2,717	74.23
Apayao	2,238	908	852	1,760	78.64
Benguet	8,806	2,762	2,626	5,388	61.19
Ifugao	4,098	1,487	1,383	2,870	70.03
Kalinga	4,549	1,634	1,491	3,125	68.70
Mountain Province	2,619	1,139	1,022	2,161	82.51
City of Baguio	5,349	2,195	2,038	4,233	79.14
**Region 1**	**96,016**	**37,908**	**36,994**	**74,902**	**78.01**
Ilocos Norte	8,912	3,675	3,717	7,392	82.94
Ilocos Sur	9,914	4,524	4,200	8,724	88.00
La Union	13,507	4,907	4,332	9,239	68.40
Pangasinan	60,360	23,696	23,712	47,408	78.54
City of Dagupan	3,323	1,106	1,033	2,139	64.37

Table 2.C.1.3 - Immunization Services for Infants and Children
Infants who completed 3 doses of Oral Polio Vaccine (OPV)
Philippines, 2023

Area	Eligible Pop (0-11 mos. old)	OPV 1			
		Male	Female	Total	%
**Region 2**	**63,967**	**24,922**	**24,350**	**49,272**	**77.03**
Batanes	240	124	90	214	89.17
Cagayan	20,786	7,535	7,153	14,688	70.66
Isabela	27,379	11,119	11,598	22,717	82.97
Nueva Vizcaya	8,798	3,289	2,916	6,205	70.53
Quirino	3,839	1,574	1,406	2,980	77.62
City of Santiago	2,925	1,281	1,187	2,468	84.38
**Region 3**	**233,544**	**94,558**	**89,712**	**184,270**	**78.90**
Aurora	4,537	1,951	1,832	3,783	83.38
Bataan	17,380	6,632	6,192	12,824	73.79
Bulacan	67,590	30,056	27,809	57,865	85.61
Nueva Ecija	43,425	16,002	15,479	31,481	72.50
Pampanga	45,651	18,156	17,599	35,755	78.32
Tarlac	29,503	11,706	11,458	23,164	78.51
Zambales	11,723	4,761	4,400	9,161	78.15
City of Angeles	9,146	3,479	3,310	6,789	74.23
City of Olongapo	4,589	1,815	1,633	3,448	75.14
**Region 4A**	**307,982**	**119,708**	**112,654**	**232,362**	**75.45**
Batangas	53,251	21,474	20,437	41,911	78.70
Cavite	77,964	31,155	29,270	60,425	77.50
Laguna	61,525	24,427	23,170	47,597	77.36
Quezon	40,810	14,967	14,007	28,974	71.00
Rizal	68,235	25,367	23,630	48,997	71.81
City of Lucena	6,197	2,318	2,140	4,458	71.94
**Region 4B**	**66,121**	**26,298**	**24,540**	**50,838**	**76.89**
Marinduque	4,144	1,665	1,441	3,106	74.95
Occidental Mindoro	11,640	4,690	4,438	9,128	78.42
Oriental Mindoro	18,943	6,933	6,424	13,357	70.51
Palawan	20,317	8,015	7,457	15,472	76.15
Romblon	5,489	2,312	2,184	4,496	81.91
City of Puerto Princesa	5,588	2,683	2,596	5,279	94.47

Table 2.C.1.3 - Immunization Services for Infants and Children
Infants who completed 3 doses of Oral Polio Vaccine (OPV)
Philippines, 2023

Area	Eligible Pop (0-11 mos. old)	OPV 1			
		Male	Female	Total	%
**Region 5**	**135,480**	**48,165**	**45,088**	**93,253**	**68.83**
Albay	27,440	9,521	9,090	18,611	67.82
Camarines Norte	14,695	5,355	4,875	10,230	69.62
Camarines Sur	43,452	13,889	13,025	26,914	61.94
Catanduanes	5,414	2,178	2,086	4,264	78.76
Masbate	22,365	8,666	8,182	16,848	75.33
Sorsogon	17,929	6,978	6,432	13,410	74.80
City of Naga	4,185	1,578	1,398	2,976	71.11
**Region 6**	**148,020**	**53,983**	**50,857**	**104,840**	**70.83**
Aklan	11,283	3,563	3,328	6,891	61.07
Antique	11,930	3,893	3,754	7,647	64.10
Capiz	13,931	5,059	4,542	9,601	68.92
Guimaras	3,351	1,120	1,144	2,264	67.56
Iloilo	36,140	13,572	12,607	26,179	72.44
Negros Occidental	52,270	19,718	18,854	38,572	73.79
City of Bacolod	10,373	3,924	3,661	7,585	73.12
City of Iloilo	8,742	3,134	2,967	6,101	69.79
**Region 7**	**165,673**	**61,708**	**58,256**	**119,964**	**72.41**
Bohol	26,235	9,025	8,615	17,640	67.24
Cebu	72,201	24,490	22,959	47,449	65.72
Negros Oriental	28,722	11,091	10,402	21,493	74.83
Siquijor	1,680	585	552	1,137	67.68
City of Cebu	19,267	8,120	7,601	15,721	81.60
City of Lapu-Lapu	10,679	5,128	5,010	10,138	94.93
City of Mandaue	6,889	3,269	3,117	6,386	92.70
**Region 8**	**93,813**	**33,512**	**31,488**	**65,000**	**69.29**
Biliran	3,373	1,577	1,478	3,055	90.57
Eastern Samar	9,624	3,532	3,350	6,882	71.51
Leyte	31,559	10,133	9,521	19,654	62.28
Northern Samar	14,056	5,172	4,759	9,931	70.65
Southern Leyte	7,406	2,451	2,325	4,776	64.49
Samar	17,011	6,800	6,401	13,201	77.60
Ormoc City	5,292	1,913	1,783	3,696	69.84
City of Tacloban	5,492	1,934	1,871	3,805	69.28

Table 2.C.1.3 - Immunization Services for Infants and Children
Infants who completed 3 doses of Oral Polio Vaccine (OPV)
Philippines, 2023

Area	Eligible Pop (0-11 mos. old)	OPV 1			
		Male	Female	Total	%
**Region 9**	**84,815**	**34,682**	**32,995**	**67,677**	**79.79**
Zamboanga del Norte	24,841	9,555	9,110	18,665	75.14
Zamboanga del Sur	23,757	8,578	8,136	16,714	70.35
Zamboanga Sibugay	13,841	5,366	5,227	10,593	76.53
City of Isabela	3,201	1,361	1,279	2,640	82.47
City of Zamboanga	19,175	9,822	9,243	19,065	99.43
**Region 10**	**109,169**	**44,730**	**47,119**	**91,849**	**84.13**
Bukidnon	33,403	14,059	17,669	31,728	94.99
Camiguin	1,727	644	695	1,339	77.53
Lanao del Norte	17,760	6,655	6,471	13,126	73.91
Misamis Occidental	12,328	6,086	5,720	11,806	95.77
Misamis Oriental	21,975	8,316	8,253	16,569	75.40
City of Cagayan De Oro	14,308	6,406	5,893	12,299	85.96
City of Iligan	7,668	2,564	2,418	4,982	64.97
**Region 11**	**107,001**	**43,997**	**40,840**	**84,837**	**79.29**
Davao de Oro	15,253	6,495	6,061	12,556	82.32
Davao del Norte	23,453	9,198	8,488	17,686	75.41
Davao Oriental	11,935	4,942	4,617	9,559	80.09
Davao del Sur	13,083	5,274	4,910	10,184	77.84
Davao Occidental	6,629	2,520	2,415	4,935	74.45
City of Davao	36,648	15,568	14,349	29,917	81.63
**Region 12**	**95,279**	**36,145**	**33,841**	**69,986**	**73.45**
Cotabato	26,521	10,662	9,883	20,545	77.47
Sarangani	13,322	5,269	4,789	10,058	75.50
South Cotabato	21,595	8,162	7,698	15,860	73.44
Sultan Kudarat	19,391	6,843	6,618	13,461	69.42
City of General Santos	14,450	5,209	4,853	10,062	69.63
**BARMM**	**132,841**	**39,020**	**38,945**	**77,965**	**58.69**
Basilan	11,636	2,768	2,754	5,522	47.46
Lanao del Sur	33,374	12,532	12,971	25,503	76.42
Maguindanao	41,650	12,563	12,420	24,983	59.98

Table 2.C.1.3 - Immunization Services for Infants and Children
Infants who completed 3 doses of Oral Polio Vaccine (OPV)
Philippines, 2023

Area	Eligible Pop (0-11 mos. old)	OPV 1			
		Male	Female	Total	%
Sulu	21,378	5,283	5,323	10,606	49.61
Tawi-Tawi	12,609	2,768	2,727	5,495	43.58
SGA	5,103	1,104	881	1,985	38.90
City of Cotabato	7,091	2,002	1,869	3,871	54.59
**CARAGA**	**55,065**	**22,879**	**21,373**	**44,252**	**80.36**
Agusan del Norte	7,286	3,033	2,823	5,856	80.37
Agusan del Sur	16,038	6,068	5,798	11,866	73.99
Surigao del Norte	10,186	4,334	3,995	8,329	81.77
Surigao del Sur	12,736	5,413	4,948	10,361	81.35
Dinagat Islands	2,134	884	836	1,720	80.60
City of Butuan	6,685	3,147	2,973	6,120	91.55

Table 2.C.1.3 - Immunization Services for Infants and Children
Infants who completed 3 doses of Oral Polio Vaccine (OPV)
Philippines, 2023

Area	Eligible Pop (0-11 mos. old)	OPV 2			
		Male	Female	Total	%
**Philippines**	**2,187,333**	**793,473**	**758,841**	**1,552,314**	**70.97**
**NCR**	**261,228**	**103,110**	**100,324**	**203,434**	**77.88**
Malabon City	7,385	2,592	2,463	5,055	68.45
Navotas City	5,210	2,310	2,342	4,652	89.29
Valenzuela City	13,477	4,919	4,742	9,661	71.69
Caloocan City	33,168	8,444	8,123	16,567	49.95
Marikina City	8,342	2,871	2,755	5,626	67.44
Pasig City	17,682	6,030	5,817	11,847	67.00
Pateros	1,116	393	371	764	68.46
Taguig City	18,992	6,190	5,666	11,856	62.43
Quezon City	59,136	32,992	33,270	66,262	112.05
Makati City	10,944	4,375	4,144	8,519	77.84
Mandaluyong City	7,922	2,940	2,973	5,913	74.64
San Juan City	2,168	531	536	1,067	49.22
Manila City	34,128	13,868	13,336	27,204	79.71
Las Piñas City	11,263	4,159	3,955	8,114	72.04
Muntinlupa City	9,881	3,714	3,425	7,139	72.25
Parañaque City	13,280	3,743	3,455	7,198	54.20
Pasay City	7,134	3,039	2,951	5,990	83.96
**CAR**	**31,319**	**10,109**	**9,536**	**19,645**	**62.73**
Abra	3,660	1,207	1,199	2,406	65.74
Apayao	2,238	764	718	1,482	66.22
Benguet	8,806	2,421	2,350	4,771	54.18
Ifugao	4,098	1,224	1,117	2,341	57.13
Kalinga	4,549	1,354	1,302	2,656	58.39
Mountain Province	2,619	1,076	910	1,986	75.83
City of Baguio	5,349	2,063	1,940	4,003	74.84
**Region 1**	**96,016**	**36,340**	**35,653**	**71,993**	**74.98**
Ilocos Norte	8,912	3,532	3,625	7,157	80.31
Ilocos Sur	9,914	4,422	4,101	8,523	85.97
La Union	13,507	5,024	4,571	9,595	71.04
Pangasinan	60,360	22,311	22,328	44,639	73.95
City of Dagupan	3,323	1,051	1,028	2,079	62.56

Table 2.C.1.3 - Immunization Services for Infants and Children
Infants who completed 3 doses of Oral Polio Vaccine (OPV)
Philippines, 2023

Area	Eligible Pop (0-11 mos. old)	OPV 2			
		Male	Female	Total	%
**Region 2**	**63,967**	**23,281**	**21,791**	**45,072**	**70.46**
Batanes	240	137	91	228	95.00
Cagayan	20,786	6,613	6,241	12,854	61.84
Isabela	27,379	11,074	10,435	21,509	78.56
Nueva Vizcaya	8,798	2,826	2,526	5,352	60.83
Quirino	3,839	1,354	1,260	2,614	68.09
City of Santiago	2,925	1,277	1,238	2,515	85.98
**Region 3**	**233,544**	**90,475**	**85,723**	**176,198**	**75.45**
Aurora	4,537	1,930	1,791	3,721	82.01
Bataan	17,380	6,325	5,898	12,223	70.33
Bulacan	67,590	29,229	27,094	56,323	83.33
Nueva Ecija	43,425	15,132	14,554	29,686	68.36
Pampanga	45,651	17,206	16,604	33,810	74.06
Tarlac	29,503	11,126	10,929	22,055	74.76
Zambales	11,723	4,716	4,373	9,089	77.53
City of Angeles	9,146	3,418	3,204	6,622	72.40
City of Olongapo	4,589	1,393	1,276	2,669	58.16
**Region 4A**	**307,982**	**117,454**	**112,147**	**229,601**	**74.55**
Batangas	53,251	21,317	20,408	41,725	78.36
Cavite	77,964	30,591	29,285	59,876	76.80
Laguna	61,525	24,097	23,007	47,104	76.56
Quezon	40,810	14,418	13,727	28,145	68.97
Rizal	68,235	24,773	23,536	48,309	70.80
City of Lucena	6,197	2,258	2,184	4,442	71.68
**Region 4B**	**66,121**	**23,696**	**22,565**	**46,261**	**69.96**
Marinduque	4,144	1,628	1,423	3,051	73.62
Occidental Mindoro	11,640	4,079	3,882	7,961	68.39
Oriental Mindoro	18,943	6,409	6,229	12,638	66.72
Palawan	20,317	6,739	6,366	13,105	64.50
Romblon	5,489	2,277	2,190	4,467	81.38
City of Puerto Princesa	5,588	2,564	2,475	5,039	90.18

Table 2.C.1.3 - Immunization Services for Infants and Children
Infants who completed 3 doses of Oral Polio Vaccine (OPV)
Philippines, 2023

Area	Eligible Pop (0-11 mos. old)	OPV 2			
		Male	Female	Total	%
**Region 5**	**135,480**	**43,042**	**40,426**	**83,468**	**61.61**
Albay	27,440	9,216	8,793	18,009	65.63
Camarines Norte	14,695	4,921	4,533	9,454	64.33
Camarines Sur	43,452	11,224	10,515	21,739	50.03
Catanduanes	5,414	2,241	2,143	4,384	80.98
Masbate	22,365	7,330	7,092	14,422	64.48
Sorsogon	17,929	6,575	5,949	12,524	69.85
City of Naga	4,185	1,535	1,401	2,936	70.16
**Region 6**	**148,020**	**51,504**	**48,429**	**99,933**	**67.51**
Aklan	11,283	3,396	3,316	6,712	59.49
Antique	11,930	3,140	3,076	6,216	52.10
Capiz	13,931	5,136	4,786	9,922	71.22
Guimaras	3,351	1,044	1,021	2,065	61.62
Iloilo	36,140	13,797	12,521	26,318	72.82
Negros Occidental	52,270	18,399	17,515	35,914	68.71
City of Bacolod	10,373	3,714	3,412	7,126	68.70
City of Iloilo	8,742	2,878	2,782	5,660	64.74
**Region 7**	**165,673**	**56,398**	**53,463**	**109,861**	**66.31**
Bohol	26,235	8,148	7,784	15,932	60.73
Cebu	72,201	21,302	20,011	41,313	57.22
Negros Oriental	28,722	9,996	9,386	19,382	67.48
Siquijor	1,680	574	559	1,133	67.44
City of Cebu	19,267	7,970	7,612	15,582	80.87
City of Lapu-Lapu	10,679	5,079	4,858	9,937	93.05
City of Mandaue	6,889	3,329	3,253	6,582	95.54
**Region 8**	**93,813**	**30,004**	**28,614**	**58,618**	**62.48**
Biliran	3,373	1,578	1,484	3,062	90.78
Eastern Samar	9,624	3,308	3,109	6,417	66.68
Leyte	31,559	8,515	8,203	16,718	52.97
Northern Samar	14,056	4,493	4,245	8,738	62.17
Southern Leyte	7,406	2,447	2,314	4,761	64.29
Samar	17,011	5,960	5,663	11,623	68.33
Ormoc City	5,292	1,851	1,770	3,621	68.42
City of Tacloban	5,492	1,852	1,826	3,678	66.97

Table 2.C.1.3 - Immunization Services for Infants and Children

Infants who completed 3 doses of Oral Polio Vaccine (OPV)

Philippines, 2023

Area	Eligible Pop (0-11 mos. old)	OPV 2			
		Male	Female	Total	%
**Region 9**	**84,815**	**34,192**	**32,516**	**66,708**	**78.65**
Zamboanga del Norte	24,841	9,419	9,164	18,583	74.81
Zamboanga del Sur	23,757	8,586	8,283	16,869	71.01
Zamboanga Sibugay	13,841	5,314	4,993	10,307	74.47
City of Isabela	3,201	1,399	1,276	2,675	83.57
City of Zamboanga	19,175	9,474	8,800	18,274	95.30
**Region 10**	**109,169**	**42,864**	**41,804**	**84,668**	**77.56**
Bukidnon	33,403	13,012	12,481	25,493	76.32
Camiguin	1,727	615	625	1,240	71.80
Lanao del Norte	17,760	6,748	6,605	13,353	75.19
Misamis Occidental	12,328	5,796	6,304	12,100	98.15
Misamis Oriental	21,975	8,194	8,041	16,235	73.88
City of Cagayan De Oro	14,308	6,406	5,745	12,151	84.92
City of Iligan	7,668	2,093	2,003	4,096	53.42
**Region 11**	**107,001**	**42,782**	**40,074**	**82,856**	**77.43**
Davao de Oro	15,253	6,179	5,919	12,098	79.32
Davao del Norte	23,453	9,134	8,450	17,584	74.98
Davao Oriental	11,935	4,777	4,583	9,360	78.42
Davao del Sur	13,083	5,217	4,828	10,045	76.78
Davao Occidental	6,629	2,354	2,237	4,591	69.26
City of Davao	36,648	15,121	14,057	29,178	79.62
**Region 12**	**95,279**	**34,293**	**32,377**	**66,670**	**69.97**
Cotabato	26,521	10,203	9,529	19,732	74.40
Sarangani	13,322	4,971	4,698	9,669	72.58
South Cotabato	21,595	7,664	7,316	14,980	69.37
Sultan Kudarat	19,391	6,425	6,185	12,610	65.03
City of General Santos	14,450	5,030	4,649	9,679	66.98
**BARMM**	**132,841**	**33,207**	**33,865**	**67,072**	**50.49**
Basilan	11,636	2,006	2,080	4,086	35.12
Lanao del Sur	33,374	12,491	13,027	25,518	76.46
Maguindanao	41,650	9,836	9,602	19,438	46.67

Table 2.C.1.3 - Immunization Services for Infants and Children
Infants who completed 3 doses of Oral Polio Vaccine (OPV)
Philippines, 2023

Area	Eligible Pop (0-11 mos. old)	OPV 2			
		Male	Female	Total	%
Sulu	21,378	4,200	4,566	8,766	41.00
Tawi-Tawi	12,609	2,536	2,496	5,032	39.91
SGA	5,103	498	480	978	19.17
City of Cotabato	7,091	1,640	1,614	3,254	45.89
**CARAGA**	**55,065**	**20,722**	**19,534**	**40,256**	**73.11**
Agusan del Norte	7,286	2,726	2,454	5,180	71.10
Agusan del Sur	16,038	5,345	5,219	10,564	65.87
Surigao del Norte	10,186	3,884	3,685	7,569	74.31
Surigao del Sur	12,736	4,736	4,374	9,110	71.53
Dinagat Islands	2,134	885	868	1,753	82.15
City of Butuan	6,685	3,146	2,934	6,080	90.95

Table 2.C.1.3 - Immunization Services for Infants and Children
Infants who completed 3 doses of Oral Polio Vaccine (OPV)
Philippines, 2023

Area	Eligible Pop (0-11 mos. old)	OPV 3			
		Male	Female	Total	%
**Philippines**	**2,187,333**	**747,537**	**714,975**	**1,462,512**	**66.86**
**NCR**	**261,228**	**101,223**	**98,938**	**200,161**	**76.62**
Malabon City	7,385	2,497	2,367	4,864	65.86
Navotas City	5,210	2,369	2,318	4,687	89.96
Valenzuela City	13,477	4,695	4,523	9,218	68.40
Caloocan City	33,168	7,340	7,435	14,775	44.55
Marikina City	8,342	2,792	2,678	5,470	65.57
Pasig City	17,682	5,865	5,625	11,490	64.98
Pateros	1,116	418	377	795	71.24
Taguig City	18,992	6,196	5,654	11,850	62.39
Quezon City	59,136	32,789	32,698	65,487	110.74
Makati City	10,944	4,479	4,333	8,812	80.52
Mandaluyong City	7,922	3,080	3,119	6,199	78.25
San Juan City	2,168	547	539	1,086	50.09
Manila City	34,128	13,947	13,741	27,688	81.13
Las Piñas City	11,263	3,914	3,703	7,617	67.63
Muntinlupa City	9,881	3,664	3,509	7,173	72.59
Parañaque City	13,280	3,715	3,468	7,183	54.09
Pasay City	7,134	2,916	2,851	5,767	80.84
**CAR**	**31,319**	**9,316**	**8,902**	**18,218**	**58.17**
Abra	3,660	1,137	1,102	2,239	61.17
Apayao	2,238	660	624	1,284	57.37
Benguet	8,806	2,305	2,246	4,551	51.68
Ifugao	4,098	1,115	1,031	2,146	52.37
Kalinga	4,549	1,264	1,242	2,506	55.09
Mountain Province	2,619	1,015	878	1,893	72.28
City of Baguio	5,349	1,820	1,779	3,599	67.28
**Region 1**	**96,016**	**34,692**	**33,837**	**68,529**	**71.37**
Ilocos Norte	8,912	3,348	3,369	6,717	75.37
Ilocos Sur	9,914	4,241	3,916	8,157	82.28
La Union	13,507	4,866	4,311	9,177	67.94
Pangasinan	60,360	21,247	21,320	42,567	70.52
City of Dagupan	3,323	990	921	1,911	57.51

Table 2.C.1.3 - Immunization Services for Infants and Children
Infants who completed 3 doses of Oral Polio Vaccine (OPV)
Philippines, 2023

Area	Eligible Pop (0-11 mos. old)	OPV 3			
		Male	Female	Total	%
**Region 2**	**63,967**	**21,789**	**20,167**	**41,956**	**65.59**
Batanes	240	133	94	227	94.58
Cagayan	20,786	5,509	5,311	10,820	52.05
Isabela	27,379	10,957	10,018	20,975	76.61
Nueva Vizcaya	8,798	2,628	2,331	4,959	56.37
Quirino	3,839	1,223	1,144	2,367	61.66
City of Santiago	2,925	1,339	1,269	2,608	89.16
**Region 3**	**233,544**	**84,934**	**80,563**	**165,497**	**70.86**
Aurora	4,537	1,839	1,714	3,553	78.31
Bataan	17,380	5,652	5,260	10,912	62.78
Bulacan	67,590	28,595	26,458	55,053	81.45
Nueva Ecija	43,425	13,294	12,647	25,941	59.74
Pampanga	45,651	15,637	15,329	30,966	67.83
Tarlac	29,503	10,905	10,702	21,607	73.24
Zambales	11,723	4,534	4,225	8,759	74.72
City of Angeles	9,146	3,050	2,951	6,001	65.61
City of Olongapo	4,589	1,428	1,277	2,705	58.95
**Region 4A**	**307,982**	**114,676**	**108,976**	**223,652**	**72.62**
Batangas	53,251	20,937	20,019	40,956	76.91
Cavite	77,964	29,602	28,313	57,915	74.28
Laguna	61,525	23,890	22,561	46,451	75.50
Quezon	40,810	13,777	12,988	26,765	65.58
Rizal	68,235	24,229	22,910	47,139	69.08
City of Lucena	6,197	2,241	2,185	4,426	71.42
**Region 4B**	**66,121**	**21,686**	**20,434**	**42,120**	**63.70**
Marinduque	4,144	1,585	1,353	2,938	70.90
Occidental Mindoro	11,640	3,647	3,447	7,094	60.95
Oriental Mindoro	18,943	6,045	5,846	11,891	62.77
Palawan	20,317	5,684	5,366	11,050	54.39
Romblon	5,489	2,146	1,992	4,138	75.39
City of Puerto Princesa	5,588	2,579	2,430	5,009	89.64

Table 2.C.1.3 - Immunization Services for Infants and Children
Infants who completed 3 doses of Oral Polio Vaccine (OPV)
Philippines, 2023

Area	Eligible Pop (0-11 mos. old)	OPV 3			
		Male	Female	Total	%
**Region 5**	**135,480**	**38,568**	**35,949**	**74,517**	**55.00**
Albay	27,440	8,661	8,200	16,861	61.45
Camarines Norte	14,695	4,209	3,869	8,078	54.97
Camarines Sur	43,452	9,648	8,861	18,509	42.60
Catanduanes	5,414	2,240	2,071	4,311	79.63
Masbate	22,365	6,474	6,172	12,646	56.54
Sorsogon	17,929	5,851	5,324	11,175	62.33
City of Naga	4,185	1,485	1,452	2,937	70.18
**Region 6**	**148,020**	**47,686**	**45,356**	**93,042**	**62.86**
Aklan	11,283	3,178	3,074	6,252	55.41
Antique	11,930	2,695	2,645	5,340	44.76
Capiz	13,931	5,095	4,805	9,900	71.06
Guimaras	3,351	1,000	950	1,950	58.19
Iloilo	36,140	13,216	12,397	25,613	70.87
Negros Occidental	52,270	16,897	16,097	32,994	63.12
City of Bacolod	10,373	3,003	2,823	5,826	56.17
City of Iloilo	8,742	2,602	2,565	5,167	59.11
**Region 7**	**165,673**	**50,604**	**48,074**	**98,678**	**59.56**
Bohol	26,235	7,047	6,765	13,812	52.65
Cebu	72,201	17,975	17,215	35,190	48.74
Negros Oriental	28,722	8,537	8,041	16,578	57.72
Siquijor	1,680	560	574	1,134	67.50
City of Cebu	19,267	7,855	7,512	15,367	79.76
City of Lapu-Lapu	10,679	5,151	4,590	9,741	91.22
City of Mandaue	6,889	3,479	3,377	6,856	99.52
**Region 8**	**93,813**	**27,713**	**26,117**	**53,830**	**57.38**
Biliran	3,373	1,506	1,465	2,971	88.08
Eastern Samar	9,624	2,940	2,839	5,779	60.05
Leyte	31,559	7,837	7,370	15,207	48.19
Northern Samar	14,056	4,011	3,770	7,781	55.36
Southern Leyte	7,406	2,293	2,178	4,471	60.37
Samar	17,011	5,480	5,093	10,573	62.15
Ormoc City	5,292	1,799	1,658	3,457	65.33
City of Tacloban	5,492	1,847	1,744	3,591	65.39

Table 2.C.1.3 - Immunization Services for Infants and Children
Infants who completed 3 doses of Oral Polio Vaccine (OPV)
Philippines, 2023

Area	Eligible Pop (0-11 mos. old)	OPV 3			
		Male	Female	Total	%
**Region 9**	**84,815**	**32,596**	**31,521**	**64,117**	**75.60**
Zamboanga del Norte	24,841	9,268	9,136	18,404	74.09
Zamboanga del Sur	23,757	8,404	8,084	16,488	69.40
Zamboanga Sibugay	13,841	4,807	4,708	9,515	68.75
City of Isabela	3,201	1,362	1,299	2,661	83.13
City of Zamboanga	19,175	8,755	8,294	17,049	88.91
**Region 10**	**109,169**	**40,815**	**39,221**	**80,036**	**73.31**
Bukidnon	33,403	11,922	11,428	23,350	69.90
Camiguin	1,727	570	592	1,162	67.28
Lanao del Norte	17,760	6,701	6,515	13,216	74.41
Misamis Occidental	12,328	5,538	5,421	10,959	88.90
Misamis Oriental	21,975	8,063	7,843	15,906	72.38
City of Cagayan De Oro	14,308	6,175	5,803	11,978	83.72
City of Iligan	7,668	1,846	1,619	3,465	45.19
**Region 11**	**107,001**	**41,416**	**39,048**	**80,464**	**75.20**
Davao de Oro	15,253	5,923	5,681	11,604	76.08
Davao del Norte	23,453	8,893	8,284	17,177	73.24
Davao Oriental	11,935	4,591	4,282	8,873	74.34
Davao del Sur	13,083	4,865	4,619	9,484	72.49
Davao Occidental	6,629	2,302	2,141	4,443	67.02
City of Davao	36,648	14,842	14,041	28,883	78.81
**Region 12**	**95,279**	**32,732**	**31,059**	**63,791**	**66.95**
Cotabato	26,521	9,935	9,307	19,242	72.55
Sarangani	13,322	4,785	4,554	9,339	70.10
South Cotabato	21,595	7,426	7,072	14,498	67.14
Sultan Kudarat	19,391	5,948	5,601	11,549	59.56
City of General Santos	14,450	4,638	4,525	9,163	63.41
**BARMM**	**132,841**	**27,767**	**28,374**	**56,141**	**42.26**
Basilan	11,636	1,536	1,549	3,085	26.51
Lanao del Sur	33,374	12,014	12,802	24,816	74.36
Maguindanao	41,650	7,324	7,232	14,556	34.95

Table 2.C.1.3 - Immunization Services for Infants and Children

Infants who completed 3 doses of Oral Polio Vaccine (OPV)

Philippines, 2023

Area	Eligible Pop (0-11 mos. old)	OPV 3			
		Male	Female	Total	%
Sulu	21,378	3,081	3,105	6,186	28.94
Tawi-Tawi	12,609	2,094	2,032	4,126	32.72
SGA	5,103	289	287	576	11.29
City of Cotabato	7,091	1,429	1,367	2,796	39.43
**CARAGA**	**55,065**	**19,324**	**18,439**	**37,763**	**68.58**
Agusan del Norte	7,286	2,409	2,231	4,640	63.68
Agusan del Sur	16,038	5,026	4,963	9,989	62.28
Surigao del Norte	10,186	3,673	3,355	7,028	69.00
Surigao del Sur	12,736	4,229	4,050	8,279	65.00
Dinagat Islands	2,134	937	889	1,826	85.57
City of Butuan	6,685	3,050	2,951	6,001	89.77

Table 2.C.1.3 - Immunization Services for Infants and Children
Infants who completed 2 doses of IPV
Philippines, 2023

Area	Eligible Pop (0-11 mos. old)	IPV 1				IPV 2 (Routine)			
		Male	Female	Total	%	Male	Female	Total	%
**Philippines**	**2,187,333**	**813,072**	**766,774**	**1,579,846**	**72.23**	**659,856**	**623,233**	**1,283,089**	**58.66**
**NCR**	**261,228**	**89,627**	**86,791**	**176,418**	**67.53**	**72,133**	**69,043**	**141,176**	**54.04**
Malabon City	7,385	2,562	2,456	5,018	67.95	2,298	2,170	4,468	60.50
Navotas City	5,210	2,154	2,132	4,286	82.26	1,934	1,879	3,813	73.19
Valenzuela City	13,477	4,755	4,531	9,286	68.90	4,437	4,141	8,578	63.65
Caloocan City	33,168	8,776	8,654	17,430	52.55	7,278	6,936	14,214	42.85
Marikina City	8,342	2,741	2,630	5,371	64.39	2,420	2,349	4,769	57.17
Pasig City	17,682	5,142	4,849	9,991	56.50	4,269	4,087	8,356	47.26
Pateros	1,116	405	382	787	70.52	391	370	761	68.19
Taguig City	18,992	5,784	5,225	11,009	57.97	4,507	4,384	8,891	46.81
Quezon City	59,136	20,591	20,645	41,236	69.73	10,071	10,057	20,128	34.04
Makati City	10,944	4,345	4,219	8,564	78.25	3,958	3,830	7,788	71.16
Mandaluyong City	7,922	3,694	3,632	7,326	92.48	2,662	2,665	5,327	67.24
San Juan City	2,168	559	540	1,099	50.69	520	462	982	45.30
Manila City	34,128	14,125	13,589	27,714	81.21	14,384	13,905	28,289	82.89
Las Piñas City	11,263	4,035	3,819	7,854	69.73	4,128	3,620	7,748	68.79
Muntinlupa City	9,881	3,494	3,287	6,781	68.63	3,264	3,063	6,327	64.03
Parañaque City	13,280	3,574	3,287	6,861	51.66	2,837	2,560	5,397	40.64
Pasay City	7,134	2,891	2,914	5,805	81.37	2,775	2,565	5,340	74.85
**CAR**	**31,319**	**11,506**	**10,907**	**22,413**	**71.56**	**10,021**	**9,448**	**19,469**	**62.16**
Abra	3,660	1,551	1,505	3,056	83.50	1,285	1,266	2,551	69.70
Apayao	2,238	872	826	1,698	75.87	749	716	1,465	65.46
Benguet	8,806	2,843	2,712	5,555	63.08	2,673	2,499	5,172	58.73
Ifugao	4,098	1,303	1,251	2,554	62.32	722	621	1,343	32.77
Kalinga	4,549	1,650	1,593	3,243	71.29	1,490	1,383	2,873	63.16
Mountain Province	2,619	1,133	971	2,104	80.34	980	919	1,899	72.51
City of Baguio	5,349	2,154	2,049	4,203	78.58	2,122	2,044	4,166	77.88
**Region 1**	**96,016**	**37,764**	**36,749**	**74,513**	**77.60**	**34,634**	**32,139**	**66,773**	**69.54**
Ilocos Norte	8,912	3,538	3,569	7,107	79.75	3,471	3,415	6,886	77.27
Ilocos Sur	9,914	4,680	4,345	9,025	91.03	4,714	4,363	9,077	91.56
La Union	13,507	5,098	4,639	9,737	72.09	4,733	4,235	8,968	66.40
Pangasinan	60,360	23,458	23,274	46,732	77.42	20,792	19,283	40,075	66.39
City of Dagupan	3,323	990	922	1,912	57.54	924	843	1,767	53.17

Table 2.C.1.3 - Immunization Services for Infants and Children
Infants who completed 2 doses of IPV
Philippines, 2023

Area	Eligible Pop (0-11 mos. old)	IPV 1				IPV 2 (Routine)			
		Male	Female	Total	%	Male	Female	Total	%
**Region 2**	**63,967**	**24,359**	**23,307**	**47,666**	**74.52**	**15,541**	**14,666**	**30,207**	**47.22**
Batanes	240	133	95	228	95.00	107	91	198	82.50
Cagayan	20,786	7,292	6,997	14,289	68.74	4,426	4,184	8,610	41.42
Isabela	27,379	11,312	10,923	22,235	81.21	8,074	7,690	15,764	57.58
Nueva Vizcaya	8,798	3,024	2,817	5,841	66.39	249	217	466	5.30
Quirino	3,839	1,261	1,212	2,473	64.42	1,226	1,241	2,467	64.26
City of Santiago	2,925	1,337	1,263	2,600	88.89	1,459	1,243	2,702	92.38
**Region 3**	**233,544**	**96,080**	**91,031**	**187,111**	**80.12**	**90,167**	**83,600**	**173,767**	**74.40**
Aurora	4,537	1,979	1,804	3,783	83.38	1,912	1,700	3,612	79.61
Bataan	17,380	6,510	6,026	12,536	72.13	6,415	5,911	12,326	70.92
Bulacan	67,590	31,586	29,285	60,871	90.06	29,193	26,663	55,856	82.64
Nueva Ecija	43,425	16,066	15,253	31,319	72.12	15,180	14,259	29,439	67.79
Pampanga	45,651	18,713	18,361	37,074	81.21	17,771	17,016	34,787	76.20
Tarlac	29,503	12,166	11,849	24,015	81.40	11,984	10,710	22,694	76.92
Zambales	11,723	4,583	4,260	8,843	75.43	3,874	3,670	7,544	64.35
City of Angeles	9,146	2,810	2,657	5,467	59.77	2,277	2,224	4,501	49.21
City of Olongapo	4,589	1,667	1,536	3,203	69.80	1,561	1,447	3,008	65.55
**Region 4A**	**307,982**	**115,454**	**109,214**	**224,668**	**72.95**	**89,681**	**84,841**	**174,522**	**56.67**
Batangas	53,251	21,564	20,561	42,125	79.11	17,345	16,273	33,618	63.13
Cavite	77,964	29,458	27,936	57,394	73.62	23,385	22,438	45,823	58.77
Laguna	61,525	24,122	22,725	46,847	76.14	19,284	18,304	37,588	61.09
Quezon	40,810	14,005	13,186	27,191	66.63	9,067	8,437	17,504	42.89
Rizal	68,235	24,073	22,630	46,703	68.44	19,021	17,837	36,858	54.02
City of Lucena	6,197	2,232	2,176	4,408	71.13	1,579	1,552	3,131	50.52
**Region 4B**	**66,121**	**23,526**	**21,869**	**45,395**	**68.65**	**16,027**	**15,167**	**31,194**	**47.18**
Marinduque	4,144	1,719	1,490	3,209	77.44	1,437	1,230	2,667	64.36
Occidental Mindoro	11,640	3,890	3,607	7,497	64.41	2,353	2,202	4,555	39.13
Oriental Mindoro	18,943	6,303	5,998	12,301	64.94	4,563	4,566	9,129	48.19
Palawan	20,317	6,830	6,312	13,142	64.68	3,886	3,574	7,460	36.72
Romblon	5,489	2,240	2,083	4,323	78.76	1,940	1,856	3,796	69.16
City of Puerto Princesa	5,588	2,544	2,379	4,923	88.10	1,848	1,739	3,587	64.19
**Region 5**	**135,480**	**47,874**	**44,143**	**92,017**	**67.92**	**35,864**	**33,643**	**69,507**	**51.30**

Table 2.C.1.3 - Immunization Services for Infants and Children

Infants who completed 2 doses of IPV

Philippines, 2023

Area	Eligible Pop (0-11 mos. old)	IPV 1				IPV 2 (Routine)			
		Male	Female	Total	%	Male	Female	Total	%
Albay	27,440	9,773	8,907	18,680	68.08	6,905	6,436	13,341	48.62
Camarines Norte	14,695	5,242	4,720	9,962	67.79	3,365	3,252	6,617	45.03
Camarines Sur	43,452	13,947	12,766	26,713	61.48	10,465	9,587	20,052	46.15
Catanduanes	5,414	2,234	2,052	4,286	79.17	1,767	1,665	3,432	63.39
Masbate	22,365	8,204	7,951	16,155	72.23	6,256	6,171	12,427	55.56
Sorsogon	17,929	7,050	6,397	13,447	75.00	5,980	5,456	11,436	63.78
City of Naga	4,185	1,424	1,350	2,774	66.28	1,126	1,076	2,202	52.62
**Region 6**	**148,020**	**54,021**	**50,862**	**104,883**	**70.86**	**44,121**	**41,107**	**85,228**	**57.58**
Aklan	11,283	3,564	3,430	6,994	61.99	2,619	2,447	5,066	44.90
Antique	11,930	4,168	4,040	8,208	68.80	1,562	1,445	3,007	25.21
Capiz	13,931	5,217	4,862	10,079	72.35	4,627	4,381	9,008	64.66
Guimaras	3,351	1,234	1,144	2,378	70.96	1,047	932	1,979	59.06
Iloilo	36,140	13,569	12,361	25,930	71.75	10,343	9,461	19,804	54.80
Negros Occidental	52,270	19,378	18,466	37,844	72.40	17,639	16,460	34,099	65.24
City of Bacolod	10,373	3,734	3,427	7,161	69.03	3,293	3,136	6,429	61.98
City of Iloilo	8,742	3,157	3,132	6,289	71.94	2,991	2,845	5,836	66.76
**Region 7**	**165,673**	**61,373**	**57,613**	**118,986**	**71.82**	**57,333**	**53,714**	**111,047**	**67.03**
Bohol	26,235	8,789	8,296	17,085	65.12	7,982	7,563	15,545	59.25
Cebu	72,201	25,097	23,472	48,569	67.27	24,076	22,599	46,675	64.65
Negros Oriental	28,722	10,384	9,730	20,114	70.03	8,931	8,200	17,131	59.64
Siquijor	1,680	567	573	1,140	67.86	514	492	1,006	59.88
City of Cebu	19,267	7,954	7,641	15,595	80.94	7,190	6,834	14,024	72.79
City of Lapu-Lapu	10,679	5,151	4,590	9,741	91.22	5,101	4,571	9,672	90.57
City of Mandaue	6,889	3,431	3,311	6,742	97.87	3,539	3,455	6,994	101.52
**Region 8**	**93,813**	**31,755**	**29,646**	**61,401**	**65.45**	**25,108**	**23,501**	**48,609**	**51.81**
Biliran	3,373	1,577	1,520	3,097	91.82	1,441	1,390	2,831	83.93
Eastern Samar	9,624	3,335	3,115	6,450	67.02	2,990	2,802	5,792	60.18
Leyte	31,559	10,234	9,452	19,686	62.38	7,371	6,866	14,237	45.11
Northern Samar	14,056	4,432	4,132	8,564	60.93	3,068	2,781	5,849	41.61
Southern Leyte	7,406	2,438	2,338	4,776	64.49	2,407	2,263	4,670	63.06
Samar	17,011	6,113	5,650	11,763	69.15	4,235	4,001	8,236	48.42
Ormoc City	5,292	1,813	1,689	3,502	66.18	1,849	1,673	3,522	66.55
City of Tacloban	5,492	1,813	1,750	3,563	64.88	1,747	1,725	3,472	63.22
**Region 9**	**84,815**	**33,292**	**32,193**	**65,485**	**77.21**	**26,942**	**26,264**	**53,206**	**62.73**

Table 2.C.1.3 - Immunization Services for Infants and Children
Infants who completed 2 doses of IPV
Philippines, 2023

Area	Eligible Pop (0-11 mos. old)	IPV 1				IPV 2 (Routine)			
		Male	Female	Total	%	Male	Female	Total	%
Zamboanga del Norte	24,841	9,234	9,060	18,294	73.64	7,159	7,083	14,242	57.33
Zamboanga del Sur	23,757	8,395	8,042	16,437	69.19	6,783	6,418	13,201	55.57
Zamboanga Sibugay	13,841	5,243	5,139	10,382	75.01	4,285	4,254	8,539	61.69
City of Isabela	3,201	1,366	1,346	2,712	84.72	1,208	1,342	2,550	79.66
City of Zamboanga	19,175	9,054	8,606	17,660	92.10	7,507	7,167	14,674	76.53
**Region 10**	**109,169**	**42,252**	**39,821**	**82,073**	**75.18**	**30,276**	**28,853**	**59,129**	**54.16**
Bukidnon	33,403	12,579	11,776	24,355	72.91	8,012	7,622	15,634	46.80
Camiguin	1,727	622	630	1,252	72.50	610	615	1,225	70.93
Lanao del Norte	17,760	6,703	6,479	13,182	74.22	5,164	5,054	10,218	57.53
Misamis Occidental	12,328	5,394	4,949	10,343	83.90	2,822	2,761	5,583	45.29
Misamis Oriental	21,975	8,050	7,760	15,810	71.95	5,384	4,978	10,362	47.15
City of Cagayan De Oro	14,308	6,408	5,934	12,342	86.26	5,910	5,495	11,405	79.71
City of Iligan	7,668	2,496	2,293	4,789	62.45	2,374	2,328	4,702	61.32
**Region 11**	**107,001**	**42,267**	**39,650**	**81,917**	**76.56**	**37,732**	**35,078**	**72,810**	**68.05**
Davao de Oro	15,253	6,191	5,859	12,050	79.00	5,706	5,417	11,123	72.92
Davao del Norte	23,453	8,959	8,365	17,324	73.87	8,498	7,931	16,429	70.05
Davao Oriental	11,935	4,823	4,490	9,313	78.03	4,331	4,016	8,347	69.94
Davao del Sur	13,083	5,078	4,787	9,865	75.40	4,571	4,176	8,747	66.86
Davao Occidental	6,629	2,422	2,241	4,663	70.34	1,780	1,717	3,497	52.75
City of Davao	36,648	14,794	13,908	28,702	78.32	12,846	11,821	24,667	67.31
**Region 12**	**95,279**	**34,274**	**32,142**	**66,416**	**69.71**	**27,972**	**26,606**	**54,578**	**57.28**
Cotabato	26,521	9,918	9,213	19,131	72.14	7,068	6,505	13,573	51.18
Sarangani	13,322	4,935	4,544	9,479	71.15	3,825	3,555	7,380	55.40
South Cotabato	21,595	8,007	7,520	15,527	71.90	7,155	6,847	14,002	64.84
Sultan Kudarat	19,391	6,791	6,391	13,182	67.98	6,394	6,204	12,598	64.97
City of General Santos	14,450	4,623	4,474	9,097	62.96	3,530	3,495	7,025	48.62
**BARMM**	**132,841**	**45,121**	**39,238**	**84,359**	**63.50**	**29,364**	**29,424**	**58,788**	**44.25**
Basilan	11,636	1,479	1,554	3,033	26.07	476	497	973	8.36
Lanao del Sur	33,374	11,642	12,290	23,932	71.71	11,060	11,568	22,628	67.80
Maguindanao	41,650	19,729	13,337	33,066	79.39	10,658	10,552	21,210	50.92
Sulu	21,378	4,732	4,846	9,578	44.80	2,055	2,132	4,187	19.59

Table 2.C.1.3 - Immunization Services for Infants and Children
Infants who completed 2 doses of IPV
Philippines, 2023

Area	Eligible Pop (0-11 mos. old)	IPV 1				IPV 2 (Routine)			
		Male	Female	Total	%	Male	Female	Total	%
Tawi-Tawi	12,609	5,052	4,945	9,997	79.28	3,195	2,805	6,000	47.59
SGA	5,103	726	595	1,321	25.89	222	245	467	9.15
City of Cotabato	7,091	1,761	1,671	3,432	48.40	1,698	1,625	3,323	46.86
**CARAGA**	**55,065**	**22,527**	**21,598**	**44,125**	**80.13**	**16,940**	**16,139**	**33,079**	**60.07**
Agusan del Norte	7,286	2,698	2,629	5,327	73.11	2,229	2,154	4,383	60.16
Agusan del Sur	16,038	6,109	5,900	12,009	74.88	4,291	4,007	8,298	51.74
Surigao del Norte	10,186	4,240	4,020	8,260	81.09	3,304	3,136	6,440	63.22
Surigao del Sur	12,736	5,330	5,018	10,348	81.25	3,586	3,405	6,991	54.89
Dinagat Islands	2,134	967	918	1,885	88.33	638	614	1,252	58.67
City of Butuan	6,685	3,183	3,113	6,296	94.18	2,892	2,823	5,715	85.49

Table 2.C.1.3 - Immunization Services for Infants and Children
Infants who completed 2 doses of IPV (Catch-up)
Philippines, 2023

Area	Eligible Pop (0-23 mos. old)	IPV 2 (Catch-up)			
		Male	Female	Total	%
**Philippines**	**4,439,744**	**37,614**	**35,364**	**72,978**	**1.64**
**NCR**	**527,611**	**4,158**	**3,985**	**8,143**	**1.54**
Malabon City	14,760	91	87	178	1.21
Navotas City	10,567	0	0	0	0.00
Valenzuela City	27,833	0	0	0	0.00
Caloocan City	68,436	146	137	283	0.41
Marikina City	17,132	113	102	215	1.25
Pasig City	34,415	707	636	1,343	3.90
Pateros	2,327	0	0	0	0.00
Taguig City	38,182	374	442	816	2.14
Quezon City	119,999	666	680	1,346	1.12
Makati City	21,851	0	0	0	0.00
Mandaluyong City	15,994	216	190	406	2.54
San Juan City	4,424	5	2	7	0.16
Manila City	69,059	1,148	1,099	2,247	3.25
Las Piñas City	22,395	239	238	477	2.13
Muntinlupa City	19,878	137	132	269	1.35
Parañaque City	26,714	272	202	474	1.77
Pasay City	13,645	44	38	82	0.60
**CAR**	**63,511**	**328**	**280**	**608**	**0.96**
Abra	7,640	73	59	132	1.73
Apayao	4,446	32	13	45	1.01
Benguet	17,815	99	99	198	1.11
Ifugao	8,282	52	43	95	1.15
Kalinga	9,140	29	29	58	0.63
Mountain Province	5,353	23	21	44	0.82
City of Baguio	10,835	20	16	36	0.33
**Region 1**	**193,398**	**1,726**	**1,560**	**3,286**	**1.70**
Ilocos Norte	18,052	156	177	333	1.84
Ilocos Sur	20,224	31	41	72	0.36
La Union	26,967	378	343	721	2.67
Pangasinan	121,427	1,046	895	1,941	1.60
City of Dagupan	6,728	115	104	219	3.26

Table 2.C.1.3 - Immunization Services for Infants and Children
Infants who completed 2 doses of IPV (Catch-up)
Philippines, 2023

Area	Eligible Pop (0-23 mos. old)	IPV 2 (Catch-up)			
		Male	Female	Total	%
**Region 2**	**129,641**	**1,143**	**1,015**	**2,158**	**1.66**
Batanes	506	0	0	0	0.00
Cagayan	42,363	617	509	1,126	2.66
Isabela	55,538	388	391	779	1.40
Nueva Vizcaya	17,957	32	24	56	0.31
Quirino	7,599	73	59	132	1.74
City of Santiago	5,678	33	32	65	1.14
**Region 3**	**477,173**	**3,782**	**3,468**	**7,250**	**1.52**
Aurora	9,275	2	1	3	0.03
Bataan	34,755	285	261	546	1.57
Bulacan	139,441	1,661	1,570	3,231	2.32
Nueva Ecija	88,324	869	721	1,590	1.80
Pampanga	94,043	302	301	603	0.64
Tarlac	58,852	427	318	745	1.27
Zambales	23,879	136	199	335	1.40
City of Angeles	19,460	8	9	17	0.09
City of Olongapo	9,144	92	88	180	1.97
**Region 4A**	**627,372**	**7,586**	**7,133**	**14,719**	**2.35**
Batangas	110,367	1,523	1,365	2,888	2.62
Cavite	159,531	2,694	2,578	5,272	3.30
Laguna	124,592	1,223	1,162	2,385	1.91
Quezon	82,870	1,269	1,193	2,462	2.97
Rizal	137,811	582	582	1,164	0.84
City of Lucena	12,201	295	253	548	4.49
**Region 4B**	**132,983**	**1,721**	**1,580**	**3,301**	**2.48**
Marinduque	9,011	29	23	52	0.58
Occidental Mindoro	23,392	292	277	569	2.43
Oriental Mindoro	37,572	546	445	991	2.64
Palawan	40,855	635	631	1,266	3.10
Romblon	11,128	68	52	120	1.08
City of Puerto Princesa	11,025	151	152	303	2.75
**Region 5**	**274,409**	**3,354**	**3,100**	**6,454**	**2.35**

Table 2.C.1.3 - Immunization Services for Infants and Children
Infants who completed 2 doses of IPV (Catch-up)
Philippines, 2023

Area	Eligible Pop (0-23 mos. old)	IPV 2 (Catch-up)			
		Male	Female	Total	%
Albay	56,272	535	467	1,002	1.78
Camarines Norte	29,420	439	427	866	2.94
Camarines Sur	87,934	1,211	1,154	2,365	2.69
Catanduanes	11,311	141	114	255	2.25
Masbate	44,856	673	615	1,288	2.87
Sorsogon	36,207	261	235	496	1.37
City of Naga	8,409	94	88	182	2.16
**Region 6**	**301,871**	**1,926**	**1,792**	**3,718**	**1.23**
Aklan	22,397	15	2	17	0.08
Antique	23,897	200	173	373	1.56
Capiz	28,314	211	191	402	1.42
Guimaras	6,891	10	12	22	0.32
Iloilo	73,411	400	414	814	1.11
Negros Occidental	107,574	743	692	1,435	1.33
City of Bacolod	21,441	295	262	557	2.60
City of Iloilo	17,946	52	46	98	0.55
**Region 7**	**332,249**	**1,099**	**976**	**2,075**	**0.62**
Bohol	52,141	239	193	432	0.83
Cebu	145,235	637	595	1,232	0.85
Negros Oriental	57,318	0	0	0	0.00
Siquijor	3,144	45	44	89	2.83
City of Cebu	39,339	117	100	217	0.55
City of Lapu-Lapu	21,428	61	44	105	0.49
City of Mandaue	13,644	0	0	0	0.00
**Region 8**	**188,647**	**1,214**	**1,146**	**2,360**	**1.25**
Biliran	6,854	197	169	366	5.34
Eastern Samar	19,231	210	165	375	1.95
Leyte	64,629	0	0	0	0.00
Northern Samar	27,748	473	516	989	3.56
Southern Leyte	15,183	16	13	29	0.19
Samar	33,750	99	75	174	0.52
Ormoc City	10,381	130	134	264	2.54
City of Tacloban	10,871	89	74	163	1.50
**Region 9**	**170,508**	**2,077**	**2,115**	**4,192**	**2.46**

Table 2.C.1.3 - Immunization Services for Infants and Children
Infants who completed 2 doses of IPV (Catch-up)
Philippines, 2023

Area	Eligible Pop (0-23 mos. old)	IPV 2 (Catch-up)			
		Male	Female	Total	%
Zamboanga del Norte	49,986	677	667	1,344	2.69
Zamboanga del Sur	46,868	427	485	912	1.95
Zamboanga Sibugay	27,895	228	213	441	1.58
City of Isabela	6,629	13	17	30	0.45
City of Zamboanga	39,130	732	733	1,465	3.74
**Region 10**	**219,683**	**1,310**	**1,256**	**2,566**	**1.17**
Bukidnon	67,677	303	339	642	0.95
Camiguin	3,443	0	3	3	0.09
Lanao del Norte	36,002	147	143	290	0.81
Misamis Occidental	24,599	227	217	444	1.80
Misamis Oriental	43,704	68	78	146	0.33
City of Cagayan De Oro	28,786	38	35	73	0.25
City of Iligan	15,472	527	441	968	6.26
**Region 11**	**216,508**	**2,457**	**2,416**	**4,873**	**2.25**
Davao de Oro	30,772	275	272	547	1.78
Davao del Norte	46,998	263	253	516	1.10
Davao Oriental	23,538	207	205	412	1.75
Davao del Sur	27,118	259	287	546	2.01
Davao Occidental	13,930	160	166	326	2.34
City of Davao	74,152	1,293	1,233	2,526	3.41
**Region 12**	**192,345**	**2,393**	**2,189**	**4,582**	**2.38**
Cotabato	54,477	869	790	1,659	3.05
Sarangani	26,213	518	473	991	3.78
South Cotabato	43,569	432	362	794	1.82
Sultan Kudarat	39,243	70	64	134	0.34
City of General Santos	28,843	504	500	1,004	3.48
**BARMM**	**280,272**	**262**	**322**	**584**	**0.21**
Basilan	24,464	18	21	39	0.16
Lanao del Sur	70,235	0	0	0	0.00
Maguindanao	86,270	174	209	383	0.44
Sulu	47,113	0	0	0	0.00
Tawi-Tawi	26,664	6	10	16	0.06

Table 2.C.1.3 - Immunization Services for Infants and Children
Infants who completed 2 doses of IPV (Catch-up)
Philippines, 2023

Area	Eligible Pop (0-23 mos. old)	IPV 2 (Catch-up)			
		Male	Female	Total	%
SGA	10,920	64	82	146	1.34
City of Cotabato	14,606	0	0	0	0.00
**CARAGA**	**111,563**	**1,078**	**1,031**	**2,109**	**1.89**
Agusan del Norte	14,769	193	209	402	2.72
Agusan del Sur	32,014	74	63	137	0.43
Surigao del Norte	20,693	281	274	555	2.68
Surigao del Sur	26,073	499	453	952	3.65
Dinagat Islands	4,556	0	0	0	0.00
City of Butuan	13,458	31	32	63	0.47

Table 2.C.1.4 - Immunization Services for Infants and Children
Infants who completed 3 doses of Pneumococcal Conjugate Vaccine (PCV)
Philippines, 2023

Area	Eligible Pop (0-11 mos. old)	PCV 1				PCV 2			
		Male	Female	Total	%	Male	Female	Total	%
**Philippines**	**2,187,333**	**862,162**	**818,266**	**1,680,428**	**76.83**	**856,679**	**818,124**	**1,674,803**	**76.57**
**NCR**	**261,228**	**98,965**	**95,103**	**194,068**	**74.29**	**98,402**	**95,871**	**194,273**	**74.37**
Malabon City	7,385	2,671	2,565	5,236	70.90	2,604	2,472	5,076	68.73
Navotas City	5,210	2,384	2,342	4,726	90.71	2,374	2,308	4,682	89.87
Valenzuela City	13,477	4,923	4,615	9,538	70.77	4,823	4,839	9,662	71.69
Caloocan City	33,168	10,104	9,497	19,601	59.10	9,570	9,215	18,785	56.64
Marikina City	8,342	2,934	2,759	5,693	68.25	2,783	2,695	5,478	65.67
Pasig City	17,682	5,883	5,653	11,536	65.24	5,845	5,645	11,490	64.98
Pateros	1,116	391	391	782	70.07	403	378	781	69.98
Taguig City	18,992	4,888	4,439	9,327	49.11	4,655	4,419	9,074	47.78
Quezon City	59,136	27,575	27,761	55,336	93.57	29,227	29,618	58,845	99.51
Makati City	10,944	4,131	4,023	8,154	74.51	4,175	3,847	8,022	73.30
Mandaluyong City	7,922	3,107	3,044	6,151	77.64	3,054	3,091	6,145	77.57
San Juan City	2,168	545	546	1,091	50.32	534	532	1,066	49.17
Manila City	34,128	14,570	13,620	28,190	82.60	13,930	13,254	27,184	79.65
Las Piñas City	11,263	4,279	4,045	8,324	73.91	4,198	3,979	8,177	72.60
Muntinlupa City	9,881	3,681	3,354	7,035	71.20	3,573	3,322	6,895	69.78
Parañaque City	13,280	3,885	3,581	7,466	56.22	3,776	3,429	7,205	54.25
Pasay City	7,134	3,014	2,868	5,882	82.45	2,878	2,828	5,706	79.98
**CAR**	**31,319**	**12,612**	**11,674**	**24,286**	**77.54**	**12,639**	**11,915**	**24,554**	**78.40**
Abra	3,660	1,588	1,518	3,106	84.86	1,608	1,507	3,115	85.11
Apayao	2,238	965	926	1,891	84.50	971	1,006	1,977	88.34
Benguet	8,806	3,015	2,855	5,870	66.66	3,037	2,929	5,966	67.75
Ifugao	4,098	1,682	1,466	3,148	76.82	1,638	1,480	3,118	76.09
Kalinga	4,549	1,904	1,766	3,670	80.68	1,898	1,793	3,691	81.14
Mountain Province	2,619	1,209	1,041	2,250	85.91	1,229	1,061	2,290	87.44
City of Baguio	5,349	2,249	2,102	4,351	81.34	2,258	2,139	4,397	82.20
**Region 1**	**96,016**	**38,141**	**35,761**	**73,902**	**76.97**	**39,446**	**37,251**	**76,697**	**79.88**
Ilocos Norte	8,912	3,834	3,890	7,724	86.67	3,824	3,921	7,745	86.91
Ilocos Sur	9,914	4,697	4,309	9,006	90.84	4,841	4,390	9,231	93.11
La Union	13,507	5,095	4,504	9,599	71.07	5,353	4,789	10,142	75.09
Pangasinan	60,360	23,353	21,952	45,305	75.06	24,287	23,070	47,357	78.46
City of Dagupan	3,323	1,162	1,106	2,268	68.25	1,141	1,081	2,222	66.87

Table 2.C.1.4 - Immunization Services for Infants and Children
Infants who completed 3 doses of Pneumococcal Conjugate Vaccine (PCV)
Philippines, 2023

Area	Eligible Pop (0-11 mos. old)	PCV 1				PCV 2			
		Male	Female	Total	%	Male	Female	Total	%
**Region 2**	**63,967**	**26,397**	**24,768**	**51,165**	**79.99**	**26,705**	**24,969**	**51,674**	**80.78**
Batanes	240	123	90	213	88.75	134	91	225	93.75
Cagayan	20,786	8,263	7,711	15,974	76.85	8,478	7,955	16,433	79.06
Isabela	27,379	11,683	11,048	22,731	83.02	11,632	11,174	22,806	83.30
Nueva Vizcaya	8,798	3,403	3,100	6,503	73.91	3,468	2,896	6,364	72.33
Quirino	3,839	1,656	1,625	3,281	85.46	1,712	1,617	3,329	86.72
City of Santiago	2,925	1,269	1,194	2,463	84.21	1,281	1,236	2,517	86.05
**Region 3**	**233,544**	**98,773**	**93,512**	**192,285**	**82.33**	**98,521**	**93,617**	**192,138**	**82.27**
Aurora	4,537	2,023	1,883	3,906	86.09	2,072	1,912	3,984	87.81
Bataan	17,380	6,774	6,324	13,098	75.36	6,818	6,325	13,143	75.62
Bulacan	67,590	31,538	29,171	60,709	89.82	31,284	29,081	60,365	89.31
Nueva Ecija	43,425	16,849	16,134	32,983	75.95	16,980	16,394	33,374	76.85
Pampanga	45,651	19,232	18,710	37,942	83.11	19,169	18,550	37,719	82.62
Tarlac	29,503	12,333	11,987	24,320	82.43	12,258	12,022	24,280	82.30
Zambales	11,723	4,749	4,421	9,170	78.22	4,778	4,486	9,264	79.02
City of Angeles	9,146	3,517	3,327	6,844	74.83	3,481	3,290	6,771	74.03
City of Olongapo	4,589	1,758	1,555	3,313	72.19	1,681	1,557	3,238	70.56
**Region 4A**	**307,982**	**121,838**	**115,362**	**237,200**	**77.02**	**120,157**	**113,984**	**234,141**	**76.02**
Batangas	53,251	21,573	20,640	42,213	79.27	21,595	20,629	42,224	79.29
Cavite	77,964	31,627	29,671	61,298	78.62	31,330	29,647	60,977	78.21
Laguna	61,525	25,196	24,657	49,853	81.03	24,864	23,651	48,515	78.85
Quezon	40,810	15,501	14,578	30,079	73.70	15,175	14,402	29,577	72.47
Rizal	68,235	25,603	23,692	49,295	72.24	24,920	23,457	48,377	70.90
City of Lucena	6,197	2,338	2,124	4,462	72.00	2,273	2,198	4,471	72.15
**Region 4B**	**66,121**	**27,946**	**25,866**	**53,812**	**81.38**	**27,019**	**25,642**	**52,661**	**79.64**
Marinduque	4,144	1,711	1,465	3,176	76.64	1,746	1,503	3,249	78.40
Occidental Mindoro	11,640	5,259	4,930	10,189	87.53	5,108	4,806	9,914	85.17
Oriental Mindoro	18,943	7,236	6,677	13,913	73.45	6,983	6,700	13,683	72.23
Palawan	20,317	8,843	8,104	16,947	83.41	8,360	7,898	16,258	80.02
Romblon	5,489	2,336	2,202	4,538	82.67	2,392	2,308	4,700	85.63
City of Puerto Princesa	5,588	2,561	2,488	5,049	90.35	2,430	2,427	4,857	86.92

Table 2.C.1.4 - Immunization Services for Infants and Children

Infants who completed 3 doses of Pneumococcal Conjugate Vaccine (PCV)

Philippines, 2023

Area	Eligible Pop (0-11 mos. old)	PCV 1				PCV 2			
		Male	Female	Total	%	Male	Female	Total	%
**Region 5**	**135,480**	**50,962**	**48,679**	**99,641**	**73.55**	**51,687**	**48,473**	**100,160**	**73.93**
Albay	27,440	9,894	9,418	19,312	70.38	9,917	9,248	19,165	69.84
Camarines Norte	14,695	5,604	5,149	10,753	73.17	5,793	5,281	11,074	75.36
Camarines Sur	43,452	15,309	14,286	29,595	68.11	15,143	13,971	29,114	67.00
Catanduanes	5,414	2,159	2,088	4,247	78.44	2,175	2,063	4,238	78.28
Masbate	22,365	9,203	9,624	18,827	84.18	9,766	9,723	19,489	87.14
Sorsogon	17,929	7,255	6,662	13,917	77.62	7,376	6,784	14,160	78.98
City of Naga	4,185	1,538	1,452	2,990	71.45	1,517	1,403	2,920	69.77
**Region 6**	**148,020**	**57,559**	**53,424**	**110,983**	**74.98**	**57,033**	**53,962**	**110,995**	**74.99**
Aklan	11,283	3,844	3,589	7,433	65.88	3,840	3,759	7,599	67.35
Antique	11,930	4,514	4,347	8,861	74.27	4,527	4,430	8,957	75.08
Capiz	13,931	5,082	4,577	9,659	69.33	5,270	4,895	10,165	72.97
Guimaras	3,351	1,244	1,226	2,470	73.71	1,291	1,243	2,534	75.62
Iloilo	36,140	14,549	12,893	27,442	75.93	13,829	12,837	26,666	73.79
Negros Occidental	52,270	20,863	19,817	40,680	77.83	20,774	19,753	40,527	77.53
City of Bacolod	10,373	4,200	3,805	8,005	77.17	4,159	3,830	7,989	77.02
City of Iloilo	8,742	3,263	3,170	6,433	73.59	3,343	3,215	6,558	75.02
**Region 7**	**165,673**	**64,901**	**60,828**	**125,729**	**75.89**	**64,778**	**61,434**	**126,212**	**76.18**
Bohol	26,235	9,556	8,948	18,504	70.53	9,572	9,230	18,802	71.67
Cebu	72,201	26,345	24,697	51,042	70.69	26,632	25,091	51,723	71.64
Negros Oriental	28,722	11,775	10,925	22,700	79.03	11,570	10,912	22,482	78.27
Siquijor	1,680	560	525	1,085	64.58	581	562	1,143	68.04
City of Cebu	19,267	8,209	7,555	15,764	81.82	7,954	7,507	15,461	80.25
City of Lapu-Lapu	10,679	5,128	5,010	10,138	94.93	5,079	4,858	9,937	93.05
City of Mandaue	6,889	3,328	3,168	6,496	94.30	3,390	3,274	6,664	96.73
**Region 8**	**93,813**	**35,561**	**33,608**	**69,169**	**73.73**	**35,553**	**33,974**	**69,527**	**74.11**
Biliran	3,373	1,592	1,500	3,092	91.67	1,620	1,510	3,130	92.80
Eastern Samar	9,624	3,743	3,651	7,394	76.83	3,890	3,720	7,610	79.07
Leyte	31,559	11,343	10,566	21,909	69.42	11,419	10,868	22,287	70.62
Northern Samar	14,056	5,290	5,132	10,422	74.15	5,455	5,269	10,724	76.29
Southern Leyte	7,406	2,581	2,403	4,984	67.30	2,588	2,478	5,066	68.40
Samar	17,011	7,135	6,659	13,794	81.09	6,789	6,450	13,239	77.83
Ormoc City	5,292	1,921	1,798	3,719	70.28	1,932	1,828	3,760	71.05
City of Tacloban	5,492	1,956	1,899	3,855	70.19	1,860	1,851	3,711	67.57

Table 2.C.1.4 - Immunization Services for Infants and Children
Infants who completed 3 doses of Pneumococcal Conjugate Vaccine (PCV)
Philippines, 2023

Area	Eligible Pop (0-11 mos. old)	PCV 1				PCV 2			
		Male	Female	Total	%	Male	Female	Total	%
**Region 9**	**84,815**	**34,629**	**33,106**	**67,735**	**79.86**	**34,453**	**33,161**	**67,614**	**79.72**
Zamboanga del Norte	24,841	9,597	9,213	18,810	75.72	9,452	9,360	18,812	75.73
Zamboanga del Sur	23,757	8,598	8,231	16,829	70.84	8,763	8,403	17,166	72.26
Zamboanga Sibugay	13,841	5,537	5,388	10,925	78.93	5,711	5,444	11,155	80.59
City of Isabela	3,201	1,374	1,258	2,632	82.22	1,370	1,267	2,637	82.38
City of Zamboanga	19,175	9,523	9,016	18,539	96.68	9,157	8,687	17,844	93.06
**Region 10**	**109,169**	**44,990**	**42,739**	**87,729**	**80.36**	**43,976**	**42,659**	**86,635**	**79.36**
Bukidnon	33,403	13,957	13,129	27,086	81.09	13,551	12,958	26,509	79.36
Camiguin	1,727	664	717	1,381	79.97	674	681	1,355	78.46
Lanao del Norte	17,760	6,837	6,654	13,491	75.96	6,682	6,827	13,509	76.06
Misamis Occidental	12,328	5,666	5,233	10,899	88.41	5,169	5,218	10,387	84.26
Misamis Oriental	21,975	8,400	8,231	16,631	75.68	8,489	8,245	16,734	76.15
City of Cagayan De Oro	14,308	6,441	5,955	12,396	86.64	6,455	5,933	12,388	86.58
City of Iligan	7,668	3,025	2,820	5,845	76.23	2,956	2,797	5,753	75.03
**Region 11**	**107,001**	**44,265**	**41,255**	**85,520**	**79.92**	**43,757**	**41,196**	**84,953**	**79.39**
Davao de Oro	15,253	6,484	6,022	12,506	81.99	6,384	6,101	12,485	81.85
Davao del Norte	23,453	9,191	8,515	17,706	75.50	9,152	8,556	17,708	75.50
Davao Oriental	11,935	4,988	4,627	9,615	80.56	5,018	4,691	9,709	81.35
Davao del Sur	13,083	5,457	5,180	10,637	81.30	5,334	5,043	10,377	79.32
Davao Occidental	6,629	2,655	2,528	5,183	78.19	2,622	2,503	5,125	77.31
City of Davao	36,648	15,490	14,383	29,873	81.51	15,247	14,302	29,549	80.63
**Region 12**	**95,279**	**37,197**	**34,758**	**71,955**	**75.52**	**36,755**	**34,903**	**71,658**	**75.21**
Cotabato	26,521	10,478	9,626	20,104	75.80	10,117	9,499	19,616	73.96
Sarangani	13,322	5,594	5,190	10,784	80.95	5,550	5,241	10,791	81.00
South Cotabato	21,595	8,590	8,094	16,684	77.26	8,525	8,029	16,554	76.66
Sultan Kudarat	19,391	7,197	6,876	14,073	72.57	7,286	7,137	14,423	74.38
City of General Santos	14,450	5,338	4,972	10,310	71.35	5,277	4,997	10,274	71.10
**BARMM**	**132,841**	**42,827**	**43,766**	**86,593**	**65.19**	**41,443**	**41,952**	**83,395**	**62.78**
Basilan	11,636	3,230	3,224	6,454	55.47	2,921	2,962	5,883	50.56
Lanao del Sur	33,374	13,440	13,942	27,382	82.05	13,551	14,240	27,791	83.27

Table 2.C.1.4 - Immunization Services for Infants and Children

Infants who completed 3 doses of Pneumococcal Conjugate Vaccine (PCV)

Philippines, 2023

Area	Eligible Pop (0-11 mos. old)	PCV 1				PCV 2			
		Male	Female	Total	%	Male	Female	Total	%
Maguindanao	41,650	14,195	14,053	28,248	67.82	13,293	13,144	26,437	63.47
Sulu	21,378	6,019	6,156	12,175	56.95	5,620	5,654	11,274	52.74
Tawi-Tawi	12,609	3,253	3,242	6,495	51.51	3,188	3,173	6,361	50.45
SGA	5,103	1,083	1,100	2,183	42.78	830	688	1,518	29.75
City of Cotabato	7,091	1,607	2,049	3,656	51.56	2,040	2,091	4,131	58.26
**CARAGA**	**55,065**	**24,599**	**24,057**	**48,656**	**88.36**	**24,355**	**23,161**	**47,516**	**86.29**
Agusan del Norte	7,286	3,249	2,952	6,201	85.11	3,179	2,927	6,106	83.80
Agusan del Sur	16,038	6,803	6,600	13,403	83.57	6,779	6,626	13,405	83.58
Surigao del Norte	10,186	4,601	5,327	9,928	97.47	4,605	4,417	9,022	88.57
Surigao del Sur	12,736	5,849	5,293	11,142	87.48	5,694	5,270	10,964	86.09
Dinagat Islands	2,134	862	852	1,714	80.32	929	881	1,810	84.82
City of Butuan	6,685	3,235	3,033	6,268	93.76	3,169	3,040	6,209	92.88

Table 2.C.1.4 - Immunization Services for Infants and Children
Infants who completed 3 doses of Pneumococcal Conjugate Vaccine (PCV)
Philippines, 2023

Area	Eligible Pop (0-11 mos. old)	PCV 3			
		Male	Female	Total	%
**Philippines**	**2,187,333**	**841,913**	**807,095**	**1,649,008**	**75.39**
**NCR**	**261,228**	**96,626**	**94,318**	**190,944**	**73.09**
Malabon City	7,385	2,489	2,456	4,945	66.96
Navotas City	5,210	2,253	2,173	4,426	84.95
Valenzuela City	13,477	4,813	4,729	9,542	70.80
Caloocan City	33,168	9,310	8,709	18,019	54.33
Marikina City	8,342	2,735	2,636	5,371	64.39
Pasig City	17,682	5,885	5,679	11,564	65.40
Pateros	1,116	416	385	801	71.77
Taguig City	18,992	4,657	4,349	9,006	47.42
Quezon City	59,136	28,176	28,483	56,659	95.81
Makati City	10,944	3,985	3,930	7,915	72.32
Mandaluyong City	7,922	3,188	3,262	6,450	81.42
San Juan City	2,168	529	472	1,001	46.17
Manila City	34,128	14,063	13,561	27,624	80.94
Las Piñas City	11,263	4,206	4,015	8,221	72.99
Muntinlupa City	9,881	3,622	3,486	7,108	71.94
Parañaque City	13,280	3,581	3,358	6,939	52.25
Pasay City	7,134	2,718	2,635	5,353	75.04
**CAR**	**31,319**	**12,789**	**12,081**	**24,870**	**79.41**
Abra	3,660	1,597	1,565	3,162	86.39
Apayao	2,238	992	973	1,965	87.80
Benguet	8,806	3,124	3,014	6,138	69.70
Ifugao	4,098	1,688	1,529	3,217	78.50
Kalinga	4,549	1,907	1,789	3,696	81.25
Mountain Province	2,619	1,248	1,067	2,315	88.39
City of Baguio	5,349	2,233	2,144	4,377	81.83
**Region 1**	**96,016**	**39,640**	**37,719**	**77,359**	**80.57**
Ilocos Norte	8,912	3,833	3,897	7,730	86.74
Ilocos Sur	9,914	4,769	4,438	9,207	92.87
La Union	13,507	5,277	4,956	10,233	75.76
Pangasinan	60,360	24,652	23,372	48,024	79.56
City of Dagupan	3,323	1,109	1,056	2,165	65.15

Table 2.C.1.4 - Immunization Services for Infants and Children
Infants who completed 3 doses of Pneumococcal Conjugate Vaccine (PCV)
Philippines, 2023

Area	Eligible Pop (0-11 mos. old)	PCV 3			
		Male	Female	Total	%
**Region 2**	**63,967**	**26,541**	**24,932**	**51,473**	**80.47**
Batanes	240	135	95	230	95.83
Cagayan	20,786	8,185	7,824	16,009	77.02
Isabela	27,379	11,940	11,050	22,990	83.97
Nueva Vizcaya	8,798	3,234	3,076	6,310	71.72
Quirino	3,839	1,698	1,626	3,324	86.59
City of Santiago	2,925	1,349	1,261	2,610	89.23
**Region 3**	**233,544**	**99,172**	**94,554**	**193,726**	**82.95**
Aurora	4,537	2,087	1,916	4,003	88.23
Bataan	17,380	6,827	6,364	13,191	75.90
Bulacan	67,590	31,766	29,728	61,494	90.98
Nueva Ecija	43,425	17,140	16,480	33,620	77.42
Pampanga	45,651	19,208	18,725	37,933	83.09
Tarlac	29,503	12,524	12,223	24,747	83.88
Zambales	11,723	4,520	4,238	8,758	74.71
City of Angeles	9,146	3,401	3,280	6,681	73.05
City of Olongapo	4,589	1,699	1,600	3,299	71.89
**Region 4A**	**307,982**	**117,375**	**111,799**	**229,174**	**74.41**
Batangas	53,251	21,266	20,335	41,601	78.12
Cavite	77,964	30,777	29,539	60,316	77.36
Laguna	61,525	24,212	23,082	47,294	76.87
Quezon	40,810	14,746	13,886	28,632	70.16
Rizal	68,235	24,200	22,839	47,039	68.94
City of Lucena	6,197	2,174	2,118	4,292	69.26
**Region 4B**	**66,121**	**25,737**	**24,747**	**50,484**	**76.35**
Marinduque	4,144	1,656	1,501	3,157	76.18
Occidental Mindoro	11,640	4,813	4,576	9,389	80.66
Oriental Mindoro	18,943	6,707	6,692	13,399	70.73
Palawan	20,317	7,866	7,450	15,316	75.39
Romblon	5,489	2,326	2,219	4,545	82.80
City of Puerto Princesa	5,588	2,369	2,309	4,678	83.72

Table 2.C.1.4 - Immunization Services for Infants and Children
Infants who completed 3 doses of Pneumococcal Conjugate Vaccine (PCV)
Philippines, 2023

Area	Eligible Pop (0-11 mos. old)	PCV 3			
		Male	Female	Total	%
**Region 5**	**135,480**	**48,717**	**46,228**	**94,945**	**70.08**
Albay	27,440	9,700	9,392	19,092	69.58
Camarines Norte	14,695	5,462	5,147	10,609	72.19
Camarines Sur	43,452	14,499	13,685	28,184	64.86
Catanduanes	5,414	2,166	2,024	4,190	77.39
Masbate	22,365	8,160	7,814	15,974	71.42
Sorsogon	17,929	7,322	6,758	14,080	78.53
City of Naga	4,185	1,408	1,408	2,816	67.29
**Region 6**	**148,020**	**56,862**	**54,059**	**110,921**	**74.94**
Aklan	11,283	3,798	3,726	7,524	66.68
Antique	11,930	4,497	4,417	8,914	74.72
Capiz	13,931	5,297	5,032	10,329	74.14
Guimaras	3,351	1,325	1,210	2,535	75.65
Iloilo	36,140	13,721	12,777	26,498	73.32
Negros Occidental	52,270	20,771	19,864	40,635	77.74
City of Bacolod	10,373	4,123	3,798	7,921	76.36
City of Iloilo	8,742	3,330	3,235	6,565	75.10
**Region 7**	**165,673**	**64,577**	**61,184**	**125,761**	**75.91**
Bohol	26,235	9,665	9,247	18,912	72.09
Cebu	72,201	26,846	25,453	52,299	72.44
Negros Oriental	28,722	11,414	10,830	22,244	77.45
Siquijor	1,680	573	559	1,132	67.38
City of Cebu	19,267	7,514	7,186	14,700	76.30
City of Lapu-Lapu	10,679	5,131	4,572	9,703	90.86
City of Mandaue	6,889	3,434	3,337	6,771	98.29
**Region 8**	**93,813**	**35,208**	**33,478**	**68,686**	**73.22**
Biliran	3,373	1,589	1,551	3,140	93.09
Eastern Samar	9,624	3,864	3,690	7,554	78.49
Leyte	31,559	11,342	10,799	22,141	70.16
Northern Samar	14,056	5,348	5,110	10,458	74.40
Southern Leyte	7,406	2,674	2,510	5,184	70.00
Samar	17,011	6,617	6,282	12,899	75.83
Ormoc City	5,292	1,933	1,779	3,712	70.14
City of Tacloban	5,492	1,841	1,757	3,598	65.51

Table 2.C.1.4 - Immunization Services for Infants and Children
Infants who completed 3 doses of Pneumococcal Conjugate Vaccine (PCV)
Philippines, 2023

Area	Eligible Pop (0-11 mos. old)	PCV 3			
		Male	Female	Total	%
**Region 9**	**84,815**	**33,446**	**32,364**	**65,810**	**77.59**
Zamboanga del Norte	24,841	9,473	9,151	18,624	74.97
Zamboanga del Sur	23,757	8,432	8,137	16,569	69.74
Zamboanga Sibugay	13,841	5,541	5,421	10,962	79.20
City of Isabela	3,201	1,347	1,319	2,666	83.29
City of Zamboanga	19,175	8,653	8,336	16,989	88.60
**Region 10**	**109,169**	**43,367**	**42,078**	**85,445**	**78.27**
Bukidnon	33,403	13,238	12,738	25,976	77.77
Camiguin	1,727	661	679	1,340	77.59
Lanao del Norte	17,760	6,901	6,798	13,699	77.13
Misamis Occidental	12,328	4,937	4,959	9,896	80.27
Misamis Oriental	21,975	8,456	8,420	16,876	76.80
City of Cagayan De Oro	14,308	6,306	5,779	12,085	84.46
City of Iligan	7,668	2,868	2,705	5,573	72.68
**Region 11**	**107,001**	**42,319**	**40,312**	**82,631**	**77.22**
Davao de Oro	15,253	6,214	6,072	12,286	80.55
Davao del Norte	23,453	8,879	8,430	17,309	73.80
Davao Oriental	11,935	4,866	4,633	9,499	79.59
Davao del Sur	13,083	5,231	4,855	10,086	77.09
Davao Occidental	6,629	2,535	2,490	5,025	75.80
City of Davao	36,648	14,594	13,832	28,426	77.56
**Region 12**	**95,279**	**36,068**	**34,030**	**70,098**	**73.57**
Cotabato	26,521	9,574	9,006	18,580	70.06
Sarangani	13,322	5,500	5,166	10,666	80.06
South Cotabato	21,595	8,459	7,990	16,449	76.17
Sultan Kudarat	19,391	7,356	6,881	14,237	73.42
City of General Santos	14,450	5,179	4,987	10,166	70.35
**BARMM**	**132,841**	**39,226**	**40,050**	**79,276**	**59.68**
Basilan	11,636	2,523	2,675	5,198	44.67
Lanao del Sur	33,374	13,504	14,131	27,635	82.80
Maguindanao	41,650	12,267	12,229	24,496	58.81

Table 2.C.1.4 - Immunization Services for Infants and Children
Infants who completed 3 doses of Pneumococcal Conjugate Vaccine (PCV)
Philippines, 2023

Area	Eligible Pop (0-11 mos. old)	PCV 3			
		Male	Female	Total	%
Sulu	21,378	5,254	5,399	10,653	49.83
Tawi-Tawi	12,609	3,082	3,035	6,117	48.51
SGA	5,103	612	586	1,198	23.48
City of Cotabato	7,091	1,984	1,995	3,979	56.11
**CARAGA**	**55,065**	**24,243**	**23,162**	**47,405**	**86.09**
Agusan del Norte	7,286	3,174	2,928	6,102	83.75
Agusan del Sur	16,038	6,876	6,611	13,487	84.09
Surigao del Norte	10,186	4,580	4,379	8,959	87.95
Surigao del Sur	12,736	5,439	5,172	10,611	83.32
Dinagat Islands	2,134	959	919	1,878	88.00
City of Butuan	6,685	3,215	3,153	6,368	95.26

Table 2.C.1.5 - Immunization Services for Infants and Children
Children vaccinated with 2 doses of Measles Containing Vaccine (MCV)
Philippines, 2023

Area	Eligible Pop (0-11 mos. old)	MCV 1			
		Male	Female	Total	%
**Philippines**	**2,187,333**	**835,153**	**784,743**	**1,619,896**	**74.06**
**NCR**	**261,228**	**106,139**	**102,757**	**208,896**	**79.97**
Malabon City	7,385	2,909	2,805	5,714	77.37
Navotas City	5,210	2,225	2,154	4,379	84.05
Valenzuela City	13,477	4,613	4,271	8,884	65.92
Caloocan City	33,168	9,794	9,758	19,552	58.95
Marikina City	8,342	2,573	2,497	5,070	60.78
Pasig City	17,682	5,135	4,908	10,043	56.80
Pateros	1,116	417	394	811	72.67
Taguig City	18,992	5,887	5,662	11,549	60.81
Quezon City	59,136	32,918	32,782	65,700	111.10
Makati City	10,944	4,602	4,353	8,955	81.83
Mandaluyong City	7,922	3,248	3,270	6,518	82.28
San Juan City	2,168	546	518	1,064	49.08
Manila City	34,128	14,375	13,712	28,087	82.30
Las Piñas City	11,263	4,292	4,159	8,451	75.03
Muntinlupa City	9,881	3,770	3,549	7,319	74.07
Parañaque City	13,280	5,907	5,264	11,171	84.12
Pasay City	7,134	2,928	2,701	5,629	78.90
**CAR**	**31,319**	**12,189**	**11,347**	**23,536**	**75.15**
Abra	3,660	1,394	1,382	2,776	75.85
Apayao	2,238	961	926	1,887	84.32
Benguet	8,806	3,183	2,922	6,105	69.33
Ifugao	4,098	1,594	1,406	3,000	73.21
Kalinga	4,549	1,894	1,718	3,612	79.40
Mountain Province	2,619	1,120	1,029	2,149	82.05
City of Baguio	5,349	2,043	1,964	4,007	74.91
**Region 1**	**96,016**	**38,498**	**35,652**	**74,150**	**77.23**
Ilocos Norte	8,912	3,792	3,724	7,516	84.34
Ilocos Sur	9,914	4,948	4,552	9,500	95.82
La Union	13,507	4,959	4,506	9,465	70.07
Pangasinan	60,360	23,652	21,800	45,452	75.30
City of Dagupan	3,323	1,147	1,070	2,217	66.72

Table 2.C.1.5 - Immunization Services for Infants and Children
Children vaccinated with 2 doses of Measles Containing Vaccine (MCV)
Philippines, 2023

Area	Eligible Pop (0-11 mos. old)	MCV 1			
		Male	Female	Total	%
**Region 2**	**63,967**	**25,761**	**24,143**	**49,904**	**78.02**
Batanes	240	125	109	234	97.50
Cagayan	20,786	7,755	7,353	15,108	72.68
Isabela	27,379	11,446	10,808	22,254	81.28
Nueva Vizcaya	8,798	3,298	2,957	6,255	71.10
Quirino	3,839	1,643	1,621	3,264	85.02
City of Santiago	2,925	1,494	1,295	2,789	95.35
**Region 3**	**233,544**	**96,979**	**90,477**	**187,456**	**80.27**
Aurora	4,537	2,079	1,822	3,901	85.98
Bataan	17,380	6,729	6,167	12,896	74.20
Bulacan	67,590	30,938	28,185	59,123	87.47
Nueva Ecija	43,425	16,771	15,628	32,399	74.61
Pampanga	45,651	18,520	17,641	36,161	79.21
Tarlac	29,503	12,655	12,216	24,871	84.30
Zambales	11,723	4,290	4,060	8,350	71.23
City of Angeles	9,146	3,400	3,247	6,647	72.68
City of Olongapo	4,589	1,597	1,511	3,108	67.73
**Region 4A**	**307,982**	**113,068**	**105,414**	**218,482**	**70.94**
Batangas	53,251	21,245	19,653	40,898	76.80
Cavite	77,964	29,779	27,927	57,706	74.02
Laguna	61,525	23,533	22,292	45,825	74.48
Quezon	40,810	13,831	12,776	26,607	65.20
Rizal	68,235	22,526	20,700	43,226	63.35
City of Lucena	6,197	2,154	2,066	4,220	68.10
**Region 4B**	**66,121**	**25,367**	**23,867**	**49,234**	**74.46**
Marinduque	4,144	1,686	1,420	3,106	74.95
Occidental Mindoro	11,640	4,776	4,463	9,239	79.37
Oriental Mindoro	18,943	6,625	6,452	13,077	69.03
Palawan	20,317	7,676	7,155	14,831	73.00
Romblon	5,489	2,117	2,030	4,147	75.55
City of Puerto Princesa	5,588	2,487	2,347	4,834	86.51

Table 2.C.1.5 - Immunization Services for Infants and Children

Children vaccinated with 2 doses of Measles Containing Vaccine (MCV)

Philippines, 2023

Area	Eligible Pop (0-11 mos. old)	MCV 1			
		Male	Female	Total	%
**Region 5**	**135,480**	**48,477**	**43,631**	**92,108**	**67.99**
Albay	27,440	9,712	8,912	18,624	67.87
Camarines Norte	14,695	5,171	4,687	9,858	67.08
Camarines Sur	43,452	13,617	12,364	25,981	59.79
Catanduanes	5,414	2,012	1,916	3,928	72.55
Masbate	22,365	9,790	8,239	18,029	80.61
Sorsogon	17,929	6,801	6,211	13,012	72.58
City of Naga	4,185	1,374	1,302	2,676	63.94
**Region 6**	**148,020**	**54,278**	**50,735**	**105,013**	**70.95**
Aklan	11,283	3,580	3,383	6,963	61.71
Antique	11,930	4,391	4,054	8,445	70.79
Capiz	13,931	4,983	4,638	9,621	69.06
Guimaras	3,351	1,284	1,154	2,438	72.75
Iloilo	36,140	13,010	11,904	24,914	68.94
Negros Occidental	52,270	19,960	18,972	38,932	74.48
City of Bacolod	10,373	3,649	3,373	7,022	67.69
City of Iloilo	8,742	3,421	3,257	6,678	76.39
**Region 7**	**165,673**	**62,939**	**58,932**	**121,871**	**73.56**
Bohol	26,235	9,008	8,412	17,420	66.40
Cebu	72,201	26,203	24,605	50,808	70.37
Negros Oriental	28,722	11,116	10,233	21,349	74.33
Siquijor	1,680	524	518	1,042	62.02
City of Cebu	19,267	7,412	7,100	14,512	75.32
City of Lapu-Lapu	10,679	5,101	4,571	9,672	90.57
City of Mandaue	6,889	3,575	3,493	7,068	102.60
**Region 8**	**93,813**	**34,406**	**31,873**	**66,279**	**70.65**
Biliran	3,373	1,607	1,493	3,100	91.91
Eastern Samar	9,624	3,751	3,470	7,221	75.03
Leyte	31,559	10,937	10,017	20,954	66.40
Northern Samar	14,056	5,141	4,712	9,853	70.10
Southern Leyte	7,406	2,613	2,368	4,981	67.26
Samar	17,011	6,307	5,979	12,286	72.22
Ormoc City	5,292	2,014	1,826	3,840	72.56
City of Tacloban	5,492	2,036	2,008	4,044	73.63

Table 2.C.1.5 - Immunization Services for Infants and Children
Children vaccinated with 2 doses of Measles Containing Vaccine (MCV)
Philippines, 2023

Area	Eligible Pop (0-11 mos. old)	MCV 1			
		Male	Female	Total	%
**Region 9**	**84,815**	**32,216**	**31,008**	**63,224**	**74.54**
Zamboanga del Norte	24,841	8,788	8,466	17,254	69.46
Zamboanga del Sur	23,757	7,921	7,400	15,321	64.49
Zamboanga Sibugay	13,841	5,222	5,162	10,384	75.02
City of Isabela	3,201	1,264	1,318	2,582	80.66
City of Zamboanga	19,175	9,021	8,662	17,683	92.22
**Region 10**	**109,169**	**45,456**	**41,556**	**87,012**	**79.70**
Bukidnon	33,403	13,975	12,976	26,951	80.68
Camiguin	1,727	617	610	1,227	71.05
Lanao del Norte	17,760	7,756	6,311	14,067	79.21
Misamis Occidental	12,328	5,290	5,027	10,317	83.69
Misamis Oriental	21,975	8,660	8,154	16,814	76.51
City of Cagayan De Oro	14,308	6,465	5,938	12,403	86.69
City of Iligan	7,668	2,693	2,540	5,233	68.24
**Region 11**	**107,001**	**42,177**	**39,143**	**81,320**	**76.00**
Davao de Oro	15,253	6,273	5,756	12,029	78.86
Davao del Norte	23,453	8,888	8,318	17,206	73.36
Davao Oriental	11,935	4,990	4,576	9,566	80.15
Davao del Sur	13,083	5,012	4,622	9,634	73.64
Davao Occidental	6,629	2,342	2,311	4,653	70.19
City of Davao	36,648	14,672	13,560	28,232	77.04
**Region 12**	**95,279**	**35,596**	**33,675**	**69,271**	**72.70**
Cotabato	26,521	9,758	9,094	18,852	71.08
Sarangani	13,322	5,407	4,908	10,315	77.43
South Cotabato	21,595	8,190	7,762	15,952	73.87
Sultan Kudarat	19,391	7,202	6,902	14,104	72.73
City of General Santos	14,450	5,039	5,009	10,048	69.54
**BARMM**	**132,841**	**38,049**	**38,517**	**76,566**	**57.64**
Basilan	11,636	2,169	2,117	4,286	36.83
Lanao del Sur	33,374	13,024	13,773	26,797	80.29
Maguindanao	41,650	12,734	12,465	25,199	60.50

Table 2.C.1.5 - Immunization Services for Infants and Children
Children vaccinated with 2 doses of Measles Containing Vaccine (MCV)
Philippines, 2023

Area	Eligible Pop (0-11 mos. old)	MCV 1			
		Male	Female	Total	%
Sulu	21,378	4,760	4,893	9,653	45.15
Tawi-Tawi	12,609	2,867	2,819	5,686	45.09
SGA	5,103	574	638	1,212	23.75
City of Cotabato	7,091	1,921	1,812	3,733	52.64
**CARAGA**	**55,065**	**23,558**	**22,016**	**45,574**	**82.76**
Agusan del Norte	7,286	2,916	2,802	5,718	78.48
Agusan del Sur	16,038	6,740	6,287	13,027	81.23
Surigao del Norte	10,186	4,385	4,041	8,426	82.72
Surigao del Sur	12,736	5,225	4,766	9,991	78.45
Dinagat Islands	2,134	858	814	1,672	78.35
City of Butuan	6,685	3,434	3,306	6,740	100.82

Table 2.C.1.5 - Immunization Services for Infants and Children
Children vaccinated with 2 doses of Measles Containing Vaccine (MCV)
Philippines, 2023

Area	Eligible Pop (0-12 mos. old)	MCV 2			
		Male	Female	Total	%
**Philippines**	**2,378,123**	**832,448**	**795,782**	**1,628,230**	**68.47**
**NCR**	**283,594**	**114,408**	**112,967**	**227,375**	**80.18**
Malabon City	7,999	2,872	2,802	5,674	70.93
Navotas City	5,658	2,684	2,753	5,437	96.09
Valenzuela City	14,677	4,671	4,929	9,600	65.41
Caloocan City	36,121	11,770	11,410	23,180	64.17
Marikina City	9,077	2,196	2,243	4,439	48.90
Pasig City	19,080	7,667	7,368	15,035	78.80
Pateros	1,220	401	434	835	68.44
Taguig City	20,594	5,648	5,420	11,068	53.74
Quezon City	64,225	33,809	34,811	68,620	106.84
Makati City	11,855	5,274	5,007	10,281	86.72
Mandaluyong City	8,593	4,303	4,244	8,547	99.46
San Juan City	2,358	629	561	1,190	50.47
Manila City	37,136	15,550	14,982	30,532	82.22
Las Piñas City	12,192	4,291	4,090	8,381	68.74
Muntinlupa City	10,714	3,654	3,515	7,169	66.91
Parañaque City	14,400	5,745	5,308	11,053	76.76
Pasay City	7,695	3,244	3,090	6,334	82.31
**CAR**	**34,010**	**12,081**	**11,336**	**23,417**	**68.85**
Abra	3,962	1,449	1,404	2,853	72.01
Apayao	2,426	989	872	1,861	76.71
Benguet	9,576	3,096	2,896	5,992	62.57
Ifugao	4,451	1,532	1,434	2,966	66.64
Kalinga	4,942	1,957	1,812	3,769	76.26
Mountain Province	2,844	1,125	1,033	2,158	75.88
City of Baguio	5,809	1,933	1,885	3,818	65.73
**Region 1**	**104,366**	**39,145**	**36,995**	**76,140**	**72.95**
Ilocos Norte	9,742	3,879	3,803	7,682	78.85
Ilocos Sur	10,746	5,153	4,938	10,091	93.90
La Union	14,697	4,864	4,514	9,378	63.81
Pangasinan	65,571	24,041	22,633	46,674	71.18
City of Dagupan	3,610	1,208	1,107	2,315	64.13

Table 2.C.1.5 - Immunization Services for Infants and Children
Children vaccinated with 2 doses of Measles Containing Vaccine (MCV)
Philippines, 2023

Area	Eligible Pop (0-12 mos. old)	MCV 2			
		Male	Female	Total	%
**Region 2**	**69,686**	**26,539**	**24,185**	**50,724**	**72.79**
Batanes	254	135	106	241	94.88
Cagayan	22,675	7,703	7,152	14,855	65.51
Isabela	29,819	11,543	11,154	22,697	76.12
Nueva Vizcaya	9,595	3,850	2,589	6,439	67.11
Quirino	4,180	1,687	1,738	3,425	81.94
City of Santiago	3,163	1,621	1,446	3,067	96.96
**Region 3**	**254,083**	**96,686**	**91,830**	**188,516**	**74.19**
Aurora	4,951	2,315	2,121	4,436	89.60
Bataan	18,839	5,777	5,334	11,111	58.98
Bulacan	73,619	31,240	29,542	60,782	82.56
Nueva Ecija	47,224	16,504	15,442	31,946	67.65
Pampanga	49,724	17,170	16,695	33,865	68.11
Tarlac	31,988	13,790	13,343	27,133	84.82
Zambales	12,759	4,575	4,413	8,988	70.44
City of Angeles	10,007	3,292	3,102	6,394	63.90
City of Olongapo	4,972	2,023	1,838	3,861	77.65
**Region 4A**	**334,903**	**109,310**	**103,946**	**213,256**	**63.68**
Batangas	58,142	20,611	19,610	40,221	69.18
Cavite	84,833	28,176	26,881	55,057	64.90
Laguna	66,806	24,848	23,856	48,704	72.90
Quezon	44,376	12,623	11,916	24,539	55.30
Rizal	74,041	20,765	19,531	40,296	54.42
City of Lucena	6,705	2,287	2,152	4,439	66.20
**Region 4B**	**71,762**	**24,584**	**23,158**	**47,742**	**66.53**
Marinduque	4,553	1,593	1,408	3,001	65.91
Occidental Mindoro	12,622	4,564	4,258	8,822	69.89
Oriental Mindoro	20,518	6,865	6,621	13,486	65.73
Palawan	22,056	7,111	6,744	13,855	62.82
Romblon	5,972	1,983	1,847	3,830	64.13
City of Puerto Princesa	6,041	2,468	2,280	4,748	78.60
**Region 5**	**147,409**	**45,478**	**41,943**	**87,421**	**59.31**

Table 2.C.1.5 - Immunization Services for Infants and Children
Children vaccinated with 2 doses of Measles Containing Vaccine (MCV)
Philippines, 2023

Area	Eligible Pop (0-12 mos. old)	MCV 2			
		Male	Female	Total	%
Albay	29,925	10,007	9,316	19,323	64.57
Camarines Norte	15,946	4,734	4,283	9,017	56.55
Camarines Sur	47,270	12,915	11,915	24,830	52.53
Catanduanes	5,919	1,860	1,702	3,562	60.18
Masbate	24,297	8,156	7,603	15,759	64.86
Sorsogon	19,511	6,524	5,882	12,406	63.58
City of Naga	4,541	1,282	1,242	2,524	55.58
**Region 6**	**161,010**	**54,855**	**52,038**	**106,893**	**66.39**
Aklan	12,238	3,397	3,222	6,619	54.09
Antique	12,949	4,263	4,072	8,335	64.37
Capiz	15,187	5,094	4,791	9,885	65.09
Guimaras	3,657	1,293	1,159	2,452	67.05
Iloilo	39,225	13,901	13,146	27,047	68.95
Negros Occidental	56,925	19,938	18,997	38,935	68.40
City of Bacolod	11,298	3,475	3,293	6,768	59.90
City of Iloilo	9,531	3,494	3,358	6,852	71.89
**Region 7**	**179,825**	**61,742**	**58,704**	**120,446**	**66.98**
Bohol	28,467	8,539	7,894	16,433	57.73
Cebu	78,406	25,582	24,354	49,936	63.69
Negros Oriental	31,173	10,245	9,678	19,923	63.91
Siquijor	1,796	565	532	1,097	61.08
City of Cebu	20,947	7,771	7,406	15,177	72.45
City of Lapu-Lapu	11,580	5,318	5,156	10,474	90.45
City of Mandaue	7,456	3,722	3,684	7,406	99.33
**Region 8**	**101,728**	**33,188**	**31,599**	**64,787**	**63.69**
Biliran	3,673	1,501	1,441	2,942	80.10
Eastern Samar	10,406	3,476	3,341	6,817	65.51
Leyte	34,410	10,713	9,995	20,708	60.18
Northern Samar	15,211	4,661	4,859	9,520	62.59
Southern Leyte	8,028	2,537	2,350	4,887	60.87
Samar	18,353	6,313	5,778	12,091	65.88
Ormoc City	5,717	1,971	1,866	3,837	67.12
City of Tacloban	5,930	2,016	1,969	3,985	67.20
**Region 9**	**92,197**	**31,467**	**30,816**	**62,283**	**67.55**

Table 2.C.1.5 - Immunization Services for Infants and Children
Children vaccinated with 2 doses of Measles Containing Vaccine (MCV)
Philippines, 2023

Area	Eligible Pop (0-12 mos. old)	MCV 2			
		Male	Female	Total	%
Zamboanga del Norte	27,032	8,718	8,585	17,303	64.01
Zamboanga del Sur	25,767	7,569	7,273	14,842	57.60
Zamboanga Sibugay	15,055	5,000	5,006	10,006	66.46
City of Isabela	3,493	1,504	1,465	2,969	85.00
City of Zamboanga	20,850	8,676	8,487	17,163	82.32
**Region 10**	**118,571**	**45,079**	**42,928**	**88,007**	**74.22**
Bukidnon	36,317	13,479	12,617	26,096	71.86
Camiguin	1,877	572	545	1,117	59.51
Lanao del Norte	19,325	7,043	6,828	13,871	71.78
Misamis Occidental	13,380	5,192	5,090	10,282	76.85
Misamis Oriental	23,838	9,122	8,839	17,961	75.35
City of Cagayan De Oro	15,513	6,902	6,441	13,343	86.01
City of Iligan	8,321	2,769	2,568	5,337	64.14
**Region 11**	**116,229**	**41,173**	**38,186**	**79,359**	**68.28**
Davao de Oro	16,570	6,237	5,764	12,001	72.43
Davao del Norte	25,431	8,412	7,861	16,273	63.99
Davao Oriental	12,918	4,874	4,566	9,440	73.08
Davao del Sur	14,284	4,542	4,002	8,544	59.82
Davao Occidental	7,244	2,141	2,088	4,229	58.38
City of Davao	39,782	14,967	13,905	28,872	72.58
**Region 12**	**103,466**	**35,302**	**34,017**	**69,319**	**67.00**
Cotabato	28,890	9,602	8,977	18,579	64.31
Sarangani	14,406	5,427	5,275	10,702	74.29
South Cotabato	23,454	8,184	7,903	16,087	68.59
Sultan Kudarat	21,064	7,284	6,838	14,122	67.04
City of General Santos	15,652	4,805	5,024	9,829	62.80
**BARMM**	**145,404**	**35,979**	**36,742**	**72,721**	**50.01**
Basilan	12,723	1,782	1,822	3,604	28.33
Lanao del Sur	36,576	12,923	13,700	26,623	72.79
Maguindanao	45,411	11,855	11,541	23,396	51.52
Sulu	23,576	4,431	4,670	9,101	38.60
Tawi-Tawi	13,801	2,685	2,706	5,391	39.06

Table 2.C.1.5 - Immunization Services for Infants and Children
Children vaccinated with 2 doses of Measles Containing Vaccine (MCV)
Philippines, 2023

Area	Eligible Pop (0-12 mos. old)	MCV 2			
		Male	Female	Total	%
SGA	5,596	435	460	895	15.99
City of Cotabato	7,721	1,868	1,843	3,711	48.06
**CARAGA**	**59,880**	**25,432**	**24,392**	**49,824**	**83.21**
Agusan del Norte	7,920	3,651	3,472	7,123	89.94
Agusan del Sur	17,393	7,422	7,293	14,715	84.60
Surigao del Norte	11,088	4,397	4,204	8,601	77.57
Surigao del Sur	13,879	5,148	4,826	9,974	71.86
Dinagat Islands	2,346	875	813	1,688	71.95
City of Butuan	7,254	3,939	3,784	7,723	106.47

Table 2.C.1.6 - Immunization Services for Infants and Children
Fully Immunized Children (FIC)
Philippines, 2023

Area	Eligible Pop (0-12 mos. old)	FIC			
		Male	Female	Total	%
**Philippines**	**2,378,123**	**756,354**	**726,130**	**1,482,484**	**62.34**
**NCR**	**283,594**	**112,399**	**110,428**	**222,827**	**78.57**
Malabon City	7,999	2,872	2,802	5,674	70.93
Navotas City	5,658	2,516	2,591	5,107	90.26
Valenzuela City	14,677	4,671	4,929	9,600	65.41
Caloocan City	36,121	11,668	11,395	23,063	63.85
Marikina City	9,077	1,910	1,954	3,864	42.57
Pasig City	19,080	7,610	7,324	14,934	78.27
Pateros	1,220	381	411	792	64.92
Taguig City	20,594	5,189	5,060	10,249	49.77
Quezon City	64,225	33,938	34,023	67,961	105.82
Makati City	11,855	5,233	4,976	10,209	86.12
Mandaluyong City	8,593	4,272	4,275	8,547	99.46
San Juan City	2,358	563	500	1,063	45.08
Manila City	37,136	15,552	14,962	30,514	82.17
Las Piñas City	12,192	4,036	3,962	7,998	65.60
Muntinlupa City	10,714	3,330	3,129	6,459	60.29
Parañaque City	14,400	5,612	5,230	10,842	75.29
Pasay City	7,695	3,046	2,905	5,951	77.34
**CAR**	**34,010**	**11,391**	**10,725**	**22,116**	**65.03**
Abra	3,962	1,425	1,390	2,815	71.05
Apayao	2,426	952	846	1,798	74.11
Benguet	9,576	2,887	2,714	5,601	58.49
Ifugao	4,451	1,430	1,356	2,786	62.59
Kalinga	4,942	1,926	1,797	3,723	75.33
Mountain Province	2,844	1,087	988	2,075	72.96
City of Baguio	5,809	1,684	1,634	3,318	57.12
**Region 1**	**104,366**	**38,229**	**36,162**	**74,391**	**71.28**
Ilocos Norte	9,742	3,875	3,803	7,678	78.81
Ilocos Sur	10,746	5,117	4,916	10,033	93.36
La Union	14,697	4,802	4,444	9,246	62.91
Pangasinan	65,571	23,567	22,218	45,785	69.83
City of Dagupan	3,610	868	781	1,649	45.68

Table 2.C.1.6 - Immunization Services for Infants and Children
Fully Immunized Children (FIC)
Philippines, 2023

Area	Eligible Pop (0-12 mos. old)	FIC			
		Male	Female	Total	%
**Region 2**	**69,686**	**24,823**	**23,258**	**48,081**	**69.00**
Batanes	254	135	106	241	94.88
Cagayan	22,675	7,227	6,635	13,862	61.13
Isabela	29,819	11,029	10,535	21,564	72.32
Nueva Vizcaya	9,595	3,124	2,803	5,927	61.77
Quirino	4,180	1,687	1,733	3,420	81.82
City of Santiago	3,163	1,621	1,446	3,067	96.96
**Region 3**	**254,083**	**92,283**	**88,056**	**180,339**	**70.98**
Aurora	4,951	2,314	2,120	4,434	89.56
Bataan	18,839	5,688	5,227	10,915	57.94
Bulacan	73,619	29,037	27,663	56,700	77.02
Nueva Ecija	47,224	15,617	14,700	30,317	64.20
Pampanga	49,724	16,599	16,211	32,810	65.98
Tarlac	31,988	13,472	13,061	26,533	82.95
Zambales	12,759	4,575	4,412	8,987	70.44
City of Angeles	10,007	2,958	2,824	5,782	57.78
City of Olongapo	4,972	2,023	1,838	3,861	77.65
**Region 4A**	**334,903**	**89,846**	**85,831**	**175,677**	**52.46**
Batangas	58,142	17,218	16,404	33,622	57.83
Cavite	84,833	22,994	21,979	44,973	53.01
Laguna	66,806	21,114	20,307	41,421	62.00
Quezon	44,376	9,710	9,392	19,102	43.05
Rizal	74,041	16,907	15,979	32,886	44.42
City of Lucena	6,705	1,903	1,770	3,673	54.78
**Region 4B**	**71,762**	**21,546**	**20,366**	**41,912**	**58.40**
Marinduque	4,553	1,451	1,281	2,732	60.00
Occidental Mindoro	12,622	3,680	3,480	7,160	56.73
Oriental Mindoro	20,518	6,444	6,280	12,724	62.01
Palawan	22,056	6,321	5,981	12,302	55.78
Romblon	5,972	1,800	1,664	3,464	58.00
City of Puerto Princesa	6,041	1,850	1,680	3,530	58.43

Table 2.C.1.6 - Immunization Services for Infants and Children
Fully Immunized Children (FIC)
Philippines, 2023

Area	Eligible Pop (0-12 mos. old)	FIC			
		Male	Female	Total	%
**Region 5**	**147,409**	**39,246**	**36,450**	**75,696**	**51.35**
Albay	29,925	9,024	8,519	17,543	58.62
Camarines Norte	15,946	3,920	3,675	7,595	47.63
Camarines Sur	47,270	10,584	9,776	20,360	43.07
Catanduanes	5,919	1,838	1,670	3,508	59.27
Masbate	24,297	6,926	6,391	13,317	54.81
Sorsogon	19,511	5,786	5,280	11,066	56.72
City of Naga	4,541	1,168	1,139	2,307	50.80
**Region 6**	**161,010**	**50,537**	**48,258**	**98,795**	**61.36**
Aklan	12,238	3,171	3,075	6,246	51.04
Antique	12,949	3,937	3,771	7,708	59.53
Capiz	15,187	4,730	4,488	9,218	60.70
Guimaras	3,657	1,250	1,113	2,363	64.62
Iloilo	39,225	13,870	13,156	27,026	68.90
Negros Occidental	56,925	17,821	17,137	34,958	61.41
City of Bacolod	11,298	2,328	2,218	4,546	40.24
City of Iloilo	9,531	3,430	3,300	6,730	70.61
**Region 7**	**179,825**	**51,731**	**49,529**	**101,260**	**56.31**
Bohol	28,467	6,529	6,067	12,596	44.25
Cebu	78,406	21,129	20,158	41,287	52.66
Negros Oriental	31,173	7,793	7,530	15,323	49.15
Siquijor	1,796	509	467	976	54.34
City of Cebu	20,947	7,251	6,955	14,206	67.82
City of Lapu-Lapu	11,580	4,917	4,802	9,719	83.93
City of Mandaue	7,456	3,603	3,550	7,153	95.94
**Region 8**	**101,728**	**30,185**	**28,786**	**58,971**	**57.97**
Biliran	3,673	1,326	1,281	2,607	70.98
Eastern Samar	10,406	3,169	3,067	6,236	59.93
Leyte	34,410	10,160	9,596	19,756	57.41
Northern Samar	15,211	3,923	3,647	7,570	49.77
Southern Leyte	8,028	2,353	2,215	4,568	56.90
Samar	18,353	5,414	5,325	10,739	58.51
Ormoc City	5,717	1,929	1,829	3,758	65.73
City of Tacloban	5,930	1,911	1,826	3,737	63.02

Table 2.C.1.6 - Immunization Services for Infants and Children
Fully Immunized Children (FIC)
Philippines, 2023

Area	Eligible Pop (0-12 mos. old)	FIC			
		Male	Female	Total	%
**Region 9**	**92,197**	**25,296**	**24,887**	**50,183**	**54.43**
Zamboanga del Norte	27,032	7,203	7,112	14,315	52.96
Zamboanga del Sur	25,767	5,572	5,373	10,945	42.48
Zamboanga Sibugay	15,055	4,142	4,212	8,354	55.49
City of Isabela	3,493	1,428	1,379	2,807	80.36
City of Zamboanga	20,850	6,951	6,811	13,762	66.00
**Region 10**	**118,571**	**42,393**	**41,080**	**83,473**	**70.40**
Bukidnon	36,317	11,997	11,399	23,396	64.42
Camiguin	1,877	517	495	1,012	53.92
Lanao del Norte	19,325	6,698	6,495	13,193	68.27
Misamis Occidental	13,380	4,950	5,253	10,203	76.26
Misamis Oriental	23,838	8,656	8,476	17,132	71.87
City of Cagayan De Oro	15,513	6,890	6,453	13,343	86.01
City of Iligan	8,321	2,685	2,509	5,194	62.42
**Region 11**	**116,229**	**38,138**	**35,342**	**73,480**	**63.22**
Davao de Oro	16,570	6,035	5,567	11,602	70.02
Davao del Norte	25,431	8,105	7,593	15,698	61.73
Davao Oriental	12,918	4,869	4,524	9,393	72.71
Davao del Sur	14,284	3,946	3,431	7,377	51.65
Davao Occidental	7,244	1,715	1,678	3,393	46.84
City of Davao	39,782	13,468	12,549	26,017	65.40
**Region 12**	**103,466**	**32,592**	**31,519**	**64,111**	**61.96**
Cotabato	28,890	8,043	7,626	15,669	54.24
Sarangani	14,406	5,311	5,152	10,463	72.63
South Cotabato	23,454	7,830	7,558	15,388	65.61
Sultan Kudarat	21,064	7,061	6,659	13,720	65.13
City of General Santos	15,652	4,347	4,524	8,871	56.68
**BARMM**	**145,404**	**31,519**	**32,143**	**63,662**	**43.78**
Basilan	12,723	1,638	1,552	3,190	25.07
Lanao del Sur	36,576	11,767	12,507	24,274	66.37
Maguindanao	45,411	9,931	9,722	19,653	43.28

Table 2.C.1.6 - Immunization Services for Infants and Children
Fully Immunized Children (FIC)
Philippines, 2023

Area	Eligible Pop (0-12 mos. old)	FIC			
		Male	Female	Total	%
Sulu	23,576	3,839	4,005	7,844	33.27
Tawi-Tawi	13,801	2,447	2,473	4,920	35.65
SGA	5,596	109	103	212	3.79
City of Cotabato	7,721	1,788	1,781	3,569	46.22
**CARAGA**	**59,880**	**24,200**	**23,310**	**47,510**	**79.34**
Agusan del Norte	7,920	3,449	3,304	6,753	85.27
Agusan del Sur	17,393	7,208	7,115	14,323	82.35
Surigao del Norte	11,088	4,108	3,932	8,040	72.51
Surigao del Sur	13,879	4,700	4,437	9,137	65.83
Dinagat Islands	2,346	811	750	1,561	66.54
City of Butuan	7,254	3,924	3,772	7,696	106.09

Table 2.C.1.6 - Immunization Services for Infants and Children
Completely Immunized Children (CIC)
Philippines, 2023

Area	Eligible Pop (13-23 mos. old)	CIC			
		Male	Female	Total	%
**Philippines**	**2,069,191**	**110,702**	**103,277**	**213,979**	**10.34**
**NCR**	**244,297**	**16,698**	**16,138**	**32,836**	**13.44**
Malabon City	6,764	1,820	1,730	3,550	52.48
Navotas City	4,913	200	203	403	8.20
Valenzuela City	13,161	215	252	467	3.55
Caloocan City	32,344	1,988	2,117	4,105	12.69
Marikina City	8,059	286	283	569	7.06
Pasig City	15,338	1,459	1,402	2,861	18.65
Pateros	1,108	44	49	93	8.39
Taguig City	17,588	1,683	1,665	3,348	19.04
Quezon City	55,804	4,445	4,338	8,783	15.74
Makati City	10,000	215	165	380	3.80
Mandaluyong City	7,404	280	251	531	7.17
San Juan City	2,068	66	61	127	6.14
Manila City	32,066	1,459	1,278	2,737	8.54
Las Piñas City	10,209	1,468	1,378	2,846	27.88
Muntinlupa City	9,166	430	402	832	9.08
Parañaque City	12,318	439	376	815	6.62
Pasay City	5,987	201	188	389	6.50
**CAR**	**29,725**	**647**	**589**	**1,236**	**4.16**
Abra	3,741	27	19	46	1.23
Apayao	2,041	27	17	44	2.16
Benguet	8,269	197	171	368	4.45
Ifugao	3,864	90	69	159	4.11
Kalinga	4,227	34	24	58	1.37
Mountain Province	2,543	38	45	83	3.26
City of Baguio	5,040	234	244	478	9.48
**Region 1**	**89,684**	**916**	**833**	**1,749**	**1.95**
Ilocos Norte	8,442	4	0	4	0.05
Ilocos Sur	9,640	36	22	58	0.60
La Union	12,392	62	70	132	1.07
Pangasinan	56,084	474	415	889	1.59
City of Dagupan	3,126	340	326	666	21.31

Table 2.C.1.6 - Immunization Services for Infants and Children
Completely Immunized Children (CIC)
Philippines, 2023

Area	Eligible Pop (13-23 mos. old)	CIC			
		Male	Female	Total	%
**Region 2**	**60,379**	**1,107**	**983**	**2,090**	**3.46**
Batanes	253	0	0	0	0.00
Cagayan	19,835	470	395	865	4.36
Isabela	25,910	382	339	721	2.78
Nueva Vizcaya	8,404	247	242	489	5.82
Quirino	3,456	8	7	15	0.43
City of Santiago	2,521	0	0	0	0.00
**Region 3**	**223,571**	**11,710**	**10,679**	**22,389**	**10.01**
Aurora	4,356	57	29	86	1.97
Bataan	15,945	1,335	1,115	2,450	15.37
Bulacan	65,915	4,472	4,197	8,669	13.15
Nueva Ecija	41,230	2,031	1,768	3,799	9.21
Pampanga	44,392	2,195	2,020	4,215	9.49
Tarlac	26,947	828	801	1,629	6.05
Zambales	11,152	136	138	274	2.46
City of Angeles	9,459	540	482	1,022	10.80
City of Olongapo	4,175	116	129	245	5.87
**Region 4A**	**293,184**	**24,415**	**22,934**	**47,349**	**16.15**
Batangas	52,431	4,275	3,933	8,208	15.65
Cavite	74,843	6,368	6,097	12,465	16.65
Laguna	57,905	5,130	4,869	9,999	17.27
Quezon	38,702	3,072	2,819	5,891	15.22
Rizal	63,797	5,043	4,737	9,780	15.33
City of Lucena	5,506	527	479	1,006	18.27
**Region 4B**	**61,472**	**3,429**	**3,266**	**6,695**	**10.89**
Marinduque	4,503	144	127	271	6.02
Occidental Mindoro	10,800	855	795	1,650	15.28
Oriental Mindoro	17,109	720	711	1,431	8.36
Palawan	18,872	923	866	1,789	9.48
Romblon	5,194	146	141	287	5.53
City of Puerto Princesa	4,994	641	626	1,267	25.37
**Region 5**	**127,639**	**7,340**	**6,602**	**13,942**	**10.92**

Table 2.C.1.6 - Immunization Services for Infants and Children
Completely Immunized Children (CIC)
Philippines, 2023

Area	Eligible Pop (13-23 mos. old)	CIC			
		Male	Female	Total	%
Albay	26,485	1,148	1,091	2,239	8.45
Camarines Norte	13,522	999	808	1,807	13.36
Camarines Sur	40,842	2,540	2,282	4,822	11.81
Catanduanes	5,445	278	242	520	9.55
Masbate	20,663	1,134	1,080	2,214	10.71
Sorsogon	16,808	925	844	1,769	10.52
City of Naga	3,874	316	255	571	14.74
**Region 6**	**141,582**	**4,695**	**4,308**	**9,003**	**6.36**
Aklan	10,207	460	462	922	9.03
Antique	11,074	252	238	490	4.42
Capiz	13,196	394	334	728	5.52
Guimaras	3,252	43	46	89	2.74
Iloilo	34,495	9	8	17	0.05
Negros Occidental	50,746	2,368	2,119	4,487	8.84
City of Bacolod	10,153	1,104	1,044	2,148	21.16
City of Iloilo	8,459	65	57	122	1.44
**Region 7**	**152,992**	**10,466**	**9,734**	**20,200**	**13.20**
Bohol	23,886	2,002	1,818	3,820	15.99
Cebu	67,026	4,888	4,615	9,503	14.18
Negros Oriental	26,246	2,486	2,292	4,778	18.20
Siquijor	1,380	49	54	103	7.46
City of Cebu	18,408	517	454	971	5.27
City of Lapu-Lapu	9,852	405	367	772	7.84
City of Mandaue	6,194	119	134	253	4.08
**Region 8**	**87,768**	**3,847**	**3,322**	**7,169**	**8.17**
Biliran	3,196	179	168	347	10.86
Eastern Samar	8,946	528	475	1,003	11.21
Leyte	30,519	1,105	909	2,014	6.60
Northern Samar	12,655	754	659	1,413	11.17
Southern Leyte	7,249	184	135	319	4.40
Samar	15,566	940	843	1,783	11.45
Ormoc City	4,676	42	38	80	1.71
City of Tacloban	4,961	115	95	210	4.23
**Region 9**	**78,653**	**5,920**	**5,641**	**11,561**	**14.70**

Table 2.C.1.6 - Immunization Services for Infants and Children
Completely Immunized Children (CIC)
Philippines, 2023

Area	Eligible Pop (13-23 mos. old)	CIC			
		Male	Female	Total	%
Zamboanga del Norte	23,089	1,401	1,326	2,727	11.81
Zamboanga del Sur	21,225	1,701	1,598	3,299	15.54
Zamboanga Sibugay	12,897	778	771	1,549	12.01
City of Isabela	3,143	72	84	156	4.96
City of Zamboanga	18,299	1,968	1,862	3,830	20.93
**Region 10**	**101,490**	**4,717**	**4,309**	**9,026**	**8.89**
Bukidnon	31,448	2,095	1,947	4,042	12.85
Camiguin	1,576	56	52	108	6.85
Lanao del Norte	16,756	830	811	1,641	9.79
Misamis Occidental	11,335	465	404	869	7.67
Misamis Oriental	19,927	688	571	1,259	6.32
City of Cagayan De Oro	13,289	271	249	520	3.91
City of Iligan	7,159	312	275	587	8.20
**Region 11**	**100,464**	**4,737**	**4,346**	**9,083**	**9.04**
Davao de Oro	14,241	258	256	514	3.61
Davao del Norte	21,607	266	237	503	2.33
Davao Oriental	10,641	267	247	514	4.83
Davao del Sur	12,873	665	632	1,297	10.08
Davao Occidental	6,704	543	486	1,029	15.35
City of Davao	34,398	2,738	2,488	5,226	15.19
**Region 12**	**89,081**	**3,626**	**3,333**	**6,959**	**7.81**
Cotabato	25,676	1,713	1,528	3,241	12.62
Sarangani	11,828	163	182	345	2.92
South Cotabato	20,160	450	421	871	4.32
Sultan Kudarat	18,220	239	199	438	2.40
City of General Santos	13,197	1,061	1,003	2,064	15.64
**BARMM**	**135,304**	**5,231**	**5,178**	**10,409**	**7.69**
Basilan	11,779	414	417	831	7.05
Lanao del Sur	33,842	1,501	1,568	3,069	9.07
Maguindanao	40,942	2,105	2,067	4,172	10.19
Sulu	23,613	675	665	1,340	5.67
Tawi-Tawi	12,899	273	238	511	3.96

Table 2.C.1.6 - Immunization Services for Infants and Children
Completely Immunized Children (CIC)
Philippines, 2023

Area	Eligible Pop (13-23 mos. old)	CIC			
		Male	Female	Total	%
SGA	5,335	185	165	350	6.56
City of Cotabato	6,894	78	58	136	1.97
**CARAGA**	**51,906**	**1,201**	**1,082**	**2,283**	**4.40**
Agusan del Norte	6,874	203	166	369	5.37
Agusan del Sur	14,668	146	137	283	1.93
Surigao del Norte	9,671	285	266	551	5.70
Surigao del Sur	12,249	460	429	889	7.26
Dinagat Islands	2,225	92	72	164	7.37
City of Butuan	6,219	15	12	27	0.43

Table 2.C.2.1 - Nutrition Services for Infants and Children
Newborns initiated on breastfeeding immediately after birth lasting for at least 90 minutes
Philippines, 2023

Area	Live Births	Newborns initiated on breastfeeding immediately after birth			
		Male	Female	Total	%
**Philippines**	**1,426,704**	**608,469**	**602,082**	**1,210,551**	**84.85**
**NCR**	**183,183**	**76,541**	**75,405**	**151,946**	**82.95**
Malabon City	1,781	1,273	1,281	2,554	143.40
Navotas City	4,103	1,956	2,147	4,103	100.00
Valenzuela City	5,569	2,398	2,342	4,740	85.11
Caloocan City	22,175	4,939	5,144	10,083	45.47
Marikina City	2,518	1,333	1,185	2,518	100.00
Pasig City	11,340	3,137	3,007	6,144	54.18
Pateros	787	403	383	786	99.87
Taguig City	12,346	5,294	5,185	10,479	84.88
Quezon City	57,393	25,885	24,927	50,812	88.53
Makati City	3,527	1,781	1,737	3,518	99.74
Mandaluyong City	7,655	3,732	3,592	7,324	95.68
San Juan City	751	354	380	734	97.74
Manila City	23,674	12,259	11,763	24,022	101.47
Las Piñas City	6,827	3,255	3,339	6,594	96.59
Muntinlupa City	7,504	1,885	1,797	3,682	49.07
Parañaque City	8,323	3,384	3,559	6,943	83.42
Pasay City	6,910	3,273	3,637	6,910	100.00
**CAR**	**24,815**	**11,595**	**10,825**	**22,420**	**90.35**
Abra	2,716	796	807	1,603	59.02
Apayao	2,782	937	1,020	1,957	70.35
Benguet	4,086	2,124	1,880	4,004	97.99
Ifugao	2,401	1,160	1,056	2,216	92.29
Kalinga	2,376	1,170	1,135	2,305	97.01
Mountain Province	3,361	1,732	1,524	3,256	96.88
City of Baguio	7,093	3,676	3,403	7,079	99.80
**Region 1**	**50,514**	**24,939**	**23,985**	**48,924**	**96.85**
Ilocos Norte	6,048	2,845	2,984	5,829	96.38
Ilocos Sur	7,661	3,962	3,697	7,659	99.97
La Union	10,613	5,044	4,449	9,493	89.45
Pangasinan	17,403	8,624	8,530	17,154	98.57
City of Dagupan	8,789	4,464	4,325	8,789	100.00

Table 2.C.2.1 - Nutrition Services for Infants and Children
Newborns initiated on breastfeeding immediately after birth lasting for at least 90 minutes
Philippines, 2023

Area	Live Births	Newborns initiated on breastfeeding immediately after birth			
		Male	Female	Total	%
**Region 2**	**36,685**	**14,340**	**13,670**	**28,010**	**76.35**
Batanes	216	122	91	213	98.61
Cagayan	8,404	3,708	3,518	7,226	85.98
Isabela	15,203	5,385	5,158	10,543	69.35
Nueva Vizcaya	7,254	2,389	2,359	4,748	65.45
Quirino	2,774	1,298	1,148	2,446	88.18
City of Santiago	2,834	1,438	1,396	2,834	100.00
**Region 3**	**152,056**	**57,062**	**56,678**	**113,740**	**74.80**
Aurora	3,171	1,309	1,328	2,637	83.16
Bataan	11,007	6,529	6,071	12,600	114.47
Bulacan	41,925	15,854	15,755	31,609	75.39
Nueva Ecija	28,807	11,603	11,316	22,919	79.56
Pampanga	25,935	7,499	7,554	15,053	58.04
Tarlac	20,924	6,510	7,250	13,760	65.76
Zambales	8,646	3,132	3,112	6,244	72.22
City of Angeles	7,506	3,423	3,224	6,647	88.56
City of Olongapo	4,135	1,203	1,068	2,271	54.92
**Region 4A**	**202,104**	**84,832**	**86,710**	**171,542**	**84.88**
Batangas	38,151	17,288	17,333	34,621	90.75
Cavite	50,543	20,751	21,284	42,035	83.17
Laguna	44,295	17,221	18,295	35,516	80.18
Quezon	24,211	10,928	10,668	21,596	89.20
Rizal	41,811	17,142	17,662	34,804	83.24
City of Lucena	3,093	1,502	1,468	2,970	96.02
**Region 4B**	**47,849**	**21,590**	**19,614**	**41,204**	**86.11**
Marinduque	2,772	1,514	1,258	2,772	100.00
Occidental Mindoro	8,414	4,124	3,915	8,039	95.54
Oriental Mindoro	15,155	6,055	4,828	10,883	71.81
Palawan	13,077	6,006	5,798	11,804	90.27
Romblon	4,355	1,955	1,902	3,857	88.56
City of Puerto Princesa	4,076	1,936	1,913	3,849	94.43

Table 2.C.2.1 - Nutrition Services for Infants and Children
Newborns initiated on breastfeeding immediately after birth lasting for at least 90 minutes
Philippines, 2023

Area	Live Births	Newborns initiated on breastfeeding immediately after birth			
		Male	Female	Total	%
**Region 5**	**89,477**	**34,779**	**34,162**	**68,941**	**77.05**
Albay	16,144	5,938	6,075	12,013	74.41
Camarines Norte	10,499	4,924	5,258	10,182	96.98
Camarines Sur	17,601	7,403	6,919	14,322	81.37
Catanduanes	4,304	2,228	2,076	4,304	100.00
Masbate	14,187	6,658	6,559	13,217	93.16
Sorsogon	13,580	6,848	6,518	13,366	98.42
City of Naga	13,162	780	757	1,537	11.68
**Region 6**	**96,610**	**44,613**	**43,408**	**88,021**	**91.11**
Aklan	6,225	2,815	2,823	5,638	90.57
Antique	8,240	4,001	3,860	7,861	95.40
Capiz	10,744	5,533	5,040	10,573	98.41
Guimaras	2,371	1,165	1,172	2,337	98.57
Iloilo	22,616	9,971	9,809	19,780	87.46
Negros Occidental	34,516	15,830	15,576	31,406	90.99
City of Bacolod	6,121	2,572	2,401	4,973	81.24
City of Iloilo	5,777	2,726	2,727	5,453	94.39
**Region 7**	**113,020**	**48,171**	**47,006**	**95,177**	**84.21**
Bohol	18,473	6,437	6,116	12,553	67.95
Cebu	41,522	17,977	17,387	35,364	85.17
Negros Oriental	19,266	8,063	8,383	16,446	85.36
Siquijor	1,014	490	504	994	98.03
City of Cebu	15,464	7,836	7,554	15,390	99.52
City of Lapu-Lapu	7,508	2,418	2,434	4,852	64.62
City of Mandaue	9,773	4,950	4,628	9,578	98.00
**Region 8**	**59,902**	**25,824**	**25,834**	**51,658**	**86.24**
Biliran	3,066	1,541	1,433	2,974	97.00
Eastern Samar	6,971	3,231	3,433	6,664	95.60
Leyte	12,199	5,609	5,622	11,231	92.06
Northern Samar	10,341	5,004	4,935	9,939	96.11
Southern Leyte	5,061	1,895	1,953	3,848	76.03
Samar	10,416	4,208	4,188	8,396	80.61
Ormoc City	6,003	1,559	1,459	3,018	50.27
City of Tacloban	5,845	2,777	2,811	5,588	95.60

Table 2.C.2.1 - Nutrition Services for Infants and Children
Newborns initiated on breastfeeding immediately after birth lasting for at least 90 minutes
Philippines, 2023

Area	Live Births	Newborns initiated on breastfeeding immediately after birth			
		Male	Female	Total	%
**Region 9**	**47,802**	**21,694**	**22,756**	**44,450**	**92.99**
Zamboanga del Norte	13,406	6,106	6,219	12,325	91.94
Zamboanga del Sur	8,244	3,404	3,823	7,227	87.66
Zamboanga Sibugay	7,392	3,467	3,407	6,874	92.99
City of Isabela	1,793	817	735	1,552	86.56
City of Zamboanga	16,967	7,900	8,572	16,472	97.08
**Region 10**	**71,506**	**30,924**	**31,030**	**61,954**	**86.64**
Bukidnon	23,203	10,236	10,414	20,650	89.00
Camiguin	1,345	679	667	1,346	100.07
Lanao del Norte	6,718	2,679	2,976	5,655	84.18
Misamis Occidental	6,248	3,235	3,004	6,239	99.86
Misamis Oriental	9,329	4,282	4,665	8,947	95.91
City of Cagayan De Oro	17,424	7,447	6,848	14,295	82.04
City of Iligan	7,239	2,366	2,456	4,822	66.61
**Region 11**	**81,198**	**37,917**	**35,890**	**73,807**	**90.90**
Davao de Oro	11,482	5,688	5,302	10,990	95.72
Davao del Norte	17,050	8,255	7,678	15,933	93.45
Davao Oriental	8,540	4,189	3,933	8,122	95.11
Davao del Sur	9,423	4,431	4,074	8,505	90.26
Davao Occidental	4,769	1,620	1,613	3,233	67.79
City of Davao	29,934	13,734	13,290	27,024	90.28
**Region 12**	**63,981**	**30,050**	**30,020**	**60,070**	**93.89**
Cotabato	17,313	7,993	7,949	15,942	92.08
Sarangani	9,930	4,964	4,804	9,768	98.37
South Cotabato	16,287	8,039	7,878	15,917	97.73
Sultan Kudarat	12,543	6,185	5,923	12,108	96.53
City of General Santos	7,908	2,869	3,466	6,335	80.11
**BARMM**	**64,968**	**23,756**	**25,454**	**49,210**	**75.74**
Basilan	4,764	1,550	1,687	3,237	67.95
Lanao del Sur	17,803	8,026	8,438	16,464	92.48
Maguindanao	19,910	5,504	6,183	11,687	58.70

Table 2.C.2.1 - Nutrition Services for Infants and Children

Newborns initiated on breastfeeding immediately after birth lasting for at least 90 minutes

Philippines, 2023

Area	Live Births	Newborns initiated on breastfeeding immediately after birth			
		Male	Female	Total	%
Sulu	11,518	4,223	4,561	8,784	76.26
Tawi-Tawi	5,994	2,343	2,386	4,729	78.90
SGA	1,431	406	469	875	61.15
City of Cotabato	3,548	1,704	1,730	3,434	96.79
**CARAGA**	**41,034**	**19,842**	**19,635**	**39,477**	**96.21**
Agusan del Norte	5,748	2,899	2,768	5,667	98.59
Agusan del Sur	10,949	5,345	5,461	10,806	98.69
Surigao del Norte	7,722	3,803	3,816	7,619	98.67
Surigao del Sur	9,741	4,949	4,690	9,639	98.95
Dinagat Islands	1,031	498	479	977	94.76
City of Butuan	5,843	2,348	2,421	4,769	81.62

Table 2.C.2.1 - Nutrition Services for Infants and Children
Infants Exclusively Breastfed until 5th month and 29 days
Philippines, 2023

Area	Eligible Pop (0-11 mos. old)	Infants Exclusively Breastfed until 5th month and 29 days			
		Male	Female	Total	%
**Philippines**	**2,187,333**	**581,355**	**574,073**	**1,155,428**	**52.82**
**NCR**	**261,228**	**73,475**	**70,237**	**143,712**	**55.01**
Malabon City	7,385	2,100	2,129	4,229	57.26
Navotas City	5,210	2,172	2,299	4,471	85.82
Valenzuela City	13,477	3,497	3,339	6,836	50.72
Caloocan City	33,168	4,437	4,554	8,991	27.11
Marikina City	8,342	1,631	1,546	3,177	38.08
Pasig City	17,682	624	679	1,303	7.37
Pateros	1,116	285	251	536	48.03
Taguig City	18,992	8,233	7,939	16,172	85.15
Quezon City	59,136	27,086	24,421	51,507	87.10
Makati City	10,944	1,253	1,245	2,498	22.83
Mandaluyong City	7,922	2,206	2,130	4,336	54.73
San Juan City	2,168	215	250	465	21.45
Manila City	34,128	9,635	9,397	19,032	55.77
Las Piñas City	11,263	3,259	3,284	6,543	58.09
Muntinlupa City	9,881	2,408	2,480	4,888	49.47
Parañaque City	13,280	2,500	2,345	4,845	36.48
Pasay City	7,134	1,934	1,949	3,883	54.43
**CAR**	**31,319**	**9,535**	**9,379**	**18,914**	**60.39**
Abra	3,660	1,257	1,259	2,516	68.74
Apayao	2,238	834	818	1,652	73.82
Benguet	8,806	2,434	2,404	4,838	54.94
Ifugao	4,098	949	918	1,867	45.56
Kalinga	4,549	1,474	1,437	2,911	63.99
Mountain Province	2,619	1,075	1,021	2,096	80.03
City of Baguio	5,349	1,512	1,522	3,034	56.72
**Region 1**	**96,016**	**31,280**	**30,009**	**61,289**	**63.83**
Ilocos Norte	8,912	3,184	3,380	6,564	73.65
Ilocos Sur	9,914	5,015	4,610	9,625	97.08
La Union	13,507	3,596	3,253	6,849	50.71
Pangasinan	60,360	18,728	18,077	36,805	60.98
City of Dagupan	3,323	757	689	1,446	43.51

Table 2.C.2.1 - Nutrition Services for Infants and Children
Infants Exclusively Breastfed until 5th month and 29 days
Philippines, 2023

Area	Eligible Pop (0-11 mos. old)	Infants Exclusively Breastfed until 5th month and 29 days			
		Male	Female	Total	%
**Region 2**	**63,967**	**20,258**	**19,677**	**39,935**	**62.43**
Batanes	240	99	69	168	70.00
Cagayan	20,786	6,115	5,741	11,856	57.04
Isabela	27,379	8,579	8,376	16,955	61.93
Nueva Vizcaya	8,798	2,955	2,944	5,899	67.05
Quirino	3,839	1,236	1,233	2,469	64.31
City of Santiago	2,925	1,274	1,314	2,588	88.48
**Region 3**	**233,544**	**54,156**	**53,883**	**108,039**	**46.26**
Aurora	4,537	1,348	1,305	2,653	58.47
Bataan	17,380	5,695	5,514	11,209	64.49
Bulacan	67,590	16,539	16,357	32,896	48.67
Nueva Ecija	43,425	10,028	9,829	19,857	45.73
Pampanga	45,651	7,749	7,958	15,707	34.41
Tarlac	29,503	8,307	8,264	16,571	56.17
Zambales	11,723	2,267	2,216	4,483	38.24
City of Angeles	9,146	1,569	1,763	3,332	36.43
City of Olongapo	4,589	654	677	1,331	29.00
**Region 4A**	**307,982**	**62,191**	**62,775**	**124,966**	**40.58**
Batangas	53,251	9,732	9,582	19,314	36.27
Cavite	77,964	13,536	14,028	27,564	35.35
Laguna	61,525	16,303	16,207	32,510	52.84
Quezon	40,810	8,705	8,575	17,280	42.34
Rizal	68,235	12,836	13,327	26,163	38.34
City of Lucena	6,197	1,079	1,056	2,135	34.45
**Region 4B**	**66,121**	**23,095**	**20,361**	**43,456**	**65.72**
Marinduque	4,144	1,358	1,174	2,532	61.10
Occidental Mindoro	11,640	3,988	3,863	7,851	67.45
Oriental Mindoro	18,943	8,163	5,883	14,046	74.15
Palawan	20,317	6,356	6,224	12,580	61.92
Romblon	5,489	2,222	2,172	4,394	80.05
City of Puerto Princesa	5,588	1,008	1,045	2,053	36.74
**Region 5**	**135,480**	**35,813**	**36,089**	**71,902**	**53.07**

Table 2.C.2.1 - Nutrition Services for Infants and Children
Infants Exclusively Breastfed until 5th month and 29 days
Philippines, 2023

Area	Eligible Pop (0-11 mos. old)	Infants Exclusively Breastfed until 5th month and 29 days			
		Male	Female	Total	%
Albay	27,440	6,846	6,958	13,804	50.31
Camarines Norte	14,695	4,228	4,245	8,473	57.66
Camarines Sur	43,452	9,763	9,899	19,662	45.25
Catanduanes	5,414	1,816	1,674	3,490	64.46
Masbate	22,365	6,649	6,970	13,619	60.89
Sorsogon	17,929	5,742	5,579	11,321	63.14
City of Naga	4,185	769	764	1,533	36.63
**Region 6**	**148,020**	**34,908**	**35,357**	**70,265**	**47.47**
Aklan	11,283	2,242	2,302	4,544	40.27
Antique	11,930	2,742	2,786	5,528	46.34
Capiz	13,931	3,565	3,413	6,978	50.09
Guimaras	3,351	1,134	1,076	2,210	65.95
Iloilo	36,140	9,238	9,548	18,786	51.98
Negros Occidental	52,270	12,588	12,685	25,273	48.35
City of Bacolod	10,373	1,385	1,488	2,873	27.70
City of Iloilo	8,742	2,014	2,059	4,073	46.59
**Region 7**	**165,673**	**47,460**	**46,979**	**94,439**	**57.00**
Bohol	26,235	6,865	6,815	13,680	52.14
Cebu	72,201	21,463	21,717	43,180	59.81
Negros Oriental	28,722	9,025	8,738	17,763	61.84
Siquijor	1,680	541	530	1,071	63.75
City of Cebu	19,267	5,384	5,048	10,432	54.14
City of Lapu-Lapu	10,679	3,015	2,981	5,996	56.15
City of Mandaue	6,889	1,167	1,150	2,317	33.63
**Region 8**	**93,813**	**22,994**	**22,684**	**45,678**	**48.69**
Biliran	3,373	1,149	1,140	2,289	67.86
Eastern Samar	9,624	2,291	2,291	4,582	47.61
Leyte	31,559	8,235	8,110	16,345	51.79
Northern Samar	14,056	3,314	3,404	6,718	47.79
Southern Leyte	7,406	1,945	1,926	3,871	52.27
Samar	17,011	3,285	3,193	6,478	38.08
Ormoc City	5,292	1,384	1,288	2,672	50.49
City of Tacloban	5,492	1,391	1,332	2,723	49.58
**Region 9**	**84,815**	**23,060**	**23,182**	**46,242**	**54.52**

Table 2.C.2.1 - Nutrition Services for Infants and Children
Infants Exclusively Breastfed until 5th month and 29 days
Philippines, 2023

Area	Eligible Pop (0-11 mos. old)	Infants Exclusively Breastfed until 5th month and 29 days			
		Male	Female	Total	%
Zamboanga del Norte	24,841	6,517	6,729	13,246	53.32
Zamboanga del Sur	23,757	5,898	5,728	11,626	48.94
Zamboanga Sibugay	13,841	3,232	3,284	6,516	47.08
City of Isabela	3,201	697	767	1,464	45.74
City of Zamboanga	19,175	6,716	6,674	13,390	69.83
**Region 10**	**109,169**	**37,233**	**37,652**	**74,885**	**68.60**
Bukidnon	33,403	11,536	11,764	23,300	69.75
Camiguin	1,727	520	604	1,124	65.08
Lanao del Norte	17,760	4,891	5,084	9,975	56.17
Misamis Occidental	12,328	4,674	4,460	9,134	74.09
Misamis Oriental	21,975	6,844	7,186	14,030	63.85
City of Cagayan De Oro	14,308	6,228	6,014	12,242	85.56
City of Iligan	7,668	2,540	2,540	5,080	66.25
**Region 11**	**107,001**	**36,192**	**34,355**	**70,547**	**65.93**
Davao de Oro	15,253	5,308	5,096	10,404	68.21
Davao del Norte	23,453	7,282	6,858	14,140	60.29
Davao Oriental	11,935	5,002	4,829	9,831	82.37
Davao del Sur	13,083	4,307	4,004	8,311	63.53
Davao Occidental	6,629	2,209	2,096	4,305	64.94
City of Davao	36,648	12,084	11,472	23,556	64.28
**Region 12**	**95,279**	**29,049**	**29,257**	**58,306**	**61.20**
Cotabato	26,521	7,177	7,135	14,312	53.96
Sarangani	13,322	5,543	5,352	10,895	81.78
South Cotabato	21,595	7,137	7,506	14,643	67.81
Sultan Kudarat	19,391	6,661	6,635	13,296	68.57
City of General Santos	14,450	2,531	2,629	5,160	35.71
**BARMM**	**132,841**	**22,391**	**23,821**	**46,212**	**34.79**
Basilan	11,636	878	985	1,863	16.01
Lanao del Sur	33,374	9,776	10,334	20,110	60.26
Maguindanao	41,650	4,685	4,950	9,635	23.13
Sulu	21,378	2,666	2,913	5,579	26.10
Tawi-Tawi	12,609	1,908	2,026	3,934	31.20

Table 2.C.2.1 - Nutrition Services for Infants and Children
Infants Exclusively Breastfed until 5th month and 29 days
Philippines, 2023

Area	Eligible Pop (0-11 mos. old)	Infants Exclusively Breastfed until 5th month and 29 days			
		Male	Female	Total	%
SGA	5,103	468	608	1,076	21.09
City of Cotabato	7,091	2,010	2,005	4,015	56.62
**CARAGA**	**55,065**	**18,265**	**18,376**	**36,641**	**66.54**
Agusan del Norte	7,286	2,384	2,437	4,821	66.17
Agusan del Sur	16,038	5,930	6,088	12,018	74.93
Surigao del Norte	10,186	2,962	3,027	5,989	58.80
Surigao del Sur	12,736	3,698	3,512	7,210	56.61
Dinagat Islands	2,134	483	507	990	46.39
City of Butuan	6,685	2,808	2,805	5,613	83.96

Table 2.C.2.1 - Nutrition Services for Infants and Children

Infants who continued breastfeeding and were introduced to complementary feeding beginning at 6 months of age

Philippines, 2023

Area	Eligible Pop (6-11 mos. old)	Infants who continued breastfeeding and were introduced to complementary feeding			
		Male	Female	Total	%
**Philippines**	**1,114,805**	**534,913**	**530,722**	**1,065,635**	**95.59**
**NCR**	**131,452**	**68,812**	**67,918**	**136,730**	**104.02**
Malabon City	3,702	1,916	1,992	3,908	105.56
Navotas City	2,614	2,070	2,194	4,264	163.12
Valenzuela City	6,752	2,726	2,668	5,394	79.89
Caloocan City	16,684	2,292	2,243	4,535	27.18
Marikina City	4,178	1,413	1,386	2,799	66.99
Pasig City	8,854	852	905	1,757	19.84
Pateros	563	231	214	445	79.04
Taguig City	9,510	6,471	6,269	12,740	133.96
Quezon City	29,642	26,634	26,686	53,320	179.88
Makati City	5,491	1,065	1,073	2,138	38.94
Mandaluyong City	3,970	1,707	1,643	3,350	84.38
San Juan City	1,092	246	230	476	43.59
Manila City	17,496	10,485	10,129	20,614	117.82
Las Piñas City	5,642	3,090	3,069	6,159	109.16
Muntinlupa City	4,946	2,964	2,773	5,737	115.99
Parañaque City	6,649	2,805	2,622	5,427	81.62
Pasay City	3,667	1,845	1,822	3,667	100.00
**CAR**	**16,268**	**9,238**	**9,083**	**18,321**	**112.62**
Abra	1,982	1,248	1,240	2,488	125.53
Apayao	1,188	750	754	1,504	126.60
Benguet	4,481	2,356	2,354	4,710	105.11
Ifugao	2,135	910	878	1,788	83.75
Kalinga	2,355	1,469	1,430	2,899	123.10
Mountain Province	1,387	1,044	989	2,033	146.58
City of Baguio	2,740	1,461	1,438	2,899	105.80
**Region 1**	**49,645**	**32,326**	**31,082**	**63,408**	**127.72**
Ilocos Norte	4,730	3,136	3,218	6,354	134.33
Ilocos Sur	5,342	5,110	4,728	9,838	184.16
La Union	7,041	3,763	3,441	7,204	102.32
Pangasinan	30,853	19,535	18,965	38,500	124.79
City of Dagupan	1,679	782	730	1,512	90.05

Table 2.C.2.1 - Nutrition Services for Infants and Children

Infants who continued breastfeeding and were introduced to complementary feeding beginning at 6 months of age

Philippines, 2023

Area	Eligible Pop (6-11 mos. old)	Infants who continued breastfeeding and were introduced to complementary feeding			
		Male	Female	Total	%
**Region 2**	**33,156**	**19,336**	**18,774**	**38,110**	**114.94**
Batanes	133	93	71	164	123.31
Cagayan	10,799	5,717	5,441	11,158	103.32
Isabela	14,209	8,595	8,355	16,950	119.29
Nueva Vizcaya	4,546	2,459	2,406	4,865	107.02
Quirino	1,988	1,201	1,193	2,394	120.42
City of Santiago	1,481	1,271	1,308	2,579	174.14
**Region 3**	**118,351**	**52,636**	**51,328**	**103,964**	**87.84**
Aurora	2,336	1,310	1,297	2,607	111.60
Bataan	8,818	5,236	5,041	10,277	116.55
Bulacan	34,076	16,358	15,753	32,111	94.23
Nueva Ecija	22,142	9,413	8,945	18,358	82.91
Pampanga	23,067	8,158	8,227	16,385	71.03
Tarlac	15,033	8,216	8,149	16,365	108.86
Zambales	5,984	2,095	2,010	4,105	68.60
City of Angeles	4,592	1,465	1,557	3,022	65.81
City of Olongapo	2,303	385	349	734	31.87
**Region 4A**	**156,001**	**59,499**	**61,061**	**120,560**	**77.28**
Batangas	27,177	11,273	11,144	22,417	82.49
Cavite	39,399	12,852	13,168	26,020	66.04
Laguna	31,107	14,571	14,392	28,963	93.11
Quezon	21,003	8,318	8,152	16,470	78.42
Rizal	34,204	11,392	13,127	24,519	71.68
City of Lucena	3,111	1,093	1,078	2,171	69.78
**Region 4B**	**33,790**	**6,123**	**6,153**	**12,276**	**36.33**
Marinduque	2,179	216	172	388	17.81
Occidental Mindoro	5,896	3,647	3,618	7,265	123.22
Oriental Mindoro	9,689	680	772	1,452	14.99
Palawan	10,346	1,334	1,318	2,652	25.63
Romblon	2,851	142	167	309	10.84
City of Puerto Princesa	2,829	104	106	210	7.42
**Region 5**	**69,494**	**34,149**	**33,934**	**68,083**	**97.97**

Table 2.C.2.1 - Nutrition Services for Infants and Children

Infants who continued breastfeeding and were introduced to complementary feeding beginning at 6 months of age

Philippines, 2023

Area	Eligible Pop (6-11 mos. old)	Infants who continued breastfeeding and were introduced to complementary feeding			
		Male	Female	Total	%
Albay	14,076	6,147	6,247	12,394	88.05
Camarines Norte	7,487	3,941	3,964	7,905	105.58
Camarines Sur	22,245	8,929	8,973	17,902	80.48
Catanduanes	2,865	1,769	1,610	3,379	117.94
Masbate	11,476	7,221	7,158	14,379	125.30
Sorsogon	9,240	5,477	5,325	10,802	116.90
City of Naga	2,105	665	657	1,322	62.80
**Region 6**	**76,022**	**33,697**	**33,811**	**67,508**	**88.80**
Aklan	5,790	2,249	2,290	4,539	78.39
Antique	6,243	2,697	2,701	5,398	86.46
Capiz	7,206	3,544	3,401	6,945	96.38
Guimaras	1,720	1,129	1,056	2,185	127.03
Iloilo	18,950	9,465	9,565	19,030	100.42
Negros Occidental	26,442	10,969	11,051	22,020	83.28
City of Bacolod	5,217	1,655	1,684	3,339	64.00
City of Iloilo	4,454	1,989	2,063	4,052	90.97
**Region 7**	**84,357**	**46,505**	**45,845**	**92,350**	**109.48**
Bohol	13,690	6,775	6,595	13,370	97.66
Cebu	36,629	19,658	19,861	39,519	107.89
Negros Oriental	14,644	8,874	8,622	17,496	119.48
Siquijor	900	538	522	1,060	117.78
City of Cebu	9,680	5,159	4,841	10,000	103.31
City of Lapu-Lapu	5,357	2,818	2,885	5,703	106.46
City of Mandaue	3,457	2,683	2,519	5,202	150.48
**Region 8**	**49,125**	**20,674**	**20,411**	**41,085**	**83.63**
Biliran	1,748	1,130	1,106	2,236	127.92
Eastern Samar	5,114	2,017	2,117	4,134	80.84
Leyte	16,469	7,377	7,154	14,531	88.23
Northern Samar	7,313	2,860	2,882	5,742	78.52
Southern Leyte	3,973	1,554	1,562	3,116	78.43
Samar	9,002	3,162	3,052	6,214	69.03
Ormoc City	2,694	1,415	1,275	2,690	99.85
City of Tacloban	2,812	1,159	1,263	2,422	86.13
**Region 9**	**43,395**	**21,010**	**21,283**	**42,293**	**97.46**

Table 2.C.2.1 - Nutrition Services for Infants and Children

Infants who continued breastfeeding and were introduced to complementary feeding beginning at 6 months of age

Philippines, 2023

Area	Eligible Pop (6-11 mos. old)	Infants who continued breastfeeding and were introduced to complementary feeding			
		Male	Female	Total	%
Zamboanga del Norte	12,781	6,148	6,397	12,545	98.15
Zamboanga del Sur	12,231	5,528	5,416	10,944	89.48
Zamboanga Sibugay	7,123	2,998	3,043	6,041	84.81
City of Isabela	1,624	640	719	1,359	83.68
City of Zamboanga	9,636	5,696	5,708	11,404	118.35
**Region 10**	**55,577**	**33,313**	**33,348**	**66,661**	**119.94**
Bukidnon	16,927	10,097	10,139	20,236	119.55
Camiguin	892	605	640	1,245	139.57
Lanao del Norte	9,100	4,376	4,555	8,931	98.14
Misamis Occidental	6,413	4,696	4,612	9,308	145.14
Misamis Oriental	11,196	5,856	5,925	11,781	105.23
City of Cagayan De Oro	7,195	5,281	5,146	10,427	144.92
City of Iligan	3,854	2,402	2,331	4,733	122.81
**Region 11**	**54,069**	**33,535**	**31,821**	**65,356**	**120.88**
Davao de Oro	7,733	4,962	4,714	9,676	125.13
Davao del Norte	11,841	7,218	6,894	14,112	119.18
Davao Oriental	6,064	4,459	4,223	8,682	143.17
Davao del Sur	6,659	4,496	4,130	8,626	129.54
Davao Occidental	3,363	1,902	1,884	3,786	112.58
City of Davao	18,409	10,498	9,976	20,474	111.22
**Region 12**	**48,192**	**27,671**	**27,380**	**55,051**	**114.23**
Cotabato	13,506	7,079	6,998	14,077	104.23
Sarangani	6,730	5,125	4,937	10,062	149.51
South Cotabato	10,901	6,287	6,562	12,849	117.87
Sultan Kudarat	9,819	6,861	6,484	13,345	135.91
City of General Santos	7,236	2,319	2,399	4,718	65.20
**BARMM**	**67,735**	**20,340**	**21,333**	**41,673**	**61.52**
Basilan	5,917	951	1,038	1,989	33.62
Lanao del Sur	17,274	8,515	9,058	17,573	101.73
Maguindanao	21,085	4,129	4,167	8,296	39.35
Sulu	10,897	2,597	2,839	5,436	49.89
Tawi-Tawi	6,413	1,678	1,795	3,473	54.16

Table 2.C.2.1 - Nutrition Services for Infants and Children

Infants who continued breastfeeding and were introduced to complementary feeding beginning at 6 months of age

Philippines, 2023

Area	Eligible Pop (6-11 mos. old)	Infants who continued breastfeeding and were introduced to complementary feeding			
		Male	Female	Total	%
SGA	2,586	403	502	905	35.00
City of Cotabato	3,563	2,067	1,934	4,001	112.29
**CARAGA**	**28,176**	**16,049**	**16,157**	**32,206**	**114.30**
Agusan del Norte	3,728	1,943	2,001	3,944	105.79
Agusan del Sur	8,175	5,373	5,549	10,922	133.60
Surigao del Norte	5,260	2,361	2,384	4,745	90.21
Surigao del Sur	6,521	3,802	3,612	7,414	113.69
Dinagat Islands	1,111	459	468	927	83.44
City of Butuan	3,381	2,111	2,143	4,254	125.82

Table 2.C.2.2- Nutrition Services for Infants and Children
Infants born preterm or with low birth weight given iron supplements
Philippines, 2023

Area	Total No. of live births with low birth weight	Infants preterm or with LBW (< 2500 grams) given Iron			
		Male	Female	Total	%
**Philippines**	**85,337**	**9,503**	**10,444**	**19,947**	**23.37**
**NCR**	**7,441**	**1,315**	**1,304**	**2,619**	**35.20**
Malabon City	28	5	7	12	42.86
Navotas City	143	48	60	108	75.52
Valenzuela City	417	94	124	218	52.28
Caloocan City	340	22	27	49	14.41
Marikina City	123	64	59	123	100.00
Pasig City	1,917	52	56	108	5.63
Pateros	19	12	7	19	100.00
Taguig City	242	142	100	242	100.00
Quezon City	1,664	139	94	233	14.00
Makati City	259	51	64	115	44.40
Mandaluyong City	138	33	27	60	43.48
San Juan City	44	20	24	44	100.00
Manila City	967	185	185	370	38.26
Las Piñas City	200	46	42	88	44.00
Muntinlupa City	170	78	92	170	100.00
Parañaque City	441	168	188	356	80.73
Pasay City	329	156	148	304	92.40
**CAR**	**2,394**	**156**	**144**	**300**	**12.53**
Abra	302	36	20	56	18.54
Apayao	242	21	36	57	23.55
Benguet	343	38	37	75	21.87
Ifugao	78	3	1	4	5.13
Kalinga	92	18	10	28	30.43
Mountain Province	278	4	12	16	5.76
City of Baguio	1,059	36	28	64	6.04
**Region 1**	**4,038**	**355**	**468**	**823**	**20.38**
Ilocos Norte	606	86	131	217	35.81
Ilocos Sur	387	54	57	111	28.68
La Union	1,146	85	96	181	15.79
Pangasinan	384	120	149	269	70.05
City of Dagupan	1,515	10	35	45	2.97

Table 2.C.2.2- Nutrition Services for Infants and Children

Infants born preterm or with low birth weight given iron supplements

Philippines, 2023

Area	Total No. of live births with low birth weight	Infants preterm or with LBW (< 2500 grams) given Iron			
		Male	Female	Total	%
**Region 2**	**2,082**	**309**	**366**	**675**	**32.42**
Batanes	28	0	0	0	0.00
Cagayan	279	87	159	246	88.17
Isabela	710	142	144	286	40.28
Nueva Vizcaya	842	55	48	103	12.23
Quirino	178	23	14	37	20.79
City of Santiago	45	2	1	3	6.67
**Region 3**	**10,259**	**790**	**829**	**1,619**	**15.78**
Aurora	306	63	54	117	38.24
Bataan	1,233	74	78	152	12.33
Bulacan	3,477	340	336	676	19.44
Nueva Ecija	1,336	149	182	331	24.78
Pampanga	1,594	139	149	288	18.07
Tarlac	408	7	11	18	4.41
Zambales	978	15	18	33	3.37
City of Angeles	567	0	0	0	0.00
City of Olongapo	360	3	1	4	1.11
**Region 4A**	**10,276**	**1,016**	**1,104**	**2,120**	**20.63**
Batangas	1,576	107	121	228	14.47
Cavite	2,558	277	304	581	22.71
Laguna	1,980	109	100	209	10.56
Quezon	1,274	145	154	299	23.47
Rizal	2,621	342	395	737	28.12
City of Lucena	267	36	30	66	24.72
**Region 4B**	**1,743**	**320**	**349**	**669**	**38.38**
Marinduque	233	41	45	86	36.91
Occidental Mindoro	384	23	17	40	10.42
Oriental Mindoro	236	100	133	233	98.73
Palawan	459	124	113	237	51.63
Romblon	278	19	26	45	16.19
City of Puerto Princesa	153	13	15	28	18.30

Table 2.C.2.2- Nutrition Services for Infants and Children

Infants born preterm or with low birth weight given iron supplements

Philippines, 2023

Area	Total No. of live births with low birth weight	Infants preterm or with LBW (< 2500 grams) given Iron			
		Male	Female	Total	%
**Region 5**	**9,417**	**691**	**692**	**1,383**	**14.69**
Albay	1,437	120	117	237	16.49
Camarines Norte	1,364	138	163	301	22.07
Camarines Sur	1,295	102	125	227	17.53
Catanduanes	510	122	94	216	42.35
Masbate	927	101	101	202	21.79
Sorsogon	1,384	79	67	146	10.55
City of Naga	2,500	29	25	54	2.16
**Region 6**	**7,839**	**683**	**757**	**1,440**	**18.37**
Aklan	391	76	70	146	37.34
Antique	1,528	27	18	45	2.95
Capiz	759	39	50	89	11.73
Guimaras	267	46	65	111	41.57
Iloilo	1,410	157	138	295	20.92
Negros Occidental	2,552	262	335	597	23.39
City of Bacolod	594	18	26	44	7.41
City of Iloilo	338	58	55	113	33.43
**Region 7**	**9,640**	**1,079**	**1,258**	**2,337**	**24.24**
Bohol	1,891	107	139	246	13.01
Cebu	3,845	176	199	375	9.75
Negros Oriental	997	78	69	147	14.74
Siquijor	117	2	1	3	2.56
City of Cebu	891	180	180	360	40.40
City of Lapu-Lapu	729	125	156	281	38.55
City of Mandaue	1,170	411	514	925	79.06
**Region 8**	**3,995**	**427**	**497**	**924**	**23.13**
Biliran	270	53	58	111	41.11
Eastern Samar	238	12	10	22	9.24
Leyte	405	128	182	310	76.54
Northern Samar	998	33	32	65	6.51
Southern Leyte	496	55	39	94	18.95
Samar	783	35	49	84	10.73
Ormoc City	773	103	118	221	28.59
City of Tacloban	32	8	9	17	53.13

Table 2.C.2.2- Nutrition Services for Infants and Children

Infants born preterm or with low birth weight given iron supplements

Philippines, 2023

Area	Total No. of live births with low birth weight	Infants preterm or with LBW (< 2500 grams) given Iron			
		Male	Female	Total	%
**Region 9**	**1,803**	**279**	**341**	**620**	**34.39**
Zamboanga del Norte	687	101	147	248	36.10
Zamboanga del Sur	370	76	92	168	45.41
Zamboanga Sibugay	540	40	40	80	14.81
City of Isabela	4	0	1	1	25.00
City of Zamboanga	202	62	61	123	60.89
**Region 10**	**4,020**	**293**	**362**	**655**	**16.29**
Bukidnon	947	146	217	363	38.33
Camiguin	66	4	3	7	10.61
Lanao del Norte	58	2	5	7	12.07
Misamis Occidental	203	51	52	103	50.74
Misamis Oriental	214	41	43	84	39.25
City of Cagayan De Oro	2,354	16	10	26	1.10
City of Iligan	178	33	32	65	36.52
**Region 11**	**4,391**	**1,011**	**1,001**	**2,012**	**45.82**
Davao de Oro	726	200	194	394	54.27
Davao del Norte	998	224	235	459	45.99
Davao Oriental	420	128	126	254	60.48
Davao del Sur	465	90	83	173	37.20
Davao Occidental	236	36	45	81	34.32
City of Davao	1,546	333	318	651	42.11
**Region 12**	**1,840**	**477**	**607**	**1,084**	**58.91**
Cotabato	621	108	111	219	35.27
Sarangani	104	22	24	46	44.23
South Cotabato	530	134	223	357	67.36
Sultan Kudarat	430	203	227	430	100.00
City of General Santos	155	10	22	32	20.65
**BARMM**	**1,678**	**78**	**90**	**168**	**10.01**
Basilan	101	1	5	6	5.94
Lanao del Sur	651	24	30	54	8.29
Maguindanao	443	21	11	32	7.22

Table 2.C.2.2- Nutrition Services for Infants and Children

Infants born preterm or with low birth weight given iron supplements

Philippines, 2023

Area	Total No. of live births with low birth weight	Infants preterm or with LBW (< 2500 grams) given Iron			
		Male	Female	Total	%
Sulu	350	9	12	21	6.00
Tawi-Tawi	61	6	15	21	34.43
SGA	2	2	0	2	100.00
City of Cotabato	70	15	17	32	45.71
**CARAGA**	**2,481**	**224**	**275**	**499**	**20.11**
Agusan del Norte	210	57	62	119	56.67
Agusan del Sur	336	5	10	15	4.46
Surigao del Norte	791	32	40	72	9.10
Surigao del Sur	735	118	140	258	35.10
Dinagat Islands	134	10	16	26	19.40
City of Butuan	275	2	7	9	3.27

Table 2.C.2.2- Nutrition Services for Infants and Children
Infants 6-11 mos. old given Vitamin A supplementation
Philippines, 2023

Area	Eligible Pop (6-11 mos. old)	Infants 6-11 mos. old Given Vitamin A			
		Male	Female	Total	%
**Philippines**	**1,114,805**	**724,508**	**710,978**	**1,435,486**	**128.77**
**NCR**	**131,452**	**101,410**	**101,043**	**202,453**	**154.01**
Malabon City	3,702	2,900	2,841	5,741	155.08
Navotas City	2,614	3,354	3,686	7,040	269.32
Valenzuela City	6,752	7,113	7,150	14,263	211.24
Caloocan City	16,684	8,250	8,305	16,555	99.23
Marikina City	4,178	2,562	2,389	4,951	118.50
Pasig City	8,854	4,659	4,427	9,086	102.62
Pateros	563	400	384	784	139.25
Taguig City	9,510	7,371	7,197	14,568	153.19
Quezon City	29,642	31,412	31,449	62,861	212.07
Makati City	5,491	2,308	2,214	4,522	82.35
Mandaluyong City	3,970	2,737	3,004	5,741	144.61
San Juan City	1,092	518	476	994	91.03
Manila City	17,496	15,079	14,621	29,700	169.75
Las Piñas City	5,642	5,087	5,009	10,096	178.94
Muntinlupa City	4,946	2,569	2,740	5,309	107.34
Parañaque City	6,649	3,101	2,921	6,022	90.57
Pasay City	3,667	1,990	2,230	4,220	115.08
**CAR**	**16,268**	**10,494**	**10,128**	**20,622**	**126.76**
Abra	1,982	1,394	1,382	2,776	140.06
Apayao	1,188	1,069	1,000	2,069	174.16
Benguet	4,481	2,387	2,269	4,656	103.91
Ifugao	2,135	1,141	1,185	2,326	108.95
Kalinga	2,355	1,235	1,106	2,341	99.41
Mountain Province	1,387	1,027	922	1,949	140.52
City of Baguio	2,740	2,241	2,264	4,505	164.42
**Region 1**	**49,645**	**24,428**	**22,779**	**47,207**	**95.09**
Ilocos Norte	4,730	2,487	2,270	4,757	100.57
Ilocos Sur	5,342	2,810	2,643	5,453	102.08
La Union	7,041	2,970	2,725	5,695	80.88
Pangasinan	30,853	15,106	14,176	29,282	94.91
City of Dagupan	1,679	1,055	965	2,020	120.31

Table 2.C.2.2- Nutrition Services for Infants and Children
Infants 6-11 mos. old given Vitamin A supplementation
Philippines, 2023

Area	Eligible Pop (6-11 mos. old)	Infants 6-11 mos. old Given Vitamin A			
		Male	Female	Total	%
**Region 2**	**33,156**	**28,001**	**26,128**	**54,129**	**163.26**
Batanes	133	59	59	118	88.72
Cagayan	10,799	8,791	7,831	16,622	153.92
Isabela	14,209	12,469	11,854	24,323	171.18
Nueva Vizcaya	4,546	4,259	4,165	8,424	185.31
Quirino	1,988	1,367	1,319	2,686	135.11
City of Santiago	1,481	1,056	900	1,956	132.07
**Region 3**	**118,351**	**77,327**	**76,060**	**153,387**	**129.60**
Aurora	2,336	2,064	2,012	4,076	174.49
Bataan	8,818	6,640	7,624	14,264	161.76
Bulacan	34,076	25,817	24,374	50,191	147.29
Nueva Ecija	22,142	11,903	11,469	23,372	105.56
Pampanga	23,067	15,973	16,038	32,011	138.77
Tarlac	15,033	6,650	6,606	13,256	88.18
Zambales	5,984	2,398	2,306	4,704	78.61
City of Angeles	4,592	4,050	3,810	7,860	171.17
City of Olongapo	2,303	1,832	1,821	3,653	158.62
**Region 4A**	**156,001**	**102,064**	**101,925**	**203,989**	**130.76**
Batangas	27,177	14,763	14,187	28,950	106.52
Cavite	39,399	25,462	25,022	50,484	128.14
Laguna	31,107	22,043	21,204	43,247	139.03
Quezon	21,003	12,183	11,543	23,726	112.96
Rizal	34,204	24,926	27,235	52,161	152.50
City of Lucena	3,111	2,687	2,734	5,421	174.25
**Region 4B**	**33,790**	**20,121**	**19,709**	**39,830**	**117.88**
Marinduque	2,179	2,490	2,352	4,842	222.21
Occidental Mindoro	5,896	2,738	2,645	5,383	91.30
Oriental Mindoro	9,689	5,389	5,302	10,691	110.34
Palawan	10,346	5,582	5,571	11,153	107.80
Romblon	2,851	1,712	1,684	3,396	119.12
City of Puerto Princesa	2,829	2,210	2,155	4,365	154.29

Table 2.C.2.2- Nutrition Services for Infants and Children
Infants 6-11 mos. old given Vitamin A supplementation
Philippines, 2023

Area	Eligible Pop (6-11 mos. old)	Infants 6-11 mos. old Given Vitamin A			
		Male	Female	Total	%
**Region 5**	**69,494**	**40,510**	**39,007**	**79,517**	**114.42**
Albay	14,076	8,227	8,015	16,242	115.39
Camarines Norte	7,487	3,435	3,244	6,679	89.21
Camarines Sur	22,245	12,087	11,458	23,545	105.84
Catanduanes	2,865	2,032	1,939	3,971	138.60
Masbate	11,476	7,189	7,169	14,358	125.11
Sorsogon	9,240	5,924	5,435	11,359	122.93
City of Naga	2,105	1,616	1,747	3,363	159.76
**Region 6**	**76,022**	**39,277**	**38,825**	**78,102**	**102.74**
Aklan	5,790	3,182	3,067	6,249	107.93
Antique	6,243	3,911	3,835	7,746	124.07
Capiz	7,206	4,739	4,295	9,034	125.37
Guimaras	1,720	1,227	1,150	2,377	138.20
Iloilo	18,950	7,479	8,244	15,723	82.97
Negros Occidental	26,442	12,593	12,154	24,747	93.59
City of Bacolod	5,217	2,832	2,802	5,634	107.99
City of Iloilo	4,454	3,314	3,278	6,592	148.00
**Region 7**	**84,357**	**52,618**	**52,444**	**105,062**	**124.54**
Bohol	13,690	7,359	7,034	14,393	105.14
Cebu	36,629	22,179	23,268	45,447	124.07
Negros Oriental	14,644	8,235	8,067	16,302	111.32
Siquijor	900	475	470	945	105.00
City of Cebu	9,680	6,961	6,344	13,305	137.45
City of Lapu-Lapu	5,357	4,119	3,972	8,091	151.04
City of Mandaue	3,457	3,290	3,289	6,579	190.31
**Region 8**	**49,125**	**29,076**	**27,752**	**56,828**	**115.68**
Biliran	1,748	1,191	1,157	2,348	134.32
Eastern Samar	5,114	2,831	2,615	5,446	106.49
Leyte	16,469	10,285	9,954	20,239	122.89
Northern Samar	7,313	3,767	3,549	7,316	100.04
Southern Leyte	3,973	2,002	1,932	3,934	99.02
Samar	9,002	6,215	5,827	12,042	133.77
Ormoc City	2,694	1,362	1,331	2,693	99.96
City of Tacloban	2,812	1,423	1,387	2,810	99.93

Table 2.C.2.2- Nutrition Services for Infants and Children
Infants 6-11 mos. old given Vitamin A supplementation
Philippines, 2023

Area	Eligible Pop (6-11 mos. old)	Infants 6-11 mos. old Given Vitamin A			
		Male	Female	Total	%
**Region 9**	**43,395**	**27,418**	**27,271**	**54,689**	**126.03**
Zamboanga del Norte	12,781	7,057	6,994	14,051	109.94
Zamboanga del Sur	12,231	7,386	7,282	14,668	119.92
Zamboanga Sibugay	7,123	4,507	4,488	8,995	126.28
City of Isabela	1,624	694	712	1,406	86.58
City of Zamboanga	9,636	7,774	7,795	15,569	161.57
**Region 10**	**55,577**	**43,785**	**42,877**	**86,662**	**155.93**
Bukidnon	16,927	12,582	12,703	25,285	149.38
Camiguin	892	428	416	844	94.62
Lanao del Norte	9,100	7,051	6,825	13,876	152.48
Misamis Occidental	6,413	3,996	3,942	7,938	123.78
Misamis Oriental	11,196	7,388	7,433	14,821	132.38
City of Cagayan De Oro	7,195	8,595	8,055	16,650	231.41
City of Iligan	3,854	3,745	3,503	7,248	188.06
**Region 11**	**54,069**	**43,251**	**40,663**	**83,914**	**155.20**
Davao de Oro	7,733	7,847	7,329	15,176	196.25
Davao del Norte	11,841	8,656	8,330	16,986	143.45
Davao Oriental	6,064	3,428	3,280	6,708	110.62
Davao del Sur	6,659	6,148	5,528	11,676	175.34
Davao Occidental	3,363	2,135	2,062	4,197	124.80
City of Davao	18,409	15,037	14,134	29,171	158.46
**Region 12**	**48,192**	**31,144**	**30,502**	**61,646**	**127.92**
Cotabato	13,506	7,147	7,093	14,240	105.43
Sarangani	6,730	5,234	4,987	10,221	151.87
South Cotabato	10,901	7,960	7,768	15,728	144.28
Sultan Kudarat	9,819	7,285	7,131	14,416	146.82
City of General Santos	7,236	3,518	3,523	7,041	97.31
**BARMM**	**67,735**	**35,609**	**36,400**	**72,009**	**106.31**
Basilan	5,917	2,225	2,249	4,474	75.61
Lanao del Sur	17,274	10,836	11,462	22,298	129.08
Maguindanao	21,085	10,289	10,234	20,523	97.33

Table 2.C.2.2- Nutrition Services for Infants and Children
Infants 6-11 mos. old given Vitamin A supplementation
Philippines, 2023

Area	Eligible Pop (6-11 mos. old)	Infants 6-11 mos. old Given Vitamin A			
		Male	Female	Total	%
Sulu	10,897	6,245	6,568	12,813	117.58
Tawi-Tawi	6,413	3,129	3,224	6,353	99.06
SGA	2,586	1,047	1,003	2,050	79.27
City of Cotabato	3,563	1,838	1,660	3,498	98.18
**CARAGA**	**28,176**	**17,975**	**17,465**	**35,440**	**125.78**
Agusan del Norte	3,728	2,443	2,476	4,919	131.95
Agusan del Sur	8,175	5,526	5,621	11,147	136.35
Surigao del Norte	5,260	1,496	1,264	2,760	52.47
Surigao del Sur	6,521	4,192	3,862	8,054	123.51
Dinagat Islands	1,111	478	551	1,029	92.62
City of Butuan	3,381	3,840	3,691	7,531	222.74

Table 2.C.2.2- Nutrition Services for Infants and Children
Children 12-59 mos. old given Vitamin A supplementation
Philippines, 2023

Area	Eligible Pop (12-59 mos. old)	Children 12-59 mos. Old Given Vitamin A			
		Male	Female	Total	%
**Philippines**	**9,146,807**	**3,131,566**	**3,041,629**	**6,173,195**	**67.49**
**NCR**	**1,048,668**	**441,271**	**436,950**	**878,221**	**83.75**
Malabon City	29,472	11,075	11,556	22,631	76.79
Navotas City	20,859	9,329	9,389	18,718	89.74
Valenzuela City	54,902	36,120	36,598	72,718	132.45
Caloocan City	138,744	91,057	82,814	173,871	125.32
Marikina City	34,632	4,906	4,758	9,664	27.90
Pasig City	65,711	27,106	26,193	53,299	81.11
Pateros	4,824	1,191	1,249	2,440	50.58
Taguig City	74,943	39,286	39,039	78,325	104.51
Quezon City	238,612	94,806	95,985	190,791	79.96
Makati City	42,518	23,674	23,991	47,665	112.11
Mandaluyong City	31,561	13,993	15,393	29,386	93.11
San Juan City	8,619	3,769	3,991	7,760	90.03
Manila City	139,020	20,861	21,199	42,060	30.25
Las Piñas City	45,251	10,325	10,619	20,944	46.28
Muntinlupa City	39,143	19,235	20,081	39,316	100.44
Parañaque City	53,316	23,372	22,295	45,667	85.65
Pasay City	26,541	11,166	11,800	22,966	86.53
**CAR**	**131,920**	**44,676**	**41,744**	**86,420**	**65.51**
Abra	16,360	6,666	6,212	12,878	78.72
Apayao	9,270	3,576	3,490	7,066	76.22
Benguet	37,084	9,908	9,388	19,296	52.03
Ifugao	17,191	7,525	6,854	14,379	83.64
Kalinga	18,590	5,519	4,977	10,496	56.46
Mountain Province	11,394	4,454	4,025	8,479	74.42
City of Baguio	22,031	7,028	6,798	13,826	62.76
**Region 1**	**391,908**	**117,550**	**116,906**	**234,456**	**59.82**
Ilocos Norte	38,194	10,797	10,541	21,338	55.87
Ilocos Sur	43,632	16,322	15,729	32,051	73.46
La Union	55,447	23,706	22,293	45,999	82.96
Pangasinan	241,204	63,312	65,013	128,325	53.20
City of Dagupan	13,431	3,413	3,330	6,743	50.20

Table 2.C.2.2- Nutrition Services for Infants and Children

Children 12-59 mos. old given Vitamin A supplementation

Philippines, 2023

Area	Eligible Pop (12-59 mos. old)	Children 12-59 mos. Old Given Vitamin A			
		Male	Female	Total	%
**Region 2**	**273,976**	**114,652**	**111,209**	**225,861**	**82.44**
Batanes	1,131	178	410	588	51.99
Cagayan	90,741	27,713	25,267	52,980	58.39
Isabela	116,264	53,389	54,422	107,811	92.73
Nueva Vizcaya	38,816	20,490	19,007	39,497	101.75
Quirino	15,691	6,399	6,161	12,560	80.05
City of Santiago	11,333	6,483	5,942	12,425	109.64
**Region 3**	**982,471**	**369,487**	**355,677**	**725,164**	**73.81**
Aurora	19,792	8,816	8,428	17,244	87.13
Bataan	70,606	31,024	29,165	60,189	85.25
Bulacan	286,564	119,788	115,017	234,805	81.94
Nueva Ecija	183,214	76,120	72,991	149,111	81.39
Pampanga	191,692	69,030	67,660	136,690	71.31
Tarlac	120,696	35,682	34,390	70,072	58.06
Zambales	49,599	9,814	9,426	19,240	38.79
City of Angeles	41,866	13,348	12,870	26,218	62.62
City of Olongapo	18,442	5,865	5,730	11,595	62.87
**Region 4A**	**1,282,073**	**333,956**	**323,314**	**657,270**	**51.27**
Batangas	228,336	71,737	67,832	139,569	61.12
Cavite	333,432	86,447	83,926	170,373	51.10
Laguna	257,644	60,193	58,490	118,683	46.06
Quezon	164,813	56,297	53,041	109,338	66.34
Rizal	273,505	49,343	50,302	99,645	36.43
City of Lucena	24,343	9,939	9,723	19,662	80.77
**Region 4B**	**273,659**	**110,355**	**106,588**	**216,943**	**79.27**
Marinduque	19,647	13,624	12,784	26,408	134.41
Occidental Mindoro	47,933	21,963	21,369	43,332	90.40
Oriental Mindoro	75,450	29,580	28,338	57,918	76.76
Palawan	84,854	22,189	22,209	44,398	52.32
Romblon	22,821	13,911	13,387	27,298	119.62
City of Puerto Princesa	22,954	9,088	8,501	17,589	76.63

Table 2.C.2.2- Nutrition Services for Infants and Children
Children 12-59 mos. old given Vitamin A supplementation
Philippines, 2023

Area	Eligible Pop (12-59 mos. old)	Children 12-59 mos. Old Given Vitamin A			
		Male	Female	Total	%
**Region 5**	**563,991**	**186,342**	**177,866**	**364,208**	**64.58**
Albay	115,352	41,098	39,370	80,468	69.76
Camarines Norte	61,493	22,526	22,136	44,662	72.63
Camarines Sur	179,598	50,500	48,230	98,730	54.97
Catanduanes	24,838	7,871	7,508	15,379	61.92
Masbate	91,183	26,546	24,726	51,272	56.23
Sorsogon	74,885	29,796	27,574	57,370	76.61
City of Naga	16,642	8,005	8,322	16,327	98.11
**Region 6**	**622,616**	**180,285**	**175,648**	**355,933**	**57.17**
Aklan	46,468	7,390	7,772	15,162	32.63
Antique	50,529	12,099	11,512	23,611	46.73
Capiz	61,188	21,936	21,541	43,477	71.05
Guimaras	14,733	4,504	4,354	8,858	60.12
Iloilo	153,196	63,230	62,355	125,585	81.98
Negros Occidental	216,379	53,395	51,393	104,788	48.43
City of Bacolod	43,741	4,470	4,356	8,826	20.18
City of Iloilo	36,382	13,261	12,365	25,626	70.44
**Region 7**	**675,230**	**189,652**	**182,177**	**371,829**	**55.07**
Bohol	108,880	41,046	38,180	79,226	72.76
Cebu	292,954	69,268	68,767	138,035	47.12
Negros Oriental	119,479	39,533	37,277	76,810	64.29
Siquijor	6,402	2,397	2,324	4,721	73.74
City of Cebu	78,931	23,305	21,549	44,854	56.83
City of Lapu-Lapu	42,039	8,371	7,861	16,232	38.61
City of Mandaue	26,545	5,732	6,219	11,951	45.02
**Region 8**	**392,835**	**115,832**	**111,613**	**227,445**	**57.90**
Biliran	15,130	5,590	5,160	10,750	71.05
Eastern Samar	39,097	14,186	13,362	27,548	70.46
Leyte	134,060	37,766	36,748	74,514	55.58
Northern Samar	58,605	21,000	20,754	41,754	71.25
Southern Leyte	32,590	6,917	6,397	13,314	40.85
Samar	70,667	18,977	18,612	37,589	53.19
Ormoc City	20,482	6,685	6,075	12,760	62.30
City of Tacloban	22,204	4,711	4,505	9,216	41.51

Table 2.C.2.2- Nutrition Services for Infants and Children

Children 12-59 mos. old given Vitamin A supplementation

Philippines, 2023

Area	Eligible Pop (12-59 mos. old)	Children 12-59 mos. Old Given Vitamin A			
		Male	Female	Total	%
**Region 9**	**352,418**	**80,504**	**78,956**	**159,460**	**45.25**
Zamboanga del Norte	98,637	29,634	29,037	58,671	59.48
Zamboanga del Sur	97,160	19,970	19,420	39,390	40.54
Zamboanga Sibugay	60,678	14,080	13,645	27,725	45.69
City of Isabela	13,695	2,190	2,218	4,408	32.19
City of Zamboanga	82,248	14,630	14,636	29,266	35.58
**Region 10**	**455,672**	**232,898**	**222,121**	**455,019**	**99.86**
Bukidnon	144,267	69,836	65,155	134,991	93.57
Camiguin	7,321	2,778	2,698	5,476	74.80
Lanao del Norte	74,627	39,555	35,457	75,012	100.52
Misamis Occidental	49,512	20,659	19,986	40,645	82.09
Misamis Oriental	88,020	52,732	50,721	103,453	117.53
City of Cagayan De Oro	59,656	39,320	40,032	79,352	133.02
City of Iligan	32,269	8,018	8,072	16,090	49.86
**Region 11**	**449,181**	**233,880**	**223,376**	**457,256**	**101.80**
Davao de Oro	66,024	42,135	38,792	80,927	122.57
Davao del Norte	97,797	54,857	53,852	108,709	111.16
Davao Oriental	48,998	19,549	18,766	38,315	78.20
Davao del Sur	59,194	29,968	27,918	57,886	97.79
Davao Occidental	31,702	11,479	11,277	22,756	71.78
City of Davao	145,466	75,892	72,771	148,663	102.20
**Region 12**	**395,854**	**141,023**	**138,414**	**279,437**	**70.59**
Cotabato	116,778	42,138	40,699	82,837	70.94
Sarangani	54,086	24,684	24,017	48,701	90.04
South Cotabato	86,294	42,822	42,520	85,342	98.90
Sultan Kudarat	78,587	22,495	22,062	44,557	56.70
City of General Santos	60,109	8,884	9,116	18,000	29.95
**BARMM**	**617,557**	**163,742**	**165,588**	**329,330**	**53.33**
Basilan	53,087	8,836	9,493	18,329	34.53
Lanao del Sur	151,974	78,558	79,631	158,189	104.09
Maguindanao	189,608	17,755	17,599	35,354	18.65

Table 2.C.2.2- Nutrition Services for Infants and Children

Children 12-59 mos. old given Vitamin A supplementation

Philippines, 2023

Area	Eligible Pop (12-59 mos. old)	Children 12-59 mos. Old Given Vitamin A			
		Male	Female	Total	%
Sulu	109,806	27,064	29,216	56,280	51.25
Tawi-Tawi	56,967	11,493	11,670	23,163	40.66
SGA	23,873	3,375	3,647	7,022	29.41
City of Cotabato	32,242	16,661	14,332	30,993	96.13
**CARAGA**	**236,778**	**75,461**	**73,482**	**148,943**	**62.90**
Agusan del Norte	32,033	9,752	9,072	18,824	58.76
Agusan del Sur	67,299	23,852	23,908	47,760	70.97
Surigao del Norte	42,408	0	0	0	0.00
Surigao del Sur	55,577	23,780	23,158	46,938	84.46
Dinagat Islands	10,955	1,864	1,912	3,776	34.47
City of Butuan	28,506	16,213	15,432	31,645	111.01

Table 2.C.2.3 - Nutrition Services for Infants and Children
Infants 6-11 mos. old who completed Micronutrient Powder supplementation
Philippines, 2023

Area	Eligible Pop (6-11 mos. old)	Infants 6-11 mos. old who completed MNP supplementation			
		Male	Female	Total	%
**Philippines**	**1,114,805**	**111,994**	**113,958**	**225,952**	**20.27**
**NCR**	**131,452**	**50,107**	**53,178**	**103,285**	**78.57**
Malabon City	3,702	1,653	1,624	3,277	88.52
Navotas City	2,614	270	345	615	23.53
Valenzuela City	6,752	745	729	1,474	21.83
Caloocan City	16,684	747	813	1,560	9.35
Marikina City	4,178	454	456	910	21.78
Pasig City	8,854	329	373	702	7.93
Pateros	563	83	72	155	27.53
Taguig City	9,510	4,628	4,692	9,320	98.00
Quezon City	29,642	32,818	35,308	68,126	229.83
Makati City	5,491	1,324	1,302	2,626	47.82
Mandaluyong City	3,970	853	947	1,800	45.34
San Juan City	1,092	433	437	870	79.67
Manila City	17,496	371	397	768	4.39
Las Piñas City	5,642	285	274	559	9.91
Muntinlupa City	4,946	2,437	2,731	5,168	104.49
Parañaque City	6,649	907	898	1,805	27.15
Pasay City	3,667	1,770	1,780	3,550	96.81
**CAR**	**16,268**	**697**	**701**	**1,398**	**8.59**
Abra	1,982	159	155	314	15.84
Apayao	1,188	27	20	47	3.96
Benguet	4,481	169	176	345	7.70
Ifugao	2,135	171	157	328	15.36
Kalinga	2,355	85	106	191	8.11
Mountain Province	1,387	42	44	86	6.20
City of Baguio	2,740	44	43	87	3.18
**Region 1**	**49,645**	**8,129**	**7,471**	**15,600**	**31.42**
Ilocos Norte	4,730	1,369	1,259	2,628	55.56
Ilocos Sur	5,342	2,452	2,232	4,684	87.68
La Union	7,041	285	289	574	8.15
Pangasinan	30,853	3,793	3,463	7,256	23.52
City of Dagupan	1,679	230	228	458	27.28

Table 2.C.2.3 - Nutrition Services for Infants and Children
Infants 6-11 mos. old who completed Micronutrient Powder supplementation
Philippines, 2023

Area	Eligible Pop (6-11 mos. old)	Infants 6-11 mos. old who completed MNP supplementation			
		Male	Female	Total	%
**Region 2**	**33,156**	**3,657**	**4,038**	**7,695**	**23.21**
Batanes	133	2	2	4	3.01
Cagayan	10,799	845	1,333	2,178	20.17
Isabela	14,209	1,844	1,817	3,661	25.77
Nueva Vizcaya	4,546	761	685	1,446	31.81
Quirino	1,988	169	171	340	17.10
City of Santiago	1,481	36	30	66	4.46
**Region 3**	**118,351**	**6,134**	**5,900**	**12,034**	**10.17**
Aurora	2,336	219	178	397	16.99
Bataan	8,818	343	361	704	7.98
Bulacan	34,076	3,179	3,097	6,276	18.42
Nueva Ecija	22,142	1,045	950	1,995	9.01
Pampanga	23,067	445	421	866	3.75
Tarlac	15,033	634	626	1,260	8.38
Zambales	5,984	71	55	126	2.11
City of Angeles	4,592	171	199	370	8.06
City of Olongapo	2,303	27	13	40	1.74
**Region 4A**	**156,001**	**9,146**	**8,947**	**18,093**	**11.60**
Batangas	27,177	836	750	1,586	5.84
Cavite	39,399	2,084	2,024	4,108	10.43
Laguna	31,107	2,341	2,183	4,524	14.54
Quezon	21,003	472	509	981	4.67
Rizal	34,204	3,201	3,306	6,507	19.02
City of Lucena	3,111	212	175	387	12.44
**Region 4B**	**33,790**	**736**	**722**	**1,458**	**4.31**
Marinduque	2,179	106	106	212	9.73
Occidental Mindoro	5,896	30	25	55	0.93
Oriental Mindoro	9,689	180	187	367	3.79
Palawan	10,346	370	363	733	7.08
Romblon	2,851	48	41	89	3.12
City of Puerto Princesa	2,829	2	0	2	0.07

Table 2.C.2.3 - Nutrition Services for Infants and Children
Infants 6-11 mos. old who completed Micronutrient Powder supplementation
Philippines, 2023

Area	Eligible Pop (6-11 mos. old)	Infants 6-11 mos. old who completed MNP supplementation			
		Male	Female	Total	%
**Region 5**	**69,494**	**6,408**	**6,199**	**12,607**	**18.14**
Albay	14,076	3,107	2,927	6,034	42.87
Camarines Norte	7,487	239	249	488	6.52
Camarines Sur	22,245	631	606	1,237	5.56
Catanduanes	2,865	188	179	367	12.81
Masbate	11,476	1,921	1,913	3,834	33.41
Sorsogon	9,240	198	214	412	4.46
City of Naga	2,105	124	111	235	11.16
**Region 6**	**76,022**	**3,426**	**3,370**	**6,796**	**8.94**
Aklan	5,790	363	352	715	12.35
Antique	6,243	143	124	267	4.28
Capiz	7,206	216	244	460	6.38
Guimaras	1,720	129	164	293	17.03
Iloilo	18,950	1,461	1,286	2,747	14.50
Negros Occidental	26,442	1,082	1,172	2,254	8.52
City of Bacolod	5,217	32	28	60	1.15
City of Iloilo	4,454	0	0	0	0.00
**Region 7**	**84,357**	**2,821**	**2,981**	**5,802**	**6.88**
Bohol	13,690	126	100	226	1.65
Cebu	36,629	816	1,046	1,862	5.08
Negros Oriental	14,644	313	326	639	4.36
Siquijor	900	26	34	60	6.67
City of Cebu	9,680	113	87	200	2.07
City of Lapu-Lapu	5,357	1,396	1,365	2,761	51.54
City of Mandaue	3,457	31	23	54	1.56
**Region 8**	**49,125**	**2,975**	**2,764**	**5,739**	**11.68**
Biliran	1,748	90	135	225	12.87
Eastern Samar	5,114	541	454	995	19.46
Leyte	16,469	1,283	1,179	2,462	14.95
Northern Samar	7,313	392	378	770	10.53
Southern Leyte	3,973	171	169	340	8.56
Samar	9,002	479	417	896	9.95
Ormoc City	2,694	19	32	51	1.89
City of Tacloban	2,812	0	0	0	0.00

Table 2.C.2.3 - Nutrition Services for Infants and Children
Infants 6-11 mos. old who completed Micronutrient Powder supplementation
Philippines, 2023

Area	Eligible Pop (6-11 mos. old)	Infants 6-11 mos. old who completed MNP supplementation			
		Male	Female	Total	%
**Region 9**	**43,395**	**2,183**	**2,112**	**4,295**	**9.90**
Zamboanga del Norte	12,781	781	749	1,530	11.97
Zamboanga del Sur	12,231	526	507	1,033	8.45
Zamboanga Sibugay	7,123	45	61	106	1.49
City of Isabela	1,624	481	440	921	56.71
City of Zamboanga	9,636	350	355	705	7.32
**Region 10**	**55,577**	**2,371**	**2,466**	**4,837**	**8.70**
Bukidnon	16,927	1,016	1,045	2,061	12.18
Camiguin	892	0	0	0	0.00
Lanao del Norte	9,100	298	336	634	6.97
Misamis Occidental	6,413	442	492	934	14.56
Misamis Oriental	11,196	98	111	209	1.87
City of Cagayan De Oro	7,195	292	269	561	7.80
City of Iligan	3,854	225	213	438	11.36
**Region 11**	**54,069**	**6,542**	**6,482**	**13,024**	**24.09**
Davao de Oro	7,733	853	823	1,676	21.67
Davao del Norte	11,841	569	719	1,288	10.88
Davao Oriental	6,064	273	301	574	9.47
Davao del Sur	6,659	649	570	1,219	18.31
Davao Occidental	3,363	134	128	262	7.79
City of Davao	18,409	4,064	3,941	8,005	43.48
**Region 12**	**48,192**	**1,846**	**1,781**	**3,627**	**7.53**
Cotabato	13,506	268	284	552	4.09
Sarangani	6,730	88	84	172	2.56
South Cotabato	10,901	677	620	1,297	11.90
Sultan Kudarat	9,819	190	167	357	3.64
City of General Santos	7,236	623	626	1,249	17.26
**BARMM**	**67,735**	**2,897**	**2,927**	**5,824**	**8.60**
Basilan	5,917	137	137	274	4.63
Lanao del Sur	17,274	1,477	1,642	3,119	18.06
Maguindanao	21,085	141	159	300	1.42

Table 2.C.2.3 - Nutrition Services for Infants and Children
Infants 6-11 mos. old who completed Micronutrient Powder supplementation
Philippines, 2023

Area	Eligible Pop (6-11 mos. old)	Infants 6-11 mos. old who completed MNP supplementation			
		Male	Female	Total	%
Sulu	10,897	0	0	0	0.00
Tawi-Tawi	6,413	326	316	642	10.01
SGA	2,586	13	20	33	1.28
City of Cotabato	3,563	803	653	1,456	40.86
**CARAGA**	**28,176**	**1,919**	**1,919**	**3,838**	**13.62**
Agusan del Norte	3,728	88	89	177	4.75
Agusan del Sur	8,175	846	781	1,627	19.90
Surigao del Norte	5,260	510	547	1,057	20.10
Surigao del Sur	6,521	202	226	428	6.56
Dinagat Islands	1,111	13	23	36	3.24
City of Butuan	3,381	260	253	513	15.17

Table 2.C.2.3 - Nutrition Services for Infants and Children
Children 12-23 mos. old who completed Micronutrient Powder supplementation
Philippines, 2023

Area	Eligible Pop (12-23 mos. old)	Children 12-23 mos. old who completed MNP supplementation			
		Male	Female	Total	%
**Philippines**	**2,252,411**	**118,119**	**116,182**	**234,301**	**10.40**
**NCR**	**266,383**	**29,438**	**29,167**	**58,605**	**22.00**
Malabon City	7,375	2,669	2,633	5,302	71.89
Navotas City	5,357	425	483	908	16.95
Valenzuela City	14,356	500	565	1,065	7.42
Caloocan City	35,268	829	770	1,599	4.53
Marikina City	8,790	284	302	586	6.67
Pasig City	16,733	284	327	611	3.65
Pateros	1,211	94	107	201	16.60
Taguig City	19,190	8,717	8,330	17,047	88.83
Quezon City	60,863	2,702	2,307	5,009	8.23
Makati City	10,907	1,365	1,390	2,755	25.26
Mandaluyong City	8,072	1,567	1,988	3,555	44.04
San Juan City	2,256	381	422	803	35.59
Manila City	34,931	188	172	360	1.03
Las Piñas City	11,132	309	312	621	5.58
Muntinlupa City	9,997	4,968	5,206	10,174	101.77
Parañaque City	13,434	748	655	1,403	10.44
Pasay City	6,511	3,408	3,198	6,606	101.46
**CAR**	**32,192**	**1,114**	**1,111**	**2,225**	**6.91**
Abra	3,980	265	249	514	12.91
Apayao	2,208	49	44	93	4.21
Benguet	9,009	297	328	625	6.94
Ifugao	4,184	276	265	541	12.93
Kalinga	4,591	88	99	187	4.07
Mountain Province	2,734	48	50	98	3.58
City of Baguio	5,486	91	76	167	3.04
**Region 1**	**97,382**	**12,006**	**12,713**	**24,719**	**25.38**
Ilocos Norte	9,140	865	847	1,712	18.73
Ilocos Sur	10,310	6,056	6,301	12,357	119.85
La Union	13,460	436	419	855	6.35
Pangasinan	61,067	3,146	3,730	6,876	11.26
City of Dagupan	3,405	1,503	1,416	2,919	85.73

Table 2.C.2.3 - Nutrition Services for Infants and Children
Children 12-23 mos. old who completed Micronutrient Powder supplementation
Philippines, 2023

Area	Eligible Pop (12-23 mos. old)	Children 12-23 mos. old who completed MNP supplementation			
		Male	Female	Total	%
**Region 2**	**65,674**	**4,799**	**4,314**	**9,113**	**13.88**
Batanes	266	0	2	2	0.75
Cagayan	21,577	804	732	1,536	7.12
Isabela	28,159	2,878	2,451	5,329	18.92
Nueva Vizcaya	9,159	843	810	1,653	18.05
Quirino	3,760	221	250	471	12.53
City of Santiago	2,753	53	69	122	4.43
**Region 3**	**243,629**	**11,104**	**10,892**	**21,996**	**9.03**
Aurora	4,738	495	483	978	20.64
Bataan	17,375	524	533	1,057	6.08
Bulacan	71,851	5,178	4,895	10,073	14.02
Nueva Ecija	44,899	1,904	1,936	3,840	8.55
Pampanga	48,392	1,177	1,182	2,359	4.87
Tarlac	29,349	705	734	1,439	4.90
Zambales	12,156	163	112	275	2.26
City of Angeles	10,314	898	954	1,852	17.96
City of Olongapo	4,555	60	63	123	2.70
**Region 4A**	**319,390**	**11,315**	**11,059**	**22,374**	**7.01**
Batangas	57,116	664	589	1,253	2.19
Cavite	81,567	2,182	2,128	4,310	5.28
Laguna	63,067	4,652	4,472	9,124	14.47
Quezon	42,060	709	728	1,437	3.42
Rizal	69,576	3,011	3,075	6,086	8.75
City of Lucena	6,004	97	67	164	2.73
**Region 4B**	**66,862**	**1,340**	**1,294**	**2,634**	**3.94**
Marinduque	4,867	412	399	811	16.66
Occidental Mindoro	11,752	26	26	52	0.44
Oriental Mindoro	18,629	322	315	637	3.42
Palawan	20,538	566	548	1,114	5.42
Romblon	5,639	14	6	20	0.35
City of Puerto Princesa	5,437	0	0	0	0.00

Table 2.C.2.3 - Nutrition Services for Infants and Children
Children 12-23 mos. old who completed Micronutrient Powder supplementation
Philippines, 2023

Area	Eligible Pop (12-23 mos. old)	Children 12-23 mos. old who completed MNP supplementation			
		Male	Female	Total	%
**Region 5**	**138,929**	**8,154**	**7,467**	**15,621**	**11.24**
Albay	28,832	1,669	1,796	3,465	12.02
Camarines Norte	14,725	172	122	294	2.00
Camarines Sur	44,482	754	751	1,505	3.38
Catanduanes	5,897	171	143	314	5.32
Masbate	22,491	4,789	4,077	8,866	39.42
Sorsogon	18,278	558	550	1,108	6.06
City of Naga	4,224	41	28	69	1.63
**Region 6**	**153,851**	**3,876**	**3,888**	**7,764**	**5.05**
Aklan	11,114	131	141	272	2.45
Antique	11,967	112	117	229	1.91
Capiz	14,383	383	395	778	5.41
Guimaras	3,540	267	314	581	16.41
Iloilo	37,271	1,393	1,226	2,619	7.03
Negros Occidental	55,304	1,555	1,650	3,205	5.80
City of Bacolod	11,068	35	45	80	0.72
City of Iloilo	9,204	0	0	0	0.00
**Region 7**	**166,576**	**4,688**	**5,032**	**9,720**	**5.84**
Bohol	25,906	237	238	475	1.83
Cebu	73,034	852	1,101	1,953	2.67
Negros Oriental	28,596	862	1,174	2,036	7.12
Siquijor	1,464	19	17	36	2.46
City of Cebu	20,072	127	133	260	1.30
City of Lapu-Lapu	10,749	2,563	2,338	4,901	45.59
City of Mandaue	6,755	28	31	59	0.87
**Region 8**	**94,834**	**4,274**	**4,242**	**8,516**	**8.98**
Biliran	3,481	314	284	598	17.18
Eastern Samar	9,607	198	220	418	4.35
Leyte	33,070	2,414	2,367	4,781	14.46
Northern Samar	13,692	551	583	1,134	8.28
Southern Leyte	7,777	415	408	823	10.58
Samar	16,739	329	305	634	3.79
Ormoc City	5,089	53	75	128	2.52
City of Tacloban	5,379	0	0	0	0.00

Table 2.C.2.3 - Nutrition Services for Infants and Children
Children 12-23 mos. old who completed Micronutrient Powder supplementation
Philippines, 2023

Area	Eligible Pop (12-23 mos. old)	Children 12-23 mos. old who completed MNP supplementation			
		Male	Female	Total	%
**Region 9**	**85,693**	**2,744**	**2,771**	**5,515**	**6.44**
Zamboanga del Norte	25,145	861	881	1,742	6.93
Zamboanga del Sur	23,111	416	438	854	3.70
Zamboanga Sibugay	14,054	35	29	64	0.46
City of Isabela	3,428	1,159	1,157	2,316	67.56
City of Zamboanga	19,955	273	266	539	2.70
**Region 10**	**110,514**	**3,907**	**3,867**	**7,774**	**7.03**
Bukidnon	34,274	1,694	1,751	3,445	10.05
Camiguin	1,716	3	2	5	0.29
Lanao del Norte	18,242	282	249	531	2.91
Misamis Occidental	12,271	471	458	929	7.57
Misamis Oriental	21,729	174	169	343	1.58
City of Cagayan De Oro	14,478	1,009	982	1,991	13.75
City of Iligan	7,804	274	256	530	6.79
**Region 11**	**109,507**	**7,213**	**7,066**	**14,279**	**13.04**
Davao de Oro	15,519	632	637	1,269	8.18
Davao del Norte	23,545	713	731	1,444	6.13
Davao Oriental	11,603	638	643	1,281	11.04
Davao del Sur	14,035	384	365	749	5.34
Davao Occidental	7,301	144	142	286	3.92
City of Davao	37,504	4,702	4,548	9,250	24.66
**Region 12**	**97,066**	**2,806**	**2,786**	**5,592**	**5.76**
Cotabato	27,956	605	627	1,232	4.41
Sarangani	12,891	221	208	429	3.33
South Cotabato	21,974	1,268	1,232	2,500	11.38
Sultan Kudarat	19,852	125	107	232	1.17
City of General Santos	14,393	587	612	1,199	8.33
**BARMM**	**147,431**	**5,599**	**4,970**	**10,569**	**7.17**
Basilan	12,828	114	142	256	2.00
Lanao del Sur	36,861	2,521	2,473	4,994	13.55
Maguindanao	44,620	344	347	691	1.55

Table 2.C.2.3 - Nutrition Services for Infants and Children
Children 12-23 mos. old who completed Micronutrient Powder supplementation
Philippines, 2023

Area	Eligible Pop (12-23 mos. old)	Children 12-23 mos. old who completed MNP supplementation			
		Male	Female	Total	%
Sulu	25,735	0	0	0	0.00
Tawi-Tawi	14,055	648	594	1,242	8.84
SGA	5,817	73	70	143	2.46
City of Cotabato	7,515	1,899	1,344	3,243	43.15
**CARAGA**	**56,498**	**3,742**	**3,543**	**7,285**	**12.89**
Agusan del Norte	7,483	166	171	337	4.50
Agusan del Sur	15,976	1,268	1,102	2,370	14.83
Surigao del Norte	10,507	1,147	1,091	2,238	21.30
Surigao del Sur	13,337	648	669	1,317	9.87
Dinagat Islands	2,422	61	86	147	6.07
City of Butuan	6,773	452	424	876	12.93

Table 2.C.2.4 - Nutrition Services for Infants and Children

Children 0-59 mos. old whose nutritional status are normal, stunted and overweight/obese

Philippines, 2023

Area	Eligible Pop (0-59 mos. old)	Normal				Stunted			
		Male	Female	Total	%	Male	Female	Total	%
**Philippines**	**11,334,140**	**3,325,356**	**3,172,769**	**6,498,125**	**57.33**	**184,085**	**158,368**	**342,453**	**3.02**
**NCR**	**1,309,896**	**123,777**	**143,620**	**267,397**	**20.41**	**818**	**812**	**1,630**	**0.12**
Malabon City	36,857			0	0.00			0	0.00
Navotas City	26,069			0	0.00			0	0.00
Valenzuela City	68,379	31,862	31,429	63,291	92.56	201	218	419	0.61
Caloocan City	171,912			0	0.00			0	0.00
Marikina City	42,974			0	0.00			0	0.00
Pasig City	83,393			0	0.00			0	0.00
Pateros	5,940			0	0.00			0	0.00
Taguig City	93,935	45,264	43,895	89,159	94.92	65	53	118	0.13
Quezon City	297,748			0	0.00			0	0.00
Makati City	53,462			0	0.00			0	0.00
Mandaluyong City	39,483	18,316	17,568	35,884	90.88	42	52	94	0.24
San Juan City	10,787	4,475	4,084	8,559	79.35	44	60	104	0.96
Manila City	173,148			0	0.00			0	0.00
Las Piñas City	56,514	23,860	46,644	70,504	124.75	466	429	895	1.58
Muntinlupa City	49,024			0	0.00			0	0.00
Parañaque City	66,596			0	0.00			0	0.00
Pasay City	33,675			0	0.00			0	0.00
**CAR**	**163,239**	**44,172**	**41,737**	**85,909**	**52.63**	**3,938**	**3,328**	**7,266**	**4.45**
Abra	20,020	7,026	6,650	13,676	68.31	510	420	930	4.65
Apayao	11,508	4,594	4,408	9,002	78.22	538	423	961	8.35
Benguet	45,890	10,515	9,736	20,251	44.13	598	499	1,097	2.39
Ifugao	21,289	8,721	8,091	16,812	78.97	360	317	677	3.18
Kalinga	23,139	5,251	5,280	10,531	45.51	1,291	1,047	2,338	10.10
Mountain Province	14,013	6,548	6,154	12,702	90.64	622	597	1,219	8.70
City of Baguio	27,380	1,517	1,418	2,935	10.72	19	25	44	0.16
**Region 1**	**487,924**	**207,277**	**192,031**	**399,308**	**81.84**	**5,754**	**4,625**	**10,379**	**2.13**
Ilocos Norte	47,106	17,003	15,808	32,811	69.65	903	666	1,569	3.33
Ilocos Sur	53,546	22,121	20,685	42,806	79.94	893	778	1,671	3.12
La Union	68,954	27,139	25,056	52,195	75.70	897	790	1,687	2.45
Pangasinan	301,564	135,385	125,241	260,626	86.42	2,567	1,996	4,563	1.51
City of Dagupan	16,754	5,629	5,241	10,870	64.88	494	395	889	5.31

Table 2.C.2.4 - Nutrition Services for Infants and Children

Children 0-59 mos. old whose nutritional status are normal, stunted and overweight/obese

Philippines, 2023

Area	Eligible Pop (0-59 mos. old)	Normal				Stunted			
		Male	Female	Total	%	Male	Female	Total	%
**Region 2**	**337,943**	**29,110**	**29,067**	**58,177**	**17.22**	**1,653**	**1,386**	**3,039**	**0.90**
Batanes	1,371	483	396	879	64.11	18	16	34	2.48
Cagayan	111,527	8,704	8,711	17,415	15.62	405	263	668	0.60
Isabela	143,643	6,330	6,323	12,653	8.81	678	634	1,312	0.91
Nueva Vizcaya	47,614	3,828	3,872	7,700	16.17	271	211	482	1.01
Quirino	19,530	9,765	9,765	19,530	100.00	281	262	543	2.78
City of Santiago	14,258	0	0	0	0.00	0	0	0	0.00
**Region 3**	**1,216,015**	**600,470**	**574,653**	**1,175,123**	**96.64**	**23,948**	**20,019**	**43,967**	**3.62**
Aurora	24,329	9,035	8,468	17,503	71.94	1,004	882	1,886	7.75
Bataan	87,986	26,352	26,665	53,017	60.26	1,083	875	1,958	2.23
Bulacan	354,154	235,266	221,021	456,287	128.84	6,567	5,404	11,971	3.38
Nueva Ecija	226,639	92,549	89,083	181,632	80.14	5,159	4,392	9,551	4.21
Pampanga	237,343	118,869	115,144	234,013	98.60	4,092	3,641	7,733	3.26
Tarlac	150,199	70,424	67,897	138,321	92.09	3,386	2,732	6,118	4.07
Zambales	61,322	20,302	19,012	39,314	64.11	2,013	1,576	3,589	5.85
City of Angeles	51,012	15,771	15,799	31,570	61.89	395	312	707	1.39
City of Olongapo	23,031	11,902	11,564	23,466	101.89	249	205	454	1.97
**Region 4A**	**1,590,055**	**277,897**	**269,111**	**547,008**	**34.40**	**27,660**	**23,070**	**50,730**	**3.19**
Batangas	281,587	60,271	57,569	117,840	41.85	2,889	2,559	5,448	1.93
Cavite	411,396	105,101	101,464	206,565	50.21	5,290	4,662	9,952	2.42
Laguna	319,169	32,928	32,536	65,464	20.51	10,112	8,291	18,403	5.77
Quezon	205,623	44,947	42,193	87,140	42.38	6,052	5,075	11,127	5.41
Rizal	341,740	34,631	35,326	69,957	20.47	1,718	1,207	2,925	0.86
City of Lucena	30,540	19	23	42	0.14	1,599	1,276	2,875	9.41
**Region 4B**	**339,780**	**150,816**	**142,849**	**293,665**	**86.43**	**17,051**	**14,788**	**31,839**	**9.37**
Marinduque	23,791	3,744	3,477	7,221	30.35	406	358	764	3.21
Occidental Mindoro	59,573	24,868	23,891	48,759	81.85	3,880	3,260	7,140	11.99
Oriental Mindoro	94,393	34,468	31,487	65,955	69.87	4,793	4,010	8,803	9.33
Palawan	105,171	42,040	40,478	82,518	78.46	5,079	4,551	9,630	9.16
Romblon	28,310	33,394	32,291	65,685	232.02	1,908	1,713	3,621	12.79
City of Puerto Princesa	28,542	12,302	11,225	23,527	82.43	985	896	1,881	6.59
**Region 5**	**699,471**	**223,217**	**199,524**	**422,741**	**60.44**	**17,906**	**15,563**	**33,469**	**4.78**

Table 2.C.2.4 - Nutrition Services for Infants and Children

Children 0-59 mos. old whose nutritional status are normal, stunted and overweight/obese

Philippines, 2023

Area	Eligible Pop (0-59 mos. old)	Normal				Stunted			
		Male	Female	Total	%	Male	Female	Total	%
Albay	142,792	30,331	28,954	59,285	41.52	2,354	2,049	4,403	3.08
Camarines Norte	76,188	22,596	21,219	43,815	57.51	2,838	2,339	5,177	6.80
Camarines Sur	223,050	58,420	56,745	115,165	51.63	3,477	3,047	6,524	2.92
Catanduanes	30,252	12,732	12,017	24,749	81.81	1,922	1,693	3,615	11.95
Masbate	113,548	56,690	40,514	97,204	85.61	5,337	4,486	9,823	8.65
Sorsogon	92,814	31,220	29,672	60,892	65.61	1,833	1,795	3,628	3.91
City of Naga	20,827	11,228	10,403	21,631	103.86	145	154	299	1.44
**Region 6**	**770,636**	**268,822**	**251,743**	**520,565**	**67.55**	**13,755**	**11,821**	**25,576**	**3.32**
Aklan	57,751	10,089	9,625	19,714	34.14	374	297	671	1.16
Antique	62,459	10,435	9,923	20,358	32.59	1,059	984	2,043	3.27
Capiz	75,119	31,810	30,335	62,145	82.73	894	737	1,631	2.17
Guimaras	18,084	7,741	7,427	15,168	83.88	592	522	1,114	6.16
Iloilo	189,336	76,431	69,242	145,673	76.94	5,775	4,790	10,565	5.58
Negros Occidental	268,649	91,998	87,476	179,474	66.81	4,166	3,732	7,898	2.94
City of Bacolod	54,114	22,496	20,599	43,095	79.64	372	325	697	1.29
City of Iloilo	45,124	17,822	17,116	34,938	77.43	523	434	957	2.12
**Region 7**	**840,903**	**182,930**	**178,495**	**361,425**	**42.98**	**9,370**	**8,225**	**17,595**	**2.09**
Bohol	135,115	13,336	12,444	25,780	19.08	525	478	1,003	0.74
Cebu	365,155	89,286	88,893	178,179	48.80	4,648	4,315	8,963	2.45
Negros Oriental	148,201	23,775	23,388	47,163	31.82	2,523	2,049	4,572	3.08
Siquijor	8,082	2,803	2,678	5,481	67.82	227	175	402	4.97
City of Cebu	98,198	38,581	36,317	74,898	76.27	704	617	1,321	1.35
City of Lapu-Lapu	52,718	227	221	448	0.85	0	0	0	0.00
City of Mandaue	33,434	14,922	14,554	29,476	88.16	743	591	1,334	3.99
**Region 8**	**486,648**	**49,409**	**51,152**	**100,561**	**20.66**	**8,561**	**7,112**	**15,673**	**3.22**
Biliran	18,503	5,296	5,192	10,488	56.68	717	609	1,326	7.17
Eastern Samar	48,721	1,303	1,179	2,482	5.09	137	110	247	0.51
Leyte	165,619	0	0	0	0.00	0	0	0	0.00
Northern Samar	72,661	22,439	21,338	43,777	60.25	4,210	3,491	7,701	10.60
Southern Leyte	39,996	7,356	6,843	14,199	35.50	1,887	1,278	3,165	7.91
Samar	87,678	6,678	6,035	12,713	14.50	401	334	735	0.84
Ormoc City	25,774	*	*	0	0.00	*	*	0	0.00
City of Tacloban	27,696	6,337	10,565	16,902	61.03	1,209	1,290	2,499	9.02

Table 2.C.2.4 - Nutrition Services for Infants and Children

Children 0-59 mos. old whose nutritional status are normal, stunted and overweight/obese

Philippines, 2023

Area	Eligible Pop (0-59 mos. old)	Normal				Stunted			
		Male	Female	Total	%	Male	Female	Total	%
**Region 9**	**437,233**	**115,538**	**112,252**	**227,790**	**52.10**	**8,485**	**7,504**	**15,989**	**3.66**
Zamboanga del Norte	123,478	51,713	49,921	101,634	82.31	5,181	4,653	9,834	7.96
Zamboanga del Sur	120,917	22,354	21,403	43,757	36.19	2,316	1,967	4,283	3.54
Zamboanga Sibugay	74,519	1,603	1,610	3,213	4.31	48	45	93	0.12
City of Isabela	16,896	10,544	10,164	20,708	122.56	293	201	494	2.92
City of Zamboanga	101,423	29,324	29,154	58,478	57.66	647	638	1,285	1.27
**Region 10**	**564,841**	**322,269**	**277,114**	**599,383**	**106.12**	**10,987**	**9,648**	**20,635**	**3.65**
Bukidnon	177,670	162,163	121,712	283,875	159.78	4,896	4,445	9,341	5.26
Camiguin	9,048	2,946	2,993	5,939	65.64	382	332	714	7.89
Lanao del Norte	92,387	40,345	40,923	81,268	87.96	2,914	2,383	5,297	5.73
Misamis Occidental	61,840	21,042	20,901	41,943	67.83	901	719	1,620	2.62
Misamis Oriental	109,995	40,724	38,003	78,727	71.57	1,748	1,653	3,401	3.09
City of Cagayan De Oro	73,964	35,251	32,612	67,863	91.75	125	93	218	0.29
City of Iligan	39,937	19,798	19,970	39,768	99.58	21	23	44	0.11
**Region 11**	**556,182**	**226,253**	**213,234**	**439,487**	**79.02**	**10,018**	**8,549**	**18,567**	**3.34**
Davao de Oro	81,277	32,757	30,026	62,783	77.25	1,664	1,493	3,157	3.88
Davao del Norte	121,250	48,729	45,311	94,040	77.56	2,004	1,888	3,892	3.21
Davao Oriental	60,933	26,523	25,074	51,597	84.68	2,226	1,698	3,924	6.44
Davao del Sur	72,277	25,692	24,251	49,943	69.10	1,272	1,029	2,301	3.18
Davao Occidental	38,331	10,327	9,931	20,258	52.85	1,965	1,644	3,609	9.42
City of Davao	182,114	82,225	78,641	160,866	88.33	887	797	1,684	0.92
**Region 12**	**491,133**	**211,142**	**199,131**	**410,273**	**83.54**	**6,628**	**5,572**	**12,200**	**2.48**
Cotabato	143,299	59,656	56,086	115,742	80.77	2,125	1,702	3,827	2.67
Sarangani	67,408	32,308	30,803	63,111	93.63	852	739	1,591	2.36
South Cotabato	107,889	46,773	43,540	90,313	83.71	1,112	991	2,103	1.95
Sultan Kudarat	97,978	41,590	39,764	81,354	83.03	1,807	1,516	3,323	3.39
City of General Santos	74,559	30,815	28,938	59,753	80.14	732	624	1,356	1.82
**BARMM**	**750,398**	**172,993**	**183,591**	**356,584**	**47.52**	**10,362**	**9,629**	**19,991**	**2.66**
Basilan	64,723	19,291	19,140	38,431	59.38	2,358	2,281	4,639	7.17
Lanao del Sur	185,348	71,124	76,064	147,188	79.41	2,092	1,898	3,990	2.15
Maguindanao	231,258	7,256	7,601	14,857	6.42	108	115	223	0.10
Sulu	131,184	50,174	55,765	105,939	80.76	3,038	2,847	5,885	4.49

Table 2.C.2.4 - Nutrition Services for Infants and Children

Children 0-59 mos. old whose nutritional status are normal, stunted and overweight/obese

Philippines, 2023

Area	Eligible Pop (0-59 mos. old)	Normal				Stunted			
		Male	Female	Total	%	Male	Female	Total	%
Tawi-Tawi	69,576	10,976	11,371	22,347	32.12	1,875	1,691	3,566	5.13
SGA	28,976	7,174	7,049	14,223	49.09	516	463	979	3.38
City of Cotabato	39,333	6,998	6,601	13,599	34.57	375	334	709	1.80
**CARAGA**	**291,843**	**119,264**	**113,465**	**232,729**	**79.74**	**7,191**	**6,717**	**13,908**	**4.77**
Agusan del Norte	39,319	18,876	17,821	36,697	93.33	908	747	1,655	4.21
Agusan del Sur	83,337	35,143	32,557	67,700	81.24	1,913	1,729	3,642	4.37
Surigao del Norte	52,594	20,850	20,683	41,533	78.97	2,140	2,149	4,289	8.15
Surigao del Sur	68,313	25,553	24,803	50,356	73.71	1,835	1,725	3,560	5.21
Dinagat Islands	13,089	1,338	1,283	2,621	20.02	83	109	192	1.47
City of Butuan	35,191	17,504	16,318	33,822	96.11	312	258	570	1.62

Table 2.C.2.4 - Nutrition Services for Infants and Children

Children 0-59 mos. old whose nutritional status are normal, stunted and overweight/obese

Philippines, 2023

Area	Eligible Pop (0-59 mos. old)	Overweight/obese			
		Male	Female	Total	%
**Philippines**	**11,334,140**	**74,043**	**60,261**	**134,304**	**1.18**
**NCR**	**1,309,896**	**599**	**550**	**1,149**	**0.09**
Malabon City	36,857			0	0.00
Navotas City	26,069			0	0.00
Valenzuela City	68,379	243	258	501	0.73
Caloocan City	171,912			0	0.00
Marikina City	42,974			0	0.00
Pasig City	83,393			0	0.00
Pateros	5,940			0	0.00
Taguig City	93,935	45	33	78	0.08
Quezon City	297,748			0	0.00
Makati City	53,462			0	0.00
Mandaluyong City	39,483	52	35	87	0.22
San Juan City	10,787	167	168	335	3.11
Manila City	173,148			0	0.00
Las Piñas City	56,514	92	56	148	0.26
Muntinlupa City	49,024			0	0.00
Parañaque City	66,596			0	0.00
Pasay City	33,675			0	0.00
**CAR**	**163,239**	**1,054**	**788**	**1,842**	**1.13**
Abra	20,020	185	172	357	1.78
Apayao	11,508	163	121	284	2.47
Benguet	45,890	255	196	451	0.98
Ifugao	21,289	144	69	213	1.00
Kalinga	23,139	126	106	232	1.00
Mountain Province	14,013	168	116	284	2.03
City of Baguio	27,380	13	8	21	0.08
**Region 1**	**487,924**	**4,377**	**3,548**	**7,925**	**1.62**
Ilocos Norte	47,106	647	529	1,176	2.50
Ilocos Sur	53,546	771	567	1,338	2.50
La Union	68,954	620	477	1,097	1.59
Pangasinan	301,564	2,011	1,669	3,680	1.22
City of Dagupan	16,754	328	306	634	3.78

Table 2.C.2.4 - Nutrition Services for Infants and Children
Children 0-59 mos. old whose nutritional status are normal, stunted and overweight/obese
Philippines, 2023

Area	Eligible Pop (0-59 mos. old)	Overweight/obese			
		Male	Female	Total	%
**Region 2**	**337,943**	**476**	**345**	**821**	**0.24**
Batanes	1,371	21	23	44	3.21
Cagayan	111,527	86	67	153	0.14
Isabela	143,643	160	118	278	0.19
Nueva Vizcaya	47,614	84	52	136	0.29
Quirino	19,530	125	85	210	1.08
City of Santiago	14,258	0	0	0	0.00
**Region 3**	**1,216,015**	**17,107**	**14,070**	**31,177**	**2.56**
Aurora	24,329	324	249	573	2.36
Bataan	87,986	747	640	1,387	1.58
Bulacan	354,154	5,019	4,008	9,027	2.55
Nueva Ecija	226,639	3,618	2,960	6,578	2.90
Pampanga	237,343	4,589	3,968	8,557	3.61
Tarlac	150,199	1,732	1,376	3,108	2.07
Zambales	61,322	697	532	1,229	2.00
City of Angeles	51,012	267	225	492	0.96
City of Olongapo	23,031	114	112	226	0.98
**Region 4A**	**1,590,055**	**14,713**	**11,635**	**26,348**	**1.66**
Batangas	281,587	2,000	1,483	3,483	1.24
Cavite	411,396	4,393	3,584	7,977	1.94
Laguna	319,169	5,238	3,961	9,199	2.88
Quezon	205,623	1,742	1,393	3,135	1.52
Rizal	341,740	1,067	987	2,054	0.60
City of Lucena	30,540	273	227	500	1.64
**Region 4B**	**339,780**	**3,216**	**2,529**	**5,745**	**1.69**
Marinduque	23,791	110	62	172	0.72
Occidental Mindoro	59,573	432	347	779	1.31
Oriental Mindoro	94,393	965	757	1,722	1.82
Palawan	105,171	754	533	1,287	1.22
Romblon	28,310	512	548	1,060	3.74
City of Puerto Princesa	28,542	443	282	725	2.54
**Region 5**	**699,471**	**5,098**	**3,945**	**9,043**	**1.29**

Table 2.C.2.4 - Nutrition Services for Infants and Children

Children 0-59 mos. old whose nutritional status are normal, stunted and overweight/obese

Philippines, 2023

Area	Eligible Pop (0-59 mos. old)	Overweight/obese			
		Male	Female	Total	%
Albay	142,792	1,121	923	2,044	1.43
Camarines Norte	76,188	552	400	952	1.25
Camarines Sur	223,050	1,421	954	2,375	1.06
Catanduanes	30,252	493	378	871	2.88
Masbate	113,548	1,100	882	1,982	1.75
Sorsogon	92,814	384	387	771	0.83
City of Naga	20,827	27	21	48	0.23
**Region 6**	**770,636**	**5,454**	**4,329**	**9,783**	**1.27**
Aklan	57,751	245	196	441	0.76
Antique	62,459	354	276	630	1.01
Capiz	75,119	615	555	1,170	1.56
Guimaras	18,084	304	215	519	2.87
Iloilo	189,336	1,961	1,560	3,521	1.86
Negros Occidental	268,649	1,480	1,152	2,632	0.98
City of Bacolod	54,114	131	92	223	0.41
City of Iloilo	45,124	364	283	647	1.43
**Region 7**	**840,903**	**3,427**	**2,726**	**6,153**	**0.73**
Bohol	135,115	174	130	304	0.22
Cebu	365,155	1,454	1,180	2,634	0.72
Negros Oriental	148,201	1,136	862	1,998	1.35
Siquijor	8,082	102	93	195	2.41
City of Cebu	98,198	325	252	577	0.59
City of Lapu-Lapu	52,718	0	0	0	0.00
City of Mandaue	33,434	236	209	445	1.33
**Region 8**	**486,648**	**2,313**	**2,137**	**4,450**	**0.91**
Biliran	18,503	227	181	408	2.21
Eastern Samar	48,721	30	25	55	0.11
Leyte	165,619	0	0	0	0.00
Northern Samar	72,661	1,174	1,005	2,179	3.00
Southern Leyte	39,996	596	430	1,026	2.57
Samar	87,678	90	76	166	0.19
Ormoc City	25,774	*	*	0	0.00
City of Tacloban	27,696	196	420	616	2.22

Table 2.C.2.4 - Nutrition Services for Infants and Children
Children 0-59 mos. old whose nutritional status are normal, stunted and overweight/obese
Philippines, 2023

Area	Eligible Pop (0-59 mos. old)	Overweight/obese			
		Male	Female	Total	%
**Region 9**	**437,233**	**1,665**	**1,390**	**3,055**	**0.70**
Zamboanga del Norte	123,478	543	439	982	0.80
Zamboanga del Sur	120,917	720	610	1,330	1.10
Zamboanga Sibugay	74,519	3	0	3	0.00
City of Isabela	16,896	169	147	316	1.87
City of Zamboanga	101,423	230	194	424	0.42
**Region 10**	**564,841**	**2,719**	**2,250**	**4,969**	**0.88**
Bukidnon	177,670	1,263	1,079	2,342	1.32
Camiguin	9,048	141	111	252	2.79
Lanao del Norte	92,387	505	470	975	1.06
Misamis Occidental	61,840	324	233	557	0.90
Misamis Oriental	109,995	453	324	777	0.71
City of Cagayan De Oro	73,964	31	33	64	0.09
City of Iligan	39,937	2	0	2	0.01
**Region 11**	**556,182**	**3,265**	**2,572**	**5,837**	**1.05**
Davao de Oro	81,277	601	415	1,016	1.25
Davao del Norte	121,250	736	576	1,312	1.08
Davao Oriental	60,933	663	554	1,217	2.00
Davao del Sur	72,277	562	446	1,008	1.39
Davao Occidental	38,331	411	353	764	1.99
City of Davao	182,114	292	228	520	0.29
**Region 12**	**491,133**	**2,810**	**2,197**	**5,007**	**1.02**
Cotabato	143,299	731	538	1,269	0.89
Sarangani	67,408	255	173	428	0.63
South Cotabato	107,889	404	329	733	0.68
Sultan Kudarat	97,978	699	567	1,266	1.29
City of General Santos	74,559	721	590	1,311	1.76
**BARMM**	**750,398**	**3,087**	**3,152**	**6,239**	**0.83**
Basilan	64,723	422	422	844	1.30
Lanao del Sur	185,348	348	359	707	0.38
Maguindanao	231,258	1,082	1,096	2,178	0.94
Sulu	131,184	632	680	1,312	1.00

Table 2.C.2.4 - Nutrition Services for Infants and Children

Children 0-59 mos. old whose nutritional status are normal, stunted and overweight/obese

Philippines, 2023

Area	Eligible Pop (0-59 mos. old)	Overweight/obese			
		Male	Female	Total	%
Tawi-Tawi	69,576	452	442	894	1.28
SGA	28,976	80	84	164	0.57
City of Cotabato	39,333	71	69	140	0.36
**CARAGA**	**291,843**	**2,663**	**2,098**	**4,761**	**1.63**
Agusan del Norte	39,319	204	155	359	0.91
Agusan del Sur	83,337	573	419	992	1.19
Surigao del Norte	52,594	713	596	1,309	2.49
Surigao del Sur	68,313	916	702	1,618	2.37
Dinagat Islands	13,089	57	63	120	0.92
City of Butuan	35,191	200	163	363	1.03

Table 2.C.2.4 - Nutrition Services for Infants and Children
Children 0-59 mos. old whose nutritional status are normal, stunted and overweight/obese
Philippines, 2023

Area	Eligible Pop (0-59 mos. old)	Wasted-MAM				Wasted-SAM			
		Male	Female	Total	%	Male	Female	Total	%
**Philippines**	**11,334,140**	**34,754**	**30,116**	**64,870**	**0.57**	**12,488**	**10,182**	**22,670**	**0.20**
**NCR**	**1,309,896**	**113**	**97**	**210**	**0.02**	**16**	**11**	**27**	**0.00**
Malabon City	36,857			0	0.00			0	0.00
Navotas City	26,069			0	0.00			0	0.00
Valenzuela City	68,379			0	0.00			0	0.00
Caloocan City	171,912			0	0.00			0	0.00
Marikina City	42,974			0	0.00			0	0.00
Pasig City	83,393			0	0.00			0	0.00
Pateros	5,940			0	0.00			0	0.00
Taguig City	93,935	55	40	95	0.10	7	3	10	0.01
Quezon City	297,748			0	0.00			0	0.00
Makati City	53,462			0	0.00			0	0.00
Mandaluyong City	39,483			0	0.00			0	0.00
San Juan City	10,787	16	7	23	0.21	0	1	1	0.01
Manila City	173,148			0	0.00			0	0.00
Las Piñas City	56,514	42	50	92	0.16	9	7	16	0.03
Muntinlupa City	49,024			0	0.00			0	0.00
Parañaque City	66,596			0	0.00			0	0.00
Pasay City	33,675			0	0.00			0	0.00
**CAR**	**163,239**	**335**	**295**	**630**	**0.39**	**93**	**87**	**180**	**0.11**
Abra	20,020	113	90	203	1.01	34	19	53	0.26
Apayao	11,508	98	114	212	1.84	26	15	41	0.36
Benguet	45,890	11	18	29	0.06	3	13	16	0.03
Ifugao	21,289	31	33	64	0.30	13	25	38	0.18
Kalinga	23,139	61	28	89	0.38	14	8	22	0.10
Mountain Province	14,013	12	3	15	0.11	2	7	9	0.06
City of Baguio	27,380	9	9	18	0.07	1	0	1	0.00
**Region 1**	**487,924**	**1,564**	**1,204**	**2,768**	**0.57**	**604**	**492**	**1,096**	**0.22**
Ilocos Norte	47,106	179	144	323	0.69	53	43	96	0.20
Ilocos Sur	53,546	166	120	286	0.53	44	31	75	0.14
La Union	68,954	178	152	330	0.48	55	46	101	0.15
Pangasinan	301,564	885	684	1,569	0.52	354	291	645	0.21
City of Dagupan	16,754	156	104	260	1.55	98	81	179	1.07

Table 2.C.2.4 - Nutrition Services for Infants and Children
Children 0-59 mos. old whose nutritional status are normal, stunted and overweight/obese
Philippines, 2023

Area	Eligible Pop (0-59 mos. old)	Wasted-MAM				Wasted-SAM			
		Male	Female	Total	%	Male	Female	Total	%
**Region 2**	**337,943**	**181**	**230**	**411**	**0.12**	**46**	**37**	**83**	**0.02**
Batanes	1,371	7	10	17	1.24	3	4	7	0.51
Cagayan	111,527	22	29	51	0.05	0	0	0	0.00
Isabela	143,643	140	171	311	0.22	40	30	70	0.05
Nueva Vizcaya	47,614	0	0	0	0.00	0	0	0	0.00
Quirino	19,530	12	20	32	0.16	3	3	6	0.03
City of Santiago	14,258	0	0	0	0.00	0	0	0	0.00
**Region 3**	**1,216,015**	**7,299**	**6,140**	**13,439**	**1.11**	**3,994**	**3,070**	**7,064**	**0.58**
Aurora	24,329	124	107	231	0.95	45	36	81	0.33
Bataan	87,986	479	418	897	1.02	87	98	185	0.21
Bulacan	354,154	1,934	1,573	3,507	0.99	913	683	1,596	0.45
Nueva Ecija	226,639	1,780	1,353	3,133	1.38	534	427	961	0.42
Pampanga	237,343	1,833	1,693	3,526	1.49	1,829	1,355	3,184	1.34
Tarlac	150,199	852	741	1,593	1.06	382	324	706	0.47
Zambales	61,322	206	186	392	0.64	138	109	247	0.40
City of Angeles	51,012	0	0	0	0.00	0	0	0	0.00
City of Olongapo	23,031	91	69	160	0.69	66	38	104	0.45
**Region 4A**	**1,590,055**	**3,915**	**3,433**	**7,348**	**0.46**	**1,454**	**1,171**	**2,625**	**0.17**
Batangas	281,587	353	302	655	0.23	99	115	214	0.08
Cavite	411,396	542	559	1,101	0.27	264	176	440	0.11
Laguna	319,169	1,533	1,291	2,824	0.88	330	305	635	0.20
Quezon	205,623	1,031	913	1,944	0.95	658	491	1,149	0.56
Rizal	341,740	300	250	550	0.16	27	24	51	0.01
City of Lucena	30,540	156	118	274	0.90	76	60	136	0.45
**Region 4B**	**339,780**	**2,002**	**1,708**	**3,710**	**1.09**	**467**	**361**	**828**	**0.24**
Marinduque	23,791	72	48	120	0.50	17	10	27	0.11
Occidental Mindoro	59,573	623	475	1,098	1.84	164	121	285	0.48
Oriental Mindoro	94,393	163	160	323	0.34	16	9	25	0.03
Palawan	105,171	992	870	1,862	1.77	229	179	408	0.39
Romblon	28,310	133	135	268	0.95	34	38	72	0.25
City of Puerto Princesa	28,542	19	20	39	0.14	7	4	11	0.04

Table 2.C.2.4 - Nutrition Services for Infants and Children

Children 0-59 mos. old whose nutritional status are normal, stunted and overweight/obese

Philippines, 2023

Area	Eligible Pop (0-59 mos. old)	Wasted-MAM				Wasted-SAM			
		Male	Female	Total	%	Male	Female	Total	%
**Region 5**	**699,471**	**3,784**	**3,156**	**6,940**	**0.99**	**870**	**740**	**1,610**	**0.23**
Albay	142,792	553	447	1,000	0.70	71	53	124	0.09
Camarines Norte	76,188	362	285	647	0.85	73	65	138	0.18
Camarines Sur	223,050	1,117	1,009	2,126	0.95	380	371	751	0.34
Catanduanes	30,252	295	226	521	1.72	63	46	109	0.36
Masbate	113,548	1,105	827	1,932	1.70	246	174	420	0.37
Sorsogon	92,814	347	357	704	0.76	37	30	67	0.07
City of Naga	20,827	5	5	10	0.05	0	1	1	0.00
**Region 6**	**770,636**	**2,760**	**2,360**	**5,120**	**0.66**	**824**	**618**	**1,442**	**0.19**
Aklan	57,751	73	56	129	0.22	33	13	46	0.08
Antique	62,459	153	167	320	0.51	52	29	81	0.13
Capiz	75,119	205	188	393	0.52	45	22	67	0.09
Guimaras	18,084	24	16	40	0.22	8	6	14	0.08
Iloilo	189,336	833	658	1,491	0.79	231	206	437	0.23
Negros Occidental	268,649	1,233	1,043	2,276	0.85	344	275	619	0.23
City of Bacolod	54,114	112	98	210	0.39	62	29	91	0.17
City of Iloilo	45,124	127	134	261	0.58	49	38	87	0.19
**Region 7**	**840,903**	**1,696**	**1,424**	**3,120**	**0.37**	**360**	**317**	**677**	**0.08**
Bohol	135,115	77	69	146	0.11	17	18	35	0.03
Cebu	365,155	743	647	1,390	0.38	231	209	440	0.12
Negros Oriental	148,201	562	460	1,022	0.69	0	0	0	0.00
Siquijor	8,082	29	30	59	0.73	5	6	11	0.14
City of Cebu	98,198	225	187	412	0.42	82	71	153	0.16
City of Lapu-Lapu	52,718	0	0	0	0.00	0	0	0	0.00
City of Mandaue	33,434	60	31	91	0.27	25	13	38	0.11
**Region 8**	**486,648**	**1,334**	**1,182**	**2,516**	**0.52**	**342**	**371**	**713**	**0.15**
Biliran	18,503	132	119	251	1.36	35	38	73	0.39
Eastern Samar	48,721	7	0	7	0.01	9	4	13	0.03
Leyte	165,619	0	0	0	0.00	0	0	0	0.00
Northern Samar	72,661	568	447	1,015	1.40	155	119	274	0.38
Southern Leyte	39,996	265	224	489	1.22	41	40	81	0.20
Samar	87,678	17	18	35	0.04	2	6	8	0.01
Ormoc City	25,774	*	*	0	0.00	*	*	0	0.00
City of Tacloban	27,696	345	374	719	2.60	100	164	264	0.95

Table 2.C.2.4 - Nutrition Services for Infants and Children

Children 0-59 mos. old whose nutritional status are normal, stunted and overweight/obese

Philippines, 2023

Area	Eligible Pop (0-59 mos. old)	Wasted-MAM				Wasted-SAM			
		Male	Female	Total	%	Male	Female	Total	%
**Region 9**	**437,233**	**1,751**	**1,685**	**3,436**	**0.79**	**700**	**588**	**1,288**	**0.29**
Zamboanga del Norte	123,478	437	482	919	0.74	177	149	326	0.26
Zamboanga del Sur	120,917	830	726	1,556	1.29	316	273	589	0.49
Zamboanga Sibugay	74,519	21	22	43	0.06	11	5	16	0.02
City of Isabela	16,896	205	179	384	2.27	108	73	181	1.07
City of Zamboanga	101,423	258	276	534	0.53	88	88	176	0.17
**Region 10**	**564,841**	**1,251**	**1,020**	**2,271**	**0.40**	**377**	**324**	**701**	**0.12**
Bukidnon	177,670	957	813	1,770	1.00	268	249	517	0.29
Camiguin	9,048	25	24	49	0.54	6	12	18	0.20
Lanao del Norte	92,387	5	5	10	0.01	0	0	0	0.00
Misamis Occidental	61,840	45	26	71	0.11	25	20	45	0.07
Misamis Oriental	109,995	147	97	244	0.22	50	26	76	0.07
City of Cagayan De Oro	73,964	16	14	30	0.04	14	5	19	0.03
City of Iligan	39,937	56	41	97	0.24	14	12	26	0.07
**Region 11**	**556,182**	**1,634**	**1,408**	**3,042**	**0.55**	**473**	**367**	**840**	**0.15**
Davao de Oro	81,277	137	99	236	0.29	32	24	56	0.07
Davao del Norte	121,250	262	245	507	0.42	37	39	76	0.06
Davao Oriental	60,933	358	281	639	1.05	93	73	166	0.27
Davao del Sur	72,277	282	200	482	0.67	62	31	93	0.13
Davao Occidental	38,331	404	365	769	2.01	180	141	321	0.84
City of Davao	182,114	191	218	409	0.22	69	59	128	0.07
**Region 12**	**491,133**	**1,542**	**1,259**	**2,801**	**0.57**	**537**	**388**	**925**	**0.19**
Cotabato	143,299	531	394	925	0.65	174	114	288	0.20
Sarangani	67,408	164	141	305	0.45	46	23	69	0.10
South Cotabato	107,889	170	150	320	0.30	50	33	83	0.08
Sultan Kudarat	97,978	452	398	850	0.87	181	151	332	0.34
City of General Santos	74,559	225	176	401	0.54	86	67	153	0.21
**BARMM**	**750,398**	**2,108**	**2,117**	**4,225**	**0.56**	**857**	**821**	**1,678**	**0.22**
Basilan	64,723	364	340	704	1.09	120	104	224	0.35
Lanao del Sur	185,348	187	250	437	0.24	140	117	257	0.14

Table 2.C.2.4 - Nutrition Services for Infants and Children

Children 0-59 mos. old whose nutritional status are normal, stunted and overweight/obese

Philippines, 2023

Area	Eligible Pop (0-59 mos. old)	Wasted-MAM				Wasted-SAM			
		Male	Female	Total	%	Male	Female	Total	%
Maguindanao	231,258	68	91	159	0.07	28	28	56	0.02
Sulu	131,184	871	860	1,731	1.32	171	174	345	0.26
Tawi-Tawi	69,576	438	425	863	1.24	371	368	739	1.06
SGA	28,976	25	21	46	0.16	11	15	26	0.09
City of Cotabato	39,333	155	130	285	0.72	16	15	31	0.08
**CARAGA**	**291,843**	**1,485**	**1,398**	**2,883**	**0.99**	**474**	**419**	**893**	**0.31**
Agusan del Norte	39,319	78	83	161	0.41	22	12	34	0.09
Agusan del Sur	83,337	393	392	785	0.94	160	134	294	0.35
Surigao del Norte	52,594	475	505	980	1.86	159	169	328	0.62
Surigao del Sur	68,313	443	336	779	1.14	130	96	226	0.33
Dinagat Islands	13,089	14	18	32	0.24	3	8	11	0.08
City of Butuan	35,191	82	64	146	0.41	0	0	0	0.00

Table 2.C.2.4 - Nutrition Services for Infants and Children

Children 0-59 mos. old whose nutritional status are normal, stunted and overweight/obese

Philippines, 2023

Area	Eligible Pop (0-59 mos. old)	Wasted			
		Male	Female	Total	%
**Philippines**	**11,334,140**	**47,242**	**40,298**	**87,540**	**0.77**
**NCR**	**1,309,896**	**129**	**108**	**237**	**0.02**
Malabon City	36,857	0	0	0	0.00
Navotas City	26,069	0	0	0	0.00
Valenzuela City	68,379	0	0	0	0.00
Caloocan City	171,912	0	0	0	0.00
Marikina City	42,974	0	0	0	0.00
Pasig City	83,393	0	0	0	0.00
Pateros	5,940	0	0	0	0.00
Taguig City	93,935	62	43	105	0.11
Quezon City	297,748	0	0	0	0.00
Makati City	53,462	0	0	0	0.00
Mandaluyong City	39,483	0	0	0	0.00
San Juan City	10,787	16	8	24	0.22
Manila City	173,148	0	0	0	0.00
Las Piñas City	56,514	51	57	108	0.19
Muntinlupa City	49,024	0	0	0	0.00
Parañaque City	66,596	0	0	0	0.00
Pasay City	33,675	0	0	0	0.00
**CAR**	**163,239**	**428**	**382**	**810**	**0.50**
Abra	20,020	147	109	256	1.28
Apayao	11,508	124	129	253	2.20
Benguet	45,890	14	31	45	0.10
Ifugao	21,289	44	58	102	0.48
Kalinga	23,139	75	36	111	0.48
Mountain Province	14,013	14	10	24	0.17
City of Baguio	27,380	10	9	19	0.07
**Region 1**	**487,924**	**2,168**	**1,696**	**3,864**	**0.79**
Ilocos Norte	47,106	232	187	419	0.89
Ilocos Sur	53,546	210	151	361	0.67
La Union	68,954	233	198	431	0.63
Pangasinan	301,564	1,239	975	2,214	0.73
City of Dagupan	16,754	254	185	439	2.62

Table 2.C.2.4 - Nutrition Services for Infants and Children
Children 0-59 mos. old whose nutritional status are normal, stunted and overweight/obese
Philippines, 2023

Area	Eligible Pop (0-59 mos. old)	Wasted			
		Male	Female	Total	%
**Region 2**	**337,943**	**227**	**267**	**494**	**0.15**
Batanes	1,371	10	14	24	1.75
Cagayan	111,527	22	29	51	0.05
Isabela	143,643	180	201	381	0.27
Nueva Vizcaya	47,614	0	0	0	0.00
Quirino	19,530	15	23	38	0.19
City of Santiago	14,258	0	0	0	0.00
**Region 3**	**1,216,015**	**11,293**	**9,210**	**20,503**	**1.69**
Aurora	24,329	169	143	312	1.28
Bataan	87,986	566	516	1,082	1.23
Bulacan	354,154	2,847	2,256	5,103	1.44
Nueva Ecija	226,639	2,314	1,780	4,094	1.81
Pampanga	237,343	3,662	3,048	6,710	2.83
Tarlac	150,199	1,234	1,065	2,299	1.53
Zambales	61,322	344	295	639	1.04
City of Angeles	51,012	0	0	0	0.00
City of Olongapo	23,031	157	107	264	1.15
**Region 4A**	**1,590,055**	**5,369**	**4,604**	**9,973**	**0.63**
Batangas	281,587	452	417	869	0.31
Cavite	411,396	806	735	1,541	0.37
Laguna	319,169	1,863	1,596	3,459	1.08
Quezon	205,623	1,689	1,404	3,093	1.50
Rizal	341,740	327	274	601	0.18
City of Lucena	30,540	232	178	410	1.34
**Region 4B**	**339,780**	**2,469**	**2,069**	**4,538**	**1.34**
Marinduque	23,791	89	58	147	0.62
Occidental Mindoro	59,573	787	596	1,383	2.32
Oriental Mindoro	94,393	179	169	348	0.37
Palawan	105,171	1,221	1,049	2,270	2.16
Romblon	28,310	167	173	340	1.20
City of Puerto Princesa	28,542	26	24	50	0.18

Table 2.C.2.4 - Nutrition Services for Infants and Children
Children 0-59 mos. old whose nutritional status are normal, stunted and overweight/obese
Philippines, 2023

Area	Eligible Pop (0-59 mos. old)	Wasted			
		Male	Female	Total	%
**Region 5**	**699,471**	**4,654**	**3,896**	**8,550**	**1.22**
Albay	142,792	624	500	1,124	0.79
Camarines Norte	76,188	435	350	785	1.03
Camarines Sur	223,050	1,497	1,380	2,877	1.29
Catanduanes	30,252	358	272	630	2.08
Masbate	113,548	1,351	1,001	2,352	2.07
Sorsogon	92,814	384	387	771	0.83
City of Naga	20,827	5	6	11	0.05
**Region 6**	**770,636**	**3,584**	**2,978**	**6,562**	**0.85**
Aklan	57,751	106	69	175	0.30
Antique	62,459	205	196	401	0.64
Capiz	75,119	250	210	460	0.61
Guimaras	18,084	32	22	54	0.30
Iloilo	189,336	1,064	864	1,928	1.02
Negros Occidental	268,649	1,577	1,318	2,895	1.08
City of Bacolod	54,114	174	127	301	0.56
City of Iloilo	45,124	176	172	348	0.77
**Region 7**	**840,903**	**2,056**	**1,741**	**3,797**	**0.45**
Bohol	135,115	94	87	181	0.13
Cebu	365,155	974	856	1,830	0.50
Negros Oriental	148,201	562	460	1,022	0.69
Siquijor	8,082	34	36	70	0.87
City of Cebu	98,198	307	258	565	0.58
City of Lapu-Lapu	52,718	0	0	0	0.00
City of Mandaue	33,434	85	44	129	0.39
**Region 8**	**486,648**	**1,676**	**1,553**	**3,229**	**0.66**
Biliran	18,503	167	157	324	1.75
Eastern Samar	48,721	16	4	20	0.04
Leyte	165,619	0	0	0	0.00
Northern Samar	72,661	723	566	1,289	1.77
Southern Leyte	39,996	306	264	570	1.43
Samar	87,678	19	24	43	0.05
Ormoc City	25,774	0	0	0	0.00
City of Tacloban	27,696	445	538	983	3.55

Table 2.C.2.4 - Nutrition Services for Infants and Children
Children 0-59 mos. old whose nutritional status are normal, stunted and overweight/obese
Philippines, 2023

Area	Eligible Pop (0-59 mos. old)	Wasted			
		Male	Female	Total	%
**Region 9**	**437,233**	**2,451**	**2,273**	**4,724**	**1.08**
Zamboanga del Norte	123,478	614	631	1,245	1.01
Zamboanga del Sur	120,917	1,146	999	2,145	1.77
Zamboanga Sibugay	74,519	32	27	59	0.08
City of Isabela	16,896	313	252	565	3.34
City of Zamboanga	101,423	346	364	710	0.70
**Region 10**	**564,841**	**1,628**	**1,344**	**2,972**	**0.53**
Bukidnon	177,670	1,225	1,062	2,287	1.29
Camiguin	9,048	31	36	67	0.74
Lanao del Norte	92,387	5	5	10	0.01
Misamis Occidental	61,840	70	46	116	0.19
Misamis Oriental	109,995	197	123	320	0.29
City of Cagayan De Oro	73,964	30	19	49	0.07
City of Iligan	39,937	70	53	123	0.31
**Region 11**	**556,182**	**2,107**	**1,775**	**3,882**	**0.70**
Davao de Oro	81,277	169	123	292	0.36
Davao del Norte	121,250	299	284	583	0.48
Davao Oriental	60,933	451	354	805	1.32
Davao del Sur	72,277	344	231	575	0.80
Davao Occidental	38,331	584	506	1,090	2.84
City of Davao	182,114	260	277	537	0.29
**Region 12**	**491,133**	**2,079**	**1,647**	**3,726**	**0.76**
Cotabato	143,299	705	508	1,213	0.85
Sarangani	67,408	210	164	374	0.55
South Cotabato	107,889	220	183	403	0.37
Sultan Kudarat	97,978	633	549	1,182	1.21
City of General Santos	74,559	311	243	554	0.74
**BARMM**	**750,398**	**2,965**	**2,938**	**5,903**	**0.79**
Basilan	64,723	484	444	928	1.43
Lanao del Sur	185,348	327	367	694	0.37
Maguindanao	231,258	96	119	215	0.09

Table 2.C.2.4 - Nutrition Services for Infants and Children
Children 0-59 mos. old whose nutritional status are normal, stunted and overweight/obese
Philippines, 2023

Area	Eligible Pop (0-59 mos. old)	Wasted			
		Male	Female	Total	%
Sulu	131,184	1,042	1,034	2,076	1.58
Tawi-Tawi	69,576	809	793	1,602	2.30
SGA	28,976	36	36	72	0.25
City of Cotabato	39,333	171	145	316	0.80
**CARAGA**	**291,843**	**1,959**	**1,817**	**3,776**	**1.29**
Agusan del Norte	39,319	100	95	195	0.50
Agusan del Sur	83,337	553	526	1,079	1.29
Surigao del Norte	52,594	634	674	1,308	2.49
Surigao del Sur	68,313	573	432	1,005	1.47
Dinagat Islands	13,089	17	26	43	0.33
City of Butuan	35,191	82	64	146	0.41

Table 2.C.3.1 -Deworming Services for Children and Adolescents
Children/Adolescents who completed 2 doses of deworming tablet
Philippines, 2023

Area	Eligible Pop (1-4 years old)	No. of children 1-4 y/o given 2 doses of deworming drugs			
		Male	Female	Total	%
**Philippines**	**9,146,807**	**2,110,476**	**2,093,461**	**4,203,937**	**45.96**
**NCR**	**1,048,668**	**181,656**	**180,316**	**361,972**	**34.52**
Malabon City	29,472	319	871	1,190	4.04
Navotas City	20,859	1,471	1,409	2,880	13.81
Valenzuela City	54,902	19,179	19,031	38,210	69.60
Caloocan City	138,744	37,262	36,125	73,387	52.89
Marikina City	34,632	1,154	1,105	2,259	6.52
Pasig City	65,711	1,081	1,092	2,173	3.31
Pateros	4,824	537	560	1,097	22.74
Taguig City	74,943	42,194	41,608	83,802	111.82
Quezon City	238,612	1,002	1,002	2,004	0.84
Makati City	42,518	10,167	9,532	19,699	46.33
Mandaluyong City	31,561	1,457	1,601	3,058	9.69
San Juan City	8,619	346	285	631	7.32
Manila City	139,020	19,401	19,456	38,857	27.95
Las Piñas City	45,251	5,274	5,308	10,582	23.39
Muntinlupa City	39,143	20,782	22,044	42,826	109.41
Parañaque City	53,316	13,424	12,914	26,338	49.40
Pasay City	26,541	6,606	6,373	12,979	48.90
**CAR**	**131,920**	**33,596**	**31,265**	**64,861**	**49.17**
Abra	16,360	6,560	6,165	12,725	77.78
Apayao	9,270	1,848	1,667	3,515	37.92
Benguet	37,084	8,021	7,429	15,450	41.66
Ifugao	17,191	5,317	4,945	10,262	59.69
Kalinga	18,590	3,240	3,061	6,301	33.89
Mountain Province	11,394	4,650	4,246	8,896	78.08
City of Baguio	22,031	3,960	3,752	7,712	35.01
**Region 1**	**391,908**	**74,270**	**75,366**	**149,636**	**38.18**
Ilocos Norte	38,194	11,228	10,508	21,736	56.91
Ilocos Sur	43,632	15,167	14,540	29,707	68.09
La Union	55,447	14,413	13,795	28,208	50.87
Pangasinan	241,204	32,678	35,768	68,446	28.38
City of Dagupan	13,431	784	755	1,539	11.46

Table 2.C.3.1 -Deworming Services for Children and Adolescents
Children/Adolescents who completed 2 doses of deworming tablet
Philippines, 2023

Area	Eligible Pop (1-4 years old)	No. of children 1-4 y/o given 2 doses of deworming drugs			
		Male	Female	Total	%
**Region 2**	**273,976**	**75,021**	**71,396**	**146,417**	**53.44**
Batanes	1,131	112	145	257	22.72
Cagayan	90,741	27,676	26,860	54,536	60.10
Isabela	116,264	18,194	17,762	35,956	30.93
Nueva Vizcaya	38,816	15,979	14,451	30,430	78.40
Quirino	15,691	7,323	7,049	14,372	91.59
City of Santiago	11,333	5,737	5,129	10,866	95.88
**Region 3**	**982,471**	**283,498**	**339,491**	**622,989**	**63.41**
Aurora	19,792	8,655	8,472	17,127	86.53
Bataan	70,606	35,997	33,900	69,897	99.00
Bulacan	286,564	73,859	69,843	143,702	50.15
Nueva Ecija	183,214	57,261	52,303	109,564	59.80
Pampanga	191,692	71,256	69,866	141,122	73.62
Tarlac	120,696	12,929	80,824	93,753	77.68
Zambales	49,599	10,992	11,647	22,639	45.64
City of Angeles	41,866	2,961	2,919	5,880	14.04
City of Olongapo	18,442	9,588	9,717	19,305	104.68
**Region 4A**	**1,282,073**	**220,583**	**209,642**	**430,225**	**33.56**
Batangas	228,336	59,082	55,646	114,728	50.25
Cavite	333,432	75,535	73,306	148,841	44.64
Laguna	257,644	34,863	31,691	66,554	25.83
Quezon	164,813	20,506	19,337	39,843	24.17
Rizal	273,505	21,828	20,900	42,728	15.62
City of Lucena	24,343	8,769	8,762	17,531	72.02
**Region 4B**	**273,659**	**84,744**	**81,068**	**165,812**	**60.59**
Marinduque	19,647	7,601	7,111	14,712	74.88
Occidental Mindoro	47,933	20,156	18,969	39,125	81.62
Oriental Mindoro	75,450	34,595	33,243	67,838	89.91
Palawan	84,854	15,598	15,065	30,663	36.14
Romblon	22,821	6,783	6,669	13,452	58.95
City of Puerto Princesa	22,954	11	11	22	0.10

Table 2.C.3.1 -Deworming Services for Children and Adolescents
Children/Adolescents who completed 2 doses of deworming tablet
Philippines, 2023

Area	Eligible Pop (1-4 years old)	No. of children 1-4 y/o given 2 doses of deworming drugs			
		Male	Female	Total	%
**Region 5**	**563,991**	**110,849**	**106,107**	**216,956**	**38.47**
Albay	115,352	16,478	15,703	32,181	27.90
Camarines Norte	61,493	7,919	7,685	15,604	25.38
Camarines Sur	179,598	41,147	38,497	79,644	44.35
Catanduanes	24,838	6,337	6,012	12,349	49.72
Masbate	91,183	22,538	22,027	44,565	48.87
Sorsogon	74,885	13,592	13,287	26,879	35.89
City of Naga	16,642	2,838	2,896	5,734	34.45
**Region 6**	**622,616**	**127,081**	**118,877**	**245,958**	**39.50**
Aklan	46,468	18,025	17,261	35,286	75.94
Antique	50,529	10,185	9,318	19,503	38.60
Capiz	61,188	25,995	25,218	51,213	83.70
Guimaras	14,733	4,580	4,248	8,828	59.92
Iloilo	153,196	20,565	19,463	40,028	26.13
Negros Occidental	216,379	36,991	36,552	73,543	33.99
City of Bacolod	43,741	560	580	1,140	2.61
City of Iloilo	36,382	10,180	6,237	16,417	45.12
**Region 7**	**675,230**	**144,563**	**132,297**	**276,860**	**41.00**
Bohol	108,880	37,436	35,256	72,692	66.76
Cebu	292,954	50,164	44,050	94,214	32.16
Negros Oriental	119,479	30,894	27,654	58,548	49.00
Siquijor	6,402	3,329	3,126	6,455	100.83
City of Cebu	78,931	15,810	14,950	30,760	38.97
City of Lapu-Lapu	42,039	4,946	4,723	9,669	23.00
City of Mandaue	26,545	1,984	2,538	4,522	17.04
**Region 8**	**392,835**	**75,658**	**72,808**	**148,466**	**37.79**
Biliran	15,130	3,006	2,779	5,785	38.24
Eastern Samar	39,097	9,501	9,947	19,448	49.74
Leyte	134,060	24,819	24,017	48,836	36.43
Northern Samar	58,605	12,581	12,569	25,150	42.91
Southern Leyte	32,590	8,876	8,213	17,089	52.44
Samar	70,667	5,017	5,154	10,171	14.39
Ormoc City	20,482	10,023	8,382	18,405	89.86
City of Tacloban	22,204	1,835	1,747	3,582	16.13

Table 2.C.3.1 -Deworming Services for Children and Adolescents
Children/Adolescents who completed 2 doses of deworming tablet
Philippines, 2023

Area	Eligible Pop (1-4 years old)	No. of children 1-4 y/o given 2 doses of deworming drugs			
		Male	Female	Total	%
**Region 9**	**352,418**	**122,402**	**125,580**	**247,982**	**70.37**
Zamboanga del Norte	98,637	31,166	34,891	66,057	66.97
Zamboanga del Sur	97,160	39,829	40,330	80,159	82.50
Zamboanga Sibugay	60,678	13,510	12,581	26,091	43.00
City of Isabela	13,695	5,541	5,568	11,109	81.12
City of Zamboanga	82,248	32,356	32,210	64,566	78.50
**Region 10**	**455,672**	**105,526**	**109,177**	**214,703**	**47.12**
Bukidnon	144,267	35,919	32,834	68,753	47.66
Camiguin	7,321	3,592	3,341	6,933	94.70
Lanao del Norte	74,627	10,405	10,492	20,897	28.00
Misamis Occidental	49,512	26,020	33,877	59,897	120.97
Misamis Oriental	88,020	5,015	4,634	9,649	10.96
City of Cagayan De Oro	59,656	9,533	9,524	19,057	31.94
City of Iligan	32,269	15,042	14,475	29,517	91.47
**Region 11**	**449,181**	**186,865**	**177,814**	**364,679**	**81.19**
Davao de Oro	66,024	28,075	26,765	54,840	83.06
Davao del Norte	97,797	42,903	41,557	84,460	86.36
Davao Oriental	48,998	21,107	20,426	41,533	84.76
Davao del Sur	59,194	22,012	20,957	42,969	72.59
Davao Occidental	31,702	4,114	4,016	8,130	25.65
City of Davao	145,466	68,654	64,093	132,747	91.26
**Region 12**	**395,854**	**125,093**	**122,198**	**247,291**	**62.47**
Cotabato	116,778	37,803	36,697	74,500	63.80
Sarangani	54,086	21,671	21,746	43,417	80.27
South Cotabato	86,294	46,603	46,264	92,867	107.62
Sultan Kudarat	78,587	17,137	15,872	33,009	42.00
City of General Santos	60,109	1,879	1,619	3,498	5.82
**BARMM**	**617,557**	**96,706**	**76,977**	**173,683**	**28.12**
Basilan	53,087	2,542	2,527	5,069	9.55
Lanao del Sur	151,974	32,901	33,583	66,484	43.75
Maguindanao	189,608	14,487	13,868	28,355	14.95

Table 2.C.3.1 -Deworming Services for Children and Adolescents
Children/Adolescents who completed 2 doses of deworming tablet
Philippines, 2023

Area	Eligible Pop (1-4 years old)	No. of children 1-4 y/o given 2 doses of deworming drugs			
		Male	Female	Total	%
Sulu	109,806	11,754	13,501	25,255	23.00
Tawi-Tawi	56,967	22,228	5,403	27,631	48.50
SGA	23,873	103	113	216	0.90
City of Cotabato	32,242	12,691	7,982	20,673	64.12
**Caraga**	**236,778**	**62,365**	**63,082**	**125,447**	**52.98**
Agusan del Norte	32,033	7,356	6,976	14,332	44.74
Agusan del Sur	67,299	17,413	19,327	36,740	54.59
Surigao del Norte	42,408	10,000	9,408	19,408	45.76
Surigao del Sur	55,577	18,990	18,336	37,326	67.16
Dinagat Islands	10,955	1,712	1,637	3,349	30.57
City of Butuan	28,506	6,894	7,398	14,292	50.14

Table 2.C.3.1 -Deworming Services for Children and Adolescents
Children/Adolescents who completed 2 doses of deworming tablet
Philippines, 2023

Area	Eligible Pop (5-9 years old)	No. of children 5-9 y/o given 2 doses of deworming drugs			
		Male	Female	Total	%
**Philippines**	**11,540,487**	**1,284,844**	**1,347,840**	**2,632,684**	**22.81**
**NCR**	**1,266,961**	**89,253**	**89,239**	**178,492**	**14.09**
Malabon City	37,412	251	626	877	2.34
Navotas City	26,458	607	609	1,216	4.60
Valenzuela City	66,781	8,000	8,731	16,731	25.05
Caloocan City	169,048	6,159	5,931	12,090	7.15
Marikina City	43,139	213	192	405	0.94
Pasig City	80,162	157	154	311	0.39
Pateros	6,024	201	230	431	7.15
Taguig City	88,058	39,484	38,075	77,559	88.08
Quezon City	282,763	232	269	501	0.18
Makati City	48,550	2,063	1,888	3,951	8.14
Mandaluyong City	37,479	575	487	1,062	2.83
San Juan City	10,144	166	160	326	3.21
Manila City	170,068	8,124	8,029	16,153	9.50
Las Piñas City	56,329	1,634	1,469	3,103	5.51
Muntinlupa City	47,189	8,720	9,050	17,770	37.66
Parañaque City	61,993	11,357	12,046	23,403	37.75
Pasay City	35,364	1,310	1,293	2,603	7.36
**CAR**	**176,217**	**10,367**	**10,145**	**20,512**	**11.64**
Abra	24,374	2,458	2,597	5,055	20.74
Apayao	12,447	104	90	194	1.56
Benguet	45,884	312	255	567	1.24
Ifugao	22,877	3,289	3,139	6,428	28.10
Kalinga	24,586	1,306	1,288	2,594	10.55
Mountain Province	16,281	2,176	2,080	4,256	26.14
City of Baguio	29,768	722	696	1,418	4.76
**Region 1**	**507,969**	**52,306**	**54,235**	**106,541**	**20.97**
Ilocos Norte	49,811	4,155	4,185	8,340	16.74
Ilocos Sur	60,631	12,158	11,699	23,857	39.35
La Union	73,826	7,407	7,363	14,770	20.01
Pangasinan	306,476	27,598	29,995	57,593	18.79
City of Dagupan	17,225	988	993	1,981	11.50

Table 2.C.3.1 -Deworming Services for Children and Adolescents
Children/Adolescents who completed 2 doses of deworming tablet
Philippines, 2023

Area	Eligible Pop (5-9 years old)	No. of children 5-9 y/o given 2 doses of deworming drugs			
		Male	Female	Total	%
**Region 2**	**368,957**	**15,655**	**15,141**	**30,796**	**8.35**
Batanes	1,547	53	52	105	6.79
Cagayan	125,812	2,956	2,900	5,856	4.65
Isabela	153,849	2,770	2,523	5,293	3.44
Nueva Vizcaya	51,830	1,752	1,708	3,460	6.68
Quirino	21,346	969	1,140	2,109	9.88
City of Santiago	14,573	7,155	6,818	13,973	95.88
**Region 3**	**1,239,937**	**133,490**	**221,705**	**355,195**	**28.65**
Aurora	25,809	3,565	3,223	6,788	26.30
Bataan	87,766	14,419	13,870	28,289	32.23
Bulacan	366,493	42,454	39,968	82,422	22.49
Nueva Ecija	231,837	25,379	23,958	49,337	21.28
Pampanga	236,484	18,469	25,116	43,585	18.43
Tarlac	155,070	14,579	101,374	115,953	74.77
Zambales	66,902	7,392	7,188	14,580	21.79
City of Angeles	46,182	1,719	1,693	3,412	7.39
City of Olongapo	23,394	5,514	5,315	10,829	46.29
**Region 4A**	**1,609,243**	**141,202**	**132,451**	**273,653**	**17.01**
Batangas	281,141	30,016	27,943	57,959	20.62
Cavite	414,716	52,391	49,286	101,677	24.52
Laguna	323,870	26,747	24,222	50,969	15.74
Quezon	215,362	13,955	13,545	27,500	12.77
Rizal	344,313	15,364	14,766	30,130	8.75
City of Lucena	29,841	2,729	2,689	5,418	18.16
**Region 4B**	**364,479**	**70,815**	**68,331**	**139,146**	**38.18**
Marinduque	25,104	6,094	5,694	11,788	46.96
Occidental Mindoro	62,765	14,582	14,223	28,805	45.89
Oriental Mindoro	100,070	36,869	35,540	72,409	72.36
Palawan	114,310	10,959	10,432	21,391	18.71
Romblon	32,739	2,311	2,442	4,753	14.52
City of Puerto Princesa	29,491	0	0	0	0.00

Table 2.C.3.1 -Deworming Services for Children and Adolescents
Children/Adolescents who completed 2 doses of deworming tablet
Philippines, 2023

Area	Eligible Pop (5-9 years old)	No. of children 5-9 y/o given 2 doses of deworming drugs			
		Male	Female	Total	%
**Region 5**	**718,752**	**95,842**	**89,239**	**185,081**	**25.75**
Albay	152,223	12,744	12,567	25,311	16.63
Camarines Norte	76,076	6,829	6,270	13,099	17.22
Camarines Sur	222,559	45,353	42,200	87,553	39.34
Catanduanes	31,531	4,480	4,152	8,632	27.38
Masbate	118,110	15,876	13,951	29,827	25.25
Sorsogon	97,373	7,696	7,113	14,809	15.21
City of Naga	20,880	2,864	2,986	5,850	28.02
**Region 6**	**799,446**	**77,792**	**77,327**	**155,119**	**19.40**
Aklan	60,105	25,004	24,732	49,736	82.75
Antique	62,837	5,684	5,516	11,200	17.82
Capiz	76,214	8,512	8,940	17,452	22.90
Guimaras	18,528	4,675	4,346	9,021	48.69
Iloilo	201,181	14,911	14,105	29,016	14.42
Negros Occidental	282,627	16,899	17,569	34,468	12.20
City of Bacolod	55,018	515	579	1,094	1.99
City of Iloilo	42,936	1,592	1,540	3,132	7.29
**Region 7**	**854,076**	**39,566**	**39,782**	**79,348**	**9.29**
Bohol	140,990	2,938	2,861	5,799	4.11
Cebu	371,546	14,948	13,629	28,577	7.69
Negros Oriental	152,055	7,394	7,411	14,805	9.74
Siquijor	9,103	2,462	2,511	4,973	54.63
City of Cebu	94,920	4,359	4,257	8,616	9.08
City of Lapu-Lapu	52,729	2,390	2,370	4,760	9.03
City of Mandaue	32,733	5,075	6,743	11,818	36.10
**Region 8**	**510,779**	**32,388**	**35,987**	**68,375**	**13.39**
Biliran	20,872	1,832	1,844	3,676	17.61
Eastern Samar	51,975	308	347	655	1.26
Leyte	172,238	2,333	2,520	4,853	2.82
Northern Samar	77,943	10,728	10,383	21,111	27.09
Southern Leyte	41,591	3,366	3,133	6,499	15.63
Samar	93,083	2,125	2,159	4,284	4.60
Ormoc City	25,503	1,648	1,698	3,346	13.12
City of Tacloban	27,574	10,048	13,903	23,951	86.86

Table 2.C.3.1 -Deworming Services for Children and Adolescents
Children/Adolescents who completed 2 doses of deworming tablet
Philippines, 2023

Area	Eligible Pop (5-9 years old)	No. of children 5-9 y/o given 2 doses of deworming drugs			
		Male	Female	Total	%
**Region 9**	**454,310**	**158,325**	**164,093**	**322,418**	**70.97**
Zamboanga del Norte	123,327	42,299	47,381	89,680	72.72
Zamboanga del Sur	123,611	46,836	47,911	94,747	76.65
Zamboanga Sibugay	82,854	16,911	16,363	33,274	40.16
City of Isabela	15,852	7,102	7,285	14,387	90.76
City of Zamboanga	108,666	45,177	45,153	90,330	83.13
**Region 10**	**586,318**	**84,508**	**89,814**	**174,322**	**29.73**
Bukidnon	193,051	27,383	25,875	53,258	27.59
Camiguin	10,230	2,957	2,649	5,606	54.80
Lanao del Norte	95,067	13,942	15,128	29,070	30.58
Misamis Occidental	63,776	19,409	25,506	44,915	70.43
Misamis Oriental	111,404	5,760	5,296	11,056	9.92
City of Cagayan De Oro	73,402	3,371	3,799	7,170	9.77
City of Iligan	39,388	11,686	11,561	23,247	59.02
**Region 11**	**565,867**	**50,591**	**47,258**	**97,849**	**17.29**
Davao de Oro	86,855	4,956	4,627	9,583	11.03
Davao del Norte	121,301	5,255	5,270	10,525	8.68
Davao Oriental	66,167	3,688	3,570	7,258	10.97
Davao del Sur	70,997	3,776	3,395	7,171	10.10
Davao Occidental	39,583	2,903	2,622	5,525	13.96
City of Davao	180,964	30,013	27,774	57,787	31.93
**Region 12**	**509,412**	**91,942**	**89,689**	**181,631**	**35.66**
Cotabato	151,702	17,673	17,563	35,236	23.23
Sarangani	69,278	18,461	18,447	36,908	53.28
South Cotabato	110,077	32,678	31,110	63,788	57.95
Sultan Kudarat	101,443	21,344	21,084	42,428	41.82
City of General Santos	76,912	1,786	1,485	3,271	4.25
**BARMM**	**683,399**	**94,812**	**79,891**	**174,703**	**25.56**
Basilan	62,390	2,860	2,637	5,497	8.81
Lanao del Sur	162,846	31,941	32,902	64,843	39.82
Maguindanao	200,131	14,501	14,907	29,408	14.69

Table 2.C.3.1 -Deworming Services for Children and Adolescents
Children/Adolescents who completed 2 doses of deworming tablet
Philippines, 2023

Area	Eligible Pop (5-9 years old)	No. of children 5-9 y/o given 2 doses of deworming drugs			
		Male	Female	Total	%
Sulu	136,276	13,377	14,184	27,561	20.22
Tawi-Tawi	58,175	20,364	6,155	26,519	45.58
SGA	26,540	34	42	76	0.29
City of Cotabato	37,041	11,735	9,064	20,799	56.15
**Caraga**	**324,365**	**45,990**	**43,513**	**89,503**	**27.59**
Agusan del Norte	44,794	6,342	6,135	12,477	27.85
Agusan del Sur	93,557	11,807	10,895	22,702	24.27
Surigao del Norte	57,169	13,175	11,960	25,135	43.97
Surigao del Sur	74,598	5,911	5,648	11,559	15.50
Dinagat Islands	14,122	1,374	1,339	2,713	19.21
City of Butuan	40,125	7,381	7,536	14,917	37.18

Table 2.C.3.1 -Deworming Services for Children and Adolescents
Children/Adolescents who completed 2 doses of deworming tablet
Philippines, 2023

Area	Eligible Pop (1-4 years old)	Eligible Pop (10-19 years old)	No. of children 10-19 y/o given 2 doses of deworming drugs			
			Male	Female	Total	%
**Philippines**	**9,146,807**	**22,092,100**	**1,840,362**	**1,841,261**	**3,681,623**	**16.66**
**NCR**	**1,048,668**	**2,350,929**	**80,007**	**78,014**	**158,021**	**6.72**
Malabon City	29,472	72,042	154	347	501	0.70
Navotas City	20,859	48,936	229	220	449	0.92
Valenzuela City	54,902	131,897	7,112	7,254	14,366	10.89
Caloocan City	138,744	315,608	3,361	3,312	6,673	2.11
Marikina City	34,632	81,236	121	130	251	0.31
Pasig City	65,711	140,495	334	321	655	0.47
Pateros	4,824	11,472	135	161	296	2.58
Taguig City	74,943	160,685	48,255	45,849	94,104	58.56
Quezon City	238,612	511,899	218	206	424	0.08
Makati City	42,518	89,568	723	875	1,598	1.78
Mandaluyong City	31,561	67,468	551	418	969	1.44
San Juan City	8,619	18,743	231	225	456	2.43
Manila City	139,020	323,671	8,313	8,219	16,532	5.11
Las Piñas City	45,251	105,511	467	474	941	0.89
Muntinlupa City	39,143	89,738	8,649	8,887	17,536	19.54
Parañaque City	53,316	111,939	133	139	272	0.24
Pasay City	26,541	70,021	1,021	977	1,998	2.85
**CAR**	**131,920**	**365,747**	**14,515**	**14,014**	**28,529**	**7.80**
Abra	16,360	51,082	2,376	2,238	4,614	9.03
Apayao	9,270	26,276	346	384	730	2.78
Benguet	37,084	90,230	344	305	649	0.72
Ifugao	17,191	45,351	5,149	5,047	10,196	22.48
Kalinga	18,590	50,417	2,965	2,952	5,917	11.74
Mountain Province	11,394	34,549	2,470	2,260	4,730	13.69
City of Baguio	22,031	67,842	865	828	1,693	2.50
**Region 1**	**391,908**	**1,033,725**	**79,132**	**83,936**	**163,068**	**15.77**
Ilocos Norte	38,194	110,610	2,125	2,164	4,289	3.88
Ilocos Sur	43,632	131,715	23,104	23,447	46,551	35.34
La Union	55,447	153,038	11,764	11,751	23,515	15.37
Pangasinan	241,204	602,900	40,366	44,853	85,219	14.13
City of Dagupan	13,431	35,462	1,773	1,721	3,494	9.85

Table 2.C.3.1 -Deworming Services for Children and Adolescents
Children/Adolescents who completed 2 doses of deworming tablet
Philippines, 2023

Area	Eligible Pop (1-4 years old)	Eligible Pop (10-19 years old)	No. of children 10-19 y/o given 2 doses of deworming drugs			
			Male	Female	Total	%
**Region 2**	**273,976**	**728,786**	**21,788**	**20,427**	**42,215**	**5.79**
Batanes	1,131	3,495	114	117	231	6.61
Cagayan	90,741	248,957	4,262	3,892	8,154	3.28
Isabela	116,264	304,267	2,961	2,666	5,627	1.85
Nueva Vizcaya	38,816	99,396	2,527	2,698	5,225	5.26
Quirino	15,691	42,435	95	80	175	0.41
City of Santiago	11,333	30,236	11,829	10,974	22,803	75.42
**Region 3**	**982,471**	**2,427,685**	**182,988**	**216,991**	**399,979**	**16.48**
Aurora	19,792	50,812	5,483	5,400	10,883	21.42
Bataan	70,606	166,813	8,315	7,985	16,300	9.77
Bulacan	286,564	719,278	45,384	81,768	127,152	17.68
Nueva Ecija	183,214	449,332	24,221	24,718	48,939	10.89
Pampanga	191,692	478,672	62,137	59,709	121,846	25.46
Tarlac	120,696	298,044	20,066	20,567	40,633	13.63
Zambales	49,599	130,070	10,053	9,819	19,872	15.28
City of Angeles	41,866	86,516	1,911	1,824	3,735	4.32
City of Olongapo	18,442	48,148	5,418	5,201	10,619	22.05
**Region 4A**	**1,282,073**	**3,096,849**	**175,354**	**167,352**	**342,706**	**11.07**
Batangas	228,336	558,191	44,527	41,658	86,185	15.44
Cavite	333,432	802,360	60,780	58,372	119,152	14.85
Laguna	257,644	613,299	34,531	32,469	67,000	10.92
Quezon	164,813	419,468	21,097	20,095	41,192	9.82
Rizal	273,505	646,427	12,021	12,125	24,146	3.74
City of Lucena	24,343	57,104	2,398	2,633	5,031	8.81
**Region 4B**	**273,659**	**713,293**	**113,543**	**109,560**	**223,103**	**31.28**
Marinduque	19,647	51,888	10,698	10,215	20,913	40.30
Occidental Mindoro	47,933	119,082	19,282	18,545	37,827	31.77
Oriental Mindoro	75,450	197,315	63,411	61,877	125,288	63.50
Palawan	84,854	213,388	16,878	15,837	32,715	15.33
Romblon	22,821	69,602	3,274	3,086	6,360	9.14
City of Puerto Princesa	22,954	62,018	0	0	0	0.00

Table 2.C.3.1 -Deworming Services for Children and Adolescents

Children/Adolescents who completed 2 doses of deworming tablet

Philippines, 2023

Area	Eligible Pop (1-4 years old)	Eligible Pop (10-19 years old)	No. of children 10-19 y/o given 2 doses of deworming drugs			
			Male	Female	Total	%
**Region 5**	**563,991**	**1,401,417**	**139,658**	**129,836**	**269,494**	**19.23**
Albay	115,352	308,726	20,613	19,377	39,990	12.95
Camarines Norte	61,493	140,206	11,618	10,700	22,318	15.92
Camarines Sur	179,598	434,240	64,470	60,433	124,903	28.76
Catanduanes	24,838	62,144	1,934	1,863	3,797	6.11
Masbate	91,183	224,156	25,377	22,942	48,319	21.56
Sorsogon	74,885	187,573	12,641	11,703	24,344	12.98
City of Naga	16,642	44,372	3,005	2,818	5,823	13.12
**Region 6**	**622,616**	**1,573,241**	**125,759**	**120,009**	**245,768**	**15.62**
Aklan	46,468	121,245	48,045	46,186	94,231	77.72
Antique	50,529	123,564	8,730	8,355	17,085	13.83
Capiz	61,188	157,150	10,898	11,023	21,921	13.95
Guimaras	14,733	35,434	9,118	8,533	17,651	49.81
Iloilo	153,196	400,929	24,766	23,649	48,415	12.08
Negros Occidental	216,379	544,526	21,592	19,669	41,261	7.58
City of Bacolod	43,741	111,224	658	584	1,242	1.12
City of Iloilo	36,382	79,169	1,952	2,010	3,962	5.00
**Region 7**	**675,230**	**1,640,051**	**36,429**	**35,730**	**72,159**	**4.40**
Bohol	108,880	289,867	1,879	1,715	3,594	1.24
Cebu	292,954	689,816	19,955	20,029	39,984	5.80
Negros Oriental	119,479	291,835	7,041	6,495	13,536	4.64
Siquijor	6,402	18,833	4,203	4,022	8,225	43.67
City of Cebu	78,931	184,895	2,123	2,296	4,419	2.39
City of Lapu-Lapu	42,039	99,001	1,217	1,157	2,374	2.40
City of Mandaue	26,545	65,804	11	16	27	0.04
**Region 8**	**392,835**	**998,652**	**51,064**	**55,737**	**106,801**	**10.69**
Biliran	15,130	39,658	1,448	1,917	3,365	8.49
Eastern Samar	39,097	106,612	468	425	893	0.84
Leyte	134,060	330,340	2,777	2,923	5,700	1.73
Northern Samar	58,605	153,658	20,845	19,853	40,698	26.49
Southern Leyte	32,590	87,160	5,415	5,256	10,671	12.24
Samar	70,667	183,123	2,922	3,029	5,951	3.25
Ormoc City	20,482	47,590	2,942	4,017	6,959	14.62
City of Tacloban	22,204	50,511	14,247	18,317	32,564	64.47

Table 2.C.3.1 -Deworming Services for Children and Adolescents
Children/Adolescents who completed 2 doses of deworming tablet
Philippines, 2023

Area	Eligible Pop (1-4 years old)	Eligible Pop (10-19 years old)	No. of children 10-19 y/o given 2 doses of deworming drugs			
			Male	Female	Total	%
**Region 9**	**352,418**	**845,152**	**267,641**	**278,574**	**546,215**	**64.63**
Zamboanga del Norte	98,637	228,135	76,510	82,535	159,045	69.72
Zamboanga del Sur	97,160	232,591	79,199	82,146	161,345	69.37
Zamboanga Sibugay	60,678	153,599	32,449	31,089	63,538	41.37
City of Isabela	13,695	28,477	10,950	10,987	21,937	77.03
City of Zamboanga	82,248	202,350	68,533	71,817	140,350	69.36
**Region 10**	**455,672**	**1,080,119**	**140,402**	**147,746**	**288,148**	**26.68**
Bukidnon	144,267	347,189	41,206	39,187	80,393	23.16
Camiguin	7,321	19,153	5,683	5,170	10,853	56.66
Lanao del Norte	74,627	168,789	25,948	26,273	52,221	30.94
Misamis Occidental	49,512	125,805	32,593	42,087	74,680	59.36
Misamis Oriental	88,020	204,355	9,751	8,991	18,742	9.17
City of Cagayan De Oro	59,656	138,975	6,196	6,504	12,700	9.14
City of Iligan	32,269	75,853	19,025	19,534	38,559	50.83
**Region 11**	**449,181**	**1,069,862**	**45,985**	**40,399**	**86,384**	**8.07**
Davao de Oro	66,024	167,660	5,544	5,256	10,800	6.44
Davao del Norte	97,797	224,538	2,715	2,703	5,418	2.41
Davao Oriental	48,998	126,834	2,908	2,648	5,556	4.38
Davao del Sur	59,194	135,874	3,428	3,165	6,593	4.85
Davao Occidental	31,702	71,106	4,829	4,594	9,423	13.25
City of Davao	145,466	343,850	26,561	22,033	48,594	14.13
**Region 12**	**395,854**	**952,092**	**145,219**	**144,570**	**289,789**	**30.44**
Cotabato	116,778	281,805	22,104	22,863	44,967	15.96
Sarangani	54,086	128,236	31,401	32,056	63,457	49.48
South Cotabato	86,294	207,259	48,546	47,976	96,522	46.57
Sultan Kudarat	78,587	192,822	40,886	39,655	80,541	41.77
City of General Santos	60,109	141,970	2,282	2,020	4,302	3.03
**BARMM**	**617,557**	**1,203,744**	**146,558**	**127,086**	**273,644**	**22.73**
Basilan	53,087	102,910	3,831	4,460	8,291	8.06
Lanao del Sur	151,974	298,349	50,885	52,669	103,554	34.71
Maguindanao	189,608	329,657	20,228	21,085	41,313	12.53

Table 2.C.3.1 -Deworming Services for Children and Adolescents
Children/Adolescents who completed 2 doses of deworming tablet
Philippines, 2023

Area	Eligible Pop (1-4 years old)	Eligible Pop (10-19 years old)	No. of children 10-19 y/o given 2 doses of deworming drugs			
			Male	Female	Total	%
Sulu	109,806	254,360	20,320	21,888	42,208	16.59
Tawi-Tawi	56,967	98,079	35,598	9,736	45,334	46.22
SGA	23,873	47,703	96	126	222	0.47
City of Cotabato	32,242	72,686	15,600	17,122	32,722	45.02
**Caraga**	**236,778**	**610,756**	**74,320**	**71,280**	**145,600**	**23.84**
Agusan del Norte	32,033	84,785	11,705	11,111	22,816	26.91
Agusan del Sur	67,299	168,869	15,062	13,786	28,848	17.08
Surigao del Norte	42,408	112,617	24,573	23,424	47,997	42.62
Surigao del Sur	55,577	138,529	8,797	8,134	16,931	12.22
Dinagat Islands	10,955	27,122	1,763	1,905	3,668	13.52
City of Butuan	28,506	78,834	12,420	12,920	25,340	32.14

Table 2.C.4.1 - Management of Sick Infants and Children

High risk infants and children with measles and/or persistent diarrhea who received Vitamin A capsule aside from routine supplementation

Philippines, 2023

Area	Sick Infants aged 6-11 mos.						
	Seen			given Vitamin A 100,000 IU			
	Male	Female	Total	Male	Female	Total	%
**Philippines**	**90,122**	**85,707**	**175,829**	**44,082**	**41,529**	**85,611**	**48.69**
**NCR**	**1,668**	**1,530**	**3,198**	**869**	**793**	**1,662**	**51.97**
Malabon City	24	23	47	24	23	47	100.00
Navotas City	98	89	187	89	69	158	84.49
Valenzuela City	0	0	0	0	0	0	0.00
Caloocan City	16	16	32	16	16	32	100.00
Marikina City	2	0	2	2	0	2	100.00
Pasig City	0	0	0	0	0	0	0.00
Pateros	14	12	26	14	12	26	100.00
Taguig City	1,104	963	2,067	346	282	628	30.38
Quezon City	59	66	125	57	57	114	91.20
Makati City	140	139	279	113	114	227	81.36
Mandaluyong City	0	0	0	0	0	0	0.00
San Juan City	0	0	0	0	0	0	0.00
Manila City	7	22	29	7	22	29	100.00
Las Piñas City	2	2	4	2	2	4	100.00
Muntinlupa City	118	129	247	118	129	247	100.00
Parañaque City	30	27	57	27	25	52	91.23
Pasay City	54	42	96	54	42	96	100.00
**CAR**	**318**	**309**	**627**	**215**	**208**	**423**	**67.46**
Abra	37	41	78	29	36	65	83.33
Apayao	72	84	156	39	39	78	50.00
Benguet	22	24	46	22	24	46	100.00
Ifugao	126	107	233	80	63	143	61.37
Kalinga	27	18	45	19	13	32	71.11
Mountain Province	0	0	0	0	0	0	0.00
City of Baguio	34	35	69	26	33	59	85.51
**Region 1**	**4,157**	**3,721**	**7,878**	**3,404**	**2,852**	**6,256**	**79.41**
Ilocos Norte	156	159	315	149	153	302	95.87
Ilocos Sur	557	565	1,122	259	223	482	42.96
La Union	183	119	302	160	102	262	86.75
Pangasinan	3,261	2,878	6,139	2,836	2,374	5,210	84.87

Table 2.C.4.1 - Management of Sick Infants and Children
High risk infants and children with measles and/or persistent diarrhea who received Vitamin A capsule aside from routine supplementation
Philippines, 2023

Area	Sick Infants aged 6-11 mos.						
	Seen			given Vitamin A 100,000 IU			
City of Dagupan	0	0	0	0	0	0	0.00
**Region 2**	**5,217**	**4,839**	**10,056**	**2,125**	**2,003**	**4,128**	**41.05**
Batanes	15	14	29	2	0	2	6.90
Cagayan	681	572	1,253	307	276	583	46.53
Isabela	4,157	3,827	7,984	1,607	1,496	3,103	38.87
Nueva Vizcaya	207	233	440	76	73	149	33.86
Quirino	157	193	350	133	158	291	83.14
City of Santiago	0	0	0	0	0	0	0.00
**Region 3**	**13,038**	**12,085**	**25,123**	**6,756**	**6,079**	**12,835**	**51.09**
Aurora	151	180	331	90	100	190	57.40
Bataan	579	531	1,110	161	161	322	29.01
Bulacan	6,071	5,539	11,610	3,302	2,929	6,231	53.67
Nueva Ecija	2,000	1,876	3,876	743	678	1,421	36.66
Pampanga	972	860	1,832	794	716	1,510	82.42
Tarlac	2,228	2,078	4,306	1,146	1,033	2,179	50.60
Zambales	537	553	1,090	188	170	358	32.84
City of Angeles	386	366	752	297	252	549	73.01
City of Olongapo	114	102	216	35	40	75	34.72
**Region 4A**	**14,490**	**13,824**	**28,314**	**3,681**	**3,484**	**7,165**	**25.31**
Batangas	451	409	860	134	111	245	28.49
Cavite	7,235	6,961	14,196	1,610	1,564	3,174	22.36
Laguna	1,185	1,032	2,217	260	213	473	21.34
Quezon	2,993	2,730	5,723	481	405	886	15.48
Rizal	2,191	2,250	4,441	761	749	1,510	34.00
City of Lucena	435	442	877	435	442	877	100.00
**Region 4B**	**1,666**	**1,678**	**3,344**	**522**	**533**	**1,055**	**31.55**
Marinduque	44	32	76	20	10	30	39.47
Occidental Mindoro	523	534	1,057	174	203	377	35.67
Oriental Mindoro	391	395	786	135	122	257	32.70
Palawan	338	345	683	159	185	344	50.37
Romblon	257	236	493	25	7	32	6.49

Table 2.C.4.1 - Management of Sick Infants and Children
High risk infants and children with measles and/or persistent diarrhea who received Vitamin A capsule aside from routine supplementation
Philippines, 2023

Area	Sick Infants aged 6-11 mos.						
	Seen			given Vitamin A 100,000 IU			
City of Puerto Princesa	113	136	249	9	6	15	6.02
**Region 5**	**4,308**	**4,263**	**8,571**	**1,710**	**1,629**	**3,339**	**38.96**
Albay	1,778	1,766	3,544	407	393	800	22.57
Camarines Norte	428	514	942	251	340	591	62.74
Camarines Sur	91	96	187	36	26	62	33.16
Catanduanes	55	65	120	6	15	21	17.50
Masbate	1,184	1,069	2,253	848	730	1,578	70.04
Sorsogon	772	753	1,525	162	125	287	18.82
City of Naga	0	0	0	0	0	0	0.00
**Region 6**	**5,889**	**5,408**	**11,297**	**2,708**	**2,593**	**5,301**	**46.92**
Aklan	647	673	1,320	380	424	804	60.91
Antique	589	501	1,090	121	150	271	24.86
Capiz	105	110	215	105	110	215	100.00
Guimaras	6	4	10	0	0	0	0.00
Iloilo	2,042	1,826	3,868	1,001	870	1,871	48.37
Negros Occidental	1,876	1,708	3,584	812	791	1,603	44.73
City of Bacolod	425	402	827	202	182	384	46.43
City of Iloilo	199	184	383	87	66	153	39.95
**Region 7**	**1,967**	**1,783**	**3,750**	**1,399**	**1,328**	**2,727**	**72.72**
Bohol	184	152	336	22	23	45	13.39
Cebu	805	762	1,567	541	555	1,096	69.94
Negros Oriental	42	38	80	17	20	37	46.25
Siquijor	6	7	13	0	0	0	0.00
City of Cebu	158	117	275	99	68	167	60.73
City of Lapu-Lapu	195	193	388	143	148	291	75.00
City of Mandaue	577	514	1,091	577	514	1,091	100.00
**Region 8**	**7,447**	**6,765**	**14,212**	**2,818**	**2,636**	**5,454**	**38.38**
Biliran	525	478	1,003	73	64	137	13.66
Eastern Samar	925	884	1,809	201	191	392	21.67
Leyte	1,420	1,350	2,770	499	489	988	35.67
Northern Samar	2,283	2,028	4,311	553	534	1,087	25.21
Southern Leyte	220	163	383	53	55	108	28.20
Samar	1,608	1,404	3,012	1,020	914	1,934	64.21

Table 2.C.4.1 - Management of Sick Infants and Children
High risk infants and children with measles and/or persistent diarrhea who received Vitamin A capsule aside from routine supplementation
Philippines, 2023

Area	Sick Infants aged 6-11 mos.						
	Seen			given Vitamin A 100,000 IU			
Ormoc City	1	0	1	1	0	1	100.00
City of Tacloban	465	458	923	418	389	807	87.43
**Region 9**	**4,807**	**4,784**	**9,591**	**3,123**	**3,050**	**6,173**	**64.36**
Zamboanga del Norte	1,625	1,577	3,202	961	948	1,909	59.62
Zamboanga del Sur	1,620	1,586	3,206	1,249	1,200	2,449	76.39
Zamboanga Sibugay	512	572	1,084	172	206	378	34.87
City of Isabela	230	190	420	164	121	285	67.86
City of Zamboanga	820	859	1,679	577	575	1,152	68.61
**Region 10**	**8,372**	**8,418**	**16,790**	**5,030**	**4,965**	**9,995**	**59.53**
Bukidnon	3,088	3,170	6,258	1,308	1,317	2,625	41.95
Camiguin	73	68	141	3	1	4	2.84
Lanao del Norte	1,447	1,610	3,057	1,086	1,157	2,243	73.37
Misamis Occidental	1,544	1,452	2,996	1,456	1,358	2,814	93.93
Misamis Oriental	538	464	1,002	134	122	256	25.55
City of Cagayan De Oro	569	496	1,065	302	254	556	52.21
City of Iligan	1,113	1,158	2,271	741	756	1,497	65.92
**Region 11**	**4,995**	**4,661**	**9,656**	**3,149**	**2,936**	**6,085**	**63.02**
Davao de Oro	591	603	1,194	288	326	614	51.42
Davao del Norte	1,370	1,273	2,643	456	429	885	33.48
Davao Oriental	1,076	955	2,031	942	815	1,757	86.51
Davao del Sur	327	296	623	165	151	316	50.72
Davao Occidental	179	216	395	80	87	167	42.28
City of Davao	1,452	1,318	2,770	1,218	1,128	2,346	84.69
**Region 12**	**4,585**	**4,473**	**9,058**	**2,723**	**2,638**	**5,361**	**59.19**
Cotabato	2,122	2,143	4,265	529	602	1,131	26.52
Sarangani	1,848	1,773	3,621	1,635	1,544	3,179	87.79
South Cotabato	227	199	426	185	150	335	78.64
Sultan Kudarat	87	89	176	86	89	175	99.43
City of General Santos	301	269	570	288	253	541	94.91

Table 2.C.4.1 - Management of Sick Infants and Children

High risk infants and children with measles and/or persistent diarrhea who received Vitamin A capsule aside from routine supplementation

Philippines, 2023

Area	Sick Infants aged 6-11 mos.						
	Seen			given Vitamin A 100,000 IU			
**BARMM**	**4,301**	**4,312**	**8,613**	**2,779**	**2,740**	**5,519**	**64.08**
Basilan	1,004	1,052	2,056	549	532	1,081	52.58
Lanao del Sur	1,246	1,192	2,438	786	744	1,530	62.76
Maguindanao	20	14	34	16	9	25	73.53
Sulu	1,499	1,474	2,973	1,134	1,132	2,266	76.22
Tawi-Tawi	286	271	557	166	163	329	59.07
SGA	54	65	119	27	38	65	54.62
City of Cotabato	192	244	436	101	122	223	51.15
**Caraga**	**2,897**	**2,854**	**5,751**	**1,071**	**1,062**	**2,133**	**37.09**
Agusan del Norte	305	316	621	68	84	152	24.48
Agusan del Sur	1,415	1,280	2,695	640	588	1,228	45.57
Surigao del Norte	971	1,034	2,005	277	275	552	27.53
Surigao del Sur	62	73	135	48	66	114	84.44
Dinagat Islands	104	109	213	9	18	27	12.68
City of Butuan	40	42	82	29	31	60	73.17

Table 2.C.4.1 - Management of Sick Infants and Children
High risk infants and children with measles and/or persistent diarrhea who received Vitamin A capsule aside from routine supplementation
Philippines, 2023

Area	Sick Children aged 12-59 mos.						
	Seen			given Vitamin A 200,000 IU			
	Male	Female	Total	Male	Female	Total	%
**Philippines**	**195,230**	**188,245**	**383,475**	**84,383**	**81,260**	**165,643**	**43.20**
**NCR**	**4,490**	**4,307**	**8,797**	**2,216**	**2,204**	**4,420**	**50.24**
Malabon City	40	39	79	37	36	73	92.41
Navotas City	219	223	442	200	193	393	88.91
Valenzuela City	0	0	0	0	0	0	0.00
Caloocan City	38	66	104	38	66	104	100.00
Marikina City	3	4	7	3	4	7	100.00
Pasig City	0	0	0	0	0	0	0.00
Pateros	29	22	51	29	22	51	100.00
Taguig City	3,398	3,271	6,669	1,205	1,254	2,459	36.87
Quezon City	103	86	189	103	86	189	100.00
Makati City	217	222	439	163	168	331	75.40
Mandaluyong City	0	0	0	0	0	0	0.00
San Juan City	0	0	0	0	0	0	0.00
Manila City	9	16	25	9	16	25	100.00
Las Piñas City	6	3	9	6	3	9	100.00
Muntinlupa City	198	178	376	198	178	376	100.00
Parañaque City	63	44	107	58	45	103	96.26
Pasay City	167	133	300	167	133	300	100.00
**CAR**	**633**	**553**	**1,186**	**427**	**323**	**750**	**63.24**
Abra	155	140	295	115	109	224	75.93
Apayao	141	157	298	32	25	57	19.13
Benguet	53	29	82	53	29	82	100.00
Ifugao	234	175	409	185	117	302	73.84
Kalinga	48	51	99	42	42	84	84.85
Mountain Province	0	0	0	0	0	0	0.00
City of Baguio	2	1	3	0	1	1	33.33
**Region 1**	**8,511**	**7,604**	**16,115**	**7,048**	**6,101**	**13,149**	**81.59**
Ilocos Norte	372	321	693	363	312	675	97.40
Ilocos Sur	1,317	1,252	2,569	572	503	1,075	41.85
La Union	1,003	1,240	2,243	978	1,219	2,197	97.95
Pangasinan	5,819	4,791	10,610	5,135	4,067	9,202	86.73

Table 2.C.4.1 - Management of Sick Infants and Children

High risk infants and children with measles and/or persistent diarrhea who received Vitamin A capsule aside from routine supplementation

Philippines, 2023

Area	Sick Children aged 12-59 mos.						
	Seen			given Vitamin A 200,000 IU			
City of Dagupan	0	0	0	0	0	0	0.00
**Region 2**	**10,926**	**10,159**	**21,085**	**4,327**	**4,061**	**8,388**	**39.78**
Batanes	111	116	227	26	36	62	27.31
Cagayan	1,156	1,095	2,251	508	406	914	40.60
Isabela	8,615	8,013	16,628	3,303	3,119	6,422	38.62
Nueva Vizcaya	599	517	1,116	118	160	278	24.91
Quirino	445	418	863	372	340	712	82.50
City of Santiago	0	0	0	0	0	0	0.00
**Region 3**	**28,130**	**27,236**	**55,366**	**11,378**	**10,917**	**22,295**	**40.27**
Aurora	569	481	1,050	291	213	504	48.00
Bataan	1,170	1,181	2,351	323	303	626	26.63
Bulacan	13,515	12,724	26,239	6,110	5,870	11,980	45.66
Nueva Ecija	4,069	3,835	7,904	955	917	1,872	23.68
Pampanga	1,931	1,832	3,763	1,541	1,525	3,066	81.48
Tarlac	4,491	4,907	9,398	1,225	1,243	2,468	26.26
Zambales	1,363	1,341	2,704	477	432	909	33.62
City of Angeles	726	676	1,402	400	358	758	54.07
City of Olongapo	296	259	555	56	56	112	20.18
**Region 4A**	**33,948**	**32,780**	**66,728**	**7,068**	**7,124**	**14,192**	**21.27**
Batangas	1,267	1,119	2,386	385	346	731	30.64
Cavite	16,737	16,462	33,199	3,039	3,161	6,200	18.68
Laguna	2,507	2,070	4,577	423	453	876	19.14
Quezon	7,190	7,091	14,281	856	833	1,689	11.83
Rizal	5,408	5,147	10,555	1,526	1,450	2,976	28.20
City of Lucena	839	891	1,730	839	881	1,720	99.42
**Region 4B**	**5,523**	**5,259**	**10,782**	**1,107**	**1,074**	**2,181**	**20.23**
Marinduque	145	183	328	91	152	243	74.09
Occidental Mindoro	2,579	2,332	4,911	269	220	489	9.96
Oriental Mindoro	749	710	1,459	208	201	409	28.03
Palawan	1,053	1,030	2,083	510	473	983	47.19
Romblon	722	764	1,486	26	26	52	3.50
City of Puerto Princesa	275	240	515	3	2	5	0.97

Table 2.C.4.1 - Management of Sick Infants and Children
High risk infants and children with measles and/or persistent diarrhea who received Vitamin A capsule aside from routine supplementation
Philippines, 2023

Area	Sick Children aged 12-59 mos.						
	Seen			given Vitamin A 200,000 IU			
**Region 5**	**8,091**	**8,074**	**16,165**	**3,407**	**3,422**	**6,829**	**42.25**
Albay	3,092	3,133	6,225	588	589	1,177	18.91
Camarines Norte	1,005	1,057	2,062	758	814	1,572	76.24
Camarines Sur	197	184	381	63	57	120	31.50
Catanduanes	193	173	366	51	39	90	24.59
Masbate	2,167	2,141	4,308	1,707	1,708	3,415	79.27
Sorsogon	1,437	1,381	2,818	240	210	450	15.97
City of Naga	0	5	5	0	5	5	100.00
**Region 6**	**14,822**	**14,097**	**28,919**	**5,373**	**5,258**	**10,631**	**36.76**
Aklan	1,653	1,584	3,237	976	941	1,917	59.22
Antique	1,271	1,217	2,488	405	353	758	30.47
Capiz	230	257	487	230	257	487	100.00
Guimaras	11	10	21	1	0	1	4.76
Iloilo	5,352	4,987	10,339	2,295	2,254	4,549	44.00
Negros Occidental	5,075	4,816	9,891	1,089	1,106	2,195	22.19
City of Bacolod	615	626	1,241	244	235	479	38.60
City of Iloilo	615	600	1,215	133	112	245	20.16
**Region 7**	**4,177**	**4,105**	**8,282**	**2,848**	**2,820**	**5,668**	**68.44**
Bohol	289	194	483	93	46	139	28.78
Cebu	2,167	2,250	4,417	1,300	1,319	2,619	59.29
Negros Oriental	25	34	59	15	24	39	66.10
Siquijor	28	20	48	1	2	3	6.25
City of Cebu	248	187	435	125	92	217	49.89
City of Lapu-Lapu	360	353	713	254	270	524	73.49
City of Mandaue	1,060	1,067	2,127	1,060	1,067	2,127	100.00
**Region 8**	**16,849**	**16,372**	**33,221**	**6,055**	**5,733**	**11,788**	**35.48**
Biliran	1,396	1,263	2,659	194	181	375	14.10
Eastern Samar	2,643	2,790	5,433	937	869	1,806	33.24
Leyte	3,246	3,243	6,489	997	1,012	2,009	30.96
Northern Samar	4,644	4,421	9,065	1,185	1,078	2,263	24.96
Southern Leyte	583	488	1,071	119	91	210	19.61
Samar	3,033	2,887	5,920	1,717	1,621	3,338	56.39

Table 2.C.4.1 - Management of Sick Infants and Children

High risk infants and children with measles and/or persistent diarrhea who received Vitamin A capsule aside from routine supplementation

Philippines, 2023

Area	Sick Children aged 12-59 mos.						
	Seen			given Vitamin A 200,000 IU			
Ormoc City	7	1	8	7	1	8	100.00
City of Tacloban	1,297	1,279	2,576	899	880	1,779	69.06
**Region 9**	**9,390**	**9,078**	**18,468**	**5,930**	**5,693**	**11,623**	**62.94**
Zamboanga del Norte	2,942	2,795	5,737	1,732	1,664	3,396	59.19
Zamboanga del Sur	3,491	3,313	6,804	2,618	2,477	5,095	74.88
Zamboanga Sibugay	1,181	1,271	2,452	324	340	664	27.08
City of Isabela	368	356	724	240	240	480	66.30
City of Zamboanga	1,408	1,343	2,751	1,016	972	1,988	72.26
**Region 10**	**16,168**	**15,715**	**31,883**	**8,238**	**8,048**	**16,286**	**51.08**
Bukidnon	6,356	6,233	12,589	2,176	2,167	4,343	34.50
Camiguin	160	149	309	1	3	4	1.29
Lanao del Norte	2,885	2,948	5,833	1,780	1,886	3,666	62.85
Misamis Occidental	2,098	1,976	4,074	1,822	1,695	3,517	86.33
Misamis Oriental	1,264	1,232	2,496	601	551	1,152	46.15
City of Cagayan De Oro	1,069	984	2,053	337	326	663	32.29
City of Iligan	2,336	2,193	4,529	1,521	1,420	2,941	64.94
**Region 11**	**10,348**	**9,638**	**19,986**	**6,477**	**6,045**	**12,522**	**62.65**
Davao de Oro	1,033	1,143	2,176	640	750	1,390	63.88
Davao del Norte	3,677	3,263	6,940	1,138	997	2,135	30.76
Davao Oriental	2,254	2,051	4,305	2,097	1,897	3,994	92.78
Davao del Sur	673	625	1,298	376	328	704	54.24
Davao Occidental	399	401	800	209	223	432	54.00
City of Davao	2,312	2,155	4,467	2,017	1,850	3,867	86.57
**Region 12**	**8,318**	**8,156**	**16,474**	**4,869**	**4,649**	**9,518**	**57.78**
Cotabato	3,691	3,729	7,420	919	921	1,840	24.80
Sarangani	3,414	3,331	6,745	2,946	2,839	5,785	85.77
South Cotabato	506	442	948	365	314	679	71.62
Sultan Kudarat	141	133	274	117	101	218	79.56
City of General Santos	566	521	1,087	522	474	996	91.63

Table 2.C.4.1 - Management of Sick Infants and Children
High risk infants and children with measles and/or persistent diarrhea who received Vitamin A capsule aside from routine supplementation
Philippines, 2023

Area	Sick Children aged 12-59 mos.						
	Seen			given Vitamin A 200,000 IU			
**BARMM**	**8,933**	**9,070**	**18,003**	**5,353**	**5,611**	**10,964**	**60.90**
Basilan	1,937	1,957	3,894	1,026	1,084	2,110	54.19
Lanao del Sur	2,476	2,539	5,015	1,588	1,679	3,267	65.14
Maguindanao	25	19	44	19	7	26	59.09
Sulu	3,172	3,194	6,366	2,231	2,385	4,616	72.51
Tawi-Tawi	795	757	1,552	312	299	611	39.37
SGA	89	71	160	39	41	80	50.00
City of Cotabato	439	533	972	138	116	254	26.13
**Caraga**	**5,973**	**6,042**	**12,015**	**2,262**	**2,177**	**4,439**	**36.95**
Agusan del Norte	732	664	1,396	209	208	417	29.87
Agusan del Sur	2,824	2,821	5,645	1,389	1,291	2,680	47.48
Surigao del Norte	2,007	2,164	4,171	472	502	974	23.35
Surigao del Sur	163	139	302	150	129	279	92.38
Dinagat Islands	232	240	472	37	42	79	16.74
City of Butuan	15	14	29	5	5	10	34.48

Table 2.C.4.2 - Management of Sick Infants and Children

Diarrhea cases 0-59 mos. old who received Oral Rehydration Salt (ORS) solution and ORS with Zinc drops/syrup

Philippines, 2023

Area	Children 0-59 mos. old with Diarrhea										
	Seen			Given ORS				Given ORS w/ zinc			
	Male	Female	Total	Male	Female	Total	%	Male	Female	Total	%
**Philippines**	**55,094**	**48,714**	**103,808**	**24,226**	**21,707**	**45,933**	**44.25**	**28,268**	**25,522**	**53,790**	**51.82**
**NCR**	**5,214**	**4,354**	**9,568**	**2,203**	**1,802**	**4,005**	**41.86**	**2,973**	**2,508**	**5,481**	**57.28**
Malabon City	182	149	331	126	103	229	69.18	47	36	83	25.08
Navotas City	331	317	648	218	209	427	65.90	113	108	221	34.10
Valenzuela City	322	248	570	258	198	456	80.00	64	50	114	20.00
Caloocan City	267	197	464	112	94	206	44.40	155	103	258	55.60
Marikina City	23	15	38	5	2	7	18.42	18	13	31	81.58
Pasig City	184	136	320	53	39	92	28.75	131	97	228	71.25
Pateros	18	15	33	15	12	27	81.82	3	3	6	18.18
Taguig City	816	761	1,577	106	108	214	13.57	687	620	1,307	82.88
Quezon City	1,029	855	1,884	549	434	983	52.18	480	421	901	47.82
Makati City	204	148	352	72	41	113	32.10	128	104	232	65.91
Mandaluyong City	76	67	143	38	42	80	55.94	38	25	63	44.06
San Juan City	131	115	246	5	6	11	4.47	126	109	235	95.53
Manila City	806	656	1,462	380	314	694	47.47	426	342	768	52.53
Las Piñas City	309	278	587	68	53	121	20.61	241	225	466	79.39
Muntinlupa City	310	230	540	136	99	235	43.52	172	133	305	56.48
Parañaque City	70	48	118	52	37	89	75.42	18	11	29	24.58
Pasay City	136	119	255	10	11	21	8.24	126	108	234	91.76
**CAR**	**2,609**	**2,064**	**4,673**	**862**	**701**	**1,563**	**33.45**	**1,716**	**1,340**	**3,056**	**65.40**
Abra	205	170	375	34	38	72	19.20	170	132	302	80.53
Apayao	290	226	516	27	24	51	9.88	261	200	461	89.34
Benguet	506	362	868	158	111	269	30.99	348	251	599	69.01
Ifugao	730	578	1,308	336	282	618	47.25	381	285	666	50.92
Kalinga	521	440	961	230	168	398	41.42	276	263	539	56.09
Mountain Province	270	225	495	76	74	150	30.30	194	150	344	69.49
City of Baguio	87	63	150	1	4	5	3.33	86	59	145	96.67
**Region 1**	**2,519**	**2,280**	**4,799**	**1,469**	**1,161**	**2,630**	**54.80**	**1,050**	**918**	**1,968**	**41.01**
Ilocos Norte	61	62	123	36	32	68	55.28	25	29	54	43.90
Ilocos Sur	644	561	1,205	78	84	162	13.44	566	477	1,043	86.56
La Union	179	351	530	38	35	73	13.77	141	116	257	48.49
Pangasinan	1,635	1,306	2,941	1,317	1,010	2,327	79.12	318	296	614	20.88
City of Dagupan	0	0	0	0	0	0	0.00	0	0	0	0.00

Table 2.C.4.2 - Management of Sick Infants and Children

Diarrhea cases 0-59 mos. old who received Oral Rehydration Salt (ORS) solution and ORS with Zinc drops/syrup

Philippines, 2023

Area	Children 0-59 mos. old with Diarrhea										
	Seen			Given ORS				Given ORS w/ zinc			
	Male	Female	Total	Male	Female	Total	%	Male	Female	Total	%
**Region 2**	**2,357**	**1,936**	**4,293**	**435**	**386**	**821**	**19.12**	**1,716**	**1,393**	**3,109**	**72.42**
Batanes	1	7	8	0	3	3	37.50	1	3	4	50.00
Cagayan	294	250	544	164	145	309	56.80	129	102	231	42.46
Isabela	1,669	1,377	3,046	54	52	106	3.48	1,421	1,178	2,599	85.33
Nueva Vizcaya	356	281	637	209	182	391	61.38	144	99	243	38.15
Quirino	37	21	58	8	4	12	20.69	21	11	32	55.17
City of Santiago	0	0	0	0	0	0	0.00	0	0	0	0.00
**Region 3**	**7,672**	**7,169**	**14,841**	**3,362**	**3,159**	**6,521**	**43.94**	**4,195**	**3,959**	**8,154**	**54.94**
Aurora	86	77	163	14	16	30	18.40	74	59	133	81.60
Bataan	746	611	1,357	355	289	644	47.46	383	330	713	52.54
Bulacan	3,601	3,323	6,924	1,354	1,176	2,530	36.54	2,183	2,092	4,275	61.74
Nueva Ecija	830	785	1,615	407	424	831	51.46	404	353	757	46.87
Pampanga	671	678	1,349	372	385	757	56.12	297	293	590	43.74
Tarlac	1,344	1,310	2,654	659	672	1,331	50.15	669	635	1,304	49.13
Zambales	207	172	379	108	87	195	51.45	92	92	184	48.55
City of Angeles	156	178	334	93	105	198	59.28	64	73	137	41.02
City of Olongapo	31	35	66	0	5	5	7.58	29	32	61	92.42
**Region 4A**	**3,154**	**3,000**	**6,154**	**2,643**	**2,465**	**5,108**	**83.00**	**1,877**	**1,795**	**3,672**	**59.67**
Batangas	215	162	377	125	79	204	54.11	146	118	264	70.03
Cavite	764	720	1,484	555	525	1,080	72.78	379	353	732	49.33
Laguna	509	536	1,045	388	381	769	73.59	229	232	461	44.11
Quezon	649	600	1,249	587	541	1,128	90.31	394	377	771	61.73
Rizal	761	717	1,478	735	679	1,414	95.67	478	459	937	63.40
City of Lucena	256	265	521	253	260	513	98.46	251	256	507	97.31
**Region 4B**	**1,217**	**1,124**	**2,341**	**397**	**391**	**788**	**33.66**	**576**	**521**	**1,097**	**46.86**
Marinduque	38	31	69	15	14	29	42.03	23	17	40	57.97
Occidental Mindoro	407	360	767	31	30	61	7.95	166	157	323	42.11
Oriental Mindoro	91	93	184	30	32	62	33.70	61	59	120	65.22
Palawan	575	533	1,108	251	237	488	44.04	306	271	577	52.08
Romblon	74	65	139	57	54	111	79.86	7	4	11	7.91
City of Puerto Princesa	32	42	74	13	24	37	50.00	13	13	26	35.14

Table 2.C.4.2 - Management of Sick Infants and Children

Diarrhea cases 0-59 mos. old who received Oral Rehydration Salt (ORS) solution and ORS with Zinc drops/syrup

Philippines, 2023

Area	Children 0-59 mos. old with Diarrhea										
	Seen			Given ORS				Given ORS w/ zinc			
	Male	Female	Total	Male	Female	Total	%	Male	Female	Total	%
**Region 5**	**1,922**	**1,671**	**3,593**	**1,291**	**1,130**	**2,421**	**67.38**	**1,085**	**902**	**1,987**	**55.30**
Albay	310	242	552	156	127	283	51.27	150	107	257	46.56
Camarines Norte	284	291	575	188	184	372	64.70	108	130	238	41.39
Camarines Sur	276	228	504	143	123	266	52.78	164	134	298	59.13
Catanduanes	102	56	158	36	22	58	36.71	65	34	99	62.66
Masbate	778	705	1,483	662	586	1,248	84.15	545	450	995	67.09
Sorsogon	170	145	315	104	87	191	60.63	53	46	99	31.43
City of Naga	2	4	6	2	1	3	50.00	0	1	1	16.67
**Region 6**	**1,944**	**1,833**	**3,777**	**1,582**	**1,480**	**3,062**	**81.07**	**1,076**	**1,037**	**2,113**	**55.94**
Aklan	153	147	300	134	130	264	88.00	100	95	195	65.00
Antique	151	155	306	84	96	180	58.82	54	55	109	35.62
Capiz	70	63	133	37	34	71	53.38	33	29	62	46.62
Guimaras	16	8	24	15	8	23	95.83	7	4	11	45.83
Iloilo	387	368	755	364	337	701	92.85	243	240	483	63.97
Negros Occidental	920	877	1,797	731	709	1,440	80.13	509	476	985	54.81
City of Bacolod	107	109	216	105	93	198	91.67	61	76	137	63.43
City of Iloilo	140	106	246	112	73	185	75.20	69	62	131	53.25
**Region 7**	**2,358**	**2,147**	**4,505**	**966**	**928**	**1,894**	**42.04**	**1,623**	**1,411**	**3,034**	**67.35**
Bohol	756	724	1,480	363	356	719	48.58	386	371	757	51.15
Cebu	553	525	1,078	311	326	637	59.09	398	322	720	66.79
Negros Oriental	602	539	1,141	0	0	0	0.00	577	508	1,085	95.09
Siquijor	11	16	27	11	15	26	96.30	0	0	0	0.00
City of Cebu	244	185	429	119	99	218	50.82	157	117	274	63.87
City of Lapu-Lapu	142	134	276	112	108	220	79.71	105	93	198	71.74
City of Mandaue	50	24	74	50	24	74	100.00	0	0	0	0.00
**Region 8**	**2,082**	**1,676**	**3,758**	**1,353**	**1,088**	**2,441**	**64.95**	**1,398**	**1,186**	**2,584**	**68.76**
Biliran	182	180	362	32	30	62	17.13	170	166	336	92.82
Eastern Samar	233	214	447	160	140	300	67.11	59	85	144	32.21
Leyte	334	280	614	263	214	477	77.69	280	234	514	83.71
Northern Samar	607	452	1,059	486	383	869	82.06	378	303	681	64.31
Southern Leyte	100	62	162	33	24	57	35.19	64	37	101	62.35
Samar	476	356	832	282	211	493	59.25	339	267	606	72.84
Ormoc City	94	88	182	74	68	142	78.02	75	68	143	78.57
City of Tacloban	56	44	100	23	18	41	41.00	33	26	59	59.00

Table 2.C.4.2 - Management of Sick Infants and Children

Diarrhea cases 0-59 mos. old who received Oral Rehydration Salt (ORS) solution and ORS with Zinc drops/syrup

Philippines, 2023

Area	Children 0-59 mos. old with Diarrhea										
	Seen			Given ORS				Given ORS w/ zinc			
	Male	Female	Total	Male	Female	Total	%	Male	Female	Total	%
**Region 9**	**2,199**	**1,989**	**4,188**	**676**	**608**	**1,284**	**30.66**	**1,465**	**1,326**	**2,791**	**66.64**
Zamboanga del Norte	834	774	1,608	248	238	486	30.22	576	526	1,102	68.53
Zamboanga del Sur	531	449	980	60	59	119	12.14	437	364	801	81.73
Zamboanga Sibugay	234	262	496	64	65	129	26.01	163	185	348	70.16
City of Isabela	229	206	435	23	21	44	10.11	204	184	388	89.20
City of Zamboanga	371	298	669	281	225	506	75.64	85	67	152	22.72
**Region 10**	**3,218**	**3,133**	**6,351**	**2,594**	**2,481**	**5,075**	**79.91**	**2,469**	**2,434**	**4,903**	**77.20**
Bukidnon	659	629	1,288	520	473	993	77.10	489	496	985	76.48
Camiguin	6	5	11	3	0	3	27.27	5	5	10	90.91
Lanao del Norte	444	423	867	366	398	764	88.12	276	254	530	61.13
Misamis Occidental	1,503	1,484	2,987	1,229	1,158	2,387	79.91	1,401	1,357	2,758	92.33
Misamis Oriental	200	225	425	167	173	340	80.00	85	104	189	44.47
City of Cagayan De Oro	158	148	306	149	133	282	92.16	121	120	241	78.76
City of Iligan	248	219	467	160	146	306	65.52	92	98	190	40.69
**Region 11**	**1,684**	**1,562**	**3,246**	**676**	**646**	**1,322**	**40.73**	**1,088**	**953**	**2,041**	**62.88**
Davao de Oro	180	203	383	90	99	189	49.35	111	121	232	60.57
Davao del Norte	362	283	645	152	136	288	44.65	264	176	440	68.22
Davao Oriental	447	375	822	119	118	237	28.83	311	240	551	67.03
Davao del Sur	154	139	293	79	60	139	47.44	75	74	149	50.85
Davao Occidental	84	97	181	65	60	125	69.06	50	58	108	59.67
City of Davao	457	465	922	171	173	344	37.31	277	284	561	60.85
**Region 12**	**8,885**	**6,919**	**15,804**	**1,088**	**956**	**2,044**	**12.93**	**481**	**445**	**926**	**5.86**
Cotabato	419	412	831	291	263	554	66.67	108	125	233	28.04
Sarangani	366	309	675	321	282	603	89.33	12	9	21	3.11
South Cotabato	261	238	499	212	193	405	81.16	133	119	252	50.50
Sultan Kudarat	7,627	5,768	13,395	151	112	263	1.96	130	110	240	1.79
City of General Santos	212	192	404	113	106	219	54.21	98	82	180	44.55
**BARMM**	**5,414**	**5,234**	**10,648**	**2,265**	**1,971**	**4,236**	**39.78**	**3,218**	**3,134**	**6,352**	**59.65**
Basilan	711	647	1,358	628	588	1,216	89.54	83	59	142	10.46

Table 2.C.4.2 - Management of Sick Infants and Children

Diarrhea cases 0-59 mos. old who received Oral Rehydration Salt (ORS) solution and ORS with Zinc drops/syrup

Philippines, 2023

Area	Children 0-59 mos. old with Diarrhea										
	Seen			Given ORS				Given ORS w/ zinc			
	Male	Female	Total	Male	Female	Total	%	Male	Female	Total	%
Lanao del Sur	1,260	1,303	2,563	689	673	1,362	53.14	561	612	1,173	45.77
Maguindanao	1,910	1,879	3,789	650	452	1,102	29.08	1,361	1,333	2,694	71.10
Sulu	1,464	1,313	2,777	297	248	545	19.63	1,150	1,047	2,197	79.11
Tawi-Tawi	0	0	0	0	0	0	0.00	0	0	0	0.00
SGA	6	12	18	1	10	11	61.11	3	4	7	38.89
City of Cotabato	63	80	143	0	0	0	0.00	60	79	139	97.20
**Caraga**	**646**	**623**	**1,269**	**364**	**354**	**718**	**56.58**	**262**	**260**	**522**	**41.13**
Agusan del Norte	35	41	76	30	39	69	90.79	1	0	1	1.32
Agusan del Sur	209	208	417	109	115	224	53.72	99	92	191	45.80
Surigao del Norte	198	195	393	142	120	262	66.67	53	76	129	32.82
Surigao del Sur	155	148	303	65	70	135	44.55	78	71	149	49.17
Dinagat Islands	45	28	73	16	7	23	31.51	29	21	50	68.49
City of Butuan	4	3	7	2	3	5	71.43	2	0	2	28.57

Table 2.C.4.2 - Management of Sick Infants and Children
Pneumonia cases among children 0-59 mos. old who received treatment
Philippines, 2023

Area	Children 0-59 mos. old with Pneumonia						
	Seen			Received treatment			
	Male	Female	Total	Male	Female	Total	%
**Philippines**	**53,934**	**48,626**	**102,560**	**52,474**	**47,322**	**99,796**	**97.30**
**NCR**	**7,951**	**7,001**	**14,952**	**7,909**	**6,950**	**14,859**	**99.38**
Malabon City	1,121	913	2,034	1,104	891	1,995	98.08
Navotas City	31	27	58	31	27	58	100.00
Valenzuela City	653	644	1,297	646	638	1,284	99.00
Caloocan City	1,165	1,036	2,201	1,165	1,036	2,201	100.00
Marikina City	13	3	16	13	3	16	100.00
Pasig City	336	311	647	336	311	647	100.00
Pateros	2	1	3	2	1	3	100.00
Taguig City	277	219	496	261	200	461	92.94
Quezon City	1,763	1,786	3,549	1,763	1,786	3,549	100.00
Makati City	323	247	570	323	247	570	100.00
Mandaluyong City	601	497	1,098	601	497	1,098	100.00
San Juan City	215	210	425	215	210	425	100.00
Manila City	749	547	1,296	749	547	1,296	100.00
Las Piñas City	74	71	145	74	71	145	100.00
Muntinlupa City	341	248	589	341	248	589	100.00
Parañaque City	32	17	49	30	13	43	87.76
Pasay City	255	224	479	255	224	479	100.00
**CAR**	**2,652**	**2,265**	**4,917**	**2,528**	**2,161**	**4,689**	**95.36**
Abra	145	149	294	142	149	291	98.98
Apayao	589	476	1,065	491	389	880	82.63
Benguet	324	285	609	320	283	603	99.01
Ifugao	681	528	1,209	666	518	1,184	97.93
Kalinga	232	195	427	231	193	424	99.30
Mountain Province	224	198	422	224	198	422	100.00
City of Baguio	457	434	891	454	431	885	99.33
**Region 1**	**1,839**	**1,636**	**3,475**	**1,800**	**1,612**	**3,412**	**98.19**
Ilocos Norte	54	46	100	54	46	100	100.00
Ilocos Sur	370	300	670	369	300	669	99.85
La Union	532	478	1,010	503	461	964	95.45
Pangasinan	882	812	1,694	873	805	1,678	99.06
City of Dagupan	1	0	1	1	0	1	100.00

Table 2.C.4.2 - Management of Sick Infants and Children

Pneumonia cases among children 0-59 mos. old who received treatment

Philippines, 2023

Area	Children 0-59 mos. old with Pneumonia						
	Seen			Received treatment			
	Male	Female	Total	Male	Female	Total	%
**Region 2**	**2,605**	**2,164**	**4,769**	**2,557**	**2,130**	**4,687**	**98.28**
Batanes	15	14	29	14	14	28	96.55
Cagayan	245	210	455	238	203	441	96.92
Isabela	1,838	1,641	3,479	1,824	1,628	3,452	99.22
Nueva Vizcaya	459	271	730	433	257	690	94.52
Quirino	48	28	76	48	28	76	100.00
City of Santiago	0	0	0	0	0	0	0.00
**Region 3**	**2,225**	**1,968**	**4,193**	**2,151**	**1,906**	**4,057**	**96.76**
Aurora	175	119	294	172	118	290	98.64
Bataan	219	175	394	216	174	390	98.98
Bulacan	565	542	1,107	550	524	1,074	97.02
Nueva Ecija	577	465	1,042	567	458	1,025	98.37
Pampanga	375	376	751	373	373	746	99.33
Tarlac	150	112	262	117	87	204	77.86
Zambales	132	138	270	126	133	259	95.93
City of Angeles	26	37	63	25	36	61	96.83
City of Olongapo	6	4	10	5	3	8	80.00
**Region 4A**	**1,973**	**1,794**	**3,767**	**1,828**	**1,668**	**3,496**	**92.81**
Batangas	137	116	253	100	100	200	79.05
Cavite	269	231	500	240	196	436	87.20
Laguna	85	83	168	77	68	145	86.31
Quezon	582	549	1,131	548	522	1,070	94.61
Rizal	835	744	1,579	798	711	1,509	95.57
City of Lucena	65	71	136	65	71	136	100.00
**Region 4B**	**676**	**617**	**1,293**	**639**	**556**	**1,195**	**92.42**
Marinduque	20	12	32	19	12	31	96.88
Occidental Mindoro	39	40	79	23	20	43	54.43
Oriental Mindoro	113	94	207	109	93	202	97.58
Palawan	311	282	593	295	263	558	94.10
Romblon	191	187	378	191	167	358	94.71
City of Puerto Princesa	2	2	4	2	1	3	75.00

Table 2.C.4.2 - Management of Sick Infants and Children

Pneumonia cases among children 0-59 mos. old who received treatment

Philippines, 2023

Area	Children 0-59 mos. old with Pneumonia						
	Seen			Received treatment			
	Male	Female	Total	Male	Female	Total	%
**Region 5**	**1,780**	**1,548**	**3,328**	**1,665**	**1,455**	**3,120**	**93.75**
Albay	124	143	267	111	108	219	82.02
Camarines Norte	176	149	325	170	142	312	96.00
Camarines Sur	320	231	551	295	218	513	93.10
Catanduanes	71	61	132	69	61	130	98.48
Masbate	850	733	1,583	787	704	1,491	94.19
Sorsogon	239	231	470	233	222	455	96.81
City of Naga	0	0	0	0	0	0	0.00
**Region 6**	**4,234**	**4,209**	**8,443**	**4,098**	**4,066**	**8,164**	**96.70**
Aklan	118	104	222	114	99	213	95.95
Antique	753	733	1,486	742	725	1,467	98.72
Capiz	500	550	1,050	500	550	1,050	100.00
Guimaras	44	41	85	44	41	85	100.00
Iloilo	1,033	992	2,025	995	939	1,934	95.51
Negros Occidental	1,260	1,319	2,579	1,247	1,295	2,542	98.57
City of Bacolod	229	219	448	161	169	330	73.66
City of Iloilo	297	251	548	295	248	543	99.09
**Region 7**	**6,114**	**5,252**	**11,366**	**6,010**	**5,164**	**11,174**	**98.31**
Bohol	148	128	276	124	97	221	80.07
Cebu	1,989	1,730	3,719	1,940	1,699	3,639	97.85
Negros Oriental	1,982	1,802	3,784	1,961	1,788	3,749	99.08
Siquijor	210	196	406	208	188	396	97.54
City of Cebu	1,673	1,291	2,964	1,665	1,287	2,952	99.60
City of Lapu-Lapu	94	90	184	94	90	184	100.00
City of Mandaue	18	15	33	18	15	33	100.00
**Region 8**	**4,501**	**4,180**	**8,681**	**4,412**	**4,077**	**8,489**	**97.79**
Biliran	434	351	785	434	339	773	98.47
Eastern Samar	906	878	1,784	879	848	1,727	96.80
Leyte	1,074	1,027	2,101	1,057	1,000	2,057	97.91
Northern Samar	423	352	775	411	342	753	97.16
Southern Leyte	102	92	194	102	92	194	100.00
Samar	735	684	1,419	717	663	1,380	97.25
Ormoc City	353	310	663	353	310	663	100.00

Table 2.C.4.2 - Management of Sick Infants and Children

Pneumonia cases among children 0-59 mos. old who received treatment

Philippines, 2023

Area	Children 0-59 mos. old with Pneumonia						
	Seen			Received treatment			
	Male	Female	Total	Male	Female	Total	%
City of Tacloban	474	486	960	459	483	942	98.13
**Region 9**	**2,976**	**2,625**	**5,601**	**2,931**	**2,569**	**5,500**	**98.20**
Zamboanga del Norte	1,630	1,481	3,111	1,624	1,465	3,089	99.29
Zamboanga del Sur	905	750	1,655	889	740	1,629	98.43
Zamboanga Sibugay	204	185	389	195	176	371	95.37
City of Isabela	116	105	221	113	98	211	95.48
City of Zamboanga	121	104	225	110	90	200	88.89
**Region 10**	**4,935**	**4,595**	**9,530**	**4,810**	**4,468**	**9,278**	**97.36**
Bukidnon	1,197	1,102	2,299	1,167	1,132	2,299	100.00
Camiguin	36	25	61	35	25	60	98.36
Lanao del Norte	817	806	1,623	817	799	1,616	99.57
Misamis Occidental	1,727	1,653	3,380	1,713	1,600	3,313	98.02
Misamis Oriental	467	394	861	405	329	734	85.25
City of Cagayan De Oro	506	452	958	491	421	912	95.20
City of Iligan	185	163	348	182	162	344	98.85
**Region 11**	**2,724**	**2,628**	**5,352**	**2,671**	**2,583**	**5,254**	**98.17**
Davao de Oro	437	612	1,049	432	607	1,039	99.05
Davao del Norte	682	632	1,314	677	631	1,308	99.54
Davao Oriental	930	793	1,723	916	785	1,701	98.72
Davao del Sur	214	195	409	207	186	393	96.09
Davao Occidental	59	39	98	59	39	98	100.00
City of Davao	402	357	759	380	335	715	94.20
**Region 12**	**2,592**	**2,363**	**4,955**	**2,530**	**2,304**	**4,834**	**97.56**
Cotabato	953	856	1,809	919	836	1,755	97.01
Sarangani	250	205	455	239	200	439	96.48
South Cotabato	823	738	1,561	811	707	1,518	97.25
Sultan Kudarat	341	368	709	336	366	702	99.01
City of General Santos	225	196	421	225	195	420	99.76
**BARMM**	**3,139**	**2,854**	**5,993**	**2,935**	**2,739**	**5,674**	**94.68**
Basilan	457	456	913	388	407	795	87.08

Table 2.C.4.2 - Management of Sick Infants and Children

Pneumonia cases among children 0-59 mos. old who received treatment

Philippines, 2023

Area	Children 0-59 mos. old with Pneumonia						
	Seen			Received treatment			
	Male	Female	Total	Male	Female	Total	%
Lanao del Sur	842	779	1,621	829	767	1,596	98.46
Maguindanao	1,330	1,234	2,564	1,305	1,172	2,477	96.61
Sulu	498	361	859	402	370	772	89.87
Tawi-Tawi	0	0	0	0	0	0	0.00
SGA	3	8	11	3	8	11	100.00
City of Cotabato	9	16	25	8	15	23	92.00
**Caraga**	**1,018**	**927**	**1,945**	**1,000**	**914**	**1,914**	**98.41**
Agusan del Norte	19	14	33	16	11	27	81.82
Agusan del Sur	151	125	276	151	125	276	100.00
Surigao del Norte	405	389	794	391	380	771	97.10
Surigao del Sur	415	377	792	415	376	791	99.87
Dinagat Islands	23	19	42	22	19	41	97.62
City of Butuan	5	3	8	5	3	8	100.00

Table 2.C.4.3 - Nutrition Services for Infants and Children
Moderately Wasted Children enrolled and cured in Supplementary Feeding Program (SFP)
Philippines, 2023

Area	Total of MAM	MAM - admitted in SFP				MAM - cured in SFP			
		Male	Female	Total	%	Male	Female	Total	%
**Philippines**	**100,800**	**15,372**	**13,906**	**29,278**	**29.05**	**5,420**	**5,034**	**10,454**	**10.37**
**NCR**	**2,702**	**1,213**	**1,201**	**2,414**	**89.34**	**560**	**640**	**1,200**	**44.41**
Malabon City	17	7	10	17	100.00	0	0	0	0.00
Navotas City	23	13	10	23	100.00	13	10	23	100.00
Valenzuela City	6	0	0	0	0.00	4	6	10	166.67
Caloocan City	195	92	103	195	100.00	10	10	20	10.26
Marikina City	1	1	0	1	100.00	0	0	0	0.00
Pasig City	204	113	85	198	97.06	27	41	68	33.33
Pateros	70	4	2	6	8.57	4	2	6	8.57
Taguig City	95	55	40	95	100.00	55	40	95	100.00
Quezon City	1,601	468	495	963	60.15	283	354	637	39.79
Makati City	129	41	35	76	58.91	23	20	43	33.33
Mandaluyong City	94	20	36	56	59.57	22	30	52	55.32
San Juan City	23	5	4	9	39.13	4	3	7	30.43
Manila City	0	332	327	659	0.00	90	79	169	0.00
Las Piñas City	92	4	12	16	17.39	4	12	16	17.39
Muntinlupa City	55	2	1	3	5.45	19	31	50	90.91
Parañaque City	97	56	41	97	100.00	2	2	4	4.12
Pasay City	0	0	0	0	0.00	0	0	0	0.00
**CAR**	**630**	**64**	**60**	**124**	**19.68**	**2**	**2**	**4**	**0.63**
Abra	203	22	14	36	17.73	0	0	0	0.00
Apayao	212	31	37	68	32.08	0	1	1	0.47
Benguet	29	0	2	2	6.90	0	0	0	0.00
Ifugao	64	2	3	5	7.81	0	0	0	0.00
Kalinga	89	3	4	7	7.87	0	0	0	0.00
Mountain Province	15	6	0	6	40.00	2	1	3	20.00
City of Baguio	18	0	0	0	0.00	0	0	0	0.00
**Region 1**	**2,394**	**601**	**397**	**998**	**41.69**	**141**	**110**	**251**	**10.48**
Ilocos Norte	666	299	203	502	75.38	80	62	142	21.32
Ilocos Sur	734	210	142	352	47.96	57	45	102	13.90
La Union	314	64	38	102	32.48	4	3	7	2.23
Pangasinan	441	28	14	42	9.52	0	0	0	0.00
City of Dagupan	239	0	0	0	0.00	0	0	0	0.00

Table 2.C.4.3 - Nutrition Services for Infants and Children
Moderately Wasted Children enrolled and cured in Supplementary Feeding Program (SFP)
Philippines, 2023

Area	Total of MAM	MAM - admitted in SFP				MAM - cured in SFP			
		Male	Female	Total	%	Male	Female	Total	%
**Region 2**	**421**	**15**	**23**	**38**	**9.03**	**8**	**6**	**14**	**3.33**
Batanes	0	0	0	0	0.00	0	0	0	0.00
Cagayan	51	0	0	0	0.00	0	0	0	0.00
Isabela	311	1	5	6	1.93	5	0	5	1.61
Nueva Vizcaya	0	0	0	0	0.00	0	0	0	0.00
Quirino	59	14	18	32	54.24	3	6	9	15.25
City of Santiago	0	0	0	0	0.00	0	0	0	0.00
**Region 3**	**13,439**	**636**	**468**	**1,104**	**8.21**	**494**	**328**	**822**	**6.12**
Aurora	231	45	35	80	34.63	7	2	9	3.90
Bataan	897	0	0	0	0.00	0	0	0	0.00
Bulacan	3,507	426	312	738	21.04	346	218	564	16.08
Nueva Ecija	3,133	112	80	192	6.13	92	70	162	5.17
Pampanga	3,526	3	2	5	0.14	0	0	0	0.00
Tarlac	1,593	0	0	0	0.00	0	0	0	0.00
Zambales	392	50	39	89	22.70	49	38	87	22.19
City of Angeles	0	0	0	0	0.00	0	0	0	0.00
City of Olongapo	160	0	0	0	0.00	0	0	0	0.00
**Region 4A**	**8,825**	**1,345**	**1,191**	**2,536**	**28.74**	**459**	**401**	**860**	**9.75**
Batangas	675	20	18	38	5.63	6	5	11	1.63
Cavite	1,562	211	187	398	25.48	176	164	340	21.77
Laguna	2,981	481	456	937	31.43	28	31	59	1.98
Quezon	2,244	472	413	885	39.44	94	91	185	8.24
Rizal	550	2	2	4	0.73	0	0	0	0.00
City of Lucena	813	159	115	274	33.70	155	110	265	32.60
**Region 4B**	**8,931**	**1,874**	**1,645**	**3,519**	**39.40**	**784**	**647**	**1,431**	**16.02**
Marinduque	127	72	48	120	94.49	37	22	59	46.46
Occidental Mindoro	1,030	495	412	907	88.06	395	306	701	68.06
Oriental Mindoro	1,673	163	160	323	19.31	15	13	28	1.67
Palawan	2,906	992	870	1,862	64.07	86	83	169	5.82
Romblon	384	133	135	268	69.79	0	0	0	0.00
City of Puerto Princesa	2,811	19	20	39	1.39	251	223	474	16.86

Table 2.C.4.3 - Nutrition Services for Infants and Children

Moderately Wasted Children enrolled and cured in Supplementary Feeding Program (SFP)

Philippines, 2023

Area	Total of MAM	MAM - admitted in SFP				MAM - cured in SFP			
		Male	Female	Total	%	Male	Female	Total	%
**Region 5**	**7,798**	**1,078**	**1,161**	**2,239**	**28.71**	**334**	**283**	**617**	**7.91**
Albay	958	109	95	204	21.29	24	20	44	4.59
Camarines Norte	271	91	72	163	60.15	59	39	98	36.16
Camarines Sur	2,070	256	305	561	27.10	42	49	91	4.40
Catanduanes	550	116	110	226	41.09	33	21	54	9.82
Masbate	3,235	481	534	1,015	31.38	160	147	307	9.49
Sorsogon	704	22	43	65	9.23	14	4	18	2.56
City of Naga	10	3	2	5	50.00	2	3	5	50.00
**Region 6**	**5,120**	**2,157**	**1,755**	**3,912**	**76.41**	**471**	**392**	**863**	**16.86**
Aklan	129	73	56	129	100.00	0	0	0	0.00
Antique	320	78	87	165	51.56	25	36	61	19.06
Capiz	393	157	142	299	76.08	66	62	128	32.57
Guimaras	40	3	2	5	12.50	1	1	2	5.00
Iloilo	1,491	1,026	793	1,819	122.00	77	55	132	8.85
Negros Occidental	2,276	581	443	1,024	44.99	166	132	298	13.09
City of Bacolod	210	112	98	210	100.00	41	26	67	31.90
City of Iloilo	261	127	134	261	100.00	95	80	175	67.05
**Region 7**	**28,430**	**193**	**158**	**351**	**1.23**	**15**	**18**	**33**	**0.12**
Bohol	781	8	6	14	1.79	1	0	1	0.13
Cebu	26,618	175	138	313	1.18	2	1	3	0.01
Negros Oriental	206	0	0	0	0.00	0	0	0	0.00
Siquijor	775	4	5	9	1.16	1	0	1	0.13
City of Cebu	50	6	9	15	30.00	11	17	28	56.00
City of Lapu-Lapu	0	0	0	0	0.00	0	0	0	0.00
City of Mandaue	0	0	0	0	0.00	0	0	0	0.00
**Region 8**	**3,090**	**674**	**663**	**1,337**	**43.27**	**279**	**265**	**544**	**17.61**
Biliran	245	30	45	75	30.61	8	19	27	11.02
Eastern Samar	0	0	0	0	0.00	0	0	0	0.00
Leyte	559	174	160	334	59.75	89	70	159	28.44
Northern Samar	934	354	333	687	73.55	169	159	328	35.12
Southern Leyte	489	44	62	106	21.68	5	4	9	1.84
Samar	144	72	63	135	93.75	8	13	21	14.58
Ormoc City	0	0	0	0	0.00	0	0	0	0.00
City of Tacloban	719	0	0	0	0.00	0	0	0	0.00

Table 2.C.4.3 - Nutrition Services for Infants and Children
Moderately Wasted Children enrolled and cured in Supplementary Feeding Program (SFP)
Philippines, 2023

Area	Total of MAM	MAM - admitted in SFP				MAM - cured in SFP			
		Male	Female	Total	%	Male	Female	Total	%
**Region 9**	**3,849**	**1,053**	**1,102**	**2,155**	**55.99**	**380**	**479**	**859**	**22.32**
Zamboanga del Norte	919	258	329	587	63.87	206	287	493	53.65
Zamboanga del Sur	1,556	350	348	698	44.86	47	47	94	6.04
Zamboanga Sibugay	456	126	123	249	54.61	6	1	7	1.54
City of Isabela	384	156	157	313	81.51	79	83	162	42.19
City of Zamboanga	534	163	145	308	57.68	42	61	103	19.29
**Region 10**	**2,450**	**923**	**869**	**1,792**	**73.14**	**386**	**318**	**704**	**28.73**
Bukidnon	1,785	728	675	1,403	78.60	257	182	439	24.59
Camiguin	4	1	3	4	100.00	0	0	0	0.00
Lanao del Norte	5	1	4	5	100.00	0	0	0	0.00
Misamis Occidental	122	0	0	0	0.00	0	0	0	0.00
Misamis Oriental	303	76	73	149	49.17	51	60	111	36.63
City of Cagayan De Oro	61	33	28	61	100.00	10	5	15	24.59
City of Iligan	170	84	86	170	100.00	68	71	139	81.76
**Region 11**	**3,032**	**561**	**581**	**1,142**	**37.66**	**281**	**330**	**611**	**20.15**
Davao de Oro	307	121	89	210	68.40	35	61	96	31.27
Davao del Norte	647	111	161	272	42.04	65	59	124	19.17
Davao Oriental	745	69	67	136	18.26	70	90	160	21.48
Davao del Sur	285	39	35	74	25.96	22	14	36	12.63
Davao Occidental	586	148	144	292	49.83	53	53	106	18.09
City of Davao	462	73	85	158	34.20	36	53	89	19.26
**Region 12**	**3,161**	**905**	**864**	**1,769**	**55.96**	**281**	**279**	**560**	**17.72**
Cotabato	1,193	486	469	955	80.05	148	131	279	23.39
Sarangani	664	179	162	341	51.36	37	40	77	11.60
South Cotabato	321	132	116	248	77.26	29	32	61	19.00
Sultan Kudarat	164	81	83	164	100.00	0	0	0	0.00
City of General Santos	819	27	34	61	7.45	67	76	143	17.46
**BARMM**	**3,900**	**1,438**	**1,304**	**2,742**	**70.31**	**259**	**293**	**552**	**14.15**
Basilan	190	34	134	168	88.42	6	13	19	10.00
Lanao del Sur	364	82	108	190	52.20	26	38	64	17.58

Table 2.C.4.3 - Nutrition Services for Infants and Children

Moderately Wasted Children enrolled and cured in Supplementary Feeding Program (SFP)

Philippines, 2023

Area	Total of MAM	MAM - admitted in SFP				MAM - cured in SFP			
		Male	Female	Total	%	Male	Female	Total	%
Maguindanao	398	134	169	303	76.13	61	75	136	34.17
Sulu	11	1	2	3	27.27	0	0	0	0.00
Tawi-Tawi	2,536	1,032	761	1,793	70.70	76	96	172	6.78
SGA	116	0	0	0	0.00	0	0	0	0.00
City of Cotabato	285	155	130	285	100.00	90	71	161	56.49
**Caraga**	**2,628**	**642**	**464**	**1,106**	**42.09**	**286**	**243**	**529**	**20.13**
Agusan del Norte	243	110	54	164	67.49	14	17	31	12.76
Agusan del Sur	764	0	0	0	0.00	0	0	0	0.00
Surigao del Norte	678	165	131	296	43.66	82	81	163	24.04
Surigao del Sur	795	367	279	646	81.26	190	145	335	42.14
Dinagat Islands	32	0	0	0	0.00	0	0	0	0.00
City of Butuan	116	0	0	0	0.00	0	0	0	0.00

Table 2.C.4.3 - Nutrition Services for Infants and Children

Moderately Wasted Children defaulted and died in Supplementary Feeding Program (SFP)

Philippines, 2023

Area	Total of MAM	MAM-defaulted in SFP				MAM-died in SFP			
		Male	Female	Total	%	Male	Female	Total	%
**Philippines**	**100,800**	**1,107**	**1,101**	**2,208**	**2.19**	**59**	**35**	**94**	**0.09**
**NCR**	**2,702**	**131**	**117**	**248**	**9.18**	**0**	**0**	**0**	**0.00**
Malabon City	17	0	0	0	0.00	0	0	0	0.00
Navotas City	23	0	0	0	0.00	0	0	0	0.00
Valenzuela City	6	0	0	0	0.00	0	0	0	0.00
Caloocan City	195	0	0	0	0.00	0	0	0	0.00
Marikina City	1	0	0	0	0.00	0	0	0	0.00
Pasig City	204	10	22	32	15.69	0	0	0	0.00
Pateros	70	0	0	0	0.00	0	0	0	0.00
Taguig City	95	0	0	0	0.00	0	0	0	0.00
Quezon City	1,601	89	78	167	10.43	0	0	0	0.00
Makati City	129	10	0	10	7.75	0	0	0	0.00
Mandaluyong City	94	2	2	4	4.26	0	0	0	0.00
San Juan City	23	1	0	1	4.35	0	0	0	0.00
Manila City	0	11	9	20	0.00	0	0	0	0.00
Las Piñas City	92	0	0	0	0.00	0	0	0	0.00
Muntinlupa City	55	2	0	2	3.64	0	0	0	0.00
Parañaque City	97	6	6	12	12.37	0	0	0	0.00
Pasay City	0	0	0	0	0.00	0	0	0	0.00
**CAR**	**630**	**1**	**0**	**1**	**0.16**	**0**	**0**	**0**	**0.00**
Abra	203	0	0	0	0.00	0	0	0	0.00
Apayao	212	0	0	0	0.00	0	0	0	0.00
Benguet	29	0	0	0	0.00	0	0	0	0.00
Ifugao	64	0	0	0	0.00	0	0	0	0.00
Kalinga	89	0	0	0	0.00	0	0	0	0.00
Mountain Province	15	1	0	1	6.67	0	0	0	0.00
City of Baguio	18	0	0	0	0.00	0	0	0	0.00
**Region 1**	**2,394**	**0**	**0**	**0**	**0.00**	**0**	**0**	**0**	**0.00**
Ilocos Norte	666	0	0	0	0.00	0	0	0	0.00
Ilocos Sur	734	0	0	0	0.00	0	0	0	0.00
La Union	314	0	0	0	0.00	0	0	0	0.00
Pangasinan	441	0	0	0	0.00	0	0	0	0.00
City of Dagupan	239	0	0	0	0.00	0	0	0	0.00

Table 2.C.4.3 - Nutrition Services for Infants and Children
Moderately Wasted Children defaulted and died in Supplementary Feeding Program (SFP)
Philippines, 2023

Area	Total of MAM	MAM-defaulted in SFP				MAM-died in SFP			
		Male	Female	Total	%	Male	Female	Total	%
**Region 2**	**421**	**6**	**9**	**15**	**3.56**	**0**	**0**	**0**	**0.00**
Batanes	0	0	0	0	0.00	0	0	0	0.00
Cagayan	51	0	0	0	0.00	0	0	0	0.00
Isabela	311	0	0	0	0.00	0	0	0	0.00
Nueva Vizcaya	0	0	0	0	0.00	0	0	0	0.00
Quirino	59	6	9	15	25.42	0	0	0	0.00
City of Santiago	0	0	0	0	0.00	0	0	0	0.00
**Region 3**	**13,439**	**62**	**42**	**104**	**0.77**	**0**	**0**	**0**	**0.00**
Aurora	231	0	0	0	0.00	0	0	0	0.00
Bataan	897	0	0	0	0.00	0	0	0	0.00
Bulacan	3,507	41	31	72	2.05	0	0	0	0.00
Nueva Ecija	3,133	20	10	30	0.96	0	0	0	0.00
Pampanga	3,526	0	0	0	0.00	0	0	0	0.00
Tarlac	1,593	0	0	0	0.00	0	0	0	0.00
Zambales	392	1	1	2	0.51	0	0	0	0.00
City of Angeles	0	0	0	0	0.00	0	0	0	0.00
City of Olongapo	160	0	0	0	0.00	0	0	0	0.00
**Region 4A**	**8,825**	**26**	**29**	**55**	**0.62**	**4**	**3**	**7**	**0.08**
Batangas	675	4	0	4	0.59	0	0	0	0.00
Cavite	1,562	14	9	23	1.47	4	0	4	0.26
Laguna	2,981	2	11	13	0.44	0	3	3	0.10
Quezon	2,244	6	9	15	0.67	0	0	0	0.00
Rizal	550	0	0	0	0.00	0	0	0	0.00
City of Lucena	813	0	0	0	0.00	0	0	0	0.00
**Region 4B**	**8,931**	**170**	**125**	**295**	**3.30**	**3**	**0**	**3**	**0.03**
Marinduque	127	0	0	0	0.00	0	0	0	0.00
Occidental Mindoro	1,030	86	54	140	13.59	1	0	1	0.10
Oriental Mindoro	1,673	1	4	5	0.30	0	0	0	0.00
Palawan	2,906	8	3	11	0.38	0	0	0	0.00
Romblon	384	0	0	0	0.00	0	0	0	0.00
City of Puerto Princesa	2,811	75	64	139	4.94	2	0	2	0.07

Table 2.C.4.3 - Nutrition Services for Infants and Children

Moderately Wasted Children defaulted and died in Supplementary Feeding Program (SFP)

Philippines, 2023

Area	Total of MAM	MAM-defaulted in SFP				MAM-died in SFP			
		Male	Female	Total	%	Male	Female	Total	%
**Region 5**	**7,798**	**215**	**306**	**521**	**6.68**	**2**	**1**	**3**	**0.04**
Albay	958	3	0	3	0.31	0	0	0	0.00
Camarines Norte	271	9	12	21	7.75	0	0	0	0.00
Camarines Sur	2,070	18	19	37	1.79	1	1	2	0.10
Catanduanes	550	12	8	20	3.64	0	0	0	0.00
Masbate	3,235	173	267	440	13.60	1	0	1	0.03
Sorsogon	704	0	0	0	0.00	0	0	0	0.00
City of Naga	10	0	0	0	0.00	0	0	0	0.00
**Region 6**	**5,120**	**15**	**14**	**29**	**0.57**	**9**	**5**	**14**	**0.27**
Aklan	129	0	0	0	0.00	0	0	0	0.00
Antique	320	3	3	6	1.88	0	0	0	0.00
Capiz	393	0	0	0	0.00	0	0	0	0.00
Guimaras	40	0	0	0	0.00	0	0	0	0.00
Iloilo	1,491	11	10	21	1.41	9	5	14	0.94
Negros Occidental	2,276	1	1	2	0.09	0	0	0	0.00
City of Bacolod	210	0	0	0	0.00	0	0	0	0.00
City of Iloilo	261	0	0	0	0.00	0	0	0	0.00
**Region 7**	**28,430**	**1**	**0**	**1**	**0.00**	**2**	**0**	**2**	**0.01**
Bohol	781	0	0	0	0.00	2	0	2	0.26
Cebu	26,618	1	0	1	0.00	0	0	0	0.00
Negros Oriental	206	0	0	0	0.00	0	0	0	0.00
Siquijor	775	0	0	0	0.00	0	0	0	0.00
City of Cebu	50	0	0	0	0.00	0	0	0	0.00
City of Lapu-Lapu	0	0	0	0	0.00	0	0	0	0.00
City of Mandaue	0	0	0	0	0.00	0	0	0	0.00
**Region 8**	**3,090**	**17**	**30**	**47**	**1.52**	**0**	**4**	**4**	**0.13**
Biliran	245	0	1	1	0.41	0	0	0	0.00
Eastern Samar	0	0	0	0	0.00	0	0	0	0.00
Leyte	559	4	1	5	0.89	0	2	2	0.36
Northern Samar	934	13	27	40	4.28	0	2	2	0.21
Southern Leyte	489	0	0	0	0.00	0	0	0	0.00
Samar	144	0	1	1	0.69	0	0	0	0.00
Ormoc City	0	0	0	0	0.00	0	0	0	0.00
City of Tacloban	719	0	0	0	0.00	0	0	0	0.00

Table 2.C.4.3 - Nutrition Services for Infants and Children

Moderately Wasted Children defaulted and died in Supplementary Feeding Program (SFP)

Philippines, 2023

Area	Total of MAM	MAM-defaulted in SFP				MAM-died in SFP			
		Male	Female	Total	%	Male	Female	Total	%
**Region 9**	**3,849**	**29**	**38**	**67**	**1.74**	**2**	**0**	**2**	**0.05**
Zamboanga del Norte	919	7	6	13	1.41	2	0	2	0.22
Zamboanga del Sur	1,556	1	1	2	0.13	0	0	0	0.00
Zamboanga Sibugay	456	6	12	18	3.95	0	0	0	0.00
City of Isabela	384	0	0	0	0.00	0	0	0	0.00
City of Zamboanga	534	15	19	34	6.37	0	0	0	0.00
**Region 10**	**2,450**	**62**	**96**	**158**	**6.45**	**0**	**0**	**0**	**0.00**
Bukidnon	1,785	31	37	68	3.81	0	0	0	0.00
Camiguin	4	0	0	0	0.00	0	0	0	0.00
Lanao del Norte	5	0	0	0	0.00	0	0	0	0.00
Misamis Occidental	122	0	0	0	0.00	0	0	0	0.00
Misamis Oriental	303	31	59	90	29.70	0	0	0	0.00
City of Cagayan De Oro	61	0	0	0	0.00	0	0	0	0.00
City of Iligan	170	0	0	0	0.00	0	0	0	0.00
**Region 11**	**3,032**	**39**	**33**	**72**	**2.37**	**24**	**14**	**38**	**1.25**
Davao de Oro	307	9	4	13	4.23	0	0	0	0.00
Davao del Norte	647	6	6	12	1.85	4	3	7	1.08
Davao Oriental	745	12	6	18	2.42	0	0	0	0.00
Davao del Sur	285	3	7	10	3.51	0	1	1	0.35
Davao Occidental	586	0	2	2	0.34	5	2	7	1.19
City of Davao	462	9	8	17	3.68	15	8	23	4.98
**Region 12**	**3,161**	**37**	**28**	**65**	**2.06**	**8**	**5**	**13**	**0.41**
Cotabato	1,193	28	23	51	4.27	8	5	13	1.09
Sarangani	664	0	0	0	0.00	0	0	0	0.00
South Cotabato	321	7	5	12	3.74	0	0	0	0.00
Sultan Kudarat	164	0	0	0	0.00	0	0	0	0.00
City of General Santos	819	2	0	2	0.24	0	0	0	0.00
**BARMM**	**3,900**	**235**	**199**	**434**	**11.13**	**5**	**3**	**8**	**0.21**
Basilan	190	4	1	5	2.63	1	1	2	1.05
Lanao del Sur	364	0	1	1	0.27	0	0	0	0.00

Table 2.C.4.3 - Nutrition Services for Infants and Children

Moderately Wasted Children defaulted and died in Supplementary Feeding Program (SFP)

Philippines, 2023

Area	Total of MAM	MAM-defaulted in SFP				MAM-died in SFP			
		Male	Female	Total	%	Male	Female	Total	%
Maguindanao	398	26	37	63	15.83	4	2	6	1.51
Sulu	11	0	0	0	0.00	0	0	0	0.00
Tawi-Tawi	2,536	196	141	337	13.29	0	0	0	0.00
SGA	116	2	3	5	4.31	0	0	0	0.00
City of Cotabato	285	7	16	23	8.07	0	0	0	0.00
**Caraga**	**2,628**	**61**	**35**	**96**	**3.65**	**0**	**0**	**0**	**0.00**
Agusan del Norte	243	0	0	0	0.00	0	0	0	0.00
Agusan del Sur	764	0	0	0	0.00	0	0	0	0.00
Surigao del Norte	678	3	1	4	0.59	0	0	0	0.00
Surigao del Sur	795	58	34	92	11.57	0	0	0	0.00
Dinagat Islands	32	0	0	0	0.00	0	0	0	0.00
City of Butuan	116	0	0	0	0.00	0	0	0	0.00

Table 2.C.4.4. Nutrition Services for Infants and Children

Severe Acute Malnutrition Children enrolled and cured in Supplementary Feeding Program (SFP)

Philippines, 2023

Area	Total of SAM	SAM-admitted in OTC				SAM-cured in OTC			
		Male	Female	Total	%	Male	Female	Total	%
**Philippines**	**51,884**	**5,109**	**4,445**	**9,554**	**18.41**	**1,632**	**1,549**	**3,181**	**6.13**
**NCR**	**1,147**	**428**	**415**	**843**	**73.50**	**193**	**269**	**462**	**40.28**
Malabon City	0	0	0	0	0.00	0	0	0	0.00
Navotas City	0	0	0	0	0.00	0	0	0	0.00
Valenzuela City	0	0	0	0	0.00	2	1	3	0.00
Caloocan City	85	38	47	85	100.00	0	0	0	0.00
Marikina City	0	0	0	0	0.00	0	0	0	0.00
Pasig City	91	54	37	91	100.00	13	12	25	27.47
Pateros	3	3	0	3	100.00	1	0	1	33.33
Taguig City	10	7	3	10	100.00	4	6	10	100.00
Quezon City	866	282	285	567	65.47	151	227	378	43.65
Makati City	39	16	13	29	74.36	6	4	10	25.64
Mandaluyong City	0	11	14	25	0.00	10	5	15	0.00
San Juan City	1	0	1	1	100.00	0	1	1	100.00
Manila City	0	0	0	0	0.00	0	0	0	0.00
Las Piñas City	16	9	7	16	100.00	1	3	4	25.00
Muntinlupa City	21	1	0	1	4.76	4	6	10	47.62
Parañaque City	15	7	8	15	100.00	1	4	5	33.33
Pasay City	0	0	0	0	0.00	0	0	0	0.00
**CAR**	**180**	**4**	**5**	**9**	**5.00**	**0**	**0**	**0**	**0.00**
Abra	53	0	0	0	0.00	0	0	0	0.00
Apayao	41	0	0	0	0.00	0	0	0	0.00
Benguet	16	1	1	2	12.50	0	0	0	0.00
Ifugao	38	0	0	0	0.00	0	0	0	0.00
Kalinga	22	2	1	3	13.64	0	0	0	0.00
Mountain Province	9	1	3	4	44.44	0	0	0	0.00
City of Baguio	1	0	0	0	0.00	0	0	0	0.00
**Region 1**	**530**	**131**	**123**	**254**	**47.92**	**20**	**12**	**32**	**6.04**
Ilocos Norte	156	74	81	155	99.36	0	1	1	0.64
Ilocos Sur	225	50	24	74	32.89	15	8	23	10.22
La Union	100	6	14	20	20.00	5	3	8	8.00
Pangasinan	49	1	4	5	10.20	0	0	0	0.00
City of Dagupan	0	0	0	0	0.00	0	0	0	0.00

Table 2.C.4.4. Nutrition Services for Infants and Children

Severe Acute Malnutrition Children enrolled and cured in Supplementary Feeding Program (SFP)

Philippines, 2023

Area	Total of SAM	SAM-admitted in OTC				SAM-cured in OTC			
		Male	Female	Total	%	Male	Female	Total	%
**Region 2**	**83**	**8**	**5**	**13**	**15.66**	**0**	**0**	**0**	**0.00**
Batanes	0	0	0	0	0.00	0	0	0	0.00
Cagayan	0	0	0	0	0.00	0	0	0	0.00
Isabela	70	0	0	0	0.00	0	0	0	0.00
Nueva Vizcaya	0	0	0	0	0.00	0	0	0	0.00
Quirino	13	8	5	13	100.00	0	0	0	0.00
City of Santiago	0	0	0	0	0.00	0	0	0	0.00
**Region 3**	**7,064**	**151**	**103**	**254**	**3.60**	**104**	**81**	**185**	**2.62**
Aurora	81	0	0	0	0.00	0	0	0	0.00
Bataan	185	0	0	0	0.00	0	0	0	0.00
Bulacan	1,596	139	97	236	14.79	95	78	173	10.84
Nueva Ecija	961	1	1	2	0.21	1	0	1	0.10
Pampanga	3,184	0	2	2	0.06	0	0	0	0.00
Tarlac	706	0	0	0	0.00	0	0	0	0.00
Zambales	247	11	3	14	5.67	8	3	11	4.45
City of Angeles	0	0	0	0	0.00	0	0	0	0.00
City of Olongapo	104	0	0	0	0.00	0	0	0	0.00
**Region 4A**	**4,182**	**885**	**771**	**1,656**	**39.60**	**169**	**134**	**303**	**7.25**
Batangas	223	2	8	10	4.48	1	3	4	1.79
Cavite	588	79	61	140	23.81	29	33	62	10.54
Laguna	1,480	222	239	461	31.15	11	14	25	1.69
Quezon	1,418	495	402	897	63.26	47	26	73	5.15
Rizal	64	11	3	14	21.88	0	0	0	0.00
City of Lucena	409	76	58	134	32.76	81	58	139	33.99
**Region 4B**	**2,330**	**453**	**389**	**842**	**36.14**	**203**	**159**	**362**	**15.54**
Marinduque	28	17	10	27	96.43	10	6	16	57.14
Occidental Mindoro	295	150	149	299	101.36	131	118	249	84.41
Oriental Mindoro	145	16	9	25	17.24	1	4	5	3.45
Palawan	675	229	179	408	60.44	21	9	30	4.44
Romblon	139	34	38	72	51.80	0	0	0	0.00
City of Puerto Princesa	1,048	7	4	11	1.05	40	22	62	5.92

Table 2.C.4.4. Nutrition Services for Infants and Children

Severe Acute Malnutrition Children enrolled and cured in Supplementary Feeding Program (SFP)

Philippines, 2023

Area	Total of SAM	SAM-admitted in OTC				SAM-cured in OTC			
		Male	Female	Total	%	Male	Female	Total	%
**Region 5**	**2,755**	**329**	**237**	**566**	**20.54**	**61**	**78**	**139**	**5.05**
Albay	144	54	41	95	65.97	14	17	31	21.53
Camarines Norte	64	28	27	55	85.94	9	15	24	37.50
Camarines Sur	736	99	83	182	24.73	28	36	64	8.70
Catanduanes	116	31	23	54	46.55	5	4	9	7.76
Masbate	1,627	108	53	161	9.90	5	5	10	0.61
Sorsogon	67	9	9	18	26.87	0	1	1	1.49
City of Naga	1	0	1	1	100.00	0	0	0	0.00
**Region 6**	**1,442**	**341**	**231**	**572**	**39.67**	**143**	**81**	**224**	**15.53**
Aklan	46	33	13	46	100.00	0	0	0	0.00
Antique	81	13	11	24	29.63	4	5	9	11.11
Capiz	67	19	26	45	67.16	28	20	48	71.64
Guimaras	14	0	0	0	0.00	0	0	0	0.00
Iloilo	437	92	65	157	35.93	24	15	39	8.92
Negros Occidental	619	73	49	122	19.71	26	18	44	7.11
City of Bacolod	91	62	29	91	100.00	21	4	25	27.47
City of Iloilo	87	49	38	87	100.00	40	19	59	67.82
**Region 7**	**24,365**	**19**	**6**	**25**	**0.10**	**7**	**7**	**14**	**0.06**
Bohol	292	2	0	2	0.68	0	0	0	0.00
Cebu	24,053	7	3	10	0.04	2	5	7	0.03
Negros Oriental	0	0	0	0	0.00	0	0	0	0.00
Siquijor	3	2	1	3	100.00	0	0	0	0.00
City of Cebu	17	8	2	10	58.82	5	2	7	41.18
City of Lapu-Lapu	0	0	0	0	0.00	0	0	0	0.00
City of Mandaue	0	0	0	0	0.00	0	0	0	0.00
**Region 8**	**1,016**	**172**	**198**	**370**	**36.42**	**103**	**128**	**231**	**22.74**
Biliran	73	12	11	23	31.51	9	5	14	19.18
Eastern Samar	0	0	0	0	0.00	0	0	0	0.00
Leyte	234	15	15	30	12.82	57	74	131	55.98
Northern Samar	307	112	138	250	81.43	32	41	73	23.78
Southern Leyte	81	9	11	20	24.69	0	0	0	0.00
Samar	57	24	23	47	82.46	5	8	13	22.81
Ormoc City	0	0	0	0	0.00	0	0	0	0.00
City of Tacloban	264	0	0	0	0.00	0	0	0	0.00

Table 2.C.4.4. Nutrition Services for Infants and Children

Severe Acute Malnutrition Children enrolled and cured in Supplementary Feeding Program (SFP)

Philippines, 2023

Area	Total of SAM	SAM-admitted in OTC				SAM-cured in OTC			
		Male	Female	Total	%	Male	Female	Total	%
**Region 9**	**1,167**	**170**	**145**	**315**	**26.99**	**121**	**101**	**222**	**19.02**
Zamboanga del Norte	326	87	74	161	49.39	91	73	164	50.31
Zamboanga del Sur	589	43	29	72	12.22	24	18	42	7.13
Zamboanga Sibugay	19	17	10	27	142.11	2	1	3	15.79
City of Isabela	57	0	0	0	0.00	0	0	0	0.00
City of Zamboanga	176	23	32	55	31.25	4	9	13	7.39
**Region 10**	**741**	**254**	**267**	**521**	**70.31**	**90**	**104**	**194**	**26.18**
Bukidnon	574	181	188	369	64.29	59	61	120	20.91
Camiguin	1	0	1	1	100.00	0	0	0	0.00
Lanao del Norte	5	2	3	5	100.00	0	0	0	0.00
Misamis Occidental	14	6	2	8	57.14	0	3	3	21.43
Misamis Oriental	67	31	27	58	86.57	22	25	47	70.15
City of Cagayan De Oro	57	24	33	57	100.00	2	5	7	12.28
City of Iligan	23	10	13	23	100.00	7	10	17	73.91
**Region 11**	**900**	**181**	**159**	**340**	**37.78**	**71**	**55**	**126**	**14.00**
Davao de Oro	112	39	17	56	50.00	8	9	17	15.18
Davao del Norte	106	16	26	42	39.62	16	7	23	21.70
Davao Oriental	223	12	25	37	16.59	14	4	18	8.07
Davao del Sur	96	17	6	23	23.96	7	6	13	13.54
Davao Occidental	245	70	56	126	51.43	21	23	44	17.96
City of Davao	118	27	29	56	47.46	5	6	11	9.32
**Region 12**	**996**	**354**	**282**	**636**	**63.86**	**78**	**78**	**156**	**15.66**
Cotabato	246	110	75	185	75.20	49	40	89	36.18
Sarangani	182	62	38	100	54.95	9	5	14	7.69
South Cotabato	58	21	32	53	91.38	12	16	28	48.28
Sultan Kudarat	27	17	10	27	100.00	0	0	0	0.00
City of General Santos	483	144	127	271	56.11	8	17	25	5.18
**BARMM**	**2,059**	**1,016**	**925**	**1,941**	**94.27**	**175**	**162**	**337**	**16.37**
Basilan	1	0	0	0	0.00	0	0	0	0.00
Lanao del Sur	295	145	146	291	98.64	39	28	67	22.71

Table 2.C.4.4. Nutrition Services for Infants and Children

Severe Acute Malnutrition Children enrolled and cured in Supplementary Feeding Program (SFP)

Philippines, 2023

Area	Total of SAM	SAM-admitted in OTC				SAM-cured in OTC			
		Male	Female	Total	%	Male	Female	Total	%
Maguindanao	235	60	62	122	51.91	14	14	28	11.91
Sulu	12	7	5	12	100.00	3	0	3	25.00
Tawi-Tawi	1,398	755	643	1,398	100.00	98	103	201	14.38
SGA	87	33	54	87	100.00	5	9	14	16.09
City of Cotabato	31	16	15	31	100.00	16	8	24	77.42
**Caraga**	**927**	**213**	**184**	**397**	**42.83**	**94**	**100**	**194**	**20.93**
Agusan del Norte	89	18	16	34	38.20	4	4	8	8.99
Agusan del Sur	317	0	0	0	0.00	0	0	0	0.00
Surigao del Norte	236	65	73	138	58.47	15	42	57	24.15
Surigao del Sur	234	130	95	225	96.15	75	54	129	55.13
Dinagat Islands	11	0	0	0	0.00	0	0	0	0.00
City of Butuan	40	0	0	0	0.00	0	0	0	0.00

Table 2.C.4.4 - Nutrition Services for Infants and Children

Severe Acute Malnutrition Children defaulted and died in Supplementary Feeding Program (SFP)

Philippines, 2023

Area	Total of SAM	SAM-defaulted in OTC				SAM-died in OTC			
		Male	Female	Total	%	Male	Female	Total	%
**Philippines**	**51,884**	**585**	**504**	**1,089**	**2.10**	**808**	**131**	**939**	**1.81**
**NCR**	**1,147**	**67**	**72**	**139**	**12.12**	**1**	**11**	**12**	**1.05**
Malabon City	0	0	0	0	0.00	0	0	0	0.00
Navotas City	0	0	0	0	0.00	0	0	0	0.00
Valenzuela City	0	0	0	0	0.00	1	0	1	0.00
Caloocan City	85	0	0	0	0.00	0	0	0	0.00
Marikina City	0	0	0	0	0.00	0	0	0	0.00
Pasig City	91	4	3	7	7.69	0	0	0	0.00
Pateros	3	0	0	0	0.00	0	0	0	0.00
Taguig City	10	0	0	0	0.00	0	0	0	0.00
Quezon City	866	56	56	112	12.93	0	0	0	0.00
Makati City	39	0	0	0	0.00	0	0	0	0.00
Mandaluyong City	0	2	8	10	0.00	0	0	0	0.00
San Juan City	1	0	0	0	0.00	0	0	0	0.00
Manila City	0	0	0	0	0.00	0	0	0	0.00
Las Piñas City	16	3	3	6	37.50	0	9	9	56.25
Muntinlupa City	21	1	1	2	9.52	0	1	1	4.76
Parañaque City	15	1	1	2	13.33	0	1	1	6.67
Pasay City	0	0	0	0	0.00	0	0	0	0.00
**CAR**	**180**	**0**	**0**	**0**	**0.00**	**0**	**0**	**0**	**0.00**
Abra	53	0	0	0	0.00	0	0	0	0.00
Apayao	41	0	0	0	0.00	0	0	0	0.00
Benguet	16	0	0	0	0.00	0	0	0	0.00
Ifugao	38	0	0	0	0.00	0	0	0	0.00
Kalinga	22	0	0	0	0.00	0	0	0	0.00
Mountain Province	9	0	0	0	0.00	0	0	0	0.00
City of Baguio	1	0	0	0	0.00	0	0	0	0.00
**Region 1**	**530**	**0**	**1**	**1**	**0.19**	**0**	**0**	**0**	**0.00**
Ilocos Norte	156	0	0	0	0.00	0	0	0	0.00
Ilocos Sur	225	0	1	1	0.44	0	0	0	0.00
La Union	100	0	0	0	0.00	0	0	0	0.00
Pangasinan	49	0	0	0	0.00	0	0	0	0.00
City of Dagupan	0	0	0	0	0.00	0	0	0	0.00

Table 2.C.4.4 - Nutrition Services for Infants and Children

Severe Acute Malnutrition Children defaulted and died in Supplementary Feeding Program (SFP)

Philippines, 2023

Area	Total of SAM	SAM-defaulted in OTC				SAM-died in OTC			
		Male	Female	Total	%	Male	Female	Total	%
**Region 2**	**83**	**3**	**2**	**5**	**6.02**	**0**	**0**	**0**	**0.00**
Batanes	0	0	0	0	0.00	0	0	0	0.00
Cagayan	0	0	0	0	0.00	0	0	0	0.00
Isabela	70	0	0	0	0.00	0	0	0	0.00
Nueva Vizcaya	0	0	0	0	0.00	0	0	0	0.00
Quirino	13	3	2	5	38.46	0	0	0	0.00
City of Santiago	0	0	0	0	0.00	0	0	0	0.00
**Region 3**	**7,064**	**38**	**22**	**60**	**0.85**	**0**	**0**	**0**	**0.00**
Aurora	81	0	0	0	0.00	0	0	0	0.00
Bataan	185	0	0	0	0.00	0	0	0	0.00
Bulacan	1,596	35	21	56	3.51	0	0	0	0.00
Nueva Ecija	961	0	1	1	0.10	0	0	0	0.00
Pampanga	3,184	0	0	0	0.00	0	0	0	0.00
Tarlac	706	0	0	0	0.00	0	0	0	0.00
Zambales	247	3	0	3	1.21	0	0	0	0.00
City of Angeles	0	0	0	0	0.00	0	0	0	0.00
City of Olongapo	104	0	0	0	0.00	0	0	0	0.00
**Region 4A**	**4,182**	**57**	**40**	**97**	**2.32**	**753**	**43**	**796**	**19.03**
Batangas	223	1	1	2	0.90	0	1	1	0.45
Cavite	588	39	24	63	10.71	0	0	0	0.00
Laguna	1,480	17	15	32	2.16	741	1	742	50.14
Quezon	1,418	0	0	0	0.00	12	41	53	3.74
Rizal	64	0	0	0	0.00	0	0	0	0.00
City of Lucena	409	0	0	0	0.00	0	0	0	0.00
**Region 4B**	**2,330**	**52**	**51**	**103**	**4.42**	**34**	**44**	**78**	**3.35**
Marinduque	28	0	0	0	0.00	0	0	0	0.00
Occidental Mindoro	295	22	27	49	16.61	1	0	1	0.34
Oriental Mindoro	145	0	0	0	0.00	2	1	3	2.07
Palawan	675	15	9	24	3.56	31	43	74	10.96
Romblon	139	0	0	0	0.00	0	0	0	0.00
City of Puerto Princesa	1,048	15	15	30	2.86	0	0	0	0.00

Table 2.C.4.4 - Nutrition Services for Infants and Children
Severe Acute Malnutrition Children defaulted and died in Supplementary Feeding Program (SFP)
Philippines, 2023

Area	Total of SAM	SAM-defaulted in OTC				SAM-died in OTC			
		Male	Female	Total	%	Male	Female	Total	%
**Region 5**	**2,755**	**153**	**116**	**269**	**9.76**	**6**	**6**	**12**	**0.44**
Albay	144	2	1	3	2.08	0	0	0	0.00
Camarines Norte	64	4	2	6	9.38	1	0	1	1.56
Camarines Sur	736	13	11	24	3.26	3	6	9	1.22
Catanduanes	116	4	5	9	7.76	2	0	2	1.72
Masbate	1,627	130	97	227	13.95	0	0	0	0.00
Sorsogon	67	0	0	0	0.00	0	0	0	0.00
City of Naga	1	0	0	0	0.00	0	0	0	0.00
**Region 6**	**1,442**	**9**	**5**	**14**	**0.97**	**9**	**5**	**14**	**0.97**
Aklan	46	0	0	0	0.00	0	0	0	0.00
Antique	81	1	2	3	3.70	0	2	2	2.47
Capiz	67	0	0	0	0.00	0	0	0	0.00
Guimaras	14	0	0	0	0.00	0	0	0	0.00
Iloilo	437	7	3	10	2.29	7	3	10	2.29
Negros Occidental	619	1	0	1	0.16	1	0	1	0.16
City of Bacolod	91	0	0	0	0.00	0	0	0	0.00
City of Iloilo	87	0	0	0	0.00	1	0	1	1.15
**Region 7**	**24,365**	**1**	**0**	**1**	**0.00**	**0**	**0**	**0**	**0.00**
Bohol	292	0	0	0	0.00	0	0	0	0.00
Cebu	24,053	1	0	1	0.00	0	0	0	0.00
Negros Oriental	0	0	0	0	0.00	0	0	0	0.00
Siquijor	3	0	0	0	0.00	0	0	0	0.00
City of Cebu	17	0	0	0	0.00	0	0	0	0.00
City of Lapu-Lapu	0	0	0	0	0.00	0	0	0	0.00
City of Mandaue	0	0	0	0	0.00	0	0	0	0.00
**Region 8**	**1,016**	**14**	**7**	**21**	**2.07**	**0**	**1**	**1**	**0.10**
Biliran	73	7	5	12	16.44	0	0	0	0.00
Eastern Samar	0	0	0	0	0.00	0	0	0	0.00
Leyte	234	1	1	2	0.85	0	0	0	0.00
Northern Samar	307	3	1	4	1.30	0	0	0	0.00
Southern Leyte	81	0	0	0	0.00	0	0	0	0.00
Samar	57	3	0	3	5.26	0	1	1	1.75
Ormoc City	0	0	0	0	0.00	0	0	0	0.00
City of Tacloban	264	0	0	0	0.00	0	0	0	0.00

Table 2.C.4.4 - Nutrition Services for Infants and Children
Severe Acute Malnutrition Children defaulted and died in Supplementary Feeding Program (SFP)
Philippines, 2023

Area	Total of SAM	SAM-defaulted in OTC				SAM-died in OTC			
		Male	Female	Total	%	Male	Female	Total	%
**Region 9**	**1,167**	**6**	**6**	**12**	**1.03**	**0**	**1**	**1**	**0.09**
Zamboanga del Norte	326	0	1	1	0.31	0	0	0	0.00
Zamboanga del Sur	589	0	0	0	0.00	0	0	0	0.00
Zamboanga Sibugay	19	4	3	7	36.84	0	1	1	5.26
City of Isabela	57	0	0	0	0.00	0	0	0	0.00
City of Zamboanga	176	2	2	4	2.27	0	0	0	0.00
**Region 10**	**741**	**12**	**9**	**21**	**2.83**	**0**	**1**	**1**	**0.13**
Bukidnon	574	9	9	18	3.14	0	0	0	0.00
Camiguin	1	0	0	0	0.00	0	0	0	0.00
Lanao del Norte	5	0	0	0	0.00	0	0	0	0.00
Misamis Occidental	14	0	0	0	0.00	0	0	0	0.00
Misamis Oriental	67	0	0	0	0.00	0	1	1	1.49
City of Cagayan De Oro	57	3	0	3	5.26	0	0	0	0.00
City of Iligan	23	0	0	0	0.00	0	0	0	0.00
**Region 11**	**900**	**10**	**15**	**25**	**2.78**	**1**	**5**	**6**	**0.67**
Davao de Oro	112	2	5	7	6.25	0	0	0	0.00
Davao del Norte	106	0	4	4	3.77	1	1	2	1.89
Davao Oriental	223	0	0	0	0.00	0	0	0	0.00
Davao del Sur	96	2	2	4	4.17	0	0	0	0.00
Davao Occidental	245	4	1	5	2.04	0	1	1	0.41
City of Davao	118	2	3	5	4.24	0	3	3	2.54
**Region 12**	**996**	**13**	**16**	**29**	**2.91**	**1**	**5**	**6**	**0.60**
Cotabato	246	13	13	26	10.57	0	3	3	1.22
Sarangani	182	0	0	0	0.00	0	0	0	0.00
South Cotabato	58	0	1	1	1.72	1	1	2	3.45
Sultan Kudarat	27	0	0	0	0.00	0	0	0	0.00
City of General Santos	483	0	2	2	0.41	0	1	1	0.21
**BARMM**	**2,059**	**132**	**124**	**256**	**12.43**	**3**	**9**	**12**	**0.58**
Basilan	1	0	0	0	0.00	0	0	0	0.00
Lanao del Sur	295	6	9	15	5.08	1	4	5	1.69

Table 2.C.4.4 - Nutrition Services for Infants and Children

Severe Acute Malnutrition Children defaulted and died in Supplementary Feeding Program (SFP)

Philippines, 2023

Area	Total of SAM	SAM-defaulted in OTC				SAM-died in OTC			
		Male	Female	Total	%	Male	Female	Total	%
Maguindanao	235	12	21	33	14.04	0	2	2	0.85
Sulu	12	1	2	3	25.00	0	1	1	8.33
Tawi-Tawi	1,398	106	90	196	14.02	2	0	2	0.14
SGA	87	3	1	4	4.60	0	1	1	1.15
City of Cotabato	31	4	1	5	16.13	0	1	1	3.23
**Caraga**	**927**	**18**	**18**	**36**	**3.88**	**0**	**0**	**0**	**0.00**
Agusan del Norte	89	1	1	2	2.25	0	0	0	0.00
Agusan del Sur	317	0	0	0	0.00	0	0	0	0.00
Surigao del Norte	236	0	0	0	0.00	0	0	0	0.00
Surigao del Sur	234	17	17	34	14.53	0	0	0	0.00
Dinagat Islands	11	0	0	0	0.00	0	0	0	0.00
City of Butuan	40	0	0	0	0.00	0	0	0	0.00

Table 2.D.1 - Oral Health Care
Children 12-59 months old who are orally fit upon oral examination or after oral rehabilitation
Philippines, 2023

Area	Eligible Population x 20%	Orally fit		Total	%
		Male	Female		
**Philippines**	**1,829,361**	**303,329**	**325,065**	**628,394**	**34.35**
**NCR**	**209,734**	**92,534**	**96,384**	**188,918**	**90.08**
Malabon City	**5,894**	1,497	1,611	**3,108**	**52.73**
Navotas City	**4,172**	1,347	1,421	**2,768**	**66.35**
Valenzuela City	**10,980**	1,248	1,301	**2,549**	**23.21**
Caloocan City	**27,749**	13,057	13,740	**26,797**	**96.57**
Marikina City	**6,926**	1,798	1,792	**3,590**	**51.83**
Pasig City	**13,142**	2,343	2,525	**4,868**	**37.04**
Pateros	**965**	364	427	**791**	**81.99**
Taguig City	**14,989**	1,943	2,015	**3,958**	**26.41**
Quezon City	**47,722**	28,898	29,700	**58,598**	**122.79**
Makati City	**8,504**	4,359	4,460	**8,819**	**103.71**
Mandaluyong City	**6,312**	1,623	1,573	**3,196**	**50.63**
San Juan City	**1,724**	411	400	**811**	**47.05**
Manila City	**27,804**	26,365	27,622	**53,987**	**194.17**
Las Piñas City	**9,050**	1,289	1,418	**2,707**	**29.91**
Muntinlupa City	**7,829**	1,145	1,175	**2,320**	**29.63**
Parañaque City	**10,663**	1,926	1,920	**3,846**	**36.07**
Pasay City	**5,308**	2,921	3,284	**6,205**	**116.89**
**CAR**	**26,384**	**5,273**	**5,505**	**10,778**	**40.85**
Abra	**3,272**	196	214	**410**	**12.53**
Apayao	**1,854**	395	399	**794**	**42.83**
Benguet	**7,417**	1,808	1,748	**3,556**	**47.95**
Ifugao	**3,438**	411	402	**813**	**23.65**
Kalinga	**3,718**	1,269	1,433	**2,702**	**72.67**
Mountain Province	**2,279**	337	388	**725**	**31.81**
Baguio City	**4,406**	857	921	**1,778**	**40.35**
**Region 1**	**78,382**	**15,757**	**16,621**	**32,378**	**41.31**
Ilocos Norte	**7,639**	2,948	3,098	**6,046**	**79.15**
Ilocos Sur	**8,726**	2,709	2,861	**5,570**	**63.83**
La Union	**11,089**	2,407	2,346	**4,753**	**42.86**
Pangasinan	**48,241**	7,656	8,249	**15,905**	**32.97**
Dagupan City	**2,686**	37	67	**104**	**3.87**

Table 2.D.1 - Oral Health Care

Children 12-59 months old who are orally fit upon oral examination or after oral rehabilitation

Philippines, 2023

Area	Eligible Population x 20%	Orally fit		Total	%
		Male	Female		
**Region 2**	**54,795**	**7,567**	**8,672**	**16,239**	**29.64**
Batanes	**226**	36	40	**76**	**33.60**
Cagayan	**18,148**	1,153	1,301	**2,454**	**13.52**
Isabela	**23,253**	4,596	5,319	**9,915**	**42.64**
Nueva Vizcaya	**7,763**	889	948	**1,837**	**23.66**
Quirino	**3,138**	778	941	**1,719**	**54.78**
Santiago City	**2,267**	115	123	**238**	**10.50**
**Region 3**	**196,494**	**39,089**	**41,658**	**80,747**	**41.09**
Aurora	**3,958**	657	707	**1,364**	**34.46**
Bataan	**14,121**	2,065	2,274	**4,339**	**30.73**
Bulacan	**57,313**	10,432	11,000	**21,432**	**37.39**
Nueva Ecija	**36,643**	11,562	12,229	**23,791**	**64.93**
Pampanga	**38,338**	5,955	6,245	**12,200**	**31.82**
Tarlac	**24,139**	5,020	5,487	**10,507**	**43.53**
Zambales	**9,920**	2,651	2,844	**5,495**	**55.39**
Angeles City	**8,373**	309	324	**633**	**7.56**
Olongapo	**3,688**	438	548	**986**	**26.73**
**Region 4A**	**256,415**	**23,986**	**26,602**	**50,588**	**19.73**
Batangas	**45,667**	5,000	5,166	**10,166**	**22.26**
Cavite	**66,686**	3,374	3,830	**7,204**	**10.80**
Laguna	**51,529**	4,769	5,270	**10,039**	**19.48**
Quezon	**32,963**	3,596	3,919	**7,515**	**22.80**
Rizal	**54,701**	7,100	8,228	**15,328**	**28.02**
Lucena City	**4,869**	147	189	**336**	**6.90**
**Region 4B**	**54,732**	**2,391**	**2,667**	**5,058**	**9.24**
Marinduque	**3,929**	121	141	**262**	**6.67**
Occidental Mindoro	**9,587**	253	316	**569**	**5.94**
Oriental Mindoro	**15,090**	792	918	**1,710**	**11.33**
Palawan	**16,971**	766	804	**1,570**	**9.25**
Romblon	**4,564**	331	359	**690**	**15.12**
Puerto Princesa City	**4,591**	128	129	**257**	**5.60**

Table 2.D.1 - Oral Health Care
Children 12-59 months old who are orally fit upon oral examination or after oral rehabilitation
Philippines, 2023

Area	Eligible Population x 20%	Orally fit		Total	%
		Male	Female		
**Region 5**	**112,798**	**21,937**	**22,820**	**44,757**	**39.68**
Albay	**23,070**	5,436	5,634	**11,070**	**47.98**
Camarines Norte	**12,299**	3,225	3,503	**6,728**	**54.71**
Camarines Sur	**35,920**	3,436	3,379	**6,815**	**18.97**
Catanduanes	**4,968**	1,662	1,668	**3,330**	**67.03**
Masbate	**18,237**	1,352	1,542	**2,894**	**15.87**
Sorsogon	**14,977**	6,655	6,856	**13,511**	**90.21**
Naga City	**3,328**	171	238	**409**	**12.29**
**Region 6**	**124,523**	**25,957**	**28,143**	**54,100**	**43.45**
Aklan	**9,294**	462	487	**949**	**10.21**
Antique	**10,106**	415	447	**862**	**8.53**
Capiz	**12,238**	2,210	2,496	**4,706**	**38.46**
Guimaras	**2,947**	507	480	**987**	**33.50**
Iloilo	**30,639**	15,235	16,225	**31,460**	**102.68**
Negros Occidental	**43,276**	2,228	2,536	**4,764**	**11.01**
Bacolod City	**8,748**	1,849	2,190	**4,039**	**46.17**
Iloilo City	**7,276**	3,051	3,282	**6,333**	**87.03**
**Region 7**	**135,046**	**16,427**	**17,744**	**34,171**	**25.30**
Bohol	**21,776**	2,450	2,658	**5,108**	**23.46**
Cebu	**58,591**	7,460	8,154	**15,614**	**26.65**
Negros Oriental	**23,896**	2,186	2,280	**4,466**	**18.69**
Siquijor	**1,280**	75	102	**177**	**13.82**
Cebu City	**15,786**	3,490	3,642	**7,132**	**45.18**
Lapu-Lapu City	**8,408**	97	105	**202**	**2.40**
Mandaue City	**5,309**	669	803	**1,472**	**27.73**
**Region 8**	**78,567**	**5,596**	**5,538**	**11,134**	**14.17**
Biliran	**3,026**	257	301	**558**	**18.44**
Eastern Samar	**7,819**	118	114	**232**	**2.97**
Northern Leyte	**26,812**	2,691	2,417	**5,108**	**19.05**
Northern Samar	**11,721**	1,164	1,265	**2,429**	**20.72**
Southern Leyte	**6,518**	419	388	**807**	**12.38**
Western Samar	**14,133**	420	521	**941**	**6.66**
Ormoc City	**4,096**	495	494	**989**	**24.14**
Tacloban City	**4,441**	32	38	**70**	**1.58**

Table 2.D.1 - Oral Health Care

Children 12-59 months old who are orally fit upon oral examination or after oral rehabilitation

Philippines, 2023

Area	Eligible Population x 20%	Orally fit		Total	%
		Male	Female		
**Region 9**	**70,484**	**5,234**	**5,887**	**11,121**	**15.78**
Zamboanga Del Norte	**19,727**	1,863	2,036	**3,899**	**19.76**
Zamboanga Del Sur	**19,432**	2,226	2,580	**4,806**	**24.73**
Zamboanga Sibugay	**12,136**	723	731	**1,454**	**11.98**
Isabela City	**2,739**	327	394	**721**	**26.32**
Zamboanga City	**16,450**	95	146	**241**	**1.47**
**Region 10**	**91,134**	**9,840**	**12,260**	**22,100**	**24.25**
Bukidnon	**28,853**	2,360	2,662	**5,022**	**17.41**
Camiguin	**1,464**	88	115	**203**	**13.86**
Lanao Del Norte	**14,925**	1,589	2,563	**4,152**	**27.82**
Misamis Occidental	**9,902**	2,884	3,487	**6,371**	**64.34**
Misamis Oriental	**17,604**	1,456	1,855	**3,311**	**18.81**
Cagayan de Oro City	**11,931**	735	817	**1,552**	**13.01**
Iligan City	**6,454**	728	761	**1,489**	**23.07**
**Region 11**	**89,836**	**7,547**	**8,432**	**15,979**	**17.79**
Davao de Oro	**13,205**	1,018	1,225	**2,243**	**16.99**
Davao del Norte	**19,559**	2,174	2,279	**4,453**	**22.77**
Davao Oriental	**9,800**	1,590	1,688	**3,278**	**33.45**
Davao del Sur	**11,839**	813	874	**1,687**	**14.25**
Davao Occidental	**6,340**	531	565	**1,096**	**17.29**
Davao City	**29,093**	1,421	1,801	**3,222**	**11.07**
**Region 12**	**79,171**	**7,949**	**9,308**	**17,257**	**21.80**
North Cotabato	**23,356**	1,729	1,755	**3,484**	**14.92**
Sarangani	**10,817**	1,256	1,405	**2,661**	**24.60**
South Cotabato	**17,259**	2,600	2,935	**5,535**	**32.07**
Sultan Kudarat	**15,717**	1,900	2,674	**4,574**	**29.10**
Gen. Santos City	**12,022**	464	539	**1,003**	**8.34**
**BARMM**	**123,511**	**11,877**	**12,205**	**24,082**	**19.50**
Basilan	**10,617**	166	155	**321**	**3.02**
Lanao Del Sur	**30,395**	1,924	2,072	**3,996**	**13.15**
Maguindanao	**37,922**	3,388	3,487	**6,875**	**18.13**
Sulu	**21,961**	5,648	5,637	**11,285**	**51.39**
Tawi-Tawi	**11,393**	406	520	**926**	**8.13**

Table 2.D.1 - Oral Health Care
Children 12-59 months old who are orally fit upon oral examination or after oral rehabilitation
Philippines, 2023

Area	Eligible Population x 20%	Orally fit		Total	%
		Male	Female		
SGA	**4,775**	*	*	*	*
Cotabato City	**6,448**	345	334	**679**	**10.53**
**CARAGA**	**47,356**	**4,368**	**4,619**	**8,987**	**18.98**
Agusan del Norte	**6,407**	687	829	**1,516**	**23.66**
Agusan del Sur	**13,460**	799	884	**1,683**	**12.50**
Surigao del Norte	**8,482**	298	286	**584**	**6.89**
Surigao del Sur	**11,115**	1,689	1,710	**3,399**	**30.58**
Province of Dinagat	**2,191**	525	514	**1,039**	**47.42**
Butuan City	**5,701**	370	396	**766**	**13.44**

Table 2.D.2 - Oral Health Care

5 years old and above with cases of decayed, missing, filled teeth (DMFT)

Philippines, 2023

Area	Total Examined	Cases of 5 y/o and above with DMFT		Total with DMFT	%
		Male	Female		
**Philippines**	**9,667,840**	**1,325,823**	**1,936,913**	**3,262,736**	**33.75**
**NCR**	**6,912,350**	**496,245**	**771,304**	**1,267,549**	**18.34**
Malabon City	**352,962**	6,322	11,666	**17,988**	**5.10**
Navotas City	**228,709**	15,037	21,386	**36,423**	**15.93**
Valenzuela City	**663,985**	13,033	22,505	**35,538**	**5.35**
Caloocan City	**85,429**	30,812	51,383	**82,195**	**96.21**
Marikina City	**25,230**	10,335	12,936	**23,271**	**92.24**
Pasig City	**73,760**	21,876	31,467	**53,343**	**72.32**
Pateros	**7,418**	3,330	4,063	**7,393**	**99.66**
Taguig City	**204,240**	15,153	32,151	**47,304**	**23.16**
Quezon City	**2,729,447**	75,044	137,313	**212,357**	**7.78**
Makati City	**58,951**	21,052	29,854	**50,906**	**86.35**
Mandaluyong City	**15,882**	6,191	8,129	**14,320**	**90.16**
San Juan City	**5,517**	1,316	2,746	**4,062**	**73.63**
Manila City	**1,716,190**	180,697	232,430	**413,127**	**24.07**
Las Piñas City	**30,056**	12,041	18,007	**30,048**	**99.97**
Muntinlupa City	**273,172**	11,109	16,692	**27,801**	**10.18**
Parañaque City	**26,230**	8,768	14,734	**23,502**	**89.60**
Pasay City	**415,172**	64,129	123,842	**187,971**	**45.28**
**CAR**	**100,753**	**18,133**	**28,463**	**46,596**	**46.25**
Abra	**4,960**	903	1,306	**2,209**	**44.54**
Apayao	**3,015**	623	973	**1,596**	**52.94**
Benguet	**25,310**	5,918	8,513	**14,431**	**57.02**
Ifugao	**6,051**	1,238	1,734	**2,972**	**49.12**
Kalinga	**33,597**	2,561	4,242	**6,803**	**20.25**
Mountain Province	**13,051**	2,966	4,111	**7,077**	**54.23**
Baguio City	**14,769**	3,924	7,584	**11,508**	**77.92**
**Region 1**	**469,258**	**50,844**	**69,231**	**120,075**	**25.59**
Ilocos Norte	**77,896**	4,817	5,490	**10,307**	**13.23**
Ilocos Sur	**90,877**	2,298	3,396	**5,694**	**6.27**
La Union	**52,498**	5,970	6,890	**12,860**	**24.50**
Pangasinan	**244,582**	36,900	51,259	**88,159**	**36.04**

Table 2.D.2 - Oral Health Care

5 years old and above with cases of decayed, missing, filled teeth (DMFT)

Philippines, 2023

Area	Total Examined	Cases of 5 y/o and above with DMFT		Total with DMFT	%
		Male	Female		
Dagupan City	**3,405**	859	2,196	**3,055**	**89.72**
**Region 2**	**155,383**	**63,887**	**91,496**	**155,383**	**100.00**
Batanes	**3**	0	3	**3**	**100.00**
Cagayan	**20,635**	7,880	12,755	**20,635**	**100.00**
Isabela	**70,915**	30,139	40,776	**70,915**	**100.00**
Nueva Vizcaya	**56,382**	22,671	33,711	**56,382**	**100.00**
Quirino	**2,880**	1,339	1,541	**2,880**	**100.00**
Santiago City	**4,568**	1,858	2,710	**4,568**	**100.00**
**Region 3**	**270,971**	**100,022**	**137,340**	**237,362**	**87.60**
Aurora	**2,688**	1,047	1,370	**2,417**	**89.92**
Bataan	**16,004**	5,753	9,971	**15,724**	**98.25**
Bulacan	**56,358**	17,500	25,789	**43,289**	**76.81**
Nueva Ecija	**85,331**	30,671	39,092	**69,763**	**81.76**
Pampanga	**46,073**	18,453	25,191	**43,644**	**94.73**
Tarlac	**47,602**	19,870	26,710	**46,580**	**97.85**
Zambales	**12,820**	5,463	7,275	**12,738**	**99.36**
Angeles City	**1,680**	559	1,121	**1,680**	**100.00**
Olongapo	**2,415**	706	821	**1,527**	**63.23**
**Region 4A**	**402,258**	**147,763**	**195,610**	**343,373**	**85.36**
Batangas	**173,712**	70,994	83,427	**154,421**	**88.89**
Cavite	**43,649**	12,715	18,482	**31,197**	**71.47**
Laguna	**92,230**	35,553	49,340	**84,893**	**92.04**
Quezon	**24,929**	6,059	9,629	**15,688**	**62.93**
Rizal	**63,510**	21,169	32,088	**53,257**	**83.86**
Lucena City	**4,228**	1,273	2,644	**3,917**	**92.64**
**Region 4B**	**127,508**	**43,441**	**72,050**	**115,491**	**90.58**
Marinduque	**2,732**	437	906	**1,343**	**49.16**
Occidental Mindoro	**87,100**	32,283	54,817	**87,100**	**100.00**
Oriental Mindoro	**17,856**	5,441	7,477	**12,918**	**72.35**
Palawan	**11,855**	3,098	5,029	**8,127**	**68.55**
Romblon	**4,408**	1,059	1,853	**2,912**	**66.06**
Puerto Princesa City	**3,557**	1,123	1,968	**3,091**	**86.90**

Table 2.D.2 - Oral Health Care

5 years old and above with cases of decayed, missing, filled teeth (DMFT)

Philippines, 2023

Area	Total Examined	Cases of 5 y/o and above with DMFT		Total with DMFT	%
		Male	Female		
**Region 5**	**145,148**	**39,776**	**65,166**	**104,942**	**72.30**
Albay	**30,711**	10,865	14,458	**25,323**	**82.46**
Camarines Norte	**20,169**	6,931	9,502	**16,433**	**81.48**
Camarines Sur	**30,984**	7,096	15,012	**22,108**	**71.35**
Catanduanes	**8,495**	3,312	4,050	**7,362**	**86.66**
Masbate	**8,684**	435	479	**914**	**10.53**
Sorsogon	**44,038**	10,861	21,353	**32,214**	**73.15**
Naga City	**2,067**	276	312	**588**	**28.45**
**Region 6**	**438,494**	**127,903**	**170,898**	**298,801**	**68.14**
Aklan	**13,700**	4,760	6,393	**11,153**	**81.41**
Antique	**11,567**	3,182	5,410	**8,592**	**74.28**
Capiz	**83,216**	36,182	44,542	**80,724**	**97.01**
Guimaras	**4,707**	958	1,691	**2,649**	**56.28**
Iloilo	**152,096**	49,481	66,746	**116,227**	**76.42**
Negros Occidental	**114,625**	18,302	25,629	**43,931**	**38.33**
Bacolod City	**29,892**	3,982	5,422	**9,404**	**31.46**
Iloilo City	**28,691**	11,056	15,065	**26,121**	**91.04**
**Region 7**	**125,609**	**47,893**	**76,663**	**124,556**	**99.16**
Bohol	**19,140**	7,806	11,334	**19,140**	**100.00**
Cebu	**31,609**	13,589	18,020	**31,609**	**100.00**
Negros Oriental	**0**	0	0	**0**	**0.00**
Siquijor	**40**	15	25	**40**	**100.00**
Cebu City	**69,040**	24,225	44,815	**69,040**	**100.00**
Lapu-Lapu City	**2,172**	1,064	1,108	**2,172**	**100.00**
Mandaue City	**3,608**	1,194	1,361	**2,555**	**70.81**
**Region 8**	**20,302**	**6,689**	**7,387**	**14,076**	**69.33**
Biliran	**3,264**	1,131	1,199	**2,330**	**71.38**
Eastern Samar	**54**	21	33	**54**	**100.00**
Northern Leyte	**6,013**	2,960	2,982	**5,942**	**98.82**
Northern Samar	**3,221**	1,364	1,660	**3,024**	**93.88**
Southern Leyte	**2,737**	330	384	**714**	**26.09**
Western Samar	**1,381**	495	616	**1,111**	**80.45**

Table 2.D.2 - Oral Health Care

5 years old and above with cases of decayed, missing, filled teeth (DMFT)

Philippines, 2023

Area	Total Examined	Cases of 5 y/o and above with DMFT		Total with DMFT	%
		Male	Female		
Ormoc City	**720**	318	393	**711**	**98.75**
Tacloban City	**2,912**	70	120	**190**	**6.52**
**Region 9**	**85,724**	**20,706**	**31,670**	**52,376**	**61.10**
Zamboanga Del Norte	**9,617**	3,019	4,095	**7,114**	**73.97**
Zamboanga Del Sur	**12,552**	5,656	6,896	**12,552**	**100.00**
Zamboanga Sibugay	**15,350**	5,421	9,656	**15,077**	**98.22**
Isabela City	**48,022**	6,568	10,882	**17,450**	**36.34**
Zamboanga City	**183**	42	141	**183**	**100.00**
**Region 10**	**151,745**	**51,909**	**73,094**	**125,003**	**82.38**
Bukidnon	**29,587**	11,642	17,945	**29,587**	**100.00**
Camiguin	**5,098**	1,191	1,085	**2,276**	**44.64**
Lanao Del Norte	**7,121**	3,000	2,794	**5,794**	**81.36**
Misamis Occidental	**42,064**	16,302	23,159	**39,461**	**93.81**
Misamis Oriental	**29,269**	6,896	10,318	**17,214**	**58.81**
Cagayan de Oro City	**21,503**	6,498	10,868	**17,366**	**80.76**
Iligan City	**17,103**	6,380	6,925	**13,305**	**77.79**
**Region 11**	**43,464**	**19,858**	**21,159**	**41,017**	**94.37**
Davao de Oro	**8,268**	3,802	4,021	**7,823**	**94.62**
Davao del Norte	**5,510**	2,312	2,449	**4,761**	**86.41**
Davao Oriental	**12,433**	5,745	6,141	**11,886**	**95.60**
Davao del Sur	**4,369**	1,987	2,199	**4,186**	**95.81**
Davao Occidental	**3,309**	1,512	1,579	**3,091**	**93.41**
Davao City	**9,575**	4,500	4,770	**9,270**	**96.81**
**Region 12**	**72,979**	**27,673**	**43,425**	**71,098**	**97.42**
North Cotabato	**21,827**	7,810	12,136	**19,946**	**91.38**
Sarangani	**4,924**	2,100	2,824	**4,924**	**100.00**
South Cotabato	**15,118**	5,771	9,347	**15,118**	**100.00**
Sultan Kudarat	**28,249**	11,475	16,774	**28,249**	**100.00**
Gen. Santos City	**2,861**	517	2,344	**2,861**	**100.00**

Table 2.D.2 - Oral Health Care

5 years old and above with cases of decayed, missing, filled teeth (DMFT)

Philippines, 2023

Area	Total Examined	Cases of 5 y/o and above with DMFT		Total with DMFT	%
		Male	Female		
**BARMM**	**91,999**	**41,624**	**49,519**	**91,143**	**99.07**
Basilan	**2,777**	1,079	1,698	**2,777**	**100.00**
Lanao Del Sur	**26,724**	11,774	14,950	**26,724**	**100.00**
Maguindanao	**20,836**	9,412	11,424	**20,836**	**100.00**
Sulu	**12,223**	6,217	6,006	**12,223**	**100.00**
Tawi-Tawi	**21,231**	9,506	11,725	**21,231**	**100.00**
SGA	**0**	0	0	**0**	**0.00**
Cotabato City	**8,208**	3,636	3,716	**7,352**	**89.57**
**CARAGA**	**53,895**	**21,457**	**32,438**	**53,895**	**100.00**
Agusan del Norte	**13,600**	5,967	7,633	**13,600**	**100.00**
Agusan del Sur	**8,736**	3,590	5,146	**8,736**	**100.00**
Surigao del Norte	**6,234**	2,200	4,034	**6,234**	**100.00**
Surigao del Sur	**22,893**	8,501	14,392	**22,893**	**100.00**
Province of Dinagat	**846**	328	518	**846**	**100.00**
Butuan City	**1,586**	871	715	**1,586**	**100.00**

Table 2.D.3 - Oral Health Care and Services

Infants 0-11 months old who received Basic Oral Health Care (BOHC)

Philippines, 2023

Area	Infants 0-11 months old who received BOHC				
	Eligible Population x 30%	Male	Female	Total	%
**Philippines**	**656,207**	**210,006**	**210,446**	**420,452**	**64.07**
**NCR**	**78,369**	**66,942**	**61,764**	**128,706**	**164.23**
Malabon City	2,216	3,103	3,095	6,198	279.69
Navotas City	1,563	535	648	1,183	75.69
Valenzuela City	4,043	2,170	2,175	4,345	107.47
Caloocan City	9,950	7,357	7,049	14,406	144.78
Marikina City	2,503	3,179	3,048	6,227	248.78
Pasig City	5,305	5,392	5,322	10,714	201.96
Pateros	335	359	413	772	230.45
Taguig City	5,698	3,870	3,741	7,611	133.57
Quezon City	17,741	7,277	7,291	14,568	82.11
Makati City	3,283	1,574	1,529	3,103	94.52
Mandaluyong City	2,377	1,676	1,711	3,387	142.49
San Juan City	650	402	398	800	123.08
Manila City	10,238	19,527	14,601	34,128	333.35
Las Piñas City	3,379	2,718	2,640	5,358	158.57
Muntinlupa City	2,964	4,169	4,186	8,355	281.88
Parañaque City	3,984	1,162	1,219	2,381	59.76
Pasay City	2,140	2,472	2,698	5,170	241.59
**CAR**	**9,396**	**3,435**	**3,629**	**7,064**	**75.18**
Abra	1,098	305	384	689	62.75
Apayao	671	271	267	538	80.18
Benguet	2,642	804	739	1,543	58.40
Ifugao	1,229	151	208	359	29.21
Kalinga	1,365	1,301	1,469	2,770	202.93
Mountain Province	786	277	253	530	67.43
Baguio City	1,605	326	309	635	39.56
**Region 1**	**28,805**	**9,156**	**9,727**	**18,883**	**65.55**
Ilocos Norte	2,674	1,304	1,374	2,678	100.15
Ilocos Sur	2,974	1,648	1,877	3,525	118.53
La Union	4,052	1,770	1,631	3,401	83.93
Pangasinan	18,108	4,316	4,697	9,013	49.77
Dagupan City	997	118	148	266	26.68

Table 2.D.3 - Oral Health Care and Services

Infants 0-11 months old who received Basic Oral Health Care (BOHC)

Philippines, 2023

Area	Infants 0-11 months old who received BOHC				
	Eligible Population x 30%	Male	Female	Total	%
**Region 2**	**19,191**	**4,794**	**5,902**	**10,696**	**55.73**
Batanes	72	18	9	27	37.50
Cagayan	6,236	536	556	1,092	17.51
Isabela	8,214	2,389	3,672	6,061	73.79
Nueva Vizcaya	2,639	816	653	1,469	55.67
Quirino	1,152	575	594	1,169	101.48
Santiago City	878	460	418	878	100.00
**Region 3**	**70,064**	**21,351**	**21,516**	**42,867**	**61.18**
Aurora	1,361	379	370	749	55.03
Bataan	5,214	545	569	1,114	21.37
Bulacan	20,277	8,136	8,238	16,374	80.75
Nueva Ecija	13,028	4,810	4,718	9,528	73.13
Pampanga	13,695	2,667	2,748	5,415	39.54
Tarlac	8,851	2,459	2,665	5,124	57.89
Zambales	3,517	1,637	1,533	3,170	90.13
Angeles City	2,744	194	161	355	12.94
Olongapo	1,377	524	514	1,038	75.38
**Region 4A**	**92,395**	**26,011**	**25,787**	**51,798**	**56.06**
Batangas	15,975	4,122	3,974	8,096	50.68
Cavite	23,389	2,377	2,380	4,757	20.34
Laguna	18,458	3,226	3,302	6,528	35.37
Quezon	12,243	2,335	2,428	4,763	38.90
Rizal	20,471	13,925	13,650	27,575	134.70
Lucena City	1,859	26	53	79	4.25
**Region 4B**	**19,836**	**1,269**	**1,175**	**2,444**	**12.32**
Marinduque	1,243	102	80	182	14.64
Occidental Mindoro	3,492	142	71	213	6.10
Oriental Mindoro	5,683	528	505	1,033	18.18
Palawan	6,095	291	307	598	9.81
Romblon	1,647	205	210	415	25.20
Puerto Princesa City	1,676	1	2	3	0.18

Table 2.D.3 - Oral Health Care and Services

Infants 0-11 months old who received Basic Oral Health Care (BOHC)

Philippines, 2023

Area	Infants 0-11 months old who received BOHC				
	Eligible Population x 30%	Male	Female	Total	%
**Region 5**	**40,646**	**5,366**	**5,213**	**10,579**	**26.03**
Albay	8,232	549	573	1,122	13.63
Camarines Norte	4,409	714	770	1,484	33.66
Camarines Sur	13,036	1,194	1,139	2,333	17.90
Catanduanes	1,624	497	378	875	53.88
Masbate	6,710	435	479	914	13.62
Sorsogon	5,379	1,877	1,772	3,649	67.84
Naga City	1,256	100	102	202	16.08
**Region 6**	**44,406**	**13,205**	**13,967**	**27,172**	**61.19**
Aklan	3,385	397	403	800	23.63
Antique	3,579	174	183	357	9.97
Capiz	4,179	2,365	2,551	4,916	117.64
Guimaras	1,005	109	148	257	25.57
Iloilo	10,842	5,020	5,523	10,543	97.24
Negros Occidental	15,681	997	1,038	2,035	12.98
Bacolod City	3,112	823	791	1,614	51.86
Iloilo City	2,623	3,320	3,330	6,650	253.53
**Region 7**	**49,703**	**17,714**	**19,137**	**36,851**	**74.14**
Bohol	7,871	484	600	1,084	13.77
Cebu	21,660	4,274	4,392	8,666	40.01
Negros Oriental	8,617	2,201	2,438	4,639	53.84
Siquijor	504	2	8	10	1.98
Cebu City	5,780	7,660	7,783	15,443	267.18
Lapu-Lapu City	3,204	0	0	0	0.00
Mandaue City	2,067	3,093	3,916	7,009	339.09
**Region 8**	**28,145**	**2,028**	**2,133**	**4,161**	**14.78**
Biliran	1,012	28	40	68	6.72
Eastern Samar	2,887	23	20	43	1.49
Northern Leyte	9,468	400	453	853	9.01
Northern Samar	4,217	354	405	759	18.00
Southern Leyte	2,222	26	28	54	2.43
Western Samar	5,103	320	256	576	11.29
Ormoc City	1,588	757	825	1,582	99.62
Tacloban City	1,648	120	106	226	13.71

Table 2.D.3 - Oral Health Care and Services

Infants 0-11 months old who received Basic Oral Health Care (BOHC)

Philippines, 2023

Area	Infants 0-11 months old who received BOHC				
	Eligible Population x 30%	Male	Female	Total	%
**Region 9**	**25,444**	**1,806**	**1,911**	**3,717**	**14.61**
Zamboanga Del Norte	7,452	465	436	901	12.09
Zamboanga Del Sur	7,127	917	1,007	1,924	27.00
Zamboanga Sibugay	4,152	253	283	536	12.91
Isabela City	960	46	62	108	11.25
Zamboanga City	5,753	125	123	248	4.31
**Region 10**	**32,750**	**6,713**	**7,403**	**14,116**	**43.10**
Bukidnon	10,021	1,228	1,464	2,692	26.86
Camiguin	518	23	33	56	10.81
Lanao Del Norte	5,328	180	172	352	6.61
Misamis Occidental	3,698	2,288	2,408	4,696	126.99
Misamis Oriental	6,593	1,439	1,576	3,015	45.73
Cagayan de Oro City	4,292	1,243	1,460	2,703	62.98
Iligan City	2,300	312	290	602	26.17
**Region 11**	**32,101**	**2,998**	**3,665**	**6,663**	**20.76**
Davao de Oro	4,576	451	548	999	21.83
Davao del Norte	7,036	641	684	1,325	18.83
Davao Oriental	3,581	543	575	1,118	31.22
Davao del Sur	3,925	333	400	733	18.68
Davao Occidental	1,989	223	233	456	22.93
Davao City	10,994	807	1,225	2,032	18.48
**Region 12**	**28,584**	**10,106**	**9,919**	**20,025**	**70.06**
North Cotabato	7,956	2,043	2,133	4,176	52.49
Sarangani	3,997	474	539	1,013	25.34
South Cotabato	6,479	3,559	3,280	6,839	105.56
Sultan Kudarat	5,817	1,614	1,559	3,173	54.55
Gen. Santos City	4,335	2,416	2,408	4,824	111.28
**BARMM**	**39,852**	**12,754**	**13,212**	**25,966**	**65.16**
Basilan	3,491	287	301	588	16.84
Lanao Del Sur	10,012	2,707	2,976	5,683	56.76
Maguindanao	12,495	3,707	3,749	7,456	59.67

Table 2.D.3 - Oral Health Care and Services

Infants 0-11 months old who received Basic Oral Health Care (BOHC)

Philippines, 2023

Area	Infants 0-11 months old who received BOHC				
	Eligible Population x 30%	Male	Female	Total	%
Sulu	6,413	5,256	5,308	10,564	164.73
Tawi-Tawi	3,783	461	482	943	24.93
SGA	1,531	0	0	0	0.00
Cotabato City	2,127	336	396	732	34.41
**CARAGA**	**16,520**	**4,358**	**4,386**	**8,744**	**52.93**
Agusan del Norte	2,186	798	899	1,697	77.63
Agusan del Sur	4,811	212	252	464	9.64
Surigao del Norte	3,056	1,450	1,353	2,803	91.72
Surigao del Sur	3,821	754	668	1,422	37.22
Province of Dinagat	640	521	524	1,045	163.28
Butuan City	2,006	623	690	1,313	65.45

Table 2.D.4 - Oral Health Care and Services

Children 1-4 years old who received Basic Oral Health Care (BOHC)

Philippines, 2023

Area	Infants 1-4 years old who received BOHC				
	Eligible Population x 30%	Male	Female	Total	%
**Philippines**	**2,744,042**	**667,689**	**724,743**	**1,392,432**	**50.74**
**NCR**	**314,600**	**130,911**	**139,044**	**269,955**	**85.81**
Malabon City	8,842	2,884	3,356	6,240	70.58
Navotas City	6,258	2,176	2,485	4,661	74.48
Valenzuela City	16,471	2,749	2,941	5,690	34.55
Caloocan City	41,623	19,631	21,468	41,099	98.74
Marikina City	10,390	2,985	3,055	6,040	58.14
Pasig City	19,713	7,245	8,056	15,301	77.62
Pateros	1,447	857	971	1,828	126.31
Taguig City	22,483	5,830	6,052	11,882	52.85
Quezon City	71,584	18,425	18,959	37,384	52.22
Makati City	12,755	5,373	5,787	11,160	87.49
Mandaluyong City	9,468	4,457	4,712	9,169	96.84
San Juan City	2,586	804	858	1,662	64.28
Manila City	41,706	40,816	42,653	83,469	200.14
Las Piñas City	13,575	4,332	4,481	8,813	64.92
Muntinlupa City	11,743	5,165	5,511	10,676	90.91
Parañaque City	15,995	2,882	3,020	5,902	36.90
Pasay City	7,962	4,300	4,679	8,979	112.77
**CAR**	**39,576**	**13,979**	**15,015**	**28,994**	**73.26**
Abra	4,908	869	851	1,720	35.04
Apayao	2,781	1,697	1,718	3,415	122.80
Benguet	11,125	3,158	3,429	6,587	59.21
Ifugao	5,157	1,249	1,251	2,500	48.47
Kalinga	5,577	3,279	4,079	7,358	131.93
Mountain Province	3,418	1,588	1,570	3,158	92.39
Baguio City	6,609	2,139	2,117	4,256	64.39
**Region 1**	**117,572**	**44,071**	**48,793**	**92,864**	**78.98**
Ilocos Norte	11,458	7,233	7,465	14,698	128.27
Ilocos Sur	13,090	7,491	7,812	15,303	116.91
La Union	16,634	8,125	8,497	16,622	99.93
Pangasinan	72,361	21,131	24,892	46,023	63.60
Dagupan City	4,029	91	127	218	5.41

Table 2.D.4 - Oral Health Care and Services

Children 1-4 years old who received Basic Oral Health Care (BOHC)

Philippines, 2023

Area	Infants 1-4 years old who received BOHC				
	Eligible Population x 30%	Male	Female	Total	%
**Region 2**	**82,193**	**26,596**	**30,340**	**56,936**	**69.27**
Batanes	339	130	162	292	86.06
Cagayan	27,222	3,897	4,123	8,020	29.46
Isabela	34,879	12,398	15,052	27,450	78.70
Nueva Vizcaya	11,645	3,587	4,174	7,761	66.65
Quirino	4,707	3,498	3,648	7,146	151.81
Santiago City	3,400	3,086	3,181	6,267	184.33
**Region 3**	**294,741**	**77,254**	**83,259**	**160,513**	**54.46**
Aurora	5,938	1,440	1,428	2,868	48.30
Bataan	21,182	4,672	6,401	11,073	52.28
Bulacan	85,969	20,233	21,781	42,014	48.87
Nueva Ecija	54,964	20,854	22,128	42,982	78.20
Pampanga	57,508	13,467	13,923	27,390	47.63
Tarlac	36,209	9,746	10,555	20,301	56.07
Zambales	14,880	4,715	4,682	9,397	63.15
Angeles City	12,560	1,012	1,161	2,173	17.30
Olongapo	5,533	1,115	1,200	2,315	41.84
**Region 4A**	**384,622**	**70,432**	**76,398**	**146,830**	**38.18**
Batangas	68,501	20,997	20,859	41,856	61.10
Cavite	100,030	8,582	9,917	18,499	18.49
Laguna	77,293	16,322	18,231	34,553	44.70
Quezon	49,444	9,943	10,380	20,323	41.10
Rizal	82,052	13,667	15,900	29,567	36.03
Lucena City	7,303	921	1,111	2,032	27.82
**Region 4B**	**82,098**	**8,193**	**9,457**	**17,650**	**21.50**
Marinduque	5,894	471	498	969	16.44
Occidental Mindoro	14,380	754	1,039	1,793	12.47
Oriental Mindoro	22,635	3,368	3,818	7,186	31.75
Palawan	25,456	2,439	2,834	5,273	20.71
Romblon	6,846	1,124	1,240	2,364	34.53
Puerto Princesa City	6,886	37	28	65	0.94

Table 2.D.4 - Oral Health Care and Services
Children 1-4 years old who received Basic Oral Health Care (BOHC)
Philippines, 2023

Area	Infants 1-4 years old who received BOHC				
	Eligible Population x 30%	Male	Female	Total	%
**Region 5**	**169,197**	**53,362**	**52,891**	**106,253**	**62.80**
Albay	34,606	10,976	10,094	21,070	60.89
Camarines Norte	18,448	5,642	5,535	11,177	60.59
Camarines Sur	53,879	12,830	13,027	25,857	47.99
Catanduanes	7,451	4,761	4,788	9,549	128.15
Masbate	27,355	2,778	3,004	5,782	21.14
Sorsogon	22,466	14,946	14,959	29,905	133.12
Naga City	4,993	1,429	1,484	2,913	58.35
**Region 6**	**186,785**	**54,670**	**58,139**	**112,809**	**60.40**
Aklan	13,940	1,862	1,869	3,731	26.76
Antique	15,159	1,461	1,540	3,001	19.80
Capiz	18,356	6,566	6,918	13,484	73.46
Guimaras	4,420	1,334	1,375	2,709	61.29
Iloilo	45,959	23,493	25,093	48,586	105.72
Negros Occidental	64,914	11,576	12,242	23,818	36.69
Bacolod City	13,122	3,830	4,140	7,970	60.74
Iloilo City	10,915	4,548	4,962	9,510	87.13
**Region 7**	**202,569**	**60,542**	**66,453**	**126,995**	**62.69**
Bohol	32,664	5,761	6,244	12,005	36.75
Cebu	87,886	21,411	23,414	44,825	51.00
Negros Oriental	35,844	10,139	10,837	20,976	58.52
Siquijor	1,921	1,422	1,280	2,702	140.69
Cebu City	23,679	14,139	16,518	30,657	129.47
Lapu-Lapu City	12,612	862	851	1,713	13.58
Mandaue City	7,964	6,808	7,309	14,117	177.27
**Region 8**	**117,851**	**13,000**	**13,640**	**26,640**	**22.60**
Biliran	4,539	655	716	1,371	30.20
Eastern Samar	11,729	222	225	447	3.81
Northern Leyte	40,218	3,465	3,450	6,915	17.19
Northern Samar	17,582	2,694	3,230	5,924	33.69
Southern Leyte	9,777	1,111	1,100	2,211	22.61
Western Samar	21,200	1,886	1,993	3,879	18.30
Ormoc City	6,145	2,698	2,626	5,324	86.65
Tacloban City	6,661	269	300	569	8.54

Table 2.D.4 - Oral Health Care and Services
Children 1-4 years old who received Basic Oral Health Care (BOHC)
Philippines, 2023

Area	Infants 1-4 years old who received BOHC				
	Eligible Population x 30%	Male	Female	Total	%
**Region 9**	**105,725**	**11,414**	**12,895**	**24,309**	**22.99**
Zamboanga Del Norte	29,591	3,735	4,113	7,848	26.52
Zamboanga Del Sur	29,148	5,206	6,182	11,388	39.07
Zamboanga Sibugay	18,203	1,515	1,566	3,081	16.93
Isabela City	4,109	810	861	1,671	40.67
Zamboanga City	24,674	148	173	321	1.30
**Region 10**	**136,702**	**22,635**	**25,219**	**47,854**	**35.01**
Bukidnon	43,280	6,631	6,904	13,535	31.27
Camiguin	2,196	551	653	1,204	54.82
Lanao Del Norte	22,388	1,429	1,659	3,088	13.79
Misamis Occidental	14,854	6,067	6,406	12,473	83.97
Misamis Oriental	26,406	5,105	6,314	11,419	43.24
Cagayan de Oro City	17,897	1,306	1,700	3,006	16.80
Iligan City	9,681	1,546	1,583	3,129	32.32
**Region 11**	**134,754**	**19,583**	**20,896**	**40,479**	**30.04**
Davao de Oro	19,807	2,276	2,580	4,856	24.52
Davao del Norte	29,339	5,586	5,329	10,915	37.20
Davao Oriental	14,699	5,158	5,389	10,547	71.75
Davao del Sur	17,758	1,833	1,982	3,815	21.48
Davao Occidental	9,511	861	1,151	2,012	21.16
Davao City	43,640	3,869	4,465	8,334	19.10
**Region 12**	**118,756**	**26,185**	**34,474**	**60,659**	**51.08**
North Cotabato	35,033	5,805	5,479	11,284	32.21
Sarangani	16,226	4,595	4,935	9,530	58.73
South Cotabato	25,888	7,591	7,925	15,516	59.93
Sultan Kudarat	23,576	4,341	11,988	16,329	69.26
Gen. Santos City	18,033	3,853	4,147	8,000	44.36
**BARMM**	**185,267**	**20,139**	**21,900**	**42,039**	**22.69**
Basilan	15,926	745	844	1,589	9.98
Lanao Del Sur	45,592	6,332	6,786	13,118	28.77

Table 2.D.4 - Oral Health Care and Services

Children 1-4 years old who received Basic Oral Health Care (BOHC)

Philippines, 2023

Area	Infants 1-4 years old who received BOHC				
	Eligible Population x 30%	Male	Female	Total	%
Maguindanao	56,882	4,498	5,184	9,682	17.02
Sulu	32,942	5,695	5,549	11,244	34.13
Tawi-Tawi	17,090	1,040	1,226	2,266	13.26
SGA	7,162	0	0	0	0.00
Cotabato City	9,673	1,829	2,311	4,140	42.80
**CARAGA**	**71,033**	**14,723**	**15,930**	**30,653**	**43.15**
Agusan del Norte	9,610	2,032	2,326	4,358	45.35
Agusan del Sur	20,190	2,510	2,985	5,495	27.22
Surigao del Norte	12,722	1,625	1,697	3,322	26.11
Surigao del Sur	16,673	5,966	6,259	12,225	73.32
Province of Dinagat	3,287	1,479	1,478	2,957	89.97
Butuan City	8,552	1,111	1,185	2,296	26.85

Table 1.D.5 - Oral Health Care and Services

Children 5-9 years old who received Basic Oral Health Care (BOHC)

Philippines, 2023

Area	Children 5-9 years old who received BOHC				
	Eligible Population x 30%	Male	Female	Total	%
**Philippines**	**3,462,146**	**566,972**	**581,747**	**1,148,719**	**33.18**
**NCR**	**380,088**	**140,901**	**140,486**	**281,387**	**74.03**
Malabon City	11,224	3,293	3,078	6,371	56.76
Navotas City	7,937	2,440	2,797	5,237	65.98
Valenzuela City	20,034	1,388	1,432	2,820	14.08
Caloocan City	50,714	6,848	7,232	14,080	27.76
Marikina City	12,942	5,553	5,542	11,095	85.73
Pasig City	24,049	12,639	12,904	25,543	106.21
Pateros	1,807	853	866	1,719	95.12
Taguig City	26,417	2,261	2,375	4,636	17.55
Quezon City	84,829	8,205	8,746	16,951	19.98
Makati City	14,565	10,817	10,514	21,331	146.45
Mandaluyong City	11,244	1,068	1,144	2,212	19.67
San Juan City	3,043	351	379	730	23.99
Manila City	51,020	67,524	65,586	133,110	260.90
Las Piñas City	16,899	6,075	5,469	11,544	68.31
Muntinlupa City	14,157	5,435	5,880	11,315	79.93
Parañaque City	18,598	3,631	3,950	7,581	40.76
Pasay City	10,609	2,520	2,592	5,112	48.18
**CAR**	**52,865**	**21,341**	**21,687**	**43,028**	**81.39**
Abra	7,312	1,149	1,228	2,377	32.51
Apayao	3,734	1,022	1,093	2,115	56.64
Benguet	13,765	3,979	4,170	8,149	59.20
Ifugao	6,863	3,189	3,194	6,383	93.00
Kalinga	7,376	3,537	3,938	7,475	101.34
Mountain Province	4,884	2,065	1,989	4,054	83.00
Baguio City	8,930	6,400	6,075	12,475	139.69
**Region 1**	**152,391**	**41,759**	**42,603**	**84,362**	**55.36**
Ilocos Norte	14,943	8,100	8,531	16,631	111.29
Ilocos Sur	18,189	9,104	9,441	18,545	101.96
La Union	22,148	7,240	6,735	13,975	63.10
Pangasinan	91,943	17,165	17,751	34,916	37.98
Dagupan City	5,168	150	145	295	5.71

Table 1.D.5 - Oral Health Care and Services

Children 5-9 years old who received Basic Oral Health Care (BOHC)

Philippines, 2023

Area	Children 5-9 years old who received BOHC				
	Eligible Population x 30%	Male	Female	Total	%
**Region 2**	**110,687**	**24,445**	**24,541**	**48,986**	**44.26**
Batanes	464	168	172	340	73.26
Cagayan	37,744	2,260	2,692	4,952	13.12
Isabela	46,155	8,219	9,089	17,308	37.50
Nueva Vizcaya	15,549	7,949	6,492	14,441	92.87
Quirino	6,404	3,905	4,058	7,963	124.35
Santiago City	4,372	1,944	2,038	3,982	91.08
**Region 3**	**371,981**	**43,536**	**45,986**	**89,522**	**24.07**
Aurora	7,743	1,125	1,094	2,219	28.66
Bataan	26,330	2,790	2,744	5,534	21.02
Bulacan	109,948	9,832	11,495	21,327	19.40
Nueva Ecija	69,551	11,175	11,734	22,909	32.94
Pampanga	70,945	6,814	6,889	13,703	19.31
Tarlac	46,521	5,178	5,326	10,504	22.58
Zambales	20,071	3,722	3,644	7,366	36.70
Angeles City	13,855	134	139	273	1.97
Olongapo	7,018	2,766	2,921	5,687	81.03
**Region 4A**	**482,773**	**61,120**	**58,011**	**119,131**	**24.68**
Batangas	84,342	23,608	18,035	41,643	49.37
Cavite	124,415	6,361	6,652	13,013	10.46
Laguna	97,161	12,712	13,138	25,850	26.61
Quezon	64,609	8,441	9,041	17,482	27.06
Rizal	103,294	9,601	10,781	20,382	19.73
Lucena City	8,952	397	364	761	8.50
**Region 4B**	**109,344**	**8,370**	**8,595**	**16,965**	**15.52**
Marinduque	7,531	439	434	873	11.59
Occidental Mindoro	18,830	863	1,049	1,912	10.15
Oriental Mindoro	30,021	3,971	3,834	7,805	26.00
Palawan	34,293	2,008	2,057	4,065	11.85
Romblon	9,822	1,029	1,136	2,165	22.04
Puerto Princesa City	8,847	60	85	145	1.64

Table 1.D.5 - Oral Health Care and Services

Children 5-9 years old who received Basic Oral Health Care (BOHC)

Philippines, 2023

Area	Children 5-9 years old who received BOHC				
	Eligible Population x 30%	Male	Female	Total	%
**Region 5**	**215,626**	**25,284**	**25,768**	**51,052**	**23.68**
Albay	45,667	6,555	6,174	12,729	27.87
Camarines Norte	22,823	3,378	3,508	6,886	30.17
Camarines Sur	66,768	4,659	4,939	9,598	14.38
Catanduanes	9,459	1,951	2,090	4,041	42.72
Masbate	35,433	2,211	2,306	4,517	12.75
Sorsogon	29,212	6,280	6,485	12,765	43.70
Naga City	6,264	250	266	516	8.24
**Region 6**	**239,834**	**43,558**	**47,021**	**90,579**	**37.77**
Aklan	18,032	1,847	2,139	3,986	22.11
Antique	18,851	1,311	1,497	2,808	14.90
Capiz	22,864	5,965	6,570	12,535	54.82
Guimaras	5,558	510	547	1,057	19.02
Iloilo	60,354	18,670	19,946	38,616	63.98
Negros Occidental	84,788	7,844	8,831	16,675	19.67
Bacolod City	16,505	3,426	3,340	6,766	40.99
Iloilo City	12,881	3,985	4,151	8,136	63.16
**Region 7**	**256,223**	**47,287**	**49,792**	**97,079**	**37.89**
Bohol	42,297	4,637	4,767	9,404	22.23
Cebu	111,464	14,959	16,088	31,047	27.85
Negros Oriental	45,617	6,662	7,277	13,939	30.56
Siquijor	2,731	245	279	524	19.19
Cebu City	28,476	5,435	4,787	10,222	35.90
Lapu-Lapu City	15,819	494	499	993	6.28
Mandaue City	9,820	14,855	16,095	30,950	315.18
**Region 8**	**153,234**	**13,205**	**13,481**	**26,686**	**17.42**
Biliran	6,262	2,502	2,450	4,952	79.09
Eastern Samar	15,593	247	311	558	3.58
Northern Leyte	51,671	4,056	3,816	7,872	15.23
Northern Samar	23,383	3,372	3,529	6,901	29.51
Southern Leyte	12,477	1,021	949	1,970	15.79
Western Samar	27,925	1,528	1,811	3,339	11.96
Ormoc City	7,651	318	393	711	9.29
Tacloban City	8,272	161	222	383	4.63

Table 1.D.5 - Oral Health Care and Services

Children 5-9 years old who received Basic Oral Health Care (BOHC)

Philippines, 2023

Area	Children 5-9 years old who received BOHC				
	Eligible Population x 30%	Male	Female	Total	%
**Region 9**	**136,293**	**8,815**	**9,596**	**18,411**	**13.51**
Zamboanga Del Norte	36,998	1,297	1,461	2,758	7.45
Zamboanga Del Sur	37,083	4,331	5,026	9,357	25.23
Zamboanga Sibugay	24,856	2,620	2,540	5,160	20.76
Isabela City	4,756	479	483	962	20.23
Zamboanga City	32,600	88	86	174	0.53
**Region 10**	**175,895**	**20,483**	**21,982**	**42,465**	**24.14**
Bukidnon	57,915	5,196	6,000	11,196	19.33
Camiguin	3,069	476	488	964	31.41
Lanao Del Norte	28,520	1,108	1,133	2,241	7.86
Misamis Occidental	19,133	6,070	6,463	12,533	65.51
Misamis Oriental	33,421	2,980	2,915	5,895	17.64
Cagayan de Oro City	22,021	1,011	1,275	2,286	10.38
Iligan City	11,816	3,642	3,708	7,350	62.20
**Region 11**	**169,760**	**14,625**	**16,938**	**31,563**	**18.59**
Davao de Oro	26,057	1,892	2,137	4,029	15.46
Davao del Norte	36,390	2,905	3,190	6,095	16.75
Davao Oriental	19,850	4,068	4,363	8,431	42.47
Davao del Sur	21,299	1,630	1,845	3,475	16.32
Davao Occidental	11,875	954	1,053	2,007	16.90
Davao City	54,289	3,176	4,350	7,526	13.86
**Region 12**	**152,824**	**14,096**	**15,706**	**29,802**	**19.50**
North Cotabato	45,511	4,355	4,900	9,255	20.34
Sarangani	20,783	1,776	2,062	3,838	18.47
South Cotabato	33,023	3,170	3,330	6,500	19.68
Sultan Kudarat	30,433	3,598	4,136	7,734	25.41
Gen. Santos City	23,074	1,197	1,278	2,475	10.73
**BARMM**	**205,020**	**25,801**	**26,965**	**52,766**	**25.74**
Basilan	18,717	843	1,129	1,972	10.54
Lanao Del Sur	48,854	8,168	8,893	17,061	34.92

Table 1.D.5 - Oral Health Care and Services

Children 5-9 years old who received Basic Oral Health Care (BOHC)

Philippines, 2023

Area	Children 5-9 years old who received BOHC				
	Eligible Population x 30%	Male	Female	Total	%
Maguindanao	60,039	6,412	6,469	12,881	21.45
Sulu	40,883	6,752	6,527	13,279	32.48
Tawi-Tawi	17,453	2,356	2,419	4,775	27.36
SGA	7,962	*	*	*	*
Cotabato City	11,112	1,270	1,528	2,798	25.18
**CARAGA**	**97,310**	**12,346**	**12,589**	**24,935**	**25.62**
Agusan del Norte	13,438	2,180	2,381	4,561	33.94
Agusan del Sur	28,067	2,438	2,631	5,069	18.06
Surigao del Norte	17,151	980	1,066	2,046	11.93
Surigao del Sur	22,379	4,876	4,728	9,604	42.91
Province of Dinagat	4,237	675	722	1,397	32.97
Butuan City	12,038	1,197	1,061	2,258	18.76

Table 1.D.6 - Oral Health Care and Services

Adolescents 10-14 years who received Basic Oral Health Care (BOHC)

Philippines, 2023

Area	Adolescents 10-14 years old who received BOHC				
	Eligible Population x 30%	Male	Female	Total	%
**Philippines**	**3,407,208**	**402,957**	**430,150**	**833,107**	**24.45**
**NCR**	**361,588**	**132,562**	**137,617**	**270,179**	**74.72**
Malabon City	11,009	2,227	2,181	4,408	40.04
Navotas City	7,530	2,507	2,838	5,345	70.98
Valenzuela City	20,278	1,089	1,101	2,190	10.80
Caloocan City	48,418	4,093	4,778	8,871	18.32
Marikina City	12,618	2,822	2,661	5,483	43.45
Pasig City	21,890	9,581	10,167	19,748	90.21
Pateros	1,789	1,225	1,087	2,312	129.24
Taguig City	25,111	1,822	2,150	3,972	15.82
Quezon City	79,322	27,094	27,696	54,790	69.07
Makati City	13,819	11,023	13,370	24,393	176.52
Mandaluyong City	10,589	989	1,111	2,100	19.83
San Juan City	2,909	289	293	582	20.01
Manila City	48,661	59,307	59,121	118,428	243.37
Las Piñas City	16,112	2,785	2,779	5,564	34.53
Muntinlupa City	13,469	1,640	1,836	3,476	25.81
Parañaque City	17,534	1,057	1,093	2,150	12.26
Pasay City	10,530	3,012	3,355	6,367	60.47
**CAR**	**54,362**	**15,589**	**16,718**	**32,307**	**59.43**
Abra	7,627	1,132	1,230	2,362	30.97
Apayao	4,023	689	816	1,505	37.41
Benguet	13,511	2,562	2,911	5,473	40.51
Ifugao	6,959	1,423	1,834	3,257	46.80
Kalinga	7,557	2,809	3,482	6,291	83.24
Mountain Province	5,170	1,306	1,430	2,736	52.92
Baguio City	9,515	5,668	5,015	10,683	112.27
**Region 1**	**157,022**	**30,535**	**31,009**	**61,544**	**39.19**
Ilocos Norte	16,772	5,074	5,132	10,206	60.85
Ilocos Sur	19,945	5,616	5,602	11,218	56.24
La Union	23,014	2,446	2,707	5,153	22.39
Pangasinan	91,993	17,275	17,415	34,690	37.71
Dagupan City	5,297	124	153	277	5.23

Table 1.D.6 - Oral Health Care and Services

Adolescents 10-14 years who received Basic Oral Health Care (BOHC)

Philippines, 2023

Area	Adolescents 10-14 years old who received BOHC				
	Eligible Population x 30%	Male	Female	Total	%
**Region 2**	**112,456**	**16,459**	**19,092**	**35,551**	**31.61**
Batanes	504	84	94	178	35.32
Cagayan	38,452	1,625	1,904	3,529	9.18
Isabela	47,114	7,115	8,176	15,291	32.46
Nueva Vizcaya	15,125	4,715	5,681	10,396	68.73
Quirino	6,589	1,576	1,773	3,349	50.83
Santiago City	4,672	1,344	1,464	2,808	60.10
**Region 3**	**373,990**	**25,479**	**26,306**	**51,785**	**13.85**
Aurora	7,878	764	820	1,584	20.11
Bataan	26,006	714	694	1,408	5.41
Bulacan	111,011	5,217	5,691	10,908	9.83
Nueva Ecija	69,863	6,734	7,227	13,961	19.98
Pampanga	72,404	4,270	3,951	8,221	11.35
Tarlac	46,590	3,951	4,035	7,986	17.14
Zambales	20,096	2,309	2,435	4,744	23.61
Angeles City	13,085	78	72	150	1.15
Olongapo	7,057	1,442	1,381	2,823	40.00
**Region 4A**	**476,163**	**36,157**	**37,801**	**73,958**	**15.53**
Batangas	83,936	16,450	16,216	32,666	38.92
Cavite	122,959	2,461	2,903	5,364	4.36
Laguna	94,907	4,398	4,975	9,373	9.88
Quezon	65,461	3,162	3,227	6,389	9.76
Rizal	100,113	9,590	10,385	19,975	19.95
Lucena City	8,786	96	95	191	2.17
**Region 4B**	**109,891**	**4,401**	**5,208**	**9,609**	**8.74**
Marinduque	7,943	152	190	342	4.31
Occidental Mindoro	18,592	545	549	1,094	5.88
Oriental Mindoro	30,539	2,124	2,467	4,591	15.03
Palawan	33,151	974	1,258	2,232	6.73
Romblon	10,573	497	610	1,107	10.47
Puerto Princesa City	9,094	109	134	243	2.67

Table 1.D.6 - Oral Health Care and Services

Adolescents 10-14 years who received Basic Oral Health Care (BOHC)

Philippines, 2023

Area	Adolescents 10-14 years old who received BOHC				
	Eligible Population x 30%	Male	Female	Total	%
**Region 5**	**214,355**	**7,633**	**8,289**	**15,922**	**7.43**
Albay	46,588	1,558	1,656	3,214	6.90
Camarines Norte	21,936	1,169	1,183	2,352	10.72
Camarines Sur	66,364	964	1,164	2,128	3.21
Catanduanes	9,423	785	728	1,513	16.06
Masbate	35,450	1,488	1,544	3,032	8.55
Sorsogon	28,112	1,607	1,943	3,550	12.63
Naga City	6,481	62	71	133	2.05
**Region 6**	**240,756**	**33,278**	**35,347**	**68,625**	**28.50**
Aklan	18,712	898	1,061	1,959	10.47
Antique	18,657	507	0	507	2.72
Capiz	23,451	5,799	6,130	11,929	50.87
Guimaras	5,446	383	526	909	16.69
Iloilo	61,263	14,352	14,586	28,938	47.24
Negros Occidental	84,371	5,715	6,772	12,487	14.80
Bacolod City	16,733	2,891	3,362	6,253	37.37
Iloilo City	12,123	2,733	2,910	5,643	46.55
**Region 7**	**250,950**	**29,843**	**32,666**	**62,509**	**24.91**
Bohol	43,388	1,993	2,441	4,434	10.22
Cebu	106,758	5,455	6,183	11,638	10.90
Negros Oriental	44,841	3,113	3,677	6,790	15.14
Siquijor	2,786	145	142	287	10.30
Cebu City	27,772	2,922	2,886	5,808	20.91
Lapu-Lapu City	15,638	627	608	1,235	7.90
Mandaue City	9,768	15,588	16,729	32,317	330.84
**Region 8**	**152,647**	**6,921**	**8,129**	**15,050**	**9.86**
Biliran	6,195	153	164	317	5.12
Eastern Samar	16,022	285	357	642	4.01
Northern Leyte	51,490	2,357	2,796	5,153	10.01
Northern Samar	23,379	2,278	2,898	5,176	22.14
Southern Leyte	13,174	131	140	271	2.06
Western Samar	27,480	1,671	1,713	3,384	12.31
Ormoc City	7,275	0	0	0	0.00
Tacloban City	7,633	46	61	107	1.40

Table 1.D.6 - Oral Health Care and Services

Adolescents 10-14 years who received Basic Oral Health Care (BOHC)

Philippines, 2023

Area	Adolescents 10-14 years old who received BOHC				
	Eligible Population x 30%	Male	Female	Total	%
**Region 9**	**131,386**	**3,552**	**4,514**	**8,066**	**6.14**
Zamboanga Del Norte	35,464	462	683	1,145	3.23
Zamboanga Del Sur	36,175	2,184	2,714	4,898	13.54
Zamboanga Sibugay	24,182	592	750	1,342	5.55
Isabela City	4,475	277	329	606	13.54
Zamboanga City	31,091	37	38	75	0.24
**Region 10**	**168,179**	**15,226**	**17,679**	**32,905**	**19.57**
Bukidnon	54,854	3,948	5,261	9,209	16.79
Camiguin	2,965	540	575	1,115	37.60
Lanao Del Norte	26,712	455	465	920	3.44
Misamis Occidental	19,095	6,441	6,845	13,286	69.58
Misamis Oriental	31,880	1,027	1,315	2,342	7.35
Cagayan de Oro City	21,166	1,109	1,416	2,525	11.93
Iligan City	11,507	1,706	1,802	3,508	30.48
**Region 11**	**167,499**	**8,235**	**9,251**	**17,486**	**10.44**
Davao de Oro	25,825	1,246	1,328	2,574	9.97
Davao del Norte	35,218	1,461	1,756	3,217	9.13
Davao Oriental	19,916	2,001	2,100	4,101	20.59
Davao del Sur	21,610	928	934	1,862	8.62
Davao Occidental	11,687	599	736	1,335	11.42
Davao City	53,245	2,000	2,397	4,397	8.26
**Region 12**	**148,377**	**9,752**	**11,326**	**21,078**	**14.21**
North Cotabato	44,185	3,064	3,528	6,592	14.92
Sarangani	20,075	1,157	1,346	2,503	12.47
South Cotabato	32,364	1,894	2,023	3,917	12.10
Sultan Kudarat	29,645	3,188	3,873	7,061	23.82
Gen. Santos City	22,108	449	556	1,005	4.55
**BARMM**	**193,458**	**18,573**	**19,572**	**38,145**	**19.72**
Basilan	17,185	198	307	505	2.94
Lanao Del Sur	47,599	4,971	5,748	10,719	22.52
Maguindanao	55,715	5,046	4,912	9,958	17.87

Table 1.D.6 - Oral Health Care and Services

Adolescents 10-14 years who received Basic Oral Health Care (BOHC)

Philippines, 2023

Area	Adolescents 10-14 years old who received BOHC				
	Eligible Population x 30%	Male	Female	Total	%
Sulu	39,411	5,629	5,360	10,989	27.88
Tawi-Tawi	15,496	2,040	2,228	4,268	27.54
SGA	7,377	0	0	0	0.00
Cotabato City	10,675	689	1,017	1,706	15.98
**CARAGA**	**94,130**	**8,762**	**9,626**	**18,388**	**19.53**
Agusan del Norte	13,260	1,531	1,771	3,302	24.90
Agusan del Sur	26,713	1,210	1,453	2,663	9.97
Surigao del Norte	16,591	882	1,215	2,097	12.64
Surigao del Sur	21,424	3,381	3,563	6,944	32.41
Province of Dinagat	4,118	354	394	748	18.17
Butuan City	12,025	1,404	1,230	2,634	21.90

Table 1.D.7 - Oral Health Care and Services
Adolescents 15- 19 years old who received Basic Oral Health Care (BOHC)
Philippines, 2023

Area	Adolescents 15-19 years old who received BOHC				
	Eligible Population x 30%	Male	Female	Total	%
**Philippines**	**3,220,422**	**319,926**	**377,060**	**696,986**	**21.64**
**NCR**	**343,691**	**79,897**	**92,202**	**172,099**	**50.07**
Malabon City	10,604	1,521	2,207	3,728	35.16
Navotas City	7,151	2,295	2,562	4,857	67.92
Valenzuela City	19,291	1,037	1,276	2,313	11.99
Caloocan City	46,264	6,090	7,407	13,497	29.17
Marikina City	11,753	654	684	1,338	11.38
Pasig City	20,258	7,080	8,989	16,069	79.32
Pateros	1,653	379	438	817	49.43
Taguig City	23,094	2,570	3,211	5,781	25.03
Quezon City	74,248	14,128	16,701	30,829	41.52
Makati City	13,051	7,060	7,757	14,817	113.53
Mandaluyong City	9,651	1,448	1,522	2,970	30.77
San Juan City	2,714	233	329	562	20.70
Manila City	48,440	28,029	30,590	58,619	121.01
Las Piñas City	15,542	1,045	1,370	2,415	15.54
Muntinlupa City	13,452	1,973	2,180	4,153	30.87
Parañaque City	16,048	981	1,057	2,038	12.70
Pasay City	10,477	3,374	3,922	7,296	69.64
**CAR**	**55,362**	**9,460**	**12,499**	**21,959**	**39.66**
Abra	7,697	1,214	1,283	2,497	32.44
Apayao	3,860	369	530	899	23.29
Benguet	13,559	1,984	2,944	4,928	36.35
Ifugao	6,646	1,037	1,323	2,360	35.51
Kalinga	7,568	2,630	3,689	6,319	83.50
Mountain Province	5,195	590	969	1,559	30.01
Baguio City	10,837	1,636	1,761	3,397	31.35
**Region 1**	**153,096**	**27,462**	**30,347**	**57,809**	**37.76**
Ilocos Norte	16,411	4,692	5,099	9,791	59.66
Ilocos Sur	19,570	4,208	4,813	9,021	46.10
La Union	22,897	1,883	2,398	4,281	18.70
Pangasinan	88,877	16,555	17,842	34,397	38.70
Dagupan City	5,342	124	195	319	5.97

Table 1.D.7 - Oral Health Care and Services

Adolescents 15- 19 years old who received Basic Oral Health Care (BOHC)

Philippines, 2023

Area	Adolescents 15-19 years old who received BOHC				
	Eligible Population x 30%	Male	Female	Total	%
**Region 2**	**106,180**	**13,656**	**17,201**	**30,857**	**29.06**
Batanes	545	156	278	434	79.71
Cagayan	36,235	1,720	2,152	3,872	10.69
Isabela	44,166	6,537	7,709	14,246	32.26
Nueva Vizcaya	14,694	2,516	3,813	6,329	43.07
Quirino	6,142	1,581	2,014	3,595	58.53
Santiago City	4,399	1,146	1,235	2,381	54.13
**Region 3**	**354,315**	**26,482**	**30,112**	**56,594**	**15.97**
Aurora	7,365	372	494	866	11.76
Bataan	24,038	417	564	981	4.08
Bulacan	104,773	5,754	6,853	12,607	12.03
Nueva Ecija	64,937	7,320	8,852	16,172	24.90
Pampanga	71,198	4,241	4,306	8,547	12.00
Tarlac	42,824	3,877	3,970	7,847	18.32
Zambales	18,925	2,564	3,113	5,677	30.00
Angeles City	12,870	82	110	192	1.49
Olongapo	7,387	1,855	1,850	3,705	50.15
**Region 4A**	**452,892**	**35,809**	**42,668**	**78,477**	**17.33**
Batangas	83,521	17,061	18,400	35,461	42.46
Cavite	117,749	4,275	5,448	9,723	8.26
Laguna	89,083	5,331	7,771	13,102	14.71
Quezon	60,380	3,349	3,958	7,307	12.10
Rizal	93,815	5,679	6,929	12,608	13.44
Lucena City	8,345	114	162	276	3.31
**Region 4B**	**104,097**	**2,959**	**4,052**	**7,011**	**6.74**
Marinduque	7,624	178	232	410	5.38
Occidental Mindoro	17,133	502	859	1,361	7.94
Oriental Mindoro	28,655	951	1,190	2,141	7.47
Palawan	30,866	709	966	1,675	5.43
Romblon	10,308	398	558	956	9.27
Puerto Princesa City	9,511	221	247	468	4.92

Table 1.D.7 - Oral Health Care and Services

Adolescents 15- 19 years old who received Basic Oral Health Care (BOHC)

Philippines, 2023

Area	Adolescents 15-19 years old who received BOHC				
	Eligible Population x 30%	Male	Female	Total	%
**Region 5**	**206,071**	**8,350**	**10,686**	**19,036**	**9.24**
Albay	46,030	2,710	2,842	5,552	12.06
Camarines Norte	20,126	1,114	1,432	2,546	12.65
Camarines Sur	63,908	853	1,350	2,203	3.45
Catanduanes	9,220	318	405	723	7.84
Masbate	31,796	1,381	1,538	2,919	9.18
Sorsogon	28,160	1,870	2,994	4,864	17.27
Naga City	6,831	104	125	229	3.35
**Region 6**	**231,216**	**25,538**	**30,477**	**56,015**	**24.23**
Aklan	17,662	952	1,207	2,159	12.22
Antique	18,413	0	0	0	0.00
Capiz	23,694	5,641	6,608	12,249	51.70
Guimaras	5,184	247	245	492	9.49
Iloilo	59,015	10,400	11,901	22,301	37.79
Negros Occidental	78,986	5,035	6,789	11,824	14.97
Bacolod City	16,634	2,041	2,274	4,315	25.94
Iloilo City	11,628	1,222	1,453	2,675	23.00
**Region 7**	**241,065**	**24,632**	**29,406**	**54,038**	**22.42**
Bohol	43,572	881	1,071	1,952	4.48
Cebu	100,187	6,087	6,860	12,947	12.92
Negros Oriental	42,710	3,230	4,251	7,481	17.52
Siquijor	2,864	160	183	343	11.98
Cebu City	27,697	3,025	4,829	7,854	28.36
Lapu-Lapu City	14,063	890	896	1,786	12.70
Mandaue City	9,973	10,359	11,316	21,675	217.34
**Region 8**	**146,949**	**8,490**	**11,424**	**19,914**	**13.55**
Biliran	5,703	813	933	1,746	30.62
Eastern Samar	15,962	141	225	366	2.29
Northern Leyte	47,612	1,373	1,558	2,931	6.16
Northern Samar	22,718	2,945	4,908	7,853	34.57
Southern Leyte	12,974	867	1,028	1,895	14.61
Western Samar	27,457	1,621	1,816	3,437	12.52
Ormoc City	7,002	655	793	1,448	20.68
Tacloban City	7,521	75	163	238	3.16

Table 1.D.7 - Oral Health Care and Services

Adolescents 15- 19 years old who received Basic Oral Health Care (BOHC)

Philippines, 2023

Area	Adolescents 15-19 years old who received BOHC				
	Eligible Population x 30%	Male	Female	Total	%
**Region 9**	**122,159**	**3,462**	**4,597**	**8,059**	**6.60**
Zamboanga Del Norte	32,976	467	702	1,169	3.54
Zamboanga Del Sur	33,602	2,229	2,882	5,111	15.21
Zamboanga Sibugay	21,898	354	464	818	3.74
Isabela City	4,069	371	480	851	20.92
Zamboanga City	29,615	41	69	110	0.37
**Region 10**	**155,857**	**14,297**	**16,682**	**30,979**	**19.88**
Bukidnon	49,303	3,839	4,993	8,832	17.91
Camiguin	2,781	635	559	1,194	42.94
Lanao Del Norte	23,925	480	589	1,069	4.47
Misamis Occidental	18,647	6,658	7,148	13,806	74.04
Misamis Oriental	29,427	989	1,313	2,302	7.82
Cagayan de Oro City	20,526	711	971	1,682	8.19
Iligan City	11,249	985	1,109	2,094	18.62
**Region 11**	**153,459**	**6,855**	**8,213**	**15,068**	**9.82**
Davao de Oro	24,473	911	1,055	1,966	8.03
Davao del Norte	32,144	1,424	1,707	3,131	9.74
Davao Oriental	18,134	1,596	1,972	3,568	19.68
Davao del Sur	19,153	684	832	1,516	7.92
Davao Occidental	9,645	411	464	875	9.07
Davao City	49,910	1,829	2,183	4,012	8.04
**Region 12**	**137,251**	**8,757**	**11,218**	**19,975**	**14.55**
North Cotabato	40,356	2,498	2,970	5,468	13.55
Sarangani	18,396	1,015	1,475	2,490	13.54
South Cotabato	29,813	1,750	2,158	3,908	13.11
Sultan Kudarat	28,202	3,178	4,002	7,180	25.46
Gen. Santos City	20,483	316	613	929	4.54
**BARMM**	**167,666**	**16,702**	**17,076**	**33,778**	**20.15**
Basilan	13,688	196	295	491	3.59
Lanao Del Sur	41,906	4,071	4,391	8,462	20.19
Maguindanao	43,183	4,306	4,233	8,539	19.77

Table 1.D.7 - Oral Health Care and Services

Adolescents 15- 19 years old who received Basic Oral Health Care (BOHC)

Philippines, 2023

Area	Adolescents 15-19 years old who received BOHC				
	Eligible Population x 30%	Male	Female	Total	%
Sulu	36,897	5,844	5,743	11,587	31.40
Tawi-Tawi	13,928	1,854	1,992	3,846	27.61
SGA	6,934	0	0	0	0.00
Cotabato City	11,131	431	422	853	7.66
**CARAGA**	**89,097**	**7,118**	**8,200**	**15,318**	**17.19**
Agusan del Norte	12,176	1,333	1,415	2,748	22.57
Agusan del Sur	23,948	1,014	1,321	2,335	9.75
Surigao del Norte	17,195	899	1,147	2,046	11.90
Surigao del Sur	20,135	2,728	3,008	5,736	28.49
Province of Dinagat	4,019	186	242	428	10.65
Butuan City	11,625	958	1,067	2,025	17.42

Table 1.D.8 - Oral Health Care and Services

Adults 20 -59 years old who received Basic Oral Health Care (BOHC)
Philippines, 2023

Area	Adults 20-59 years old who received BOHC				
	Eligible Population x30%	Male	Female	Total	%
**Philippines**	**17,163,471**	**727,143**	**1,161,366**	**1,888,509**	**11.00**
**NCR**	**2,336,002**	**140,249**	**232,049**	**372,298**	**15.94**
Malabon City	63,917	3,255	7,464	10,719	16.77
Navotas City	40,378	7,045	8,883	15,928	39.45
Valenzuela City	124,139	4,472	7,631	12,103	9.75
Caloocan City	279,002	13,759	21,011	34,770	12.46
Marikina City	76,244	1,573	2,307	3,880	5.09
Pasig City	138,005	13,712	27,867	41,579	30.13
Pateros	11,088	947	1,342	2,289	20.64
Taguig City	155,824	7,247	13,305	20,552	13.19
Quezon City	508,060	16,021	30,032	46,053	9.06
Makati City	115,235	7,491	13,340	20,831	18.08
Mandaluyong City	76,937	2,251	4,031	6,282	8.17
San Juan City	22,333	686	1,520	2,206	9.88
Manila City	319,034	42,755	67,865	110,620	34.67
Las Piñas City	104,763	1,634	3,650	5,284	5.04
Muntinlupa City	96,341	4,761	7,514	12,275	12.74
Parañaque City	121,825	1,737	2,307	4,044	3.32
Pasay City	82,879	10,903	11,980	22,883	27.61
**CAR**	**292,389**	**24,528**	**46,129**	**70,657**	**24.17**
Abra	38,362	4,361	5,644	10,005	26.08
Apayao	19,679	902	2,212	3,114	15.82
Benguet	75,969	5,867	10,482	16,349	21.52
Ifugao	32,497	1,975	4,816	6,791	20.90
Kalinga	35,911	7,688	13,538	21,226	59.11
Mountain Province	24,372	1,638	3,797	5,435	22.30
Baguio City	65,600	2,097	5,640	7,737	11.79
**Region 1**	**831,674**	**79,742**	**106,471**	**186,213**	**22.39**
Ilocos Norte	99,341	9,848	11,181	21,029	21.17
Ilocos Sur	113,221	15,679	21,135	36,814	32.52
La Union	132,987	6,676	13,385	20,061	15.08
Pangasinan	458,418	47,023	59,807	106,830	23.30

Table 1.D.8 - Oral Health Care and Services

Adults 20 -59 years old who received Basic Oral Health Care (BOHC)
Philippines, 2023

Area	Adults 20-59 years old who received BOHC				
	Eligible Population x30%	Male	Female	Total	%
Dagupan City	27,707	516	963	1,479	5.34
**Region 2**	**589,525**	**40,646**	**65,129**	**105,775**	**17.94**
Batanes	3,086	404	563	967	31.33
Cagayan	200,810	3,835	8,463	12,298	6.12
Isabela	248,905	18,469	28,931	47,400	19.04
Nueva Vizcaya	79,759	6,644	13,532	20,176	25.30
Quirino	32,738	7,720	8,938	16,658	50.88
Santiago City	24,227	3,574	4,702	8,276	34.16
**Region 3**	**2,010,334**	**71,996**	**102,687**	**174,683**	**8.69**
Aurora	36,375	1,183	1,444	2,627	7.22
Bataan	137,297	2,028	4,257	6,285	4.58
Bulacan	609,405	18,862	33,410	52,272	8.58
Nueva Ecija	367,443	17,090	19,746	36,836	10.02
Pampanga	401,283	10,689	14,413	25,102	6.26
Tarlac	234,977	10,418	14,166	24,584	10.46
Zambales	102,207	6,395	9,948	16,343	15.99
Angeles City	77,576	375	615	990	1.28
Olongapo	43,771	4,956	4,688	9,644	22.03
**Region 4A**	**2,687,066**	**111,444**	**176,274**	**287,718**	**10.71**
Batangas	475,745	56,498	73,487	129,985	27.32
Cavite	744,050	15,660	25,880	41,540	5.58
Laguna	579,901	18,826	38,071	56,897	9.81
Quezon	292,741	7,085	13,119	20,204	6.90
Rizal	550,946	12,827	24,587	37,414	6.79
Lucena City	43,683	548	1,130	1,678	3.84
**Region 4B**	**482,062**	**7,517**	**13,603**	**21,120**	**4.38**
Marinduque	33,564	394	978	1,372	4.09
Occidental Mindoro	76,916	897	845	1,742	2.26
Oriental Mindoro	136,360	2,789	4,732	7,521	5.52
Palawan	138,858	1,888	3,994	5,882	4.24
Romblon	44,840	1,019	1,922	2,941	6.56
Puerto Princesa City	51,524	530	1,132	1,662	3.23

Table 1.D.8 - Oral Health Care and Services

Adults 20 -59 years old who received Basic Oral Health Care (BOHC)
Philippines, 2023

Area	Adults 20-59 years old who received BOHC				
	Eligible Population x30%	Male	Female	Total	%
**Region 5**	**867,155**	**19,490**	**46,946**	**66,436**	**7.66**
Albay	204,109	6,035	12,427	18,462	9.05
Camarines Norte	91,491	3,157	5,167	8,324	9.10
Camarines Sur	261,065	1,895	11,029	12,924	4.95
Catanduanes	38,468	781	1,945	2,726	7.09
Masbate	119,955	2,767	4,788	7,555	6.30
Sorsogon	118,783	4,609	10,888	15,497	13.05
Naga City	33,284	246	702	948	2.85
**Region 6**	**1,216,572**	**59,765**	**94,797**	**154,562**	**12.70**
Aklan	96,053	3,542	5,160	8,702	9.06
Antique	90,631	1,674	3,402	5,076	5.60
Capiz	125,513	16,342	19,515	35,857	28.57
Guimaras	29,485	747	2,325	3,072	10.42
Iloilo	315,863	14,821	23,795	38,616	12.23
Negros Occidental	389,828	15,386	28,917	44,303	11.36
Bacolod City	95,152	3,887	6,204	10,091	10.61
Iloilo City	74,048	3,366	5,479	8,845	11.94
**Region 7**	**1,264,042**	**48,100**	**87,587**	**135,687**	**10.73**
Bohol	207,329	2,190	4,951	7,141	3.44
Cebu	509,584	15,004	27,202	42,206	8.28
Negros Oriental	218,252	7,672	12,776	20,448	9.37
Siquijor	15,995	615	996	1,611	10.07
Cebu City	161,227	10,488	29,040	39,528	24.52
Lapu-Lapu City	85,518	1,049	1,061	2,110	2.47
Mandaue City	66,138	11,082	11,561	22,643	34.24
**Region 8**	**660,476**	**10,942**	**17,592**	**28,534**	**4.32**
Biliran	25,601	369	620	989	3.86
Eastern Samar	69,233	451	986	1,437	2.08
Northern Leyte	225,385	4,315	6,802	11,117	4.93
Northern Samar	87,950	1,256	1,794	3,050	3.47
Southern Leyte	63,557	1,083	1,933	3,016	4.75
Western Samar	113,373	2,244	2,746	4,990	4.40
Ormoc City	35,539	1,140	1,926	3,066	8.63

Table 1.D.8 - Oral Health Care and Services

Adults 20 -59 years old who received Basic Oral Health Care (BOHC)
Philippines, 2023

Area	Adults 20-59 years old who received BOHC				
	Eligible Population x30%	Male	Female	Total	%
Tacloban City	39,838	84	785	869	2.18
**Region 9**	**575,294**	**10,766**	**16,894**	**27,660**	**4.81**
Zamboanga Del Norte	151,213	1,826	3,162	4,988	3.30
Zamboanga Del Sur	154,191	5,796	8,430	14,226	9.23
Zamboanga Sibugay	96,976	1,510	2,459	3,969	4.09
Isabela City	18,644	1,575	2,351	3,926	21.06
Zamboanga City	154,270	59	492	551	0.36
**Region 10**	**765,629**	**32,408**	**49,688**	**82,096**	**10.72**
Bukidnon	232,826	8,158	13,127	21,285	9.14
Camiguin	13,583	1,093	1,537	2,630	19.36
Lanao Del Norte	102,709	849	1,212	2,061	2.01
Misamis Occidental	93,944	15,253	21,912	37,165	39.56
Misamis Oriental	143,669	2,391	4,284	6,675	4.65
Cagayan de Oro City	122,907	3,210	5,879	9,089	7.40
Iligan City	55,991	1,454	1,737	3,191	5.70
**Region 11**	**824,854**	**16,004**	**18,388**	**34,392**	**4.17**
Davao de Oro	117,824	2,258	2,163	4,421	3.75
Davao del Norte	178,171	3,036	4,950	7,986	4.48
Davao Oriental	85,582	3,002	4,312	7,314	8.55
Davao del Sur	106,420	1,663	1,629	3,292	3.09
Davao Occidental	44,141	917	945	1,862	4.22
Davao City	292,716	5,128	4,389	9,517	3.25
**Region 12**	**671,925**	**21,401**	**39,295**	**60,696**	**9.03**
North Cotabato	192,763	5,264	8,677	13,941	7.23
Sarangani	83,398	1,638	3,482	5,120	6.14
South Cotabato	151,644	4,857	8,803	13,660	9.01
Sultan Kudarat	129,077	8,327	12,010	20,337	15.76
Gen. Santos City	115,043	1,315	6,323	7,638	6.64
**BARMM**	**669,715**	**20,561**	**26,100**	**46,661**	**6.97**
Basilan	55,564	531	1,167	1,698	3.06
Lanao Del Sur	158,087	5,276	7,697	12,973	8.21

Table 1.D.8 - Oral Health Care and Services

Adults 20 -59 years old who received Basic Oral Health Care (BOHC)
Philippines, 2023

Area	Adults 20-59 years old who received BOHC				
	Eligible Population x30%	Male	Female	Total	%
Maguindanao	177,439	3,508	4,945	8,453	4.76
Sulu	135,429	6,917	6,870	13,787	10.18
Tawi-Tawi	60,252	3,759	4,497	8,256	13.70
SGA	31,847	0	0	0	0.00
Cotabato City	51,097	570	924	1,494	2.92
**CARAGA**	**418,760**	**11,584**	**21,737**	**33,321**	**7.96**
Agusan del Norte	57,486	2,918	6,012	8,930	15.53
Agusan del Sur	107,198	1,937	3,542	5,479	5.11
Surigao del Norte	81,976	1,188	2,319	3,507	4.28
Surigao del Sur	95,588	3,821	6,464	10,285	10.76
Province of Dinagat	18,168	350	613	963	5.30
Butuan City	58,344	1,370	2,787	4,157	7.13

Table 1.D.9 - Oral Health Care and Services

Senior citizens 60 years old and above who received Basic Oral Health Care (BOHC)

Philippines, 2023

Area	Senior citizens 60 years old and above who received BOHC				
	Eligible Population x 30%	Male	Female	Total	%
**Philippines**	**2,838,076**	**236,502**	**301,364**	**537,866**	**18.95**
**NCR**	**326,363**	**54,460**	**72,170**	**126,630**	**38.80**
Malabon City	9,135	1,036	1,730	2,766	30.28
Navotas City	5,617	1,684	2,082	3,766	67.05
Valenzuela City	15,454	1,342	2,033	3,375	21.84
Caloocan City	36,541	5,997	7,367	13,364	36.57
Marikina City	13,040	674	800	1,474	11.30
Pasig City	18,395	5,348	7,568	12,916	70.22
Pateros	1,765	299	419	718	40.68
Taguig City	16,553	1,294	1,693	2,987	18.05
Quezon City	72,375	12,925	18,259	31,184	43.09
Makati City	18,652	1,459	1,830	3,289	17.63
Mandaluyong City	10,227	945	1,160	2,105	20.58
San Juan City	4,001	248	502	750	18.75
Manila City	47,702	13,669	16,575	30,244	63.40
Las Piñas City	15,390	2,432	3,827	6,259	40.67
Muntinlupa City	13,972	1,847	2,501	4,348	31.12
Parañaque City	17,488	867	1,061	1,928	11.02
Pasay City	10,058	2,394	2,763	5,157	51.28
**CAR**	**51,552**	**6,787**	**9,391**	**16,178**	**31.38**
Abra	9,063	1,292	1,621	2,913	32.14
Apayao	3,702	231	376	607	16.40
Benguet	11,624	1,502	2,120	3,622	31.16
Ifugao	5,583	630	974	1,604	28.73
Kalinga	6,336	2,238	3,087	5,325	84.05
Mountain Province	5,067	441	533	974	19.22
Baguio City	10,177	453	680	1,133	11.13
**Region 1**	**176,507**	**22,827**	**27,296**	**50,123**	**28.40**
Ilocos Norte	23,448	4,956	5,327	10,283	43.85
Ilocos Sur	27,038	4,340	5,683	10,023	37.07
La Union	28,916	2,166	3,164	5,330	18.43
Pangasinan	92,046	11,282	12,989	24,271	26.37

Table 1.D.9 - Oral Health Care and Services

Senior citizens 60 years old and above who received Basic Oral Health Care (BOHC)

Philippines, 2023

Area	Senior citizens 60 years old and above who received BOHC				
	Eligible Population x 30%	Male	Female	Total	%
Dagupan City	5,058	83	133	216	4.27
**Region 2**	**110,029**	**16,362**	**22,967**	**39,329**	**35.74**
Batanes	722	111	161	272	37.67
Cagayan	40,221	2,137	3,271	5,408	13.45
Isabela	44,570	7,895	10,705	18,600	41.73
Nueva Vizcaya	14,852	1,830	2,724	4,554	30.66
Quirino	5,612	1,391	1,954	3,345	59.60
Santiago City	4,052	2,998	4,152	7,150	176.48
**Region 3**	**336,293**	**20,499**	**24,671**	**45,170**	**13.43**
Aurora	6,291	597	626	1,223	19.44
Bataan	22,737	1,023	1,403	2,426	10.67
Bulacan	95,283	4,840	6,227	11,067	11.61
Nueva Ecija	66,164	3,993	4,527	8,520	12.88
Pampanga	65,530	3,273	3,466	6,739	10.28
Tarlac	43,648	3,609	4,306	7,915	18.13
Zambales	19,760	1,878	2,794	4,672	23.64
Angeles City	9,743	33	60	93	0.95
Olongapo	7,136	1,253	1,262	2,515	35.24
**Region 4A**	**397,856**	**27,339**	**32,671**	**60,010**	**15.08**
Batangas	78,705	12,814	13,798	26,612	33.81
Cavite	102,722	3,173	3,903	7,076	6.89
Laguna	80,834	4,544	5,502	10,046	12.43
Quezon	53,924	3,086	4,541	7,627	14.14
Rizal	74,797	3,510	4,729	8,239	11.02
Lucena City	6,874	212	198	410	5.96
**Region 4B**	**83,810**	**4,365**	**5,959**	**10,324**	**12.32**
Marinduque	8,936	90	172	262	2.93
Occidental Mindoro	12,518	2,896	3,800	6,696	53.49
Oriental Mindoro	24,103	525	685	1,210	5.02
Palawan	20,996	516	791	1,307	6.23
Romblon	10,337	260	424	684	6.62
Puerto Princesa City	6,920	78	87	165	2.38

Table 1.D.9 - Oral Health Care and Services

Senior citizens 60 years old and above who received Basic Oral Health Care (BOHC)

Philippines, 2023

Area	Senior citizens 60 years old and above who received BOHC				
	Eligible Population x 30%	Male	Female	Total	%
**Region 5**	**166,729**	**4,707**	**6,804**	**11,511**	**6.90**
Albay	39,492	1,376	1,518	2,894	7.33
Camarines Norte	16,865	392	577	969	5.75
Camarines Sur	49,819	556	1,170	1,726	3.46
Catanduanes	8,248	394	575	969	11.75
Masbate	22,967	1,025	1,373	2,398	10.44
Sorsogon	23,726	924	1,509	2,433	10.25
Naga City	5,611	40	82	122	2.17
**Region 6**	**254,781**	**17,279**	**24,344**	**41,623**	**16.34**
Aklan	20,211	827	1,129	1,956	9.68
Antique	20,075	351	685	1,036	5.16
Capiz	27,290	3,434	4,411	7,845	28.75
Guimaras	6,327	202	427	629	9.94
Iloilo	71,018	6,901	9,077	15,978	22.50
Negros Occidental	77,257	3,921	5,866	9,787	12.67
Bacolod City	18,282	815	1,696	2,511	13.74
Iloilo City	14,321	828	1,053	1,881	13.13
**Region 7**	**226,963**	**19,141**	**24,029**	**43,170**	**19.02**
Bohol	49,325	523	822	1,345	2.73
Cebu	88,494	3,273	4,771	8,044	9.09
Negros Oriental	45,465	1,623	2,195	3,818	8.40
Siquijor	4,421	89	63	152	3.44
Cebu City	23,271	2,309	3,265	5,574	23.95
Lapu-Lapu City	8,483	480	482	962	11.34
Mandaue City	7,503	10,844	12,431	23,275	310.20
**Region 8**	**136,097**	**4,100**	**4,623**	**8,723**	**6.41**
Biliran	5,817	109	111	220	3.78
Eastern Samar	15,096	135	179	314	2.08
Northern Leyte	48,919	2,675	2,785	5,460	11.16
Northern Samar	16,457	97	159	256	1.56
Southern Leyte	15,890	230	316	546	3.44
Western Samar	21,569	552	685	1,237	5.74

Table 1.D.9 - Oral Health Care and Services

Senior citizens 60 years old and above who received Basic Oral Health Care (BOHC)

Philippines, 2023

Area	Senior citizens 60 years old and above who received BOHC				
	Eligible Population x 30%	Male	Female	Total	%
Ormoc City	6,218	183	179	362	5.82
Tacloban City	6,132	119	209	328	5.35
**Region 9**	**96,045**	**2,039**	**2,747**	**4,786**	**4.98**
Zamboanga Del Norte	28,480	390	635	1,025	3.60
Zamboanga Del Sur	26,747	1,297	1,634	2,931	10.96
Zamboanga Sibugay	15,706	213	303	516	3.29
Isabela City	2,541	106	112	218	8.58
Zamboanga City	22,572	33	63	96	0.43
**Region 10**	**122,671**	**9,666**	**12,998**	**22,664**	**18.48**
Bukidnon	33,641	1,929	2,833	4,762	14.16
Camiguin	3,222	493	572	1,065	33.05
Lanao Del Norte	13,477	199	246	445	3.30
Misamis Occidental	20,486	3,958	5,054	9,012	43.99
Misamis Oriental	24,836	1,506	2,099	3,605	14.52
Cagayan de Oro City	17,963	1,004	1,518	2,522	14.04
Iligan City	9,045	577	676	1,253	13.85
**Region 11**	**131,792**	**2,046**	**2,644**	**4,690**	**3.56**
Davao de Oro	19,113	246	306	552	2.89
Davao del Norte	28,468	354	574	928	3.26
Davao Oriental	15,686	621	786	1,407	8.97
Davao del Sur	19,108	219	273	492	2.57
Davao Occidental	6,786	86	116	202	2.98
Davao City	42,631	520	589	1,109	2.60
**Region 12**	**94,571**	**8,324**	**9,687**	**18,011**	**19.04**
North Cotabato	29,467	2,088	2,946	5,034	17.08
Sarangani	11,084	918	1,209	2,127	19.19
South Cotabato	22,589	1,432	2,117	3,549	15.71
Sultan Kudarat	17,562	3,669	2,871	6,540	37.24
Gen. Santos City	13,868	217	544	761	5.49

Table 1.D.9 - Oral Health Care and Services

Senior citizens 60 years old and above who received Basic Oral Health Care (BOHC)

Philippines, 2023

Area	Senior citizens 60 years old and above who received BOHC				
	Eligible Population x 30%	Male	Female	Total	%
**BARMM**	**50,981**	**12,720**	**13,261**	**25,981**	**50.96**
Basilan	4,780	103	157	260	5.44
Lanao Del Sur	11,054	1,643	2,024	3,667	33.17
Maguindanao	11,560	1,906	2,367	4,273	36.96
Sulu	9,870	6,886	6,712	13,598	137.77
Tawi-Tawi	4,829	1,182	1,232	2,414	49.99
SGA	4,260	0	0	0	0.00
Cotabato City	4,629	1,000	769	1,769	38.22
**CARAGA**	**75,036**	**3,841**	**5,102**	**8,943**	**11.92**
Agusan del Norte	10,668	930	1,295	2,225	20.86
Agusan del Sur	16,902	758	892	1,650	9.76
Surigao del Norte	15,017	377	490	867	5.77
Surigao del Sur	18,340	1,081	1,373	2,454	13.38
Province of Dinagat	4,270	164	263	427	10.00
Butuan City	9,839	531	789	1,320	13.42

Table 2.D.10 - Oral Health Care

Pregnant women 10-14, 15-19, 20-49 years old who received Basic Oral Health Care (BOHC)
Philippines, 2023

Area	Eligible Pop.	Pregnant Women 10-14 y/o received BOHC		Pregnant Women 15-19 y/o received BOHC		Pregnant Women 20-49 y/o received BOHC	
		No.	%	No.	%	No.	%
**Philippines**	**2,187,333**	**6,717**	**0.31**	**74,599**	**3.41**	**422,162**	**19.30**
**NCR**	**261,228**	**551**	**0.21**	**16,276**	**6.23**	**111,969**	**42.86**
Malabon City	7,385	37	0.50	560	7.58	3,549	48.06
Navotas City	5,210	32	0.61	512	9.83	2,883	55.34
Valenzuela City	13,477	13	0.10	239	1.77	2,238	16.61
Caloocan City	33,168	108	0.33	2,715	8.19	9,311	28.07
Marikina City	8,342	11	0.13	201	2.41	1,783	21.37
Pasig City	17,682	26	0.15	1,091	6.17	7,010	39.64
Pateros	1,116	0	0.00	86	7.71	390	34.95
Taguig City	18,992	31	0.16	865	4.55	5,641	29.70
Quezon City	59,136	88	0.15	3,013	5.10	37,072	62.69
Makati City	10,944	16	0.15	291	2.66	3,593	32.83
Mandaluyong City	7,922	15	0.19	330	4.17	1,744	22.01
San Juan City	2,168	1	0.05	53	2.44	633	29.20
Manila City	34,128	100	0.29	3,623	10.62	21,849	64.02
Las Piñas City	11,263	12	0.11	431	3.83	3,798	33.72
Muntinlupa City	9,881	35	0.35	632	6.40	4,312	43.64
Parañaque City	13,280	9	0.07	876	6.60	2,534	19.08
Pasay City	7,134	17	0.24	758	10.63	3,629	50.87
**CAR**	**31,319**	**74**	**0.24**	**1,067**	**3.41**	**8,045**	**25.69**
Abra	3,660	1	0.03	128	3.50	777	21.23
Apayao	2,238	17	0.76	134	5.99	649	29.00
Benguet	8,806	11	0.12	185	2.10	1,513	17.18
Ifugao	4,098	5	0.12	68	1.66	723	17.64
Kalinga	4,549	10	0.22	368	8.09	2,891	63.55
Mountain Province	2,619	25	0.95	69	2.63	387	14.78
Baguio City	5,349	5	0.09	115	2.15	1,105	20.66
**Region 1**	**96,016**	**83**	**0.09**	**3,061**	**3.19**	**23,724**	**24.71**
Ilocos Norte	8,912	4	0.04	434	4.87	2,571	28.85
Ilocos Sur	9,914	2	0.02	379	3.82	4,876	49.18
La Union	13,507	8	0.06	577	4.27	3,180	23.54

Table 2.D.10 - Oral Health Care

Pregnant women 10-14, 15-19, 20-49 years old who received Basic Oral Health Care (BOHC)
Philippines, 2023

Area	Eligible Pop.	Pregnant Women 10-14 y/o received BOHC		Pregnant Women 15-19 y/o received BOHC		Pregnant Women 20-49 y/o received BOHC	
		No.	%	No.	%	No.	%
Pangasinan	60,360	66	0.11	1,519	2.52	12,433	20.60
Dagupan City	3,323	3	0.09	152	4.57	664	19.98
**Region 2**	**63,967**	**693**	**1.08**	**5,991**	**9.37**	**20,587**	**32.18**
Batanes	240	0	0.00	7	2.92	66	27.50
Cagayan	20,786	17	0.08	566	2.72	3,500	16.84
Isabela	27,379	74	0.27	1,863	6.80	9,613	35.11
Nueva Vizcaya	8,798	592	6.73	3,035	34.50	4,246	48.26
Quirino	3,839	5	0.13	150	3.91	855	22.27
Santiago City	2,925	5	0.17	370	12.65	2,307	78.87
**Region 3**	**233,544**	**466**	**0.20**	**8,290**	**3.55**	**40,155**	**17.19**
Aurora	4,537	2	0.04	104	2.29	1,008	22.22
Bataan	17,380	20	0.12	266	1.53	1,306	7.51
Bulacan	67,590	87	0.13	2,508	3.71	15,911	23.54
Nueva Ecija	43,425	132	0.30	1,774	4.09	5,832	13.43
Pampanga	45,651	66	0.14	1,541	3.38	5,960	13.06
Tarlac	29,503	142	0.48	1,156	3.92	5,346	18.12
Zambales	11,723	7	0.06	616	5.25	2,291	19.54
Angeles City	9,146	5	0.05	40	0.44	403	4.41
Olongapo	4,589	5	0.11	285	6.21	2,098	45.72
**Region 4A**	**307,982**	**594**	**0.19**	**7,536**	**2.45**	**49,231**	**15.99**
Batangas	53,251	160	0.30	1,779	3.34	12,772	23.98
Cavite	77,964	175	0.22	771	0.99	4,394	5.64
Laguna	61,525	90	0.15	1,245	2.02	9,830	15.98
Quezon	40,810	42	0.10	1,311	3.21	9,495	23.27
Rizal	68,235	126	0.18	2,356	3.45	12,046	17.65
Lucena City	6,197	1	0.02	74	1.19	694	11.20
**Region 4B**	**66,121**	**37**	**0.06**	**518**	**0.78**	**3,514**	**5.31**
Marinduque	4,144	1	0.02	33	0.80	256	6.18
Occidental Mindoro	11,640	0	0.00	13	0.11	71	0.61
Oriental Mindoro	18,943	2	0.01	142	0.75	1,020	5.38

Table 2.D.10 - Oral Health Care

Pregnant women 10-14, 15-19, 20-49 years old who received Basic Oral Health Care (BOHC)
Philippines, 2023

Area	Eligible Pop.	Pregnant Women 10-14 y/o received BOHC		Pregnant Women 15-19 y/o received BOHC		Pregnant Women 20-49 y/o received BOHC	
		No.	%	No.	%	No.	%
Palawan	20,317	12	0.06	213	1.05	1,263	6.22
Romblon	5,489	21	0.38	84	1.53	724	13.19
Puerto Princesa City	5,588	1	0.02	33	0.59	180	3.22
**Region 5**	**135,480**	**2,428**	**1.79**	**2,653**	**1.96**	**16,846**	**12.43**
Albay	27,440	3	0.01	257	0.94	2,562	9.34
Camarines Norte	14,695	21	0.14	456	3.10	2,292	15.60
Camarines Sur	43,452	2,377	5.47	396	0.91	2,028	4.67
Catanduanes	5,414	4	0.07	204	3.77	1,101	20.34
Masbate	22,365	5	0.02	339	1.52	2,099	9.39
Sorsogon	17,929	17	0.09	960	5.35	6,479	36.14
Naga City	4,185	1	0.02	41	0.98	285	6.81
**Region 6**	**148,020**	**118**	**0.08**	**3,628**	**2.45**	**25,740**	**17.39**
Aklan	11,283	0	0.00	199	1.76	1,078	9.55
Antique	11,930	3	0.03	114	0.96	996	8.35
Capiz	13,931	7	0.05	323	2.32	2,471	17.74
Guimaras	3,351	4	0.12	117	3.49	1,097	32.74
Iloilo	36,140	20	0.06	771	2.13	6,530	18.07
Negros Occidental	52,270	53	0.10	1,262	2.41	7,205	13.78
Bacolod City	10,373	9	0.09	402	3.88	2,821	27.20
Iloilo City	8,742	22	0.25	440	5.03	3,542	40.52
**Region 7**	**165,673**	**211**	**0.13**	**8,458**	**5.11**	**33,691**	**20.34**
Bohol	26,235	10	0.04	287	1.09	1,694	6.46
Cebu	72,201	55	0.08	1,275	1.77	11,823	16.38
Negros Oriental	28,722	17	0.06	708	2.47	4,538	15.80
Siquijor	1,680	0	0.00	4	0.24	152	9.05
Cebu City	19,267	37	0.19	1,400	7.27	8,387	43.53
Lapu-Lapu City	10,679	1	0.01	22	0.21	83	0.78
Mandaue City	6,889	91	1.32	4,762	69.12	7,014	101.81
**Region 8**	**93,813**	**316**	**0.34**	**906**	**0.97**	**9,960**	**10.62**
Biliran	3,373	1	0.03	33	0.98	290	8.60

Table 2.D.10 - Oral Health Care

Pregnant women 10-14, 15-19, 20-49 years old who received Basic Oral Health Care (BOHC)
Philippines, 2023

Area	Eligible Pop.	Pregnant Women 10-14 y/o received BOHC		Pregnant Women 15-19 y/o received BOHC		Pregnant Women 20-49 y/o received BOHC	
		No.	%	No.	%	No.	%
Eastern Samar	9,624	32	0.33	176	1.83	800	8.31
Northern Leyte	31,559	214	0.68	250	0.79	1,752	5.55
Northern Samar	14,056	0	0.00	30	0.21	2,829	20.13
Southern Leyte	7,406	13	0.18	44	0.59	273	3.69
Western Samar	17,011	54	0.32	174	1.02	1,088	6.40
Ormoc City	5,292	0	0.00	0	0.00	1,977	37.36
Tacloban City	5,492	2	0.04	199	3.62	951	17.32
**Region 9**	**84,815**	**47**	**0.06**	**691**	**0.81**	**4,451**	**5.25**
Zamboanga Del Norte	24,841	1	0.00	93	0.37	603	2.43
Zamboanga Del Sur	23,757	7	0.03	246	1.04	1,996	8.40
Zamboanga Sibugay	13,841	31	0.22	207	1.50	996	7.20
Isabela City	3,201	3	0.09	40	1.25	298	9.31
Zamboanga City	19,175	5	0.03	105	0.55	558	2.91
**Region 10**	**109,169**	**207**	**0.19**	**3,051**	**2.79**	**16,218**	**14.86**
Bukidnon	33,403	84	0.25	1,365	4.09	4,840	14.49
Camiguin	1,727	0	0.00	22	1.27	76	4.40
Lanao Del Norte	17,760	12	0.07	125	0.70	935	5.26
Misamis Occidental	12,328	40	0.32	533	4.32	6,670	54.10
Misamis Oriental	21,975	39	0.18	633	2.88	2,122	9.66
Cagayan de Oro City	14,308	22	0.15	283	1.98	1,436	10.04
Iligan City	7,668	10	0.13	90	1.17	139	1.81
**Region 11**	**107,001**	**69**	**0.06**	**3,729**	**3.49**	**15,809**	**14.77**
Davao de Oro	15,253	6	0.04	645	4.23	1,702	11.16
Davao del Norte	23,453	28	0.12	1,012	4.32	4,529	19.31
Davao Oriental	11,935	18	0.15	651	5.45	3,489	29.23
Davao del Sur	13,083	4	0.03	379	2.90	1,340	10.24
Davao Occidental	6,629	6	0.09	190	2.87	742	11.19
Davao City	36,648	7	0.02	852	2.32	4,007	10.93
**Region 12**	**95,279**	**244**	**0.26**	**4,451**	**4.67**	**23,346**	**24.50**

Table 2.D.10 - Oral Health Care

Pregnant women 10-14, 15-19, 20-49 years old who received Basic Oral Health Care (BOHC)
Philippines, 2023

Area	Eligible Pop.	Pregnant Women 10-14 y/o received BOHC		Pregnant Women 15-19 y/o received BOHC		Pregnant Women 20-49 y/o received BOHC	
		No.	%	No.	%	No.	%
North Cotabato	26,521	147	0.55	844	3.18	4,017	15.15
Sarangani	13,322	15	0.11	700	5.25	3,250	24.40
South Cotabato	21,595	38	0.18	1,406	6.51	7,494	34.70
Sultan Kudarat	19,391	34	0.18	898	4.63	4,938	25.47
Gen. Santos City	14,450	10	0.07	603	4.17	3,647	25.24
**BARMM**	**132,841**	**524**	**0.39**	**2,400**	**1.81**	**10,333**	**7.78**
Basilan	11,636	2	0.02	91	0.78	550	4.73
Lanao Del Sur	33,374	41	0.12	964	2.89	2,891	8.66
Maguindanao	41,650	140	0.34	773	1.86	4,878	11.71
Sulu	21,378	2	0.01	96	0.45	475	2.22
Tawi-Tawi	12,609	20	0.16	101	0.80	783	6.21
SGA	5,103	0	0.00	0	0.00	0	0.00
Cotabato City	7,091	319	4.50	375	5.29	756	10.66
**CARAGA**	**55,065**	**55**	**0.10**	**1,893**	**3.44**	**8,543**	**15.51**
Agusan del Norte	7,286	4	0.05	111	1.52	801	10.99
Agusan del Sur	16,038	16	0.10	282	1.76	1,554	9.69
Surigao del Norte	10,186	6	0.06	446	4.38	900	8.84
Surigao del Sur	12,736	22	0.17	764	6.00	3,548	27.86
Province of Dinagat	2,134	2	0.09	99	4.64	622	29.15
Butuan City	6,685	5	0.07	191	2.86	1,118	16.72

Table 2.E.1 - Filariasis Prevention and Control
Case Detection Rate (CDR) and Clinical Rate of Lymphatic Filariasis
Philippines, 2023

Area	Total Examined for lymphatic filariasis	Number positive cases found				Lymphatic filariasis cases examined with disabilities/deformities			
		Male	Female	Total	Case Det. Rate	Male	Female	Total	Clinical Rate
**Philippines**	**11,310**	**172**	**117**	**289**	**2.56**	**7**	**7**	**14**	**4.84**
**NCR**	**0**	**0**	**0**	**0**	**0.00**	**0**	**0**	**0**	**0.00**
City of Malabon	0	0	0	0	0.00	0	0	0	0.00
City of Navotas	0	0	0	0	0.00	0	0	0	0.00
City of Valenzuela	0	0	0	0	0.00	0	0	0	0.00
City of Caloocan	0	0	0	0	0.00	0	0	0	0.00
City of Marikina	0	0	0	0	0.00	0	0	0	0.00
City of Pasig	0	0	0	0	0.00	0	0	0	0.00
Pateros	0	0	0	0	0.00	0	0	0	0.00
City of Taguig	0	0	0	0	0.00	0	0	0	0.00
Quezon City	0	0	0	0	0.00	0	0	0	0.00
City of Makati	0	0	0	0	0.00	0	0	0	0.00
City of Mandaluyong	0	0	0	0	0.00	0	0	0	0.00
City of San Juan	0	0	0	0	0.00	0	0	0	0.00
City of Manila	0	0	0	0	0.00	0	0	0	0.00
City of Las Piñas	0	0	0	0	0.00	0	0	0	0.00
City of Muntinlupa	0	0	0	0	0.00	0	0	0	0.00
City of Parañaque	0	0	0	0	0.00	0	0	0	0.00
City of Pasay	0	0	0	0	0.00	0	0	0	0.00
**CAR**	**0**	**0**	**0**	**0**	**0.00**	**0**	**0**	**0**	**0.00**
Abra	0	0	0	0	0.00	0	0	0	0.00
Apayao	0	0	0	0	0.00	0	0	0	0.00
Benguet	0	0	0	0	0.00	0	0	0	0.00
Ifugao	0	0	0	0	0.00	0	0	0	0.00
Kalinga	0	0	0	0	0.00	0	0	0	0.00
Mountain Province	0	0	0	0	0.00	0	0	0	0.00
City of Baguio	0	0	0	0	0.00	0	0	0	0.00
**Region 1**	**0**	**0**	**0**	**0**	**0.00**	**0**	**0**	**0**	**0.00**
Ilocos Norte	0	0	0	0	0.00	0	0	0	0.00
Ilocos Sur	0	0	0	0	0.00	0	0	0	0.00
La Union	0	0	0	0	0.00	0	0	0	0.00
Pangasinan	0	0	0	0	0.00	0	0	0	0.00

Table 2.E.1 - Filariasis Prevention and Control
Case Detection Rate (CDR) and Clinical Rate of Lymphatic Filariasis
Philippines, 2023

Area	Total Examined for lymphatic filariasis	Number positive cases found				Lymphatic filariasis cases examined with disabilities/deformities			
		Male	Female	Total	Case Det. Rate	Male	Female	Total	Clinical Rate
City of Dagupan	0	0	0	0	0.00	0	0	0	0.00
**Region 2**	**0**	**0**	**0**	**0**	**0.00**	**0**	**0**	**0**	**0.00**
Batanes	0	0	0	0	0.00	0	0	0	0.00
Cagayan	0	0	0	0	0.00	0	0	0	0.00
Isabela	0	0	0	0	0.00	0	0	0	0.00
Nueva Vizcaya	0	0	0	0	0.00	0	0	0	0.00
Quirino	0	0	0	0	0.00	0	0	0	0.00
City of Santiago	0	0	0	0	0.00	0	0	0	0.00
								0	0.00
**Region 3**	**0**	**0**	**0**	**0**	**0.00**	**0**	**0**	**0**	**0.00**
Aurora	0	0	0	0	0.00	0	0	0	0.00
Bataan	0	0	0	0	0.00	0	0	0	0.00
Bulacan	0	0	0	0	0.00	0	0	0	0.00
Nueva Ecija	0	0	0	0	0.00	0	0	0	0.00
Pampanga	0	0	0	0	0.00	0	0	0	0.00
Tarlac	0	0	0	0	0.00	0	0	0	0.00
Zambales	0	0	0	0	0.00	0	0	0	0.00
City of Angeles	0	0	0	0	0.00	0	0	0	0.00
City of Olongapo	0	0	0	0	0.00	0	0	0	0.00
**Region 4A**	**40**	**0**	**0**	**0**	**0.00**	**0**	**0**	**0**	**0.00**
Batangas	2	0	0	0	0.00	0	0	0	0.00
Cavite	3	0	0	0	0.00	0	0	0	0.00
Laguna	0	0	0	0	0.00	0	0	0	0.00
Quezon	35	0	0	0	0.00	0	0	0	0.00
Rizal	0	0	0	0	0.00	0	0	0	0.00
City of Lucena	0	0	0	0	0.00	0	0	0	0.00
**Region 4B**	**2,009**	**47**	**43**	**90**	**4.48**	**0**	**0**	**0**	**0.00**
Marinduque	0	0	0	0	0.00	0	0	0	0.00
Occidental Mindoro	0	0	0	0	0.00	0	0	0	0.00
Oriental Mindoro	2,009	47	43	90	4.48	0	0	0	0.00
Palawan	0	0	0	0	0.00	0	0	0	0.00
Romblon	0	0	0	0	0.00	0	0	0	0.00
City of Puerto Princesa	0	0	0	0	0.00	0	0	0	0.00

Table 2.E.1 - Filariasis Prevention and Control
Case Detection Rate (CDR) and Clinical Rate of Lymphatic Filariasis
Philippines, 2023

Area	Total Examined for lymphatic filariasis	Number positive cases found				Lymphatic filariasis cases examined with disabilities/deformities			
		Male	Female	Total	Case Det. Rate	Male	Female	Total	Clinical Rate
**Region 5**	**120**	**7**	**7**	**14**	**11.67**	**7**	**7**	**14**	**100.00**
Albay	0	0	0	0	0.00	0	0	0	0.00
Camarines Norte	120	7	7	14	11.67	7	7	14	100.00
Camarines Sur	0	0	0	0	0.00	0	0	0	0.00
Catanduanes	0	0	0	0	0.00	0	0	0	0.00
Masbate	0	0	0	0	0.00	0	0	0	0.00
Sorsogon	0	0	0	0	0.00	0	0	0	0.00
City of Naga	0	0	0	0	0.00	0	0	0	0.00
**Region 6**	**0**	**0**	**0**	**0**	**0.00**	**0**	**0**	**0**	**0.00**
Aklan	0	0	0	0	0.00	0	0	0	0.00
Antique	0	0	0	0	0.00	0	0	0	0.00
Capiz	0	0	0	0	0.00	0	0	0	0.00
Guimaras	0	0	0	0	0.00	0	0	0	0.00
Iloilo	0	0	0	0	0.00	0	0	0	0.00
Negros Occidental	0	0	0	0	0.00	0	0	0	0.00
City of Bacolod	0	0	0	0	0.00	0	0	0	0.00
City of Iloilo	0	0	0	0	0.00	0	0	0	0.00
**Region 7**	**0**	**0**	**0**	**0**	**0.00**	**0**	**0**	**0**	**0.00**
Bohol	0	0	0	0	0.00	0	0	0	0.00
Cebu	0	0	0	0	0.00	0	0	0	0.00
Negros Oriental	0	0	0	0	0.00	0	0	0	0.00
Siquijor	0	0	0	0	0.00	0	0	0	0.00
City of Cebu	0	0	0	0	0.00	0	0	0	0.00
City of Lapu-Lapu	0	0	0	0	0.00	0	0	0	0.00
City of Mandaue	0	0	0	0	0.00	0	0	0	0.00
**Region 8**	**386**	**3**	**1**	**4**	**1.04**	**0**	**0**	**0**	**0.00**
Biliran	0	0	0	0	0.00	0	0	0	0.00
Eastern Samar	386	3	1	4	1.04	0	0	0	0.00
Leyte	0	0	0	0	0.00	0	0	0	0.00
Northern Samar	0	0	0	0	0.00	0	0	0	0.00
Southern Leyte	0	0	0	0	0.00	0	0	0	0.00
Samar	0	0	0	0	0.00	0	0	0	0.00
Ormoc City	0	0	0	0	0.00	0	0	0	0.00

Table 2.E.1 - Filariasis Prevention and Control
Case Detection Rate (CDR) and Clinical Rate of Lymphatic Filariasis
Philippines, 2023

Area	Total Examined for lymphatic filariasis	Number positive cases found				Lymphatic filariasis cases examined with disabilities/deformities			
		Male	Female	Total	Case Det. Rate	Male	Female	Total	Clinical Rate
City of Tacloban	0	0	0	0	0.00	0	0	0	0.00
**Region 9**	**409**	**22**	**6**	**28**	**6.85**	**0**	**0**	**0**	**0.00**
Zamboanga del Norte	409	22	6	28	6.85	0	0	0	0.00
Zamboanga del Sur	0	0	0	0	0.00	0	0	0	0.00
Zamboanga Sibugay	0	0	0	0	0.00	0	0	0	0.00
City of Isabela	0	0	0	0	0.00	0	0	0	0.00
City of Zamboanga	0	0	0	0	0.00	0	0	0	0.00
**Region 10**	**3,818**	**2**	**1**	**3**	**0.08**	**0**	**0**	**0**	**0.00**
Bukidnon	0	0	0	0	0.00	0	0	0	0.00
Camiguin	0	0	0	0	0.00	0	0	0	0.00
Lanao del Norte	0	0	0	0	0.00	0	0	0	0.00
Misamis Occidental	0	0	0	0	0.00	0	0	0	0.00
Misamis Oriental	3,818	2	1	3	0.08	0	0	0	0.00
City of Cagayan De Oro	0	0	0	0	0.00	0	0	0	0.00
City of Iligan	0	0	0	0	0.00	0	0	0	0.00
**Region 11**	**2,808**	**91**	**58**	**149**	**5.31**	**0**	**0**	**0**	**0.00**
Davao de Oro	0	0	0	0	0.00	0	0	0	0.00
Davao del Norte	0	0	0	0	0.00	0	0	0	0.00
Davao Oriental	0	0	0	0	0.00	0	0	0	0.00
Davao del Sur	0	0	0	0	0.00	0	0	0	0.00
Davao Occidental	2417	91	58	149	6.16	0	0	0	0.00
City of Davao	391	0	0	0	0.00	0	0	0	0.00
**Region 12**	**775**	**0**	**0**	**0**	**0.00**	**0**	**0**	**0**	**0.00**
Cotabato	0	0	0	0	0.00	0	0	0	0.00
Sarangani	299	0	0	0	0.00	0	0	0	0.00
South Cotabato	476	0	0	0	0.00	0	0	0	0.00
Sultan Kudarat	0	0	0	0	0.00	0	0	0	0.00
City of General Santos	0	0	0	0	0.00	0	0	0	0.00
**BARMM**	**945**	**0**	**1**	**1**	**0.11**	**0**	**0**	**0**	**0.00**

Table 2.E.1 - Filariasis Prevention and Control
Case Detection Rate (CDR) and Clinical Rate of Lymphatic Filariasis
Philippines, 2023

Area	Total Examined for lymphatic filariasis	Number positive cases found				Lymphatic filariasis cases examined with disabilities/deformities			
		Male	Female	Total	Case Det. Rate	Male	Female	Total	Clinical Rate
Basilan	945	0	1	1	0.11	0	0	0	0.00
Lanao del Sur	0	0	0	0	0.00	0	0	0	0.00
Maguindanao	0	0	0	0	0.00	0	0	0	0.00
Sulu	0	0	0	0	0.00	**0**	**0**	0	0.00
Tawi-Tawi	0	0	0	0	0.00	0	0	0	0.00
SGA	0	0	0	0	0.00	0	0	0	0.00
City of Cotabato	0	0	0	0	0.00	0	0	0	0.00
**Caraga**	**0**	**0**	**0**	**0**	**0.00**	**0**	**0**	**0**	**0.00**
Agusan del Norte	0	0	0	0	0.00	0	0	0	0.00
Agusan del Sur	0	0	0	0	0.00	0	0	0	0.00
Surigao del Norte		0	0	0	0.00	0	0	0	0.00
Surigao del Sur	0	0	0	0	0.00	0	0	0	0.00
Dinagat Islands	0	0	0	0	0.00	0	0	0	0.00
City of Butuan	0	0	0	0	0.00	0	0	0	0.00

Table 2.E.2 - HIV-AIDS/STI Prevention and Control Services

Pregnant Women Screened for HIV and Screened and Positive with Syphilis

Philippines, 2023

Area	Eligible Pop.	Pregnant Women					
		Screened for Syphilis	%	Positive for Syphilis	%	Screened for HIV	%
**Philippines**	**2,187,333**	**650,225**	**29.73**	**6,502**	**1.00**	**570,963**	**26.10**
**NCR**	**261,228**	**128,388**	**49.15**	**589**	**0.46**	**132,181**	**50.60**
City of Malabon	7,385	1,805	24.44	14	0.78	2,281	30.89
City of Navotas	5,210	3,135	60.17	10	0.32	2,994	57.47
City of Valenzuela	13,477	4,667	34.63	103	2.21	6,136	45.53
City of Caloocan	33,168	11,663	35.16	50	0.43	11,558	34.85
City of Marikina	8,342	3,355	40.22	7	0.21	2,528	30.30
City of Pasig	17,682	12,432	70.31	22	0.18	12,423	70.26
Pateros	1,116	286	25.63	0	0.00	361	32.35
City of Taguig	18,992	9,543	50.25	129	1.35	9,553	50.30
Quezon City	59,136	33,724	57.03	44	0.13	34,288	57.98
City of Makati	10,944	2,716	24.82	8	0.29	3,194	29.18
City of Mandaluyong	7,922	6,991	88.25	5	0.07	6,910	87.23
City of San Juan	2,168	875	40.36	2	0.23	876	40.41
City of Manila	34,128	18,514	54.25	70	0.38	18,982	55.62
City of Las Piñas	11,263	3,548	31.50	15	0.42	4,386	38.94
City of Muntinlupa	9,881	3,509	35.51	15	0.43	3,989	40.37
City of Parañaque	13,280	6,209	46.75	13	0.21	5,948	44.79
City of Pasay	7,134	5,416	75.92	82	1.51	5,774	80.94
**CAR**	**31,319**	**21,083**	**67.32**	**56**	**0.27**	**13,922**	**44.45**
Abra	3,660	1,401	38.28	6	0.43	1,319	36.04
Apayao	2,238	2,635	117.74	24	0.91	867	38.74
Benguet	8,806	7,141	81.09	1	0.01	3,951	44.87
Ifugao	4,098	1,866	45.53	1	0.05	1,284	31.33
Kalinga	4,549	1,661	36.51	1	0.06	1,490	32.75
Mountain Province	2,619	3,328	127.07	5	0.15	2,027	77.40
City of Baguio	5,349	3,051	57.04	18	0.59	2,984	55.79
**Region 1**	**96,016**	**31,638**	**32.95**	**107**	**0.34**	**21,098**	**21.97**
Ilocos Norte	8,912	5,071	56.90	7	0.14	4,154	46.61
Ilocos Sur	9,914	6,066	61.19	2	0.03	4,139	41.75
La Union	13,507	6,166	45.65	21	0.34	4,683	34.67

Table 2.E.2 - HIV-AIDS/STI Prevention and Control Services

Pregnant Women Screened for HIV and Screened and Positive with Syphilis

Philippines, 2023

Area	Eligible Pop.	Pregnant Women					
		Screened for Syphilis	%	Positive for Syphilis	%	Screened for HIV	%
Pangasinan	60,360	14,077	23.32	75	0.53	7,671	12.71
City of Dagupan	3,323	258	7.76	2	0.78	451	13.57
**Region 2**	**63,967**	**16,033**	**25.06**	**308**	**1.92**	**10,178**	**15.91**
Batanes	240	124	51.67	1	0.81	105	43.75
Cagayan	20,786	1,589	7.64	37	2.33	1,231	5.92
Isabela	27,379	9,906	36.18	227	2.29	5,336	19.49
Nueva Vizcaya	8,798	2,567	29.18	31	1.21	1,518	17.25
Quirino	3,839	1,597	41.60	7	0.44	1,637	42.64
City of Santiago	2,925	250	8.55	5	2.00	351	12.00
**Region 3**	**233,544**	**84,338**	**36.11**	**717**	**0.85**	**80,799**	**34.60**
Aurora	4,537	2,082	45.89	11	0.53	2,436	53.69
Bataan	17,380	8,836	50.84	47	0.53	9,059	52.12
Bulacan	67,590	34,960	51.72	63	0.18	33,521	49.59
Nueva Ecija	43,425	9,010	20.75	300	3.33	8,315	19.15
Pampanga	45,651	15,707	34.41	219	1.39	14,631	32.05
Tarlac	29,503	7,331	24.85	44	0.60	5,843	19.80
Zambales	11,723	3,301	28.16	18	0.55	4,008	34.19
City of Angeles	9,146	1,583	17.31	4	0.25	1,518	16.60
City of Olongapo	4,589	1,528	33.30	11	0.72	1,468	31.99
**Region 4A**	**307,982**	**81,253**	**26.38**	**1,099**	**1.35**	**73,302**	**23.80**
Batangas	53,251	10,066	18.90	75	0.75	6,498	12.20
Cavite	77,964	19,255	24.70	235	1.22	17,644	22.63
Laguna	61,525	22,211	36.10	353	1.59	18,291	29.73
Quezon	40,810	7,531	18.45	141	1.87	7,323	17.94
Rizal	68,235	20,038	29.37	246	1.23	22,091	32.37
City of Lucena	6,197	2,152	34.73	49	2.28	1,455	23.48
**Region 4B**	**66,121**	**19,673**	**29.75**	**221**	**1.12**	**17,228**	**26.06**
Marinduque	4,144	1,261	30.43	7	0.56	1,073	25.89
Occidental Mindoro	11,640	2,664	22.89	48	1.80	375	3.22

Table 2.E.2 - HIV-AIDS/STI Prevention and Control Services

Pregnant Women Screened for HIV and Screened and Positive with Syphilis

Philippines, 2023

Area	Eligible Pop.	Pregnant Women					
		Screened for Syphilis	%	Positive for Syphilis	%	Screened for HIV	%
Oriental Mindoro	18,943	6,870	36.27	32	0.47	6,050	31.94
Palawan	20,317	4,512	22.21	65	1.44	5,768	28.39
Romblon	5,489	1,949	35.51	28	1.44	1,302	23.72
City of Puerto Princesa	5,588	2,417	43.25	41	1.70	2,660	47.60
**Region 5**	**135,480**	**25,841**	**19.07**	**295**	**1.14**	**19,897**	**14.69**
Albay	27,440	7,892	28.76	63	0.80	6,768	24.66
Camarines Norte	14,695	2,973	20.23	74	2.49	754	5.13
Camarines Sur	43,452	3,741	8.61	32	0.86	4,082	9.39
Catanduanes	5,414	2,444	45.14	9	0.37	2,315	42.76
Masbate	22,365	5,003	22.37	79	1.58	3,301	14.76
Sorsogon	17,929	2,822	15.74	30	1.06	1,227	6.84
City of Naga	4,185	966	23.08	8	0.83	1,450	34.65
**Region 6**	**148,020**	**45,489**	**30.73**	**638**	**1.40**	**29,301**	**19.80**
Aklan	11,283	3,917	34.72	23	0.59	2,303	20.41
Antique	11,930	3,556	29.81	63	1.77	2,027	16.99
Capiz	13,931	3,301	23.70	40	1.21	1,548	11.11
Guimaras	3,351	2,117	63.18	10	0.47	1,902	56.76
Iloilo	36,140	14,381	39.79	129	0.90	6,200	17.16
Negros Occidental	52,270	10,393	19.88	103	0.99	9,059	17.33
City of Bacolod	10,373	4,724	45.54	263	5.57	3,660	35.28
City of Iloilo	8,742	3,100	35.46	7	0.23	2,602	29.76
**Region 7**	**165,673**	**68,724**	**41.48**	**1,019**	**1.48**	**68,238**	**41.19**
Bohol	26,235	13,310	50.73	137	1.03	10,462	39.88
Cebu	72,201	16,356	22.65	539	3.30	20,444	28.32
Negros Oriental	28,722	4,427	15.41	49	1.11	2,489	8.67
Siquijor	1,680	890	52.98	5	0.56	870	51.79
City of Cebu	19,267	8,963	46.52	158	1.76	8,881	46.09
City of Lapu-Lapu	10,679	5,423	50.78	116	2.14	5,809	54.40
City of Mandaue	6,889	19,355	280.96	15	0.08	19,283	279.91

Table 2.E.2 - HIV-AIDS/STI Prevention and Control Services

Pregnant Women Screened for HIV and Screened and Positive with Syphilis

Philippines, 2023

Area	Eligible Pop.	Pregnant Women					
		Screened for Syphilis	%	Positive for Syphilis	%	Screened for HIV	%
**Region 8**	**93,813**	**17,279**	**18.42**	**305**	**1.77**	**20,083**	**21.41**
Biliran	3,373	1,471	43.61	5	0.34	1,844	54.67
Eastern Samar	9,624	2,008	20.86	20	1.00	2,320	24.11
Leyte	31,559	3,109	9.85	147	4.73	3,472	11.00
Northern Samar	14,056	4,630	32.94	46	0.99	4,729	33.64
Southern Leyte	7,406	479	6.47	1	0.21	1,306	17.63
Samar	17,011	1,462	8.59	71	4.86	1,961	11.53
Ormoc City	5,292	1,593	30.10	9	0.56	1,753	33.13
City of Tacloban	5,492	2,527	46.01	6	0.24	2,698	49.13
**Region 9**	**84,815**	**5,772**	**6.81**	**33**	**0.57**	**2,427**	**2.86**
Zamboanga del Norte	24,841	578	2.33	2	0.35	704	2.83
Zamboanga del Sur	23,757	1,088	4.58	13	1.19	226	0.95
Zamboanga Sibugay	13,841	3,078	22.24	15	0.49	587	4.24
City of Isabela	19,175	226	1.18	0	0.00	226	1.18
City of Zamboanga	3,201	802	25.05	3	0.37	684	21.37
**Region 10**	**109,169**	**21,237**	**19.45**	**210**	**0.99**	**17,770**	**16.28**
Bukidnon	33,403	4,030	12.06	50	1.24	2,832	8.48
Camiguin	1,727	468	27.10	0	0.00	220	12.74
Lanao del Norte	17,760	1,071	6.03	0	0.00	1,689	9.51
Misamis Occidental	12,328	1,823	14.79	0	0.00	3,419	27.73
Misamis Oriental	21,975	6,037	27.47	36	0.60	6,055	27.55
City of Cagayan De Oro	14,308	6,702	46.84	124	1.85	1,782	12.45
City of Iligan	7,668	1,106	14.42	0	0.00	1,773	23.12
**Region 11**	**107,001**	**26,586**	**24.85**	**146**	**0.55**	**21,034**	**19.66**
Davao de Oro	15,253	356	2.33	7	1.97	953	6.25
Davao del Norte	23,453	5,753	24.53	32	0.56	4,005	17.08
Davao Oriental	11,935	343	2.87	5	1.46	459	3.85
Davao del Sur	13,083	1,586	12.12	10	0.63	2,434	18.60
Davao Occidental	6,629	190	2.87	2	1.05	1,479	22.31
City of Davao	36,648	18,358	50.09	90	0.49	11,704	31.94

Table 2.E.2 - HIV-AIDS/STI Prevention and Control Services

Pregnant Women Screened for HIV and Screened and Positive with Syphilis

Philippines, 2023

Area	Eligible Pop.	Pregnant Women					
		Screened for Syphilis	%	Positive for Syphilis	%	Screened for HIV	%
**Region 12**	**95,279**	**28,215**	**29.61**	**607**	**2.15**	**15,873**	**16.66**
Cotabato	26,521	4,063	15.32	158	3.89	3,043	11.47
Sarangani	13,322	5,621	42.19	177	3.15	1,978	14.85
South Cotabato	21,595	7,683	35.58	126	1.64	7,034	32.57
Sultan Kudarat	19,391	7,827	40.36	69	0.88	1,727	8.91
City of General Santos	14,450	3,021	20.91	77	2.55	2,091	14.47
**BARMM**	**132,841**	**16,866**	**12.70**	**52**	**0.31**	**17,276**	**13.01**
Basilan	11,636	101	0.87	0	0.00	34	0.29
Lanao del Sur	33,374	14,440	43.27	0	0.00	13,613	40.79
Maguindanao	41,650	272	0.65	16	5.88	599	1.44
Sulu	21,378	251	1.17	9	3.59	252	1.18
Tawi-Tawi	12,609	65	0.52	1	1.54	392	3.11
SGA	5,103	0	0.00	0	0.00	0	0.00
City of Cotabato	7,091	1,737	24.50	26	1.50	2,386	33.65
**Caraga**	**55,065**	**11,810**	**21.45**	**100**	**0.85**	**10,356**	**18.81**
Agusan del Norte	7,286	1,578	21.66	23	1.46	1,418	19.46
Agusan del Sur	16,038	2,271	14.16	46	2.03	1,169	7.29
Surigao del Norte	10,186	2,216	21.76	16	0.72	2,145	21.06
Surigao del Sur	12,736	870	6.83	3	0.34	667	5.24
Dinagat Islands	2,134	908	42.55	9	0.99	938	43.96
City of Butuan	6,685	3,967	59.34	3	0.08	4,019	60.12

Table 2.E.3 Leprosy

Prevalence and Detection Rate

Philippines, 2023

Area	Population	Leprosy Cases undergoing Treatment			Prev. Rate	Newly Detected Case			Detection Rate
		Male	Female	Total		Male	Female	Total	
**Philippines**	**111,638,549**	**644**	**268**	**912**	**0.08**	**696**	**390**	**1,086**	**0.97**
**NCR**	**13,802,335**	**4**	**0**	**4**	**0.00**	**5**	**0**	**5**	**0.04**
City of Malabon	389,819	0	0	0	0.00	0	0	0	0.00
City of Navotas	254,778	0	0	0	0.00	0	0	0	0.00
City of Valenzuela	732,364	0	0	0	0.00	2	0	2	0.27
City of Caloocan	1,708,376	0	0	0	0.00	0	0	0	0.00
City of Marikina	464,962	0	0	0	0.00	0	0	0	0.00
City of Pasig	825,380	1	0	1	0.01	2	0	2	0.24
Pateros	66,279	0	0	0	0.00	0	0	0	0.00
City of Taguig	917,267	3	0	3	0.03	1	0	1	0.11
Quezon City	3,027,195	0	0	0	0.00	0	0	0	0.00
City of Makati	637,870	0	0	0	0.00	0	0	0	0.00
City of Mandaluyong	434,978	0	0	0	0.00	0	0	0	0.00
City of San Juan	127,452	0	0	0	0.00	0	0	0	0.00
City of Manila	1,889,338	0	0	0	0.00	0	0	0	0.00
City of Las Piñas	618,866	0	0	0	0.00	0	0	0	0.00
City of Muntinlupa	553,660	0	0	0	0.00	0	0	0	0.00
City of Parañaque	704,904	0	0	0	0.00	0	0	0	0.00
City of Pasay	448,847	0	0	0	0.00	0	0	0	0.00
**CAR**	**1,851,672**	**13**	**10**	**23**	**0.12**	**12**	**6**	**18**	**0.97**
Abra	253,559	3	1	4	0.16	3	0	3	1.18
Apayao	128,167	3	1	4	0.31	4	0	4	3.12
Benguet	473,982	0	1	1	0.02	0	0	0	0.00
Ifugao	216,448	1	0	1	0.05	1	0	1	0.46
Kalinga	238,965	0	0	0	0.00	0	0	0	0.00
Mountain Province	162,971	0	1	1	0.06	2	0	2	1.23
City of Baguio	377,580	6	6	12	0.32	2	6	8	2.12
**Region 1**	**5,390,222**	**72**	**18**	**90**	**0.17**	**72**	**27**	**99**	**1.84**
Ilocos Norte	616,826	14	3	17	0.28	13	2	15	2.43
Ilocos Sur	713,424	9	6	15	0.21	5	11	16	2.24
La Union	835,494	5	1	6	0.07	4	0	4	0.48
Pangasinan	3,045,822	43	8	51	0.17	47	14	61	2.00

Table 2.E.3 Leprosy

Prevalence and Detection Rate

Philippines, 2023

Area	Population	Leprosy Cases undergoing Treatment			Prev. Rate	Newly Detected Case			Detection Rate
		Male	Female	Total		Male	Female	Total	
City of Dagupan	178,656	1	0	1	0.06	3	0	3	1.68
**Region 2**	**3,767,534**	**5**	**8**	**13**	**0.03**	**12**	**6**	**18**	**0.48**
Batanes	19,107	0	0	0	0.00	0	0	0	0.00
Cagayan	1,289,735	0	3	3	0.02	6	2	8	0.62
Isabela	1,580,009	0	0	0	0.00	3	1	4	0.25
Nueva Vizcaya	514,210	3	2	5	0.10	1	2	3	0.58
Quirino	211,146	1	2	3	0.14	2	1	3	1.42
City of Santiago	153,327	1	1	2	0.13	0	0	0	0.00
**Region 3**	**12,705,726**	**71**	**31**	**102**	**0.08**	**67**	**31**	**98**	**0.77**
Aurora	243,172	6	0	6	0.25	3	0	3	1.23
Bataan	876,011	3	1	4	0.05	2	0	2	0.23
Bulacan	3,788,883	2	2	4	0.01	1	1	2	0.05
Nueva Ecija	2,353,166	10	3	13	0.06	17	8	25	1.06
Pampanga	2,508,544	10	3	13	0.05	13	1	14	0.56
Tarlac	1,532,063	27	14	41	0.27	20	15	35	2.28
Zambales	664,850	5	1	6	0.09	6	1	7	1.05
City of Angeles	474,775	6	6	12	0.25	5	5	10	2.11
City of Olongapo	264,262	2	1	3	0.11	0	0	0	0.00
**Region 4A**	**16,579,218**	**9**	**7**	**16**	**0.01**	**20**	**11**	**31**	**0.19**
Batangas	2,969,087	0	0	0	0.00	3	1	4	0.13
Cavite	4,451,043	1	1	2	0.00	4	1	5	0.11
Laguna	3,458,788	2	0	2	0.01	0	0	0	0.00
Quezon	1,996,004	1	4	5	0.03	3	7	10	0.50
Rizal	3,418,289	2	2	4	0.01	5	0	5	0.15
City of Lucena	286,007	3	0	3	0.10	5	2	7	2.45
**Region 4B**	**3,303,791**	**10**	**4**	**14**	**0.04**	**10**	**11**	**21**	**0.64**
Marinduque	242,451	0	0	0	0.00	0	0	0	0.00
Occidental Mindoro	539,533	5	1	6	0.11	5	1	6	1.11
Oriental Mindoro	926,654	0	0	0	0.00	1	6	7	0.76
Palawan	965,716	1	3	4	0.04	3	1	4	0.41

Table 2.E.3 Leprosy

Prevalence and Detection Rate

Philippines, 2023

Area	Population	Leprosy Cases undergoing Treatment			Prev. Rate	Newly Detected Case			Detection Rate
		Male	Female	Total		Male	Female	Total	
Romblon	314,575	0	0	0	0.00	0	0	0	0.00
City of Puerto Princesa	314,862	4	0	4	0.13	1	3	4	1.27
**Region 5**	**6,265,917**	**4**	**3**	**7**	**0.01**	**4**	**3**	**7**	**0.11**
Albay	1,415,746	0	1	1	0.01	0	1	1	0.07
Camarines Norte	653,656	0	0	0	0.00	0	0	0	0.00
Camarines Sur	1,916,128	0	0	0	0.00	0	0	0	0.00
Catanduanes	279,648	1	0	1	0.04	0	0	0	0.00
Masbate	932,221	3	1	4	0.04	1	1	2	0.21
Sorsogon	852,791	0	1	1	0.01	3	1	4	0.47
City of Naga	215,727	0	0	0	0.00	0	0	0	0.00
**Region 6**	**8,047,833**	**27**	**24**	**51**	**0.06**	**43**	**44**	**87**	**1.08**
Aklan	626,648	1	0	1	0.02	1	0	1	0.16
Antique	617,880	2	0	2	0.03	3	2	5	0.81
Capiz	817,827	0	0	0	0.00	1	0	1	0.12
Guimaras	191,419	0	0	0	0.00	0	0	0	0.00
Iloilo	2,081,051	10	10	20	0.10	27	34	61	2.93
Negros Occidental	2,652,749	11	12	23	0.09	8	6	14	0.53
City of Bacolod	598,468	3	2	5	0.08	3	2	5	0.84
City of Iloilo	461,791	0	0	0	0.00	0	0	0	0.00
**Region 7**	**8,305,046**	**10**	**2**	**12**	**0.01**	**32**	**4**	**36**	**0.43**
Bohol	1,421,485	6	2	8	0.06	16	2	18	1.27
Cebu	3,420,109	4	0	4	0.01	13	2	15	0.44
Negros Oriental	1,471,146	0	0	0	0.00	0	0	0	0.00
Siquijor	104,070	0	0	0	0.00	0	0	0	0.00
City of Cebu	993,006	-1	0	-1	-0.01	2	0	2	0.20
City of Lapu-Lapu	517,787	0	0	0	0.00	0	0	0	0.00
City of Mandaue	377,443	1	0	1	0.03	1	0	1	0.26
**Region 8**	**4,651,322**	**109**	**46**	**155**	**0.33**	**99**	**49**	**148**	**3.18**
Biliran	183,761	2	1	3	0.16	3	1	4	2.18
Eastern Samar	488,406	11	2	13	0.27	9	2	11	2.25

Table 2.E.3 Leprosy

Prevalence and Detection Rate

Philippines, 2023

Area	Population	Leprosy Cases undergoing Treatment			Prev. Rate	Newly Detected Case			Detecti on Rate
		Male	Female	Total		Male	Female	Total	
Leyte	1,582,542	39	23	62	0.39	37	30	67	4.23
Northern Samar	652,283	16	6	22	0.34	7	6	13	1.99
Southern Leyte	433,569	10	0	10	0.23	12	0	12	2.77
Samar	813,690	25	12	37	0.45	26	7	33	4.06
Ormoc City	238,056	3	1	4	0.17	2	1	3	1.26
City of Tacloban	259,015	3	1	4	0.15	3	2	5	1.93
**Region 9**	**3,974,491**	**43**	**23**	**66**	**0.17**	**120**	**85**	**205**	**5.16**
Zamboanga del Norte	1,073,915	27	17	44	0.41	96	71	167	15.55
Zamboanga del Sur	1,080,246	5	0	5	0.05	11	7	18	1.67
Zamboanga Sibugay	686,576	10	6	16	0.23	9	6	15	2.18
City of Isabela	131,842	0	0	0	0.00	4	1	5	3.79
City of Zamboanga	1,001,912	1	0	1	0.01	0	0	0	0.00
**Region 10**	**5,192,278**	**65**	**36**	**101**	**0.19**	**55**	**39**	**94**	**1.81**
Bukidnon	1,606,133	21	8	29	0.18	21	8	29	1.81
Camiguin	94,450	0	0	0	0.00	0	0	0	0.00
Lanao del Norte	743,529	0	9	9	0.12	8	3	11	1.48
Misamis Occidental	632,855	0	2	2	0.03	3	0		0.00
Misamis Oriental	987,437	16	8	24	0.24	7	4	11	1.11
								0	
City of Cagayan De Oro	755,908	15	3	18	0.24	10	8		0.00
City of Iligan	371,966	13	6	19	0.51	6	16		0.00
**Region 11**	**5,380,729**	**76**	**0**	**76**	**0.14**	**35**	**11**	**46**	**0.85**
Davao de Oro	792,250	4		4	0.05	3	0	3	0.38
Davao del Norte	1,155,884	9		9	0.08	6	0	6	0.52
Davao Oriental	591,494	29		29	0.49	10	5	15	2.54
Davao del Sur	697,574	17		17	0.24	7	3	10	1.43
Davao Occidental	318,776	8		8	0.25	4	1	5	1.57
City of Davao	1,824,751	9		9	0.05	5	2	7	0.38
**Region 12**	**4,507,622**	**22**	**9**	**31**	**0.07**	**34**	**20**	**54**	**1.20**
Cotabato	1,317,572	1	2	3	0.02	2	2	4	0.30
Sarangani	579,863	8	0	8	0.14	14	5	19	3.28

Table 2.E.3 Leprosy

Prevalence and Detection Rate

Philippines, 2023

Area	Population	Leprosy Cases undergoing Treatment			Prev. Rate	Newly Detected Case			Detection Rate
		Male	Female	Total		Male	Female	Total	
South Cotabato	1,006,003	4	2	6	0.06	4	3	7	0.70
Sultan Kudarat	881,039	0	0	0	0.00	4	3	7	0.79
City of General Santos	723,145	9	5	14	0.19	10	7	17	2.35
**BARMM**	**5,039,862**	**28**	**22**	**50**	**0.10**	**23**	**23**	**46**	**0.91**
Basilan	431,169	6	7	13	0.30	1	3	4	0.93
Lanao del Sur	1,210,346	0	0	0	0.00	2	2	4	0.33
Maguindanao	1,391,043	6	0	6	0.04	4	0	4	0.29
Sulu	1,006,150	4	10	14	0.14	6	16	22	2.19
Tawi-Tawi	442,766	8	2	10	0.23	8	2	10	2.26
SGA	223,577	0	0	0	0.00	0	0	0	0.00
City of Cotabato	334,811	4	3	7	0.21	2	0	2	0.60
**Caraga**	**2,872,951**	**76**	**25**	**101**	**0.35**	**53**	**20**	**73**	**2.54**
Agusan del Norte	396,078	0	0	0	0.00	1	0	1	0.25
Agusan del Sur	759,431	15	6	21	0.28	7	3	10	1.32
Surigao del Norte	545,689	45	12	57	1.04	42	11	53	9.71
Surigao del Sur	661,201	1	0	1	0.02	0	0	0	0.00
Dinagat Islands	129,127	1	3	4	0.31	1	3	4	3.10
City of Butuan	381,425	14	4	18	0.47	2	3	5	1.31

Table 2.E.4 - Rabies
Cases of Animal Bites and Deaths Due to Rabies
Philippines, 2023

Area	Animal Bites			Death due to rabies			Rate
	Male	Female	Total	Male	Female	Total	
**Philippines**	**706,562**	**801,532**	**1,508,094**	**241**	**64**	**305**	**0.02**
**NCR**	**120,531**	**146,962**	**267,493**	**16**	**2**	**18**	**0.01**
City of Malabon	5,161	5,966	11,127	0	0	0	0.00
City of Navotas	3,189	3,712	6,901	2	0	2	0.03
City of Valenzuela	4,460	5,306	9,766	2	1	3	0.03
City of Caloocan	5,212	6,211	11,423	4	0	4	0.04
City of Marikina	3,936	4,869	8,805	0	0	0	0.00
City of Pasig	16,761	21,176	37,937	1	0	1	0.00
Pateros	1,350	1,787	3,137	0	0	0	0.00
City of Taguig	28,155	33,855	62,010	1	0	1	0.00
Quezon City	22,468	26,920	49,388	5	0	5	0.01
City of Makati	2,231	2,908	5,139	0	0	0	0.00
City of Mandaluyong	5,315	7,337	12,652	0	1	1	0.01
City of San Juan	2,118	2,652	4,770	0	0	0	0.00
City of Manila	5,030	5,943	10,973	0	0	0	0.00
City of Las Piñas	2,668	3,534	6,202	0	0	0	0.00
City of Muntinlupa	4,620	5,509	10,129	1	0	1	0.01
City of Parañaque	4,021	4,830	8,851	0	0	0	0.00
Pasay City	3,836	4,447	8,283	0	0	0	0.00
**CAR**	**23,001**	**21,413**	**44,414**	**3**	**1**	**4**	**0.01**
Abra	2,334	2,354	4,688	2	0	2	0.04
Apayao	1,951	1,898	3,849	1	0	1	0.03
Benguet	2,988	2,817	5,805	0	0	0	0.00
Ifugao	2,250	1,882	4,132	0	0	0	0.00
Kalinga	2,407	1,995	4,402	0	0	0	0.00
Mountain Province	1,776	1,477	3,253	0	1	1	0.03
City of Baguio	9,295	8,990	18,285	0	0	0	0.00
**Region 1**	**41,463**	**46,531**	**87,994**	**25**	**3**	**28**	**0.03**
Ilocos Norte	6,078	6,494	12,572	13	1	14	0.11
Ilocos Sur	7,894	9,226	17,120	2	1	3	0.02
La Union	13,255	15,037	28,292	2	1	3	0.01
Pangasinan	10,912	12,009	22,921	8	0	8	0.03
City of Dagupan	3,324	3,765	7,089	0	0	0	0.00
**Region 2**	**42,393**	**43,600**	**85,993**	**8**	**1**	**9**	**0.01**
Batanes	9	6	15	0	0	0	0.00

Table 2.E.4 - Rabies
Cases of Animal Bites and Deaths Due to Rabies
Philippines, 2023

Area	Animal Bites			Death due to rabies			Rate
	Male	Female	Total	Male	Female	Total	
Cagayan	4,466	4,270	8,736	1	0	1	0.01
Isabela	24,916	25,999	50,915	7	1	8	0.02
Nueva Vizcaya	7,774	8,614	16,388	0	0	0	0.00
Quirino	4,428	3,803	8,231	0	0	0	0.00
City of Santiago	800	908	1,708	0	0	0	0.00
**Region 3**	**74,429**	**87,425**	**161,854**	**38**	**16**	**54**	**0.03**
Aurora	3,827	4,254	8,081	2	0	2	0.02
Bataan	6,310	7,366	13,676	3	0	3	0.02
Bulacan	22,187	26,328	48,515	16	7	23	0.05
Nueva Ecija	20,579	23,908	44,487	7	5	12	0.03
Pampanga	3,796	4,334	8,130	3	1	4	0.05
Tarlac	11,987	14,192	26,179	2	0	2	0.01
Zambales	4,002	4,995	8,997	5	2	7	0.08
City of Angeles	892	977	1,869	0	1	1	0.05
City of Olongapo	849	1,071	1,920	0	0	0	0.00
**Region 4A**	**103,059**	**120,055**	**223,114**	**19**	**6**	**25**	**0.01**
Batangas	13,617	16,115	29,732	0	0	0	0.00
Cavite	17,696	19,355	37,051	1	1	2	0.01
Laguna	19,872	23,752	43,624	4	4	8	0.02
Quezon	9,077	11,016	20,093	3	1	4	0.02
Rizal	40,763	47,547	88,310	2	0	2	0.00
City of Lucena	2,034	2,270	4,304	9	0	9	0.21
**Region 4B**	**21,515**	**24,047**	**45,562**	**4**	**3**	**7**	**0.02**
Marinduque	3,093	3,966	7,059	1	0	1	0.01
Occidental Mindoro	6,341	6,776	13,117	0	1	1	0.01
Oriental Mindoro	4,040	4,632	8,672	0	0	0	0.00
Palawan	3,833	3,890	7,723	1	0	1	0.01
Romblon	1,399	1,701	3,100	2	2	4	0.13
City of Puerto Princesa	2,809	3,082	5,891	0	0	0	0.00
**Region 5**	**19,962**	**23,170**	**43,132**	**18**	**6**	**24**	**0.06**
Albay	3,772	3,916	7,688	4	1	5	0.07
Camarines Norte	479	531	1,010	0	0	0	0.00
Camarines Sur	4,841	5,683	10,524	10	3	13	0.12
Catanduanes	2,599	3,531	6,130	0	0	0	0.00
Masbate	192	204	396	1	0	1	0.25
Sorsogon	6,655	7,614	14,269	3	1	4	0.03

Table 2.E.4 - Rabies
Cases of Animal Bites and Deaths Due to Rabies
Philippines, 2023

Area	Animal Bites			Death due to rabies			Rate
	Male	Female	Total	Male	Female	Total	
City of Naga	1,424	1,691	3,115	0	1	1	0.03
**Region 6**	**54,462**	**61,582**	**116,044**	**27**	**2**	**29**	**0.02**
Aklan	7,603	8,926	16,529	4	0	4	0.02
Antique	756	850	1,606	2	0	2	0.12
Capiz	10,945	12,454	23,399	5	1	6	0.03
Guimaras	1,898	2,189	4,087	1	0	1	0.02
Iloilo	9,238	10,727	19,965	5	0	5	0.03
Negros Occidental	14,983	15,800	30,783	5	1	6	0.02
City of Bacolod	6,577	7,658	14,235	2	0	2	0.01
City of Iloilo	2,462	2,978	5,440	3	0	3	0.06
**Region 7**	**53,246**	**59,601**	**112,847**	**10**	**2**	**12**	**0.01**
Bohol	7,485	8,395	15,880	5	1	6	0.04
Cebu	18,288	20,485	38,773	2	0	2	0.01
Negros Oriental	4,345	4,793	9,138	1	1	2	0.02
Siquijor	0	0	0	0	0	0	0.00
City of Cebu	8,401	9,562	17,963	1	0	1	0.01
City of Lapu-Lapu	8,568	9,593	18,161	1	0	1	0.01
City of Mandaue	6,159	6,773	12,932	0	0	0	0.00
**Region 8**	**14,768**	**15,821**	**30,589**	**9**	**1**	**10**	**0.03**
Biliran	1,577	1,447	3,024	0	0	0	0.00
Eastern Samar	278	289	567	3	0	3	0.53
Leyte	2,292	2,319	4,611	2	0	2	0.04
Northern Samar	172	182	354	1	1	2	0.56
Southern Leyte	2,492	2,812	5,304	0	0	0	0.00
Samar	1,069	1,110	2,179	0	0	0	0.00
City of Ormoc	4,728	5,242	9,970	0	0	0	0.00
City of Tacloban	2,160	2,420	4,580	3	0	3	0.07
**Region 9**	**12,494**	**13,812**	**26,306**	**9**	**3**	**12**	**0.05**
Zamboanga del Norte	2,003	2,280	4,283	5	1	6	0.14
Zamboanga del Sur	3,439	3,850	7,289	2	0	2	0.03
Zamboanga Sibugay	6,346	6,933	13,279	0	1	1	0.01
City of Isabela	524	550	1,074	0	0	0	0.00
City of Zamboanga	182	199	381	2	1	3	0.79

Table 2.E.4 - Rabies
Cases of Animal Bites and Deaths Due to Rabies
Philippines, 2023

Area	Animal Bites			Death due to rabies			Rate
	Male	Female	Total	Male	Female	Total	
**Region 10**	**31,449**	**33,511**	**64,960**	**11**	**5**	**16**	**0.02**
Bukidnon	5,569	5,913	11,482	5	1	6	0.05
Camiguin	854	786	1,640	0	0	0	0.00
Lanao del Norte	1,925	1,947	3,872	0	0	0	0.00
Misamis Occidental	2,004	2,080	4,084	4	0	4	0.10
Misamis Oriental	7,461	7,570	15,031	0	3	3	0.02
City of Cagayan De Oro	8,319	9,510	17,829	1	1	2	0.01
City of Iligan	5,317	5,705	11,022	1	0	1	0.01
**Region 11**	**56,565**	**65,302**	**121,867**	**14**	**10**	**24**	**0.02**
Davao de Oro	6,565	7,097	13,662	4	2	6	0.04
Davao del Norte	9,492	12,759	22,251	1	2	3	0.01
Davao Oriental	5,311	6,046	11,357	1	3	4	0.04
Davao del Sur	5,910	6,559	12,469	5	1	6	0.05
Davao Occidental	1,236	1,369	2,605	0	1	1	0.04
City of Davao	28,051	31,472	59,523	3	1	4	0.01
**Region 12**	**23,557**	**24,418**	**47,975**	**26**	**2**	**28**	**0.06**
Cotabato	3,664	4,242	7,906	6	0	6	0.08
Sarangani	1,827	1,994	3,821	4	0	4	0.10
South Cotabato	6,350	6,732	13,082	8	1	9	0.07
Sultan Kudarat	9,051	8,910	17,961	5	0	5	0.03
City of General Santos	2,665	2,540	5,205	3	1	4	0.08
**BARMM**	**2,415**	**2,329**	**4,744**	**3**	**1**	**4**	**0.08**
Basilan	245	254	499	1	1	2	0.40
Lanao del Sur	350	418	768	0	0	0	0.00
Maguindanao	384	377	761	2	0	2	0.26
Sulu	234	130	364	0	0	0	0.00
Tawi-Tawi	435	325	760	0	0	0	0.00
SGA	0	0	0	0	0	0	0.00
City of Cotabato	767	825	1,592	0	0	0	0.00
**Caraga**	**11,253**	**11,953**	**23,206**	**1**	**0**	**1**	**0.00**
Agusan del Norte	370	421	791	0	0	0	0.00
Agusan del Sur	359	312	671	1	0	1	0.15
Surigao del Norte	5,509	6,215	11,724	0	0	0	0.00
Surigao del Sur	4,484	4,424	8,908	0	0	0	0.00
Dinagat Islands	509	563	1,072	0	0	0	0.00

Table 2.E.4 - Rabies
Cases of Animal Bites and Deaths Due to Rabies
Philippines, 2023

Area	Animal Bites			Death due to rabies			Rate
	Male	Female	Total	Male	Female	Total	
City of Butuan	22	18	40	0	0	0	0.00

Table 1.E.5.1 - Schistosomiasis Prevention and Control

Suspected Schistosomiasis cases seen, Acute clinically diagnosed cases seen and Acute Confirmed Cases by Stool Examination

Philippines, 2023

Area	Total Patients seen	Number of Suspected Cases				No. of Acute Clinically Diagnosed Cases				No. of Acute Cases Confirmed Positive by Stool Examination			
		Male	Female	Total	%	Male	Female	Total	%	Male	Female	Total	%
**Philippines**	**90,453**	**17,009**	**16,546**	**33,555**	**37.10**	**858**	**548**	**1,406**	**4.19**	**299**	**179**	**478**	**34.00**
**NCR**	**0**	**0**	**0**	**0**	**0.00**	**0**	**0**	**0**	**0.00**	**0**	**0**	**0**	**0.00**
City of Malabon	0	0	0	0	0.00	0	0	0	0.00	0	0	0	0.00
City of Navotas	0	0	0	0	0.00	0	0	0	0.00	0	0	0	0.00
City of Valenzuela	0	0	0	0	0.00	0	0	0	0.00	0	0	0	0.00
City of Caloocan	0	0	0	0	0.00	0	0	0	0.00	0	0	0	0.00
City of Marikina	0	0	0	0	0.00	0	0	0	0.00	0	0	0	0.00
City of Pasig	0	0	0	0	0.00	0	0	0	0.00	0	0	0	0.00
Pateros	0	0	0	0	0.00	0	0	0	0.00	0	0	0	0.00
City of Taguig	0	0	0	0	0.00	0	0	0	0.00	0	0	0	0.00
Quezon City	0	0	0	0	0.00	0	0	0	0.00	0	0	0	0.00
City of Makati	0	0	0	0	0.00	0	0	0	0.00	0	0	0	0.00
City of Mandaluyong	0	0	0	0	0.00	0	0	0	0.00	0	0	0	0.00
City of San Juan	0	0	0	0	0.00	0	0	0	0.00	0	0	0	0.00
City of Manila	0	0	0	0	0.00	0	0	0	0.00	0	0	0	0.00
City of Las Piñas	0	0	0	0	0.00	0	0	0	0.00	0	0	0	0.00
City of Muntinlupa	0	0	0	0	0.00	0	0	0	0.00	0	0	0	0.00
City of Parañaque	0	0	0	0	0.00	0	0	0	0.00	0	0	0	0.00
City of Pasay	0	0	0	0	0.00	0	0	0	0.00	0	0	0	0.00
**CAR**	**0**	**0**	**0**	**0**	**0.00**	**0**	**0**	**0**	**0.00**	**0**	**0**	**0**	**0.00**
Abra	0	0	0	0	0.00	0	0	0	0.00	0	0	0	0.00
Apayao	0	0	0	0	0.00	0	0	0	0.00	0	0	0	0.00
Benguet	0	0	0	0	0.00	0	0	0	0.00	0	0	0	0.00
Ifugao	0	0	0	0	0.00	0	0	0	0.00	0	0	0	0.00
Kalinga	0	0	0	0	0.00	0	0	0	0.00	0	0	0	0.00
Mountain Province	0	0	0	0	0.00	0	0	0	0.00	0	0	0	0.00
City of Baguio	0	0	0	0	0.00	0	0	0	0.00	0	0	0	0.00
**Region 1**	**0**	**0**	**0**	**0**	**0.00**	**0**	**0**	**0**	**0.00**	**0**	**0**	**0**	**0.00**
Ilocos Norte	0	0	0	0	0.00	0	0	0	0.00	0	0	0	0.00

Table 1.E.5.1 - Schistosomiasis Prevention and Control

Suspected Schistosomiasis cases seen, Acute clinically diagnosed cases seen and Acute Confirmed Cases by Stool Examination

Philippines, 2023

Area	Total Patients seen	Number of Suspected Cases				No. of Acute Clinically Diagnosed Cases				No. of Acute Cases Confirmed Positive by Stool Examination			
		Male	Female	Total	%	Male	Female	Total	%	Male	Female	Total	%
Ilocos Sur	0	0	0	0	0.00	0	0	0	0.00	0	0	0	0.00
La Union	0	0	0	0	0.00	0	0	0	0.00	0	0	0	0.00
Pangasinan	0	0	0	0	0.00	0	0	0	0.00	0	0	0	0.00
City of Dagupan	0	0	0	0	0.00	0	0	0	0.00	0	0	0	0.00
**Region 2**	**0**	**0**	**0**	**0**	**0.00**	**0**	**0**	**0**	**0.00**	**0**	**0**	**0**	**0.00**
Batanes	0	0	0	0	0.00	0	0	0	0.00	0	0	0	0.00
Cagayan	0	0	0	0	0.00	0	0	0	0.00	0	0	0	0.00
Isabela	0	0	0	0	0.00	0	0	0	0.00	0	0	0	0.00
Nueva Vizcaya	0	0	0	0	0.00	0	0	0	0.00	0	0	0	0.00
Quirino	0	0	0	0	0.00	0	0	0	0.00	0	0	0	0.00
City of Santiago	0	0	0	0	0.00	0	0	0	0.00	0	0	0	0.00
**Region 3**	**0**	**0**	**0**	**0**	**0.00**	**0**	**0**	**0**	**0.00**	**0**	**0**	**0**	**0.00**
Aurora	0	0	0	0	0.00	0	0	0	0.00	0	0	0	0.00
Bataan	0	0	0	0	0.00	0	0	0	0.00	0	0	0	0.00
Bulacan	0	0	0	0	0.00	0	0	0	0.00	0	0	0	0.00
Nueva Ecija	0	0	0	0	0.00	0	0	0	0.00	0	0	0	0.00
Pampanga	0	0	0	0	0.00	0	0	0	0.00	0	0	0	0.00
Tarlac	0	0	0	0	0.00	0	0	0	0.00	0	0	0	0.00
Zambales	0	0	0	0	0.00	0	0	0	0.00	0	0	0	0.00
City of Angeles	0	0	0	0	0.00	0	0	0	0.00	0	0	0	0.00
City of Olongapo	0	0	0	0	0.00	0	0	0	0.00	0	0	0	0.00
**Region 4A**	**244**	**19**	**16**	**35**	**14.34**	**0**	**0**	**0**	**0.00**	**0**	**0**	**0**	**0.00**
Batangas	2	0	0	0	0.00	0	0	0	0.00	0	0	0	0.00
Cavite	24	1	0	1	68.57	0	0	0	0.00	0	0	0	0.00
Laguna	0	0	0	0	0.00	0	0	0	0.00	0	0	0	0.00
Quezon	127	14	15	29	27.61	0	0	0	0.00	0	0	0	0.00
Rizal	91	4	1	5	5.43	0	0	0	0.00	0	0	0	0.00
City of Lucena	0	0	0	0	0.00	0	0	0	0.00	0	0	0	0.00

Table 1.E.5.1 - Schistosomiasis Prevention and Control

Suspected Schistosomiasis cases seen, Acute clinically diagnosed cases seen and Acute Confirmed Cases by Stool Examination

Philippines, 2023

Area	Total Patients seen	Number of Suspected Cases				No. of Acute Clinically Diagnosed Cases				No. of Acute Cases Confirmed Positive by Stool Examination			
		Male	Female	Total	%	Male	Female	Total	%	Male	Female	Total	%
**Region 4B**	**816**	**11**	**7**	**18**	**2.21**	**1**	**0**	**1**	**5.56**	**0**	**0**	**0**	**0.00**
Marinduque	2	0	0	0	0.00	0	0	0	0.00	0	0	0	0.00
Occidental Mindoro	0	0	0	0	0.00	0	0	0	0.00	0	0	0	0.00
Oriental Mindoro	811	10	7	17	2.10	1	0	1	5.88	0	0	0	0.00
Palawan	1	0	0	0	0.00	0	0	0	0.00	0	0	0	0.00
Romblon	2	1	0	1	50.00	0	0	0	0.00	0	0	0	0.00
City of Puerto Princesa	0	0	0	0	0.00	0	0	0	0.00	0	0	0	0.00
**Region 5**	**22**	**16**	**6**	**22**	**100.00**	**0**	**0**	**0**	**0.00**	**0**	**0**	**0**	**0.00**
Albay	2	2	0	2	100.00	0	0	0	0.00	0	0	0	0.00
Camarines Norte	0	0	0	0	0.00	0	0	0	0.00	0	0	0	0.00
Camarines Sur	0	0	0	0	0.00	0	0	0	0.00	0	0	0	0.00
Catanduanes	0	0	0	0	0.00	0	0	0	0.00	0	0	0	0.00
Masbate	0	0	0	0	0.00	0	0	0	0.00	0	0	0	0.00
Sorsogon	20	14	6	20	100.00	0	0	0	0.00	0	0	0	0.00
City of Naga	0	0	0	0	0.00	0	0	0	0.00	0	0	0	0.00
**Region 6**	**0**	**0**	**0**	**0**	**0.00**	**0**	**0**	**0**	**0.00**	**0**	**0**	**0**	**0.00**
Aklan	0	0	0	0	0.00	0	0	0	0.00	0	0	0	0.00
Antique	0	0	0	0	0.00	0	0	0	0.00	0	0	0	0.00
Capiz	0	0	0	0	0.00	0	0	0	0.00	0	0	0	0.00
Guimaras	0	0	0	0	0.00	0	0	0	0.00	0	0	0	0.00
Iloilo	0	0	0	0	0.00	0	0	0	0.00	0	0	0	0.00
Negros Occidental	0	0	0	0	0.00	0	0	0	0.00	0	0	0	0.00
City of Bacolod	0	0	0	0	0.00	0	0	0	0.00	0	0	0	0.00
City of Iloilo	0	0	0	0	0.00	0	0	0	0.00	0	0	0	0.00
**Region 7**	**21**	**10**	**10**	**20**	**95.24**	**1**	**0**	**1**	**5.00**	**0**	**0**	**0**	**0.00**
Bohol	20	10	10	20	100.00	0	0	0	0.00	0	0	0	0.00
Cebu	0	0	0	0	0.00	0	0	0	0.00	0	0	0	0.00
Negros Oriental	1	0	0	0	0.00	1	0	1	0.00	0	0	0	0.00
Siquijor	0	0	0	0	0.00	0	0	0	0.00	0	0	0	0.00

Table 1.E.5.1 - Schistosomiasis Prevention and Control

Suspected Schistosomiasis cases seen, Acute clinically diagnosed cases seen and Acute Confirmed Cases by Stool Examination

Philippines, 2023

Area	Total Patients seen	Number of Suspected Cases				No. of Acute Clinically Diagnosed Cases				No. of Acute Cases Confirmed Positive by Stool Examination			
		Male	Female	Total	%	Male	Female	Total	%	Male	Female	Total	%
City of Cebu	0	0	0	0	0.00	0	0	0	0.00	0	0	0	0.00
City of Lapu-Lapu	0	0	0	0	0.00	0	0	0	0.00	0	0	0	0.00
City of Mandaue	0	0	0	0	0.00	0	0	0	0.00	0	0	0	0.00
**Region 8**	**59,087**	**8,630**	**8,490**	**17,120**	**28.97**	**94**	**76**	**170**	**0.99**	**82**	**48**	**130**	**76.47**
Biliran	0	0	0	0	0.00	0	0	0	0.00	0	0	0	0.00
Eastern Samar	22,596	20	13	33	0.15	18	11	29	87.88	16	8	24	82.76
Leyte	7,204	65	40	105	1.46	1	1	2	1.90	1	1	2	100.00
Northern Samar	18,178	3,736	3,848	7,584	41.72	62	56	118	1.56	54	31	85	72.03
Southern Leyte	0	0	0	0	0.00	0	0	0	0.00	0	0	0	0.00
Samar	10,394	4,441	4,242	8,683	83.54	8	4	12	0.14	6	4	10	83.33
Ormoc City	0	0	0	0	0.00	0	0	0	0.00	0	0	0	0.00
City of Tacloban	715	368	347	715	100.00	5	4	9	1.26	5	4	9	100.00
**Region 9**	**204**	**131**	**49**	**180**	**88.24**	**130**	**49**	**179**	**99.44**	**7**	**8**	**15**	**8.38**
Zamboanga del Norte	8	2	6	8	100.00	2	6	8	100.00	2	6	8	100.00
Zamboanga del Sur	194	128	42	170	87.63	128	42	170	100.00	5	2	7	4.12
Zamboanga Sibugay	2	1	1	2	100.00	0	1	1	50.00	0	0	0	0.00
City of Zamboanga	0	0	0	0	0.00	0	0	0	0.00	0	0	0	0.00
City of Isabela	0	0	0	0	0.00	0	0	0	0.00	0	0	0	0.00
**Region 10**	**2,582**	**1,390**	**1,146**	**2,536**	**98.22**	**421**	**309**	**730**	**28.79**	**104**	**51**	**155**	**21.23**
Bukidnon	798	479	280	759	95.11	323	187	510	67.19	74	31	105	20.59
Camiguin	0	0	0	0	0.00	0	0	0	0.00	0	0	0	0.00
Lanao del Norte	1,736	882	854	1,736	100.00	84	116	200	11.52	19	16	35	17.50
Misamis Occidental	48	29	12	41	85.42	14	6	20	48.78	11	4	15	75.00
Misamis Oriental	0	0	0	0	0.00	0	0	0	0.00	0	0	0	0.00
City of Cagayan De Oro	0	0	0	0	0.00	0	0	0	0.00	0	0	0	0.00
City of Iligan	0	0	0	0	0.00	0	0	0	0.00	0	0	0	0.00

Table 1.E.5.1 - Schistosomiasis Prevention and Control

Suspected Schistosomiasis cases seen, Acute clinically diagnosed cases seen and Acute Confirmed Cases by Stool Examination

Philippines, 2023

Area	Total Patients seen	Number of Suspected Cases				No. of Acute Clinically Diagnosed Cases				No. of Acute Cases Confirmed Positive by Stool Examination			
		Male	Female	Total	%	Male	Female	Total	%	Male	Female	Total	%
**Region 11**	**3,632**	**366**	**440**	**806**	**22.19**	**46**	**32**	**78**	**9.68**	**21**	**15**	**36**	**46.15**
Davao de Oro	358	150	139	289	80.73	31	18	49	16.96	11	6	17	34.69
Davao del Norte	2,462	50	30	80	3.25	6	3	9	11.25	1	0	1	11.11
Davao Oriental	0	0	0	0	0.00	0	0	0	0.00	0	0	0	0.00
Davao del Sur	1	0	1	1	100.00	0	1	1	100.0 0	0	0	0	0.00
Davao Occidental	89	12	15	27	30.34	9	9	18	66.67	9	9	18	100.00
City of Davao	722	154	255	409	56.65	0	1	1	0.24	0	0	0	0.00
**Region 12**	**229**	**44**	**33**	**77**	**33.62**	**17**	**13**	**30**	**38.96**	**15**	**12**	**27**	**90.00**
Cotabato	65	19	10	29	44.62	14	9	23	79.31	12	8	20	86.96
Sarangani	0	0	0	0	0.00	0	0	0	0.00	0	0	0	0.00
South Cotabato	164	25	23	48	29.27	3	4	7	14.58	3	4	7	100.00
Sultan Kudarat	0	0	0	0	0.00	0	0	0	0.00	0	0	0	0.00
City of General Santos	0	0	0	0	0.00	0	0	0	0.00	0	0	0	0.00
**BARMM**	**1,526**	**820**	**706**	**1,526**	**100.00**	**10**	**9**	**19**	**1.25**	**3**	**8**	**11**	**57.89**
Basilan	0	0	0	0	0.00	0	0	0	0.00	0	0	0	0.00
Lanao del Sur	1,516	813	703	1,516	100.00	3	7	10	0.66	3	7	10	100.00
Maguindanao	9	7	2	9	100.00	7	2	9	100.0 0	0	0	0	0.00
Sulu	0	0	0	0	0.00	0	0	0	0.00	0	0	0	0.00
Tawi-Tawi	0	0	0	0	0.00	0	0	0	0.00	0	0	0	0.00
SGA	1	0	1	1	100.00	0	0	0	0.00	0	1	1	0.00
City of Cotabato	0	0	0	0	0.00	0	0	0	0.00	0	0	0	0.00
**Caraga**	**22,090**	**5,572**	**5,643**	**11,215**	**50.77**	**138**	**60**	**198**	**1.77**	**67**	**37**	**104**	**52.53**
Agusan del Norte	1,427	14	3	17	1.19	5	1	6	35.29	1	0	1	16.67
Agusan del Sur	19,678	5,535	5,628	11,163	56.73	110	49	159	1.42	48	27	75	47.17
Surigao del Norte	628	4	2	6	0.96	4	2	6	100.0 0	2	2	4	66.67
Surigao del Sur	0	0	0	0	0.00	0	0	0	0.00	0	0	0	0.00
Dinagat Islands	2	2	0	2	100.00	2	0	2	100.0 0	2	0	2	100.00

Table 1.E.5.1 - Schistosomiasis Prevention and Control

Suspected Schistosomiasis cases seen, Acute clinically diagnosed cases seen and Acute Confirmed Cases by Stool Examination

Philippines, 2023

Area	Total Patients seen	Number of Suspected Cases				No. of Acute Clinically Diagnosed Cases				No. of Acute Cases Confirmed Positive by Stool Examination			
		Male	Female	Total	%	Male	Female	Total	%	Male	Female	Total	%
City of Butuan	355	17	10	27	7.61	17	8	25	92.59	14	8	22	88.00

Table 1.E.5.2 - Schistosomiasis Prevention and Control

Chronic Clinically Diagnosed Cases and Chronic Clinically diagnosed cases confirmed by stool examination

Philippines, 2023

Area	No. of Clinically Diagnosed Chronic Cases				No. of chronic clinically diagnosed cases confirmed by stool examination (KKT) or rectal biopsy			
	Male	Female	Total	%	Male	Female	Total	%
**Philippines**	**460**	**283**	**743**	**2.21**	**116**	**83**	**199**	**26.78**
**NCR**	**0**	**0**	**0**	**0.00**	**0**	**0**	**0**	**0.00**
City of Malabon	0	0	0	0.00	0	0	0	0.00
City of Navotas	0	0	0	0.00	0	0	0	0.00
City of Valenzuela	0	0	0	0.00	0	0	0	0.00
City of Caloocan	0	0	0	0.00	0	0	0	0.00
City of Marikina	0	0	0	0.00	0	0	0	0.00
City of Pasig	0	0	0	0.00	0	0	0	0.00
Pateros	0	0	0	0.00	0	0	0	0.00
City of Taguig	0	0	0	0.00	0	0	0	0.00
Quezon City	0	0	0	0.00	0	0	0	0.00
City of Makati	0	0	0	0.00	0	0	0	0.00
City of Mandaluyong	0	0	0	0.00	0	0	0	0.00
City of San Juan	0	0	0	0.00	0	0	0	0.00
City of Manila	0	0	0	0.00	0	0	0	0.00
City of Las Piñas	0	0	0	0.00	0	0	0	0.00
City of Muntinlupa	0	0	0	0.00	0	0	0	0.00
City of Parañaque	0	0	0	0.00	0	0	0	0.00
City of Pasay	0	0	0	0.00	0	0	0	0.00
**CAR**	**0**	**0**	**0**	**0.00**	**0**	**0**	**0**	**0.00**
Abra	0	0	0	0.00	0	0	0	0.00
Apayao	0	0	0	0.00	0	0	0	0.00
Benguet	0	0	0	0.00	0	0	0	0.00
Ifugao	0	0	0	0.00	0	0	0	0.00
Kalinga	0	0	0	0.00	0	0	0	0.00
Mountain Province	0	0	0	0.00	0	0	0	0.00
City of Baguio	0	0	0	0.00	0	0	0	0.00
**Region 1**	**0**	**0**	**0**	**0.00**	**0**	**0**	**0**	**0.00**
Ilocos Norte	0	0	0	0.00	0	0	0	0.00

Table 1.E.5.2 - Schistosomiasis Prevention and Control

Chronic Clinically Diagnosed Cases and Chronic Clinically diagnosed cases confirmed by stool examination

Philippines, 2023

Area	No. of Clinically Diagnosed Chronic Cases				No. of chronic clinically diagnosed cases confirmed by stool examination (KKT) or rectal biopsy			
	Male	Female	Total	%	Male	Female	Total	%
Ilocos Sur	0	0	0	0.00	0	0	0	0.00
La Union	0	0	0	0.00	0	0	0	0.00
Pangasinan	0	0	0	0.00	0	0	0	0.00
City of Dagupan	0	0	0	0.00	0	0	0	0.00
**Region 2**	**0**	**0**	**0**	**0.00**	**0**	**0**	**0**	**0.00**
Batanes	0	0	0	0.00	0	0	0	0.00
Cagayan	0	0	0	0.00	0	0	0	0.00
Isabela	0	0	0	0.00	0	0	0	0.00
Nueva Vizcaya	0	0	0	0.00	0	0	0	0.00
Quirino	0	0	0	0.00	0	0	0	0.00
City of Santiago	0	0	0	0.00	0	0	0	0.00
**Region 3**	**0**	**0**	**0**	**0.00**	**0**	**0**	**0**	**0.00**
Aurora	0	0	0	0.00	0	0	0	0.00
Bataan	0	0	0	0.00	0	0	0	0.00
Bulacan	0	0	0	0.00	0	0	0	0.00
Nueva Ecija	0	0	0	0.00	0	0	0	0.00
Pampanga	0	0	0	0.00	0	0	0	0.00
Tarlac	0	0	0	0.00	0	0	0	0.00
Zambales	0	0	0	0.00	0	0	0	0.00
City of Angeles	0	0	0	0.00	0	0	0	0.00
City of Olongapo	0	0	0	0.00	0	0	0	0.00
**Region 4A**	**0**	**0**	0	**0.00**	**0**	**0**	**0**	**0.00**
Batangas	0	0	0	0.00	0	0	0	0.00
Cavite	0	0	0	0.00	0	0	0	0.00
Laguna	0	0	0	0.00	0	0	0	0.00
Quezon	0	0	0	0.00	0	0	0	0.00
Rizal	0	0	0	0.00	0	0	0	0.00
City of Lucena	0	0	0	0.00	0	0	0	0.00

Table 1.E.5.2 - Schistosomiasis Prevention and Control

Chronic Clinically Diagnosed Cases and Chronic Clinically diagnosed cases confirmed by stool examination

Philippines, 2023

Area	No. of Clinically Diagnosed Chronic Cases				No. of chronic clinically diagnosed cases confirmed by stool examination (KKT) or rectal biopsy			
	Male	Female	Total	%	Male	Female	Total	%
**Region 4B**	**1**	**0**	**1**	**5.56**	**1**	**0**	**1**	**100.00**
Marinduque	0	0	0	0.00	0	0	0	0.00
Occidental Mindoro	0	0	0	0.00	0	0	0	0.00
Oriental Mindoro	0	0	0	0.00	0	0	0	0.00
Palawan	1	0	1	0.00	1	0	1	100.00
Romblon	0	0	0	0.00	0	0	0	0.00
City of Puerto Princesa	0	0	0	0.00	0	0	0	0.00
**Region 5**	**2**	**1**	**3**	**13.64**	**0**	**0**	**0**	**0.00**
Albay	0	0	0	0.00	0	0	0	0.00
Camarines Norte	0	0	0	0.00	0	0	0	0.00
Camarines Sur	0	0	0	0.00	0	0	0	0.00
Catanduanes	0	0	0	0.00	0	0	0	0.00
Masbate	0	0	0	0.00	0	0	0	0.00
Sorsogon	2	1	3	15.00	0	0	0	0.00
City of Naga	0	0	0	0.00	0	0	0	0.00
**Region 6**	**0**	**0**	**0**	**0.00**	**0**	**0**	**0**	**0.00**
Aklan	0	0	0	0.00	0	0	0	0.00
Antique	0	0	0	0.00	0	0	0	0.00
Capiz	0	0	0	0.00	0	0	0	0.00
Guimaras	0	0	0	0.00	0	0	0	0.00
Iloilo	0	0	0	0.00	0	0	0	0.00
Negros Occidental	0	0	0	0.00	0	0	0	0.00
City of Bacolod	0	0	0	0.00	0	0	0	0.00
City of Iloilo	0	0	0	0.00	0	0	0	0.00
**Region 7**	**0**	**0**	**0**	**0.00**	**0**	**0**	**0**	**0.00**
Bohol	0	0	0	0.00	0	0	0	0.00
Cebu	0	0	0	0.00	0	0	0	0.00
Negros Oriental	0	0	0	0.00	0	0	0	0.00
Siquijor	0	0	0	0.00	0	0	0	0.00

Table 1.E.5.2 - Schistosomiasis Prevention and Control

Chronic Clinically Diagnosed Cases and Chronic Clinically diagnosed cases confirmed by stool examination

Philippines, 2023

Area	No. of Clinically Diagnosed Chronic Cases				No. of chronic clinically diagnosed cases confirmed by stool examination (KKT) or rectal biopsy			
	Male	Female	Total	%	Male	Female	Total	%
City of Cebu	0	0	0	0.00	0	0	0	0.00
City of Lapu-Lapu	0	0	0	0.00	0	0	0	0.00
City of Mandaue	0	0	0	0.00	0	0	0	0.00
**Region 8**	**142**	**98**	**240**	**1.40**	**17**	**9**	**26**	**10.83**
Biliran	0	0	0	0.00	0	0	0	0.00
Eastern Samar	1	2	3	9.09	0	2	2	66.67
Leyte	0	0	0	0.00	0	0	0	0.00
Northern Samar	135	94	229	3.02	11	5	16	6.99
Southern Leyte	0	0	0	0.00	0	0	0	0.00
Samar	5	1	6	0.07	5	1	6	100.00
Ormoc City	0	0	0	0.00	0	0	0	0.00
City of Tacloban	1	1	2	0.28	1	1	2	100.00
**Region 9**	**0**	**0**	**0**	**0.00**	**0**	**0**	**0**	**0.00**
Zamboanga del Norte	0	0	0	0.00	0	0	0	0.00
Zamboanga del Sur	0	0	0	0.00	0	0	0	0.00
Zamboanga Sibugay	0	0	0	0.00	0	0	0	0.00
City of Zamboanga	0	0	0	0.00	0	0	0	0.00
City of Isabela	0	0	0	0.00	0	0	0	0.00
**Region 10**	**171**	**84**	**255**	**10.06**	**33**	**30**	**63**	**24.71**
Bukidnon	151	76	227	29.91	16	24	40	17.62
Camiguin	0	0	0	0.00	0	0	0	0.00
Lanao del Norte	3	1	4	0.23	3	1	4	100.00
Misamis Occidental	17	7	24	58.54	14	5	19	79.17
Misamis Oriental	0	0	0	0.00	0	0	0	0.00
City of Cagayan De Oro	0	0	0	0.00	0	0	0	0.00
City of Iligan	0	0	0	0.00	0	0	0	0.00

Table 1.E.5.2 - Schistosomiasis Prevention and Control

Chronic Clinically Diagnosed Cases and Chronic Clinically diagnosed cases confirmed by stool examination

Philippines, 2023

Area	No. of Clinically Diagnosed Chronic Cases				No. of chronic clinically diagnosed cases confirmed by stool examination (KKT) or rectal biopsy			
	Male	Female	Total	%	Male	Female	Total	%
**Region 11**	**38**	**28**	**66**	**8.19**	**38**	**28**	**66**	**100.00**
Davao de Oro	6	3	9	3.11	6	3	9	100.00
Davao del Norte	32	25	57	71.25	32	25	57	100.00
Davao Oriental	0	0	0	0.00	0	0	0	0.00
Davao del Sur	0	0	0	0.00	0	0	0	0.00
Davao Occidental	0	0	0	0.00	0	0	0	0.00
City of Davao	0	0	0	0.00	0	0	0	0.00
**Region 12**	**5**	**3**	**8**	**10.39**	**5**	**3**	**8**	**100.00**
Cotabato	5	2	7	24.14	5	2	7	100.00
Sarangani	0	0	0	0.00	0	0	0	0.00
South Cotabato	0	1	1	2.08	0	1	1	100.00
Sultan Kudarat	0	0	0	0.00	0	0	0	0.00
City of General Santos	0	0	0	0.00	0	0	0	0.00
**BARMM**	**10**	**9**	**19**	**1.25**	**3**	**7**	**10**	**52.63**
Basilan	0	0	0	0.00	0	0	0	0.00
Lanao del Sur	3	7	10	0.66	3	7	10	100.00
Maguindanao	7	2	9	100.00	0	0	0	0.00
Sulu	0	0	0	0.00	0	0	0	0.00
Tawi-Tawi	0	0	0	0.00	0	0	0	0.00
SGA	0	0	0	0.00	0	0	0	0.00
City of Cotabato	0	0	0	0.00	0	0	0	0.00
**Caraga**	**91**	**60**	**151**	**1.35**	**19**	**6**	**25**	**16.56**
Agusan del Norte	6	2	8	47.06	3	1	4	50.00
Agusan del Sur	84	56	140	1.25	16	4	20	14.29
Surigao del Norte	1	0	1	16.67	0	0	0	0.00
Surigao del Sur	0	0	0	0.00	0	0	0	0.00
Dinagat Islands	0	0	0	0.00	0	0	0	0.00
City of Butuan	0	2	2	7.41	0	1	1	50.00

Table 1.E.5.3 - Schistosomiasis Prevention and Control

Chronic Clinically Diagnosed cases treated in the Health Facility and Confirmed Chronic Cases referred to a Hospital Facility

Philippines, 2023

Area	No. of Chronic Clinically Diagnosed cases treated in the Health Facility				No. of Confirmed Chronic Cases referred to Hospital Facility			
	Male	Female	Total	%	Male	Female	Total	%
**Philippines**	**241**	**123**	**364**	**53.77**	**14**	**3**	**17**	**8.54**
**NCR**	**0**	**0**	**0**	**0.00**	**0**	**0**	**0**	**0.00**
City of Malabon	0	0	0	0.00	0	0	0	0.00
City of Navotas	0	0	0	0.00	0	0	0	0.00
City of Valenzuela	0	0	0	0.00	0	0	0	0.00
City of Caloocan	0	0	0	0.00	0	0	0	0.00
City of Marikina	0	0	0	0.00	0	0	0	0.00
City of Pasig	0	0	0	0.00	0	0	0	0.00
Pateros	0	0	0	0.00	0	0	0	0.00
City of Taguig	0	0	0	0.00	0	0	0	0.00
Quezon City	0	0	0	0.00	0	0	0	0.00
City of Makati	0	0	0	0.00	0	0	0	0.00
City of Mandaluyong	0	0	0	0.00	0	0	0	0.00
City of San Juan	0	0	0	0.00	0	0	0	0.00
City of Manila	0	0	0	0.00	0	0	0	0.00
City of Las Piñas	0	0	0	0.00	0	0	0	0.00
City of Muntinlupa	0	0	0	0.00	0	0	0	0.00
City of Parañaque	0	0	0	0.00	0	0	0	0.00
City of Pasay	0	0	0	0.00	0	0	0	0.00
**CAR**	**0**	**0**	**0**	**0.00**	**0**	**0**	**0**	**0.00**
Abra	0	0	0	0.00	0	0	0	0.00
Apayao	0	0	0	0.00	0	0	0	0.00
Benguet	0	0	0	0.00	0	0	0	0.00
Ifugao	0	0	0	0.00	0	0	0	0.00
Kalinga	0	0	0	0.00	0	0	0	0.00
Mountain Province	0	0	0	0.00	0	0	0	0.00
City of Baguio	0	0	0	0.00	0	0	0	0.00
**Region 1**	**0**	**0**	**0**	**0.00**	**0**	**0**	**0**	**0.00**
Ilocos Norte	0	0	0	0.00	0	0	0	0.00
Ilocos Sur	0	0	0	0.00	0	0	0	0.00
La Union	0	0	0	0.00	0	0	0	0.00
Pangasinan	0	0	0	0.00	0	0	0	0.00
City of Dagupan	0	0	0	0.00	0	0	0	0.00

Table 1.E.5.3 - Schistosomiasis Prevention and Control
Chronic Clinically Diagnosed cases treated in the Health Facility and Confirmed Chronic Cases referred to a Hospital Facility
Philippines, 2023

Area	No. of Chronic Clinically Diagnosed cases treated in the Health Facility				No. of Confirmed Chronic Cases referred to Hospital Facility			
	Male	Female	Total	%	Male	Female	Total	%
**Region 2**	**0**	**0**	**0**	**0.00**	**0**	**0**	**0**	**0.00**
Batanes	0	0	0	0.00	0	0	0	0.00
Cagayan	0	0	0	0.00	0	0	0	0.00
Isabela	0	0	0	0.00	0	0	0	0.00
Nueva Vizcaya	0	0	0	0.00	0	0	0	0.00
Quirino	0	0	0	0.00	0	0	0	0.00
City of Santiago	0	0	0	0.00	0	0	0	0.00
**Region 3**	**0**	**0**	**0**	**0.00**	**0**	**0**	**0**	**0.00**
Aurora	0	0	0	0.00	0	0	0	0.00
Bataan	0	0	0	0.00	0	0	0	0.00
Bulacan	0	0	0	0.00	0	0	0	0.00
Nueva Ecija	0	0	0	0.00	0	0	0	0.00
Pampanga	0	0	0	0.00	0	0	0	0.00
Tarlac	0	0	0	0.00	0	0	0	0.00
Zambales	0	0	0	0.00	0	0	0	0.00
City of Angeles	0	0	0	0.00	0	0	0	0.00
City of Olongapo	0	0	0	0.00	0	0	0	0.00
**Region 4A**	**0**	**0**	**0**	**0.00**	**0**	**0**	**0**	**0.00**
Batangas	0	0	0	0.00	0	0	0	0.00
Cavite	0	0	0	0.00	0	0	0	0.00
Laguna	0	0	0	0.00	0	0	0	0.00
Quezon	0	0	8	88.89	0	0	0	0.00
Rizal	0	0	0	0.00	0	0	0	0.00
City of Lucena	0	0	0	0.00	0	0	0	0.00
**Region 4B**	**0**	**0**	**0**	**0.00**	**1**	**0**	**1**	**100.00**
Marinduque	0	0	0	0.00	0	0	0	0.00
Occidental Mindoro	0	0	0	0.00	0	0	0	0.00
Oriental Mindoro	0	0	0	0.00	0	0	0	0.00
Palawan	0	0	0	0.00	1	0	1	100.00
Romblon	0	0	0	0.00	0	0	0	0.00
City of Puerto Princesa	0	0	0	0.00	0	0	0	0.00

Table 1.E.5.3 - Schistosomiasis Prevention and Control

Chronic Clinically Diagnosed cases treated in the Health Facility and Confirmed Chronic Cases referred to a Hospital Facility

Philippines, 2023

Area	No. of Chronic Clinically Diagnosed cases treated in the Health Facility				No. of Confirmed Chronic Cases referred to Hospital Facility			
	Male	Female	Total	%	Male	Female	Total	%
**Region 5**	**0**	**0**	**0**	**0.00**	**0**	**0**	**0**	**0.00**
Albay	0	0	0	0.00	0	0	0	0.00
Camarines Norte	0	0	0	0.00	0	0	0	0.00
Camarines Sur	0	0	0	0.00	0	0	0	0.00
Catanduanes	0	0	0	0.00	0	0	0	0.00
Masbate	0	0	0	0.00	0	0	0	0.00
Sorsogon	0	0	0	0.00	0	0	0	0.00
City of Naga	0	0	0	0.00	0	0	0	0.00
**Region 6**	**0**	**0**	**0**	**0.00**	**0**	**0**	**0**	**0.00**
Aklan	0	0	0	0.00	0	0	0	0.00
Antique	0	0	0	0.00	0	0	0	0.00
Capiz	0	0	0	0.00	0	0	0	0.00
Guimaras	0	0	0	0.00	0	0	0	0.00
Iloilo	0	0	0	0.00	0	0	0	0.00
Negros Occidental	0	0	0	0.00	0	0	0	0.00
City of Bacolod	0	0	0	0.00	0	0	0	0.00
City of Iloilo	0	0	0	0.00	0	0	0	0.00
**Region 7**	**0**	**0**	**0**	**0.00**	**0**	**0**	**0**	**0.00**
Bohol	0	0	01	0.00	0	0	0	0.00
Cebu	0	0	0	0.00	0	0	0	0.00
Negros Oriental	0	0	0	0.00	0	0	0	0.00
Siquijor	0	0	0	0.00	0	0	0	0.00
City of Cebu	0	0	0	0.00	0	0	0	0.00
City of Lapu-Lapu	0	0	0	0.00	0	0	0	0.00
City of Mandaue	0	0	0	0.00	0	0	0	0.00
**Region 8**	**65**	**35**	**100**	**64.10**	**3**	**0**	**3**	**11.54**
Biliran	0	0	0	0.00	0	0	0	0.00
Eastern Samar	2	1	3	11.54	0	0	0	0.00
Leyte	0	0	0	0.00	0	0	0	0.00
Northern Samar	52	28	80	79.21	1	0	1	6.25
Southern Leyte	0	0	0	0.00	0	0	0	0.00
Samar	5	1	6	37.50	2	0	2	33.33
Ormoc City	0	0	0	0.00	0	0	0	0.00
City of Tacloban	6	5	11	100.00	0	0	0	0.00

Table 1.E.5.3 - Schistosomiasis Prevention and Control
Chronic Clinically Diagnosed cases treated in the Health Facility and Confirmed Chronic Cases referred to a Hospital Facility
Philippines, 2023

Area	No. of Chronic Clinically Diagnosed cases treated in the Health Facility				No. of Confirmed Chronic Cases referred to Hospital Facility			
	Male	Female	Total	%	Male	Female	Total	%
**Region 9**	**0**	**0**	**0**	**0.00**	**0**	**0**	**0**	**0.00**
Zamboanga del Norte	0	0	0	0.00	0	0	0	0.00
Zamboanga del Sur	0	0	0	0.00	0	0	0	0.00
Zamboanga Sibugay	0	0	0	0.00	0	0	0	0.00
City of Zamboanga	0	0	0	0.00	0	0	0	0.00
City of Isabela	0	0	0	0.00	0	0	0	0.00
**Region 10**	**89**	**37**	**126**	**57.80**	**1**	**0**	**1**	**1.59**
Bukidnon	77	30	107	73.79	1	0	1	2.50
Camiguin	0	0	0	0.00	0	0	0	0.00
Lanao del Norte	5	3	8	20.51	0	0	0	0.00
Misamis Occidental	7	4	11	32.35	0	0	0	0.00
Misamis Oriental	0	0	0	0.00	0	0	0	0.00
City of Cagayan De Oro	0	0	0	0.00	0	0	0	0.00
City of Iligan	0	0	0	0.00	0	0	0	0.00
**Region 11**	**39**	**29**	**68**	**66.67**	**5**	**3**	**8**	**12.12**
Davao de Oro	6	2	8	30.77	1	0	1	11.11
Davao del Norte	33	25	58	100.00	4	3	7	12.28
Davao Oriental	0	0	0	0.00	0	0	0	0.00
Davao del Sur	0	1	1	0.00	0	0	0	0.00
Davao Occidental	0	1	1	5.56	0	0	0	0.00
City of Davao	0	0	0	0.00	0	0	0	0.00
**Region 12**	**18**	**10**	**28**	**80.00**	**2**	**0**	**2**	**25.00**
Cotabato	15	5	20	74.07	2	0	2	28.57
Sarangani	0	0	0	0.00	0	0	0	0.00
South Cotabato	3	5	8	100.00	0	0	0	0.00
Sultan Kudarat	0	0	0	0.00	0	0	0	0.00
City of General Santos	0	0	0	0.00	0	0	0	0.00
**BARMM**	**4**	**1**	**5**	**23.81**	**0**	**0**	**0**	**0.00**
Basilan	0	0	0	0.00	0	0	0	0.00
Lanao del Sur	0	0	0	0.00	0	0	0	0.00

Table 1.E.5.3 - Schistosomiasis Prevention and Control

Chronic Clinically Diagnosed cases treated in the Health Facility and Confirmed Chronic Cases referred to a Hospital Facility
Philippines, 2023

Area	No. of Chronic Clinically Diagnosed cases treated in the Health Facility				No. of Confirmed Chronic Cases referred to Hospital Facility			
	Male	Female	Total	%	Male	Female	Total	%
Maguindanao	4	1	5	0.00	0	0	0	0.00
Sulu	0	0	0	0.00	0	0	0	0.00
Tawi-Tawi	0	0	0	0.00	0	0	0	0.00
SGA	0	0	0	0.00	0	0	0	0.00
City of Cotabato	0	0	0	0.00	0	0	0	0.00
**Caraga**	**26**	**10**	**36**	**27.91**	**2**	**0**	**2**	**8.00**
Agusan del Norte	4	1	5	100.00	1	0	1	25.00
Agusan del Sur	16	6	22	23.16	0	0	0	0.00
Surigao del Norte	1	1	2	50.00	1	0	1	0.00
Surigao del Sur	0	0	0	0.00	0	0	0	0.00
Dinagat Islands	2	0	2	100.00	0	0	0	0.00
City of Butuan	3	2	5	21.74	0	0	0	0.00

Table 2.E.6.1 - Soil Transmitted Helminthiasis Prevention and Control

45.6Children 1-4 who completed 2 doses of deworming tablets

Philippines, 2023

Area	Elig. Pop.	Number 1-4 yrs. Old who completed 2 doses of deworming tablets			
		Male	Female	Total	%
**Philippines**	**9,146,807**	**2,093,642**	**2,077,508**	**4,171,150**	**45.60**
**NCR**	**1,048,668**	**181,656**	**180,316**	**361,972**	**34.52**
City of Malabon	29,472	319	871	1,190	4.04
City of Navotas	20,859	1,471	1,409	2,880	13.81
City of Valenzuela	54,902	19,179	19,031	38,210	69.60
City of Caloocan	138,744	37,262	36,125	73,387	52.89
City of Marikina	34,632	1,154	1,105	2,259	6.52
City of Pasig	65,711	1,081	1,092	2,173	3.31
Pateros	4,824	537	560	1,097	22.74
City of Taguig	74,943	42,194	41,608	83,802	111.82
Quezon City	238,612	1,002	1,002	2,004	0.84
City of Makati	42,518	10,167	9,532	19,699	46.33
City of Mandaluyong	31,561	1,457	1,601	3,058	9.69
City of San Juan	8,619	346	285	631	7.32
City of Manila	139,020	19,401	19,456	38,857	27.95
City of Las Piñas	45,251	5,274	5,308	10,582	23.39
City of Muntinlupa	39,143	20,782	22,044	42,826	109.41
City of Parañaque	53,316	13,424	12,914	26,338	49.40
Pasay City	26,541	6,606	6,373	12,979	48.90
**CAR**	**131,920**	**33,596**	**31,265**	**64,861**	**49.17**
Abra	16,360	6,560	6,165	12,725	77.78
Apayao	9,270	1,848	1,667	3,515	37.92
Benguet	37,084	8,021	7,429	15,450	41.66
Ifugao	17,191	5,317	4,945	10,262	59.69
Kalinga	18,590	3,240	3,061	6,301	33.89
Mountain Province	11,394	4,650	4,246	8,896	78.08
City of Baguio	22,031	3,960	3,752	7,712	35.01
**Region 1**	**391,908**	**81,713**	**82,334**	**164,047**	**41.86**
Ilocos Norte	38,194	12,138	11,423	23,561	61.69
Ilocos Sur	43,632	15,221	14,585	29,806	68.31

Table 2.E.6.1 - Soil Transmitted Helminthiasis Prevention and Control

45.6Children 1-4 who completed 2 doses of deworming tablets

Philippines, 2023

Area	Elig. Pop.	Number 1-4 yrs. Old who completed 2 doses of deworming tablets			
		Male	Female	Total	%
La Union	55,447	20,892	19,803	40,695	73.39
Pangasinan	241,204	32,678	35,768	68,446	28.38
City of Dagupan	13,431	784	755	1,539	11.46
**Region 2**	**273,976**	**75,021**	**71,396**	**146,417**	**53.44**
Batanes	1,131	112	145	257	22.72
Cagayan	90,741	27,676	26,860	54,536	60.10
Isabela	116,264	18,194	17,762	35,956	30.93
Nueva Vizcaya	38,816	15,979	14,451	30,430	78.40
Quirino	15,691	7,323	7,049	14,372	91.59
City of Santiago	11,333	5,737	5,129	10,866	95.88
**Region 3**	**982,471**	**283,498**	**339,491**	**622,989**	**63.41**
Aurora	19,792	8,655	8,472	17,127	86.53
Bataan	70,606	35,997	33,900	69,897	99.00
Bulacan	286,564	73,859	69,843	143,702	50.15
Nueva Ecija	183,214	57,261	52,303	109,564	59.80
Pampanga	191,692	71,256	69,866	141,122	73.62
Tarlac	120,696	12,929	80,824	93,753	77.68
Zambales	49,599	10,992	11,647	22,639	45.64
City of Angeles	41,866	2,961	2,919	5,880	14.04
City of Olongapo	18,442	9,588	9,717	19,305	104.68
**Region 4A**	**1,282,073**	**220,583**	**209,642**	**430,225**	**33.56**
Batangas	228,336	59,082	55,646	114,728	50.25
Cavite	333,432	75,535	73,306	148,841	44.64
Laguna	257,644	34,863	31,691	66,554	25.83
Quezon	164,813	20,506	19,337	39,843	24.17
Rizal	273,505	21,828	20,900	42,728	15.62
City of Lucena	24,343	8,769	8,762	17,531	72.02
**Region 4B**	**273,659**	**78,330**	**74,882**	**153,212**	**55.99**
Marinduque	19,647	7,601	7,111	14,712	74.88

Table 2.E.6.1 - Soil Transmitted Helminthiasis Prevention and Control

45.6Children 1-4 who completed 2 doses of deworming tablets

Philippines, 2023

Area	Elig. Pop.	Number 1-4 yrs. Old who completed 2 doses of deworming tablets			
		Male	Female	Total	%
Occidental Mindoro	47,933	20,156	18,969	39,125	81.62
Oriental Mindoro	75,450	29,805	28,631	58,436	77.45
Palawan	84,854	14,337	13,882	28,219	33.26
Romblon	22,821	6,420	6,278	12,698	55.64
City of Puerto Princesa	22,954	11	11	22	0.10
**Region 5**	**563,991**	**110,849**	**106,107**	**216,956**	**38.47**
Albay	115,352	16,478	15,703	32,181	27.90
Camarines Norte	61,493	7,919	7,685	15,604	25.38
Camarines Sur	179,598	41,147	38,497	79,644	44.35
Catanduanes	24,838	6,337	6,012	12,349	49.72
Masbate	91,183	22,538	22,027	44,565	48.87
Sorsogon	74,885	13,592	13,287	26,879	35.89
City of Naga	16,642	2,838	2,896	5,734	34.45
**Region 6**	**622,616**	**127,081**	**118,877**	**245,958**	**39.50**
Aklan	46,468	18,025	17,261	35,286	75.94
Antique	50,529	10,185	9,318	19,503	38.60
Capiz	61,188	25,995	25,218	51,213	83.70
Guimaras	14,733	4,580	4,248	8,828	59.92
Iloilo	153,196	20,565	19,463	40,028	26.13
Negros Occidental	216,379	36,991	36,552	73,543	33.99
City of Bacolod	43,741	560	580	1,140	2.61
City of Iloilo	36,382	10,180	6,237	16,417	45.12
**Region 7**	**675,230**	**144,563**	**132,297**	**276,860**	**41.00**
Bohol	108,880	37,436	35,256	72,692	66.76
Cebu	292,954	50,164	44,050	94,214	32.16
Negros Oriental	119,479	30,894	27,654	58,548	49.00
Siquijor	6,402	3,329	3,126	6,455	100.83
City of Cebu	78,931	15,810	14,950	30,760	38.97
City of Lapu-Lapu	42,039	4,946	4,723	9,669	23.00
City of Mandaue	26,545	1,984	2,538	4,522	17.04

Table 2.E.6.1 - Soil Transmitted Helminthiasis Prevention and Control

45.6Children 1-4 who completed 2 doses of deworming tablets

Philippines, 2023

Area	Elig. Pop.	Number 1-4 yrs. Old who completed 2 doses of deworming tablets			
		Male	Female	Total	%
**Region 8**	**392,835**	**75,658**	**72,808**	**148,466**	**37.79**
Biliran	15,130	3,006	2,779	5,785	38.24
Eastern Samar	39,097	9,501	9,947	19,448	49.74
Leyte	134,060	24,819	24,017	48,836	36.43
Northern Samar	58,605	12,581	12,569	25,150	42.91
Southern Leyte	32,590	8,876	8,213	17,089	52.44
Samar	70,667	5,017	5,154	10,171	14.39
City of Ormoc	20,482	10,023	8,382	18,405	89.86
City of Tacloban	22,204	1,835	1,747	3,582	16.13
**Region 9**	**352,418**	**116,742**	**120,824**	**237,566**	**67.41**
Zamboanga del Norte	98,637	32,184	35,699	67,883	68.82
Zamboanga del Sur	97,160	39,829	40,330	80,159	82.50
Zamboanga Sibugay	60,678	4,817	4,654	9,471	15.61
City of Isabela	13,695	5,541	5,569	11,110	81.12
City of Zamboanga	82,248	34,371	34,572	68,943	83.82
**Region 10**	**455,672**	**90,572**	**93,979**	**184,551**	**40.50**
Bukidnon	144,267	29,895	26,554	56,449	39.13
Camiguin	7,321	3,592	3,341	6,933	94.70
Lanao del Norte	74,627	10,405	10,492	20,897	28.00
Misamis Occidental	49,512	26,020	33,877	59,897	120.97
Misamis Oriental	88,020	5,015	4,634	9,649	10.96
City of Cagayan De Oro	59,656	603	606	1,209	2.03
City of Iligan	32,269	15,042	14,475	29,517	91.47
**Region 11**	**449,181**	**186,865**	**177,814**	**364,679**	**81.19**
Davao de Oro	66,024	28,075	26,765	54,840	83.06
Davao del Norte	97,797	42,903	41,557	84,460	86.36
Davao Oriental	48,998	21,107	20,426	41,533	84.76
Davao del Sur	59,194	22,012	20,957	42,969	72.59
Davao Occidental	31,702	4,114	4,016	8,130	25.65

Table 2.E.6.1 - Soil Transmitted Helminthiasis Prevention and Control

45.6Children 1-4 who completed 2 doses of deworming tablets

Philippines, 2023

Area	Elig. Pop.	Number 1-4 yrs. Old who completed 2 doses of deworming tablets			
		Male	Female	Total	%
City of Davao	145,466	68,654	64,093	132,747	91.26
**Region 12**	**395,854**	**127,940**	**125,321**	**253,261**	**63.98**
Cotabato	116,778	37,803	36,697	74,500	63.80
Sarangani	54,086	21,671	21,746	43,417	80.27
South Cotabato	86,294	49,450	49,387	98,837	114.54
Sultan Kudarat	78,587	17,137	15,872	33,009	42.00
City of General Santos	60,109	1,879	1,619	3,498	5.82
**BARMM**	**617,557**	**96,610**	**77,073**	**173,683**	**28.12**
Basilan	53,087	2,542	2,527	5,069	9.55
Lanao del Sur	151,974	32,901	33,583	66,484	43.75
Maguindanao	189,608	14,487	13,868	28,355	14.95
Sulu	109,806	11,754	13,501	25,255	23.00
Tawi-Tawi	56,967	22,228	5,403	27,631	48.50
SGA	23,873	103	113	216	0.90
City of Cotabato	32,242	12,595	8,078	20,673	64.12
**Caraga**	**236,778**	**62,365**	**63,082**	**125,447**	**52.98**
Agusan del Norte	32,033	7,356	6,976	14,332	44.74
Agusan del Sur	67,299	17,413	19,327	36,740	54.59
Surigao del Norte	42,408	10,000	9,408	19,408	45.76
Surigao del Sur	55,577	18,990	18,336	37,326	67.16
Dinagat Islands	10,955	1,712	1,637	3,349	30.57
City of Butuan	28,506	6,894	7,398	14,292	50.14

Table 2.E.5.1 - Soil Transmitted Helminthiasis Prevention and Control

Children 5-9 years old who completed 2 doses of deworming tablets

Philippines, 2023

Area	Elig. Pop.	Number 5-9 yrs. Old who completed 2 doses of deworming tablets			
		Male	Female	Total	%
**Philippines**	**11,540,487**	**1,326,552**	**1,392,991**	**2,719,543**	**23.57**
**NCR**	**1,266,961**	**89,253**	**89,239**	**178,492**	**14.09**
City of Malabon	37,412	251	626	877	2.34
City of Navotas	26,458	607	609	1,216	4.60
City of Valenzuela	66,781	8,000	8,731	16,731	25.05
City of Caloocan	169,048	6,159	5,931	12,090	7.15
City of Marikina	43,139	213	192	405	0.94
City of Pasig	80,162	157	154	311	0.39
Pateros	6,024	201	230	431	7.15
City of Taguig	88,058	39,484	38,075	77,559	88.08
Quezon City	282,763	232	269	501	0.18
City of Makati	48,550	2,063	1,888	3,951	8.14
City of Mandaluyong	37,479	575	487	1,062	2.83
City of San Juan	10,144	166	160	326	3.21
City of Manila	170,068	8,124	8,029	16,153	9.50
City of Las Piñas	56,329	1,634	1,469	3,103	5.51
City of Muntinlupa	47,189	8,720	9,050	17,770	37.66
City of Parañaque	61,993	11,357	12,046	23,403	37.75
Pasay City	35,364	1,310	1,293	2,603	7.36
**CAR**	**176,217**	**10,367**	**10,145**	**20,512**	**11.64**
Abra	24,374	2,458	2,597	5,055	20.74
Apayao	12,447	104	90	194	1.56
Benguet	45,884	312	255	567	1.24
Ifugao	22,877	3,289	3,139	6,428	28.10
Kalinga	24,586	1,306	1,288	2,594	10.55
Mountain Province	16,281	2,176	2,080	4,256	26.14
City of Baguio	29,768	722	696	1,418	4.76
**Region 1**	**507,969**	**54,890**	**56,524**	**111,414**	**21.93**
Ilocos Norte	49,811	3,805	3,754	7,559	15.18

Table 2.E.5.1 - Soil Transmitted Helminthiasis Prevention and Control

Children 5-9 years old who completed 2 doses of deworming tablets

Philippines, 2023

Area	Elig. Pop.	Number 5-9 yrs. Old who completed 2 doses of deworming tablets			
		Male	Female	Total	%
Ilocos Sur	60,631	12,332	11,876	24,208	39.93
La Union	73,826	10,167	9,906	20,073	27.19
Pangasinan	306,476	27,598	29,995	57,593	18.79
City of Dagupan	17,225	988	993	1,981	11.50
**Region 2**	**368,957**	**15,655**	**15,141**	**30,796**	**8.35**
Batanes	1,547	53	52	105	6.79
Cagayan	125,812	2,956	2,900	5,856	4.65
Isabela	153,849	2,770	2,523	5,293	3.44
Nueva Vizcaya	51,830	1,752	1,708	3,460	6.68
Quirino	21,346	969	1,140	2,109	9.88
City of Santiago	14,573	7,155	6,818	13,973	95.88
**Region 3**	**1,239,937**	**181,620**	**273,601**	**455,221**	**36.71**
Aurora	25,809	8,241	7,368	15,609	60.48
Bataan	87,766	15,229	14,632	29,861	34.02
Bulacan	366,493	42,792	46,284	89,076	24.30
Nueva Ecija	231,837	31,912	30,811	62,723	27.05
Pampanga	236,484	47,388	52,524	99,912	42.25
Tarlac	155,070	14,579	101,374	115,953	74.77
Zambales	66,902	14,246	13,600	27,846	41.62
City of Angeles	46,182	1,719	1,693	3,412	7.39
City of Olongapo	23,394	5,514	5,315	10,829	46.29
**Region 4A**	**1,609,243**	**141,202**	**132,451**	**273,653**	**17.01**
Batangas	281,141	30,016	27,943	57,959	20.62
Cavite	414,716	52,391	49,286	101,677	24.52
Laguna	323,870	26,747	24,222	50,969	15.74
Quezon	215,362	13,955	13,545	27,500	12.77
Rizal	344,313	15,364	14,766	30,130	8.75
City of Lucena	29,841	2,729	2,689	5,418	18.16
**Region 4B**	**364,479**	**65,071**	**62,672**	**127,743**	**35.05**

Table 2.E.5.1 - Soil Transmitted Helminthiasis Prevention and Control

Children 5-9 years old who completed 2 doses of deworming tablets

Philippines, 2023

Area	Elig. Pop.	Number 5-9 yrs. Old who completed 2 doses of deworming tablets			
		Male	Female	Total	%
Marinduque	25,104	6,094	5,694	11,788	46.96
Occidental Mindoro	62,765	14,582	14,223	28,805	45.89
Oriental Mindoro	100,070	31,871	30,606	62,477	62.43
Palawan	114,310	10,217	9,708	19,925	17.43
Romblon	32,739	2,307	2,441	4,748	14.50
City of Puerto Princesa	29,491	0	0	0	0.00
**Region 5**	**718,752**	**95,842**	**89,239**	**185,081**	**25.75**
Albay	152,223	12,744	12,567	25,311	16.63
Camarines Norte	76,076	6,829	6,270	13,099	17.22
Camarines Sur	222,559	45,353	42,200	87,553	39.34
Catanduanes	31,531	4,480	4,152	8,632	27.38
Masbate	118,110	15,876	13,951	29,827	25.25
Sorsogon	97,373	7,696	7,113	14,809	15.21
City of Naga	20,880	2,864	2,986	5,850	28.02
**Region 6**	**799,446**	**77,792**	**77,327**	**155,119**	**19.40**
Aklan	60,105	25,004	24,732	49,736	82.75
Antique	62,837	5,684	5,516	11,200	17.82
Capiz	76,214	8,512	8,940	17,452	22.90
Guimaras	18,528	4,675	4,346	9,021	48.69
Iloilo	201,181	14,911	14,105	29,016	14.42
Negros Occidental	282,627	16,899	17,569	34,468	12.20
City of Bacolod	55,018	515	579	1,094	1.99
City of Iloilo	42,936	1,592	1,540	3,132	7.29
**Region 7**	**854,076**	**39,566**	**39,782**	**79,348**	**9.29**
Bohol	140,990	2,938	2,861	5,799	4.11
Cebu	371,546	14,948	13,629	28,577	7.69
Negros Oriental	152,055	7,394	7,411	14,805	9.74
Siquijor	9,103	2,462	2,511	4,973	54.63
City of Cebu	94,920	4,359	4,257	8,616	9.08
City of Lapu-Lapu	52,729	2,390	2,370	4,760	9.03
City of Mandaue	32,733	5,075	6,743	11,818	36.10

Table 2.E.5.1 - Soil Transmitted Helminthiasis Prevention and Control

Children 5-9 years old who completed 2 doses of deworming tablets

Philippines, 2023

Area	Elig. Pop.	Number 5-9 yrs. Old who completed 2 doses of deworming tablets			
		Male	Female	Total	%
**Region 8**	**510,779**	**32,388**	**35,987**	**68,375**	**13.39**
Biliran	20,872	1,832	1,844	3,676	17.61
Eastern Samar	51,975	308	347	655	1.26
Leyte	172,238	2,333	2,520	4,853	2.82
Northern Samar	77,943	10,728	10,383	21,111	27.09
Southern Leyte	41,591	3,366	3,133	6,499	15.63
Samar	93,083	2,125	2,159	4,284	4.60
City of Ormoc	25,503	1,648	1,698	3,346	13.12
City of Tacloban	27,574	10,048	13,903	23,951	86.86
**Region 9**	**454,310**	**161,490**	**167,222**	**328,712**	**72.35**
Zamboanga del Norte	123,327	43,549	48,647	92,196	74.76
Zamboanga del Sur	123,611	46,836	47,911	94,747	76.65
Zamboanga Sibugay	82,854	13,144	13,289	26,433	31.90
City of Isabela	15,852	7,104	7,286	14,390	90.78
City of Zamboanga	108,666	50,857	50,089	100,946	92.90
**Region 10**	**586,318**	**75,739**	**80,688**	**156,427**	**26.68**
Bukidnon	193,051	21,674	20,252	41,926	21.72
Camiguin	10,230	2,957	2,649	5,606	54.80
Lanao del Norte	95,067	13,942	15,128	29,070	30.58
Misamis Occidental	63,776	19,409	25,506	44,915	70.43
Misamis Oriental	111,404	5,760	5,296	11,056	9.92
City of Cagayan De Oro	73,402	311	296	607	0.83
City of Iligan	39,388	11,686	11,561	23,247	59.02
**Region 11**	**565,867**	**50,591**	**47,258**	**97,849**	**17.29**
Davao de Oro	86,855	4,956	4,627	9,583	11.03
Davao del Norte	121,301	5,255	5,270	10,525	8.68
Davao Oriental	66,167	3,688	3,570	7,258	10.97
Davao del Sur	70,997	3,776	3,395	7,171	10.10
Davao Occidental	39,583	2,903	2,622	5,525	13.96

Table 2.E.5.1 - Soil Transmitted Helminthiasis Prevention and Control

Children 5-9 years old who completed 2 doses of deworming tablets

Philippines, 2023

Area	Elig. Pop.	Number 5-9 yrs. Old who completed 2 doses of deworming tablets			
		Male	Female	Total	%
City of Davao	180,964	30,013	27,774	57,787	31.93
**Region 12**	**509,412**	**94,416**	**92,179**	**186,595**	**36.63**
Cotabato	151,702	17,673	17,563	35,236	23.23
Sarangani	69,278	18,461	18,447	36,908	53.28
South Cotabato	110,077	35,152	33,600	68,752	62.46
Sultan Kudarat	101,443	21,344	21,084	42,428	41.82
City of General Santos	76,912	1,786	1,485	3,271	4.25
**BARMM**	**683,399**	**94,680**	**80,023**	**174,703**	**25.56**
Basilan	62,390	2,860	2,637	5,497	8.81
Lanao del Sur	162,846	31,941	32,902	64,843	39.82
Maguindanao	200,131	14,501	14,907	29,408	14.69
Sulu	136,276	13,377	14,184	27,561	20.22
Tawi-Tawi	58,175	20,364	6,155	26,519	45.58
SGA	26,540	34	42	76	0.29
City of Cotabato	37,041	11,603	9,196	20,799	56.15
**Caraga**	**324,365**	**45,990**	**43,513**	**89,503**	**27.59**
Agusan del Norte	44,794	6,342	6,135	12,477	27.85
Agusan del Sur	93,557	11,807	10,895	22,702	24.27
Surigao del Norte	57,169	13,175	11,960	25,135	43.97
Surigao del Sur	74,598	5,911	5,648	11,559	15.50
Dinagat Islands	14,122	1,374	1,339	2,713	19.21
City of Butuan	40,125	7,381	7,536	14,917	37.18

Table 2.E.6.2 - Soil Transmitted Helminthiasis Prevention and Control

Adolescents 10-19 and Women 20-49 years old who completed 2 doses of deworming tablets

Philippines, 2023

Area	Elig. Pop.	Number 10-19 yrs. Old who completed 2 doses of deworming tablets				Elig. Pop.	Women 20 - 49 yrs. Old who completed 2 doses of deworming tablets	%
		Male	Female	Total	%			
**Philippines**	**22,092,100**	**1,890,257**	**1,867,812**	**3,758,069**	**17.01**	**23,333,149**	**275,603**	**1.18**
**NCR**	**2,350,929**	**80,007**	**78,014**	**158,021**	**6.72**	**3,252,030**	**13,418**	**0.41**
City of Malabon	72,042	154	347	501	0.70	87,491	27	0.03
City of Navotas	48,936	229	220	449	0.92	54,683	0	0.00
City of Valenzuela	131,897	7,112	7,254	14,366	10.89	170,610	0	0.00
City of Caloocan	315,608	3,361	3,312	6,673	2.11	385,390	4,473	1.16
City of Marikina	81,236	121	130	251	0.31	105,549	436	0.41
City of Pasig	140,495	334	321	655	0.47	196,152	0	0.00
Pateros	11,472	135	161	296	2.58	15,343	1	0.01
City of Taguig	160,685	48,255	45,849	94,104	58.56	220,771	1,998	0.91
Quezon City	511,899	218	206	424	0.08	707,952	1927	0.27
City of Makati	89,568	723	875	1,598	1.78	162,810	1,186	0.73
City of Mandaluyong	67,468	551	418	969	1.44	110,035	46	0.04
City of San Juan	18,743	231	225	456	2.43	31,411	0	0.00
City of Manila	323,671	8,313	8,219	16,532	5.11	440,950	3,267	0.74
City of Las Piñas	105,511	467	474	941	0.89	147,463	0	0.00
City of Muntinlupa	89,738	8,649	8,887	17,536	19.54	127,632	0	0.00
City of Parañaque	111,939	133	139	272	0.24	170,182	57	0.03
Pasay City	70,021	1,021	977	1,998	2.85	117,606	0	0.00
**CAR**	**365,747**	**14,515**	**14,014**	**28,529**	**7.80**	**389,772**	**0**	**0.00**
Abra	51,082	2,376	2,238	4,614	9.03	49,943	0	0.00
Apayao	26,276	346	384	730	2.78	25,741	0	0.00
Benguet	90,230	344	305	649	0.72	100,367	0	0.00
Ifugao	45,351	5,149	5,047	10,196	22.48	42,673	0	0.00
Kalinga	50,417	2,965	2,952	5,917	11.74	47,705	0	0.00
Mountain Province	34,549	2,470	2,260	4,730	13.69	31,322	0	0.00
City of Baguio	67,842	865	828	1,693	2.50	92,021	0	0.00

Table 2.E.6.2 - Soil Transmitted Helminthiasis Prevention and Control
Adolescents 10-19 and Women 20-49 years old who completed 2 doses of deworming tablets
Philippines, 2023

Area	Elig. Pop.	Number 10-19 yrs. Old who completed 2 doses of deworming tablets				Elig. Pop.	Women 20 - 49 yrs. Old who completed 2 doses of deworming tablets	%
		Male	Female	Total	%			
**Region 1**	**1,033,725**	**84,876**	**89,137**	**174,013**	**16.83**	**1,101,925**	**10,080**	**0.91**
Ilocos Norte	110,610	2,125	2,164	4,289	3.88	130,434	728	0.56
Ilocos Sur	131,715	23,378	23,671	47,049	35.72	147,642	6,228	4.22
La Union	153,038	17,306	16,835	34,141	22.31	175,550	2,837	1.62
Pangasinan	602,900	40,294	44,746	85,040	14.11	611,270	287	0.05
City of Dagupan	35,462	1,773	1,721	3,494	9.85	37,029	0	0.00
**Region 2**	**728,786**	**21,788**	**20,427**	**42,215**	**5.79**	**781,565**	**12,007**	**1.54**
Batanes	3,495	114	117	231	6.61	3,803	4	0.11
Cagayan	248,957	4,262	3,892	8,154	3.28	267,668	405	0.15
Isabela	304,267	2,961	2,666	5,627	1.85	330,456	0	0.00
Nueva Vizcaya	99,396	2,527	2,698	5,225	5.26	103,814	0	0.00
Quirino	42,435	95	80	175	0.41	42,961	0	0.00
City of Santiago	30,236	11,829	10,974	22,803	75.42	32,863	11,598	35.29
**Region 3**	**2,427,685**	**247,595**	**256,577**	**504,172**	**20.77**	**2,723,213**	**23,624**	**0.87**
Aurora	50,812	10,116	9,939	20,055	39.47	48,357	11	0.02
Bataan	166,813	9,100	8,792	17,892	10.73	186,777	0	0.00
Bulacan	719,278	65,313	81,768	147,081	20.45	829,793	20,378	2.46
Nueva Ecija	449,332	35,847	31,512	67,359	14.99	491,945	726	0.15
Pampanga	478,672	84,343	81,914	166,257	34.73	544,315	523	0.10
Tarlac	298,044	20,066	20,567	40,633	13.63	313,441	3	0.00
Zambales	130,070	15,481	15,060	30,541	23.48	136,198	1,976	1.45
City of Angeles	86,516	1,911	1,824	3,735	4.32	111,726	7	0.01
City of Olongapo	48,148	5,418	5,201	10,619	22.05	60,661	0	0.00
**Region 4A**	**3,096,849**	**175,354**	**167,352**	**342,706**	**11.07**	**3,714,166**	**3,706**	**0.10**
Batangas	558,191	44,527	41,658	86,185	15.44	657,327	437	0.07
Cavite	802,360	60,780	58,372	119,152	14.85	1,035,486	747	0.07
Laguna	613,299	34,531	32,469	67,000	10.92	810,512	1,654	0.20
Quezon	419,468	21,097	20,095	41,192	9.82	387,179	40	0.01
Rizal	646,427	12,021	12,125	24,146	3.74	763,636	548	0.07

Table 2.E.6.2 - Soil Transmitted Helminthiasis Prevention and Control

Adolescents 10-19 and Women 20-49 years old who completed 2 doses of deworming tablets

Philippines, 2023

Area	Elig. Pop.	Number 10-19 yrs. Old who completed 2 doses of deworming tablets				Elig. Pop.	Women 20 - 49 yrs. Old who completed 2 doses of deworming tablets	%
		Male	Female	Total	%			
City of Lucena	57,104	2,398	2,633	5,031	8.81	60,026	280	0.47
**Region 4B**	**713,293**	**105,649**	**102,132**	**207,781**	**29.13**	**646,146**	**22,864**	**3.54**
Marinduque	51,888	10,698	10,215	20,913	40.30	43,352	0	0.00
Occidental Mindoro	119,082	19,282	18,545	37,827	31.77	102,103	0	0.00
Oriental Mindoro	197,315	56,641	55,507	112,148	56.84	184,492	22,700	12.30
Palawan	213,388	15,758	14,781	30,539	14.31	186,141	164	0.09
Romblon	69,602	3,270	3,084	6,354	9.13	57,995	0	0.00
City of Puerto Princesa	62,018	0	0	0	0.00	72,063	0	0.00
**Region 5**	**1,401,417**	**139,658**	**129,836**	**269,494**	**19.23**	**1,158,144**	**4,912**	**0.42**
Albay	308,726	20,613	19,377	39,990	12.95	274,245	1,141	0.42
Camarines Norte	140,206	11,618	10,700	22,318	15.92	121,500	753	0.62
Camarines Sur	434,240	64,470	60,433	124,903	28.76	348,071	2,113	0.61
Catanduanes	62,144	1,934	1,863	3,797	6.11	50,147	310	0.62
Masbate	224,156	25,377	22,942	48,319	21.56	160,298	0	0.00
Sorsogon	187,573	12,641	11,703	24,344	12.98	156,964	541	0.34
City of Naga	44,372	3,005	2,818	5,823	13.12	46,919	54	0.12
**Region 6**	**1,573,241**	**125,759**	**120,009**	**245,768**	**15.62**	**1,596,545**	**35,103**	**2.20**
Aklan	121,245	48,045	46,186	94,231	77.72	126,494	16,612	13.13
Antique	123,564	8,730	8,355	17,085	13.83	117,381	36	0.03
Capiz	157,150	10,898	11,023	21,921	13.95	163,635	2,729	1.67
Guimaras	35,434	9,118	8,533	17,651	49.81	38,508	10	0.03
Iloilo	400,929	24,766	23,649	48,415	12.08	412,021	2,244	0.54
Negros Occidental	544,526	21,592	19,669	41,261	7.58	508,032	13,370	2.63
City of Bacolod	111,224	658	584	1,242	1.12	129,677	102	0.08
City of Iloilo	79,169	1,952	2,010	3,962	5.00	100,797	0	0.00
**Region 7**	**1,640,051**	**36,429**	**35,730**	**72,159**	**4.40**	**1,713,885**	**18,049**	**1.05**
Bohol	289,867	1,879	1,715	3,594	1.24	270,329	365	0.14

Table 2.E.6.2 - Soil Transmitted Helminthiasis Prevention and Control

Adolescents 10-19 and Women 20-49 years old who completed 2 doses of deworming tablets

Philippines, 2023

Area	Elig. Pop.	Number 10-19 yrs. Old who completed 2 doses of deworming tablets				Elig. Pop.	Women 20 - 49 yrs. Old who completed 2 doses of deworming tablets	%
		Male	Female	Total	%			
Cebu	689,816	19,955	20,029	39,984	5.80	691,568	30	0.00
Negros Oriental	291,835	7,041	6,495	13,536	4.64	287,222	1,347	0.47
Siquijor	18,833	4,203	4,022	8,225	43.67	20,724	11,054	53.34
City of Cebu	184,895	2,123	2,296	4,419	2.39	226,860	4,555	2.01
City of Lapu-Lapu	99,001	1,217	1,157	2,374	2.40	124,825	357	0.29
City of Mandaue	65,804	11	16	27	0.04	92,357	341	0.37
**Region 8**	**998,652**	**51,064**	**55,737**	**106,801**	**10.69**	**863,980**	**14,325**	**1.66**
Biliran	39,658	1,448	1,917	3,365	8.49	33,281	0	0.00
Eastern Samar	106,612	468	425	893	0.84	89,302	2,720	3.05
Leyte	330,340	2,777	2,923	5,700	1.73	291,718	129	0.04
Northern Samar	153,658	20,845	19,853	40,698	26.49	116,473	10,776	9.25
Southern Leyte	87,160	5,415	5,256	10,671	12.24	82,250	74	0.09
Samar	183,123	2,922	3,029	5,951	3.25	148,370	396	0.27
Ormoc City	47,590	2,942	4,017	6,959	14.62	48,110	0	0.00
City of Tacloban	50,511	14,247	18,317	32,564	64.47	54,476	230	0.42
**Region 9**	**845,152**	**262,676**	**275,874**	**538,550**	**63.72**	**775,559**	**2,203**	**0.28**
Zamboanga del Norte	228,135	78,762	84,876	163,638	71.73	200,171	800	0.40
Zamboanga del Sur	232,591	79,199	82,146	161,345	69.37	205,556	264	0.13
Zamboanga Sibugay	153,599	18,204	18,301	36,505	23.77	130,593	10	0.01
City of Isabela	28,477	10,950	10,987	21,937	77.03	26,481	250	0.94
City of Zamboanga	202,350	75,561	79,564	155,125	76.66	212,758	879	0.41
**Region 10**	**1,080,119**	**129,491**	**136,325**	**265,816**	**24.61**	**1,033,116**	**11,384**	**1.10**
Bukidnon	347,189	35,827	33,650	69,477	20.01	309,473	472	0.15
Camiguin	19,153	5,683	5,170	10,853	56.66	17,449	0	0.00
Lanao del Norte	168,789	25,948	26,273	52,221	30.94	146,337	63	0.04
Misamis Occidental	125,805	32,593	42,087	74,680	59.36	122,665	524	0.43
Misamis Oriental	204,355	9,751	8,991	18,742	9.17	190,618	6	0.00
City of Cagayan De Oro	138,975	664	620	1,284	0.92	169,407	0	0.00
City of Iligan	75,853	19,025	19,534	38,559	50.83	77,167	10,319	13.37

Table 2.E.6.2 - Soil Transmitted Helminthiasis Prevention and Control

Adolescents 10-19 and Women 20-49 years old who completed 2 doses of deworming tablets

Philippines, 2023

Area	Elig. Pop.	Number 10-19 yrs. Old who completed 2 doses of deworming tablets				Elig. Pop.	Women 20 - 49 yrs. Old who completed 2 doses of deworming tablets	%
		Male	Female	Total	%			
**Region 11**	**1,069,862**	**45,985**	**40,399**	**86,384**	**8.07**	**1,104,925**	**20,101**	**1.82**
Davao de Oro	167,660	5,544	5,256	10,800	6.44	152,210	3,830	2.52
Davao del Norte	224,538	2,715	2,703	5,418	2.41	238,752	2,143	0.90
Davao Oriental	126,834	2,908	2,648	5,556	4.38	110,127	1,560	1.42
Davao del Sur	135,874	3,428	3,165	6,593	4.85	140,675	2,926	2.08
Davao Occidental	71,106	4,829	4,594	9,423	13.25	58,144	3,825	6.58
City of Davao	343,850	26,561	22,033	48,594	14.13	405,017	5,817	1.44
**Region 12**	**952,092**	**148,628**	**147,788**	**296,416**	**31.13**	**915,349**	**80,317**	**8.77**
Cotabato	281,805	22,104	22,863	44,967	15.96	260,975	333	0.13
Sarangani	128,236	31,401	32,056	63,457	49.48	112,127	3,544	3.16
South Cotabato	207,259	51,955	51,194	103,149	49.77	205,661	3,547	1.72
Sultan Kudarat	192,822	40,886	39,655	80,541	41.77	176,963	72,607	41.03
City of General Santos	141,970	2,282	2,020	4,302	3.03	159,623	286	0.18
**BARMM**	**1,203,744**	**146,463**	**127,181**	**273,644**	**22.73**	**1,012,036**	**287**	**0.03**
Basilan	102,910	3,831	4,460	8,291	8.06	83,314	0	0.00
Lanao del Sur	298,349	50,885	52,669	103,554	34.71	243,371	0	0.00
Maguindanao	329,657	20,228	21,085	41,313	12.53	267,846	125	0.05
Sulu	254,360	20,320	21,888	42,208	16.59	206,019	0	0.00
Tawi-Tawi	98,079	35,598	9,736	45,334	46.22	89,777	0	0.00
SGA	47,703	96	126	222	0.47	45,679	0	0.00
City of Cotabato	72,686	15,505	17,217	32,722	45.02	76,030	162	0.21
**Caraga**	**610,756**	**74,320**	**71,280**	**145,600**	**23.84**	**550,793**	**3,223**	**0.59**
Agusan del Norte	84,785	11,705	11,111	22,816	26.91	75,598	0	0.00
Agusan del Sur	168,869	15,062	13,786	28,848	17.08	139,914	0	0.00
Surigao del Norte	112,617	24,573	23,424	47,997	42.62	108,437	3,165	2.92
Surigao del Sur	138,529	8,797	8,134	16,931	12.22	124,366	0	0.00

Table 2.E.6.2 - Soil Transmitted Helminthiasis Prevention and Control

Adolescents 10-19 and Women 20-49 years old who completed 2 doses of deworming tablets

Philippines, 2023

Area	Elig. Pop.	Number 10-19 yrs. Old who completed 2 doses of deworming tablets				Elig. Pop.	Women 20 - 49 yrs. Old who completed 2 doses of deworming tablets	%
		Male	Female	Total	%			
Dinagat Islands	27,122	1,763	1,905	3,668	13.52	23,864	58	0.24
City of Butuan	78,834	12,420	12,920	25,340	32.14	78,614	0	0.00

Table 2.E6.3 - Soil Transmitted Helminthiasis Prevention and Control

Pregnant Women who completed 1 dose of deworming tablets

Philippines, 2023

Area	Elig. Pop	Pregnant Women who completed 1 dose of deworming tablets	
		Number	%
**Philippines**	**2,187,333**	**240,598**	**11.00**
**NCR**	**261,228**	**14,765**	**5.65**
City of Malabon	7,385	13	0.18
City of Navotas	5,210	7	0.13
City of Valenzuela	13,477	103	0.76
City of Caloocan	33,168	0	0.00
City of Marikina	8,342	1,462	17.53
City of Pasig	17,682	12	0.07
Pateros	1,116	82	7.35
City of Taguig	18,992	4,983	26.24
City of Quezon	59,136	6,131	10.37
City of Makati	10,944	145	1.32
City of Mandaluyong	7,922	0	0.00
City of San Juan	2,168	18	0.83
City of Manila	34,128	7	0.02
City of Las Piñas	11,263	409	3.63
City of Muntinlupa	9,881	0	0.00
City of Parañaque	13,280	940	7.08
City of Pasay	7,134	453	6.35
**CAR**	**31,319**	**5,635**	**17.99**
Abra	3,660	384	10.49
Apayao	2,238	928	41.47
Benguet	8,806	213	2.42
Ifugao	4,098	1,427	34.82
Kalinga	4,549	1,446	31.79
Mountain Province	2,619	102	3.89
City of Baguio	5,349	1,135	21.22
**Region 1**	**96,016**	**12,668**	**13.19**
Ilocos Norte	8,912	2,279	25.57
Ilocos Sur	9,914	669	6.75
La Union	13,507	2,330	17.25

Table 2.E6.3 - Soil Transmitted Helminthiasis Prevention and Control

Pregnant Women who completed 1 dose of deworming tablets

Philippines, 2023

Area	Elig. Pop	Pregnant Women who completed 1 dose of deworming tablets	
		Number	%
Pangasinan	60,360	7,390	12.24
City of Dagupan	3,323	0	0.00
**Region 2**	**63,967**	**7,514**	**11.75**
Batanes	240	37	15.42
Cagayan	20,786	1,697	8.16
Isabela	27,379	3,691	13.48
Nueva Vizcaya	8,798	1,282	14.57
Quirino	3,839	7	0.18
City of Santiago	2,925	800	27.35
**Region 3**	**233,544**	**5,817**	**2.49**
Aurora	4,537	11	0.24
Bataan	17,380	0	0.00
Bulacan	67,590	200	0.30
Nueva Ecija	43,425	282	0.65
Pampanga	45,651	9	0.02
Tarlac	29,503	5,052	17.12
Zambales	11,723	261	2.23
City of Angeles	9,146	2	0.02
City of Olongapo	4,589	0	0.00
**Region 4A**	**307,982**	**12,030**	**3.91**
Batangas	53,251	307	0.58
Cavite	77,964	1,622	2.08
Laguna	61,525	7,344	11.94
Quezon	40,810	1,226	3.00
Rizal	68,235	1,481	2.17
City of Lucena	6,197	50	0.81
**Region 4B**	**66,121**	**6,237**	**9.43**
Marinduque	4,144	537	12.96
Occidental Mindoro	11,640	432	3.71

Table 2.E6.3 - Soil Transmitted Helminthiasis Prevention and Control

Pregnant Women who completed 1 dose of deworming tablets

Philippines, 2023

Area	Elig. Pop	Pregnant Women who completed 1 dose of deworming tablets	
		Number	%
Oriental Mindoro	18,943	1,977	10.44
Palawan	20,317	2,784	13.70
Romblon	5,489	507	9.24
City of Puerto Princesa	5,588	0	0.00
**Region 5**	**135,480**	**25,016**	**18.46**
Albay	27,440	2,651	9.66
Camarines Norte	14,695	4,649	31.64
Camarines Sur	43,452	6,962	16.02
Catanduanes	5,414	1,548	28.59
Masbate	22,365	4,530	20.25
Sorsogon	17,929	4,113	22.94
City of Naga	4,185	563	13.45
**Region 6**	**148,020**	**11,627**	**7.86**
Aklan	11,283	1,322	11.72
Antique	11,930	2,785	23.34
Capiz	13,931	728	5.23
Guimaras	3,351	3,275	97.73
Iloilo	36,140	3,502	9.69
Negros Occidental	52,270	0	0.00
City of Bacolod	10,373	15	0.14
City of Iloilo	8,742	0	0.00
**Region 7**	**165,673**	**35,506**	**21.43**
Bohol	26,235	4,830	18.41
Cebu	72,201	13,148	18.21
Negros Oriental	28,722	2,014	7.01
Siquijor	1,680	623	37.08
City of Cebu	19,267	8,909	46.24
City of Lapu-Lapu	10,679	1,031	9.65
City of Mandaue	6,889	4,951	71.87

Table 2.E6.3 - Soil Transmitted Helminthiasis Prevention and Control

Pregnant Women who completed 1 dose of deworming tablets

Philippines, 2023

Area	Elig. Pop	Pregnant Women who completed 1 dose of deworming tablets	
		Number	%
**Region 8**	**93,813**	**11,944**	**12.73**
Biliran	3,373	1,496	44.35
Eastern Samar	9,624	1,992	20.70
Leyte	31,559	1,156	3.66
Northern Samar	14,056	1,321	9.40
Southern Leyte	7,406	1,834	24.76
Samar	17,011	1,812	10.65
Ormoc City	5,292	1,917	36.22
City of Tacloban	5,492	416	7.57
**Region 9**	**84,815**	**10,949**	**12.91**
Zamboanga del Norte	24,841	3,104	12.50
Zamboanga del Sur	23,757	1,062	4.47
Zamboanga Sibugay	13,841	1,217	8.79
City of Isabela	19,175	697	3.63
City of Zamboanga	3,201	4,869	152.11
**Region 10**	**109,169**	**11,063**	**10.13**
Bukidnon	33,403	4,459	13.35
Camiguin	1,727	261	15.11
Lanao del Norte	17,760	1,556	8.76
Misamis Occidental	12,328	618	5.01
Misamis Oriental	21,975	717	3.26
City of Cagayan De Oro	14,308	0	0.00
City of Iligan	7,668	3,452	45.02
**Region 11**	**107,001**	**27,258**	**25.47**
Davao de Oro	15,253	6,190	40.58
Davao del Norte	23,453	6,136	26.16
Davao Oriental	11,935	4,257	35.67
Davao del Sur	13,083	1,440	11.01
Davao Occidental	6,629	2,567	38.72
City of Davao	36,648	6,668	18.19

Table 2.E6.3 - Soil Transmitted Helminthiasis Prevention and Control

Pregnant Women who completed 1 dose of deworming tablets

Philippines, 2023

Area	Elig. Pop	Pregnant Women who completed 1 dose of deworming tablets	
		Number	%
**Region 12**	**95,279**	**31,178**	**32.72**
Cotabato	26,521	4,005	15.10
Sarangani	13,322	8,661	65.01
South Cotabato	21,595	6,760	31.30
Sultan Kudarat	19,391	10,025	51.70
City of General Santos	14,450	1,727	11.95
**BARMM**	**132,841**	**8,692**	**6.54**
Basilan	11,636	246	2.11
Lanao del Sur	33,374	3,475	10.41
Maguindanao	41,650	1,643	3.94
Sulu	21,378	1,618	7.57
Tawi-Tawi	12,609	993	7.88
SGA	5,103	57	1.12
City of Cotabato	7,091	660	9.31
**Caraga**	**55,065**	**2,699**	**4.90**
Agusan del Norte	7,286	1	0.01
Agusan del Sur	16,038	404	2.52
Surigao del Norte	10,186	925	9.08
Surigao del Sur	12,736	1,231	9.67
Dinagat Islands	2,134	125	5.86
City of Butuan	6,685	13	0.19

Table 2.E.7.1 - Tuberculosis
Treatment Success Rate DSTB - All Forms
PHILIPPINES, 2023

Area	TB all forms cured and Completely Treated			Registered TB all forms Registered during Specified Period			Treatment Success Rate
	Male	Female	Total	Male	Female	Total	
**Philippines**	**229,982**	**128,690**	**358,672**	**249,208**	**137,873**	**387,081**	**92.66**
**N C R**	**37,699**	**21,804**	**59,503**	**41,252**	**23,450**	**64,702**	**91.96**
Malabon City	1,631	879	2,510	1,880	988	2,868	87.52
Navotas City	881	464	1,345	987	502	1,489	90.33
Valenzuela City	2,090	1,281	3,371	2,336	1,359	3,695	91.23
Caloocan City	3,868	2,550	6,418	4,200	2,732	6,932	92.59
Marikina City	1,249	670	1,919	1,379	742	2,121	90.48
Pasig City	2,060	1,374	3,434	2,333	1,534	3,867	88.80
Pateros	127	71	198	143	75	218	90.83
Taguig City	2,903	1,764	4,667	3,036	1,836	4,872	95.79
Quezon City	7,815	4,593	12,408	8,412	4,936	13,348	92.96
Makati City	1,271	790	2,061	1,371	847	2,218	92.92
Mandaluyong City	1,384	648	2,032	1,558	714	2,272	89.44
San Juan City	242	146	388	275	157	432	89.81
Manila City	6,678	3,769	10,447	7,228	3,991	11,219	93.12
Las Piñas City	1,106	640	1,746	1,250	695	1,945	89.77
Muntinlupa City	1,721	579	2,300	1,973	644	2,617	87.89
Parañaque City	1,517	849	2,366	1,623	897	2,520	93.89
Pasay City	1,156	737	1,893	1,268	801	2,069	91.49
**CAR**	**2,085**	**1,014**	**3,099**	**2,276**	**1,113**	**3,389**	91.44
Abra	501	202	703	543	222	765	91.90
Apayao	211	106	317	232	115	347	91.35
Benguet	244	97	341	261	110	371	91.91
Ifugao	215	113	328	221	118	339	96.76
Kalinga	331	151	482	355	160	515	93.59
Mt. Province	179	104	283	198	112	310	91.29
Baguio City	404	241	645	466	276	742	86.93
**Region 1**	**9,980**	**13,173**	**23,153**	**10,543**	**13,866**	**24,409**	94.85
Ilocos Norte	734	301	1,035	815	330	1,145	90.39
Ilocos Sur	1,027	596	1,623	1,086	623	1,709	94.97
La Union	1,756	988	2,744	1,898	1,044	2,942	93.27
Pangasinan	5,920	3,242	9,162	6,162	3,356	9,518	96.26
Dagupan City	543	282	825	582	302	884	93.33

Table 2.E.7.1 - Tuberculosis
Treatment Success Rate DSTB - All Forms
PHILIPPINES, 2023

Area	TB all forms cured and Completely Treated			Registered TB all forms Registered during Specified Period			Treatment Success Rate
	Male	Female	Total	Male	Female	Total	
**Region 2**	**7,150**	**4,111**	**11,261**	**7,640**	**4,343**	**11,983**	93.97
Batanes	28	29	57	36	31	67	85.07
Cagayan	2,350	1,373	3,723	2,491	1,427	3,918	95.02
Isabela	3,278	1,922	5,200	3,506	2,044	5,550	93.69
Nueva Vizcaya	663	329	992	736	366	1,102	90.02
Quirino	308	175	483	326	184	510	94.71
Santiago City	523	283	806	545	291	836	96.41
**Region 3**	**25,344**	**13,738**	**39,082**	**27,272**	**14,683**	**41,955**	93.15
Aurora	641	325	966	680	344	1,024	94.34
Bataan	2,425	1,311	3,736	2,560	1,364	3,924	95.21
Bulacan	8,263	4,738	13,001	8,856	5,054	13,910	93.47
Nueva Ecija	4,011	1,977	5,988	4,248	2,079	6,327	94.64
Pampanga	4,265	2,295	6,560	4,624	2,455	7,079	92.67
Tarlac	2,566	1,188	3,754	2,726	1,274	4,000	93.85
Zambales	1,797	1,180	2,977	2,026	1,316	3,342	89.08
Angeles City	721	**341**	1,062	779	**361**	**1,140**	93.16
Olongapo City	655	383	1,038	773	436	1,209	85.86
**Region 4A**	**34,746**	**19,002**	**53,748**	**37,290**	**20,219**	**57,509**	93.46
Batangas	6,143	3,442	9,585	6,511	3,586	10,097	94.93
Cavite	9,111	4,945	14,056	9,788	5,282	15,070	93.27
Laguna	7,599	4,178	11,777	7,992	4,370	12,362	95.27
Quezon	3,934	1,979	5,913	4,292	2,157	6,449	91.69
Rizal	7,254	4,115	11,369	7,878	4,415	12,293	92.48
Lucena City	705	343	1,048	829	409	1,238	84.65
**Region 4B**	**8,060**	**4,378**	**12,438**	**8,954**	**4,832**	**13,786**	90.22
Marinduque	695	415	1,110	804	478	1,282	86.58
Mindoro Occidental	1,326	637	1,963	1,571	772	2,343	83.78
Mindoro Oriental	2,655	1,536	4,191	2,790	1,598	4,388	95.51
Palawan	1,805	1,009	2,814	2,057	1,126	3,183	88.41
Romblon	775	406	1,181	874	459	1,333	88.60
Puerto Princesa City	804	375	1,179	858	399	1,257	93.79
**Region 5**	**15,797**	**8,284**	**24,081**	**17,349**	**9,045**	**26,394**	91.24
Albay	3,738	**1,945**	5,683	4,280	2,253	6,533	86.99
Camarines Norte	1,735	692	2,427	1,928	752	2,680	90.56

Table 2.E.7.1 - Tuberculosis
Treatment Success Rate DSTB - All Forms
PHILIPPINES, 2023

Area	TB all forms cured and Completely Treated			Registered TB all forms Registered during Specified Period			Treatment Success Rate
	Male	Female	Total	Male	Female	Total	
Camarines Sur	4,781	2,622	7,403	5,184	2,828	8,012	92.40
Catanduanes	684	362	1,046	742	376	1,118	93.56
Masbate	2,146	1,207	3,353	2,323	1,296	3,619	92.65
Sorsogon	2,131	1,098	3,229	2,272	1,162	3,434	94.03
Naga City	582	358	940	620	378	998	94.19
**Region 6**	**21,783**	**10,430**	**32,213**	**22,949**	**10,908**	**33,857**	95.14
Aklan	1,389	704	2,093	1,510	748	2,258	92.69
Antique	1,582	849	2,431	1,698	897	2,595	93.68
Capiz	1,901	997	2,898	1,991	1,048	3,039	95.36
Guimaras	367	180	547	375	185	560	97.68
Iloilo	4,677	2,085	6,762	4,899	2,185	7,084	95.45
Negros Occidental	8,300	3,914	12,214	8,661	4,043	12,704	96.14
Bacolod City	1,994	948	2,942	2,153	1,007	3,160	93.10
Iloilo City	1,573	753	2,326	1,662	795	2,457	94.67
**Region 7**	**15,423**	**7,623**	**23,046**	**17,114**	**8,332**	**25,446**	90.57
Bohol	2,117	1,130	3,247	2,297	1,206	3,503	92.69
Cebu	5,826	2,804	8,630	6,300	3,009	9,309	92.71
Negros Oriental	2,673	1,332	4,005	2,911	1,429	4,340	92.28
Siquijor	211	78	289	229	81	310	93.23
Cebu City	2,634	**1,312**	3,946	3,125	1,516	4,641	85.02
Lapu-Lapu City	1,139	611	1,750	1,292	687	1,979	88.43
Mandaue City	823	356	1,179	960	404	1,364	86.44
**Region 8**	**8,067**	**4,293**	**12,360**	**8,889**	**4,693**	**13,582**	91.00
Biliran	454	204	658	489	218	707	93.07
Eastern Samar	718	386	1,104	768	415	1,183	93.32
Northern Leyte	2,629	1,257	3,886	2,919	1,379	4,298	90.41
Northern Samar	1,090	652	1,742	1,219	722	1,941	89.75
Southern Leyte	702	**386**	1,088	743	411	1,154	94.28
Western Samar	1,245	704	1,949	1,365	767	2,132	91.42
Ormoc City	455	229	684	533	264	797	85.82
Tacloban City	774	475	1,249	853	517	1,370	91.17
**Region 9**	**7,176**	**3,177**	**10,353**	**7,797**	**3,385**	**11,182**	92.59
Zamboanga del Norte	1,638	746	2,384	1,739	776	2,515	94.79
Zamboanga del Sur	1,818	814	2,632	2,076	877	2,953	89.13
Zamboanga Sibugay	1,175	479	1,654	1,293	531	1,824	90.68

Table 2.E.7.1 - Tuberculosis
Treatment Success Rate DSTB - All Forms
PHILIPPINES, 2023

Area	TB all forms cured and Completely Treated			Registered TB all forms Registered during Specified Period			Treatment Success Rate
	Male	Female	Total	Male	Female	Total	
Isabela City	148	77	225	159	82	241	93.36
Zamboanga City	2,397	**1,061**	3,458	2,530	1,119	3,649	94.77
**Region 10**	**8,410**	**3,846**	**12,256**	**9,008**	**4,093**	**13,101**	93.55
Bukidnon	1,629	729	2,358	1,737	778	2,515	93.76
Camiguin	124	47	171	136	48	184	92.93
Lanao del Norte	1,190	622	1,812	1,236	648	1,884	96.18
Misamis Occidental	927	392	1,319	1,030	441	1,471	89.67
Misamis Oriental	1,873	771	2,644	1,956	798	2,754	96.01
Cagayan de Oro City	1,602	817	2,419	1,743	869	2,612	92.61
Iligan City	1,065	468	1,533	1,170	511	1,681	91.20
**Region 11**	**9,414**	**4,051**	**13,465**	**10,498**	**4,437**	**14,935**	90.16
Davao de Oro	1,268	417	1,685	1,395	445	1,840	91.58
Davao del Norte	2,414	943	3,357	2,720	1,050	3,770	89.05
Davao Oriental	1,191	525	1,716	1,262	548	1,810	94.81
Davao del Sur	1,016	455	1,471	1,057	465	1,522	96.65
Davao Occidental	391	189	580	416	197	613	94.62
Davao City	3,134	1,522	4,656	3,648	1,732	5,380	86.54
**Region 12**	**9,929**	**4,675**	**14,604**	**10,595**	**4,908**	**15,503**	94.20
North Cotabato	2,480	1,230	3,710	2,705	1,305	4,010	92.52
Sarangani	969	498	1,467	1,027	525	1,552	94.52
South Cotabato	2,823	1,222	4,045	2,996	1,281	4,277	94.58
Sultan Kudarat	1,941	991	2,932	2,004	1,018	3,022	97.02
Gen. Santos City	1,716	734	2,450	1,863	779	2,642	92.73
**BARMM**	**4,673**	**2,992**	**7,665**	**5,378**	**3,401**	**8,779**	87.31
Basilan	432	273	705	460	293	753	93.63
Lanao del Sur	922	861	1,783	953	895	1,848	96.48
Maguindanao	1,536	754	2,290	1,571	770	2,341	97.82
Sulu	672	**394**	1,066	760	433	1,193	89.35
Tawi-Tawi	380	241	621	433	265	698	88.97
SGU	*	*	*	*	*	*	*
Cotabato City	731	469	1,200	1,201	745	1,946	61.66

Table 2.E.7.1 - Tuberculosis

Treatment Success Rate DSTB - All Forms

PHILIPPINES, 2023

Area	TB all forms cured and Completely Treated			Registered TB all forms Registered during Specified Period			Treatment Success Rate
	Male	Female	Total	Male	Female	Total	
**Caraga**	**6,331**	**3,113**	**9,444**	**6,680**	**3,278**	**9,958**	94.84
Agusan del Norte	820	365	1,185	863	380	1,243	95.33
Agusan del Sur	1,564	686	2,250	1,633	712	2,345	95.95
Surigao del Norte	1,292	652	1,944	1,391	703	2,094	92.84
Surigao del Sur	1,604	853	2,457	1,698	909	2,607	94.25
Province of Dinagat	**197**	**96**	293	206	100	306	95.75
Butuan City	854	461	1,315	889	474	1,363	96.48

Table 2.E.7.2 - Tuberculosis
Treatment Success Rate - MDRTB
Philippines, 2023

Area	Registered Bacteriologically Confirmed Drug Resistant Cured and Completely Treated			Registered Bacteriologically Confirmed Drug Resistant during Specified Period			Treatment Success Rate
	Male	Female	Total	Male	Female	Total	
**Philippines**	**3,233**	**1,316**	**4,549**	**4,102**	**1,740**	**5,842**	**77.87**
**N C R**	**702**	**326**	**1,028**	**917**	**440**	**1,357**	**75.76**
Malabon City	27	12	39	34	14	48	81.25
Navotas City	29	3	32	34	6	40	80.00
Valenzuela City	27	18	45	42	20	62	72.58
Caloocan City	74	34	108	99	47	146	73.97
Marikina City	25	11	36	35	15	50	72.00
Pasig City	30	16	46	39	25	64	71.88
Pateros	0	0	0	0	0	0	0.00
Taguig City	73	21	94	88	26	114	82.46
Quezon City	119	78	197	150	98	248	79.44
Makati City	25	13	38	32	19	51	74.51
Mandaluyong City	23	13	36	35	14	49	73.47
San Juan City	13	14	27	17	17	34	79.41
Manila City	133	56	189	179	89	268	70.52
Las Piñas City	19	10	29	25	17	42	69.05
Muntinlupa City	52	8	60	62	10	72	83.33
Parañaque City	20	9	29	30	11	41	70.73
Pasay City	13	10	23	16	12	28	82.14
**CAR**	**25**	**9**	**34**	**34**	**9**	**43**	**79.07**
Abra	7	0	7	7	0	7	100.00
Apayao	5	3	8	7	3	10	80.00
Benguet	0	0	0	0	0	0	0.00
Ifugao	4	1	5	4	1	5	100.00
Kalinga	4	3	7	6	3	9	77.78
Mt. Province	0	0	0	0	0	0	0.00
Baguio City	5	2	7	10	2	12	58.33
**Region 1**	**148**	**62**	**210**	**179**	**78**	**257**	**81.71**
Ilocos Norte	8	5	13	11	5	16	81.25
Ilocos Sur	9	2	11	13	2	15	73.33
La Union	18	12	30	25	15	40	75.00
Pangasinan	86	33	119	99	42	141	84.40
Dagupan City	27	10	37	31	14	45	82.22

Table 2.E.7.2 - Tuberculosis
Treatment Success Rate - MDRTB
Philippines, 2023

Area	Registered Bacteriologically Confirmed Drug Resistant Cured and Completely Treated			Registered Bacteriologically Confirmed Drug Resistant during Specified Period			Treatment Success Rate
	Male	Female	Total	Male	Female	Total	
**Region 2**	**58**	**18**	**76**	**82**	**19**	**101**	**75.25**
Batanes	0	0	0	0	0	0	0.00
Cagayan	31	8	39	43	8	51	76.47
Isabela	0	0	0	0	0	0	0.00
Nueva Vizcaya	2	5	7	4	5	9	77.78
Quirino	4	1	5	5	2	7	71.43
Santiago City	21	4	25	30	4	34	73.53
**Region 3**	**396**	**178**	**574**	**511**	**230**	**741**	**77.46**
Aurora	5	4	9	6	5	11	81.82
Bataan	25	9	34	40	10	50	68.00
Bulacan	144	56	200	182	68	250	80.00
Nueva Ecija	45	22	67	59	34	93	72.04
Pampanga	72	36	108	96	48	144	75.00
Tarlac	41	24	65	47	27	74	87.84
Zambales	23	12	35	35	18	53	66.04
Angeles City	30	9	39	34	12	46	84.78
Olongapo City	11	6	17	12	8	20	85.00
**Region 4A**	**557**	**232**	**789**	**710**	**301**	**1,011**	**78.04**
Batangas	93	29	122	114	35	149	81.88
Cavite	115	59	174	145	78	223	78.03
Laguna	150	53	203	180	67	247	82.19
Quezon	51	19	70	62	27	89	78.65
Rizal	126	57	183	181	77	258	70.93
Lucena City	22	15	37	28	17	45	82.22
**Region 4B**	**97**	**28**	**125**	**108**	**42**	**150**	**83.33**
Marinduque	8	3	11	11	4	15	73.33
Mindoro Occidental	24	5	29	26	8	34	85.29
Mindoro Oriental	35	8	43	38	14	52	82.69
Palawan	14	7	21	16	9	25	84.00
Romblon	10	3	13	10	4	14	92.86
Puerto Princesa City	6	2	8	7	3	10	80.00
**Region 5**	**261**	**89**	**350**	**307**	**118**	**425**	**82.35**
Albay	63	29	92	80	36	116	79.31
Camarines Norte	50	17	67	58	20	78	85.90
Camarines Sur	10	2	12	14	4	18	66.67

Table 2.E.7.2 - Tuberculosis
Treatment Success Rate - MDRTB
Philippines, 2023

Area	Registered Bacteriologically Confirmed Drug Resistant Cured and Completely Treated			Registered Bacteriologically Confirmed Drug Resistant during Specified Period			Treatment Success Rate
	Male	Female	Total	Male	Female	Total	
Catanduanes	12	4	16	12	8	20	80.00
Masbate	8	4	12	8	5	13	92.31
Sorsogon	51	11	62	56	16	72	86.11
Naga City	67	22	89	79	29	108	82.41
**Region 6**	**289**	**116**	**405**	**344**	**131**	**475**	**85.26**
Aklan	20	8	28	29	11	40	70.00
Antique	20	9	29	21	9	30	96.67
Capiz	26	10	36	27	11	38	94.74
Guimaras	0	0	0	0	0	0	0.00
Iloilo	30	15	45	33	17	50	90.00
Negros Occidental	106	38	144	126	42	168	85.71
Bacolod City	39	21	60	49	24	73	82.19
Iloilo City	48	15	63	59	17	76	82.89
**Region 7**	**122**	**27**	**149**	**211**	**65**	**276**	**53.99**
Bohol	28	7	35	30	10	40	87.50
Cebu	0	0	0	0	0	0	0.00
Negros Oriental	0	0	0	41	3	44	0.00
Siquijor	0	0	0	0	0	0	0.00
Cebu City	59	17	76	87	27	114	66.67
Lapu-Lapu City	0	0	0	0	0	0	0.00
Mandaue City	35	3	38	53	25	78	48.72
**Region 8**	**110**	**50**	**160**	**131**	**59**	**190**	**84.21**
Biliran	0	0	0	0	0	0	0.00
Eastern Samar	9	10	19	11	10	21	90.48
Northern Leyte	61	22	83	74	24	98	84.69
Northern Samar	12	7	19	17	8	25	76.00
Southern Leyte	16	4	20	16	6	22	90.91
Western Samar	12	7	19	13	11	24	79.17
Ormoc City	0	0	0	0	0	0	0.00
Tacloban City	0	0	0	0	0	0	0.00
**Region 9**	**78**	**18**	**96**	**91**	**27**	**118**	**81.36**
Zamboanga del Norte	20	1	21	23	2	25	84.00
Zamboanga del Sur	10	9	19	13	10	23	82.61
Zamboanga Sibugay	12	3	15	15	9	24	62.50
Isabela City	2	1	3	2	1	3	100.00

Table 2.E.7.2 - Tuberculosis
Treatment Success Rate - MDRTB
Philippines, 2023

Area	Registered Bacteriologically Confirmed Drug Resistant Cured and Completely Treated			Registered Bacteriologically Confirmed Drug Resistant during Specified Period			Treatment Success Rate
	Male	Female	Total	Male	Female	Total	
Zamboanga City	34	4	38	38	5	43	88.37
**Region 10**	**79**	**29**	**108**	**98**	**39**	**137**	**78.83**
Bukidnon	3	0	3	5	0	5	60.00
Camiguin	0	0	0	0	0	0	0.00
Lanao del Norte	14	6	20	16	8	24	83.33
Misamis Occidental	11	3	14	15	4	19	73.68
Misamis Oriental	7	3	10	7	5	12	83.33
Cagayan de Oro City	27	8	35	32	9	41	85.37
Iligan City	17	9	26	23	13	36	72.22
**Region 11**	**120**	**30**	**150**	**141**	**44**	**185**	**81.08**
Davao de Oro	0	0	0	0	0	0	0.00
Davao del Norte	60	10	70	69	14	83	84.34
Davao Oriental	13	3	16	17	3	20	80.00
Davao del Sur	15	2	17	16	2	18	94.44
Davao Occidental	3	0	3	3	0	3	100.00
Davao City	29	15	44	36	25	61	72.13
**Region 12**	**84**	**38**	**122**	**107**	**44**	**151**	**80.79**
North Cotabato	28	9	37	35	12	47	78.72
Sarangani	5	3	8	6	3	9	88.89
South Cotabato	16	9	25	23	11	34	73.53
Sultan Kudarat	13	6	19	15	6	21	90.48
Gen. Santos City	22	11	33	28	12	40	82.50
**BARMM**	**45**	**34**	**79**	**55**	**49**	**104**	**75.96**
Basilan	3	3	6	3	3	6	100.00
Lanao del Sur	16	12	28	20	19	39	71.79
Maguindanao	5	8	13	8	9	17	76.47
Sulu	3	1	4	3	2	5	80.00
Tawi-Tawi	2	1	3	2	2	4	75.00
SGU	*	*	*	*	*	*	*
Cotabato City	16	9	25	19	14	33	75.76
**Caraga**	**62**	**32**	**94**	**76**	**39**	**115**	**81.74**
Agusan del Norte	4	1	5	5	4	9	55.56
Agusan del Sur	10	4	14	12	5	17	82.35
Surigao del Norte	19	13	32	22	15	37	86.49

Table 2.E.7.2 - Tuberculosis
Treatment Success Rate - MDRTB
Philippines, 2023

Area	Registered Bacteriologically Confirmed Drug Resistant Cured and Completely Treated			Registered Bacteriologically Confirmed Drug Resistant during Specified Period			Treatment Success Rate
	Male	Female	Total	Male	Female	Total	
Surigao del Sur	20	14	34	25	15	40	85.00
Province of Dinagat	1	0	1	1	0	1	100.00
Butuan City	8	0	8	11	0	11	72.73

Table 2.E.7.3 - Tuberculosis
Case Notification Rate (All forms)
PHILIPPINES, 2023

Area	Total Population	Notified TB cases all forms				CNR (All Forms)
		Male	Female	Unknown	Total	
**PHILIPPINES**	**111,638,549**	**365,836**	**209,931**	**3**	**575,770**	**515.74**
**N C R**	**13,802,335**	**67,632**	**43,596**	**0**	**111,228**	**805.86**
Malabon City	389,819	2,535	1,565	0	4,100	1051.77
Navotas City	254,778	1,539	822	0	2,361	926.69
Valenzuela City	732,364	3,339	2,120	0	5,459	745.39
Caloocan City	1,708,376	7,222	4,575	0	11,797	690.54
Marikina City	464,962	1,969	1,170	0	3,139	675.11
Pasig City	825,380	3,891	2,647	0	6,538	792.12
Pateros	66,279	372	280	0	652	983.72
Taguig City	917,267	4,081	2,699	0	6,780	739.15
Quezon City	3,027,195	16,170	10,267	0	26,437	873.32
Makati City	637,870	2,838	2,211	0	5,049	791.54
Mandaluyong City	434,978	2,232	1,305	0	3,537	813.14
San Juan City	127,452	685	493	0	1,178	924.27
Manila City	1,889,338	10,712	7,846	0	18,558	982.25
Las Piñas City	618,866	2,523	1,534	0	4,057	655.55
Muntinlupa City	553,660	2,785	1,118	0	3,903	704.95
Parañaque City	704,904	2,589	1,564	0	4,153	589.16
Pasay City	448,847	2,150	1,380	0	3,530	786.46
**CAR**	**1,851,672**	**3,079**	**1,616**	**0**	**4,695**	**253.55**
Abra	253,559	694	384	0	1,078	425.15
Apayao	128,167	243	149	0	392	305.85
Benguet	473,982	424	144	0	568	119.84
Ifugao	216,448	220	129	0	349	161.24
Kalinga	238,965	464	245	0	709	296.70
Mt. Province	162,971	181	104	0	285	174.88
Baguio City	377,580	853	461	0	1,314	348.01
**Region 1**	**5,390,222**	**13,858**	**7,574**	**0**	**21,432**	**397.61**
Ilocos Norte	616,826	852	449	0	1,301	210.92
Ilocos Sur	713,424	1,691	888	0	2,579	361.50
La Union	835,494	2,419	1,382	0	3,801	454.94

Table 2.E.7.3 - Tuberculosis
Case Notification Rate (All forms)
PHILIPPINES, 2023

Area	Total Population	Notified TB cases all forms				CNR (All Forms)
		Male	Female	Unknown	Total	
Pangasinan	3,045,822	7,831	4,242	0	12,073	396.38
Dagupan City	178,656	1,065	613	0	1,678	939.24
**Region 2**	**3,767,534**	**10,851**	**6,549**	**0**	**17,400**	**461.84**
Batanes	19,107	18	16	0	34	177.95
Cagayan	1,289,735	3,433	2,017	0	5,450	422.57
Isabela	1,580,009	5,132	3,247	0	8,379	530.31
Nueva Vizcaya	514,210	832	426	0	1,258	244.65
Quirino	211,146	472	331	0	803	380.31
Santiago City	153,327	964	512	0	1,476	962.65
**Region 3**	**12,705,726**	**43,207**	**25,298**	**2**	**68,507**	**539.18**
Aurora	243,172	693	336	0	1,029	423.16
Bataan	876,011	3,326	1,908	1	5,234	597.48
Bulacan	3,788,883	14,914	9,204	0	24,118	636.55
Nueva Ecija	2,353,166	6,779	3,937	0	10,716	455.39
Pampanga	2,508,544	8,000	4,359	0	12,359	492.68
Tarlac	1,532,063	4,392	2,256	1	6,648	433.92
Zambales	664,850	2,454	1,646	0	4,100	616.68
Angeles City	474,775	1,419	923	0	2,342	493.29
Olongapo City	264,262	1,230	729	0	1,959	741.31
**Region 4A**	**16,579,218**	**61,655**	**38,359**	**1**	**100,015**	**603.26**
Batangas	2,969,087	12,702	8,194	0	20,896	703.79
Cavite	4,451,043	15,051	9,248	0	24,299	545.92
Laguna	3,458,788	13,981	8,545	1	22,526	651.27
Quezon	1,996,004	6,864	4,072	0	10,936	547.89
Rizal	3,418,289	11,938	7,550	0	19,488	570.11
Lucena City	286,007	1,119	750	0	1,869	653.48
**Region 4B**	**3,303,791**	**10,777**	**5,944**	**0**	**16,721**	**506.12**
Marinduque	242,451	998	576	0	1,574	649.20
Mindoro Occidental	539,533	1,807	1,026	0	2,833	525.08
Mindoro Oriental	926,654	3,443	1,930	0	5,373	579.83
Palawan	965,716	2,180	1,208	0	3,388	350.83
Romblon	314,575	977	517	0	1,494	474.93

Table 2.E.7.3 - Tuberculosis
Case Notification Rate (All forms)
PHILIPPINES, 2023

Area	Total Population	Notified TB cases all forms				CNR (All Forms)
		Male	Female	Unknown	Total	
Puerto Princesa City	314,862	1,372	687	0	2,059	653.94
**Region 5**	**6,265,917**	**21,493**	**11,992**	**0**	**33,485**	**534.40**
Albay	1,415,746	5,523	3,041	0	8,564	604.91
Camarines Norte	653,656	2,063	945	0	3,008	460.18
Camarines Sur	1,916,128	5,810	3,293	0	9,103	475.07
Catanduanes	279,648	1,053	609	0	1,662	594.32
Masbate	932,221	2,781	1,522	0	4,303	461.59
Sorsogon	852,791	2,544	1,408	0	3,952	463.42
Naga City	215,727	1,719	1,174	0	2,893	1341.05
**Region 6**	**8,047,833**	**30,055**	**15,235**	**0**	**45,290**	**562.76**
Aklan	626,648	1,661	776	0	2,437	388.89
Antique	617,880	2,444	1,223	0	3,667	593.48
Capiz	817,827	2,603	1,409	0	4,012	490.57
Guimaras	191,419	485	209	0	694	362.56
Iloilo	2,081,051	6,105	2,859	0	8,964	430.74
Negros Occidental	2,652,749	10,287	4,996	0	15,283	576.12
Bacolod City	598,468	3,593	2,097	0	5,690	950.76
Iloilo City	461,791	2,877	1,666	0	4,543	983.78
**Region 7**	**8,305,046**	**24,150**	**12,276**	**0**	**36,426**	**438.60**
Bohol	1,421,485	2,927	1,497	0	4,424	311.22
Cebu	3,420,109	7,826	3,994	0	11,820	345.60
Negros Oriental	1,471,146	4,448	2,186	0	6,634	450.94
Siquijor	104,070	165	58	0	223	214.28
Cebu City	993,006	5,121	2,682	0	7,803	785.80
Lapu-Lapu City	517,787	2,039	1,024	0	3,063	591.56
Mandaue City	377,443	1,624	835	0	2,459	651.49
**Region 8**	**4,651,322**	**11,099**	**6,177**	**0**	**17,276**	**371.42**
Biliran	183,761	440	239	0	679	369.50
Eastern Samar	488,406	1,130	626	0	1,756	359.54
Northern Leyte	1,582,542	3,419	1,679	0	5,098	322.14
Northern Samar	652,283	1,667	943	0	2,610	400.13
Southern Leyte	433,569	919	497	0	1,416	326.59
Western Samar	813,690	1,739	999	0	2,738	336.49

Table 2.E.7.3 - Tuberculosis
Case Notification Rate (All forms)
PHILIPPINES, 2023

Area	Total Population	Notified TB cases all forms				CNR (All Forms)
		Male	Female	Unknown	Total	
Ormoc City	238,056	410	236	0	646	271.36
Tacloban City	259,015	1,375	958	0	2,333	900.72
**Region 9**	**3,974,491**	**10,657**	**5,210**	**0**	**15,867**	**399.22**
Zamboanga del Norte	1,073,915	2,305	1,042	0	3,347	311.66
Zamboanga del Sur	1,080,246	2,782	1,285	0	4,067	376.49
Zamboanga Sibugay	686,576	1,686	763	0	2,449	356.70
Isabela City	131,842	323	213	0	536	406.55
Zamboanga City	1,001,912	3,561	1,907	0	5,468	545.76
**Region 10**	**5,192,278**	**13,176**	**7,260**	**0**	**20,436**	**393.58**
Bukidnon	1,606,133	2,062	937	0	2,999	186.72
Camiguin	94,450	120	59	0	179	189.52
Lanao del Norte	743,529	1,565	951	0	2,516	338.39
Misamis Occidental	632,855	1,829	824	0	2,653	419.21
Misamis Oriental	987,437	2,420	1,272	0	3,692	373.90
Cagayan de Oro City	755,908	3,185	2,042	0	5,227	691.49
Iligan City	371,966	1,995	1,175	0	3,170	852.23
**Region 11**	**5,380,729**	**14,496**	**6,648**	**0**	**21,144**	**392.96**
Davao de Oro	792,250	1,866	833	0	2,699	340.68
Davao del Norte	1,155,884	3,490	1,506	0	4,996	432.22
Davao Oriental	591,494	1,285	483	0	1,768	298.90
Davao del Sur	697,574	1,771	783	0	2,554	366.13
Davao Occidental	318,776	497	252	0	749	234.96
Davao City	1,824,751	5,587	2,791	0	8,378	459.13
**Region 12**	**4,507,622**	**14,494**	**7,070**	**0**	**21,564**	**478.39**
North Cotabato	1,317,572	3,938	1,945	0	5,883	446.50
Sarangani	579,863	1,254	759	0	2,013	347.15
South Cotabato	1,006,003	3,714	1,593	0	5,307	527.53
Sultan Kudarat	881,039	2,474	1,226	0	3,700	419.96
Gen. Santos City	723,145	3,114	1,547	0	4,661	644.55

Table 2.E.7.3 - Tuberculosis

Case Notification Rate (All forms)

PHILIPPINES, 2023

Area	Total Population	Notified TB cases all forms				CNR (All Forms)
		Male	Female	Unknown	Total	
**BARMM**	**5,039,862**	**6,883**	**4,830**	**0**	**11,713**	**232.41**
Basilan	431,169	497	422	0	919	213.14
Lanao del Sur	1,210,346	1,343	1,199	0	2,542	210.02
Maguindanao	1,391,043	1,720	1,050	0	2,770	199.13
Sulu	1,006,150	963	689	0	1,652	164.19
Tawi-Tawi	442,766	439	316	0	755	170.52
SGU	223,577	*	*		*	*
						0.00
Cotabato City	334,811	1,921	1,154	0	3,075	918.43
**CARAGA**	**2,872,951**	**8,274**	**4,297**	**0**	**12,571**	**437.56**
Agusan del Norte	396,078	882	380	0	1,262	318.62
Agusan del Sur	759,431	1,771	828	0	2,599	342.23
Surigao del Norte	545,689	2,019	1,108	0	3,127	573.04
Surigao del Sur	661,201	2,016	1,057	0	3,073	464.76
Province of Dinagat	129,127	244	141	0	385	298.16
Butuan City	381,425	1,342	783	0	2,125	557.12

Table 2.E.7.4 - Tuberculosis
Case Notification Rate (DRTB)
PHILIPPINES, 2023

Area	Registered Bacteriologically Confirmed Drug Resistant			Estimated MDR among New & Retreatment			CNR (DRTB)
	Male	Female	Total	Male	Female	Total	
**PHILIPPINES**	**5,551**	**2,236**	**7,787**	**12,691**	**6,821**	**19,512**	**39.9**
**N C R**	**1,296**	**550**	**1,846**	**2,441**	**1,428**	**3,869**	**47.7**
Malabon City	47	23	70	105	61	166	42.2
Navotas City	36	13	49	60	30	90	54.5
Valenzuela City	49	33	82	119	70	189	43.4
Caloocan City	157	73	230	259	149	408	56.4
Marikina City	43	16	59	71	40	111	53.1
Pasig City	60	27	87	134	84	218	39.9
Pateros	6	0	6	11	6	17	35.6
Taguig City	106	27	133	145	88	233	57.1
Quezon City	246	130	376	558	331	890	42.3
Makati City	35	14	49	91	65	156	31.4
Mandaluyong City	39	12	51	78	40	118	43.3
San Juan City	13	5	18	20	13	33	54.3
Manila City	275	100	375	432	259	692	54.2
Las Piñas City	38	21	59	75	43	117	50.2
Muntinlupa City	92	16	108	101	39	140	77.2
Parañaque City	33	26	59	89	54	143	41.3
Pasay City	21	14	35	93	56	149	23.4
**CAR**	**41**	**17**	**58**	**89**	**45**	**135**	**43.0**
Abra	8	1	9	21	11	32	28.5
Apayao	6	3	9	6	5	11	85.1
Benguet	0	0	0	11	3	15	0.0
Ifugao	2	1	3	6	3	9	32.4
Kalinga	8	2	10	15	8	23	43.4
Mt. Province	2	1	3	6	4	10	30.0
Baguio City	15	9	24	24	12	36	67.0
**Region 1**	**239**	**80**	**319**	**446**	**232**	**677**	**47.1**
Ilocos Norte	7	3	10	27	13	40	24.7
Ilocos Sur	21	7	28	48	25	73	38.3
La Union	41	15	56	77	39	116	48.3
Pangasinan	144	48	192	258	137	395	48.6
Dagupan City	26	7	33	35	18	53	62.2

Table 2.E.7.4 - Tuberculosis
Case Notification Rate (DRTB)
PHILIPPINES, 2023

Area	Registered Bacteriologically Confirmed Drug Resistant			Estimated MDR among New & Retreatment			CNR (DRTB)
	Male	Female	Total	Male	Female	Total	
**Region 2**	**82**	**42**	**124**	**340**	**194**	**534**	**23.2**
Batanes	1	0	1	1	0	1	111.4
Cagayan	36	23	59	104	60	164	35.9
Isabela	2	0	2	171	99	270	0.7
Nueva Vizcaya	8	3	11	27	14	41	27.0
Quirino	5	0	5	12	9	21	23.7
Santiago City	30	16	46	25	12	37	123.4
**Region 3**	**747**	**334**	**1,081**	**1,479**	**818**	**2,297**	**47.1**
Aurora	15	3	18	26	11	37	48.3
Bataan	58	25	83	123	67	190	43.6
Bulacan	277	112	389	490	285	776	50.1
Nueva Ecija	104	50	154	230	124	354	43.5
Pampanga	133	69	202	265	139	404	50.0
Tarlac	70	39	109	159	75	234	46.5
Zambales	47	24	71	97	65	162	43.9
Angeles City	31	8	39	46	28	74	52.8
Olongapo City	12	4	16	43	23	66	24.3
**Region 4A**	**962**	**412**	**1,374**	**2,095**	**1,214**	**3,309**	**41.5**
Batangas	163	47	210	409	239	648	32.4
Cavite	221	102	323	485	279	765	42.2
Laguna	232	95	327	473	271	744	44.0
Quezon	64	31	95	249	138	387	24.6
Rizal	241	116	357	435	260	695	51.3
Lucena City	41	21	62	44	27	71	87.7
**Region 4B**	**219**	**69**	**288**	**428**	**228**	**656**	**43.9**
Marinduque	13	3	16	38	21	58	27.5
Mindoro Occidental	54	16	70	66	33	99	70.9
Mindoro Oriental	76	18	94	147	82	228	41.1
Palawan	39	15	54	91	49	140	38.7
Romblon	19	12	31	43	24	67	45.9
Puerto Princesa City	18	5	23	44	19	63	36.4
**Region 5**	**408**	**149**	**557**	**791**	**422**	**1,214**	**45.9**

Table 2.E.7.4 - Tuberculosis
Case Notification Rate (DRTB)
PHILIPPINES, 2023

Area	Registered Bacteriologically Confirmed Drug Resistant			Estimated MDR among New & Retreatment			CNR (DRTB)
	Male	Female	Total	Male	Female	Total	
Albay	95	35	130	211	112	323	40.2
Camarines Norte	78	25	103	68	26	95	108.6
Camarines Sur	118	39	157	230	129	359	43.7
Catanduanes	19	10	29	44	24	69	42.2
Masbate	41	17	58	104	55	159	36.5
Sorsogon	37	10	47	89	48	137	34.3
Naga City	20	13	33	44	27	72	46.1
**Region 6**	**341**	**113**	**454**	**1,187**	**561**	**1,748**	**26.0**
Aklan	24	5	29	59	27	86	33.9
Antique	16	15	31	94	48	142	21.8
Capiz	24	9	33	82	42	124	26.5
Guimaras	5	0	5	13	5	18	28.3
Iloilo	59	17	76	231	103	334	22.7
Negros Occidental	148	52	200	479	216	695	28.8
Bacolod City	46	12	58	135	69	205	28.3
Iloilo City	19	3	22	94	50	144	15.2
**Region 7**	**287**	**99**	**386**	**839**	**393**	**1,232**	**31.3**
Bohol	41	13	54	99	45	144	37.6
Cebu	1	1	2	250	120	370	0.5
Negros Oriental	58	8	66	168	79	247	26.8
Siquijor	0	0	0	6	2	8	0.0
Cebu City	107	46	153	199	88	287	53.3
Lapu-Lapu City	1	0	1	69	36	105	1.0
Mandaue City	79	31	110	48	23	71	154.4
**Region 8**	**156**	**61**	**217**	**346**	**189**	**534**	**40.6**
Biliran	0	0	0	13	7	21	0.0
Eastern Samar	22	7	29	39	22	61	47.8
Northern Leyte (Leyte)	82	28	110	104	50	154	71.4
Northern Samar	18	13	31	53	32	85	36.6
Southern Leyte	17	10	27	33	17	51	53.2
Western Samar	17	3	20	48	27	76	26.4
Ormoc City	0	0	0	17	9	26	0.0
Tacloban City	0	0	0	38	24	62	0.0

Table 2.E.7.4 - Tuberculosis
Case Notification Rate (DRTB)
PHILIPPINES, 2023

Area	Registered Bacteriologically Confirmed Drug Resistant			Estimated MDR among New & Retreatment			CNR (DRTB)
	Male	Female	Total	Male	Female	Total	
**Region 9**	**127**	**43**	**170**	**341**	**158**	**500**	**34.0**
Zamboanga del Norte	9	6	15	72	31	102	14.6
Zamboanga del Sur	37	10	47	91	40	131	35.9
Zamboanga Sibugay	46	13	59	63	26	89	66.5
Isabela City	8	3	11	9	6	15	73.8
Zamboanga City	27	11	38	107	56	163	23.4
**Region 10**	**127**	**62**	**189**	**415**	**221**	**636**	**29.7**
Bukidnon	20	9	29	62	28	90	32.3
Camiguin	1	0	1	3	2	5	22.0
Lanao del Norte	15	10	25	61	36	96	26.0
Misamis Occidental	16	11	27	56	25	81	33.3
Misamis Oriental	14	5	19	89	46	135	14.0
Cagayan de Oro City	43	17	60	86	52	139	43.3
Iligan City	18	10	28	57	33	90	31.0
**Region 11**	**179**	**50**	**229**	**465**	**198**	**663**	**34.6**
Davao de Oro	0	0	0	62	25	88	0.0
Davao del Norte	77	13	90	106	43	150	60.1
Davao Oriental	21	1	22	49	17	66	33.5
Davao del Sur	21	8	29	57	23	80	36.2
Davao Occidental	6	3	9	16	9	25	36.5
Davao City	54	25	79	175	80	255	31.0
**Region 12**	**116**	**40**	**156**	**499**	**232**	**731**	**21.3**
North Cotabato	38	14	52	124	62	186	28.0
Sarangani	3	2	5	51	32	83	6.0
South Cotabato	36	9	45	119	48	167	26.9
Sultan Kudarat	12	6	18	79	36	115	15.6
Gen. Santos City	27	9	36	125	55	180	20.0
**BARMM**	**105**	**68**	**173**	**215**	**150**	**365**	**47.4**
Basilan	5	2	7	16	12	28	24.7
Lanao del Sur	20	16	36	43	39	82	43.8
Maguindanao	33	24	57	56	36	92	61.9
Sulu	11	8	19	25	18	43	44.2

Table 2.E.7.4 - Tuberculosis
Case Notification Rate (DRTB)
PHILIPPINES, 2023

Area	Registered Bacteriologically Confirmed Drug Resistant			Estimated MDR among New & Retreatment			CNR (DRTB)
	Male	Female	Total	Male	Female	Total	
Tawi-Tawi	5	1	6	13	9	22	27.9
SGU	*	*		*	*	*	0.0
Cotabato City	31	17	48	63	35	98	49.1
**CARAGA**	**119**	**47**	**166**	**276**	**137**	**413**	**40.2**
Agusan del Norte	11	5	16	29	12	42	38.3
Agusan del Sur	19	5	24	64	30	94	25.6
Surigao del Norte	32	12	44	68	35	103	42.7
Surigao del Sur	42	17	59	76	37	113	52.0
Province of Dinagat	4	4	8	8	4	12	64.5
Butuan City	11	4	15	30	18	48	31.2

Table 2.F.1.1 -Lifestyle Related Diseases (Risk Assessment)
Adults aged 20 yrs old and above who were risk-assessed using the PhilPEN protocol
Philippines, 2023

Area	Eligible Pop. (20 y/o & above)	Adults who were risk-assessed			
		Male	Female	Total	%
**Philippines**	**66,671,822**	**6,240,772**	**7,400,052**	**13,640,824**	**20.46**
**NCR**	**8,874,549**	**388,833**	**674,967**	**1,063,800**	**11.99**
Malabon City	**243,508**	13,627	26,657	**40,284**	**16.54**
Navotas City	**153,315**	12,283	23,602	**35,885**	**23.41**
Valenzuela City	**465,307**	37,152	60,729	**97,881**	**21.04**
Caloocan City	**1,051,808**	32,075	73,583	**105,658**	**10.05**
Marikina City	**297,613**	3,730	7,092	**10,822**	**3.64**
Pasig City	**521,330**	35,820	58,678	**94,498**	**18.13**
Pateros	**42,843**	1,668	3,319	**4,987**	**11.64**
Taguig City	**574,589**	54,362	91,885	**146,247**	**25.45**
Quezon City	**1,934,785**	44,502	80,127	**124,629**	**6.44**
Makati City	**446,290**	13,294	30,395	**43,689**	**9.79**
Mandaluyong City	**290,548**	16,607	30,424	**47,031**	**16.19**
San Juan City	**87,778**	3,652	6,374	**10,026**	**11.42**
Manila City	**1,222,451**	62,293	92,326	**154,619**	**12.65**
Las Piñas City	**400,512**	2,865	3,483	**6,348**	**1.58**
Muntinlupa City	**367,709**	8,879	16,162	**25,041**	**6.81**
Parañaque City	**464,376**	16,737	33,045	**49,782**	**10.72**
Pasay City	**309,787**	29,287	37,086	**66,373**	**21.43**
**CAR**	**1,146,469**	**123,301**	**185,357**	**308,658**	**26.92**
Abra	**158,083**	28,680	35,719	**64,399**	**40.74**
Apayao	**77,936**	12,258	22,093	**34,351**	**44.08**
Benguet	**291,978**	16,017	25,993	**42,010**	**14.39**
Ifugao	**126,931**	11,580	24,332	**35,912**	**28.29**
Kalinga	**140,823**	30,272	40,129	**70,401**	**49.99**
Mountain Province	**98,128**	12,714	13,167	**25,881**	**26.37**
Baguio City	**252,590**	11,780	23,924	**35,704**	**14.14**
**Region 1**	**3,360,604**	**406,769**	**445,834**	**852,603**	**25.37**
Ilocos Norte	**409,299**	26,363	29,275	**55,638**	**13.59**
Ilocos Sur	**467,532**	83,814	99,978	**183,792**	**39.31**
La Union	**539,676**	89,140	106,174	**195,314**	**36.19**
Pangasinan	**1,834,882**	199,067	202,406	**401,473**	**21.88**
Dagupan City	**109,215**	8,385	8,001	**16,386**	**15.00**

Table 2.F.1.1 -Lifestyle Related Diseases (Risk Assessment)

Adults aged 20 yrs old and above who were risk-assessed using the PhilPEN protocol

Philippines, 2023

Area	Eligible Pop. (20 y/o & above)	Adults who were risk-assessed			
		Male	Female	Total	%
**Region 2**	**2,331,848**	**384,121**	**481,383**	**865,504**	**37.12**
Batanes	**12,694**	2,159	2,013	**4,172**	**32.87**
Cagayan	**803,439**	79,086	90,571	**169,657**	**21.12**
Isabela	**978,250**	191,728	259,568	**451,296**	**46.13**
Nueva Vizcaya	**315,370**	22,936	34,897	**57,833**	**18.34**
Quirino	**127,835**	43,623	47,423	**91,046**	**71.22**
Santiago City	**94,260**	44,589	46,911	**91,500**	**97.07**
**Region 3**	**7,822,089**	**1,231,731**	**1,477,537**	**2,709,268**	**34.64**
Aurora	**142,222**	55,941	63,523	**119,464**	**84.00**
Bataan	**533,446**	66,092	89,497	**155,589**	**29.17**
Bulacan	**2,348,958**	501,430	591,114	**1,092,544**	**46.51**
Nueva Ecija	**1,445,358**	202,132	234,339	**436,471**	**30.20**
Pampanga	**1,556,045**	181,344	231,694	**413,038**	**26.54**
Tarlac	**928,750**	98,549	98,783	**197,332**	**21.25**
Zambales	**406,556**	98,304	128,222	**226,526**	**55.72**
Angeles City	**291,065**	10,620	14,470	**25,090**	**8.62**
Olongapo	**169,689**	17,319	25,895	**43,214**	**25.47**
**Region 4A**	**10,283,071**	**324,665**	**369,959**	**694,624**	**6.76**
Batangas	**1,848,168**	132,544	97,350	**229,894**	**12.44**
Cavite	**2,822,571**	89,312	127,446	**216,758**	**7.68**
Laguna	**2,202,450**	34,268	50,244	**84,512**	**3.84**
Quezon	**1,155,551**	35,849	48,298	**84,147**	**7.28**
Rizal	**2,085,809**	28,075	37,687	**65,762**	**3.15**
Lucena City	**168,522**	4,617	8,934	**13,551**	**8.04**
**Region 4B**	**1,886,239**	**124,287**	**65,859**	**190,146**	**10.08**
Marinduque	**141,668**	4,400	4,170	**8,570**	**6.05**
Occidental Mindoro	**298,113**	46,115	5,259	**51,374**	**17.23**
Oriental Mindoro	**534,876**	16,237	10,051	**26,288**	**4.91**
Palawan	**532,847**	49,031	32,350	**81,381**	**15.27**
Romblon	**183,924**	6,872	10,803	**17,675**	**9.61**
Puerto Princesa City	**194,811**	1,632	3,226	**4,858**	**2.49**
**Region 5**	**3,446,277**	**1,114,377**	**1,290,455**	**2,404,832**	**69.78**
Albay	**812,005**	216,410	256,160	**472,570**	**58.20**
Camarines Norte	**361,186**	101,986	128,397	**230,383**	**63.79**

Table 2.F.1.1 -Lifestyle Related Diseases (Risk Assessment)
Adults aged 20 yrs old and above who were risk-assessed using the PhilPEN protocol
Philippines, 2023

Area	Eligible Pop. (20 y/o & above)	Adults who were risk-assessed			
		Male	Female	Total	%
Camarines Sur	**1,036,279**	333,683	386,923	**720,606**	**69.54**
Catanduanes	**155,721**	58,676	62,227	**120,903**	**77.64**
Masbate	**476,407**	228,233	247,378	**475,611**	**99.83**
Sorsogon	**475,031**	147,939	169,368	**317,307**	**66.80**
Naga City	**129,648**	27,450	40,002	**67,452**	**52.03**
**Region 6**	**4,904,510**	**525,398**	**557,668**	**1,083,066**	**22.08**
Aklan	**387,547**	40,809	53,762	**94,571**	**24.40**
Antique	**369,020**	25,856	37,459	**63,315**	**17.16**
Capiz	**509,344**	56,558	73,009	**129,567**	**25.44**
Guimaras	**119,373**	28,402	37,314	**65,716**	**55.05**
Iloilo	**1,289,605**	175,503	163,461	**338,964**	**26.28**
Negros Occidental	**1,556,947**	165,253	143,003	**308,256**	**19.80**
Bacolod City	**378,112**	5,162	13,773	**18,935**	**5.01**
Iloilo City	**294,562**	27,855	35,887	**63,742**	**21.64**
**Region 7**	**4,970,016**	**241,570**	**352,457**	**594,027**	**11.95**
Bohol	**855,513**	46,017	58,509	**104,526**	**12.22**
Cebu	**1,993,592**	122,395	171,095	**293,490**	**14.72**
Negros Oriental	**879,055**	17,466	33,458	**50,924**	**5.79**
Siquijor	**68,052**	3,172	5,585	**8,757**	**12.87**
Cebu City	**614,993**	9,748	18,352	**28,100**	**4.57**
Lapu-Lapu City	**313,339**	19,075	22,659	**41,734**	**13.32**
Mandaue City	**245,472**	23,697	42,799	**66,496**	**27.09**
**Region 8**	**2,655,243**	**200,750**	**238,087**	**438,837**	**16.53**
Biliran	**104,728**	8,617	11,546	**20,163**	**19.25**
Eastern Samar	**281,098**	43,731	55,492	**99,223**	**35.30**
Northern Leyte	**914,345**	26,702	37,243	**63,945**	**6.99**
Northern Samar	**348,021**	17,512	18,611	**36,123**	**10.38**
Southern Leyte	**264,822**	27,134	28,481	**55,615**	**21.00**
Western Samar	**449,806**	41,078	48,145	**89,223**	**19.84**
Ormoc City	**139,189**	8,975	10,685	**19,660**	**14.12**
Tacloban City	**153,234**	27,001	27,884	**54,885**	**35.82**
**Region 9**	**2,237,796**	**137,168**	**170,761**	**307,929**	**13.76**
Zamboanga Del Norte	**598,975**	29,776	33,447	**63,223**	**10.56**
Zamboanga Del Sur	**603,127**	22,173	29,771	**51,944**	**8.61**

Table 2.F.1.1 -Lifestyle Related Diseases (Risk Assessment)
Adults aged 20 yrs old and above who were risk-assessed using the PhilPEN protocol
Philippines, 2023

Area	Eligible Pop. (20 y/o & above)	Adults who were risk-assessed			
		Male	Female	Total	%
Zamboanga Sibugay	**375,604**	77,905	92,187	**170,092**	**45.28**
Isabela City	**70,617**	1,150	418	**1,568**	**2.22**
Zamboanga City	**589,473**	6,164	14,938	**21,102**	**3.58**
**Region 10**	**2,961,000**	**246,242**	**186,380**	**432,622**	**14.61**
Bukidnon	**888,223**	65,253	30,472	**95,725**	**10.78**
Camiguin	**56,019**	2,298	2,683	**4,981**	**8.89**
Lanao Del Norte	**387,286**	17,124	17,229	**34,353**	**8.87**
Misamis Occidental	**381,434**	86,901	77,121	**164,022**	**43.00**
Misamis Oriental	**561,683**	43,596	44,128	**87,724**	**15.62**
Cagayan de Oro City	**469,567**	24,288	10,032	**34,320**	**7.31**
Iligan City	**216,788**	6,782	4,715	**11,497**	**5.30**
**Region 11**	**3,188,818**	**360,406**	**436,828**	**797,234**	**25.00**
Davao de Oro	**456,458**	72,162	87,864	**160,026**	**35.06**
Davao del Norte	**688,795**	88,707	106,294	**195,001**	**28.31**
Davao Oriental	**337,560**	73,349	77,664	**151,013**	**44.74**
Davao del Sur	**418,426**	30,407	37,365	**67,772**	**16.20**
Davao Occidental	**169,756**	13,226	14,331	**27,557**	**16.23**
Davao City	**1,117,823**	82,555	113,310	**195,865**	**17.52**
**Region 12**	**2,554,985**	**178,591**	**212,995**	**391,586**	**15.33**
North Cotabato	**740,766**	58,914	69,550	**128,464**	**17.34**
Sarangani	**314,941**	23,895	29,164	**53,059**	**16.85**
South Cotabato	**580,778**	43,876	52,086	**95,962**	**16.52**
Sultan Kudarat	**488,796**	48,669	57,721	**106,390**	**21.77**
Gen. Santos City	**429,704**	3,237	4,474	**7,711**	**1.79**
**BARMM**	**2,402,321**	**112,670**	**118,116**	**230,786**	**9.61**
Basilan	**201,146**	9,783	7,230	**17,013**	**8.46**
Lanao Del Sur	**563,803**	57,290	66,601	**123,891**	**21.97**
Maguindanao	**629,997**	22,350	21,620	**43,970**	**6.98**
Sulu	**484,330**	3,754	2,931	**6,685**	**1.38**
Tawi-Tawi	**216,936**	12,658	12,775	**25,433**	**11.72**
SGA	**120,358**	*	*	*****	*****
Cotabato City	**185,751**	6,835	6,959	**13,794**	**7.43**

Table 2.F.1.1 -Lifestyle Related Diseases (Risk Assessment)

Adults aged 20 yrs old and above who were risk-assessed using the PhilPEN protocol

Philippines, 2023

Area	Eligible Pop. (20 y/o & above)	Adults who were risk-assessed			
		Male	Female	Total	%
**Caraga**	**1,645,987**	**139,893**	**135,409**	**275,302**	**16.73**
Agusan del Norte	**227,180**	15,865	17,503	**33,368**	**14.69**
Agusan del Sur	**413,668**	47,802	35,064	**82,866**	**20.03**
Surigao del Norte	**323,309**	14,332	11,736	**26,068**	**8.06**
Surigao del Sur	**379,761**	27,919	30,203	**58,122**	**15.30**
Province of Dinagat	**74,794**	3,181	3,210	**6,391**	**8.54**
Butuan City	**227,275**	30,794	37,693	**68,487**	**30.13**

Table 2.F.1.2 -Lifestyle Related Diseases (Tobacco Use, Harmful Use of Alcohol)
Adults aged 20 yrs old and above who are current smokers, binge drinkers
Philippines, 2023

Area	Total No. of clients ≥ 20 y/o risk-assessed	Current Smokers				Binge Drinkers			
		Male	Female	Total	%	Male	Female	Total	%
**Philippines**	**13,640,824**	**1,861,931**	**488,925**	**2,350,856**	**17.23**	**1,660,028**	**630,490**	**2,290,518**	**16.79**
**N C R**	**1,063,800**	**61,453**	**37,003**	**98,456**	**9.26**	**75,657**	**62,016**	**137,673**	**12.94**
Malabon City	**40,284**	2,343	1,720	**4,063**	**10.09**	2,712	2,829	**5,541**	**13.75**
Navotas City	**35,885**	2,655	1,860	**4,515**	**12.58**	2,988	2,152	**5,140**	**14.32**
Valenzuela City	**97,881**	5,262	1,956	**7,218**	**7.37**	3,994	2,612	**6,606**	**6.75**
Caloocan City	**105,658**	4,829	3,621	**8,450**	**8.00**	7,300	8,696	**15,996**	**15.14**
Marikina City	**10,822**	975	409	**1,384**	**12.79**	1,039	627	**1,666**	**15.39**
Pasig City	**94,498**	5,029	2,199	**7,228**	**7.65**	7,257	4,986	**12,243**	**12.96**
Pateros	**4,987**	415	277	**692**	**13.88**	474	392	**866**	**17.37**
Taguig City	**146,247**	9,317	4,704	**14,021**	**9.59**	16,109	13,881	**29,990**	**20.51**
Quezon City	**124,629**	8,128	5,518	**13,646**	**10.95**	11,702	9,217	**20,919**	**16.79**
Makati City	**43,689**	2,253	2,479	**4,732**	**10.83**	1,581	1,106	**2,687**	**6.15**
Mandaluyong City	**47,031**	3,010	1,572	**4,582**	**9.74**	2,659	1,997	**4,656**	**9.90**
San Juan City	**10,026**	729	383	**1,112**	**11.09**	751	495	**1,246**	**12.43**
Manila City	**154,619**	7,612	4,485	**12,097**	**7.82**	7,605	5,512	**13,117**	**8.48**
Las Piñas City	**6,348**	363	124	**487**	**7.67**	447	197	**644**	**10.14**
Muntinlupa City	**25,041**	1,360	617	**1,977**	**7.90**	1,225	1,005	**2,230**	**8.91**
Parañaque City	**49,782**	2,461	1,545	**4,006**	**8.05**	2,827	2,169	**4,996**	**10.04**
Pasay City	**66,373**	4,712	3,534	**8,246**	**12.42**	4,987	4,143	**9,130**	**13.76**
**C A R**	**308,658**	**23,827**	**7,370**	**31,197**	**10.11**	**34,727**	**11,163**	**45,890**	**14.87**
Abra	**64,399**	7,216	2,613	**9,829**	**15.26**	8,785	3,394	**12,179**	**18.91**
Apayao	**34,351**	2,935	900	**3,835**	**11.16**	4,286	1,405	**5,691**	**16.57**
Benguet	**42,010**	2,194	623	**2,817**	**6.71**	3,459	1,127	**4,586**	**10.92**
Ifugao	**35,912**	511	249	**760**	**2.12**	1,460	691	**2,151**	**5.99**
Kalinga	**70,401**	6,378	1,387	**7,765**	**11.03**	8,473	1,224	**9,697**	**13.77**
Mt. Province	**25,881**	1,978	234	**2,212**	**8.55**	4,047	371	**4,418**	**17.07**
Baguio City	**35,704**	2,615	1,364	**3,979**	**11.14**	4,217	2,951	**7,168**	**20.08**
**Region 1**	**852,603**	**70,997**	**22,251**	**93,248**	**10.94**	**68,138**	**25,976**	**94,114**	**11.04**
Ilocos Norte	**55,638**	4,516	778	**5,294**	**9.52**	2,258	856	**3,114**	**5.60**
Ilocos Sur	**183,792**	13,039	2,875	**15,914**	**8.66**	11,839	2,494	**14,333**	**7.80**
La Union	**195,314**	15,433	3,645	**19,078**	**9.77**	18,021	4,890	**22,911**	**11.73**
Pangasinan	**401,473**	37,124	14,485	**51,609**	**12.85**	35,161	17,307	**52,468**	**13.07**

Table 2.F.1.2 -Lifestyle Related Diseases (Tobacco Use, Harmful Use of Alcohol)
Adults aged 20 yrs old and above who are current smokers, binge drinkers
Philippines, 2023

Area	Total No. of clients ≥ 20 y/o risk-assessed	Current Smokers				Binge Drinkers			
		Male	Female	Total	%	Male	Female	Total	%
Dagupan City	**16,386**	885	468	**1,353**	**8.26**	859	429	**1,288**	**7.86**
**Region 2**	**865,504**	**72,365**	**15,879**	**88,244**	**10.20**	**87,542**	**18,725**	**106,267**	**12.28**
Batanes	**4,172**	408	89	**497**	**11.91**	1,381	573	**1,954**	**46.84**
Cagayan	**169,657**	15,910	4,240	**20,150**	**11.88**	15,779	3,361	**19,140**	**11.28**
Isabela	**451,296**	34,572	6,603	**41,175**	**9.12**	42,268	7,353	**49,621**	**11.00**
Nueva Vizcaya	**57,833**	3,322	1,363	**4,685**	**8.10**	3,797	1,669	**5,466**	**9.45**
Quirino	**91,046**	7,162	1,014	**8,176**	**8.98**	9,526	1,186	**10,712**	**11.77**
Santiago City	**91,500**	10,991	2,570	**13,561**	**14.82**	14,791	4,583	**19,374**	**21.17**
**Region 3**	**2,709,268**	**269,314**	**97,359**	**366,673**	**13.53**	**285,719**	**129,669**	**415,388**	**15.33**
Aurora	**119,464**	7,408	2,081	**9,489**	**7.94**	11,652	5,488	**17,140**	**14.35**
Bataan	**155,589**	9,430	2,453	**11,883**	**7.64**	7,288	2,677	**9,965**	**6.40**
Bulacan	**1,092,544**	116,328	49,519	**165,847**	**15.18**	132,482	72,668	**205,150**	**18.78**
Nueva Ecija	**436,471**	61,536	16,322	**77,858**	**17.84**	65,855	22,523	**88,378**	**20.25**
Pampanga	**413,038**	38,990	11,531	**50,521**	**12.23**	40,287	13,172	**53,459**	**12.94**
Tarlac	**197,332**	11,152	4,524	**15,676**	**7.94**	8,938	3,778	**12,716**	**6.44**
Zambales	**226,526**	18,153	7,190	**25,343**	**11.19**	13,945	5,922	**19,867**	**8.77**
Angeles City	**25,090**	3,645	2,105	**5,750**	**22.92**	3,105	2,073	**5,178**	**20.64**
Olongapo	**43,214**	2,672	1,634	**4,306**	**9.96**	2,167	1,368	**3,535**	**8.18**
**Region 4A**	**694,624**	**88,133**	**21,794**	**109,927**	**15.83**	**85,624**	**36,431**	**122,055**	**17.57**
Batangas	**229,894**	44,085	5,913	**49,998**	**21.75**	38,202	6,416	**44,618**	**19.41**
Cavite	**216,758**	15,898	6,006	**21,904**	**10.11**	17,265	8,847	**26,112**	**12.05**
Laguna	**84,512**	8,753	2,568	**11,321**	**13.40**	9,166	4,306	**13,472**	**15.94**
Quezon	**84,147**	12,919	4,251	**17,170**	**20.40**	15,062	12,162	**27,224**	**32.35**
Rizal	**65,762**	5,738	2,720	**8,458**	**12.86**	5,193	3,952	**9,145**	**13.91**
Lucena City	**13,551**	740	336	**1,076**	**7.94**	736	748	**1,484**	**10.95**
**Region 4B**	**190,146**	**72,287**	**10,358**	**82,645**	**43.46**	**29,013**	**7,546**	**36,559**	**19.23**
Marinduque	**8,570**	2,446	186	**2,632**	**30.71**	135	77	**212**	**2.47**
Occidental Mindoro	**51,374**	40,739	3,809	**44,548**	**86.71**	2,258	704	**2,962**	**5.77**
Oriental Mindoro	**26,288**	6,094	854	**6,948**	**26.43**	8,256	1,647	**9,903**	**37.67**
Palawan	**81,381**	20,917	4,911	**25,828**	**31.74**	15,242	4,060	**19,302**	**23.72**
Romblon	**17,675**	1,759	442	**2,201**	**12.45**	2,883	859	**3,742**	**21.17**
Puerto Princesa City	**4,858**	332	156	**488**	**10.05**	239	199	**438**	**9.02**

Table 2.F.1.2 -Lifestyle Related Diseases (Tobacco Use, Harmful Use of Alcohol)
Adults aged 20 yrs old and above who are current smokers, binge drinkers
Philippines, 2023

Area	Total No. of clients ≥ 20 y/o risk-assessed	Current Smokers				Binge Drinkers			
		Male	Female	Total	%	Male	Female	Total	%
**Region 5**	**2,404,832**	**408,254**	**94,895**	**503,149**	**20.92**	**316,223**	**118,674**	**434,897**	**18.08**
Albay	**472,570**	81,479	7,342	**88,821**	**18.80**	51,952	11,859	**63,811**	**13.50**
Camarines Norte	**230,383**	37,557	13,790	**51,347**	**22.29**	39,200	15,181	**54,381**	**23.60**
Camarines Sur	**720,606**	115,863	18,215	**134,078**	**18.61**	98,426	37,723	**136,149**	**18.89**
Catanduanes	**120,903**	22,800	3,025	**25,825**	**21.36**	23,016	9,633	**32,649**	**27.00**
Masbate	**475,611**	93,774	43,891	**137,665**	**28.94**	50,750	26,379	**77,129**	**16.22**
Sorsogon	**317,307**	47,864	5,366	**53,230**	**16.78**	45,468	14,193	**59,661**	**18.80**
Naga City	**67,452**	8,917	3,266	**12,183**	**18.06**	7,411	3,706	**11,117**	**16.48**
**Region 6**	**1,083,066**	**240,554**	**36,112**	**276,666**	**25.54**	**234,378**	**37,873**	**272,251**	**25.14**
Aklan	**94,571**	4,333	815	**5,148**	**5.44**	5,888	1,709	**7,597**	**8.03**
Antique	**63,315**	5,978	1,809	**7,787**	**12.30**	6,186	1,166	**7,352**	**11.61**
Capiz	**129,567**	11,481	1,430	**12,911**	**9.96**	15,865	3,362	**19,227**	**14.84**
Guimaras	**65,716**	5,296	433	**5,729**	**8.72**	6,502	819	**7,321**	**11.14**
Iloilo	**338,964**	144,309	18,991	**163,300**	**48.18**	121,921	14,834	**136,755**	**40.34**
Negros Occidental	**308,256**	65,387	11,225	**76,612**	**24.85**	73,814	14,145	**87,959**	**28.53**
Bacolod City	**18,935**	1,007	548	**1,555**	**8.21**	1,723	984	**2,707**	**14.30**
Iloilo City	**63,742**	2,763	861	**3,624**	**5.69**	2,479	854	**3,333**	**5.23**
**Region 7**	**594,027**	**55,084**	**16,834**	**71,918**	**12.11**	**66,538**	**30,522**	**97,060**	**16.34**
Bohol	**104,526**	7,289	2,313	**9,602**	**9.19**	9,711	4,313	**14,024**	**13.42**
Cebu	**293,490**	38,160	11,529	**49,689**	**16.93**	45,453	20,860	**66,313**	**22.59**
Negros Oriental	**50,924**	5,160	1,367	**6,527**	**12.82**	5,811	1,992	**7,803**	**15.32**
Siquijor	**8,757**	548	110	**658**	**7.51**	842	480	**1,322**	**15.10**
Cebu City	**28,100**	3,045	1,335	**4,380**	**15.59**	3,652	2,537	**6,189**	**22.02**
Lapu-Lapu City	**41,734**	613	120	**733**	**1.76**	630	184	**814**	**1.95**
Mandaue City	**66,496**	269	60	**329**	**0.49**	439	156	**595**	**0.89**
**Region 8**	**438,837**	**55,246**	**12,961**	**68,207**	**15.54**	**62,851**	**36,250**	**99,101**	**22.58**
Biliran	**20,163**	1,688	359	**2,047**	**10.15**	2,463	1,416	**3,879**	**19.24**
Eastern Samar	**99,223**	12,860	2,812	**15,672**	**15.79**	15,764	8,540	**24,304**	**24.49**
Northern Leyte	**63,945**	10,906	1,602	**12,508**	**19.56**	8,163	4,761	**12,924**	**20.21**
Northern Samar	**36,123**	6,667	2,444	**9,111**	**25.22**	8,828	5,068	**13,896**	**38.47**
Southern Leyte	**55,615**	9,898	1,635	**11,533**	**20.74**	8,995	3,766	**12,761**	**22.95**
Western Samar	**89,223**	7,553	2,036	**9,589**	**10.75**	8,071	5,170	**13,241**	**14.84**
Ormoc City	**19,660**	456	116	**572**	**2.91**	573	374	**947**	**4.82**

Table 2.F.1.2 -Lifestyle Related Diseases (Tobacco Use, Harmful Use of Alcohol)
Adults aged 20 yrs old and above who are current smokers, binge drinkers
Philippines, 2023

Area	Total No. of clients ≥ 20 y/o risk-assessed	Current Smokers				Binge Drinkers			
		Male	Female	Total	%	Male	Female	Total	%
Tacloban City	**54,885**	5,218	1,957	**7,175**	**13.07**	9,994	7,155	**17,149**	**31.25**
**Region 9**	**307,929**	**37,260**	**7,695**	**44,955**	**14.60**	**32,006**	**9,194**	**41,200**	**13.38**
Zamboanga del Norte	**63,223**	8,767	2,457	**11,224**	**17.75**	7,098	2,724	**9,822**	**15.54**
Zamboanga del Sur	**51,944**	6,111	1,382	**7,493**	**14.43**	5,659	2,108	**7,767**	**14.95**
Zamboanga Sibugay	**170,092**	19,075	3,006	**22,081**	**12.98**	17,178	3,884	**21,062**	**12.38**
Isabela City	**1,568**	772	124	**896**	**57.14**	288	37	**325**	**20.73**
Zamboanga City	**21,102**	2,535	726	**3,261**	**15.45**	1,783	441	**2,224**	**10.54**
**Region 10**	**432,622**	**114,241**	**33,932**	**148,173**	**34.25**	**94,875**	**39,708**	**134,583**	**31.11**
Bukidnon	**95,725**	35,585	15,391	**50,976**	**53.25**	33,807	14,206	**48,013**	**50.16**
Camiguin	**4,981**	140	8	**148**	**2.97**	167	37	**204**	**4.10**
Lanao del Norte	**34,353**	8,074	3,054	**11,128**	**32.39**	5,332	2,323	**7,655**	**22.28**
Misamis Occidental	**164,022**	41,540	8,216	**49,756**	**30.33**	26,483	14,222	**40,705**	**24.82**
Misamis Oriental	**87,724**	13,279	2,314	**15,593**	**17.78**	13,409	4,166	**17,575**	**20.03**
Cagayan de Oro City	**34,320**	10,824	3,605	**14,429**	**42.04**	11,531	3,665	**15,196**	**44.28**
Iligan City	**11,497**	4,799	1,344	**6,143**	**53.43**	4,146	1,089	**5,235**	**45.53**
**Region 11**	**797,234**	**134,741**	**33,090**	**167,831**	**21.05**	**102,407**	**40,509**	**142,916**	**17.93**
Davao de Oro	**160,026**	29,377	6,469	**35,846**	**22.40**	23,590	7,910	**31,500**	**19.68**
Davao del Norte	**195,001**	35,156	5,751	**40,907**	**20.98**	24,056	8,064	**32,120**	**16.47**
Davao Oriental	**151,013**	41,958	9,812	**51,770**	**34.28**	31,067	12,606	**43,673**	**28.92**
Davao del Sur	**67,772**	15,133	3,830	**18,963**	**27.98**	10,977	3,956	**14,933**	**22.03**
Davao Occidental	**27,557**	2,395	566	**2,961**	**10.75**	2,124	628	**2,752**	**9.99**
Davao City	**195,865**	10,722	6,662	**17,384**	**8.88**	10,593	7,345	**17,938**	**9.16**
**Region 12**	**391,586**	**48,150**	**11,520**	**59,670**	**15.24**	**33,908**	**7,325**	**41,233**	**10.53**
North Cotabato	**128,464**	20,546	4,395	**24,941**	**19.41**	14,200	2,388	**16,588**	**12.91**
Sarangani	**53,059**	5,313	1,472	**6,785**	**12.79**	3,474	955	**4,429**	**8.35**
South Cotabato	**95,962**	8,207	803	**9,010**	**9.39**	6,503	1,407	**7,910**	**8.24**
Sultan Kudarat	**106,390**	13,482	4,715	**18,197**	**17.10**	9,140	2,363	**11,503**	**10.81**
Gen. Santos City	**7,711**	602	135	**737**	**9.56**	591	212	**803**	**10.41**
**BARMM**	**230,786**	**52,067**	**14,250**	**66,317**	**28.74**	**5,660**	**922**	**6,582**	**2.85**

Table 2.F.1.2 -Lifestyle Related Diseases (Tobacco Use, Harmful Use of Alcohol)
Adults aged 20 yrs old and above who are current smokers, binge drinkers
Philippines, 2023

Area	Total No. of clients ≥ 20 y/o risk-assessed	Current Smokers				Binge Drinkers			
		Male	Female	Total	%	Male	Female	Total	%
Basilan	**17,013**	6,557	1,224	**7,781**	**45.74**	253	26	**279**	**1.64**
Lanao del Sur	**123,891**	20,779	9,117	**29,896**	**24.13**	1,633	364	**1,997**	**1.61**
Maguindanao	**43,970**	11,744	2,278	**14,022**	**31.89**	1,406	251	**1,657**	**3.77**
Sulu	**6,685**	2,999	1,083	**4,082**	**61.06**	64	1	**65**	**0.97**
Tawi-Tawi	**25,433**	8,836	316	**9,152**	**35.98**	1,832	59	**1,891**	**7.44**
SGA	*	*	*	*	*	*	*	*	*
Cotabato City	**13,794**	1,152	232	**1,384**	**10.03**	472	221	**693**	**5.02**
**Caraga**	**275,302**	**57,958**	**15,622**	**73,580**	**26.73**	**44,762**	**17,987**	**62,749**	**22.79**
Agusan del Norte	**33,368**	6,953	3,451	**10,404**	**31.18**	3,752	1,526	**5,278**	**15.82**
Agusan del Sur	**82,866**	23,811	4,474	**28,285**	**34.13**	19,993	5,808	**25,801**	**31.14**
Surigao del Norte	**26,068**	4,880	638	**5,518**	**21.17**	4,324	1,687	**6,011**	**23.06**
Surigao del Sur	**58,122**	13,773	4,423	**18,196**	**31.31**	8,620	3,824	**12,444**	**21.41**
Province of Dinagat	**6,391**	832	200	**1,032**	**16.15**	1,512	588	**2,100**	**32.86**
Butuan City	**68,487**	7,709	2,436	**10,145**	**14.81**	6,561	4,554	**11,115**	**16.23**

Table 2.F.1.2 -Lifestyle Related Diseases (Unhealthy Diet)
Adults aged 20 yrs old and above who are
Philippines, 2023

Area	Total No. of clients ≥ 20 y/o risk-assessed	Overweight or obese			
		Male	Female	Total	%
**Philippines**	**13,640,824**	**1,070,052**	**1,468,127**	**2,538,179**	**18.61**
**N C R**	**1,063,800**	**143,940**	**269,047**	**412,987**	**38.82**
Malabon City	**40,284**	5,892	12,091	**17,983**	**44.64**
Navotas City	**35,885**	5,162	10,661	**15,823**	**44.09**
Valenzuela City	**97,881**	10,436	17,753	**28,189**	**28.80**
Caloocan City	**105,658**	10,481	28,458	**38,939**	**36.85**
Marikina City	**10,822**	1,871	3,982	**5,853**	**54.08**
Pasig City	**94,498**	12,100	18,751	**30,851**	**32.65**
Pateros	**4,987**	948	2,046	**2,994**	**60.04**
Taguig City	**146,247**	23,664	44,412	**68,076**	**46.55**
Quezon City	**124,629**	16,094	28,435	**44,529**	**35.73**
Makati City	**43,689**	7,995	18,230	**26,225**	**60.03**
Mandaluyong City	**47,031**	7,468	13,833	**21,301**	**45.29**
San Juan City	**10,026**	2,360	4,218	**6,578**	**65.61**
Manila City	**154,619**	22,114	35,555	**57,669**	**37.30**
Las Piñas City	**6,348**	438	756	**1,194**	**18.81**
Muntinlupa City	**25,041**	4,257	8,091	**12,348**	**49.31**
Parañaque City	**49,782**	4,681	8,807	**13,488**	**27.09**
Pasay City	**66,373**	7,979	12,968	**20,947**	**31.56**
**C A R**	**308,658**	**26,359**	**44,440**	**70,799**	**22.94**
Abra	**64,399**	3,027	4,376	**7,403**	**11.50**
Apayao	**34,351**	2,231	3,723	**5,954**	**17.33**
Benguet	**42,010**	5,701	9,403	**15,104**	**35.95**
Ifugao	**35,912**	1,410	2,573	**3,983**	**11.09**
Kalinga	**70,401**	6,147	8,608	**14,755**	**20.96**
Mt. Province	**25,881**	1,998	3,402	**5,400**	**20.86**
Baguio City	**35,704**	5,845	12,355	**18,200**	**50.97**
**Region 1**	**852,603**	**41,669**	**52,472**	**94,141**	**11.04**
Ilocos Norte	**55,638**	2,460	2,846	**5,306**	**9.54**
Ilocos Sur	**183,792**	8,073	11,014	**19,087**	**10.39**
La Union	**195,314**	17,654	20,250	**37,904**	**19.41**
Pangasinan	**401,473**	12,967	17,872	**30,839**	**7.68**

Table 2.F.1.2 -Lifestyle Related Diseases (Unhealthy Diet)
Adults aged 20 yrs old and above who are
Philippines, 2023

Area	Total No. of clients ≥ 20 y/o risk-assessed	Overweight or obese			
		Male	Female	Total	%
Dagupan City	**16,386**	515	490	**1,005**	**6.13**
**Region 2**	**865,504**	**31,196**	**39,728**	**70,924**	**8.19**
Batanes	**4,172**	882	973	**1,855**	**44.46**
Cagayan	**169,657**	2,488	3,839	**6,327**	**3.73**
Isabela	**451,296**	17,170	22,560	**39,730**	**8.80**
Nueva Vizcaya	**57,833**	2,056	3,490	**5,546**	**9.59**
Quirino	**91,046**	2,578	3,144	**5,722**	**6.28**
Santiago City	**91,500**	6,022	5,722	**11,744**	**12.83**
**Region 3**	**2,709,268**	**142,239**	**189,139**	**331,378**	**12.23**
Aurora	**119,464**	4,833	5,988	**10,821**	**9.06**
Bataan	**155,589**	5,632	9,528	**15,160**	**9.74**
Bulacan	**1,092,544**	68,078	88,278	**156,356**	**14.31**
Nueva Ecija	**436,471**	26,716	36,006	**62,722**	**14.37**
Pampanga	**413,038**	22,125	28,660	**50,785**	**12.30**
Tarlac	**197,332**	6,483	7,996	**14,479**	**7.34**
Zambales	**226,526**	6,723	9,714	**16,437**	**7.26**
Angeles City	**25,090**	1,033	1,945	**2,978**	**11.87**
Olongapo	**43,214**	616	1,024	**1,640**	**3.80**
**Region 4A**	**694,624**	**31,222**	**56,147**	**87,369**	**12.58**
Batangas	**229,894**	3,504	4,730	**8,234**	**3.58**
Cavite	**216,758**	13,532	24,290	**37,822**	**17.45**
Laguna	**84,512**	3,342	6,039	**9,381**	**11.10**
Quezon	**84,147**	5,308	10,836	**16,144**	**19.19**
Rizal	**65,762**	4,486	7,809	**12,295**	**18.70**
Lucena City	**13,551**	1,050	2,443	**3,493**	**25.78**
**Region 4B**	**190,146**	**6,486**	**9,739**	**16,225**	**8.53**
Marinduque	**8,570**	336	715	**1,051**	**12.26**
Occidental Mindoro	**51,374**	371	780	**1,151**	**2.24**
Oriental Mindoro	**26,288**	844	1,066	**1,910**	**7.27**
Palawan	**81,381**	3,148	4,272	**7,420**	**9.12**
Romblon	**17,675**	1,786	2,906	**4,692**	**26.55**
Puerto Princesa City	**4,858**	1	0	**1**	**0.02**

Table 2.F.1.2 -Lifestyle Related Diseases (Unhealthy Diet)

Adults aged 20 yrs old and above who are

Philippines, 2023

Area	Total No. of clients ≥ 20 y/o risk-assessed	Overweight or obese			
		Male	Female	Total	%
**Region 5**	**2,404,832**	**368,689**	**425,821**	**794,510**	**33.04**
Albay	**472,570**	69,823	72,829	**142,652**	**30.19**
Camarines Norte	**230,383**	32,674	33,921	**66,595**	**28.91**
Camarines Sur	**720,606**	106,343	123,989	**230,332**	**31.96**
Catanduanes	**120,903**	22,273	27,314	**49,587**	**41.01**
Masbate	**475,611**	87,715	104,059	**191,774**	**40.32**
Sorsogon	**317,307**	40,178	52,375	**92,553**	**29.17**
Naga City	**67,452**	9,683	11,334	**21,017**	**31.16**
**Region 6**	**1,083,066**	**59,367**	**74,375**	**133,742**	**12.35**
Aklan	**94,571**	3,170	3,899	**7,069**	**7.47**
Antique	**63,315**	1,841	2,931	**4,772**	**7.54**
Capiz	**129,567**	6,852	10,230	**17,082**	**13.18**
Guimaras	**65,716**	4,053	5,616	**9,669**	**14.71**
Iloilo	**338,964**	25,924	26,677	**52,601**	**15.52**
Negros Occidental	**308,256**	15,185	19,905	**35,090**	**11.38**
Bacolod City	**18,935**	1,038	3,173	**4,211**	**22.24**
Iloilo City	**63,742**	1,304	1,944	**3,248**	**5.10**
**Region 7**	**594,027**	**42,913**	**65,085**	**107,998**	**18.18**
Bohol	**104,526**	8,817	12,093	**20,910**	**20.00**
Cebu	**293,490**	27,092	39,195	**66,287**	**22.59**
Negros Oriental	**50,924**	2,821	6,590	**9,411**	**18.48**
Siquijor	**8,757**	615	1,051	**1,666**	**19.02**
Cebu City	**28,100**	3,081	5,520	**8,601**	**30.61**
Lapu-Lapu City	**41,734**	291	360	**651**	**1.56**
Mandaue City	**66,496**	196	276	**472**	**0.71**
**Region 8**	**438,837**	**15,681**	**19,616**	**35,297**	**8.04**
Biliran	**20,163**	1,404	1,710	**3,114**	**15.44**
Eastern Samar	**99,223**	3,427	3,192	**6,619**	**6.67**
Northern Leyte	**63,945**	1,210	1,638	**2,848**	**4.45**
Northern Samar	**36,123**	1,785	2,712	**4,497**	**12.45**
Southern Leyte	**55,615**	5,496	6,906	**12,402**	**22.30**
Western Samar	**89,223**	965	1,936	**2,901**	**3.25**
Ormoc City	**19,660**	355	471	**826**	**4.20**
Tacloban City	**54,885**	1,039	1,051	**2,090**	**3.81**

Table 2.F.1.2 -Lifestyle Related Diseases (Unhealthy Diet)

Adults aged 20 yrs old and above who are

Philippines, 2023

Area	Total No. of clients ≥ 20 y/o risk-assessed	Overweight or obese			
		Male	Female	Total	%
**Region 9**	**307,929**	**11,556**	**16,029**	**27,585**	**8.96**
Zamboanga del Norte	**63,223**	2,491	3,633	**6,124**	**9.69**
Zamboanga del Sur	**51,944**	1,895	3,104	**4,999**	**9.62**
Zamboanga Sibugay	**170,092**	6,412	7,859	**14,271**	**8.39**
Isabela City	**1,568**	8	17	**25**	**1.59**
Zamboanga City	**21,102**	750	1,416	**2,166**	**10.26**
**Region 10**	**432,622**	**19,717**	**26,666**	**46,383**	**10.72**
Bukidnon	**95,725**	5,384	8,880	**14,264**	**14.90**
Camiguin	**4,981**	61	121	**182**	**3.65**
Lanao del Norte	**34,353**	2,373	2,369	**4,742**	**13.80**
Misamis Occidental	**164,022**	3,639	4,518	**8,157**	**4.97**
Misamis Oriental	**87,724**	6,336	8,860	**15,196**	**17.32**
Cagayan de Oro City	**34,320**	1,411	1,367	**2,778**	**8.09**
Iligan City	**11,497**	513	551	**1,064**	**9.25**
**Region 11**	**797,234**	**87,025**	**122,338**	**209,363**	**26.26**
Davao de Oro	**160,026**	23,755	31,690	**55,445**	**34.65**
Davao del Norte	**195,001**	23,321	30,002	**53,323**	**27.34**
Davao Oriental	**151,013**	20,937	27,301	**48,238**	**31.94**
Davao del Sur	**67,772**	4,131	8,136	**12,267**	**18.10**
Davao Occidental	**27,557**	2,280	3,446	**5,726**	**20.78**
Davao City	**195,865**	12,601	21,763	**34,364**	**17.54**
**Region 12**	**391,586**	**19,205**	**26,270**	**45,475**	**11.61**
North Cotabato	**128,464**	6,047	8,055	**14,102**	**10.98**
Sarangani	**53,059**	5,266	7,436	**12,702**	**23.94**
South Cotabato	**95,962**	3,381	4,994	**8,375**	**8.73**
Sultan Kudarat	**106,390**	4,120	5,278	**9,398**	**8.83**
Gen. Santos City	**7,711**	391	507	**898**	**11.65**
**BARMM**	**230,786**	**5,220**	**6,971**	**12,191**	**5.28**
Basilan	**17,013**	128	189	**317**	**1.86**
Lanao del Sur	**123,891**	2,480	3,251	**5,731**	**4.63**
Maguindanao	**43,970**	1,470	1,886	**3,356**	**7.63**

Table 2.F.1.2 -Lifestyle Related Diseases (Unhealthy Diet)

Adults aged 20 yrs old and above who are

Philippines, 2023

Area	Total No. of clients ≥ 20 y/o risk-assessed	Overweight or obese			
		Male	Female	Total	%
Sulu	**6,685**	114	320	**434**	**6.49**
Tawi-Tawi	**25,433**	669	907	**1,576**	**6.20**
SGA	*	*	*	*	*
Cotabato City	**13,794**	359	418	**777**	**5.63**
**Caraga**	**275,302**	**17,568**	**24,244**	**41,812**	**15.19**
Agusan del Norte	**33,368**	1,346	2,223	**3,569**	**10.70**
Agusan del Sur	**82,866**	4,409	4,790	**9,199**	**11.10**
Surigao del Norte	**26,068**	1,829	2,973	**4,802**	**18.42**
Surigao del Sur	**58,122**	4,160	6,365	**10,525**	**18.11**
Province of Dinagat	**6,391**	343	590	**933**	**14.60**
Butuan City	**68,487**	5,481	7,303	**12,784**	**18.67**

Table 2.F.2.1 -Cancer Prevention and Control (Cervical Cancer Screening)
Women aged 20 yrs old and above screened for Cervical Cancer
Philippines, 2023

Area	Eligible Pop. (20 y/o and above female)	Women screened for Cervical Cancer		Women found positive or suspected for Cervical Cancer using Visual Inspection with Acetic Acid or Pap Smear	
		Total	%	Total	%
**Philippines**	**33,225,012**	**199,270**	**0.60**	**4,611**	**2.31**
**NCR**	**4,516,591**	**95,123**	**2.11**	**1,946**	**2.05**
Malabon City	**121,986**	1,746	**1.43**	23	**1.32**
Navotas City	**75,797**	5,425	**7.16**	75	**1.38**
Valenzuela City	**230,344**	6,700	**2.91**	129	**1.93**
Caloocan City	**530,000**	17,028	**3.21**	125	**0.73**
Marikina City	**153,502**	1,738	**1.13**	5	**0.29**
Pasig City	**268,051**	1,056	**0.39**	41	**3.88**
Pateros	**21,910**	981	**4.48**	10	**1.02**
Taguig City	**288,153**	15,630	**5.42**	411	**2.63**
Quezon City	**992,183**	9,024	**0.91**	124	**1.37**
Makati City	**232,541**	4,993	**2.15**	277	**5.55**
Mandaluyong City	**150,874**	2,195	**1.45**	48	**2.19**
San Juan City	**46,537**	392	**0.84**	21	**5.36**
Manila City	**625,460**	21,893	**3.50**	495	**2.26**
Las Piñas City	**205,687**	458	**0.22**	26	**5.68**
Muntinlupa City	**178,508**	461	**0.26**	75	**16.27**
Parañaque City	**238,355**	2,481	**1.04**	35	**1.41**
Pasay City	**156,703**	2,922	**1.86**	26	**0.89**
**CAR**	**560,344**	**3,861**	**0.69**	**196**	**5.08**
Abra	**78,001**	26	**0.03**	1	**3.85**
Apayao	**37,562**	2	**0.01**	0	**0.00**
Benguet	**139,356**	1,380	**0.99**	56	**4.06**
Ifugao	**60,502**	126	**0.21**	53	**42.06**
Kalinga	**68,059**	18	**0.03**	0	**0.00**
Mountain Province	**46,991**	499	**1.06**	13	**2.61**
Baguio City	**129,873**	1,810	**1.39**	73	**4.03**
**Region 1**	**1,680,611**	**1,395**	**0.08**	**35**	**2.51**
Ilocos Norte	**206,676**	90	**0.04**	0	**0.00**
Ilocos Sur	**234,240**	486	**0.21**	32	**6.58**
La Union	**270,291**	325	**0.12**	3	**0.92**
Pangasinan	**914,755**	494	**0.05**	0	**0.00**
Dagupan City	**54,649**	0	**0.00**	0	**0.00**
**Region 2**	**1,152,694**	**2,166**	**0.19**	**86**	**3.97**
Batanes	**6,155**	0	**0.00**	0	**0.00**
Cagayan	**401,718**	536	**0.13**	5	**0.93**
Isabela	**483,225**	717	**0.15**	26	**3.63**
Nueva Vizcaya	**153,001**	560	**0.37**	6	**1.07**
Quirino	**61,463**	5	**0.01**	0	**0.00**

Table 2.F.2.1 -Cancer Prevention and Control (Cervical Cancer Screening)
Women aged 20 yrs old and above screened for Cervical Cancer
Philippines, 2023

Area	Eligible Pop. (20 y/o and above female)	Women screened for Cervical Cancer		Women found positive or suspected for Cervical Cancer using Visual Inspection with Acetic Acid or Pap Smear	
		Total	%	Total	%
Santiago City	**47,132**	348	**0.74**	49	**14.08**
**Region 3**	**3,906,413**	**26,203**	**0.67**	**250**	**0.95**
Aurora	**69,556**	20	**0.03**	0	**0.00**
Bataan	**267,239**	320	**0.12**	6	**1.88**
Bulacan	**1,177,120**	22,186	**1.88**	219	**0.99**
Nueva Ecija	**721,713**	721	**0.10**	10	**1.39**
Pampanga	**772,254**	1,052	**0.14**	0	**0.00**
Tarlac	**461,120**	143	**0.03**	6	**4.20**
Zambales	**203,085**	287	**0.14**	4	**1.39**
Angeles City	**147,661**	1,456	**0.99**	5	**0.34**
Olongapo	**86,665**	18	**0.02**	0	**0.00**
**Region 4A**	**5,184,464**	**10,834**	**0.21**	**690**	**6.37**
Batangas	**934,932**	2,748	**0.29**	187	**6.80**
Cavite	**1,429,336**	2,086	**0.15**	122	**5.85**
Laguna	**1,114,012**	2,291	**0.21**	81	**3.54**
Quezon	**572,906**	2,780	**0.49**	258	**9.28**
Rizal	**1,047,431**	909	**0.09**	42	**4.62**
Lucena City	**85,847**	20	**0.02**	0	**0.00**
**Region 4B**	**926,088**	**1,500**	**0.16**	**243**	**16.20**
Marinduque	**71,559**	0	**0.00**	0	**0.00**
Occidental Mindoro	**144,369**	109	**0.08**	16	**14.68**
Oriental Mindoro	**267,473**	35	**0.01**	11	**31.43**
Palawan	**254,167**	1,308	**0.51**	216	**16.51**
Romblon	**92,261**	0	**0.00**	0	**0.00**
Puerto Princesa City	**96,259**	48	**0.05**	0	**0.00**
**Region 5**	**1,722,705**	**10,766**	**0.62**	**77**	**0.72**
Albay	**408,218**	2,846	**0.70**	8	**0.28**
Camarines Norte	**179,680**	34	**0.02**	0	**0.00**
Camarines Sur	**515,360**	1,796	**0.35**	52	**2.90**
Catanduanes	**76,996**	153	**0.20**	0	**0.00**
Masbate	**237,742**	5,314	**2.24**	5	**0.09**
Sorsogon	**237,288**	451	**0.19**	12	**2.66**
Naga City	**67,421**	172	**0.26**	0	**0.00**
**Region 6**	**2,436,731**	**4,965**	**0.20**	**58**	**1.17**
Aklan	**191,910**	251	**0.13**	13	**5.18**
Antique	**182,428**	128	**0.07**	3	**2.34**
Capiz	**252,932**	156	**0.06**	0	**0.00**
Guimaras	**58,302**	35	**0.06**	7	**20.00**
Iloilo	**639,588**	1,729	**0.27**	4	**0.23**
Negros Occidental	**766,545**	2,414	**0.31**	27	**1.12**

Table 2.F.2.1 -Cancer Prevention and Control (Cervical Cancer Screening)
Women aged 20 yrs old and above screened for Cervical Cancer
Philippines, 2023

Area	Eligible Pop. (20 y/o and above female)	Women screened for Cervical Cancer		Women found positive or suspected for Cervical Cancer using Visual Inspection with Acetic Acid or Pap Smear	
		Total	%	Total	%
Bacolod City	**193,833**	64	**0.03**	0	**0.00**
Iloilo City	**151,193**	188	**0.12**	4	**2.13**
**Region 7**	**2,472,579**	**10,383**	**0.42**	**218**	**2.10**
Bohol	**424,533**	74	**0.02**	1	**1.35**
Cebu	**988,347**	8,690	**0.88**	189	**2.17**
Negros Oriental	**433,965**	120	**0.03**	4	**3.33**
Siquijor	**33,994**	0	**0.00**	0	**0.00**
Cebu City	**312,024**	722	**0.23**	24	**3.32**
Lapu-Lapu City	**158,178**	41	**0.03**	0	**0.00**
Mandaue City	**121,538**	736	**0.61**	0	**0.00**
**Region 8**	**1,300,762**	**752**	**0.06**	**26**	**3.46**
Biliran	**52,056**	0	**0.00**	0	**0.00**
Eastern Samar	**135,401**	186	**0.14**	0	**0.00**
Northern Leyte	**446,771**	192	**0.04**	9	**4.69**
Northern Samar	**170,665**	168	**0.10**	14	**8.33**
Southern Leyte	**131,926**	146	**0.11**	3	**2.05**
Western Samar	**218,518**	60	**0.03**	0	**0.00**
Ormoc City	**68,865**	0	**0.00**	0	**0.00**
Tacloban City	**76,560**	0	**0.00**	0	**0.00**
**Region 9**	**1,100,888**	**679**	**0.06**	**9**	**1.33**
Zamboanga Del Norte	**292,931**	19	**0.01**	1	**5.26**
Zamboanga Del Sur	**295,398**	126	**0.04**	1	**0.79**
Zamboanga Sibugay	**183,637**	320	**0.17**	0	**0.00**
Isabela City	**35,475**	0	**0.00**	0	**0.00**
Zamboanga City	**293,447**	214	**0.07**	7	**3.27**
**Region 10**	**1,454,924**	**1,512**	**0.10**	**35**	**2.31**
Bukidnon	**423,470**	947	**0.22**	18	**1.90**
Camiguin	**27,832**	0	**0.00**	0	**0.00**
Lanao Del Norte	**193,774**	4	**0.00**	0	**0.00**
Misamis Occidental	**190,495**	63	**0.03**	9	**14.29**
Misamis Oriental	**275,499**	454	**0.16**	6	**1.32**
Cagayan de Oro City	**234,169**	37	**0.02**	1	**2.70**

Table 2.F.2.1 -Cancer Prevention and Control (Cervical Cancer Screening)
Women aged 20 yrs old and above screened for Cervical Cancer
Philippines, 2023

Area	Eligible Pop. (20 y/o and above female)	Women screened for Cervical Cancer		Women found positive or suspected for Cervical Cancer using Visual Inspection with Acetic Acid or Pap Smear	
		Total	%	Total	%
Iligan City	**109,685**	7	**0.01**	1	**14.29**
**Region 11**	**1,549,867**	**22,633**	**1.46**	**580**	**2.56**
Davao de Oro	**215,685**	5,907	**2.74**	115	**1.95**
Davao del Norte	**333,371**	3,652	**1.10**	159	**4.35**
Davao Oriental	**161,962**	1,909	**1.18**	71	**3.72**
Davao del Sur	**203,889**	3,489	**1.71**	171	**4.90**
Davao Occidental	**80,642**	539	**0.67**	30	**5.57**
Davao City	**554,318**	7,137	**1.29**	34	**0.48**
**Region 12**	**1,249,878**	**2,460**	**0.20**	**24**	**0.98**
North Cotabato	**362,376**	199	**0.05**	16	**8.04**
Sarangani	**150,401**	961	**0.64**	6	**0.62**
South Cotabato	**285,335**	349	**0.12**	1	**0.29**
Sultan Kudarat	**238,325**	933	**0.39**	1	**0.11**
Gen. Santos City	**213,441**	18	**0.01**	0	**0.00**
**BARMM**	**1,206,769**	**1,438**	**0.12**	**47**	**3.27**
Basilan	**101,641**	0	**0.00**	0	**0.00**
Lanao Del Sur	**287,277**	503	**0.18**	40	**7.95**
Maguindanao	**312,630**	838	**0.27**	7	**0.84**
Sulu	**244,710**	0	**0.00**	0	**0.00**
Tawi-Tawi	**107,435**	0	**0.00**	0	**0.00**
SGA	**59,943**	*	*	*	*
Cotabato City	**93,133**	97	**0.10**	0	**0.00**
**Caraga**	**802,704**	**2,600**	**0.32**	**91**	**3.50**
Agusan del Norte	**111,036**	348	**0.31**	0	**0.00**
Agusan del Sur	**197,532**	277	**0.14**	15	**5.42**
Surigao del Norte	**159,623**	535	**0.34**	1	**0.19**
Surigao del Sur	**184,479**	906	**0.49**	39	**4.30**
Province of Dinagat	**36,965**	494	**1.34**	36	**7.29**
Butuan City	**113,069**	40	**0.04**	0	**0.00**

Table 2.F.2.1 -Cancer Prevention and Control (Breast Cancer Screening)
Women aged 20 yrs old and above screened for Breast Cancer
Philippines, 2023

Area	Eligible Pop. (20 y/o and above female)	Women screened for breast mass		Women with suspicious breast mass	
		Total	%	Total	%
**Philippines**	**33,225,012**	**830,691**	**2.50**	**12,288**	**1.48**
**NCR**	**4,516,591**	**177,232**	**3.92**	**2,534**	**1.43**
Malabon City	**121,986**	1,815	**1.49**	14	**0.77**
Navotas City	**75,797**	5,395	**7.12**	9	**0.17**
Valenzuela City	**230,344**	3,432	**1.49**	40	**1.17**
Caloocan City	**530,000**	16,801	**3.17**	78	**0.46**
Marikina City	**153,502**	2,049	**1.33**	43	**2.10**
Pasig City	**268,051**	2,516	**0.94**	79	**3.14**
Pateros	**21,910**	891	**4.07**	28	**3.14**
Taguig City	**288,153**	20,975	**7.28**	1,019	**4.86**
Quezon City	**992,183**	24,867	**2.51**	144	**0.58**
Makati City	**232,541**	7,070	**3.04**	43	**0.61**
Mandaluyong City	**150,874**	2,289	**1.52**	66	**2.88**
San Juan City	**46,537**	224	**0.48**	2	**0.89**
Manila City	**625,460**	66,629	**10.65**	743	**1.12**
Las Piñas City	**205,687**	1,297	**0.63**	28	**2.16**
Muntinlupa City	**178,508**	1,089	**0.61**	58	**5.33**
Parañaque City	**238,355**	6,468	**2.71**	124	**1.92**
Pasay City	**156,703**	13,425	**8.57**	16	**0.12**
**CAR**	**560,344**	**30,921**	**5.52**	**477**	**1.54**
Abra	**78,001**	1,777	**2.28**	15	**0.84**
Apayao	**37,562**	4,520	**12.03**	18	**0.40**
Benguet	**139,356**	10,420	**7.48**	264	**2.53**
Ifugao	**60,502**	872	**1.44**	13	**1.49**
Kalinga	**68,059**	1,173	**1.72**	19	**1.62**
Mountain Province	**46,991**	2,704	**5.75**	18	**0.67**
Baguio City	**129,873**	9,455	**7.28**	130	**1.37**
**Region 1**	**1,680,611**	**18,169**	**1.08**	**78**	**0.43**
Ilocos Norte	**206,676**	377	**0.18**	6	**1.59**
Ilocos Sur	**234,240**	9,301	**3.97**	32	**0.34**
La Union	**270,291**	6,646	**2.46**	8	**0.12**
Pangasinan	**914,755**	1,844	**0.20**	31	**1.68**
Dagupan City	**54,649**	1	**0.00**	1	**100.00**

Table 2.F.2.1 -Cancer Prevention and Control (Breast Cancer Screening)
Women aged 20 yrs old and above screened for Breast Cancer
Philippines, 2023

Area	Eligible Pop. (20 y/o and above female)	Women screened for breast mass		Women with suspicious breast mass	
		Total	%	Total	%
**Region 2**	**1,152,694**	**32,154**	**2.79**	**373**	**1.16**
Batanes	**6,155**	0	**0.00**	0	**0.00**
Cagayan	**401,718**	3,621	**0.90**	16	**0.44**
Isabela	**483,225**	12,011	**2.49**	289	**2.41**
Nueva Vizcaya	**153,001**	6,095	**3.98**	32	**0.53**
Quirino	**61,463**	1,640	**2.67**	11	**0.67**
Santiago City	**47,132**	8,787	**18.64**	25	**0.28**
**Region 3**	**3,906,413**	**186,676**	**4.78**	**1,021**	**0.55**
Aurora	**69,556**	2,972	**4.27**	18	**0.61**
Bataan	**267,239**	7,025	**2.63**	142	**2.02**
Bulacan	**1,177,120**	154,752	**13.15**	490	**0.32**
Nueva Ecija	**721,713**	2,866	**0.40**	246	**8.58**
Pampanga	**772,254**	14,309	**1.85**	32	**0.22**
Tarlac	**461,120**	559	**0.12**	33	**5.90**
Zambales	**203,085**	1,811	**0.89**	39	**2.15**
Angeles City	**147,661**	2,145	**1.45**	14	**0.65**
Olongapo	**86,665**	237	**0.27**	7	**2.95**
**Region 4A**	**5,184,464**	**21,804**	**0.42**	**689**	**3.16**
Batangas	**934,932**	2,963	**0.32**	77	**2.60**
Cavite	**1,429,336**	2,909	**0.20**	96	**3.30**
Laguna	**1,114,012**	7,969	**0.72**	212	**2.66**
Quezon	**572,906**	5,370	**0.94**	164	**3.05**
Rizal	**1,047,431**	2,446	**0.23**	134	**5.48**
Lucena City	**85,847**	147	**0.17**	6	**4.08**
**Region 4B**	**926,088**	**5,378**	**0.58**	**160**	**2.98**
Marinduque	**71,559**	6	**0.01**	2	**33.33**
Occidental Mindoro	**144,369**	3,060	**2.12**	29	**0.95**
Oriental Mindoro	**267,473**	21	**0.01**	3	**14.29**
Palawan	**254,167**	2,268	**0.89**	120	**5.29**
Romblon	**92,261**	11	**0.01**	6	**54.55**
Puerto Princesa City	**96,259**	12	**0.01**	0	**0.00**
**Region 5**	**1,722,705**	**73,843**	**4.29**	**2,821**	**3.82**
Albay	**408,218**	16,935	**4.15**	539	**3.18**

Table 2.F.2.1 -Cancer Prevention and Control (Breast Cancer Screening)
Women aged 20 yrs old and above screened for Breast Cancer
Philippines, 2023

Area	Eligible Pop. (20 y/o and above female)	Women screened for breast mass		Women with suspicious breast mass	
		Total	%	Total	%
Camarines Norte	**179,680**	1,858	**1.03**	9	**0.48**
Camarines Sur	**515,360**	30,322	**5.88**	430	**1.42**
Catanduanes	**76,996**	30	**0.04**	17	**56.67**
Masbate	**237,742**	9,116	**3.83**	1,545	**16.95**
Sorsogon	**237,288**	14,552	**6.13**	279	**1.92**
Naga City	**67,421**	1,030	**1.53**	2	**0.19**
**Region 6**	**2,436,731**	**78,044**	**3.20**	**682**	**0.87**
Aklan	**191,910**	283	**0.15**	2	**0.71**
Antique	**182,428**	3,881	**2.13**	28	**0.72**
Capiz	**252,932**	16,652	**6.58**	139	**0.83**
Guimaras	**58,302**	3,498	**6.00**	31	**0.89**
Iloilo	**639,588**	22,211	**3.47**	153	**0.69**
Negros Occidental	**766,545**	11,871	**1.55**	288	**2.43**
Bacolod City	**193,833**	571	**0.29**	27	**4.73**
Iloilo City	**151,193**	19,077	**12.62**	14	**0.07**
**Region 7**	**2,472,579**	**48,589**	**1.97**	**580**	**1.19**
Bohol	**424,533**	125	**0.03**	14	**11.20**
Cebu	**988,347**	34,454	**3.49**	257	**0.75**
Negros Oriental	**433,965**	637	**0.15**	63	**9.89**
Siquijor	**33,994**	777	**2.29**	5	**0.64**
Cebu City	**312,024**	11,326	**3.63**	93	**0.82**
Lapu-Lapu City	**158,178**	392	**0.25**	2	**0.51**
Mandaue City	**121,538**	878	**0.72**	146	**16.63**
**Region 8**	**1,300,762**	**3,596**	**0.28**	**348**	**9.68**
Biliran	**52,056**	971	**1.87**	26	**2.68**
Eastern Samar	**135,401**	197	**0.15**	3	**1.52**
Northern Leyte	**446,771**	467	**0.10**	15	**3.21**
Northern Samar	**170,665**	156	**0.09**	33	**21.15**
Southern Leyte	**131,926**	69	**0.05**	7	**10.14**
Western Samar	**218,518**	157	**0.07**	55	**35.03**
Ormoc City	**68,865**	0	**0.00**	0	**0.00**
Tacloban City	**76,560**	1,579	**2.06**	209	**13.24**
**Region 9**	**1,100,888**	**4,023**	**0.37**	**115**	**2.86**
Zamboanga Del Norte	**292,931**	767	**0.26**	23	**3.00**

Table 2.F.2.1 -Cancer Prevention and Control (Breast Cancer Screening)
Women aged 20 yrs old and above screened for Breast Cancer
Philippines, 2023

Area	Eligible Pop. (20 y/o and above female)	Women screened for breast mass		Women with suspicious breast mass	
		Total	%	Total	%
Zamboanga Del Sur	**295,398**	221	**0.07**	58	**26.24**
Zamboanga Sibugay	**183,637**	512	**0.28**	13	**2.54**
Isabela City	**35,475**	0	**0.00**	0	**0.00**
Zamboanga City	**293,447**	2,523	**0.86**	21	**0.83**
**Region 10**	**1,454,924**	**5,540**	**0.38**	**278**	**5.02**
Bukidnon	**423,470**	1,358	**0.32**	59	**4.34**
Camiguin	**27,832**	1	**0.00**	1	**100.00**
Lanao Del Norte	**193,774**	1,797	**0.93**	10	**0.56**
Misamis Occidental	**190,495**	346	**0.18**	133	**38.44**
Misamis Oriental	**275,499**	1,553	**0.56**	43	**2.77**
Cagayan de Oro City	**234,169**	405	**0.17**	6	**1.48**
Iligan City	**109,685**	80	**0.07**	26	**32.50**
**Region 11**	**1,549,867**	**78,369**	**5.06**	**912**	**1.16**
Davao de Oro	**215,685**	24,293	**11.26**	291	**1.20**
Davao del Norte	**333,371**	11,664	**3.50**	119	**1.02**
Davao Oriental	**161,962**	7,767	**4.80**	80	**1.03**
Davao del Sur	**203,889**	7,044	**3.45**	189	**2.68**
Davao Occidental	**80,642**	2,485	**3.08**	64	**2.58**
Davao City	**554,318**	25,116	**4.53**	169	**0.67**
**Region 12**	**1,249,878**	**45,729**	**3.66**	**723**	**1.58**
North Cotabato	**362,376**	5,256	**1.45**	122	**2.32**
Sarangani	**150,401**	1,582	**1.05**	1	**0.06**
South Cotabato	**285,335**	4,644	**1.63**	143	**3.08**
Sultan Kudarat	**238,325**	31,366	**13.16**	448	**1.43**
Gen. Santos City	**213,441**	2,881	**1.35**	9	**0.31**
**BARMM**	**1,206,769**	**12,450**	**1.03**	**297**	**2.39**
Basilan	**101,641**	2	**0.00**	0	**0.00**
Lanao Del Sur	**287,277**	3,756	**1.31**	252	**6.71**
Maguindanao	**312,630**	8,087	**2.59**	44	**0.54**
Sulu	**244,710**	0	**0.00**	0	**0.00**
Tawi-Tawi	**107,435**	399	**0.37**	0	**0.00**
SGA	**59,943**	*	*	*	*
Cotabato City	**93,133**	206	**0.22**	1	**0.49**

Table 2.F.2.1 -Cancer Prevention and Control (Breast Cancer Screening)

Women aged 20 yrs old and above screened for Breast Cancer

Philippines, 2023

Area	Eligible Pop. (20 y/o and above female)	Women screened for breast mass		Women with suspicious breast mass	
		Total	%	Total	%
**Caraga**	**802,704**	**8,174**	**1.02**	**200**	**2.45**
Agusan del Norte	**111,036**	3,530	**3.18**	54	**1.53**
Agusan del Sur	**197,532**	706	**0.36**	28	**3.97**
Surigao del Norte	**159,623**	630	**0.39**	38	**6.03**
Surigao del Sur	**184,479**	2,925	**1.59**	61	**2.09**
Province of Dinagat	**36,965**	202	**0.55**	18	**8.91**
Butuan City	**113,069**	181	**0.16**	1	**0.55**

Table 2.F.3.1 -Cardiovascular Disease and Diabetes Mellitus Prevention and Control

Identified 20 yrs old and above adults with Hypertension and Type 2 Diabetes Mellitus

Philippines, 2023

Area	Total No. of clients ≥ 20 y/o risk-assessed	Identified Hypertensive Adults				Identified with Type 2 Diabetes Mellitus			
		Male	Female	Total	%	Male	Female	Total	%
**Philippines**	**13,640,824**	**771,369**	**1,015,186**	**1,786,555**	**13.10**	**165,776**	**220,870**	**386,646**	**2.83**
**NCR**	**1,063,800**	**81,302**	**117,732**	**199,034**	**18.71**	**26,412**	**37,331**	**63,743**	**5.99**
Malabon City	**40,284**	3,823	5,566	**9,389**	**23.31**	1,341	2,014	**3,355**	**8.33**
Navotas City	**35,885**	2,158	3,854	**6,012**	**16.75**	657	952	**1,609**	**4.48**
Valenzuela City	**97,881**	7,999	12,199	**20,198**	**20.64**	2,818	4,040	**6,858**	**7.01**
Caloocan City	**105,658**	5,904	9,652	**15,556**	**14.72**	1,847	3,230	**5,077**	**4.81**
Marikina City	**10,822**	979	1,546	**2,525**	**23.33**	245	347	**592**	**5.47**
Pasig City	**94,498**	2,797	3,951	**6,748**	**7.14**	1,101	1,655	**2,756**	**2.92**
Pateros	**4,987**	721	1,203	**1,924**	**38.58**	267	503	**770**	**15.44**
Taguig City	**146,247**	10,960	14,933	**25,893**	**17.70**	3,297	4,388	**7,685**	**5.25**
Quezon City	**124,629**	13,808	21,377	**35,185**	**28.23**	4,144	5,605	**9,749**	**7.82**
Makati City	**43,689**	1,263	1,872	**3,135**	**7.18**	751	1,286	**2,037**	**4.66**
Mandaluyong City	**47,031**	6,789	7,759	**14,548**	**30.93**	1,655	1,787	**3,442**	**7.32**
San Juan City	**10,026**	1,493	2,462	**3,955**	**39.45**	494	836	**1,330**	**13.27**
Manila City	**154,619**	11,335	15,746	**27,081**	**17.51**	2,330	3,156	**5,486**	**3.55**
Las Piñas City	**6,348**	1,304	1,951	**3,255**	**51.28**	698	896	**1,594**	**25.11**
Muntinlupa City	**25,041**	1,047	1,270	**2,317**	**9.25**	317	414	**731**	**2.92**
Parañaque City	**49,782**	6,974	9,731	**16,705**	**33.56**	3,885	5,359	**9,244**	**18.57**
Pasay City	**66,373**	1,948	2,660	**4,608**	**6.94**	565	863	**1,428**	**2.15**
**CAR**	**308,658**	**10,818**	**16,227**	**27,045**	**8.76**	**1,058**	**1,902**	**2,960**	**0.96**
Abra	**64,399**	1,078	1,352	**2,430**	**3.77**	93	171	**264**	**0.41**
Apayao	**34,351**	289	433	**722**	**2.10**	49	91	**140**	**0.41**
Benguet	**42,010**	2,305	3,875	**6,180**	**14.71**	354	609	**963**	**2.29**
Ifugao	**35,912**	935	1,509	**2,444**	**6.81**	97	131	**228**	**0.63**
Kalinga	**70,401**	3,250	4,641	**7,891**	**11.21**	210	509	**719**	**1.02**
Mountain Province	**25,881**	1,793	2,630	**4,423**	**17.09**	147	248	**395**	**1.53**
Baguio City	**35,704**	1,168	1,787	**2,955**	**8.28**	108	143	**251**	**0.70**
**Region 1**	**852,603**	**53,237**	**71,982**	**125,219**	**14.69**	**13,056**	**17,471**	**30,527**	**3.58**
Ilocos Norte	**55,638**	7,818	11,328	**19,146**	**34.41**	1,780	2,641	**4,421**	**7.95**
Ilocos Sur	**183,792**	17,207	23,548	**40,755**	**22.17**	3,288	4,789	**8,077**	**4.39**
La Union	**195,314**	10,958	12,992	**23,950**	**12.26**	2,955	4,034	**6,989**	**3.58**
Pangasinan	**401,473**	16,501	23,532	**40,033**	**9.97**	4,625	5,671	**10,296**	**2.56**

Table 2.F.3.1 -Cardiovascular Disease and Diabetes Mellitus Prevention and Control

Identified 20 yrs old and above adults with Hypertension and Type 2 Diabetes Mellitus

Philippines, 2023

Area	Total No. of clients ≥ 20 y/o risk-assessed	Identified Hypertensive Adults				Identified with Type 2 Diabetes Mellitus			
		Male	Female	Total	%	Male	Female	Total	%
Dagupan City	**16,386**	753	582	**1,335**	**8.15**	408	336	**744**	**4.54**
**Region 2**	**865,504**	**36,883**	**47,792**	**84,675**	**9.78**	**4,879**	**7,119**	**11,998**	**1.39**
Batanes	**4,172**	39	45	**84**	**2.01**	31	31	**62**	**1.49**
Cagayan	**169,657**	1,129	1,346	**2,475**	**1.46**	206	349	**555**	**0.33**
Isabela	**451,296**	24,638	31,871	**56,509**	**12.52**	3,142	4,240	**7,382**	**1.64**
Nueva Vizcaya	**57,833**	1,080	1,233	**2,313**	**4.00**	143	234	**377**	**0.65**
Quirino	**91,046**	9,666	12,869	**22,535**	**24.75**	1,219	2,060	**3,279**	**3.60**
Santiago City	**91,500**	331	428	**759**	**0.83**	138	205	**343**	**0.37**
**Region 3**	**2,709,268**	**122,298**	**164,358**	**286,656**	**10.58**	**33,413**	**43,588**	**77,001**	**2.84**
Aurora	**119,464**	1,842	2,259	**4,101**	**3.43**	121	195	**316**	**0.26**
Bataan	**155,589**	2,591	3,241	**5,832**	**3.75**	610	829	**1,439**	**0.92**
Bulacan	**1,092,544**	61,967	85,090	**147,057**	**13.46**	19,172	26,046	**45,218**	**4.14**
Nueva Ecija	**436,471**	15,887	22,211	**38,098**	**8.73**	3,543	4,928	**8,471**	**1.94**
Pampanga	**413,038**	22,356	30,206	**52,562**	**12.73**	5,478	7,287	**12,765**	**3.09**
Tarlac	**197,332**	7,614	7,969	**15,583**	**7.90**	2,953	2,217	**5,170**	**2.62**
Zambales	**226,526**	8,531	11,408	**19,939**	**8.80**	1,131	1,470	**2,601**	**1.15**
Angeles City	**25,090**	385	539	**924**	**3.68**	130	193	**323**	**1.29**
Olongapo	**43,214**	1,125	1,435	**2,560**	**5.92**	275	423	**698**	**1.62**
**Region 4A**	**694,624**	**48,940**	**64,512**	**113,452**	**16.33**	**13,140**	**16,085**	**29,225**	**4.21**
Batangas	**229,894**	12,140	13,349	**25,489**	**11.09**	4,436	4,674	**9,110**	**3.96**
Cavite	**216,758**	14,882	20,485	**35,367**	**16.32**	3,985	4,856	**8,841**	**4.08**
Laguna	**84,512**	7,122	10,107	**17,229**	**20.39**	1,721	2,368	**4,089**	**4.84**
Quezon	**84,147**	3,669	6,045	**9,714**	**11.54**	579	897	**1,476**	**1.75**
Rizal	**65,762**	9,883	12,675	**22,558**	**34.30**	2,185	3,010	**5,195**	**7.90**
Lucena City	**13,551**	1,244	1,851	**3,095**	**22.84**	234	280	**514**	**3.79**
**Region 4B**	**190,146**	**6,283**	**9,209**	**15,492**	**8.15**	**1,085**	**1,827**	**2,912**	**1.53**
Marinduque	**8,570**	526	775	**1,301**	**15.18**	101	159	**260**	**3.03**
Occidental Mindoro	**51,374**	990	1,482	**2,472**	**4.81**	193	301	**494**	**0.96**
Oriental Mindoro	**26,288**	1,971	2,843	**4,814**	**18.31**	318	485	**803**	**3.05**
Palawan	**81,381**	1,458	1,909	**3,367**	**4.14**	172	339	**511**	**0.63**
Romblon	**17,675**	496	722	**1,218**	**6.89**	47	93	**140**	**0.79**

Table 2.F.3.1 -Cardiovascular Disease and Diabetes Mellitus Prevention and Control

Identified 20 yrs old and above adults with Hypertension and Type 2 Diabetes Mellitus

Philippines, 2023

Area	Total No. of clients ≥ 20 y/o risk-assessed	Identified Hypertensive Adults				Identified with Type 2 Diabetes Mellitus			
		Male	Female	Total	%	Male	Female	Total	%
Puerto Princesa City	**4,858**	842	1,478	**2,320**	**47.76**	254	450	**704**	**14.49**
**Region 5**	**2,404,832**	**87,540**	**94,367**	**181,907**	**7.56**	**5,119**	**5,936**	**11,055**	**0.46**
Albay	**472,570**	26,999	25,257	**52,256**	**11.06**	1,392	1,633	**3,025**	**0.64**
Camarines Norte	**230,383**	6,560	5,331	**11,891**	**5.16**	262	271	**533**	**0.23**
Camarines Sur	**720,606**	11,513	14,666	**26,179**	**3.63**	563	697	**1,260**	**0.17**
Catanduanes	**120,903**	2,291	2,831	**5,122**	**4.24**	168	247	**415**	**0.34**
Masbate	**475,611**	26,625	32,572	**59,197**	**12.45**	1,701	2,360	**4,061**	**0.85**
Sorsogon	**317,307**	5,049	4,463	**9,512**	**3.00**	466	503	**969**	**0.31**
Naga City	**67,452**	8,503	9,247	**17,750**	**26.32**	567	225	**792**	**1.17**
**Region 6**	**1,083,066**	**92,067**	**120,025**	**212,092**	**19.58**	**24,842**	**29,598**	**54,440**	**5.03**
Aklan	**94,571**	**10,047**	**12,867**	**22,914**	**24.23**	**4,161**	**4,733**	**8,894**	**9.40**
Antique	**63,315**	**7,147**	**10,157**	**17,304**	**27.33**	**1,726**	**2,253**	**3,979**	**6.28**
Capiz	**129,567**	**13,674**	**20,557**	**34,231**	**26.42**	**2,832**	**3,893**	**6,725**	**5.19**
Guimaras	**65,716**	**4,317**	**5,476**	**9,793**	**14.90**	**630**	**891**	**1,521**	**2.31**
Iloilo	**338,964**	**32,790**	**37,603**	**70,393**	**20.77**	**10,606**	**11,107**	**21,713**	**6.41**
Negros Occidental	**308,256**	**15,304**	**21,270**	**36,574**	**11.86**	**1,844**	**2,632**	**4,476**	**1.45**
Bacolod City	**18,935**	**830**	**1,658**	**2,488**	**13.14**	**213**	**413**	**626**	**3.31**
Iloilo City	**63,742**	**7,958**	**10,437**	**18,395**	**28.86**	**2,830**	**3,676**	**6,506**	**10.21**
**Region 7**	**594,027**	**35,732**	**48,524**	**84,256**	**14.18**	**4,883**	**7,445**	**12,328**	**2.08**
Bohol	**104,526**	2,748	3,358	**6,106**	**5.84**	300	397	**697**	**0.67**
Cebu	**293,490**	27,211	36,399	**63,610**	**21.67**	3,180	4,900	**8,080**	**2.75**
Negros Oriental	**50,924**	1,134	1,806	**2,940**	**5.77**	401	692	**1,093**	**2.15**
Siquijor	**8,757**	688	1,263	**1,951**	**22.28**	116	216	**332**	**3.79**
Cebu City	**28,100**	2,652	4,048	**6,700**	**23.84**	698	1,026	**1,724**	**6.14**
Lapu-Lapu City	**41,734**	500	625	**1,125**	**2.70**	92	89	**181**	**0.43**
Mandaue City	**66,496**	799	1,025	**1,824**	**2.74**	96	125	**221**	**0.33**
**Region 8**	**438,837**	**18,522**	**25,923**	**44,445**	**10.13**	**2,562**	**3,496**	**6,058**	**1.38**
Biliran	**20,163**	637	820	**1,457**	**7.23**	120	117	**237**	**1.18**
Eastern Samar	**99,223**	2,328	3,803	**6,131**	**6.18**	401	674	**1,075**	**1.08**
Northern Leyte	**63,945**	2,884	5,149	**8,033**	**12.56**	437	621	**1,058**	**1.65**
Northern Samar	**36,123**	924	1,276	**2,200**	**6.09**	141	239	**380**	**1.05**
Southern Leyte	**55,615**	604	686	**1,290**	**2.32**	129	177	**306**	**0.55**
Western Samar	**89,223**	5,850	8,325	**14,175**	**15.89**	772	956	**1,728**	**1.94**

Table 2.F.3.1 -Cardiovascular Disease and Diabetes Mellitus Prevention and Control

Identified 20 yrs old and above adults with Hypertension and Type 2 Diabetes Mellitus

Philippines, 2023

Area	Total No. of clients ≥ 20 y/o risk-assessed	Identified Hypertensive Adults				Identified with Type 2 Diabetes Mellitus			
		Male	Female	Total	%	Male	Female	Total	%
Ormoc City	**19,660**	4,981	5,429	**10,410**	**52.95**	535	659	**1,194**	**6.07**
Tacloban City	**54,885**	314	435	**749**	**1.36**	27	53	**80**	**0.15**
**Region 9**	**307,929**	**8,014**	**10,457**	**18,471**	**6.00**	**1,376**	**1,867**	**3,243**	**1.05**
Zamboanga Del Norte	**63,223**	3,011	3,771	**6,782**	**10.73**	518	708	**1,226**	**1.94**
Zamboanga Del Sur	**51,944**	1,313	1,834	**3,147**	**6.06**	236	327	**563**	**1.08**
Zamboanga Sibugay	**170,092**	2,520	3,277	**5,797**	**3.41**	397	533	**930**	**0.55**
Isabela City	**1,568**	77	113	**190**	**12.12**	22	38	**60**	**3.83**
Zamboanga City	**21,102**	1,093	1,462	**2,555**	**12.11**	203	261	**464**	**2.20**
**Region 10**	**432,622**	**34,403**	**45,943**	**80,346**	**18.57**	**5,545**	**7,738**	**13,283**	**3.07**
Bukidnon	**95,725**	6,228	7,411	**13,639**	**14.25**	590	864	**1,454**	**1.52**
Camiguin	**4,981**	63	102	**165**	**3.31**	12	21	**33**	**0.66**
Lanao Del Norte	**34,353**	1,252	1,756	**3,008**	**8.76**	317	422	**739**	**2.15**
Misamis Occidental	**164,022**	16,483	23,325	**39,808**	**24.27**	2,119	3,088	**5,207**	**3.17**
Misamis Oriental	**87,724**	6,896	9,427	**16,323**	**18.61**	1,505	2,058	**3,563**	**4.06**
Cagayan de Oro City	**34,320**	1,470	1,651	**3,121**	**9.09**	500	659	**1,159**	**3.38**
Iligan City	**11,497**	2,011	2,271	**4,282**	**37.24**	502	626	**1,128**	**9.81**
**Region 11**	**797,234**	**58,653**	**80,076**	**138,729**	**17.40**	**15,285**	**21,613**	**36,898**	**4.63**
Davao de Oro	**160,026**	15,406	23,598	**39,004**	**24.37**	2,998	4,968	**7,966**	**4.98**
Davao del Norte	**195,001**	11,602	15,513	**27,115**	**13.91**	3,011	4,161	**7,172**	**3.68**
Davao Oriental	**151,013**	7,045	8,517	**15,562**	**10.31**	2,035	2,892	**4,927**	**3.26**
Davao del Sur	**67,772**	7,798	10,309	**18,107**	**26.72**	1,299	2,196	**3,495**	**5.16**
Davao Occidental	**27,557**	1,925	2,691	**4,616**	**16.75**	325	436	**761**	**2.76**
Davao City	**195,865**	14,877	19,448	**34,325**	**17.52**	5,617	6,960	**12,577**	**6.42**
**Region 12**	**391,586**	**12,890**	**16,079**	**28,969**	**7.40**	**3,356**	**4,027**	**7,383**	**1.89**
North Cotabato	**128,464**	5,471	7,286	**12,757**	**9.93**	772	1,066	**1,838**	**1.43**
Sarangani	**53,059**	1,628	2,344	**3,972**	**7.49**	281	437	**718**	**1.35**
South Cotabato	**95,962**	2,483	2,970	**5,453**	**5.68**	431	618	**1,049**	**1.09**
Sultan Kudarat	**106,390**	2,790	2,763	**5,553**	**5.22**	1,715	1,693	**3,408**	**3.20**
Gen. Santos City	**7,711**	518	716	**1,234**	**16.00**	157	213	**370**	**4.80**

Table 2.F.3.1 -Cardiovascular Disease and Diabetes Mellitus Prevention and Control

Identified 20 yrs old and above adults with Hypertension and Type 2 Diabetes Mellitus

Philippines, 2023

Area	Total No. of clients ≥ 20 y/o risk-assessed	Identified Hypertensive Adults				Identified with Type 2 Diabetes Mellitus			
		Male	Female	Total	%	Male	Female	Total	%
**BARMM**	**230,786**	**41,944**	**57,316**	**99,260**	**43.01**	**5,497**	**7,968**	**13,465**	**5.83**
Basilan	**17,013**	4,645	6,588	**11,233**	**66.03**	654	849	**1,503**	**8.83**
Lanao Del Sur	**123,891**	26,173	35,399	**61,572**	**49.70**	3,035	4,403	**7,438**	**6.00**
Maguindanao	**43,970**	4,299	5,548	**9,847**	**22.39**	752	1,099	**1,851**	**4.21**
Sulu	**6,685**	920	1,607	**2,527**	**37.80**	165	258	**423**	**6.33**
Tawi-Tawi	**25,433**	3,662	4,508	**8,170**	**32.12**	318	523	**841**	**3.31**
SGA	*	*	*	*	*	*	*	*	*
Cotabato City	**13,794**	2,245	3,666	**5,911**	**42.85**	573	836	**1,409**	**10.21**
**Caraga**	**275,302**	21,843	24,664	**46,507**	**16.89**	4,268	5,859	**10,127**	**3.68**
Agusan del Norte	**33,368**	3,954	4,802	**8,756**	**26.24**	802	1,092	**1,894**	**5.68**
Agusan del Sur	**82,866**	7,274	8,128	**15,402**	**18.59**	1,160	1,552	**2,712**	**3.27**
Surigao del Norte	**26,068**	3,352	3,450	**6,802**	**26.09**	743	957	**1,700**	**6.52**
Surigao del Sur	**58,122**	6,124	6,917	**13,041**	**22.44**	1,284	1,879	**3,163**	**5.44**
Province of Dinagat	**6,391**	237	301	**538**	**8.42**	39	96	**135**	**2.11**
Butuan City	**68,487**	902	1,066	**1,968**	**2.87**	240	283	**523**	**0.76**

Table 2.F.4.1 -Blindness Prevention Program

Senior citizens 60 yrs old and above screened for visual acuity and diagnosed with eye diseases

Philippines, 2023

Area	Eligible Pop.(60 y/o and above)	Screened for Visual Acuity				Diagnosed with eye diseases			
		Male	Female	Total	%	Male	Female	Total	%
**Philippines**	**9,460,253**	**149,123**	**213,853**	**362,976**	**3.84**	**28,399**	**39,162**	**67,561**	**18.61**
**NCR**	**1,087,877**	**51,309**	**75,476**	**126,785**	**11.65**	**6,783**	**9,708**	**16,491**	**13.01**
City of Malabon	**30,451**	1,476	1,823	**3,299**	**10.83**	70	81	**151**	**4.58**
City of Navotas	**18,722**	465	669	**1,134**	**6.06**	13	7	**20**	**1.76**
City of Valenzuela	**51,512**	2,173	3,062	**5,235**	**10.16**	195	301	**496**	**9.47**
City of Caloocan	**121,803**	3,470	5,089	**8,559**	**7.03**	119	200	**319**	**3.73**
City of Marikina	**43,467**	499	989	**1,488**	**3.42**	0	0	**0**	**0.00**
City of Pasig	**61,315**	6,116	9,385	**15,501**	**25.28**	1,565	2,481	**4,046**	**26.10**
Pateros	**5,884**	1,101	1,663	**2,764**	**46.97**	0	0	**0**	**0.00**
City of Taguig	**55,176**	6,278	10,571	**16,849**	**30.54**	431	663	**1,094**	**6.49**
Quezon City	**241,251**	1,016	1,682	**2,698**	**1.12**	282	461	**743**	**27.54**
City of Makati	**62,174**	1,028	1,579	**2,607**	**4.19**	176	386	**562**	**21.56**
City of Mandaluyong	**34,090**	139	238	**377**	**1.11**	18	13	**31**	**8.22**
City of San Juan	**13,335**	0	0	**0**	**0.00**	0	0	**0**	**0.00**
City of Manila	**159,005**	23,217	31,962	**55,179**	**34.70**	2,717	3,332	**6,049**	**10.96**
City of Las Piñas	**51,301**	324	439	**763**	**1.49**	295	414	**709**	**92.92**
City of Muntinlupa	**46,573**	90	110	**200**	**0.43**	51	64	**115**	**57.50**
City of Parañaque	**58,293**	59	122	**181**	**0.31**	46	106	**152**	**83.98**
Pasay City	**33,525**	3,858	6,093	**9,951**	**29.68**	805	1,199	**2,004**	**20.14**
**CAR**	**171,839**	**5,575**	**8,293**	**13,868**	**8.07**	**1,276**	**1,554**	**2,830**	**20.41**
Abra	**30,211**	2,562	3,388	**5,950**	**19.69**	294	332	**626**	**10.52**
Apayao	**12,341**	171	300	**471**	**3.82**	22	63	**85**	**18.05**
Benguet	**38,747**	1,074	1,718	**2,792**	**7.21**	169	249	**418**	**14.97**
Ifugao	**18,609**	751	784	**1,535**	**8.25**	663	652	**1,315**	**85.67**
Kalinga	**21,119**	56	85	**141**	**0.67**	30	50	**80**	**56.74**
Mountain Province	**16,889**	466	899	**1,365**	**8.08**	21	30	**51**	**3.74**
Baguio City	**33,923**	495	1,119	**1,614**	**4.76**	77	178	**255**	**15.80**
**Region 1**	**588,356**	**7,168**	**11,145**	**18,313**	**3.11**	**319**	**471**	**790**	**4.31**
Ilocos Norte	**78,161**	403	526	**929**	**1.19**	4	2	**6**	**0.65**
Ilocos Sur	**90,128**	4,123	7,135	**11,258**	**12.49**	122	226	**348**	**3.09**
La Union	**96,387**	455	700	**1,155**	**1.20**	89	87	**176**	**15.24**
Pangasinan	**306,821**	2,187	2,784	**4,971**	**1.62**	104	156	**260**	**5.23**

Table 2.F.4.1 -Blindness Prevention Program
Senior citizens 60 yrs old and above screened for visual acuity and diagnosed with eye diseases
Philippines, 2023

Area	Eligible Pop.(60 y/o and above)	Screened for Visual Acuity				Diagnosed with eye diseases			
		Male	Female	Total	%	Male	Female	Total	%
Dagupan City	**16,859**	0	0	**0**	**0.00**	0	0	**0**	**0.00**
**Region 2**	**366,764**	**9,008**	**12,783**	**21,791**	**5.94**	**1,039**	**1,655**	**2,694**	**12.36**
Batanes	**2,407**	0	3	**3**	**0.12**	0	3	**3**	**100.00**
Cagayan	**134,071**	1,170	1,567	**2,737**	**2.04**	44	58	**102**	**3.73**
Isabela	**148,566**	3,889	5,464	**9,353**	**6.30**	644	1,071	**1,715**	**18.34**
Nueva Vizcaya	**49,508**	172	508	**680**	**1.37**	15	43	**58**	**8.53**
Quirino	**18,707**	409	511	**920**	**4.92**	152	240	**392**	**42.61**
Santiago City	**13,505**	3,368	4,730	**8,098**	**59.96**	184	240	**424**	**5.24**
**Region 3**	**1,120,977**	**17,444**	**25,830**	**43,274**	**3.86**	**2,260**	**3,591**	**5,851**	**13.52**
Aurora	**20,971**	28	44	**72**	**0.34**	14	30	**44**	**61.11**
Bataan	**75,791**	147	192	**339**	**0.45**	56	84	**140**	**41.30**
Bulacan	**317,609**	13,269	20,256	**33,525**	**10.56**	1,543	2,624	**4,167**	**12.43**
Nueva Ecija	**220,548**	2,416	3,505	**5,921**	**2.68**	561	713	**1,274**	**21.52**
Pampanga	**218,434**	1,186	1,372	**2,558**	**1.17**	23	31	**54**	**2.11**
Tarlac	**145,493**	350	416	**766**	**0.53**	62	107	**169**	**22.06**
Zambales	**65,866**	29	11	**40**	**0.06**	1	2	**3**	**7.50**
Angeles City	**32,478**	16	30	**46**	**0.14**	0	0	**0**	**0.00**
Olongapo	**23,787**	3	4	**7**	**0.03**	0	0	**0**	**0.00**
**Region 4A**	**1,326,186**	**4,661**	**6,425**	**11,086**	**0.84**	**1,311**	**1,733**	**3,044**	**27.46**
Batangas	**262,350**	439	644	**1,083**	**0.41**	103	107	**210**	**19.39**
Cavite	**342,406**	1,286	1,947	**3,233**	**0.94**	236	320	**556**	**17.20**
Laguna	**269,448**	921	1,337	**2,258**	**0.84**	342	479	**821**	**36.36**
Quezon	**179,747**	540	890	**1,430**	**0.80**	112	219	**331**	**23.15**
Rizal	**249,323**	1,197	1,281	**2,478**	**0.99**	298	364	**662**	**26.72**
Lucena City	**22,912**	278	326	**604**	**2.64**	220	244	**464**	**76.82**
**Region 4B**	**279,367**	**381**	**555**	**936**	**0.34**	**85**	**151**	**236**	**25.21**
Marinduque	**29,788**	6	10	**16**	**0.05**	0	2	**2**	**12.50**
Occidental Mindoro	**41,728**	153	196	**349**	**0.84**	6	19	**25**	**7.16**
Oriental Mindoro	**80,343**	18	30	**48**	**0.06**	2	5	**7**	**14.58**
Palawan	**69,986**	193	289	**482**	**0.69**	70	103	**173**	**35.89**
Romblon	**34,457**	10	29	**39**	**0.11**	6	21	**27**	**69.23**

Table 2.F.4.1 -Blindness Prevention Program

Senior citizens 60 yrs old and above screened for visual acuity and diagnosed with eye diseases

Philippines, 2023

Area	Eligible Pop.(60 y/o and above)	Screened for Visual Acuity				Diagnosed with eye diseases			
		Male	Female	Total	%	Male	Female	Total	%
Puerto Princesa City	**23,065**	1	1	**2**	**0.01**	1	1	**2**	**100.00**
**Region 5**	**555,762**	**17,249**	**23,105**	**40,354**	**7.26**	**1,921**	**2,601**	**4,522**	**11.21**
Albay	**131,641**	1,290	1,573	**2,863**	**2.17**	319	421	**740**	**25.85**
Camarines Norte	**56,217**	205	334	**539**	**0.96**	9	11	**20**	**3.71**
Camarines Sur	**166,064**	6,418	8,532	**14,950**	**9.00**	1,035	1,273	**2,308**	**15.44**
Catanduanes	**27,493**	874	886	**1,760**	**6.40**	85	194	**279**	**15.85**
Masbate	**76,557**	6,051	8,055	**14,106**	**18.43**	274	402	**676**	**4.79**
Sorsogon	**79,087**	2,248	3,429	**5,677**	**7.18**	194	295	**489**	**8.61**
Naga City	**18,703**	163	296	**459**	**2.45**	5	5	**10**	**2.18**
**Region 6**	**849,270**	**8,096**	**12,200**	**20,296**	**2.39**	**1,335**	**1,946**	**3,281**	**16.17**
Aklan	**67,371**	318	384	**702**	**1.04**	45	61	**106**	**15.10**
Antique	**66,916**	151	254	**405**	**0.61**	105	182	**287**	**70.86**
Capiz	**90,968**	1,451	2,377	**3,828**	**4.21**	76	81	**157**	**4.10**
Guimaras	**21,090**	1,076	1,688	**2,764**	**13.11**	29	65	**94**	**3.40**
Iloilo	**236,728**	1,656	1,718	**3,374**	**1.43**	504	523	**1,027**	**30.44**
Negros Occidental	**257,522**	3,159	5,408	**8,567**	**3.33**	543	993	**1,536**	**17.93**
Bacolod City	**60,939**	149	154	**303**	**0.50**	2	4	**6**	**1.98**
Iloilo City	**47,736**	136	217	**353**	**0.74**	31	37	**68**	**19.26**
**Region 7**	**756,543**	**4,836**	**6,793**	**11,629**	**1.54**	**4,280**	**5,955**	**10,235**	**88.01**
Bohol	**164,418**	168	159	**327**	**0.20**	35	65	**100**	**30.58**
Cebu	**294,979**	4,409	6,307	**10,716**	**3.63**	4,018	5,635	**9,653**	**90.08**
Negros Oriental	**151,550**	167	162	**329**	**0.22**	155	136	**291**	**88.45**
Siquijor	**14,737**	0	0	**0**	**0.00**	0	0	**0**	**0.00**
Cebu City	**77,570**	45	72	**117**	**0.15**	27	31	**58**	**49.57**
Lapu-Lapu City	**28,278**	2	5	**7**	**0.02**	0	0	**0**	**0.00**
Mandaue City	**25,011**	45	88	**133**	**0.53**	45	88	**133**	**100.00**
**Region 8**	**453,658**	**868**	**954**	**1,822**	**0.40**	**381**	**464**	**845**	**46.38**
Biliran	**19,390**	14	23	**37**	**0.19**	0	5	**5**	**13.51**
Eastern Samar	**50,320**	5	14	**19**	**0.04**	4	6	**10**	**52.63**
Northern Leyte	**163,063**	277	305	**582**	**0.36**	199	180	**379**	**65.12**
Northern Samar	**54,856**	430	396	**826**	**1.51**	121	175	**296**	**35.84**
Southern Leyte	**52,966**	63	104	**167**	**0.32**	4	6	**10**	**5.99**
Western Samar	**71,897**	69	103	**172**	**0.24**	46	83	**129**	**75.00**

Table 2.F.4.1 -Blindness Prevention Program

Senior citizens 60 yrs old and above screened for visual acuity and diagnosed with eye diseases

Philippines, 2023

Area	Eligible Pop.(60 y/o and above)	Screened for Visual Acuity				Diagnosed with eye diseases			
		Male	Female	Total	%	Male	Female	Total	%
Ormoc City	**20,726**	0	0	**0**	**0.00**	0	0	**0**	**0.00**
Tacloban City	**20,440**	10	9	**19**	**0.09**	7	9	**16**	**84.21**
**Region 9**	**320,151**	**381**	**566**	**947**	**0.30**	**120**	**226**	**346**	**36.54**
Zamboanga Del Norte	**94,932**	174	214	**388**	**0.41**	54	95	**149**	**38.40**
Zamboanga Del Sur	**89,158**	36	59	**95**	**0.11**	7	17	**24**	**25.26**
Zamboanga Sibugay	**52,352**	77	144	**221**	**0.42**	48	100	**148**	**66.97**
Isabela City	**8,469**	0	0	**0**	**0.00**	0	0	**0**	**0.00**
Zamboanga City	**75,240**	94	149	**243**	**0.32**	11	14	**25**	**10.29**
**Region 10**	**408,904**	**7,375**	**8,923**	**16,298**	**3.99**	**3,057**	**3,493**	**6,550**	**40.19**
Bukidnon	**112,138**	175	303	**478**	**0.43**	41	79	**120**	**25.10**
Camiguin	**10,741**	17	8	**25**	**0.23**	1	1	**2**	**8.00**
Lanao Del Norte	**44,922**	817	922	**1,739**	**3.87**	184	213	**397**	**22.83**
Misamis Occidental	**68,288**	5,999	7,313	**13,312**	**19.49**	2,765	3,102	**5,867**	**44.07**
Misamis Oriental	**82,786**	264	244	**508**	**0.61**	21	39	**60**	**11.81**
Cagayan de Oro City	**59,878**	12	16	**28**	**0.05**	9	12	**21**	**75.00**
Iligan City	**30,151**	91	117	**208**	**0.69**	36	47	**83**	**39.90**
**Region 11**	**439,306**	**8,335**	**11,950**	**20,285**	**4.62**	**2,691**	**3,554**	**6,245**	**30.79**
Davao de Oro	**63,711**	1,493	2,202	**3,695**	**5.80**	302	347	**649**	**17.56**
Davao del Norte	**94,892**	1,206	1,526	**2,732**	**2.88**	704	799	**1,503**	**55.01**
Davao Oriental	**52,287**	1,694	2,134	**3,828**	**7.32**	641	809	**1,450**	**37.88**
Davao del Sur	**63,692**	486	825	**1,311**	**2.06**	170	236	**406**	**30.97**
Davao Occidental	**22,620**	389	482	**871**	**3.85**	132	170	**302**	**34.67**
Davao City	**142,104**	3,067	4,781	**7,848**	**5.52**	742	1,193	**1,935**	**24.66**
**Region 12**	**315,236**	**4,758**	**6,375**	**11,133**	**3.53**	**1,082**	**1,441**	**2,523**	**22.66**
North Cotabato	**98,224**	1,300	2,022	**3,322**	**3.38**	348	499	**847**	**25.50**
Sarangani	**36,948**	1,443	1,730	**3,173**	**8.59**	184	213	**397**	**12.51**
South Cotabato	**75,297**	424	542	**966**	**1.28**	108	114	**222**	**22.98**
Sultan Kudarat	**58,539**	1,556	2,000	**3,556**	**6.07**	439	613	**1,052**	**29.58**
Gen. Santos City	**46,228**	35	81	**116**	**0.25**	3	2	**5**	**4.31**

Table 2.F.4.1 -Blindness Prevention Program

Senior citizens 60 yrs old and above screened for visual acuity and diagnosed with eye diseases

Philippines, 2023

Area	Eligible Pop.(60 y/o and above)	Screened for Visual Acuity				Diagnosed with eye diseases			
		Male	Female	Total	%	Male	Female	Total	%
**BARMM**	**169,938**	**840**	**1,215**	**2,055**	**1.21**	**297**	**370**	**667**	**32.46**
Basilan	**15,932**	119	176	**295**	**1.85**	48	50	**98**	**33.22**
Lanao Del Sur	**36,847**	137	234	**371**	**1.01**	98	116	**214**	**57.68**
Maguindanao	**38,534**	438	518	**956**	**2.48**	142	185	**327**	**34.21**
Sulu	**32,901**	0	0	**0**	**0.00**	0	0	**0**	**0.00**
Tawi-Tawi	**16,095**	44	75	**119**	**0.74**	0	0	**0**	**0.00**
SGA	**14,200**	12	33	**45**	**0.32**	0	0	**0**	**0.00**
Cotabato City	**15,429**	90	179	**269**	**1.74**	9	19	**28**	**10.41**
**Caraga**	**250,119**	**839**	**1265**	**2104**	**0.84**	**162**	**249**	**411**	**19.53**
Agusan del Norte	**35,560**	18	20	**38**	**0.11**	1	0	**1**	**2.63**
Agusan del Sur	**56,340**	180	208	**388**	**0.69**	7	27	**34**	**8.76**
Surigao del Norte	**50,056**	122	163	**285**	**0.57**	41	69	**110**	**38.60**
Surigao del Sur	**61,134**	378	630	**1,008**	**1.65**	110	149	**259**	**25.69**
Province of Dinagat	**14,233**	131	237	**368**	**2.59**	1	2	**3**	**0.82**
Butuan City	**32,796**	10	7	**17**	**0.05**	2	2	**4**	**23.53**

Table 2.F.5.1 -Immunization for Senior Citizen

Senior citizen 60 yrs old and above who received one (1) dose of Pneumococcal Polysaccharide Vaccine (PPV) and one (1) dose of Influenza Vaccine

Philippines, 2023

Area	Eligible Pop. (60 y/o & above)	Received one (1) dose of PPV				Received one (1) dose of Influenza Vaccine			
		Male	Female	Total	%	Male	Female	Total	%
**Philippines**	**9,460,253**	**259,488**	**401,816**	**661,304**	**6.99**	**545,216**	**878,549**	**1,423,765**	**15.05**
**NCR**	**1,087,877**	**43,943**	**70,025**	**113,968**	**10.48**	**82,884**	**137,579**	**220,463**	**20.27**
Malabon City	**30,451**	1,707	2,793	**4,500**	**14.78**	5,343	8,657	**14,000**	**45.98**
Navotas City	**18,722**	743	1,292	**2,035**	**10.87**	2,750	4,638	**7,388**	**39.46**
Valenzuela City	**51,512**	1,059	1,919	**2,978**	**5.78**	3,640	5,448	**9,088**	**17.64**
Caloocan City	**121,803**	3,840	6,303	**10,143**	**8.33**	12,719	20,817	**33,536**	**27.53**
Marikina City	**43,467**	2,165	3,281	**5,446**	**12.53**	5,027	8,108	**13,135**	**30.22**
Pasig City	**61,315**	566	834	**1,400**	**2.28**	2,545	4,302	**6,847**	**11.17**
Pateros	**5,884**	259	465	**724**	**12.30**	594	997	**1,591**	**27.04**
Taguig City	**55,176**	2,270	4,008	**6,278**	**11.38**	3,275	5,884	**9,159**	**16.60**
Quezon City	**241,251**	10,723	18,155	**28,878**	**11.97**	14,142	22,813	**36,955**	**15.32**
Makati City	**62,174**	3,471	1,828	**5,299**	**8.52**	5,767	10,132	**15,899**	**25.57**
Mandaluyong City	**34,090**	1,098	1,407	**2,505**	**7.35**	2,090	2,972	**5,062**	**14.85**
San Juan City	**13,335**	547	927	**1,474**	**11.05**	1,867	3,128	**4,995**	**37.46**
Manila City	**159,005**	6,961	11,427	**18,388**	**11.56**	9,209	15,284	**24,493**	**15.40**
Las Piñas City	**51,301**	2,428	4,524	**6,952**	**13.55**	4,709	8,568	**13,277**	**25.88**
Muntinlupa City	**46,573**	1,790	3,412	**5,202**	**11.17**	3,135	5,724	**8,859**	**19.02**
Parañaque City	**58,293**	2,858	5,535	**8,393**	**14.40**	4,670	8,019	**12,689**	**21.77**
Pasay City	**33,525**	1,458	1,915	**3,373**	**10.06**	1,402	2,088	**3,490**	**10.41**
**CAR**	**171,839**	**3,254**	**5,257**	**8,511**	**4.95**	**12,391**	**20,798**	**33,189**	**19.31**
Abra	**30,211**	913	1,238	**2,151**	**7.12**	4,960	7,008	**11,968**	**39.61**
Apayao	**12,341**	218	295	**513**	**4.16**	611	1,062	**1,673**	**13.56**
Benguet	**38,747**	410	805	**1,215**	**3.14**	2,023	3,605	**5,628**	**14.52**
Ifugao	**18,609**	380	626	**1,006**	**5.41**	1,084	1,785	**2,869**	**15.42**
Kalinga	**21,119**	586	893	**1,479**	**7.00**	1,279	2,153	**3,432**	**16.25**
Mountain Province	**16,889**	247	527	**774**	**4.58**	749	1,801	**2,550**	**15.10**
Baguio City	**33,923**	500	873	**1,373**	**4.05**	1,685	3,384	**5,069**	**14.94**
**Region 1**	**588,356**	**14,109**	**20,565**	**34,674**	**5.89**	**40,937**	**69,080**	**110,017**	**18.70**
Ilocos Norte	**78,161**	1,801	2,594	**4,395**	**5.62**	4,777	6,357	**11,134**	**14.24**
Ilocos Sur	**90,128**	2,895	3,760	**6,655**	**7.38**	9,174	12,938	**22,112**	**24.53**
La Union	**96,387**	3,277	4,690	**7,967**	**8.27**	9,730	14,876	**24,606**	**25.53**
Pangasinan	**306,821**	5,025	8,584	**13,609**	**4.44**	15,560	33,065	**48,625**	**15.85**

Table 2.F.5.1 -Immunization for Senior Citizen

Senior citizen 60 yrs old and above who received one (1) dose of Pneumococcal Polysaccharide Vaccine (PPV) and one (1) dose of Influenza Vaccine

Philippines, 2023

Area	Eligible Pop. (60 y/o & above)	Received one (1) dose of PPV				Received one (1) dose of Influenza Vaccine			
		Male	Female	Total	%	Male	Female	Total	%
Dagupan City	**16,859**	1,111	937	**2,048**	**12.15**	1,696	1,844	**3,540**	**21.00**
**Region 2**	**366,764**	**9,802**	**15,979**	**25,781**	**7.03**	**25,626**	**44,804**	**70,430**	**19.20**
Batanes	**2,407**	48	59	**107**	**4.45**	343	668	**1,011**	**42.00**
Cagayan	**134,071**	2,860	4,599	**7,459**	**5.56**	6,852	12,330	**19,182**	**14.31**
Isabela	**148,566**	4,874	7,895	**12,769**	**8.59**	12,580	21,604	**34,184**	**23.01**
Nueva Vizcaya	**49,508**	934	1,877	**2,811**	**5.68**	2,674	5,379	**8,053**	**16.27**
Quirino	**18,707**	679	916	**1,595**	**8.53**	1,420	1,840	**3,260**	**17.43**
Santiago City	**13,505**	407	633	**1,040**	**7.70**	1,757	2,983	**4,740**	**35.10**
**Region 3**	**1,120,977**	**45,769**	**66,940**	**112,709**	**10.05**	**113,395**	**165,922**	**279,317**	**24.92**
Aurora	**20,971**	435	716	**1,151**	**5.49**	1,688	2,621	**4,309**	**20.55**
Bataan	**75,791**	4,581	6,791	**11,372**	**15.00**	9,360	14,242	**23,602**	**31.14**
Bulacan	**317,609**	13,746	20,756	**34,502**	**10.86**	34,894	52,989	**87,883**	**27.67**
Nueva Ecija	**220,548**	8,549	13,573	**22,122**	**10.03**	25,829	38,653	**64,482**	**29.24**
Pampanga	**218,434**	8,568	10,988	**19,556**	**8.95**	17,447	21,843	**39,290**	**17.99**
Tarlac	**145,493**	7,015	9,380	**16,395**	**11.27**	15,978	22,259	**38,237**	**26.28**
Zambales	**65,866**	2,062	3,648	**5,710**	**8.67**	2,453	5,080	**7,533**	**11.44**
Angeles City	**32,478**	732	939	**1,671**	**5.15**	3,742	5,657	**9,399**	**28.94**
Olongapo	**23,787**	81	149	**230**	**0.97**	2,004	2,578	**4,582**	**19.26**
**Region 4A**	**1,326,186**	**47,954**	**72,007**	**119,961**	**9.05**	**73,072**	**118,368**	**191,440**	**14.44**
Batangas	**262,350**	13,002	20,595	**33,597**	**12.81**	18,772	31,155	**49,927**	**19.03**
Cavite	**342,406**	14,356	21,830	**36,186**	**10.57**	24,919	38,910	**63,829**	**18.64**
Laguna	**269,448**	7,470	11,088	**18,558**	**6.89**	14,466	22,768	**37,234**	**13.82**
Quezon	**179,747**	5,352	6,735	**12,087**	**6.72**	4,178	7,487	**11,665**	**6.49**
Rizal	**249,323**	6,518	10,459	**16,977**	**6.81**	9,354	15,087	**24,441**	**9.80**
Lucena City	**22,912**	1,256	1,300	**2,556**	**11.16**	1,383	2,961	**4,344**	**18.96**
**Region 4B**	**279,367**	**4,412**	**7,351**	**11,763**	**4.21**	**7,206**	**13,241**	**20,447**	**7.32**
Marinduque	**29,788**	274	561	**835**	**2.80**	1,116	2,542	**3,658**	**12.28**
Occidental Mindoro	**41,728**	430	813	**1,243**	**2.98**	1,207	2,128	**3,335**	**7.99**
Oriental Mindoro	**80,343**	740	1,311	**2,051**	**2.55**	1,845	3,264	**5,109**	**6.36**
Palawan	**69,986**	2,024	3,105	**5,129**	**7.33**	1,642	2,858	**4,500**	**6.43**
Romblon	**34,457**	200	414	**614**	**1.78**	465	1,007	**1,472**	**4.27**
Puerto Princesa City	**23,065**	744	1,147	**1,891**	**8.20**	931	1,442	**2,373**	**10.29**

Table 2.F.5.1 -Immunization for Senior Citizen

Senior citizen 60 yrs old and above who received one (1) dose of Pneumococcal Polysaccharide Vaccine (PPV) and one (1) dose of Influenza Vaccine

Philippines, 2023

Area	Eligible Pop. (60 y/o & above)	Received one (1) dose of PPV				Received one (1) dose of Influenza Vaccine			
		Male	Female	Total	%	Male	Female	Total	%
**Region 5**	**555,762**	**11,987**	**21,464**	**33,451**	**6.02**	**26,413**	**51,365**	**77,778**	**13.99**
Albay	**131,641**	2,557	4,617	**7,174**	**5.45**	6,699	13,020	**19,719**	**14.98**
Camarines Norte	**56,217**	1,430	2,491	**3,921**	**6.97**	1,937	3,029	**4,966**	**8.83**
Camarines Sur	**166,064**	2,589	4,840	**7,429**	**4.47**	6,504	13,665	**20,169**	**12.15**
Catanduanes	**27,493**	1,238	1,571	**2,809**	**10.22**	3,619	5,569	**9,188**	**33.42**
Masbate	**76,557**	1,174	2,085	**3,259**	**4.26**	2,000	4,528	**6,528**	**8.53**
Sorsogon	**79,087**	2,463	4,729	**7,192**	**9.09**	4,497	9,086	**13,583**	**17.17**
Naga City	**18,703**	536	1,131	**1,667**	**8.91**	1,157	2,468	**3,625**	**19.38**
**Region 6**	**849,270**	**17,294**	**28,873**	**46,167**	**5.44**	**41,507**	**68,295**	**109,802**	**12.93**
Aklan	**67,371**	994	1,686	**2,680**	**3.98**	1,023	1,733	**2,756**	**4.09**
Antique	**66,916**	659	1,344	**2,003**	**2.99**	733	1,505	**2,238**	**3.34**
Capiz	**90,968**	1,114	2,175	**3,289**	**3.62**	3,010	4,754	**7,764**	**8.53**
Guimaras	**21,090**	801	1,329	**2,130**	**10.10**	950	1,687	**2,637**	**12.50**
Iloilo	**236,728**	5,577	7,731	**13,308**	**5.62**	20,054	29,190	**49,244**	**20.80**
Negros Occidental	**257,522**	5,711	10,574	**16,285**	**6.32**	12,127	23,067	**35,194**	**13.67**
Bacolod City	**60,939**	1,476	2,623	**4,099**	**6.73**	1,508	3,015	**4,523**	**7.42**
Iloilo City	**47,736**	962	1,411	**2,373**	**4.97**	2,102	3,344	**5,446**	**11.41**
**Region 7**	**756,543**	**5,042**	**9,154**	**14,196**	**1.88**	**10,969**	**20,233**	**31,202**	**4.12**
Bohol	**164,418**	864	1,721	**2,585**	**1.57**	3,583	6,736	**10,319**	**6.28**
Cebu	**294,979**	738	1,255	**1,993**	**0.68**	1,872	3,662	**5,534**	**1.88**
Negros Oriental	**151,550**	1,091	1,815	**2,906**	**1.92**	1,643	3,164	**4,807**	**3.17**
Siquijor	**14,737**	83	194	**277**	**1.88**	111	261	**372**	**2.52**
Cebu City	**77,570**	1,932	3,622	**5,554**	**7.16**	3,065	5,293	**8,358**	**10.77**
Lapu-Lapu City	**28,278**	27	24	**51**	**0.18**	193	324	**517**	**1.83**
Mandaue City	**25,011**	307	523	**830**	**3.32**	502	793	**1,295**	**5.18**
**Region 8**	**453,658**	**3,864**	**6,228**	**10,092**	**2.22**	**8,825**	**14,283**	**23,108**	**5.09**
Biliran	**19,390**	326	639	**965**	**4.98**	610	1,408	**2,018**	**10.41**
Eastern Samar	**50,320**	191	362	**553**	**1.10**	336	661	**997**	**1.98**
Northern Leyte	**163,063**	759	1,335	**2,094**	**1.28**	669	1,184	**1,853**	**1.14**
Northern Samar	**54,856**	625	897	**1,522**	**2.77**	1,271	2,526	**3,797**	**6.92**
Southern Leyte	**52,966**	361	681	**1,042**	**1.97**	1,206	2,286	**3,492**	**6.59**
Western Samar	**71,897**	710	1,058	**1,768**	**2.46**	853	1,409	**2,262**	**3.15**
Ormoc City	**20,726**	146	188	**334**	**1.61**	1,866	2,066	**3,932**	**18.97**
Tacloban City	**20,440**	746	1,068	**1,814**	**8.87**	2,014	2,743	**4,757**	**23.27**

Table 2.F.5.1 -Immunization for Senior Citizen

Senior citizen 60 yrs old and above who received one (1) dose of Pneumococcal Polysaccharide Vaccine (PPV) and one (1) dose of Influenza Vaccine

Philippines, 2023

Area	Eligible Pop. (60 y/o & above)	Received one (1) dose of PPV				Received one (1) dose of Influenza Vaccine			
		Male	Female	Total	%	Male	Female	Total	%
**Region 9**	**320,151**	**5,498**	**8,932**	**14,430**	**4.51**	**19,287**	**30,482**	**49,769**	**15.55**
Zamboanga Del Norte	**94,932**	2,161	3,149	**5,310**	**5.59**	5,692	8,897	**14,589**	**15.37**
Zamboanga Del Sur	**89,158**	1,161	2,033	**3,194**	**3.58**	6,525	9,562	**16,087**	**18.04**
Zamboanga Sibugay	**52,352**	720	1,255	**1,975**	**3.77**	1,853	3,007	**4,860**	**9.28**
Isabela City	**8,469**	116	160	**276**	**3.26**	354	522	**876**	**10.34**
Zamboanga City	**75,240**	1,340	2,335	**3,675**	**4.88**	4,863	8,494	**13,357**	**17.75**
**Region 10**	**408,904**	**11,027**	**16,242**	**27,269**	**6.67**	**23,008**	**33,209**	**56,217**	**13.75**
Bukidnon	**112,138**	1,945	2,969	**4,914**	**4.38**	4,032	6,307	**10,339**	**9.22**
Camiguin	**10,741**	414	541	**955**	**8.89**	678	1,122	**1,800**	**16.76**
Lanao Del Norte	**44,922**	1,400	1,912	**3,312**	**7.37**	1,936	2,440	**4,376**	**9.74**
Misamis Occidental	**68,288**	2,265	3,663	**5,928**	**8.68**	5,480	8,575	**14,055**	**20.58**
Misamis Oriental	**82,786**	2,359	3,383	**5,742**	**6.94**	5,687	7,505	**13,192**	**15.94**
Cagayan de Oro City	**59,878**	1,344	1,900	**3,244**	**5.42**	2,187	3,201	**5,388**	**9.00**
Iligan City	**30,151**	1,300	1,874	**3,174**	**10.53**	3,008	4,059	**7,067**	**23.44**
**Region 11**	**439,306**	**13,472**	**20,371**	**33,843**	**7.70**	**21,369**	**31,951**	**53,320**	**12.14**
Davao de Oro	**63,711**	1,795	2,714	**4,509**	**7.08**	2,431	3,667	**6,098**	**9.57**
Davao del Norte	**94,892**	2,885	4,390	**7,275**	**7.67**	3,893	5,910	**9,803**	**10.33**
Davao Oriental	**52,287**	1,184	1,746	**2,930**	**5.60**	2,797	4,172	**6,969**	**13.33**
Davao del Sur	**63,692**	1,063	1,674	**2,737**	**4.30**	2,234	3,949	**6,183**	**9.71**
Davao Occidental	**22,620**	737	1,144	**1,881**	**8.32**	932	1,497	**2,429**	**10.74**
Davao City	**142,104**	5,808	8,703	**14,511**	**10.21**	9,082	12,756	**21,838**	**15.37**
**Region 12**	**315,236**	**12,667**	**18,715**	**31,382**	**9.96**	**21,469**	**32,220**	**53,689**	**17.03**
North Cotabato	**98,224**	4,318	6,829	**11,147**	**11.35**	6,594	10,529	**17,123**	**17.43**
Sarangani	**36,948**	2,233	3,024	**5,257**	**14.23**	4,873	6,414	**11,287**	**30.55**
South Cotabato	**75,297**	2,176	3,420	**5,596**	**7.43**	4,430	7,399	**11,829**	**15.71**
Sultan Kudarat	**58,539**	3,562	4,650	**8,212**	**14.03**	4,453	6,114	**10,567**	**18.05**
Gen. Santos City	**46,228**	378	792	**1,170**	**2.53**	1,119	1,764	**2,883**	**6.24**
**BARMM**	**169,938**	**3,233**	**4,278**	**7,511**	**4.42**	**4,095**	**6,442**	**10,537**	**6.20**
Basilan	**15,932**	147	181	**328**	**2.06**	142	227	**369**	**2.32**
Lanao Del Sur	**36,847**	130	149	**279**	**0.76**	178	189	**367**	**1.00**
Maguindanao	**38,534**	2,408	2,977	**5,385**	**13.97**	3,163	5,008	**8,171**	**21.20**

Table 2.F.5.1 -Immunization for Senior Citizen

Senior citizen 60 yrs old and above who received one (1) dose of Pneumococcal Polysaccharide Vaccine (PPV) and one (1) dose of Influenza Vaccine

Philippines, 2023

Area	Eligible Pop. (60 y/o & above)	Received one (1) dose of PPV				Received one (1) dose of Influenza Vaccine			
		Male	Female	Total	%	Male	Female	Total	%
Sulu	**32,901**	0	0	**0**	**0.00**	0	0	**0**	**0.00**
Tawi-Tawi	**16,095**	184	355	**539**	**3.35**	201	265	**466**	**2.90**
SGA	**14,200**	117	178	**295**	**2.08**	131	196	**327**	**2.30**
Cotabato City	**15,429**	247	438	**685**	**4.44**	280	557	**837**	**5.42**
**Caraga**	**250,119**	**6,161**	**9,435**	**15,596**	**6.24**	**12,763**	**20,277**	**33,040**	**13.21**
Agusan del Norte	**35,560**	796	1,009	**1,805**	**5.08**	1,239	2,051	**3,290**	**9.25**
Agusan del Sur	**56,340**	1,414	1,960	**3,374**	**5.99**	2,485	3,635	**6,120**	**10.86**
Surigao del Norte	**50,056**	1,083	1,912	**2,995**	**5.98**	1,901	3,463	**5,364**	**10.72**
Surigao del Sur	**61,134**	1,873	3,007	**4,880**	**7.98**	4,989	8,112	**13,101**	**21.43**
Province of Dinagat	**14,233**	160	291	**451**	**3.17**	789	1,130	**1,919**	**13.48**
Butuan City	**32,796**	835	1,256	**2,091**	**6.38**	1,360	1,886	**3,246**	**9.90**

2.G. Environmental Health and Sanitation Services
Households (HHs) with access to basic safe water supply and manage drinking water
Philippines, 2023

Area	Projected No. of HHs	HHs with Access to Basic Safe Water Supply			
		Level 1		Level 2	
		No.	%	No.	%
**Philippines**	**27,025,049**	**5,488,950**	**22.08**	**3,067,006**	**12.34**
**NCR**	**3,582,003**	**11,806**	**0.34**	**60,467**	**1.74**
City of Malabon	96,542	0	0.00	0	0.00
City of Navotas	65,013	0	0.00	0	0.00
City of Valenzuela	197,719	0	0.00	0	0.00
City of Caloocan	415,628	0	0.00	0	0.00
City of Marikina	106,453	0	0.00	0	0.00
City of Pasig	218,789	0	0.00	0	0.00
Pateros	16,094	0	0.00	1030	6.01
City of Taguig	255,377	0	0.00	0	0.00
City of Quezon	755,486	0	0.00	0	0.00
City of Makati	188,824	0	0.00	0	0.00
City of Mandaluyong	119,486	0	0.00	0	0.00
City of San Juan	31,796	0	0.00	0	0.00
City of Manila	497,563	8,262	1.85	40083	8.95
City of Las Piñas	160,153	0	0.00	814	0.52
City of Muntinlupa	140,933	3,544	2.53	1786	1.28
City of Parañaque	186,153	0	0.00	16754	9.00
City of Pasay	129,994	0	0.00	0	
**CAR**	**452,303**	**90,167**	**20.64**	**83,230**	**19.06**
Abra	59,546	11,409	18.91	17,737	29.40
Apayao	29,739	17,859	70.28	3,409	13.41
Benguet	120,091	20,150	18.29	12,115	11.00
Ifugao	50,865	13,460	27.81	19,693	40.69
Kalinga	50,063	13,616	26.74	14,226	27.93
Mt. Province	38,714	8,265	21.29	4,824	12.43
City of Baguio	103,285	5,408	5.26	11,226	10.93
**Region 1**	**1,328,091**	**603,980**	**46.02**	**65,207**	**4.97**
Ilocos Norte	154,790	41,723	27.40	13,386	8.79
Ilocos Sur	173,863	72,615	40.26	11,591	6.43

2.G. Environmental Health and Sanitation Services
Households (HHs) with access to basic safe water supply and manage drinking water
Philippines, 2023

Area	Projected No. of HHs	HHs with Access to Basic Safe Water Supply			
		Level 1		Level 2	
		No.	%	No.	%
La Union	208,309	125,992	60.35	24,032	11.51
Pangasinan	748,061	362,161	49.69	16,198	2.22
City of Dagupan	43,068	1,489	3.52	0	0.00
**Region 2**	**927,620**	**499,880**	**56.78**	**54,966**	**6.24**
Batanes	5,631	2	0.04	10	0.18
Cagayan	306,503	152,639	52.30	21,277	7.29
Isabela	394,188	247,830	65.26	6,529	1.72
Nueva Vizcaya	132,487	56,301	48.79	19,957	17.30
Quirino	51,317	32,374	64.43	7,193	14.31
City of Santiago	37,494	10,734	28.63	0	0.00
**Region 3**	**3,109,563**	**635,696**	**20.95**	**32,385**	**1.07**
Aurora	58,836	28,818	46.81	6,843	11.11
Bataan	214,501	33,284	15.95	2,578	1.24
Bulacan	940,427	69,946	7.50	0	0.00
Nueva Ecija	583,231	197,804	35.47	13,359	2.40
Pampanga	590,627	77,232	13.89	568	0.10
Tarlac	366,272	144,033	40.49	1,250	0.35
Zambales	168,891	81,967	46.14	7,787	4.38
City of Angeles	119,320	2,612	2.20	0	0.00
City of Olongapo	67,458	0	0.00	0	0.00
**Region 4A**	**4,159,056**	**387,672**	**9.87**	**220,974**	**5.63**
Batangas	731,131	70,134	10.28	26,491	3.88
Cavite	1,122,831	26,281	2.42	1,517	0.14
Laguna	936,359	98,440	11.04	24,595	2.76
Quezon	494,967	161,303	36.50	93,473	21.15
Rizal	805,162	31,514	4.17	70,206	9.29
City of Lucena	68,606	0	0.00	4,692	6.84

2.G. Environmental Health and Sanitation Services
Households (HHs) with access to basic safe water supply and manage drinking water
Philippines, 2023

Area	Projected No. of HHs	HHs with Access to Basic Safe Water Supply			
		Level 1		Level 2	
		No.	%	No.	%
**Region 4B**	**811,290**	**300,503**	**40.14**	**120,817**	**16.14**
Marinduque	61,366	12,679	19.25	10,906	16.56
Mindoro Occidental	130,025	71,177	60.17	14,539	12.29
Mindoro Oriental	219,399	113,010	52.05	30,561	14.07
Palawan	237,247	84,792	42.18	39,350	19.58
Romblon	79,038	14,958	20.41	14,507	19.79
City of Puerto Princesa	84,215	3,887	5.33	10,954	15.01
**Region 5**	**1,406,129**	**328,790**	**28.64**	**315,389**	**27.47**
Albay	320,720	89,224	30.14	80,722	27.27
Camarines Norte	149,644	22,286	16.40	46,937	34.54
Camarines Sur	421,818	66,284	30.40	68,657	31.48
Catanduanes	62,062	12,891	21.52	12,293	20.52
Masbate	211,595	95,008	49.78	49,773	26.08
Sorsogon	192,865	42,977	21.49	55,955	27.98
City of Naga	47,425	120	0.25	1,052	2.22
**Region 6**	**1,962,744**	**799,045**	**42.79**	**252,246**	**13.51**
Aklan	154,126	51,372	35.09	25,144	17.17
Antique	148,873	35,777	23.34	46,661	30.44
Capiz	205,260	114,730	57.18	12,667	6.31
Guimaras	48,377	22,546	45.23	3,559	7.14
Iloilo	513,388	288,337	58.95	62,177	12.71
Negros Occidental	645,071	246,596	41.54	97,966	16.50
City of Bacolod	142,385	10,410	8.05	4,072	3.15
City of Iloilo	105,264	29,277	27.80	0	0.00
**Region 7**	**2,021,190**	**274,572**	**15.61**	**332,882**	**18.92**
Bohol	328,287	39,264	13.78	30,294	10.63
Cebu	822,220	117,178	15.28	183,404	23.92
Negros Oriental	357,223	44,084	13.33	76,243	23.06
Siquijor	25,976	942	3.54	4,585	17.21

2.G. Environmental Health and Sanitation Services
Households (HHs) with access to basic safe water supply and manage drinking water
Philippines, 2023

Area	Projected No. of HHs	HHs with Access to Basic Safe Water Supply			
		Level 1		Level 2	
		No.	%	No.	%
City of Cebu	245,447	7,048	5.29	15,094	11.32
City of Lapu-Lapu	134,909	55,312	42.93	12,550	9.74
City of Mandaue	107,128	10,744	12.24	10,712	12.20
**Region 8**	**1,106,829**	**271,168**	**26.27**	**320,330**	**31.04**
Biliran	44,107	2,419	5.56	10,671	24.53
Eastern Samar	118,486	37,418	33.76	46,773	42.20
Northern Leyte	396,570	96,490	26.37	114,535	31.30
Northern Samar	142,773	83,264	64.04	35,618	27.39
Southern Leyte	101,814	4,312	4.45	9,718	10.04
Western Samar	186,449	38,500	22.52	69,668	40.75
City of Ormoc	57,759	2,708	4.69	9,472	16.41
City of Tacloban	58,871	6,057	10.75	23,875	42.38
**Region 9**	**918,762**	**131,430**	**19.09**	**187,055**	**27.16**
Zamboanga del Norte	254,162	69,708	30.85	68,406	30.28
Zamboanga del Sur	245,986	31,496	16.19	66,672	34.27
Zamboanga Sibugay	158,578	6,729	24.12	12,128	43.46
City of Isabela	26,946	3,914	18.08	5,895	27.24
City of Zamboanga	233,090	19,583	8.96	33,954	15.53
**Region 10**	**1,238,184**	**158,191**	**13.93**	**233,563**	**20.57**
Bukidnon	372,160	47,665	13.85	102,777	29.87
Camiguin	22,677	172	0.81	1,366	6.43
Lanao del Norte	166,135	47,829	38.64	22,683	18.33
Misamis Occidental	152,910	28,513	18.68	35,321	23.15
Misamis Oriental	237,526	24,350	10.71	50,528	22.22
City of Cagayan de Oro	197,409	2,305	1.19	13,944	7.20
City of Iligan	89,367	7,357	10.09	6,944	9.52
**Region 11**	**1,372,765**	**279,935**	**23.78**	**252,118**	**21.42**
Davao de Oro	194,995	65,778	34.87	61,575	32.64
Davao del Norte	279,110	96,910	41.39	20,961	8.95
Davao Oriental	143,732	51,837	34.78	37,523	25.18

2.G. Environmental Health and Sanitation Services

Households (HHs) with access to basic safe water supply and manage drinking water

Philippines, 2023

Area	Projected No. of HHs	HHs with Access to Basic Safe Water Supply			
		Level 1		Level 2	
		No.	%	No.	%
Davao del Sur	187,241	34,179	19.98	38,257	22.37
Davao Occidental	78,592	19,227	27.87	39,948	57.90
City of Davao	489,095	12,004	3.29	53,854	14.75
**Region 12**	**1,101,285**	**348,237**	**33.76**	**192,714**	**18.68**
North Cotabato	318,661	88,109	29.50	63,666	21.32
Sarangani	138,873	55,629	44.47	39,810	31.83
South Cotabato	252,646	64,393	24.72	55,543	21.32
Sultan Kudarat	209,264	123,363	65.61	29,635	15.76
City of General Santos	181,841	16,743	10.52	4,060	2.55
**BARMM**	**849,357**	**243,440**	**42.23**	**183,826**	**31.89**
Basilan	74,283	22,794	43.76	19,339	37.12
Lanao del Sur	185,938	64,091	35.66	64,229	35.74
Maguindanao	231,980	100,925	52.50	60,228	31.33
Sulu	167,120	23,768	28.99	31,633	38.59
Tawi-Tawi	75,188	30,556	81.29	5,060	13.46
SGA	49,498	0	0.00	0	0.00
City of Cotabato	65,350	1,306	3.98	3,337	10.17
**CARAGA**	**677,878**	**124,438**	**19.64**	**158,837**	**25.07**
Agusan del Norte	93,026	20,877	22.98	24,617	27.10
Agusan del Sur	178,710	57,644	34.87	45,003	27.22
Surigao del Norte	130,077	10,677	7.82	42,257	30.96
Surigao del Sur	154,978	19,600	14.00	31,874	22.78
Province of Dinagat	29,632	3,011	10.17	8,615	29.10
City of Butuan	91,455	12,629	17.68	6,471	9.06

2.G. Environmental Health and Sanitation Services
Households (HHs) with access to basic safe water supply and manage drinking water
Philippines, 2023

Area	HHs with Access to Basic Safe Water Supply				HHs using Safely Managed Drinking Water Services	
	Level 3		Total	%		
	No.	%			No.	%
**Philippines**	**16,301,875**	**65.58**	**24,857,831**	**91.98**	**18,209,154**	**67.38**
**NCR**	**3,396,000**	**97.92**	**3,468,273**	**96.82**	**3,351,424**	**93.56**
City of Malabon	96,542	100.00	96,542	100.00	96,542	100.00
City of Navotas	64,168	100.00	64,168	98.70	64,168	98.70
City of Valenzuela	166,278	100.00	166,278	84.10	166,278	84.10
City of Caloocan	415,636	100.00	415,636	100.00	365,980	88.05
City of Marikina	106,453	100.00	106,453	100.00	106,453	100.00
City of Pasig	218,789	100.00	218,789	100.00	218,789	100.00
Pateros	16,094	93.99	17,124	106.40	15,030	93.39
City of Taguig	255,122	100.00	255,122	99.90	255,122	99.90
City of Quezon	740,608	100.00	740,608	98.03	740,608	98.03
City of Makati	188,824	100.00	188,824	100.00	188,824	100.00
City of Mandaluyong	119,486	100.00	119,486	100.00	119,486	100.00
City of San Juan	31,796	100.00	31,796	100.00	31,796	100.00
City of Manila	399,444	89.20	447,789	90.00	399,444	80.28
City of Las Piñas	154,305	99.48	155,119	96.86	155,119	96.86
City of Muntinlupa	134,656	96.19	139,986	99.33	139,986	99.33
City of Parañaque	169,399	91.00	186,153	100.00	169,399	91.00
City of Pasay	118,400	100.00	118,400	91.08	118,400	91.08
**CAR**	**263,376**	**60.30**	**436,773**	**96.57**	**282,867**	**62.54**
Abra	31,186	51.69	60,332	101.32	26,525	44.55
Apayao	4,145	16.31	25,413	85.45	3,378	11.36
Benguet	77,885	70.71	110,150	91.72	76,217	63.47
Ifugao	15,239	31.49	48,392	95.14	32,306	63.51
Kalinga	23,085	45.33	50,927	101.73	31,845	63.61
Mt. Province	25,729	66.28	38,818	100.27	14,008	36.18
City of Baguio	86,107	83.81	102,741	99.47	98,588	95.45
**Region 1**	**643,328**	**49.01**	**1,312,515**	**98.83**	**829,295**	**62.44**
Ilocos Norte	97,145	63.80	152,254	98.36	114,231	73.80
Ilocos Sur	96,166	53.32	180,372	103.74	103,487	59.52

2.G. Environmental Health and Sanitation Services
Households (HHs) with access to basic safe water supply and manage drinking water
Philippines, 2023

Area	HHs with Access to Basic Safe Water Supply				HHs using Safely Managed Drinking Water Services	
	Level 3		Total	%		
	No.	%			No.	%
La Union	58,734	28.13	208,758	100.22	71,349	34.25
Pangasinan	350,482	48.09	728,841	97.43	499,427	66.76
City of Dagupan	40,801	96.48	42,290	98.19	40,801	94.74
**Region 2**	**325,510**	**36.97**	**880,356**	**94.90**	**550,672**	**59.36**
Batanes	5,619	99.79	5,631	100.00	67	1.19
Cagayan	117,912	40.40	291,828	95.21	131,144	42.79
Isabela	125,412	33.02	379,771	96.34	286,521	72.69
Nueva Vizcaya	39,126	33.91	115,384	87.09	91,670	69.19
Quirino	10,681	21.26	50,248	97.92	14,510	28.28
City of Santiago	26,760	71.37	37,494	100.00	26,760	71.37
**Region 3**	**2,365,950**	**77.98**	**3,034,031**	**97.57**	**2,803,798**	**90.17**
Aurora	25,906	42.08	61,567	104.64	50,443	85.73
Bataan	172,871	82.82	208,733	97.31	194,912	90.87
Bulacan	862,265	92.50	932,211	99.13	859,852	91.43
Nueva Ecija	346,538	62.14	557,701	95.62	475,623	81.55
Pampanga	478,271	86.01	556,071	94.15	550,773	93.25
Tarlac	210,408	59.15	355,691	97.11	331,872	90.61
Zambales	87,896	49.48	177,650	105.19	155,938	92.33
City of Angeles	115,883	97.80	118,495	99.31	118,495	99.31
City of Olongapo	65,912	100.00	65,912	97.71	65,890	97.68
**Region 4A**	**3,318,970**	**84.50**	**3,927,616**	**94.44**	**3,440,872**	**82.73**
Batangas	585,643	85.84	682,268	93.32	637,647	87.21
Cavite	1,059,830	97.44	1,087,628	96.86	1,054,708	93.93
Laguna	768,347	86.20	891,382	95.20	747,714	79.85
Quezon	187,097	42.34	441,873	89.27	256,981	51.92
Rizal	654,139	86.54	755,859	93.88	679,908	84.44
City of Lucena	63,914	93.16	68,606	100.00	63,914	93.16

2.G. Environmental Health and Sanitation Services

Households (HHs) with access to basic safe water supply and manage drinking water

Philippines, 2023

Area	HHs with Access to Basic Safe Water Supply				HHs using Safely Managed Drinking Water Services	
	Level 3		Total	%		
	No.	%			No.	%
**Region 4B**	**327,245**	**43.72**	**748,565**	**92.27**	**400,661**	**49.39**
Marinduque	42,265	64.18	65,850	107.31	42,676	69.54
Mindoro Occidental	32,576	27.54	118,292	90.98	33,077	25.44
Mindoro Oriental	73,566	33.88	217,137	98.97	118,152	53.85
Palawan	76,859	38.24	201,001	84.72	116,803	49.23
Romblon	43,838	59.80	73,303	92.74	27,925	35.33
City of Puerto Princesa	58,141	79.66	72,982	86.66	62,028	73.65
**Region 5**	**504,030**	**43.90**	**1,148,209**	**81.66**	**665,646**	**47.34**
Albay	126,099	42.59	296,045	92.31	202,992	63.29
Camarines Norte	66,668	49.06	135,891	90.81	88,954	59.44
Camarines Sur	83,132	38.12	218,073	51.70	61,696	14.63
Catanduanes	34,732	57.97	59,916	96.54	29,057	46.82
Masbate	46,076	24.14	190,857	90.20	132,099	62.43
Sorsogon	101,070	50.53	200,002	103.70	104,475	54.17
City of Naga	46,253	97.53	47,425	100.00	46,373	97.78
**Region 6**	**816,286**	**43.71**	**1,867,577**	**95.15**	**1,078,261**	**54.94**
Aklan	69,894	47.74	146,410	94.99	101,797	66.05
Antique	70,869	46.23	153,307	102.98	80,560	54.11
Capiz	73,254	36.51	200,651	97.75	109,601	53.40
Guimaras	23,742	47.63	49,847	103.04	29,414	60.80
Iloilo	138,567	28.33	489,081	95.27	211,959	41.29
Negros Occidental	249,119	41.96	593,681	92.03	364,880	56.56
City of Bacolod	114,817	88.80	129,299	90.81	100,595	70.65
City of Iloilo	76,024	72.20	105,301	100.04	79,455	75.48
**Region 7**	**1,151,536**	**65.47**	**1,758,990**	**87.03**	**1,225,213**	**60.62**
Bohol	215,313	75.58	284,871	86.77	130,109	39.63
Cebu	466,278	60.80	766,860	93.27	583,408	70.96
Negros Oriental	210,351	63.61	330,678	92.57	223,847	62.66
Siquijor	21,111	79.25	26,638	102.55	7,864	30.27

2.G. Environmental Health and Sanitation Services
Households (HHs) with access to basic safe water supply and manage drinking water
Philippines, 2023

Area	HHs with Access to Basic Safe Water Supply				HHs using Safely Managed Drinking Water Services	
	Level 3		Total	%		
	No.	%			No.	%
City of Cebu	111,181	83.39	133,323	54.32	111,181	45.30
City of Lapu-Lapu	60,980	47.33	128,842	95.50	91,738	68.00
City of Mandaue	66,322	75.56	87,778	81.94	77,066	71.94
**Region 8**	**440,648**	**42.69**	**1,032,146**	**93.25**	**538,878**	**48.69**
Biliran	30,405	69.90	43,495	98.61	31,919	72.37
Eastern Samar	26,650	24.04	110,841	93.55	12,175	10.28
Northern Leyte	154,943	42.34	365,968	92.28	191,766	48.36
Northern Samar	11,136	8.56	130,018	91.07	51,989	36.41
Southern Leyte	82,789	85.51	96,819	95.09	82,791	81.32
Western Samar	62,776	36.72	170,944	91.68	93,581	50.19
City of Ormoc	45,546	78.90	57,726	99.94	48,254	83.54
City of Tacloban	26,403	46.87	56,335	95.69	26,403	44.85
**Region 9**	**370,139**	**53.75**	**688,624**	**74.95**	**435,026**	**47.35**
Zamboanga del Norte	87,808	38.87	225,922	88.89	146,889	57.79
Zamboanga del Sur	96,382	49.54	194,550	79.09	100,596	40.90
Zamboanga Sibugay	9,046	32.42	27,903	17.60	14,269	9.00
City of Isabela	11,834	54.68	21,643	80.32	11,834	43.92
City of Zamboanga	165,069	75.51	218,606	93.79	161,438	69.26
**Region 10**	**743,933**	**65.51**	**1,135,687**	**91.72**	**633,954**	**51.20**
Bukidnon	193,609	56.27	344,051	92.45	170,523	45.82
Camiguin	19,700	92.76	21,238	93.65	16,640	73.38
Lanao del Norte	53,262	43.03	123,774	74.50	29,735	17.90
Misamis Occidental	88,766	58.17	152,600	99.80	107,867	70.54
Misamis Oriental	152,550	67.08	227,428	95.75	103,490	43.57
City of Cagayan de Oro	177,437	91.61	193,686	98.11	158,659	80.37
City of Iligan	58,609	80.39	72,910	81.58	47,040	52.64
**Region 11**	**645,030**	**54.80**	**1,177,083**	**85.75**	**885,308**	**64.49**
Davao de Oro	61,289	32.49	188,642	96.74	145,410	74.57
Davao del Norte	116,292	49.66	234,163	83.90	227,381	81.47
Davao Oriental	59,682	40.04	149,042	103.69	79,283	55.16

2.G. Environmental Health and Sanitation Services

Households (HHs) with access to basic safe water supply and manage drinking water

Philippines, 2023

Area	HHs with Access to Basic Safe Water Supply				HHs using Safely Managed Drinking Water Services	
	Level 3		Total	%		
	No.	%			No.	%
Davao del Sur	98,614	57.65	171,050	91.35	107,329	57.32
Davao Occidental	9,818	14.23	68,993	87.79	26,570	33.81
City of Davao	299,335	81.97	365,193	74.67	299,335	61.20
**Region 12**	**490,444**	**47.55**	**1,031,395**	**93.65**	**556,097**	**50.50**
North Cotabato	146,888	49.18	298,663	93.72	158,703	49.80
Sarangani	29,646	23.70	125,085	90.07	84,603	60.92
South Cotabato	140,535	53.95	260,471	103.10	178,359	70.60
Sultan Kudarat	35,020	18.63	188,018	89.85	52,559	25.12
City of General Santos	138,355	86.93	159,158	87.53	81,873	45.02
**BARMM**	**149,130**	**25.87**	**576,396**	**67.86**	**161,125**	**18.97**
Basilan	9,960	19.12	52,093	70.13	14,804	19.93
Lanao del Sur	51,390	28.60	179,710	96.65	63,219	34.00
Maguindanao	31,075	16.17	192,228	82.86	30,526	13.16
Sulu	26,573	32.42	81,974	49.05	23,111	13.83
Tawi-Tawi	1,973	5.25	37,589	49.99	0	0.00
SGA	0	0.00	0	0.00	0	0.00
City of Cotabato	28,159	85.85	32,802	50.19	29,465	45.09
**CARAGA**	**350,320**	**55.29**	**633,595**	**93.47**	**370,057**	**54.59**
Agusan del Norte	45,344	49.92	90,838	97.65	61,901	66.54
Agusan del Sur	62,661	37.91	165,308	92.50	66,003	36.93
Surigao del Norte	83,547	61.22	136,481	104.92	87,885	67.56
Surigao del Sur	88,477	63.22	139,951	90.30	91,071	58.76
Province of Dinagat	17,976	60.73	29,602	99.90	11,167	37.69
City of Butuan	52,315	73.25	71,415	78.09	52,030	56.89

2.G. Environmental Health and Sanitation Services
Households (HHs) with access to basic sanitation facility
Philippines, 2023

Area	Projected No. of HHs	HHs with Basic Sanitation Facility			
		Total	%	Pour/Flush Toilet	
				Septic Tank	
				No.	%
**Philippines**	**27,025,049**	**22,972,827**	**85.01**	**21,558,019**	**93.84**
**NCR**	**3,582,003**	**3,222,685**	**89.97**	**2,879,027**	**89.34**
City of Malabon	96,542	93,577	96.93	87,784	93.81
City of Navotas	65,013	59,812	92.00	59,812	100.00
City of Valenzuela	197,719	166,135	84.03	166,135	100.00
City of Caloocan	415,628	365,980	88.05	365,980	100.00
					0.00
City of Marikina	106,453	106,453	100.00	104,453	98.12
City of Pasig	218,789	38,536	17.61	33,644	87.31
Pateros	16,094	16,065	99.82	16,065	100.00
City of Taguig	255,377	252,108	98.72	222,638	88.31
City of Quezon	755,486	736,580	97.50	735,738	99.89
City of Makati	188,824	187,937	99.53	163,877	87.20
City of Mandaluyong	119,486	119,486	100.00	119,486	100.00
City of San Juan	31,796	31,682	99.64	31,214	98.52
City of Manila	497,563	437,879	88.00	199,025	45.45
					0.00
City of Las Piñas	160,153	155,512	97.10	155,162	99.77
City of Muntinlupa	140,933	140,914	99.99	127,675	90.60
City of Parañque	186,153	186,153	100.00	162,463	87.27
City of Pasay	129,994	127,876	98.37	127,876	100.00
**CAR**	**452,303**	**423,820**	**93.70**	**372,283**	**87.84**
Abra	59,546	58,947	98.99	54,219	91.98
Apayao	29,739	25,537	85.87	19,625	76.85
Benguet	120,091	106,750	88.89	102,860	96.36
Ifugao	50,865	45,607	89.66	37,522	82.27
Kalinga	50,063	47,871	95.62	38,622	80.68
Mt. Province	38,714	36,320	93.82	28,025	77.16
City of Baguio	103,285	102,788	99.52	91,410	88.93

2.G. Environmental Health and Sanitation Services
Households (HHs) with access to basic sanitation facility
Philippines, 2023

Area	Projected No. of HHs	HHs with Basic Sanitation Facility			
		Total	%	Pour/Flush Toilet	
				Septic Tank	
				No.	%
**Region 1**	**1,328,091**	**1,255,448**	**94.53**	**1,227,174**	**97.75**
Ilocos Norte	154,790	149,399	96.52	148,306	99.27
Ilocos Sur	173,863	174,590	100.42	174,590	100.00
La Union	208,309	199,646	95.84	176,051	88.18
Pangasinan	748,061	692,137	92.52	688,551	99.48
City of Dagupan	43,068	39,676	92.12	39,676	100.00
**Region 2**	**927,620**	**850,652**	**91.70**	**821,661**	**96.59**
Batanes	5,631	5,631	100.00	5,631	100.00
Cagayan	306,503	273,134	89.11	265,016	97.03
Isabela	394,188	369,805	93.81	355,997	96.27
Nueva Vizcaya	132,487	114,516	86.44	109,699	95.79
Quirino	51,317	50,072	97.57	47,824	95.51
City of Santiago	37,494	37,494	100.00	37,494	100.00
**Region 3**	**3,109,563**	**2,939,464**	**94.53**	**2,901,723**	**98.72**
Aurora	58,836	55,574	94.46	54,061	97.28
Bataan	214,501	205,019	95.58	204,967	99.97
Bulacan	940,427	909,926	96.76	906,789	99.66
Nueva Ecija	583,231	535,249	91.77	517,792	96.74
Pampanga	590,627	543,726	92.06	540,900	99.48
Tarlac	366,272	351,413	95.94	350,924	99.86
Zambales	168,891	153,851	91.09	141,624	92.05
City of Angeles	119,320	117,713	98.65	117,713	100.00
City of Olongapo	67,458	66,993	99.31	66,953	99.94
**Region 4A**	**4,159,056**	**3,833,979**	**92.18**	**3,760,567**	**98.09**
Batangas	731,131	674,217	92.22	653,686	96.95
Cavite	1,122,831	1,074,548	95.70	1,074,173	99.97
Laguna	936,359	872,828	93.22	871,148	99.81
Quezon	494,967	409,719	82.78	375,758	91.71

2.G. Environmental Health and Sanitation Services
Households (HHs) with access to basic sanitation facility
Philippines, 2023

Area	Projected No. of HHs	HHs with Basic Sanitation Facility			
		Total	%	Pour/Flush Toilet	
				Septic Tank	
				No.	%
Rizal	805,162	734,062	91.17	721,888	98.34
City of Lucena	68,606	68,605	100.00	63,914	93.16
**Region 4B**	**811,290**	**555,651**	**68.49**	**541,466**	**97.45**
Marinduque	61,366	61,863	100.81	61,863	100.00
Mindoro Occidental	130,025	108,221	83.23	107,032	98.90
Mindoro Oriental	219,399	152,758	69.63	151,864	99.41
Palawan	237,247	120,532	50.80	111,173	92.24
Romblon	79,038	66,301	83.88	65,419	98.67
City of Puerto Princesa	84,215	45,976	54.59	44115	95.95
**Region 5**	**1,406,129**	**1,014,492**	**72.15**	**962,301**	**94.86**
Albay	320,720	268,237	83.64	233689	87.12
Camarines Norte	149,644	128,115	85.61	123626	96.50
Camarines Sur	421,818	181,887	43.12	173,029	95.13
Catanduanes	62,062	55,539	89.49	55,411	99.77
Masbate	211,595	152,471	72.06	150,143	98.47
Sorsogon	192,865	180,827	93.76	178,987	98.98
City of Naga	47,425	47,416	99.98	47,416	100.00
**Region 6**	**1,962,744**	**1,789,256**	**91.16**	**1,697,947**	**94.90**
Aklan	154,126	146,853	95.28	134,680	91.71
Antique	148,873	146,401	98.34	143,346	97.91
Capiz	205,260	193,991	94.51	165,927	85.53
Guimaras	48,377	50,046	103.45	48,767	97.44
Iloilo	513,388	506,446	98.65	477,340	94.25
Negros Occidental	645,071	517,356	80.20	503,615	97.34
City of Bacolod	142,385	127,007	89.20	123,248	97.04
City of Iloilo	105,264	101,156	96.10	101,024	99.87

2.G. Environmental Health and Sanitation Services
Households (HHs) with access to basic sanitation facility
Philippines, 2023

Area	Projected No. of HHs	HHs with Basic Sanitation Facility			
		Total	%	Pour/Flush Toilet	
				Septic Tank	
				No.	%
**Region 7**	**2,021,190**	**1,650,396**	**81.65**	**1,626,103**	**98.53**
Bohol	328,287	280,853	85.55	272,133	96.90
Cebu	822,220	781,197	95.01	767,375	98.23
Negros Oriental	357,223	318,289	89.10	316,767	99.52
Siquijor	25,976	25,926	99.81	25,702	99.14
City of Cebu	245,447	50,392	20.53	50,387	99.99
City of Lapu-Lapu	134,909	114,504	84.87	114,504	100.00
City of Mandaue	107,128	79,235	73.96	79,235	100.00
**Region 8**	**1,106,829**	**917,061**	**82.85**	**782,512**	**85.33**
Biliran	44,107	38,899	88.19	38,867	99.92
Eastern Samar	118,486	94,329	79.61	93,540	99.16
Northern Leyte	396,570	330,588	83.36	323,867	97.97
Northern Samar	142,773	118,667	83.12	105,786	89.15
Southern Leyte	101,814	96,822	95.10	23,522	24.29
Western Samar	186,449	129,692	69.56	120,103	92.61
City of Ormoc	57,759	56,004	96.96	38,267	68.33
City of Tacloban	58,871	52,060	88.43	38,560	74.07
**Region 9**	**918,762**	**528,063**	**57.48**	**437,459**	**82.84**
Zamboanga del Norte	254,162	184,463	72.58	168,098	91.13
Zamboanga del Sur	245,986	105,336	42.82	67,114	63.71
Zamboanga Sibugay	158,578	12,342	7.78	2,867	23.23
City of Isabela	26,946	15,801	58.64	15,563	98.49
City of Zamboanga	233,090	210,121	90.15	183,817	87.48
**Region 10**	**1,238,184**	**976,725**	**78.88**	**721,692**	**73.89**
Bukidnon	372,160	319,897	85.96	221,505	69.24
Camiguin	22,677	19,928	87.88	19649	98.60
Lanao del Norte	166,135	38,450	23.14	29,525	76.79
Misamis Occidental	152,910	134,510	87.97	110,096	81.85

2.G. Environmental Health and Sanitation Services
Households (HHs) with access to basic sanitation facility
Philippines, 2023

Area	Projected No. of HHs	HHs with Basic Sanitation Facility			
		Total	%	Pour/Flush Toilet	
				Septic Tank	
				No.	%
Misamis Oriental	237,526	217,810	91.70	107,380	49.30
City of Cagayan de Oro	197,409	187,224	94.84	186,528	99.63
City of Iligan	89,367	58,906	65.91	47,009	79.80
**Region 11**	**1,372,765**	**1,134,414**	**82.64**	**1,094,153**	**96.45**
Davao de Oro	194,995	194,772	99.89	179,595	92.21
Davao del Norte	279,110	261,910	93.84	252,838	96.54
Davao Oriental	143,732	143,325	99.72	137,965	96.26
Davao del Sur	187,241	143,678	76.73	141,861	98.74
Davao Occidental	78,592	54,308	69.10	45,473	83.73
City of Davao	489,095	336,421	68.78	336,421	100.00
**Region 12**	**1,101,285**	**897,080**	**81.46**	**852,940**	**95.08**
North Cotabato	318,661	265,072	83.18	252,442	95.24
Sarangani	138,873	103,737	74.70	94,180	90.79
South Cotabato	252,646	230,531	91.25	224,305	97.30
Sultan Kudarat	209,264	139,693	66.75	128,626	92.08
City of General Santos	181,841	158,047	86.91	153,387	97.05
**BARMM**	**849,357**	**404,778**	**47.66**	**317,468**	**78.43**
Basilan	74,283	29,739	40.03	28,457	95.69
Lanao del Sur	185,938	107,913	58.04	73,819	68.41
Maguindanao	231,980	196,097	84.53	161,500	82.36
Sulu	167,120	38,743	23.18	22,374	57.75
Tawi-Tawi	75,188	8,605	11.44	7,637	88.75
SGA	49,498	-	0.00	0	0.00
City of Cotabato	65,350	23,681	36.24	23,681	100.00
**CARAGA**	**677,878**	**578,863**	**85.39**	**561,543**	**97.01**
Agusan del Norte	93,026	85,330	91.73	84,775	99.35

2.G. Environmental Health and Sanitation Services
Households (HHs) with access to basic sanitation facility
Philippines, 2023

Area	Projected No. of HHs	HHs with Basic Sanitation Facility			
		Total	%	Pour/Flush Toilet	
				Septic Tank	
				No.	%
Agusan del Sur	178,710	166,499	93.17	160,267	96.26
Surigao del Norte	130,077	120,610	92.72	119,486	99.07
Surigao del Sur	154,978	129,071	83.28	119,915	92.91
Province of Dinagat	29,632	24,665	83.24	24,665	100.00
City of Butuan	91,455	52,688	57.61	52,435	99.52

2.G. Environmental Health and Sanitation Services
Households (HHs) with access to basic sanitation facility
Philippines, 2023

Area	HHs with Basic Sanitation Facility				HHs using Safely Managed Sanitation Service	
	Pour/Flush Toilet					
	Community sewer/sewerage system		Ventilated improved Pit Latrine (VIP)			
	No.	%	No.	%	No.	%
**Philippines**	**448,530**	**1.95**	**966,278**	**4.21**	**17,208,867**	**63.68**
**NCR**	**331,195**	**10.28**	**12,463**	**0.39**	**2,513,362**	**70.17**
City of Malabon	5,793	6.19	0	0.00	86,888	90.00
City of Navotas	0	0.00	0	0.00	59,812	92.00
City of Valenzuela	0	0.00	0	0.00	166,135	84.03
City of Caloocan	0	0.00	0	0.00	365,980	88.05
		0.00		0.00		
City of Marikina	2,000	1.88	0	0.00	106,453	100.00
City of Pasig	4,892	12.69	0	0.00	38,271	17.49
Pateros	0	0.00	0	0.00	15,030	93.39
City of Taguig	29,470	11.69	0	0.00	252,108	98.72
City of Quezon	842	0.11	0	0.00	364,391	48.23
				0.00		
City of Makati	24,060	12.80	0	0.00	152,425	80.72
City of Mandaluyong	0	0.00	0	0.00	53,769	45.00
City of San Juan	468	1.48	0	0.00	2,760	8.68
City of Manila	226,391	51.70	12,463	2.85	425,416	85.50
		0.00		0.00		
City of Las Piñas	350	0.23	0	0.00	155,512	97.10
City of Muntinlupa	13,239	9.40	0	0.00	139,900	99.27
City of Parañque	23,690	12.73	0	0.00	11,907	6.40
City of Pasay	0	0.00	0	0.00	116,605	89.70
**CAR**	**11,364**	**2.68**	**40,173**	**9.48**	**408,127**	**90.23**
Abra	0	0.00	4,728	8.02	56,136	94.27
Apayao	0	0.00	5,912	23.15	24,637	82.84
Benguet	0	0.00	3,890	3.64	104,521	87.03
Ifugao	0	0.00	8,085	17.73	44,012	86.53
Kalinga	0	0.00	9,249	19.32	42,159	84.21
Mt. Province	0	0.00	8,295	22.84	34,676	89.57
City of Baguio	11,364	11.06	14	0.01	101,986	98.74

2.G. Environmental Health and Sanitation Services
Households (HHs) with access to basic sanitation facility
Philippines, 2023

Area	HHs with Basic Sanitation Facility				HHs using Safely Managed Sanitation Service	
	Pour/Flush Toilet					
	Community sewer/sewerage system		Ventilated improved Pit Latrine (VIP)			
	No.	%	No.	%	No.	%
**Region 1**	**13,401**	**1.07**	**14,873**	**1.18**	**460,044**	**34.64**
Ilocos Norte	0	0.00	1,093	0.73	91,786	59.30
Ilocos Sur	0	0.00	0	0.00	65,743	37.81
La Union	13,116	6.57	10,479	5.25	23,595	11.33
Pangasinan	285	0.04	3,301	0.48	271,627	36.31
City of Dagupan	0	0.00	0	0.00	7,293	16.93
**Region 2**	**0**	**0.00**	**28,991**	**3.41**	**794,606**	**85.66**
Batanes	0	0.00	0	0.00	5,631	100.00
Cagayan	0	0.00	8118	2.97	249,437	81.38
Isabela	0	0.00	13808	3.73	345,537	87.66
Nueva Vizcaya	0	0.00	4817	4.21	112,843	85.17
Quirino	0	0.00	2248	4.49	47,824	93.19
City of Santiago	0	0.00	0	0.00	33,334	88.90
**Region 3**	**10,315**	**0.35**	**27,426**	**0.93**	**2,796,882**	**89.94**
Aurora	0	0.00	1,513	2.72	55,063	93.59
Bataan	0	0.00	52	0.03	200,283	93.37
Bulacan	305	0.03	2,832	0.31	820,026	87.20
Nueva Ecija	0	0.00	17,457	3.26	498,075	85.40
Pampanga	14	0.00	2,812	0.52	536,490	90.83
Tarlac	0	0.00	489	0.14	348,698	95.20
Zambales	9,996	6.50	2,231	1.45	153,667	90.99
City of Angeles	0	0.00	0	0.00	117,713	98.65
City of Olongapo	0	0.00	40	0.06	66,867	99.12
**Region 4A**	**9,780**	**0.26**	**63,632**	**1.66**	**3,420,133**	**82.23**
Batangas	2,360	0.35	18,171	2.70	646,181	88.38
Cavite	363	0.03	12	0.00	1,062,356	94.61
Laguna	0	0.00	1,680	0.19	703,431	75.12
Quezon	3,183	0.78	30,778	7.51	342,357	69.17
Rizal	3,874	0.53	8,300	1.13	601,894	74.75

2.G. Environmental Health and Sanitation Services
Households (HHs) with access to basic sanitation facility
Philippines, 2023

Area	HHs with Basic Sanitation Facility				HHs using Safely Managed Sanitation Service	
	Pour/Flush Toilet					
	Community sewer/sewerage system		Ventilated improved Pit Latrine (VIP)			
	No.	%	No.	%	No.	%
City of Lucena	0	0.00	4,691	6.84	63,914	93.16
**Region 4B**	**2,559**	**0.46**	**11,626**	**2.09**	**341,449**	**42.09**
Marinduque	0	0.00	0	0.00	0	0.00
Mindoro Occidental	0	0.00	1,189	1.10	62,872	48.35
Mindoro Oriental	121	0.08	773	0.51	124,455	56.73
Palawan	2,438	2.02	6,921	5.74	61,745	26.03
Romblon	0	0.00	882	1.33	44,293	56.04
City of Puerto Princesa	0	0.00	1,861	4.05	48,084	57.10
**Region 5**	**29,940**	**2.95**	**22,251**	**2.19**	**806,070**	**57.33**
Albay	26,247	9.79	8,301	3.09	236,472	73.73
Camarines Norte	1,746	1.36	2,743	2.14	124,896	83.46
Camarines Sur	1,630	0.90	7,228	3.97	44,179	10.47
Catanduanes	0	0.00	128	0.23	48,259	77.76
Masbate	280	0.18	2,048	1.34	132,460	62.60
Sorsogon	37	0.02	1,803	1.00	172,388	89.38
City of Naga	0	0.00	0	0.00	47,416	99.98
**Region 6**	**12,972**	**0.72**	**78,337**	**4.38**	**1,369,605**	**69.78**
Aklan	665	0.45	11,508	7.84	99,532	64.58
Antique	0	0.00	3,055	2.09	137,855	92.60
Capiz	0	0.00	28,064	14.47	173,704	84.63
Guimaras	0	0.00	1,279	2.56	44,560	92.11
Iloilo	6,234	1.23	22,872	4.52	275,367	53.64
Negros Occidental	6,053	1.17	7,688	1.49	430,288	66.70
City of Bacolod	0	0.00	3,759	2.96	107,287	75.35
City of Iloilo	20	0.02	112	0.11	101,012	95.96

2.G. Environmental Health and Sanitation Services
Households (HHs) with access to basic sanitation facility
Philippines, 2023

Area	HHs with Basic Sanitation Facility				HHs using Safely Managed Sanitation Service	
	Pour/Flush Toilet					
	Community sewer/sewerage system		Ventilated improved Pit Latrine (VIP)			
	No.	%	No.	%	No.	%
**Region 7**	**10,636**	**0.64**	**13,657**	**0.83**	**1,488,299**	**73.63**
Bohol	0	0.00	8,720	3.10	246,413	75.06
Cebu	9,923	1.27	3,899	0.50	658,542	80.09
Negros Oriental	713	0.22	809	0.25	318,289	89.10
Siquijor	0	0.00	224	0.86	22,047	84.87
City of Cebu	0	0.00	5	0.01	49,269	20.07
City of Lapu-Lapu	0	0.00	0	0.00	114,504	84.87
City of Mandaue	0	0.00	0	0.00	79,235	73.96
**Region 8**	**482**	**0.05**	**134,067**	**14.62**	**243,161**	**21.97**
Biliran	0	0.00	32	0.08	0	0.00
Eastern Samar	139	0.15	650	0.69	12,515	10.56
Northern Leyte	279	0.08	6,442	1.95	50	0.01
Northern Samar	14	0.01	12,867	10.84	44,809	31.38
Southern Leyte	0	0.00	73,300	75.71	23,522	23.10
Western Samar	50	0.04	9,539	7.36	60,570	32.49
City of Ormoc	0	0.00	17,737	31.67	50,231	86.97
City of Tacloban	0	0.00	13,500	25.93	51,464	87.42
**Region 9**	**6,049**	**1.15**	**84,555**	**16.01**	**433,711**	**47.21**
Zamboanga del Norte	3,897	2.11	12,468	6.76	160,627	63.20
Zamboanga del Sur	2,152	2.04	36,070	34.24	82,181	33.41
Zamboanga Sibugay	0	0.00	9,475	76.77	11,006	6.94
City of Isabela	0	0.00	238	1.51	10,269	38.11
City of Zamboanga	0	0.00	26,304	12.52	169,628	72.77
**Region 10**	**0**	**0.00**	**255,033**	**26.11**	**220,720**	**17.83**
Bukidnon	0	0.00	98,392	30.76	80,112	21.53
Camiguin	0	0.00	279	1.40	17840	78.67
Lanao del Norte	0	0.00	8,925	23.21	2,206	1.33
Misamis Occidental	0	0.00	24,414	18.15	101,178	66.17
Misamis Oriental	0	0.00	110,430	50.70	2,802	1.18

2.G. Environmental Health and Sanitation Services
Households (HHs) with access to basic sanitation facility
Philippines, 2023

Area	HHs with Basic Sanitation Facility				HHs using Safely Managed Sanitation Service	
	Pour/Flush Toilet					
	Community sewer/sewerage system		Ventilated improved Pit Latrine (VIP)			
	No.	%	No.	%	No.	%
City of Cagayan de Oro	0	0.00	696	0.37	14,210	7.20
City of Iligan	0	0.00	11,897	20.20	2372	2.65
**Region 11**	**5,647**	**0.50**	**34,614**	**3.05**	**1,058,367**	**77.10**
Davao de Oro	1	0.00	15,176	7.79	182,080	93.38
Davao del Norte	5,646	2.16	3,426	1.31	248,135	88.90
Davao Oriental	0	0.00	5,360	3.74	113,838	79.20
Davao del Sur	0	0.00	1,817	1.26	136,802	73.06
Davao Occidental	0	0.00	8,835	16.27	41,091	52.28
City of Davao	0	0.00	0	0.00	336,421	68.78
**Region 12**	**4,190**	**0.47**	**39,950**	**4.45**	**775,784**	**70.44**
North Cotabato	0	0.00	12,630	4.76	245,055	76.90
Sarangani	0	0.00	9,557	9.21	79,405	57.18
South Cotabato	0	0.00	6,226	2.70	169,321	67.02
Sultan Kudarat	435	0.31	10,632	7.61	137,994	65.94
City of General Santos	3755	2.38	905	0.57	144,009	79.20
**BARMM**	**0**	**0.00**	**87,310**	**21.57**	**78,547**	**9.25**
Basilan	0	0.00	1,282	4.31	0	0.00
Lanao del Sur	0	0.00	34,094	31.59	42,980	23.12
Maguindanao	0	0.00	34,597	17.64	0	0.00
Sulu	0	0.00	16,369	42.25	32,524	19.46
Tawi-Tawi	0	0.00	968	11.25	2,224	2.96
SGA	0	0.00	0	0.00	0	0.00
City of Cotabato	0	0.00	0	0.00	819	1.25
**CARAGA**	**0**	**0.00**	**17,320**	**2.99**	**0**	**0.00**
Agusan del Norte	0	0.00	555	0.65	0	0.00
Agusan del Sur	0	0.00	6,232	3.74	0	0.00
Surigao del Norte	0	0.00	1,124	0.93	0	0.00

2.G. Environmental Health and Sanitation Services
Households (HHs) with access to basic sanitation facility
Philippines, 2023

Area	HHs with Basic Sanitation Facility				HHs using Safely Managed Sanitation Service	
	Pour/Flush Toilet					
	Community sewer/sewerage system		Ventilated improved Pit Latrine (VIP)			
	No.	%	No.	%	No.	%
Surigao del Sur	0	0.00	9,156	7.09	0	0.00
Province of Dinagat	0	0.00	0	0.00	0	0.00
City of Butuan	0	0.00	253	0.48	0	0.00

2.G. Environmental Health and Sanitation Services

Proportion of Municipalities, Cities and Chartered Cities Certified as ZOD

Philippines, 2023

Area	Total No. of Municipalities, Cities and Chartered Cities per Province	No. of Municipalities, Cities and Chartered Cities Certified as ZOD	
		No.	%
**Philippines**	**1,634**	**569**	**34.82**
**N C R**	**17**	**13**	**76.47**
City of Malabon	1	1	100.00
City of Navotas	1	0	0.00
City of Valenzuela	1	0	0.00
City of Caloocan	1	0	0.00
City of Marikina	1	1	100.00
City of Pasig	1	1	100.00
Pateros	1	1	100.00
City of Taguig	1	1	100.00
City of Quezon	1	1	100.00
City of Makati	1	1	100.00
City of Mandaluyong	1	1	100.00
City of San Juan	1	1	100.00
City of Manila	1	0	0.00
City of Las Piñas	1	1	100.00
City of Muntinlupa	1	1	100.00
City of Parañaque	1	1	100.00
City of Pasay	1	1	100.00
**CAR**	**77**	**45**	**58.44**
Abra	27	22	81.48
Apayao	7	1	14.29
Benguet	13	4	30.77
Ifugao	11	5	45.45
Kalinga	8	6	75.00
Mt. Province	10	6	60.00
City of Baguio	1	1	100.00

2.G. Environmental Health and Sanitation Services

Proportion of Municipalities, Cities and Chartered Cities Certified as ZOD

Philippines, 2023

Area	Total No. of Municipalities, Cities and Chartered Cities per Province	No. of Municipalities, Cities and Chartered Cities Certified as ZOD	
		No.	%
**Region 1**	**125**	**105**	**84.00**
Ilocos Norte	23	17	73.91
Ilocos Sur	34	30	88.24
La Union	20	20	100.00
Pangasinan	47	38	80.85
City of Dagupan	1	0	0.00
**Region 2**	**93**	**15**	**16.13**
Batanes	6	6	100.00
Cagayan	29	0	0.00
Isabela	36	7	19.44
Nueva Vizcaya	15	0	0.00
Quirino	6	2	33.33
City of Santiago	1	0	0.00
**Region 3**	**130**	**83**	**63.85**
Aurora	8	4	50.00
Bataan	12	8	66.67
Bulacan	24	17	70.83
Nueva Ecija	32	20	62.50
Pampanga	21	9	42.86
Tarlac	18	15	83.33
Zambales	13	8	61.54
City of Angeles	1	1	100.00
City of Olongapo	1	1	100.00
**Region 4A**	**142**	**62**	**43.66**
Batangas	34	21	61.76
Cavite	23	18	78.26
Laguna	30	8	26.67
Quezon	40	9	22.50
Rizal	14	5	35.71

2.G. Environmental Health and Sanitation Services

Proportion of Municipalities, Cities and Chartered Cities Certified as ZOD

Philippines, 2023

Area	Total No. of Municipalities, Cities and Chartered Cities per Province	No. of Municipalities, Cities and Chartered Cities Certified as ZOD	
		No.	%
City of Lucena	1	1	100.00
**Region 4B**	**72**	**18**	**25.00**
Marinduque	6	5	83.33
Mindoro Occidental	11	1	9.09
Mindoro Oriental	14	5	35.71
Palawan	23	4	17.39
Romblon	17	3	17.65
City of Puerto Princesa	1	0	0.00
**Region 5**	**114**	**7**	**6.14**
Albay	18	1	5.56
Camarines Norte	12	0	0.00
Camarines Sur	36	1	2.78
Catanduanes	11	0	0.00
Masbate	21	2	9.52
Sorsogon	15	2	13.33
City of Naga	1	1	100.00
**Region 6**	**133**	**75**	**56.39**
Aklan	17	13	76.47
Antique	18	1	5.56
Capiz	17	15	88.24
Guimaras	5	1	20.00
Iloilo	43	43	100.00
Negros Occidental	31	2	6.45
City of Bacolod	1	0	0.00
City of Iloilo	1	0	0.00

2.G. Environmental Health and Sanitation Services

Proportion of Municipalities, Cities and Chartered Cities Certified as ZOD

Philippines, 2023

Area	Total No. of Municipalities, Cities and Chartered Cities per Province	No. of Municipalities, Cities and Chartered Cities Certified as ZOD	
		No.	%
**Region 7**	**132**	**5**	**3.79**
Bohol	48	2	4.17
Cebu	50	3	6.00
Negros Oriental	25	0	0.00
Siquijor	6	0	0.00
City of Cebu	1	0	0.00
City of Lapu-Lapu	1	0	0.00
City of Mandaue	1	0	0.00
**Region 8**	**143**	**47**	**32.87**
Biliran	8	2	25.00
Eastern Samar	23	5	21.74
Northern Leyte	41	17	41.46
Northern Samar	24	5	20.83
Southern Leyte	19	10	52.63
Western Samar	26	8	30.77
City of Ormoc	1	0	0.00
City of Tacloban	1	0	0.00
**Region 9**	**72**	**4**	**5.56**
Zamboanga del Norte	27	4	14.81
Zamboanga del Sur	27	*	0.00
Zamboanga Sibugay	16	*	0.00
City of Isabela	1	0	0.00
City of Zamboanga	1	*	0.00
**Region 10**	**93**	**20**	**21.51**
Bukidnon	22	0	0.00
Camiguin	5	1	20.00
Lanao del Norte	22	4	18.18
Misamis Occidental	17	4	23.53
Misamis Oriental	25	11	44.00

2.G. Environmental Health and Sanitation Services

Proportion of Municipalities, Cities and Chartered Cities Certified as ZOD

Philippines, 2023

Area	Total No. of Municipalities, Cities and Chartered Cities per Province	No. of Municipalities, Cities and Chartered Cities Certified as ZOD	
		No.	%
City of Cagayan de Oro	1	0	0.00
City of Iligan	1	0	0.00
**Region 11**	**49**	**6**	**12.24**
Davao de Oro	11	5	45.45
Davao del Norte	11	1	9.09
Davao Oriental	11	0	0.00
Davao del Sur	10	0	0.00
Davao Occidental	5	0	0.00
City of Davao	1	0	0.00
**Region 12**	**49**	**11**	**22.45**
North Cotabato	18	5	27.78
Sarangani	7	3	42.86
South Cotabato	11	0	0.00
Sultan Kudarat	12	3	25.00
City of General Santos	1	0	0.00
**BARMM**	**120**	**35**	**29.17**
Basilan	12	0	0.00
Lanao del Sur	40	1	2.50
Maguindanao	36	33	91.67
Sulu	19	0	0.00
Tawi-Tawi	11	0	0.00
SGA	1	0	0.00
City of Cotabato	1	1	0.00

2.G. Environmental Health and Sanitation Services

Proportion of Municipalities, Cities and Chartered Cities Certified as ZOD

Philippines, 2023

Area	Total No. of Municipalities, Cities and Chartered Cities per Province	No. of Municipalities, Cities and Chartered Cities Certified as ZOD	
		No.	%
**CARAGA**	**73**	**18**	**24.66**
Agusan del Norte	11	2	18.18
Agusan del Sur	14	4	28.57
Surigao del Norte	21	4	19.05
Surigao del Sur	19	8	42.11
Province of Dinagat	7	0	0.00
City of Butuan	1	0	0.00

Table 2.H.2.1 - Acute Bloody Diarrhea

By Age group, Sex, Provinces and Cities

Philippines, 2023

Region/Province/City	0 - 6 days		7 - 28 days		29 days - 11 mos.		1 - 4 yrs.		5 - 9 yrs.		10 -14 yrs.		15 - 19 yrs.	
	M	F	M	F	M	F	M	F	M	F	M	F	M	F
**Philippines**	**3**	**5**	**6**	**3**	**131**	**110**	**473**	**426**	**283**	**174**	**109**	**86**	**73**	**96**
**NCR**	**0**	**0**	**1**	**1**	**2**	**2**	**4**	**7**	**5**	**2**	**0**	**2**	**1**	**0**
Malabon City	0	0	0	0	0	0	0	0	0	0	0	0	0	0
Navotas City	0	0	0	0	0	0	0	0	0	0	0	0	0	0
Valenzuela City	0	0	0	0	0	0	0	0	0	0	0	0	0	0
Caloocan City	0	0	1	1	1	1	2	0	0	1	0	1	0	0
Marikina City	0	0	0	0	0	0	0	0	0	0	0	0	0	0
Pasig City	0	0	0	0	0	0	0	0	0	0	0	0	0	0
Pateros	0	0	0	0	0	0	0	0	0	0	0	0	0	0
Taguig City	0	0	0	0	0	0	0	0	0	0	0	0	0	0
Quezon City	0	0	0	0	0	0	0	0	0	0	0	0	0	0
Makati City	0	0	0	0	1	0	2	2	4	0	0	0	1	0
Mandaluyong City	0	0	0	0	0	1	0	5	1	1	0	1	0	0
San Juan City	0	0	0	0	0	0	0	0	0	0	0	0	0	0
Manila City	0	0	0	0	0	0	0	0	0	0	0	0	0	0
Las Piñas City	0	0	0	0	0	0	0	0	0	0	0	0	0	0
Muntinlupa City	0	0	0	0	0	0	0	0	0	0	0	0	0	0
Parañaque City	0	0	0	0	0	0	0	0	0	0	0	0	0	0
Pasay City	0	0	0	0	0	0	0	0	0	0	0	0	0	0
**CAR**	**0**	**3**	**2**	**2**	**45**	**34**	**183**	**155**	**98**	**52**	**50**	**27**	**24**	**36**
Abra	0	0	0	0	2	0	15	3	1	0	2	0	0	1
Apayao	0	0	0	0	0	1	3	1	1	1	0	0	0	2
Benguet	0	0	1	0	0	2	1	1	1	2	1	0	0	2
Ifugao	0	0	0	0	26	15	102	90	67	28	26	14	18	19
Kalinga	0	3	1	2	11	12	50	49	13	15	13	7	5	7
Mt. Province	0	0	0	0	0	0	1	0	1	0	2	0	0	0
City of Baguio	0	0	0	0	6	4	11	11	14	6	6	6	1	5
**Region 1**	**0**	**0**	**0**	**0**	**1**	**2**	**1**	**1**	**1**	**0**	**0**	**0**	**0**	**0**
Ilocos Norte	0	0	0	0	0	0	0	0	0	0	0	0	0	0
Ilocos Sur	0	0	0	0	1	2	1	1	1	0	0	0	0	0
La Union	0	0	0	0	0	0	0	0	0	0	0	0	0	0

Table 2.H.2.1 - Acute Bloody Diarrhea

By Age group, Sex, Provinces and Cities

Philippines, 2023

Region/Province/City	0 - 6 days		7 - 28 days		29 days - 11 mos.		1 - 4 yrs.		5 - 9 yrs.		10 -14 yrs.		15 - 19 yrs.	
	M	F	M	F	M	F	M	F	M	F	M	F	M	F
Pangasinan	0	0	0	0	0	0	0	0	0	0	0	0	0	0
City of Dagupan	0	0	0	0	0	0	0	0	0	0	0	0	0	0
**Region 2**	**0**	**0**	**0**	**0**	**26**	**14**	**60**	**80**	**49**	**44**	**13**	**18**	**10**	**19**
Batanes	0	0	0	0	0	0	0	0	0	0	0	0	0	0
Cagayan	0	0	0	0	1	5	16	42	21	18	7	10	9	14
Isabela	0	0	0	0	22	9	40	35	24	21	5	7	1	5
Nueva Vizcaya	0	0	0	0	3	0	4	3	4	5	1	1	0	0
Quirino	0	0	0	0	0	0	0	0	0	0	0	0	0	0
City of Santiago	0	0	0	0	0	0	0	0	0	0	0	0	0	0
**Region 3**	**1**	**0**	**0**	**0**	**5**	**2**	**17**	**18**	**10**	**9**	**6**	**4**	**5**	**4**
Aurora	0	0	0	0	0	0	0	1	0	0	0	0	0	0
Bataan	0	0	0	0	0	0	0	0	0	0	0	0	0	0
Bulacan	1	0	0	0	4	2	13	12	3	4	1	0	1	1
Nueva Ecija	0	0	0	0	1	0	0	0	2	1	0	0	0	0
Pampanga	0	0	0	0	0	0	0	0	2	2	4	3	3	2
Tarlac	0	0	0	0	0	0	0	0	2	0	0	0	0	0
Zambales	0	0	0	0	0	0	4	3	1	1	0	0	1	1
City of Angeles	0	0	0	0	0	0	0	2	0	0	0	0	0	0
City of Olongapo	0	0	0	0	0	0	0	0	0	1	1	1	0	0
**Region 4A**	**0**	**0**	**0**	**0**	**4**	**11**	**53**	**32**	**28**	**20**	**10**	**10**	**5**	**4**
Batangas	0	0	0	0	2	2	23	10	12	9	3	1	3	1
Cavite	0	0	0	0	0	2	1	1	0	2	0	1	0	1
Laguna	0	0	0	0	0	6	22	14	11	7	4	5	2	2
Quezon	0	0	0	0	1	1	2	4	3	1	2	3	0	0
Rizal	0	0	0	0	1	0	5	3	2	1	1	0	0	0
City of Lucena	0	0	0	0	0	0	0	0	0	0	0	0	0	0
**Region 4B**	**0**	**0**	**1**	**0**	**3**	**4**	**28**	**18**	**17**	**11**	**5**	**6**	**2**	**2**
Marinduque	0	0	0	0	0	0	0	0	0	0	0	0	0	0
Mindoro Occidental	0	0	0	0	1	0	3	3	0	0	0	0	0	0
Mindoro Oriental	0	0	0	0	0	0	0	0	0	0	0	0	0	0

Table 2.H.2.1 - Acute Bloody Diarrhea

By Age group, Sex, Provinces and Cities

Philippines, 2023

Region/Province/City	0 - 6 days		7 - 28 days		29 days - 11 mos.		1 - 4 yrs.		5 - 9 yrs.		10 -14 yrs.		15 - 19 yrs.	
	M	F	M	F	M	F	M	F	M	F	M	F	M	F
Palawan	0	0	1	0	2	4	25	14	17	11	5	6	2	2
Romblon	0	0	0	0	0	0	0	1	0	0	0	0	0	0
City of Puerto Princesa	0	0	0	0	0	0	0	0	0	0	0	0	0	0
**Region 5**	**0**	**0**	**0**	**0**	**0**	**0**	**2**	**2**	**0**	**0**	**1**	**1**	**0**	**1**
Albay	0	0	0	0	0	0	0	0	0	0	0	0	0	0
Camarines Norte	0	0	0	0	0	0	0	0	0	0	0	0	0	0
Camarines Sur	0	0	0	0	0	0	0	0	0	0	0	0	0	0
Catanduanes	0	0	0	0	0	0	1	0	0	0	0	1	0	1
Masbate	0	0	0	0	0	0	0	0	0	0	0	0	0	0
Sorsogon	0	0	0	0	0	0	1	2	0	0	1	0	0	0
City of Naga	0	0	0	0	0	0	0	0	0	0	0	0	0	0
**Region 6**	**0**	**1**	**0**	**0**	**2**	**1**	**5**	**8**	**5**	**3**	**0**	**0**	**1**	**1**
Aklan	0	0	0	0	0	0	0	0	0	0	0	0	0	0
Antique	0	0	0	0	0	0	0	0	0	0	0	0	0	0
Capiz	0	0	0	0	0	0	0	0	0	0	0	0	0	0
Guimaras	0	0	0	0	0	0	0	0	0	0	0	0	0	0
Iloilo	0	0	0	0	0	0	0	0	0	0	0	0	0	0
Negros Occidental	0	1	0	0	2	1	5	7	5	3	0	0	1	0
City of Bacolod	0	0	0	0	0	0	0	1	0	0	0	0	0	1
City of Iloilo	0	0	0	0	0	0	0	0	0	0	0	0	0	0
**Region 7**	**0**	**0**	**0**	**0**	**1**	**0**	**1**	**5**	**1**	**0**	**1**	**0**	**1**	**2**
Bohol	0	0	0	0	0	0	0	0	0	0	0	0	0	0
Cebu	0	0	0	0	1	0	0	5	1	0	1	0	0	2
Negros Oriental	0	0	0	0	0	0	0	0	0	0	0	0	0	0
Siquijor	0	0	0	0	0	0	0	0	0	0	0	0	0	0
City of Cebu	0	0	0	0	0	0	0	0	0	0	0	0	1	0
City of Lapu-Lapu	0	0	0	0	0	0	1	0	0	0	0	0	0	0
City of Mandaue	0	0	0	0	0	0	0	0	0	0	0	0	0	0
**Region 8**	**0**	**0**	**2**	**0**	**11**	**5**	**57**	**44**	**32**	**15**	**7**	**2**	**4**	**8**
Biliran	0	0	1	0	1	0	15	4	8	9	2	1	2	3

Table 2.H.2.1 - Acute Bloody Diarrhea

By Age group, Sex, Provinces and Cities

Philippines, 2023

Region/Province/City	0 - 6 days		7 - 28 days		29 days - 11 mos.		1 - 4 yrs.		5 - 9 yrs.		10 -14 yrs.		15 - 19 yrs.	
	M	F	M	F	M	F	M	F	M	F	M	F	M	F
Eastern Samar	0	0	0	0	1	1	2	3	2	0	0	0	0	0
Northern Leyte	0	0	0	0	1	0	12	12	8	3	1	0	1	1
Northern Samar	0	0	0	0	2	3	18	11	6	0	1	0	0	1
Southern Leyte	0	0	0	0	1	0	3	2	0	0	1	1	1	1
Western Samar	0	0	1	0	1	0	2	10	6	1	1	0	0	0
City of Ormoc	0	0	0	0	4	1	5	2	2	2	1	0	0	1
City of Tacloban	0	0	0	0	0	0	0	0	0	0	0	0	0	1
**Region 9**	**0**	**0**	**0**	**0**	**4**	**2**	**5**	**3**	**3**	**0**	**2**	**2**	**2**	**1**
Zamboanga del Norte	0	0	0	0	0	0	0	0	0	0	0	0	1	0
Zamboanga del Sur	0	0	0	0	0	0	0	0	0	0	0	0	0	0
Zamboanga Sibugay	0	0	0	0	3	2	5	3	3	0	2	2	1	1
City of Isabela	0	0	0	0	0	0	0	0	0	0	0	0	0	0
City of Zamboanga	0	0	0	0	1	0	0	0	0	0	0	0	0	0
**Region 10**	**0**	**0**	**0**	**0**	**14**	**18**	**23**	**17**	**11**	**8**	**4**	**3**	**3**	**6**
Bukidnon	0	0	0	0	5	14	8	8	3	3	1	1	2	4
Camiguin	0	0	0	0	0	0	0	0	0	0	0	0	0	0
Lanao del Norte	0	0	0	0	0	0	0	0	0	0	0	0	0	0
Misamis Occidental	0	0	0	0	1	0	6	0	1	1	0	0	0	0
Misamis Oriental	0	0	0	0	0	1	2	0	3	2	0	0	0	2
City of Cagayan de Oro	0	0	0	0	0	0	2	2	0	0	0	0	0	0
City of Iligan	0	0	0	0	8	3	5	7	4	2	3	2	1	0
**Region 11**	**2**	**1**	**0**	**0**	**8**	**5**	**18**	**17**	**7**	**4**	**4**	**4**	**5**	**8**
Davao de Oro	0	0	0	0	3	1	1	6	1	0	0	1	1	0
Davao del Norte	0	0	0	0	0	0	2	1	0	0	0	1	0	1
Davao Oriental	0	0	0	0	1	0	0	0	2	1	2	0	0	1
Davao del Sur	0	0	0	0	1	0	0	0	0	0	1	0	0	0
Davao Occidental	0	1	0	0	3	2	8	5	1	2	0	1	0	0
City of Davao	2	0	0	0	0	2	7	5	3	1	1	1	4	6
**Region 12**	**0**	**0**	**0**	**0**	**3**	**4**	**6**	**4**	**6**	**1**	**1**	**0**	**0**	**0**
North Cotabato	0	0	0	0	0	0	0	0	0	0	0	0	0	0

Table 2.H.2.1 - Acute Bloody Diarrhea

By Age group, Sex, Provinces and Cities

Philippines, 2023

Region/Province/City	0 - 6 days		7 - 28 days		29 days - 11 mos.		1 - 4 yrs.		5 - 9 yrs.		10 -14 yrs.		15 - 19 yrs.	
	M	F	M	F	M	F	M	F	M	F	M	F	M	F
Sarangani	0	0	0	0	0	0	1	0	2	0	0	0	0	0
South Cotabato	0	0	0	0	3	3	5	4	4	1	1	0	0	0
Sultan Kudarat	0	0	0	0	0	1	0	0	0	0	0	0	0	0
City of General Santos	0	0	0	0	0	0	0	0	0	0	0	0	0	0
**BARMM**	**0**	**0**	**0**	**0**	**0**	**0**	**2**	**2**	**0**	**0**	**0**	**0**	**2**	**0**
Basilan	0	0	0	0	0	0	0	1	0	0	0	0	0	0
Lanao del Sur	0	0	0	0	0	0	1	0	0	0	0	0	0	0
Maguindanao	0	0	0	0	0	0	0	0	0	0	0	0	0	0
Sulu	0	0	0	0	0	0	0	0	0	0	0	0	0	0
Tawi-Tawi	0	0	0	0	0	0	0	0	0	0	0	0	0	0
SGA	0	0	0	0	0	0	0	0	0	0	0	0	0	0
City of Cotabato	0	0	0	0	0	0	1	1	0	0	0	0	2	0
**Caraga**	**0**	**0**	**0**	**0**	**2**	**6**	**8**	**13**	**10**	**5**	**5**	**7**	**8**	**4**
Agusan del Norte	0	0	0	0	0	0	0	0	0	0	0	0	0	0
Agusan del Sur	0	0	0	0	0	0	3	5	8	1	5	4	7	3
Surigao del Norte	0	0	0	0	0	2	1	2	0	2	0	0	1	0
Surigao del Sur	0	0	0	0	2	4	4	6	2	2	0	3	0	1
Province of Dinagat	0	0	0	0	0	0	0	0	0	0	0	0	0	0
City of Butuan	0	0	0	0	0	0	0	0	0	0	0	0	0	0

Table 2.H.2.1 - Acute Bloody Diarrhea

By Age group, Sex, Provinces and Cities

Philippines, 2023

Region/Province/City	20 - 24 yrs.		25 - 29 yrs.		30 - 34 yrs.		35 -39 yrs.		40 - 44 yrs.		45 -49 yrs.		50 -54 yrs.	
	M	F	M	F	M	F	M	F	M	F	M	F	M	F
**Philippines**	**62**	**104**	**46**	**92**	**53**	**77**	**49**	**57**	**47**	**56**	**44**	**67**	**37**	**84**
**NCR**	**0**	**1**	**0**	**1**	**2**	**1**	**0**	**0**	**0**	**5**	**1**	**0**	**4**	**6**
Malabon City	0	0	0	0	0	0	0	0	0	0	0	0	0	0
Navotas City	0	0	0	0	0	0	0	0	0	0	0	0	0	0
Valenzuela City	0	0	0	0	0	0	0	0	0	0	0	0	0	0
Caloocan City	0	0	0	0	0	0	0	0	0	0	0	0	0	0
Marikina City	0	0	0	0	0	0	0	0	0	0	0	0	0	0
Pasig City	0	0	0	0	0	0	0	0	0	0	0	0	0	0
Pateros	0	0	0	0	0	0	0	0	0	0	0	0	0	0
Taguig City	0	0	0	0	0	0	0	0	0	0	0	0	0	0
Quezon City	0	0	0	0	0	0	0	0	0	0	0	0	0	0
Makati City	0	1	0	1	0	1	0	0	0	0	0	0	0	0
Mandaluyong City	0	0	0	0	0	0	0	0	0	0	0	0	0	0
San Juan City	0	0	0	0	0	0	0	0	0	0	0	0	0	0
Manila City	0	0	0	0	0	0	0	0	0	0	0	0	0	0
Las Piñas City	0	0	0	0	2	0	0	0	0	5	1	0	4	6
Muntinlupa City	0	0	0	0	0	0	0	0	0	0	0	0	0	0
Parañaque City	0	0	0	0	0	0	0	0	0	0	0	0	0	0
Pasay City	0	0	0	0	0	0	0	0	0	0	0	0	0	0
**CAR**	**13**	**20**	**8**	**18**	**19**	**17**	**8**	**20**	**12**	**7**	**12**	**16**	**7**	**18**
Abra	0	1	1	1	1	2	2	2	0	0	0	1	0	1
Apayao	0	0	0	0	0	1	0	0	0	0	1	2	0	0
Benguet	0	0	0	0	0	0	0	0	1	0	0	1	0	0
Ifugao	9	7	3	7	9	8	3	8	8	3	7	3	4	8
Kalinga	2	11	4	7	6	4	3	5	3	1	2	7	2	7
Mt. Province	0	0	0	0	1	0	0	0	0	0	0	1	0	0
City of Baguio	2	1	0	3	2	2	0	5	0	3	2	1	1	2
**Region 1**	**3**	**0**	**0**	**0**	**0**	**0**	**0**	**0**	**0**	**3**	**0**	**0**	**0**	**0**
Ilocos Norte	0	0	0	0	0	0	0	0	0	0	0	0	0	0
Ilocos Sur	3	0	0	0	0	0	0	0	0	3	0	0	0	0
La Union	0	0	0	0	0	0	0	0	0	0	0	0	0	0
Pangasinan	0	0	0	0	0	0	0	0	0	0	0	0	0	0

Table 2.H.2.1 - Acute Bloody Diarrhea

By Age group, Sex, Provinces and Cities

Philippines, 2023

Region/Province/City	20 - 24 yrs.		25 - 29 yrs.		30 - 34 yrs.		35 -39 yrs.		40 - 44 yrs.		45 -49 yrs.		50 -54 yrs.	
	M	F	M	F	M	F	M	F	M	F	M	F	M	F
City of Dagupan	0	0	0	0	0	0	0	0	0	0	0	0	0	0
**Region 2**	**10**	**32**	**7**	**29**	**12**	**15**	**9**	**14**	**12**	**15**	**8**	**22**	**10**	**25**
Batanes	0	0	0	0	0	0	0	0	0	0	0	0	0	0
Cagayan	6	24	3	21	8	2	7	5	6	5	6	11	9	16
Isabela	2	6	3	7	4	12	2	9	6	10	2	10	1	9
Nueva Vizcaya	1	2	1	1	0	1	0	0	0	0	0	1	0	0
Quirino	0	0	0	0	0	0	0	0	0	0	0	0	0	0
City of Santiago	1	0	0	0	0	0	0	0	0	0	0	0	0	0
**Region 3**	**2**	**10**	**2**	**4**	**4**	**6**	**4**	**3**	**3**	**7**	**4**	**6**	**3**	**5**
Aurora	0	0	0	0	0	0	0	0	0	0	0	0	0	0
Bataan	0	6	0	0	0	0	0	0	0	0	0	0	0	0
Bulacan	0	1	1	1	1	0	1	0	0	2	1	1	0	0
Nueva Ecija	0	0	0	0	0	0	0	0	0	0	0	0	0	0
Pampanga	2	1	1	3	3	4	2	3	2	5	3	5	3	2
Tarlac	0	0	0	0	0	0	0	0	0	0	0	0	0	0
Zambales	0	2	0	0	0	0	1	0	1	0	0	0	0	1
City of Angeles	0	0	0	0	0	0	0	0	0	0	0	0	0	0
City of Olongapo	0	0	0	0	0	2	0	0	0	0	0	0	0	2
**Region 4A**	**9**	**10**	**4**	**9**	**3**	**2**	**2**	**3**	**4**	**2**	**0**	**2**	**2**	**0**
Batangas	4	5	1	1	0	0	1	1	2	1	0	2	2	0
Cavite	0	1	0	0	0	0	0	0	0	0	0	0	0	0
Laguna	5	3	3	7	2	2	1	2	1	1	0	0	0	0
Quezon	0	0	0	1	1	0	0	0	0	0	0	0	0	0
Rizal	0	1	0	0	0	0	0	0	1	0	0	0	0	0
City of Lucena	0	0	0	0	0	0	0	0	0	0	0	0	0	0
**Region 4B**	**3**	**7**	**4**	**4**	**2**	**7**	**4**	**2**	**2**	**0**	**3**	**4**	**1**	**4**
Marinduque	0	0	0	0	0	0	0	0	0	0	0	0	0	0
Mindoro Occidental	0	0	0	0	0	0	0	0	0	0	0	0	0	0
Mindoro Oriental	0	0	0	0	0	0	0	0	0	0	0	0	0	0
Palawan	3	7	4	4	2	7	4	2	2	0	2	4	1	3

Table 2.H.2.1 - Acute Bloody Diarrhea

By Age group, Sex, Provinces and Cities

Philippines, 2023

Region/Province/City	20 - 24 yrs.		25 - 29 yrs.		30 - 34 yrs.		35 -39 yrs.		40 - 44 yrs.		45 -49 yrs.		50 -54 yrs.	
	M	F	M	F	M	F	M	F	M	F	M	F	M	F
Romblon	0	0	0	0	0	0	0	0	0	0	1	0	0	1
City of Puerto Princesa	0	0	0	0	0	0	0	0	0	0	0	0	0	0
**Region 5**	**0**	**1**	**2**	**0**	**0**	**0**	**0**	**0**	**1**	**0**	**0**	**0**	**0**	**0**
Albay	0	0	0	0	0	0	0	0	0	0	0	0	0	0
Camarines Norte	0	0	0	0	0	0	0	0	0	0	0	0	0	0
Camarines Sur	0	0	0	0	0	0	0	0	0	0	0	0	0	0
Catanduanes	0	0	0	0	0	0	0	0	0	0	0	0	0	0
Masbate	0	0	0	0	0	0	0	0	0	0	0	0	0	0
Sorsogon	0	1	2	0	0	0	0	0	1	0	0	0	0	0
City of Naga	0	0	0	0	0	0	0	0	0	0	0	0	0	0
**Region 6**	**2**	**0**	**1**	**1**	**1**	**0**	**0**	**1**	**1**	**1**	**1**	**0**	**0**	**1**
Aklan	0	0	0	0	0	0	0	0	0	0	0	0	0	0
Antique	0	0	0	0	0	0	0	0	0	0	0	0	0	0
Capiz	0	0	0	0	0	0	0	0	0	0	0	0	0	0
Guimaras	0	0	0	0	0	0	0	0	0	0	0	0	0	0
Iloilo	0	0	1	0	0	0	0	0	0	0	0	0	0	0
Negros Occidental	1	0	0	1	1	0	0	1	1	1	1	0	0	1
City of Bacolod	1	0	0	0	0	0	0	0	0	0	0	0	0	0
City of Iloilo	0	0	0	0	0	0	0	0	0	0	0	0	0	0
**Region 7**	**1**	**1**	**1**	**1**	**0**	**2**	**0**	**2**	**0**	**0**	**0**	**0**	**0**	**0**
Bohol	0	0	0	0	0	0	0	0	0	0	0	0	0	0
Cebu	1	0	1	1	0	2	0	2	0	0	0	0	0	0
Negros Oriental	0	0	0	0	0	0	0	0	0	0	0	0	0	0
Siquijor	0	0	0	0	0	0	0	0	0	0	0	0	0	0
City of Cebu	0	1	0	0	0	0	0	0	0	0	0	0	0	0
City of Lapu lapu	0	0	0	0	0	0	0	0	0	0	0	0	0	0
City of Mandaue	0	0	0	0	0	0	0	0	0	0	0	0	0	0
**Region 8**	**4**	**11**	**8**	**10**	**4**	**9**	**9**	**1**	**3**	**7**	**8**	**9**	**6**	**9**
Biliran	4	6	5	2	1	1	6	0	1	1	3	1	2	3
Eastern Samar	0	0	0	0	0	0	1	0	0	0	0	1	0	1

Table 2.H.2.1 - Acute Bloody Diarrhea

By Age group, Sex, Provinces and Cities

Philippines, 2023

Region/Province/City	20 - 24 yrs.		25 - 29 yrs.		30 - 34 yrs.		35 -39 yrs.		40 - 44 yrs.		45 -49 yrs.		50 -54 yrs.	
	M	F	M	F	M	F	M	F	M	F	M	F	M	F
Northern Leyte	0	0	1	5	2	4	1	1	0	0	2	3	4	2
Northern Samar	0	1	0	0	0	1	1	0	0	1	0	1	0	0
Southern Leyte	0	1	0	0	1	1	0	0	0	1	0	1	0	0
Western Samar	0	1	2	3	0	1	0	0	0	3	2	0	0	2
City of Ormoc	0	2	0	0	0	1	0	0	2	1	1	2	0	1
City of Tacloban	0	0	0	0	0	0	0	0	0	0	0	0	0	0
**Region 9**	**0**	**0**	**1**	**1**	**0**	**0**	**1**	**0**	**0**	**1**	**0**	**1**	**0**	**0**
Zamboanga del Norte	0	0	0	0	0	0	0	0	0	0	0	0	0	0
Zamboanga del Sur	0	0	0	0	0	0	0	0	0	0	0	0	0	0
Zamboanga Sibugay	0	0	1	1	0	0	1	0	0	1	0	1	0	0
City of Isabela	0	0	0	0	0	0	0	0	0	0	0	0	0	0
City of Zamboanga	0	0	0	0	0	0	0	0	0	0	0	0	0	0
**Region 10**	**3**	**2**	**2**	**4**	**2**	**1**	**2**	**3**	**0**	**2**	**3**	**1**	**0**	**2**
Bukidnon	1	1	0	3	1	0	0	3	0	0	2	0	0	1
Camiguin	0	0	0	0	0	0	0	0	0	0	0	0	0	0
Lanao del Norte	0	0	0	0	0	0	0	0	0	0	0	0	0	0
Misamis Occidental	0	0	0	0	0	0	0	0	0	1	0	0	0	0
Misamis Oriental	0	0	1	1	1	0	1	0	0	1	0	0	0	0
City of Cagayan de Oro	0	0	0	0	0	0	0	0	0	0	0	0	0	0
City of Iligan	2	1	1	0	0	1	1	0	0	0	1	1	0	1
**Region 11**	**1**	**3**	**4**	**3**	**0**	**11**	**2**	**5**	**2**	**2**	**1**	**1**	**1**	**6**
Davao de Oro	0	0	0	0	0	3	1	0	0	0	0	0	0	2
Davao del Norte	0	0	1	1	0	2	1	1	1	0	0	0	0	0
Davao Oriental	1	0	0	0	0	0	0	0	0	0	1	0	0	0
Davao del Sur	0	1	0	0	0	1	0	0	0	0	0	0	0	0
Davao Occidental	0	0	2	0	0	3	0	3	0	1	0	0	0	0
City of Davao	0	2	1	2	0	2	0	1	1	1	0	1	1	4
**Region 12**	**0**	**1**	**0**	**1**	**0**	**0**	**1**	**0**	**1**	**0**	**1**	**0**	**0**	**0**
North Cotabato	0	0	0	0	0	0	0	0	0	0	0	0	0	0

Table 2.H.2.1 - Acute Bloody Diarrhea

By Age group, Sex, Provinces and Cities

Philippines, 2023

Region/Province/City	20 - 24 yrs.		25 - 29 yrs.		30 - 34 yrs.		35 -39 yrs.		40 - 44 yrs.		45 -49 yrs.		50 -54 yrs.	
	M	F	M	F	M	F	M	F	M	F	M	F	M	F
Sarangani	0	0	0	0	0	0	0	0	0	0	0	0	0	0
South Cotabato	0	1	0	1	0	0	1	0	1	0	1	0	0	0
Sultan Kudarat	0	0	0	0	0	0	0	0	0	0	0	0	0	0
City of General Santos	0	0	0	0	0	0	0	0	0	0	0	0	0	0
**BARMM**	**2**	**0**	**0**	**0**	**0**	**0**	**0**	**1**	**0**	**0**	**0**	**0**	**1**	**0**
Basilan	1	0	0	0	0	0	0	0	0	0	0	0	0	0
Lanao del Sur	1	0	0	0	0	0	0	0	0	0	0	0	0	0
Maguindanao	0	0	0	0	0	0	0	0	0	0	0	0	0	0
Sulu	0	0	0	0	0	0	0	0	0	0	0	0	0	0
Tawi-Tawi	0	0	0	0	0	0	0	0	0	0	0	0	0	0
SGA	0	0	0	0	0	0	0	0	0	0	0	0	0	0
City of Cotabato	0	0	0	0	0	0	0	1	0	0	0	0	1	0
**Caraga**	**9**	**5**	**2**	**6**	**4**	**6**	**7**	**2**	**6**	**4**	**2**	**5**	**2**	**8**
Agusan del Norte	0	0	0	0	1	0	0	0	0	0	0	0	0	0
Agusan del Sur	6	1	2	5	2	4	4	2	6	3	2	3	2	5
Surigao del Norte	0	1	0	0	0	0	0	0	0	0	0	1	0	0
Surigao del Sur	3	3	0	1	1	2	3	0	0	1	0	1	0	3
Province of Dinagat	0	0	0	0	0	0	0	0	0	0	0	0	0	0
City of Butuan	0	0	0	0	0	0	0	0	0	0	0	0	0	0

Table 2.H.2.1 Acute Bloody Diarrhea

By Age group, Sex, Provinces and Cities

Philippines, 2023

Region/Province/City	55 - 59 yrs.		60 - 64. yrs.		65 - 69 yrs.		70 yrs above		Total		Total Both Sexes	
	M	F	M	F	M	F	M	F	M	F	Number	Rate
**Philippines**	**35**	**69**	**69**	**98**	**51**	**100**	**76**	**151**	**1,647**	**1,855**	**3,502**	**3.14**
**NCR**	**4**	**13**	**14**	**32**	**14**	**36**	**24**	**56**	**76**	**165**	**241**	**1.75**
Malabon City	0	0	0	0	0	0	0	0	0	0	0	0.00
Navotas City	0	0	0	0	0	0	0	0	0	0	0	0.00
Valenzuela City	0	0	0	0	0	0	0	0	0	0	0	0.00
Caloocan City	0	0	0	0	0	0	0	0	4	4	8	0.47
Marikina City	0	0	0	0	0	0	0	0	0	0	0	0.00
Pasig City	0	0	0	0	0	0	0	0	0	0	0	0.00
Pateros	0	0	0	0	0	0	0	0	0	0	0	0.00
Taguig City	0	0	0	0	0	0	0	0	0	0	0	0.00
Quezon City	0	0	0	0	0	0	0	0	0	0	0	0.00
Makati City	0	0	0	0	0	0	0	2	8	7	15	2.35
Mandaluyong City	0	0	0	0	0	0	0	0	1	8	9	2.07
San Juan City	0	0	0	0	0	0	0	0	0	0	0	0.00
Manila City	0	0	0	0	0	0	0	0	0	0	0	0.00
Las Piñas City	4	13	14	32	14	36	24	54	63	146	209	33.77
Muntinlupa City	0	0	0	0	0	0	0	0	0	0	0	0.00
Parañaque City	0	0	0	0	0	0	0	0	0	0	0	0.00
Pasay City	0	0	0	0	0	0	0	0	0	0	0	0.00
**CAR**	**7**	**19**	**12**	**19**	**16**	**22**	**19**	**22**	**535**	**507**	**1,042**	**56.27**
Abra	0	0	2	3	3	1	1	1	30	17	47	18.54
Apayao	0	0	0	0	0	0	0	0	5	8	13	10.14
Benguet	0	1	0	1	1	0	0	0	6	10	16	3.38
Ifugao	5	11	5	6	7	14	12	12	311	253	564	260.57
Kalinga	2	6	4	5	5	6	6	5	132	159	291	121.78
Mt. Province	0	0	0	1	0	0	0	0	5	2	7	4.30
City of Baguio	0	1	1	3	0	1	0	4	46	58	104	27.54
**Region 1**	**0**	**0**	**1**	**0**	**0**	**0**	**0**	**3**	**7**	**9**	**16**	**0.30**
Ilocos Norte	0	0	0	0	0	0	0	0	0	0	0	0.00
Ilocos Sur	0	0	1	0	0	0	0	3	7	9	16	2.24
La Union	0	0	0	0	0	0	0	0	0	0	0	0.00
Pangasinan	0	0	0	0	0	0	0	0	0	0	0	0.00

Table 2.H.2.1 Acute Bloody Diarrhea

By Age group, Sex, Provinces and Cities

Philippines, 2023

Region/Province/City	55 - 59 yrs.		60 - 64. yrs.		65 - 69 yrs.		70 yrs above		Total		Total Both Sexes	
	M	F	M	F	M	F	M	F	M	F	Number	Rate
City of Dagupan	0	0	0	0	0	0	0	0	0	0	0	0.00
**Region 2**	**4**	**13**	**21**	**19**	**8**	**24**	**13**	**46**	**272**	**429**	**701**	**18.61**
Batanes	0	0	0	0	0	0	0	0	0	0	0	0.00
Cagayan	2	7	13	7	3	13	5	25	122	225	347	26.90
Isabela	2	6	7	12	5	11	6	21	132	190	322	20.38
Nueva Vizcaya	0	0	1	0	0	0	2	0	17	14	31	6.03
Quirino	0	0	0	0	0	0	0	0	0	0	0	0.00
City of Santiago	0	0	0	0	0	0	0	0	1	0	1	0.65
**Region 3**	**5**	**5**	**1**	**3**	**0**	**0**	**0**	**2**	**72**	**88**	**160**	**1.26**
Aurora	0	0	0	0	0	0	0	0	0	1	1	0.41
Bataan	0	0	0	0	0	0	0	0	0	6	6	0.68
Bulacan	1	0	0	0	0	0	0	1	28	25	53	1.40
Nueva Ecija	0	0	0	0	0	0	0	0	3	1	4	0.17
Pampanga	4	3	1	3	0	0	0	1	30	37	67	2.67
Tarlac	0	0	0	0	0	0	0	0	2	0	2	0.13
Zambales	0	0	0	0	0	0	0	0	8	8	16	2.41
City of Angeles	0	0	0	0	0	0	0	0	0	2	2	0.42
City of Olongapo	0	2	0	0	0	0	0	0	1	8	9	3.41
**Region 4A**	**0**	**3**	**0**	**1**	**1**	**0**	**0**	**1**	**125**	**110**	**235**	**1.42**
Batangas	0	2	0	0	0	0	0	1	53	36	89	3.00
Cavite	0	0	0	0	0	0	0	0	1	8	9	0.20
Laguna	0	0	0	1	0	0	0	0	51	50	101	2.92
Quezon	0	1	0	0	1	0	0	0	10	11	21	1.05
Rizal	0	0	0	0	0	0	0	0	10	5	15	0.44
City of Lucena	0	0	0	0	0	0	0	0	0	0	0	0.00
**Region 4B**	**4**	**1**	**3**	**3**	**3**	**4**	**1**	**5**	**86**	**82**	**168**	**5.09**
Marinduque	0	0	0	0	0	0	0	1	0	1	1	0.41
Mindoro Occidental	0	0	0	0	0	0	0	0	4	3	7	1.30
Mindoro Oriental	0	0	0	0	0	0	0	0	0	0	0	0.00
Palawan	4	1	3	3	2	4	1	4	80	76	156	16.15

Table 2.H.2.1 Acute Bloody Diarrhea

By Age group, Sex, Provinces and Cities

Philippines, 2023

Region/Province/City	55 - 59 yrs.		60 - 64. yrs.		65 - 69 yrs.		70 yrs above		Total		Total Both Sexes	
	M	F	M	F	M	F	M	F	M	F	Number	Rate
Romblon	0	0	0	0	1	0	0	0	2	2	4	1.27
City of Puerto Princesa	0	0	0	0	0	0	0	0	0	0	0	0.00
**Region 5**	**0**	**0**	**0**	**0**	**0**	**0**	**0**	**0**	**6**	**5**	**11**	**0.18**
Albay	0	0	0	0	0	0	0	0	0	0	0	0.00
Camarines Norte	0	0	0	0	0	0	0	0	0	0	0	0.00
Camarines Sur	0	0	0	0	0	0	0	0	0	0	0	0.00
Catanduanes	0	0	0	0	0	0	0	0	1	2	3	1.07
Masbate	0	0	0	0	0	0	0	0	0	0	0	0.00
Sorsogon	0	0	0	0	0	0	0	0	5	3	8	0.94
City of Naga	0	0	0	0	0	0	0	0	0	0	0	0.00
**Region 6**	**0**	**0**	**1**	**1**	**0**	**0**	**1**	**0**	**21**	**19**	**40**	**0.50**
Aklan	0	0	0	0	0	0	0	0	0	0	0	0.00
Antique	0	0	0	0	0	0	0	0	0	0	0	0.00
Capiz	0	0	0	0	0	0	0	0	0	0	0	0.00
Guimaras	0	0	0	0	0	0	0	0	0	0	0	0.00
Iloilo	0	0	0	0	0	0	0	0	1	0	1	0.05
Negros Occidental	0	0	1	1	0	0	0	0	18	17	35	1.32
City of Bacolod	0	0	0	0	0	0	1	0	2	2	4	0.67
City of Iloilo	0	0	0	0	0	0	0	0	0	0	0	0.00
**Region 7**	**0**	**0**	**0**	**0**	**3**	**0**	**0**	**1**	**10**	**14**	**24**	**0.29**
Bohol	0	0	0	0	0	0	0	0	0	0	0	0.00
Cebu	0	0	0	0	2	0	0	1	7	13	20	0.58
Negros Oriental	0	0	0	0	0	0	0	0	0	0	0	0.00
Siquijor	0	0	0	0	0	0	0	0	0	0	0	0.00
City of Cebu	0	0	0	0	1	0	0	0	2	1	3	0.30
City of Lapu-Lapu	0	0	0	0	0	0	0	0	1	0	1	0.19
City of Mandaue	0	0	0	0	0	0	0	0	0	0	0	0.00
**Region 8**	**5**	**7**	**9**	**8**	**1**	**4**	**11**	**8**	**181**	**157**	**338**	**7.27**
Biliran	0	0	0	3	0	1	1	0	52	35	87	47.34
Eastern Samar	0	0	0	0	0	0	0	1	6	7	13	2.66

Table 2.H.2.1 Acute Bloody Diarrhea

By Age group, Sex, Provinces and Cities

Philippines, 2023

Region/Province/City	55 - 59 yrs.		60 - 64. yrs.		65 - 69 yrs.		70 yrs above		Total		Total Both Sexes	
	M	F	M	F	M	F	M	F	M	F	Number	Rate
Northern Leyte	2	2	2	4	0	0	4	2	41	39	80	5.06
Northern Samar	0	1	1	0	0	0	2	1	31	21	52	7.97
Southern Leyte	0	0	0	0	0	0	0	0	7	8	15	3.46
Western Samar	3	4	6	1	0	1	2	3	26	30	56	6.88
City of Ormoc	0	0	0	0	1	2	2	1	18	16	34	14.28
City of Tacloban	0	0	0	0	0	0	0	0	0	1	1	0.39
**Region 9**	**0**	**0**	**0**	**1**	**0**	**1**	**0**	**2**	**18**	**15**	**33**	**0.83**
Zamboanga del Norte	0	0	0	0	0	0	0	0	1	0	1	0.09
Zamboanga del Sur	0	0	0	0	0	0	0	0	0	0	0	0.00
Zamboanga Sibugay	0	0	0	1	0	1	0	2	16	15	31	4.52
City of Isabela	0	0	0	0	0	0	0	0	0	0	0	0.00
City of Zamboanga	0	0	0	0	0	0	0	0	1	0	1	0.10
**Region 10**	**1**	**0**	**4**	**2**	**0**	**5**	**0**	**2**	**72**	**76**	**148**	**2.85**
Bukidnon	0	0	2	2	0	2	0	2	25	44	69	4.30
Camiguin	0	0	0	0	0	0	0	0	0	0	0	0.00
Lanao del Norte	0	0	0	0	0	0	0	0	0	0	0	0.00
Misamis Occidental	0	0	0	0	0	0	0	0	8	2	10	1.58
Misamis Oriental	0	0	2	0	0	3	0	0	10	10	20	2.03
City of Cagayan de Oro	0	0	0	0	0	0	0	0	2	2	4	0.53
City of Iligan	1	0	0	0	0	0	0	0	27	18	45	12.10
**Region 11**	**3**	**2**	**2**	**2**	**0**	**1**	**3**	**2**	**63**	**77**	**140**	**2.60**
Davao de Oro	1	0	0	0	0	0	0	1	8	14	22	2.78
Davao del Norte	2	0	0	0	0	0	0	0	7	7	14	1.21
Davao Oriental	0	0	0	0	0	0	1	0	8	2	10	1.69
Davao del Sur	0	0	2	1	0	0	0	1	4	4	8	1.15
Davao Occidental	0	0	0	0	0	1	1	0	15	19	34	10.67
City of Davao	0	2	0	1	0	0	1	0	21	31	52	2.85
**Region 12**	**1**	**2**	**0**	**0**	**0**	**0**	**0**	**0**	**20**	**13**	**33**	**0.73**
North Cotabato	0	0	0	0	0	0	0	0	0	0	0	0.00
Sarangani	1	1	0	0	0	0	0	0	4	1	5	0.86

Table 2.H.2.1 Acute Bloody Diarrhea

By Age group, Sex, Provinces and Cities

Philippines, 2023

Region/Province/City	55 - 59 yrs.		60 - 64. yrs.		65 - 69 yrs.		70 yrs above		Total		Total Both Sexes	
	M	F	M	F	M	F	M	F	M	F	Number	Rate
South Cotabato	0	1	0	0	0	0	0	0	16	11	27	2.68
Sultan Kudarat	0	0	0	0	0	0	0	0	0	1	1	0.11
City of General Santos	0	0	0	0	0	0	0	0	0	0	0	0.00
**BARMM**	**1**	**0**	**0**	**0**	**0**	**1**	**1**	**0**	**9**	**4**	**13**	**0.26**
Basilan	0	0	0	0	0	0	0	0	1	1	2	0.46
Lanao del Sur	0	0	0	0	0	0	0	0	2	0	2	0.17
Maguindanao	0	0	0	0	0	0	0	0	0	0	0	0.00
Sulu	0	0	0	0	0	0	0	0	0	0	0	0.00
Tawi-Tawi	0	0	0	0	0	0	0	0	0	0	0	0.00
SGA	0	0	0	0	0	0	0	0	0	0	0	
City of Cotabato	1	0	0	0	0	1	1	0	6	3	9	2.69
**Caraga**	**0**	**4**	**1**	**7**	**5**	**2**	**3**	**1**	**74**	**85**	**159**	**5.53**
Agusan del Norte	0	0	0	0	0	0	0	0	1	0	1	0.25
Agusan del Sur	0	3	1	5	2	2	3	0	53	46	99	13.04
Surigao del Norte	0	0	0	0	0	0	0	0	2	8	10	1.83
Surigao del Sur	0	1	0	2	3	0	0	1	18	31	49	7.41
Province of Dinagat	0	0	0	0	0	0	0	0	0	0	0	0.00
City of Butuan	0	0	0	0	0	0	0	0	0	0	0	0.00

Table 2.H.2.2 Acute Febrile Illness

By Age group, Sex, Provinces and Cities

Philippines, 2023

Region/Province/City	0 - 6 days		7 - 28 days		29 days - 11 mos.		1 - 4 yrs.		5 - 9 yrs.		10 -14 yrs.		15 - 19 yrs.	
	M	F	M	F	M	F	M	F	M	F	M	F	M	F
**PHILIPPINES**	**76**	**64**	**204**	**278**	**2,772**	**2,853**	**8,003**	**7,783**	**6,547**	**6,226**	**3,189**	**2,860**	**1,532**	**1,508**
**NCR**	**9**	**0**	**1**	**0**	**4**	**11**	**34**	**21**	**20**	**17**	**14**	**12**	**21**	**14**
Malabon City	0	0	0	0	0	0	0	0	0	0	0	0	0	0
Navotas City	0	0	0	0	0	0	0	0	0	0	0	0	0	0
Valenzuela City	0	0	0	0	0	0	0	0	1	0	3	2	0	0
Caloocan City	1	0	1	0	0	1	0	3	1	1	1	0	0	0
Marikina City	0	0	0	0	0	0	0	0	0	0	0	0	0	0
Pasig City	0	0	0	0	1	4	5	2	3	2	0	0	0	1
Pateros	0	0	0	0	0	0	0	0	0	0	0	0	0	0
Taguig City	8	0	0	0	0	0	0	0	0	0	0	0	0	0
Quezon City	0	0	0	0	0	0	0	0	0	0	0	0	0	0
Makati City	0	0	0	0	2	2	3	0	3	1	3	1	0	4
Mandaluyong City	0	0	0	0	0	0	0	0	0	0	0	0	0	0
San Juan City	0	0	0	0	0	0	1	0	0	0	0	0	0	0
Manila City	0	0	0	0	0	0	0	0	0	0	0	0	0	0
Las Piñas City	0	0	0	0	1	4	25	16	12	13	7	9	21	9
Muntinlupa City	0	0	0	0	0	0	0	0	0	0	0	0	0	0
Parañaque City	0	0	0	0	0	0	0	0	0	0	0	0	0	0
Pasay City	0	0	0	0	0	0	0	0	0	0	0	0	0	0
**CAR**	**0**	**3**	**7**	**6**	**73**	**51**	**176**	**232**	**158**	**118**	**53**	**37**	**18**	**22**
Abra	0	0	0	0	0	1	1	1	0	1	0	2	0	0
Apayao	0	0	0	0	0	0	0	0	0	0	0	0	0	0
Benguet	0	0	0	0	0	0	0	0	0	0	0	0	0	0
Ifugao	0	0	0	0	0	0	0	0	0	0	0	0	0	0
Kalinga	0	0	0	0	0	0	0	0	0	0	0	0	0	0
Mt. Province	0	3	7	6	71	49	173	224	157	116	51	35	18	22
City of Baguio	0	0	0	0	2	1	2	7	1	1	2	0	0	0
**Region 1**	**0**	**0**	**0**	**0**	**10**	**16**	**59**	**56**	**60**	**63**	**23**	**12**	**17**	**12**
Ilocos Norte	0	0	0	0	0	0	0	0	0	0	0	0	0	0
Ilocos Sur	0	0	0	0	10	14	54	44	54	55	22	11	14	10
La Union	0	0	0	0	0	2	5	12	6	8	1	1	3	2

Table 2.H.2.2 Acute Febrile Illness

By Age group, Sex, Provinces and Cities

Philippines, 2023

Region/Province/City	0 - 6 days		7 - 28 days		29 days - 11 mos.		1 - 4 yrs.		5 - 9 yrs.		10 -14 yrs.		15 - 19 yrs.	
	M	F	M	F	M	F	M	F	M	F	M	F	M	F
Pangasinan	0	0	0	0	0	0	0	0	0	0	0	0	0	0
City of Dagupan	0	0	0	0	0	0	0	0	0	0	0	0	0	0
**Region 2**	**0**	**0**	**0**	**0**	**23**	**26**	**70**	**83**	**103**	**117**	**98**	**69**	**34**	**43**
Batanes	0	0	0	0	0	1	3	8	5	3	2	4	0	0
Cagayan	0	0	0	0	0	0	0	0	0	0	0	0	0	0
Isabela	0	0	0	0	20	19	49	60	89	100	89	63	24	39
Nueva Vizcaya	0	0	0	0	1	2	10	6	4	5	2	0	4	1
Quirino	0	0	0	0	2	3	6	4	4	4	3	2	4	2
City of Santiago	0	0	0	0	0	1	2	5	1	5	2	0	2	1
**Region 3**	**15**	**11**	**74**	**104**	**784**	**829**	**2,158**	**2,060**	**1,889**	**1,723**	**1,057**	**971**	**543**	**544**
Aurora	0	0	0	0	6	4	11	17	14	16	8	8	0	0
Bataan	10	6	31	24	72	81	160	164	152	122	108	86	47	27
Bulacan	3	2	27	43	451	460	1,214	1,187	1,148	1,077	622	590	314	323
Nueva Ecija	0	0	4	21	115	128	279	177	171	135	100	68	58	31
Pampanga	1	0	8	12	51	41	118	129	94	40	35	37	36	24
Tarlac	1	0	0	0	6	19	36	64	66	86	70	72	38	65
Zambales	0	3	3	3	67	76	274	273	197	215	96	94	37	56
City of Angeles	0	0	0	0	11	15	22	18	15	15	7	4	7	9
City of Olongapo	0	0	1	1	5	5	44	31	32	17	11	12	6	9
**Region 4A**	**38**	**42**	**91**	**129**	**1,447**	**1,478**	**3,968**	**3,889**	**3,138**	**3,074**	**1,378**	**1,251**	**626**	**612**
Batangas	2	2	3	9	247	288	796	806	735	664	299	275	141	127
Cavite	1	1	9	6	221	234	634	642	443	414	210	161	105	82
Laguna	4	1	25	28	330	316	746	702	619	598	255	209	118	115
Quezon	26	33	46	73	461	464	1,348	1,283	1,027	1,061	446	413	181	186
Rizal	0	0	8	9	131	110	342	358	263	283	155	177	72	93
City of Lucena	5	5	0	4	57	66	102	98	51	54	13	16	9	9
**Region 4B**	**0**	**1**	**2**	**5**	**57**	**67**	**351**	**346**	**289**	**248**	**127**	**95**	**69**	**52**
Marinduque	0	0	0	0	5	3	24	20	24	23	3	6	3	5
Mindoro Occidental	0	0	2	2	5	4	5	3	2	0	4	0	1	0
Mindoro Oriental	0	0	0	0	0	0	0	0	0	0	0	0	0	0

Table 2.H.2.2 Acute Febrile Illness

By Age group, Sex, Provinces and Cities

Philippines, 2023

Region/Province/City	0 - 6 days		7 - 28 days		29 days - 11 mos.		1 - 4 yrs.		5 - 9 yrs.		10 -14 yrs.		15 - 19 yrs.	
	M	F	M	F	M	F	M	F	M	F	M	F	M	F
Palawan	0	0	0	2	30	39	192	187	160	128	72	54	37	27
Romblon	0	1	0	0	3	4	18	15	10	15	8	13	6	3
City of Puerto Princesa	0	0	0	1	14	17	112	121	93	82	40	22	22	17
**Region 5**	**0**	**0**	**2**	**0**	**61**	**75**	**271**	**258**	**207**	**197**	**94**	**74**	**45**	**46**
Albay	0	0	0	0	30	25	124	124	86	69	38	31	19	19
Camarines Norte	0	0	1	0	5	8	31	26	21	22	19	13	8	3
Camarines Sur	0	0	1	0	20	30	90	92	81	87	33	27	16	20
Catanduanes	0	0	0	0	0	3	2	2	4	3	1	1	0	0
Masbate	0	0	0	0	4	6	6	7	5	6	1	0	2	3
Sorsogon	0	0	0	0	1	3	16	6	8	9	2	2	0	1
City of Naga	0	0	0	0	1	0	2	1	2	1	0	0	0	0
**Region 6**	**0**	**0**	**0**	**0**	**12**	**17**	**20**	**19**	**15**	**11**	**5**	**4**	**0**	**2**
Aklan	0	0	0	0	0	0	0	0	0	0	0	0	0	0
Antique	0	0	0	0	0	0	0	0	0	0	0	0	0	0
Capiz	0	0	0	0	1	3	3	2	0	2	0	0	0	0
Guimaras	0	0	0	0	2	5	2	5	4	3	1	3	0	0
Iloilo	0	0	0	0	0	0	0	1	0	0	0	0	0	0
Negros Occidental	0	0	0	0	9	9	15	11	11	6	4	1	0	2
City of Bacolod	0	0	0	0	0	0	0	0	0	0	0	0	0	0
City of Iloilo	0	0	0	0	0	0	0	0	0	0	0	0	0	0
**Region 7**	**5**	**1**	**12**	**12**	**99**	**89**	**348**	**346**	**278**	**295**	**136**	**120**	**70**	**68**
Bohol	0	0	0	0	1	0	2	0	0	1	0	0	0	0
Cebu	0	0	0	0	1	0	4	1	2	0	2	0	0	0
Negros Oriental	5	1	12	12	92	83	342	338	273	291	134	119	70	68
Siquijor	0	0	0	0	0	0	0	1	0	0	0	1	0	0
City of Cebu	0	0	0	0	3	4	0	4	2	2	0	0	0	0
City of Lapu-Lapu	0	0	0	0	2	2	0	2	1	1	0	0	0	0
City of Mandaue	0	0	0	0	0	0	0	0	0	0	0	0	0	0
**Region 8**	**0**	**0**	**0**	**0**	**2**	**2**	**1**	**3**	**0**	**4**	**1**	**0**	**0**	**1**
Biliran	0	0	0	0	1	0	0	0	0	0	0	0	0	0

Table 2.H.2.2 Acute Febrile Illness

By Age group, Sex, Provinces and Cities

Philippines, 2023

Region/Province/City	0 - 6 days		7 - 28 days		29 days - 11 mos.		1 - 4 yrs.		5 - 9 yrs.		10 -14 yrs.		15 - 19 yrs.	
	M	F	M	F	M	F	M	F	M	F	M	F	M	F
Eastern Samar	0	0	0	0	1	2	0	3	0	2	1	0	0	0
Northern Leyte	0	0	0	0	0	0	1	0	0	2	0	0	0	1
Northern Samar	0	0	0	0	0	0	0	0	0	0	0	0	0	0
Southern Leyte	0	0	0	0	0	0	0	0	0	0	0	0	0	0
Western Samar	0	0	0	0	0	0	0	0	0	0	0	0	0	0
City of Ormoc	0	0	0	0	0	0	0	0	0	0	0	0	0	0
City of Tacloban	0	0	0	0	0	0	0	0	0	0	0	0	0	0
**Region 9**	**0**	**0**	**0**	**0**	**0**	**0**	**0**	**0**	**0**	**0**	**0**	**0**	**0**	**0**
Zamboanga del Norte	0	0	0	0	0	0	0	0	0	0	0	0	0	0
Zamboanga del Sur	0	0	0	0	0	0	0	0	0	0	0	0	0	0
Zamboanga Sibugay	0	0	0	0	0	0	0	0	0	0	0	0	0	0
City of Isabela	0	0	0	0	0	0	0	0	0	0	0	0	0	0
City of Zamboanga	0	0	0	0	0	0	0	0	0	0	0	0	0	0
**Region 10**	**7**	**6**	**9**	**16**	**144**	**157**	**385**	**327**	**275**	**243**	**136**	**164**	**51**	**67**
Bukidnon	6	6	8	11	93	97	169	137	137	117	65	59	32	29
Camiguin	0	0	0	0	0	0	0	0	0	0	0	0	0	0
Lanao del Norte	0	0	0	0	9	29	100	75	48	49	35	68	6	22
Misamis Occidental	0	0	0	0	23	12	56	46	42	26	16	12	4	4
Misamis Oriental	0	0	0	1	1	2	26	33	27	32	10	13	3	5
City of Cagayan de Oro	1	0	1	4	18	17	34	36	21	19	10	12	6	7
City of Iligan	0	0	0	0	0	0	0	0	0	0	0	0	0	0
**Region 11**	**1**	**0**	**4**	**2**	**36**	**22**	**110**	**96**	**72**	**73**	**43**	**28**	**15**	**9**
Davao de Oro	0	0	0	0	1	3	3	6	3	3	4	3	2	1
Davao del Norte	1	0	0	0	13	6	49	31	22	15	4	8	5	3
Davao Oriental	0	0	0	0	6	1	7	10	13	14	14	8	0	0
Davao del Sur	0	0	0	0	1	1	1	1	1	1	0	0	0	0
Davao Occidental	0	0	0	2	6	6	16	18	9	8	8	5	5	3
City of Davao	0	0	4	0	9	5	34	30	24	32	13	4	3	2
**Region 12**	**0**	**0**	**1**	**4**	**8**	**6**	**38**	**31**	**28**	**32**	**19**	**20**	**21**	**15**
North Cotabato	0	0	0	0	0	0	0	0	0	0	0	0	0	0

Table 2.H.2.2 Acute Febrile Illness

By Age group, Sex, Provinces and Cities

Philippines, 2023

Region/Province/City	0 - 6 days		7 - 28 days		29 days - 11 mos.		1 - 4 yrs.		5 - 9 yrs.		10 -14 yrs.		15 - 19 yrs.	
	M	F	M	F	M	F	M	F	M	F	M	F	M	F
Sarangani	0	0	0	0	0	0	0	0	0	0	0	0	0	0
South Cotabato	0	0	0	0	0	0	0	0	0	0	0	0	0	0
Sultan Kudarat	0	0	1	4	8	6	38	31	28	32	19	20	21	15
City of General Santos	0	0	0	0	0	0	0	0	0	0	0	0	0	0
**BARMM**	**1**	**0**	**0**	**0**	**11**	**7**	**12**	**15**	**15**	**10**	**5**	**2**	**2**	**1**
Basilan	0	0	0	0	1	2	0	0	2	3	2	1	0	0
Lanao del Sur	0	0	0	0	6	4	7	8	9	3	2	0	2	0
Maguindanao	0	0	0	0	0	0	0	0	0	0	0	0	0	0
Sulu	0	0	0	0	0	0	0	0	0	0	0	0	0	0
Tawi-Tawi	0	0	0	0	0	0	0	0	0	0	0	0	0	0
SGA	0	0	0	0	0	0	0	0	0	0	0	0	0	0
City of Cotabato	1	0	0	0	4	1	5	7	4	4	1	1	0	1
**Caraga**	**0**	**0**	**1**	**0**	**1**	**0**	**2**	**1**	**0**	**1**	**0**	**1**	**0**	**0**
Agusan del Norte	0	0	0	0	0	0	0	0	0	0	0	0	0	0
Agusan del Sur	0	0	0	0	0	0	0	0	0	0	0	0	0	0
Surigao del Norte	0	0	0	0	0	0	0	0	0	0	0	0	0	0
Surigao del Sur	0	0	0	0	0	0	0	0	0	0	0	0	0	0
Province of Dinagat	0	0	1	0	1	0	2	1	0	1	0	1	0	0
City of Butuan	0	0	0	0	0	0	0	0	0	0	0	0	0	0

Table 2.H.2.2 Acute Febrile Illness

By Age group, Sex, Provinces and Cities

Philippines, 2023

Region/Province/City	20 - 24 yrs.		25 - 29 yrs.		30 - 34 yrs.		35 -39 yrs.		40 - 44 yrs.		45 -49 yrs.		50 -54 yrs.	
	M	F	M	F	M	F	M	F	M	F	M	F	M	F
**PHILIPPINES**	**1,162**	**1,313**	**982**	**1,174**	**844**	**1,041**	**682**	**871**	**584**	**815**	**614**	**721**	**444**	**612**
**NCR**	**3**	**12**	**3**	**8**	**2**	**2**	**3**	**4**	**5**	**3**	**1**	**3**	**6**	**0**
Malabon City	0	0	0	0	0	0	0	0	0	0	0	0	0	0
Navotas City	0	0	0	0	0	0	0	0	0	0	0	0	0	0
Valenzuela City	0	2	2	0	0	0	0	0	1	0	0	0	0	0
Caloocan City	1	0	0	1	1	2	0	1	0	2	1	0	0	0
Marikina City	0	0	0	0	0	0	0	0	0	0	0	0	0	0
Pasig City	1	0	0	0	0	0	0	1	0	0	0	0	0	0
Pateros	0	0	0	0	0	0	0	0	0	0	0	0	0	0
Taguig City	0	0	0	0	0	0	0	0	0	0	0	0	0	0
Quezon City	0	0	0	0	0	0	0	0	0	0	0	0	0	0
Makati City	1	1	1	4	1	0	0	2	1	0	0	0	0	0
Mandaluyong City	0	0	0	0	0	0	0	0	0	0	0	0	0	0
San Juan City	0	0	0	0	0	0	0	0	0	0	0	0	0	0
Manila City	0	0	0	0	0	0	0	0	0	0	0	0	0	0
Las Piñas City	0	9	0	3	0	0	3	0	3	1	0	3	6	0
Muntinlupa City	0	0	0	0	0	0	0	0	0	0	0	0	0	0
Parañaque City	0	0	0	0	0	0	0	0	0	0	0	0	0	0
Pasay City	0	0	0	0	0	0	0	0	0	0	0	0	0	0
**CAR**	**7**	**13**	**7**	**6**	**5**	**4**	**2**	**2**	**3**	**5**	**4**	**9**	**1**	**6**
Abra	0	0	0	0	0	0	0	0	0	0	0	0	0	0
Apayao	0	0	0	0	0	0	0	0	0	0	0	0	0	0
Benguet	0	0	0	0	0	0	0	0	0	0	0	0	0	0
Ifugao	0	0	0	0	0	0	0	0	0	0	0	0	0	0
Kalinga	0	0	0	0	0	0	0	0	0	0	0	0	0	0
Mt. Province	7	13	7	6	5	4	2	2	3	5	4	9	1	6
City of Baguio	0	0	0	0	0	0	0	0	0	0	0	0	0	0
**Region 1**	**8**	**9**	**3**	**10**	**3**	**0**	**3**	**5**	**4**	**1**	**3**	**6**	**0**	**2**
Ilocos Norte	0	0	0	0	0	0	0	0	0	0	0	0	0	0
Ilocos Sur	6	9	1	6	2	0	2	3	4	1	2	2	0	1
La Union	2	0	2	4	1	0	1	2	0	0	1	4	0	1
Pangasinan	0	0	0	0	0	0	0	0	0	0	0	0	0	0

Table 2.H.2.2 Acute Febrile Illness

By Age group, Sex, Provinces and Cities

Philippines, 2023

Region/Province/City	20 - 24 yrs.		25 - 29 yrs.		30 - 34 yrs.		35 -39 yrs.		40 - 44 yrs.		45 -49 yrs.		50 -54 yrs.	
	M	F	M	F	M	F	M	F	M	F	M	F	M	F
City of Dagupan	0	0	0	0	0	0	0	0	0	0	0	0	0	0
**Region 2**	**20**	**25**	**23**	**32**	**26**	**9**	**23**	**27**	**18**	**13**	**17**	**31**	**5**	**19**
Batanes	0	0	0	0	0	0	0	0	0	0	0	0	0	0
Cagayan	0	0	0	0	0	0	0	0	0	1	0	0	0	0
Isabela	20	24	23	32	22	8	21	21	15	10	14	23	1	15
Nueva Vizcaya	0	1	0	0	2	1	1	1	1	0	0	0	0	0
Quirino	0	0	0	0	0	0	1	4	1	2	3	8	4	4
City of Santiago	0	0	0	0	2	0	0	1	1	0	0	0	0	0
**Region 3**	**456**	**512**	**397**	**433**	**328**	**375**	**269**	**332**	**262**	**278**	**284**	**230**	**143**	**214**
Aurora	0	0	0	0	0	4	2	0	0	4	0	0	0	0
Bataan	32	45	19	41	25	30	39	36	25	29	89	30	20	32
Bulacan	243	284	225	237	189	220	144	167	144	150	117	123	81	110
Nueva Ecija	29	30	32	35	17	20	22	40	27	25	23	29	18	22
Pampanga	32	31	28	20	24	18	17	18	10	11	16	8	12	18
Tarlac	50	48	38	32	25	26	18	29	22	20	8	20	2	16
Zambales	50	50	37	45	29	42	15	27	25	28	23	16	9	12
City of Angeles	10	8	10	6	3	4	8	4	4	2	5	1	0	3
City of Olongapo	10	16	8	17	16	11	4	11	5	9	3	3	1	1
**Region 4A**	**432**	**439**	**367**	**429**	**284**	**437**	**216**	**310**	**184**	**331**	**168**	**292**	**149**	**217**
Batangas	79	106	81	112	66	149	57	95	53	124	50	111	35	68
Cavite	76	70	47	71	31	53	32	45	18	29	19	18	18	12
Laguna	79	83	81	66	47	46	48	42	32	49	30	55	19	32
Quezon	129	127	111	126	81	122	50	98	48	87	52	90	63	92
Rizal	66	48	41	51	55	62	26	27	30	39	16	18	14	13
City of Lucena	3	5	6	3	4	5	3	3	3	3	1	0	0	0
**Region 4B**	**45**	**66**	**41**	**35**	**54**	**37**	**12**	**39**	**17**	**26**	**19**	**18**	**12**	**27**
Marinduque	2	3	1	0	2	5	3	4	2	6	2	4	4	3
Mindoro Occidental	0	2	1	1	0	0	0	0	0	0	0	0	0	0
Mindoro Oriental	0	0	0	0	0	0	0	0	0	0	0	0	0	0
Palawan	25	39	21	19	28	18	7	22	9	13	9	8	4	13

Table 2.H.2.2 Acute Febrile Illness

By Age group, Sex, Provinces and Cities

Philippines, 2023

Region/Province/City	20 - 24 yrs.		25 - 29 yrs.		30 - 34 yrs.		35 -39 yrs.		40 - 44 yrs.		45 -49 yrs.		50 -54 yrs.	
	M	F	M	F	M	F	M	F	M	F	M	F	M	F
Romblon	2	6	0	3	0	1	0	1	1	1	0	1	1	0
City of Puerto Princesa	16	16	18	12	24	13	2	12	5	6	8	5	3	11
**Region 5**	**35**	**45**	**16**	**29**	**24**	**45**	**19**	**20**	**19**	**26**	**19**	**22**	**15**	**22**
Albay	11	23	6	12	6	20	8	11	5	12	5	10	7	13
Camarines Norte	5	7	3	2	1	11	4	5	2	1	0	5	2	2
Camarines Sur	18	10	7	15	15	13	6	4	10	13	11	2	6	5
Catanduanes	0	4	0	0	0	0	0	0	0	0	0	1	0	0
Masbate	1	1	0	0	0	0	1	0	0	0	0	0	0	0
Sorsogon	0	0	0	0	2	1	0	0	2	0	3	3	0	2
City of Naga	0	0	0	0	0	0	0	0	0	0	0	1	0	0
**Region 6**	**2**	**2**	**2**	**1**	**0**	**1**	**0**	**1**	**2**	**0**	**0**	**2**	**0**	**0**
Aklan	0	0	0	0	0	0	0	0	0	0	0	0	0	0
Antique	0	0	0	0	0	0	0	0	0	0	0	0	0	0
Capiz	0	0	1	0	0	0	0	0	0	0	0	0	0	0
Guimaras	1	0	1	0	0	0	0	0	2	0	0	1	0	0
Iloilo	0	0	0	0	0	0	0	0	0	0	0	0	0	0
Negros Occidental	1	2	0	1	0	1	0	1	0	0	0	1	0	0
City of Bacolod	0	0	0	0	0	0	0	0	0	0	0	0	0	0
City of Iloilo	0	0	0	0	0	0	0	0	0	0	0	0	0	0
**Region 7**	**54**	**64**	**41**	**76**	**28**	**38**	**25**	**43**	**24**	**41**	**22**	**44**	**23**	**36**
Bohol	0	0	0	1	0	0	0	0	0	0	0	0	0	0
Cebu	0	0	0	0	0	0	0	0	0	0	0	1	0	0
Negros Oriental	54	64	41	75	28	38	25	43	24	41	22	43	23	36
Siquijor	0	0	0	0	0	0	0	0	0	0	0	0	0	0
City of Cebu	0	0	0	0	0	0	0	0	0	0	0	0	0	0
City of Lapu-Lapu	0	0	0	0	0	0	0	0	0	0	0	0	0	0
City of Mandaue	0	0	0	0	0	0	0	0	0	0	0	0	0	0
**Region 8**	**1**	**0**	**2**	**0**	**0**	**0**	**0**	**0**	**0**	**0**	**0**	**1**	**1**	**1**
Biliran	0	0	0	0	0	0	0	0	0	0	0	0	0	0
Eastern Samar	1	0	1	0	0	0	0	0	0	0	0	1	1	1

Table 2.H.2.2 Acute Febrile Illness

By Age group, Sex, Provinces and Cities

Philippines, 2023

Region/Province/City	20 - 24 yrs.		25 - 29 yrs.		30 - 34 yrs.		35 -39 yrs.		40 - 44 yrs.		45 -49 yrs.		50 -54 yrs.	
	M	F	M	F	M	F	M	F	M	F	M	F	M	F
Northern Leyte	0	0	1	0	0	0	0	0	0	0	0	0	0	0
Northern Samar	0	0	0	0	0	0	0	0	0	0	0	0	0	0
Southern Leyte	0	0	0	0	0	0	0	0	0	0	0	0	0	0
Western Samar	0	0	0	0	0	0	0	0	0	0	0	0	0	0
City of Ormoc	0	0	0	0	0	0	0	0	0	0	0	0	0	0
City of Tacloban	0	0	0	0	0	0	0	0	0	0	0	0	0	0
**Region 9**	**0**	**0**	**0**	**0**	**0**	**0**	**0**	**0**	**0**	**0**	**0**	**0**	**0**	**0**
Zamboanga del Norte	0	0	0	0	0	0	0	0	0	0	0	0	0	0
Zamboanga del Sur	0	0	0	0	0	0	0	0	0	0	0	0	0	0
Zamboanga Sibugay	0	0	0	0	0	0	0	0	0	0	0	0	0	0
City of Isabela	0	0	0	0	0	0	0	0	0	0	0	0	0	0
City of Zamboanga	0	0	0	0	0	0	0	0	0	0	0	0	0	0
**Region 10**	**55**	**74**	**42**	**79**	**62**	**50**	**90**	**50**	**22**	**64**	**52**	**35**	**69**	**34**
Bukidnon	30	33	19	36	13	31	23	23	16	30	15	20	19	18
Camiguin	0	0	0	0	0	0	0	0	0	0	0	0	0	0
Lanao del Norte	8	30	3	14	41	14	55	20	5	27	32	3	47	10
Misamis Occidental	6	7	2	3	2	1	1	0	0	2	2	3	1	3
Misamis Oriental	5	4	10	17	2	1	8	4	1	2	2	6	1	3
City of Cagayan de Oro	6	0	8	9	4	3	3	3	0	3	1	3	1	0
City of Iligan	0	0	0	0	0	0	0	0	0	0	0	0	0	0
**Region 11**	**17**	**28**	**22**	**17**	**9**	**21**	**5**	**17**	**2**	**7**	**4**	**11**	**4**	**11**
Davao de Oro	0	1	0	0	0	4	3	1	0	0	1	1	0	2
Davao del Norte	6	3	3	1	5	3	2	2	0	1	0	1	0	0
Davao Oriental	6	13	8	6	0	6	0	6	1	1	0	0	0	0
Davao del Sur	0	0	0	0	0	0	0	0	0	0	0	0	0	0
Davao Occidental	5	2	3	1	2	4	0	1	0	3	0	1	0	1
City of Davao	0	9	8	9	2	4	0	7	1	2	3	8	4	8
**Region 12**	**22**	**21**	**13**	**17**	**16**	**22**	**14**	**18**	**21**	**16**	**21**	**17**	**16**	**22**
North Cotabato	0	0	0	0	0	0	0	0	0	0	0	0	0	0
Sarangani	0	0	0	0	0	0	0	0	0	0	0	0	0	0

Table 2.H.2.2 Acute Febrile Illness

By Age group, Sex, Provinces and Cities

Philippines, 2023

Region/Province/City	20 - 24 yrs.		25 - 29 yrs.		30 - 34 yrs.		35 -39 yrs.		40 - 44 yrs.		45 -49 yrs.		50 -54 yrs.	
	M	F	M	F	M	F	M	F	M	F	M	F	M	F
South Cotabato	0	0	0	0	0	0	0	0	0	0	0	0	0	0
Sultan Kudarat	22	21	13	17	16	22	14	18	21	16	21	17	16	22
City of General Santos	0	0	0	0	0	0	0	0	0	0	0	0	0	0
**BARMM**	**4**	**3**	**3**	**2**	**3**	**0**	**0**	**3**	**1**	**4**	**0**	**0**	**0**	**1**
Basilan	1	0	0	0	1	0	0	0	0	0	0	0	0	0
Lanao del Sur	2	1	1	2	2	0	0	1	0	3	0	0	0	0
Maguindanao	0	0	0	0	0	0	0	0	0	0	0	0	0	0
Sulu	0	0	0	0	0	0	0	0	0	0	0	0	0	0
Tawi-Tawi	0	0	0	0	0	0	0	0	0	0	0	0	0	0
SGA	0	0	0	0	0	0	0	0	0	0	0	0	0	0
City of Cotabato	1	2	2	0	0	0	0	2	1	1	0	0	0	1
**Caraga**	**1**	**0**	**0**	**0**	**0**	**0**	**1**	**0**	**0**	**0**	**0**	**0**	**0**	**0**
Agusan del Norte	0	0	0	0	0	0	0	0	0	0	0	0	0	0
Agusan del Sur	0	0	0	0	0	0	0	0	0	0	0	0	0	0
Surigao del Norte	0	0	0	0	0	0	0	0	0	0	0	0	0	0
Surigao del Sur	0	0	0	0	0	0	0	0	0	0	0	0	0	0
Province of Dinagat	1	0	0	0	0	0	1	0	0	0	0	0	0	0
City of Butuan	0	0	0	0	0	0	0	0	0	0	0	0	0	0

Table 2.H.2.2 Acute Febrile Illness

By Age group, Sex, Provinces and Cities

Philippines, 2023

Region/Province/City	55 - 59 yrs.		60 - 64. yrs.		65 - 69 yrs.		70 yrs above		Total		Total Both Sexes	
	M	F	M	F	M	F	M	F	M	F	Number	Rate
**PHILIPPINES**	**393**	**508**	**338**	**476**	**1,316**	**374**	**252**	**396**	**29,934**	**29,873**	**59,807**	**53.57**
**NCR**	**0**	**9**	**1**	**2**	**4**	**3**	**0**	**2**	**131**	**123**	**254**	**1.84**
Malabon City	0	0	0	0	0	0	0	0	0	0	0	0.00
Navotas City	0	0	0	0	0	0	0	0	0	0	0	0.00
Valenzuela City	0	0	0	0	0	0	0	0	7	4	11	1.50
Caloocan City	0	0	1	1	1	0	0	0	9	12	21	1.23
Marikina City	0	0	0	0	0	0	0	0	0	0	0	0.00
Pasig City	0	0	0	0	0	0	0	0	10	10	20	2.42
Pateros	0	0	0	0	0	0	0	0	0	0	0	0.00
Taguig City	0	0	0	0	0	0	0	0	8	0	8	0.87
Quezon City	0	0	0	0	0	0	0	0	0	0	0	0.00
Makati City	0	0	0	1	0	0	0	0	15	16	31	4.86
Mandaluyong City	0	0	0	0	0	0	0	0	0	0	0	0.00
San Juan City	0	0	0	0	0	0	0	0	1	0	1	0.78
Manila City	0	0	0	0	0	0	0	0	0	0	0	0.00
Las Piñas City	0	9	0	0	3	3	0	2	81	81	162	26.18
Muntinlupa City	0	0	0	0	0	0	0	0	0	0	0	0.00
Parañaque City	0	0	0	0	0	0	0	0	0	0	0	0.00
Pasay City	0	0	0	0	0	0	0	0	0	0	0	0.00
**CAR**	**3**	**6**	**2**	**4**	**10**	**17**	**0**	**15**	**529**	**556**	**1,085**	**58.60**
Abra	0	0	0	0	0	0	0	0	1	5	6	2.37
Apayao	0	0	0	0	0	0	0	0	0	0	0	0.00
Benguet	0	0	0	0	0	0	0	0	0	0	0	0.00
Ifugao	0	0	0	0	0	0	0	0	0	0	0	0.00
Kalinga	0	0	0	0	0	0	0	0	0	0	0	0.00
Mt. Province	3	6	2	4	10	17	0	15	521	542	1,063	652.26
City of Baguio	0	0	0	0	0	0	0	0	7	9	16	4.24
**Region 1**	**2**	**4**	**4**	**1**	**2**	**1**	**4**	**9**	**205**	**207**	**412**	**7.64**
Ilocos Norte	0	0	0	0	0	0	0	0	0	0	0	0.00
Ilocos Sur	2	2	2	1	0	1	4	1	179	161	340	47.66
La Union	0	2	2	0	2	0	0	8	26	46	72	8.62

Table 2.H.2.2 Acute Febrile Illness
By Age group, Sex, Provinces and Cities
Philippines, 2023

Region/Province/City	55 - 59 yrs.		60 - 64. yrs.		65 - 69 yrs.		70 yrs above		Total		Total Both Sexes	
	M	F	M	F	M	F	M	F	M	F	Number	Rate
Pangasinan	0	0	0	0	0	0	0	0	0	0	0	0.00
City of Dagupan	0	0	0	0	0	0	0	0	0	0	0	0.00
**Region 2**	**10**	**12**	**18**	**12**	**12**	**11**	**5**	**13**	**505**	**542**	**1,047**	**27.79**
Batanes	1	0	0	0	0	0	1	0	12	16	28	146.54
Cagayan	0	0	0	0	0	0	0	0	0	1	1	0.08
Isabela	7	9	16	7	9	7	0	9	419	446	865	54.75
Nueva Vizcaya	0	0	0	0	0	0	0	0	25	17	42	8.17
Quirino	2	3	2	5	3	4	4	4	39	49	88	41.68
City of Santiago	0	0	0	0	0	0	0	0	10	13	23	15.00
**Region 3**	**161**	**191**	**123**	**142**	**111**	**120**	**107**	**146**	**9,161**	**9,215**	**18,376**	**144.63**
Aurora	0	0	0	0	0	0	2	5	43	58	101	41.53
Bataan	15	21	17	31	21	17	30	29	912	851	1,763	201.25
Bulacan	81	97	55	64	54	58	27	41	5,139	5,233	10,372	273.75
Nueva Ecija	25	37	18	13	11	16	12	29	961	856	1,817	77.22
Pampanga	16	5	3	3	2	2	2	0	505	417	922	36.75
Tarlac	5	10	9	6	3	8	3	0	400	521	921	60.12
Zambales	16	17	19	24	17	17	28	40	942	1,038	1,980	297.81
City of Angeles	0	0	0	0	0	0	0	0	102	89	191	40.23
City of Olongapo	3	4	2	1	3	2	3	2	157	152	309	116.93
**Region 4A**	**137**	**176**	**115**	**199**	**1,104**	**146**	**86**	**135**	**13,928**	**13,586**	**27,514**	**165.95**
Batangas	24	52	28	57	35	59	34	47	2,765	3,151	5,916	199.25
Cavite	19	13	11	5	4	5	4	7	1,902	1,868	3,770	84.70
Laguna	20	24	17	26	1,014	17	21	25	3,505	2,434	5,939	171.71
Quezon	63	58	45	77	36	57	22	49	4,235	4,496	8,731	437.42
Rizal	9	25	13	31	15	8	3	6	1,259	1,358	2,617	76.56
City of Lucena	2	4	1	3	0	0	2	1	262	279	541	189.16
**Region 4B**	**8**	**11**	**7**	**9**	**21**	**3**	**8**	**21**	**1,139**	**1,106**	**2,245**	**67.95**
Marinduque	1	1	1	2	0	1	1	11	78	97	175	72.18
Mindoro Occidental	0	0	0	0	0	0	0	0	20	12	32	5.93
Mindoro Oriental	0	0	0	0	0	0	0	0	0	0	0	0.00

Table 2.H.2.2 Acute Febrile Illness
By Age group, Sex, Provinces and Cities
Philippines, 2023

Region/Province/City	55 - 59 yrs.		60 - 64. yrs.		65 - 69 yrs.		70 yrs above		Total		Total Both Sexes	
	M	F	M	F	M	F	M	F	M	F	Number	Rate
Palawan	4	6	4	5	12	2	4	5	618	587	1,205	124.78
Romblon	0	1	0	0	0	0	0	4	49	69	118	37.51
City of Puerto Princesa	3	3	2	2	9	0	3	1	374	341	715	227.08
**Region 5**	**8**	**15**	**15**	**13**	**12**	**16**	**9**	**17**	**871**	**920**	**1,791**	**28.58**
Albay	2	6	7	8	5	10	2	8	361	401	762	53.82
Camarines Norte	0	5	1	0	2	2	0	3	105	115	220	33.66
Camarines Sur	5	3	5	4	4	2	2	4	330	331	661	34.50
Catanduanes	0	0	0	0	0	0	0	0	7	14	21	7.51
Masbate	0	1	0	0	0	0	2	0	22	24	46	4.93
Sorsogon	1	0	2	1	1	2	3	2	41	32	73	8.56
City of Naga	0	0	0	0	0	0	0	0	5	3	8	3.71
**Region 6**	**0**	**0**	**0**	**0**	**1**	**1**	**0**	**0**	**59**	**61**	**120**	**1.49**
Aklan	0	0	0	0	0	0	0	0	0	0	0	0.00
Antique	0	0	0	0	0	0	0	0	0	0	0	0.00
Capiz	0	0	0	0	0	0	0	0	5	7	12	1.47
Guimaras	0	0	0	0	0	0	0	0	13	17	30	15.67
Iloilo	0	0	0	0	0	0	0	0	0	1	1	0.05
Negros Occidental	0	0	0	0	1	1	0	0	41	36	77	2.90
City of Bacolod	0	0	0	0	0	0	0	0	0	0	0	0.00
City of Iloilo	0	0	0	0	0	0	0	0	0	0	0	0.00
**Region 7**	**19**	**21**	**12**	**32**	**6**	**20**	**11**	**10**	**1,213**	**1,356**	**2,569**	**30.93**
Bohol	0	0	0	0	0	0	0	0	3	2	5	0.35
Cebu	0	0	0	0	0	0	0	0	9	2	11	0.32
Negros Oriental	19	21	12	32	6	20	11	10	1,193	1,335	2,528	171.84
Siquijor	0	0	0	0	0	0	0	0	0	2	2	1.92
City of Cebu	0	0	0	0	0	0	0	0	5	10	15	1.51
City of Lapu-Lapu	0	0	0	0	0	0	0	0	3	5	8	1.55
City of Mandaue	0	0	0	0	0	0	0	0	0	0	0	0.00
**Region 8**	**0**	**1**	**0**	**1**	**0**	**0**	**0**	**0**	**8**	**14**	**22**	**0.47**
Biliran	0	0	0	0	0	0	0	0	1	0	1	0.54

Table 2.H.2.2 Acute Febrile Illness

By Age group, Sex, Provinces and Cities

Philippines, 2023

Region/Province/City	55 - 59 yrs.		60 - 64. yrs.		65 - 69 yrs.		70 yrs above		Total		Total Both Sexes	
	M	F	M	F	M	F	M	F	M	F	Number	Rate
Eastern Samar	0	1	0	1	0	0	0	0	5	11	16	3.28
Northern Leyte	0	0	0	0	0	0	0	0	2	3	5	0.32
Northern Samar	0	0	0	0	0	0	0	0	0	0	0	0.00
Southern Leyte	0	0	0	0	0	0	0	0	0	0	0	0.00
Western Samar	0	0	0	0	0	0	0	0	0	0	0	0.00
City of Ormoc	0	0	0	0	0	0	0	0	0	0	0	0.00
City of Tacloban	0	0	0	0	0	0	0	0	0	0	0	0.00
**Region 9**	**0**	**0**	**0**	**0**	**0**	**0**	**0**	**0**	**0**	**0**	**0**	**0.00**
Zamboanga del Norte	0	0	0	0	0	0	0	0	0	0	0	0.00
Zamboanga del Sur	0	0	0	0	0	0	0	0	0	0	0	0.00
Zamboanga Sibugay	0	0	0	0	0	0	0	0	0	0	0	0.00
City of Isabela	0	0	0	0	0	0	0	0	0	0	0	0.00
City of Zamboanga	0	0	0	0	0	0	0	0	0	0	0	0.00
**Region 10**	**22**	**38**	**20**	**33**	**13**	**12**	**9**	**20**	**1,463**	**1,469**	**2,932**	**56.47**
Bukidnon	15	13	12	15	7	9	4	11	683	695	1,378	85.80
Camiguin	0	0	0	0	0	0	0	0	0	0	0	0.00
Lanao del Norte	0	9	2	12	2	1	1	1	394	384	778	104.64
Misamis Occidental	3	3	3	1	2	1	1	0	164	124	288	45.51
Misamis Oriental	1	6	0	2	1	0	1	3	99	134	233	23.60
City of Cagayan de Oro	3	7	3	3	1	1	2	5	123	132	255	33.73
City of Iligan	0	0	0	0	0	0	0	0	0	0	0	0.00
**Region 11**	**4**	**9**	**1**	**4**	**5**	**12**	**4**	**7**	**358**	**374**	**732**	**13.60**
Davao de Oro	1	0	0	0	0	1	0	0	18	26	44	5.55
Davao del Norte	1	3	0	1	1	1	1	0	113	79	192	16.61
Davao Oriental	0	1	0	1	0	0	0	1	55	68	123	20.79
Davao del Sur	0	0	0	0	0	0	0	0	3	3	6	0.86
Davao Occidental	2	5	1	1	1	2	1	0	59	63	122	38.27
City of Davao	0	0	0	1	3	8	2	6	110	135	245	13.43
**Region 12**	**18**	**15**	**19**	**22**	**14**	**12**	**9**	**1**	**298**	**291**	**589**	**13.07**
North Cotabato	0	0	0	0	0	0	0	0	0	0	0	0.00

Table 2.H.2.2 Acute Febrile Illness

By Age group, Sex, Provinces and Cities

Philippines, 2023

Region/Province/City	55 - 59 yrs.		60 - 64. yrs.		65 - 69 yrs.		70 yrs above		Total		Total Both Sexes	
	M	F	M	F	M	F	M	F	M	F	Number	Rate
Sarangani	0	0	0	0	0	0	0	0	0	0	0	0.00
South Cotabato	0	0	0	0	0	0	0	0	0	0	0	0.00
Sultan Kudarat	18	15	19	22	14	12	9	1	298	291	589	66.85
City of General Santos	0	0	0	0	0	0	0	0	0	0	0	0.00
**BARMM**	**1**	**0**	**1**	**2**	**1**	**0**	**0**	**0**	**60**	**50**	**110**	**2.18**
Basilan	0	0	0	0	0	0	0	0	7	6	13	3.02
Lanao del Sur	1	0	0	1	1	0	0	0	33	23	56	4.63
Maguindanao	0	0	0	0	0	0	0	0	0	0	0	0.00
Sulu	0	0	0	0	0	0	0	0	0	0	0	0.00
Tawi-Tawi	0	0	0	0	0	0	0	0	0	0	0	0.00
SGA	0	0	0	0	0	0	0	0	0	0	0	
City of Cotabato	0	0	1	1	0	0	0	0	20	21	41	12.25
**Caraga**	**0**	**0**	**0**	**0**	**0**	**0**	**0**	**0**	**6**	**3**	**9**	**0.31**
Agusan del Norte	0	0	0	0	0	0	0	0	0	0	0	0.00
Agusan del Sur	0	0	0	0	0	0	0	0	0	0	0	0.00
Surigao del Norte	0	0	0	0	0	0	0	0	0	0	0	0.00
Surigao del Sur	0	0	0	0	0	0	0	0	0	0	0	0.00
Province of Dinagat	0	0	0	0	0	0	0	0	6	3	9	6.97
City of Butuan	0	0	0	0	0	0	0	0	0	0	0	0.00

Table 2.H.2.3 Acute Flaccid Paralysis

By Age group, Sex, Provinces and Cities

Philippines, 2023

Region/Province/City	0 - 6 days		7 - 28 days		29 days - 11 mos.		1 - 4 yrs.		5 - 9 yrs.		10 -14 yrs.		15 - 19 yrs.	
	M	F	M	F	M	F	M	F	M	F	M	F	M	F
**Philippines**	**0**	**0**	**0**	**0**	**14**	**4**	**26**	**16**	**15**	**15**	**15**	**9**	**7**	**7**
**NCR**	**0**	**0**	**0**	**0**	**0**	**0**	**0**	**0**	**0**	**0**	**0**	**1**	**0**	**0**
Malabon City	0	0	0	0	0	0	0	0	0	0	0	0	0	0
Navotas City	0	0	0	0	0	0	0	0	0	0	0	0	0	0
Valenzuela City	0	0	0	0	0	0	0	0	0	0	0	1	0	0
Caloocan City	0	0	0	0	0	0	0	0	0	0	0	0	0	0
Marikina City	0	0	0	0	0	0	0	0	0	0	0	0	0	0
Pasig City	0	0	0	0	0	0	0	0	0	0	0	0	0	0
Pateros	0	0	0	0	0	0	0	0	0	0	0	0	0	0
Taguig City	0	0	0	0	0	0	0	0	0	0	0	0	0	0
Quezon City	0	0	0	0	0	0	0	0	0	0	0	0	0	0
Makati City	0	0	0	0	0	0	0	0	0	0	0	0	0	0
Mandaluyong City	0	0	0	0	0	0	0	0	0	0	0	0	0	0
San Juan City	0	0	0	0	0	0	0	0	0	0	0	0	0	0
Manila City	0	0	0	0	0	0	0	0	0	0	0	0	0	0
Las Piñas City	0	0	0	0	0	0	0	0	0	0	0	0	0	0
Muntinlupa City	0	0	0	0	0	0	0	0	0	0	0	0	0	0
Parañaque City	0	0	0	0	0	0	0	0	0	0	0	0	0	0
Pasay City	0	0	0	0	0	0	0	0	0	0	0	0	0	0
**CAR**	**0**	**0**	**0**	**0**	**0**	**0**	**3**	**1**	**2**	**2**	**1**	**1**	**0**	**0**
Abra	0	0	0	0	0	0	0	0	0	1	0	0	0	0
Apayao	0	0	0	0	0	0	0	0	0	0	0	0	0	0
Benguet	0	0	0	0	0	0	1	0	0	0	0	0	0	0
Ifugao	0	0	0	0	0	0	0	0	0	0	0	0	0	0
Kalinga	0	0	0	0	0	0	0	0	0	0	0	0	0	0
Mt. Province	0	0	0	0	0	0	0	0	0	0	0	0	0	0
City of Baguio	0	0	0	0	0	0	2	1	2	1	1	1	0	0
**Region 1**	**0**	**0**	**0**	**0**	**0**	**0**	**0**	**0**	**0**	**0**	**0**	**0**	**0**	**0**
Ilocos Norte	0	0	0	0	0	0	0	0	0	0	0	0	0	0
Ilocos Sur	0	0	0	0	0	0	0	0	0	0	0	0	0	0
La Union	0	0	0	0	0	0	0	0	0	0	0	0	0	0

Table 2.H.2.3 Acute Flaccid Paralysis
By Age group, Sex, Provinces and Cities
Philippines, 2023

Region/Province/City	0 - 6 days		7 - 28 days		29 days - 11 mos.		1 - 4 yrs.		5 - 9 yrs.		10 -14 yrs.		15 - 19 yrs.	
	M	F	M	F	M	F	M	F	M	F	M	F	M	F
Pangasinan	0	0	0	0	0	0	0	0	0	0	0	0	0	0
City of Dagupan	0	0	0	0	0	0	0	0	0	0	0	0	0	0
**Region 2**	**0**	**0**	**0**	**0**	**0**	**0**	**0**	**0**	**1**	**0**	**1**	**0**	**0**	**0**
Batanes	0	0	0	0	0	0	0	0	0	0	0	0	0	0
Cagayan	0	0	0	0	0	0	0	0	1	0	0	0	0	0
Isabela	0	0	0	0	0	0	0	0	0	0	1	0	0	0
Nueva Vizcaya	0	0	0	0	0	0	0	0	0	0	0	0	0	0
Quirino	0	0	0	0	0	0	0	0	0	0	0	0	0	0
City of Santiago	0	0	0	0	0	0	0	0	0	0	0	0	0	0
**Region 3**	**0**	**0**	**0**	**0**	**0**	**0**	**0**	**0**	**1**	**1**	**2**	**0**	**1**	**0**
Aurora	0	0	0	0	0	0	0	0	0	0	0	0	0	0
Bataan	0	0	0	0	0	0	0	0	0	0	0	0	0	0
Bulacan	0	0	0	0	0	0	0	0	0	1	2	0	1	0
Nueva Ecija	0	0	0	0	0	0	0	0	0	0	0	0	0	0
Pampanga	0	0	0	0	0	0	0	0	0	0	0	0	0	0
Tarlac	0	0	0	0	0	0	0	0	0	0	0	0	0	0
Zambales	0	0	0	0	0	0	0	0	1	0	0	0	0	0
City of Angeles	0	0	0	0	0	0	0	0	0	0	0	0	0	0
City of Olongapo	0	0	0	0	0	0	0	0	0	0	0	0	0	0
**Region 4A**	**0**	**0**	**0**	**0**	**2**	**1**	**2**	**0**	**1**	**2**	**0**	**1**	**0**	**0**
Batangas	0	0	0	0	1	0	0	0	0	0	0	0	0	0
Cavite	0	0	0	0	0	0	0	0	0	0	0	0	0	0
Laguna	0	0	0	0	0	0	0	0	0	0	0	0	0	0
Quezon	0	0	0	0	0	0	0	0	0	0	0	0	0	0
Rizal	0	0	0	0	1	1	2	0	1	2	0	1	0	0
City of Lucena	0	0	0	0	0	0	0	0	0	0	0	0	0	0
**Region 4B**	**0**	**0**	**0**	**0**	**6**	**1**	**15**	**9**	**9**	**7**	**9**	**2**	**4**	**3**
Marinduque	0	0	0	0	0	0	0	0	0	0	0	0	0	0
Mindoro Occidental	0	0	0	0	0	0	1	0	0	0	0	0	0	0
Mindoro Oriental	0	0	0	0	0	0	0	0	0	0	0	0	0	0

Table 2.H.2.3 Acute Flaccid Paralysis

By Age group, Sex, Provinces and Cities

Philippines, 2023

Region/Province/City	0 - 6 days		7 - 28 days		29 days - 11 mos.		1 - 4 yrs.		5 - 9 yrs.		10 -14 yrs.		15 - 19 yrs.	
	M	F	M	F	M	F	M	F	M	F	M	F	M	F
Palawan	0	0	0	0	6	1	14	9	9	7	9	2	4	3
Romblon	0	0	0	0	0	0	0	0	0	0	0	0	0	0
City of Puerto Princesa	0	0	0	0	0	0	0	0	0	0	0	0	0	0
**Region 5**	**0**	**0**	**0**	**0**	**0**	**0**	**0**	**0**	**0**	**0**	**0**	**0**	**0**	**0**
Albay	0	0	0	0	0	0	0	0	0	0	0	0	0	0
Camarines Norte	0	0	0	0	0	0	0	0	0	0	0	0	0	0
Camarines Sur	0	0	0	0	0	0	0	0	0	0	0	0	0	0
Catanduanes	0	0	0	0	0	0	0	0	0	0	0	0	0	0
Masbate	0	0	0	0	0	0	0	0	0	0	0	0	0	0
Sorsogon	0	0	0	0	0	0	0	0	0	0	0	0	0	0
City of Naga	0	0	0	0	0	0	0	0	0	0	0	0	0	0
**Region 6**	**0**	**0**	**0**	**0**	**0**	**0**	**0**	**0**	**0**	**0**	**0**	**1**	**1**	**0**
Aklan	0	0	0	0	0	0	0	0	0	0	0	0	0	0
Antique	0	0	0	0	0	0	0	0	0	0	0	0	0	0
Capiz	0	0	0	0	0	0	0	0	0	0	0	0	0	0
Guimaras	0	0	0	0	0	0	0	0	0	0	0	0	1	0
Iloilo	0	0	0	0	0	0	0	0	0	0	0	1	0	0
Negros Occidental	0	0	0	0	0	0	0	0	0	0	0	0	0	0
City of Bacolod	0	0	0	0	0	0	0	0	0	0	0	0	0	0
City of Iloilo	0	0	0	0	0	0	0	0	0	0	0	0	0	0
**Region 7**	**0**	**0**	**0**	**0**	**0**	**0**	**0**	**0**	**0**	**0**	**0**	**0**	**0**	**0**
Bohol	0	0	0	0	0	0	0	0	0	0	0	0	0	0
Cebu	0	0	0	0	0	0	0	0	0	0	0	0	0	0
Negros Oriental	0	0	0	0	0	0	0	0	0	0	0	0	0	0
Siquijor	0	0	0	0	0	0	0	0	0	0	0	0	0	0
City of Cebu	0	0	0	0	0	0	0	0	0	0	0	0	0	0
City of Lapu-Lapu	0	0	0	0	0	0	0	0	0	0	0	0	0	0
City of Mandaue	0	0	0	0	0	0	0	0	0	0	0	0	0	0
**Region 8**	**0**	**0**	**0**	**0**	**3**	**2**	**5**	**4**	**0**	**3**	**0**	**2**	**0**	**2**
Biliran	0	0	0	0	0	0	0	0	0	0	0	0	0	0

Table 2.H.2.3 Acute Flaccid Paralysis

By Age group, Sex, Provinces and Cities

Philippines, 2023

Region/Province/City	0 - 6 days		7 - 28 days		29 days - 11 mos.		1 - 4 yrs.		5 - 9 yrs.		10 -14 yrs.		15 - 19 yrs.	
	M	F	M	F	M	F	M	F	M	F	M	F	M	F
Eastern Samar	0	0	0	0	0	2	3	4	0	3	0	2	0	2
Northern Leyte	0	0	0	0	0	0	0	0	0	0	0	0	0	0
Northern Samar	0	0	0	0	0	0	0	0	0	0	0	0	0	0
Southern Leyte	0	0	0	0	0	0	0	0	0	0	0	0	0	0
Western Samar	0	0	0	0	0	0	0	0	0	0	0	0	0	0
City of Ormoc	0	0	0	0	3	0	2	0	0	0	0	0	0	0
City of Tacloban	0	0	0	0	0	0	0	0	0	0	0	0	0	0
**Region 9**	**0**	**0**	**0**	**0**	**0**	**0**	**1**	**0**	**0**	**0**	**0**	**0**	**0**	**0**
Zamboanga del Norte	0	0	0	0	0	0	0	0	0	0	0	0	0	0
Zamboanga del Sur	0	0	0	0	0	0	0	0	0	0	0	0	0	0
Zamboanga Sibugay	0	0	0	0	0	0	1	0	0	0	0	0	0	0
City of Isabela	0	0	0	0	0	0	0	0	0	0	0	0	0	0
City of Zamboanga	0	0	0	0	0	0	0	0	0	0	0	0	0	0
**Region 10**	**0**	**0**	**0**	**0**	**0**	**0**	**0**	**0**	**0**	**0**	**0**	**0**	**0**	**0**
Bukidnon	0	0	0	0	0	0	0	0	0	0	0	0	0	0
Camiguin	0	0	0	0	0	0	0	0	0	0	0	0	0	0
Lanao del Norte	0	0	0	0	0	0	0	0	0	0	0	0	0	0
Misamis Occidental	0	0	0	0	0	0	0	0	0	0	0	0	0	0
Misamis Oriental	0	0	0	0	0	0	0	0	0	0	0	0	0	0
City of Cagayan de Oro	0	0	0	0	0	0	0	0	0	0	0	0	0	0
City of Iligan	0	0	0	0	0	0	0	0	0	0	0	0	0	0
**Region 11**	**0**	**0**	**0**	**0**	**2**	**0**	**0**	**1**	**0**	**0**	**1**	**0**	**0**	**1**
Davao de Oro	0	0	0	0	2	0	0	0	0	0	1	0	0	0
Davao del Norte	0	0	0	0	0	0	0	1	0	0	0	0	0	1
Davao Oriental	0	0	0	0	0	0	0	0	0	0	0	0	0	0
Davao del Sur	0	0	0	0	0	0	0	0	0	0	0	0	0	0
Davao Occidental	0	0	0	0	0	0	0	0	0	0	0	0	0	0
City of Davao	0	0	0	0	0	0	0	0	0	0	0	0	0	0
**Region 12**	**0**	**0**	**0**	**0**	**0**	**0**	**0**	**1**	**0**	**0**	**1**	**1**	**1**	**0**
North Cotabato	0	0	0	0	0	0	0	0	0	0	0	0	0	0

Table 2.H.2.3 Acute Flaccid Paralysis

By Age group, Sex, Provinces and Cities

Philippines, 2023

Region/Province/City	0 - 6 days		7 - 28 days		29 days - 11 mos.		1 - 4 yrs.		5 - 9 yrs.		10 -14 yrs.		15 - 19 yrs.	
	M	F	M	F	M	F	M	F	M	F	M	F	M	F
Sarangani	0	0	0	0	0	0	0	0	0	0	0	0	0	0
South Cotabato	0	0	0	0	0	0	0	0	0	0	1	1	0	0
Sultan Kudarat	0	0	0	0	0	0	0	1	0	0	0	0	1	0
City of General Santos	0	0	0	0	0	0	0	0	0	0	0	0	0	0
**BARMM**	**0**	**0**	**0**	**0**	**1**	**0**	**0**	**0**	**1**	**0**	**0**	**0**	**0**	**1**
Basilan	0	0	0	0	0	0	0	0	0	0	0	0	0	0
Lanao del Sur	0	0	0	0	0	0	0	0	1	0	0	0	0	1
Maguindanao	0	0	0	0	0	0	0	0	0	0	0	0	0	0
Sulu	0	0	0	0	0	0	0	0	0	0	0	0	0	0
Tawi-Tawi	0	0	0	0	0	0	0	0	0	0	0	0	0	0
SGA	0	0	0	0	0	0	0	0	0	0	0	0	0	0
City of Cotabato	0	0	0	0	1	0	0	0	0	0	0	0	0	0
**Caraga**	**0**	**0**	**0**	**0**	**0**	**0**	**0**	**0**	**0**	**0**	**0**	**0**	**0**	**0**
Agusan del Norte	0	0	0	0	0	0	0	0	0	0	0	0	0	0
Agusan del Sur	0	0	0	0	0	0	0	0	0	0	0	0	0	0
Surigao del Norte	0	0	0	0	0	0	0	0	0	0	0	0	0	0
Surigao del Sur	0	0	0	0	0	0	0	0	0	0	0	0	0	0
[truncated: 1,851,576 more chars]
