# Supplementary material for: Neglected Tropical Diseases Elimination in the Philippines: Challenges and Gaps
Source: Trop Med Infect Dis. 2026 Apr 17;11(4):106. doi: 10.3390/tropicalmed11040106 (PMC13120366; doi:10.3390/tropicalmed11040106)
Supplement: Supplementary file 1 [file tropicalmed-11-00106-s001.zip › Supplementary File S6. FHSIS 2024.pdf]

FIELD HEALTH SERVICES INFORMATION SYSTEM

ANNUAL REPORT

2024

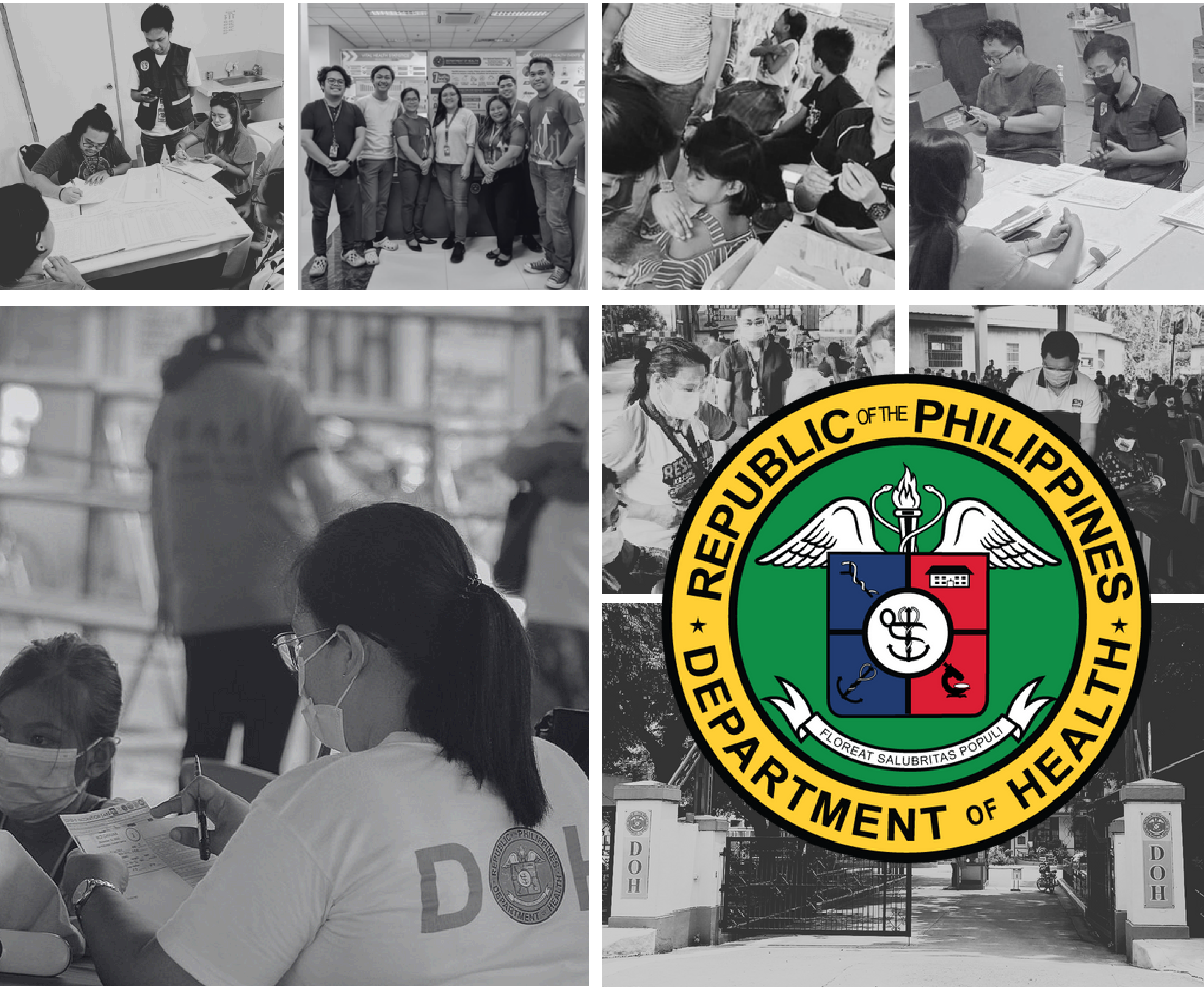

EPIDEMIOLOGY BUREAU
MONITORING AND EVALUATION DIVISION
HEALTH STATISTICS SECTION

fhsis@doh.gov.ph
8651-7800 loc. 2928/2953

Republic of the Philippines
DEPARTMENT OF HEALTH
Office of the Secretary

BAGONG PILIPINAS

MESSAGE

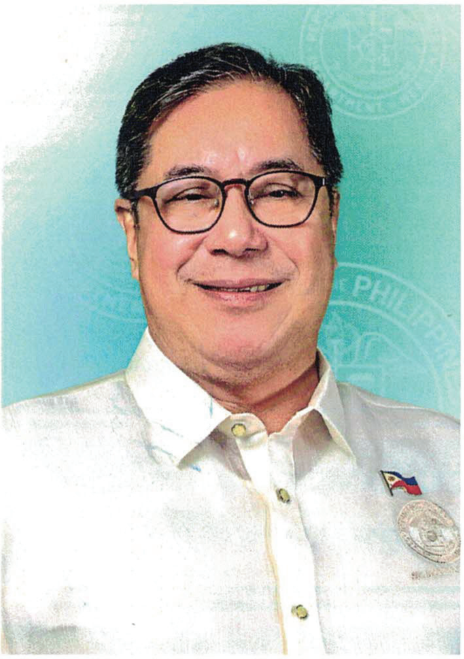

As one Department of Health (DOH), we proudly present the **CY 2024 Field Health Services Information System (FHSIS) Annual Report**. Designated by Executive Order No. 352 s. 1996, the FHSIS serves as our official statistical system to generate critical data on various public health programs and services, health status of the population (morbidity), and health systems capacity (demographics) to support more responsive and evidence-informed decisions in line with the **DOH's 8-Point Action Agenda (8PAA)** and the Universal Health Care Act.

Since its launch in 1989, the FHSIS has been a key data source not only for the Department but also for various stakeholders, including other national government agencies, non-government organizations, academic and research institutions, and legislative bodies. While challenges persist due to the sector's evolving data needs, we remain committed to enhancing the system's relevance and responsiveness in today's dynamic information management landscape.

This year's FHSIS report highlights the accomplishments of select priority programs outlined in the 8PAA, including immunization, maternal health, water, sanitation, and hygiene (WASH), and non-communicable disease prevention and control. Additionally, it presents the performance of municipalities and cities across the country in the Seal of Good Local Governance (SGLG) and the Local Government Unit Health Scorecard (LGU HSC).

We extend our heartfelt gratitude to the health workers, data managers, coordinators, program managers, and other partners at all levels of the health system—from barangay to municipal, city, provincial, regional, and national—for their tireless efforts in data collection, reporting, management, and generation, culminating in the release of the CY 2024 FHSIS Annual Report. We also appreciate the contributions of local government units, Centers for Health Development, the Ministry of Health - Bangsamoro Autonomous Region in Muslim Mindanao, the Disease Prevention and Control Bureau, and the Epidemiology Bureau for leading this endeavor. Furthermore, we acknowledge the Public Health Services Cluster for its overall management and oversight of public health programs and services.

Finally, we encourage everyone to promote and utilize this health statistical product as we continue strengthening and expanding primary healthcare services for Filipino families at the grassroots level. Together, let us enhance health outcomes, build stronger health systems, and ensure healthcare access for all Filipinos – ***dahil sa Bagong Pilipinas, Bawat Buhay Mahalaga*!**

TEODORO J. HERBOSA, MD
Secretary, Department of Health

Republic of the Philippines
DEPARTMENT OF HEALTH

MARY ANN PALERMO-MAESTRAL, MD, MBA-HA, FPPS, CHA, FPCHA
Undersecretary of Health, Public Health Services Cluster

FARWA M. HOMBRE, RN, MBA, PhD
Assistant Secretary of Health, Public Health Services Cluster

GERNA M. MANATAD, MD, PHSAE, MDM, CESO IV
Director IV, Epidemiology Bureau

ALFONSO MIGUEL R. REGALA, MD, MBA
Medical Officer V, Monitoring and Evaluation Division

HEALTH STATISTICS SECTION

MARIEL A. DEJESA, MD, MPM
Medical Officer IV

KARLA MAE C. RAMIREZ
Senior Health Program Officer

ROMCY A. SAGUCIO
Senior Health Program Officer

JANE I. GALO
Statistician II

JOSE PAOLO UNGSON
Supervising Health Program Officer

DEXTER JAY B. FLORES
Senior Health Program Officer

JULIUS T. CASTRO
Senior Health Program Officer

AARON B. DECENA
Senior Health Program Officer

HERNAN T. TAYER
Computer Programmer II

TABLE OF CONTENTS
Field Health Services Information System
2024 Annual Report

CHAPTER 1 – MAIN REPORT ANALYSIS

**8 Priority Health Outcome (PHO)**		
	2024 Key Findings	**1**
1.1	Fully Immunized Children	**3-4**
1.2	Safely Managed Drinking Water Services	**5**
1.3	Maternal Deaths	**6-7**
1.4	Hypertension and Type II Diabetes Mellitus Management	**8-9**
1.5	Cervical and Breast Cancer Screening	**10-12**
**1.A Family Planning (FP) Services**		
	2024 Key Findings	**13**
1.A.1.	Modern Family Planning Services	**14-19**
1.A.2.	Vital Statistics (Live Births and Adolescent Birth Rate)	**20-22**
**1.B Maternal Health**		
	2024 Key Findings	**23**
1.B.1.	Atenatal Care Services	**24-28**
1.B.2.	Intrapartum Care and Delivery Outcomes	**29-32**
1.B.3.	Postnatal Care Services	**33-36**
**1.C Child Health**		
	2024 Key Findings	**37**
1.C.1	Immunization Services for Infants and Children	**38- 42**
1.C.2	Nutrition Services for Infants and Children	**43-51**
1.C.3	Management of Sick Infants and Children	**52-56**
1.C.4	Child Mortality	**57-58**
**1.D Oral Health Care and Services**		
	2024 Key Findings	**59**
1.D.1	Basic Oral Health Care (BOHC)	**60-65**
**1.E Non-Communicable Diseases**		
	2024 Key Findings	**66**
1.E.1	Risk-Assessment, Hypertension and Type II Diabetes Mellitus	**67-71**
1.E.2	Cervical Cancer	**72-73**
1.E.3	Pneumococcal Polysaccharide Vaccine (PPV) and Influenza Vaccine	**74**
**1.F.A Infectious Diseases**		
	2024 Key Findings	**75**
1.F.A.1	Filariasis	**76-77**
1.F.A.2	HIV-AIDS/STI	**78-79**
1.F.A.3	Schistosomiasis	**80-82**
1.F.A.4	Soil-Transmitted Helminthiasis	**83-86**
**1.F.B Infectious Diseases**		
	2024 Key Findings	**87**
1.F.B.1	Tuberculosis	**88-92**
1.F.B.2	Leprosy	**93-94**
1.F.B.3	Rabies	**95-97**
**1.G Morbidity**		
	Leading Causes of Morbidity<br>Top 10 Diseases Based on Increase in Cases	**98-99**

CHAPTER 1 – MAIN REPORT ANALYSIS

8 Priority Health Outcome (PHO)		
**1.H Environmental Health and Sanitation**		
	2024 Key Findings	**100**
1.H.1	Basic Safe Water Supply (BSWS)	**102-103**
	Safely Managed Drinking Water Services (SMDWS)	
1.H.2	Basic Sanitation Facility (BSF)	**104**
	Safely Managed Sanitation Services (SMSS)	
1.H.3	Zero Open Defecation	**105**
**1.I Demographics**		
	2024 Key Findings	**106**
1.I.1	Public Health Workers	**107-111**
1.I.2	Barangay Health Station	
1.I.3	Health Centers	

CHAPTER 2 – SUMMARY TABLES

**2.A Family Planning Services**		
Table 2.A.1	New Acceptors	**112-132**
Table 2.A.2	Other Acceptors	**133-153**
Table 2.A.3	Drop-outs	**154-174**
Table 2.A.4	Current Users	**175-195**
Table 2.A.5	Demand Satisfied	**196-198**
**2.B Maternal Care and Services**		
2.B.1 Prenatal Care		
Table 2.B.1.1	Women who gave birth with at least 4 Prenatal Check-ups	**199-212**
Table 2.B.1.2	Pregnant Women seen according to their Nutritional Status	**213-214**
Table 2.B.1.3	Pregnant Women for the 1st time given at least 2 doses of Tetanus Diphtheria (Td) vaccination	**215-217**
Table 2.B.1.4	Pregnant Women for the 2nd time or more given at least 3 doses of Tetanus Diphtheria (Td) vaccination	**218-220**
Table 2.B.1.5	Pregnant Women who completed Iron with Folic Acid/Multiple Micronutrient Supplementation	**221-223**
Table 2.B.1.6	Pregnant Women who completed doses of Calcium Carbonate Supplementation	**224-226**
Table 2.B.1.7	Pregnant Women given 1 dose of Deworming tablet	**227-229**
Table 2.B.1.8	Pregnant Women screened for Hepatitis B	**230-232**
Table 2.B.1.9	Pregnant Women tested positive for Hepatitis B	**233-235**
Table 2.B.1.10	Pregnant Women tested for Complete Blood Count (CBC) or Hemoglobin (Hgb) & Hematocrit (Hct) Count	**236-238**
Table 2.B.1.11	Pregnant Women tested for Complete Blood Count or Hgb & Hct count diagnosed with Anemia	**239-241**
Table 2.B.1.12	Pregnant Women screened for Gestational Diabetes	**242-244**
Table 2.B.1.13	Pregnant Women tested positive for Gestational Diabetes	**245-247**
2.B.2 Intrapartum Care and Delivery Outcome		
Table 2.B.2.1	Women who Delivered a Live Baby or Stillbirth/Fetal Death	**248-253**
	Deliveries Attended by Skilled Health Professionals	**248-250**
	Facility Based Delivery	**251-253**
Table 2.B.2.2	Delivery by Type (Vaginal and Cesarean)	**254-257**
Table 2.B.2.3	Pregnancy by Outcome (Full Term and Pre-term)	**258-260**
Table 2.B.2.4	Pregnancy by Outcome (Fetal Deaths and Abortion)	**261-263**
Table 2.B.2.5	Live Births by Birth Weight	**264-266**
2.B.3 Postpartum and Newborn Care		
Table 2.B.3.1	Postpartum Women together with their Newborn who completed at least 2 Postpartum Check-ups	**267-268**
Table 2.B.3.2	Postpartum Women who completed Iron with Folic Acid Supplementation	**269-271**
Table 2.B.3.3	Postpartum Women who completed Vitamin A supplementation	**272-274**
**2.C Child Care and Services**		
2.C.1 Immunization Services for Infants and Children		
Table 2.C.1.1	Newborn or Infants Vaccinated with BCG	**275-276**
	Newborn Vaccinated with Hepatitis B antigen within 24 hours after birth	**275-276**
	Children Protected at Birth (CPAB) from Neonatal Tetanus	**275-276**
Table 2.C.1.2	3 doses of DPT-HiB-HepB antigen	**277-278**
Table 2.C.1.3	3 doses of Oral Polio Vaccine (OPV)	**279-280**
Table 2.C.1.4	2 doses of Inactivated Polio Vaccine (IPV)	**281-283**
Table 2.C.1.5	3 doses of Pneumococcal Conjugate Vaccine (PCV)	**283-285**
Table 2.C.1.6	2 doses of Measles Containing Vaccine (MCV)	**286-288**
Table 2.C.1.7	Fully Immunized Children (FIC)	**289-315**

CHAPTER 2 – SUMMARY TABLES

	Completely Immunized Children (CIC)	**316-317**
2.C.2 School-Based Immunization Services for Children		
Table 2.C.2.1	Grade 1 learners given one dose of Tetanus diphtheria toxoid (Td) vaccine	**318-319**
	Grade 7 learners given one dose of Tetanus diphtheria toxoid (Td) vaccine	**318-319**
Table 2.C.2.2	Grade 1 learners given one dose of Measles Rubella (MR) vaccine	**320-321**
	Grade 7 learners given one dose of Measles Rubella (MR) vaccine	**320-321**
2.C.3 Nutrition Services for Infants and Children		
Table 2.C.3.1	Initiated on Breastfeeding within 1 hour after birth	**322-323**
	Infants Exclusively Breastfed until 5th month and 29 days	**322-323**
	Infants who Continued Breastfeeding and were Introduced to Complementary Feeding beginning at 6 months of age	**322-323**
Table 2.C.3.2	Infants Born Preterm or with Low Birth Weight Given Iron Supplements	**324-325**
	Infants 6-11 months old who completed Vitamin A Supplementation	**324-325**
	Children 12-59 months old who completed Vitamin A Supplementation	**324-325**
Table 2.C.3.3	Infants 6-11 months old who completed Micronutrient Powder (MNP) or Lipid-Based Nutrient Supplement – Small Quantity (LNS-SQ) Supplementation	**326-328**
	Children 12-23 months old who completed Micronutrient Powder (MNP) or Lipid-Based Nutrient Supplement – Small Quantity (LNS-SQ) Supplementation	**326-328**
Table 2.C.3.4	Children 0-59 months old whose Nutritional Status are Normal, Stunted and Overweight/Obese	**329-330**
	Children 0-59 months old whose Nutritional Status are Wasted-MAM, Wasted-SAM and Wasted	**331-332**
Table 2.C.3.5	Moderate Acute Malnutrition (MAM) Children admitted, cured, defaulted and died in Supplementary Feeding Program (SFP)	**333-334**
Table 2.C.3.6	Severe Acute Malnutrition (SAM) Children without complication admitted, cured, defaulted and died in Outpatient Therapeutic Care (OTC)	**335-336**
2.C.4 Management of Sick Infants and Children		
Table 2.C.4.1	High Risk Infants 6-11 months old with Measles and/or Persistent Diarrhea who received Vitamin A capsule aside from routine supplementation	**337-338**
	High Risk Children 12-59 months old with Measles and/or Persistent Diarrhea who received Vitamin A capsule aside from routine supplementation	**337-338**
Table 2.C.4.2	Diarrhea cases seen (0-59 months old) who received Oral Rehydration Salt Solution (ORS) and ORS with Zinc drops or syrup	**339-340**
	Pneumonia cases seen (0-59 months old) and received treatment	**339-340**
**2.D Oral Health Care and Services**		
Table 2.D.1	Children 12-59 months old who are orally fit upon oral examination or after oral rehabilitation	**341-343**
Table 2.D.2	Children 5 years old and above with new cases of Decayed-Missing Filled Teeth (DMFT)	**344-346**
Table 2.D.3	Basic Oral Health Care (BOHC) Infants 0-11 months	**347-348**
Table 2.D.4	BOHC Children 1-4 years old	**349-351**
Table 2.D.5	BOHC Children 5-9 years old	**352354**
Table 2.D.6	BOHC Adolescents 10-14 years old	**355-357**
Table 2.D.7	BOHC Adolescents 15-19 years old	**358-360**
Table 2.D.8	BOHC Adolescents 20-59 years old	**361-363**
Table 2.D.9	BOHC Senior Citizen 60 years old and above	**364-365**
Table 2.D.10	BOHC Pregnant Women (10-14, 15-19, and 20-49 years old)	**366-368**
**2.E Infectious Disease Prevention and Control Services**		
2.E.1 Filariasis Prevention and Control		
Table 2.E.1.1	Case Detection Rate	**369-371**
	Lymphatic Filariasis Cases and Clinical Rate	**369-371**

CHAPTER 2 – SUMMARY TABLES

2.E.2 Schistosomiasis Prevention and Control		
Table 2.E.2.1	Suspected Schistosomiasis Cases Seen	**372-374**
	Acute Clinically Diagnosed Cases Seen	**372-374**
	Acute Confirmed Cases	**372-374**
Table 2.E.2.2	Chronic Clinically Diagnosed Cases Seen	**375-377**
	Chronic Clinically Diagnosed Cases Confirmed by Stool Examination	**375-377**
	Chronic Clinically Diagnosed Cases Treated in the Health Facility	**375-377**
	Confirmed Chronic Cases referred to a Hospital Facility	**375-377**
2.E.3 Soil Transmitted Helminthiasis Prevention and Control		
Table 2.E.3.1	Preschool-Aged Children (PSAC) who completed 2 doses of Deworming Tablets	**378-380**
	School Aged Children (SAC) who completed 2 doses of Deworming Tablets	**378-380**
Table 2.E.3.2	Adolescents 10-19 years old who completed 2 doses of Deworming Tablet	**381-383**
Table 2.E.3.3	Women of Reproductive Age (WRA) 20-49 years old who completed 2 doses of Deworming Tablet	**383-386**
	Pregnant Women who completed 1 dose of Deworming Tablet	**383-386**
2.E.4 HIV-AIDS/STI Prevention and Control		
Table 2.E.4.1	Pregnant Women Screened for Syphilis	**387-391**
	Pregnant Women Positive for Syphilis	**387-391**
	Pregnant Women Screened for HIV	**387-391**
2.E.5 Leprosy Prevention and Control		
Table 2.E.5.1	Prevalence Rate	**392-394**
	Case Detection Rate	**392-394**
2.E.6 Rabies		
Table 2.E.6.1	Deaths due to Rabies	**395-397**
2.E.7 Tuberculosis Prevention and Control *(source: Integrated Tuberculosis Information System)*		
Table 2.E.7.1	Case Notification Rate (CNR), all forms	**398-411**
	Case Notification Rate (DRTB)	**398-411**
Table 2.E.7.2	Treatment Success Rate (TSR), DSTB-all forms	**412-425**
	Treatment Success Rate (TSR), MDRTB	**412-425**
**2.F Non-Communicable Disease Prevention and Control Services**		
2.F.1 Lifestyle Related Diseases (Risk Assessment Using PhilPEN protocol)		
Table 2.F.1.1	Adults 20-59 years old who were Risk-assessed using the PhilPEN Protocol	**426-443**
Table 2.F.1.2	Adults 20-59 years old who are Current Smokers based on the PhilPEN Protocol	**444-446**
	Adults 20-59 years old who are Binge Drinkers based on the PhilPEN Protocol	**444-446**
	Adults 20-59 years old who are Overweight/Obese based on the PhilPEN Protocol	**444-446**
Table 2.F.1.3	Senior Citizens who were Risk-assessed using the PhilPEN Protocol	**447-464**
Table 2.F.1.4	Senior Citizens who are Current Smokers based on the PhilPEN Protocol	**465-467**
	Senior Citizens who are Binge Drinkers based on the PhilPEN Protocol	**465-467**
	Senior Citizens who are Overweight/Obese based on the PhilPEN Protocol	**465-467**
2.F.2 Cardiovascular Disease and Diabetes Mellitus Prevention and Control		
Table 2.F.2.1	Adults (20-59 years old) Identified as Hypertensive	**468-470**
	Adults (20-59 years old) Identified as Hypertensive and provided with antihypertensive medications	**468-470**
Table 2.F.2.2	Adults (20-59 years old) Identified with Type 2 Diabetes Mellitus	**471-473**
	Adults (20-59 years old) Identified with Type 2 Diabetes Mellitus and provided with antidiabetic medications	**471-473**
Table 2.F.2.3	Senior Citizens Identified as Hypertensive	**474-476**

CHAPTER 2 – SUMMARY TABLES

	Senior Citizens Identified as Hypertensive and provided with antihypertensive medications	**474-476**
Table 2.F.2.4	Senior Citizens Identified with Type 2 Diabetes Mellitus	**477-479**
	Senior Citizens Identified with Type 2 Diabetes Mellitus and provided with antidiabetic medications	**477-479**
2.F.3 Cancer Prevention and Control		
Table 2.F.3.1	Women 20 years old and above Screened for Cervical Cancer using Visual Inspection with Acetic Acid (VIA) or Pap Smear	**480-482**
	Women 20 years old and above Found Positive or Suspected for Cervical Cancer	**480-482**
	Women 20 years old and above Screened for Breast Mass	**480-482**
	Women 20 years old and above with Suspicious Breast Mass	**480-482**
Table 2.F.3.2	Women 30-65 years old Screened for Cervical Cancer using Visual Inspection with Acetic Acid (VIA) or Pap Smear or HPV DNA	**483-485**
	Women 30-65 years old Found Positive or Suspected for Cervical Cancer	**483-485**
	Women 30-65 years old Screened for Breast Mass	**483-485**
	Women 30-65 years old with Suspicious Breast Mass	**483-485**
2.F.4 Blindness Prevention Program		
Table 2.F.4.1	Senior Citizens Screened for Visual Acuity	**486-488**
	Senior Citizens Diagnosed with Eye Disease/s	**486-488**
2.F.5 Immunization for Senior Citizens		
Table 2.F.5.1	Senior Citizens Received 1 dose of Pneumococcal Polysaccharide Vaccine (PPV)	**489-491**
	Senior Citizens Received 1 dose of Influenza Vaccine	**489-491**
**2.G Water, Sanitation and Hygiene**		
Table 2.G.1	Households with Access to Basic Safe Water Supply (Level I, II, III)	**492-494**
Table 2.G.2	Households using Safely Managed Drinking-Water Services	**495-512**
Table 2.G.3	Households with Basic Sanitation Facility	**513-515**
Table 2.G.4	Households using Safely Managed Sanitation Service	**516-533**
Table 2.G.5	Municipality/City declared zero open defecation (ZOD)	**534-535**
**2.H Morbidity**		
2.H.1 Top Ten Leading Causes of Morbidity		
Table 2.H.1.1	Top Ten Leading Causes of Morbidity	**534**
2.H.2 Morbidity Rate (by Type of Disease, Sex and Age Group)		
Table 2.H.2.1	Acute Bloody Diarrhea	**535-537**
Table 2.H.2.2	Acute Febrile Illness	**538-540**
Table 2.H.2.3	Acute Flaccid Paralysis	**541-542**
Table 2.H.2.4	Acute Hemorrhagic Fever	**543-544**
Table 2.H.2.5	Acute Lower Respiratory Tract Infection	**545-546**
Table 2.H.2.6	Acute Respiratory Infection (less than 5 years old)	**547-549**
Table 2.H.2.7	Acute Respiratory Infection (more than 5 years old)	**550-551**
Table 2.H.2.8	Acute Watery Diarrhea	**552-553**
Table 2.H.2.9	Animal Bites	**554-555**
Table 2.H.2.10	Bronchitis	**556-557**
Table 2.H.2.11	Cholera	**558-560**
Table 2.H.2.12	Chronic Obstructive Pulmonary Disease	**561-562**
Table 2.H.2.13	Diphtheria	**563-564**
Table 2.H.2.14	Diseases of the Heart	**565-566**
Table 2.H.2.15	Filariasis	**567-569**
Table 2.H.2.16	Fever of Unknown Origin	**570-572**

Table 2.H.2.17	Genital Ulcer	**573-575**
Table 2.H.2.18	Gonorrhea	**576-578**
Table 2.H.2.19	Hypertension	**579-581**
Table 2.H.2.20	Influenza-Like Illness	**582-584**
Table 2.H.2.21	Influenza	**585-587**
Table 2.H.2.22	Leprosy	**588-590**
Table 2.H.2.23	Leptospirosis	**591-593**
Table 2.H.2.24	Malaria	**594-596**
Table 2.H.2.25	Measles	**597-599**
Table 2.H.2.26	Meningococcemia	**600-602**
Table 2.H.2.27	Neonatal Tetanus	**603-605**
Table 2.H.2.28	Non-Neonatal Tetanus	**606-608**
Table 2.H.2.29	Pneumonia	**609-611**
Table 2.H.2.30	Rabies (Human)	**612-614**
Table 2.H.2.31	Paralytic Shellfish Poisoning	**615-617**
Table 2.H.2.32	Schistosomiasis	**618-620**
Table 2.H.2.33	Skin Diseases	**621-623**
Table 2.H.2.34	Syphilis	**624-626**
Table 2.H.2.35	Tuberculosis (All Forms)	**627-629**
Table 2.H.2.36	Typhoid and Paratyphoid Fever	**630-632**
Table 2.H.2.37	Urethral Discharge	**633-635**
Table 2.H.2.38	Urinary Tract Infection	**636-638**
Table 2.H.2.39	Viral Encephalitis	**639-641**
Table 2.H.2.40	Viral Hepatitis	**642-644**
Table 2.H.2.41	Viral Meningitis	**645-647**
Table 2.H.2.42	Whooping Cough	**648-650**
	**2.I Mortality**	
Table 2.I.1	Maternal Mortality Ratio (MMR)	**651-653**
Table 2.I.2	Infant Mortality Rate (IMR) and Neonatal Mortality Rate (NMR)	**654-656**
Table 2.I.2	Perinatal Mortality Rate (PMR)	**654-656**
	Under Five Mortality Rate (UFMR)	**657-659**
	**2.J Natality**	
Table 2.J.1	Live Births	**660-661**
	Crude Birth Rate	**660-661**
	Adolescent Birth Rate	**660-661**
	**2.K Demographic Data**	
Table 2.K.1	Number and Ratio of Barangays	**662-663**
	Number and Ratio of Health Centers (HC)	**662-663**
	Number and Ratio of Barangay Health Stations (BHS)	**662-663**
Table 2.K.2	Number and Ratio of Doctors	**664-665**
	Number and Ratio of Nurses	**664-665**
	Number and Ratio of Midwives	**664-665**
	Number and Ratio of Dentists	**664-665**
Table 2.K.3	Number and Ratio of Medical Technologists	**666-667**
	Number and Ratio of Nutritionists	**666-667**
	Number and Ratio of Sanitary Engineers	**666-667**
	Number and Ratio of Sanitary Inspectors	**668-669**
	Number and Ratio of Active Barangay Health Workers (BHWs)	**668-669**

CHAPTER I
Main Report Analysis

8 Priority Health Outcomes

2024 KEY FINDINGS

64.85% Fully Immunized Children (FIC)

5 out of 17 regions have surpassed the target coverage level of **73.62%** for safely managed drinking water services

64.85 Maternal Mortality Ratio (MMR)

78.35% Adults & 83.45% Senior Citizens of Hypertensives provided with antihypertensive medications

75.43% Adults & 84.09% Senior Citizens of Type II Diabetics provided with antidiabetic medications

0.40% **1st Sem** (20 & above)
0.89% **2nd Sem** (30-65 y/o)
of women were screened for Cervical Cancer

1.50% **1st Sem** (20 y/o & above)
2.90% **2nd Sem** (30-65 y/o)
of women were screened for Breast Mass

8 PRIORITY HEALTH OUTCOMES

In line with the Philippine Development Plan 2023-2028, the 8-Point Action Agenda (8PAA)[1] outlines a people-oriented approach to healthcare reforms, highlights the involvement of the community in health promotion, and acknowledges the vital role of health workers in the health sector.[2] Guiding stakeholders in monitoring and evaluating the 8PAA indicators, the Department of Health's 8 Priority Health Outcomes serve as select tracer outcome measures for primary care service delivery.[3]

The Eight Priority Health Outcomes include:

1. **Immunization:** Achieve 95% Fully immunized Children from 72%,
2. **Nutrition (First 1,000 Days):** Decrease stunting to 13.5% from 27%,
3. **Water Supply, Sanitation, and Hygiene (WASH) :** Increase percentage of population with access to safe water from 88% to 100%,
4. **Maternal Health**: Decrease maternal deaths to <111 per live births from 154 per 100,000 live births,
5. **Tuberculosis and HIV:** Zero TB Cases mortality rate from 34 per 100,000 population and HIV 85% of PLHIV should be diagnosed, 85% of those diagnosed with HIV should be receiving antiretroviral therapy (ART) and 85% of all those receive ART should achieve viral suppression,
6. **Road Safety:** Decrease death rate attributed to road injuries to 4 from 8 per 100,000 population,
7. **Noncommunicable Diseases, specifically Hypertension (HPN) and Diabetes Mellitus (DM):** increase HPN and DM management by 50%, and
8. **Noncommunicable Diseases, specifically Cancer:** increase screening, diagnosis and treatment of cancer by 50%

While these eight (8) areas represent the comprehensive goals of the health sector, the FHSIS only reports the indicators for **immunization, maternal deaths, safely managed drinking water (under WASH), noncommunicable diseases such as HPN and DM management,** and **cancer.** The remaining indicators on nutrition, tuberculosis and HIV, and road safety are monitored and reported through other surveillance systems and program based reporting tools.

This chapter examines the available FHSIS data corresponding to these five covered outcomes. It aims to assess trends, identify gaps, and provide evidence-based insights to guide local and national health policy. By focusing on what is currently measurable through FHSIS, this analysis also underscores the need for strengthening data systems to fully capture and monitor the broader set of priority health concerns.

---

[1] *Department of Health AO No. 2023-0015 or the "Adoption of the 8-Point Action Agenda as the Medium-Term Strategy of the Health Sector for 2023 to 2028"*
[2] *8 Point Agenda. (n.d.). Republic of the Philippines Department of Health. Retrieved June 5, 2025*
[3] *Department of Health AO No. 2024-0014 or the "Guidelines on the Implementation of the 8-Point Action Agenda, Monitoring, Evaluation, Accountability, and Learning System.*

1. FULLY IMMUNIZED CHILDREN

Immunization is one of the most effective and cost-efficient public health interventions available, preventing millions of deaths each year. A Fully Immunized Child (FIC) as defined by the World Health Organization (WHO) as a child who has received all essential vaccines within the first year of life, represents a critical benchmark for national and global health systems. These vaccines include protection against diseases such as tuberculosis, diphtheria, tetanus, pertussis, polio, hepatitis B, and measles. Ensuring that every child is fully immunized not only safeguards individual health but also contributes to community-wide protection through herd immunity. Strengthening FIC rates is vital in reducing child mortality, preventing disease outbreaks, and achieving broader health equity goals.

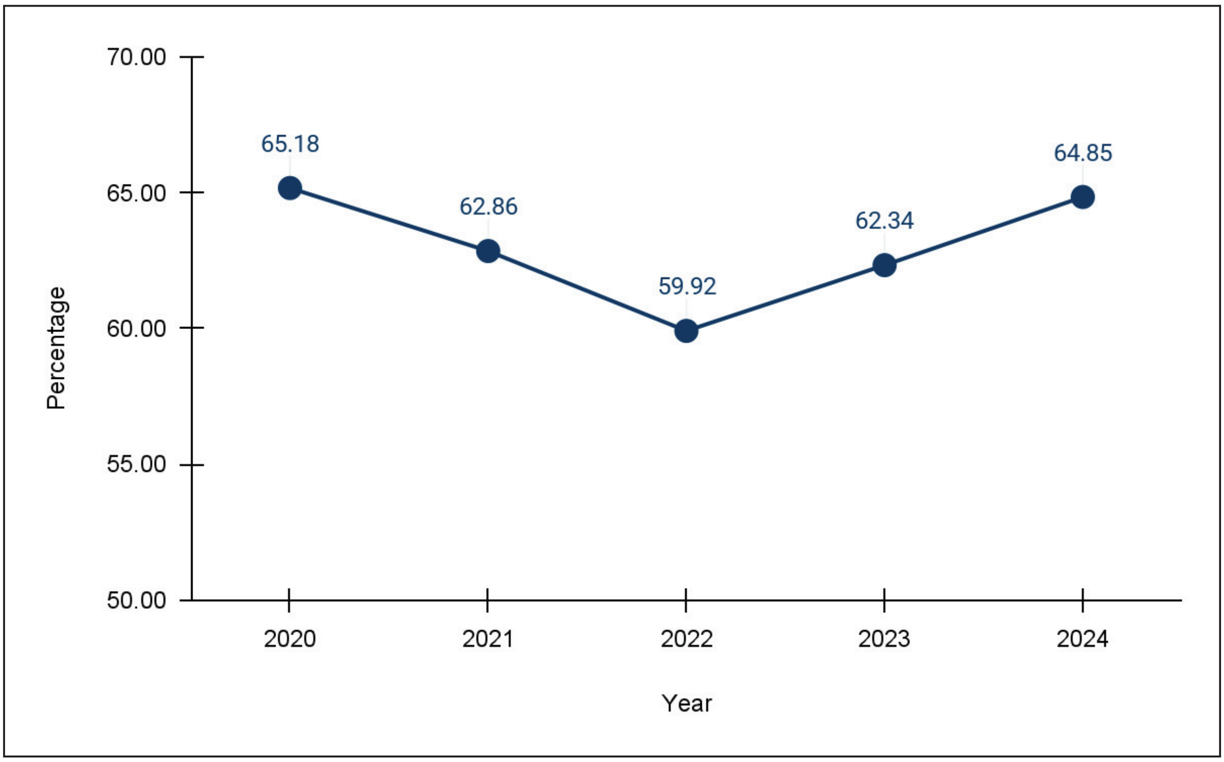

Figure 1.1: Fully Immunized Children, Philippines, 2020 to 2024

Following the decline in the proportion of fully immunized children (FIC) from 2020 to 2022, a positive shift was observed in 2023 and sustained through 2024, reaching a total of 1,551,537 FIC and increasing the national coverage to 64.85%. This resulted in a 5% increase in the FIC coverage since 2022 *[Figure 1.1]*.

Despite this progress, around 30% or 718,000 children still need to be fully immunized to achieve the national target of 95%. Reaching this target at both national and regional level is critical to protect children from vaccine-preventable diseases, improve their chances of survival, and help them in developing into healthy adults.

The regional FIC coverage ranged from 47.84% in Region 5 to 79.92% in CARAGA. CARAGA remained as the leading region in terms of FIC coverage, followed

by NCR at 78.80%, Region 3 at 76.04% and Region 1 at 75.10%. Region 3, NCR, and Region 4A were the most densely populated regions and also recorded the highest number of FIC. Meanwhile, 10 out of the 17 regions were below the national average and those with below 60% were Region 4B (58.87%), Region 5 (47.84%), Region 8 (47.86%), and BARMM (51.68%) *[Figure 1.2]*.

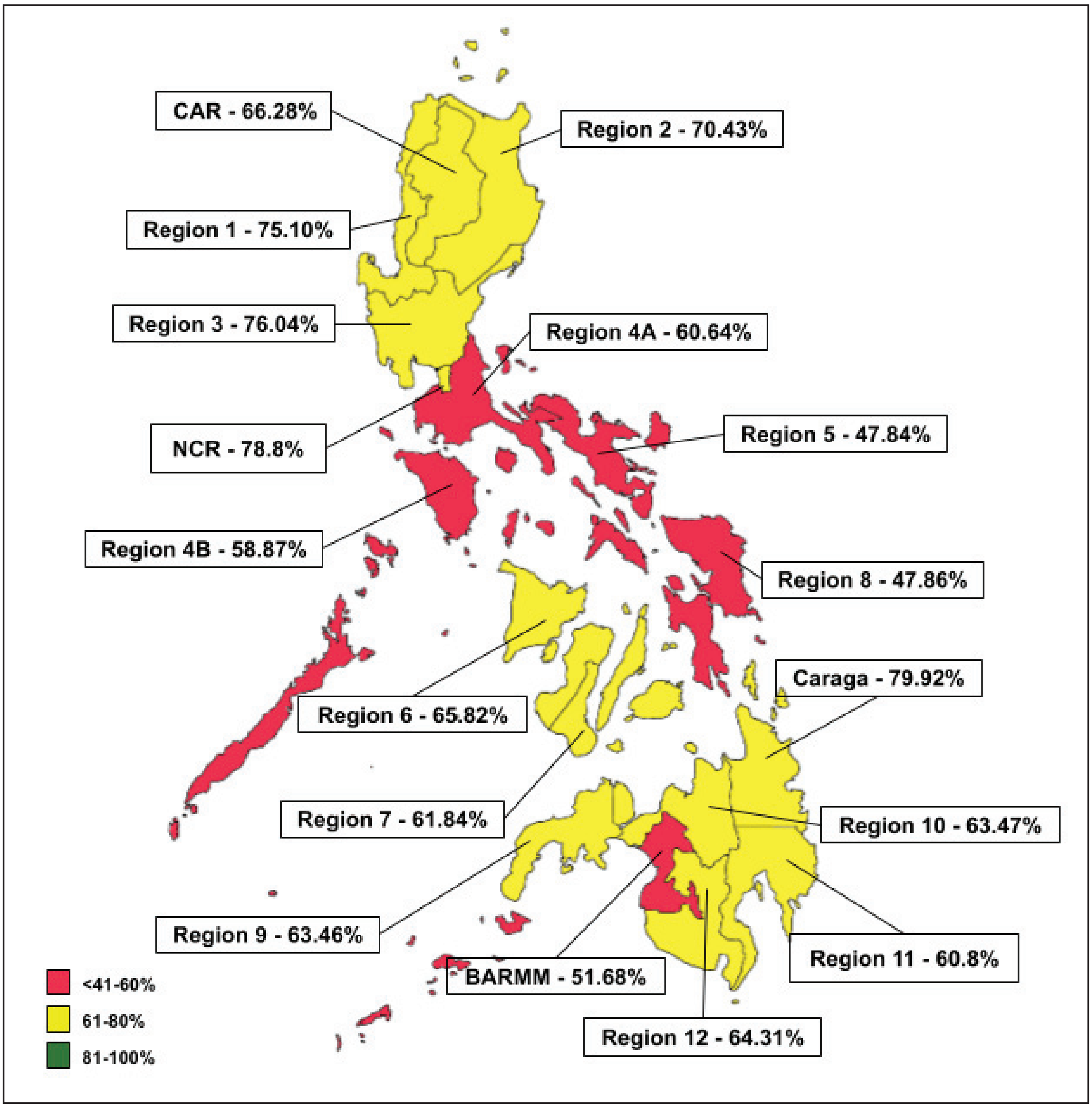

Figure 1.2: Fully Immunized Children by Region, 2024

2. SAFELY MANAGED DRINKING WATER SERVICES

Safely managed drinking water services are essential for preventing waterborne diseases and promoting overall health and well-being. According to the World Health Organization, access to safe and reliable drinking water significantly reduces the incidence of diarrheal diseases and supports the achievement of multiple sustainable development goals related to health and equity.

Out of the 17 regions in the country, 6 regions have surpassed the target coverage level of 73.62% for safely managed drinking water services. The National Capital Region (NCR) had the highest SMDWS coverage, with 94.13% of households having access, followed by Region 3 (92.53%), and Region 4A (85.22%). Other regions that exceeded the target were Region 1 (76.09%), Region 11 (78.59%), and CAR (67.84%), although CAR fell slightly short of the target *[Figure 2.1]*.

On the other hand, several regions had notably lower coverage. BARMM had the lowest proportion of households with access to safely managed drinking water, at only 20.13%, followed by Region 4B (42.19%), and Region 5 (54.06%) *[Figure 2.1]*.

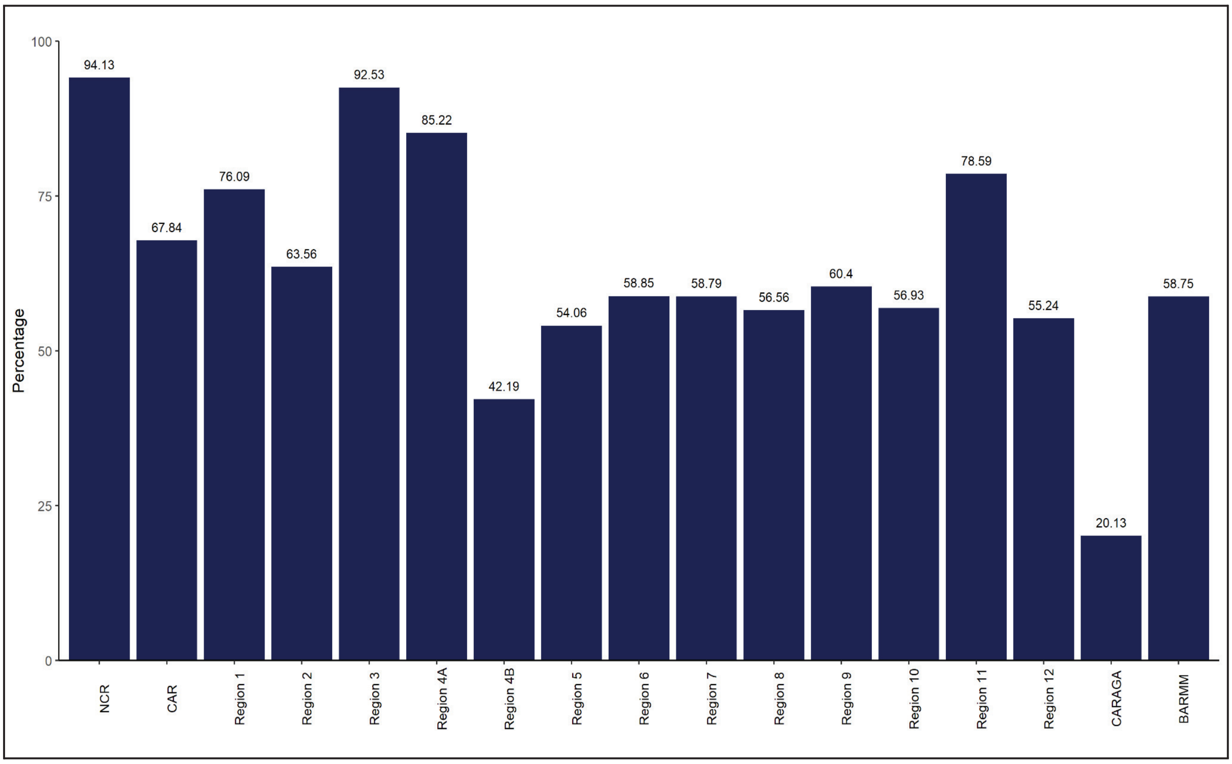

Figure 2.1: Safely Managed Drinking Water Services, by Region, 2024

3. MATERNAL DEATHS

The Maternal Mortality Ratio (MMR) is a key indicator of the quality and responsiveness of a country's health system, reflecting the capacity to deliver timely and effective maternal health services. It is defined as the number of maternal deaths per 100,000 live births and serves as a global benchmark for tracking progress in maternal health outcomes. A declining MMR indicates improvements in areas such as antenatal care, skilled birth attendance, emergency obstetric care, and postnatal services. In the Philippines, the government has prioritized maternal health through various policy frameworks, including the Sustainable Development Goals (SDG 3), the 8 Priority Health Outcomes, and the Universal Health Care (UHC) Law, which is operationalized through the Omnibus Health Guidelines.

Over the past five years, the MMR in the Philippines has shown a fluctuating pattern. In 2020, the MMR was recorded at 70.7. This increased notably in 2021 to 84.9, likely as a result of the COVID-19 pandemic, which severely disrupted access to essential maternal and reproductive health services. The pandemic strained health facilities and deterred many women from seeking timely care due to lockdowns and fears of infection. In the years that followed, a downward trend emerged. By 2022, the MMR had declined significantly to 64.7, suggesting that recovery efforts and health system adaptations were beginning to take effect *[Figure 3.1]*.

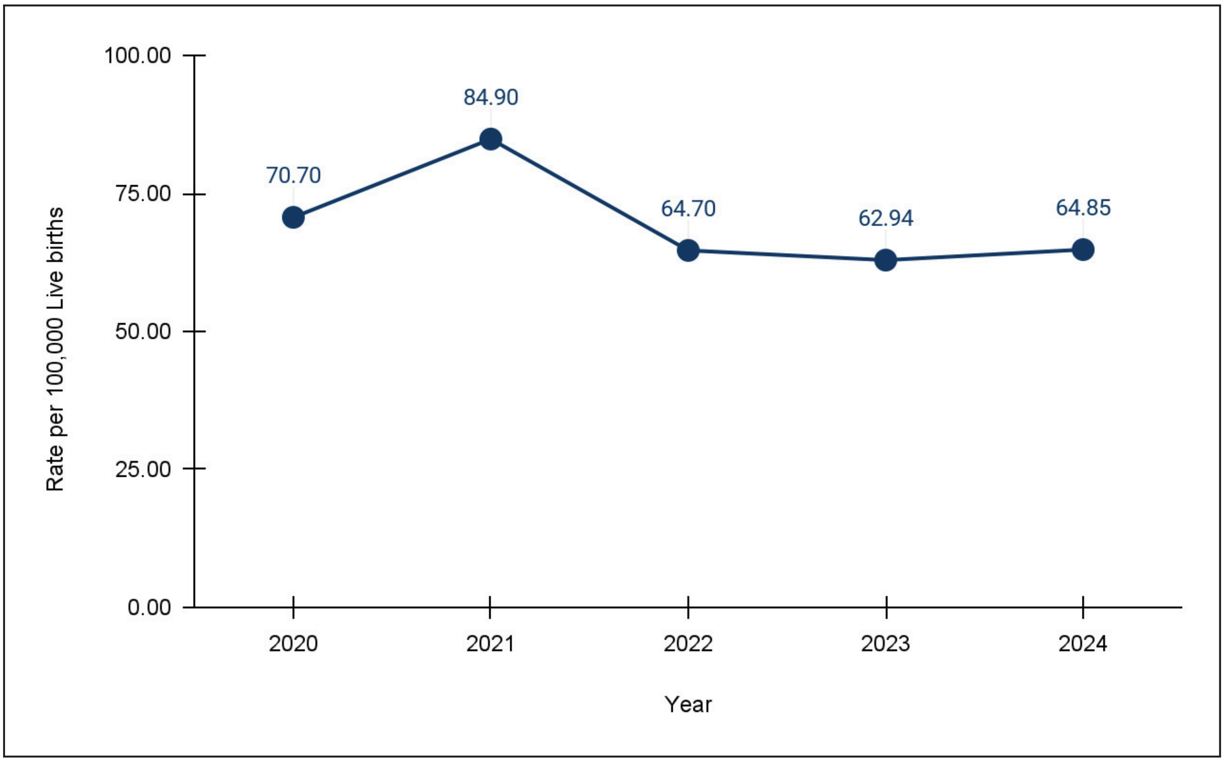

Figure 3.1: Maternal Mortality Ratio, Philippines, 2020-2024

Regionally, the 2024 data indicate that nearly all Philippine regions reported maternal mortality rates below the SDG 3.1 benchmark of 70 maternal deaths per 100,000 live births. NCR, CAR, and Regions 1 through 10 , recorded MMRs ranging between 57 and 59 per 100,000 live births, demonstrating stable and consistent performance in maternal health service delivery *[Figure 3.2]*. Notably, BARMM reported the lowest MMR at 39.16, highlighting significant progress in a region historically challenged by limited healthcare access. On the other hand, CARAGA reported the highest MMR at 68.88 *[Figure 3.2].* While this figure remains below the SDG threshold, it is nearing the limit and signals a need for more focused support and intervention. Region 11 also reported a relatively elevated MMR of 64.86, suggesting that certain localities within the region may benefit from strengthened maternal health programs and services *[Figure 3.2]*.

Taken together, the national and regional data for 2024 reflect considerable progress in improving maternal health outcomes in the Philippines. The overall national trend points to successful efforts in reducing maternal mortality, although variations between regions underscore the importance of localized strategies. Sustaining current achievements while addressing gaps in higher-risk areas such as CARAGA and Region 11 will be essential to ensuring equitable maternal health outcomes across the country *[Figure 3.2]*.

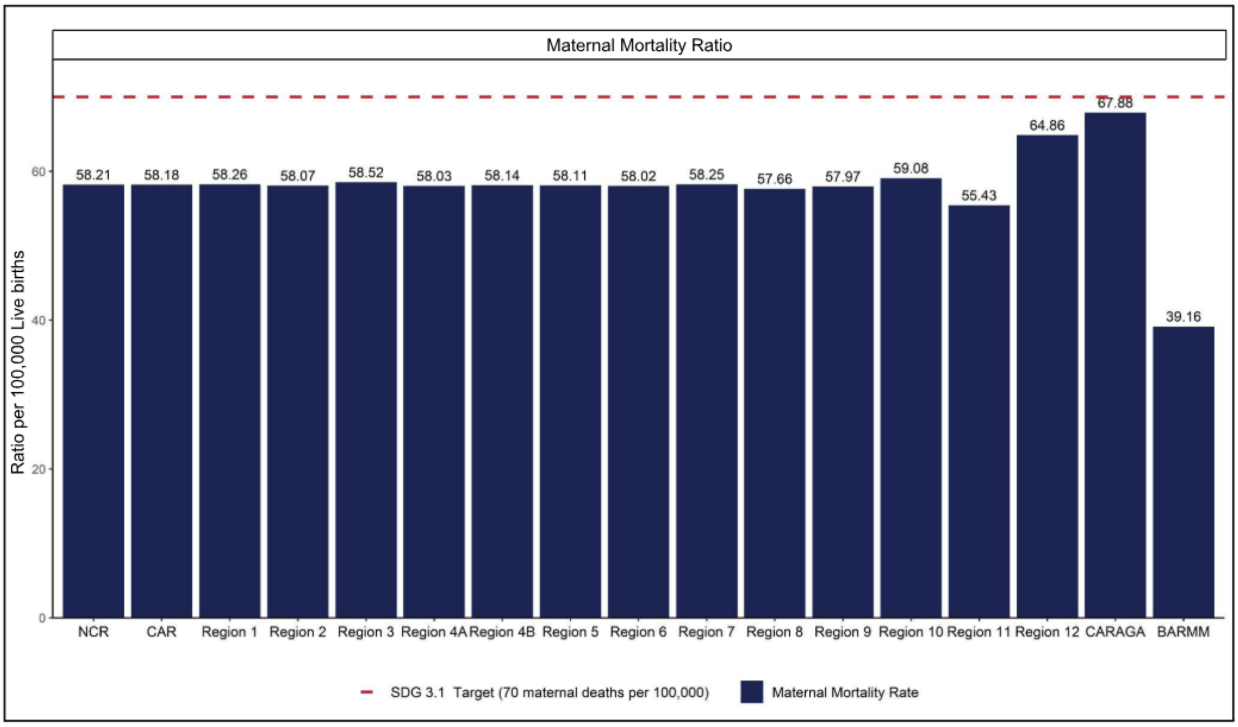

Figure 3.2: Maternal Mortality Ratio by Regions, 2024

4. HYPERTENSION AND TYPE II DIABETES MANAGEMENT

Effective management of hypertension and type 2 diabetes mellitus is crucial to prevent serious complications such as heart disease, stroke, kidney failure, and premature death. According to the World Health Organization, proper control of these conditions through medication and lifestyle interventions significantly reduces the risk of cardiovascular diseases, which remain the leading cause of death globally.

The provision of medication to identified hypertensive patients in Primary Care Facilities (PCFs) varies across regions for both adults and senior citizens. Among adults, the highest coverage rates were observed in the National Capital Region (NCR) at 101.8%, followed by CAR with 94.4%, Region 4B at 91.08%, Region 1 with 89.73%, and CARAGA at 86.3%. On the other end of the spectrum, Region 5 reported a significantly lower coverage of just 27.53%, more than 40 percentage points lower than the next-lowest region, Region 4A (68.71%).

For senior citizens with hypertension, the highest medication coverage was recorded in CAR (96.53%), NCR (93.11%), Region 1 (91.49%), Region 9 (91.2%), and Region 4B (90.12%). The lowest coverage was noted in Region 4A (0%), Region 5 (37.86%), and BARMM (48.59%).

For adults identified with type 2 diabetes, medication provision varied significantly across regions. The highest coverage was recorded in CAR at 107.6%, followed by NCR (100.51%), Region 4B (96.59%), CARAGA (85.85%), and Region 11 (84.03%), indicating strong performance in these areas. At the lower end, BARMM and Region 5 stood out with notably low coverage rates at 23% and 24.95%, respectively. These figures are less than half the coverage rate of Region 1 (64.19%), which ranks next-lowest.

Among senior citizens with type 2 diabetes, the highest coverage was found in CAR (121.04%), NCR (97.33%), Region 1 (95.36%), Region 4B (92.67%), and Region 8 (86.63%). The lowest coverage rates were recorded in Region 4A (0%), BARMM (35.67%), and Region 5 (43.59%).

Overall, the data indicate regional variation in medication provision for both hypertensive and diabetic patients across adult and senior citizen populations in PCFs. It is important to note that the data quality for the 2024 medication data may have limitations, as this represents the initial year of implementation.

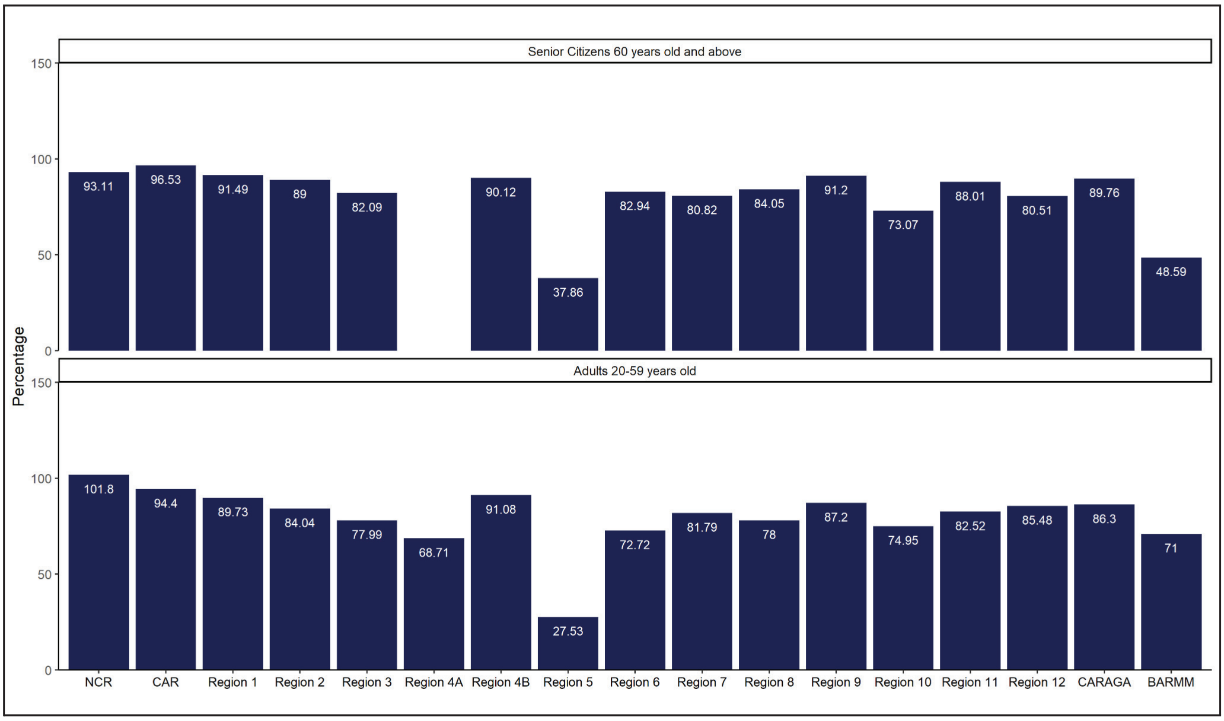

Figure 4.1: Hypertensives Provided with Antihypertensive Medication, by Region, 2024

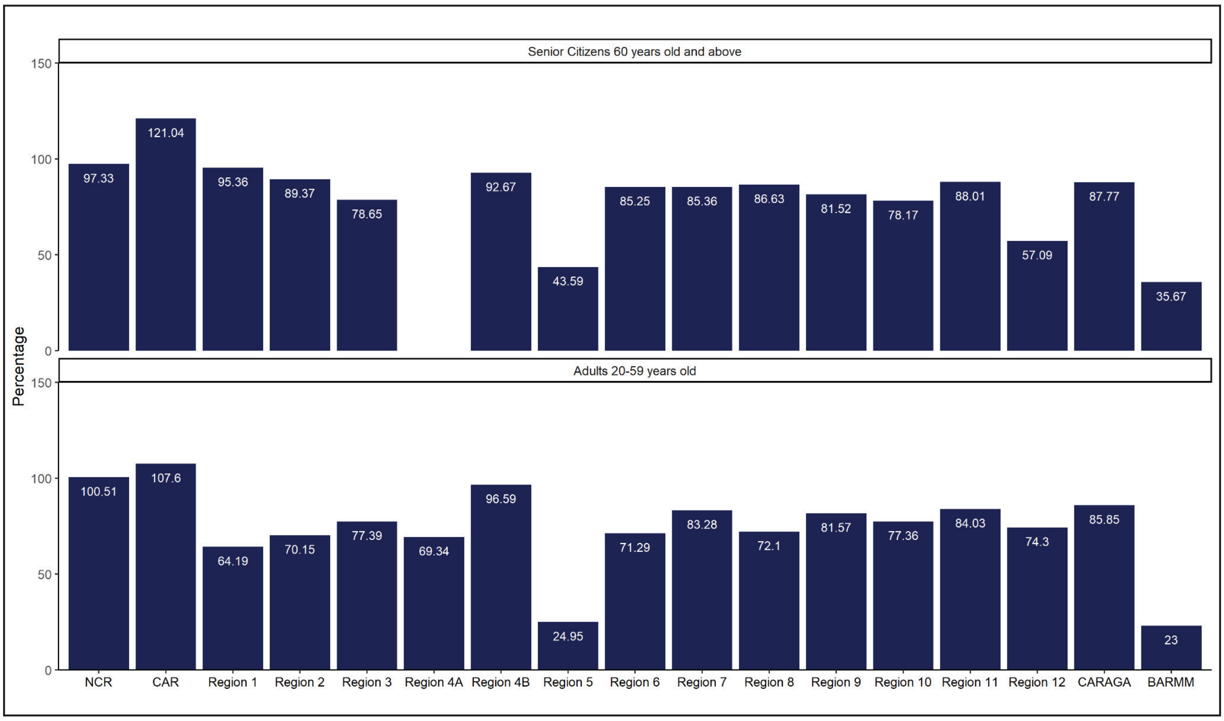

Figure 4.2: Type II Diabetics Provided with Antidiabetic Medication, by Region, 2024

5. CERVICAL AND BREAST CANCER SCREENING

According to the World Health Organization, early diagnosis significantly improves cancer outcomes by enabling treatment at the earliest possible stage, making it a key public health strategy across all settings. For cervical cancer specifically, the U.S. National Cancer Institute notes that when diagnosed early, the five-year relative survival rate can be as high as 91%, underscoring the life-saving potential of regular screening.

In 2024, the Philippines strengthened its efforts to detect cervical and breast cancer early through targeted routine screening services integrated into primary care. During the first semester, cervical and breast cancer screening targeted women aged 20 years and above. In the second semester, the eligible age group was narrowed to women aged 30 to 65 years, in accordance with the updated program guidelines outlined in Department Memorandum (DM) 2024-0187, titled “Guidelines on the Revision of Cancer-Related Indicators in the FHSIS,” and its subsequent amendment, DM 2024-0187-A. Screening methods for cervical cancer included visual inspection with acetic acid (VIA) and Pap smear, while clinical breast examination (CBE) was used for breast cancer screening. These services are delivered through public health facilities nationwide as part of the country’s broader strategy to reduce the burden of women’s cancers through early detection and timely care.

In the first semester, cervical and breast cancer screening was conducted among women aged 20 years and above. Screening rates across regions were generally low, with most regions reporting less than 1% of women screened for cervical cancer. The National Capital Region (NCR) and Region 11 recorded the highest cervical cancer screening rates at 1.12% and 1.15%, respectively. Similarly, for breast cancer, Region 11 had the highest coverage at 3.73%, followed by NCR at 2.94%, and CAR at 2.68%. The rest of the regions reported breast cancer screening rates below 3%, with the lowest recorded in Region 4A (0.33%), Region 4B (0.45%), and Region 9 (0.33%).

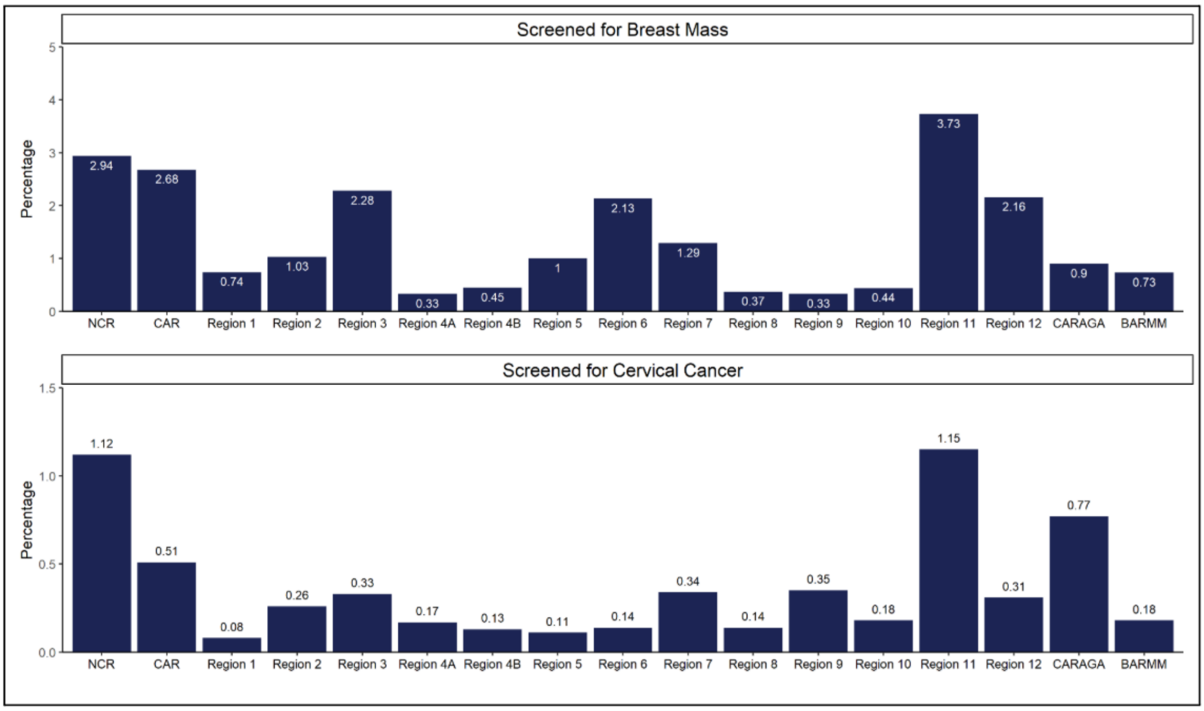

Figure 5.1: Screened for Cervical Cancer and Breast Mass, by Region, 1st Semester 2024

In the second semester, the screening rates increased in nearly all regions for both cervical and breast cancer. However, it should be noted that the screening in the second semester focused on a narrower age group: women aged 30 to 65 years. CARAGA demonstrated the most notable improvement in cervical cancer screening, rising from 0.77% in the first semester to 3.82% in the second semester. Similarly, Region 11 increased from 1.15% to 1.92%, Region 3 from 0.33% to 1.58%, and Region 8 from 0.14% to 1.20%.

Overall, the data reflect a general upward trend in cancer screening coverage across most regions during the second semester. However, variations among regions remain, highlighting the need for continued efforts to ensure equitable access to cancer screening services nationwide.

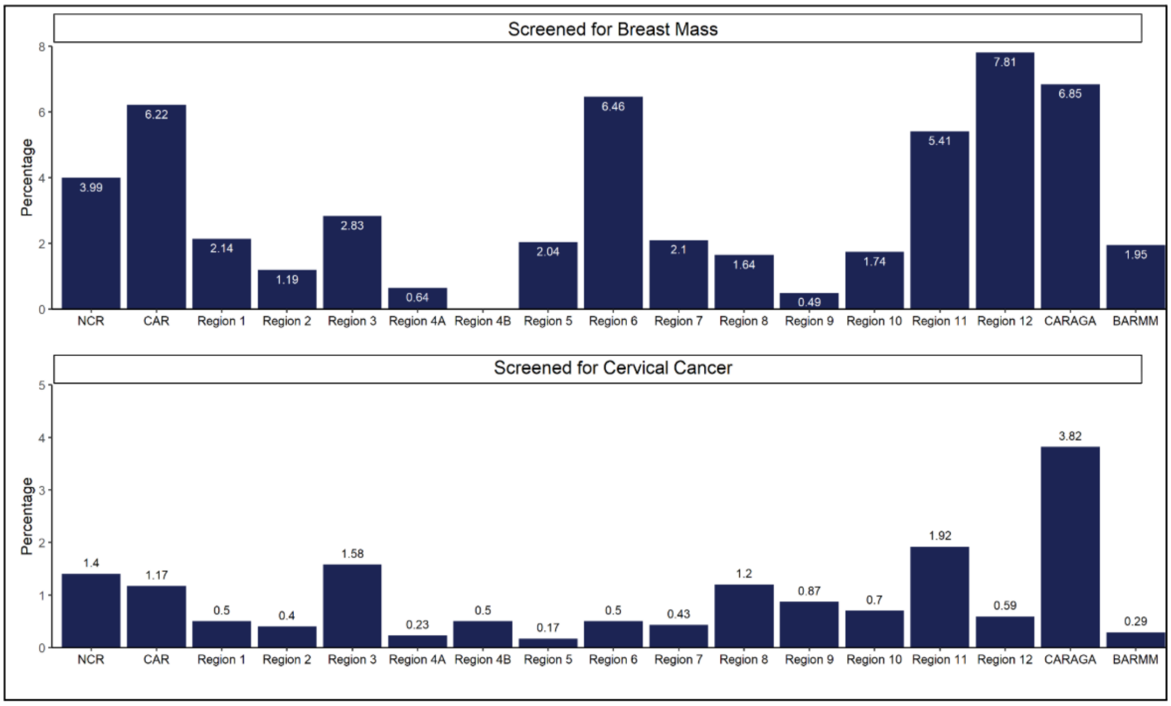

Figure 5.2: Screened for Cervical Cancer and Breast Mass, by Region, 2nd Semester 2024

1.A Family Planning Services

2024
KEY
FINDINGS

MODERN METHODS OF FAMILY PLANNING

9,031,012
women aged 10-49 years old were using modern FP methods with an increase of 4% from 2023

Pills-Combined Oral Contraceptives and injectables
remain the most commonly used methods, with injectables increasing by 7.52% and COC by 0.76%

Long-acting reversible contraceptives (LARCs)
Implant use increase by 15.41%, while postpartum IUD use also increased by 12.62%

5.71% Declining New Acceptors
mainly due to reductions in tubal ligation (-24.78%), postpartum IUD (-18.82%), and interval IUD (-40.91%)

71.88% of women of reproductive age (WRA) with Demand Satisfied of FP
The highest coverage is in Region 10 (91.07%), while the lowest is in Region 4A (46.01%)

ADOLESCENT BIRTH

24.40 births per 1,000 women
The ABR dropped down from 25.20 in 2023, with 128,717 live births recorded among adolescent mothers

1.A.1. MODERN FAMILY PLANNING SERVICES

Formula:

Current User of Modern Family Planning

CU End = CU of previous month + New Acceptor of previous month + Other acceptors of present month - Drop-out of present month

New Acceptors of Modern Family Planning

Sum of all women of reproductive age (WRA) who are new acceptors of FP method

Demand satisfied with Modern Family Planning (MFP) Methods

Numerator:	Number of WRA (15-49 years old) who are currently using, or whose partner is currently using, any MFP method
Denominator:	Total Demand (Td) for FP which refers to the number of WRA (15–49 years old) with demand for FP

The Philippines upholds the Universal Health Care (UHC) Law and the Responsible Parenthood and Reproductive Health (RPRH) Law to ensure that all Filipinos have access to affordable, high-quality healthcare services, including family planning (FP). In alignment with the Department of Health's (DOH) Eight-Point Action Agenda (8PAA), these laws reinforce the integration of reproductive health services into the healthcare system, empowering individuals and couples to make informed choices about family planning.

Expanding access to modern FP methods remains a crucial public health priority. To sustain progress, it is essential to address barriers such as contraceptive availability, quality of services, and demand generation.[1] This chapter presents the latest family planning service statistics provided by public health facilities, highlighting trends in modern contraceptive use among women of reproductive age (15-49 years).

From 2020 to 2024, the number of current users of modern FP methods in the Philippines exhibited a steady upward trend. In 2020, there were 7,366,659 users, which increased each year to reach 9,031,012 in 2024. The most notable growth occurred between 2021 and 2022, with an increase of nearly 770,000 users. While the rate of growth slowed slightly in subsequent years, the overall trend suggests a continuous adoption of modern contraceptive methods across the country *[Figure 1.A.1.1]*.

This increase reflects ongoing efforts to improve access to reproductive health services, strengthen service delivery, and enhance awareness of family planning options. Moving forward, sustaining this momentum will require a comprehensive approach, ensuring that all eligible individuals particularly those in underserved communities can access and utilize modern contraceptive methods.

---

[1] *Exploring Opportunities for mCPR Growth in Philippines, Track20, December 2022*

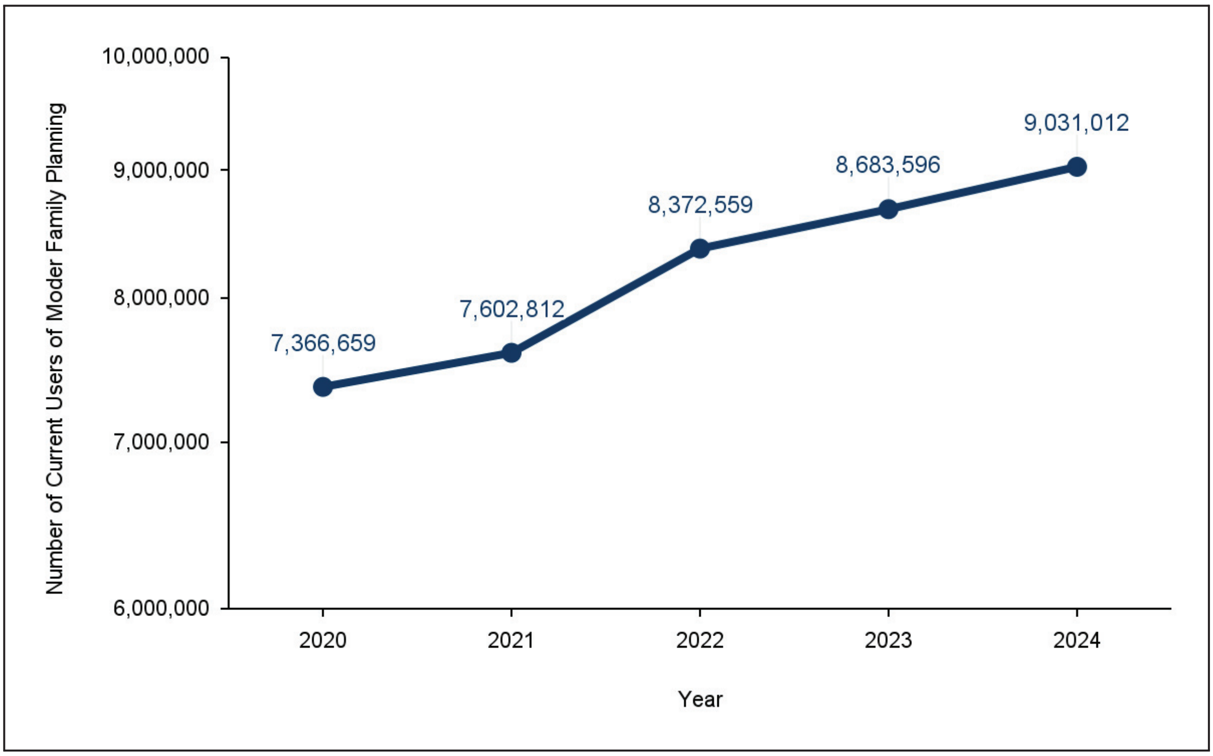

Figure 1.A.1.1: Current Users of Modern Family Planning, Philippines 2020 to 2024

Modern methods of FP use in the Philippines for 2024 shows an overall increase of 4% compared to 2023, with a total of 9,031,012 users. The most commonly used methods remain Combined Oral Contraceptive Pills (COC) and Injectables, which together account for over half of all users. COC use increased slightly by 0.76%, while Injectables saw a higher increase of 7.52% *[Table 1.A.1.1]*.

Among the methods with the highest growth, Implants recorded a 15.41% rise, and postpartum IUD use increased by 12.62%. Condom use also showed a notable increase of 12.53%. In contrast, interval IUD use declined by 6.09%. The World Health Organization (WHO) also highlighted implants in preventing pregnancies, stating that although oral pills, which are commonly used by women, may lead to 7 pregnancies per 100 women, using implants will only result in 0.1 pregnancies per 100 women[2].

While overall use of modern contraceptive methods continues to demonstrate an upward trend, a closer examination reveals a redistribution in method-specific uptake, particularly among NFP methods. Lactational Amenorrhea Method (LAM) has seen a reduction of 1.56%, Cervical Mucus Method (CCM) has decreased by 4.37%, Basal Body Temperature (BBT) use has fallen by 12.60%, and the Symptothermal Method (STM) has experienced a 5.45% drop. Additionally, male sterilization (NSV), already characterized by low uptake, has further declined by 0.61% *[Table 1.A.1.1]*.

These changes may be driven by evolving user preferences, changing perceptions of the effectiveness of these methods, or a shift in the types of family

[2] *Family planning/contraception methods Fact Sheet, World Health Organization, 2023*

planning services available. As users' needs and priorities evolve, they may choose different methods based on factors like convenience, accessibility, or perceived effectiveness. Additionally, the broader availability of diverse family planning options may encourage individuals to explore alternatives rather than discontinue family planning altogether.

Table 1.A.1.1 : Modern Contraception Use by Method, Philippines, 2024

Method	2024		2023	% change 2023 vs. 2024
	Number of Users	Percent	Number of Users	
PILLS-COC	3,016,910	33.41	2,994,278	0.76 (↑)
INJECTABLES	1,929,425	21.36	1,794,406	7.52 (↑)
IMPLANTS	911,983	10.10	790,220	15.41 (↑)
FSTR/BTL	835,637	9.25	822,843	1.55 (↑)
NFP-LAM	628,621	6.96	638,588	-1.56 (↓)
CONDOM	479,759	5.31	426,324	12.53 (↑)
PILLS-POP	475,600	5.27	457,336	3.99 (↑)
IUD-INTERVAL	390,342	4.32	415,634	-6.09 (↓)
IUD-POSTPARTUM	187,911	2.08	166,849	12.62 (↑)
NFP-SDM	120,845	1.34	120,665	0.15 (↑)
NFP-CCM	41,637	0.46	43,540	-4.37 (↓)
MSTR/NSV	7,453	0.08	7,499	-0.61 (↓)
NFP-BBT	2,809	0.03	3,214	-12.60 (↓)
NFP-STM	2,080	0.02	2,200	-5.45 (↓)
**TOTAL**	**9,031,012**	**100.00**	**8,683,596**	**4.00 (↑)**

In parallel, the number of new acceptors individuals initiating modern FP use for the first time totaled 1,016,971 in 2024. This marks a slight drop from 1,078,501 in 2023. This decrease could be attributed to several factors, including shifting preferences for specific methods, socio-economic barriers to access, and regional disparities in service delivery. The decline in first-time users may also be influenced by gaps in outreach programs and less-than-ideal accessibility of family planning services in certain areas *[Figure 1.A.1.2]*.

The continued adoption of long-acting reversible contraceptives (LARCs) such as implants and intrauterine devices (IUDs), which are over 99% effective with typical use, likely contributed to maintaining a high number of acceptors. These methods require minimal follow-up, making them a preferred choice for individuals seeking reliable, long-term contraception *[Figure 1.A.1.2]*.

Despite the slight drop from 2023, the overall trend suggests sustained interest in modern family planning, with a notable preference for highly effective and long-acting methods. The fluctuations highlight the importance of ensuring continuous access to a range of contraceptive options to support individuals in making informed choices *[Figure 1.A.1.2]*.

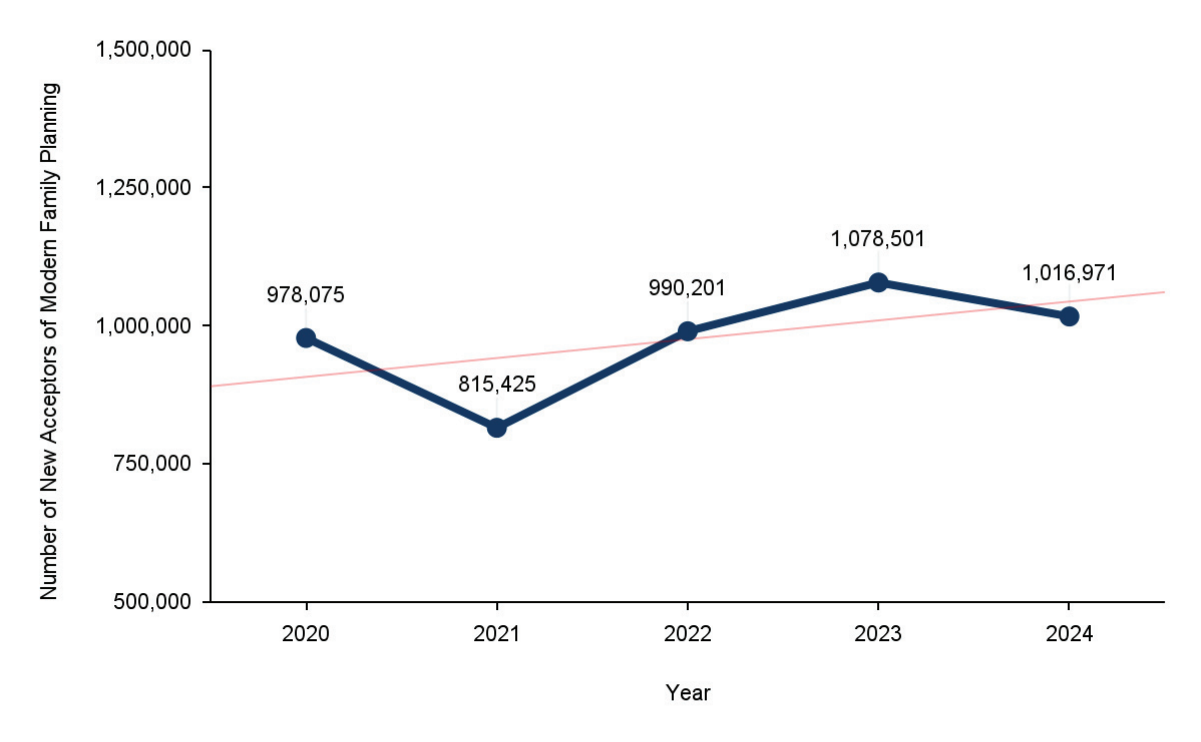

Figure 1.A.1.2 : New Acceptors of Modern Family Planning, Philippines 2020 to 2024

In 2024, the total number of new acceptors of family planning methods was 1,016,971, a 5.71% decrease from 2023. NFP-LAM remained the most used method, accounting for 38.94% of acceptors, though it declined by 3.59%. Injectables had a slight decrease of 1.12%, while oral contraceptive pills (COC) and implants dropped by 12.89% and 12.50%, respectively.

Permanent methods declined significantly, with tubal ligation decreasing by 24.78% and postpartum IUD insertions by 18.82%. Interval IUD insertions had the largest reduction at 40.91%. In contrast, condom use increased by 9.61%, and natural methods such as NFP-SDM and NFP-STM rose by 27.06% and 47.55%, respectively. Male sterilization recorded the highest increase at 52.84% *[Table 1.A.1.2]*.

Table 1.A.1.2 : New Acceptors of Modern Contraception by Method, Philippines, 2024

Method	2024		2023	% change 2023 vs. 2024
	Number of Users	Percent	Number of Users	
NFP-LAM	396,017	38.94	410,749	-3.59 (↓)
INJECTABLES	202,563	19.92	204,866	-1.12 (↓)
PILLS-COC	124,911	12.28	143,393	-12.89 (↓)
IMPLANTS	91,600	9.01	104,684	-12.50 (↓)
PILLS-POP	84,906	8.35	90,084	-5.75 (↓)
CONDOM	68,739	6.76	62,711	9.61 (↑)
FSTR/BTL	18,275	1.80	24,294	-24.78 (↓)
IUD-POSTPARTUM	14,817	1.46	18,251	-18.82 (↓)
IUD-INTERVAL	7,544	0.74	12,766	-40.91 (↓)
NFP-SDM	4,884	0.48	3,844	27.06 (↑)
NFP-CCM	1,085	0.11	1,381	-21.43 (↓)

Method	2024		2023	% change 2023 vs. 2024
	Number of Users	Percent	Number of Users	
NFP-BBT	629	0.06	816	-22.92 (↓)
MSTR/NSV	700	0.07	458	52.84 (↑)
NFP-STM	301	0.03	204	47.55 (↑)
**TOTAL**	**1,016,971**	**100.00**	**1,078,501**	**-5.71** (↓)

As we examine the shifts in contraceptive method use and the trends in new acceptors, it becomes crucial to also assess the extent to which demand for modern family planning methods is being satisfied. The concept of demand satisfied measures the proportion of individuals who desire contraception and are able to access and use modern methods effectively.

Since 2023, the Proportion of the Demand for Family Planning Satisfied by Modern Methods has been included as an indicator in the Field Health Services Information System (FHSIS). This indicator provides a more precise measure than the modern contraceptive prevalence rate, as it specifically accounts for women with an identified need for modern family planning[3]. Additionally, it requires an estimate of total demand for family planning, which is derived by applying the total demand factor to the total Women of Reproductive Age (WRA) population. This computation follows the Department Memorandum 2024-0214, titled *"Annual Targets for Maternal Health Indicators, Demand Satisfied with Modern Family Planning Methods, and Adolescent Birth Rate."*

For CY 2024, this indicator remains a key metric for assessing the extent to which the need for modern family planning is met, ensuring data-driven decision-making in family planning programs. In the Philippines, 71.88% of women of reproductive age (WRA) are currently using family planning methods, with 9,023,602 users out of a total demand factor of 12,554,137. The highest percentage of users is recorded in Region 10 at 91.07%, followed by CARAGA at 90.71% and NCR at 85.32. Other regions with relatively high percentages include CAR at 85.32%, Region 1 at 84.49%, Region 9 at 84.67%, Region 12 at 84.09% and Region 11 at 80.53%. Several regions, such as Region 4A (79.54%), Region 3 (74.95%), Region 5 (71.38%), Region 2 (73.51%), and Region 6 (73.46%), surpasses the national average *[Table 1.A.1.3]*.

Region 4A has the lowest percentage of family planning users at 46.01%, followed by Region 7 at 50.14%. These regions have the lowest recorded percentages among all areas. Other regions, such as Region 8 (69.28%) and BARMM (70.03%), have higher recorded percentages. The distribution of family planning users varies across regions, with some areas showing significantly higher or lower percentages than the national average *[Table 1.A.1.3]*.

[3] *Guideline on the New Indicator Demand Satisfied with Modern Family Planning Methods for the Program Expenditure Classification (PREXC) for Family Planning (Department Memorandum 2022-0417)*

Table 1.A.1.3. Demand for Family Planning Satisfied by Modern Methods, by Region, 2024

Area	Estimated Demand for Family Planning (Total WRA x Total Demand Factor)	Current Users of Modern FP (WRA only)	%Demand Satisfied by Modern FP
**PHILIPPINES**	**12,554,137**	**9,023,602**	**71.88**
NCR	1,419,597	1,211,268	85.32
CAR	201,786	172,024	85.25
Region 1	603,589	509,950	84.49
Region 2	480,684	352,474	73.51
Region 3	1,422,512	1,066,107	74.95
Region 4A	1,982,927	912,403	46.01
Region 4B	370,463	294,677	79.54
Region 5	669,763	478,059	71.38
Region 6	910,199	668,596	73.46
Region 7	968,334	485,526	50.14
Region 8	493,882	342,180	69.28
Region 9	444,737	376,574	84.67
Region 10	594,090	541,053	91.07
Region 11	671,512	540,780	80.53
Region 12	566,630	476,475	84.09
CARAGA	328,137	297,641	90.71
BARMM	425,296	297,815	70.03

Note: This only considers women of reproductive age (15 to 49 years old)

1.A.2. VITAL STATISTICS

Formula:
Number of live births

Sum of all live births in a given year

Adolescent Birth Rate

Numerator: Number of live births to adolescent women
Denominator: Projected population 10-14, 15-19, 20-49 years old (female only)

Adolescent pregnancies remain a significant public health concern, influencing both the well-being of young mothers and the broader societal outcomes. Monitoring trends in the Adolescent Birth Rate (ABR) is crucial to understanding shifts in reproductive health patterns and evaluating the effectiveness of interventions. This chapter hereby presents the service statistics for Adolescent birth rate.

In 2024, the Adolescent Birth Rate (ABR) stands at 24.40 births per 1,000 women aged 15 to 19, showing a decrease of 0.80 from the 25.20 recorded in 2023. The number of live births among 15-19 years old in 2024 is 128,717, lower than the 132,157 reported in 2023. Between 2022 and 2023, the ABR increased by 0.84, rising from 24.36 in 2022 to 25.20 in 2023. In contrast, the period from 2021 to 2022 saw a larger increase of 2.05, with the ABR moving from 22.31 in 2021 to 24.36 in 2022. The largest recorded decrease occurred from 2020 to 2021, when the ABR dropped by 1.77, from 24.08 in 2020 to 22.31 in 2021 *[Figure 1.A.2.1]*.

The Omnibus Health Guidelines stress the need for accessible and effective family planning services to reduce adolescent pregnancies[4]. The fluctuations in the ABR between 2020 and 2024 highlight changes in adolescent reproductive health trends. They also emphasize the importance of regularly assessing the effectiveness of family planning programs. The decline in ABR from 2023 to 2024 reflects improvements in contraceptive access and reproductive health services. However, the earlier increases point to varying levels of service coverage, awareness, or accessibility.

Adolescent pregnancies remain a critical issue under Sustainable Development Goal (SDG) 3.7, which aims to ensure universal access to reproductive health services, including contraception and education, by 2030[5]. Monitoring annual changes in the adolescent birth rate is essential for tracking progress toward national and global reproductive health goals. Reducing adolescent pregnancies is key to achieving the targets of SDG 3.7 and enhancing the effectiveness of family planning initiatives.

---

[4] *Omnibus Health Guidelines for Adults Version 2023*
[5] *Sustainable Development Goal (SDG) Target 3.1 2015*

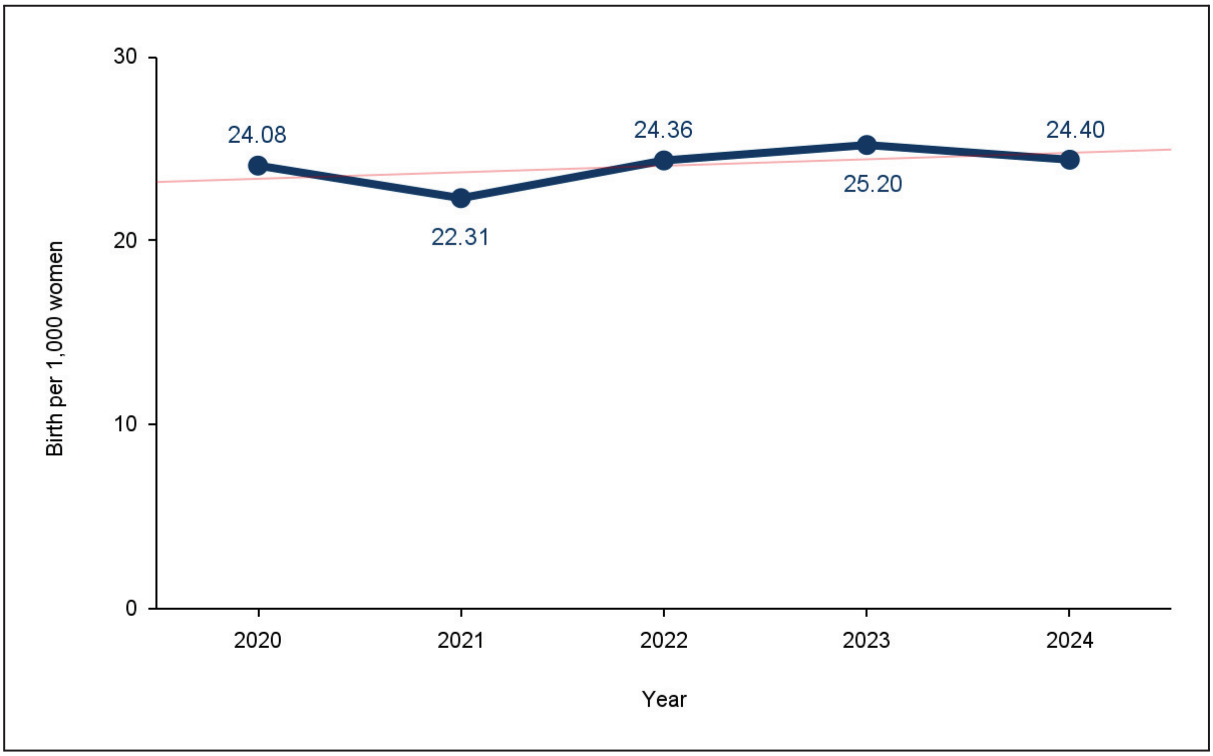

Figure 1.A.2.1. Adolescent Birth Rate, Philippines, 2020 to 2024

The total number of live births among adolescent women aged 15-19 in the Philippines is 128,717. Among all the regions, Region 3 recorded the highest absolute number of adolescent live births, with 13,093 cases. This was closely followed by Region 4A, which had 12,594 live births, and Region 7, with 11,136. The National Capital Region (NCR) also had a notable number, with 11,459 cases *[Table 1.A.4]*.

Regions with relatively lower counts include the Cordillera Administrative Region (CAR) with 2,122 live births, Region 2 with 3,617, and Region 9 with 4,019. Caraga and BARMM also had relatively fewer live births, with 4,745 and 5,126, respectively. Other regions showed moderate numbers, such as Region 10 with 8,725, Region 11 with 10,524, and Region 12 with 8,896. The count in Region 6 reached 7,249, while Region 8 had 6,887 live births. Region 5 recorded 8,575 cases, and Region 4B had 5,642 *[Table 1.A.2.1]*.

Table 1.A.2.1. Adolescent Birth Rate, by Region, 2024

Area	Livebirth among 15-19 yrs old	ABR per 1,000 women
**PHILIPPINES**	**128,717**	**24.40**
NCR	11,459	20.19
CAR	2,122	23.37
Region 1	4,308	17.46
Region 2	3,617	21.05
Region 3	13,093	22.67
Region 4A	12,594	17.02
Region 4B	5,642	33.07
Region 5	8,575	25.41

Area	Livebirth among 15-19 yrs old	ABR per 1,000 women
Region 6	7,249	19.35
Region 7	11,136	28.19
Region 8	6,887	28.93
Region 9	4,019	19.99
Region 10	8,725	33.88
Region 11	10,524	41.63
Region 12	8,896	39.10
Caraga	4,745	32.65
BARMM	5,126	18.22

1.B Maternal Health

2024 KEY FINDINGS

ANTENATAL CARE SERVICES

84.52%
of women who gave birth had at least four (4) Antenatal care (ANC) visits

22.22%
of women who were pregnant for the first time completed Tetanus-Diphtheria vaccination

33.93%
of women with repeat pregnancies completed Tetanus-Diphtheria (Td2 Plus) vaccination

INTRAPARTUM AND POSTNATAL CARE SERVICES

94.00%
of deliveries occurred in Health Facility (FBD)

95.07%
of deliveries were attended by Skilled Health Professionals

93.99%
of postpartum women had at least two (2) postnatal care (PNC) visit

MATERNAL DEATHS

64.85
Deaths per 100,000 live births
Maternal Mortality Ratio (MMR)

1.B.1. ANTENATAL CARE SERVICES

Formula:

Women who gave birth with at least 4 antenatal check-ups

Numerator: Number of women who gave birth with at least 4 or more antenatal check-ups
Denominator: Total number of deliveries

Women pregnant for the first time given Tetanus diphtheria (Td) vaccination

Numerator: Number of pregnant women for the first time given at least 2 doses of Td vaccine
Denominator: Eligible population 0-11 mos

Pregnant women with repeat pregnancy given Tetanus Diphtheria (Td) Vaccination

Numerator: Number of pregnant women for the 2nd or more times given at least 3 doses of Td vaccine (Td2 Plus)
Denominator: Eligible population 0-11 mos

Maternal health services are essential in ensuring the safety and well-being of women during pregnancy, childbirth, and the postpartum period. Many maternal deaths are preventable through timely medical care, skilled birth attendance, and proper antenatal and postnatal support. Strengthening health systems and improving access to quality maternal care remain crucial in reducing risks and complications.

The Omnibus Health Guidelines for Adult emphasize the need for early detection and management of high-risk pregnancies, essential vaccinations, and access to emergency obstetric and newborn care[1]. Comprehensive family planning services, improved referral systems, and financial risk protection through PhilHealth further support safe motherhood.

The Philippines is committed to reducing maternal mortality to fewer than 70 deaths per 100,000 live births by 2030[2], following the Eight-Point Action Agenda (8PAA). This includes expanding antenatal care, by increasing facility-based deliveries, strengthening emergency obstetric care, and ensuring skilled health providers.

This chapter presents national data on antenatal care visits, tetanus-diphtheria vaccination, skilled birth attendance, delivery outcomes, and postnatal care. Timely and high-quality care is critical in preventing complications, ensuring safe pregnancies, and improving maternal and newborn health.

In the Philippines, the percentage of women who gave birth after receiving at least four antenatal check-ups has fluctuated over the years, reaching a low of 76.68% in 2023 and peaking at 85.28% in 2021. The most recent data from 2024 shows an Antenatal Care (ANC) coverage of 84.53%, surpassing the 2021 level, suggesting a potential recovery from the decline observed in 2022 and 2023.

---

[1] *Omnibus Health Guidelines for Adults Version 2023*
[2] *Sustainable Development Goal (SDG) Target 3.1 2015*

Td vaccination coverage among first-time pregnant women showed a consistent downward trend, decreasing from 25.67% in 2020 to 19.85% in 2023 before slightly increasing to 22.22% in 2024. Similarly, the vaccination accomplishment among women with repeated pregnancies declined from 40.64% in 2020 to 32.16% in 2023, with a slight increase to 33.93% in 2024. This pattern suggests that Td vaccine coverage has generally been decreasing over time, despite fluctuations in ANC coverage *[Figure 1.B.1.1]*.

A potential relationship emerges between ANC visits and Td vaccination coverage. Although a higher percentage of women received at least four ANC check-ups in 2021 (85.28%), Td vaccination coverage for both first-time and repeated pregnancies were lower than in 2020. A similar pattern is observed in 2024, where ANC coverage increased from 76.68% in 2023 to 84.53%. However, Td vaccination coverage did not rise proportionally. While Td vaccination in women pregnant for the first time increased slightly from 19.85% to 22.22%, and coverage in women with repeated pregnancy increased from 32.16% to 33.93%, these gains remain modest. This suggests that while more women are attending ANC visits, Td vaccine coverage is not necessarily improving at the same rate *[Figure 1.B.1.1]*.

The declining Td vaccination coverage, despite relatively stable ANC coverage, may indicate gaps in Td vaccine administration during antenatal visits. Possible reasons for this discrepancy include vaccine stockouts and missed opportunities during ANC consultations. Additionally, alternative vaccines, such as Tdap, may be administered in some cases, particularly in private healthcare facilities[3]. These instances may not be fully captured in the FHSIS indicator for CY 2024.

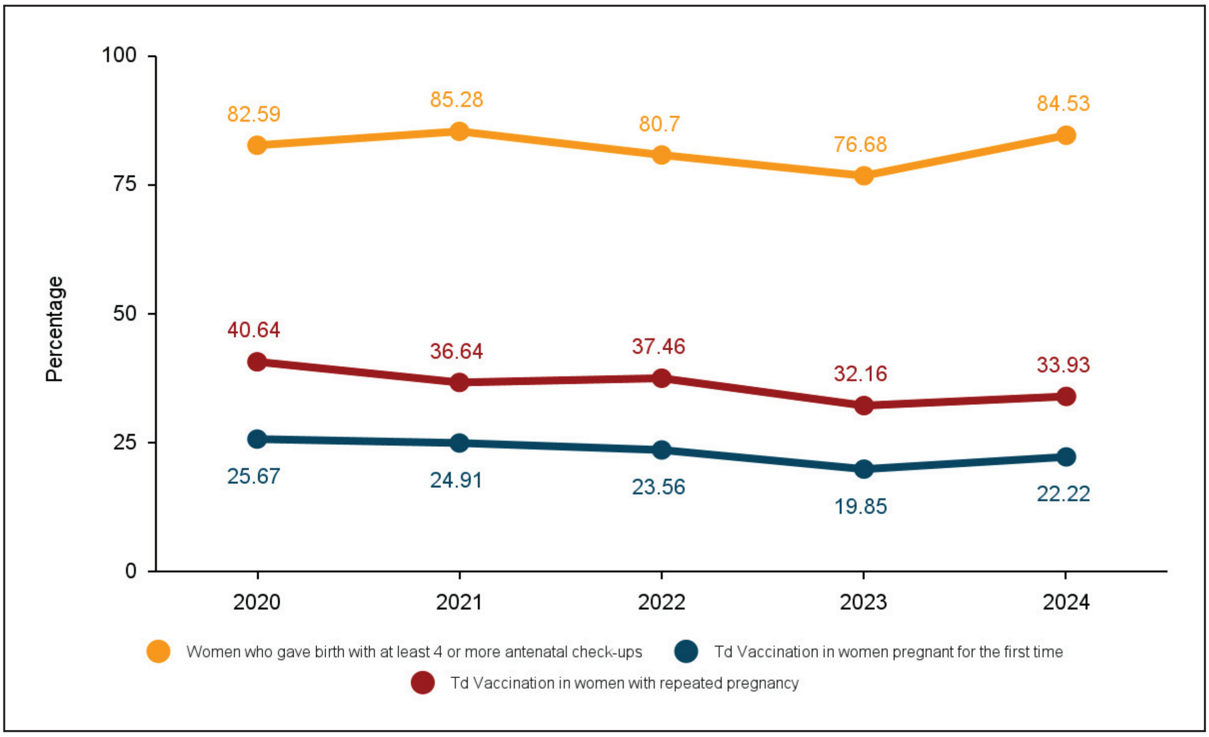

Figure 1.B.1.1: Antenatal Care Indicators, Philippines, 2020 to 2024

[3] *American College of Obstetricians and Gynecologists. ACOG Committee Opinion No. 718: Update on Immunization and Pregnancy: Tetanus, Diphtheria, and Pertussis Vaccination. Obstet Gynecol. 2017;130(3):e153–e157. Reaffirmed 2020*

In 2024, Regional data on Antenatal care indicators show significant variations in antenatal care and tetanus-diphtheria (Td) vaccination coverage across different areas. The percentage of women who gave birth after receiving at least four antenatal check-ups fluctuates widely, with Region 1 (113.64%) and Region 3 (104.30%) reporting figures exceeding 100%, which may indicate the inclusion of non-resident patients. The lowest recorded percentage is in Region 7 (62.01%). This indicator is included in the Local Government Unit (LGU) Health Scorecard to assess the accessibility and utilization of maternal healthcare services. Ensuring that pregnant women receive at least four antenatal visits is crucial for monitoring pregnancy progress, detecting complications early, and providing necessary interventions to improve maternal and neonatal health outcomes *[Figure 1.B.1.2]*.

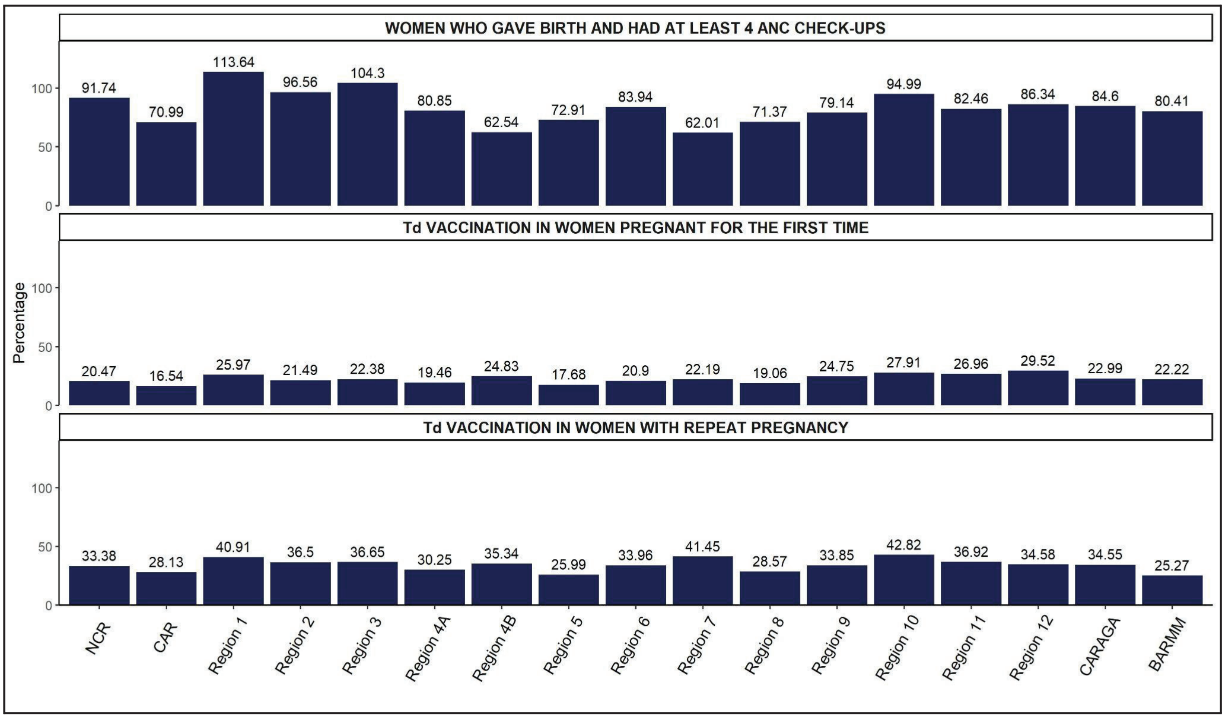

Figure 1.B.1.2: Antenatal Care Indicators, by Region, 2024

Additionally, the data on Td vaccination coverage for women pregnant for the first time and those with repeated pregnancies also highlight regional disparities. The highest recorded vaccination coverage for first-time pregnancies is in Region 12 (29.52%), which is noticeably higher than other regions, where coverage ranges between 16.54% and 29.52%. Vaccination status for women with repeated pregnancies are relatively higher, with Region 10 (42.82%) and Region 1 (40.91%) showing the highest coverage. This trend suggests that prior exposure to maternal healthcare services may contribute to better vaccination uptake among women with previous pregnancies, where coverage ranges between 25.27% and 42.82%. The variations in Td vaccination coverage emphasize the need for strengthened immunization efforts, particularly for first-time pregnancies, to ensure adequate protection against maternal and neonatal tetanus *[Figure 1.B.1.2]*.

While Td vaccination coverage offers valuable insights into preventive maternal health interventions, it is equally important to examine broader maternal health outcomes. In particular, understanding the relationship between antenatal care (ANC) utilization and maternal mortality provides a more comprehensive view of

the healthcare system's effectiveness in safeguarding mothers during pregnancy and childbirth

In the Philippines, maternal mortality rate (MMR) is recognized as one of the eight (8) priority health outcomes, reflecting its critical role in assessing Philippines overall health system performance, and two important indicators to measure it are the coverage of women who had at least four antenatal check-ups before giving birth and the maternal mortality rate (MMR), which represents the number of mothers who died per 100,000 live births. These indicators are directly linked to SDG 3, which aims to ensure healthy lives and promote well-being for all at all ages, particularly through the reduction of maternal mortality. Examining data from 2020 to 2024 reveals some notable trends in these indicators

Generally, having more antenatal check-ups is expected to help reduce maternal deaths by enabling the early detection and management of pregnancy-related risks. The Omnibus Health Guidelines for Adults (OHG) highlights the importance of routine antenatal check-ups for early detection of pregnancy risks, including screening for infectious diseases, providing nutritional supplements, and delivering health education key strategies for reducing maternal mortality[4]. However, the data does not always follow this expected pattern. In 2021, the highest accomplishment of women (85.28%) received at least four check-ups, yet the maternal mortality rate was also at its highest (84.86%). Meanwhile, in 2023, fewer women (76.68%) received sufficient antenatal care, but the MMR was at its lowest 62.94% *[Figure 1.B.1.3]*.

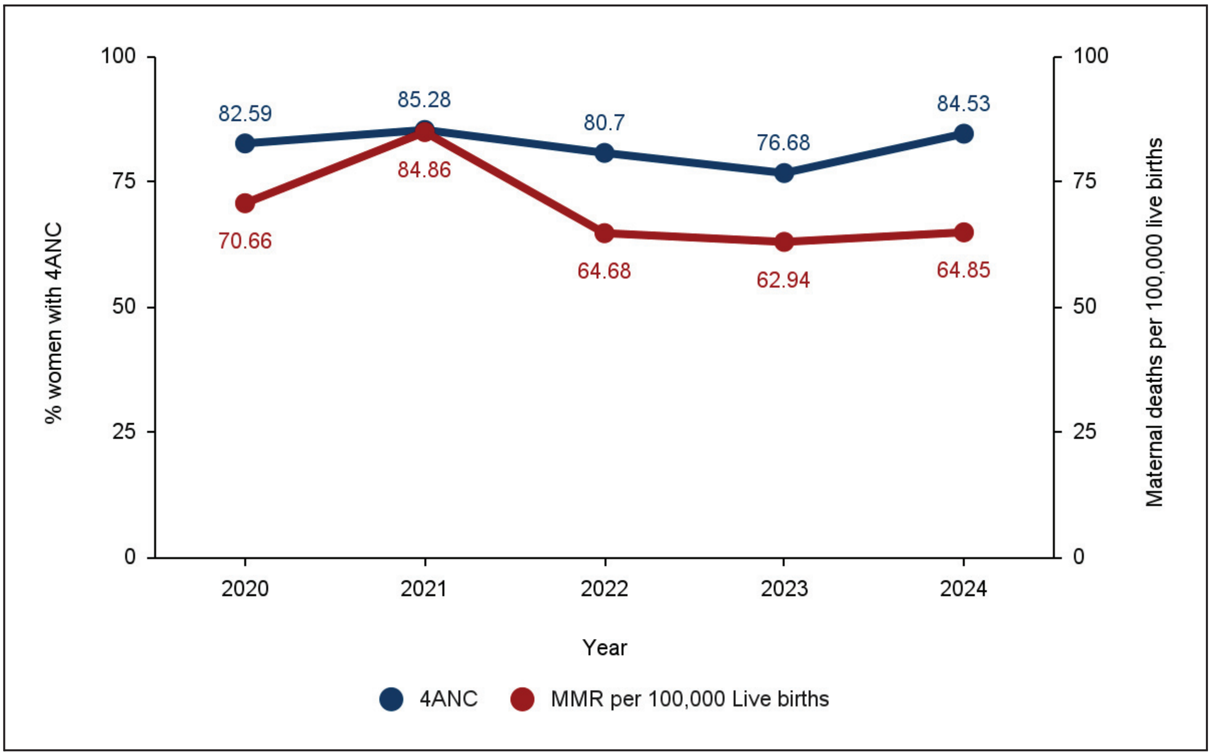

Figure 1.B.1.3: Antenatal Care Coverage and Maternal Mortality Rate, Philippines, 2020 to 2024

This suggests that while antenatal care is essential, it is not the only factor affecting maternal deaths. Other elements, such as the availability of skilled health

[4] *Department of Health (DOH). Omnibus Health Guidelines for Adults, 2023*

workers, the quality of hospital services, and the ability to manage pregnancy complications, also play a major role. Even if many women receive regular check-ups, lack of access to quality and timely health care can still lead to high maternal deaths.

1.B.2. INTRAPARTUM CARE AND DELIVERY OUTCOMES

Formula:

Facility-based Delivery (FBD)

Numerator: Number of deliveries in health facilities (clinics, BHS, RHUs, lying-ins, hospitals, etc.)
Denominator: Total number of deliveries

Births attended by Skilled Health Professionals (SHP)

Numerator: Number of deliveries attended by skilled health professionals (licensed midwives, doctors, or nurses)
Denominator: Total number of deliveries

Intrapartum care is essential for ensuring safe childbirth and improving maternal and neonatal outcomes. Many birth-related complications can be prevented or managed through skilled healthcare providers, continuous monitoring, and timely interventions. Quality intrapartum care reduces maternal and neonatal morbidity and mortality, emphasizing the need for well-equipped facilities and adherence to evidence-based practices

This chapter hereby presents data on facility-based deliveries (FBD), skilled health professionals (SHP), and live births with low birth weights (LBW) across the country.

In the Philippines, maternal intrapartum care has shown improvements over the years, as seen in the increasing numbers of births attended by SHP and FBD from 2020 to 2024. Births attended by SHP rose from 90.15% in 2020 to 95.07% in 2024, while FBD increased from 89.41% to 94.00% during the same period. These trends reflect efforts to enhance maternal intrapartum and newborn care services, ensuring that more women give birth under safe conditions *[Figure 1.B.2.1]*.

However, antenatal care coverage, measured by the percentage of women receiving at least four check-ups before delivery, has fluctuated. It peaked at 85.28% in 2021, but dropped significantly to 76.68% in 2023 before recovering to 84.53% in 2024 *[Figure 1.B.2.1]*.

Completing at least four antenatal check-ups plays a crucial role in increasing FBD and SHP. Health professionals emphasize safe delivery practices during each antenatal visit, educate mothers on the importance of giving birth in a healthcare facility, and identify potential pregnancy complications early. The decline in antenatal check-ups in 2023 coincided with a slower increase in FBD from 91.69% in 2022 to 92.96% in 2023 and SHP from 92.95% to 93.97% *[Figure 1.B.2.1]*.

Ensuring consistent access to antenatal care is essential in preventing maternal and fetal deaths. Women who attend regular check-ups are more likely to receive proper medical guidance and interventions that can reduce pregnancy-related complications. Strengthening maternal health programs and addressing barriers to antenatal care and safe delivery will be key to sustaining

these positive developments and further improving maternal and newborn health outcomes in the country.

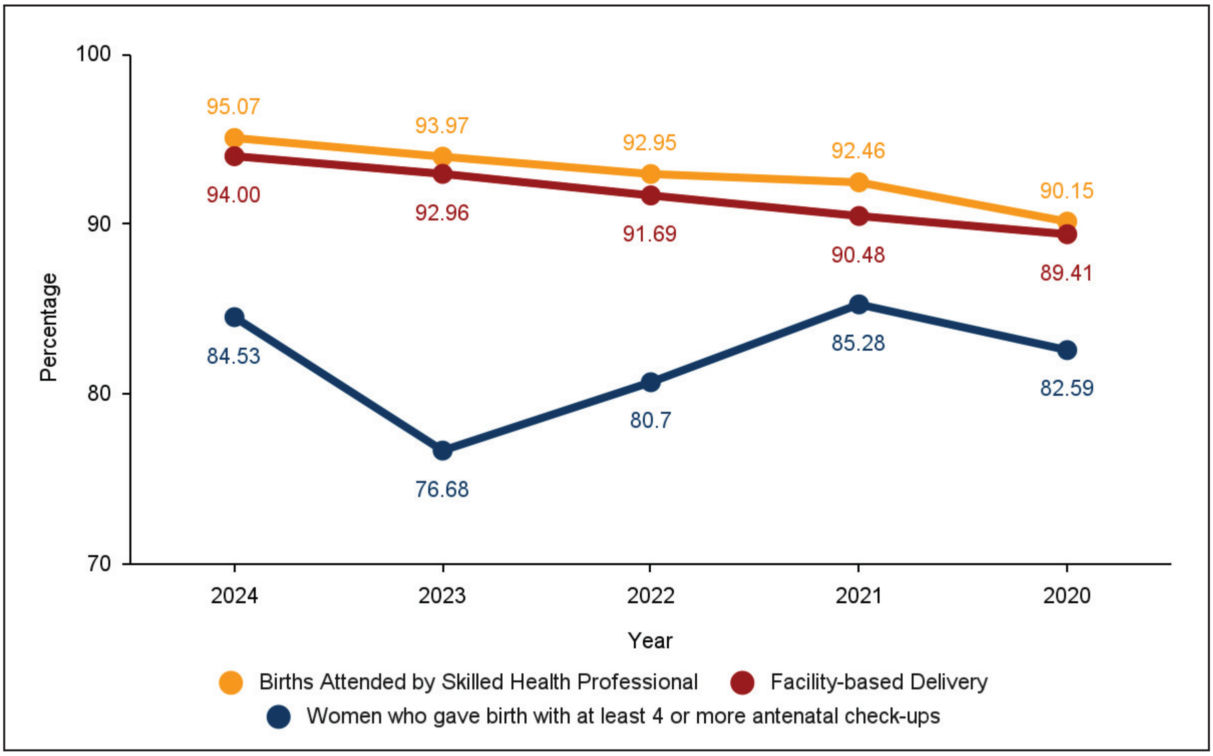

Figure 1.B.2.1: 4ANC, Facility-based Deliveries and Skilled Health Professional, Philippines, 2020 to 2024

In 2024, regional data showed varying performance in deliveries attended by SHP and FBD across the country. With an annual target of 95% for both indicators, several regions met or exceeded this benchmark for skilled birth attendance, including Region 1 (99.87%), NCR (99.00%), Region 3 (98.75%), Region 2 (98.64%), CAR (98.15%), CARAGA (97.47%), Region 5 (96.78%), Region 8 (96.40%), Region 11 (95.90%), and Region 6 (95.75%). These figures suggest adequate availability of professional healthcare services during childbirth in those areas *[Table 1.B.2.1]*.

For facility-based deliveries, most regions also demonstrated high coverage, though typically lower than their skilled health professional percentage, which is consistent with expected patterns as some skilled births may occur outside formal health facilities (e.g. home deliveries). Region 1 reported the highest FBD coverage at 99.83%, followed closely by NCR at 98.85% Region 2 at 98.35% and Region 3 at 98.27%, all surpassing the national target *[Table 1.B.2.1]*.

It should be noted that BARMM is lagging behind both in terms of SHP and FBD possibly reflecting challenges in health infrastructure and human resources for health (HRH)[5] as well as socio-cultural barriers[6]

[5] The health system in BARMM faces challenges including a shortage of health workers, with a low ratio of health personnel per capita due to migration, inadequate job opportunities, and limited career development (HRH2030, 2021)
[6] Geographic and socio-cultural barriers, such as governance issues and cultural norms, further impede health service delivery and utilization in the region (World Bank, 2020; University Research Co., LLC, 2023).

Table 1.B.2.1: Facility-based Deliveries and Skilled Health Professional, by Region, 2024

Region	Total Deliveries	Skilled Health Professional	Facility-based Delivery
N C R	183,911	99.00%	98.85%
C A R	23,991	98.15%	96.99%
Region 1	51,470	99.87%	99.83%
Region 2	33,193	98.64%	98.35%
Region 3	141,448	98.75%	98.27%
Region 4A	199,246	94.91%	93.67%
Region 4B	42,540	87.10%	86.23%
Region 5	85,695	96.78%	96.25%
Region 6	85,521	95.75%	95.58%
Region 7	107,656	96.63%	96.32%
Region 8	58,689	96.40%	96.09%
Region 9	31,545	82.32%	82.10%
Region 10	63,866	93.80%	91.07%
Region 11	73,746	95.90%	95.74%
Region 12	61,770	94.30%	94.23%
BARMM	60,678	72.27%	60.64%
CARAGA	38,638	97.47%	97.31%

These high coverage of facility-based deliveries contribute significantly to ensuring safer births and improved health outcomes for both mothers and newborns. One such outcome that is closely monitored is birth weight, a critical indicator of maternal and newborn health[7].

Birth weight refers to the weight of live-born newborns measured within the first hour of life classified as greater than 2500 grams (normal), less than 2500 grams (low), or unknown. It is a key indicator of newborn health and reflects the quality of maternal and child health services.

In 2024, most regions reported high proportions of normal birth weights, ranging from 82.33% to 96.47%. NCR had the highest at 96.47%, followed by Region 2 (94.13%) and Region 12 (93.82%). Region 9 had the lowest at 82.33%, linked to a higher accomplishment of unknown weights *[Table 1.B.2.2]*.

Low birth weight among all regions, CAR recorded the highest proportion of LBW at 10.82%. This is closely followed by Region 5 and 6 at 9.99% and 8,26%. Additionally, the lowest proportion of LBW were observed in BARMM, Region 12 and NCR. While NCR's figure may reflect better access to health services and higher urban healthcare infrastructure, BARMM's exceptionally low LBW percentage may warrant further examination, especially considering it also has a relatively high percentage of births with unknown weight (7.88%). This could suggest underreporting or data gaps rather than truly low incidence, highlighting a potential issue with data quality or coverage in that region *[Table 1.B.2.2]*.

Unknown birth weights varied widely across the regions, indicating differences in data recording and reporting. Region 9 reported the highest proportion of unknown birth weights at 12.61%, followed by BARMM at 7.88%, while Region 1 and Region 2 had the lowest at 0.02% and 0.11%, respectively. These discrepancies suggest the

[7] *Pediatric Health, Medicine and Therapeutics 2021*

need to improve data recording and monitoring across regions. Despite most births being within the normal weight range, the presence of high proportions of unknown birth weights in some areas highlights persistent gaps in both health outcomes and data quality *[Table 1.B.2.2]*.

Table 1.B.2.2: Live births Birth weight, by Region, 2024

Region	Total Live births	Normal Birth Weight (≥ 2500 grams)	Low Birth Weight (< 2500 grams)	Unknown Birth Weight
NCR	183,878	96.47%	3.20	0.33
CAR	23,873	89.10%	10.82	0.08
Region 1	51,383	92.76%	7.21	0.02
Region 2	33,145	94.13%	5.77%	0.11%
Region 3	140,769	92.60%	7.01%	0.39%
Region 4A	196,166	93.03%	5.08%	1.88%
Region 4B	42,233	90.49%	6.08%	3.43%
Region 5	85,536	88.24%	9.99%	1.78%
Region 6	85,513	90.83%	8.26%	0.91%
Region 7	107,859	90.77%	8.00%	1.22%
Region 8	58,580	90.24%	7.73%	2.03%
Region 9	31,451	82.33%	5.06%	12.61%
Region 10	63,659	93.26%	3.84%	2.89%
Region 11	73,703	90.97%	6.00%	3.03%
Region 12	61,644	93.82%	3.50%	2.67%
BARMM	60,588	90.48%	1.64%	7.88%
CARAGA	38,525	92.59%	5.64%	1.77%

1.B.3. POSTNATAL CARE SERVICES

Formula:

Women together with their newborn who completed at least 2 postpartum check-ups

Numerator: Number of postpartum women and their newborn who completed at least 2 postnatal check-ups
Denominator: Total number of deliveries

Postpartum women who completed Iron with Folic Acid supplementation

Numerator: Number of postpartum women who completed iron with folic acid supplementation
Denominator: Eligible population 0-11 mos

Postpartum women who completed Vitamin A supplementation

Numerator: Number of postpartum women who completed Vitamin A supplementation
Denominator: Eligible population 0-11 mos

Most maternal and infant deaths occur within the first six (6) weeks after delivery and are often due to preventable causes. Therefore, providing timely and comprehensive care during the postpartum period is crucial to prevent and manage complications, safeguard the health of both mother and child, and support birth planning[8]. The first postnatal check-up should be conducted within 24 hours after delivery, followed by a second check-up within seven (7) days. Women who experience stillbirths are also required to undergo at least two (2) postnatal check-ups. These early postnatal visits are critical opportunities to assess the mother's physical and emotional well-being, promote newborn care practices, and identify any warning signs. Ensuring access to quality postnatal services is essential for improving maternal and newborn outcomes and reducing preventable mortality.

This chapter presents the coverage of postpartum women who completed at least two (2) postnatal check-ups, along with the coverage of vitamin A and/or iron supplementation among postpartum women.

The coverage of postnatal care (PNC) services improved steadily from 2020 to 2024. The coverage increased from 85.63% to 90.86% between 2020 and 2021 indicating a significant rise in the provision or utilization of postnatal care services. While the coverage decreased slightly to 88.69% in 2022, it recovered to 90.58% in 2023 and continued to increase to its highest point to date at 93.99% in 2024 *[Figure 1.B.3.1]*.

This upward trend suggests that there have been successful efforts to improve access to and utilization of postnatal care services. While there were minor fluctuations in 2022 and 2023, the overall increase in coverage highlights ongoing progress in strengthening postnatal healthcare services *[Figure 1.B.3.1]*.

---

[8] *WHO Maternal Mortality Factsheets 2025*

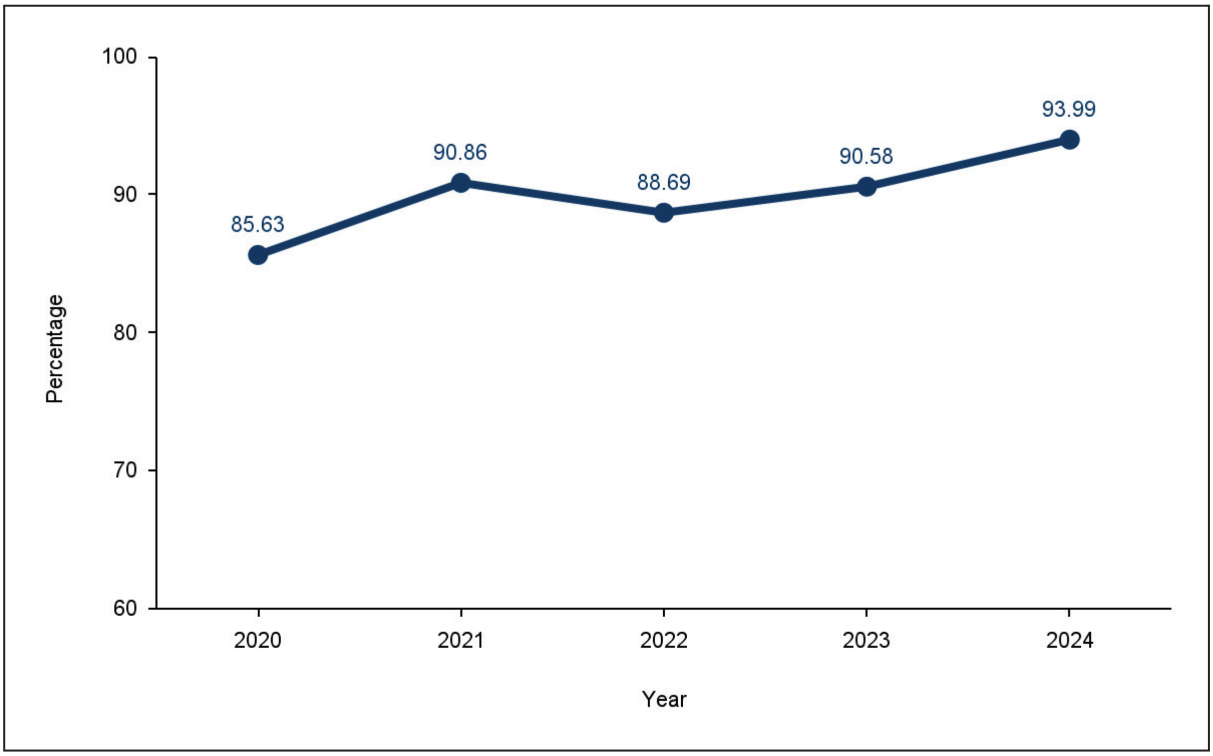

Figure 1.B.3.1: Women with at least 2 Postnatal Check-ups, Philippines, 2020 to 2024

While national-level statistics reveal sustained growth in 2PNC coverage, the regional breakdown offers a more nuanced understanding of the progress and remaining gaps across the country. In 2024, some regions recorded 2PNC coverage above the national target of 95%. Region 1 (113.4%) and Region 3 (105.74%) reported the highest coverage, followed by NCR (98.58%), Region 10 (97.29%), Region 11 (95.25%), and Region 12 (96.32%), all meeting or surpassing the set national benchmark. These figures indicate that more than half of the regions have reached or exceeded the target.

Other regions showed moderate coverage but remained below the national target. Regions such as Region 2 (93.91%), Region 6 (93.78%), Region 9 (92.38%), Region 7 (90.29%), Region 4A (89.65%), Region 5 (88.29%), Region 4B (88.09%), and BARMM (89.79%) are close to the target but did not meet the 95% national target *[Figure 1.B.3.2]*

Meanwhile, a few regions reported notably lower coverage levels. CAR (73.29%), Region 8 (81.96%), and CARAGA (83.23%) posted the lowest figures in 2024, highlighting a wider gap from the national target compared to other regions *[Figure 1.B.3.2]*. However, it also emphasizes the need for continued monitoring and focused efforts to ensure that all regions are able to meet the national target of 95% coverage.

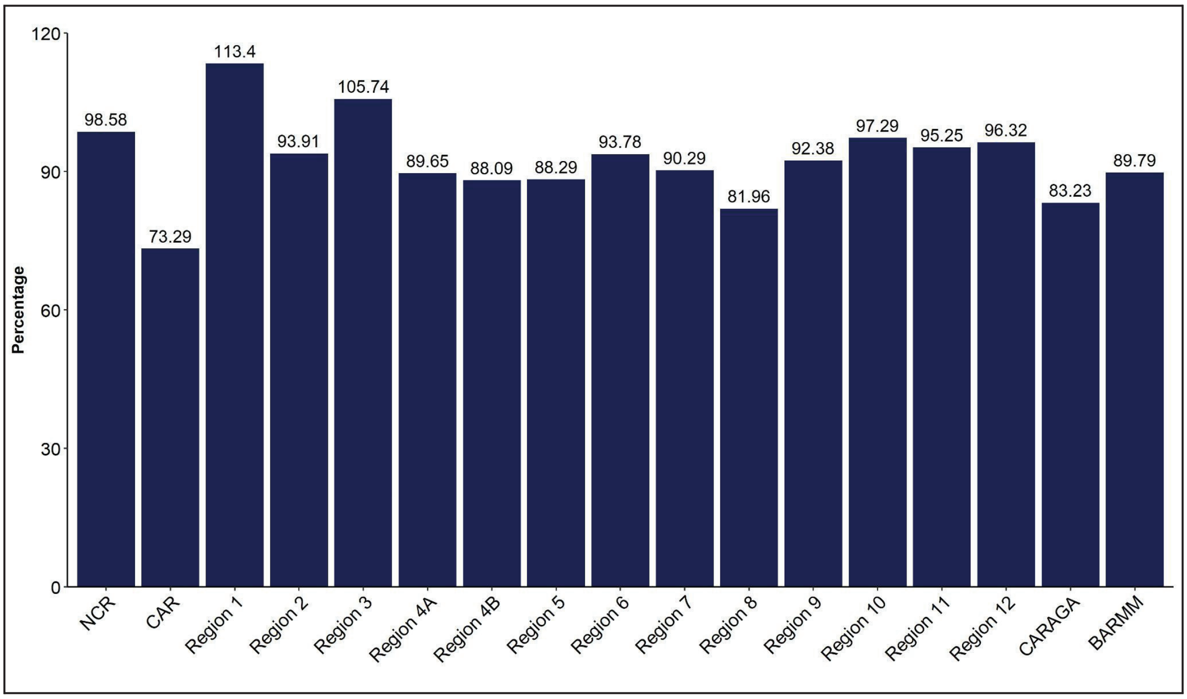

Figure 1.B.3.2: Women with at least 2 Postnatal Check-ups, by Region, 2024

While efforts to improve PNC coverage have yielded positive trends nationally and regional, attention must also be directed toward the quality and continuity of postpartum care particularly in terms of nutritional supplementation[9]. One key aspect of this is the completion of Iron and Folic Acid (IFA) and Vitamin A supplementation among postpartum women[10]. Tracking data from 2020 to 2024 provides valuable insight into how these essential interventions have progressed over time

From 2020 to 2021, the completion rate for IFA supplementation declined from 52.62% to 49.95% while Vitamin A supplementation declined from 53.11% to 50.93%. However, both indicators showed consistent improvement over the next two yearsIn 2020, the completion rate for IFA supplementation was 52.62%, slightly lower than the 53.11% recorded for Vitamin A supplementation. A decline is observed in both supplements in 2021, where IFA supplementation dropped to 49.95%, and Vitamin A supplementation to 50.93%, marking the lowest rates in the five-year period *[Figure 1.B.3.3]*.

By 2022, the IFA completion rate increased to 53.44% and Vitamin A rose to 55.48%. The upward trend continued in 2023, reaching 56.42% for IFA and 57.43% for Vitamin A—the highest in the dataset. In 2024, there was a slight decrease in both indicators, with IFA dropping to 55.28% and Vitamin A to 55.4%. Throughout all five years, the completion rate for Vitamin A supplementation remained slightly higher than that of IFA supplementation. The data reflects a general trend of improvement over time, despite minor fluctuations *[Figure 1.B.3.3]*.

---

[9] *WHO. (2022). Postnatal care for mothers and newborns: Highlights from the World Health Organization 2022 guidelines.*
[10] *WHO. (2011). Guideline: Vitamin A supplementation for postpartum women*

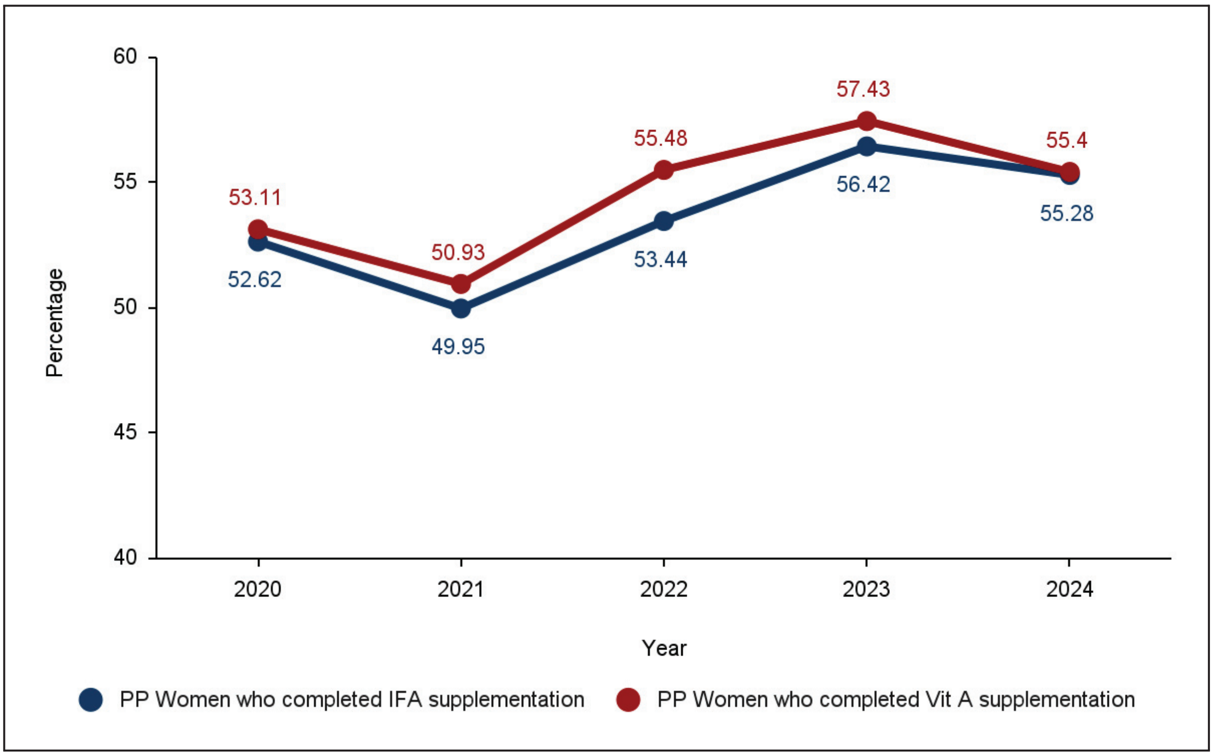

Figure 1.B.3.3: Postpartum Supplementation, Philippines, 2020 to 2024

1.C Child Health

2024 KEY FINDINGS

INFANTS & CHILDREN IMMUNIZATION

1,551,537 of children (**64.85%**) have been fully immunized

62.50% BCG

78.21% OPV 3

58.63% DPT-HiB-HepB 3

70.54% MCV 2

INFANT & CHILD NUTRITION

87.02% of newborns have been initiated on breastfeeding within 1 hour after birth

49.78% of infants have been Exclusively breastfed

27.08% Infants preterm or with LBW (< 2500 grams) given Iron

69.77% Children 12-59 months were given Vitamin A supplementation

CHILD MORTALITY

6.89 deaths per 1,000 live births
Neonatal Mortality Rate (NMR)

11.46 deaths per 1,000 live births
Infant Mortality Rate (IMR)

15.41 deaths per 1,000 live births
Under Five Mortality Rate (UFMR)

1.C.1 IMMUNIZATION SERVICES FOR INFANTS AND CHILDREN

Formula:

Bacillus Calmette–Guérin (BCG)

Numerator: Number of newborn / infants vaccinated with BCG antigen

Denominator: Eligible population 0-11 months

Children Protected at Birth (CPAB)

Numerator: Number of newborns whose mothers pregnant are for the first time who have received at least 2 doses of TT/Td vaccination at least a month prior to delivery PLUS number of newborns whose mothers are pregnant for the 2nd or more times and received at least 3 doses of TT/Td

Denominator: Eligible population aged 0-11 months

Oral Polio Vaccine (OPV)

Numerator: Number of infants vaccinated with OPV 1, 2, 3

Denominator: Eligible population aged 0-11 months

Pneumococcal Conjugate Vaccine (PCV)

Numerator: Number of infants vaccinated with PCV 1, 2, 3

Denominator: Eligible population aged 0-11 months

Hepatitis B within 24 hours after birth

Numerator: Number of newborn vaccinated with birth dose of HepB antigen within 24hr

Denominator: Eligible population aged 0-11 months

Pentavalent vaccine (DPT-HiB-HepB)

Numerator: Number of infants vaccinated with DPT-HiB-HepB 1, 2, 3

Denominator: Eligible population aged 0-11 months

Inactivated Polio Vaccine (IPV) 1

Numerator: Number of infants vaccinated with IPV1

Denominator: Eligible population 0-11 months

Measles Containing Vaccine (MCV) 2

Numerator: Number of children vaccinated with MCV2

Denominator: Eligible Population aged 0-12 months

Fully-Immunized Children (FIC)

Numerator: Number of FIC

Denominator: Eligible population aged 0-12 months

Completely Immunized Children (CIC)

Numerator: Number of CIC

Denominator: Eligible population aged 13-23 months

Vaccination remains a cornerstone of disease prevention, especially for young children who are most vulnerable to serious illnesses. In the Philippines, the routine immunization schedule includes vaccines such as Bacillus Calmette-Guérin (BCG) for Tuberculosis; the Pentavalent vaccine for Diphtheria, Pertussis (whooping cough), Tetanus, Haemophilus influenzae type B, and Hepatitis B (DPT-HiB-HepB); Oral and Inactivated Polio Vaccines; Pneumococcal Conjugate Vaccine (PCV); and Measles-Containing Vaccine (MCV). To further safeguard newborns, expectant mothers are also provided with tetanus toxoid immunization to prevent neonatal tetanus.

Timely administration of vaccines is crucial to ensure their full effectiveness in protecting children against preventable diseases. A child is deemed fully immunized upon receiving one dose of BCG, three doses each of Oral Polio Vaccine (OPV) and the Pentavalent vaccine (DPT-HiB-HepB), and two doses of the MCV—MCV1 at 9 months and MCV2 at 12 months. Children who may have missed any of these scheduled doses still have the opportunity to catch up and complete their vaccinations before reaching two years of age and they are classified as Completely Immunized Children (CIC).

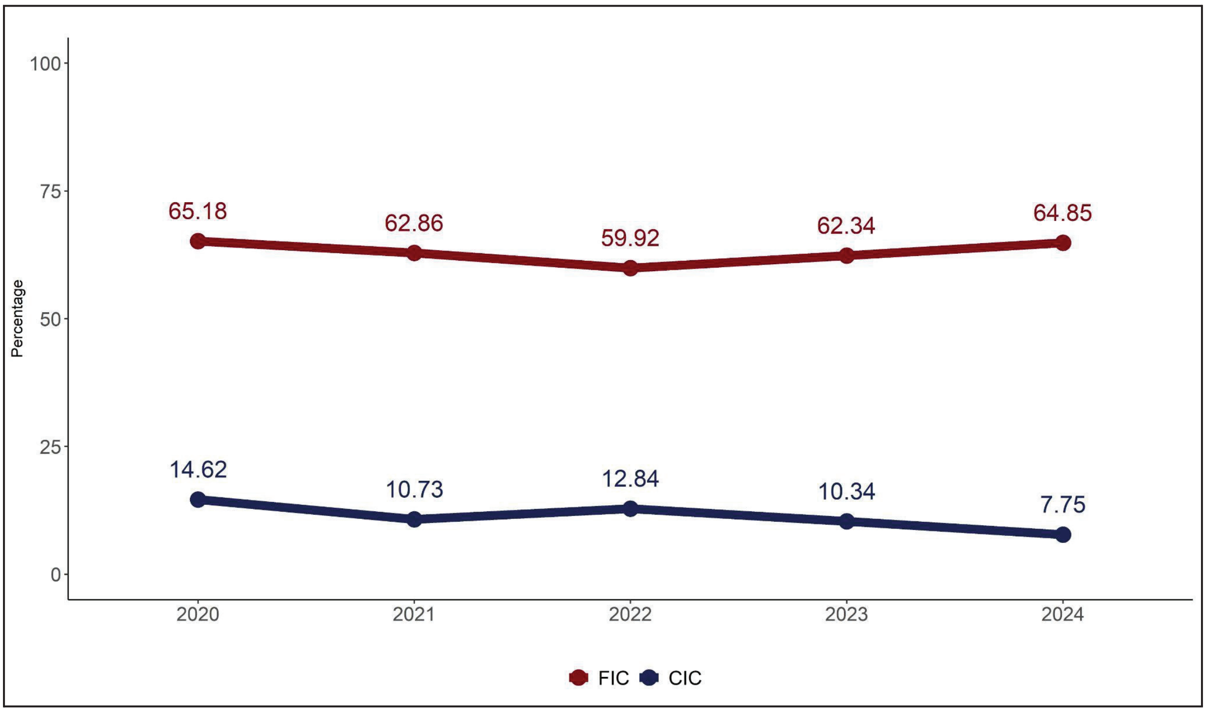

Figure 1.C.1. FIC and CIC, Philippines, 2020 to 2024

Following the decline in the proportion of fully immunized children (FIC) from 2020 to 2022, a positive shift was observed in 2023 and sustained through 2024, reaching a total of 1,551,537 FIC and increasing the national coverage to 64.85%. This resulted in a 5% increase in the FIC coverage since 2022.

Despite this progress, around 30% or 718,000 children still need to be fully immunized to achieve the national target of 95%. Reaching this target at both national and regional level is critical to protect children from vaccine-preventable diseases, improve their chances of survival, and help them in developing into healthy adults.

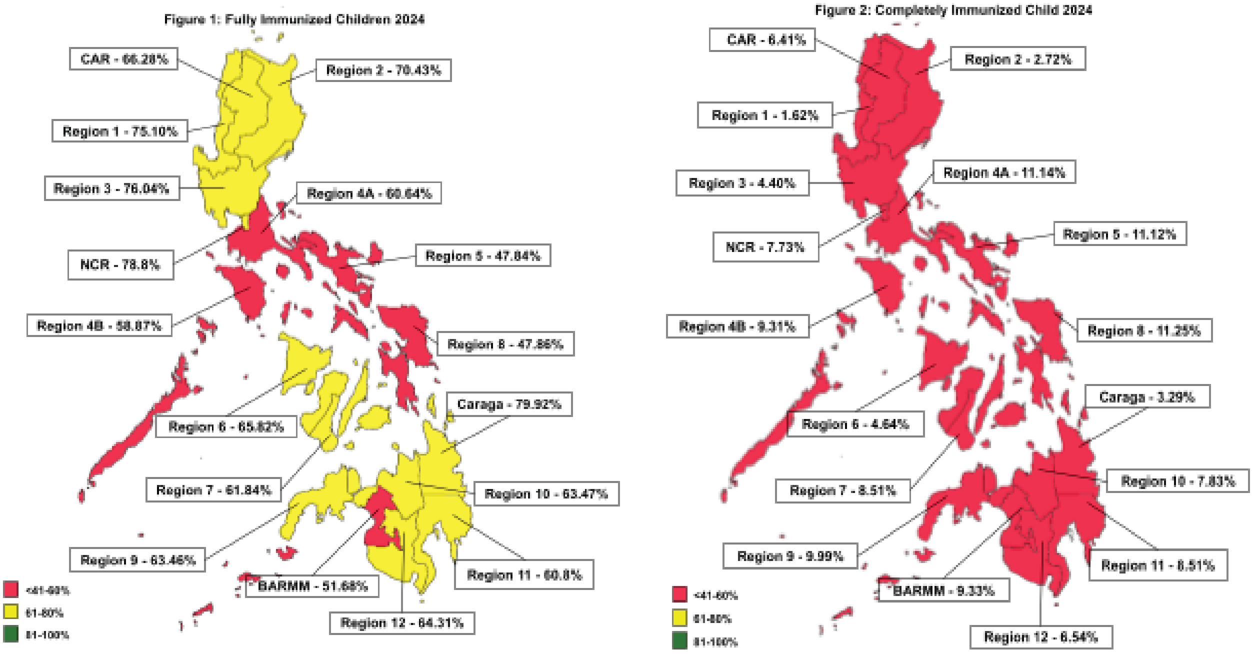

Figure 1.C.2: FIC and CIC, Philippines, 2024

The regional FIC coverage ranged from 47.84% in Region 5 to 79.92% in CARAGA. CARAGA remained as the leading region in terms of FIC coverage, followed by NCR at 78.80%, Region 3 at 76.04% and Region 1 at 75.10%. Region 3, NCR, and Region 4A were the most densely populated regions and also recorded the highest number of FIC. Meanwhile, 10 out of the 17 regions were below the national average and those with below 60% were Region 4B (58.87%), Region 5 (47.84%), Region 8 (47.86%), and BARMM (51.68%).

In terms of CIC, 161,402 children were able to complete their immunization before reaching two years of age, accounting for 7.75% of the previous eligible population. Region 4A was able to completely immunize the highest at 32,908, followed by NCR, Region 5 and Region 7.

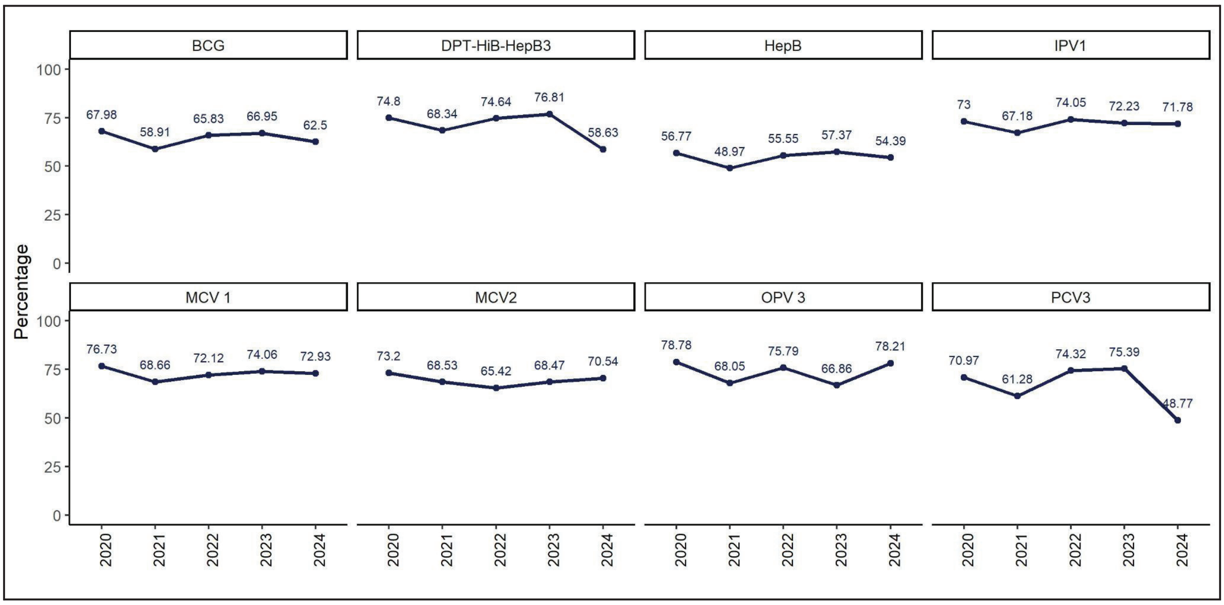

Figure 1.C.1.2. Immunization in Children, Philippines, 2020 to 2024

Analyzing the immunization trends across different vaccines revealed varying coverage rates, ranging from 48.77% for PCV3 to 78.21% for OPV3 in 2024. Despite an overall increase in FIC coverage, most vaccines showed a decline in coverage, with the highest decline observed for PCV and Pentavalent doses. Notably, the drop-out rates for PCV and Pentavalent doses significantly increased from 1.55% and 1.87% in 2023 to 14.52% and 17.88% in 2024, respectively. This coincides with the reported stock-outs of these vaccines in 2024.

In contrast, coverages for MCV2, IPV2, OPV2 and OPV3 increased in 2024 which shows efforts in reducing missed doses or drop-out rates. The upward trend across OPV doses suggests additional coverage for later doses. However, to achieve the target FIC coverage, it is essential to boost the coverage across all the vaccines, particularly for BCG (62.50%) and Pentavalent doses (with DPT-HiB-HepB3 at only 58.63%).

In terms of vaccine coverage by region, NCR recorded the highest coverages for BCG (84.43%), Hepatitis B within 24 hours (80.14%), CPAB (67.82%), and MCV1 (83.13%). However, its coverages for PCV doses were notably low, with PCV3 at 37.60%, and a relatively high drop-out rate of 32.27%. Meanwhile, Region 2 also showed strong performance across several vaccines, recording the highest among the regions for DPT-HiB-HepB3 (74.51%), OPV3 (88.56%), IPV1 (80.90%), and PCV doses, with PCV3 at 73.21%. It also posted the lowest drop-out rates for PCV doses (1.46%) and Pentavalent doses (-0.13%), indicating strong follow-through for multi-dose vaccines. CARAGA, on the other hand, reported the highest coverages among the regions for OPV2 (82.51%) and MCV2 (82.95%).

In contrast, Region 8 reported low coverages for its Pentavalent doses, with DPT-HiB-HepB3 at only 33.18 - the lowest among all regions. It exhibited the highest Pentavalent drop-out rate at 45.55% or 26,015 children missed completing the required doses. Region 9 had the lowest coverages among the regions for BCG (38.93%), Hepatitis B within 24 hours (30.87%), and OPV3 (71.26%). Similarly, Region 5 and Region 10 are also among the regions with the lowest DPT-HiB-HepB3 coverages at 35.52% and 39.06%, respectively, with high drop-out rates of 38.35% and 37.92%. Region 3 posted the highest PCV drop-out rate at 32.29%. In addition, BARMM still needs to improve their immunization efforts, having the lowest coverage for CPAB (38.00%) and IPV1 (65.14%), MCV1 (63.61%) and MCV2 (58.77%). Among the vaccine coverages in BARMM, the lowest was Hepatitis B within 24 hours at just 35.15%.

The data provides critical insights into the Philippines' immunization performance, highlighting the need for targeted interventions. These include addressing regional disparities through effective service delivery interventions, increasing coverage of underperforming vaccines through catch-up campaigns, improved follow-through on multi-dose vaccines, and consistent supply.

Table 1.C.1.2: Immunization in Children, by Region, 2024

Region	BCG	HepB	DPT-HiB-HepB 3	OPV3	IPV1	MCV2	PCV3	CPAB
NCR	84.43	80.14	68.00	78.44	75.65	81.25	37.60	67.82
CAR	73.38	69.11	67.09	84.90	71.64	71.91	55.22	50.31
Region 1	63.22	59.29	73.37	80.73	78.61	76.40	59.77	59.78
Region 2	70.92	68.93	74.51	88.56	80.90	72.65	73.21	64.73
Region 3	63.19	46.65	59.83	82.28	71.69	76.71	38.51	56.91
Region 4A	62.70	56.69	62.44	72.62	69.19	68.03	52.27	54.04
Region 4B	62.90	47.68	49.36	83.58	78.32	69.04	34.48	51.37
Region 5	49.29	43.67	35.52	78.57	66.29	58.86	35.30	42.58
Region 6	53.01	51.52	57.16	77.69	70.87	69.41	54.22	52.74
Region 7	60.31	50.07	58.04	83.12	73.67	68.72	63.72	53.21
Region 8	59.81	52.63	33.18	79.29	73.62	61.85	41.63	44.38
Region 9	38.93	30.87	59.93	71.26	69.49	72.15	45.52	46.77
Region 10	53.76	45.57	39.06	72.75	68.72	71.08	51.82	54.73
Region 11	66.83	62.09	61.72	72.04	69.36	67.04	56.93	63.48
Region 12	60.57	55.04	68.02	75.74	71.62	68.75	57.03	58.32
CARAGA	67.33	60.74	53.48	82.73	76.19	82.95	66.63	57.26
BARMM	57.06	35.15	65.68	79.66	65.14	58.77	40.47	38.00

1.C.2. NUTRITION SERVICES FOR INFANTS AND CHILDREN

Formula

Proportion of newborns who were initiated on breastfeeding within 1 hour after birth

Numerator: Number of newborns who were initiated on breastfeeding within 1 hour after birth

Denominator: Total number of live births

Proportion of infants born preterm or with low birth weight given iron supplements

Numerator: Number of preterm infants and/or infants with low birth weight (less than 2500 grams) given iron supplement starting 1 month until 3 months

Denominator: Total number of live births with low birth weight (taken from Maternal Care TCL)

Proportion of infants exclusively breastfed until 5th month and 29 days

Numerator: Number of infants age 5 months and 29 days old who have been exclusively breastfed from birth until 5th month and 1 day before the child turns 6 months

Denominator: Eligible population 6 months

Proportion of infants who continued breastfeeding and were introduced to complementary feeding beginning at 6 months of age

Numerator: Number of infants who continued breastfeeding and were introduced to complementary feeding beginning at 6 months of age

Denominator: Eligible population 6-11 months

Proportion of infants / children who completed Vitamin A supplementation

Numerator:
a. Number of Infant 6-11 months old who completed 1 dose of Vitamin A supplementation
b. Number of children with ages 12-59 months old who completed 2 doses Vitamin A Supplementation

Denominator:
a. Eligible population 6-11 months
b. Eligible population 12-59 months

Proportion of infants 6-11 months old and children 12-23 months old who completed Micronutrient Powder (MNP) or Lipid-Based Nutrient Supplement – Small Quantity (LNS-SQ) supplementation

Numerator:
a. Number of infants 6-11 months old who completed MNP or Lipid-Based Nutrient Supplement – Small Quantity (LNS-SQ) supplementation.
b. Number of children 12-23 months old who completed MNP or Lipid-Based Nutrient Supplement – Small Quantity (LNS-SQ) supplementation

Denominator:
a. Eligible population 6-11 months
b. Eligible population 12-23 months

Proportion of 0-59 months old who are: Stunted, Wasted-MAM, Wasted-SAM, Overweight/ Obese and Normal

Numerator:
a. Number of infants and children 0-59 months old who are stunted
b. Number of infants and children 0-59 months old who have moderate acute malnutrition (MAM)
c. Number of infants and children 0-59 months old who have severe acute malnutrition (SAM)

d. Number of infants and children 0-59 months old who are overweight/obese
e. Number of infants and children 0-59 months old who have normal weight for length/height for age measurement

Denominator: Total number of children 0-59 months old seen during the reporting period at health facilities

---

Breastfeeding is one the most effective practices to ensure child survival, optimal growth and development.[1] This is because breast milk contains the nutrients that infants need in their first months of life and antibodies that protect from childhood diseases. The World Health Organization (WHO) and the United Nations International Children's Emergency Fund (UNICEF) recommend the initiation of breastfeeding within the first hour of life of the child and exclusive breastfeeding for the first six months.[2] Beyond six months, children can be provided with complimentary food with continued breastfeeding. However, the child should receive breastfeeding as often as the child desires.

The Philippines supports breastfeeding practices through Executive Order (EO) No. 51 ("Milk Code") and the Expanded Breastfeeding Promotion Act of 2009 (Republic Act (RA) 10028). The Milk Code mandates the promotion and protection of breastfeeding by prohibiting the marketing of formula milk in hospitals and donations of milk samples to health providers.[3] The Milk Code also requires formula milk to have special labels. On the other hand, RA 10028 mandates the support of breastfeeding in the workplace as well as the promotion of breastfeeding education.[4] The Philippines also highlights the importance of health and nutrition for children through the "Kalusugan at Nutrisyon ng Mag-Nanay Act" or "First 1,000 Days Law" (RA 11148).[5] This law mandates efforts to strengthen maternal, neonatal, and child health and nutrition during the first 1,000 days of a child's life.

In 2024, breastfeeding was initiated within the first hour of life among nine in 10 live births in the Philippines. However, the annual target of 95 percent was not achieved in 2024. It should be noted that there were variations across the regions. Specifically, Region 1 (99.61%), CAR (96.85%) and Region 10 (94.39%) had the highest proportion of newborns who were breastfed within one hour after birth. On the other hand, the regions with the lowest reported accomplishment for the indicator are Region 5 (76.99%), BARMM (77.38%) and Region 8 (82.1%). It should be noted that about 15% of deliveries in the Philippines were Cesarean sections. Immediate breastfeeding can be challenging for women who delivered through Cesarean section because of limited mobility and pain after surgery, effects of anesthesia, and ongoing management of medical complications.[6] *[Table 1.C.2.1]*.

---

[1] *World Health Organization: WHO. (2019, November 11). Breastfeeding.*
[2] *United Nations International Children's Emergency Fund (UNICEF). (2018). Breastfeeding: A Mother's Gift, for Every Child.*
[3] *Executive Order No. 51. (October 20, 1986)*
[4] *Republic Act No. 10028 (March 16, 2010)*
[5] *Republic Act No. 11148 (July 23, 2018)*
[6] *Getaneh T, Negesse A, Dessie G, Desta M, Temesgen H, Getu T, Gelaye K. Impact of cesarean section on timely initiation of breastfeeding in Ethiopia: a systematic review and meta-analysis. Int Breastfeed J. 2021 Jul 5;16(1):51. doi: 10.1186/s13006-021-00399-9. PMID: 34225731; PMCID: PMC8259022.*

Table 1.C.2.1. Proportion of Newborns who were Initiated on Breastfeeding within 1 hour After Birth, by Region, Philippines, 2024

Area	Live births	Newborns initiated on breastfeeding within 1 hour after birth	
		No.	%
**Philippines**	**1,338,505**	**1,164,731**	**87.02**
NCR	183,878	156,670	85.20
CAR	23,873	23,120	96.85
Region 1	51,383	51,184	99.61
Region 2	33,145	28,346	85.52
Region 3	140,769	116,465	82.73
Region 4A	196,166	177,077	90.27
Region 4B	42,233	36,990	87.59
Region 5	85,536	65,856	76.99
Region 6	85,513	79,403	92.85
Region 7	107,859	88,716	82.25
Region 8	58,580	48,093	82.10
Region 9	31,451	26,188	83.27
Region 10	63,659	60,089	94.39
Region 11	73,703	67,315	91.33
Region 12	61,644	56,838	92.20
CARAGA	38,525	35,501	92.15
BARMM	60,588	46,880	77.38

In 2024, less than 50% of the infants aged 11 months and below were exclusively breastfed until the 5th month and 29 days after their birth in the Philippines. This is lower than the accomplishment in 2023 of 52.82% and the annual target of 95%. The proportion of exclusive breastfeeding among infants aged 11 months and below also declined across the regions with the exception of BARMM and Region 8. Regions with the highest coverage are Region 10 (63.52%), Region 1 (62.74%) and CARAGA (57.59%). On the other hand, regions with the lowest coverage are Region 4A (38.61), BARMM (41.22%) and Region 3 (45.1%). These results may also reflect challenges in exclusive breastfeeding such as the woman's need to return to work and lactation issues.[7] To address these challenges, targeted interventions designed to address factors for early cessation of exclusive breastfeeding as well as improved education on breastfeeding techniques should be explored *[Table 1.C.2.2].*

Table 1.C.2.2. Proportion of Infants Exclusively Breastfed Until 5th month and 29 days, by Region, Philippines, 2024

Area	Eligible Population (0-11mos. old)	Infants Exclusively Breastfed until 5th month and 29 days		
		No.	%	% Change from 2023
**Philippines**	**2,200,865**	**1,095,590**	**49.78**	**-5.76 (↓)**
NCR	263,248	136,521	51.86	-5.73 (↓)
CAR	31,490	17,846	56.67	-6.16 (↓)
Region 1	96,024	60,249	62.74	-1.70 (↓)
Region 2	64,034	35,138	54.87	-12.10 (↓)
Region 3	235,313	106,126	45.10	-2.51 (↓)
Region 4A	310,150	119,762	38.61	-4.84 (↓)

[7] *Olalere, O., & Harley, C. (2024, December 2). Why women discontinue exclusive breastfeeding: a scoping review. British Journal of Midwifery.*

Area	Eligible Population (0-11mos. old)	Infants Exclusively Breastfed until 5th month and 29 days		
		No.	%	% Change from 2023
Region 4B	66,508	37,466	56.33	-14.28 (↓)
Region 5	136,611	67,128	49.14	-7.41 (↓)
Region 6	147,934	68,860	46.55	-1.94 (↓)
Region 7	167,016	87,085	52.14	-8.52 (↓)
Region 8	93,713	47,738	50.94	4.62 (↑)
Region 9	85,348	41,181	48.25	-11.50 (↓)
Region 10	110,290	70,052	63.52	-7.41 (↓)
Region 11	107,934	60,127	55.71	-15.51 (↓)
Region 12	95,296	52,947	55.56	-9.21 (↓)
CARAGA	55,371	31,890	57.59	-13.45 (↓)
BARMM	134,585	55,474	41.22	18.48 (↑)

In 2024, More than nine in 10 infants aged six to 11 months continued breastfeeding and were introduced to complementary feeding. This indicates that the annual target of at least 50 percent was achieved. All the regions reported accomplishments that are greater than the annual national target. However, it should be noted that eight out of the 17 regions had accomplishments greater than the projected eligible population. While the data suggest good program performance, it may also suggest the need for data validation and review of the eligible population computation methodology *[Table 1.C.2.3]*.

Table 1.C.2.3. Proportion of Infants who Continued Breastfeeding and were Introduced to Complementary Feeding Beginning at 6 months of Age, by Region, Philippines, 2024

Area	Eligible Population (6-11 mos. old)	Infants who continued breastfeeding and were introduced to complementary feeding	
		No.	%
**Philippines**	**1,121,239**	**1,054,968**	**94.09**
NCR	132,468	131,173	99.02
CAR	16,337	17,422	106.64
Region 1	49,634	62,391	125.70
Region 2	33,164	31,936	96.30
Region 3	119,190	101,455	85.12
Region 4A	157,056	118,936	75.73
Region 4B	33,988	34,311	100.95
Region 5	70,057	65,242	93.13
Region 6	75,942	66,211	87.19
Region 7	84,979	85,918	101.10
Region 8	49,025	44,132	90.02
Region 9	43,592	40,725	93.42
Region 10	56,153	63,587	113.24
Region 11	54,553	57,709	105.79
Region 12	48,212	50,587	104.93
CARAGA	28,326	29,496	104.13
BARMM	68,563	53,737	78.38

Iron is necessary to carry Oxygen in the hemoglobin as well the development of the immune and neural systems, and in muscle metabolism.[8] Thought to be the leading

[8] *Guideline: Daily iron supplementation in infants and children. Geneva: World Health Organization; 2016.*

cause of anemia, Iron deficiency is caused by inadequate intake or absorption of dietary Iron. It should be noted that iron deficiency in children below two years old can result in significant and irreversible effects on brain development. In 2011, WHO estimated that 300 million children were affected by anemia. To address the global magnitude of anemia, the WHO recommended oral Iron supplementation to reduce the prevalence of anemia.

In the Philippines, the proportion of infants born preterm or with low birth weight given iron supplements increased to 27.08% in 2024 from 23.37% in 2023. However, the accomplishment in 2024 is still far from the annual target of 95 percent. Coverage remained low across the regions where Region 1 reported the highest coverage (55.75%) while Region 7 reported the lowest coverage (12.94%) *[Table 1.C.2.4]*.

Table 1.C.2.4. Proportion of Infants Born Preterm or with Low Birth Weight Given Iron Supplements, by Region, Philippines, 2024

Area	Total No. of live births with low birth weight	Infants preterm or with LBW (< 2500 grams) given Iron		
		Total	%	St% Change from 2023
**Philippines**	**79,047**	**21,408**	**27.08**	**15.89 (↑)**
NCR	5,884	2,658	45.17	28.33 (↑)
CAR	2,584	298	11.53	-7.96 (↓)
Region 1	3,706	2,066	55.75	173.54 (↑)
Region 2	1,912	632	33.05	1.96 (↑)
Region 3	9,868	2,489	25.22	59.84 (↑)
Region 4A	9,975	2,357	23.63	14.54 (↑)
Region 4B	2,567	789	30.74	-19.92 (↓)
Region 5	8,543	1,313	15.37	4.62 (↑)
Region 6	7,066	1,517	21.47	16.87 (↑)
Region 7	8,631	1,117	12.94	-46.61 (↓)
Region 8	4,529	933	20.60	-10.94 (↓)
Region 9	1,590	530	33.33	-3.07 (↓)
Region 10	2,447	822	33.59	106.21 (↑)
Region 11	4,421	1,903	43.04	-6.06 (↓)
Region 12	2,160	1,014	46.94	-20.31 (↓)
CARAGA	2,173	640	29.45	46.46 (↑)
BARMM	991	330	33.30	232.66 (↑)

Vitamin A is essential for healthy growth and development of children and the functioning of the immune system.[9] While vitamin A is generally obtained through a healthy diet, vitamin A deficiency remains the leading cause of preventable childhood blindness. Additionally, vitamin A deficiency exacerbates the risk of death from common childhood infections. Affecting about one in three children aged 6 to 59 months globally, WHO identified vitamin A deficiency as a public health problem.

[9] *World Health Organization: WHO. Vitamin A. (n.d.).*

In the Philippines, the number of infants six to 11 months old who were given vitamin A exceeded the projected eligible population as well as the annual target. Coverage remained high across the regions where the eligible populations were also exceeded with the exception of Region 2 and Region 6. While the data suggest good program performance, it may also suggest the need to strengthen data validation procedures of the reported data and to revisit the eligible population *[Table 1.C.2.5]*.

Table 1.C.2.5. Proportion of Infants aged 6-11 months who Completed Vitamin A Supplementation, by Region, Philippines, 2024

Area	Eligible Population (6-11 mos. old)	Infants 6-11 mos. old Given Vitamin A		
		Total	%	% change from 2023
**Philippines**	**1,121,239**	**1,466,792**	**130.82**	**1.59** (↑)
NCR	132,468	193,890	146.37	-4.96 (↓)
CAR	16,337	21,356	130.72	3.13 (↑)
Region 1	49,634	77,067	155.27	63.29 (↑)
Region 2	33,164	32,124	96.86	-40.67 (↓)
Region 3	119,190	174,040	146.02	12.67 (↑)
Region 4A	157,056	208,016	132.45	1.29 (↑)
Region 4B	33,988	46,304	136.24	15.57 (↑)
Region 5	70,057	83,962	119.85	4.74 (↑)
Region 6	75,942	70,569	92.92	-9.55 (↓)
Region 7	84,979	107,095	126.03	1.19 (↑)
Region 8	49,025	60,230	122.86	6.20 (↑)
Region 9	43,592	56,155	128.82	2.21 (↑)
Region 10	56,153	79,532	141.63	-9.17 (↓)
Region 11	54,553	76,697	140.59	-9.41 (↓)
Region 12	48,212	58,183	120.68	-5.66 (↓)
CARAGA	28,326	43,427	153.31	21.89 (↑)
BARMM	68,563	78,145	113.98	7.21 (↑)

The number of children aged 12 to 59 months who were given vitamin A increased from 67.49% in 2023 to 69.77% in 2024. Despite the increase in coverage, the annual target of 95% was not achieved in 2024. Looking at regional coverage, only Region 4B (102.51%) and Region 11 (95.3%) have coverage rates that exceed the annual national target. On the other hand, Region 9 (51.42%) and Region 4A (52.21%) had the lowest coverage *[Table 1.C.2.6]*.

Table 1.C.2.6. Children 12-59 months who Completed Vit. A supplementation, by Region, Philippines 2024

Area	Eligible Population (12-59 mos. old)	Children 12-59 mos. Old Given Vitamin A		
		Total	%	% change from 2023
**Philippines**	**9,205,720**	**6,422,489**	**69.77**	**3.37** (↑)
NCR	1,057,004	814,759	77.08	-7.96 (↓)
CAR	132,709	87,903	66.24	1.11 (↑)
Region 1	391,947	292,363	74.59	24.69 (↑)
Region 2	274,420	219,943	80.15	-2.78 (↓)
Region 3	989,808	812,687	82.11	11.24 (↑)
Region 4A	1,291,246	674,098	52.21	1.82 (↑)
Region 4B	275,267	282,165	102.51	29.31 (↑)
Region 5	569,012	306,534	53.87	-16.58 (↓)

Area	Eligible Population (12-59 mos. old)	Children 12-59 mos. Old Given Vitamin A		
		Total	%	% change from 2023
Region 6	622,304	330,308	53.08	-7.16 (↓)
Region 7	680,875	388,596	57.07	3.64 (↑)
Region 8	392,682	248,450	63.27	9.27 (↑)
Region 9	354,698	182,388	51.42	13.64 (↑)
Region 10	460,481	432,510	93.93	-5.94 (↓)
Region 11	453,316	432,298	95.36	-6.32 (↓)
Region 12	396,809	329,623	83.07	17.68 (↑)
CARAGA	238,285	178,457	74.89	19.07 (↑)
BARMM	624,857	409,407	65.52	22.86 (↑)

While needed in very small amounts, micronutrients are of critical importance to the body's health.[10] Micronutrients are needed for the body to produce enzymes, hormones and other substances needed for growth and development. Deficiency in any micronutrient can result in severe and potentially fatal conditions. According to the WHO, the burden of micronutrient deficiencies are disproportionately concentrated in low- and middle-income countries.

Data for 2024 show that the Philippines has low MNP or LNS-SQ supplementation coverage among infants and children, and the annual target of 95% was not achieved. Among infants aged six to 11 months, only 16.77% completed MNP or LNS-SQ supplementation. Coverage was also low across the regions where coverage is between 47% (NCR) and 7.11% (BARMM) *[Table 1.C.2.7]*.

Table 1.C.2.7. Proportion of Infants 6-11 months old who Completed Micronutrient Powder (MNP) or Lipid-Based Nutrient Supplement – Small Quantity (LNS-SQ) supplementation, by Region, Philippines, 2024

Area	Eligible Population (6-11 mos. old)	Infants 6-11 mos. old who completed MNP or LNS-SQ supplementation	
		No.	%
**Philippines**	**1,121,239**	**188,004**	**16.77**
NCR	132,468	62,256	47.00
CAR	16,337	2,234	13.67
Region 1	49,634	12,176	24.53
Region 2	33,164	8,824	26.61
Region 3	119,190	16,005	13.43
Region 4A	157,056	15,689	9.99
Region 4B	33,988	4,297	12.64
Region 5	70,057	11,975	17.09
Region 6	75,942	6,672	8.79
Region 7	84,979	7,868	9.26
Region 8	49,025	5,913	12.06
Region 9	43,592	3,106	7.13
Region 10	56,153	6,577	11.71
Region 11	54,553	8,333	15.28
Region 12	48,212	3,851	7.99
CARAGA	28,326	7,355	25.97
BARMM	68,563	4,873	7.11

[10] *World Health Organization: WHO. Micronutrients. (n.d.).*

Data for 2024 also showed that only 12.61% of children aged between 12 and 23 months completed MNP or LNS-SQ supplementation in the Philippines. Coverage was also low across the regions where coverage is only between 36.51% (NCR) and 4.71% (Region 9) *[Table 1.C.2.8]*.

Table 1.C.2.8. Proportion of Children 12-23 months old who Completed Micronutrient Powder (MNP) or Lipid-Based Nutrient Supplement – Small Quantity (LNS-SQ) supplementation, by Region, Philippines, 2024

Area	Eligible Population (12-23 mos. old)	Children 12-23 mos. old who completed MNP or LNS-SQ supplementation	
		No.	%
**Philippines**	**2,266,749**	**285,728**	**12.61**
NCR	268,478	98,013	36.51
CAR	32,402	2,112	6.52
Region 1	97,386	18,104	18.59
Region 2	65,768	11,313	17.20
Region 3	245,405	30,507	12.43
Region 4A	321,682	17,086	5.31
Region 4B	67,274	10,403	15.46
Region 5	140,132	16,410	11.71
Region 6	153,816	11,510	7.48
Region 7	167,951	8,444	5.03
Region 8	94,774	8,540	9.01
Region 9	86,199	4,064	4.71
Region 10	111,675	12,656	11.33
Region 11	110,500	12,080	10.93
Region 12	97,218	6,198	6.38
CARAGA	56,849	10,862	19.11
BARMM	149,240	7,426	4.98

According to the World Bank, malnutrition has always been a problem in the Philippines where one in three children below five years old suffered from stunting in 2019.[11] Additionally, the national stunting levels were exceeded in BARMM (45%), in Region 4B (41%), Region 5 (40%), Region 6 (40%), and in Region 12 (40%). On the other hand, "wasting" or acute malnutrition for children under five years in the P has gradually increased from 6.9% in 2009 to 7.9% in 2013.[12] Through the implementation of Integrated Management of Acute Malnutrition (IMAM), an intervention recognized by UNICEF and WHO, acute malnutrition has been managed, globally reducing death rates to less than 10%.[13]

It is in this context that the DOH issued Administrative Order (AO) No. 2015-0055 or the National Guidelines on the Management of Acute Malnutrition for Children under 5 years. AO No. 2015-0055 provided the policies and strategic framework to guide a multi-sectoral approach in the adoption and implementation of the Philippine Integrated Management of Acute Malnutrition (PIMAM).

---

[11] *Mbuya, Nkosinathi V. N., Gabriel Demombynes, Sharon Faye A. Piza, and Ann Jillian V. Adona. Undernutrition in the Philippines: Scale, Scope, and Opportunities for Nutrition Policy and Programming. International Development in Focus. Washington, DC: World Bank. doi:10.1596/978-1-4648-1701-4. License: Creative Commons Attribution CC BY 3.0 IGO*

[12] *Food and Nutrition Research Institute, Department of Science and Technology (Philippines). Philippines National Nutrition Survey 2013-2014.*

[13] *DOH AO No. 2015-0055*

In 2024, 85.7% of children zero to 59 months old who were seen at health facilities had normal weight for length/height for age measurement. On the other hand, less than a tenth were either wasted, stunted, overweight or obese. At the regional level, it can be noted that Region 3 had the highest proportion of children zero to 59 months old who were wasted (2.19%) or overweight/obese (2.91%). On the other hand, the regions with the highest proportion of children zero to 59 months old who were stunted were Region 4B (7.8%) and Region 8 (7.78%) *[Table 1.C.2.9]*.

Table 1.C.2.9. Children 0-59 mos. Old whose Nutritional Status are Normal, Stunted, Overweight/Obese, Wasted-MAM, Wasted-SAM, Wasted, by Region, Philippines, 2024

Area	0-59 months old seen	Wasted	Wasted-SAM	Wasted-MAM	Overweight/ Obese	Stunted	% Normal
**Philippines**	**9,840,251**	**1.14**	**0.29**	**0.85**	**1.55**	**4.15**	**85.70**
NCR	1,091,445	0.44	0.13	0.31	0.92	1.88	95.92
CAR	91,978	0.76	0.16	0.60	1.83	6.67	90.74
Region 1	411,055	0.85	0.26	0.60	1.71	2.53	96.99
Region 2	278,200	1.12	0.31	0.81	2.18	4.39	86.14
Region 3	1,093,763	2.19	0.66	1.53	2.91	3.74	92.74
Region 4A	1,601,396	0.76	0.15	0.60	1.47	2.91	67.91
Region 4B	452,319	1.11	0.25	0.86	1.36	7.80	89.73
Region 5	521,729	1.98	0.52	1.46	2.03	7.16	88.78
Region 6	770,238	1.42	0.12	1.30	1.44	4.65	75.95
Region 7	503,043	1.06	0.17	0.89	1.44	4.56	92.95
Region 8	229,233	1.43	0.36	1.07	2.64	7.78	88.53
Region 9	376,175	1.37	0.36	1.01	1.09	3.87	92.33
Region 10	492,914	1.20	0.55	0.65	0.78	3.31	92.66
Region 11	489,093	0.74	0.18	0.56	1.53	4.98	85.67
Region 12	456,137	0.58	0.13	0.44	0.89	2.60	91.56
CARAGA	256,594	1.93	0.53	1.40	1.70	5.50	90.41
BARMM	724,939	0.91	0.25	0.66	0.99	5.72	77.97

In line with the inclusion of nutrition to the 8 Priority Health Outcomes, it is imperative to develop catch up plans to address the low coverage of micronutrient supplementation relative to the annual targets. However, regional disparities across all child nutrition indicators suggest the need for targeted interventions prioritizing underserved areas and reforms on nutrition governance especially at the local level.

1.C.3. CHILD CARE MANAGEMENT OF SICK INFANTS AND CHILDREN

Proportion of high-risk infants and children with measles and/or persistent diarrhea who received Vitamin A capsule aside from routine supplementation

Numerator:
a. Number of sick infants 6-11 months old who received Vitamin A capsule aside from routine supplementation
b. Number of sick children 12-59 months old who received Vitamin A capsule aside from routine supplementation

Denominator:
a. Total number of sick infants 6-11 months old seen
b. Total number of sick children 12-59 months old seen

Proportion of diarrhea cases 0-59 months old who received oral rehydration salt solution (ORS) and ORS with zinc drops/syrup

Numerator:
a. Number of diarrhea cases 0-59 months old who received oral rehydration salt solution (ORS)
b. Number of diarrhea cases 0-59 months old who received oral rehydration salt solution (ORS) with zinc drops/syrup

Denominator: For a,b: Total number of acute diarrhea cases 0-59 months old seen

Proportion of pneumonia cases among children 0-59 months old who received treatment

Numerator: Number of pneumonia cases 0-59 months old who received treatment

Denominator: Total number of pneumonia cases 0-59 months old seen

---

Over 80% deaths below five years of age were caused by neonatal conditions and infectious diseases such as pneumonia, diarrhea, and measles. These conditions are often exacerbated by malnutrition.[14] Most childhood deaths can be prevented through the implementation of interventions that are effective even in resource constrained settings. It is in this context that WHO and UNICEF developed the Integrated Management of Childhood Illness (IMCI) to strengthen prevention and management of common childhood illnesses. IMCI was designed to improve the skills of health workers, the health system as well as family and community practices.

In the Philippines, the DOH issued AO No. 119 s. 2003 or the “Updated Guidelines on Micronutrient Supplementation (Vitamin A, Iron, and Iodine)” on December 2, 2023. AO No. 119 s. 2003 mandates vitamin A supplementation to high risk children aiming to reestablish body reserves that were drained through repeated and chronic infectious diseases and protect against the severity of subsequent infections.

In 2024, the proportion of sick infants aged six to 11 years old who received Vitamin A continued to decline from 2021. Similarly, the proportion of sick children aged 12 to 59 months declined further in 2024. Based on the data in 2024, the annual target of 100% for both sick infants aged six to 11 years old and sick children aged 12 to 59 months were not achieved *[Figure 1.C.3.1].*

---

[14] *Integrated management of childhood illness. (n.d.).*

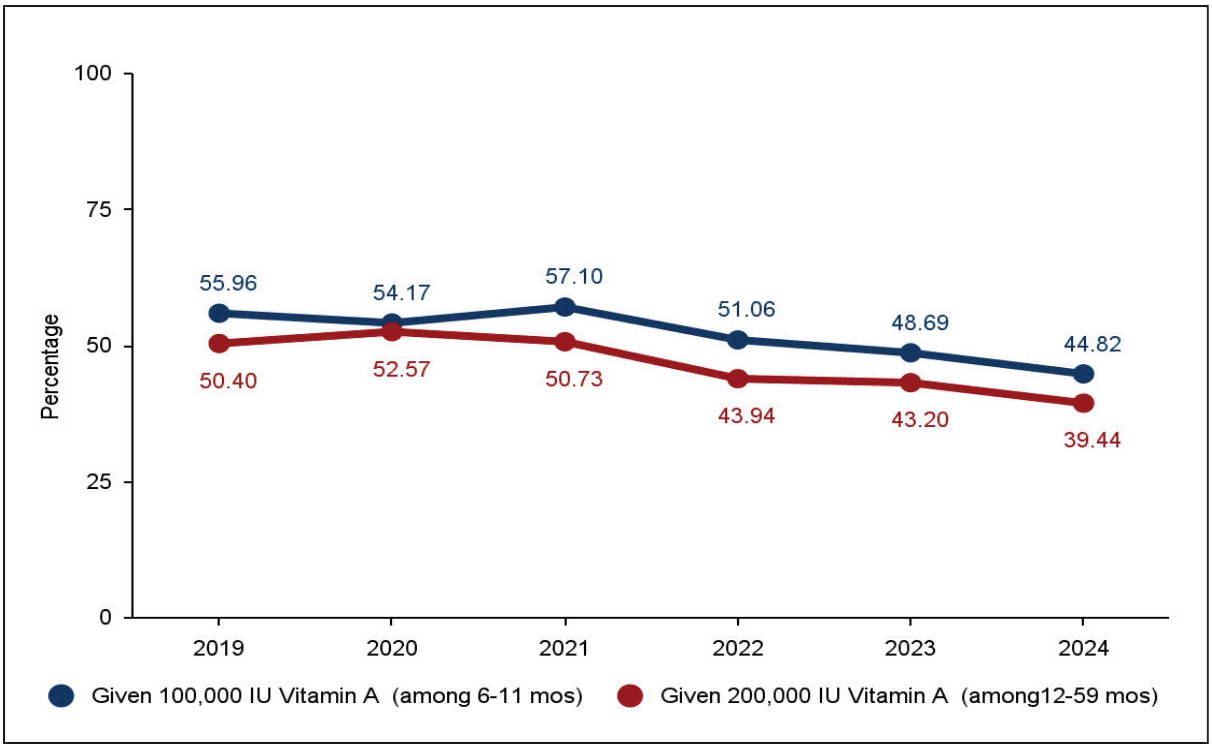

Figure 1.C.3.1. Proportion of High-risk Infants and Children with Measles and/or Persistent Diarrhea Who Received Vitamin A Capsule Aside from Routine Supplementation, Philippines, 2019-2024

Coverage rates varied across the regions for both sick infants aged six to 11 years old and sick children aged 12 to 59 months. NCR has the highest proportion (88.7%) of sick infants aged six to 11 years old who received vitamin A while Region 4A had the lowest (25.11%). For vitamin A supplementation among children aged 12 to 59 months, NCR also had the highest coverage (80.19%) while Region 4A also had the lowest coverage (19.58%) *[Table 1.C.3.2].*

Table 1.C.3.2. Proportion of High-risk Infants and Children with Measles and/or Persistent Diarrhea who Received Vitamin A Capsule Aside from Routine Supplementation, by Region,Philippines, 2024

Area	Sick Infants aged 6-11 mos.			Sick Children aged 12-59 mos.		
	Seen	Given Vitamin A (100,000 IU)		Seen	Given Vitamin A (200,000 IU)	
		Total	%		Total	%
**Philippines**	**151,650**	**67,966**	**44.82**	**343,246**	**135,363**	**39.44**
NCR	469	416	88.70	1,040	834	80.19
CAR	281	231	82.21	585	390	66.67
Region 1	208	166	79.81	420	315	75.00
Region 2	11,928	4,761	39.91	26,692	9,096	34.08
Region 3	24,245	11,715	48.32	55,249	21,552	39.01
Region 4A	32,994	8,285	25.11	79,495	15,567	19.58
Region 4B	2,874	1,282	44.61	6,179	2,287	37.01
Region 5	6,273	2,425	38.66	12,672	5,532	43.66
Region 6	10,326	4,253	41.19	26,749	9,250	34.58
Region 7	2,236	680	30.41	6,515	1,440	22.10
Region 8	12,506	5,915	47.30	31,706	14,615	46.10
Region 9	7,056	5,185	73.48	11,257	8,048	71.49

Area	Sick Infants aged 6-11 mos.			Sick Children aged 12-59 mos.		
	Seen	Given Vitamin A (100,000 IU)		Seen	Given Vitamin A (200,000 IU)	
		Total	%		Total	%
Region 10	13,590	6,786	49.93	27,161	13,334	49.09
Region 11	6,564	3,993	60.83	16,751	9,531	56.90
Region 12	6,762	4,107	60.74	13,073	7,841	59.98
CARAGA	4,036	1,580	39.15	8,426	3,455	41.00
BARMM	9,302	6,186	66.50	19,276	12,276	63.69

The proportion of diarrhea cases 0-59 months old who received ORS increased from 44.25% in 2023 to 53.72% in 2024 following the sharp decline between 2022 and 2023. On the other hand, the proportion who received ORS with zinc drops/syrup also increased from 51.82% in 2023 to 56.86% in 2024. It should be noted that the coverage rates for ORS with zinc drops/syrup increased steadily between 2019 to 2022 but declined between 2022 and 2023. The data for 2024 showed that the annual targets of 100% for those receiving ORS and ORS with zinc drops/syrup were not achieved *[Figure 1.C.3.2].*

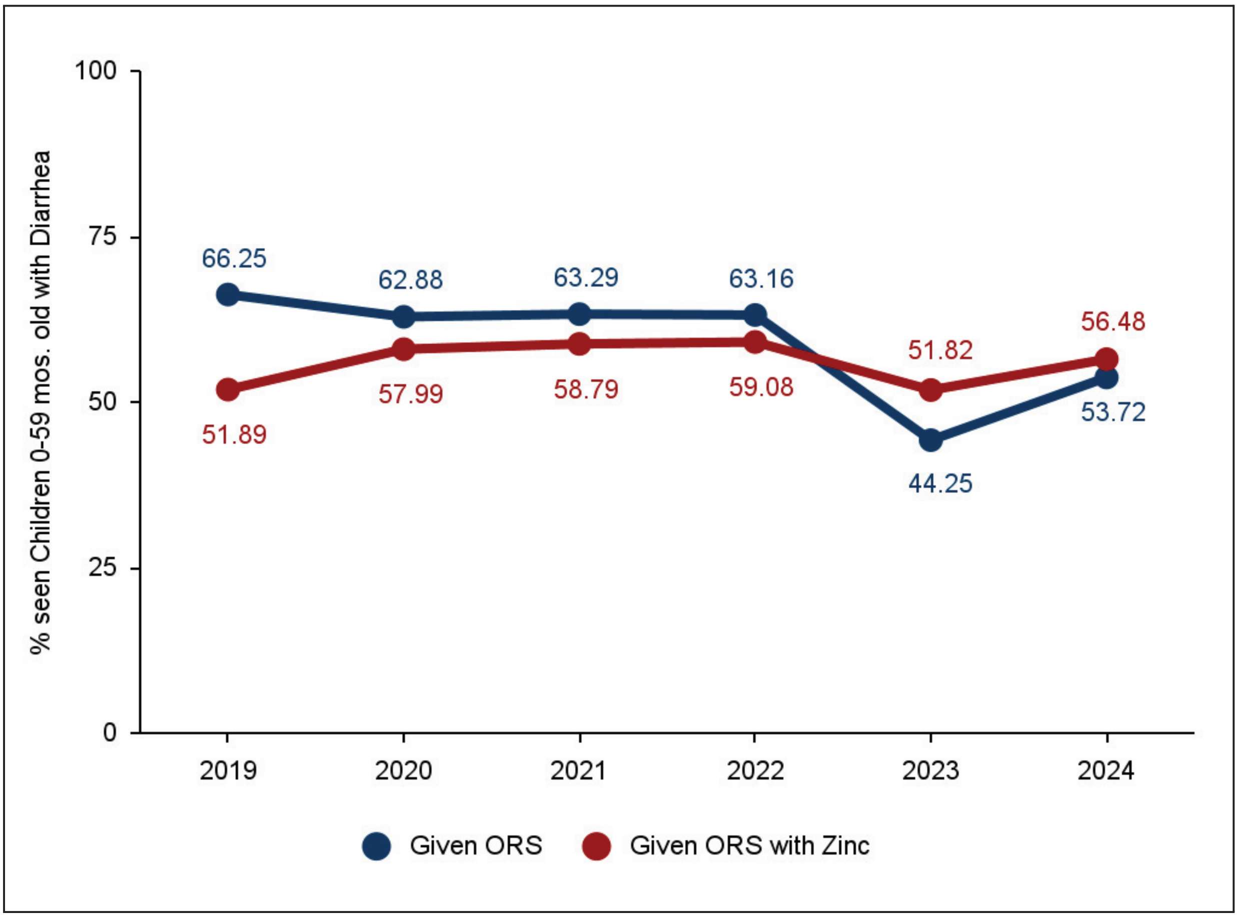

Figure 1.C.3.2. Proportion of Diarrhea cases 0-59 months old Who Received Oral Rehydration Salt Solution (ORS) and ORS with Zinc Drops/Syrup, Philippines, 2019-2024

The data for 2024 also showed some variation across the regions for both diarrhea cases aged zero to 59 months who were given ORS and those who were given ORS with zinc drops/syrup. Region 4A had the highest proportion (02.15%) of diarrhea cases aged between zero to 59 months old who received ORS while Region 11 had the lowest coverage. For those given ORS with zinc drops/syrup, Region 2 had the highest coverage (76.71%) while CARAGA had the lowest coverage (26.05%) *[Table 1.C.3.3].*

Table 1.C.3.3. Proportion of Diarrhea Cases 0-59 months old Who Received Oral Rehydration Salt Solution (ORS) and ORS with Zinc Drops/Syrup, by Region,Philippines, 2024

Area	Children 0-59 mos. old with Diarrhea				
	Seen	Given ORS		Given ORS w/ zinc	
		Total	%	Total	%
**Philippines**	**88,703**	**47,655**	**53.72**	**50,102**	**56.48**
NCR	11,471	4,911	42.81	6,515	56.80
CAR	4,363	1,179	27.02	3,116	71.42
Region 1	3,548	1,940	54.68	1,608	45.32
Region 2	4,044	1,730	42.78	3,102	76.71
Region 3	13,758	6,337	46.06	7,290	52.99
Region 4A	7,496	6,158	82.15	5,021	66.98
Region 4B	2,467	1,451	58.82	903	36.60
Region 5	3,860	1,542	39.95	2,258	58.50
Region 6	4,000	2,753	68.83	1,804	45.10
Region 7	4,519	2,023	44.77	2,452	54.26
Region 8	4,249	3,245	76.37	3,187	75.01
Region 9	4,188	1,282	30.61	2,860	68.29
Region 10	3,881	3,012	77.61	2,499	64.39
Region 11	2,415	647	26.79	1,698	70.31
Region 12	2,166	1,527	70.50	620	28.62
CARAGA	2,100	1,537	73.19	547	26.05
BARMM	10,178	6,381	62.69	4,622	45.41

While the coverage for pneumonia treatment among children zero to 59 months old declined slightly between 2023 (97.3%) and 2024 (96.84%), the coverage rate remained consistently above 95%. However, the annual target of 100% coverage was not achieved *[Figure 1.C.3.3]*.

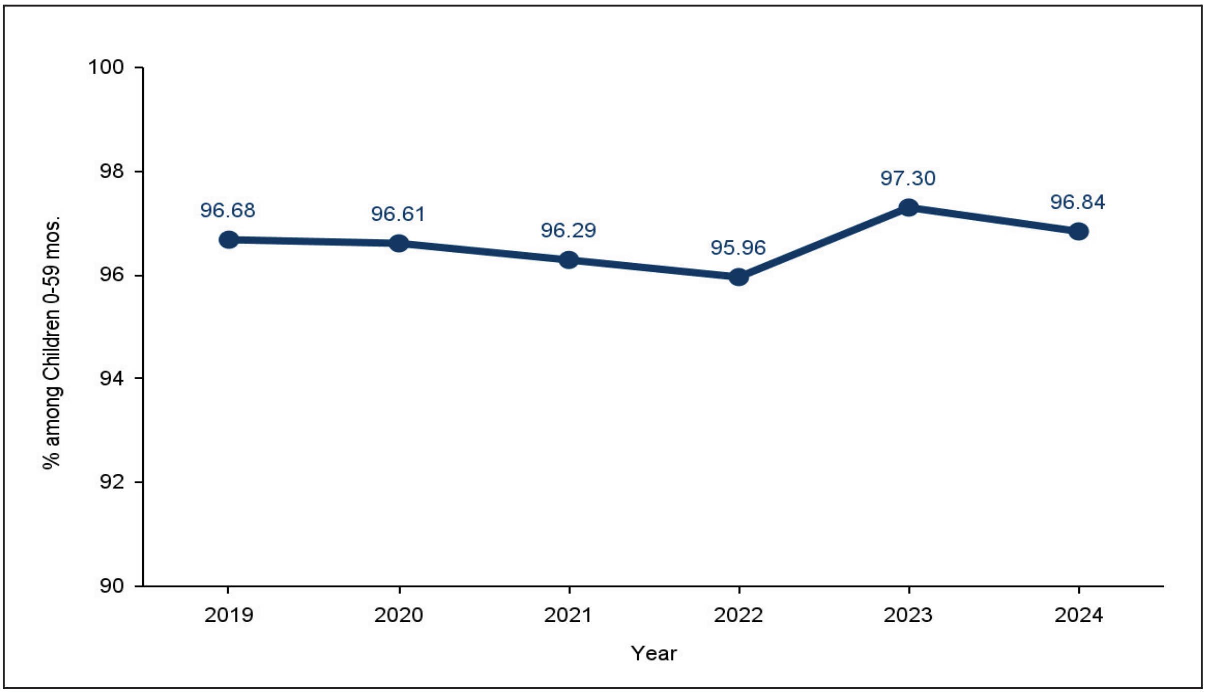

Figure 1.C.3.3. Proportion of Pneumonia Cases Among Children 0-59 months Old Who Received Treatment, Philippines, 2019-2024

Regional data for 2024 showed that only Region achieved 100% coverage rate. It should be noted that the rest of the regions had coverage rates above 90% with the exception of BARMM (89.93%) and Region 4A (84.65%) *[Table 1.C.3.4]*.

Table 1.C.3.4. Proportion of Pneumonia Cases Among Children 0-59 months old Who Received Treatment, by Region, Philippines, 2024

Area	Children 0-59 mos. old with Pneumonia		
		Received Treatment	
	Seen	Total	%
**Philippines**	**104,429**	**101,132**	**96.84**
NCR	16,734	16,700	99.80
CAR	4,085	4,059	99.36
Region 1	4,818	4,818	100.00
Region 2	7,353	6,950	94.52
Region 3	4,847	4,705	97.07
Region 4A	3,954	3,347	84.65
Region 4B	1,300	1,285	98.85
Region 5	3,911	3,846	98.34
Region 6	10,134	9,796	96.66
Region 7	9,672	8,892	91.94
Region 8	10,137	10,021	98.86
Region 9	4,365	4,269	97.80
Region 10	7,306	7,271	99.52
Region 11	4,403	4,355	98.91
Region 12	2,775	2,690	96.94
CARAGA	3,797	3,777	99.47
BARMM	4,838	4,351	89.93

1.C.4 CHILD MORTALITY

Article 24 of the United Nations Convention on the Rights of the Child recognizes that no child shall be deprived of his or her right of access to health care services, and that all States shall take appropriate measures to diminish infant and child mortality[15]. Consequently, the SDGs aim to end preventable deaths in children under-five years of age globally by 2030. Specifically, the under-five mortality rate is targeted to be lowered to 25 deaths per 1,000 live births, while neonatal mortality is aimed to be as low as 12 deaths per 1,000 live births.

Monitoring mortality in children is essential in assessing the effectiveness of the existing child care programs. As the health of the child is heavily dependent on the mother's health, indicators of child mortality may also be linked to maternal health care programs. This chapter presents data on under-five mortality, as well as infant (under 1 year old), neonatal (0 to 28 days), and perinatal mortality. Note, however, that the official deaths statistics shall come from the PSA and numbers in this chapter were from reports submitted in the FHSIS.

From 2020 to 2024, all four mortality indicators show a consistent upward trend, highlighting critical challenges in maternal and child health. Infant mortality increased from 7.34 to 11.46 per 1,000 live births, indicating potential gaps in postnatal care, early childhood nutrition, immunization, and broader social influences *[Figure 1.C.4.1]*.

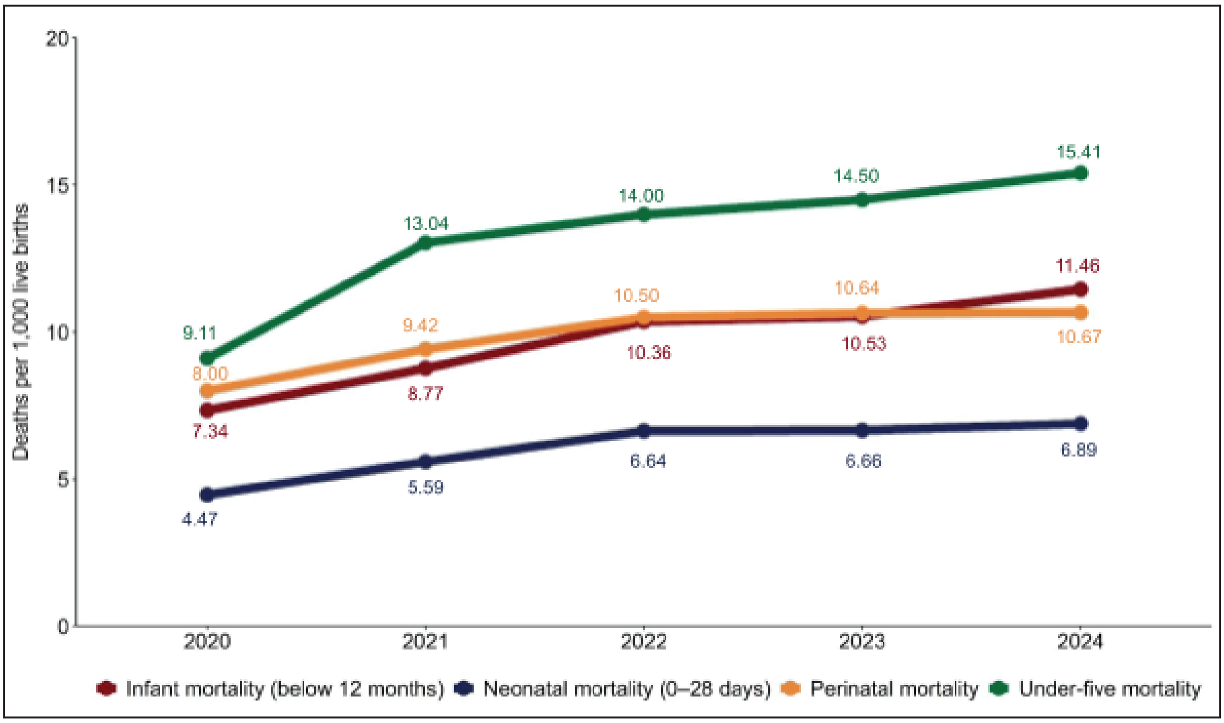

Figure 1.C.4.1: Child Mortality, Philippines, 2020 to 2024

[15] *How the Convention on the Rights of the Child works, UNICEF*

Neonatal mortality, covering deaths within the first 28 days, rose from 4.47 to 6.89 per 1,000 livebirths, reflecting ongoing risks like preterm birth, low birth weight, and birth-related trauma, emphasizing the need for high-quality care during delivery and the immediate postpartum period *[Figure 1.C.4.1]*.

Perinatal mortality, including stillbirths and early neonatal deaths, climbed from 8.00 to 10.67 per 1,000 livebirths, suggesting challenges in managing maternal complications and ensuring safe deliveries *[Figure 1.C.4.1]*.

Under-five mortality jumped from 9.11 to 15.41 per 1,000 livebirths, pointing to broader health issues beyond the neonatal period, including preventable infections, malnutrition, and healthcare access, reflecting the cumulative impact of various factors on child survival *[Figure 1.C.4.1]*.

1.D Oral Health Care and Services

2024
KEY
FINDINGS

BOHC COVERAGE AMONG SELECTED AGE GROUP

65.63%
Infants 0-11 months old

51.87%
Children 1-4 years old

33.39%
School Aged Children 5-9 years old

25.42%
Adolescents 10-19 years old

12.91%
Adults 20-59 years old

21.26%
Senior Citizens

BOHC COVERAGE AMONG PREGNANT WOMEN

0.20%
Pregnant Women 10-14 years old

4.27%
Pregnant Women 15-19 years old

21.36%
Pregnant Women 20-49 years old

Formula:

Basic Oral Health Care (BOHC) proportion specified age groups

Numerator: Total number of clients per specified age groups who received BOHC

Denominator: Eligible population of the specified age group x 30% (service target)

BOHC proportion among pregnant women

Numerator: Total number of pregnant women in specified age groups who received BOHC

Denominator: Eligible population of under 1 year old

Oral health is a fundamental pillar of overall health and well-being. Guided by Republic Act No. 11223, or the Universal Health Care (UHC) Act, the Department of Health (DOH) continues to strengthen the delivery of accessible, quality, and comprehensive oral health services, particularly for vulnerable and underserved populations.

Globally, oral diseases are among the most prevalent noncommunicable diseases, affecting nearly 3.5 billion people, with major impacts on quality of life and economic productivity[1]. Conditions such as dental caries, periodontal disease, and oral cancers can lead to pain, discomfort, and social and functional limitations. The Centers for Disease Control and Prevention (CDC) emphasize that oral health is essential to general health and well-being, noting that poor oral health can interfere with eating, speaking, learning, and socializing, and is associated with systemic conditions such as diabetes, heart disease, and stroke[2].

Recognizing these impacts, the National Oral Health Program—anchored on Administrative Order No. 2007-0007—adopts a lifecycle approach to providing preventive, promotive, and curative dental services. Central to this effort is the delivery of BOHC, a package of essential services integrated into primary care through Rural Health Units (RHUs) and Main Health Centers (MHCs). BOHC aims to instill lifelong oral hygiene habits and reduce the burden of oral disease from early childhood through adulthood.

Service coverage is routinely monitored through the Field Health Services Information System (FHSIS), which tracks the delivery of BOHC across various life stages. The program targets 100% coverage for children and other specified age groups, and at least 50% coverage among pregnant women. These indicators focus exclusively on routine oral health services provided in public Primary Care Facilities (PCFs), excluding dental missions, private sector services, and non-routine activities, to enable consistent evaluation of program implementation.

---

[1] *Noncommunicable Diseases Fact Sheet – World Health Organization (WHO), 2022*

[2] *Oral Health: Fast Facts – Centers for Disease Control and Prevention (CDC), 2023*

[3] *Pourat N, Finocchio L, Snyder JE, Garro N. Impact of Well-Baby Visits on the Timing of First Dental Checkups. Academic Pediatrics, 2017;17(1):84–90. Available from: https://www.ncbi.nlm.nih.gov/pmc/articles/PMC5734461*

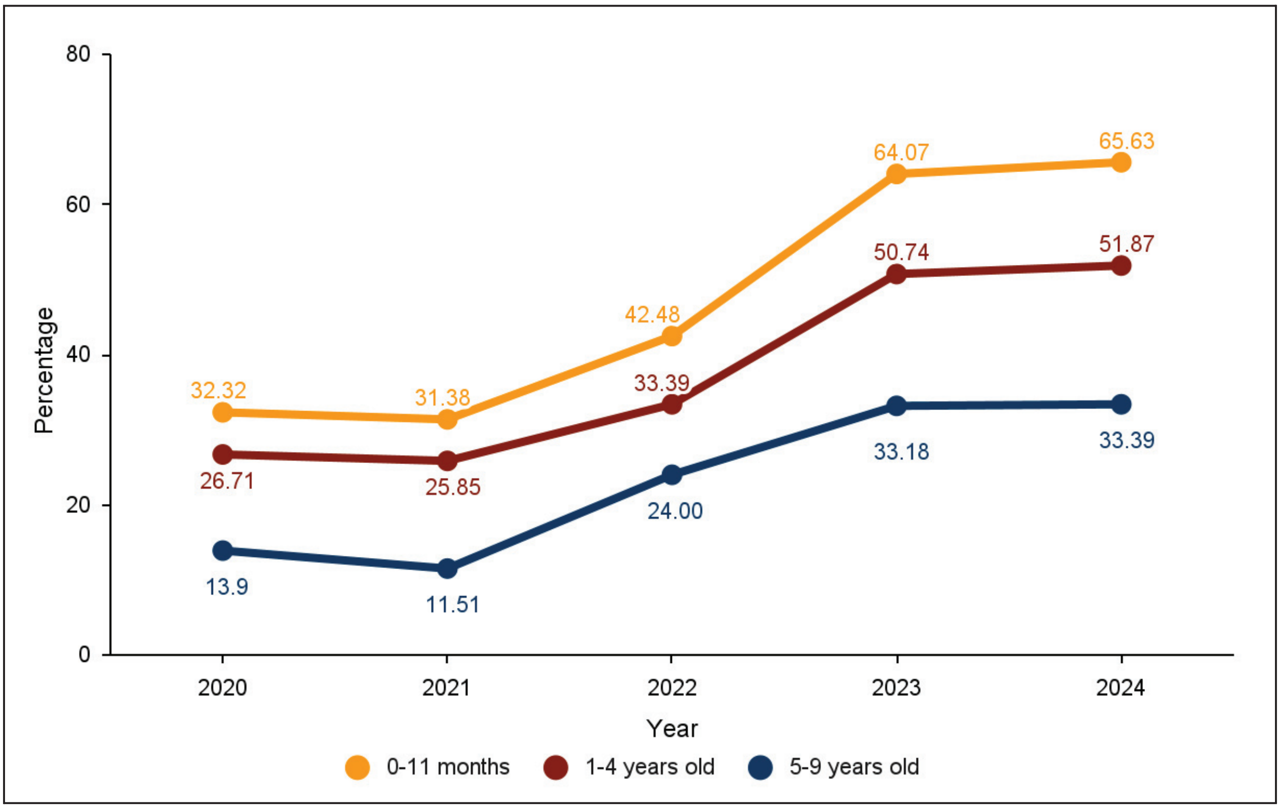

Figure 1.D.1.1: Basic Oral Health Care among Infants and Children, Philippines, 2020-2024

The 2024 BOHC data show a continuing trend of highest service reach among the youngest age group (0–11 months), with 65.63% of the target infants receiving BOHC. This steady rise from previous years suggests that oral health interventions are being well-integrated into maternal and child health programs, particularly during routine immunization and well-baby visits[3]. Among children aged 1–4 and 5–9 years, BOHC coverage also improved, reaching 51.87% and 33.39% in 2024, respectively. These figures may reflect successful efforts to embed oral health education and preventive services within early childhood care programs *[Figure 1.D.1.1]*.

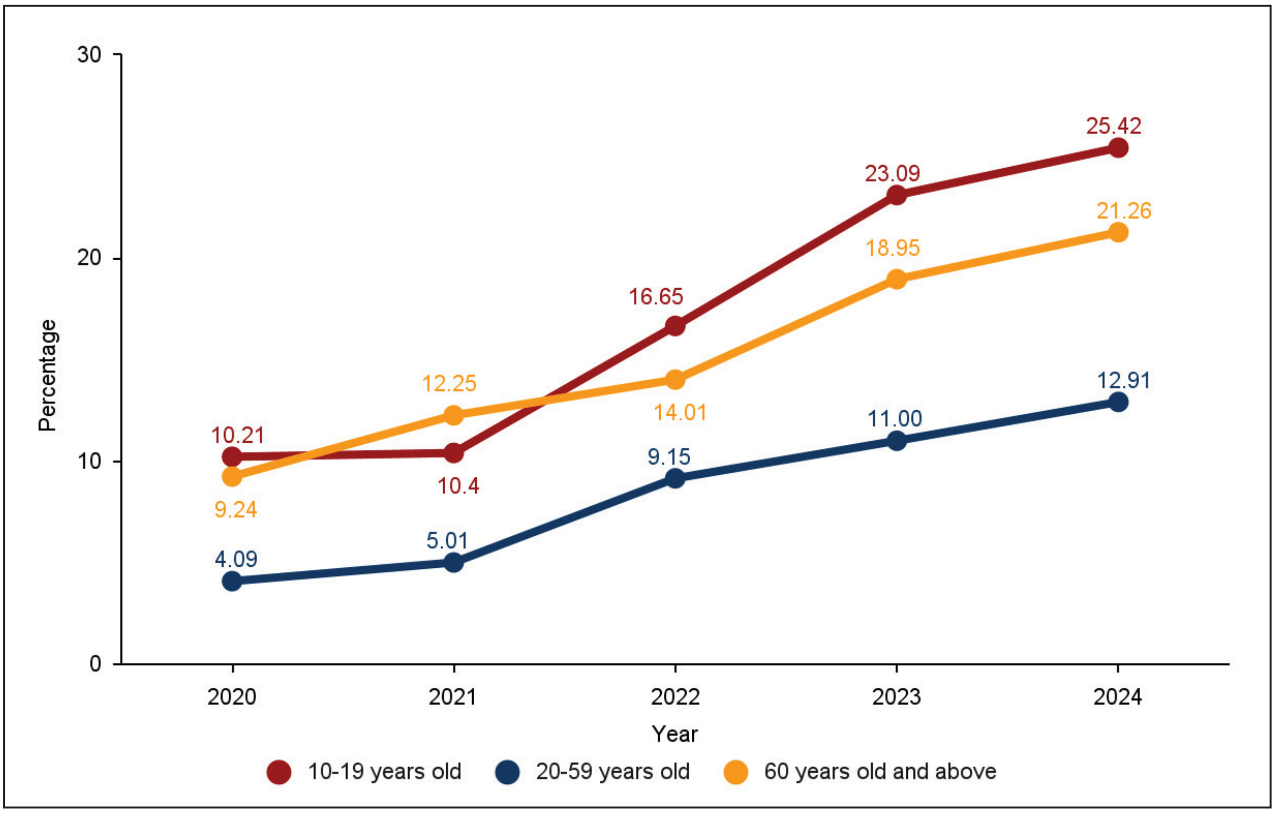

Figure 1.D.1.2: Basic Oral Health Care among Adolescent, Adults and Elderly, Philippines, 2020-2024

Coverage among adolescents (10–19 years) rose to 25.42% in 2024, continuing a gradual upward trajectory since 2020. However, coverage in this group remains below service target (100%). Despite gains in most groups, working-age adults (20–59 years) again recorded the lowest BOHC coverage, at 12.91%. While this reflects a modest increase from the prior year, the low uptake remains a concern. Competing demands such as employment and household responsibilities may hinder this group from accessing preventive care. On the other hand, coverage among senior citizens (60 years and above) rose to 21.26%, marking a steady improvement from previous years. This suggests growing responsiveness to the oral health needs of senior citizens, who are at higher risk for conditions such as periodontal disease and tooth loss *[Figure 1.D.1.2]*.

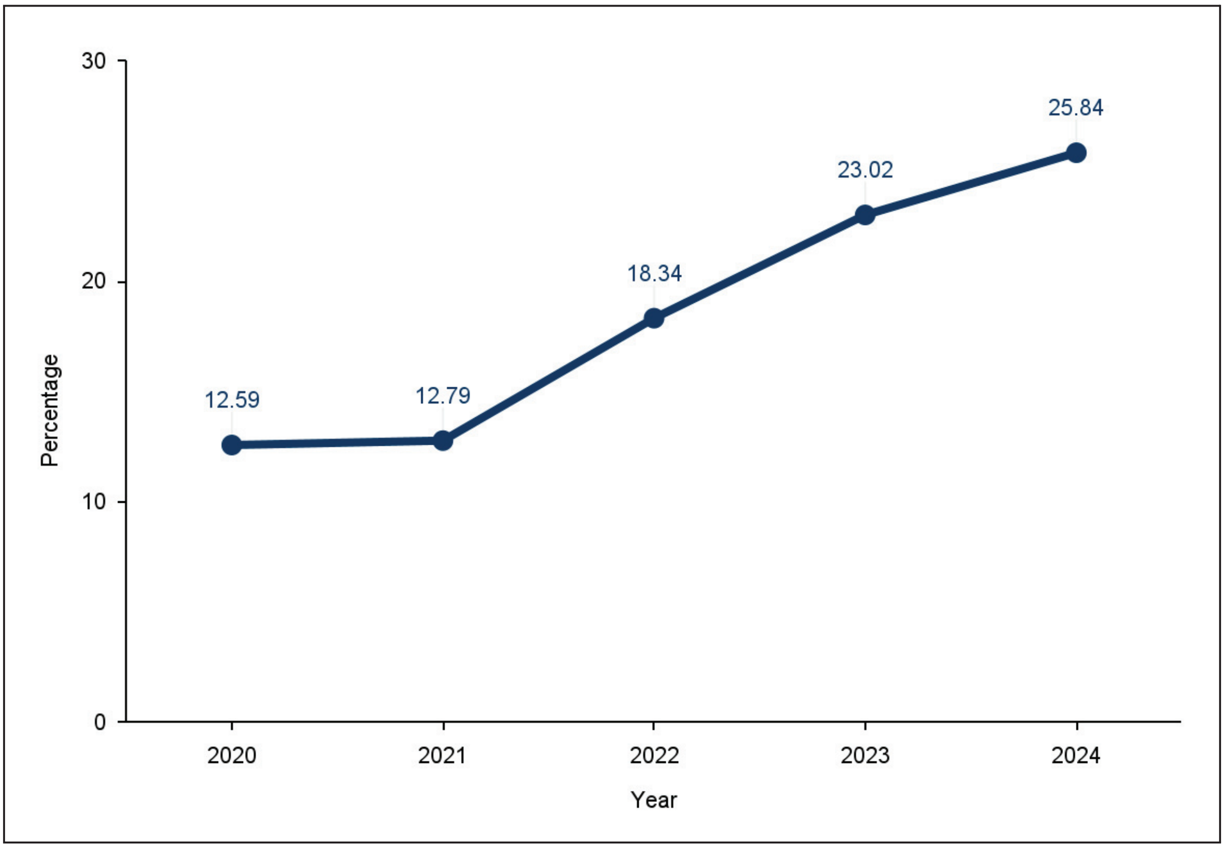

Figure 1.D.1.2: Basic Oral Health Care among Pregnant Women, Philippines, 2020-2024

In 2024, the BOHC coverage among pregnant women aged 10–49 years rose to 25.84%, continuing a steady upward trend over the past five years. While this shows progress compared to 23.02% in 2023 and nearly double the 2020 baseline (12.59%), the coverage remains significantly below the 50% national target *[Figure 1.D.1.2]*.

Low BOHC uptake in this population may indicate barriers such as lack of awareness about the importance of oral health during pregnancy, competing maternal health priorities, or logistical challenges in accessing dental services during prenatal care. Given the increased vulnerability of pregnant women to oral health problems—such as gingivitis and periodontal disease—this gap could have wider implications for both maternal and neonatal health outcomes.

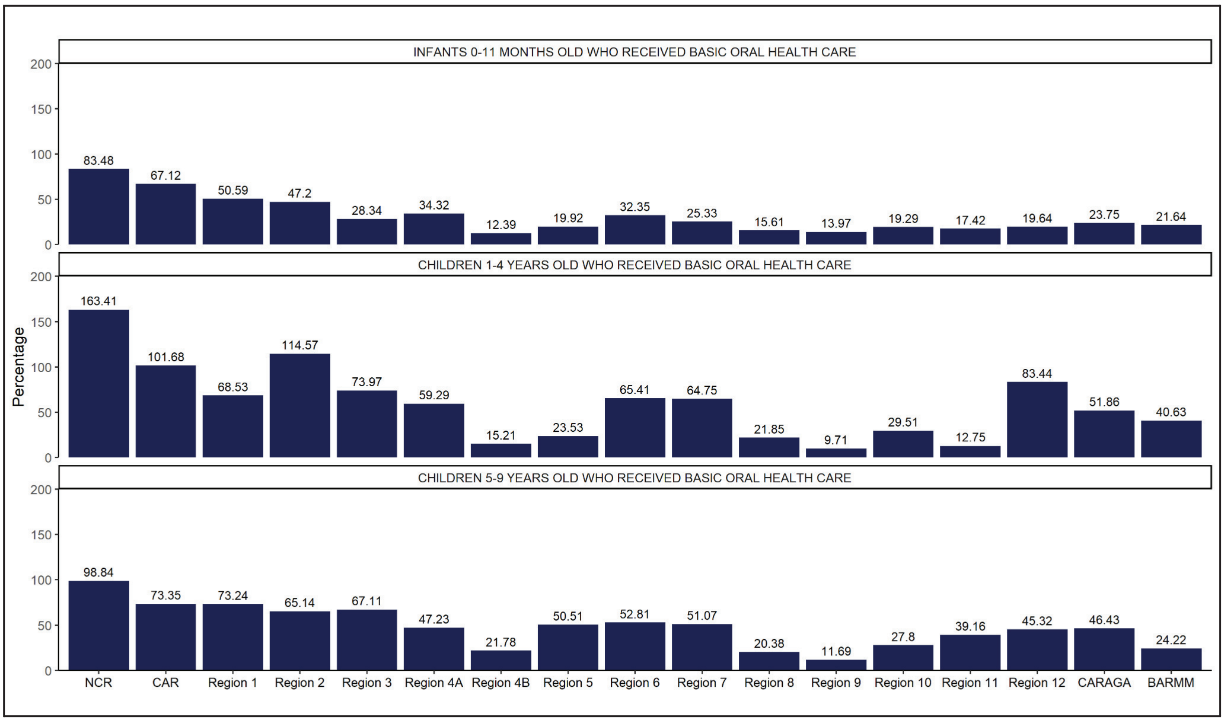

Figure 1.D.1.3: Basic Oral Health Care among Infants and Children, by Region, 2024

In 2024, only three of the 17 regions (NCR, CAR, and Region 2) surpassed the BOHC service target for infants, with average coverage above 100%. Across all regions, infant BOHC coverage spanned from a high of 163.41% in NCR to a low of 9.71% in Region 9 [Figure 1.D.3]. Yet many regions remain below 50% coverage, highlighting the urgent need to strengthen BOHC advocacy among infants to secure their lifelong oral health.

No region met the BOHC service target (100%) for children aged 1-4 years or 5-9 years. Among 1-4 year olds, coverage ranged from 11.69% in Region 9 to 98.84% in NCR; among 5-9 year olds, it ranged from 12.39% in Region 4B to 83.48% in NCR [Figure 1.D.3]. For adolescents and adults [Figure 1.D.4], BOHC coverage is the highest at 74.53% for adolescents in NCR and 26.74% for adults in CAR, while Region 9 had the lowest rates at 8.07% for adolescents and 4.84% for adults. Among senior citizens [Figure 1.D.5], coverage was highest in NCR at 45.83% and lowest in Region 9 at 5.37%. These figures highlight a notable coverage gap across age groups and across the regions., Specifically, BOHC coverage among adults is considerably lower compared to other age groups while the BOHC coverage in Region 9 is the lowest across the regions for all age-groups.

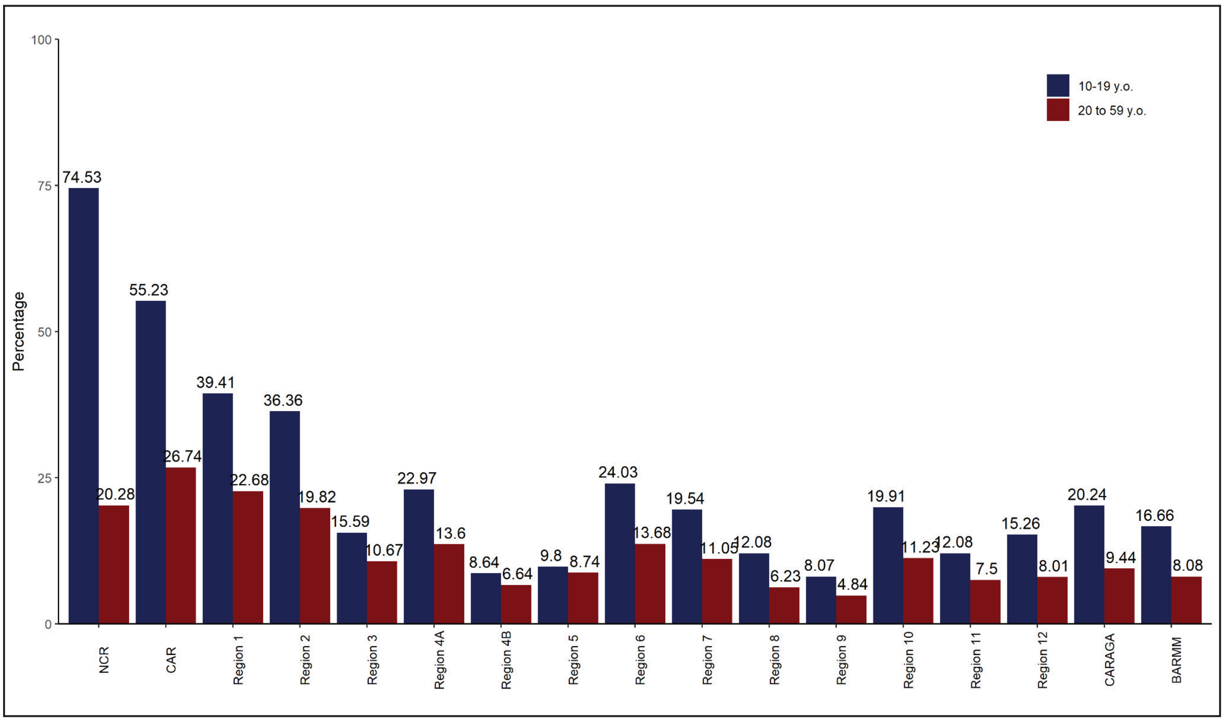

Figure 1.D.1.4: Basic Oral Health Care among Adolescents and Adults, by Region, 2024

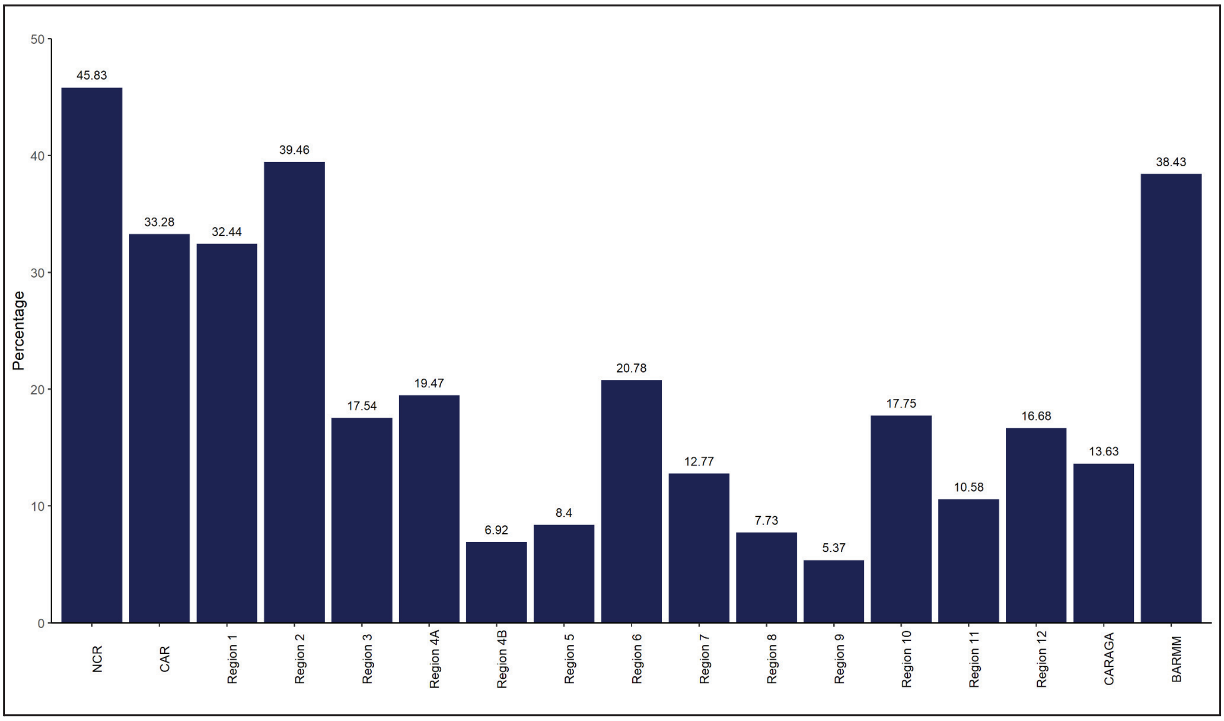

Figure 1.D.1.5: Basic Oral Health Care among Senior Citizens, Philippines, 2024

1.E Non Communicable Diseases

2024 KEY FINDINGS

Hypertension and Diabetes Mellitus Type II

26.31%
Adults were assessed using the PhilPEN Protocol

35.87%
Senior Citizens were assessed using the PhilPEN Protocol

13.34%
Adults were identified hypertensives among risk-assessed

32.47%
Senior Citizens were identified hypertensives among risk-assessed

4.34%
Adults were identified with Diabetes Mellitus Type 2 among risk-assessed

9.58%
Senior Citizens were identified with Diabetes Mellitus Type 2 among risk-assessed

Cancer Prevention and Control

0.89%
of women aged 20 years and above were screened for Cervical Cancer

2.90%
of women aged 20 years and above were screened for Breast Mass

2.62%
of women who were screened were found positive for or suspected with Cervical Cancer

1.01%
of women who were screened were found with suspicious breast mass

Formula:
Adults and Elderly who were risk assessed using the PhilPEN protocol

Numerator 1: Number of adults who were risk assessed using the PhilPEN protocol
Denominator 1: Eligible Population 20-59 years old
Numerator 2: Number of elderly who were risk assessed using the PhilPEN protocol
Denominator 2: Eligible Population 60 years old and above

Identified Hypertensive Adults and Elderly

Numerator 1: Number of adults identified as hypertensive
Denominator 1: Total number of adults 20-59 years old who were risk assessed using the PhilPEN protocol
Numerator 2: Number of elderly identified as hypertensive
Denominator 2: Total number of adults 60 years old who were risk assessed using the PhilPEN protocol

Identified Adults and Elderly with Type 2 Diabetes

Numerator 1: Number of adults identified with type II diabetes mellitus
Denominator 1: Total number of adults 20-59 years old who were risk assessed using the PhilPEN protocol
Numerator 2: Number of elderly identified with type II diabetes mellitus
Denominator 2: Total number of adults 60 years old who were risk assessed using the PhilPEN protocol

Women Screened for Cervical Cancer using Visual Inspection with Acetic Acid (VIA) or Pap Smear

Numerator 1: Number of women 20 years old and above screened for cervical cancer using VIA or pap smear
Denominator 1: Eligible population 20 years old and above (female only)
Numerator 2: Number of women 30-65 years old screened for cervical cancer using VIA or pap smear
Denominator 2: Eligible population 30-65 years old (female only)

Women with Suspicious Breast Mass

Numerator: Number of women 20 years old and above with suspicious breast mass
Denominator: Total number of women 20 years old and above screened for breast mass

Elderly who Received 1 Dose of Pneumococcal Polysaccharide Vaccine (PPV)

Numerator: Number of senior citizens 60 years old and above who received 1 dose of PPV
Denominator: Eligible population 60 years old and above

Noncommunicable diseases (NCDs) are a major public health challenge worldwide, responsible for the majority of deaths and disabilities. According to the World Health Organization (WHO), NCDs account for approximately 74% of all deaths

globally[1]. These diseases, including cardiovascular diseases (CVDs), diabetes, chronic respiratory diseases, and cancers, are characterized by their prolonged duration and slow progression. Unlike infectious diseases, NCDs stem from a combination of genetic, physiological, environmental, and behavioral factors. Key modifiable risk factors such as tobacco use, physical inactivity, unhealthy diets, and excessive alcohol consumption significantly contribute to NCD-related morbidity and mortality[2]. Early identification of these risk factors allows for the implementation of preventive measures that can reduce the overall disease burden. Furthermore, systematic tracking of NCD indicators ensures accountability and informs policy decisions, strengthening the overall health system response to these diseases[1].

In the Philippines, the Department of Health (DOH) has recognized the critical need to address NCDs through policies such as Administrative Order No. 2012-0029, which institutionalizes the Philippine Package of Essential NCD Interventions (PhilPEN)[3]. This initiative, adapted from the WHO's WHO PEN, facilitates the integration of hypertension and diabetes management into primary healthcare settings. Local Government Units (LGUs) play a crucial role in implementing PhilPEN, ensuring that services and products are accessible within primary healthcare facilities. Furthermore, the Universal Health Care (UHC) Law, or Republic Act No. 11223, strengthens the integration of NCD interventions within the broader healthcare system by guaranteeing equitable access to preventive, diagnostic, and treatment services[4].

Cancer prevention also plays a pivotal role in NCD control. Among women, cervical and breast cancers are among the most preventable types of cancer. Routine screening and early detection significantly enhance treatment outcomes and reduce mortality rates[5]. Likewise, elderly populations are particularly vulnerable to vaccine-preventable diseases such as influenza and pneumococcal infections due to age-related declines in immunity[1]. Republic Act 9994, also known as the "Expanded Senior Citizens Act," mandates the DOH to provide free influenza and pneumococcal immunization for indigent senior citizens, highlighting the government's commitment to safeguarding this vulnerable group[6].

In 2024, the Philippines continued to strengthen its response to NCDs, as reflected in improved national screening coverage and age-disaggregated reporting. The proportion of individuals aged 20 years and above who underwent risk assessment using the PhilPEN protocol rose to 27.65%, a marked increase from 20.90% in 2023. While this still fell slightly short of the national target of 30%, the age-specific data paint a more encouraging picture. Senior citizens demonstrated the highest screening uptake at 35.87%, compared to 26.31% among adults aged 20–59. This disparity may reflect greater health utilization among older populations, possibly due to factors such as

having greater flexibility in their daily schedules, increased health awareness, or more frequent interactions with healthcare providers *[Figure 1.E.1.1 and Figure 1.E.1.2].*

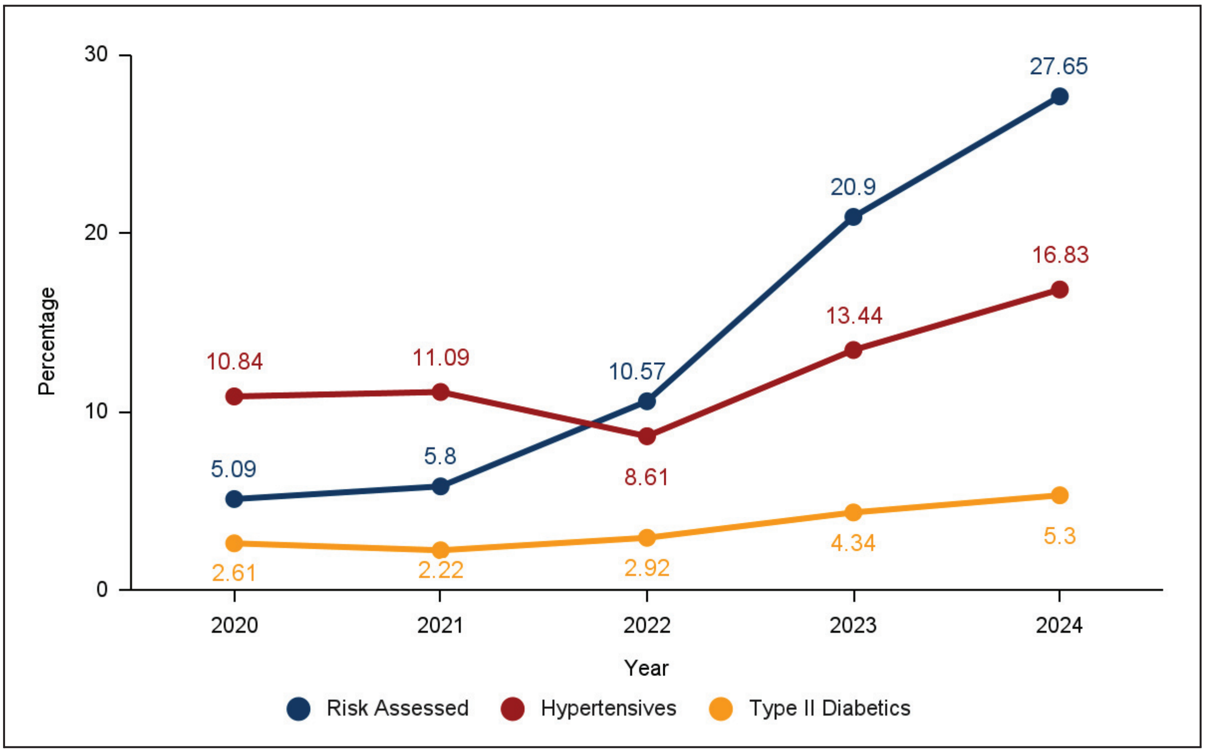

Figure 1.E.1.1: Risk Assessment, Hypertensives and Type II Diabetics, Philippines, 2020 to 2024

Hypertension and diabetes indicators followed expected age-related patterns. The prevalence of hypertension among adults aged 20 and above increased to 16.83% in 2024 from 13.44% of the previous year, continuing an overall upward trend over the past five years. This is the highest recorded prevalence within the period, more than doubling the rate observed in 2022 (8.61%) and significantly higher than 2020 levels (10.84%). This sustained increase may be attributed to both improved case detection and an actual rise in disease burden, particularly among senior citizens.

Similarly, the prevalence of type II diabetes also showed a steady increase, reaching 5.30% in 2024 from 4.34% in 2023. The prevalence in 2022 (2.92%) and 2021 (2.22%) was notably lower, suggesting a consistent upward trajectory that warrants close monitoring and stronger prevention strategies *[Figure 1.E.1.1].*

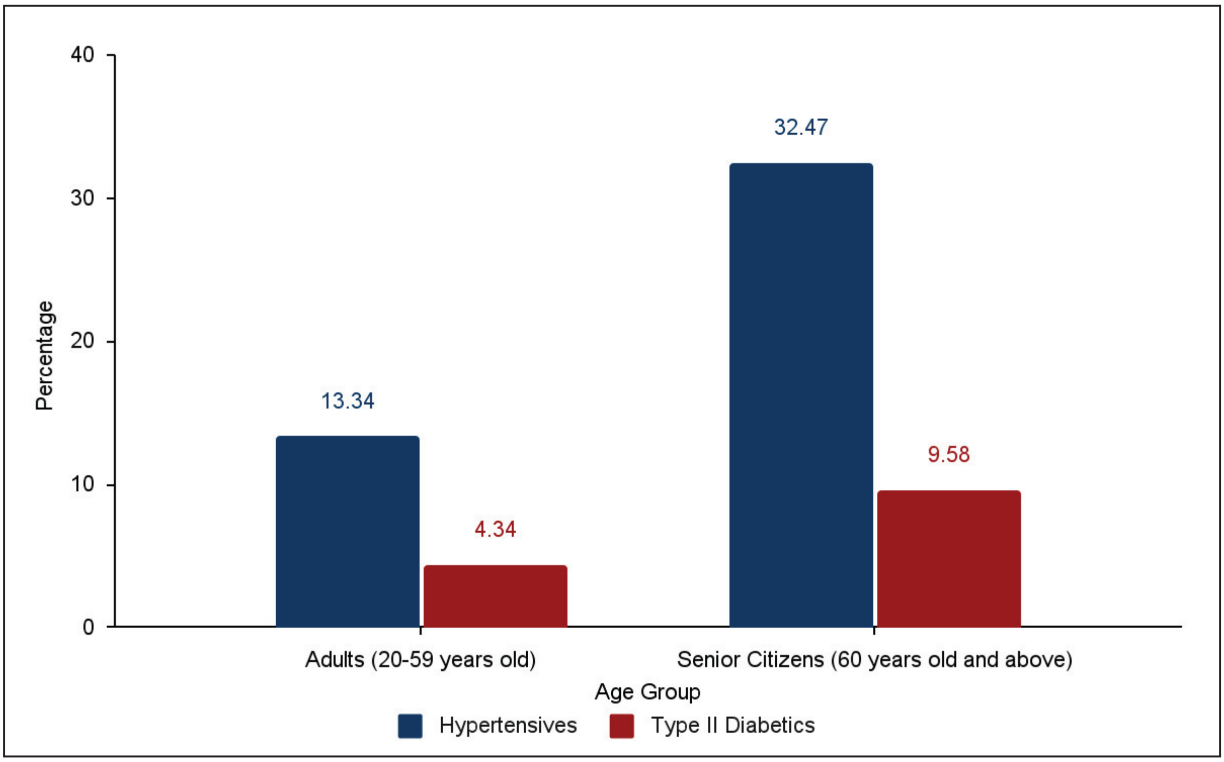

Figure 1.E.1.2: Hypertensives and Type II Diabetics, Philippines, 2024

For 2024, the age group classification was refined, splitting adults into 20–59 years and 60 years and above to better capture differences in prevalence. When disaggregated, the data revealed that hypertension was far more common among senior citizens, with a prevalence rate of 32.47%, compared to just 13.34% among adults. This distribution is consistent with global evidence showing that blood pressure tends to rise with age. According to the World Health Organization, more than 60% of people over the age of 60 are hypertensive, underscoring the need for intensified case detection and management efforts in older populations[7]. Similarly, diabetes prevalence also increased, reaching 5.30% among adults aged 20 and above. The rate was more than twice as high among senior citizens (9.58%) compared to adults aged 20–59 (4.34%) *[Figure 1.E.1.2].*

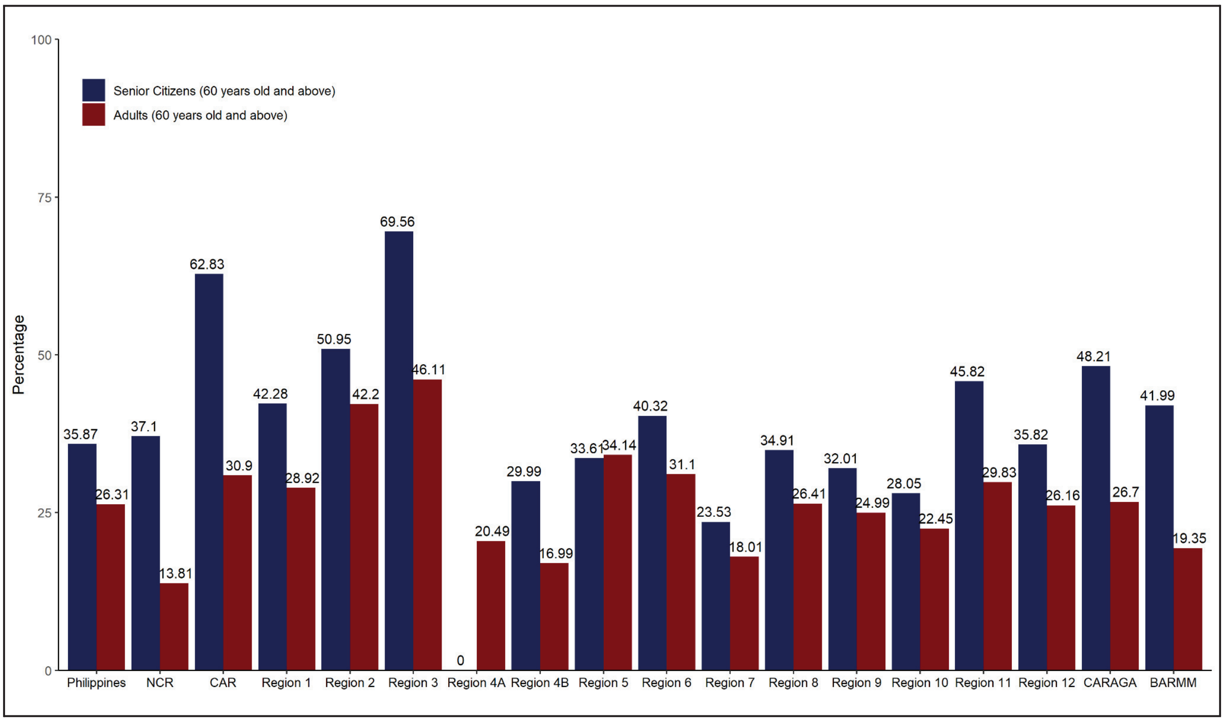

Figure 1.E.1.3: Risk Assessment, by Region, 2024

In 2024, the implementation of NCD risk assessments varied widely across regions and age groups. Among adults aged 20–59 years, the highest coverage was recorded in Region 3 (46.11%), followed by Region 2 (42.20%) and Region 5 (34.14%). In contrast, the lowest adult screening rates were recorded in NCR (13.81%) and Region 4B (16.99%).

For senior citizens (60 years old and above), coverage exceeded the national target of 30% in most regions. The highest coverage was observed in Region 3 (69.56%), CAR (62.83%), and Region 2 (50.95%). However, some regions such as Region 7 (23.53%) and Region 10 (28.05%) fell below the target. While Region 4A had no available data on senior citizens due to the lack of appropriate disaggregation in the collected data. These variations highlight regional differences in reaching the elderly population. Nevertheless, persistent regional disparities indicate the need for more equitable and targeted implementation across all age groups *[Figure 1.E.1.3].*

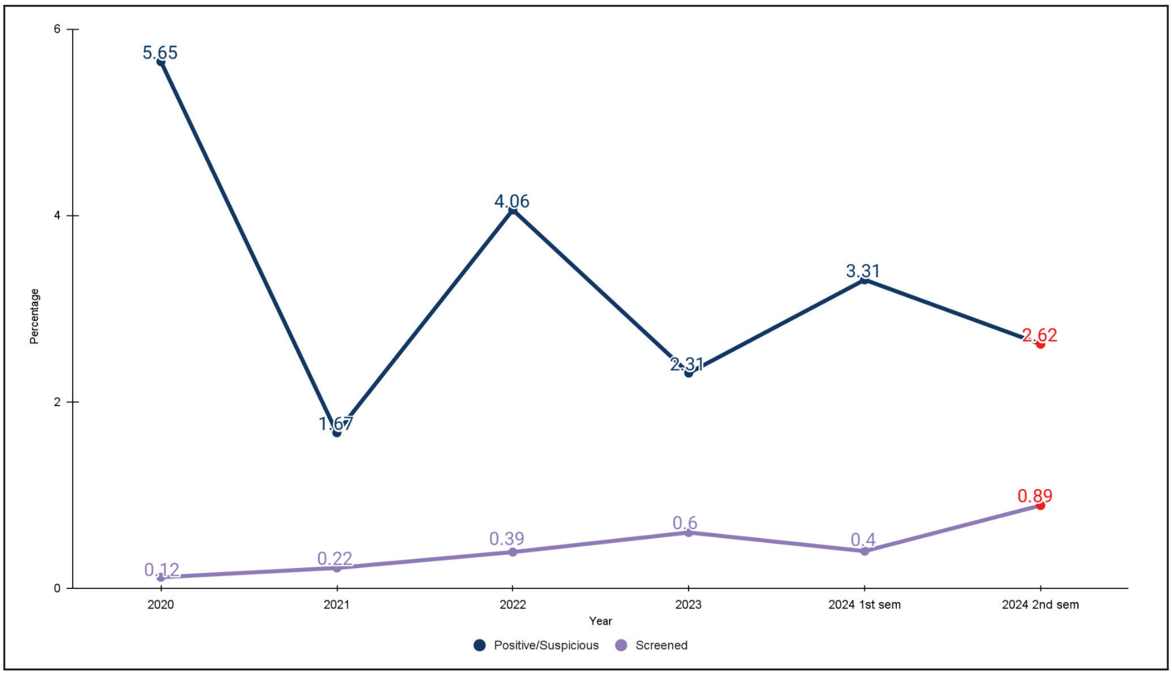

Figure 1.E.2.1: Cervical Cancer Screening and Outcome (Positive/Suspicious), Philippines 2020-2024

Cervical cancer screening coverage among Filipino women aged 20 years and above has remained limited from 2020 to the first semester of 2024, ranging between 0.12% and 0.6%, with a slight decline to 0.4% in early 2024. Cancer indicators for the second semester of 2024 changed, focusing on the target age group of 30 to 65 years, cervical cancer showed coverage at 0.89%.

The proportion of positive or suspicious cervical cancer cases among those screened has fluctuated over time, peaking at 5.65% in 2020. In the first half of 2024, the positive or suspicious cervical cancer cases was 3.31%, while for the 30 to 65 age group in the second semester of 2024, it declined to 2.62% *[Figure 1.E.2.1].*

In 2024, cervical cancer screening coverage remained well below the 70% target in every region. Only six regions managed to screen at least 1% of their eligible women: CARAGA (3.82%), Region 11 (1.92%), Region 3 (1.58%), NCR (1.40%), CAR (1.17%), and Region 8 (1.20%), against a national average of just 0.89%. Among those screened, the highest rates of positive or suspicious VIA/Pap results were seen in Region 9 (6.11%), Region 4A (6.03%) and CARAGA (4.81%), while Region 8 had the lowest positivity rate at 0.81% *[Figure 1.E.2.2 and Figure 1.E.2.3].*

Breast mass screening followed a similar trend of underutilization: only five regions reached or exceeded 5% coverage, Region 12 (7.81%), CARAGA (6.85%), Region 6 (6.46%), CAR (6.22%), and Region 11 (5.41%), with a national average of 2.90% and Region 4B with no reports at all. Of those screened, Region 4A again had

the highest proportion of suspicious findings (5.73%), followed by CAR (1.5%) and CARAGA (1.36%), compared to a national mean of 1.01% *[Figure 1.E.2.2 and Figure 1.E.2.3].*

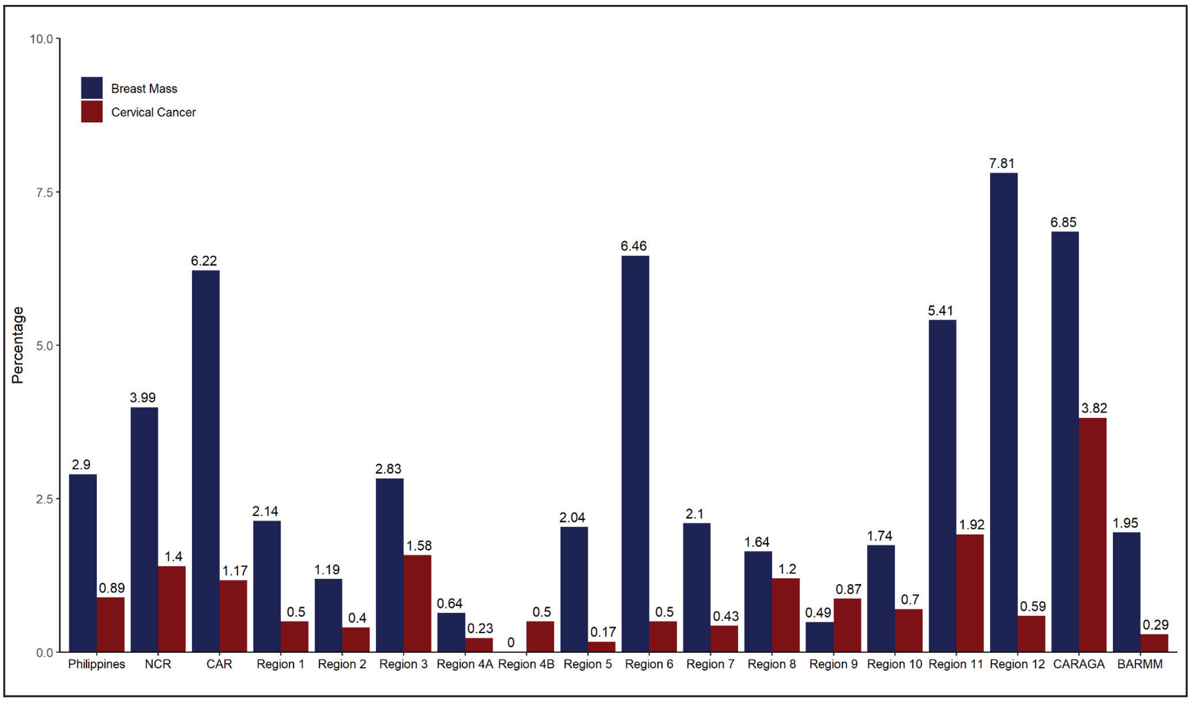

Figure 1.E.2.2: Cervical Cancer Screening, by Region, 2024

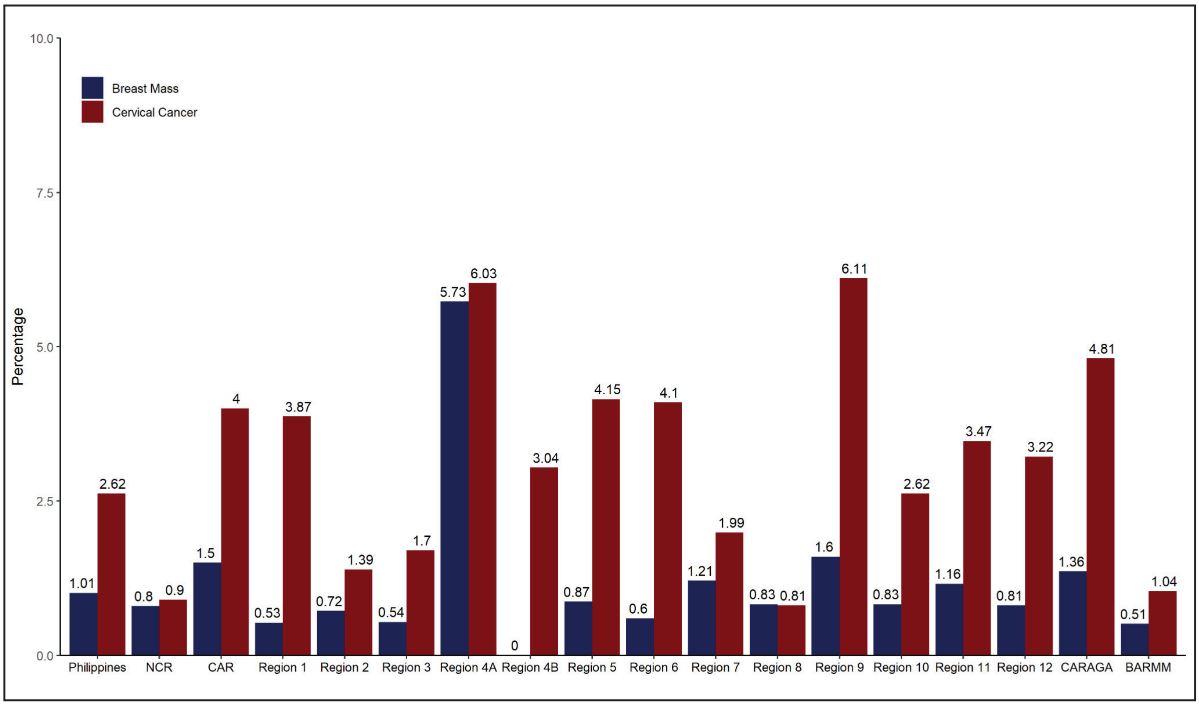

Figure 1.E.2.3: Suspicious Cervical Cancer and Breast Mass among Screened, by Region, 2024

Nationally, the average coverage among senior citizens was 6.71% for PPV and 8.92% for the influenza vaccine. These figures reflect persistently low vaccination rates, indicating the continued need to strengthen immunization efforts for senior citizens. Given the increased risk of complications and mortality among seniors due to age-related health vulnerabilities such as immunosenescence and the higher prevalence of chronic conditions ensuring that they receive timely and adequate protection against vaccine-preventable diseases remains a public health priority *[Figure 1.E.3.1]*.[8]

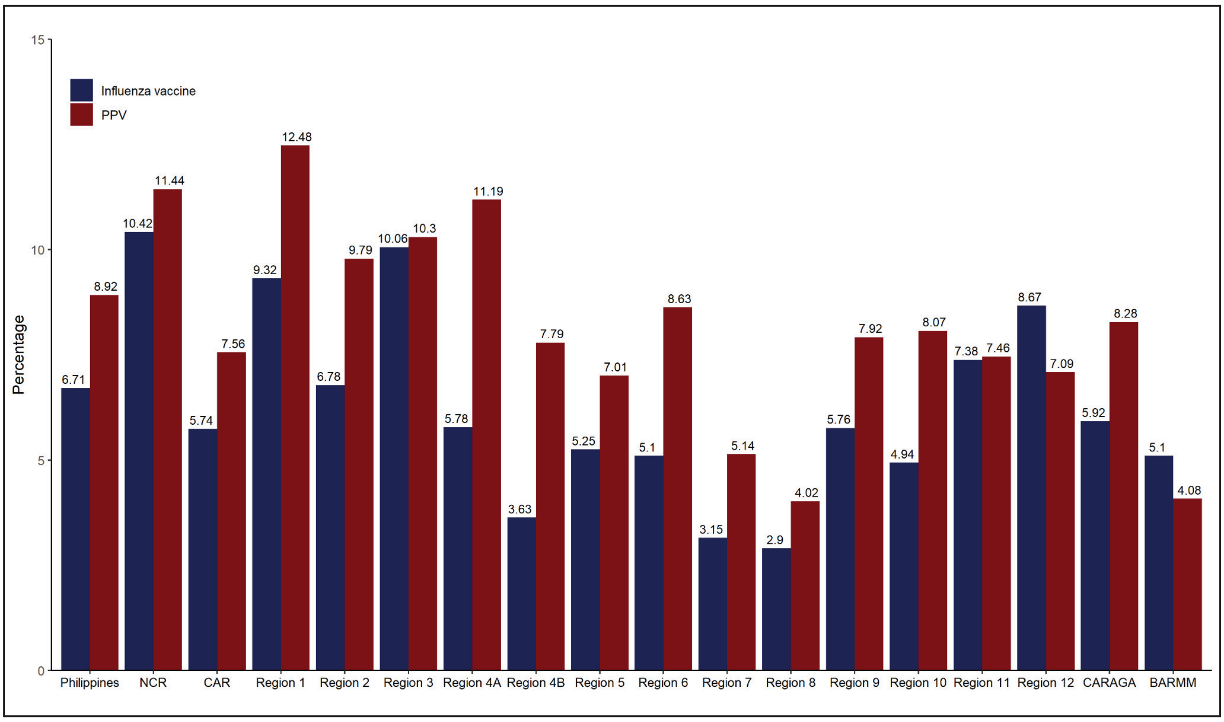

Figure 1.E.3.1 PPV and Influenza Vaccination in Senior Citizens, by Region, 2024

[1]*Noncommunicable Diseases Fact Sheet – World Health Organization (WHO), 2022*

[2]*Noncommunicable Diseases – Centers for Disease Control and Prevention (CDC), 2023*

[3]*Administrative Order No. 2012-0029: Implementing Guidelines on the Institutionalization of the Philippine Package of Essential NCD Interventions (PhilPEN) – Department of Health (DOH), 2012*

[4] *Republic Act No. 11223: Universal Health Care Law – Republic of the Philippines, 2019*

[5] *Cancer Prevention – World Health Organization (WHO), 2021*

[6] *Republic Act No. 9994: Expanded Senior Citizens Act – Republic of the Philippines, 2010*

[7]*World Health Organization. Hypertension: Key Facts. Geneva: WHO; 2023. Available from:* [8] *https://www.who.int/news-room/fact-sheets/detail/hypertension*

[8]*Vaccines for the elderly: current use and future challenges – Weinberger B., Immunity & Ageing, 2018*

1.F.A Infectious Diseases

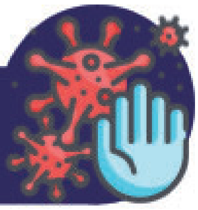

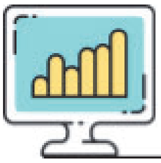

2024 KEY FINDINGS

FILARIASIS

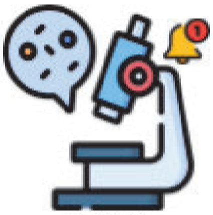
DETECTION

2.30
Case Detection Rate (CDR)

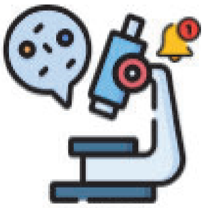
CLINICAL

3.13
Clinical Rate (CR)

HIV-AIDS/STI

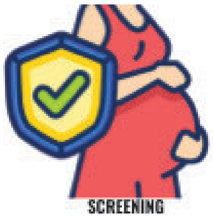
SCREENING

30.10%
Pregnant Women Screened for Syphilis

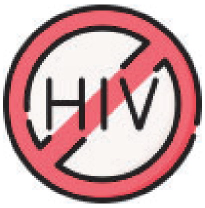

0.75%
Tested Positive for Syphilis

SCHISTOSOMIASIS

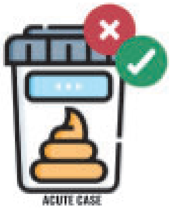
ACUTE CASE

83.59%
Acute Cases Confirmed Positive by Stool Examination

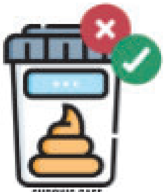
CHRONIC CASE

55.01%
Chronic Clinically Diagnosed Cases confirmed by Stool Examination

SOIL-TRANSMITTED HELMINTHIASIS

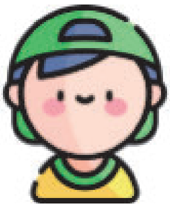

57.06%
PSAC (1-4 years old)

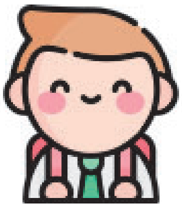

31.84%
SAC (5-9 years old)

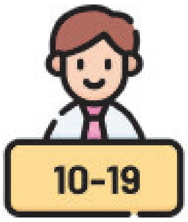

24.04 %
Adolescent (10-19 years old)

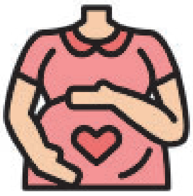

12.24 %
Pregnant Women

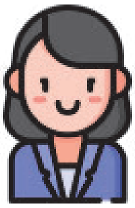

1.05 %
Women Reproductive Age (WRA)

1.F.A.1. FILARIASIS

Formula:

Case Detection Rate of lymphatic filariasis in endemic provinces

Numerator:	Number of individuals examined found positive for lymphatic filariasis
Denominator:	Total no. of individuals examined for lymphatic filariasis

Clinical Rate of lymphatic filariasis in endemic provinces

Numerator:	Number of lymphatic filariasis cases examined with disabilities /deformities
Denominator:	Total no. of cases with lymphatic filariasis

Filariasis is a tropical disease caused by parasitic worms known as filariae, which are transmitted to humans through bites of infected mosquitoes. The most common form is called *lymphatic filariasis*, caused by *Wucheria bancrofti, Brugia malayi and Brugia timori,* which affects the lymphatic system and can lead to a painful swelling in the limbs and other parts of the body, causing long term disability.[1]

This chapter presents the latest data on filariasis including case detection and clinical rates in endemic provinces captured in the FHSIS within primary health care. In the Philippines, lymphatic filariasis was historically endemic in many provinces. Through sustained public health interventions – including mass drug administration (MDA), vector control, health education, and morbidity management, substantial progress has been achieved. And as of 2023, 44 out of 46 previously endemic provinces have been declared as filaria-free.[2] Below is the five (5) year national trend (2020-2024) for case detection rate and clinical rate of lymphatic filariasis *[Figure 1.F.A.1.1]*.

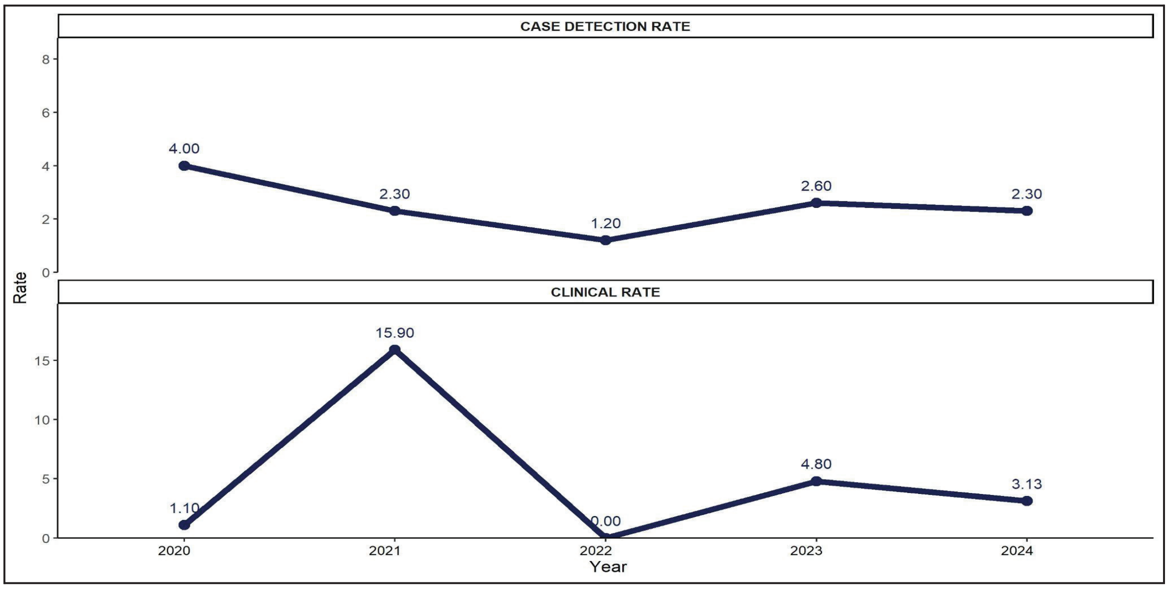

Figure 1.F.A.1.1: Five Year Trend Case Detection Rate (CDR) and Clinical Rate of Lymphatic Filariasis, Philippines, 2020 to 2024

---

[1] *Lymphatic filariasis*, World Health Organization, 2023
[2] *Field Health Information Services, 2023*

The case detection rate (CDR) of lymphatic filariasis in the Philippines has generally declined from 4.00 in 2020 to 2.30 in 2024 with a slight increase in 2023 at 2.60, – the 2024 CDR represents a 8.60% decrease in the past previous years. However, the 2024 CDR of 2.30 remains above the CDR target of less than 1% prevalence, indicating that the Philippines has not yet met its goal of eliminating filariasis in the country.

In contrast, the Clinical Rate (CR) showed more variability, with a spike in 2021 (15.90) and a drop to zero in 2022. The CR in 2024 was 3.13, which is 42.66 % lower than the levels seen across 2020 to 2023 *[Figure 1.F.A.1.1].*

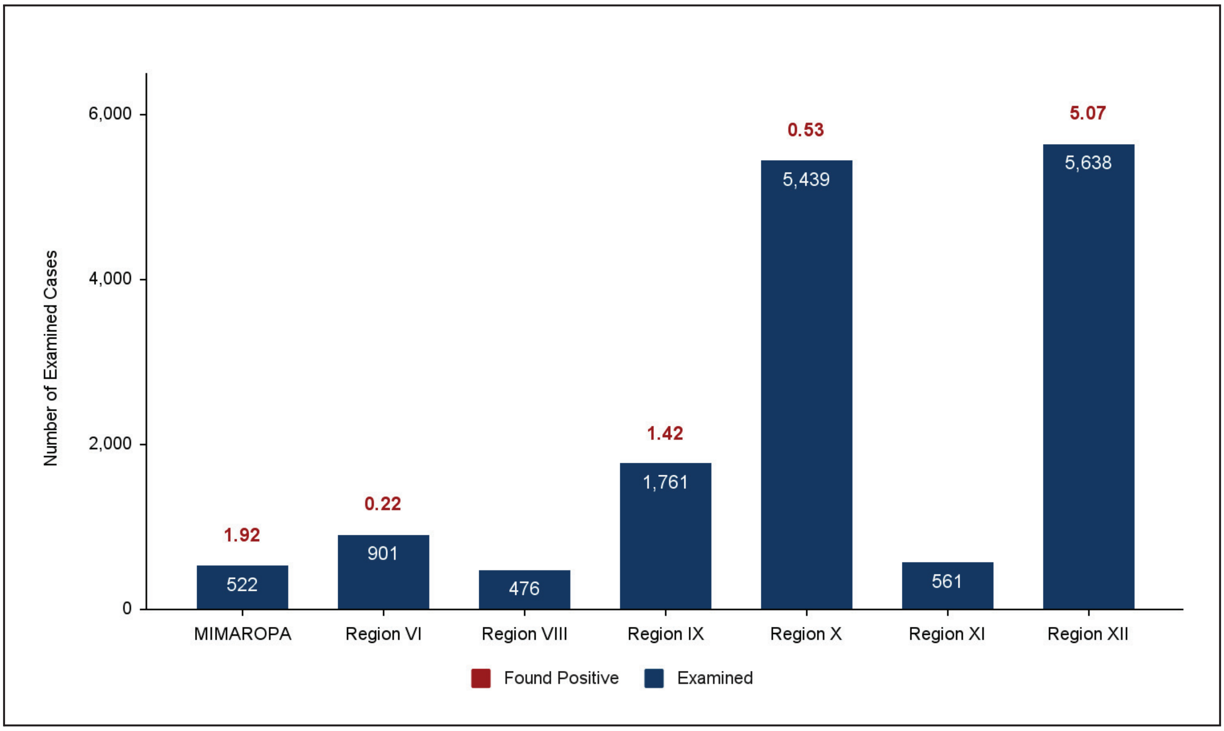

Figure 1.F.A.1.2. Regional Distribution of Examined and Positive Cases for Lymphatic Filariasis by Region, 2020 to 2024

In 2024, a total of 15,298 were examined for lymphatic filariasis across several endemic regions in the Philippines, with 352 confirmed positive cases, resulting in an overall 2.30 Case Detection Rate (CDR) *[Figure 1.F.A.1.2]*.

Region XII reported the highest number of positive cases at 5.07 (286 out of 5638 examined), followed by MIMAROPA with 1.92 CDR (10 out of 522 examined) and Region 9 with 1.42 % CDR (25 out of 1761 examined).On the other hand, Region X, despite having the second highest examined cases of 5,439, had a relatively low CDR of 0.53% (29 out of 5439 examined) while Region VIII and XI reported zero positive cases *[Figure 1.F.A.1.2]*.

1.F.A.2. HIV-AIDS/STI Prevention and Control Services

Formula:

Proportion of Pregnant women screened for Syphilis

Numerator: No. of pregnant women screened for syphilis

Proportion of Pregnant women positive for Syphilis

Numerator: No. of pregnant women tested positive for syphilis

Denominator: Total no. of pregnant women screened for syphilis

Proportion of Pregnant women screened for HIV

Numerator: No. of pregnant women screened for HIV

Denominator: Under 1 Eligible Population

Under the Infectious Disease Program of Field Health Information System (FHSIS), two key indicators are being monitored to track maternal infection control efforts: (1) Proportion of Pregnant women who were screened and tested positive for Syphilis, and the (2) Proportion of Pregnant women screened for HIV. These indicators are essential for assessing the effectiveness of antenatal screening in preventing vertical transmission of infections.

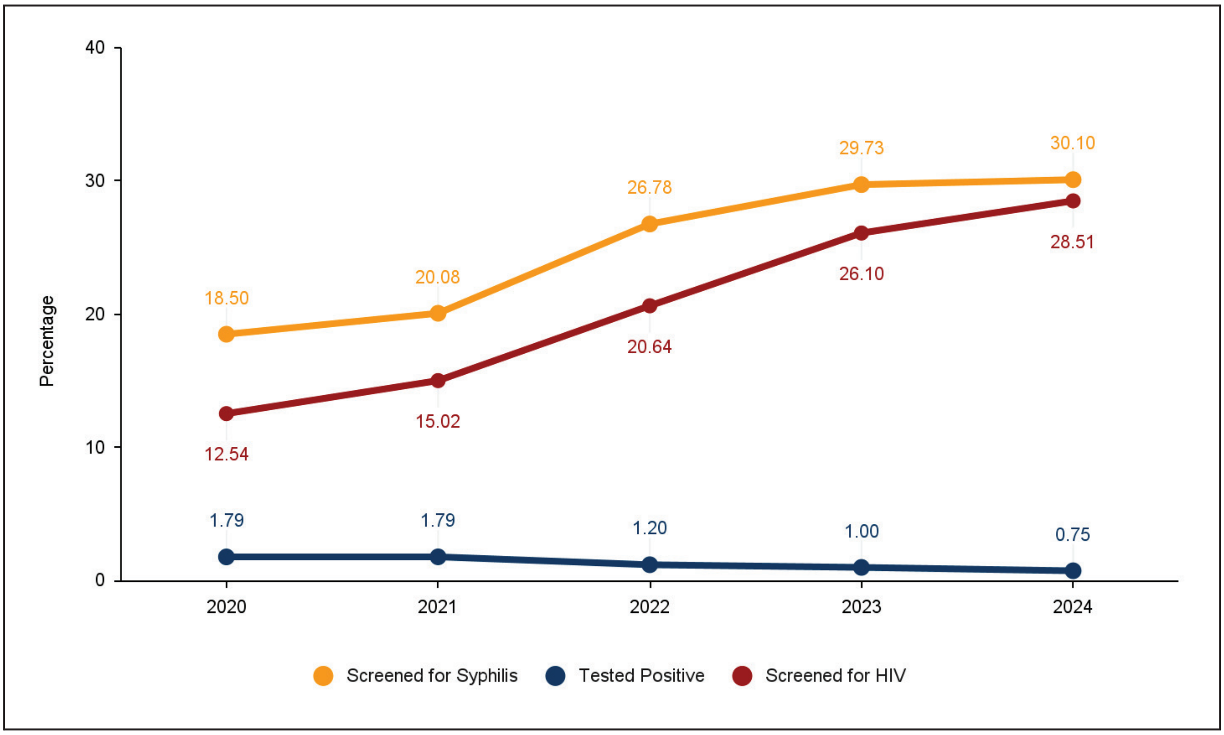

Figure 1.F.A.2.1: Screening Coverage for Syphilis and HIV Among Pregnant Women, Philippines, 2020- 2024

As shown in Figure 1.F.A.2.1. *Screening Coverage for Syphilis and HIV Among Pregnant Women*, there was a marked increase in screening coverage for both indicators. From 2020 to 2024, the proportion of pregnant women screened for syphilis rose from 18.50% to 30.10%, while HIV screening increased from 12.54% to 28.51%. This upward trend indicates improved integration of infectious disease screening into routine prenatal care.

In contrast, the percentage of pregnant women who tested positive for syphilis showed a consistent decline over the same period – from 1.79% in 2020-2021 to 0.75% in 2024.

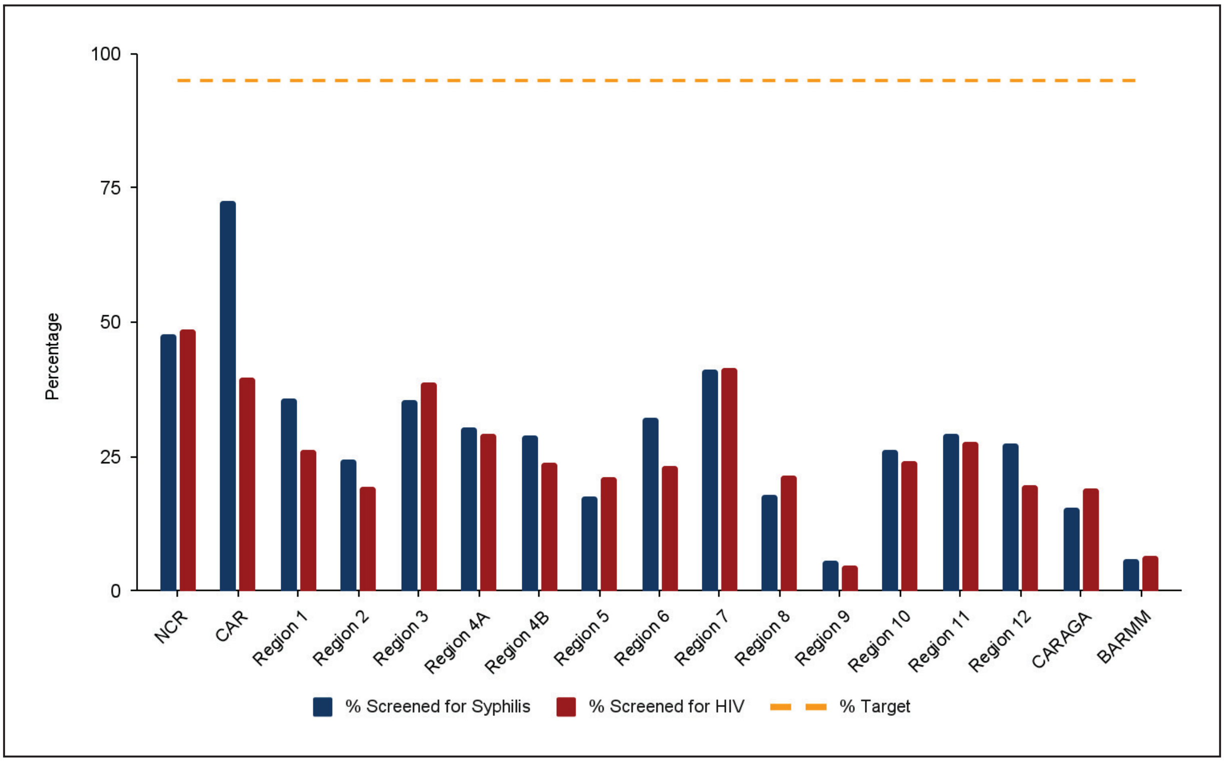

Figure 1.F.A.2.2: Screening Coverage for Syphilis and HIV Among Pregnant Women, By Region, 2024

In 2024, screening coverage for syphilis and HIV among regions in the Philippines remained significantly below the national target of 95%. The Cordillera Administrative Region (CAR) led all regions with the highest syphilis screening rate at 72.52%, while the National Capital Region (NCR) recorded the highest HIV screening rate at 48.58%. Despite these figures, no region achieved the target coverage and wide disparities were evident across the country. Regions such as BARMM, Region 9, and CARAGA reported the lowest screening rates at less than 20%, for both syphilis and HIV. Notably, most regions showed higher screening rates for syphilis than HIV, except for NCR and Region 7 where HIV screening slightly exceeded screening for syphilis.

1.F.A.3. SCHISTOSOMIASIS- PREVENTION CONTROL PROGRAM

Formula:

Proportion of Acute Confirmed Cases

Numerator:	No. of acute cases confirmed positive by stool examination (Kato-Katz technique) in the health facility
Denominator:	Total number of acute clinically diagnosed cases

Proportion of Confirmed Chronic Cases

Numerator:	No. of chronic clinically diagnosed cases confirmed by stool examination (KKT) or rectal biopsy
Denominator:	Total number of chronic clinically diagnosed cases

Proportion of chronic clinically diagnosed cases treated in health facility

Numerator:	No. of chronic clinically diagnosed cases treated in health facility
Denominator:	Total no. of confirmed cases (acute and chronic)

Schistosomiasis, also known as bilharzia, is a parasitic disease caused by blood flukes of the genus Schistosoma. In the Philippines, it remains endemic in certain rural areas where water contact activities and the presence of infected snail vectors contribute to sustained transmission. The disease can lead to both acute and chronic health conditions, affecting the liver, intestines, and other organs if left untreated.[3]

To address the burden of schistosomiasis, the Department of Health (DOH) implements a control program focused on early detection, clinical management and regular mass drug administration (MDA) in endemic areas. Monitoring of the said disease was integrated into the Field Health Information System (FHSIS), which ensures consistent reporting and monitoring of program indicators.

Health facilities collect and report data on clinical and laboratory-confirmed cases using diagnostic methods such as the Kato-Katz technique and rectal biopsy. Schistosomiasis cases that were treated within the health facility were also tracked to ensure that confirmed cases received appropriate care. These indicators will guide decision makers in assessing the effectiveness of the interventions and in directing resources to high-risk communities.

This section presents an analysis of trends in acute and chronic clinically diagnosed confirmed cases over time, which will help to identify progress in case detection and treatment coverage.

---

[3] World Health Organization. (2022). Schistosomiasis: Key facts. Retrieved from https://www.who.int/news-room/fact-sheets/detail/schistosomiasis

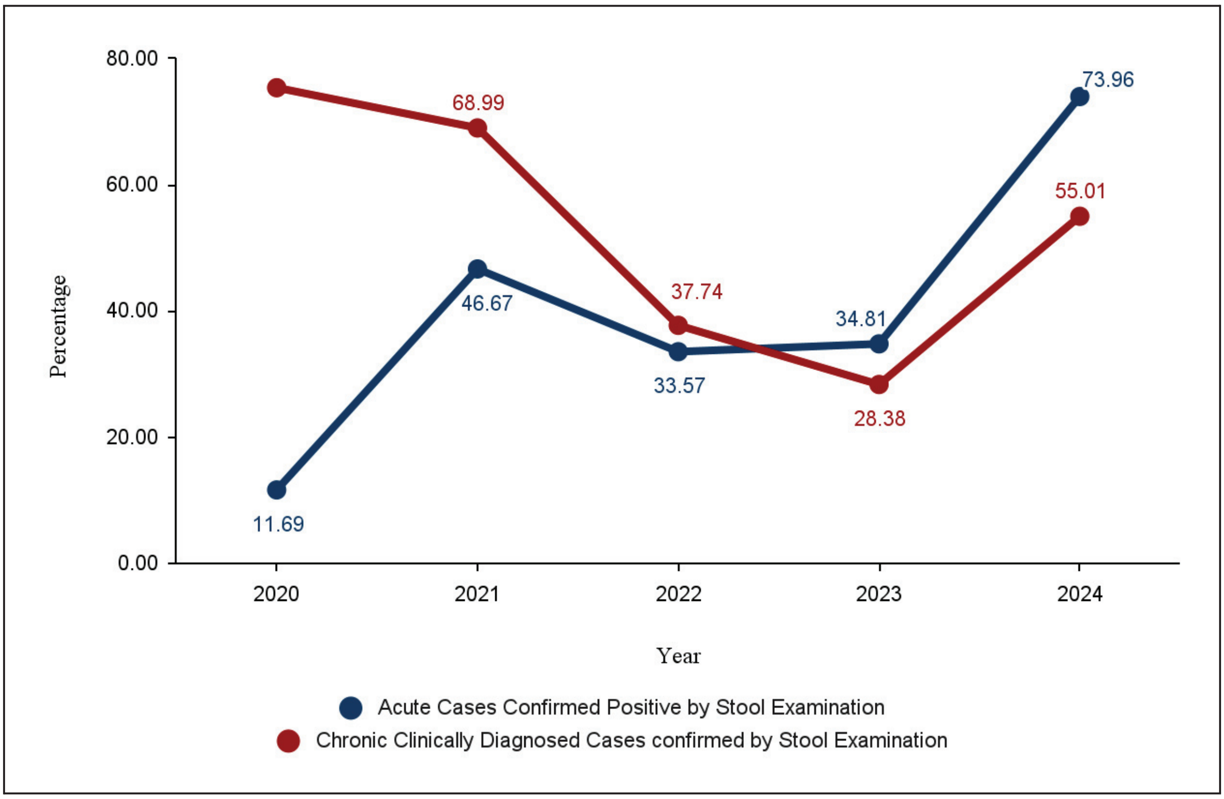

Figure 1.F.A.3.1: Trend in Schistosomiasis Diagnosis : Acute and Chronically Confirmed Cases, Philippines, 2020-2024

The proportion of acute cases confirmed positive by stool examination exhibited an overall increasing pattern with some fluctuations. Starting at 11.69% in 2020, the figures rose significantly to 46.67% in 2021. Although there was a slight decline to 33.57%. In 2022, the graph shows an increase to 34.81% in 2023, followed by a notable spike in 2024, reaching 83.58% – the highest percentage of acute cases confirmed positive by stool examination recorded in the five year period.This pattern shows a possible resurgence of acute infections, indicating that active disease transmission remains a public health concern in affected areas *[Figure 1.F.A.3.4].*

Meanwhile, the proportion of chronic clinically diagnosed cases confirmed by stool examination, shows a decreasing trend from 2020 with 75.36% to 28.38% in 2023. It increases significantly in 2024 at 55.01% with a decrease of 4.56% change compared with the 4-year average data from 2020-2023.

With the trends showing a possible resurgence of acute and chronic confirmed cases in 2024, it is crucial to further analyze the increasing data on treated cases . Below is the treatment coverage, among the acute and chronic cases across the region.

Table 1.F.A.3.1: Treatment Coverage and Case Distribution of Acute and Chronic Confirmed Cases, by Region, 2024

Region	No. of Acute Confirmed Cases	No. of Chronic Clinically Diagnosed Cases Confirmed	Number of Chronic Clinically Diagnosed Cases Treated in the Health Facility among Acute and Chronic Cases	%
NCR	0	0	0	0.00
CAR	0	0	0	0.00
Region I	0	0	0	0.00
Region II	0	0	0	0.00
Region III	0	0	0	0.00
Region IV-A	0	0	0	0.00
Region IV-B	0	0	0	0.00
Region V	6	0	6	100.00
Region VI	0	0	0	0.00
Region VII	0	0	0	0.00
Region VIII	748	261	559	55.40
Region IX	30	274	223	73.36
Region X	167	76	164	67.49
Region XI	30	76	76	71.70
Region XII	92	72	101	61.59
CARAGA	667	130	99	12.42
BARMM	78	71	1	0.67

*Note: 0 = zero cases; * = no report*

The acute and chronic cases remains highly concentrated in Region 8, CARAGA, Region 9 and Region 10 – with Region 8 reporting the highest number of 748 acute confirmed cases and a treatment coverage of 55.4% (559 treated cases among acute and chronic confirmed cases) relative to its target (1,009). CARAGA also poses a pressing concern for public health intervention, with a record of 667 acute confirmed cases but a critically low treatment coverage of only 12% (99 out of 797). Similar to this, BARMM recorded the lowest treatment coverage of 0.67% (1 out of 149) , respectively *[Figure 1.F.A.3.2.]*.

In contrast, several regions reported zero cases such as NCR, CAR, Region 1, Region 2, Region 3, Region 4A, Region 6 and Region 7, which could indicate that these areas are not part of endemic zones. While Region 5, 9, 10, 11, and 12, reported moderate treatment coverage but still fell short of their targets, indicating the need for strengthened case management and follow up strategies.

These findings emphasize the concentration of schistosomiasis burden in specific endemic areas and the ongoing need to close treatment gaps in high burden areas.

1.F.A.4 SOIL TRANSMITTED-HELMINTHIASIS

Formula:

Proportion of PSAC, 12-59 months old, who completed 2 doses of deworming tablet

Numerator:	No. of 1-4 years old (12-59 months old) who completed 2 doses of deworming tablet in a year
Denominator:	Eligible population 1-4 years old

Proportion of SAC, 5-9 years old, who completed 2 doses of deworming tablet

Numerator:	No. of 5-9 years old who completed 2 doses of deworming tablet in a year
Denominator:	Eligible population 5-9 years old

Proportion of Adolescents, 10-19 years old, who completed 2 doses of deworming tablet

Numerator:	No. of SAC, 10-19 years old who completed 2 doses of deworming tablet in a year
Denominator:	Eligible population 10-19 years old

Proportion of WRA, 20-49 years old, who completed 2 doses of deworming tablet

Numerator:	No. of WRA 20-49 years old who completed 2 doses of deworming tablet in a year
Denominator:	Eligible population 20-49 years old (female only)

Proportion of pregnant women who completed 1 dose of deworming tablet

Numerator:	Number of pregnant women who completed at least 1 dose of deworming tablet
Denominator:	Eligible population 10-19 years old

Soil-Transmitted Helminthiasis (STH) is a parasitic infection caused by intestinal worms that predominantly affects vulnerable groups such as young children and women of reproductive age. Monitoring deworming coverage through health information systems like the Field Health Services Information System (FHSIS) is essential to reducing disease burden and morbidity in endemic areas. The program emphasizes administering recommended doses of deworming tablets to targeted age groups to control transmission and improve health outcomes.[4]

The set of indicators measures the proportion of preschool-aged children (12-59 months), school-aged children (5-9 years), adolescents (10-19 years), women of reproductive age (WRA), and pregnant women who have completed the appropriate doses of deworming tablets within a year.

Monitoring these indicators regularly through the Field Health Services Information System (FHSIS) allows health authorities to continuously assess program effectiveness in the Philippines and ensure that deworming interventions reach the intended populations, and improve health outcomes and achieve the national and global disease control targets.

[4] World Health Organization. (2022). Soil-transmitted helminth infections.

The analysis below shows the five (5) year trend of the deworming coverage per target group.

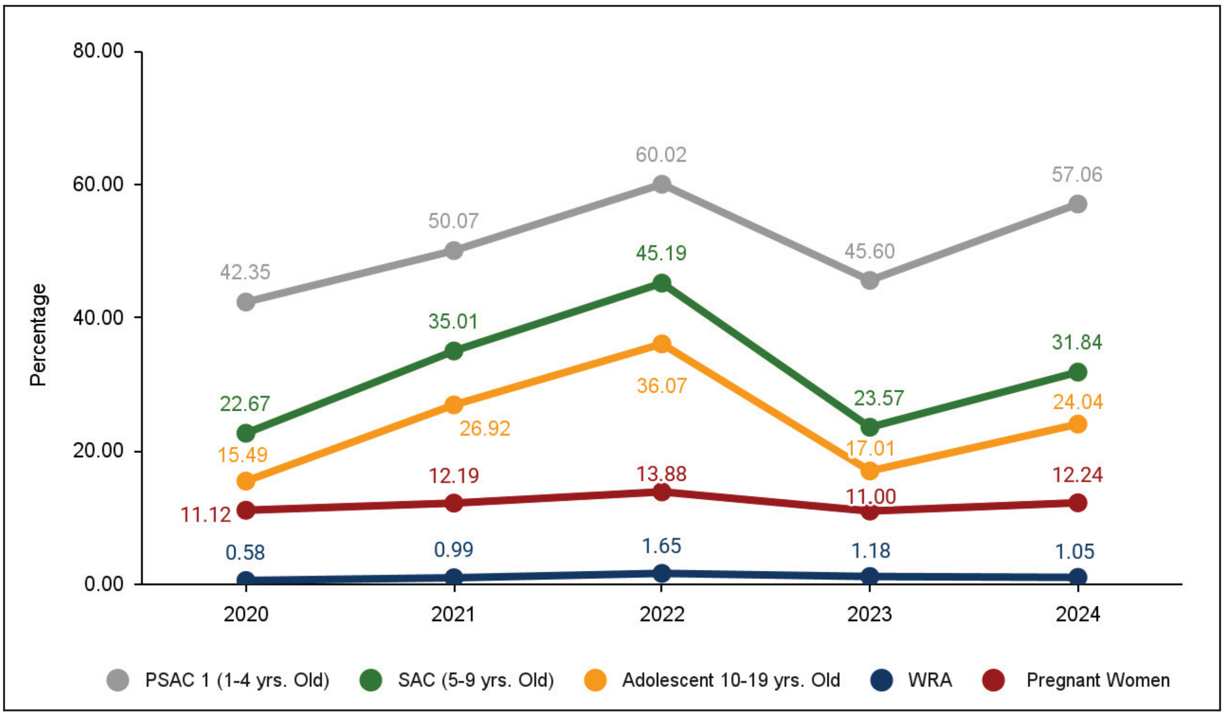

Figure 1.F.A.4.1: Five-Year Trend of Deworming Coverage, Philippines, 2020-2024

Between 2020 and 2024, deworming coverage among pre-school aged children (1-4 years old) consistently remained the highest across all target age groups. Coverage steadily increased from 42.35% in 2020, reaching its highest peak at 60.02% in 2022. However, a notable decline occurred in 2023, with its coverage dropping to 45.60%.This was followed by a recovery in 2024, with coverage rising to 57.06%.

In comparison, school-aged children (5-9 years old) coverage showed a generally positive trend with some variability. Starting at 22.67% in 2020, coverage increased substantially to 45.19% in 2022, with a sharp decline to 23.57% in 2023, before partially recovering to 31.84% in 2024. A similar trend was observed among adolescents (10-19 years old), whose coverage increased from 15.49% in 2020 to 36.07% in 2022, followed by a significant drop to 17.01% in 2023 and slight rebound to 24.04% in 2024.

Meanwhile, pregnant women who completed one (1) dose of deworming tablets demonstrated a more stable coverage throughout the five-year period. Starting at 11.20% in 2020, coverage peaked at 13.88% in 2022, slightly dipped to 11.00% in 2023, and an increase to 12.24%, indicating a relatively consistent performance on this target group.In contrast, women of reproductive age (WRA) (20-49 years old) had the lowest coverage throughout the period. Starting at only 0.58% in 2020, coverage then

gradually increased to 1.65% – which is the highest recorded deworming coverage among WRA in 2022. Then followed by a slight decrease to 1.18% in 2023 and further to 1.05% in 2024.

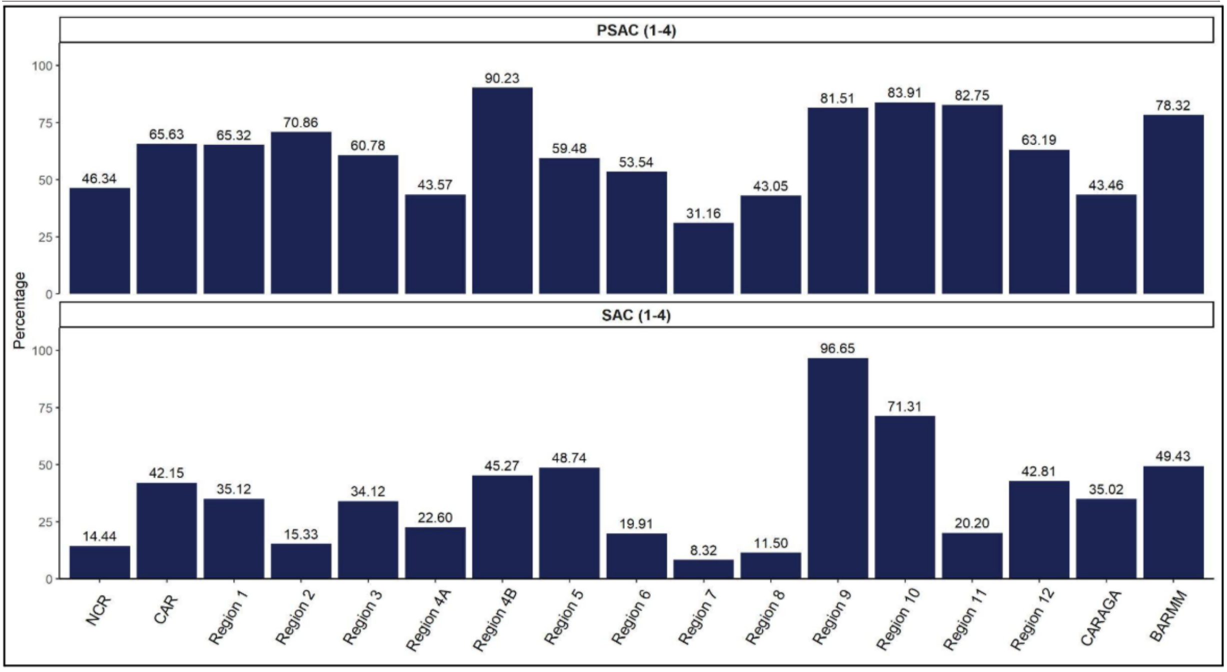

Figure 1.F.A.4.2: Proportion of 1 to 4 years old (PSAC and SAC) Completed Their Doses of Deworming Tablets by Region, 2024

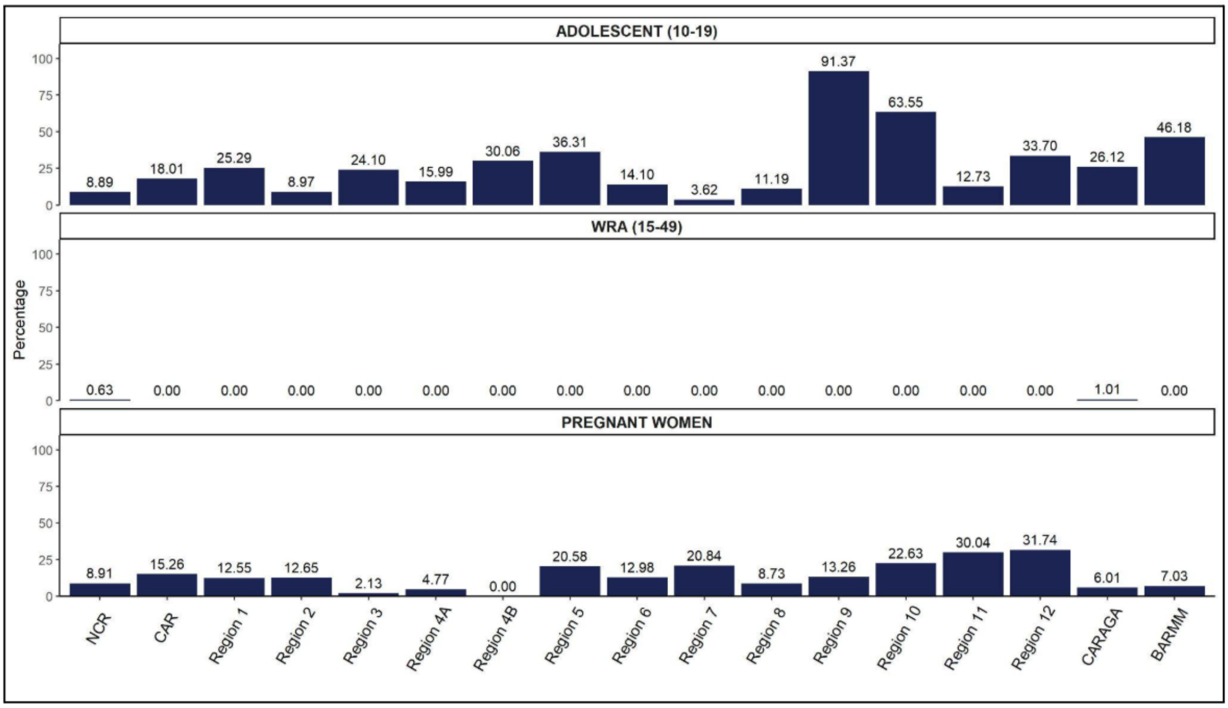

Figure 1.F.A.4.3: 5-9 years old, 10-19 years old, 20-49 years old and Pregnant Women Who Completed Their Doses of Deworming Tablets by Region, 2024

This reveals significant variations in deworming services across the targeted age groups. While the national target for deworming services stands firm at 85%, the journey towards the benchmark remains bumpy across regions. Region 4B was the only area that went beyond the target for 1-4 years old, reaching 90.23%. While Regions 9 (81.51%) and Region 10 (83.91%) had high coverage in this group, they still fell behind the target. Among the 5-9 and 10-19 years old groups, Region 9 led all the regions reaching a 96.65% and 91.37% deworming coverage respectively. It only shows strong deworming efforts among school aged-children and adolescents. Region 10 also showed good results in the same group, with 71.31% for 5-9 years old and 63.55% for 10-19 years old, although they were still unable to reach the target *[Figure 1.F.A.4.2]*.

In contrast, several regions such as NCR, Region 7 and 8 lagged considerably behind the national target across all age groups, with deworming coverage rates as low as 8.32% among 5-9 years olds in Region 7. This indicates that there may be several challenges in reaching and sustaining deworming activities in these areas. While deworming services among WRA (20-49 years) and pregnant women was generally low across the nation – with less than 1% coverage for WRA and below 31% for pregnant women. These figures highlight a critical gap in extending deworming interventions specially among WRA and pregnant women, – with most regions reporting low coverage of deworming is primarily due to the limited supply of deworming tablets and the prioritization of children during mass drug administration activities. Several regions indicated that the available stock was allocated mainly to preschool and school-aged children, resulting in minimal reach for WRA. This approach, while effective in boosting child coverage, leaves other vulnerable groups underserved. *[Figure 1.F.A.4.3]*.

1.F.B Infectious Diseases

2024 KEY FINDINGS

INFECTIOUS TUBERCULOSIS PREVENTION AND CONTROL

486.21
Case per 100,000 population
TB Case Notification Rate (All Forms)

93.31%
TB treatment Success Rate (All forms)

9.11
Case per 100,000 population
TB Case Notification Rate (DRTB)

75.69%
TB treatment Success Rate (DRTB)

LEPROSY PREVENTION AND CONTROL

1.14
per 10,000 population
Newly detected leprosy cases

0.44
per 100,000 population
Leprosy cases undergoing treatment

RABIES PREVENTION AND CONTROL

0.01%
Deaths are due to Rabies

2,456,422
Animal bite cases reported

1.F.B.1. INFECTIOUS TUBERCULOSIS

Formula:

TB Case Notification, All Forms

Numerator: Number of registered new and relapse TB cases (and cases with unknown previous TB treatment history), all forms (bacteriologically confirmed plus clinically diagnosed, pulmonary and extrapulmonary) in the reporting period

Denominator: Total population

Drug Resistant TB Notifications

Numerator: Number of registered bacteriologically confirmed drug resistant TB (RR/MDR-TB Cases)

Denominator: Total population

Treatment Success Rate (TSR), DSTB-all forms

Numerator: Total Number of TB, all forms that are cured and completely treated

Denominator: Total Number of TB, all forms registered during a specified period

Treatment Success Rate (TSR), MDR TB

Numerator: Total Number of registered bacteriology confirmed drug resistant TB Cases (RR/MDR-TB) cured and completed treatment

Denominator: Total Number of registered bacteriologically confirmed drug resistant TB cases (RR/MDR-TB) during a specified period

---

Tuberculosis (TB) is an infectious disease that is spread through the air when individuals with the disease cough, sneeze or spit. While TB is preventable and curable, about one in four globally have been infected with TB, and around five to 10% of those infected with TB will develop the disease.[1] In 2023, the WHO estimated that about 10.8 million individuals (6 million men, 3.6 million women and 1.3 million children) fell ill due to TB.

Timely and accurate diagnosis followed by uninterrupted treatment according to international standards reduces deaths and improves the health of people who develop TB.[2] In the Philippines, the TB Case Notification Rate (CNR) of all forms of TB per 100,000 decreased between 2023 (515.74) and 2024 (486.21) following the steady rise between 2019 to 2023. However, the data shows that the target of 10% increase (567.31) from the previous year was not achieved in 2024 *[Figure 1.F.B.1.1]*.

[1] *World Health Organization: WHO & World Health Organization: WHO. (2025, March 14). Tuberculosis.*
[2] *World Health Organization. 3.1 notifications. (n.d.)*

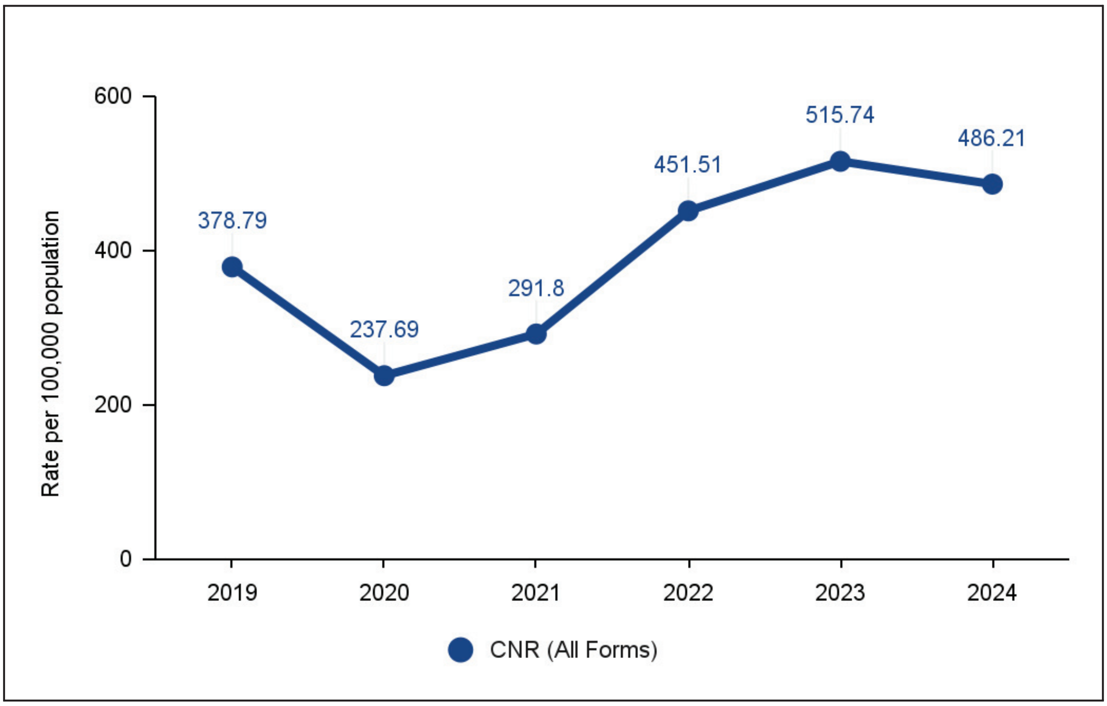

Figure 1.F.B.1.1: Case Notification Rate (All forms), Philippines, 2019 to 2024

In 2024, the CNR for drug-resistant Tuberculosis (DRTB) per 100,000 decreased by 77% between 2023 (39.9) and 2024 (9.11). This indicates that the target of 10% increase (43.89) from the previous year was not achieved in 2024. It should be noted that while some fluctuations can be observed, the values between 2019 and 2024 suggest that the CNR for DRTB is generally declining *[Figure 1.F.B.1.2].*

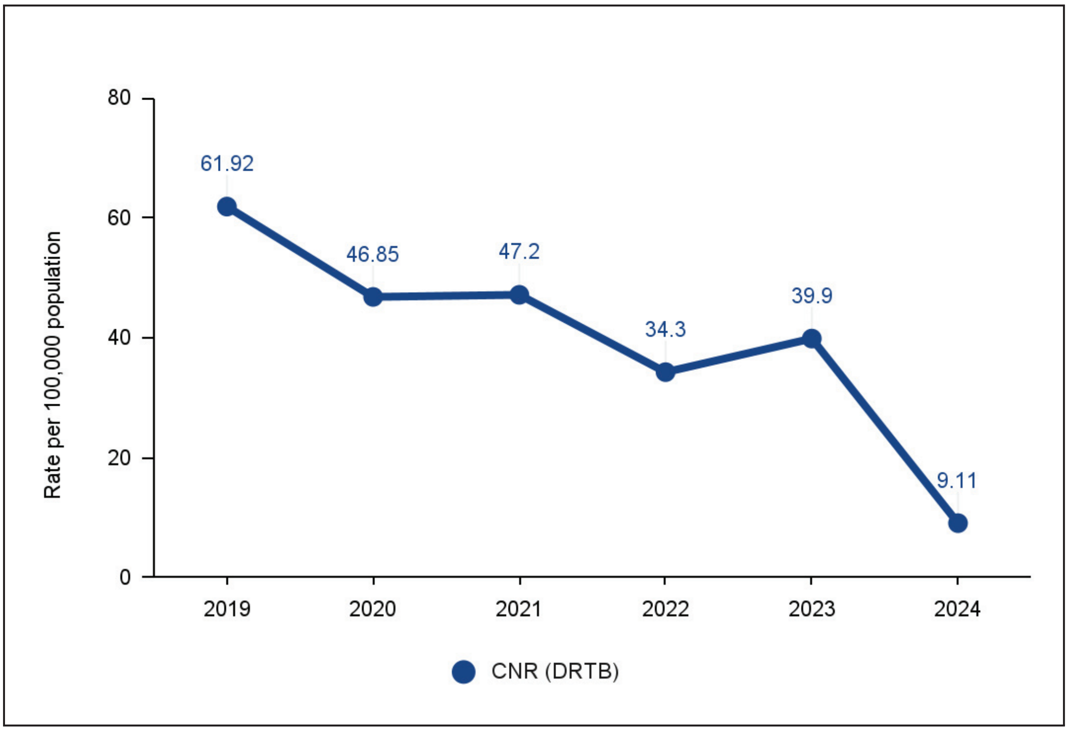

Figure 1.F.B.1.2: Case Notification Rate (Drug Resistant Tuberculosis), Philippines, 2019 to 2024

The regions with the highest CNR of all forms of TB per 100,000 are Region 6 (983.39), NCR (721.65) and Region 4A (537.77). On the other hand, CNR of all forms of TB per 100,000 are the lowest in BARMM (252.21), CAR (317.21) and Region 8 (354.22) *[Figure 1.F.B.1.3].*

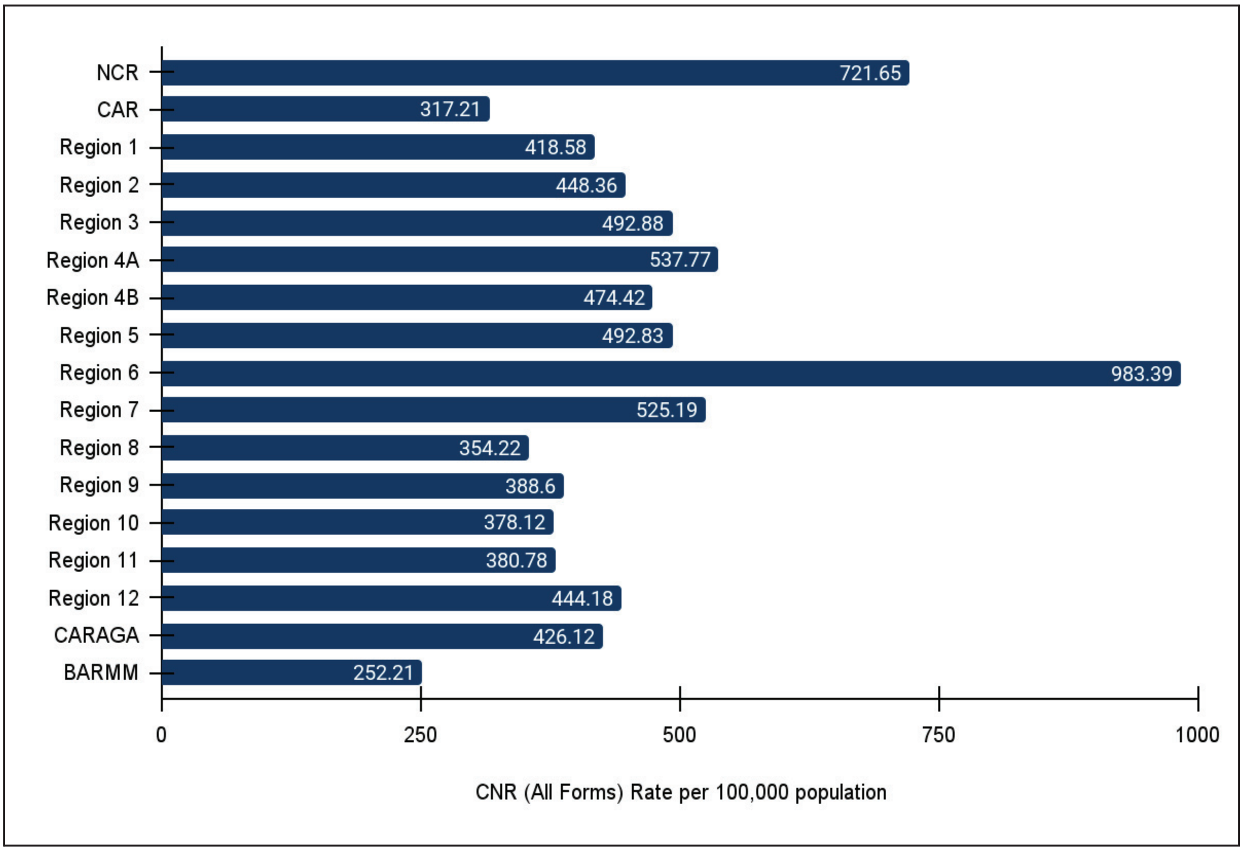

Figure 1.F.B.1.3: Case Notification Rate (All Forms), by Region, Philippines 2019 to 2024

The regions with the highest CNR for DRTB per 100,000 are Region 6 (16.62), NCR (15.92) and Region 5 (11.98). On the other hand, the regions with the lowest CNR for DRTB per 100,000 in 2024 are Region 12 (4.13), CAR (4.47) and Region 2 (4.68). While low or declining CNR can indicate good program performance resulting in lower disease transmission, it can also be the result of poor case finding.[3] These findings warrant further investigation since an artificially low CNR may suggest the need to intensify case finding activities *[Figure 1.F.B.1.4].*

[3] *Philippine Business for Social Progress. Analysis of National TB Program Indicators. (n.d.).*

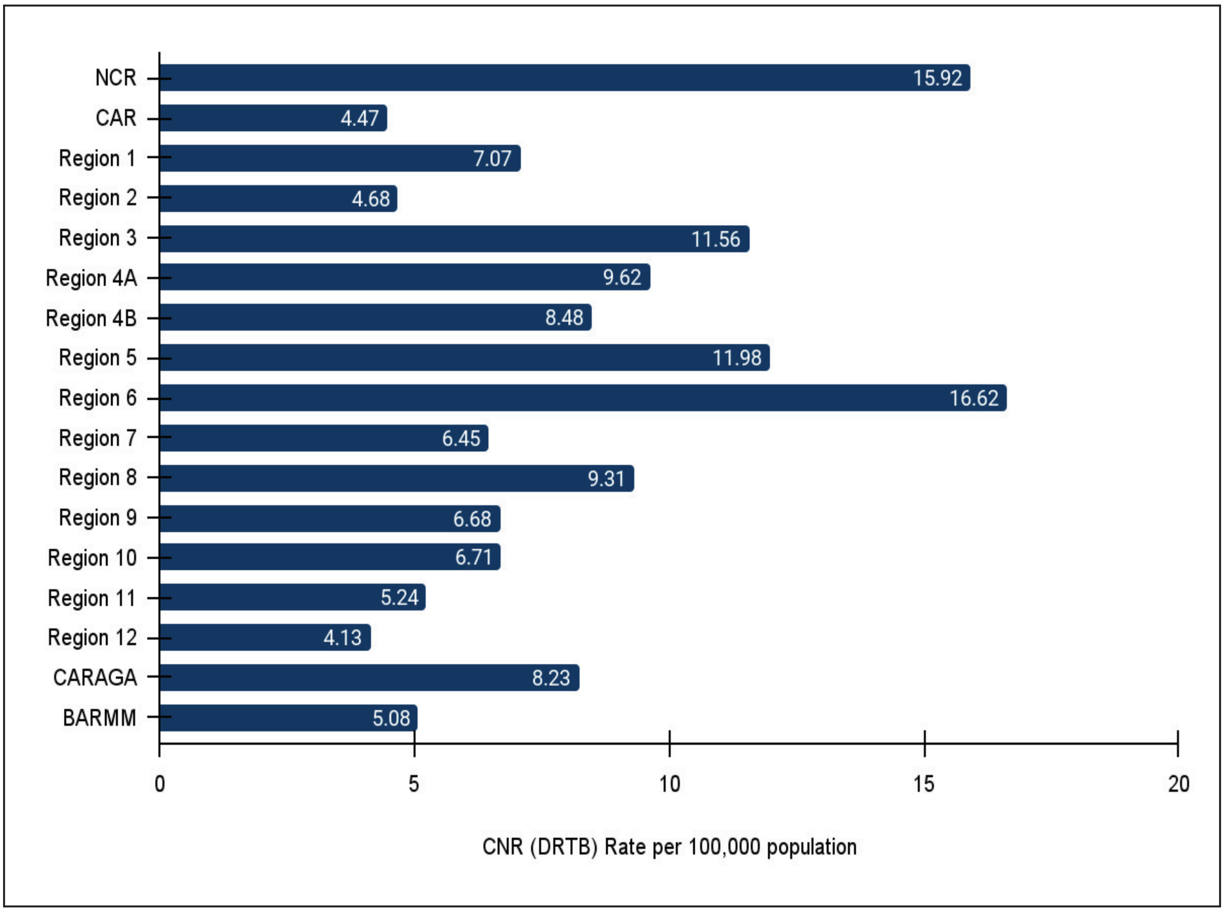

Figure 1.F.B.1.4: Case Notification Rate (Drug Resistant Tuberculosis), by Region, Philippines 2019 to 2024

The Treatment Success Rate (TSR) for all forms of TB in 2024 is 93.31% indicating that the national target of 90% was achieved. The region with the highest TSR for all forms of TB in 2024 is Region 1 (95.75%). However, it should be noted that the rest of the regions have values greater than the national target of 90% with the exception of Region 7 (89.51%) and BARMM (84.36%) *[Figure 1.F.B.1.5].*

The TSR for DRTB in 2024 is 75.69% indicating that the national target of 90% was not achieved. The regions with the highest TSR for DRTB in 2024 are Region 4B (86.53%), Region 1 (83.48%) and Region 6 (81.38%). On the other hand, the regions with the lowest TSR for DRTB in 2024 are Region 7 (68.28%), NCR (70.02%) and Region 11 (72.49%). These results reflect the challenges in treatment adherence including, but to limited to, patient concerns on drug reaction, fear of being stigmatized and lack of family support[4] *[Figure 1.F.B.1.5].*

[4] *Yutaka Endo, Jahn Jaramillo, Rajendra Prasad Hubraj Yadav. Patient- and Health-System-Related Barriers to Treatment Adherence for Patients with Drug-Resistant Tuberculosis in the Philippines: A Mixed-Methods Study. Tuberculosis Research and Treatment. 2022 November. DOI: 10.1155/2022/6466960*

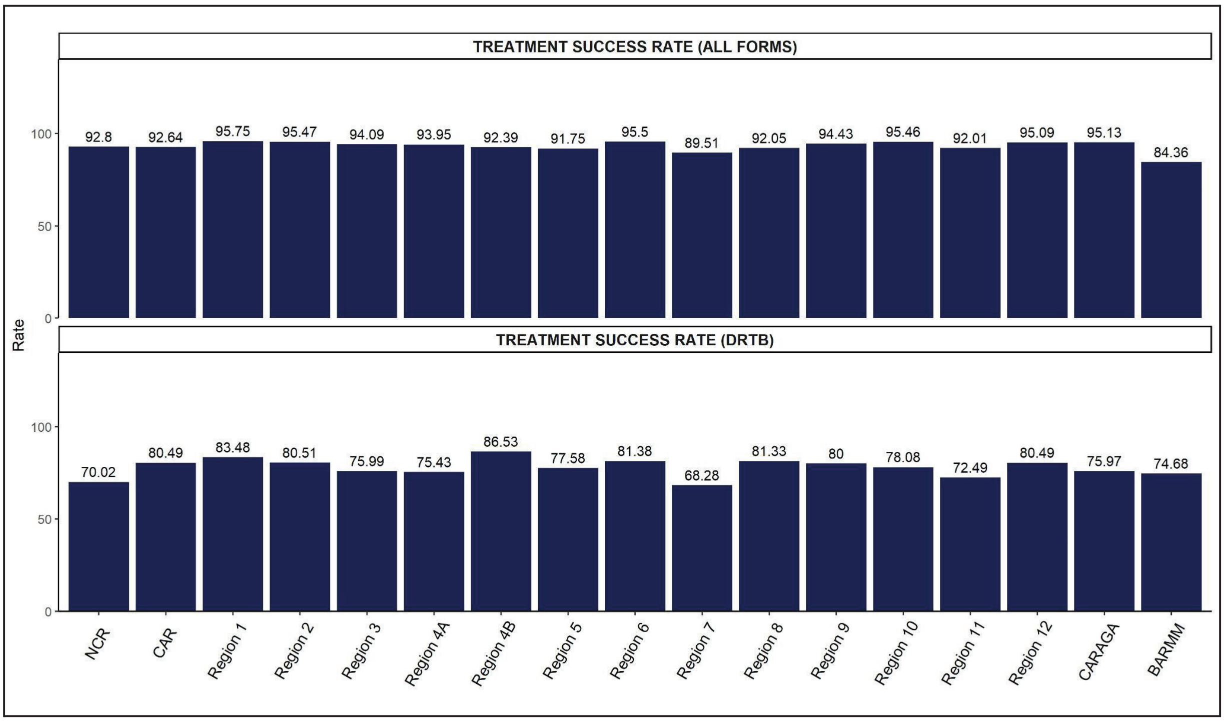

Figure 1.F.B.1.5: Treatment Success Rate, All Forms and Drug Resistant Tuberculosis by Region, Philippines, 2019 to 2024

The inclusion of Tuberculosis and HIV Tuberculosis in the 8 Priority Health Outcomes highlights the importance of diagnosing, treating and curing individuals with Tuberculosis. Challenges in case notification for all types of TB and DRTB may indicate the need to intensify casefinding activities and improve private sector reporting. On the other hand, results on TSR for DRTB may reflect the documented challenges in treatment adherence including, but not limited to, patient concerns on drug reaction, fear of being stigmatized and lack of family support.[5] Results also suggest that interventions aiming to address those challenges should be prioritized.

---

[5] *Yutaka Endo, Jahn Jaramillo, Rajendra Prasad Hubraj Yadav. Patient- and Health-System-Related Barriers to Treatment Adherence for Patients with Drug-Resistant Tuberculosis in the Philippines: A Mixed-Methods Study. Tuberculosis Research and Treatment. 2022 November. DOI: 10.1155/2022/6466960*

1.F.B.2. Leprosy

Formula:

Case Detection Rate

Numerator: Number of newly detected cases during reporting period
Denominator: Total Population

Leprosy Prevalence Rate

Numerator: Total number of leprosy cases on treatment during reporting period
Denominator: Total Population

According to the WHO, Leprosy is a neglected tropical disease with more than 200,000 cases annually from 120 countries.[6] While Leprosy is reported from all six (6) WHO regions, the majority of the new cases are detected from South-East Asia including the Philippines.

The Leprosy Prevalence Rate fluctuated from 2019 to 2024 with a sharp decrease between 2020 and 2021 and a sharp increase between 2023 and 2024. Despite the fluctuations in Leprosy Prevalence Rate, the national target of one per 10,000 was achieved annually from 2019 to 2024 *[Figure 1.F.B.2.1]*.

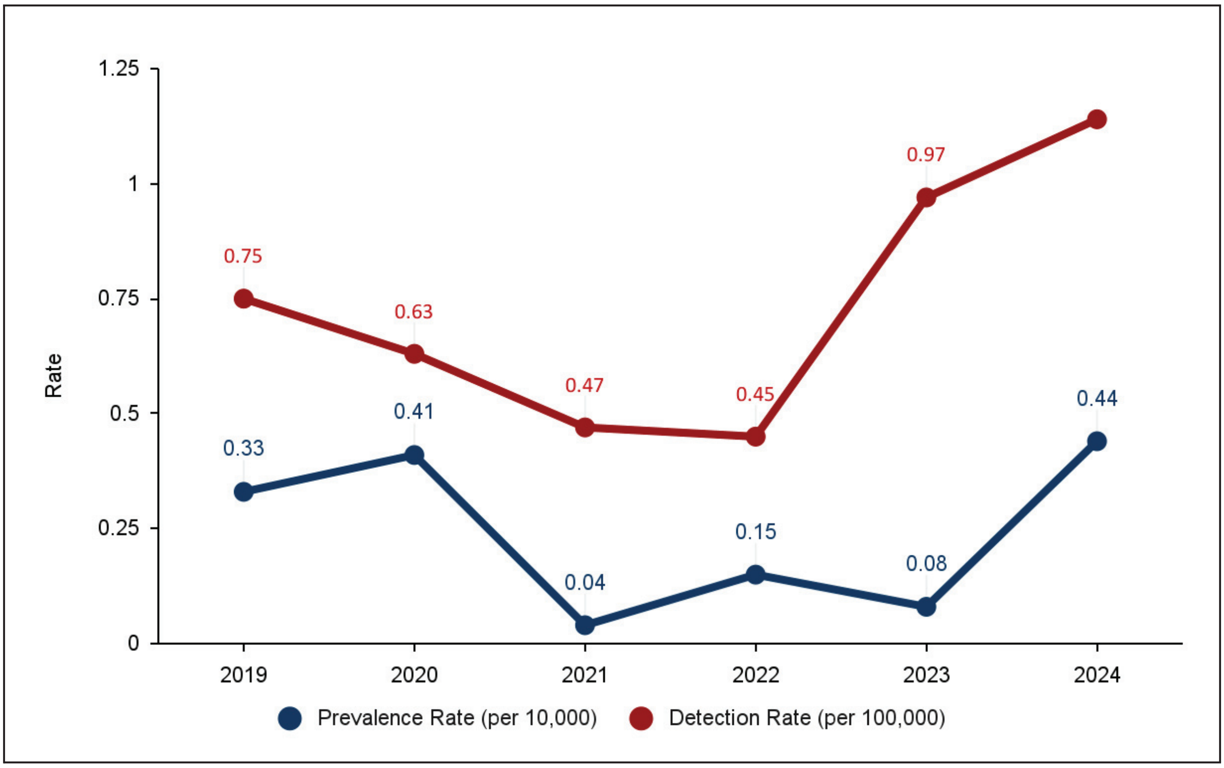

Figure 1.F.B.2.1: Prevalence and Detection Rate for Leprosy, Philippines, 2019 to 2024

[6] *World Health Organization: WHO. (2025, January 24). Leprosy.*

On the other hand, the Case Detection Rate for Leprosy increased between 2022 and 2024 with a notable sharp increase in 2023. This is in contrast to the declining trend of case detection rate between 2019 to 2022. However, the Case Detection Rate for Leprosy target of less than 5% from the 2023 accomplishment was not achieved. *[Figure 1.F.B.2.1].*

In 2024, the regions with the highest Leprosy Prevalence Rate per 10,000 are Region 9 (2.86), Region 8 (1.31) and Region 1 (0.98). On the other hand, the regions with the lowest Leprosy Prevalence Rate per 10,000 are NCR (0.02), Region 4A (0.05) and Region 5 (0.08). In the same year, the regions with the highest Case Detection Rate for Leprosy per 100,000 are Region 9 (11.33) and Region 8 (3.20). On the other hand, the regions with the lowest Case Detection Rate for Leprosy per 100,000 are Region 4A (0.05) and NCR (0.02) *[Figure 1.F.B.2.2]*.

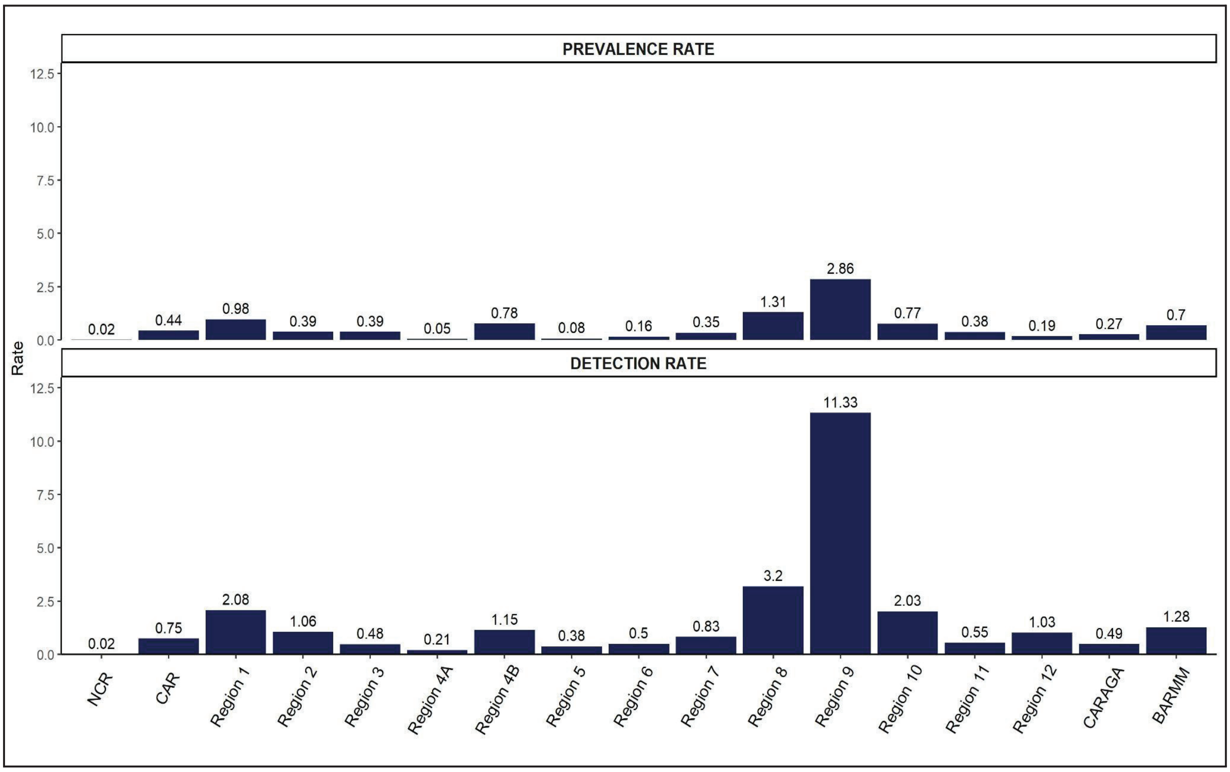

Figure 1.F.B.2.2: Prevalence and Detection Rate for Leprosy by Region, Philippines, 2024

1.F.B.3. Rabies

Formula:

Proportion of deaths due to Rabies

Numerator: Number of deaths due to rabies
Denominator: Total number of Animal Bites

Leprosy Prevalence Rate

Numerator: Total number of leprosy cases on treatment during reporting period
Denominator: Total Population

Rabies is a viral zoonotic disease affecting the central nervous system. According to the WHO,[7] dogs account for 99% of human rabies cases and the frequent victims are children between five and 14 years old. Additionally, Rabies causes tens of thousands of deaths annually despite being a vaccine-preventable disease.

Between 2019 to 2024, the proportion of deaths due to rabies declined steadily. These reflect efforts of the country to address rabies such as advocacy and awareness campaigns[8] and the establishment of animal bite centers increasing access to rabies Post Exposure Prophylaxis (PEP)[9] *[Figure 1.F.B.3.1].*

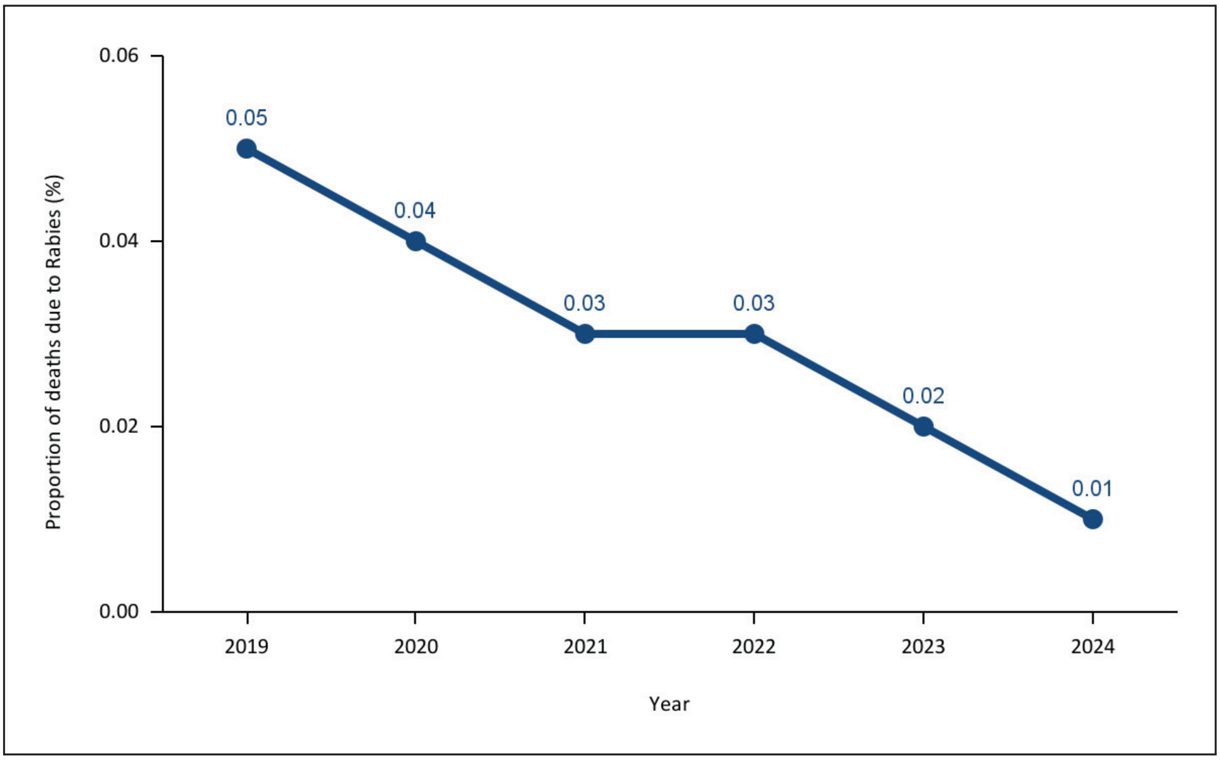

Figure 1.F.B.3.1: Proportion of Deaths due to Rabies, Philippines, 2019 to 2024

---

[7] *World Health Organization: WHO. (2024, June 5). Rabies*
[8] *Philippine News Agency. Join the campaign for rabies-free Philippines. (2023, March 6)*
[9] *Department of Health. National Rabies Prevention and Control Program Strategic Plan 2020 - 2025*

The regions with the highest proportion of deaths due to rabies in 2024 are Region 4B and BARMM (0.06%), and Region 12 (0.05%). On the other hand, the regions with the lowest reported proportion of deaths due to rabies in 2024 (less than 0.01%) are CAR, NCR and Region 7 *[Figure 1.F.B.3.2]*.

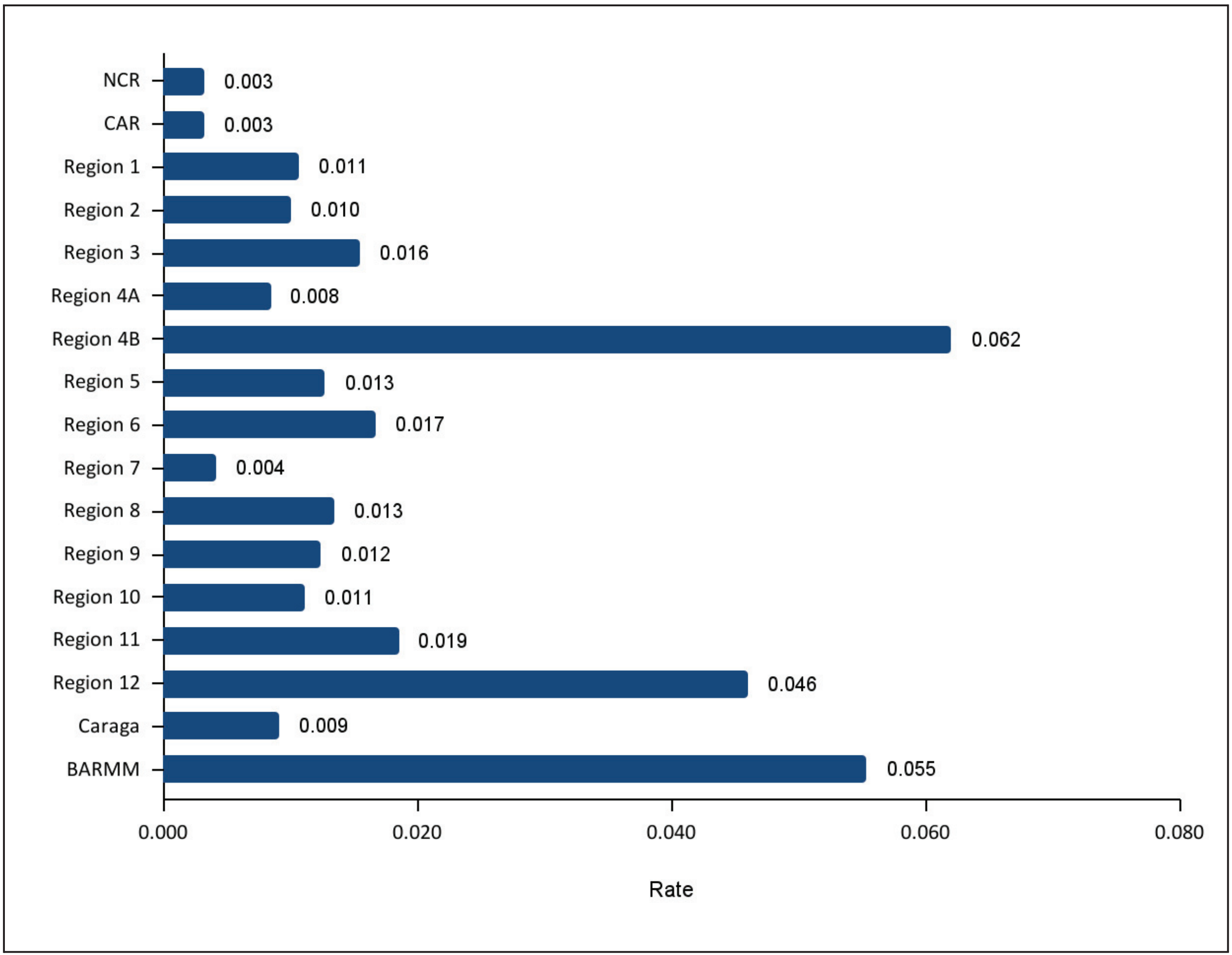

Figure 1.F.B.3.2: Proportion of Deaths due to Rabies by Region, Philippines, 2024

In 2024, a total of 2,456,422 animal bite cases were reported in the Philippines. The regions with the highest number of animal bite cases in 2024 are NCR (461,862), Region 4A (319,023) and Region 3 (251,137). Collectively, NCR, Region 4A and Region 3 account for about two in five of all animal bite cases in the Philippines.

On the other hand, the regions with the lowest number of animal bite cases in 2024 are BARMM (9,025), Region 4B (46,712) and Region 8 (59,334). *[Table 1.F.B.3.1]*

Table 1.F.B.3.1: Animal Bite Cases, by Region, 2024

Regions	Animal Bite Cases	% deaths due to rabies
NCR	461,862	0.003
CAR	62,112	0.003
Region 1	177,797	0.011
Region 2	129,226	0.010
Region 3	251,137	0.016
Region 4A	319,023	0.008
Region 4B	46,712	0.062
Region 5	62,726	0.013
Region 6	137,654	0.017
Region 7	214,251	0.004
Region 8	59,334	0.013
Region 9	64,168	0.012
Region 10	116,693	0.011
Region 11	194,200	0.019
Region 12	84,693	0.046
Caraga	65,809	0.009
BARMM	9,025	0.055

1.G Morbidity

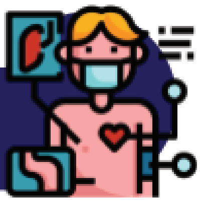

The Field Health Service Information System (FHSIS) collects morbidity data from public primary care facilities using a predefined list of 42 diseases, which are submitted monthly to the Department of Health-Epidemiology Bureau (DOH-EB) and consolidated for the annual report. While this chapter does not provide a complete representation of national morbidity statistics, the DOH-EB is actively exploring mechanisms to expand coverage to include all diseases and health-related conditions. This chapter focuses on the top 10 leading causes of morbidity within the predefined disease list and identifies diseases with the highest year-on-year increases in cases. These data aid in tracking trends, particularly for priority diseases, supporting evidence-based public health decision-making, strategic planning, and resource allocation.

Table 1.G.1: Leading Causes of Morbidity, Philippines, 2020 to 2024

Rank				2024<br>Top 10 Causes of Morbidity	2024		
2020	2021	2022	2023		Count	Rate per 100k population:	% change vs. 2023
1	1	1	1	1. Acute Respiratory Infection (ARI)	2,467,264	2,201.34	30.00% ↑
2	2	2	2	2. Hypertension	1,226,831	1,094.60	42.48% ↑
4	3	3	3	3. Animal Bites	997,421	889.92	64.31% ↑
3	4	4	4	4. Urinary Tract Infection	534,405	476.81	36.18% ↑
7	6	7	5	5. Pneumonia	361,372	322.42	48.57% ↑
5	5	6	6	6. Skin Diseases	342,515	305.60	33.33% ↑
6	7	5	7	7. Acute Lower Respiratory Tract Infection	283,265	252.74	-7.10%
10	8	9	8	8. Tuberculosis (All Forms)	175,696	156.76	20.83% ↑
9	>10	>10	9	9. Bronchitis	146,792	130.97	62.80% ↑
>10	9	10	10	10. Fever of Unknown Origin	121,774	108.65	40.77% ↑

The 2024 morbidity data shows that while the top 10 diseases ranking in 2023 did not change in 2024, the overall incidence rates of these diseases increased except for Acute Lower Respiratory Tract Infection. For instance, ARI (2,467,264 cases; 2,201.34 per 100k) and Hypertension (1,226,831 cases; 1,094.60 per 100k) have consistently ranked first and second not only between 2023 and 2024 but also for the past five years. Both diseases increased substantially from 2023 to 2024: ARI increased by 30.00% while hypertension increased by 42.48%.

Among the top 10 causes of morbidity, Animal Bites (997,421 cases; 889.92 per 100k) had the largest percentage increase at 64.31%. This was followed by Bronchitis (146.792 cases; 130.97 per 100k) at 62.80%, then Pneumonia (361,372 cases; 322.42 per 100k) and Hypertension (1,226,831 cases; 1,094.60 per 100k) with observed percentage increase by 48.57% and 42.48%, respectively. Acute Lower Respiratory Tract Infection (283,265 cases; 252.74 per 100k) maintained the same rank as in 2023, although both its case count and rate declined by 7.10%. It should be noted that respiratory conditions made up five of the top ten cases of morbidity, led by ARI, followed by Pneumonia (361,372; 322.42/100k), Acute Lower Respiratory Tract Infection, Tuberculosis (175,696; 156.76/100k), and Bronchitis (146,792; 130.97/100k) *[Table 1.G.1]*.

Lastly, Table 1.G.2 highlights the diseases, out of the 42 pre-selected morbidities, that showed the largest increase in cases compared to the previous year. Consistent monitoring of notifiable diseases, even those with relatively lower case counts, is essential to support effective prevention and control efforts by decision-makers, program managers, and implementers at all levels. In 2024, Non-Neonatal Tetanus recorded the highest percentage rise at 478.57% (from 126 to 729 cases), followed by Filariasis with a 459.62% increase (104 to 582 cases) and Rabies with 179.34% (121 to 338 cases). Acute Hemorrhagic Fever cases rose by 179.34% 129.78% (20,562 to 47,247 cases), while Meningococcemia more than doubled, up 105.71% (35 to 72 cases). Notable growth was also observed in Genital Ulcer, Acute Flaccid Paralysis, and Measles, and Bronchitis with increases ranging from 62.80% to 98.55%. Animal Bites remained the most reported condition, rising 64.31% from 607,031 to 997,421 cases, underscoring continued public health risks *[Table 1.G.2]*.

Table 1.G.2: Top 10 Diseases Based on Increase in Cases, Philippines, 2024

Diseases	Number of Cases (2023)	Number of Cases (2024)	% change
1. Non-Neonatal Tetanus	126	729	478.57%
2. Filariasis	104	582	459.62%
3. Rabies	121	338	179.34%
4. Acute Hemorrhagic Fever	20,562	47,247	129.78%
5. Meningoccocemia	35	72	105.71%
6. Genital Ulcer	207	411	98.55%
7. Acute Flaccid Paralysis	234	428	82.91%
8. Measles	1,110	1,887	70.00%
9. Animal Bites	607,031	997,421	64.31%
10. Bronchitis	90,165	146,792	62.80%

1.H Environmental Health and Sanitation Services

2024 KEY FINDINGS

ACCESS AND OF SAFE WATER SUPPLY AND SANITATION FACILITIES

27,130,697
Projected Number of Households (HHs)

94.22%
HHs with access to Basic Safe Water Supply (BSWS)

88.35%
HHs with Basic Sanitation Facility (BSF)

71.40%
HHs using Safely Managed Water Services (SMDWS)

68.96%
HHs using Safely Managed Sanitation Services (SMSS)

ZERO OPEN DEFECATION

48.16%
Municipalities/ Cities certified as ZOD

Formula:

Proportion of Households with Basic Safe Water Supply (BSWS)

Numerator:	Total number of households with access to improved water supply such as Level I, II and III
Denominator:	Projected number of Households for the given year

Proportion of Households using Safely Managed Drinking Water Services (SMDWS)

Numerator:	Total number of households using drinking water from an improved water source that is accessible on premises, available when needed and free from fecal and/or priority chemical contamination
Denominator:	Projected number of Households for the given year

Proportion of Households with Basic Sanitation Facility (BSF)

Numerator:	Total number of households with improved facilities which are not shared with other households
Denominator:	Projected number of Households for the given year

Proportion of Households using Safely Managed Sanitation Services (SMSS)

Numerator:	Total number of households using improved facilities that are not shared with other households and where excreta are safely disposed of in situ or removed and treated offsite
Denominator:	Projected number of Households for the given year

Proportion of Municipalities/Cities Certified as Zero Open Defecation (ZOD) Area

Numerator:	Number of municipalities and chartered cities certified as ZOD area
Denominator:	Total number of municipalities and chartered cities

---

Health is inevitably linked to the environment such that good health requires clean air, a stable climate, a preserved natural environment, and access to adequate water, sanitation and hygiene.[1] The United Nations General Assembly and Human Rights Council recognized the human right to safe drinking water in 2010.[2] On the other hand, the United Nations General Assembly explicitly recognized the human right to sanitation in 2015.

In 2010, the Department of Health (DOH) issued Administrative Order (AO) No. 2010-0021 declaring sustainable sanitation as a national policy and program priority. In 2019, the DOH issued AO No. 2019-0054 setting the Guidelines on the Implementation of the Philippine Approach to Sustainable Sanitation (PhATSS). Through the issuance of guidelines to operationalize the national policy on sustainable sanitation, the Philippines aims to reduce risks and diseases related to environmental sanitation in line with the Sustainable Development Goal 6 (SDG 6) on sanitation.[3]

[1] *World Health Organization: WHO. (2020, July 2). Environmental health.*
[2] *Un-Water. (n.d.). Human Rights to water and Sanitation | UN-Water. UN-Water.*
[3] *SDG 6 mandates the availability and sustainable management of water and sanitation for all.*

A Basic Safe Water Supply (BSWS) is an improved source of drinking-water such as Point Source (Level 1), Communal Faucet System or Stand Post (Level 2) and Waterworks System or Individual House Connection (Level 3). A BSWS is a Safely Managed Drinking Water service (SMDWS) if it is (1) located inside the household/within the premises, (2) available at least 12 hours per day and (3) the water supplied is free from fecal contamination.

Out of the projected 27,130,697 households in the Philippines in 2024, 94.22%(23,957,667 households) had access to BSWS while 71.4% (19,371,323 households) were using SMDWS. It should be noted that the annual target of $\geq$ 92.49% for the proportion of households with access to BSWS was achieved while the annual target of $\geq$ 73.62% for the proportion of households using SMDWS was not achieved in 2024. While the values of both indicators increased from 2019 to 2024, there is a noticeable gap between the proportion of households with access to BSWS and the proportion of households with access to SMDWS.However, it should be noted that the gap between the values of the two indicators also decreased between 2023 and 2024. *[Figure 1.H.1].*

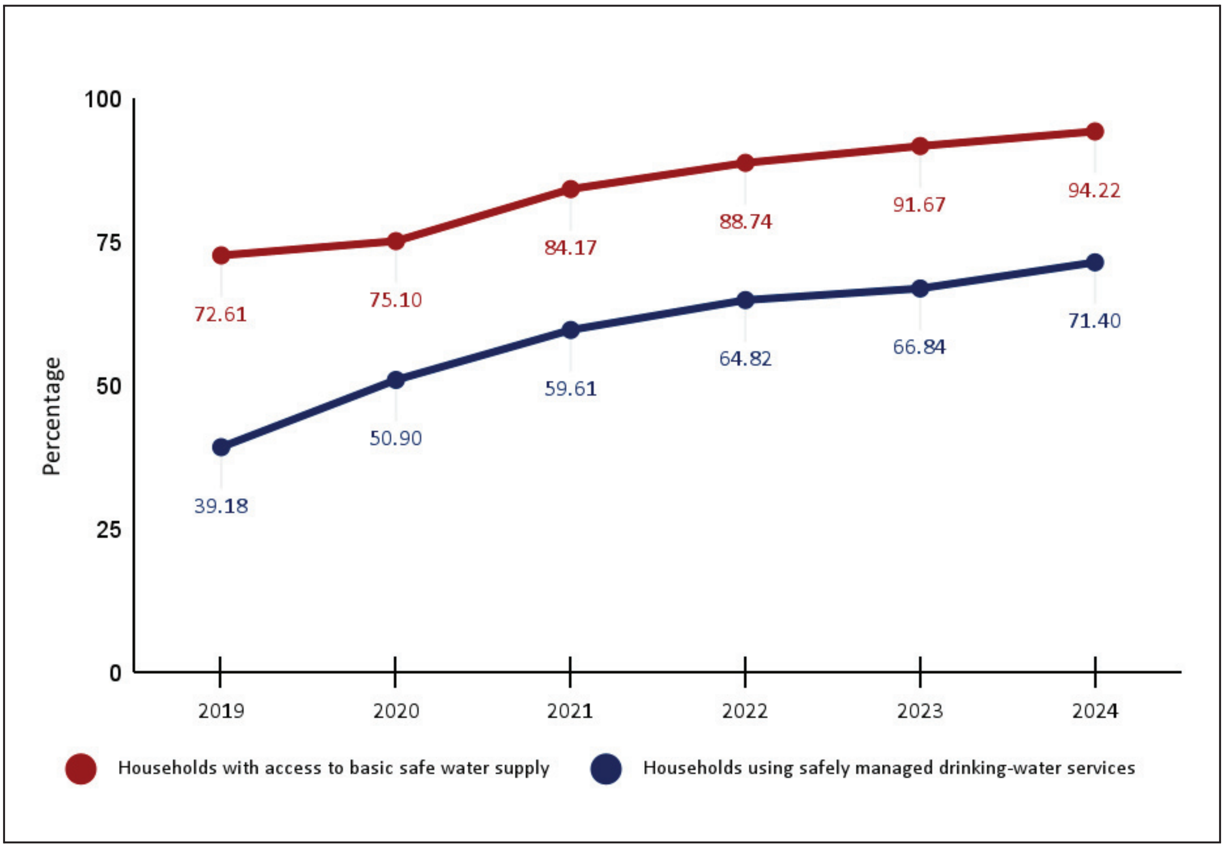

Figure 1H.1: Access to Basic Safe Water Supply and Safely Managed Drinking Water Services, Philippines, 2019 to 2024

In 15 out of 17 regions, more than 90% of the total projected households had access to BSWS in 2024. The regions with the highest access to BSWS are Region 1 (99.44%), Region 3 (98.79%) and CAR (98.56%). The regions where less than 90% of the total projected households had access to BSWS are BARMM (68.86%) and Region 5 (81.32%). In terms of SMDWS, NCR (94.13%) had the highest coverage while BARMM had the lowest coverage (20.13%) *[Table 1.H.1]*.

Table 1.H.1: Access to Basic Safe Water Supply and Safely Managed Drinking Water Services, Region, 2024

Area	Projected No. of HHs	Basic Safe Water Supply		Safely Managed Drinking Water Services	
		No. of HH	%	No. of HH	%
NCR	3,607,432	3,503,846	97.13	3,395,682	94.13
CAR	453,727	447,214	98.56	307,787	67.84
Region 1	1,323,286	1,315,840	99.44	1,006,908	76.09
Region 2	925,795	898,601	97.06	588,433	63.56
Region 3	3,127,682	3,089,890	98.79	2,894,138	92.53
Region 4A	4,183,053	4,000,104	95.63	3,564,723	85.22
Region 4B	813,179	750,751	92.32	343,050	42.19
Region 5	1,410,201	1,146,793	81.32	762,365	54.06
Region 6	1,957,331	1,881,194	96.11	1,151,941	58.85
Region 7	2,032,874	1,843,154	90.67	1,195,075	58.79
Region 8	1,100,629	1,066,273	96.88	622,550	56.56
Region 9	920,356	852,038	92.58	555,928	60.40
Region 10	1,247,577	1,162,045	93.14	710,223	56.93
Region 11	1,382,920	1,337,519	96.72	1,086,861	78.59
Region 12	1,112,953	1,024,737	92.07	614,816	55.24
CARAGA	679,544	655,701	96.49	399,266	58.75
BARMM	852,158	586,754	68.86	171,577	20.13

Along with safe drinking-water, sanitation services are necessary to reduce the risk of malnutrition, diarrhea, schistosomiasis and the spread of intestinal worms. A Basic Sanitation Facility (BSF) refers to improved facilities which are not shared with other households. A BSF is considered a Safely Managed Sanitation Service (SMSS) if it is an improved facility that is not shared with other households and where excreta are safely disposed of in situ or removed and treated offsite.

Out of the projected number of households in 2024, 88.3% or 23,957,667 households have access to BSFs while 68.93% or 18,701,394 households were using SMSSs. The results in 2024 indicate that the annual target of ≥ 89.09% for the proportion of households with access to BSFs and ≥ 74.58% for the proportion of households using SMDWS were not achieved *[Table 1.H.2]*.

In terms of access to BSF, CAR (96.32%), Region 3 (96.27%) and Region 1 (95.93%) had the highest coverage while BARMM (55.45%), Region 5 (75.44%) and Region 7 (78.16%) had the lowest coverage in 2024. In terms of SMSS, the regions with highest SMSS coverage are Region 3 (92.58%), NCR (91.46%) and (91.42%) CAR

while CARAGA (7.37%), Region 4B (9.12%) and BARMM (14.98) had the lowest coverage in 2024. It should be noted that across the regions, there is a gap between the households with access to BSF and the households who are using SMSS *[Table 1.H.2].*

Table 1.H.2: Access to Basic Sanitation Facility and Safely Managed Sanitation Services, by Region, Philippines,2024

Area	Projected No. of HHs	HH with Basic Sanitation Facility		HHs using Safely Managed Sanitation Service	
		No.	%	No.	%
**Philippines**	**27,130,697**	**23,957,667**	**88.30**	**18,701,394**	**68.93**
NCR	3,607,432	3,304,987	91.62	3,299,498	91.46
CAR	453,727	437,014	96.32	414,798	91.42
Region 1	1,323,286	1,269,370	95.93	475,416	35.93
Region 2	925,795	866,590	93.60	827,942	89.43
Region 3	3,127,682	3,011,057	96.27	2,895,680	92.58
Region 4A	4,183,053	3,914,959	93.59	3,498,911	83.64
Region 4B	813,179	675,993	83.13	74,125	9.12
Region 5	1,410,201	1,063,868	75.44	955,658	67.77
Region 6	1,957,331	1,798,035	91.86	1,483,691	75.80
Region 7	2,032,874	1,588,821	78.16	1,417,946	69.75
Region 8	1,100,629	951,930	86.49	294,112	26.72
Region 9	920,356	770,658	83.73	624,363	67.84
Region 10	1,247,577	1,035,963	83.04	205,349	16.46
Region 11	1,382,920	1,259,690	91.09	1,232,222	89.10
Region 12	1,112,953	918,211	82.50	823,939	74.03
CARAGA	679,544	617,983	90.94	50,116	7.37
BARMM	852,158	472,538	55.45	127,628	14.98

In line with the Department Memorandum 2022-0511 or "Redefining the Zero Open Defecation (ZOD) Grade 1 Level under the PhATSS and the reporting of ZOD data in the DOH-FHSIS", municipalities or cities were used as the units of analysis for ZOD instead of barangays. Municipalities or cities are classified as ZOD areas if at least 95% of its total households have BSF or if it has undergone the ZOD certification process.

In 2024, 48.16% or 787 of the 1,634 municipalities and cities in the Philippines were classified as ZOD areas. While ZOD areas increased nationally by 38.07% between 2023 and 2024, the annual target of ≥ 89.09% for the Proportion of Municipality/City declared ZOD was not achieved. Regions with the highest proportion of ZOD areas are Region 1 (92.00%), NCR (82.35%) and Region 3 (75.38%). On the other hand, Region 5 (17.54%), Region 7 (21.21%), and Region 9 (23.61) had the lowest proportion of ZOD areas in 2024 *[Table 1.H.3]*.

Table 1.H.3: ZOD Municipalities/Cities, by Region, Philippines,2023 and 2024

Area	ZOD Municipalities, Cities, and Chartered Cities			
	2023		2024	
	No.	%	No.	%
**PHILIPPINES**	**570**	**34.88**	**787**	**48.16**
NCR	13	76.47	14	82.35
CAR	45	58.44	55	71.43

Region 1	105	84.00	115	92.00
Region 2	16	17.20	45	48.39
Region 3	83	63.85	98	75.38
Region 4A	62	43.66	67	47.18
Region 4B	18	25.00	29	39.73
Region 5	7	6.14	20	17.54
Region 6	75	56.39	79	59.40
Region 7	5	3.79	28	21.21
Region 8	47	32.87	65	45.45
Region 9	4	5.56	17	23.61
Region 10	20	21.51	31	33.33
Region 11	6	12.24	29	59.18
Region 12	11	22.45	19	38.78
CARAGA	18	24.66	40	54.79
BARMM	35	29.17	36	30.25

Recognizing its key role in health, the Philippines included Water, Sanitation and Hygiene (WASH) in the 8 Priority Health Outcomes with the aim of increasing the access of Filipinos to safe water. While there is a general increasing trend for BWS and SMDWS, regional disparities in both the water and sanitation indicators may indicate the need to prioritize underserved areas. Especially because a safe and sufficient water supply and sanitation are crucial in preventing neglected tropical diseases (NTDs) such as trachoma, soil-transmitted helminths and schistosomiasis.[4] For instance, the data for 2024 shows that while CARAGA has the lowest coverage of SMSS, it is also one of the regions where Schistosomiasis cases are highly concentrated (667 acute cases and 130 chronic cases).

[4] World Health Organization: WHO. (2019, November 7). Water, sanitation and hygiene (WASH).

1.1 Demographics

2024 KEY FINDINGS

1 : 21,981
Doctor to Population Ratio

1 : 5,402
Nurses to Population Ratio

1 : 4,490
Midwives to Population Ratio

1 : 55,103
Dentist to Population Ratio

1 : 125,369
Nutritionist to Population Ratio

1 : 33,648
Medical Technologist to Population Ratio

1 : 3,113,329
Sanitary Engineers to Population Ratio

1 : 40,995
Sanitation Inspectors to Population Ratio

HEALTH FACILITY TO POPULATION RATIO
Health Center (HC) and Barangay Health Station (BHS)

1 : 41,206
Health Center (HC) to Population Ratio

1 : 4,390
Barangay Health Station (BHS) to Population Ratio

Formula:

Barangay to Population Ratio
Numerator: Estimated total population
Denominator: Total number of barangays

BHS to Population Ratio
Numerator: Estimated total population
Denominator: Total number of BHS

Health Center (HC) to Population Ratio
Numerator: Estimated total population
Denominator: Total number of health centers

Active Barangay Health Worker (BHW) to HH Ratio
Numerator: Estimated total number of households
Denominator: Total number of active BHW

Public Health Worker to Population Ratio
Numerator: Estimated total population
Denominator: Total number of public health workers (per group)

The Field Health Services Information System (FHSIS) continues to track and monitor community- or population-based data, particularly demographic information that is reported annually. This demographic data includes details such as the total catchment population, number of households, barangays, existing health facilities (e.g., health centers and barangay health stations), and healthcare workers. Ensuring the provision of quality healthcare services relies heavily on the equitable distribution of both health facilities and healthcare personnel.

At the Local Government Unit (LGU) level, the Department of Health (DOH) establishes ideal distribution ratios of health professionals relative to the catchment population of primary care facilities (PCFs). These ratios serve as benchmarks for both the DOH and LGUs in identifying staffing needs and evaluating progress toward achieving target healthcare worker (HCW) levels at PCFs.

To further support human resource planning and development, the DOH, in collaboration with the Human Resources for Health Network Philippines, developed the National Human Resources for Health Master Plan (NHRHMP) 2020–2040. This long-term strategic plan aligns HRH development with the goals of Universal Health Care[1]. Below are the recommended HCW-to-population ratio:

[1] *https://hhrdb.doh.gov.ph/hrh-network/*

Physician/Doctor	1:20,000	Nutritionist	1:20,000
Dentist	1:50,000	Medical Technologist	1:50,000
Public Health Nurse	1:10,000	Sanitary Inspector	1:20,000
Midwife	1:5,000	Sanitary Engineer	-

This chapter presents selected demographic data for the Philippines in 2024, focusing specifically on the ratios of health facilities and healthcare workers (HCWs) to the estimated population. The data presented have been submitted by the Centers for Health Development (CHDs) and the Ministry of Health of the Bangsamoro Autonomous Region in Muslim Mindanao (MOH-BARMM).

Over the past five years, the country's population has continued to grow, albeit at a slower rate. Between 2020 and 2024, the total population increased by 3.04%, rising from 108,771,978 to 112,079,845. During the same period, a slight decline in the number of barangays reporting to the Field Health Services Information System (FHSIS) was observed, from 42,134 in 2020 to 41,590 in 2024. It is important to note that official statistics on geographic areas in the Philippines are provided by the Philippine Statistics Authority (PSA).

Meanwhile, the number of BHS has generally increased over the years *[Figure 1.I.1]*. In 2024, a total of 25,532 BHS were reported to the FHSIS, resulting in a BHS to population ratio of 1:4,390. This essentially means that on the average, one (1) BHS caters to approximately 4,390 individuals. In addition, there were 2,720 HC in 2024, which translated to an HC to population ratio of 1:41,206.

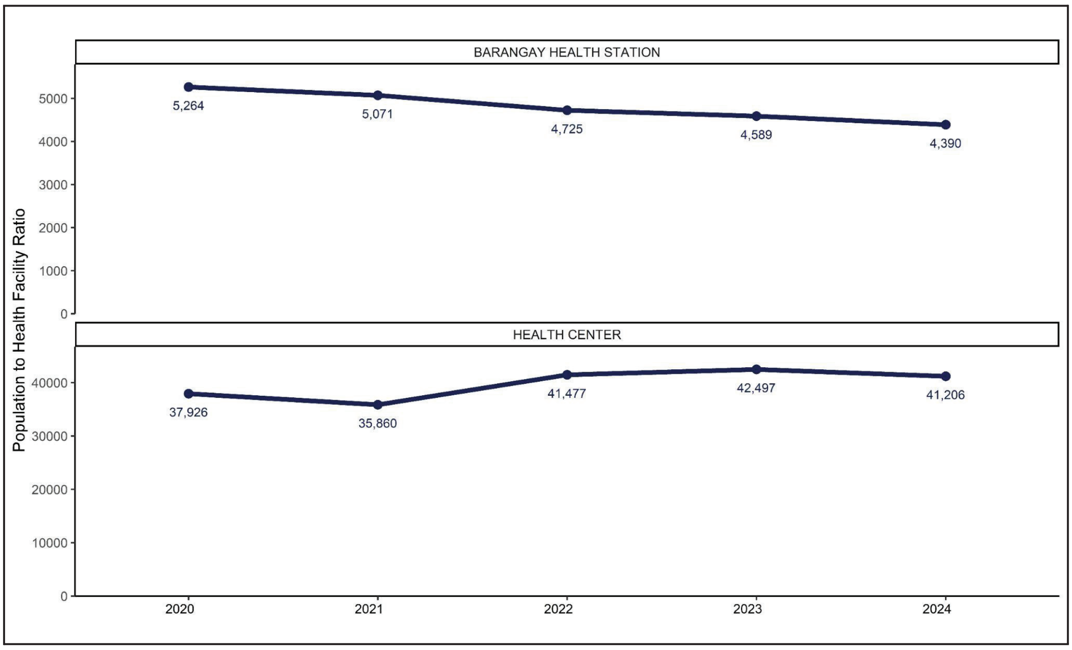

Figure 1.I.1: Health Facility to Population Ratio, Philippines, 2020 to 2024

In terms of HCW, the ratio of the population to HCW was decreasing for doctors, dentists, public health nurses (PHN's), midwives, nutritionists and sanitary engineers. On the contrary, it was increasing for medical technologists and sanitary inspectors. The increase was more prominent for the Sanitary Inspector, in which the ratio grew to 40,995 from 37,908 in 2023.

Note that this only covers personnel hired by either the DOH or the LGUs. Nonetheless, this means that the growth in the number of several HCWs in PCFs hired by the government is not proportional to the growth in the population. The increase in the population has slowed down in recent years which may be an opportunity to hone and grow the workforce, especially in the health sector. Ensuring that every Filipino has access to quality health care does not only entail improvement in terms of facilities, but also includes the people who will man these facilities.

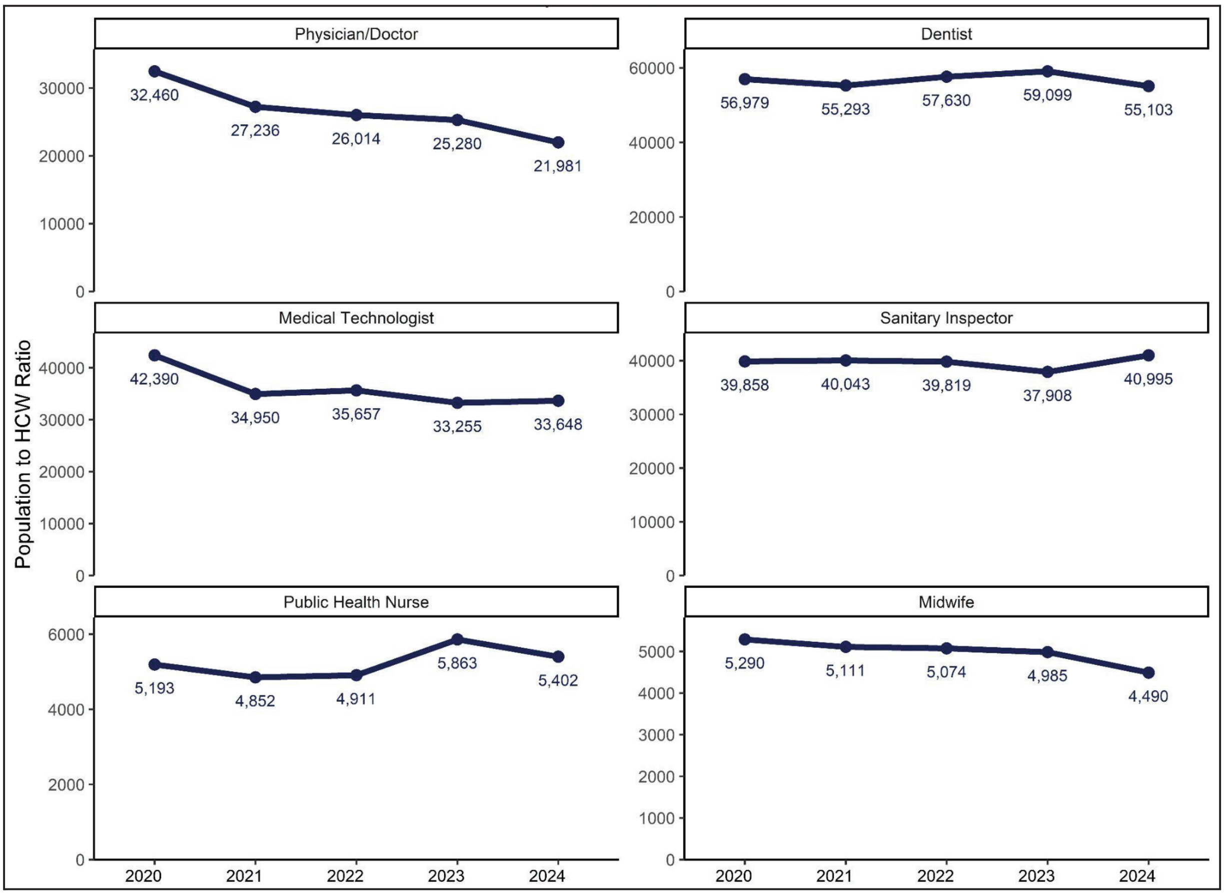

Figure 1.I.2: Health Care Worker to Population Ratio, Philippines, 2020 to 2024

Looking at the regional statistics, Region 4A was the most populous region in the country with an estimated population of 16,675,819, while CAR had the least population of 1,857,123. Consequently, Region 4A had the highest number of households (4,183,053) and CAR had the lowest (453,727). Meanwhile, the total number of barangays was highest in Region 8 (4,365) and lowest in Region 12 (1,105).

The number of HCs was highest in NCR (444) with a 1:31,311 HC to population ratio and lowest in Region 12 (67 HC) with 1:67,994 HC to population ratio. As to the number of BHS, Region 4A had the most with 2,921 BHS or a 1:5,709 BHS to population ratio, while NCR had the least with only 35 BHS resulting in a 1:397,203 BHS to population ratio *[Table 1.I.1]*.

Table 1.I.1: Health Facility to Population Ratio, by Region, 2024

Region	No. of BHS	BHS to Population Ratio	No. of Health Centers	HC to Population Ratio
NCR	35	397,203	444	31,311
CAR	933	1,990	99	18,759
Region 1	1,652	3,252	170	31,600
Region 2	1,693	2,221	98	38,374
Region 3	2,317	5,516	318	40,192
Region 4A	2,921	5,709	302	55,218
Region 4B	1,292	2,563	109	30,384
Region 5	2,663	2,360	141	44,581
Region 6	2,191	3,664	146	54,978
Region 7	2,322	3,597	158	52,866
Region 8	897	5,158	175	26,440
Region 9	987	4,035	100	39,823
Region 10	1,355	3,861	137	38,192
Region 11	1,275	4,252	80	67,766
Region 12	1,132	4,024	67	67,994
CARAGA	1,211	2,378	90	32,002
BARMM	656	7,706	86	58,782

For the public healthcare workforce in PCFs, only CAR met the recommended HCW to population ratio across the following professions: doctor, dentist, PHN, midwife, medical technologist, and sanitary inspector. The NCR met the recommended ratios for almost the same professions as CAR, with the exception of the ratio for midwives and sanity inspectors *[Table 1.I.2]*.

Meanwhile, all regions met the recommended 1 PHN per 10,000 population. Notably, several regions still fell short of the recommended ratios for midwives and medical technologists despite the national ratios being within the recommendations. In the NCR, for example, 1 midwife had a catchment population of 11,104 or double the recommended 5,000. A complete table found in *[Table 1.I.2]* would show that all regions had very few nutritionists, sanitary engineers, and sanity inspectors. Further, Region 6, Region 7, Region 8, CARAGA, and BARMM reported zero sanitary engineers.

Table 1.I.2: Health Care Worker to Population Ratio, by Region, 2024

Region	HCW to Population Ratio							
	Doctors	Dentists	Public Health Nurse	Midwives	Medical Technologists	Sanitary Engineers	Sanitation Inspectors	Nutritionist
**RECOMMENDED RATIO**	**20,000**	**50,000**	**10,000**	**5,000**	**50,000**	**20,000**	**20,000**	**20,000**
NCR	13,958	23,404	8,173	11,104	29,642	2,780,422	43,994	86,349
CAR	15,476	36,414	2,684	2,008	16,883	619,041	16,010	71,428
Region 1	24,756	57,148	5,936	4,956	25,827	1,790,652	28,423	268,598
Region 2	22,792	110,606	5,187	3,431	24,262	3,760,607	33,879	221,212
Region 3	31,636	64,227	8,337	6,798	38,848	6,390,564	47,513	311,735
Region 4A	34,312	74,115	7,392	8,371	58,718	3,335,164	59,985	282,641
Region 4B	6,202	18,817	1,863	1,351	27,371	473,120	174,307	23,656
Region 5	28,572	73,092	3,921	3,547	34,162	3,142,947	35,919	96,706
Region 6	18,976	60,809	6,396	3,609	25,977	0	32,236	211,231
Region 7	23,332	94,918	6,650	4,661	39,965	0	40,352	128,504
Region 8	19,859	69,061	4,098	3,527	23,252	0	23,608	308,472
Region 9	29,498	97,128	4,314	3,803	37,926	3,982,251	33,464	76,582
Region 10	25,648	91,794	4,247	3,634	34,651	5,232,243	41,858	87,204
Region 11	35,902	79,724	5,024	5,767	34,530	5,421,241	52,127	108,425
Region 12	20,247	96,928	5,334	2,388	24,894	911,120	43,387	81,350
CARAGA	24,203	64,004	2,769	3,251	26,424	0	27,963	151,588
BARMM	46,378	168,508	6,440	5,271	103,168	0	95,382	505,523

CHAPTER II

Summary Tables

Table 2.A.1 - Modern Methods of Family Planning
New Acceptors
Philippines, 2024

Area	Total Current Users	FSTR/BTL			Total	MSTR/NSV			Total
		Age group				Age group			
		10-14	15-19	20-49		10-14	15-19	20-49	
**PHILIPPINES**	**8,664,035**	**1**	**64**	**18,210**	**18,275**	**0**	**20**	**680**	**700**
**NCR**	**1,041,531**	**0**	**1**	**3,095**	**3,096**	**0**	**2**	**409**	**411**
City of Malabon	26,264	0	0	18	18	0	1	3	4
City of Navotas	23,816	0	0	71	71	0	0	0	0
City of Valenzuela	40,454	0	0	187	187	0	0	22	22
City of Caloocan	73,676	0	0	108	108	0	0	0	0
City of Marikina	24,847	0	0	14	14	0	0	1	1
City of Pasig	58,937	0	0	257	257	0	0	142	142
Pateros	4,204	0	0	12	12	0	0	1	1
City of Taguig	92,053	0	0	16	16	0	0	2	2
Quezon City	338,677	0	0	247	247	0	0	2	2
City of Makati	28,572	0	0	167	167	0	0	1	1
City of Mandaluyong	27,196	0	0	105	105	0	0	5	5
City of San Juan	6,139	0	0	0	0	0	0	0	0
City of Manila	138,102	0	0	1,392	1,392	0	0	221	221
City of Las Piñas	26,980	0	0	66	66	0	0	0	0
City of Muntinlupa	44,186	0	1	299	300	0	1	8	9
City of Parañaque	47,100	0	0	120	120	0	0	1	1
Pasay City	40,328	0	0	16	16	0	0	0	0
**CAR**	**165,663**	**0**	**0**	**169**	**169**	**0**	**0**	**10**	**10**
Abra	19,755	0	0	16	16	0	0	0	0
Apayao	24,752	0	0	0	0	0	0	0	0
Benguet	37,253	0	0	0	0	0	0	0	0
Ifugao	21,120	0	0	0	0	0	0	0	0
Kalinga	18,267	0	0	126	126	0	0	0	0
Mountain Province	13,957	0	0	2	2	0	0	0	0
City of Baguio	30,559	0	0	25	25	0	0	10	10
**Region 1**	**498,204**	**0**	**2**	**885**	**887**	**0**	**0**	**14**	**14**
Ilocos Norte	52,824	0	0	48	48	0	0	13	13
Ilocos Sur	78,125	0	1	308	309	0	0	0	0
La Union	67,784	0	1	76	77	0	0	1	1
Pangasinan	292,353	0	0	453	453	0	0	0	0
City of Dagupan	7,118	0	0	0	0	0	0	0	0
**Region 2**	**343,378**	**0**	**0**	**303**	**303**	**0**	**0**	**27**	**27**
Batanes	955	0	0	0	0	0	0	0	0
Cagayan	108,930	0	0	57	57	0	0	0	0
Isabela	146,131	0	0	75	75	0	0	27	27
Nueva Vizcaya	52,095	0	0	149	149	0	0	0	0
Quirino	21,240	0	0	22	22	0	0	0	0
City of Santiago	14,027	0	0	0	0	0	0	0	0
**Region 3**	**985,325**	**1**	**5**	**5,766**	**5,772**	**0**	**2**	**39**	**41**
Aurora	24,562	0	0	1	1	0	0	0	0
Bataan	65,716	0	0	116	116	0	1	0	1
Bulacan	302,904	0	4	1,606	1,610	0	0	7	7
Nueva Ecija	200,897	1	0	295	296	0	0	6	6
Pampanga	167,294	0	1	2,173	2,174	0	0	6	6
Tarlac	118,444	0	0	366	366	0	1	13	14
Zambales	54,326	0	0	558	558	0	0	3	3
City of Angeles	30,345	0	0	419	419	0	0	0	0
City of Olongapo	20,837	0	0	232	232	0	0	4	4
**Region 4A**	**888,692**	**0**	**7**	**1,812**	**1,819**	**0**	**9**	**61**	**70**
Batangas	158,569	0	2	224	226	0	0	11	11
Cavite	175,332	0	1	449	450	0	9	11	20
Laguna	286,016	0	1	621	622	0	0	18	18
Quezon	106,773	0	0	72	72	0	0	0	0
Rizal	156,554	0	3	440	443	0	0	21	21
City of Lucena	5,448	0	0	6	6	0	0	0	0
**Region 4B**	**291,730**	**0**	**7**	**344**	**351**	**0**	**0**	**7**	**7**
Marinduque	16,436	0	0	52	52	0	0	0	0
Occidental Mindoro	48,902	0	0	1	1	0	0	0	0
Oriental Mindoro	68,962	0	0	144	144	0	0	5	5
Palawan	100,239	0	0	55	55	0	0	2	2

Table 2.A.1 - Modern Methods of Family Planning
New Acceptors
Philippines, 2024

Area	Total Current Users	FSTR/BTL Age group 10-14	FSTR/BTL Age group 15-19	FSTR/BTL Age group 20-49	Total	MSTR/NSV Age group 10-14	MSTR/NSV Age group 15-19	MSTR/NSV Age group 20-49	Total
Romblon	22,467	0	7	73	80	0	0	0	0
City of Puerto Princesa	34,724	0	0	19	19	0	0	0	0
**Region 5**	**491,006**	**0**	**0**	**312**	**312**	**0**	**1**	**1**	**2**
Albay	115,867	0	0	159	159	0	0	1	1
Camarines Norte	46,839	0	0	6	6	0	1	0	1
Camarines Sur	107,464	0	0	4	4	0	0	0	0
Catanduanes	24,326	0	0	18	18	0	0	0	0
Masbate	96,672	0	0	37	37	0	0	0	0
Sorsogon	66,956	0	0	19	19	0	0	0	0
City of Naga	32,882	0	0	69	69	0	0	0	0
**Region 6**	**667,635**	**0**	**2**	**972**	**974**	**0**	**0**	**40**	**40**
Aklan	48,553	0	1	68	69	0	0	1	1
Antique	49,316	0	0	248	248	0	0	17	17
Capiz	77,942	0	0	74	74	0	0	1	1
Guimaras	17,943	0	0	31	31	0	0	0	0
Iloilo	182,130	0	0	87	87	0	0	0	0
Negros Occidental	206,889	0	1	228	229	0	0	21	21
City of Bacolod	30,421	0	0	146	146	0	0	0	0
City of Iloilo	54,441	0	0	90	90	0	0	0	0
**Region 7**	**483,438**	**0**	**1**	**785**	**786**	**0**	**1**	**0**	**1**
Bohol	83,324	0	0	162	162	0	0	0	0
Cebu	191,250	0	1	403	404	0	1	0	1
Negros Oriental	98,214	0	0	62	62	0	0	0	0
Siquijor	9,225	0	0	0	0	0	0	0	0
City of Cebu	34,897	0	0	37	37	0	0	0	0
City of Lapu-Lapu	36,794	0	0	0	0	0	0	0	0
City of Mandaue	29,734	0	0	121	121	0	0	0	0
**Region 8**	**340,613**	**0**	**0**	**686**	**686**	**0**	**0**	**8**	**8**
Biliran	12,714	0	0	16	16	0	0	0	0
Eastern Samar	39,982	0	0	1	1	0	0	0	0
Leyte	125,631	0	0	517	517	0	0	7	7
Northern Samar	48,697	0	0	15	15	0	0	0	0
Southern Leyte	27,251	0	0	11	11	0	0	0	0
Samar	42,957	0	0	19	19	0	0	1	1
Ormoc City	20,171	0	0	1	1	0	0	0	0
City of Tacloban	23,210	0	0	106	106	0	0	0	0
**Region 9**	**354,267**	**0**	**0**	**190**	**190**	**0**	**0**	**1**	**1**
Zamboanga del Norte	126,112	0	0	0	0	0	0	0	0
Zamboanga del Sur	102,687	0	0	24	24	0	0	0	0
Zamboanga Sibugay	47,839	0	0	0	0	0	0	0	0
City of Isabela	7,167	0	0	0	0	0	0	0	0
City of Zamboanga	70,462	0	0	166	166	0	0	1	1
**Region 10**	**511,701**	**0**	**13**	**910**	**923**	**0**	**0**	**3**	**3**
Bukidnon	167,011	0	1	95	96	0	0	2	2
Camiguin	6,884	0	0	7	7	0	0	0	0
Lanao del Norte	73,898	0	0	74	74	0	0	0	0
Misamis Occidental	60,887	0	0	42	42	0	0	0	0
Misamis Oriental	111,011	0	2	194	196	0	0	0	0
City of Cagayan De Oro	57,799	0	10	128	138	0	0	1	1
City of Iligan	34,211	0	0	370	370	0	0	0	0
**Region 11**	**535,532**	**0**	**12**	**1,188**	**1,200**	**0**	**1**	**38**	**39**
Davao de Oro	84,560	0	0	232	232	0	1	1	2
Davao del Norte	112,914	0	0	224	224	0	0	9	9
Davao Oriental	59,172	0	5	31	36	0	0	7	7
Davao del Sur	67,054	0	4	87	91	0	0	2	2
Davao Occidental	33,334	0	3	49	52	0	0	0	0
City of Davao	178,498	0	0	565	565	0	0	19	19
**Region 12**	**476,219**	**0**	**14**	**369**	**383**	**0**	**4**	**16**	**20**
Cotabato	136,508	0	11	201	212	0	1	9	10
Sarangani	76,390	0	2	28	30	0	0	0	0
South Cotabato	102,265	0	0	86	86	0	3	0	3

Table 2.A.1 - Modern Methods of Family Planning
New Acceptors
Philippines, 2024

Area	Total Current Users	FSTR/BTL Age group 10-14	FSTR/BTL Age group 15-19	FSTR/BTL Age group 20-49	Total	MSTR/NSV Age group 10-14	MSTR/NSV Age group 15-19	MSTR/NSV Age group 20-49	Total
Sultan Kudarat	104,518	0	1	28	29	0	0	6	6
City of General Santos	56,538	0	0	26	26	0	0	1	1
**Caraga**	**292,511**	**0**	**0**	**355**	**355**	**0**	**0**	**3**	**3**
Agusan del Norte	40,148	0	0	115	115	0	0	3	3
Agusan del Sur	86,721	0	0	9	9	0	0	0	0
Surigao del Norte	53,159	0	0	191	191	0	0	0	0
Surigao del Sur	60,298	0	0	6	6	0	0	0	0
Dinagat Islands	9,606	0	0	0	0	0	0	0	0
City of Butuan	42,579	0	0	34	34	0	0	0	0
**BARMM**	**296,590**	**0**	**0**	**69**	**69**	**0**	**0**	**3**	**3**
Basilan	19,309	0	0	0	0	0	0	0	0
Lanao del Sur	39,800	0	0	7	7	0	0	0	0
Maguindanao del Norte	36,321	0	0	0	0	0	0	0	0
Maguindanao del Sur	51,529	0	0	36	36	0	0	0	0
Sulu	80,817	0	0	1	1	0	0	0	0
Tawi-Tawi	19,107	0	0	2	2	0	0	0	0
SGA	7,550	0	0	0	0	0	0	0	0
City of Cotabato	42,157	0	0	23	23	0	0	3	3

Table 2.A.1 - Modern Methods of Family Planning
New Acceptors
Philippines, 2024

Area	Total Current Users	CONDOM			Total	IUD-INTERVAL			Total
		Age group				Age group			
		10-14	15-19	20-49		10-14	15-19	20-49	
**PHILIPPINES**	**8,664,035**	**179**	**4,053**	**64,507**	**68,739**	**13**	**632**	**6,899**	**7,544**
**NCR**	**1,041,531**	**65**	**1,708**	**34,352**	**36,125**	**0**	**75**	**1,099**	**1,174**
City of Malabon	26,264	2	36	346	384	0	2	15	17
City of Navotas	23,816	0	304	623	927	0	13	23	36
City of Valenzuela	40,454	0	17	752	769	0	1	11	12
City of Caloocan	73,676	2	72	516	590	0	3	26	29
City of Marikina	24,847	0	6	78	84	0	2	6	8
City of Pasig	58,937	9	100	1,023	1,132	0	3	91	94
Pateros	4,204	0	0	35	35	0	0	11	11
City of Taguig	92,053	1	172	2,556	2,729	0	4	85	89
Quezon City	338,677	1	159	2,882	3,042	0	15	135	150
City of Makati	28,572	0	18	2,196	2,214	0	1	146	147
City of Mandaluyong	27,196	0	3	313	316	0	1	37	38
City of San Juan	6,139	0	0	19	19	0	0	52	52
City of Manila	138,102	48	347	16,421	16,816	0	11	207	218
City of Las Piñas	26,980	0	83	3,717	3,800	0	1	17	18
City of Muntinlupa	44,186	0	14	185	199	0	2	43	45
City of Parañaque	47,100	2	294	2,235	2,531	0	4	33	37
Pasay City	40,328	0	83	455	538	0	12	161	173
**CAR**	**165,663**	**1**	**14**	**241**	**256**	**0**	**3**	**24**	**27**
Abra	19,755	0	4	44	48	0	0	4	4
Apayao	24,752	0	3	13	16	0	0	2	2
Benguet	37,253	0	4	61	65	0	2	14	16
Ifugao	21,120	0	0	4	4	0	1	2	3
Kalinga	18,267	0	0	29	29	0	0	0	0
Mountain Province	13,957	1	0	4	5	0	0	0	0
City of Baguio	30,559	0	3	86	89	0	0	2	2
**Region 1**	**498,204**	**89**	**44**	**653**	**786**	**0**	**32**	**509**	**541**
Ilocos Norte	52,824	0	1	57	58	0	0	14	14
Ilocos Sur	78,125	0	15	145	160	0	2	153	155
La Union	67,784	1	7	47	55	0	6	26	32
Pangasinan	292,353	88	21	394	503	0	24	315	339
City of Dagupan	7,118	0	0	10	10	0	0	1	1
**Region 2**	**343,378**	**0**	**8**	**176**	**184**	**4**	**33**	**126**	**163**
Batanes	955	0	0	0	0	0	0	0	0
Cagayan	108,930	0	0	100	100	1	21	85	107
Isabela	146,131	0	4	47	51	3	10	37	50
Nueva Vizcaya	52,095	0	3	19	22	0	2	4	6
Quirino	21,240	0	1	10	11	0	0	0	0
City of Santiago	14,027	0	0	0	0	0	0	0	0
**Region 3**	**985,325**	**3**	**544**	**10,350**	**10,897**	**1**	**26**	**410**	**437**
Aurora	24,562	0	0	6	6	0	0	0	0
Bataan	65,716	0	119	1,374	1,493	1	1	21	23
Bulacan	302,904	1	246	5,241	5,488	0	12	184	196
Nueva Ecija	200,897	1	50	627	678	0	2	35	37
Pampanga	167,294	0	45	1,634	1,679	0	7	147	154
Tarlac	118,444	0	63	890	953	0	3	19	22
Zambales	54,326	0	14	385	399	0	0	3	3
City of Angeles	30,345	1	0	41	42	0	0	0	0
City of Olongapo	20,837	0	7	152	159	0	1	1	2
**Region 4A**	**888,692**	**5**	**470**	**5,981**	**6,456**	**4**	**46**	**452**	**502**
Batangas	158,569	0	19	445	464	0	2	57	59
Cavite	175,332	0	35	387	422	0	3	71	74
Laguna	286,016	4	382	2,305	2,691	0	27	131	158
Quezon	106,773	1	17	119	137	0	5	58	63
Rizal	156,554	0	17	2,711	2,728	0	9	135	144
City of Lucena	5,448	0	0	14	14	4	0	0	4
**Region 4B**	**291,730**	**0**	**26**	**277**	**303**	**0**	**9**	**138**	**147**
Marinduque	16,436	0	0	8	8	0	0	2	2
Occidental Mindoro	48,902	0	1	14	15	0	0	0	0
Oriental Mindoro	68,962	0	8	67	75	0	2	83	85
Palawan	100,239	0	11	123	134	0	7	40	47

Table 2.A.1 - Modern Methods of Family Planning
New Acceptors
Philippines, 2024

Area	Total Current Users	CONDOM			Total	IUD-INTERVAL			Total
		Age group				Age group			
		10-14	15-19	20-49		10-14	15-19	20-49	
Romblon	22,467	0	1	29	30	0	0	11	11
City of Puerto Princesa	34,724	0	5	36	41	0	0	2	2
**Region 5**	**491,006**	**1**	**77**	**1,544**	**1,622**	**0**	**12**	**104**	**116**
Albay	115,867	0	13	404	417	0	2	34	36
Camarines Norte	46,839	0	21	91	112	0	0	3	3
Camarines Sur	107,464	0	16	593	609	0	6	20	26
Catanduanes	24,326	1	9	26	36	0	0	0	0
Masbate	96,672	0	15	108	123	0	3	33	36
Sorsogon	66,956	0	3	38	41	0	0	0	0
City of Naga	32,882	0	0	284	284	0	1	14	15
**Region 6**	**667,635**	**2**	**159**	**1,375**	**1,536**	**0**	**75**	**630**	**705**
Aklan	48,553	0	13	197	210	0	3	9	12
Antique	49,316	1	6	109	116	0	0	9	9
Capiz	77,942	0	5	96	101	0	6	79	85
Guimaras	17,943	0	1	15	16	0	0	2	2
Iloilo	182,130	0	26	251	277	0	30	164	194
Negros Occidental	206,889	1	99	553	653	0	15	232	247
City of Bacolod	30,421	0	4	101	105	0	11	66	77
City of Iloilo	54,441	0	5	53	58	0	10	69	79
**Region 7**	**483,438**	**2**	**168**	**1,672**	**1,842**	**0**	**94**	**1,116**	**1,210**
Bohol	83,324	0	17	195	212	0	23	246	269
Cebu	191,250	2	64	805	871	0	45	499	544
Negros Oriental	98,214	0	44	212	256	0	11	106	117
Siquijor	9,225	0	1	20	21	0	0	1	1
City of Cebu	34,897	0	6	158	164	0	7	136	143
City of Lapu-Lapu	36,794	0	30	186	216	0	2	16	18
City of Mandaue	29,734	0	6	96	102	0	6	112	118
**Region 8**	**340,613**	**0**	**88**	**640**	**728**	**0**	**23**	**327**	**350**
Biliran	12,714	0	3	18	21	0	0	4	4
Eastern Samar	39,982	0	4	75	79	0	0	5	5
Leyte	125,631	0	4	173	177	0	6	168	174
Northern Samar	48,697	0	5	67	72	0	1	1	2
Southern Leyte	27,251	0	2	38	40	0	1	22	23
Samar	42,957	0	7	121	128	0	0	37	37
Ormoc City	20,171	0	2	9	11	0	5	7	12
City of Tacloban	23,210	0	61	139	200	0	10	83	93
**Region 9**	**354,267**	**0**	**137**	**611**	**748**	**0**	**31**	**223**	**254**
Zamboanga del Norte	126,112	0	24	81	105	0	3	65	68
Zamboanga del Sur	102,687	0	6	127	133	0	10	78	88
Zamboanga Sibugay	47,839	0	45	117	162	0	16	43	59
City of Isabela	7,167	0	61	156	217	0	0	0	0
City of Zamboanga	70,462	0	1	130	131	0	2	37	39
**Region 10**	**511,701**	**1**	**171**	**1,978**	**2,150**	**1**	**78**	**668**	**747**
Bukidnon	167,011	0	40	197	237	1	17	84	102
Camiguin	6,884	0	0	9	9	0	0	0	0
Lanao del Norte	73,898	0	20	860	880	0	4	101	105
Misamis Occidental	60,887	0	13	157	170	0	1	39	40
Misamis Oriental	111,011	1	13	96	110	0	10	102	112
City of Cagayan De Oro	57,799	0	63	483	546	0	37	246	283
City of Iligan	34,211	0	22	176	198	0	9	96	105
**Region 11**	**535,532**	**7**	**158**	**2,398**	**2,563**	**2**	**37**	**561**	**600**
Davao de Oro	84,560	0	9	114	123	0	2	83	85
Davao del Norte	112,914	0	9	480	489	0	11	113	124
Davao Oriental	59,172	3	3	43	49	0	1	36	37
Davao del Sur	67,054	1	19	68	88	2	8	101	111
Davao Occidental	33,334	2	10	27	39	0	4	22	26
City of Davao	178,498	1	108	1,666	1,775	0	11	206	217
**Region 12**	**476,219**	**2**	**129**	**704**	**835**	**1**	**26**	**258**	**285**
Cotabato	136,508	2	24	306	332	0	10	114	124
Sarangani	76,390	0	15	39	54	0	1	13	14
South Cotabato	102,265	0	26	134	160	0	6	51	57

Table 2.A.1 - Modern Methods of Family Planning
New Acceptors
Philippines, 2024

Area	Total Current Users	CONDOM			Total	IUD-INTERVAL			Total
		Age group				Age group			
		10-14	15-19	20-49		10-14	15-19	20-49	
Sultan Kudarat	104,518	0	46	61	107	0	3	22	25
City of General Santos	56,538	0	18	164	182	1	6	58	65
**Caraga**	**292,511**	**1**	**57**	**439**	**497**	**0**	**28**	**195**	**223**
Agusan del Norte	40,148	1	14	75	90	0	11	46	57
Agusan del Sur	86,721	0	20	66	86	0	4	69	73
Surigao del Norte	53,159	0	5	155	160	0	5	47	52
Surigao del Sur	60,298	0	10	49	59	0	4	17	21
Dinagat Islands	9,606	0	0	18	18	0	0	9	9
City of Butuan	42,579	0	8	76	84	0	4	7	11
**BARMM**	**296,590**	**0**	**95**	**1,116**	**1,211**	**0**	**4**	**59**	**63**
Basilan	19,309	0	2	28	30	0	1	4	5
Lanao del Sur	39,800	0	6	298	304	0	1	8	9
Maguindanao del Norte	36,321	0	5	154	159	0	0	3	3
Maguindanao del Sur	51,529	0	35	252	287	0	0	0	0
Sulu	80,817	0	39	171	210	0	0	7	7
Tawi-Tawi	19,107	0	0	6	6	0	0	0	0
SGA	7,550	0	1	11	12	0	0	0	0
City of Cotabato	42,157	0	7	196	203	0	2	37	39

Table 2.A.1 - Modern Methods of Family Planning
New Acceptors
Philippines, 2024

Area	Total Current Users	IUD-POSTPARTUM			Total	PILLS-POP			Total
		Age group				Age group			
		10-14	15-19	20-49		10-14	15-19	20-49	
**PHILIPPINES**	**8,664,035**	**141**	**3,073**	**11,603**	**14,817**	**325**	**10,166**	**74,415**	**84,906**
**NCR**	**1,041,531**	**36**	**875**	**4,502**	**5,413**	**60**	**1,326**	**21,692**	**23,078**
City of Malabon	26,264	0	0	10	10	6	62	493	561
City of Navotas	23,816	0	0	0	0	0	188	614	802
City of Valenzuela	40,454	0	0	82	82	0	16	752	768
City of Caloocan	73,676	14	251	533	798	0	89	740	829
City of Marikina	24,847	7	132	740	879	1	7	168	176
City of Pasig	58,937	5	96	498	599	2	21	745	768
Pateros	4,204	0	0	1	1	0	1	22	23
City of Taguig	92,053	0	26	220	246	0	116	1,359	1,475
Quezon City	338,677	8	220	902	1,130	2	211	2,647	2,860
City of Makati	28,572	0	38	417	455	0	12	648	660
City of Mandaluyong	27,196	0	24	119	143	0	7	219	226
City of San Juan	6,139	0	0	0	0	0	10	115	125
City of Manila	138,102	2	78	724	804	48	408	9,763	10,219
City of Las Piñas	26,980	0	5	39	44	1	63	1,565	1,629
City of Muntinlupa	44,186	0	0	0	0	0	42	333	375
City of Parañaque	47,100	0	3	9	12	0	52	877	929
Pasay City	40,328	0	2	208	210	0	21	632	653
**CAR**	**165,663**	**0**	**5**	**22**	**27**	**0**	**57**	**572**	**629**
Abra	19,755	0	0	0	0	0	6	50	56
Apayao	24,752	0	0	0	0	0	13	31	44
Benguet	37,253	0	4	14	18	0	17	177	194
Ifugao	21,120	0	0	0	0	0	2	7	9
Kalinga	18,267	0	0	2	2	0	8	41	49
Mountain Province	13,957	0	0	0	0	0	1	14	15
City of Baguio	30,559	0	1	6	7	0	10	252	262
**Region 1**	**498,204**	**0**	**29**	**243**	**272**	**3**	**209**	**1,593**	**1,805**
Ilocos Norte	52,824	0	1	6	7	0	19	174	193
Ilocos Sur	78,125	0	1	4	5	0	40	571	611
La Union	67,784	0	6	29	35	0	12	68	80
Pangasinan	292,353	0	21	204	225	3	135	765	903
City of Dagupan	7,118	0	0	0	0	0	3	15	18
**Region 2**	**343,378**	**5**	**45**	**114**	**164**	**2**	**235**	**1,095**	**1,332**
Batanes	955	0	0	0	0	0	0	0	0
Cagayan	108,930	2	33	50	85	1	80	458	539
Isabela	146,131	0	4	19	23	1	141	554	696
Nueva Vizcaya	52,095	3	8	44	55	0	5	60	65
Quirino	21,240	0	0	1	1	0	9	23	32
City of Santiago	14,027	0	0	0	0	0	0	0	0
**Region 3**	**985,325**	**22**	**266**	**905**	**1,193**	**31**	**1,267**	**7,607**	**8,905**
Aurora	24,562	0	1	1	2	0	1	7	8
Bataan	65,716	0	0	8	8	1	188	895	1,084
Bulacan	302,904	0	67	204	271	2	365	2,974	3,341
Nueva Ecija	200,897	10	104	215	329	25	483	1,579	2,087
Pampanga	167,294	12	93	461	566	1	123	944	1,068
Tarlac	118,444	0	0	4	4	1	52	672	725
Zambales	54,326	0	1	5	6	0	27	215	242
City of Angeles	30,345	0	0	7	7	1	14	143	158
City of Olongapo	20,837	0	0	0	0	0	14	178	192
**Region 4A**	**888,692**	**3**	**48**	**302**	**353**	**12**	**1,259**	**9,122**	**10,393**
Batangas	158,569	0	3	54	57	3	87	851	941
Cavite	175,332	1	9	52	62	0	154	1,484	1,638
Laguna	286,016	0	7	59	66	2	626	4,064	4,692
Quezon	106,773	1	3	14	18	4	151	657	812
Rizal	156,554	1	26	123	150	0	214	1,926	2,140
City of Lucena	5,448	0	0	0	0	3	27	140	170
**Region 4B**	**291,730**	**1**	**37**	**141**	**179**	**7**	**222**	**957**	**1,186**
Marinduque	16,436	0	0	0	0	0	12	96	108
Occidental Mindoro	48,902	0	0	0	0	2	25	59	86
Oriental Mindoro	68,962	0	36	132	168	2	50	352	404
Palawan	100,239	1	1	1	3	1	97	245	343

Table 2.A.1 - Modern Methods of Family Planning
New Acceptors
Philippines, 2024

Area	Total Current Users	IUD-POSTPARTUM Age group 10-14	15-19	20-49	Total	PILLS-POP Age group 10-14	15-19	20-49	Total
Romblon	22,467	0	0	7	7	0	9	95	104
City of Puerto Princesa	34,724	0	0	1	1	2	29	110	141
**Region 5**	**491,006**	**0**	**18**	**59**	**77**	**0**	**256**	**1,905**	**2,161**
Albay	115,867	0	2	15	17	0	52	557	609
Camarines Norte	46,839	0	6	9	15	0	40	164	204
Camarines Sur	107,464	0	6	17	23	0	107	792	899
Catanduanes	24,326	0	0	0	0	0	1	3	4
Masbate	96,672	0	1	1	2	0	35	256	291
Sorsogon	66,956	0	1	0	1	0	13	47	60
City of Naga	32,882	0	2	17	19	0	8	86	94
**Region 6**	**667,635**	**6**	**127**	**601**	**734**	**4**	**545**	**4,156**	**4,705**
Aklan	48,553	0	1	9	10	1	22	243	266
Antique	49,316	0	2	13	15	0	9	178	187
Capiz	77,942	0	0	14	14	0	31	404	435
Guimaras	17,943	1	2	9	12	0	5	66	71
Iloilo	182,130	1	15	126	142	0	85	1,498	1,583
Negros Occidental	206,889	4	60	221	285	3	379	1,572	1,954
City of Bacolod	30,421	0	42	204	246	0	12	189	201
City of Iloilo	54,441	0	5	5	10	0	2	6	8
**Region 7**	**483,438**	**12**	**318**	**1,375**	**1,705**	**3**	**706**	**4,981**	**5,690**
Bohol	83,324	7	108	474	589	0	72	721	793
Cebu	191,250	5	166	681	852	1	268	1,568	1,837
Negros Oriental	98,214	0	4	11	15	2	164	563	729
Siquijor	9,225	0	0	0	0	0	4	48	52
City of Cebu	34,897	0	11	56	67	0	120	1,139	1,259
City of Lapu-Lapu	36,794	0	0	0	0	0	58	325	383
City of Mandaue	29,734	0	29	153	182	0	20	617	637
**Region 8**	**340,613**	**0**	**36**	**275**	**311**	**0**	**180**	**1,143**	**1,323**
Biliran	12,714	0	0	6	6	0	8	57	65
Eastern Samar	39,982	0	1	4	5	0	30	121	151
Leyte	125,631	0	8	163	171	0	42	450	492
Northern Samar	48,697	0	5	12	17	0	25	119	144
Southern Leyte	27,251	0	2	10	12	0	9	60	69
Samar	42,957	0	1	20	21	0	17	153	170
Ormoc City	20,171	0	0	0	0	0	18	25	43
City of Tacloban	23,210	0	19	60	79	0	31	158	189
**Region 9**	**354,267**	**11**	**414**	**630**	**1,055**	**0**	**276**	**1,421**	**1,697**
Zamboanga del Norte	126,112	0	6	10	16	0	15	185	200
Zamboanga del Sur	102,687	11	380	510	901	0	82	273	355
Zamboanga Sibugay	47,839	0	24	48	72	0	48	278	326
City of Isabela	7,167	0	0	0	0	0	26	166	192
City of Zamboanga	70,462	0	4	62	66	0	105	519	624
**Region 10**	**511,701**	**38**	**631**	**1,329**	**1,998**	**2**	**670**	**3,202**	**3,874**
Bukidnon	167,011	0	10	55	65	1	271	495	767
Camiguin	6,884	0	3	2	5	0	1	5	6
Lanao del Norte	73,898	0	3	29	32	0	38	812	850
Misamis Occidental	60,887	0	0	2	2	0	30	231	261
Misamis Oriental	111,011	15	230	525	770	0	77	289	366
City of Cagayan De Oro	57,799	15	239	247	501	1	163	964	1,128
City of Iligan	34,211	8	146	469	623	0	90	406	496
**Region 11**	**535,532**	**3**	**39**	**386**	**428**	**38**	**1,321**	**7,031**	**8,390**
Davao de Oro	84,560	0	6	11	17	6	210	671	887
Davao del Norte	112,914	0	10	90	100	4	197	908	1,109
Davao Oriental	59,172	0	0	4	4	4	74	385	463
Davao del Sur	67,054	1	3	16	20	11	230	685	926
Davao Occidental	33,334	1	3	7	11	2	49	103	154
City of Davao	178,498	1	17	258	276	11	561	4,279	4,851
**Region 12**	**476,219**	**0**	**34**	**235**	**269**	**150**	**633**	**2,711**	**3,494**
Cotabato	136,508	0	10	87	97	2	268	1,206	1,476
Sarangani	76,390	0	0	18	18	0	97	188	285
South Cotabato	102,265	0	1	32	33	6	88	611	705

Table 2.A.1 - Modern Methods of Family Planning
New Acceptors
Philippines, 2024

Area	Total Current Users	IUD-POSTPARTUM Age group			Total	PILLS-POP Age group			Total
		10-14	15-19	20-49		10-14	15-19	20-49	
Sultan Kudarat	104,518	0	6	28	34	142	90	233	465
City of General Santos	56,538	0	17	70	87	0	90	473	563
**Caraga**	**292,511**	**3**	**107**	**307**	**417**	**7**	**635**	**2,202**	**2,844**
Agusan del Norte	40,148	2	27	25	54	2	147	495	644
Agusan del Sur	86,721	1	12	15	28	4	144	365	513
Surigao del Norte	53,159	0	61	180	241	0	189	663	852
Surigao del Sur	60,298	0	0	56	56	1	78	403	482
Dinagat Islands	9,606	0	0	1	1	0	2	40	42
City of Butuan	42,579	0	7	30	37	0	75	236	311
**BARMM**	**296,590**	**1**	**44**	**177**	**222**	**6**	**369**	**3,025**	**3,400**
Basilan	19,309	0	2	8	10	1	52	186	239
Lanao del Sur	39,800	0	0	6	6	4	25	290	319
Maguindanao del Norte	36,321	0	0	3	3	0	50	469	519
Maguindanao del Sur	51,529	0	1	0	1	0	80	591	671
Sulu	80,817	0	1	2	3	0	102	915	1,017
Tawi-Tawi	19,107	0	0	0	0	1	13	210	224
SGA	7,550	0	11	21	32	0	24	107	131
City of Cotabato	42,157	1	29	137	167	0	23	257	280

Table 2.A.1 - Modern Methods of Family Planning
New Acceptors
Philippines, 2024

Area	Total Current Users	PILLS-COC Age group 10-14	15-19	20-49	Total	INJECTABLES Age group 10-14	15-19	20-49	Total
**PHILIPPINES**	**8,664,035**	**186**	**7,897**	**116,828**	**124,911**	**344**	**24,545**	**177,674**	**202,563**
**NCR**	**1,041,531**	**107**	**928**	**27,292**	**28,327**	**81**	**3,152**	**37,989**	**41,222**
City of Malabon	26,264	0	11	308	319	4	89	666	759
City of Navotas	23,816	0	258	532	790	1	330	1,062	1,393
City of Valenzuela	40,454	1	32	2,036	2,069	0	248	3,637	3,885
City of Caloocan	73,676	1	85	976	1,062	1	383	2,719	3,103
City of Marikina	24,847	0	8	119	127	3	170	1,201	1,374
City of Pasig	58,937	0	19	1,356	1,375	1	152	2,018	2,171
Pateros	4,204	0	0	53	53	0	3	130	133
City of Taguig	92,053	4	84	1,793	1,881	5	319	4,365	4,689
Quezon City	338,677	0	86	2,609	2,695	3	293	3,192	3,488
City of Makati	28,572	0	10	866	876	0	20	1,141	1,161
City of Mandaluyong	27,196	0	6	409	415	0	26	773	799
City of San Juan	6,139	0	0	109	109	0	15	303	318
City of Manila	138,102	101	184	11,512	11,797	58	528	9,761	10,347
City of Las Piñas	26,980	0	36	2,530	2,566	3	166	3,167	3,336
City of Muntinlupa	44,186	0	32	387	419	2	244	1,861	2,107
City of Parañaque	47,100	0	36	1,251	1,287	0	117	1,272	1,389
Pasay City	40,328	0	41	446	487	0	49	721	770
**CAR**	**165,663**	**0**	**46**	**422**	**468**	**3**	**116**	**879**	**998**
Abra	19,755	0	7	79	86	1	8	51	60
Apayao	24,752	0	11	18	29	0	37	31	68
Benguet	37,253	0	8	160	168	0	20	260	280
Ifugao	21,120	0	1	18	19	0	1	13	14
Kalinga	18,267	0	6	43	49	0	6	46	52
Mountain Province	13,957	0	6	7	13	1	7	15	23
City of Baguio	30,559	0	7	97	104	1	37	463	501
**Region 1**	**498,204**	**1**	**186**	**4,600**	**4,787**	**7**	**443**	**4,261**	**4,711**
Ilocos Norte	52,824	0	12	407	419	0	11	167	178
Ilocos Sur	78,125	0	49	1,078	1,127	0	58	771	829
La Union	67,784	0	16	176	192	1	56	375	432
Pangasinan	292,353	1	107	2,894	3,002	6	306	2,770	3,082
City of Dagupan	7,118	0	2	45	47	0	12	178	190
**Region 2**	**343,378**	**1**	**125**	**1,015**	**1,141**	**9**	**381**	**1,334**	**1,724**
Batanes	955	0	1	2	3	0	0	2	2
Cagayan	108,930	1	51	581	633	1	104	509	614
Isabela	146,131	0	64	357	421	8	255	699	962
Nueva Vizcaya	52,095	0	6	62	68	0	12	94	106
Quirino	21,240	0	3	13	16	0	10	30	40
City of Santiago	14,027	0	0	0	0	0	0	0	0
**Region 3**	**985,325**	**12**	**1,433**	**21,171**	**22,616**	**31**	**3,818**	**28,489**	**32,338**
Aurora	24,562	0	1	19	20	0	5	66	71
Bataan	65,716	1	76	1,503	1,580	4	476	2,710	3,190
Bulacan	302,904	4	502	8,706	9,212	4	1,067	9,647	10,718
Nueva Ecija	200,897	5	301	3,200	3,506	11	737	4,322	5,070
Pampanga	167,294	2	239	2,541	2,782	6	746	5,076	5,828
Tarlac	118,444	0	85	2,105	2,190	1	247	2,702	2,950
Zambales	54,326	0	28	754	782	4	287	1,683	1,974
City of Angeles	30,345	0	41	508	549	0	128	1,288	1,416
City of Olongapo	20,837	0	160	1,835	1,995	1	125	995	1,121
**Region 4A**	**888,692**	**5**	**952**	**14,190**	**15,147**	**22**	**3,645**	**27,625**	**31,292**
Batangas	158,569	0	58	1,831	1,889	2	276	2,934	3,212
Cavite	175,332	2	131	2,207	2,340	2	728	6,737	7,467
Laguna	286,016	0	515	4,425	4,940	4	901	7,424	8,329
Quezon	106,773	0	86	853	939	5	551	2,423	2,979
Rizal	156,554	3	153	4,831	4,987	7	1,085	7,757	8,849
City of Lucena	5,448	0	9	43	52	2	104	350	456
**Region 4B**	**291,730**	**3**	**172**	**1,383**	**1,558**	**17**	**1,018**	**3,226**	**4,261**
Marinduque	16,436	0	2	110	112	0	27	193	220
Occidental Mindoro	48,902	0	13	64	77	1	47	92	140
Oriental Mindoro	68,962	0	48	592	640	0	83	588	671
Palawan	100,239	1	89	298	388	10	650	1,597	2,257

Table 2.A.1 - Modern Methods of Family Planning
New Acceptors
Philippines, 2024

Area	Total Current Users	PILLS-COC Age group 10-14	15-19	20-49	Total	INJECTABLES Age group 10-14	15-19	20-49	Total
Romblon	22,467	0	12	192	204	0	43	229	272
City of Puerto Princesa	34,724	2	8	127	137	6	168	527	701
**Region 5**	**491,006**	**1**	**228**	**4,867**	**5,096**	**3**	**662**	**6,540**	**7,205**
Albay	115,867	1	47	1,388	1,436	1	200	2,159	2,360
Camarines Norte	46,839	0	21	161	182	0	98	460	558
Camarines Sur	107,464	0	88	1,874	1,962	1	196	2,226	2,423
Catanduanes	24,326	0	2	35	37	1	11	83	95
Masbate	96,672	0	47	616	663	0	112	946	1,058
Sorsogon	66,956	0	17	139	156	0	30	209	239
City of Naga	32,882	0	6	654	660	0	15	457	472
**Region 6**	**667,635**	**4**	**614**	**8,264**	**8,882**	**10**	**1,551**	**10,851**	**12,412**
Aklan	48,553	0	27	466	493	1	124	969	1,094
Antique	49,316	1	24	394	419	1	164	1,041	1,206
Capiz	77,942	0	28	599	627	2	143	1,120	1,265
Guimaras	17,943	1	12	268	281	0	23	137	160
Iloilo	182,130	0	150	2,661	2,811	0	266	2,814	3,080
Negros Occidental	206,889	2	294	2,636	2,932	5	685	3,463	4,153
City of Bacolod	30,421	0	33	836	869	1	114	1,030	1,145
City of Iloilo	54,441	0	46	404	450	0	32	277	309
**Region 7**	**483,438**	**0**	**378**	**6,154**	**6,532**	**23**	**1,613**	**11,604**	**13,240**
Bohol	83,324	0	15	549	564	1	135	1,141	1,277
Cebu	191,250	0	143	2,687	2,830	12	545	4,366	4,923
Negros Oriental	98,214	0	69	834	903	3	387	1,933	2,323
Siquijor	9,225	0	2	44	46	0	13	83	96
City of Cebu	34,897	0	96	1,325	1,421	6	313	2,238	2,557
City of Lapu-Lapu	36,794	0	45	335	380	0	143	1,039	1,182
City of Mandaue	29,734	0	8	380	388	1	77	804	882
**Region 8**	**340,613**	**7**	**423**	**4,158**	**4,588**	**9**	**705**	**4,776**	**5,490**
Biliran	12,714	0	7	69	76	0	21	156	177
Eastern Samar	39,982	4	38	280	322	1	134	452	587
Leyte	125,631	1	185	1,354	1,540	3	145	1,473	1,621
Northern Samar	48,697	0	63	537	600	0	114	704	818
Southern Leyte	27,251	0	9	104	113	0	10	133	143
Samar	42,957	2	67	1,185	1,254	5	201	1,269	1,475
Ormoc City	20,171	0	9	79	88	0	7	30	37
City of Tacloban	23,210	0	45	550	595	0	73	559	632
**Region 9**	**354,267**	**3**	**313**	**2,142**	**2,458**	**10**	**861**	**4,607**	**5,478**
Zamboanga del Norte	126,112	0	33	398	431	2	126	678	806
Zamboanga del Sur	102,687	0	136	508	644	1	142	807	950
Zamboanga Sibugay	47,839	2	39	263	304	4	210	898	1,112
City of Isabela	7,167	0	11	224	235	0	54	403	457
City of Zamboanga	70,462	1	94	749	844	3	329	1,821	2,153
**Region 10**	**511,701**	**7**	**548**	**6,238**	**6,793**	**13**	**941**	**5,177**	**6,131**
Bukidnon	167,011	0	200	1,061	1,261	7	338	1,012	1,357
Camiguin	6,884	1	1	9	11	0	2	6	8
Lanao del Norte	73,898	1	69	1,366	1,436	1	107	1,130	1,238
Misamis Occidental	60,887	1	42	454	497	0	42	438	480
Misamis Oriental	111,011	1	30	475	506	0	104	489	593
City of Cagayan De Oro	57,799	3	161	2,058	2,222	5	269	1,557	1,831
City of Iligan	34,211	0	45	815	860	0	79	545	624
**Region 11**	**535,532**	**17**	**699**	**6,517**	**7,233**	**56**	**1,622**	**8,136**	**9,814**
Davao de Oro	84,560	2	55	514	571	19	183	927	1,129
Davao del Norte	112,914	0	65	1,076	1,141	8	264	1,166	1,438
Davao Oriental	59,172	0	63	497	560	7	58	351	416
Davao del Sur	67,054	1	100	433	534	3	222	856	1,081
Davao Occidental	33,334	4	73	266	343	7	320	606	933
City of Davao	178,498	10	343	3,731	4,084	12	575	4,230	4,817
**Region 12**	**476,219**	**9**	**459**	**3,698**	**4,166**	**12**	**1,386**	**6,140**	**7,538**
Cotabato	136,508	5	136	1,254	1,395	4	470	2,154	2,628
Sarangani	76,390	0	74	590	664	0	218	714	932
South Cotabato	102,265	1	81	711	793	2	267	1,117	1,386

Table 2.A.1 - Modern Methods of Family Planning
New Acceptors
Philippines, 2024

Area	Total Current Users	PILLS-COC			Total	INJECTABLES			Total
		Age group				Age group			
		10-14	15-19	20-49		10-14	15-19	20-49	
Sultan Kudarat	104,518	2	75	396	473	2	181	647	830
City of General Santos	56,538	1	93	747	841	4	250	1,508	1,762
**Caraga**	**292,511**	**5**	**154**	**1,756**	**1,915**	**13**	**845**	**2,902**	**3,760**
Agusan del Norte	40,148	1	32	335	368	3	115	468	586
Agusan del Sur	86,721	4	44	286	334	5	133	529	667
Surigao del Norte	53,159	0	27	476	503	3	409	1,049	1,461
Surigao del Sur	60,298	0	25	236	261	2	80	259	341
Dinagat Islands	9,606	0	5	100	105	0	23	143	166
City of Butuan	42,579	0	21	323	344	0	85	454	539
**BARMM**	**296,590**	**4**	**239**	**2,961**	**3,204**	**25**	**1,786**	**13,138**	**14,949**
Basilan	19,309	4	29	252	285	8	290	1,368	1,666
Lanao del Sur	39,800	0	10	235	245	0	70	865	935
Maguindanao del Norte	36,321	0	32	289	321	1	266	1,786	2,053
Maguindanao del Sur	51,529	0	39	376	415	4	503	3,011	3,518
Sulu	80,817	0	102	1,189	1,291	7	360	3,352	3,719
Tawi-Tawi	19,107	0	12	167	179	1	89	694	784
SGA	7,550	0	6	75	81	1	87	722	810
City of Cotabato	42,157	0	9	378	387	3	121	1,340	1,464

Table 2.A.1 - Modern Methods of Family Planning
New Acceptors
Philippines, 2024

Area	Total Current Users	IMPLANTS Age group 10-14	IMPLANTS Age group 15-19	IMPLANTS Age group 20-49	Total	NFP-CCM Age group 10-14	NFP-CCM Age group 15-19	NFP-CCM Age group 20-49	Total
**PHILIPPINES**	**8,664,035**	**484**	**16,085**	**75,031**	**91,600**	**0**	**76**	**1,009**	**1,085**
**NCR**	**1,041,531**	**153**	**2,962**	**17,752**	**20,867**	**0**	**0**	**94**	**94**
City of Malabon	26,264	2	88	244	334	0	0	0	0
City of Navotas	23,816	5	190	509	704	0	0	0	0
City of Valenzuela	40,454	0	103	757	860	0	0	2	2
City of Caloocan	73,676	7	495	2,161	2,663	0	0	0	0
City of Marikina	24,847	6	108	636	750	0	0	0	0
City of Pasig	58,937	11	197	1,495	1,703	0	0	0	0
Pateros	4,204	0	4	57	61	0	0	0	0
City of Taguig	92,053	3	77	1,249	1,329	0	0	0	0
Quezon City	338,677	9	286	1,732	2,027	0	0	3	3
City of Makati	28,572	0	11	460	471	0	0	0	0
City of Mandaluyong	27,196	1	54	645	700	0	0	59	59
City of San Juan	6,139	0	20	201	221	0	0	0	0
City of Manila	138,102	83	872	5,227	6,182	0	0	0	0
City of Las Piñas	26,980	20	355	1,186	1,561	0	0	0	0
City of Muntinlupa	44,186	0	20	296	316	0	0	0	0
City of Parañaque	47,100	4	74	562	640	0	0	0	0
Pasay City	40,328	2	8	335	345	0	0	30	30
**CAR**	**165,663**	**1**	**112**	**449**	**562**	**0**	**1**	**10**	**11**
Abra	19,755	0	6	60	66	0	0	0	0
Apayao	24,752	0	24	58	82	0	0	0	0
Benguet	37,253	0	28	113	141	0	1	10	11
Ifugao	21,120	0	2	11	13	0	0	0	0
Kalinga	18,267	0	28	64	92	0	0	0	0
Mountain Province	13,957	1	8	20	29	0	0	0	0
City of Baguio	30,559	0	16	123	139	0	0	0	0
**Region 1**	**498,204**	**4**	**188**	**1,337**	**1,529**	**0**	**2**	**26**	**28**
Ilocos Norte	52,824	0	23	99	122	0	0	0	0
Ilocos Sur	78,125	0	21	311	332	0	2	23	25
La Union	67,784	4	56	157	217	0	0	3	3
Pangasinan	292,353	0	87	762	849	0	0	0	0
City of Dagupan	7,118	0	1	8	9	0	0	0	0
**Region 2**	**343,378**	**6**	**191**	**566**	**763**	**0**	**0**	**0**	**0**
Batanes	955	0	0	1	1	0	0	0	0
Cagayan	108,930	0	40	198	238	0	0	0	0
Isabela	146,131	5	123	259	387	0	0	0	0
Nueva Vizcaya	52,095	0	21	86	107	0	0	0	0
Quirino	21,240	1	7	22	30	0	0	0	0
City of Santiago	14,027	0	0	0	0	0	0	0	0
**Region 3**	**985,325**	**65**	**2,057**	**9,244**	**11,366**	**0**	**4**	**38**	**42**
Aurora	24,562	0	2	5	7	0	0	0	0
Bataan	65,716	32	352	717	1,101	0	0	0	0
Bulacan	302,904	5	457	3,046	3,508	0	2	9	11
Nueva Ecija	200,897	17	407	1,433	1,857	0	2	7	9
Pampanga	167,294	7	633	2,178	2,818	0	0	20	20
Tarlac	118,444	1	61	726	788	0	0	0	0
Zambales	54,326	1	72	439	512	0	0	2	2
City of Angeles	30,345	2	36	490	528	0	0	0	0
City of Olongapo	20,837	0	37	210	247	0	0	0	0
**Region 4A**	**888,692**	**29**	**979**	**7,334**	**8,342**	**0**	**5**	**15**	**20**
Batangas	158,569	3	97	904	1,004	0	2	5	7
Cavite	175,332	4	179	1,245	1,428	0	0	0	0
Laguna	286,016	8	289	2,151	2,448	0	0	0	0
Quezon	106,773	5	97	663	765	0	3	1	4
Rizal	156,554	9	317	2,351	2,677	0	0	9	9
City of Lucena	5,448	0	0	20	20	0	0	0	0
**Region 4B**	**291,730**	**3**	**406**	**1,495**	**1,904**	**0**	**4**	**138**	**142**
Marinduque	16,436	1	51	181	233	0	0	0	0
Occidental Mindoro	48,902	0	45	98	143	0	1	0	1
Oriental Mindoro	68,962	1	72	299	372	0	0	2	2
Palawan	100,239	0	161	578	739	0	1	3	4

Table 2.A.1 - Modern Methods of Family Planning
New Acceptors
Philippines, 2024

Area	Total Current Users	IMPLANTS			Total	NFP-CCM			Total
		Age group				Age group			
		10-14	15-19	20-49		10-14	15-19	20-49	
Romblon	22,467	0	54	241	295	0	2	133	135
City of Puerto Princesa	34,724	1	23	98	122	0	0	0	0
**Region 5**	**491,006**	**9**	**455**	**2,937**	**3,401**	**0**	**18**	**256**	**274**
Albay	115,867	3	100	851	954	0	0	8	8
Camarines Norte	46,839	0	18	88	106	0	0	0	0
Camarines Sur	107,464	4	237	1,406	1,647	0	0	1	1
Catanduanes	24,326	1	32	103	136	0	0	4	4
Masbate	96,672	0	31	198	229	0	7	231	238
Sorsogon	66,956	1	10	70	81	0	11	12	23
City of Naga	32,882	0	27	221	248	0	0	0	0
**Region 6**	**667,635**	**5**	**462**	**2,817**	**3,284**	**0**	**6**	**151**	**157**
Aklan	48,553	0	20	173	193	0	0	0	0
Antique	49,316	0	41	142	183	0	6	108	114
Capiz	77,942	0	12	169	181	0	0	0	0
Guimaras	17,943	0	19	61	80	0	0	0	0
Iloilo	182,130	5	131	1,051	1,187	0	0	42	42
Negros Occidental	206,889	0	204	850	1,054	0	0	1	1
City of Bacolod	30,421	0	23	233	256	0	0	0	0
City of Iloilo	54,441	0	12	138	150	0	0	0	0
**Region 7**	**483,438**	**30**	**1,052**	**5,683**	**6,765**	**0**	**0**	**3**	**3**
Bohol	83,324	1	242	839	1,082	0	0	0	0
Cebu	191,250	24	413	2,287	2,724	0	0	0	0
Negros Oriental	98,214	2	98	401	501	0	0	3	3
Siquijor	9,225	0	2	15	17	0	0	0	0
City of Cebu	34,897	1	160	1,092	1,253	0	0	0	0
City of Lapu-Lapu	36,794	1	47	284	332	0	0	0	0
City of Mandaue	29,734	1	90	765	856	0	0	0	0
**Region 8**	**340,613**	**8**	**408**	**2,667**	**3,083**	**0**	**1**	**14**	**15**
Biliran	12,714	1	45	121	167	0	0	0	0
Eastern Samar	39,982	2	74	293	369	0	0	5	5
Leyte	125,631	1	94	725	820	0	0	0	0
Northern Samar	48,697	1	44	240	285	0	1	3	4
Southern Leyte	27,251	0	16	135	151	0	0	0	0
Samar	42,957	3	47	540	590	0	0	6	6
Ormoc City	20,171	0	13	19	32	0	0	0	0
City of Tacloban	23,210	0	75	594	669	0	0	0	0
**Region 9**	**354,267**	**31**	**1,612**	**4,149**	**5,792**	**0**	**13**	**30**	**43**
Zamboanga del Norte	126,112	3	219	755	977	0	13	30	43
Zamboanga del Sur	102,687	4	707	1,103	1,814	0	0	0	0
Zamboanga Sibugay	47,839	3	226	558	787	0	0	0	0
City of Isabela	7,167	3	102	320	425	0	0	0	0
City of Zamboanga	70,462	18	358	1,413	1,789	0	0	0	0
**Region 10**	**511,701**	**13**	**846**	**3,700**	**4,559**	**0**	**14**	**191**	**205**
Bukidnon	167,011	4	271	801	1,076	0	6	59	65
Camiguin	6,884	0	1	8	9	0	0	0	0
Lanao del Norte	73,898	0	77	825	902	0	8	130	138
Misamis Occidental	60,887	0	40	318	358	0	0	0	0
Misamis Oriental	111,011	2	169	435	606	0	0	2	2
City of Cagayan De Oro	57,799	5	186	740	931	0	0	0	0
City of Iligan	34,211	2	102	573	677	0	0	0	0
**Region 11**	**535,532**	**53**	**992**	**3,973**	**5,018**	**0**	**0**	**13**	**13**
Davao de Oro	84,560	3	83	255	341	0	0	2	2
Davao del Norte	112,914	15	190	693	898	0	0	3	3
Davao Oriental	59,172	4	39	264	307	0	0	0	0
Davao del Sur	67,054	10	147	360	517	0	0	2	2
Davao Occidental	33,334	0	81	231	312	0	0	0	0
City of Davao	178,498	21	452	2,170	2,643	0	0	6	6
**Region 12**	**476,219**	**32**	**1,472**	**4,515**	**6,019**	**0**	**6**	**18**	**24**
Cotabato	136,508	11	412	1,536	1,959	0	0	2	2
Sarangani	76,390	0	165	547	712	0	5	2	7
South Cotabato	102,265	11	326	1,001	1,338	0	1	14	15

Table 2.A.1 - Modern Methods of Family Planning
New Acceptors
Philippines, 2024

Area	Total Current Users	IMPLANTS Age group			Total	NFP-CCM Age group			Total
		10-14	15-19	20-49		10-14	15-19	20-49	
Sultan Kudarat	104,518	6	291	454	751	0	0	0	0
City of General Santos	56,538	4	278	977	1,259	0	0	0	0
**Caraga**	**292,511**	**30**	**970**	**2,521**	**3,521**	**0**	**1**	**6**	**7**
Agusan del Norte	40,148	11	158	381	550	0	0	0	0
Agusan del Sur	86,721	7	211	580	798	0	0	4	4
Surigao del Norte	53,159	2	380	811	1,193	0	0	2	2
Surigao del Sur	60,298	7	106	476	589	0	0	0	0
Dinagat Islands	9,606	0	23	110	133	0	0	0	0
City of Butuan	42,579	3	92	163	258	0	1	0	1
**BARMM**	**296,590**	**12**	**921**	**3,892**	**4,825**	**0**	**1**	**6**	**7**
Basilan	19,309	4	146	480	630	0	1	5	6
Lanao del Sur	39,800	0	25	291	316	0	0	0	0
Maguindanao del Norte	36,321	3	144	484	631	0	0	0	0
Maguindanao del Sur	51,529	3	226	644	873	0	0	0	0
Sulu	80,817	1	162	661	824	0	0	0	0
Tawi-Tawi	19,107	1	149	872	1,022	0	0	0	0
SGA	7,550	0	6	20	26	0	0	0	0
City of Cotabato	42,157	0	63	440	503	0	0	1	1

Table 2.A.1 - Modern Methods of Family Planning
New Acceptors
Philippines, 2024

Area	Total Current Users	NFP-BBT Age group 10-14	15-19	20-49	Total	NFP-STM Age group 10-14	15-19	20-49	Total
**PHILIPPINES**	**8,664,035**	**4**	**97**	**528**	**629**	**0**	**49**	**252**	**301**
**NCR**	**1,041,531**	**0**	**13**	**56**	**69**	**0**	**0**	**5**	**5**
City of Malabon	26,264	0	0	0	0	0	0	0	0
City of Navotas	23,816	0	0	0	0	0	0	0	0
City of Valenzuela	40,454	0	0	4	4	0	0	0	0
City of Caloocan	73,676	0	0	0	0	0	0	0	0
City of Marikina	24,847	0	0	0	0	0	0	0	0
City of Pasig	58,937	0	0	0	0	0	0	0	0
Pateros	4,204	0	0	0	0	0	0	0	0
City of Taguig	92,053	0	0	0	0	0	0	0	0
Quezon City	338,677	0	12	30	42	0	0	0	0
City of Makati	28,572	0	0	0	0	0	0	0	0
City of Mandaluyong	27,196	0	0	14	14	0	0	5	5
City of San Juan	6,139	0	0	0	0	0	0	0	0
City of Manila	138,102	0	1	8	9	0	0	0	0
City of Las Piñas	26,980	0	0	0	0	0	0	0	0
City of Muntinlupa	44,186	0	0	0	0	0	0	0	0
City of Parañaque	47,100	0	0	0	0	0	0	0	0
Pasay City	40,328	0	0	0	0	0	0	0	0
**CAR**	**165,663**	**0**	**7**	**18**	**25**	**0**	**0**	**0**	**0**
Abra	19,755	0	0	1	1	0	0	0	0
Apayao	24,752	0	0	0	0	0	0	0	0
Benguet	37,253	0	0	0	0	0	0	0	0
Ifugao	21,120	0	2	10	12	0	0	0	0
Kalinga	18,267	0	1	1	2	0	0	0	0
Mountain Province	13,957	0	4	6	10	0	0	0	0
City of Baguio	30,559	0	0	0	0	0	0	0	0
**Region 1**	**498,204**	**0**	**4**	**7**	**11**	**0**	**7**	**33**	**40**
Ilocos Norte	52,824	0	0	0	0	0	0	0	0
Ilocos Sur	78,125	0	0	4	4	0	0	0	0
La Union	67,784	0	0	0	0	0	0	0	0
Pangasinan	292,353	0	4	3	7	0	7	33	40
City of Dagupan	7,118	0	0	0	0	0	0	0	0
**Region 2**	**343,378**	**0**	**11**	**23**	**34**	**0**	**0**	**0**	**0**
Batanes	955	0	0	0	0	0	0	0	0
Cagayan	108,930	0	0	3	3	0	0	0	0
Isabela	146,131	0	8	16	24	0	0	0	0
Nueva Vizcaya	52,095	0	3	2	5	0	0	0	0
Quirino	21,240	0	0	2	2	0	0	0	0
City of Santiago	14,027	0	0	0	0	0	0	0	0
**Region 3**	**985,325**	**0**	**8**	**48**	**56**	**0**	**0**	**10**	**10**
Aurora	24,562	0	1	1	2	0	0	0	0
Bataan	65,716	0	1	16	17	0	0	8	8
Bulacan	302,904	0	0	0	0	0	0	0	0
Nueva Ecija	200,897	0	0	0	0	0	0	0	0
Pampanga	167,294	0	0	0	0	0	0	2	2
Tarlac	118,444	0	0	3	3	0	0	0	0
Zambales	54,326	0	0	0	0	0	0	0	0
City of Angeles	30,345	0	6	22	28	0	0	0	0
City of Olongapo	20,837	0	0	6	6	0	0	0	0
**Region 4A**	**888,692**	**2**	**10**	**34**	**46**	**0**	**25**	**2**	**27**
Batangas	158,569	0	0	4	4	0	0	2	2
Cavite	175,332	0	0	4	4	0	0	0	0
Laguna	286,016	0	3	16	19	0	0	0	0
Quezon	106,773	0	4	2	6	0	25	0	25
Rizal	156,554	0	3	8	11	0	0	0	0
City of Lucena	5,448	2	0	0	2	0	0	0	0
**Region 4B**	**291,730**	**0**	**2**	**7**	**9**	**0**	**0**	**0**	**0**
Marinduque	16,436	0	0	1	1	0	0	0	0
Occidental Mindoro	48,902	0	0	0	0	0	0	0	0
Oriental Mindoro	68,962	0	2	4	6	0	0	0	0
Palawan	100,239	0	0	0	0	0	0	0	0

Table 2.A.1 - Modern Methods of Family Planning
New Acceptors
Philippines, 2024

Area	Total Current Users	NFP-BBT Age group 10-14	NFP-BBT 15-19	NFP-BBT 20-49	Total	NFP-STM Age group 10-14	NFP-STM 15-19	NFP-STM 20-49	Total
Romblon	22,467	0	0	2	2	0	0	0	0
City of Puerto Princesa	34,724	0	0	0	0	0	0	0	0
**Region 5**	**491,006**	**0**	**5**	**36**	**41**	**0**	**0**	**13**	**13**
Albay	115,867	0	1	2	3	0	0	3	3
Camarines Norte	46,839	0	0	2	2	0	0	0	0
Camarines Sur	107,464	0	2	24	26	0	0	1	1
Catanduanes	24,326	0	2	5	7	0	0	0	0
Masbate	96,672	0	0	2	2	0	0	0	0
Sorsogon	66,956	0	0	0	0	0	0	8	8
City of Naga	32,882	0	0	1	1	0	0	1	1
**Region 6**	**667,635**	**0**	**2**	**40**	**42**	**0**	**0**	**3**	**3**
Aklan	48,553	0	2	16	18	0	0	0	0
Antique	49,316	0	0	0	0	0	0	0	0
Capiz	77,942	0	0	3	3	0	0	0	0
Guimaras	17,943	0	0	0	0	0	0	0	0
Iloilo	182,130	0	0	20	20	0	0	3	3
Negros Occidental	206,889	0	0	1	1	0	0	0	0
City of Bacolod	30,421	0	0	0	0	0	0	0	0
City of Iloilo	54,441	0	0	0	0	0	0	0	0
**Region 7**	**483,438**	**0**	**2**	**8**	**10**	**0**	**0**	**7**	**7**
Bohol	83,324	0	0	0	0	0	0	0	0
Cebu	191,250	0	0	0	0	0	0	0	0
Negros Oriental	98,214	0	0	1	1	0	0	7	7
Siquijor	9,225	0	0	0	0	0	0	0	0
City of Cebu	34,897	0	2	7	9	0	0	0	0
City of Lapu-Lapu	36,794	0	0	0	0	0	0	0	0
City of Mandaue	29,734	0	0	0	0	0	0	0	0
**Region 8**	**340,613**	**0**	**6**	**28**	**34**	**0**	**0**	**8**	**8**
Biliran	12,714	0	2	5	7	0	0	0	0
Eastern Samar	39,982	0	0	0	0	0	0	0	0
Leyte	125,631	0	0	4	4	0	0	8	8
Northern Samar	48,697	0	0	0	0	0	0	0	0
Southern Leyte	27,251	0	0	1	1	0	0	0	0
Samar	42,957	0	4	18	22	0	0	0	0
Ormoc City	20,171	0	0	0	0	0	0	0	0
City of Tacloban	23,210	0	0	0	0	0	0	0	0
**Region 9**	**354,267**	**0**	**0**	**0**	**0**	**0**	**0**	**0**	**0**
Zamboanga del Norte	126,112	0	0	0	0	0	0	0	0
Zamboanga del Sur	102,687	0	0	0	0	0	0	0	0
Zamboanga Sibugay	47,839	0	0	0	0	0	0	0	0
City of Isabela	7,167	0	0	0	0	0	0	0	0
City of Zamboanga	70,462	0	0	0	0	0	0	0	0
**Region 10**	**511,701**	**2**	**16**	**150**	**168**	**0**	**9**	**154**	**163**
Bukidnon	167,011	2	8	13	23	0	0	0	0
Camiguin	6,884	0	0	1	1	0	0	0	0
Lanao del Norte	73,898	0	5	111	116	0	9	154	163
Misamis Occidental	60,887	0	0	3	3	0	0	0	0
Misamis Oriental	111,011	0	1	1	2	0	0	0	0
City of Cagayan De Oro	57,799	0	2	17	19	0	0	0	0
City of Iligan	34,211	0	0	4	4	0	0	0	0
**Region 11**	**535,532**	**0**	**4**	**9**	**13**	**0**	**0**	**11**	**11**
Davao de Oro	84,560	0	0	4	4	0	0	0	0
Davao del Norte	112,914	0	4	4	8	0	0	1	1
Davao Oriental	59,172	0	0	0	0	0	0	0	0
Davao del Sur	67,054	0	0	1	1	0	0	0	0
Davao Occidental	33,334	0	0	0	0	0	0	0	0
City of Davao	178,498	0	0	0	0	0	0	10	10
**Region 12**	**476,219**	**0**	**5**	**27**	**32**	**0**	**1**	**4**	**5**
Cotabato	136,508	0	1	15	16	0	0	0	0
Sarangani	76,390	0	0	0	0	0	1	4	5
South Cotabato	102,265	0	2	6	8	0	0	0	0

Table 2.A.1 - Modern Methods of Family Planning
New Acceptors
Philippines, 2024

Area	Total Current Users	NFP-BBT			Total	NFP-STM			Total
		Age group				Age group			
		10-14	15-19	20-49		10-14	15-19	20-49	
Sultan Kudarat	104,518	0	0	1	1	0	0	0	0
City of General Santos	56,538	0	2	5	7	0	0	0	0
**Caraga**	**292,511**	**0**	**2**	**37**	**39**	**0**	**7**	**2**	**9**
Agusan del Norte	40,148	0	0	3	3	0	5	0	5
Agusan del Sur	86,721	0	0	32	32	0	2	0	2
Surigao del Norte	53,159	0	2	0	2	0	0	2	2
Surigao del Sur	60,298	0	0	0	0	0	0	0	0
Dinagat Islands	9,606	0	0	0	0	0	0	0	0
City of Butuan	42,579	0	0	2	2	0	0	0	0
**BARMM**	**296,590**	**0**	**0**	**0**	**0**	**0**	**0**	**0**	**0**
Basilan	19,309	0	0	0	0	0	0	0	0
Lanao del Sur	39,800	0	0	0	0	0	0	0	0
Maguindanao del Norte	36,321	0	0	0	0	0	0	0	0
Maguindanao del Sur	51,529	0	0	0	0	0	0	0	0
Sulu	80,817	0	0	0	0	0	0	0	0
Tawi-Tawi	19,107	0	0	0	0	0	0	0	0
SGA	7,550	0	0	0	0	0	0	0	0
City of Cotabato	42,157	0	0	0	0	0	0	0	0

Table 2.A.1 - Modern Methods of Family Planning
New Acceptors
Philippines, 2024

Area	Total Current Users	NFP-SDM			Total	NFP-LAM			Total
		Age group				Age group			
		10-14	15-19	20-49		10-14	15-19	20-49	
**PHILIPPINES**	**8,664,035**	**36**	**292**	**4,556**	**4,884**	**1,215**	**58,961**	**335,841**	**396,017**
**NCR**	**1,041,531**	**0**	**7**	**350**	**357**	**108**	**6,301**	**73,194**	**79,603**
City of Malabon	26,264	0	0	0	0	5	132	546	683
City of Navotas	23,816	0	0	0	0	2	315	2,506	2,823
City of Valenzuela	40,454	0	0	191	191	2	72	1,705	1,779
City of Caloocan	73,676	0	0	1	1	10	590	3,472	4,072
City of Marikina	24,847	0	0	0	0	6	135	865	1,006
City of Pasig	58,937	0	0	0	0	3	138	3,000	3,141
Pateros	4,204	0	0	0	0	0	7	641	648
City of Taguig	92,053	0	1	4	5	40	1,468	15,660	17,168
Quezon City	338,677	0	0	16	16	17	1,121	10,161	11,299
City of Makati	28,572	0	0	0	0	0	54	871	925
City of Mandaluyong	27,196	0	0	6	6	5	259	4,363	4,627
City of San Juan	6,139	0	0	0	0	0	41	1,157	1,198
City of Manila	138,102	0	6	132	138	14	723	12,668	13,405
City of Las Piñas	26,980	0	0	0	0	1	268	4,472	4,741
City of Muntinlupa	44,186	0	0	0	0	1	225	2,776	3,002
City of Parañaque	47,100	0	0	0	0	2	539	5,798	6,339
Pasay City	40,328	0	0	0	0	0	214	2,533	2,747
**CAR**	**165,663**	**0**	**0**	**13**	**13**	**26**	**889**	**3,557**	**4,472**
Abra	19,755	0	0	5	5	2	184	768	954
Apayao	24,752	0	0	0	0	2	111	306	419
Benguet	37,253	0	0	5	5	4	113	790	907
Ifugao	21,120	0	0	1	1	5	135	429	569
Kalinga	18,267	0	0	0	0	5	220	805	1,030
Mountain Province	13,957	0	0	2	2	8	125	316	449
City of Baguio	30,559	0	0	0	0	0	1	143	144
**Region 1**	**498,204**	**0**	**3**	**538**	**541**	**86**	**2,587**	**19,984**	**22,657**
Ilocos Norte	52,824	0	0	6	6	4	182	3,177	3,363
Ilocos Sur	78,125	0	3	499	502	8	363	4,379	4,750
La Union	67,784	0	0	27	27	7	407	2,238	2,652
Pangasinan	292,353	0	0	6	6	65	1,562	9,629	11,256
City of Dagupan	7,118	0	0	0	0	2	73	561	636
**Region 2**	**343,378**	**0**	**1**	**3**	**4**	**48**	**1,827**	**6,538**	**8,413**
Batanes	955	0	0	0	0	0	16	51	67
Cagayan	108,930	0	0	0	0	8	492	2,444	2,944
Isabela	146,131	0	0	0	0	25	722	2,233	2,980
Nueva Vizcaya	52,095	0	0	2	2	8	282	966	1,256
Quirino	21,240	0	1	1	2	3	64	217	284
City of Santiago	14,027	0	0	0	0	4	251	627	882
**Region 3**	**985,325**	**0**	**19**	**414**	**433**	**68**	**4,085**	**32,897**	**37,050**
Aurora	24,562	0	1	0	1	6	214	522	742
Bataan	65,716	0	11	18	29	15	679	3,564	4,258
Bulacan	302,904	0	4	142	146	10	885	9,828	10,723
Nueva Ecija	200,897	0	0	3	3	17	1,048	4,840	5,905
Pampanga	167,294	0	0	238	238	12	446	6,294	6,752
Tarlac	118,444	0	3	7	10	3	426	4,444	4,873
Zambales	54,326	0	0	3	3	3	284	1,390	1,677
City of Angeles	30,345	0	0	2	2	2	79	1,496	1,577
City of Olongapo	20,837	0	0	1	1	0	24	519	543
**Region 4A**	**888,692**	**2**	**24**	**385**	**411**	**79**	**4,880**	**38,439**	**43,398**
Batangas	158,569	0	0	19	19	7	448	3,722	4,177
Cavite	175,332	0	0	0	0	9	788	8,387	9,184
Laguna	286,016	0	23	314	337	27	1,486	12,695	14,208
Quezon	106,773	0	1	46	47	17	797	3,020	3,834
Rizal	156,554	0	0	6	6	15	1,357	10,602	11,974
City of Lucena	5,448	2	0	0	2	4	4	13	21
**Region 4B**	**291,730**	**0**	**4**	**67**	**71**	**60**	**2,884**	**8,509**	**11,453**
Marinduque	16,436	0	0	0	0	1	41	332	374
Occidental Mindoro	48,902	0	0	0	0	30	1,019	1,682	2,731
Oriental Mindoro	68,962	0	0	1	1	13	372	1,458	1,843
Palawan	100,239	0	4	6	10	10	1,089	2,913	4,012

Table 2.A.1 - Modern Methods of Family Planning
New Acceptors
Philippines, 2024

Area	Total Current Users	NFP-SDM			Total	NFP-LAM			Total
		Age group				Age group			
		10-14	15-19	20-49		10-14	15-19	20-49	
Romblon	22,467	0	0	56	56	6	268	1,869	2,143
City of Puerto Princesa	34,724	0	0	4	4	0	95	255	350
**Region 5**	**491,006**	**3**	**156**	**1,451**	**1,610**	**76**	**5,177**	**19,767**	**25,020**
Albay	115,867	0	15	440	455	10	502	4,521	5,033
Camarines Norte	46,839	0	0	0	0	15	585	2,016	2,616
Camarines Sur	107,464	0	0	193	193	17	1,439	5,428	6,884
Catanduanes	24,326	1	1	25	27	4	262	699	965
Masbate	96,672	2	134	720	856	14	1,382	3,466	4,862
Sorsogon	66,956	0	6	25	31	16	970	2,689	3,675
City of Naga	32,882	0	0	48	48	0	37	948	985
**Region 6**	**667,635**	**0**	**15**	**355**	**370**	**92**	**4,359**	**22,509**	**26,960**
Aklan	48,553	0	0	9	9	5	317	2,234	2,556
Antique	49,316	0	4	117	121	1	185	1,416	1,602
Capiz	77,942	0	0	0	0	1	188	2,061	2,250
Guimaras	17,943	0	0	0	0	6	163	612	781
Iloilo	182,130	0	2	72	74	24	801	6,116	6,941
Negros Occidental	206,889	0	9	156	165	54	2,612	9,426	12,092
City of Bacolod	30,421	0	0	0	0	1	91	638	730
City of Iloilo	54,441	0	0	1	1	0	2	6	8
**Region 7**	**483,438**	**0**	**1**	**62**	**63**	**85**	**4,589**	**24,611**	**29,285**
Bohol	83,324	0	0	3	3	4	334	1,660	1,998
Cebu	191,250	0	0	2	2	22	1,516	9,480	11,018
Negros Oriental	98,214	0	0	1	1	32	1,276	4,511	5,819
Siquijor	9,225	0	0	56	56	1	33	131	165
City of Cebu	34,897	0	1	0	1	23	1,076	6,817	7,916
City of Lapu-Lapu	36,794	0	0	0	0	3	321	1,750	2,074
City of Mandaue	29,734	0	0	0	0	0	33	262	295
**Region 8**	**340,613**	**0**	**4**	**99**	**103**	**42**	**2,256**	**10,800**	**13,098**
Biliran	12,714	0	0	3	3	2	111	543	656
Eastern Samar	39,982	0	2	11	13	11	318	1,357	1,686
Leyte	125,631	0	2	66	68	11	599	3,794	4,404
Northern Samar	48,697	0	0	2	2	4	300	1,494	1,798
Southern Leyte	27,251	0	0	10	10	0	178	685	863
Samar	42,957	0	0	0	0	11	306	1,596	1,913
Ormoc City	20,171	0	0	7	7	0	194	400	594
City of Tacloban	23,210	0	0	0	0	3	250	931	1,184
**Region 9**	**354,267**	**0**	**1**	**22**	**23**	**43**	**3,175**	**7,946**	**11,164**
Zamboanga del Norte	126,112	0	0	18	18	16	1,110	2,461	3,587
Zamboanga del Sur	102,687	0	0	3	3	5	526	1,006	1,537
Zamboanga Sibugay	47,839	0	1	1	2	4	372	871	1,247
City of Isabela	7,167	0	0	0	0	2	118	492	612
City of Zamboanga	70,462	0	0	0	0	16	1,049	3,116	4,181
**Region 10**	**511,701**	**31**	**50**	**532**	**613**	**134**	**5,745**	**20,834**	**26,713**
Bukidnon	167,011	22	4	78	104	76	2,497	4,134	6,707
Camiguin	6,884	0	0	0	0	0	85	205	290
Lanao del Norte	73,898	0	7	136	143	20	510	4,237	4,767
Misamis Occidental	60,887	9	36	269	314	2	376	2,129	2,507
Misamis Oriental	111,011	0	3	15	18	19	837	2,560	3,416
City of Cagayan De Oro	57,799	0	0	31	31	12	1,184	6,286	7,482
City of Iligan	34,211	0	0	3	3	5	256	1,283	1,544
**Region 11**	**535,532**	**0**	**1**	**155**	**156**	**113**	**2,283**	**11,711**	**14,107**
Davao de Oro	84,560	0	0	11	11	4	75	345	424
Davao del Norte	112,914	0	0	3	3	27	355	1,603	1,985
Davao Oriental	59,172	0	0	120	120	41	642	1,485	2,168
Davao del Sur	67,054	0	1	5	6	7	187	705	899
Davao Occidental	33,334	0	0	11	11	7	203	266	476
City of Davao	178,498	0	0	5	5	27	821	7,307	8,155
**Region 12**	**476,219**	**0**	**1**	**10**	**11**	**57**	**3,614**	**12,571**	**16,242**
Cotabato	136,508	0	0	4	4	16	649	2,726	3,391
Sarangani	76,390	0	0	5	5	0	1,028	2,436	3,464
South Cotabato	102,265	0	1	0	1	14	821	2,674	3,509

Table 2.A.1 - Modern Methods of Family Planning
New Acceptors
Philippines, 2024

Area	Total Current Users	NFP-SDM			Total	NFP-LAM			Total
		Age group				Age group			
		10-14	15-19	20-49		10-14	15-19	20-49	
Sultan Kudarat	104,518	0	0	0	0	23	806	2,029	2,858
City of General Santos	56,538	0	0	1	1	4	310	2,706	3,020
**Caraga**	**292,511**	**0**	**5**	**86**	**91**	**38**	**1,741**	**7,118**	**8,897**
Agusan del Norte	40,148	0	1	14	15	3	292	1,199	1,494
Agusan del Sur	86,721	0	0	5	5	9	488	1,064	1,561
Surigao del Norte	53,159	0	3	36	39	5	186	1,570	1,761
Surigao del Sur	60,298	0	1	14	15	15	391	2,154	2,560
Dinagat Islands	9,606	0	0	17	17	0	47	195	242
City of Butuan	42,579	0	0	0	0	6	337	936	1,279
**BARMM**	**296,590**	**0**	**0**	**14**	**14**	**60**	**2,569**	**14,856**	**17,485**
Basilan	19,309	0	0	0	0	0	159	540	699
Lanao del Sur	39,800	0	0	9	9	30	507	4,763	5,300
Maguindanao del Norte	36,321	0	0	0	0	8	355	2,033	2,396
Maguindanao del Sur	51,529	0	0	0	0	13	844	3,042	3,899
Sulu	80,817	0	0	0	0	1	165	945	1,111
Tawi-Tawi	19,107	0	0	0	0	2	274	1,037	1,313
SGA	7,550	0	0	0	0	0	61	504	565
City of Cotabato	42,157	0	0	5	5	6	204	1,992	2,202

Table 2.A.2 - Modern Methods of Family Planning
Other Acceptors
Philippines, 2024

Area	Total Current Users	FSTR/BTL			Total	MSTR/NSV			Total
		Age group				Age group			
		10-14	15-19	20-49		10-14	15-19	20-49	
**PHILIPPINES**	**8,664,035**	**11**	**339**	**72,865**	**73,215**	**0**	**11**	**1,116**	**1,127**
**N C R**	**1,041,531**	**0**	**4**	**11,874**	**11,878**	**0**	**1**	**192**	**193**
City of Malabon	26,264	0	0	796	796	0	0	13	13
City of Navotas	23,816	0	0	44	44	0	0	0	0
City of Valenzuela	40,454	0	0	1,757	1,757	0	0	92	92
City of Caloocan	73,676	0	1	1,093	1,094	0	0	0	0
City of Marikina	24,847	0	0	284	284	0	0	0	0
City of Pasig	58,937	0	0	417	417	0	0	3	3
Pateros	4,204	0	0	12	12	0	0	0	0
City of Taguig	92,053	0	1	757	758	0	0	2	2
Quezon City	338,677	0	0	4,126	4,126	0	0	34	34
City of Makati	28,572	0	0	30	30	0	0	0	0
City of Mandaluyong	27,196	0	0	56	56	0	0	2	2
City of San Juan	6,139	0	0	0	0	0	0	0	0
City of Manila	138,102	0	0	1,184	1,184	0	1	35	36
City of Las Piñas	26,980	0	0	624	624	0	0	5	5
City of Muntinlupa	44,186	0	0	36	36	0	0	0	0
City of Parañaque	47,100	0	0	51	51	0	0	1	1
Pasay City	40,328	0	2	607	609	0	0	5	5
**C A R**	**165,663**	**0**	**0**	**2,599**	**2,599**	**0**	**0**	**45**	**45**
Abra	19,755	0	0	18	18	0	0	0	0
Apayao	24,752	0	0	9	9	0	0	0	0
Benguet	37,253	0	0	320	320	0	0	37	37
Ifugao	21,120	0	0	326	326	0	0	3	3
Kalinga	18,267	0	0	117	117	0	0	0	0
Mountain Province	13,957	0	0	453	453	0	0	0	0
City of Baguio	30,559	0	0	1,356	1,356	0	0	5	5
**Region 1**	**498,204**	**0**	**9**	**3,710**	**3,719**	**0**	**0**	**29**	**29**
Ilocos Norte	52,824	0	8	1,151	1,159	0	0	6	6
Ilocos Sur	78,125	0	0	117	117	0	0	0	0
La Union	67,784	0	0	668	668	0	0	16	16
Pangasinan	292,353	0	1	1,755	1,756	0	0	7	7
City of Dagupan	7,118	0	0	19	19	0	0	0	0
**Region 2**	**343,378**	**0**	**40**	**3,644**	**3,684**	**0**	**0**	**12**	**12**
Batanes	955	0	0	21	21	0	0	0	0
Cagayan	108,930	0	0	992	992	0	0	1	1
Isabela	146,131	0	35	1,372	1,407	0	0	1	1
Nueva Vizcaya	52,095	0	0	954	954	0	0	10	10
Quirino	21,240	0	5	273	278	0	0	0	0
City of Santiago	14,027	0	0	32	32	0	0	0	0
**Region 3**	**985,325**	**4**	**11**	**18,341**	**18,356**	**0**	**0**	**40**	**40**
Aurora	24,562	0	0	164	164	0	0	0	0
Bataan	65,716	0	0	903	903	0	0	0	0
Bulacan	302,904	0	0	5,190	5,190	0	0	10	10
Nueva Ecija	200,897	4	1	4,579	4,584	0	0	1	1
Pampanga	167,294	0	4	3,957	3,961	0	0	19	19
Tarlac	118,444	0	6	2,127	2,133	0	0	2	2
Zambales	54,326	0	0	1,231	1,231	0	0	0	0
City of Angeles	30,345	0	0	144	144	0	0	3	3
City of Olongapo	20,837	0	0	46	46	0	0	5	5
**Region 4A**	**888,692**	**0**	**43**	**6,294**	**6,337**	**0**	**7**	**168**	**175**
Batangas	158,569	0	2	1,293	1,295	0	0	100	100
Cavite	175,332	0	33	1,775	1,808	0	0	25	25
Laguna	286,016	0	6	1,196	1,202	0	7	33	40
Quezon	106,773	0	1	852	853	0	0	4	4
Rizal	156,554	0	1	1,129	1,130	0	0	6	6
City of Lucena	5,448	0	0	49	49	0	0	0	0
**Region 4B**	**291,730**	**7**	**86**	**2,482**	**2,575**	**0**	**1**	**21**	**22**
Marinduque	16,436	0	0	101	101	0	0	1	1
Occidental Mindoro	48,902	3	0	298	301	0	0	1	1
Oriental Mindoro	68,962	4	82	1,285	1,371	0	0	15	15
Palawan	100,239	0	0	350	350	0	0	3	3

Table 2.A.2 - Modern Methods of Family Planning
Other Acceptors
Philippines, 2024

Area	Total Current Users	FSTR/BTL Age group 10-14	15-19	20-49	Total	MSTR/NSV Age group 10-14	15-19	20-49	Total
Romblon	22,467	0	4	140	144	0	0	1	1
City of Puerto Princesa	34,724	0	0	308	308	0	1	0	1
**Region 5**	**491,006**	**0**	**5**	**1,582**	**1,587**	**0**	**0**	**12**	**12**
Albay	115,867	0	2	428	430	0	0	0	0
Camarines Norte	46,839	0	1	23	24	0	0	0	0
Camarines Sur	107,464	0	0	4	4	0	0	0	0
Catanduanes	24,326	0	0	366	366	0	0	6	6
Masbate	96,672	0	0	188	188	0	0	0	0
Sorsogon	66,956	0	2	494	496	0	0	4	4
City of Naga	32,882	0	0	79	79	0	0	2	2
**Region 6**	**667,635**	**0**	**2**	**3,302**	**3,304**	**0**	**0**	**39**	**39**
Aklan	48,553	0	0	131	131	0	0	0	0
Antique	49,316	0	0	246	246	0	0	2	2
Capiz	77,942	0	0	201	201	0	0	2	2
Guimaras	17,943	0	0	125	125	0	0	0	0
Iloilo	182,130	0	0	302	302	0	0	0	0
Negros Occidental	206,889	0	2	1,376	1,378	0	0	30	30
City of Bacolod	30,421	0	0	541	541	0	0	3	3
City of Iloilo	54,441	0	0	380	380	0	0	2	2
**Region 7**	**483,438**	**0**	**2**	**6,909**	**6,911**	**0**	**0**	**334**	**334**
Bohol	83,324	0	0	286	286	0	0	2	2
Cebu	191,250	0	0	5,709	5,709	0	0	280	280
Negros Oriental	98,214	0	0	477	477	0	0	10	10
Siquijor	9,225	0	0	0	0	0	0	0	0
City of Cebu	34,897	0	2	427	429	0	0	37	37
City of Lapu-Lapu	36,794	0	0	0	0	0	0	5	5
City of Mandaue	29,734	0	0	10	10	0	0	0	0
**Region 8**	**340,613**	**0**	**13**	**1,491**	**1,504**	**0**	**1**	**33**	**34**
Biliran	12,714	0	0	63	63	0	0	2	2
Eastern Samar	39,982	0	0	55	55	0	0	1	1
Leyte	125,631	0	0	300	300	0	0	13	13
Northern Samar	48,697	0	4	46	50	0	0	1	1
Southern Leyte	27,251	0	0	484	484	0	1	0	1
Samar	42,957	0	0	105	105	0	0	2	2
Ormoc City	20,171	0	6	170	176	0	0	13	13
City of Tacloban	23,210	0	3	268	271	0	0	1	1
**Region 9**	**354,267**	**0**	**0**	**611**	**611**	**0**	**0**	**0**	**0**
Zamboanga del Norte	126,112	0	0	207	207	0	0	0	0
Zamboanga del Sur	102,687	0	0	293	293	0	0	0	0
Zamboanga Sibugay	47,839	0	0	3	3	0	0	0	0
City of Isabela	7,167	0	0	0	0	0	0	0	0
City of Zamboanga	70,462	0	0	108	108	0	0	0	0
**Region 10**	**511,701**	**0**	**19**	**3,101**	**3,120**	**0**	**0**	**52**	**52**
Bukidnon	167,011	0	4	1,344	1,348	0	0	12	12
Camiguin	6,884	0	0	48	48	0	0	0	0
Lanao del Norte	73,898	0	0	42	42	0	0	0	0
Misamis Occidental	60,887	0	0	249	249	0	0	1	1
Misamis Oriental	111,011	0	15	960	975	0	0	30	30
City of Cagayan De Oro	57,799	0	0	337	337	0	0	8	8
City of Iligan	34,211	0	0	121	121	0	0	1	1
**Region 11**	**535,532**	**0**	**7**	**2,776**	**2,783**	**0**	**0**	**40**	**40**
Davao de Oro	84,560	0	1	401	402	0	0	2	2
Davao del Norte	112,914	0	0	588	588	0	0	3	3
Davao Oriental	59,172	0	3	379	382	0	0	16	16
Davao del Sur	67,054	0	1	595	596	0	0	1	1
Davao Occidental	33,334	0	2	190	192	0	0	5	5
City of Davao	178,498	0	0	623	623	0	0	13	13
**Region 12**	**476,219**	**0**	**96**	**2,028**	**2,124**	**0**	**1**	**83**	**84**
Cotabato	136,508	0	3	770	773	0	0	21	21
Sarangani	76,390	0	0	219	219	0	0	15	15
South Cotabato	102,265	0	0	468	468	0	0	11	11

Table 2.A.2 - Modern Methods of Family Planning
Other Acceptors
Philippines, 2024

Area	Total Current Users	FSTR/BTL Age group 10-14	FSTR/BTL Age group 15-19	FSTR/BTL Age group 20-49	Total	MSTR/NSV Age group 10-14	MSTR/NSV Age group 15-19	MSTR/NSV Age group 20-49	Total
Sultan Kudarat	104,518	0	93	311	404	0	1	36	37
City of General Santos	56,538	0	0	260	260	0	0	0	0
**Caraga**	**292,511**	**0**	**1**	**1,749**	**1,750**	**0**	**0**	**11**	**11**
Agusan del Norte	40,148	0	1	210	211	0	0	1	1
Agusan del Sur	86,721	0	0	676	676	0	0	7	7
Surigao del Norte	53,159	0	0	403	403	0	0	2	2
Surigao del Sur	60,298	0	0	324	324	0	0	1	1
Dinagat Islands	9,606	0	0	28	28	0	0	0	0
City of Butuan	42,579	0	0	108	108	0	0	0	0
**BARMM**	**296,590**	**0**	**1**	**372**	**373**	**0**	**0**	**5**	**5**
Basilan	19,309	0	0	35	35	0	0	0	0
Lanao del Sur	39,800	0	0	196	196	0	0	4	4
Maguindanao del Norte	36,321	0	0	16	16	0	0	0	0
Maguindanao del Sur	51,529	0	1	57	58	0	0	0	0
Sulu	80,817	0	0	5	5	0	0	0	0
Tawi-Tawi	19,107	0	0	26	26	0	0	0	0
SGA	7,550	0	0	0	0	0	0	0	0
City of Cotabato	42,157	0	0	37	37	0	0	1	1

Table 2.A.2 - Modern Methods of Family Planning
Other Acceptors
Philippines, 2024

Area	Total Current Users	CONDOM Age group 10-14	CONDOM Age group 15-19	CONDOM Age group 20-49	Total	IUD-INTERVAL Age group 10-14	IUD-INTERVAL Age group 15-19	IUD-INTERVAL Age group 20-49	Total
**PHILIPPINES**	**8,664,035**	**148**	**3,999**	**82,966**	**87,113**	**134**	**1,966**	**27,480**	**29,580**
**N C R**	**1,041,531**	**30**	**1,270**	**26,711**	**28,011**	**7**	**274**	**4,122**	**4,403**
City of Malabon	26,264	1	163	919	1,083	0	24	317	341
City of Navotas	23,816	6	335	603	944	0	27	46	73
City of Valenzuela	40,454	5	7	848	860	0	6	291	297
City of Caloocan	73,676	0	19	785	804	4	96	835	935
City of Marikina	24,847	0	0	176	176	0	0	53	53
City of Pasig	58,937	0	4	568	572	0	3	210	213
Pateros	4,204	0	0	21	21	0	0	19	19
City of Taguig	92,053	0	24	361	385	0	11	217	228
Quezon City	338,677	3	304	15,424	15,731	0	16	909	925
City of Makati	28,572	0	5	463	468	0	0	10	10
City of Mandaluyong	27,196	0	7	359	366	0	0	107	107
City of San Juan	6,139	0	0	13	13	0	0	0	0
City of Manila	138,102	11	278	4,552	4,841	0	29	335	364
City of Las Piñas	26,980	0	2	172	174	1	0	106	107
City of Muntinlupa	44,186	0	0	22	22	0	0	14	14
City of Parañaque	47,100	0	33	296	329	0	1	18	19
Pasay City	40,328	4	89	1,129	1,222	2	61	635	698
**C A R**	**165,663**	**0**	**35**	**1,611**	**1,646**	**0**	**13**	**486**	**499**
Abra	19,755	0	5	139	144	0	0	5	5
Apayao	24,752	0	8	66	74	0	3	183	186
Benguet	37,253	0	4	532	536	0	4	183	187
Ifugao	21,120	0	2	237	239	0	0	39	39
Kalinga	18,267	0	9	116	125	0	3	38	41
Mountain Province	13,957	0	2	84	86	0	3	28	31
City of Baguio	30,559	0	5	437	442	0	0	10	10
**Region 1**	**498,204**	**0**	**40**	**1,425**	**1,465**	**0**	**39**	**625**	**664**
Ilocos Norte	52,824	0	3	86	89	0	13	10	23
Ilocos Sur	78,125	0	2	113	115	0	4	22	26
La Union	67,784	0	6	195	201	0	15	100	115
Pangasinan	292,353	0	29	1,028	1,057	0	7	486	493
City of Dagupan	7,118	0	0	3	3	0	0	7	7
**Region 2**	**343,378**	**0**	**42**	**1,228**	**1,270**	**3**	**146**	**1,384**	**1,533**
Batanes	955	0	0	5	5	0	0	0	0
Cagayan	108,930	0	1	400	401	1	32	669	702
Isabela	146,131	0	18	429	447	1	78	444	523
Nueva Vizcaya	52,095	0	18	300	318	1	27	205	233
Quirino	21,240	0	5	91	96	0	8	43	51
City of Santiago	14,027	0	0	3	3	0	1	23	24
**Region 3**	**985,325**	**83**	**625**	**8,251**	**8,959**	**103**	**149**	**940**	**1,192**
Aurora	24,562	0	5	851	856	0	2	39	41
Bataan	65,716	0	75	572	647	1	2	86	89
Bulacan	302,904	0	99	4,075	4,174	0	11	342	353
Nueva Ecija	200,897	0	24	614	638	0	8	224	232
Pampanga	167,294	81	271	868	1,220	101	124	119	344
Tarlac	118,444	2	118	516	636	1	1	101	103
Zambales	54,326	0	28	607	635	0	1	13	14
City of Angeles	30,345	0	2	49	51	0	0	16	16
City of Olongapo	20,837	0	3	99	102	0	0	0	0
**Region 4A**	**888,692**	**3**	**236**	**5,658**	**5,897**	**9**	**92**	**2,186**	**2,287**
Batangas	158,569	0	5	690	695	0	4	318	322
Cavite	175,332	0	61	1,091	1,152	1	33	537	571
Laguna	286,016	0	135	1,224	1,359	2	15	377	394
Quezon	106,773	3	13	723	739	0	24	598	622
Rizal	156,554	0	19	1,869	1,888	2	16	344	362
City of Lucena	5,448	0	3	61	64	4	0	12	16
**Region 4B**	**291,730**	**2**	**245**	**1,749**	**1,996**	**1**	**36**	**803**	**840**
Marinduque	16,436	0	0	14	14	0	0	16	16
Occidental Mindoro	48,902	2	6	238	246	1	3	62	66
Oriental Mindoro	68,962	0	149	634	783	0	19	486	505
Palawan	100,239	0	83	485	568	0	11	170	181

Table 2.A.2 - Modern Methods of Family Planning
Other Acceptors
Philippines, 2024

Area	Total Current Users	CONDOM			Total	IUD-INTERVAL			Total
		Age group				Age group			
		10-14	15-19	20-49		10-14	15-19	20-49	
Romblon	22,467	0	0	50	50	0	1	9	10
City of Puerto Princesa	34,724	0	7	328	335	0	2	60	62
**Region 5**	**491,006**	**0**	**163**	**3,484**	**3,647**	**0**	**14**	**297**	**311**
Albay	115,867	0	26	630	656	0	1	45	46
Camarines Norte	46,839	0	15	318	333	0	6	122	128
Camarines Sur	107,464	0	32	1,137	1,169	0	1	27	28
Catanduanes	24,326	0	16	269	285	0	0	23	23
Masbate	96,672	0	51	555	606	0	3	33	36
Sorsogon	66,956	0	18	404	422	0	2	37	39
City of Naga	32,882	0	5	171	176	0	1	10	11
**Region 6**	**667,635**	**2**	**221**	**4,591**	**4,814**	**2**	**129**	**2,278**	**2,409**
Aklan	48,553	0	8	292	300	0	1	28	29
Antique	49,316	0	2	238	240	0	1	32	33
Capiz	77,942	0	1	281	282	0	0	96	96
Guimaras	17,943	0	4	334	338	0	0	20	20
Iloilo	182,130	1	103	809	913	1	21	326	348
Negros Occidental	206,889	1	85	1,961	2,047	0	19	924	943
City of Bacolod	30,421	0	12	162	174	1	49	337	387
City of Iloilo	54,441	0	6	514	520	0	38	515	553
**Region 7**	**483,438**	**1**	**207**	**11,577**	**11,785**	**1**	**648**	**5,140**	**5,789**
Bohol	83,324	0	12	430	442	0	23	1,716	1,739
Cebu	191,250	0	158	9,918	10,076	1	606	1,877	2,484
Negros Oriental	98,214	1	20	726	747	0	10	648	658
Siquijor	9,225	0	3	44	47	0	0	175	175
City of Cebu	34,897	0	5	255	260	0	9	615	624
City of Lapu-Lapu	36,794	0	9	157	166	0	0	65	65
City of Mandaue	29,734	0	0	47	47	0	0	44	44
**Region 8**	**340,613**	**5**	**196**	**1,845**	**2,046**	**0**	**71**	**949**	**1,020**
Biliran	12,714	0	0	101	101	0	5	55	60
Eastern Samar	39,982	0	1	284	285	0	3	38	41
Leyte	125,631	0	38	275	313	0	21	225	246
Northern Samar	48,697	3	2	419	424	0	0	65	65
Southern Leyte	27,251	0	10	251	261	0	1	131	132
Samar	42,957	2	72	116	190	0	1	63	64
Ormoc City	20,171	0	35	314	349	0	4	140	144
City of Tacloban	23,210	0	38	85	123	0	36	232	268
**Region 9**	**354,267**	**3**	**120**	**1,831**	**1,954**	**0**	**22**	**1,262**	**1,284**
Zamboanga del Norte	126,112	0	34	589	623	0	4	280	284
Zamboanga del Sur	102,687	3	22	634	659	0	11	725	736
Zamboanga Sibugay	47,839	0	41	312	353	0	6	229	235
City of Isabela	7,167	0	19	201	220	0	0	7	7
City of Zamboanga	70,462	0	4	95	99	0	1	21	22
**Region 10**	**511,701**	**17**	**225**	**4,322**	**4,564**	**7**	**138**	**2,954**	**3,099**
Bukidnon	167,011	2	103	1,614	1,719	1	80	1,595	1,676
Camiguin	6,884	0	3	36	39	0	1	8	9
Lanao del Norte	73,898	0	21	644	665	0	2	44	46
Misamis Occidental	60,887	0	17	401	418	0	3	112	115
Misamis Oriental	111,011	15	32	489	536	6	39	720	765
City of Cagayan De Oro	57,799	0	34	969	1,003	0	10	266	276
City of Iligan	34,211	0	15	169	184	0	3	209	212
**Region 11**	**535,532**	**1**	**83**	**2,302**	**2,386**	**0**	**46**	**1,266**	**1,312**
Davao de Oro	84,560	0	4	366	370	0	7	232	239
Davao del Norte	112,914	0	16	898	914	0	16	372	388
Davao Oriental	59,172	0	7	186	193	0	6	146	152
Davao del Sur	67,054	0	14	183	197	0	5	219	224
Davao Occidental	33,334	0	4	34	38	0	5	111	116
City of Davao	178,498	1	38	635	674	0	7	186	193
**Region 12**	**476,219**	**1**	**103**	**2,713**	**2,817**	**0**	**40**	**1,275**	**1,315**
Cotabato	136,508	1	28	1,536	1,565	0	16	492	508
Sarangani	76,390	0	5	199	204	0	0	82	82
South Cotabato	102,265	0	14	417	431	0	6	372	378

Table 2.A.2 - Modern Methods of Family Planning
Other Acceptors
Philippines, 2024

Area	Total Current Users	CONDOM			Total	IUD-INTERVAL			Total
		Age group				Age group			
		10-14	15-19	20-49		10-14	15-19	20-49	
Sultan Kudarat	104,518	0	44	301	345	0	13	244	257
City of General Santos	56,538	0	12	260	272	0	5	85	90
**Caraga**	**292,511**	**0**	**135**	**2,454**	**2,589**	**1**	**84**	**1,378**	**1,463**
Agusan del Norte	40,148	0	18	352	370	0	5	389	394
Agusan del Sur	86,721	0	26	694	720	0	21	374	395
Surigao del Norte	53,159	0	14	335	349	1	5	231	237
Surigao del Sur	60,298	0	54	535	589	0	48	271	319
Dinagat Islands	9,606	0	2	124	126	0	3	68	71
City of Butuan	42,579	0	21	414	435	0	2	45	47
**BARMM**	**296,590**	**0**	**53**	**1,214**	**1,267**	**0**	**25**	**135**	**160**
Basilan	19,309	0	0	19	19	0	0	16	16
Lanao del Sur	39,800	0	23	610	633	0	0	22	22
Maguindanao del Norte	36,321	0	2	159	161	0	14	50	64
Maguindanao del Sur	51,529	0	14	198	212	0	0	6	6
Sulu	80,817	0	13	76	89	0	0	5	5
Tawi-Tawi	19,107	0	0	12	12	0	0	6	6
SGA	7,550	0	0	38	38	0	0	0	0
City of Cotabato	42,157	0	1	102	103	0	11	30	41

Table 2.A.2 - Modern Methods of Family Planning
Other Acceptors
Philippines, 2024

Area	Total Current Users	IUD-POSTPARTUM Age group 10-14	IUD-POSTPARTUM 15-19	IUD-POSTPARTUM 20-49	Total	PILLS-POP Age group 10-14	PILLS-POP 15-19	PILLS-POP 20-49	Total
**PHILIPPINES**	**8,664,035**	**611**	**4,199**	**31,131**	**35,941**	**165**	**9,430**	**120,532**	**130,127**
**N C R**	**1,041,531**	**5**	**252**	**7,087**	**7,344**	**15**	**866**	**18,025**	**18,906**
City of Malabon	26,264	0	2	78	80	2	40	906	948
City of Navotas	23,816	0	0	0	0	6	194	531	731
City of Valenzuela	40,454	0	1	241	242	0	7	1,346	1,353
City of Caloocan	73,676	1	39	1,113	1,153	0	36	1,021	1,057
City of Marikina	24,847	0	2	118	120	0	1	98	99
City of Pasig	58,937	0	3	163	166	0	8	423	431
Pateros	4,204	0	0	2	2	0	1	27	28
City of Taguig	92,053	1	7	121	129	0	24	668	692
Quezon City	338,677	3	155	3,758	3,916	2	251	7,529	7,782
City of Makati	28,572	0	1	39	40	0	7	140	147
City of Mandaluyong	27,196	0	4	51	55	0	9	239	248
City of San Juan	6,139	0	0	0	0	0	2	45	47
City of Manila	138,102	0	10	136	146	5	211	3,176	3,392
City of Las Piñas	26,980	0	0	12	12	0	10	204	214
City of Muntinlupa	44,186	0	0	0	0	0	5	31	36
City of Parañaque	47,100	0	0	3	3	0	32	350	382
Pasay City	40,328	0	28	1,252	1,280	0	28	1,291	1,319
**C A R**	**165,663**	**0**	**15**	**255**	**270**	**1**	**208**	**2,976**	**3,185**
Abra	19,755	0	2	7	9	1	19	311	331
Apayao	24,752	0	1	10	11	0	39	346	385
Benguet	37,253	0	6	94	100	0	28	658	686
Ifugao	21,120	0	3	13	16	0	43	382	425
Kalinga	18,267	0	0	17	17	0	38	319	357
Mountain Province	13,957	0	3	48	51	0	29	294	323
City of Baguio	30,559	0	0	66	66	0	12	666	678
**Region 1**	**498,204**	**1**	**90**	**881**	**972**	**4**	**838**	**15,199**	**16,041**
Ilocos Norte	52,824	0	9	18	27	0	36	1,239	1,275
Ilocos Sur	78,125	0	1	6	7	0	16	401	417
La Union	67,784	1	40	110	151	0	26	213	239
Pangasinan	292,353	0	39	737	776	4	760	13,346	14,110
City of Dagupan	7,118	0	1	10	11	0	0	0	0
**Region 2**	**343,378**	**8**	**151**	**790**	**949**	**8**	**559**	**5,370**	**5,937**
Batanes	955	0	0	1	1	0	1	13	14
Cagayan	108,930	1	17	149	167	0	106	1,234	1,340
Isabela	146,131	1	29	299	329	7	332	2,948	3,287
Nueva Vizcaya	52,095	6	102	324	432	1	83	837	921
Quirino	21,240	0	3	17	20	0	37	338	375
City of Santiago	14,027	0	0	0	0	0	0	0	0
**Region 3**	**985,325**	**560**	**1,528**	**721**	**2,809**	**39**	**1,276**	**7,526**	**8,841**
Aurora	24,562	0	1	11	12	0	21	325	346
Bataan	65,716	0	1	22	23	0	40	904	944
Bulacan	302,904	0	17	269	286	1	173	2,385	2,559
Nueva Ecija	200,897	1	36	333	370	0	224	1,463	1,687
Pampanga	167,294	559	1,473	55	2,087	37	674	1,110	1,821
Tarlac	118,444	0	0	22	22	0	112	929	1,041
Zambales	54,326	0	0	3	3	0	19	192	211
City of Angeles	30,345	0	0	6	6	0	4	62	66
City of Olongapo	20,837	0	0	0	0	1	9	156	166
**Region 4A**	**888,692**	**4**	**71**	**1,064**	**1,139**	**6**	**594**	**9,364**	**9,964**
Batangas	158,569	1	23	405	429	0	28	923	951
Cavite	175,332	1	9	211	221	1	118	2,496	2,615
Laguna	286,016	0	18	108	126	0	168	2,004	2,172
Quezon	106,773	0	7	113	120	3	118	1,656	1,777
Rizal	156,554	2	14	221	237	1	111	2,017	2,129
City of Lucena	5,448	0	0	6	6	1	51	268	320
**Region 4B**	**291,730**	**16**	**32**	**291**	**339**	**19**	**402**	**4,150**	**4,571**
Marinduque	16,436	0	0	2	2	0	1	57	58
Occidental Mindoro	48,902	0	2	77	79	5	114	1,078	1,197
Oriental Mindoro	68,962	16	28	167	211	0	83	795	878
Palawan	100,239	0	1	13	14	11	133	1,234	1,378

Table 2.A.2 - Modern Methods of Family Planning
Other Acceptors
Philippines, 2024

Area	Total Current Users	IUD-POSTPARTUM			Total	PILLS-POP			Total
		Age group				Age group			
		10-14	15-19	20-49		10-14	15-19	20-49	
Romblon	22,467	0	1	14	15	2	10	126	138
City of Puerto Princesa	34,724	0	0	18	18	1	61	860	922
**Region 5**	**491,006**	**0**	**13**	**154**	**167**	**3**	**466**	**7,192**	**7,661**
Albay	115,867	0	3	33	36	1	42	1,837	1,880
Camarines Norte	46,839	0	0	0	0	0	81	887	968
Camarines Sur	107,464	0	5	70	75	1	85	1,570	1,656
Catanduanes	24,326	0	3	14	17	0	8	122	130
Masbate	96,672	0	1	16	17	1	111	1,889	2,001
Sorsogon	66,956	0	0	8	8	0	138	858	996
City of Naga	32,882	0	1	13	14	0	1	29	30
**Region 6**	**667,635**	**2**	**79**	**1,131**	**1,212**	**9**	**383**	**4,395**	**4,787**
Aklan	48,553	0	2	2	4	0	10	453	463
Antique	49,316	0	0	12	12	0	14	367	381
Capiz	77,942	0	0	11	11	0	6	244	250
Guimaras	17,943	0	1	10	11	0	5	172	177
Iloilo	182,130	1	4	45	50	9	106	356	471
Negros Occidental	206,889	0	31	543	574	0	226	2,256	2,482
City of Bacolod	30,421	1	18	326	345	0	13	500	513
City of Iloilo	54,441	0	23	182	205	0	3	47	50
**Region 7**	**483,438**	**5**	**1,470**	**11,621**	**13,096**	**18**	**482**	**9,380**	**9,880**
Bohol	83,324	3	38	1,129	1,170	0	40	615	655
Cebu	191,250	2	1,427	10,177	11,606	0	175	5,818	5,993
Negros Oriental	98,214	0	1	58	59	17	153	1,419	1,589
Siquijor	9,225	0	0	4	4	0	2	97	99
City of Cebu	34,897	0	4	238	242	1	75	1,013	1,089
City of Lapu-Lapu	36,794	0	0	8	8	0	36	277	313
City of Mandaue	29,734	0	0	7	7	0	1	141	142
**Region 8**	**340,613**	**1**	**50**	**373**	**424**	**0**	**193**	**2,390**	**2,583**
Biliran	12,714	0	2	188	190	0	10	117	127
Eastern Samar	39,982	0	5	14	19	0	13	255	268
Leyte	125,631	1	3	78	82	0	20	491	511
Northern Samar	48,697	0	0	12	12	0	32	397	429
Southern Leyte	27,251	0	2	21	23	0	20	261	281
Samar	42,957	0	0	12	12	0	13	255	268
Ormoc City	20,171	0	4	10	14	0	62	445	507
City of Tacloban	23,210	0	34	38	72	0	23	169	192
**Region 9**	**354,267**	**0**	**68**	**1,537**	**1,605**	**3**	**313**	**3,665**	**3,981**
Zamboanga del Norte	126,112	0	4	201	205	3	83	719	805
Zamboanga del Sur	102,687	0	52	1,236	1,288	0	110	1,281	1,391
Zamboanga Sibugay	47,839	0	12	81	93	0	72	1,071	1,143
City of Isabela	7,167	0	0	6	6	0	1	140	141
City of Zamboanga	70,462	0	0	13	13	0	47	454	501
**Region 10**	**511,701**	**6**	**182**	**2,720**	**2,908**	**13**	**707**	**5,319**	**6,039**
Bukidnon	167,011	0	11	161	172	3	402	1,987	2,392
Camiguin	6,884	0	0	6	6	0	3	25	28
Lanao del Norte	73,898	0	0	22	22	0	28	424	452
Misamis Occidental	60,887	0	2	28	30	0	37	341	378
Misamis Oriental	111,011	6	134	1,802	1,942	9	121	1,335	1,465
City of Cagayan De Oro	57,799	0	23	410	433	0	60	659	719
City of Iligan	34,211	0	12	291	303	1	56	548	605
**Region 11**	**535,532**	**1**	**22**	**487**	**510**	**5**	**598**	**7,209**	**7,812**
Davao de Oro	84,560	0	1	23	24	0	101	1,339	1,440
Davao del Norte	112,914	0	1	193	194	1	106	1,239	1,346
Davao Oriental	59,172	0	0	14	14	2	118	981	1,101
Davao del Sur	67,054	1	2	21	24	0	54	568	622
Davao Occidental	33,334	0	2	22	24	0	25	257	282
City of Davao	178,498	0	16	214	230	2	194	2,825	3,021
**Region 12**	**476,219**	**1**	**35**	**590**	**626**	**14**	**461**	**5,973**	**6,448**
Cotabato	136,508	0	6	173	179	5	142	1,619	1,766
Sarangani	76,390	0	0	48	48	0	72	502	574
South Cotabato	102,265	0	15	99	114	2	89	832	923

Table 2.A.2 - Modern Methods of Family Planning
Other Acceptors
Philippines, 2024

Area	Total Current Users	IUD-POSTPARTUM Age group			Total	PILLS-POP Age group			Total
		10-14	15-19	20-49		10-14	15-19	20-49	
Sultan Kudarat	104,518	1	12	196	209	7	111	2,562	2,680
City of General Santos	56,538	0	2	74	76	0	47	458	505
**Caraga**	**292,511**	**1**	**110**	**1,149**	**1,260**	**8**	**778**	**6,342**	**7,128**
Agusan del Norte	40,148	1	10	106	117	0	92	979	1,071
Agusan del Sur	86,721	0	17	241	258	7	290	2,016	2,313
Surigao del Norte	53,159	0	66	325	391	0	166	967	1,133
Surigao del Sur	60,298	0	15	440	455	1	170	1,643	1,814
Dinagat Islands	9,606	0	0	11	11	0	3	94	97
City of Butuan	42,579	0	2	26	28	0	57	643	700
**BARMM**	**296,590**	**0**	**31**	**280**	**311**	**0**	**306**	**6,057**	**6,363**
Basilan	19,309	0	0	10	10	0	27	197	224
Lanao del Sur	39,800	0	3	43	46	0	39	1,196	1,235
Maguindanao del Norte	36,321	0	7	23	30	0	65	970	1,035
Maguindanao del Sur	51,529	0	1	9	10	0	78	1,116	1,194
Sulu	80,817	0	0	4	4	0	62	1,428	1,490
Tawi-Tawi	19,107	0	0	0	0	0	27	910	937
SGA	7,550	0	0	0	0	0	3	126	129
City of Cotabato	42,157	0	20	191	211	0	5	114	119

Table 2.A.2 - Modern Methods of Family Planning
Other Acceptors
Philippines, 2024

Area	Total Current Users	PILLS-COC			Total	INJECTABLES			Total
		Age group				Age group			
		10-14	15-19	20-49		10-14	15-19	20-49	
**PHILIPPINES**	**8,664,035**	**179**	**15,205**	**347,712**	**363,096**	**322**	**19,445**	**309,030**	**328,797**
**N C R**	**1,041,531**	**9**	**1,598**	**42,567**	**44,174**	**39**	**2,449**	**42,999**	**45,487**
City of Malabon	26,264	1	63	2,003	2,067	4	169	2,671	2,844
City of Navotas	23,816	0	295	796	1,091	9	417	1,241	1,667
City of Valenzuela	40,454	0	59	4,541	4,600	2	196	4,340	4,538
City of Caloocan	73,676	1	88	2,719	2,808	1	208	6,048	6,257
City of Marikina	24,847	0	5	335	340	0	22	616	638
City of Pasig	58,937	0	20	1,259	1,279	0	41	1,153	1,194
Pateros	4,204	0	0	60	60	0	1	71	72
City of Taguig	92,053	0	39	1,727	1,766	1	46	1,220	1,267
Quezon City	338,677	0	639	19,891	20,530	1	519	13,220	13,740
City of Makati	28,572	0	12	423	435	0	13	571	584
City of Mandaluyong	27,196	0	12	249	261	0	7	276	283
City of San Juan	6,139	0	0	109	109	0	4	197	201
City of Manila	138,102	7	289	5,595	5,891	21	660	7,321	8,002
City of Las Piñas	26,980	0	14	540	554	0	32	857	889
City of Muntinlupa	44,186	0	20	612	632	0	34	718	752
City of Parañaque	47,100	0	25	557	582	0	42	625	667
Pasay City	40,328	0	18	1,151	1,169	0	38	1,854	1,892
**C A R**	**165,663**	**6**	**260**	**8,286**	**8,552**	**20**	**409**	**7,212**	**7,641**
Abra	19,755	0	45	1,023	1,068	0	32	483	515
Apayao	24,752	3	54	919	976	9	60	636	705
Benguet	37,253	1	33	1,839	1,873	0	41	1,522	1,563
Ifugao	21,120	1	38	1,521	1,560	1	58	883	942
Kalinga	18,267	1	50	1,083	1,134	9	97	994	1,100
Mountain Province	13,957	0	33	676	709	1	67	737	805
City of Baguio	30,559	0	7	1,225	1,232	0	54	1,957	2,011
**Region 1**	**498,204**	**1**	**465**	**11,581**	**12,047**	**5**	**462**	**8,076**	**8,543**
Ilocos Norte	52,824	0	16	1,109	1,125	0	21	512	533
Ilocos Sur	78,125	0	15	892	907	1	18	494	513
La Union	67,784	0	103	1,510	1,613	0	87	1,659	1,746
Pangasinan	292,353	1	330	8,060	8,391	4	336	5,365	5,705
City of Dagupan	7,118	0	1	10	11	0	0	46	46
**Region 2**	**343,378**	**2**	**1,069**	**16,904**	**17,975**	**9**	**931**	**9,791**	**10,731**
Batanes	955	0	0	57	57	0	10	123	133
Cagayan	108,930	0	134	4,597	4,731	1	207	2,249	2,457
Isabela	146,131	2	716	7,520	8,238	7	460	5,037	5,504
Nueva Vizcaya	52,095	0	140	3,321	3,461	1	129	1,353	1,483
Quirino	21,240	0	46	701	747	0	57	530	587
City of Santiago	14,027	0	33	708	741	0	68	499	567
**Region 3**	**985,325**	**45**	**1,655**	**34,244**	**35,944**	**95**	**2,201**	**30,449**	**32,745**
Aurora	24,562	0	71	1,262	1,333	0	62	911	973
Bataan	65,716	1	90	1,785	1,876	2	184	3,340	3,526
Bulacan	302,904	0	460	13,756	14,216	0	596	10,114	10,710
Nueva Ecija	200,897	0	297	5,917	6,214	3	313	5,415	5,731
Pampanga	167,294	40	441	3,179	3,660	81	577	3,405	4,063
Tarlac	118,444	4	199	3,831	4,034	6	250	3,206	3,462
Zambales	54,326	0	49	1,589	1,638	1	150	2,398	2,549
City of Angeles	30,345	0	23	868	891	0	17	527	544
City of Olongapo	20,837	0	25	2,057	2,082	2	52	1,133	1,187
**Region 4A**	**888,692**	**18**	**1,026**	**34,298**	**35,342**	**29**	**1,947**	**40,786**	**42,762**
Batangas	158,569	0	54	8,403	8,457	4	83	3,123	3,210
Cavite	175,332	1	271	8,211	8,483	3	496	12,328	12,827
Laguna	286,016	1	317	4,778	5,096	1	290	5,500	5,791
Quezon	106,773	14	185	6,900	7,099	18	507	8,789	9,314
Rizal	156,554	2	158	5,727	5,887	3	471	10,292	10,766
City of Lucena	5,448	0	41	279	320	0	100	754	854
**Region 4B**	**291,730**	**16**	**613**	**14,895**	**15,524**	**15**	**1,106**	**15,459**	**16,580**
Marinduque	16,436	0	1	280	281	0	6	188	194
Occidental Mindoro	48,902	6	129	3,672	3,807	5	182	3,172	3,359
Oriental Mindoro	68,962	6	131	4,272	4,409	1	143	2,377	2,521
Palawan	100,239	2	287	4,274	4,563	3	563	6,489	7,055

Table 2.A.2 - Modern Methods of Family Planning
Other Acceptors
Philippines, 2024

Area	Total Current Users	PILLS-COC			Total	INJECTABLES			Total
		Age group				Age group			
		10-14	15-19	20-49		10-14	15-19	20-49	
Romblon	22,467	0	21	627	648	0	37	519	556
City of Puerto Princesa	34,724	2	44	1,770	1,816	6	175	2,714	2,895
**Region 5**	**491,006**	**5**	**624**	**23,767**	**24,396**	**10**	**851**	**16,752**	**17,613**
Albay	115,867	0	55	3,463	3,518	1	68	2,183	2,252
Camarines Norte	46,839	0	59	2,817	2,876	0	140	2,495	2,635
Camarines Sur	107,464	3	106	6,338	6,447	4	207	5,598	5,809
Catanduanes	24,326	0	18	1,171	1,189	0	95	1,575	1,670
Masbate	96,672	1	315	6,590	6,906	0	112	946	1,058
Sorsogon	66,956	1	61	2,978	3,040	5	217	3,712	3,934
City of Naga	32,882	0	10	410	420	0	12	243	255
**Region 6**	**667,635**	**22**	**1,264**	**27,426**	**28,712**	**12**	**1,044**	**18,610**	**19,666**
Aklan	48,553	0	58	1,593	1,651	0	69	1,495	1,564
Antique	49,316	1	44	1,444	1,489	0	81	1,995	2,076
Capiz	77,942	0	27	1,262	1,289	0	27	630	657
Guimaras	17,943	0	27	1,382	1,409	0	20	423	443
Iloilo	182,130	12	473	4,823	5,308	7	232	2,675	2,914
Negros Occidental	206,889	9	551	13,990	14,550	5	525	9,389	9,919
City of Bacolod	30,421	0	11	953	964	0	36	1,298	1,334
City of Iloilo	54,441	0	73	1,979	2,052	0	54	705	759
**Region 7**	**483,438**	**4**	**541**	**18,541**	**19,086**	**8**	**915**	**18,839**	**19,762**
Bohol	83,324	0	85	2,559	2,644	0	155	2,642	2,797
Cebu	191,250	2	161	5,847	6,010	2	263	6,723	6,988
Negros Oriental	98,214	2	209	5,963	6,174	4	305	5,863	6,172
Siquijor	9,225	0	4	472	476	0	15	389	404
City of Cebu	34,897	0	59	2,840	2,899	1	112	2,041	2,154
City of Lapu-Lapu	36,794	0	21	601	622	0	52	769	821
City of Mandaue	29,734	0	2	259	261	1	13	412	426
**Region 8**	**340,613**	**14**	**1,001**	**11,697**	**12,712**	**9**	**692**	**9,892**	**10,593**
Biliran	12,714	0	26	433	459	0	22	465	487
Eastern Samar	39,982	0	62	1,464	1,526	0	72	1,292	1,364
Leyte	125,631	0	108	2,207	2,315	0	92	2,053	2,145
Northern Samar	48,697	8	56	1,672	1,736	2	116	1,699	1,817
Southern Leyte	27,251	0	51	1,359	1,410	0	63	975	1,038
Samar	42,957	6	534	2,273	2,813	7	134	1,826	1,967
Ormoc City	20,171	0	104	1,832	1,936	0	153	1,352	1,505
City of Tacloban	23,210	0	60	457	517	0	40	230	270
**Region 9**	**354,267**	**3**	**644**	**13,976**	**14,623**	**3**	**829**	**14,952**	**15,784**
Zamboanga del Norte	126,112	2	213	4,055	4,270	2	281	3,849	4,132
Zamboanga del Sur	102,687	0	251	5,543	5,794	1	260	4,774	5,035
Zamboanga Sibugay	47,839	1	91	2,230	2,322	0	156	3,381	3,537
City of Isabela	7,167	0	10	313	323	0	23	421	444
City of Zamboanga	70,462	0	79	1,835	1,914	0	109	2,527	2,636
**Region 10**	**511,701**	**14**	**1,448**	**26,130**	**27,592**	**22**	**1,217**	**14,178**	**15,417**
Bukidnon	167,011	5	843	12,519	13,367	8	779	6,936	7,723
Camiguin	6,884	0	4	133	137	0	4	79	83
Lanao del Norte	73,898	0	122	1,722	1,844	1	54	1,194	1,249
Misamis Occidental	60,887	1	107	2,453	2,561	0	54	1,076	1,130
Misamis Oriental	111,011	7	239	2,714	2,960	13	177	2,064	2,254
City of Cagayan De Oro	57,799	0	84	4,569	4,653	0	97	1,654	1,751
City of Iligan	34,211	1	49	2,020	2,070	0	52	1,175	1,227
**Region 11**	**535,532**	**4**	**853**	**17,845**	**18,702**	**7**	**1,067**	**13,085**	**14,159**
Davao de Oro	84,560	0	91	3,587	3,678	0	85	1,810	1,895
Davao del Norte	112,914	1	172	3,772	3,945	0	194	2,734	2,928
Davao Oriental	59,172	2	179	2,964	3,145	0	182	1,604	1,786
Davao del Sur	67,054	0	94	1,794	1,888	1	111	1,484	1,596
Davao Occidental	33,334	1	125	1,948	2,074	5	232	2,126	2,363
City of Davao	178,498	0	192	3,780	3,972	1	263	3,327	3,591
**Region 12**	**476,219**	**9**	**1,111**	**20,464**	**21,584**	**15**	**1,246**	**15,153**	**16,414**
Cotabato	136,508	3	259	6,933	7,195	8	309	4,132	4,449
Sarangani	76,390	0	167	2,991	3,158	0	277	2,454	2,731
South Cotabato	102,265	2	175	4,634	4,811	2	246	3,203	3,451

Table 2.A.2 - Modern Methods of Family Planning
Other Acceptors
Philippines, 2024

Area	Total Current Users	PILLS-COC			Total	INJECTABLES			Total
		Age group				Age group			
		10-14	15-19	20-49		10-14	15-19	20-49	
Sultan Kudarat	104,518	4	417	4,335	4,756	5	277	2,705	2,987
City of General Santos	56,538	0	93	1,571	1,664	0	137	2,659	2,796
**Caraga**	**292,511**	**5**	**723**	**15,586**	**16,314**	**22**	**949**	**10,546**	**11,517**
Agusan del Norte	40,148	1	101	2,119	2,221	5	124	1,517	1,646
Agusan del Sur	86,721	2	203	4,029	4,234	2	207	2,762	2,971
Surigao del Norte	53,159	0	144	3,362	3,506	0	332	2,152	2,484
Surigao del Sur	60,298	2	186	3,085	3,273	14	179	2,096	2,289
Dinagat Islands	9,606	0	8	584	592	0	28	440	468
City of Butuan	42,579	0	81	2,407	2,488	1	79	1,579	1,659
**BARMM**	**296,590**	**2**	**310**	**9,505**	**9,817**	**2**	**1,130**	**22,251**	**23,383**
Basilan	19,309	0	41	754	795	1	124	1,466	1,591
Lanao del Sur	39,800	0	25	1,040	1,065	0	74	2,373	2,447
Maguindanao del Norte	36,321	1	71	664	736	0	211	2,541	2,752
Maguindanao del Sur	51,529	0	27	810	837	1	287	4,677	4,965
Sulu	80,817	1	114	4,726	4,841	0	320	8,588	8,908
Tawi-Tawi	19,107	0	11	1,067	1,078	0	32	1,205	1,237
SGA	7,550	0	9	164	173	0	40	938	978
City of Cotabato	42,157	0	12	280	292	0	42	463	505

Table 2.A.2 - Modern Methods of Family Planning
Other Acceptors
Philippines, 2024

Area	Total Current Users	IMPLANTS Age group 10-14	15-19	20-49	Total	NFP-CCM Age group 10-14	15-19	20-49	Total
**PHILIPPINES**	**8,664,035**	**331**	**12,927**	**162,136**	**175,394**	**15**	**212**	**6,426**	**6,653**
**N C R**	**1,041,531**	**23**	**1,880**	**29,044**	**30,947**	**1**	**0**	**66**	**67**
City of Malabon	26,264	3	126	1,856	1,985	0	0	0	0
City of Navotas	23,816	1	193	706	900	0	0	0	0
City of Valenzuela	40,454	0	103	1,997	2,100	0	0	2	2
City of Caloocan	73,676	5	243	4,113	4,361	0	0	0	0
City of Marikina	24,847	1	17	497	515	0	0	0	0
City of Pasig	58,937	1	35	1,277	1,313	0	0	0	0
Pateros	4,204	0	0	30	30	0	0	0	0
City of Taguig	92,053	1	13	794	808	0	0	0	0
Quezon City	338,677	3	371	9,255	9,629	0	0	0	0
City of Makati	28,572	0	3	145	148	0	0	0	0
City of Mandaluyong	27,196	0	17	430	447	0	0	50	50
City of San Juan	6,139	0	1	37	38	0	0	0	0
City of Manila	138,102	5	498	4,887	5,390	0	0	9	9
City of Las Piñas	26,980	3	186	1,834	2,023	0	0	0	0
City of Muntinlupa	44,186	0	0	141	141	0	0	0	0
City of Parañaque	47,100	0	35	400	435	0	0	0	0
Pasay City	40,328	0	39	645	684	1	0	5	6
**C A R**	**165,663**	**17**	**318**	**3,510**	**3,845**	**1**	**29**	**646**	**676**
Abra	19,755	0	24	319	343	0	0	2	2
Apayao	24,752	9	54	333	396	0	0	0	0
Benguet	37,253	2	45	727	774	0	0	12	12
Ifugao	21,120	2	36	455	493	0	1	128	129
Kalinga	18,267	0	35	539	574	1	28	483	512
Mountain Province	13,957	4	103	523	630	0	0	21	21
City of Baguio	30,559	0	21	614	635	0	0	0	0
**Region 1**	**498,204**	**3**	**253**	**3,213**	**3,469**	**0**	**18**	**1,062**	**1,080**
Ilocos Norte	52,824	0	35	270	305	0	12	843	855
Ilocos Sur	78,125	0	3	147	150	0	6	157	163
La Union	67,784	3	118	717	838	0	0	62	62
Pangasinan	292,353	0	97	2,065	2,162	0	0	0	0
City of Dagupan	7,118	0	0	14	14	0	0	0	0
**Region 2**	**343,378**	**8**	**492**	**4,693**	**5,193**	**0**	**0**	**89**	**89**
Batanes	955	0	1	19	20	0	0	18	18
Cagayan	108,930	0	78	1,334	1,412	0	0	7	7
Isabela	146,131	7	277	2,202	2,486	0	0	1	1
Nueva Vizcaya	52,095	0	75	724	799	0	0	60	60
Quirino	21,240	1	45	283	329	0	0	3	3
City of Santiago	14,027	0	16	131	147	0	0	0	0
**Region 3**	**985,325**	**139**	**1,334**	**10,525**	**11,998**	**0**	**0**	**42**	**42**
Aurora	24,562	1	44	399	444	0	0	4	4
Bataan	65,716	0	86	1,236	1,322	0	0	3	3
Bulacan	302,904	0	137	2,978	3,115	0	0	3	3
Nueva Ecija	200,897	5	216	2,170	2,391	0	0	0	0
Pampanga	167,294	132	739	1,817	2,688	0	0	32	32
Tarlac	118,444	1	54	1,050	1,105	0	0	0	0
Zambales	54,326	0	44	514	558	0	0	0	0
City of Angeles	30,345	0	9	265	274	0	0	0	0
City of Olongapo	20,837	0	5	96	101	0	0	0	0
**Region 4A**	**888,692**	**21**	**877**	**14,322**	**15,220**	**0**	**0**	**89**	**89**
Batangas	158,569	1	80	1,439	1,520	0	0	0	0
Cavite	175,332	4	207	3,318	3,529	0	0	0	0
Laguna	286,016	4	216	3,002	3,222	0	0	28	28
Quezon	106,773	5	144	2,999	3,148	0	0	60	60
Rizal	156,554	7	215	3,470	3,692	0	0	1	1
City of Lucena	5,448	0	15	94	109	0	0	0	0
**Region 4B**	**291,730**	**14**	**643**	**8,225**	**8,882**	**12**	**25**	**474**	**511**
Marinduque	16,436	0	6	211	217	0	0	2	2
Occidental Mindoro	48,902	4	222	2,345	2,571	0	22	298	320
Oriental Mindoro	68,962	2	75	1,172	1,249	0	0	33	33
Palawan	100,239	7	258	3,539	3,804	12	1	45	58

Table 2.A.2 - Modern Methods of Family Planning
Other Acceptors
Philippines, 2024

Area	Total Current Users	IMPLANTS			Total	NFP-CCM			Total
		Age group				Age group			
		10-14	15-19	20-49		10-14	15-19	20-49	
Romblon	22,467	0	32	249	281	0	2	96	98
City of Puerto Princesa	34,724	1	50	709	760	0	0	0	0
**Region 5**	**491,006**	**5**	**478**	**8,626**	**9,109**	**0**	**91**	**2,544**	**2,635**
Albay	115,867	0	49	1,066	1,115	0	3	157	160
Camarines Norte	46,839	0	27	423	450	0	1	57	58
Camarines Sur	107,464	3	180	3,482	3,665	0	0	11	11
Catanduanes	24,326	0	97	1,052	1,149	0	15	523	538
Masbate	96,672	1	41	1,192	1,234	0	30	1,135	1,165
Sorsogon	66,956	1	77	1,245	1,323	0	42	650	692
City of Naga	32,882	0	7	166	173	0	0	11	11
**Region 6**	**667,635**	**7**	**428**	**5,698**	**6,133**	**0**	**3**	**306**	**309**
Aklan	48,553	0	20	244	264	0	0	1	1
Antique	49,316	0	38	649	687	0	3	258	261
Capiz	77,942	0	3	216	219	0	0	21	21
Guimaras	17,943	0	12	319	331	0	0	0	0
Iloilo	182,130	5	98	1,064	1,167	0	0	3	3
Negros Occidental	206,889	2	191	2,217	2,410	0	0	17	17
City of Bacolod	30,421	0	39	584	623	0	0	0	0
City of Iloilo	54,441	0	27	405	432	0	0	6	6
**Region 7**	**483,438**	**8**	**675**	**12,483**	**13,166**	**0**	**3**	**91**	**94**
Bohol	83,324	2	187	2,202	2,391	0	0	0	0
Cebu	191,250	1	247	5,746	5,994	0	0	4	4
Negros Oriental	98,214	2	116	1,724	1,842	0	3	87	90
Siquijor	9,225	0	10	336	346	0	0	0	0
City of Cebu	34,897	3	81	1,336	1,420	0	0	0	0
City of Lapu-Lapu	36,794	0	20	536	556	0	0	0	0
City of Mandaue	29,734	0	14	603	617	0	0	0	0
**Region 8**	**340,613**	**5**	**463**	**5,958**	**6,426**	**0**	**2**	**25**	**27**
Biliran	12,714	1	53	446	500	0	2	4	6
Eastern Samar	39,982	2	60	869	931	0	0	6	6
Leyte	125,631	0	77	870	947	0	0	0	0
Northern Samar	48,697	2	40	887	929	0	0	8	8
Southern Leyte	27,251	0	46	645	691	0	0	6	6
Samar	42,957	0	43	1,078	1,121	0	0	1	1
Ormoc City	20,171	0	111	861	972	0	0	0	0
City of Tacloban	23,210	0	33	302	335	0	0	0	0
**Region 9**	**354,267**	**6**	**893**	**12,146**	**13,045**	**0**	**0**	**15**	**15**
Zamboanga del Norte	126,112	4	336	3,301	3,641	0	0	15	15
Zamboanga del Sur	102,687	0	334	4,484	4,818	0	0	0	0
Zamboanga Sibugay	47,839	1	129	2,390	2,520	0	0	0	0
City of Isabela	7,167	0	14	372	386	0	0	0	0
City of Zamboanga	70,462	1	80	1,599	1,680	0	0	0	0
**Region 10**	**511,701**	**18**	**1,018**	**9,613**	**10,649**	**0**	**24**	**475**	**499**
Bukidnon	167,011	6	648	4,944	5,598	0	14	236	250
Camiguin	6,884	0	1	73	74	0	0	0	0
Lanao del Norte	73,898	0	36	746	782	0	0	80	80
Misamis Occidental	60,887	0	38	622	660	0	0	0	0
Misamis Oriental	111,011	12	224	1,799	2,035	0	10	156	166
City of Cagayan De Oro	57,799	0	35	645	680	0	0	1	1
City of Iligan	34,211	0	36	784	820	0	0	2	2
**Region 11**	**535,532**	**10**	**589**	**6,522**	**7,121**	**0**	**0**	**35**	**35**
Davao de Oro	84,560	0	47	943	990	0	0	12	12
Davao del Norte	112,914	1	119	1,678	1,798	0	0	3	3
Davao Oriental	59,172	4	97	1,044	1,145	0	0	5	5
Davao del Sur	67,054	5	70	735	810	0	0	9	9
Davao Occidental	33,334	0	96	884	980	0	0	5	5
City of Davao	178,498	0	160	1,238	1,398	0	0	1	1
**Region 12**	**476,219**	**15**	**1,370**	**13,122**	**14,507**	**1**	**13**	**187**	**201**
Cotabato	136,508	1	321	3,584	3,906	0	0	96	96
Sarangani	76,390	0	244	2,297	2,541	0	1	68	69
South Cotabato	102,265	2	335	2,618	2,955	1	0	5	6

Table 2.A.2 - Modern Methods of Family Planning
Other Acceptors
Philippines, 2024

Area	Total Current Users	IMPLANTS			Total	NFP-CCM			Total
		Age group				Age group			
		10-14	15-19	20-49		10-14	15-19	20-49	
Sultan Kudarat	104,518	8	376	2,897	3,281	0	12	18	30
City of General Santos	56,538	4	94	1,726	1,824	0	0	0	0
**Caraga**	**292,511**	**14**	**766**	**8,498**	**9,278**	**0**	**4**	**279**	**283**
Agusan del Norte	40,148	7	166	1,415	1,588	0	0	0	0
Agusan del Sur	86,721	2	184	2,257	2,443	0	3	138	141
Surigao del Norte	53,159	1	172	1,426	1,599	0	1	22	23
Surigao del Sur	60,298	3	144	2,226	2,373	0	0	119	119
Dinagat Islands	9,606	0	27	382	409	0	0	0	0
City of Butuan	42,579	1	73	792	866	0	0	0	0
**BARMM**	**296,590**	**18**	**450**	**5,938**	**6,406**	**0**	**0**	**1**	**1**
Basilan	19,309	3	82	776	861	0	0	1	1
Lanao del Sur	39,800	0	24	615	639	0	0	0	0
Maguindanao del Norte	36,321	7	96	965	1,068	0	0	0	0
Maguindanao del Sur	51,529	2	67	947	1,016	0	0	0	0
Sulu	80,817	4	58	1,072	1,134	0	0	0	0
Tawi-Tawi	19,107	2	73	1,195	1,270	0	0	0	0
SGA	7,550	0	6	57	63	0	0	0	0
City of Cotabato	42,157	0	44	311	355	0	0	0	0

Table 2.A.2 - Modern Methods of Family Planning
Other Acceptors
Philippines, 2024

Area	Total Current Users	NFP-BBT			Total	NFP-STM			Total
		Age group				Age group			
		10-14	15-19	20-49		10-14	15-19	20-49	
**PHILIPPINES**	**8,664,035**	**5**	**55**	**1,156**	**1,216**	**0**	**13**	**591**	**604**
**N C R**	**1,041,531**	**0**	**4**	**152**	**156**	**0**	**0**	**35**	**35**
City of Malabon	26,264	0	0	0	0	0	0	0	0
City of Navotas	23,816	0	0	0	0	0	0	0	0
City of Valenzuela	40,454	0	1	120	121	0	0	29	29
City of Caloocan	73,676	0	0	0	0	0	0	0	0
City of Marikina	24,847	0	0	0	0	0	0	0	0
City of Pasig	58,937	0	0	0	0	0	0	0	0
Pateros	4,204	0	0	0	0	0	0	0	0
City of Taguig	92,053	0	0	0	0	0	0	0	0
Quezon City	338,677	0	0	2	2	0	0	6	6
City of Makati	28,572	0	0	0	0	0	0	0	0
City of Mandaluyong	27,196	0	0	0	0	0	0	0	0
City of San Juan	6,139	0	0	0	0	0	0	0	0
City of Manila	138,102	0	0	0	0	0	0	0	0
City of Las Piñas	26,980	0	0	0	0	0	0	0	0
City of Muntinlupa	44,186	0	0	0	0	0	0	0	0
City of Parañaque	47,100	0	3	30	33	0	0	0	0
Pasay City	40,328	0	0	0	0	0	0	0	0
**C A R**	**165,663**	**0**	**2**	**88**	**90**	**0**	**0**	**3**	**3**
Abra	19,755	0	0	0	0	0	0	0	0
Apayao	24,752	0	0	0	0	0	0	0	0
Benguet	37,253	0	0	0	0	0	0	0	0
Ifugao	21,120	0	0	4	4	0	0	2	2
Kalinga	18,267	0	2	83	85	0	0	0	0
Mountain Province	13,957	0	0	1	1	0	0	0	0
City of Baguio	30,559	0	0	0	0	0	0	1	1
**Region 1**	**498,204**	**0**	**0**	**109**	**109**	**0**	**0**	**128**	**128**
Ilocos Norte	52,824	0	0	0	0	0	0	0	0
Ilocos Sur	78,125	0	0	98	98	0	0	37	37
La Union	67,784	0	0	0	0	0	0	10	10
Pangasinan	292,353	0	0	11	11	0	0	81	81
City of Dagupan	7,118	0	0	0	0	0	0	0	0
**Region 2**	**343,378**	**0**	**0**	**67**	**67**	**0**	**3**	**19**	**22**
Batanes	955	0	0	0	0	0	0	0	0
Cagayan	108,930	0	0	4	4	0	0	0	0
Isabela	146,131	0	0	0	0	0	0	0	0
Nueva Vizcaya	52,095	0	0	63	63	0	3	19	22
Quirino	21,240	0	0	0	0	0	0	0	0
City of Santiago	14,027	0	0	0	0	0	0	0	0
**Region 3**	**985,325**	**0**	**2**	**11**	**13**	**0**	**3**	**10**	**13**
Aurora	24,562	0	0	4	4	0	0	5	5
Bataan	65,716	0	2	5	7	0	0	0	0
Bulacan	302,904	0	0	0	0	0	0	0	0
Nueva Ecija	200,897	0	0	0	0	0	0	0	0
Pampanga	167,294	0	0	2	2	0	1	1	2
Tarlac	118,444	0	0	0	0	0	2	4	6
Zambales	54,326	0	0	0	0	0	0	0	0
City of Angeles	30,345	0	0	0	0	0	0	0	0
City of Olongapo	20,837	0	0	0	0	0	0	0	0
**Region 4A**	**888,692**	**3**	**15**	**45**	**63**	**0**	**0**	**32**	**32**
Batangas	158,569	0	0	3	3	0	0	0	0
Cavite	175,332	0	14	11	25	0	0	0	0
Laguna	286,016	0	0	14	14	0	0	20	20
Quezon	106,773	0	1	11	12	0	0	12	12
Rizal	156,554	0	0	6	6	0	0	0	0
City of Lucena	5,448	3	0	0	3	0	0	0	0
**Region 4B**	**291,730**	**0**	**7**	**92**	**99**	**0**	**4**	**62**	**66**
Marinduque	16,436	0	0	0	0	0	0	0	0
Occidental Mindoro	48,902	0	1	79	80	0	0	23	23
Oriental Mindoro	68,962	0	6	2	8	0	0	27	27
Palawan	100,239	0	0	4	4	0	4	12	16

Table 2.A.2 - Modern Methods of Family Planning
Other Acceptors
Philippines, 2024

Area	Total Current Users	NFP-BBT			Total	NFP-STM			Total
		Age group				Age group			
		10-14	15-19	20-49		10-14	15-19	20-49	
Romblon	22,467	0	0	4	4	0	0	0	0
City of Puerto Princesa	34,724	0	0	3	3	0	0	0	0
**Region 5**	**491,006**	**0**	**3**	**105**	**108**	**0**	**2**	**86**	**88**
Albay	115,867	0	0	25	25	0	0	10	10
Camarines Norte	46,839	0	0	2	2	0	0	13	13
Camarines Sur	107,464	0	2	63	65	0	0	26	26
Catanduanes	24,326	0	0	10	10	0	0	19	19
Masbate	96,672	0	1	1	2	0	2	12	14
Sorsogon	66,956	0	0	4	4	0	0	5	5
City of Naga	32,882	0	0	0	0	0	0	1	1
**Region 6**	**667,635**	**0**	**0**	**5**	**5**	**0**	**0**	**24**	**24**
Aklan	48,553	0	0	0	0	0	0	0	0
Antique	49,316	0	0	1	1	0	0	0	0
Capiz	77,942	0	0	1	1	0	0	0	0
Guimaras	17,943	0	0	0	0	0	0	0	0
Iloilo	182,130	0	0	0	0	0	0	18	18
Negros Occidental	206,889	0	0	3	3	0	0	6	6
City of Bacolod	30,421	0	0	0	0	0	0	0	0
City of Iloilo	54,441	0	0	0	0	0	0	0	0
**Region 7**	**483,438**	**0**	**4**	**48**	**52**	**0**	**0**	**0**	**0**
Bohol	83,324	0	0	0	0	0	0	0	0
Cebu	191,250	0	0	0	0	0	0	0	0
Negros Oriental	98,214	0	4	48	52	0	0	0	0
Siquijor	9,225	0	0	0	0	0	0	0	0
City of Cebu	34,897	0	0	0	0	0	0	0	0
City of Lapu-Lapu	36,794	0	0	0	0	0	0	0	0
City of Mandaue	29,734	0	0	0	0	0	0	0	0
**Region 8**	**340,613**	**0**	**5**	**53**	**58**	**0**	**0**	**36**	**36**
Biliran	12,714	0	0	0	0	0	0	0	0
Eastern Samar	39,982	0	0	2	2	0	0	0	0
Leyte	125,631	0	1	29	30	0	0	32	32
Northern Samar	48,697	0	2	11	13	0	0	4	4
Southern Leyte	27,251	0	0	0	0	0	0	0	0
Samar	42,957	0	0	9	9	0	0	0	0
Ormoc City	20,171	0	2	2	4	0	0	0	0
City of Tacloban	23,210	0	0	0	0	0	0	0	0
**Region 9**	**354,267**	**0**	**0**	**8**	**8**	**0**	**0**	**0**	**0**
Zamboanga del Norte	126,112	0	0	7	7	0	0	0	0
Zamboanga del Sur	102,687	0	0	1	1	0	0	0	0
Zamboanga Sibugay	47,839	0	0	0	0	0	0	0	0
City of Isabela	7,167	0	0	0	0	0	0	0	0
City of Zamboanga	70,462	0	0	0	0	0	0	0	0
**Region 10**	**511,701**	**2**	**8**	**193**	**203**	**0**	**1**	**106**	**107**
Bukidnon	167,011	0	5	93	98	0	1	49	50
Camiguin	6,884	0	0	1	1	0	0	0	0
Lanao del Norte	73,898	0	2	49	51	0	0	43	43
Misamis Occidental	60,887	0	0	20	20	0	0	5	5
Misamis Oriental	111,011	2	1	30	33	0	0	9	9
City of Cagayan De Oro	57,799	0	0	0	0	0	0	0	0
City of Iligan	34,211	0	0	0	0	0	0	0	0
**Region 11**	**535,532**	**0**	**0**	**38**	**38**	**0**	**0**	**15**	**15**
Davao de Oro	84,560	0	0	1	1	0	0	0	0
Davao del Norte	112,914	0	0	13	13	0	0	1	1
Davao Oriental	59,172	0	0	13	13	0	0	4	4
Davao del Sur	67,054	0	0	0	0	0	0	0	0
Davao Occidental	33,334	0	0	11	11	0	0	9	9
City of Davao	178,498	0	0	0	0	0	0	1	1
**Region 12**	**476,219**	**0**	**3**	**83**	**86**	**0**	**0**	**20**	**20**
Cotabato	136,508	0	3	53	56	0	0	18	18
Sarangani	76,390	0	0	0	0	0	0	0	0
South Cotabato	102,265	0	0	6	6	0	0	2	2

Table 2.A.2 - Modern Methods of Family Planning
Other Acceptors
Philippines, 2024

Area	Total Current Users	NFP-BBT			Total	NFP-STM			Total
		Age group				Age group			
		10-14	15-19	20-49		10-14	15-19	20-49	
Sultan Kudarat	104,518	0	0	12	12	0	0	0	0
City of General Santos	56,538	0	0	12	12	0	0	0	0
**Caraga**	**292,511**	**0**	**2**	**56**	**58**	**0**	**0**	**15**	**15**
Agusan del Norte	40,148	0	1	10	11	0	0	0	0
Agusan del Sur	86,721	0	0	32	32	0	0	0	0
Surigao del Norte	53,159	0	0	9	9	0	0	15	15
Surigao del Sur	60,298	0	0	5	5	0	0	0	0
Dinagat Islands	9,606	0	0	0	0	0	0	0	0
City of Butuan	42,579	0	1	0	1	0	0	0	0
**BARMM**	**296,590**	**0**	**0**	**3**	**3**	**0**	**0**	**0**	**0**
Basilan	19,309	0	0	0	0	0	0	0	0
Lanao del Sur	39,800	0	0	3	3	0	0	0	0
Maguindanao del Norte	36,321	0	0	0	0	0	0	0	0
Maguindanao del Sur	51,529	0	0	0	0	0	0	0	0
Sulu	80,817	0	0	0	0	0	0	0	0
Tawi-Tawi	19,107	0	0	0	0	0	0	0	0
SGA	7,550	0	0	0	0	0	0	0	0
City of Cotabato	42,157	0	0	0	0	0	0	0	0

Table 2.A.2 - Modern Methods of Family Planning
Other Acceptors
Philippines, 2024

Area	Total Current Users	NFP-SDM Age group 10-14	15-19	20-49	Total	NFP-LAM Age group 10-14	15-19	20-49	Total
**PHILIPPINES**	**8,664,035**	**11**	**1,032**	**22,984**	**24,027**	**255**	**16,039**	**330,077**	**346,371**
**N C R**	**1,041,531**	**7**	**35**	**1,483**	**1,525**	**9**	**1,228**	**36,668**	**37,905**
City of Malabon	26,264	0	0	1	1	5	34	943	982
City of Navotas	23,816	0	0	0	0	1	83	406	490
City of Valenzuela	40,454	0	33	1,365	1,398	0	177	2,408	2,585
City of Caloocan	73,676	0	0	3	3	0	134	3,565	3,699
City of Marikina	24,847	0	0	1	1	0	1	130	131
City of Pasig	58,937	0	0	0	0	0	6	260	266
Pateros	4,204	0	0	0	0	0	0	85	85
City of Taguig	92,053	0	0	0	0	1	32	490	523
Quezon City	338,677	0	1	43	44	2	408	24,697	25,107
City of Makati	28,572	0	0	0	0	0	1	44	45
City of Mandaluyong	27,196	0	0	37	37	0	9	294	303
City of San Juan	6,139	0	0	0	0	0	0	45	45
City of Manila	138,102	4	1	1	6	0	21	471	492
City of Las Piñas	26,980	0	0	0	0	0	1	19	20
City of Muntinlupa	44,186	0	0	0	0	0	14	148	162
City of Parañaque	47,100	0	0	0	0	0	69	308	377
Pasay City	40,328	3	0	32	35	0	238	2,355	2,593
**C A R**	**165,663**	**0**	**30**	**2,310**	**2,340**	**6**	**302**	**8,877**	**9,185**
Abra	19,755	0	5	64	69	1	64	1,399	1,464
Apayao	24,752	0	0	1	1	0	32	645	677
Benguet	37,253	0	7	1,213	1,220	0	56	1,934	1,990
Ifugao	21,120	0	17	835	852	4	58	1,500	1,562
Kalinga	18,267	0	0	45	45	0	41	2,031	2,072
Mountain Province	13,957	0	1	149	150	1	50	1,105	1,156
City of Baguio	30,559	0	0	3	3	0	1	263	264
**Region 1**	**498,204**	**0**	**32**	**842**	**874**	**10**	**799**	**18,024**	**18,833**
Ilocos Norte	52,824	0	0	38	38	1	91	1,111	1,203
Ilocos Sur	78,125	0	2	165	167	0	36	1,284	1,320
La Union	67,784	0	27	519	546	3	246	3,789	4,038
Pangasinan	292,353	0	3	120	123	6	426	11,815	12,247
City of Dagupan	7,118	0	0	0	0	0	0	25	25
**Region 2**	**343,378**	**0**	**5**	**281**	**286**	**26**	**846**	**13,314**	**14,186**
Batanes	955	0	0	1	1	0	1	120	121
Cagayan	108,930	0	0	0	0	1	153	3,072	3,226
Isabela	146,131	0	3	27	30	13	310	4,245	4,568
Nueva Vizcaya	52,095	0	2	253	255	10	303	3,311	3,624
Quirino	21,240	0	0	0	0	2	46	706	754
City of Santiago	14,027	0	0	0	0	0	33	1,860	1,893
**Region 3**	**985,325**	**0**	**6**	**547**	**553**	**15**	**1,033**	**18,297**	**19,345**
Aurora	24,562	0	0	2	2	0	96	1,824	1,920
Bataan	65,716	0	0	1	1	1	44	1,395	1,440
Bulacan	302,904	0	5	428	433	0	208	4,625	4,833
Nueva Ecija	200,897	0	0	1	1	8	236	4,149	4,393
Pampanga	167,294	0	1	105	106	4	120	1,408	1,532
Tarlac	118,444	0	0	6	6	1	251	3,173	3,425
Zambales	54,326	0	0	3	3	1	40	1,399	1,440
City of Angeles	30,345	0	0	0	0	0	31	101	132
City of Olongapo	20,837	0	0	1	1	0	7	223	230
**Region 4A**	**888,692**	**0**	**22**	**619**	**641**	**21**	**1,200**	**30,220**	**31,441**
Batangas	158,569	0	1	87	88	3	146	5,894	6,043
Cavite	175,332	0	0	5	5	4	251	6,192	6,447
Laguna	286,016	0	21	370	391	5	262	4,474	4,741
Quezon	106,773	0	0	156	156	5	247	5,571	5,823
Rizal	156,554	0	0	1	1	2	292	8,074	8,368
City of Lucena	5,448	0	0	0	0	2	2	15	19
**Region 4B**	**291,730**	**2**	**150**	**1,117**	**1,269**	**10**	**910**	**15,836**	**16,756**
Marinduque	16,436	0	0	2	2	0	5	263	268
Occidental Mindoro	48,902	2	5	354	361	3	248	5,637	5,888
Oriental Mindoro	68,962	0	143	287	430	1	297	4,112	4,410
Palawan	100,239	0	1	69	70	6	275	4,023	4,304

Table 2.A.2 - Modern Methods of Family Planning
Other Acceptors
Philippines, 2024

Area	Total Current Users	NFP-SDM			Total	NFP-LAM			Total
		Age group				Age group			
		10-14	15-19	20-49		10-14	15-19	20-49	
Romblon	22,467	0	1	335	336	0	64	1,135	1,199
City of Puerto Princesa	34,724	0	0	70	70	0	21	666	687
**Region 5**	**491,006**	**0**	**311**	**6,874**	**7,185**	**8**	**1,469**	**33,715**	**35,192**
Albay	115,867	0	4	683	687	1	131	3,084	3,216
Camarines Norte	46,839	0	0	99	99	0	299	3,207	3,506
Camarines Sur	107,464	0	9	420	429	4	327	9,559	9,890
Catanduanes	24,326	0	31	1,188	1,219	0	98	2,171	2,269
Masbate	96,672	0	256	3,295	3,551	1	409	8,236	8,646
Sorsogon	66,956	0	11	1,112	1,123	2	200	7,180	7,382
City of Naga	32,882	0	0	77	77	0	5	278	283
**Region 6**	**667,635**	**0**	**54**	**2,040**	**2,094**	**16**	**812**	**19,909**	**20,737**
Aklan	48,553	0	0	73	73	0	71	1,702	1,773
Antique	49,316	0	0	296	296	1	17	991	1,009
Capiz	77,942	0	0	12	12	0	49	928	977
Guimaras	17,943	0	0	5	5	1	28	1,181	1,210
Iloilo	182,130	0	18	595	613	5	184	4,009	4,198
Negros Occidental	206,889	0	36	1,013	1,049	9	420	10,519	10,948
City of Bacolod	30,421	0	0	0	0	0	29	478	507
City of Iloilo	54,441	0	0	46	46	0	14	101	115
**Region 7**	**483,438**	**0**	**4**	**213**	**217**	**3**	**777**	**22,090**	**22,870**
Bohol	83,324	0	0	14	14	1	91	2,824	2,916
Cebu	191,250	0	1	77	78	0	223	7,098	7,321
Negros Oriental	98,214	0	0	80	80	2	294	7,154	7,450
Siquijor	9,225	0	3	37	40	0	5	213	218
City of Cebu	34,897	0	0	5	5	0	76	2,861	2,937
City of Lapu-Lapu	36,794	0	0	0	0	0	87	1,853	1,940
City of Mandaue	29,734	0	0	0	0	0	1	87	88
**Region 8**	**340,613**	**0**	**62**	**588**	**650**	**12**	**593**	**11,374**	**11,979**
Biliran	12,714	0	0	20	20	0	34	660	694
Eastern Samar	39,982	0	1	303	304	0	119	1,419	1,538
Leyte	125,631	0	57	71	128	6	136	2,625	2,767
Northern Samar	48,697	0	0	3	3	5	93	2,120	2,218
Southern Leyte	27,251	0	0	61	61	0	64	1,407	1,471
Samar	42,957	0	0	0	0	0	17	974	991
Ormoc City	20,171	0	4	130	134	0	108	1,541	1,649
City of Tacloban	23,210	0	0	0	0	1	22	628	651
**Region 9**	**354,267**	**0**	**46**	**592**	**638**	**3**	**977**	**17,491**	**18,471**
Zamboanga del Norte	126,112	0	46	511	557	1	376	6,244	6,621
Zamboanga del Sur	102,687	0	0	2	2	1	191	3,612	3,804
Zamboanga Sibugay	47,839	0	0	79	79	1	102	2,195	2,298
City of Isabela	7,167	0	0	0	0	0	15	519	534
City of Zamboanga	70,462	0	0	0	0	0	293	4,921	5,214
**Region 10**	**511,701**	**2**	**201**	**2,993**	**3,196**	**44**	**1,747**	**24,248**	**26,039**
Bukidnon	167,011	0	42	1,406	1,448	34	1,009	10,413	11,456
Camiguin	6,884	0	0	0	0	0	19	628	647
Lanao del Norte	73,898	2	34	153	189	0	129	3,109	3,238
Misamis Occidental	60,887	0	110	1,067	1,177	2	157	2,967	3,126
Misamis Oriental	111,011	0	15	342	357	7	258	3,960	4,225
City of Cagayan De Oro	57,799	0	0	1	1	1	76	1,496	1,573
City of Iligan	34,211	0	0	24	24	0	99	1,675	1,774
**Region 11**	**535,532**	**0**	**33**	**502**	**535**	**17**	**578**	**9,109**	**9,704**
Davao de Oro	84,560	0	0	9	9	0	30	450	480
Davao del Norte	112,914	0	0	44	44	0	76	1,784	1,860
Davao Oriental	59,172	0	18	133	151	11	244	3,481	3,736
Davao del Sur	67,054	0	0	5	5	0	10	202	212
Davao Occidental	33,334	0	15	300	315	0	60	639	699
City of Davao	178,498	0	0	11	11	6	158	2,553	2,717
**Region 12**	**476,219**	**0**	**28**	**257**	**285**	**20**	**1,394**	**15,186**	**16,600**
Cotabato	136,508	0	0	42	42	3	142	2,294	2,439
Sarangani	76,390	0	16	114	130	0	376	4,053	4,429
South Cotabato	102,265	0	0	84	84	7	259	3,187	3,453

Table 2.A.2 - Modern Methods of Family Planning
Other Acceptors
Philippines, 2024

Area	Total Current Users	NFP-SDM			Total	NFP-LAM			Total
		Age group				Age group			
		10-14	15-19	20-49		10-14	15-19	20-49	
Sultan Kudarat	104,518	0	12	16	28	10	310	4,179	4,499
City of General Santos	56,538	0	0	1	1	0	307	1,473	1,780
**Caraga**	**292,511**	**0**	**13**	**1,659**	**1,672**	**34**	**710**	**13,287**	**14,031**
Agusan del Norte	40,148	0	3	106	109	0	89	1,693	1,782
Agusan del Sur	86,721	0	1	405	406	0	284	3,475	3,759
Surigao del Norte	53,159	0	7	857	864	0	32	771	803
Surigao del Sur	60,298	0	0	100	100	33	144	4,656	4,833
Dinagat Islands	9,606	0	2	189	191	0	16	271	287
City of Butuan	42,579	0	0	2	2	1	145	2,421	2,567
**BARMM**	**296,590**	**0**	**0**	**67**	**67**	**1**	**664**	**22,432**	**23,097**
Basilan	19,309	0	0	0	0	0	43	692	735
Lanao del Sur	39,800	0	0	57	57	1	139	7,329	7,469
Maguindanao del Norte	36,321	0	0	0	0	0	165	3,777	3,942
Maguindanao del Sur	51,529	0	0	0	0	0	165	4,857	5,022
Sulu	80,817	0	0	0	0	0	90	3,718	3,808
Tawi-Tawi	19,107	0	0	8	8	0	40	1,644	1,684
SGA	7,550	0	0	0	0	0	5	129	134
City of Cotabato	42,157	0	0	2	2	0	17	286	303

Table 2.A.3 - Modern Methods of Family Planning
Drop Outs
Philippines, 2024

Area	Total Current Users	FSTR/BTL Age group 10-14	FSTR/BTL Age group 15-19	FSTR/BTL Age group 20-49	Total	MSTR/NSV Age group 10-14	MSTR/NSV Age group 15-19	MSTR/NSV Age group 20-49	Total
**PHILIPPINES**	**8,664,035**	**5**	**214**	**78,407**	**78,626**	**0**	**30**	**1,873**	**1,903**
**N C R**	**1,041,531**	**0**	**12**	**7,640**	**7,652**	**0**	**1**	**142**	**143**
City of Malabon	26,264	0	0	667	667	0	1	3	4
City of Navotas	23,816	0	0	72	72	0	0	0	0
City of Valenzuela	40,454	0	0	1,856	1,856	0	0	110	110
City of Caloocan	73,676	0	1	413	414	0	0	2	2
City of Marikina	24,847	0	0	145	145	0	0	0	0
City of Pasig	58,937	0	1	119	120	0	0	11	11
Pateros	4,204	0	0	31	31	0	0	1	1
City of Taguig	92,053	0	0	58	58	0	0	1	1
Quezon City	338,677	0	1	2,495	2,496	0	0	7	7
City of Makati	28,572	0	0	7	7	0	0	0	0
City of Mandaluyong	27,196	0	9	336	345	0	0	1	1
City of San Juan	6,139	0	0	52	52	0	0	2	2
City of Manila	138,102	0	0	351	351	0	0	2	2
City of Las Piñas	26,980	0	0	110	110	0	0	0	0
City of Muntinlupa	44,186	0	0	8	8	0	0	0	0
City of Parañaque	47,100	0	0	36	36	0	0	0	0
Pasay City	40,328	0	0	884	884	0	0	2	2
**C A R**	**165,663**	**0**	**0**	**2,273**	**2,273**	**0**	**0**	**14**	**14**
Abra	19,755	0	0	214	214	0	0	0	0
Apayao	24,752	0	0	40	40	0	0	0	0
Benguet	37,253	0	0	1,135	1,135	0	0	2	2
Ifugao	21,120	0	0	231	231	0	0	5	5
Kalinga	18,267	0	0	62	62	0	0	1	1
Mountain Province	13,957	0	0	253	253	0	0	0	0
City of Baguio	30,559	0	0	338	338	0	0	6	6
**Region 1**	**498,204**	**0**	**12**	**5,384**	**5,396**	**0**	**0**	**13**	**13**
Ilocos Norte	52,824	0	8	1,747	1,755	0	0	3	3
Ilocos Sur	78,125	0	0	510	510	0	0	1	1
La Union	67,784	0	3	1,118	1,121	0	0	3	3
Pangasinan	292,353	0	1	1,837	1,838	0	0	5	5
City of Dagupan	7,118	0	0	172	172	0	0	1	1
**Region 2**	**343,378**	**0**	**6**	**3,501**	**3,507**	**0**	**0**	**22**	**22**
Batanes	955	0	0	19	19	0	0	0	0
Cagayan	108,930	0	0	1,116	1,116	0	0	5	5
Isabela	146,131	0	5	1,208	1,213	0	0	8	8
Nueva Vizcaya	52,095	0	1	956	957	0	0	9	9
Quirino	21,240	0	0	140	140	0	0	0	0
City of Santiago	14,027	0	0	62	62	0	0	0	0
**Region 3**	**985,325**	**5**	**8**	**11,593**	**11,606**	**0**	**2**	**35**	**37**
Aurora	24,562	0	0	295	295	0	0	1	1
Bataan	65,716	0	0	845	845	0	0	13	13
Bulacan	302,904	0	1	2,626	2,627	0	1	6	7
Nueva Ecija	200,897	5	3	2,079	2,087	0	0	0	0
Pampanga	167,294	0	4	2,514	2,518	0	0	0	0
Tarlac	118,444	0	0	2,418	2,418	0	1	4	5
Zambales	54,326	0	0	272	272	0	0	4	4
City of Angeles	30,345	0	0	512	512	0	0	7	7
City of Olongapo	20,837	0	0	32	32	0	0	0	0
**Region 4A**	**888,692**	**0**	**29**	**10,815**	**10,844**	**0**	**7**	**67**	**74**
Batangas	158,569	0	0	2,355	2,355	0	0	32	32
Cavite	175,332	0	16	2,941	2,957	0	0	2	2
Laguna	286,016	0	7	3,143	3,150	0	7	21	28
Quezon	106,773	0	1	1,669	1,670	0	0	9	9
Rizal	156,554	0	5	655	660	0	0	3	3
City of Lucena	5,448	0	0	52	52	0	0	0	0
**Region 4B**	**291,730**	**0**	**28**	**3,706**	**3,734**	**0**	**8**	**124**	**132**
Marinduque	16,436	0	0	99	99	0	0	12	12
Occidental Mindoro	48,902	0	0	728	728	0	0	1	1
Oriental Mindoro	68,962	0	21	1,713	1,734	0	0	72	72
Palawan	100,239	0	4	422	426	0	8	33	41

Table 2.A.3 - Modern Methods of Family Planning
Drop Outs
Philippines, 2024

Area	Total Current Users	FSTR/BTL Age group			Total	MSTR/NSV Age group			Total
		10-14	15-19	20-49		10-14	15-19	20-49	
Romblon	22,467	0	1	421	422	0	0	3	3
City of Puerto Princesa	34,724	0	2	323	325	0	0	3	3
**Region 5**	**491,006**	**0**	**7**	**3,135**	**3,142**	**0**	**0**	**69**	**69**
Albay	115,867	0	0	455	455	0	0	5	5
Camarines Norte	46,839	0	1	316	317	0	0	0	0
Camarines Sur	107,464	0	0	632	632	0	0	8	8
Catanduanes	24,326	0	4	341	345	0	0	25	25
Masbate	96,672	0	1	428	429	0	0	7	7
Sorsogon	66,956	0	1	538	539	0	0	6	6
City of Naga	32,882	0	0	425	425	0	0	18	18
**Region 6**	**667,635**	**0**	**9**	**6,793**	**6,802**	**0**	**5**	**678**	**683**
Aklan	48,553	0	2	593	595	0	0	0	0
Antique	49,316	0	1	438	439	0	0	9	9
Capiz	77,942	0	0	528	528	0	0	54	54
Guimaras	17,943	0	0	124	124	0	0	0	0
Iloilo	182,130	0	0	1,172	1,172	0	0	51	51
Negros Occidental	206,889	0	4	3,080	3,084	0	5	558	563
City of Bacolod	30,421	0	1	129	130	0	0	2	2
City of Iloilo	54,441	0	1	729	730	0	0	4	4
**Region 7**	**483,438**	**0**	**5**	**5,422**	**5,427**	**0**	**2**	**225**	**227**
Bohol	83,324	0	0	1,127	1,127	0	0	73	73
Cebu	191,250	0	3	2,134	2,137	0	1	80	81
Negros Oriental	98,214	0	0	207	207	0	0	16	16
Siquijor	9,225	0	0	142	142	0	0	0	0
City of Cebu	34,897	0	2	425	427	0	1	54	55
City of Lapu-Lapu	36,794	0	0	1,356	1,356	0	0	2	2
City of Mandaue	29,734	0	0	31	31	0	0	0	0
**Region 8**	**340,613**	**0**	**10**	**2,367**	**2,377**	**0**	**0**	**56**	**56**
Biliran	12,714	0	0	63	63	0	0	8	8
Eastern Samar	39,982	0	0	406	406	0	0	15	15
Leyte	125,631	0	0	1,071	1,071	0	0	19	19
Northern Samar	48,697	0	4	208	212	0	0	1	1
Southern Leyte	27,251	0	0	101	101	0	0	2	2
Samar	42,957	0	0	176	176	0	0	2	2
Ormoc City	20,171	0	6	121	127	0	0	9	9
City of Tacloban	23,210	0	0	221	221	0	0	0	0
**Region 9**	**354,267**	**0**	**0**	**919**	**919**	**0**	**0**	**5**	**5**
Zamboanga del Norte	126,112	0	0	456	456	0	0	2	2
Zamboanga del Sur	102,687	0	0	154	154	0	0	2	2
Zamboanga Sibugay	47,839	0	0	120	120	0	0	0	0
City of Isabela	7,167	0	0	45	45	0	0	1	1
City of Zamboanga	70,462	0	0	144	144	0	0	0	0
**Region 10**	**511,701**	**0**	**29**	**2,630**	**2,659**	**0**	**0**	**82**	**82**
Bukidnon	167,011	0	9	1,007	1,016	0	0	28	28
Camiguin	6,884	0	0	9	9	0	0	0	0
Lanao del Norte	73,898	0	0	177	177	0	0	2	2
Misamis Occidental	60,887	0	0	345	345	0	0	2	2
Misamis Oriental	111,011	0	10	619	629	0	0	45	45
City of Cagayan De Oro	57,799	0	10	219	229	0	0	0	0
City of Iligan	34,211	0	0	254	254	0	0	5	5
**Region 11**	**535,532**	**0**	**19**	**4,533**	**4,552**	**0**	**0**	**148**	**148**
Davao de Oro	84,560	0	0	897	897	0	0	24	24
Davao del Norte	112,914	0	9	834	843	0	0	20	20
Davao Oriental	59,172	0	4	548	552	0	0	25	25
Davao del Sur	67,054	0	6	450	456	0	0	21	21
Davao Occidental	33,334	0	0	415	415	0	0	14	14
City of Davao	178,498	0	0	1,389	1,389	0	0	44	44
**Region 12**	**476,219**	**0**	**21**	**4,632**	**4,653**	**0**	**5**	**158**	**163**
Cotabato	136,508	0	3	1,066	1,069	0	1	29	30
Sarangani	76,390	0	12	353	365	0	0	21	21
South Cotabato	102,265	0	0	1,097	1,097	0	3	57	60

Table 2.A.3 - Modern Methods of Family Planning
Drop Outs
Philippines, 2024

Area	Total Current Users	FSTR/BTL			Total	MSTR/NSV			Total
		Age group				Age group			
		10-14	15-19	20-49		10-14	15-19	20-49	
Sultan Kudarat	104,518	0	4	922	926	0	1	41	42
City of General Santos	56,538	0	2	1,194	1,196	0	0	10	10
**Caraga**	**292,511**	**0**	**19**	**2,156**	**2,175**	**0**	**0**	**30**	**30**
Agusan del Norte	40,148	0	0	334	334	0	0	4	4
Agusan del Sur	86,721	0	1	967	968	0	0	5	5
Surigao del Norte	53,159	0	6	399	405	0	0	5	5
Surigao del Sur	60,298	0	0	182	182	0	0	13	13
Dinagat Islands	9,606	0	11	34	45	0	0	1	1
City of Butuan	42,579	0	1	240	241	0	0	2	2
**BARMM**	**296,590**	**0**	**0**	**908**	**908**	**0**	**0**	**5**	**5**
Basilan	19,309	0	0	46	46	0	0	0	0
Lanao del Sur	39,800	0	0	167	167	0	0	0	0
Maguindanao del Norte	36,321	0	0	239	239	0	0	0	0
Maguindanao del Sur	51,529	0	0	232	232	0	0	1	1
Sulu	80,817	0	0	12	12	0	0	0	0
Tawi-Tawi	19,107	0	0	26	26	0	0	0	0
SGA	7,550	0	0	38	38	0	0	0	0
City of Cotabato	42,157	0	0	148	148	0	0	4	4

Table 2.A.3 - Modern Methods of Family Planning
Drop Outs
Philippines, 2024

Area	Total Current Users	CONDOM			Total	IUD-INTERVAL			Total
		Age group				Age group			
		10-14	15-19	20-49		10-14	15-19	20-49	
**PHILIPPINES**	**8,664,035**	**169**	**6,806**	**95,593**	**102,568**	**25**	**1,986**	**60,246**	**62,257**
**N C R**	**1,041,531**	**80**	**2,124**	**25,567**	**27,771**	**4**	**193**	**4,223**	**4,420**
City of Malabon	26,264	4	280	1,884	2,168	0	48	295	343
City of Navotas	23,816	0	401	1,253	1,654	1	25	91	117
City of Valenzuela	40,454	10	18	805	833	0	4	246	250
City of Caloocan	73,676	0	73	1,283	1,356	3	47	140	190
City of Marikina	24,847	0	2	71	73	0	1	47	48
City of Pasig	58,937	3	51	704	758	0	4	165	169
Pateros	4,204	0	0	34	34	0	0	15	15
City of Taguig	92,053	0	8	382	390	0	0	44	44
Quezon City	338,677	9	875	12,830	13,714	0	32	1,486	1,518
City of Makati	28,572	0	9	424	433	0	0	3	3
City of Mandaluyong	27,196	0	28	478	506	0	4	111	115
City of San Juan	6,139	0	0	16	16	0	0	90	90
City of Manila	138,102	52	146	2,742	2,940	0	7	205	212
City of Las Piñas	26,980	0	49	1,295	1,344	0	0	43	43
City of Muntinlupa	44,186	0	0	67	67	0	0	346	346
City of Parañaque	47,100	2	105	359	466	0	19	97	116
Pasay City	40,328	0	79	940	1,019	0	2	799	801
**C A R**	**165,663**	**1**	**29**	**1,512**	**1,542**	**0**	**8**	**476**	**484**
Abra	19,755	0	6	92	98	0	0	5	5
Apayao	24,752	0	6	52	58	0	1	22	23
Benguet	37,253	0	8	627	635	0	4	226	230
Ifugao	21,120	0	1	141	142	0	1	38	39
Kalinga	18,267	1	4	124	129	0	1	61	62
Mountain Province	13,957	0	3	110	113	0	1	54	55
City of Baguio	30,559	0	1	366	367	0	0	70	70
**Region 1**	**498,204**	**1**	**120**	**2,318**	**2,439**	**0**	**57**	**1,437**	**1,494**
Ilocos Norte	52,824	0	5	395	400	0	10	75	85
Ilocos Sur	78,125	0	12	260	272	0	1	70	71
La Union	67,784	0	21	224	245	0	10	180	190
Pangasinan	292,353	1	81	1,393	1,475	0	36	1,107	1,143
City of Dagupan	7,118	0	1	46	47	0	0	5	5
**Region 2**	**343,378**	**1**	**47**	**1,086**	**1,134**	**6**	**129**	**1,993**	**2,128**
Batanes	955	0	0	9	9	0	0	2	2
Cagayan	108,930	0	1	114	115	1	56	920	977
Isabela	146,131	1	24	453	478	4	52	580	636
Nueva Vizcaya	52,095	0	13	405	418	1	19	392	412
Quirino	21,240	0	9	96	105	0	0	75	75
City of Santiago	14,027	0	0	9	9	0	2	24	26
**Region 3**	**985,325**	**10**	**489**	**9,836**	**10,335**	**2**	**28**	**1,638**	**1,668**
Aurora	24,562	0	7	158	165	0	3	62	65
Bataan	65,716	6	27	1,065	1,098	1	4	44	49
Bulacan	302,904	0	133	2,862	2,995	1	5	573	579
Nueva Ecija	200,897	1	38	681	720	0	6	333	339
Pampanga	167,294	2	119	2,881	3,002	0	6	379	385
Tarlac	118,444	1	143	1,095	1,239	0	4	197	201
Zambales	54,326	0	20	813	833	0	0	33	33
City of Angeles	30,345	0	1	78	79	0	0	15	15
City of Olongapo	20,837	0	1	203	204	0	0	2	2
**Region 4A**	**888,692**	**5**	**1,464**	**12,978**	**14,447**	**3**	**104**	**4,506**	**4,613**
Batangas	158,569	1	11	1,492	1,504	0	9	496	505
Cavite	175,332	0	41	1,355	1,396	2	26	707	735
Laguna	286,016	3	1,355	8,613	9,971	1	53	1,177	1,231
Quezon	106,773	1	35	842	878	0	13	1,952	1,965
Rizal	156,554	0	20	573	593	0	3	160	163
City of Lucena	5,448	0	2	103	105	0	0	14	14
**Region 4B**	**291,730**	**3**	**88**	**1,664**	**1,755**	**0**	**38**	**1,769**	**1,807**
Marinduque	16,436	0	0	48	48	0	0	63	63
Occidental Mindoro	48,902	0	7	395	402	0	7	510	517
Oriental Mindoro	68,962	2	8	445	455	0	19	922	941
Palawan	100,239	0	46	385	431	0	5	169	174

Table 2.A.3 - Modern Methods of Family Planning
Drop Outs
Philippines, 2024

Area	Total Current Users	CONDOM			Total	IUD-INTERVAL			Total
		Age group				Age group			
		10-14	15-19	20-49		10-14	15-19	20-49	
Romblon	22,467	0	4	61	65	0	0	57	57
City of Puerto Princesa	34,724	1	23	330	354	0	7	48	55
**Region 5**	**491,006**	**14**	**195**	**6,735**	**6,944**	**0**	**36**	**1,330**	**1,366**
Albay	115,867	0	39	903	942	0	1	225	226
Camarines Norte	46,839	1	35	574	610	0	10	329	339
Camarines Sur	107,464	0	33	1,598	1,631	0	6	312	318
Catanduanes	24,326	13	9	356	378	0	0	30	30
Masbate	96,672	0	59	612	671	0	14	336	350
Sorsogon	66,956	0	14	497	511	0	0	46	46
City of Naga	32,882	0	6	2,195	2,201	0	5	52	57
**Region 6**	**667,635**	**2**	**429**	**6,536**	**6,967**	**3**	**317**	**4,477**	**4,797**
Aklan	48,553	0	131	787	918	0	0	62	62
Antique	49,316	0	8	199	207	0	0	74	74
Capiz	77,942	0	5	424	429	0	4	547	551
Guimaras	17,943	0	3	297	300	0	0	31	31
Iloilo	182,130	1	29	1,145	1,175	1	44	444	489
Negros Occidental	206,889	1	194	2,827	3,022	0	150	2,780	2,930
City of Bacolod	30,421	0	22	179	201	2	85	225	312
City of Iloilo	54,441	0	37	678	715	0	34	314	348
**Region 7**	**483,438**	**3**	**233**	**4,183**	**4,419**	**1**	**294**	**16,342**	**16,637**
Bohol	83,324	0	50	710	760	0	134	2,657	2,791
Cebu	191,250	2	99	1,766	1,867	1	97	9,286	9,384
Negros Oriental	98,214	0	39	693	732	0	16	742	758
Siquijor	9,225	0	3	86	89	0	2	91	93
City of Cebu	34,897	0	5	207	212	0	21	659	680
City of Lapu-Lapu	36,794	1	28	602	631	0	22	2,871	2,893
City of Mandaue	29,734	0	9	119	128	0	2	36	38
**Region 8**	**340,613**	**4**	**176**	**2,566**	**2,746**	**0**	**83**	**2,165**	**2,248**
Biliran	12,714	0	4	102	106	0	1	54	55
Eastern Samar	39,982	1	18	427	446	0	1	124	125
Leyte	125,631	0	45	625	670	0	19	952	971
Northern Samar	48,697	3	19	535	557	0	17	105	122
Southern Leyte	27,251	0	1	193	194	0	2	310	312
Samar	42,957	0	3	178	181	0	12	109	121
Ormoc City	20,171	0	40	311	351	0	12	236	248
City of Tacloban	23,210	0	46	195	241	0	19	275	294
**Region 9**	**354,267**	**0**	**118**	**2,119**	**2,237**	**0**	**33**	**2,360**	**2,393**
Zamboanga del Norte	126,112	0	26	790	816	0	7	1,003	1,010
Zamboanga del Sur	102,687	0	21	518	539	0	15	712	727
Zamboanga Sibugay	47,839	0	22	277	299	0	8	333	341
City of Isabela	7,167	0	45	277	322	0	0	1	1
City of Zamboanga	70,462	0	4	257	261	0	3	311	314
**Region 10**	**511,701**	**15**	**373**	**4,489**	**4,877**	**1**	**223**	**5,394**	**5,618**
Bukidnon	167,011	3	126	1,323	1,452	0	122	2,642	2,764
Camiguin	6,884	0	3	19	22	0	2	27	29
Lanao del Norte	73,898	0	81	671	752	0	6	288	294
Misamis Occidental	60,887	3	66	1,134	1,203	0	9	260	269
Misamis Oriental	111,011	9	35	524	568	1	35	1,484	1,520
City of Cagayan De Oro	57,799	0	5	117	122	0	8	206	214
City of Iligan	34,211	0	57	701	758	0	41	487	528
**Region 11**	**535,532**	**24**	**283**	**4,355**	**4,662**	**2**	**84**	**4,940**	**5,026**
Davao de Oro	84,560	0	18	601	619	0	27	869	896
Davao del Norte	112,914	0	41	877	918	0	18	706	724
Davao Oriental	59,172	0	5	268	273	0	2	354	356
Davao del Sur	67,054	0	15	362	377	0	13	831	844
Davao Occidental	33,334	0	10	92	102	0	4	294	298
City of Davao	178,498	24	194	2,155	2,373	2	20	1,886	1,908
**Region 12**	**476,219**	**5**	**261**	**2,825**	**3,091**	**0**	**132**	**3,259**	**3,391**
Cotabato	136,508	1	39	976	1,016	0	31	1,370	1,401
Sarangani	76,390	0	18	310	328	0	6	303	309
South Cotabato	102,265	4	73	695	772	0	58	710	768

Table 2.A.3 - Modern Methods of Family Planning
Drop Outs
Philippines, 2024

Area	Total Current Users	CONDOM			Total	IUD-INTERVAL			Total
		Age group				Age group			
		10-14	15-19	20-49		10-14	15-19	20-49	
Sultan Kudarat	104,518	0	91	643	734	0	20	747	767
City of General Santos	56,538	0	40	201	241	0	17	129	146
**Caraga**	**292,511**	**1**	**232**	**3,129**	**3,362**	**3**	**200**	**3,490**	**3,693**
Agusan del Norte	40,148	1	36	399	436	1	69	536	606
Agusan del Sur	86,721	0	105	939	1,044	1	49	1,522	1,572
Surigao del Norte	53,159	0	6	317	323	1	17	372	390
Surigao del Sur	60,298	0	55	755	810	0	56	598	654
Dinagat Islands	9,606	0	12	122	134	0	2	113	115
City of Butuan	42,579	0	18	597	615	0	7	349	356
**BARMM**	**296,590**	**0**	**145**	**3,695**	**3,840**	**0**	**27**	**447**	**474**
Basilan	19,309	0	2	110	112	0	6	30	36
Lanao del Sur	39,800	0	50	1,855	1,905	0	0	68	68
Maguindanao del Norte	36,321	0	23	359	382	0	11	104	115
Maguindanao del Sur	51,529	0	45	564	609	0	0	43	43
Sulu	80,817	0	3	145	148	0	0	3	3
Tawi-Tawi	19,107	0	1	68	69	0	0	3	3
SGA	7,550	0	7	59	66	0	0	10	10
City of Cotabato	42,157	0	14	535	549	0	10	186	196

Table 2.A.3 - Modern Methods of Family Planning
Drop Outs
Philippines, 2024

Area	Total Current Users	IUD-POSTPARTUM			Total	PILLS-POP			Total
		Age group				Age group			
		10-14	15-19	20-49		10-14	15-19	20-49	
**PHILIPPINES**	**8,664,035**	**126**	**3,923**	**25,686**	**29,735**	**463**	**17,404**	**157,708**	**175,575**
**N C R**	**1,041,531**	**35**	**364**	**4,851**	**5,250**	**65**	**2,266**	**23,157**	**25,488**
City of Malabon	26,264	0	29	256	285	7	114	1,475	1,596
City of Navotas	23,816	0	0	3	3	0	414	1,261	1,675
City of Valenzuela	40,454	24	4	231	259	0	26	1,551	1,577
City of Caloocan	73,676	9	163	952	1,124	1	209	1,723	1,933
City of Marikina	24,847	0	1	57	58	0	18	112	130
City of Pasig	58,937	0	5	68	73	2	19	876	897
Pateros	4,204	0	0	0	0	0	1	12	13
City of Taguig	92,053	0	0	12	12	6	29	542	577
Quezon City	338,677	2	89	1,011	1,102	5	779	7,962	8,746
City of Makati	28,572	0	3	24	27	0	5	304	309
City of Mandaluyong	27,196	0	23	149	172	0	78	628	706
City of San Juan	6,139	0	0	0	0	0	6	156	162
City of Manila	138,102	0	33	680	713	44	350	3,935	4,329
City of Las Piñas	26,980	0	5	71	76	0	54	1,168	1,222
City of Muntinlupa	44,186	0	6	14	20	0	14	216	230
City of Parañaque	47,100	0	0	11	11	0	96	292	388
Pasay City	40,328	0	3	1,312	1,315	0	54	944	998
**C A R**	**165,663**	**0**	**8**	**178**	**186**	**1**	**270**	**3,216**	**3,487**
Abra	19,755	0	0	27	27	1	30	413	444
Apayao	24,752	0	0	5	5	0	59	370	429
Benguet	37,253	0	4	65	69	0	46	613	659
Ifugao	21,120	0	1	8	9	0	22	495	517
Kalinga	18,267	0	3	3	6	0	31	389	420
Mountain Province	13,957	0	0	38	38	0	54	280	334
City of Baguio	30,559	0	0	32	32	0	28	656	684
**Region 1**	**498,204**	**0**	**59**	**233**	**292**	**4**	**416**	**6,835**	**7,255**
Ilocos Norte	52,824	0	3	46	49	0	20	460	480
Ilocos Sur	78,125	0	0	19	19	0	53	702	755
La Union	67,784	0	49	59	108	1	85	332	418
Pangasinan	292,353	0	4	102	106	3	255	5,311	5,569
City of Dagupan	7,118	0	3	7	10	0	3	30	33
**Region 2**	**343,378**	**4**	**143**	**682**	**829**	**6**	**637**	**4,820**	**5,463**
Batanes	955	0	0	1	1	0	0	13	13
Cagayan	108,930	0	16	137	153	0	154	1,088	1,242
Isabela	146,131	0	30	146	176	5	361	2,119	2,485
Nueva Vizcaya	52,095	4	95	379	478	0	90	1,304	1,394
Quirino	21,240	0	2	19	21	1	32	296	329
City of Santiago	14,027	0	0	0	0	0	0	0	0
**Region 3**	**985,325**	**3**	**77**	**2,056**	**2,136**	**50**	**1,659**	**12,098**	**13,807**
Aurora	24,562	0	2	19	21	0	28	278	306
Bataan	65,716	1	7	130	138	2	182	1,385	1,569
Bulacan	302,904	1	8	110	119	1	479	3,819	4,299
Nueva Ecija	200,897	0	22	122	144	34	461	2,341	2,836
Pampanga	167,294	1	19	1,491	1,511	11	258	1,792	2,061
Tarlac	118,444	0	19	159	178	2	169	1,553	1,724
Zambales	54,326	0	0	17	17	0	30	385	415
City of Angeles	30,345	0	0	8	8	0	23	134	157
City of Olongapo	20,837	0	0	0	0	0	29	411	440
**Region 4A**	**888,692**	**9**	**170**	**1,121**	**1,300**	**6**	**2,044**	**24,524**	**26,574**
Batangas	158,569	1	79	320	400	3	71	4,416	4,490
Cavite	175,332	4	26	238	268	1	141	2,480	2,622
Laguna	286,016	2	30	286	318	2	1,311	14,083	15,396
Quezon	106,773	0	19	119	138	0	233	1,589	1,822
Rizal	156,554	2	16	149	167	0	217	1,673	1,890
City of Lucena	5,448	0	0	9	9	0	71	283	354
**Region 4B**	**291,730**	**9**	**41**	**512**	**562**	**54**	**668**	**5,508**	**6,230**
Marinduque	16,436	0	0	1	1	0	4	236	240
Occidental Mindoro	48,902	0	0	28	28	2	123	1,329	1,454
Oriental Mindoro	68,962	0	33	403	436	2	95	1,390	1,487
Palawan	100,239	9	5	23	37	41	272	1,537	1,850

Table 2.A.3 - Modern Methods of Family Planning
Drop Outs
Philippines, 2024

Area	Total Current Users	IUD-POSTPARTUM				PILLS-POP			
		Age group			Total	Age group			Total
		10-14	15-19	20-49		10-14	15-19	20-49	
Romblon	22,467	0	2	31	33	5	23	395	423
City of Puerto Princesa	34,724	0	1	26	27	4	151	621	776
**Region 5**	**491,006**	**0**	**49**	**211**	**260**	**0**	**646**	**10,935**	**11,581**
Albay	115,867	0	10	68	78	0	71	2,219	2,290
Camarines Norte	46,839	0	29	16	45	0	81	2,351	2,432
Camarines Sur	107,464	0	8	60	68	0	216	2,585	2,801
Catanduanes	24,326	0	0	14	14	0	30	310	340
Masbate	96,672	0	1	35	36	0	170	2,507	2,677
Sorsogon	66,956	0	0	9	9	0	72	858	930
City of Naga	32,882	0	1	9	10	0	6	105	111
**Region 6**	**667,635**	**14**	**364**	**1,916**	**2,294**	**7**	**1,352**	**9,377**	**10,736**
Aklan	48,553	0	4	104	108	1	89	737	827
Antique	49,316	0	0	10	10	0	46	530	576
Capiz	77,942	0	1	26	27	0	55	747	802
Guimaras	17,943	0	0	4	4	0	25	373	398
Iloilo	182,130	2	92	188	282	3	134	1,617	1,754
Negros Occidental	206,889	9	181	1,077	1,267	1	948	4,683	5,632
City of Bacolod	30,421	3	72	28	103	2	29	503	534
City of Iloilo	54,441	0	14	479	493	0	26	187	213
**Region 7**	**483,438**	**17**	**1,264**	**5,621**	**6,902**	**12**	**1,080**	**9,197**	**10,289**
Bohol	83,324	2	212	1,090	1,304	0	176	1,278	1,454
Cebu	191,250	15	1,001	3,909	4,925	7	352	2,641	3,000
Negros Oriental	98,214	0	15	47	62	4	276	1,229	1,509
Siquijor	9,225	0	1	97	98	0	18	111	129
City of Cebu	34,897	0	9	276	285	1	87	1,000	1,088
City of Lapu-Lapu	36,794	0	21	186	207	0	147	2,702	2,849
City of Mandaue	29,734	0	5	16	21	0	24	236	260
**Region 8**	**340,613**	**0**	**110**	**591**	**701**	**1**	**387**	**3,588**	**3,976**
Biliran	12,714	0	0	9	9	0	19	231	250
Eastern Samar	39,982	0	6	32	38	0	48	404	452
Leyte	125,631	0	42	228	270	0	61	896	957
Northern Samar	48,697	0	18	82	100	1	65	547	613
Southern Leyte	27,251	0	1	63	64	0	19	252	271
Samar	42,957	0	0	13	13	0	38	444	482
Ormoc City	20,171	0	8	25	33	0	89	453	542
City of Tacloban	23,210	0	35	139	174	0	48	361	409
**Region 9**	**354,267**	**3**	**471**	**1,955**	**2,429**	**2**	**381**	**3,877**	**4,260**
Zamboanga del Norte	126,112	0	3	217	220	0	70	994	1,064
Zamboanga del Sur	102,687	3	456	1,594	2,053	0	106	1,145	1,251
Zamboanga Sibugay	47,839	0	6	28	34	0	56	706	762
City of Isabela	7,167	0	0	0	0	0	46	387	433
City of Zamboanga	70,462	0	6	116	122	2	103	645	750
**Region 10**	**511,701**	**18**	**457**	**2,224**	**2,699**	**11**	**1,253**	**8,299**	**9,563**
Bukidnon	167,011	0	24	188	212	3	582	2,209	2,794
Camiguin	6,884	0	0	3	3	0	5	13	18
Lanao del Norte	73,898	0	14	79	93	0	207	1,557	1,764
Misamis Occidental	60,887	0	1	63	64	0	103	1,796	1,899
Misamis Oriental	111,011	12	301	1,378	1,691	2	155	1,470	1,627
City of Cagayan De Oro	57,799	0	52	371	423	0	52	536	588
City of Iligan	34,211	6	65	142	213	6	149	718	873
**Region 11**	**535,532**	**7**	**60**	**1,035**	**1,102**	**56**	**1,145**	**8,510**	**9,711**
Davao de Oro	84,560	0	5	107	112	9	233	1,303	1,545
Davao del Norte	112,914	0	14	208	222	13	194	1,452	1,659
Davao Oriental	59,172	0	0	54	54	4	129	734	867
Davao del Sur	67,054	1	5	92	98	4	148	813	965
Davao Occidental	33,334	1	3	35	39	2	84	349	435
City of Davao	178,498	5	33	539	577	24	357	3,859	4,240
**Region 12**	**476,219**	**0**	**96**	**1,086**	**1,182**	**167**	**1,226**	**8,358**	**9,751**
Cotabato	136,508	0	24	242	266	8	429	2,719	3,156
Sarangani	76,390	0	3	42	45	0	152	592	744
South Cotabato	102,265	0	43	206	249	10	188	1,430	1,628

Table 2.A.3 - Modern Methods of Family Planning
Drop Outs
Philippines, 2024

Area	Total Current Users	IUD-POSTPARTUM Age group 10-14	IUD-POSTPARTUM Age group 15-19	IUD-POSTPARTUM Age group 20-49	Total	PILLS-POP Age group 10-14	PILLS-POP Age group 15-19	PILLS-POP Age group 20-49	Total
Sultan Kudarat	104,518	0	20	529	549	148	314	2,905	3,367
City of General Santos	56,538	0	6	67	73	1	143	712	856
**Caraga**	**292,511**	**7**	**107**	**1,069**	**1,183**	**18**	**1,337**	**8,894**	**10,249**
Agusan del Norte	40,148	1	20	156	177	2	304	1,468	1,774
Agusan del Sur	86,721	1	32	343	376	13	490	2,176	2,679
Surigao del Norte	53,159	0	28	191	219	0	106	1,986	2,092
Surigao del Sur	60,298	5	24	338	367	3	278	1,749	2,030
Dinagat Islands	9,606	0	2	10	12	0	8	137	145
City of Butuan	42,579	0	1	31	32	0	151	1,378	1,529
**BARMM**	**296,590**	**0**	**83**	**345**	**428**	**3**	**637**	**6,515**	**7,155**
Basilan	19,309	0	25	52	77	0	76	378	454
Lanao del Sur	39,800	0	4	31	35	0	84	1,874	1,958
Maguindanao del Norte	36,321	0	10	189	199	0	102	875	977
Maguindanao del Sur	51,529	0	1	10	11	0	133	995	1,128
Sulu	80,817	0	0	7	7	1	131	1,377	1,509
Tawi-Tawi	19,107	0	0	0	0	0	9	489	498
SGA	7,550	0	0	0	0	0	72	289	361
City of Cotabato	42,157	0	43	56	99	2	30	238	270

Table 2.A.3 - Modern Methods of Family Planning
Drop Outs
Philippines, 2024

Area	Total Current Users	PILLS-COC			Total	INJECTABLES			Total
		Age group				Age group			
		10-14	15-19	20-49		10-14	15-19	20-49	
**PHILIPPINES**	**8,664,035**	**292**	**24,796**	**440,602**	**465,690**	**485**	**34,113**	**360,850**	**395,448**
**N C R**	**1,041,531**	**128**	**2,536**	**42,719**	**45,383**	**118**	**4,367**	**49,318**	**53,803**
City of Malabon	26,264	1	147	3,895	4,043	9	349	3,800	4,158
City of Navotas	23,816	0	616	2,280	2,896	1	691	2,300	2,992
City of Valenzuela	40,454	10	117	5,559	5,686	4	427	6,381	6,812
City of Caloocan	73,676	0	195	3,456	3,651	0	648	6,593	7,241
City of Marikina	24,847	0	4	239	243	0	76	762	838
City of Pasig	58,937	0	37	2,254	2,291	3	80	3,275	3,358
Pateros	4,204	0	0	92	92	0	1	72	73
City of Taguig	92,053	2	40	663	705	3	81	1,529	1,613
Quezon City	338,677	1	986	17,657	18,644	4	1,302	13,110	14,416
City of Makati	28,572	0	5	345	350	0	23	674	697
City of Mandaluyong	27,196	0	81	996	1,077	0	127	1,227	1,354
City of San Juan	6,139	0	3	218	221	1	19	444	464
City of Manila	138,102	109	95	2,065	2,269	90	168	2,447	2,705
City of Las Piñas	26,980	0	38	1,131	1,169	2	172	2,481	2,655
City of Muntinlupa	44,186	5	37	395	437	1	80	2,463	2,544
City of Parañaque	47,100	0	76	371	447	0	97	549	646
Pasay City	40,328	0	59	1,103	1,162	0	26	1,211	1,237
**C A R**	**165,663**	**2**	**226**	**7,449**	**7,677**	**2**	**437**	**7,554**	**7,993**
Abra	19,755	0	24	751	775	1	44	532	577
Apayao	24,752	1	56	919	976	0	89	689	778
Benguet	37,253	0	46	1,539	1,585	0	59	1,324	1,383
Ifugao	21,120	1	29	1,168	1,198	0	25	858	883
Kalinga	18,267	0	27	1,043	1,070	0	80	1,075	1,155
Mountain Province	13,957	0	31	794	825	1	63	847	911
City of Baguio	30,559	0	13	1,235	1,248	0	77	2,229	2,306
**Region 1**	**498,204**	**4**	**1,031**	**23,801**	**24,836**	**6**	**831**	**7,994**	**8,831**
Ilocos Norte	52,824	0	55	3,077	3,132	0	34	850	884
Ilocos Sur	78,125	0	68	1,694	1,762	0	39	887	926
La Union	67,784	0	100	2,469	2,569	1	175	1,774	1,950
Pangasinan	292,353	4	800	16,179	16,983	5	567	4,199	4,771
City of Dagupan	7,118	0	8	382	390	0	16	284	300
**Region 2**	**343,378**	**2**	**850**	**15,369**	**16,221**	**16**	**969**	**10,513**	**11,498**
Batanes	955	0	4	45	49	1	12	134	147
Cagayan	108,930	2	244	4,862	5,108	1	201	2,288	2,490
Isabela	146,131	0	366	6,722	7,088	14	516	5,336	5,866
Nueva Vizcaya	52,095	0	154	2,314	2,468	0	132	1,866	1,998
Quirino	21,240	0	65	1,114	1,179	0	89	614	703
City of Santiago	14,027	0	17	312	329	0	19	275	294
**Region 3**	**985,325**	**11**	**2,503**	**37,580**	**40,094**	**39**	**4,289**	**40,153**	**44,481**
Aurora	24,562	0	63	1,756	1,819	0	64	1,070	1,134
Bataan	65,716	1	112	1,790	1,903	2	461	4,574	5,037
Bulacan	302,904	0	862	11,858	12,720	6	1,405	12,525	13,936
Nueva Ecija	200,897	3	443	6,312	6,758	12	731	5,612	6,355
Pampanga	167,294	2	451	6,265	6,718	8	721	6,581	7,310
Tarlac	118,444	5	393	4,771	5,169	6	516	5,026	5,548
Zambales	54,326	0	111	2,572	2,683	4	294	3,104	3,402
City of Angeles	30,345	0	31	993	1,024	0	60	520	580
City of Olongapo	20,837	0	37	1,263	1,300	1	37	1,141	1,179
**Region 4A**	**888,692**	**7**	**2,809**	**45,590**	**48,406**	**25**	**4,514**	**49,841**	**54,380**
Batangas	158,569	1	124	6,351	6,476	1	192	4,747	4,940
Cavite	175,332	3	259	9,938	10,200	7	743	12,933	13,683
Laguna	286,016	0	1,887	18,160	20,047	3	1,851	12,731	14,585
Quezon	106,773	3	264	6,542	6,809	8	700	8,531	9,239
Rizal	156,554	0	231	4,105	4,336	5	839	9,994	10,838
City of Lucena	5,448	0	44	494	538	1	189	905	1,095
**Region 4B**	**291,730**	**25**	**747**	**16,685**	**17,457**	**64**	**1,545**	**14,963**	**16,572**
Marinduque	16,436	0	3	289	292	0	9	267	276
Occidental Mindoro	48,902	0	153	4,836	4,989	1	184	3,347	3,532
Oriental Mindoro	68,962	1	124	4,644	4,769	0	148	2,907	3,055
Palawan	100,239	21	348	4,053	4,422	55	760	5,307	6,122

Table 2.A.3 - Modern Methods of Family Planning
Drop Outs
Philippines, 2024

Area	Total Current Users	PILLS-COC Age group 10-14	PILLS-COC Age group 15-19	PILLS-COC Age group 20-49	Total	INJECTABLES Age group 10-14	INJECTABLES Age group 15-19	INJECTABLES Age group 20-49	Total
Romblon	22,467	2	19	722	743	1	47	633	681
City of Puerto Princesa	34,724	1	100	2,141	2,242	7	397	2,502	2,906
**Region 5**	**491,006**	**1**	**993**	**31,135**	**32,129**	**7**	**1,308**	**20,955**	**22,270**
Albay	115,867	1	123	4,634	4,758	0	177	2,976	3,153
Camarines Norte	46,839	0	101	3,205	3,306	1	225	2,459	2,685
Camarines Sur	107,464	0	257	8,570	8,827	1	346	6,492	6,839
Catanduanes	24,326	0	17	1,183	1,200	3	106	1,465	1,574
Masbate	96,672	0	382	6,951	7,333	0	258	4,029	4,287
Sorsogon	66,956	0	95	3,307	3,402	2	183	2,897	3,082
City of Naga	32,882	0	18	3,285	3,303	0	13	637	650
**Region 6**	**667,635**	**15**	**2,264**	**31,271**	**33,550**	**28**	**2,614**	**23,191**	**25,833**
Aklan	48,553	3	171	1,259	1,433	4	437	1,343	1,784
Antique	49,316	0	98	1,340	1,438	1	149	1,773	1,923
Capiz	77,942	0	84	1,808	1,892	0	138	1,344	1,482
Guimaras	17,943	0	51	1,026	1,077	0	32	428	460
Iloilo	182,130	3	257	5,834	6,094	1	300	4,355	4,656
Negros Occidental	206,889	8	1,343	16,342	17,693	13	1,192	12,052	13,257
City of Bacolod	30,421	1	108	728	837	9	277	892	1,178
City of Iloilo	54,441	0	152	2,934	3,086	0	89	1,004	1,093
**Region 7**	**483,438**	**5**	**1,520**	**37,663**	**39,188**	**29**	**3,227**	**27,772**	**31,028**
Bohol	83,324	0	228	3,226	3,454	1	482	3,311	3,794
Cebu	191,250	3	662	24,754	25,419	11	1,531	12,154	13,696
Negros Oriental	98,214	1	321	5,104	5,426	12	688	5,885	6,585
Siquijor	9,225	0	19	915	934	0	70	656	726
City of Cebu	34,897	1	216	2,468	2,685	4	285	2,995	3,284
City of Lapu-Lapu	36,794	0	66	1,018	1,084	0	122	2,340	2,462
City of Mandaue	29,734	0	8	178	186	1	49	431	481
**Region 8**	**340,613**	**9**	**1,070**	**18,796**	**19,875**	**11**	**991**	**13,911**	**14,913**
Biliran	12,714	0	30	571	601	1	28	462	491
Eastern Samar	39,982	3	120	3,399	3,522	0	152	2,114	2,266
Leyte	125,631	1	314	5,138	5,453	5	208	3,318	3,531
Northern Samar	48,697	0	149	2,120	2,269	2	126	2,403	2,531
Southern Leyte	27,251	0	36	1,199	1,235	0	41	929	970
Samar	42,957	5	102	2,570	2,677	3	146	2,035	2,184
Ormoc City	20,171	0	150	2,021	2,171	0	152	1,221	1,373
City of Tacloban	23,210	0	169	1,778	1,947	0	138	1,429	1,567
**Region 9**	**354,267**	**4**	**581**	**13,612**	**14,197**	**15**	**670**	**10,771**	**11,456**
Zamboanga del Norte	126,112	0	207	4,370	4,577	1	209	3,010	3,220
Zamboanga del Sur	102,687	1	132	3,179	3,312	2	103	2,270	2,375
Zamboanga Sibugay	47,839	1	80	2,685	2,766	0	131	2,111	2,242
City of Isabela	7,167	0	17	228	245	0	25	336	361
City of Zamboanga	70,462	2	145	3,150	3,297	12	202	3,044	3,258
**Region 10**	**511,701**	**17**	**1,798**	**20,689**	**22,504**	**13**	**1,479**	**12,177**	**13,669**
Bukidnon	167,011	5	939	11,226	12,170	8	699	5,389	6,096
Camiguin	6,884	1	14	140	155	0	3	75	78
Lanao del Norte	73,898	2	335	1,954	2,291	1	183	1,245	1,429
Misamis Occidental	60,887	1	151	1,864	2,016	0	146	964	1,110
Misamis Oriental	111,011	0	159	2,870	3,029	0	224	1,999	2,223
City of Cagayan De Oro	57,799	7	12	612	631	2	35	497	534
City of Iligan	34,211	1	188	2,023	2,212	2	189	2,008	2,199
**Region 11**	**535,532**	**35**	**1,533**	**28,306**	**29,874**	**36**	**1,611**	**17,175**	**18,822**
Davao de Oro	84,560	13	181	5,734	5,928	3	225	3,145	3,373
Davao del Norte	112,914	5	304	4,722	5,031	4	245	2,431	2,680
Davao Oriental	59,172	0	164	3,106	3,270	0	140	1,607	1,747
Davao del Sur	67,054	1	125	2,399	2,525	6	196	1,958	2,160
Davao Occidental	33,334	6	313	2,938	3,257	6	373	1,960	2,339
City of Davao	178,498	10	446	9,407	9,863	17	432	6,074	6,523
**Region 12**	**476,219**	**10**	**1,868**	**30,031**	**31,909**	**31**	**2,143**	**18,820**	**20,994**
Cotabato	136,508	3	530	7,833	8,366	6	559	4,280	4,845
Sarangani	76,390	0	268	4,751	5,019	0	244	2,844	3,088
South Cotabato	102,265	6	450	6,625	7,081	13	491	4,664	5,168

Table 2.A.3 - Modern Methods of Family Planning
Drop Outs
Philippines, 2024

Area	Total Current Users	PILLS-COC			Total	INJECTABLES			Total
		Age group				Age group			
		10-14	15-19	20-49		10-14	15-19	20-49	
Sultan Kudarat	104,518	1	414	9,338	9,753	9	599	4,995	5,603
City of General Santos	56,538	0	206	1,484	1,690	3	250	2,037	2,290
**Caraga**	**292,511**	**15**	**1,370**	**16,439**	**17,824**	**28**	**998**	**10,338**	**11,364**
Agusan del Norte	40,148	1	152	1,944	2,097	7	180	1,625	1,812
Agusan del Sur	86,721	10	377	5,114	5,501	7	252	2,695	2,954
Surigao del Norte	53,159	0	238	2,071	2,309	0	151	1,383	1,534
Surigao del Sur	60,298	4	287	3,075	3,366	9	199	1,937	2,145
Dinagat Islands	9,606	0	26	536	562	0	28	339	367
City of Butuan	42,579	0	290	3,699	3,989	5	188	2,359	2,552
**BARMM**	**296,590**	**2**	**1,097**	**23,467**	**24,566**	**17**	**2,120**	**25,404**	**27,541**
Basilan	19,309	1	116	1,301	1,418	5	246	1,737	1,988
Lanao del Sur	39,800	0	44	2,365	2,409	0	77	2,681	2,758
Maguindanao del Norte	36,321	0	123	2,688	2,811	3	341	2,405	2,749
Maguindanao del Sur	51,529	0	475	6,877	7,352	3	749	7,447	8,199
Sulu	80,817	0	163	5,325	5,488	2	368	7,280	7,650
Tawi-Tawi	19,107	0	47	2,027	2,074	1	68	1,181	1,250
SGA	7,550	0	61	1,307	1,368	0	107	1,432	1,539
City of Cotabato	42,157	1	68	1,577	1,646	3	164	1,241	1,408

Table 2.A.3 - Modern Methods of Family Planning
Drop Outs
Philippines, 2024

Area	Total Current Users	IMPLANTS			Total	NFP-CCM			Total
		Age group				Age group			
		10-14	15-19	20-49		10-14	15-19	20-49	
**PHILIPPINES**	**8,664,035**	**389**	**14,255**	**131,540**	**146,184**	**34**	**291**	**9,207**	**9,532**
**N C R**	**1,041,531**	**84**	**1,730**	**17,023**	**18,837**	**0**	**0**	**206**	**206**
City of Malabon	26,264	7	142	983	1,132	0	0	0	0
City of Navotas	23,816	3	239	669	911	0	0	0	0
City of Valenzuela	40,454	3	114	2,358	2,475	0	0	11	11
City of Caloocan	73,676	5	262	1,363	1,630	0	0	0	0
City of Marikina	24,847	0	7	176	183	0	0	1	1
City of Pasig	58,937	0	96	894	990	0	0	0	0
Pateros	4,204	0	0	20	20	0	0	0	0
City of Taguig	92,053	0	5	349	354	0	0	0	0
Quezon City	338,677	5	324	4,143	4,472	0	0	4	4
City of Makati	28,572	0	1	130	131	0	0	0	0
City of Mandaluyong	27,196	1	42	650	693	0	0	181	181
City of San Juan	6,139	0	0	76	76	0	0	0	0
City of Manila	138,102	58	361	3,529	3,948	0	0	9	9
City of Las Piñas	26,980	2	104	685	791	0	0	0	0
City of Muntinlupa	44,186	0	0	194	194	0	0	0	0
City of Parañaque	47,100	0	28	313	341	0	0	0	0
Pasay City	40,328	0	5	491	496	0	0	0	0
**C A R**	**165,663**	**1**	**197**	**2,304**	**2,502**	**1**	**8**	**505**	**514**
Abra	19,755	0	21	217	238	0	0	60	60
Apayao	24,752	0	34	159	193	0	0	0	0
Benguet	37,253	1	38	370	409	0	0	2	2
Ifugao	21,120	0	17	320	337	0	0	95	95
Kalinga	18,267	0	17	266	283	1	8	314	323
Mountain Province	13,957	0	43	267	310	0	0	33	33
City of Baguio	30,559	0	27	705	732	0	0	1	1
**Region 1**	**498,204**	**1**	**194**	**2,343**	**2,538**	**0**	**9**	**468**	**477**
Ilocos Norte	52,824	0	17	177	194	0	4	56	60
Ilocos Sur	78,125	0	14	137	151	0	4	131	135
La Union	67,784	1	75	487	563	0	0	262	262
Pangasinan	292,353	0	87	1,505	1,592	0	1	19	20
City of Dagupan	7,118	0	1	37	38	0	0	0	0
**Region 2**	**343,378**	**5**	**308**	**3,129**	**3,442**	**0**	**0**	**91**	**91**
Batanes	955	0	2	5	7	0	0	14	14
Cagayan	108,930	0	66	789	855	0	0	4	4
Isabela	146,131	5	186	1,725	1,916	0	0	3	3
Nueva Vizcaya	52,095	0	39	431	470	0	0	67	67
Quirino	21,240	0	11	139	150	0	0	3	3
City of Santiago	14,027	0	4	40	44	0	0	0	0
**Region 3**	**985,325**	**23**	**855**	**8,322**	**9,200**	**0**	**26**	**187**	**213**
Aurora	24,562	0	32	393	425	0	0	129	129
Bataan	65,716	16	239	1,154	1,409	0	0	25	25
Bulacan	302,904	1	184	2,109	2,294	0	0	10	10
Nueva Ecija	200,897	2	168	1,286	1,456	0	0	0	0
Pampanga	167,294	2	110	1,607	1,719	0	26	18	44
Tarlac	118,444	2	66	848	916	0	0	0	0
Zambales	54,326	0	54	585	639	0	0	5	5
City of Angeles	30,345	0	2	152	154	0	0	0	0
City of Olongapo	20,837	0	0	188	188	0	0	0	0
**Region 4A**	**888,692**	**17**	**827**	**8,195**	**9,039**	**0**	**2**	**435**	**437**
Batangas	158,569	3	49	552	604	0	0	5	5
Cavite	175,332	4	130	2,143	2,277	0	1	48	49
Laguna	286,016	4	295	2,821	3,120	0	1	193	194
Quezon	106,773	4	202	976	1,182	0	0	186	186
Rizal	156,554	2	145	1,603	1,750	0	0	3	3
City of Lucena	5,448	0	6	100	106	0	0	0	0
**Region 4B**	**291,730**	**72**	**578**	**5,335**	**5,985**	**12**	**63**	**810**	**885**
Marinduque	16,436	0	11	167	178	0	0	6	6
Occidental Mindoro	48,902	2	97	950	1,049	0	60	413	473
Oriental Mindoro	68,962	1	92	791	884	0	0	187	187
Palawan	100,239	66	268	2,668	3,002	12	1	74	87

Table 2.A.3 - Modern Methods of Family Planning
Drop Outs
Philippines, 2024

Area	Total Current Users	IMPLANTS			Total	NFP-CCM			Total
		Age group				Age group			
		10-14	15-19	20-49		10-14	15-19	20-49	
Romblon	22,467	0	45	182	227	0	2	125	127
City of Puerto Princesa	34,724	3	65	577	645	0	0	5	5
**Region 5**	**491,006**	**13**	**703**	**8,888**	**9,604**	**21**	**99**	**3,858**	**3,978**
Albay	115,867	2	79	1,028	1,109	0	5	337	342
Camarines Norte	46,839	0	10	736	746	0	1	213	214
Camarines Sur	107,464	5	315	3,048	3,368	0	16	520	536
Catanduanes	24,326	5	75	790	870	0	15	442	457
Masbate	96,672	0	144	2,113	2,257	21	35	1,404	1,460
Sorsogon	66,956	1	73	1,001	1,075	0	27	937	964
City of Naga	32,882	0	7	172	179	0	0	5	5
**Region 6**	**667,635**	**10**	**847**	**7,940**	**8,797**	**0**	**9**	**401**	**410**
Aklan	48,553	2	24	479	505	0	0	32	32
Antique	49,316	0	56	707	763	0	7	339	346
Capiz	77,942	1	22	400	423	0	0	12	12
Guimaras	17,943	0	11	57	68	0	0	2	2
Iloilo	182,130	0	174	1,746	1,920	0	2	7	9
Negros Occidental	206,889	6	344	3,101	3,451	0	0	8	8
City of Bacolod	30,421	1	178	1,109	1,288	0	0	0	0
City of Iloilo	54,441	0	38	341	379	0	0	1	1
**Region 7**	**483,438**	**18**	**997**	**11,455**	**12,470**	**0**	**4**	**130**	**134**
Bohol	83,324	3	286	2,643	2,932	0	0	1	1
Cebu	191,250	7	336	5,436	5,779	0	0	25	25
Negros Oriental	98,214	5	176	1,024	1,205	0	4	80	84
Siquijor	9,225	0	17	81	98	0	0	0	0
City of Cebu	34,897	2	99	1,239	1,340	0	0	24	24
City of Lapu-Lapu	36,794	1	48	498	547	0	0	0	0
City of Mandaue	29,734	0	35	534	569	0	0	0	0
**Region 8**	**340,613**	**4**	**589**	**5,523**	**6,116**	**0**	**4**	**280**	**284**
Biliran	12,714	0	43	176	219	0	0	15	15
Eastern Samar	39,982	0	54	608	662	0	0	43	43
Leyte	125,631	1	102	1,598	1,701	0	3	71	74
Northern Samar	48,697	3	60	891	954	0	0	115	115
Southern Leyte	27,251	0	23	384	407	0	0	32	32
Samar	42,957	0	56	788	844	0	1	4	5
Ormoc City	20,171	0	105	599	704	0	0	0	0
City of Tacloban	23,210	0	146	479	625	0	0	0	0
**Region 9**	**354,267**	**4**	**637**	**9,846**	**10,487**	**0**	**0**	**1**	**1**
Zamboanga del Norte	126,112	2	152	2,180	2,334	0	0	1	1
Zamboanga del Sur	102,687	1	273	4,263	4,537	0	0	0	0
Zamboanga Sibugay	47,839	0	94	2,163	2,257	0	0	0	0
City of Isabela	7,167	0	20	195	215	0	0	0	0
City of Zamboanga	70,462	1	98	1,045	1,144	0	0	0	0
**Region 10**	**511,701**	**20**	**1,289**	**8,661**	**9,970**	**0**	**48**	**796**	**844**
Bukidnon	167,011	13	808	5,066	5,887	0	38	203	241
Camiguin	6,884	0	3	21	24	0	0	0	0
Lanao del Norte	73,898	2	115	1,027	1,144	0	5	54	59
Misamis Occidental	60,887	0	30	378	408	0	0	24	24
Misamis Oriental	111,011	3	210	1,067	1,280	0	5	286	291
City of Cagayan De Oro	57,799	0	59	678	737	0	0	0	0
City of Iligan	34,211	2	64	424	490	0	0	229	229
**Region 11**	**535,532**	**31**	**911**	**8,761**	**9,703**	**0**	**2**	**330**	**332**
Davao de Oro	84,560	3	120	1,332	1,455	0	0	37	37
Davao del Norte	112,914	5	230	1,627	1,862	0	1	19	20
Davao Oriental	59,172	2	48	660	710	0	0	186	186
Davao del Sur	67,054	5	68	764	837	0	0	58	58
Davao Occidental	33,334	4	150	982	1,136	0	0	5	5
City of Davao	178,498	12	295	3,396	3,703	0	1	25	26
**Region 12**	**476,219**	**36**	**1,656**	**9,646**	**11,338**	**0**	**14**	**269**	**283**
Cotabato	136,508	4	402	2,526	2,932	0	0	119	119
Sarangani	76,390	0	144	1,452	1,596	0	2	44	46
South Cotabato	102,265	11	526	2,206	2,743	0	0	90	90

Table 2.A.3 - Modern Methods of Family Planning
Drop Outs
Philippines, 2024

Area	Total Current Users	IMPLANTS			Total	NFP-CCM			Total
		Age group				Age group			
		10-14	15-19	20-49		10-14	15-19	20-49	
Sultan Kudarat	104,518	18	457	2,381	2,856	0	12	16	28
City of General Santos	56,538	3	127	1,081	1,211	0	0	0	0
**Caraga**	**292,511**	**42**	**1,260**	**7,416**	**8,718**	**0**	**3**	**383**	**386**
Agusan del Norte	40,148	23	315	1,282	1,620	0	0	17	17
Agusan del Sur	86,721	12	351	2,476	2,839	0	1	263	264
Surigao del Norte	53,159	0	215	1,104	1,319	0	0	24	24
Surigao del Sur	60,298	7	175	1,434	1,616	0	1	79	80
Dinagat Islands	9,606	0	35	367	402	0	0	0	0
City of Butuan	42,579	0	169	753	922	0	1	0	1
**BARMM**	**296,590**	**8**	**677**	**6,753**	**7,438**	**0**	**0**	**57**	**57**
Basilan	19,309	1	155	796	952	0	0	0	0
Lanao del Sur	39,800	2	24	592	618	0	0	0	0
Maguindanao del Norte	36,321	1	112	622	735	0	0	0	0
Maguindanao del Sur	51,529	1	195	1,440	1,636	0	0	0	0
Sulu	80,817	1	94	2,068	2,163	0	0	0	0
Tawi-Tawi	19,107	0	68	932	1,000	0	0	0	0
SGA	7,550	0	6	103	109	0	0	0	0
City of Cotabato	42,157	2	23	200	225	0	0	57	57

Table 2.A.3 - MODERN METHOD OF FAMILY PLANNING
Drop Outs
Philippines, 2024

Area	Total Current Users	NFP-BBT Age group 10-14	NFP-BBT Age group 15-19	NFP-BBT Age group 20-49	Total	NFP-STM Age group 10-14	NFP-STM Age group 15-19	NFP-STM Age group 20-49	Total
**PHILIPPINES**	**8,664,035**	**7**	**240**	**2,035**	**2,282**	**0**	**33**	**1,042**	**1,075**
**N C R**	**1,041,531**	**0**	**49**	**269**	**318**	**0**	**7**	**87**	**94**
City of Malabon	26,264	0	0	0	0	0	0	0	0
City of Navotas	23,816	0	0	0	0	0	0	0	0
City of Valenzuela	40,454	0	8	137	145	0	0	0	0
City of Caloocan	73,676	0	0	0	0	0	0	0	0
City of Marikina	24,847	0	0	0	0	0	0	0	0
City of Pasig	58,937	0	0	0	0	0	0	0	0
Pateros	4,204	0	0	0	0	0	0	0	0
City of Taguig	92,053	0	0	0	0	0	0	0	0
Quezon City	338,677	0	12	23	35	0	0	6	6
City of Makati	28,572	0	0	0	0	0	0	0	0
City of Mandaluyong	27,196	0	0	1	1	0	0	81	81
City of San Juan	6,139	0	0	0	0	0	0	0	0
City of Manila	138,102	0	29	16	45	0	0	0	0
City of Las Piñas	26,980	0	0	0	0	0	0	0	0
City of Muntinlupa	44,186	0	0	0	0	0	0	0	0
City of Parañaque	47,100	0	0	0	0	0	7	0	7
Pasay City	40,328	0	0	92	92	0	0	0	0
**C A R**	**165,663**	**0**	**4**	**41**	**45**	**0**	**1**	**32**	**33**
Abra	19,755	0	0	25	25	0	1	26	27
Apayao	24,752	0	0	0	0	0	0	0	0
Benguet	37,253	0	0	0	0	0	0	0	0
Ifugao	21,120	0	0	1	1	0	0	6	6
Kalinga	18,267	0	0	1	1	0	0	0	0
Mountain Province	13,957	0	4	7	11	0	0	0	0
City of Baguio	30,559	0	0	7	7	0	0	0	0
**Region 1**	**498,204**	**1**	**6**	**163**	**170**	**0**	**0**	**179**	**179**
Ilocos Norte	52,824	0	1	5	6	0	0	41	41
Ilocos Sur	78,125	0	2	125	127	0	0	19	19
La Union	67,784	0	0	14	14	0	0	3	3
Pangasinan	292,353	1	3	17	21	0	0	116	116
City of Dagupan	7,118	0	0	2	2	0	0	0	0
**Region 2**	**343,378**	**0**	**10**	**33**	**43**	**0**	**0**	**14**	**14**
Batanes	955	0	0	0	0	0	0	0	0
Cagayan	108,930	0	0	1	1	0	0	6	6
Isabela	146,131	0	7	29	36	0	0	3	3
Nueva Vizcaya	52,095	0	3	3	6	0	0	5	5
Quirino	21,240	0	0	0	0	0	0	0	0
City of Santiago	14,027	0	0	0	0	0	0	0	0
**Region 3**	**985,325**	**0**	**5**	**58**	**63**	**0**	**3**	**10**	**13**
Aurora	24,562	0	1	6	7	0	0	5	5
Bataan	65,716	0	2	24	26	0	0	0	0
Bulacan	302,904	0	0	0	0	0	0	0	0
Nueva Ecija	200,897	0	0	0	0	0	0	0	0
Pampanga	167,294	0	0	8	8	0	1	0	1
Tarlac	118,444	0	0	2	2	0	2	5	7
Zambales	54,326	0	0	0	0	0	0	0	0
City of Angeles	30,345	0	2	18	20	0	0	0	0
City of Olongapo	20,837	0	0	0	0	0	0	0	0
**Region 4A**	**888,692**	**0**	**7**	**214**	**221**	**0**	**0**	**37**	**37**
Batangas	158,569	0	0	26	26	0	0	0	0
Cavite	175,332	0	0	18	18	0	0	0	0
Laguna	286,016	0	5	39	44	0	0	33	33
Quezon	106,773	0	2	124	126	0	0	2	2
Rizal	156,554	0	0	7	7	0	0	2	2
City of Lucena	5,448	0	0	0	0	0	0	0	0
**Region 4B**	**291,730**	**0**	**9**	**80**	**89**	**0**	**0**	**31**	**31**
Marinduque	16,436	0	0	0	0	0	0	0	0
Occidental Mindoro	48,902	0	1	19	20	0	0	13	13
Oriental Mindoro	68,962	0	8	51	59	0	0	15	15
Palawan	100,239	0	0	1	1	0	0	2	2

Table 2.A.3 - Modern Methods of Family Planning
Drop Outs
Philippines, 2024

Area	Total Current Users	NFP-BBT			Total	NFP-STM			Total
		Age group				Age group			
		10-14	15-19	20-49		10-14	15-19	20-49	
Romblon	22,467	0	0	9	9	0	0	1	1
City of Puerto Princesa	34,724	0	0	0	0	0	0	0	0
**Region 5**	**491,006**	**1**	**8**	**283**	**292**	**0**	**6**	**409**	**415**
Albay	115,867	0	4	36	40	0	1	11	12
Camarines Norte	46,839	0	1	6	7	0	0	16	16
Camarines Sur	107,464	0	3	200	203	0	3	268	271
Catanduanes	24,326	1	0	36	37	0	0	63	63
Masbate	96,672	0	0	3	3	0	2	12	14
Sorsogon	66,956	0	0	0	0	0	0	34	34
City of Naga	32,882	0	0	2	2	0	0	5	5
**Region 6**	**667,635**	**0**	**1**	**54**	**55**	**0**	**3**	**23**	**26**
Aklan	48,553	0	0	9	9	0	3	21	24
Antique	49,316	0	0	3	3	0	0	1	1
Capiz	77,942	0	0	10	10	0	0	0	0
Guimaras	17,943	0	0	6	6	0	0	0	0
Iloilo	182,130	0	0	1	1	0	0	0	0
Negros Occidental	206,889	0	1	22	23	0	0	1	1
City of Bacolod	30,421	0	0	0	0	0	0	0	0
City of Iloilo	54,441	0	0	3	3	0	0	0	0
**Region 7**	**483,438**	**0**	**47**	**197**	**244**	**0**	**0**	**2**	**2**
Bohol	83,324	0	0	0	0	0	0	0	0
Cebu	191,250	0	44	177	221	0	0	0	0
Negros Oriental	98,214	0	1	11	12	0	0	2	2
Siquijor	9,225	0	0	0	0	0	0	0	0
City of Cebu	34,897	0	2	9	11	0	0	0	0
City of Lapu-Lapu	36,794	0	0	0	0	0	0	0	0
City of Mandaue	29,734	0	0	0	0	0	0	0	0
**Region 8**	**340,613**	**0**	**9**	**94**	**103**	**0**	**0**	**7**	**7**
Biliran	12,714	0	0	0	0	0	0	0	0
Eastern Samar	39,982	0	0	9	9	0	0	0	0
Leyte	125,631	0	0	37	37	0	0	3	3
Northern Samar	48,697	0	3	19	22	0	0	4	4
Southern Leyte	27,251	0	0	22	22	0	0	0	0
Samar	42,957	0	4	6	10	0	0	0	0
Ormoc City	20,171	0	2	1	3	0	0	0	0
City of Tacloban	23,210	0	0	0	0	0	0	0	0
**Region 9**	**354,267**	**0**	**0**	**8**	**8**	**0**	**0**	**3**	**3**
Zamboanga del Norte	126,112	0	0	8	8	0	0	1	1
Zamboanga del Sur	102,687	0	0	0	0	0	0	2	2
Zamboanga Sibugay	47,839	0	0	0	0	0	0	0	0
City of Isabela	7,167	0	0	0	0	0	0	0	0
City of Zamboanga	70,462	0	0	0	0	0	0	0	0
**Region 10**	**511,701**	**3**	**26**	**236**	**265**	**0**	**6**	**151**	**157**
Bukidnon	167,011	1	14	83	98	0	1	34	35
Camiguin	6,884	0	0	4	4	0	0	0	0
Lanao del Norte	73,898	0	4	59	63	0	1	55	56
Misamis Occidental	60,887	0	0	7	7	0	0	27	27
Misamis Oriental	111,011	2	5	46	53	0	4	35	39
City of Cagayan De Oro	57,799	0	2	17	19	0	0	0	0
City of Iligan	34,211	0	1	20	21	0	0	0	0
**Region 11**	**535,532**	**0**	**22**	**76**	**98**	**0**	**1**	**48**	**49**
Davao de Oro	84,560	0	0	0	0	0	0	0	0
Davao del Norte	112,914	0	12	23	35	0	0	13	13
Davao Oriental	59,172	0	0	12	12	0	0	1	1
Davao del Sur	67,054	0	0	0	0	0	0	13	13
Davao Occidental	33,334	0	10	0	10	0	0	8	8
City of Davao	178,498	0	0	41	41	0	1	13	14
**Region 12**	**476,219**	**2**	**30**	**87**	**119**	**0**	**0**	**6**	**6**
Cotabato	136,508	0	1	14	15	0	0	1	1
Sarangani	76,390	0	5	14	19	0	0	4	4
South Cotabato	102,265	2	3	10	15	0	0	0	0

Table 2.A.3 - Modern Methods of Family Planning
Drop Outs
Philippines, 2024

Area	Total Current Users	NFP-BBT			Total	NFP-STM			Total
		Age group				Age group			
		10-14	15-19	20-49		10-14	15-19	20-49	
Sultan Kudarat	104,518	0	0	20	20	0	0	0	0
City of General Santos	56,538	0	21	29	50	0	0	1	1
**Caraga**	**292,511**	**0**	**7**	**128**	**135**	**0**	**6**	**3**	**9**
Agusan del Norte	40,148	0	0	8	8	0	5	0	5
Agusan del Sur	86,721	0	0	64	64	0	0	0	0
Surigao del Norte	53,159	0	2	41	43	0	1	2	3
Surigao del Sur	60,298	0	2	10	12	0	0	1	1
Dinagat Islands	9,606	0	0	1	1	0	0	0	0
City of Butuan	42,579	0	3	4	7	0	0	0	0
**BARMM**	**296,590**	**0**	**0**	**14**	**14**	**0**	**0**	**0**	**0**
Basilan	19,309	0	0	0	0	0	0	0	0
Lanao del Sur	39,800	0	0	3	3	0	0	0	0
Maguindanao del Norte	36,321	0	0	0	0	0	0	0	0
Maguindanao del Sur	51,529	0	0	0	0	0	0	0	0
Sulu	80,817	0	0	0	0	0	0	0	0
Tawi-Tawi	19,107	0	0	0	0	0	0	0	0
SGA	7,550	0	0	0	0	0	0	0	0
City of Cotabato	42,157	0	0	11	11	0	0	0	0

Table 2.A.3 - Modern Methods of Family Planning
Drop Outs
Philippines, 2024

Area	Total Current Users	NFP-SDM			Total	NFP-LAM			Total
		Age group				Age group			
		10-14	15-19	20-49		10-14	15-19	20-49	
**PHILIPPINES**	**8,664,035**	**175**	**1,267**	**27,286**	**28,728**	**1,308**	**74,223**	**678,121**	**753,652**
**N C R**	**1,041,531**	**86**	**39**	**1,200**	**1,325**	**172**	**7,634**	**101,375**	**109,181**
City of Malabon	26,264	0	0	1	1	9	177	1,679	1,865
City of Navotas	23,816	0	0	0	0	0	381	2,956	3,337
City of Valenzuela	40,454	86	32	906	1,024	61	192	3,880	4,133
City of Caloocan	73,676	0	0	4	4	7	846	7,354	8,207
City of Marikina	24,847	0	0	0	0	1	17	407	425
City of Pasig	58,937	0	0	6	6	18	504	2,615	3,137
Pateros	4,204	0	0	0	0	0	4	784	788
City of Taguig	92,053	0	0	0	0	7	825	9,825	10,657
Quezon City	338,677	0	0	74	74	18	1,804	35,035	36,857
City of Makati	28,572	0	0	0	0	0	19	956	975
City of Mandaluyong	27,196	0	0	29	29	7	304	5,053	5,364
City of San Juan	6,139	0	0	0	0	0	48	1,191	1,239
City of Manila	138,102	0	7	143	150	35	614	10,167	10,816
City of Las Piñas	26,980	0	0	12	12	5	227	4,494	4,726
City of Muntinlupa	44,186	0	0	0	0	1	186	2,698	2,885
City of Parañaque	47,100	0	0	20	20	3	1,082	6,744	7,829
Pasay City	40,328	0	0	5	5	0	404	5,537	5,941
**C A R**	**165,663**	**2**	**12**	**1,230**	**1,244**	**30**	**1,166**	**12,533**	**13,729**
Abra	19,755	0	2	86	88	4	266	2,226	2,496
Apayao	24,752	0	0	1	1	2	152	1,006	1,160
Benguet	37,253	0	5	500	505	5	175	2,778	2,958
Ifugao	21,120	2	3	366	371	6	202	1,774	1,982
Kalinga	18,267	0	0	105	105	4	218	2,817	3,039
Mountain Province	13,957	0	2	143	145	9	151	1,445	1,605
City of Baguio	30,559	0	0	29	29	0	2	487	489
**Region 1**	**498,204**	**0**	**13**	**870**	**883**	**46**	**2,915**	**36,829**	**39,790**
Ilocos Norte	52,824	0	0	352	352	3	246	4,592	4,841
Ilocos Sur	78,125	0	9	252	261	5	368	5,513	5,886
La Union	67,784	0	4	159	163	10	613	6,169	6,792
Pangasinan	292,353	0	0	107	107	27	1,602	19,967	21,596
City of Dagupan	7,118	0	0	0	0	1	86	588	675
**Region 2**	**343,378**	**0**	**3**	**288**	**291**	**72**	**2,468**	**19,590**	**22,130**
Batanes	955	0	0	3	3	0	18	170	188
Cagayan	108,930	0	0	4	4	4	512	5,094	5,610
Isabela	146,131	0	0	8	8	44	884	6,362	7,290
Nueva Vizcaya	52,095	0	2	246	248	19	681	4,554	5,254
Quirino	21,240	0	1	1	2	2	135	960	1,097
City of Santiago	14,027	0	0	26	26	3	238	2,450	2,691
**Region 3**	**985,325**	**0**	**9**	**620**	**629**	**77**	**5,026**	**50,255**	**55,358**
Aurora	24,562	0	1	6	7	4	328	2,311	2,643
Bataan	65,716	0	1	71	72	16	578	5,274	5,868
Bulacan	302,904	0	3	165	168	6	1,014	11,927	12,947
Nueva Ecija	200,897	0	1	27	28	27	1,342	8,752	10,121
Pampanga	167,294	0	0	323	323	13	654	8,254	8,921
Tarlac	118,444	0	3	7	10	2	642	8,470	9,114
Zambales	54,326	0	0	11	11	7	333	2,829	3,169
City of Angeles	30,345	0	0	8	8	1	89	1,376	1,466
City of Olongapo	20,837	0	0	2	2	1	46	1,062	1,109
**Region 4A**	**888,692**	**0**	**29**	**1,282**	**1,311**	**82**	**5,516**	**67,740**	**73,338**
Batangas	158,569	0	1	272	273	8	463	9,546	10,017
Cavite	175,332	0	8	18	26	7	814	15,150	15,971
Laguna	286,016	0	18	541	559	22	1,806	19,891	21,719
Quezon	106,773	0	2	445	447	30	1,013	9,112	10,155
Rizal	156,554	0	0	6	6	13	1,414	14,002	15,429
City of Lucena	5,448	0	0	0	0	2	6	39	47
**Region 4B**	**291,730**	**0**	**2**	**399**	**401**	**102**	**4,028**	**28,699**	**32,829**
Marinduque	16,436	0	0	5	5	1	27	698	726
Occidental Mindoro	48,902	0	2	68	70	26	1,547	9,947	11,520
Oriental Mindoro	68,962	0	0	53	53	13	581	5,662	6,256
Palawan	100,239	0	0	69	69	51	1,388	8,498	9,937

Table 2.A.3 - Modern Methods of Family Planning
Drop Outs
Philippines, 2024

Area	Total Current Users	NFP-SDM			Total	NFP-LAM			Total
		Age group				Age group			
		10-14	15-19	20-49		10-14	15-19	20-49	
Romblon	22,467	0	0	169	169	9	319	2,835	3,163
City of Puerto Princesa	34,724	0	0	35	35	2	166	1,059	1,227
**Region 5**	**491,006**	**54**	**521**	**12,332**	**12,907**	**68**	**6,472**	**57,991**	**64,531**
Albay	115,867	0	26	1,912	1,938	6	503	7,382	7,891
Camarines Norte	46,839	0	29	578	607	3	784	5,445	6,232
Camarines Sur	107,464	0	38	4,012	4,050	11	1,703	15,664	17,378
Catanduanes	24,326	1	7	862	870	9	407	4,401	4,817
Masbate	96,672	53	394	3,810	4,257	19	1,919	12,168	14,106
Sorsogon	66,956	0	27	789	816	20	1,141	10,709	11,870
City of Naga	32,882	0	0	369	369	0	15	2,222	2,237
**Region 6**	**667,635**	**0**	**60**	**1,459**	**1,519**	**111**	**5,314**	**45,808**	**51,233**
Aklan	48,553	0	2	158	160	9	423	4,482	4,914
Antique	49,316	0	1	102	103	2	185	2,365	2,552
Capiz	77,942	0	0	0	0	1	99	2,197	2,297
Guimaras	17,943	0	0	15	15	5	218	1,932	2,155
Iloilo	182,130	0	11	284	295	34	937	10,437	11,408
Negros Occidental	206,889	0	45	858	903	58	3,299	22,949	26,306
City of Bacolod	30,421	0	0	0	0	2	139	1,288	1,429
City of Iloilo	54,441	0	1	42	43	0	14	158	172
**Region 7**	**483,438**	**0**	**6**	**677**	**683**	**116**	**6,643**	**53,497**	**60,256**
Bohol	83,324	0	0	66	66	6	472	4,706	5,184
Cebu	191,250	0	2	455	457	49	3,003	20,186	23,238
Negros Oriental	98,214	0	1	71	72	39	1,452	11,751	13,242
Siquijor	9,225	0	2	83	85	0	80	423	503
City of Cebu	34,897	0	1	2	3	20	1,098	9,467	10,585
City of Lapu-Lapu	36,794	0	0	0	0	2	518	6,657	7,177
City of Mandaue	29,734	0	0	0	0	0	20	307	327
**Region 8**	**340,613**	**0**	**85**	**1,269**	**1,354**	**44**	**2,644**	**20,794**	**23,482**
Biliran	12,714	0	0	117	117	1	171	1,320	1,492
Eastern Samar	39,982	0	2	503	505	10	384	3,060	3,454
Leyte	125,631	0	69	355	424	16	564	4,810	5,390
Northern Samar	48,697	0	9	182	191	8	343	4,040	4,391
Southern Leyte	27,251	0	1	32	33	0	232	2,026	2,258
Samar	42,957	0	0	0	0	7	325	2,131	2,463
Ormoc City	20,171	0	4	80	84	0	377	2,127	2,504
City of Tacloban	23,210	0	0	0	0	2	248	1,280	1,530
**Region 9**	**354,267**	**0**	**16**	**488**	**504**	**34**	**3,851**	**25,607**	**29,492**
Zamboanga del Norte	126,112	0	16	384	400	18	1,402	8,438	9,858
Zamboanga del Sur	102,687	0	0	0	0	2	509	3,896	4,407
Zamboanga Sibugay	47,839	0	0	75	75	3	416	2,949	3,368
City of Isabela	7,167	0	0	29	29	0	142	1,118	1,260
City of Zamboanga	70,462	0	0	0	0	11	1,382	9,206	10,599
**Region 10**	**511,701**	**32**	**394**	**2,894**	**3,320**	**112**	**6,889**	**45,448**	**52,449**
Bukidnon	167,011	22	101	1,508	1,631	53	2,939	13,977	16,969
Camiguin	6,884	0	0	18	18	0	86	754	840
Lanao del Norte	73,898	0	6	78	84	10	728	6,844	7,582
Misamis Occidental	60,887	9	255	355	619	13	409	5,198	5,620
Misamis Oriental	111,011	1	18	347	366	14	970	6,372	7,356
City of Cagayan De Oro	57,799	0	0	61	61	15	1,241	7,929	9,185
City of Iligan	34,211	0	14	527	541	7	516	4,374	4,897
**Region 11**	**535,532**	**0**	**7**	**889**	**896**	**87**	**2,586**	**21,293**	**23,966**
Davao de Oro	84,560	0	0	5	5	2	107	1,311	1,420
Davao del Norte	112,914	0	3	128	131	10	388	3,463	3,861
Davao Oriental	59,172	0	2	664	666	32	823	4,820	5,675
Davao del Sur	67,054	0	1	22	23	6	164	981	1,151
Davao Occidental	33,334	0	1	27	28	7	233	905	1,145
City of Davao	178,498	0	0	43	43	30	871	9,813	10,714
**Region 12**	**476,219**	**0**	**30**	**308**	**338**	**68**	**4,940**	**29,618**	**34,626**
Cotabato	136,508	0	0	17	17	20	836	5,500	6,356
Sarangani	76,390	0	17	151	168	0	1,574	7,134	8,708
South Cotabato	102,265	0	1	72	73	11	1,011	6,198	7,220

Table 2.A.3 - Modern Methods of Family Planning
Drop Outs
Philippines, 2024

Area	Total Current Users	NFP-SDM			Total	NFP-LAM			Total
		Age group				Age group			
		10-14	15-19	20-49		10-14	15-19	20-49	
Sultan Kudarat	104,518	0	12	68	80	33	1,013	6,388	7,434
City of General Santos	56,538	0	0	0	0	4	506	4,398	4,908
**Caraga**	**292,511**	**1**	**41**	**1,019**	**1,061**	**56**	**2,633**	**21,781**	**24,470**
Agusan del Norte	40,148	0	4	78	82	2	345	2,622	2,969
Agusan del Sur	86,721	0	18	300	318	13	748	5,021	5,782
Surigao del Norte	53,159	1	13	446	460	2	283	2,589	2,874
Surigao del Sur	60,298	0	0	139	139	28	590	6,740	7,358
Dinagat Islands	9,606	0	6	56	62	0	66	486	552
City of Butuan	42,579	0	0	0	0	11	601	4,323	4,935
**BARMM**	**296,590**	**0**	**0**	**62**	**62**	**31**	**3,498**	**39,263**	**42,792**
Basilan	19,309	0	0	4	4	2	208	1,228	1,438
Lanao del Sur	39,800	0	0	44	44	3	589	13,503	14,095
Maguindanao del Norte	36,321	0	0	0	0	9	508	5,942	6,459
Maguindanao del Sur	51,529	0	0	0	0	7	1,319	8,342	9,668
Sulu	80,817	0	0	0	0	0	276	4,869	5,145
Tawi-Tawi	19,107	0	0	2	2	0	298	2,949	3,247
SGA	7,550	0	0	0	0	0	59	615	674
City of Cotabato	42,157	0	0	12	12	10	241	1,815	2,066

Table 2.A.4 - Modern Methods of Family Planning
Current User (Ending)
Philippines, 2024

Area	Total Current Users	FSTR/BTL Age group 10-14	FSTR/BTL Age group 15-19	FSTR/BTL Age group 20-49	Total	MSTR/NSV Age group 10-14	MSTR/NSV Age group 15-19	MSTR/NSV Age group 20-49	Total
**PHILIPPINES**	**9,031,012**	**7**	**889**	**834,741**	**835,637**	**0**	**36**	**7,417**	**7,453**
**N C R**	**1,212,532**	**0**	**76**	**102,782**	**102,858**	**0**	**10**	**1,363**	**1,373**
City of Malabon	24,231	0	0	3,489	3,489	0	0	16	16
City of Navotas	23,645	0	1	606	607	0	0	5	5
City of Valenzuela	45,887	0	0	6,524	6,524	0	0	92	92
City of Caloocan	83,350	0	0	10,379	10,379	0	0	10	10
City of Marikina	29,479	0	0	2,500	2,500	0	0	201	201
City of Pasig	64,363	0	0	5,655	5,655	0	0	182	182
Pateros	4,444	0	0	402	402	0	0	7	7
City of Taguig	113,829	0	2	5,116	5,118	0	6	52	58
Quezon City	365,159	0	67	36,572	36,639	0	0	258	258
City of Makati	34,624	0	0	2,047	2,047	0	0	40	40
City of Mandaluyong	26,244	0	0	2,166	2,166	0	0	45	45
City of San Juan	6,312	0	0	181	181	0	0	7	7
City of Manila	210,914	0	2	10,877	10,879	0	1	364	365
City of Las Piñas	37,215	0	0	4,417	4,417	0	0	20	20
City of Muntinlupa	46,022	0	2	4,064	4,066	0	2	27	29
City of Parañaque	52,957	0	0	3,325	3,325	0	1	32	33
Pasay City	43,857	0	2	4,462	4,464	0	0	5	5
**C A R**	**172,183**	**0**	**2**	**33,337**	**33,339**	**0**	**0**	**249**	**249**
Abra	19,945	0	0	3,117	3,117	0	0	2	2
Apayao	25,169	0	0	997	997	0	0	2	2
Benguet	38,784	0	2	7,246	7,248	0	0	67	67
Ifugao	22,540	0	0	2,974	2,974	0	0	64	64
Kalinga	19,221	0	0	3,327	3,327	0	0	2	2
Mountain Province	14,288	0	0	5,683	5,683	0	0	4	4
City of Baguio	32,236	0	0	9,993	9,993	0	0	108	108
**Region 1**	**510,193**	**0**	**30**	**66,424**	**66,454**	**0**	**0**	**114**	**114**
Ilocos Norte	51,601	0	11	9,709	9,720	0	0	49	49
Ilocos Sur	80,076	0	3	10,260	10,263	0	0	3	3
La Union	67,429	0	1	11,305	11,306	0	0	25	25
Pangasinan	304,595	0	15	34,470	34,485	0	0	36	36
City of Dagupan	6,492	0	0	680	680	0	0	1	1
**Region 2**	**352,751**	**0**	**112**	**45,386**	**45,498**	**0**	**0**	**109**	**109**
Batanes	967	0	0	169	169	0	0	0	0
Cagayan	112,004	0	66	13,147	13,213	0	0	32	32
Isabela	151,442	0	40	18,594	18,634	0	0	37	37
Nueva Vizcaya	52,387	0	0	8,774	8,774	0	0	34	34
Quirino	21,116	0	6	3,427	3,433	0	0	6	6
City of Santiago	14,835	0	0	1,275	1,275	0	0	0	0
**Region 3**	**1,067,691**	**0**	**12**	**189,302**	**189,314**	**0**	**1**	**237**	**238**
Aurora	24,504	0	0	2,182	2,182	0	0	3	3
Bataan	71,353	0	0	8,391	8,391	0	1	7	8
Bulacan	341,316	0	3	54,315	54,318	0	0	64	64
Nueva Ecija	216,078	0	0	40,413	40,413	0	0	12	12
Pampanga	178,397	0	3	47,394	47,397	0	0	35	35
Tarlac	120,786	0	6	18,723	18,729	0	0	16	16
Zambales	57,286	0	0	8,934	8,934	0	0	13	13
City of Angeles	33,168	0	0	6,263	6,263	0	0	75	75
City of Olongapo	24,803	0	0	2,687	2,687	0	0	12	12
**Region 4A**	**913,336**	**0**	**77**	**94,882**	**94,959**	**0**	**15**	**423**	**438**
Batangas	162,127	0	12	20,145	20,157	0	0	104	104
Cavite	185,925	0	35	23,071	23,106	0	9	103	112
Laguna	258,745	0	13	27,475	27,488	0	0	123	123
Quezon	111,585	0	10	9,440	9,450	0	6	17	23
Rizal	189,319	0	7	14,561	14,568	0	0	73	73
City of Lucena	5,635	0	0	190	190	0	0	3	3
**Region 4B**	**294,862**	**7**	**127**	**23,469**	**23,603**	**0**	**1**	**187**	**188**
Marinduque	16,756	0	0	2,350	2,350	0	0	7	7
Occidental Mindoro	45,599	3	0	2,057	2,060	0	0	6	6
Oriental Mindoro	69,825	4	82	7,586	7,672	0	0	38	38
Palawan	104,000	0	0	5,880	5,880	0	0	84	84

Table 2.A.4 - Modern Methods of Family Planning
Current User (Ending)
Philippines, 2024

Area	Total Current Users	FSTR/BTL Age group 10-14	15-19	20-49	Total	MSTR/NSV Age group 10-14	15-19	20-49	Total
Romblon	23,163	0	10	3,136	3,146	0	0	39	39
City of Puerto Princesa	35,519	0	35	2,460	2,495	0	1	13	14
**Region 5**	**478,179**	**0**	**7**	**26,974**	**26,981**	**0**	**1**	**228**	**229**
Albay	118,150	0	4	7,623	7,627	0	0	62	62
Camarines Norte	44,180	0	0	1,172	1,172	0	1	5	6
Camarines Sur	104,506	0	0	3,946	3,946	0	0	9	9
Catanduanes	23,525	0	2	3,247	3,249	0	0	53	53
Masbate	92,606	0	0	2,711	2,711	0	0	25	25
Sorsogon	67,474	0	1	6,242	6,243	0	0	37	37
City of Naga	27,738	0	0	2,033	2,033	0	0	37	37
**Region 6**	**668,982**	**0**	**8**	**49,905**	**49,913**	**0**	**1**	**1,280**	**1,281**
Aklan	48,366	0	1	3,125	3,126	0	0	3	3
Antique	51,842	0	2	6,663	6,665	0	1	85	86
Capiz	78,489	0	0	4,306	4,306	0	0	277	277
Guimaras	18,806	0	0	1,318	1,318	0	0	6	6
Iloilo	185,570	0	1	11,907	11,908	0	0	212	212
Negros Occidental	198,892	0	4	13,384	13,388	0	0	615	615
City of Bacolod	33,573	0	0	4,310	4,310	0	0	71	71
City of Iloilo	53,444	0	0	4,892	4,892	0	0	11	11
**Region 7**	**485,713**	**0**	**23**	**25,988**	**26,011**	**0**	**0**	**854**	**854**
Bohol	82,389	0	0	8,140	8,140	0	0	145	145
Cebu	189,570	0	1	11,386	11,387	0	0	202	202
Negros Oriental	104,439	0	20	3,380	3,400	0	0	190	190
Siquijor	8,591	0	0	295	295	0	0	1	1
City of Cebu	41,141	0	2	1,967	1,969	0	0	266	266
City of Lapu-Lapu	26,667	0	0	371	371	0	0	50	50
City of Mandaue	32,916	0	0	449	449	0	0	0	0
**Region 8**	**342,292**	**0**	**44**	**19,104**	**19,148**	**0**	**1**	**238**	**239**
Biliran	13,195	0	0	1,426	1,426	0	0	29	29
Eastern Samar	37,602	0	40	1,896	1,936	0	0	35	35
Leyte	124,892	0	0	9,627	9,627	0	0	89	89
Northern Samar	48,081	0	1	1,023	1,024	0	0	40	40
Southern Leyte	28,645	0	0	939	939	0	1	11	12
Samar	46,978	0	0	1,633	1,633	0	0	12	12
Ormoc City	20,250	0	0	1,296	1,296	0	0	15	15
City of Tacloban	22,649	0	3	1,264	1,267	0	0	7	7
**Region 9**	**376,798**	**0**	**6**	**14,656**	**14,662**	**0**	**0**	**19**	**19**
Zamboanga del Norte	129,763	0	0	5,110	5,110	0	0	1	1
Zamboanga del Sur	113,598	0	3	4,200	4,203	0	0	6	6
Zamboanga Sibugay	52,229	0	0	1,659	1,659	0	0	7	7
City of Isabela	8,454	0	1	532	533	0	0	1	1
City of Zamboanga	72,754	0	2	3,155	3,157	0	0	4	4
**Region 10**	**541,549**	**0**	**9**	**33,372**	**33,381**	**0**	**0**	**193**	**193**
Bukidnon	174,789	0	1	10,761	10,762	0	0	89	89
Camiguin	7,102	0	0	1,101	1,101	0	0	5	5
Lanao del Norte	77,655	0	0	1,144	1,144	0	0	17	17
Misamis Occidental	61,818	0	0	3,399	3,399	0	0	8	8
Misamis Oriental	114,743	0	8	10,441	10,449	0	0	43	43
City of Cagayan De Oro	71,604	0	0	4,111	4,111	0	0	16	16
City of Iligan	33,838	0	0	2,415	2,415	0	0	15	15
**Region 11**	**541,328**	**0**	**37**	**42,511**	**42,548**	**0**	**2**	**1,110**	**1,112**
Davao de Oro	81,619	0	1	6,787	6,788	0	1	97	98
Davao del Norte	116,452	0	0	11,485	11,485	0	0	135	135
Davao Oriental	60,788	0	8	5,326	5,334	0	0	125	125
Davao del Sur	67,988	0	23	4,427	4,450	0	0	80	80
Davao Occidental	33,573	0	5	3,399	3,404	0	0	53	53
City of Davao	180,908	0	0	11,087	11,087	0	1	620	621
**Region 12**	**476,809**	**0**	**311**	**38,852**	**39,163**	**0**	**4**	**683**	**687**
Cotabato	141,578	0	22	9,729	9,751	0	0	100	100
Sarangani	76,320	0	192	4,272	4,464	0	4	145	149
South Cotabato	100,488	0	0	9,052	9,052	0	0	311	311

Table 2.A.4 - Modern Methods of Family Planning
Current User (Ending)
Philippines, 2024

Area	Total Current Users	FSTR/BTL			Total	MSTR/NSV			Total
		Age group				Age group			
		10-14	15-19	20-49		10-14	15-19	20-49	
Sultan Kudarat	97,463	0	94	8,372	8,466	0	0	84	84
City of General Santos	60,960	0	3	7,427	7,430	0	0	43	43
**Caraga**	**297,799**	**0**	**5**	**19,854**	**19,859**	**0**	**0**	**108**	**108**
Agusan del Norte	41,712	0	4	3,261	3,265	0	0	1	1
Agusan del Sur	84,822	0	0	6,234	6,234	0	0	37	37
Surigao del Norte	59,436	0	0	3,239	3,239	0	0	25	25
Surigao del Sur	62,409	0	1	4,123	4,124	0	0	31	31
Dinagat Islands	10,221	0	0	377	377	0	0	0	0
City of Butuan	39,199	0	0	2,620	2,620	0	0	14	14
**BARMM**	**298,015**	**0**	**3**	**7,943**	**7,946**	**0**	**0**	**22**	**22**
Basilan	20,641	0	0	506	506	0	0	0	0
Lanao del Sur	37,006	0	0	1,780	1,780	0	0	22	22
Maguindanao del Norte	37,544	0	0	637	637	0	0	0	0
Maguindanao del Sur	45,670	0	3	979	982	0	0	0	0
Sulu	87,159	0	0	1,304	1,304	0	0	0	0
Tawi-Tawi	20,726	0	0	336	336	0	0	0	0
SGA	6,557	0	0	103	103	0	0	0	0
City of Cotabato	42,712	0	0	2,298	2,298	0	0	0	0

Table 2.A.4 - Modern Methods of Family Planning
Current User (Ending)
Philippines, 2024

Area	Total Current Users	CONDOM			Total	IUD-INTERVAL			Total
		Age group				Age group			
		10-14	15-19	20-49		10-14	15-19	20-49	
**PHILIPPINES**	**9,031,012**	**398**	**17,113**	**462,248**	**479,759**	**201**	**8,181**	**381,960**	**390,342**
**N C R**	**1,212,532**	**90**	**5,961**	**143,301**	**149,352**	**15**	**1,414**	**42,563**	**43,992**
City of Malabon	24,231	0	38	955	993	0	25	1,342	1,367
City of Navotas	23,645	6	568	1,073	1,647	0	40	79	119
City of Valenzuela	45,887	0	10	1,601	1,611	0	3	324	327
City of Caloocan	83,350	2	158	3,233	3,393	4	306	4,894	5,204
City of Marikina	29,479	0	18	1,064	1,082	0	53	1,238	1,291
City of Pasig	64,363	15	173	3,712	3,900	0	57	2,591	2,648
Pateros	4,444	0	1	305	306	0	2	497	499
City of Taguig	113,829	16	781	11,123	11,920	1	97	2,759	2,857
Quezon City	365,159	1	1,363	61,943	63,307	1	430	17,362	17,793
City of Makati	34,624	1	56	5,673	5,730	2	16	1,019	1,037
City of Mandaluyong	26,244	0	31	2,279	2,310	1	13	696	710
City of San Juan	6,312	0	0	168	168	0	3	1,309	1,312
City of Manila	210,914	40	1,160	34,059	35,259	1	153	1,630	1,784
City of Las Piñas	37,215	3	104	5,122	5,229	2	16	583	601
City of Muntinlupa	46,022	0	78	1,360	1,438	1	70	2,449	2,520
City of Parañaque	52,957	2	534	6,669	7,205	0	30	1,091	1,121
Pasay City	43,857	4	888	2,962	3,854	2	100	2,700	2,802
**C A R**	**172,183**	**3**	**229**	**7,380**	**7,612**	**0**	**79**	**5,665**	**5,744**
Abra	19,945	0	106	1,477	1,583	0	2	17	19
Apayao	25,169	0	18	195	213	0	3	417	420
Benguet	38,784	0	13	2,834	2,847	0	18	2,441	2,459
Ifugao	22,540	0	6	628	634	0	3	963	966
Kalinga	19,221	0	21	535	556	0	9	636	645
Mountain Province	14,288	1	3	446	450	0	4	381	385
City of Baguio	32,236	2	62	1,265	1,329	0	40	810	850
**Region 1**	**510,193**	**91**	**339**	**20,859**	**21,289**	**7**	**260**	**10,181**	**10,448**
Ilocos Norte	51,601	0	16	1,316	1,332	0	20	579	599
Ilocos Sur	80,076	0	63	2,626	2,689	0	9	597	606
La Union	67,429	2	44	1,081	1,127	0	66	1,505	1,571
Pangasinan	304,595	89	216	15,681	15,986	7	162	7,419	7,588
City of Dagupan	6,492	0	0	155	155	0	3	81	84
**Region 2**	**352,751**	**1**	**92**	**3,818**	**3,911**	**8**	**411**	**16,095**	**16,514**
Batanes	967	0	0	12	12	0	0	6	6
Cagayan	112,004	1	41	844	886	5	216	10,600	10,821
Isabela	151,442	0	19	1,713	1,732	2	122	3,620	3,744
Nueva Vizcaya	52,387	0	22	910	932	0	41	903	944
Quirino	21,116	0	9	304	313	0	24	693	717
City of Santiago	14,835	0	1	35	36	1	8	273	282
**Region 3**	**1,067,691**	**97**	**2,014**	**51,211**	**53,322**	**109**	**402**	**16,234**	**16,745**
Aurora	24,504	0	17	1,874	1,891	0	2	244	246
Bataan	71,353	4	270	3,646	3,920	1	40	538	579
Bulacan	341,316	1	582	22,728	23,311	5	96	9,870	9,971
Nueva Ecija	216,078	0	255	4,754	5,009	1	70	2,847	2,918
Pampanga	178,397	83	540	8,995	9,618	101	162	924	1,187
Tarlac	120,786	2	267	5,261	5,530	1	18	872	891
Zambales	57,286	6	54	2,073	2,133	0	3	558	561
City of Angeles	33,168	1	14	633	648	0	3	295	298
City of Olongapo	24,803	0	15	1,247	1,262	0	8	86	94
**Region 4A**	**913,336**	**45**	**2,516**	**44,956**	**47,517**	**31**	**522**	**27,389**	**27,942**
Batangas	162,127	10	322	9,318	9,650	1	24	3,384	3,409
Cavite	185,925	3	275	6,806	7,084	5	123	5,332	5,460
Laguna	258,745	21	1,636	16,368	18,025	2	178	7,427	7,607
Quezon	111,585	9	54	2,453	2,516	12	86	6,850	6,948
Rizal	189,319	2	227	9,965	10,194	3	111	4,377	4,491
City of Lucena	5,635	0	2	46	48	8	0	19	27
**Region 4B**	**294,862**	**2**	**312**	**7,406**	**7,720**	**1**	**66**	**5,755**	**5,822**
Marinduque	16,756	0	0	309	309	0	0	342	342
Occidental Mindoro	45,599	2	11	995	1,008	1	3	825	829
Oriental Mindoro	69,825	0	164	1,848	2,012	0	33	2,493	2,526
Palawan	104,000	0	102	2,505	2,607	0	24	1,045	1,069

Table 2.A.4 - Modern Methods of Family Planning
Current User (Ending)
Philippines, 2024

Area	Total Current Users	CONDOM				IUD-INTERVAL			
		Age group			Total	Age group			Total
		10-14	15-19	20-49		10-14	15-19	20-49	
Romblon	23,163	0	1	567	568	0	3	705	708
City of Puerto Princesa	35,519	0	34	1,182	1,216	0	3	345	348
**Region 5**	**478,179**	**0**	**402**	**27,290**	**27,692**	**0**	**106**	**5,124**	**5,230**
Albay	118,150	0	109	9,887	9,996	0	11	979	990
Camarines Norte	44,180	0	85	1,907	1,992	0	60	951	1,011
Camarines Sur	104,506	0	71	5,249	5,320	0	13	1,030	1,043
Catanduanes	23,525	0	21	592	613	0	1	50	51
Masbate	92,606	0	88	2,784	2,872	0	3	1,525	1,528
Sorsogon	67,474	0	21	2,333	2,354	0	2	209	211
City of Naga	27,738	0	7	4,538	4,545	0	16	380	396
**Region 6**	**668,982**	**3**	**725**	**32,310**	**33,038**	**9**	**467**	**34,233**	**34,709**
Aklan	48,366	0	71	3,361	3,432	0	6	609	615
Antique	51,842	1	17	1,588	1,606	0	2	591	593
Capiz	78,489	0	30	3,757	3,787	0	25	6,312	6,337
Guimaras	18,806	0	6	1,224	1,230	0	1	302	303
Iloilo	185,570	1	287	9,509	9,797	7	158	8,892	9,057
Negros Occidental	198,892	1	277	6,723	7,001	0	149	11,783	11,932
City of Bacolod	33,573	0	6	1,526	1,532	2	45	3,317	3,364
City of Iloilo	53,444	0	31	4,622	4,653	0	81	2,427	2,508
**Region 7**	**485,713**	**2**	**466**	**23,262**	**23,730**	**1**	**1,256**	**47,370**	**48,627**
Bohol	82,389	0	20	4,787	4,807	0	112	11,157	11,269
Cebu	189,570	1	123	9,442	9,566	1	909	18,800	19,710
Negros Oriental	104,439	1	146	3,400	3,547	0	139	9,398	9,537
Siquijor	8,591	0	4	398	402	0	2	535	537
City of Cebu	41,141	0	11	1,054	1,065	0	64	3,558	3,622
City of Lapu-Lapu	26,667	0	147	1,893	2,040	0	19	1,269	1,288
City of Mandaue	32,916	0	15	2,288	2,303	0	11	2,653	2,664
**Region 8**	**342,292**	**7**	**487**	**12,070**	**12,564**	**0**	**207**	**21,110**	**21,317**
Biliran	13,195	0	16	362	378	0	19	689	708
Eastern Samar	37,602	0	37	2,522	2,559	0	10	446	456
Leyte	124,892	2	125	3,805	3,932	0	85	11,163	11,248
Northern Samar	48,081	3	35	1,264	1,302	0	4	1,725	1,729
Southern Leyte	28,645	0	25	1,066	1,091	0	28	3,957	3,985
Samar	46,978	2	91	798	891	0	13	776	789
Ormoc City	20,250	0	9	979	988	0	0	1,091	1,091
City of Tacloban	22,649	0	149	1,274	1,423	0	48	1,263	1,311
**Region 9**	**376,798**	**3**	**411**	**11,166**	**11,580**	**0**	**362**	**25,037**	**25,399**
Zamboanga del Norte	129,763	0	106	5,284	5,390	0	62	9,537	9,599
Zamboanga del Sur	113,598	3	61	3,029	3,093	0	176	9,472	9,648
Zamboanga Sibugay	52,229	0	170	1,857	2,027	0	83	3,846	3,929
City of Isabela	8,454	0	52	299	351	0	2	86	88
City of Zamboanga	72,754	0	22	697	719	0	39	2,096	2,135
**Region 10**	**541,549**	**30**	**892**	**24,240**	**25,162**	**11**	**1,110**	**44,303**	**45,424**
Bukidnon	174,789	3	161	4,426	4,590	4	407	13,627	14,038
Camiguin	7,102	0	6	268	274	0	2	697	699
Lanao del Norte	77,655	0	134	7,498	7,632	0	33	2,888	2,921
Misamis Occidental	61,818	17	197	3,887	4,101	0	22	2,687	2,709
Misamis Oriental	114,743	10	149	3,255	3,414	7	272	14,950	15,229
City of Cagayan De Oro	71,604	0	172	3,234	3,406	0	289	6,533	6,822
City of Iligan	33,838	0	73	1,672	1,745	0	85	2,921	3,006
**Region 11**	**541,328**	**21**	**1,118**	**20,430**	**21,569**	**2**	**612**	**33,864**	**34,478**
Davao de Oro	81,619	0	50	2,941	2,991	0	43	5,474	5,517
Davao del Norte	116,452	0	123	4,903	5,026	0	104	7,488	7,592
Davao Oriental	60,788	3	27	1,353	1,383	0	12	2,808	2,820
Davao del Sur	67,988	1	76	1,709	1,786	2	144	7,038	7,184
Davao Occidental	33,573	3	24	229	256	0	44	1,692	1,736
City of Davao	180,908	14	818	9,295	10,127	0	265	9,364	9,629
**Region 12**	**476,809**	**2**	**583**	**13,471**	**14,056**	**6**	**446**	**24,150**	**24,602**
Cotabato	141,578	2	125	4,578	4,705	1	143	10,493	10,637
Sarangani	76,320	0	133	2,275	2,408	0	43	2,023	2,066
South Cotabato	100,488	0	83	2,818	2,901	0	91	4,910	5,001

Table 2.A.4 - Modern Methods of Family Planning
Current User (Ending)
Philippines, 2024

Area	Total Current Users	CONDOM			Total	IUD-INTERVAL			Total
		Age group				Age group			
		10-14	15-19	20-49		10-14	15-19	20-49	
Sultan Kudarat	97,463	0	172	2,081	2,253	0	101	3,362	3,463
City of General Santos	60,960	0	70	1,719	1,789	5	68	3,362	3,435
**Caraga**	**297,799**	**0**	**253**	**10,407**	**10,660**	**0**	**362**	**20,074**	**20,436**
Agusan del Norte	41,712	0	38	1,523	1,561	0	132	3,978	4,110
Agusan del Sur	84,822	0	49	2,964	3,013	0	99	6,965	7,064
Surigao del Norte	59,436	0	52	1,864	1,916	0	49	2,770	2,819
Surigao del Sur	62,409	0	63	2,037	2,100	0	57	4,338	4,395
Dinagat Islands	10,221	0	3	559	562	0	12	644	656
City of Butuan	39,199	0	48	1,460	1,508	0	13	1,379	1,392
**BARMM**	**298,015**	**1**	**313**	**8,671**	**8,985**	**1**	**99**	**2,813**	**2,913**
Basilan	20,641	0	12	243	255	1	30	278	309
Lanao del Sur	37,006	0	71	3,084	3,155	0	24	685	709
Maguindanao del Norte	37,544	0	37	1,178	1,215	0	15	602	617
Maguindanao del Sur	45,670	1	58	1,082	1,141	0	0	155	155
Sulu	87,159	0	81	588	669	0	0	321	321
Tawi-Tawi	20,726	0	0	373	373	0	0	4	4
SGA	6,557	0	7	241	248	0	0	2	2
City of Cotabato	42,712	0	47	1,882	1,929	0	30	766	796

Table 2.A.4 - Modern Methods of Family Planning
Current User (Ending)
Philippines, 2024

Area	Total Current Users	IUD-POSTPARTUM Age group 10-14	15-19	20-49	Total	PILLS-POP Age group 10-14	15-19	20-49	Total
**PHILIPPINES**	**9,031,012**	**1,025**	**17,332**	**169,554**	**187,911**	**684**	**39,448**	**435,468**	**475,600**
**N C R**	**1,212,532**	**171**	**4,689**	**42,137**	**46,997**	**135**	**6,168**	**80,428**	**86,731**
City of Malabon	24,231	0	7	88	95	1	189	776	966
City of Navotas	23,645	0	2	5	7	12	523	1,195	1,730
City of Valenzuela	45,887	0	25	899	924	1	19	1,284	1,304
City of Caloocan	83,350	12	311	1,974	2,297	0	238	3,338	3,576
City of Marikina	29,479	37	795	4,342	5,174	1	53	1,033	1,087
City of Pasig	64,363	25	599	2,767	3,391	3	253	2,441	2,697
Pateros	4,444	0	0	4	4	0	1	113	114
City of Taguig	113,829	5	84	1,014	1,103	16	1,249	7,258	8,523
Quezon City	365,159	76	1,691	23,815	25,582	63	1,643	25,663	27,369
City of Makati	34,624	0	134	1,832	1,966	0	79	3,364	3,443
City of Mandaluyong	26,244	7	221	1,091	1,319	0	89	1,672	1,761
City of San Juan	6,312	0	0	0	0	0	10	170	180
City of Manila	210,914	8	201	1,884	2,093	29	952	20,185	21,166
City of Las Piñas	37,215	0	2	107	109	1	116	2,332	2,449
City of Muntinlupa	46,022	0	1	23	24	7	318	1,411	1,736
City of Parañaque	52,957	1	502	709	1,212	1	378	4,433	4,812
Pasay City	43,857	0	114	1,583	1,697	0	58	3,760	3,818
**C A R**	**172,183**	**0**	**39**	**6,685**	**6,724**	**14**	**727**	**8,271**	**9,012**
Abra	19,945	0	3	11	14	0	121	1,168	1,289
Apayao	25,169	0	10	297	307	3	132	973	1,108
Benguet	38,784	0	10	585	595	0	97	1,638	1,735
Ifugao	22,540	0	4	92	96	4	64	1,711	1,779
Kalinga	19,221	0	3	52	55	0	102	459	561
Mountain Province	14,288	0	8	90	98	0	27	476	503
City of Baguio	32,236	0	1	5,558	5,559	7	184	1,846	2,037
**Region 1**	**510,193**	**3**	**166**	**1,594**	**1,763**	**5**	**972**	**16,247**	**17,224**
Ilocos Norte	51,601	0	16	121	137	1	102	2,755	2,858
Ilocos Sur	80,076	0	5	236	241	0	151	3,839	3,990
La Union	67,429	3	78	312	393	0	67	780	847
Pangasinan	304,595	0	56	839	895	4	640	8,800	9,444
City of Dagupan	6,492	0	11	86	97	0	12	73	85
**Region 2**	**352,751**	**11**	**285**	**1,836**	**2,132**	**20**	**1,586**	**15,557**	**17,163**
Batanes	967	0	0	0	0	0	2	16	18
Cagayan	112,004	3	150	816	969	5	594	5,194	5,793
Isabela	151,442	2	64	627	693	12	778	7,891	8,681
Nueva Vizcaya	52,387	5	69	367	441	2	148	1,851	2,001
Quirino	21,116	1	2	26	29	1	64	605	670
City of Santiago	14,835	0	0	0	0	0	0	0	0
**Region 3**	**1,067,691**	**625**	**3,100**	**10,842**	**14,567**	**71**	**4,263**	**33,187**	**37,521**
Aurora	24,504	1	3	78	82	1	31	860	892
Bataan	71,353	0	13	415	428	0	240	2,547	2,787
Bulacan	341,316	0	141	2,287	2,428	3	872	10,119	10,994
Nueva Ecija	216,078	53	1,310	3,972	5,335	27	1,090	6,841	7,958
Pampanga	178,397	571	1,602	3,300	5,473	37	1,322	5,440	6,799
Tarlac	120,786	0	8	256	264	1	428	4,635	5,064
Zambales	57,286	0	8	227	235	0	155	1,448	1,603
City of Angeles	33,168	0	2	180	182	1	55	577	633
City of Olongapo	24,803	0	13	127	140	1	70	720	791
**Region 4A**	**913,336**	**27**	**371**	**12,285**	**12,683**	**90**	**4,446**	**64,799**	**69,335**
Batangas	162,127	0	114	4,074	4,188	29	708	9,126	9,863
Cavite	185,925	0	39	918	957	11	543	6,834	7,388
Laguna	258,745	20	88	5,117	5,225	33	1,985	23,095	25,113
Quezon	111,585	3	6	401	410	7	405	6,191	6,603
Rizal	189,319	4	124	1,773	1,901	6	730	19,006	19,742
City of Lucena	5,635	0	0	2	2	4	75	547	626
**Region 4B**	**294,862**	**20**	**110**	**3,345**	**3,475**	**17**	**1,288**	**14,575**	**15,880**
Marinduque	16,756	0	1	5	6	0	57	2,346	2,403
Occidental Mindoro	45,599	0	4	71	75	5	184	2,065	2,254
Oriental Mindoro	69,825	19	98	2,362	2,479	2	179	1,783	1,964
Palawan	104,000	1	4	86	91	2	605	5,227	5,834

Table 2.A.4 - Modern Methods of Family Planning
Current User (Ending)
Philippines, 2024

Area	Total Current Users	IUD-POSTPARTUM			Total	PILLS-POP			Total
		Age group				Age group			
		10-14	15-19	20-49		10-14	15-19	20-49	
Romblon	23,163	0	2	771	773	2	31	574	607
City of Puerto Princesa	35,519	0	1	50	51	6	232	2,580	2,818
**Region 5**	**478,179**	**0**	**63**	**930**	**993**	**3**	**1,163**	**19,772**	**20,938**
Albay	118,150	0	8	390	398	1	227	5,713	5,941
Camarines Norte	44,180	0	27	83	110	0	235	2,187	2,422
Camarines Sur	104,506	0	13	239	252	1	219	4,566	4,786
Catanduanes	23,525	0	3	46	49	0	3	177	180
Masbate	92,606	0	2	46	48	1	241	3,929	4,171
Sorsogon	67,474	0	2	40	42	0	193	2,359	2,552
City of Naga	27,738	0	8	86	94	0	45	841	886
**Region 6**	**668,982**	**6**	**597**	**11,852**	**12,455**	**145**	**1,913**	**18,786**	**20,844**
Aklan	48,366	0	4	63	67	3	104	2,797	2,904
Antique	51,842	0	2	145	147	0	75	1,463	1,538
Capiz	78,489	0	0	66	66	0	94	1,459	1,553
Guimaras	18,806	1	4	50	55	0	21	457	478
Iloilo	185,570	4	268	4,509	4,781	7	648	5,399	6,054
Negros Occidental	198,892	0	241	3,920	4,161	6	881	6,076	6,963
City of Bacolod	33,573	1	37	2,443	2,481	129	68	873	1,070
City of Iloilo	53,444	0	41	656	697	0	22	262	284
**Region 7**	**485,713**	**13**	**1,694**	**16,372**	**18,079**	**27**	**1,867**	**21,052**	**22,946**
Bohol	82,389	9	337	4,811	5,157	0	71	1,943	2,014
Cebu	189,570	4	1,302	9,890	11,196	3	787	7,354	8,144
Negros Oriental	104,439	0	0	519	519	21	412	3,606	4,039
Siquijor	8,591	0	0	17	17	0	14	338	352
City of Cebu	41,141	0	19	724	743	1	224	3,012	3,237
City of Lapu-Lapu	26,667	0	1	89	90	0	263	3,079	3,342
City of Mandaue	32,916	0	35	322	357	2	96	1,720	1,818
**Region 8**	**342,292**	**1**	**227**	**2,946**	**3,174**	**3**	**861**	**11,988**	**12,852**
Biliran	13,195	0	7	275	282	0	31	419	450
Eastern Samar	37,602	0	11	88	99	0	66	697	763
Leyte	124,892	1	60	1,264	1,325	2	305	6,309	6,616
Northern Samar	48,081	0	8	390	398	0	102	986	1,088
Southern Leyte	28,645	0	22	392	414	0	40	860	900
Samar	46,978	0	51	470	521	1	161	1,011	1,173
Ormoc City	20,250	0	0	32	32	0	68	661	729
City of Tacloban	22,649	0	68	35	103	0	88	1,045	1,133
**Region 9**	**376,798**	**39**	**1,775**	**16,228**	**18,042**	**16**	**1,034**	**12,830**	**13,880**
Zamboanga del Norte	129,763	0	84	3,614	3,698	13	229	4,547	4,789
Zamboanga del Sur	113,598	27	1,085	7,846	8,958	2	271	3,662	3,935
Zamboanga Sibugay	52,229	0	70	501	571	0	254	2,565	2,819
City of Isabela	8,454	0	0	8	8	0	50	311	361
City of Zamboanga	72,754	12	536	4,259	4,807	1	230	1,745	1,976
**Region 10**	**541,549**	**79**	**2,595**	**18,289**	**20,963**	**23**	**2,769**	**28,513**	**31,305**
Bukidnon	174,789	0	62	900	962	5	1,048	5,247	6,300
Camiguin	7,102	0	5	53	58	0	7	67	74
Lanao del Norte	77,655	0	20	594	614	0	235	10,850	11,085
Misamis Occidental	61,818	0	33	436	469	0	302	3,153	3,455
Misamis Oriental	114,743	46	1,553	8,442	10,041	16	493	3,503	4,012
City of Cagayan De Oro	71,604	26	631	6,471	7,128	1	433	3,772	4,206
City of Iligan	33,838	7	291	1,393	1,691	1	251	1,921	2,173
**Region 11**	**541,328**	**11**	**292**	**4,770**	**5,073**	**60**	**4,368**	**35,896**	**40,324**
Davao de Oro	81,619	0	21	425	446	6	604	4,546	5,156
Davao del Norte	116,452	1	60	1,157	1,218	6	611	5,939	6,556
Davao Oriental	60,788	0	2	224	226	3	279	2,831	3,113
Davao del Sur	67,988	2	39	651	692	13	647	3,916	4,576
Davao Occidental	33,573	1	7	91	99	7	134	612	753
City of Davao	180,908	7	163	2,222	2,392	25	2,093	18,052	20,170
**Region 12**	**476,809**	**8**	**385**	**8,764**	**9,157**	**22**	**2,688**	**20,066**	**22,776**
Cotabato	141,578	0	64	1,418	1,482	3	642	7,036	7,681
Sarangani	76,320	0	6	174	180	0	901	3,760	4,661
South Cotabato	100,488	0	82	1,897	1,979	8	530	4,234	4,772

Table 2.A.4 - Modern Methods of Family Planning
Current User (Ending)
Philippines, 2024

Area	Total Current Users	IUD-POSTPARTUM Age group 10-14	IUD-POSTPARTUM Age group 15-19	IUD-POSTPARTUM Age group 20-49	Total	PILLS-POP Age group 10-14	PILLS-POP Age group 15-19	PILLS-POP Age group 20-49	Total
Sultan Kudarat	97,463	1	104	3,642	3,747	6	256	2,508	2,770
City of General Santos	60,960	7	129	1,633	1,769	5	359	2,528	2,892
**Caraga**	**297,799**	**6**	**649**	**7,776**	**8,431**	**10**	**1,841**	**17,234**	**19,085**
Agusan del Norte	41,712	4	103	858	965	0	242	2,579	2,821
Agusan del Sur	84,822	1	70	1,126	1,197	5	484	4,291	4,780
Surigao del Norte	59,436	1	270	1,541	1,812	0	437	3,629	4,066
Surigao del Sur	62,409	0	34	3,410	3,444	5	380	4,240	4,625
Dinagat Islands	10,221	0	0	27	27	0	16	284	300
City of Butuan	39,199	0	172	814	986	0	282	2,211	2,493
**BARMM**	**298,015**	**5**	**295**	**2,903**	**3,203**	**23**	**1,494**	**16,267**	**17,784**
Basilan	20,641	0	70	375	445	1	82	841	924
Lanao del Sur	37,006	0	5	337	342	7	197	3,043	3,247
Maguindanao del Norte	37,544	0	16	224	240	0	193	2,027	2,220
Maguindanao del Sur	45,670	0	9	53	62	0	192	2,653	2,845
Sulu	87,159	3	2	115	120	14	661	4,449	5,124
Tawi-Tawi	20,726	0	5	19	24	1	57	957	1,015
SGA	6,557	0	11	23	34	0	45	987	1,032
City of Cotabato	42,712	2	177	1,757	1,936	0	67	1,310	1,377

Table 2.A.4 - Modern Methods of Family Planning
Current User (Ending)
Philippines, 2024

Area	Total Current Users	PILLS-COC			Total	INJECTABLES			Total
		Age group				Age group			
		10-14	15-19	20-49		10-14	15-19	20-49	
**PHILIPPINES**	**9,031,012**	**573**	**73,890**	**2,942,447**	**3,016,910**	**938**	**102,081**	**1,826,406**	**1,929,425**
**N C R**	**1,212,532**	**70**	**7,487**	**237,401**	**244,958**	**147**	**15,802**	**248,906**	**264,855**
City of Malabon	24,231	0	336	4,701	5,037	2	553	5,812	6,367
City of Navotas	23,645	7	1,018	4,595	5,620	21	1,438	7,809	9,268
City of Valenzuela	45,887	1	277	9,210	9,488	0	609	9,760	10,369
City of Caloocan	83,350	5	348	9,399	9,752	7	1,315	23,524	24,846
City of Marikina	29,479	0	44	2,450	2,494	9	676	6,759	7,444
City of Pasig	64,363	4	415	11,011	11,430	8	1,219	15,855	17,082
Pateros	4,444	0	4	1,408	1,412	0	13	1,180	1,193
City of Taguig	113,829	18	951	18,001	18,970	12	1,240	24,218	25,470
Quezon City	365,159	10	1,529	90,642	92,181	12	2,029	47,092	49,133
City of Makati	34,624	0	78	5,357	5,435	0	198	8,060	8,258
City of Mandaluyong	26,244	0	127	4,043	4,170	0	129	5,159	5,288
City of San Juan	6,312	0	2	1,769	1,771	0	20	1,927	1,947
City of Manila	210,914	20	996	38,136	39,152	65	2,690	51,285	54,040
City of Las Piñas	37,215	0	116	6,446	6,562	4	368	7,108	7,480
City of Muntinlupa	46,022	5	700	12,881	13,586	6	1,769	8,042	9,817
City of Parañaque	52,957	0	460	8,639	9,099	1	1,412	11,857	13,270
Pasay City	43,857	0	86	8,713	8,799	0	124	13,459	13,583
**C A R**	**172,183**	**14**	**958**	**50,209**	**51,181**	**59**	**1,492**	**21,959**	**23,510**
Abra	19,945	6	241	7,846	8,093	15	170	2,287	2,472
Apayao	25,169	2	277	16,961	17,240	11	206	2,103	2,320
Benguet	38,784	1	100	10,099	10,200	1	164	5,881	6,046
Ifugao	22,540	4	91	4,799	4,894	3	153	3,161	3,317
Kalinga	19,221	1	101	5,719	5,821	11	161	2,748	2,920
Mountain Province	14,288	0	69	2,327	2,396	3	107	1,364	1,474
City of Baguio	32,236	0	79	2,458	2,537	15	531	4,415	4,961
**Region 1**	**510,193**	**5**	**2,695**	**200,856**	**203,556**	**19**	**3,325**	**107,410**	**110,754**
Ilocos Norte	51,601	2	137	20,081	20,220	0	123	7,980	8,103
Ilocos Sur	80,076	0	442	30,602	31,044	2	338	14,289	14,629
La Union	67,429	0	369	26,727	27,096	3	483	15,741	16,227
Pangasinan	304,595	3	1,729	121,309	123,041	14	2,318	67,404	69,736
City of Dagupan	6,492	0	18	2,137	2,155	0	63	1,996	2,059
**Region 2**	**352,751**	**23**	**2,999**	**171,450**	**174,472**	**67**	**2,662**	**50,555**	**53,284**
Batanes	967	0	2	186	188	0	12	269	281
Cagayan	112,004	13	653	55,005	55,671	22	657	13,758	14,437
Isabela	151,442	4	1,503	77,908	79,415	14	1,285	21,702	23,001
Nueva Vizcaya	52,387	4	381	23,534	23,919	4	326	6,559	6,889
Quirino	21,116	2	336	8,864	9,202	27	143	4,449	4,619
City of Santiago	14,835	0	124	5,953	6,077	0	239	3,818	4,057
**Region 3**	**1,067,691**	**74**	**10,857**	**336,423**	**347,354**	**156**	**16,035**	**262,013**	**278,204**
Aurora	24,504	0	137	11,446	11,583	0	166	3,874	4,040
Bataan	71,353	8	663	16,443	17,114	12	1,941	23,344	25,297
Bulacan	341,316	6	3,039	108,631	111,676	11	4,240	83,722	87,973
Nueva Ecija	216,078	6	2,804	81,401	84,211	13	2,887	45,010	47,910
Pampanga	178,397	41	1,521	41,231	42,793	87	2,904	40,083	43,074
Tarlac	120,786	6	1,499	42,510	44,015	16	1,559	32,732	34,307
Zambales	57,286	6	425	17,132	17,563	7	1,257	17,063	18,327
City of Angeles	33,168	1	327	9,552	9,880	3	484	9,807	10,294
City of Olongapo	24,803	0	442	8,077	8,519	7	597	6,378	6,982
**Region 4A**	**913,336**	**137**	**7,212**	**251,678**	**259,027**	**114**	**12,232**	**226,298**	**238,644**
Batangas	162,127	47	702	56,137	56,886	15	1,099	27,319	28,433
Cavite	185,925	25	1,182	53,616	54,823	30	2,390	51,453	53,873
Laguna	258,745	36	3,465	68,428	71,929	23	3,485	51,569	55,077
Quezon	111,585	11	568	36,714	37,293	18	1,622	31,146	32,786
Rizal	189,319	18	1,266	35,678	36,962	25	3,466	61,623	65,114
City of Lucena	5,635	0	29	1,105	1,134	3	170	3,188	3,361
**Region 4B**	**294,862**	**21**	**2,105**	**98,855**	**100,981**	**35**	**4,039**	**68,521**	**72,595**
Marinduque	16,756	0	13	4,937	4,950	1	92	2,956	3,049
Occidental Mindoro	45,599	7	333	15,297	15,637	6	385	8,878	9,269
Oriental Mindoro	69,825	6	391	30,886	31,283	1	445	11,324	11,770
Palawan	104,000	5	1,156	31,097	32,258	14	2,121	29,020	31,155

Table 2.A.4 - Modern Methods of Family Planning
Current User (Ending)
Philippines, 2024

Area	Total Current Users	PILLS-COC			Total	INJECTABLES			Total
		Age group				Age group			
		10-14	15-19	20-49		10-14	15-19	20-49	
Romblon	23,163	0	36	6,269	6,305	1	120	3,592	3,713
City of Puerto Princesa	35,519	3	176	10,369	10,548	12	876	12,751	13,639
**Region 5**	**478,179**	**10**	**2,404**	**177,050**	**179,464**	**12**	**2,830**	**82,803**	**85,645**
Albay	118,150	1	568	43,346	43,915	4	642	25,839	26,485
Camarines Norte	44,180	0	405	18,327	18,732	0	555	8,398	8,953
Camarines Sur	104,506	4	384	36,727	37,115	5	639	19,935	20,579
Catanduanes	23,525	1	21	4,107	4,129	0	131	4,624	4,755
Masbate	92,606	2	831	40,755	41,588	0	394	8,310	8,704
Sorsogon	67,474	2	159	23,277	23,438	3	394	11,888	12,285
City of Naga	27,738	0	36	10,511	10,547	0	75	3,809	3,884
**Region 6**	**668,982**	**32**	**4,286**	**280,136**	**284,454**	**29**	**4,999**	**129,696**	**134,724**
Aklan	48,366	0	217	17,498	17,715	2	420	13,948	14,370
Antique	51,842	2	172	15,533	15,707	1	453	13,809	14,263
Capiz	78,489	2	385	36,028	36,415	2	604	18,464	19,070
Guimaras	18,806	1	61	11,240	11,302	0	53	2,293	2,346
Iloilo	185,570	22	1,101	74,936	76,059	14	1,301	35,132	36,447
Negros Occidental	198,892	5	2,064	87,132	89,201	7	1,948	33,058	35,013
City of Bacolod	33,573	0	83	7,601	7,684	3	56	6,075	6,134
City of Iloilo	53,444	0	203	30,168	30,371	0	164	6,917	7,081
**Region 7**	**485,713**	**5**	**1,881**	**143,169**	**145,055**	**29**	**4,184**	**106,238**	**110,451**
Bohol	82,389	0	93	23,202	23,295	1	301	13,290	13,592
Cebu	189,570	1	508	55,825	56,334	11	1,247	36,033	37,291
Negros Oriental	104,439	4	704	37,520	38,228	7	1,322	28,138	29,467
Siquijor	8,591	0	15	3,078	3,093	0	51	2,477	2,528
City of Cebu	41,141	0	308	8,888	9,196	6	575	8,209	8,790
City of Lapu-Lapu	26,667	0	212	5,448	5,660	1	485	8,941	9,427
City of Mandaue	32,916	0	41	9,208	9,249	3	203	9,150	9,356
**Region 8**	**342,292**	**23**	**2,905**	**128,042**	**130,970**	**14**	**2,860**	**59,681**	**62,555**
Biliran	13,195	0	59	4,757	4,816	0	74	1,741	1,815
Eastern Samar	37,602	1	299	14,439	14,739	2	400	7,965	8,367
Leyte	124,892	0	970	43,941	44,911	0	817	19,446	20,263
Northern Samar	48,081	8	274	18,483	18,765	2	410	10,301	10,713
Southern Leyte	28,645	0	77	11,596	11,673	0	211	3,817	4,028
Samar	46,978	14	868	19,762	20,644	10	559	9,229	9,798
Ormoc City	20,250	0	105	8,971	9,076	0	139	2,212	2,351
City of Tacloban	22,649	0	253	6,093	6,346	0	250	4,970	5,220
**Region 9**	**376,798**	**7**	**2,725**	**119,775**	**122,507**	**21**	**3,491**	**66,951**	**70,463**
Zamboanga del Norte	129,763	5	833	45,484	46,322	7	996	21,943	22,946
Zamboanga del Sur	113,598	0	759	37,278	38,037	0	708	16,284	16,992
Zamboanga Sibugay	52,229	2	530	14,418	14,950	7	629	9,879	10,515
City of Isabela	8,454	0	64	1,628	1,692	0	108	1,893	2,001
City of Zamboanga	72,754	0	539	20,967	21,506	7	1,050	16,952	18,009
**Region 10**	**541,549**	**32**	**6,656**	**185,798**	**192,486**	**40**	**4,525**	**72,279**	**76,844**
Bukidnon	174,789	7	2,383	72,572	74,962	16	1,603	15,236	16,855
Camiguin	7,102	0	42	2,756	2,798	0	12	703	715
Lanao del Norte	77,655	9	655	20,728	21,392	2	637	18,281	18,920
Misamis Occidental	61,818	1	1,352	23,429	24,782	0	384	8,217	8,601
Misamis Oriental	114,743	10	1,140	34,107	35,257	18	730	12,631	13,379
City of Cagayan De Oro	71,604	3	743	21,607	22,353	3	755	10,554	11,312
City of Iligan	33,838	2	341	10,599	10,942	1	404	6,657	7,062
**Region 11**	**541,328**	**33**	**7,208**	**211,127**	**218,368**	**101**	**7,144**	**80,171**	**87,416**
Davao de Oro	81,619	2	955	40,825	41,782	22	621	10,227	10,870
Davao del Norte	116,452	1	910	51,140	52,051	12	1,051	14,992	16,055
Davao Oriental	60,788	2	646	29,113	29,761	7	393	6,669	7,069
Davao del Sur	67,988	2	1,147	29,000	30,149	12	1,218	10,566	11,796
Davao Occidental	33,573	2	740	12,345	13,087	13	1,077	7,246	8,336
City of Davao	180,908	24	2,810	48,704	51,538	35	2,784	30,471	33,290
**Region 12**	**476,809**	**70**	**6,492**	**172,605**	**179,167**	**37**	**7,695**	**87,608**	**95,340**
Cotabato	141,578	43	1,190	56,745	57,978	10	1,378	24,612	26,000
Sarangani	76,320	0	1,677	29,242	30,919	0	1,607	14,297	15,904
South Cotabato	100,488	10	1,193	39,411	40,614	5	1,329	15,532	16,866

Table 2.A.4 - Modern Methods of Family Planning
Current User (Ending)
Philippines, 2024

Area	Total Current Users	PILLS-COC			Total	INJECTABLES			Total
		Age group				Age group			
		10-14	15-19	20-49		10-14	15-19	20-49	
Sultan Kudarat	97,463	10	1,843	36,363	38,216	11	2,273	18,821	21,105
City of General Santos	60,960	7	589	10,844	11,440	11	1,108	14,346	15,465
**Caraga**	**297,799**	**8**	**2,110**	**111,241**	**113,359**	**23**	**2,666**	**38,401**	**41,090**
Agusan del Norte	41,712	1	339	15,112	15,452	5	364	5,707	6,076
Agusan del Sur	84,822	4	638	32,745	33,387	3	618	9,760	10,381
Surigao del Norte	59,436	0	332	21,378	21,710	3	809	9,948	10,760
Surigao del Sur	62,409	3	400	22,332	22,735	11	395	4,730	5,136
Dinagat Islands	10,221	0	35	3,679	3,714	0	72	1,423	1,495
City of Butuan	39,199	0	366	15,995	16,361	1	408	6,833	7,242
**BARMM**	**298,015**	**9**	**2,910**	**66,632**	**69,551**	**35**	**6,100**	**116,916**	**123,051**
Basilan	20,641	5	231	3,553	3,789	17	617	6,746	7,380
Lanao del Sur	37,006	0	188	7,174	7,362	4	565	9,841	10,410
Maguindanao del Norte	37,544	2	253	7,245	7,500	1	854	15,032	15,887
Maguindanao del Sur	45,670	0	279	7,421	7,700	5	1,585	22,308	23,898
Sulu	87,159	2	1,397	23,410	24,809	6	1,377	39,753	41,136
Tawi-Tawi	20,726	0	191	4,789	4,980	1	289	6,465	6,755
SGA	6,557	0	54	723	777	1	161	3,397	3,559
City of Cotabato	42,712	0	317	12,317	12,634	0	652	13,374	14,026

Table 2.A.4 - Modern Methods of Family Planning
Current User (Ending)
Philippines, 2024

Area	Total Current Users	IMPLANTS Age group 10-14	15-19	20-49	Total	NFP-CCM Age group 10-14	15-19	20-49	Total
**PHILIPPINES**	**9,031,012**	**1,432**	**75,041**	**835,510**	**911,983**	**154**	**867**	**40,616**	**41,637**
**N C R**	**1,212,532**	**378**	**15,554**	**137,318**	**153,250**	**1**	**2**	**586**	**589**
City of Malabon	24,231	3	519	4,242	4,764	0	0	0	0
City of Navotas	23,645	5	629	2,089	2,723	0	0	0	0
City of Valenzuela	45,887	2	1,590	10,048	11,640	0	0	1	1
City of Caloocan	83,350	20	1,574	16,042	17,636	0	0	0	0
City of Marikina	29,479	18	561	4,940	5,519	0	2	13	15
City of Pasig	64,363	28	704	8,515	9,247	0	0	11	11
Pateros	4,444	0	7	343	350	0	0	0	0
City of Taguig	113,829	8	248	6,684	6,940	0	0	0	0
Quezon City	365,159	86	3,173	34,203	37,462	0	0	12	12
City of Makati	34,624	0	83	2,809	2,892	0	0	0	0
City of Mandaluyong	26,244	4	184	3,374	3,562	0	0	316	316
City of San Juan	6,312	0	35	412	447	0	0	0	0
City of Manila	210,914	159	4,322	28,080	32,561	0	0	1	1
City of Las Piñas	37,215	35	1,169	7,013	8,217	0	0	0	0
City of Muntinlupa	46,022	1	139	2,174	2,314	0	0	0	0
City of Parañaque	52,957	7	451	3,283	3,741	0	0	0	0
Pasay City	43,857	2	166	3,067	3,235	1	0	232	233
**C A R**	**172,183**	**40**	**992**	**13,966**	**14,998**	**0**	**112**	**2,264**	**2,376**
Abra	19,945	2	105	1,439	1,546	0	1	56	57
Apayao	25,169	13	129	1,320	1,462	0	0	1	1
Benguet	38,784	1	114	2,393	2,508	0	1	26	27
Ifugao	22,540	5	93	1,587	1,685	0	9	808	817
Kalinga	19,221	0	134	1,937	2,071	0	101	1,239	1,340
Mountain Province	14,288	8	146	1,193	1,347	0	0	133	133
City of Baguio	32,236	11	271	4,097	4,379	0	0	1	1
**Region 1**	**510,193**	**10**	**1,270**	**21,671**	**22,951**	**0**	**38**	**2,875**	**2,913**
Ilocos Norte	51,601	3	92	1,192	1,287	0	16	1,221	1,237
Ilocos Sur	80,076	0	71	1,296	1,367	0	22	1,506	1,528
La Union	67,429	6	342	3,014	3,362	0	0	148	148
Pangasinan	304,595	1	748	15,688	16,437	0	0	0	0
City of Dagupan	6,492	0	17	481	498	0	0	0	0
**Region 2**	**352,751**	**28**	**1,210**	**16,734**	**17,972**	**0**	**1**	**285**	**286**
Batanes	967	0	1	57	58	0	0	75	75
Cagayan	112,004	9	271	4,445	4,725	0	0	11	11
Isabela	151,442	16	659	7,603	8,278	0	1	3	4
Nueva Vizcaya	52,387	1	163	3,133	3,297	0	0	178	178
Quirino	21,116	2	76	857	935	0	0	18	18
City of Santiago	14,835	0	40	639	679	0	0	0	0
**Region 3**	**1,067,691**	**254**	**7,580**	**68,759**	**76,593**	**123**	**6**	**144**	**273**
Aurora	24,504	1	112	1,788	1,901	0	0	6	6
Bataan	71,353	29	1,138	6,509	7,676	0	0	13	13
Bulacan	341,316	13	1,179	22,855	24,047	0	2	14	16
Nueva Ecija	216,078	53	2,081	13,245	15,379	0	2	7	9
Pampanga	178,397	144	1,968	10,101	12,213	0	2	84	86
Tarlac	120,786	1	166	4,730	4,897	0	0	0	0
Zambales	57,286	3	366	4,353	4,722	0	0	20	20
City of Angeles	33,168	3	153	2,864	3,020	0	0	0	0
City of Olongapo	24,803	7	417	2,314	2,738	123	0	0	123
**Region 4A**	**913,336**	**134**	**4,536**	**71,308**	**75,978**	**27**	**31**	**1,267**	**1,325**
Batangas	162,127	10	288	5,245	5,543	0	2	0	2
Cavite	185,925	19	1,138	19,958	21,115	0	0	109	109
Laguna	258,745	71	1,469	23,385	24,925	27	26	365	418
Quezon	111,585	7	393	7,788	8,188	0	3	187	190
Rizal	189,319	27	1,235	14,753	16,015	0	0	606	606
City of Lucena	5,635	0	13	179	192	0	0	0	0
**Region 4B**	**294,862**	**24**	**2,357**	**34,521**	**36,902**	**0**	**79**	**5,768**	**5,847**
Marinduque	16,756	4	139	1,817	1,960	0	0	0	0
Occidental Mindoro	45,599	4	485	6,257	6,746	0	61	2,923	2,984
Oriental Mindoro	69,825	3	301	4,493	4,797	0	0	88	88
Palawan	104,000	9	1,111	16,723	17,843	0	7	641	648

Table 2.A.4 - Modern Methods of Family Planning
Current User (Ending)
Philippines, 2024

Area	Total Current Users	IMPLANTS Age group 10-14	IMPLANTS 15-19	IMPLANTS 20-49	Total	NFP-CCM Age group 10-14	NFP-CCM 15-19	NFP-CCM 20-49	Total
Romblon	23,163	1	129	2,070	2,200	0	11	2,115	2,126
City of Puerto Princesa	35,519	3	192	3,161	3,356	0	0	1	1
**Region 5**	**478,179**	**17**	**2,122**	**42,211**	**44,350**	**2**	**312**	**14,899**	**15,213**
Albay	118,150	8	344	7,814	8,166	0	3	1,250	1,253
Camarines Norte	44,180	0	256	2,838	3,094	0	1	170	171
Camarines Sur	104,506	6	781	17,107	17,894	0	0	514	514
Catanduanes	23,525	1	188	3,580	3,769	0	15	1,563	1,578
Masbate	92,606	1	285	4,769	5,055	2	225	6,958	7,185
Sorsogon	67,474	1	200	4,865	5,066	0	68	4,382	4,450
City of Naga	27,738	0	68	1,238	1,306	0	0	62	62
**Region 6**	**668,982**	**31**	**2,250**	**34,858**	**37,139**	**0**	**90**	**5,084**	**5,174**
Aklan	48,366	0	85	1,596	1,681	0	1	29	30
Antique	51,842	0	93	2,444	2,537	0	86	4,811	4,897
Capiz	78,489	0	90	2,434	2,524	0	1	70	71
Guimaras	18,806	0	25	681	706	0	0	0	0
Iloilo	185,570	12	599	10,842	11,453	0	0	102	102
Negros Occidental	198,892	7	1,040	12,222	13,269	0	2	37	39
City of Bacolod	33,573	12	224	2,054	2,290	0	0	29	29
City of Iloilo	53,444	0	94	2,585	2,679	0	0	6	6
**Region 7**	**485,713**	**57**	**3,325**	**53,637**	**57,019**	**0**	**4**	**478**	**482**
Bohol	82,389	10	880	10,279	11,169	0	0	18	18
Cebu	189,570	38	1,261	22,452	23,751	0	0	0	0
Negros Oriental	104,439	5	328	5,500	5,833	0	4	460	464
Siquijor	8,591	0	26	715	741	0	0	0	0
City of Cebu	41,141	2	482	6,794	7,278	0	0	0	0
City of Lapu-Lapu	26,667	1	114	1,964	2,079	0	0	0	0
City of Mandaue	32,916	1	234	5,933	6,168	0	0	0	0
**Region 8**	**342,292**	**18**	**1,936**	**37,151**	**39,105**	**0**	**16**	**1,649**	**1,665**
Biliran	13,195	2	203	2,028	2,233	0	2	94	96
Eastern Samar	37,602	4	239	3,595	3,838	0	0	207	207
Leyte	124,892	5	491	11,773	12,269	0	4	78	82
Northern Samar	48,081	0	184	5,069	5,253	0	1	6	7
Southern Leyte	28,645	0	128	2,756	2,884	0	0	184	184
Samar	46,978	6	285	6,303	6,594	0	0	1,078	1,078
Ormoc City	20,250	0	160	2,805	2,965	0	9	2	11
City of Tacloban	22,649	1	246	2,822	3,069	0	0	0	0
**Region 9**	**376,798**	**91**	**7,155**	**65,576**	**72,822**	**0**	**13**	**111**	**124**
Zamboanga del Norte	129,763	16	1,678	18,630	20,324	0	13	104	117
Zamboanga del Sur	113,598	14	2,559	21,762	24,335	0	0	0	0
Zamboanga Sibugay	52,229	7	1,162	11,726	12,895	0	0	7	7
City of Isabela	8,454	18	331	2,129	2,478	0	0	0	0
City of Zamboanga	72,754	36	1,425	11,329	12,790	0	0	0	0
**Region 10**	**541,549**	**64**	**5,416**	**52,183**	**57,663**	**0**	**93**	**2,392**	**2,485**
Bukidnon	174,789	37	2,928	24,760	27,725	0	21	549	570
Camiguin	7,102	0	10	239	249	0	0	0	0
Lanao del Norte	77,655	1	295	5,675	5,971	0	3	184	187
Misamis Occidental	61,818	0	201	2,994	3,195	0	21	147	168
Misamis Oriental	114,743	19	937	9,365	10,321	0	48	1,507	1,555
City of Cagayan De Oro	71,604	6	833	6,129	6,968	0	0	1	1
City of Iligan	33,838	1	212	3,021	3,234	0	0	4	4
**Region 11**	**541,328**	**104**	**5,419**	**54,359**	**59,882**	**0**	**18**	**800**	**818**
Davao de Oro	81,619	7	444	6,791	7,242	0	3	107	110
Davao del Norte	116,452	17	1,068	11,959	13,044	0	0	51	51
Davao Oriental	60,788	9	331	5,092	5,432	0	1	162	163
Davao del Sur	67,988	18	663	5,572	6,253	0	0	103	103
Davao Occidental	33,573	0	520	3,903	4,423	0	0	0	0
City of Davao	180,908	53	2,393	21,042	23,488	0	14	377	391
**Region 12**	**476,809**	**73**	**6,825**	**60,891**	**67,789**	**1**	**11**	**707**	**719**
Cotabato	141,578	16	1,323	18,372	19,711	0	1	542	543
Sarangani	76,320	0	887	8,592	9,479	0	5	63	68
South Cotabato	100,488	22	1,934	11,914	13,870	1	5	79	85

Table 2.A.4 - Modern Methods of Family Planning
Current User (Ending)
Philippines, 2024

Area	Total Current Users	IMPLANTS Age group 10-14	IMPLANTS Age group 15-19	IMPLANTS Age group 20-49	Total	NFP-CCM Age group 10-14	NFP-CCM Age group 15-19	NFP-CCM Age group 20-49	Total
Sultan Kudarat	97,463	21	1,488	11,374	12,883	0	0	17	17
City of General Santos	60,960	14	1,193	10,639	11,846	0	0	6	6
**Caraga**	**297,799**	**51**	**3,810**	**37,972**	**41,833**	**0**	**40**	**1,298**	**1,338**
Agusan del Norte	41,712	8	511	4,893	5,412	0	0	14	14
Agusan del Sur	84,822	20	1,253	11,253	12,526	0	39	1,024	1,063
Surigao del Norte	59,436	7	923	7,439	8,369	0	1	30	31
Surigao del Sur	62,409	10	579	8,292	8,881	0	0	216	216
Dinagat Islands	10,221	0	124	2,144	2,268	0	0	0	0
City of Butuan	39,199	6	420	3,951	4,377	0	0	14	14
**BARMM**	**298,015**	**58**	**3,284**	**32,395**	**35,737**	**0**	**1**	**9**	**10**
Basilan	20,641	14	545	3,519	4,078	0	1	9	10
Lanao del Sur	37,006	6	208	2,994	3,208	0	0	0	0
Maguindanao del Norte	37,544	12	591	4,571	5,174	0	0	0	0
Maguindanao del Sur	45,670	9	597	3,936	4,542	0	0	0	0
Sulu	87,159	12	651	8,918	9,581	0	0	0	0
Tawi-Tawi	20,726	5	413	5,429	5,847	0	0	0	0
SGA	6,557	0	14	179	193	0	0	0	0
City of Cotabato	42,712	0	265	2,849	3,114	0	0	0	0

Table 2.A.4 - Modern Methods of Family Planning
Current User (Ending)
Philippines, 2024

Area	Total Current Users	NFP-BBT			Total	NFP-STM			Total
		Age group				Age group			
		10-14	15-19	20-49		10-14	15-19	20-49	
**PHILIPPINES**	**9,031,012**	**131**	**186**	**2,492**	**2,809**	**19**	**117**	**1,944**	**2,080**
**N C R**	**1,212,532**	**0**	**7**	**195**	**202**	**0**	**0**	**107**	**107**
City of Malabon	24,231	0	0	0	0	0	0	0	0
City of Navotas	23,645	0	0	0	0	0	0	0	0
City of Valenzuela	45,887	0	2	66	68	0	0	30	30
City of Caloocan	83,350	0	0	0	0	0	0	0	0
City of Marikina	29,479	0	0	30	30	0	0	0	0
City of Pasig	64,363	0	0	0	0	0	0	0	0
Pateros	4,444	0	0	0	0	0	0	0	0
City of Taguig	113,829	0	0	0	0	0	0	0	0
Quezon City	365,159	0	0	9	9	0	0	0	0
City of Makati	34,624	0	0	2	2	0	0	0	0
City of Mandaluyong	26,244	0	0	14	14	0	0	62	62
City of San Juan	6,312	0	0	0	0	0	0	0	0
City of Manila	210,914	0	2	4	6	0	0	9	9
City of Las Piñas	37,215	0	0	0	0	0	0	0	0
City of Muntinlupa	46,022	0	0	0	0	0	0	0	0
City of Parañaque	52,957	0	3	33	36	0	0	0	0
Pasay City	43,857	0	0	37	37	0	0	6	6
**C A R**	**172,183**	**0**	**7**	**170**	**177**	**0**	**0**	**40**	**40**
Abra	19,945	0	0	12	12	0	0	13	13
Apayao	25,169	0	0	0	0	0	0	0	0
Benguet	38,784	0	0	0	0	0	0	0	0
Ifugao	22,540	0	2	13	15	0	0	5	5
Kalinga	19,221	0	5	136	141	0	0	19	19
Mountain Province	14,288	0	0	5	5	0	0	0	0
City of Baguio	32,236	0	0	4	4	0	0	3	3
**Region 1**	**510,193**	**0**	**2**	**167**	**169**	**0**	**16**	**155**	**171**
Ilocos Norte	51,601	0	0	17	17	0	0	23	23
Ilocos Sur	80,076	0	0	137	137	0	9	118	127
La Union	67,429	0	0	0	0	0	0	11	11
Pangasinan	304,595	0	2	13	15	0	7	3	10
City of Dagupan	6,492	0	0	0	0	0	0	0	0
**Region 2**	**352,751**	**0**	**13**	**122**	**135**	**1**	**7**	**82**	**90**
Batanes	967	0	0	0	0	0	0	0	0
Cagayan	112,004	0	3	22	25	0	0	0	0
Isabela	151,442	0	10	28	38	0	0	0	0
Nueva Vizcaya	52,387	0	0	62	62	1	7	82	90
Quirino	21,116	0	0	10	10	0	0	0	0
City of Santiago	14,835	0	0	0	0	0	0	0	0
**Region 3**	**1,067,691**	**0**	**7**	**166**	**173**	**0**	**9**	**21**	**30**
Aurora	24,504	0	0	4	4	0	0	0	0
Bataan	71,353	0	3	16	19	0	0	8	8
Bulacan	341,316	0	0	4	4	0	0	0	0
Nueva Ecija	216,078	0	0	0	0	0	0	0	0
Pampanga	178,397	0	0	4	4	0	9	8	17
Tarlac	120,786	0	0	1	1	0	0	0	0
Zambales	57,286	0	0	0	0	0	0	5	5
City of Angeles	33,168	0	4	4	8	0	0	0	0
City of Olongapo	24,803	0	0	133	133	0	0	0	0
**Region 4A**	**913,336**	**65**	**32**	**157**	**254**	**18**	**27**	**357**	**402**
Batangas	162,127	33	0	16	49	0	0	6	6
Cavite	185,925	0	15	5	20	0	2	1	3
Laguna	258,745	27	3	58	88	18	0	0	18
Quezon	111,585	0	8	20	28	0	25	24	49
Rizal	189,319	0	6	58	64	0	0	326	326
City of Lucena	5,635	5	0	0	5	0	0	0	0
**Region 4B**	**294,862**	**0**	**3**	**101**	**104**	**0**	**4**	**62**	**66**
Marinduque	16,756	0	0	3	3	0	0	0	0
Occidental Mindoro	45,599	0	0	60	60	0	0	22	22
Oriental Mindoro	69,825	0	3	3	6	0	0	19	19
Palawan	104,000	0	0	30	30	0	4	21	25

Table 2.A.4 - Modern Methods of Family Planning
Current User (Ending)
Philippines, 2024

Area	Total Current Users	NFP-BBT			Total	NFP-STM			Total
		Age group				Age group			
		10-14	15-19	20-49		10-14	15-19	20-49	
Romblon	23,163	0	0	2	2	0	0	0	0
City of Puerto Princesa	35,519	0	0	3	3	0	0	0	0
**Region 5**	**478,179**	**0**	**20**	**240**	**260**	**0**	**31**	**253**	**284**
Albay	118,150	0	1	63	64	0	0	47	47
Camarines Norte	44,180	0	0	19	19	0	29	110	139
Camarines Sur	104,506	0	1	57	58	0	0	40	40
Catanduanes	23,525	0	14	32	46	0	0	6	6
Masbate	92,606	0	4	27	31	0	2	22	24
Sorsogon	67,474	0	0	5	5	0	0	3	3
City of Naga	27,738	0	0	37	37	0	0	25	25
**Region 6**	**668,982**	**60**	**17**	**146**	**223**	**0**	**0**	**68**	**68**
Aklan	48,366	0	2	16	18	0	0	4	4
Antique	51,842	0	0	3	3	0	0	5	5
Capiz	78,489	0	1	4	5	0	0	0	0
Guimaras	18,806	0	0	1	1	0	0	0	0
Iloilo	185,570	0	2	48	50	0	0	53	53
Negros Occidental	198,892	0	4	36	40	0	0	6	6
City of Bacolod	33,573	60	8	34	102	0	0	0	0
City of Iloilo	53,444	0	0	4	4	0	0	0	0
**Region 7**	**485,713**	**0**	**4**	**48**	**52**	**0**	**2**	**26**	**28**
Bohol	82,389	0	0	0	0	0	0	0	0
Cebu	189,570	0	0	0	0	0	0	0	0
Negros Oriental	104,439	0	4	48	52	0	2	26	28
Siquijor	8,591	0	0	0	0	0	0	0	0
City of Cebu	41,141	0	0	0	0	0	0	0	0
City of Lapu-Lapu	26,667	0	0	0	0	0	0	0	0
City of Mandaue	32,916	0	0	0	0	0	0	0	0
**Region 8**	**342,292**	**0**	**8**	**142**	**150**	**0**	**0**	**96**	**96**
Biliran	13,195	0	3	10	13	0	0	0	0
Eastern Samar	37,602	0	0	16	16	0	0	14	14
Leyte	124,892	0	3	87	90	0	0	77	77
Northern Samar	48,081	0	0	3	3	0	0	4	4
Southern Leyte	28,645	0	0	3	3	0	0	1	1
Samar	46,978	0	2	21	23	0	0	0	0
Ormoc City	20,250	0	0	2	2	0	0	0	0
City of Tacloban	22,649	0	0	0	0	0	0	0	0
**Region 9**	**376,798**	**0**	**0**	**12**	**12**	**0**	**0**	**27**	**27**
Zamboanga del Norte	129,763	0	0	7	7	0	0	11	11
Zamboanga del Sur	113,598	0	0	1	1	0	0	0	0
Zamboanga Sibugay	52,229	0	0	4	4	0	0	16	16
City of Isabela	8,454	0	0	0	0	0	0	0	0
City of Zamboanga	72,754	0	0	0	0	0	0	0	0
**Region 10**	**541,549**	**6**	**34**	**398**	**438**	**0**	**12**	**414**	**426**
Bukidnon	174,789	1	9	88	98	0	0	105	105
Camiguin	7,102	0	0	8	8	0	0	1	1
Lanao del Norte	77,655	5	4	129	138	0	12	199	211
Misamis Occidental	61,818	0	3	36	39	0	0	7	7
Misamis Oriental	114,743	0	18	126	144	0	0	100	100
City of Cagayan De Oro	71,604	0	0	10	10	0	0	0	0
City of Iligan	33,838	0	0	1	1	0	0	2	2
**Region 11**	**541,328**	**0**	**7**	**235**	**242**	**0**	**0**	**138**	**138**
Davao de Oro	81,619	0	0	14	14	0	0	3	3
Davao del Norte	116,452	0	0	4	4	0	0	11	11
Davao Oriental	60,788	0	0	68	68	0	0	104	104
Davao del Sur	67,988	0	4	99	103	0	0	3	3
Davao Occidental	33,573	0	0	11	11	0	0	1	1
City of Davao	180,908	0	3	39	42	0	0	16	16
**Region 12**	**476,809**	**0**	**14**	**132**	**146**	**0**	**6**	**71**	**77**
Cotabato	141,578	0	3	88	91	0	0	17	17
Sarangani	76,320	0	0	0	0	0	1	10	11
South Cotabato	100,488	0	0	31	31	0	0	29	29

Table 2.A.4 - Modern Methods of Family Planning
Current User (Ending)
Philippines, 2024

Area	Total Current Users	NFP-BBT			Total	NFP-STM			Total
		Age group				Age group			
		10-14	15-19	20-49		10-14	15-19	20-49	
Sultan Kudarat	97,463	0	0	6	6	0	0	0	0
City of General Santos	60,960	0	11	7	18	0	5	15	20
**Caraga**	**297,799**	**0**	**7**	**59**	**66**	**0**	**3**	**24**	**27**
Agusan del Norte	41,712	0	1	8	9	0	0	0	0
Agusan del Sur	84,822	0	0	0	0	0	2	0	2
Surigao del Norte	59,436	0	3	17	20	0	0	20	20
Surigao del Sur	62,409	0	2	34	36	0	1	4	5
Dinagat Islands	10,221	0	0	0	0	0	0	0	0
City of Butuan	39,199	0	1	0	1	0	0	0	0
**BARMM**	**298,015**	**0**	**4**	**2**	**6**	**0**	**0**	**3**	**3**
Basilan	20,641	0	0	1	1	0	0	0	0
Lanao del Sur	37,006	0	0	0	0	0	0	1	1
Maguindanao del Norte	37,544	0	0	0	0	0	0	0	0
Maguindanao del Sur	45,670	0	0	0	0	0	0	0	0
Sulu	87,159	0	0	0	0	0	0	2	2
Tawi-Tawi	20,726	0	0	0	0	0	0	0	0
SGA	6,557	0	0	0	0	0	0	0	0
City of Cotabato	42,712	0	4	1	5	0	0	0	0

Table 2.A.4 - Modern Methods of Family Planning
Current User (Ending)
Philippines, 2024

Area	Total Current Users	NFP-SDM			Total	NFP-LAM			Total
		Age group				Age group			
		10-14	15-19	20-49		10-14	15-19	20-49	
**PHILIPPINES**	**9,031,012**	**43**	**3,944**	**116,858**	**120,845**	**1,805**	**67,506**	**559,310**	**628,621**
**N C R**	**1,212,532**	**8**	**36**	**2,531**	**2,575**	**249**	**9,927**	**104,517**	**114,693**
City of Malabon	24,231	0	0	1	1	1	134	1,001	1,136
City of Navotas	23,645	0	0	0	0	8	127	1,784	1,919
City of Valenzuela	45,887	0	2	652	654	0	203	2,652	2,855
City of Caloocan	83,350	0	7	76	83	4	676	5,494	6,174
City of Marikina	29,479	0	0	4	4	9	365	2,264	2,638
City of Pasig	64,363	0	0	32	32	3	278	7,807	8,088
Pateros	4,444	0	0	0	0	0	4	153	157
City of Taguig	113,829	1	1	14	16	62	2,905	29,887	32,854
Quezon City	365,159	0	19	1,543	1,562	25	1,222	12,605	13,852
City of Makati	34,624	0	0	0	0	0	144	3,630	3,774
City of Mandaluyong	26,244	0	0	110	110	3	121	4,287	4,411
City of San Juan	6,312	0	0	0	0	0	9	290	299
City of Manila	210,914	4	2	5	11	114	1,034	12,440	13,588
City of Las Piñas	37,215	0	0	7	7	1	169	1,954	2,124
City of Muntinlupa	46,022	0	0	0	0	10	1,621	8,861	10,492
City of Parañaque	52,957	0	5	53	58	1	416	8,628	9,045
Pasay City	43,857	3	0	34	37	8	499	780	1,287
**C A R**	**172,183**	**0**	**54**	**7,961**	**8,015**	**29**	**1,100**	**8,077**	**9,206**
Abra	19,945	0	6	276	282	7	215	1,224	1,446
Apayao	25,169	0	0	8	8	5	131	955	1,091
Benguet	38,784	0	5	3,174	3,179	0	144	1,729	1,873
Ifugao	22,540	0	40	3,121	3,161	10	221	1,902	2,133
Kalinga	19,221	0	2	390	392	3	258	1,110	1,371
Mountain Province	14,288	0	1	964	965	4	90	751	845
City of Baguio	32,236	0	0	28	28	0	41	406	447
**Region 1**	**510,193**	**0**	**146**	**7,031**	**7,177**	**103**	**4,501**	**40,606**	**45,210**
Ilocos Norte	51,601	0	0	917	917	3	252	4,847	5,102
Ilocos Sur	80,076	0	61	4,106	4,167	10	587	8,688	9,285
La Union	67,429	0	40	1,361	1,401	6	449	3,460	3,915
Pangasinan	304,595	0	45	616	661	82	3,150	23,029	26,261
City of Dagupan	6,492	0	0	31	31	2	63	582	647
**Region 2**	**352,751**	**0**	**6**	**683**	**689**	**118**	**2,728**	**17,650**	**20,496**
Batanes	967	0	0	13	13	0	11	136	147
Cagayan	112,004	0	1	154	155	26	874	4,366	5,266
Isabela	151,442	0	3	234	237	19	848	6,081	6,948
Nueva Vizcaya	52,387	0	2	282	284	13	376	4,153	4,542
Quirino	21,116	0	0	0	0	5	192	967	1,164
City of Santiago	14,835	0	0	0	0	55	427	1,947	2,429
**Region 3**	**1,067,691**	**6**	**37**	**4,527**	**4,570**	**69**	**4,334**	**44,384**	**48,787**
Aurora	24,504	0	0	9	9	4	202	1,459	1,665
Bataan	71,353	0	11	68	79	13	752	4,269	5,034
Bulacan	341,316	0	18	3,542	3,560	7	771	12,176	12,954
Nueva Ecija	216,078	0	1	15	16	11	914	5,983	6,908
Pampanga	178,397	0	1	748	749	8	516	8,428	8,952
Tarlac	120,786	0	2	14	16	4	570	6,482	7,056
Zambales	57,286	0	2	43	45	7	358	2,760	3,125
City of Angeles	33,168	6	2	77	85	14	174	1,594	1,782
City of Olongapo	24,803	0	0	11	11	1	77	1,233	1,311
**Region 4A**	**913,336**	**22**	**1,515**	**21,781**	**23,318**	**223**	**6,500**	**54,791**	**61,514**
Batangas	162,127	0	289	18,622	18,911	89	347	4,490	4,926
Cavite	185,925	0	15	411	426	32	1,095	10,322	11,449
Laguna	258,745	10	89	2,118	2,217	59	2,896	17,537	20,492
Quezon	111,585	10	1,122	551	1,683	8	690	4,720	5,418
Rizal	189,319	0	0	79	79	31	1,441	17,712	19,184
City of Lucena	5,635	2	0	0	2	4	31	10	45
**Region 4B**	**294,862**	**2**	**163**	**2,928**	**3,093**	**56**	**2,610**	**15,920**	**18,586**
Marinduque	16,756	0	0	37	37	1	99	1,240	1,340
Occidental Mindoro	45,599	2	8	861	871	22	589	3,167	3,778
Oriental Mindoro	69,825	0	143	293	436	8	553	4,174	4,735
Palawan	104,000	0	9	602	611	19	1,013	4,833	5,865

Table 2.A.4 - Modern Methods of Family Planning
Current User (Ending)
Philippines, 2024

Area	Total Current Users	NFP-SDM Age group 10-14	NFP-SDM Age group 15-19	NFP-SDM Age group 20-49	Total	NFP-LAM Age group 10-14	NFP-LAM Age group 15-19	NFP-LAM Age group 20-49	Total
Romblon	23,163	0	3	924	927	3	245	1,801	2,049
City of Puerto Princesa	35,519	0	0	211	211	3	111	705	819
**Region 5**	**478,179**	**1**	**1,055**	**30,866**	**31,922**	**75**	**4,355**	**34,548**	**38,978**
Albay	118,150	0	16	5,373	5,389	19	582	7,216	7,817
Camarines Norte	44,180	0	27	1,434	1,461	25	709	4,164	4,898
Camarines Sur	104,506	0	17	4,205	4,222	14	1,057	7,657	8,728
Catanduanes	23,525	0	30	3,389	3,419	0	187	1,441	1,628
Masbate	92,606	1	936	10,095	11,032	12	1,087	6,533	7,632
Sorsogon	67,474	0	26	5,208	5,234	5	660	4,889	5,554
City of Naga	27,738	0	3	1,162	1,165	0	73	2,648	2,721
**Region 6**	**668,982**	**2**	**116**	**10,114**	**10,232**	**69**	**4,144**	**40,515**	**44,728**
Aklan	48,366	0	0	221	221	4	381	3,795	4,180
Antique	51,842	0	3	700	703	2	220	2,870	3,092
Capiz	78,489	0	0	15	15	1	359	3,703	4,063
Guimaras	18,806	0	0	31	31	3	84	943	1,030
Iloilo	185,570	2	39	4,797	4,838	26	1,321	13,412	14,759
Negros Occidental	198,892	0	74	4,138	4,212	32	1,672	11,348	13,052
City of Bacolod	33,573	0	0	41	41	1	95	4,369	4,465
City of Iloilo	53,444	0	0	171	171	0	12	75	87
**Region 7**	**485,713**	**0**	**1**	**1,147**	**1,148**	**53**	**3,447**	**27,731**	**31,231**
Bohol	82,389	0	0	487	487	3	223	2,070	2,296
Cebu	189,570	0	0	24	24	10	1,366	10,589	11,965
Negros Oriental	104,439	0	0	172	172	30	1,027	7,906	8,963
Siquijor	8,591	0	1	459	460	1	18	146	165
City of Cebu	41,141	0	0	5	5	8	519	4,443	4,970
City of Lapu-Lapu	26,667	0	0	0	0	1	280	2,039	2,320
City of Mandaue	32,916	0	0	0	0	0	14	538	552
**Region 8**	**342,292**	**0**	**148**	**2,820**	**2,968**	**46**	**3,406**	**32,037**	**35,489**
Biliran	13,195	0	0	137	137	2	60	750	812
Eastern Samar	37,602	0	136	1,695	1,831	6	379	2,357	2,742
Leyte	124,892	0	4	124	128	1	1,323	12,911	14,235
Northern Samar	48,081	0	0	199	199	1	484	7,071	7,556
Southern Leyte	28,645	0	7	256	263	0	260	2,008	2,268
Samar	46,978	0	0	0	0	32	551	3,239	3,822
Ormoc City	20,250	0	1	409	410	0	111	1,173	1,284
City of Tacloban	22,649	0	0	0	0	4	238	2,528	2,770
**Region 9**	**376,798**	**0**	**91**	**2,491**	**2,582**	**47**	**3,312**	**21,320**	**24,679**
Zamboanga del Norte	129,763	0	90	2,196	2,286	17	1,048	8,098	9,163
Zamboanga del Sur	113,598	0	0	10	10	5	587	3,788	4,380
Zamboanga Sibugay	52,229	0	1	285	286	4	434	2,106	2,544
City of Isabela	8,454	0	0	0	0	3	157	781	941
City of Zamboanga	72,754	0	0	0	0	18	1,086	6,547	7,651
**Region 10**	**541,549**	**2**	**447**	**14,108**	**14,557**	**209**	**5,893**	**34,120**	**40,222**
Bukidnon	174,789	0	62	5,358	5,420	126	2,474	9,713	12,313
Camiguin	7,102	0	0	56	56	0	129	935	1,064
Lanao del Norte	77,655	2	35	434	471	27	544	6,381	6,952
Misamis Occidental	61,818	0	286	4,309	4,595	6	741	5,543	6,290
Misamis Oriental	114,743	0	58	3,660	3,718	29	1,148	5,904	7,081
City of Cagayan De Oro	71,604	0	0	34	34	16	641	4,580	5,237
City of Iligan	33,838	0	6	257	263	5	216	1,064	1,285
**Region 11**	**541,328**	**0**	**80**	**2,122**	**2,202**	**216**	**2,892**	**24,050**	**27,158**
Davao de Oro	81,619	0	0	43	43	3	61	495	559
Davao del Norte	116,452	0	1	173	174	24	416	2,610	3,050
Davao Oriental	60,788	0	21	1,176	1,197	41	601	3,351	3,993
Davao del Sur	67,988	0	32	41	73	6	128	606	740
Davao Occidental	33,573	0	22	509	531	5	198	680	883
City of Davao	180,908	0	4	180	184	137	1,488	16,308	17,933
**Region 12**	**476,809**	**0**	**6**	**577**	**583**	**115**	**3,838**	**18,594**	**22,547**
Cotabato	141,578	0	0	130	130	7	346	2,399	2,752
Sarangani	76,320	0	0	105	105	0	1,084	4,822	5,906
South Cotabato	100,488	0	6	290	296	25	827	3,829	4,681

Table 2.A.4 - Modern Methods of Family Planning
Current User (Ending)
Philippines, 2024

Area	Total Current Users	NFP-SDM			Total	NFP-LAM			Total
		Age group				Age group			
		10-14	15-19	20-49		10-14	15-19	20-49	
Sultan Kudarat	97,463	0	0	42	42	30	966	3,415	4,411
City of General Santos	60,960	0	0	10	10	53	615	4,129	4,797
**Caraga**	**297,799**	**0**	**42**	**4,929**	**4,971**	**60**	**1,962**	**14,514**	**16,536**
Agusan del Norte	41,712	0	3	237	240	4	253	1,529	1,786
Agusan del Sur	84,822	0	6	1,102	1,108	12	582	3,436	4,030
Surigao del Norte	59,436	0	20	2,232	2,252	3	192	2,202	2,397
Surigao del Sur	62,409	0	11	788	799	39	612	5,231	5,882
Dinagat Islands	10,221	0	0	451	451	0	53	318	371
City of Butuan	39,199	0	2	119	121	2	270	1,798	2,070
**BARMM**	**298,015**	**0**	**1**	**242**	**243**	**68**	**2,557**	**25,936**	**28,561**
Basilan	20,641	0	0	32	32	11	181	2,720	2,912
Lanao del Sur	37,006	0	1	83	84	30	593	6,063	6,686
Maguindanao del Norte	37,544	0	0	0	0	2	283	3,769	4,054
Maguindanao del Sur	45,670	0	0	0	0	7	472	3,866	4,345
Sulu	87,159	0	0	20	20	14	416	3,643	4,073
Tawi-Tawi	20,726	0	0	47	47	3	204	1,138	1,345
SGA	6,557	0	0	0	0	0	77	532	609
City of Cotabato	42,712	0	0	60	60	1	331	4,205	4,537

Table 2.A.5 - Modern Family Planning

Number and proportion of women of reproductive age (WRA) 15-49 years old who have demand for Family Planning (FP) and currently using, or whose partner is currently using, any modern FP methods

Philippines, 2024

Area	WRA x Total Demand Factor	FP Current User Ending (15-49 y.o.)	%
**PHILIPPINES**	**12,554,137**	**9,023,602**	**71.88**
**N C R**	**1,419,597**	**1,211,268**	**85.32**
City of Malabon	**39,071**	24,224	**62.00**
City of Navotas	**24,783**	23,586	**95.17**
City of Valenzuela	**75,443**	45,883	**60.82**
City of Caloocan	**172,059**	83,296	**48.41**
City of Marikina	**47,747**	29,405	**61.59**
City of Pasig	**88,078**	64,277	**72.98**
Pateros	**6,525**	4,444	**68.11**
City of Taguig	**129,358**	113,690	**87.89**
Quezon City	**318,054**	364,885	**114.72**
City of Makati	**31,659**	34,621	**109.36**
City of Mandaluyong	**48,263**	26,229	**54.35**
City of San Juan	**13,652**	6,312	**46.24**
City of Manila	**187,951**	210,474	**111.98**
City of Las Piñas	**62,725**	37,169	**59.26**
City of Muntinlupa	**54,265**	45,992	**84.75**
City of Parañaque	**71,293**	52,944	**74.26**
Pasay City	**48,672**	43,837	**90.07**
**C A R**	**201,786**	**172,024**	**85.25**
Abra	**29,664**	19,915	**67.14**
Apayao	**16,207**	25,135	**155.09**
Benguet	**44,450**	38,781	**87.25**
Ifugao	**21,557**	22,514	**104.44**
Kalinga	**28,404**	19,206	**67.62**
Mountain Province	**17,050**	14,272	**83.70**
City of Baguio	**44,455**	32,201	**72.44**
**Region 1**	**603,589**	**509,950**	**84.49**
Ilocos Norte	**76,043**	51,592	**67.85**
Ilocos Sur	**72,814**	80,064	**109.96**
La Union	**101,277**	67,409	**66.56**
Pangasinan	**332,188**	304,395	**91.63**
City of Dagupan	**21,268**	6,490	**30.52**
**Region 2**	**480,684**	**352,474**	**73.51**
Batanes	**2,411**	967	**40.11**
Cagayan	**172,063**	111,920	**65.05**
Isabela	**192,129**	151,373	**78.79**
Nueva Vizcaya	**69,505**	52,357	**75.33**
Quirino	**23,900**	21,078	**88.19**
City of Santiago	**20,675**	14,779	**75.95**
**Region 3**	**1,422,512**	**1,066,107**	**74.95**
Aurora	**27,835**	24,497	**88.01**
Bataan	**97,827**	71,286	**72.87**
Bulacan	**463,483**	341,270	**73.63**
Nueva Ecija	**275,278**	215,914	**78.43**
Pampanga	**279,929**	177,325	**63.35**
Tarlac	**130,535**	120,755	**92.51**
Zambales	**63,569**	57,257	**90.07**

Table 2.A.5 - Modern Family Planning

Number and proportion of women of reproductive age (WRA) 15-49 years old who have demand for Family Planning (FP) and currently using, or whose partner is currently using, any modern FP methods

Philippines, 2024

Area	WRA x Total Demand Factor	FP Current User Ending (15-49 y.o.)	%
City of Angeles	**56,311**	33,139	**58.85**
City of Olongapo	**27,746**	24,664	**88.89**
**Region 4A**	**1,982,927**	**912,403**	**46.01**
Batangas	**341,939**	161,893	**47.35**
Cavite	**506,915**	185,800	**36.65**
Laguna	**461,316**	258,398	**56.01**
Quezon	**232,864**	111,500	**47.88**
Rizal	**407,122**	189,203	**46.47**
City of Lucena	**32,771**	5,609	**17.12**
**Region 4B**	**370,463**	**294,677**	**79.54**
Marinduque	**22,081**	16,750	**75.86**
Occidental Mindoro	**64,282**	45,547	**70.85**
Oriental Mindoro	**92,471**	69,782	**75.46**
Palawan	**116,562**	103,950	**89.18**
Romblon	**33,428**	23,156	**69.27**
City of Puerto Princesa	**41,639**	35,492	**85.24**
**Region 5**	**669,763**	**478,059**	**71.38**
Albay	**154,367**	118,117	**76.52**
Camarines Norte	**76,437**	44,155	**57.77**
Camarines Sur	**199,475**	104,476	**52.38**
Catanduanes	**28,246**	23,523	**83.28**
Masbate	**97,457**	92,587	**95.00**
Sorsogon	**87,241**	67,463	**77.33**
City of Naga	**26,539**	27,738	**104.52**
**Region 6**	**910,199**	**668,596**	**73.46**
Aklan	**72,689**	48,357	**66.53**
Antique	**64,388**	51,836	**80.51**
Capiz	**102,682**	78,484	**76.43**
Guimaras	**20,586**	18,801	**91.33**
Iloilo	**226,482**	185,475	**81.89**
Negros Occidental	**293,228**	198,834	**67.81**
City of Bacolod	**73,917**	33,365	**45.14**
City of Iloilo	**56,226**	53,444	**95.05**
**Region 7**	**968,334**	**485,526**	**50.14**
Bohol	**145,899**	82,366	**56.45**
Cebu	**395,354**	189,501	**47.93**
Negros Oriental	**168,857**	104,371	**61.81**
Siquijor	**11,604**	8,590	**74.03**
City of Cebu	**126,857**	41,124	**32.42**
City of Lapu-Lapu	**69,091**	26,664	**38.59**
City of Mandaue	**50,672**	32,910	**64.95**
**Region 8**	**493,882**	**342,180**	**69.28**
Biliran	**19,890**	13,191	**66.32**
Eastern Samar	**55,689**	37,589	**67.50**
Leyte	**157,672**	124,881	**79.20**
Northern Samar	**68,490**	48,067	**70.18**
Southern Leyte	**53,238**	28,645	**53.81**

Table 2.A.5 - Modern Family Planning

Number and proportion of women of reproductive age (WRA) 15-49 years old who have demand for Family Planning (FP) and currently using, or whose partner is currently using, any modern FP methods

Philippines, 2024

Area	WRA x Total Demand Factor	FP Current User Ending (15-49 y.o.)	%
Samar	**80,447**	46,913	**58.32**
Ormoc City	**27,549**	20,250	**73.50**
City of Tacloban	**30,906**	22,644	**73.27**
**Region 9**	**444,737**	**376,574**	**84.67**
Zamboanga del Norte	**111,390**	129,705	**116.44**
Zamboanga del Sur	**114,665**	113,547	**99.03**
Zamboanga Sibugay	**84,869**	52,209	**61.52**
City of Isabela	**14,957**	8,433	**56.38**
City of Zamboanga	**118,856**	72,680	**61.15**
**Region 10**	**594,090**	**541,053**	**91.07**
Bukidnon	**205,239**	174,590	**85.07**
Camiguin	**10,495**	7,102	**67.67**
Lanao del Norte	**63,475**	77,609	**122.27**
Misamis Occidental	**70,173**	61,794	**88.06**
Misamis Oriental	**105,432**	114,588	**108.68**
City of Cagayan De Oro	**94,786**	71,549	**75.48**
City of Iligan	**44,491**	33,821	**76.02**
**Region 11**	**671,512**	**540,780**	**80.53**
Davao de Oro	**96,769**	81,579	**84.30**
Davao del Norte	**138,097**	116,391	**84.28**
Davao Oriental	**67,537**	60,723	**89.91**
Davao del Sur	**86,290**	67,932	**78.73**
Davao Occidental	**36,825**	33,542	**91.08**
City of Davao	**245,994**	180,613	**73.42**
**Region 12**	**566,630**	**476,475**	**84.09**
Cotabato	**178,109**	141,496	**79.44**
Sarangani	**80,701**	76,320	**94.57**
South Cotabato	**120,892**	100,417	**83.06**
Sultan Kudarat	**92,693**	97,384	**105.06**
City of General Santos	**94,235**	60,858	**64.58**
**Caraga**	**328,137**	**297,641**	**90.71**
Agusan del Norte	**43,026**	41,690	**96.90**
Agusan del Sur	**89,757**	84,777	**94.45**
Surigao del Norte	**61,181**	59,422	**97.12**
Surigao del Sur	**72,543**	62,341	**85.94**
Dinagat Islands	**14,444**	10,221	**70.76**
City of Butuan	**47,187**	39,190	**83.05**
**BARMM**	**425,296**	**297,815**	**70.03**
Basilan	**32,752**	20,592	**62.87**
Lanao del Sur	**103,138**	36,959	**35.83**
Maguindanao del Norte	**61,048**	37,527	**61.47**
Maguindanao del Sur	**68,814**	45,648	**66.34**
Sulu	**77,757**	87,108	**112.03**
Tawi-Tawi	**34,894**	20,716	**59.37**
SGA	**18,056**	6,556	**36.31**
City of Cotabato	**28,839**	42,709	**148.09**

2.B.1.1 - Prenatal Care
Women who gave birth with at least 4 Prenatal Check-ups
Philippines, 2024

PSGC10	Area	Total Deliveries				Women who gave birth with at least four or more prenatal check-ups (4ANC)							
		10-14 y.o.	15-19 y.o.	20-49 y.o.	Total	10-14 y.o.	%	15-19 y.o.	%	20-49 y.o.	%	Total	%
	**PHILIPPINES**	**3,285**	**137,240**	**1,203,053**	**1,343,578**	**2,448**	**74.52**	**112,763**	**82.16**	**1,020,442**	**84.82**	**1,135,653**	**84.52**
**1300000000**	**National Capital Region (NCR)**	**297**	**11,284**	**172,330**	**183,911**	**235**	**79.12**	**10,896**	**96.56**	**157,584**	**91.44**	**168,715**	**91.74**
**1380100000**	**City of Caloocan**	**57**	**2,129**	**21,982**	**24,168**	**57**	**100.00**	**2,129**	**100.00**	**21,982**	**100.00**	**24,168**	**100.00**
**1380200000**	**City of Las Piñas**	**10**	**432**	**5,914**	**6,356**	**10**	**100.00**	**349**	**80.79**	**5,051**	**85.41**	**5,410**	**85.12**
**1380300000**	**City of Makati**	**3**	**112**	**1,395**	**1,510**	**3**	**100.00**	**111**	**99.11**	**1,336**	**95.77**	**1,450**	**96.03**
**1380400000**	**City of Malabon**	**12**	**332**	**2,685**	**3,029**	**8**	**66.67**	**248**	**74.70**	**2,149**	**80.04**	**2,405**	**79.40**
**1380500000**	**City of Mandaluyong**	**11**	**283**	**7,411**	**7,705**	**11**	**100.00**	**279**	**98.59**	**7,383**	**99.62**	**7,673**	**99.58**
**1380600000**	**City of Manila**	**28**	**1,543**	**22,420**	**23,991**	**8**	**28.57**	**1,390**	**90.08**	**16,194**	**72.23**	**17,592**	**73.33**
**1380700000**	**City of Marikina**	**9**	**226**	**2,441**	**2,676**	**9**	**100.00**	**226**	**100.00**	**2,441**	**100.00**	**2,676**	**100.00**
**1380800000**	**City of Muntinlupa**	**15**	**565**	**7,068**	**7,648**	**13**	**86.67**	**592**	**104.78**	**7,495**	**106.04**	**8,100**	**105.91**
**1380900000**	**City of Navotas**	**6**	**308**	**3,632**	**3,946**	**5**	**83.33**	**298**	**96.75**	**3,589**	**98.82**	**3,892**	**98.63**
**1381000000**	**City of Parañaque**	**16**	**560**	**7,521**	**8,097**	**10**	**62.50**	**438**	**78.21**	**5,229**	**69.53**	**5,677**	**70.11**
**1381100000**	**Pasay City**	**8**	**265**	**6,620**	**6,893**	**6**	**75.00**	**262**	**98.87**	**6,365**	**96.15**	**6,633**	**96.23**
**1381200000**	**City of Pasig**	**36**	**818**	**10,086**	**10,940**	**17**	**47.22**	**668**	**81.66**	**8,813**	**87.38**	**9,498**	**86.82**
**1381300000**	**Quezon City**	**51**	**2,225**	**56,245**	**58,521**	**42**	**82.35**	**2,100**	**94.38**	**53,632**	**95.35**	**55,774**	**95.31**
**1381400000**	**City of San Juan**	**0**	**35**	**734**	**769**	**0**	**0.00**	**34**	**97.14**	**698**	**95.10**	**732**	**95.19**
**1381500000**	**City of Taguig**	**22**	**1,050**	**11,222**	**12,294**	**23**	**104.55**	**998**	**95.05**	**9,891**	**88.14**	**10,912**	**88.76**
**1381600000**	**City of Valenzuela**	**13**	**364**	**4,237**	**4,614**	**13**	**100.00**	**748**	**205.49**	**4,712**	**111.21**	**5,473**	**118.62**
**1381701000**	**Pateros**	**0**	**37**	**717**	**754**	**0**	**0.00**	**26**	**70.27**	**624**	**87.03**	**650**	**86.21**
**1400000000**	**Cordillera Administrative Region (CAR)**	**55**	**2,118**	**21,818**	**23,991**	**20**	**36.36**	**1,393**	**65.77**	**15,619**	**71.59**	**17,032**	**70.99**
**1430300000**	**City of Baguio**	18	377	6,298	**6,693**	2	11.11	151	40.05	2,660	42.24	**2,813**	**42.03**
**1400100000**	**Abra**	3	311	2,339	**2,653**	3	100.00	233	74.92	2,095	89.57	**2,331**	**87.86**
1400101000	Bangued	3	275	2,098	**2,376**	0	0.00	48	17.45	462	22.02	**510**	**21.46**
1400102000	Boliney	0	1	6	**7**	0	0.00	1	100.00	19	316.67	**20**	**285.71**
1400103000	Bucay	0	6	28	**34**	0	0.00	22	366.67	147	525.00	**169**	**497.06**
1400104000	Bucloc	0	0	3	**3**	0	0.00	0	0.00	17	566.67	**17**	**566.67**
1400105000	Daguioman	0	0	0	**0**	0	0.00	1	0.00	18	0.00	**19**	**0.00**
1400106000	Danglas	0	0	0	**0**	0	0.00	4	0.00	53	0.00	**57**	**0.00**
1400107000	Dolores	0	2	30	**32**	1	0.00	12	600.00	87	290.00	**100**	**312.50**
1400108000	La Paz	0	0	1	**1**	0	0.00	12	0.00	167	16,700.00	**179**	**17,900.00**
1400109000	Lacub	0	1	9	**10**	0	0.00	1	100.00	32	355.56	**33**	**330.00**
1400110000	Lagangilang	0	5	5	**10**	0	0.00	18	360.00	116	2,320.00	**134**	**1,340.00**
1400111000	Lagayan	0	2	4	**6**	0	0.00	3	150.00	44	1,100.00	**47**	**783.33**
1400112000	Langiden	0	0	5	**5**	0	0.00	5	0.00	32	640.00	**37**	**740.00**
1400113000	Licuan-Baay	0	0	8	**8**	0	0.00	0	0.00	11	137.50	**11**	**137.50**
1400114000	Luba	0	1	7	**8**	0	0.00	4	400.00	44	628.57	**48**	**600.00**
1400115000	Malibcong	0	0	5	**5**	0	0.00	0	0.00	18	360.00	**18**	**360.00**
1400116000	Manabo	0	1	2	**3**	0	0.00	17	1,700.00	87	4,350.00	**104**	**3,466.67**
1400117000	Peñarrubia	0	0	10	**10**	0	0.00	16	0.00	79	790.00	**95**	**950.00**
1400118000	Pidigan	0	0	1	**1**	0	0.00	11	0.00	122	12,200.00	**133**	**13,300.00**
1400119000	Pilar	0	8	32	**40**	2	0.00	8	100.00	107	334.38	**117**	**292.50**
1400120000	Sallapadan	0	0	6	**6**	0	0.00	3	0.00	54	900.00	**57**	**950.00**
1400121000	San Isidro	0	0	1	**1**	0	0.00	7	0.00	42	4,200.00	**49**	**4,900.00**
1400122000	San Juan	0	3	24	**27**	0	0.00	20	666.67	91	379.17	**111**	**411.11**
1400123000	San Quintin	0	0	0	**0**	0	0.00	2	0.00	45	0.00	**47**	**0.00**
1400124000	Tayum	0	0	13	**13**	0	0.00	10	0.00	105	807.69	**115**	**884.62**
1400125000	Tineg	0	2	24	**26**	0	0.00	2	100.00	18	75.00	**20**	**76.92**
1400126000	Tubo	0	3	17	**20**	0	0.00	3	100.00	39	229.41	**42**	**210.00**
1400127000	Villaviciosa	0	1	0	**1**	0	0.00	3	300.00	39	0.00	**42**	**4,200.00**
**1401100000**	**Benguet**	2	227	3,447	**3,676**	2	100.00	168	74.01	2,767	80.27	**2,937**	**79.90**
1401101000	Atok	0	19	207	**226**	0	0.00	0	0.00	61	29.47	**61**	**26.99**
1401103000	Bakun	0	2	22	**24**	0	0.00	2	100.00	21	95.45	**23**	**95.83**
1401104000	Bokod	0	14	146	**160**	0	0.00	14	100.00	136	93.15	**150**	**93.75**
1401105000	Buguias	0	6	154	**160**	0	0.00	4	66.67	51	33.12	**55**	**34.38**
1401106000	Itogon	0	1	26	**27**	0	0.00	1	100.00	9	34.62	**10**	**37.04**
1401107000	Kabayan	0	1	8	**9**	0	0.00	1	100.00	8	100.00	**9**	**100.00**
1401108000	Kapangan	0	1	82	**83**	0	0.00	1	100.00	77	93.90	**78**	**93.98**
1401109000	Kibungan	0	5	65	**70**	0	0.00	4	80.00	58	89.23	**62**	**88.57**
1401110000	La Trinidad	2	177	2,674	**2,853**	2	100.00	141	79.66	2,300	86.01	**2,443**	**85.63**
1401111000	Mankayan	0	1	21	**22**	0	0.00	0	0.00	5	23.81	**5**	**22.73**
1401112000	Sablan	0	0	2	**2**	0	0.00	0	0.00	2	100.00	**2**	**100.00**
1401113000	Tuba	0	0	32	**32**	0	0.00	0	0.00	31	96.88	**31**	**96.88**
1401114000	Tublay	0	0	8	**8**	0	0.00	0	0.00	8	100.00	**8**	**100.00**
**1402700000**	**Ifugao**	4	192	1,743	**1,939**	0	0.00	149	77.60	1,446	82.96	**1,595**	**82.26**
1402701000	Banaue	0	6	80	**86**	0	0.00	5	83.33	68	85.00	**73**	**84.88**
1402702000	Hungduan	0	5	70	**75**	0	0.00	4	80.00	52	74.29	**56**	**74.67**
1402703000	Kiangan	0	2	54	**56**	0	0.00	2	100.00	54	100.00	**56**	**100.00**
1402704000	Lagawe	2	39	388	**429**	0	0.00	22	56.41	229	59.02	**251**	**58.51**
1402705000	Lamut	1	58	429	**488**	0	0.00	40	68.97	340	79.25	**380**	**77.87**
1402706000	Mayoyao	0	4	93	**97**	0	0.00	4	100.00	93	100.00	**97**	**100.00**
1402707000	Alfonso Lista	0	50	320	**370**	0	0.00	46	92.00	307	95.94	**353**	**95.41**
1402708000	Aguinaldo	0	8	125	**133**	0	0.00	8	100.00	123	98.40	**131**	**98.50**
1402709000	Hingyon	0	1	5	**6**	0	0.00	1	100.00	4	80.00	**5**	**83.33**
1402710000	Tinoc	1	16	117	**134**	0	0.00	14	87.50	116	99.15	**130**	**97.01**
1402711000	Asipulo	0	3	62	**65**	0	0.00	3	100.00	60	96.77	**63**	**96.92**
**1403200000**	**Kalinga**	5	155	2,032	**2,192**	4	80.00	153	98.71	1,959	96.41	**2,116**	**96.53**
1403201000	Balbalan	0	2	38	**40**	0	0.00	1	50.00	33	86.84	**34**	**85.00**
1403206000	Lubuagan	0	1	71	**72**	0	0.00	1	100.00	28	39.44	**29**	**40.28**
1403208000	Pasil	0	0	8	**8**	0	0.00	0	0.00	8	100.00	**8**	**100.00**
1403209000	Pinukpuk	0	11	92	**103**	0	0.00	9	81.82	82	89.13	**91**	**88.35**
1403211000	Rizal	0	9	183	**192**	0	0.00	9	100.00	171	93.44	**180**	**93.75**
1403213000	City of Tabuk	5	132	1,602	**1,739**	4	80.00	132	100.00	1,602	100.00	**1,738**	**99.94**
1403214000	Tanudan	0	0	5	**5**	0	0.00	0	0.00	4	80.00	**4**	**80.00**
1403215000	Tinglayan	0	0	33	**33**	0	0.00	1	0.00	31	93.94	**32**	**96.97**
**1404400000**	**Mountain Province**	9	330	2,917	**3,256**	3	33.33	265	80.30	2,654	90.98	**2,922**	**89.74**
1404401000	Barlig	0	0	8	**8**	0	0.00	0	0.00	6	75.00	**6**	**75.00**
1404402000	Bauko	1	192	1,826	**2,019**	1	100.00	185	96.35	1,794	98.25	**1,980**	**98.07**
1404403000	Besao	1	10	89	**100**	0	0.00	7	70.00	76	85.39	**83**	**83.00**
1404404000	Bontoc	2	45	582	**629**	1	50.00	30	66.67	498	85.57	**529**	**84.10**
1404405000	Natonin	1	4	35	**40**	0	0.00	3	75.00	28	80.00	**31**	**77.50**
1404406000	Paracelis	3	75	296	**374**	1	33.33	37	49.33	178	60.14	**216**	**57.75**
1404407000	Sabangan	0	0	3	**3**	0	0.00	0	0.00	3	100.00	**3**	**100.00**
1404408000	Sadanga	0	0	24	**24**	0	0.00	0	0.00	24	100.00	**24**	**100.00**
1404409000	Sagada	1	3	48	**52**	0	0.00	2	66.67	43	89.58	**45**	**86.54**
1404410000	Tadian	0	1	6	**7**	0	0.00	1	100.00	4	66.67	**5**	**71.43**
**1408100000**	**Apayao**	14	526	3,042	**3,582**	6	42.86	274	52.09	2,038	67.00	**2,318**	**64.71**
1408101000	Calanasan	0	11	42	**53**	0	0.00	5	45.45	20	47.62	**25**	**47.17**
1408102000	Conner	1	93	627	**721**	0	0.00	48	51.61	344	54.86	**392**	**54.37**
1408103000	Flora	0	17	83	**100**	0	0.00	13	76.47	72	86.75	**85**	**85.00**
1408104000	Kabugao	1	17	75	**93**	1	100.00	15	88.24	60	80.00	**76**	**81.72**
1408105000	Luna	12	377	2,150	**2,539**	5	41.67	183	48.54	1,489	69.26	**1,677**	**66.05**
1408106000	Pudtol	0	6	51	**57**	0	0.00	6	100.00	40	78.43	**46**	**80.70**
1408107000	Santa Marcela	0	5	14	**19**	0	0.00	4	80.00	13	92.86	**17**	**89.47**
**100000000**	**Region I (Ilocos Region)**	**169**	**4,304**	**46,997**	**51,470**	**116**	**68.64**	**4,870**	**113.15**	**53,502**	**113.84**	**58,488**	**113.64**
**102800000**	**Ilocos Norte**	13	314	5,428	**5,755**	11	84.62	428	136.31	6,072	111.86	**6,511**	**113.14**
102801000	Adams	0	0	0	**0**	0	0.00	1	0.00	13	0.00	**14**	**0.00**
102802000	Bacarra	0	0	10	**10**	0	0.00	23	0.00	271	2,710.00	**294**	**2,940.00**
102803000	Badoc	0	0	16	**16**	1	0.00	42	0.00	270	1,687.50	**313**	**1,956.25**
102804000	Bangui	2	22	236	**260**	0	0.00	13	59.09	132	55.93	**145**	**55.77**
102805000	City of Batac	10	261	2,877	**3,148**	0	0.00	33	12.64	490	17.03	**523**	**16.61**
102806000	Burgos	0	0	0	**0**	0	0.00	4	0.00	92	0.00	**96**	**0.00**
102807000	Carasi	0	0	0	**0**	0	0.00	0	0.00	10	0.00	**10**	**0.00**
102808000	Currimao	0	0	0	**0**	3	0.00	15	0.00	99	0.00	**117**	**0.00**
102809000	Dingras	0	0	7	**7**	0	0.00	19	0.00	230	3,285.71	**249**	**3,557.14**
102810000	Dumalneg	0	0	0	**0**	0	0.00	3	0.00	25	0.00	**28**	**0.00**
102811000	Banna	0	0	9	**9**	0	0.00	20	0.00	103	1,144.44	**123**	**1,366.67**
102812000	City of Laoag	0	21	1,912	**1,933**	0	0.00	21	100.00	1,913	100.05	**1,934**	**100.05**
102813000	Marcos	0	4	14	**18**	0	0.00	20	500.00	154	1,100.00	**174**	**966.67**
102814000	Nueva Era	0	1	12	**13**	0	0.00	14	1,400.00	106	883.33	**120**	**923.08**
102815000	Pagudpud	0	0	6	**6**	3	0.00	21	0.00	201	3,350.00	**225**	**3,750.00**
102816000	Paoay	0	0	0	**0**	1	0.00	42	0.00	195	0.00	**238**	**0.00**
102817000	Pasuquin	0	1	10	**11**	2	0.00	18	1,800.00	109	1,090.00	**129**	**1,172.73**
102818000	Piddig	0	0	109	**109**	0	0.00	26	0.00	365	334.86	**391**	**358.72**
102819000	Pinili	0	0	0	**0**	0	0.00	9	0.00	157	0.00	**166**	**0.00**
102820000	San Nicolas	1	2	162	**165**	0	0.00	22	1,100.00	450	277.78	**472**	**286.06**
102821000	Sarrat	0	0	10	**10**	0	0.00	18	0.00	233	2,330.00	**251**	**2,510.00**
102822000	Solsona	0	2	30	**32**	1	0.00	13	650.00	194	646.67	**208**	**650.00**

PSGC10	Area	Total Deliveries				Women who gave birth with at least four or more prenatal check-ups (4ANC)							
		10-14 y.o.	15-19 y.o.	20-49 y.o.	Total	10-14 y.o.	%	15-19 y.o.	%	20-49 y.o.	%	Total	%
102823000	Vintar	0	0	8	8	0	0.00	31	0.00	260	3,250.00	291	3,637.50
**102900000**	**Ilocos Sur**	14	467	6,911	7,392	8	57.14	506	108.35	7,817	113.11	8,331	112.70
102901000	Alilem	0	0	2	2	0	0.00	2	0.00	50	2,500.00	52	2,600.00
102902000	Banayoyo	0	0	0	0	0	0.00	9	0.00	85	0.00	94	0.00
102903000	Bantay	0	8	525	533	0	0.00	16	200.00	793	151.05	809	151.78
102904000	Burgos	0	1	1	2	0	0.00	17	1,700.00	116	11,600.00	133	6,650.00
102905000	Cabugao	0	8	188	196	0	0.00	12	150.00	291	154.79	303	154.59
102906000	City of Candon	0	31	1,746	1,777	0	0.00	31	100.00	924	52.92	955	53.74
102907000	Caoayan	0	0	0	0	1	0.00	6	0.00	309	0.00	316	0.00
102908000	Cervantes	0	3	23	26	0	0.00	13	433.33	180	782.61	193	742.31
102909000	Galimuyod	0	0	2	2	0	0.00	3	0.00	77	3,850.00	80	4,000.00
102910000	Gregorio del Pilar	0	0	2	2	0	0.00	1	0.00	37	1,850.00	38	1,900.00
102911000	Lidlidda	0	0	0	0	0	0.00	3	0.00	41	0.00	44	0.00
102912000	Magsingal	0	8	391	399	0	0.00	11	137.50	450	115.09	461	115.54
102913000	Nagbukel	0	0	1	1	1	0.00	10	0.00	47	4,700.00	58	5,800.00
102914000	Narvacan	2	110	1,107	1,219	1	50.00	65	59.09	427	38.57	493	40.44
102915000	Quirino	0	3	25	28	0	0.00	11	366.67	91	364.00	102	364.29
102916000	Salcedo	0	0	14	14	1	0.00	8	0.00	86	614.29	95	678.57
102917000	San Emilio	0	1	0	1	0	0.00	7	700.00	63	0.00	70	7,000.00
102918000	San Esteban	0	0	0	0	0	0.00	3	0.00	76	0.00	79	0.00
102919000	San Ildefonso	0	1	2	3	0	0.00	7	700.00	71	3,550.00	78	2,600.00
102920000	San Juan	0	0	0	0	0	0.00	8	0.00	228	0.00	236	0.00
102921000	San Vicente	0	0	1	1	0	0.00	15	0.00	117	11,700.00	132	13,200.00
102922000	Santa	0	0	16	16	0	0.00	7	0.00	145	906.25	152	950.00
102923000	Santa Catalina	1	0	2	3	0	0.00	3	0.00	273	13,650.00	276	9,200.00
102924000	Santa Cruz	0	0	1	1	1	0.00	16	0.00	391	39,100.00	408	40,800.00
102925000	Santa Lucia	0	7	182	189	1	0.00	9	128.57	382	209.89	392	207.41
102926000	Santa Maria	0	0	6	6	0	0.00	24	0.00	256	4,266.67	280	4,666.67
102927000	Santiago	0	0	0	0	1	0.00	18	0.00	194	0.00	213	0.00
102928000	Santo Domingo	0	3	20	23	0	0.00	18	600.00	311	1,555.00	329	1,430.43
102929000	Sigay	0	0	1	1	0	0.00	2	0.00	19	1,900.00	21	2,100.00
102930000	Sinait	1	83	759	843	0	0.00	43	51.81	279	36.76	322	38.20
102931000	Sugpon	0	0	0	0	0	0.00	4	0.00	30	0.00	34	0.00
102932000	Suyo	0	0	4	4	0	0.00	4	0.00	109	2,725.00	113	2,825.00
102933000	Tagudin	1	29	346	376	1	100.00	29	100.00	346	100.00	376	100.00
102934000	City of Vigan	9	171	1,544	1,724	0	0.00	71	41.52	523	33.87	594	34.45
**103300000**	**La Union**	32	691	7,039	7,762	34	106.25	702	101.59	7,275	103.35	8,011	103.21
103301000	Agoo	4	61	565	630	3	75.00	56	91.80	552	97.70	611	96.98
103302000	Aringay	0	5	58	63	1	0.00	41	820.00	394	679.31	436	692.06
103303000	Bacnotan	0	19	265	284	0	0.00	32	168.42	443	167.17	475	167.25
103304000	Bagulin	0	2	14	16	0	0.00	17	850.00	121	864.29	138	862.50
103305000	Balaoan	0	46	393	439	0	0.00	34	73.91	350	89.06	384	87.47
103306000	Bangar	0	1	21	22	2	0.00	42	4,200.00	332	1,580.95	376	1,709.09
103307000	Bauang	0	3	20	23	3	0.00	71	2,366.67	530	2,650.00	604	2,626.09
103308000	Burgos	0	0	2	2	0	0.00	4	0.00	77	3,850.00	81	4,050.00
103309000	Caba	0	1	53	54	1	0.00	15	1,500.00	223	420.75	239	442.59
103310000	Luna	0	0	5	5	0	0.00	27	0.00	273	5,460.00	300	6,000.00
103311000	Naguilian	1	54	441	496	3	300.00	57	105.56	496	112.47	556	112.10
103312000	Pugo	0	0	0	0	0	0.00	12	0.00	137	0.00	149	0.00
103313000	Rosario	0	7	152	159	4	0.00	52	742.86	485	319.08	541	340.25
103314000	City of San Fernando	27	486	4,905	5,418	8	29.63	106	21.81	1,063	21.67	1,177	21.72
103315000	San Gabriel	0	3	44	47	2	0.00	13	433.33	246	559.09	261	555.32
103316000	San Juan	0	0	0	0	3	0.00	29	0.00	392	0.00	424	0.00
103317000	Santo Tomas	0	1	32	33	2	0.00	49	4,900.00	293	915.63	344	1,042.42
103318000	Santol	0	1	14	15	1	0.00	10	1,000.00	126	900.00	137	913.33
103319000	Sudipen	0	0	1	1	0	0.00	6	0.00	200	20,000.00	206	20,600.00
103320000	Tubao	0	1	54	55	1	0.00	29	2,900.00	542	1,003.70	572	1,040.00
**105500000**	**Pangasinan**	88	2,268	20,860	23,216	55	62.50	3,019	133.11	30,345	145.47	33,419	143.95
105501000	Agno	0	3	33	36	1	0.00	42	1,400.00	314	951.52	357	991.67
105502000	Aguilar	0	0	175	175	0	0.00	25	0.00	384	219.43	409	233.71
105503000	City of Alaminos	0	110	1,134	1,244	0	0.00	121	110.00	1,116	98.41	1,237	99.44
105504000	Alcala	0	0	4	4	1	0.00	51	0.00	462	11,550.00	514	12,850.00
105505000	Anda	1	13	202	216	0	0.00	49	376.92	368	182.18	417	193.06
105506000	Asingan	0	0	139	139	0	0.00	53	0.00	548	394.24	601	432.37
105507000	Balungao	0	2	11	13	0	0.00	10	500.00	102	927.27	112	861.54
105508000	Bani	0	7	111	118	0	0.00	7	100.00	111	100.00	118	100.00
105509000	Basista	0	0	45	45	0	0.00	42	0.00	611	1,357.78	653	1,451.11
105510000	Bautista	0	17	107	124	0	0.00	46	270.59	618	577.57	664	535.48
105511000	Bayambang	3	239	1,714	1,956	9	300.00	189	79.08	1,775	103.56	1,973	100.87
105512000	Binalonan	0	0	15	15	3	0.00	44	0.00	748	4,986.67	795	5,300.00
105513000	Binmaley	0	1	154	155	0	0.00	16	1,600.00	447	290.26	463	298.71
105514000	Bolinao	0	37	388	425	1	0.00	191	516.22	1,025	264.18	1,217	286.35
105515000	Bugallon	0	4	174	178	1	0.00	80	2,000.00	857	492.53	938	526.97
105516000	Burgos	0	0	12	12	1	0.00	45	0.00	383	3,191.67	429	3,575.00
105517000	Calasiao	1	17	305	323	1	100.00	125	735.29	1,225	401.64	1,351	418.27
105519000	Dasol	0	45	251	296	0	0.00	33	73.33	438	174.50	471	159.12
105520000	Infanta	1	9	140	150	0	0.00	12	133.33	164	117.14	176	117.33
105521000	Labrador	0	1	42	43	0	0.00	32	3,200.00	228	542.86	260	604.65
105522000	Lingayen	12	205	311	528	0	0.00	88	42.93	1,025	329.58	1,113	210.80
105523000	Mabini	0	0	0	0	1	0.00	40	0.00	454	0.00	495	0.00
105524000	Malasiqui	0	28	532	560	1	0.00	89	317.86	1,158	217.67	1,248	222.86
105525000	Manaoag	0	12	324	336	2	0.00	50	416.67	761	234.88	813	241.96
105526000	Mangaldan	0	11	337	348	5	0.00	103	936.36	1,350	400.59	1,458	418.97
105527000	Mangatarem	0	62	701	763	3	0.00	86	138.71	709	101.14	798	104.59
105528000	Mapandan	0	7	175	182	1	0.00	31	442.86	335	191.43	367	201.65
105529000	Natividad	0	0	3	3	2	0.00	13	0.00	246	8,200.00	261	8,700.00
105530000	Pozorrubio	0	6	147	153	4	0.00	72	1,200.00	724	492.52	800	522.88
105531000	Rosales	6	201	1,734	1,941	1	16.67	63	31.34	562	32.41	626	32.25
105532000	City of San Carlos	61	864	8,135	9,060	7	11.48	191	22.11	2,388	29.35	2,586	28.54
105533000	San Fabian	0	7	91	98	0	0.00	143	2,042.86	839	921.98	982	1,002.04
105534000	San Jacinto	0	0	0	0	1	0.00	49	0.00	506	0.00	556	0.00
105535000	San Manuel	0	6	135	141	2	0.00	77	1,283.33	546	404.44	625	443.26
105536000	San Nicolas	0	29	254	283	0	0.00	20	68.97	252	99.21	272	96.11
105537000	San Quintin	0	4	66	70	1	0.00	47	1,175.00	315	477.27	363	518.57
105538000	Santa Barbara	0	7	75	82	1	0.00	94	1,342.86	1,004	1,338.67	1,099	1,340.24
105539000	Santa Maria	0	1	13	14	0	0.00	54	5,400.00	309	2,376.92	363	2,592.86
105540000	Santo Tomas	0	0	1	1	0	0.00	0	0.00	59	5,900.00	59	5,900.00
105541000	Sison	0	0	9	9	1	0.00	44	0.00	602	6,688.89	647	7,188.89
105542000	Sual	0	3	14	17	0	0.00	51	1,700.00	308	2,200.00	359	2,111.76
105543000	Tayug	1	39	413	453	0	0.00	41	105.13	358	86.68	399	88.08
105544000	Umingan	0	24	512	536	1	0.00	63	262.50	840	164.06	904	168.66
105545000	Urbiztondo	0	5	81	86	0	0.00	73	1,460.00	591	729.63	664	772.09
105546000	City of Urdaneta	2	242	1,625	1,869	2	100.00	183	75.62	1,235	76.00	1,420	75.98
105547000	Villasis	0	0	16	16	1	0.00	25	0.00	502	3,137.50	528	3,300.00
105548000	Laoac	0	0	0	0	0	0.00	16	0.00	443	0.00	459	0.00
105518000	**City of Dagupan**	22	564	6,759	7,345	8	36.36	215	38.12	1,993	29.49	2,216	30.17
**200000000**	**Region II (Cagayan Valley)**	**93**	**3,613**	**29,487**	**33,193**	**84**	**90.32**	**3,502**	**96.93**	**28,464**	**96.53**	**32,050**	**96.56**
**203135000**	**City of Santiago**	3	274	2,565	2,842	3	100.00	274	100.00	2,565	100.00	2,842	100.00
**200900000**	**Batanes**	0	15	187	202	0	0.00	5	33.33	130	69.52	135	66.83
200901000	Basco	0	15	117	132	0	0.00	5	33.33	78	66.67	83	62.88
200902000	Itbayat	0	0	20	20	0	0.00	0	0.00	17	85.00	17	85.00
200903000	Ivana	0	0	16	16	0	0.00	0	0.00	13	81.25	13	81.25
200904000	Mahatao	0	0	12	12	0	0.00	0	0.00	10	83.33	10	83.33
200905000	Sabtang	0	0	12	12	0	0.00	0	0.00	2	16.67	2	16.67
200906000	Uyugan	0	0	10	10	0	0.00	0	0.00	10	100.00	10	100.00
**201500000**	**Cagayan**	16	812	7,045	7,873	11	68.75	757	93.23	6,386	90.65	7,154	90.87
201501000	Abulug	0	0	10	10	0	0.00	0	0.00	9	90.00	9	90.00
201502000	Alcala	0	16	134	150	0	0.00	16	100.00	134	100.00	150	100.00
201503000	Allacapan	0	9	114	123	0	0.00	7	77.78	105	92.11	112	91.06
201504000	Amulung	0	26	203	229	0	0.00	26	100.00	203	100.00	229	100.00
201505000	Aparri	0	138	960	1,098	0	0.00	137	99.28	958	99.79	1,095	99.73
201506000	Baggao	1	60	480	541	1	100.00	57	95.00	453	94.38	511	94.45
201507000	Ballesteros	0	20	127	147	0	0.00	19	95.00	126	99.21	145	98.64
201508000	Buguey	0	3	20	23	0	0.00	2	66.67	19	95.00	21	91.30
201509000	Calayan	0	25	126	151	0	0.00	20	80.00	107	84.92	127	84.11
201510000	Camalaniugan	0	37	468	505	0	0.00	37	100.00	468	100.00	505	100.00
201511000	Claveria	0	4	18	22	0	0.00	0	0.00	20	111.11	20	90.91

2.B.1.1 - Prenatal Care

Women who gave birth with at least 4 Prenatal Check-ups

Philippines, 2024

PSGC10	Area	Total Deliveries				Women who gave birth with at least four or more prenatal check-ups (4ANC)							
		10-14 y.o.	15-19 y.o.	20-49 y.o.	Total	10-14 y.o.	%	15-19 y.o.	%	20-49 y.o.	%	Total	%
201512000	Enrile	0	5	69	74	0	0.00	5	100.00	69	100.00	74	100.00
201513000	Gattaran	1	46	316	363	0	0.00	42	91.30	285	90.19	327	90.08
201514000	Gonzaga	4	94	466	564	3	75.00	112	119.15	428	91.85	543	96.28
201515000	Iguig	0	2	89	91	0	0.00	2	100.00	89	100.00	91	100.00
201516000	Lal-Lo	0	0	9	9	0	0.00	0	0.00	9	100.00	9	100.00
201517000	Lasam	1	38	182	221	1	100.00	26	68.42	116	63.74	143	64.71
201518000	Pamplona	0	2	43	45	0	0.00	2	100.00	41	95.35	43	95.56
201519000	Peñablanca	0	22	159	181	0	0.00	19	86.36	162	101.89	181	100.00
201520000	Piat	0	23	138	161	0	0.00	17	73.91	144	104.35	161	100.00
201521000	Rizal	0	0	18	18	0	0.00	0	0.00	18	100.00	18	100.00
201522000	Sanchez-Mira	0	8	116	124	0	0.00	8	100.00	116	100.00	124	100.00
201523000	Santa Ana	2	17	209	228	2	100.00	17	100.00	190	90.91	209	91.67
201524000	Santa Praxedes	0	0	13	13	0	0.00	0	0.00	9	69.23	9	69.23
201525000	Santa Teresita	0	1	31	32	0	0.00	1	100.00	31	100.00	32	100.00
201526000	Santo Niño	0	12	96	108	0	0.00	12	100.00	88	91.67	100	92.59
201527000	Solana	1	47	398	446	1	100.00	45	95.74	390	97.99	436	97.76
201528000	Tuao	2	65	492	559	2	100.00	65	100.00	492	100.00	559	100.00
201529000	Tuguegarao City	4	92	1,541	1,637	1	25.00	63	68.48	1,107	71.84	1,171	71.53
**203100000**	**Isabela**	26	1,370	11,631	13,027	23	88.46	1,327	96.86	11,335	97.46	12,685	97.37
203101000	Alicia	1	62	750	813	1	100.00	56	90.32	697	92.93	754	92.74
203102000	Angadanan	1	23	146	170	1	100.00	23	100.00	146	100.00	170	100.00
203103000	Aurora	0	8	89	97	0	0.00	8	100.00	89	100.00	97	100.00
203104000	Benito Soliven	1	2	23	26	1	100.00	2	100.00	23	100.00	26	100.00
203105000	Burgos	0	1	28	29	0	0.00	1	100.00	28	100.00	29	100.00
203106000	Cabagan	1	33	416	450	1	100.00	33	100.00	414	99.52	448	99.56
203107000	Cabatuan	0	14	176	190	0	0.00	14	100.00	176	100.00	190	100.00
203108000	City of Cauayan	8	139	1,215	1,362	5	62.50	124	89.21	1,105	90.95	1,234	90.60
203109000	Cordon	0	33	279	312	0	0.00	33	100.00	279	100.00	312	100.00
203110000	Dinapigue	0	14	83	97	0	0.00	13	92.86	80	96.39	93	95.88
203111000	Divilacan	0	6	18	24	0	0.00	6	100.00	18	100.00	24	100.00
203112000	Echague	1	113	1,079	1,193	1	100.00	111	98.23	1,072	99.35	1,184	99.25
203113000	Gamu	0	0	15	15	0	0.00	0	0.00	15	100.00	15	100.00
203114000	City of Ilagan	5	579	4,448	5,032	5	100.00	579	100.00	4,432	99.64	5,016	99.68
203115000	Jones	1	27	198	226	1	100.00	27	100.00	198	100.00	226	100.00
203116000	Luna	0	0	7	7	0	0.00	0	0.00	6	85.71	6	85.71
203117000	Maconacon	0	3	12	15	0	0.00	1	33.33	8	66.67	9	60.00
203118000	Delfin Albano	0	5	126	131	0	0.00	5	100.00	126	100.00	131	100.00
203119000	Mallig	0	2	88	90	0	0.00	3	150.00	86	97.73	89	98.89
203120000	Naguilian	0	6	108	114	0	0.00	6	100.00	108	100.00	114	100.00
203121000	Palanan	2	19	113	134	2	100.00	19	100.00	113	100.00	134	100.00
203122000	Quezon	0	4	79	83	0	0.00	4	100.00	76	96.20	80	96.39
203123000	Quirino	0	2	8	10	0	0.00	2	100.00	7	87.50	9	90.00
203124000	Ramon	0	15	216	231	0	0.00	15	100.00	180	83.33	195	84.42
203125000	Reina Mercedes	0	0	8	8	0	0.00	0	0.00	8	100.00	8	100.00
203126000	Roxas	2	73	475	550	2	100.00	68	93.15	458	96.42	528	96.00
203127000	San Agustin	0	3	45	48	0	0.00	3	100.00	45	100.00	48	100.00
203128000	San Guillermo	0	10	93	103	0	0.00	10	100.00	91	97.85	101	98.06
203129000	San Isidro	0	0	1	1	0	0.00	0	0.00	1	100.00	1	100.00
203130000	San Manuel	0	2	14	16	0	0.00	2	100.00	14	100.00	16	100.00
203131000	San Mariano	1	97	384	482	1	100.00	97	100.00	382	99.48	480	99.59
203132000	San Mateo	2	25	261	288	2	100.00	23	92.00	255	97.70	280	97.22
203133000	San Pablo	0	2	4	6	0	0.00	2	100.00	4	100.00	6	100.00
203134000	Santa Maria	0	15	132	147	0	0.00	4	26.67	101	76.52	105	71.43
203136000	Santo Tomas	0	1	2	3	0	0.00	1	100.00	2	100.00	3	100.00
203137000	Tumauini	0	32	492	524	0	0.00	32	100.00	492	100.00	524	100.00
**205000000**	**Nueva Vizcaya**	34	806	6,025	6,865	34	100.00	806	100.00	6,025	100.00	6,865	100.00
205001000	Ambaguio	0	5	40	45	0	0.00	5	100.00	40	100.00	45	100.00
205002000	Aritao	0	2	40	42	0	0.00	2	100.00	40	100.00	42	100.00
205003000	Bagabag	1	0	8	9	1	100.00	0	0.00	8	100.00	9	100.00
205004000	Bambang	8	186	1,416	1,610	8	100.00	186	100.00	1,416	100.00	1,610	100.00
205005000	Bayombong	24	545	3,805	4,374	24	100.00	545	100.00	3,805	100.00	4,374	100.00
205006000	Diadi	0	5	60	65	0	0.00	5	100.00	60	100.00	65	100.00
205007000	Dupax del Norte	0	7	67	74	0	0.00	7	100.00	67	100.00	74	100.00
205008000	Dupax del Sur	0	3	64	67	0	0.00	3	100.00	64	100.00	67	100.00
205009000	Kasibu	1	20	157	178	1	100.00	20	100.00	157	100.00	178	100.00
205010000	Kayapa	0	19	131	150	0	0.00	19	100.00	131	100.00	150	100.00
205011000	Quezon	0	4	22	26	0	0.00	4	100.00	22	100.00	26	100.00
205012000	Santa Fe	0	1	14	15	0	0.00	1	100.00	14	100.00	15	100.00
205013000	Solano	0	0	95	95	0	0.00	0	0.00	95	100.00	95	100.00
205014000	Villaverde	0	2	45	47	0	0.00	2	100.00	45	100.00	47	100.00
205015000	Alfonso Castaneda	0	7	61	68	0	0.00	7	100.00	61	100.00	68	100.00
**205700000**	**Quirino**	14	336	2,034	2,384	13	92.86	333	99.11	2,023	99.46	2,369	99.37
205701000	Aglipay	0	0	7	7	0	0.00	0	0.00	7	100.00	7	100.00
205702000	Cabarroguis	14	309	1,402	1,725	13	92.86	306	99.03	1,392	99.29	1,711	99.19
205703000	Diffun	0	1	370	371	0	0.00	1	100.00	370	100.00	371	100.00
205704000	Maddela	0	25	204	229	0	0.00	25	100.00	204	100.00	229	100.00
205705000	Saguday	0	1	2	3	0	0.00	1	100.00	2	100.00	3	100.00
205706000	Nagtipunan	0	0	49	49	0	0.00	0	0.00	48	97.96	48	97.96
**300000000**	**Region III (Central Luzon)**	**307**	**11,146**	**120,543**	**131,996**	**423**	**137.79**	**14,781**	**132.61**	**130,289**	**108.09**	**145,493**	**110.23**
**330100000**	**City of Angeles**	15	434	6,990	**7,439**	27	180.00	1,018	234.56	6,769	96.84	**7,814**	**105.04**
**331400000**	**City of Olongapo**	6	221	3,483	**3,710**	3	50.00	218	98.64	2,394	68.73	**2,615**	**70.49**
**300800000**	**Bataan**	61	1,357	9,945	**11,363**	46	75.41	1,028	75.76	7,395	74.36	**8,469**	**74.53**
300801000	Abucay	0	0	18	**18**	4	0.00	67	0.00	276	1,533.33	**347**	**1,927.78**
300802000	Bagac	0	12	110	**122**	2	0.00	47	391.67	203	184.55	**252**	**206.56**
300803000	City of Balanga	51	949	6,156	**7,156**	3	5.88	100	10.54	770	12.51	**873**	**12.20**
300804000	Dinalupihan	6	182	1,637	**1,825**	9	150.00	189	103.85	1,493	91.20	**1,691**	**92.66**
300805000	Hermosa	0	0	21	**21**	5	0.00	115	0.00	957	4,557.14	**1,077**	**5,128.57**
300806000	Limay	0	12	127	**139**	3	0.00	83	691.67	469	369.29	**555**	**399.28**
300807000	Mariveles	1	57	621	**679**	8	800.00	146	256.14	931	149.92	**1,085**	**159.79**
300808000	Morong	0	19	163	**182**	0	0.00	21	110.53	180	110.43	**201**	**110.44**
300809000	Orani	3	123	904	**1,030**	3	100.00	77	62.60	898	99.34	**978**	**94.95**
300810000	Orion	0	0	57	**57**	3	0.00	88	0.00	625	1,096.49	**716**	**1,256.14**
300811000	Pilar	0	2	129	**131**	2	0.00	43	2,150.00	256	198.45	**301**	**229.77**
300812000	Samal	0	1	2	**3**	4	0.00	52	5,200.00	337	16,850.00	**393**	**13,100.00**
**301400000**	**Bulacan**	30	1,519	30,061	**31,610**	142	473.33	4,554	299.80	46,195	153.67	**50,891**	**161.00**
	**Hospitals**	62	1,954	7,436	**9,452**	4	6.45	404	20.68	1,628	21.89	**2,036**	**21.54**
301401000	Angat	0	23	361	**384**	4	0.00	108	469.57	824	228.25	**936**	**243.75**
301402000	Balagtas	0	14	328	**342**	3	0.00	122	871.43	652	198.78	**777**	**227.19**
301403000	City of Baliwag	0	5	1,767	**1,772**	5	0.00	250	5,000.00	3,082	174.42	**3,337**	**188.32**
301404000	Bocaue	2	66	2,655	**2,723**	4	200.00	171	259.09	1,408	53.03	**1,583**	**58.13**
301405000	Bulacan	1	35	248	**284**	5	500.00	80	228.57	902	363.71	**987**	**347.54**
301406000	Bustos	1	27	775	**803**	11	1,100.00	127	470.37	769	99.23	**907**	**112.95**
301407000	Calumpit	0	10	326	**336**	2	0.00	51	510.00	1,079	330.98	**1,132**	**336.90**
301408000	Guiguinto	1	16	576	**593**	4	400.00	118	737.50	1,609	279.34	**1,731**	**291.91**
301409000	Hagonoy	0	13	378	**391**	13	0.00	283	2,176.92	2,069	547.35	**2,365**	**604.86**
301410000	City of Malolos	1	88	4,407	**4,496**	9	900.00	254	288.64	2,503	56.80	**2,766**	**61.52**
301411000	Marilao	0	19	1,079	**1,098**	9	0.00	175	921.05	1,730	160.33	**1,914**	**174.32**
301412000	City of Meycauayan	1	36	2,115	**2,152**	4	400.00	290	805.56	2,956	139.76	**3,250**	**151.02**
301413000	Norzagaray	0	61	535	**596**	2	0.00	189	309.84	1,182	220.93	**1,373**	**230.37**
301414000	Obando	0	4	121	**125**	0	0.00	61	1,525.00	883	729.75	**944**	**755.20**
301415000	Pandi	0	87	1,250	**1,337**	2	0.00	120	137.93	1,212	96.96	**1,334**	**99.78**
301416000	Paombong	0	0	66	**66**	1	0.00	45	0.00	294	445.45	**340**	**515.15**
301417000	Plaridel	0	63	1,412	**1,475**	3	0.00	184	292.06	1,517	107.44	**1,704**	**115.53**
301418000	Pulilan	0	2	282	**284**	0	0.00	66	3,300.00	1,192	422.70	**1,258**	**442.96**
301419000	San Ildefonso	1	58	1,682	**1,741**	1	100.00	152	262.07	1,320	78.48	**1,473**	**84.61**
301420000	City of San Jose Del Monte	19	825	7,443	**8,287**	35	184.21	1,062	128.73	13,380	179.77	**14,477**	**174.70**
301421000	San Miguel	1	23	428	**452**	3	300.00	119	517.39	1,308	305.61	**1,430**	**316.37**
301422000	San Rafael	0	12	386	**398**	0	0.00	84	700.00	745	193.01	**829**	**208.29**
301423000	Santa Maria	2	26	1,238	**1,266**	16	800.00	387	1,488.46	3,245	262.12	**3,648**	**288.15**
301424000	Doña Remedios Trinidad	0	6	203	**209**	6	0.00	56	933.33	334	164.53	**396**	**189.47**
**304900000**	**Nueva Ecija**	90	2,543	22,151	**24,784**	74	82.22	2,605	102.44	18,726	84.54	**21,405**	**86.37**
304901000	Aliaga	0	0	0	**0**	0	0.00	25	0.00	760	0.00	**785**	**0.00**
304902000	Bongabon	1	15	179	**195**	2	200.00	73	486.67	490	273.74	**565**	**289.74**
304903000	City of Cabanatuan	68	1,739	11,377	**13,184**	10	14.71	466	26.80	3,010	26.46	**3,486**	**26.44**
304904000	Cabiao	1	44	493	**538**	4	400.00	89	202.27	681	138.13	**774**	**143.87**
304905000	Carranglan	1	16	110	**127**	0	0.00	40	250.00	287	260.91	**327**	**257.48**
304906000	Cuyapo	1	17	163	**181**	0	0.00	70	411.76	548	336.20	**618**	**341.44**

2.B.1.1 - Prenatal Care
Women who gave birth with at least 4 Prenatal Check-ups
Philippines, 2024

PSGC10	Area	Total Deliveries				Women who gave birth with at least four or more prenatal check-ups (4ANC)							
		10-14 y.o.	15-19 y.o.	20-49 y.o.	Total	10-14 y.o.	%	15-19 y.o.	%	20-49 y.o.	%	Total	%
304907000	Gabaldon	0	15	160	**175**	1	0.00	80	533.33	421	263.13	**502**	**286.86**
304908000	City of Gapan	5	62	586	**653**	3	60.00	61	98.39	562	95.90	**626**	**95.87**
304909000	General Mamerto Natividad	0	6	119	**125**	1	0.00	34	566.67	327	274.79	**362**	**289.60**
304910000	General Tinio	0	9	121	**130**	2	0.00	125	1,388.89	464	383.47	**591**	**454.62**
304911000	Guimba	2	21	313	**336**	4	200.00	149	709.52	566	180.83	**719**	**213.99**
304912000	Jaen	0	2	32	**34**	1	0.00	66	3,300.00	887	2,771.88	**954**	**2,805.88**
304913000	Laur	0	5	28	**33**	3	0.00	63	1,260.00	343	1,225.00	**409**	**1,239.39**
304914000	Licab	0	4	54	**58**	0	0.00	33	825.00	256	474.07	**289**	**498.28**
304915000	Llanera	0	0	8	**8**	0	0.00	29	0.00	205	2,562.50	**234**	**2,925.00**
304916000	Lupao	0	2	27	**29**	2	0.00	53	2,650.00	358	1,325.93	**413**	**1,424.14**
304917000	Science City of Muñoz	0	8	138	**146**	1	0.00	105	1,312.50	691	500.72	**797**	**545.89**
304918000	Nampicuan	0	0	23	**23**	1	0.00	8	0.00	97	421.74	**106**	**460.87**
304919000	City of Palayan	0	4	31	**35**	1	0.00	52	1,300.00	326	1,051.61	**379**	**1,082.86**
304920000	Pantabangan	0	1	60	**61**	2	0.00	17	1,700.00	265	441.67	**284**	**465.57**
304921000	Peñaranda	0	1	14	**15**	1	0.00	39	3,900.00	243	1,735.71	**283**	**1,886.67**
304922000	Quezon	0	9	50	**59**	6	0.00	83	922.22	391	782.00	**480**	**813.56**
304923000	Rizal	0	69	341	**410**	3	0.00	59	85.51	381	111.73	**443**	**108.05**
304924000	San Antonio	1	37	267	**305**	2	200.00	102	275.68	714	267.42	**818**	**268.20**
304925000	San Isidro	1	44	329	**374**	1	100.00	68	154.55	322	97.87	**391**	**104.55**
304926000	San Jose City	6	281	1,845	**2,132**	6	100.00	273	97.15	1,801	97.62	**2,080**	**97.56**
304927000	San Leonardo	1	56	3,922	**3,979**	1	100.00	66	117.86	458	11.68	**525**	**13.19**
304928000	Santa Rosa	0	0	70	**70**	1	0.00	52	0.00	542	774.29	**595**	**850.00**
304929000	Santo Domingo	0	1	34	**35**	2	0.00	101	10,100.00	642	1,888.24	**745**	**2,128.57**
304930000	Talavera	2	66	1,125	**1,193**	2	100.00	66	100.00	1,154	102.58	**1,222**	**102.43**
304931000	Talugtug	0	3	39	**42**	0	0.00	23	766.67	121	310.26	**144**	**342.86**
304932000	Zaragoza	0	6	93	**99**	11	0.00	35	583.33	413	444.09	**459**	**463.64**
**305400000**	**Pampanga**	67	2,425	21,406	**23,898**	85	126.87	2,598	107.13	23,525	109.90	**26,208**	**109.67**
305402000	Apalit	1	30	1,157	**1,188**	2	200.00	90	300.00	971	83.92	**1,063**	**89.48**
305403000	Arayat	8	299	1,995	**2,302**	5	62.50	227	75.92	1,368	68.57	**1,600**	**69.50**
305404000	Bacolor	0	1	25	**26**	1	0.00	34	3,400.00	243	972.00	**278**	**1,069.23**
305405000	Candaba	0	16	71	**87**	4	0.00	143	893.75	1,070	1,507.04	**1,217**	**1,398.85**
305406000	Floridablanca	2	182	1,619	**1,803**	6	300.00	98	53.85	1,417	87.52	**1,521**	**84.36**
305407000	Guagua	4	138	1,557	**1,699**	1	25.00	136	98.55	1,678	107.77	**1,815**	**106.83**
305408000	Lubao	3	251	1,930	**2,184**	5	166.67	163	64.94	1,736	89.95	**1,904**	**87.18**
305409000	Mabalacat City	4	164	2,698	**2,866**	13	325.00	335	204.27	3,885	144.00	**4,233**	**147.70**
305410000	Macabebe	6	211	1,245	**1,462**	3	50.00	72	34.12	577	46.35	**652**	**44.60**
305411000	Magalang	6	147	1,306	**1,459**	6	100.00	129	87.76	969	74.20	**1,104**	**75.67**
305412000	Masantol	0	1	1	**2**	2	0.00	37	3,700.00	162	16,200.00	**201**	**10,050.00**
305413000	Mexico	9	85	1,637	**1,731**	0	0.00	115	135.29	1,007	61.51	**1,122**	**64.82**
305414000	Minalin	0	2	4	**6**	1	0.00	64	3,200.00	509	12,725.00	**574**	**9,566.67**
305415000	Porac	3	263	1,259	**1,525**	7	233.33	253	96.20	1,498	118.98	**1,758**	**115.28**
305416000	City of San Fernando	17	437	3,751	**4,205**	24	141.18	415	94.97	3,812	101.63	**4,251**	**101.09**
305417000	San Luis	4	192	980	**1,176**	2	50.00	71	36.98	628	64.08	**701**	**59.61**
305418000	San Simon	0	0	13	**13**	0	0.00	65	0.00	581	4,469.23	**646**	**4,969.23**
305419000	Santa Ana	0	1	3	**4**	0	0.00	12	1,200.00	337	11,233.33	**349**	**8,725.00**
305420000	Santa Rita	0	0	1	**1**	2	0.00	56	0.00	380	38,000.00	**438**	**43,800.00**
305421000	Sto. Tomas	0	5	154	**159**	1	0.00	42	840.00	318	206.49	**361**	**227.04**
305422000	Sasmuan	0	0	0	**0**	0	0.00	41	0.00	379	0.00	**420**	**0.00**
**306900000**	**Tarlac**	17	1,474	18,469	**19,960**	39	229.41	1,770	120.08	18,268	98.91	**20,077**	**100.59**
306901000	Anao	0	1	7	**8**	0	0.00	12	1,200.00	82	1,171.43	**94**	**1,175.00**
306902000	Bamban	0	4	275	**279**	5	0.00	186	4,650.00	859	312.36	**1,050**	**376.34**
306903000	Camiling	0	0	173	**173**	1	0.00	49	0.00	736	425.43	**786**	**454.34**
306904000	Capas	0	283	2,398	**2,681**	16	0.00	341	120.49	2,779	115.89	**3,136**	**116.97**
306905000	Concepcion	3	114	1,633	**1,750**	3	100.00	165	144.74	2,093	128.17	**2,261**	**129.20**
306906000	Gerona	0	3	87	**90**	0	0.00	75	2,500.00	738	848.28	**813**	**903.33**
306907000	La Paz	0	37	530	**567**	0	0.00	71	191.89	574	108.30	**645**	**113.76**
306908000	Mayantoc	0	1	28	**29**	1	0.00	11	1,100.00	137	489.29	**149**	**513.79**
306909000	Moncada	0	0	570	**570**	1	0.00	24	0.00	787	138.07	**812**	**142.46**
306910000	Paniqui	0	53	534	**587**	1	0.00	60	113.21	713	133.52	**774**	**131.86**
306911000	Pura	0	2	31	**33**	0	0.00	20	1,000.00	229	738.71	**249**	**754.55**
306912000	Ramos	0	2	49	**51**	0	0.00	15	750.00	256	522.45	**271**	**531.37**
306913000	San Clemente	0	1	5	**6**	0	0.00	13	1,300.00	91	1,820.00	**104**	**1,733.33**
306914000	San Manuel	0	4	150	**154**	0	0.00	19	475.00	275	183.33	**294**	**190.91**
306915000	Santa Ignacia	0	7	218	**225**	0	0.00	42	600.00	687	315.14	**729**	**324.00**
306916000	City of Tarlac	12	887	11,018	**11,917**	7	58.33	528	59.53	6,197	56.24	**6,732**	**56.49**
306917000	Victoria	2	54	398	**454**	4	200.00	95	175.93	456	114.57	**555**	**122.25**
306918000	San Jose	0	21	365	**386**	0	0.00	44	209.52	579	158.63	**623**	**161.40**
**307100000**	**Zambales**	14	845	5,855	**6,714**	5	35.71	662	78.34	4,616	78.84	**5,283**	**78.69**
307101000	Botolan	0	41	336	**377**	0	0.00	108	263.41	668	198.81	**776**	**205.84**
307102000	Cabangan	0	10	124	**134**	0	0.00	33	330.00	246	198.39	**279**	**208.21**
307103000	Candelaria	1	37	331	**369**	1	100.00	30	81.08	265	80.06	**296**	**80.22**
307104000	Castillejos	0	1	19	**20**	1	0.00	58	5,800.00	455	2,394.74	**514**	**2,570.00**
307105000	Iba	8	328	2,162	**2,498**	1	12.50	54	16.46	486	22.48	**541**	**21.66**
307106000	Masinloc	0	16	166	**182**	1	0.00	19	118.75	267	160.84	**287**	**157.69**
307108000	Palauig	0	7	18	**25**	0	0.00	42	600.00	339	1,883.33	**381**	**1,524.00**
307109000	San Antonio	0	1	9	**10**	0	0.00	40	4,000.00	279	3,100.00	**319**	**3,190.00**
307110000	San Felipe	0	0	21	**21**	0	0.00	44	0.00	232	1,104.76	**276**	**1,314.29**
307111000	San Marcelino	5	372	2,163	**2,540**	1	20.00	66	17.74	349	16.13	**416**	**16.38**
307112000	San Narciso	0	0	2	**2**	0	0.00	16	0.00	124	6,200.00	**140**	**7,000.00**
307113000	Santa Cruz	0	27	274	**301**	0	0.00	40	148.15	339	123.72	**379**	**125.91**
307114000	Subic	0	5	230	**235**	0	0.00	112	2,240.00	567	246.52	**679**	**288.94**
**307700000**	**Aurora**	7	328	2,183	**2,518**	2	28.57	328	100.00	2,401	109.99	**2,731**	**108.46**
307701000	Baler	7	263	1,659	**1,929**	1	14.29	53	20.15	418	25.20	**472**	**24.47**
307702000	Casiguran	0	23	210	**233**	0	0.00	30	130.43	264	125.71	**294**	**126.18**
307703000	Dilasag	0	3	21	**24**	1	0.00	34	1,133.33	200	952.38	**235**	**979.17**
307704000	Dinalungan	0	3	13	**16**	0	0.00	24	800.00	118	907.69	**142**	**887.50**
307705000	Dingalan	0	16	117	**133**	0	0.00	47	293.75	288	246.15	**335**	**251.88**
307706000	Dipaculao	0	6	53	**59**	0	0.00	45	750.00	379	715.09	**424**	**718.64**
307707000	Maria Aurora	0	6	74	**80**	0	0.00	62	1,033.33	446	602.70	**508**	**635.00**
307708000	San Luis	0	8	36	**44**	0	0.00	33	412.50	288	800.00	**321**	**729.55**
**400000000**	**Region IV-A (CALABARZON)**	**390**	**17,923**	**180,933**	**199,246**	**319**	**81.79**	**14,238**	**79.44**	**146,537**	**80.99**	**161,094**	**80.85**
**431200000**	**City of Lucena**	**6**	**323**	**2,562**	**2,891**	**5**	**83.33**	**314**	**97.21**	**2,505**	**97.78**	**2,824**	**97.68**
**401000000**	**Batangas**	**70**	**2,689**	**33,636**	**36,395**	**56**	**80.00**	**2,201**	**81.85**	**28,214**	**83.88**	**30,471**	**83.72**
401001000	Agoncillo	0	47	466	**513**	0	0.00	47	100.00	466	100.00	**513**	**100.00**
401002000	Alitagtag	0	25	240	**265**	0	0.00	24	96.00	236	98.33	**260**	**98.11**
401003000	Balayan	0	89	1,028	**1,117**	0	0.00	88	98.88	1,012	98.44	**1,100**	**98.48**
401004000	Balete	0	14	244	**258**	0	0.00	14	100.00	230	94.26	**244**	**94.57**
401005000	Batangas City	7	324	3,343	**3,674**	7	100.00	265	81.79	3,020	90.34	**3,292**	**89.60**
401006000	Bauan	2	101	1,323	**1,426**	2	100.00	99	98.02	1,325	100.15	**1,426**	**100.00**
401007000	City of Calaca	0	74	1,536	**1,610**	0	0.00	74	100.00	1,536	100.00	**1,610**	**100.00**
401008000	Calatagan	3	83	626	**712**	3	100.00	60	72.29	535	85.46	**598**	**83.99**
401009000	Cuenca	0	29	383	**412**	0	0.00	28	96.55	377	98.43	**405**	**98.30**
401010000	Ibaan	1	49	627	**677**	0	0.00	13	26.53	162	25.84	**175**	**25.85**
401011000	Laurel	2	46	529	**577**	0	0.00	31	67.39	324	61.25	**355**	**61.53**
401012000	Lemery	8	89	979	**1,076**	8	100.00	87	97.75	957	97.75	**1,052**	**97.77**
401013000	Lian	1	52	620	**673**	1	100.00	51	98.08	620	100.00	**672**	**99.85**
401014000	City of Lipa	12	320	4,550	**4,882**	5	41.67	199	62.19	3,398	74.68	**3,602**	**73.78**
401015000	Lobo	0	61	392	**453**	0	0.00	43	70.49	298	76.02	**341**	**75.28**
401016000	Mabini	1	54	440	**495**	1	100.00	54	100.00	425	96.59	**480**	**96.97**
401017000	Malvar	4	56	763	**823**	2	50.00	15	26.79	233	30.54	**250**	**30.38**
401018000	Mataasnakahoy	1	29	302	**332**	1	100.00	28	96.55	290	96.03	**319**	**96.08**
401019000	Nasugbu	5	187	1,461	**1,653**	5	100.00	143	76.47	1,294	88.57	**1,442**	**87.24**
401020000	Padre Garcia	0	59	584	**643**	0	0.00	59	100.00	544	93.15	**603**	**93.78**
401021000	Rosario	0	97	1,523	**1,620**	0	0.00	37	38.14	895	58.77	**932**	**57.53**
401022000	San Jose	6	106	1,125	**1,237**	6	100.00	106	100.00	1,125	100.00	**1,237**	**100.00**
401023000	San Juan	2	125	1,309	**1,436**	1	50.00	110	88.00	1,129	86.25	**1,240**	**86.35**
401024000	San Luis	1	25	332	**358**	1	100.00	25	100.00	332	100.00	**358**	**100.00**
401025000	San Nicolas	0	7	175	**182**	0	0.00	6	85.71	167	95.43	**173**	**95.05**
401026000	San Pascual	2	39	648	**689**	2	100.00	40	102.56	647	99.85	**689**	**100.00**
401027000	Santa Teresita	0	16	198	**214**	0	0.00	16	100.00	198	100.00	**214**	**100.00**
401028000	City of Sto. Tomas	2	166	1,973	**2,141**	2	100.00	155	93.37	1,904	96.50	**2,061**	**96.26**
401029000	Taal	7	59	660	**726**	7	100.00	59	100.00	656	99.39	**722**	**99.45**
401030000	Talisay	2	33	469	**504**	1	50.00	30	90.91	391	83.37	**422**	**83.73**
401031000	City of Tanauan	0	134	3,708	**3,842**	0	0.00	114	85.07	2,493	67.23	**2,607**	**67.86**
401032000	Taysan	0	44	477	**521**	0	0.00	42	95.45	474	99.37	**516**	**99.04**
401033000	Tingloy	0	15	120	**135**	0	0.00	5	33.33	46	38.33	**51**	**37.78**
401034000	Tuy	1	35	483	**519**	1	100.00	34	97.14	475	98.34	**510**	**98.27**

2.B.1.1 - Prenatal Care
Women who gave birth with at least 4 Prenatal Check-ups
Philippines, 2024

PSGC10	Area	Total Deliveries				Women who gave birth with at least four or more prenatal check-ups (4ANC)							
		10-14 y.o.	15-19 y.o.	20-49 y.o.	Total	10-14 y.o.	%	15-19 y.o.	%	20-49 y.o.	%	Total	%
**402100000**	**Cavite**	**73**	**3,418**	**44,751**	**48,242**	**52**	**71.23**	**2,804**	**82.04**	**33,361**	**74.55**	**36,217**	**75.07**
402101000	Alfonso	2	46	627	**675**	1	50.00	33	71.74	577	92.03	**611**	**90.52**
402102000	Amadeo	3	35	428	**466**	1	33.33	21	60.00	239	55.84	**261**	**56.01**
402103000	City of Bacoor	2	418	6,095	**6,515**	4	200.00	415	99.28	2,946	48.33	**3,365**	**51.65**
402104000	Carmona	1	41	969	**1,011**	1	100.00	42	102.44	956	98.66	**999**	**98.81**
402105000	City of Cavite	4	120	712	**836**	2	50.00	64	53.33	398	55.90	**464**	**55.50**
402106000	City of Dasmariñas	9	532	7,687	**8,228**	9	100.00	499	93.80	7,111	92.51	**7,619**	**92.60**
402107000	General Emilio Aguinaldo	1	33	240	**274**	1	100.00	25	75.76	208	86.67	**234**	**85.40**
402108000	City of General Trias	2	224	3,704	**3,930**	2	100.00	204	91.07	3,326	89.79	**3,532**	**89.87**
402109000	City of Imus	2	148	3,671	**3,821**	1	50.00	88	59.46	1,665	45.36	**1,754**	**45.90**
402110000	Indang	1	50	748	**799**	0	0.00	37	74.00	452	60.43	**489**	**61.20**
402111000	Kawit	2	107	1,115	**1,224**	0	0.00	96	89.72	938	84.13	**1,034**	**84.48**
402112000	Magallanes	1	26	317	**344**	1	100.00	15	57.69	276	87.07	**292**	**84.88**
402113000	Maragondon	1	40	490	**531**	1	100.00	37	92.50	415	84.69	**453**	**85.31**
402114000	Mendez	0	42	336	**378**	0	0.00	42	100.00	334	99.40	**376**	**99.47**
402115000	Naic	2	205	2,179	**2,386**	0	0.00	82	40.00	679	31.16	**761**	**31.89**
402116000	Noveleta	0	47	639	**686**	0	0.00	46	97.87	632	98.90	**678**	**98.83**
402117000	Rosario	7	170	1,120	**1,297**	5	71.43	127	74.71	740	66.07	**872**	**67.23**
402118000	Silang	7	291	3,165	**3,463**	3	42.86	211	72.51	2,389	75.48	**2,603**	**75.17**
402119000	City of Tagaytay	2	61	1,451	**1,514**	2	100.00	61	100.00	1,446	99.66	**1,509**	**99.67**
402120000	Tanza	7	317	4,243	**4,567**	4	57.14	235	74.13	3,149	74.22	**3,388**	**74.18**
402121000	Ternate	2	43	341	**386**	1	50.00	38	88.37	297	87.10	**336**	**87.05**
402122000	City of Trece Martires	7	216	2,511	**2,734**	6	85.71	200	92.59	2,397	95.46	**2,603**	**95.21**
402123000	Gen. Mariano Alvarez	8	206	1,963	**2,177**	7	87.50	186	90.29	1,791	91.24	**1,984**	**91.13**
**403400000**	**Laguna**	**95**	**3,953**	**39,146**	**43,194**	**90**	**94.74**	**3,421**	**86.54**	**35,764**	**91.36**	**39,275**	**90.93**
403401000	Alaminos	2	76	596	**674**	2	100.00	72	94.74	577	96.81	**651**	**96.59**
403402000	Bay	1	86	512	**599**	1	100.00	53	61.63	316	61.72	**370**	**61.77**
403403000	City of Biñan	1	228	4,924	**5,153**	2	200.00	171	75.00	4,862	98.74	**5,035**	**97.71**
403404000	City of Cabuyao	3	288	4,825	**5,116**	3	100.00	322	111.81	4,686	97.12	**5,011**	**97.95**
403405000	City of Calamba	19	679	6,106	**6,804**	19	100.00	651	95.88	5,774	94.56	**6,444**	**94.71**
403406000	Calauan	2	167	899	**1,068**	2	100.00	154	92.22	847	94.22	**1,003**	**93.91**
403407000	Cavinti	1	32	259	**292**	1	100.00	28	87.50	236	91.12	**265**	**90.75**
403408000	Famy	0	12	131	**143**	0	0.00	11	91.67	124	94.66	**135**	**94.41**
403409000	Kalayaan	2	44	302	**348**	2	100.00	43	97.73	288	95.36	**333**	**95.69**
403410000	Liliw	4	57	359	**420**	4	100.00	51	89.47	312	86.91	**367**	**87.38**
403411000	Los Baños	3	118	864	**985**	3	100.00	111	94.07	839	97.11	**953**	**96.75**
403412000	Luisiana	1	14	192	**207**	0	0.00	5	35.71	142	73.96	**147**	**71.01**
403413000	Lumban	4	46	319	**369**	5	125.00	44	95.65	303	94.98	**352**	**95.39**
403414000	Mabitac	2	42	254	**298**	1	50.00	36	85.71	218	85.83	**255**	**85.57**
403415000	Magdalena	0	48	296	**344**	0	0.00	24	50.00	204	68.92	**228**	**66.28**
403416000	Majayjay	2	42	284	**328**	2	100.00	32	76.19	250	88.03	**284**	**86.59**
403417000	Nagcarlan	2	62	587	**651**	2	100.00	60	96.77	561	95.57	**623**	**95.70**
403418000	Paete	0	28	244	**272**	0	0.00	32	114.29	240	98.36	**272**	**100.00**
403419000	Pagsanjan	1	55	429	**485**	0	0.00	40	72.73	369	86.01	**409**	**84.33**
403420000	Pakil	0	28	195	**223**	0	0.00	16	57.14	158	81.03	**174**	**78.03**
403421000	Pangil	0	48	274	**322**	1	0.00	36	75.00	213	77.74	**250**	**77.64**
403422000	Pila	2	71	502	**575**	1	50.00	39	54.93	324	64.54	**364**	**63.30**
403423000	Rizal	0	27	170	**197**	0	0.00	13	48.15	103	60.59	**116**	**58.88**
403424000	City of San Pablo	10	396	3,105	**3,511**	9	90.00	366	92.42	2,889	93.04	**3,264**	**92.96**
403425000	City of San Pedro	9	469	4,371	**4,849**	7	77.78	288	61.41	3,337	76.34	**3,632**	**74.90**
403426000	Santa Cruz	4	130	1,186	**1,320**	4	100.00	96	73.85	947	79.85	**1,047**	**79.32**
403427000	Santa Maria	0	25	323	**348**	0	0.00	24	96.00	323	100.00	**347**	**99.71**
403428000	City of Santa Rosa	18	568	5,831	**6,417**	17	94.44	552	97.18	5,641	96.74	**6,210**	**96.77**
403429000	Siniloan	0	37	380	**417**	1	0.00	21	56.76	275	72.37	**297**	**71.22**
403430000	Victoria	2	30	427	**459**	1	50.00	30	100.00	406	95.08	**437**	**95.21**
**405600000**	**Quezon**	**78**	**3,059**	**20,713**	**23,850**	**51**	**65.38**	**2,162**	**70.68**	**16,215**	**78.28**	**18,428**	**77.27**
405601000	Agdangan	1	20	137	**158**	0	0.00	21	105.00	129	94.16	**150**	**94.94**
405602000	Alabat	1	36	172	**209**	1	100.00	23	63.89	139	80.81	**163**	**77.99**
405603000	Atimonan	1	79	695	**775**	0	0.00	36	45.57	437	62.88	**473**	**61.03**
405605000	Buenavista	1	78	322	**401**	1	100.00	48	61.54	181	56.21	**230**	**57.36**
405606000	Burdeos	3	75	287	**365**	2	66.67	44	58.67	172	59.93	**218**	**59.73**
405607000	Calauag	1	89	606	**696**	2	200.00	62	69.66	436	71.95	**500**	**71.84**
405608000	Candelaria	5	160	1,440	**1,605**	5	100.00	138	86.25	1,394	96.81	**1,537**	**95.76**
405610000	Catanauan	2	161	817	**980**	0	0.00	67	41.61	396	48.47	**463**	**47.24**
405615000	Dolores	1	45	323	**369**	1	100.00	43	95.56	325	100.62	**369**	**100.00**
405616000	General Luna	0	19	201	**220**	0	0.00	17	89.47	202	100.50	**219**	**99.55**
405617000	General Nakar	3	44	400	**447**	1	33.33	32	72.73	322	80.50	**355**	**79.42**
405618000	Guinayangan	1	53	331	**385**	0	0.00	34	64.15	227	68.58	**261**	**67.79**
405619000	Gumaca	0	94	774	**868**	0	0.00	62	65.96	616	79.59	**678**	**78.11**
405620000	Infanta	2	74	829	**905**	1	50.00	64	86.49	674	81.30	**739**	**81.66**
405621000	Jomalig	0	26	70	**96**	0	0.00	21	80.77	91	130.00	**112**	**116.67**
405622000	Lopez	4	160	892	**1,056**	2	50.00	131	81.88	789	88.45	**922**	**87.31**
405623000	Lucban	0	33	308	**341**	0	0.00	23	69.70	288	93.51	**311**	**91.20**
405625000	Macalelon	3	40	270	**313**	2	66.67	27	67.50	232	85.93	**261**	**83.39**
405627000	Mauban	3	169	872	**1,044**	3	100.00	159	94.08	839	96.22	**1,001**	**95.88**
405628000	Mulanay	3	133	689	**825**	3	100.00	56	42.11	432	62.70	**491**	**59.52**
405629000	Padre Burgos	2	30	213	**245**	2	100.00	24	80.00	185	86.85	**211**	**86.12**
405630000	Pagbilao	0	136	880	**1,016**	0	0.00	120	88.24	648	73.64	**768**	**75.59**
405631000	Panukulan	0	9	74	**83**	0	0.00	9	100.00	58	78.38	**67**	**80.72**
405632000	Patnanungan	0	37	152	**189**	0	0.00	9	24.32	46	30.26	**55**	**29.10**
405633000	Perez	2	30	190	**222**	1	50.00	24	80.00	114	60.00	**139**	**62.61**
405634000	Pitogo	0	32	262	**294**	0	0.00	31	96.88	254	96.95	**285**	**96.94**
405635000	Plaridel	0	11	112	**123**	0	0.00	3	27.27	86	76.79	**89**	**72.36**
405636000	Polillo	2	63	358	**423**	0	0.00	30	47.62	255	71.23	**285**	**67.38**
405637000	Quezon	1	28	165	**194**	0	0.00	11	39.29	93	56.36	**104**	**53.61**
405638000	Real	1	57	458	**516**	0	0.00	57	100.00	349	76.20	**406**	**78.68**
405639000	Sampaloc	1	12	122	**135**	0	0.00	8	66.67	92	75.41	**100**	**74.07**
405640000	San Andres	1	100	468	**569**	0	0.00	61	61.00	329	70.30	**390**	**68.54**
405641000	San Antonio	0	37	452	**489**	0	0.00	34	91.89	454	100.44	**488**	**99.80**
405642000	San Francisco	6	185	775	**966**	4	66.67	73	39.46	342	44.13	**419**	**43.37**
405644000	San Narciso	7	127	634	**768**	6	85.71	96	75.59	441	69.56	**543**	**70.70**
405645000	Sariaya	9	200	1,692	**1,901**	9	100.00	199	99.50	1,692	100.00	**1,900**	**99.95**
405646000	Tagkawayan	2	89	665	**756**	0	0.00	36	40.45	329	49.47	**365**	**48.28**
405647000	City of Tayabas	2	121	1,253	**1,376**	2	100.00	120	99.17	1,222	97.53	**1,344**	**97.67**
405648000	Tiaong	5	128	1,098	**1,231**	1	20.00	72	56.25	661	60.20	**734**	**59.63**
405649000	Unisan	2	39	255	**296**	2	100.00	37	94.87	244	95.69	**283**	**95.61**
**405800000**	**Rizal**	**68**	**4,481**	**40,125**	**44,674**	**65**	**95.59**	**3,336**	**74.45**	**30,478**	**75.96**	**33,879**	**75.84**
405801000	Angono	4	214	1,811	**2,029**	4	100.00	213	99.53	1,806	99.72	**2,023**	**99.70**
405802000	City of Antipolo	7	1,409	10,834	**12,250**	17	242.86	817	57.98	7,802	72.01	**8,636**	**70.50**
405803000	Baras	3	169	972	**1,144**	2	66.67	146	86.39	922	94.86	**1,070**	**93.53**
405804000	Binangonan	15	462	4,124	**4,601**	7	46.67	355	76.84	3,583	86.88	**3,945**	**85.74**
405805000	Cainta	8	230	3,983	**4,221**	9	112.50	234	101.74	3,476	87.27	**3,719**	**88.11**
405806000	Cardona	4	88	531	**623**	2	50.00	80	90.91	519	97.74	**601**	**96.47**
405807000	Jala-Jala	1	59	399	**459**	1	100.00	55	93.22	359	89.97	**415**	**90.41**
405808000	Rodriguez	6	569	5,286	**5,861**	3	50.00	292	51.32	1,942	36.74	**2,237**	**38.17**
405809000	Morong	5	121	924	**1,050**	5	100.00	117	96.69	905	97.94	**1,027**	**97.81**
405810000	Pililla	1	70	901	**972**	0	0.00	81	115.71	773	85.79	**854**	**87.86**
405811000	San Mateo	4	287	2,929	**3,220**	7	175.00	228	79.44	1,441	49.20	**1,676**	**52.05**
405812000	Tanay	3	241	1,519	**1,763**	2	66.67	194	80.50	1,298	85.45	**1,494**	**84.74**
405813000	Taytay	2	450	4,912	**5,364**	2	100.00	450	100.00	4,885	99.45	**5,337**	**99.50**
405814000	Teresa	5	112	1,000	**1,117**	4	80.00	74	66.07	767	76.70	**845**	**75.65**
**1700000000**	**Region IV-B (MIMAROPA)**	**126**	**5,691**	**36,723**	**42,540**	**72**	**57.14**	**3,401**	**59.76**	**23,133**	**62.99**	**26,606**	**62.54**
**1731500000**	**City of Puerto Princesa**	12	465	3,410	**3,887**	4	33.33	107	23.01	990	29.03	**1,101**	**28.33**
**1704000000**	**Marinduque**	6	280	2,421	**2,707**	3	50.00	201	71.79	1,869	77.20	**2,073**	**76.58**
1704001000	Boac	5	197	1,463	**1,665**	2	40.00	126	63.96	1,016	69.45	**1,144**	**68.71**
1704002000	Buenavista	0	15	142	**157**	0	0.00	11	73.33	113	79.58	**124**	**78.98**
1704003000	Gasan	0	2	121	**123**	0	0.00	2	100.00	101	83.47	**103**	**83.74**
1704004000	Mogpog	0	0	4	**4**	0	0.00	0	0.00	1	25.00	**1**	**25.00**
1704005000	Santa Cruz	1	43	439	**483**	1	100.00	43	100.00	417	94.99	**461**	**95.45**
1704006000	Torrijos	0	23	252	**275**	0	0.00	19	82.61	221	87.70	**240**	**87.27**
**1705100000**	**Occidental Mindoro**	27	1,216	7,039	**8,282**	15	55.56	752	61.84	4,773	67.81	**5,540**	**66.89**
1705101000	Abra De Ilog	6	125	362	**493**	1	16.67	52	41.60	159	43.92	**212**	**43.00**
1705102000	Calintaan	0	24	165	**189**	0	0.00	10	41.67	94	56.97	**104**	**55.03**
1705103000	Looc	0	1	12	**13**	0	0.00	0	0.00	10	83.33	**10**	**76.92**
1705104000	Lubang	0	11	90	**101**	0	0.00	11	100.00	89	98.89	**100**	**99.01**
1705105000	Magsaysay	4	51	349	**404**	2	50.00	21	41.18	236	67.62	**259**	**64.11**
1705106000	Mamburao	4	255	1,549	**1,808**	1	25.00	89	34.90	498	32.15	**588**	**32.52**

PSGC10	Area	Total Deliveries				Women who gave birth with at least four or more prenatal check-ups (4ANC)							
		10-14 y.o.	15-19 y.o.	20-49 y.o.	Total	10-14 y.o.	%	15-19 y.o.	%	20-49 y.o.	%	Total	%
1705107000	Paluan	2	68	238	308	1	50.00	44	64.71	167	70.17	212	68.83
1705108000	Rizal	0	21	220	241	0	0.00	4	19.05	74	33.64	78	32.37
1705109000	Sablayan	5	233	1,519	1,757	5	100.00	232	99.57	1,518	99.93	1,755	99.89
1705110000	San Jose	6	388	2,203	2,597	5	83.33	264	68.04	1,650	74.90	1,919	73.89
1705111000	Santa Cruz	0	39	332	371	0	0.00	25	64.10	278	83.73	303	81.67
**1705200000**	**Oriental Mindoro**	28	1,260	10,272	11,560	21	75.00	635	50.40	5,734	55.82	6,390	55.28
1705201000	Baco	0	2	67	69	0	0.00	2	100.00	61	91.04	63	91.30
1705202000	Bansud	2	8	129	139	2	100.00	8	100.00	123	95.35	133	95.68
1705203000	Bongabong	0	60	528	588	0	0.00	51	85.00	500	94.70	551	93.71
1705204000	Bulalacao	1	125	558	684	6	600.00	48	38.40	251	44.98	305	44.59
1705205000	City of Calapan	16	513	3,844	4,373	10	62.50	294	57.31	2,024	52.65	2,328	53.24
1705206000	Gloria	0	1	90	91	0	0.00	1	100.00	75	83.33	76	83.52
1705207000	Mansalay	0	48	231	279	0	0.00	30	62.50	155	67.10	185	66.31
1705208000	Naujan	0	12	343	355	0	0.00	10	83.33	329	95.92	339	95.49
1705209000	Pinamalayan	3	301	1,825	2,129	1	33.33	22	7.31	153	8.38	176	8.27
1705210000	Pola	0	2	14	16	0	0.00	2	100.00	10	71.43	12	75.00
1705211000	Puerto Galera	1	20	220	241	1	100.00	16	80.00	195	88.64	212	87.97
1705212000	Roxas	5	135	1,601	1,741	0	0.00	131	97.04	1,575	98.38	1,706	97.99
1705213000	San Teodoro	0	11	52	63	0	0.00	3	27.27	21	40.38	24	38.10
1705214000	Socorro	0	10	544	554	1	0.00	10	100.00	83	15.26	94	16.97
1705215000	Victoria	0	12	226	238	0	0.00	7	58.33	179	79.20	186	78.15
**1705300000**	**Palawan**	47	2,041	9,904	11,992	28	59.57	1,469	71.97	7,377	74.49	8,874	74.00
1705301000	Aborlan	2	68	405	475	0	0.00	16	23.53	115	28.40	131	27.58
1705302000	Agutaya	0	15	71	86	0	0.00	11	73.33	65	91.55	76	88.37
1705303000	Araceli	1	2	40	43	0	0.00	1	50.00	38	95.00	39	90.70
1705304000	Balabac	0	37	319	356	0	0.00	0	0.00	0	0.00	0	0.00
1705305000	Bataraza	5	287	1,242	1,534	5	100.00	243	84.67	1,115	89.77	1,363	88.85
1705306000	Brooke'S Point	9	200	981	1,190	8	88.89	161	80.50	759	77.37	928	77.98
1705307000	Busuanga	0	11	145	156	0	0.00	5	45.45	76	52.41	81	51.92
1705308000	Cagayancillo	0	4	36	40	0	0.00	1	25.00	28	77.78	29	72.50
1705309000	Coron	1	77	672	750	1	100.00	80	103.90	676	100.60	757	100.93
1705310000	Cuyo	0	41	198	239	0	0.00	17	41.46	80	40.40	97	40.59
1705311000	Dumaran	2	33	139	174	0	0.00	24	72.73	102	73.38	126	72.41
1705312000	El Nido	3	65	355	423	2	66.67	38	58.46	261	73.52	301	71.16
1705313000	Linapacan	0	22	131	153	0	0.00	13	59.09	70	53.44	83	54.25
1705314000	Magsaysay	0	2	43	45	0	0.00	2	100.00	28	65.12	30	66.67
1705315000	Narra	4	157	865	1,026	2	50.00	151	96.18	873	100.92	1,026	100.00
1705317000	Quezon	0	175	771	946	0	0.00	143	81.71	579	75.10	722	76.32
1705318000	Roxas	5	168	807	980	3	60.00	127	75.60	623	77.20	753	76.84
1705319000	San Vicente	2	56	202	260	1	50.00	39	69.64	147	72.77	187	71.92
1705320000	Taytay	6	270	1,073	1,349	2	33.33	126	46.67	571	53.22	699	51.82
1705321000	Kalayaan	0	0	0	0	0	0.00	0	0.00	0	0.00	0	0.00
1705322000	Culion	0	57	249	306	0	0.00	51	89.47	228	91.57	279	91.18
1705323000	Rizal	4	187	729	920	1	25.00	122	65.24	524	71.88	647	70.33
1705324000	Sofronio Española	3	107	431	541	3	100.00	98	91.59	419	97.22	520	96.12
**1705900000**	**Romblon**	6	429	3,677	4,112	1	16.67	237	55.24	2,390	65.00	2,628	63.91
1705901000	Alcantara	0	0	21	21	0	0.00	0	0.00	19	90.48	19	90.48
1705902000	Banton	0	0	20	20	0	0.00	0	0.00	20	100.00	20	100.00
1705903000	Cajidiocan	0	46	248	294	0	0.00	35	76.09	167	67.34	202	68.71
1705904000	Calatrava	0	3	27	30	0	0.00	1	33.33	17	62.96	18	60.00
1705905000	Concepcion	0	2	11	13	0	0.00	0	0.00	10	90.91	10	76.92
1705906000	Corcuera	0	7	33	40	0	0.00	3	42.86	16	48.48	19	47.50
1705907000	Looc	3	73	434	510	0	0.00	46	63.01	328	75.58	374	73.33
1705908000	Magdiwang	0	22	186	208	0	0.00	11	50.00	98	52.69	109	52.40
1705909000	Odiongan	3	120	1,204	1,327	1	33.33	63	52.50	757	62.87	821	61.87
1705910000	Romblon	0	77	653	730	0	0.00	35	45.45	405	62.02	440	60.27
1705911000	San Agustin	0	13	260	273	0	0.00	12	92.31	225	86.54	237	86.81
1705912000	San Andres	0	34	213	247	0	0.00	21	61.76	116	54.46	137	55.47
1705913000	San Fernando	0	4	149	153	0	0.00	2	50.00	101	67.79	103	67.32
1705914000	San Jose	0	25	118	143	0	0.00	7	28.00	33	27.97	40	27.97
1705915000	Santa Fe	0	1	53	54	0	0.00	1	100.00	41	77.36	42	77.78
1705916000	Ferrol	0	1	1	2	0	0.00	0	0.00	0	0.00	0	0.00
1705917000	Santa Maria	0	1	46	47	0	0.00	0	0.00	37	80.43	37	78.72
**500000000**	**Region V (Bicol Region)**	139	9,086	76,470	85,695	78	56.12	6,122	67.38	56,276	73.59	62,476	72.91
**500500000**	**Albay**	16	1,203	14,538	15,757	8	50.00	985	81.88	12,648	87.00	13,641	86.57
500501000	Bacacay	0	31	517	548	0	0.00	31	100.00	515	99.61	546	99.64
500502000	Camalig	0	11	333	344	0	0.00	11	100.00	307	92.19	318	92.44
500503000	Daraga	5	187	2,832	3,024	1	20.00	58	31.02	1,390	49.08	1,449	47.92
500504000	Guinobatan	1	22	458	481	0	0.00	20	90.91	449	98.03	469	97.51
500505000	Jovellar	0	3	61	64	0	0.00	3	100.00	61	100.00	64	100.00
500506000	City of Legazpi	1	145	2,966	3,112	1	100.00	145	100.00	2,966	100.00	3,112	100.00
500507000	Libon	1	34	438	473	0	0.00	20	58.82	387	88.36	407	86.05
500508000	City of Ligao	7	356	2,408	2,771	6	85.71	356	100.00	2,408	100.00	2,770	99.96
500509000	Malilipot	0	8	436	444	0	0.00	8	100.00	423	97.02	431	97.07
500510000	Malinao	0	6	263	269	0	0.00	6	100.00	263	100.00	269	100.00
500511000	Manito	0	27	251	278	0	0.00	23	85.19	226	90.04	249	89.57
500512000	Oas	1	0	9	10	0	0.00	0	0.00	9	100.00	9	90.00
500513000	Pio Duran	0	83	619	702	0	0.00	47	56.63	559	90.31	606	86.32
500514000	Polangui	0	49	758	807	0	0.00	47	95.92	690	91.03	737	91.33
500515000	Rapu-Rapu	0	7	50	57	0	0.00	3	42.86	32	64.00	35	61.40
500516000	Santo Domingo	0	1	114	115	0	0.00	1	100.00	112	98.25	113	98.26
500517000	City of Tabaco	0	206	1,434	1,640	0	0.00	186	90.29	1,371	95.61	1,557	94.94
500518000	Tiwi	0	27	591	618	0	0.00	20	74.07	480	81.22	500	80.91
**501600000**	**Camarines Norte**	17	1,154	8,983	10,154	17	100.00	1,117	96.79	8,812	98.10	9,946	97.95
501601000	Basud	0	0	55	55	0	0.00	0	0.00	53	96.36	53	96.36
501602000	Capalonga	0	39	266	305	0	0.00	36	92.31	257	96.62	293	96.07
501603000	Daet	16	891	5,858	6,765	16	100.00	891	100.00	5,854	99.93	6,761	99.94
501604000	San Lorenzo Ruiz	0	0	3	3	0	0.00	0	0.00	0	0.00	0	0.00
501605000	Jose Panganiban	0	33	350	383	0	0.00	29	87.88	352	100.57	381	99.48
501606000	Labo	1	79	963	1,043	1	100.00	69	87.34	952	98.86	1,022	97.99
501607000	Mercedes	0	8	202	210	0	0.00	5	62.50	169	83.66	174	82.86
501608000	Paracale	0	14	213	227	0	0.00	11	78.57	195	91.55	206	90.75
501609000	San Vicente	0	4	56	60	0	0.00	4	100.00	56	100.00	60	100.00
501610000	Santa Elena	0	56	443	499	0	0.00	47	83.93	395	89.16	442	88.58
501611000	Talisay	0	0	144	144	0	0.00	0	0.00	144	100.00	144	100.00
501612000	Vinzons	0	30	430	460	0	0.00	25	83.33	385	89.53	410	89.13
**501700000**	**Camarines Sur**	10	1,312	14,898	16,220	6	60.00	837	63.80	10,574	70.98	11,417	70.39
501701000	Baao	0	48	685	733	0	0.00	24	50.00	351	51.24	375	51.16
501702000	Balatan	0	42	522	564	0	0.00	42	100.00	522	100.00	564	100.00
501703000	Bato	0	22	488	510	0	0.00	16	72.73	423	86.68	439	86.08
501704000	Bombon	0	5	327	332	0	0.00	5	100.00	287	87.77	292	87.95
501705000	Buhi	0	112	909	1,021	0	0.00	80	71.43	614	67.55	694	67.97
501706000	Bula	0	40	484	524	0	0.00	32	80.00	443	91.53	475	90.65
501707000	Cabusao	0	0	25	25	0	0.00	0	0.00	11	44.00	11	44.00
501708000	Calabanga	0	13	279	292	0	0.00	3	23.08	121	43.37	124	42.47
501709000	Camaligan	0	0	2	2	0	0.00	0	0.00	2	100.00	2	100.00
501710000	Canaman	0	0	39	39	0	0.00	0	0.00	31	79.49	31	79.49
501711000	Caramoan	0	74	516	590	0	0.00	23	31.08	235	45.54	258	43.73
501712000	Del Gallego	2	49	379	430	2	100.00	48	97.96	353	93.14	403	93.72
501713000	Gainza	0	0	1	1	0	0.00	0	0.00	0	0.00	0	0.00
501714000	Garchitorena	1	72	377	450	0	0.00	9	12.50	95	25.20	104	23.11
501715000	Goa	0	94	887	981	0	0.00	83	88.30	817	92.11	900	91.74
501716000	City of Iriga	0	82	1,512	1,594	0	0.00	63	76.83	1,241	82.08	1,304	81.81
501717000	Lagonoy	2	55	627	684	2	100.00	50	90.91	567	90.43	619	90.50
501718000	Libmanan	0	45	524	569	0	0.00	22	48.89	281	53.63	303	53.25
501719000	Lupi	0	3	41	44	0	0.00	2	66.67	35	85.37	37	84.09
501720000	Magarao	0	7	123	130	0	0.00	5	71.43	113	91.87	118	90.77
501721000	Milaor	0	1	10	11	0	0.00	1	100.00	2	20.00	3	27.27
501721000	Minalabac	0	14	247	261	0	0.00	7	50.00	118	47.77	125	47.89
501723000	Nabua	0	17	534	551	0	0.00	10	58.82	270	50.56	280	50.82
501725000	Ocampo	0	29	299	328	0	0.00	9	31.03	120	40.13	129	39.33
501726000	Pamplona	0	16	376	392	0	0.00	13	81.25	364	96.81	377	96.17
501727000	Pasacao	0	26	313	339	0	0.00	11	42.31	155	49.52	166	48.97
501728000	Pili	0	16	438	454	0	0.00	10	62.50	275	62.79	285	62.78
501729000	Presentacion	0	11	78	89	0	0.00	4	36.36	51	65.38	55	61.80
501730000	Ragay	1	74	609	684	1	100.00	42	56.76	442	72.58	485	70.91
501731000	Sagñay	0	29	266	295	0	0.00	24	82.76	177	66.54	201	68.14

2.B.1.1 - Prenatal Care
Women who gave birth with at least 4 Prenatal Check-ups
Philippines, 2024

PSGC10	Area	Total Deliveries				Women who gave birth with at least four or more prenatal check-ups (4ANC)							
		10-14 y.o.	15-19 y.o.	20-49 y.o.	Total	10-14 y.o.	%	15-19 y.o.	%	20-49 y.o.	%	Total	%
501732000	San Fernando	0	3	104	107	0	0.00	2	66.67	102	98.08	104	97.20
501733000	San Jose	0	24	397	421	0	0.00	24	100.00	395	99.50	419	99.52
501734000	Sipocot	0	38	661	699	0	0.00	31	81.58	518	78.37	549	78.54
501735000	Siruma	1	21	149	171	0	0.00	9	42.86	46	30.87	55	32.16
501736000	Tigaon	2	90	751	843	1	50.00	61	67.78	490	65.25	552	65.48
501737000	Tinambac	1	140	919	1,060	0	0.00	72	51.43	507	55.17	579	54.62
**502000000**	**Catanduanes**	**9**	**458**	**3,352**	**3,819**	**5**	**55.56**	**364**	**79.48**	**2,885**	**86.07**	**3,254**	**85.21**
502001000	Bagamanoc	0	0	1	1	0	0.00	0	0.00	0	0.00	0	0.00
502002000	Baras	0	0	1	1	0	0.00	0	0.00	1	100.00	1	100.00
502003000	Bato	0	10	184	194	0	0.00	8	80.00	170	92.39	178	91.75
502004000	Caramoran	1	16	137	154	1	100.00	13	81.25	119	86.86	133	86.36
502005000	Gigmoto	1	12	83	96	0	0.00	4	33.33	60	72.29	64	66.67
502006000	Pandan	1	94	367	462	1	100.00	88	93.62	354	96.46	443	95.89
502007000	Panganiban	0	1	4	5	0	0.00	1	100.00	4	100.00	5	100.00
502008000	San Andres	0	39	273	312	0	0.00	29	74.36	246	90.11	275	88.14
502009000	San Miguel	0	0	1	1	0	0.00	0	0.00	1	100.00	1	100.00
502010000	Viga	0	50	331	381	0	0.00	48	96.00	319	96.37	367	96.33
502011000	Virac	6	236	1,970	2,212	3	50.00	173	73.31	1,611	81.78	1,787	80.79
**504100000**	**Masbate**	**23**	**2,159**	**12,529**	**14,711**	**15**	**65.22**	**1,555**	**72.02**	**9,542**	**76.16**	**11,112**	**75.54**
504101000	Aroroy	6	277	1,259	1,542	3	50.00	208	75.09	1,041	82.68	1,252	81.19
504102000	Baleno	0	62	333	395	0	0.00	42	67.74	278	83.48	320	81.01
504103000	Balud	1	78	548	627	1	100.00	56	71.79	391	71.35	448	71.45
504104000	Batuan	0	22	64	86	0	0.00	18	81.82	51	79.69	69	80.23
504105000	Cataingan	0	57	594	651	0	0.00	35	61.40	435	73.23	470	72.20
504106000	Cawayan	2	209	1,162	1,373	2	100.00	199	95.22	1,108	95.35	1,309	95.34
504107000	Claveria	1	93	445	539	0	0.00	47	50.54	284	63.82	331	61.41
504108000	Dimasalang	0	44	295	339	0	0.00	34	77.27	274	92.88	308	90.86
504109000	Esperanza	1	31	181	213	0	0.00	22	70.97	138	76.24	160	75.12
504110000	Mandaon	0	119	594	713	0	0.00	61	51.26	381	64.14	442	61.99
504111000	City of Masbate	8	417	2,873	3,298	6	75.00	299	71.70	1,911	66.52	2,216	67.19
504112000	Milagros	0	162	872	1,034	0	0.00	158	97.53	876	100.46	1,034	100.00
504113000	Mobo	0	86	352	438	0	0.00	80	93.02	333	94.60	413	94.29
504114000	Monreal	1	13	114	128	1	100.00	19	146.15	160	140.35	180	140.63
504115000	Palanas	0	66	317	383	0	0.00	55	83.33	295	93.06	350	91.38
504116000	Pio V. Corpuz	0	39	277	316	0	0.00	29	74.36	208	75.09	237	75.00
504117000	Placer	0	106	733	839	0	0.00	90	84.91	634	86.49	724	86.29
504118000	San Fernando	0	3	45	48	1	0.00	10	333.33	59	131.11	70	145.83
504119000	San Jacinto	1	91	425	517	0	0.00	27	29.67	161	37.88	188	36.36
504120000	San Pascual	1	58	451	510	0	0.00	15	25.86	205	45.45	220	43.14
504121000	Uson	1	126	595	722	1	100.00	51	40.48	319	53.61	371	51.39
**506200000**	**Sorsogon**	**28**	**1,313**	**11,083**	**12,424**	**24**	**85.71**	**1,108**	**84.39**	**10,168**	**91.74**	**11,300**	**90.95**
506202000	Barcelona	0	0	47	47	0	0.00	0	0.00	46	97.87	46	97.87
506203000	Bulan	1	33	640	674	1	100.00	29	87.88	594	92.81	624	92.58
506204000	Bulusan	0	1	0	1	0	0.00	0	0.00	0	0.00	0	0.00
506205000	Casiguran	0	33	277	310	0	0.00	25	75.76	247	89.17	272	87.74
506206000	Castilla	0	87	753	840	0	0.00	36	41.38	480	63.75	516	61.43
506207000	Donsol	0	124	705	829	0	0.00	113	91.13	655	92.91	768	92.64
506208000	Gubat	1	82	667	750	0	0.00	64	78.05	558	83.66	622	82.93
506209000	Irosin	9	326	1,437	1,772	7	77.78	238	73.01	1,191	82.88	1,436	81.04
506210000	Juban	0	18	345	363	0	0.00	17	94.44	335	97.10	352	96.97
506211000	Magallanes	1	33	305	339	1	100.00	22	66.67	253	82.95	276	81.42
506212000	Matnog	1	26	275	302	1	100.00	20	76.92	242	88.00	263	87.09
506213000	Pilar	0	19	478	497	0	0.00	19	100.00	472	98.74	491	98.79
506214000	Prieto Diaz	0	4	84	88	0	0.00	0	0.00	54	64.29	54	61.36
506215000	Santa Magdalena	0	4	24	28	0	0.00	4	100.00	24	100.00	28	100.00
506216000	City of Sorsogon	15	523	5,046	5,584	14	93.33	521	99.62	5,017	99.43	5,552	99.43
**501724000**	**City of Naga**	**36**	**1,487**	**11,087**	**12,610**	**3**	**8.33**	**156**	**10.49**	**1,647**	**14.86**	**1,806**	**14.32**
**600000000**	**Region VI (Western Visayas)**	**183**	**9,328**	**76,010**	**85,521**	**121**	**66.12**	**7,466**	**80.04**	**64,203**	**84.47**	**71,790**	**83.94**
**630200000**	**City of Bacolod**	2	271	4,420	**4,693**	1	50.00	148	54.61	2,385	53.96	**2,534**	**54.00**
**631000000**	**City of Iloilo**	15	456	4,611	**5,082**	10	66.67	412	90.35	4,420	95.86	**4,842**	**95.28**
**600400000**	**Aklan**	8	612	5,360	**5,980**	5	62.50	520	84.97	4,869	90.84	**5,394**	**90.20**
600401000	Altavas	1	35	175	211	0	0.00	20	57.14	129	73.71	149	70.62
600402000	Balete	0	33	212	245	0	0.00	30	90.91	215	101.42	245	100.00
600403000	Banga	0	25	263	288	0	0.00	19	76.00	253	96.20	272	94.44
600404000	Batan	0	30	214	244	0	0.00	29	96.67	181	84.58	210	86.07
600405000	Buruanga	0	29	174	203	0	0.00	29	100.00	161	92.53	190	93.60
600406000	Ibajay	3	40	503	546	1	33.33	19	47.50	376	74.75	396	72.53
600407000	Kalibo	0	86	723	809	0	0.00	79	91.86	690	95.44	769	95.06
600408000	Lezo	0	15	143	158	0	0.00	15	100.00	141	98.60	156	98.73
600409000	Libacao	0	35	175	210	0	0.00	12	34.29	96	54.86	108	51.43
600410000	Madalag	1	22	186	209	1	100.00	22	100.00	186	100.00	209	100.00
600411000	Makato	0	12	335	347	0	0.00	17	141.67	323	96.42	340	97.98
600412000	Malay	0	88	561	649	0	0.00	79	89.77	498	88.77	577	88.91
600413000	Malinao	1	14	208	223	1	100.00	17	121.43	177	85.10	195	87.44
600414000	Nabas	1	44	460	505	1	100.00	49	111.36	443	96.30	493	97.62
600415000	New Washington	1	55	483	539	1	100.00	43	78.18	483	100.00	527	97.77
600416000	Numancia	0	30	309	339	0	0.00	27	90.00	300	97.09	327	96.46
600417000	Tangalan	0	19	236	255	0	0.00	14	73.68	217	91.95	231	90.59
**600600000**	**Antique**	11	731	6,726	**7,468**	8	72.73	475	64.98	5,001	74.35	**5,484**	**73.43**
600601000	Anini-Y	0	2	60	62	0	0.00	5	250.00	133	221.67	138	222.58
600602000	Barbaza	0	17	150	167	1	0.00	12	70.59	174	116.00	187	111.98
600603000	Belison	2	2	32	36	0	0.00	6	300.00	93	290.63	99	275.00
600604000	Bugasong	0	10	176	186	0	0.00	23	230.00	257	146.02	280	150.54
600605000	Caluya	0	36	475	511	0	0.00	74	205.56	674	141.89	748	146.38
600606000	Culasi	3	96	667	766	2	66.67	47	48.96	368	55.17	417	54.44
600607000	Tobias Fornier	0	11	127	138	0	0.00	10	90.91	122	96.06	132	95.65
600608000	Hamtic	0	7	163	170	0	0.00	36	514.29	440	269.94	476	280.00
600609000	Laua-An	0	15	162	177	1	0.00	11	73.33	164	101.23	176	99.44
600610000	Libertad	0	2	64	66	0	0.00	10	500.00	156	243.75	166	251.52
600611000	Pandan	1	32	296	329	0	0.00	25	78.13	291	98.31	316	96.05
600612000	Patnongon	1	14	115	130	0	0.00	23	164.29	250	217.39	273	210.00
600613000	San Jose	4	373	3,112	3,489	3	75.00	56	15.01	542	17.42	601	17.23
600614000	San Remigio	0	17	162	179	0	0.00	54	317.65	365	225.31	419	234.08
600615000	Sebaste	0	15	204	219	0	0.00	16	106.67	140	68.63	156	71.23
600616000	Sibalom	0	48	471	519	0	0.00	35	72.92	571	121.23	606	116.76
600617000	Tibiao	0	10	101	111	1	0.00	21	210.00	117	115.84	139	125.23
600618000	Valderrama	0	24	189	213	0	0.00	11	45.83	144	76.19	155	72.77
**601900000**	**Capiz**	14	703	6,764	**7,481**	12	85.71	651	92.60	6,490	95.95	**7,153**	**95.62**
601901000	Cuartero	0	20	239	259	0	0.00	12	60.00	175	73.22	187	72.20
601902000	Dao	0	19	441	460	0	0.00	19	100.00	441	100.00	460	100.00
601903000	Dumalag	0	24	214	238	0	0.00	23	95.83	213	99.53	236	99.16
601904000	Dumarao	0	36	348	384	0	0.00	36	100.00	348	100.00	384	100.00
601905000	Ivisan	0	15	218	233	0	0.00	15	100.00	218	100.00	233	100.00
601906000	Jamindan	0	20	244	264	0	0.00	20	100.00	244	100.00	264	100.00
601907000	Ma-Ayon	0	53	341	394	0	0.00	51	96.23	339	99.41	390	98.98
601908000	Mambusao	0	19	262	281	0	0.00	19	100.00	262	100.00	281	100.00
601909000	Panay	1	40	326	367	1	100.00	40	100.00	326	100.00	367	100.00
601910000	Panitan	1	32	293	326	0	0.00	18	56.25	235	80.20	253	77.61
601911000	Pilar	2	44	433	479	2	100.00	44	100.00	432	99.77	478	99.79
601912000	Pontevedra	1	73	371	445	1	100.00	73	100.00	371	100.00	445	100.00
601913000	President Roxas	0	19	180	199	0	0.00	19	100.00	176	97.78	195	97.99
601914000	City of Roxas	4	186	1,957	2,147	3	75.00	177	95.16	1,877	95.91	2,057	95.81
601915000	Sapi-An	2	33	225	260	2	100.00	29	87.88	205	91.11	236	90.77
601916000	Sigma	1	9	190	200	1	100.00	9	100.00	190	100.00	200	100.00
601917000	Tapaz	2	61	482	545	2	100.00	47	77.05	438	90.87	487	89.36
**603000000**	**Iloilo**	32	1,966	17,747	**19,745**	24	75.00	1,774	90.23	16,690	94.04	**18,488**	**93.63**
603001000	Ajuy	1	62	459	522	1	100.00	62	100.00	458	99.78	521	99.81
603002000	Alimodian	1	33	364	398	1	100.00	21	63.64	306	84.07	328	82.41
603003000	Anilao	0	47	293	340	0	0.00	45	95.74	291	99.32	336	98.82
603004000	Badiangan	0	16	167	183	0	0.00	9	56.25	114	68.26	123	67.21
603005000	Balasan	2	44	388	434	2	100.00	44	100.00	388	100.00	434	100.00
603006000	Banate	1	35	290	326	1	100.00	34	97.14	290	100.00	325	99.69
603007000	Barotac Nuevo	1	51	732	784	1	100.00	51	100.00	732	100.00	784	100.00
603008000	Barotac Viejo	1	57	425	483	1	100.00	57	100.00	425	100.00	483	100.00
603009000	Batad	0	32	170	202	0	0.00	31	96.88	168	98.82	199	98.51
603010000	Bingawan	0	30	144	174	0	0.00	25	83.33	126	87.50	151	86.78
603012000	Cabatuan	0	67	778	845	0	0.00	67	100.00	759	97.56	826	97.75

2.B.1.1 - Prenatal Care
Women who gave birth with at least 4 Prenatal Check-ups
Philippines, 2024

PSGC10	Area	Total Deliveries				Women who gave birth with at least four or more prenatal check-ups (4ANC)							
		10-14 y.o.	15-19 y.o.	20-49 y.o.	Total	10-14 y.o.	%	15-19 y.o.	%	20-49 y.o.	%	Total	%
603013000	Calinog	1	78	587	**666**	1	100.00	66	84.62	579	98.64	**646**	**97.00**
603014000	Carles	0	97	953	**1,050**	0	0.00	94	96.91	853	89.51	**947**	**90.19**
603015000	Concepcion	3	33	425	**461**	1	33.33	11	33.33	277	65.18	**289**	**62.69**
603016000	Dingle	2	26	363	**391**	2	100.00	26	100.00	363	100.00	**391**	**100.00**
603017000	Dueñas	1	35	272	**308**	1	100.00	34	97.14	268	98.53	**303**	**98.38**
603018000	Dumangas	1	97	588	**686**	1	100.00	89	91.75	587	99.83	**677**	**98.69**
603019000	Estancia	0	4	121	**125**	0	0.00	4	100.00	118	97.52	**122**	**97.60**
603020000	Guimbal	0	25	353	**378**	0	0.00	25	100.00	352	99.72	**377**	**99.74**
603021000	Igbaras	0	33	261	**294**	0	0.00	22	66.67	216	82.76	**238**	**80.95**
603023000	Janiuay	1	48	566	**615**	0	0.00	37	77.08	512	90.46	**549**	**89.27**
603025000	Lambunao	0	76	776	**852**	0	0.00	76	100.00	747	96.26	**823**	**96.60**
603026000	Leganes	1	23	287	**311**	0	0.00	9	39.13	261	90.94	**270**	**86.82**
603027000	Lemery	1	70	277	**348**	0	0.00	63	90.00	277	100.00	**340**	**97.70**
603028000	Leon	0	21	298	**319**	0	0.00	19	90.48	267	89.60	**286**	**89.66**
603029000	Maasin	0	45	405	**450**	0	0.00	32	71.11	345	85.19	**377**	**83.78**
603030000	Miagao	0	38	492	**530**	0	0.00	33	86.84	489	99.39	**522**	**98.49**
603031000	Mina	0	36	193	**229**	0	0.00	34	94.44	175	90.67	**209**	**91.27**
603032000	New Lucena	0	17	157	**174**	0	0.00	12	70.59	135	85.99	**147**	**84.48**
603034000	Oton	2	55	538	**595**	2	100.00	55	100.00	538	100.00	**595**	**100.00**
603035000	City of Passi	1	126	843	**970**	0	0.00	112	88.89	745	88.37	**857**	**88.35**
603036000	Pavia	0	12	439	**451**	0	0.00	12	100.00	431	98.18	**443**	**98.23**
603037000	Pototan	0	70	649	**719**	0	0.00	70	100.00	648	99.85	**718**	**99.86**
603038000	San Dionisio	0	48	324	**372**	0	0.00	37	77.08	232	71.60	**269**	**72.31**
603039000	San Enrique	1	59	472	**532**	1	100.00	59	100.00	472	100.00	**532**	**100.00**
603040000	San Joaquin	2	49	443	**494**	1	50.00	33	67.35	389	87.81	**423**	**85.63**
603041000	San Miguel	1	10	199	**210**	1	100.00	8	80.00	125	62.81	**134**	**63.81**
603042000	San Rafael	4	31	148	**183**	3	75.00	31	100.00	148	100.00	**182**	**99.45**
603043000	Santa Barbara	1	38	574	**613**	1	100.00	38	100.00	570	99.30	**609**	**99.35**
603044000	Sara	0	76	497	**573**	0	0.00	76	100.00	495	99.60	**571**	**99.65**
603045000	Tigbauan	2	79	674	**755**	2	100.00	79	100.00	670	99.41	**751**	**99.47**
603046000	Tubungan	0	27	189	**216**	0	0.00	23	85.19	183	96.83	**206**	**95.37**
603047000	Zarraga	0	10	174	**184**	0	0.00	9	90.00	166	95.40	**175**	**95.11**
**604500000**	**Negros Occidental**	90	4,369	28,431	**32,890**	53	58.89	3,280	75.07	22,446	78.95	**25,779**	**78.38**
604502000	City of Bago	7	191	1,697	**1,895**	7	100.00	190	99.48	1,649	97.17	**1,846**	**97.41**
604503000	Binalbagan	4	124	823	**951**	2	50.00	67	54.03	595	72.30	**664**	**69.82**
604504000	City of Cadiz	6	293	1,664	**1,963**	5	83.33	246	83.96	1,468	88.22	**1,719**	**87.57**
604505000	Calatrava	4	159	928	**1,091**	1	25.00	70	44.03	496	53.45	**567**	**51.97**
604506000	Candoni	2	54	230	**286**	0	0.00	33	61.11	176	76.52	**209**	**73.08**
604507000	Cauayan	1	187	1,121	**1,309**	1	100.00	69	36.90	425	37.91	**495**	**37.82**
604508000	Enrique B. Magalona	1	82	592	**675**	1	100.00	50	60.98	454	76.69	**505**	**74.81**
604509000	City of Escalante	3	176	1,207	**1,386**	1	33.33	155	88.07	1,030	85.34	**1,186**	**85.57**
604510000	City of Himamaylan	8	240	1,530	**1,778**	8	100.00	240	100.00	1,530	100.00	**1,778**	**100.00**
604511000	Hinigaran	5	102	803	**910**	0	0.00	59	57.84	513	63.89	**572**	**62.86**
604512000	Hinoba-an	3	109	722	**834**	3	100.00	107	98.17	702	97.23	**812**	**97.36**
604513000	Ilog	1	96	644	**741**	1	100.00	94	97.92	631	97.98	**726**	**97.98**
604514000	Isabela	0	104	744	**848**	0	0.00	100	96.15	559	75.13	**659**	**77.71**
604515000	City of Kabankalan	7	346	2,119	**2,472**	1	14.29	166	47.98	1,101	51.96	**1,268**	**51.29**
604516000	City of La Carlota	2	89	567	**658**	0	0.00	71	79.78	538	94.89	**609**	**92.55**
604517000	La Castellana	4	178	919	**1,101**	0	0.00	149	83.71	723	78.67	**872**	**79.20**
604518000	Manapla	1	70	575	**646**	1	100.00	62	88.57	527	91.65	**590**	**91.33**
604519000	Moises Padilla	3	118	510	**631**	2	66.67	87	73.73	401	78.63	**490**	**77.65**
604520000	Murcia	1	148	962	**1,111**	1	100.00	100	67.57	636	66.11	**737**	**66.34**
604521000	Pontevedra	0	63	506	**569**	0	0.00	38	60.32	419	82.81	**457**	**80.32**
604522000	Pulupandan	0	29	251	**280**	0	0.00	15	51.72	158	62.95	**173**	**61.79**
604523000	City of Sagay	7	230	1,635	**1,872**	4	57.14	202	87.83	1,460	89.30	**1,666**	**89.00**
604524000	City of San Carlos	5	272	1,625	**1,902**	1	20.00	152	55.88	916	56.37	**1,069**	**56.20**
604525000	San Enrique	0	19	244	**263**	0	0.00	12	63.16	209	85.66	**221**	**84.03**
604526000	City of Silay	6	177	1,546	**1,729**	5	83.33	171	96.61	1,496	96.77	**1,672**	**96.70**
604527000	City of Sipalay	2	171	946	**1,119**	2	100.00	136	79.53	825	87.21	**963**	**86.06**
604528000	City of Talisay	2	151	1,145	**1,298**	2	100.00	151	100.00	1,145	100.00	**1,298**	**100.00**
604529000	Toboso	2	107	587	**696**	2	100.00	89	83.18	489	83.30	**580**	**83.33**
604530000	Valladolid	1	48	402	**451**	1	100.00	38	79.17	377	93.78	**416**	**92.24**
604531000	City of Victorias	0	136	823	**959**	0	0.00	80	58.82	501	60.87	**581**	**60.58**
604532000	Salvador Benedicto	2	100	364	**466**	1	50.00	81	81.00	297	81.59	**379**	**81.33**
**607900000**	**Guimaras**	11	220	1,951	**2,182**	8	72.73	206	93.64	1,902	97.49	**2,116**	**96.98**
607901000	Buenavista	2	48	505	**555**	2	100.00	45	93.75	484	95.84	**531**	**95.68**
607902000	Jordan	0	55	455	**510**	0	0.00	54	98.18	444	97.58	**498**	**97.65**
607903000	Nueva Valencia	7	52	412	**471**	5	71.43	48	92.31	414	100.49	**467**	**99.15**
607904000	San Lorenzo	0	45	321	**366**	0	0.00	45	100.00	315	98.13	**360**	**98.36**
607905000	Sibunag	2	20	258	**280**	1	50.00	14	70.00	245	94.96	**260**	**92.86**
**700000000**	**Region VII (Central Visayas)**	**230**	**11,162**	**96,264**	**107,656**	**98**	**42.61**	**6,251**	**56.00**	**60,409**	**62.75**	**66,758**	**62.01**
**730600000**	**City of Cebu**	29	1,437	12,529	**13,995**	24	82.76	1,161	80.79	9,464	75.54	**10,649**	**76.09**
**731100000**	**City of Lapu-Lapu**	24	584	6,311	**6,919**	24	100.00	551	94.35	6,268	99.32	**6,843**	**98.90**
**731300000**	**City of Mandaue**	23	698	8,494	**9,215**	3	13.04	134	19.20	1,775	20.90	**1,912**	**20.75**
**701200000**	**Bohol**	**22**	**1,518**	**15,537**	**17,077**	**8**	**36.36**	**805**	**53.03**	**9,334**	**60.08**	**10,147**	**59.42**
701201000	Alburquerque	0	0	0	**0**	0	0.00	0	0.00	0	0.00	**0**	**0.00**
701202000	Alicia	0	6	98	**104**	0	0.00	4	66.67	65	66.33	**69**	**66.35**
701203000	Anda	0	5	114	**119**	0	0.00	5	100.00	114	100.00	**119**	**100.00**
701204000	Antequera	0	0	19	**19**	0	0.00	0	0.00	8	42.11	**8**	**42.11**
701205000	Baclayon	0	1	40	**41**	0	0.00	0	0.00	32	80.00	**32**	**78.05**
701206000	Balilihan	0	1	24	**25**	0	0.00	0	0.00	8	33.33	**8**	**32.00**
701207000	Batuan	0	0	15	**15**	0	0.00	0	0.00	13	86.67	**13**	**86.67**
701208000	Bilar	0	10	74	**84**	0	0.00	6	60.00	60	81.08	**66**	**78.57**
701209000	Buenavista	0	8	153	**161**	0	0.00	8	100.00	151	98.69	**159**	**98.76**
701210000	Calape	0	18	271	**289**	0	0.00	18	100.00	271	100.00	**289**	**100.00**
701211000	Candijay	1	11	163	**175**	0	0.00	7	63.64	148	90.80	**155**	**88.57**
701212000	Carmen	0	90	824	**914**	0	0.00	9	10.00	229	27.79	**238**	**26.04**
701213000	Catigbian	0	13	174	**187**	0	0.00	12	92.31	160	91.95	**172**	**91.98**
701214000	Clarin	0	8	122	**130**	0	0.00	7	87.50	86	70.49	**93**	**71.54**
701215000	Corella	0	0	21	**21**	0	0.00	0	0.00	21	100.00	**21**	**100.00**
701216000	Cortes	0	1	31	**32**	0	0.00	0	0.00	14	45.16	**14**	**43.75**
701217000	Dagohoy	0	2	59	**61**	0	0.00	2	100.00	54	91.53	**56**	**91.80**
701218000	Danao	0	6	115	**121**	0	0.00	3	50.00	95	82.61	**98**	**80.99**
701219000	Dauis	0	4	121	**125**	0	0.00	3	75.00	109	90.08	**112**	**89.60**
701220000	Dimiao	0	2	36	**38**	0	0.00	0	0.00	30	83.33	**30**	**78.95**
701221000	Duero	0	0	10	**10**	0	0.00	0	0.00	5	50.00	**5**	**50.00**
701222000	Garcia Hernandez	0	3	36	**39**	0	0.00	2	66.67	29	80.56	**31**	**79.49**
701223000	Guindulman	0	5	43	**48**	0	0.00	4	80.00	38	88.37	**42**	**87.50**
701224000	Inabanga	0	29	234	**263**	0	0.00	23	79.31	209	89.32	**232**	**88.21**
701225000	Jagna	3	101	1,015	**1,119**	1	33.33	38	37.62	467	46.01	**506**	**45.22**
701226000	Getafe	0	22	257	**279**	0	0.00	5	22.73	153	59.53	**158**	**56.63**
701227000	Lila	0	4	9	**13**	0	0.00	1	25.00	9	100.00	**10**	**76.92**
701228000	Loay	0	1	13	**14**	0	0.00	0	0.00	5	38.46	**5**	**35.71**
701229000	Loboc	0	0	33	**33**	0	0.00	0	0.00	32	96.97	**32**	**96.97**
701230000	Loon	0	14	195	**209**	0	0.00	5	35.71	162	83.08	**167**	**79.90**
701231000	Mabini	0	17	182	**199**	0	0.00	12	70.59	161	88.46	**173**	**86.93**
701232000	Maribojoc	0	3	49	**52**	0	0.00	3	100.00	48	97.96	**51**	**98.08**
701233000	Panglao	0	2	217	**219**	0	0.00	2	100.00	207	95.39	**209**	**95.43**
701234000	Pilar	1	10	151	**162**	0	0.00	5	50.00	103	68.21	**108**	**66.67**
701235000	President Carlos P. Garcia	0	23	171	**194**	0	0.00	13	56.52	98	57.31	**111**	**57.22**
701236000	Sagbayan	0	0	46	**46**	0	0.00	0	0.00	44	95.65	**44**	**95.65**
701237000	San Isidro	0	0	28	**28**	0	0.00	0	0.00	28	100.00	**28**	**100.00**
701238000	San Miguel	0	13	166	**179**	0	0.00	11	84.62	153	92.17	**164**	**91.62**
701239000	Sevilla	0	0	19	**19**	0	0.00	0	0.00	17	89.47	**17**	**89.47**
701240000	Sierra Bullones	0	2	100	**102**	0	0.00	2	100.00	73	73.00	**75**	**73.53**
701241000	Sikatuna	0	0	14	**14**	0	0.00	0	0.00	12	85.71	**12**	**85.71**
701242000	City of Tagbilaran	8	522	5,892	**6,422**	4	50.00	301	57.66	3,212	54.51	**3,517**	**54.76**
701243000	Talibon	0	150	913	**1,063**	0	0.00	141	94.00	805	88.17	**946**	**88.99**
701244000	Trinidad	0	7	124	**131**	0	0.00	1	14.29	74	59.68	**75**	**57.25**
701245000	Tubigon	0	11	407	**418**	0	0.00	9	81.82	389	95.58	**398**	**95.22**
701246000	Ubay	9	362	2,482	**2,853**	3	33.33	119	32.87	889	35.82	**1,011**	**35.44**
701247000	Valencia	0	8	83	**91**	0	0.00	5	62.50	76	91.57	**81**	**89.01**
701248000	Bien Unido	0	23	174	**197**	0	0.00	19	82.61	168	96.55	**187**	**94.92**
**702200000**	**Cebu**	80	4,543	36,259	**40,882**	19	23.75	2,370	52.17	22,329	61.58	**24,718**	**60.46**
702201000	Alcantara	0	16	101	**117**	0	0.00	1	6.25	12	11.88	**13**	**11.11**
702202000	Alcoy	0	10	143	**153**	0	0.00	0	0.00	48	33.57	**48**	**31.37**
702203000	Alegria	0	9	124	**133**	0	0.00	2	22.22	75	60.48	**77**	**57.89**

2.B.1.1 - Prenatal Care
Women who gave birth with at least 4 Prenatal Check-ups
Philippines, 2024

PSGC10	Area	Total Deliveries				Women who gave birth with at least four or more prenatal check-ups (4ANC)							
		10-14 y.o.	15-19 y.o.	20-49 y.o.	Total	10-14 y.o.	%	15-19 y.o.	%	20-49 y.o.	%	Total	%
702204000	Aloguinsan	0	7	96	**103**	0	0.00	6	85.71	80	83.33	**86**	**83.50**
702205000	Argao	0	30	366	**396**	0	0.00	12	40.00	251	68.58	**263**	**66.41**
702206000	Asturias	0	3	220	**223**	0	0.00	1	33.33	195	88.64	**196**	**87.89**
702207000	Badian	1	56	378	**435**	0	0.00	54	96.43	365	96.56	**419**	**96.32**
702208000	Balamban	5	468	2,503	**2,976**	1	20.00	277	59.19	1,527	61.01	**1,805**	**60.65**
702209000	Bantayan	0	106	812	**918**	0	0.00	52	49.06	605	74.51	**657**	**71.57**
702210000	Barili	1	38	532	**571**	0	0.00	16	42.11	191	35.90	**207**	**36.25**
702211000	City of Bogo	12	539	3,858	**4,409**	2	16.67	129	23.93	2,250	58.32	**2,381**	**54.00**
702212000	Boljoon	0	12	87	**99**	0	0.00	7	58.33	66	75.86	**73**	**73.74**
702213000	Borbon	0	2	144	**146**	0	0.00	2	100.00	143	99.31	**145**	**99.32**
702214000	City of Carcar	11	791	4,546	**5,348**	1	9.09	554	70.04	3,821	84.05	**4,376**	**81.82**
702215000	Carmen	0	7	135	**142**	0	0.00	4	57.14	122	90.37	**126**	**88.73**
702216000	Catmon	0	4	83	**87**	0	0.00	4	100.00	60	72.29	**64**	**73.56**
702218000	Compostela	0	1	183	**184**	0	0.00	0	0.00	169	92.35	**169**	**91.85**
702219000	Consolacion	0	18	1,031	**1,049**	0	0.00	18	100.00	697	67.60	**715**	**68.16**
702220000	Cordova	0	7	460	**467**	0	0.00	5	71.43	439	95.43	**444**	**95.07**
702221000	Daanbantayan	1	32	709	**742**	0	0.00	13	40.63	158	22.28	**171**	**23.05**
702222000	Dalaguete	0	35	373	**408**	0	0.00	21	60.00	189	50.67	**210**	**51.47**
702223000	Danao City	21	587	4,131	**4,739**	4	19.05	128	21.81	1,216	29.44	**1,348**	**28.44**
702224000	Dumanjug	0	19	225	**244**	0	0.00	19	100.00	159	70.67	**178**	**72.95**
702225000	Ginatilan	0	2	41	**43**	0	0.00	2	100.00	31	75.61	**33**	**76.74**
702227000	Liloan	0	0	344	**344**	0	0.00	0	0.00	254	73.84	**254**	**73.84**
702228000	Madridejos	0	7	223	**230**	0	0.00	4	57.14	155	69.51	**159**	**69.13**
702229000	Malabuyoc	0	16	191	**207**	0	0.00	4	25.00	107	56.02	**111**	**53.62**
702231000	Medellin	0	0	104	**104**	0	0.00	0	0.00	104	100.00	**104**	**100.00**
702232000	Minglanilla	0	33	1,111	**1,144**	0	0.00	33	100.00	1,070	96.31	**1,103**	**96.42**
702233000	Moalboal	0	4	155	**159**	0	0.00	0	0.00	110	70.97	**110**	**69.18**
702234000	City of Naga	2	105	1,124	**1,231**	0	0.00	52	49.52	633	56.32	**685**	**55.65**
702235000	Oslob	0	20	151	**171**	0	0.00	7	35.00	49	32.45	**56**	**32.75**
702236000	Pilar	0	0	40	**40**	0	0.00	0	0.00	23	57.50	**23**	**57.50**
702237000	Pinamungajan	0	3	253	**256**	0	0.00	2	66.67	239	94.47	**241**	**94.14**
702238000	Poro	0	1	0	**1**	0	0.00	0	0.00	0	0.00	**0**	**0.00**
702239000	Ronda	0	3	83	**86**	0	0.00	0	0.00	49	59.04	**49**	**56.98**
702240000	Samboan	0	3	67	**70**	0	0.00	0	0.00	50	74.63	**50**	**71.43**
702241000	San Fernando	0	15	454	**469**	0	0.00	15	100.00	450	99.12	**465**	**99.15**
702242000	San Francisco	0	156	900	**1,056**	0	0.00	140	89.74	837	93.00	**977**	**92.52**
702243000	San Remigio	0	0	148	**148**	0	0.00	0	0.00	125	84.46	**125**	**84.46**
702244000	Santa Fe	0	6	220	**226**	0	0.00	2	33.33	152	69.09	**154**	**68.14**
702245000	Santander	0	4	64	**68**	0	0.00	1	25.00	47	73.44	**48**	**70.59**
702246000	Sibonga	0	3	378	**381**	0	0.00	2	66.67	230	60.85	**232**	**60.89**
702247000	Sogod	0	10	240	**250**	0	0.00	9	90.00	184	76.67	**193**	**77.20**
702248000	Tabogon	0	0	16	**16**	0	0.00	0	0.00	14	87.50	**14**	**87.50**
702249000	Tabuelan	0	17	293	**310**	0	0.00	11	64.71	213	72.70	**224**	**72.26**
702250000	City of Talisay	20	958	5,966	**6,944**	11	55.00	754	78.71	4,241	71.09	**5,006**	**72.09**
702251000	City of Toledo	5	351	2,124	**2,480**	0	0.00	6	1.71	59	2.78	**65**	**2.62**
702252000	Tuburan	1	29	328	**358**	0	0.00	1	3.45	64	19.51	**65**	**18.16**
702253000	Tudela	0	0	1	**1**	0	0.00	0	0.00	1	100.00	**1**	**100.00**
**704600000**	**Negros Oriental**	**51**	**2,307**	**16,213**	**18,571**	**19**	**37.25**	**1,174**	**50.89**	**10,459**	**64.51**	**11,652**	**62.74**
704601000	Amlan	0	8	112	**120**	0	0.00	4	50.00	72	64.29	**76**	**63.33**
704602000	Ayungon	1	40	188	**229**	0	0.00	18	45.00	121	64.36	**139**	**60.70**
704603000	Bacong	0	0	146	**146**	0	0.00	0	0.00	76	52.05	**76**	**52.05**
704604000	City of Bais	0	76	1,022	**1,098**	0	0.00	23	30.26	323	31.60	**346**	**31.51**
704605000	Basay	1	42	236	**279**	1	100.00	42	100.00	232	98.31	**275**	**98.57**
704606000	City of Bayawan	6	321	1,669	**1,996**	2	33.33	141	43.93	858	51.41	**1,001**	**50.15**
704607000	Bindoy	1	57	549	**607**	0	0.00	11	19.30	163	29.69	**174**	**28.67**
704608000	City of Canlaon	0	97	501	**598**	0	0.00	73	75.26	438	87.43	**511**	**85.45**
704609000	Dauin	0	1	93	**94**	0	0.00	0	0.00	67	72.04	**67**	**71.28**
704610000	City of Dumaguete	31	983	6,967	**7,981**	14	45.16	590	60.02	6,187	88.80	**6,791**	**85.09**
704611000	City of Guihulngan	4	248	1,413	**1,665**	1	25.00	92	37.10	320	22.65	**413**	**24.80**
704612000	Jimalalud	0	44	220	**264**	0	0.00	8	18.18	29	13.18	**37**	**14.02**
704613000	La Libertad	0	22	203	**225**	0	0.00	7	31.82	99	48.77	**106**	**47.11**
704614000	Mabinay	2	85	648	**735**	1	50.00	38	44.71	384	59.26	**423**	**57.55**
704615000	Manjuyod	0	6	44	**50**	0	0.00	0	0.00	0	0.00	**0**	**0.00**
704616000	Pamplona	0	4	48	**52**	0	0.00	4	100.00	45	93.75	**49**	**94.23**
704617000	San Jose	0	2	41	**43**	0	0.00	1	50.00	31	75.61	**32**	**74.42**
704618000	Santa Catalina	0	59	694	**753**	0	0.00	15	25.42	269	38.76	**284**	**37.72**
704619000	Siaton	3	110	641	**754**	0	0.00	75	68.18	431	67.24	**506**	**67.11**
704620000	Sibulan	0	12	48	**60**	0	0.00	0	0.00	0	0.00	**0**	**0.00**
704621000	City of Tanjay	0	20	99	**119**	0	0.00	2	10.00	23	23.23	**25**	**21.01**
704622000	Tayasan	1	35	245	**281**	0	0.00	11	31.43	61	24.90	**72**	**25.62**
704623000	Valencia	0	3	47	**50**	0	0.00	0	0.00	29	61.70	**29**	**58.00**
704624000	Vallehermoso	1	20	234	**255**	0	0.00	12	60.00	170	72.65	**182**	**71.37**
704625000	Zamboanguita	0	12	105	**117**	0	0.00	7	58.33	31	29.52	**38**	**32.48**
**706100000**	**Siquijor**	**1**	**75**	**921**	**997**	**1**	**100.00**	**56**	**74.67**	**780**	**84.69**	**837**	**83.95**
706101000	Enrique Villanueva	0	0	3	**3**	0	0.00	0	0.00	3	100.00	**3**	**100.00**
706102000	Larena	0	0	4	**4**	0	0.00	0	0.00	3	75.00	**3**	**75.00**
706103000	Lazi	0	3	20	**23**	0	0.00	2	66.67	8	40.00	**10**	**43.48**
706104000	Maria	0	0	23	**23**	0	0.00	0	0.00	21	91.30	**21**	**91.30**
706105000	San Juan	0	0	6	**6**	0	0.00	0	0.00	6	100.00	**6**	**100.00**
706106000	Siquijor	1	72	865	**938**	1	100.00	54	75.00	739	85.43	**794**	**84.65**
**800000000**	**Region VIII (Eastern Visayas)**	**116**	**6,954**	**51,619**	**58,689**	**65**	**56.03**	**4,459**	**64.12**	**37,361**	**72.38**	**41,885**	**71.37**
**803738000**	**Ormoc City**	**15**	**626**	**4,728**	**5,369**	**9**	**60.00**	**498**	**79.55**	**3,511**	**74.26**	**4,018**	**74.84**
**831600000**	**City of Tacloban**	**10**	**490**	**5,448**	**5,948**	**9**	**90.00**	**480**	**97.96**	**5,382**	**98.79**	**5,871**	**98.71**
**802600000**	**Eastern Samar**	**17**	**858**	**5,498**	**6,373**	**15**	**88.24**	**739**	**86.13**	**5,064**	**92.11**	**5,818**	**91.29**
802601000	Arteche	0	51	244	**295**	0	0.00	50	98.04	240	98.36	**290**	**98.31**
802602000	Balangiga	4	29	159	**192**	4	100.00	29	100.00	159	100.00	**192**	**100.00**
802603000	Balangkayan	1	27	101	**129**	1	100.00	20	74.07	83	82.18	**104**	**80.62**
802604000	City of Borongan	1	112	879	**992**	1	100.00	112	100.00	873	99.32	**986**	**99.40**
802605000	Can-Avid	1	56	253	**310**	0	0.00	32	57.14	194	76.68	**226**	**72.90**
802606000	Dolores	4	97	596	**697**	4	100.00	87	89.69	549	92.11	**640**	**91.82**
802607000	General Macarthur	0	12	109	**121**	0	0.00	12	100.00	105	96.33	**117**	**96.69**
802608000	Giporlos	0	17	95	**112**	0	0.00	17	100.00	95	100.00	**112**	**100.00**
802609000	Guiuan	1	59	546	**606**	0	0.00	53	89.83	530	97.07	**583**	**96.20**
802610000	Hernani	0	7	70	**77**	0	0.00	7	100.00	66	94.29	**73**	**94.81**
802611000	Jipapad	0	44	120	**164**	0	0.00	15	34.09	64	53.33	**79**	**48.17**
802612000	Lawaan	0	20	160	**180**	0	0.00	17	85.00	136	85.00	**153**	**85.00**
802613000	Llorente	0	38	229	**267**	0	0.00	37	97.37	213	93.01	**250**	**93.63**
802614000	Maslog	0	19	93	**112**	0	0.00	19	100.00	92	98.92	**111**	**99.11**
802615000	Maydolong	2	40	218	**260**	2	100.00	40	100.00	208	95.41	**250**	**96.15**
802616000	Mercedes	0	8	72	**80**	0	0.00	8	100.00	72	100.00	**80**	**100.00**
802617000	Oras	0	72	460	**532**	0	0.00	48	66.67	398	86.52	**446**	**83.83**
802618000	Quinapondan	2	26	190	**218**	2	100.00	24	92.31	178	93.68	**204**	**93.58**
802619000	Salcedo	1	32	227	**260**	1	100.00	32	100.00	225	99.12	**258**	**99.23**
802620000	San Julian	0	18	132	**150**	0	0.00	13	72.22	94	71.21	**107**	**71.33**
802621000	San Policarpo	0	23	170	**193**	0	0.00	18	78.26	138	81.18	**156**	**80.83**
802622000	Sulat	0	22	167	**189**	0	0.00	20	90.91	144	86.23	**164**	**86.77**
802623000	Taft	0	29	208	**237**	0	0.00	29	100.00	208	100.00	**237**	**100.00**
**803700000**	**Leyte**	**21**	**1,366**	**10,058**	**11,445**	**10**	**47.62**	**822**	**60.18**	**7,533**	**74.90**	**8,365**	**73.09**
803701000	Abuyog	2	115	698	**815**	2	100.00	93	80.87	585	83.81	**680**	**83.44**
803702000	Alangalang	0	14	284	**298**	0	0.00	14	100.00	283	99.65	**297**	**99.66**
803703000	Albuera	1	45	462	**508**	1	100.00	40	88.89	394	85.28	**435**	**85.63**
803705000	Babatngon	0	7	237	**244**	0	0.00	7	100.00	230	97.05	**237**	**97.13**
803706000	Barugo	0	23	216	**239**	0	0.00	20	86.96	216	100.00	**236**	**98.74**
803707000	Bato	0	0	95	**95**	0	0.00	0	0.00	61	64.21	**61**	**64.21**
803708000	City of Baybay	3	148	1,089	**1,240**	0	0.00	47	31.76	675	61.98	**722**	**58.23**
803710000	Burauen	2	146	440	**588**	1	50.00	122	83.56	405	92.05	**528**	**89.80**
803713000	Calubian	0	50	330	**380**	0	0.00	42	84.00	327	99.09	**369**	**97.11**
803714000	Capoocan	0	16	217	**233**	0	0.00	9	56.25	117	53.92	**126**	**54.08**
803715000	Carigara	3	233	711	**947**	0	0.00	19	8.15	271	38.12	**290**	**30.62**
803717000	Dagami	0	12	225	**237**	0	0.00	12	100.00	225	100.00	**237**	**100.00**
803718000	Dulag	0	12	251	**263**	0	0.00	6	50.00	109	43.43	**115**	**43.73**
803719000	Hilongos	2	87	613	**702**	0	0.00	14	16.09	203	33.12	**217**	**30.91**
803720000	Hindang	0	5	87	**92**	0	0.00	2	40.00	53	60.92	**55**	**59.78**
803721000	Inopacan	0	0	42	**42**	0	0.00	0	0.00	31	73.81	**31**	**73.81**
803722000	Isabel	0	7	220	**227**	0	0.00	7	100.00	198	90.00	**205**	**90.31**
803723000	Jaro	0	0	201	**201**	0	0.00	0	0.00	113	56.22	**113**	**56.22**
803724000	Javier	0	12	90	**102**	0	0.00	12	100.00	87	96.67	**99**	**97.06**

2.B.1.1 - Prenatal Care
Women who gave birth with at least 4 Prenatal Check-ups
Philippines, 2024

PSGC10	Area	Total Deliveries				Women who gave birth with at least four or more prenatal check-ups (4ANC)							
		10-14 y.o.	15-19 y.o.	20-49 y.o.	Total	10-14 y.o.	%	15-19 y.o.	%	20-49 y.o.	%	Total	%
803725000	Julita	0	0	0	0	0	0.00	0	0.00	0	0.00	0	0.00
803726000	Kananga	0	26	246	272	0	0.00	26	100.00	242	98.37	268	98.53
803728000	La Paz	0	5	50	55	0	0.00	1	20.00	30	60.00	31	56.36
803729000	Leyte	0	19	201	220	0	0.00	14	73.68	194	96.52	208	94.55
803730000	Macarthur	0	0	3	3	0	0.00	0	0.00	2	66.67	2	66.67
803731000	Mahaplag	0	1	49	50	0	0.00	1	100.00	49	100.00	50	100.00
803733000	Matag-Ob	0	12	146	158	0	0.00	9	75.00	143	97.95	152	96.20
803734000	Matalom	0	4	99	103	0	0.00	3	75.00	98	98.99	101	98.06
803735000	Mayorga	0	9	299	308	0	0.00	8	88.89	184	61.54	192	62.34
803736000	Merida	1	37	252	290	1	100.00	21	56.76	170	67.46	192	66.21
803739000	Palo	3	153	676	832	3	100.00	132	86.27	510	75.44	645	77.52
803740000	Palompon	3	52	240	295	1	33.33	38	73.08	174	72.50	213	72.20
803741000	Pastrana	0	1	64	65	0	0.00	0	0.00	55	85.94	55	84.62
803742000	San Isidro	0	7	141	148	0	0.00	4	57.14	88	62.41	92	62.16
803743000	San Miguel	0	6	85	91	0	0.00	6	100.00	84	98.82	90	98.90
803744000	Santa Fe	0	2	83	85	0	0.00	2	100.00	83	100.00	85	100.00
803745000	Tabango	0	56	305	361	0	0.00	49	87.50	268	87.87	317	87.81
803746000	Tabontabon	0	1	37	38	0	0.00	1	100.00	37	100.00	38	100.00
803748000	Tanauan	0	12	195	207	0	0.00	10	83.33	169	86.67	179	86.47
803749000	Tolosa	0	10	86	96	0	0.00	10	100.00	86	100.00	96	100.00
803750000	Tunga	0	0	35	35	0	0.00	0	0.00	26	74.29	26	74.29
803751000	Villaba	1	21	258	280	1	100.00	21	100.00	258	100.00	280	100.00
**804800000**	**Northern Samar**	**19**	**1,345**	**8,891**	**10,255**	**5**	**26.32**	**451**	**33.53**	**3,887**	**43.72**	**4,343**	**42.35**
804801000	Allen	3	201	1,257	1,461	0	0.00	11	5.47	151	12.01	162	11.09
804802000	Biri	0	15	98	113	0	0.00	13	86.67	89	90.82	102	90.27
804803000	Bobon	0	12	112	124	0	0.00	12	100.00	110	98.21	122	98.39
804804000	Capul	0	20	97	117	0	0.00	12	60.00	83	85.57	95	81.20
804805000	Catarman	10	592	3,712	4,314	0	0.00	72	12.16	658	17.73	730	16.92
804806000	Catubig	2	94	442	538	1	50.00	14	14.89	232	52.49	247	45.91
804807000	Gamay	0	94	318	412	0	0.00	45	47.87	205	64.47	250	60.68
804808000	Laoang	1	132	713	846	1	100.00	117	88.64	555	77.84	673	79.55
804809000	Lapinig	0	26	148	174	0	0.00	24	92.31	144	97.30	168	96.55
804810000	Las Navas	0	23	292	315	0	0.00	22	95.65	251	85.96	273	86.67
804811000	Lavezares	0	2	22	24	0	0.00	0	0.00	14	63.64	14	58.33
804812000	Mapanas	0	5	60	65	0	0.00	3	60.00	53	88.33	56	86.15
804813000	Mondragon	0	20	381	401	0	0.00	20	100.00	369	96.85	389	97.01
804814000	Palapag	0	20	362	382	0	0.00	20	100.00	362	100.00	382	100.00
804815000	Pambujan	0	26	255	281	0	0.00	14	53.85	138	54.12	152	54.09
804816000	Rosario	0	5	56	61	0	0.00	5	100.00	56	100.00	61	100.00
804817000	San Antonio	3	11	48	62	3	100.00	10	90.91	44	91.67	57	91.94
804818000	San Isidro	0	2	24	26	0	0.00	2	100.00	24	100.00	26	100.00
804819000	San Jose	0	10	48	58	0	0.00	10	100.00	44	91.67	54	93.10
804820000	San Roque	0	13	189	202	0	0.00	9	69.23	170	89.95	179	88.61
804821000	San Vicente	0	5	27	32	0	0.00	5	100.00	27	100.00	32	100.00
804822000	Silvino Lobos	0	11	143	154	0	0.00	8	72.73	67	46.85	75	48.70
804823000	Victoria	0	1	27	28	0	0.00	1	100.00	15	55.56	16	57.14
804824000	Lope De Vega	0	5	60	65	0	0.00	2	40.00	26	43.33	28	43.08
**806000000**	**Samar**	**23**	**1,455**	**10,434**	**11,912**	**10**	**43.48**	**964**	**66.25**	**7,433**	**71.24**	**8,407**	**70.58**
806001000	Almagro	0	1	17	18	0	0.00	1	100.00	11	64.71	12	66.67
806002000	Basey	3	126	1,119	1,248	2	66.67	77	61.11	645	57.64	724	58.01
806003000	City of Calbayog	6	224	1,826	2,056	1	16.67	149	66.52	1,049	57.45	1,199	58.32
806004000	Calbiga	1	1	81	83	0	0.00	0	0.00	37	45.68	37	44.58
806005000	City of Catbalogan	11	876	5,146	6,033	7	63.64	605	69.06	4,114	79.95	4,726	78.34
806006000	Daram	0	4	194	198	0	0.00	1	25.00	72	37.11	73	36.87
806007000	Gandara	0	61	442	503	0	0.00	25	40.98	270	61.09	295	58.65
806008000	Hinabangan	0	3	43	46	0	0.00	2	66.67	40	93.02	42	91.30
806009000	Jiabong	0	2	108	110	0	0.00	2	100.00	108	100.00	110	100.00
806010000	Marabut	0	4	47	51	0	0.00	4	100.00	47	100.00	51	100.00
806011000	Matuguinao	1	9	85	95	0	0.00	0	0.00	38	44.71	38	40.00
806012000	Motiong	0	15	122	137	0	0.00	13	86.67	112	91.80	125	91.24
806013000	Pinabacdao	0	16	101	117	0	0.00	6	37.50	89	88.12	95	81.20
806014000	San Jose De Buan	1	12	68	81	0	0.00	7	58.33	32	47.06	39	48.15
806015000	San Sebastian	0	4	46	50	0	0.00	3	75.00	44	95.65	47	94.00
806016000	Santa Margarita	0	6	35	41	0	0.00	2	33.33	27	77.14	29	70.73
806017000	Santa Rita	0	25	226	251	0	0.00	18	72.00	156	69.03	174	69.32
806018000	Santo Niño	0	5	50	55	0	0.00	4	80.00	30	60.00	34	61.82
806019000	Talalora	0	6	71	77	0	0.00	3	50.00	42	59.15	45	58.44
806020000	Tarangnan	0	12	169	181	0	0.00	10	83.33	104	61.54	114	62.98
806021000	Villareal	0	17	146	163	0	0.00	10	58.82	101	69.18	111	68.10
806022000	Paranas	0	11	148	159	0	0.00	8	72.73	143	96.62	151	94.97
806023000	Zumarraga	0	2	43	45	0	0.00	2	100.00	41	95.35	43	95.56
806024000	Tagapul-An	0	5	44	49	0	0.00	4	80.00	25	56.82	29	59.18
806025000	San Jorge	0	7	43	50	0	0.00	7	100.00	43	100.00	50	100.00
806026000	Pagsanghan	0	1	14	15	0	0.00	1	100.00	13	92.86	14	93.33
**806400000**	**Southern Leyte**	**5**	**429**	**3,896**	**4,330**	**5**	**100.00**	**314**	**73.19**	**2,746**	**70.48**	**3,065**	**70.79**
806401000	Anahawan	1	58	288	347	1	100.00	57	98.28	285	98.96	343	98.85
806402000	Bontoc	0	0	14	14	0	0.00	0	0.00	14	100.00	14	100.00
806403000	Hinunangan	0	7	184	191	0	0.00	7	100.00	184	100.00	191	100.00
806404000	Hinundayan	0	1	6	7	0	0.00	1	100.00	6	100.00	7	100.00
806405000	Libagon	0	1	6	7	0	0.00	1	100.00	6	100.00	7	100.00
806406000	Liloan	0	36	148	184	0	0.00	32	88.89	146	98.65	178	96.74
806407000	City of Maasin	0	172	1,691	1,863	0	0.00	65	37.79	571	33.77	636	34.14
806408000	Macrohon	0	0	7	7	0	0.00	0	0.00	7	100.00	7	100.00
806409000	Malitbog	0	0	4	4	0	0.00	0	0.00	4	100.00	4	100.00
806410000	Padre Burgos	0	0	12	12	0	0.00	0	0.00	12	100.00	12	100.00
806411000	Pintuyan	0	6	117	123	0	0.00	4	66.67	94	80.34	98	79.67
806412000	Saint Bernard	0	3	102	105	0	0.00	3	100.00	102	100.00	105	100.00
806413000	San Francisco	0	0	4	4	0	0.00	0	0.00	4	100.00	4	100.00
806414000	San Juan	0	0	25	25	0	0.00	0	0.00	23	92.00	23	92.00
806415000	San Ricardo	0	0	0	0	0	0.00	0	0.00	0	0.00	0	0.00
806416000	Silago	0	3	115	118	0	0.00	3	100.00	115	100.00	118	100.00
806417000	Sogod	4	142	1,150	1,296	4	100.00	141	99.30	1,150	100.00	1,295	99.92
806418000	Tomas Oppus	0	0	16	16	0	0.00	0	0.00	16	100.00	16	100.00
806419000	Limasawa	0	0	7	7	0	0.00	0	0.00	7	100.00	7	100.00
**807800000**	**Biliran**	**6**	**385**	**2,666**	**3,057**	**2**	**33.33**	**191**	**49.61**	**1,805**	**67.70**	**1,998**	**65.36**
807801000	Almeria	0	4	56	60	0	0.00	2	50.00	48	85.71	50	83.33
807802000	Biliran	0	2	77	79	0	0.00	2	100.00	70	90.91	72	91.14
807803000	Cabucgayan	0	7	148	155	0	0.00	5	71.43	129	87.16	134	86.45
807804000	Caibiran	0	17	157	174	0	0.00	15	88.24	147	93.63	162	93.10
807805000	Culaba	0	22	111	133	0	0.00	20	90.91	107	96.40	127	95.49
807806000	Kawayan	0	7	103	110	0	0.00	4	57.14	82	79.61	86	78.18
807807000	Maripipi	0	6	38	44	0	0.00	6	100.00	37	97.37	43	97.73
807808000	Naval	6	320	1,976	2,302	2	33.33	137	42.81	1,185	59.97	1,324	57.52
**900000000**	**Region IX (Zamboanga Peninsula)**	**68**	**4,040**	**27,437**	**31,545**	**43**	**63.24**	**3,034**	**75.10**	**21,882**	**79.75**	**24,959**	**79.12**
**931700000**	**City of Zamboanga**	3	332	3,061	**3,396**	3	100.00	283	85.24	2,750	89.84	**3,036**	**89.40**
**990101000**	**City of Isabela**	2	299	2,095	**2,396**	1	50.00	213	71.24	1,571	74.99	**1,785**	**74.50**
**907200000**	**Zamboanga del Norte**	26	1,694	10,453	**12,173**	18	69.23	1,428	84.30	9,173	87.75	**10,619**	**87.23**
907201000	City of Dapitan	2	127	1,401	1,530	2	100.00	119	93.70	1,338	95.50	1,459	95.36
907202000	City of Dipolog	7	265	1,916	2,188	7	100.00	265	100.00	1,794	93.63	2,066	94.42
907203000	Katipunan	0	13	144	157	0	0.00	13	100.00	144	100.00	157	100.00
907204000	La Libertad	0	2	39	41	0	0.00	2	100.00	39	100.00	41	100.00
907205000	Labason	3	169	738	910	3	100.00	135	79.88	582	78.86	720	79.12
907206000	Liloy	5	171	742	918	0	0.00	147	85.96	709	95.55	856	93.25
907207000	Manukan	1	55	347	403	1	100.00	44	80.00	277	79.83	322	79.90
907208000	Mutia	0	0	7	7	0	0.00	0	0.00	4	57.14	4	57.14
907209000	Piñan	0	21	207	228	0	0.00	19	90.48	203	98.07	222	97.37
907210000	Polanco	0	9	94	103	0	0.00	9	100.00	94	100.00	103	100.00
907211000	Pres. Manuel A. Roxas	0	16	159	175	0	0.00	10	62.50	156	98.11	166	94.86
907212000	Rizal	0	3	43	46	0	0.00	2	66.67	24	55.81	26	56.52
907213000	Salug	0	9	129	138	0	0.00	4	44.44	118	91.47	122	88.41
907214000	Sergio Osmeña Sr.	3	63	328	394	2	66.67	21	33.33	138	42.07	161	40.86
907215000	Siayan	1	104	433	538	0	0.00	92	88.46	358	82.68	450	83.64
907216000	Sibuco	0	43	332	375	0	0.00	30	69.77	216	65.06	246	65.60
907217000	Sibutad	0	7	101	108	0	0.00	7	100.00	101	100.00	108	100.00
907218000	Sindangan	2	268	1,229	1,499	2	100.00	213	79.48	1,099	89.42	1,314	87.66
907219000	Siocon	0	86	458	544	0	0.00	84	97.67	455	99.34	539	99.08
907220000	Sirawai	1	37	233	271	0	0.00	26	70.27	199	85.41	225	83.03

PSGC10	Area	Total Deliveries				Women who gave birth with at least four or more prenatal check-ups (4ANC)							
		10-14 y.o.	15-19 y.o.	20-49 y.o.	Total	10-14 y.o.	%	15-19 y.o.	%	20-49 y.o.	%	Total	%
907221000	Tampilisan	0	29	199	**228**	0	0.00	17	58.62	115	57.79	**132**	**57.89**
907222000	Jose Dalman	0	69	286	**355**	0	0.00	54	78.26	236	82.52	**290**	**81.69**
907223000	Gutalac	0	8	170	**178**	0	0.00	7	87.50	153	90.00	**160**	**89.89**
907224000	Baliguian	0	14	94	**108**	0	0.00	14	100.00	94	100.00	**108**	**100.00**
907225000	Godod	0	6	74	**80**	0	0.00	3	50.00	53	71.62	**56**	**70.00**
907226000	Bacungan	1	64	321	**386**	1	100.00	58	90.63	272	84.74	**331**	**85.75**
907227000	Kalawit	0	36	229	**265**	0	0.00	33	91.67	202	88.21	**235**	**88.68**
**907300000**	**Zamboanga del Sur**	22	955	6,596	**7,573**	9	40.91	582	60.94	4,517	68.48	**5,108**	**67.45**
907302000	Aurora	0	12	447	**459**	0	0.00	1	8.33	155	34.68	**156**	**33.99**
907303000	Bayog	2	61	225	**288**	1	50.00	36	59.02	136	60.44	**173**	**60.07**
907305000	Dimataling	3	13	156	**172**	1	33.33	12	92.31	151	96.79	**164**	**95.35**
907306000	Dinas	0	14	127	**141**	0	0.00	6	42.86	51	40.16	**57**	**40.43**
907307000	Dumalinao	0	4	16	**20**	0	0.00	2	50.00	13	81.25	**15**	**75.00**
907308000	Dumingag	3	114	648	**765**	1	33.33	56	49.12	392	60.49	**449**	**58.69**
907311000	Kumalarang	0	31	129	**160**	0	0.00	6	19.35	21	16.28	**27**	**16.88**
907312000	Labangan	0	8	95	**103**	0	0.00	7	87.50	88	92.63	**95**	**92.23**
907313000	Lapuyan	0	21	114	**135**	0	0.00	4	19.05	14	12.28	**18**	**13.33**
907315000	Mahayag	1	21	159	**181**	0	0.00	19	90.48	141	88.68	**160**	**88.40**
907317000	Margosatubig	3	73	297	**373**	0	0.00	36	49.32	145	48.82	**181**	**48.53**
907318000	Midsalip	0	50	287	**337**	0	0.00	20	40.00	160	55.75	**180**	**53.41**
907319000	Molave	0	27	230	**257**	0	0.00	27	100.00	228	99.13	**255**	**99.22**
907322000	City of Pagadian	8	285	2,162	**2,455**	5	62.50	196	68.77	1,740	80.48	**1,941**	**79.06**
907323000	Ramon Magsaysay	0	2	100	**102**	0	0.00	2	100.00	94	94.00	**96**	**94.12**
907324000	San Miguel	0	4	62	**66**	0	0.00	2	50.00	31	50.00	**33**	**50.00**
907325000	San Pablo	0	12	147	**159**	0	0.00	5	41.67	29	19.73	**34**	**21.38**
907327000	Tabina	0	25	144	**169**	0	0.00	5	20.00	40	27.78	**45**	**26.63**
907328000	Tambulig	0	15	191	**206**	0	0.00	10	66.67	147	76.96	**157**	**76.21**
907330000	Tukuran	0	28	248	**276**	0	0.00	24	85.71	236	95.16	**260**	**94.20**
907333000	Lakewood	0	14	89	**103**	0	0.00	7	50.00	72	80.90	**79**	**76.70**
907337000	Josefina	0	14	70	**84**	0	0.00	12	85.71	68	97.14	**80**	**95.24**
907338000	Pitogo	0	58	229	**287**	0	0.00	57	98.28	214	93.45	**271**	**94.43**
907340000	Sominot	0	19	68	**87**	0	0.00	18	94.74	66	97.06	**84**	**96.55**
907341000	Vincenzo A. Sagun	1	7	29	**37**	0	0.00	7	100.00	28	96.55	**35**	**94.59**
907343000	Guipos	1	9	40	**50**	1	100.00	2	22.22	21	52.50	**24**	**48.00**
907344000	Tigbao	0	14	87	**101**	0	0.00	3	21.43	36	41.38	**39**	**38.61**
**908300000**	**Zamboanga Sibugay**	**15**	**760**	**5,232**	**6,007**	**12**	**80.00**	**528**	**69.47**	**3,871**	**73.99**	**4,411**	**73.43**
908301000	Alicia	1	24	334	**359**	1	100.00	15	62.50	250	74.85	**266**	**74.09**
908302000	Buug	0	26	203	**229**	0	0.00	23	88.46	197	97.04	**220**	**96.07**
908303000	Diplahan	0	75	322	**397**	0	0.00	74	98.67	322	100.00	**396**	**99.75**
908304000	Imelda	0	14	154	**168**	0	0.00	13	92.86	154	100.00	**167**	**99.40**
908305000	Ipil	4	240	1,094	**1,338**	3	75.00	115	47.92	633	57.86	**751**	**56.13**
908306000	Kabasalan	1	17	340	**358**	1	100.00	14	82.35	247	72.65	**262**	**73.18**
908307000	Mabuhay	1	17	150	**168**	1	100.00	8	47.06	99	66.00	**108**	**64.29**
908308000	Malangas	1	42	287	**330**	1	100.00	40	95.24	284	98.95	**325**	**98.48**
908309000	Naga	0	31	303	**334**	0	0.00	29	93.55	248	81.85	**277**	**82.93**
908310000	Olutanga	2	51	240	**293**	2	100.00	49	96.08	224	93.33	**275**	**93.86**
908311000	Payao	0	30	202	**232**	0	0.00	25	83.33	141	69.80	**166**	**71.55**
908312000	Roseller Lim	1	57	492	**550**	0	0.00	34	59.65	281	57.11	**315**	**57.27**
908313000	Siay	1	31	301	**333**	0	0.00	26	83.87	242	80.40	**268**	**80.48**
908314000	Talusan	0	6	58	**64**	0	0.00	3	50.00	47	81.03	**50**	**78.13**
908315000	Titay	2	34	316	**352**	2	100.00	20	58.82	211	66.77	**233**	**66.19**
908316000	Tungawan	1	65	436	**502**	1	100.00	40	61.54	291	66.74	**332**	**66.14**
**1000000000**	**Region X (Northern Mindanao)**	**204**	**8,959**	**54,703**	**63,866**	**183**	**89.71**	**8,400**	**93.76**	**52,085**	**95.21**	**60,668**	**94.99**
**1030500000**	**City of Cagayan De Oro**	34	1,588	9,857	**11,479**	33	97.06	1,584	99.75	9,798	99.40	**11,415**	**99.44**
**1030900000**	**City of Iligan**	14	655	5,686	**6,355**	14	100.00	651	99.39	5,573	98.01	**6,238**	**98.16**
**1001300000**	**Bukidnon**	93	3,693	16,418	**20,204**	79	84.95	3,317	89.82	15,033	91.56	**18,429**	**91.21**
1001301000	Baungon	0	17	159	**176**	0	0.00	17	100.00	156	98.11	**173**	**98.30**
1001302000	Damulog	0	29	135	**164**	0	0.00	23	79.31	109	80.74	**132**	**80.49**
1001303000	Dangcagan	2	68	286	**356**	2	100.00	64	94.12	283	98.95	**349**	**98.03**
1001304000	Don Carlos	2	206	968	**1,176**	2	100.00	196	95.15	952	98.35	**1,150**	**97.79**
1001305000	Impasug-ong	0	80	388	**468**	0	0.00	60	75.00	303	78.09	**363**	**77.56**
1001306000	Kadingilan	1	62	346	**409**	0	0.00	58	93.55	333	96.24	**391**	**95.60**
1001307000	Kalilangan	0	67	504	**571**	0	0.00	57	85.07	447	88.69	**504**	**88.27**
1001308000	Kibawe	7	124	482	**613**	7	100.00	107	86.29	420	87.14	**534**	**87.11**
1001309000	Kitaotao	6	147	476	**629**	5	83.33	103	70.07	350	73.53	**458**	**72.81**
1001310000	Lantapan	4	190	762	**956**	4	100.00	184	96.84	755	99.08	**943**	**98.64**
1001311000	Libona	3	155	612	**770**	1	33.33	145	93.55	568	92.81	**714**	**92.73**
1001312000	City of Malaybalay	7	443	2,371	**2,821**	7	100.00	430	97.07	2,330	98.27	**2,767**	**98.09**
1001313000	Malitbog	3	98	386	**487**	3	100.00	80	81.63	334	86.53	**417**	**85.63**
1001314000	Manolo Fortich	5	271	1,435	**1,711**	5	100.00	268	98.89	1,414	98.54	**1,687**	**98.60**
1001315000	Maramag	7	333	1,530	**1,870**	6	85.71	256	76.88	1,263	82.55	**1,525**	**81.55**
1001316000	Pangantucan	9	162	602	**773**	7	77.78	129	79.63	498	82.72	**634**	**82.02**
1001317000	Quezon	19	387	1,377	**1,783**	18	94.74	370	95.61	1,316	95.57	**1,704**	**95.57**
1001318000	San Fernando	2	194	607	**803**	1	50.00	171	88.14	512	84.35	**684**	**85.18**
1001319000	Sumilao	1	66	275	**342**	0	0.00	45	68.18	243	88.36	**288**	**84.21**
1001320000	Talakag	9	334	1,041	**1,384**	8	88.89	334	100.00	1,035	99.42	**1,377**	**99.49**
1001321000	City of Valencia	2	156	1,219	**1,377**	1	50.00	138	88.46	1,020	83.68	**1,159**	**84.17**
1001322000	Cabanglasan	4	104	457	**565**	2	50.00	82	78.85	392	85.78	**476**	**84.25**
**1001800000**	**Camiguin**	3	153	1,097	**1,253**	1	33.33	85	55.56	864	78.76	**950**	**75.82**
1001801000	Catarman	1	42	191	**234**	1	100.00	23	54.76	118	61.78	**142**	**60.68**
1001802000	Guinsiliban	1	11	73	**85**	0	0.00	4	36.36	60	82.19	**64**	**75.29**
1001803000	Mahinog	0	18	145	**163**	0	0.00	11	61.11	119	82.07	**130**	**79.75**
1001804000	Mambajao	0	49	508	**557**	0	0.00	37	75.51	415	81.69	**452**	**81.15**
1001805000	Sagay	1	33	180	**214**	0	0.00	10	30.30	152	84.44	**162**	**75.70**
**1003500000**	**Lanao del Norte**	27	893	9,016	**9,936**	26	96.30	876	98.10	8,667	96.13	**9,569**	**96.31**
1003501000	Bacolod	1	8	135	**144**	1	100.00	8	100.00	135	100.00	**144**	**100.00**
1003502000	Baloi	2	50	430	**482**	2	100.00	50	100.00	430	100.00	**482**	**100.00**
1003503000	Baroy	7	270	1,730	**2,007**	7	100.00	270	100.00	1,730	100.00	**2,007**	**100.00**
1003505000	Kapatagan	12	306	2,051	**2,369**	12	100.00	306	100.00	2,051	100.00	**2,369**	**100.00**
1003506000	Sultan Naga Dimaporo	0	34	307	**341**	0	0.00	31	91.18	165	53.75	**196**	**57.48**
1003507000	Kauswagan	0	12	169	**181**	0	0.00	8	66.67	139	82.25	**147**	**81.22**
1003508000	Kolambugan	0	53	440	**493**	0	0.00	51	96.23	426	96.82	**477**	**96.75**
1003509000	Lala	0	1	46	**47**	0	0.00	1	100.00	46	100.00	**47**	**100.00**
1003510000	Linamon	0	0	11	**11**	0	0.00	0	0.00	11	100.00	**11**	**100.00**
1003511000	Magsaysay	0	0	248	**248**	0	0.00	0	0.00	242	97.58	**242**	**97.58**
1003512000	Maigo	1	18	86	**105**	1	100.00	18	100.00	79	91.86	**98**	**93.33**
1003513000	Matungao	0	4	98	**102**	0	0.00	4	100.00	98	100.00	**102**	**100.00**
1003514000	Munai	2	27	732	**761**	1	50.00	25	92.59	676	92.35	**702**	**92.25**
1003515000	Nunungan	0	6	506	**512**	0	0.00	5	83.33	494	97.63	**499**	**97.46**
1003516000	Pantao Ragat	0	5	310	**315**	0	0.00	5	100.00	310	100.00	**315**	**100.00**
1003517000	Poona Piagapo	0	33	412	**445**	0	0.00	33	100.00	409	99.27	**442**	**99.33**
1003518000	Salvador	0	2	93	**95**	0	0.00	2	100.00	84	90.32	**86**	**90.53**
1003519000	Sapad	1	19	109	**129**	1	100.00	18	94.74	109	100.00	**128**	**99.22**
1003520000	Tagoloan	0	17	230	**247**	0	0.00	15	88.24	203	88.26	**218**	**88.26**
1003521000	Tangcal	1	6	532	**539**	1	100.00	6	100.00	532	100.00	**539**	**100.00**
1003522000	Tubod	0	5	16	**21**	0	0.00	3	60.00	16	100.00	**19**	**90.48**
1003523000	Pantar	0	17	325	**342**	0	0.00	17	100.00	282	86.77	**299**	**87.43**
**1004200000**	**Misamis Occidental**	6	639	4,309	**4,954**	6	100.00	616	96.40	4,155	96.43	**4,777**	**96.43**
1004201000	Aloran	0	0	1	**1**	0	0.00	0	0.00	1	100.00	**1**	**100.00**
1004202000	Baliangao	0	0	1	**1**	0	0.00	0	0.00	0	0.00	**0**	**0.00**
1004203000	Bonifacio	0	19	79	**98**	0	0.00	15	78.95	64	81.01	**79**	**80.61**
1004204000	Calamba	3	100	700	**803**	3	100.00	100	100.00	694	99.14	**797**	**99.25**
1004205000	Clarin	0	0	0	**0**	0	0.00	0	0.00	0	0.00	**0**	**0.00**
1004206000	Concepcion	1	2	23	**26**	1	100.00	1	50.00	19	82.61	**21**	**80.77**
1004207000	Jimenez	0	13	129	**142**	0	0.00	13	100.00	129	100.00	**142**	**100.00**
1004208000	Lopez Jaena	0	0	1	**1**	0	0.00	0	0.00	0	0.00	**0**	**0.00**
1004209000	City of Oroquieta	1	86	762	**849**	1	100.00	76	88.37	731	95.93	**808**	**95.17**
1004210000	City of Ozamiz	1	216	1,500	**1,717**	1	100.00	214	99.07	1,493	99.53	**1,708**	**99.48**
1004211000	Panaon	0	0	0	**0**	0	0.00	0	0.00	13	0.00	**13**	**0.00**
1004212000	Plaridel	0	8	36	**44**	0	0.00	7	87.50	31	86.11	**38**	**86.36**
1004213000	Sapang Dalaga	0	6	22	**28**	0	0.00	6	100.00	22	100.00	**28**	**100.00**
1004214000	Sinacaban	0	5	59	**64**	0	0.00	5	100.00	59	100.00	**64**	**100.00**
1004215000	City of Tangub	0	147	841	**988**	0	0.00	142	96.60	765	90.96	**907**	**91.80**
1004216000	Tudela	0	5	77	**82**	0	0.00	5	100.00	56	72.73	**61**	**74.39**
1004217000	Don Victoriano Chiongbian	0	32	78	**110**	0	0.00	32	100.00	78	100.00	**110**	**100.00**
**1004300000**	**Misamis Oriental**	27	1,338	8,320	**9,685**	24	88.89	1,271	94.99	7,995	96.09	**9,290**	**95.92**
1004301000	Alubijid	0	14	146	**160**	0	0.00	14	100.00	146	100.00	**160**	**100.00**

2.B.1.1 - Prenatal Care
Women who gave birth with at least 4 Prenatal Check-ups
Philippines, 2024

PSGC10	Area	Total Deliveries				Women who gave birth with at least four or more prenatal check-ups (4ANC)							
		10-14 y.o.	15-19 y.o.	20-49 y.o.	Total	10-14 y.o.	%	15-19 y.o.	%	20-49 y.o.	%	Total	%
1004302000	Balingasag	0	111	624	735	0	0.00	111	100.00	624	100.00	735	100.00
1004303000	Balingoan	0	0	6	6	0	0.00	0	0.00	6	100.00	6	100.00
1004304000	Binuangan	0	1	2	3	0	0.00	0	0.00	2	100.00	2	66.67
1004306000	Claveria	3	100	462	565	3	100.00	100	100.00	462	100.00	565	100.00
1004307000	City of El Salvador	1	66	390	457	1	100.00	50	75.76	336	86.15	387	84.68
1004308000	City of Gingoog	11	370	1,852	2,233	11	100.00	354	95.68	1,805	97.46	2,170	97.18
1004309000	Gitagum	0	3	4	7	0	0.00	0	0.00	1	25.00	1	14.29
1004310000	Initao	1	57	344	402	1	100.00	56	98.25	344	100.00	401	99.75
1004311000	Jasaan	0	62	712	774	0	0.00	61	98.39	705	99.02	766	98.97
1004312000	Kinoguitan	0	0	1	1	0	0.00	0	0.00	1	100.00	1	100.00
1004313000	Lagonglong	0	8	30	38	0	0.00	8	100.00	30	100.00	38	100.00
1004314000	Laguindingan	0	1	3	4	0	0.00	1	100.00	3	100.00	4	100.00
1004315000	Libertad	0	0	0	0	0	0.00	0	0.00	0	0.00	0	0.00
1004316000	Lugait	0	0	4	4	0	0.00	0	0.00	3	75.00	3	75.00
1004317000	Magsaysay	0	27	228	255	0	0.00	27	100.00	228	100.00	255	100.00
1004318000	Manticao	3	104	446	553	1	33.33	89	85.58	391	87.67	481	86.98
1004319000	Medina	0	19	221	240	0	0.00	19	100.00	221	100.00	240	100.00
1004320000	Naawan	0	0	0	0	0	0.00	0	0.00	0	0.00	0	0.00
1004321000	Opol	3	79	640	722	3	100.00	79	100.00	640	100.00	722	100.00
1004322000	Salay	0	2	67	69	0	0.00	2	100.00	67	100.00	69	100.00
1004323000	Sugbongcogon	0	0	4	4	0	0.00	1	0.00	3	75.00	4	100.00
1004324000	Tagoloan	5	193	1,112	1,310	4	80.00	193	100.00	1,104	99.28	1,301	99.31
1004325000	Talisayan	0	45	395	440	0	0.00	30	66.67	246	62.28	276	62.73
1004326000	Villanueva	0	76	627	703	0	0.00	76	100.00	627	100.00	703	100.00
**1100000000**	**Region XI (Davao Region)**	395	10,313	63,038	73,746	264	66.84	7,883	76.44	52,664	83.54	60,811	82.46
**1130700000**	**City of Davao**	69	2,705	24,093	26,867	47	68.12	2,194	81.11	21,181	87.91	23,422	87.18
**1102300000**	**Davao del Norte**	99	2,168	13,220	15,487	65	65.66	1,606	74.08	10,991	83.14	12,662	81.76
1102301000	Asuncion	7	137	661	805	5	71.43	113	82.48	538	81.39	656	81.49
1102303000	Carmen	14	182	1,013	1,209	10	71.43	95	52.20	585	57.75	690	57.07
1102305000	Kapalong	9	229	940	1,178	4	44.44	118	51.53	647	68.83	769	65.28
1102314000	New Corella	4	120	614	738	3	75.00	108	90.00	555	90.39	666	90.24
1102315000	City of Panabo	14	357	2,346	2,717	12	85.71	285	79.83	2,010	85.68	2,307	84.91
1102317000	Island Garden City of Samal	9	227	1,427	1,663	6	66.67	212	93.39	1,357	95.09	1,575	94.71
1102318000	Santo Tomas	6	215	1,368	1,589	3	50.00	137	63.72	1,036	75.73	1,176	74.01
1102319000	City of Tagum	21	472	3,971	4,464	14	66.67	441	93.43	3,818	96.15	4,273	95.72
1102322000	Talaingod	8	113	330	451	2	25.00	21	18.58	93	28.18	116	25.72
1102323000	Braulio E. Dujali	6	38	301	345	5	83.33	25	65.79	195	64.78	225	65.22
1102324000	San Isidro	1	78	249	328	1	100.00	51	65.38	157	63.05	209	63.72
**1102400000**	**Davao del Sur**	62	1,432	7,187	8,681	36	58.06	975	68.09	5,063	70.45	6,074	69.97
1102401000	Bansalan	1	107	592	700	0	0.00	52	48.60	408	68.92	460	65.71
1102403000	City of Digos	20	315	1,995	2,330	4	20.00	136	43.17	1,053	52.78	1,193	51.20
1102404000	Hagonoy	8	108	567	683	6	75.00	90	83.33	509	89.77	605	88.58
1102406000	Kiblawan	7	110	511	628	6	85.71	97	88.18	449	87.87	552	87.90
1102407000	Magsaysay	5	131	558	694	4	80.00	103	78.63	460	82.44	567	81.70
1102408000	Malalag	6	118	440	564	6	100.00	108	91.53	402	91.36	516	91.49
1102410000	Matanao	2	142	646	790	1	50.00	90	63.38	458	70.90	549	69.49
1102411000	Padada	2	41	259	302	1	50.00	29	70.73	233	89.96	263	87.09
1102412000	Santa Cruz	7	280	1,265	1,552	5	71.43	196	70.00	781	61.74	982	63.27
1102414000	Sulop	4	80	354	438	3	75.00	74	92.50	310	87.57	387	88.36
**1102500000**	**Davao Oriental**	56	1,272	6,591	7,919	39	69.64	1,065	83.73	5,681	86.19	6,785	85.68
1102501000	Baganga	5	127	680	812	2	40.00	90	70.87	503	73.97	595	73.28
1102502000	Banaybanay	5	102	519	626	5	100.00	101	99.02	518	99.81	624	99.68
1102503000	Boston	0	26	154	180	0	0.00	21	80.77	133	86.36	154	85.56
1102504000	Caraga	4	65	372	441	2	50.00	54	83.08	337	90.59	393	89.12
1102505000	Cateel	6	128	533	667	5	83.33	99	77.34	440	82.55	544	81.56
1102506000	Governor Generoso	11	175	663	849	9	81.82	146	83.43	542	81.75	697	82.10
1102507000	Lupon	5	144	732	881	5	100.00	132	91.67	686	93.72	823	93.42
1102508000	Manay	5	61	408	474	1	20.00	42	68.85	344	84.31	387	81.65
1102509000	City of Mati	12	311	1,828	2,151	9	75.00	279	89.71	1,611	88.13	1,899	88.28
1102510000	San Isidro	0	64	356	420	0	0.00	51	79.69	311	87.36	362	86.19
1102511000	Tarragona	3	69	346	418	1	33.33	50	72.46	256	73.99	307	73.44
**1108200000**	**Davao de Oro**	61	1,670	8,779	10,510	48	78.69	1,490	89.22	8,154	92.88	9,692	92.22
1108201000	Compostela	7	173	881	1,061	6	85.71	157	90.75	842	95.57	1,005	94.72
1108202000	Laak	11	230	869	1,110	9	81.82	196	85.22	718	82.62	923	83.15
1108203000	Mabini	0	92	521	613	0	0.00	92	100.00	515	98.85	607	99.02
1108204000	Maco	8	169	1,007	1,184	8	100.00	169	100.00	1,007	100.00	1,184	100.00
1108205000	Maragusan	5	118	823	946	2	40.00	89	75.42	688	83.60	779	82.35
1108206000	Mawab	1	79	406	486	1	100.00	79	100.00	404	99.51	484	99.59
1108207000	Monkayo	10	222	1,336	1,568	9	90.00	217	97.75	1,313	98.28	1,539	98.15
1108208000	Montevista	2	107	447	556	2	100.00	100	93.46	421	94.18	523	94.06
1108209000	Nabunturan	1	152	889	1,042	0	0.00	144	94.74	858	96.51	1,002	96.16
1108210000	New Bataan	4	78	493	575	4	100.00	75	96.15	490	99.39	569	98.96
1108211000	Pantukan	12	250	1,107	1,369	7	58.33	172	68.80	898	81.12	1,077	78.67
**1108600000**	**Davao Occidental**	48	1,066	3,168	4,282	29	60.42	553	51.88	1,594	50.32	2,176	50.82
1108601000	Don Marcelino	5	147	392	544	4	80.00	113	76.87	294	75.00	411	75.55
1108602000	Jose Abad Santos	7	255	866	1,128	3	42.86	77	30.20	255	29.45	335	29.70
1108603000	Malita	26	434	1,226	1,686	15	57.69	229	52.76	656	53.51	900	53.38
1108604000	Santa Maria	7	169	509	685	5	71.43	121	71.60	338	66.40	464	67.74
1108605000	Sarangani	3	61	175	239	2	66.67	13	21.31	51	29.14	66	27.62
**1200000000**	**Region XII (SOCCSKSARGEN)**	282	8,938	52,550	61,770	209	74.11	7,483	83.72	45,640	86.85	53,332	86.34
**1230800000**	**City of General Santos**	15	792	8,430	9,237	10	66.67	637	80.43	6,261	74.27	6,908	74.79
**1204700000**	**Cotabato**	78	2,383	14,247	16,708	59	75.64	2,078	87.20	12,931	90.76	15,068	90.18
1204701000	Alamada	5	172	978	1,155	3	60.00	153	88.95	908	92.84	1,064	92.12
1204702000	Carmen	11	244	1,024	1,279	6	54.55	146	59.84	684	66.80	836	65.36
1204703000	Kabacan	5	99	913	1,017	3	60.00	102	103.03	685	75.03	790	77.68
1204704000	City of Kidapawan	8	253	1,756	2,017	7	87.50	232	91.70	1,727	98.35	1,966	97.47
1204705000	Libungan	6	102	610	718	6	100.00	97	95.10	582	95.41	685	95.40
1204706000	Magpet	1	130	683	814	0	0.00	116	89.23	617	90.34	733	90.05
1204707000	Makilala	7	179	991	1,177	4	57.14	161	89.94	897	90.51	1,062	90.23
1204708000	Matalam	2	122	856	980	2	100.00	122	100.00	855	99.88	979	99.90
1204709000	Midsayap	1	119	1,052	1,172	1	100.00	116	97.48	987	93.82	1,104	94.20
1204710000	M'Lang	2	106	1,042	1,150	2	100.00	106	100.00	1,042	100.00	1,150	100.00
1204711000	Pigkawayan	3	42	468	513	3	100.00	42	100.00	447	95.51	492	95.91
1204712000	Pikit	2	149	827	978	0	0.00	118	79.19	713	86.22	831	84.97
1204713000	President Roxas	7	111	473	591	7	100.00	109	98.20	466	98.52	582	98.48
1204714000	Tulunan	1	146	698	845	1	100.00	132	90.41	673	96.42	806	95.38
1204715000	Antipas	2	56	276	334	2	100.00	51	91.07	265	96.01	318	95.21
1204716000	Banisilan	10	124	603	737	9	90.00	116	93.55	584	96.85	709	96.20
1204717000	Aleosan	3	54	395	452	2	66.67	43	79.63	336	85.06	381	84.29
1204718000	Arakan	2	175	602	779	1	50.00	116	66.29	463	76.91	580	74.45
**1206300000**	**South Cotabato**	81	2,204	12,261	14,546	62	76.54	1,894	85.93	11,053	90.15	13,009	89.43
1206302000	Banga	3	169	990	1,162	2	66.67	156	92.31	913	92.22	1,071	92.17
1206306000	City of Koronadal	14	332	2,322	2,668	12	85.71	297	89.46	2,145	92.38	2,454	91.98
1206311000	Norala	1	83	929	1,013	1	100.00	70	84.34	898	96.66	969	95.66
1206312000	Polomolok	21	317	2,189	2,527	13	61.90	241	76.03	1,857	84.83	2,111	83.54
1206313000	Surallah	6	210	1,196	1,412	6	100.00	208	99.05	1,191	99.58	1,405	99.50
1206314000	Tampakan	8	108	423	539	5	62.50	92	85.19	370	87.47	467	86.64
1206315000	Tantangan	3	101	479	583	3	100.00	98	97.03	475	99.16	576	98.80
1206316000	T'Boli	11	384	1,339	1,734	8	72.73	333	86.72	1,219	91.04	1,560	89.97
1206317000	Tupi	3	187	1,035	1,225	2	66.67	148	79.14	834	80.58	984	80.33
1206318000	Santo Niño	2	46	390	438	2	100.00	45	97.83	387	99.23	434	99.09
1206319000	Lake Sebu	9	267	969	1,245	8	88.89	206	77.15	764	78.84	978	78.55
**1206500000**	**Sultan Kudarat**	60	1,781	9,990	11,831	48	80.00	1,537	86.30	9,103	91.12	10,688	90.34
1206501000	Bagumbayan	11	211	1,022	1,244	11	100.00	210	99.53	1,018	99.61	1,239	99.60
1206502000	Columbio	1	104	420	525	1	100.00	94	90.38	395	94.05	490	93.33
1206503000	Esperanza	6	149	691	846	5	83.33	134	89.93	654	94.65	793	93.74
1206504000	Isulan	4	208	1,222	1,434	4	100.00	208	100.00	1,222	100.00	1,434	100.00
1206505000	Kalamansig	6	164	669	839	3	50.00	110	67.07	516	77.13	629	74.97
1206506000	Lebak	6	191	982	1,179	3	50.00	109	57.07	676	68.84	788	66.84
1206507000	Lutayan	5	124	999	1,128	5	100.00	118	95.16	979	98.00	1,102	97.70
1206508000	Lambayong	1	142	945	1,088	1	100.00	140	98.59	947	100.21	1,088	100.00
1206509000	Palimbang	5	130	772	907	5	100.00	115	88.46	600	77.72	720	79.38
1206510000	President Quirino	1	67	536	604	1	100.00	63	94.03	532	99.25	596	98.68
1206511000	City of Tacurong	2	128	1,215	1,345	2	100.00	129	100.78	1,197	98.52	1,328	98.74
1206512000	Sen. Ninoy Aquino	12	163	517	692	7	58.33	107	65.64	367	70.99	481	69.51
**1208000000**	**Sarangani**	48	1,778	7,622	9,448	30	62.50	1,337	75.20	6,292	82.55	7,659	81.06
1208001000	Alabel	8	271	1,051	1,330	6	75.00	170	62.73	812	77.26	988	74.29

PSGC10	Area	Total Deliveries				Women who gave birth with at least four or more prenatal check-ups (4ANC)							
		10-14 y.o.	15-19 y.o.	20-49 y.o.	Total	10-14 y.o.	%	15-19 y.o.	%	20-49 y.o.	%	Total	%
1208002000	Glan	5	360	1,269	1,634	2	40.00	298	82.78	1,130	89.05	1,430	87.52
1208003000	Kiamba	6	197	882	1,085	4	66.67	132	67.01	697	79.02	833	76.77
1208004000	Maasim	9	258	977	1,244	6	66.67	226	87.60	909	93.04	1,141	91.72
1208005000	Maitum	10	179	668	857	8	80.00	128	71.51	563	84.28	699	81.56
1208006000	Malapatan	4	216	1,535	1,755	1	25.00	178	82.41	1,183	77.07	1,362	77.61
1208007000	Malungon	6	297	1,240	1,543	3	50.00	205	69.02	998	80.48	1,206	78.16
**1600000000**	**Region XIII (Caraga)**	99	5,072	33,467	38,638	62	62.63	4,029	79.44	28,596	85.45	32,687	84.60
**1630400000**	**City of Butuan**	10	569	5,181	5,760	8	80.00	531	93.32	5,048	97.43	5,587	97.00
**1600200000**	**Agusan del Norte**	20	799	4,769	5,588	18	90.00	746	93.37	4,573	95.89	5,337	95.51
1600201000	Buenavista	0	24	509	533	0	0.00	22	91.67	506	99.41	528	99.06
1600203000	City of Cabadbaran	4	239	1,407	1,650	3	75.00	199	83.26	1,258	89.41	1,460	88.48
1600204000	Carmen	0	28	232	260	0	0.00	28	100.00	231	99.57	259	99.62
1600205000	Jabonga	1	25	181	207	0	0.00	20	80.00	177	97.79	197	95.17
1600206000	Kitcharao	0	17	157	174	0	0.00	13	76.47	132	84.08	145	83.33
1600207000	Las Nieves	0	10	92	102	0	0.00	9	90.00	86	93.48	95	93.14
1600208000	Magallanes	0	2	18	20	0	0.00	1	50.00	17	94.44	18	90.00
1600209000	Nasipit	0	55	321	376	0	0.00	55	100.00	319	99.38	374	99.47
1600210000	Santiago	0	2	7	9	0	0.00	2	100.00	7	100.00	9	100.00
1600211000	Tubay	0	1	5	6	0	0.00	1	100.00	2	40.00	3	50.00
1600212000	Remedios T. Romualdez	0	0	2	2	0	0.00	0	0.00	0	0.00	0	0.00
	**Agusan del Norte Provincial Hospital (ADNPH)**	15	396	1,838	2,249	15	100.00	396	100.00	1,838	100.00	2,249	100.00
**1600300000**	**Agusan del Sur**	37	1,357	8,490	9,884	18	48.65	1,213	89.39	8,114	95.57	9,345	94.55
1600301000	City of Bayugan	0	52	858	910	0	0.00	49	94.23	841	98.02	890	97.80
1600302000	Bunawan	10	264	804	1,078	10	100.00	264	100.00	804	100.00	1,078	100.00
1600303000	Esperanza	0	49	301	350	0	0.00	30	61.22	240	79.73	270	77.14
1600304000	La Paz	0	45	313	358	0	0.00	45	100.00	313	100.00	358	100.00
1600305000	Loreto	2	85	346	433	2	100.00	59	69.41	282	81.50	343	79.21
1600306000	Prosperidad	21	635	3,569	4,225	2	9.52	553	87.09	3,380	94.70	3,935	93.14
1600307000	Rosario	0	25	217	242	0	0.00	24	96.00	206	94.93	230	95.04
1600308000	San Francisco	0	1	552	553	0	0.00	1	100.00	552	100.00	553	100.00
1600309000	San Luis	1	25	216	242	1	100.00	23	92.00	205	94.91	229	94.63
1600310000	Santa Josefa	0	9	114	123	0	0.00	9	100.00	114	100.00	123	100.00
1600311000	Talacogon	2	77	377	456	2	100.00	72	93.51	371	98.41	445	97.59
1600312000	Trento	1	61	609	671	1	100.00	56	91.80	593	97.37	650	96.87
1600313000	Veruela	0	6	49	55	0	0.00	6	100.00	48	97.96	54	98.18
1600314000	Sibagat	0	23	165	188	0	0.00	22	95.65	165	100.00	187	99.47
**1606700000**	**Surigao del Norte**	4	854	6,499	7,357	0	0.00	397	46.49	3,753	57.75	4,150	56.41
1606701000	Alegria	0	0	21	21	0	0.00	0	0.00	17	80.95	17	80.95
1606702000	Bacuag	0	0	1	1	0	0.00	0	0.00	0	0.00	0	0.00
1606704000	Burgos	0	1	2	3	0	0.00	1	100.00	1	50.00	2	66.67
1606706000	Claver	0	8	183	191	0	0.00	6	75.00	173	94.54	179	93.72
1606707000	Dapa	0	205	1,122	1,327	0	0.00	68	33.17	486	43.32	554	41.75
1606708000	Del Carmen	0	0	3	3	0	0.00	0	0.00	2	66.67	2	66.67
1606710000	General Luna	0	0	6	6	0	0.00	0	0.00	3	50.00	3	50.00
1606711000	Gigaquit	0	4	64	68	0	0.00	4	100.00	62	96.88	66	97.06
1606714000	Mainit	0	6	115	121	0	0.00	6	100.00	110	95.65	116	95.87
1606715000	Malimono	0	4	77	81	0	0.00	4	100.00	77	100.00	81	100.00
1606716000	Pilar	0	1	1	2	0	0.00	0	0.00	0	0.00	0	0.00
1606717000	Placer	0	38	437	475	0	0.00	33	86.84	362	82.84	395	83.16
1606718000	San Benito	0	0	5	5	0	0.00	0	0.00	3	60.00	3	60.00
1606719000	San Francisco	0	0	2	2	0	0.00	0	0.00	2	100.00	2	100.00
1606720000	San Isidro	0	0	0	0	0	0.00	0	0.00	0	0.00	0	0.00
1606721000	Santa Monica	0	0	2	2	0	0.00	0	0.00	1	50.00	1	50.00
1606722000	Sison	0	0	48	48	0	0.00	0	0.00	48	100.00	48	100.00
1606723000	Socorro	0	27	198	225	0	0.00	25	92.59	188	94.95	213	94.67
1606724000	City of Surigao	4	558	4,160	4,722	0	0.00	248	44.44	2,166	52.07	2,414	51.12
1606725000	Tagana-An	0	1	20	21	0	0.00	1	100.00	20	100.00	21	100.00
1606727000	Tubod	0	1	32	33	0	0.00	1	100.00	32	100.00	33	100.00
**1606800000**	**Surigao del Sur**	27	1,388	7,771	9,186	17	62.96	1,046	75.36	6,393	82.27	7,456	81.17
1606801000	Barobo	1	28	405	434	0	0.00	20	71.43	280	69.14	300	69.12
1606802000	Bayabas	0	0	14	14	0	0.00	0	0.00	9	64.29	9	64.29
1606803000	City of Bislig	10	214	1,369	1,593	5	50.00	172	80.37	1,264	92.33	1,441	90.46
1606804000	Cagwait	0	9	99	108	0	0.00	6	66.67	95	95.96	101	93.52
1606805000	Cantilan	0	2	42	44	0	0.00	2	100.00	42	100.00	44	100.00
1606806000	Carmen	0	0	2	2	0	0.00	0	0.00	1	50.00	1	50.00
1606807000	Carrascal	0	13	188	201	0	0.00	7	53.85	161	85.64	168	83.58
1606808000	Cortes	0	6	122	128	0	0.00	6	100.00	122	100.00	128	100.00
1606809000	Hinatuan	1	100	521	622	1	100.00	83	83.00	507	97.31	591	95.02
1606810000	Lanuza	0	3	18	21	0	0.00	0	0.00	11	61.11	11	52.38
1606811000	Lianga	2	151	455	608	2	100.00	59	39.07	241	52.97	302	49.67
1606812000	Lingig	0	19	153	172	0	0.00	16	84.21	138	90.20	154	89.53
1606813000	Madrid	0	100	533	633	0	0.00	97	97.00	512	96.06	609	96.21
1606814000	Marihatag	1	95	429	525	0	0.00	16	16.84	83	19.35	99	18.86
1606815000	San Agustin	0	2	73	75	0	0.00	2	100.00	69	94.52	71	94.67
1606816000	San Miguel	2	42	270	314	1	50.00	23	54.76	231	85.56	255	81.21
1606817000	Tagbina	0	2	75	77	0	0.00	2	100.00	57	76.00	59	76.62
1606818000	Tago	0	1	31	32	0	0.00	0	0.00	19	61.29	19	59.38
1606819000	City of Tandag	10	601	2,972	3,583	8	80.00	535	89.02	2,551	85.83	3,094	86.35
**1608500000**	**Dinagat Islands**	1	105	757	863	1	100.00	96	91.43	715	94.45	812	94.09
1608501000	Basilisa	0	0	8	8	0	0.00	0	0.00	6	75.00	6	75.00
1608502000	Cagdianao	0	0	5	5	0	0.00	0	0.00	5	100.00	5	100.00
1608503000	Dinagat	0	77	405	482	0	0.00	73	94.81	389	96.05	462	95.85
1608504000	Libjo	1	17	192	210	1	100.00	17	100.00	189	98.44	207	98.57
1608505000	Loreto	0	8	88	96	0	0.00	4	50.00	82	93.18	86	89.58
1608506000	San Jose	0	3	58	61	0	0.00	2	66.67	43	74.14	45	73.77
1608507000	Tubajon	0	0	1	1	0	0.00	0	0.00	1	100.00	1	100.00
**1900000000**	**BARMM**	70	5,354	55,229	60,653	52	74.29	4,152	77.55	44,569	80.70	48,773	80.41
**1908703000**	**City of Cotabato**	3	382	3,208	3,593	3	100.00	322	84.29	2,793	87.06	3,118	86.78
**1900700000**	**Basilan**	10	829	4,195	5,034	8	80.00	459	55.37	2,492	59.40	2,959	58.78
1900702000	City of Lamitan	6	330	1,311	1,647	5	83.33	117	35.45	565	43.10	687	41.71
1900703000	Lantawan	1	36	146	183	1	100.00	24	66.67	97	66.44	122	66.67
1900704000	Maluso	0	59	506	565	0	0.00	34	57.63	251	49.60	285	50.44
1900705000	Sumisip	0	86	551	637	0	0.00	66	76.74	384	69.69	450	70.64
1900706000	Tipo-Tipo	1	83	252	336	1	100.00	73	87.95	168	66.67	242	72.02
1900707000	Tuburan	0	50	402	452	0	0.00	38	76.00	350	87.06	388	85.84
1900708000	Akbar	0	10	51	61	0	0.00	6	60.00	37	72.55	43	70.49
1900709000	Al-Barka	0	38	162	200	0	0.00	44	115.79	200	123.46	244	122.00
1900710000	Hadji Mohammad Ajul	2	39	168	209	1	50.00	22	56.41	149	88.69	172	82.30
1900711000	Ungkaya Pukan	0	16	100	116	0	0.00	12	75.00	72	72.00	84	72.41
1900712000	Hadji Muhtamad	0	34	202	236	0	0.00	13	38.24	110	54.46	123	52.12
1900713000	Tabuan-Lasa	0	48	344	392	0	0.00	10	20.83	109	31.69	119	30.36
**1903600000**	**Lanao del Sur**	11	605	13,410	14,026	11	100.00	750	123.97	12,364	92.20	13,125	93.58
1903601000	Bacolod-Kalawi	0	0	118	118	0	0.00	0	0.00	95	80.51	95	80.51
1903602000	Balabagan	0	4	61	65	0	0.00	4	100.00	60	98.36	64	98.46
1903603000	Balindong	0	24	329	353	0	0.00	24	100.00	328	99.70	352	99.72
1903604000	Bayang	0	8	114	122	0	0.00	8	100.00	114	100.00	122	100.00
1903605000	Binidayan	0	4	210	214	0	0.00	4	100.00	195	92.86	199	92.99
1903606000	Bubong	0	11	134	145	0	0.00	11	100.00	134	100.00	145	100.00
1903607000	Butig	0	11	340	351	0	0.00	9	81.82	307	90.29	316	90.03
1903609000	Ganassi	0	2	160	162	0	0.00	0	0.00	85	53.13	85	52.47
1903610000	Kapai	0	1	207	208	0	0.00	1	100.00	207	100.00	208	100.00
1903611000	Lumba-Bayabao	0	1	385	386	0	0.00	1	100.00	385	100.00	386	100.00
1903612000	Lumbatan	2	7	206	215	2	100.00	7	100.00	176	85.44	185	86.05
1903613000	Madalum	0	12	472	484	0	0.00	12	100.00	472	100.00	484	100.00
1903614000	Madamba	0	6	301	307	0	0.00	5	83.33	289	96.01	294	95.77
1903615000	Malabang	3	103	920	1,026	3	100.00	103	100.00	920	100.00	1,026	100.00
1903616000	Marantao	0	0	104	104	0	0.00	0	0.00	103	99.04	103	99.04
1903617000	City of Marawi	1	95	4,418	4,514	1	100.00	304	320.00	4,098	92.76	4,403	97.54
1903618000	Masiu	0	7	388	395	0	0.00	7	100.00	388	100.00	395	100.00
1903619000	Mulondo	0	3	117	120	0	0.00	3	100.00	116	99.15	119	99.17
1903620000	Pagayawan	0	1	121	122	0	0.00	1	100.00	121	100.00	122	100.00
1903621000	Piagapo	0	16	467	483	0	0.00	16	100.00	465	99.57	481	99.59
1903622000	Poona Bayabao	0	5	374	379	0	0.00	5	100.00	373	99.73	378	99.74
1903623000	Pualas	0	13	168	181	0	0.00	12	92.31	166	98.81	178	98.34
1903624000	Ditsaan-Ramain	0	3	138	141	0	0.00	3	100.00	138	100.00	141	100.00
1903625000	Saguiaran	0	4	208	212	0	0.00	1	25.00	133	63.94	134	63.21
1903626000	Tamparan	0	10	358	368	0	0.00	3	30.00	301	84.08	304	82.61

2.B.1.1 - Prenatal Care
Women who gave birth with at least 4 Prenatal Check-ups
Philippines, 2024

PSGC10	Area	Total Deliveries				Women who gave birth with at least four or more prenatal check-ups (4ANC)							
		10-14 y.o.	15-19 y.o.	20-49 y.o.	Total	10-14 y.o.	%	15-19 y.o.	%	20-49 y.o.	%	Total	%
1903627000	Taraka	0	5	142	147	0	0.00	5	100.00	130	91.55	135	91.84
1903628000	Tubaran	0	16	240	256	0	0.00	16	100.00	240	100.00	256	100.00
1903629000	Tugaya	0	6	101	107	0	0.00	3	50.00	70	69.31	73	68.22
1903630000	Wao	2	81	388	471	2	100.00	80	98.77	382	98.45	464	98.51
1903631000	Marogong	0	46	260	306	0	0.00	16	34.78	79	30.38	95	31.05
1903632000	Calanogas	0	7	279	286	0	0.00	4	57.14	228	81.72	232	81.12
1903633000	Buadiposo-Buntong	1	7	100	108	1	100.00	7	100.00	100	100.00	108	100.00
1903634000	Maguing	0	6	58	64	0	0.00	6	100.00	58	100.00	64	100.00
1903635000	Picong	0	28	142	170	0	0.00	18	64.29	83	58.45	101	59.41
1903636000	Lumbayanague	1	9	191	201	1	100.00	9	100.00	188	98.43	198	98.51
1903637000	Amai Manabilang	0	1	32	33	0	0.00	1	100.00	29	90.63	30	90.91
1903638000	Tagoloan Ii	0	14	162	176	0	0.00	14	100.00	162	100.00	176	100.00
1903639000	Kapatagan	0	13	192	205	0	0.00	12	92.31	169	88.02	181	88.29
1903640000	Sultan Dumalondong	1	14	101	116	1	100.00	14	100.00	95	94.06	110	94.83
1903641000	Lumbaca-Unayan	0	1	204	205	0	0.00	1	100.00	182	89.22	183	89.27
**1906600000**	**Sulu**	**6**	**739**	**9,655**	**10,400**	**7**	**116.67**	**746**	**100.95**	**9,333**	**96.66**	**10,086**	**96.98**
1906601000	Indanan	0	30	941	971	2	0.00	33	110.00	843	89.59	878	90.42
1906602000	Jolo	1	124	861	986	1	100.00	161	129.84	1,197	139.02	1,359	137.83
1906603000	Kalingalan Caluang	0	64	614	678	0	0.00	51	79.69	567	92.35	618	91.15
1906604000	Luuk	0	19	415	434	0	0.00	16	84.21	360	86.75	376	86.64
1906605000	Maimbung	0	64	559	623	0	0.00	93	145.31	657	117.53	750	120.39
1906606000	Hadji Panglima Tahil	1	33	208	242	1	100.00	26	78.79	162	77.88	189	78.10
1906607000	Old Panamao	1	10	478	489	0	0.00	11	110.00	458	95.82	469	95.91
1906608000	Pangutaran	3	66	415	484	3	100.00	44	66.67	224	53.98	271	55.99
1906609000	Parang	0	41	597	638	0	0.00	55	134.15	817	136.85	872	136.68
1906610000	Pata	0	16	138	154	0	0.00	11	68.75	120	86.96	131	85.06
1906611000	Patikul	0	43	733	776	0	0.00	22	51.16	440	60.03	462	59.54
1906612000	Siasi	0	52	867	919	0	0.00	52	100.00	866	99.88	918	99.89
1906613000	Talipao	0	41	523	564	0	0.00	58	141.46	598	114.34	656	116.31
1906614000	Tapul	0	9	312	321	0	0.00	9	100.00	308	98.72	317	98.75
1906615000	Tongkil	0	24	405	429	0	0.00	26	108.33	409	100.99	435	101.40
1906616000	Panglima Estino	0	32	572	604	0	0.00	22	68.75	570	99.65	592	98.01
1906617000	Lugus	0	12	182	194	0	0.00	12	100.00	123	67.58	135	69.59
1906618000	Pandami	0	24	490	514	0	0.00	16	66.67	342	69.80	358	69.65
1906619000	Omar	0	35	345	380	0	0.00	28	80.00	272	78.84	300	78.95
**1907000000**	**Tawi-Tawi**	**10**	**516**	**4,843**	**5,369**	**6**	**60.00**	**283**	**54.84**	**3,447**	**71.17**	**3,736**	**69.58**
1907001000	Panglima Sugala	1	60	367	428	1	100.00	36	60.00	195	53.13	232	54.21
1907002000	Bongao	3	232	1,606	1,841	2	66.67	75	32.33	659	41.03	736	39.98
1907003000	Mapun	0	21	209	230	0	0.00	19	90.48	146	69.86	165	71.74
1907004000	Simunul	0	15	235	250	0	0.00	9	60.00	202	85.96	211	84.40
1907005000	Sitangkai	0	75	709	784	0	0.00	70	93.33	676	95.35	746	95.15
1907006000	South Ubian	0	21	285	306	0	0.00	10	47.62	249	87.37	259	84.64
1907007000	Tandubas	0	3	454	457	0	0.00	3	100.00	454	100.00	457	100.00
1907008000	Turtle Islands	2	13	73	88	0	0.00	8	61.54	47	64.38	55	62.50
1907009000	Languyan	1	7	290	298	0	0.00	3	42.86	259	89.31	262	87.92
1907010000	Sapa-Sapa	0	14	279	293	0	0.00	14	100.00	279	100.00	293	100.00
1907011000	Sibutu	3	55	336	394	3	100.00	36	65.45	281	83.63	320	81.22
**1908700000**	**Maguindanao del Norte**	**7**	**690**	**7,149**	**7,846**	**6**	**85.71**	**465**	**67.39**	**5,057**	**70.74**	**5,528**	**70.46**
1908701000	Barira	0	6	446	452	0	0.00	7	116.67	327	73.32	334	73.89
1908702000	Buldon	0	23	465	488	0	0.00	19	82.61	439	94.41	458	93.85
1908704000	Datu Blah T. Sinsuat	0	44	410	454	0	0.00	28	63.64	325	79.27	353	77.75
1908705000	Datu Odin Sinsuat	0	117	1,143	1,260	1	0.00	88	75.21	694	60.72	783	62.14
1908706000	Kabuntalan	0	16	557	573	0	0.00	15	93.75	522	93.72	537	93.72
1908707000	Matanog	1	17	203	221	0	0.00	12	70.59	153	75.37	165	74.66
1908708000	Northern Kabuntalan	0	3	141	144	0	0.00	3	100.00	112	79.43	115	79.86
1908709000	Parang	0	85	1,252	1,337	0	0.00	27	31.76	469	37.46	496	37.10
1908710000	Sultan Kudarat	0	84	1,191	1,275	2	0.00	62	73.81	1,068	89.67	1,132	88.78
1908711000	Sultan Mastura	4	57	414	475	3	75.00	34	59.65	286	69.08	323	68.00
1908712000	Talitay	1	33	196	230	0	0.00	29	87.88	174	88.78	203	88.26
1908713000	Upi	1	205	731	937	0	0.00	141	68.78	488	66.76	629	67.13
**1908800000**	**Maguindanao del Sur**	**22**	**1,369**	**11,452**	**12,843**	**11**	**50.00**	**892**	**65.16**	**7,928**	**69.23**	**8,831**	**68.76**
1908801000	Ampatuan	0	31	282	313	0	0.00	20	64.52	252	89.36	272	86.90
1908802000	Buluan	3	224	1,174	1,401	0	0.00	100	44.64	484	41.23	584	41.68
1908803000	Datu Abdullah Sangki	1	66	534	601	0	0.00	47	71.21	403	75.47	450	74.88
1908804000	Datu Anggal Midtimbang	0	3	220	223	0	0.00	4	133.33	183	83.18	187	83.86
1908805000	Datu Hoffer Ampatuan	4	199	1,002	1,205	4	100.00	163	81.91	893	89.12	1,060	87.97
1908806000	Datu Paglas	0	35	583	618	0	0.00	34	97.14	554	95.03	588	95.15
1908807000	Datu Piang	1	49	479	529	1	100.00	34	69.39	374	78.08	409	77.32
1908808000	Datu Salibo	0	22	453	475	0	0.00	16	72.73	310	68.43	326	68.63
1908809000	Datu Saudi Ampatuan	1	66	482	549	2	200.00	50	75.76	295	61.20	347	63.21
1908810000	Datu Unsay	1	23	223	247	0	0.00	11	47.83	174	78.03	185	74.90
1908811000	Gen. S.K. Pendatun	4	93	538	635	0	0.00	41	44.09	278	51.67	319	50.24
1908812000	Guindulungan	0	18	389	407	0	0.00	15	83.33	372	95.63	387	95.09
1908813000	Mamasapano	0	28	372	400	0	0.00	8	28.57	212	56.99	220	55.00
1908814000	Mangudadatu	0	18	104	122	0	0.00	13	72.22	62	59.62	75	61.48
1908815000	Pagagawan	0	78	615	693	0	0.00	67	85.90	526	85.53	593	85.57
1908816000	Pagalungan	0	21	645	666	0	0.00	17	80.95	520	80.62	537	80.63
1908817000	Paglat	2	19	144	165	1	50.00	18	94.74	106	73.61	125	75.76
1908818000	Pandag	0	13	131	144	0	0.00	9	69.23	109	83.21	118	81.94
1908819000	Rajah Buayan	0	18	797	815	0	0.00	10	55.56	479	60.10	489	60.00
1908820000	Shariff Aguak	1	66	462	529	0	0.00	51	77.27	322	69.70	373	70.51
1908821000	Shariff Saydona Mustapha	2	47	509	558	2	100.00	23	48.94	208	40.86	233	41.76
1908822000	South Upi	0	166	642	808	0	0.00	94	56.63	314	48.91	408	50.50
1908823000	Sultan Sa Barongis	1	47	300	348	0	0.00	28	59.57	126	42.00	154	44.25
1908824000	Talayan	1	19	372	392	1	100.00	19	100.00	372	100.00	392	100.00
**1999900000**	**SGU**	**1**	**224**	**1,317**	**1,542**	**0**	**0.00**	**235**	**104.91**	**1,155**	**87.70**	**1,390**	**90.14**
1999901000	Carmen Cluster	1	41	183	225	0	0.00	21	51.22	86	46.99	107	47.56
1999902000	Kabacan Cluster	0	34	163	197	0	0.00	39	114.71	107	65.64	146	74.11
1999903000	Midsayap Cluster I	0	18	78	96	0	0.00	11	61.11	84	107.69	95	98.96
1999904000	Midsayap Cluster II	0	4	164	168	0	0.00	13	325.00	148	90.24	161	95.83
1999905000	Pigcawayan Cluster	0	13	83	96	0	0.00	7	53.85	167	201.20	174	181.25
1999906000	Pikit Cluster I	0	17	177	194	0	0.00	14	82.35	154	87.01	168	86.60
1999907000	Pikit Cluster II	0	72	176	248	0	0.00	96	133.33	277	157.39	373	150.40
1999908000	Pikit Cluster III	0	25	293	318	0	0.00	34	136.00	132	45.05	166	52.20

Table 2.B.1.2 - Prenatal Care
Pregnant women seen according to their nutritional status
Philippines, 2024

Area	Eligible Population (0-11 months)	Pregnant women seen during the 1st trimester according to their Body Mass Index (BMI)														
		Normal BMI					Low BMI					High BMI				
		Age Group			Total	%	Age Group			Total	%	Age Group			Total	%
		10-14	15-19	20-49			10-14	15-19	20-49			10-14	15-19	20-49		
**PHILIPPINES**	**2,200,865**	**3,060**	**100,192**	**710,692**	**813,944**	**36.98**	**730**	**13,115**	**34,341**	**48,186**	**2.19**	**396**	**9,540**	**138,257**	**148,193**	**6.73**
**N C R**	**263,248**	**220**	**5,277**	**74,798**	**80,295**	**30.50**	**64**	**1,704**	**3,963**	**5,731**	**2.18**	**26**	**879**	**19,458**	**20,363**	**7.74**
City of Malabon	7,447	7	226	1,318	**1,551**	**20.83**	9	86	253	**348**	**4.67**	1	78	1,046	**1,125**	**15.11**
City of Navotas	5,263	18	362	2,562	**2,942**	**55.90**	0	1	125	**126**	**2.39**	0	0	252	**252**	**4.79**
City of Valenzuela	13,601	14	285	2,887	**3,186**	**23.42**	2	41	200	**243**	**1.79**	0	48	1,321	**1,369**	**10.07**
City of Caloocan	33,499	27	794	6,207	**7,028**	**20.98**	9	159	403	**571**	**1.70**	0	78	1,506	**1,584**	**4.73**
City of Marikina	8,399	1	53	471	**525**	**6.25**	1	17	55	**73**	**0.87**	0	20	436	**456**	**5.43**
City of Pasig	17,856	57	254	3,052	**3,363**	**18.83**	3	65	185	**253**	**1.42**	10	49	1,376	**1,435**	**8.04**
Pateros	1,120	0	35	366	**401**	**35.80**	0	1	6	**7**	**0.63**	0	2	53	**55**	**4.91**
City of Taguig	26,021	8	409	3,004	**3,421**	**13.15**	3	117	452	**572**	**2.20**	2	120	2,839	**2,961**	**11.38**
Quezon City	59,607	14	453	29,762	**30,229**	**50.71**	20	745	631	**1,396**	**2.34**	5	53	1,539	**1,597**	**2.68**
City of Makati	4,196	1	24	319	**344**	**8.20**	1	13	61	**75**	**1.79**	0	22	562	**584**	**13.92**
City of Mandaluyong	7,989	8	213	4,605	**4,826**	**60.41**	0	11	48	**59**	**0.74**	1	25	440	**466**	**5.83**
City of San Juan	2,173	0	20	324	**344**	**15.83**	1	2	12	**15**	**0.69**	0	4	210	**214**	**9.85**
City of Manila	34,268	29	1,086	8,773	**9,888**	**28.85**	7	145	541	**693**	**2.02**	1	75	1,894	**1,970**	**5.75**
City of Las Piñas	11,346	11	208	2,151	**2,370**	**20.89**	2	93	265	**360**	**3.17**	1	96	1,701	**1,798**	**15.85**
City of Muntinlupa	9,949	7	308	2,381	**2,696**	**27.10**	2	82	241	**325**	**3.27**	1	110	2,199	**2,310**	**23.22**
City of Parañaque	13,385	7	299	3,570	**3,876**	**28.96**	2	71	259	**332**	**2.48**	2	53	1,010	**1,065**	**7.96**
Pasay City	7,129	11	248	3,046	**3,305**	**46.36**	2	55	226	**283**	**3.97**	2	46	1,074	**1,122**	**15.74**
**C A R**	**31,490**	**40**	**1,479**	**12,886**	**14,405**	**45.74**	**21**	**127**	**376**	**524**	**1.66**	**10**	**197**	**3,615**	**3,822**	**12.14**
Abra	3,639	5	220	1,393	**1,618**	**44.46**	2	25	79	**106**	**2.91**	0	14	259	**273**	**7.50**
Apayao	2,222	4	150	881	**1,035**	**46.58**	1	21	56	**78**	**3.51**	0	26	273	**299**	**13.46**
Benguet	8,900	6	359	4,264	**4,629**	**52.01**	1	16	58	**75**	**0.84**	3	73	1,259	**1,335**	**15.00**
Ifugao	4,145	7	236	1,700	**1,943**	**46.88**	9	9	40	**58**	**1.40**	0	22	393	**415**	**10.01**
Kalinga	4,582	9	198	1,723	**1,930**	**42.12**	1	25	65	**91**	**1.99**	0	13	501	**514**	**11.22**
Mountain Province	2,615	3	175	1,528	**1,706**	**65.24**	3	9	31	**43**	**1.64**	0	18	443	**461**	**17.63**
City of Baguio	5,387	6	141	1,397	**1,544**	**28.66**	4	22	47	**73**	**1.36**	7	31	487	**525**	**9.75**
**Region 1**	**96,024**	**89**	**3,547**	**36,468**	**40,104**	**41.76**	**8**	**240**	**1,164**	**1,412**	**1.47**	**3**	**163**	**2,575**	**2,741**	**2.85**
Ilocos Norte	8,809	8	330	4,949	**5,287**	**60.02**	0	15	94	**109**	**1.24**	0	7	247	**254**	**2.88**
Ilocos Sur	9,834	14	502	6,093	**6,609**	**67.21**	1	9	52	**62**	**0.63**	0	11	221	**232**	**2.36**
La Union	13,436	19	587	5,364	**5,970**	**44.43**	1	54	154	**209**	**1.56**	0	40	597	**637**	**4.74**
Pangasinan	60,591	45	2,002	19,456	**21,503**	**35.49**	6	160	851	**1,017**	**1.68**	3	104	1,501	**1,608**	**2.65**
City of Dagupan	3,354	3	126	606	**735**	**21.91**	0	2	13	**15**	**0.45**	0	1	9	**10**	**0.30**
**Region 2**	**64,034**	**124**	**3,511**	**23,291**	**26,926**	**42.05**	**19**	**333**	**899**	**1,251**	**1.95**	**7**	**425**	**4,135**	**4,567**	**7.13**
Batanes	241	1	4	81	**86**	**35.68**	0	0	0	**0**	**0.00**	0	1	68	**69**	**28.63**
Cagayan	20,723	23	863	5,872	**6,758**	**32.61**	2	85	260	**347**	**1.67**	1	48	913	**962**	**4.64**
Isabela	27,362	62	1,578	10,011	**11,651**	**42.58**	12	155	409	**576**	**2.11**	4	223	2,135	**2,362**	**8.63**
Nueva Vizcaya	8,880	22	521	3,467	**4,010**	**45.16**	4	63	170	**237**	**2.67**	2	132	738	**872**	**9.82**
Quirino	3,865	8	256	1,662	**1,926**	**49.83**	1	25	43	**69**	**1.79**	0	17	202	**219**	**5.67**
City of Santiago	2,963	8	289	2,198	**2,495**	**84.21**	0	5	17	**22**	**0.74**	0	4	79	**83**	**2.80**
**Region 3**	**235,313**	**497**	**12,278**	**87,081**	**99,856**	**42.44**	**66**	**1,133**	**2,337**	**3,536**	**1.50**	**33**	**869**	**10,873**	**11,775**	**5.00**
Aurora	4,578	10	271	1,682	**1,963**	**42.88**	1	46	89	**136**	**2.97**	2	19	211	**232**	**5.07**
Bataan	17,556	34	789	4,796	**5,619**	**32.01**	5	119	299	**423**	**2.41**	4	116	1,786	**1,906**	**10.86**
Bulacan	68,115	228	4,012	30,493	**34,733**	**50.99**	26	513	848	**1,387**	**2.04**	16	332	4,417	**4,765**	**7.00**
Nueva Ecija	43,626	92	2,099	12,351	**14,542**	**33.33**	9	151	287	**447**	**1.02**	2	125	1,278	**1,405**	**3.22**
Pampanga	46,146	50	2,017	15,244	**17,311**	**37.51**	12	137	345	**494**	**1.07**	6	120	1,763	**1,889**	**4.09**
Tarlac	29,655	25	1,266	11,878	**13,169**	**44.41**	4	49	180	**233**	**0.79**	2	60	467	**529**	**1.78**
Zambales	11,796	15	683	3,362	**4,060**	**34.42**	7	57	111	**175**	**1.48**	1	43	506	**550**	**4.66**
City of Angeles	9,232	38	974	6,249	**7,261**	**78.65**	1	23	79	**103**	**1.12**	0	8	91	**99**	**1.07**
City of Olongapo	4,609	5	167	1,026	**1,198**	**25.99**	1	38	99	**138**	**2.99**	0	46	354	**400**	**8.68**
**Region 4A**	**310,150**	**294**	**10,133**	**84,590**	**95,017**	**30.64**	**103**	**1,896**	**6,116**	**8,115**	**2.62**	**45**	**1,136**	**18,315**	**19,496**	**6.29**
Batangas	53,484	34	1,474	17,068	**18,576**	**34.73**	13	227	680	**920**	**1.72**	5	141	2,646	**2,792**	**5.22**
Cavite	78,589	57	2,024	17,342	**19,423**	**24.71**	24	372	1,471	**1,867**	**2.38**	12	267	4,498	**4,777**	**6.08**
Laguna	61,967	64	2,268	21,621	**23,953**	**38.65**	27	582	1,694	**2,303**	**3.72**	16	334	5,477	**5,827**	**9.40**
Quezon	40,954	66	1,883	11,091	**13,040**	**31.84**	15	370	1,014	**1,399**	**3.42**	5	195	2,980	**3,180**	**7.76**
Rizal	68,903	70	2,187	15,830	**18,087**	**26.25**	15	306	1,199	**1,520**	**2.21**	7	185	2,532	**2,724**	**3.95**
City of Lucena	6,253	3	297	1,638	**1,938**	**30.99**	9	39	58	**106**	**1.70**	0	14	182	**196**	**3.13**
**Region 4B**	**66,508**	**107**	**3,298**	**20,683**	**24,088**	**36.22**	**16**	**541**	**1,531**	**2,088**	**3.14**	**9**	**279**	**3,336**	**3,624**	**5.45**
Marinduque	4,124	4	0	1,212	**1,216**	**29.49**	1	32	65	**98**	**2.38**	0	15	327	**342**	**8.29**
Occidental Mindoro	11,748	25	699	3,693	**4,417**	**37.60**	7	108	292	**407**	**3.46**	2	56	684	**742**	**6.32**
Oriental Mindoro	19,007	31	827	6,362	**7,220**	**37.99**	3	96	293	**392**	**2.06**	3	32	422	**457**	**2.40**
Palawan	20,503	42	1,503	7,168	**8,713**	**42.50**	2	234	675	**911**	**4.44**	1	90	998	**1,089**	**5.31**
Romblon	5,488	3	172	1,654	**1,829**	**33.33**	1	43	151	**195**	**3.55**	1	11	256	**268**	**4.88**
City of Puerto Princesa	5,638	2	97	594	**693**	**12.29**	2	28	55	**85**	**1.51**	2	75	649	**726**	**12.88**
**Region 5**	**136,611**	**97**	**5,239**	**38,169**	**43,505**	**31.85**	**49**	**950**	**2,939**	**3,938**	**2.88**	**8**	**569**	**9,370**	**9,947**	**7.28**
Albay	27,665	12	952	11,195	**12,159**	**43.95**	2	111	468	**581**	**2.10**	0	36	1,001	**1,037**	**3.75**
Camarines Norte	14,894	17	654	4,024	**4,695**	**31.52**	20	183	542	**745**	**5.00**	0	110	1,268	**1,378**	**9.25**
Camarines Sur	43,855	25	1,322	9,296	**10,643**	**24.27**	10	232	704	**946**	**2.16**	3	140	2,605	**2,748**	**6.27**
Catanduanes	5,395	5	229	1,568	**1,802**	**33.40**	1	64	187	**252**	**4.67**	0	35	593	**628**	**11.64**
Masbate	22,495	18	1,262	6,404	**7,684**	**34.16**	10	139	400	**549**	**2.44**	1	107	1,410	**1,518**	**6.75**
Sorsogon	18,065	19	718	4,777	**5,514**	**30.52**	5	200	605	**810**	**4.48**	4	127	2,334	**2,465**	**13.65**
City of Naga	4,242	1	102	905	**1,008**	**23.76**	1	21	33	**55**	**1.30**	0	14	159	**173**	**4.08**
**Region 6**	**147,934**	**131**	**5,544**	**41,384**	**47,059**	**31.81**	**30**	**840**	**2,437**	**3,307**	**2.24**	**7**	**402**	**7,464**	**7,873**	**5.32**
Aklan	11,281	5	347	3,076	**3,428**	**30.39**	1	58	189	**248**	**2.20**	0	28	841	**869**	**7.70**
Antique	11,899	29	446	3,808	**4,283**	**35.99**	7	89	312	**408**	**3.43**	0	20	606	**626**	**5.26**
Capiz	13,872	7	334	3,091	**3,432**	**24.74**	1	27	106	**134**	**0.97**	0	26	383	**409**	**2.95**
Guimaras	3,361	8	146	1,195	**1,349**	**40.14**	2	28	86	**116**	**3.45**	0	26	455	**481**	**14.31**
Iloilo	35,981	23	1,334	11,738	**13,095**	**36.39**	1	108	362	**471**	**1.31**	2	32	655	**689**	**1.91**

Table 2.B.1.2 - Prenatal Care
Pregnant women seen according to their nutritional status
Philippines, 2024

Area	Eligible Population (0-11 months)	Pregnant women seen during the 1st trimester according to their Body Mass Index (BMI)														
		Normal BMI					Low BMI					High BMI				
		Age Group			Total	%	Age Group			Total	%	Age Group			Total	%
		10-14	15-19	20-49			10-14	15-19	20-49			10-14	15-19	20-49		
Negros Occidental	52,445	51	2,571	15,147	**17,769**	**33.88**	17	466	1,126	**1,609**	**3.07**	4	215	3,386	**3,605**	**6.87**
City of Bacolod	10,361	2	118	1,219	**1,339**	**12.92**	0	48	174	**222**	**2.14**	0	39	730	**769**	**7.42**
City of Iloilo	8,734	6	248	2,110	**2,364**	**27.07**	1	16	82	**99**	**1.13**	1	16	408	**425**	**4.87**
**Region 7**	**167,016**	**131**	**7,582**	**51,608**	**59,321**	**35.52**	**24**	**920**	**2,557**	**3,501**	**2.10**	**12**	**789**	**13,498**	**14,299**	**8.56**
Bohol	26,210	11	698	6,889	**7,598**	**28.99**	1	93	320	**414**	**1.58**	0	68	1,728	**1,796**	**6.85**
Cebu	72,875	49	3,764	22,108	**25,921**	**35.57**	8	383	1,139	**1,530**	**2.10**	3	328	5,125	**5,456**	**7.49**
Negros Oriental	28,980	35	1,333	7,469	**8,837**	**30.49**	6	220	516	**742**	**2.56**	6	158	2,381	**2,545**	**8.78**
Siquijor	1,671	1	31	303	**335**	**20.05**	0	10	44	**54**	**3.23**	0	2	294	**296**	**17.71**
City of Cebu	19,466	18	911	5,566	**6,495**	**33.37**	8	134	353	**495**	**2.54**	2	188	2,881	**3,071**	**15.78**
City of Lapu-Lapu	10,833	9	532	5,187	**5,728**	**52.88**	1	29	98	**128**	**1.18**	1	23	492	**516**	**4.76**
City of Mandaue	6,981	8	313	4,086	**4,407**	**63.13**	0	51	87	**138**	**1.98**	0	22	597	**619**	**8.87**
**Region 8**	**93,713**	**94**	**4,640**	**30,280**	**35,014**	**37.36**	**21**	**474**	**1,319**	**1,814**	**1.94**	**10**	**255**	**3,671**	**3,936**	**4.20**
Biliran	3,378	4	200	1,307	**1,511**	**44.73**	2	38	95	**135**	**4.00**	1	31	343	**375**	**11.10**
Eastern Samar	9,594	16	680	3,593	**4,289**	**44.71**	5	56	136	**197**	**2.05**	0	26	280	**306**	**3.19**
Leyte	31,504	33	1,495	10,668	**12,196**	**38.71**	7	110	417	**534**	**1.70**	2	60	824	**886**	**2.81**
Northern Samar	14,053	16	589	3,926	**4,531**	**32.24**	0	65	152	**217**	**1.54**	2	36	464	**502**	**3.57**
Southern Leyte	7,368	4	253	2,303	**2,560**	**34.74**	0	42	165	**207**	**2.81**	1	22	483	**506**	**6.87**
Samar	16,946	14	738	4,421	**5,173**	**30.53**	5	92	208	**305**	**1.80**	3	34	670	**707**	**4.17**
Ormoc City	5,344	5	384	2,098	**2,487**	**46.54**	2	67	115	**184**	**3.44**	1	41	557	**599**	**11.21**
City of Tacloban	5,526	2	301	1,964	**2,267**	**41.02**	0	4	31	**35**	**0.63**	0	5	50	**55**	**1.00**
**Region 9**	**85,348**	**125**	**5,403**	**28,109**	**33,637**	**39.41**	**16**	**448**	**1,017**	**1,481**	**1.74**	**5**	**235**	**2,724**	**2,964**	**3.47**
Zamboanga del Norte	24,977	34	1,425	7,841	**9,300**	**37.23**	7	124	223	**354**	**1.42**	0	35	489	**524**	**2.10**
Zamboanga del Sur	23,905	25	1,348	6,377	**7,750**	**32.42**	2	105	238	**345**	**1.44**	2	33	534	**569**	**2.38**
Zamboanga Sibugay	13,903	26	894	4,353	**5,273**	**37.93**	4	85	198	**287**	**2.06**	1	73	771	**845**	**6.08**
City of Isabela	3,209	2	246	1,307	**1,555**	**48.46**	0	10	24	**34**	**1.06**	0	6	44	**50**	**1.56**
City of Zamboanga	19,354	38	1,490	8,231	**9,759**	**50.42**	3	124	334	**461**	**2.38**	2	88	886	**976**	**5.04**
**Region 10**	**110,290**	**203**	**8,762**	**45,043**	**54,008**	**48.97**	**22**	**685**	**1,310**	**2,017**	**1.83**	**26**	**692**	**7,645**	**8,363**	**7.58**
Bukidnon	33,913	93	3,455	12,382	**15,930**	**46.97**	7	353	547	**907**	**2.67**	11	328	3,100	**3,439**	**10.14**
Camiguin	1,727	2	85	577	**664**	**38.45**	0	7	20	**27**	**1.56**	1	2	43	**46**	**2.66**
Lanao del Norte	17,881	18	637	7,020	**7,675**	**42.92**	1	12	45	**58**	**0.32**	1	6	148	**155**	**0.87**
Misamis Occidental	12,342	17	821	6,069	**6,907**	**55.96**	0	16	45	**61**	**0.49**	0	9	152	**161**	**1.30**
Misamis Oriental	22,184	41	1,666	7,893	**9,600**	**43.27**	6	105	275	**386**	**1.74**	2	49	583	**634**	**2.86**
City of Cagayan De Oro	14,503	26	1,712	8,392	**10,130**	**69.85**	6	166	281	**453**	**3.12**	11	276	3,213	**3,500**	**24.13**
City of Iligan	7,740	6	386	2,710	**3,102**	**40.08**	2	26	97	**125**	**1.61**	0	22	406	**428**	**5.53**
**Region 11**	**107,934**	**396**	**7,282**	**35,661**	**43,339**	**40.15**	**112**	**1,266**	**2,581**	**3,959**	**3.67**	**82**	**1,505**	**17,925**	**19,512**	**18.08**
Davao de Oro	15,434	72	1,311	4,837	**6,220**	**40.30**	20	264	418	**702**	**4.55**	20	397	4,144	**4,561**	**29.55**
Davao del Norte	23,685	89	1,465	7,422	**8,976**	**37.90**	25	296	612	**933**	**3.94**	20	328	4,486	**4,834**	**20.41**
Davao Oriental	12,025	54	898	3,555	**4,507**	**37.48**	8	163	305	**476**	**3.96**	14	196	2,253	**2,463**	**20.48**
Davao del Sur	13,178	60	891	3,535	**4,486**	**34.04**	14	205	417	**636**	**4.83**	13	169	1,967	**2,149**	**16.31**
Davao Occidental	6,606	26	563	1,200	**1,789**	**27.08**	6	73	77	**156**	**2.36**	3	55	481	**539**	**8.16**
City of Davao	37,006	95	2,154	15,112	**17,361**	**46.91**	39	265	752	**1,056**	**2.85**	12	360	4,594	**4,966**	**13.42**
**Region 12**	**95,296**	**349**	**7,912**	**36,096**	**44,357**	**46.55**	**138**	**832**	**1,688**	**2,658**	**2.79**	**101**	**667**	**7,234**	**8,002**	**8.40**
Cotabato	25,696	70	2,037	9,232	**11,339**	**44.13**	11	162	288	**461**	**1.79**	5	132	1,477	**1,614**	**6.28**
Sarangani	13,501	44	1,683	6,135	**7,862**	**58.23**	14	161	223	**398**	**2.95**	6	147	1,355	**1,508**	**11.17**
South Cotabato	21,842	70	1,980	9,397	**11,447**	**52.41**	13	205	462	**680**	**3.11**	5	119	1,906	**2,030**	**9.29**
Sultan Kudarat	19,613	149	1,338	6,123	**7,610**	**38.80**	94	116	267	**477**	**2.43**	85	122	1,356	**1,563**	**7.97**
City of General Santos	14,644	16	874	5,209	**6,099**	**41.65**	6	188	448	**642**	**4.38**	0	147	1,140	**1,287**	**8.79**
**CARAGA**	**55,371**	**82**	**3,785**	**22,064**	**25,931**	**46.83**	**16**	**377**	**855**	**1,248**	**2.25**	**4**	**264**	**3,399**	**3,667**	**6.62**
Agusan del Norte	7,331	11	504	2,631	**3,146**	**42.91**	4	71	96	**171**	**2.33**	1	21	443	**465**	**6.34**
Agusan del Sur	16,180	27	1,316	7,221	**8,564**	**52.93**	3	80	181	**264**	**1.63**	2	69	700	**771**	**4.77**
Surigao del Norte	10,168	12	657	4,197	**4,866**	**47.86**	0	91	177	**268**	**2.64**	0	68	759	**827**	**8.13**
Surigao del Sur	12,834	19	694	4,101	**4,814**	**37.51**	5	82	246	**333**	**2.59**	1	75	1,044	**1,120**	**8.73**
Dinagat Islands	2,117	2	101	715	**818**	**38.64**	2	15	45	**62**	**2.93**	0	9	172	**181**	**8.55**
City of Butuan	6,741	11	513	3,199	**3,723**	**55.23**	2	38	110	**150**	**2.23**	0	22	281	**303**	**4.49**
**BARMM**	**134,585**	**81**	**4,520**	**42,481**	**47,082**	**34.98**	**5**	**349**	**1,252**	**1,606**	**1.19**	**8**	**214**	**3,020**	**3,242**	**2.41**
Basilan	11,653	14	397	2,198	**2,609**	**22.39**	2	85	218	**305**	**2.62**	5	23	111	**139**	**1.19**
Lanao del Sur	33,339	14	1,031	14,560	**15,605**	**46.81**	1	21	253	**275**	**0.82**	0	13	334	**347**	**1.04**
Maguindanao del Norte	18,923	17	712	5,783	**6,512**	**34.41**	1	56	164	**221**	**1.17**	1	54	803	**858**	**4.53**
Maguindanao del Sur	23,214	12	978	6,841	**7,831**	**33.73**	0	41	97	**138**	**0.59**	0	42	452	**494**	**2.13**
Sulu	21,381	6	500	6,934	**7,440**	**34.80**	0	76	262	**338**	**1.58**	0	41	704	**745**	**3.48**
Tawi-Tawi	12,602	6	270	2,653	**2,929**	**23.24**	1	51	208	**260**	**2.06**	1	28	426	**455**	**3.61**
SGA	6,305	3	302	1,254	**1,559**	**24.73**	0	8	13	**21**	**0.33**	0	2	32	**34**	**0.54**
City of Cotabato	7,168	9	330	2,258	**2,597**	**36.23**	0	11	37	**48**	**0.67**	1	11	158	**170**	**2.37**

*Legend: * - No Report*

Table 2.B.1.3 - Prenatal Care
Pregnant Women for the 1st time given at least 2 doses of Tetanus Diphtheria (Td) vaccination
Philippines, 2024

Area	Eligible Population (0-11 months)	Pregnant women for the 1st time given at least two (2) doses of Td Vaccine							
		Age Group						Total	%
		10-14 yrs old		15-19 yrs old		20-49 yrs old			
		No.	%	No.	%	No.	%		
**PHILIPPINES**	**2,200,865**	**2,802**	**0.13**	**98,046**	**4.45**	**388,253**	**17.64**	**489,101**	**22.22**
**N C R**	**263,248**	**174**	**0.07**	**6,456**	**2.45**	**47,269**	**17.96**	**53,899**	**20.47**
City of Malabon	**7,447**	6	**0.08**	142	**1.91**	807	**10.84**	**955**	**12.82**
City of Navotas	**5,263**	2	**0.04**	131	**2.49**	923	**17.54**	**1,056**	**20.06**
City of Valenzuela	**13,601**	25	**0.18**	602	**4.43**	6,523	**47.96**	**7,150**	**52.57**
City of Caloocan	**33,499**	22	**0.07**	956	**2.85**	5,768	**17.22**	**6,746**	**20.14**
City of Marikina	**8,399**	6	**0.07**	181	**2.16**	612	**7.29**	**799**	**9.51**
City of Pasig	**17,856**	16	**0.09**	258	**1.44**	3,417	**19.14**	**3,691**	**20.67**
Pateros	**1,120**	0	**0.00**	30	**2.68**	215	**19.20**	**245**	**21.88**
City of Taguig	**26,021**	10	**0.04**	640	**2.46**	2,906	**11.17**	**3,556**	**13.67**
Quezon City	**59,607**	33	**0.06**	1,174	**1.97**	10,449	**17.53**	**11,656**	**19.55**
City of Makati	**4,196**	3	**0.07**	73	**1.74**	445	**10.61**	**521**	**12.42**
City of Mandaluyong	**7,989**	2	**0.03**	156	**1.95**	1,765	**22.09**	**1,923**	**24.07**
City of San Juan	**2,173**	0	**0.00**	8	**0.37**	181	**8.33**	**189**	**8.70**
City of Manila	**34,268**	26	**0.08**	1,033	**3.01**	5,897	**17.21**	**6,956**	**20.30**
City of Las Piñas	**11,346**	5	**0.04**	247	**2.18**	2,230	**19.65**	**2,482**	**21.88**
City of Muntinlupa	**9,949**	6	**0.06**	330	**3.32**	1,876	**18.86**	**2,212**	**22.23**
City of Parañaque	**13,385**	7	**0.05**	282	**2.11**	1,885	**14.08**	**2,174**	**16.24**
Pasay City	**7,129**	5	**0.07**	213	**2.99**	1,370	**19.22**	**1,588**	**22.28**
**C A R**	**31,490**	**39**	**0.12**	**887**	**2.82**	**4,282**	**13.60**	**5,208**	**16.54**
Abra	**3,639**	2	**0.05**	126	**3.46**	584	**16.05**	**712**	**19.57**
Apayao	**2,222**	13	**0.59**	146	**6.57**	264	**11.88**	**423**	**19.04**
Benguet	**8,900**	4	**0.04**	133	**1.49**	1,026	**11.53**	**1,163**	**13.07**
Ifugao	**4,145**	6	**0.14**	130	**3.14**	757	**18.26**	**893**	**21.54**
Kalinga	**4,582**	7	**0.15**	221	**4.82**	714	**15.58**	**942**	**20.56**
Mountain Province	**2,615**	0	**0.00**	93	**3.56**	341	**13.04**	**434**	**16.60**
City of Baguio	**5,387**	7	**0.13**	38	**0.71**	596	**11.06**	**641**	**11.90**
**Region 1**	**96,024**	**69**	**0.07**	**3,448**	**3.59**	**21,420**	**22.31**	**24,937**	**25.97**
Ilocos Norte	**8,809**	4	**0.05**	252	**2.86**	2,909	**33.02**	**3,165**	**35.93**
Ilocos Sur	**9,834**	3	**0.03**	371	**3.77**	3,004	**30.55**	**3,378**	**34.35**
La Union	**13,436**	18	**0.13**	535	**3.98**	2,670	**19.87**	**3,223**	**23.99**
Pangasinan	**60,591**	40	**0.07**	2,162	**3.57**	12,391	**20.45**	**14,593**	**24.08**
City of Dagupan	**3,354**	4	**0.12**	128	**3.82**	446	**13.30**	**578**	**17.23**
**Region 2**	**64,034**	**90**	**0.14**	**3,130**	**4.89**	**10,538**	**16.46**	**13,758**	**21.49**
Batanes	**241**	0	**0.00**	7	**2.90**	39	**16.18**	**46**	**19.09**
Cagayan	**20,723**	20	**0.10**	902	**4.35**	2,996	**14.46**	**3,918**	**18.91**
Isabela	**27,362**	43	**0.16**	1,377	**5.03**	4,504	**16.46**	**5,924**	**21.65**
Nueva Vizcaya	**8,880**	18	**0.20**	436	**4.91**	1,621	**18.25**	**2,075**	**23.37**
Quirino	**3,865**	4	**0.10**	205	**5.30**	666	**17.23**	**875**	**22.64**
City of Santiago	**2,963**	5	**0.17**	203	**6.85**	712	**24.03**	**920**	**31.05**
			**0.00**		**0.00**		**0.00**		
**Region 3**	**235,313**	**349**	**0.15**	**9,923**	**4.22**	**42,389**	**18.01**	**52,661**	**22.38**
Aurora	**4,578**	8	**0.17**	243	**5.31**	849	**18.55**	**1,100**	**24.03**
Bataan	**17,556**	44	**0.25**	808	**4.60**	2,216	**12.62**	**3,068**	**17.48**
Bulacan	**68,115**	122	**0.18**	3,659	**5.37**	17,091	**25.09**	**20,872**	**30.64**
Nueva Ecija	**43,626**	64	**0.15**	1,926	**4.41**	5,993	**13.74**	**7,983**	**18.30**
Pampanga	**46,146**	66	**0.14**	1,466	**3.18**	7,749	**16.79**	**9,281**	**20.11**
Tarlac	**29,655**	27	**0.09**	1,034	**3.49**	5,126	**17.29**	**6,187**	**20.86**
Zambales	**11,796**	5	**0.04**	522	**4.43**	1,392	**11.80**	**1,919**	**16.27**
City of Angeles	**9,232**	12	**0.13**	171	**1.85**	1,393	**15.09**	**1,576**	**17.07**
City of Olongapo	**4,609**	1	**0.02**	94	**2.04**	580	**12.58**	**675**	**14.65**
**Region 4A**	**310,150**	**239**	**0.08**	**9,389**	**3.03**	**50,728**	**16.36**	**60,356**	**19.46**
Batangas	**53,484**	32	**0.06**	1,600	**2.99**	12,177	**22.77**	**13,809**	**25.82**
Cavite	**78,589**	49	**0.06**	1,860	**2.37**	11,129	**14.16**	**13,038**	**16.59**
Laguna	**61,967**	58	**0.09**	2,282	**3.68**	13,785	**22.25**	**16,125**	**26.02**
Quezon	**40,954**	64	**0.16**	1,982	**4.84**	5,883	**14.36**	**7,929**	**19.36**
Rizal	**68,903**	30	**0.04**	1,423	**2.07**	6,885	**9.99**	**8,338**	**12.10**

Table 2.B.1.3 - Prenatal Care
Pregnant Women for the 1st time given at least 2 doses of Tetanus Diphtheria (Td) vaccination
Philippines, 2024

Area	Eligible Population (0-11 months)	Pregnant women for the 1st time given at least two (2) doses of Td Vaccine							
		Age Group						Total	%
		10-14 yrs old		15-19 yrs old		20-49 yrs old			
		No.	%	No.	%	No.	%		
City of Lucena	6,253	6	0.10	242	3.87	869	13.90	1,117	17.86
**Region 4B**	66,508	83	0.12	4,073	6.12	12,360	18.58	16,516	24.83
Marinduque	4,124	4	0.10	193	4.68	701	17.00	898	21.77
Occidental Mindoro	11,748	30	0.26	920	7.83	1,854	15.78	2,804	23.87
Oriental Mindoro	19,007	13	0.07	803	4.22	3,850	20.26	4,666	24.55
Palawan	20,503	25	0.12	1,463	7.14	3,509	17.11	4,997	24.37
Romblon	5,488	7	0.13	256	4.66	1,048	19.10	1,311	23.89
City of Puerto Princesa	5,638	4	0.07	438	7.77	1,398	24.80	1,840	32.64
**Region 5**	136,611	78	0.06	5,056	3.70	19,025	13.93	24,159	17.68
Albay	27,665	11	0.04	694	2.51	4,507	16.29	5,212	18.84
Camarines Norte	14,894	8	0.05	545	3.66	1,909	12.82	2,462	16.53
Camarines Sur	43,855	15	0.03	1,156	2.64	4,797	10.94	5,968	13.61
Catanduanes	5,395	4	0.07	291	5.39	816	15.13	1,111	20.59
Masbate	22,495	28	0.12	1,371	6.09	3,461	15.39	4,860	21.60
Sorsogon	18,065	9	0.05	891	4.93	2,821	15.62	3,721	20.60
City of Naga	4,242	3	0.07	108	2.55	714	16.83	825	19.45
**Region 6**	147,934	120	0.08	6,159	4.16	24,633	16.65	30,912	20.90
Aklan	11,281	5	0.04	446	3.95	1,953	17.31	2,404	21.31
Antique	11,899	14	0.12	423	3.55	2,226	18.71	2,663	22.38
Capiz	13,872	7	0.05	367	2.65	1,773	12.78	2,147	15.48
Guimaras	3,361	8	0.24	162	4.82	686	20.41	856	25.47
Iloilo	35,981	24	0.07	1,218	3.39	6,690	18.59	7,932	22.04
Negros Occidental	52,445	51	0.10	3,115	5.94	8,429	16.07	11,595	22.11
City of Bacolod	10,361	3	0.03	214	2.07	1,479	14.27	1,696	16.37
City of Iloilo	8,734	8	0.09	214	2.45	1,397	15.99	1,619	18.54
**Region 7**	167,016	183	0.11	8,590	5.14	28,293	16.94	37,066	22.19
Bohol	26,210	22	0.08	1,127	4.30	4,965	18.94	6,114	23.33
Cebu	72,875	59	0.08	3,528	4.84	10,444	14.33	14,031	19.25
Negros Oriental	28,980	49	0.17	1,906	6.58	4,934	17.03	6,889	23.77
Siquijor	1,671	0	0.00	57	3.41	264	15.80	321	19.21
City of Cebu	19,466	26	0.13	1,009	5.18	3,379	17.36	4,414	22.68
City of Lapu-Lapu	10,833	8	0.07	416	3.84	2,402	22.17	2,826	26.09
City of Mandaue	6,981	19	0.27	547	7.84	1,905	27.29	2,471	35.40
**Region 8**	93,713	80	0.09	3,800	4.05	13,982	14.92	17,862	19.06
Biliran	3,378	7	0.21	224	6.63	698	20.66	929	27.50
Eastern Samar	9,594	17	0.18	520	5.42	1,563	16.29	2,100	21.89
Leyte	31,504	28	0.09	1,006	3.19	4,345	13.79	5,379	17.07
Northern Samar	14,053	11	0.08	642	4.57	2,195	15.62	2,848	20.27
Southern Leyte	7,368	5	0.07	261	3.54	1,414	19.19	1,680	22.80
Samar	16,946	6	0.04	570	3.36	1,986	11.72	2,562	15.12
Ormoc City	5,344	5	0.09	311	5.82	829	15.51	1,145	21.43
City of Tacloban	5,526	1	0.02	266	4.81	952	17.23	1,219	22.06
**Region 9**	85,348	111	0.13	5,315	6.23	15,701	18.40	21,127	24.75
Zamboanga del Norte	24,977	32	0.13	1,531	6.13	5,233	20.95	6,796	27.21
Zamboanga del Sur	23,905	27	0.11	1,374	5.75	3,585	15.00	4,986	20.86
Zamboanga Sibugay	13,903	20	0.14	1,009	7.26	2,618	18.83	3,647	26.23
City of Isabela	3,209	0	0.00	159	4.95	815	25.40	974	30.35
City of Zamboanga	19,354	32	0.17	1,242	6.42	3,450	17.83	4,724	24.41
**Region 10**	110,290	206	0.19	8,515	7.72	22,059	20.00	30,780	27.91
Bukidnon	33,913	85	0.25	3,187	9.40	5,077	14.97	8,349	24.62
Camiguin	1,727	1	0.06	73	4.23	186	10.77	260	15.06
Lanao del Norte	17,881	27	0.15	618	3.46	3,769	21.08	4,414	24.69
Misamis Occidental	12,342	3	0.02	526	4.26	2,440	19.77	2,969	24.06
Misamis Oriental	22,184	50	0.23	1,402	6.32	3,562	16.06	5,014	22.60
City of Cagayan De Oro	14,503	34	0.23	2,285	15.76	5,509	37.99	7,828	53.98
City of Iligan	7,740	6	0.08	424	5.48	1,516	19.59	1,946	25.14

Table 2.B.1.3 - Prenatal Care

Pregnant Women for the 1st time given at least 2 doses of Tetanus Diphtheria (Td) vaccination

Philippines, 2024

Area	Eligible Population (0-11 months)	Pregnant women for the 1st time given at least two (2) doses of Td Vaccine							
		Age Group						Total	%
		10-14 yrs old		15-19 yrs old		20-49 yrs old			
		No.	%	No.	%	No.	%		
**Region 11**	**107,934**	**510**	**0.47**	**8,374**	**7.76**	**20,210**	**18.72**	**29,094**	**26.96**
Davao de Oro	**15,434**	109	**0.71**	1,363	**8.83**	2,485	**16.10**	**3,957**	**25.64**
Davao del Norte	**23,685**	117	**0.49**	1,747	**7.38**	3,977	**16.79**	**5,841**	**24.66**
Davao Oriental	**12,025**	58	**0.48**	975	**8.11**	1,727	**14.36**	**2,760**	**22.95**
Davao del Sur	**13,178**	74	**0.56**	1,226	**9.30**	2,005	**15.21**	**3,305**	**25.08**
Davao Occidental	**6,606**	54	**0.82**	849	**12.85**	798	**12.08**	**1,701**	**25.75**
City of Davao	**37,006**	98	**0.26**	2,214	**5.98**	9,218	**24.91**	**11,530**	**31.16**
**Region 12**	**95,296**	**326**	**0.34**	**7,263**	**7.62**	**20,538**	**21.55**	**28,127**	**29.52**
Cotabato	**25,696**	55	**0.21**	1,794	**6.98**	6,126	**23.84**	**7,975**	**31.04**
Sarangani	**13,501**	27	**0.20**	1,410	**10.44**	3,320	**24.59**	**4,757**	**35.23**
South Cotabato	**21,842**	59	**0.27**	1,888	**8.64**	4,925	**22.55**	**6,872**	**31.46**
Sultan Kudarat	**19,613**	163	**0.83**	1,446	**7.37**	3,658	**18.65**	**5,267**	**26.85**
City of General Santos	**14,644**	22	**0.15**	725	**4.95**	2,509	**17.13**	**3,256**	**22.23**
**CARAGA**	**55,371**	**81**	**0.15**	**3,423**	**6.18**	**9,227**	**16.66**	**12,731**	**22.99**
Agusan del Norte	**7,331**	16	**0.22**	459	**6.26**	1,170	**15.96**	**1,645**	**22.44**
Agusan del Sur	**16,180**	23	**0.14**	1,081	**6.68**	2,530	**15.64**	**3,634**	**22.46**
Surigao del Norte	**10,168**	12	**0.12**	574	**5.65**	1,726	**16.97**	**2,312**	**22.74**
Surigao del Sur	**12,834**	20	**0.16**	754	**5.88**	2,253	**17.55**	**3,027**	**23.59**
Dinagat Islands	**2,117**	3	**0.14**	95	**4.49**	331	**15.64**	**429**	**20.26**
City of Butuan	**6,741**	7	**0.10**	460	**6.82**	1,217	**18.05**	**1,684**	**24.98**
**BARMM**	**134,585**	**64**	**0.05**	**4,245**	**3.15**	**25,599**	**19.02**	**29,908**	**22.22**
Basilan	**11,653**	10	**0.09**	354	**3.04**	1,060	**9.10**	**1,424**	**12.22**
Lanao del Sur	**33,339**	7	**0.02**	498	**1.49**	7,548	**22.64**	**8,053**	**24.15**
Maguindanao del Norte	**18,923**	15	**0.08**	870	**4.60**	3,667	**19.38**	**4,552**	**24.06**
Maguindanao del Sur	**23,214**	16	**0.07**	1,161	**5.00**	4,085	**17.60**	**5,262**	**22.67**
Sulu	**21,381**	7	**0.03**	459	**2.15**	4,201	**19.65**	**4,667**	**21.83**
Tawi-Tawi	**12,602**	3	**0.02**	411	**3.26**	1,961	**15.56**	**2,375**	**18.85**
SGA	**6,305**	2	**0.03**	241	**3.82**	1,023	**16.23**	**1,266**	**20.08**
City of Cotabato	**7,168**	4	**0.06**	251	**3.50**	2,054	**28.66**	**2,309**	**32.21**

*Legend: *- No Report*

Table 2.B.1.4 - Prenatal Care
Pregnant Women for the 2nd time or more given at least 3 doses of Td vaccination (Td2 Plus)
Philippines, 2024

Area	Eligible Population (0-11 months)	Pregnant women for the 2nd or more times given at least 3 doses of Td vaccination (Td2 Plus)							
		Age Group						Total	%
		10-14 yrs old		15-19 yrs old		20-49 yrs old			
		No.	%	No.	%	No.	%		
**PHILIPPINES**	**2,200,865**	**814**	**0.04**	**45,705**	**2.08**	**700,230**	**31.82**	**746,749**	**33.93**
**N C R**	**263,248**	**29**	**0.01**	**2,231**	**0.85**	**85,617**	**32.52**	**87,877**	**33.38**
City of Malabon	**7,447**	0	**0.00**	54	**0.73**	1,231	**16.53**	**1,285**	**17.26**
City of Navotas	**5,263**	3	**0.06**	126	**2.39**	1,136	**21.58**	**1,265**	**24.04**
City of Valenzuela	**13,601**	2	**0.01**	172	**1.26**	1,830	**13.45**	**2,004**	**14.73**
City of Caloocan	**33,499**	1	**0.00**	137	**0.41**	7,772	**23.20**	**7,910**	**23.61**
City of Marikina	**8,399**	0	**0.00**	34	**0.40**	1,162	**13.83**	**1,196**	**14.24**
City of Pasig	**17,856**	4	**0.02**	114	**0.64**	4,071	**22.80**	**4,189**	**23.46**
Pateros	**1,120**	2	**0.18**	3	**0.27**	291	**25.98**	**296**	**26.43**
City of Taguig	**26,021**	7	**0.03**	354	**1.36**	5,642	**21.68**	**6,003**	**23.07**
Quezon City	**59,607**	2	**0.00**	207	**0.35**	38,232	**64.14**	**38,441**	**64.49**
City of Makati	**4,196**	0	**0.00**	20	**0.48**	664	**15.82**	**684**	**16.30**
City of Mandaluyong	**7,989**	0	**0.00**	82	**1.03**	3,071	**38.44**	**3,153**	**39.47**
City of San Juan	**2,173**	0	**0.00**	3	**0.14**	142	**6.53**	**145**	**6.67**
City of Manila	**34,268**	3	**0.01**	264	**0.77**	9,026	**26.34**	**9,293**	**27.12**
City of Las Piñas	**11,346**	1	**0.01**	165	**1.45**	3,512	**30.95**	**3,678**	**32.42**
City of Muntinlupa	**9,949**	0	**0.00**	182	**1.83**	2,792	**28.06**	**2,974**	**29.89**
City of Parañaque	**13,385**	2	**0.01**	197	**1.47**	2,884	**21.55**	**3,083**	**23.03**
Pasay City	**7,129**	2	**0.03**	117	**1.64**	2,159	**30.28**	**2,278**	**31.95**
**C A R**	**31,490**	**18**	**0.06**	**555**	**1.76**	**8,284**	**26.31**	**8,857**	**28.13**
Abra	**3,639**	2	**0.05**	141	**3.87**	1,116	**30.67**	**1,259**	**34.60**
Apayao	**2,222**	9	**0.41**	59	**2.66**	673	**30.29**	**741**	**33.35**
Benguet	**8,900**	0	**0.00**	42	**0.47**	1,792	**20.13**	**1,834**	**20.61**
Ifugao	**4,145**	0	**0.00**	57	**1.38**	1,168	**28.18**	**1,225**	**29.55**
Kalinga	**4,582**	5	**0.11**	187	**4.08**	2,106	**45.96**	**2,298**	**50.15**
Mountain Province	**2,615**	0	**0.00**	40	**1.53**	775	**29.64**	**815**	**31.17**
City of Baguio	**5,387**	2	**0.04**	29	**0.54**	654	**12.14**	**685**	**12.72**
**Region 1**	**96,024**	**32**	**0.03**	**2,009**	**2.09**	**37,239**	**38.78**	**39,280**	**40.91**
Ilocos Norte	**8,809**	2	**0.02**	114	**1.29**	3,666	**41.62**	**3,782**	**42.93**
Ilocos Sur	**9,834**	2	**0.02**	166	**1.69**	4,976	**50.60**	**5,144**	**52.31**
La Union	**13,436**	4	**0.03**	289	**2.15**	5,165	**38.44**	**5,458**	**40.62**
Pangasinan	**60,591**	24	**0.04**	1,418	**2.34**	22,686	**37.44**	**24,128**	**39.82**
City of Dagupan	**3,354**	0	**0.00**	22	**0.66**	746	**22.24**	**768**	**22.90**
**Region 2**	**64,034**	**35**	**0.05**	**1,505**	**2.35**	**21,833**	**34.10**	**23,373**	**36.50**
Batanes	**241**	0	**0.00**	1	**0.41**	50	**20.75**	**51**	**21.16**
Cagayan	**20,723**	9	**0.04**	309	**1.49**	6,085	**29.36**	**6,403**	**30.90**
Isabela	**27,362**	17	**0.06**	746	**2.73**	9,719	**35.52**	**10,482**	**38.31**
Nueva Vizcaya	**8,880**	2	**0.02**	149	**1.68**	2,708	**30.50**	**2,859**	**32.20**
Quirino	**3,865**	2	**0.05**	63	**1.63**	1,208	**31.25**	**1,273**	**32.94**
City of Santiago	**2,963**	5	**0.17**	237	**8.00**	2,063	**69.63**	**2,305**	**77.79**
**Region 3**	**235,313**	**111**	**0.05**	**5,814**	**2.47**	**80,313**	**34.13**	**86,238**	**36.65**
Aurora	**4,578**	0	**0.00**	49	**1.07**	1,398	**30.54**	**1,447**	**31.61**
Bataan	**17,556**	4	**0.02**	266	**1.52**	5,284	**30.10**	**5,554**	**31.64**
Bulacan	**68,115**	51	**0.07**	2,140	**3.14**	30,137	**44.24**	**32,328**	**47.46**
Nueva Ecija	**43,626**	17	**0.04**	938	**2.15**	12,583	**28.84**	**13,538**	**31.03**
Pampanga	**46,146**	22	**0.05**	1,176	**2.55**	13,585	**29.44**	**14,783**	**32.04**
Tarlac	**29,655**	4	**0.01**	607	**2.05**	10,422	**35.14**	**11,033**	**37.20**
Zambales	**11,796**	1	**0.01**	207	**1.75**	3,152	**26.72**	**3,360**	**28.48**
City of Angeles	**9,232**	12	**0.13**	398	**4.31**	2,715	**29.41**	**3,125**	**33.85**
City of Olongapo	**4,609**	0	**0.00**	33	**0.72**	1,037	**22.50**	**1,070**	**23.22**
**Region 4A**	**310,150**	**81**	**0.03**	**5,174**	**1.67**	**88,571**	**28.56**	**93,826**	**30.25**
Batangas	**53,484**	7	**0.01**	631	**1.18**	20,443	**38.22**	**21,081**	**39.42**
Cavite	**78,589**	18	**0.02**	1,302	**1.66**	19,200	**24.43**	**20,520**	**26.11**
Laguna	**61,967**	19	**0.03**	1,061	**1.71**	21,373	**34.49**	**22,453**	**36.23**
Quezon	**40,954**	16	**0.04**	887	**2.17**	12,231	**29.87**	**13,134**	**32.07**
Rizal	**68,903**	20	**0.03**	1,133	**1.64**	13,761	**19.97**	**14,914**	**21.64**

Table 2.B.1.4 - Prenatal Care
Pregnant Women for the 2nd time or more given at least 3 doses of Td vaccination (Td2 Plus)
Philippines, 2024

Area	Eligible Population (0-11 months)	Pregnant women for the 2nd or more times given at least 3 doses of Td vaccination (Td2 Plus)							
		Age Group						Total	%
		10-14 yrs old		15-19 yrs old		20-49 yrs old			
		No.	%	No.	%	No.	%		
City of Lucena	**6,253**	1	**0.02**	160	**2.56**	1,563	**25.00**	**1,724**	**27.57**
**Region 4B**	**66,508**	**35**	**0.05**	**1,510**	**2.27**	**21,958**	**33.02**	**23,503**	**35.34**
									**0.00**
Marinduque	**4,124**	2	**0.05**	33	**0.80**	1,350	**32.74**	**1,385**	**33.58**
Occidental Mindoro	**11,748**	7	**0.06**	278	**2.37**	4,151	**35.33**	**4,436**	**37.76**
Oriental Mindoro	**19,007**	22	**0.12**	574	**3.02**	7,027	**36.97**	**7,623**	**40.11**
Palawan	**20,503**	3	**0.01**	453	**2.21**	5,677	**27.69**	**6,133**	**29.91**
Romblon	**5,488**	1	**0.02**	126	**2.30**	2,225	**40.54**	**2,352**	**42.86**
City of Puerto Princesa	**5,638**	0	**0.00**	46	**0.82**	1,528	**27.10**	**1,574**	**27.92**
**Region 5**	**136,611**	**28**	**0.02**	**2,181**	**1.60**	**33,299**	**24.38**	**35,508**	**25.99**
Albay	**27,665**	3	**0.01**	256	**0.93**	7,127	**25.76**	**7,386**	**26.70**
Camarines Norte	**14,894**	1	**0.01**	255	**1.71**	4,025	**27.02**	**4,281**	**28.74**
Camarines Sur	**43,855**	2	**0.00**	280	**0.64**	6,545	**14.92**	**6,827**	**15.57**
Catanduanes	**5,395**	1	**0.02**	152	**2.82**	1,519	**28.16**	**1,672**	**30.99**
Masbate	**22,495**	13	**0.06**	768	**3.41**	6,967	**30.97**	**7,748**	**34.44**
Sorsogon	**18,065**	8	**0.04**	450	**2.49**	6,388	**35.36**	**6,846**	**37.90**
City of Naga	**4,242**	0	**0.00**	20	**0.47**	728	**17.16**	**748**	**17.63**
**Region 6**	**147,934**	**46**	**0.03**	**3,385**	**2.29**	**46,809**	**31.64**	**50,240**	**33.96**
Aklan	**11,281**	3	**0.03**	178	**1.58**	3,142	**27.85**	**3,323**	**29.46**
Antique	**11,899**	2	**0.02**	206	**1.73**	3,754	**31.55**	**3,962**	**33.30**
Capiz	**13,872**	2	**0.01**	249	**1.79**	4,476	**32.27**	**4,727**	**34.08**
Guimaras	**3,361**	3	**0.09**	82	**2.44**	1,326	**39.45**	**1,411**	**41.98**
Iloilo	**35,981**	7	**0.02**	807	**2.24**	11,402	**31.69**	**12,216**	**33.95**
Negros Occidental	**52,445**	25	**0.05**	1,712	**3.26**	18,405	**35.09**	**20,142**	**38.41**
City of Bacolod	**10,361**	0	**0.00**	57	**0.55**	2,192	**21.16**	**2,249**	**21.71**
City of Iloilo	**8,734**	4	**0.05**	94	**1.08**	2,112	**24.18**	**2,210**	**25.30**
**Region 7**	**167,016**	**65**	**0.04**	**4,952**	**2.96**	**64,210**	**38.45**	**69,227**	**41.45**
Bohol	**26,210**	9	**0.03**	640	**2.44**	9,321	**35.56**	**9,970**	**38.04**
Cebu	**72,875**	18	**0.02**	1,733	**2.38**	24,230	**33.25**	**25,981**	**35.65**
Negros Oriental	**28,980**	11	**0.04**	805	**2.78**	10,873	**37.52**	**11,689**	**40.33**
Siquijor	**1,671**	0	**0.00**	22	**1.32**	571	**34.17**	**593**	**35.49**
City of Cebu	**19,466**	15	**0.08**	1,040	**5.34**	9,203	**47.28**	**10,258**	**52.70**
City of Lapu-Lapu	**10,833**	3	**0.03**	259	**2.39**	4,365	**40.29**	**4,627**	**42.71**
City of Mandaue	**6,981**	9	**0.13**	453	**6.49**	5,647	**80.89**	**6,109**	**87.51**
**Region 8**	**93,713**	**17**	**0.02**	**1,452**	**1.55**	**25,305**	**27.00**	**26,774**	**28.57**
Biliran	**3,378**	2	**0.06**	105	**3.11**	1,594	**47.19**	**1,701**	**50.36**
Eastern Samar	**9,594**	1	**0.01**	188	**1.96**	2,828	**29.48**	**3,017**	**31.45**
Leyte	**31,504**	5	**0.02**	547	**1.74**	9,003	**28.58**	**9,555**	**30.33**
Northern Samar	**14,053**	4	**0.03**	169	**1.20**	3,136	**22.32**	**3,309**	**23.55**
Southern Leyte	**7,368**	1	**0.01**	58	**0.79**	1,805	**24.50**	**1,864**	**25.30**
Samar	**16,946**	1	**0.01**	178	**1.05**	3,589	**21.18**	**3,768**	**22.24**
Ormoc City	**5,344**	2	**0.04**	84	**1.57**	1,630	**30.50**	**1,716**	**32.11**
City of Tacloban	**5,526**	1	**0.02**	123	**2.23**	1,720	**31.13**	**1,844**	**33.37**
**Region 9**	**85,348**	**31**	**0.04**	**2,363**	**2.77**	**26,493**	**31.04**	**28,887**	**33.85**
Zamboanga del Norte	**24,977**	11	**0.04**	662	**2.65**	7,662	**30.68**	**8,335**	**33.37**
Zamboanga del Sur	**23,905**	3	**0.01**	373	**1.56**	5,550	**23.22**	**5,926**	**24.79**
Zamboanga Sibugay	**13,903**	7	**0.05**	423	**3.04**	4,513	**32.46**	**4,943**	**35.55**
City of Isabela	**3,209**	0	**0.00**	36	**1.12**	576	**17.95**	**612**	**19.07**
City of Zamboanga	**19,354**	10	**0.05**	869	**4.49**	8,192	**42.33**	**9,071**	**46.87**
**Region 10**	**110,290**	**37**	**0.03**	**4,278**	**3.88**	**42,908**	**38.90**	**47,223**	**42.82**
Bukidnon	**33,913**	17	**0.05**	1,471	**4.34**	12,782	**37.69**	**14,270**	**42.08**
Camiguin	**1,727**	0	**0.00**	43	**2.49**	531	**30.75**	**574**	**33.24**
Lanao del Norte	**17,881**	0	**0.00**	186	**1.04**	5,074	**28.38**	**5,260**	**29.42**
Misamis Occidental	**12,342**	0	**0.00**	244	**1.98**	4,557	**36.92**	**4,801**	**38.90**
Misamis Oriental	**22,184**	7	**0.03**	700	**3.16**	6,711	**30.25**	**7,418**	**33.44**
City of Cagayan De Oro	**14,503**	11	**0.08**	1,371	**9.45**	10,503	**72.42**	**11,885**	**81.95**

Table 2.B.1.4 - Prenatal Care
Pregnant Women for the 2nd time or more given at least 3 doses of Td vaccination (Td2 Plus)
Philippines, 2024

Area	Eligible Population (0-11 months)	Pregnant women for the 2nd or more times given at least 3 doses of Td vaccination (Td2 Plus)							
		Age Group						Total	%
		10-14 yrs old		15-19 yrs old		20-49 yrs old			
		No.	%	No.	%	No.	%		
City of Iligan	**7,740**	2	**0.03**	263	**3.40**	2,750	**35.53**	**3,015**	**38.95**
**Region 11**	**107,934**	**55**	**0.05**	**2,089**	**1.94**	**37,706**	**34.93**	**39,850**	**36.92**
Davao de Oro	**15,434**	6	**0.04**	375	**2.43**	6,240	**40.43**	**6,621**	**42.90**
Davao del Norte	**23,685**	12	**0.05**	410	**1.73**	8,563	**36.15**	**8,985**	**37.94**
Davao Oriental	**12,025**	4	**0.03**	319	**2.65**	4,584	**38.12**	**4,907**	**40.81**
Davao del Sur	**13,178**	7	**0.05**	295	**2.24**	4,910	**37.26**	**5,212**	**39.55**
Davao Occidental	**6,606**	7	**0.11**	206	**3.12**	2,210	**33.45**	**2,423**	**36.68**
City of Davao	**37,006**	19	**0.05**	484	**1.31**	11,199	**30.26**	**11,702**	**31.62**
**Region 12**	**95,296**	**167**	**0.18**	**3,575**	**3.75**	**29,208**	**30.65**	**32,950**	**34.58**
Cotabato	**25,696**	23	**0.09**	816	**3.18**	8,043	**31.30**	**8,882**	**34.57**
Sarangani	**13,501**	7	**0.05**	443	**3.28**	4,228	**31.32**	**4,678**	**34.65**
South Cotabato	**21,842**	24	**0.11**	1,164	**5.33**	9,026	**41.32**	**10,214**	**46.76**
Sultan Kudarat	**19,613**	103	**0.53**	681	**3.47**	3,677	**18.75**	**4,461**	**22.75**
City of General Santos	**14,644**	10	**0.07**	471	**3.22**	4,234	**28.91**	**4,715**	**32.20**
**CARAGA**	**55,371**	**23**	**0.04**	**1,156**	**2.09**	**17,949**	**32.42**	**19,128**	**34.55**
Agusan del Norte	**7,331**	0	**0.00**	131	**1.79**	2,054	**28.02**	**2,185**	**29.80**
Agusan del Sur	**16,180**	4	**0.02**	316	**1.95**	5,169	**31.95**	**5,489**	**33.92**
Surigao del Norte	**10,168**	6	**0.06**	164	**1.61**	3,011	**29.61**	**3,181**	**31.28**
Surigao del Sur	**12,834**	8	**0.06**	366	**2.85**	4,506	**35.11**	**4,880**	**38.02**
Dinagat Islands	**2,117**	1	**0.05**	18	**0.85**	530	**25.04**	**549**	**25.93**
City of Butuan	**6,741**	4	**0.06**	161	**2.39**	2,679	**39.74**	**2,844**	**42.19**
**BARMM**	**134,585**	**4**	**0.00**	**1,476**	**1.10**	**32,528**	**24.17**	**34,008**	**25.27**
Basilan	**11,653**	1	**0.01**	189	**1.62**	1,372	**11.77**	**1,562**	**13.40**
Lanao del Sur	**33,339**	0	**0.00**	358	**1.07**	9,385	**28.15**	**9,743**	**29.22**
Maguindanao del Norte	**18,923**	1	**0.01**	217	**1.15**	5,948	**31.43**	**6,166**	**32.58**
Maguindanao del Sur	**23,214**	1	**0.00**	234	**1.01**	6,304	**27.16**	**6,539**	**28.17**
Sulu	**21,381**	0	**0.00**	177	**0.83**	4,995	**23.36**	**5,172**	**24.19**
Tawi-Tawi	**12,602**	0	**0.00**	139	**1.10**	2,449	**19.43**	**2,588**	**20.54**
SGA	**6,305**	0	**0.00**	53	**0.84**	861	**13.66**	**914**	**14.50**
City of Cotabato	**7,168**	1	**0.01**	109	**1.52**	1,214	**16.94**	**1,324**	**18.47**

*Legend: * - No Report*

Table 2.B.1.5 - Prenatal Care
Pregnant Women who completed Iron with Folic Acid/Multiple Micronutrient Supplementation
Philippines,

Area	Eligible Population (0-11 months)	Iron with Folic Acid/Multiple Micronutrient Supplementation							
		Age Group in Year						Total	%
		10-14		15-19		20-49			
		No.	%	No.	%	No.	%		
PHILIPPINES	2,200,865	2,816	0.13	118,375	5.38	1,010,043	45.89	1,131,234	51.40
N C R	263,248	309	0.12	11,247	4.27	136,382	51.81	147,938	56.20
City of Malabon	7,447	13	0.17	383	5.14	3,024	40.61	3,420	45.92
City of Navotas	5,263	8	0.15	335	6.37	3,443	65.42	3,786	71.94
City of Valenzuela	13,601	12	0.09	362	2.66	4,801	35.30	5,175	38.05
City of Caloocan	33,499	60	0.18	1,952	5.83	20,690	61.76	22,702	67.77
City of Marikina	8,399	2	0.02	215	2.56	2,204	26.24	2,421	28.82
City of Pasig	17,856	48	0.27	626	3.51	8,643	48.40	9,317	52.18
Pateros	1,120	0	0.00	50	4.46	610	54.46	660	58.93
City of Taguig	26,021	24	0.09	1,062	4.08	10,724	41.21	11,810	45.39
Quezon City	59,607	51	0.09	1,634	2.74	31,385	52.65	33,070	55.48
City of Makati	4,196	3	0.07	114	2.72	1,361	32.44	1,478	35.22
City of Mandaluyong	7,989	9	0.11	272	3.40	6,316	79.06	6,597	82.58
City of San Juan	2,173	0	0.00	31	1.43	617	28.39	648	29.82
City of Manila	34,268	33	0.10	2,075	6.06	16,553	48.30	18,661	54.46
City of Las Piñas	11,346	6	0.05	444	3.91	5,568	49.07	6,018	53.04
City of Muntinlupa	9,949	16	0.16	760	7.64	8,768	88.13	9,544	95.93
City of Parañaque	13,385	12	0.09	610	4.56	6,643	49.63	7,265	54.28
Pasay City	7,129	12	0.17	322	4.52	5,032	70.58	5,366	75.27
C A R	31,490	39	0.12	1,565	4.97	18,274	58.03	19,878	63.12
Abra	3,639	5	0.14	213	5.85	2,040	56.06	2,258	62.05
Apayao	2,222	12	0.54	168	7.56	957	43.07	1,137	51.17
Benguet	8,900	3	0.03	369	4.15	5,797	65.13	6,169	69.31
Ifugao	4,145	3	0.07	162	3.91	1,918	46.27	2,083	50.25
Kalinga	4,582	4	0.09	226	4.93	2,541	55.46	2,771	60.48
Mountain Province	2,615	7	0.27	280	10.71	2,716	103.86	3,003	114.84
City of Baguio	5,387	5	0.09	147	2.73	2,305	42.79	2,457	45.61
Region 1	96,024	94	0.10	4,407	4.59	50,876	52.98	55,377	57.67
Ilocos Norte	8,809	4	0.05	359	4.08	5,697	64.67	6,060	68.79
Ilocos Sur	9,834	9	0.09	469	4.77	7,360	74.84	7,838	79.70
La Union	13,436	16	0.12	583	4.34	6,682	49.73	7,281	54.19
Pangasinan	60,591	59	0.10	2,808	4.63	29,408	48.54	32,275	53.27
City of Dagupan	3,354	6	0.18	188	5.61	1,729	51.55	1,923	57.33
Region 2	64,034	100	0.16	3,754	5.86	30,389	47.46	34,243	53.48
Batanes	241	0	0.00	4	1.66	93	38.59	97	40.25
Cagayan	20,723	13	0.06	850	4.10	7,763	37.46	8,626	41.63
Isabela	27,362	56	0.20	1,737	6.35	13,116	47.94	14,909	54.49
Nueva Vizcaya	8,880	22	0.25	623	7.02	4,671	52.60	5,316	59.86
Quirino	3,865	6	0.16	266	6.88	2,183	56.48	2,455	63.52
City of Santiago	2,963	3	0.10	274	9.25	2,563	86.50	2,840	95.85
Region 3	235,313	402	0.17	15,180	6.45	131,412	55.85	146,994	62.47
Aurora	4,578	2	0.04	281	6.14	2,071	45.24	2,354	51.42
Bataan	17,556	34	0.19	948	5.40	8,288	47.21	9,270	52.80
Bulacan	68,115	162	0.24	5,398	7.92	48,496	71.20	54,056	79.36
Nueva Ecija	43,626	58	0.13	2,473	5.67	17,897	41.02	20,428	46.83
Pampanga	46,146	60	0.13	2,359	5.11	21,881	47.42	24,300	52.66
Tarlac	29,655	28	0.09	1,684	5.68	17,640	59.48	19,352	65.26
Zambales	11,796	5	0.04	678	5.75	4,609	39.07	5,292	44.86
City of Angeles	9,232	48	0.52	1,074	11.63	8,305	89.96	9,427	102.11
City of Olongapo	4,609	5	0.11	285	6.18	2,225	48.28	2,515	54.57
Region 4A	310,150	274	0.09	12,817	4.13	126,973	40.94	140,064	45.16
Batangas	53,484	37	0.07	1,949	3.64	25,669	47.99	27,655	51.71
Cavite	78,589	33	0.04	2,218	2.82	24,612	31.32	26,863	34.18
Laguna	61,967	83	0.13	3,078	4.97	31,998	51.64	35,159	56.74
Quezon	40,954	42	0.10	1,839	4.49	14,479	35.35	16,360	39.95
Rizal	68,903	73	0.11	3,391	4.92	28,233	40.97	31,697	46.00
City of Lucena	6,253	6	0.10	342	5.47	1,982	31.70	2,330	37.26
Region 4B	66,508	92	0.14	4,327	6.51	28,549	42.93	32,968	49.57
Marinduque	4,124	3	0.07	184	4.46	1,953	47.36	2,140	51.89
Occidental Mindoro	11,748	25	0.21	1,052	8.95	6,142	52.28	7,219	61.45

Table 2.B.1.5 - Prenatal Care
Pregnant Women who completed Iron with Folic Acid/Multiple Micronutrient Supplementation
Philippines,

Area	Eligible Population (0-11 months)	Iron with Folic Acid/Multiple Micronutrient Supplementation							
		Age Group in Year						Total	%
		10-14		15-19		20-49			
		No.	%	No.	%	No.	%		
Oriental Mindoro	**19,007**	27	**0.14**	957	**5.03**	8,142	**42.84**	**9,126**	**48.01**
Palawan	**20,503**	30	**0.15**	1,613	**7.87**	8,334	**40.65**	**9,977**	**48.66**
Romblon	**5,488**	5	**0.09**	270	**4.92**	2,642	**48.14**	**2,917**	**53.15**
City of Puerto Princesa	**5,638**	2	**0.04**	251	**4.45**	1,336	**23.70**	**1,589**	**28.18**
**Region 5**	**136,611**	**60**	**0.04**	**5,497**	**4.02**	**49,706**	**36.39**	**55,263**	**40.45**
Albay	**27,665**	5	**0.02**	712	**2.57**	11,729	**42.40**	**12,446**	**44.99**
Camarines Norte	**14,894**	5	**0.03**	669	**4.49**	5,301	**35.59**	**5,975**	**40.12**
Camarines Sur	**43,855**	15	**0.03**	1,139	**2.60**	11,483	**26.18**	**12,637**	**28.82**
Catanduanes	**5,395**	2	**0.04**	322	**5.97**	2,533	**46.95**	**2,857**	**52.96**
Masbate	**22,495**	16	**0.07**	1,673	**7.44**	9,762	**43.40**	**11,451**	**50.90**
Sorsogon	**18,065**	14	**0.08**	830	**4.59**	7,568	**41.89**	**8,412**	**46.57**
City of Naga	**4,242**	3	**0.07**	152	**3.58**	1,330	**31.35**	**1,485**	**35.01**
**Region 6**	**147,934**	**109**	**0.07**	**6,687**	**4.52**	**58,259**	**39.38**	**65,055**	**43.98**
Aklan	**11,281**	5	**0.04**	518	**4.59**	4,996	**44.29**	**5,519**	**48.92**
Antique	**11,899**	3	**0.03**	375	**3.15**	4,519	**37.98**	**4,897**	**41.15**
Capiz	**13,872**	11	**0.08**	487	**3.51**	5,189	**37.41**	**5,687**	**41.00**
Guimaras	**3,361**	10	**0.30**	198	**5.89**	1,781	**52.99**	**1,989**	**59.18**
Iloilo	**35,981**	27	**0.08**	1,420	**3.95**	14,122	**39.25**	**15,569**	**43.27**
Negros Occidental	**52,445**	47	**0.09**	3,178	**6.06**	21,406	**40.82**	**24,631**	**46.97**
City of Bacolod	**10,361**	0	**0.00**	134	**1.29**	2,275	**21.96**	**2,409**	**23.25**
City of Iloilo	**8,734**	6	**0.07**	377	**4.32**	3,971	**45.47**	**4,354**	**49.85**
**Region 7**	**167,016**	**183**	**0.11**	**9,228**	**5.53**	**76,833**	**46.00**	**86,244**	**51.64**
Bohol	**26,210**	21	**0.08**	1,016	**3.88**	11,101	**42.35**	**12,138**	**46.31**
Cebu	**72,875**	42	**0.06**	3,897	**5.35**	29,744	**40.82**	**33,683**	**46.22**
Negros Oriental	**28,980**	58	**0.20**	1,783	**6.15**	12,062	**41.62**	**13,903**	**47.97**
Siquijor	**1,671**	1	**0.06**	46	**2.75**	796	**47.64**	**843**	**50.45**
City of Cebu	**19,466**	24	**0.12**	1,227	**6.30**	9,906	**50.89**	**11,157**	**57.32**
City of Lapu-Lapu	**10,833**	13	**0.12**	552	**5.10**	6,530	**60.28**	**7,095**	**65.49**
City of Mandaue	**6,981**	24	**0.34**	707	**10.13**	6,694	**95.89**	**7,425**	**106.36**
**Region 8**	**93,713**	**83**	**0.09**	**4,876**	**5.20**	**38,868**	**41.48**	**43,827**	**46.77**
Biliran	**3,378**	5	**0.15**	186	**5.51**	1,829	**54.14**	**2,020**	**59.80**
Eastern Samar	**9,594**	17	**0.18**	693	**7.22**	4,603	**47.98**	**5,313**	**55.38**
Leyte	**31,504**	29	**0.09**	1,657	**5.26**	13,228	**41.99**	**14,914**	**47.34**
Northern Samar	**14,053**	9	**0.06**	558	**3.97**	4,341	**30.89**	**4,908**	**34.92**
Southern Leyte	**7,368**	2	**0.03**	300	**4.07**	3,448	**46.80**	**3,750**	**50.90**
Samar	**16,946**	10	**0.06**	807	**4.76**	6,594	**38.91**	**7,411**	**43.73**
Ormoc City	**5,344**	6	**0.11**	357	**6.68**	2,516	**47.08**	**2,879**	**53.87**
City of Tacloban	**5,526**	5	**0.09**	318	**5.75**	2,309	**41.78**	**2,632**	**47.63**
**Region 9**	**85,348**	**83**	**0.10**	**5,094**	**5.97**	**31,915**	**37.39**	**37,092**	**43.46**
Zamboanga del Norte	**24,977**	34	**0.14**	1,372	**5.49**	8,894	**35.61**	**10,300**	**41.24**
Zamboanga del Sur	**23,905**	11	**0.05**	1,104	**4.62**	6,509	**27.23**	**7,624**	**31.89**
Zamboanga Sibugay	**13,903**	9	**0.06**	740	**5.32**	4,601	**33.09**	**5,350**	**38.48**
City of Isabela	**3,209**	1	**0.03**	202	**6.29**	1,310	**40.82**	**1,513**	**47.15**
City of Zamboanga	**19,354**	28	**0.14**	1,676	**8.66**	10,601	**54.77**	**12,305**	**63.58**
**Region 10**	**110,290**	**184**	**0.17**	**9,811**	**8.90**	**59,212**	**53.69**	**69,207**	**62.75**
Bukidnon	**33,913**	91	**0.27**	4,007	**11.82**	16,820	**49.60**	**20,918**	**61.68**
Camiguin	**1,727**	1	**0.06**	128	**7.41**	1,041	**60.28**	**1,170**	**67.75**
Lanao del Norte	**17,881**	13	**0.07**	942	**5.27**	10,299	**57.60**	**11,254**	**62.94**
Misamis Occidental	**12,342**	6	**0.05**	881	**7.14**	8,113	**65.73**	**9,000**	**72.92**
Misamis Oriental	**22,184**	31	**0.14**	1,695	**7.64**	9,330	**42.06**	**11,056**	**49.84**
City of Cagayan De Oro	**14,503**	34	**0.23**	1,664	**11.47**	9,566	**65.96**	**11,264**	**77.67**
City of Iligan	**7,740**	8	**0.10**	494	**6.38**	4,043	**52.24**	**4,545**	**58.72**
**Region 11**	**107,934**	**406**	**0.38**	**8,845**	**8.19**	**57,182**	**52.98**	**66,433**	**61.55**
Davao de Oro	**15,434**	53	**0.34**	1,567	**10.15**	8,460	**54.81**	**10,080**	**65.31**
Davao del Norte	**23,685**	125	**0.53**	1,770	**7.47**	11,560	**48.81**	**13,455**	**56.81**
Davao Oriental	**12,025**	59	**0.49**	1,196	**9.95**	6,246	**51.94**	**7,501**	**62.38**
Davao del Sur	**13,178**	62	**0.47**	1,145	**8.69**	5,906	**44.82**	**7,113**	**53.98**
Davao Occidental	**6,606**	39	**0.59**	678	**10.26**	1,957	**29.62**	**2,674**	**40.48**
City of Davao	**37,006**	68	**0.18**	2,489	**6.73**	23,053	**62.30**	**25,610**	**69.20**

Table 2.B.1.5 - Prenatal Care
Pregnant Women who completed Iron with Folic Acid/Multiple Micronutrient Supplementation
Philippines, 2024

Area	Eligible Population (0-11 months)	Iron with Folic Acid/Multiple Micronutrient Supplementation							
		Age Group in Year						Total	%
		10-14		15-19		20-49			
		No.	%	No.	%	No.	%		
**Region 12**	95,296	310	0.33	8,119	8.52	46,874	49.19	55,303	58.03
Cotabato	25,696	51	0.20	2,019	7.86	12,534	48.78	14,604	56.83
Sarangani	13,501	36	0.27	1,608	11.91	7,036	52.11	8,680	64.29
South Cotabato	21,842	55	0.25	1,923	8.80	11,134	50.98	13,112	60.03
Sultan Kudarat	19,613	152	0.77	1,567	7.99	8,738	44.55	10,457	53.32
City of General Santos	14,644	16	0.11	1,002	6.84	7,432	50.75	8,450	57.70
**CARAGA**	55,371	45	0.08	3,079	5.56	23,404	42.27	26,528	47.91
Agusan del Norte	7,331	8	0.11	342	4.67	2,359	32.18	2,709	36.95
Agusan del Sur	16,180	16	0.10	1,092	6.75	7,433	45.94	8,541	52.79
Surigao del Norte	10,168	3	0.03	456	4.48	4,175	41.06	4,634	45.57
Surigao del Sur	12,834	12	0.09	627	4.89	4,701	36.63	5,340	41.61
Dinagat Islands	2,117	1	0.05	76	3.59	675	31.88	752	35.52
City of Butuan	6,741	5	0.07	486	7.21	4,061	60.24	4,552	67.53
**BARMM**	134,585	43	0.03	3,842	2.85	44,935	33.39	48,820	36.27
Basilan	11,653	5	0.04	369	3.17	2,122	18.21	2,496	21.42
Lanao del Sur	33,339	7	0.02	765	2.29	15,205	45.61	15,977	47.92
Maguindanao del Norte	18,923	7	0.04	681	3.60	7,387	39.04	8,075	42.67
Maguindanao del Sur	23,214	7	0.03	790	3.40	6,278	27.04	7,075	30.48
Sulu	21,381	2	0.01	541	2.53	7,807	36.51	8,350	39.05
Tawi-Tawi	12,602	10	0.08	286	2.27	3,038	24.11	3,334	26.46
SGA	6,305	0	0.00	167	2.65	1,122	17.80	1,289	20.44
City of Cotabato	7,168	5	0.07	243	3.39	1,976	27.57	2,224	31.03

*Legend: * - No Report*

Table 2.B.1.6 - Prenatal Care
Pregnant women who completed the dose of Calcium Carbonate Supplementation
Philippines, 2024

Area	Eligible Population (0-11 months)	Calcium Carbonate Supplementation							
		Age Group in Year						Total	%
		10-14		15-19		20-49			
		No.	%	No.	%	No.	%		
PHILIPPINES	2,200,865	2,089	0.09	85,258	3.87	741,260	33.68	828,607	37.65
N C R	263,248	190	0.07	8,342	3.17	94,850	36.03	103,382	39.27
City of Malabon	7,447	3	0.04	200	2.69	1,564	21.00	1,767	23.73
City of Navotas	5,263	5	0.10	292	5.55	3,475	66.03	3,772	71.67
City of Valenzuela	13,601	16	0.12	250	1.84	2,869	21.09	3,135	23.05
City of Caloocan	33,499	43	0.13	1,781	5.32	19,005	56.73	20,829	62.18
City of Marikina	8,399	3	0.04	218	2.60	2,199	26.18	2,420	28.81
City of Pasig	17,856	15	0.08	543	3.04	6,748	37.79	7,306	40.92
Pateros	1,120	0	0.00	42	3.75	598	53.39	640	57.14
City of Taguig	26,021	22	0.08	1,062	4.08	11,025	42.37	12,109	46.54
Quezon City	59,607	7	0.01	284	0.48	6,810	11.42	7,101	11.91
City of Makati	4,196	3	0.07	112	2.67	1,395	33.25	1,510	35.99
City of Mandaluyong	7,989	5	0.06	180	2.25	5,026	62.91	5,211	65.23
City of San Juan	2,173	0	0.00	35	1.61	615	28.30	650	29.91
City of Manila	34,268	32	0.09	1,682	4.91	13,490	39.37	15,204	44.37
City of Las Piñas	11,346	5	0.04	341	3.01	4,239	37.36	4,585	40.41
City of Muntinlupa	9,949	14	0.14	653	6.56	6,462	64.95	7,129	71.66
City of Parañaque	13,385	10	0.07	384	2.87	4,420	33.02	4,814	35.97
Pasay City	7,129	7	0.10	283	3.97	4,910	68.87	5,200	72.94
C A R	31,490	25	0.08	1,411	4.48	16,169	51.35	17,605	55.91
Abra	3,639	2	0.05	156	4.29	1,522	41.82	1,680	46.17
Apayao	2,222	5	0.23	154	6.93	1,039	46.76	1,198	53.92
Benguet	8,900	3	0.03	337	3.79	5,704	64.09	6,044	67.91
Ifugao	4,145	4	0.10	125	3.02	1,334	32.18	1,463	35.30
Kalinga	4,582	3	0.07	279	6.09	2,465	53.80	2,747	59.95
Mountain Province	2,615	1	0.04	213	8.15	2,154	82.37	2,368	90.55
City of Baguio	5,387	7	0.13	147	2.73	1,951	36.22	2,105	39.08
Region 1	96,024	96	0.10	4,316	4.49	48,332	50.33	52,744	54.93
Ilocos Norte	8,809	8	0.09	345	3.92	5,527	62.74	5,880	66.75
Ilocos Sur	9,834	9	0.09	463	4.71	7,147	72.68	7,619	77.48
La Union	13,436	15	0.11	609	4.53	6,661	49.58	7,285	54.22
Pangasinan	60,591	58	0.10	2,710	4.47	27,267	45.00	30,035	49.57
City of Dagupan	3,354	6	0.18	189	5.64	1,730	51.58	1,925	57.39
Region 2	64,034	104	0.16	3,531	5.51	28,659	44.76	32,294	50.43
Batanes	241	0	0.00	0	0.00	42	17.43	42	17.43
Cagayan	20,723	11	0.05	673	3.25	6,195	29.89	6,879	33.20
Isabela	27,362	64	0.23	1,725	6.30	13,112	47.92	14,901	54.46
Nueva Vizcaya	8,880	20	0.23	617	6.95	4,639	52.24	5,276	59.41
Quirino	3,865	6	0.16	244	6.31	2,125	54.98	2,375	61.45
City of Santiago	2,963	3	0.10	272	9.18	2,546	85.93	2,821	95.21
Region 3	235,313	354	0.15	12,225	5.20	107,371	45.63	119,950	50.97
Aurora	4,578	2	0.04	193	4.22	1,525	33.31	1,720	37.57
Bataan	17,556	23	0.13	520	2.96	4,333	24.68	4,876	27.77
Bulacan	68,115	149	0.22	4,868	7.15	43,955	64.53	48,972	71.90
Nueva Ecija	43,626	60	0.14	2,159	4.95	15,622	35.81	17,841	40.90
Pampanga	46,146	78	0.17	1,969	4.27	19,324	41.88	21,371	46.31
Tarlac	29,655	22	0.07	1,237	4.17	12,975	43.75	14,234	48.00
Zambales	11,796	3	0.03	278	2.36	1,738	14.73	2,019	17.12
City of Angeles	9,232	12	0.13	792	8.58	6,534	70.78	7,338	79.48
City of Olongapo	4,609	5	0.11	209	4.53	1,365	29.62	1,579	34.26
Region 4A	310,150	211	0.07	9,329	3.01	90,098	29.05	99,638	32.13
Batangas	53,484	16	0.03	578	1.08	8,068	15.08	8,662	16.20
Cavite	78,589	28	0.04	1,810	2.30	19,887	25.31	21,725	27.64
Laguna	61,967	73	0.12	2,668	4.31	27,878	44.99	30,619	49.41
Quezon	40,954	34	0.08	1,418	3.46	10,877	26.56	12,329	30.10
Rizal	68,903	55	0.08	2,585	3.75	21,871	31.74	24,511	35.57
City of Lucena	6,253	5	0.08	270	4.32	1,517	24.26	1,792	28.66
Region 4B	66,508	56	0.08	2,757	4.15	19,496	29.31	22,309	33.54
Marinduque	4,124	4	0.10	153	3.71	1,591	38.58	1,748	42.39
Occidental Mindoro	11,748	23	0.20	988	8.41	5,645	48.05	6,656	56.66

Table 2.B.1.6 - Prenatal Care
Pregnant women who completed the dose of Calcium Carbonate Supplementation
Philippines, 2024

Area	Eligible Population (0-11 months)	Calcium Carbonate Supplementation							
		Age Group in Year						Total	%
		10-14		15-19		20-49			
		No.	%	No.	%	No.	%		
Oriental Mindoro	**19,007**	13	**0.07**	679	**3.57**	6,363	**33.48**	**7,055**	**37.12**
Palawan	**20,503**	14	**0.07**	668	**3.26**	3,597	**17.54**	**4,279**	**20.87**
Romblon	**5,488**	1	**0.02**	206	**3.75**	1,889	**34.42**	**2,096**	**38.19**
City of Puerto Princesa	**5,638**	1	**0.02**	63	**1.12**	411	**7.29**	**475**	**8.42**
**Region 5**	**136,611**	**47**	**0.03**	**4,341**	**3.18**	**40,383**	**29.56**	**44,771**	**32.77**
Albay	**27,665**	7	**0.03**	675	**2.44**	10,668	**38.56**	**11,350**	**41.03**
Camarines Norte	**14,894**	4	**0.03**	553	**3.71**	4,158	**27.92**	**4,715**	**31.66**
Camarines Sur	**43,855**	13	**0.03**	929	**2.12**	9,283	**21.17**	**10,225**	**23.32**
Catanduanes	**5,395**	1	**0.02**	270	**5.00**	2,213	**41.02**	**2,484**	**46.04**
Masbate	**22,495**	15	**0.07**	1,265	**5.62**	7,508	**33.38**	**8,788**	**39.07**
Sorsogon	**18,065**	7	**0.04**	583	**3.23**	5,691	**31.50**	**6,281**	**34.77**
City of Naga	**4,242**	0	**0.00**	66	**1.56**	862	**20.32**	**928**	**21.88**
**Region 6**	**147,934**	**78**	**0.05**	**4,467**	**3.02**	**39,465**	**26.68**	**44,010**	**29.75**
Aklan	**11,281**	6	**0.05**	398	**3.53**	4,095	**36.30**	**4,499**	**39.88**
Antique	**11,899**	4	**0.03**	211	**1.77**	2,491	**20.93**	**2,706**	**22.74**
Capiz	**13,872**	7	**0.05**	418	**3.01**	4,302	**31.01**	**4,727**	**34.08**
Guimaras	**3,361**	9	**0.27**	170	**5.06**	1,581	**47.04**	**1,760**	**52.37**
Iloilo	**35,981**	18	**0.05**	1,052	**2.92**	10,714	**29.78**	**11,784**	**32.75**
Negros Occidental	**52,445**	34	**0.06**	2,151	**4.10**	14,783	**28.19**	**16,968**	**32.35**
City of Bacolod	**10,361**	0	**0.00**	63	**0.61**	1,424	**13.74**	**1,487**	**14.35**
City of Iloilo	**8,734**	0	**0.00**	4	**0.05**	75	**0.86**	**79**	**0.90**
**Region 7**	**167,016**	**101**	**0.06**	**5,834**	**3.49**	**51,151**	**30.63**	**57,086**	**34.18**
Bohol	**26,210**	13	**0.05**	564	**2.15**	6,215	**23.71**	**6,792**	**25.91**
Cebu	**72,875**	27	**0.04**	2,354	**3.23**	17,726	**24.32**	**20,107**	**27.59**
Negros Oriental	**28,980**	13	**0.04**	460	**1.59**	4,081	**14.08**	**4,554**	**15.71**
Siquijor	**1,671**	0	**0.00**	24	**1.44**	459	**27.47**	**483**	**28.90**
City of Cebu	**19,466**	23	**0.12**	1,182	**6.07**	9,555	**49.09**	**10,760**	**55.28**
City of Lapu-Lapu	**10,833**	11	**0.10**	554	**5.11**	6,350	**58.62**	**6,915**	**63.83**
City of Mandaue	**6,981**	14	**0.20**	696	**9.97**	6,765	**96.91**	**7,475**	**107.08**
**Region 8**	**93,713**	**55**	**0.06**	**3,038**	**3.24**	**24,747**	**26.41**	**27,840**	**29.71**
Biliran	**3,378**	2	**0.06**	177	**5.24**	1,586	**46.95**	**1,765**	**52.25**
Eastern Samar	**9,594**	16	**0.17**	538	**5.61**	3,637	**37.91**	**4,191**	**43.68**
Leyte	**31,504**	21	**0.07**	733	**2.33**	6,244	**19.82**	**6,998**	**22.21**
Northern Samar	**14,053**	5	**0.04**	238	**1.69**	2,066	**14.70**	**2,309**	**16.43**
Southern Leyte	**7,368**	1	**0.01**	262	**3.56**	3,046	**41.34**	**3,309**	**44.91**
Samar	**16,946**	4	**0.02**	570	**3.36**	4,736	**27.95**	**5,310**	**31.33**
Ormoc City	**5,344**	2	**0.04**	276	**5.16**	1,802	**33.72**	**2,080**	**38.92**
City of Tacloban	**5,526**	4	**0.07**	244	**4.42**	1,630	**29.50**	**1,878**	**33.98**
**Region 9**	**85,348**	**34**	**0.04**	**2,563**	**3.00**	**16,293**	**19.09**	**18,890**	**22.13**
Zamboanga del Norte	**24,977**	10	**0.04**	704	**2.82**	4,547	**18.20**	**5,261**	**21.06**
Zamboanga del Sur	**23,905**	9	**0.04**	653	**2.73**	3,695	**15.46**	**4,357**	**18.23**
Zamboanga Sibugay	**13,903**	3	**0.02**	327	**2.35**	2,033	**14.62**	**2,363**	**17.00**
City of Isabela	**3,209**	1	**0.03**	24	**0.75**	101	**3.15**	**126**	**3.93**
City of Zamboanga	**19,354**	11	**0.06**	855	**4.42**	5,917	**30.57**	**6,783**	**35.05**
**Region 10**	**110,290**	**162**	**0.15**	**7,204**	**6.53**	**44,614**	**40.45**	**51,980**	**47.13**
Bukidnon	**33,913**	67	**0.20**	2,519	**7.43**	11,002	**32.44**	**13,588**	**40.07**
Camiguin	**1,727**	0	**0.00**	79	**4.57**	700	**40.53**	**779**	**45.11**
Lanao del Norte	**17,881**	13	**0.07**	784	**4.38**	7,649	**42.78**	**8,446**	**47.23**
Misamis Occidental	**12,342**	3	**0.02**	682	**5.53**	5,740	**46.51**	**6,425**	**52.06**
Misamis Oriental	**22,184**	22	**0.10**	1,186	**5.35**	7,299	**32.90**	**8,507**	**38.35**
City of Cagayan De Oro	**14,503**	34	**0.23**	1,485	**10.24**	8,488	**58.53**	**10,007**	**69.00**
City of Iligan	**7,740**	23	**0.30**	469	**6.06**	3,736	**48.27**	**4,228**	**54.63**
**Region 11**	**107,934**	**264**	**0.24**	**6,960**	**6.45**	**48,823**	**45.23**	**56,047**	**51.93**
Davao de Oro	**15,434**	62	**0.40**	1,400	**9.07**	7,526	**48.76**	**8,988**	**58.24**
Davao del Norte	**23,685**	60	**0.25**	1,439	**6.08**	10,166	**42.92**	**11,665**	**49.25**
Davao Oriental	**12,025**	39	**0.32**	874	**7.27**	4,908	**40.81**	**5,821**	**48.41**
Davao del Sur	**13,178**	36	**0.27**	870	**6.60**	4,571	**34.69**	**5,477**	**41.56**
Davao Occidental	**6,606**	12	**0.18**	197	**2.98**	599	**9.07**	**808**	**12.23**
City of Davao	**37,006**	55	**0.15**	2,180	**5.89**	21,053	**56.89**	**23,288**	**62.93**

Table 2.B.1.6 - Prenatal Care

Pregnant women who completed the dose of Calcium Carbonate Supplementation

Philippines, 2024

Area	Eligible Population (0-11 months)	Calcium Carbonate Supplementation							
		Age Group in Year						Total	%
		10-14		15-19		20-49			
		No.	%	No.	%	No.	%		
**Region 12**	**95,296**	**253**	**0.27**	**5,050**	**5.30**	**29,701**	**31.17**	**35,004**	**36.73**
Cotabato	**25,696**	47	**0.18**	1,369	**5.33**	8,485	**33.02**	**9,901**	**38.53**
Sarangani	**13,501**	18	**0.13**	880	**6.52**	3,794	**28.10**	**4,692**	**34.75**
South Cotabato	**21,842**	34	**0.16**	952	**4.36**	5,715	**26.17**	**6,701**	**30.68**
Sultan Kudarat	**19,613**	150	**0.76**	1,226	**6.25**	6,903	**35.20**	**8,279**	**42.21**
City of General Santos	**14,644**	4	**0.03**	623	**4.25**	4,804	**32.81**	**5,431**	**37.09**
**CARAGA**	**55,371**	**33**	**0.06**	**2,154**	**3.89**	**16,569**	**29.92**	**18,756**	**33.87**
Agusan del Norte	**7,331**	6	**0.08**	235	**3.21**	1,551	**21.16**	**1,792**	**24.44**
Agusan del Sur	**16,180**	11	**0.07**	818	**5.06**	5,792	**35.80**	**6,621**	**40.92**
Surigao del Norte	**10,168**	2	**0.02**	364	**3.58**	3,300	**32.45**	**3,666**	**36.05**
Surigao del Sur	**12,834**	11	**0.09**	406	**3.16**	2,921	**22.76**	**3,338**	**26.01**
Dinagat Islands	**2,117**	1	**0.05**	59	**2.79**	555	**26.22**	**615**	**29.05**
City of Butuan	**6,741**	2	**0.03**	272	**4.04**	2,450	**36.34**	**2,724**	**40.41**
**BARMM**	**134,585**	**26**	**0.02**	**1,736**	**1.29**	**24,539**	**18.23**	**26,301**	**19.54**
Basilan	**11,653**	2	**0.02**	111	**0.95**	605	**5.19**	**718**	**6.16**
Lanao del Sur	**33,339**	9	**0.03**	370	**1.11**	10,067	**30.20**	**10,446**	**31.33**
Maguindanao del Norte	**18,923**	4	**0.02**	398	**2.10**	5,007	**26.46**	**5,409**	**28.58**
Maguindanao del Sur	**23,214**	8	**0.03**	410	**1.77**	3,683	**15.87**	**4,101**	**17.67**
Sulu	**21,381**	0	**0.00**	112	**0.52**	1,948	**9.11**	**2,060**	**9.63**
Tawi-Tawi	**12,602**	0	**0.00**	75	**0.60**	819	**6.50**	**894**	**7.09**
SGA	**6,305**	0	**0.00**	82	**1.30**	604	**9.58**	**686**	**10.88**
City of Cotabato	**7,168**	3	**0.04**	178	**2.48**	1,806	**25.20**	**1,987**	**27.72**

*Legend: * - No Report*

Table 2.B.1.7 - Prenatal Care
Pregnant Women given 1 dose of Deworming tablet
Philippines, 2024

Area	Eligible Population (0-11 months)	Pregnant women given one (1) dose of Deworming Tablet							
		Age Group						Total	%
		10-14 yrs old		15-19 yrs old		20-49 yrs old			
		No.	%	No.	%	No.	%		
PHILIPPINES	2,200,865	1,317	0.06	34,689	1.58	247,108	11.23	283,114	12.86
N C R	263,248	28	0.01	1,684	0.64	21,765	8.27	23,477	8.92
City of Malabon	7,447	0	**0.00**	2	**0.03**	23	**0.31**	**25**	**0.34**
City of Navotas	5,263	0	**0.00**	0	**0.00**	0	**0.00**	**0**	**0.00**
City of Valenzuela	13,601	0	**0.00**	11	**0.08**	182	**1.34**	**193**	**1.42**
City of Caloocan	33,499	0	**0.00**	0	**0.00**	0	**0.00**	**0**	**0.00**
City of Marikina	8,399	7	**0.08**	214	**2.55**	2,226	**26.50**	**2,447**	**29.13**
City of Pasig	17,856	1	**0.01**	11	**0.06**	85	**0.48**	**97**	**0.54**
Pateros	1,120	0	**0.00**	6	**0.54**	56	**5.00**	**62**	**5.54**
City of Taguig	26,021	9	**0.03**	761	**2.92**	7,580	**29.13**	**8,350**	**32.09**
Quezon City	59,607	7	**0.01**	406	**0.68**	8,120	**13.62**	**8,533**	**14.32**
City of Makati	4,196	0	**0.00**	14	**0.33**	119	**2.84**	**133**	**3.17**
City of Mandaluyong	7,989	0	**0.00**	0	**0.00**	0	**0.00**	**0**	**0.00**
City of San Juan	2,173	0	**0.00**	2	**0.09**	94	**4.33**	**96**	**4.42**
City of Manila	34,268	0	**0.00**	0	**0.00**	6	**0.02**	**6**	**0.02**
City of Las Piñas	11,346	1	**0.01**	107	**0.94**	1,564	**13.78**	**1,672**	**14.74**
City of Muntinlupa	9,949	0	**0.00**	3	**0.03**	34	**0.34**	**37**	**0.37**
City of Parañaque	13,385	0	**0.00**	33	**0.25**	447	**3.34**	**480**	**3.59**
Pasay City	7,129	3	**0.04**	114	**1.60**	1,229	**17.24**	**1,346**	**18.88**
C A R	31,490	5	0.02	415	1.32	4,386	13.93	4,806	15.26
Abra	3,639	1	**0.03**	39	**1.07**	431	**11.84**	**471**	**12.94**
Apayao	2,222	1	**0.05**	161	**7.25**	807	**36.32**	**969**	**43.61**
Benguet	8,900	0	**0.00**	11	**0.12**	261	**2.93**	**272**	**3.06**
Ifugao	4,145	3	**0.07**	70	**1.69**	1,091	**26.32**	**1,164**	**28.08**
Kalinga	4,582	0	**0.00**	77	**1.68**	897	**19.58**	**974**	**21.26**
Mountain Province	2,615	0	**0.00**	9	**0.34**	161	**6.16**	**170**	**6.50**
City of Baguio	5,387	0	**0.00**	48	**0.89**	738	**13.70**	**786**	**14.59**
Region 1	96,024	30	0.03	991	1.03	11,033	11.49	12,054	12.55
Ilocos Norte	8,809	1	**0.01**	131	**1.49**	2,469	**28.03**	**2,601**	**29.53**
Ilocos Sur	9,834	2	**0.02**	59	**0.60**	1,024	**10.41**	**1,085**	**11.03**
La Union	13,436	4	**0.03**	130	**0.97**	1,761	**13.11**	**1,895**	**14.10**
Pangasinan	60,591	23	**0.04**	650	**1.07**	5,669	**9.36**	**6,342**	**10.47**
City of Dagupan	3,354	0	**0.00**	21	**0.63**	110	**3.28**	**131**	**3.91**
Region 2	64,034	19	0.03	939	1.47	7,141	11.15	8,099	12.65
Batanes	241	0	**0.00**	1	**0.41**	35	**14.52**	**36**	**14.94**
Cagayan	20,723	4	**0.02**	283	**1.37**	2,026	**9.78**	**2,313**	**11.16**
Isabela	27,362	10	**0.04**	477	**1.74**	3,509	**12.82**	**3,996**	**14.60**
Nueva Vizcaya	8,880	2	**0.02**	115	**1.30**	899	**10.12**	**1,016**	**11.44**
Quirino	3,865	2	**0.05**	2	**0.05**	13	**0.34**	**17**	**0.44**
City of Santiago	2,963	1	**0.03**	61	**2.06**	659	**22.24**	**721**	**24.33**
Region 3	235,313	475	0.20	902	0.38	3,640	1.55	5,017	2.13
Aurora	4,578	0	**0.00**	0	**0.00**	0	**0.00**	**0**	**0.00**
Bataan	17,556	0	**0.00**	0	**0.00**	0	**0.00**	**0**	**0.00**
Bulacan	68,115	467	**0.69**	540	**0.79**	549	**0.81**	**1,556**	**2.28**
Nueva Ecija	43,626	4	**0.01**	68	**0.16**	318	**0.73**	**390**	**0.89**
Pampanga	46,146	0	**0.00**	60	**0.13**	312	**0.68**	**372**	**0.81**
Tarlac	29,655	2	**0.01**	157	**0.53**	1,823	**6.15**	**1,982**	**6.68**
Zambales	11,796	2	**0.02**	20	**0.17**	200	**1.70**	**222**	**1.88**
City of Angeles	9,232	0	**0.00**	0	**0.00**	0	**0.00**	**0**	**0.00**
City of Olongapo	4,609	0	**0.00**	57	**1.24**	438	**9.50**	**495**	**10.74**
Region 4A	310,150	35	0.01	1,403	0.45	13,367	4.31	14,805	4.77
Batangas	53,484	0	**0.00**	70	**0.13**	1,084	**2.03**	**1,154**	**2.16**
Cavite	78,589	4	**0.01**	172	**0.22**	1,409	**1.79**	**1,585**	**2.02**
Laguna	61,967	19	**0.03**	715	**1.15**	7,835	**12.64**	**8,569**	**13.83**
Quezon	40,954	8	**0.02**	246	**0.60**	1,591	**3.88**	**1,845**	**4.51**
Rizal	68,903	4	**0.01**	200	**0.29**	1,444	**2.10**	**1,648**	**2.39**
City of Lucena	6,253	0	**0.00**	0	**0.00**	4	**0.06**	**4**	**0.06**
Region 4B	66,508	20	0.03	834	1.25	5,684	8.55	6,538	9.83
Marinduque	4,124	1	**0.02**	75	**1.82**	658	**15.96**	**734**	**17.80**
Occidental Mindoro	11,748	3	**0.03**	109	**0.93**	628	**5.35**	**740**	**6.30**
Oriental Mindoro	19,007	3	**0.02**	204	**1.07**	1,649	**8.68**	**1,856**	**9.76**
Palawan	20,503	12	**0.06**	385	**1.88**	2,136	**10.42**	**2,533**	**12.35**

Table 2.B.1.7 - Prenatal Care
Pregnant Women given 1 dose of Deworming tablet
Philippines, 2024

Area	Eligible Population (0-11 months)	Pregnant women given one (1) dose of Deworming Tablet							
		Age Group						Total	%
		10-14 yrs old		15-19 yrs old		20-49 yrs old			
		No.	%	No.	%	No.	%		
Romblon	5,488	1	**0.02**	61	**1.11**	612	**11.15**	**674**	**12.28**
City of Puerto Princesa	5,638	0	**0.00**	0	**0.00**	1	**0.02**	**1**	**0.02**
**Region 5**	**136,611**	**37**	**0.03**	**3,177**	**2.33**	**24,907**	**18.23**	**28,121**	**20.58**
Albay	27,665	1	**0.00**	205	**0.74**	3,067	**11.09**	**3,273**	**11.83**
Camarines Norte	14,894	6	**0.04**	522	**3.50**	4,014	**26.95**	**4,542**	**30.50**
Camarines Sur	43,855	13	**0.03**	937	**2.14**	7,211	**16.44**	**8,161**	**18.61**
Catanduanes	5,395	3	**0.06**	233	**4.32**	1,854	**34.37**	**2,090**	**38.74**
Masbate	22,495	10	**0.04**	707	**3.14**	4,096	**18.21**	**4,813**	**21.40**
Sorsogon	18,065	4	**0.02**	509	**2.82**	4,077	**22.57**	**4,590**	**25.41**
City of Naga	4,242	0	**0.00**	64	**1.51**	588	**13.86**	**652**	**15.37**
**Region 6**	**147,934**	**25**	**0.02**	**2,174**	**1.47**	**16,997**	**11.49**	**19,196**	**12.98**
Aklan	11,281	0	**0.00**	152	**1.35**	1,156	**10.25**	**1,308**	**11.59**
Antique	11,899	3	**0.03**	191	**1.61**	1,596	**13.41**	**1,790**	**15.04**
Capiz	13,872	5	**0.04**	294	**2.12**	2,641	**19.04**	**2,940**	**21.19**
Guimaras	3,361	3	**0.09**	91	**2.71**	804	**23.92**	**898**	**26.72**
Iloilo	35,981	1	**0.00**	409	**1.14**	3,448	**9.58**	**3,858**	**10.72**
Negros Occidental	52,445	10	**0.02**	863	**1.65**	5,144	**9.81**	**6,017**	**11.47**
City of Bacolod	10,361	3	**0.03**	174	**1.68**	2,208	**21.31**	**2,385**	**23.02**
City of Iloilo	8,734	0	**0.00**	0	**0.00**	0	**0.00**	**0**	**0.00**
**Region 7**	**167,016**	**62**	**0.04**	**3,537**	**2.12**	**31,211**	**18.69**	**34,810**	**20.84**
Bohol	26,210	12	**0.05**	452	**1.72**	4,935	**18.83**	**5,399**	**20.60**
Cebu	72,875	21	**0.03**	1,321	**1.81**	10,277	**14.10**	**11,619**	**15.94**
Negros Oriental	28,980	4	**0.01**	352	**1.21**	2,295	**7.92**	**2,651**	**9.15**
Siquijor	1,671	0	**0.00**	44	**2.63**	509	**30.46**	**553**	**33.09**
City of Cebu	19,466	20	**0.10**	971	**4.99**	8,870	**45.57**	**9,861**	**50.66**
City of Lapu-Lapu	10,833	0	**0.00**	113	**1.04**	1,171	**10.81**	**1,284**	**11.85**
City of Mandaue	6,981	5	**0.07**	284	**4.07**	3,154	**45.18**	**3,443**	**49.32**
**Region 8**	**93,713**	**25**	**0.03**	**1,247**	**1.33**	**9,533**	**10.17**	**10,805**	**11.53**
Biliran	3,378	6	**0.18**	194	**5.74**	1,498	**44.35**	**1,698**	**50.27**
Eastern Samar	9,594	5	**0.05**	122	**1.27**	883	**9.20**	**1,010**	**10.53**
Leyte	31,504	2	**0.01**	199	**0.63**	1,696	**5.38**	**1,897**	**6.02**
Northern Samar	14,053	7	**0.05**	103	**0.73**	771	**5.49**	**881**	**6.27**
Southern Leyte	7,368	4	**0.05**	161	**2.19**	1,423	**19.31**	**1,588**	**21.55**
Samar	16,946	1	**0.01**	157	**0.93**	1,381	**8.15**	**1,539**	**9.08**
Ormoc City	5,344	0	**0.00**	291	**5.45**	1,676	**31.36**	**1,967**	**36.81**
City of Tacloban	5,526	0	**0.00**	20	**0.36**	205	**3.71**	**225**	**4.07**
**Region 9**	**85,348**	**26**	**0.03**	**1,694**	**1.98**	**9,596**	**11.24**	**11,316**	**13.26**
Zamboanga del Norte	24,977	10	**0.04**	506	**2.03**	3,047	**12.20**	**3,563**	**14.27**
Zamboanga del Sur	23,905	3	**0.01**	180	**0.75**	916	**3.83**	**1,099**	**4.60**
Zamboanga Sibugay	13,903	1	**0.01**	218	**1.57**	1,017	**7.31**	**1,236**	**8.89**
City of Isabela	3,209	0	**0.00**	63	**1.96**	535	**16.67**	**598**	**18.64**
City of Zamboanga	19,354	12	**0.06**	727	**3.76**	4,081	**21.09**	**4,820**	**24.90**
**Region 10**	**110,290**	**62**	**0.06**	**3,785**	**3.43**	**21,116**	**19.15**	**24,963**	**22.63**
Bukidnon	33,913	36	**0.11**	1,642	**4.84**	6,961	**20.53**	**8,639**	**25.47**
Camiguin	1,727	1	**0.06**	77	**4.46**	592	**34.28**	**670**	**38.80**
Lanao del Norte	17,881	4	**0.02**	212	**1.19**	2,164	**12.10**	**2,380**	**13.31**
Misamis Occidental	12,342	1	**0.01**	71	**0.58**	443	**3.59**	**515**	**4.17**
Misamis Oriental	22,184	4	**0.02**	184	**0.83**	1,075	**4.85**	**1,263**	**5.69**
City of Cagayan De Oro	14,503	10	**0.07**	1,222	**8.43**	6,678	**46.05**	**7,910**	**54.54**
City of Iligan	7,740	6	**0.08**	377	**4.87**	3,203	**41.38**	**3,586**	**46.33**
**Region 11**	**107,934**	**270**	**0.25**	**5,280**	**4.89**	**26,875**	**24.90**	**32,425**	**30.04**
Davao de Oro	15,434	52	**0.34**	1,072	**6.95**	5,441	**35.25**	**6,565**	**42.54**
Davao del Norte	23,685	76	**0.32**	1,263	**5.33**	7,073	**29.86**	**8,412**	**35.52**
Davao Oriental	12,025	48	**0.40**	798	**6.64**	3,714	**30.89**	**4,560**	**37.92**
Davao del Sur	13,178	21	**0.16**	425	**3.23**	1,770	**13.43**	**2,216**	**16.82**
Davao Occidental	6,606	35	**0.53**	774	**11.72**	2,066	**31.27**	**2,875**	**43.52**
City of Davao	37,006	38	**0.10**	948	**2.56**	6,811	**18.41**	**7,797**	**21.07**
**Region 12**	**95,296**	**172**	**0.18**	**5,119**	**5.37**	**24,980**	**26.21**	**30,271**	**31.77**
Cotabato	25,696	26	**0.10**	774	**3.01**	3,847	**14.97**	**4,647**	**18.08**
Sarangani	13,501	43	**0.32**	1,582	**11.72**	6,263	**46.39**	**7,888**	**58.43**
South Cotabato	21,842	30	**0.14**	968	**4.43**	5,584	**25.57**	**6,582**	**30.13**

Table 2.B.1.7 - Prenatal Care
Pregnant Women given 1 dose of Deworming tablet
Philippines, 2024

Area	Eligible Population (0-11 months)	Pregnant women given one (1) dose of Deworming Tablet							
		Age Group						Total	%
		10-14 yrs old		15-19 yrs old		20-49 yrs old			
		No.	%	No.	%	No.	%		
Sultan Kudarat	19,613	71	**0.36**	1,683	**8.58**	8,432	**42.99**	**10,186**	**51.93**
City of General Santos	14,644	2	**0.01**	112	**0.76**	854	**5.83**	**968**	**6.61**
**CARAGA**	**55,371**	**10**	**0.02**	**493**	**0.89**	**3,213**	**5.80**	**3,716**	**6.71**
Agusan del Norte	7,331	0	**0.00**	5	**0.07**	32	**0.44**	**37**	**0.50**
Agusan del Sur	16,180	2	**0.01**	126	**0.78**	793	**4.90**	**921**	**5.69**
Surigao del Norte	10,168	4	**0.04**	146	**1.44**	1,087	**10.69**	**1,237**	**12.17**
Surigao del Sur	12,834	4	**0.03**	210	**1.64**	1,249	**9.73**	**1,463**	**11.40**
Dinagat Islands	2,117	0	**0.00**	6	**0.28**	52	**2.46**	**58**	**2.74**
City of Butuan	6,741	0	**0.00**	0	**0.00**	0	**0.00**	**0**	**0.00**
**BARMM**	**134,585**	**16**	**0.01**	**1,015**	**0.75**	**11,664**	**8.67**	**12,695**	**9.43**
Basilan	11,653	0	**0.00**	48	**0.41**	202	**1.73**	**250**	**2.15**
Lanao del Sur	33,339	1	**0.00**	86	**0.26**	2,337	**7.01**	**2,424**	**7.27**
Maguindanao del Norte	18,923	4	**0.02**	134	**0.71**	1,322	**6.99**	**1,460**	**7.72**
Maguindanao del Sur	23,214	7	**0.03**	376	**1.62**	3,728	**16.06**	**4,111**	**17.71**
Sulu	21,381	3	**0.01**	126	**0.59**	2,222	**10.39**	**2,351**	**11.00**
Tawi-Tawi	12,602	1	**0.01**	137	**1.09**	1,032	**8.19**	**1,170**	**9.28**
SGA	6,305	0	**0.00**	17	**0.27**	165	**2.62**	**182**	**2.89**
City of Cotabato	7,168	0	**0.00**	91	**1.27**	656	**9.15**	**747**	**10.42**

*Legend: * - No Report*

Table 2.B.1.8 - Prenatal Care
Pregnant women screened for Hepatitis B
Philippines, 2024

Area	Eligible Population (0-11 months)	Total No. of pregnant women Screened for Hepatitis B							
		Age Group						Total	%
		10-14 yrs old		15-19 yrs old		20-49 yrs old			
		No.	%	No.	%	No.	%		
**PHILIPPINES**	**2,200,865**	**2,651**	**0.12**	**83,901**	**3.81**	**682,665**	**31.02**	**769,217**	**34.95**
**N C R**	**263,248**	**309**	**0.12**	**10,231**	**3.89**	**118,967**	**45.19**	**129,507**	**49.20**
City of Malabon	7,447	10	0.13	230	3.09	1,939	26.04	**2,179**	**29.26**
City of Navotas	5,263	18	0.34	376	7.14	3,101	58.92	**3,495**	**66.41**
City of Valenzuela	13,601	17	0.12	470	3.46	4,908	36.09	**5,395**	**39.67**
City of Caloocan	33,499	52	0.16	1,782	5.32	16,359	48.83	**18,193**	**54.31**
City of Marikina	8,399	1	0.01	159	1.89	1,957	23.30	**2,117**	**25.21**
City of Pasig	17,856	29	0.16	623	3.49	9,088	50.90	**9,740**	**54.55**
Pateros	1,120	0	0.00	32	2.86	246	21.96	**278**	**24.82**
City of Taguig	26,021	16	0.06	956	3.67	8,919	34.28	**9,891**	**38.01**
Quezon City	59,607	58	0.10	1,905	3.20	32,971	55.31	**34,934**	**58.61**
City of Makati	4,196	3	0.07	108	2.57	1,393	33.20	**1,504**	**35.84**
City of Mandaluyong	7,989	8	0.10	284	3.55	5,878	73.58	**6,170**	**77.23**
City of San Juan	2,173	1	0.05	50	2.30	694	31.94	**745**	**34.28**
City of Manila	34,268	61	0.18	1,883	5.49	15,722	45.88	**17,666**	**51.55**
City of Las Piñas	11,346	5	0.04	259	2.28	3,770	33.23	**4,034**	**35.55**
City of Muntinlupa	9,949	7	0.07	305	3.07	2,874	28.89	**3,186**	**32.02**
City of Parañaque	13,385	12	0.09	512	3.83	5,298	39.58	**5,822**	**43.50**
Pasay City	7,129	11	0.15	297	4.17	3,850	54.00	**4,158**	**58.33**
**C A R**	**31,490**	**59**	**0.19**	**2,244**	**7.13**	**19,920**	**63.26**	**22,223**	**70.57**
Abra	3,639	2	0.05	176	4.84	1,075	29.54	**1,253**	**34.43**
Apayao	2,222	11	0.50	419	18.86	2,423	109.05	**2,853**	**128.40**
Benguet	8,900	7	0.08	580	6.52	6,669	74.93	**7,256**	**81.53**
Ifugao	4,145	15	0.36	247	5.96	1,929	46.54	**2,191**	**52.86**
Kalinga	4,582	4	0.09	194	4.23	1,217	26.56	**1,415**	**30.88**
Mountain Province	2,615	14	0.54	409	15.64	3,965	151.63	**4,388**	**167.80**
City of Baguio	5,387	6	0.11	219	4.07	2,642	49.04	**2,867**	**53.22**
**Region 1**	**96,024**	**75**	**0.08**	**3,156**	**3.29**	**32,690**	**34.04**	**35,921**	**37.41**
Ilocos Norte	8,809	4	0.05	391	4.44	5,430	61.64	**5,825**	**66.13**
Ilocos Sur	9,834	11	0.11	509	5.18	6,412	65.20	**6,932**	**70.49**
La Union	13,436	19	0.14	613	4.56	5,408	40.25	**6,040**	**44.95**
Pangasinan	60,591	38	0.06	1,557	2.57	14,751	24.35	**16,346**	**26.98**
City of Dagupan	3,354	3	0.09	86	2.56	689	20.54	**778**	**23.20**
**Region 2**	**64,034**	**89**	**0.14**	**2,113**	**3.30**	**17,634**	**27.54**	**19,836**	**30.98**
Batanes	241	0	0.00	4	1.66	110	45.64	**114**	**47.30**
Cagayan	20,723	14	0.07	450	2.17	2,784	13.43	**3,248**	**15.67**
Isabela	27,362	47	0.17	733	2.68	9,275	33.90	**10,055**	**36.75**
Nueva Vizcaya	8,880	21	0.24	635	7.15	3,730	42.00	**4,386**	**49.39**
Quirino	3,865	6	0.16	241	6.24	1,365	35.32	**1,612**	**41.71**
City of Santiago	2,963	1	0.03	50	1.69	370	12.49	**421**	**14.21**
**Region 3**	**235,313**	**906**	**0.39**	**12,263**	**5.21**	**89,633**	**38.09**	**102,802**	**43.69**
Aurora	4,578	8	0.17	249	5.44	1,611	35.19	**1,868**	**40.80**
Bataan	17,556	46	0.26	1,188	6.77	7,766	44.24	**9,000**	**51.26**
Bulacan	68,115	656	0.96	5,057	7.42	40,884	60.02	**46,597**	**68.41**
Nueva Ecija	43,626	76	0.17	1,740	3.99	10,581	24.25	**12,397**	**28.42**
Pampanga	46,146	60	0.13	1,721	3.73	12,519	27.13	**14,300**	**30.99**
Tarlac	29,655	23	0.08	1,252	4.22	10,198	34.39	**11,473**	**38.69**
Zambales	11,796	9	0.08	725	6.15	3,881	32.90	**4,615**	**39.12**
City of Angeles	9,232	23	0.25	159	1.72	952	10.31	**1,134**	**12.28**
City of Olongapo	4,609	5	0.11	172	3.73	1,241	26.93	**1,418**	**30.77**
**Region 4A**	**310,150**	**259**	**0.08**	**10,266**	**3.31**	**98,506**	**31.76**	**109,031**	**35.15**
Batangas	53,484	28	0.05	1,062	1.99	14,588	27.28	**15,678**	**29.31**
Cavite	78,589	53	0.07	2,247	2.86	22,733	28.93	**25,033**	**31.85**
Laguna	61,967	74	0.12	2,486	4.01	26,390	42.59	**28,950**	**46.72**
Quezon	40,954	39	0.10	1,453	3.55	10,939	26.71	**12,431**	**30.35**
Rizal	68,903	56	0.08	2,692	3.91	21,993	31.92	**24,741**	**35.91**
City of Lucena	6,253	9	0.14	326	5.21	1,863	29.79	**2,198**	**35.15**
**Region 4B**	**66,508**	**65**	**0.10**	**2,891**	**4.35**	**18,994**	**28.56**	**21,950**	**33.00**
Marinduque	4,124	5	0.12	122	2.96	1,333	32.32	**1,460**	**35.40**
Occidental Mindoro	11,748	6	0.05	623	5.30	3,605	30.69	**4,234**	**36.04**
Oriental Mindoro	19,007	12	0.06	511	2.69	4,613	24.27	**5,136**	**27.02**
Palawan	20,503	26	0.13	1,017	4.96	5,329	25.99	**6,372**	**31.08**

Table 2.B.1.8 - Prenatal Care
Pregnant women screened for Hepatitis B
Philippines, 2024

Area	Eligible Population (0-11 months)	Total No. of pregnant women Screened for Hepatitis B							
		Age Group						Total	%
		10-14 yrs old		15-19 yrs old		20-49 yrs old			
		No.	%	No.	%	No.	%		
Romblon	5,488	10	0.18	230	4.19	1,970	35.90	**2,210**	**40.27**
City of Puerto Princesa	5,638	6	0.11	388	6.88	2,144	38.03	**2,538**	**45.02**
**Region 5**	**136,611**	**52**	**0.04**	**3,295**	**2.41**	**29,251**	**21.41**	**32,598**	**23.86**
Albay	27,665	4	0.01	634	2.29	8,557	30.93	**9,195**	**33.24**
Camarines Norte	14,894	9	0.06	366	2.46	2,857	19.18	**3,232**	**21.70**
Camarines Sur	43,855	10	0.02	441	1.01	5,149	11.74	**5,600**	**12.77**
Catanduanes	5,395	7	0.13	320	5.93	1,966	36.44	**2,293**	**42.50**
Masbate	22,495	11	0.05	885	3.93	4,879	21.69	**5,775**	**25.67**
Sorsogon	18,065	9	0.05	599	3.32	5,251	29.07	**5,859**	**32.43**
City of Naga	4,242	2	0.05	50	1.18	592	13.96	**644**	**15.18**
**Region 6**	**147,934**	**97**	**0.07**	**6,078**	**4.11**	**48,272**	**32.63**	**54,447**	**36.80**
Aklan	11,281	4	0.04	375	3.32	3,543	31.41	**3,922**	**34.77**
Antique	11,899	2	0.02	393	3.30	3,281	27.57	**3,676**	**30.89**
Capiz	13,872	11	0.08	390	2.81	3,777	27.23	**4,178**	**30.12**
Guimaras	3,361	10	0.30	199	5.92	1,734	51.59	**1,943**	**57.81**
Iloilo	35,981	21	0.06	1,537	4.27	13,526	37.59	**15,084**	**41.92**
Negros Occidental	52,445	40	0.08	2,545	4.85	16,231	30.95	**18,816**	**35.88**
City of Bacolod	10,361	2	0.02	390	3.76	3,499	33.77	**3,891**	**37.55**
City of Iloilo	8,734	7	0.08	249	2.85	2,681	30.70	**2,937**	**33.63**
**Region 7**	**167,016**	**190**	**0.11**	**9,139**	**5.47**	**69,131**	**41.39**	**78,460**	**46.98**
Bohol	26,210	25	0.10	1,036	3.95	9,744	37.18	**10,805**	**41.22**
Cebu	72,875	53	0.07	3,096	4.25	19,843	27.23	**22,992**	**31.55**
Negros Oriental	28,980	27	0.09	1,254	4.33	7,922	27.34	**9,203**	**31.76**
Siquijor	1,671	1	0.06	55	3.29	681	40.75	**737**	**44.11**
City of Cebu	19,466	17	0.09	1,072	5.51	8,509	43.71	**9,598**	**49.31**
City of Lapu-Lapu	10,833	3	0.03	436	4.02	5,256	48.52	**5,695**	**52.57**
City of Mandaue	6,981	64	0.92	2,190	31.37	17,176	246.04	**19,430**	**278.33**
**Region 8**	**93,713**	**53**	**0.06**	**3,362**	**3.59**	**25,491**	**27.20**	**28,906**	**30.85**
Biliran	3,378	3	0.09	229	6.78	1,611	47.69	**1,843**	**54.56**
Eastern Samar	9,594	10	0.10	417	4.35	2,523	26.30	**2,950**	**30.75**
Leyte	31,504	20	0.06	908	2.88	7,771	24.67	**8,699**	**27.61**
Northern Samar	14,053	9	0.06	585	4.16	3,775	26.86	**4,369**	**31.09**
Southern Leyte	7,368	3	0.04	250	3.39	2,717	36.88	**2,970**	**40.31**
Samar	16,946	2	0.01	357	2.11	2,861	16.88	**3,220**	**19.00**
Ormoc City	5,344	2	0.04	288	5.39	1,783	33.36	**2,073**	**38.79**
City of Tacloban	5,526	4	0.07	328	5.94	2,450	44.34	**2,782**	**50.34**
**Region 9**	**85,348**	**15**	**0.02**	**1,004**	**1.18**	**6,164**	**7.22**	**7,183**	**8.42**
Zamboanga del Norte	24,977	3	0.01	208	0.83	1,324	5.30	**1,535**	**6.15**
Zamboanga del Sur	23,905	9	0.04	220	0.92	1,177	4.92	**1,406**	**5.88**
Zamboanga Sibugay	13,903	2	0.01	221	1.59	1,208	8.69	**1,431**	**10.29**
City of Isabela	3,209	0	0.00	89	2.77	475	14.80	**564**	**17.58**
City of Zamboanga	19,354	1	0.01	266	1.37	1,980	10.23	**2,247**	**11.61**
**Region 10**	**110,290**	**127**	**0.12**	**5,755**	**5.22**	**32,172**	**29.17**	**38,054**	**34.50**
Bukidnon	33,913	39	0.12	1,652	4.87	7,508	22.14	**9,199**	**27.13**
Camiguin	1,727	2	0.12	118	6.83	949	54.95	**1,069**	**61.90**
Lanao del Norte	17,881	15	0.08	443	2.48	3,286	18.38	**3,744**	**20.94**
Misamis Occidental	12,342	2	0.02	328	2.66	2,805	22.73	**3,135**	**25.40**
Misamis Oriental	22,184	44	0.20	1,613	7.27	8,497	38.30	**10,154**	**45.77**
City of Cagayan De Oro	14,503	22	0.15	1,394	9.61	7,572	52.21	**8,988**	**61.97**
City of Iligan	7,740	3	0.04	207	2.67	1,555	20.09	**1,765**	**22.80**
**Region 11**	**107,934**	**171**	**0.16**	**4,143**	**3.84**	**27,735**	**25.70**	**32,049**	**29.69**
Davao de Oro	15,434	8	0.05	180	1.17	1,004	6.51	**1,192**	**7.72**
Davao del Norte	23,685	34	0.14	730	3.08	4,924	20.79	**5,688**	**24.02**
Davao Oriental	12,025	6	0.05	112	0.93	824	6.85	**942**	**7.83**
Davao del Sur	13,178	21	0.16	522	3.96	2,812	21.34	**3,355**	**25.46**
Davao Occidental	6,606	13	0.20	345	5.22	877	13.28	**1,235**	**18.70**
City of Davao	37,006	89	0.24	2,254	6.09	17,294	46.73	**19,637**	**53.06**
**Region 12**	**95,296**	**145**	**0.15**	**5,596**	**5.87**	**30,003**	**31.48**	**35,744**	**37.51**
Cotabato	25,696	36	0.14	1,325	5.16	7,458	29.02	**8,819**	**34.32**
Sarangani	13,501	23	0.17	1,209	8.95	4,977	36.86	**6,209**	**45.99**
South Cotabato	21,842	50	0.23	1,377	6.30	7,562	34.62	**8,989**	**41.15**

Table 2.B.1.8 - Prenatal Care
Pregnant women screened for Hepatitis B
Philippines, 2024

Area	Eligible Population (0-11 months)	Total No. of pregnant women Screened for Hepatitis B							
		Age Group						Total	%
		10-14 yrs old		15-19 yrs old		20-49 yrs old			
		No.	%	No.	%	No.	%		
Sultan Kudarat	19,613	28	0.14	1,089	5.55	6,120	31.20	**7,237**	**36.90**
City of General Santos	14,644	8	0.05	596	4.07	3,886	26.54	**4,490**	**30.66**
**CARAGA**	**55,371**	**30**	**0.05**	**1,964**	**3.55**	**12,748**	**23.02**	**14,742**	**26.62**
Agusan del Norte	7,331	8	0.11	339	4.62	2,053	28.00	**2,400**	**32.74**
Agusan del Sur	16,180	5	0.03	338	2.09	2,022	12.50	**2,365**	**14.62**
Surigao del Norte	10,168	6	0.06	475	4.67	3,086	30.35	**3,567**	**35.08**
Surigao del Sur	12,834	2	0.02	203	1.58	1,361	10.60	**1,566**	**12.20**
Dinagat Islands	2,117	2	0.09	135	6.38	842	39.77	**979**	**46.24**
City of Butuan	6,741	7	0.10	474	7.03	3,384	50.20	**3,865**	**57.34**
**BARMM**	**134,585**	**9**	**0.01**	**401**	**0.30**	**5,354**	**3.98**	**5,764**	**4.28**
Basilan	11,653	5	0.04	53	0.45	181	1.55	**239**	**2.05**
Lanao del Sur	33,339	0	0.00	64	0.19	2,455	7.36	**2,519**	**7.56**
Maguindanao del Norte	18,923	1	0.01	31	0.16	343	1.81	**375**	**1.98**
Maguindanao del Sur	23,214	1	0.00	71	0.31	812	3.50	**884**	**3.81**
Sulu	21,381	1	0.00	85	0.40	608	2.84	**694**	**3.25**
Tawi-Tawi	12,602	0	0.00	12	0.10	43	0.34	**55**	**0.44**
SGA	6,305	0	0.00	13	0.21	74	1.17	**87**	**1.38**
City of Cotabato	7,168	1	0.01	72	1.00	838	11.69	**911**	**12.71**

*Legend: - * - No Report*

Table 2.B.1.9 - Prenatal Care
Pregnant women tested positive for Hepatitis B
Philippines, 2024

Area	Eligible Population (0-11 months)	Pregnant women Tested Positive for Hepatitis B							
		Age Group						Total	%
		10-14 yrs old		15-19 yrs old		20-49 yrs old			
		No.	%	No.	%	No.	%		
**PHILIPPINES**	**2,200,865**	**46**	**1.74**	**1,032**	**1.23**	**9,790**	**1.43**	**10,868**	**1.41**
**N C R**	**263,248**	**1**	**0.32**	**77**	**0.75**	**1,176**	**0.99**	**1,254**	**0.97**
City of Malabon	7,447	0	**0.00**	1	**0.43**	31	**1.60**	**32**	**1.47**
City of Navotas	5,263	0	**0.00**	0	**0.00**	16	**0.52**	**16**	**0.46**
City of Valenzuela	13,601	0	**0.00**	1	**0.21**	47	**0.96**	**48**	**0.89**
City of Caloocan	33,499	0	**0.00**	22	**1.23**	247	**1.51**	**269**	**1.48**
City of Marikina	8,399	0	**0.00**	2	**1.26**	14	**0.72**	**16**	**0.76**
City of Pasig	17,856	0	**0.00**	6	**0.96**	93	**1.02**	**99**	**1.02**
Pateros	1,120	0	**0.00**	0	**0.00**	1	**0.41**	**1**	**0.36**
City of Taguig	26,021	0	**0.00**	6	**0.63**	138	**1.55**	**144**	**1.46**
Quezon City	59,607	1	**1.72**	14	**0.73**	249	**0.76**	**264**	**0.76**
City of Makati	4,196	0	**0.00**	1	**0.93**	32	**2.30**	**33**	**2.19**
City of Mandaluyong	7,989	0	**0.00**	1	**0.35**	12	**0.20**	**13**	**0.21**
City of San Juan	2,173	0	**0.00**	0	**0.00**	2	**0.29**	**2**	**0.27**
City of Manila	34,268	0	**0.00**	10	**0.53**	71	**0.45**	**81**	**0.46**
City of Las Piñas	11,346	0	**0.00**	2	**0.77**	39	**1.03**	**41**	**1.02**
City of Muntinlupa	9,949	0	**0.00**	7	**2.30**	59	**2.05**	**66**	**2.07**
City of Parañaque	13,385	0	**0.00**	3	**0.59**	64	**1.21**	**67**	**1.15**
Pasay City	7,129	0	**0.00**	1	**0.34**	61	**1.58**	**62**	**1.49**
**C A R**	**31,490**	**1**	**1.69**	**13**	**0.58**	**115**	**0.58**	**129**	**0.58**
Abra	3,639	0	**0.00**	1	**0.57**	8	**0.74**	**9**	**0.72**
Apayao	2,222	0	**0.00**	4	**0.95**	34	**1.40**	**38**	**1.33**
Benguet	8,900	1	**14.29**	0	**0.00**	8	**0.12**	**9**	**0.12**
Ifugao	4,145	0	**0.00**	4	**1.62**	31	**1.61**	**35**	**1.60**
Kalinga	4,582	0	**0.00**	3	**1.55**	15	**1.23**	**18**	**1.27**
Mountain Province	2,615	0	**0.00**	0	**0.00**	6	**0.15**	**6**	**0.14**
City of Baguio	5,387	0	**0.00**	1	**0.46**	13	**0.49**	**14**	**0.49**
**Region 1**	**96,024**	**0**	**0.00**	**13**	**0.41**	**199**	**0.61**	**212**	**0.59**
Ilocos Norte	8,809	0	**0.00**	1	**0.26**	4	**0.07**	**5**	**0.09**
Ilocos Sur	9,834	0	**0.00**	0	**0.00**	5	**0.08**	**5**	**0.07**
La Union	13,436	0	**0.00**	3	**0.49**	12	**0.22**	**15**	**0.25**
Pangasinan	60,591	0	**0.00**	9	**0.58**	168	**1.14**	**177**	**1.08**
City of Dagupan	3,354	0	**0.00**	0	**0.00**	10	**1.45**	**10**	**1.29**
**Region 2**	**64,034**	**0**	**0.00**	**61**	**2.89**	**392**	**2.22**	**453**	**2.28**
Batanes	241	0	**0.00**	0	**0.00**	1	**0.91**	**1**	**0.88**
Cagayan	20,723	0	**0.00**	6	**1.33**	20	**0.72**	**26**	**0.80**
Isabela	27,362	0	**0.00**	33	**4.50**	202	**2.18**	**235**	**2.34**
Nueva Vizcaya	8,880	0	**0.00**	18	**2.83**	151	**4.05**	**169**	**3.85**
Quirino	3,865	0	**0.00**	4	**1.66**	16	**1.17**	**20**	**1.24**
City of Santiago	2,963	0	**0.00**	0	**0.00**	2	**0.54**	**2**	**0.48**
**Region 3**	**235,313**	**3**	**0.33**	**97**	**0.79**	**853**	**0.95**	**953**	**0.93**
Aurora	4,578	0	**0.00**	0	**0.00**	18	**1.12**	**18**	**0.96**
Bataan	17,556	0	**0.00**	7	**0.59**	108	**1.39**	**115**	**1.28**
Bulacan	68,115	1	**0.15**	23	**0.45**	294	**0.72**	**318**	**0.68**
Nueva Ecija	43,626	1	**1.32**	38	**2.18**	181	**1.71**	**220**	**1.77**
Pampanga	46,146	1	**1.67**	15	**0.87**	128	**1.02**	**144**	**1.01**
Tarlac	29,655	0	**0.00**	8	**0.64**	47	**0.46**	**55**	**0.48**
Zambales	11,796	0	**0.00**	5	**0.69**	53	**1.37**	**58**	**1.26**
City of Angeles	9,232	0	**0.00**	1	**0.63**	6	**0.63**	**7**	**0.62**
City of Olongapo	4,609	0	**0.00**	0	**0.00**	18	**1.45**	**18**	**1.27**
**Region 4A**	**310,150**	**19**	**7.34**	**121**	**1.18**	**1,420**	**1.44**	**1,560**	**1.43**
Batangas	53,484	7	**25.00**	4	**0.38**	170	**1.17**	**181**	**1.15**
Cavite	78,589	2	**3.77**	18	**0.80**	309	**1.36**	**329**	**1.31**
Laguna	61,967	3	**4.05**	46	**1.85**	420	**1.59**	**469**	**1.62**
Quezon	40,954	7	**17.95**	32	**2.20**	311	**2.84**	**350**	**2.82**
Rizal	68,903	0	**0.00**	17	**0.63**	189	**0.86**	**206**	**0.83**
City of Lucena	6,253	0	**0.00**	4	**1.23**	21	**1.13**	**25**	**1.14**
**Region 4B**	**66,508**	**2**	**3.08**	**29**	**1.00**	**312**	**1.64**	**343**	**1.56**
Marinduque	4,124	0	**0.00**	1	**0.82**	21	**1.58**	**22**	**1.51**
Occidental Mindoro	11,748	0	**0.00**	6	**0.96**	45	**1.25**	**51**	**1.20**
Oriental Mindoro	19,007	0	**0.00**	8	**1.57**	57	**1.24**	**65**	**1.27**
Palawan	20,503	2	**7.69**	11	**1.08**	119	**2.23**	**132**	**2.07**

Table 2.B.1.9 - Prenatal Care
Pregnant women tested positive for Hepatitis B
Philippines, 2024

Area	Eligible Population (0-11 months)	Pregnant women Tested Positive for Hepatitis B							
		Age Group						Total	%
		10-14 yrs old		15-19 yrs old		20-49 yrs old			
		No.	%	No.	%	No.	%		
Romblon	5,488	0	**0.00**	1	**0.43**	45	**2.28**	**46**	**2.08**
City of Puerto Princesa	5,638	0	**0.00**	2	**0.52**	25	**1.17**	**27**	**1.06**
**Region 5**	**136,611**	**0**	**0.00**	**48**	**1.46**	**843**	**2.88**	**891**	**2.73**
Albay	27,665	0	**0.00**	4	**0.63**	110	**1.29**	**114**	**1.24**
Camarines Norte	14,894	0	**0.00**	5	**1.37**	60	**2.10**	**65**	**2.01**
Camarines Sur	43,855	0	**0.00**	3	**0.68**	270	**5.24**	**273**	**4.88**
Catanduanes	5,395	0	**0.00**	6	**1.88**	89	**4.53**	**95**	**4.14**
Masbate	22,495	0	**0.00**	20	**2.26**	141	**2.89**	**161**	**2.79**
Sorsogon	18,065	0	**0.00**	8	**1.34**	167	**3.18**	**175**	**2.99**
City of Naga	4,242	0	**0.00**	2	**4.00**	6	**1.01**	**8**	**1.24**
**Region 6**	**147,934**	**2**	**2.06**	**80**	**1.32**	**795**	**1.65**	**877**	**1.61**
Aklan	11,281	0	**0.00**	9	**2.40**	106	**2.99**	**115**	**2.93**
Antique	11,899	2	**100.00**	11	**2.80**	96	**2.93**	**109**	**2.97**
Capiz	13,872	0	**0.00**	6	**1.54**	62	**1.64**	**68**	**1.63**
Guimaras	3,361	0	**0.00**	6	**3.02**	38	**2.19**	**44**	**2.26**
Iloilo	35,981	0	**0.00**	22	**1.43**	210	**1.55**	**232**	**1.54**
Negros Occidental	52,445	0	**0.00**	22	**0.86**	214	**1.32**	**236**	**1.25**
City of Bacolod	10,361	0	**0.00**	4	**1.03**	48	**1.37**	**52**	**1.34**
City of Iloilo	8,734	0	**0.00**	0	**0.00**	21	**0.78**	**21**	**0.72**
**Region 7**	**167,016**	**0**	**0.00**	**78**	**0.85**	**645**	**0.93**	**723**	**0.92**
Bohol	26,210	0	**0.00**	26	**2.51**	207	**2.12**	**233**	**2.16**
Cebu	72,875	0	**0.00**	13	**0.42**	220	**1.11**	**233**	**1.01**
Negros Oriental	28,980	0	**0.00**	22	**1.75**	141	**1.78**	**163**	**1.77**
Siquijor	1,671	0	**0.00**	0	**0.00**	12	**1.76**	**12**	**1.63**
City of Cebu	19,466	0	**0.00**	0	**0.00**	26	**0.31**	**26**	**0.27**
City of Lapu-Lapu	10,833	0	**0.00**	15	**3.44**	33	**0.63**	**48**	**0.84**
City of Mandaue	6,981	0	**0.00**	2	**0.09**	6	**0.03**	**8**	**0.04**
**Region 8**	**93,713**	**3**	**5.66**	**49**	**1.46**	**464**	**1.82**	**516**	**1.79**
Biliran	3,378	1	**33.33**	4	**1.75**	30	**1.86**	**35**	**1.90**
Eastern Samar	9,594	0	**0.00**	1	**0.24**	29	**1.15**	**30**	**1.02**
Leyte	31,504	0	**0.00**	10	**1.10**	193	**2.48**	**203**	**2.33**
Northern Samar	14,053	2	**22.22**	20	**3.42**	114	**3.02**	**136**	**3.11**
Southern Leyte	7,368	0	**0.00**	4	**1.60**	24	**0.88**	**28**	**0.94**
Samar	16,946	0	**0.00**	6	**1.68**	47	**1.64**	**53**	**1.65**
Ormoc City	5,344	0	**0.00**	4	**1.39**	26	**1.46**	**30**	**1.45**
City of Tacloban	5,526	0	**0.00**	0	**0.00**	1	**0.04**	**1**	**0.04**
**Region 9**	**85,348**	**0**	**0.00**	**10**	**1.00**	**121**	**1.96**	**131**	**1.82**
Zamboanga del Norte	24,977	0	**0.00**	0	**0.00**	24	**1.81**	**24**	**1.56**
Zamboanga del Sur	23,905	0	**0.00**	6	**2.73**	30	**2.55**	**36**	**2.56**
Zamboanga Sibugay	13,903	0	**0.00**	2	**0.90**	32	**2.65**	**34**	**2.38**
City of Isabela	3,209	0	**0.00**	1	**1.12**	8	**1.68**	**9**	**1.60**
City of Zamboanga	19,354	0	**0.00**	1	**0.38**	27	**1.36**	**28**	**1.25**
**Region 10**	**110,290**	**2**	**1.57**	**123**	**2.14**	**581**	**1.81**	**706**	**1.86**
Bukidnon	33,913	1	**2.56**	53	**3.21**	252	**3.36**	**306**	**3.33**
Camiguin	1,727	0	**0.00**	1	**0.85**	1	**0.11**	**2**	**0.19**
Lanao del Norte	17,881	0	**0.00**	2	**0.45**	12	**0.37**	**14**	**0.37**
Misamis Occidental	12,342	0	**0.00**	4	**1.22**	26	**0.93**	**30**	**0.96**
Misamis Oriental	22,184	0	**0.00**	31	**1.92**	118	**1.39**	**149**	**1.47**
City of Cagayan De Oro	14,503	1	**4.55**	25	**1.79**	136	**1.80**	**162**	**1.80**
City of Iligan	7,740	0	**0.00**	7	**3.38**	36	**2.32**	**43**	**2.44**
**Region 11**	**107,934**	**9**	**5.26**	**45**	**1.09**	**299**	**1.08**	**353**	**1.10**
Davao de Oro	15,434	0	**0.00**	0	**0.00**	7	**0.70**	**7**	**0.59**
Davao del Norte	23,685	1	**2.94**	5	**0.68**	62	**1.26**	**68**	**1.20**
Davao Oriental	12,025	0	**0.00**	2	**1.79**	17	**2.06**	**19**	**2.02**
Davao del Sur	13,178	1	**4.76**	5	**0.96**	43	**1.53**	**49**	**1.46**
Davao Occidental	6,606	0	**0.00**	19	**5.51**	66	**7.53**	**85**	**6.88**
City of Davao	37,006	7	**7.87**	14	**0.62**	104	**0.60**	**125**	**0.64**
**Region 12**	**95,296**	**4**	**2.76**	**150**	**2.68**	**1,203**	**4.01**	**1,357**	**3.80**
Cotabato	25,696	1	**2.78**	32	**2.42**	246	**3.30**	**279**	**3.16**
Sarangani	13,501	2	**8.70**	50	**4.14**	240	**4.82**	**292**	**4.70**
South Cotabato	21,842	0	**0.00**	19	**1.38**	105	**1.39**	**124**	**1.38**

Table 2.B.1.9 - Prenatal Care
Pregnant women tested positive for Hepatitis B
Philippines, 2024

Area	Eligible Population (0-11 months)	Pregnant women Tested Positive for Hepatitis B							
		Age Group						Total	%
		10-14 yrs old		15-19 yrs old		20-49 yrs old			
		No.	%	No.	%	No.	%		
Sultan Kudarat	19,613	1	**3.57**	21	**1.93**	176	**2.88**	**198**	**2.74**
City of General Santos	14,644	0	**0.00**	28	**4.70**	436	**11.22**	**464**	**10.33**
**CARAGA**	**55,371**	**0**	**0.00**	**29**	**1.48**	**294**	**2.31**	**323**	**2.19**
Agusan del Norte	7,331	0	**0.00**	5	**1.47**	63	**3.07**	**68**	**2.83**
Agusan del Sur	16,180	0	**0.00**	6	**1.78**	51	**2.52**	**57**	**2.41**
Surigao del Norte	10,168	0	**0.00**	6	**1.26**	80	**2.59**	**86**	**2.41**
Surigao del Sur	12,834	0	**0.00**	8	**3.94**	65	**4.78**	**73**	**4.66**
Dinagat Islands	2,117	0	**0.00**	0	**0.00**	13	**1.54**	**13**	**1.33**
City of Butuan	6,741	0	**0.00**	4	**0.84**	22	**0.65**	**26**	**0.67**
**BARMM**	**134,585**	**0**	**0.00**	**9**	**2.24**	**78**	**1.46**	**87**	**1.51**
Basilan	11,653	0	**0.00**	0	**0.00**	7	**3.87**	**7**	**2.93**
Lanao del Sur	33,339	0	**0.00**	0	**0.00**	14	**0.57**	**14**	**0.56**
Maguindanao del Norte	18,923	0	**0.00**	2	**6.45**	16	**4.66**	**18**	**4.80**
Maguindanao del Sur	23,214	0	**0.00**	4	**5.63**	15	**1.85**	**19**	**2.15**
Sulu	21,381	0	**0.00**	3	**3.53**	17	**2.80**	**20**	**2.88**
Tawi-Tawi	12,602	0	**0.00**	0	**0.00**	4	**9.30**	**4**	**7.27**
SGA	6,305	0	**0.00**	0	**0.00**	2	**2.70**	**2**	**2.30**
City of Cotabato	7,168	0	**0.00**	0	**0.00**	3	**0.36**	**3**	**0.33**

*Legend: - * - No Report*

Table 2.B.1.10 - Prenatal Care
Pregnant Women tested for Complete Blood Count (CBC) or Hemoglobin (Hgb) & Hematocrit (Hct) Count
Philippines, 2024

Area	Eligible Population (0-11 months)	Total no. of screened pregnant women tested for CBC or Hgb or Hct count							
		Age Group						Total	%
		10-14 yrs old		15-19 yrs old		20-49 yrs old			
		No.	%	No.	%	No.	%		
**PHILIPPINES**	**2,200,865**	**3,133**	**0.14**	**101,967**	**4.63**	**772,992**	**35.12**	**878,092**	**39.90**
**N C R**	**263,248**	**291**	**0.11**	**9,849**	**3.74**	**112,800**	**42.85**	**122,940**	**46.70**
City of Malabon	7,447	10	0.13	269	3.61	2,023	27.17	**2,302**	**30.91**
City of Navotas	5,263	20	0.38	373	7.09	3,098	58.86	**3,491**	**66.33**
City of Valenzuela	13,601	20	0.15	532	3.91	5,518	40.57	**6,070**	**44.63**
City of Caloocan	33,499	39	0.12	1,129	3.37	12,433	37.11	**13,601**	**40.60**
City of Marikina	8,399	4	0.05	162	1.93	2,247	26.75	**2,413**	**28.73**
City of Pasig	17,856	30	0.17	640	3.58	9,202	51.53	**9,872**	**55.29**
Pateros	1,120	1	0.09	55	4.91	652	58.21	**708**	**63.21**
City of Taguig	26,021	17	0.07	985	3.79	9,071	34.86	**10,073**	**38.71**
Quezon City	59,607	59	0.10	1,930	3.24	29,251	49.07	**31,240**	**52.41**
City of Makati	4,196	3	0.07	177	4.22	1,419	33.82	**1,599**	**38.11**
City of Mandaluyong	7,989	10	0.13	275	3.44	5,737	71.81	**6,022**	**75.38**
City of San Juan	2,173	1	0.05	39	1.79	638	29.36	**678**	**31.20**
City of Manila	34,268	55	0.16	1,845	5.38	15,473	45.15	**17,373**	**50.70**
City of Las Piñas	11,346	2	0.02	230	2.03	3,507	30.91	**3,739**	**32.95**
City of Muntinlupa	9,949	6	0.06	311	3.13	2,827	28.41	**3,144**	**31.60**
City of Parañaque	13,385	0	0.00	446	3.33	4,401	32.88	**4,847**	**36.21**
Pasay City	7,129	14	0.20	451	6.33	5,303	74.39	**5,768**	**80.91**
**C A R**	**31,490**	**73**	**0.23**	**2,394**	**7.60**	**21,116**	**67.06**	**23,583**	**74.89**
Abra	3,639	2	0.05	156	4.29	908	24.95	**1,066**	**29.29**
Apayao	2,222	16	0.72	490	22.05	2,589	116.52	**3,095**	**139.29**
Benguet	8,900	11	0.12	529	5.94	6,529	73.36	**7,069**	**79.43**
Ifugao	4,145	17	0.41	307	7.41	2,383	57.49	**2,707**	**65.31**
Kalinga	4,582	8	0.17	282	6.15	2,033	44.37	**2,323**	**50.70**
Mountain Province	2,615	15	0.57	418	15.98	4,003	153.08	**4,436**	**169.64**
City of Baguio	5,387	4	0.07	212	3.94	2,671	49.58	**2,887**	**53.59**
**Region 1**	**96,024**	**105**	**0.11**	**3,723**	**3.88**	**38,899**	**40.51**	**42,727**	**44.50**
Ilocos Norte	8,809	4	0.05	388	4.40	4,948	56.17	**5,340**	**60.62**
Ilocos Sur	9,834	12	0.12	455	4.63	6,564	66.75	**7,031**	**71.50**
La Union	13,436	23	0.17	681	5.07	6,045	44.99	**6,749**	**50.23**
Pangasinan	60,591	63	0.10	2,133	3.52	20,780	34.30	**22,976**	**37.92**
City of Dagupan	3,354	3	0.09	66	1.97	562	16.76	**631**	**18.81**
**Region 2**	**64,034**	**117**	**0.18**	**4,445**	**6.94**	**24,086**	**37.61**	**28,648**	**44.74**
Batanes	241	0	0.00	4	1.66	106	43.98	**110**	**45.64**
Cagayan	20,723	34	0.16	1,023	4.94	7,051	34.02	**8,108**	**39.13**
Isabela	27,362	56	0.20	2,342	8.56	10,920	39.91	**13,318**	**48.67**
Nueva Vizcaya	8,880	21	0.24	625	7.04	3,688	41.53	**4,334**	**48.81**
Quirino	3,865	5	0.13	402	10.40	1,884	48.75	**2,291**	**59.28**
City of Santiago	2,963	1	0.03	49	1.65	437	14.75	**487**	**16.44**
**Region 3**	**235,313**	**809**	**0.34**	**11,376**	**4.83**	**83,107**	**35.32**	**95,292**	**40.50**
Aurora	4,578	7	0.15	275	6.01	1,605	35.06	**1,887**	**41.22**
Bataan	17,556	41	0.23	1,057	6.02	7,197	40.99	**8,295**	**47.25**
Bulacan	68,115	588	0.86	4,996	7.33	39,817	58.46	**45,401**	**66.65**
Nueva Ecija	43,626	82	0.19	1,965	4.50	11,230	25.74	**13,277**	**30.43**
Pampanga	46,146	36	0.08	1,096	2.38	8,738	18.94	**9,870**	**21.39**
Tarlac	29,655	19	0.06	1,131	3.81	9,470	31.93	**10,620**	**35.81**
Zambales	11,796	9	0.08	583	4.94	3,024	25.64	**3,616**	**30.65**
City of Angeles	9,232	23	0.25	135	1.46	906	9.81	**1,064**	**11.53**
City of Olongapo	4,609	4	0.09	138	2.99	1,120	24.30	**1,262**	**27.38**
**Region 4A**	**310,150**	**281**	**0.09**	**10,602**	**3.42**	**102,225**	**32.96**	**113,108**	**36.47**
Batangas	53,484	27	0.05	1,250	2.34	16,861	31.53	**18,138**	**33.91**
Cavite	78,589	52	0.07	2,181	2.78	22,531	28.67	**24,764**	**31.51**
Laguna	61,967	77	0.12	2,418	3.90	26,047	42.03	**28,542**	**46.06**
Quezon	40,954	51	0.12	1,736	4.24	12,712	31.04	**14,499**	**35.40**
Rizal	68,903	62	0.09	2,650	3.85	22,055	32.01	**24,767**	**35.94**

Table 2.B.1.10 - Prenatal Care
Pregnant Women tested for Complete Blood Count (CBC) or Hemoglobin (Hgb) & Hematocrit (Hct) Count
Philippines, 2024

Area	Eligible Population (0-11 months)	Total no. of screened pregnant women tested for CBC or Hgb or Hct count							
		Age Group						Total	%
		10-14 yrs old		15-19 yrs old		20-49 yrs old			
		No.	%	No.	%	No.	%		
City of Lucena	6,253	12	0.19	367	5.87	2,019	32.29	**2,398**	**38.35**
**Region 4B**	**66,508**	**46**	**0.07**	**2,686**	**4.04**	**17,625**	**26.50**	**20,357**	**30.61**
Marinduque	4,124	8	0.19	125	3.03	1,263	30.63	**1,396**	**33.85**
Occidental Mindoro	11,748	7	0.06	539	4.59	3,146	26.78	**3,692**	**31.43**
Oriental Mindoro	19,007	10	0.05	469	2.47	4,134	21.75	**4,613**	**24.27**
Palawan	20,503	16	0.08	982	4.79	5,061	24.68	**6,059**	**29.55**
Romblon	5,488	4	0.07	195	3.55	1,738	31.67	**1,937**	**35.30**
City of Puerto Princesa	5,638	1	0.02	376	6.67	2,283	40.49	**2,660**	**47.18**
**Region 5**	**136,611**	**65**	**0.05**	**3,964**	**2.90**	**33,137**	**24.26**	**37,166**	**27.21**
Albay	27,665	6	0.02	701	2.53	9,755	35.26	**10,462**	**37.82**
Camarines Norte	14,894	13	0.09	595	3.99	4,207	28.25	**4,815**	**32.33**
Camarines Sur	43,855	11	0.03	462	1.05	4,666	10.64	**5,139**	**11.72**
Catanduanes	5,395	7	0.13	408	7.56	2,668	49.45	**3,083**	**57.15**
Masbate	22,495	11	0.05	883	3.93	4,661	20.72	**5,555**	**24.69**
Sorsogon	18,065	17	0.09	900	4.98	7,037	38.95	**7,954**	**44.03**
City of Naga	4,242	0	0.00	15	0.35	143	3.37	**158**	**3.72**
**Region 6**	**147,934**	**105**	**0.07**	**6,439**	**4.35**	**50,570**	**34.18**	**57,114**	**38.61**
Aklan	11,281	6	0.05	406	3.60	3,978	35.26	**4,390**	**38.91**
Antique	11,899	5	0.04	417	3.50	3,285	27.61	**3,707**	**31.15**
Capiz	13,872	10	0.07	435	3.14	4,205	30.31	**4,650**	**33.52**
Guimaras	3,361	9	0.27	197	5.86	1,784	53.08	**1,990**	**59.21**
Iloilo	35,981	20	0.06	1,467	4.08	13,365	37.14	**14,852**	**41.28**
Negros Occidental	52,445	47	0.09	2,864	5.46	17,702	33.75	**20,613**	**39.30**
City of Bacolod	10,361	1	0.01	391	3.77	3,448	33.28	**3,840**	**37.06**
City of Iloilo	8,734	7	0.08	262	3.00	2,803	32.09	**3,072**	**35.17**
**Region 7**	**167,016**	**188**	**0.11**	**9,672**	**5.79**	**73,652**	**44.10**	**83,512**	**50.00**
Bohol	26,210	30	0.11	1,077	4.11	9,913	37.82	**11,020**	**42.05**
Cebu	72,875	36	0.05	3,295	4.52	22,520	30.90	**25,851**	**35.47**
Negros Oriental	28,980	31	0.11	1,514	5.22	9,523	32.86	**11,068**	**38.19**
Siquijor	1,671	1	0.06	44	2.63	571	34.17	**616**	**36.86**
City of Cebu	19,466	16	0.08	1,025	5.27	8,399	43.15	**9,440**	**48.49**
City of Lapu-Lapu	10,833	10	0.09	544	5.02	5,606	51.75	**6,160**	**56.86**
City of Mandaue	6,981	64	0.92	2,173	31.13	17,120	245.24	**19,357**	**277.28**
**Region 8**	**93,713**	**59**	**0.06**	**3,721**	**3.97**	**28,715**	**30.64**	**32,495**	**34.68**
Biliran	3,378	2	0.06	232	6.87	1,693	50.12	**1,927**	**57.05**
Eastern Samar	9,594	9	0.09	378	3.94	2,468	25.72	**2,855**	**29.76**
Leyte	31,504	25	0.08	1,102	3.50	8,973	28.48	**10,100**	**32.06**
Northern Samar	14,053	9	0.06	664	4.72	4,471	31.82	**5,144**	**36.60**
Southern Leyte	7,368	7	0.10	333	4.52	3,202	43.46	**3,542**	**48.07**
Samar	16,946	1	0.01	423	2.50	3,416	20.16	**3,840**	**22.66**
Ormoc City	5,344	2	0.04	257	4.81	1,815	33.96	**2,074**	**38.81**
City of Tacloban	5,526	4	0.07	332	6.01	2,677	48.44	**3,013**	**54.52**
**Region 9**	**85,348**	**45**	**0.05**	**2,945**	**3.45**	**15,641**	**18.33**	**18,631**	**21.83**
Zamboanga del Norte	24,977	9	0.04	721	2.89	3,946	15.80	**4,676**	**18.72**
Zamboanga del Sur	23,905	18	0.08	806	3.37	4,199	17.57	**5,023**	**21.01**
Zamboanga Sibugay	13,903	14	0.10	763	5.49	3,710	26.68	**4,487**	**32.27**
City of Isabela	3,209	0	0.00	202	6.29	1,170	36.46	**1,372**	**42.75**
City of Zamboanga	19,354	4	0.02	453	2.34	2,616	13.52	**3,073**	**15.88**
**Region 10**	**110,290**	**174**	**0.16**	**7,726**	**7.01**	**42,575**	**38.60**	**50,475**	**45.77**
Bukidnon	33,913	86	0.25	3,251	9.59	13,369	39.42	**16,706**	**49.26**
Camiguin	1,727	2	0.12	117	6.77	959	55.53	**1,078**	**62.42**
Lanao del Norte	17,881	14	0.08	450	2.52	3,175	17.76	**3,639**	**20.35**
Misamis Occidental	12,342	2	0.02	639	5.18	5,173	41.91	**5,814**	**47.11**
Misamis Oriental	22,184	38	0.17	1,809	8.15	9,412	42.43	**11,259**	**50.75**
City of Cagayan De Oro	14,503	22	0.15	1,148	7.92	7,922	54.62	**9,092**	**62.69**
City of Iligan	7,740	10	0.13	312	4.03	2,565	33.14	**2,887**	**37.30**

Table 2.B.1.10 - Prenatal Care
Pregnant Women tested for Complete Blood Count (CBC) or Hemoglobin (Hgb) & Hematocrit (Hct) Count
Philippines, 2024

Area	Eligible Population (0-11 months)	Total no. of screened pregnant women tested for CBC or Hgb or Hct count							
		Age Group						Total	%
		10-14 yrs old		15-19 yrs old		20-49 yrs old			
		No.	%	No.	%	No.	%		
**Region 11**	**107,934**	**441**	**0.41**	**9,757**	**9.04**	**55,230**	**51.17**	**65,428**	**60.62**
Davao de Oro	15,434	93	0.60	1,714	11.11	8,771	56.83	**10,578**	**68.54**
Davao del Norte	23,685	97	0.41	2,117	8.94	12,346	52.13	**14,560**	**61.47**
Davao Oriental	12,025	60	0.50	1,153	9.59	5,983	49.75	**7,196**	**59.84**
Davao del Sur	13,178	67	0.51	1,279	9.71	6,063	46.01	**7,409**	**56.22**
Davao Occidental	6,606	29	0.44	769	11.64	2,029	30.71	**2,827**	**42.79**
City of Davao	37,006	95	0.26	2,725	7.36	20,038	54.15	**22,858**	**61.77**
**Region 12**	**95,296**	**240**	**0.25**	**7,385**	**7.75**	**38,094**	**39.97**	**45,719**	**47.98**
Cotabato	25,696	55	0.21	1,828	7.11	10,053	39.12	**11,936**	**46.45**
Sarangani	13,501	43	0.32	1,620	12.00	6,548	48.50	**8,211**	**60.82**
South Cotabato	21,842	68	0.31	1,799	8.24	9,396	43.02	**11,263**	**51.57**
Sultan Kudarat	19,613	40	0.20	1,262	6.43	6,583	33.56	**7,885**	**40.20**
City of General Santos	14,644	34	0.23	876	5.98	5,514	37.65	**6,424**	**43.87**
**CARAGA**	**55,371**	**73**	**0.13**	**4,011**	**7.24**	**23,702**	**42.81**	**27,786**	**50.18**
Agusan del Norte	7,331	7	0.10	386	5.27	2,428	33.12	**2,821**	**38.48**
Agusan del Sur	16,180	34	0.21	1,594	9.85	8,165	50.46	**9,793**	**60.53**
Surigao del Norte	10,168	9	0.09	582	5.72	3,623	35.63	**4,214**	**41.44**
Surigao del Sur	12,834	14	0.11	865	6.74	5,414	42.18	**6,293**	**49.03**
Dinagat Islands	2,117	2	0.09	132	6.24	887	41.90	**1,021**	**48.23**
City of Butuan	6,741	7	0.10	452	6.71	3,185	47.25	**3,644**	**54.06**
**BARMM**	**134,585**	**21**	**0.02**	**1,272**	**0.95**	**11,818**	**8.78**	**13,111**	**9.74**
Basilan	11,653	4	0.03	108	0.93	417	3.58	**529**	**4.54**
Lanao del Sur	33,339	7	0.02	329	0.99	4,328	12.98	**4,664**	**13.99**
Maguindanao del Norte	18,923	6	0.03	262	1.38	2,193	11.59	**2,461**	**13.01**
Maguindanao del Sur	23,214	1	0.00	292	1.26	2,332	10.05	**2,625**	**11.31**
Sulu	21,381	1	0.00	110	0.51	894	4.18	**1,005**	**4.70**
Tawi-Tawi	12,602	0	0.00	19	0.15	90	0.71	**109**	**0.86**
SGA	6,305	1	0.02	49	0.78	353	5.60	**403**	**6.39**
City of Cotabato	7,168	1	0.01	103	1.44	1,211	16.89	**1,315**	**18.35**

*Legend: * - No Report*

Table 2.B.1.11 - Prenatal Care
Pregnant Women tested for Complete Blood Count or Hgb & Hct count diagnosed with Anemia
Philippines, 2024

Area	Eligible Population (0-11 months)	Pregnant women tested for CBC/Hgb and Hct diagnosed with anemia							
		Age Group						Total	%
		10-14 yrs old		15-19 yrs old		20-49 yrs old			
		No.	%	No.	%	No.	%		
**PHILIPPINES**	2,200,865	452	14.43	13,729	13.46	68,617	8.88	82,798	9.43
**N C R**	263,248	27	9.28	1,062	10.78	8,371	7.42	9,460	7.69
City of Malabon	7,447	0	0.00	21	7.81	90	4.45	111	4.82
City of Navotas	5,263	0	0.00	0	0.00	69	2.23	69	1.98
City of Valenzuela	13,601	2	10.00	52	9.77	378	6.85	432	7.12
City of Caloocan	33,499	4	10.26	197	17.45	1,422	11.44	1,623	11.93
City of Marikina	8,399	0	0.00	14	8.64	146	6.50	160	6.63
City of Pasig	17,856	3	10.00	100	15.63	727	7.90	830	8.41
Pateros	1,120	0	0.00	2	3.64	24	3.68	26	3.67
City of Taguig	26,021	3	17.65	157	15.94	920	10.14	1,080	10.72
Quezon City	59,607	6	10.17	265	13.73	2,144	7.33	2,415	7.73
City of Makati	4,196	1	33.33	7	3.95	98	6.91	106	6.63
City of Mandaluyong	7,989	1	10.00	24	8.73	168	2.93	193	3.20
City of San Juan	2,173	0	0.00	1	2.56	11	1.72	12	1.77
City of Manila	34,268	0	0.00	43	2.33	403	2.60	446	2.57
City of Las Piñas	11,346	0	0.00	20	8.70	320	9.12	340	9.09
City of Muntinlupa	9,949	4	66.67	39	12.54	378	13.37	421	13.39
City of Parañaque	13,385	1	0.00	41	9.19	512	11.63	554	11.43
Pasay City	7,129	2	14.29	79	17.52	561	10.58	642	11.13
**C A R**	31,490	12	16.44	229	9.57	1,201	5.69	1,442	6.11
Abra	3,639	0	0.00	8	5.13	35	3.85	43	4.03
Apayao	2,222	2	12.50	42	8.57	162	6.26	206	6.66
Benguet	8,900	1	9.09	20	3.78	252	3.86	273	3.86
Ifugao	4,145	5	29.41	66	21.50	263	11.04	334	12.34
Kalinga	4,582	2	25.00	82	29.08	452	22.23	536	23.07
Mountain Province	2,615	1	6.67	7	1.67	27	0.67	35	0.79
City of Baguio	5,387	1	25.00	4	1.89	10	0.37	15	0.52
**Region 1**	96,024	8	7.62	280	7.52	1,665	4.28	1,953	4.57
Ilocos Norte	8,809	0	0.00	6	1.55	36	0.73	42	0.79
Ilocos Sur	9,834	0	0.00	22	4.84	109	1.66	131	1.86
La Union	13,436	1	4.35	26	3.82	142	2.35	169	2.50
Pangasinan	60,591	6	9.52	189	8.86	1,026	4.94	1,221	5.31
City of Dagupan	3,354	1	33.33	37	56.06	352	62.63	390	61.81
**Region 2**	64,034	13	11.11	601	13.52	1,847	7.67	2,461	8.59
Batanes	241	0	0.00	0	0.00	0	0.00	0	0.00
Cagayan	20,723	3	8.82	113	11.05	590	8.37	706	8.71
Isabela	27,362	6	10.71	376	16.05	783	7.17	1,165	8.75
Nueva Vizcaya	8,880	4	19.05	72	11.52	336	9.11	412	9.51
Quirino	3,865	0	0.00	33	8.21	105	5.57	138	6.02
City of Santiago	2,963	0	0.00	7	14.29	33	7.55	40	8.21
**Region 3**	235,313	33	4.08	880	7.74	4,072	4.90	4,985	5.23
Aurora	4,578	0	0.00	25	9.09	96	5.98	121	6.41
Bataan	17,556	7	17.07	135	12.77	644	8.95	786	9.48
Bulacan	68,115	17	2.89	486	9.73	2,140	5.37	2,643	5.82
Nueva Ecija	43,626	4	4.88	75	3.82	396	3.53	475	3.58
Pampanga	46,146	1	2.78	36	3.28	227	2.60	264	2.67
Tarlac	29,655	3	15.79	81	7.16	364	3.84	448	4.22
Zambales	11,796	1	11.11	20	3.43	78	2.58	99	2.74
City of Angeles	9,232	0	0.00	7	5.19	30	3.31	37	3.48
City of Olongapo	4,609	0	0.00	15	10.87	97	8.66	112	8.87
**Region 4A**	310,150	52	18.51	1,392	13.13	8,532	8.35	9,976	8.82
Batangas	53,484	15	55.56	99	7.92	814	4.83	928	5.12
Cavite	78,589	8	15.38	269	12.33	1,756	7.79	2,033	8.21
Laguna	61,967	17	22.08	411	17.00	2,463	9.46	2,891	10.13
Quezon	40,954	10	19.61	313	18.03	1,664	13.09	1,987	13.70
Rizal	68,903	2	3.23	243	9.17	1,595	7.23	1,840	7.43

Table 2.B.1.11 - Prenatal Care
Pregnant Women tested for Complete Blood Count or Hgb & Hct count diagnosed with Anemia
Philippines, 2024

Area	Eligible Population (0-11 months)	Pregnant women tested for CBC/Hgb and Hct diagnosed with anemia							
		Age Group						Total	%
		10-14 yrs old		15-19 yrs old		20-49 yrs old			
		No.	%	No.	%	No.	%		
City of Lucena	6,253	0	**0.00**	57	**15.53**	240	**11.89**	**297**	**12.39**
**Region 4B**	**66,508**	**9**	**19.57**	**308**	**11.47**	**1,513**	**8.58**	**1,830**	**8.99**
Marinduque	4,124	2	**25.00**	18	**14.40**	80	**6.33**	**100**	**7.16**
Occidental Mindoro	11,748	2	**28.57**	57	**10.58**	349	**11.09**	**408**	**11.05**
Oriental Mindoro	19,007	1	**10.00**	20	**4.26**	218	**5.27**	**239**	**5.18**
Palawan	20,503	4	**25.00**	158	**16.09**	550	**10.87**	**712**	**11.75**
Romblon	5,488	0	**0.00**	22	**11.28**	121	**6.96**	**143**	**7.38**
City of Puerto Princesa	5,638	0	**0.00**	33	**8.78**	195	**8.54**	**228**	**8.57**
**Region 5**	**136,611**	**11**	**16.92**	**491**	**12.39**	**3,213**	**9.70**	**3,715**	**10.00**
Albay	27,665	2	**33.33**	50	**7.13**	565	**5.79**	**617**	**5.90**
Camarines Norte	14,894	2	**15.38**	130	**21.85**	740	**17.59**	**872**	**18.11**
Camarines Sur	43,855	0	**0.00**	19	**4.11**	229	**4.91**	**248**	**4.83**
Catanduanes	5,395	0	**0.00**	25	**6.13**	156	**5.85**	**181**	**5.87**
Masbate	22,495	3	**27.27**	129	**14.61**	630	**13.52**	**762**	**13.72**
Sorsogon	18,065	4	**23.53**	138	**15.33**	888	**12.62**	**1,030**	**12.95**
City of Naga	4,242	0	**0.00**	0	**0.00**	5	**3.50**	**5**	**3.16**
**Region 6**	**147,934**	**13**	**12.38**	**586**	**9.10**	**3,101**	**6.13**	**3,700**	**6.48**
Aklan	11,281	0	**0.00**	77	**18.97**	358	**9.00**	**435**	**9.91**
Antique	11,899	2	**40.00**	73	**17.51**	360	**10.96**	**435**	**11.73**
Capiz	13,872	1	**10.00**	32	**7.36**	233	**5.54**	**266**	**5.72**
Guimaras	3,361	1	**11.11**	19	**9.64**	183	**10.26**	**203**	**10.20**
Iloilo	35,981	4	**20.00**	78	**5.32**	362	**2.71**	**444**	**2.99**
Negros Occidental	52,445	5	**10.64**	260	**9.08**	1,154	**6.52**	**1,419**	**6.88**
City of Bacolod	10,361	0	**0.00**	33	**8.44**	299	**8.67**	**332**	**8.65**
City of Iloilo	8,734	0	**0.00**	14	**5.34**	152	**5.42**	**166**	**5.40**
**Region 7**	**167,016**	**29**	**15.43**	**956**	**9.88**	**5,152**	**7.00**	**6,137**	**7.35**
Bohol	26,210	1	**3.33**	126	**11.70**	797	**8.04**	**924**	**8.38**
Cebu	72,875	13	**36.11**	461	**13.99**	2,051	**9.11**	**2,525**	**9.77**
Negros Oriental	28,980	7	**22.58**	222	**14.66**	1,152	**12.10**	**1,381**	**12.48**
Siquijor	1,671	0	**0.00**	3	**6.82**	23	**4.03**	**26**	**4.22**
City of Cebu	19,466	3	**18.75**	57	**5.56**	471	**5.61**	**531**	**5.63**
City of Lapu-Lapu	10,833	0	**0.00**	80	**14.71**	572	**10.20**	**652**	**10.58**
City of Mandaue	6,981	5	**7.81**	7	**0.32**	86	**0.50**	**98**	**0.51**
**Region 8**	**93,713**	**6**	**10.17**	**308**	**8.28**	**2,043**	**7.11**	**2,357**	**7.25**
Biliran	3,378	1	**50.00**	64	**27.59**	272	**16.07**	**337**	**17.49**
Eastern Samar	9,594	1	**11.11**	29	**7.67**	142	**5.75**	**172**	**6.02**
Leyte	31,504	3	**12.00**	71	**6.44**	643	**7.17**	**717**	**7.10**
Northern Samar	14,053	0	**0.00**	72	**10.84**	386	**8.63**	**458**	**8.90**
Southern Leyte	7,368	1	**14.29**	29	**8.71**	225	**7.03**	**255**	**7.20**
Samar	16,946	0	**0.00**	24	**5.67**	251	**7.35**	**275**	**7.16**
Ormoc City	5,344	0	**0.00**	16	**6.23**	121	**6.67**	**137**	**6.61**
City of Tacloban	5,526	0	**0.00**	3	**0.90**	3	**0.11**	**6**	**0.20**
**Region 9**	**85,348**	**5**	**11.11**	**407**	**13.82**	**1,560**	**9.97**	**1,972**	**10.58**
Zamboanga del Norte	24,977	3	**33.33**	71	**9.85**	280	**7.10**	**354**	**7.57**
Zamboanga del Sur	23,905	1	**5.56**	129	**16.00**	578	**13.77**	**708**	**14.10**
Zamboanga Sibugay	13,903	1	**7.14**	191	**25.03**	563	**15.18**	**755**	**16.83**
City of Isabela	3,209	0	**0.00**	7	**3.47**	28	**2.39**	**35**	**2.55**
City of Zamboanga	19,354	0	**0.00**	9	**1.99**	111	**4.24**	**120**	**3.90**
**Region 10**	**110,290**	**21**	**12.07**	**963**	**12.46**	**3,569**	**8.38**	**4,553**	**9.02**
Bukidnon	33,913	17	**19.77**	610	**18.76**	1,997	**14.94**	**2,624**	**15.71**
Camiguin	1,727	0	**0.00**	11	**9.40**	21	**2.19**	**32**	**2.97**
Lanao del Norte	17,881	0	**0.00**	10	**2.22**	99	**3.12**	**109**	**3.00**
Misamis Occidental	12,342	0	**0.00**	19	**2.97**	165	**3.19**	**184**	**3.16**
Misamis Oriental	22,184	2	**5.26**	158	**8.73**	598	**6.35**	**758**	**6.73**
City of Cagayan De Oro	14,503	2	**9.09**	111	**9.67**	394	**4.97**	**507**	**5.58**
City of Iligan	7,740	0	**0.00**	44	**14.10**	295	**11.50**	**339**	**11.74**

Table 2.B.1.11 - Prenatal Care
Pregnant Women tested for Complete Blood Count or Hgb & Hct count diagnosed with Anemia
Philippines, 2024

Area	Eligible Population (0-11 months)	Pregnant women tested for CBC/Hgb and Hct diagnosed with anemia							
		Age Group						Total	%
		10-14 yrs old		15-19 yrs old		20-49 yrs old			
		No.	%	No.	%	No.	%		
**Region 11**	**107,934**	**170**	**38.55**	**3,254**	**33.35**	**14,111**	**25.55**	**17,535**	**26.80**
Davao de Oro	15,434	38	**40.86**	554	**32.32**	2,269	**25.87**	**2,861**	**27.05**
Davao del Norte	23,685	22	**22.68**	497	**23.48**	1,973	**15.98**	**2,492**	**17.12**
Davao Oriental	12,025	28	**46.67**	456	**39.55**	1,852	**30.95**	**2,336**	**32.46**
Davao del Sur	13,178	27	**40.30**	476	**37.22**	1,961	**32.34**	**2,464**	**33.26**
Davao Occidental	6,606	6	**20.69**	211	**27.44**	573	**28.24**	**790**	**27.94**
City of Davao	37,006	49	**51.58**	1,060	**38.90**	5,483	**27.36**	**6,592**	**28.84**
**Region 12**	**95,296**	**26**	**10.83**	**917**	**12.42**	**3,930**	**10.32**	**4,873**	**10.66**
Cotabato	25,696	4	**7.27**	170	**9.30**	930	**9.25**	**1,104**	**9.25**
Sarangani	13,501	7	**16.28**	208	**12.84**	778	**11.88**	**993**	**12.09**
South Cotabato	21,842	8	**11.76**	150	**8.34**	684	**7.28**	**842**	**7.48**
Sultan Kudarat	19,613	3	**7.50**	83	**6.58**	439	**6.67**	**525**	**6.66**
City of General Santos	14,644	4	**11.76**	306	**34.93**	1,099	**19.93**	**1,409**	**21.93**
**CARAGA**	**55,371**	**16**	**21.92**	**948**	**23.64**	**3,969**	**16.75**	**4,933**	**17.75**
Agusan del Norte	7,331	3	**42.86**	99	**25.65**	364	**14.99**	**466**	**16.52**
Agusan del Sur	16,180	6	**17.65**	459	**28.80**	1,691	**20.71**	**2,156**	**22.02**
Surigao del Norte	10,168	0	**0.00**	78	**13.40**	413	**11.40**	**491**	**11.65**
Surigao del Sur	12,834	4	**28.57**	246	**28.44**	1,202	**22.20**	**1,452**	**23.07**
Dinagat Islands	2,117	1	**50.00**	15	**11.36**	93	**10.48**	**109**	**10.68**
City of Butuan	6,741	2	**28.57**	51	**11.28**	206	**6.47**	**259**	**7.11**
**BARMM**	**134,585**	**1**	**4.76**	**147**	**11.56**	**768**	**6.50**	**916**	**6.99**
Basilan	11,653	0	**0.00**	10	**9.26**	34	**8.15**	**44**	**8.32**
Lanao del Sur	33,339	0	**0.00**	3	**0.91**	81	**1.87**	**84**	**1.80**
Maguindanao del Norte	18,923	1	**16.67**	49	**18.70**	225	**10.26**	**275**	**11.17**
Maguindanao del Sur	23,214	0	**0.00**	46	**15.75**	198	**8.49**	**244**	**9.30**
Sulu	21,381	0	**0.00**	22	**20.00**	134	**14.99**	**156**	**15.52**
Tawi-Tawi	12,602	0	**0.00**	2	**10.53**	10	**11.11**	**12**	**11.01**
SGA	6,305	0	**0.00**	7	**14.29**	30	**8.50**	**37**	**9.18**
City of Cotabato	7,168	0	**0.00**	8	**7.77**	56	**4.62**	**64**	**4.87**

*Legend: * - No Report*

Table 2.B.1.12 - Prenatal Care
Pregnant Women screened for Gestational Diabetes
Philippines, 2024

Area	Eligible Population (0-11 months)	Total No. of pregnant women screened for Gestational Diabetes							
		Age Group						Total	%
		10-14 yrs old		15-19 yrs old		20-49 yrs old			
		No.	%	No.	%	No.	%		
**PHILIPPINES**	**2,200,865**	**1,453**	**0.07**	**41,397**	**1.88**	**376,036**	**17.09**	**418,886**	**19.03**
**N C R**	**263,248**	**171**	**0.06**	**5,935**	**2.25**	**81,861**	**31.10**	**87,967**	**33.42**
City of Malabon	7,447	3	0.04	61	0.82	518	6.96	**582**	**7.82**
City of Navotas	5,263	16	0.30	286	5.43	2,564	48.72	**2,866**	**54.46**
City of Valenzuela	13,601	5	0.04	99	0.73	1,650	12.13	**1,754**	**12.90**
City of Caloocan	33,499	16	0.05	588	1.76	7,579	22.62	**8,183**	**24.43**
City of Marikina	8,399	5	0.06	178	2.12	2,062	24.55	**2,245**	**26.73**
City of Pasig	17,856	15	0.08	415	2.32	6,941	38.87	**7,371**	**41.28**
Pateros	1,120	1	0.09	41	3.66	499	44.55	**541**	**48.30**
City of Taguig	26,021	20	0.08	715	2.75	7,424	28.53	**8,159**	**31.36**
Quezon City	59,607	11	0.02	507	0.85	20,248	33.97	**20,766**	**34.84**
City of Makati	4,196	3	0.07	106	2.53	1,093	26.05	**1,202**	**28.65**
City of Mandaluyong	7,989	6	0.08	235	2.94	5,227	65.43	**5,468**	**68.44**
City of San Juan	2,173	1	0.05	37	1.70	534	24.57	**572**	**26.32**
City of Manila	34,268	57	0.17	1,831	5.34	15,448	45.08	**17,336**	**50.59**
City of Las Piñas	11,346	2	0.02	93	0.82	2,024	17.84	**2,119**	**18.68**
City of Muntinlupa	9,949	3	0.03	171	1.72	1,790	17.99	**1,964**	**19.74**
City of Parañaque	13,385	1	0.01	346	2.58	3,359	25.10	**3,706**	**27.69**
Pasay City	7,129	6	0.08	226	3.17	2,901	40.69	**3,133**	**43.95**
**C A R**	**31,490**	**28**	**0.09**	**789**	**2.51**	**8,565**	**27.20**	**9,382**	**29.79**
Abra	3,639	0	0.00	17	0.47	153	4.20	**170**	**4.67**
Apayao	2,222	6	0.27	135	6.08	864	38.88	**1,005**	**45.23**
Benguet	8,900	8	0.09	304	3.42	3,947	44.35	**4,259**	**47.85**
Ifugao	4,145	10	0.24	74	1.79	621	14.98	**705**	**17.01**
Kalinga	4,582	1	0.02	47	1.03	336	7.33	**384**	**8.38**
Mountain Province	2,615	0	0.00	8	0.31	95	3.63	**103**	**3.94**
City of Baguio	5,387	3	0.06	204	3.79	2,549	47.32	**2,756**	**51.16**
**Region 1**	**96,024**	**46**	**0.05**	**2,061**	**2.15**	**21,880**	**22.79**	**23,987**	**24.98**
Ilocos Norte	8,809	1	0.01	175	1.99	2,040	23.16	**2,216**	**25.16**
Ilocos Sur	9,834	10	0.10	356	3.62	5,342	54.32	**5,708**	**58.04**
La Union	13,436	15	0.11	467	3.48	4,365	32.49	**4,847**	**36.07**
Pangasinan	60,591	20	0.03	1,060	1.75	10,110	16.69	**11,190**	**18.47**
City of Dagupan	3,354	0	0.00	3	0.09	23	0.69	**26**	**0.78**
**Region 2**	**64,034**	**35**	**0.05**	**1,042**	**1.63**	**7,350**	**11.48**	**8,427**	**13.16**
Batanes	241	0	0.00	2	0.83	62	25.73	**64**	**26.56**
Cagayan	20,723	4	0.02	110	0.53	707	3.41	**821**	**3.96**
Isabela	27,362	19	0.07	460	1.68	3,236	11.83	**3,715**	**13.58**
Nueva Vizcaya	8,880	7	0.08	312	3.51	2,640	29.73	**2,959**	**33.32**
Quirino	3,865	5	0.13	144	3.73	607	15.71	**756**	**19.56**
City of Santiago	2,963	0	0.00	14	0.47	98	3.31	**112**	**3.78**
**Region 3**	**235,313**	**582**	**0.25**	**5,550**	**2.36**	**44,415**	**18.87**	**50,547**	**21.48**
Aurora	4,578	2	0.04	65	1.42	373	8.15	**440**	**9.61**
Bataan	17,556	16	0.09	430	2.45	3,296	18.77	**3,742**	**21.31**
Bulacan	68,115	513	0.75	3,166	4.65	26,802	39.35	**30,481**	**44.75**
Nueva Ecija	43,626	29	0.07	784	1.80	5,024	11.52	**5,837**	**13.38**
Pampanga	46,146	13	0.03	477	1.03	4,515	9.78	**5,005**	**10.85**
Tarlac	29,655	3	0.01	427	1.44	2,977	10.04	**3,407**	**11.49**
Zambales	11,796	5	0.04	111	0.94	644	5.46	**760**	**6.44**
City of Angeles	9,232	0	0.00	32	0.35	263	2.85	**295**	**3.20**
City of Olongapo	4,609	1	0.02	58	1.26	521	11.30	**580**	**12.58**
**Region 4A**	**310,150**	**123**	**0.04**	**5,472**	**1.76**	**56,740**	**18.29**	**62,335**	**20.10**
Batangas	53,484	10	0.02	557	1.04	7,103	13.28	**7,670**	**14.34**
Cavite	78,589	24	0.03	1,282	1.63	13,806	17.57	**15,112**	**19.23**
Laguna	61,967	34	0.05	1,149	1.85	14,759	23.82	**15,942**	**25.73**
Quezon	40,954	13	0.03	532	1.30	4,333	10.58	**4,878**	**11.91**
Rizal	68,903	37	0.05	1,806	2.62	15,676	22.75	**17,519**	**25.43**
City of Lucena	6,253	5	0.08	146	2.33	1,063	17.00	**1,214**	**19.41**
**Region 4B**	**66,508**	**18**	**0.03**	**1,039**	**1.56**	**6,961**	**10.47**	**8,018**	**12.06**
Marinduque	4,124	3	0.07	58	1.41	712	17.26	**773**	**18.74**
Occidental Mindoro	11,748	1	0.01	130	1.11	752	6.40	**883**	**7.52**
Oriental Mindoro	19,007	4	0.02	147	0.77	1,454	7.65	**1,605**	**8.44**
Palawan	20,503	10	0.05	575	2.80	2,865	13.97	**3,450**	**16.83**
Romblon	5,488	0	0.00	91	1.66	892	16.25	**983**	**17.91**

Table 2.B.1.12 - Prenatal Care
Pregnant Women screened for Gestational Diabetes
Philippines, 2024

Area	Eligible Population (0-11 months)	Total No. of pregnant women screened for Gestational Diabetes							
		Age Group						Total	%
		10-14 yrs old		15-19 yrs old		20-49 yrs old			
		No.	%	No.	%	No.	%		
City of Puerto Princesa	5,638	0	0.00	38	0.67	286	5.07	**324**	**5.75**
**Region 5**	**136,611**	**19**	**0.01**	**1,247**	**0.91**	**12,663**	**9.27**	**13,929**	**10.20**
Albay	27,665	3	0.01	284	1.03	4,713	17.04	**5,000**	**18.07**
Camarines Norte	14,894	2	0.01	86	0.58	923	6.20	**1,011**	**6.79**
Camarines Sur	43,855	7	0.02	277	0.63	2,415	5.51	**2,699**	**6.15**
Catanduanes	5,395	1	0.02	57	1.06	484	8.97	**542**	**10.05**
Masbate	22,495	2	0.01	325	1.44	1,857	8.26	**2,184**	**9.71**
Sorsogon	18,065	4	0.02	214	1.18	2,171	12.02	**2,389**	**13.22**
City of Naga	4,242	0	0.00	4	0.09	100	2.36	**104**	**2.45**
**Region 6**	**147,934**	**81**	**0.05**	**4,309**	**2.91**	**36,968**	**24.99**	**41,358**	**27.96**
Aklan	11,281	1	0.01	234	2.07	2,471	21.90	**2,706**	**23.99**
Antique	11,899	2	0.02	158	1.33	1,291	10.85	**1,451**	**12.19**
Capiz	13,872	5	0.04	311	2.24	2,916	21.02	**3,232**	**23.30**
Guimaras	3,361	6	0.18	156	4.64	1,466	43.62	**1,628**	**48.44**
Iloilo	35,981	18	0.05	1,160	3.22	10,712	29.77	**11,890**	**33.05**
Negros Occidental	52,445	40	0.08	1,726	3.29	12,665	24.15	**14,431**	**27.52**
City of Bacolod	10,361	2	0.02	367	3.54	3,176	30.65	**3,545**	**34.21**
City of Iloilo	8,734	7	0.08	197	2.26	2,271	26.00	**2,475**	**28.34**
**Region 7**	**167,016**	**85**	**0.05**	**3,253**	**1.95**	**25,465**	**15.25**	**28,803**	**17.25**
Bohol	26,210	3	0.01	189	0.72	2,335	8.91	**2,527**	**9.64**
Cebu	72,875	16	0.02	1,262	1.73	8,871	12.17	**10,149**	**13.93**
Negros Oriental	28,980	6	0.02	265	0.91	1,962	6.77	**2,233**	**7.71**
Siquijor	1,671	0	0.00	10	0.60	130	7.78	**140**	**8.38**
City of Cebu	19,466	4	0.02	457	2.35	4,227	21.71	**4,688**	**24.08**
City of Lapu-Lapu	10,833	1	0.01	216	1.99	2,406	22.21	**2,623**	**24.21**
City of Mandaue	6,981	55	0.79	854	12.23	5,534	79.27	**6,443**	**92.29**
**Region 8**	**93,713**	**29**	**0.03**	**1,113**	**1.19**	**8,768**	**9.36**	**9,910**	**10.57**
Biliran	3,378	0	0.00	13	0.38	131	3.88	**144**	**4.26**
Eastern Samar	9,594	3	0.03	116	1.21	934	9.74	**1,053**	**10.98**
Leyte	31,504	16	0.05	263	0.83	1,992	6.32	**2,271**	**7.21**
Northern Samar	14,053	6	0.04	233	1.66	1,458	10.38	**1,697**	**12.08**
Southern Leyte	7,368	0	0.00	84	1.14	766	10.40	**850**	**11.54**
Samar	16,946	1	0.01	119	0.70	1,028	6.07	**1,148**	**6.77**
Ormoc City	5,344	1	0.02	105	1.96	808	15.12	**914**	**17.10**
City of Tacloban	5,526	2	0.04	180	3.26	1,651	29.88	**1,833**	**33.17**
**Region 9**	**85,348**	**9**	**0.01**	**521**	**0.61**	**3,030**	**3.55**	**3,560**	**4.17**
Zamboanga del Norte	24,977	2	0.01	100	0.40	722	2.89	**824**	**3.30**
Zamboanga del Sur	23,905	5	0.02	98	0.41	509	2.13	**612**	**2.56**
Zamboanga Sibugay	13,903	0	0.00	31	0.22	109	0.78	**140**	**1.01**
City of Isabela	3,209	0	0.00	140	4.36	760	23.68	**900**	**28.05**
City of Zamboanga	19,354	2	0.01	152	0.79	930	4.81	**1,084**	**5.60**
**Region 10**	**110,290**	**57**	**0.05**	**3,043**	**2.76**	**19,012**	**17.24**	**22,112**	**20.05**
Bukidnon	33,913	19	0.06	662	1.95	3,199	9.43	**3,880**	**11.44**
Camiguin	1,727	2	0.12	111	6.43	874	50.61	**987**	**57.15**
Lanao del Norte	17,881	1	0.01	49	0.27	523	2.92	**573**	**3.20**
Misamis Occidental	12,342	0	0.00	259	2.10	2,330	18.88	**2,589**	**20.98**
Misamis Oriental	22,184	20	0.09	924	4.17	5,022	22.64	**5,966**	**26.89**
City of Cagayan De Oro	14,503	13	0.09	946	6.52	6,243	43.05	**7,202**	**49.66**
City of Iligan	7,740	2	0.03	92	1.19	821	10.61	**915**	**11.82**
**Region 11**	**107,934**	**87**	**0.08**	**2,204**	**2.04**	**16,504**	**15.29**	**18,795**	**17.41**
Davao de Oro	15,434	2	0.01	60	0.39	551	3.57	**613**	**3.97**
Davao del Norte	23,685	4	0.02	218	0.92	2,435	10.28	**2,657**	**11.22**
Davao Oriental	12,025	1	0.01	43	0.36	338	2.81	**382**	**3.18**
Davao del Sur	13,178	9	0.07	179	1.36	1,054	8.00	**1,242**	**9.42**
Davao Occidental	6,606	19	0.29	414	6.27	1,140	17.26	**1,573**	**23.81**
City of Davao	37,006	52	0.14	1,290	3.49	10,986	29.69	**12,328**	**33.31**
**Region 12**	**95,296**	**63**	**0.07**	**2,414**	**2.53**	**13,800**	**14.48**	**16,277**	**17.08**
Cotabato	25,696	16	0.06	524	2.04	3,482	13.55	**4,022**	**15.65**
Sarangani	13,501	12	0.09	614	4.55	2,794	20.69	**3,420**	**25.33**
South Cotabato	21,842	15	0.07	609	2.79	3,351	15.34	**3,975**	**18.20**
Sultan Kudarat	19,613	15	0.08	427	2.18	2,364	12.05	**2,806**	**14.31**

Table 2.B.1.12 - Prenatal Care
Pregnant Women screened for Gestational Diabetes
Philippines, 2024

Area	Eligible Population (0-11 months)	Total No. of pregnant women screened for Gestational Diabetes							
		Age Group						Total	%
		10-14 yrs old		15-19 yrs old		20-49 yrs old			
		No.	%	No.	%	No.	%		
City of General Santos	14,644	5	0.03	240	1.64	1,809	12.35	**2,054**	**14.03**
**CARAGA**	**55,371**	**14**	**0.03**	**875**	**1.58**	**5,800**	**10.47**	**6,689**	**12.08**
Agusan del Norte	7,331	2	0.03	98	1.34	629	8.58	**729**	**9.94**
Agusan del Sur	16,180	5	0.03	162	1.00	1,181	7.30	**1,348**	**8.33**
Surigao del Norte	10,168	3	0.03	239	2.35	1,468	14.44	**1,710**	**16.82**
Surigao del Sur	12,834	1	0.01	131	1.02	780	6.08	**912**	**7.11**
Dinagat Islands	2,117	0	0.00	56	2.65	434	20.50	**490**	**23.15**
City of Butuan	6,741	3	0.04	189	2.80	1,308	19.40	**1,500**	**22.25**
**BARMM**	**134,585**	**6**	**0.00**	**530**	**0.39**	**6,254**	**4.65**	**6,790**	**5.05**
Basilan	11,653	0	0.00	32	0.27	91	0.78	**123**	**1.06**
Lanao del Sur	33,339	0	0.00	24	0.07	2,025	6.07	**2,049**	**6.15**
Maguindanao del Norte	18,923	0	0.00	88	0.47	1,112	5.88	**1,200**	**6.34**
Maguindanao del Sur	23,214	4	0.02	170	0.73	1,277	5.50	**1,451**	**6.25**
Sulu	21,381	1	0.00	86	0.40	662	3.10	**749**	**3.50**
Tawi-Tawi	12,602	0	0.00	3	0.02	6	0.05	**9**	**0.07**
SGA	6,305	0	0.00	9	0.14	40	0.63	**49**	**0.78**
City of Cotabato	7,168	1	0.01	118	1.65	1,041	14.52	**1,160**	**16.18**

*Legend: * - No Report*

Table 2.B.1.13 - Prenatal Care
Pregnant Women tested positive for Gestational Diabetes
Philippines, 2024

Area	Eligible Population (0-11 months)	Pregnant women tested positive for Gestational Diabetes							
		Age Group						Total	%
		10-14 yrs old		15-19 yrs old		20-49 yrs old			
		No.	%	No.	%	No.	%		
**PHILIPPINES**	**2,200,865**	**31**	**2.13**	**684**	**1.65**	**10,022**	**2.67**	**10,737**	**2.56**
**N C R**	**263,248**	**2**	**1.17**	**116**	**1.95**	**3,178**	**3.88**	**3,296**	**3.75**
City of Malabon	7,447	0	**0.00**	0	**0.00**	30	**5.79**	**30**	**5.15**
City of Navotas	5,263	0	**0.00**	0	**0.00**	89	**3.47**	**89**	**3.11**
City of Valenzuela	13,601	0	**0.00**	1	**1.01**	158	**9.58**	**159**	**9.06**
City of Caloocan	33,499	1	**6.25**	62	**10.54**	979	**12.92**	**1,042**	**12.73**
City of Marikina	8,399	0	**0.00**	1	**0.56**	17	**0.82**	**18**	**0.80**
City of Pasig	17,856	0	**0.00**	19	**4.58**	622	**8.96**	**641**	**8.70**
Pateros	1,120	0	**0.00**	0	**0.00**	15	**3.01**	**15**	**2.77**
City of Taguig	26,021	1	**5.00**	19	**2.66**	330	**4.45**	**350**	**4.29**
Quezon City	59,607	0	**0.00**	4	**0.79**	489	**2.42**	**493**	**2.37**
City of Makati	4,196	0	**0.00**	1	**0.94**	52	**4.76**	**53**	**4.41**
City of Mandaluyong	7,989	0	**0.00**	0	**0.00**	20	**0.38**	**20**	**0.37**
City of San Juan	2,173	0	**0.00**	0	**0.00**	11	**2.06**	**11**	**1.92**
City of Manila	34,268	0	**0.00**	0	**0.00**	72	**0.47**	**72**	**0.42**
City of Las Piñas	11,346	0	**0.00**	1	**1.08**	60	**2.96**	**61**	**2.88**
City of Muntinlupa	9,949	0	**0.00**	3	**1.75**	51	**2.85**	**54**	**2.75**
City of Parañaque	13,385	0	**0.00**	4	**1.16**	116	**3.45**	**120**	**3.24**
Pasay City	7,129	0	**0.00**	1	**0.44**	67	**2.31**	**68**	**2.17**
**C A R**	**31,490**	**0**	**0.00**	**14**	**1.77**	**238**	**2.78**	**252**	**2.69**
Abra	3,639	0	**0.00**	0	**0.00**	6	**3.92**	**6**	**3.53**
Apayao	2,222	0	**0.00**	2	**1.48**	65	**7.52**	**67**	**6.67**
Benguet	8,900	0	**0.00**	11	**3.62**	132	**3.34**	**143**	**3.36**
Ifugao	4,145	0	**0.00**	0	**0.00**	13	**2.09**	**13**	**1.84**
Kalinga	4,582	0	**0.00**	0	**0.00**	4	**1.19**	**4**	**1.04**
Mountain Province	2,615	0	**0.00**	0	**0.00**	10	**10.53**	**10**	**9.71**
City of Baguio	5,387	0	**0.00**	1	**0.49**	8	**0.31**	**9**	**0.33**
**Region 1**	**96,024**	**0**	**0.00**	**3**	**0.15**	**195**	**0.89**	**198**	**0.83**
Ilocos Norte	8,809	0	**0.00**	0	**0.00**	23	**1.13**	**23**	**1.04**
Ilocos Sur	9,834	0	**0.00**	0	**0.00**	28	**0.52**	**28**	**0.49**
La Union	13,436	0	**0.00**	0	**0.00**	52	**1.19**	**52**	**1.07**
Pangasinan	60,591	0	**0.00**	3	**0.28**	92	**0.91**	**95**	**0.85**
City of Dagupan	3,354	0	**0.00**	0	**0.00**	0	**0.00**	**0**	**0.00**
**Region 2**	**64,034**	**0**	**0.00**	**60**	**5.76**	**450**	**6.12**	**510**	**6.05**
Batanes	241	0	**0.00**	0	**0.00**	2	**3.23**	**2**	**3.13**
Cagayan	20,723	0	**0.00**	0	**0.00**	24	**3.39**	**24**	**2.92**
Isabela	27,362	0	**0.00**	37	**8.04**	123	**3.80**	**160**	**4.31**
Nueva Vizcaya	8,880	0	**0.00**	22	**7.05**	285	**10.80**	**307**	**10.38**
Quirino	3,865	0	**0.00**	1	**0.69**	13	**2.14**	**14**	**1.85**
City of Santiago	2,963	0	**0.00**	0	**0.00**	3	**3.06**	**3**	**2.68**
**Region 3**	**235,313**	**5**	**0.86**	**35**	**0.63**	**781**	**1.76**	**821**	**1.62**
Aurora	4,578	0	**0.00**	0	**0.00**	9	**2.41**	**9**	**2.05**
Bataan	17,556	1	**6.25**	10	**2.33**	135	**4.10**	**146**	**3.90**
Bulacan	68,115	4	**0.78**	15	**0.47**	409	**1.53**	**428**	**1.40**
Nueva Ecija	43,626	0	**0.00**	5	**0.64**	55	**1.09**	**60**	**1.03**
Pampanga	46,146	0	**0.00**	2	**0.42**	99	**2.19**	**101**	**2.02**
Tarlac	29,655	0	**0.00**	3	**0.70**	45	**1.51**	**48**	**1.41**
Zambales	11,796	0	**0.00**	0	**0.00**	15	**2.33**	**15**	**1.97**
City of Angeles	9,232	0	**0.00**	0	**0.00**	5	**1.90**	**5**	**1.69**
City of Olongapo	4,609	0	**0.00**	0	**0.00**	9	**1.73**	**9**	**1.55**
**Region 4A**	**310,150**	**6**	**4.88**	**122**	**2.23**	**1,619**	**2.85**	**1,747**	**2.80**
Batangas	53,484	0	**0.00**	6	**1.08**	124	**1.75**	**130**	**1.69**
Cavite	78,589	0	**0.00**	17	**1.33**	247	**1.79**	**264**	**1.75**
Laguna	61,967	0	**0.00**	28	**2.44**	528	**3.58**	**556**	**3.49**
Quezon	40,954	0	**0.00**	7	**1.32**	203	**4.68**	**210**	**4.31**
Rizal	68,903	6	**16.22**	61	**3.38**	504	**3.22**	**571**	**3.26**
City of Lucena	6,253	0	**0.00**	3	**2.05**	13	**1.22**	**16**	**1.32**
**Region 4B**	**66,508**	**2**	**11.11**	**9**	**0.87**	**145**	**2.08**	**156**	**1.95**
Marinduque	4,124	0	**0.00**	0	**0.00**	4	**0.56**	**4**	**0.52**
Occidental Mindoro	11,748	0	**0.00**	2	**1.54**	33	**4.39**	**35**	**3.96**
Oriental Mindoro	19,007	0	**0.00**	2	**1.36**	28	**1.93**	**30**	**1.87**
Palawan	20,503	2	**20.00**	4	**0.70**	51	**1.78**	**57**	**1.65**
Romblon	5,488	0	**0.00**	1	**1.10**	16	**1.79**	**17**	**1.73**

Table 2.B.1.13 - Prenatal Care
Pregnant Women tested positive for Gestational Diabetes
Philippines, 2024

Area	Eligible Population (0-11 months)	Pregnant women tested positive for Gestational Diabetes							
		Age Group						Total	%
		10-14 yrs old		15-19 yrs old		20-49 yrs old			
		No.	%	No.	%	No.	%		
City of Puerto Princesa	5,638	0	**0.00**	0	**0.00**	13	**4.55**	**13**	**4.01**
**Region 5**	**136,611**	**0**	**0.00**	**16**	**1.28**	**333**	**2.63**	**349**	**2.51**
Albay	27,665	0	**0.00**	2	**0.70**	97	**2.06**	**99**	**1.98**
Camarines Norte	14,894	0	**0.00**	2	**2.33**	32	**3.47**	**34**	**3.36**
Camarines Sur	43,855	0	**0.00**	0	**0.00**	72	**2.98**	**72**	**2.67**
Catanduanes	5,395	0	**0.00**	0	**0.00**	18	**3.72**	**18**	**3.32**
Masbate	22,495	0	**0.00**	1	**0.31**	30	**1.62**	**31**	**1.42**
Sorsogon	18,065	0	**0.00**	11	**5.14**	82	**3.78**	**93**	**3.89**
City of Naga	4,242	0	**0.00**	0	**0.00**	2	**2.00**	**2**	**1.92**
**Region 6**	**147,934**	**0**	**0.00**	**106**	**2.46**	**911**	**2.46**	**1,017**	**2.46**
Aklan	11,281	0	**0.00**	1	**0.43**	78	**3.16**	**79**	**2.92**
Antique	11,899	0	**0.00**	12	**7.59**	81	**6.27**	**93**	**6.41**
Capiz	13,872	0	**0.00**	3	**0.96**	33	**1.13**	**36**	**1.11**
Guimaras	3,361	0	**0.00**	0	**0.00**	13	**0.89**	**13**	**0.80**
Iloilo	35,981	0	**0.00**	32	**2.76**	219	**2.04**	**251**	**2.11**
Negros Occidental	52,445	0	**0.00**	14	**0.81**	176	**1.39**	**190**	**1.32**
City of Bacolod	10,361	0	**0.00**	44	**11.99**	261	**8.22**	**305**	**8.60**
City of Iloilo	8,734	0	**0.00**	0	**0.00**	50	**2.20**	**50**	**2.02**
**Region 7**	**167,016**	**3**	**3.53**	**59**	**1.81**	**676**	**2.65**	**738**	**2.56**
Bohol	26,210	0	**0.00**	13	**6.88**	145	**6.21**	**158**	**6.25**
Cebu	72,875	3	**18.75**	35	**2.77**	330	**3.72**	**368**	**3.63**
Negros Oriental	28,980	0	**0.00**	5	**1.89**	43	**2.19**	**48**	**2.15**
Siquijor	1,671	0	**0.00**	0	**0.00**	2	**1.54**	**2**	**1.43**
City of Cebu	19,466	0	**0.00**	1	**0.22**	47	**1.11**	**48**	**1.02**
City of Lapu-Lapu	10,833	0	**0.00**	5	**2.31**	109	**4.53**	**114**	**4.35**
City of Mandaue	6,981	0	**0.00**	0	**0.00**	0	**0.00**	**0**	**0.00**
**Region 8**	**93,713**	**1**	**3.45**	**17**	**1.53**	**120**	**1.37**	**138**	**1.39**
Biliran	3,378	0	**0.00**	0	**0.00**	8	**6.11**	**8**	**5.56**
Eastern Samar	9,594	0	**0.00**	0	**0.00**	17	**1.82**	**17**	**1.61**
Leyte	31,504	0	**0.00**	7	**2.66**	36	**1.81**	**43**	**1.89**
Northern Samar	14,053	0	**0.00**	3	**1.29**	11	**0.75**	**14**	**0.82**
Southern Leyte	7,368	0	**0.00**	0	**0.00**	11	**1.44**	**11**	**1.29**
Samar	16,946	0	**0.00**	4	**3.36**	14	**1.36**	**18**	**1.57**
Ormoc City	5,344	1	**100.00**	3	**2.86**	23	**2.85**	**27**	**2.95**
City of Tacloban	5,526	0	**0.00**	0	**0.00**	0	**0.00**	**0**	**0.00**
**Region 9**	**85,348**	**0**	**0.00**	**7**	**1.34**	**57**	**1.88**	**64**	**1.80**
Zamboanga del Norte	24,977	0	**0.00**	2	**2.00**	13	**1.80**	**15**	**1.82**
Zamboanga del Sur	23,905	0	**0.00**	3	**3.06**	26	**5.11**	**29**	**4.74**
Zamboanga Sibugay	13,903	0	**0.00**	1	**3.23**	3	**2.75**	**4**	**2.86**
City of Isabela	3,209	0	**0.00**	0	**0.00**	6	**0.79**	**6**	**0.67**
City of Zamboanga	19,354	0	**0.00**	1	**0.66**	9	**0.97**	**10**	**0.92**
**Region 10**	**110,290**	**2**	**3.51**	**30**	**0.99**	**354**	**1.86**	**386**	**1.75**
Bukidnon	33,913	1	**5.26**	17	**2.57**	96	**3.00**	**114**	**2.94**
Camiguin	1,727	0	**0.00**	0	**0.00**	3	**0.34**	**3**	**0.30**
Lanao del Norte	17,881	0	**0.00**	0	**0.00**	7	**1.34**	**7**	**1.22**
Misamis Occidental	12,342	0	**0.00**	0	**0.00**	28	**1.20**	**28**	**1.08**
Misamis Oriental	22,184	1	**5.00**	9	**0.97**	101	**2.01**	**111**	**1.86**
City of Cagayan De Oro	14,503	0	**0.00**	4	**0.42**	111	**1.78**	**115**	**1.60**
City of Iligan	7,740	0	**0.00**	0	**0.00**	8	**0.97**	**8**	**0.87**
**Region 11**	**107,934**	**3**	**3.45**	**19**	**0.86**	**328**	**1.99**	**350**	**1.86**
Davao de Oro	15,434	0	**0.00**	2	**3.33**	37	**6.72**	**39**	**6.36**
Davao del Norte	23,685	0	**0.00**	1	**0.46**	83	**3.41**	**84**	**3.16**
Davao Oriental	12,025	0	**0.00**	0	**0.00**	43	**12.72**	**43**	**11.26**
Davao del Sur	13,178	1	**11.11**	1	**0.56**	32	**3.04**	**34**	**2.74**
Davao Occidental	6,606	2	**10.53**	5	**1.21**	25	**2.19**	**32**	**2.03**
City of Davao	37,006	0	**0.00**	10	**0.78**	108	**0.98**	**118**	**0.96**
**Region 12**	**95,296**	**7**	**11.11**	**40**	**1.66**	**335**	**2.43**	**382**	**2.35**
Cotabato	25,696	3	**18.75**	5	**0.95**	79	**2.27**	**87**	**2.16**
Sarangani	13,501	0	**0.00**	9	**1.47**	64	**2.29**	**73**	**2.13**
South Cotabato	21,842	0	**0.00**	4	**0.66**	44	**1.31**	**48**	**1.21**
Sultan Kudarat	19,613	1	**6.67**	5	**1.17**	55	**2.33**	**61**	**2.17**

Table 2.B.1.13 - Prenatal Care
Pregnant Women tested positive for Gestational Diabetes
Philippines, 2024

Area	Eligible Population (0-11 months)	Pregnant women tested positive for Gestational Diabetes							
		Age Group						Total	%
		10-14 yrs old		15-19 yrs old		20-49 yrs old			
		No.	%	No.	%	No.	%		
City of General Santos	14,644	3	**60.00**	17	**7.08**	93	**5.14**	**113**	**5.50**
**CARAGA**	**55,371**	**0**	**0.00**	**16**	**1.83**	**192**	**3.31**	**208**	**3.11**
Agusan del Norte	7,331	0	**0.00**	0	**0.00**	13	**2.07**	**13**	**1.78**
Agusan del Sur	16,180	0	**0.00**	3	**1.85**	28	**2.37**	**31**	**2.30**
Surigao del Norte	10,168	0	**0.00**	6	**2.51**	55	**3.75**	**61**	**3.57**
Surigao del Sur	12,834	0	**0.00**	6	**4.58**	76	**9.74**	**82**	**8.99**
Dinagat Islands	2,117	0	**0.00**	1	**1.79**	13	**3.00**	**14**	**2.86**
City of Butuan	6,741	0	**0.00**	0	**0.00**	7	**0.54**	**7**	**0.47**
**BARMM**	**134,585**	**0**	**0.00**	**15**	**2.83**	**110**	**1.76**	**125**	**1.84**
Basilan	11,653	0	**0.00**	0	**0.00**	4	**4.40**	**4**	**3.25**
Lanao del Sur	33,339	0	**0.00**	0	**0.00**	8	**0.40**	**8**	**0.39**
Maguindanao del Norte	18,923	0	**0.00**	2	**2.27**	23	**2.07**	**25**	**2.08**
Maguindanao del Sur	23,214	0	**0.00**	7	**4.12**	27	**2.11**	**34**	**2.34**
Sulu	21,381	0	**0.00**	6	**6.98**	22	**3.32**	**28**	**3.74**
Tawi-Tawi	12,602	0	**0.00**	0	**0.00**	0	**0.00**	**0**	**0.00**
SGA	6,305	0	**0.00**	0	**0.00**	0	**0.00**	**0**	**0.00**
City of Cotabato	7,168	0	**0.00**	0	**0.00**	26	**2.50**	**26**	**2.24**

*Legend: * - No Report*

Table 2.B.2.1 - Intrapartum Care and Delivery Outcome
Women who delivered a live baby or stillbirth/fetal death, deliveries Attended by Skilled Health Professionals deliveries in health facilities (FBD)
Philippines, 2024

Area	Total Deliveries				Skilled Health Professional							
	Total Number women who delivered				Deliveries attended by skilled health professional							
	Age Group (in Years)			Total	Doctors	%	Nurses	%	Midwives	%	Total	%
	10-14	15-19	20-49									
**PHILIPPINES**	**3,285**	**137,240**	**1,203,053**	**1,343,578**	**865,813**	**64.44**	**13,568**	**1.01**	**397,995**	**29.62**	**1,277,376**	**95.07**
**N C R**	**297**	**11,285**	**172,329**	**183,911**	**139,503**	**75.85**	**142**	**0.08**	**42,425**	**23.07**	**182,070**	**99.00**
City of Malabon	12	332	2,685	**3,029**	2,347	**77.48**	1	**0.03**	619	**20.44**	**2,967**	**97.95**
City of Navotas	6	308	3,632	**3,946**	2,601	**65.91**	1	**0.03**	1,344	**34.06**	**3,946**	**100.00**
City of Valenzuela	13	364	4,237	**4,614**	3,651	**79.13**	5	**0.11**	921	**19.96**	**4,577**	**99.20**
City of Caloocan	57	2,129	21,982	**24,168**	13,060	**54.04**	19	**0.08**	11,000	**45.51**	**24,079**	**99.63**
City of Marikina	9	226	2,441	**2,676**	1,317	**49.22**	0	**0.00**	1,350	**50.45**	**2,667**	**99.66**
City of Pasig	36	818	10,086	**10,940**	10,251	**93.70**	4	**0.04**	591	**5.40**	**10,846**	**99.14**
Pateros	0	37	717	**754**	464	**61.54**	0	**0.00**	286	**37.93**	**750**	**99.47**
City of Taguig	22	1,051	11,221	**12,294**	8,413	**68.43**	19	**0.15**	3,440	**27.98**	**11,872**	**96.57**
Quezon City	51	2,225	56,245	**58,521**	47,192	**80.64**	0	**0.00**	11,124	**19.01**	**58,316**	**99.65**
City of Makati	3	112	1,395	**1,510**	1,402	**92.85**	1	**0.07**	104	**6.89**	**1,507**	**99.80**
City of Mandaluyong	11	283	7,411	**7,705**	7,087	**91.98**	0	**0.00**	618	**8.02**	**7,705**	**100.00**
City of San Juan	0	35	734	**769**	762	**99.09**	1	**0.13**	4	**0.52**	**767**	**99.74**
City of Manila	28	1,543	22,420	**23,991**	22,352	**93.17**	4	**0.02**	1,488	**6.20**	**23,844**	**99.39**
City of Las Piñas	10	432	5,914	**6,356**	2,573	**40.48**	48	**0.76**	3,597	**56.59**	**6,218**	**97.83**
City of Muntinlupa	15	565	7,068	**7,648**	4,021	**52.58**	3	**0.04**	3,304	**43.20**	**7,328**	**95.82**
City of Parañaque	16	560	7,521	**8,097**	6,266	**77.39**	21	**0.26**	1,511	**18.66**	**7,798**	**96.31**
Pasay City	8	265	6,620	**6,893**	5,744	**83.33**	15	**0.22**	1,124	**16.31**	**6,883**	**99.85**
**C A R**	**55**	**2,118**	**21,818**	**23,991**	**21,731**	**90.58**	**309**	**1.29**	**1,508**	**6.29**	**23,548**	**98.15**
Abra	3	311	2,339	**2,653**	2,464	**92.88**	53	**2.00**	93	**3.51**	**2,610**	**98.38**
Apayao	14	526	3,042	**3,582**	3,315	**92.55**	19	**0.53**	58	**1.62**	**3,392**	**94.70**
Benguet	2	227	3,447	**3,676**	3,461	**94.15**	11	**0.30**	86	**2.34**	**3,558**	**96.79**
Ifugao	4	192	1,743	**1,939**	1,422	**73.34**	32	**1.65**	440	**22.69**	**1,894**	**97.68**
Kalinga	5	155	2,032	**2,192**	1,405	**64.10**	63	**2.87**	717	**32.71**	**2,185**	**99.68**
Mountain Province	9	330	2,917	**3,256**	3,005	**92.29**	131	**4.02**	99	**3.04**	**3,235**	**99.36**
City of Baguio	18	377	6,298	**6,693**	6,659	**99.49**	0	**0.00**	15	**0.22**	**6,674**	**99.72**
**Region 1**	**169**	**4,304**	**46,997**	**51,470**	**45,064**	**87.55**	**49**	**0.10**	**6,292**	**12.22**	**51,405**	**99.87**
Ilocos Norte	13	314	5,428	**5,755**	5,652	**98.21**	2	**0.03**	100	**1.74**	**5,754**	**99.98**
Ilocos Sur	14	467	6,911	**7,392**	7,297	**98.71**	23	**0.31**	72	**0.97**	**7,392**	**100.00**
La Union	32	691	7,039	**7,762**	7,389	**95.19**	1	**0.01**	360	**4.64**	**7,750**	**99.85**
Pangasinan	88	2,268	20,860	**23,216**	18,963	**81.68**	23	**0.10**	4,199	**18.09**	**23,185**	**99.87**
City of Dagupan	22	564	6,759	**7,345**	5,763	**78.46**	0	**0.00**	1,561	**21.25**	**7,324**	**99.71**
**Region 2**	**93**	**3,613**	**29,487**	**33,193**	**24,881**	**74.96**	**76**	**0.23**	**7,786**	**23.46**	**32,743**	**98.64**
Batanes	0	15	187	**202**	197	**97.52**	3	**1.49**	2	**0.99**	**202**	**100.00**
Cagayan	16	812	7,045	**7,873**	5,327	**67.66**	29	**0.37**	2,400	**30.48**	**7,756**	**98.51**
Isabela	26	1,370	11,631	**13,027**	9,585	**73.58**	20	**0.15**	3,264	**25.06**	**12,869**	**98.79**
Nueva Vizcaya	34	806	6,025	**6,865**	6,382	**92.96**	24	**0.35**	292	**4.25**	**6,698**	**97.57**
Quirino	14	336	2,034	**2,384**	2,058	**86.33**	0	**0.00**	320	**13.42**	**2,378**	**99.75**
City of Santiago	3	274	2,565	**2,842**	1,332	**46.87**	0	**0.00**	1,508	**53.06**	**2,840**	**99.93**
**Region 3**	**369**	**13,100**	**127,979**	**141,448**	**104,902**	**74.16**	**507**	**0.36**	**34,275**	**24.23**	**139,684**	**98.75**
Aurora	7	328	2,183	**2,518**	2,146	**85.23**	1	**0.04**	337	**13.38**	**2,484**	**98.65**
Bataan	61	1,357	9,945	**11,363**	10,498	**92.39**	5	**0.04**	810	**7.13**	**11,313**	**99.56**
Bulacan	92	3,473	37,497	**41,062**	28,513	**69.44**	6	**0.01**	12,188	**29.68**	**40,707**	**99.14**
Nueva Ecija	90	2,543	22,151	**24,784**	19,837	**80.04**	120	**0.48**	4,484	**18.09**	**24,441**	**98.62**
Pampanga	67	2,425	21,406	**23,898**	18,781	**78.59**	177	**0.74**	4,365	**18.27**	**23,323**	**97.59**
Tarlac	17	1,474	18,469	**19,960**	13,096	**65.61**	114	**0.57**	6,647	**33.30**	**19,857**	**99.48**
Zambales	14	845	5,855	**6,714**	5,197	**77.41**	81	**1.21**	1,244	**18.53**	**6,522**	**97.14**
City of Angeles	15	434	6,990	**7,439**	3,537	**47.55**	3	**0.04**	3,882	**52.18**	**7,422**	**99.77**
City of Olongapo	6	221	3,483	**3,710**	3,297	**88.87**	0	**0.00**	318	**8.57**	**3,615**	**97.44**
**Region 4A**	**390**	**17,923**	**180,933**	**199,246**	**119,906**	**60.18**	**1,118**	**0.56**	**68,081**	**34.17**	**189,105**	**94.91**
Batangas	70	2,689	33,636	**36,395**	27,984	**76.89**	59	**0.16**	6,922	**19.02**	**34,965**	**96.07**
Cavite	73	3,418	44,751	**48,242**	24,276	**50.32**	370	**0.77**	22,182	**45.98**	**46,828**	**97.07**
Laguna	95	3,953	39,146	**43,194**	23,222	**53.76**	300	**0.69**	18,238	**42.22**	**41,760**	**96.68**
Quezon	78	3,059	20,713	**23,850**	13,514	**56.66**	128	**0.54**	8,224	**34.48**	**21,866**	**91.68**
Rizal	68	4,481	40,125	**44,674**	28,984	**64.88**	252	**0.56**	11,599	**25.96**	**40,835**	**91.41**
City of Lucena	6	323	2,562	**2,891**	1,926	**66.62**	9	**0.31**	916	**31.68**	**2,851**	**98.62**
**Region 4B**	**126**	**5,691**	**36,723**	**42,540**	**27,525**	**64.70**	**804**	**1.89**	**8,723**	**20.51**	**37,052**	**87.10**
Marinduque	6	280	2,421	**2,707**	2,408	**88.95**	45	**1.66**	234	**8.64**	**2,687**	**99.26**
Occidental Mindoro	27	1,216	7,039	**8,282**	5,015	**60.55**	27	**0.33**	1,734	**20.94**	**6,776**	**81.82**
Oriental Mindoro	28	1,260	10,272	**11,560**	9,459	**81.83**	193	**1.67**	993	**8.59**	**10,645**	**92.08**
Palawan	47	2,041	9,904	**11,992**	5,303	**44.22**	426	**3.55**	3,389	**28.26**	**9,118**	**76.03**
Romblon	6	429	3,677	**4,112**	3,455	**84.02**	59	**1.43**	550	**13.38**	**4,064**	**98.83**
City of Puerto Princesa	12	465	3,410	**3,887**	1,885	**48.49**	54	**1.39**	1,823	**46.90**	**3,762**	**96.78**
**Region 5**	**139**	**9,086**	**76,470**	**85,695**	**38,248**	**44.63**	**3,572**	**4.17**	**41,119**	**47.98**	**82,939**	**96.78**
Albay	16	1,203	14,538	**15,757**	7,542	**47.86**	604	**3.83**	7,431	**47.16**	**15,577**	**98.86**
Camarines Norte	17	1,154	8,983	**10,154**	2,041	**20.10**	1,120	**11.03**	6,828	**67.24**	**9,989**	**98.38**
Camarines Sur	10	1,312	14,898	**16,220**	3,132	**19.31**	1,005	**6.20**	10,661	**65.73**	**14,798**	**91.23**

Table 2.B.2.1 - Intrapartum Care and Delivery Outcome
Women who delivered a live baby or stillbirth/fetal death, deliveries Attended by Skilled Health Professionals deliveries in health facilities (FBD)
Philippines, 2024

Area	Total Deliveries				Skilled Health Professional							
	Total Number women who delivered				Deliveries attended by skilled health professional							
	Age Group (in Years)			Total	Doctors	%	Nurses	%	Midwives	%	Total	%
	10-14	15-19	20-49									
Catanduanes	9	458	3,352	**3,819**	2,737	**71.67**	120	**3.14**	899	**23.54**	**3,756**	**98.35**
Masbate	23	2,159	12,529	**14,711**	2,290	**15.57**	437	**2.97**	11,252	**76.49**	**13,979**	**95.02**
Sorsogon	28	1,313	11,083	**12,424**	9,032	**72.70**	286	**2.30**	3,013	**24.25**	**12,331**	**99.25**
City of Naga	36	1,487	11,087	**12,610**	11,474	**90.99**	0	**0.00**	1,035	**8.21**	**12,509**	**99.20**
**Region 6**	**183**	**9,328**	**76,010**	**85,521**	**60,315**	**70.53**	**258**	**0.30**	**21,316**	**24.92**	**81,889**	**95.75**
Aklan	8	612	5,360	**5,980**	4,981	**83.29**	12	**0.20**	614	**10.27**	**5,607**	**93.76**
Antique	11	731	6,726	**7,468**	5,070	**67.89**	26	**0.35**	1,906	**25.52**	**7,002**	**93.76**
Capiz	14	703	6,764	**7,481**	5,284	**70.63**	7	**0.09**	1,933	**25.84**	**7,224**	**96.56**
Guimaras	11	220	1,951	**2,182**	2,108	**96.61**	0	**0.00**	72	**3.30**	**2,180**	**99.91**
Iloilo	32	1,966	17,747	**19,745**	17,890	**90.61**	84	**0.43**	1,478	**7.49**	**19,452**	**98.52**
Negros Occidental	90	4,369	28,431	**32,890**	16,635	**50.58**	129	**0.39**	13,977	**42.50**	**30,741**	**93.47**
City of Bacolod	2	271	4,420	**4,693**	3,879	**82.66**	0	**0.00**	727	**15.49**	**4,606**	**98.15**
City of Iloilo	15	456	4,611	**5,082**	4,468	**87.92**	0	**0.00**	609	**11.98**	**5,077**	**99.90**
**Region 7**	**230**	**11,162**	**96,264**	**107,656**	**68,029**	**63.19**	**784**	**0.73**	**35,211**	**32.71**	**104,024**	**96.63**
Bohol	22	1,518	15,537	**17,077**	11,693	**68.47**	194	**1.14**	5,086	**29.78**	**16,973**	**99.39**
Cebu	80	4,543	36,259	**40,882**	21,731	**53.16**	258	**0.63**	17,716	**43.33**	**39,705**	**97.12**
Negros Oriental	51	2,307	16,213	**18,571**	12,010	**64.67**	194	**1.04**	4,374	**23.55**	**16,578**	**89.27**
Siquijor	1	75	921	**997**	924	**92.68**	0	**0.00**	53	**5.32**	**977**	**97.99**
City of Cebu	29	1,437	12,529	**13,995**	10,171	**72.68**	39	**0.28**	3,615	**25.83**	**13,825**	**98.79**
City of Lapu-Lapu	24	584	6,311	**6,919**	3,681	**53.20**	82	**1.19**	3,112	**44.98**	**6,875**	**99.36**
City of Mandaue	23	698	8,494	**9,215**	7,819	**84.85**	17	**0.18**	1,255	**13.62**	**9,091**	**98.65**
**Region 8**	**116**	**6,954**	**51,619**	**58,689**	**39,597**	**67.47**	**646**	**1.10**	**16,333**	**27.83**	**56,576**	**96.40**
Biliran	6	385	2,666	**3,057**	1,983	**64.87**	16	**0.52**	1,040	**34.02**	**3,039**	**99.41**
Eastern Samar	17	858	5,498	**6,373**	5,093	**79.92**	23	**0.36**	1,051	**16.49**	**6,167**	**96.77**
Leyte	21	1,366	10,058	**11,445**	4,006	**35.00**	254	**2.22**	7,074	**61.81**	**11,334**	**99.03**
Northern Samar	19	1,345	8,891	**10,255**	7,107	**69.30**	43	**0.42**	2,119	**20.66**	**9,269**	**90.39**
Southern Leyte	5	429	3,896	**4,330**	3,942	**91.04**	3	**0.07**	376	**8.68**	**4,321**	**99.79**
Samar	23	1,455	10,434	**11,912**	8,681	**72.88**	156	**1.31**	2,293	**19.25**	**11,130**	**93.44**
Ormoc City	15	626	4,728	**5,369**	3,365	**62.67**	151	**2.81**	1,852	**34.49**	**5,368**	**99.98**
City of Tacloban	10	490	5,448	**5,948**	5,420	**91.12**	0	**0.00**	528	**8.88**	**5,948**	**100.00**
**Region 9**	**68**	**4,040**	**27,437**	**31,545**	**12,395**	**39.29**	**369**	**1.17**	**13,205**	**41.86**	**25,969**	**82.32**
Zamboanga del Norte	26	1,694	10,453	**12,173**	6,834	**56.14**	129	**1.06**	4,004	**32.89**	**10,967**	**90.09**
Zamboanga del Sur	22	955	6,596	**7,573**	1,669	**22.04**	44	**0.58**	4,713	**62.23**	**6,426**	**84.85**
Zamboanga Sibugay	15	760	5,232	**6,007**	1,787	**29.75**	129	**2.15**	3,360	**55.93**	**5,276**	**87.83**
City of Isabela	2	299	2,095	**2,396**	1,683	**70.24**	0	**0.00**	12	**0.50**	**1,695**	**70.74**
City of Zamboanga	3	332	3,061	**3,396**	422	**12.43**	67	**1.97**	1,116	**32.86**	**1,605**	**47.26**
**Region 10**	**204**	**8,959**	**54,703**	**63,866**	**41,294**	**64.66**	**752**	**1.18**	**17,862**	**27.97**	**59,908**	**93.80**
Bukidnon	93	3,693	16,418	**20,204**	11,326	**56.06**	40	**0.20**	6,832	**33.82**	**18,198**	**90.07**
Camiguin	3	153	1,097	**1,253**	1,172	**93.54**	1	**0.08**	79	**6.30**	**1,252**	**99.92**
Lanao del Norte	27	893	9,016	**9,936**	5,068	**51.01**	68	**0.68**	3,404	**34.26**	**8,540**	**85.95**
Misamis Occidental	6	639	4,309	**4,954**	3,658	**73.84**	310	**6.26**	858	**17.32**	**4,826**	**97.42**
Misamis Oriental	27	1,338	8,320	**9,685**	6,015	**62.11**	330	**3.41**	3,136	**32.38**	**9,481**	**97.89**
City of Cagayan De Oro	34	1,588	9,857	**11,479**	8,134	**70.86**	2	**0.02**	3,262	**28.42**	**11,398**	**99.29**
City of Iligan	14	655	5,686	**6,355**	5,921	**93.17**	1	**0.02**	291	**4.58**	**6,213**	**97.77**
**Region 11**	**395**	**10,313**	**63,038**	**73,746**	**51,664**	**70.06**	**254**	**0.34**	**18,805**	**25.50**	**70,723**	**95.90**
Davao de Oro	61	1,670	8,779	**10,510**	8,231	**78.32**	45	**0.43**	2,116	**20.13**	**10,392**	**98.88**
Davao del Norte	99	2,168	13,220	**15,487**	11,751	**75.88**	50	**0.32**	3,191	**20.60**	**14,992**	**96.80**
Davao Oriental	56	1,272	6,591	**7,919**	6,727	**84.95**	35	**0.44**	1,008	**12.73**	**7,770**	**98.12**
Davao del Sur	62	1,432	7,187	**8,681**	5,452	**62.80**	20	**0.23**	2,995	**34.50**	**8,467**	**97.53**
Davao Occidental	48	1,066	3,168	**4,282**	2,455	**57.33**	27	**0.63**	744	**17.38**	**3,226**	**75.34**
City of Davao	69	2,705	24,093	**26,867**	17,048	**63.45**	77	**0.29**	8,751	**32.57**	**25,876**	**96.31**
**Region 12**	**282**	**8,938**	**52,550**	**61,770**	**34,494**	**55.84**	**372**	**0.60**	**23,382**	**37.85**	**58,248**	**94.30**
Cotabato	78	2,383	14,247	**16,708**	10,796	**64.62**	125	**0.75**	4,158	**24.89**	**15,079**	**90.25**
Sarangani	48	1,778	7,622	**9,448**	3,433	**36.34**	48	**0.51**	5,454	**57.73**	**8,935**	**94.57**
South Cotabato	81	2,204	12,261	**14,546**	10,427	**71.68**	89	**0.61**	3,345	**23.00**	**13,861**	**95.29**
Sultan Kudarat	60	1,781	9,990	**11,831**	6,699	**56.62**	21	**0.18**	4,644	**39.25**	**11,364**	**96.05**
City of General Santos	15	792	8,430	**9,237**	3,139	**33.98**	89	**0.96**	5,781	**62.59**	**9,009**	**97.53**
**CARAGA**	**99**	**5,072**	**33,467**	**38,638**	**26,134**	**67.64**	**707**	**1.83**	**10,819**	**28.00**	**37,660**	**97.47**
Agusan del Norte	20	799	4,769	**5,588**	3,856	**69.01**	0	**0.00**	1,632	**29.21**	**5,488**	**98.21**
Agusan del Sur	37	1,357	8,490	**9,884**	5,825	**58.93**	56	**0.57**	3,406	**34.46**	**9,287**	**93.96**
Surigao del Norte	4	854	6,499	**7,357**	5,791	**78.71**	266	**3.62**	1,237	**16.81**	**7,294**	**99.14**
Surigao del Sur	27	1,388	7,771	**9,186**	6,455	**70.27**	355	**3.86**	2,182	**23.75**	**8,992**	**97.89**
Dinagat Islands	1	105	757	**863**	754	**87.37**	27	**3.13**	71	**8.23**	**852**	**98.73**
City of Butuan	10	569	5,181	**5,760**	3,453	**59.95**	3	**0.05**	2,291	**39.77**	**5,747**	**99.77**
**BARMM**	**70**	**5,354**	**55,229**	**60,653**	**10,131**	**16.70**	**2,849**	**4.70**	**30,853**	**50.87**	**43,833**	**72.27**
Basilan	10	829	4,195	**5,034**	229	**4.55**	982	**19.51**	1,703	**33.83**	**2,914**	**57.89**
Lanao del Sur	11	605	13,410	**14,026**	3,266	**23.29**	690	**4.92**	7,684	**54.78**	**11,640**	**82.99**

Table 2.B.2.1 - Intrapartum Care and Delivery Outcome

Women who delivered a live baby or stillbirth/fetal death, deliveries Attended by Skilled Health Professionals deliveries in health facilities (FBD)

Philippines, 2024

Area	Total Deliveries				Skilled Health Professional							
	Total Number women who delivered				Deliveries attended by skilled health professional							
	Age Group (in Years)			Total	Doctors	%	Nurses	%	Midwives	%	Total	%
	10-14	15-19	20-49									
Maguindanao del Norte	7	690	7,149	**7,846**	1,581	**20.15**	170	**2.17**	5,023	**64.02**	**6,774**	**86.34**
Maguindanao del Sur	22	1,369	11,452	**12,843**	1,088	**8.47**	398	**3.10**	7,899	**61.50**	**9,385**	**73.07**
Sulu	6	739	9,655	**10,400**	366	**3.52**	225	**2.16**	4,610	**44.33**	**5,201**	**50.01**
Tawi-Tawi	10	516	4,843	**5,369**	889	**16.56**	382	**7.11**	3,055	**56.90**	**4,326**	**80.57**
SGA	1	224	1,317	**1,542**	31	**2.01**	0	**0.00**	165	**10.70**	**196**	**12.71**
City of Cotabato	3	382	3,208	**3,593**	2,681	**74.62**	2	**0.06**	714	**19.87**	**3,397**	**94.54**

*Legend: * - No Report*

Table 2.B.2.1 - Intrapartum Care and Delivery Outcome

Women who delivered a live baby or stillbirth/fetal death, deliveries Attended by Skilled Health Professionals deliveries in health facilities (FBD)

Philippines, 2024

Area	Total Deliveries				Facility Based Delivery					
	Total Number women who delivered				Type of Health Facility				Total	%
	Age Group (in Years)			Total	Public	%	Private	%		
	10-14	15-19	20-49							
**PHILIPPINES**	**3,285**	**137,240**	**1,203,053**	**1,343,578**	**896,256**	**66.71**	**366,703**	**27.29**	**1,262,959**	**94.00**
**N C R**	**297**	**11,285**	**172,329**	**183,911**	**125,510**	**68.24**	**56,279**	**30.60**	**181,789**	**98.85**
City of Malabon	12	332	2,685	**3,029**	2,276	**75.14**	683	**22.55**	**2,959**	**97.69**
City of Navotas	6	308	3,632	**3,946**	2,658	**67.36**	1,286	**32.59**	**3,944**	**99.95**
City of Valenzuela	13	364	4,237	**4,614**	3,609	**78.22**	905	**19.61**	**4,514**	**97.83**
City of Caloocan	57	2,129	21,982	**24,168**	11,617	**48.07**	12,454	**51.53**	**24,071**	**99.60**
City of Marikina	9	226	2,441	**2,676**	1,022	**38.19**	1,642	**61.36**	**2,664**	**99.55**
City of Pasig	36	818	10,086	**10,940**	8,024	**73.35**	2,768	**25.30**	**10,792**	**98.65**
Pateros	0	37	717	**754**	332	**44.03**	405	**53.71**	**737**	**97.75**
City of Taguig	22	1,051	11,221	**12,294**	8,682	**70.62**	3,164	**25.74**	**11,846**	**96.36**
Quezon City	51	2,225	56,245	**58,521**	38,977	**66.60**	19,325	**33.02**	**58,302**	**99.63**
City of Makati	3	112	1,395	**1,510**	1,328	**87.95**	176	**11.66**	**1,504**	**99.60**
City of Mandaluyong	11	283	7,411	**7,705**	6,951	**90.21**	753	**9.77**	**7,704**	**99.99**
City of San Juan	0	35	734	**769**	716	**93.11**	51	**6.63**	**767**	**99.74**
City of Manila	28	1,543	22,420	**23,991**	20,712	**86.33**	3,086	**12.86**	**23,798**	**99.20**
City of Las Piñas	10	432	5,914	**6,356**	4,187	**65.87**	2,015	**31.70**	**6,202**	**97.58**
City of Muntinlupa	15	565	7,068	**7,648**	4,349	**56.86**	2,969	**38.82**	**7,318**	**95.69**
City of Parañaque	16	560	7,521	**8,097**	5,512	**68.07**	2,272	**28.06**	**7,784**	**96.13**
Pasay City	8	265	6,620	**6,893**	4,558	**66.13**	2,325	**33.73**	**6,883**	**99.85**
**C A R**	**55**	**2,118**	**21,818**	**23,991**	**19,742**	**82.29**	**3,527**	**14.70**	**23,269**	**96.99**
Abra	3	311	2,339	**2,653**	2,222	**83.75**	325	**12.25**	**2,547**	**96.00**
Apayao	14	526	3,042	**3,582**	3,349	**93.50**	31	**0.87**	**3,380**	**94.36**
Benguet	2	227	3,447	**3,676**	2,942	**80.03**	554	**15.07**	**3,496**	**95.10**
Ifugao	4	192	1,743	**1,939**	1,619	**83.50**	222	**11.45**	**1,841**	**94.95**
Kalinga	5	155	2,032	**2,192**	1,555	**70.94**	609	**27.78**	**2,164**	**98.72**
Mountain Province	9	330	2,917	**3,256**	3,119	**95.79**	51	**1.57**	**3,170**	**97.36**
City of Baguio	18	377	6,298	**6,693**	4,936	**73.75**	1,735	**25.92**	**6,671**	**99.67**
**Region 1**	**169**	**4,304**	**46,997**	**51,470**	**39,213**	**76.19**	**12,167**	**23.64**	**51,380**	**99.83**
Ilocos Norte	13	314	5,428	**5,755**	5,458	**94.84**	296	**5.14**	**5,754**	**99.98**
Ilocos Sur	14	467	6,911	**7,392**	4,304	**58.23**	3,086	**41.75**	**7,390**	**99.97**
La Union	32	691	7,039	**7,762**	7,001	**90.20**	747	**9.62**	**7,748**	**99.82**
Pangasinan	88	2,268	20,860	**23,216**	18,510	**79.73**	4,664	**20.09**	**23,174**	**99.82**
City of Dagupan	22	564	6,759	**7,345**	3,940	**53.64**	3,374	**45.94**	**7,314**	**99.58**
**Region 2**	**93**	**3,613**	**29,487**	**33,193**	**26,302**	**79.24**	**6,344**	**19.11**	**32,646**	**98.35**
Batanes	0	15	187	**202**	200	**99.01**	0	**0.00**	**200**	**99.01**
Cagayan	16	812	7,045	**7,873**	6,854	**87.06**	860	**10.92**	**7,714**	**97.98**
Isabela	26	1,370	11,631	**13,027**	9,257	**71.06**	3,559	**27.32**	**12,816**	**98.38**
Nueva Vizcaya	34	806	6,025	**6,865**	6,605	**96.21**	93	**1.35**	**6,698**	**97.57**
Quirino	14	336	2,034	**2,384**	2,098	**88.00**	280	**11.74**	**2,378**	**99.75**
City of Santiago	3	274	2,565	**2,842**	1,288	**45.32**	1,552	**54.61**	**2,840**	**99.93**
**Region 3**	**369**	**13,100**	**127,979**	**141,448**	**80,303**	**56.77**	**58,697**	**41.50**	**139,000**	**98.27**
Aurora	7	328	2,183	**2,518**	2,162	**85.86**	310	**12.31**	**2,472**	**98.17**
Bataan	61	1,357	9,945	**11,363**	8,999	**79.20**	2,260	**19.89**	**11,259**	**99.08**
Bulacan	92	3,473	37,497	**41,062**	15,689	**38.21**	24,965	**60.80**	**40,654**	**99.01**
Nueva Ecija	90	2,543	22,151	**24,784**	14,928	**60.23**	9,318	**37.60**	**24,246**	**97.83**
Pampanga	67	2,425	21,406	**23,898**	15,536	**65.01**	7,580	**31.72**	**23,116**	**96.73**
Tarlac	17	1,474	18,469	**19,960**	11,991	**60.08**	7,811	**39.13**	**19,802**	**99.21**
Zambales	14	845	5,855	**6,714**	6,324	**94.19**	148	**2.20**	**6,472**	**96.40**
City of Angeles	15	434	6,990	**7,439**	2,247	**30.21**	5,153	**69.27**	**7,400**	**99.48**
City of Olongapo	6	221	3,483	**3,710**	2,427	**65.42**	1,152	**31.05**	**3,579**	**96.47**
**Region 4A**	**390**	**17,923**	**180,933**	**199,246**	**103,008**	**51.70**	**83,627**	**41.97**	**186,635**	**93.67**
Batangas	70	2,689	33,636	**36,395**	15,591	**42.84**	18,847	**51.78**	**34,438**	**94.62**
Cavite	73	3,418	44,751	**48,242**	16,543	**34.29**	29,860	**61.90**	**46,403**	**96.19**
Laguna	95	3,953	39,146	**43,194**	24,069	**55.72**	17,097	**39.58**	**41,166**	**95.30**
Quezon	78	3,059	20,713	**23,850**	16,454	**68.99**	5,150	**21.59**	**21,604**	**90.58**
Rizal	68	4,481	40,125	**44,674**	28,230	**63.19**	11,944	**26.74**	**40,174**	**89.93**
City of Lucena	6	323	2,562	**2,891**	2,121	**73.37**	729	**25.22**	**2,850**	**98.58**
**Region 4B**	**126**	**5,691**	**36,723**	**42,540**	**32,534**	**76.48**	**4,147**	**9.75**	**36,681**	**86.23**
Marinduque	6	280	2,421	**2,707**	2,683	**99.11**	0	**0.00**	**2,683**	**99.11**
Occidental Mindoro	27	1,216	7,039	**8,282**	6,356	**76.74**	384	**4.64**	**6,740**	**81.38**
Oriental Mindoro	28	1,260	10,272	**11,560**	9,051	**78.30**	1,518	**13.13**	**10,569**	**91.43**
Palawan	47	2,041	9,904	**11,992**	8,207	**68.44**	689	**5.75**	**8,896**	**74.18**
Romblon	6	429	3,677	**4,112**	3,862	**93.92**	194	**4.72**	**4,056**	**98.64**
City of Puerto Princesa	12	465	3,410	**3,887**	2,375	**61.10**	1,362	**35.04**	**3,737**	**96.14**
**Region 5**	**139**	**9,086**	**76,470**	**85,695**	**58,544**	**68.32**	**23,940**	**27.94**	**82,484**	**96.25**
Albay	16	1,203	14,538	**15,757**	9,974	**63.30**	5,486	**34.82**	**15,460**	**98.12**
Camarines Norte	17	1,154	8,983	**10,154**	7,873	**77.54**	2,115	**20.83**	**9,988**	**98.37**
Camarines Sur	10	1,312	14,898	**16,220**	4,422	**27.26**	10,340	**63.75**	**14,762**	**91.01**

Table 2.B.2.1 - Intrapartum Care and Delivery Outcome
Women who delivered a live baby or stillbirth/fetal death, deliveries Attended by Skilled Health Professionals deliveries in health facilities (FBD)
Philippines, 2024

Area	Total Deliveries				Facility Based Delivery					
	Total Number women who delivered				Type of Health Facility				Total	%
	Age Group (in Years)			Total	Public	%	Private	%		
	10-14	15-19	20-49							
Catanduanes	9	458	3,352	3,819	3,468	90.81	286	7.49	3,754	98.30
Masbate	23	2,159	12,529	14,711	12,133	82.48	1,657	11.26	13,790	93.74
Sorsogon	28	1,313	11,083	12,424	10,531	84.76	1,783	14.35	12,314	99.11
City of Naga	36	1,487	11,087	12,610	10,143	80.44	2,273	18.03	12,416	98.46
**Region 6**	183	9,328	76,010	85,521	69,778	81.59	11,959	13.98	81,737	95.58
Aklan	8	612	5,360	5,980	5,064	84.68	501	8.38	5,565	93.06
Antique	11	731	6,726	7,468	6,528	87.41	449	6.01	6,977	93.43
Capiz	14	703	6,764	7,481	4,520	60.42	2,693	36.00	7,213	96.42
Guimaras	11	220	1,951	2,182	2,047	93.81	129	5.91	2,176	99.73
Iloilo	32	1,966	17,747	19,745	18,434	93.36	993	5.03	19,427	98.39
Negros Occidental	90	4,369	28,431	32,890	26,562	80.76	4,138	12.58	30,700	93.34
City of Bacolod	2	271	4,420	4,693	2,813	59.94	1,790	38.14	4,603	98.08
City of Iloilo	15	456	4,611	5,082	3,810	74.97	1,266	24.91	5,076	99.88
**Region 7**	230	11,162	96,264	107,656	76,491	71.05	27,207	25.27	103,698	96.32
Bohol	22	1,518	15,537	17,077	14,743	86.33	2,186	12.80	16,929	99.13
Cebu	80	4,543	36,259	40,882	30,233	73.95	9,383	22.95	39,616	96.90
Negros Oriental	51	2,307	16,213	18,571	13,669	73.60	2,836	15.27	16,505	88.88
Siquijor	1	75	921	997	976	97.89	0	0.00	976	97.89
City of Cebu	29	1,437	12,529	13,995	8,967	64.07	4,815	34.41	13,782	98.48
City of Lapu-Lapu	24	584	6,311	6,919	3,072	44.40	3,762	54.37	6,834	98.77
City of Mandaue	23	698	8,494	9,215	4,831	52.43	4,225	45.85	9,056	98.27
**Region 8**	116	6,954	51,619	58,689	41,683	71.02	14,710	25.06	56,393	96.09
Biliran	6	385	2,666	3,057	3,032	99.18	0	0.00	3,032	99.18
Eastern Samar	17	858	5,498	6,373	2,918	45.79	3,204	50.27	6,122	96.06
Leyte	21	1,366	10,058	11,445	6,995	61.12	4,293	37.51	11,288	98.63
Northern Samar	19	1,345	8,891	10,255	8,755	85.37	497	4.85	9,252	90.22
Southern Leyte	5	429	3,896	4,330	3,285	75.87	1,022	23.60	4,307	99.47
Samar	23	1,455	10,434	11,912	9,204	77.27	1,909	16.03	11,113	93.29
Ormoc City	15	626	4,728	5,369	2,075	38.65	3,266	60.83	5,341	99.48
City of Tacloban	10	490	5,448	5,948	5,419	91.11	519	8.73	5,938	99.83
**Region 9**	68	4,040	27,437	31,545	23,692	75.11	2,208	7.00	25,900	82.10
Zamboanga del Norte	26	1,694	10,453	12,173	10,924	89.74	32	0.26	10,956	90.00
Zamboanga del Sur	22	955	6,596	7,573	4,623	61.05	1,797	23.73	6,420	84.77
Zamboanga Sibugay	15	760	5,232	6,007	5,032	83.77	237	3.95	5,269	87.71
City of Isabela	2	299	2,095	2,396	1,564	65.28	128	5.34	1,692	70.62
City of Zamboanga	3	332	3,061	3,396	1,549	45.61	14	0.41	1,563	46.02
**Region 10**	204	8,959	54,703	63,866	50,460	79.01	7,700	12.06	58,160	91.07
Bukidnon	93	3,693	16,418	20,204	14,633	72.43	3,490	17.27	18,123	89.70
Camiguin	3	153	1,097	1,253	1,187	94.73	64	5.11	1,251	99.84
Lanao del Norte	27	893	9,016	9,936	6,834	68.78	141	1.42	6,975	70.20
Misamis Occidental	6	639	4,309	4,954	4,265	86.09	507	10.23	4,772	96.33
Misamis Oriental	27	1,338	8,320	9,685	7,836	80.91	1,596	16.48	9,432	97.39
City of Cagayan De Oro	34	1,588	9,857	11,479	10,351	90.17	1,047	9.12	11,398	99.29
City of Iligan	14	655	5,686	6,355	5,354	84.25	855	13.45	6,209	97.70
**Region 11**	395	10,313	63,038	73,746	48,496	65.76	22,108	29.98	70,604	95.74
Davao de Oro	61	1,670	8,779	10,510	8,970	85.35	1,412	13.43	10,382	98.78
Davao del Norte	99	2,168	13,220	15,487	11,320	73.09	3,659	23.63	14,979	96.72
Davao Oriental	56	1,272	6,591	7,919	7,624	96.27	119	1.50	7,743	97.78
Davao del Sur	62	1,432	7,187	8,681	3,341	38.49	5,110	58.86	8,451	97.35
Davao Occidental	48	1,066	3,168	4,282	2,516	58.76	680	15.88	3,196	74.64
City of Davao	69	2,705	24,093	26,867	14,725	54.81	11,128	41.42	25,853	96.23
**Region 12**	282	8,938	52,550	61,770	41,953	67.92	16,255	26.32	58,208	94.23
Cotabato	78	2,383	14,247	16,708	10,083	60.35	4,964	29.71	15,047	90.06
Sarangani	48	1,778	7,622	9,448	8,029	84.98	901	9.54	8,930	94.52
South Cotabato	81	2,204	12,261	14,546	11,332	77.90	2,527	17.37	13,859	95.28
Sultan Kudarat	60	1,781	9,990	11,831	8,940	75.56	2,423	20.48	11,363	96.04
City of General Santos	15	792	8,430	9,237	3,569	38.64	5,440	58.89	9,009	97.53
**CARAGA**	99	5,072	33,467	38,638	28,520	73.81	9,077	23.49	37,597	97.31
Agusan del Norte	20	799	4,769	5,588	3,587	64.19	1,898	33.97	5,485	98.16
Agusan del Sur	37	1,357	8,490	9,884	6,629	67.07	2,629	26.60	9,258	93.67
Surigao del Norte	4	854	6,499	7,357	6,686	90.88	592	8.05	7,278	98.93
Surigao del Sur	27	1,388	7,771	9,186	8,609	93.72	372	4.05	8,981	97.77
Dinagat Islands	1	105	757	863	848	98.26	0	0.00	848	98.26
City of Butuan	10	569	5,181	5,760	2,161	37.52	3,586	62.26	5,747	99.77
**BARMM**	70	5,354	55,229	60,653	30,027	49.51	6,751	11.13	36,778	60.64
Basilan	10	829	4,195	5,034	1,570	31.19	70	1.39	1,640	32.58
Lanao del Sur	11	605	13,410	14,026	7,769	55.39	2,443	17.42	10,212	72.81

Table 2.B.2.1 - Intrapartum Care and Delivery Outcome
Women who delivered a live baby or stillbirth/fetal death, deliveries Attended by Skilled Health Professionals deliveries in health facilities (FBD)
Philippines, 2024

Area	Total Deliveries				Facility Based Delivery					
	Total Number women who delivered				Type of Health Facility				Total	%
	Age Group (in Years)			Total	Public	%	Private	%		
	10-14	15-19	20-49							
Maguindanao del Norte	7	690	7,149	**7,846**	3,037	**38.71**	1,182	**15.07**	**4,219**	**53.77**
Maguindanao del Sur	22	1,369	11,452	**12,843**	5,470	**42.59**	2,054	**15.99**	**7,524**	**58.58**
Sulu	6	739	9,655	**10,400**	5,554	**53.40**	147	**1.41**	**5,701**	**54.82**
Tawi-Tawi	10	516	4,843	**5,369**	3,871	**72.10**	49	**0.91**	**3,920**	**73.01**
SGA	1	224	1,317	**1,542**	0	**0.00**	165	**10.70**	**165**	**10.70**
City of Cotabato	3	382	3,208	**3,593**	2,756	**76.70**	641	**17.84**	**3,397**	**94.54**

*Legend: * - No Report*

Table 2.B.2.2 - Intrapartum Care and Delivery Outcome
Delivery by Type (Vaginal and Cesarean)
Philippines, 2024

Area	Total number of Deliveries by age group				Delivery by Type							
	Age Group in Year			Total	Vaginal							
					Age Group in Year						Total	%
	10-14	15-19	20-49		10-14	%	15-19	%	20-49	%		
**PHILIPPINES**	**3,285**	**137,240**	**1,203,053**	**1,343,578**	**2,937**	**89.41**	**124,590**	**90.78**	**1,012,339**	**84.15**	**1,139,866**	**84.84**
**N C R**	**297**	**11,285**	**172,329**	**183,911**	**271**	**91.25**	**10,336**	**91.59**	**152,816**	**88.68**	**163,423**	**88.86**
City of Malabon	12	332	2,685	**3,029**	12	**100.00**	283	**85.24**	2,124	**79.11**	**2,419**	**79.86**
City of Navotas	6	308	3,632	**3,946**	5	**83.33**	266	**86.36**	2,903	**79.93**	**3,174**	**80.44**
City of Valenzuela	13	364	4,237	**4,614**	12	**92.31**	317	**87.09**	3,245	**76.59**	**3,574**	**77.46**
City of Caloocan	57	2,129	21,982	**24,168**	52	**91.23**	2,001	**93.99**	20,564	**93.55**	**22,617**	**93.58**
City of Marikina	9	226	2,441	**2,676**	7	**77.78**	193	**85.40**	1,966	**80.54**	**2,166**	**80.94**
City of Pasig	36	818	10,086	**10,940**	35	**97.22**	788	**96.33**	9,307	**92.28**	**10,130**	**92.60**
Pateros	0	37	717	**754**	0	**0.00**	35	**94.59**	610	**85.08**	**645**	**85.54**
City of Taguig	22	1,051	11,221	**12,294**	17	**77.27**	976	**92.86**	9,521	**84.85**	**10,514**	**85.52**
Quezon City	51	2,225	56,245	**58,521**	47	**92.16**	2,124	**95.46**	52,642	**93.59**	**54,813**	**93.66**
City of Makati	3	112	1,395	**1,510**	3	**100.00**	88	**78.57**	1,030	**73.84**	**1,121**	**74.24**
City of Mandaluyong	11	283	7,411	**7,705**	10	**90.91**	259	**91.52**	6,643	**89.64**	**6,912**	**89.71**
City of San Juan	0	35	734	**769**	0	**0.00**	28	**80.00**	557	**75.89**	**585**	**76.07**
City of Manila	28	1,543	22,420	**23,991**	28	**100.00**	1,348	**87.36**	19,178	**85.54**	**20,554**	**85.67**
City of Las Piñas	10	432	5,914	**6,356**	9	**90.00**	389	**90.05**	5,158	**87.22**	**5,556**	**87.41**
City of Muntinlupa	15	565	7,068	**7,648**	12	**80.00**	516	**91.33**	5,923	**83.80**	**6,451**	**84.35**
City of Parañaque	16	560	7,521	**8,097**	15	**93.75**	500	**89.29**	5,941	**78.99**	**6,456**	**79.73**
Pasay City	8	265	6,620	**6,893**	7	**87.50**	225	**84.91**	5,504	**83.14**	**5,736**	**83.21**
**C A R**	**55**	**2,118**	**21,818**	**23,991**	**50**	**90.91**	**1,680**	**79.32**	**14,600**	**66.92**	**16,330**	**68.07**
Abra	3	311	2,339	**2,653**	2	**66.67**	248	**79.74**	1,427	**61.01**	**1,677**	**63.21**
Apayao	14	526	3,042	**3,582**	13	**92.86**	452	**85.93**	2,203	**72.42**	**2,668**	**74.48**
Benguet	2	227	3,447	**3,676**	2	**100.00**	174	**76.65**	2,620	**76.01**	**2,796**	**76.06**
Ifugao	4	192	1,743	**1,939**	4	**100.00**	170	**88.54**	1,509	**86.57**	**1,683**	**86.80**
Kalinga	5	155	2,032	**2,192**	5	**100.00**	133	**85.81**	1,648	**81.10**	**1,786**	**81.48**
Mountain Province	9	330	2,917	**3,256**	8	**88.89**	260	**78.79**	1,906	**65.34**	**2,174**	**66.77**
City of Baguio	18	377	6,298	**6,693**	16	**88.89**	243	**64.46**	3,287	**52.19**	**3,546**	**52.98**
**Region 1**	**169**	**4,304**	**46,997**	**51,470**	**140**	**82.84**	**3,835**	**89.10**	**40,755**	**86.72**	**44,730**	**86.90**
Ilocos Norte	13	314	5,428	**5,755**	5	**38.46**	224	**71.34**	4,215	**77.65**	**4,444**	**77.22**
Ilocos Sur	14	467	6,911	**7,392**	12	**85.71**	357	**76.45**	5,341	**77.28**	**5,710**	**77.25**
La Union	32	691	7,039	**7,762**	24	**75.00**	617	**89.29**	6,472	**91.94**	**7,113**	**91.64**
Pangasinan	88	2,268	20,860	**23,216**	79	**89.77**	2,080	**91.71**	18,107	**86.80**	**20,266**	**87.29**
City of Dagupan	22	564	6,759	**7,345**	20	**90.91**	557	**98.76**	6,620	**97.94**	**7,197**	**97.99**
**Region 2**	**93**	**3,613**	**29,487**	**33,193**	**80**	**86.02**	**3,018**	**83.53**	**22,415**	**76.02**	**25,513**	**76.86**
Batanes	0	15	187	**202**	0	**0.00**	12	**80.00**	138	**73.80**	**150**	**74.26**
Cagayan	16	812	7,045	**7,873**	16	**100.00**	755	**92.98**	6,149	**87.28**	**6,920**	**87.90**
Isabela	26	1,370	11,631	**13,027**	20	**76.92**	1,142	**83.36**	8,741	**75.15**	**9,903**	**76.02**
Nueva Vizcaya	34	806	6,025	**6,865**	29	**85.29**	622	**77.17**	3,977	**66.01**	**4,628**	**67.41**
Quirino	14	336	2,034	**2,384**	12	**85.71**	267	**79.46**	1,521	**74.78**	**1,800**	**75.50**
City of Santiago	3	274	2,565	**2,842**	3	**100.00**	220	**80.29**	1,889	**73.65**	**2,112**	**74.31**
**Region 3**	**369**	**13,100**	**127,979**	**141,448**	**279**	**75.61**	**10,166**	**77.60**	**84,593**	**66.10**	**95,038**	**67.19**
Aurora	7	328	2,183	**2,518**	6	**85.71**	310	**94.51**	1,855	**84.97**	**2,171**	**86.22**
Bataan	61	1,357	9,945	**11,363**	46	**75.41**	1,106	**81.50**	6,329	**63.64**	**7,481**	**65.84**
Bulacan	92	3,473	37,497	**41,062**	75	**81.52**	2,824	**81.31**	24,094	**64.26**	**26,993**	**65.74**
Nueva Ecija	90	2,543	22,151	**24,784**	67	**74.44**	2,045	**80.42**	15,542	**70.16**	**17,654**	**71.23**
Pampanga	67	2,425	21,406	**23,898**	43	**64.18**	1,539	**63.46**	11,204	**52.34**	**12,786**	**53.50**
Tarlac	17	1,474	18,469	**19,960**	11	**64.71**	1,023	**69.40**	13,326	**72.15**	**14,360**	**71.94**
Zambales	14	845	5,855	**6,714**	13	**92.86**	690	**81.66**	3,933	**67.17**	**4,636**	**69.05**
City of Angeles	15	434	6,990	**7,439**	12	**80.00**	409	**94.24**	4,859	**69.51**	**5,280**	**70.98**
City of Olongapo	6	221	3,483	**3,710**	6	**100.00**	220	**99.55**	3,451	**99.08**	**3,677**	**99.11**
**Region 4A**	**390**	**17,923**	**180,933**	**199,246**	**353**	**90.51**	**16,074**	**89.68**	**142,351**	**78.68**	**158,778**	**79.69**
Batangas	70	2,689	33,636	**36,395**	58	**82.86**	2,172	**80.77**	21,280	**63.27**	**23,510**	**64.60**
Cavite	73	3,418	44,751	**48,242**	64	**87.67**	3,073	**89.91**	36,002	**80.45**	**39,139**	**81.13**
Laguna	95	3,953	39,146	**43,194**	86	**90.53**	3,606	**91.22**	32,580	**83.23**	**36,272**	**83.97**
Quezon	78	3,059	20,713	**23,850**	75	**96.15**	2,907	**95.03**	17,891	**86.38**	**20,873**	**87.52**
Rizal	68	4,481	40,125	**44,674**	64	**94.12**	4,007	**89.42**	32,364	**80.66**	**36,435**	**81.56**
City of Lucena	6	323	2,562	**2,891**	6	**100.00**	309	**95.67**	2,234	**87.20**	**2,549**	**88.17**
**Region 4B**	**126**	**5,691**	**36,723**	**42,540**	**117**	**92.86**	**5,427**	**95.36**	**32,905**	**89.60**	**38,449**	**90.38**
Marinduque	6	280	2,421	**2,707**	4	**66.67**	248	**88.57**	1,996	**82.45**	**2,248**	**83.04**
Occidental Mindoro	27	1,216	7,039	**8,282**	27	**100.00**	1,165	**95.81**	6,364	**90.41**	**7,556**	**91.23**
Oriental Mindoro	28	1,260	10,272	**11,560**	22	**78.57**	1,176	**93.33**	8,662	**84.33**	**9,860**	**85.29**
Palawan	47	2,041	9,904	**11,992**	47	**100.00**	1,984	**97.21**	9,462	**95.54**	**11,493**	**95.84**
Romblon	6	429	3,677	**4,112**	6	**100.00**	416	**96.97**	3,369	**91.62**	**3,791**	**92.19**
City of Puerto Princesa	12	465	3,410	**3,887**	11	**91.67**	438	**94.19**	3,052	**89.50**	**3,501**	**90.07**
**Region 5**	**139**	**9,086**	**76,470**	**85,695**	**121**	**87.05**	**8,354**	**91.94**	**68,974**	**90.20**	**77,449**	**90.38**
Albay	16	1,203	14,538	**15,757**	15	**93.75**	1,143	**95.01**	13,575	**93.38**	**14,733**	**93.50**
Camarines Norte	17	1,154	8,983	**10,154**	16	**94.12**	959	**83.10**	7,539	**83.93**	**8,514**	**83.85**
Camarines Sur	10	1,312	14,898	**16,220**	10	**100.00**	1,295	**98.70**	14,416	**96.76**	**15,721**	**96.92**
Catanduanes	9	458	3,352	**3,819**	8	**88.89**	421	**91.92**	2,605	**77.71**	**3,034**	**79.44**
Masbate	23	2,159	12,529	**14,711**	23	**100.00**	2,112	**97.82**	12,302	**98.19**	**14,437**	**98.14**
Sorsogon	28	1,313	11,083	**12,424**	27	**96.43**	1,231	**93.75**	9,355	**84.41**	**10,613**	**85.42**
City of Naga	36	1,487	11,087	**12,610**	22	**61.11**	1,193	**80.23**	9,182	**82.82**	**10,397**	**82.45**
**Region 6**	**183**	**9,328**	**76,010**	**85,521**	**171**	**93.44**	**8,841**	**94.78**	**66,448**	**87.42**	**75,460**	**88.24**
Aklan	8	612	5,360	**5,980**	8	**100.00**	571	**93.30**	4,663	**87.00**	**5,242**	**87.66**
Antique	11	731	6,726	**7,468**	8	**72.73**	706	**96.58**	5,939	**88.30**	**6,653**	**89.09**
Capiz	14	703	6,764	**7,481**	14	**100.00**	657	**93.46**	5,660	**83.68**	**6,331**	**84.63**
Guimaras	11	220	1,951	**2,182**	10	**90.91**	201	**91.36**	1,644	**84.26**	**1,855**	**85.01**
Iloilo	32	1,966	17,747	**19,745**	30	**93.75**	1,848	**94.00**	15,730	**88.63**	**17,608**	**89.18**

Table 2.B.2.2 - Intrapartum Care and Delivery Outcome
Delivery by Type (Vaginal and Cesarean)
Philippines, 2024

Area	Total number of Deliveries by age group				Delivery by Type							
	Age Group in Year			Total	Vaginal							
					Age Group in Year						Total	%
	10-14	15-19	20-49		10-14	%	15-19	%	20-49	%		
Negros Occidental	90	4,369	28,431	**32,890**	86	**95.56**	4,174	**95.54**	25,133	**88.40**	**29,393**	**89.37**
City of Bacolod	2	271	4,420	**4,693**	2	**100.00**	271	**100.00**	3,880	**87.78**	**4,153**	**88.49**
City of Iloilo	15	456	4,611	**5,082**	13	**86.67**	413	**90.57**	3,799	**82.39**	**4,225**	**83.14**
**Region 7**	**230**	**11,162**	**96,264**	**107,656**	**215**	**93.48**	**10,497**	**94.04**	**85,648**	**88.97**	**96,360**	**89.51**
Bohol	22	1,518	15,537	**17,077**	20	**90.91**	1,485	**97.83**	14,666	**94.39**	**16,171**	**94.69**
Cebu	80	4,543	36,259	**40,882**	76	**95.00**	4,267	**93.92**	32,679	**90.13**	**37,022**	**90.56**
Negros Oriental	51	2,307	16,213	**18,571**	50	**98.04**	2,242	**97.18**	14,751	**90.98**	**17,043**	**91.77**
Siquijor	1	75	921	**997**	1	**100.00**	67	**89.33**	766	**83.17**	**834**	**83.65**
City of Cebu	29	1,437	12,529	**13,995**	23	**79.31**	1,323	**92.07**	11,032	**88.05**	**12,378**	**88.45**
City of Lapu-Lapu	24	584	6,311	**6,919**	22	**91.67**	467	**79.97**	5,333	**84.50**	**5,822**	**84.15**
City of Mandaue	23	698	8,494	**9,215**	23	**100.00**	646	**92.55**	6,421	**75.59**	**7,090**	**76.94**
**Region 8**	**116**	**6,954**	**51,619**	**58,689**	**108**	**93.10**	**6,414**	**92.23**	**45,262**	**87.68**	**51,784**	**88.23**
Biliran	6	385	2,666	**3,057**	6	**100.00**	379	**98.44**	2,430	**91.15**	**2,815**	**92.08**
Eastern Samar	17	858	5,498	**6,373**	13	**76.47**	729	**84.97**	4,380	**79.67**	**5,122**	**80.37**
Leyte	21	1,366	10,058	**11,445**	20	**95.24**	1,302	**95.31**	9,570	**95.15**	**10,892**	**95.17**
Northern Samar	19	1,345	8,891	**10,255**	18	**94.74**	1,273	**94.65**	7,981	**89.76**	**9,272**	**90.41**
Southern Leyte	5	429	3,896	**4,330**	4	**80.00**	366	**85.31**	3,063	**78.62**	**3,433**	**79.28**
Samar	23	1,455	10,434	**11,912**	23	**100.00**	1,375	**94.50**	9,017	**86.42**	**10,415**	**87.43**
Ormoc City	15	626	4,728	**5,369**	14	**93.33**	549	**87.70**	3,766	**79.65**	**4,329**	**80.63**
City of Tacloban	10	490	5,448	**5,948**	10	**100.00**	441	**90.00**	5,055	**92.79**	**5,506**	**92.57**
**Region 9**	**68**	**4,040**	**27,437**	**31,545**	**64**	**94.12**	**3,953**	**97.85**	**26,471**	**96.48**	**30,488**	**96.65**
Zamboanga del Norte	26	1,694	10,453	**12,173**	25	**96.15**	1,658	**97.87**	10,136	**96.97**	**11,819**	**97.09**
Zamboanga del Sur	22	955	6,596	**7,573**	20	**90.91**	933	**97.70**	6,421	**97.35**	**7,374**	**97.37**
Zamboanga Sibugay	15	760	5,232	**6,007**	15	**100.00**	747	**98.29**	4,987	**95.32**	**5,749**	**95.71**
City of Isabela	2	299	2,095	**2,396**	1	**50.00**	288	**96.32**	1,895	**90.45**	**2,184**	**91.15**
City of Zamboanga	3	332	3,061	**3,396**	3	**100.00**	327	**98.49**	3,032	**99.05**	**3,362**	**99.00**
**Region 10**	**204**	**8,959**	**54,703**	**63,866**	**179**	**87.75**	**8,368**	**93.40**	**49,766**	**90.97**	**58,313**	**91.31**
Bukidnon	93	3,693	16,418	**20,204**	86	**92.47**	3,491	**94.53**	14,793	**90.10**	**18,370**	**90.92**
Camiguin	3	153	1,097	**1,253**	3	**100.00**	140	**91.50**	927	**84.50**	**1,070**	**85.40**
Lanao del Norte	27	893	9,016	**9,936**	17	**62.96**	755	**84.55**	8,534	**94.65**	**9,306**	**93.66**
Misamis Occidental	6	639	4,309	**4,954**	6	**100.00**	613	**95.93**	3,943	**91.51**	**4,562**	**92.09**
Misamis Oriental	27	1,338	8,320	**9,685**	25	**92.59**	1,263	**94.39**	7,807	**93.83**	**9,095**	**93.91**
City of Cagayan De Oro	34	1,588	9,857	**11,479**	33	**97.06**	1,498	**94.33**	8,794	**89.22**	**10,325**	**89.95**
City of Iligan	14	655	5,686	**6,355**	9	**64.29**	608	**92.82**	4,968	**87.37**	**5,585**	**87.88**
**Region 11**	**395**	**10,313**	**63,038**	**73,746**	**358**	**90.63**	**9,281**	**89.99**	**51,064**	**81.01**	**60,703**	**82.31**
Davao de Oro	61	1,670	8,779	**10,510**	56	**91.80**	1,541	**92.28**	7,327	**83.46**	**8,924**	**84.91**
Davao del Norte	99	2,168	13,220	**15,487**	89	**89.90**	1,927	**88.88**	10,317	**78.04**	**12,333**	**79.63**
Davao Oriental	56	1,272	6,591	**7,919**	53	**94.64**	1,195	**93.95**	5,745	**87.16**	**6,993**	**88.31**
Davao del Sur	62	1,432	7,187	**8,681**	56	**90.32**	1,348	**94.13**	6,010	**83.62**	**7,414**	**85.40**
Davao Occidental	48	1,066	3,168	**4,282**	45	**93.75**	1,013	**95.03**	2,857	**90.18**	**3,915**	**91.43**
City of Davao	69	2,705	24,093	**26,867**	59	**85.51**	2,257	**83.44**	18,808	**78.06**	**21,124**	**78.62**
**Region 12**	**282**	**8,938**	**52,550**	**61,770**	**268**	**95.04**	**8,395**	**93.92**	**45,697**	**86.96**	**54,360**	**88.00**
Cotabato	78	2,383	14,247	**16,708**	73	**93.59**	2,215	**92.95**	12,347	**86.66**	**14,635**	**87.59**
Sarangani	48	1,778	7,622	**9,448**	46	**95.83**	1,706	**95.95**	7,021	**92.11**	**8,773**	**92.86**
South Cotabato	81	2,204	12,261	**14,546**	78	**96.30**	2,073	**94.06**	10,452	**85.25**	**12,603**	**86.64**
Sultan Kudarat	60	1,781	9,990	**11,831**	56	**93.33**	1,696	**95.23**	8,627	**86.36**	**10,379**	**87.73**
City of General Santos	15	792	8,430	**9,237**	15	**100.00**	705	**89.02**	7,250	**86.00**	**7,970**	**86.28**
**CARAGA**	**99**	**5,072**	**33,467**	**38,638**	**97**	**97.98**	**4,754**	**93.73**	**29,119**	**87.01**	**33,970**	**87.92**
Agusan del Norte	20	799	4,769	**5,588**	20	**100.00**	739	**92.49**	3,945	**82.72**	**4,704**	**84.18**
Agusan del Sur	37	1,357	8,490	**9,884**	36	**97.30**	1,290	**95.06**	7,491	**88.23**	**8,817**	**89.20**
Surigao del Norte	4	854	6,499	**7,357**	3	**75.00**	803	**94.03**	5,776	**88.88**	**6,582**	**89.47**
Surigao del Sur	27	1,388	7,771	**9,186**	27	**100.00**	1,280	**92.22**	6,986	**89.90**	**8,293**	**90.28**
Dinagat Islands	1	105	757	**863**	1	**100.00**	105	**100.00**	757	**100.00**	**863**	**100.00**
City of Butuan	10	569	5,181	**5,760**	10	**100.00**	537	**94.38**	4,164	**80.37**	**4,711**	**81.79**
**BARMM**	**70**	**5,354**	**55,229**	**60,653**	**66**	**94.29**	**5,197**	**97.07**	**53,455**	**96.79**	**58,718**	**96.81**
Basilan	10	829	4,195	**5,034**	10	**100.00**	823	**99.28**	4,171	**99.43**	**5,004**	**99.40**
Lanao del Sur	11	605	13,410	**14,026**	11	**100.00**	572	**94.55**	12,795	**95.41**	**13,378**	**95.38**
Maguindanao del Norte	7	690	7,149	**7,846**	6	**85.71**	687	**99.57**	7,088	**99.15**	**7,781**	**99.17**
Maguindanao del Sur	22	1,369	11,452	**12,843**	20	**90.91**	1,309	**95.62**	11,027	**96.29**	**12,356**	**96.21**
Sulu	6	739	9,655	**10,400**	6	**100.00**	739	**100.00**	9,655	**100.00**	**10,400**	**100.00**
Tawi-Tawi	10	516	4,843	**5,369**	10	**100.00**	515	**99.81**	4,798	**99.07**	**5,323**	**99.14**
SGA	1	224	1,317	**1,542**	1	**100.00**	224	**100.00**	1,317	**100.00**	**1,542**	**100.00**
City of Cotabato	3	382	3,208	**3,593**	2	**66.67**	328	**85.86**	2,604	**81.17**	**2,934**	**81.66**

Note: Put asterisk (*) for No Report and Zero (0) for No Case

Deliveries should be reported by place of occurrence.

Vaginal - deliver

Table 2.B.2.2 - Intrapartum Care and Delivery Outcome
Delivery by Type (Vaginal and Cesarean)
Philippines, 2024

Area	Total number of Deliveries by age group				Cesarian Section							
	Age Group in Year			Total	Age Group in Year						Total	%
	10-14	15-19	20-49		10-14	%	15-19	%	20-49	%		
**PHILIPPINES**	**3,285**	**137,240**	**1,203,053**	**1,343,578**	**344**	**10.47**	**12,018**	**8.76**	**185,792**	**15.44**	**198,154**	**14.75**
**N C R**	**297**	**11,285**	**172,329**	**183,911**	**24**	**8.08**	**915**	**8.11**	**19,549**	**11.34**	**20,488**	**11.14**
City of Malabon	12	332	2,685	**3,029**	0	**0.00**	49	**14.76**	561	**20.89**	**610**	**20.14**
City of Navotas	6	308	3,632	**3,946**	1	**16.67**	42	**13.64**	729	**20.07**	**772**	**19.56**
City of Valenzuela	13	364	4,237	**4,614**	1	**7.69**	47	**12.91**	992	**23.41**	**1,040**	**22.54**
City of Caloocan	57	2,129	21,982	**24,168**	5	**8.77**	126	**5.92**	1,420	**6.46**	**1,551**	**6.42**
City of Marikina	9	226	2,441	**2,676**	2	**22.22**	28	**12.39**	480	**19.66**	**510**	**19.06**
City of Pasig	36	818	10,086	**10,940**	1	**2.78**	30	**3.67**	779	**7.72**	**810**	**7.40**
Pateros	0	37	717	**754**	0	**0.00**	2	**5.41**	107	**14.92**	**109**	**14.46**
City of Taguig	22	1,051	11,221	**12,294**	3	**13.64**	49	**4.66**	1,728	**15.40**	**1,780**	**14.48**
Quezon City	51	2,225	56,245	**58,521**	4	**7.84**	101	**4.54**	3,603	**6.41**	**3,708**	**6.34**
City of Makati	3	112	1,395	**1,510**	0	**0.00**	24	**21.43**	365	**26.16**	**389**	**25.76**
City of Mandaluyong	11	283	7,411	**7,705**	1	**9.09**	24	**8.48**	768	**10.36**	**793**	**10.29**
City of San Juan	0	35	734	**769**	0	**0.00**	7	**20.00**	177	**24.11**	**184**	**23.93**
City of Manila	28	1,543	22,420	**23,991**	0	**0.00**	194	**12.57**	3,243	**14.46**	**3,437**	**14.33**
City of Las Piñas	10	432	5,914	**6,356**	1	**10.00**	43	**9.95**	756	**12.78**	**800**	**12.59**
City of Muntinlupa	15	565	7,068	**7,648**	3	**20.00**	49	**8.67**	1,145	**16.20**	**1,197**	**15.65**
City of Parañaque	16	560	7,521	**8,097**	1	**6.25**	60	**10.71**	1,580	**21.01**	**1,641**	**20.27**
Pasay City	8	265	6,620	**6,893**	1	**12.50**	40	**15.09**	1,116	**16.86**	**1,157**	**16.79**
**C A R**	**55**	**2,118**	**21,818**	**23,991**	**5**	**9.09**	**438**	**20.68**	**7,218**	**33.08**	**7,661**	**31.93**
Abra	3	311	2,339	**2,653**	1	**33.33**	63	**20.26**	912	**38.99**	**976**	**36.79**
Apayao	14	526	3,042	**3,582**	1	**7.14**	74	**14.07**	839	**27.58**	**914**	**25.52**
Benguet	2	227	3,447	**3,676**	0	**0.00**	53	**23.35**	827	**23.99**	**880**	**23.94**
Ifugao	4	192	1,743	**1,939**	0	**0.00**	22	**11.46**	234	**13.43**	**256**	**13.20**
Kalinga	5	155	2,032	**2,192**	0	**0.00**	22	**14.19**	384	**18.90**	**406**	**18.52**
Mountain Province	9	330	2,917	**3,256**	1	**11.11**	70	**21.21**	1,011	**34.66**	**1,082**	**33.23**
City of Baguio	18	377	6,298	**6,693**	2	**11.11**	134	**35.54**	3,011	**47.81**	**3,147**	**47.02**
**Region 1**	**169**	**4,304**	**46,997**	**51,470**	**29**	**17.16**	**469**	**10.90**	**6,242**	**13.28**	**6,740**	**13.10**
Ilocos Norte	13	314	5,428	**5,755**	8	**61.54**	90	**28.66**	1,213	**22.35**	**1,311**	**22.78**
Ilocos Sur	14	467	6,911	**7,392**	2	**14.29**	110	**23.55**	1,570	**22.72**	**1,682**	**22.75**
La Union	32	691	7,039	**7,762**	8	**25.00**	74	**10.71**	567	**8.06**	**649**	**8.36**
Pangasinan	88	2,268	20,860	**23,216**	9	**10.23**	188	**8.29**	2,753	**13.20**	**2,950**	**12.71**
City of Dagupan	22	564	6,759	**7,345**	2	**9.09**	7	**1.24**	139	**2.06**	**148**	**2.01**
**Region 2**	**93**	**3,613**	**29,487**	**33,193**	**13**	**13.98**	**582**	**16.11**	**7,085**	**24.03**	**7,680**	**23.14**
Batanes	0	15	187	**202**	0	**0.00**	3	**20.00**	49	**26.20**	**52**	**25.74**
Cagayan	16	812	7,045	**7,873**	0	**0.00**	44	**5.42**	909	**12.90**	**953**	**12.10**
Isabela	26	1,370	11,631	**13,027**	6	**23.08**	228	**16.64**	2,890	**24.85**	**3,124**	**23.98**
Nueva Vizcaya	34	806	6,025	**6,865**	5	**14.71**	184	**22.83**	2,048	**33.99**	**2,237**	**32.59**
Quirino	14	336	2,034	**2,384**	2	**14.29**	69	**20.54**	513	**25.22**	**584**	**24.50**
City of Santiago	3	274	2,565	**2,842**	0	**0.00**	54	**19.71**	676	**26.35**	**730**	**25.69**
**Region 3**	**369**	**13,100**	**127,979**	**141,448**	**88**	**23.85**	**2,390**	**18.24**	**38,352**	**29.97**	**40,830**	**28.87**
Aurora	7	328	2,183	**2,518**	1	**14.29**	18	**5.49**	328	**15.03**	**347**	**13.78**
Bataan	61	1,357	9,945	**11,363**	15	**24.59**	251	**18.50**	3,616	**36.36**	**3,882**	**34.16**
Bulacan	92	3,473	37,497	**41,062**	17	**18.48**	649	**18.69**	13,403	**35.74**	**14,069**	**34.26**
Nueva Ecija	90	2,543	22,151	**24,784**	23	**25.56**	498	**19.58**	6,609	**29.84**	**7,130**	**28.77**
Pampanga	67	2,425	21,406	**23,898**	22	**32.84**	342	**14.10**	5,168	**24.14**	**5,532**	**23.15**
Tarlac	17	1,474	18,469	**19,960**	6	**35.29**	451	**30.60**	5,143	**27.85**	**5,600**	**28.06**
Zambales	14	845	5,855	**6,714**	1	**7.14**	155	**18.34**	1,922	**32.83**	**2,078**	**30.95**
City of Angeles	15	434	6,990	**7,439**	3	**20.00**	25	**5.76**	2,131	**30.49**	**2,159**	**29.02**
City of Olongapo	6	221	3,483	**3,710**	0	**0.00**	1	**0.45**	32	**0.92**	**33**	**0.89**
**Region 4A**	**390**	**17,923**	**180,933**	**199,246**	**37**	**9.49**	**1,849**	**10.32**	**38,582**	**21.32**	**40,468**	**20.31**
Batangas	70	2,689	33,636	**36,395**	12	**17.14**	517	**19.23**	12,356	**36.73**	**12,885**	**35.40**
Cavite	73	3,418	44,751	**48,242**	9	**12.33**	345	**10.09**	8,749	**19.55**	**9,103**	**18.87**
Laguna	95	3,953	39,146	**43,194**	9	**9.47**	347	**8.78**	6,566	**16.77**	**6,922**	**16.03**
Quezon	78	3,059	20,713	**23,850**	3	**3.85**	152	**4.97**	2,822	**13.62**	**2,977**	**12.48**
Rizal	68	4,481	40,125	**44,674**	4	**5.88**	474	**10.58**	7,761	**19.34**	**8,239**	**18.44**
City of Lucena	6	323	2,562	**2,891**	0	**0.00**	14	**4.33**	328	**12.80**	**342**	**11.83**
**Region 4B**	**126**	**5,691**	**36,723**	**42,540**	**9**	**7.14**	**264**	**4.64**	**3,818**	**10.40**	**4,091**	**9.62**
Marinduque	6	280	2,421	**2,707**	2	**33.33**	32	**11.43**	425	**17.55**	**459**	**16.96**
Occidental Mindoro	27	1,216	7,039	**8,282**	0	**0.00**	51	**4.19**	675	**9.59**	**726**	**8.77**
Oriental Mindoro	28	1,260	10,272	**11,560**	6	**21.43**	84	**6.67**	1,610	**15.67**	**1,700**	**14.71**
Palawan	47	2,041	9,904	**11,992**	0	**0.00**	57	**2.79**	442	**4.46**	**499**	**4.16**
Romblon	6	429	3,677	**4,112**	0	**0.00**	13	**3.03**	308	**8.38**	**321**	**7.81**
City of Puerto Princesa	12	465	3,410	**3,887**	1	**8.33**	27	**5.81**	358	**10.50**	**386**	**9.93**
**Region 5**	**139**	**9,086**	**76,470**	**85,695**	**18**	**12.95**	**731**	**8.05**	**7,497**	**9.80**	**8,246**	**9.62**
Albay	16	1,203	14,538	**15,757**	1	**6.25**	60	**4.99**	963	**6.62**	**1,024**	**6.50**
Camarines Norte	17	1,154	8,983	**10,154**	1	**5.88**	195	**16.90**	1,444	**16.07**	**1,640**	**16.15**
Camarines Sur	10	1,312	14,898	**16,220**	0	**0.00**	17	**1.30**	482	**3.24**	**499**	**3.08**
Catanduanes	9	458	3,352	**3,819**	1	**11.11**	38	**8.30**	746	**22.26**	**785**	**20.56**
Masbate	23	2,159	12,529	**14,711**	0	**0.00**	45	**2.08**	229	**1.83**	**274**	**1.86**
Sorsogon	28	1,313	11,083	**12,424**	1	**3.57**	82	**6.25**	1,728	**15.59**	**1,811**	**14.58**
City of Naga	36	1,487	11,087	**12,610**	14	**38.89**	294	**19.77**	1,905	**17.18**	**2,213**	**17.55**
**Region 6**	**183**	**9,328**	**76,010**	**85,521**	**10**	**5.46**	**479**	**5.14**	**9,572**	**12.59**	**10,061**	**11.76**
Aklan	8	612	5,360	**5,980**	0	**0.00**	39	**6.37**	699	**13.04**	**738**	**12.34**
Antique	11	731	6,726	**7,468**	1	**9.09**	26	**3.56**	788	**11.72**	**815**	**10.91**
Capiz	14	703	6,764	**7,481**	0	**0.00**	46	**6.54**	1,104	**16.32**	**1,150**	**15.37**
Guimaras	11	220	1,951	**2,182**	1	**9.09**	19	**8.64**	307	**15.74**	**327**	**14.99**
Iloilo	32	1,966	17,747	**19,745**	2	**6.25**	114	**5.80**	2,021	**11.39**	**2,137**	**10.82**

Table 2.B.2.2 - Intrapartum Care and Delivery Outcome
Delivery by Type (Vaginal and Cesarean)
Philippines, 2024

Area	Total number of Deliveries by age group				Cesarian Section							
	Age Group in Year			Total	Age Group in Year						Total	%
	10-14	15-19	20-49		10-14	%	15-19	%	20-49	%		
Negros Occidental	90	4,369	28,431	**32,890**	4	**4.44**	192	**4.39**	3,301	**11.61**	**3,497**	**10.63**
City of Bacolod	2	271	4,420	**4,693**	0	**0.00**	0	**0.00**	540	**12.22**	**540**	**11.51**
City of Iloilo	15	456	4,611	**5,082**	2	**13.33**	43	**9.43**	812	**17.61**	**857**	**16.86**
**Region 7**	**230**	**11,162**	**96,264**	**107,656**	**15**	**6.52**	**665**	**5.96**	**10,616**	**11.03**	**11,296**	**10.49**
Bohol	22	1,518	15,537	**17,077**	2	**9.09**	33	**2.17**	871	**5.61**	**906**	**5.31**
Cebu	80	4,543	36,259	**40,882**	4	**5.00**	276	**6.08**	3,580	**9.87**	**3,860**	**9.44**
Negros Oriental	51	2,307	16,213	**18,571**	1	**1.96**	65	**2.82**	1,462	**9.02**	**1,528**	**8.23**
Siquijor	1	75	921	**997**	0	**0.00**	8	**10.67**	155	**16.83**	**163**	**16.35**
City of Cebu	29	1,437	12,529	**13,995**	6	**20.69**	114	**7.93**	1,497	**11.95**	**1,617**	**11.55**
City of Lapu-Lapu	24	584	6,311	**6,919**	2	**8.33**	117	**20.03**	978	**15.50**	**1,097**	**15.85**
City of Mandaue	23	698	8,494	**9,215**	0	**0.00**	52	**7.45**	2,073	**24.41**	**2,125**	**23.06**
**Region 8**	**116**	**6,954**	**51,619**	**58,689**	**9**	**7.76**	**539**	**7.75**	**6,357**	**12.32**	**6,905**	**11.77**
Biliran	6	385	2,666	**3,057**	0	**0.00**	6	**1.56**	236	**8.85**	**242**	**7.92**
Eastern Samar	17	858	5,498	**6,373**	4	**23.53**	126	**14.69**	1,121	**20.39**	**1,251**	**19.63**
Leyte	21	1,366	10,058	**11,445**	2	**9.52**	64	**4.69**	487	**4.84**	**553**	**4.83**
Northern Samar	19	1,345	8,891	**10,255**	1	**5.26**	74	**5.50**	908	**10.21**	**983**	**9.59**
Southern Leyte	5	429	3,896	**4,330**	1	**20.00**	63	**14.69**	833	**21.38**	**897**	**20.72**
Samar	23	1,455	10,434	**11,912**	0	**0.00**	80	**5.50**	1,417	**13.58**	**1,497**	**12.57**
Ormoc City	15	626	4,728	**5,369**	1	**6.67**	77	**12.30**	962	**20.35**	**1,040**	**19.37**
City of Tacloban	10	490	5,448	**5,948**	0	**0.00**	49	**10.00**	393	**7.21**	**442**	**7.43**
**Region 9**	**68**	**4,040**	**27,437**	**31,545**	**5**	**7.35**	**87**	**2.15**	**965**	**3.52**	**1,057**	**3.35**
Zamboanga del Norte	26	1,694	10,453	**12,173**	1	**3.85**	36	**2.13**	317	**3.03**	**354**	**2.91**
Zamboanga del Sur	22	955	6,596	**7,573**	3	**13.64**	17	**1.78**	179	**2.71**	**199**	**2.63**
Zamboanga Sibugay	15	760	5,232	**6,007**	0	**0.00**	13	**1.71**	245	**4.68**	**258**	**4.29**
City of Isabela	2	299	2,095	**2,396**	1	**50.00**	16	**5.35**	195	**9.31**	**212**	**8.85**
City of Zamboanga	3	332	3,061	**3,396**	0	**0.00**	5	**1.51**	29	**0.95**	**34**	**1.00**
**Region 10**	**204**	**8,959**	**54,703**	**63,866**	**25**	**12.25**	**571**	**6.37**	**4,957**	**9.06**	**5,553**	**8.69**
Bukidnon	93	3,693	16,418	**20,204**	7	**7.53**	202	**5.47**	1,625	**9.90**	**1,834**	**9.08**
Camiguin	3	153	1,097	**1,253**	0	**0.00**	13	**8.50**	170	**15.50**	**183**	**14.60**
Lanao del Norte	27	893	9,016	**9,936**	10	**37.04**	138	**15.45**	482	**5.35**	**630**	**6.34**
Misamis Occidental	6	639	4,309	**4,954**	0	**0.00**	22	**3.44**	370	**8.59**	**392**	**7.91**
Misamis Oriental	27	1,338	8,320	**9,685**	2	**7.41**	75	**5.61**	513	**6.17**	**590**	**6.09**
City of Cagayan De Oro	34	1,588	9,857	**11,479**	1	**2.94**	74	**4.66**	1,079	**10.95**	**1,154**	**10.05**
City of Iligan	14	655	5,686	**6,355**	5	**35.71**	47	**7.18**	718	**12.63**	**770**	**12.12**
**Region 11**	**395**	**10,313**	**63,038**	**73,746**	**37**	**9.37**	**1,033**	**10.02**	**11,970**	**18.99**	**13,040**	**17.68**
Davao de Oro	61	1,670	8,779	**10,510**	5	**8.20**	129	**7.72**	1,452	**16.54**	**1,586**	**15.09**
Davao del Norte	99	2,168	13,220	**15,487**	10	**10.10**	241	**11.12**	2,904	**21.97**	**3,155**	**20.37**
Davao Oriental	56	1,272	6,591	**7,919**	3	**5.36**	77	**6.05**	846	**12.84**	**926**	**11.69**
Davao del Sur	62	1,432	7,187	**8,681**	6	**9.68**	84	**5.87**	1,177	**16.38**	**1,267**	**14.60**
Davao Occidental	48	1,066	3,168	**4,282**	3	**6.25**	54	**5.07**	306	**9.66**	**363**	**8.48**
City of Davao	69	2,705	24,093	**26,867**	10	**14.49**	448	**16.56**	5,285	**21.94**	**5,743**	**21.38**
**Region 12**	**282**	**8,938**	**52,550**	**61,770**	**14**	**4.96**	**545**	**6.10**	**6,851**	**13.04**	**7,410**	**12.00**
Cotabato	78	2,383	14,247	**16,708**	5	**6.41**	168	**7.05**	1,900	**13.34**	**2,073**	**12.41**
Sarangani	48	1,778	7,622	**9,448**	2	**4.17**	72	**4.05**	601	**7.89**	**675**	**7.14**
South Cotabato	81	2,204	12,261	**14,546**	3	**3.70**	131	**5.94**	1,809	**14.75**	**1,943**	**13.36**
Sultan Kudarat	60	1,781	9,990	**11,831**	4	**6.67**	87	**4.88**	1,361	**13.62**	**1,452**	**12.27**
City of General Santos	15	792	8,430	**9,237**	0	**0.00**	87	**10.98**	1,180	**14.00**	**1,267**	**13.72**
**CARAGA**	**99**	**5,072**	**33,467**	**38,638**	**2**	**2.02**	**318**	**6.27**	**4,348**	**12.99**	**4,668**	**12.08**
Agusan del Norte	20	799	4,769	**5,588**	0	**0.00**	60	**7.51**	824	**17.28**	**884**	**15.82**
Agusan del Sur	37	1,357	8,490	**9,884**	1	**2.70**	67	**4.94**	999	**11.77**	**1,067**	**10.80**
Surigao del Norte	4	854	6,499	**7,357**	1	**25.00**	51	**5.97**	723	**11.12**	**775**	**10.53**
Surigao del Sur	27	1,388	7,771	**9,186**	0	**0.00**	108	**7.78**	785	**10.10**	**893**	**9.72**
Dinagat Islands	1	105	757	**863**	0	**0.00**	0	**0.00**	0	**0.00**	**0**	**0.00**
City of Butuan	10	569	5,181	**5,760**	0	**0.00**	32	**5.62**	1,017	**19.63**	**1,049**	**18.21**
**BARMM**	**70**	**5,354**	**55,229**	**60,653**	**4**	**5.71**	**143**	**2.67**	**1,813**	**3.28**	**1,960**	**3.23**
Basilan	10	829	4,195	**5,034**	0	**0.00**	6	**0.72**	24	**0.57**	**30**	**0.60**
Lanao del Sur	11	605	13,410	**14,026**	0	**0.00**	32	**5.29**	641	**4.78**	**673**	**4.80**
Maguindanao del Norte	7	690	7,149	**7,846**	1	**14.29**	3	**0.43**	61	**0.85**	**65**	**0.83**
Maguindanao del Sur	22	1,369	11,452	**12,843**	2	**9.09**	60	**4.38**	425	**3.71**	**487**	**3.79**
Sulu	6	739	9,655	**10,400**	0	**0.00**	0	**0.00**	0	**0.00**	**0**	**0.00**
Tawi-Tawi	10	516	4,843	**5,369**	0	**0.00**	1	**0.19**	45	**0.93**	**46**	**0.86**
SGA	1	224	1,317	**1,542**	0	**0.00**	0	**0.00**	0	**0.00**	**0**	**0.00**
City of Cotabato	3	382	3,208	**3,593**	1	**33.33**	41	**10.73**	617	**19.23**	**659**	**18.34**

Note: Put asterisk (*) for No Report and Zero (0) for No Case

ies by NSD, Vaccum and Forcep

Deliveries should be reported by place of occurrence.

Table 2.B.2.3 - Intrapartum Care and Delivery Outcome
Pregnancy by Outcome (Full Term and Pre-term)
Philippines, 2024

Area	Total number of Deliveries	Pregnancy Outcome									
		Full term (37-42 weeks AOG)					Pre-term (22-36 weeks AOG)				
		Age Group in Year			Total	%	Age Group in Year			Total	%
		10-14	15-19	20-49			10-14	15-19	20-49		
**PHILIPPINES**	**1,343,578**	**3,119**	**132,222**	**1,174,570**	**1,309,911**	**97.49**	**155**	**3,518**	**17,128**	**20,801**	**1.55**
**N C R**	**183,911**	**291**	**11,115**	**170,968**	**182,374**	**99.16**	**6**	**123**	**1,113**	**1,242**	**0.68**
City of Malabon	3,029	11	324	2,633	**2,968**	**97.99**	1	5	42	**48**	**1.58**
City of Navotas	3,946	6	300	3,563	**3,869**	**98.05**	0	5	67	**72**	**1.82**
City of Valenzuela	4,614	13	351	4,158	**4,522**	**98.01**	0	12	76	**88**	**1.91**
City of Caloocan	24,168	57	2,121	21,924	**24,102**	**99.73**	0	6	49	**55**	**0.23**
City of Marikina	2,676	9	223	2,417	**2,649**	**98.99**	0	2	23	**25**	**0.93**
City of Pasig	10,940	35	790	9,660	**10,485**	**95.84**	1	17	318	**336**	**3.07**
Pateros	754	0	32	707	**739**	**98.01**	0	1	4	**5**	**0.66**
City of Taguig	12,294	22	1,035	11,146	**12,203**	**99.26**	0	6	62	**68**	**0.55**
Quezon City	58,521	49	2,212	56,105	**58,366**	**99.74**	2	11	113	**126**	**0.22**
City of Makati	1,510	3	105	1,345	**1,453**	**96.23**	0	6	41	**47**	**3.11**
City of Mandaluyong	7,705	11	279	7,352	**7,642**	**99.18**	0	4	30	**34**	**0.44**
City of San Juan	769	0	34	715	**749**	**97.40**	0	1	13	**14**	**1.82**
City of Manila	23,991	28	1,531	22,293	**23,852**	**99.42**	0	12	117	**129**	**0.54**
City of Las Piñas	6,356	10	423	5,872	**6,305**	**99.20**	0	6	32	**38**	**0.60**
City of Muntinlupa	7,648	14	553	7,031	**7,598**	**99.35**	1	8	32	**41**	**0.54**
City of Parañaque	8,097	16	543	7,479	**8,038**	**99.27**	0	17	42	**59**	**0.73**
Pasay City	6,893	7	259	6,568	**6,834**	**99.14**	1	4	52	**57**	**0.83**
**C A R**	**23,991**	**51**	**1,976**	**20,721**	**22,748**	**94.82**	**4**	**115**	**895**	**1,014**	**4.23**
Abra	2,653	2	270	2,186	**2,458**	**92.65**	1	32	120	**153**	**5.77**
Apayao	3,582	13	502	2,932	**3,447**	**96.23**	1	13	76	**90**	**2.51**
Benguet	3,676	2	208	3,243	**3,453**	**93.93**	0	19	187	**206**	**5.60**
Ifugao	1,939	3	186	1,725	**1,914**	**98.71**	1	2	12	**15**	**0.77**
Kalinga	2,192	5	151	1,993	**2,149**	**98.04**	0	4	25	**29**	**1.32**
Mountain Province	3,256	9	315	2,809	**3,133**	**96.22**	0	12	77	**89**	**2.73**
City of Baguio	6,693	17	344	5,833	**6,194**	**92.54**	1	33	398	**432**	**6.45**
**Region 1**	**51,470**	**156**	**4,179**	**45,744**	**50,079**	**97.30**	**12**	**112**	**999**	**1,123**	**2.18**
Ilocos Norte	5,755	11	275	5,025	**5,311**	**92.28**	2	39	373	**414**	**7.19**
Ilocos Sur	7,392	8	422	6,586	**7,016**	**94.91**	6	45	314	**365**	**4.94**
La Union	7,762	30	679	6,773	**7,482**	**96.39**	2	9	199	**210**	**2.71**
Pangasinan	23,216	86	2,243	20,705	**23,034**	**99.22**	2	19	95	**116**	**0.50**
City of Dagupan	7,345	21	560	6,655	**7,236**	**98.52**	0	0	18	**18**	**0.25**
**Region 2**	**33,193**	**87**	**3,477**	**28,803**	**32,367**	**97.51**	**5**	**111**	**535**	**651**	**1.96**
Batanes	202	0	15	180	**195**	**96.53**	0	0	6	**6**	**2.97**
Cagayan	7,873	14	789	6,859	**7,662**	**97.32**	1	17	152	**170**	**2.16**
Isabela	13,027	25	1,300	11,395	**12,720**	**97.64**	1	62	189	**252**	**1.93**
Nueva Vizcaya	6,865	33	770	5,805	**6,608**	**96.26**	1	25	164	**190**	**2.77**
Quirino	2,384	13	333	2,015	**2,361**	**99.04**	1	3	8	**12**	**0.50**
City of Santiago	2,842	2	270	2,549	**2,821**	**99.26**	1	4	16	**21**	**0.74**
**Region 3**	**141,448**	**328**	**11,965**	**119,739**	**132,032**	**93.34**	**36**	**528**	**2,576**	**3,140**	**2.22**
Aurora	2,518	7	321	2,137	**2,465**	**97.90**	0	4	22	**26**	**1.03**
Bataan	11,363	57	1,272	9,453	**10,782**	**94.89**	4	73	415	**492**	**4.33**
Bulacan	41,062	82	3,293	36,732	**40,107**	**97.67**	9	162	596	**767**	**1.87**
Nueva Ecija	24,784	83	2,474	21,444	**24,001**	**96.84**	6	59	565	**630**	**2.54**
Pampanga	23,898	52	1,775	15,933	**17,760**	**74.32**	13	95	373	**481**	**2.01**
Tarlac	19,960	16	1,418	18,091	**19,525**	**97.82**	0	52	279	**331**	**1.66**
Zambales	6,714	12	786	5,611	**6,409**	**95.46**	2	55	200	**257**	**3.83**
City of Angeles	7,439	13	407	6,862	**7,282**	**97.89**	2	27	121	**150**	**2.02**
City of Olongapo	3,710	6	219	3,476	**3,701**	**99.76**	0	1	5	**6**	**0.16**
**Region 4A**	**199,246**	**368**	**17,530**	**178,253**	**196,151**	**98.45**	**18**	**316**	**2,067**	**2,401**	**1.21**
Batangas	36,395	66	2,608	33,107	**35,781**	**98.31**	4	58	369	**431**	**1.18**
Cavite	48,242	67	3,346	44,163	**47,576**	**98.62**	5	64	528	**597**	**1.24**
Laguna	43,194	93	3,863	38,519	**42,475**	**98.34**	2	72	433	**507**	**1.17**
Quezon	23,850	72	2,983	20,324	**23,379**	**98.03**	3	52	230	**285**	**1.19**
Rizal	44,674	64	4,407	39,599	**44,070**	**98.65**	4	70	490	**564**	**1.26**
City of Lucena	2,891	6	323	2,541	**2,870**	**99.27**	0	0	17	**17**	**0.59**

Table 2.B.2.3 - Intrapartum Care and Delivery Outcome
Pregnancy by Outcome (Full Term and Pre-term)
Philippines, 2024

Area	Total number of Deliveries	Pregnancy Outcome									
		Full term (37-42 weeks AOG)					Pre-term (22-36 weeks AOG)				
		Age Group in Year			Total	%	Age Group in Year			Total	%
		10-14	15-19	20-49			10-14	15-19	20-49		
**Region 4B**	**42,540**	**117**	**5,507**	**35,832**	**41,456**	**97.45**	**4**	**122**	**499**	**625**	**1.47**
Marinduque	2,707	6	272	2,368	**2,646**	**97.75**	0	6	25	**31**	**1.15**
Occidental Mindoro	8,282	24	1,175	6,881	**8,080**	**97.56**	2	31	80	**113**	**1.36**
Oriental Mindoro	11,560	26	1,215	10,008	**11,249**	**97.31**	0	28	183	**211**	**1.83**
Palawan	11,992	45	1,982	9,683	**11,710**	**97.65**	0	30	99	**129**	**1.08**
Romblon	4,112	5	415	3,560	**3,980**	**96.79**	1	13	80	**94**	**2.29**
City of Puerto Princesa	3,887	11	448	3,332	**3,791**	**97.53**	1	14	32	**47**	**1.21**
**Region 5**	**85,695**	**127**	**8,500**	**74,065**	**82,692**	**96.50**	**6**	**477**	**1,749**	**2,232**	**2.60**
Albay	15,757	15	1,161	14,407	**15,583**	**98.90**	1	39	103	**143**	**0.91**
Camarines Norte	10,154	14	814	7,729	**8,557**	**84.27**	3	299	1,181	**1,483**	**14.61**
Camarines Sur	16,220	9	1,287	14,789	**16,085**	**99.17**	0	15	49	**64**	**0.39**
Catanduanes	3,819	7	450	3,301	**3,758**	**98.40**	1	4	9	**14**	**0.37**
Masbate	14,711	21	2,096	12,312	**14,429**	**98.08**	0	39	78	**117**	**0.80**
Sorsogon	12,424	26	1,219	10,677	**11,922**	**95.96**	1	80	252	**333**	**2.68**
City of Naga	12,610	35	1,473	10,850	**12,358**	**98.00**	0	1	77	**78**	**0.62**
**Region 6**	**85,521**	**176**	**8,974**	**74,142**	**83,292**	**97.39**	**4**	**284**	**1,365**	**1,653**	**1.93**
Aklan	5,980	8	586	5,257	**5,851**	**97.84**	0	17	77	**94**	**1.57**
Antique	7,468	9	682	6,388	**7,079**	**94.79**	0	40	262	**302**	**4.04**
Capiz	7,481	14	686	6,644	**7,344**	**98.17**	0	11	74	**85**	**1.14**
Guimaras	2,182	10	213	1,879	**2,102**	**96.33**	1	6	59	**66**	**3.02**
Iloilo	19,745	31	1,905	17,427	**19,363**	**98.07**	1	49	242	**292**	**1.48**
Negros Occidental	32,890	88	4,208	27,779	**32,075**	**97.52**	1	129	415	**545**	**1.66**
City of Bacolod	4,693	2	256	4,225	**4,483**	**95.53**	0	14	174	**188**	**4.01**
City of Iloilo	5,082	14	438	4,543	**4,995**	**98.29**	1	18	62	**81**	**1.59**
**Region 7**	**107,656**	**213**	**10,720**	**93,790**	**104,723**	**97.28**	**15**	**376**	**1,846**	**2,237**	**2.08**
Bohol	17,077	20	1,490	15,282	**16,792**	**98.33**	2	22	142	**166**	**0.97**
Cebu	40,882	73	4,327	35,368	**39,768**	**97.28**	5	186	624	**815**	**1.99**
Negros Oriental	18,571	46	2,216	15,712	**17,974**	**96.79**	5	71	371	**447**	**2.41**
Siquijor	997	1	71	895	**967**	**96.99**	0	4	19	**23**	**2.31**
City of Cebu	13,995	29	1,397	12,375	**13,801**	**98.61**	0	37	138	**175**	**1.25**
City of Lapu-Lapu	6,919	24	561	6,060	**6,645**	**96.04**	0	20	223	**243**	**3.51**
City of Mandaue	9,215	20	658	8,098	**8,776**	**95.24**	3	36	329	**368**	**3.99**
**Region 8**	**58,689**	**130**	**6,624**	**50,316**	**57,070**	**97.24**	**4**	**253**	**937**	**1,194**	**2.03**
Biliran	3,057	6	374	2,580	**2,960**	**96.83**	0	7	35	**42**	**1.37**
Eastern Samar	6,373	17	831	5,358	**6,206**	**97.38**	0	21	89	**110**	**1.73**
Leyte	11,445	38	1,315	9,941	**11,294**	**98.68**	0	34	76	**110**	**0.96**
Northern Samar	10,255	18	1,227	8,465	**9,710**	**94.69**	1	100	316	**417**	**4.07**
Southern Leyte	4,330	5	422	3,864	**4,291**	**99.10**	0	5	25	**30**	**0.69**
Samar	11,912	21	1,374	10,231	**11,626**	**97.60**	3	59	170	**232**	**1.95**
Ormoc City	5,369	15	595	4,489	**5,099**	**94.97**	0	23	184	**207**	**3.86**
City of Tacloban	5,948	10	486	5,388	**5,884**	**98.92**	0	4	42	**46**	**0.77**
**Region 9**	**31,545**	**65**	**3,949**	**27,130**	**31,144**	**98.73**	**3**	**59**	**152**	**214**	**0.68**
Zamboanga del Norte	12,173	25	1,657	10,315	**11,997**	**98.55**	1	22	68	**91**	**0.75**
Zamboanga del Sur	7,573	22	935	6,542	**7,499**	**99.02**	0	15	36	**51**	**0.67**
Zamboanga Sibugay	6,007	13	732	5,147	**5,892**	**98.09**	2	20	46	**68**	**1.13**
City of Isabela	2,396	2	296	2,071	**2,369**	**98.87**	0	0	0	**0**	**0.00**
City of Zamboanga	3,396	3	329	3,055	**3,387**	**99.73**	0	2	2	**4**	**0.12**
**Region 10**	**63,866**	**198**	**8,764**	**53,973**	**62,935**	**98.54**	**5**	**135**	**470**	**610**	**0.96**
Bukidnon	20,204	91	3,604	16,143	**19,838**	**98.19**	2	65	162	**229**	**1.13**
Camiguin	1,253	2	149	1,076	**1,227**	**97.92**	0	3	14	**17**	**1.36**
Lanao del Norte	9,936	27	881	8,972	**9,880**	**99.44**	0	6	25	**31**	**0.31**
Misamis Occidental	4,954	6	623	4,259	**4,888**	**98.67**	0	10	38	**48**	**0.97**
Misamis Oriental	9,685	26	1,324	8,246	**9,596**	**99.08**	1	10	38	**49**	**0.51**
City of Cagayan De Oro	11,479	34	1,538	9,735	**11,307**	**98.50**	0	33	94	**127**	**1.11**
City of Iligan	6,355	12	645	5,542	**6,199**	**97.55**	2	8	99	**109**	**1.72**
**Region 11**	**73,746**	**377**	**10,066**	**61,789**	**72,232**	**97.95**	**20**	**186**	**751**	**957**	**1.30**
Davao de Oro	10,510	56	1,640	8,546	**10,242**	**97.45**	3	31	145	**179**	**1.70**

Table 2.B.2.3 - Intrapartum Care and Delivery Outcome
Pregnancy by Outcome (Full Term and Pre-term)
Philippines, 2024

Area	Total number of Deliveries	Pregnancy Outcome									
		Full term (37-42 weeks AOG)					Pre-term (22-36 weeks AOG)				
		Age Group in Year			Total	%	Age Group in Year			Total	%
		10-14	15-19	20-49			10-14	15-19	20-49		
Davao del Norte	15,487	90	2,115	12,809	**15,014**	**96.95**	10	44	261	**315**	**2.03**
Davao Oriental	7,919	55	1,227	6,466	**7,748**	**97.84**	0	31	52	**83**	**1.05**
Davao del Sur	8,681	58	1,385	7,017	**8,460**	**97.45**	4	35	110	**149**	**1.72**
Davao Occidental	4,282	46	1,032	3,072	**4,150**	**96.92**	1	16	48	**65**	**1.52**
City of Davao	26,867	72	2,667	23,879	**26,618**	**99.07**	2	29	135	**166**	**0.62**
**Region 12**	**61,770**	**272**	**8,702**	**51,546**	**60,520**	**97.98**	**9**	**165**	**620**	**794**	**1.29**
Cotabato	16,708	75	2,318	13,958	**16,351**	**97.86**	2	47	177	**226**	**1.35**
Sarangani	9,448	47	1,748	7,525	**9,320**	**98.65**	1	21	64	**86**	**0.91**
South Cotabato	14,546	79	2,128	11,979	**14,186**	**97.53**	2	49	161	**212**	**1.46**
Sultan Kudarat	11,831	56	1,734	9,747	**11,537**	**97.52**	4	32	137	**173**	**1.46**
City of General Santos	9,237	15	774	8,337	**9,126**	**98.80**	0	16	81	**97**	**1.05**
**CARAGA**	**38,638**	**95**	**4,925**	**32,856**	**37,876**	**98.03**	**3**	**92**	**351**	**446**	**1.15**
Agusan del Norte	5,588	19	764	4,740	**5,523**	**98.84**	0	7	13	**20**	**0.36**
Agusan del Sur	9,884	37	1,345	8,409	**9,791**	**99.06**	0	3	17	**20**	**0.20**
Surigao del Norte	7,357	4	849	6,463	**7,316**	**99.44**	0	2	14	**16**	**0.22**
Surigao del Sur	9,186	24	1,301	7,485	**8,810**	**95.91**	3	76	194	**273**	**2.97**
Dinagat Islands	863	1	104	753	**858**	**99.42**	0	1	3	**4**	**0.46**
City of Butuan	5,760	10	562	5,006	**5,578**	**96.84**	0	3	110	**113**	**1.96**
**BARMM**	**60,653**	**68**	**5,249**	**54,903**	**60,220**	**99.29**	**1**	**64**	**203**	**268**	**0.44**
Basilan	5,034	10	800	4,117	**4,927**	**97.87**	0	20	38	**58**	**1.15**
Lanao del Sur	14,026	10	596	13,409	**14,015**	**99.92**	0	3	13	**16**	**0.11**
Maguindanao del Norte	7,846	7	685	7,136	**7,828**	**99.77**	0	3	8	**11**	**0.14**
Maguindanao del Sur	12,843	21	1,340	11,326	**12,687**	**98.79**	1	20	79	**100**	**0.78**
Sulu	10,400	6	733	9,640	**10,379**	**99.80**	0	1	12	**13**	**0.13**
Tawi-Tawi	5,369	10	503	4,798	**5,311**	**98.92**	0	9	22	**31**	**0.58**
SGA	1,542	1	224	1,314	**1,539**	**99.81**	0	0	2	**2**	**0.13**
City of Cotabato	3,593	3	368	3,163	**3,534**	**98.36**	0	8	29	**37**	**1.03**

*Legend: * - No Report*

Table 2.B.2.3 - Intrapartum Care and Delivery Outcome
Pregnancy by Outcome (Full Term and Pre-term)
Philippines, 2024

Area	Total number of Deliveries	Pregnancy Outcome								
		Fetal Death					Abortion (Counts Only)			
		Age Group			Total	%	Age Group			Total
		10-14	15-19	20-49			10-14	15-19	20-49	
**PHILIPPINES**	**1,343,578**	**32**	**863**	**6,431**	**7,326**	**0.55**	**78**	**1,231**	**9,927**	**11,236**
**N C R**	**183,911**	**0**	**25**	**270**	**295**	**0.16**	**2**	**42**	**582**	**626**
City of Malabon	3,029	0	0	13	**13**	**0.43**	1	11	91	**103**
City of Navotas	3,946	0	1	4	**5**	**0.13**	0	0	0	**0**
City of Valenzuela	4,614	0	1	3	**4**	**0.09**	0	2	52	**54**
City of Caloocan	24,168	0	2	9	**11**	**0.05**	0	1	32	**33**
City of Marikina	2,676	0	0	2	**2**	**0.07**	0	0	7	**7**
City of Pasig	10,940	0	11	108	**119**	**1.09**	1	3	71	**75**
Pateros	754	0	3	7	**10**	**1.33**	0	1	13	**14**
City of Taguig	12,294	0	2	21	**23**	**0.19**	0	7	50	**57**
Quezon City	58,521	0	2	27	**29**	**0.05**	0	2	54	**56**
City of Makati	1,510	0	0	10	**10**	**0.66**	0	1	42	**43**
City of Mandaluyong	7,705	0	0	29	**29**	**0.38**	0	0	6	**6**
City of San Juan	769	0	0	6	**6**	**0.78**	0	5	14	**19**
City of Manila	23,991	0	0	10	**10**	**0.04**	0	5	93	**98**
City of Las Piñas	6,356	0	3	10	**13**	**0.20**	0	1	7	**8**
City of Muntinlupa	7,648	0	0	9	**9**	**0.12**	0	3	37	**40**
City of Parañaque	8,097	0	0	0	**0**	**0.00**	0	0	4	**4**
Pasay City	6,893	0	0	2	**2**	**0.03**	0	0	9	**9**
**C A R**	**23,991**	**0**	**27**	**202**	**229**	**0.95**	**3**	**63**	**1,136**	**1,202**
Abra	2,653	0	9	33	**42**	**1.58**	0	8	126	**134**
Apayao	3,582	0	11	34	**45**	**1.26**	2	17	182	**201**
Benguet	3,676	0	0	17	**17**	**0.46**	0	16	206	**222**
Ifugao	1,939	0	4	6	**10**	**0.52**	0	2	83	**85**
Kalinga	2,192	0	0	14	**14**	**0.64**	0	0	32	**32**
Mountain Province	3,256	0	3	31	**34**	**1.04**	0	13	210	**223**
City of Baguio	6,693	0	0	67	**67**	**1.00**	1	7	297	**305**
**Region 1**	**51,470**	**1**	**12**	**255**	**268**	**0.52**	**1**	**8**	**90**	**99**
Ilocos Norte	5,755	0	0	30	**30**	**0.52**	0	2	10	**12**
Ilocos Sur	7,392	0	1	10	**11**	**0.15**	0	1	12	**13**
La Union	7,762	0	1	69	**70**	**0.90**	0	2	45	**47**
Pangasinan	23,216	0	6	60	**66**	**0.28**	1	3	23	**27**
City of Dagupan	7,345	1	4	86	**91**	**1.24**	0	0	0	**0**
**Region 2**	**33,193**	**0**	**10**	**165**	**175**	**0.53**	**4**	**63**	**705**	**772**
Batanes	202	0	0	1	**1**	**0.50**	0	0	16	**16**
Cagayan	7,873	0	4	37	**41**	**0.52**	1	7	118	**126**
Isabela	13,027	0	4	51	**55**	**0.42**	1	23	148	**172**
Nueva Vizcaya	6,865	0	2	65	**67**	**0.98**	1	25	345	**371**
Quirino	2,384	0	0	11	**11**	**0.46**	1	8	78	**87**
City of Santiago	2,842	0	0	0	**0**	**0.00**	0	0	0	**0**
**Region 3**	**141,448**	**4**	**64**	**637**	**705**	**0.50**	**9**	**129**	**1,193**	**1,331**
Aurora	2,518	0	3	24	**27**	**1.07**	0	0	6	**6**
Bataan	11,363	0	12	77	**89**	**0.78**	2	35	504	**541**
Bulacan	41,062	1	18	169	**188**	**0.46**	6	76	498	**580**
Nueva Ecija	24,784	1	10	142	**153**	**0.62**	0	4	37	**41**
Pampanga	23,898	1	12	72	**85**	**0.36**	1	11	119	**131**
Tarlac	19,960	1	4	99	**104**	**0.52**	0	0	7	**7**
Zambales	6,714	0	4	44	**48**	**0.71**	0	2	22	**24**
City of Angeles	7,439	0	0	7	**7**	**0.09**	0	0	0	**0**
City of Olongapo	3,710	0	1	3	**4**	**0.11**	0	1	0	**1**
**Region 4A**	**199,246**	**4**	**77**	**613**	**694**	**0.35**	**3**	**111**	**688**	**802**
Batangas	36,395	0	23	160	**183**	**0.50**	0	30	169	**199**
Cavite	48,242	1	8	60	**69**	**0.14**	2	30	108	**140**
Laguna	43,194	0	18	194	**212**	**0.49**	1	26	208	**235**
Quezon	23,850	3	24	159	**186**	**0.78**	0	20	154	**174**
Rizal	44,674	0	4	36	**40**	**0.09**	0	5	46	**51**
City of Lucena	2,891	0	0	4	**4**	**0.14**	0	0	3	**3**

Table 2.B.2.3 - Intrapartum Care and Delivery Outcome
Pregnancy by Outcome (Full Term and Pre-term)
Philippines, 2024

Area	Total number of Deliveries	Pregnancy Outcome								
		Fetal Death					Abortion (Counts Only)			
		Age Group			Total	%	Age Group			Total
		10-14	15-19	20-49			10-14	15-19	20-49	
**Region 4B**	**42,540**	**5**	**62**	**392**	**459**	**1.08**	**1**	**55**	**317**	**373**
Marinduque	2,707	0	2	28	**30**	**1.11**	0	5	23	**28**
Occidental Mindoro	8,282	1	10	78	**89**	**1.07**	0	6	69	**75**
Oriental Mindoro	11,560	2	17	81	**100**	**0.87**	0	2	36	**38**
Palawan	11,992	2	29	122	**153**	**1.28**	1	39	130	**170**
Romblon	4,112	0	1	37	**38**	**0.92**	0	1	41	**42**
City of Puerto Princesa	3,887	0	3	46	**49**	**1.26**	0	2	18	**20**
**Region 5**	**85,695**	**6**	**72**	**696**	**774**	**0.90**	**4**	**112**	**1,132**	**1,248**
Albay	15,757	0	3	28	**31**	**0.20**	0	7	95	**102**
Camarines Norte	10,154	0	8	106	**114**	**1.12**	0	6	148	**154**
Camarines Sur	16,220	1	10	63	**74**	**0.46**	2	6	96	**104**
Catanduanes	3,819	1	4	42	**47**	**1.23**	1	7	35	**43**
Masbate	14,711	2	20	143	**165**	**1.12**	0	27	150	**177**
Sorsogon	12,424	1	14	154	**169**	**1.36**	1	58	562	**621**
City of Naga	12,610	1	13	160	**174**	**1.38**	0	1	46	**47**
**Region 6**	**85,521**	**3**	**70**	**503**	**576**	**0.67**	**7**	**100**	**855**	**962**
Aklan	5,980	0	9	26	**35**	**0.59**	1	4	73	**78**
Antique	7,468	2	9	76	**87**	**1.16**	1	10	78	**89**
Capiz	7,481	0	6	46	**52**	**0.70**	0	4	32	**36**
Guimaras	2,182	0	1	13	**14**	**0.64**	1	4	43	**48**
Iloilo	19,745	0	12	78	**90**	**0.46**	1	34	246	**281**
Negros Occidental	32,890	1	32	237	**270**	**0.82**	3	38	316	**357**
City of Bacolod	4,693	0	1	21	**22**	**0.47**	0	4	43	**47**
City of Iloilo	5,082	0	0	6	**6**	**0.12**	0	2	24	**26**
**Region 7**	**107,656**	**2**	**66**	**628**	**696**	**0.65**	**2**	**132**	**936**	**1,070**
Bohol	17,077	0	6	113	**119**	**0.70**	1	5	79	**85**
Cebu	40,882	2	30	267	**299**	**0.73**	1	95	453	**549**
Negros Oriental	18,571	0	20	130	**150**	**0.81**	0	13	149	**162**
Siquijor	997	0	0	7	**7**	**0.70**	0	3	29	**32**
City of Cebu	13,995	0	3	16	**19**	**0.14**	0	4	25	**29**
City of Lapu-Lapu	6,919	0	3	28	**31**	**0.45**	0	0	50	**50**
City of Mandaue	9,215	0	4	67	**71**	**0.77**	0	12	151	**163**
**Region 8**	**58,689**	**0**	**55**	**371**	**426**	**0.73**	**0**	**32**	**243**	**275**
Biliran	3,057	0	4	51	**55**	**1.80**	0	0	5	**5**
Eastern Samar	6,373	0	6	51	**57**	**0.89**	0	13	71	**84**
Leyte	11,445	0	4	37	**41**	**0.36**	0	3	37	**40**
Northern Samar	10,255	0	18	110	**128**	**1.25**	0	6	48	**54**
Southern Leyte	4,330	0	2	7	**9**	**0.21**	0	1	26	**27**
Samar	11,912	0	14	41	**55**	**0.46**	0	8	52	**60**
Ormoc City	5,369	0	7	56	**63**	**1.17**	0	0	0	**0**
City of Tacloban	5,948	0	0	18	**18**	**0.30**	0	1	4	**5**
**Region 9**	**31,545**	**0**	**32**	**155**	**187**	**0.59**	**3**	**32**	**198**	**233**
Zamboanga del Norte	12,173	0	15	70	**85**	**0.70**	1	15	74	**90**
Zamboanga del Sur	7,573	0	4	19	**23**	**0.30**	2	4	40	**46**
Zamboanga Sibugay	6,007	0	8	39	**47**	**0.78**	0	12	58	**70**
City of Isabela	2,396	0	4	23	**27**	**1.13**	0	0	0	**0**
City of Zamboanga	3,396	0	1	4	**5**	**0.15**	0	1	26	**27**
**Region 10**	**63,866**	**1**	**43**	**277**	**321**	**0.50**	**2**	**61**	**223**	**286**
Bukidnon	20,204	0	24	113	**137**	**0.68**	2	36	94	**132**
Camiguin	1,253	1	1	7	**9**	**0.72**	0	1	3	**4**
Lanao del Norte	9,936	0	3	22	**25**	**0.25**	0	0	3	**3**
Misamis Occidental	4,954	0	3	15	**18**	**0.36**	0	2	19	**21**
Misamis Oriental	9,685	0	5	35	**40**	**0.41**	0	10	35	**45**
City of Cagayan De Oro	11,479	0	5	40	**45**	**0.39**	0	12	59	**71**
City of Iligan	6,355	0	2	45	**47**	**0.74**	0	0	10	**10**
**Region 11**	**73,746**	**4**	**82**	**473**	**559**	**0.76**	**19**	**115**	**738**	**872**
Davao de Oro	10,510	0	11	78	**89**	**0.85**	6	37	173	**216**

Table 2.B.2.3 - Intrapartum Care and Delivery Outcome
Pregnancy by Outcome (Full Term and Pre-term)
Philippines, 2024

Area	Total number of Deliveries	Pregnancy Outcome								
		Fetal Death					Abortion (Counts Only)			
		Age Group			Total	%	Age Group			Total
		10-14	15-19	20-49			10-14	15-19	20-49	
Davao del Norte	15,487	2	16	141	**159**	**1.03**	4	25	253	**282**
Davao Oriental	7,919	1	14	74	**89**	**1.12**	0	6	80	**86**
Davao del Sur	8,681	0	12	60	**72**	**0.83**	5	22	97	**124**
Davao Occidental	4,282	1	18	48	**67**	**1.56**	2	16	55	**73**
City of Davao	26,867	0	11	72	**83**	**0.31**	2	9	80	**91**
**Region 12**	**61,770**	**1**	**80**	**375**	**456**	**0.74**	**16**	**118**	**521**	**655**
Cotabato	16,708	1	18	112	**131**	**0.78**	2	39	196	**237**
Sarangani	9,448	0	9	33	**42**	**0.44**	0	11	54	**65**
South Cotabato	14,546	0	27	121	**148**	**1.02**	12	37	139	**188**
Sultan Kudarat	11,831	0	24	97	**121**	**1.02**	2	28	115	**145**
City of General Santos	9,237	0	2	12	**14**	**0.15**	0	3	17	**20**
**CARAGA**	**38,638**	**1**	**55**	**260**	**316**	**0.82**	**1**	**29**	**168**	**198**
Agusan del Norte	5,588	1	28	16	**45**	**0.81**	0	1	9	**10**
Agusan del Sur	9,884	0	9	64	**73**	**0.74**	0	5	36	**41**
Surigao del Norte	7,357	0	3	22	**25**	**0.34**	1	4	31	**36**
Surigao del Sur	9,186	0	11	92	**103**	**1.12**	0	12	65	**77**
Dinagat Islands	863	0	0	1	**1**	**0.12**	0	6	17	**23**
City of Butuan	5,760	0	4	65	**69**	**1.20**	0	1	10	**11**
**BARMM**	**60,653**	**0**	**31**	**159**	**190**	**0.31**	**1**	**29**	**202**	**232**
Basilan	5,034	0	10	39	**49**	**0.97**	1	12	60	**73**
Lanao del Sur	14,026	0	5	15	**20**	**0.14**	0	9	50	**59**
Maguindanao del Norte	7,846	0	2	5	**7**	**0.09**	0	1	9	**10**
Maguindanao del Sur	12,843	0	9	47	**56**	**0.44**	0	3	32	**35**
Sulu	10,400	0	1	7	**8**	**0.08**	0	3	18	**21**
Tawi-Tawi	5,369	0	3	24	**27**	**0.50**	0	0	26	**26**
SGA	1,542	0	0	1	**1**	**0.06**	0	0	1	**1**
City of Cotabato	3,593	0	1	21	**22**	**0.61**	0	1	6	**7**

*Legend: * - No Report*

Table 2.B.2.5 - Intrapartum Care and Delivery Outcome
Number and proportion of livebirths by birth weight
Philippines, 2024

Area	Total number of live births	Normal Birth Weight (≥ 2500 grams)		Low Birth Weight (< 2500 grams)		Unknown Birth Weight	
		No.	%	No.	%	No.	%
**PHILIPPINES**	**1,338,505**	**1,233,144**	**92.13**	**79,047**	**5.91**	**26,314**	**1.97**
**N C R**	**183,878**	**177,381**	**96.47**	**5,884**	**3.20**	**613**	**0.33**
City of Malabon	**3,029**	2,880	**95.08**	138	**4.56**	11	**0.36**
City of Navotas	**3,949**	3,824	**96.83**	124	**3.14**	1	**0.03**
City of Valenzuela	**4,610**	4,289	**93.04**	319	**6.92**	2	**0.04**
City of Caloocan	**24,159**	23,848	**98.71**	299	**1.24**	12	**0.05**
City of Marikina	**2,680**	2,540	**94.78**	139	**5.19**	1	**0.04**
City of Pasig	**10,914**	8,981	**82.29**	1,925	**17.64**	8	**0.07**
Pateros	**747**	720	**96.39**	27	**3.61**	0	**0.00**
City of Taguig	**12,299**	11,942	**97.10**	272	**2.21**	85	**0.69**
Quezon City	**58,494**	57,942	**99.06**	524	**0.90**	28	**0.05**
City of Makati	**1,505**	1,387	**92.16**	116	**7.71**	2	**0.13**
City of Mandaluyong	**7,708**	7,575	**98.27**	133	**1.73**	0	**0.00**
City of San Juan	**767**	723	**94.26**	43	**5.61**	1	**0.13**
City of Manila	**24,013**	23,296	**97.01**	694	**2.89**	23	**0.10**
City of Las Piñas	**6,343**	6,126	**96.58**	164	**2.59**	53	**0.84**
City of Muntinlupa	**7,640**	7,343	**96.11**	204	**2.67**	93	**1.22**
City of Parañaque	**8,119**	7,386	**90.97**	446	**5.49**	287	**3.53**
Pasay City	**6,902**	6,579	**95.32**	317	**4.59**	6	**0.09**
**C A R**	**23,873**	**21,270**	**89.10**	**2,584**	**10.82**	**19**	**0.08**
Abra	**2,629**	2,304	**87.64**	324	**12.32**	1	**0.04**
Apayao	**3,571**	3,182	**89.11**	384	**10.75**	5	**0.14**
Benguet	**3,681**	3,336	**90.63**	333	**9.05**	12	**0.33**
Ifugao	**1,934**	1,877	**97.05**	56	**2.90**	1	**0.05**
Kalinga	**2,187**	2,092	**95.66**	95	**4.34**	0	**0.00**
Mountain Province	**3,239**	2,912	**89.90**	327	**10.10**	0	**0.00**
City of Baguio	**6,632**	5,567	**83.94**	1,065	**16.06**	0	**0.00**
**Region 1**	**51,383**	**47,665**	**92.76**	**3,706**	**7.21**	**12**	**0.02**
Ilocos Norte	**5,761**	5,198	**90.23**	563	**9.77**	0	**0.00**
Ilocos Sur	**7,393**	6,925	**93.67**	468	**6.33**	0	**0.00**
La Union	**7,714**	6,804	**88.20**	908	**11.77**	2	**0.03**
Pangasinan	**23,195**	22,718	**97.94**	467	**2.01**	10	**0.04**
City of Dagupan	**7,320**	6,020	**82.24**	1,300	**17.76**	0	**0.00**
**Region 2**	**33,145**	**31,198**	**94.13**	**1,912**	**5.77**	**35**	**0.11**
Batanes	**203**	178	**87.68**	25	**12.32**	0	**0.00**
Cagayan	**7,853**	7,519	**95.75**	323	**4.11**	11	**0.14**
Isabela	**13,002**	12,417	**95.50**	561	**4.31**	24	**0.18**
Nueva Vizcaya	**6,850**	6,039	**88.16**	811	**11.84**	0	**0.00**
Quirino	**2,383**	2,225	**93.37**	158	**6.63**	0	**0.00**
City of Santiago	**2,854**	2,820	**98.81**	34	**1.19**	0	**0.00**
**Region 3**	**140,769**	**130,359**	**92.60**	**9,868**	**7.01**	**542**	**0.39**
Aurora	**2,493**	2,254	**90.41**	235	**9.43**	4	**0.16**
Bataan	**11,051**	9,651	**87.33**	1,388	**12.56**	12	**0.11**
Bulacan	**40,887**	37,776	**92.39**	2,942	**7.20**	169	**0.41**
Nueva Ecija	**24,785**	22,871	**92.28**	1,893	**7.64**	21	**0.08**
Pampanga	**23,850**	21,985	**92.18**	1,661	**6.96**	204	**0.86**
Tarlac	**19,860**	19,465	**98.01**	317	**1.60**	78	**0.39**
Zambales	**6,697**	5,974	**89.20**	677	**10.11**	46	**0.69**
City of Angeles	**7,433**	7,083	**95.29**	350	**4.71**	0	**0.00**
City of Olongapo	**3,713**	3,300	**88.88**	405	**10.91**	8	**0.22**
**Region 4A**	**196,166**	**182,494**	**93.03**	**9,975**	**5.08**	**3,697**	**1.88**
Batangas	**36,374**	34,091	**93.72**	1,921	**5.28**	362	**1.00**
Cavite	**47,820**	44,112	**92.25**	2,793	**5.84**	915	**1.91**
Laguna	**42,719**	40,471	**94.74**	1,710	**4.00**	538	**1.26**

Table 2.B.2.5 - Intrapartum Care and Delivery Outcome
Number and proportion of livebirths by birth weight
Philippines, 2024

Area	Total number of live births	Normal Birth Weight (≥ 2500 grams)		Low Birth Weight (< 2500 grams)		Unknown Birth Weight	
		No.	%	No.	%	No.	%
Quezon	**23,818**	22,039	**92.53**	1,228	**5.16**	551	**2.31**
Rizal	**42,541**	39,072	**91.85**	2,154	**5.06**	1,315	**3.09**
City of Lucena	**2,894**	2,709	**93.61**	169	**5.84**	16	**0.55**
**Region 4B**	**42,233**	**38,218**	**90.49**	**2,567**	**6.08**	**1,448**	**3.43**
Marinduque	**2,702**	2,485	**91.97**	213	**7.88**	4	**0.15**
Occidental Mindoro	**8,238**	7,206	**87.47**	557	**6.76**	475	**5.77**
Oriental Mindoro	**11,484**	10,532	**91.71**	448	**3.90**	504	**4.39**
Palawan	**11,857**	10,732	**90.51**	695	**5.86**	430	**3.63**
Romblon	**4,093**	3,734	**91.23**	351	**8.58**	8	**0.20**
City of Puerto Princesa	**3,859**	3,529	**91.45**	303	**7.85**	27	**0.70**
**Region 5**	**85,536**	**75,474**	**88.24**	**8,543**	**9.99**	**1,519**	**1.78**
Albay	**15,792**	14,492	**91.77**	1,218	**7.71**	82	**0.52**
Camarines Norte	**10,040**	8,859	**88.24**	1,178	**11.73**	3	**0.03**
Camarines Sur	**16,172**	14,141	**87.44**	1,103	**6.82**	928	**5.74**
Catanduanes	**3,792**	3,315	**87.42**	477	**12.58**	0	**0.00**
Masbate	**14,700**	13,337	**90.73**	863	**5.87**	500	**3.40**
Sorsogon	**12,269**	10,961	**89.34**	1,306	**10.64**	2	**0.02**
City of Naga	**12,771**	10,369	**81.19**	2,398	**18.78**	4	**0.03**
**Region 6**	**85,513**	**77,672**	**90.83**	**7,066**	**8.26**	**775**	**0.91**
Aklan	**5,996**	5,471	**91.24**	396	**6.60**	129	**2.15**
Antique	**7,433**	5,974	**80.37**	1,385	**18.63**	74	**1.00**
Capiz	**7,460**	7,002	**93.86**	421	**5.64**	37	**0.50**
Guimaras	**2,183**	1,928	**88.32**	255	**11.68**	0	**0.00**
Iloilo	**19,712**	18,405	**93.37**	1,270	**6.44**	37	**0.19**
Negros Occidental	**32,946**	29,832	**90.55**	2,631	**7.99**	483	**1.47**
City of Bacolod	**4,678**	4,176	**89.27**	490	**10.47**	12	**0.26**
City of Iloilo	**5,105**	4,884	**95.67**	218	**4.27**	3	**0.06**
**Region 7**	**107,859**	**97,907**	**90.77**	**8,631**	**8.00**	**1,321**	**1.22**
Bohol	**17,051**	15,308	**89.78**	1,733	**10.16**	10	**0.06**
Cebu	**40,609**	37,431	**92.17**	3,063	**7.54**	115	**0.28**
Negros Oriental	**19,156**	16,868	**88.06**	1,100	**5.74**	1,188	**6.20**
Siquijor	**998**	866	**86.77**	132	**13.23**	0	**0.00**
City of Cebu	**14,009**	13,193	**94.18**	808	**5.77**	8	**0.06**
City of Lapu-Lapu	**6,892**	6,292	**91.29**	600	**8.71**	0	**0.00**
City of Mandaue	**9,144**	7,949	**86.93**	1,195	**13.07**	0	**0.00**
**Region 8**	**58,580**	**52,864**	**90.24**	**4,529**	**7.73**	**1,187**	**2.03**
Biliran	**3,024**	2,721	**89.98**	303	**10.02**	0	**0.00**
Eastern Samar	**6,337**	5,947	**93.85**	282	**4.45**	108	**1.70**
Leyte	**11,412**	10,722	**93.95**	547	**4.79**	143	**1.25**
Northern Samar	**10,189**	8,662	**85.01**	970	**9.52**	557	**5.47**
Southern Leyte	**4,325**	3,893	**90.01**	432	**9.99**	0	**0.00**
Samar	**11,901**	10,684	**89.77**	838	**7.04**	379	**3.18**
Ormoc City	**5,423**	4,587	**84.58**	836	**15.42**	0	**0.00**
City of Tacloban	**5,969**	5,648	**94.62**	321	**5.38**	0	**0.00**
**Region 9**	**31,451**	**25,894**	**82.33**	**1,590**	**5.06**	**3,967**	**12.61**
Zamboanga del Norte	**12,119**	10,391	**85.74**	777	**6.41**	951	**7.85**
Zamboanga del Sur	**7,571**	6,537	**86.34**	231	**3.05**	803	**10.61**
Zamboanga Sibugay	**5,982**	5,396	**90.20**	454	**7.59**	132	**2.21**
City of Isabela	**2,386**	1,932	**80.97**	111	**4.65**	343	**14.38**
City of Zamboanga	**3,393**	1,638	**48.28**	17	**0.50**	1,738	**51.22**
**Region 10**	**63,659**	**59,371**	**93.26**	**2,447**	**3.84**	**1,841**	**2.89**
Bukidnon	**20,120**	18,531	**92.10**	855	**4.25**	734	**3.65**
Camiguin	**1,253**	1,171	**93.46**	79	**6.30**	3	**0.24**

Table 2.B.2.5 - Intrapartum Care and Delivery Outcome
Number and proportion of livebirths by birth weight
Philippines, 2024

Area	Total number of live births	Normal Birth Weight (≥ 2500 grams)		Low Birth Weight (< 2500 grams)		Unknown Birth Weight	
		No.	%	No.	%	No.	%
Lanao del Norte	**9,910**	8,761	**88.41**	282	**2.85**	867	**8.75**
Misamis Occidental	**4,958**	4,725	**95.30**	168	**3.39**	65	**1.31**
Misamis Oriental	**9,659**	9,289	**96.17**	291	**3.01**	79	**0.82**
City of Cagayan De Oro	**11,463**	10,838	**94.55**	617	**5.38**	8	**0.07**
City of Iligan	**6,296**	6,056	**96.19**	155	**2.46**	85	**1.35**
**Region 11**	**73,703**	**67,048**	**90.97**	**4,421**	**6.00**	**2,234**	**3.03**
Davao de Oro	**10,504**	9,737	**92.70**	675	**6.43**	92	**0.88**
Davao del Norte	**15,420**	13,979	**90.65**	1,100	**7.13**	341	**2.21**
Davao Oriental	**7,883**	7,435	**94.32**	396	**5.02**	52	**0.66**
Davao del Sur	**8,695**	7,971	**91.67**	486	**5.59**	238	**2.74**
Davao Occidental	**4,254**	3,367	**79.15**	230	**5.41**	657	**15.44**
City of Davao	**26,947**	24,559	**91.14**	1,534	**5.69**	854	**3.17**
**Region 12**	**61,644**	**57,837**	**93.82**	**2,160**	**3.50**	**1,647**	**2.67**
Cotabato	**16,676**	14,920	**89.47**	737	**4.42**	1,019	**6.11**
Sarangani	**9,447**	9,100	**96.33**	166	**1.76**	181	**1.92**
South Cotabato	**14,510**	13,820	**95.24**	533	**3.67**	157	**1.08**
Sultan Kudarat	**11,779**	11,156	**94.71**	458	**3.89**	165	**1.40**
City of General Santos	**9,232**	8,841	**95.76**	266	**2.88**	125	**1.35**
**CARAGA**	**38,525**	**35,672**	**92.59**	**2,173**	**5.64**	**680**	**1.77**
Agusan del Norte	**5,584**	5,268	**94.34**	237	**4.24**	79	**1.41**
Agusan del Sur	**9,857**	9,277	**94.12**	201	**2.04**	379	**3.84**
Surigao del Norte	**7,386**	6,570	**88.95**	775	**10.49**	41	**0.56**
Surigao del Sur	**9,106**	8,388	**92.12**	551	**6.05**	167	**1.83**
Dinagat Islands	**865**	766	**88.55**	94	**10.87**	5	**0.58**
City of Butuan	**5,727**	5,403	**94.34**	315	**5.50**	9	**0.16**
**BARMM**	**60,588**	**54,820**	**90.48**	**991**	**1.64**	**4,777**	**7.88**
Basilan	**4,995**	4,076	**81.60**	208	**4.16**	711	**14.23**
Lanao del Sur	**14,028**	12,805	**91.28**	96	**0.68**	1,127	**8.03**
Maguindanao del Norte	**7,850**	7,325	**93.31**	139	**1.77**	386	**4.92**
Maguindanao del Sur	**12,814**	11,925	**93.06**	263	**2.05**	626	**4.89**
Sulu	**10,405**	9,408	**90.42**	93	**0.89**	904	**8.69**
Tawi-Tawi	**5,358**	5,023	**93.75**	102	**1.90**	233	**4.35**
SGA	**1,542**	815	**52.85**	7	**0.45**	720	**46.69**
City of Cotabato	**3,596**	3,443	**95.75**	83	**2.31**	70	**1.95**

*Legend: * - No Report*

Table 2.B.3.1 - Postpartum and Newborn Care
Postpartum Women together with their Newborn who completed at least 2 Postpartum Check-ups
Philippines, 2024

Area	Total Deliveries				Completed at least 2 postpartum check-ups							
					Age Group in Year						Total	%
					10-14 y.o.		15-19 y.o.		20-49 y.o.			
	10-14 y.o.	15-19 y.o.	20-49 y.o.	Total	No.	%	No.	%	No.	%		
**PHILIPPINES**	**3,285**	**137,240**	**1,203,053**	**1,343,578**	**2,926**	**89.07**	**126,695**	**92.32**	**1,133,258**	**94.20**	**1,262,879**	**93.99**
**N C R**	**297**	**11,285**	**172,329**	**183,911**	**270**	**90.91**	**11,046**	**97.88**	**169,985**	**98.64**	**181,301**	**98.58**
City of Malabon	12	332	2,685	**3,029**	12	**100.00**	330	**99.40**	2,664	**99.22**	**3,006**	**99.24**
City of Navotas	6	308	3,632	**3,946**	6	**100.00**	308	**100.00**	3,632	**100.00**	**3,946**	**100.00**
City of Valenzuela	13	364	4,237	**4,614**	13	**100.00**	399	**109.62**	4,784	**112.91**	**5,196**	**112.61**
City of Caloocan	57	2,129	21,982	**24,168**	57	**100.00**	2,125	**99.81**	21,926	**99.75**	**24,108**	**99.75**
City of Marikina	9	226	2,441	**2,676**	7	**77.78**	211	**93.36**	2,283	**93.53**	**2,501**	**93.46**
City of Pasig	36	818	10,086	**10,940**	21	**58.33**	720	**88.02**	10,165	**100.78**	**10,906**	**99.69**
Pateros	0	37	717	**754**	0	**0.00**	35	**94.59**	711	**99.16**	**746**	**98.94**
City of Taguig	22	1,051	11,221	**12,294**	20	**90.91**	1,048	**99.71**	11,161	**99.47**	**12,229**	**99.47**
Quezon City	51	2,225	56,245	**58,521**	49	**96.08**	2,163	**97.21**	55,082	**97.93**	**57,294**	**97.90**
City of Makati	3	112	1,395	**1,510**	3	**100.00**	112	**100.00**	1,394	**99.93**	**1,509**	**99.93**
City of Mandaluyong	11	283	7,411	**7,705**	11	**100.00**	283	**100.00**	7,399	**99.84**	**7,693**	**99.84**
City of San Juan	0	35	734	**769**	0	**0.00**	35	**100.00**	698	**95.10**	**733**	**95.32**
City of Manila	28	1,543	22,420	**23,991**	28	**100.00**	1,541	**99.87**	22,399	**99.91**	**23,968**	**99.90**
City of Las Piñas	10	432	5,914	**6,356**	8	**80.00**	401	**92.82**	5,805	**98.16**	**6,214**	**97.77**
City of Muntinlupa	15	565	7,068	**7,648**	15	**100.00**	565	**100.00**	7,067	**99.99**	**7,647**	**99.99**
City of Parañaque	16	560	7,521	**8,097**	12	**75.00**	506	**90.36**	6,205	**82.50**	**6,723**	**83.03**
Pasay City	8	265	6,620	**6,893**	8	**100.00**	264	**99.62**	6,610	**99.85**	**6,882**	**99.84**
**C A R**	**55**	**2,118**	**21,818**	**23,991**	**41**	**74.55**	**1,447**	**68.32**	**16,096**	**73.77**	**17,584**	**73.29**
Abra	3	311	2,339	**2,653**	5	**166.67**	269	**86.50**	2,248	**96.11**	**2,522**	**95.06**
Apayao	14	526	3,042	**3,582**	9	**64.29**	310	**58.94**	2,072	**68.11**	**2,391**	**66.75**
Benguet	2	227	3,447	**3,676**	2	**100.00**	166	**73.13**	2,891	**83.87**	**3,059**	**83.22**
Ifugao	4	192	1,743	**1,939**	8	**200.00**	191	**99.48**	1,949	**111.82**	**2,148**	**110.78**
Kalinga	5	155	2,032	**2,192**	5	**100.00**	155	**100.00**	2,023	**99.56**	**2,183**	**99.59**
Mountain Province	9	330	2,917	**3,256**	8	**88.89**	197	**59.70**	1,903	**65.24**	**2,108**	**64.74**
City of Baguio	18	377	6,298	**6,693**	4	**22.22**	159	**42.18**	3,010	**47.79**	**3,173**	**47.41**
**Region 1**	**169**	**4,304**	**46,997**	**51,470**	**138**	**81.66**	**5,017**	**116.57**	**53,211**	**113.22**	**58,366**	**113.40**
Ilocos Norte	13	314	5,428	**5,755**	11	**84.62**	455	**144.90**	6,216	**114.52**	**6,682**	**116.11**
Ilocos Sur	14	467	6,911	**7,392**	12	**85.71**	543	**116.27**	7,880	**114.02**	**8,435**	**114.11**
La Union	32	691	7,039	**7,762**	37	**115.63**	749	**108.39**	7,569	**107.53**	**8,355**	**107.64**
Pangasinan	88	2,268	20,860	**23,216**	70	**79.55**	3,055	**134.70**	29,554	**141.68**	**32,679**	**140.76**
City of Dagupan	22	564	6,759	**7,345**	8	**36.36**	215	**38.12**	1,992	**29.47**	**2,215**	**30.16**
**Region 2**	**93**	**3,613**	**29,487**	**33,193**	**80**	**86.02**	**3,347**	**92.64**	**27,744**	**94.09**	**31,171**	**93.91**
Batanes	0	15	187	**202**	0	**0.00**	15	**100.00**	185	**98.93**	**200**	**99.01**
Cagayan	16	812	7,045	**7,873**	15	**93.75**	663	**81.65**	6,378	**90.53**	**7,056**	**89.62**
Isabela	26	1,370	11,631	**13,027**	24	**92.31**	1,343	**98.03**	11,482	**98.72**	**12,849**	**98.63**
Nueva Vizcaya	34	806	6,025	**6,865**	24	**70.59**	716	**88.83**	5,111	**84.83**	**5,851**	**85.23**
Quirino	14	336	2,034	**2,384**	14	**100.00**	336	**100.00**	2,023	**99.46**	**2,373**	**99.54**
City of Santiago	3	274	2,565	**2,842**	3	**100.00**	274	**100.00**	2,565	**100.00**	**2,842**	**100.00**
**Region 3**	**369**	**13,100**	**127,979**	**141,448**	**373**	**101.08**	**14,291**	**109.09**	**134,906**	**105.41**	**149,570**	**105.74**
Aurora	7	328	2,183	**2,518**	3	**42.86**	341	**103.96**	2,412	**110.49**	**2,756**	**109.45**
Bataan	61	1,357	9,945	**11,363**	48	**78.69**	1,117	**82.31**	8,630	**86.78**	**9,795**	**86.20**
Bulacan	92	3,473	37,497	**41,062**	119	**129.35**	4,416	**127.15**	47,484	**126.63**	**52,019**	**126.68**
Nueva Ecija	90	2,543	22,151	**24,784**	75	**83.33**	2,465	**96.93**	18,702	**84.43**	**21,242**	**85.71**
Pampanga	67	2,425	21,406	**23,898**	80	**119.40**	2,485	**102.47**	23,891	**111.61**	**26,456**	**110.70**
Tarlac	17	1,474	18,469	**19,960**	33	**194.12**	1,977	**134.12**	19,556	**105.89**	**21,566**	**108.05**
Zambales	14	845	5,855	**6,714**	7	**50.00**	732	**86.63**	5,536	**94.55**	**6,275**	**93.46**
City of Angeles	15	434	6,990	**7,439**	7	**46.67**	608	**140.09**	5,943	**85.02**	**6,558**	**88.16**
City of Olongapo	6	221	3,483	**3,710**	1	**16.67**	150	**67.87**	2,752	**79.01**	**2,903**	**78.25**
**Region 4A**	**390**	**17,923**	**180,933**	**199,246**	**364**	**93.33**	**15,584**	**86.95**	**162,668**	**89.91**	**178,616**	**89.65**
Batangas	70	2,689	33,636	**36,395**	62	**88.57**	2,527	**93.98**	31,233	**92.86**	**33,822**	**92.93**
Cavite	73	3,418	44,751	**48,242**	65	**89.04**	2,972	**86.95**	37,846	**84.57**	**40,883**	**84.75**
Laguna	95	3,953	39,146	**43,194**	87	**91.58**	3,404	**86.11**	36,053	**92.10**	**39,544**	**91.55**
Quezon	78	3,059	20,713	**23,850**	80	**102.56**	2,820	**92.19**	19,635	**94.80**	**22,535**	**94.49**
Rizal	68	4,481	40,125	**44,674**	64	**94.12**	3,539	**78.98**	35,343	**88.08**	**38,946**	**87.18**
City of Lucena	6	323	2,562	**2,891**	6	**100.00**	322	**99.69**	2,558	**99.84**	**2,886**	**99.83**
**Region 4B**	**126**	**5,691**	**36,723**	**42,540**	**105**	**83.33**	**4,977**	**87.45**	**32,392**	**88.21**	**37,474**	**88.09**
Marinduque	6	280	2,421	**2,707**	1	**16.67**	216	**77.14**	2,123	**87.69**	**2,340**	**86.44**
Occidental Mindoro	27	1,216	7,039	**8,282**	23	**85.19**	1,002	**82.40**	5,861	**83.26**	**6,886**	**83.14**
Oriental Mindoro	28	1,260	10,272	**11,560**	21	**75.00**	979	**77.70**	8,715	**84.84**	**9,715**	**84.04**
Palawan	47	2,041	9,904	**11,992**	43	**91.49**	1,986	**97.31**	9,641	**97.34**	**11,670**	**97.31**
Romblon	6	429	3,677	**4,112**	5	**83.33**	397	**92.54**	3,290	**89.48**	**3,692**	**89.79**
City of Puerto Princesa	12	465	3,410	**3,887**	12	**100.00**	397	**85.38**	2,762	**81.00**	**3,171**	**81.58**
**Region 5**	**139**	**9,086**	**76,470**	**85,695**	**105**	**75.54**	**8,074**	**88.86**	**67,479**	**88.24**	**75,658**	**88.29**
Albay	16	1,203	14,538	**15,757**	11	**68.75**	1,192	**99.09**	14,549	**100.08**	**15,752**	**99.97**
Camarines Norte	17	1,154	8,983	**10,154**	14	**82.35**	848	**73.48**	6,798	**75.68**	**7,660**	**75.44**
Camarines Sur	10	1,312	14,898	**16,220**	26	**260.00**	1,907	**145.35**	17,149	**115.11**	**19,082**	**117.64**
Catanduanes	9	458	3,352	**3,819**	3	**33.33**	455	**99.34**	3,230	**96.36**	**3,688**	**96.57**
Masbate	23	2,159	12,529	**14,711**	20	**86.96**	2,170	**100.51**	12,568	**100.31**	**14,758**	**100.32**
Sorsogon	28	1,313	11,083	**12,424**	23	**82.14**	1,193	**90.86**	10,034	**90.54**	**11,250**	**90.55**
City of Naga	36	1,487	11,087	**12,610**	8	**22.22**	309	**20.78**	3,151	**28.42**	**3,468**	**27.50**
**Region 6**	**183**	**9,328**	**76,010**	**85,521**	**169**	**92.35**	**8,776**	**94.08**	**71,260**	**93.75**	**80,205**	**93.78**
Aklan	8	612	5,360	**5,980**	7	**87.50**	582	**95.10**	5,194	**96.90**	**5,783**	**96.71**
Antique	11	731	6,726	**7,468**	12	**109.09**	619	**84.68**	5,769	**85.77**	**6,400**	**85.70**
Capiz	14	703	6,764	**7,481**	13	**92.86**	661	**94.03**	6,553	**96.88**	**7,227**	**96.60**
Guimaras	11	220	1,951	**2,182**	9	**81.82**	215	**97.73**	1,915	**98.15**	**2,139**	**98.03**
Iloilo	32	1,966	17,747	**19,745**	30	**93.75**	1,846	**93.90**	17,035	**95.99**	**18,911**	**95.78**

Table 2.B.3.1 - Postpartum and Newborn Care
Postpartum Women together with their Newborn who completed at least 2 Postpartum Check-ups
Philippines, 2024

Area	Total Deliveries				Completed at least 2 postpartum check-ups							
					Age Group in Year						Total	%
					10-14 y.o.		15-19 y.o.		20-49 y.o.			
	10-14 y.o.	15-19 y.o.	20-49 y.o.	Total	No.	%	No.	%	No.	%		
Negros Occidental	90	4,369	28,431	**32,890**	80	**88.89**	4,023	**92.08**	26,380	**92.79**	**30,483**	**92.68**
City of Bacolod	2	271	4,420	**4,693**	3	**150.00**	375	**138.38**	3,814	**86.29**	**4,192**	**89.32**
City of Iloilo	15	456	4,611	**5,082**	15	**100.00**	455	**99.78**	4,600	**99.76**	**5,070**	**99.76**
**Region 7**	**230**	**11,162**	**96,264**	**107,656**	**171**	**74.35**	**9,661**	**86.55**	**87,369**	**90.76**	**97,201**	**90.29**
Bohol	22	1,518	15,537	**17,077**	18	**81.82**	1,100	**72.46**	12,288	**79.09**	**13,406**	**78.50**
Cebu	80	4,543	36,259	**40,882**	54	**67.50**	3,899	**85.82**	33,544	**92.51**	**37,497**	**91.72**
Negros Oriental	51	2,307	16,213	**18,571**	38	**74.51**	2,022	**87.65**	14,726	**90.83**	**16,786**	**90.39**
Siquijor	1	75	921	**997**	0	**0.00**	74	**98.67**	878	**95.33**	**952**	**95.49**
City of Cebu	29	1,437	12,529	**13,995**	26	**89.66**	1,413	**98.33**	12,299	**98.16**	**13,738**	**98.16**
City of Lapu-Lapu	24	584	6,311	**6,919**	14	**58.33**	540	**92.47**	6,089	**96.48**	**6,643**	**96.01**
City of Mandaue	23	698	8,494	**9,215**	21	**91.30**	613	**87.82**	7,545	**88.83**	**8,179**	**88.76**
**Region 8**	**116**	**6,954**	**51,619**	**58,689**	**92**	**79.31**	**5,263**	**75.68**	**42,746**	**82.81**	**48,101**	**81.96**
Biliran	6	385	2,666	**3,057**	4	**66.67**	283	**73.51**	2,213	**83.01**	**2,500**	**81.78**
Eastern Samar	17	858	5,498	**6,373**	18	**105.88**	842	**98.14**	5,428	**98.73**	**6,288**	**98.67**
Leyte	21	1,366	10,058	**11,445**	18	**85.71**	1,245	**91.14**	9,630	**95.74**	**10,893**	**95.18**
Northern Samar	19	1,345	8,891	**10,255**	13	**68.42**	665	**49.44**	5,609	**63.09**	**6,287**	**61.31**
Southern Leyte	5	429	3,896	**4,330**	7	**140.00**	442	**103.03**	3,877	**99.51**	**4,326**	**99.91**
Samar	23	1,455	10,434	**11,912**	19	**82.61**	908	**62.41**	7,998	**76.65**	**8,925**	**74.92**
Ormoc City	15	626	4,728	**5,369**	5	**33.33**	411	**65.65**	2,674	**56.56**	**3,090**	**57.55**
City of Tacloban	10	490	5,448	**5,948**	8	**80.00**	467	**95.31**	5,317	**97.60**	**5,792**	**97.38**
**Region 9**	**68**	**4,040**	**27,437**	**31,545**	**58**	**85.29**	**3,663**	**90.67**	**25,420**	**92.65**	**29,141**	**92.38**
Zamboanga del Norte	26	1,694	10,453	**12,173**	18	**69.23**	1,530	**90.32**	9,653	**92.35**	**11,201**	**92.02**
Zamboanga del Sur	22	955	6,596	**7,573**	21	**95.45**	877	**91.83**	6,109	**92.62**	**7,007**	**92.53**
Zamboanga Sibugay	15	760	5,232	**6,007**	15	**100.00**	729	**95.92**	5,107	**97.61**	**5,851**	**97.40**
City of Isabela	2	299	2,095	**2,396**	1	**50.00**	214	**71.57**	1,620	**77.33**	**1,835**	**76.59**
City of Zamboanga	3	332	3,061	**3,396**	3	**100.00**	313	**94.28**	2,931	**95.75**	**3,247**	**95.61**
**Region 10**	**204**	**8,959**	**54,703**	**63,866**	**186**	**91.18**	**8,710**	**97.22**	**53,240**	**97.33**	**62,136**	**97.29**
Bukidnon	93	3,693	16,418	**20,204**	86	**92.47**	3,585	**97.08**	15,975	**97.30**	**19,646**	**97.24**
Camiguin	3	153	1,097	**1,253**	3	**100.00**	151	**98.69**	1,083	**98.72**	**1,237**	**98.72**
Lanao del Norte	27	893	9,016	**9,936**	26	**96.30**	883	**98.88**	8,658	**96.03**	**9,567**	**96.29**
Misamis Occidental	6	639	4,309	**4,954**	5	**83.33**	622	**97.34**	4,214	**97.80**	**4,841**	**97.72**
Misamis Oriental	27	1,338	8,320	**9,685**	23	**85.19**	1,296	**96.86**	8,073	**97.03**	**9,392**	**96.97**
City of Cagayan De Oro	34	1,588	9,857	**11,479**	30	**88.24**	1,550	**97.61**	9,711	**98.52**	**11,291**	**98.36**
City of Iligan	14	655	5,686	**6,355**	13	**92.86**	623	**95.11**	5,526	**97.19**	**6,162**	**96.96**
**Region 11**	**395**	**10,313**	**63,038**	**73,746**	**359**	**90.89**	**9,691**	**93.97**	**60,191**	**95.48**	**70,241**	**95.25**
Davao de Oro	61	1,670	8,779	**10,510**	57	**93.44**	1,658	**99.28**	8,647	**98.50**	**10,362**	**98.59**
Davao del Norte	99	2,168	13,220	**15,487**	87	**87.88**	2,079	**95.89**	12,784	**96.70**	**14,950**	**96.53**
Davao Oriental	56	1,272	6,591	**7,919**	51	**91.07**	1,252	**98.43**	6,471	**98.18**	**7,774**	**98.17**
Davao del Sur	62	1,432	7,187	**8,681**	60	**96.77**	1,333	**93.09**	6,669	**92.79**	**8,062**	**92.87**
Davao Occidental	48	1,066	3,168	**4,282**	41	**85.42**	813	**76.27**	2,331	**73.58**	**3,185**	**74.38**
City of Davao	69	2,705	24,093	**26,867**	63	**91.30**	2,556	**94.49**	23,289	**96.66**	**25,908**	**96.43**
**Region 12**	**282**	**8,938**	**52,550**	**61,770**	**271**	**96.10**	**8,586**	**96.06**	**50,640**	**96.37**	**59,497**	**96.32**
Cotabato	78	2,383	14,247	**16,708**	77	**98.72**	2,280	**95.68**	13,819	**97.00**	**16,176**	**96.82**
Sarangani	48	1,778	7,622	**9,448**	43	**89.58**	1,724	**96.96**	7,419	**97.34**	**9,186**	**97.23**
South Cotabato	81	2,204	12,261	**14,546**	80	**98.77**	2,128	**96.55**	11,912	**97.15**	**14,120**	**97.07**
Sultan Kudarat	60	1,781	9,990	**11,831**	59	**98.33**	1,751	**98.32**	9,805	**98.15**	**11,615**	**98.17**
City of General Santos	15	792	8,430	**9,237**	12	**80.00**	703	**88.76**	7,685	**91.16**	**8,400**	**90.94**
**CARAGA**	**99**	**5,072**	**33,467**	**38,638**	**78**	**78.79**	**3,997**	**78.81**	**28,083**	**83.91**	**32,158**	**83.23**
Agusan del Norte	20	799	4,769	**5,588**	18	**90.00**	752	**94.12**	4,587	**96.18**	**5,357**	**95.87**
Agusan del Sur	37	1,357	8,490	**9,884**	34	**91.89**	1,298	**95.65**	8,249	**97.16**	**9,581**	**96.93**
Surigao del Norte	4	854	6,499	**7,357**	0	**0.00**	534	**62.53**	4,492	**69.12**	**5,026**	**68.32**
Surigao del Sur	27	1,388	7,771	**9,186**	17	**62.96**	765	**55.12**	5,056	**65.06**	**5,838**	**63.55**
Dinagat Islands	1	105	757	**863**	1	**100.00**	104	**99.05**	740	**97.75**	**845**	**97.91**
City of Butuan	10	569	5,181	**5,760**	8	**80.00**	544	**95.61**	4,959	**95.72**	**5,511**	**95.68**
**BARMM**	**70**	**5,354**	**55,229**	**60,653**	**66**	**94.29**	**4,565**	**85.26**	**49,828**	**90.22**	**54,459**	**89.79**
Basilan	10	829	4,195	**5,034**	18	**180.00**	657	**79.25**	3,371	**80.36**	**4,046**	**80.37**
Lanao del Sur	11	605	13,410	**14,026**	11	**100.00**	550	**90.91**	12,073	**90.03**	**12,634**	**90.08**
Maguindanao del Norte	7	690	7,149	**7,846**	3	**42.86**	535	**77.54**	6,027	**84.31**	**6,565**	**83.67**
Maguindanao del Sur	22	1,369	11,452	**12,843**	17	**77.27**	1,058	**77.28**	9,654	**84.30**	**10,729**	**83.54**
Sulu	6	739	9,655	**10,400**	4	**66.67**	785	**106.22**	9,977	**103.34**	**10,766**	**103.52**
Tawi-Tawi	10	516	4,843	**5,369**	10	**100.00**	514	**99.61**	4,695	**96.94**	**5,219**	**97.21**
SGA	1	224	1,317	**1,542**	0	**0.00**	144	**64.29**	1,163	**88.31**	**1,307**	**84.76**
City of Cotabato	3	382	3,208	**3,593**	3	**100.00**	322	**84.29**	2,868	**89.40**	**3,193**	**88.87**

*Leegen: * - No Report*

Table 2.B.3.2 - Postpartum and Newborn Care
Postpartum Women who completed Iron with Folic Acid Supplementation
Philippines, 2024

Area	Eligible Population (0-11 months)	Iron with Folic Supplementation							
		Age Group in Year						Total	%
		10-14		15-19		20-49			
		No.	%	No.	%	No.	%		
**PHILIPPINES**	**2,200,865**	**8,892**	**0.40**	**126,430**	**5.74**	**1,081,349**	**49.13**	**1,216,671**	**55.28**
**NCR**	**263,248**	**231**	**0.09**	**9,798**	**3.72**	**141,749**	**53.85**	**151,778**	**57.66**
City of Malabon	**7,447**	12	**0.16**	326	**4.38**	2,638	**35.42**	**2,976**	**39.96**
City of Navotas	**5,263**	6	**0.11**	308	**5.85**	3,632	**69.01**	**3,946**	**74.98**
City of Valenzuela	**13,601**	13	**0.10**	399	**2.93**	4,784	**35.17**	**5,196**	**38.20**
City of Caloocan	**33,499**	44	**0.13**	1,604	**4.79**	21,216	**63.33**	**22,864**	**68.25**
City of Marikina	**8,399**	9	**0.11**	220	**2.62**	2,394	**28.50**	**2,623**	**31.23**
City of Pasig	**17,856**	22	**0.12**	594	**3.33**	7,836	**43.88**	**8,452**	**47.33**
Pateros	**1,120**	0	**0.00**	19	**1.70**	700	**62.50**	**719**	**64.20**
City of Taguig	**26,021**	20	**0.08**	1,043	**4.01**	11,104	**42.67**	**12,167**	**46.76**
Quezon City	**59,607**	30	**0.05**	1,517	**2.55**	30,737	**51.57**	**32,284**	**54.16**
City of Makati	**4,196**	4	**0.10**	111	**2.65**	1,322	**31.51**	**1,437**	**34.25**
City of Mandaluyong	**7,989**	11	**0.14**	274	**3.43**	7,468	**93.48**	**7,753**	**97.05**
City of San Juan	**2,173**	0	**0.00**	27	**1.24**	665	**30.60**	**692**	**31.85**
City of Manila	**34,268**	17	**0.05**	1,617	**4.72**	21,666	**63.23**	**23,300**	**67.99**
City of Las Piñas	**11,346**	7	**0.06**	405	**3.57**	5,782	**50.96**	**6,194**	**54.59**
City of Muntinlupa	**9,949**	16	**0.16**	566	**5.69**	6,996	**70.32**	**7,578**	**76.17**
City of Parañaque	**13,385**	12	**0.09**	506	**3.78**	6,205	**46.36**	**6,723**	**50.23**
Pasay City	**7,129**	8	**0.11**	262	**3.68**	6,604	**92.64**	**6,874**	**96.42**
**CAR**	**31,490**	**22**	**0.07**	**1,488**	**4.73**	**17,806**	**56.54**	**19,316**	**61.34**
Abra	**3,639**	4	**0.11**	265	**7.28**	2,253	**61.91**	**2,522**	**69.30**
Apayao	**2,222**	3	**0.14**	194	**8.73**	1,274	**57.34**	**1,471**	**66.20**
Benguet	**8,900**	1	**0.01**	352	**3.96**	5,388	**60.54**	**5,741**	**64.51**
Ifugao	**4,145**	4	**0.10**	166	**4.00**	1,875	**45.24**	**2,045**	**49.34**
Kalinga	**4,582**	2	**0.04**	208	**4.54**	2,474	**53.99**	**2,684**	**58.58**
Mountain Province	**2,615**	5	**0.19**	146	**5.58**	1,506	**57.59**	**1,657**	**63.37**
City of Baguio	**5,387**	3	**0.06**	157	**2.91**	3,036	**56.36**	**3,196**	**59.33**
**Region 1**	**96,024**	**86**	**0.09**	**4,541**	**4.73**	**49,821**	**51.88**	**54,448**	**56.70**
Ilocos Norte	**8,809**	7	**0.08**	331	**3.76**	5,534	**62.82**	**5,872**	**66.66**
Ilocos Sur	**9,834**	13	**0.13**	523	**5.32**	7,915	**80.49**	**8,451**	**85.94**
La Union	**13,436**	16	**0.12**	643	**4.79**	6,454	**48.04**	**7,113**	**52.94**
Pangasinan	**60,591**	43	**0.07**	2,817	**4.65**	27,898	**46.04**	**30,758**	**50.76**
City of Dagupan	**3,354**	7	**0.21**	227	**6.77**	2,020	**60.23**	**2,254**	**67.20**
**Region 2**	**64,034**	**121**	**0.19**	**3,775**	**5.90**	**31,837**	**49.72**	**35,733**	**55.80**
Batanes	**241**	0	**0.00**	11	**4.56**	170	**70.54**	**181**	**75.10**
Cagayan	**20,723**	35	**0.17**	956	**4.61**	8,820	**42.56**	**9,811**	**47.34**
Isabela	**27,362**	55	**0.20**	1,589	**5.81**	13,686	**50.02**	**15,330**	**56.03**
Nueva Vizcaya	**8,880**	24	**0.27**	726	**8.18**	4,598	**51.78**	**5,348**	**60.23**
Quirino	**3,865**	5	**0.13**	230	**5.95**	2,013	**52.08**	**2,248**	**58.16**
City of Santiago	**2,963**	2	**0.07**	263	**8.88**	2,550	**86.06**	**2,815**	**95.01**
**Region 3**	**235,313**	**6,669**	**2.83**	**21,463**	**9.12**	**145,770**	**61.95**	**173,902**	**73.90**
Aurora	**4,578**	1	**0.02**	284	**6.20**	2,062	**45.04**	**2,347**	**51.27**
Bataan	**17,556**	40	**0.23**	1,115	**6.35**	8,523	**48.55**	**9,678**	**55.13**
Bulacan	**68,115**	115	**0.17**	4,863	**7.14**	49,198	**72.23**	**54,176**	**79.54**
Nueva Ecija	**43,626**	49	**0.11**	2,243	**5.14**	17,772	**40.74**	**20,064**	**45.99**
Pampanga	**46,146**	6,414	**13.90**	9,514	**20.62**	34,919	**75.67**	**50,847**	**110.19**
Tarlac	**29,655**	30	**0.10**	1,897	**6.40**	19,213	**64.79**	**21,140**	**71.29**
Zambales	**11,796**	7	**0.06**	703	**5.96**	5,300	**44.93**	**6,010**	**50.95**
City of Angeles	**9,232**	12	**0.13**	695	**7.53**	5,993	**64.92**	**6,700**	**72.57**
City of Olongapo	**4,609**	1	**0.02**	149	**3.23**	2,790	**60.53**	**2,940**	**63.79**
**Region 4A**	**310,150**	**288**	**0.09**	**13,448**	**4.34**	**148,288**	**47.81**	**162,024**	**52.24**
Batangas	**53,484**	42	**0.08**	2,094	**3.92**	29,004	**54.23**	**31,140**	**58.22**
Cavite	**78,589**	51	**0.06**	2,439	**3.10**	32,406	**41.23**	**34,896**	**44.40**
Laguna	**61,967**	72	**0.12**	3,052	**4.93**	34,045	**54.94**	**37,169**	**59.98**
Quezon	**40,954**	40	**0.10**	2,110	**5.15**	16,917	**41.31**	**19,067**	**46.56**
Rizal	**68,903**	76	**0.11**	3,478	**5.05**	33,788	**49.04**	**37,342**	**54.20**
City of Lucena	**6,253**	7	**0.11**	275	**4.40**	2,128	**34.03**	**2,410**	**38.54**
**Region 4B**	**66,508**	**107**	**0.16**	**4,686**	**7.05**	**31,719**	**47.69**	**36,512**	**54.90**
Marinduque	**4,124**	4	**0.10**	187	**4.53**	2,251	**54.58**	**2,442**	**59.21**
Occidental Mindoro	**11,748**	27	**0.23**	1,174	**9.99**	7,169	**61.02**	**8,370**	**71.25**
Oriental Mindoro	**19,007**	23	**0.12**	830	**4.37**	7,904	**41.58**	**8,757**	**46.07**
Palawan	**20,503**	39	**0.19**	1,860	**9.07**	9,349	**45.60**	**11,248**	**54.86**
Romblon	**5,488**	6	**0.11**	366	**6.67**	3,120	**56.85**	**3,492**	**63.63**

Table 2.B.3.2 - Postpartum and Newborn Care
Postpartum Women who completed Iron with Folic Acid Supplementation
Philippines, 2024

Area	Eligible Population (0-11 months)	Iron with Folic Supplementation							
		Age Group in Year						Total	%
		10-14		15-19		20-49			
		No.	%	No.	%	No.	%		
City of Puerto Princesa	5,638	8	0.14	269	4.77	1,926	34.16	2,203	39.07
**Region 5**	136,611	72	0.05	6,346	4.65	57,534	42.12	63,952	46.81
Albay	27,665	8	0.03	913	3.30	12,906	46.65	13,827	49.98
Camarines Norte	14,894	9	0.06	756	5.08	6,309	42.36	7,074	47.50
Camarines Sur	43,855	16	0.04	1,502	3.42	13,815	31.50	15,333	34.96
Catanduanes	5,395	4	0.07	313	5.80	2,899	53.73	3,216	59.61
Masbate	22,495	15	0.07	1,726	7.67	10,649	47.34	12,390	55.08
Sorsogon	18,065	19	0.11	956	5.29	9,228	51.08	10,203	56.48
City of Naga	4,242	1	0.02	180	4.24	1,728	40.74	1,909	45.00
**Region 6**	147,934	119	0.08	7,647	5.17	65,775	44.46	73,541	49.71
Aklan	11,281	4	0.04	483	4.28	4,889	43.34	5,376	47.66
Antique	11,899	13	0.11	513	4.31	5,127	43.09	5,653	47.51
Capiz	13,872	8	0.06	537	3.87	5,814	41.91	6,359	45.84
Guimaras	3,361	4	0.12	165	4.91	1,878	55.88	2,047	60.90
Iloilo	35,981	18	0.05	1,525	4.24	14,913	41.45	16,456	45.74
Negros Occidental	52,445	62	0.12	3,562	6.79	23,999	45.76	27,623	52.67
City of Bacolod	10,361	2	0.02	451	4.35	4,824	46.56	5,277	50.93
City of Iloilo	8,734	8	0.09	411	4.71	4,331	49.59	4,750	54.39
**Region 7**	167,016	150	0.09	8,576	5.13	80,404	48.14	89,130	53.37
Bohol	26,210	12	0.05	909	3.47	11,311	43.16	12,232	46.67
Cebu	72,875	53	0.07	3,322	4.56	30,032	41.21	33,407	45.84
Negros Oriental	28,980	27	0.09	1,903	6.57	13,930	48.07	15,860	54.73
Siquijor	1,671	0	0.00	65	3.89	878	52.54	943	56.43
City of Cebu	19,466	25	0.13	1,329	6.83	11,924	61.26	13,278	68.21
City of Lapu-Lapu	10,833	22	0.20	569	5.25	6,622	61.13	7,213	66.58
City of Mandaue	6,981	11	0.16	479	6.86	5,707	81.75	6,197	88.77
**Region 8**	93,713	86	0.09	4,820	5.14	38,992	41.61	43,898	46.84
Biliran	3,378	4	0.12	261	7.73	2,081	61.60	2,346	69.45
Eastern Samar	9,594	13	0.14	735	7.66	5,078	52.93	5,826	60.73
Leyte	31,504	25	0.08	1,480	4.70	10,423	33.08	11,928	37.86
Northern Samar	14,053	14	0.10	525	3.74	4,688	33.36	5,227	37.19
Southern Leyte	7,368	5	0.07	306	4.15	3,295	44.72	3,606	48.94
Samar	16,946	7	0.04	728	4.30	6,530	38.53	7,265	42.87
Ormoc City	5,344	10	0.19	346	6.47	2,594	48.54	2,950	55.20
City of Tacloban	5,526	8	0.14	439	7.94	4,303	77.87	4,750	85.96
**Region 9**	85,348	81	0.09	5,457	6.39	35,720	41.85	41,258	48.34
Zamboanga del Norte	24,977	22	0.09	1,526	6.11	9,805	39.26	11,353	45.45
Zamboanga del Sur	23,905	18	0.08	1,215	5.08	7,531	31.50	8,764	36.66
Zamboanga Sibugay	13,903	19	0.14	928	6.67	5,676	40.83	6,623	47.64
City of Isabela	3,209	0	0.00	289	9.01	1,448	45.12	1,737	54.13
City of Zamboanga	19,354	22	0.11	1,499	7.75	11,260	58.18	12,781	66.04
**Region 10**	110,290	186	0.17	9,439	8.56	55,675	50.48	65,300	59.21
Bukidnon	33,913	92	0.27	4,091	12.06	18,088	53.34	22,271	65.67
Camiguin	1,727	2	0.12	153	8.86	1,081	62.59	1,236	71.57
Lanao del Norte	17,881	23	0.13	841	4.70	8,966	50.14	9,830	54.97
Misamis Occidental	12,342	10	0.08	769	6.23	5,812	47.09	6,591	53.40
Misamis Oriental	22,184	28	0.13	1,677	7.56	9,052	40.80	10,757	48.49
City of Cagayan De Oro	14,503	24	0.17	1,481	10.21	8,687	59.90	10,192	70.28
City of Iligan	7,740	7	0.09	427	5.52	3,989	51.54	4,423	57.14
**Region 11**	107,934	360	0.33	9,216	8.54	57,619	53.38	67,195	62.26
Davao de Oro	15,434	55	0.36	1,613	10.45	8,438	54.67	10,106	65.48
Davao del Norte	23,685	85	0.36	2,103	8.88	12,876	54.36	15,064	63.60
Davao Oriental	12,025	54	0.45	1,151	9.57	6,144	51.09	7,349	61.11
Davao del Sur	13,178	63	0.48	1,166	8.85	5,842	44.33	7,071	53.66
Davao Occidental	6,606	39	0.59	730	11.05	2,196	33.24	2,965	44.88
City of Davao	37,006	64	0.17	2,453	6.63	22,123	59.78	24,640	66.58
**Region 12**	95,296	223	0.23	8,339	8.75	48,070	50.44	56,632	59.43
Cotabato	25,696	51	0.20	2,067	8.04	13,151	51.18	15,269	59.42
Sarangani	13,501	39	0.29	1,652	12.24	7,235	53.59	8,926	66.11
South Cotabato	21,842	66	0.30	2,088	9.56	12,068	55.25	14,222	65.11
Sultan Kudarat	19,613	56	0.29	1,871	9.54	8,970	45.73	10,897	55.56

Table 2.B.3.2 - Postpartum and Newborn Care
Postpartum Women who completed Iron with Folic Acid Supplementation
Philippines, 2024

Area	Eligible Population (0-11 months)	Iron with Folic Supplementation							
		Age Group in Year						Total	%
		10-14		15-19		20-49			
		No.	%	No.	%	No.	%		
City of General Santos	**14,644**	11	**0.08**	661	**4.51**	6,646	**45.38**	**7,318**	**49.97**
**CARAGA**	**55,371**	**43**	**0.08**	**3,296**	**5.95**	**25,054**	**45.25**	**28,393**	**51.28**
Agusan del Norte	**7,331**	1	**0.01**	367	**5.01**	2,234	**30.47**	**2,602**	**35.49**
Agusan del Sur	**16,180**	15	**0.09**	988	**6.11**	7,423	**45.88**	**8,426**	**52.08**
Surigao del Norte	**10,168**	3	**0.03**	681	**6.70**	5,228	**51.42**	**5,912**	**58.14**
Surigao del Sur	**12,834**	15	**0.12**	606	**4.72**	4,886	**38.07**	**5,507**	**42.91**
Dinagat Islands	**2,117**	3	**0.14**	136	**6.42**	952	**44.97**	**1,091**	**51.54**
City of Butuan	**6,741**	6	**0.09**	518	**7.68**	4,331	**64.25**	**4,855**	**72.02**
**BARMM**	**134,585**	**48**	**0.04**	**4,095**	**3.04**	**49,516**	**36.79**	**53,659**	**39.87**
Basilan	**11,653**	20	**0.17**	658	**5.65**	3,283	**28.17**	**3,961**	**33.99**
Lanao del Sur	**33,339**	6	**0.02**	512	**1.54**	15,003	**45.00**	**15,521**	**46.56**
Maguindanao del Norte	**18,923**	9	**0.05**	760	**4.02**	8,364	**44.20**	**9,133**	**48.26**
Maguindanao del Sur	**23,214**	5	**0.02**	900	**3.88**	7,803	**33.61**	**8,708**	**37.51**
Sulu	**21,381**	0	**0.00**	477	**2.23**	7,521	**35.18**	**7,998**	**37.41**
Tawi-Tawi	**12,602**	4	**0.03**	343	**2.72**	3,914	**31.06**	**4,261**	**33.81**
SGA	**6,305**	0	**0.00**	136	**2.16**	978	**15.51**	**1,114**	**17.67**
City of Cotabato	**7,168**	4	**0.06**	309	**4.31**	2,650	**36.97**	**2,963**	**41.34**

*Legend: * - No Report*

Table 2.B.3.3 - Postpartum and Newborn Care
Postpartum Women who completed Vitamin A supplementation
Philippines, 2024

Area	Eligible Population (0-11 months)	Vitamin A Supplementation							
		Age Group in Year						Total	%
		10-14		15-19		20-49			
		No.	%	No.	%	No.	%		
**PHILIPPINES**	**2,200,865**	**3,069**	**0.14**	**124,359**	**5.65**	**1,091,882**	**49.61**	**1,219,310**	**55.40**
**NCR**	**263,248**	**276**	**0.10**	**10,861**	**4.13**	**167,796**	**63.74**	**178,933**	**67.97**
City of Malabon	**7,447**	12	**0.16**	329	**4.42**	2,638	**35.42**	**2,979**	**40.00**
City of Navotas	**5,263**	6	**0.11**	308	**5.85**	3,632	**69.01**	**3,946**	**74.98**
City of Valenzuela	**13,601**	13	**0.10**	399	**2.93**	4,784	**35.17**	**5,196**	**38.20**
City of Caloocan	**33,499**	57	**0.17**	2,109	**6.30**	21,890	**65.35**	**24,056**	**71.81**
City of Marikina	**8,399**	8	**0.10**	219	**2.61**	2,360	**28.10**	**2,587**	**30.80**
City of Pasig	**17,856**	19	**0.11**	508	**2.84**	7,257	**40.64**	**7,784**	**43.59**
Pateros	**1,120**	0	**0.00**	33	**2.95**	706	**63.04**	**739**	**65.98**
City of Taguig	**26,021**	22	**0.08**	1,042	**4.00**	11,139	**42.81**	**12,203**	**46.90**
Quezon City	**59,607**	52	**0.09**	2,211	**3.71**	56,064	**94.06**	**58,327**	**97.85**
City of Makati	**4,196**	3	**0.07**	111	**2.65**	1,393	**33.20**	**1,507**	**35.92**
City of Mandaluyong	**7,989**	11	**0.14**	268	**3.35**	7,291	**91.26**	**7,570**	**94.76**
City of San Juan	**2,173**	0	**0.00**	36	**1.66**	712	**32.77**	**748**	**34.42**
City of Manila	**34,268**	28	**0.08**	1,539	**4.49**	22,378	**65.30**	**23,945**	**69.88**
City of Las Piñas	**11,346**	9	**0.08**	421	**3.71**	5,842	**51.49**	**6,272**	**55.28**
City of Muntinlupa	**9,949**	16	**0.16**	567	**5.70**	6,985	**70.21**	**7,568**	**76.07**
City of Parañaque	**13,385**	12	**0.09**	506	**3.78**	6,205	**46.36**	**6,723**	**50.23**
Pasay City	**7,129**	8	**0.11**	255	**3.58**	6,520	**91.46**	**6,783**	**95.15**
**CAR**	**31,490**	**48**	**0.15**	**1,620**	**5.14**	**17,847**	**56.68**	**19,515**	**61.97**
Abra	**3,639**	4	**0.11**	266	**7.31**	2,252	**61.89**	**2,522**	**69.30**
Apayao	**2,222**	8	**0.36**	230	**10.35**	1,194	**53.74**	**1,432**	**64.45**
Benguet	**8,900**	13	**0.15**	220	**2.47**	3,742	**42.04**	**3,975**	**44.66**
Ifugao	**4,145**	8	**0.19**	197	**4.75**	2,070	**49.94**	**2,275**	**54.89**
Kalinga	**4,582**	8	**0.17**	222	**4.85**	2,458	**53.64**	**2,688**	**58.66**
Mountain Province	**2,615**	4	**0.15**	328	**12.54**	3,116	**119.16**	**3,448**	**131.85**
City of Baguio	**5,387**	3	**0.06**	157	**2.91**	3,015	**55.97**	**3,175**	**58.94**
**Region 1**	**96,024**	**116**	**0.12**	**4,329**	**4.51**	**45,730**	**47.62**	**50,175**	**52.25**
Ilocos Norte	**8,809**	10	**0.11**	345	**3.92**	4,899	**55.61**	**5,254**	**59.64**
Ilocos Sur	**9,834**	12	**0.12**	493	**5.01**	7,239	**73.61**	**7,744**	**78.75**
La Union	**13,436**	31	**0.23**	696	**5.18**	7,039	**52.39**	**7,766**	**57.80**
Pangasinan	**60,591**	55	**0.09**	2,570	**4.24**	24,576	**40.56**	**27,201**	**44.89**
City of Dagupan	**3,354**	8	**0.24**	225	**6.71**	1,977	**58.94**	**2,210**	**65.89**
**Region 2**	**64,034**	**111**	**0.17**	**3,705**	**5.79**	**30,149**	**47.08**	**33,965**	**53.04**
Batanes	**241**	0	**0.00**	15	**6.22**	186	**77.18**	**201**	**83.40**
Cagayan	**20,723**	33	**0.16**	983	**4.74**	8,404	**40.55**	**9,420**	**45.46**
Isabela	**27,362**	48	**0.18**	1,516	**5.54**	12,216	**44.65**	**13,780**	**50.36**
Nueva Vizcaya	**8,880**	20	**0.23**	704	**7.93**	4,954	**55.79**	**5,678**	**63.94**
Quirino	**3,865**	7	**0.18**	213	**5.51**	1,824	**47.19**	**2,044**	**52.88**
City of Santiago	**2,963**	3	**0.10**	274	**9.25**	2,565	**86.57**	**2,842**	**95.92**
**Region 3**	**235,313**	**335**	**0.14**	**11,946**	**5.08**	**112,650**	**47.87**	**124,931**	**53.09**
Aurora	**4,578**	2	**0.04**	291	**6.36**	2,096	**45.78**	**2,389**	**52.18**
Bataan	**17,556**	39	**0.22**	1,089	**6.20**	8,446	**48.11**	**9,574**	**54.53**
Bulacan	**68,115**	106	**0.16**	3,931	**5.77**	41,843	**61.43**	**45,880**	**67.36**
Nueva Ecija	**43,626**	50	**0.11**	1,791	**4.11**	13,758	**31.54**	**15,599**	**35.76**
Pampanga	**46,146**	100	**0.22**	2,153	**4.67**	21,771	**47.18**	**24,024**	**52.06**
Tarlac	**29,655**	20	**0.07**	1,493	**5.03**	14,665	**49.45**	**16,178**	**54.55**
Zambales	**11,796**	5	**0.04**	613	**5.20**	4,515	**38.28**	**5,133**	**43.51**
City of Angeles	**9,232**	12	**0.13**	440	**4.77**	3,154	**34.16**	**3,606**	**39.06**
City of Olongapo	**4,609**	1	**0.02**	145	**3.15**	2,402	**52.12**	**2,548**	**55.28**
**Region 4A**	**310,150**	**375**	**0.12**	**15,124**	**4.88**	**157,418**	**50.76**	**172,917**	**55.75**
Batangas	**53,484**	62	**0.12**	2,444	**4.57**	29,863	**55.84**	**32,369**	**60.52**
Cavite	**78,589**	71	**0.09**	2,971	**3.78**	37,649	**47.91**	**40,691**	**51.78**
Laguna	**61,967**	85	**0.14**	3,348	**5.40**	35,248	**56.88**	**38,681**	**62.42**
Quezon	**40,954**	67	**0.16**	2,688	**6.56**	19,238	**46.97**	**21,993**	**53.70**
Rizal	**68,903**	83	**0.12**	3,353	**4.87**	32,860	**47.69**	**36,296**	**52.68**
City of Lucena	**6,253**	7	**0.11**	320	**5.12**	2,560	**40.94**	**2,887**	**46.17**
**Region 4B**	**66,508**	**107**	**0.16**	**4,439**	**6.67**	**30,020**	**45.14**	**34,566**	**51.97**
Marinduque	**4,124**	1	**0.02**	246	**5.97**	2,278	**55.24**	**2,525**	**61.23**
Occidental Mindoro	**11,748**	30	**0.26**	1,103	**9.39**	6,466	**55.04**	**7,599**	**64.68**
Oriental Mindoro	**19,007**	20	**0.11**	933	**4.91**	8,385	**44.12**	**9,338**	**49.13**
Palawan	**20,503**	35	**0.17**	1,424	**6.95**	7,188	**35.06**	**8,647**	**42.17**
Romblon	**5,488**	9	**0.16**	334	**6.09**	2,882	**52.51**	**3,225**	**58.76**

Table 2.B.3.3 - Postpartum and Newborn Care
Postpartum Women who completed Vitamin A supplementation
Philippines, 2024

Area	Eligible Population (0-11 months)	Vitamin A Supplementation							
		Age Group in Year						Total	%
		10-14		15-19		20-49			
		No.	%	No.	%	No.	%		
City of Puerto Princesa	**5,638**	12	**0.21**	399	**7.08**	2,821	**50.04**	**3,232**	**57.33**
**Region 5**	**136,611**	**131**	**0.10**	**7,544**	**5.52**	**60,421**	**44.23**	**68,096**	**49.85**
Albay	**27,665**	5	**0.02**	867	**3.13**	11,808	**42.68**	**12,680**	**45.83**
Camarines Norte	**14,894**	15	**0.10**	876	**5.88**	6,622	**44.46**	**7,513**	**50.44**
Camarines Sur	**43,855**	24	**0.05**	1,705	**3.89**	15,296	**34.88**	**17,025**	**38.82**
Catanduanes	**5,395**	39	**0.72**	670	**12.42**	2,952	**54.72**	**3,661**	**67.86**
Masbate	**22,495**	21	**0.09**	2,072	**9.21**	11,794	**52.43**	**13,887**	**61.73**
Sorsogon	**18,065**	24	**0.13**	1,187	**6.57**	10,314	**57.09**	**11,525**	**63.80**
City of Naga	**4,242**	3	**0.07**	167	**3.94**	1,635	**38.54**	**1,805**	**42.55**
**Region 6**	**147,934**	**171**	**0.12**	**8,500**	**5.75**	**68,279**	**46.16**	**76,950**	**52.02**
Aklan	**11,281**	11	**0.10**	540	**4.79**	4,823	**42.75**	**5,374**	**47.64**
Antique	**11,899**	14	**0.12**	602	**5.06**	5,713	**48.01**	**6,329**	**53.19**
Capiz	**13,872**	13	**0.09**	658	**4.74**	6,491	**46.79**	**7,162**	**51.63**
Guimaras	**3,361**	10	**0.30**	218	**6.49**	1,939	**57.69**	**2,167**	**64.47**
Iloilo	**35,981**	26	**0.07**	1,792	**4.98**	16,175	**44.95**	**17,993**	**50.01**
Negros Occidental	**52,445**	79	**0.15**	3,939	**7.51**	25,228	**48.10**	**29,246**	**55.77**
City of Bacolod	**10,361**	3	**0.03**	340	**3.28**	3,770	**36.39**	**4,113**	**39.70**
City of Iloilo	**8,734**	15	**0.17**	411	**4.71**	4,140	**47.40**	**4,566**	**52.28**
**Region 7**	**167,016**	**157**	**0.09**	**8,614**	**5.16**	**77,729**	**46.54**	**86,500**	**51.79**
Bohol	**26,210**	15	**0.06**	1,027	**3.92**	11,940	**45.56**	**12,982**	**49.53**
Cebu	**72,875**	48	**0.07**	3,048	**4.18**	26,468	**36.32**	**29,564**	**40.57**
Negros Oriental	**28,980**	41	**0.14**	1,980	**6.83**	14,592	**50.35**	**16,613**	**57.33**
Siquijor	**1,671**	0	**0.00**	75	**4.49**	876	**52.42**	**951**	**56.91**
City of Cebu	**19,466**	24	**0.12**	1,470	**7.55**	11,992	**61.60**	**13,486**	**69.28**
City of Lapu-Lapu	**10,833**	14	**0.13**	580	**5.35**	6,354	**58.65**	**6,948**	**64.14**
City of Mandaue	**6,981**	15	**0.21**	434	**6.22**	5,507	**78.89**	**5,956**	**85.32**
**Region 8**	**93,713**	**98**	**0.10**	**5,112**	**5.45**	**40,806**	**43.54**	**46,016**	**49.10**
Biliran	**3,378**	3	**0.09**	278	**8.23**	2,161	**63.97**	**2,442**	**72.29**
Eastern Samar	**9,594**	17	**0.18**	815	**8.49**	5,314	**55.39**	**6,146**	**64.06**
Leyte	**31,504**	26	**0.08**	1,325	**4.21**	10,045	**31.88**	**11,396**	**36.17**
Northern Samar	**14,053**	13	**0.09**	552	**3.93**	5,373	**38.23**	**5,938**	**42.25**
Southern Leyte	**7,368**	7	**0.10**	354	**4.80**	3,204	**43.49**	**3,565**	**48.38**
Samar	**16,946**	20	**0.12**	990	**5.84**	8,215	**48.48**	**9,225**	**54.44**
Ormoc City	**5,344**	5	**0.09**	421	**7.88**	2,679	**50.13**	**3,105**	**58.10**
City of Tacloban	**5,526**	7	**0.13**	377	**6.82**	3,815	**69.04**	**4,199**	**75.99**
**Region 9**	**85,348**	**94**	**0.11**	**5,388**	**6.31**	**36,074**	**42.27**	**41,556**	**48.69**
Zamboanga del Norte	**24,977**	26	**0.10**	1,621	**6.49**	10,521	**42.12**	**12,168**	**48.72**
Zamboanga del Sur	**23,905**	26	**0.11**	1,187	**4.97**	7,946	**33.24**	**9,159**	**38.31**
Zamboanga Sibugay	**13,903**	16	**0.12**	918	**6.60**	5,709	**41.06**	**6,643**	**47.78**
City of Isabela	**3,209**	0	**0.00**	208	**6.48**	1,408	**43.88**	**1,616**	**50.36**
City of Zamboanga	**19,354**	26	**0.13**	1,454	**7.51**	10,490	**54.20**	**11,970**	**61.85**
**Region 10**	**110,290**	**192**	**0.17**	**9,113**	**8.26**	**54,537**	**49.45**	**63,842**	**57.89**
Bukidnon	**33,913**	93	**0.27**	4,004	**11.81**	17,718	**52.25**	**21,815**	**64.33**
Camiguin	**1,727**	2	**0.12**	153	**8.86**	1,083	**62.71**	**1,238**	**71.69**
Lanao del Norte	**17,881**	26	**0.15**	918	**5.13**	9,280	**51.90**	**10,224**	**57.18**
Misamis Occidental	**12,342**	11	**0.09**	782	**6.34**	5,723	**46.37**	**6,516**	**52.80**
Misamis Oriental	**22,184**	20	**0.09**	1,225	**5.52**	6,982	**31.47**	**8,227**	**37.09**
City of Cagayan De Oro	**14,503**	32	**0.22**	1,569	**10.82**	9,737	**67.14**	**11,338**	**78.18**
City of Iligan	**7,740**	8	**0.10**	462	**5.97**	4,014	**51.86**	**4,484**	**57.93**
**Region 11**	**107,934**	**373**	**0.35**	**10,078**	**9.34**	**61,404**	**56.89**	**71,855**	**66.57**
Davao de Oro	**15,434**	57	**0.37**	1,659	**10.75**	8,688	**56.29**	**10,404**	**67.41**
Davao del Norte	**23,685**	92	**0.39**	2,130	**8.99**	12,960	**54.72**	**15,182**	**64.10**
Davao Oriental	**12,025**	53	**0.44**	1,265	**10.52**	6,503	**54.08**	**7,821**	**65.04**
Davao del Sur	**13,178**	56	**0.42**	1,405	**10.66**	6,979	**52.96**	**8,440**	**64.05**
Davao Occidental	**6,606**	50	**0.76**	981	**14.85**	2,843	**43.04**	**3,874**	**58.64**
City of Davao	**37,006**	65	**0.18**	2,638	**7.13**	23,431	**63.32**	**26,134**	**70.62**
**Region 12**	**95,296**	**268**	**0.28**	**8,246**	**8.65**	**45,246**	**47.48**	**53,760**	**56.41**
Cotabato	**25,696**	68	**0.26**	1,934	**7.53**	11,212	**43.63**	**13,214**	**51.42**
Sarangani	**13,501**	46	**0.34**	1,730	**12.81**	7,461	**55.26**	**9,237**	**68.42**
South Cotabato	**21,842**	78	**0.36**	2,124	**9.72**	11,846	**54.23**	**14,048**	**64.32**
Sultan Kudarat	**19,613**	59	**0.30**	1,955	**9.97**	9,637	**49.14**	**11,651**	**59.40**

Table 2.B.3.3 - Postpartum and Newborn Care
Postpartum Women who completed Vitamin A supplementation
Philippines, 2024

Area	Eligible Population (0-11 months)	Vitamin A Supplementation							
		Age Group in Year						Total	%
		10-14		15-19		20-49			
		No.	%	No.	%	No.	%		
City of General Santos	**14,644**	17	**0.12**	503	**3.43**	5,090	**34.76**	**5,610**	**38.31**
**CARAGA**	**55,371**	**101**	**0.18**	**4,184**	**7.56**	**27,362**	**49.42**	**31,647**	**57.15**
Agusan del Norte	**7,331**	5	**0.07**	403	**5.50**	2,440	**33.28**	**2,848**	**38.85**
Agusan del Sur	**16,180**	43	**0.27**	1,555	**9.61**	9,239	**57.10**	**10,837**	**66.98**
Surigao del Norte	**10,168**	3	**0.03**	553	**5.44**	4,387	**43.15**	**4,943**	**48.61**
Surigao del Sur	**12,834**	40	**0.31**	970	**7.56**	5,969	**46.51**	**6,979**	**54.38**
Dinagat Islands	**2,117**	1	**0.05**	159	**7.51**	973	**45.96**	**1,133**	**53.52**
City of Butuan	**6,741**	9	**0.13**	544	**8.07**	4,354	**64.59**	**4,907**	**72.79**
**BARMM**	**134,585**	**116**	**0.09**	**5,556**	**4.13**	**58,414**	**43.40**	**64,086**	**47.62**
Basilan	**11,653**	42	**0.36**	701	**6.02**	3,680	**31.58**	**4,423**	**37.96**
Lanao del Sur	**33,339**	20	**0.06**	747	**2.24**	15,697	**47.08**	**16,464**	**49.38**
Maguindanao del Norte	**18,923**	14	**0.07**	1,024	**5.41**	9,811	**51.85**	**10,849**	**57.33**
Maguindanao del Sur	**23,214**	16	**0.07**	1,423	**6.13**	11,431	**49.24**	**12,870**	**55.44**
Sulu	**21,381**	5	**0.02**	801	**3.75**	9,960	**46.58**	**10,766**	**50.35**
Tawi-Tawi	**12,602**	17	**0.13**	397	**3.15**	4,062	**32.23**	**4,476**	**35.52**
SGA	**6,305**	0	**0.00**	129	**2.05**	1,067	**16.92**	**1,196**	**18.97**
City of Cotabato	**7,168**	2	**0.03**	334	**4.66**	2,706	**37.75**	**3,042**	**42.44**

*Legend: * - No Report*

Table 2.C.1.1 - Immunization Services for Infants and Children
Newborns or Infants Vaccinated with BCG, Hepatitis B within 24 hours after birth, and Children Protected at Birth (CPAB) from Neonatal Tetanus
Philippines, 2024

Area	Eligible Population (0-11mos. old)	BCG				HepaB within 24 hours after birth				CPAB from Neonatal Tetanus			
		Male	Female	Total	%	Male	Female	Total	%	Male	Female	Total	%
**PHILIPPINES**	**2,200,865**	**701,633**	**673,853**	**1,375,486**	**62.50**	**609,621**	**587,332**	**1,196,953**	**54.39**	**608,817**	**593,950**	**1,202,767**	**54.65**
**NCR**	**263,248**	**112,550**	**109,720**	**222,270**	**84.43**	**106,838**	**104,142**	**210,980**	**80.14**	**89,931**	**88,593**	**178,524**	**67.82**
City of Malabon	7,447	2,040	1,865	3,905	52.44	1,963	1,805	3,768	50.60	1,977	1,855	3,832	51.46
City of Navotas	5,263	2,733	2,793	5,526	105.00	2,683	2,746	5,429	103.15	2,311	2,410	4,721	89.70
City of Valenzuela	13,601	3,942	3,819	7,761	57.06	3,749	3,618	7,367	54.17	2,836	2,694	5,530	40.66
City of Caloocan	33,499	9,959	9,728	19,687	58.77	8,776	8,568	17,344	51.77	6,530	6,243	12,773	38.13
City of Marikina	8,399	4,092	3,956	8,048	95.82	3,955	3,861	7,816	93.06	1,455	1,426	2,881	34.30
City of Pasig	17,856	9,306	8,903	18,209	101.98	8,969	8,546	17,515	98.09	5,009	4,999	10,008	56.05
Pateros	1,120	386	424	810	72.32	371	399	770	68.75	375	402	777	69.38
City of Taguig	26,021	4,751	4,638	9,389	36.08	4,005	3,946	7,951	30.56	5,547	5,426	10,973	42.17
Quezon City	59,607	33,894	33,747	67,641	113.48	33,348	33,242	66,590	111.72	32,534	32,656	65,190	109.37
City of Makati	4,196	2,149	2,216	4,365	104.03	2,123	2,161	4,284	102.10	1,624	1,727	3,351	79.86
City of Mandaluyong	7,989	3,195	3,150	6,345	79.42	2,343	2,327	4,670	58.46	2,959	2,962	5,921	74.11
City of San Juan	2,173	556	453	1,009	46.43	532	438	970	44.64	269	281	550	25.31
City of Manila	34,268	16,073	15,118	31,191	91.02	15,819	14,881	30,700	89.59	14,361	13,584	27,945	81.55
City of Las Piñas	11,346	4,297	4,176	8,473	74.68	3,790	3,690	7,480	65.93	2,485	2,620	5,105	44.99
City of Muntinlupa	9,949	3,656	3,480	7,136	71.73	3,282	3,029	6,311	63.43	2,647	2,686	5,333	53.60
City of Parañaque	13,385	6,935	6,948	13,883	103.72	6,678	6,667	13,345	99.70	2,979	2,862	5,841	43.64
Pasay City	7,129	4,586	4,306	8,892	124.73	4,452	4,218	8,670	121.62	4,033	3,760	7,793	109.31
**CAR**	**31,490**	**11,832**	**11,275**	**23,107**	**73.38**	**11,170**	**10,594**	**21,764**	**69.11**	**8,116**	**7,727**	**15,843**	**50.31**
Abra	3,639	1,005	934	1,939	53.28	1,380	1,313	2,693	74.00	1,196	1,071	2,267	62.30
Apayao	2,222	1,789	1,664	3,453	155.40	1,108	1,015	2,123	95.54	623	681	1,304	58.69
Benguet	8,900	2,002	1,930	3,932	44.18	1,799	1,736	3,535	39.72	1,996	1,962	3,958	44.47
Ifugao	4,145	902	925	1,827	44.08	838	797	1,635	39.45	923	856	1,779	42.92
Kalinga	4,582	1,141	1,077	2,218	48.41	1,159	1,079	2,238	48.84	1,227	1,122	2,349	51.27
Mountain Province	2,615	1,586	1,545	3,131	119.73	1,572	1,571	3,143	120.19	872	833	1,705	65.20
City of Baguio	5,387	3,407	3,200	6,607	122.65	3,314	3,083	6,397	118.75	1,279	1,202	2,481	46.06
**Region 1**	**96,024**	**31,055**	**29,653**	**60,708**	**63.22**	**29,032**	**27,903**	**56,935**	**59.29**	**29,267**	**28,133**	**57,400**	**59.78**
Ilocos Norte	8,809	4,169	4,120	8,289	94.10	4,025	3,994	8,019	91.03	3,661	3,714	7,375	83.72
Ilocos Sur	9,834	4,376	4,227	8,603	87.48	4,341	4,179	8,520	86.64	4,470	4,247	8,717	88.64
La Union	13,436	4,349	4,127	8,476	63.08	4,175	3,968	8,143	60.61	4,311	4,099	8,410	62.59
Pangasinan	60,591	17,111	16,197	33,308	54.97	15,456	14,787	30,243	49.91	16,001	15,262	31,263	51.60
City of Dagupan	3,354	1,050	982	2,032	60.58	1,035	975	2,010	59.93	824	811	1,635	48.75
**Region 2**	**64,034**	**23,399**	**22,012**	**45,411**	**70.92**	**22,730**	**21,407**	**44,137**	**68.93**	**21,175**	**20,275**	**41,450**	**64.73**
Batanes	241	89	94	183	75.93	94	107	201	83.40	94	103	197	81.74
Cagayan	20,723	5,739	5,225	10,964	52.91	5,400	4,967	10,367	50.03	5,151	4,917	10,068	48.58
Isabela	27,362	10,710	10,311	21,021	76.83	10,517	10,092	20,609	75.32	10,447	10,013	20,460	74.78
Nueva Vizcaya	8,880	3,866	3,600	7,466	84.08	3,739	3,471	7,210	81.19	2,821	2,720	5,541	62.40
Quirino	3,865	1,520	1,403	2,923	75.63	1,510	1,391	2,901	75.06	1,190	1,143	2,333	60.36
City of Santiago	2,963	1,475	1,379	2,854	96.32	1,470	1,379	2,849	96.15	1,472	1,379	2,851	96.22
**Region 3**	**235,313**	**75,865**	**72,835**	**148,700**	**63.19**	**55,388**	**54,379**	**109,767**	**46.65**	**67,930**	**65,998**	**133,928**	**56.91**
Aurora	4,578	901	911	1,812	39.58	742	754	1,496	32.68	1,350	1,312	2,662	58.15
Bataan	17,556	5,785	5,629	11,414	65.01	4,225	4,195	8,420	47.96	4,697	4,456	9,153	52.14
Bulacan	68,115	29,871	28,800	58,671	86.14	21,986	21,303	43,289	63.55	19,865	19,813	39,678	58.25
Nueva Ecija	43,626	11,179	10,835	22,014	50.46	7,424	7,194	14,618	33.51	11,437	10,745	22,182	50.85
Pampanga	46,146	12,611	11,745	24,356	52.78	9,713	8,660	18,373	39.81	13,089	12,517	25,606	55.49
Tarlac	29,655	6,617	6,550	13,167	44.40	4,215	4,192	8,407	28.35	7,650	7,775	15,425	52.01
Zambales	11,796	3,029	2,951	5,980	50.70	1,496	1,455	2,951	25.02	2,970	2,933	5,903	50.04
City of Angeles	9,232	4,406	4,096	8,502	92.09	3,967	3,644	7,611	82.44	4,928	4,582	9,510	103.01
City of Olongapo	4,609	1,466	1,318	2,784	60.40	1,620	2,982	4,602	99.85	1,944	1,865	3,809	82.64
**Region 4A**	**310,150**	**99,606**	**94,869**	**194,475**	**62.70**	**89,935**	**85,885**	**175,820**	**56.69**	**85,359**	**82,237**	**167,596**	**54.04**
Batangas	53,484	17,463	16,500	33,963	63.50	16,416	15,499	31,915	59.67	17,000	16,115	33,115	61.92
Cavite	78,589	26,149	24,986	51,135	65.07	24,682	23,774	48,456	61.66	20,842	20,167	41,009	52.18
Laguna	61,967	21,131	20,281	41,412	66.83	18,345	17,544	35,889	57.92	19,140	18,480	37,620	60.71
Quezon	40,954	11,391	10,802	22,193	54.19	9,300	8,794	18,094	44.18	10,484	9,872	20,356	49.70
Rizal	68,903	22,037	20,850	42,887	62.24	19,759	18,828	38,587	56.00	16,462	16,155	32,617	47.34
City of Lucena	6,253	1,435	1,450	2,885	46.14	1,433	1,446	2,879	46.04	1,431	1,448	2,879	46.04
**Region 4B**	**66,508**	**21,339**	**20,492**	**41,831**	**62.90**	**16,122**	**15,589**	**31,711**	**47.68**	**17,381**	**16,781**	**34,162**	**51.37**
Marinduque	4,124	1,373	1,371	2,744	66.54	1,391	1,368	2,759	66.90	1,409	1,332	2,741	66.46
Occidental Mindoro	11,748	4,262	3,894	8,156	69.42	3,311	2,945	6,256	53.25	3,157	2,929	6,086	51.80
Oriental Mindoro	19,007	5,546	5,366	10,912	57.41	4,364	4,359	8,723	45.89	4,276	4,098	8,374	44.06
Palawan	20,503	6,513	6,275	12,788	62.37	3,753	3,677	7,430	36.24	4,968	4,924	9,892	48.25
Romblon	5,488	1,906	1,827	3,733	68.02	1,807	1,682	3,489	63.58	1,808	1,715	3,523	64.19
City of Puerto Princesa	5,638	1,739	1,759	3,498	62.04	1,496	1,558	3,054	54.17	1,763	1,783	3,546	62.89
**Region 5**	**136,611**	**34,496**	**32,838**	**67,334**	**49.29**	**30,246**	**29,417**	**59,663**	**43.67**	**29,583**	**28,580**	**58,163**	**42.58**
Albay	27,665	8,708	8,321	17,029	61.55	8,574	8,093	16,667	60.25	7,429	7,130	14,559	52.63
Camarines Norte	14,894	3,835	3,657	7,492	50.30	2,503	2,490	4,993	33.52	3,103	3,175	6,278	42.15
Camarines Sur	43,855	6,817	6,460	13,277	30.27	6,228	5,893	12,121	27.64	5,359	5,081	10,440	23.81
Catanduanes	5,395	1,679	1,668	3,347	62.04	1,490	1,493	2,983	55.29	1,745	1,715	3,460	64.13
Masbate	22,495	7,219	6,769	13,988	62.18	6,010	6,252	12,262	54.51	5,920	5,620	11,540	51.30
Sorsogon	18,065	5,114	4,807	9,921	54.92	4,296	4,008	8,304	45.97	5,249	5,029	10,278	56.89
City of Naga	4,242	1,124	1,156	2,280	53.75	1,145	1,188	2,333	55.00	778	830	1,608	37.91
**Region 6**	**147,934**	**40,597**	**37,830**	**78,427**	**53.01**	**39,332**	**36,885**	**76,217**	**51.52**	**39,880**	**38,143**	**78,023**	**52.74**
Aklan	11,281	2,939	2,729	5,668	50.24	2,354	2,281	4,635	41.09	2,788	2,675	5,463	48.43
Antique	11,899	2,713	2,499	5,212	43.80	3,625	3,347	6,972	58.59	3,211	2,869	6,080	51.10
Capiz	13,872	3,983	3,707	7,690	55.44	4,422	4,137	8,559	61.70	3,695	3,481	7,176	51.73

Table 2.C.1.1 - Immunization Services for Infants and Children
Newborns or Infants Vaccinated with BCG, Hepatitis B within 24 hours after birth, and Children Protected at Birth (CPAB) from Neonatal Tetanus
Philippines, 2024

Area	Eligible Population (0-11mos. old)	BCG				HepaB within 24 hours after birth				CPAB from Neonatal Tetanus			
		Male	Female	Total	%	Male	Female	Total	%	Male	Female	Total	%
Guimaras	3,361	1,073	1,024	2,097	62.39	617	546	1,163	34.60	1,087	1,065	2,152	64.03
Iloilo	35,981	7,882	7,378	15,260	42.41	7,247	6,958	14,205	39.48	9,789	9,259	19,048	52.94
Negros Occidental	52,445	16,360	15,213	31,573	60.20	15,435	14,343	29,778	56.78	14,687	14,258	28,945	55.19
City of Bacolod	10,361	3,177	2,906	6,083	58.71	3,152	2,896	6,048	58.37	2,164	2,216	4,380	42.27
City of Iloilo	8,734	2,470	2,374	4,844	55.46	2,480	2,377	4,857	55.61	2,459	2,320	4,779	54.72
**Region 7**	**167,016**	**51,757**	**48,975**	**100,732**	**60.31**	**42,973**	**40,656**	**83,629**	**50.07**	**44,948**	**43,923**	**88,871**	**53.21**
Bohol	26,210	7,850	7,326	15,176	57.90	7,831	7,402	15,233	58.12	5,920	5,894	11,814	45.07
Cebu	72,875	20,813	19,863	40,676	55.82	17,938	17,120	35,058	48.11	17,783	17,389	35,172	48.26
Negros Oriental	28,980	8,550	8,062	16,612	57.32	5,859	5,560	11,419	39.40	6,975	6,997	13,972	48.21
Siquijor	1,671	510	443	953	57.03	597	521	1,118	66.91	491	448	939	56.19
City of Cebu	19,466	7,052	6,619	13,671	70.23	7,096	6,631	13,727	70.52	7,160	6,640	13,800	70.89
City of Lapu-Lapu	10,833	3,254	3,151	6,405	59.12	549	524	1,073	9.90	3,849	3,788	7,637	70.50
City of Mandaue	6,981	3,728	3,511	7,239	103.70	3,103	2,898	6,001	85.96	2,770	2,767	5,537	79.32
**Region 8**	**93,713**	**28,824**	**27,222**	**56,046**	**59.81**	**25,414**	**23,908**	**49,322**	**52.63**	**21,155**	**20,435**	**41,590**	**44.38**
Biliran	3,378	1,604	1,410	3,014	89.22	1,602	1,410	3,012	89.17	1,219	1,096	2,315	68.53
Eastern Samar	9,594	3,286	2,967	6,253	65.18	2,358	2,141	4,499	46.89	3,097	2,860	5,957	62.09
Leyte	31,504	8,102	7,584	15,686	49.79	7,286	6,915	14,201	45.08	7,229	7,139	14,368	45.61
Northern Samar	14,053	5,057	4,703	9,760	69.45	4,498	4,233	8,731	62.13	2,181	2,235	4,416	31.42
Southern Leyte	7,368	1,852	1,789	3,641	49.42	1,767	1,700	3,467	47.05	1,889	1,790	3,679	49.93
Samar	16,946	5,037	4,867	9,904	58.44	3,695	3,449	7,144	42.16	2,726	2,655	5,381	31.75
Ormoc City	5,344	1,572	1,420	2,992	55.99	1,620	1,498	3,118	58.35	1,471	1,378	2,849	53.31
City of Tacloban	5,526	2,314	2,482	4,796	86.79	2,588	2,562	5,150	93.20	1,343	1,282	2,625	47.50
**Region 9**	**85,348**	**16,883**	**16,343**	**33,226**	**38.93**	**13,355**	**12,990**	**26,345**	**30.87**	**19,807**	**20,109**	**39,916**	**46.77**
Zamboanga del Norte	24,977	6,452	6,130	12,582	50.37	5,199	5,096	10,295	41.22	6,214	6,280	12,494	50.02
Zamboanga del Sur	23,905	4,159	3,947	8,106	33.91	3,412	3,345	6,757	28.27	4,065	4,059	8,124	33.98
Zamboanga Sibugay	13,903	3,148	3,147	6,295	45.28	2,905	2,778	5,683	40.88	3,321	3,272	6,593	47.42
City of Isabela	3,209	1,047	967	2,014	62.76	824	748	1,572	48.99	707	667	1,374	42.82
City of Zamboanga	19,354	2,077	2,152	4,229	21.85	1,015	1,023	2,038	10.53	5,500	5,831	11,331	58.55
**Region 10**	**110,290**	**29,893**	**29,397**	**59,290**	**53.76**	**25,436**	**24,824**	**50,260**	**45.57**	**30,424**	**29,933**	**60,357**	**54.73**
Bukidnon	33,913	10,193	10,003	20,196	59.55	8,009	7,885	15,894	46.87	9,521	9,289	18,810	55.47
Camiguin	1,727	628	598	1,226	70.99	643	608	1,251	72.44	631	599	1,230	71.22
Lanao del Norte	17,881	4,914	5,019	9,933	55.55	2,875	2,751	5,626	31.46	3,622	3,680	7,302	40.84
Misamis Occidental	12,342	3,073	2,902	5,975	48.41	2,817	2,666	5,483	44.43	3,508	3,399	6,907	55.96
Misamis Oriental	22,184	5,912	5,843	11,755	52.99	5,900	5,819	11,719	52.83	6,085	6,073	12,158	54.81
City of Cagayan De Oro	14,503	3,153	2,990	6,143	42.36	3,441	3,265	6,706	46.24	5,223	5,012	10,235	70.57
City of Iligan	7,740	2,020	2,042	4,062	52.48	1,751	1,830	3,581	46.27	1,834	1,881	3,715	48.00
**Region 11**	**107,934**	**36,919**	**35,212**	**72,131**	**66.83**	**34,427**	**32,585**	**67,012**	**62.09**	**35,253**	**33,264**	**68,517**	**63.48**
Davao de Oro	15,434	5,337	5,054	10,391	67.33	5,036	4,784	9,820	63.63	5,242	4,984	10,226	66.26
Davao del Norte	23,685	7,730	7,113	14,843	62.67	7,583	7,022	14,605	61.66	7,726	7,185	14,911	62.96
Davao Oriental	12,025	3,957	3,768	7,725	64.24	3,811	3,593	7,404	61.57	3,688	3,472	7,160	59.54
Davao del Sur	13,178	4,197	4,187	8,384	63.62	4,154	3,998	8,152	61.86	3,901	3,763	7,664	58.16
Davao Occidental	6,606	1,834	1,769	3,603	54.54	1,486	1,347	2,833	42.89	1,821	1,710	3,531	53.45
City of Davao	37,006	13,864	13,321	27,185	73.46	12,357	11,841	24,198	65.39	12,875	12,150	25,025	67.62
**Region 12**	**95,296**	**29,299**	**28,424**	**57,723**	**60.57**	**26,543**	**25,906**	**52,449**	**55.04**	**28,275**	**27,299**	**55,574**	**58.32**
Cotabato	25,696	8,432	8,080	16,512	64.26	6,837	6,604	13,441	52.31	7,557	7,347	14,904	58.00
Sarangani	13,501	4,640	4,450	9,090	67.33	4,453	4,235	8,688	64.35	4,264	4,086	8,350	61.85
South Cotabato	21,842	7,133	6,909	14,042	64.29	6,751	6,525	13,276	60.78	7,175	6,880	14,055	64.35
Sultan Kudarat	19,613	5,936	5,856	11,792	60.12	5,591	5,588	11,179	57.00	5,604	5,450	11,054	56.36
City of General Santos	14,644	3,158	3,129	6,287	42.93	2,911	2,954	5,865	40.05	3,675	3,536	7,211	49.24
**CARAGA**	**55,371**	**19,031**	**18,252**	**37,283**	**67.33**	**17,119**	**16,515**	**33,634**	**60.74**	**16,067**	**15,638**	**31,705**	**57.26**
Agusan del Norte	7,331	3,063	2,914	5,977	81.53	2,799	2,640	5,439	74.19	1,857	1,894	3,751	51.17
Agusan del Sur	16,180	4,755	4,686	9,441	58.35	3,233	3,214	6,447	39.85	4,011	3,918	7,929	49.00
Surigao del Norte	10,168	3,972	3,648	7,620	74.94	3,882	3,637	7,519	73.95	3,013	2,973	5,986	58.87
Surigao del Sur	12,834	4,449	4,317	8,766	68.30	4,501	4,369	8,870	69.11	4,141	3,924	8,065	62.84
Dinagat Islands	2,117	478	457	935	44.17	399	416	815	38.50	510	463	973	45.96
City of Butuan	6,741	2,314	2,230	4,544	67.41	2,305	2,239	4,544	67.41	2,535	2,466	5,001	74.19
**BARMM**	**134,585**	**38,288**	**38,504**	**76,792**	**57.06**	**23,561**	**23,747**	**47,308**	**35.15**	**24,266**	**26,882**	**51,148**	**38.00**
Basilan	11,653	2,435	2,425	4,860	41.71	1,726	1,649	3,375	28.96	1,423	1,408	2,831	24.29
Lanao del Sur	33,339	9,710	9,533	19,243	57.72	8,204	8,182	16,386	49.15	7,261	7,334	14,595	43.78
Maguindanao del Norte	18,923	6,973	7,064	14,037	74.18	2,806	2,838	5,644	29.83	3,008	5,116	8,124	42.93
Maguindanao del Sur	23,214	8,198	8,206	16,404	70.66	3,916	4,055	7,971	34.34	4,173	4,174	8,347	35.96
Sulu	21,381	5,272	5,446	10,718	50.13	3,240	3,310	6,550	30.63	4,044	4,343	8,387	39.23
Tawi-Tawi	12,602	2,512	2,647	5,159	40.94	2,440	2,554	4,994	39.63	2,209	2,385	4,594	36.45
SGA	6,305	1,362	1,432	2,794	44.31	274	277	551	8.74	684	701	1,385	21.97
City of Cotabato	7,168	1,826	1,751	3,577	49.90	955	882	1,837	25.63	1,464	1,421	2,885	40.25

* = No Report

Table 2.C.1.2 - Immunization Services for Infants
3 doses of DPT-HiB-HepB antigen
Philippines, 2024

Area	Eligible Population (0-11mos. old)	DPT-HiB-HepB 1				DPT-HiB-HepB 2				DPT-HiB-HepB 3			
		Male	Female	Total	%	Male	Female	Total	%	Male	Female	Total	%
**PHILIPPINES**	**2,200,865**	**774,257**	**735,331**	**1,509,588**	**68.59**	**727,720**	**696,235**	**1,423,955**	**64.70**	**658,128**	**632,227**	**1,290,355**	**58.63**
**NCR**	**263,248**	**102,141**	**99,076**	**201,217**	**76.44**	**95,419**	**93,037**	**188,456**	**71.59**	**90,499**	**88,504**	**179,003**	**68.00**
City of Malabon	7,447	2,717	2,507	5,224	70.15	2,631	2,426	5,057	67.91	2,221	2,050	4,271	57.35
City of Navotas	5,263	2,791	2,740	5,531	105.09	2,774	2,772	5,546	105.38	2,688	2,769	5,457	103.69
City of Valenzuela	13,601	3,714	3,438	7,152	52.58	3,614	3,283	6,897	50.71	3,180	2,984	6,164	45.32
City of Caloocan	33,499	11,150	10,689	21,839	65.19	10,609	10,221	20,830	62.18	10,118	9,910	20,028	59.79
City of Marikina	8,399	2,436	2,455	4,891	58.23	2,392	2,325	4,717	56.16	2,157	2,075	4,232	50.39
City of Pasig	17,856	7,190	6,839	14,029	78.57	6,931	6,755	13,686	76.65	6,812	6,673	13,485	75.52
Pateros	1,120	423	467	890	79.46	427	463	890	79.46	415	418	833	74.38
City of Taguig	26,021	5,442	5,267	10,709	41.16	5,019	4,829	9,848	37.85	4,182	4,072	8,254	31.72
Quezon City	59,607	26,530	26,288	52,818	88.61	23,170	22,898	46,068	77.29	21,920	21,607	43,527	73.02
City of Makati	4,196	2,121	2,076	4,197	100.02	2,101	1,987	4,088	97.43	2,029	1,927	3,956	94.28
City of Mandaluyong	7,989	4,336	4,266	8,602	107.67	4,384	4,278	8,662	108.42	4,489	4,363	8,852	110.80
City of San Juan	2,173	654	596	1,250	57.52	599	576	1,175	54.07	574	556	1,130	52.00
City of Manila	34,268	15,904	15,084	30,988	90.43	14,844	14,436	29,280	85.44	14,874	14,104	28,978	84.56
City of Las Piñas	11,346	3,643	3,648	7,291	64.26	3,573	3,617	7,190	63.37	3,381	3,340	6,721	59.24
City of Muntinlupa	9,949	2,967	2,880	5,847	58.77	2,791	2,768	5,559	55.87	2,381	2,372	4,753	47.77
City of Parañaque	13,385	6,672	6,636	13,308	99.42	6,456	6,487	12,943	96.70	6,126	6,580	12,706	94.93
Pasay City	7,129	3,451	3,200	6,651	93.29	3,104	2,916	6,020	84.44	2,952	2,704	5,656	79.34
**CAR**	**31,490**	**11,711**	**11,003**	**22,714**	**72.13**	**11,510**	**10,811**	**22,321**	**70.88**	**10,961**	**10,166**	**21,127**	**67.09**
Abra	3,639	1,473	1,322	2,795	76.81	1,497	1,366	2,863	78.68	1,492	1,356	2,848	78.26
Apayao	2,222	893	857	1,750	78.76	796	756	1,552	69.85	770	702	1,472	66.25
Benguet	8,900	2,682	2,449	5,131	57.65	2,601	2,450	5,051	56.75	2,435	2,289	4,724	53.08
Ifugao	4,145	1,419	1,329	2,748	66.30	1,470	1,334	2,804	67.65	1,345	1,251	2,596	62.63
Kalinga	4,582	1,866	1,723	3,589	78.33	1,925	1,724	3,649	79.64	1,834	1,602	3,436	74.99
Mountain Province	2,615	1,014	972	1,986	75.95	986	952	1,938	74.11	937	888	1,825	69.79
City of Baguio	5,387	2,364	2,351	4,715	87.53	2,235	2,229	4,464	82.87	2,148	2,078	4,226	78.45
**Region 1**	**96,024**	**36,299**	**34,555**	**70,854**	**73.79**	**36,624**	**34,772**	**71,396**	**74.35**	**36,062**	**34,388**	**70,450**	**73.37**
Ilocos Norte	8,809	3,935	3,938	7,873	89.37	3,836	3,932	7,768	88.18	3,826	3,963	7,789	88.42
Ilocos Sur	9,834	4,771	4,581	9,352	95.10	4,720	4,551	9,271	94.27	4,905	4,726	9,631	97.94
La Union	13,436	4,828	4,539	9,367	69.72	4,972	4,665	9,637	71.73	5,087	4,670	9,757	72.62
Pangasinan	60,591	21,705	20,491	42,196	69.64	22,069	20,671	42,740	70.54	21,246	20,084	41,330	68.21
City of Dagupan	3,354	1,060	1,006	2,066	61.60	1,027	953	1,980	59.03	998	945	1,943	57.93
**Region 2**	**64,034**	**24,651**	**22,997**	**47,648**	**74.41**	**24,740**	**23,497**	**48,237**	**75.33**	**24,341**	**23,371**	**47,712**	**74.51**
Batanes	241	94	93	187	77.59	87	89	176	73.03	81	85	166	68.88
Cagayan	20,723	7,765	7,276	15,041	72.58	7,773	7,399	15,172	73.21	7,599	7,304	14,903	71.92
Isabela	27,362	10,532	9,748	20,280	74.12	10,638	10,078	20,716	75.71	10,513	9,990	20,503	74.93
Nueva Vizcaya	8,880	3,226	3,074	6,300	70.95	3,226	3,057	6,283	70.75	3,087	3,133	6,220	70.05
Quirino	3,865	1,533	1,429	2,962	76.64	1,542	1,433	2,975	76.97	1,595	1,472	3,067	79.35
City of Santiago	2,963	1,501	1,377	2,878	97.13	1,474	1,441	2,915	98.38	1,466	1,387	2,853	96.29
**Region 3**	**235,313**	**84,641**	**80,912**	**165,553**	**70.35**	**79,937**	**76,719**	**156,656**	**66.57**	**71,571**	**69,223**	**140,794**	**59.83**
Aurora	4,578	1,697	1,669	3,366	73.53	1,699	1,680	3,379	73.81	1,598	1,584	3,182	69.51
Bataan	17,556	5,972	5,658	11,630	66.25	5,818	5,544	11,362	64.72	5,310	5,078	10,388	59.17
Bulacan	68,115	27,131	26,005	53,136	78.01	26,620	25,456	52,076	76.45	25,119	24,238	49,357	72.46
Nueva Ecija	43,626	14,430	13,640	28,070	64.34	13,677	13,045	26,722	61.25	12,109	11,670	23,779	54.51
Pampanga	46,146	16,799	15,634	32,433	70.28	15,159	14,487	29,646	64.24	12,784	12,267	25,051	54.29
Tarlac	29,655	9,867	9,953	19,820	66.84	9,155	9,072	18,227	61.46	8,249	8,241	16,490	55.61
Zambales	11,796	3,897	3,871	7,768	65.85	3,606	3,416	7,022	59.53	2,906	2,725	5,631	47.74
City of Angeles	9,232	2,972	2,757	5,729	62.06	2,400	2,332	4,732	51.26	1,837	1,883	3,720	40.29
City of Olongapo	4,609	1,876	1,725	3,601	78.13	1,803	1,687	3,490	75.72	1,659	1,537	3,196	69.34
**Region 4A**	**310,150**	**112,770**	**105,754**	**218,524**	**70.46**	**107,471**	**101,805**	**209,276**	**67.48**	**99,026**	**94,620**	**193,646**	**62.44**
Batangas	53,484	18,869	17,509	36,378	68.02	17,947	16,744	34,691	64.86	15,646	14,890	30,536	57.09
Cavite	78,589	30,587	28,644	59,231	75.37	29,724	28,263	57,987	73.79	28,168	27,094	55,262	70.32
Laguna	61,967	23,768	22,565	46,333	74.77	22,345	21,390	43,735	70.58	20,680	20,004	40,684	65.65
Quezon	40,954	13,620	12,731	26,351	64.34	12,417	11,672	24,089	58.82	10,705	9,992	20,697	50.54
Rizal	68,903	23,898	22,403	46,301	67.20	23,166	21,955	45,121	65.48	22,218	21,119	43,337	62.90
City of Lucena	6,253	2,028	1,902	3,930	62.85	1,872	1,781	3,653	58.42	1,609	1,521	3,130	50.06
**Region 4B**	**66,508**	**23,177**	**21,588**	**44,765**	**67.31**	**20,570**	**19,280**	**39,850**	**59.92**	**16,820**	**16,008**	**32,828**	**49.36**
Marinduque	4,124	1,537	1,448	2,985	72.38	1,430	1,433	2,863	69.42	1,357	1,319	2,676	64.89
Occidental Mindoro	11,748	4,439	4,006	8,445	71.88	4,041	3,550	7,591	64.62	3,609	3,304	6,913	58.84
Oriental Mindoro	19,007	6,347	6,012	12,359	65.02	6,091	5,693	11,784	62.00	5,122	4,920	10,042	52.83
Palawan	20,503	6,742	6,275	13,017	63.49	5,353	5,172	10,525	51.33	3,843	3,653	7,496	36.56
Romblon	5,488	2,115	1,998	4,113	74.95	2,036	1,890	3,926	71.54	1,742	1,695	3,437	62.63
City of Puerto Princesa	5,638	1,997	1,849	3,846	68.22	1,619	1,542	3,161	56.07	1,147	1,117	2,264	40.16
**Region 5**	**136,611**	**40,648**	**38,055**	**78,703**	**57.61**	**33,209**	**31,449**	**64,658**	**47.33**	**24,870**	**23,653**	**48,523**	**35.52**
Albay	27,665	8,394	7,999	16,393	59.26	7,310	6,843	14,153	51.16	6,076	5,871	11,947	43.18
Camarines Norte	14,894	3,620	3,469	7,089	47.60	3,011	2,805	5,816	39.05	2,054	1,975	4,029	27.05
Camarines Sur	43,855	12,084	11,100	23,184	52.87	9,459	9,002	18,461	42.10	6,432	6,044	12,476	28.45
Catanduanes	5,395	1,840	1,767	3,607	66.86	1,827	1,745	3,572	66.21	1,693	1,646	3,339	61.89
Masbate	22,495	7,014	6,562	13,576	60.35	4,543	4,377	8,920	39.65	2,824	2,553	5,377	23.90
Sorsogon	18,065	6,454	6,004	12,458	68.96	5,900	5,607	11,507	63.70	4,788	4,665	9,453	52.33
City of Naga	4,242	1,242	1,154	2,396	56.48	1,159	1,070	2,229	52.55	1,003	899	1,902	44.84

Table 2.C.1.2 - Immunization Services for Infants
3 doses of DPT-HiB-HepB antigen
Philippines, 2024

Area	Eligible Population (0-11mos. old)	DPT-HiB-HepB 1				DPT-HiB-HepB 2				DPT-HiB-HepB 3			
		Male	Female	Total	%	Male	Female	Total	%	Male	Female	Total	%
**Region 6**	**147,934**	**49,658**	**46,604**	**96,262**	**65.07**	**48,034**	**45,566**	**93,600**	**63.27**	**43,333**	**41,230**	**84,563**	**57.16**
Aklan	11,281	3,703	3,389	7,092	62.87	3,515	3,202	6,717	59.54	3,051	2,817	5,868	52.02
Antique	11,899	4,166	3,859	8,025	67.44	4,013	3,756	7,769	65.29	3,307	3,184	6,491	54.55
Capiz	13,872	4,368	4,201	8,569	61.77	4,535	4,406	8,941	64.45	4,214	3,938	8,152	58.77
Guimaras	3,361	1,141	1,111	2,252	67.00	1,104	1,113	2,217	65.96	987	983	1,970	58.61
Iloilo	35,981	12,385	11,451	23,836	66.25	12,434	12,082	24,516	68.14	12,177	11,710	23,887	66.39
Negros Occidental	52,445	17,501	16,517	34,018	64.86	16,122	15,070	31,192	59.48	13,470	12,909	26,379	50.30
City of Bacolod	10,361	3,498	3,274	6,772	65.36	3,327	3,117	6,444	62.19	3,148	2,885	6,033	58.23
City of Iloilo	8,734	2,896	2,802	5,698	65.24	2,984	2,820	5,804	66.45	2,979	2,804	5,783	66.21
**Region 7**	**167,016**	**57,865**	**53,283**	**111,148**	**66.55**	**55,619**	**52,308**	**107,927**	**64.62**	**50,132**	**46,808**	**96,940**	**58.04**
Bohol	26,210	8,453	7,864	16,317	62.25	8,134	7,701	15,835	60.42	7,367	6,982	14,349	54.75
Cebu	72,875	24,209	22,137	46,346	63.60	23,256	21,947	45,203	62.03	20,688	19,296	39,984	54.87
Negros Oriental	28,980	10,758	10,060	20,818	71.84	10,382	9,749	20,131	69.47	9,280	8,764	18,044	62.26
Siquijor	1,671	550	501	1,051	62.90	546	513	1,059	63.38	540	498	1,038	62.12
City of Cebu	19,466	7,148	6,429	13,577	69.75	6,677	6,140	12,817	65.84	5,961	5,486	11,447	58.81
City of Lapu-Lapu	10,833	3,934	3,704	7,638	70.51	3,879	3,678	7,557	69.76	3,572	3,231	6,803	62.80
City of Mandaue	6,981	2,813	2,588	5,401	77.37	2,745	2,580	5,325	76.28	2,724	2,551	5,275	75.56
**Region 8**	**93,713**	**29,739**	**27,370**	**57,109**	**60.94**	**24,333**	**22,864**	**47,197**	**50.36**	**15,917**	**15,177**	**31,094**	**33.18**
Biliran	3,378	1,449	1,458	2,907	86.06	1,352	1,261	2,613	77.35	1,067	992	2,059	60.95
Eastern Samar	9,594	3,390	3,001	6,391	66.61	2,824	2,542	5,366	55.93	1,663	1,548	3,211	33.47
Leyte	31,504	9,365	8,556	17,921	56.88	7,389	6,988	14,377	45.64	4,727	4,430	9,157	29.07
Northern Samar	14,053	4,737	4,477	9,214	65.57	3,945	3,857	7,802	55.52	2,482	2,457	4,939	35.15
Southern Leyte	7,368	2,259	2,023	4,282	58.12	2,127	1,919	4,046	54.91	1,607	1,472	3,079	41.79
Samar	16,946	5,396	4,935	10,331	60.96	3,827	3,610	7,437	43.89	2,064	2,130	4,194	24.75
Ormoc City	5,344	1,706	1,511	3,217	60.20	1,618	1,448	3,066	57.37	1,268	1,142	2,410	45.10
City of Tacloban	5,526	1,437	1,409	2,846	51.50	1,251	1,239	2,490	45.06	1,039	1,006	2,045	37.01
**Region 9**	**85,348**	**29,511**	**28,218**	**57,729**	**67.64**	**28,122**	**27,424**	**55,546**	**65.08**	**26,072**	**25,080**	**51,152**	**59.93**
Zamboanga del Norte	24,977	8,570	7,954	16,524	66.16	8,216	7,859	16,075	64.36	7,772	7,293	15,065	60.32
Zamboanga del Sur	23,905	7,379	7,121	14,500	60.66	7,198	6,908	14,106	59.01	6,545	6,327	12,872	53.85
Zamboanga Sibugay	13,903	4,503	4,455	8,958	64.43	4,173	4,391	8,564	61.60	3,954	3,883	7,837	56.37
City of Isabela	3,209	1,127	1,070	2,197	68.46	1,128	1,114	2,242	69.87	1,110	1,060	2,170	67.62
City of Zamboanga	19,354	7,932	7,618	15,550	80.35	7,407	7,152	14,559	75.22	6,691	6,517	13,208	68.24
**Region 10**	**110,290**	**35,058**	**34,334**	**69,392**	**62.92**	**29,175**	**28,203**	**57,378**	**52.02**	**21,874**	**21,203**	**43,077**	**39.06**
Bukidnon	33,913	12,027	11,501	23,528	69.38	9,637	9,146	18,783	55.39	6,809	6,377	13,186	38.88
Camiguin	1,727	616	598	1,214	70.30	544	533	1,077	62.36	395	362	757	43.83
Lanao del Norte	17,881	4,965	5,136	10,101	56.49	4,462	4,459	8,921	49.89	3,515	3,602	7,117	39.80
Misamis Occidental	12,342	3,876	3,685	7,561	61.26	3,309	3,126	6,435	52.14	2,548	2,455	5,003	40.54
Misamis Oriental	22,184	6,358	6,254	12,612	56.85	5,302	5,149	10,451	47.11	4,150	4,136	8,286	37.35
City of Cagayan De Oro	14,503	4,865	4,812	9,677	66.72	3,983	3,828	7,811	53.86	2,919	2,751	5,670	39.10
City of Iligan	7,740	2,351	2,348	4,699	60.71	1,938	1,962	3,900	50.39	1,538	1,520	3,058	39.51
**Region 11**	**107,934**	**38,150**	**36,194**	**74,344**	**68.88**	**36,350**	**34,677**	**71,027**	**65.81**	**34,288**	**32,334**	**66,622**	**61.72**
Davao de Oro	15,434	5,664	5,428	11,092	71.87	5,167	5,034	10,201	66.09	4,518	4,344	8,862	57.42
Davao del Norte	23,685	8,147	7,686	15,833	66.85	8,010	7,620	15,630	65.99	7,999	7,577	15,576	65.76
Davao Oriental	12,025	4,311	4,071	8,382	69.70	4,290	3,928	8,218	68.34	4,010	3,648	7,658	63.68
Davao del Sur	13,178	4,810	4,676	9,486	71.98	4,709	4,655	9,364	71.06	4,602	4,371	8,973	68.09
Davao Occidental	6,606	2,283	2,166	4,449	67.35	2,313	2,147	4,460	67.51	2,226	2,088	4,314	65.30
City of Davao	37,006	12,935	12,167	25,102	67.83	11,861	11,293	23,154	62.57	10,933	10,306	21,239	57.39
**Region 12**	**95,296**	**33,958**	**32,258**	**66,216**	**69.48**	**34,036**	**32,070**	**66,106**	**69.37**	**33,239**	**31,580**	**64,819**	**68.02**
Cotabato	25,696	10,001	9,413	19,414	75.55	10,035	9,509	19,544	76.06	9,971	9,411	19,382	75.43
Sarangani	13,501	5,015	4,696	9,711	71.93	5,021	4,557	9,578	70.94	4,866	4,576	9,442	69.94
South Cotabato	21,842	7,493	7,107	14,600	66.84	7,546	7,171	14,717	67.38	7,455	7,079	14,534	66.54
Sultan Kudarat	19,613	6,666	6,355	13,021	66.39	6,638	6,380	13,018	66.37	6,475	6,329	12,804	65.28
City of General Santos	14,644	4,783	4,687	9,470	64.67	4,796	4,453	9,249	63.16	4,472	4,185	8,657	59.12
**CARAGA**	**55,371**	**20,010**	**18,888**	**38,898**	**70.25**	**18,065**	**17,229**	**35,294**	**63.74**	**15,176**	**14,435**	**29,611**	**53.48**
Agusan del Norte	7,331	2,803	2,672	5,475	74.68	2,740	2,619	5,359	73.10	2,505	2,468	4,973	67.84
Agusan del Sur	16,180	5,732	5,511	11,243	69.49	5,284	5,062	10,346	63.94	4,662	4,462	9,124	56.39
Surigao del Norte	10,168	3,718	3,363	7,081	69.64	3,368	3,145	6,513	64.05	2,738	2,564	5,302	52.14
Surigao del Sur	12,834	4,481	4,244	8,725	67.98	3,692	3,586	7,278	56.71	2,792	2,564	5,356	41.73
Dinagat Islands	2,117	735	666	1,401	66.18	663	618	1,281	60.51	583	529	1,112	52.53
City of Butuan	6,741	2,541	2,432	4,973	73.77	2,318	2,199	4,517	67.01	1,896	1,848	3,744	55.54
**BARMM**	**134,585**	**44,270**	**44,242**	**88,512**	**65.77**	**44,506**	**44,524**	**89,030**	**66.15**	**43,947**	**44,447**	**88,394**	**65.68**
Basilan	11,653	3,065	2,902	5,967	51.21	3,114	2,873	5,987	51.38	2,671	2,637	5,308	45.55
Lanao del Sur	33,339	11,504	11,409	22,913	68.73	11,607	11,477	23,084	69.24	11,535	11,545	23,080	69.23
Maguindanao del Norte	18,923	7,896	7,760	15,656	82.74	7,943	7,958	15,901	84.03	8,160	8,137	16,297	86.12
Maguindanao del Sur	23,214	8,542	8,492	17,034	73.38	8,586	8,570	17,156	73.90	8,594	8,617	17,211	74.14
Sulu	21,381	6,388	6,516	12,904	60.35	6,135	6,424	12,559	58.74	5,943	6,302	12,245	57.27
Tawi-Tawi	12,602	2,919	3,298	6,217	49.33	3,216	3,372	6,588	52.28	3,164	3,307	6,471	51.35
SGA	6,305	1,732	1,781	3,513	55.72	1,681	1,716	3,397	53.88	1,638	1,680	3,318	52.62
City of Cotabato	7,168	2,224	2,084	4,308	60.10	2,224	2,134	4,358	60.80	2,242	2,222	4,464	62.28

* = No Report

Table 2.C.1.3 - Immunization Services for Infants and Children
3 doses of Oral Polio Vaccine (OPV)
Philippines, 2024

Area	Eligible Population (0-11mos. old)	OPV 1				OPV 2				OPV 3			
		Male	Female	Total	%	Male	Female	Total	%	Male	Female	Total	%
**PHILIPPINES**	**2,200,865**	**825,603**	**783,193**	**1,608,796**	**73.10**	**855,364**	**816,503**	**1,671,867**	**75.96**	**879,740**	**841,513**	**1,721,253**	**78.21**
**NCR**	**263,248**	**108,035**	**104,530**	**212,565**	**80.75**	**105,716**	**102,197**	**207,913**	**78.98**	**104,712**	**101,772**	**206,484**	**78.44**
City of Malabon	7,447	2,674	2,399	5,073	68.12	2,687	2,434	5,121	68.77	2,497	2,317	4,814	64.64
City of Navotas	5,263	2,760	2,720	5,480	104.12	2,742	2,723	5,465	103.84	2,763	2,802	5,565	105.74
City of Valenzuela	13,601	4,125	3,906	8,031	59.05	4,074	3,791	7,865	57.83	4,165	3,844	8,009	58.89
City of Caloocan	33,499	10,796	10,183	20,979	62.63	11,143	10,710	21,853	65.23	11,250	10,981	22,231	66.36
City of Marikina	8,399	2,527	2,514	5,041	60.02	2,468	2,432	4,900	58.34	2,392	2,276	4,668	55.58
City of Pasig	17,856	7,024	6,613	13,637	76.37	6,805	6,700	13,505	75.63	6,877	6,819	13,696	76.70
Pateros	1,120	451	468	919	82.05	476	456	932	83.21	488	459	947	84.55
City of Taguig	26,021	6,132	6,025	12,157	46.72	5,933	5,755	11,688	44.92	5,786	5,727	11,513	44.25
Quezon City	59,607	30,362	29,954	60,316	101.19	28,749	28,279	57,028	95.67	28,418	27,872	56,290	94.44
City of Makati	4,196	2,108	2,055	4,163	99.21	2,087	2,007	4,094	97.57	2,123	2,029	4,152	98.95
City of Mandaluyong	7,989	4,475	4,386	8,861	110.92	4,422	4,319	8,741	109.41	4,449	4,330	8,779	109.89
City of San Juan	2,173	657	596	1,253	57.66	607	588	1,195	54.99	614	574	1,188	54.67
City of Manila	34,268	16,011	15,138	31,149	90.90	14,926	14,578	29,504	86.10	15,053	14,314	29,367	85.70
City of Las Piñas	11,346	4,101	4,012	8,113	71.51	4,200	4,137	8,337	73.48	4,282	4,276	8,558	75.43
City of Muntinlupa	9,949	3,408	3,201	6,609	66.43	4,138	3,250	7,388	74.26	3,387	3,273	6,660	66.94
City of Parañaque	13,385	6,787	7,031	13,818	103.23	6,880	6,911	13,791	103.03	6,867	6,896	13,763	102.82
Pasay City	7,129	3,637	3,329	6,966	97.71	3,379	3,127	6,506	91.26	3,301	2,983	6,284	88.15
**CAR**	**31,490**	**12,292**	**11,505**	**23,797**	**75.57**	**13,311**	**12,485**	**25,796**	**81.92**	**13,802**	**12,933**	**26,735**	**84.90**
Abra	3,639	1,554	1,422	2,976	81.78	1,748	1,625	3,373	92.69	1,752	1,661	3,413	93.79
Apayao	2,222	930	841	1,771	79.70	936	877	1,813	81.59	1,045	973	2,018	90.82
Benguet	8,900	2,856	2,684	5,540	62.25	3,190	2,966	6,156	69.17	3,308	3,071	6,379	71.67
Ifugao	4,145	1,519	1,444	2,963	71.48	1,663	1,504	3,167	76.41	1,638	1,548	3,186	76.86
Kalinga	4,582	1,932	1,705	3,637	79.38	2,219	1,973	4,192	91.49	2,366	2,075	4,441	96.92
Mountain Province	2,615	1,084	1,024	2,108	80.61	1,151	1,116	2,267	86.69	1,188	1,120	2,308	88.26
City of Baguio	5,387	2,417	2,385	4,802	89.14	2,404	2,424	4,828	89.62	2,505	2,485	4,990	92.63
**Region 1**	**96,024**	**36,402**	**34,548**	**70,950**	**73.89**	**37,625**	**36,043**	**73,668**	**76.72**	**39,721**	**37,798**	**77,519**	**80.73**
Ilocos Norte	8,809	3,844	3,869	7,713	87.56	3,908	3,973	7,881	89.47	4,151	4,272	8,423	95.62
Ilocos Sur	9,834	4,649	4,438	9,087	92.40	4,690	4,578	9,268	94.24	5,009	4,818	9,827	99.93
La Union	13,436	4,761	4,432	9,193	68.42	4,897	4,590	9,487	70.61	5,150	4,694	9,844	73.27
Pangasinan	60,591	22,063	20,790	42,853	70.73	23,019	21,869	44,888	74.08	24,241	22,912	47,153	77.82
City of Dagupan	3,354	1,085	1,019	2,104	62.73	1,111	1,033	2,144	63.92	1,170	1,102	2,272	67.74
**Region 2**	**64,034**	**24,975**	**23,333**	**48,308**	**75.44**	**26,983**	**25,564**	**52,547**	**82.06**	**29,073**	**27,633**	**56,706**	**88.56**
Batanes	241	94	95	189	78.42	89	90	179	74.27	99	91	190	78.84
Cagayan	20,723	8,027	7,406	15,433	74.47	8,869	8,456	17,325	83.60	10,148	9,705	19,853	95.80
Isabela	27,362	10,534	9,815	20,349	74.37	11,158	10,531	21,689	79.27	11,750	11,089	22,839	83.47
Nueva Vizcaya	8,880	3,306	3,193	6,499	73.19	3,683	3,522	7,205	81.14	3,717	3,609	7,326	82.50
Quirino	3,865	1,533	1,426	2,959	76.56	1,719	1,553	3,272	84.66	1,872	1,766	3,638	94.13
City of Santiago	2,963	1,481	1,398	2,879	97.17	1,465	1,412	2,877	97.10	1,487	1,373	2,860	96.52
**Region 3**	**235,313**	**91,010**	**86,503**	**177,513**	**75.44**	**93,325**	**89,408**	**182,733**	**77.66**	**98,675**	**94,930**	**193,605**	**82.28**
Aurora	4,578	1,765	1,697	3,462	75.62	1,864	1,829	3,693	80.67	1,955	1,930	3,885	84.86
Bataan	17,556	6,219	5,945	12,164	69.29	6,513	6,207	12,720	72.45	7,057	6,685	13,742	78.28
Bulacan	68,115	28,509	27,065	55,574	81.59	29,139	27,905	57,044	83.75	30,363	29,124	59,487	87.33
Nueva Ecija	43,626	15,400	14,467	29,867	68.46	16,240	15,503	31,743	72.76	17,855	17,206	35,061	80.37
Pampanga	46,146	18,335	17,046	35,381	76.67	18,337	17,430	35,767	77.51	19,250	18,562	37,812	81.94
Tarlac	29,655	11,423	11,447	22,870	77.12	11,847	11,713	23,560	79.45	12,761	12,546	25,307	85.34
Zambales	11,796	4,277	4,160	8,437	71.52	4,326	4,130	8,456	71.69	4,238	4,007	8,245	69.90
City of Angeles	9,232	3,410	3,132	6,542	70.86	3,279	3,065	6,344	68.72	3,372	3,205	6,577	71.24
City of Olongapo	4,609	1,672	1,544	3,216	69.78	1,780	1,626	3,406	73.90	1,824	1,665	3,489	75.70
**Region 4A**	**310,150**	**117,179**	**109,728**	**226,907**	**73.16**	**116,729**	**110,476**	**227,205**	**73.26**	**115,122**	**110,106**	**225,228**	**72.62**
Batangas	53,484	20,120	18,653	38,773	72.49	20,479	19,152	39,631	74.10	20,111	19,219	39,330	73.54
Cavite	78,589	30,800	28,896	59,696	75.96	30,783	29,378	60,161	76.55	30,809	29,500	60,309	76.74
Laguna	61,967	25,528	24,003	49,531	79.93	25,112	23,935	49,047	79.15	24,620	24,060	48,680	78.56
Quezon	40,954	14,440	13,362	27,802	67.89	14,462	13,573	28,035	68.45	14,235	13,288	27,523	67.20
Rizal	68,903	24,056	22,730	46,786	67.90	23,708	22,431	46,139	66.96	23,259	22,085	45,344	65.81
City of Lucena	6,253	2,235	2,084	4,319	69.07	2,185	2,007	4,192	67.04	2,088	1,954	4,042	64.64
**Region 4B**	**66,508**	**26,409**	**24,704**	**51,113**	**76.85**	**27,861**	**25,987**	**53,848**	**80.96**	**28,584**	**27,003**	**55,587**	**83.58**
Marinduque	4,124	1,563	1,444	3,007	72.91	1,530	1,535	3,065	74.32	1,528	1,500	3,028	73.42
Occidental Mindoro	11,748	5,209	4,739	9,948	84.68	5,621	5,103	10,724	91.28	5,872	5,415	11,287	96.08
Oriental Mindoro	19,007	6,650	6,247	12,897	67.85	6,953	6,444	13,397	70.48	6,917	6,576	13,493	70.99
Palawan	20,503	8,481	7,981	16,462	80.29	9,167	8,559	17,726	86.46	9,627	9,123	18,750	91.45
Romblon	5,488	2,145	2,021	4,166	75.91	2,216	2,089	4,305	78.44	2,246	2,082	4,328	78.86
City of Puerto Princesa	5,638	2,361	2,272	4,633	82.17	2,374	2,257	4,631	82.14	2,394	2,307	4,701	83.38
**Region 5**	**136,611**	**47,047**	**44,089**	**91,136**	**66.71**	**52,711**	**49,028**	**101,739**	**74.47**	**55,409**	**51,921**	**107,330**	**78.57**
Albay	27,665	9,435	8,894	18,329	66.25	10,131	9,607	19,738	71.35	10,589	10,233	20,822	75.26
Camarines Norte	14,894	4,688	4,315	9,003	60.45	5,161	4,859	10,020	67.28	5,465	5,122	10,587	71.08
Camarines Sur	43,855	15,007	14,055	29,062	66.27	17,499	16,248	33,747	76.95	18,110	17,017	35,127	80.10
Catanduanes	5,395	1,849	1,786	3,635	67.38	1,864	1,802	3,666	67.95	1,896	1,845	3,741	69.34
Masbate	22,495	7,893	7,457	15,350	68.24	9,453	8,411	17,864	79.41	10,076	8,899	18,975	84.35
Sorsogon	18,065	6,754	6,306	13,060	72.29	7,219	6,823	14,042	77.73	7,894	7,520	15,414	85.33
City of Naga	4,242	1,421	1,276	2,697	63.58	1,384	1,278	2,662	62.75	1,379	1,285	2,664	62.80

Table 2.C.1.3 - Immunization Services for Infants and Children
3 doses of Oral Polio Vaccine (OPV)
Philippines, 2024

Area	Eligible Population (0-11mos. old)	OPV 1				OPV 2				OPV 3			
		Male	Female	Total	%	Male	Female	Total	%	Male	Female	Total	%
**Region 6**	**147,934**	**52,803**	**49,428**	**102,231**	**69.11**	**56,204**	**53,361**	**109,565**	**74.06**	**59,142**	**55,786**	**114,928**	**77.69**
Aklan	11,281	3,885	3,514	7,399	65.59	4,143	3,791	7,934	70.33	4,215	3,927	8,142	72.17
Antique	11,899	4,567	4,203	8,770	73.70	5,215	4,993	10,208	85.79	5,503	5,256	10,759	90.42
Capiz	13,872	4,371	4,217	8,588	61.91	4,605	4,571	9,176	66.15	4,846	4,560	9,406	67.81
Guimaras	3,361	1,200	1,173	2,373	70.60	1,386	1,350	2,736	81.40	1,430	1,393	2,823	83.99
Iloilo	35,981	12,442	11,591	24,033	66.79	12,915	12,362	25,277	70.25	13,017	12,445	25,462	70.77
Negros Occidental	52,445	19,631	18,394	38,025	72.50	20,579	19,372	39,951	76.18	21,953	20,584	42,537	81.11
City of Bacolod	10,361	3,752	3,499	7,251	69.98	4,003	3,763	7,766	74.95	4,551	4,160	8,711	84.07
City of Iloilo	8,734	2,955	2,837	5,792	66.32	3,358	3,159	6,517	74.62	3,627	3,461	7,088	81.15
**Region 7**	**167,016**	**61,460**	**57,471**	**118,931**	**71.21**	**66,880**	**62,973**	**129,853**	**77.75**	**71,698**	**67,129**	**138,827**	**83.12**
Bohol	26,210	8,940	8,386	17,326	66.10	9,937	9,352	19,289	73.59	11,039	10,344	21,383	81.58
Cebu	72,875	25,917	24,364	50,281	69.00	29,550	27,875	57,425	78.80	32,501	30,329	62,830	86.22
Negros Oriental	28,980	11,422	10,732	22,154	76.45	12,263	11,693	23,956	82.66	13,106	12,451	25,557	88.19
Siquijor	1,671	551	495	1,046	62.60	541	506	1,047	62.66	552	522	1,074	64.27
City of Cebu	19,466	7,358	6,649	14,007	71.96	7,321	6,629	13,950	71.66	7,223	6,647	13,870	71.25
City of Lapu-Lapu	10,833	4,504	4,298	8,802	81.25	4,502	4,281	8,783	81.08	4,424	4,122	8,546	78.89
City of Mandaue	6,981	2,768	2,547	5,315	76.14	2,766	2,637	5,403	77.40	2,853	2,714	5,567	79.75
**Region 8**	**93,713**	**34,296**	**31,828**	**66,124**	**70.56**	**37,109**	**35,048**	**72,157**	**77.00**	**38,021**	**36,280**	**74,301**	**79.29**
Biliran	3,378	1,501	1,394	2,895	85.70	1,578	1,432	3,010	89.11	1,605	1,452	3,057	90.50
Eastern Samar	9,594	3,685	3,366	7,051	73.49	3,945	3,675	7,620	79.42	4,115	3,920	8,035	83.75
Leyte	31,504	11,437	10,525	21,962	69.71	12,960	12,218	25,178	79.92	13,332	12,519	25,851	82.06
Northern Samar	14,053	5,258	4,996	10,254	72.97	5,688	5,540	11,228	79.90	5,841	5,880	11,721	83.41
Southern Leyte	7,368	2,314	2,046	4,360	59.17	2,550	2,253	4,803	65.19	2,772	2,543	5,315	72.14
Samar	16,946	6,466	6,124	12,590	74.29	6,694	6,445	13,139	77.53	6,564	6,383	12,947	76.40
Ormoc City	5,344	1,810	1,628	3,438	64.33	1,906	1,685	3,591	67.20	1,995	1,822	3,817	71.43
City of Tacloban	5,526	1,825	1,749	3,574	64.68	1,788	1,800	3,588	64.93	1,797	1,761	3,558	64.39
**Region 9**	**85,348**	**30,411**	**29,272**	**59,683**	**69.93**	**30,246**	**29,417**	**59,663**	**69.91**	**30,843**	**29,975**	**60,818**	**71.26**
Zamboanga del Norte	24,977	8,420	7,975	16,395	65.64	8,210	7,711	15,921	63.74	8,148	7,849	15,997	64.05
Zamboanga del Sur	23,905	7,453	7,113	14,566	60.93	7,531	7,431	14,962	62.59	7,627	7,420	15,047	62.94
Zamboanga Sibugay	13,903	4,839	4,855	9,694	69.73	4,827	4,919	9,746	70.10	5,325	5,269	10,594	76.20
City of Isabela	3,209	1,238	1,190	2,428	75.66	1,265	1,252	2,517	78.44	1,344	1,263	2,607	81.24
City of Zamboanga	19,354	8,461	8,139	16,600	85.77	8,413	8,104	16,517	85.34	8,399	8,174	16,573	85.63
**Region 10**	**110,290**	**39,785**	**38,303**	**78,088**	**70.80**	**40,327**	**39,060**	**79,387**	**71.98**	**40,916**	**39,320**	**80,236**	**72.75**
Bukidnon	33,913	13,358	12,590	25,948	76.51	13,189	12,644	25,833	76.17	12,946	12,082	25,028	73.80
Camiguin	1,727	657	649	1,306	75.62	700	689	1,389	80.43	742	676	1,418	82.11
Lanao del Norte	17,881	6,106	6,067	12,173	68.08	6,304	6,230	12,534	70.10	6,336	6,348	12,684	70.94
Misamis Occidental	12,342	4,251	4,082	8,333	67.52	4,358	4,219	8,577	69.49	4,637	4,341	8,978	72.74
Misamis Oriental	22,184	6,906	6,625	13,531	60.99	7,106	6,936	14,042	63.30	7,509	7,328	14,837	66.88
City of Cagayan De Oro	14,503	5,776	5,600	11,376	78.44	5,733	5,499	11,232	77.45	5,746	5,563	11,309	77.98
City of Iligan	7,740	2,731	2,690	5,421	70.04	2,937	2,843	5,780	74.68	3,000	2,982	5,982	77.29
**Region 11**	**107,934**	**39,236**	**37,591**	**76,827**	**71.18**	**39,410**	**37,576**	**76,986**	**71.33**	**40,097**	**37,663**	**77,760**	**72.04**
Davao de Oro	15,434	5,803	5,570	11,373	73.69	5,924	5,708	11,632	75.37	5,937	5,709	11,646	75.46
Davao del Norte	23,685	8,084	7,646	15,730	66.41	8,054	7,712	15,766	66.57	8,146	7,693	15,839	66.87
Davao Oriental	12,025	4,407	4,118	8,525	70.89	4,568	4,245	8,813	73.29	4,712	4,250	8,962	74.53
Davao del Sur	13,178	4,814	4,664	9,478	71.92	4,844	4,699	9,543	72.42	5,072	4,790	9,862	74.84
Davao Occidental	6,606	2,321	2,197	4,518	68.39	2,496	2,343	4,839	73.25	2,435	2,307	4,742	71.78
City of Davao	37,006	13,807	13,396	27,203	73.51	13,524	12,869	26,393	71.32	13,795	12,914	26,709	72.17
**Region 12**	**95,296**	**34,518**	**32,751**	**67,269**	**70.59**	**36,643**	**34,455**	**71,098**	**74.61**	**37,040**	**35,133**	**72,173**	**75.74**
Cotabato	25,696	10,036	9,403	19,439	75.65	10,298	9,752	20,050	78.03	10,409	9,638	20,047	78.02
Sarangani	13,501	5,200	4,804	10,004	74.10	5,569	5,015	10,584	78.39	5,541	5,200	10,741	79.56
South Cotabato	21,842	7,752	7,299	15,051	68.91	8,388	7,855	16,243	74.37	8,374	8,021	16,395	75.06
Sultan Kudarat	19,613	6,760	6,475	13,235	67.48	7,259	6,958	14,217	72.49	7,582	7,394	14,976	76.36
City of General Santos	14,644	4,770	4,770	9,540	65.15	5,129	4,875	10,004	68.31	5,134	4,880	10,014	68.38
**CARAGA**	**55,371**	**21,837**	**20,285**	**42,122**	**76.07**	**23,495**	**22,194**	**45,689**	**82.51**	**23,514**	**22,293**	**45,807**	**82.73**
Agusan del Norte	7,331	2,966	2,747	5,713	77.93	3,215	2,977	6,192	84.46	3,352	3,199	6,551	89.36
Agusan del Sur	16,180	6,359	5,988	12,347	76.31	6,909	6,778	13,687	84.59	6,539	6,380	12,919	79.85
Surigao del Norte	10,168	3,981	3,684	7,665	75.38	4,305	4,023	8,328	81.90	4,521	4,224	8,745	86.01
Surigao del Sur	12,834	5,038	4,661	9,699	75.57	5,457	5,107	10,564	82.31	5,418	5,122	10,540	82.13
Dinagat Islands	2,117	766	664	1,430	67.55	856	780	1,636	77.28	855	803	1,658	78.32
City of Butuan	6,741	2,727	2,541	5,268	78.15	2,753	2,529	5,282	78.36	2,829	2,565	5,394	80.02
**BARMM**	**134,585**	**47,908**	**47,324**	**95,232**	**70.76**	**50,789**	**51,231**	**102,020**	**75.80**	**53,371**	**53,838**	**107,209**	**79.66**
Basilan	11,653	3,242	3,133	6,375	54.71	3,596	3,586	7,182	61.63	3,577	3,563	7,140	61.27
Lanao del Sur	33,339	11,965	11,830	23,795	71.37	12,114	11,955	24,069	72.19	12,298	12,107	24,405	73.20
Maguindanao del Norte	18,923	8,475	8,245	16,720	88.36	9,531	9,500	19,031	100.57	10,222	10,141	20,363	107.61
Maguindanao del Sur	23,214	9,298	9,050	18,348	79.04	10,006	9,917	19,923	85.82	10,548	10,802	21,350	91.97
Sulu	21,381	6,672	6,898	13,570	63.47	6,969	7,394	14,363	67.18	7,606	7,912	15,518	72.58
Tawi-Tawi	12,602	3,884	4,070	7,954	63.12	4,153	4,461	8,614	68.35	4,522	4,828	9,350	74.19
SGA	6,305	2,086	1,976	4,062	64.43	2,010	2,041	4,051	64.25	1,911	1,880	3,791	60.13
City of Cotabato	7,168	2,286	2,122	4,408	61.50	2,410	2,377	4,787	66.78	2,687	2,605	5,292	73.83

** = No Report*

Table 2.C.1.4 - Immunization Services for Infants and Children
2 doses of Inactivated Polio Vaccine (IPV)
Philippines, 2024

Area	Eligible Population (0-11mos. old)	IPV 1				IPV 2			
		Male	Female	Total	%	Male	Female	Total	%
**Philippines**	**2,200,865**	**809,931**	**769,943**	**1,579,874**	**71.78**	**698,647**	**663,500**	**1,362,147**	**61.89**
**NCR**	**263,248**	**101,194**	**97,951**	**199,145**	**75.65**	**97,615**	**94,431**	**192,046**	**72.95**
City of Malabon	7,447	2,602	2,368	4,970	66.74	2,321	2,175	4,496	60.37
City of Navotas	5,263	2,291	2,239	4,530	86.07	2,461	2,429	4,890	92.91
City of Valenzuela	13,601	4,012	3,627	7,639	56.16	3,941	3,463	7,404	54.44
City of Caloocan	33,499	11,258	10,803	22,061	65.86	10,499	10,324	20,823	62.16
City of Marikina	8,399	2,428	2,313	4,741	56.45	2,298	2,085	4,383	52.18
City of Pasig	17,856	6,461	6,247	12,708	71.17	5,940	5,868	11,808	66.13
Pateros	1,120	468	439	907	80.98	467	436	903	80.63
City of Taguig	26,021	5,425	5,288	10,713	41.17	5,043	4,755	9,798	37.65
Quezon City	59,607	26,932	26,636	53,568	89.87	25,319	25,010	50,329	84.43
City of Makati	4,196	2,065	2,005	4,070	97.00	1,952	1,925	3,877	92.40
City of Mandaluyong	7,989	4,232	4,132	8,364	104.69	4,338	4,161	8,499	106.38
City of San Juan	2,173	585	554	1,139	52.42	527	548	1,075	49.47
City of Manila	34,268	15,081	14,344	29,425	85.87	16,611	15,710	32,321	94.32
City of Las Piñas	11,346	3,954	3,878	7,832	69.03	3,860	3,795	7,655	67.47
City of Muntinlupa	9,949	3,274	3,277	6,551	65.85	2,961	2,973	5,934	59.64
City of Parañaque	13,385	6,726	6,725	13,451	100.49	6,021	6,008	12,029	89.87
Pasay City	7,129	3,400	3,076	6,476	90.84	3,056	2,766	5,822	81.67
**CAR**	**31,490**	**11,667**	**10,894**	**22,561**	**71.64**	**9,693**	**8,873**	**18,566**	**58.96**
Abra	3,639	1,529	1,454	2,983	81.97	1,415	1,324	2,739	75.27
Apayao	2,222	941	858	1,799	80.96	763	658	1,421	63.95
Benguet	8,900	2,708	2,513	5,221	58.66	2,419	2,232	4,651	52.26
Ifugao	4,145	1,335	1,252	2,587	62.41	865	799	1,664	40.14
Kalinga	4,582	1,908	1,669	3,577	78.07	1,568	1,362	2,930	63.95
Mountain Province	2,615	1,034	980	2,014	77.02	876	802	1,678	64.17
City of Baguio	5,387	2,212	2,168	4,380	81.31	1,787	1,696	3,483	64.66
**Region 1**	**96,024**	**38,745**	**36,736**	**75,481**	**78.61**	**36,370**	**34,369**	**70,739**	**73.67**
Ilocos Norte	8,809	4,025	4,101	8,126	92.25	4,364	4,394	8,758	99.42
Ilocos Sur	9,834	4,821	4,607	9,428	95.87	4,689	4,425	9,114	92.68
La Union	13,436	5,174	4,688	9,862	73.40	4,658	4,178	8,836	65.76
Pangasinan	60,591	23,573	22,248	45,821	75.62	21,493	20,288	41,781	68.96
City of Dagupan	3,354	1,152	1,092	2,244	66.91	1,166	1,084	2,250	67.08
**Region 2**	**64,034**	**26,596**	**25,209**	**51,805**	**80.90**	**22,919**	**21,518**	**44,437**	**69.40**
Batanes	241	98	91	189	78.42	105	94	199	82.57
Cagayan	20,723	8,568	8,193	16,761	80.88	7,405	6,867	14,272	68.87
Isabela	27,362	11,227	10,610	21,837	79.81	10,043	9,491	19,534	71.39
Nueva Vizcaya	8,880	3,510	3,330	6,840	77.03	2,487	2,380	4,867	54.81
Quirino	3,865	1,711	1,612	3,323	85.98	1,405	1,298	2,703	69.94
City of Santiago	2,963	1,482	1,373	2,855	96.36	1,474	1,388	2,862	96.59
**Region 3**	**235,313**	**86,368**	**82,317**	**168,685**	**71.69**	**72,438**	**69,149**	**141,587**	**60.17**
Aurora	4,578	1,887	1,866	3,753	81.98	1,918	1,876	3,794	82.87
Bataan	17,556	6,275	5,939	12,214	69.57	5,490	5,146	10,636	60.58
Bulacan	68,115	26,611	25,161	51,772	76.01	22,079	20,827	42,906	62.99
Nueva Ecija	43,626	15,656	14,795	30,451	69.80	12,139	11,916	24,055	55.14
Pampanga	46,146	16,516	15,848	32,364	70.13	14,566	13,921	28,487	61.73
Tarlac	29,655	11,588	11,358	22,946	77.38	10,566	10,181	20,747	69.96
Zambales	11,796	3,864	3,650	7,514	63.70	3,292	3,072	6,364	53.95
City of Angeles	9,232	2,628	2,493	5,121	55.47	1,445	1,381	2,826	30.61
City of Olongapo	4,609	1,343	1,207	2,550	55.33	943	829	1,772	38.45
**Region 4A**	**310,150**	**110,173**	**104,435**	**214,608**	**69.19**	**94,858**	**90,524**	**185,382**	**59.77**
Batangas	53,484	19,428	18,286	37,714	70.51	16,118	15,536	31,654	59.18
Cavite	78,589	29,239	28,160	57,399	73.04	26,634	25,853	52,487	66.79
Laguna	61,967	24,174	23,158	47,332	76.38	21,923	20,861	42,784	69.04
Quezon	40,954	13,386	12,412	25,798	62.99	9,853	9,164	19,017	46.44
Rizal	68,903	21,960	20,546	42,506	61.69	18,611	17,461	36,072	52.35
City of Lucena	6,253	1,986	1,873	3,859	61.71	1,719	1,649	3,368	53.86
**Region 4B**	**66,508**	**26,992**	**25,097**	**52,089**	**78.32**	**18,710**	**17,553**	**36,263**	**54.52**
Marinduque	4,124	1,474	1,390	2,864	69.45	1,344	1,200	2,544	61.69
Occidental Mindoro	11,748	5,533	5,091	10,624	90.43	3,610	3,261	6,871	58.49
Oriental Mindoro	19,007	6,750	6,219	12,969	68.23	4,930	4,807	9,737	51.23
Palawan	20,503	8,682	8,078	16,760	81.74	4,999	4,603	9,602	46.83
Romblon	5,488	2,194	2,032	4,226	77.00	1,887	1,791	3,678	67.02

Table 2.C.1.4 - Immunization Services for Infants and Children
2 doses of Inactivated Polio Vaccine (IPV)
Philippines, 2024

Area	Eligible Population (0-11mos. old)	IPV 1				IPV 2			
		Male	Female	Total	%	Male	Female	Total	%
City of Puerto Princesa	5,638	2,359	2,287	4,646	82.41	1,940	1,891	3,831	67.95
**Region 5**	**136,611**	**46,709**	**43,845**	**90,554**	**66.29**	**34,469**	**32,716**	**67,185**	**49.18**
Albay	27,665	9,590	8,798	18,388	66.47	6,532	6,484	13,016	47.05
Camarines Norte	14,894	4,660	4,512	9,172	61.58	3,613	3,355	6,968	46.78
Camarines Sur	43,855	14,117	13,173	27,290	62.23	10,007	9,389	19,396	44.23
Catanduanes	5,395	1,815	1,761	3,576	66.28	1,545	1,439	2,984	55.31
Masbate	22,495	8,568	8,003	16,571	73.67	5,771	5,488	11,259	50.05
Sorsogon	18,065	6,596	6,318	12,914	71.49	5,767	5,421	11,188	61.93
City of Naga	4,242	1,363	1,280	2,643	62.31	1,234	1,140	2,374	55.96
**Region 6**	**147,934**	**54,017**	**50,819**	**104,836**	**70.87**	**47,765**	**44,756**	**92,521**	**62.54**
Aklan	11,281	3,837	3,502	7,339	65.06	3,137	2,911	6,048	53.61
Antique	11,899	4,432	4,086	8,518	71.59	3,278	3,081	6,359	53.44
Capiz	13,872	4,866	4,501	9,367	67.52	4,680	4,247	8,927	64.35
Guimaras	3,361	1,242	1,236	2,478	73.73	1,168	1,167	2,335	69.47
Iloilo	35,981	12,665	12,190	24,855	69.08	11,731	10,742	22,473	62.46
Negros Occidental	52,445	20,025	18,781	38,806	73.99	17,412	16,513	33,925	64.69
City of Bacolod	10,361	3,920	3,653	7,573	73.09	3,428	3,187	6,615	63.85
City of Iloilo	8,734	3,030	2,870	5,900	67.55	2,931	2,908	5,839	66.85
**Region 7**	**167,016**	**63,462**	**59,583**	**123,045**	**73.67**	**57,425**	**53,318**	**110,743**	**66.31**
Bohol	26,210	9,578	8,931	18,509	70.62	8,422	7,831	16,253	62.01
Cebu	72,875	26,446	24,915	51,361	70.48	24,441	22,533	46,974	64.46
Negros Oriental	28,980	12,520	11,818	24,338	83.98	9,796	9,051	18,847	65.03
Siquijor	1,671	560	525	1,085	64.93	558	519	1,077	64.45
City of Cebu	19,466	7,108	6,485	13,593	69.83	6,742	6,179	12,921	66.38
City of Lapu-Lapu	10,833	4,356	4,122	8,478	78.26	4,332	4,123	8,455	78.05
City of Mandaue	6,981	2,894	2,787	5,681	81.38	3,134	3,082	6,216	89.04
**Region 8**	**93,713**	**35,511**	**33,485**	**68,996**	**73.62**	**26,960**	**25,404**	**52,364**	**55.88**
Biliran	3,378	1,581	1,416	2,997	88.72	1,380	1,225	2,605	77.12
Eastern Samar	9,594	3,907	3,636	7,543	78.62	2,898	2,748	5,646	58.85
Leyte	31,504	11,610	10,920	22,530	71.51	8,590	8,038	16,628	52.78
Northern Samar	14,053	5,465	5,246	10,711	76.22	3,769	3,606	7,375	52.48
Southern Leyte	7,368	2,665	2,413	5,078	68.92	2,243	2,044	4,287	58.18
Samar	16,946	6,493	6,300	12,793	75.49	4,598	4,401	8,999	53.10
Ormoc City	5,344	1,981	1,812	3,793	70.98	1,653	1,575	3,228	60.40
City of Tacloban	5,526	1,809	1,742	3,551	64.26	1,829	1,767	3,596	65.07
**Region 9**	**85,348**	**30,212**	**29,099**	**59,311**	**69.49**	**28,039**	**26,727**	**54,766**	**64.17**
Zamboanga del Norte	24,977	8,283	7,924	16,207	64.89	7,578	7,287	14,865	59.51
Zamboanga del Sur	23,905	7,598	7,345	14,943	62.51	6,945	6,713	13,658	57.13
Zamboanga Sibugay	13,903	5,023	4,900	9,923	71.37	4,579	4,261	8,840	63.58
City of Isabela	3,209	1,219	1,172	2,391	74.51	1,153	1,045	2,198	68.49
City of Zamboanga	19,354	8,089	7,758	15,847	81.88	7,784	7,421	15,205	78.56
**Region 10**	**110,290**	**38,757**	**37,033**	**75,790**	**68.72**	**29,906**	**28,461**	**58,367**	**52.92**
Bukidnon	33,913	12,150	11,115	23,265	68.60	9,000	8,384	17,384	51.26
Camiguin	1,727	716	669	1,385	80.20	655	636	1,291	74.75
Lanao del Norte	17,881	6,076	6,020	12,096	67.65	4,942	4,882	9,824	54.94
Misamis Occidental	12,342	4,276	4,139	8,415	68.18	3,684	3,557	7,241	58.67
Misamis Oriental	22,184	7,207	7,010	14,217	64.09	5,592	5,360	10,952	49.37
City of Cagayan De Oro	14,503	5,685	5,448	11,133	76.76	3,876	3,644	7,520	51.85
City of Iligan	7,740	2,647	2,632	5,279	68.20	2,157	1,998	4,155	53.68
**Region 11**	**107,934**	**38,740**	**36,125**	**74,865**	**69.36**	**35,275**	**33,138**	**68,413**	**63.38**
Davao de Oro	15,434	5,806	5,551	11,357	73.58	5,310	5,131	10,441	67.65
Davao del Norte	23,685	8,108	7,592	15,700	66.29	8,408	7,658	16,066	67.83
Davao Oriental	12,025	4,389	3,958	8,347	69.41	3,857	3,590	7,447	61.93
Davao del Sur	13,178	4,833	4,469	9,302	70.59	4,385	4,118	8,503	64.52
Davao Occidental	6,606	2,354	2,171	4,525	68.50	2,027	1,875	3,902	59.07
City of Davao	37,006	13,250	12,384	25,634	69.27	11,288	10,766	22,054	59.60
**Region 12**	**95,296**	**35,073**	**33,178**	**68,251**	**71.62**	**31,203**	**29,256**	**60,459**	**63.44**
Cotabato	25,696	10,129	9,388	19,517	75.95	8,553	7,925	16,478	64.13
Sarangani	13,501	5,241	4,899	10,140	75.11	4,364	4,104	8,468	62.72
South Cotabato	21,842	7,947	7,603	15,550	71.19	7,525	6,972	14,497	66.37
Sultan Kudarat	19,613	7,038	6,830	13,868	70.71	6,534	6,340	12,874	65.64

Table 2.C.1.4 - Immunization Services for Infants and Children
2 doses of Inactivated Polio Vaccine (IPV)
Philippines, 2024

Area	Eligible Population (0-11mos. old)	IPV 1				IPV 2			
		Male	Female	Total	%	Male	Female	Total	%
City of General Santos	14,644	4,718	4,458	9,176	62.66	4,227	3,915	8,142	55.60
**CARAGA**	**55,371**	**21,841**	**20,347**	**42,188**	**76.19**	**19,359**	**17,886**	**37,245**	**67.26**
Agusan del Norte	7,331	3,152	2,927	6,079	82.92	2,775	2,473	5,248	71.59
Agusan del Sur	16,180	6,303	6,014	12,317	76.12	5,237	4,977	10,214	63.13
Surigao del Norte	10,168	3,963	3,634	7,597	74.71	3,706	3,353	7,059	69.42
Surigao del Sur	12,834	4,751	4,453	9,204	71.72	4,196	3,889	8,085	63.00
Dinagat Islands	2,117	854	795	1,649	77.89	783	692	1,475	69.67
City of Butuan	6,741	2,818	2,524	5,342	79.25	2,662	2,502	5,164	76.61
**BARMM**	**134,585**	**43,874**	**43,790**	**87,664**	**65.14**	**35,643**	**35,421**	**71,064**	**52.80**
Basilan	11,653	2,835	2,705	5,540	47.54	1,663	1,556	3,219	27.62
Lanao del Sur	33,339	11,036	11,138	22,174	66.51	9,984	9,987	19,971	59.90
Maguindanao del Norte	18,923	7,695	7,559	15,254	80.61	6,444	6,366	12,810	67.70
Maguindanao del Sur	23,214	8,293	8,310	16,603	71.52	7,125	6,979	14,104	60.76
Sulu	21,381	6,244	6,341	12,585	58.86	4,504	4,698	9,202	43.04
Tawi-Tawi	12,602	3,890	3,946	7,836	62.18	3,009	3,069	6,078	48.23
SGA	6,305	1,427	1,404	2,831	44.90	1,024	951	1,975	31.32
City of Cotabato	7,168	2,454	2,387	4,841	67.54	1,890	1,815	3,705	51.69

** = No Report*

Table 2.C.1.5 - Immunization Services for Infants and Children
Number and Proportion of Infants who completed 3 doses of Pneumococcal Conjugate Vaccine (PCV)
Philippines, 2024

Area	Eligible Population (0-11mos. old)	PCV 1				PCV 2				PCV 3			
		Male	Female	Total	%	Male	Female	Total	%	Male	Female	Total	%
**Philippines**	**2,200,865**	**671,022**	**636,058**	**1,307,080**	**59.39**	**566,764**	**541,932**	**1,108,696**	**50.38**	**547,727**	**525,579**	**1,073,306**	**48.77**
**NCR**	**263,248**	**74,330**	**71,801**	**146,131**	**55.51**	**52,844**	**51,746**	**104,590**	**39.73**	**50,187**	**48,785**	**98,972**	**37.60**
City of Malabon	7,447	2,498	2,255	4,753	63.82	1,986	1,776	3,762	50.52	1,967	1,731	3,698	49.66
City of Navotas	5,263	2,219	2,145	4,364	82.92	1,989	1,987	3,976	75.55	2,061	2,144	4,205	79.90
City of Valenzuela	13,601	3,189	2,982	6,171	45.37	1,697	1,471	3,168	23.29	1,381	1,242	2,623	19.29
City of Caloocan	33,499	7,642	7,204	14,846	44.32	3,882	3,849	7,731	23.08	3,100	3,040	6,140	18.33
City of Marikina	8,399	2,322	2,230	4,552	54.20	1,696	1,638	3,334	39.70	1,671	1,544	3,215	38.28
City of Pasig	17,856	5,209	4,914	10,123	56.69	4,128	4,073	8,201	45.93	4,141	4,107	8,248	46.19
Pateros	1,120	411	445	856	76.43	371	376	747	66.70	396	385	781	69.73
City of Taguig	26,021	4,948	4,815	9,763	37.52	2,965	2,702	5,667	21.78	2,220	2,025	4,245	16.31
Quezon City	59,607	15,519	15,190	30,709	51.52	9,804	10,054	19,858	33.31	9,415	9,467	18,882	31.68
City of Makati	4,196	2,066	2,004	4,070	97.00	2,011	1,893	3,904	93.04	1,967	1,873	3,840	91.52
City of Mandaluyong	7,989	4,405	4,336	8,741	109.41	4,234	4,162	8,396	105.09	4,471	4,200	8,671	108.54
City of San Juan	2,173	625	597	1,222	56.24	528	521	1,049	48.27	505	529	1,034	47.58
City of Manila	34,268	8,538	8,066	16,604	48.45	5,901	5,618	11,519	33.61	5,423	5,182	10,605	30.95
City of Las Piñas	11,346	3,670	3,665	7,335	64.65	3,313	3,334	6,647	58.58	3,351	3,294	6,645	58.57
City of Muntinlupa	9,949	2,010	2,080	4,090	41.11	938	971	1,909	19.19	893	877	1,770	17.79
City of Parañaque	13,385	6,196	6,216	12,412	92.73	5,052	5,145	10,197	76.18	5,037	5,099	10,136	75.73
Pasay City	7,129	2,863	2,657	5,520	77.43	2,349	2,176	4,525	63.47	2,188	2,046	4,234	59.39
**CAR**	**31,490**	**10,430**	**9,770**	**20,200**	**64.15**	**9,119**	**8,581**	**17,700**	**56.21**	**9,053**	**8,336**	**17,389**	**55.22**
Abra	3,639	1,224	1,103	2,327	63.95	972	937	1,909	52.46	913	815	1,728	47.49
Apayao	2,222	719	656	1,375	61.88	589	588	1,177	52.97	634	561	1,195	53.78
Benguet	8,900	2,225	2,070	4,295	48.26	1,898	1,773	3,671	41.25	1,930	1,799	3,729	41.90
Ifugao	4,145	1,299	1,167	2,466	59.49	1,224	1,082	2,306	55.63	1,152	1,062	2,214	53.41
Kalinga	4,582	1,650	1,504	3,154	68.83	1,570	1,383	2,953	64.45	1,612	1,365	2,977	64.97
Mountain Province	2,615	882	848	1,730	66.16	576	545	1,121	42.87	594	527	1,121	42.87
City of Baguio	5,387	2,431	2,422	4,853	90.09	2,290	2,273	4,563	84.70	2,218	2,207	4,425	82.14
**Region 1**	**96,024**	**32,099**	**30,884**	**62,983**	**65.59**	**29,771**	**28,306**	**58,077**	**60.48**	**29,312**	**28,077**	**57,389**	**59.77**
Ilocos Norte	8,809	3,824	3,870	7,694	87.34	3,734	3,805	7,539	85.58	3,652	3,759	7,411	84.13
Ilocos Sur	9,834	4,561	4,339	8,900	90.50	4,555	4,424	8,979	91.31	4,701	4,517	9,218	93.74
La Union	13,436	4,498	4,204	8,702	64.77	4,254	4,035	8,289	61.69	4,444	3,974	8,418	62.65
Pangasinan	60,591	18,425	17,731	36,156	59.67	16,606	15,479	32,085	52.95	15,898	15,225	31,123	51.37
City of Dagupan	3,354	791	740	1,531	45.65	622	563	1,185	35.33	617	602	1,219	36.34
**Region 2**	**64,034**	**24,586**	**22,988**	**47,574**	**74.29**	**24,865**	**23,598**	**48,463**	**75.68**	**24,055**	**22,824**	**46,879**	**73.21**
Batanes	241	95	95	190	78.84	90	91	181	75.10	98	94	192	79.67
Cagayan	20,723	7,823	7,312	15,135	73.03	7,914	7,568	15,482	74.71	7,612	7,321	14,933	72.06
Isabela	27,362	10,374	9,600	19,974	73.00	10,599	9,968	20,567	75.17	10,375	9,707	20,082	73.39
Nueva Vizcaya	8,880	3,258	3,158	6,416	72.25	3,186	3,123	6,309	71.05	3,008	2,861	5,869	66.09
Quirino	3,865	1,551	1,427	2,978	77.05	1,609	1,452	3,061	79.20	1,486	1,454	2,940	76.07
City of Santiago	2,963	1,485	1,396	2,881	97.23	1,467	1,396	2,863	96.63	1,476	1,387	2,863	96.63
**Region 3**	**235,313**	**68,457**	**65,351**	**133,808**	**56.86**	**50,171**	**48,397**	**98,568**	**41.89**	**46,015**	**44,593**	**90,608**	**38.51**
Aurora	4,578	1,604	1,576	3,180	69.46	1,326	1,398	2,724	59.50	1,328	1,309	2,637	57.60
Bataan	17,556	4,754	4,532	9,286	52.89	2,689	2,567	5,256	29.94	1,834	1,768	3,602	20.52
Bulacan	68,115	20,484	19,594	40,078	58.84	16,251	15,689	31,940	46.89	16,509	16,042	32,551	47.79
Nueva Ecija	43,626	13,660	12,900	26,560	60.88	10,888	10,639	21,527	49.34	9,446	9,123	18,569	42.56
Pampanga	46,146	12,600	11,896	24,496	53.08	7,729	7,369	15,098	32.72	6,543	6,295	12,838	27.82
Tarlac	29,655	7,626	7,494	15,120	50.99	5,151	4,876	10,027	33.81	4,687	4,599	9,286	31.31
Zambales	11,796	3,815	3,659	7,474	63.36	3,219	3,106	6,325	53.62	3,188	3,096	6,284	53.27
City of Angeles	9,232	1,991	1,943	3,934	42.61	1,011	946	1,957	21.20	581	601	1,182	12.80
City of Olongapo	4,609	1,923	1,757	3,680	79.84	1,907	1,807	3,714	80.58	1,899	1,760	3,659	79.39
**Region 4A**	**310,150**	**98,547**	**92,115**	**190,662**	**61.47**	**83,927**	**79,209**	**163,136**	**52.60**	**82,804**	**79,312**	**162,116**	**52.27**
Batangas	53,484	14,214	13,390	27,604	51.61	10,933	10,271	21,204	39.65	11,365	10,856	22,221	41.55
Cavite	78,589	25,629	23,856	49,485	62.97	21,183	20,090	41,273	52.52	20,478	19,745	40,223	51.18
Laguna	61,967	22,675	21,458	44,133	71.22	20,887	19,710	40,597	65.51	20,733	20,094	40,827	65.89
Quezon	40,954	11,798	10,964	22,762	55.58	10,545	9,945	20,490	50.03	10,306	9,692	19,998	48.83
Rizal	68,903	22,137	20,518	42,655	61.91	18,521	17,450	35,971	52.21	18,095	17,287	35,382	51.35
City of Lucena	6,253	2,094	1,929	4,023	64.34	1,858	1,743	3,601	57.59	1,827	1,638	3,465	55.41
**Region 4B**	**66,508**	**17,016**	**15,991**	**33,007**	**49.63**	**12,674**	**11,865**	**24,539**	**36.90**	**11,812**	**11,117**	**22,929**	**34.48**
Marinduque	4,124	1,451	1,360	2,811	68.16	1,212	1,188	2,400	58.20	1,143	1,090	2,233	54.15
Occidental Mindoro	11,748	3,217	2,984	6,201	52.78	3,089	2,750	5,839	49.70	3,018	2,805	5,823	49.57
Oriental Mindoro	19,007	5,004	4,721	9,725	51.17	3,739	3,470	7,209	37.93	3,526	3,254	6,780	35.67
Palawan	20,503	3,865	3,643	7,508	36.62	2,117	2,066	4,183	20.40	1,728	1,720	3,448	16.82
Romblon	5,488	1,701	1,576	3,277	59.71	1,179	1,147	2,326	42.38	1,128	1,087	2,215	40.36
City of Puerto Princesa	5,638	1,778	1,707	3,485	61.81	1,338	1,244	2,582	45.80	1,269	1,161	2,430	43.10
**Region 5**	**136,611**	**33,812**	**31,500**	**65,312**	**47.81**	**24,963**	**23,620**	**48,583**	**35.56**	**24,582**	**23,645**	**48,227**	**35.30**
Albay	27,665	7,124	6,029	13,153	47.54	3,560	3,489	7,049	25.48	3,927	3,984	7,911	28.60
Camarines Norte	14,894	4,014	3,885	7,899	53.03	3,048	2,999	6,047	40.60	3,067	2,873	5,940	39.88
Camarines Sur	43,855	7,668	7,176	14,844	33.85	6,676	6,175	12,851	29.30	6,405	6,097	12,502	28.51
Catanduanes	5,395	1,406	1,327	2,733	50.66	1,020	944	1,964	36.40	1,179	1,095	2,274	42.15
Masbate	22,495	6,521	6,465	12,986	57.73	5,135	4,851	9,986	44.39	4,618	4,430	9,048	40.22
Sorsogon	18,065	5,925	5,560	11,485	63.58	4,577	4,330	8,907	49.31	4,514	4,326	8,840	48.93
City of Naga	4,242	1,154	1,058	2,212	52.15	947	832	1,779	41.94	872	840	1,712	40.36

Table 2.C.1.5 - Immunization Services for Infants and Children
Number and Proportion of Infants who completed 3 doses of Pneumococcal Conjugate Vaccine (PCV)
Philippines, 2024

Area	Eligible Population (0-11mos. old)	PCV 1				PCV 2				PCV 3			
		Male	Female	Total	%	Male	Female	Total	%	Male	Female	Total	%
**Region 6**	**147,934**	**47,788**	**44,611**	**92,399**	**62.46**	**42,857**	**40,545**	**83,402**	**56.38**	**41,160**	**39,057**	**80,217**	**54.22**
Aklan	11,281	3,445	3,177	6,622	58.70	3,145	2,888	6,033	53.48	2,947	2,781	5,728	50.78
Antique	11,899	3,396	3,205	6,601	55.48	3,162	2,970	6,132	51.53	3,254	3,002	6,256	52.58
Capiz	13,872	4,175	3,988	8,163	58.85	4,079	3,984	8,063	58.12	4,102	3,833	7,935	57.20
Guimaras	3,361	1,148	1,121	2,269	67.51	1,173	1,177	2,350	69.92	1,181	1,202	2,383	70.90
Iloilo	35,981	11,872	10,979	22,851	63.51	11,477	10,850	22,327	62.05	11,085	10,702	21,787	60.55
Negros Occidental	52,445	17,267	16,018	33,285	63.47	13,892	13,105	26,997	51.48	12,720	12,016	24,736	47.17
City of Bacolod	10,361	3,593	3,330	6,923	66.82	2,878	2,676	5,554	53.60	2,724	2,575	5,299	51.14
City of Iloilo	8,734	2,892	2,793	5,685	65.09	3,051	2,895	5,946	68.08	3,147	2,946	6,093	69.76
**Region 7**	**167,016**	**57,279**	**53,683**	**110,962**	**66.44**	**55,460**	**52,390**	**107,850**	**64.57**	**54,791**	**51,631**	**106,422**	**63.72**
Bohol	26,210	8,223	7,733	15,956	60.88	7,599	7,233	14,832	56.59	7,393	6,914	14,307	54.59
Cebu	72,875	23,618	22,220	45,838	62.90	23,071	21,904	44,975	61.72	23,396	21,768	45,164	61.97
Negros Oriental	28,980	10,724	10,173	20,897	72.11	10,444	9,929	20,373	70.30	9,897	9,494	19,391	66.91
Siquijor	1,671	551	491	1,042	62.36	543	509	1,052	62.96	538	520	1,058	63.32
City of Cebu	19,466	7,200	6,442	13,642	70.08	7,031	6,405	13,436	69.02	6,822	6,472	13,294	68.29
City of Lapu-Lapu	10,833	4,159	4,032	8,191	75.61	3,975	3,728	7,703	71.11	3,875	3,656	7,531	69.52
City of Mandaue	6,981	2,804	2,592	5,396	77.30	2,797	2,682	5,479	78.48	2,870	2,807	5,677	81.32
**Region 8**	**93,713**	**25,361**	**23,720**	**49,081**	**52.37**	**20,355**	**19,344**	**39,699**	**42.36**	**19,811**	**19,201**	**39,012**	**41.63**
Biliran	3,378	1,301	1,146	2,447	72.44	942	857	1,799	53.26	871	791	1,662	49.20
Eastern Samar	9,594	2,568	2,399	4,967	51.77	1,924	1,779	3,703	38.60	1,878	1,838	3,716	38.73
Leyte	31,504	9,128	8,352	17,480	55.49	7,739	7,271	15,010	47.64	7,881	7,486	15,367	48.78
Northern Samar	14,053	4,230	4,054	8,284	58.95	3,329	3,312	6,641	47.26	3,032	3,106	6,138	43.68
Southern Leyte	7,368	1,986	1,809	3,795	51.51	1,526	1,355	2,881	39.10	1,546	1,441	2,987	40.54
Samar	16,946	3,502	3,485	6,987	41.23	2,919	2,903	5,822	34.36	2,750	2,788	5,538	32.68
Ormoc City	5,344	1,457	1,324	2,781	52.04	1,059	925	1,984	37.13	932	840	1,772	33.16
City of Tacloban	5,526	1,189	1,151	2,340	42.35	917	942	1,859	33.64	921	911	1,832	33.15
**Region 9**	**85,348**	**24,835**	**23,655**	**48,490**	**56.81**	**19,772**	**19,214**	**38,986**	**45.68**	**19,736**	**19,111**	**38,847**	**45.52**
Zamboanga del Norte	24,977	7,015	6,495	13,510	54.09	5,820	5,611	11,431	45.77	5,863	5,647	11,510	46.08
Zamboanga del Sur	23,905	5,868	5,587	11,455	47.92	4,814	4,740	9,554	39.97	4,825	4,702	9,527	39.85
Zamboanga Sibugay	13,903	3,991	3,932	7,923	56.99	3,211	3,207	6,418	46.16	3,225	3,153	6,378	45.87
City of Isabela	3,209	1,002	989	1,991	62.04	794	763	1,557	48.52	826	815	1,641	51.14
City of Zamboanga	19,354	6,959	6,652	13,611	70.33	5,133	4,893	10,026	51.80	4,997	4,794	9,791	50.59
**Region 10**	**110,290**	**35,149**	**33,948**	**69,097**	**62.65**	**30,646**	**29,691**	**60,337**	**54.71**	**29,013**	**28,136**	**57,149**	**51.82**
Bukidnon	33,913	12,187	11,669	23,856	70.34	10,378	9,825	20,203	59.57	9,504	9,014	18,518	54.60
Camiguin	1,727	641	637	1,278	74.00	655	655	1,310	75.85	638	601	1,239	71.74
Lanao del Norte	17,881	5,317	5,313	10,630	59.45	5,150	5,080	10,230	57.21	5,045	5,107	10,152	56.78
Misamis Occidental	12,342	3,662	3,458	7,120	57.69	3,448	3,354	6,802	55.11	3,567	3,343	6,910	55.99
Misamis Oriental	22,184	6,001	5,707	11,708	52.78	5,077	4,913	9,990	45.03	4,656	4,593	9,249	41.69
City of Cagayan De Oro	14,503	5,009	4,801	9,810	67.64	4,014	3,906	7,920	54.61	3,687	3,589	7,276	50.17
City of Iligan	7,740	2,332	2,363	4,695	60.66	1,924	1,958	3,882	50.16	1,916	1,889	3,805	49.16
**Region 11**	**107,934**	**35,222**	**33,642**	**68,864**	**63.80**	**31,946**	**30,214**	**62,160**	**57.59**	**31,690**	**29,756**	**61,446**	**56.93**
Davao de Oro	15,434	5,653	5,349	11,002	71.28	5,559	5,296	10,855	70.33	5,411	5,149	10,560	68.42
Davao del Norte	23,685	6,974	6,612	13,586	57.36	6,213	5,848	12,061	50.92	6,282	5,875	12,157	51.33
Davao Oriental	12,025	3,968	3,739	7,707	64.09	3,338	3,107	6,445	53.60	3,257	2,964	6,221	51.73
Davao del Sur	13,178	4,452	4,353	8,805	66.82	3,736	3,645	7,381	56.01	3,596	3,382	6,978	52.95
Davao Occidental	6,606	1,725	1,698	3,423	51.82	1,760	1,693	3,453	52.27	1,834	1,692	3,526	53.38
City of Davao	37,006	12,450	11,891	24,341	65.78	11,340	10,625	21,965	59.36	11,310	10,694	22,004	59.46
**Region 12**	**95,296**	**30,620**	**28,765**	**59,385**	**62.32**	**28,295**	**26,753**	**55,048**	**57.77**	**27,827**	**26,523**	**54,350**	**57.03**
Cotabato	25,696	8,226	7,721	15,947	62.06	7,031	6,684	13,715	53.37	7,084	6,628	13,712	53.36
Sarangani	13,501	4,065	3,688	7,753	57.43	3,677	3,388	7,065	52.33	3,565	3,416	6,981	51.71
South Cotabato	21,842	7,107	6,684	13,791	63.14	6,882	6,485	13,367	61.20	6,731	6,365	13,096	59.96
Sultan Kudarat	19,613	6,404	6,047	12,451	63.48	6,216	5,877	12,093	61.66	6,170	5,990	12,160	62.00
City of General Santos	14,644	4,818	4,625	9,443	64.48	4,489	4,319	8,808	60.15	4,277	4,124	8,401	57.37
**CARAGA**	**55,371**	**20,122**	**18,665**	**38,787**	**70.05**	**19,249**	**18,131**	**37,380**	**67.51**	**18,829**	**18,063**	**36,892**	**66.63**
Agusan del Norte	7,331	2,891	2,667	5,558	75.82	2,918	2,807	5,725	78.09	2,869	2,779	5,648	77.04
Agusan del Sur	16,180	5,338	5,003	10,341	63.91	4,665	4,514	9,179	56.73	4,526	4,487	9,013	55.70
Surigao del Norte	10,168	3,864	3,574	7,438	73.15	3,948	3,620	7,568	74.43	3,769	3,639	7,408	72.86
Surigao del Sur	12,834	4,686	4,362	9,048	70.50	4,480	4,227	8,707	67.84	4,394	4,166	8,560	66.70
Dinagat Islands	2,117	787	735	1,522	71.89	805	794	1,599	75.53	830	779	1,609	76.00
City of Butuan	6,741	2,556	2,324	4,880	72.39	2,433	2,169	4,602	68.27	2,441	2,213	4,654	69.04
**BARMM**	**134,585**	**35,369**	**34,969**	**70,338**	**52.26**	**29,850**	**30,328**	**60,178**	**44.71**	**27,050**	**27,412**	**54,462**	**40.47**
Basilan	11,653	1,658	1,628	3,286	28.20	1,055	1,055	2,110	18.11	861	913	1,774	15.22
Lanao del Sur	33,339	8,949	8,855	17,804	53.40	8,400	8,261	16,661	49.97	7,995	7,983	15,978	47.93
Maguindanao del Norte	18,923	5,431	5,200	10,631	56.18	3,606	3,608	7,214	38.12	2,636	2,499	5,135	27.14
Maguindanao del Sur	23,214	6,085	6,053	12,138	52.29	4,415	4,363	8,778	37.81	3,633	3,672	7,305	31.47
Sulu	21,381	6,077	6,213	12,290	57.48	5,709	5,971	11,680	54.63	5,527	5,826	11,353	53.10
Tawi-Tawi	12,602	3,562	3,733	7,295	57.89	3,425	3,758	7,183	57.00	3,220	3,324	6,544	51.93
SGA	6,305	1,229	1,218	2,447	38.81	1,084	1,054	2,138	33.91	1,040	965	2,005	31.80
City of Cotabato	7,168	2,378	2,069	4,447	62.04	2,156	2,258	4,414	61.58	2,138	2,230	4,368	60.94

* = No Data
* = No Report

Table 2.C.1.6 - Immunization Services for Infants and Children
Number and Proportion of Children vaccinated with 2 doses of Measles Containing Vaccine (MCV)
Philippines, 2024

Area	Eligible Population (0-11mos. old)	MCV 1				Eligible Population (0-12mos. old)	MCV 2			
		Male	Female	Total	%		Male	Female	Total	%
**Philippines**	**2,200,865**	**826,274**	**778,887**	**1,605,161**	**72.93**	**2,392,392**	**863,948**	**823,597**	**1,687,545**	**70.54**
**NCR**	**263,248**	**111,565**	**107,277**	**218,842**	**83.13**	**285,798**	**117,672**	**114,535**	**232,207**	**81.25**
City of Malabon	7,447	2,662	2,409	5,071	68.09	8,070	3,188	3,183	6,371	78.95
City of Navotas	5,263	2,776	2,693	5,469	103.91	5,718	2,769	2,799	5,568	97.38
City of Valenzuela	13,601	4,371	3,617	7,988	58.73	14,813	4,168	3,902	8,070	54.48
City of Caloocan	33,499	11,791	11,443	23,234	69.36	36,488	13,516	13,016	26,532	72.71
City of Marikina	8,399	2,302	2,129	4,431	52.76	9,135	2,031	1,970	4,001	43.80
City of Pasig	17,856	6,582	6,387	12,969	72.63	19,265	8,346	8,177	16,523	85.77
Pateros	1,120	472	452	924	82.50	1,221	487	427	914	74.86
City of Taguig	26,021	5,458	5,113	10,571	40.62	28,175	5,980	5,495	11,475	40.73
Quezon City	59,607	34,065	33,519	67,584	113.38	64,737	34,031	33,776	67,807	104.74
City of Makati	4,196	2,180	2,119	4,299	102.45	4,580	2,207	2,112	4,319	94.30
City of Mandaluyong	7,989	4,479	4,292	8,771	109.79	8,672	4,455	4,474	8,929	102.96
City of San Juan	2,173	563	547	1,110	51.08	2,363	626	646	1,272	53.83
City of Manila	34,268	16,799	15,914	32,713	95.46	37,288	18,091	17,433	35,524	95.27
City of Las Piñas	11,346	4,162	3,974	8,136	71.71	12,284	4,511	4,393	8,904	72.48
City of Muntinlupa	9,949	3,358	3,292	6,650	66.84	10,788	3,293	3,153	6,446	59.75
City of Parañaque	13,385	6,345	6,446	12,791	95.56	14,517	6,399	6,293	12,692	87.43
Pasay City	7,129	3,200	2,931	6,131	86.00	7,684	3,574	3,286	6,860	89.28
**CAR**	**31,490**	**11,945**	**11,026**	**22,971**	**72.95**	**34,199**	**12,851**	**11,740**	**24,591**	**71.91**
Abra	3,639	1,499	1,420	2,919	80.21	3,951	1,672	1,564	3,236	81.90
Apayao	2,222	914	825	1,739	78.26	2,416	917	858	1,775	73.47
Benguet	8,900	2,868	2,682	5,550	62.36	9,666	2,933	2,740	5,673	58.69
Ifugao	4,145	1,521	1,342	2,863	69.07	4,502	1,710	1,530	3,240	71.97
Kalinga	4,582	1,986	1,764	3,750	81.84	4,979	2,186	1,966	4,152	83.39
Mountain Province	2,615	1,069	982	2,051	78.43	2,838	1,168	997	2,165	76.29
City of Baguio	5,387	2,088	2,011	4,099	76.09	5,847	2,265	2,085	4,350	74.40
**Region 1**	**96,024**	**38,282**	**36,092**	**74,374**	**77.45**	**104,281**	**40,855**	**38,813**	**79,668**	**76.40**
Ilocos Norte	8,809	4,060	4,062	8,122	92.20	9,590	4,429	4,291	8,720	90.93
Ilocos Sur	9,834	4,867	4,530	9,397	95.56	10,656	5,494	5,391	10,885	102.15
La Union	13,436	5,135	4,571	9,706	72.24	14,588	5,226	4,655	9,881	67.73
Pangasinan	60,591	23,123	21,891	45,014	74.29	65,801	24,513	23,342	47,855	72.73
City of Dagupan	3,354	1,097	1,038	2,135	63.66	3,646	1,193	1,134	2,327	63.82
**Region 2**	**64,034**	**25,116**	**23,616**	**48,732**	**76.10**	**69,664**	**26,133**	**24,476**	**50,609**	**72.65**
Batanes	241	107	92	199	82.57	259	128	90	218	84.17
Cagayan	20,723	7,918	7,334	15,252	73.60	22,583	7,876	7,397	15,273	67.63
Isabela	27,362	10,829	10,227	21,056	76.95	29,775	11,482	10,814	22,296	74.88
Nueva Vizcaya	8,880	3,150	3,052	6,202	69.84	9,661	3,341	3,095	6,436	66.62
Quirino	3,865	1,624	1,519	3,143	81.32	4,186	1,727	1,542	3,269	78.09
City of Santiago	2,963	1,488	1,392	2,880	97.20	3,200	1,579	1,538	3,117	97.41
**Region 3**	**235,313**	**93,655**	**88,292**	**181,947**	**77.32**	**256,011**	**100,366**	**96,032**	**196,398**	**76.71**
Aurora	4,578	1,944	1,877	3,821	83.46	4,977	2,349	2,224	4,573	91.88
Bataan	17,556	6,251	5,811	12,062	68.71	19,041	6,238	5,873	12,111	63.60
Bulacan	68,115	30,271	28,205	58,476	85.85	74,192	32,446	30,516	62,962	84.86
Nueva Ecija	43,626	15,508	14,914	30,422	69.73	47,474	16,334	15,737	32,071	67.55
Pampanga	46,146	17,920	17,031	34,951	75.74	50,255	18,244	17,688	35,932	71.50
Tarlac	29,655	12,522	11,962	24,484	82.56	32,147	14,764	14,510	29,274	91.06
Zambales	11,796	4,470	4,109	8,579	72.73	12,830	4,927	4,541	9,468	73.80
City of Angeles	9,232	3,228	2,996	6,224	67.42	10,104	3,216	3,161	6,377	63.11
City of Olongapo	4,609	1,541	1,387	2,928	63.53	4,991	1,848	1,782	3,630	72.73
**Region 4A**	**310,150**	**115,018**	**108,238**	**223,256**	**71.98**	**337,182**	**117,655**	**111,746**	**229,401**	**68.03**
Batangas	53,484	20,616	19,348	39,964	74.72	58,339	19,520	18,393	37,913	64.99
Cavite	78,589	30,245	28,924	59,169	75.29	85,491	30,601	29,530	60,131	70.34
Laguna	61,967	25,236	23,893	49,129	79.28	67,298	26,399	25,279	51,678	76.79
Quezon	40,954	13,907	12,584	26,491	64.68	44,516	13,591	12,810	26,401	59.31
Rizal	68,903	22,952	21,495	44,447	64.51	74,779	24,953	23,155	48,108	64.33
City of Lucena	6,253	2,062	1,994	4,056	64.86	6,759	2,591	2,579	5,170	76.49
**Region 4B**	**66,508**	**25,304**	**23,306**	**48,610**	**73.09**	**72,212**	**26,002**	**23,855**	**49,857**	**69.04**
Marinduque	4,124	1,468	1,364	2,832	68.67	4,531	1,597	1,380	2,977	65.70
Occidental Mindoro	11,748	4,934	4,453	9,387	79.90	12,749	5,009	4,604	9,613	75.40
Oriental Mindoro	19,007	6,493	5,986	12,479	65.65	20,606	7,018	6,394	13,412	65.09
Palawan	20,503	7,919	7,308	15,227	74.27	22,257	7,822	7,192	15,014	67.46
Romblon	5,488	2,169	2,010	4,179	76.15	5,966	2,225	2,044	4,269	71.56
City of Puerto Princesa	5,638	2,321	2,185	4,506	79.92	6,103	2,331	2,241	4,572	74.91

Table 2.C.1.6 - Immunization Services for Infants and Children
Number and Proportion of Children vaccinated with 2 doses of Measles Containing Vaccine (MCV)
Philippines, 2024

Area	Eligible Population (0-11mos. old)	MCV 1				Eligible Population (0-12mos. old)	MCV 2			
		Male	Female	Total	%		Male	Female	Total	%
**Region 5**	**136,611**	**45,995**	**43,178**	**89,173**	**65.28**	**148,559**	**44,945**	**42,496**	**87,441**	**58.86**
Albay	27,665	9,552	8,890	18,442	66.66	30,159	10,248	9,742	19,990	66.28
Camarines Norte	14,894	4,633	4,384	9,017	60.54	16,163	4,396	4,064	8,460	52.34
Camarines Sur	43,855	13,754	12,806	26,560	60.56	47,677	12,467	11,704	24,171	50.70
Catanduanes	5,395	2,025	1,847	3,872	71.77	5,886	2,083	1,930	4,013	68.18
Masbate	22,495	8,095	7,780	15,875	70.57	24,426	7,952	7,588	15,540	63.62
Sorsogon	18,065	6,580	6,220	12,800	70.86	19,647	6,449	6,219	12,668	64.48
City of Naga	4,242	1,356	1,251	2,607	61.46	4,601	1,350	1,249	2,599	56.49
**Region 6**	**147,934**	**53,876**	**49,907**	**103,783**	**70.15**	**160,897**	**57,527**	**54,146**	**111,673**	**69.41**
Aklan	11,281	3,670	3,339	7,009	62.13	12,236	3,852	3,529	7,381	60.32
Antique	11,899	4,378	4,059	8,437	70.91	12,896	4,564	4,322	8,886	68.91
Capiz	13,872	5,085	4,557	9,642	69.51	15,118	5,536	4,990	10,526	69.63
Guimaras	3,361	1,204	1,222	2,426	72.18	3,663	1,259	1,277	2,536	69.23
Iloilo	35,981	13,228	12,135	25,363	70.49	39,077	14,885	13,893	28,778	73.64
Negros Occidental	52,445	19,413	18,118	37,531	71.56	57,118	20,511	19,518	40,029	70.08
City of Bacolod	10,361	3,692	3,360	7,052	68.06	11,280	3,469	3,205	6,674	59.17
City of Iloilo	8,734	3,206	3,117	6,323	72.40	9,509	3,451	3,412	6,863	72.17
**Region 7**	**167,016**	**61,138**	**56,886**	**118,024**	**70.67**	**181,246**	**64,227**	**60,331**	**124,558**	**68.72**
Bohol	26,210	9,023	8,307	17,330	66.12	28,418	9,228	8,723	17,951	63.17
Cebu	72,875	26,088	24,084	50,172	68.85	79,137	27,297	25,461	52,758	66.67
Negros Oriental	28,980	10,683	10,040	20,723	71.51	31,449	10,638	10,022	20,660	65.69
Siquijor	1,671	566	515	1,081	64.69	1,778	580	522	1,102	61.98
City of Cebu	19,466	7,047	6,471	13,518	69.44	21,163	7,436	6,827	14,263	67.40
City of Lapu-Lapu	10,833	4,534	4,357	8,891	82.07	11,745	5,407	5,230	10,637	90.57
City of Mandaue	6,981	3,197	3,112	6,309	90.37	7,556	3,641	3,546	7,187	95.12
**Region 8**	**93,713**	**31,959**	**30,044**	**62,003**	**66.16**	**101,678**	**32,158**	**30,726**	**62,884**	**61.85**
Biliran	3,378	1,457	1,312	2,769	81.97	3,679	1,415	1,389	2,804	76.22
Eastern Samar	9,594	3,416	3,235	6,651	69.32	10,364	3,378	3,336	6,714	64.78
Leyte	31,504	10,576	9,850	20,426	64.84	34,367	10,662	10,138	20,800	60.52
Northern Samar	14,053	4,732	4,613	9,345	66.50	15,211	4,823	4,617	9,440	62.06
Southern Leyte	7,368	2,427	2,152	4,579	62.15	8,024	2,520	2,272	4,792	59.72
Samar	16,946	5,776	5,471	11,247	66.37	18,286	5,585	5,454	11,039	60.37
Ormoc City	5,344	1,703	1,607	3,310	61.94	5,774	1,839	1,711	3,550	61.48
City of Tacloban	5,526	1,872	1,804	3,676	66.52	5,973	1,936	1,809	3,745	62.70
**Region 9**	**85,348**	**31,505**	**29,531**	**61,036**	**71.51**	**92,714**	**34,143**	**32,752**	**66,895**	**72.15**
Zamboanga del Norte	24,977	8,652	8,110	16,762	67.11	27,153	9,117	8,792	17,909	65.96
Zamboanga del Sur	23,905	7,862	7,442	15,304	64.02	25,904	8,441	8,159	16,600	64.08
Zamboanga Sibugay	13,903	5,195	4,814	10,009	71.99	15,123	5,545	5,238	10,783	71.30
City of Isabela	3,209	1,227	1,080	2,307	71.89	3,494	1,384	1,274	2,658	76.07
City of Zamboanga	19,354	8,569	8,085	16,654	86.05	21,040	9,656	9,289	18,945	90.04
**Region 10**	**110,290**	**40,734**	**38,775**	**79,509**	**72.09**	**119,709**	**43,357**	**41,735**	**85,092**	**71.08**
Bukidnon	33,913	12,900	11,820	24,720	72.89	36,852	13,560	12,689	26,249	71.23
Camiguin	1,727	653	643	1,296	75.04	1,878	678	659	1,337	71.19
Lanao del Norte	17,881	6,413	6,239	12,652	70.76	19,447	7,035	6,939	13,974	71.86
Misamis Occidental	12,342	4,517	4,358	8,875	71.91	13,379	4,939	4,595	9,534	71.26
Misamis Oriental	22,184	7,559	7,291	14,850	66.94	24,027	7,825	7,722	15,547	64.71
City of Cagayan De Oro	14,503	6,083	5,916	11,999	82.73	15,725	6,625	6,518	13,143	83.58
City of Iligan	7,740	2,609	2,508	5,117	66.11	8,401	2,695	2,613	5,308	63.18
**Region 11**	**107,934**	**40,473**	**37,499**	**77,972**	**72.24**	**117,243**	**40,431**	**38,173**	**78,604**	**67.04**
Davao de Oro	15,434	5,825	5,550	11,375	73.70	16,758	5,643	5,476	11,119	66.35
Davao del Norte	23,685	8,707	7,978	16,685	70.45	25,693	8,795	8,138	16,933	65.91
Davao Oriental	12,025	4,662	4,270	8,932	74.28	13,017	4,799	4,414	9,213	70.78
Davao del Sur	13,178	4,966	4,621	9,587	72.75	14,382	4,893	4,698	9,591	66.69
Davao Occidental	6,606	2,388	2,219	4,607	69.74	7,224	2,339	2,189	4,528	62.68
City of Davao	37,006	13,925	12,861	26,786	72.38	40,169	13,962	13,258	27,220	67.76
**Region 12**	**95,296**	**34,248**	**31,863**	**66,111**	**69.37**	**103,515**	**36,743**	**34,425**	**71,168**	**68.75**
Cotabato	25,696	9,844	9,054	18,898	73.54	28,029	10,533	9,881	20,414	72.83
Sarangani	13,501	5,035	4,642	9,677	71.68	14,599	5,496	5,144	10,640	72.88
South Cotabato	21,842	7,877	7,197	15,074	69.01	23,727	8,508	7,782	16,290	68.66
Sultan Kudarat	19,613	6,865	6,595	13,460	68.63	21,299	7,617	7,364	14,981	70.34
City of General Santos	14,644	4,627	4,375	9,002	61.47	15,861	4,589	4,254	8,843	55.75

Table 2.C.1.6 - Immunization Services for Infants and Children

Number and Proportion of Children vaccinated with 2 doses of Measles Containing Vaccine (MCV)

Philippines, 2024

Area	Eligible Population (0-11mos. old)	MCV 1				Eligible Population (0-12mos. old)	MCV 2			
		Male	Female	Total	%		Male	Female	Total	%
**CARAGA**	**55,371**	**22,404**	**20,807**	**43,211**	**78.04**	**60,237**	**25,625**	**24,343**	**49,968**	**82.95**
Agusan del Norte	7,331	3,231	2,816	6,047	82.49	7,979	3,840	3,598	7,438	93.22
Agusan del Sur	16,180	6,216	5,918	12,134	74.99	17,563	7,398	7,203	14,601	83.13
Surigao del Norte	10,168	4,090	3,797	7,887	77.57	11,072	4,532	4,313	8,845	79.89
Surigao del Sur	12,834	4,888	4,536	9,424	73.43	13,988	5,122	4,720	9,842	70.36
Dinagat Islands	2,117	856	736	1,592	75.20	2,322	945	860	1,805	77.73
City of Butuan	6,741	3,123	3,004	6,127	90.89	7,313	3,788	3,649	7,437	101.70
**BARMM**	**134,585**	**43,057**	**42,550**	**85,607**	**63.61**	**147,247**	**43,258**	**43,273**	**86,531**	**58.77**
Basilan	11,653	2,386	2,474	4,860	41.71	12,747	2,479	2,338	4,817	37.79
Lanao del Sur	33,339	11,700	11,747	23,447	70.33	36,535	11,679	11,742	23,421	64.11
Maguindanao del Norte	18,923	7,695	7,414	15,109	79.84	20,607	7,936	7,755	15,691	76.14
Maguindanao del Sur	23,214	8,031	7,882	15,913	68.55	25,329	8,213	8,394	16,607	65.57
Sulu	21,381	5,485	5,505	10,990	51.40	23,547	5,385	5,509	10,894	46.26
Tawi-Tawi	12,602	3,704	3,663	7,367	58.46	13,789	3,690	3,609	7,299	52.93
SGA	6,305	1,896	1,754	3,650	57.89	6,889	1,705	1,670	3,375	48.99
City of Cotabato	7,168	2,160	2,111	4,271	59.58	7,804	2,171	2,256	4,427	56.73

0 = No Data

* = No Report

2.C.1.7 Immunization Services for Infants and Children
Number and Proportion of Fully Immunized Children
Philippines, 2024

Area	Eligible Population (0-12 years old)	Fully Immunized Children (FIC)			
		Male	Female	Total	%
**NCR**	**285,798**	**114,085**	**111,135**	**225,220**	**78.80**
**City of Caloocan**	**36,488**	13,255	12,774	**26,029**	**71.34**
**City of Las Piñas**	**12,284**	4,967	4,860	**9,827**	**80.00**
**City of Makati**	**4,580**	2,202	2,109	**4,311**	**94.13**
**City of Malabon**	**8,070**	3,116	3,148	**6,264**	**77.62**
**City of Mandaluyong**	**8,672**	4,190	4,164	**8,354**	**96.33**
**City of Manila**	**37,288**	18,085	17,445	**35,530**	**95.29**
**City of Marikina**	**9,135**	1,698	1,627	**3,325**	**36.40**
**City of Muntinlupa**	**10,788**	2,962	2,934	**5,896**	**54.65**
**City of Navotas**	**5,718**	2,776	2,666	**5,442**	**95.17**
**City of Parañaque**	**14,517**	6,273	6,191	**12,464**	**85.86**
**Pasay City**	**7,684**	3,202	2,975	**6,177**	**80.39**
**City of Pasig**	**19,265**	8,317	8,145	**16,462**	**85.45**
**Quezon City**	**64,737**	32,933	32,672	**65,605**	**101.34**
**City of San Juan**	**2,363**	535	562	**1,097**	**46.42**
**City of Taguig**	**28,175**	5,321	4,909	**10,230**	**36.31**
**City of Valenzuela**	**14,813**	3,795	3,559	**7,354**	**49.65**
**Pateros**	**1,221**	458	395	**853**	**69.86**
**CAR**	**34,199**	**11,838**	**10,830**	**22,668**	**66.28**
**City of Baguio**	**5,847**	1,916	1,775	**3,691**	**63.13**
**Abra**	**3,951**	1,651	1,542	**3,193**	**80.81**
Bangued	808	378	368	**746**	**92.33**
Boliney	79	15	23	**38**	**48.10**
Bucay	279	138	117	**255**	**91.40**
Bucloc	30	7	6	**13**	**43.33**
Daguioman	34	16	9	**25**	**73.53**
Danglas	60	33	27	**60**	**100.00**
Dolores	193	92	81	**173**	**89.64**
La Paz	268	125	145	**270**	**100.75**
Lacub	67	12	13	**25**	**37.31**
Lagangilang	243	100	97	**197**	**81.07**
Lagayan	64	37	24	**61**	**95.31**
Langiden	58	17	12	**29**	**50.00**
Licuan-Baay	49	20	18	**38**	**77.55**
Luba	96	32	38	**70**	**72.92**
Malibcong	63	9	9	**18**	**28.57**
Manabo	193	92	81	**173**	**89.64**
Peñarrubia	107	71	52	**123**	**114.95**
Pidigan	185	94	59	**153**	**82.70**
Pilar	130	65	39	**104**	**80.00**
Sallapadan	98	20	39	**59**	**60.20**
San Isidro	60	40	32	**72**	**120.00**
San Juan	201	45	59	**104**	**51.74**
San Quintin	96	28	45	**73**	**76.04**
Tayum	237	70	54	**124**	**52.32**
Tineg	96	29	31	**60**	**62.50**
Tubo	66	34	32	**66**	**100.00**
Villaviciosa	91	32	32	**64**	**70.33**
**Benguet**	**9,666**	2,570	2,410	**4,980**	**51.52**
Atok	364	98	92	**190**	**52.20**
Bakun	326	75	82	**157**	**48.16**
Bokod	317	88	93	**181**	**57.10**
Buguias	1,045	311	257	**568**	**54.35**
Itogon	1,208	243	219	**462**	**38.25**
Kabayan	340	103	105	**208**	**61.18**
Kapangan	294	100	77	**177**	**60.20**
Kibungan	411	101	103	**204**	**49.64**
La Trinidad	2,934	751	718	**1,469**	**50.07**
Mankayan	936	250	259	**509**	**54.38**
Sablan	182	64	55	**119**	**65.38**
Tuba	911	191	188	**379**	**41.60**
Tublay	398	195	162	**357**	**89.70**
**Ifugao**	**4,502**	1,584	1,402	**2,986**	**66.33**
Banaue	368	110	113	**223**	**60.60**
Hungduan	187	53	44	**97**	**51.87**
Kiangan	336	117	117	**234**	**69.64**

2.C.1.7 Immunization Services for Infants and Children
Number and Proportion of Fully Immunized Children
Philippines, 2024

Area	Eligible Population (0-12 years old)	Fully Immunized Children (FIC)			
		Male	Female	Total	%
Lagawe	373	130	119	**249**	**66.76**
Lamut	531	194	185	**379**	**71.37**
Mayoyao	320	84	58	**142**	**44.38**
Alfonso Lista	705	321	267	**588**	**83.40**
Aguinaldo	556	207	156	**363**	**65.29**
Hingyon	174	62	44	**106**	**60.92**
Tinoc	484	176	176	**352**	**72.73**
Asipulo	468	130	123	**253**	**54.06**
**Kalinga**	**4,979**	2,146	1,927	**4,073**	**81.80**
Balbalan	198	75	77	**152**	**76.77**
Lubuagan	181	56	74	**130**	**71.82**
Pasil	207	67	54	**121**	**58.45**
Pinukpuk	739	199	189	**388**	**52.50**
Rizal	419	176	122	**298**	**71.12**
City of Tabuk	2,809	1,440	1,263	**2,703**	**96.23**
Tanudan	151	69	59	**128**	**84.77**
Tinglayan	275	64	89	**153**	**55.64**
**Mountain Province**	**2,838**	1,079	939	**2,018**	**71.11**
Barlig	81	29	19	**48**	**59.26**
Bauko	605	283	244	**527**	**87.11**
Besao	124	35	39	**74**	**59.68**
Bontoc	405	159	137	**296**	**73.09**
Natonin	183	63	73	**136**	**74.32**
Paracelis	712	227	187	**414**	**58.15**
Sabangan	160	53	41	**94**	**58.75**
Sadanga	125	70	53	**123**	**98.40**
Sagada	168	57	47	**104**	**61.90**
Tadian	275	103	99	**202**	**73.45**
**Apayao**	**2,416**	892	835	**1,727**	**71.48**
Calanasan	215	88	65	**153**	**71.16**
Conner	621	201	191	**392**	**63.12**
Flora	351	101	104	**205**	**58.40**
Kabugao	291	125	127	**252**	**86.60**
Luna	440	138	140	**278**	**63.18**
Pudtol	266	137	111	**248**	**93.23**
Santa Marcela	232	102	97	**199**	**85.78**
**Region 1**	**104,281**	**40,150**	**38,168**	**78,318**	**75.10**
**Ilocos Norte**	**9,590**	4,429	4,291	**8,720**	**90.93**
Adams	59	7	13	**20**	**33.90**
Bacarra	472	195	172	**367**	**77.75**
Badoc	467	208	173	**381**	**81.58**
Bangui	189	108	92	**200**	**105.82**
City of Batac	874	400	376	**776**	**88.79**
Burgos	146	50	63	**113**	**77.40**
Carasi	31	14	10	**24**	**77.42**
Currimao	179	100	71	**171**	**95.53**
Dingras	667	363	339	**702**	**105.25**
Dumalneg	56	26	28	**54**	**96.43**
Banna	257	112	106	**218**	**84.82**
City of Laoag	1,965	912	994	**1,906**	**97.00**
Marcos	310	165	112	**277**	**89.35**
Nueva Era	247	120	115	**235**	**95.14**
Pagudpud	430	183	182	**365**	**84.88**
Paoay	342	131	157	**288**	**84.21**
Pasuquin	436	216	192	**408**	**93.58**
Piddig	403	196	192	**388**	**96.28**
Pinili	241	116	132	**248**	**102.90**
San Nicolas	639	233	238	**471**	**73.71**
Sarrat	339	168	156	**324**	**95.58**
Solsona	361	195	180	**375**	**103.88**
Vintar	480	211	198	**409**	**85.21**
**Ilocos Sur**	**10,656**	5,494	5,391	**10,885**	**102.15**
Alilem	95	46	50	**96**	**101.05**
Banayoyo	127	50	50	**100**	**78.74**
Bantay	603	400	347	**747**	**123.88**
Burgos	183	89	87	**176**	**96.17**

2.C.1.7 Immunization Services for Infants and Children

Number and Proportion of Fully Immunized Children

Philippines, 2024

Area	Eligible Population (0-12 years old)	Fully Immunized Children (FIC)			
		Male	Female	Total	%
Cabugao	639	244	240	**484**	**75.74**
City of Candon	1,088	606	617	**1,223**	**112.41**
Caoayan	306	200	174	**374**	**122.22**
Cervantes	338	165	132	**297**	**87.87**
Galimuyod	120	45	56	**101**	**84.17**
Gregorio del Pilar	66	37	20	**57**	**86.36**
Lidlidda	59	23	28	**51**	**86.44**
Magsingal	486	272	262	**534**	**109.88**
Nagbukel	77	31	24	**55**	**71.43**
Narvacan	723	424	385	**809**	**111.89**
Quirino	121	59	78	**137**	**113.22**
Salcedo	117	58	62	**120**	**102.56**
San Emilio	82	52	44	**96**	**117.07**
San Esteban	110	42	46	**88**	**80.00**
San Ildefonso	125	58	42	**100**	**80.00**
San Juan	356	162	176	**338**	**94.94**
San Vicente	224	101	85	**186**	**83.04**
Santa	192	109	90	**199**	**103.65**
Santa Catalina	235	120	126	**246**	**104.68**
Santa Cruz	595	278	287	**565**	**94.96**
Santa Lucia	385	284	341	**625**	**162.34**
Santa Maria	409	127	120	**247**	**60.39**
Santiago	224	118	118	**236**	**105.36**
Santo Domingo	356	198	197	**395**	**110.96**
Sigay	35	13	12	**25**	**71.43**
Sinait	327	213	210	**423**	**129.36**
Sugpon	83	22	18	**40**	**48.19**
Suyo	148	77	61	**138**	**93.24**
Tagudin	739	367	368	**735**	**99.46**
City of Vigan	883	404	438	**842**	**95.36**
**La Union**	**14,588**	5,208	4,643	**9,851**	**67.53**
Agoo	1,262	393	333	**726**	**57.53**
Aringay	909	336	309	**645**	**70.96**
Bacnotan	753	261	255	**516**	**68.53**
Bagulin	292	85	85	**170**	**58.22**
Balaoan	593	239	209	**448**	**75.55**
Bangar	597	217	198	**415**	**69.51**
Bauang	1,479	476	454	**930**	**62.88**
Burgos	157	44	70	**114**	**72.61**
Caba	399	146	111	**257**	**64.41**
Luna	575	232	239	**471**	**81.91**
Naguilian	895	380	297	**677**	**75.64**
Pugo	362	144	106	**250**	**69.06**
Rosario	1,159	364	352	**716**	**61.78**
City of San Fernando	2,337	896	742	**1,638**	**70.09**
San Gabriel	348	127	113	**240**	**68.97**
San Juan	704	241	200	**441**	**62.64**
Santo Tomas	753	257	238	**495**	**65.74**
Santol	245	93	70	**163**	**66.53**
Sudipen	232	83	71	**154**	**66.38**
Tubao	537	194	191	**385**	**71.69**
**Pangasinan**	**65,801**	**24,065**	**22,966**	**47,031**	**71.47**
Agno	653	264	245	**509**	**77.95**
Aguilar	1,078	289	296	**585**	**54.27**
City of Alaminos	2,181	667	668	**1,335**	**61.21**
Alcala	1,045	561	531	**1,092**	**104.50**
Anda	872	473	461	**934**	**107.11**
Asingan	1,104	454	437	**891**	**80.71**
Balungao	603	213	219	**432**	**71.64**
Bani	1,108	525	536	**1,061**	**95.76**
Basista	887	454	401	**855**	**96.39**
Bautista	916	259	257	**516**	**56.33**
Bayambang	2,953	1,053	1,012	**2,065**	**69.93**
Binalonan	1,017	500	495	**995**	**97.84**
Binmaley	2,031	628	588	**1,216**	**59.87**
Bolinao	1,932	612	616	**1,228**	**63.56**

2.C.1.7 Immunization Services for Infants and Children
Number and Proportion of Fully Immunized Children
Philippines, 2024

Area	Eligible Population (0-12 years old)	Fully Immunized Children (FIC)			
		Male	Female	Total	%
Bugallon	1,858	640	577	**1,217**	**65.50**
Burgos	453	229	197	**426**	**94.04**
Calasiao	2,131	769	695	**1,464**	**68.70**
Dasol	670	276	307	**583**	**87.01**
Infanta	616	243	255	**498**	**80.84**
Labrador	590	131	146	**277**	**46.95**
Lingayen	2,289	1,008	806	**1,814**	**79.25**
Mabini	509	275	292	**567**	**111.39**
Malasiqui	3,258	1,042	1,085	**2,127**	**65.29**
Manaoag	1,685	713	735	**1,448**	**85.93**
Mangaldan	2,725	884	817	**1,701**	**62.42**
Mangatarem	1,650	511	478	**989**	**59.94**
Mapandan	886	302	277	**579**	**65.35**
Natividad	499	201	179	**380**	**76.15**
Pozorrubio	1,613	636	597	**1,233**	**76.44**
Rosales	1,393	528	432	**960**	**68.92**
City of San Carlos	4,804	1,606	1,517	**3,123**	**65.01**
San Fabian	2,034	670	715	**1,385**	**68.09**
San Jacinto	1,014	233	247	**480**	**47.34**
San Manuel	1,155	462	414	**876**	**75.84**
San Nicolas	729	245	218	**463**	**63.51**
San Quintin	735	253	267	**520**	**70.75**
Santa Barbara	2,066	1,027	947	**1,974**	**95.55**
Santa Maria	649	205	183	**388**	**59.78**
Santo Tomas	235	86	57	**143**	**60.85**
Sison	1,079	429	386	**815**	**75.53**
Sual	902	329	277	**606**	**67.18**
Tayug	1,023	234	236	**470**	**45.94**
Umingan	1,600	488	514	**1,002**	**62.63**
Urbiztondo	1,426	725	675	**1,400**	**98.18**
City of Urdaneta	3,165	1,032	949	**1,981**	**62.59**
Villasis	1,327	340	364	**704**	**53.05**
Laoac	653	361	363	**724**	**110.87**
**City of Dagupan**	**3,646**	**954**	**877**	**1,831**	**50.22**
**Region 2**	**69,664**	**25,334**	**23,729**	**49,063**	**70.43**
**City of Santiago**	**3200**	1,579	1,538	3,117	97.41
**Batanes**	**259**	127	88	215	83.01
Basco	159	78	54	132	83.02
Itbayat	36	25	7	32	88.89
Ivana	15	7	8	15	100.00
Mahatao	20	7	7	14	70.00
Sabtang	16	5	4	9	56.25
Uyugan	13	5	8	13	100.00
**Cagayan**	**22,583**	7,583	7,079	14,662	64.92
Abulug	668	142	142	284	42.51
Alcala	640	177	194	371	57.97
Allacapan	633	206	198	404	63.82
Amulung	965	319	318	637	66.01
Aparri	1,284	383	344	727	56.62
Baggao	1,519	513	542	1,055	69.45
Ballesteros	725	241	234	475	65.52
Buguey	510	174	183	357	70.00
Calayan	309	92	98	190	61.49
Camalaniugan	398	151	136	287	72.11
Claveria	474	129	122	251	52.95
Enrile	611	233	240	473	77.41
Gattaran	1,065	383	314	697	65.45
Gonzaga	808	299	262	561	69.43
Iguig	573	195	151	346	60.38
Lal-Lo	914	341	344	685	74.95
Lasam	739	231	238	469	63.46
Pamplona	439	128	109	237	53.99
Peñablanca	978	302	309	611	62.47
Piat	423	138	110	248	58.63
Rizal	350	118	109	227	64.86
Sanchez-Mira	411	148	113	261	63.50

2.C.1.7 Immunization Services for Infants and Children
Number and Proportion of Fully Immunized Children
Philippines, 2024

Area	Eligible Population (0-12 years old)	Fully Immunized Children (FIC)			
		Male	Female	Total	%
Santa Ana	797	253	249	502	62.99
Santa Praxedes	61	13	25	38	62.30
Santa Teresita	339	140	119	259	76.40
Santo Niño	516	197	150	347	67.25
Solana	1,622	654	512	1,166	71.89
Tuao	1,038	400	409	809	77.94
Tuguegarao City	2,774	883	805	1,688	60.85
**Isabela**	**29,775**	11,201	10,596	21,797	73.21
Alicia	1,269	517	498	1,015	79.98
Angadanan	789	381	373	754	95.56
Aurora	616	302	295	597	96.92
Benito Soliven	541	193	190	383	70.79
Burgos	490	155	137	292	59.59
Cabagan	1,035	422	430	852	82.32
Cabatuan	686	276	256	532	77.55
City of Cauayan	2,890	919	874	1,793	62.04
Cordon	980	379	301	680	69.39
Dinapigue	142	62	74	136	95.77
Divilacan	111	26	26	52	46.85
Echague	1,891	644	625	1,269	67.11
Gamu	536	185	191	376	70.15
City of Ilagan	3,060	1,221	1,176	2,397	78.33
Jones	761	295	288	583	76.61
Luna	376	121	113	234	62.23
Maconacon	81	20	26	46	56.79
Delfin Albano	503	127	147	274	54.47
Mallig	633	210	208	418	66.03
Naguilian	524	239	254	493	94.08
Palanan	326	138	126	264	80.98
Quezon	503	178	166	344	68.39
Quirino	430	138	123	261	60.70
Ramon	1,226	428	409	837	68.27
Reina Mercedes	531	203	169	372	70.06
Roxas	1,323	463	405	868	65.61
San Agustin	325	128	97	225	69.23
San Guillermo	423	198	145	343	81.09
San Isidro	519	192	164	356	68.59
San Manuel	656	204	213	417	63.57
San Mariano	1,241	535	487	1,022	82.35
San Mateo	1,182	488	404	892	75.47
San Pablo	531	212	198	410	77.21
Santa Maria	565	206	220	426	75.40
Santo Tomas	477	147	137	284	59.54
Tumauini	1,603	649	651	1,300	81.10
**Nueva Vizcaya**	**9,661**	3,125	2,899	6,024	62.35
Ambaguio	409	110	107	217	53.06
Aritao	794	277	267	544	68.51
Bagabag	649	194	189	383	59.01
Bambang	1,002	415	362	777	77.54
Bayombong	1,252	444	400	844	67.41
Diadi	375	144	129	273	72.80
Dupax del Norte	610	159	148	307	50.33
Dupax del Sur	415	103	125	228	54.94
Kasibu	1,010	234	225	459	45.45
Kayapa	621	153	137	290	46.70
Quezon	502	197	188	385	76.69
Santa Fe	345	132	100	232	67.25
Solano	1,118	379	306	685	61.27
Villaverde	397	122	144	266	67.00
Alfonso Castaneda	162	62	72	134	82.72
**Quirino**	**4,186**	1,719	1,529	3,248	77.59
Aglipay	616	198	144	342	55.52
Cabarroguis	646	288	234	522	80.80
Diffun	1,214	593	574	1,167	96.13
Maddela	816	284	240	524	64.22
Saguday	316	144	142	286	90.51

2.C.1.7 Immunization Services for Infants and Children
Number and Proportion of Fully Immunized Children
Philippines, 2024

Area	Eligible Population (0-12 years old)	Fully Immunized Children (FIC)			
		Male	Female	Total	%
Nagtipunan	578	212	195	407	70.42
**Region 3**	**256,011**	**99,456**	**95,223**	**194,679**	**76.04**
**City of Angeles**	**10,104**	3,152	3,096	**6,248**	**61.84**
**City of Olongapo**	**4,991**	1,846	1,781	**3,627**	**72.67**
**Bataan**	**19,041**	6,211	5,844	**12,055**	**63.31**
Abucay	929	318	308	**626**	**67.38**
Bagac	694	237	206	**443**	**63.83**
City of Balanga	2,255	731	617	**1,348**	**59.78**
Dinalupihan	2,624	998	882	**1,880**	**71.65**
Hermosa	1,797	597	578	**1,175**	**65.39**
Limay	1,964	543	549	**1,092**	**55.60**
Mariveles	3,188	1,029	1,057	**2,086**	**65.43**
Morong	835	275	241	**516**	**61.80**
Orani	1,575	487	447	**934**	**59.30**
Orion	1,367	410	352	**762**	**55.74**
Pilar	1,014	266	304	**570**	**56.21**
Samal	799	320	303	**623**	**77.97**
**Bulacan**	**74,192**	31,932	30,098	**62,030**	**83.61**
**Hospitals**		0	0	**0**	**0.00**
Angat	1,281	636	628	**1,264**	**98.67**
Balagtas	1,433	649	581	**1,230**	**85.83**
City of Baliwag	3,496	1,746	1,650	**3,396**	**97.14**
Bocaue	3,063	893	840	**1,733**	**56.58**
Bulacan	1,419	702	601	**1,303**	**91.83**
Bustos	1,443	619	542	**1,161**	**80.46**
Calumpit	2,408	958	929	**1,887**	**78.36**
Guiguinto	2,157	1,041	1,021	**2,062**	**95.60**
Hagonoy	2,758	1,352	1,363	**2,715**	**98.44**
City of Malolos	4,297	1,652	1,435	**3,087**	**71.84**
Marilao	5,427	1,911	1,900	**3,811**	**70.22**
City of Meycauayan	4,925	1,965	1,825	**3,790**	**76.95**
Norzagaray	2,955	1,110	1,021	**2,131**	**72.12**
Obando	1,015	532	533	**1,065**	**104.93**
Pandi	3,603	1,044	1,008	**2,052**	**56.95**
Paombong	1,032	341	314	**655**	**63.47**
Plaridel	1,970	1,077	893	**1,970**	**100.00**
Pulilan	2,089	1,107	1,018	**2,125**	**101.72**
San Ildefonso	2,425	873	878	**1,751**	**72.21**
City of San Jose Del Monte	12,946	6,291	6,095	**12,386**	**95.67**
San Miguel	3,577	1,377	1,261	**2,638**	**73.75**
San Rafael	1,922	685	632	**1,317**	**68.52**
Santa Maria	5,722	3,057	2,788	**5,845**	**102.15**
Doña Remedios Trinidad	829	314	342	**656**	**79.13**
**Nueva Ecija**	**47,474**	16,280	15,665	**31,945**	**67.29**
Aliaga	1,428	470	444	**914**	**64.01**
Bongabon	1,433	471	474	**945**	**65.95**
City of Cabanatuan	6,631	2,101	1,963	**4,064**	**61.29**
Cabiao	1,799	758	719	**1,477**	**82.10**
Carranglan	917	286	311	**597**	**65.10**
Cuyapo	1,276	426	374	**800**	**62.70**
Gabaldon	930	350	332	**682**	**73.33**
City of Gapan	2,487	745	779	**1,524**	**61.28**
General Mamerto Natividad	996	296	318	**614**	**61.65**
General Tinio	1,227	397	378	**775**	**63.16**
Guimba	2,555	817	763	**1,580**	**61.84**
Jaen	1,625	616	568	**1,184**	**72.86**
Laur	760	274	263	**537**	**70.66**
Licab	548	196	209	**405**	**73.91**
Llanera	920	370	433	**803**	**87.28**
Lupao	910	264	292	**556**	**61.10**
Science City of Muñoz	1,616	653	592	**1,245**	**77.04**
Nampicuan	276	92	75	**167**	**60.51**
City of Palayan	1,181	352	321	**673**	**56.99**
Pantabangan	642	287	275	**562**	**87.54**
Peñaranda	611	250	202	**452**	**73.98**
Quezon	809	266	304	**570**	**70.46**

2.C.1.7 Immunization Services for Infants and Children
Number and Proportion of Fully Immunized Children
Philippines, 2024

Area	Eligible Population (0-12 years old)	Fully Immunized Children (FIC)			
		Male	Female	Total	%
Rizal	1,365	528	474	**1,002**	**73.41**
San Antonio	1,662	624	600	**1,224**	**73.65**
San Isidro	1,117	357	352	**709**	**63.47**
San Jose City	3,381	1,279	1,211	**2,490**	**73.65**
San Leonardo	1,408	429	449	**878**	**62.36**
Santa Rosa	1,522	473	450	**923**	**60.64**
Santo Domingo	1,156	395	375	**770**	**66.61**
Talavera	2,748	941	891	**1,832**	**66.67**
Talugtug	458	159	126	**285**	**62.23**
Zaragoza	1,080	358	348	**706**	**65.37**
**Pampanga**	**50,255**	18,054	17,516	**35,570**	**70.78**
Apalit	2,327	833	785	**1,618**	**69.53**
Arayat	3,260	1,037	1,063	**2,100**	**64.42**
Bacolor	917	223	202	**425**	**46.35**
Candaba	2,434	799	788	**1,587**	**65.20**
Floridablanca	2,841	1,085	1,046	**2,131**	**75.01**
Guagua	2,425	1,078	1,133	**2,211**	**91.18**
Lubao	3,865	1,482	1,328	**2,810**	**72.70**
Mabalacat City	5,887	2,060	2,063	**4,123**	**70.04**
Macabebe	1,681	425	454	**879**	**52.29**
Magalang	2,613	789	714	**1,503**	**57.52**
Masantol	1,371	318	324	**642**	**46.83**
Mexico	3,533	1,202	1,124	**2,326**	**65.84**
Minalin	1,131	345	330	**675**	**59.68**
Porac	3,052	1,126	997	**2,123**	**69.56**
City of San Fernando	7,130	2,935	3,048	**5,983**	**83.91**
San Luis	1,007	502	431	**933**	**92.65**
San Simon	1,298	477	402	**879**	**67.72**
Santa Ana	1,175	460	400	**860**	**73.19**
Santa Rita	935	338	324	**662**	**70.80**
Sto. Tomas	807	265	278	**543**	**67.29**
Sasmuan	566	275	282	**557**	**98.41**
**Tarlac**	**32,147**	14,732	14,482	**29,214**	**90.88**
Anao	155	82	90	**172**	**110.97**
Bamban	1,774	752	690	**1,442**	**81.29**
Camiling	1,565	700	712	**1,412**	**90.22**
Capas	3,736	1,881	1,852	**3,733**	**99.92**
Concepcion	3,945	1,724	1,692	**3,416**	**86.59**
Gerona	1,802	805	715	**1,520**	**84.35**
La Paz	1,465	649	639	**1,288**	**87.92**
Mayantoc	534	262	264	**526**	**98.50**
Moncada	1,169	560	606	**1,166**	**99.74**
Paniqui	2,012	853	760	**1,613**	**80.17**
Pura	420	202	160	**362**	**86.19**
Ramos	462	199	187	**386**	**83.55**
San Clemente	193	99	76	**175**	**90.67**
San Manuel	588	273	288	**561**	**95.41**
Santa Ignacia	968	518	578	**1,096**	**113.22**
City of Tarlac	8,885	4,073	4,047	**8,120**	**91.39**
Victoria	1,462	606	650	**1,256**	**85.91**
San Jose	1,012	494	476	**970**	**95.85**
**Zambales**	**12,830**	4,901	4,518	**9,419**	**73.41**
Botolan	1,514	532	505	**1,037**	**68.49**
Cabangan	610	229	221	**450**	**73.77**
Candelaria	624	247	210	**457**	**73.24**
Castillejos	1,371	504	457	**961**	**70.09**
Iba	1,056	474	409	**883**	**83.62**
Masinloc	1,145	494	456	**950**	**82.97**
Palauig	769	286	266	**552**	**71.78**
San Antonio	646	257	274	**531**	**82.20**
San Felipe	412	167	170	**337**	**81.80**
San Marcelino	646	263	255	**518**	**80.19**
San Narciso	449	221	184	**405**	**90.20**
Santa Cruz	1,223	533	429	**962**	**78.66**
Subic	2,365	694	682	**1,376**	**58.18**
**Aurora**	**4,977**	2,348	2,223	**4,571**	**91.84**

2.C.1.7 Immunization Services for Infants and Children
Number and Proportion of Fully Immunized Children
Philippines, 2024

Area	Eligible Population (0-12 years old)	Fully Immunized Children (FIC)			
		Male	Female	Total	%
Baler	909	471	431	**902**	**99.23**
Casiguran	591	235	231	**466**	**78.85**
Dilasag	412	171	175	**346**	**83.98**
Dinalungan	243	130	111	**241**	**99.18**
Dingalan	569	273	273	**546**	**95.96**
Dipaculao	775	387	373	**760**	**98.06**
Maria Aurora	969	386	388	**774**	**79.88**
San Luis	509	295	241	**536**	**105.30**
**Region 4A**	**337,182**	**104,690**	**99,762**	**204,452**	**60.64**
**City of Lucena**	**6,759**	**2,484**	**2,407**	**4,891**	**72.36**
**Batangas**	**58,339**	**18,051**	**17,149**	**35,200**	**60.34**
Agoncillo	737	287	302	**589**	**79.92**
Alitagtag	563	143	131	**274**	**48.67**
Balayan	1,836	333	328	**661**	**36.00**
Balete	568	118	123	**241**	**42.43**
Batangas City	6,751	2,046	1,932	**3,978**	**58.92**
Bauan	1,529	854	778	**1,632**	**106.74**
City of Calaca	1,834	868	839	**1,707**	**93.08**
Calatagan	1,172	357	273	**630**	**53.75**
Cuenca	714	170	189	**359**	**50.28**
Ibaan	1,203	213	213	**426**	**35.41**
Laurel	937	158	176	**334**	**35.65**
Lemery	1,784	526	489	**1,015**	**56.89**
Lian	1,156	161	150	**311**	**26.90**
City of Lipa	7,893	2,292	2,148	**4,440**	**56.25**
Lobo	751	224	251	**475**	**63.25**
Mabini	929	283	240	**523**	**56.30**
Malvar	1,347	485	453	**938**	**69.64**
Mataasnakahoy	549	237	209	**446**	**81.24**
Nasugbu	3,182	1,025	1,025	**2,050**	**64.42**
Padre Garcia	1,074	357	326	**683**	**63.59**
Rosario	2,564	724	753	**1,477**	**57.61**
San Jose	1,550	728	659	**1,387**	**89.48**
San Juan	2,320	740	743	**1,483**	**63.92**
San Luis	626	140	112	**252**	**40.26**
San Nicolas	410	169	156	**325**	**79.27**
San Pascual	1,383	427	364	**791**	**57.19**
Santa Teresita	385	102	103	**205**	**53.25**
City of Sto. Tomas	4,590	1,197	1,134	**2,331**	**50.78**
Taal	1,132	494	473	**967**	**85.42**
Talisay	908	172	163	**335**	**36.89**
City of Tanauan	3,933	1,406	1,328	**2,734**	**69.51**
Taysan	759	338	295	**633**	**83.40**
Tingloy	360	71	72	**143**	**39.72**
Tuy	910	206	219	**425**	**46.70**
**Cavite**	**85,491**	**27,309**	**26,286**	**53,595**	**62.69**
Alfonso	1,018	311	305	**616**	**60.51**
Amadeo	738	218	222	**440**	**59.62**
City of Bacoor	13,038	2,591	2,550	**5,141**	**39.43**
Carmona	2,216	1,023	929	**1,952**	**88.09**
City of Cavite	1,853	439	448	**887**	**47.87**
City of Dasmariñas	13,153	5,054	4,889	**9,943**	**75.59**
General Emilio Aguinaldo	463	142	183	**325**	**70.19**
City of General Trias	9,091	2,849	2,672	**5,521**	**60.73**
City of Imus	8,391	2,024	1,856	**3,880**	**46.24**
Indang	1,123	408	412	**820**	**73.02**
Kawit	2,140	604	510	**1,114**	**52.06**
Magallanes	505	154	142	**296**	**58.61**
Maragondon	873	335	341	**676**	**77.43**
Mendez	651	211	206	**417**	**64.06**
Naic	3,400	1,422	1,265	**2,687**	**79.03**
Noveleta	909	267	281	**548**	**60.29**
Rosario	2,085	712	690	**1,402**	**67.24**
Silang	6,278	2,597	2,425	**5,022**	**79.99**
City of Tagaytay	1,722	853	828	**1,681**	**97.62**
Tanza	6,739	2,127	2,124	**4,251**	**63.08**

2.C.1.7 Immunization Services for Infants and Children
Number and Proportion of Fully Immunized Children
Philippines, 2024

Area	Eligible Population (0-12 years old)	Fully Immunized Children (FIC)			
		Male	Female	Total	%
Ternate	500	162	183	**345**	**69.00**
City of Trece Martires	4,761	1,639	1,669	**3,308**	**69.48**
Gen. Mariano Alvarez	3,844	1,167	1,156	**2,323**	**60.43**
**Laguna**	**67,298**	**23,989**	**23,092**	**47,081**	**69.96**
Alaminos	1,015	302	275	**577**	**56.85**
Bay	1,484	255	207	**462**	**31.13**
City of Biñan	7,006	3,368	3,353	**6,721**	**95.93**
City of Cabuyao	6,963	3,374	3,319	**6,693**	**96.12**
City of Calamba	10,936	4,097	3,866	**7,963**	**72.81**
Calauan	1,708	645	675	**1,320**	**77.28**
Cavinti	444	121	103	**224**	**50.45**
Famy	412	99	115	**214**	**51.94**
Kalayaan	565	222	194	**416**	**73.63**
Liliw	690	207	219	**426**	**61.74**
Los Baños	2,111	493	480	**973**	**46.09**
Luisiana	404	81	78	**159**	**39.36**
Lumban	686	196	188	**384**	**55.98**
Mabitac	506	157	137	**294**	**58.10**
Magdalena	621	159	143	**302**	**48.63**
Majayjay	588	107	87	**194**	**32.99**
Nagcarlan	1,288	478	424	**902**	**70.03**
Paete	526	164	156	**320**	**60.84**
Pagsanjan	947	247	224	**471**	**49.74**
Pakil	497	86	89	**175**	**35.21**
Pangil	564	154	153	**307**	**54.43**
Pila	1,185	358	301	**659**	**55.61**
Rizal	357	101	89	**190**	**53.22**
City of San Pablo	5,803	1,764	1,692	**3,456**	**59.56**
City of San Pedro	6,398	2,167	2,041	**4,208**	**65.77**
Santa Cruz	2,457	358	384	**742**	**30.20**
Santa Maria	693	176	146	**322**	**46.46**
City of Santa Rosa	8,521	3,627	3,592	**7,219**	**84.72**
Siniloan	924	232	176	**408**	**44.16**
Victoria	999	194	186	**380**	**38.04**
**Quezon**	**44,516**	**11,238**	**10,528**	**21,766**	**48.89**
Agdangan	301	50	68	**118**	**39.20**
Alabat	221	98	73	**171**	**77.38**
Atimonan	1,308	329	315	**644**	**49.24**
Buenavista	673	220	226	**446**	**66.27**
Burdeos	578	119	104	**223**	**38.58**
Calauag	1,611	319	281	**600**	**37.24**
Candelaria	3,108	909	824	**1,733**	**55.76**
Catanauan	1,759	343	358	**701**	**39.85**
Dolores	672	194	181	**375**	**55.80**
General Luna	563	149	127	**276**	**49.02**
General Nakar	1,012	286	220	**506**	**50.00**
Guinayangan	896	158	165	**323**	**36.05**
Gumaca	1,635	439	406	**845**	**51.68**
Infanta	1,798	397	375	**772**	**42.94**
Jomalig	229	50	44	**94**	**41.05**
Lopez	2,169	620	574	**1,194**	**55.05**
Lucban	1,024	285	265	**550**	**53.71**
Macalelon	586	156	154	**310**	**52.90**
Mauban	1,812	524	479	**1,003**	**55.35**
Mulanay	1,313	298	287	**585**	**44.55**
Padre Burgos	450	150	146	**296**	**65.78**
Pagbilao	1,746	508	545	**1,053**	**60.31**
Panukulan	461	72	58	**130**	**28.20**
Patnanungan	423	39	36	**75**	**17.73**
Perez	279	78	81	**159**	**56.99**
Pitogo	444	135	123	**258**	**58.11**
Plaridel	167	44	42	**86**	**51.50**
Polillo	826	218	223	**441**	**53.39**
Quezon	306	94	92	**186**	**60.78**
Real	956	326	336	**662**	**69.25**
Sampaloc	286	67	70	**137**	**47.90**

2.C.1.7 Immunization Services for Infants and Children
Number and Proportion of Fully Immunized Children
Philippines, 2024

Area	Eligible Population (0-12 years old)	Fully Immunized Children (FIC)			
		Male	Female	Total	%
San Andres	954	214	206	**420**	**44.03**
San Antonio	785	243	242	**485**	**61.78**
San Francisco	1,683	315	277	**592**	**35.18**
San Narciso	1,325	214	218	**432**	**32.60**
Sariaya	3,673	1,117	975	**2,092**	**56.96**
Tagkawayan	1,222	218	201	**419**	**34.29**
City of Tayabas	2,378	584	541	**1,125**	**47.31**
Tiaong	2,365	505	457	**962**	**40.68**
Unisan	519	154	133	**287**	**55.30**
**Rizal**	**74,779**	**21,619**	**20,300**	**41,919**	**56.06**
Angono	2,869	917	881	**1,798**	**62.67**
City of Antipolo	20,926	5,452	5,105	**10,557**	**50.45**
Baras	2,289	981	914	**1,895**	**82.79**
Binangonan	6,825	2,031	1,904	**3,935**	**57.66**
Cainta	7,291	2,199	2,156	**4,355**	**59.73**
Cardona	1,099	376	369	**745**	**67.79**
Jala-Jala	780	237	215	**452**	**57.95**
Rodriguez	10,412	2,311	2,134	**4,445**	**42.69**
Morong	1,434	543	512	**1,055**	**73.57**
Pililla	1,750	600	564	**1,164**	**66.51**
San Mateo	5,730	1,184	1,178	**2,362**	**41.22**
Tanay	3,517	1,083	992	**2,075**	**59.00**
Taytay	8,404	3,125	2,835	**5,960**	**70.92**
Teresa	1,453	580	541	**1,121**	**77.15**
**Region 4B**	**72,212**	**22,176**	**20,336**	**42,512**	**58.87**
**City of Puerto Princesa**	**6,103**	1,682	1,620	**3,302**	**54.10**
**Marinduque**	**4,531**	1,491	1,274	**2,765**	**61.02**
Boac	1,011	305	256	**561**	**55.49**
Buenavista	516	211	182	**393**	**76.16**
Gasan	691	223	193	**416**	**60.20**
Mogpog	636	220	162	**382**	**60.06**
Santa Cruz	997	312	286	**598**	**59.98**
Torrijos	680	220	195	**415**	**61.03**
**Occidental Mindoro**	**12,749**	4,248	3,947	**8,195**	**64.28**
Abra De Ilog	933	192	183	**375**	**40.19**
Calintaan	664	286	246	**532**	**80.12**
Looc	146	36	43	**79**	**54.11**
Lubang	259	75	76	**151**	**58.30**
Magsaysay	1,078	156	127	**283**	**26.25**
Mamburao	1,096	380	368	**748**	**68.25**
Paluan	556	200	154	**354**	**63.67**
Rizal	990	189	163	**352**	**35.56**
Sablayan	2,285	1,071	1,029	**2,100**	**91.90**
San Jose	3,688	1,393	1,292	**2,685**	**72.80**
Santa Cruz	1,054	270	266	**536**	**50.85**
**Oriental Mindoro**	**20,606**	6,565	6,007	**12,572**	**61.01**
Baco	863	293	293	**586**	**67.90**
Bansud	971	313	300	**613**	**63.13**
Bongabong	2,082	519	540	**1,059**	**50.86**
Bulalacao	1,242	416	401	**817**	**65.78**
City of Calapan	2,989	1,020	919	**1,939**	**64.87**
Gloria	1,145	342	293	**635**	**55.46**
Mansalay	1,638	421	407	**828**	**50.55**
Naujan	2,344	761	692	**1,453**	**61.99**
Pinamalayan	1,815	541	512	**1,053**	**58.02**
Pola	648	200	188	**388**	**59.88**
Puerto Galera	966	272	251	**523**	**54.14**
Roxas	1,427	674	634	**1,308**	**91.66**
San Teodoro	432	100	80	**180**	**41.67**
Socorro	881	238	203	**441**	**50.06**
Victoria	1,163	455	294	**749**	**64.40**
**Palawan**	**22,257**	6,062	5,538	**11,600**	**52.12**
Aborlan	771	279	243	**522**	**67.70**
Agutaya	248	48	53	**101**	**40.73**
Araceli	337	94	84	**178**	**52.82**
Balabac	1,372	105	96	**201**	**14.65**

2.C.1.7 Immunization Services for Infants and Children
Number and Proportion of Fully Immunized Children
Philippines, 2024

Area	Eligible Population (0-12 years old)	Fully Immunized Children (FIC)			
		Male	Female	Total	%
Bataraza	2,224	748	691	**1,439**	**64.70**
Brooke'S Point	1,550	592	572	**1,164**	**75.10**
Busuanga	725	150	126	**276**	**38.07**
Cagayancillo	119	23	21	**44**	**36.97**
Coron	1,802	682	678	**1,360**	**75.47**
Cuyo	489	130	112	**242**	**49.49**
Dumaran	529	143	144	**287**	**54.25**
El Nido	1,254	356	326	**682**	**54.39**
Linapacan	390	119	92	**211**	**54.10**
Magsaysay	212	83	69	**152**	**71.70**
Narra	1,560	389	403	**792**	**50.77**
Quezon	1,634	347	298	**645**	**39.47**
Roxas	1,533	324	278	**602**	**39.27**
San Vicente	771	291	189	**480**	**62.26**
Taytay	1,808	496	476	**972**	**53.76**
Kalayaan	0	2	1	**3**	**0.00**
Culion	570	72	43	**115**	**20.18**
Rizal	1,513	315	290	**605**	**39.99**
Sofronio Española	846	274	253	**527**	**62.29**
**Romblon**	**5,966**	2,128	1,950	**4,078**	**68.35**
Alcantara	333	114	112	**226**	**67.87**
Banton	75	23	10	**33**	**44.00**
Cajidiocan	495	128	129	**257**	**51.92**
Calatrava	207	73	61	**134**	**64.73**
Concepcion	50	10	8	**18**	**36.00**
Corcuera	114	44	52	**96**	**84.21**
Looc	386	151	149	**300**	**77.72**
Magdiwang	328	114	91	**205**	**62.50**
Odiongan	853	335	311	**646**	**75.73**
Romblon	827	286	261	**547**	**66.14**
San Agustin	466	178	182	**360**	**77.25**
San Andres	368	139	125	**264**	**71.74**
San Fernando	482	205	171	**376**	**78.01**
San Jose	237	88	71	**159**	**67.09**
Santa Fe	365	126	88	**214**	**58.63**
Ferrol	190	53	69	**122**	**64.21**
Santa Maria	190	61	60	**121**	**63.68**
**Region 5**	**148,559**	**36,519**	**34,551**	**71,070**	**47.84**
**Albay**	**30,159**	**8,773**	**8,437**	**17,210**	**57.06**
Bacacay	1,625	338	372	**710**	**43.69**
Camalig	1,527	600	543	**1,143**	**74.85**
Daraga	2,761	1,106	1,075	**2,181**	**78.99**
Guinobatan	1,864	608	598	**1,206**	**64.70**
Jovellar	350	85	92	**177**	**50.57**
City of Legazpi	4,800	1,388	1,311	**2,699**	**56.23**
Libon	1,758	306	334	**640**	**36.41**
City of Ligao	2,635	1,062	1,075	**2,137**	**81.10**
Malilipot	914	240	228	**468**	**51.20**
Malinao	1,033	184	183	**367**	**35.53**
Manito	644	143	122	**265**	**41.15**
Oas	1,374	374	352	**726**	**52.84**
Pio Duran	1,092	282	265	**547**	**50.09**
Polangui	1,789	465	457	**922**	**51.54**
Rapu-Rapu	881	156	139	**295**	**33.48**
Santo Domingo	707	268	241	**509**	**71.99**
City of Tabaco	3,120	794	715	**1,509**	**48.37**
Tiwi	1,285	374	335	**709**	**55.18**
**Camarines Norte**	**16,163**	**3,167**	**2,925**	**6,092**	**37.69**
Basud	1,108	336	298	**634**	**57.22**
Capalonga	855	110	100	**210**	**24.56**
Daet	2,404	648	573	**1,221**	**50.79**
San Lorenzo Ruiz	360	33	32	**65**	**18.06**
Jose Panganiban	1,867	232	249	**481**	**25.76**
Labo	2,885	445	441	**886**	**30.71**
Mercedes	1,511	343	302	**645**	**42.69**
Paracale	1,792	361	331	**692**	**38.62**

2.C.1.7 Immunization Services for Infants and Children
Number and Proportion of Fully Immunized Children
Philippines, 2024

Area	Eligible Population (0-12 years old)	Fully Immunized Children (FIC)			
		Male	Female	Total	%
San Vicente	277	84	64	**148**	**53.43**
Santa Elena	1,127	216	216	**432**	**38.33**
Talisay	648	119	98	**217**	**33.49**
Vinzons	1,329	240	221	**461**	**34.69**
**Camarines Sur**	**47,677**	**9,704**	**9,117**	**18,821**	**39.48**
Baao	1,316	158	193	**351**	**26.67**
Balatan	879	139	132	**271**	**30.83**
Bato	1,378	106	93	**199**	**14.44**
Bombon	440	137	122	**259**	**58.86**
Buhi	2,056	563	502	**1,065**	**51.80**
Bula	1,938	343	337	**680**	**35.09**
Cabusao	513	93	89	**182**	**35.48**
Calabanga	2,333	546	520	**1,066**	**45.69**
Camaligan	557	153	134	**287**	**51.53**
Canaman	768	139	123	**262**	**34.11**
Caramoan	1,404	208	182	**390**	**27.78**
Del Gallego	679	114	103	**217**	**31.96**
Gainza	292	60	57	**117**	**40.07**
Garchitorena	817	127	140	**267**	**32.68**
Goa	1,846	583	575	**1,158**	**62.73**
City of Iriga	2,584	616	586	**1,202**	**46.52**
Lagonoy	1,551	369	345	**714**	**46.03**
Libmanan	2,775	725	670	**1,395**	**50.27**
Lupi	829	156	163	**319**	**38.48**
Magarao	608	125	136	**261**	**42.93**
Milaor	852	152	132	**284**	**33.33**
Minalabac	1,322	297	253	**550**	**41.60**
Nabua	2,044	481	459	**940**	**45.99**
Ocampo	1,386	211	205	**416**	**30.01**
Pamplona	1,059	155	127	**282**	**26.63**
Pasacao	1,391	237	240	**477**	**34.29**
Pili	2,558	397	405	**802**	**31.35**
Presentacion	574	81	103	**184**	**32.06**
Ragay	1,571	311	306	**617**	**39.27**
Sagñay	960	246	215	**461**	**48.02**
San Fernando	1,017	200	200	**400**	**39.33**
San Jose	1,201	263	258	**521**	**43.38**
Sipocot	1,738	519	410	**929**	**53.45**
Siruma	489	73	57	**130**	**26.58**
Tigaon	1,757	314	293	**607**	**34.55**
Tinambac	2,195	307	252	**559**	**25.47**
**Catanduanes**	**5,886**	**2,028**	**1,893**	**3,921**	**66.62**
Bagamanoc	244	82	68	**150**	**61.48**
Baras	266	99	102	**201**	**75.56**
Bato	434	162	141	**303**	**69.82**
Caramoran	833	272	262	**534**	**64.11**
Gigmoto	180	60	50	**110**	**61.11**
Pandan	453	194	151	**345**	**76.16**
Panganiban	174	47	65	**112**	**64.37**
San Andres	844	276	221	**497**	**58.89**
San Miguel	321	90	107	**197**	**61.37**
Viga	503	181	179	**360**	**71.57**
Virac	1,634	565	547	**1,112**	**68.05**
**Masbate**	**24,426**	**6,113**	**5,856**	**11,969**	**49.00**
Aroroy	2,740	572	541	**1,113**	**40.62**
Baleno	696	173	178	**351**	**50.43**
Balud	1,011	269	275	**544**	**53.81**
Batuan	356	123	113	**236**	**66.29**
Cataingan	1,277	338	298	**636**	**49.80**
Cawayan	1,738	473	426	**899**	**51.73**
Claveria	1,041	333	306	**639**	**61.38**
Dimasalang	588	110	114	**224**	**38.10**
Esperanza	398	74	74	**148**	**37.19**
Mandaon	1,280	394	382	**776**	**60.63**
City of Masbate	3,190	993	912	**1,905**	**59.72**
Milagros	1,682	364	359	**723**	**42.98**

2.C.1.7 Immunization Services for Infants and Children
Number and Proportion of Fully Immunized Children
Philippines, 2024

Area	Eligible Population (0-12 years old)	Fully Immunized Children (FIC)			
		Male	Female	Total	%
Mobo	1,045	154	177	**331**	**31.67**
Monreal	622	160	190	**350**	**56.27**
Palanas	639	186	154	**340**	**53.21**
Pio V. Corpuz	544	109	109	**218**	**40.07**
Placer	1,637	300	302	**602**	**36.77**
San Fernando	439	144	130	**274**	**62.41**
San Jacinto	763	236	204	**440**	**57.67**
San Pascual	1,152	320	306	**626**	**54.34**
Uson	1,588	288	306	**594**	**37.41**
**Sorsogon**	**19,647**	**5,549**	**5,250**	**10,799**	**54.97**
Barcelona	376	147	131	**278**	**73.94**
Bulan	2,629	862	846	**1,708**	**64.97**
Bulusan	484	84	77	**161**	**33.26**
Casiguran	907	192	202	**394**	**43.44**
Castilla	1,467	332	372	**704**	**47.99**
Donsol	1,324	429	421	**850**	**64.20**
Gubat	1,194	361	347	**708**	**59.30**
Irosin	1,550	407	363	**770**	**49.68**
Juban	926	217	189	**406**	**43.84**
Magallanes	946	323	302	**625**	**66.07**
Matnog	984	307	259	**566**	**57.52**
Pilar	1,746	310	344	**654**	**37.46**
Prieto Diaz	468	151	116	**267**	**57.05**
Santa Magdalena	328	109	101	**210**	**64.02**
City of Sorsogon	4,318	1,318	1,180	**2,498**	**57.85**
**City of Naga**	**4,601**	**1,185**	**1,073**	**2,258**	**49.08**
**Region 6**	**160,897**	**54,470**	**51,433**	**105,903**	**65.82**
**City of Bacolod**	**11,280**	2,747	2,521	**5,268**	**46.70**
**City of Iloilo**	**9,509**	3,423	3,375	**6,798**	**71.49**
**Aklan**	**12,236**	3,817	3,527	**7,344**	**60.02**
Altavas	499	104	98	**202**	**40.48**
Balete	539	172	167	**339**	**62.89**
Banga	679	182	184	**366**	**53.90**
Batan	528	147	144	**291**	**55.11**
Buruanga	337	104	109	**213**	**63.20**
Ibajay	1,090	318	268	**586**	**53.76**
Kalibo	1,770	717	717	**1,434**	**81.02**
Lezo	265	100	94	**194**	**73.21**
Libacao	596	104	87	**191**	**32.05**
Madalag	342	109	119	**228**	**66.67**
Makato	595	270	228	**498**	**83.70**
Malay	1,443	409	407	**816**	**56.55**
Malinao	440	97	72	**169**	**38.41**
Nabas	973	318	287	**605**	**62.18**
New Washington	1,036	342	260	**602**	**58.11**
Numancia	655	170	158	**328**	**50.08**
Tangalan	449	154	128	**282**	**62.81**
**Antique**	**12,896**	4,355	4,161	**8,516**	**66.04**
Anini-Y	374	110	101	**211**	**56.42**
Barbaza	375	138	149	**287**	**76.53**
Belison	257	57	64	**121**	**47.08**
Bugasong	758	231	205	**436**	**57.52**
Caluya	1,185	443	416	**859**	**72.49**
Culasi	961	346	299	**645**	**67.12**
Tobias Fornier	630	162	146	**308**	**48.89**
Hamtic	993	385	372	**757**	**76.23**
Laua-An	568	186	199	**385**	**67.78**
Libertad	367	118	138	**256**	**69.75**
Pandan	701	216	223	**439**	**62.62**
Patnongon	788	212	228	**440**	**55.84**
San Jose	1,231	496	462	**958**	**77.82**
San Remigio	800	328	317	**645**	**80.63**
Sebaste	404	116	101	**217**	**53.71**
Sibalom	1,365	475	401	**876**	**64.18**
Tibiao	668	169	187	**356**	**53.29**
Valderrama	471	167	153	**320**	**67.94**

2.C.1.7 Immunization Services for Infants and Children
Number and Proportion of Fully Immunized Children
Philippines, 2024

Area	Eligible Population (0-12 years old)	Fully Immunized Children (FIC)			
		Male	Female	Total	%
**Capiz**	**15,118**	5,234	4,767	**10,001**	**66.15**
Cuartero	445	149	121	**270**	**60.67**
Dao	541	226	178	**404**	**74.68**
Dumalag	538	190	140	**330**	**61.34**
Dumarao	891	295	281	**576**	**64.65**
Ivisan	591	202	177	**379**	**64.13**
Jamindan	818	267	225	**492**	**60.15**
Ma-Ayon	811	264	267	**531**	**65.47**
Mambusao	707	214	164	**378**	**53.47**
Panay	835	286	270	**556**	**66.59**
Panitan	726	238	200	**438**	**60.33**
Pilar	1,057	286	266	**552**	**52.22**
Pontevedra	988	334	305	**639**	**64.68**
President Roxas	653	195	197	**392**	**60.03**
City of Roxas	3,373	1,263	1,214	**2,477**	**73.44**
Sapi-An	534	254	237	**491**	**91.95**
Sigma	535	229	206	**435**	**81.31**
Tapaz	1,075	342	319	**661**	**61.49**
**Iloilo**	**39,077**	14,858	13,875	**28,733**	**73.53**
Ajuy	1,155	403	367	**770**	**66.67**
Alimodian	730	169	176	**345**	**47.26**
Anilao	584	203	182	**385**	**65.92**
Badiangan	463	141	101	**242**	**52.27**
Balasan	689	256	231	**487**	**70.68**
Banate	647	251	227	**478**	**73.88**
Barotac Nuevo	1,125	582	512	**1,094**	**97.24**
Barotac Viejo	1,058	384	326	**710**	**67.11**
Batad	361	172	137	**309**	**85.60**
Bingawan	295	85	72	**157**	**53.22**
Cabatuan	1,047	526	507	**1,033**	**98.66**
Calinog	1,227	423	381	**804**	**65.53**
Carles	1,639	617	576	**1,193**	**72.79**
Concepcion	971	279	297	**576**	**59.32**
Dingle	737	243	227	**470**	**63.77**
Dueñas	525	212	189	**401**	**76.38**
Dumangas	1,337	465	457	**922**	**68.96**
Estancia	1,203	253	279	**532**	**44.22**
Guimbal	559	211	226	**437**	**78.18**
Igbaras	508	194	158	**352**	**69.29**
Janiuay	1,294	407	384	**791**	**61.13**
Lambunao	1,657	612	518	**1,130**	**68.20**
Leganes	641	215	237	**452**	**70.51**
Lemery	595	226	174	**400**	**67.23**
Leon	866	232	246	**478**	**55.20**
Maasin	826	251	225	**476**	**57.63**
Miagao	1,267	633	577	**1,210**	**95.50**
Mina	370	135	135	**270**	**72.97**
New Lucena	380	113	117	**230**	**60.53**
Oton	1,749	658	713	**1,371**	**78.39**
City of Passi	1,871	649	573	**1,222**	**65.31**
Pavia	1,262	591	571	**1,162**	**92.08**
Pototan	1,459	625	542	**1,167**	**79.99**
San Dionisio	869	339	322	**661**	**76.06**
San Enrique	791	426	408	**834**	**105.44**
San Joaquin	822	348	343	**691**	**84.06**
San Miguel	529	171	142	**313**	**59.17**
San Rafael	379	118	105	**223**	**58.84**
Santa Barbara	1,249	576	545	**1,121**	**89.75**
Sara	1,185	511	497	**1,008**	**85.06**
Tigbauan	1,250	642	563	**1,205**	**96.40**
Tubungan	406	141	122	**263**	**64.78**
Zarraga	500	170	188	**358**	**71.60**
**Negros Occidental**	**57,118**	18,811	17,959	**36,770**	**64.38**
City of Bago	4,013	1,642	1,608	**3,250**	**80.99**
Binalbagan	1,460	443	401	**844**	**57.81**
City of Cadiz	3,499	1,218	1,065	**2,283**	**65.25**

2.C.1.7 Immunization Services for Infants and Children
Number and Proportion of Fully Immunized Children
Philippines, 2024

Area	Eligible Population (0-12 years old)	Fully Immunized Children (FIC)			
		Male	Female	Total	%
Calatrava	1,777	487	467	**954**	**53.69**
Candoni	536	194	168	**362**	**67.54**
Cauayan	2,728	623	637	**1,260**	**46.19**
Enrique B. Magalona	1,379	397	389	**786**	**57.00**
City of Escalante	2,319	805	838	**1,643**	**70.85**
City of Himamaylan	2,571	992	928	**1,920**	**74.68**
Hinigaran	1,853	607	526	**1,133**	**61.14**
Hinoba-an	1,514	442	407	**849**	**56.08**
Ilog	1,356	470	459	**929**	**68.51**
Isabela	1,377	382	387	**769**	**55.85**
City of Kabankalan	4,626	1,400	1,397	**2,797**	**60.46**
City of La Carlota	1,357	459	470	**929**	**68.46**
La Castellana	1,916	487	537	**1,024**	**53.44**
Manapla	1,193	299	338	**637**	**53.39**
Moises Padilla	1,032	290	312	**602**	**58.33**
Murcia	1,949	624	601	**1,225**	**62.85**
Pontevedra	1,045	301	311	**612**	**58.56**
Pulupandan	496	152	140	**292**	**58.87**
City of Sagay	3,361	1,140	1,109	**2,249**	**66.91**
City of San Carlos	2,815	823	739	**1,562**	**55.49**
San Enrique	432	87	95	**182**	**42.13**
City of Silay	2,500	1,114	1,048	**2,162**	**86.48**
City of Sipalay	1,732	685	586	**1,271**	**73.38**
City of Talisay	2,129	896	808	**1,704**	**80.04**
Toboso	901	312	253	**565**	**62.71**
Valladolid	791	234	210	**444**	**56.13**
City of Victorias	1,801	600	539	**1,139**	**63.24**
Salvador Benedicto	660	206	186	**392**	**59.39**
**Guimaras**	**3,663**	1,225	1,248	**2,473**	**67.51**
Buenavista	792	299	299	**598**	**75.51**
Jordan	860	288	271	**559**	**65.00**
Nueva Valencia	797	279	285	**564**	**70.77**
San Lorenzo	730	186	235	**421**	**57.67**
Sibunag	484	173	158	**331**	**68.39**
**Region 7**	**181,246**	**57,604**	**54,476**	**112,080**	**61.84**
**City of Cebu**	**21,163**	7,160	6,617	**13,777**	**65.10**
**City of Lapu-Lapu**	**11,745**	5,168	4,978	**10,146**	**86.39**
**City of Mandaue**	**7,556**	3,575	3,480	**7,055**	**93.37**
**Bohol**	**28,418**	7,769	7,419	**15,188**	**53.44**
Alburquerque	152	45	36	**81**	**53.29**
Alicia	541	136	139	**275**	**50.83**
Anda	309	116	108	**224**	**72.49**
Antequera	245	73	86	**159**	**64.90**
Baclayon	416	87	101	**188**	**45.19**
Balilihan	333	97	87	**184**	**55.26**
Batuan	270	99	79	**178**	**65.93**
Bilar	359	86	88	**174**	**48.47**
Buenavista	663	224	179	**403**	**60.78**
Calape	642	169	169	**338**	**52.65**
Candijay	630	186	151	**337**	**53.49**
Carmen	1,121	271	249	**520**	**46.39**
Catigbian	482	109	106	**215**	**44.61**
Clarin	400	97	107	**204**	**51.00**
Corella	154	50	50	**100**	**64.94**
Cortes	317	69	69	**138**	**43.53**
Dagohoy	419	116	110	**226**	**53.94**
Danao	481	134	106	**240**	**49.90**
Dauis	1,054	336	268	**604**	**57.31**
Dimiao	254	61	56	**117**	**46.06**
Duero	323	85	84	**169**	**52.32**
Garcia Hernandez	408	106	96	**202**	**49.51**
Guindulman	671	162	168	**330**	**49.18**
Inabanga	990	273	298	**571**	**57.68**
Jagna	640	174	166	**340**	**53.13**
Getafe	936	180	178	**358**	**38.25**
Lila	193	32	30	**62**	**32.12**

2.C.1.7 Immunization Services for Infants and Children
Number and Proportion of Fully Immunized Children
Philippines, 2024

Area	Eligible Population (0-12 years old)	Fully Immunized Children (FIC)			
		Male	Female	Total	%
Loay	290	79	79	**158**	**54.48**
Loboc	293	89	84	**173**	**59.04**
Loon	730	220	219	**439**	**60.14**
Mabini	600	164	161	**325**	**54.17**
Maribojoc	373	119	111	**230**	**61.66**
Panglao	888	215	184	**399**	**44.93**
Pilar	599	159	147	**306**	**51.09**
President Carlos P. Garcia	544	126	147	**273**	**50.18**
Sagbayan	551	122	116	**238**	**43.19**
San Isidro	182	35	55	**90**	**49.45**
San Miguel	604	133	135	**268**	**44.37**
Sevilla	190	54	47	**101**	**53.16**
Sierra Bullones	549	158	138	**296**	**53.92**
Sikatuna	84	41	44	**85**	**101.19**
City of Tagbilaran	1,871	751	795	**1,546**	**82.63**
Talibon	1,741	540	466	**1,006**	**57.78**
Trinidad	808	212	192	**404**	**50.00**
Tubigon	909	322	311	**633**	**69.64**
Ubay	2,162	390	349	**739**	**34.18**
Valencia	468	110	122	**232**	**49.57**
Bien Unido	579	157	153	**310**	**53.54**
**Cebu**	**79,137**	**24,396**	**23,001**	**47,397**	**59.89**
Alcantara	406	94	82	**176**	**43.35**
Alcoy	392	111	106	**217**	**55.36**
Alegria	584	169	168	**337**	**57.71**
Aloguinsan	821	302	238	**540**	**65.77**
Argao	1,630	433	432	**865**	**53.07**
Asturias	1,325	379	378	**757**	**57.13**
Badian	1,066	307	296	**603**	**56.57**
Balamban	2,255	619	619	**1,238**	**54.90**
Bantayan	1,758	560	508	**1,068**	**60.75**
Barili	1,908	426	410	**836**	**43.82**
City of Bogo	2,212	636	669	**1,305**	**59.00**
Boljoon	304	120	85	**205**	**67.43**
Borbon	938	411	382	**793**	**84.54**
City of Carcar	3,484	965	969	**1,934**	**55.51**
Carmen	1,473	481	415	**896**	**60.83**
Catmon	767	215	190	**405**	**52.80**
Compostela	1,365	697	609	**1,306**	**95.68**
Consolacion	3,808	928	942	**1,870**	**49.11**
Cordova	1,830	676	548	**1,224**	**66.89**
Daanbantayan	2,076	739	697	**1,436**	**69.17**
Dalaguete	1,493	410	382	**792**	**53.05**
Danao City	4,007	1,230	1,241	**2,471**	**61.67**
Dumanjug	1,364	338	332	**670**	**49.12**
Ginatilan	316	44	59	**103**	**32.59**
Liloan	3,440	893	786	**1,679**	**48.81**
Madridejos	874	251	201	**452**	**51.72**
Malabuyoc	381	144	120	**264**	**69.29**
Medellin	1,504	456	417	**873**	**58.05**
Minglanilla	3,335	1,597	1,447	**3,044**	**91.27**
Moalboal	889	210	193	**403**	**45.33**
City of Naga	3,532	950	908	**1,858**	**52.60**
Oslob	637	170	138	**308**	**48.35**
Pilar	198	49	38	**87**	**43.94**
Pinamungajan	1,832	612	573	**1,185**	**64.68**
Poro	501	173	150	**323**	**64.47**
Ronda	403	109	95	**204**	**50.62**
Samboan	415	86	69	**155**	**37.35**
San Fernando	1,812	485	505	**990**	**54.64**
San Francisco	1,557	340	293	**633**	**40.66**
San Remigio	1,400	563	495	**1,058**	**75.57**
Santa Fe	830	245	209	**454**	**54.70**
Santander	427	108	107	**215**	**50.35**
Sibonga	1,226	431	427	**858**	**69.98**
Sogod	959	272	258	**530**	**55.27**

2.C.1.7 Immunization Services for Infants and Children
Number and Proportion of Fully Immunized Children
Philippines, 2024

Area	Eligible Population (0-12 years old)	Fully Immunized Children (FIC)			
		Male	Female	Total	%
Tabogon	1,070	245	232	**477**	**44.58**
Tabuelan	684	254	227	**481**	**70.32**
City of Talisay	6,041	2,498	2,534	**5,032**	**83.30**
City of Toledo	5,834	1,407	1,356	**2,763**	**47.36**
Tuburan	1,539	513	430	**943**	**61.27**
Tudela	235	45	36	**81**	**34.47**
**Negros Oriental**	**31,449**	**8,971**	**8,482**	**17,453**	**55.50**
Amlan	517	138	139	**277**	**53.58**
Ayungon	1,080	332	315	**647**	**59.91**
Bacong	889	226	224	**450**	**50.62**
City of Bais	1,923	308	274	**582**	**30.27**
Basay	753	182	190	**372**	**49.40**
City of Bayawan	2,842	935	808	**1,743**	**61.33**
Bindoy	941	313	340	**653**	**69.39**
City of Canlaon	1,377	441	449	**890**	**64.63**
Dauin	522	205	217	**422**	**80.84**
City of Dumaguete	2,339	936	834	**1,770**	**75.67**
City of Guihulngan	2,546	488	499	**987**	**38.77**
Jimalalud	841	170	175	**345**	**41.02**
La Libertad	967	278	241	**519**	**53.67**
Mabinay	2,048	683	703	**1,386**	**67.68**
Manjuyod	959	210	183	**393**	**40.98**
Pamplona	821	254	232	**486**	**59.20**
San Jose	479	127	116	**243**	**50.73**
Santa Catalina	1,842	475	471	**946**	**51.36**
Siaton	1,888	530	475	**1,005**	**53.23**
Sibulan	1,234	377	373	**750**	**60.78**
City of Tanjay	1,585	464	420	**884**	**55.77**
Tayasan	846	254	220	**474**	**56.03**
Valencia	685	267	236	**503**	**73.43**
Vallehermoso	893	198	210	**408**	**45.69**
Zamboanguita	632	180	138	**318**	**50.32**
**Siquijor**	**1,778**	**565**	**499**	**1,064**	**59.84**
Enrique Villanueva	111	38	33	**71**	**63.96**
Larena	225	72	50	**122**	**54.22**
Lazi	396	138	122	**260**	**65.66**
Maria	261	85	69	**154**	**59.00**
San Juan	263	81	84	**165**	**62.74**
Siquijor	522	151	141	**292**	**55.94**
**Region 8**	**101,678**	**24,835**	**23,830**	**48,665**	**47.86**
**Ormoc City**	**5,774**	**1,816**	**1,677**	**3,493**	**60.50**
**City of Tacloban**	**5,973**	**1,736**	**1,650**	**3,386**	**56.69**
**Eastern Samar**	**10,364**	**2,671**	**2,597**	**5,268**	**50.83**
Arteche	334	98	94	**192**	**57.49**
Balangiga	312	107	104	**211**	**67.63**
Balangkayan	214	52	64	**116**	**54.21**
City of Borongan	1,508	453	469	**922**	**61.14**
Can-Avid	563	157	144	**301**	**53.46**
Dolores	1,203	247	247	**494**	**41.06**
General Macarthur	326	82	86	**168**	**51.53**
Giporlos	278	59	54	**113**	**40.65**
Guiuan	1,142	261	330	**591**	**51.75**
Hernani	132	54	40	**94**	**71.21**
Jipapad	201	39	44	**83**	**41.29**
Lawaan	276	105	85	**190**	**68.84**
Llorente	397	92	74	**166**	**41.81**
Maslog	159	28	51	**79**	**49.69**
Maydolong	377	65	52	**117**	**31.03**
Mercedes	104	40	23	**63**	**60.58**
Oras	811	194	152	**346**	**42.66**
Quinapondan	271	81	78	**159**	**58.67**
Salcedo	403	86	96	**182**	**45.16**
San Julian	278	65	57	**122**	**43.88**
San Policarpo	350	89	73	**162**	**46.29**
Sulat	298	106	76	**182**	**61.07**
Taft	427	111	104	**215**	**50.35**

2.C.1.7 Immunization Services for Infants and Children
Number and Proportion of Fully Immunized Children
Philippines, 2024

Area	Eligible Population (0-12 years old)	Fully Immunized Children (FIC)			
		Male	Female	Total	%
**Leyte**	**34,367**	**8,271**	**7,938**	**16,209**	**47.16**
Abuyog	1,278	258	269	**527**	**41.24**
Alangalang	1,303	402	399	**801**	**61.47**
Albuera	1,179	282	311	**593**	**50.30**
Babatngon	644	129	136	**265**	**41.15**
Barugo	724	225	219	**444**	**61.33**
Bato	926	137	121	**258**	**27.86**
City of Baybay	2,223	688	608	**1,296**	**58.30**
Burauen	1,223	315	304	**619**	**50.61**
Calubian	557	179	136	**315**	**56.55**
Capoocan	898	192	186	**378**	**42.09**
Carigara	1,179	340	333	**673**	**57.08**
Dagami	770	157	124	**281**	**36.49**
Dulag	1,063	285	256	**541**	**50.89**
Hilongos	1,403	312	332	**644**	**45.90**
Hindang	388	103	129	**232**	**59.79**
Inopacan	395	104	86	**190**	**48.10**
Isabel	1,040	233	252	**485**	**46.63**
Jaro	997	295	271	**566**	**56.77**
Javier	546	157	142	**299**	**54.76**
Julita	297	66	68	**134**	**45.12**
Kananga	1,563	292	296	**588**	**37.62**
La Paz	351	82	69	**151**	**43.02**
Leyte	1,029	322	319	**641**	**62.29**
Macarthur	455	126	114	**240**	**52.75**
Mahaplag	589	87	83	**170**	**28.86**
Matag-Ob	374	103	129	**232**	**62.03**
Matalom	629	178	160	**338**	**53.74**
Mayorga	436	132	120	**252**	**57.80**
Merida	718	102	105	**207**	**28.83**
Palo	1,947	435	388	**823**	**42.27**
Palompon	1,138	238	223	**461**	**40.51**
Pastrana	408	122	130	**252**	**61.76**
San Isidro	723	129	103	**232**	**32.09**
San Miguel	433	94	74	**168**	**38.80**
Santa Fe	515	104	93	**197**	**38.25**
Tabango	780	132	135	**267**	**34.23**
Tabontabon	256	86	75	**161**	**62.89**
Tanauan	1,369	306	313	**619**	**45.22**
Tolosa	526	97	101	**198**	**37.64**
Tunga	140	49	43	**92**	**65.71**
Villaba	955	196	183	**379**	**39.69**
**Northern Samar**	**15,211**	**3,405**	**3,236**	**6,641**	**43.66**
Allen	568	193	126	**319**	**56.16**
Biri	297	79	74	**153**	**51.52**
Bobon	611	207	236	**443**	**72.50**
Capul	264	77	101	**178**	**67.42**
Catarman	2,427	366	312	**678**	**27.94**
Catubig	709	129	116	**245**	**34.56**
Gamay	415	137	104	**241**	**58.07**
Laoang	1,353	283	250	**533**	**39.39**
Lapinig	330	66	60	**126**	**38.18**
Las Navas	945	151	149	**300**	**31.75**
Lavezares	668	151	137	**288**	**43.11**
Mapanas	336	100	95	**195**	**58.04**
Mondragon	1,097	332	335	**667**	**60.80**
Palapag	782	172	184	**356**	**45.52**
Pambujan	765	138	145	**283**	**36.99**
Rosario	281	66	78	**144**	**51.25**
San Antonio	181	64	48	**112**	**61.88**
San Isidro	684	178	200	**378**	**55.26**
San Jose	435	145	140	**285**	**65.52**
San Roque	862	122	115	**237**	**27.49**
San Vicente	149	36	34	**70**	**46.98**
Silvino Lobos	336	71	63	**134**	**39.88**
Victoria	335	66	57	**123**	**36.72**

2.C.1.7 Immunization Services for Infants and Children
Number and Proportion of Fully Immunized Children
Philippines, 2024

Area	Eligible Population (0-12 years old)	Fully Immunized Children (FIC)			
		Male	Female	Total	%
Lope De Vega	381	76	77	**153**	**40.16**
**Samar**	**18,286**	**3,565**	**3,515**	**7,080**	**38.72**
Almagro	145	18	26	**44**	**30.34**
Basey	1,308	260	210	**470**	**35.93**
City of Calbayog	4,471	852	880	**1,732**	**38.74**
Calbiga	496	114	109	**223**	**44.96**
City of Catbalogan	2,653	659	630	**1,289**	**48.59**
Daram	850	84	103	**187**	**22.00**
Gandara	822	106	101	**207**	**25.18**
Hinabangan	252	57	53	**110**	**43.65**
Jiabong	461	142	135	**277**	**60.09**
Marabut	384	64	65	**129**	**33.59**
Matuguinao	189	27	35	**62**	**32.80**
Motiong	345	55	62	**117**	**33.91**
Pinabacdao	369	38	54	**92**	**24.93**
San Jose De Buan	208	30	26	**56**	**26.92**
San Sebastian	223	44	30	**74**	**33.18**
Santa Margarita	654	161	165	**326**	**49.85**
Santa Rita	1,081	248	275	**523**	**48.38**
Santo Niño	238	42	42	**84**	**35.29**
Talalora	162	33	37	**70**	**43.21**
Tarangnan	644	89	79	**168**	**26.09**
Villareal	509	119	122	**241**	**47.35**
Paranas	759	157	121	**278**	**36.63**
Zumarraga	374	57	44	**101**	**27.01**
Tagapul-An	152	46	35	**81**	**53.29**
San Jorge	400	34	43	**77**	**19.25**
Pagsanghan	137	29	33	**62**	**45.26**
**Southern Leyte**	**8,024**	**2,156**	**2,001**	**4,157**	**51.81**
Anahawan	139	40	35	**75**	**53.96**
Bontoc	513	187	174	**361**	**70.37**
Hinunangan	447	173	156	**329**	**73.60**
Hinundayan	197	66	66	**132**	**67.01**
Libagon	333	77	81	**158**	**47.45**
Liloan	459	114	126	**240**	**52.29**
City of Maasin	1,668	380	371	**751**	**45.02**
Macrohon	452	116	117	**233**	**51.55**
Malitbog	450	87	102	**189**	**42.00**
Padre Burgos	194	37	30	**67**	**34.54**
Pintuyan	157	53	50	**103**	**65.61**
Saint Bernard	549	185	140	**325**	**59.20**
San Francisco	243	51	56	**107**	**44.03**
San Juan	262	66	59	**125**	**47.71**
San Ricardo	212	38	39	**77**	**36.32**
Silago	247	114	106	**220**	**89.07**
Sogod	1,005	249	198	**447**	**44.48**
Tomas Oppus	392	91	68	**159**	**40.56**
Limasawa	105	32	27	**59**	**56.19**
**Biliran**	**3,679**	**1,215**	**1,216**	**2,431**	**66.08**
Almeria	316	123	99	**222**	**70.25**
Biliran	400	105	87	**192**	**48.00**
Cabucgayan	471	150	144	**294**	**62.42**
Caibiran	569	180	167	**347**	**60.98**
Culaba	267	87	114	**201**	**75.28**
Kawayan	342	120	123	**243**	**71.05**
Maripipi	103	30	24	**54**	**52.43**
Naval	1,211	420	458	**878**	**72.50**
**Region 9**	**92,714**	**30,018**	**28,818**	**58,836**	**63.46**
**City of Zamboanga**	**21,040**	8,565	8,247	**16,812**	**79.90**
**City of Isabela**	**3,494**	1,342	1,228	**2,570**	**73.55**
**Zamboanga del Norte**	**27,153**	8,015	7,717	**15,732**	**57.94**
City of Dapitan	1,999	764	839	**1,603**	**80.19**
City of Dipolog	3,157	1,003	980	**1,983**	**62.81**
Katipunan	1,186	337	332	**669**	**56.41**
La Libertad	180	41	39	**80**	**44.44**
Labason	1,122	406	395	**801**	**71.39**

2.C.1.7 Immunization Services for Infants and Children
Number and Proportion of Fully Immunized Children
Philippines, 2024

Area	Eligible Population (0-12 years old)	Fully Immunized Children (FIC)			
		Male	Female	Total	%
Liloy	1,001	254	254	**508**	**50.75**
Manukan	868	234	224	**458**	**52.76**
Mutia	285	68	84	**152**	**53.33**
Piñan	467	172	162	**334**	**71.52**
Polanco	1,087	470	428	**898**	**82.61**
Pres. Manuel A. Roxas	941	255	202	**457**	**48.57**
Rizal	332	122	135	**257**	**77.41**
Salug	918	335	255	**590**	**64.27**
Sergio Osmeña Sr.	936	221	180	**401**	**42.84**
Siayan	1,122	325	318	**643**	**57.31**
Sibuco	1,074	146	152	**298**	**27.75**
Sibutad	381	119	114	**233**	**61.15**
Sindangan	3,021	844	802	**1,646**	**54.49**
Siocon	1,338	237	238	**475**	**35.50**
Sirawai	864	197	183	**380**	**43.98**
Tampilisan	647	113	142	**255**	**39.41**
Jose Dalman	861	246	221	**467**	**54.24**
Gutalac	929	372	336	**708**	**76.21**
Baliguian	596	142	146	**288**	**48.32**
Godod	469	132	112	**244**	**52.03**
Bacungan	767	266	229	**495**	**64.54**
Kalawit	605	194	215	**409**	**67.60**
**Zamboanga del Sur**	**25,904**	7,030	6,846	**13,876**	**53.57**
Aurora	1,292	300	305	**605**	**46.83**
Bayog	842	272	249	**521**	**61.88**
Dimataling	776	229	195	**424**	**54.64**
Dinas	1,033	164	198	**362**	**35.04**
Dumalinao	761	268	293	**561**	**73.72**
Dumingag	1,366	549	530	**1,079**	**78.99**
Kumalarang	695	142	130	**272**	**39.14**
Labangan	1,171	333	316	**649**	**55.42**
Lapuyan	657	137	126	**263**	**40.03**
Mahayag	1,135	252	244	**496**	**43.70**
Margosatubig	897	198	216	**414**	**46.15**
Midsalip	800	164	140	**304**	**38.00**
Molave	1,320	430	434	**864**	**65.45**
City of Pagadian	5,249	1,707	1,701	**3,408**	**64.93**
Ramon Magsaysay	602	193	208	**401**	**66.61**
San Miguel	514	140	137	**277**	**53.89**
San Pablo	681	144	152	**296**	**43.47**
Tabina	641	82	76	**158**	**24.65**
Tambulig	949	193	149	**342**	**36.04**
Tukuran	996	214	175	**389**	**39.06**
Lakewood	434	140	144	**284**	**65.44**
Josefina	296	117	126	**243**	**82.09**
Pitogo	766	105	132	**237**	**30.94**
Sominot	477	70	68	**138**	**28.93**
Vincenzo A. Sagun	585	188	129	**317**	**54.19**
Guipos	404	158	146	**304**	**75.25**
Tigbao	565	141	127	**268**	**47.43**
**Zamboanga Sibugay**	**15,123**	**5,066**	**4,780**	**9,846**	**65.11**
Alicia	988	417	397	**814**	**82.39**
Buug	824	242	247	**489**	**59.34**
Diplahan	650	245	252	**497**	**76.46**
Imelda	635	247	238	**485**	**76.38**
Ipil	1,877	736	697	**1,433**	**76.35**
Kabasalan	1,150	326	321	**647**	**56.26**
Mabuhay	967	120	131	**251**	**25.96**
Malangas	601	307	257	**564**	**93.84**
Naga	1,012	301	265	**566**	**55.93**
Olutanga	862	198	172	**370**	**42.92**
Payao	942	325	330	**655**	**69.53**
Roseller Lim	901	346	338	**684**	**75.92**
Siay	928	331	293	**624**	**67.24**
Talusan	640	118	126	**244**	**38.13**
Titay	1,117	401	372	**773**	**69.20**

2.C.1.7 Immunization Services for Infants and Children
Number and Proportion of Fully Immunized Children
Philippines, 2024

Area	Eligible Population (0-12 years old)	Fully Immunized Children (FIC)			
		Male	Female	Total	%
Tungawan	1,029	406	344	**750**	**72.89**
**Region 10**	**119,709**	**38,922**	**37,063**	**75,985**	**63.47**
**City of Cagayan De Oro**	**15,725**	6,561	6,309	**12,870**	**81.84**
**City of Iligan**	**8,401**	2,574	2,460	**5,034**	**59.92**
**Bukidnon**	**36,852**	11,886	10,901	**22,787**	**61.83**
Baungon	805	296	313	**609**	**75.65**
Damulog	864	132	121	**253**	**29.28**
Dangcagan	577	180	177	**357**	**61.87**
Don Carlos	1,748	693	618	**1,311**	**75.00**
Impasug-ong	1,395	339	291	**630**	**45.16**
Kadingilan	739	250	231	**481**	**65.09**
Kalilangan	1,026	310	275	**585**	**57.02**
Kibawe	927	225	225	**450**	**48.54**
Kitaotao	1,270	208	185	**393**	**30.94**
Lantapan	1,528	644	594	**1,238**	**81.02**
Libona	1,142	455	467	**922**	**80.74**
City of Malaybalay	4,504	1,247	1,225	**2,472**	**54.88**
Malitbog	465	396	375	**771**	**165.81**
Manolo Fortich	2,591	1,127	1,079	**2,206**	**85.14**
Maramag	2,521	731	661	**1,392**	**55.22**
Pangantucan	1,329	509	423	**932**	**70.13**
Quezon	2,638	526	453	**979**	**37.11**
San Fernando	1,647	286	240	**526**	**31.94**
Sumilao	698	231	213	**444**	**63.61**
Talakag	2,164	1,199	1,079	**2,278**	**105.27**
City of Valencia	5,296	1,666	1,445	**3,111**	**58.74**
Cabanglasan	978	236	211	**447**	**45.71**
**Camiguin**	**1,878**	634	610	**1,244**	**66.24**
Catarman	319	124	110	**234**	**73.35**
Guinsiliban	140	44	47	**91**	**65.00**
Mahinog	291	70	77	**147**	**50.52**
Mambajao	855	294	260	**554**	**64.80**
Sagay	273	102	116	**218**	**79.85**
**Lanao del Norte**	**19,447**	5,861	5,772	**11,633**	**59.82**
Bacolod	525	161	157	**318**	**60.57**
Baloi	2,092	858	899	**1,757**	**83.99**
Baroy	527	144	137	**281**	**53.32**
Kapatagan	1,499	414	355	**769**	**51.30**
Sultan Naga Dimaporo	1,807	220	245	**465**	**25.73**
Kauswagan	641	232	230	**462**	**72.07**
Kolambugan	595	275	248	**523**	**87.90**
Lala	1,947	831	757	**1,588**	**81.56**
Linamon	468	201	200	**401**	**85.68**
Magsaysay	567	186	164	**350**	**61.73**
Maigo	534	147	183	**330**	**61.80**
Matungao	428	79	89	**168**	**39.25**
Munai	1,117	333	341	**674**	**60.34**
Nunungan	537	253	266	**519**	**96.65**
Pantao Ragat	915	141	158	**299**	**32.68**
Poona Piagapo	1,051	266	272	**538**	**51.19**
Salvador	678	190	181	**371**	**54.72**
Sapad	533	247	248	**495**	**92.87**
Tagoloan	419	84	86	**170**	**40.57**
Tangcal	606	136	123	**259**	**42.74**
Tubod	1,197	410	377	**787**	**65.75**
Pantar	764	53	56	**109**	**14.27**
**Misamis Occidental**	**13,379**	4,499	4,156	**8,655**	**64.69**
Aloran	488	209	198	**407**	**83.40**
Baliangao	392	107	103	**210**	**53.57**
Bonifacio	845	228	229	**457**	**54.08**
Calamba	471	248	207	**455**	**96.60**
Clarin	832	281	299	**580**	**69.71**
Concepcion	183	31	20	**51**	**27.87**
Jimenez	548	279	244	**523**	**95.44**
Lopez Jaena	514	172	170	**342**	**66.54**
City of Oroquieta	1,461	536	432	**968**	**66.26**

2.C.1.7 Immunization Services for Infants and Children
Number and Proportion of Fully Immunized Children
Philippines, 2024

Area	Eligible Population (0-12 years old)	Fully Immunized Children (FIC)			
		Male	Female	Total	%
City of Ozamiz	3,334	986	896	**1,882**	**56.45**
Panaon	178	32	41	**73**	**41.01**
Plaridel	748	334	344	**678**	**90.64**
Sapang Dalaga	437	82	94	**176**	**40.27**
Sinacaban	412	205	203	**408**	**99.03**
City of Tangub	1,688	477	411	**888**	**52.61**
Tudela	563	173	165	**338**	**60.04**
Don Victoriano Chiongbian	285	119	100	**219**	**76.84**
**Misamis Oriental**	**24,027**	6,907	6,855	**13,762**	**57.28**
Alubijid	711	219	198	**417**	**58.65**
Balingasag	2,006	474	451	**925**	**46.11**
Balingoan	268	83	64	**147**	**54.85**
Binuangan	139	41	31	**72**	**51.80**
Claveria	1,424	309	299	**608**	**42.70**
City of El Salvador	1,524	382	377	**759**	**49.80**
City of Gingoog	3,592	1,056	1,007	**2,063**	**57.43**
Gitagum	402	100	94	**194**	**48.26**
Initao	704	229	243	**472**	**67.05**
Jasaan	1,334	389	389	**778**	**58.32**
Kinoguitan	351	97	87	**184**	**52.42**
Lagonglong	686	173	135	**308**	**44.90**
Laguindingan	600	182	202	**384**	**64.00**
Libertad	295	59	48	**107**	**36.27**
Lugait	460	114	110	**224**	**48.70**
Magsaysay	1,017	210	219	**429**	**42.18**
Manticao	736	175	196	**371**	**50.41**
Medina	855	194	170	**364**	**42.57**
Naawan	461	127	122	**249**	**54.01**
Opol	1,683	488	537	**1,025**	**60.90**
Salay	709	218	268	**486**	**68.55**
Sugbongcogon	245	96	89	**185**	**75.51**
Tagoloan	2,079	849	909	**1,758**	**84.56**
Talisayan	666	156	150	**306**	**45.95**
Villanueva	1,080	487	460	**947**	**87.69**
**Region 11**	**117,243**	**36,729**	**34,550**	**71,279**	**60.80**
**City of Davao**	**40,169**	11,971	11,314	**23,285**	**57.97**
**Davao del Norte**	**25,693**	8,502	7,865	**16,367**	**63.70**
Asuncion	1,473	451	363	**814**	**55.26**
Carmen	1,994	604	548	**1,152**	**57.77**
Kapalong	2,071	558	550	**1,108**	**53.50**
New Corella	1,232	419	373	**792**	**64.29**
City of Panabo	4,683	1,558	1,492	**3,050**	**65.13**
Island Garden City of Samal	2,611	959	875	**1,834**	**70.24**
Santo Tomas	2,974	867	837	**1,704**	**57.30**
City of Tagum	6,710	2,562	2,360	**4,922**	**73.35**
Talaingod	831	190	188	**378**	**45.49**
Braulio E. Dujali	608	137	106	**243**	**39.97**
San Isidro	506	197	173	**370**	**73.12**
**Davao del Sur**	**14,382**	4,147	3,965	**8,112**	**56.40**
Bansalan	1,235	350	286	**636**	**51.50**
City of Digos	3,948	1,132	1,149	**2,281**	**57.78**
Hagonoy	1,162	379	336	**715**	**61.53**
Kiblawan	989	315	330	**645**	**65.22**
Magsaysay	1,197	344	356	**700**	**58.48**
Malalag	933	313	289	**602**	**64.52**
Matanao	1,288	358	342	**700**	**54.35**
Padada	549	194	176	**370**	**67.40**
Santa Cruz	2,422	562	521	**1,083**	**44.72**
Sulop	659	200	180	**380**	**57.66**
**Davao Oriental**	**13,017**	4,676	4,308	**8,984**	**69.02**
Baganga	1,352	461	412	**873**	**64.57**
Banaybanay	947	399	308	**707**	**74.66**
Boston	352	117	107	**224**	**63.64**
Caraga	678	218	235	**453**	**66.81**
Cateel	1,035	379	346	**725**	**70.05**
Governor Generoso	1,364	488	459	**947**	**69.43**

2.C.1.7 Immunization Services for Infants and Children
Number and Proportion of Fully Immunized Children
Philippines, 2024

Area	Eligible Population (0-12 years old)	Fully Immunized Children (FIC)			
		Male	Female	Total	%
Lupon	1,481	522	466	**988**	**66.71**
Manay	827	256	262	**518**	**62.64**
City of Mati	3,583	1,370	1,260	**2,630**	**73.40**
San Isidro	789	282	244	**526**	**66.67**
Tarragona	609	184	209	**393**	**64.53**
**Davao de Oro**	**16,758**	5,379	5,138	**10,517**	**62.76**
Compostela	1,920	681	644	**1,325**	**69.01**
Laak	1,883	569	487	**1,056**	**56.08**
Mabini	993	351	336	**687**	**69.18**
Maco	1,780	708	629	**1,337**	**75.11**
Maragusan	1,395	428	415	**843**	**60.43**
Mawab	836	259	264	**523**	**62.56**
Monkayo	2,029	737	757	**1,494**	**73.63**
Montevista	899	285	220	**505**	**56.17**
Nabunturan	1,830	561	536	**1,097**	**59.95**
New Bataan	1,033	310	323	**633**	**61.28**
Pantukan	2,160	490	527	**1,017**	**47.08**
**Davao Occidental**	**7,224**	2,054	1,960	**4,014**	**55.56**
Don Marcelino	1,007	267	287	**554**	**55.01**
Jose Abad Santos	1,774	491	474	**965**	**54.40**
Malita	2,666	799	761	**1,560**	**58.51**
Santa Maria	1,264	353	323	**676**	**53.48**
Sarangani	513	144	115	**259**	**50.49**
**Region 12**	**103,515**	**34,276**	**32,291**	**66,567**	**64.31**
**City of General Santos**	**15,861**	4,010	3,800	**7,810**	**49.24**
**Cotabato**	**28,029**	9,730	9,118	**18,848**	**67.24**
Alamada	1,774	583	576	**1,159**	**65.33**
Carmen	1,988	706	687	**1,393**	**70.07**
Kabacan	1,830	602	521	**1,123**	**61.37**
City of Kidapawan	3,531	1,243	1,140	**2,383**	**67.49**
Libungan	1,142	412	358	**770**	**67.43**
Magpet	1,126	362	382	**744**	**66.07**
Makilala	1,870	728	671	**1,399**	**74.81**
Matalam	1,700	620	580	**1,200**	**70.59**
Midsayap	2,244	739	653	**1,392**	**62.03**
M'Lang	1,902	677	559	**1,236**	**64.98**
Pigkawayan	1,072	392	365	**757**	**70.62**
Pikit	1,790	468	473	**941**	**52.57**
President Roxas	1,026	364	381	**745**	**72.61**
Tulunan	1,427	611	535	**1,146**	**80.31**
Antipas	495	165	185	**350**	**70.71**
Banisilan	1,199	384	407	**791**	**65.97**
Aleosan	850	266	265	**531**	**62.47**
Arakan	1,063	408	380	**788**	**74.13**
**South Cotabato**	**23,727**	7,830	7,232	**15,062**	**63.48**
Banga	2,030	683	642	**1,325**	**65.27**
City of Koronadal	4,325	1,395	1,290	**2,685**	**62.08**
Norala	985	529	507	**1,036**	**105.18**
Polomolok	4,072	1,272	1,129	**2,401**	**58.96**
Surallah	2,088	837	806	**1,643**	**78.69**
Tampakan	992	333	274	**607**	**61.19**
Tantangan	1,143	382	339	**721**	**63.08**
T'Boli	3,087	888	852	**1,740**	**56.37**
Tupi	1,756	590	535	**1,125**	**64.07**
Santo Niño	877	313	281	**594**	**67.73**
Lake Sebu	2,372	608	577	**1,185**	**49.96**
**Sultan Kudarat**	**21,299**	7,405	7,171	**14,576**	**68.44**
Bagumbayan	1,794	704	700	**1,404**	**78.26**
Columbio	945	320	309	**629**	**66.56**
Esperanza	1,623	577	530	**1,107**	**68.21**
Isulan	2,336	1,007	899	**1,906**	**81.59**
Kalamansig	1,273	448	427	**875**	**68.74**
Lebak	2,129	684	692	**1,376**	**64.63**
Lutayan	1,647	667	676	**1,343**	**81.54**
Lambayong	2,191	705	598	**1,303**	**59.47**
Palimbang	2,812	673	707	**1,380**	**49.08**

2.C.1.7 Immunization Services for Infants and Children
Number and Proportion of Fully Immunized Children
Philippines, 2024

Area	Eligible Population (0-12 years old)	Fully Immunized Children (FIC)			
		Male	Female	Total	%
President Quirino	1,061	449	488	**937**	**88.31**
City of Tacurong	2,341	785	784	**1,569**	**67.02**
Sen. Ninoy Aquino	1,147	386	361	**747**	**65.13**
**Sarangani**	**14,599**	5,301	4,970	**10,271**	**70.35**
Alabel	2,151	707	690	**1,397**	**64.95**
Glan	2,857	1,057	1,010	**2,067**	**72.35**
Kiamba	1,782	622	540	**1,162**	**65.21**
Maasim	1,788	735	671	**1,406**	**78.64**
Maitum	1,210	447	445	**892**	**73.72**
Malapatan	2,093	890	822	**1,712**	**81.80**
Malungon	2,718	843	792	**1,635**	**60.15**
**Caraga**	**60,237**	**24,635**	**23,504**	**48,139**	**79.92**
**City of Butuan**	**7,313**	3,760	3,623	**7,383**	**100.96**
**Agusan del Norte**	**7,979**	3,722	3,523	**7,245**	**90.80**
Buenavista	1,392	709	713	**1,422**	**102.16**
City of Cabadbaran	1,545	585	574	**1,159**	**75.02**
Carmen	489	264	234	**498**	**101.84**
Jabonga	518	226	200	**426**	**82.24**
Kitcharao	441	199	220	**419**	**95.01**
Las Nieves	677	350	308	**658**	**97.19**
Magallanes	431	199	155	**354**	**82.13**
Nasipit	812	407	377	**784**	**96.55**
Santiago	719	344	340	**684**	**95.13**
Tubay	616	268	267	**535**	**86.85**
Remedios T. Romualdez	339	171	135	**306**	**90.27**
**Agusan del Norte Provincial Hospital (ADNPH)**		0	0	**0**	**0.00**
**Agusan del Sur**	**17,563**	**7,268**	**7,066**	**14,334**	**81.61**
City of Bayugan	2,446	921	947	**1,868**	**76.37**
Bunawan	1,080	486	502	**988**	**91.48**
Esperanza	1,559	509	469	**978**	**62.73**
La Paz	952	381	402	**783**	**82.25**
Loreto	1,104	526	451	**977**	**88.50**
Prosperidad	2,008	902	806	**1,708**	**85.06**
Rosario	1,223	471	457	**928**	**75.88**
San Francisco	1,862	817	797	**1,614**	**86.68**
San Luis	940	352	384	**736**	**78.30**
Santa Josefa	567	279	293	**572**	**100.88**
Talacogon	1,039	350	347	**697**	**67.08**
Trento	1,118	529	452	**981**	**87.75**
Veruela	921	438	464	**902**	**97.94**
Sibagat	744	307	295	**602**	**80.91**
**Surigao del Norte**	**11,072**	4,356	4,163	**8,519**	**76.94**
Alegria	326	158	161	**319**	**97.85**
Bacuag	267	102	108	**210**	**78.65**
Burgos	94	31	36	**67**	**71.28**
Claver	808	344	330	**674**	**83.42**
Dapa	587	266	273	**539**	**91.82**
Del Carmen	455	128	139	**267**	**58.68**
General Luna	514	155	134	**289**	**56.23**
Gigaquit	423	170	170	**340**	**80.38**
Mainit	594	269	246	**515**	**86.70**
Malimono	302	119	114	**233**	**77.15**
Pilar	233	79	64	**143**	**61.37**
Placer	602	226	223	**449**	**74.58**
San Benito	120	53	46	**99**	**82.50**
San Francisco	252	101	114	**215**	**85.32**
San Isidro	181	44	44	**88**	**48.62**
Santa Monica	187	60	53	**113**	**60.43**
Sison	285	146	136	**282**	**98.95**
Socorro	842	218	219	**437**	**51.90**
City of Surigao	3,414	1,425	1,286	**2,711**	**79.41**
Tagana-An	329	133	141	**274**	**83.28**
Tubod	257	129	126	**255**	**99.22**
**Surigao del Sur**	**13,988**	**4,672**	**4,341**	**9,013**	**64.43**
Barobo	1,128	507	436	**943**	**83.60**
Bayabas	193	61	65	**126**	**65.28**

2.C.1.7 Immunization Services for Infants and Children
Number and Proportion of Fully Immunized Children
Philippines, 2024

Area	Eligible Population (0-12 years old)	Fully Immunized Children (FIC)			
		Male	Female	Total	%
City of Bislig	2,081	696	708	**1,404**	**67.47**
Cagwait	477	148	110	**258**	**54.09**
Cantilan	648	276	277	**553**	**85.34**
Carmen	223	80	61	**141**	**63.23**
Carrascal	519	200	161	**361**	**69.56**
Cortes	355	120	105	**225**	**63.38**
Hinatuan	957	307	262	**569**	**59.46**
Lanuza	333	99	75	**174**	**52.25**
Lianga	755	215	201	**416**	**55.10**
Lingig	804	298	264	**562**	**69.90**
Madrid	317	95	99	**194**	**61.20**
Marihatag	473	138	129	**267**	**56.45**
San Agustin	484	113	94	**207**	**42.77**
San Miguel	1,096	309	301	**610**	**55.66**
Tagbina	919	276	248	**524**	**57.02**
Tago	846	256	228	**484**	**57.21**
City of Tandag	1,380	478	517	**995**	**72.10**
**Dinagat Islands**	**2,322**	**857**	**788**	**1,645**	**70.84**
Basilisa	682	122	127	**249**	**36.51**
Cagdianao	330	174	159	**333**	**100.91**
Dinagat	222	80	99	**179**	**80.63**
Libjo	308	176	161	**337**	**109.42**
Loreto	149	84	61	**145**	**97.32**
San Jose	480	151	145	**296**	**61.67**
Tubajon	151	70	36	**106**	**70.20**
**BARMM**	**147,247**	**38,075**	**38,026**	**76,101**	**51.68**
**City of Cotabato**	**7,804**	**2,135**	**2,168**	**4,303**	**55.14**
**Basilan**	**12,747**	**1,985**	**1,968**	**3,953**	**31.01**
City of Lamitan	2,866	537	536	1,073	37.44
Lantawan	978	137	123	260	26.58
Maluso	1,323	243	244	487	36.81
Sumisip	1,544	307	271	578	37.44
Tipo-Tipo	713	107	113	220	30.86
Tuburan	803	210	230	440	54.79
Akbar	539	13	21	34	6.31
Al-Barka	841	84	84	168	19.98
Hadji Mohammad Ajul	843	34	48	82	9.73
Ungkaya Pukan	958	97	101	198	20.67
Hadji Muhtamad	770	89	85	174	22.60
Tabuan-Lasa	569	127	112	239	42.00
**Lanao del Sur**	**36,535**	**10,256**	**10,256**	**20,512**	**56.14**
Bacolod-Kalawi	646	177	171	348	53.87
Balabagan	841	133	135	268	31.87
Balindong	880	393	343	736	83.64
Bayang	392	86	80	166	42.35
Binidayan	808	138	145	283	35.02
Bubong	796	114	97	211	26.51
Butig	854	104	138	242	28.34
Ganassi	645	139	119	258	40.00
Kapai	564	115	118	233	41.31
Lumba-Bayabao	1,026	368	357	725	70.66
Lumbatan	709	103	102	205	28.91
Madalum	817	239	224	463	56.67
Madamba	721	230	219	449	62.27
Malabang	1,460	500	507	1,007	68.97
Marantao	1,239	308	329	637	51.41
City of Marawi	6,851	3,240	3,263	6,503	94.92
Masiu	1,049	25	31	56	5.34
Mulondo	788	165	151	316	40.10
Pagayawan	534	21	36	57	10.67
Piagapo	1,062	468	470	938	88.32
Poona Bayabao	1,141	244	288	532	46.63
Pualas	495	118	112	230	46.46
Ditsaan-Ramain	766	129	109	238	31.07
Saguiaran	870	168	170	338	38.85
Tamparan	882	261	298	559	63.38

2.C.1.7 Immunization Services for Infants and Children
Number and Proportion of Fully Immunized Children
Philippines, 2024

Area	Eligible Population (0-12 years old)	Fully Immunized Children (FIC)			
		Male	Female	Total	%
Taraka	600	174	176	350	58.33
Tubaran	499	83	66	149	29.86
Tugaya	719	149	129	278	38.66
Wao	1,304	413	434	847	64.95
Marogong	1,213	82	75	157	12.94
Calanogas	519	182	157	339	65.32
Buadiposo-Buntong	447	176	175	351	78.52
Maguing	1,030	55	54	109	10.58
Picong	426	99	110	209	49.06
Lumbayanague	710	98	102	200	28.17
Amai Manabilang	458	205	191	396	86.46
Tagoloan Ii	262	91	90	181	69.08
Kapatagan	725	241	253	494	68.14
Sultan Dumalondong	477	151	163	314	65.83
Lumbaca-Unayan	310	71	69	140	45.16
**Sulu**	**23,547**	**5,150**	**5,274**	**10,424**	**44.27**
Indanan	2,493	678	621	1,299	52.11
Jolo	3,094	811	772	1,583	51.16
Kalingalan Caluang	689	282	268	550	79.83
Luuk	805	176	181	357	44.35
Maimbung	1,609	275	307	582	36.17
Hadji Panglima Tahil	277	39	33	72	25.99
Old Panamao	899	212	214	426	47.39
Pangutaran	1,259	147	147	294	23.35
Parang	1,593	428	496	924	58.00
Pata	551	76	86	162	29.40
Patikul	1,751	308	334	642	36.66
Siasi	1,531	328	321	649	42.39
Talipao	2,157	407	406	813	37.69
Tapul	468	119	132	251	53.63
Tongkil	1,306	77	85	162	12.40
Panglima Estino	769	211	267	478	62.16
Lugus	794	102	81	183	23.05
Pandami	858	241	274	515	60.02
Omar	644	233	249	482	74.84
**Tawi-Tawi**	**13,789**	**2,770**	**2,713**	**5,483**	**39.76**
Panglima Sugala	1,543	114	147	261	16.92
Bongao	3,813	862	802	1,664	43.64
Mapun	759	217	175	392	51.65
Simunul	836	206	241	447	53.47
Sitangkai	1,048	345	340	685	65.36
South Ubian	728	171	150	321	44.09
Tandubas	884	266	255	521	58.94
Turtle Islands	157	50	53	103	65.61
Languyan	1,588	95	98	193	12.15
Sapa-Sapa	1,397	213	201	414	29.63
Sibutu	1,036	231	251	482	46.53
**Maguindanao del Norte**	**20,607**	**7,352**	**7,096**	**14,448**	**70.11**
Barira	1,156	412	406	818	70.76
Buldon	1,541	681	620	1,301	84.43
Datu Blah T. Sinsuat	910	269	263	532	58.46
Datu Odin Sinsuat	3,916	1,239	1,163	2,402	61.34
Kabuntalan	883	363	366	729	82.56
Matanog	1,439	396	394	790	54.90
Northern Kabuntalan	569	268	251	519	91.21
Parang	3,172	918	876	1,794	56.56
Sultan Kudarat	3,528	1,705	1,614	3,319	94.08
Sultan Mastura	1,002	327	335	662	66.07
Talitay	460	208	231	439	95.43
Upi	2,031	566	577	1,143	56.28
**Maguindanao del Sur**	**25,329**	**7,341**	**7,449**	**14,790**	**58.39**
Ampatuan	863	287	274	561	65.01
Buluan	1,775	262	220	482	27.15
Datu Abdullah Sangki	1,133	213	221	434	38.31
Datu Anggal Midtimbang	917	212	201	413	45.04
Datu Hoffer Ampatuan	836	163	199	362	43.30

2.C.1.7 Immunization Services for Infants and Children
Number and Proportion of Fully Immunized Children
Philippines, 2024

Area	Eligible Population (0-12 years old)	Fully Immunized Children (FIC)			
		Male	Female	Total	%
Datu Paglas	1,205	527	540	1,067	88.55
Datu Piang	772	321	350	671	86.92
Datu Salibo	837	223	249	472	56.39
Datu Saudi Ampatuan	983	249	225	474	48.22
Datu Unsay	309	127	134	261	84.47
Gen. S.K. Pendatun	1,248	332	351	683	54.73
Guindulungan	846	328	312	640	75.65
Mamasapano	952	299	336	635	66.70
Mangudadatu	1,121	228	221	449	40.05
Pagagawan	1,170	461	421	882	75.38
Pagalungan	1,644	623	585	1,208	73.48
Paglat	773	163	141	304	39.33
Pandag	1,258	263	245	508	40.38
Rajah Buayan	1,382	372	421	793	57.38
Shariff Aguak	1,249	288	336	624	49.96
Shariff Saydona Mustapha	829	244	233	477	57.54
South Upi	1,528	557	653	1,210	79.19
Sultan Sa Barongis	899	226	214	440	48.94
Talayan	800	373	367	740	92.50
**SGU**	**6,889**	**1,086**	**1,102**	**2,188**	**31.76**
Carmen Cluster	1,066	159	186	345	32.36
Kabacan Cluster	510	90	70	160	31.37
Midsayap Cluster I	914	86	89	175	19.15
Midsayap Cluster II	852	94	103	197	23.12
Pigcawayan Cluster	613	148	139	287	46.82
Pikit Cluster I	1,122	181	166	347	30.93
Pikit Cluster II	957	192	183	375	39.18
Pikit Cluster III	855	136	166	302	35.32

Legend:
0 = No Data
* = No Report

Table 2.C.1.7 Immunization Services for Infants and Children
Number and Proportion of Completely Immunized Children
Philippines, 2024

Area	Eligible Population (13-23mos. old)	CIC			
		Male	Female	Total	%
**Philippines**	**2,082,671**	**83,535**	**77,867**	**161,402**	**7.75**
**NCR**	**246,251**	**9,785**	**9,247**	**19,032**	**7.73**
City of Malabon	6,821	939	979	1,918	28.12
City of Navotas	4,966	124	130	254	5.11
City of Valenzuela	13,281	468	396	864	6.51
City of Caloocan	32,675	424	332	756	2.31
City of Marikina	8,116	346	327	673	8.29
City of Pasig	15,498	859	786	1,645	10.61
Pateros	1,118	49	35	84	7.51
City of Taguig	23,653	1472	1360	2,832	11.97
Quezon City	56,256	1850	1827	3,677	6.54
City of Makati	4,206	56	47	103	2.45
City of Mandaluyong	7,459	271	304	575	7.71
City of San Juan	2,073	91	84	175	8.44
City of Manila	32,206	342	332	674	2.09
City of Las Piñas	10,286	989	951	1,940	18.86
City of Muntinlupa	9,227	566	534	1,100	11.92
City of Parañaque	12,412	575	520	1,095	8.82
Pasay City	5,998	364	303	667	11.12
**CAR**	**29,904**	**1,006**	**912**	**1,918**	**6.41**
Abra	3,692	23	24	47	1.27
Apayao	2,046	13	17	30	1.47
Benguet	8,353	342	328	670	8.02
Ifugao	3,912	108	102	210	5.37
Kalinga	4,268	29	29	58	1.36
Mountain Province	2,536	86	48	134	5.28
City of Baguio	5,097	405	364	769	15.09
**Region 1**	**89,709**	**755**	**699**	**1,454**	**1.62**
Ilocos Norte	8,391	2	2	4	0.05
Ilocos Sur	9,535	21	18	39	0.41
La Union	12,344	54	57	111	0.90
Pangasinan	56,289	443	369	812	1.44
City of Dagupan	3,150	235	253	488	15.49
**Region 2**	**60,543**	**846**	**803**	**1,649**	**2.72**
Batanes	253	1	2	3	1.19
Cagayan	19,808	367	368	735	3.71
Isabela	25,923	271	237	508	1.96
Nueva Vizcaya	8,514	191	176	367	4.31
Quirino	3,490	11	18	29	0.83
City of Santiago	2,555	5	2	7	0.27
**Region 3**	**225,265**	**5,203**	**4,717**	**9,920**	**4.40**
Aurora	4,412	40	25	65	1.47
Bataan	16,092	557	457	1,014	6.30
Bulacan	66,424	2282	2047	4,329	6.52
Nueva Ecija	41,411	500	482	982	2.37
Pampanga	44,863	1040	994	2,034	4.53
Tarlac	27,097	438	388	826	3.05
Zambales	11,224	91	77	168	1.50
City of Angeles	9,545	189	197	386	4.04
City of Olongapo	4,197	66	50	116	2.76
**Region 4A**	**295,286**	**17,205**	**15,703**	**32,908**	**11.14**
Batangas	52,710	3216	2937	6,153	11.67
Cavite	75,463	4231	3817	8,048	10.66
Laguna	58,293	3506	3212	6,718	11.52
Quezon	38,858	2408	2245	4,653	11.97
Rizal	64,401	3523	3134	6,657	10.34
City of Lucena	5,561	321	358	679	12.21
**Region 4B**	**61,818**	**3,057**	**2,697**	**5,754**	**9.31**
Marinduque	4,472	106	106	212	4.74
Occidental Mindoro	10,898	719	615	1,334	12.24
Oriental Mindoro	17,176	466	398	864	5.03
Palawan	19,049	1137	1005	2,142	11.24
Romblon	5,198	74	64	138	2.65
City of Puerto Princesa	5,025	555	509	1,064	21.17
**Region 5**	**128,789**	**7,400**	**6,917**	**14,317**	**11.12**
Albay	26,743	1323	1230	2,553	9.55
Camarines Norte	13,721	953	907	1,860	13.56
Camarines Sur	41,247	2341	2196	4,537	11.00
Catanduanes	5,458	89	73	162	2.97
Masbate	20,790	1214	1092	2,306	11.09
Sorsogon	16,911	1241	1224	2,465	14.58
City of Naga	3,919	239	195	434	11.07

Table 2.C.1.7 Immunization Services for Infants and Children
Number and Proportion of Completely Immunized Children
Philippines, 2024

Area	Eligible Population (13-23mos. old)	CIC			
		Male	Female	Total	%
**Region 6**	**141,586**	**3,423**	**3,144**	**6,567**	**4.64**
Aklan	10,225	320	301	621	6.07
Antique	11,046	237	224	461	4.17
Capiz	13,203	307	224	531	4.02
Guimaras	3,272	34	29	63	1.93
Iloilo	34,281	59	65	124	0.36
Negros Occidental	50,948	1713	1598	3,311	6.50
City of Bacolod	10,156	725	666	1,391	13.70
City of Iloilo	8,455	28	37	65	0.77
**Region 7**	**154,295**	**6,922**	**6,203**	**13,125**	**8.51**
Bohol	23,892	1444	1268	2,712	11.35
Cebu	67,665	3113	2807	5,920	8.75
Negros Oriental	26,491	1779	1607	3,386	12.78
Siquijor	1,370	15	23	38	2.77
City of Cebu	18,602	276	207	483	2.60
City of Lapu-Lapu	10,001	229	225	454	4.54
City of Mandaue	6,274	66	66	132	2.10
**Region 8**	**87,673**	**5,057**	**4,810**	**9,867**	**11.25**
Biliran	3,225	206	172	378	11.72
Eastern Samar	8,898	518	495	1,013	11.38
Leyte	30,488	1787	1706	3,493	11.46
Northern Samar	12,601	849	795	1,644	13.05
Southern Leyte	7,191	284	217	501	6.97
Samar	15,549	1272	1288	2,560	16.46
Ormoc City	4,729	15	20	35	0.74
City of Tacloban	4,992	126	117	243	4.87
**Region 9**	**79,177**	**4,104**	**3,807**	**7,911**	**9.99**
Zamboanga del Norte	23,222	1061	1015	2,076	8.94
Zamboanga del Sur	21,361	1313	1199	2,512	11.76
Zamboanga Sibugay	12,975	463	413	876	6.75
City of Isabela	3,156	39	39	78	2.47
City of Zamboanga	18,463	1228	1141	2,369	12.83
**Region 10**	**102,612**	**4,085**	**3,945**	**8,030**	**7.83**
Bukidnon	31,940	1672	1621	3,293	10.31
Camiguin	1,580	42	49	91	5.76
Lanao del Norte	16,888	727	741	1,468	8.69
Misamis Occidental	11,344	302	272	574	5.06
Misamis Oriental	20,179	819	758	1,577	7.82
City of Cagayan De Oro	13,460	246	222	468	3.48
City of Iligan	7,221	277	282	559	7.74
**Region 11**	**101,368**	**4,436**	**4,186**	**8,622**	**8.51**
Davao de Oro	14,391	273	267	540	3.75
Davao del Norte	21,839	297	277	574	2.63
Davao Oriental	10,749	149	143	292	2.72
Davao del Sur	12,975	767	761	1,528	11.78
Davao Occidental	6,688	301	286	587	8.78
City of Davao	34,726	2649	2452	5,101	14.69
**Region 12**	**89,192**	**2,992**	**2,843**	**5,835**	**6.54**
Cotabato	24,980	1072	1059	2,131	8.53
Sarangani	12,003	206	196	402	3.35
South Cotabato	20,384	743	625	1,368	6.71
Sultan Kudarat	18,454	222	204	426	2.31
City of General Santos	13,371	749	759	1,508	11.28
**CARAGA**	**52,238**	**928**	**789**	**1,717**	**3.29**
Agusan del Norte	6,917	116	73	189	2.73
Agusan del Sur	14,804	147	169	316	2.13
Surigao del Norte	9,674	156	138	294	3.04
Surigao del Sur	12,356	383	310	693	5.61
Dinagat Islands	2,221	95	75	170	7.65
City of Butuan	6,266	31	24	55	0.88
**BARMM**	**136,965**	**6,331**	**6,445**	**12,776**	**9.33**
Basilan	11,794	555	535	1,090	9.24
Lanao del Sur	33,825	1596	1733	3,329	9.84
Maguindanao del Norte	18,407	998	1014	2,012	10.93
Maguindanao del Sur	23,108	1476	1560	3,036	13.14
Sulu	23,613	707	616	1,323	5.60
Tawi-Tawi	12,872	243	247	490	3.81
SGA	6,380	667	650	1,317	20.64
City of Cotabato	6,966	89	90	179	2.57

0 = No Data
* = No Report

Table 2.C.2.1 Immunization Services for School-Aged Children
Number and Proportion of Grade 1 and Grade 7 learners given one dose of Tetanus diphtheria toxoid (Td) vaccine
Philippines, 2024

Area	Enrolled Grade 1 learners	Grade 1 learners given Td				Enrolled Grade 7 learners	Grade 7 learners given Td			
		Male	Female	Total	%		Male	Female	Total	%
**Philippines**	**1,382,242**	**421,336**	**423,720**	**845,056**	**61.14**	**1,415,645**	**342,700**	**378,776**	**721,476**	**50.96**
**NCR**	**159,664**	**20,716**	**21,797**	**42,513**	**26.63**	**148,610**	**16,146**	**17,923**	**34,069**	**22.93**
City of Malabon	6,471	761	768	1,529	23.63	5,784	577	593	1,170	20.23
City of Navotas	0	0	0	0	0.00	0	0	0	0	0.00
City of Valenzuela	0	0	0	0	0.00	0	0	0	0	0.00
City of Caloocan	25,760	3,020	3,003	6,023	23.38	20,878	3098	3011	6,109	29.26
City of Marikina	6,748	886	955	1,841	27.28	7,994	933	1085	2,018	25.24
City of Pasig	9,931	1,530	1,572	3,102	31.24	11,748	1541	1552	3,093	26.33
Pateros	726	105	117	222	30.58	660	92	109	201	30.45
City of Taguig	15,448	2,447	2,604	5,051	32.70	14,424	1922	2407	4,329	30.01
Quezon City	37,748	3,491	3,722	7,213	19.11	31,789	2002	2245	4,247	13.36
City of Makati	7,896	784	786	1,570	19.88	8,388	489	623	1,112	13.26
City of Mandaluyong	6,998	1,240	1,333	2,573	36.77	4,809	1530	1032	2,562	53.28
City of San Juan	1,073	114	124	238	22.18	872	61	83	144	16.51
City of Manila	13,029	2,253	2,268	4,521	34.70	14,268	992	1355	2,347	16.45
City of Las Piñas	7,896	1,412	1,381	2,793	35.37	8,388	994	1256	2,250	26.82
City of Muntinlupa	6,998	1,048	1,092	2,140	30.58	4,809	593	754	1,347	28.01
City of Parañaque	7,936	929	1,394	2,323	29.27	9,039	746	1119	1,865	20.63
Pasay City	5,006	696	678	1,374	27.45	4,760	576	699	1,275	26.79
**CAR**	**21,570**	**7,258**	**7,120**	**14,378**	**66.66**	**22,048**	**5,962**	**6,636**	**12,598**	**57.14**
Abra	3,649	1,718	1,674	3,392	92.96	4,315	1847	1863	3,710	85.98
Apayao	199	104	95	199	100.00	230	0	0	0	0.00
Benguet	4,180	2,153	2,016	4,169	99.74	3,175	1392	1777	3,169	99.81
Ifugao	574	295	278	573	99.83	308	155	153	308	100.00
Kalinga	4,379	1,598	1,753	3,351	76.52	3,827	1148	1337	2,485	64.93
Mountain Province	2,545	1,252	1,166	2,418	95.01	2,938	1305	1389	2,694	91.70
City of Baguio	6,044	138	138	276	4.57	7,255	115	117	232	3.20
**Region 1**	**2,979**	**5,139**	**5,208**	**10,347**	**347.33**	**2,781**	**5,969**	**5,898**	**11,867**	**426.72**
Ilocos Norte	*	*	*	0	0.00	*	*	*	0	0.00
Ilocos Sur	*	4,287	4,124	8,411	0.00	*	5231	5031	10,262	0.00
La Union	*	*	*	0	0.00	*	*	*	0	0.00
Pangasinan	*	*	*	0	0.00	*	*	*	0	0.00
City of Dagupan	2,979	852	1,084	1,936	64.99	2,781	738	867	1,605	57.71
**Region 2**	**57,231**	**24,123**	**20,369**	**44,492**	**77.74**	**59,017**	**22,280**	**18,372**	**40,652**	**68.88**
Batanes	112	55	57	112	100.00	132	70	62	132	100.00
Cagayan	18,456	6,767	6,991	13,758	74.54	13,897	5787	6718	12,505	89.98
Isabela	24,874	12,729	8,168	20,897	84.01	28,794	13137	7521	20,658	71.74
Nueva Vizcaya	8,049	2,216	2,757	4,973	61.78	9,967	1459	1945	3,404	34.15
Quirino	3,346	1,153	1,259	2,412	72.09	3,729	880	1073	1,953	52.37
City of Santiago	2,394	1,203	1,137	2,340	97.74	2,498	947	1053	2,000	80.06
**Region 3**	**202,031**	**65,142**	**64,539**	**129,681**	**64.19**	**218,646**	**44,833**	**50,593**	**95,426**	**43.64**
Aurora	4,357	1,866	1,759	3,625	83.20	5,144	1442	1596	3,038	59.06
Bataan	14,716	4,210	4,088	8,298	56.39	16,125	2727	3303	6,030	37.40
Bulacan	59,659	14,618	14,516	29,134	48.83	62,644	8884	9565	18,449	29.45
Nueva Ecija	36,267	15,167	15,150	30,317	83.59	42,062	11677	13576	25,253	60.04
Pampanga	38,153	11,841	11,491	23,332	61.15	40,501	7822	8527	16,349	40.37
Tarlac	24,785	11,146	11,541	22,687	91.54	28,127	8253	9179	17,432	61.98
Zambales	11,678	3,646	3,429	7,075	60.58	12,906	2638	3189	5,827	45.15
City of Angeles	8,194	2,091	2,013	4,104	50.09	6,975	1147	1328	2,475	35.48
City of Olongapo	4,222	557	552	1,109	26.27	4,162	243	330	573	13.77
**Region 4A**	**126,270**	**38,207**	**38,321**	**76,528**	**60.61**	**118,369**	**24,765**	**29,388**	**54,153**	**45.75**
Batangas	33,430	13,376	13,143	26,519	79.33	24,699	7865	9299	17,164	69.49
Cavite	40,782	10,429	10,725	21,154	51.87	41,758	7529	8850	16,379	39.22
Laguna	19,416	5,787	5,645	11,432	58.88	22,776	3817	4331	8,148	35.77
Quezon	15,783	5,693	5,748	11,441	72.49	12,518	3781	4895	8,676	69.31
Rizal	15,293	2,115	2,302	4,417	28.88	16,183	1588	1763	3,351	20.71
City of Lucena	1,566	807	758	1,565	99.94	435	185	250	435	100.00
**Region 4B**	**0**	**0**	**0**	**0**	**0.00**	**0**	**0**	**0**	**0**	**0.00**
Marinduque	*	*	*	0	0.00	*	*	*	0	0.00
Occidental Mindoro	*	*	*	0	0.00	*	*	*	0	0.00
Oriental Mindoro	*	*	*	0	0.00	*	*	*	0	0.00
Palawan	*	*	*	0	0.00	*	*	*	0	0.00
Romblon	*	*	*	0	0.00	*	*	*	0	0.00
City of Puerto Princes	*	*	*	0	0.00	*	*	*	0	0.00
**Region 5**	**126,158**	**40,175**	**38,524**	**78,699**	**62.38**	**142,581**	**34,585**	**36,568**	**71,153**	**49.90**
Albay	25,983	7,308	7,150	14,458	55.64	31,040	6768	6855	13,623	43.89
Camarines Norte	12,977	3,594	3,531	7,125	54.90	15,619	3357	3719	7,076	45.30
Camarines Sur	39,899	12,009	11,184	23,193	58.13	43,055	9803	10881	20,684	48.04
Catanduanes	5,559	2,782	2,604	5,386	96.89	6,335	2910	3038	5,948	93.89
Masbate	20,668	6,217	6,085	12,302	59.52	22,338	4341	4599	8,940	40.02
Sorsogon	16,797	7,570	7,274	14,844	88.37	19,198	6419	6174	12,593	65.60
City of Naga	4,275	695	696	1,391	32.54	4,996	987	1302	2,289	45.82

Table 2.C.2.1 Immunization Services for School-Aged Children

Number and Proportion of Grade 1 and Grade 7 learners given one dose of Tetanus diphtheria toxoid (Td) vaccine

Philippines, 2024

Area	Enrolled Grade 1 learners	Grade 1 learners given Td				Enrolled Grade 7 learners	Grade 7 learners given Td			
		Male	Female	Total	%		Male	Female	Total	%
**Region 6**	**128,759**	**39,473**	**40,982**	**80,455**	**62.48**	**147,865**	**36,386**	**40,083**	**76,469**	**51.72**
Aklan	8,823	3,373	3,146	6,519	73.89	11,272	2778	2484	5,262	46.68
Antique	10,201	4,774	4,297	9,071	88.92	12,370	4857	4691	9,548	77.19
Capiz	11,369	4,599	4,696	9,295	81.76	12,661	4529	4536	9,065	71.60
Guimaras	2,762	1,334	1,259	2,593	93.88	3,777	1395	1534	2,929	77.55
Iloilo	30,353	5,385	5,581	10,966	36.13	38,717	6463	6470	12,933	33.40
Negros Occidental	50,434	17,422	18,574	35,996	71.37	52,507	14346	17077	31,423	59.85
City of Bacolod	8,970	783	1,578	2,361	26.32	9,731	534	1548	2,082	21.40
City of Iloilo	5,847	1,803	1,851	3,654	62.49	6,830	1484	1743	3,227	47.25
**Region 7**	**106,964**	**25,707**	**26,523**	**52,230**	**48.83**	**107,376**	**21,453**	**25,383**	**46,836**	**43.62**
Bohol	14,573	6,444	6,424	12,868	88.30	13,886	4569	5577	10,146	73.07
Cebu	59,469	11,725	12,488	24,213	40.72	60,237	10518	12122	22,640	37.58
Negros Oriental	12,952	3,913	3,876	7,789	60.14	12,810	3041	3862	6,903	53.89
Siquijor	1,199	437	403	840	70.06	1,098	316	398	714	65.03
City of Cebu	10,774	1,999	2,021	4,020	37.31	10,970	1773	2333	4,106	37.43
City of Lapu-Lapu	1,053	184	177	361	34.28	1,087	94	85	179	16.47
City of Mandaue	6,944	1,005	1,134	2,139	30.80	7,288	1142	1006	2,148	29.47
**Region 8**	**61,477**	**13,661**	**14,037**	**27,698**	**45.05**	**51,906**	**10,139**	**12,156**	**22,295**	**42.95**
Biliran	2,147	1,085	1,049	2,134	99.39	1,519	729	790	1,519	100.00
Eastern Samar	9,604	2,933	2,932	5,865	61.07	8,816	2401	2501	4,902	55.60
Leyte	25,563	4,189	4,323	8,512	33.30	17,283	3192	4219	7,411	42.88
Northern Samar	4,784	1,293	1,505	2,798	58.49	3,149	936	1200	2,136	67.83
Southern Leyte	1,344	601	492	1,093	81.32	1,332	483	422	905	67.94
Samar	10,093	2,027	2,206	4,233	41.94	10,034	1524	1753	3,277	32.66
Ormoc City	3,960	1,030	987	2,017	50.93	4,829	636	884	1,520	31.48
City of Tacloban	3,982	503	543	1,046	26.27	4,944	238	387	625	12.64
**Region 9**	**92,999**	**35,960**	**36,581**	**72,541**	**78.00**	**90,334**	**28,432**	**31,456**	**59,888**	**66.30**
Zamboanga del Norte	19,463	7,681	7,939	15,620	80.25	19,293	6534	7424	13,958	72.35
Zamboanga del Sur	36,088	13,749	13,669	27,418	75.98	34,209	10983	11492	22,475	65.70
Zamboanga Sibugay	13,275	5,619	6,004	11,623	87.56	14,914	4941	5670	10,611	71.15
City of Isabela	2,770	839	1,060	1,899	68.56	2,528	590	695	1,285	50.83
City of Zamboanga	21,403	8,072	7,909	15,981	74.67	19,390	5384	6175	11,559	59.61
**Region 10**	**96,367**	**38,665**	**37,323**	**75,988**	**78.85**	**94,622**	**34,732**	**35,902**	**70,634**	**74.65**
Bukidnon	33,825	12,611	11,891	24,502	72.44	29,411	9291	10014	19,305	65.64
Camiguin	1,552	735	719	1,454	93.69	1,785	771	813	1,584	88.74
Lanao del Norte	13,749	6,099	6,106	12,205	88.77	12,562	4887	5192	10,079	80.23
Misamis Occidental	11,165	1,391	1,496	2,887	25.86	10,036	1705	2100	3,805	37.91
Misamis Oriental	17,748	9,546	7,913	17,459	98.37	20,799	10822	8822	19,644	94.45
City of Cagayan De C	11,867	5,636	6,241	11,877	100.08	13,029	4799	6003	10,802	82.91
City of Iligan	6,461	2,647	2,957	5,604	86.74	7,000	2457	2958	5,415	77.36
**Region 11**	**76,372**	**20,363**	**26,721**	**47,084**	**61.65**	**77,472**	**17,512**	**25,756**	**43,268**	**55.85**
Davao de Oro	11,160	3,917	3,847	7,764	69.57	10,812	3234	3907	7,141	66.05
Davao del Norte	21,149	6,620	6,580	13,200	62.41	22,375	6297	7002	13,299	59.44
Davao Oriental	8,944	3,370	3,281	6,651	74.36	8,685	2806	3509	6,315	72.71
Davao del Sur	12,717	488	7,068	7,556	59.42	12,442	388	5475	5,863	47.12
Davao Occidental	6,530	2,316	2,308	4,624	70.81	5,601	1724	2083	3,807	67.97
City of Davao	15,872	3,652	3,637	7,289	45.92	17,557	3063	3780	6,843	38.98
**Region 12**	**67,198**	**19,883**	**20,103**	**39,986**	**59.50**	**72,604**	**15,875**	**19,006**	**34,881**	**48.04**
Cotabato	10,739	4,343	4,389	8,732	81.31	12,569	3711	4391	8,102	64.46
Sarangani	12,066	3,520	3,459	6,979	57.84	10,223	2485	2957	5,442	53.23
South Cotabato	17,383	4,576	4,532	9,108	52.40	20,823	4007	4660	8,667	41.62
Sultan Kudarat	14,507	5,278	5,581	10,859	74.85	16,662	3898	4403	8,301	49.82
City of General Santo	12,503	2,166	2,142	4,308	34.46	12,327	1774	2595	4,369	35.44
**CARAGA**	**56,203**	**26,864**	**25,572**	**52,436**	**93.30**	**61,414**	**23,631**	**23,656**	**47,287**	**77.00**
Agusan del Norte	7,263	3,777	3,553	7,330	100.92	8,663	3879	3779	7,658	88.40
Agusan del Sur	15,544	7,782	7,476	15,258	98.16	16,782	6929	6658	13,587	80.96
Surigao del Norte	10,498	4,680	4,523	9,203	87.66	11,010	3775	3858	7,633	69.33
Surigao del Sur	13,225	5,779	5,548	11,327	85.65	12,870	4401	4714	9,115	70.82
Dinagat Islands	2,153	1,013	953	1,966	91.31	2,431	858	847	1,705	70.14
City of Butuan	7,520	3,833	3,519	7,352	97.77	9,658	3789	3800	7,589	78.58
**BARMM**	**0**	**0**	**0**	**0**	**0.00**	**0**	**0**	**0**	**0**	**0.00**
Basilan	*	*	*	0	0.00	*	*	*	0	0.00
Lanao del Sur	*	*	*	0	0.00	*	*	*	0	0.00
Maguindanao del Norte	*	*	*	0	0.00	*	*	*	0	0.00
Maguindanao del Sur	*	*	*	0	0.00	*	*	*	0	0.00
Sulu	*	*	*	0	0.00	*	*	*	0	0.00
Tawi-Tawi	*	*	*	0	0.00	*	*	*	0	0.00
SGA	*	*	*	0	0.00	*	*	*	0	0.00
City of Cotabato	*	*	*	0	0.00	*	*	*	0	0.00

0 = No Data

* = No Report

Table 2.C.2.2 Immunization Services for School-Aged Children
Number and Proportion of Grade 1 and Grade 7 learners given one dose of Measles Rubella (MR) vaccine
Philippines, 2024

Area	Enrolled Grade 1 learners	Grade 1 learners given MR				Enrolled Grade 7 learners	Grade 7 learners given MR			
		Male	Female	Total	%		Male	Female	Total	%
**Philippines**	**1,381,426**	**420,123**	**419,976**	**840,099**	**60.81**	**1,417,032**	**334,158**	**376,023**	**710,181**	**50.12**
**NCR**	**159,664**	**20,004**	**20,937**	**40,941**	**25.64**	**150,820**	**15,015**	**17,890**	**32,905**	**21.82**
City of Malabon	6,471	885	953	1,838	28.40	7,994	933	1,084	2,017	25.23
City of Navotas	0	0	0	0	0.00	0	0	0	0	0.00
City of Valenzuela	0	0	0	0	0.00	0	0	0	0	0.00
City of Caloocan	25,760	3,020	3,005	6,025	23.39	20,878	3,098	3,011	6,109	29.26
City of Marikina	6,748	885	953	1,838	27.24	7,994	933	1,085	2,018	25.24
City of Pasig	9,931	1,530	1,572	3,102	31.24	11,748	1,541	1,553	3,094	26.34
Pateros	726	105	117	222	30.58	660	92	102	194	29.39
City of Taguig	15,448	2,448	2,610	5,058	32.74	14,424	1,922	2,407	4,329	30.01
Quezon City	37,748	2,254	2,268	4,522	11.98	31,789	992	1,343	2,335	7.35
City of Makati	7,896	1,404	1,376	2,780	35.21	8,388	995	1,255	2,250	26.82
City of Mandaluyong	6,998	1,034	1,117	2,151	30.74	4,809	592	753	1,345	27.97
City of San Juan	1,073	114	124	238	22.18	872	61	83	144	16.51
City of Manila	13,029	2,254	2,268	4,522	34.71	14,268	922	1,343	2,265	15.87
City of Las Piñas	7,896	1,404	1,376	2,780	35.21	8,388	995	1,255	2,250	26.82
City of Muntinlupa	6,998	1,034	1,117	2,151	30.74	4,809	592	753	1,345	27.97
City of Parañaque	7,936	934	1,403	2,337	29.45	9,039	771	1,164	1,935	21.41
Pasay City	5,006	699	678	1,377	27.51	4,760	576	699	1,275	26.79
**CAR**	**21,570**	**7,121**	**6,999**	**14,120**	**65.46**	**22,048**	**5,922**	**6,620**	**12,542**	**56.88**
Abra	3,649	1,683	1,646	3,329	91.23	4,315	1,794	1,824	3,618	83.85
Apayao	199	0	0	0	0.00	230	113	117	230	100.00
Benguet	4,180	2,159	2,019	4,178	99.95	3,175	1,393	1,780	3,173	99.94
Ifugao	574	296	276	572	99.65	308	154	153	307	99.68
Kalinga	4,379	1,599	1,753	3,352	76.55	3,827	1,148	1,337	2,485	64.93
Mountain Province	2,545	1,253	1,167	2,420	95.09	2,938	1,305	1,389	2,694	91.70
City of Baguio	6,044	131	138	269	4.45	7,255	15	20	35	0.48
**Region 1**	**2,979**	**5,139**	**5,208**	**10,347**	**347.33**	**2,781**	**5,969**	**5,898**	**11,867**	**426.72**
Ilocos Norte	*	*	*	0	0.00	*	*	*	0	0.00
Ilocos Sur	*	4,287	4,124	8,411	0.00	*	5,231	5,031	10,262	0.00
La Union	*	*	*	0	0.00	*	*	*	0	0.00
Pangasinan	*	*	*	0	0.00	*	*	*	0	0.00
City of Dagupan	2,979	852	1,084	1,936	64.99	2,781	738	867	1,605	57.71
**Region 2**	**56,415**	**24,858**	**18,975**	**43,833**	**77.70**	**58,276**	**15,980**	**17,629**	**33,609**	**57.67**
Batanes	112	55	57	112	100.00	132	70	62	132	100.00
Cagayan	18,456	6,792	7,013	13,805	74.80	13,897	5,486	6,777	12,263	88.24
Isabela	24,874	13,482	6,997	20,479	82.33	28,794	7,210	6,807	14,017	48.68
Nueva Vizcaya	8,049	2,219	2,759	4,978	61.85	9,967	1,459	1,947	3,406	34.17
Quirino	2,530	1,022	1,097	2,119	83.75	2,988	808	983	1,791	59.94
City of Santiago	2,394	1,288	1,052	2,340	97.74	2,498	947	1,053	2,000	80.06
**Region 3**	**202,031**	**66,334**	**65,395**	**131,729**	**65.20**	**218,646**	**45,180**	**51,032**	**96,212**	**44.00**
Aurora	4,357	1,867	1,759	3,626	83.22	5,144	1,462	1,624	3,086	59.99
Bataan	14,716	4,285	4,145	8,430	57.28	16,125	2,733	3,410	6,143	38.10
Bulacan	59,659	15,450	15,235	30,685	51.43	62,644	9,393	10,023	19,416	30.99
Nueva Ecija	36,267	15,178	15,152	30,330	83.63	42,062	11,453	13,468	24,921	59.25
Pampanga	38,153	12,081	11,606	23,687	62.08	40,501	7,934	8,666	16,600	40.99
Tarlac	24,785	11,187	11,487	22,674	91.48	28,127	8,263	9,170	17,433	61.98
Zambales	11,678	3,625	3,450	7,075	60.58	12,906	2,542	3,018	5,560	43.08
City of Angeles	8,194	2,102	2,009	4,111	50.17	6,975	1,159	1,323	2,482	35.58
City of Olongapo	4,222	559	552	1,111	26.31	4,162	241	330	571	13.72
**Region 4A**	**126,270**	**38,045**	**38,009**	**76,054**	**60.23**	**118,369**	**24,633**	**29,154**	**53,787**	**45.44**
Batangas	33,430	13,387	13,144	26,531	79.36	24,699	7,827	9,300	17,127	69.34
Cavite	40,782	10,317	10,338	20,655	50.65	41,758	7,520	8,767	16,287	39.00
Laguna	19,416	5,847	5,629	11,476	59.11	22,776	3,825	4,334	8,159	35.82
Quezon	15,783	5,685	5,880	11,565	73.28	12,518	3,681	4,736	8,417	67.24
Rizal	15,293	2,002	2,262	4,264	27.88	16,183	1,595	1,767	3,362	20.77
City of Lucena	1,566	807	756	1,563	99.81	435	185	250	435	100.00
**Region 4B**	**0**	**0**	**0**	**0**	**0.00**	**0**	**0**	**0**	**0**	**0.00**
Marinduque	*	*	*	0	0.00	*	*	*	0	0.00
Occidental Mindoro	*	*	*	0	0.00	*	*	*	0	0.00
Oriental Mindoro	*	*	*	0	0.00	*	*	*	0	0.00
Palawan	*	*	*	0	0.00	*	*	*	0	0.00
Romblon	*	*	*	0	0.00	*	*	*	0	0.00
City of Puerto Princesa	*	*	*	0	0.00	*	*	*	0	0.00
**Region 5**	**126,158**	**40,262**	**38,389**	**78,651**	**62.34**	**142,581**	**34,854**	**36,717**	**71,571**	**50.20**
Albay	25,983	7,384	7,033	14,417	55.49	31,040	6,842	7,041	13,883	44.73
Camarines Norte	12,977	3,586	3,516	7,102	54.73	15,619	3,371	3,627	6,998	44.80
Camarines Sur	39,899	11,851	11,072	22,923	57.45	43,055	9,858	10,919	20,777	48.26
Catanduanes	5,559	2,782	2,604	5,386	96.89	6,335	2,910	3,038	5,948	93.89
Masbate	20,668	6,422	6,250	12,672	61.31	22,338	4,459	4,612	9,071	40.61
Sorsogon	16,797	7,542	7,258	14,800	88.11	19,198	6,425	6,178	12,603	65.65
City of Naga	4,275	695	656	1,351	31.60	4,996	989	1,302	2,291	45.86

Table 2.C.2.2 Immunization Services for School-Aged Children
Number and Proportion of Grade 1 and Grade 7 learners given one dose of Measles Rubella (MR) vaccine
Philippines, 2024

Area	Enrolled Grade 1 learners	Grade 1 learners given MR				Enrolled Grade 7 learners	Grade 7 learners given MR			
		Male	Female	Total	%		Male	Female	Total	%
**Region 6**	**128,759**	**39,434**	**41,208**	**80,642**	**62.63**	**147,865**	**36,657**	**39,953**	**76,610**	**51.81**
Aklan	8,823	3,386	3,159	6,545	74.18	11,272	2,789	2,495	5,284	46.88
Antique	10,201	4,773	4,313	9,086	89.07	12,370	4,908	4,722	9,630	77.85
Capiz	11,369	4,601	4,697	9,298	81.78	12,661	4,533	4,541	9,074	71.67
Guimaras	2,762	1,336	1,259	2,595	93.95	3,777	1,393	1,534	2,927	77.50
Iloilo	30,353	5,400	5,584	10,984	36.19	38,717	6,464	6,471	12,935	33.41
Negros Occidental	50,434	17,339	18,753	36,092	71.56	52,507	14,551	16,899	31,450	59.90
City of Bacolod	8,970	784	1,579	2,363	26.34	9,731	535	1,548	2,083	21.41
City of Iloilo	5,847	1,815	1,864	3,679	62.92	6,830	1,484	1,743	3,227	47.25
**Region 7**	**106,964**	**24,696**	**25,362**	**50,058**	**46.80**	**107,376**	**20,311**	**24,282**	**44,593**	**41.53**
Bohol	14,573	6,443	6,414	12,857	88.22	13,886	4,574	5,554	10,128	72.94
Cebu	59,469	11,737	12,498	24,235	40.75	60,237	10,566	12,134	22,700	37.68
Negros Oriental	12,952	3,913	3,876	7,789	60.14	12,810	2,997	3,780	6,777	52.90
Siquijor	1,199	439	407	846	70.56	1,098	314	397	711	64.75
City of Cebu	10,774	2,004	2,013	4,017	37.28	10,970	1,773	2,332	4,105	37.42
City of Lapu-Lapu	1,053	160	154	314	29.82	1,087	87	85	172	15.82
City of Mandaue	6,944	0	0	0	0.00	7,288	0	0	0	0.00
**Region 8**	**61,477**	**12,532**	**12,878**	**25,410**	**41.33**	**51,906**	**9,451**	**11,146**	**20,597**	**39.68**
Biliran	2,147	1,092	1,055	2,147	100.00	1,519	729	790	1,519	100.00
Eastern Samar	9,604	2,882	2,891	5,773	60.11	8,816	2,315	2,453	4,768	54.08
Leyte	25,563	3,981	4,112	8,093	31.66	17,283	3,184	4,218	7,402	42.83
Northern Samar	4,784	413	563	976	20.40	3,149	251	382	633	20.10
Southern Leyte	1,344	601	494	1,095	81.47	1,332	427	422	849	63.74
Samar	10,093	2,028	2,224	4,252	42.13	10,034	1,670	1,929	3,599	35.87
Ormoc City	3,960	1,032	997	2,029	51.24	4,829	637	565	1,202	24.89
City of Tacloban	3,982	503	542	1,045	26.24	4,944	238	387	625	12.64
**Region 9**	**92,999**	**35,925**	**36,708**	**72,633**	**78.10**	**90,252**	**28,384**	**31,634**	**60,018**	**66.50**
Zamboanga del Norte	19,463	7,692	7,938	15,630	80.31	19,211	6,539	7,422	13,961	72.67
Zamboanga del Sur	36,088	13,741	13,664	27,405	75.94	34,209	10,957	11,514	22,471	65.69
Zamboanga Sibugay	13,275	5,553	6,130	11,683	88.01	14,914	4,949	5,905	10,854	72.78
City of Isabela	2,770	839	1,060	1,899	68.56	2,528	590	695	1,285	50.83
City of Zamboanga	21,403	8,100	7,916	16,016	74.83	19,390	5,349	6,098	11,447	59.04
**Region 10**	**96,367**	**38,601**	**37,394**	**75,995**	**78.86**	**94,622**	**34,763**	**35,922**	**70,685**	**74.70**
Bukidnon	33,825	12,611	11,880	24,491	72.41	29,411	9,314	9,996	19,310	65.66
Camiguin	1,552	735	719	1,454	93.69	1,785	771	813	1,584	88.74
Lanao del Norte	13,749	6,100	6,105	12,205	88.77	12,562	4,887	5,192	10,079	80.23
Misamis Occidental	11,165	1,391	1,496	2,887	25.86	10,036	1,705	2,126	3,831	38.17
Misamis Oriental	17,748	9,543	7,912	17,455	98.35	20,799	10,822	8,818	19,640	94.43
City of Cagayan De Oro	11,867	5,574	6,325	11,899	100.27	13,029	4,807	6,019	10,826	83.09
City of Iligan	6,461	2,647	2,957	5,604	86.74	7,000	2,457	2,958	5,415	77.36
**Region 11**	**76,372**	**20,363**	**26,721**	**47,084**	**61.65**	**77,472**	**17,512**	**25,756**	**43,268**	**55.85**
Davao de Oro	11,160	3,917	3,847	7,764	69.57	10,812	3,234	3,907	7,141	66.05
Davao del Norte	21,149	6,620	6,580	13,200	62.41	22,375	6,297	7,002	13,299	59.44
Davao Oriental	8,944	3,370	3,281	6,651	74.36	8,685	2,806	3,509	6,315	72.71
Davao del Sur	12,717	488	7,068	7,556	59.42	12,442	388	5,475	5,863	47.12
Davao Occidental	6,530	2,316	2,308	4,624	70.81	5,601	1,724	2,083	3,807	67.97
City of Davao	15,872	3,652	3,637	7,289	45.92	17,557	3,063	3,780	6,843	38.98
**Region 12**	**67,198**	**19,889**	**20,373**	**40,262**	**59.92**	**72,604**	**15,795**	**18,911**	**34,706**	**47.80**
Cotabato	10,739	4,306	4,353	8,659	80.63	12,569	3,669	4,331	8,000	63.65
Sarangani	12,066	3,524	3,464	6,988	57.91	10,223	2,490	2,952	5,442	53.23
South Cotabato	17,383	4,616	4,563	9,179	52.80	20,823	3,981	4,646	8,627	41.43
Sultan Kudarat	14,507	5,277	5,581	10,858	74.85	16,662	3,881	4,387	8,268	49.62
City of General Santos	12,503	2,166	2,412	4,578	36.62	12,327	1,774	2,595	4,369	35.44
**CARAGA**	**56,203**	**26,920**	**25,420**	**52,340**	**93.13**	**61,414**	**23,732**	**23,479**	**47,211**	**76.87**
Agusan del Norte	7,263	3,784	3,556	7,340	101.06	8,663	3,879	3,779	7,658	88.40
Agusan del Sur	15,544	7,886	7,279	15,165	97.56	16,782	7,022	6,482	13,504	80.47
Surigao del Norte	10,498	4,632	4,578	9,210	87.73	11,010	3,781	3,854	7,635	69.35
Surigao del Sur	13,225	5,776	5,550	11,326	85.64	12,870	4,402	4,715	9,117	70.84
Dinagat Islands	2,153	1,007	940	1,947	90.43	2,431	859	849	1,708	70.26
City of Butuan	7,520	3,835	3,517	7,352	97.77	9,658	3,789	3,800	7,589	78.58
**BARMM**	**0**	**0**	**0**	**0**	**0.00**	**0**	**0**	**0**	**0**	**0.00**
Basilan	*	*	*	0	0.00	*	*	*	0	0.00
Lanao del Sur	*	*	*	0	0.00	*	*	*	0	0.00
Maguindanao del Norte	*	*	*	0	0.00	*	*	*	0	0.00
Maguindanao del Sur	*	*	*	0	0.00	*	*	*	0	0.00
Sulu	*	*	*	0	0.00	*	*	*	0	0.00
Tawi-Tawi	*	*	*	0	0.00	*	*	*	0	0.00
SGA	*	*	*	0	0.00	*	*	*	0	0.00
City of Cotabato	*	*	*	0	0.00	*	*	*	0	0.00

0 = No Data
* = No Report

Table 2.C.3.1 - Nutrition Services for Infants and Children

Numbers and Proportion of newborns initiated on breastfeeding within 1 hour after birth, infants exclusively breastfed until 5th month and 29 days,
and infants who continued breastfeeding and were introduced to complementary feeding beginning at 6 months of age
Philippines, 2024

Area	Livebirths	Newborns initiated on breastfeeding within 1 hour after birth				Eligible Population (0-11mos. old)	Infants Exclusively Breastfed until 5th month and 29 days				Eligible Population (6-11mos. old)	Infants who continued breastfeeding and were introduced to complementary feeding			
		Male	Female	Total	%		Male	Female	Total	%		Male	Female	Total	%
**Philippines**	**1,338,505**	**584,929**	**579,802**	**1,164,731**	**87.02**	**2,200,865**	**548,744**	**546,846**	**1,095,590**	**49.78**	**1,121,239**	**530,128**	**524,840**	**1,054,968**	**94.09**
**NCR**	**183,878**	**77,550**	**79,120**	**156,670**	**85.20**	**263,248**	**68,452**	**68,069**	**136,521**	**51.86**	**132,468**	**65,968**	**65,205**	**131,173**	**99.02**
City of Malabon	3,029	1,518	1,480	2,998	98.98	7,447	1,855	1,843	3,698	49.66	3,734	1,730	1,567	3,297	88.30
City of Navotas	3,949	1,921	2,028	3,949	100.00	5,263	2,245	2,283	4,528	86.03	2,640	1,802	1,852	3,654	138.41
City of Valenzuela	4,610	2,358	2,603	4,961	107.61	13,601	3,075	2,958	6,033	44.36	6,816	2,813	2,689	5,502	80.72
City of Caloocan	24,159	4,278	4,589	8,867	36.70	33,499	4,953	5,105	10,058	30.02	16,846	3,523	3,451	6,974	41.40
City of Marikina	2,680	1,232	1,385	2,617	97.65	8,399	1,601	1,695	3,296	39.24	4,207	1,350	1,408	2,758	65.56
City of Pasig	10,914	3,294	3,237	6,531	59.84	17,856	845	923	1,768	9.90	8,945	1,011	1,058	2,069	23.13
Pateros	747	354	393	747	100.00	1,120	131	147	278	24.82	564	141	142	283	50.18
City of Taguig	12,299	4,770	5,294	10,064	81.83	26,021	5,445	5,764	11,209	43.08	13,030	4,777	5,198	9,975	76.55
Quezon City	58,494	29,036	29,458	58,494	100.00	59,607	28,183	28,118	56,301	94.45	29,872	28,656	28,695	57,351	191.99
City of Makati	1,505	772	732	1,504	99.93	4,196	605	609	1,214	28.93	2,109	513	513	1,026	48.65
City of Mandaluyong	7,708	3,524	3,418	6,942	90.06	7,989	2,246	2,135	4,381	54.84	4,011	1,993	1,914	3,907	97.41
City of San Juan	767	366	372	738	96.22	2,173	233	261	494	22.73	1,096	228	288	516	47.08
City of Manila	24,013	12,710	11,850	24,560	102.28	34,268	6,709	6,027	12,736	37.17	17,580	7,136	6,555	13,691	77.88
City of Las Piñas	6,343	3,130	3,302	6,432	101.40	11,346	3,008	3,047	6,055	53.37	5,682	2,769	2,754	5,523	97.20
City of Muntinlupa	7,640	1,648	1,753	3,401	44.52	9,949	2,026	1,943	3,969	39.89	4,979	2,524	2,350	4,874	97.89
City of Parañaque	8,119	3,398	3,565	6,963	85.76	13,385	3,223	3,300	6,523	48.73	6,698	2,934	2,826	5,760	86.00
Pasay City	6,902	3,241	3,661	6,902	100.00	7,129	2,069	1,911	3,980	55.83	3,659	2,068	1,945	4,013	109.67
**CAR**	**23,873**	**11,628**	**11,492**	**23,120**	**96.85**	**31,490**	**9,169**	**8,677**	**17,846**	**56.67**	**16,337**	**8,959**	**8,463**	**17,422**	**106.64**
Abra	2,629	1,126	1,456	2,582	98.21	3,639	1,281	1,182	2,463	67.68	1,967	1,263	1,165	2,428	123.44
Apayao	3,571	1,722	1,622	3,344	93.64	2,222	727	748	1,475	66.38	1,174	664	673	1,337	113.88
Benguet	3,681	1,828	1,759	3,587	97.45	8,900	2,337	2,199	4,536	50.97	4,524	2,317	2,184	4,501	99.49
Ifugao	1,934	828	806	1,634	84.49	4,145	953	943	1,896	45.74	2,157	923	910	1,833	84.98
Kalinga	2,187	1,047	1,088	2,135	97.62	4,582	1,541	1,274	2,815	61.44	2,370	1,502	1,244	2,746	115.86
Mountain Province	3,239	1,664	1,557	3,221	99.44	2,615	969	947	1,916	73.27	1,389	967	936	1,903	137.01
City of Baguio	6,632	3,413	3,204	6,617	99.77	5,387	1,361	1,384	2,745	50.96	2,756	1,323	1,351	2,674	97.02
**Region 1**	**51,383**	**26,056**	**25,128**	**51,184**	**99.61**	**96,024**	**30,610**	**29,639**	**60,249**	**62.74**	**49,634**	**31,535**	**30,856**	**62,391**	**125.70**
Ilocos Norte	5,761	2,802	2,822	5,624	97.62	8,809	3,339	3,425	6,764	76.79	4,676	3,255	3,408	6,663	142.49
Ilocos Sur	7,393	3,778	3,614	7,392	99.99	9,834	4,878	4,510	9,388	95.46	5,294	5,653	5,316	10,969	207.20
La Union	7,714	3,979	3,735	7,714	100.00	13,436	4,393	4,164	8,557	63.69	7,001	4,225	4,013	8,238	117.67
Pangasinan	23,195	11,673	11,461	23,134	99.74	60,591	17,550	17,120	34,670	57.22	30,970	17,867	17,657	35,524	114.70
City of Dagupan	7,320	3,824	3,496	7,320	100.00	3,354	450	420	870	25.94	1,693	535	462	997	58.89
**Region 2**	**33,145**	**14,239**	**14,107**	**28,346**	**85.52**	**64,034**	**17,654**	**17,484**	**35,138**	**54.87**	**33,164**	**15,982**	**15,954**	**31,936**	**96.30**
Batanes	203	94	106	200	98.52	241	69	75	144	59.75	134	65	68	133	99.25
Cagayan	7,853	3,968	3,705	7,673	97.71	20,723	5,872	5,573	11,445	55.23	10,775	5,444	5,270	10,714	99.43
Isabela	13,002	3,864	4,527	8,391	64.54	27,362	7,101	7,184	14,285	52.21	14,175	6,732	6,877	13,609	96.01
Nueva Vizcaya	6,850	3,578	3,267	6,845	99.93	8,880	2,779	2,745	5,524	62.21	4,574	1,978	2,072	4,050	88.54
Quirino	2,383	1,260	1,123	2,383	100.00	3,865	1,014	1,015	2,029	52.50	2,005	1,046	892	1,938	96.66
City of Santiago	2,854	1,475	1,379	2,854	100.00	2,963	819	892	1,711	57.75	1,501	717	775	1,492	99.40
**Region 3**	**140,769**	**58,562**	**57,903**	**116,465**	**82.73**	**235,313**	**52,887**	**53,239**	**106,126**	**45.10**	**119,190**	**50,585**	**50,870**	**101,455**	**85.12**
Aurora	2,493	1,268	1,265	2,533	101.60	4,578	1,168	1,225	2,393	52.27	2,367	1,079	1,114	2,193	92.65
Bataan	11,051	7,091	7,007	14,098	127.57	17,556	4,887	5,002	9,889	56.33	8,893	4,638	4,696	9,334	104.96
Bulacan	40,887	15,034	14,868	29,902	73.13	68,115	16,685	16,671	33,356	48.97	34,332	15,967	15,693	31,660	92.22
Nueva Ecija	24,785	12,328	12,035	24,363	98.30	43,626	10,594	10,602	21,196	48.59	22,222	10,437	10,591	21,028	94.63
Pampanga	23,850	6,306	6,169	12,475	52.31	46,146	7,124	7,217	14,341	31.08	23,330	7,436	7,668	15,104	64.74
Tarlac	19,860	6,888	7,357	14,245	71.73	29,655	7,812	7,769	15,581	52.54	15,094	6,599	6,559	13,158	87.17
Zambales	6,697	3,382	3,280	6,662	99.48	11,796	2,437	2,530	4,967	42.11	6,008	2,408	2,496	4,904	81.62
City of Angeles	7,433	5,098	4,820	9,918	133.43	9,232	1,441	1,515	2,956	32.02	4,631	1,502	1,599	3,101	66.96
City of Olongapo	3,713	1,167	1,102	2,269	61.11	4,609	739	708	1,447	31.40	2,313	519	454	973	42.07
**Region 4A**	**196,166**	**88,310**	**88,767**	**177,077**	**90.27**	**310,150**	**59,729**	**60,033**	**119,762**	**38.61**	**157,056**	**59,662**	**59,274**	**118,936**	**75.73**
Batangas	36,374	17,204	16,691	33,895	93.18	53,484	9,254	9,066	18,320	34.25	27,268	10,899	10,697	21,596	79.20
Cavite	47,820	21,225	21,399	42,624	89.13	78,589	14,519	14,928	29,447	37.47	39,699	14,196	14,271	28,467	71.71
Laguna	42,719	18,954	19,659	38,613	90.39	61,967	13,840	13,639	27,479	44.34	31,323	13,916	13,570	27,486	87.75
Quezon	23,818	10,973	10,684	21,657	90.93	40,954	8,445	8,013	16,458	40.19	21,080	8,625	8,269	16,894	80.14
Rizal	42,541	18,524	18,893	37,417	87.96	68,903	12,235	12,984	25,219	36.60	34,543	10,959	11,302	22,261	64.44
City of Lucena	2,894	1,430	1,441	2,871	99.21	6,253	1,436	1,403	2,839	45.40	3,143	1,067	1,165	2,232	71.01
**Region 4B**	**42,233**	**18,609**	**18,381**	**36,990**	**87.59**	**66,508**	**18,896**	**18,570**	**37,466**	**56.33**	**33,988**	**17,401**	**16,910**	**34,311**	**100.95**
Marinduque	2,702	1,358	1,323	2,681	99.22	4,124	1,134	970	2,104	51.02	2,163	1,041	845	1,886	87.19
Occidental Mindoro	8,238	4,168	3,938	8,106	98.40	11,748	4,186	3,873	8,059	68.60	5,966	3,865	3,547	7,412	124.24
Oriental Mindoro	11,484	3,904	3,888	7,792	67.85	19,007	4,418	4,499	8,917	46.91	9,724	3,999	4,025	8,024	82.52
Palawan	11,857	5,540	5,579	11,119	93.78	20,503	6,336	6,392	12,728	62.08	10,426	6,002	6,067	12,069	115.76
Romblon	4,093	1,853	1,748	3,601	87.98	5,488	1,470	1,502	2,972	54.15	2,859	1,397	1,364	2,761	96.57
City of Puerto Princesa	3,859	1,786	1,905	3,691	95.65	5,638	1,352	1,334	2,686	47.64	2,850	1,097	1,062	2,159	75.75
**Region 5**	**85,536**	**33,287**	**32,569**	**65,856**	**76.99**	**136,611**	**33,533**	**33,595**	**67,128**	**49.14**	**70,057**	**32,511**	**32,731**	**65,242**	**93.13**
Albay	15,792	5,920	5,931	11,851	75.04	27,665	5,772	5,981	11,753	42.48	14,204	5,970	6,243	12,213	85.98
Camarines Norte	10,040	4,795	4,958	9,753	97.14	14,894	3,910	3,977	7,887	52.95	7,585	3,663	3,749	7,412	97.72
Camarines Sur	16,172	6,786	6,570	13,356	82.59	43,855	8,974	8,888	17,862	40.73	22,457	8,259	8,161	16,420	73.12
Catanduanes	3,792	1,720	1,704	3,424	90.30	5,395	1,528	1,473	3,001	55.63	2,846	1,431	1,410	2,841	99.82
Masbate	14,700	6,594	6,473	13,067	88.89	22,495	7,373	7,423	14,796	65.77	11,515	7,743	7,829	15,572	135.23
Sorsogon	12,269	6,318	5,824	12,142	98.96	18,065	5,122	4,993	10,115	55.99	9,317	4,643	4,507	9,150	98.21
City of Naga	12,771	1,154	1,109	2,263	17.72	4,242	854	860	1,714	40.41	2,133	802	832	1,634	76.61
**Region 6**	**85,513**	**40,472**	**38,931**	**79,403**	**92.85**	**147,934**	**34,622**	**34,238**	**68,860**	**46.55**	**75,942**	**33,535**	**32,676**	**66,211**	**87.19**
Aklan	5,996	2,908	2,707	5,615	93.65	11,281	2,235	2,157	4,392	38.93	5,792	2,253	2,108	4,361	75.29
Antique	7,433	3,802	3,505	7,307	98.30	11,899	2,625	2,555	5,180	43.53	6,237	2,521	2,470	4,991	80.02
Capiz	7,460	3,796	3,657	7,453	99.91	13,872	3,041	3,006	6,047	43.59	7,164	2,906	2,881	5,787	80.78
Guimaras	2,183	1,079	1,066	2,145	98.26	3,361	1,014	1,006	2,020	60.10	1,723	1,004	1,009	2,013	116.83
Iloilo	19,712	9,738	8,944	18,682	94.77	35,981	8,304	8,621	16,925	47.04	18,837	8,698	8,553	17,251	91.58
Negros Occidental	32,946	15,433	15,417	30,850	93.64	52,445	13,885	13,331	27,216	51.89	26,516	12,385	11,939	24,324	91.73
City of Bacolod	4,678	1,142	1,167	2,309	49.36	10,361	1,482	1,470	2,952	28.49	5,215	1,755	1,692	3,447	66.10
City of Iloilo	5,105	2,574	2,468	5,042	98.77	8,734	2,036	2,092	4,128	47.26	4,458	2,013	2,024	4,037	90.56
**Region 7**	**107,859**	**45,374**	**43,342**	**88,716**	**82.25**	**167,016**	**44,117**	**42,968**	**87,085**	**52.14**	**84,979**	**43,201**	**42,717**	**85,918**	**101.10**
Bohol	17,051	8,139	7,745	15,884	93.16	26,210	6,364	6,154	12,518	47.76	13,650	6,411	6,192	12,603	92.33
Cebu	40,609	17,041	16,209	33,250	81.88	72,875	19,659	18,914	38,573	52.93	36,964	19,437	19,449	38,886	105.20
Negros Oriental	19,156	5,853	5,651	11,504	60.05	28,980	8,736	8,646	17,382	59.98	14,755	8,331	8,188	16,519	111.96
Siquijor	998	520	465	985	98.70	1,671	487	472	959	57.39	901	491	470	961	106.66
City of Cebu	14,009	6,921	6,506	13,427	95.85	19,466	5,213	5,007	10,220	52.50	9,771	4,870	4,711	9,581	98.06
City of Lapu-Lapu	6,892	2,286	2,549	4,835	70.15	10,833	2,242	2,281	4,523	41.75	5,432	2,638	2,684	5,322	97.97
City of Mandaue	9,144	4,614	4,217	8,831	96.58	6,981	1,416	1,494	2,910	41.68	3,506	1,023	1,023	2,046	58.36

Table 2.C.3.1 - Nutrition Services for Infants and Children

Numbers and Proportion of newborns initiated on breastfeeding within 1 hour after birth, infants exclusively breastfed until 5th month and 29 days, and infants who continued breastfeeding and were introduced to complementary feeding beginning at 6 months of age

Philippines, 2024

Area	Livebirths	Newborns initiated on breastfeeding within 1 hour after birth				Eligible Population (0-11mos. old)	Infants Exclusively Breastfed until 5th month and 29 days				Eligible Population (6-11mos. old)	Infants who continued breastfeeding and were introduced to complementary feeding			
		Male	Female	Total	%		Male	Female	Total	%		Male	Female	Total	%
**Region 8**	**58,580**	**24,074**	**24,019**	**48,093**	**82.10**	**93,713**	**23,634**	**24,104**	**47,738**	**50.94**	**49,025**	**22,325**	**21,807**	**44,132**	**90.02**
Biliran	3,024	1,451	1,311	2,762	91.34	3,378	1,045	949	1,994	59.03	1,748	1,016	883	1,899	108.64
Eastern Samar	6,337	3,203	3,019	6,222	98.19	9,594	2,174	2,164	4,338	45.22	5,093	2,095	2,015	4,110	80.70
Leyte	11,412	4,879	5,082	9,961	87.29	31,504	7,976	8,690	16,666	52.90	16,445	7,525	7,192	14,717	89.49
Northern Samar	10,189	4,070	4,127	8,197	80.45	14,053	3,866	3,836	7,702	54.81	7,302	3,373	3,451	6,824	93.45
Southern Leyte	4,325	1,880	1,882	3,762	86.98	7,368	1,679	1,587	3,266	44.33	3,927	1,620	1,663	3,283	83.60
Samar	11,901	4,080	4,333	8,413	70.69	16,946	4,018	4,049	8,067	47.60	8,959	4,061	3,959	8,020	89.52
Ormoc City	5,423	1,443	1,364	2,807	51.76	5,344	1,310	1,174	2,484	46.48	2,723	1,345	1,221	2,566	94.23
City of Tacloban	5,969	3,068	2,901	5,969	100.00	5,526	1,566	1,655	3,221	58.29	2,828	1,290	1,423	2,713	95.93
**Region 9**	**31,451**	**12,868**	**13,320**	**26,188**	**83.27**	**85,348**	**20,373**	**20,808**	**41,181**	**48.25**	**43,592**	**20,343**	**20,382**	**40,725**	**93.42**
Zamboanga del Norte	12,119	5,557	5,632	11,189	92.33	24,977	5,207	5,158	10,365	41.50	12,815	5,750	5,574	11,324	88.37
Zamboanga del Sur	7,571	3,246	3,351	6,597	87.14	23,905	5,775	5,856	11,631	48.66	12,284	5,365	5,330	10,695	87.06
Zamboanga Sibugay	5,982	2,661	2,834	5,495	91.86	13,903	3,087	3,208	6,295	45.28	7,147	3,027	3,109	6,136	85.85
City of Isabela	2,386	713	692	1,405	58.89	3,209	217	249	466	14.52	1,622	713	773	1,486	91.62
City of Zamboanga	3,393	691	811	1,502	44.27	19,354	6,087	6,337	12,424	64.19	9,724	5,488	5,596	11,084	113.99
**Region 10**	**63,659**	**30,200**	**29,889**	**60,089**	**94.39**	**110,290**	**34,729**	**35,323**	**70,052**	**63.52**	**56,153**	**31,895**	**31,692**	**63,587**	**113.24**
Bukidnon	20,120	9,910	9,622	19,532	97.08	33,913	11,350	11,260	22,610	66.67	17,180	10,167	9,876	20,043	116.66
Camiguin	1,253	642	610	1,252	99.92	1,727	503	543	1,046	60.57	894	580	614	1,194	133.56
Lanao del Norte	9,910	4,522	4,395	8,917	89.98	17,881	4,934	5,144	10,078	56.36	9,185	4,407	4,516	8,923	97.15
Misamis Occidental	4,958	2,575	2,382	4,957	99.98	12,342	4,407	4,548	8,955	72.56	6,416	4,161	4,201	8,362	130.33
Misamis Oriental	9,659	4,576	4,857	9,433	97.66	22,184	5,525	5,798	11,323	51.04	11,297	5,059	5,093	10,152	89.86
City of Cagayan De Oro	11,463	5,760	5,699	11,459	99.97	14,503	5,784	5,779	11,563	79.73	7,290	5,361	5,325	10,686	146.58
City of Iligan	6,296	2,215	2,324	4,539	72.09	7,740	2,226	2,251	4,477	57.84	3,891	2,160	2,067	4,227	108.64
**Region 11**	**73,703**	**34,694**	**32,621**	**67,315**	**91.33**	**107,934**	**30,829**	**29,298**	**60,127**	**55.71**	**54,553**	**29,982**	**27,727**	**57,709**	**105.79**
Davao de Oro	10,504	5,224	4,954	10,178	96.90	15,434	5,312	5,140	10,452	67.72	7,836	4,445	4,298	8,743	111.57
Davao del Norte	15,420	7,560	7,022	14,582	94.57	23,685	6,553	6,198	12,751	53.84	11,955	6,516	6,166	12,682	106.08
Davao Oriental	7,883	3,835	3,614	7,449	94.49	12,025	4,030	3,649	7,679	63.86	6,106	3,988	3,548	7,536	123.42
Davao del Sur	8,695	4,075	3,843	7,918	91.06	13,178	3,744	3,638	7,382	56.02	6,702	3,814	3,711	7,525	112.28
Davao Occidental	4,254	1,599	1,462	3,061	71.96	6,606	1,829	1,685	3,514	53.19	3,353	1,740	1,630	3,370	100.51
City of Davao	26,947	12,401	11,726	24,127	89.54	37,006	9,361	8,988	18,349	49.58	18,601	9,479	8,374	17,853	95.98
**Region 12**	**61,644**	**28,405**	**28,433**	**56,838**	**92.20**	**95,296**	**26,823**	**26,124**	**52,947**	**55.56**	**48,212**	**25,373**	**25,214**	**50,587**	**104.93**
Cotabato	16,676	7,468	7,626	15,094	90.51	25,696	7,714	7,633	15,347	59.73	13,104	7,055	7,085	14,140	107.91
Sarangani	9,447	4,669	4,615	9,284	98.27	13,501	4,835	4,557	9,392	69.57	6,813	4,648	4,466	9,114	133.77
South Cotabato	14,510	7,235	6,949	14,184	97.75	21,842	6,115	6,150	12,265	56.15	11,022	5,450	5,594	11,044	100.20
Sultan Kudarat	11,779	5,766	5,694	11,460	97.29	19,613	6,011	5,543	11,554	58.91	9,939	6,097	5,900	11,997	120.71
City of General Santos	9,232	3,267	3,549	6,816	73.83	14,644	2,148	2,241	4,389	29.97	7,334	2,123	2,169	4,292	58.52
**CARAGA**	**38,525**	**17,959**	**17,542**	**35,501**	**92.15**	**55,371**	**15,970**	**15,920**	**31,890**	**57.59**	**28,326**	**14,729**	**14,767**	**29,496**	**104.13**
Agusan del Norte	5,584	1,927	1,889	3,816	68.34	7,331	2,172	2,191	4,363	59.51	3,746	1,934	1,947	3,881	103.60
Agusan del Sur	9,857	4,906	4,876	9,782	99.24	16,180	5,189	5,149	10,338	63.89	8,244	4,745	4,755	9,500	115.24
Surigao del Norte	7,386	3,718	3,506	7,224	97.81	10,168	2,616	2,566	5,182	50.96	5,240	2,323	2,301	4,624	88.24
Surigao del Sur	9,106	4,559	4,457	9,016	99.01	12,834	3,270	3,279	6,549	51.03	6,570	3,642	3,577	7,219	109.88
Dinagat Islands	865	422	429	851	98.38	2,117	421	415	836	39.49	1,112	335	319	654	58.81
City of Butuan	5,727	2,427	2,385	4,812	84.02	6,741	2,302	2,320	4,622	68.57	3,414	1,750	1,868	3,618	105.98
**BARMM**	**60,588**	**22,642**	**24,238**	**46,880**	**77.38**	**134,585**	**26,717**	**28,757**	**55,474**	**41.22**	**68,563**	**26,142**	**27,595**	**53,737**	**78.38**
Basilan	4,995	1,529	1,737	3,266	65.39	11,653	815	993	1,808	15.52	5,932	1,057	1,287	2,344	39.51
Lanao del Sur	14,028	5,971	6,200	12,171	86.76	33,339	7,547	8,244	15,791	47.36	17,227	6,988	7,458	14,446	83.86
Maguindanao del Norte	7,850	4,834	5,055	9,889	125.97	18,923	6,357	6,904	13,261	70.08	9,574	6,084	6,446	12,530	130.88
Maguindanao del Sur	12,814	2,473	2,646	5,119	39.95	23,214	3,168	3,166	6,334	27.29	11,750	3,504	3,486	6,990	59.49
Sulu	10,405	3,626	4,131	7,757	74.55	21,381	3,994	4,344	8,338	39.00	10,899	3,804	4,008	7,812	71.68
Tawi-Tawi	5,358	1,933	2,091	4,024	75.10	12,602	1,925	2,037	3,962	31.44	6,401	1,727	1,775	3,502	54.71
SGA	1,542	495	639	1,134	73.54	6,305	774	901	1,675	26.57	3,181	824	989	1,813	56.99
City of Cotabato	3,596	1,781	1,739	3,520	97.89	7,168	2,137	2,168	4,305	60.06	3,599	2,154	2,146	4,300	119.48

0 = No Data

* = No Report

Table 2.C.3.2 - Nutrition Services for Infants and Children

Number of Infants born preterm or with low birth weight given iron supplements and infants 6-11 mos. old and children 12-59 mos. old given Vitamin A supplementation
Philippines, 2024

Area	Total No. of live births with low	Infants preterm or with LBW				Eligible Population	Infants 6-11 mos. old Given Vitamin A				Eligible Population	Children 12-59 mos. Old Given Vitamin A			
		Male	Female	Total	%		Male	Female	Total	%		Male	Female	Total	%
**Philippines**	**79,047**	**10,173**	**11,235**	**21,408**	**27.08**	**1,121,239**	**742,736**	**724,056**	**1,466,792**	**130.82**	**9,205,720**	**3,258,061**	**3,164,428**	**6,422,489**	**69.77**
**NCR**	**5,884**	**1,178**	**1,480**	**2,658**	**45.17**	**132,468**	**97,946**	**95,944**	**193,890**	**146.37**	**1,057,004**	**406,008**	**408,751**	**814,759**	**77.08**
City of Malabon	138	63	75	138	100.00	3,734	2,637	2,372	5,009	134.15	29,737	11,394	10,827	22,221	74.73
City of Navotas	124	39	58	97	78.23	2,640	3,091	3,105	6,196	234.70	21,089	7,060	7,490	14,550	68.99
City of Valenzuela	319	56	64	120	37.62	6,816	5,917	6,389	12,306	180.55	55,396	37,027	41,607	78,634	141.95
City of Caloocan	299	21	23	44	14.72	16,846	7,822	7,587	15,409	91.47	140,123	39,014	38,075	77,089	55.02
City of Marikina	139	57	69	126	90.65	4,207	2,283	2,161	4,444	105.63	34,881	5,604	5,438	11,042	31.66
City of Pasig	1,925	181	259	440	22.86	8,945	3,906	3,753	7,659	85.62	66,370	28,295	27,560	55,855	84.16
Pateros	27	6	17	23	85.19	564	354	340	694	123.05	4,848	1,008	976	1,984	40.92
City of Taguig	272	64	53	117	43.01	13,030	6,445	6,366	12,811	98.32	100,735	44,962	44,687	89,649	88.99
Quezon City	524	106	105	211	40.27	29,872	33,698	33,472	67,170	224.86	240,612	102,201	102,977	205,178	85.27
City of Makati	116	21	26	47	40.52	2,109	1,437	1,404	2,841	134.71	17,867	14,891	13,917	28,808	161.24
City of Mandaluyong	133	62	71	133	100.00	4,011	2,805	2,761	5,566	138.77	31,813	12,137	12,411	24,548	77.16
City of San Juan	43	15	22	37	86.05	1,096	767	702	1,469	134.03	8,633	4,186	3,596	7,782	90.14
City of Manila	694	72	109	181	26.08	17,580	13,664	12,855	26,519	150.85	139,634	31,436	30,867	62,303	44.62
City of Las Piñas	164	9	22	31	18.90	5,682	4,609	4,421	9,030	158.92	45,597	7,977	8,874	16,851	36.96
City of Muntinlupa	204	83	121	204	100.00	4,979	2,557	2,597	5,154	103.51	39,414	19,275	20,479	39,754	100.86
City of Parañaque	446	181	229	410	91.93	6,698	3,990	3,752	7,742	115.59	53,742	27,641	26,686	54,327	101.09
Pasay City	317	142	157	299	94.32	3,659	1,964	1,907	3,871	105.79	26,513	11,900	12,284	24,184	91.22
**CAR**	**2,584**	**128**	**170**	**298**	**11.53**	**16,337**	**10,985**	**10,371**	**21,356**	**130.72**	**132,709**	**44,750**	**43,153**	**87,903**	**66.24**
Abra	324	25	33	58	17.90	1,967	1,534	1,500	3,034	154.25	16,211	5,272	5,020	10,292	63.49
Apayao	384	24	26	50	13.02	1,174	939	889	1,828	155.71	9,250	2,810	2,600	5,410	58.49
Benguet	333	22	25	47	14.11	4,524	2,638	2,475	5,113	113.02	37,415	12,273	12,544	24,817	66.33
Ifugao	56	1	1	2	3.57	2,157	1,063	965	2,028	94.02	17,379	6,864	6,362	13,226	76.10
Kalinga	95	28	27	55	57.89	2,370	2,190	1,984	4,174	176.12	18,770	6,101	5,535	11,636	61.99
Mountain Province	327	17	16	33	10.09	1,389	994	906	1,900	136.79	11,403	4,309	4,027	8,336	73.10
City of Baguio	1,065	11	42	53	4.98	2,756	1,627	1,652	3,279	118.98	22,281	7,121	7,065	14,186	63.67
**Region 1**	**3,706**	**1,062**	**1,004**	**2,066**	**55.75**	**49,634**	**39,651**	**37,416**	**77,067**	**155.27**	**391,947**	**149,508**	**142,855**	**292,363**	**74.59**
Ilocos Norte	563	85	71	156	27.71	4,676	5,667	5,526	11,193	239.37	37,839	20,771	19,636	40,407	106.79
Ilocos Sur	468	62	72	134	28.63	5,294	7,178	6,611	13,789	260.46	43,294	26,220	24,727	50,947	117.68
La Union	908	100	114	214	23.57	7,001	5,369	4,801	10,170	145.26	55,151	21,972	20,486	42,458	76.99
Pangasinan	467	131	131	262	56.10	30,970	19,935	19,114	39,049	126.09	242,115	73,124	70,783	143,907	59.44
City of Dagupan	1,300	684	616	1,300	100.00	1,693	1,502	1,364	2,866	169.29	13,548	7,421	7,223	14,644	108.09
**Region 2**	**1,912**	**310**	**322**	**632**	**33.05**	**33,164**	**16,010**	**16,114**	**32,124**	**96.86**	**274,420**	**112,498**	**107,445**	**219,943**	**80.15**
Batanes	25	0	4	4	16.00	134	72	61	133	99.25	1,134	558	576	1,134	100.00
Cagayan	323	79	87	166	51.39	10,775	5,289	4,983	10,272	95.33	90,515	44,246	40,337	84,583	93.45
Isabela	561	122	103	225	40.11	14,175	6,902	7,062	13,964	98.51	116,237	40,281	39,554	79,835	68.68
Nueva Vizcaya	811	33	40	73	9.00	4,574	2,013	2,315	4,328	94.62	39,228	16,453	16,332	32,785	83.58
Quirino	158	74	84	158	100.00	2,005	994	1,011	2,005	100.00	15,836	5,468	5,505	10,973	69.29
City of Santiago	34	2	4	6	17.65	1,501	740	682	1,422	94.74	11,470	5,492	5,141	10,633	92.70
**Region 3**	**9,868**	**1,139**	**1,350**	**2,489**	**25.22**	**119,190**	**88,246**	**85,794**	**174,040**	**146.02**	**989,808**	**414,824**	**397,863**	**812,687**	**82.11**
Aurora	235	36	36	72	30.64	2,367	1,917	1,862	3,779	159.65	19,981	8,261	7,622	15,883	79.49
Bataan	1,388	75	66	141	10.16	8,893	6,632	6,172	12,804	143.98	71,268	40,345	37,876	78,221	109.76
Bulacan	2,942	216	290	506	17.20	34,332	27,586	27,222	54,808	159.64	288,765	124,064	118,765	242,829	84.09
Nueva Ecija	1,893	502	491	993	52.46	22,222	12,592	12,410	25,002	112.51	184,039	76,775	72,858	149,633	81.31
Pampanga	1,661	174	240	414	24.92	23,330	16,536	16,073	32,609	139.77	193,801	64,958	61,002	125,960	64.99
Tarlac	317	10	11	21	6.62	15,094	14,179	13,585	27,764	183.94	121,300	66,872	66,127	132,999	109.64
Zambales	677	15	38	53	7.83	6,008	3,403	3,338	6,741	112.20	49,856	16,989	17,028	34,017	68.23
City of Angeles	350	108	173	281	80.29	4,631	3,468	3,272	6,740	145.54	42,263	7,500	7,500	15,000	35.49
City of Olongapo	405	3	5	8	1.98	2,313	1,933	1,860	3,793	163.99	18,535	9,060	9,085	18,145	97.90
**Region 4A**	**9,975**	**1,159**	**1,198**	**2,357**	**23.63**	**157,056**	**105,518**	**102,498**	**208,016**	**132.45**	**1,291,246**	**343,955**	**330,143**	**674,098**	**52.21**
Batangas	1,921	125	112	237	12.34	27,268	17,784	17,055	34,839	127.77	229,526	74,545	69,746	144,291	62.86
Cavite	2,793	434	454	888	31.79	39,699	30,165	29,770	59,935	150.97	336,133	100,445	97,422	197,867	58.87
Laguna	1,710	197	195	392	22.92	31,323	21,787	21,601	43,388	138.52	259,461	75,488	72,135	147,623	56.90
Quezon	1,228	140	124	264	21.50	21,080	12,873	12,106	24,979	118.50	165,406	54,709	51,671	106,380	64.31
Rizal	2,154	251	302	553	25.67	34,543	20,540	19,671	40,211	116.41	276,151	32,484	32,900	65,384	23.68
City of Lucena	169	12	11	23	13.61	3,143	2,369	2,295	4,664	148.39	24,569	6,284	6,269	12,553	51.09
**Region 4B**	**2,567**	**399**	**390**	**789**	**30.74**	**33,988**	**23,580**	**22,724**	**46,304**	**136.24**	**275,267**	**145,736**	**136,429**	**282,165**	**102.51**
Marinduque	213	87	80	167	78.40	2,163	2,058	2,043	4,101	189.60	19,560	12,073	11,195	23,268	118.96
Occidental Mindoro	557	66	73	139	24.96	5,966	4,048	3,748	7,796	130.67	48,406	31,814	27,638	59,452	122.82
Oriental Mindoro	448	95	98	193	43.08	9,724	6,965	6,537	13,502	138.85	75,762	45,447	42,916	88,363	116.63
Palawan	695	118	111	229	32.95	10,426	6,183	6,143	12,326	118.22	85,588	33,236	32,691	65,927	77.03
Romblon	351	17	16	33	9.40	2,859	1,965	1,954	3,919	137.08	22,810	14,237	13,682	27,919	122.40
City of Puerto Princesa	303	16	12	28	9.24	2,850	2,361	2,299	4,660	163.51	23,141	8,929	8,307	17,236	74.48
**Region 5**	**8,543**	**572**	**741**	**1,313**	**15.37**	**70,057**	**42,300**	**41,662**	**83,962**	**119.85**	**569,012**	**157,336**	**149,198**	**306,534**	**53.87**
Albay	1,218	134	145	279	22.91	14,204	9,086	8,652	17,738	124.88	116,415	48,427	46,085	94,512	81.19
Camarines Norte	1,178	69	79	148	12.56	7,585	3,720	3,773	7,493	98.79	62,374	12,586	11,622	24,208	38.81
Camarines Sur	1,103	109	135	244	22.12	22,457	12,136	11,482	23,618	105.17	181,346	50,386	48,001	98,387	54.25
Catanduanes	477	99	161	260	54.51	2,846	1,998	1,853	3,851	135.31	24,805	7,581	7,117	14,698	59.25
Masbate	863	64	77	141	16.34	11,515	8,662	9,573	18,235	158.36	91,826	23,885	22,884	46,769	50.93
Sorsogon	1,306	78	109	187	14.32	9,317	5,630	5,292	10,922	117.23	75,406	13,063	12,174	25,237	33.47
City of Naga	2,398	19	35	54	2.25	2,133	1,068	1,037	2,105	98.69	16,840	1,408	1,315	2,723	16.17
**Region 6**	**7,066**	**737**	**780**	**1,517**	**21.47**	**75,942**	**35,927**	**34,642**	**70,569**	**92.92**	**622,304**	**167,012**	**163,296**	**330,308**	**53.08**
Aklan	396	80	87	167	42.17	5,792	2,796	2,732	5,528	95.44	46,520	7,344	7,083	14,427	31.01
Antique	1,385	27	29	56	4.04	6,237	3,941	3,871	7,812	125.25	50,356	13,191	12,204	25,395	50.43
Capiz	421	75	106	181	42.99	7,164	3,889	3,852	7,741	108.05	61,057	21,805	20,899	42,704	69.94
Guimaras	255	52	62	114	44.71	1,723	1,143	1,126	2,269	131.69	14,780	5,425	5,253	10,678	72.25
Iloilo	1,270	158	165	323	25.43	18,837	8,107	7,704	15,811	83.94	152,421	65,677	65,506	131,183	86.07
Negros Occidental	2,631	271	271	542	20.60	26,516	10,626	10,191	20,817	78.51	217,118	44,153	42,966	87,119	40.13
City of Bacolod	490	52	43	95	19.39	5,215	2,489	2,330	4,819	92.41	43,705	4,676	4,784	9,460	21.65
City of Iloilo	218	22	17	39	17.89	4,458	2,936	2,836	5,772	129.48	36,347	4,741	4,601	9,342	25.70
**Region 7**	**8,631**	**534**	**583**	**1,117**	**12.94**	**84,979**	**54,544**	**52,551**	**107,095**	**126.03**	**680,875**	**198,716**	**189,880**	**388,596**	**57.07**
Bohol	1,733	67	86	153	8.83	13,650	8,192	7,733	15,925	116.67	108,897	40,028	37,491	77,519	71.19
Cebu	3,063	155	164	319	10.41	36,964	24,107	23,416	47,523	128.57	295,731	80,809	77,485	158,294	53.53
Negros Oriental	1,100	71	81	152	13.82	14,755	8,084	8,043	16,127	109.30	120,536	41,116	39,763	80,879	67.10
Siquijor	132	4	5	9	6.82	901	575	522	1,097	121.75	6,371	2,014	1,985	3,999	62.77
	0														
City of Cebu	808	193	204	397	49.13	9,771	6,664	6,278	12,942	132.45	79,781	21,161	19,959	41,120	51.54
City of Lapu-Lapu	600	44	43	87	14.50	5,432	3,846	3,795	7,641	140.67	42,661	6,879	6,691	13,570	31.81
City of Mandaue	1,195	0	0	0	0.00	3,506	3,076	2,764	5,840	166.57	26,898	6,709	6,506	13,215	49.13

Table 2.C.3.2 - Nutrition Services for Infants and Children

Number of Infants born preterm or with low birth weight given iron supplements and infants 6-11 mos. old and children 12-59 mos. old given Vitamin A supplementation
Philippines, 2024

Area	Total No. of live births with low	Infants preterm or with LBW				Eligible Population	Infants 6-11 mos. old Given Vitamin A				Eligible Population	Children 12-59 mos. Old Given Vitamin A			
		Male	Female	Total	%		Male	Female	Total	%		Male	Female	Total	%
**Region 8**	**4,529**	**430**	**503**	**933**	**20.60**	**49,025**	**30,556**	**29,674**	**60,230**	**122.86**	**392,682**	**126,704**	**121,746**	**248,450**	**63.27**
Biliran	303	42	42	84	27.72	1,748	1,198	1,069	2,267	129.69	15,168	5,835	7,578	13,413	88.43
Eastern Samar	282	22	17	39	13.83	5,093	2,852	2,834	5,686	111.64	38,988	15,592	14,827	30,419	78.02
Leyte	547	32	71	103	18.83	16,445	11,029	10,709	21,738	132.19	133,983	46,460	41,560	88,020	65.69
Northern Samar	970	22	40	62	6.39	7,302	3,555	3,748	7,303	100.01	58,551	16,320	15,979	32,299	55.16
Southern Leyte	432	27	29	56	12.96	3,927	1,904	1,840	3,744	95.34	32,412	7,160	6,854	14,014	43.24
Samar	838	37	68	105	12.53	8,959	7,111	6,856	13,967	155.90	70,476	26,295	26,158	52,453	74.43
Ormoc City	836	109	131	240	28.71	2,723	1,409	1,294	2,703	99.27	20,712	2,585	2,371	4,956	23.93
City of Tacloban	321	139	105	244	76.01	2,828	1,498	1,324	2,822	99.79	22,392	6,457	6,419	12,876	57.50
**Region 9**	**1,590**	**234**	**296**	**530**	**33.33**	**43,592**	**28,249**	**27,906**	**56,155**	**128.82**	**354,698**	**91,377**	**91,011**	**182,388**	**51.42**
Zamboanga del Norte	777	128	167	295	37.97	12,815	7,610	7,513	15,123	118.01	99,129	34,165	35,056	69,221	69.83
Zamboanga del Sur	231	53	94	147	63.64	12,284	7,164	7,096	14,260	116.09	97,821	21,871	21,457	43,328	44.29
Zamboanga Sibugay	454	51	34	85	18.72	7,147	4,674	4,734	9,408	131.64	61,013	20,055	19,614	39,669	65.02
	0														
City of Isabela	111	2	1	3	2.70	1,622	721	690	1,411	86.99	13,714	1,449	1,373	2,822	20.58
City of Zamboanga	17	0	0	0	0.00	9,724	8,080	7,873	15,953	164.06	83,021	13,837	13,511	27,348	32.94
**Region 10**	**2,447**	**395**	**427**	**822**	**33.59**	**56,153**	**40,159**	**39,373**	**79,532**	**141.63**	**460,481**	**219,027**	**213,483**	**432,510**	**93.93**
Bukidnon	855	181	218	399	46.67	17,180	12,247	11,717	23,964	139.49	146,496	70,681	68,261	138,942	94.84
Camiguin	79	7	5	12	15.19	894	664	743	1,407	157.38	7,322	5,589	5,108	10,697	146.09
Lanao del Norte	282	28	41	69	24.47	9,185	7,274	7,245	14,519	158.07	75,128	37,943	38,132	76,075	101.26
Misamis Occidental	168	75	56	131	77.98	6,416	4,992	4,932	9,924	154.68	49,597	29,502	28,848	58,350	117.65
Misamis Oriental	291	38	47	85	29.21	11,297	5,746	5,636	11,382	100.75	88,919	42,421	41,119	83,540	93.95
City of Cagayan De Oro	617	37	34	71	11.51	7,290	6,163	6,061	12,224	167.68	60,460	21,293	20,389	41,682	68.94
City of Iligan	155	29	26	55	35.48	3,891	3,073	3,039	6,112	157.08	32,559	11,598	11,626	23,224	71.33
**Region 11**	**4,421**	**998**	**905**	**1,903**	**43.04**	**54,553**	**39,287**	**37,410**	**76,697**	**140.59**	**453,316**	**219,680**	**212,618**	**432,298**	**95.36**
Davao de Oro	675	130	134	264	39.11	7,836	6,604	6,271	12,875	164.31	66,781	46,590	44,092	90,682	135.79
Davao del Norte	1,100	265	243	508	46.18	11,955	8,712	8,424	17,136	143.34	98,839	45,704	47,268	92,972	94.06
Davao Oriental	396	76	62	138	34.85	6,106	5,037	4,709	9,746	159.61	49,438	23,529	22,212	45,741	92.52
Davao del Sur	486	104	109	213	43.83	6,702	5,794	5,629	11,423	170.44	59,693	29,118	27,367	56,485	94.63
Davao Occidental	230	63	41	104	45.22	3,353	2,251	2,122	4,373	130.42	31,655	12,080	11,663	23,743	75.01
City of Davao	1,534	360	316	676	44.07	18,601	10,889	10,255	21,144	113.67	146,910	62,659	60,016	122,675	83.50
**Region 12**	**2,160**	**460**	**554**	**1,014**	**46.94**	**48,212**	**29,491**	**28,692**	**58,183**	**120.68**	**396,809**	**170,608**	**159,015**	**329,623**	**83.07**
Cotabato	737	149	153	302	40.98	13,104	8,226	7,987	16,213	123.73	114,236	50,375	47,926	98,301	86.05
Sarangani	166	37	47	84	50.60	6,813	4,459	4,335	8,794	129.08	54,830	24,974	24,180	49,154	89.65
South Cotabato	533	175	255	430	80.68	11,022	7,655	7,545	15,200	137.91	87,302	47,546	45,624	93,170	106.72
Sultan Kudarat	458	87	79	166	36.24	9,939	5,658	5,507	11,165	112.34	79,530	40,913	34,929	75,842	95.36
City of General Santos	266	12	20	32	12.03	7,334	3,493	3,318	6,811	92.87	60,911	6,800	6,356	13,156	21.60
**CARAGA**	**2,173**	**298**	**342**	**640**	**29.45**	**28,326**	**22,054**	**21,373**	**43,427**	**153.31**	**238,285**	**89,852**	**88,605**	**178,457**	**74.89**
Agusan del Norte	237	77	61	138	58.23	3,746	3,523	3,377	6,900	184.20	32,247	12,847	12,479	25,326	78.54
Agusan del Sur	201	11	19	30	14.93	8,244	5,769	5,995	11,764	142.70	67,902	24,814	26,295	51,109	75.27
Surigao del Norte	775	34	52	86	11.10	5,240	3,301	3,165	6,466	123.40	42,410	10,410	9,967	20,377	48.05
Surigao del Sur	551	108	137	245	44.46	6,570	4,454	4,190	8,644	131.57	56,102	21,725	20,464	42,189	75.20
Dinagat Islands	94	24	22	46	48.94	1,112	677	593	1,270	114.21	10,888	2,705	2,808	5,513	50.63
City of Butuan	315	44	51	95	30.16	3,414	4,330	4,053	8,383	245.55	28,736	17,351	16,592	33,943	118.12
**BARMM**	**991**	**140**	**190**	**330**	**33.30**	**68,563**	**38,233**	**39,912**	**78,145**	**113.98**	**624,857**	**200,470**	**208,937**	**409,407**	**65.52**
Basilan	208	11	12	23	11.06	5,932	2,683	2,802	5,485	92.46	53,188	9,851	9,897	19,748	37.13
Lanao del Sur	96	41	55	96	100.00	17,227	7,969	8,322	16,291	94.57	151,859	47,871	46,448	94,319	62.11
Maguindanao del Norte	139	18	26	44	31.65	9,574	5,247	5,228	10,475	109.41	82,095	36,300	40,462	76,762	93.50
Maguindanao del Sur	263	25	24	49	18.63	11,750	7,660	8,241	15,901	135.33	110,119	38,753	39,491	78,244	71.05
Sulu	93	8	15	23	24.73	10,899	7,290	8,060	15,350	140.84	109,879	29,128	32,077	61,205	55.70
Tawi-Tawi	102	18	40	58	56.86	6,401	2,438	2,545	4,983	77.85	56,912	7,331	7,448	14,779	25.97
SGA	7	4	3	7	100.00	3,181	1,785	1,895	3,680	115.69	28,215	7,152	7,988	15,140	53.66
City of Cotabato	83	15	15	30	36.14	3,599	3,161	2,819	5,980	166.16	32,590	24,084	25,126	49,210	151.00

0 = No Data
* = No Report

Table 2.C.3.3 - Nutrition Services for Infants and Children

Number and Proportion of infants 6-11 mos. old and children 12-23 mos. old who completed Micronutrient Powder (MNP) or Lipid-Based Nutrient Supplement – Small Quantity (LNS-SQ) supplementation

Philippines, 2024

Area	Eligible Population (6-11 mos. old)	Infants 6-11 mos. old who completed MNP or LNS-SQ supplementation				Eligible Population (12-23 mos. old)	Children 12-23 mos. old who completed MNP or LNS-SQ supplementation			
		Male	Female	Total	%		Male	Female	Total	%
**Philippines**	**1,121,239**	**93,672**	**94,332**	**188,004**	**16.77**	**2,266,749**	**143,951**	**141,777**	**285,728**	**12.61**
**NCR**	**132,468**	**31,332**	**30,924**	**62,256**	**47.00**	**268,478**	**49,259**	**48,754**	**98,013**	**36.51**
City of Malabon	3,734	929	896	1,825	48.88	7,441	774	747	1,521	20.44
City of Navotas	2,640	2,213	2,276	4,489	170.04	5,416	3,808	4,096	7,904	145.94
City of Valenzuela	6,816	1,048	951	1,999	29.33	14,485	1,722	1,753	3,475	23.99
City of Caloocan	16,846	2,074	2,037	4,111	24.40	35,617	2,446	2,532	4,978	13.98
City of Marikina	4,207	1,453	1,382	2,835	67.39	8,850	1,114	1,003	2,117	23.92
City of Pasig	8,945	1,094	1,091	2,185	24.43	16,904	1,319	1,374	2,693	15.93
Pateros	564	24	20	44	7.80	1,218	9	5	14	1.15
City of Taguig	9,628	6,113	6,010	12,123	125.91	25,803	11,677	11,133	22,810	88.40
Quezon City	29,872	2,275	2,405	4,680	15.67	61,365	3,109	3,046	6,155	10.03
City of Makati	5,511	1,571	1,483	3,054	55.42	4,588	2,014	1,909	3,923	85.51
City of Mandaluyong	4,011	241	257	498	12.42	8,136	369	372	741	9.11
City of San Juan	1,096	603	605	1,208	110.22	2,256	1,395	1,161	2,556	113.30
City of Manila	17,580	113	104	217	1.23	35,058	87	109	196	0.56
City of Las Piñas	5,682	1,757	1,676	3,433	60.42	11,222	1,967	2,055	4,022	35.84
City of Muntinlupa	4,979	2,663	2,840	5,503	110.52	10,064	6,172	6,210	12,382	123.03
City of Parañaque	6,698	5,129	4,888	10,017	149.55	13,542	8,182	8,216	16,398	121.09
Pasay City	3,659	2,032	2,003	4,035	110.28	6,513	3,095	3,033	6,128	94.09
**CAR**	**16,337**	**1,130**	**1,104**	**2,234**	**13.67**	**32,402**	**1,044**	**1,068**	**2,112**	**6.52**
Abra	1,967	129	122	251	12.76	3,952	116	141	257	6.50
Apayao	1,174	66	96	162	13.80	2,210	37	29	66	2.99
Benguet	4,524	584	576	1,160	25.64	9,098	394	409	803	8.83
Ifugao	2,157	80	97	177	8.21	4,228	153	147	300	7.10
Kalinga	2,370	40	35	75	3.16	4,637	212	197	409	8.82
Mountain Province	1,389	193	148	341	24.55	2,730	84	84	168	6.15
City of Baguio	2,756	38	30	68	2.47	5,547	48	61	109	1.97
**Region 1**	**49,634**	**6,276**	**5,900**	**12,176**	**24.53**	**97,386**	**9,157**	**8,947**	**18,104**	**18.59**
Ilocos Norte	4,676	635	586	1,221	26.11	9,059	1,566	1,486	3,052	33.69
Ilocos Sur	5,294	932	888	1,820	34.38	10,228	2,708	2,786	5,494	53.72
La Union	7,001	1,030	967	1,997	28.52	13,384	1,010	930	1,940	14.49
Pangasinan	30,970	3,466	3,281	6,747	21.79	61,282	3,750	3,619	7,369	12.02
City of Dagupan	1,693	213	178	391	23.10	3,433	123	126	249	7.25
**Region 2**	**33,164**	**3,610**	**5,214**	**8,824**	**26.61**	**65,768**	**5,567**	**5,746**	**11,313**	**17.20**
Batanes	134	5	10	15	11.19	267	9	10	19	7.12
Cagayan	10,775	505	2,007	2,512	23.31	21,515	822	841	1,663	7.73
Isabela	14,175	1,808	1,934	3,742	26.40	28,156	2,024	2,226	4,250	15.09
Nueva Vizcaya	4,574	760	792	1,552	33.93	9,251	2,033	2,015	4,048	43.76
Quirino	2,005	151	128	279	13.92	3,791	223	166	389	10.26
City of Santiago	1,501	381	343	724	48.23	2,788	456	488	944	33.86
**Region 3**	**119,190**	**7,913**	**8,092**	**16,005**	**13.43**	**245,405**	**15,416**	**15,091**	**30,507**	**12.43**
Aurora	2,367	54	51	105	4.44	4,781	87	82	169	3.53
Bataan	8,893	231	254	485	5.45	17,541	422	481	903	5.15
Bulacan	34,332	4,184	4,517	8,701	25.34	72,406	6,798	6,688	13,486	18.63
Nueva Ecija	22,222	947	922	1,869	8.41	45,088	1,715	1,684	3,399	7.54
Pampanga	23,330	945	821	1,766	7.57	48,891	910	915	1,825	3.73
Tarlac	15,094	1,296	1,304	2,600	17.23	29,490	5,107	4,875	9,982	33.85
Zambales	6,008	40	34	74	1.23	12,221	74	46	120	0.98
City of Angeles	4,631	120	113	233	5.03	10,410	177	175	352	3.38
City of Olongapo	2,313	96	76	172	7.44	4,577	126	145	271	5.92
**Region 4A**	**157,056**	**7,900**	**7,789**	**15,689**	**9.99**	**321,682**	**8,707**	**8,379**	**17,086**	**5.31**
Batangas	27,268	891	879	1,770	6.49	57,394	1,266	1,185	2,451	4.27
Cavite	39,699	3,110	3,133	6,243	15.73	82,229	2,255	2,159	4,414	5.37
Laguna	31,323	2,250	2,275	4,525	14.45	63,529	2,338	2,344	4,682	7.37
Quezon	21,080	652	545	1,197	5.68	42,222	1,038	932	1,970	4.67
Rizal	34,543	956	927	1,883	5.45	70,245	1,782	1,743	3,525	5.02
City of Lucena	3,143	41	30	71	2.26	6,063	28	16	44	0.73

Table 2.C.3.3 - Nutrition Services for Infants and Children

Number and Proportion of infants 6-11 mos. old and children 12-23 mos. old who completed Micronutrient Powder (MNP) or Lipid-Based Nutrient Supplement – Small Quantity (LNS-SQ) supplementation

Philippines, 2024

Area	Eligible Population (6-11 mos. old)	Infants 6-11 mos. old who completed MNP or LNS-SQ supplementation				Eligible Population (12-23 mos. old)	Children 12-23 mos. old who completed MNP or LNS-SQ supplementation			
		Male	Female	Total	%		Male	Female	Total	%
**Region 4B**	**33,988**	**2,163**	**2,134**	**4,297**	**12.64**	**67,274**	**5,396**	**5,007**	**10,403**	**15.46**
Marinduque	2,163	24	42	66	3.05	4,843	33	17	50	1.03
Occidental Mindoro	5,966	163	148	311	5.21	11,874	307	297	604	5.09
Oriental Mindoro	9,724	962	884	1,846	18.98	18,711	1,805	1,692	3,497	18.69
Palawan	10,426	711	733	1,444	13.85	20,731	1,285	1,294	2,579	12.44
Romblon	2,859	256	274	530	18.54	5,640	1,961	1,706	3,667	65.02
City of Puerto Princesa	2,850	47	53	100	3.51	5,475	5	1	6	0.11
**Region 5**	**70,057**	**6,103**	**5,872**	**11,975**	**17.09**	**140,132**	**8,424**	**7,986**	**16,410**	**11.71**
Albay	14,204	1,940	1,843	3,783	26.63	29,099	1,881	2,030	3,911	13.44
Camarines Norte	7,585	847	674	1,521	20.05	14,936	136	108	244	1.63
Camarines Sur	22,457	665	636	1,301	5.79	44,900	595	617	1,212	2.70
Catanduanes	2,846	30	42	72	2.53	5,895	91	105	196	3.32
Masbate	11,515	2,465	2,515	4,980	43.25	22,646	5,460	4,840	10,300	45.48
Sorsogon	9,317	149	160	309	3.32	18,383	189	229	418	2.27
City of Naga	2,133	7	2	9	0.42	4,273	72	57	129	3.02
**Region 6**	**75,942**	**3,364**	**3,308**	**6,672**	**8.79**	**153,816**	**5,758**	**5,752**	**11,510**	**7.48**
Aklan	5,792	209	191	400	6.91	11,119	175	171	346	3.11
Antique	6,237	84	93	177	2.84	11,939	136	133	269	2.25
Capiz	7,164	347	374	721	10.06	14,363	521	571	1,092	7.60
Guimaras	1,723	66	56	122	7.08	3,556	37	53	90	2.53
Iloilo	18,837	1,040	1,045	2,085	11.07	37,068	2,333	2,263	4,596	12.40
Negros Occidental	26,516	1,157	1,067	2,224	8.39	55,510	1,935	1,905	3,840	6.92
City of Bacolod	5,215	125	137	262	5.02	11,067	283	307	590	5.33
City of Iloilo	4,458	336	345	681	15.28	9,194	338	349	687	7.47
**Region 7**	**84,979**	**3,927**	**3,941**	**7,868**	**9.26**	**167,951**	**4,268**	**4,176**	**8,444**	**5.03**
Bohol	13,650	995	934	1,929	14.13	25,897	775	654	1,429	5.52
Cebu	36,964	1,318	1,338	2,656	7.19	73,718	993	1,064	2,057	2.79
Negros Oriental	14,755	396	421	817	5.54	28,843	1,171	1,105	2,276	7.89
Siquijor	901	9	7	16	1.78	1,455	4	9	13	0.89
City of Cebu	9,771	100	99	199	2.04	20,286	111	90	201	0.99
City of Lapu-Lapu	5,432	901	927	1,828	33.65	10,908	870	866	1,736	15.91
City of Mandaue	3,506	208	215	423	12.07	6,844	344	388	732	10.70
**Region 8**	**49,025**	**3,021**	**2,892**	**5,913**	**12.06**	**94,774**	**4,317**	**4,223**	**8,540**	**9.01**
Biliran	1,748	161	160	321	18.36	3,499	619	591	1,210	34.58
Eastern Samar	5,093	110	128	238	4.67	9,571	273	274	547	5.72
Leyte	16,445	1,279	1,237	2,516	15.30	33,031	1,430	1,382	2,812	8.51
Northern Samar	7,302	130	113	243	3.33	13,659	186	224	410	3.00
Southern Leyte	3,927	75	82	157	4.00	7,732	82	88	170	2.20
Samar	8,959	275	281	556	6.21	16,715	558	566	1,124	6.72
Ormoc City	2,723	224	218	442	16.23	5,145	278	234	512	9.95
City of Tacloban	2,828	767	673	1,440	50.92	5,422	891	864	1,755	32.37
**Region 9**	**43,592**	**1,407**	**1,699**	**3,106**	**7.13**	**86,199**	**2,040**	**2,024**	**4,064**	**4.71**
Zamboanga del Norte	12,815	399	415	814	6.35	25,274	609	683	1,292	5.11
Zamboanga del Sur	12,284	318	370	688	5.60	23,237	193	193	386	1.66
Zamboanga Sibugay	7,147	8	14	22	0.31	14,116	48	38	86	0.61
City of Isabela	1,622	9	9	18	1.11	3,435	27	19	46	1.34
City of Zamboanga	9,724	673	891	1,564	16.08	20,137	1,163	1,091	2,254	11.19
**Region 10**	**56,153**	**3,428**	**3,149**	**6,577**	**11.71**	**111,675**	**6,437**	**6,219**	**12,656**	**11.33**
Bukidnon	17,180	1,811	1,598	3,409	19.84	34,801	3,898	3,764	7,662	22.02
Camiguin	894	29	29	58	6.49	1,718	53	55	108	6.29
Lanao del Norte	9,185	241	225	466	5.07	18,370	155	192	347	1.89
Misamis Occidental	6,416	266	251	517	8.06	12,280	403	414	817	6.65
Misamis Oriental	11,297	85	57	142	1.26	21,954	333	331	664	3.02
City of Cagayan De Or	7,290	551	581	1,132	15.53	14,677	1,026	956	1,982	13.50
City of Iligan	3,891	445	408	853	21.92	7,875	569	507	1,076	13.66

Table 2.C.3.3 - Nutrition Services for Infants and Children
Number and Proportion of infants 6-11 mos. old and children 12-23 mos. old who completed Micronutrient Powder (MNP) or Lipid-Based Nutrient Supplement – Small Quantity (LNS-SQ) supplementation
Philippines, 2024

Area	Eligible Population (6-11 mos. old)	Infants 6-11 mos. old who completed MNP or LNS-SQ supplementation				Eligible Population (12-23 mos. old)	Children 12-23 mos. old who completed MNP or LNS-SQ supplementation			
		Male	Female	Total	%		Male	Female	Total	%
**Region 11**	**54,553**	**4,243**	**4,090**	**8,333**	**15.28**	**110,500**	**6,052**	**6,028**	**12,080**	**10.93**
Davao de Oro	7,836	611	613	1,224	15.62	15,685	816	795	1,611	10.27
Davao del Norte	11,955	635	479	1,114	9.32	23,807	948	1,041	1,989	8.35
Davao Oriental	6,106	222	253	475	7.78	11,712	532	506	1,038	8.86
Davao del Sur	6,702	180	167	347	5.18	14,143	221	236	457	3.23
Davao Occidental	3,353	107	135	242	7.22	7,287	177	191	368	5.05
City of Davao	18,601	2,488	2,443	4,931	26.51	37,866	3,358	3,259	6,617	17.47
**Region 12**	**48,212**	**1,934**	**1,917**	**3,851**	**7.99**	**97,218**	**3,085**	**3,113**	**6,198**	**6.38**
Cotabato	13,104	752	767	1,519	11.59	27,235	1,493	1,668	3,161	11.61
Sarangani	6,813	248	232	480	7.05	13,076	706	692	1,398	10.69
South Cotabato	11,022	219	230	449	4.07	22,225	552	460	1,012	4.55
Sultan Kudarat	9,939	250	228	478	4.81	20,095	59	56	115	0.57
City of General Santos	7,334	465	460	925	12.61	14,587	275	237	512	3.51
**CARAGA**	**28,326**	**3,670**	**3,685**	**7,355**	**25.97**	**56,849**	**5,671**	**5,191**	**10,862**	**19.11**
Agusan del Norte	3,746	379	369	748	19.97	7,530	287	218	505	6.71
Agusan del Sur	8,244	2,556	2,587	5,143	62.38	16,119	4,267	3,775	8,042	49.89
Surigao del Norte	5,240	187	194	381	7.27	10,510	205	203	408	3.88
Surigao del Sur	6,570	375	368	743	11.31	13,458	799	878	1,677	12.46
Dinagat Islands	1,112	23	29	52	4.68	2,405	43	45	88	3.66
City of Butuan	3,414	150	138	288	8.44	6,827	70	72	142	2.08
**BARMM**	**68,563**	**2,251**	**2,622**	**4,873**	**7.11**	**149,240**	**3,353**	**4,073**	**7,426**	**4.98**
Basilan	5,932	80	92	172	2.90	12,857	110	120	230	1.79
Lanao del Sur	17,227	915	1,256	2,171	12.60	36,827	1,308	1,444	2,752	7.47
Maguindanao del Norte	9,574	225	263	488	5.10	20,061	272	286	558	2.78
Maguindanao del Sur	11,750	141	137	278	2.37	25,181	107	118	225	0.89
Sulu	10,899	153	151	304	2.79	25,726	65	559	624	2.43
Tawi-Tawi	6,401	53	47	100	1.56	14,035	75	74	149	1.06
SGA	3,181	89	99	188	5.91	6,955	107	95	202	2.90
City of Cotabato	3,599	595	577	1,172	32.56	7,598	1,309	1,377	2,686	35.35

0 = No Data
* = No Report

Table 2.C.3.4 - Nutrition Services for Infants and Children
Number and Proportion of children 0-59 mos. old whose Nutritional Status are Normal, Stunted, Overweight/Obese
Philippines, 2024

Area	Total number of children 0-59 months old seen during the reporting period at health facilities	Normal				Stunted				Overweight/Obese			
		Male	Female	Total	%	Male	Female	Total	%	Male	Female	Total	%
**Philippines**	**9,840,251**	**4,356,117**	**4,076,815**	**8,432,932**	**85.70**	**220,249**	**188,441**	**408,690**	**4.15**	**83,713**	**68,603**	**152,316**	**1.55**
**NCR**	**1,091,445**	**529,017**	**517,851**	**1,046,868**	**95.92**	**11,162**	**9,346**	**20,508**	**1.88**	**5,471**	**4,560**	**10,031**	**0.92**
City of Malabon	30,277	15,360	14,413	29,773	98.34	512	442	954	3.15	145	108	253	0.84
City of Navotas	26,716	13,003	13,241	26,244	98.23	68	62	130	0.49	172	159	331	1.24
City of Valenzuela	55,377	27,565	26,554	54,119	97.73	205	212	417	0.75	229	190	419	0.76
City of Caloocan	122,060	61,367	58,857	120,224	98.50	700	571	1,271	1.04	501	441	942	0.77
City of Marikina	38,361	19,134	19,020	38,154	99.46	88	75	163	0.42	102	101	203	0.53
City of Pasig	64,858	31,665	31,113	62,778	96.79	672	500	1,172	1.81	317	284	601	0.93
Pateros	1,921	889	884	1,773	92.30	28	44	72	3.75	27	27	54	2.81
City of Taguig	107,338	54,317	52,794	107,111	99.79	113	141	254	0.24	53	41	94	0.09
Quezon City	250,687	123,349	125,182	248,531	99.14	845	781	1,626	0.65	694	584	1,278	0.51
City of Makati	21,143	10,568	10,458	21,026	99.45	24	33	57	0.27	66	40	106	0.50
City of Mandaluyong	37,417	18,881	18,337	37,218	99.47	42	40	82	0.22	46	34	80	0.21
City of San Juan	8,847	4,410	4,117	8,527	96.38	94	48	142	1.61	175	152	327	3.70
City of Manila	128,157	56,442	54,086	110,528	86.24	6017	4931	10,948	8.54	2348	1919	4,267	3.33
City of Las Piñas	56,943	23,210	22,334	45,544	79.98	468	382	850	1.49	114	98	212	0.37
City of Muntinlupa	49,363	22,964	22,357	45,321	91.81	112	121	233	0.47	36	36	72	0.15
City of Parañaque	63,181	31,408	30,156	61,564	97.44	936	773	1,709	2.70	338	265	603	0.95
Pasay City	28,799	14,485	13,948	28,433	98.73	238	190	428	1.49	108	81	189	0.66
**CAR**	**91,978**	**42,743**	**40,714**	**83,457**	**90.74**	**3,186**	**2,952**	**6,138**	**6.67**	**1,021**	**666**	**1,687**	**1.83**
Abra	14,832	6,861	6,458	13,319	89.80	534	453	987	6.65	183	163	346	2.33
Apayao	10,028	4,323	4,581	8,904	88.79	435	392	827	8.25	100	74	174	1.74
Benguet	22,001	10,074	9,336	19,410	88.22	889	853	1,742	7.92	396	227	623	2.83
Ifugao	4,525	2,166	2,008	4,174	92.24	128	83	211	4.66	73	38	111	2.45
Kalinga	27,658	13,073	12,562	25,635	92.69	817	826	1,643	5.94	169	106	275	0.99
Mountain Province	8,618	4,044	3,710	7,754	89.97	363	325	688	7.98	95	56	151	1.75
City of Baguio	4,316	2,202	2,059	4,261	98.73	20	20	40	0.93	5	2	7	0.16
**Region 1**	**411,055**	**206,120**	**192,562**	**398,682**	**96.99**	**5,686**	**4,723**	**10,409**	**2.53**	**3,898**	**3,118**	**7,016**	**1.71**
Ilocos Norte	34,084	16,692	15,856	32,548	95.49	1050	842	1,892	5.55	605	508	1,113	3.27
Ilocos Sur	43,842	22,093	20,419	42,512	96.97	687	605	1,292	2.95	669	516	1,185	2.70
La Union	54,626	26,524	24,846	51,370	94.04	961	823	1,784	3.27	564	431	995	1.82
Pangasinan	265,777	134,640	125,545	260,185	97.90	2610	2107	4,717	1.77	1839	1474	3,313	1.25
City of Dagupan	12,726	6,171	5,896	12,067	94.82	378	346	724	5.69	221	189	410	3.22
**Region 2**	**278,200**	**121,886**	**117,754**	**239,640**	**86.14**	**6,665**	**5,559**	**12,224**	**4.39**	**3,429**	**2,646**	**6,075**	**2.18**
Batanes	975	476	448	924	94.77	18	15	33	3.38	11	7	18	1.85
Cagayan	88,169	42,174	40,168	82,342	93.39	1964	1637	3,601	4.08	946	631	1,577	1.79
Isabela	109,774	50,507	50,390	100,897	91.91	3447	2856	6,303	5.74	2125	1754	3,879	3.53
Nueva Vizcaya	47,614	12,654	11,836	24,490	51.43	784	648	1,432	3.01	241	168	409	0.86
Quirino	17,665	8,796	8,264	17,060	96.58	429	389	818	4.63	87	73	160	0.91
City of Santiago	14,003	7,279	6,648	13,927	99.46	23	14	37	0.26	19	13	32	0.23
**Region 3**	**1,093,763**	**518,673**	**495,659**	**1,014,332**	**92.74**	**22,374**	**18,582**	**40,956**	**3.74**	**17,375**	**14,404**	**31,779**	**2.91**
Aurora	20,840	9,629	8,988	18,617	89.33	996	838	1,834	8.80	342	243	585	2.81
Bataan	75,891	36,050	33,263	69,313	91.33	1547	1251	2,798	3.69	1246	997	2,243	2.96
Bulacan	305,056	150,134	142,050	292,184	95.78	6010	5161	11,171	3.66	4039	3436	7,475	2.45
Nueva Ecija	213,701	99,665	96,120	195,785	91.62	4965	4298	9,263	4.33	4428	3801	8,229	3.85
Pampanga	218,943	99,009	95,711	194,720	88.94	4246	3487	7,733	3.53	4819	3965	8,784	4.01
Tarlac	149,169	72,991	69,222	142,213	95.34	1910	1516	3,426	2.30	1253	936	2,189	1.47
Zambales	53,880	26,077	24,786	50,863	94.40	1967	1435	3,402	6.31	760	594	1,354	2.51
City of Angeles	32,017	14,752	15,197	29,949	93.54	386	304	690	2.16	341	308	649	2.03
City of Olongapo	24,266	10,366	10,322	20,688	85.26	347	292	639	2.63	147	124	271	1.12
**Region 4A**	**1,601,396**	**549,198**	**538,279**	**1,087,477**	**67.91**	**25,999**	**20,595**	**46,594**	**2.91**	**12,810**	**10,721**	**23,531**	**1.47**
Batangas	283,010	76,420	72,867	149,287	52.75	4675	2929	7,604	2.69	2172	1788	3,960	1.40
Cavite	414,722	104,686	103,085	207,771	50.10	4754	3933	8,687	2.09	3977	3588	7,565	1.82
Laguna	321,428	212,032	198,110	410,142	127.60	5721	4943	10,664	3.32	2541	1895	4,436	1.38
Quezon	206,360	84,327	84,059	168,386	81.60	8252	6493	14,745	7.15	2065	1580	3,645	1.77
Rizal	345,054	71,733	80,158	151,891	44.02	2597	2297	4,894	1.42	2055	1870	3,925	1.14
City of Lucena	30,822	*	*	0	0.00	*	*	0	0.00	*	*	0	0.00
**Region 4B**	**452,319**	**278,093**	**127,765**	**405,858**	**89.73**	**18,952**	**16,327**	**35,279**	**7.80**	**3,489**	**2,668**	**6,157**	**1.36**
Marinduque	16,790	7,711	7,040	14,751	87.86	605	505	1,110	6.61	122	106	228	1.36
Occidental Mindoro	29,132	12,466	12,222	24,688	84.75	2020	1719	3,739	12.83	227	139	366	1.26
Oriental Mindoro	235,374	181,939	36,154	218,093	92.66	7724	6472	14,196	6.03	1415	1002	2,417	1.03
Palawan	79,898	34,146	32,434	66,580	83.33	5228	4681	9,909	12.40	590	493	1,083	1.36
Romblon	84,039	38,693	36,889	75,582	89.94	3181	2752	5,933	7.06	909	682	1,591	1.89
City of Puerto Princesa	7,086	3,138	3,026	6,164	86.99	194	198	392	5.53	226	246	472	6.66
**Region 5**	**521,729**	**236,926**	**226,274**	**463,200**	**88.78**	**19,693**	**17,675**	**37,368**	**7.16**	**5,854**	**4,759**	**10,613**	**2.03**
Albay	121,735	58,232	54,853	113,085	92.89	2888	2551	5,439	4.47	1274	985	2,259	1.86
Camarines Norte	64,268	29,347	27,300	56,647	88.14	3199	2638	5,837	9.08	677	489	1,166	1.81
Camarines Sur	149,164	69,036	65,900	134,936	90.46	4666	4200	8,866	5.94	1395	1060	2,455	1.65
Catanduanes	20,667	9,066	8,479	17,545	84.89	1237	1132	2,369	11.46	243	175	418	2.02
Masbate	94,458	37,232	38,061	75,293	79.71	5903	5570	11,473	12.15	1414	1243	2,657	2.81
Sorsogon	45,633	21,022	20,025	41,047	89.95	1588	1362	2,950	6.46	590	452	1,042	2.28
City of Naga	25,804	12,991	11,656	24,647	95.52	212	222	434	1.68	261	355	616	2.39
**Region 6**	**770,238**	**300,111**	**284,860**	**584,971**	**75.95**	**19,344**	**16,484**	**35,828**	**4.65**	**6,233**	**4,821**	**11,054**	**1.44**
Aklan	57,801	23,522	22,974	46,496	80.44	1179	1031	2,210	3.82	497	403	900	1.56
Antique	62,255	13,855	13,157	27,012	43.39	1547	1260	2,807	4.51	335	265	600	0.96
Capiz	74,929	29,821	28,931	58,752	78.41	794	666	1,460	1.95	787	731	1,518	2.03
Guimaras	18,141	7,297	6,973	14,270	78.66	307	293	600	3.31	230	187	417	2.30
Iloilo	188,402	77,097	72,398	149,495	79.35	9138	7659	16,797	8.92	1779	1379	3,158	1.68
Negros Occidental	269,563	103,352	98,646	201,998	74.94	3597	3098	6,695	2.48	1352	1034	2,386	0.89
City of Bacolod	54,066	27,389	24,797	52,186	96.52	2404	2138	4,542	8.40	952	618	1,570	2.90
City of Iloilo	45,081	17,778	16,984	34,762	77.11	378	339	717	1.59	301	204	505	1.12

Table 2.C.3.4 - Nutrition Services for Infants and Children

Number and Proportion of children 0-59 mos. old whose Nutritional Status are Normal, Stunted, Overweight/Obese

Philippines, 2024

Area	Total number of children 0-59 months old seen during the reporting period at health facilities	Normal				Stunted				Overweight/Obese			
		Male	Female	Total	%	Male	Female	Total	%	Male	Female	Total	%
**Region 7**	**503,043**	**238,431**	**229,133**	**467,564**	**92.95**	**12,278**	**10,648**	**22,926**	**4.56**	**3,867**	**3,374**	**7,241**	**1.44**
Bohol	49,783	24,285	22,812	47,097	94.60	873	821	1,694	3.40	365	283	648	1.30
Cebu	241,010	113,079	109,888	222,967	92.51	6348	5709	12,057	5.00	1887	1664	3,551	1.47
Negros Oriental	68,615	29,392	28,368	57,760	84.18	3923	3187	7,110	10.36	1090	1006	2,096	3.05
Siquijor	5,854	2,686	2,621	5,307	90.66	174	145	319	5.45	90	89	179	3.06
City of Cebu	87,427	43,867	41,519	85,386	97.67	472	416	888	1.02	344	251	595	0.68
City of Lapu-Lapu	50,325	25,122	23,925	49,047	97.46	488	370	858	1.70	91	81	172	0.34
City of Mandaue	29	0	0	0	0.00	0	0	0	0.00	0	0	0	0.00
**Region 8**	**229,233**	**103,735**	**99,200**	**202,935**	**88.53**	**9,743**	**8,088**	**17,831**	**7.78**	**3,252**	**2,807**	**6,059**	**2.64**
Biliran	17,634	7,710	7,186	14,896	84.47	960	803	1,763	10.00	315	253	568	3.22
Eastern Samar	15,585	6,610	5,782	12,392	79.51	650	509	1,159	7.44	346	271	617	3.96
Leyte	45,854	21,973	20,576	42,549	92.79	2073	1820	3,893	8.49	626	578	1,204	2.63
Northern Samar	47,092	21,226	20,328	41,554	88.24	2814	2295	5,109	10.85	824	817	1,641	3.48
Southern Leyte	14,092	6,265	5,946	12,211	86.65	678	519	1,197	8.49	328	244	572	4.06
Samar	49,705	23,391	23,224	46,615	93.78	1235	1103	2,338	4.70	316	272	588	1.18
Ormoc City	21,528	8,747	8,554	17,301	80.37	1198	929	2,127	9.88	442	323	765	3.55
City of Tacloban	17,743	7,813	7,604	15,417	86.89	135	110	245	1.38	55	49	104	0.59
**Region 9**	**376,175**	**175,959**	**171,354**	**347,313**	**92.33**	**7,902**	**6,648**	**14,550**	**3.87**	**2,314**	**1,802**	**4,116**	**1.09**
Zamboanga del Norte	143,135	68,708	66,308	135,016	94.33	2941	2230	5,171	3.61	768	615	1,383	0.97
Zamboanga del Sur	64,194	28,752	27,766	56,518	88.04	2644	2299	4,943	7.70	712	552	1,264	1.97
Zamboanga Sibugay	46,530	19,890	19,244	39,134	84.10	549	475	1,024	2.20	149	94	243	0.52
City of Isabela	19,178	9,091	8,764	17,855	93.10	681	628	1,309	6.83	116	123	239	1.25
City of Zamboanga	103,138	49,518	49,272	98,790	95.78	1087	1016	2,103	2.04	569	418	987	0.96
**Region 10**	**492,914**	**231,953**	**224,774**	**456,727**	**92.66**	**8,541**	**7,796**	**16,337**	**3.31**	**2,131**	**1,736**	**3,867**	**0.78**
Bukidnon	159,172	80,446	75,427	155,873	97.93	3719	3539	7,258	4.56	745	592	1,337	0.84
Camiguin	9,049	2,848	2,747	5,595	61.83	264	224	488	5.39	96	89	185	2.04
Lanao del Norte	86,561	41,395	42,967	84,362	97.46	2072	1860	3,932	4.54	462	420	882	1.02
Misamis Occidental	43,170	20,584	19,292	39,876	92.37	1280	1085	2,365	5.48	433	349	782	1.81
Misamis Oriental	87,122	32,645	30,978	63,623	73.03	1071	952	2,023	2.32	373	271	644	0.74
City of Cagayan De Oro	67,541	34,563	32,760	67,323	99.68	92	92	184	0.27	20	14	34	0.05
City of Iligan	40,299	19,472	20,603	40,075	99.44	43	44	87	0.22	2	1	3	0.01
**Region 11**	**489,093**	**213,775**	**205,233**	**419,008**	**85.67**	**12,710**	**11,645**	**24,355**	**4.98**	**4,139**	**3,357**	**7,496**	**1.53**
Davao de Oro	104,602	47,840	46,361	94,201	90.06	2627	2634	5,261	5.03	666	472	1,138	1.09
Davao del Norte	135,817	66,691	63,206	129,897	95.64	1705	1660	3,365	2.48	607	476	1,083	0.80
Davao Oriental	61,544	23,192	21,840	45,032	73.17	2021	1819	3,840	6.24	621	445	1,066	1.73
Davao del Sur	58,332	25,936	24,501	50,437	86.47	2274	1886	4,160	7.13	790	658	1,448	2.48
Davao Occidental	38,261	10,435	10,109	20,544	53.69	2238	1896	4,134	10.80	511	497	1,008	2.63
City of Davao	90,537	39,681	39,216	78,897	87.14	1845	1750	3,595	3.97	944	809	1,753	1.94
**Region 12**	**456,137**	**214,969**	**202,676**	**417,645**	**91.56**	**6,415**	**5,423**	**11,838**	**2.60**	**2,352**	**1,728**	**4,080**	**0.89**
Cotabato	140,807	59,975	56,560	116,535	82.76	1627	1391	3,018	2.14	567	436	1,003	0.71
Sarangani	63,967	32,218	30,809	63,027	98.53	693	646	1,339	2.09	250	170	420	0.66
South Cotabato	91,813	47,048	43,877	90,925	99.03	1054	954	2,008	2.19	320	236	556	0.61
Sultan Kudarat	83,995	41,667	40,228	81,895	97.50	1902	1503	3,405	4.05	684	508	1,192	1.42
City of General Santos	75,555	34,061	31,202	65,263	86.38	1139	929	2,068	2.74	531	378	909	1.20
**CARAGA**	**256,594**	**119,557**	**112,430**	**231,987**	**90.41**	**7,753**	**6,356**	**14,109**	**5.50**	**2,505**	**1,851**	**4,356**	**1.70**
Agusan del Norte	39,578	18,163	17,015	35,178	88.88	609	527	1,136	2.87	220	156	376	0.95
Agusan del Sur	84,082	37,324	35,518	72,842	86.63	2392	1839	4,231	5.03	717	497	1,214	1.44
Surigao del Norte	47,714	21,946	20,835	42,781	89.66	2213	1795	4,008	8.40	736	570	1,306	2.74
Surigao del Sur	53,699	26,347	24,274	50,621	94.27	2126	1885	4,011	7.47	676	523	1,199	2.23
Dinagat Islands	3,961	1,809	1,675	3,484	87.96	261	199	460	11.61	89	47	136	3.43
City of Butuan	27,560	13,968	13,113	27,081	98.26	152	111	263	0.95	67	58	125	0.45
**BARMM**	**724,939**	**274,971**	**290,297**	**565,268**	**77.97**	**21,846**	**19,594**	**41,440**	**5.72**	**3,573**	**3,585**	**7,158**	**0.99**
Basilan	41,829	19,835	20,205	40,040	95.72	4468	4078	8,546	20.43	453	454	907	2.17
Lanao del Sur	218,919	79,440	83,016	162,456	74.21	4565	4038	8,603	3.93	831	847	1,678	0.77
Maguindanao del Norte	140,776	45,544	47,958	93,502	66.42	2220	2033	4,253	3.02	314	310	624	0.44
Maguindanao del Sur	140,776	45,544	47,958	93,502	66.42	2220	2033	4,253	3.02	314	310	624	0.44
Sulu	118,883	54,590	60,691	115,281	96.97	4718	4235	8,953	7.53	781	872	1,653	1.39
Tawi-Tawi	27,209	12,195	12,876	25,071	92.14	2441	2174	4,615	16.96	487	442	929	3.41
SGA	19,530	9,609	9,509	19,118	97.89	707	598	1,305	6.68	102	104	206	1.05
City of Cotabato	17,017	8,214	8,084	16,298	95.77	507	405	912	5.36	291	246	537	3.16

0 = No Data

* = No Report

Table 2.C.3.4 - Nutrition Services for Infants and Children
Number and Proportion of children 0-59 mos. old whose Nutritional Status are Wasted-MAM, Wasted-SAM, Wasted
Philippines, 2024

Area	Total number of children 0-59 months old seen during the reporting period at health facilities	Wasted-MAM				Wasted-SAM				Wasted			
		Male	Female	Total	%	Male	Female	Total	%	Male	Female	Total	%
**Philippines**	**9,840,251**	**44,992**	**38,646**	**83,638**	**0.85**	**15,395**	**12,834**	**28,229**	**0.29**	**60,387**	**51,480**	**111,867**	**1.14**
**NCR**	**1,091,445**	**1,856**	**1,501**	**3,357**	**0.31**	**760**	**653**	**1,413**	**0.13**	**2,616**	**2,154**	**4,770**	**0.44**
City of Malabon	30,277	109	74	183	0.60	42	26	68	0.22	151	100	251	0.83
City of Navotas	26,716	7	1	8	0.03	2	1	3	0.01	9	2	11	0.04
City of Valenzuela	55,377	0	0	0	0.00	0	0	0	0.00	0	0	0	0.00
City of Caloocan	122,060	154	135	289	0.24	29	37	66	0.05	183	172	355	0.29
City of Marikina	38,361	61	56	117	0.30	21	21	42	0.11	82	77	159	0.41
City of Pasig	64,858	134	106	240	0.37	38	29	67	0.10	172	135	307	0.47
Pateros	1,921	4	12	16	0.83	4	2	6	0.31	8	14	22	1.15
City of Taguig	107,338	61	59	120	0.11	10	3	13	0.01	71	62	133	0.12
Quezon City	250,687	316	273	589	0.23	155	134	289	0.12	471	407	878	0.35
City of Makati	21,143	3	5	8	0.04	2	1	3	0.01	5	6	11	0.05
City of Mandaluyong	37,417	26	22	48	0.13	6	10	16	0.04	32	32	64	0.17
City of San Juan	8,847	17	13	30	0.34	0	0	0	0.00	17	13	30	0.34
City of Manila	128,157	827	640	1,467	1.14	425	368	793	0.62	1,252	1,008	2,260	1.76
City of Las Piñas	56,943	46	33	79	0.14	8	8	16	0.03	54	41	95	0.17
City of Muntinlupa	49,363	29	25	54	0.11	10	5	15	0.03	39	30	69	0.14
City of Parañaque	63,181	41	26	67	0.11	8	8	16	0.03	49	34	83	0.13
Pasay City	28,799	21	21	42	0.15	0	0	0	0.00	21	21	42	0.15
**CAR**	**91,978**	**303**	**249**	**552**	**0.60**	**77**	**67**	**144**	**0.16**	**380**	**316**	**696**	**0.76**
Abra	14,832	79	67	146	0.98	22	12	34	0.23	101	79	180	1.21
Apayao	10,028	48	53	101	1.01	10	12	22	0.22	58	65	123	1.23
Benguet	22,001	93	65	158	0.72	40	28	68	0.31	133	93	226	1.03
Ifugao	4,525	13	13	26	0.57	2	1	3	0.07	15	14	29	0.64
Kalinga	27,658	51	39	90	0.33	3	12	15	0.05	54	51	105	0.38
Mountain Province	8,618	14	9	23	0.27	0	2	2	0.02	14	11	25	0.29
City of Baguio	4,316	5	3	8	0.19	0	0	0	0.00	5	3	8	0.19
**Region 1**	**411,055**	**1,307**	**1,141**	**2,448**	**0.60**	**595**	**464**	**1,059**	**0.26**	**1,902**	**1,605**	**3,507**	**0.85**
Ilocos Norte	34,084	152	149	301	0.88	64	59	123	0.36	216	208	424	1.24
Ilocos Sur	43,842	61	55	116	0.26	16	13	29	0.07	77	68	145	0.33
La Union	54,626	155	150	305	0.56	61	42	103	0.19	216	192	408	0.75
Pangasinan	265,777	894	752	1,646	0.62	363	274	637	0.24	1,257	1,026	2,283	0.86
City of Dagupan	12,726	45	35	80	0.63	91	76	167	1.31	136	111	247	1.94
**Region 2**	**278,200**	**1,191**	**1,067**	**2,258**	**0.81**	**478**	**376**	**854**	**0.31**	**1,669**	**1,443**	**3,112**	**1.12**
Batanes	975	8	5	13	1.33	0	0	0	0.00	8	5	13	1.33
Cagayan	88,169	236	256	492	0.56	85	72	157	0.18	321	328	649	0.74
Isabela	109,774	868	753	1,621	1.48	376	294	670	0.61	1,244	1,047	2,291	2.09
Nueva Vizcaya	47,614	46	40	86	0.18	13	6	19	0.04	59	46	105	0.22
Quirino	17,665	33	13	46	0.26	4	4	8	0.05	37	17	54	0.31
City of Santiago	14,003	0	0	0	0.00	0	0	0	0.00	0	0	0	0.00
**Region 3**	**1,093,763**	**9,219**	**7,533**	**16,752**	**1.53**	**4,009**	**3,236**	**7,245**	**0.66**	**13,228**	**10,769**	**23,997**	**2.19**
Aurora	20,840	93	69	162	0.78	29	34	63	0.30	122	103	225	1.08
Bataan	75,891	656	503	1,159	1.53	213	165	378	0.50	869	668	1,537	2.03
Bulacan	305,056	1,866	1,497	3,363	1.10	761	667	1,428	0.47	2,627	2,164	4,791	1.57
Nueva Ecija	213,701	2,226	1,804	4,030	1.89	805	639	1,444	0.68	3,031	2,443	5,474	2.56
Pampanga	218,943	2,815	2,372	5,187	2.37	1445	1074	2,519	1.15	4,260	3,446	7,706	3.52
Tarlac	149,169	810	697	1,507	1.01	372	320	692	0.46	1,182	1,017	2,199	1.47
Zambales	53,880	272	208	480	0.89	108	82	190	0.35	380	290	670	1.24
City of Angeles	32,017	397	336	733	2.29	234	217	451	1.41	631	553	1,184	3.70
City of Olongapo	24,266	84	47	131	0.54	42	38	80	0.33	126	85	211	0.87
**Region 4A**	**1,601,396**	**5,219**	**4,428**	**9,647**	**0.60**	**1,334**	**1,115**	**2,449**	**0.15**	**6,553**	**5,543**	**12,096**	**0.76**
Batangas	283,010	599	468	1,067	0.38	193	168	361	0.13	792	636	1,428	0.50
Cavite	414,722	1,154	1,051	2,205	0.53	332	302	634	0.15	1,486	1,353	2,839	0.68
Laguna	321,428	737	518	1,255	0.39	210	152	362	0.11	947	670	1,617	0.50
Quezon	206,360	1,666	1,340	3,006	1.46	447	342	789	0.38	2,113	1,682	3,795	1.84
Rizal	345,054	964	926	1,890	0.55	152	151	303	0.09	1,116	1,077	2,193	0.64
City of Lucena	30,822	99	125	224	0.73	*	*	0	0.00	99	125	224	0.73
**Region 4B**	**452,319**	**2,055**	**1,837**	**3,892**	**0.86**	**671**	**462**	**1,133**	**0.25**	**2,726**	**2,299**	**5,025**	**1.11**
Marinduque	16,790	332	279	611	3.64	70	20	90	0.54	402	299	701	4.18
Occidental Mindoro	29,132	144	109	253	0.87	48	38	86	0.30	192	147	339	1.16
Oriental Mindoro	235,374	274	225	499	0.21	99	70	169	0.07	373	295	668	0.28
Palawan	79,898	921	816	1,737	2.17	325	264	589	0.74	1,246	1,080	2,326	2.91
Romblon	84,039	356	393	749	0.89	119	65	184	0.22	475	458	933	1.11
City of Puerto Princesa	7,086	28	15	43	0.61	10	5	15	0.21	38	20	58	0.82
**Region 5**	**521,729**	**3,991**	**3,621**	**7,612**	**1.46**	**1,459**	**1,241**	**2,700**	**0.52**	**5,450**	**4,862**	**10,312**	**1.98**
Albay	121,735	431	412	843	0.69	52	57	109	0.09	483	469	952	0.78
Camarines Norte	64,268	277	206	483	0.75	71	64	135	0.21	348	270	618	0.96
Camarines Sur	149,164	1,144	1,066	2,210	1.48	391	306	697	0.47	1,535	1,372	2,907	1.95
Catanduanes	20,667	150	140	290	1.40	26	19	45	0.22	176	159	335	1.62
Masbate	94,458	1,659	1,495	3,154	3.34	880	765	1,645	1.74	2,539	2,260	4,799	5.08
Sorsogon	45,633	276	276	552	1.21	27	15	42	0.09	303	291	594	1.30
City of Naga	25,804	54	26	80	0.31	12	15	27	0.10	66	41	107	0.41
**Region 6**	**770,238**	**5,331**	**4,674**	**10,005**	**1.30**	**508**	**392**	**900**	**0.12**	**5,839**	**5,066**	**10,905**	**1.42**
Aklan	57,801	122	91	213	0.37	50	40	90	0.16	172	131	303	0.52
Antique	62,255	205	198	403	0.65	51	40	91	0.15	256	238	494	0.79
Capiz	74,929	113	93	206	0.27	80	35	115	0.15	193	128	321	0.43
Guimaras	18,141	38	34	72	0.40	7	7	14	0.08	45	41	86	0.47
Iloilo	188,402	3,987	3,474	7,461	3.96	38	35	73	0.04	4,025	3,509	7,534	4.00
Negros Occidental	269,563	511	447	958	0.36	192	166	358	0.13	703	613	1,316	0.49
City of Bacolod	54,066	262	256	518	0.96	61	45	106	0.20	323	301	624	1.15
City of Iloilo	45,081	93	81	174	0.39	29	24	53	0.12	122	105	227	0.50

Table 2.C.3.4 - Nutrition Services for Infants and Children
Number and Proportion of children 0-59 mos. old whose Nutritional Status are Wasted-MAM, Wasted-SAM, Wasted
Philippines, 2024

Area	Total number of children 0-59 months old seen during the reporting period at health facilities	Wasted-MAM				Wasted-SAM				Wasted			
		Male	Female	Total	%	Male	Female	Total	%	Male	Female	Total	%
**Region 7**	**503,043**	**2,447**	**2,031**	**4,478**	**0.89**	**445**	**389**	**834**	**0.17**	**2,892**	**2,420**	**5,312**	**1.06**
Bohol	49,783	129	142	271	0.54	37	36	73	0.15	166	178	344	0.69
Cebu	241,010	1,108	819	1,927	0.80	262	246	508	0.21	1,370	1,065	2,435	1.01
Negros Oriental	68,615	891	758	1,649	2.40	0	0	0	0.00	891	758	1,649	2.40
Siquijor	5,854	18	22	40	0.68	3	6	9	0.15	21	28	49	0.84
City of Cebu	87,427	176	185	361	0.41	118	79	197	0.23	294	264	558	0.64
City of Lapu-Lapu	50,325	111	97	208	0.41	21	19	40	0.08	132	116	248	0.49
City of Mandaue	29	14	8	22	75.86	4	3	7	24.14	18	11	29	100.00
**Region 8**	**229,233**	**1,377**	**1,072**	**2,449**	**1.07**	**457**	**371**	**828**	**0.36**	**1,834**	**1,443**	**3,277**	**1.43**
Biliran	17,634	187	190	377	2.14	57	50	107	0.61	244	240	484	2.74
Eastern Samar	15,585	95	75	170	1.09	48	39	87	0.56	143	114	257	1.65
Leyte	45,854	228	169	397	0.87	99	82	181	0.39	327	251	578	1.26
Northern Samar	47,092	411	258	669	1.42	105	80	185	0.39	516	338	854	1.81
Southern Leyte	14,092	58	54	112	0.79	7	2	9	0.06	65	56	121	0.86
Samar	49,705	58	64	122	0.25	20	22	42	0.08	78	86	164	0.33
Ormoc City	21,528	244	173	417	1.94	113	88	201	0.93	357	261	618	2.87
City of Tacloban	17,743	96	89	185	1.04	8	8	16	0.09	104	97	201	1.13
**Region 9**	**376,175**	**2,039**	**1,765**	**3,804**	**1.01**	**739**	**619**	**1,358**	**0.36**	**2,778**	**2,384**	**5,162**	**1.37**
Zamboanga del Norte	143,135	626	604	1,230	0.86	183	152	335	0.23	809	756	1,565	1.09
Zamboanga del Sur	64,194	610	442	1,052	1.64	243	174	417	0.65	853	616	1,469	2.29
Zamboanga Sibugay	46,530	100	103	203	0.44	35	38	73	0.16	135	141	276	0.59
City of Isabela	19,178	241	166	407	2.12	99	88	187	0.98	340	254	594	3.10
City of Zamboanga	103,138	462	450	912	0.88	179	167	346	0.34	641	617	1,258	1.22
**Region 10**	**492,914**	**1,665**	**1,538**	**3,203**	**0.65**	**1,337**	**1,353**	**2,690**	**0.55**	**3,002**	**2,891**	**5,893**	**1.20**
Bukidnon	159,172	438	395	833	0.52	126	103	229	0.14	564	498	1,062	0.67
Camiguin	9,049	50	41	91	1.01	24	24	48	0.53	74	65	139	1.54
Lanao del Norte	86,561	557	574	1,131	1.31	1040	1083	2,123	2.45	1,597	1,657	3,254	3.76
Misamis Occidental	43,170	189	142	331	0.77	63	66	129	0.30	252	208	460	1.07
Misamis Oriental	87,122	329	279	608	0.70	61	52	113	0.13	390	331	721	0.83
City of Cagayan De Oro	67,541	92	92	184	0.27	22	24	46	0.07	114	116	230	0.34
City of Iligan	40,299	10	15	25	0.06	1	1	2	0.00	11	16	27	0.07
**Region 11**	**489,093**	**1,431**	**1,328**	**2,759**	**0.56**	**453**	**406**	**859**	**0.18**	**1,884**	**1,734**	**3,618**	**0.74**
Davao de Oro	104,602	151	123	274	0.26	27	40	67	0.06	178	163	341	0.33
Davao del Norte	135,817	226	273	499	0.37	30	30	60	0.04	256	303	559	0.41
Davao Oriental	61,544	186	146	332	0.54	66	75	141	0.23	252	221	473	0.77
Davao del Sur	58,332	223	196	419	0.72	80	83	163	0.28	303	279	582	1.00
Davao Occidental	38,261	406	363	769	2.01	177	136	313	0.82	583	499	1,082	2.83
City of Davao	90,537	239	227	466	0.51	73	42	115	0.13	312	269	581	0.64
**Region 12**	**456,137**	**1,121**	**905**	**2,026**	**0.44**	**350**	**254**	**604**	**0.13**	**1,471**	**1,159**	**2,630**	**0.58**
Cotabato	140,807	362	324	686	0.49	132	84	216	0.15	494	408	902	0.64
Sarangani	63,967	142	97	239	0.37	42	30	72	0.11	184	127	311	0.49
South Cotabato	91,813	148	114	262	0.29	36	34	70	0.08	184	148	332	0.36
Sultan Kudarat	83,995	388	319	707	0.84	116	93	209	0.25	504	412	916	1.09
City of General Santos	75,555	81	51	132	0.17	24	13	37	0.05	105	64	169	0.22
**CARAGA**	**256,594**	**1,920**	**1,673**	**3,593**	**1.40**	**744**	**616**	**1,360**	**0.53**	**2,664**	**2,289**	**4,953**	**1.93**
Agusan del Norte	39,578	63	50	113	0.29	12	13	25	0.06	75	63	138	0.35
Agusan del Sur	84,082	304	256	560	0.67	85	73	158	0.19	389	329	718	0.85
Surigao del Norte	47,714	1,151	1,037	2,188	4.59	523	442	965	2.02	1,674	1,479	3,153	6.61
Surigao del Sur	53,699	324	274	598	1.11	103	70	173	0.32	427	344	771	1.44
Dinagat Islands	3,961	43	26	69	1.74	7	6	13	0.33	50	32	82	2.07
City of Butuan	27,560	35	30	65	0.24	14	12	26	0.09	49	42	91	0.33
**BARMM**	**724,939**	**2,520**	**2,283**	**4,803**	**0.66**	**979**	**820**	**1,799**	**0.25**	**3,499**	**3,103**	**6,602**	**0.91**
Basilan	41,829	342	336	678	1.62	104	100	204	0.49	446	436	882	2.11
Lanao del Sur	218,919	373	291	664	0.30	109	105	214	0.10	482	396	878	0.40
Maguindanao del Norte	140,776	232	209	441	0.31	119	88	207	0.15	351	297	648	0.46
Maguindanao del Sur	140,776	232	209	441	0.31	119	88	207	0.15	351	297	648	0.46
Sulu	118,883	742	705	1,447	1.22	261	241	502	0.42	1,003	946	1,949	1.64
Tawi-Tawi	27,209	417	405	822	3.02	217	170	387	1.42	634	575	1,209	4.44
SGA	19,530	97	77	174	0.89	22	10	32	0.16	119	87	206	1.05
City of Cotabato	17,017	85	51	136	0.80	28	18	46	0.27	113	69	182	1.07

0 = No Data
* = No Report

Table 2.C.3.5 - Nutrition Services for Infants and Children

Number and Proportion of Moderate Acute Malnutrition (MAM) Children admitted, cured, defaulted and died in Supplementary Feeding Program (SFP)

Philippines, 2024

Area	Total of MAM	MAM-admited in SFP				MAM-cured in SFP				MAM-defaulted in SFP				MAM-died in SFP			
		Male	Female	Total	%	Male	Female	Total	%	Male	Female	Total	%	Male	Female	Total	%
**Philippines**	**81,671**	**16,007**	**14,616**	**30,623**	**37.50**	**5,877**	**5,189**	**11,066**	**13.55**	**1,253**	**1,071**	**2,324**	**2.85**	**38**	**36**	**74**	**0.09**
**NCR**	**1,381**	**839**	**731**	**1,570**	**113.69**	**486**	**424**	**910**	**65.89**	**68**	**60**	**128**	**9.27**	**0**	**0**	**0**	**0.00**
City of Malabon	0	0	0	0	0.00	0	0	0	0.00	0	0	0	0.00	0	0	0	0.00
City of Navotas	8	7	1	8	100.00	5	0	5	62.50	0	0	0	0.00	0	0	0	0.00
City of Valenzuela	3	2	3	5	166.67	0	0	0	0.00	0	0	0	0.00	0	0	0	0.00
City of Caloocan	27	18	24	42	155.56	24	25	49	181.48	0	0	0	0.00	0	0	0	0.00
City of Marikina	25	10	16	26	104.00	0	0	0	0.00	0	0	0	0.00	0	0	0	0.00
City of Pasig	261	135	107	242	92.72	78	68	146	55.94	19	11	30	11.49	0	0	0	0.00
Pateros	1	3	4	7	700.00	0	0	0	0.00	0	0	0	0.00	0	0	0	0.00
City of Taguig	120	61	59	120	100.00	54	57	111	92.50	0	0	0	0.00	0	0	0	0.00
Quezon City	0	316	273	589	0.00	268	228	496	0.00	26	23	49	0.00	0	0	0	0.00
City of Makati	0	0	0	0	0.00	3	4	7	0.00	0	1	1	0.00	0	0	0	0.00
City of Mandaluyonc	3	2	1	3	100.00	4	1	5	166.67	3	2	5	166.67	0	0	0	0.00
City of San Juan	31	4	0	4	12.90	7	3	10	32.26	7	10	17	54.84	0	0	0	0.00
City of Manila	694	123	105	228	32.85	9	8	17	2.45	10	12	22	3.17	0	0	0	0.00
City of Las Piñas	0	41	50	91	0.00	21	13	34	0.00	3	1	4	0.00	0	0	0	0.00
City of Muntinlupa	47	26	36	62	131.91	13	17	30	63.83	0	0	0	0.00	0	0	0	0.00
City of Parañaque	142	79	45	124	87.32	0	0	0	0.00	0	0	0	0.00	0	0	0	0.00
Pasay City	19	12	7	19	100.00	0	0	0	0.00	0	0	0	0.00	0	0	0	0.00
**CAR**	**143**	**80**	**55**	**135**	**94.41**	**4**	**4**	**8**	**5.59**	**0**	**0**	**0**	**0.00**	**0**	**0**	**0**	**0.00**
Abra	1	1	0	1	100.00	0	0	0	0.00	0	0	0	0.00	0	0	0	0.00
Apayao	60	25	27	52	86.67	4	4	8	13.33	0	0	0	0.00	0	0	0	0.00
Benguet	6	5	1	6	100.00	0	0	0	0.00	0	0	0	0.00	0	0	0	0.00
Ifugao	10	5	5	10	100.00	0	0	0	0.00	0	0	0	0.00	0	0	0	0.00
Kalinga	54	35	19	54	100.00	0	0	0	0.00	0	0	0	0.00	0	0	0	0.00
Mountain Province	9	6	3	9	100.00	0	0	0	0.00	0	0	0	0.00	0	0	0	0.00
City of Baguio	3	3	0	3	100.00	0	0	0	0.00	0	0	0	0.00	0	0	0	0.00
**Region 1**	**2,522**	**676**	**624**	**1,300**	**51.55**	**123**	**142**	**265**	**10.51**	**1**	**1**	**2**	**0.08**	**0**	**1**	**1**	**0.04**
Ilocos Norte	717	241	194	435	60.67	4	6	10	1.39	0	0	0	0.00	0	0	0	0.00
Ilocos Sur	377	114	96	210	55.70	16	14	30	7.96	0	0	0	0.00	0	1	1	0.27
La Union	603	269	280	549	91.04	23	28	51	8.46	1	1	2	0.33	0	0	0	0.00
Pangasinan	355	52	54	106	29.86	80	94	174	49.01	0	0	0	0.00	0	0	0	0.00
City of Dagupan	470	0	0	0	0.00	0	0	0	0.00	0	0	0	0.00	0	0	0	0.00
**Region 2**	**1,739**	**746**	**636**	**1,382**	**79.47**	**35**	**44**	**79**	**4.54**	**18**	**14**	**32**	**1.84**	**0**	**0**	**0**	**0.00**
Batanes	0	0	0	0	0.00	0	0	0	0.00	0	0	0	0.00	0	0	0	0.00
Cagayan	170	75	48	123	72.35	2	3	5	2.94	0	0	0	0.00	0	0	0	0.00
Isabela	866	307	270	577	66.63	9	14	23	2.66	0	0	0	0.00	0	0	0	0.00
Nueva Vizcaya	661	352	309	661	100.00	23	23	46	6.96	15	12	27	4.08	0	0	0	0.00
Quirino	42	12	9	21	50.00	1	4	5	11.90	3	2	5	11.90	0	0	0	0.00
City of Santiago	0	0	0	0	0.00	0	0	0	0.00	0	0	0	0.00	0	0	0	0.00
**Region 3**	**16,752**	**544**	**456**	**1,000**	**5.97**	**341**	**301**	**642**	**3.83**	**6**	**8**	**14**	**0.08**	**0**	**1**	**1**	**0.01**
Aurora	162	26	10	36	22.22	26	10	36	22.22	0	0	0	0.00	0	0	0	0.00
Bataan	1,159	0	0	0	0.00	0	0	0	0.00	0	0	0	0.00	0	0	0	0.00
Bulacan	3,363	309	276	585	17.40	278	262	540	16.06	6	8	14	0.42	0	0	0	0.00
Nueva Ecija	4,030	125	92	217	5.38	0	0	0	0.00	0	0	0	0.00	0	0	0	0.00
Pampanga	5,187	25	36	61	1.18	1	1	2	0.04	0	0	0	0.00	0	1	1	0.02
Tarlac	1,507	0	0	0	0.00	0	0	0	0.00	0	0	0	0.00	0	0	0	0.00
Zambales	480	59	42	101	21.04	36	28	64	13.33	0	0	0	0.00	0	0	0	0.00
City of Angeles	733	0	0	0	0.00	0	0	0	0.00	0	0	0	0.00	0	0	0	0.00
City of Olongapo	131	0	0	0	0.00	0	0	0	0.00	0	0	0	0.00	0	0	0	0.00
**Region 4A**	**11,792**	**1,826**	**1,641**	**3,467**	**29.40**	**762**	**623**	**1,385**	**11.75**	**27**	**27**	**54**	**0.46**	**4**	**4**	**8**	**0.07**
Batangas	1,255	113	120	233	18.57	43	40	83	6.61	14	17	31	2.47	1	1	2	0.16
Cavite	3,086	473	427	900	29.16	469	411	880	28.52	7	7	14	0.45	0	0	0	0.00
Laguna	1,402	248	202	450	32.10	48	24	72	5.14	2	2	4	0.29	2	2	4	0.29
Quezon	3,414	485	403	888	26.01	155	122	277	8.11	3	0	3	0.09	1	1	2	0.06
Rizal	2,411	507	489	996	41.31	47	26	73	3.03	1	1	2	0.08	0	0	0	0.00
City of Lucena	224	0	0	0	0.00	0	0	0	0.00	0	0	0	0.00	0	0	0	0.00
**Region 4B**	**4,930**	**613**	**492**	**1,105**	**22.41**	**163**	**122**	**285**	**5.78**	**44**	**49**	**93**	**1.89**	**22**	**22**	**44**	**0.89**
Marinduque	146	29	18	47	32.19	11	10	21	14.38	0	0	0	0.00	0	0	0	0.00
Occidental Mindoro	655	211	178	389	59.39	73	68	141	21.53	36	42	78	11.91	0	0	0	0.00
Oriental Mindoro	1,459	113	93	206	14.12	0	0	0	0.00	0	0	0	0.00	22	22	44	3.02
Palawan	1,841	112	78	190	10.32	11	9	20	1.09	5	7	12	0.65	0	0	0	0.00
Romblon	267	89	95	184	68.91	12	5	17	6.37	0	0	0	0.00	0	0	0	0.00
City of Puerto Prince	562	59	30	89	15.84	56	30	86	15.30	3	0	3	0.53	0	0	0	0.00
**Region 5**	**7,852**	**1,708**	**1,851**	**3,559**	**45.33**	**719**	**639**	**1,358**	**17.29**	**95**	**79**	**174**	**2.22**	**4**	**2**	**6**	**0.08**
Albay	843	219	195	414	49.11	126	124	250	29.66	22	17	39	4.63	1	0	1	0.12
Camarines Norte	495	149	132	281	56.77	82	60	142	28.69	24	25	49	9.90	0	0	0	0.00
Camarines Sur	2,263	335	374	709	31.33	59	64	123	5.44	22	12	34	1.50	3	2	5	0.22
Catanduanes	290	78	70	148	51.03	30	21	51	17.59	4	2	6	2.07	0	0	0	0.00
Masbate	3,334	748	921	1,669	50.06	412	364	776	23.28	23	23	46	1.38	0	0	0	0.00
Sorsogon	552	137	129	266	48.19	5	4	9	1.63	0	0	0	0.00	0	0	0	0.00
City of Naga	75	42	30	72	96.00	5	2	7	9.33	0	0	0	0.00	0	0	0	0.00
**Region 6**	**10,005**	**638**	**529**	**1,167**	**11.66**	**300**	**252**	**552**	**5.52**	**638**	**529**	**1,167**	**11.66**	**0**	**0**	**0**	**0.00**
Aklan	213	72	38	110	51.64	16	8	24	11.27	72	38	110	51.64	0	0	0	0.00
Antique	403	55	60	115	28.54	8	7	15	3.72	55	60	115	28.54	0	0	0	0.00
Capiz	206	94	74	168	81.55	99	74	173	83.98	94	74	168	81.55	0	0	0	0.00
Guimaras	72	2	2	4	5.56	0	0	0	0.00	2	2	4	5.56	0	0	0	0.00
Iloilo	7,461	81	69	150	2.01	28	31	59	0.79	81	69	150	2.01	0	0	0	0.00
Negros Occidental	958	213	173	386	40.29	98	85	183	19.10	213	173	386	40.29	0	0	0	0.00
City of Bacolod	518	28	32	60	11.58	1	3	4	0.77	28	32	60	11.58	0	0	0	0.00
City of Iloilo	174	93	81	174	100.00	50	44	94	54.02	93	81	174	100.00	0	0	0	0.00
**Region 7**	**2,176**	**1,075**	**888**	**1,963**	**90.21**	**80**	**70**	**150**	**6.89**	**34**	**29**	**63**	**2.90**	**0**	**0**	**0**	**0.00**
Bohol	0	0	0	0	0.00	0	0	0	0.00	0	0	0	0.00	0	0	0	0.00
Cebu	2,032	990	836	1,826	89.86	76	67	143	7.04	34	29	63	3.10	0	0	0	0.00
Negros Oriental	0	0	0	0	0.00	0	0	0	0.00	0	0	0	0.00	0	0	0	0.00
Siquijor	21	12	9	21	100.00	0	0	0	0.00	0	0	0	0.00	0	0	0	0.00
City of Cebu	101	59	35	94	93.07	4	3	7	6.93	0	0	0	0.00	0	0	0	0.00
City of Lapu-Lapu	0	0	0	0	0.00	0	0	0	0.00	0	0	0	0.00	0	0	0	0.00
City of Mandaue	22	14	8	22	100.00	0	0	0	0.00	0	0	0	0.00	0	0	0	0.00

Table 2.C.3.5 - Nutrition Services for Infants and Children
Number and Proportion of Moderate Acute Malnutrition (MAM) Children admitted, cured, defaulted and died in Supplementary Feeding Program (SFP)
Philippines, 2024

Area	Total of MAM	MAM-admited in SFP				MAM-cured in SFP				MAM-defaulted in SFP				MAM-died in SFP			
		Male	Female	Total	%	Male	Female	Total	%	Male	Female	Total	%	Male	Female	Total	%
**Region 8**	**4,164**	**1,070**	**958**	**2,028**	**48.70**	**360**	**351**	**711**	**17.07**	**40**	**25**	**65**	**1.56**	**0**	**0**	**0**	**0.00**
Biliran	377	24	35	59	15.65	12	24	36	9.55	7	5	12	3.18	0	0	0	0.00
Eastern Samar	272	83	95	178	65.44	14	6	20	7.35	0	0	0	0.00	0	0	0	0.00
Leyte	1,193	459	403	862	72.25	170	144	314	26.32	1	1	2	0.17	0	0	0	0.00
Northern Samar	817	234	168	402	49.20	37	35	72	8.81	0	2	2	0.24	0	0	0	0.00
Southern Leyte	112	0	0	0	0.00	0	0	0	0.00	0	0	0	0.00	0	0	0	0.00
Samar	336	102	119	221	65.77	68	73	141	41.96	11	10	21	6.25	0	0	0	0.00
Ormoc City	872	99	79	178	20.41	55	60	115	13.19	21	7	28	3.21	0	0	0	0.00
City of Tacloban	185	69	59	128	69.19	4	9	13	7.03	0	0	0	0.00	0	0	0	0.00
**Region 9**	**3,806**	**812**	**749**	**1,561**	**41.01**	**510**	**453**	**963**	**25.30**	**40**	**37**	**77**	**2.02**	**1**	**0**	**1**	**0.03**
Zamboanga del Norte	1,232	259	273	532	43.18	147	160	307	24.92	0	0	0	0.00	0	0	0	0.00
Zamboanga del Sur	1,052	211	164	375	35.65	87	78	165	15.68	0	2	2	0.19	0	0	0	0.00
Zamboanga Sibugay	203	24	24	48	23.65	4	5	9	4.43	5	8	13	6.40	0	0	0	0.00
City of Isabela	407	171	123	294	72.24	171	123	294	72.24	0	0	0	0.00	0	0	0	0.00
City of Zamboanga	912	147	165	312	34.21	101	87	188	20.61	35	27	62	6.80	1	0	1	0.11
**Region 10**	**1,817**	**897**	**827**	**1,724**	**94.88**	**186**	**231**	**417**	**22.95**	**26**	**28**	**54**	**2.97**	**0**	**0**	**0**	**0.00**
Bukidnon	1,356	678	619	1,297	95.65	104	116	220	16.22	4	1	5	0.37	0	0	0	0.00
Camiguin	43	23	20	43	100.00	8	1	9	20.93	14	17	31	72.09	0	0	0	0.00
Lanao del Norte	158	97	61	158	100.00	40	51	91	57.59	4	9	13	8.23	0	0	0	0.00
Misamis Occidental	99	23	54	77	77.78	6	20	26	26.26	0	0	0	0.00	0	0	0	0.00
Misamis Oriental	86	42	32	74	86.05	7	9	16	18.60	4	1	5	5.81	0	0	0	0.00
City of Cagayan De	50	24	26	50	100.00	11	19	30	60.00	0	0	0	0.00	0	0	0	0.00
City of Iligan	25	10	15	25	100.00	10	15	25	100.00	0	0	0	0.00	0	0	0	0.00
**Region 11**	**2,759**	**755**	**760**	**1,515**	**54.91**	**445**	**369**	**814**	**29.50**	**38**	**27**	**65**	**2.36**	**1**	**0**	**1**	**0.04**
Davao de Oro	274	54	43	97	35.40	17	11	28	10.22	2	2	4	1.46	0	0	0	0.00
Davao del Norte	499	165	223	388	77.76	61	43	104	20.84	5	2	7	1.40	0	0	0	0.00
Davao Oriental	332	7	8	15	4.52	0	1	1	0.30	0	0	0	0.00	0	0	0	0.00
Davao del Sur	419	42	34	76	18.14	17	9	26	6.21	1	0	1	0.24	0	0	0	0.00
Davao Occidental	769	388	347	735	95.58	302	265	567	73.73	28	20	48	6.24	0	0	0	0.00
City of Davao	466	99	105	204	43.78	48	40	88	18.88	2	3	5	1.07	1	0	1	0.21
**Region 12**	**2,015**	**961**	**842**	**1,803**	**89.48**	**478**	**434**	**912**	**45.26**	**88**	**82**	**170**	**8.44**	**4**	**4**	**8**	**0.40**
Cotabato	882	422	394	816	92.52	347	306	653	74.04	79	78	157	17.80	0	0	0	0.00
Sarangani	185	80	66	146	78.92	77	71	148	80.00	2	1	3	1.62	0	0	0	0.00
South Cotabato	225	84	77	161	71.56	26	31	57	25.33	5	2	7	3.11	0	0	0	0.00
Sultan Kudarat	516	276	240	516	100.00	11	10	21	4.07	0	1	1	0.19	0	0	0	0.00
City of General Sant	207	99	65	164	79.23	17	16	33	15.94	2	0	2	0.97	4	4	8	3.86
**CARAGA**	**3,684**	**1,346**	**1,033**	**2,379**	**64.58**	**495**	**326**	**821**	**22.29**	**23**	**17**	**40**	**1.09**	**0**	**0**	**0**	**0.00**
Agusan del Norte	353	230	123	353	100.00	195	77	272	77.05	0	0	0	0.00	0	0	0	0.00
Agusan del Sur	411	186	152	338	82.24	100	102	202	49.15	0	0	0	0.00	0	0	0	0.00
Surigao del Norte	2,025	648	528	1,176	58.07	46	36	82	4.05	22	17	39	1.93	0	0	0	0.00
Surigao del Sur	773	232	194	426	55.11	128	97	225	29.11	1	0	1	0.13	0	0	0	0.00
Dinagat Islands	57	15	6	21	36.84	14	11	25	43.86	0	0	0	0.00	0	0	0	0.00
City of Butuan	65	35	30	65	100.00	12	3	15	23.08	0	0	0	0.00	0	0	0	0.00
**BARMM**	**4,134**	**1,421**	**1,544**	**2,965**	**71.72**	**390**	**404**	**794**	**19.21**	**67**	**59**	**126**	**3.05**	**2**	**2**	**4**	**0.10**
Basilan	677	208	267	475	70.16	45	33	78	11.52	4	8	12	1.77	1	0	1	0.15
Lanao del Sur	562	87	104	191	33.99	35	40	75	13.35	7	10	17	3.02	0	0	0	0.00
Maguindanao del Norte	187	61	56	117	62.57	25	26	51	27.27	3	4	7	3.74	0	0	0	0.00
Maguindanao del Sur	302	95	114	209	69.21	62	78	140	46.36	15	10	25	8.28	0	0	0	0.00
Sulu	253	38	46	84	33.20	4	11	15	5.93	0	2	2	0.79	0	0	0	0.00
Tawi-Tawi	1,904	888	919	1,807	94.91	204	208	412	21.64	37	25	62	3.26	1	2	3	0.16
SGA	217	24	26	50	23.04	0	0	0	0.00	0	0	0	0.00	0	0	0	0.00
City of Cotabato	32	20	12	32	100.00	15	8	23	71.88	1	0	1	3.13	0	0	0	0.00

* = No Report

Table 2.C.3.6 - Nutrition Services for Infants and Children

Number and Proportion of Severe Acute Malnutrition (SAM) Children without complication admitted, cured, defaulted and died in Outpatient Therapeutic Care (OTC)

Philippines, 2024

Area	Total of SAM	SAM-admited in OTC				SAM-cured in OTC				SAM-defaulted in OTC				SAM-died in OTC			
		Male	Female	Total	%	Male	Female	Total	%	Male	Female	Total	%	Male	Female	Total	%
**Philippines**	**26,741**	**5,135**	**4,554**	**9,689**	**36.23**	**1,798**	**1,591**	**3,389**	**12.67**	**344**	**281**	**625**	**2.34**	**47**	**49**	**96**	**0.36**
**NCR**	**599**	**287**	**236**	**523**	**87.31**	**143**	**114**	**257**	**42.90**	**24**	**35**	**59**	**9.85**	**0**	**3**	**3**	**0.50**
City of Malabon	0	0	0	0	0.00	0	0	0	0.00	0	0	0	0.00	0	0	0	0.00
City of Navotas	3	2	1	3	100.00	2	1	3	100.00	0	0	0	0.00	0	0	0	0.00
City of Valenzuela	21	0	0	0	0.00	0	0	0	0.00	0	0	0	0.00	0	3	3	14.29
City of Caloocan	7	11	18	29	414.29	3	8	11	157.14	2	8	10	142.86	0	0	0	0.00
City of Marikina	4	4	4	8	200.00	0	0	0	0.00	1	0	1	25.00	0	0	0	0.00
City of Pasig	87	38	29	67	77.01	15	15	30	34.48	5	7	12	13.79	0	0	0	0.00
Pateros	2	4	2	6	300.00	1	0	1	50.00	0	0	0	0.00	0	0	0	0.00
City of Taguig	13	10	3	13	100.00	9	2	11	84.62	0	0	0	0.00	0	0	0	0.00
Quezon City	0	155	134	289	0.00	98	75	173	0.00	7	9	16	0.00	0	0	0	0.00
City of Makati	0	0	0	0	0.00	1	1	2	0.00	0	1	1	0.00	0	0	0	0.00
City of Mandaluyonc	5	1	3	4	80.00	1	1	2	40.00	0	0	0	0.00	0	0	0	0.00
City of San Juan	0	0	0	0	0.00	0	0	0	0.00	0	0	0	0.00	0	0	0	0.00
City of Manila	433	39	31	70	16.17	6	8	14	3.23	4	2	6	1.39	0	0	0	0.00
City of Las Piñas	0	5	1	6	0.00	0	2	2	0.00	2	4	6	0.00	0	0	0	0.00
City of Muntinlupa	13	12	5	17	130.77	5	1	6	46.15	3	2	5	38.46	0	0	0	0.00
City of Parañaque	11	6	5	11	100.00	2	0	2	18.18	0	2	2	18.18	0	0	0	0.00
Pasay City	0	0	0	0	0.00	0	0	0	0.00	0	0	0	0.00	0	0	0	0.00
**CAR**	**2**	**2**	**0**	**2**	**100.00**	**0**	**0**	**0**	**0.00**	**0**	**0**	**0**	**0.00**	**0**	**0**	**0**	**0.00**
Abra	0	0	0	0	0.00	0	0	0	0.00	0	0	0	0.00	0	0	0	0.00
Apayao	0	0	0	0	0.00	0	0	0	0.00	0	0	0	0.00	0	0	0	0.00
Benguet	2	2	0	2	100.00	0	0	0	0.00	0	0	0	0.00	0	0	0	0.00
Ifugao	0	0	0	0	0.00	0	0	0	0.00	0	0	0	0.00	0	0	0	0.00
Kalinga	0	0	0	0	0.00	0	0	0	0.00	0	0	0	0.00	0	0	0	0.00
Mountain Province	0	0	0	0	0.00	0	0	0	0.00	0	0	0	0.00	0	0	0	0.00
City of Baguio	0	0	0	0	0.00	0	0	0	0.00	0	0	0	0.00	0	0	0	0.00
**Region 1**	**937**	**174**	**136**	**310**	**33.08**	**20**	**17**	**37**	**3.95**	**3**	**1**	**4**	**0.43**	**0**	**0**	**0**	**0.00**
Ilocos Norte	219	66	43	109	49.77	0	0	0	0.00	0	0	0	0.00	0	0	0	0.00
Ilocos Sur	80	43	25	68	85.00	7	5	12	15.00	1	0	1	1.25	0	0	0	0.00
La Union	197	15	11	26	13.20	8	7	15	7.61	0	0	0	0.00	0	0	0	0.00
Pangasinan	132	50	57	107	81.06	5	5	10	7.58	2	1	3	2.27	0	0	0	0.00
City of Dagupan	309	0	0	0	0.00	0	0	0	0.00	0	0	0	0.00	0	0	0	0.00
**Region 2**	**1,139**	**379**	**374**	**753**	**66.11**	**197**	**172**	**369**	**32.40**	**0**	**1**	**1**	**0.09**	**0**	**0**	**0**	**0.00**
Batanes	0	0	0	0	0.00	0	0	0	0.00	0	0	0	0.00	0	0	0	0.00
Cagayan	47	24	12	36	76.60	0	1	1	2.13	0	1	1	2.13	0	0	0	0.00
Isabela	1,034	323	337	660	63.83	197	171	368	35.59	0	0	0	0.00	0	0	0	0.00
Nueva Vizcaya	51	29	22	51	100.00	0	0	0	0.00	0	0	0	0.00	0	0	0	0.00
Quirino	7	3	3	6	85.71	0	0	0	0.00	0	0	0	0.00	0	0	0	0.00
City of Santiago	0	0	0	0	0.00	0	0	0	0.00	0	0	0	0.00	0	0	0	0.00
**Region 3**	**7,245**	**102**	**107**	**209**	**2.88**	**82**	**89**	**171**	**2.36**	**3**	**3**	**6**	**0.08**	**1**	**0**	**1**	**0.01**
Aurora	63	0	0	0	0.00	0	0	0	0.00	0	0	0	0.00	0	0	0	0.00
Bataan	378	0	0	0	0.00	0	0	0	0.00	0	0	0	0.00	0	0	0	0.00
Bulacan	1,428	94	105	199	13.94	70	86	156	10.92	3	3	6	0.42	1	0	1	0.07
Nueva Ecija	1,444	1	1	2	0.14	0	0	0	0.00	0	0	0	0.00	0	0	0	0.00
Pampanga	2,519	0	0	0	0.00	0	0	0	0.00	0	0	0	0.00	0	0	0	0.00
Tarlac	692	0	0	0	0.00	0	0	0	0.00	0	0	0	0.00	0	0	0	0.00
Zambales	190	7	1	8	4.21	12	3	15	7.89	0	0	0	0.00	0	0	0	0.00
City of Angeles	451	0	0	0	0.00	0	0	0	0.00	0	0	0	0.00	0	0	0	0.00
City of Olongapo	80	0	0	0	0.00	0	0	0	0.00	0	0	0	0.00	0	0	0	0.00
**Region 4A**	**3,215**	**471**	**371**	**842**	**26.19**	**263**	**197**	**460**	**14.31**	**17**	**10**	**27**	**0.84**	**26**	**24**	**50**	**1.56**
Batangas	589	19	15	34	5.77	5	5	10	1.70	12	6	18	3.06	1	1	2	0.34
Cavite	971	201	166	367	37.80	189	148	337	34.71	0	0	0	0.00	5	3	8	0.82
Laguna	437	84	50	134	30.66	16	7	23	5.26	5	4	9	2.06	19	20	39	8.92
Quezon	875	84	58	142	16.23	34	19	53	6.06	0	0	0	0.00	1	0	1	0.11
Rizal	343	83	82	165	48.10	19	18	37	10.79	0	0	0	0.00	0	0	0	0.00
City of Lucena	0	0	0	0	0.00	0	0	0	0.00	0	0	0	0.00	0	0	0	0.00
**Region 4B**	**1,289**	**142**	**94**	**236**	**18.31**	**58**	**27**	**85**	**6.59**	**15**	**13**	**28**	**2.17**	**2**	**3**	**5**	**0.39**
Marinduque	44	9	3	12	27.27	7	2	9	20.45	0	0	0	0.00	0	0	0	0.00
Occidental Mindoro	184	63	36	99	53.80	29	13	42	22.83	12	9	21	11.41	1	3	4	2.17
Oriental Mindoro	236	9	11	20	8.47	0	0	0	0.00	0	0	0	0.00	0	0	0	0.00
Palawan	663	22	17	39	5.88	4	0	4	0.60	1	1	2	0.30	0	0	0	0.00
Romblon	45	22	14	36	80.00	3	1	4	8.89	1	1	2	4.44	0	0	0	0.00
City of Puerto Prince	117	17	13	30	25.64	15	11	26	22.22	1	2	3	2.56	1	0	1	0.85
**Region 5**	**2,480**	**468**	**380**	**848**	**34.19**	**124**	**123**	**247**	**9.96**	**66**	**16**	**82**	**3.31**	**4**	**1**	**5**	**0.20**
Albay	109	34	38	72	66.06	13	14	27	24.77	8	4	12	11.01	1	0	1	0.92
Camarines Norte	96	46	38	84	87.50	6	6	12	12.50	4	1	5	5.21	1	1	2	2.08
Camarines Sur	727	143	94	237	32.60	31	10	41	5.64	8	5	13	1.79	1	0	1	0.14
Catanduanes	45	23	16	39	86.67	5	4	9	20.00	3	2	5	11.11	1	0	1	2.22
Masbate	1,441	193	183	376	26.09	64	87	151	10.48	43	4	47	3.26	0	0	0	0.00
Sorsogon	41	19	8	27	65.85	3	1	4	9.76	0	0	0	0.00	0	0	0	0.00
City of Naga	21	10	3	13	61.90	2	1	3	14.29	0	0	0	0.00	0	0	0	0.00
**Region 6**	**900**	**168**	**129**	**297**	**33.00**	**125**	**76**	**201**	**22.33**	**16**	**10**	**26**	**2.89**	**0**	**1**	**1**	**0.11**
Aklan	90	27	23	50	55.56	6	5	11	12.22	0	0	0	0.00	0	0	0	0.00
Antique	91	18	13	31	34.07	7	7	14	15.38	0	0	0	0.00	0	0	0	0.00
Capiz	115	57	28	85	73.91	70	32	102	88.70	6	3	9	7.83	0	0	0	0.00
Guimaras	14	0	0	0	0.00	0	0	0	0.00	0	0	0	0.00	0	0	0	0.00
Iloilo	73	5	9	14	19.18	6	7	13	17.81	0	1	1	1.37	0	1	1	1.37
Negros Occidental	358	28	29	57	15.92	25	19	44	12.29	0	0	0	0.00	0	0	0	0.00
City of Bacolod	106	4	3	7	6.60	1	0	1	0.94	0	0	0	0.00	0	0	0	0.00
City of Iloilo	53	29	24	53	100.00	10	6	16	30.19	10	6	16	30.19	0	0	0	0.00
**Region 7**	**517**	**272**	**218**	**490**	**94.78**	**10**	**6**	**16**	**3.09**	**7**	**3**	**10**	**1.93**	**0**	**1**	**1**	**0.19**
Bohol	0	0	0	0	0.00	0	0	0	0.00	0	0	0	0.00	0	0	0	0.00
Cebu	481	251	209	460	95.63	7	4	11	2.29	7	3	10	2.08	0	0	0	0.00
Negros Oriental	0	0	0	0	0.00	0	0	0	0.00	0	0	0	0.00	0	0	0	0.00
Siquijor	0	0	0	0	0.00	0	0	0	0.00	0	0	0	0.00	0	0	0	0.00
City of Cebu	29	17	6	23	79.31	3	2	5	17.24	0	0	0	0.00	0	1	1	3.45
City of Lapu-Lapu	0	0	0	0	0.00	0	0	0	0.00	0	0	0	0.00	0	0	0	0.00
City of Mandaue	7	4	3	7	100.00	0	0	0	0.00	0	0	0	0.00	0	0	0	0.00

Table 2.C.3.6 - Nutrition Services for Infants and Children

Number and Proportion of Severe Acute Malnutrition (SAM) Children without complication admitted, cured, defaulted and died in Outpatient Therapeutic Care (OTC)

Philippines, 2024

Area	Total of SAM	SAM-admited in OTC				SAM-cured in OTC				SAM-defaulted in OTC				SAM-died in OTC			
		Male	Female	Total	%	Male	Female	Total	%	Male	Female	Total	%	Male	Female	Total	%
**Region 8**	**1,320**	**519**	**440**	**959**	**72.65**	**49**	**49**	**98**	**7.42**	**11**	**13**	**24**	**1.82**	**0**	**1**	**1**	**0.08**
Biliran	107	5	9	14	13.08	3	0	3	2.80	1	5	6	5.61	0	0	0	0.00
Eastern Samar	94	30	27	57	60.64	6	6	12	12.77	0	0	0	0.00	0	0	0	0.00
Leyte	213	75	72	147	69.01	30	33	63	29.58	8	7	15	7.04	0	0	0	0.00
Northern Samar	269	65	62	127	47.21	6	3	9	3.35	2	1	3	1.12	0	0	0	0.00
Southern Leyte	9	0	0	0	0.00	0	0	0	0.00	0	0	0	0.00	0	0	0	0.00
Samar	66	23	29	52	78.79	4	7	11	16.67	0	0	0	0.00	0	1	1	1.52
Ormoc City	546	313	233	546	100.00	0	0	0	0.00	0	0	0	0.00	0	0	0	0.00
City of Tacloban	16	8	8	16	100.00	0	0	0	0.00	0	0	0	0.00	0	0	0	0.00
**Region 9**	**1,359**	**246**	**212**	**458**	**33.70**	**88**	**79**	**167**	**12.29**	**11**	**8**	**19**	**1.40**	**1**	**3**	**4**	**0.29**
Zamboanga del Norte	336	84	94	178	52.98	39	45	84	25.00	3	1	4	1.19	1	1	2	0.60
Zamboanga del Sur	417	113	72	185	44.36	29	19	48	11.51	0	2	2	0.48	0	0	0	0.00
Zamboanga Sibugay	73	13	6	19	26.03	6	3	9	12.33	3	1	4	5.48	0	1	1	1.37
City of Isabela	187	0	0	0	0.00	0	0	0	0.00	0	0	0	0.00	0	0	0	0.00
City of Zamboanga	346	36	40	76	21.97	14	12	26	7.51	5	4	9	2.60	0	1	1	0.29
**Region 10**	**417**	**175**	**191**	**366**	**87.77**	**47**	**60**	**107**	**25.66**	**1**	**4**	**5**	**1.20**	**0**	**0**	**0**	**0.00**
Bukidnon	149	63	73	136	91.28	9	12	21	14.09	1	2	3	2.01	0	0	0	0.00
Camiguin	27	13	14	27	100.00	0	0	0	0.00	0	0	0	0.00	0	0	0	0.00
Lanao del Norte	117	57	60	117	100.00	28	28	56	47.86	0	0	0	0.00	0	0	0	0.00
Misamis Occidental	65	13	16	29	44.62	2	7	9	13.85	0	0	0	0.00	0	0	0	0.00
Misamis Oriental	14	5	7	12	85.71	2	1	3	21.43	0	2	2	14.29	0	0	0	0.00
City of Cagayan De	43	23	20	43	100.00	6	12	18	41.86	0	0	0	0.00	0	0	0	0.00
City of Iligan	2	1	1	2	100.00	0	0	0	0.00	0	0	0	0.00	0	0	0	0.00
**Region 11**	**859**	**284**	**213**	**497**	**57.86**	**162**	**131**	**293**	**34.11**	**22**	**18**	**40**	**4.66**	**3**	**1**	**4**	**0.47**
Davao de Oro	67	14	4	18	26.87	6	2	8	11.94	0	5	5	7.46	0	0	0	0.00
Davao del Norte	60	26	26	52	86.67	3	4	7	11.67	0	0	0	0.00	1	0	1	1.67
Davao Oriental	141	6	12	18	12.77	1	0	1	0.71	0	0	0	0.00	0	0	0	0.00
Davao del Sur	163	31	16	47	28.83	1	1	2	1.23	2	0	2	1.23	0	0	0	0.00
Davao Occidental	313	177	133	310	99.04	143	112	255	81.47	19	12	31	9.90	1	1	2	0.64
City of Davao	115	30	22	52	45.22	8	12	20	17.39	1	1	2	1.74	1	0	1	0.87
**Region 12**	**456**	**218**	**183**	**401**	**87.94**	**131**	**110**	**241**	**52.85**	**24**	**19**	**43**	**9.43**	**0**	**3**	**3**	**0.66**
Cotabato	230	127	97	224	97.39	88	66	154	66.96	22	15	37	16.09	0	0	0	0.00
Sarangani	74	33	29	62	83.78	26	22	48	64.86	0	1	1	1.35	0	1	1	1.35
South Cotabato	47	16	22	38	80.85	3	3	6	12.77	0	2	2	4.26	0	1	1	2.13
Sultan Kudarat	30	13	14	27	90.00	2	7	9	30.00	1	1	2	6.67	0	1	1	3.33
City of General Sant	75	29	21	50	66.67	12	12	24	32.00	1	0	1	1.33	0	0	0	0.00
**CARAGA**	**1,911**	**363**	**371**	**734**	**38.41**	**93**	**124**	**217**	**11.36**	**14**	**9**	**23**	**1.20**	**3**	**1**	**4**	**0.21**
Agusan del Norte	109	33	75	108	99.08	22	62	84	77.06	0	0	0	0.00	0	0	0	0.00
Agusan del Sur	88	41	46	87	98.86	21	13	34	38.64	0	0	0	0.00	0	1	1	1.14
Surigao del Norte	1,478	191	176	367	24.83	16	24	40	2.71	9	7	16	1.08	1	0	1	0.07
Surigao del Sur	210	84	62	146	69.52	34	25	59	28.10	5	2	7	3.33	2	0	2	0.95
Dinagat Islands	0	0	0	0	0.00	0	0	0	0.00	0	0	0	0.00	0	0	0	0.00
City of Butuan	26	14	12	26	100.00	0	0	0	0.00	0	0	0	0.00	0	0	0	0.00
**BARMM**	**2,096**	**865**	**899**	**1,764**	**84.16**	**206**	**217**	**423**	**20.18**	**110**	**118**	**228**	**10.88**	**7**	**7**	**14**	**0.67**
Basilan	139	64	52	116	83.45	9	12	21	15.11	3	3	6	4.32	0	0	0	0.00
Lanao del Sur	302	116	120	236	78.15	24	24	48	15.89	13	16	29	9.60	0	0	0	0.00
Maguindanao del Norte	108	28	38	66	61.11	15	14	29	26.85	7	13	20	18.52	0	0	0	0.00
Maguindanao del Sur	228	71	99	170	74.56	17	26	43	18.86	24	33	57	25.00	1	0	1	0.44
Sulu	229	55	80	135	58.95	40	34	74	32.31	15	13	28	12.23	2	2	4	1.75
Tawi-Tawi	923	472	430	902	97.72	76	72	148	16.03	43	33	76	8.23	3	4	7	0.76
SGA	143	49	66	115	80.42	25	31	56	39.16	4	3	7	4.90	0	0	0	0.00
City of Cotabato	24	10	14	24	100.00	0	4	4	16.67	1	4	5	20.83	1	1	2	8.33

* = No Report

Table 2.C.4.1 - Management of Sick Infants and Children
Number of high risk infants and children with measles and persistent diarrhea who received Vitamin A capsule aside from routine supplementation
Philippines, 2024

Area	Sick Infants aged 6-11 mos.							Sick Children aged 12-59 mos.						
	Seen			given Vitamin A 100,000 IU				Seen			given Vitamin A 200,000 IU			
	Male	Female	Total	Male	Female	Total	%	Male	Female	Total	Male	Female	Total	%
**Philippines**	**77,979**	**73,671**	**151,650**	**34,537**	**33,429**	**67,966**	**44.82**	**174,778**	**168,468**	**343,246**	**69,062**	**66,301**	**135,363**	**39.44**
**NCR**	**252**	**217**	**469**	**231**	**185**	**416**	**88.70**	**561**	**479**	**1,040**	**456**	**378**	**834**	**80.19**
City of Malabon	24	26	50	24	26	50	100.00	34	35	69	34	35	69	100.00
City of Navotas	1	2	3	1	2	3	100.00	4	1	5	4	1	5	100.00
City of Valenzuela	0	0	0	0	0	0	0.00	0	0	0	0	0	0	0.00
City of Caloocan	1	1	2	1	1	2	100.00	1	0	1	1	0	1	100.00
City of Marikina	1	1	2	1	0	1	50.00	2	6	8	1	1	2	25.00
City of Pasig	0	0	0	0	0	0	0.00	0	0	0	0	0	0	0.00
Pateros	15	6	21	15	6	21	100.00	23	14	37	23	14	37	100.00
City of Taguig	47	50	97	47	48	95	97.94	108	91	199	108	91	199	100.00
Quezon City	11	11	22	11	11	22	100.00	21	24	45	21	24	45	100.00
City of Makati	37	46	83	16	17	33	39.76	135	111	246	31	20	51	20.73
City of Mandaluyong	1	1	2	1	1	2	100.00	0	0	0	0	0	0	0.00
City of San Juan	0	0	0	0	0	0	0.00	0	0	0	0	0	0	0.00
City of Manila	0	0	0	0	0	0	0.00	0	0	0	0	0	0	0.00
City of Las Piñas	1	0	1	1	0	1	100.00	1	1	2	1	1	2	100.00
City of Muntinlupa	54	32	86	54	32	86	100.00	60	52	112	60	52	112	100.00
City of Parañaque	36	21	57	36	21	57	100.00	123	106	229	123	101	224	97.82
Pasay City	23	20	43	23	20	43	100.00	49	38	87	49	38	87	100.00
**CAR**	**154**	**127**	**281**	**133**	**98**	**231**	**82.21**	**308**	**277**	**585**	**205**	**185**	**390**	**66.67**
Abra	15	8	23	15	8	23	100.00	33	47	80	28	42	70	87.50
Apayao	34	37	71	27	23	50	70.42	90	73	163	38	34	72	44.17
Benguet	24	22	46	23	18	41	89.13	25	29	54	25	29	54	100.00
Ifugao	74	54	128	63	46	109	85.16	146	117	263	106	77	183	69.58
Kalinga	5	2	7	5	2	7	100.00	7	6	13	6	3	9	69.23
Mountain Province	0	0	0	0	0	0	0.00	0	0	0	0	0	0	0.00
City of Baguio	2	4	6	0	1	1	16.67	7	5	12	2	0	2	16.67
**Region 1**	**98**	**110**	**208**	**76**	**90**	**166**	**79.81**	**204**	**216**	**420**	**153**	**162**	**315**	**75.00**
Ilocos Norte	16	30	46	13	27	40	86.96	59	51	110	48	40	88	80.00
Ilocos Sur	73	71	144	54	54	108	75.00	117	132	249	77	89	166	66.67
La Union	0	0	0	0	0	0	0.00	0	0	0	0	0	0	0.00
Pangasinan	0	0	0	0	0	0	0.00	0	0	0	0	0	0	0.00
City of Dagupan	9	9	18	9	9	18	100.00	28	33	61	28	33	61	100.00
**Region 2**	**6,196**	**5,732**	**11,928**	**2374**	**2,387**	**4,761**	**39.91**	**13,495**	**13,197**	**26,692**	**4570**	**4,526**	**9,096**	**34.08**
Batanes	23	18	41	0	3	3	7.32	95	116	211	18	16	34	16.11
Cagayan	988	945	1,933	662	652	1,314	67.98	2,324	2,137	4,461	1,509	1,399	2,908	65.19
Isabela	2,998	2,792	5,790	1,382	1,372	2,754	47.56	6,371	6,539	12,910	2,663	2,772	5,435	42.10
Nueva Vizcaya	2,114	1,923	4,037	263	311	574	14.22	4,569	4,279	8,848	262	232	494	5.58
Quirino	73	54	127	67	49	116	91.34	136	126	262	118	107	225	85.88
City of Santiago	0	0	0	0	0	0	0.00	0	0	0	0	0	0	0.00
**Region 3**	**12,482**	**11,763**	**24,245**	**5944**	**5,771**	**11,715**	**48.32**	**28,134**	**27,115**	**55,249**	**10856**	**10,696**	**21,552**	**39.01**
Aurora	237	232	469	118	101	219	46.70	715	638	1,353	302	235	537	39.69
Bataan	428	373	801	134	113	247	30.84	890	805	1,695	195	147	342	20.18
Bulacan	6,519	6,081	12,600	3,005	2,902	5,907	46.88	14,677	13,873	28,550	5,492	5,369	10,861	38.04
Nueva Ecija	1,710	1,634	3,344	649	633	1,282	38.34	4,485	4,323	8,808	1,686	1,655	3,341	37.93
Pampanga	896	844	1,740	642	642	1,284	73.79	1,559	1,721	3,280	1,124	1,230	2,354	71.77
Tarlac	1,423	1,444	2,867	911	928	1,839	64.14	2,847	2,921	5,768	1,137	1,131	2,268	39.32
Zambales	692	670	1,362	148	160	308	22.61	1,944	1,888	3,832	407	423	830	21.66
City of Angeles	411	335	746	295	255	550	73.73	667	656	1,323	382	380	762	57.60
City of Olongapo	166	150	316	42	37	79	25.00	350	290	640	131	126	257	40.16
**Region 4A**	**17,029**	**15,965**	**32,994**	**4215**	**4,070**	**8,285**	**25.11**	**40,343**	**39,152**	**79,495**	**7990**	**7,577**	**15,567**	**19.58**
Batangas	858	836	1,694	161	169	330	19.48	2,319	2,126	4,445	377	341	718	16.15
Cavite	8,142	7,643	15,785	1,633	1,527	3,160	20.02	19,388	18,740	38,128	3,163	2,959	6,122	16.06
Laguna	1,263	1,107	2,370	262	206	468	19.75	2,537	2,362	4,899	329	322	651	13.29
Quezon	3,467	3,120	6,587	507	488	995	15.11	8,459	8,007	16,466	1,052	986	2,038	12.38
Rizal	2,696	2,616	5,312	1,052	1,039	2,091	39.36	6,593	6,856	13,449	2,026	1,913	3,939	29.29
City of Lucena	603	643	1,246	600	641	1,241	99.60	1,047	1,061	2,108	1,043	1,056	2,099	99.57
**Region 4B**	**1,509**	**1,365**	**2,874**	**664**	**618**	**1,282**	**44.61**	**3,148**	**3,031**	**6,179**	**1199**	**1,088**	**2,287**	**37.01**
Marinduque	59	55	114	52	48	100	87.72	158	144	302	139	129	268	88.74
Occidental Mindoro	539	498	1,037	237	196	433	41.76	1,105	1,018	2,123	448	413	861	40.56
Oriental Mindoro	344	280	624	187	138	325	52.08	641	695	1,336	197	184	381	28.52
Palawan	220	199	419	130	128	258	61.58	454	428	882	341	302	643	72.90
Romblon	278	218	496	10	17	27	5.44	646	564	1,210	27	18	45	3.72
City of Puerto Princesa	69	115	184	48	91	139	75.54	144	182	326	47	42	89	27.30
**Region 5**	**3,135**	**3,138**	**6,273**	**1206**	**1,219**	**2,425**	**38.66**	**6,395**	**6,277**	**12,672**	**2875**	**2,657**	**5,532**	**43.66**
Albay	953	917	1,870	256	222	478	25.56	2,077	2,042	4,119	451	389	840	20.39
Camarines Norte	312	293	605	149	151	300	49.59	555	529	1,084	324	302	626	57.75
Camarines Sur	170	180	350	26	23	49	14.00	208	158	366	89	63	152	41.53
Catanduanes	17	13	30	6	5	11	36.67	21	19	40	18	15	33	82.50
Masbate	1,084	1,164	2,248	611	697	1,308	58.19	2,474	2,501	4,975	1,822	1,746	3,568	71.72
Sorsogon	598	571	1,169	158	121	279	23.87	1,058	1,028	2,086	169	142	311	14.91
City of Naga	1	0	1	0	0	0	0.00	2	0	2	2	0	2	100.00

Table 2.C.4.1 - Management of Sick Infants and Children

Number of high risk infants and children with measles and persistent diarrhea who received Vitamin A capsule aside from routine supplementation

Philippines, 2024

Area	Sick Infants aged 6-11 mos.							Sick Children aged 12-59 mos.						
	Seen			given Vitamin A 100,000 IU				Seen			given Vitamin A 200,000 IU			
	Male	Female	Total	Male	Female	Total	%	Male	Female	Total	Male	Female	Total	%
**Region 6**	**5,387**	**4,939**	**10,326**	**2124**	**2,129**	**4,253**	**41.19**	**13,691**	**13,058**	**26,749**	**4663**	**4,587**	**9,250**	**34.58**
Aklan	324	318	642	214	189	403	62.77	818	839	1,657	506	545	1,051	63.43
Antique	340	325	665	80	97	177	26.62	856	883	1,739	164	171	335	19.26
Capiz	6	5	11	6	5	11	100.00	3	5	8	3	5	8	100.00
Guimaras	2	0	2	0	0	0	0.00	3	1	4	0	0	0	0.00
Iloilo	2,107	1,875	3,982	842	838	1,680	42.19	5,494	5,219	10,713	2,343	2,302	4,645	43.36
Negros Occidental	1,912	1,807	3,719	734	773	1,507	40.52	5,281	5,028	10,309	1,274	1,278	2,552	24.76
City of Bacolod	696	609	1,305	248	227	475	36.40	1,236	1,083	2,319	373	286	659	28.42
City of Iloilo	0	0	0	0	0	0	0.00	0	0	0	0	0	0	0.00
**Region 7**	**1,168**	**1,068**	**2,236**	**357**	**323**	**680**	**30.41**	**3,422**	**3,093**	**6,515**	**781**	**659**	**1,440**	**22.10**
Bohol	70	73	143	11	11	22	15.38	113	99	212	35	41	76	35.85
Cebu	790	709	1,499	114	98	212	14.14	2,645	2,459	5,104	220	185	405	7.93
Negros Oriental	17	22	39	12	12	24	61.54	48	39	87	17	22	39	44.83
Siquijor	0	0	0	0	0	0	0.00	1	9	10	0	0	0	0.00
City of Cebu	75	74	149	43	43	86	57.72	97	88	185	88	76	164	88.65
City of Lapu-Lapu	166	141	307	132	111	243	79.15	288	215	503	200	155	355	70.58
City of Mandaue	50	49	99	45	48	93	93.94	230	184	414	221	180	401	96.86
**Region 8**	**6,433**	**6,073**	**12,506**	**3080**	**2,835**	**5,915**	**47.30**	**16,136**	**15,570**	**31,706**	**7447**	**7,168**	**14,615**	**46.10**
Biliran	476	431	907	107	101	208	22.93	1,215	1,254	2,469	284	248	532	21.55
Eastern Samar	742	779	1,521	239	207	446	29.32	2,357	2,282	4,639	578	576	1,154	24.88
Leyte	1,999	1,800	3,799	1,221	1,062	2,283	60.09	5,366	5,095	10,461	3,365	3,296	6,661	63.67
Northern Samar	1,445	1,372	2,817	457	439	896	31.81	3,049	3,044	6,093	1,009	945	1,954	32.07
Southern Leyte	36	46	82	12	25	37	45.12	81	74	155	13	17	30	19.35
Samar	1,390	1,360	2,750	838	829	1,667	60.62	3,197	2,911	6,108	1,878	1,762	3,640	59.59
Ormoc City	12	8	20	12	8	20	100.00	6	2	8	6	2	8	100.00
City of Tacloban	333	277	610	194	164	358	58.69	865	908	1,773	314	322	636	35.87
**Region 9**	**3,521**	**3,535**	**7,056**	**2603**	**2,582**	**5,185**	**73.48**	**5,771**	**5,486**	**11,257**	**4149**	**3,899**	**8,048**	**71.49**
Zamboanga del Norte	1,095	1,103	2,198	698	715	1,413	64.29	1,687	1,556	3,243	1,027	954	1,981	61.09
Zamboanga del Sur	680	662	1,342	578	549	1,127	83.98	1,635	1,550	3,185	1,345	1,266	2,611	81.98
Zamboanga Sibugay	492	489	981	326	316	642	65.44	591	648	1,239	247	238	485	39.14
City of Isabela	491	461	952	474	446	920	96.64	796	765	1,561	731	704	1,435	91.93
City of Zamboanga	763	820	1,583	527	556	1,083	68.41	1,062	967	2,029	799	737	1,536	75.70
**Region 10**	**7,026**	**6,564**	**13,590**	**3443**	**3,343**	**6,786**	**49.93**	**13,946**	**13,215**	**27,161**	**6923**	**6,411**	**13,334**	**49.09**
Bukidnon	2,414	2,378	4,792	865	895	1,760	36.73	4,749	4,527	9,276	1,430	1,312	2,742	29.56
Camiguin	47	60	107	0	0	0	0.00	160	124	284	0	0	0	0.00
Lanao del Norte	1,241	1,134	2,375	1,085	963	2,048	86.23	2,566	2,389	4,955	2,424	2,208	4,632	93.48
Misamis Occidental	481	461	942	362	378	740	78.56	1,012	957	1,969	903	862	1,765	89.64
Misamis Oriental	1,286	1,080	2,366	252	244	496	20.96	2,269	2,203	4,472	583	517	1,100	24.60
City of Cagayan De Oro	640	538	1,178	290	269	559	47.45	1,185	1,119	2,304	428	427	855	37.11
City of Iligan	917	913	1,830	589	594	1,183	64.64	2,005	1,896	3,901	1,155	1,085	2,240	57.42
**Region 11**	**3,413**	**3,151**	**6,564**	**2107**	**1,886**	**3,993**	**60.83**	**8,710**	**8,041**	**16,751**	**4938**	**4,593**	**9,531**	**56.90**
Davao de Oro	353	380	733	302	325	627	85.54	842	815	1,657	722	708	1,430	86.30
Davao del Norte	1,100	1,027	2,127	281	230	511	24.02	3,608	3,314	6,922	726	708	1,434	20.72
Davao Oriental	565	510	1,075	515	475	990	92.09	1,732	1,578	3,310	1,633	1,481	3,114	94.08
Davao del Sur	343	290	633	182	152	334	52.76	589	535	1,124	327	277	604	53.74
Davao Occidental	177	140	317	104	73	177	55.84	331	267	598	202	180	382	63.88
City of Davao	875	804	1,679	723	631	1,354	80.64	1,608	1,532	3,140	1,328	1,239	2,567	81.75
**Region 12**	**3,371**	**3,391**	**6,762**	**2058**	**2,049**	**4,107**	**60.74**	**6,617**	**6,456**	**13,073**	**3913**	**3,928**	**7,841**	**59.98**
Cotabato	1,294	1,316	2,610	479	467	946	36.25	2,657	2,628	5,285	813	880	1,693	32.03
Sarangani	1,248	1,245	2,493	1,120	1,110	2,230	89.45	2,669	2,578	5,247	2,283	2,233	4,516	86.07
South Cotabato	207	195	402	138	141	279	69.40	446	368	814	270	225	495	60.81
Sultan Kudarat	381	447	828	173	192	365	44.08	335	349	684	207	229	436	63.74
City of General Santos	241	188	429	148	139	287	66.90	510	533	1,043	340	361	701	67.21
**CARAGA**	**2,052**	**1,984**	**4,036**	**783**	**797**	**1,580**	**39.15**	**4,191**	**4,235**	**8,426**	**1803**	**1,652**	**3,455**	**41.00**
Agusan del Norte	182	146	328	77	68	145	44.21	313	317	630	57	56	113	17.94
Agusan del Sur	1,297	1,300	2,597	535	581	1,116	42.97	2,798	2,698	5,496	1,485	1,337	2,822	51.35
Surigao del Norte	377	371	748	51	44	95	12.70	780	945	1,725	81	102	183	10.61
Surigao del Sur	93	83	176	89	81	170	96.59	149	126	275	128	113	241	87.64
Dinagat Islands	79	58	137	13	6	19	13.87	109	111	220	19	16	35	15.91
City of Butuan	24	26	50	18	17	35	70.00	42	38	80	33	28	61	76.25
**BARMM**	**4,753**	**4,549**	**9,302**	**3139**	**3,047**	**6,186**	**66.50**	**9,706**	**9,570**	**19,276**	**6141**	**6,135**	**12,276**	**63.69**
Basilan	1,656	1,610	3,266	805	832	1,637	50.12	3,614	3,694	7,308	1,184	1,391	2,575	35.24
Lanao del Sur	758	727	1,485	598	565	1,163	78.32	1,358	1,267	2,625	1,301	1,210	2,511	95.66
Maguindanao del Norte	758	727	1,485	598	565	1,163	78.32	1,358	1,267	2,625	1,301	1,210	2,511	95.66
Maguindanao del Sur	8	7	15	5	6	11	73.33	9	7	16	8	7	15	93.75
Sulu	746	654	1,400	458	434	892	63.71	1,742	1,679	3,421	1,149	1,089	2,238	65.42
Tawi-Tawi	254	249	503	196	195	391	77.73	621	595	1,216	418	420	838	68.91
SGA	329	321	650	298	277	575	88.46	564	549	1,113	489	493	982	88.23
City of Cotabato	244	254	498	181	173	354	71.08	440	512	952	291	315	606	63.66

* = No Report

* = No Report

Table 1.C.4.2 - Management of Sick Infants and Children

Number and proportion of diarrhea cases 0-59 mos. old who received Oral Rehydration Salt (ORS) solution and ORS with Zinc drops/syrup and Pneumonia cases among

Philippines, 2024

Area	Children 0-59 mos. old with Diarrhea											Children 0-59 mos. old with Pneumonia						
	Seen			Given ORS				Given ORS w/ zinc				Seen			Received treatment			
	Male	Female	Total	Male	Female	Total	%	Male	Female	Total	%	Male	Female	Total	Male	Female	Total	%
**Philippines**	**47,109**	**41,594**	**88,703**	**25,234**	**22,421**	**47,655**	**53.72**	**26,631**	**23,471**	**50,102**	**56.48**	**55,255**	**49,174**	**104,429**	**53,602**	**47,530**	**101,132**	**96.84**
**NCR**	**6,245**	**5,226**	**11,471**	**2,666**	**2,245**	**4,911**	**42.81**	**3,562**	**2,953**	**6,515**	**56.80**	**9,136**	**7,598**	**16,734**	**9,122**	**7,578**	**16,700**	**99.80**
City of Malabon	123	123	246	96	98	194	78.86	24	19	43	17.48	587	441	1,028	584	440	1,024	99.61
City of Navotas	252	241	493	94	84	178	36.11	158	157	315	63.89	20	33	53	18	33	51	96.23
City of Valenzuela	443	320	763	218	183	401	52.56	225	137	362	47.44	728	603	1,331	728	603	1,331	100.00
City of Caloocan	741	641	1,382	741	641	1,382	100.00	0	0	0	0.00	2,309	1,922	4,231	2,309	1,922	4,231	100.00
City of Marikina	29	27	56	2	2	4	7.14	27	25	52	92.86	31	27	58	31	27	58	100.00
City of Pasig	334	255	589	103	64	167	28.35	231	191	422	71.65	573	450	1,023	573	450	1,023	100.00
Pateros	20	12	32	19	12	31	96.88	1	0	1	3.13	7	5	12	7	5	12	100.00
City of Taguig	922	808	1,730	164	133	297	17.17	745	662	1,407	81.33	292	285	577	292	285	577	100.00
Quezon City	1,266	1,003	2,269	642	502	1,144	50.42	624	501	1,125	49.58	2,165	1,827	3,992	2,166	1,826	3,992	100.00
City of Makati	251	247	498	46	45	91	18.27	202	195	397	79.72	229	183	412	229	183	412	100.00
City of Mandaluyong	72	60	132	27	22	49	37.12	45	38	83	62.88	261	209	470	261	209	470	100.00
City of San Juan	127	84	211	1	2	3	1.42	126	82	208	98.58	180	155	335	180	155	335	100.00
City of Manila	685	566	1,251	295	271	566	45.24	390	295	685	54.76	929	787	1,716	919	777	1,696	98.83
City of Las Piñas	250	238	488	32	33	65	13.32	218	205	423	86.68	80	44	124	80	44	124	100.00
City of Muntinlupa	557	415	972	152	111	263	27.06	407	302	709	72.94	411	327	738	411	327	738	100.00
City of Parañaque	49	56	105	21	26	47	44.76	28	30	58	55.24	54	47	101	54	39	93	92.08
Pasay City	124	130	254	13	16	29	11.42	111	114	225	88.58	280	253	533	280	253	533	100.00
**CAR**	**2,441**	**1,922**	**4,363**	**664**	**515**	**1,179**	**27.02**	**1746**	**1,370**	**3,116**	**71.42**	**2,291**	**1,794**	**4,085**	**2277**	**1,782**	**4,059**	**99.36**
Abra	156	167	323	51	45	96	29.72	105	122	227	70.28	124	84	208	122	84	206	99.04
Apayao	340	295	635	24	24	48	7.56	306	256	562	88.50	427	339	766	427	339	766	100.00
Benguet	390	321	711	105	90	195	27.43	284	229	513	72.15	170	124	294	169	121	290	98.64
Ifugao	802	613	1,415	182	146	328	23.18	614	464	1,078	76.18	887	714	1,601	881	706	1,587	99.13
Kalinga	449	288	737	245	159	404	54.82	190	116	306	41.52	170	108	278	170	108	278	100.00
Mountain Province	215	170	385	54	46	100	25.97	161	123	284	73.77	111	110	221	111	110	221	100.00
City of Baguio	89	68	157	3	5	8	5.10	86	60	146	92.99	402	315	717	397	314	711	99.16
**Region 1**	**1,841**	**1,707**	**3,548**	**1011**	**929**	**1,940**	**54.68**	**830**	**778**	**1,608**	**45.32**	**2,558**	**2,260**	**4,818**	**2558**	**2,260**	**4,818**	**100.00**
Ilocos Norte	65	46	111	32	19	51	45.95	33	27	60	54.05	128	130	258	128	130	258	100.00
Ilocos Sur	218	218	436	35	32	67	15.37	183	186	369	84.63	654	584	1,238	654	584	1,238	100.00
La Union	184	150	334	26	20	46	13.77	158	130	288	86.23	687	623	1,310	687	623	1,310	100.00
Pangasinan	1,268	1,195	2,463	918	858	1,776	72.11	350	337	687	27.89	965	837	1,802	965	837	1,802	100.00
City of Dagupan	106	98	204	0	0	0	0.00	106	98	204	100.00	124	86	210	124	86	210	100.00
**Region 2**	**2,185**	**1,859**	**4,044**	**920**	**810**	**1,730**	**42.78**	**1684**	**1,418**	**3,102**	**76.71**	**3,778**	**3,575**	**7,353**	**3575**	**3,375**	**6,950**	**94.52**
Batanes	6	3	9	3	3	6	66.67	4	1	5	55.56	22	23	45	22	23	45	100.00
Cagayan	355	281	636	298	248	546	85.85	261	203	464	72.96	303	245	548	301	243	544	99.27
Isabela	1,166	999	2,165	342	307	649	29.98	1,046	882	1,928	89.05	1,277	1,284	2,561	1,273	1,281	2,554	99.73
Nueva Vizcaya	635	554	1,189	267	241	508	42.72	360	321	681	57.28	2,147	2,007	4,154	1,951	1,812	3,763	90.59
Quirino	23	22	45	10	11	21	46.67	13	11	24	53.33	29	16	45	28	16	44	97.78
City of Santiago	0	0	0	0	0	0	0.00	0	0	0	0.00	0	0	0	0	0	0	0.00
**Region 3**	**7,249**	**6,509**	**13,758**	**3382**	**2,955**	**6,337**	**46.06**	**3803**	**3,487**	**7,290**	**52.99**	**2,598**	**2,249**	**4,847**	**2528**	**2,177**	**4,705**	**97.07**
Aurora	127	121	248	53	54	107	43.15	74	67	141	56.85	241	164	405	241	164	405	100.00
Bataan	843	761	1,604	441	403	844	52.62	389	346	735	45.82	194	172	366	194	172	366	100.00
Bulacan	3,536	3,057	6,593	1,380	1,154	2,534	38.43	2,141	1,888	4,029	61.11	791	721	1,512	783	720	1,503	99.40
Nueva Ecija	727	657	1,384	381	322	703	50.79	330	322	652	47.11	796	649	1,445	784	640	1,424	98.55
Pampanga	462	429	891	284	252	536	60.16	176	176	352	39.51	171	182	353	165	174	339	96.03
Tarlac	1,083	1,038	2,121	574	530	1,104	52.05	493	483	976	46.02	212	174	386	176	126	302	78.24
Zambales	296	278	574	186	153	339	59.06	110	125	235	40.94	168	161	329	161	157	318	96.66
City of Angeles	135	128	263	82	84	166	63.12	52	43	95	36.12	22	20	42	22	20	42	100.00
City of Olongapo	40	40	80	1	3	4	5.00	38	37	75	93.75	3	6	9	2	4	6	66.67
**Region 4A**	**3,954**	**3,542**	**7,496**	**3265**	**2,893**	**6,158**	**82.15**	**2628**	**2,393**	**5,021**	**66.98**	**2,104**	**1,850**	**3,954**	**1792**	**1,555**	**3,347**	**84.65**
Batangas	338	273	611	220	172	392	64.16	210	174	384	62.85	239	247	486	205	198	403	82.92
Cavite	948	867	1,815	660	576	1,236	68.10	466	433	899	49.53	506	437	943	313	260	573	60.76
Laguna	612	542	1,154	496	443	939	81.37	399	330	729	63.17	155	117	272	139	103	242	88.97
Quezon	781	652	1,433	658	542	1,200	83.74	434	373	807	56.32	526	443	969	495	419	914	94.32
Rizal	929	838	1,767	885	790	1,675	94.79	773	713	1,486	84.10	618	540	1,158	580	509	1,089	94.04
City of Lucena	346	370	716	346	370	716	100.00	346	370	716	100.00	60	66	126	60	66	126	100.00
**Region 4B**	**1,395**	**1,072**	**2,467**	**824**	**627**	**1,451**	**58.82**	**523**	**380**	**903**	**36.60**	**710**	**590**	**1,300**	**701**	**584**	**1,285**	**98.85**
Marinduque	43	37	80	12	16	28	35.00	30	21	51	63.75	30	30	60	30	30	60	100.00
Occidental Mindoro	376	327	703	228	233	461	65.58	126	74	200	28.45	80	81	161	80	81	161	100.00
Oriental Mindoro	95	67	162	42	26	68	41.98	52	40	92	56.79	89	77	166	88	77	165	99.40
Palawan	720	524	1,244	425	259	684	54.98	288	234	522	41.96	363	277	640	357	273	630	98.44
Romblon	142	105	247	115	92	207	83.81	18	9	27	10.93	136	120	256	136	118	254	99.22
City of Puerto Prince	19	12	31	2	1	3	9.68	9	2	11	35.48	12	5	17	10	5	15	88.24
**Region 5**	**2,080**	**1,780**	**3,860**	**856**	**686**	**1,542**	**39.95**	**1195**	**1,063**	**2,258**	**58.50**	**2,036**	**1,875**	**3,911**	**1997**	**1,849**	**3,846**	**98.34**
Albay	319	291	610	202	195	397	65.08	107	96	203	33.28	194	170	364	189	166	355	97.53
Camarines Norte	297	246	543	161	127	288	53.04	133	112	245	45.12	111	84	195	108	80	188	96.41
Camarines Sur	375	279	654	111	71	182	27.83	258	206	464	70.95	311	306	617	292	298	590	95.62
Catanduanes	107	97	204	24	17	41	20.10	83	80	163	79.90	87	86	173	86	86	172	99.42
Masbate	802	728	1,530	263	209	472	30.85	531	502	1,033	67.52	936	901	1,837	928	891	1,819	99.02
Sorsogon	175	137	312	91	65	156	50.00	82	67	149	47.76	190	147	337	187	147	334	99.11
City of Naga	5	2	7	4	2	6	85.71	1	0	1	14.29	207	181	388	207	181	388	100.00
**Region 6**	**2,092**	**1,908**	**4,000**	**1403**	**1,350**	**2,753**	**68.83**	**970**	**834**	**1,804**	**45.10**	**5,203**	**4,931**	**10,134**	**5109**	**4,687**	**9,796**	**96.66**
Aklan	75	79	154	60	63	123	79.87	15	16	31	20.13	125	120	245	125	120	245	100.00
Antique	88	81	169	51	51	102	60.36	15	6	21	12.43	455	412	867	453	401	854	98.50
Capiz	100	100	200	48	50	98	49.00	52	50	102	51.00	572	550	1,122	572	550	1,122	100.00
Guimaras	15	14	29	0	0	0	0.00	15	14	29	100.00	97	79	176	97	79	176	100.00
Iloilo	539	508	1,047	509	482	991	94.65	358	344	702	67.05	1,102	1,075	2,177	1,092	1,022	2,114	97.11
Negros Occidental	999	915	1,914	596	576	1,172	61.23	392	330	722	37.72	1,605	1,546	3,151	1,597	1,466	3,063	97.21
City of Bacolod	145	124	269	124	114	238	88.48	9	5	14	5.20	413	409	822	340	309	649	78.95
City of Iloilo	131	87	218	15	14	29	13.30	114	69	183	83.94	834	740	1,574	833	740	1,573	99.94

Table 1.C.4.2 - Management of Sick Infants and Children

Number and proportion of diarrhea cases 0-59 mos. old who received Oral Rehydration Salt (ORS) solution and ORS with Zinc drops/syrup and Pneumonia cases among

Philippines, 2024

Area	Children 0-59 mos. old with Diarrhea											Children 0-59 mos. old with Pneumonia						
	Seen			Given ORS				Given ORS w/ zinc				Seen			Received treatment			
	Male	Female	Total	Male	Female	Total	%	Male	Female	Total	%	Male	Female	Total	Male	Female	Total	%
**Region 7**	**2,393**	**2,126**	**4,519**	**1089**	**934**	**2,023**	**44.77**	**1308**	**1,144**	**2,452**	**54.26**	**5,195**	**4,477**	**9,672**	**4766**	**4,126**	**8,892**	**91.94**
Bohol	730	692	1,422	433	436	869	61.11	297	253	550	38.68	103	66	169	86	56	142	84.02
Cebu	598	502	1,100	421	307	728	66.18	137	126	263	23.91	1,531	1,279	2,810	1,202	1,021	2,223	79.11
Negros Oriental	571	546	1,117	0	0	0	0.00	549	522	1,071	95.88	1,613	1,477	3,090	1,564	1,430	2,994	96.89
Siquijor	11	10	21	7	6	13	61.90	8	3	11	52.38	107	97	204	107	97	204	100.00
City of Cebu	229	173	402	32	27	59	14.68	201	148	349	86.82	1,634	1,368	3,002	1,612	1,343	2,955	98.43
City of Lapu-Lapu	188	150	338	130	105	235	69.53	116	92	208	61.54	138	115	253	126	104	230	90.91
City of Mandaue	66	53	119	66	53	119	100.00	0	0	0	0.00	69	75	144	69	75	144	100.00
**Region 8**	**2,237**	**2,012**	**4,249**	**1708**	**1,537**	**3,245**	**76.37**	**1678**	**1,509**	**3,187**	**75.01**	**5,327**	**4,810**	**10,137**	**5262**	**4,759**	**10,021**	**98.86**
Biliran	188	154	342	101	83	184	53.80	146	103	249	72.81	294	259	553	294	259	553	100.00
Eastern Samar	220	180	400	155	131	286	71.50	136	106	242	60.50	792	711	1,503	778	681	1,459	97.07
Leyte	467	442	909	366	349	715	78.66	385	379	764	84.05	2,141	2,038	4,179	2,104	2,030	4,134	98.92
Northern Samar	684	627	1,311	642	576	1,218	92.91	470	436	906	69.11	602	562	1,164	595	555	1,150	98.80
Southern Leyte	57	51	108	20	12	32	29.63	37	37	74	68.52	56	48	104	55	47	102	98.08
Samar	413	394	807	255	250	505	62.58	307	303	610	75.59	568	441	1,009	562	436	998	98.91
Ormoc City	168	126	294	157	121	278	94.56	168	126	294	100.00	469	421	890	469	421	890	100.00
City of Tacloban	40	38	78	12	15	27	34.62	29	19	48	61.54	405	330	735	405	330	735	100.00
**Region 9**	**2,215**	**1,973**	**4,188**	**667**	**615**	**1,282**	**30.61**	**1527**	**1,333**	**2,860**	**68.29**	**2,244**	**2,121**	**4,365**	**2197**	**2,072**	**4,269**	**97.80**
Zamboanga del Norte	611	569	1,180	185	188	373	31.61	423	373	796	67.46	1,064	1,039	2,103	1,060	1,034	2,094	99.57
Zamboanga del Sur	473	425	898	74	90	164	18.26	389	325	714	79.51	538	518	1,056	506	487	993	94.03
Zamboanga Sibugay	221	183	404	72	66	138	34.16	144	112	256	63.37	115	113	228	113	107	220	96.49
City of Isabela	459	433	892	40	40	80	8.97	418	393	811	90.92	375	333	708	375	331	706	99.72
City of Zamboanga	451	363	814	296	231	527	64.74	153	130	283	34.77	152	118	270	143	113	256	94.81
**Region 10**	**2,047**	**1,834**	**3,881**	**1569**	**1,443**	**3,012**	**77.61**	**1337**	**1,162**	**2,499**	**64.39**	**3,773**	**3,533**	**7,306**	**3752**	**3,519**	**7,271**	**99.52**
Bukidnon	672	561	1,233	579	470	1,049	85.08	473	404	877	71.13	1,008	920	1,928	1,002	913	1,915	99.33
Camiguin	12	10	22	10	9	19	86.36	1	0	1	4.55	43	33	76	43	33	76	100.00
Lanao del Norte	520	459	979	344	329	673	68.74	418	325	743	75.89	1,039	902	1,941	1,041	911	1,952	100.57
Misamis Occidental	280	301	581	132	184	316	54.39	183	155	338	58.18	644	684	1,328	644	684	1,328	100.00
Misamis Oriental	163	153	316	140	132	272	86.08	114	108	222	70.25	478	447	925	478	446	924	99.89
City of Cagayan De	198	171	369	178	156	334	90.51	107	119	226	61.25	402	378	780	399	371	770	98.72
City of Iligan	202	179	381	186	163	349	91.60	41	51	92	24.15	159	169	328	145	161	306	93.29
**Region 11**	**1,267**	**1,148**	**2,415**	**342**	**305**	**647**	**26.79**	**890**	**808**	**1,698**	**70.31**	**2,294**	**2,109**	**4,403**	**2270**	**2,085**	**4,355**	**98.91**
Davao de Oro	165	138	303	88	70	158	52.15	76	66	142	46.86	519	603	1,122	510	589	1,099	97.95
Davao del Norte	268	276	544	51	51	102	18.75	211	215	426	78.31	687	597	1,284	680	597	1,277	99.45
Davao Oriental	222	163	385	40	30	70	18.18	176	130	306	79.48	470	436	906	468	436	904	99.78
Davao del Sur	156	132	288	62	58	120	41.67	93	71	164	56.94	236	188	424	234	185	419	98.82
Davao Occidental	65	56	121	22	27	49	40.50	40	28	68	56.20	38	21	59	36	19	55	93.22
City of Davao	391	383	774	79	69	148	19.12	294	298	592	76.49	344	264	608	342	259	601	98.85
**Region 12**	**1,157**	**1,009**	**2,166**	**813**	**714**	**1,527**	**70.50**	**339**	**281**	**620**	**28.62**	**1,462**	**1,313**	**2,775**	**1426**	**1,264**	**2,690**	**96.94**
Cotabato	496	417	913	355	283	638	69.88	141	129	270	29.57	544	483	1,027	525	460	985	95.91
Sarangani	207	224	431	197	213	410	95.13	8	9	17	3.94	173	142	315	168	138	306	97.14
South Cotabato	217	153	370	141	104	245	66.22	76	49	125	33.78	396	372	768	394	369	763	99.35
Sultan Kudarat	77	67	144	59	54	113	78.47	20	11	31	21.53	18	29	47	17	29	46	97.87
City of General Sant	160	148	308	61	60	121	39.29	94	83	177	57.47	331	287	618	322	268	590	95.47
**CARAGA**	**1,147**	**953**	**2,100**	**845**	**692**	**1,537**	**73.19**	**291**	**256**	**547**	**26.05**	**2,016**	**1,781**	**3,797**	**2009**	**1,768**	**3,777**	**99.47**
Agusan del Norte	49	47	96	43	42	85	88.54	6	5	11	11.46	118	134	252	116	129	245	97.22
Agusan del Sur	224	225	449	99	101	200	44.54	119	119	238	53.01	291	265	556	291	265	556	100.00
Surigao del Norte	121	98	219	81	73	154	70.32	33	28	61	27.85	229	217	446	227	213	440	98.65
Surigao del Sur	698	550	1,248	593	456	1,049	84.05	100	90	190	15.22	1,360	1,142	2,502	1,358	1,141	2,499	99.88
Dinagat Islands	38	23	61	12	10	22	36.07	31	14	45	73.77	15	18	33	15	18	33	100.00
City of Butuan	17	10	27	17	10	27	100.00	2	0	2	7.41	3	5	8	2	2	4	50.00
**BARMM**	**5,164**	**5,014**	**10,178**	**3210**	**3,171**	**6,381**	**62.69**	**2320**	**2,302**	**4,622**	**45.41**	**2,530**	**2,308**	**4,838**	**2261**	**2,090**	**4,351**	**89.93**
Basilan	998	939	1,937	578	602	1,180	60.92	552	544	1,096	56.58	493	418	911	329	290	619	67.95
Lanao del Sur	1,551	1,535	3,086	1,147	1,148	2,295	74.37	511	493	1,004	32.53	888	817	1,705	802	732	1,534	89.97
Maguindanao del Norte	654	637	1,291	135	131	266	20.60	519	505	1,024	79.32	243	241	484	241	240	481	99.38
Maguindanao del Sur	651	606	1,257	468	441	909	72.32	176	168	344	27.37	303	266	569	299	262	561	98.59
Sulu	651	606	1,257	468	441	909	72.32	176	168	344	27.37	303	266	569	299	262	561	98.59
Tawi-Tawi	267	305	572	137	149	286	50.00	229	270	499	87.24	198	210	408	195	216	411	100.74
SGA	220	217	437	204	195	399	91.30	37	32	69	15.79	64	53	117	64	53	117	100.00
City of Cotabato	172	169	341	73	64	137	40.18	120	122	242	70.97	38	37	75	32	35	67	89.33

* = No Report

* = No Report

Table 2.D.1 - ORAL HEALTH CARE
Children 12-59 months old who are orally fit upon oral examination or after oral rehabilitation
Philippines, 2024

Area	Eligible Population x 20%	Orally fit		Total	%
		Male	Female		
**Philippines**	**1,841,144**	**338,807**	**363,577**	**702,384**	**38.15**
**NCR**	**211,401**	**95,619**	**101,276**	**196,895**	**93.14**
City of Malabon	**5,947**	1,837	2,038	**3,875**	**65.15**
City of Navotas	**4,218**	1,589	1,794	**3,383**	**80.21**
City of Valenzuela	**11,079**	931	1,003	**1,934**	**17.46**
City of Caloocan	**28,025**	9,795	10,674	**20,469**	**73.04**
City of Marikina	**6,976**	1,764	1,751	**3,515**	**50.39**
City of Pasig	**13,274**	2,467	2,884	**5,351**	**40.31**
Pateros	**970**	268	314	**582**	**60.02**
City of Taguig	**15,179**	2,872	3,375	**6,247**	**41.16**
Quezon City	**48,122**	34,364	35,010	**69,374**	**144.16**
City of Makati	**8,541**	1,661	1,837	**3,498**	**40.95**
City of Mandaluyong	**6,363**	1,185	1,327	**2,512**	**39.48**
City of San Juan	**1,727**	304	321	**625**	**36.20**
City of Manila	**27,927**	28,437	30,063	**58,500**	**209.48**
City of Las Piñas	**9,119**	1,877	1,908	**3,785**	**41.50**
City of Muntinlupa	**7,883**	1,263	1,544	**2,807**	**35.61**
City of Parañaque	**10,748**	2,676	2,791	**5,467**	**50.86**
Pasay City	**5,303**	2,329	2,642	**4,971**	**93.75**
**CAR**	**26,542**	**4,317**	**4,899**	**9,216**	**34.72**
Abra	**3,242**	85	140	**225**	**6.94**
Apayao	**1,850**	299	344	**643**	**34.76**
Benguet	**7,483**	1,269	1,391	**2,660**	**35.55**
Ifugao	**3,476**	754	782	**1,536**	**44.19**
Kalinga	**3,754**	1,065	1,357	**2,422**	**64.52**
Mountain Province	**2,281**	171	194	**365**	**16.00**
City of Baguio	**4,456**	674	691	**1,365**	**30.63**
**Region 1**	**78,389**	**20,211**	**21,693**	**41,904**	**53.46**
Ilocos Norte	**7,568**	1,918	2,084	**4,002**	**52.88**
Ilocos Sur	**8,659**	4,149	4,227	**8,376**	**96.73**
La Union	**11,030**	2,652	2,570	**5,222**	**47.34**
Pangasinan	**48,423**	11,259	12,539	**23,798**	**49.15**
City of Dagupan	**2,710**	233	273	**506**	**18.67**
**Region 2**	**54,884**	**7,805**	**8,882**	**16,687**	**30.40**
Batanes	**227**	121	104	**225**	**99.21**
Cagayan	**18,103**	965	1,085	**2,050**	**11.32**
Isabela	**23,247**	4,582	5,214	**9,796**	**42.14**
Nueva Vizcaya	**7,846**	942	1,188	**2,130**	**27.15**
Quirino	**3,167**	1,161	1,259	**2,420**	**76.41**
City of Santiago	**2,294**	34	32	**66**	**2.88**
**Region 3**	**197,962**	**53,814**	**57,870**	**111,684**	**56.42**
Aurora	**3,996**	1,118	1,166	**2,284**	**57.15**
Bataan	**14,254**	1,774	2,000	**3,774**	**26.48**
Bulacan	**57,753**	12,393	13,014	**25,407**	**43.99**
Nueva Ecija	**36,808**	11,782	12,829	**24,611**	**66.86**
Pampanga	**38,760**	9,968	10,722	**20,690**	**53.38**
Tarlac	**24,260**	11,338	12,484	**23,822**	**98.19**
Zambales	**9,971**	4,367	4,421	**8,788**	**88.13**
City of Angeles	**8,453**	333	450	**783**	**9.26**
City of Olongapo	**3,707**	741	784	**1,525**	**41.14**

Table 2.D.1 - ORAL HEALTH CARE
Children 12-59 months old who are orally fit upon oral examination or after oral rehabilitation
Philippines, 2024

Area	Eligible Population x 20%	Orally fit		Total	%
		Male	Female		
**Region 4A**	**258,249**	**53,124**	**55,125**	**108,249**	**41.92**
Batangas	**45,905**	7,589	7,862	**15,451**	**33.66**
Cavite	**67,227**	5,502	6,008	**11,510**	**17.12**
Laguna	**51,892**	28,865	28,936	**57,801**	**111.39**
Quezon	**33,081**	4,277	4,424	**8,701**	**26.30**
Rizal	**55,230**	6,715	7,727	**14,442**	**26.15**
City of Lucena	**4,914**	176	168	**344**	**7.00**
**Region 4B**	**55,053**	**2,316**	**3,239**	**5,555**	**10.09**
Marinduque	**3,912**	172	226	**398**	**10.17**
Occidental Mindoro	**9,681**	559	608	**1,167**	**12.05**
Oriental Mindoro	**15,152**	525	602	**1,127**	**7.44**
Palawan	**17,118**	749	872	**1,621**	**9.47**
Romblon	**4,562**	248	857	**1,105**	**24.22**
City of Puerto Princesa	**4,628**	63	74	**137**	**2.96**
**Region 5**	**113,802**	**17,515**	**18,486**	**36,001**	**31.63**
Albay	**23,283**	4,357	4,420	**8,777**	**37.70**
Camarines Norte	**12,475**	1,711	1,933	**3,644**	**29.21**
Camarines Sur	**36,269**	2,448	2,560	**5,008**	**13.81**
Catanduanes	**4,961**	605	635	**1,240**	**24.99**
Masbate	**18,365**	1,190	1,388	**2,578**	**14.04**
Sorsogon	**15,081**	6,469	6,663	**13,132**	**87.08**
City of Naga	**3,368**	735	887	**1,622**	**48.16**
**Region 6**	**124,461**	**23,486**	**25,986**	**49,472**	**39.75**
Aklan	**9,304**	491	468	**959**	**10.31**
Antique	**10,071**	390	466	**856**	**8.50**
Capiz	**12,211**	1,959	2,366	**4,325**	**35.42**
Guimaras	**2,956**	217	245	**462**	**15.63**
Iloilo	**30,484**	13,059	14,231	**27,290**	**89.52**
Negros Occidental	**43,424**	4,429	4,738	**9,167**	**21.11**
City of Bacolod	**8,741**	604	725	**1,329**	**15.20**
City of Iloilo	**7,269**	2,337	2,747	**5,084**	**69.94**
**Region 7**	**136,175**	**13,618**	**14,722**	**28,340**	**20.81**
Bohol	**21,779**	1,850	1,830	**3,680**	**16.90**
Cebu	**59,146**	5,106	5,874	**10,980**	**18.56**
Negros Oriental	**24,107**	1,961	2,178	**4,139**	**17.17**
Siquijor	**1,274**	146	161	**307**	**24.09**
City of Cebu	**15,956**	3,033	3,023	**6,056**	**37.95**
City of Lapu-Lapu	**8,532**	1,016	1,136	**2,152**	**25.22**
City of Mandaue	**5,380**	506	520	**1,026**	**19.07**
**Region 8**	**78,536**	**4,674**	**4,536**	**9,210**	**11.73**
Biliran	**3,034**	274	228	**502**	**16.55**
Eastern Samar	**7,798**	417	350	**767**	**9.84**
Leyte	**26,797**	1,773	1,676	**3,449**	**12.87**
Northern Samar	**11,710**	1,122	1,201	**2,323**	**19.84**
Southern Leyte	**6,482**	108	95	**203**	**3.13**
Samar	**14,095**	333	260	**593**	**4.21**
Ormoc City	**4,142**	253	287	**540**	**13.04**
City of Tacloban	**4,478**	394	439	**833**	**18.60**

Table 2.D.1 - ORAL HEALTH CARE
Children 12-59 months old who are orally fit upon oral examination or after oral rehabilitation
Philippines, 2024

Area	Eligible Population x 20%	Orally fit		Total	%
		Male	Female		
**Region 9**	**70,940**	**2,744**	**2,989**	**5,733**	**8.08**
Zamboanga del Norte	**19,826**	361	417	**778**	**3.92**
Zamboanga del Sur	**19,564**	1,597	1,799	**3,396**	**17.36**
Zamboanga Sibugay	**12,203**	539	487	**1,026**	**8.41**
City of Isabela	**2,743**	196	210	**406**	**14.80**
City of Zamboanga	**16,604**	51	76	**127**	**0.76**
**Region 10**	**92,096**	**7,210**	**8,806**	**16,016**	**17.39**
Bukidnon	**29,299**	3,580	4,703	**8,283**	**28.27**
Camiguin	**1,464**	0	0	**0**	**0.00**
Lanao del Norte	**15,026**	244	303	**547**	**3.64**
Misamis Occidental	**9,919**	1,972	2,128	**4,100**	**41.33**
Misamis Oriental	**17,784**	1,097	1,317	**2,414**	**13.57**
City of Cagayan De Oro	**12,092**	234	247	**481**	**3.98**
City of Iligan	**6,512**	83	108	**191**	**2.93**
**Region 11**	**90,663**	**11,414**	**11,801**	**23,215**	**25.61**
Davao de Oro	**13,356**	1,035	1,126	**2,161**	**16.18**
Davao del Norte	**19,768**	1,635	1,826	**3,461**	**17.51**
Davao Oriental	**9,888**	3,496	3,611	**7,107**	**71.88**
Davao del Sur	**11,939**	1,170	1,351	**2,521**	**21.12**
Davao Occidental	**6,331**	2,885	2,626	**5,511**	**87.05**
City of Davao	**29,382**	1,193	1,261	**2,454**	**8.35**
**Region 12**	**79,362**	**6,847**	**7,613**	**14,460**	**18.22**
Cotabato	**22,847**	1,409	1,512	**2,921**	**12.78**
Sarangani	**10,966**	1,148	1,284	**2,432**	**22.18**
South Cotabato	**17,460**	2,441	2,730	**5,171**	**29.62**
Sultan Kudarat	**15,906**	1,073	1,203	**2,276**	**14.31**
City of General Santos	**12,182**	776	884	**1,660**	**13.63**
**CARAGA**	**47,657**	**4,816**	**5,460**	**10,276**	**21.56**
Agusan del Norte	**6,449**	808	863	**1,671**	**25.91**
Agusan del Sur	**13,580**	1,509	1,712	**3,221**	**23.72**
Surigao del Norte	**8,482**	963	1,122	**2,085**	**24.58**
Surigao del Sur	**11,220**	1,153	1,335	**2,488**	**22.17**
Dinagat Islands	**2,178**	38	40	**78**	**3.58**
City of Butuan	**5,747**	345	388	**733**	**12.75**
**BARMM**	**124,971**	**9,277**	**10,194**	**19,471**	**15.58**
Basilan	**10,638**	318	379	**697**	**6.55**
Lanao del Sur	**30,372**	2,114	2,251	**4,365**	**14.37**
Maguindanao del Norte	**16,419**	977	1,088	**2,065**	**12.58**
Maguindanao del Sur	**22,024**	956	1,009	**1,965**	**8.92**
Sulu	**21,976**	4,469	4,874	**9,343**	**42.51**
Tawi-Tawi	**11,382**	281	358	**639**	**5.61**
SGA	**5,643**	0	0	**0**	**0.00**
City of Cotabato	**6,518**	162	235	**397**	**6.09**

0 = No Data
* = No Report

Table 2.D.2 - ORAL HEALTH CARE
Children 5 years old and above with cases of Decayed, Missing, Filled Teeth (DMFT)
Philippines, 2024

Area	Total Examined	Cases of 5 y/o and above with DMFT		Total with DMFT	%
		Male	Female		
**Philippines**	5,110,964	1,567,474	2,295,367	3,862,841	75.58
**NCR**	1,714,734	587,123	828,385	1,415,508	82.55
City of Malabon	62,484	20,270	29,558	49,828	79.75
City of Navotas	53,016	22,282	29,374	51,656	97.43
City of Valenzuela	37,103	12,252	20,532	32,784	88.36
City of Caloocan	76,733	26,334	45,895	72,229	94.13
City of Marikina	21,994	8,086	11,929	20,015	91.00
City of Pasig	75,914	27,151	40,011	67,162	88.47
Pateros	7,678	3,245	4,266	7,511	97.82
City of Taguig	50,322	15,518	28,095	43,613	86.67
Quezon City	349,594	105,782	185,587	291,369	83.34
City of Makati	63,420	19,696	23,837	43,533	68.64
City of Mandaluyong	24,500	8,027	13,030	21,057	85.95
City of San Juan	6,255	2,069	3,429	5,498	87.90
City of Manila	580,552	221,335	266,258	487,593	83.99
City of Las Piñas	72,112	25,799	33,888	59,687	82.77
City of Muntinlupa	72,173	24,304	34,431	58,735	81.38
City of Parañaque	38,188	13,485	20,349	33,834	88.60
Pasay City	122,696	31,488	37,916	69,404	56.57
**CAR**	126,829	21,424	32,358	53,782	42.41
Abra	6,982	2,342	3,127	5,469	78.33
Apayao	1,168	353	380	733	62.76
Benguet	34,821	9,009	11,320	20,329	58.38
Ifugao	12,201	1,573	2,562	4,135	33.89
Kalinga	46,831	2,273	3,711	5,984	12.78
Mountain Province	9,091	1,732	3,208	4,940	54.34
City of Baguio	15,735	4,142	8,050	12,192	77.48
**Region 1**	441,061	50,895	67,996	118,891	26.96
Ilocos Norte	35,337	1,710	2,190	3,900	11.04
Ilocos Sur	100,834	2,714	3,229	5,943	5.89
La Union	41,736	1,623	2,056	3,679	8.81
Pangasinan	251,545	41,945	54,587	96,532	38.38
City of Dagupan	11,609	2,903	5,934	8,837	76.12
**Region 2**	200,512	68,397	103,718	172,115	85.84
Batanes	7,521	3,584	3,937	7,521	100.00
Cagayan	22,199	7,931	13,154	21,085	94.98
Isabela	61,459	23,993	37,466	61,459	100.00
Nueva Vizcaya	78,040	30,596	45,596	76,192	97.63
Quirino	10,853	349	410	759	6.99
City of Santiago	20,440	1,944	3,155	5,099	24.95
**Region 3**	348,888	132,162	192,774	324,936	93.13
Aurora	3,613	1,391	2,091	3,482	96.37
Bataan	23,299	7,775	12,891	20,666	88.70
Bulacan	82,529	28,489	47,293	75,782	91.82
Nueva Ecija	62,251	25,189	36,098	61,287	98.45
Pampanga	54,794	19,888	28,918	48,806	89.07
Tarlac	97,219	41,061	53,975	95,036	97.75
Zambales	16,567	6,604	9,260	15,864	95.76
City of Angeles	1,609	630	979	1,609	100.00
City of Olongapo	7,007	1,135	1,269	2,404	34.31
**Region 4A**	576,258	207,524	318,000	525,524	91.20
Batangas	181,002	69,533	84,066	153,599	84.86
Cavite	59,746	19,306	28,500	47,806	80.02
Laguna	255,499	92,044	163,455	255,499	100.00
Quezon	26,346	6,540	11,265	17,805	67.58
Rizal	52,373	19,580	29,986	49,566	94.64
City of Lucena	1,292	521	728	1,249	96.67

Table 2.D.2 - ORAL HEALTH CARE
Children 5 years old and above with cases of Decayed, Missing, Filled Teeth (DMFT)
Philippines, 2024

Area	Total Examined	Cases of 5 y/o and above with DMFT		Total with DMFT	%
		Male	Female		
**Region 4B**	68,493	17,930	31,002	48,932	71.44
Marinduque	5,654	840	1,327	2,167	38.33
Occidental Mindoro	9,581	1,923	3,327	5,250	54.80
Oriental Mindoro	20,269	7,218	10,750	17,968	88.65
Palawan	17,792	3,947	7,933	11,880	66.77
Romblon	7,140	1,398	2,295	3,693	51.72
City of Puerto Princesa	8,057	2,604	5,370	7,974	98.97
**Region 5**	156,987	30,674	55,202	85,876	54.70
Albay	43,499	1,638	1,681	3,319	7.63
Camarines Norte	17,984	6,375	9,253	15,628	86.90
Camarines Sur	30,461	7,323	17,904	25,227	82.82
Catanduanes	4,021	1,382	1,745	3,127	77.77
Masbate	11,015	4,060	5,533	9,593	87.09
Sorsogon	45,547	9,503	18,251	27,754	60.93
City of Naga	4,460	393	835	1,228	27.53
**Region 6**	445,499	137,887	197,763	335,650	75.34
Aklan	17,458	5,676	8,589	14,265	81.71
Antique	15,933	3,830	6,781	10,611	66.60
Capiz	69,532	20,483	27,488	47,971	68.99
Guimaras	3,499	277	731	1,008	28.81
Iloilo	174,743	63,230	84,787	148,017	84.71
Negros Occidental	97,995	23,152	36,760	59,912	61.14
City of Bacolod	20,046	5,101	8,604	13,705	68.37
City of Iloilo	46,293	16,138	24,023	40,161	86.75
**Region 7**	486,240	114,405	181,631	296,036	60.88
Bohol	23,287	4,973	8,625	13,598	58.39
Cebu	91,018	16,946	25,489	42,435	46.62
Negros Oriental	209,541	61,408	96,144	157,552	75.19
Siquijor	1,187	91	161	252	21.23
City of Cebu	70,568	23,844	44,634	68,478	97.04
City of Lapu-Lapu	54,421	4,771	4,058	8,829	16.22
City of Mandaue	36,218	2,372	2,520	4,892	13.51
**Region 8**	31,709	10,797	14,543	25,340	79.91
Biliran	4,812	1,197	1,110	2,307	47.94
Eastern Samar	1,580	738	842	1,580	100.00
Leyte	12,414	4,743	7,671	12,414	100.00
Northern Samar	5,349	2,160	2,251	4,411	82.46
Southern Leyte	650	263	274	537	82.62
Samar	1,318	188	244	432	32.78
Ormoc City	5,044	1,338	1,983	3,321	65.84
City of Tacloban	542	170	168	338	62.36
**Region 9**	57,583	17,875	26,534	44,409	77.12
Zamboanga del Norte	9,681	2,466	4,402	6,868	70.94
Zamboanga del Sur	13,557	5,623	6,536	12,159	89.69
Zamboanga Sibugay	27,524	9,583	15,338	24,921	90.54
City of Isabela	6,709	170	179	349	5.20
City of Zamboanga	112	33	79	112	100.00
**Region 10**	143,061	46,753	70,961	117,714	82.28
Bukidnon	27,081	8,681	12,474	21,155	78.12
Camiguin	2,400	743	1,265	2,008	83.67
Lanao del Norte	2,611	1,021	1,433	2,454	93.99
Misamis Occidental	51,052	20,135	28,468	48,603	95.20
Misamis Oriental	31,530	8,056	11,997	20,053	63.60
City of Cagayan De Oro	19,713	5,499	11,710	17,209	87.30
City of Iligan	8,674	2,618	3,614	6,232	71.85

Table 2.D.2 - ORAL HEALTH CARE
Children 5 years old and above with cases of Decayed, Missing, Filled Teeth (DMFT)
Philippines, 2024

Area	Total Examined	Cases of 5 y/o and above with DMFT		Total with DMFT	%
		Male	Female		
**Region 11**	**47,272**	**18,795**	**21,250**	**40,045**	**84.71**
Davao de Oro	8,051	2,744	3,307	6,051	75.16
Davao del Norte	10,220	3,634	4,401	8,035	78.62
Davao Oriental	20,001	9,093	9,603	18,696	93.48
Davao del Sur	3,973	1,387	1,608	2,995	75.38
Davao Occidental	1,668	679	839	1,518	91.01
City of Davao	3,359	1,258	1,492	2,750	81.87
**Region 12**	**95,693**	**36,870**	**56,210**	**93,080**	**97.27**
Cotabato	24,568	8,983	13,651	22,634	92.13
Sarangani	5,619	2,164	2,776	4,940	87.92
South Cotabato	30,040	12,706	17,334	30,040	100.00
Sultan Kudarat	26,162	10,937	15,225	26,162	100.00
City of General Santos	9,304	2,080	7,224	9,304	100.00
**CARAGA**	**71,536**	**26,720**	**43,543**	**70,263**	**98.22**
Agusan del Norte	11,011	3,969	6,381	10,350	94.00
Agusan del Sur	14,271	5,918	8,353	14,271	100.00
Surigao del Norte	10,254	4,142	6,112	10,254	100.00
Surigao del Sur	32,561	11,444	21,117	32,561	100.00
Dinagat Islands	2,038	541	885	1,426	69.97
City of Butuan	1,401	706	695	1,401	100.00
**BARMM**	**98,609**	**41,243**	**53,497**	**94,740**	**96.08**
Basilan	3,339	1,306	2,033	3,339	100.00
Lanao del Sur	27,967	12,107	15,860	27,967	100.00
Maguindanao del Norte	6,234	2,803	3,410	6,213	99.66
Maguindanao del Sur	4,541	2,034	2,364	4,398	96.85
Sulu	10,577	5,238	5,339	10,577	100.00
Tawi-Tawi	23,276	10,223	13,053	23,276	100.00
SGA	0	0	0	0	0.00
City of Cotabato	22,675	7,532	11,438	18,970	83.66

0 = No Data
* = No Report

Table 2.D.3 - ORAL HEALTH CARE

Infants 0-11 months old who received Basic Oral Health Care (BOHC)
Philippines, 2024

Area	Infants 0-11 months old who received BOHC				
	Eligible Population x 30%	Male	Female	Total	%
**Philippines**	**660,260**	**214,490**	**218,851**	**433,341**	**65.63**
**NCR**	**78,974**	**64,852**	**64,203**	**129,055**	**163.41**
City of Malabon	**2,234**	4,215	3,928	**8,143**	**364.49**
City of Navotas	**1,579**	629	752	**1,381**	**87.47**
City of Valenzuela	**4,080**	2,308	2,147	**4,455**	**109.18**
City of Caloocan	**10,050**	5,588	5,627	**11,215**	**111.60**
City of Marikina	**2,520**	3,159	3,175	**6,334**	**251.38**
City of Pasig	**5,357**	4,893	5,053	**9,946**	**185.67**
Pateros	**336**	292	326	**618**	**183.93**
City of Taguig	**5,768**	4,709	4,538	**9,247**	**160.32**
Quezon City	**17,882**	12,255	12,299	**24,554**	**137.31**
City of Makati	**3,297**	983	920	**1,903**	**57.71**
City of Mandaluyong	**2,397**	2,386	2,178	**4,564**	**190.43**
City of San Juan	**652**	398	356	**754**	**115.66**
City of Manila	**10,280**	14,613	14,346	**28,959**	**281.69**
City of Las Piñas	**3,404**	2,903	2,884	**5,787**	**170.02**
City of Muntinlupa	**2,985**	2,314	2,297	**4,611**	**154.49**
City of Parañaque	**4,016**	1,434	1,450	**2,884**	**71.82**
Pasay City	**2,139**	1,773	1,927	**3,700**	**173.00**
**CAR**	**9,447**	**4,793**	**4,813**	**9,606**	**101.68**
Abra	**1,092**	323	353	**676**	**61.92**
Apayao	**667**	116	98	**214**	**32.10**
Benguet	**2,670**	2,415	2,072	**4,487**	**168.05**
Ifugao	**1,244**	386	439	**825**	**66.34**
Kalinga	**1,375**	896	1,238	**2,134**	**155.25**
Mountain Province	**785**	424	357	**781**	**99.55**
City of Baguio	**1,616**	233	256	**489**	**30.26**
**Region 1**	**28,807**	**9,454**	**10,287**	**19,741**	**68.53**
Ilocos Norte	**2,643**	644	670	**1,314**	**49.72**
Ilocos Sur	**2,950**	2,016	1,982	**3,998**	**135.52**
La Union	**4,031**	1,539	1,587	**3,126**	**77.55**
Pangasinan	**18,177**	5,033	5,842	**10,875**	**59.83**
City of Dagupan	**1,006**	222	206	**428**	**42.54**
**Region 2**	**19,210**	**10,930**	**11,080**	**22,010**	**114.57**
Batanes	**72**	36	36	**72**	**99.59**
Cagayan	**6,217**	1,082	1,030	**2,112**	**33.97**
Isabela	**8,209**	5,917	6,067	**11,984**	**145.99**
Nueva Vizcaya	**2,664**	1,522	1,547	**3,069**	**115.20**
Quirino	**1,160**	931	1,006	**1,937**	**167.05**
City of Santiago	**889**	1,442	1,394	**2,836**	**319.05**
**Region 3**	**70,594**	**26,116**	**26,103**	**52,219**	**73.97**
Aurora	**1,373**	314	320	**634**	**46.16**
Bataan	**5,267**	1,746	1,665	**3,411**	**64.76**
Bulacan	**20,435**	8,310	8,630	**16,940**	**82.90**
Nueva Ecija	**13,088**	4,445	4,395	**8,840**	**67.54**
Pampanga	**13,844**	2,992	2,809	**5,801**	**41.90**
Tarlac	**8,897**	4,665	4,815	**9,480**	**106.56**
Zambales	**3,539**	2,293	2,191	**4,484**	**126.71**
City of Angeles	**2,770**	729	619	**1,348**	**48.67**
City of Olongapo	**1,383**	622	659	**1,281**	**92.64**
**Region 4A**	**93,045**	**27,065**	**28,099**	**55,164**	**59.29**
Batangas	**16,045**	3,558	3,768	**7,326**	**45.66**
Cavite	**23,577**	4,166	4,665	**8,831**	**37.46**
Laguna	**18,590**	3,618	3,842	**7,460**	**40.13**
Quezon	**12,286**	2,775	2,833	**5,608**	**45.64**
Rizal	**20,671**	12,917	12,963	**25,880**	**125.20**
City of Lucena	**1,876**	31	28	**59**	**3.15**
**Region 4B**	**19,952**	**1,557**	**1,477**	**3,034**	**15.21**
Marinduque	**1,237**	68	54	**122**	**9.86**
Occidental Mindoro	**3,524**	146	170	**316**	**8.97**
Oriental Mindoro	**5,702**	466	445	**911**	**15.98**
Palawan	**6,151**	464	438	**902**	**14.66**
Romblon	**1,646**	413	369	**782**	**47.50**
City of Puerto Princesa	**1,691**	0	1	**1**	**0.06**
**Region 5**	**40,983**	**4,729**	**4,916**	**9,645**	**23.53**
Albay	**8,300**	549	611	**1,160**	**13.98**
Camarines Norte	**4,468**	486	663	**1,149**	**25.72**
Camarines Sur	**13,157**	1,309	1,282	**2,591**	**19.69**
Catanduanes	**1,619**	267	282	**549**	**33.92**
Masbate	**6,749**	387	428	**815**	**12.08**
Sorsogon	**5,420**	1,621	1,553	**3,174**	**58.57**
City of Naga	**1,273**	110	97	**207**	**16.27**
**Region 6**	**44,380**	**14,109**	**14,919**	**29,028**	**65.41**
Aklan	**3,384**	794	722	**1,516**	**44.80**
Antique	**3,570**	239	283	**522**	**14.62**
Capiz	**4,162**	1,606	1,757	**3,363**	**80.81**
Guimaras	**1,008**	186	126	**312**	**30.94**
Iloilo	**10,794**	5,701	6,484	**12,185**	**112.88**
Negros Occidental	**15,734**	2,174	2,090	**4,264**	**27.10**
City of Bacolod	**3,108**	1,233	1,122	**2,355**	**75.76**
City of Iloilo	**2,620**	2,176	2,335	**4,511**	**172.16**

Table 2.D.3 - ORAL HEALTH CARE
Infants 0-11 months old who received Basic Oral Health Care (BOHC)
Philippines, 2024

Area	Infants 0-11 months old who received BOHC				
	Eligible Population x 30%	Male	Female	Total	%
**Region 7**	**50,105**	**16,042**	**16,401**	**32,443**	**64.75**
Bohol	**7,863**	807	891	**1,698**	**21.59**
Cebu	**21,863**	4,221	4,354	**8,575**	**39.22**
Negros Oriental	**8,694**	2,065	2,322	**4,387**	**50.46**
Siquijor	**501**	18	24	**42**	**8.38**
City of Cebu	**5,840**	4,880	4,764	**9,644**	**165.14**
City of Lapu-Lapu	**3,250**	3,011	2,957	**5,968**	**183.64**
City of Mandaue	**2,094**	1,040	1,089	**2,129**	**101.66**
**Region 8**	**28,114**	**3,101**	**3,041**	**6,142**	**21.85**
Biliran	**1,013**	11	7	**18**	**1.78**
Eastern Samar	**2,878**	150	166	**316**	**10.98**
Leyte	**9,451**	1,072	979	**2,051**	**21.70**
Northern Samar	**4,216**	217	214	**431**	**10.22**
Southern Leyte	**2,210**	0	0	**0**	**0.00**
Samar	**5,084**	420	457	**877**	**17.25**
Ormoc City	**1,603**	912	902	**1,814**	**113.15**
City of Tacloban	**1,658**	319	316	**635**	**38.30**
**Region 9**	**25,604**	**1,269**	**1,218**	**2,487**	**9.71**
Zamboanga del Norte	**7,493**	172	168	**340**	**4.54**
Zamboanga del Sur	**7,172**	569	582	**1,151**	**16.05**
Zamboanga Sibugay	**4,171**	271	230	**501**	**12.01**
City of Isabela	**963**	48	45	**93**	**9.66**
City of Zamboanga	**5,806**	209	193	**402**	**6.92**
**Region 10**	**33,087**	**4,468**	**5,297**	**9,765**	**29.51**
Bukidnon	**10,174**	1,756	2,019	**3,775**	**37.10**
Camiguin	**518**	0	0	**0**	**0.00**
Lanao del Norte	**5,364**	86	117	**203**	**3.78**
Misamis Occidental	**3,703**	1,613	1,958	**3,571**	**96.45**
Misamis Oriental	**6,655**	530	596	**1,126**	**16.92**
City of Cagayan De Oro	**4,351**	362	501	**863**	**19.83**
City of Iligan	**2,322**	121	106	**227**	**9.78**
**Region 11**	**32,380**	**1,991**	**2,137**	**4,128**	**12.75**
Davao de Oro	**4,630**	131	139	**270**	**5.83**
Davao del Norte	**7,106**	117	114	**231**	**3.25**
Davao Oriental	**3,608**	682	792	**1,474**	**40.86**
Davao del Sur	**3,953**	146	145	**291**	**7.36**
Davao Occidental	**1,982**	470	461	**931**	**46.98**
City of Davao	**11,102**	445	486	**931**	**8.39**
**Region 12**	**28,589**	**11,883**	**11,972**	**23,855**	**83.44**
Cotabato	**7,709**	2,788	2,817	**5,605**	**72.71**
Sarangani	**4,050**	878	887	**1,765**	**43.58**
South Cotabato	**6,553**	4,259	4,052	**8,311**	**126.84**
Sultan Kudarat	**5,884**	2,067	2,295	**4,362**	**74.13**
City of General Santos	**4,393**	1,891	1,921	**3,812**	**86.77**
**CARAGA**	**16,611**	**4,197**	**4,417**	**8,614**	**51.86**
Agusan del Norte	**2,199**	917	1,062	**1,979**	**89.98**
Agusan del Sur	**4,854**	792	734	**1,526**	**31.44**
Surigao del Norte	**3,050**	1,407	1,446	**2,853**	**93.53**
Surigao del Sur	**3,850**	583	644	**1,227**	**31.87**
Dinagat Islands	**635**	32	39	**71**	**11.18**
City of Butuan	**2,022**	466	492	**958**	**47.37**
**BARMM**	**40,376**	**7,934**	**8,471**	**16,405**	**40.63**
Basilan	**3,496**	921	890	**1,811**	**51.80**
Lanao del Sur	**10,002**	2,731	3,120	**5,851**	**58.50**
Maguindanao del Norte	**5,677**	575	636	**1,211**	**21.33**
Maguindanao del Sur	**6,964**	919	1,027	**1,946**	**27.94**
Sulu	**6,414**	1,761	1,738	**3,499**	**54.55**
Tawi-Tawi	**3,781**	362	402	**764**	**20.21**
SGA	**1,892**	0	0	**0**	**0.00**
City of Cotabato	**2,150**	665	658	**1,323**	**61.52**

0 = No Data
* = No Report

Table 2.D.4 - ORAL HEALTH CARE
Children 1-4 years old who received Basic Oral Health Care (BOHC)
Philippines, 2024

Area	Children 1-4 years old who received BOHC				
	Eligible Population x 30%	Male	Female	Total	%
**Philippines**	**2,761,716**	**695,329**	**737,077**	**1,432,406**	**51.87**
**NCR**	**317,101**	**151,596**	**161,839**	**313,435**	**98.84**
City of Malabon	**8,921**	3,937	4,323	**8,260**	**92.59**
City of Navotas	**6,327**	2,680	2,954	**5,634**	**89.05**
City of Valenzuela	**16,619**	1,880	1,941	**3,821**	**22.99**
City of Caloocan	**42,037**	17,744	19,517	**37,261**	**88.64**
City of Marikina	**10,464**	3,251	3,324	**6,575**	**62.83**
City of Pasig	**19,911**	7,753	8,911	**16,664**	**83.69**
Pateros	**1,454**	801	836	**1,637**	**112.56**
City of Taguig	**22,769**	9,077	9,678	**18,755**	**82.37**
Quezon City	**72,184**	40,503	41,886	**82,389**	**114.14**
City of Makati	**12,812**	2,259	2,393	**4,652**	**36.31**
City of Mandaluyong	**9,544**	3,536	3,704	**7,240**	**75.86**
City of San Juan	**2,590**	679	721	**1,400**	**54.06**
City of Manila	**41,890**	36,899	39,024	**75,923**	**181.24**
City of Las Piñas	**13,679**	5,725	5,765	**11,490**	**84.00**
City of Muntinlupa	**11,824**	5,457	6,189	**11,646**	**98.49**
City of Parañaque	**16,123**	4,569	5,638	**10,207**	**63.31**
Pasay City	**7,954**	4,846	5,035	**9,881**	**124.23**
**CAR**	**39,813**	**13,901**	**15,302**	**29,203**	**73.35**
Abra	**4,863**	354	397	**751**	**15.44**
Apayao	**2,775**	1,019	1,078	**2,097**	**75.57**
Benguet	**11,225**	5,122	5,500	**10,622**	**94.63**
Ifugao	**5,214**	1,539	1,723	**3,262**	**62.57**
Kalinga	**5,631**	3,066	3,757	**6,823**	**121.17**
Mountain Province	**3,421**	1,046	1,085	**2,131**	**62.29**
City of Baguio	**6,684**	1,755	1,762	**3,517**	**52.62**
**Region 1**	**117,584**	**41,907**	**44,210**	**86,117**	**73.24**
Ilocos Norte	**11,352**	4,985	5,278	**10,263**	**90.41**
Ilocos Sur	**12,988**	6,968	7,346	**14,314**	**110.21**
La Union	**16,545**	7,909	7,978	**15,887**	**96.02**
Pangasinan	**72,635**	21,440	22,973	**44,413**	**61.15**
City of Dagupan	**4,064**	605	635	**1,240**	**30.51**
**Region 2**	**82,326**	**25,679**	**27,952**	**53,631**	**65.14**
Batanes	**340**	160	179	**339**	**99.65**
Cagayan	**27,155**	2,764	2,940	**5,704**	**21.01**
Isabela	**34,871**	13,223	14,697	**27,920**	**80.07**
Nueva Vizcaya	**11,768**	4,430	4,955	**9,385**	**79.75**
Quirino	**4,751**	3,039	3,185	**6,224**	**131.01**
City of Santiago	**3,441**	2,063	1,996	**4,059**	**117.96**
**Region 3**	**296,942**	**97,646**	**101,645**	**199,291**	**67.11**
Aurora	**5,994**	1,073	1,137	**2,210**	**36.87**
Bataan	**21,380**	4,428	4,671	**9,099**	**42.56**
Bulacan	**86,630**	21,260	22,741	**44,001**	**50.79**
Nueva Ecija	**55,212**	27,108	27,374	**54,482**	**98.68**
Pampanga	**58,140**	17,432	17,801	**35,233**	**60.60**
Tarlac	**36,390**	16,430	17,425	**33,855**	**93.03**
Zambales	**14,957**	7,552	8,157	**15,709**	**105.03**
City of Angeles	**12,679**	977	1,073	**2,050**	**16.17**
City of Olongapo	**5,561**	1,386	1,266	**2,652**	**47.69**
**Region 4A**	**387,374**	**88,698**	**94,275**	**182,973**	**47.23**
Batangas	**68,858**	24,164	24,281	**48,445**	**70.36**
Cavite	**100,840**	16,134	18,085	**34,219**	**33.93**
Laguna	**77,838**	24,143	25,555	**49,698**	**63.85**
Quezon	**49,622**	10,173	10,708	**20,881**	**42.08**
Rizal	**82,845**	13,258	14,839	**28,097**	**33.92**
City of Lucena	**7,371**	826	807	**1,633**	**22.16**

Table 2.D.4 - ORAL HEALTH CARE
Children 1-4 years old who received Basic Oral Health Care (BOHC)
Philippines, 2024

Area	Eligible Population x 30%	Children 1-4 years old who received BOHC			
		Male	Female	Total	%
**Region 4B**	**82,580**	**8,850**	**9,137**	**17,987**	**21.78**
Marinduque	**5,868**	669	649	**1,318**	**22.46**
Occidental Mindoro	**14,522**	1,724	1,875	**3,599**	**24.78**
Oriental Mindoro	**22,729**	2,045	2,245	**4,290**	**18.87**
Palawan	**25,676**	2,776	2,741	**5,517**	**21.49**
Romblon	**6,843**	1,392	1,374	**2,766**	**40.42**
City of Puerto Princesa	**6,942**	244	253	**497**	**7.16**
**Region 5**	**170,704**	**42,750**	**43,464**	**86,214**	**50.51**
Albay	**34,925**	8,845	9,005	**17,850**	**51.11**
Camarines Norte	**18,712**	4,440	4,483	**8,923**	**47.69**
Camarines Sur	**54,404**	9,400	9,875	**19,275**	**35.43**
Catanduanes	**7,442**	2,033	2,097	**4,130**	**55.50**
Masbate	**27,548**	2,224	2,541	**4,765**	**17.30**
Sorsogon	**22,622**	14,385	13,839	**28,224**	**124.76**
City of Naga	**5,052**	1,423	1,624	**3,047**	**60.31**
**Region 6**	**186,691**	**47,972**	**50,615**	**98,587**	**52.81**
Aklan	**13,956**	1,479	1,531	**3,010**	**21.57**
Antique	**15,107**	925	991	**1,916**	**12.68**
Capiz	**18,317**	5,409	5,924	**11,333**	**61.87**
Guimaras	**4,434**	646	683	**1,329**	**29.97**
Iloilo	**45,726**	21,733	23,290	**45,023**	**98.46**
Negros Occidental	**65,135**	11,014	10,692	**21,706**	**33.32**
City of Bacolod	**13,112**	1,628	1,772	**3,400**	**25.93**
City of Iloilo	**10,904**	5,138	5,732	**10,870**	**99.69**
**Region 7**	**204,263**	**50,838**	**53,488**	**104,326**	**51.07**
Bohol	**32,669**	4,688	4,780	**9,468**	**28.98**
Cebu	**88,719**	19,306	20,199	**39,505**	**44.53**
Negros Oriental	**36,161**	6,832	7,874	**14,706**	**40.67**
Siquijor	**1,911**	2,380	2,144	**4,524**	**236.70**
City of Cebu	**23,934**	10,547	11,488	**22,035**	**92.06**
City of Lapu-Lapu	**12,798**	3,929	3,744	**7,673**	**59.95**
City of Mandaue	**8,069**	3,156	3,259	**6,415**	**79.50**
**Region 8**	**117,805**	**11,794**	**12,209**	**24,003**	**20.38**
Biliran	**4,550**	336	330	**666**	**14.64**
Eastern Samar	**11,696**	769	857	**1,626**	**13.90**
Leyte	**40,195**	3,834	3,854	**7,688**	**19.13**
Northern Samar	**17,565**	3,479	3,801	**7,280**	**41.45**
Southern Leyte	**9,724**	523	525	**1,048**	**10.78**
Samar	**21,143**	946	821	**1,767**	**8.36**
Ormoc City	**6,214**	1,498	1,550	**3,048**	**49.05**
City of Tacloban	**6,718**	409	471	**880**	**13.10**
**Region 9**	**106,409**	**6,005**	**6,435**	**12,440**	**11.69**
Zamboanga del Norte	**29,739**	1,155	1,281	**2,436**	**8.19**
Zamboanga del Sur	**29,346**	3,047	3,407	**6,454**	**21.99**
Zamboanga Sibugay	**18,304**	1,185	1,144	**2,329**	**12.72**
City of Isabela	**4,114**	357	343	**700**	**17.01**
City of Zamboanga	**24,906**	261	260	**521**	**2.09**
**Region 10**	**138,144**	**18,004**	**20,395**	**38,399**	**27.80**
Bukidnon	**43,949**	7,182	8,460	**15,642**	**35.59**
Camiguin	**2,197**	12	7	**19**	**0.86**
Lanao del Norte	**22,538**	745	847	**1,592**	**7.06**
Misamis Occidental	**14,879**	5,879	6,141	**12,020**	**80.78**
Misamis Oriental	**26,676**	2,928	3,526	**6,454**	**24.19**
City of Cagayan De Oro	**18,138**	801	923	**1,724**	**9.50**
City of Iligan	**9,768**	457	491	**948**	**9.71**

Table 2.D.4 - ORAL HEALTH CARE
Children 1-4 years old who received Basic Oral Health Care (BOHC)
Philippines, 2024

Area	Children 1-4 years old who received BOHC				
	Eligible Population x 30%	Male	Female	Total	%
**Region 11**	**135,995**	**26,093**	**27,156**	**53,249**	**39.16**
Davao de Oro	**20,034**	3,595	3,568	**7,163**	**35.75**
Davao del Norte	**29,652**	6,726	6,769	**13,495**	**45.51**
Davao Oriental	**14,831**	6,685	7,000	**13,685**	**92.27**
Davao del Sur	**17,908**	3,429	3,705	**7,134**	**39.84**
Davao Occidental	**9,497**	1,762	1,889	**3,651**	**38.45**
City of Davao	**44,073**	3,896	4,225	**8,121**	**18.43**
**Region 12**	**119,043**	**26,059**	**27,893**	**53,952**	**45.32**
Cotabato	**34,271**	3,543	4,021	**7,564**	**22.07**
Sarangani	**16,449**	5,915	6,200	**12,115**	**73.65**
South Cotabato	**26,191**	9,803	10,467	**20,270**	**77.39**
Sultan Kudarat	**23,859**	2,924	3,208	**6,132**	**25.70**
City of General Santos	**18,273**	3,874	3,997	**7,871**	**43.07**
**CARAGA**	**71,486**	**15,967**	**17,223**	**33,190**	**46.43**
Agusan del Norte	**9,674**	2,232	2,439	**4,671**	**48.28**
Agusan del Sur	**20,371**	4,256	4,632	**8,888**	**43.63**
Surigao del Norte	**12,723**	3,207	3,486	**6,693**	**52.61**
Surigao del Sur	**16,831**	4,628	4,984	**9,612**	**57.11**
Dinagat Islands	**3,266**	138	157	**295**	**9.03**
City of Butuan	**8,621**	1,506	1,525	**3,031**	**35.16**
**BARMM**	**187,457**	**21,570**	**23,839**	**45,409**	**24.22**
Basilan	**15,956**	1,468	1,504	**2,972**	**18.63**
Lanao del Sur	**45,558**	5,999	6,783	**12,782**	**28.06**
Maguindanao del Norte	**24,629**	1,433	2,073	**3,506**	**14.24**
Maguindanao del Sur	**33,036**	1,808	1,965	**3,773**	**11.42**
Sulu	**32,964**	4,786	5,073	**9,859**	**29.91**
Tawi-Tawi	**17,074**	1,378	1,655	**3,033**	**17.76**
SGA	**8,465**	0	0	**0**	**0.00**
City of Cotabato	**9,777**	4,698	4,786	**9,484**	**97.00**

0 = No Data
* = No Report

Table 2.D.5 - ORAL HEALTH CARE
Children 5-9 years old who received Basic Oral Health Care (BOHC)
Philippines, 2024

Area	Eligible Population x 30%	Children 5-9 years old who received BOHC			
		Male	Female	Total	%
**Philippines**	**3,484,339**	**570,185**	**597,201**	**1,163,449**	**33.39**
**NCR**	**383,107**	**158,859**	**160,940**	**319,799**	**83.48**
City of Malabon	**11,325**	7,290	7,624	**14,914**	**131.69**
City of Navotas	**8,021**	3,508	3,837	**7,345**	**91.57**
City of Valenzuela	**20,214**	2,679	2,790	**5,469**	**27.06**
City of Caloocan	**51,207**	6,389	6,928	**13,317**	**26.01**
City of Marikina	**13,031**	4,262	4,312	**8,574**	**65.79**
City of Pasig	**24,294**	9,253	9,409	**18,662**	**76.82**
Pateros	**1,817**	645	617	**1,262**	**69.45**
City of Taguig	**26,748**	3,109	3,272	**6,381**	**23.86**
Quezon City	**85,538**	11,423	11,827	**23,250**	**27.18**
City of Makati	**14,637**	7,437	7,304	**14,741**	**100.71**
City of Mandaluyong	**11,333**	2,009	2,467	**4,476**	**39.50**
City of San Juan	**3,050**	360	343	**703**	**23.05**
City of Manila	**51,260**	80,509	79,448	**159,957**	**312.05**
City of Las Piñas	**17,024**	8,060	8,037	**16,097**	**94.56**
City of Muntinlupa	**14,254**	5,174	5,217	**10,391**	**72.90**
City of Parañaque	**18,746**	3,457	3,638	**7,095**	**37.85**
Pasay City	**10,610**	3,295	3,870	**7,165**	**67.53**
**CAR**	**53,173**	**17,160**	**18,530**	**35,690**	**67.12**
Abra	**7,253**	362	444	**806**	**11.11**
Apayao	**3,731**	1,133	1,307	**2,440**	**65.41**
Benguet	**13,895**	4,774	4,997	**9,771**	**70.32**
Ifugao	**6,929**	2,418	2,538	**4,956**	**71.52**
Kalinga	**7,455**	3,686	4,231	**7,917**	**106.20**
Mountain Province	**4,889**	698	837	**1,535**	**31.39**
City of Baguio	**9,022**	4,089	4,176	**8,265**	**91.61**
**Region 1**	**152,416**	**37,683**	**39,421**	**77,104**	**50.59**
Ilocos Norte	**14,799**	3,206	3,455	**6,661**	**45.01**
Ilocos Sur	**18,036**	9,611	9,697	**19,308**	**107.05**
La Union	**22,041**	4,322	4,432	**8,754**	**39.72**
Pangasinan	**92,321**	19,923	21,105	**41,028**	**44.44**
City of Dagupan	**5,220**	621	732	**1,353**	**25.92**
**Region 2**	**110,941**	**24,675**	**27,691**	**52,366**	**47.20**
Batanes	**467**	168	196	**364**	**77.98**
Cagayan	**37,672**	2,962	3,397	**6,359**	**16.88**
Isabela	**46,180**	7,726	8,870	**16,596**	**35.94**
Nueva Vizcaya	**15,725**	9,340	10,621	**19,961**	**126.94**
Quirino	**6,471**	3,193	3,240	**6,433**	**99.42**
City of Santiago	**4,427**	1,286	1,367	**2,653**	**59.93**
**Region 3**	**374,750**	**51,507**	**54,683**	**106,190**	**28.34**
Aurora	**7,814**	1,209	1,327	**2,536**	**32.45**
Bataan	**26,569**	1,584	1,602	**3,186**	**11.99**
Bulacan	**110,829**	13,941	16,530	**30,471**	**27.49**
Nueva Ecija	**69,835**	10,219	10,491	**20,710**	**29.66**
Pampanga	**71,728**	7,297	7,797	**15,094**	**21.04**
Tarlac	**46,748**	8,935	9,143	**18,078**	**38.67**
Zambales	**20,190**	4,492	4,373	**8,865**	**43.91**
City of Angeles	**13,986**	561	550	**1,111**	**7.94**
City of Olongapo	**7,052**	3,269	2,870	**6,139**	**87.05**
**Region 4A**	**486,211**	**82,203**	**84,682**	**166,885**	**34.32**
Batangas	**84,757**	20,128	19,637	**39,765**	**46.92**
Cavite	**125,417**	8,613	9,665	**18,278**	**14.57**
Laguna	**97,851**	38,866	40,145	**79,011**	**80.75**
Quezon	**64,862**	7,725	7,694	**15,419**	**23.77**
Rizal	**104,288**	6,533	7,231	**13,764**	**13.20**
City of Lucena	**9,037**	338	310	**648**	**7.17**
**Region 4B**	**110,008**	**6,625**	**7,008**	**13,633**	**12.39**
Marinduque	**7,492**	586	591	**1,177**	**15.71**
Occidental Mindoro	**18,995**	711	803	**1,514**	**7.97**
Oriental Mindoro	**30,146**	2,299	2,228	**4,527**	**15.02**
Palawan	**34,620**	1,776	2,085	**3,861**	**11.15**
Romblon	**9,841**	1,005	1,039	**2,044**	**20.77**
City of Puerto Princesa	**8,915**	248	262	**510**	**5.72**

Table 2.D.5 - ORAL HEALTH CARE

Children 5-9 years old who received Basic Oral Health Care (BOHC)
Philippines, 2024

Area	Eligible Population x 30%	Children 5-9 years old who received BOHC			
		Male	Female	Total	%
**Region 5**	**217,566**	**21,671**	**21,678**	**43,349**	**19.92**
Albay	**46,096**	6,727	6,498	**13,225**	**28.69**
Camarines Norte	**23,148**	3,290	3,031	**6,321**	**27.31**
Camarines Sur	**67,407**	3,775	3,941	**7,716**	**11.45**
Catanduanes	**9,464**	665	639	**1,304**	**13.78**
Masbate	**35,686**	1,662	1,785	**3,447**	**9.66**
Sorsogon	**29,430**	5,208	5,387	**10,595**	**36.00**
City of Naga	**6,335**	344	397	**741**	**11.70**
**Region 6**	**239,762**	**37,670**	**39,892**	**77,562**	**32.35**
Aklan	**18,046**	1,451	1,711	**3,162**	**17.52**
Antique	**18,789**	1,188	1,451	**2,639**	**14.05**
Capiz	**22,805**	5,586	6,083	**11,669**	**51.17**
Guimaras	**5,581**	208	231	**439**	**7.87**
Iloilo	**60,092**	16,951	17,776	**34,727**	**57.79**
Negros Occidental	**85,100**	5,923	6,158	**12,081**	**14.20**
City of Bacolod	**16,485**	1,139	1,196	**2,335**	**14.16**
City of Iloilo	**12,863**	5,224	5,286	**10,510**	**81.70**
**Region 7**	**258,312**	**31,887**	**33,547**	**65,434**	**25.33**
Bohol	**42,280**	2,093	2,236	**4,329**	**10.24**
Cebu	**112,505**	9,679	10,924	**20,603**	**18.31**
Negros Oriental	**46,013**	5,586	6,641	**12,227**	**26.57**
Siquijor	**2,714**	282	370	**652**	**24.03**
City of Cebu	**28,788**	5,624	5,007	**10,631**	**36.93**
City of Lapu-Lapu	**16,057**	4,853	4,623	**9,476**	**59.02**
City of Mandaue	**9,955**	3,770	3,746	**7,516**	**75.50**
**Region 8**	**153,199**	**11,688**	**12,221**	**23,909**	**15.61**
Biliran	**6,284**	543	674	**1,217**	**19.37**
Eastern Samar	**15,531**	610	690	**1,300**	**8.37**
Leyte	**51,654**	4,368	4,301	**8,669**	**16.78**
Northern Samar	**23,376**	3,748	4,095	**7,843**	**33.55**
Southern Leyte	**12,410**	300	392	**692**	**5.58**
Samar	**27,874**	1,003	954	**1,957**	**7.02**
Ormoc City	**7,731**	857	884	**1,741**	**22.52**
City of Tacloban	**8,339**	259	231	**490**	**5.88**
**Region 9**	**137,164**	**9,514**	**9,651**	**19,165**	**13.97**
Zamboanga del Norte	**37,177**	1,063	1,150	**2,213**	**5.95**
Zamboanga del Sur	**37,313**	2,741	3,200	**5,941**	**15.92**
Zamboanga Sibugay	**25,008**	3,974	3,647	**7,621**	**30.47**
City of Isabela	**4,760**	292	281	**573**	**12.04**
City of Zamboanga	**32,906**	1,444	1,373	**2,817**	**8.56**
**Region 10**	**177,782**	**16,323**	**17,976**	**34,299**	**19.29**
Bukidnon	**58,813**	6,799	7,258	**14,057**	**23.90**
Camiguin	**3,065**	96	106	**202**	**6.59**
Lanao del Norte	**28,736**	684	741	**1,425**	**4.96**
Misamis Occidental	**19,155**	5,388	6,204	**11,592**	**60.52**
Misamis Oriental	**33,768**	1,558	1,732	**3,290**	**9.74**
City of Cagayan De Oro	**22,320**	1,180	1,239	**2,419**	**10.84**
City of Iligan	**11,924**	618	696	**1,314**	**11.02**
**Region 11**	**171,326**	**14,136**	**15,710**	**29,846**	**17.42**
Davao de Oro	**26,355**	2,234	2,787	**5,021**	**19.05**
Davao del Norte	**36,788**	3,062	3,313	**6,375**	**17.33**
Davao Oriental	**20,033**	5,973	6,354	**12,327**	**61.54**
Davao del Sur	**21,455**	1,115	1,246	**2,361**	**11.00**
Davao Occidental	**11,862**	338	403	**741**	**6.25**
City of Davao	**54,834**	1,414	1,607	**3,021**	**5.51**
**Region 12**	**154,221**	**13,924**	**16,362**	**30,286**	**19.64**
Cotabato	**45,567**	2,610	3,526	**6,136**	**13.47**
Sarangani	**21,068**	1,575	1,751	**3,326**	**15.79**
South Cotabato	**33,406**	6,448	7,403	**13,851**	**41.46**
Sultan Kudarat	**30,793**	2,370	2,566	**4,936**	**16.03**
City of General Santos	**23,388**	921	1,116	**2,037**	**8.71**

Table 2.D.5 - ORAL HEALTH CARE

Children 5-9 years old who received Basic Oral Health Care (BOHC)

Philippines, 2024

Area	Children 5-9 years old who received BOHC				
	Eligible Population x 30%	Male	Female	Total	%
**CARAGA**	**97,963**	**11,252**	**12,010**	**23,262**	**23.75**
Agusan del Norte	**13,537**	1,418	1,670	**3,088**	**22.81**
Agusan del Sur	**28,331**	2,774	2,968	**5,742**	**20.27**
Surigao del Norte	**17,161**	1,787	2,119	**3,906**	**22.76**
Surigao del Sur	**22,592**	3,108	3,139	**6,247**	**27.65**
Dinagat Islands	**4,207**	312	304	**616**	**14.64**
City of Butuan	**12,136**	1,853	1,810	**3,663**	**30.18**
**BARMM**	**206,436**	**23,408**	**25,199**	**44,670**	**21.64**
Basilan	**18,752**	1,238	1,305	**2,543**	**13.56**
Lanao del Sur	**48,738**	6,946	7,708	**14,654**	**30.07**
Maguindanao del Norte	**27,177**	2,033	2,211	**4,244**	**15.62**
Maguindanao del Sur	**33,656**	1,866	2,071		
Sulu	**40,877**	4,205	4,350	**8,555**	**20.93**
Tawi-Tawi	**17,418**	2,086	2,584	**4,670**	**26.81**
SGA	**8,584**	0	0	**0**	**0.00**
City of Cotabato	**11,234**	5,034	4,970	**10,004**	**89.05**

0 = No Data

* = No Report

Table 2.D.6 - ORAL HEALTH CARE
Adolescents 10-14 years who received Basic Oral Health Care (BOHC)
Philippines, 2024

Area	Eligible Population x 30%	Adolescents 10-14 years old who received BOHC			
		Male	Female	Total	%
**Philippines**	**3,428,567**	**424,533**	**455,848**	**880,381**	**25.68**
**NCR**	**364,350**	**152,462**	**156,764**	**309,226**	**84.87**
City of Malabon	**11,102**	5,831	5,851	**11,682**	**105.22**
City of Navotas	**7,607**	3,361	3,518	**6,879**	**90.43**
City of Valenzuela	**20,454**	1,430	1,755	**3,185**	**15.57**
City of Caloocan	**48,884**	3,156	3,548	**6,704**	**13.71**
City of Marikina	**12,707**	2,027	1,950	**3,977**	**31.30**
City of Pasig	**22,109**	7,368	7,703	**15,071**	**68.17**
Pateros	**1,798**	908	948	**1,856**	**103.21**
City of Taguig	**25,421**	2,179	2,416	**4,595**	**18.08**
Quezon City	**79,967**	28,634	30,772	**59,406**	**74.29**
City of Makati	**13,882**	5,895	6,058	**11,953**	**86.10**
City of Mandaluyong	**10,672**	2,521	2,744	**5,265**	**49.34**
City of San Juan	**2,912**	558	511	**1,069**	**36.71**
City of Manila	**48,849**	70,192	69,689	**139,881**	**286.36**
City of Las Piñas	**16,230**	7,173	7,381	**14,554**	**89.68**
City of Muntinlupa	**13,560**	6,449	6,551	**13,000**	**95.87**
City of Parañaque	**17,672**	1,307	1,455	**2,762**	**15.63**
Pasay City	**10,524**	3,473	3,914	**7,387**	**70.19**
**CAR**	**54,692**	**15,910**	**18,026**	**33,936**	**62.05**
Abra	**7,567**	597	669	**1,266**	**16.73**
Apayao	**4,024**	782	779	**1,561**	**38.79**
Benguet	**13,633**	3,857	4,267	**8,124**	**59.59**
Ifugao	**7,047**	2,006	2,328	**4,334**	**61.50**
Kalinga	**7,637**	3,099	3,899	**6,998**	**91.63**
Mountain Province	**5,172**	706	985	**1,691**	**32.69**
City of Baguio	**9,612**	4,863	5,099	**9,962**	**103.64**
**Region 1**	**157,013**	**28,604**	**30,304**	**58,908**	**37.52**
Ilocos Norte	**16,624**	2,667	2,700	**5,367**	**32.28**
Ilocos Sur	**19,776**	6,496	6,612	**13,108**	**66.28**
La Union	**22,913**	2,442	2,625	**5,067**	**22.11**
Pangasinan	**92,355**	16,574	17,841	**34,415**	**37.26**
City of Dagupan	**5,345**	425	526	**951**	**17.79**
**Region 2**	**112,706**	**18,752**	**23,089**	**41,841**	**37.12**
Batanes	**509**	96	73	**169**	**33.22**
Cagayan	**38,402**	2,434	2,693	**5,127**	**13.35**
Isabela	**47,119**	7,759	9,009	**16,768**	**35.59**
Nueva Vizcaya	**15,299**	5,454	7,933	**13,387**	**87.50**
Quirino	**6,654**	1,732	1,983	**3,715**	**55.83**
City of Santiago	**4,723**	1,277	1,398	**2,675**	**56.64**
**Region 3**	**376,723**	**27,933**	**28,914**	**56,847**	**15.09**
Aurora	**7,953**	739	816	**1,555**	**19.55**
Bataan	**26,240**	664	699	**1,363**	**5.19**
Bulacan	**111,865**	5,791	6,808	**12,599**	**11.26**
Nueva Ecija	**70,161**	4,829	5,592	**10,421**	**14.85**
Pampanga	**73,184**	4,256	4,266	**8,522**	**11.64**
Tarlac	**46,806**	6,739	5,898	**12,637**	**27.00**
Zambales	**20,214**	2,432	2,702	**5,134**	**25.40**
City of Angeles	**13,210**	401	201	**602**	**4.56**
City of Olongapo	**7,090**	2,082	1,932	**4,014**	**56.61**
**Region 4A**	**479,524**	**44,551**	**47,443**	**91,994**	**19.18**
Batangas	**84,352**	17,983	18,051	**36,034**	**42.72**
Cavite	**123,936**	4,390	5,331	**9,721**	**7.84**
Laguna	**95,597**	10,786	11,401	**22,187**	**23.21**
Quezon	**65,711**	3,733	4,498	**8,231**	**12.53**
Rizal	**101,061**	7,516	8,019	**15,535**	**15.37**
City of Lucena	**8,868**	143	143	**286**	**3.22**

Table 2.D.6 - ORAL HEALTH CARE
Adolescents 10-14 years who received Basic Oral Health Care (BOHC)
Philippines, 2024

Area	Adolescents 10-14 years old who received BOHC				
	Eligible Population x 30%	Male	Female	Total	%
**Region 4B**	**110,559**	**4,378**	**5,760**	**10,138**	**9.17**
Marinduque	**7,905**	318	323	**641**	**8.11**
Occidental Mindoro	**18,756**	452	826	**1,278**	**6.81**
Oriental Mindoro	**30,675**	1,717	2,014	**3,731**	**12.16**
Palawan	**33,456**	1,088	1,469	**2,557**	**7.64**
Romblon	**10,602**	582	792	**1,374**	**12.96**
City of Puerto Princesa	**9,166**	221	336	**557**	**6.08**
**Region 5**	**216,293**	**9,059**	**9,374**	**18,433**	**8.52**
Albay	**47,041**	2,460	2,575	**5,035**	**10.70**
Camarines Norte	**22,249**	1,011	956	**1,967**	**8.84**
Camarines Sur	**66,991**	1,378	1,449	**2,827**	**4.22**
Catanduanes	**9,416**	691	507	**1,198**	**12.72**
Masbate	**35,708**	1,249	1,354	**2,603**	**7.29**
Sorsogon	**28,332**	2,111	2,309	**4,420**	**15.60**
City of Naga	**6,556**	159	224	**383**	**5.84**
**Region 6**	**240,658**	**29,982**	**31,011**	**60,993**	**25.34**
Aklan	**18,732**	1,603	1,604	**3,207**	**17.12**
Antique	**18,592**	708	833	**1,541**	**8.29**
Capiz	**23,403**	4,058	4,358	**8,416**	**35.96**
Guimaras	**5,464**	103	145	**248**	**4.54**
Iloilo	**61,015**	14,052	14,279	**28,331**	**46.43**
Negros Occidental	**84,642**	4,702	4,463	**9,165**	**10.83**
City of Bacolod	**16,710**	964	1,272	**2,236**	**13.38**
City of Iloilo	**12,100**	3,792	4,057	**7,849**	**64.87**
**Region 7**	**253,018**	**20,993**	**23,928**	**44,921**	**17.75**
Bohol	**43,369**	1,412	1,600	**3,012**	**6.95**
Cebu	**107,789**	5,038	6,200	**11,238**	**10.43**
Negros Oriental	**45,225**	3,347	4,148	**7,495**	**16.57**
Siquijor	**2,772**	189	216	**405**	**14.61**
City of Cebu	**28,085**	2,895	2,996	**5,891**	**20.98**
City of Lapu-Lapu	**15,874**	5,945	6,470	**12,415**	**78.21**
City of Mandaue	**9,903**	2,167	2,298	**4,465**	**45.09**
**Region 8**	**152,594**	**6,869**	**7,946**	**14,815**	**9.71**
Biliran	**6,215**	229	277	**506**	**8.14**
Eastern Samar	**15,958**	375	448	**823**	**5.16**
Leyte	**51,449**	1,698	1,779	**3,477**	**6.76**
Northern Samar	**23,382**	2,688	3,295	**5,983**	**25.59**
Southern Leyte	**13,115**	55	88	**143**	**1.09**
Samar	**27,436**	941	883	**1,824**	**6.65**
Ormoc City	**7,351**	750	1,034	**1,784**	**24.27**
City of Tacloban	**7,690**	133	142	**275**	**3.58**
**Region 9**	**132,224**	**4,778**	**5,522**	**10,300**	**7.79**
Zamboanga del Norte	**35,621**	661	751	**1,412**	**3.96**
Zamboanga del Sur	**36,405**	2,347	2,921	**5,268**	**14.47**
Zamboanga Sibugay	**24,347**	1,029	1,074	**2,103**	**8.64**
City of Isabela	**4,476**	282	357	**639**	**14.28**
City of Zamboanga	**31,375**	459	419	**878**	**2.80**
**Region 10**	**169,929**	**14,878**	**17,301**	**32,179**	**18.94**
Bukidnon	**55,709**	5,314	6,525	**11,839**	**21.25**
Camiguin	**2,963**	136	167	**303**	**10.23**
Lanao del Norte	**26,880**	331	441	**772**	**2.87**
Misamis Occidental	**19,109**	6,542	6,926	**13,468**	**70.48**
Misamis Oriental	**32,204**	1,197	1,525	**2,722**	**8.45**
City of Cagayan De Oro	**21,458**	870	1,156	**2,026**	**9.44**
City of Iligan	**11,607**	488	561	**1,049**	**9.04**
**Region 11**	**169,077**	**9,113**	**11,098**	**20,211**	**11.95**
Davao de Oro	**26,128**	2,105	2,733	**4,838**	**18.52**
Davao del Norte	**35,597**	1,666	2,463	**4,129**	**11.60**
Davao Oriental	**20,105**	3,797	3,949	**7,746**	**38.53**
Davao del Sur	**21,785**	710	865	**1,575**	**7.23**
Davao Occidental	**11,675**	362	501	**863**	**7.39**
City of Davao	**53,787**	473	587	**1,060**	**1.97**

Table 2.D.6 - ORAL HEALTH CARE
Adolescents 10-14 years who received Basic Oral Health Care (BOHC)
Philippines, 2024

Area	Adolescents 10-14 years old who received BOHC				
	Eligible Population x 30%	Male	Female	Total	%
**Region 12**	**150,075**	**11,006**	**12,199**	**23,205**	**15.46**
Cotabato	**44,575**	2,523	2,906	**5,429**	**12.18**
Sarangani	**20,348**	1,133	1,164	**2,297**	**11.29**
South Cotabato	**32,750**	4,454	4,566	**9,020**	**27.54**
Sultan Kudarat	**29,994**	1,955	2,598	**4,553**	**15.18**
City of General Santos	**22,409**	941	965	**1,906**	**8.51**
**CARAGA**	**94,745**	**10,194**	**11,189**	**21,383**	**22.57**
Agusan del Norte	**13,356**	1,586	1,875	**3,461**	**25.91**
Agusan del Sur	**26,957**	1,638	1,816	**3,454**	**12.81**
Surigao del Norte	**16,594**	1,302	1,551	**2,853**	**17.19**
Surigao del Sur	**21,621**	3,646	3,729	**7,375**	**34.11**
Dinagat Islands	**4,088**	313	367	**680**	**16.63**
City of Butuan	**12,130**	1,709	1,851	**3,560**	**29.35**
**BARMM**	**194,387**	**15,071**	**15,980**	**31,051**	**15.97**
Basilan	**17,207**	528	660	**1,188**	**6.90**
Lanao del Sur	**47,441**	5,156	5,635	**10,791**	**22.75**
Maguindanao del Norte	**25,442**	1,090	1,096	**2,186**	**8.59**
Maguindanao del Sur	**31,001**	1,168	1,219	**2,387**	**7.70**
Sulu	**39,411**	3,422	3,103	**6,525**	**16.56**
Tawi-Tawi	**15,456**	2,175	2,405	**4,580**	**29.63**
SGA	**7,637**	0	0	**0**	**0.00**
City of Cotabato	**10,792**	1,532	1,862	**3,394**	**31.45**

0 = No Data
* = No Report

Table 2.D.7 - ORAL HEALTH CARE
Adolescents 15- 19 years old who received Basic Oral Health Care (BOHC)
Philippines, 2024

Area	Adolescents 15-19 years old who received BOHC				
	Eligible Population x 30%	Male	Female	Total	%
**Philippines**	**3,239,614**	**361,228**	**453,369**	**814,597**	**25.14**
**NCR**	**346,240**	**104,271**	**116,114**	**220,385**	**63.65**
City of Malabon	**10,691**	4,544	5,105	**9,649**	**90.26**
City of Navotas	**7,225**	2,917	3,347	**6,264**	**86.70**
City of Valenzuela	**19,457**	1,900	2,504	**4,404**	**22.64**
City of Caloocan	**46,693**	4,297	5,381	**9,678**	**20.73**
City of Marikina	**11,832**	579	705	**1,284**	**10.85**
City of Pasig	**20,453**	7,670	8,588	**16,258**	**79.49**
Pateros	**1,660**	648	644	**1,292**	**77.84**
City of Taguig	**23,378**	3,148	3,718	**6,866**	**29.37**
Quezon City	**74,843**	19,738	22,868	**42,606**	**56.93**
City of Makati	**13,103**	4,927	5,227	**10,154**	**77.49**
City of Mandaluyong	**9,726**	856	1,173	**2,029**	**20.86**
City of San Juan	**2,716**	295	329	**624**	**22.98**
City of Manila	**48,617**	35,750	38,164	**73,914**	**152.03**
City of Las Piñas	**15,656**	6,761	7,740	**14,501**	**92.62**
City of Muntinlupa	**13,543**	5,401	5,299	**10,700**	**79.01**
City of Parañaque	**16,171**	1,040	1,151	**2,191**	**13.55**
Pasay City	**10,477**	3,800	4,171	**7,971**	**76.08**
**CAR**	**55,703**	**11,275**	**15,760**	**27,035**	**48.53**
Abra	**7,631**	551	616	**1,167**	**15.29**
Apayao	**3,860**	522	745	**1,267**	**32.82**
Benguet	**13,679**	2,424	3,550	**5,974**	**43.67**
Ifugao	**6,729**	1,302	1,772	**3,074**	**45.68**
Kalinga	**7,648**	2,846	3,901	**6,747**	**88.22**
Mountain Province	**5,204**	616	957	**1,573**	**30.23**
City of Baguio	**10,952**	3,014	4,219	**7,233**	**66.04**
**Region 1**	**153,100**	**29,831**	**33,477**	**63,308**	**41.35**
Ilocos Norte	**16,260**	2,807	3,012	**5,819**	**35.79**
Ilocos Sur	**19,415**	5,244	6,092	**11,336**	**58.39**
La Union	**22,816**	2,271	2,737	**5,008**	**21.95**
Pangasinan	**89,219**	19,061	21,050	**40,111**	**44.96**
City of Dagupan	**5,390**	448	586	**1,034**	**19.18**
**Region 2**	**106,346**	**17,052**	**20,757**	**37,809**	**35.55**
Batanes	**554**	174	198	**372**	**67.17**
Cagayan	**36,162**	2,326	2,912	**5,238**	**14.48**
Isabela	**44,140**	7,030	8,115	**15,145**	**34.31**
Nueva Vizcaya	**14,844**	4,686	6,042	**10,728**	**72.27**
Quirino	**6,199**	1,769	2,350	**4,119**	**66.45**
City of Santiago	**4,448**	1,067	1,140	**2,207**	**49.61**
**Region 3**	**356,860**	**26,291**	**31,207**	**57,498**	**16.11**
Aurora	**7,436**	518	593	**1,111**	**14.94**
Bataan	**24,258**	646	819	**1,465**	**6.04**
Bulacan	**105,578**	6,124	7,652	**13,776**	**13.05**
Nueva Ecija	**65,167**	4,722	5,123	**9,845**	**15.11**
Pampanga	**71,975**	3,443	3,660	**7,103**	**9.87**
Tarlac	**43,007**	5,880	7,714	**13,594**	**31.61**
Zambales	**19,028**	2,139	2,925	**5,064**	**26.61**
City of Angeles	**12,989**	183	201	**384**	**2.96**
City of Olongapo	**7,423**	2,636	2,520	**5,156**	**69.46**
**Region 4A**	**456,047**	**45,922**	**77,000**	**122,922**	**26.95**
Batangas	**83,937**	18,900	19,483	**38,383**	**45.73**
Cavite	**118,662**	6,654	8,448	**15,102**	**12.73**
Laguna	**89,732**	11,456	37,835	**49,291**	**54.93**
Quezon	**60,589**	3,383	4,458	**7,841**	**12.94**
Rizal	**94,705**	5,425	6,638	**12,063**	**12.74**
City of Lucena	**8,422**	104	138	**242**	**2.87**

Table 2.D.7 - ORAL HEALTH CARE
Adolescents 15- 19 years old who received Basic Oral Health Care (BOHC)
Philippines, 2024

Area	Adolescents 15-19 years old who received BOHC				
	Eligible Population x 30%	Male	Female	Total	%
**Region 4B**	**104,673**	**3,546**	**4,905**	**8,451**	**8.07**
Marinduque	**7,578**	247	368	**615**	**8.12**
Occidental Mindoro	**17,298**	468	691	**1,159**	**6.70**
Oriental Mindoro	**28,748**	1,086	1,336	**2,422**	**8.43**
Palawan	**31,137**	966	1,336	**2,302**	**7.39**
Romblon	**10,329**	403	511	**914**	**8.85**
City of Puerto Princesa	**9,584**	376	663	**1,039**	**10.84**
**Region 5**	**207,824**	**10,121**	**13,000**	**23,121**	**11.13**
Albay	**46,456**	3,531	3,972	**7,503**	**16.15**
Camarines Norte	**20,413**	1,516	1,729	**3,245**	**15.90**
Camarines Sur	**64,497**	1,227	1,586	**2,813**	**4.36**
Catanduanes	**9,218**	333	596	**929**	**10.08**
Masbate	**31,975**	1,287	1,472	**2,759**	**8.63**
Sorsogon	**28,357**	2,049	3,325	**5,374**	**18.95**
City of Naga	**6,908**	178	320	**498**	**7.21**
**Region 6**	**231,005**	**23,274**	**29,059**	**52,333**	**22.65**
Aklan	**17,658**	1,026	1,381	**2,407**	**13.63**
Antique	**18,335**	512	678	**1,190**	**6.49**
Capiz	**23,653**	3,972	5,065	**9,037**	**38.21**
Guimaras	**5,195**	137	128	**265**	**5.10**
Iloilo	**58,718**	10,812	12,525	**23,337**	**39.74**
Negros Occidental	**79,235**	4,236	5,773	**10,009**	**12.63**
City of Bacolod	**16,605**	1,011	1,486	**2,497**	**15.04**
City of Iloilo	**11,605**	1,568	2,023	**3,591**	**30.94**
**Region 7**	**243,046**	**23,300**	**28,709**	**52,009**	**21.40**
Bohol	**43,547**	1,242	1,533	**2,775**	**6.37**
Cebu	**101,180**	5,895	7,451	**13,346**	**13.19**
Negros Oriental	**43,073**	3,288	4,361	**7,649**	**17.76**
Siquijor	**2,840**	182	259	**441**	**15.53**
City of Cebu	**28,012**	2,855	4,872	**7,727**	**27.58**
City of Lapu-Lapu	**14,280**	7,204	7,151	**14,355**	**100.53**
City of Mandaue	**10,115**	2,634	3,082	**5,716**	**56.51**
**Region 8**	**146,793**	**8,556**	**12,789**	**21,345**	**14.54**
Biliran	**5,715**	188	312	**500**	**8.75**
Eastern Samar	**15,908**	338	468	**806**	**5.07**
Leyte	**47,512**	2,347	3,741	**6,088**	**12.81**
Northern Samar	**22,708**	3,227	5,541	**8,768**	**38.61**
Southern Leyte	**12,917**	656	927	**1,583**	**12.25**
Samar	**27,396**	1,246	1,176	**2,422**	**8.84**
Ormoc City	**7,068**	467	439	**906**	**12.82**
City of Tacloban	**7,569**	87	185	**272**	**3.59**
**Region 9**	**122,842**	**4,413**	**5,859**	**10,272**	**8.36**
Zamboanga del Norte	**33,082**	656	909	**1,565**	**4.73**
Zamboanga del Sur	**33,785**	2,379	3,111	**5,490**	**16.25**
Zamboanga Sibugay	**22,016**	960	1,142	**2,102**	**9.55**
City of Isabela	**4,069**	255	417	**672**	**16.51**
City of Zamboanga	**29,890**	163	280	**443**	**1.48**
**Region 10**	**157,434**	**14,564**	**18,433**	**32,997**	**20.96**
Bukidnon	**50,061**	5,031	6,960	**11,991**	**23.95**
Camiguin	**2,783**	121	139	**260**	**9.34**
Lanao del Norte	**24,062**	267	427	**694**	**2.88**
Misamis Occidental	**18,649**	6,533	7,347	**13,880**	**74.43**
Misamis Oriental	**29,714**	1,392	1,683	**3,075**	**10.35**
City of Cagayan De Oro	**20,817**	879	1,385	**2,264**	**10.88**
City of Iligan	**11,348**	341	492	**833**	**7.34**
**Region 11**	**154,851**	**8,169**	**10,742**	**18,911**	**12.21**
Davao de Oro	**24,755**	2,108	2,800	**4,908**	**19.83**
Davao del Norte	**32,486**	1,723	2,330	**4,053**	**12.48**
Davao Oriental	**18,289**	2,630	3,249	**5,879**	**32.15**
Davao del Sur	**19,276**	745	1,008	**1,753**	**9.09**
Davao Occidental	**9,632**	288	387	**675**	**7.01**
City of Davao	**50,414**	675	968	**1,643**	**3.26**

Table 2.D.7 - ORAL HEALTH CARE
Adolescents 15- 19 years old who received Basic Oral Health Care (BOHC)
Philippines, 2024

Area	Adolescents 15-19 years old who received BOHC				
	Eligible Population x 30%	Male	Female	Total	%
**Region 12**	**138,715**	**9,184**	**11,666**	**20,850**	**15.03**
Cotabato	**40,615**	2,082	2,575	**4,657**	**11.47**
Sarangani	**18,645**	891	1,209	**2,100**	**11.26**
South Cotabato	**30,160**	3,697	4,503	**8,200**	**27.19**
Sultan Kudarat	**28,535**	2,039	2,556	**4,595**	**16.10**
City of General Santos	**20,761**	475	823	**1,298**	**6.25**
**CARAGA**	**89,635**	**7,356**	**8,586**	**15,942**	**17.79**
Agusan del Norte	**12,257**	805	1,106	**1,911**	**15.59**
Agusan del Sur	**24,157**	1,365	1,539	**2,904**	**12.02**
Surigao del Norte	**17,185**	1,275	1,506	**2,781**	**16.18**
Surigao del Sur	**20,311**	2,267	2,933	**5,200**	**25.60**
Dinagat Islands	**3,995**	111	177	**288**	**7.21**
City of Butuan	**11,731**	1,533	1,325	**2,858**	**24.36**
**BARMM**	**168,501**	**14,103**	**15,306**	**29,409**	**17.45**
Basilan	**13,703**	549	791	**1,340**	**9.78**
Lanao del Sur	**41,756**	4,515	4,608	**9,123**	**21.85**
Maguindanao del Norte	**20,427**	843	931	**1,774**	**8.68**
Maguindanao del Sur	**23,322**	1,015	1,162	**2,177**	**9.33**
Sulu	**36,899**	4,701	4,838	**9,539**	**25.85**
Tawi-Tawi	**13,892**	2,044	2,198	**4,242**	**30.53**
SGA	**7,241**	0	0	**0**	**0.00**
City of Cotabato	**11,261**	436	778	**1,214**	**10.78**

0 = No Data
* = No Report

ORAL HEALTH CARE
Adult 20 -59 years old who received Basic Oral Health Care (BOHC)
Philippines, 2024

Area	Adults 20-59 years old who received BOHC				
	Eligible Population x30%	Male	Female	Total	%
**Philippines**	**17,227,668**	**837,492**	**1,386,204**	**2,223,696**	**12.91**
**NCR**	**2,353,452**	**189,005**	**288,267**	**477,272**	**20.28**
City of Malabon	**64,505**	7,176	14,205	**21,381**	**33.15**
City of Navotas	**40,812**	10,234	12,441	**22,675**	**55.56**
City of Valenzuela	**125,255**	5,620	9,274	**14,894**	**11.89**
City of Caloocan	**281,732**	9,331	16,160	**25,491**	**9.05**
City of Marikina	**76,814**	1,483	2,475	**3,958**	**5.15**
City of Pasig	**139,421**	11,560	25,539	**37,099**	**26.61**
Pateros	**11,145**	941	1,431	**2,372**	**21.28**
City of Taguig	**157,832**	9,891	17,565	**27,456**	**17.40**
Quezon City	**512,403**	18,498	34,972	**53,470**	**10.44**
City of Makati	**115,814**	8,479	10,957	**19,436**	**16.78**
City of Mandaluyong	**77,573**	3,117	5,243	**8,360**	**10.78**
City of San Juan	**22,374**	989	1,760	**2,749**	**12.29**
City of Manila	**319,473**	63,950	88,382	**152,332**	**47.68**
City of Las Piñas	**105,584**	4,182	7,088	**11,270**	**10.67**
City of Muntinlupa	**97,033**	7,432	10,531	**17,963**	**18.51**
City of Parañaque	**122,820**	2,125	3,403	**5,528**	**4.50**
Pasay City	**82,864**	23,997	26,841	**50,838**	**61.35**
**CAR**	**293,355**	**25,984**	**52,446**	**78,430**	**26.74**
Abra	**37,989**	1,828	3,523	**5,351**	**14.09**
Apayao	**19,478**	1,470	3,839	**5,309**	**27.26**
Benguet	**76,566**	6,551	10,923	**17,474**	**22.82**
Ifugao	**32,670**	2,809	6,689	**9,498**	**29.07**
Kalinga	**36,123**	7,060	14,676	**21,736**	**60.17**
Mountain Province	**24,303**	1,758	3,994	**5,752**	**23.67**
City of Baguio	**66,227**	4,508	8,802	**13,310**	**20.10**
**Region 1**	**828,428**	**81,819**	**106,079**	**187,898**	**22.68**
Ilocos Norte	**98,318**	4,343	5,426	**9,769**	**9.94**
Ilocos Sur	**112,195**	15,927	23,463	**39,390**	**35.11**
La Union	**131,915**	5,893	10,565	**16,458**	**12.48**
Pangasinan	**458,045**	54,277	63,819	**118,096**	**25.78**
City of Dagupan	**27,954**	1,379	2,806	**4,185**	**14.97**
**Region 2**	**588,110**	**44,198**	**72,351**	**116,549**	**19.82**
Batanes	**3,115**	384	525	**909**	**29.18**
Cagayan	**199,581**	6,040	11,575	**17,615**	**8.83**
Isabela	**247,748**	17,922	29,434	**47,356**	**19.11**
Nueva Vizcaya	**80,302**	11,134	16,136	**27,270**	**33.96**
Quirino	**32,889**	5,670	10,648	**16,318**	**49.61**
City of Santiago	**24,476**	3,048	4,033	**7,081**	**28.93**
**Region 3**	**2,023,075**	**86,409**	**129,401**	**215,810**	**10.67**
Aurora	**36,380**	1,021	1,360	**2,381**	**6.54**
Bataan	**138,407**	3,162	5,732	**8,894**	**6.43**
Bulacan	**613,966**	22,647	40,079	**62,726**	**10.22**
Nueva Ecija	**367,995**	11,757	18,133	**29,890**	**8.12**
Pampanga	**405,484**	12,536	18,059	**30,595**	**7.55**
Tarlac	**235,944**	21,383	26,195	**47,578**	**20.16**
Zambales	**102,577**	8,915	14,614	**23,529**	**22.94**
City of Angeles	**78,312**	373	630	**1,003**	**1.28**
City of Olongapo	**44,012**	4,615	4,599	**9,214**	**20.94**
**Region 4A**	**2,703,384**	**129,359**	**238,276**	**367,635**	**13.60**
Batangas	**477,087**	62,597	83,105	**145,702**	**30.54**
Cavite	**749,631**	20,456	36,376	**56,832**	**7.58**
Laguna	**583,741**	23,289	75,305	**98,594**	**16.89**
Quezon	**292,583**	9,996	19,112	**29,108**	**9.95**
Rizal	**556,246**	12,585	23,479	**36,064**	**6.48**
City of Lucena	**44,098**	436	899	**1,335**	**3.03**

ORAL HEALTH CARE
Adult 20 -59 years old who received Basic Oral Health Care (BOHC)
Philippines, 2024

Area	Adults 20-59 years old who received BOHC				
	Eligible Population x30%	Male	Female	Total	%
**Region 4B**	**482,970**	**10,893**	**21,161**	**32,054**	**6.64**
Marinduque	**33,254**	727	1,570	**2,297**	**6.91**
Occidental Mindoro	**77,554**	1,657	3,162	**4,819**	**6.21**
Oriental Mindoro	**136,158**	3,448	5,587	**9,035**	**6.64**
Palawan	**139,647**	2,678	5,827	**8,505**	**6.09**
Romblon	**44,471**	847	1,653	**2,500**	**5.62**
City of Puerto Princesa	**51,886**	1,536	3,362	**4,898**	**9.44**
**Region 5**	**868,309**	**24,725**	**51,182**	**75,907**	**8.74**
Albay	**204,647**	10,983	18,465	**29,448**	**14.39**
Camarines Norte	**92,406**	3,624	4,899	**8,523**	**9.22**
Camarines Sur	**261,480**	2,394	9,395	**11,789**	**4.51**
Catanduanes	**37,964**	449	2,322	**2,771**	**7.30**
Masbate	**119,429**	2,253	4,082	**6,335**	**5.30**
Sorsogon	**118,719**	4,428	10,406	**14,834**	**12.50**
City of Naga	**33,665**	594	1,613	**2,207**	**6.56**
**Region 6**	**1,212,881**	**63,758**	**102,168**	**165,926**	**13.68**
Aklan	**95,772**	2,701	4,170	**6,871**	**7.17**
Antique	**90,134**	2,388	4,354	**6,742**	**7.48**
Capiz	**124,841**	12,555	16,867	**29,422**	**23.57**
Guimaras	**29,385**	320	1,047	**1,367**	**4.65**
Iloilo	**313,231**	21,429	32,809	**54,238**	**17.32**
Negros Occidental	**390,596**	15,185	25,814	**40,999**	**10.50**
City of Bacolod	**95,110**	3,873	6,698	**10,571**	**11.11**
City of Iloilo	**73,813**	5,307	10,409	**15,716**	**21.29**
**Region 7**	**1,271,290**	**49,297**	**91,202**	**140,499**	**11.05**
Bohol	**206,287**	3,240	7,377	**10,617**	**5.15**
Cebu	**512,947**	12,999	24,793	**37,792**	**7.37**
Negros Oriental	**219,367**	7,961	12,872	**20,833**	**9.50**
Siquijor	**15,906**	873	1,434	**2,307**	**14.50**
City of Cebu	**162,980**	11,152	30,317	**41,469**	**25.44**
City of Lapu-Lapu	**86,767**	7,710	8,377	**16,087**	**18.54**
City of Mandaue	**67,037**	5,362	6,032	**11,394**	**17.00**
**Region 8**	**655,550**	**13,963**	**26,867**	**40,830**	**6.23**
Biliran	**25,483**	508	1,056	**1,564**	**6.14**
Eastern Samar	**68,346**	847	1,419	**2,266**	**3.32**
Leyte	**223,519**	4,354	12,273	**16,627**	**7.44**
Northern Samar	**87,181**	1,559	2,595	**4,154**	**4.76**
Southern Leyte	**63,035**	1,377	2,233	**3,610**	**5.73**
Samar	**112,176**	3,644	3,413	**7,057**	**6.29**
Ormoc City	**35,789**	1,431	2,549	**3,980**	**11.12**
City of Tacloban	**40,022**	243	1,329	**1,572**	**3.93**
**Region 9**	**575,530**	**10,712**	**17,137**	**27,849**	**4.84**
Zamboanga del Norte	**150,639**	1,881	3,518	**5,399**	**3.58**
Zamboanga del Sur	**153,857**	5,048	7,832	**12,880**	**8.37**
Zamboanga Sibugay	**96,770**	2,211	2,859	**5,070**	**5.24**
City of Isabela	**18,575**	1,444	2,533	**3,977**	**21.41**
City of Zamboanga	**155,689**	128	395	**523**	**0.34**
**Region 10**	**771,159**	**31,969**	**54,634**	**86,603**	**11.23**
Bukidnon	**236,054**	8,927	15,934	**24,861**	**10.53**
Camiguin	**13,478**	319	895	**1,214**	**9.01**
Lanao del Norte	**102,678**	762	1,373	**2,135**	**2.08**
Misamis Occidental	**93,491**	15,289	21,600	**36,889**	**39.46**
Misamis Oriental	**144,425**	3,005	5,860	**8,865**	**6.14**
City of Cagayan De Oro	**124,560**	2,665	6,562	**9,227**	**7.41**
City of Iligan	**56,474**	1,002	2,410	**3,412**	**6.04**
**Region 11**	**831,052**	**23,006**	**39,282**	**62,288**	**7.50**
Davao de Oro	**118,931**	4,901	8,239	**13,140**	**11.05**
Davao del Norte	**179,746**	4,305	9,196	**13,501**	**7.51**
Davao Oriental	**86,051**	9,754	12,982	**22,736**	**26.42**
Davao del Sur	**106,797**	1,453	3,559	**5,012**	**4.69**
Davao Occidental	**44,019**	751	1,421	**2,172**	**4.93**
City of Davao	**295,508**	1,842	3,885	**5,727**	**1.94**

ORAL HEALTH CARE
Adult 20 -59 years old who received Basic Oral Health Care (BOHC)
Philippines, 2024

Area	Adults 20-59 years old who received BOHC				
	Eligible Population x30%	Male	Female	Total	%
**Region 12**	**680,809**	**18,283**	**36,244**	**54,527**	**8.01**
Cotabato	**195,904**	3,672	6,606	**10,278**	**5.25**
Sarangani	**84,499**	2,142	4,053	**6,195**	**7.33**
South Cotabato	**153,330**	6,356	10,758	**17,114**	**11.16**
Sultan Kudarat	**130,444**	4,550	6,293	**10,843**	**8.31**
City of General Santos	**116,633**	1,563	8,534	**10,097**	**8.66**
**CARAGA**	**419,391**	**12,127**	**27,455**	**39,582**	**9.44**
Agusan del Norte	**57,520**	1,879	6,124	**8,003**	**13.91**
Agusan del Sur	**107,537**	2,307	3,912	**6,219**	**5.78**
Surigao del Norte	**81,632**	2,415	4,299	**6,714**	**8.22**
Surigao del Sur	**95,898**	3,800	8,420	**12,220**	**12.74**
Dinagat Islands	**18,038**	155	492	**647**	**3.59**
City of Butuan	**58,765**	1,571	4,208	**5,779**	**9.83**
**BARMM**	**668,921**	**21,985**	**32,052**	**54,037**	**8.08**
Basilan	**55,317**	1,089	2,088	**3,177**	**5.74**
Lanao del Sur	**156,894**	6,638	8,336	**14,974**	**9.54**
Maguindanao del Norte	**84,702**	1,272	1,459	**2,731**	**3.22**
Maguindanao del Sur	**94,272**	1,524	2,983	**4,507**	**4.78**
Sulu	**135,232**	5,246	5,705	**10,951**	**8.10**
Tawi-Tawi	**60,048**	4,140	4,971	**9,111**	**15.17**
SGA	**30,790**	0	0	**0**	**0.00**
City of Cotabato	**51,666**	2,076	6,510	**8,586**	**16.62**

0 = No Data
* = No Report

Table 2.D.9 - ORAL HEALTH CARE

Senior citizens 60 years old and above who received Basic Oral Health Care (BOHC)

Philippines, 2024

Area	Eligible Population x 30%	Senior citizens 60 years old and above who received BOHC			
		Male	Female	Total	%
**Philippines**	**2,821,790**	**260,680**	**339,159**	**599,839**	**21.26**
**NCR**	**327,409**	**64,827**	**85,232**	**150,059**	**45.83**
City of Malabon	**9,207**	2,137	2,946	**5,083**	**55.21**
City of Navotas	**5,670**	2,157	2,591	**4,748**	**83.74**
City of Valenzuela	**15,570**	1,591	2,142	**3,733**	**23.98**
City of Caloocan	**36,714**	4,880	6,034	**10,914**	**29.73**
City of Marikina	**13,143**	681	839	**1,520**	**11.56**
City of Pasig	**18,580**	4,640	6,791	**11,431**	**61.52**
Pateros	**1,759**	501	689	**1,190**	**67.64**
City of Taguig	**16,767**	2,436	2,970	**5,406**	**32.24**
Quezon City	**72,893**	16,124	22,368	**38,492**	**52.81**
City of Makati	**18,720**	1,866	2,276	**4,142**	**22.13**
City of Mandaluyong	**10,287**	719	1,212	**1,931**	**18.77**
City of San Juan	**3,982**	199	346	**545**	**13.69**
City of Manila	**46,959**	16,999	20,767	**37,766**	**80.42**
City of Las Piñas	**15,515**	3,783	5,586	**9,369**	**60.39**
City of Muntinlupa	**14,074**	2,180	2,890	**5,070**	**36.02**
City of Parañaque	**17,628**	944	1,169	**2,113**	**11.99**
Pasay City	**9,939**	2,990	3,616	**6,606**	**66.47**
**CAR**	**50,953**	**7,211**	**9,748**	**16,959**	**33.28**
Abra	**8,978**	637	882	**1,519**	**16.92**
Apayao	**3,619**	427	548	**975**	**26.94**
Benguet	**11,560**	1,901	2,303	**4,204**	**36.37**
Ifugao	**5,441**	1,041	1,552	**2,593**	**47.66**
Kalinga	**6,241**	1,950	2,916	**4,866**	**77.97**
Mountain Province	**4,979**	406	385	**791**	**15.89**
City of Baguio	**10,135**	849	1,162	**2,011**	**19.84**
**Region 1**	**174,239**	**25,413**	**31,105**	**56,518**	**32.44**
Ilocos Norte	**23,178**	2,874	3,173	**6,047**	**26.09**
Ilocos Sur	**26,700**	6,420	8,560	**14,980**	**56.10**
La Union	**28,486**	2,458	3,555	**6,013**	**21.11**
Pangasinan	**90,805**	13,017	14,696	**27,713**	**30.52**
City of Dagupan	**5,069**	644	1,121	**1,765**	**34.82**
**Region 2**	**108,542**	**18,191**	**24,639**	**42,830**	**39.46**
Batanes	**723**	44	99	**143**	**19.78**
Cagayan	**39,733**	2,520	3,396	**5,916**	**14.89**
Isabela	**43,830**	9,352	12,346	**21,698**	**49.51**
Nueva Vizcaya	**14,678**	2,575	3,499	**6,074**	**41.38**
Quirino	**5,513**	1,529	2,495	**4,024**	**72.99**
City of Santiago	**4,066**	2,171	2,804	**4,975**	**122.36**
**Region 3**	**335,394**	**26,313**	**32,524**	**58,837**	**17.54**
Aurora	**6,176**	548	531	**1,079**	**17.47**
Bataan	**22,655**	1,381	1,739	**3,120**	**13.77**
Bulacan	**95,261**	6,639	8,080	**14,719**	**15.45**
Nueva Ecija	**65,634**	3,534	4,269	**7,803**	**11.89**
Pampanga	**65,534**	3,832	4,117	**7,949**	**12.13**
Tarlac	**43,535**	6,343	7,337	**13,680**	**31.42**
Zambales	**19,617**	2,519	4,390	**6,909**	**35.22**
City of Angeles	**9,815**	93	151	**244**	**2.49**
City of Olongapo	**7,167**	1,424	1,910	**3,334**	**46.52**
**Region 4A**	**397,161**	**35,637**	**41,689**	**77,326**	**19.47**
Batangas	**77,984**	13,789	15,279	**29,068**	**37.27**
Cavite	**102,697**	4,561	5,623	**10,184**	**9.92**
Laguna	**80,928**	11,118	12,654	**23,772**	**29.37**
Quezon	**53,279**	3,098	3,931	**7,029**	**13.19**
Rizal	**75,372**	3,000	4,108	**7,108**	**9.43**
City of Lucena	**6,901**	71	94	**165**	**2.39**
**Region 4B**	**82,809**	**2,292**	**3,441**	**5,733**	**6.92**
Marinduque	**8,817**	162	345	**507**	**5.75**
Occidental Mindoro	**12,435**	333	421	**754**	**6.06**
Oriental Mindoro	**23,724**	709	987	**1,696**	**7.15**
Palawan	**20,726**	637	942	**1,579**	**7.62**
Romblon	**10,178**	207	366	**573**	**5.63**
City of Puerto Princesa	**6,930**	244	380	**624**	**9.00**
**Region 5**	**164,088**	**5,436**	**8,340**	**13,776**	**8.40**
Albay	**38,878**	2,009	2,271	**4,280**	**11.01**
Camarines Norte	**16,720**	564	793	**1,357**	**8.12**
Camarines Sur	**48,976**	737	2,088	**2,825**	**5.77**
Catanduanes	**8,046**	364	565	**929**	**11.55**
Masbate	**22,511**	688	920	**1,608**	**7.14**
Sorsogon	**23,299**	981	1,533	**2,514**	**10.79**
City of Naga	**5,658**	93	170	**263**	**4.65**

Table 2.D.9 - ORAL HEALTH CARE
Senior citizens 60 years old and above who received Basic Oral Health Care (BOHC)
Philippines, 2024

Area	Eligible Population x 30%	Senior citizens 60 years old and above who received BOHC			
		Male	Female	Total	%
**Region 6**	**252,658**	**22,230**	**30,281**	**52,511**	**20.78**
Aklan	**19,973**	477	746	**1,223**	**6.12**
Antique	**19,898**	552	784	**1,336**	**6.71**
Capiz	**26,993**	3,651	4,591	**8,242**	**30.53**
Guimaras	**6,245**	67	225	**292**	**4.68**
Iloilo	**70,055**	11,236	14,106	**25,342**	**36.17**
Negros Occidental	**76,958**	3,641	5,435	**9,076**	**11.79**
City of Bacolod	**18,314**	857	1,581	**2,438**	**13.31**
City of Iloilo	**14,223**	1,749	2,813	**4,562**	**32.07**
**Region 7**	**225,802**	**12,144**	**16,686**	**28,830**	**12.77**
Bohol	**48,620**	988	1,566	**2,554**	**5.25**
Cebu	**87,996**	3,067	4,972	**8,039**	**9.14**
Negros Oriental	**45,140**	1,546	2,239	**3,785**	**8.38**
Siquijor	**4,375**	148	239	**387**	**8.85**
City of Cebu	**23,473**	1,891	2,959	**4,850**	**20.66**
City of Lapu-Lapu	**8,595**	1,060	1,028	**2,088**	**24.29**
City of Mandaue	**7,603**	3,444	3,683	**7,127**	**93.74**
**Region 8**	**134,066**	**4,217**	**6,140**	**10,357**	**7.73**
Biliran	**5,719**	114	161	**275**	**4.81**
Eastern Samar	**14,826**	300	472	**772**	**5.21**
Leyte	**47,983**	1,801	3,120	**4,921**	**10.26**
Northern Samar	**16,189**	704	853	**1,557**	**9.62**
Southern Leyte	**15,732**	302	421	**723**	**4.60**
Samar	**21,276**	689	707	**1,396**	**6.56**
Ormoc City	**6,191**	240	270	**510**	**8.24**
City of Tacloban	**6,150**	67	136	**203**	**3.30**
**Region 9**	**94,902**	**2,192**	**2,901**	**5,093**	**5.37**
Zamboanga del Norte	**28,003**	537	760	**1,297**	**4.63**
Zamboanga del Sur	**26,299**	1,097	1,412	**2,509**	**9.54**
Zamboanga Sibugay	**15,403**	383	476	**859**	**5.58**
City of Isabela	**2,509**	131	148	**279**	**11.12**
City of Zamboanga	**22,689**	44	105	**149**	**0.66**
**Region 10**	**122,137**	**8,858**	**12,819**	**21,677**	**17.75**
Bukidnon	**33,572**	2,755	4,122	**6,877**	**20.48**
Camiguin	**3,173**	61	141	**202**	**6.37**
Lanao del Norte	**13,323**	125	169	**294**	**2.21**
Misamis Occidental	**20,174**	3,899	5,411	**9,310**	**46.15**
Misamis Oriental	**24,605**	901	1,347	**2,248**	**9.14**
City of Cagayan De Oro	**18,202**	990	1,461	**2,451**	**13.47**
City of Iligan	**9,088**	127	168	**295**	**3.25**
**Region 11**	**131,691**	**5,809**	**8,122**	**13,931**	**10.58**
Davao de Oro	**19,005**	842	1,116	**1,958**	**10.30**
Davao del Norte	**28,455**	659	944	**1,603**	**5.63**
Davao Oriental	**15,565**	2,554	3,302	**5,856**	**37.62**
Davao del Sur	**19,026**	379	666	**1,045**	**5.49**
Davao Occidental	**6,762**	552	828	**1,380**	**20.41**
City of Davao	**42,878**	823	1,266	**2,089**	**4.87**
**Region 12**	**95,228**	**6,747**	**9,138**	**15,885**	**16.68**
Cotabato	**30,158**	2,094	2,778	**4,872**	**16.16**
Sarangani	**11,029**	441	655	**1,096**	**9.94**
South Cotabato	**22,565**	2,853	3,740	**6,593**	**29.22**
Sultan Kudarat	**17,423**	1,109	1,374	**2,483**	**14.25**
City of General Santos	**14,054**	250	591	**841**	**5.98**
**CARAGA**	**74,221**	**4,228**	**5,885**	**10,113**	**13.63**
Agusan del Norte	**10,515**	602	789	**1,391**	**13.23**
Agusan del Sur	**16,741**	1,066	1,352	**2,418**	**14.44**
Surigao del Norte	**14,805**	681	990	**1,671**	**11.29**
Surigao del Sur	**18,116**	1,420	2,002	**3,422**	**18.89**
Dinagat Islands	**4,227**	63	118	**181**	**4.28**
City of Butuan	**9,816**	396	634	**1,030**	**10.49**
**BARMM**	**50,489**	**8,935**	**10,469**	**19,404**	**38.43**
Basilan	**4,756**	297	511	**808**	**16.99**
Lanao del Sur	**11,574**	2,253	2,667	**4,920**	**42.51**
Maguindanao del Norte	**5,987**	665	884	**1,549**	**25.87**
Maguindanao del Sur	**5,488**	512	873	**1,385**	**25.24**
Sulu	**9,975**	3,094	3,100	**6,194**	**62.10**
Tawi-Tawi	**4,844**	1,460	1,514	**2,974**	**61.40**
SGA	**3,234**	0	0	**0**	**0.00**
City of Cotabato	**4,632**	654	920	**1,574**	**33.98**

0 = No Data
* = No Report

Table 2.D.10 - ORAL HEALTH CARE
Pregnant women 10-14, 15-19, 20-49 years old who received Basic Oral Health Care (BOHC)
Philippines, 2024

Area	Eligible Population (0-11 months)	Pregnant Women 10-14 years old received BOHC		Pregnant Women 15-19 years old received BOHC		Pregnant Women 20-49 years old received BOHC	
		No.	%	No.	%	No.	%
**Philippines**	**2,200,865**	**4,503**	**0.20**	**94,063**	**4.27**	**470,094**	**21.36**
**NCR**	**263,248**	**438**	**0.17**	**12,715**	**4.83**	**106,834**	**40.58**
City of Malabon	**7,447**	34	**0.46**	490	**6.58**	2,989	**40.14**
City of Navotas	**5,263**	18	**0.34**	614	**11.67**	3,230	**61.37**
City of Valenzuela	**13,601**	12	**0.09**	296	**2.18**	2,988	**21.97**
City of Caloocan	**33,499**	71	**0.21**	2,303	**6.87**	9,343	**27.89**
City of Marikina	**8,399**	8	**0.10**	270	**3.21**	2,412	**28.72**
City of Pasig	**17,856**	26	**0.15**	1,109	**6.21**	7,114	**39.84**
Pateros	**1,120**	0	**0.00**	55	**4.91**	298	**26.61**
City of Taguig	**19,226**	16	**0.08**	779	**4.05**	6,221	**32.36**
Quezon City	**59,607**	64	**0.11**	2,590	**4.35**	40,713	**68.30**
City of Makati	**10,991**	4	**0.04**	136	**1.24**	1,805	**16.42**
City of Mandaluyong	**7,989**	20	**0.25**	196	**2.45**	2,014	**25.21**
City of San Juan	**2,173**	0	**0.00**	49	**2.25**	516	**23.75**
City of Manila	**34,268**	73	**0.21**	1,914	**5.59**	15,030	**43.86**
City of Las Piñas	**11,346**	13	**0.11**	459	**4.05**	3,704	**32.65**
City of Muntinlupa	**9,949**	58	**0.58**	542	**5.45**	3,767	**37.86**
City of Parañaque	**13,385**	8	**0.06**	414	**3.09**	1,684	**12.58**
Pasay City	**7,129**	13	**0.18**	499	**7.00**	3,006	**42.17**
**CAR**	**31,490**	**49**	**0.16**	**989**	**3.14**	**6,827**	**21.68**
Abra	**3,639**	7	**0.19**	239	**6.57**	633	**17.39**
Apayao	**2,222**	7	**0.32**	74	**3.33**	412	**18.54**
Benguet	**8,900**	4	**0.04**	152	**1.71**	1,394	**15.66**
Ifugao	**4,145**	9	**0.22**	98	**2.36**	781	**18.84**
Kalinga	**4,582**	3	**0.07**	247	**5.39**	2,311	**50.44**
Mountain Province	**2,615**	6	**0.23**	57	**2.18**	296	**11.32**
City of Baguio	**5,387**	13	**0.24**	122	**2.26**	1,000	**18.56**
**Region 1**	**96,024**	**538**	**0.56**	**3,463**	**3.61**	**27,163**	**28.29**
Ilocos Norte	**8,809**	13	**0.15**	233	**2.65**	3,334	**37.85**
Ilocos Sur	**9,834**	7	**0.07**	352	**3.58**	4,836	**49.18**
La Union	**13,436**	22	**0.16**	583	**4.34**	3,981	**29.63**
Pangasinan	**60,591**	489	**0.81**	2,116	**3.49**	14,215	**23.46**
City of Dagupan	**3,354**	7	**0.21**	179	**5.34**	797	**23.76**
**Region 2**	**64,034**	**488**	**0.76**	**3,425**	**5.35**	**18,270**	**28.53**
Batanes	**241**	0	**0.00**	0	**0.00**	1	**0.41**
Cagayan	**20,723**	71	**0.34**	708	**3.42**	3,534	**17.05**
Isabela	**27,362**	146	**0.53**	1,648	**6.02**	8,438	**30.84**
Nueva Vizcaya	**8,880**	13	**0.15**	337	**3.80**	2,641	**29.74**
Quirino	**3,865**	248	**6.42**	421	**10.89**	1,359	**35.16**
City of Santiago	**2,963**	10	**0.34**	311	**10.50**	2,297	**77.52**
**Region 3**	**235,313**	**1,042**	**0.44**	**9,830**	**4.18**	**52,817**	**22.45**
Aurora	**4,578**	1	**0.02**	136	**2.97**	908	**19.83**
Bataan	**17,556**	13	**0.07**	395	**2.25**	2,653	**15.11**
Bulacan	**68,115**	111	**0.16**	2,686	**3.94**	19,368	**28.43**
Nueva Ecija	**43,626**	128	**0.29**	1,560	**3.58**	6,576	**15.07**
Pampanga	**46,146**	702	**1.52**	2,406	**5.21**	9,028	**19.56**
Tarlac	**29,655**	67	**0.23**	1,167	**3.94**	8,466	**28.55**
Zambales	**11,796**	13	**0.11**	948	**8.04**	2,826	**23.96**
City of Angeles	**9,232**	1	**0.01**	48	**0.52**	689	**7.46**
City of Olongapo	**4,609**	6	**0.13**	484	**10.50**	2,303	**49.97**
**Region 4A**	**310,150**	**368**	**0.12**	**30,456**	**9.82**	**80,832**	**26.06**
Batangas	**53,484**	46	**0.09**	1,244	**2.33**	12,558	**23.48**
Cavite	**78,589**	142	**0.18**	1,080	**1.37**	7,268	**9.25**
Laguna	**61,967**	62	**0.10**	24,941	**40.25**	40,834	**65.90**
Quezon	**40,954**	38	**0.09**	1,224	**2.99**	9,350	**22.83**
Rizal	**68,903**	74	**0.11**	1,803	**2.62**	9,611	**13.95**
City of Lucena	**6,253**	6	**0.10**	164	**2.62**	1,211	**19.37**

Table 2.D.10 - ORAL HEALTH CARE
Pregnant women 10-14, 15-19, 20-49 years old who received Basic Oral Health Care (BOHC)
Philippines, 2024

Area	Eligible Population (0-11 months)	Pregnant Women 10-14 years old received BOHC		Pregnant Women 15-19 years old received BOHC		Pregnant Women 20-49 years old received BOHC	
		No.	%	No.	%	No.	%
**Region 4B**	**66,508**	**61**	**0.09**	**758**	**1.14**	**3,975**	**5.98**
Marinduque	**4,124**	6	**0.15**	41	**0.99**	230	**5.58**
Occidental Mindoro	**11,748**	23	**0.20**	138	**1.17**	729	**6.21**
Oriental Mindoro	**19,007**	4	**0.02**	135	**0.71**	806	**4.24**
Palawan	**20,503**	16	**0.08**	308	**1.50**	1,244	**6.07**
Romblon	**5,488**	11	**0.20**	92	**1.68**	582	**10.60**
City of Puerto Princesa	**5,638**	1	**0.02**	44	**0.78**	384	**6.81**
**Region 5**	**136,611**	**82**	**0.06**	**2,776**	**2.03**	**17,322**	**12.68**
Albay	**27,665**	15	**0.05**	408	**1.47**	3,967	**14.34**
Camarines Norte	**14,894**	34	**0.23**	413	**2.77**	1,950	**13.09**
Camarines Sur	**43,855**	14	**0.03**	426	**0.97**	2,775	**6.33**
Catanduanes	**5,395**	0	**0.00**	148	**2.74**	810	**15.01**
Masbate	**22,495**	1	**0.00**	534	**2.37**	2,085	**9.27**
Sorsogon	**18,065**	16	**0.09**	796	**4.41**	5,234	**28.97**
City of Naga	**4,242**	2	**0.05**	51	**1.20**	501	**11.81**
**Region 6**	**147,934**	**223**	**0.15**	**3,985**	**2.69**	**27,655**	**18.69**
Aklan	**11,281**	0	**0.00**	192	**1.70**	1,033	**9.16**
Antique	**11,899**	28	**0.24**	56	**0.47**	1,088	**9.14**
Capiz	**13,872**	32	**0.23**	403	**2.91**	2,311	**16.66**
Guimaras	**3,361**	2	**0.06**	83	**2.47**	516	**15.35**
Iloilo	**35,981**	22	**0.06**	977	**2.72**	7,769	**21.59**
Negros Occidental	**52,445**	87	**0.17**	1,475	**2.81**	8,197	**15.63**
City of Bacolod	**10,361**	31	**0.30**	429	**4.14**	3,564	**34.40**
City of Iloilo	**8,734**	21	**0.24**	370	**4.24**	3,177	**36.38**
**Region 7**	**167,016**	**162**	**0.10**	**5,521**	**3.31**	**29,543**	**17.69**
Bohol	**26,210**	14	**0.05**	271	**1.03**	1,475	**5.63**
Cebu	**72,875**	20	**0.03**	947	**1.30**	7,316	**10.04**
Negros Oriental	**28,980**	33	**0.11**	829	**2.86**	4,672	**16.12**
Siquijor	**1,671**	1	**0.06**	46	**2.75**	665	**39.80**
City of Cebu	**19,466**	46	**0.24**	1,042	**5.35**	6,911	**35.50**
City of Lapu-Lapu	**10,833**	3	**0.03**	454	**4.19**	5,225	**48.23**
City of Mandaue	**6,981**	45	**0.64**	1,932	**27.68**	3,279	**46.97**
**Region 8**	**93,713**	**108**	**0.12**	**2,571**	**2.74**	**15,595**	**16.64**
Biliran	**3,378**	12	**0.36**	146	**4.32**	438	**12.97**
Eastern Samar	**9,594**	0	**0.00**	258	**2.69**	671	**6.99**
Leyte	**31,504**	33	**0.10**	1,768	**5.61**	8,009	**25.42**
Northern Samar	**14,053**	17	**0.12**	114	**0.81**	3,057	**21.75**
Southern Leyte	**7,368**	0	**0.00**	39	**0.53**	350	**4.75**
Samar	**16,946**	46	**0.27**	155	**0.91**	711	**4.20**
Ormoc City	**5,344**	0	**0.00**	0	**0.00**	1,921	**35.95**
City of Tacloban	**5,526**	0	**0.00**	91	**1.65**	438	**7.93**
**Region 9**	**85,348**	**16**	**0.02**	**766**	**0.90**	**3,845**	**4.51**
Zamboanga del Norte	**24,977**	1	**0.00**	90	**0.36**	387	**1.55**
Zamboanga del Sur	**23,905**	8	**0.03**	361	**1.51**	1,768	**7.40**
Zamboanga Sibugay	**13,903**	2	**0.01**	143	**1.03**	691	**4.97**
City of Isabela	**3,209**	1	**0.03**	67	**2.09**	394	**12.28**
City of Zamboanga	**19,354**	4	**0.02**	105	**0.54**	605	**3.13**
**Region 10**	**110,290**	**87**	**0.08**	**2,440**	**2.21**	**13,917**	**12.62**
Bukidnon	**33,913**	33	**0.10**	1,054	**3.11**	4,108	**12.11**
Camiguin	**1,727**	1	**0.06**	13	**0.75**	120	**6.95**
Lanao del Norte	**17,881**	1	**0.01**	76	**0.43**	427	**2.39**
Misamis Occidental	**12,342**	22	**0.18**	641	**5.19**	6,352	**51.47**
Misamis Oriental	**22,184**	21	**0.09**	477	**2.15**	1,929	**8.70**
City of Cagayan De Oro	**14,503**	9	**0.06**	162	**1.12**	884	**6.10**
City of Iligan	**7,740**	0	**0.00**	17	**0.22**	97	**1.25**
**Region 11**	**107,934**	**278**	**0.26**	**5,760**	**5.34**	**24,423**	**22.63**
Davao de Oro	**15,434**	55	**0.36**	1,171	**7.59**	4,932	**31.96**
Davao del Norte	**23,685**	47	**0.20**	1,041	**4.40**	5,037	**21.27**
Davao Oriental	**12,025**	51	**0.42**	1,010	**8.40**	4,455	**37.05**
Davao del Sur	**13,178**	38	**0.29**	1,030	**7.82**	4,001	**30.36**
Davao Occidental	**6,606**	54	**0.82**	518	**7.84**	1,178	**17.83**
City of Davao	**37,006**	33	**0.09**	990	**2.68**	4,820	**13.02**

Table 2.D.10 - ORAL HEALTH CARE
Pregnant women 10-14, 15-19, 20-49 years old who received Basic Oral Health Care (BOHC)
Philippines, 2024

Area	Eligible Population (0-11 months)	Pregnant Women 10-14 years old received BOHC		Pregnant Women 15-19 years old received BOHC		Pregnant Women 20-49 years old received BOHC	
		No.	%	No.	%	No.	%
**Region 12**	**95,296**	**136**	**0.14**	**4,390**	**4.61**	**21,326**	**22.38**
Cotabato	**25,696**	21	**0.08**	653	**2.54**	3,172	**12.34**
Sarangani	**13,501**	21	**0.16**	955	**7.07**	3,734	**27.66**
South Cotabato	**21,842**	59	**0.27**	1,538	**7.04**	7,561	**34.62**
Sultan Kudarat	**19,613**	8	**0.04**	636	**3.24**	3,184	**16.23**
City of General Santos	**14,644**	27	**0.18**	608	**4.15**	3,675	**25.10**
**CARAGA**	**55,371**	**123**	**0.22**	**1,538**	**2.78**	**8,447**	**15.26**
Agusan del Norte	**7,331**	2	**0.03**	173	**2.36**	968	**13.20**
Agusan del Sur	**16,180**	15	**0.09**	428	**2.65**	2,421	**14.96**
Surigao del Norte	**10,168**	73	**0.72**	187	**1.84**	1,279	**12.58**
Surigao del Sur	**12,834**	32	**0.25**	645	**5.03**	2,863	**22.31**
Dinagat Islands	**2,117**	0	**0.00**	7	**0.33**	45	**2.13**
City of Butuan	**6,741**	1	**0.01**	98	**1.45**	871	**12.92**
**BARMM**	**134,585**	**304**	**0.23**	**2,680**	**1.99**	**11,303**	**8.40**
Basilan	**11,653**	15	**0.13**	256	**2.20**	925	**7.94**
Lanao del Sur	**33,339**	75	**0.22**	1,060	**3.18**	3,489	**10.47**
Maguindanao del Norte	**18,923**	4	**0.02**	357	**1.89**	1,989	**10.51**
Maguindanao del Sur	**23,214**	17	**0.07**	434	**1.87**	2,815	**12.13**
Sulu	**21,381**	3	**0.01**	125	**0.58**	326	**1.52**
Tawi-Tawi	**12,602**	1	**0.01**	47	**0.37**	444	**3.52**
SGA	**6,305**	0	**0.00**	0	**0.00**	0	**0.00**
City of Cotabato	**7,168**	189	**2.64**	401	**5.59**	1,315	**18.35**

0 = No Data
* = No Report

Table 2.E.1.1 - Filariasis Prevention and Control
Case Detection Rate (CDR) and Clinical Rate of Lymphatic Filariasis
Philippines, 2024

Area	Total Examined for lymphatic filariasis	Number positive cases found				Lymphatic filariasis cases examined with disabilities/deformities			
		Male	Female	Total	Case Det. Rate	Male	Female	Total	Clinical Rate
**PHILIPPINES**	**15,298**	**203**	**149**	**352**	**2.30**	**5**	**6**	**11**	**3.13**
**N C R**	**0**	**0**	**0**	**0**	**0.00**	**0**	**0**	**0**	**0.00**
City of Malabon	0	0	0	0	0.00	0	0	0	0.00
City of Navotas	0	0	0	0	0.00	0	0	0	0.00
City of Valenzuela	0	0	0	0	0.00	0	0	0	0.00
City of Caloocan	0	0	0	0	0.00	0	0	0	0.00
City of Marikina	0	0	0	0	0.00	0	0	0	0.00
City of Pasig	0	0	0	0	0.00	0	0	0	0.00
Pateros	0	0	0	0	0.00	0	0	0	0.00
City of Taguig	0	0	0	0	0.00	0	0	0	0.00
Quezon City	0	0	0	0	0.00	0	0	0	0.00
City of Makati	0	0	0	0	0.00	0	0	0	0.00
City of Mandaluyong	0	0	0	0	0.00	0	0	0	0.00
City of San Juan	0	0	0	0	0.00	0	0	0	0.00
City of Manila	0	0	0	0	0.00	0	0	0	0.00
City of Las Piñas	0	0	0	0	0.00	0	0	0	0.00
City of Muntinlupa	0	0	0	0	0.00	0	0	0	0.00
City of Parañaque	0	0	0	0	0.00	0	0	0	0.00
Pasay City	0	0	0	0	0.00	0	0	0	0.00
**C A R**	**0**	**0**	**0**	**0**	**0.00**	**0**	**0**	**0**	**0.00**
Abra	0	0	0	0	0.00	0	0	0	0.00
Apayao	0	0	0	0	0.00	0	0	0	0.00
Benguet	0	0	0	0	0.00	0	0	0	0.00
Ifugao	0	0	0	0	0.00	0	0	0	0.00
Kalinga	0	0	0	0	0.00	0	0	0	0.00
Mountain Province	0	0	0	0	0.00	0	0	0	0.00
City of Baguio	0	0	0	0	0.00	0	0	0	0.00
**Region 1**	**0**	**0**	**0**	**0**	**0.00**	**0**	**0**	**0**	**0.00**
Ilocos Norte	0	0	0	0	0.00	0	0	0	0.00
Ilocos Sur	0	0	0	0	0.00	0	0	0	0.00
La Union	0	0	0	0	0.00	0	0	0	0.00
Pangasinan	0	0	0	0	0.00	0	0	0	0.00
City of Dagupan	0	0	0	0	0.00	0	0	0	0.00
**Region 2**	**0**	**0**	**0**	**0**	**0.00**	**0**	**0**	**0**	**0.00**
Batanes	0	0	0	0	0.00	0	0	0	0.00
Cagayan	0	0	0	0	0.00	0	0	0	0.00
Isabela	0	0	0	0	0.00	0	0	0	0.00
Nueva Vizcaya	0	0	0	0	0.00	0	0	0	0.00
Quirino	0	0	0	0	0.00	0	0	0	0.00
City of Santiago	0	0	0	0	0.00	0	0	0	0.00
								0	0.00
**Region 3**	**0**	**0**	**0**	**0**	**0.00**	**0**	**0**	**0**	**0.00**
Aurora	0	0	0	0	0.00	0	0	0	0.00
Bataan	0	0	0	0	0.00	0	0	0	0.00
Bulacan	0	0	0	0	0.00	0	0	0	0.00
Nueva Ecija	0	0	0	0	0.00	0	0	0	0.00
Pampanga	0	0	0	0	0.00	0	0	0	0.00
Tarlac	0	0	0	0	0.00	0	0	0	0.00
Zambales	0	0	0	0	0.00	0	0	0	0.00
City of Angeles	0	0	0	0	0.00	0	0	0	0.00
City of Olongapo	0	0	0	0	0.00	0	0	0	0.00
**Region 4A**	**0**	**0**	**0**	**0**	**0.00**	**0**	**0**	**0**	**0.00**
Batangas	0	0	0	0	0.00	0	0	0	0.00
Cavite	0	0	0	0	0.00	0	0	0	0.00
Laguna	0	0	0	0	0.00	0	0	0	0.00
Quezon	0	0	0	0	0.00	0	0	0	0.00
Rizal	0	0	0	0	0.00	0	0	0	0.00
City of Lucena	0	0	0	0	0.00	0	0	0	0.00

Table 2.E.1.1 - Filariasis Prevention and Control
Case Detection Rate (CDR) and Clinical Rate of Lymphatic Filariasis
Philippines, 2024

Area	Total Examined for lymphatic filariasis	Number positive cases found				Lymphatic filariasis cases examined with disabilities/deformities			
		Male	Female	Total	Case Det. Rate	Male	Female	Total	Clinical Rate
**Region 4B**	**522**	**4**	**6**	**10**	**1.92**	**4**	**6**	**10**	**100.00**
Marinduque	0	0	0	0	0.00	0	0	0	0.00
Occidental Mindoro	0	0	0	0	0.00	0	0	0	0.00
Oriental Mindoro	481	4	6	10	2.08	4	6	10	100.00
Palawan	41	0	0	0	0.00	0	0	0	0.00
Romblon	0	0	0	0	0.00	0	0	0	0.00
City of Puerto Princesa	0	0	0	0	0.00	0	0	0	0.00
**Region 5**	**0**	**0**	**0**	**0**	**0.00**	**0**	**0**	**0**	**0.00**
Albay	0	0	0	0	0.00	0	0	0	0.00
Camarines Norte	0	0	0	0	0.00	0	0	0	0.00
Camarines Sur	0	0	0	0	0.00	0	0	0	0.00
Catanduanes	0	0	0	0	0.00	0	0	0	0.00
Masbate	0	0	0	0	0.00	0	0	0	0.00
Sorsogon	0	0	0	0	0.00	0	0	0	0.00
City of Naga	0	0	0	0	0.00	0	0	0	0.00
**Region 6**	**901**	**1**	**1**	**2**	**0.22**	**0**	**0**	**0**	**0.00**
Aklan	0	0	0	0	0.00	0	0	0	0.00
Antique	513	0	0	0	0.00	0	0	0	0.00
Capiz	0	0	0	0	0.00	0	0	0	0.00
Guimaras	0	0	0	0	0.00	0	0	0	0.00
Iloilo	388	1	1	2	0.52	0	0	0	0.00
Negros Occidental	0	0	0	0	0.00	0	0	0	0.00
City of Bacolod	0	0	0	0	0.00	0	0	0	0.00
City of Iloilo	0	0	0	0	0.00	0	0	0	0.00
**Region 7**	**0**	**0**	**0**	**0**	**0.00**	**0**	**0**	**0**	**0.00**
Bohol	0	0	0	0	0.00	0	0	0	0.00
Cebu	0	0	0	0	0.00	0	0	0	0.00
Negros Oriental	0	0	0	0	0.00	0	0	0	0.00
Siquijor	0	0	0	0	0.00	0	0	0	0.00
City of Cebu	0	0	0	0	0.00	0	0	0	0.00
City of Lapu-Lapu	0	0	0	0	0.00	0	0	0	0.00
City of Mandaue	0	0	0	0	0.00	0	0	0	0.00
**Region 8**	**476**	**0**	**0**	**0**	**0.00**	**0**	**0**	**0**	**0.00**
Biliran	0	0	0	0	0.00	0	0	0	0.00
Eastern Samar	0	0	0	0	0.00	0	0	0	0.00
Leyte	0	0	0	0	0.00	0	0	0	0.00
Northern Samar	0	0	0	0	0.00	0	0	0	0.00
Southern Leyte	476	0	0	0	0.00	0	0	0	0.00
Samar	0	0	0	0	0.00	0	0	0	0.00
Ormoc City	0	0	0	0	0.00	0	0	0	0.00
City of Tacloban	0	0	0	0	0.00	0	0	0	0.00
**Region 9**	**1,761**	**23**	**2**	**25**	**1.42**	**0**	**0**	**0**	**0.00**
Zamboanga del Norte	1761	23	2	25	1.42	0	0	0	0.00
Zamboanga del Sur	0	0	0	0	0.00	0	0	0	0.00
Zamboanga Sibugay	0	0	0	0	0.00	0	0	0	0.00
City of Isabela	0	0	0	0	0.00	0	0	0	0.00
City of Zamboanga	0	0	0	0	0.00	0	0	0	0.00
**Region 10**	**5,439**	**17**	**12**	**29**	**0.53**	**0**	**0**	**0**	**0.00**
Bukidnon	0	0	0	0	0.00	0	0	0	0.00
Camiguin	0	0	0	0	0.00	0	0	0	0.00
Lanao del Norte	0	0	0	0	0.00	0	0	0	0.00
Misamis Occidental	0	0	0	0	0.00	0	0	0	0.00
Misamis Oriental	5439	17	12	29	0.53	0	0	0	0.00
City of Cagayan De Oro	0	0	0	0	0.00	0	0	0	0.00
City of Iligan	0	0	0	0	0.00	0	0	0	0.00

Table 2.E.1.1 - Filariasis Prevention and Control
Case Detection Rate (CDR) and Clinical Rate of Lymphatic Filariasis
Philippines, 2024

Area	Total Examined for lymphatic filariasis	Number positive cases found				Lymphatic filariasis cases examined with disabilities/deformities			
		Male	Female	Total	Case Det. Rate	Male	Female	Total	Clinical Rate
**Region 11**	**561**	**0**	**0**	**0**	**0.00**	**1**	**0**	**1**	**0.00**
Davao de Oro	0	0	0	0	0.00	0	0	0	0.00
Davao del Norte	0	0	0	0	0.00	0	0	0	0.00
Davao Oriental	0	0	0	0	0.00	0	0	0	0.00
Davao del Sur	335	0	0	0	0.00	0	0	0	0.00
Davao Occidental	226	0	0	0	0.00	1	0	1	0.00
City of Davao	0	0	0	0	0.00	0	0	0	0.00
**Region 12**	**5,638**	**158**	**128**	286	5.07	**0**	**0**	0	0.00
Cotabato	0.00	0	0	0	0.00	0	0	0	0.00
Sarangani	2,054.00	33	25	58	2.82	0	0	0	0.00
South Cotabato	191.00	1	0	1	0.52	0	0	0	0.00
Sultan Kudarat	3,393.00	124	103	227	6.69	0	0	0	0.00
City of General Santos	0.00	0	0	0	0.00	0	0	0	0.00
**CARAGA**	**0**	**0**	**0**	**0**	**0.00**	**0**	**0**	**0**	**0.00**
Agusan del Norte	0	0	0	0	0.00	0	0	0	0.00
Agusan del Sur	0	0	0	0	0.00	0	0	0	0.00
Surigao del Norte	0	0	0	0	0.00	0	0	0	0.00
Surigao del Sur	0	0	0	0	0.00	0	0	0	0.00
Dinagat Islands	0	0	0	0	0.00	0	0	0	0.00
City of Butuan	0	0	0	0	0.00	0	0	0	0.00
**BARMM**	**0**	**0**	**0**	**0**	**0.00**	**0**	**0**	**0**	**0.00**
Basilan	0	0	0	0	0.00	0	0	0	0.00
Lanao del Sur	0	0	0	0	0.00	0	0	0	0.00
Maguindanao del Norte	0	0	0	0	0.00	0	0	0	0.00
Maguindanao del Sur	0	0	0	0	0.00	0	0	0	0.00
Sulu	0	0	0	0	0.00	0	0	0	0.00
Tawi-Tawi	0	0	0	0	0.00	0	0	0	0.00
SGA									
City of Cotabato	0	0	0	0	0.00	0	0	0	0.00

Legend:
0 = No Case
** = No Report*

Table 2.E.2.1 - Schistosomiasis Prevention and Control

Number and proportion of suspected schistosomiasis cases seen, acute clinically diagnosed cases seen, and acute confirmed cases by stool examination

Philippines, 2024

Area	Total Patients seen	Number of Suspected Cases				Number of Acute Clinically Diagnosed Cases				Number of Acute Confirmed Case Positive by Stool Examination			
		Male	Female	Total	%	Male	Female	Total	%	Male	Female	Total	%
**Philippines**	**127,154**	**11,731**	**9,096**	**20,827**	**16.38**	**1,390**	**785**	**2,175**	**10.44**	**1,178**	**640**	**1,818**	**83.59**
**NCR**	**0**	**0**	**0**	**0**	**0.00**	**0**	**0**	**0**	**0.00**	**0**	**0**	**0**	**0.00**
City of Malabon	0	0	0	0	0.00	0	0	0	0.00	0	0	0	0.00
City of Navotas	0	0	0	0	0.00	0	0	0	0.00	0	0	0	0.00
City of Valenzuela	0	0	0	0	0.00	0	0	0	0.00	0	0	0	0.00
City of Caloocan	0	0	0	0	0.00	0	0	0	0.00	0	0	0	0.00
City of Marikina	0	0	0	0	0.00	0	0	0	0.00	0	0	0	0.00
City of Pasig	0	0	0	0	0.00	0	0	0	0.00	0	0	0	0.00
Pateros	0	0	0	0	0.00	0	0	0	0.00	0	0	0	0.00
City of Taguig	0	0	0	0	0.00	0	0	0	0.00	0	0	0	0.00
Quezon City	0	0	0	0	0.00	0	0	0	0.00	0	0	0	0.00
City of Makati	0	0	0	0	0.00	0	0	0	0.00	0	0	0	0.00
City of Mandaluyong	0	0	0	0	0.00	0	0	0	0.00	0	0	0	0.00
City of San Juan	0	0	0	0	0.00	0	0	0	0.00	0	0	0	0.00
City of Manila	0	0	0	0	0.00	0	0	0	0.00	0	0	0	0.00
City of Las Piñas	0	0	0	0	0.00	0	0	0	0.00	0	0	0	0.00
City of Muntinlupa	0	0	0	0	0.00	0	0	0	0.00	0	0	0	0.00
City of Parañaque	0	0	0	0	0.00	0	0	0	0.00	0	0	0	0.00
Pasay City	0	0	0	0	0.00	0	0	0	0.00	0	0	0	0.00
**CAR**	**0**	**0**	**0**	**0**	**0.00**	**0**	**0**	**0**	**0.00**	**0**	**0**	**0**	**0.00**
Abra	0	0	0	0	0.00	0	0	0	0.00	0	0	0	0.00
Apayao	0	0	0	0	0.00	0	0	0	0.00	0	0	0	0.00
Benguet	0	0	0	0	0.00	0	0	0	0.00	0	0	0	0.00
Ifugao	0	0	0	0	0.00	0	0	0	0.00	0	0	0	0.00
Kalinga	0	0	0	0	0.00	0	0	0	0.00	0	0	0	0.00
Mountain Province	0	0	0	0	0.00	0	0	0	0.00	0	0	0	0.00
City of Baguio	0	0	0	0	0.00	0	0	0	0.00	0	0	0	0.00
**Region 1**	**0**	**0**	**0**	**0**	**0.00**	**0**	**0**	**0**	**0.00**	**0**	**0**	**0**	**0.00**
Ilocos Norte	0	0	0	0	0.00	0	0	0	0.00	0	0	0	0.00
Ilocos Sur	0	0	0	0	0.00	0	0	0	0.00	0	0	0	0.00
La Union	0	0	0	0	0.00	0	0	0	0.00	0	0	0	0.00
Pangasinan	0	0	0	0	0.00	0	0	0	0.00	0	0	0	0.00
City of Dagupan	0	0	0	0	0.00	0	0	0	0.00	0	0	0	0.00
**Region 2**	**0**	**0**	**0**	**0**	**0.00**	**0**	**0**	**0**	**0.00**	**0**	**0**	**0**	**0.00**
Batanes	0	0	0	0	0.00	0	0	0	0.00	0	0	0	0.00
Cagayan	0	0	0	0	0.00	0	0	0	0.00	0	0	0	0.00
Isabela	0	0	0	0	0.00	0	0	0	0.00	0	0	0	0.00
Nueva Vizcaya	0	0	0	0	0.00	0	0	0	0.00	0	0	0	0.00
Quirino	0	0	0	0	0.00	0	0	0	0.00	0	0	0	0.00
City of Santiago	0	0	0	0	0.00	0	0	0	0.00	0	0	0	0.00
**Region 3**	**5**	**3**	**0**	**3**	**60.00**	**0**	**0**	**0**	**0.00**	**0**	**0**	**0**	**0.00**
Aurora	0	0	0	0	0.00	0	0	0	0.00	0	0	0	0.00
Bataan	0	0	0	0	0.00	0	0	0	0.00	0	0	0	0.00
Bulacan	5	3	0	3	60.00	0	0	0	0.00	0	0	0	0.00
Nueva Ecija	0	0	0	0	0.00	0	0	0	0.00	0	0	0	0.00
Pampanga	0	0	0	0	0.00	0	0	0	0.00	0	0	0	0.00
Tarlac	0	0	0	0	0.00	0	0	0	0.00	0	0	0	0.00
Zambales	0	0	0	0	0.00	0	0	0	0.00	0	0	0	0.00
City of Angeles	0	0	0	0	0.00	0	0	0	0.00	0	0	0	0.00
City of Olongapo	0	0	0	0	0.00	0	0	0	0.00	0	0	0	0.00

Table 2.E.2.1 - Schistosomiasis Prevention and Control
Number and proportion of suspected schistosomiasis cases seen, acute clinically diagnosed cases seen, and acute confirmed cases by stool examination
Philippines, 2024

Area	Total Patients seen	Number of Suspected Cases				Number of Acute Clinically Diagnosed Cases				Number of Acute Confirmed Case Positive by Stool Examination			
		Male	Female	Total	%	Male	Female	Total	%	Male	Female	Total	%
**Region 4A**	**309**	**30**	**9**	**39**	**12.62**	**0**	**0**	**0**	**0.00**	**0**	**0**	**0**	**0.00**
Batangas	1	0	1	1	100.00	0	0	0	0.00	0	0	0	0.00
Cavite	80	16	6	22	27.50	0	0	0	0.00	0	0	0	0.00
Laguna	0	0	0	0	0.00	0	0	0	0.00	0	0	0	0.00
Quezon	39	14	1	15	38.46	0	0	0	0.00	0	0	0	0.00
Rizal	189	0	1	1	0.53	0	0	0	0.00	0	0	0	0.00
City of Lucena	0	0	0	0	0.00	0	0	0	0.00	0	0	0	0.00
**Region 4B**	**1,899**	**0**	**0**	**0**	**0.00**	**0**	**0**	**0**	**0.00**	**0**	**0**	**0**	**0.00**
Marinduque	0	0	0	0	0.00	0	0	0	0.00	0	0	0	0.00
Occidental Mindoro	12	0	0	0	0.00	0	0	0	0.00	0	0	0	0.00
Oriental Mindoro	1,880	0	0	0	0.00	0	0	0	0.00	0	0	0	0.00
Palawan	7	0	0	0	0.00	0	0	0	0.00	0	0	0	0.00
Romblon	0	0	0	0	0.00	0	0	0	0.00	0	0	0	0.00
City of Puerto Princesa	0	0	0	0	0.00	0	0	0	0.00	0	0	0	0.00
**Region 5**	**32**	**22**	**10**	**32**	**100.00**	**5**	**1**	**6**	**18.75**	**5**	**1**	**6**	**100.00**
Albay	1	1	0	1	100.00	0	0	0	0.00	0	0	0	0.00
Camarines Norte	0	0	0	0	0.00	0	0	0	0.00	0	0	0	0.00
Camarines Sur	0	0	0	0	0.00	0	0	0	0.00	0	0	0	0.00
Catanduanes	0	0	0	0	0.00	0	0	0	0.00	0	0	0	0.00
Masbate	0	0	0	0	0.00	0	0	0	0.00	0	0	0	0.00
Sorsogon	31	21	10	31	100.00	5	1	6	19.35	5	1	6	100.00
City of Naga	0	0	0	0	0.00	0	0	0	0.00	0	0	0	0.00
**Region 6**	**0**	**0**	**0**	**0**	**0.00**	**0**	**0**	**0**	**0.00**	**0**	**0**	**0**	**0.00**
Aklan	0	0	0	0	0.00	0	0	0	0.00	0	0	0	0.00
Antique	0	0	0	0	0.00	0	0	0	0.00	0	0	0	0.00
Capiz	0	0	0	0	0.00	0	0	0	0.00	0	0	0	0.00
Guimaras	0	0	0	0	0.00	0	0	0	0.00	0	0	0	0.00
Iloilo	0	0	0	0	0.00	0	0	0	0.00	0	0	0	0.00
Negros Occidental	0	0	0	0	0.00	0	0	0	0.00	0	0	0	0.00
City of Bacolod	0	0	0	0	0.00	0	0	0	0.00	0	0	0	0.00
City of Iloilo	0	0	0	0	0.00	0	0	0	0.00	0	0	0	0.00
**Region 7**	**9**	**7**	**2**	**9**	**100.00**	**0**	**0**	**0**	**0.00**	**0**	**0**	**0**	**0.00**
Bohol	9	7	2	9	100.00	0	0	0	0.00	0	0	0	0.00
Cebu	0	0	0	0	0.00	0	0	0	0.00	0	0	0	0.00
Negros Oriental	0	0	0	0	0.00	0	0	0	0.00	0	0	0	0.00
Siquijor	0	0	0	0	0.00	0	0	0	0.00	0	0	0	0.00
City of Cebu	0	0	0	0	0.00	0	0	0	0.00	0	0	0	0.00
City of Lapu-Lapu	0	0	0	0	0.00	0	0	0	0.00	0	0	0	0.00
City of Mandaue	0	0	0	0	0.00	0	0	0	0.00	0	0	0	0.00
**Region 8**	**31,393**	**5,386**	**3,592**	**8,978**	**28.60**	**500**	**289**	**789**	**8.79**	**481**	**267**	**748**	**94.80**
Biliran	0	0	0	0	0.00	0	0	0	0.00	0	0	0	0.00
Eastern Samar	4,180	68	63	131	3.13	7	5	12	9.16	7	4	11	91.67
Leyte	15,218	375	168	543	3.57	228	82	310	57.09	226	82	308	99.35
Northern Samar	3,013	498	338	836	27.75	260	195	455	54.43	243	175	418	91.87
Southern Leyte	0	0	0	0	0.00	0	0	0	0.00	0	0	0	0.00
Samar	8,957	4,432	3,011	7,443	83.10	4	5	9	0.12	4	4	8	88.89
Ormoc City	0	0	0	0	0.00	0	0	0	0.00	0	0	0	0.00
City of Tacloban	25	13	12	25	100.00	1	2	3	12.00	1	2	3	100.00
**Region 9**	**8,261**	**222**	**82**	**304**	**3.68**	**26**	**4**	**30**	**9.87**	**26**	**4**	**30**	**100.00**
Zamboanga del Norte	426	2	0	2	0.47	2	0	2	100.00	2	0	2	100.00
Zamboanga del Sur	7,828	218	81	299	3.82	22	3	25	8.36	22	3	25	100.00
Zamboanga Sibugay	7	2	1	3	42.86	2	1	3	100.00	2	1	3	100.00
City of Isabela	0	0	0	0	0.00	0	0	0	0.00	0	0	0	0.00
City of Zamboanga	0	0	0	0	0.00	0	0	0	0.00	0	0	0	0.00

Table 2.E.2.1 - Schistosomiasis Prevention and Control

Number and proportion of suspected schistosomiasis cases seen, acute clinically diagnosed cases seen, and acute confirmed cases by stool examination

Philippines, 2024

Area	Total Patients seen	Number of Suspected Cases				Number of Acute Clinically Diagnosed Cases				Number of Acute Confirmed Case Positive by Stool Examination			
		Male	Female	Total	%	Male	Female	Total	%	Male	Female	Total	%
**Region 10**	**1,866**	**1,005**	**690**	**1,695**	**90.84**	**261**	**188**	**449**	**26.49**	**103**	**64**	**167**	**37.19**
Bukidnon	650	297	184	481	74.00	216	146	362	75.26	74	44	118	32.60
Camiguin	1	0	1	1	100.00	0	0	0	0.00	0	0	0	0.00
Lanao del Norte	1,129	657	472	1,129	100.00	20	20	40	3.54	19	18	37	92.50
Misamis Occidental	86	51	33	84	97.67	25	22	47	55.95	10	2	12	25.53
Misamis Oriental	0	0	0	0	0.00	0	0	0	0.00	0	0	0	0.00
City of Cagayan De Oro	0	0	0	0	0.00	0	0	0	0.00	0	0	0	0.00
City of Iligan	0	0	0	0	0.00	0	0	0	0.00	0	0	0	0.00
**Region 11**	**7,428**	**99**	**76**	**175**	**2.36**	**37**	**24**	**61**	**34.86**	**18**	**12**	**30**	**49.18**
Davao de Oro	3,720	46	26	72	1.94	21	11	32	44.44	11	2	13	40.63
Davao del Norte	2,604	52	50	102	3.92	15	13	28	27.45	7	10	17	60.71
Davao Oriental	311	0	0	0	0.00	0	0	0	0.00	0	0	0	0.00
Davao del Sur	1	1	0	1	100.00	1	0	1	100.00	0	0	0	0.00
Davao Occidental	532	0	0	0	0.00	0	0	0	0.00	0	0	0	0.00
City of Davao	260	0	0	0	0.00	0	0	0	0.00	0	0	0	0.00
**Region 12**	**312**	**116**	**53**	**169**	**54.17**	**71**	**24**	**95**	**56.21**	**69**	**23**	**92**	**96.84**
Cotabato	24	13	11	24	100.00	10	10	20	83.33	8	9	17	85.00
Sarangani	0	0	0	0	0.00	0	0	0	0.00	0	0	0	0.00
South Cotabato	90	55	27	82	91.11	23	4	27	32.93	23	4	27	100.00
Sultan Kudarat	197	47	15	62	31.47	38	10	48	77.42	38	10	48	100.00
City of General Santos	1	1	0	1	100.00	0	0	0	0.00	0	0	0	0.00
**Caraga**	**74,665**	**4,342**	**4,106**	**8,448**	**11.31**	**479**	**253**	**732**	**8.66**	**440**	**227**	**667**	**91.12**
Agusan del Norte	3,492	13	3	16	0.46	4	0	4	25.00	1	0	1	25.00
Agusan del Sur	69,365	4,003	3,787	7,790	11.23	380	199	579	7.43	354	186	540	93.26
Surigao del Norte	1,753	312	309	621	35.42	81	48	129	20.77	73	35	108	83.72
Surigao del Sur	15	7	1	8	53.33	7	1	8	100.00	5	1	6	75.00
Dinagat Islands	0	0	0	0	0.00	0	0	0	0.00	0	0	0	0.00
City of Butuan	40	7	6	13	32.50	7	5	12	92.31	7	5	12	100.00
**BARMM**	**975**	**499**	**476**	**975**	**100.00**	**11**	**2**	**13**	**1.33**	**36**	**42**	**78**	**600.00**
Basilan	0	0	0	0	0.00	0	0	0	0.00	0	0	0	0.00
Lanao del Sur	896	465	431	896	100.00	4	2	6	0.67	4	2	6	100.00
Maguindanao del Norte	14	4	10	14	100.00	0	0	0	0.00	4	10	14	0.00
Maguindanao del Sur	63	28	35	63	100.00	5	0	5	7.94	28	30	58	1160.00
Sulu	0	0	0	0	0.00	0	0	0	0.00	0	0	0	0.00
Tawi-Tawi	0	0	0	0	0.00	0	0	0	0.00	0	0	0	0.00
SGA	2	2	0	2	100.00	2	0	2	100.00	0	0	0	0.00
City of Cotabato	0	0	0	0	0.00	0	0	0	0.00	0	0	0	0.00

0 = No Case

* = No Report

Table 2.E.2.2 - Schistosomiasis Prevention and Control
Number and proportion of chronic clinically diagnosed cases seen, confirmed by stool examination, treated in the health facility, and referred to a hospital facility
Philippines, 2024

Area	Number of Chronic Clinically Diagnosed Cases				Number of Chronic Clinically Diagnosed Cases Confirmed by Stool Examination				Number of Chronic Clinically Diagnosed Cases Treated in the Health Facility among Acute and Chronic Cases				Number of Confirmed Chronic Cases referred to Hospital Facility			
	Male	Female	Total	%	Male	Female	Total	%	Male	Female	Total	%	Male	Female	Total	%
**Philippines**	**1,139**	**606**	**1,745**	**8.38**	**626**	**334**	**960**	**55.01**	**803**	**427**	**1,230**	**44.28**	**19**	**11**	**30**	**3.13**
**NCR**	**0**	**0**	**0**	**0.00**	**0**	**0**	**0**	**0.00**	**0**	**0**	**0**	**0.00**	**0**	**0**	**0**	**0.00**
City of Malabon	0	0	0	0.00	0	0	0	0.00	0	0	0	0.00	0	0	0	0.00
City of Navotas	0	0	0	0.00	0	0	0	0.00	0	0	0	0.00	0	0	0	0.00
City of Valenzuela	0	0	0	0.00	0	0	0	0.00	0	0	0	0.00	0	0	0	0.00
City of Caloocan	0	0	0	0.00	0	0	0	0.00	0	0	0	0.00	0	0	0	0.00
								0.00								
City of Marikina	0	0	0	0.00	0	0	0	0.00	0	0	0	0.00	0	0	0	0.00
City of Pasig	0	0	0	0.00	0	0	0	0.00	0	0	0	0.00	0	0	0	0.00
Pateros	0	0	0	0.00	0	0	0	0.00	0	0	0	0.00	0	0	0	0.00
City of Taguig	0	0	0	0.00	0	0	0	0.00	0	0	0	0.00	0	0	0	0.00
City of Quezon	0	0	0	0.00	0	0	0	0.00	0	0	0	0.00	0	0	0	0.00
								0.00								
City of Makati	0	0	0	0.00	0	0	0	0.00	0	0	0	0.00	0	0	0	0.00
City of Mandaluyong	0	0	0	0.00	0	0	0	0.00	0	0	0	0.00	0	0	0	0.00
City of San Juan	0	0	0	0.00	0	0	0	0.00	0	0	0	0.00	0	0	0	0.00
City of Manila	0	0	0	0.00	0	0	0	0.00	0	0	0	0.00	0	0	0	0.00
City of Las Piñas	0	0	0	0.00	0	0	0	0.00	0	0	0	0.00	0	0	0	0.00
City of Muntinlupa	0	0	0	0.00	0	0	0	0.00	0	0	0	0.00	0	0	0	0.00
City of Parañaque	0	0	0	0.00	0	0	0	0.00	0	0	0	0.00	0	0	0	0.00
City of Pasay	0	0	0	0.00	0	0	0	0.00	0	0	0	0.00	0	0	0	0.00
**CAR**	**0**	**0**	**0**	**0.00**	**0**	**0**	**0**	**0.00**	**0**	**0**	**0**	**0.00**	**0**	**0**	**0**	**0.00**
Abra	0	0	0	0.00	0	0	0	0.00	0	0	0	0.00	0	0	0	0.00
Apayao	0	0	0	0.00	0	0	0	0.00	0	0	0	0.00	0	0	0	0.00
Benguet	0	0	0	0.00	0	0	0	0.00	0	0	0	0.00	0	0	0	0.00
Ifugao	0	0	0	0.00	0	0	0	0.00	0	0	0	0.00	0	0	0	0.00
Kalinga	0	0	0	0.00	0	0	0	0.00	0	0	0	0.00	0	0	0	0.00
Mountain Province	0	0	0	0.00	0	0	0	0.00	0	0	0	0.00	0	0	0	0.00
City of Baguio	0	0	0	0.00	0	0	0	0.00	0	0	0	0.00	0	0	0	0.00
**Region 1**	**0**	**0**	**0**	**0.00**	**0**	**0**	**0**	**0.00**	**0**	**0**	**0**	**0.00**	**0**	**0**	**0**	**0.00**
Ilocos Norte	0	0	0	0.00	0	0	0	0.00	0	0	0	0.00	0	0	0	0.00
Ilocos Sur	0	0	0	0.00	0	0	0	0.00	0	0	0	0.00	0	0	0	0.00
La Union	0	0	0	0.00	0	0	0	0.00	0	0	0	0.00	0	0	0	0.00
Pangasinan	0	0	0	0.00	0	0	0	0.00	0	0	0	0.00	0	0	0	0.00
City of Dagupan	0	0	0	0.00	0	0	0	0.00	0	0	0	0.00	0	0	0	0.00
**Region 2**	**0**	**0**	**0**	**0.00**	**0**	**0**	**0**	**0.00**	**0**	**0**	**0**	**0.00**	**0**	**0**	**0**	**0.00**
Batanes	0	0	0	0.00	0	0	0	0.00	0	0	0	0.00	0	0	0	0.00
Cagayan	0	0	0	0.00	0	0	0	0.00	0	0	0	0.00	0	0	0	0.00
Isabela	0	0	0	0.00	0	0	0	0.00	0	0	0	0.00	0	0	0	0.00
Nueva Vizcaya	0	0	0	0.00	0	0	0	0.00	0	0	0	0.00	0	0	0	0.00
Quirino	0	0	0	0.00	0	0	0	0.00	0	0	0	0.00	0	0	0	0.00
												0.00				
City of Santiago	0	0	0	0.00	0	0	0	0.00	0	0	0	0.00	0	0	0	0.00
**Region 3**	**0**	**0**	**0**	**0.00**	**0**	**0**	**0**	**0.00**	**0**	**0**	**0**	**0.00**	**0**	**0**	**0**	**0.00**
Aurora	0	0	0	0.00	0	0	0	0.00	0	0	0	0.00	0	0	0	0.00
Bataan	0	0	0	0.00	0	0	0	0.00	0	0	0	0.00	0	0	0	0.00
Bulacan	0	0	0	0.00	0	0	0	0.00	0	0	0	0.00	0	0	0	0.00
Nueva Ecija	0	0	0	0.00	0	0	0	0.00	0	0	0	0.00	0	0	0	0.00
Pampanga	0	0	0	0.00	0	0	0	0.00	0	0	0	0.00	0	0	0	0.00
Tarlac	0	0	0	0.00	0	0	0	0.00	0	0	0	0.00	0	0	0	0.00
Zambales	0	0	0	0.00	0	0	0	0.00	0	0	0	0.00	0	0	0	0.00
City of Angeles	0	0	0	0.00	0	0	0	0.00	0	0	0	0.00	0	0	0	0.00
City of Olongapo	0	0	0	0.00	0	0	0	0.00	0	0	0	0.00	0	0	0	0.00
**Region 4A**	**0**	**0**	**0**	**0.00**	**0**	**0**	**0**	**0.00**	**0**	**0**	**0**	**0.00**	**0**	**0**	**0**	**0.00**
Batangas	0	0	0	0.00	0	0	0	0.00	0	0	0	0.00	0	0	0	0.00
Cavite	0	0	0	0.00	0	0	0	0.00	0	0	0	0.00	0	0	0	0.00
Laguna	0	0	0	0.00	0	0	0	0.00	0	0	0	0.00	0	0	0	0.00
Quezon	0	0	0	0.00	0	0	0	0.00	0	0	0	0.00	0	0	0	0.00
Rizal	0	0	0	0.00	0	0	0	0.00	0	0	0	0.00	0	0	0	0.00
City of Lucena	0	0	0	0.00	0	0	0	0.00	0	0	0	0.00	0	0	0	0.00
**Region 4B**	**0**	**0**	**0**	**0.00**	**0**	**0**	**0**	**0.00**	**1**	**0**	**1**	**0.00**	**0**	**3**	**3**	**0.00**
Marinduque	0	0	0	0.00	0	0	0	0.00	0	0	0	0.00	0	0	0	0.00
Occidental Mindoro	0	0	0	0.00	0	0	0	0.00	0	0	0	0.00	0	0	0	0.00
Oriental Mindoro	0	0	0	0.00	0	0	0	0.00	0	0	0	0.00	0	3	3	0.00
Palawan	0	0	0	0.00	0	0	0	0.00	1	0	1	0.00	0	0	0	0.00
Romblon	0	0	0	0.00	0	0	0	0.00	0	0	0	0.00	0	0	0	0.00
City of Puerto Princesa	0	0	0	0.00	0	0	0	0.00	0	0	0	0.00	0	0	0	0.00

Table 2.E.2.2 - Schistosomiasis Prevention and Control
Number and proportion of chronic clinically diagnosed cases seen, confirmed by stool examination, treated in the health facility, and referred to a hospital facility
Philippines, 2024

Area	Number of Chronic Clinically Diagnosed Cases				Number of Chronic Clinically Diagnosed Cases Confirmed by Stool Examination				Number of Chronic Clinically Diagnosed Cases Treated in the Health Facility among Acute and Chronic Cases				Number of Confirmed Chronic Cases referred to Hospital Facility			
	Male	Female	Total	%	Male	Female	Total	%	Male	Female	Total	%	Male	Female	Total	%
**Region 5**	**0**	**0**	**0**	**0.00**	**0**	**0**	**0**	**0.00**	**5**	**1**	**6**	**100.00**	**0**	**0**	**0**	**0.00**
Albay	0	0	0	0.00	0	0	0	0.00	0	1	1	0.00	0	0	0	0.00
Camarines Norte	0	0	0	0.00	0	0	0	0.00	0	0	0	0.00	0	0	0	0.00
Camarines Sur	0	0	0	0.00	0	0	0	0.00	0	0	0	0.00	0	0	0	0.00
Catanduanes	0	0	0	0.00	0	0	0	0.00	0	0	0	0.00	0	0	0	0.00
Masbate	0	0	0	0.00	0	0	0	0.00	0	0	0	0.00	0	0	0	0.00
Sorsogon	0	0	0	0.00	0	0	0	0.00	5	0	5	83.33	0	0	0	0.00
City of Naga	0	0	0	0.00	0	0	0	0.00	0	0	0	0.00	0	0	0	0.00
**Region 6**	**0**	**0**	**0**	**0.00**	**0**	**0**	**0**	**0.00**	**0**	**0**	**0**	**0.00**	**0**	**0**	**0**	**0.00**
Aklan	0	0	0	0.00	0	0	0	0.00	0	0	0	0.00	0	0	0	0.00
Antique	0	0	0	0.00	0	0	0	0.00	0	0	0	0.00	0	0	0	0.00
Capiz	0	0	0	0.00	0	0	0	0.00	0	0	0	0.00	0	0	0	0.00
Guimaras	0	0	0	0.00	0	0	0	0.00	0	0	0	0.00	0	0	0	0.00
Iloilo	0	0	0	0.00	0	0	0	0.00	0	0	0	0.00	0	0	0	0.00
Negros Occidental	0	0	0	0.00	0	0	0	0.00	0	0	0	0.00	0	0	0	0.00
City of Bacolod	0	0	0	0.00	0	0	0	0.00	0	0	0	0.00	0	0	0	0.00
City of Iloilo	0	0	0	0.00	0	0	0	0.00	0	0	0	0.00	0	0	0	0.00
								0.00								
**Region 7**	**0**	**0**	**0**	**0.00**	**0**	**0**	**0**	**0.00**	**0**	**0**	**0**	**0.00**	**0**	**0**	**0**	**0.00**
								0.00								
Bohol	0	0	0	0.00	0	0	0	0.00	0	0	0	0.00	0	0	0	0.00
Cebu	0	0	0	0.00	0	0	0	0.00	0	0	0	0.00	0	0	0	0.00
Negros Oriental	0	0	0	0.00	0	0	0	0.00	0	0	0	0.00	0	0	0	0.00
Siquijor	0	0	0	0.00	0	0	0	0.00	0	0	0	0.00	0	0	0	0.00
City of Cebu	0	0	0	0.00	0	0	0	0.00	0	0	0	0.00	0	0	0	0.00
City of Lapu-Lapu	0	0	0	0.00	0	0	0	0.00	0	0	0	0.00	0	0	0	0.00
City of Mandaue	0	0	0	0.00	0	0	0	0.00	0	0	0	0.00	0	0	0	0.00
								0.00								
**Region 8**	**262**	**149**	**411**	**4.58**	**170**	**91**	**261**	**63.50**	**324**	**235**	**559**	**55.40**	**6**	**4**	**10**	**3.83**
								0.00								
Biliran	0	0	0	0.00	0	0	0	0.00	0	0	0	0.00	0	0	0	0.00
Eastern Samar	6	5	11	8.40	3	2	5	45.45	6	5	11	68.75	0	0	0	0.00
Leyte	151	60	211	38.86	85	34	119	56.40	17	8	25	5.85	5	4	9	7.56
Northern Samar	95	75	170	20.33	72	48	120	70.59	290	212	502	93.31	0	0	0	0.00
Southern Leyte	0	0	0	0.00	0	0	0	0.00	0	0	0	0.00	0	0	0	0.00
Samar	10	9	19	0.26	10	7	17	89.47	11	10	21	84.00	1	0	1	5.88
								0.00								
Ormoc City	0	0	0	0.00	0	0	0	0.00	0	0	0	0.00	0	0	0	0.00
City of Tacloban	0	0	0	0.00	0	0	0	0.00	0	0	0	0.00	0	0	0	0.00
**Region 9**	**196**	**78**	**274**	**90.13**	**196**	**78**	**274**	**100.00**	**164**	**59**	**223**	**73.36**	**0**	**0**	**0**	**0.00**
Zamboanga del Norte	0	0	0	0.00	0	0	0	0.00	0	0	0	0.00	0	0	0	0.00
Zamboanga del Sur	196	78	274	91.64	196	78	274	100.00	164	59	223	74.58	0	0	0	0.00
Zamboanga Sibugay	0	0	0	0.00	0	0	0	0.00	0	0	0	0.00	0	0	0	0.00
				0.00				0.00								
City of Isabela	0	0	0	0.00	0	0	0	0.00	0	0	0	0.00	0	0	0	0.00
City of Zamboanga	0	0	0	0.00	0	0	0	0.00	0	0	0	0.00	0	0	0	0.00
**Region 10**	**485**	**270**	**755**	**44.54**	**45**	**31**	**76**	**10.07**	**113**	**51**	**164**	**67.49**	**3**	**0**	**3**	**3.95**
								0.00								
Bukidnon	42	24	66	13.72	19	9	28	42.42	78	31	109	74.66	1	0	1	3.57
Camiguin	0	0	0	0.00	0	0	0	0.00	0	0	0	0.00	0	0	0	0.00
Lanao del Norte	424	237	661	58.55	17	18	35	5.30	22	15	37	51.39	0	0	0	0.00
Misamis Occidental	19	9	28	33.33	9	4	13	46.43	13	5	18	72.00	2	0	2	15.38
Misamis Oriental	0	0	0	0.00	0	0	0	0.00	0	0	0	0.00	0	0	0	0.00
City of Cagayan De Oro	0	0	0	0.00	0	0	0	0.00	0	0	0	0.00	0	0	0	0.00
City of Iligan	0	0	0	0.00	0	0	0	0.00	0	0	0	0.00	0	0	0	0.00
**Region 11**	**51**	**35**	**86**	**49.14**	**48**	**28**	**76**	**88.37**	**49**	**27**	**76**	**71.70**	**2**	**0**	**2**	**2.63**
Davao de Oro	16	3	19	26.39	14	3	17	89.47	16	3	19	63.33	0	0	0	0.00
Davao del Norte	35	32	67	65.69	34	25	59	88.06	33	24	57	75.00	2	0	2	3.39
Davao Oriental	0	0	0	0.00	0	0	0	0.00	0	0	0	0.00	0	0	0	0.00
Davao del Sur	0	0	0	0.00	0	0	0	0.00	0	0	0	0.00	0	0	0	0.00
Davao Occidental	0	0	0	0.00	0	0	0	0.00	0	0	0	0.00	0	0	0	0.00
City of Davao	0	0	0	0.00	0	0	0	0.00	0	0	0	0.00	0	0	0	0.00
**Region 12**	**53**	**20**	**73**	**43.20**	**52**	**20**	**72**	**98.63**	**76**	**25**	**101**	**61.59**	**4**	**1**	**5**	**6.94**
Cotabato	4	1	5	20.83	3	1	4	80.00	4	2	6	28.57	3	1	4	100.00
Sarangani	0	0	0	0.00	0	0	0	0.00	0	0	0	0.00	0	0	0	0.00
South Cotabato	3	4	7	8.54	3	4	7	100.00	26	8	34	100.00	0	0	0	0.00
Sultan Kudarat	46	15	61	98.39	46	15	61	100.00	46	15	61	55.96	1	0	1	1.64
City of General Santos	0	0	0	0.00	0	0	0	0.00	0	0	0	0.00	0	0	0	0.00

Table 2.E.2.2 - Schistosomiasis Prevention and Control

Number and proportion of chronic clinically diagnosed cases seen, confirmed by stool examination, treated in the health facility, and referred to a hospital facility

Philippines, 2024

Area	Number of Chronic Clinically Diagnosed Cases				Number of Chronic Clinically Diagnosed Cases Confirmed by Stool Examination				Number of Chronic Clinically Diagnosed Cases Treated in the Health Facility among Acute and Chronic Cases				Number of Confirmed Chronic Cases referred to Hospital Facility			
	Male	Female	Total	%	Male	Female	Total	%	Male	Female	Total	%	Male	Female	Total	%
**Caraga**	**92**	**54**	**146**	**1.73**	**84**	**46**	**130**	**89.04**	**70**	**29**	**99**	**12.42**	**4**	**3**	**7**	**5.38**
Agusan del Norte	6	2	8	50.00	5	1	6	75.00	5	2	7	100.00	1	0	1	16.67
Agusan del Sur	81	50	131	1.68	74	43	117	89.31	51	22	73	11.11	0	2	2	1.71
Surigao del Norte	1	0	1	0.16	1	0	1	100.00	7	1	8	7.34	0	0	0	0.00
Surigao del Sur	3	1	4	50.00	3	1	4	100.00	6	3	9	90.00	3	1	4	100.00
Dinagat Islands	0	0	0	0.00	0	0	0	0.00	0	0	0	0.00	0	0	0	0.00
City of Butuan	1	1	2	15.38	1	1	2	100.00	1	1	2	14.29	0	0	0	0.00
**BARMM**	**0**	**0**	**0**	**0.00**	**31**	**40**	**71**	**0.00**	**1**	**0**	**1**	**0.67**	**0**	**0**	**0**	**0.00**
Basilan	0	0	0	0.00	0	0	0	0.00	0	0	0	0.00	0	0	0	0.00
Lanao del Sur	0	0	0	0.00	0	0	0	0.00	0	0	0	0.00	0	0	0	0.00
Maguindanao del Norte	0	0	0	0.00	4	10	14	0.00	0	0	0	0.00	0	0	0	0.00
Maguindanao del Sur	0	0	0	0.00	27	30	57	0.00	1	0	1	0.87	0	0	0	0.00
Sulu	0	0	0	0.00	0	0	0	0.00	0	0	0	0.00	0	0	0	0.00
Tawi-Tawi	0	0	0	0.00	0	0	0	0.00	0	0	0	0.00	0	0	0	0.00
SGA	0	0	0	0.00	0	0	0	0.00	0	0	0	0.00	0	0	0	0.00
City of Cotabato	0	0	0	0.00	0	0	0	0.00	0	0	0	0.00	0	0	0	0.00

0 = No Case

* = No Report

Table 2.E.3.1 Soil Transmitted Helminthiasis Prevention and Control

Preschool-age children (PSAC) 1-4 years old and school-age children (SAC) 5-9 years old who completed 2 doses of deworming tablets

Philippines, 2024

Area	Eligible Population (1-4 years old)	PSAC who completed 2 doses of deworming tablets				Eligible Population (5-9 years old)	SAC who completed 2 doses of deworming tablets			
		Male	Female	Total	%		Male	Female	Total	%
**Philippines**	**9,205,720**	**2,649,137**	**2,603,282**	**5,252,419**	**57.06**	**11,614,464**	**1,871,428**	**1,826,538**	**221,190**	**1.90**
**N C R**	**1,057,004**	**250,362**	**239,446**	**489,808**	**46.34**	**1,277,024**	**94,662**	**89,721**	**184,383**	**14.44**
City of Malabon	29,737	1,266	1,769	3,035	10.21	37,749	1,052	1,583	2,635	6.98
City of Navotas	21,089	92	76	168	0.80	26,737	58	66	124	0.46
City of Valenzuela	55,396	20,989	20,697	41,686	75.25	67,379	12,583	11,770	24,353	36.14
City of Caloocan	140,123	18,813	17,828	36,641	26.15	170,689	4,443	4,282	8,725	5.11
City of Marikina	34,881	905	896	1,801	5.16	43,438	491	479	970	2.23
City of Pasig	66,370	7,916	6,834	14,750	22.22	80,981	2,654	2,324	4,978	6.15
Pateros	4,848	578	616	1,194	24.63	6,057	56	65	121	2.00
City of Taguig	75,895	43,922	41,978	85,900	113.18	89,161	40,416	38,562	78,978	88.58
Quezon City	240,612	60,628	62,272	122,900	51.08	285,126	2,271	2,161	4,432	1.55
City of Makati	42,707	6,662	5,961	12,623	29.56	48,789	1,704	1,425	3,129	6.41
City of Mandaluyong	31,813	187	184	371	1.17	37,775	150	144	294	0.78
City of San Juan	8,633	686	736	1,422	16.47	10,166	454	416	870	8.56
City of Manila	139,634	25,385	25,027	50,412	36.10	170,866	6,778	6,289	13,067	7.65
City of Las Piñas	45,597	5,042	4,992	10,034	22.01	56,746	3,169	3,140	6,309	11.12
City of Muntinlupa	39,414	14,687	13,715	28,402	72.06	47,513	10,440	9,491	19,931	41.95
City of Parañaque	53,742	27,034	21,169	48,203	89.69	62,485	4,650	3,962	8,612	13.78
Pasay City	26,513	15,570	14,696	30,266	114.16	35,367	3,293	3,562	6,855	19.38
**C A R**	**132,709**	**44,625**	**42,474**	**87,099**	**65.63**	**177,244**	**38,866**	**35,841**	**74,707**	**42.15**
Abra	16,211	5,890	5,671	11,561	71.32	24,175	1,901	1,750	3,651	15.10
Apayao	9,250	3,105	2,857	5,962	64.45	12,435	154	201	355	2.85
Benguet	37,415	13,240	12,632	25,872	69.15	46,315	27,679	25,572	53,251	114.98
Ifugao	17,379	7,352	6,802	14,154	81.44	23,098	5,120	4,561	9,681	41.91
Kalinga	18,770	3,475	3,252	6,727	35.84	24,850	876	879	1,755	7.06
Mountain Province	11,403	4,642	4,280	8,922	78.24	16,298	2,311	2,115	4,426	27.16
City of Baguio	22,281	6,921	6,980	13,901	62.39	30,073	825	763	1,588	5.28
**Region 1**	**391,947**	**131,040**	**124,962**	**256,002**	**65.32**	**508,054**	**91,979**	**86,427**	**178,406**	**35.12**
Ilocos Norte	37,839	13,207	11,277	24,484	64.71	49,329	5,560	5,247	10,807	21.91
Ilocos Sur	43,294	18,840	18,320	37,160	85.83	60,119	22,076	21,093	43,169	71.81
La Union	55,151	13,286	11,866	25,152	45.61	73,471	10,843	10,487	21,330	29.03
Pangasinan	242,115	82,137	80,027	162,164	66.98	307,736	51,735	47,506	99,241	32.25
		0	0	0	0.00					
City of Dagupan	13,548	3,570	3,472	7,042	51.98	17,399	1,765	2,094	3,859	22.18
**Region 2**	**274,420**	**96,230**	**98,229**	**194,459**	**70.86**	**369,804**	**29,153**	**27,524**	**56,677**	**15.33**
Batanes	1,134	575	559	1,134	100.00	1,556	0	0	0	0.00
Cagayan	90,515	30,312	28,082	58,394	64.51	125,574	5,295	5,068	10,363	8.25
Isabela	116,237	40,599	45,264	85,863	73.87	153,934	10,569	10,017	20,586	13.37
Nueva Vizcaya	39,228	12,827	12,518	25,345	64.61	52,416	4,022	3,555	7,577	14.46
Quirino	15,836	6,434	6,544	12,978	81.95	21,569	5,032	4,863	9,895	45.88
City of Santiago	11,470	5,483	5,262	10,745	93.68	14,755	4,235	4,021	8,256	55.95
**Region 3**	**989,808**	**305,749**	**295,825**	**601,574**	**60.78**	**1,249,167**	**217,806**	**208,388**	**426,194**	**34.12**
Aurora	19,981	8,958	8,342	17,300	86.58	26,047	7,973	7,298	15,271	58.63
Bataan	71,268	22,639	21,593	44,232	62.06	88,562	19,138	18,874	38,012	42.92
Bulacan	288,765	68,869	65,746	134,615	46.62	369,430	15,377	15,232	30,609	8.29
Nueva Ecija	184,039	53,398	50,750	104,148	56.59	232,784	45,107	43,695	88,802	38.15
Pampanga	193,801	58,904	58,965	117,869	60.82	239,092	54,573	51,920	106,493	44.54
Tarlac	121,300	70,529	68,460	138,989	114.58	155,825	55,266	51,881	107,147	68.76
Zambales	49,856	14,542	13,718	28,260	56.68	67,301	16,512	15,482	31,994	47.54
City of Angeles	42,263	4,992	4,981	9,973	23.60	46,619	2,195	2,283	4,478	9.61
City of Olongapo	18,535	2,918	3,270	6,188	33.39	23,507	1,665	1,723	3,388	14.41

Table 2.E.3.1 Soil Transmitted Helminthiasis Prevention and Control
Preschool-age children (PSAC) 1-4 years old and school-age children (SAC) 5-9 years old who completed 2 doses of deworming tablets
Philippines, 2024

Area	Eligible Population (1-4 years old)	PSAC who completed 2 doses of deworming tablets				Eligible Population (5-9 years old)	SAC who completed 2 doses of deworming tablets			
		Male	Female	Total	%		Male	Female	Total	%
**Region 4A**	**1,291,246**	**288,563**	**274,049**	**562,612**	**43.57**	**1,620,703**	**186,949**	**179,389**	**366,338**	**22.60**
Batangas	229,526	65,928	62,279	128,207	55.86	282,523	25,063	23,859	48,922	17.32
Cavite	336,133	97,281	91,765	189,046	56.24	418,056	77,778	73,831	151,609	36.27
Laguna	259,461	60,160	58,112	118,272	45.58	326,169	41,147	39,620	80,767	24.76
Quezon	165,406	43,515	40,720	84,235	50.93	216,206	26,736	25,751	52,487	24.28
Rizal	276,151	18,988	18,530	37,518	13.59	347,625	15,179	15,286	30,465	8.76
City of Lucena	24,569	2,691	2,643	5,334	21.71	30,124	1,046	1,042	2,088	6.93
**Region 4B**	**275,267**	**127,119**	**121,261**	**248,380**	**90.23**	**366,693**	**85,101**	**80,903**	**166,004**	**45.27**
Marinduque	19,560	11,946	11,036	22,982	117.49	24,972	7,220	6,534	13,754	55.08
Occidental Mindoro	48,406	28,861	27,978	56,839	117.42	63,316	19,798	18,661	38,459	60.74
Oriental Mindoro	75,762	46,558	43,771	90,329	119.23	100,488	39,303	37,722	77,025	76.65
Palawan	85,588	25,324	24,486	49,810	58.20	115,399	8,934	8,567	17,501	15.17
Romblon	22,810	8,626	8,608	17,234	75.55	32,802	4,562	4,476	9,038	27.55
City of Puerto Princesa	23,141	5,804	5,382	11,186	48.34	29,716	5,284	4,943	10,227	34.42
**Region 5**	**569,012**	**172,443**	**166,031**	**338,474**	**59.48**	**725,221**	**181,124**	**172,379**	**353,503**	**48.74**
Albay	116,415	41,604	40,701	82,305	70.70	153,652	51,048	48,096	99,144	64.53
Camarines Norte	62,374	12,494	12,683	25,177	40.36	77,159	15,476	15,262	30,738	39.84
Camarines Sur	181,346	56,988	53,804	110,792	61.09	224,691	54,333	51,681	106,014	47.18
Catanduanes	24,805	8,816	8,605	17,421	70.23	31,548	12,387	12,154	24,541	77.79
Masbate	91,826	27,005	25,939	52,944	57.66	118,954	16,760	16,306	33,066	27.80
Sorsogon	75,406	24,457	23,344	47,801	63.39	98,100	30,503	28,286	58,789	59.93
City of Naga	16,840	1,079	955	2,034	12.08	21,117	617	594	1,211	5.73
**Region 6**	**622,304**	**169,829**	**163,380**	**333,209**	**53.54**	**799,206**	**81,505**	**77,589**	**159,094**	**19.91**
Aklan	46,520	11,760	11,428	23,188	49.85	60,154	19,108	18,385	37,493	62.33
Antique	50,356	15,084	14,062	29,146	57.88	62,629	8,635	8,303	16,938	27.04
Capiz	61,057	21,850	21,634	43,484	71.22	76,018	4,734	4,971	9,705	12.77
Guimaras	14,780	5,083	4,784	9,867	66.76	18,603	4,038	3,578	7,616	40.94
Iloilo	152,421	62,910	59,255	122,165	80.15	200,308	26,192	23,853	50,045	24.98
Negros Occidental	217,118	49,590	48,610	98,200	45.23	283,665	17,054	16,623	33,677	11.87
City of Bacolod	43,705	2,772	2,756	5,528	12.65	54,951	1,482	1,649	3,131	5.70
City of Iloilo	36,347	780	851	1,631	4.49	42,878	262	227	489	1.14
**Region 7**	**680,875**	**108,844**	**103,350**	**212,194**	**31.16**	**861,040**	**37,438**	**34,201**	**71,639**	**8.32**
Bohol	108,897	17,186	15,859	33,045	30.35	140,934	1,541	1,647	3,188	2.26
Cebu	295,731	31,649	30,592	62,241	21.05	375,018	8,051	7,772	15,823	4.22
Negros Oriental	120,536	26,185	24,959	51,144	42.43	153,375	4,122	4,346	8,468	5.52
Siquijor	6,371	2,292	1,893	4,185	65.69	9,046	2,467	2,407	4,874	53.88
City of Cebu	79,781	19,461	18,657	38,118	47.78	95,961	5,291	4,837	10,128	10.55
City of Lapu-Lapu	42,661	6,670	6,324	12,994	30.46	53,522	2,522	2,540	5,062	9.46
City of Mandaue	26,898	5,401	5,066	10,467	38.91	33,184	13,444	10,652	24,096	72.61
**Region 8**	**392,682**	**84,541**	**84,527**	**169,068**	**43.05**	**510,664**	**30,479**	**28,271**	**58,750**	**11.50**
Biliran	15,168	7,615	7,145	14,760	97.31	20,948	956	860	1,816	8.67
Eastern Samar	38,988	10,486	10,065	20,551	52.71	51,769	291	241	532	1.03
Leyte	133,983	21,921	21,341	43,262	32.29	172,181	13,989	12,862	26,851	15.59
Northern Samar	58,551	15,892	17,222	33,114	56.56	77,920	6,662	6,515	13,177	16.91
Southern Leyte	32,412	6,543	6,553	13,096	40.40	41,366	2,156	1,892	4,048	9.79
Samar	70,476	14,139	14,510	28,649	40.65	92,914	3,187	2,920	6,107	6.57
Ormoc City	20,712	7,822	7,562	15,384	74.28	25,769	3,228	2,974	6,202	24.07
City of Tacloban	22,392	123	129	252	1.13	27,797	10	7	17	0.06

Table 2.E.3.1 Soil Transmitted Helminthiasis Prevention and Control

Preschool-age children (PSAC) 1-4 years old and school-age children (SAC) 5-9 years old who completed 2 doses of deworming tablets

Philippines, 2024

Area	Eligible Population (1-4 years old)	PSAC who completed 2 doses of deworming tablets				Eligible Population (5-9 years old)	SAC who completed 2 doses of deworming tablets			
		Male	Female	Total	%		Male	Female	Total	%
**Region 9**	**354,698**	**143,229**	**145,878**	**289,107**	**81.51**	**457,214**	**217,585**	**224,295**	**441,880**	**96.65**
Zamboanga del Norte	99,129	40,983	45,083	86,066	86.82	123,922	59,376	61,683	121,059	97.69
Zamboanga del Sur	97,821	35,301	34,586	69,887	71.44	124,378	51,166	56,720	107,886	86.74
Zamboanga Sibugay	61,013	21,513	20,791	42,304	69.34	83,361	28,743	27,956	56,699	68.02
City of Isabela	13,714	5,546	5,737	11,283	82.27	15,867	7,738	7,629	15,367	96.85
City of Zamboanga	83,021	39,886	39,681	79,567	95.84	109,686	70,562	70,307	140,869	128.43
**Region 10**	**460,481**	**187,531**	**198,843**	**386,374**	**83.91**	**592,607**	**206,618**	**215,952**	**422,570**	**71.31**
Bukidnon	146,496	55,186	54,510	109,696	74.88	196,043	63,134	62,072	125,206	63.87
Camiguin	7,322	2,823	2,654	5,477	74.80	10,218	4,149	3,631	7,780	76.14
Lanao del Norte	75,128	21,698	21,896	43,594	58.03	95,786	31,163	28,516	59,679	62.30
Misamis Occidental	49,597	16,812	16,359	33,171	66.88	63,851	22,001	20,884	42,885	67.16
Misamis Oriental	88,919	36,683	35,453	72,136	81.13	112,561	40,590	40,022	80,612	71.62
City of Cagayan De Oro	60,460	35,930	49,024	84,954	140.51	74,400	33,192	47,920	81,112	109.02
City of Iligan	32,559	18,399	18,947	37,346	114.70	39,748	12,389	12,907	25,296	63.64
**Region 11**	**453,316**	**190,427**	**184,701**	**375,128**	**82.75**	**571,088**	**58,855**	**56,500**	**115,355**	**20.20**
Davao de Oro	66,781	36,649	34,164	70,813	106.04	87,851	16,380	15,737	32,117	36.56
Davao del Norte	98,839	43,338	41,908	85,246	86.25	122,627	10,335	9,956	20,291	16.55
Davao Oriental	49,438	23,088	22,054	45,142	91.31	66,775	15,389	14,531	29,920	44.81
Davao del Sur	59,693	23,499	24,664	48,163	80.68	71,517	1,014	1,036	2,050	2.87
Davao Occidental	31,655	7,310	6,843	14,153	44.71	39,539	4,646	4,596	9,242	23.37
City of Davao	146,910	56,543	55,068	111,611	75.97	182,779	11,091	10,644	21,735	11.89
**Region 12**	**396,809**	**128,864**	**121,878**	**250,742**	**63.19**	**514,071**	**111,783**	**108,298**	**220,081**	**42.81**
Cotabato	114,236	36,586	33,046	69,632	60.95	151,889	17,845	17,693	35,538	23.40
Sarangani	54,830	20,822	20,643	41,465	75.62	70,226	13,692	13,490	27,182	38.71
South Cotabato	87,302	38,131	36,658	74,789	85.67	111,352	17,118	17,084	34,202	30.72
Sultan Kudarat	79,530	28,746	27,695	56,441	70.97	102,644	32,911	32,354	65,265	63.58
City of General Santos	60,911	4,579	3,836	8,415	13.82	77,960	30,217	27,677	57,894	74.26
**Caraga**	**238,285**	**85,621**	**101,013**	**186,634**	**78.32**	**326,544**	**83,180**	**78,231**	**161,411**	**49.43**
Agusan del Norte	32,247	15,560	15,102	30,662	95.08	45,122	18,756	17,571	36,327	80.51
Agusan del Sur	67,902	20,109	38,086	58,195	85.70	94,437	17,983	17,068	35,051	37.12
Surigao del Norte	42,410	17,066	16,545	33,611	79.25	57,203	18,856	18,083	36,939	64.58
Surigao del Sur	56,102	22,419	21,322	43,741	77.97	75,307	15,081	14,383	29,464	39.13
Dinagat Islands	10,888	2,128	2,085	4,213	38.69	14,022	2,357	2,149	4,506	32.14
City of Butuan	28,736	8,339	7,873	16,212	56.42	40,453	10,147	8,977	19,124	47.27
**BARMM**	**624,857**	**134,120**	**137,435**	**271,555**	**43.46**	**688,120**	**118,345**	**122,629**	**240,974**	**35.02**
Basilan	53,188	3,791	3,660	7,451	14.01	62,506	3,731	4,036	7,767	12.43
Lanao del Sur	151,859	30,069	30,887	60,956	40.14	162,460	14,624	15,866	30,490	18.77
Maguindanao del Norte	82,095	31,199	31,174	62,373	75.98	90,590	31,642	31,566	63,208	69.77
Maguindanao del Sur	110,119	38,305	39,298	77,603	70.47	112,187	36,446	36,880	73,326	65.36
Sulu	109,879	13,241	14,810	28,051	25.53	136,257	14,550	17,150	31,700	23.26
Tawi-Tawi	56,912	4,753	5,266	10,019	17.60	58,060	4,886	4,906	9,792	16.87
SGA	28,215	3,598	3,723	7,321	25.95	28,612	2,422	2,250	4,672	16.33
City of Cotabato	32,590	9,164	8,617	17,781	54.56	37,448	10,044	9,975	20,019	53.46

Legend:
0 = No Case
** = No Report*

Table 2.E.3.2 Soil Transmitted Helminthiasis Prevention and Control
Adolescents 10-19 years old who completed 2 doses of deworming tablets
Philippines, 2024

Area	Eligible Population (10-19 years old)	Adolescents who completed 2 doses of deworming tablets			
		Male	Female	Total	%
**Philippines**	**22,227,272**	**2,672,302**	**2,670,408**	**5,342,710**	**24.04**
**N C R**	**2,368,633**	**107,622**	**102,902**	**210,524**	**8.89**
City of Malabon	72,642	905	1,357	2,262	3.11
City of Navotas	49,440	49	57	106	0.21
City of Valenzuela	133,035	14,550	10,628	25,178	18.93
City of Caloocan	318,592	1,650	1,711	3,361	1.05
City of Marikina	81,797	350	371	721	0.88
City of Pasig	141,874	2,688	2,719	5,407	3.81
Pateros	11,527	8	18	26	0.23
City of Taguig	162,666	49,009	48,925	97,934	60.21
Quezon City	516,031	951	931	1,882	0.36
City of Makati	89,951	776	711	1,487	1.65
City of Mandaluyong	67,992	80	101	181	0.27
City of San Juan	18,759	408	403	811	4.32
City of Manila	324,886	3,942	4,412	8,354	2.57
City of Las Piñas	106,285	1,727	1,760	3,487	3.28
City of Muntinlupa	90,344	16,008	13,779	29,787	32.97
City of Parañaque	112,810	11,178	10,536	21,714	19.25
Pasay City	70,002	3,343	4,483	7,826	11.18
**C A R**	**367,985**	**33,714**	**32,543**	**66,257**	**18.01**
Abra	50,658	2,867	2,695	5,562	10.98
Apayao	26,281	102	108	210	0.80
Benguet	91,042	19,819	19,813	39,632	43.53
Ifugao	45,920	5,520	4,912	10,432	22.72
Kalinga	50,950	551	547	1,098	2.16
Mountain Province	34,588	4,180	3,906	8,086	23.38
City of Baguio	68,546	675	562	1,237	1.80
**Region 1**	**1,033,709**	**135,110**	**126,298**	**261,408**	**25.29**
Ilocos Norte	109,612	5,819	5,312	11,131	10.15
Ilocos Sur	130,638	43,416	41,061	84,477	64.66
La Union	152,428	19,952	18,554	38,506	25.26
Pangasinan	605,247	64,538	59,923	124,461	20.56
City of Dagupan	35,784	1,385	1,448	2,833	7.92
**Region 2**	**730,174**	**33,280**	**32,253**	**65,533**	**8.97**
Batanes	3,542	0	0	0	0.00
Cagayan	248,544	5,419	4,960	10,379	4.18
Isabela	304,197	8,599	8,064	16,663	5.48
Nueva Vizcaya	100,476	3,304	3,303	6,607	6.58
Quirino	42,843	8,485	8,671	17,156	40.04
City of Santiago	30,572	7,473	7,255	14,728	48.17
**Region 3**	**2,445,276**	**295,727**	**293,508**	**589,235**	**24.10**
Aurora	51,295	10,730	10,597	21,327	41.58
Bataan	168,327	17,703	19,147	36,850	21.89
Bulacan	724,809	70,755	70,567	141,322	19.50
Nueva Ecija	451,094	36,740	35,590	72,330	16.03
Pampanga	483,862	59,762	57,699	117,461	24.28
Tarlac	299,375	77,774	76,801	154,575	51.63
Zambales	130,808	20,356	20,121	40,477	30.94
City of Angeles	87,329	220	1,169	1,389	1.59
City of Olongapo	48,377	1,687	1,817	3,504	7.24

Table 2.E.3.2 Soil Transmitted Helminthiasis Prevention and Control
Adolescents 10-19 years old who completed 2 doses of deworming tablets
Philippines, 2024

Area	Eligible Population (10-19 years old)	Adolescents who completed 2 doses of deworming tablets			
		Male	Female	Total	%
**Region 4A**	**3,118,570**	**255,299**	**243,351**	**498,650**	**15.99**
Batangas	560,963	34,898	32,828	67,726	12.07
Cavite	808,659	100,946	95,937	196,883	24.35
Laguna	617,761	59,342	56,712	116,054	18.79
Quezon	421,000	41,982	39,611	81,593	19.38
Rizal	652,554	17,173	17,347	34,520	5.29
City of Lucena	57,633	958	916	1,874	3.25
**Region 4B**	**717,440**	**110,252**	**105,420**	**215,672**	**30.06**
Marinduque	51,609	8,053	7,599	15,652	30.33
Occidental Mindoro	120,180	30,500	29,244	59,744	49.71
Oriental Mindoro	198,074	54,120	51,736	105,856	53.44
Palawan	215,308	10,215	9,679	19,894	9.24
Romblon	69,770	2,156	1,927	4,083	5.85
City of Puerto Princesa	62,499	5,208	5,235	10,443	16.71
**Region 5**	**1,413,724**	**262,793**	**250,561**	**513,354**	**36.31**
Albay	311,655	61,547	58,855	120,402	38.63
Camarines Norte	142,206	29,198	30,004	59,202	41.63
Camarines Sur	438,292	73,342	69,599	142,941	32.61
Catanduanes	62,115	23,953	22,475	46,428	74.75
Masbate	225,612	21,124	20,399	41,523	18.40
Sorsogon	188,964	53,145	48,619	101,764	53.85
City of Naga	44,880	484	610	1,094	2.44
**Region 6**	**1,572,207**	**111,673**	**110,009**	**221,682**	**14.10**
Aklan	121,300	32,868	32,759	65,627	54.10
Antique	123,091	10,369	10,507	20,876	16.96
Capiz	156,852	6,132	6,041	12,173	7.76
Guimaras	35,529	7,260	6,667	13,927	39.20
Iloilo	399,111	42,727	41,853	84,580	21.19
Negros Occidental	546,258	10,431	10,248	20,679	3.79
City of Bacolod	111,052	1,610	1,664	3,274	2.95
City of Iloilo	79,014	276	270	546	0.69
**Region 7**	**1,653,547**	**29,991**	**29,950**	**59,941**	**3.62**
Bohol	289,722	817	766	1,583	0.55
Cebu	696,564	11,062	10,774	21,836	3.13
Negros Oriental	294,326	3,137	2,879	6,016	2.04
Siquijor	18,706	4,812	4,643	9,455	50.55
City of Cebu	186,990	3,427	3,441	6,868	3.67
City of Lapu-Lapu	100,512	1,017	1,829	2,846	2.83
City of Mandaue	66,727	5,719	5,618	11,337	16.99
**Region 8**	**997,958**	**56,680**	**53,854**	**110,534**	**11.08**
Biliran	39,765	203	210	413	1.04
Eastern Samar	106,220	10,370	9,954	20,324	19.13
Leyte	329,868	21,399	19,878	41,277	12.51
Northern Samar	153,633	12,805	12,681	25,486	16.59
Southern Leyte	86,773	3,032	2,840	5,872	6.77
Samar	182,773	7,010	6,241	13,251	7.25
Ormoc City	48,063	1,844	2,048	3,892	8.10
City of Tacloban	50,863	17	2	19	0.04

Table 2.E.3.2 Soil Transmitted Helminthiasis Prevention and Control
Adolescents 10-19 years old who completed 2 doses of deworming tablets
Philippines, 2024

Area	Eligible Population (10-19 years old)	Adolescents who completed 2 doses of deworming tablets			
		Male	Female	Total	%
**Region 9**	**850,219**	**377,536**	**399,349**	**776,885**	**91.37**
Zamboanga del Norte	229,010	106,429	112,128	218,557	95.44
Zamboanga del Sur	233,964	96,881	107,679	204,560	87.43
Zamboanga Sibugay	154,543	55,596	53,548	109,144	70.62
City of Isabela	28,485	12,511	13,250	25,761	90.44
City of Zamboanga	204,217	106,119	112,744	218,863	107.17
**Region 10**	**1,091,211**	**328,478**	**365,040**	**693,518**	**63.55**
Bukidnon	352,566	102,055	101,372	203,427	57.70
Camiguin	19,151	5,053	5,142	10,195	53.23
Lanao del Norte	169,805	37,578	50,211	87,789	51.70
Misamis Occidental	125,860	40,329	37,508	77,837	61.84
Misamis Oriental	206,393	69,289	67,029	136,318	66.05
City of Cagayan De Oro	140,917	58,810	86,669	145,479	103.24
City of Iligan	76,519	15,364	17,109	32,473	42.44
**Region 11**	**1,079,760**	**70,281**	**67,170**	**137,451**	**12.73**
Davao de Oro	169,609	19,360	18,423	37,783	22.28
Davao del Norte	226,943	9,403	9,221	18,624	8.21
Davao Oriental	127,980	26,030	24,741	50,771	39.67
Davao del Sur	136,869	384	394	778	0.57
Davao Occidental	71,022	8,122	7,686	15,808	22.26
City of Davao	347,337	6,982	6,705	13,687	3.94
**Region 12**	**962,632**	**163,880**	**160,504**	**324,384**	**33.70**
Cotabato	283,965	26,998	27,272	54,270	19.11
Sarangani	129,975	19,201	19,066	38,267	29.44
South Cotabato	209,697	20,325	20,166	40,491	19.31
Sultan Kudarat	195,096	61,244	58,493	119,737	61.37
City of General Santos	143,899	36,112	35,507	71,619	49.77
**Caraga**	**614,600**	**144,224**	**139,616**	**283,840**	**46.18**
Agusan del Norte	85,374	32,807	31,416	64,223	75.23
Agusan del Sur	170,379	32,194	31,491	63,685	37.38
Surigao del Norte	112,595	36,355	34,334	70,689	62.78
Surigao del Sur	139,773	23,314	22,340	45,654	32.66
Dinagat Islands	26,945	4,328	4,088	8,416	31.23
City of Butuan	79,534	15,226	15,947	31,173	39.19
**BARMM**	**1,209,627**	**155,762**	**158,080**	**313,842**	**25.95**
Basilan	103,033	6,561	6,696	13,257	12.87
Lanao del Sur	297,325	9,536	9,292	18,828	6.33
Maguindanao del Norte	152,897	41,081	41,216	82,297	53.83
Maguindanao del Sur	181,076	49,543	50,200	99,743	55.08
Sulu	254,366	22,018	22,789	44,807	17.62
Tawi-Tawi	97,827	5,895	6,447	12,342	12.62
SGA	49,593	1,087	1,010	2,097	4.23
City of Cotabato	73,510	21,128	21,440	42,568	57.91

Legend:
0 = No Case
* = No Report

Table 2.E.3.3 Soil Transmitted Helminthiasis Prevention and Control
Women of reproductive age (WRA) 20-49 years old, and pregnant women who completed 2 doses of deworming tablets
Philippines, 2024

Area	Eligible Population (Female 20-49 years old)	WRA who completed 2 doses of deworming tablets		Eligible Population (0-11 months)	Pregnant Women who completed 1 dose of deworming tablets	
		Number	%		Number	%
**Philippines**	**23,430,619**	**247,112**	**1.05**	**2,200,865**	**269,353**	**12.24**
**N C R**	**3,277,053**	**20,767**	**0.63**	**263,248**	**23,453**	**8.91**
City of Malabon	88,290	0	0.00	7,447	25	0.34
City of Navotas	55,272	0	0.00	5,263	0	0.00
City of Valenzuela	172,146	1,114	0.00	13,601	193	1.42
City of Caloocan	389,214	1,955	0.00	33,499	0	0.00
City of Marikina	106,333	1,271	0.00	8,399	2,447	29.13
City of Pasig	198,149	254	0.00	17,856	97	0.54
Pateros	15,427	13	0.00	1,120	62	5.54
City of Taguig	223,609	11,494	0.00	19,226	8,350	43.43
Quezon City	713,970	149	0.00	59,607	8,533	14.32
City of Makati	163,631	977	0.00	10,991	133	1.21
City of Mandaluyong	110,920	300	0.00	7,989	0	0.00
City of San Juan	31,489	0	0.00	2,173	96	4.42
City of Manila	442,163	1,983	0.00	34,268	6	0.02
City of Las Piñas	148,622	447	0.00	11,346	1,672	14.74
City of Muntinlupa	128,540	0	0.00	9,949	13	0.13
City of Parañaque	171,558	34	0.00	13,385	480	3.59
Pasay City	117,720	776	0.00	7,129	1,346	18.88
**C A R**	**391,382**	**874**	**0.00**	**31,490**	**4,806**	**15.26**
Abra	49,449	14	0.00	3,639	471	12.94
Apayao	25,508	661	0.00	2,222	969	43.61
Benguet	101,149	0	0.00	8,900	272	3.06
Ifugao	42,999	5	0.00	4,145	1,164	28.08
Kalinga	47,996	0	0.00	4,582	974	21.26
Mountain Province	31,256	21	0.00	2,615	170	6.50
City of Baguio	93,025	173	0.00	5,387	786	14.59
**Region 1**	**1,098,314**	**8,790**	**0.00**	**96,024**	**12,054**	**12.55**
Ilocos Norte	129,095	875	0.00	8,809	2,601	29.53
Ilocos Sur	146,319	6,090	0.00	9,834	1,085	11.04
La Union	174,227	1,825	0.00	13,436	1,895	14.10
Pangasinan	611,297	0	0.00	60,591	6,342	10.47
City of Dagupan	37,376	0	0.00	3,354	131	3.91
**Region 2**	**780,177**	**6,703**	**0.00**	**64,034**	**8,099**	**12.65**
Batanes	3,829	0	0.00	241	36	14.94
Cagayan	266,198	115	0.00	20,723	2,313	11.16
Isabela	329,187	852	0.00	27,362	3,996	14.60
Nueva Vizcaya	104,567	303	0.00	8,880	1,016	11.44
Quirino	43,185	1,617	0.00	3,865	17	0.44
City of Santiago	33,211	3,816	0.00	2,963	721	24.33
**Region 3**	**2,741,173**	**23,285**	**0.00**	**235,313**	**5,017**	**2.13**
Aurora	48,407	0	0.00	4,578	0	0.00
Bataan	188,341	0	0.00	17,556	0	0.00
Bulacan	836,121	13,191	0.00	68,115	1,556	2.28
Nueva Ecija	492,879	48	0.00	43,626	390	0.89
Pampanga	550,125	6,200	0.00	46,146	372	0.81
Tarlac	314,780	415	0.00	29,655	1,982	6.68
Zambales	136,755	553	0.00	11,796	222	1.88
City of Angeles	112,766	1,634	0.00	9,232	0	0.00
City of Olongapo	60,999	1,244	0.00	4,609	495	10.74

Table 2.E.3.3 Soil Transmitted Helminthiasis Prevention and Control
Women of reproductive age (WRA) 20-49 years old, and pregnant women who completed 2 doses of deworming tablets
Philippines, 2024

Area	Eligible Population (Female 20-49 years old)	WRA who completed 2 doses of deworming tablets		Eligible Population (0-11 months)	Pregnant Women who completed 1 dose of deworming tablets	
		Number	%		Number	%
**Region 4A**	**3,737,811**	**4,745**	**0.00**	**310,150**	**14,805**	**4.77**
Batangas	659,547	0	0.00	53,484	1,154	2.16
Cavite	1,043,392	3,505	0.00	78,589	1,585	2.02
Laguna	815,988	1,002	0.00	61,967	8,569	13.83
Quezon	387,306	197	0.00	40,954	1,845	4.51
Rizal	770,966	41	0.00	68,903	1,648	2.39
City of Lucena	60,612	0	0.00	6,253	4	0.06
**Region 4B**	**647,741**	**5,601**	**0.00**	**66,508**	**0**	**0.00**
Marinduque	42,955	0	0.00	4,124	0	0.00
Occidental Mindoro	103,056	0	0.00	11,748	0	0.00
Oriental Mindoro	184,415	4,062	0.00	19,007	0	0.00
Palawan	187,216	52	0.00	20,503	0	0.00
Romblon	57,530	0	0.00	5,488	0	0.00
City of Puerto Princesa	72,569	1,487	0.00	5,638	0	0.00
**Region 5**	**1,160,699**	**23,164**	**0.00**	**136,611**	**28,121**	**20.58**
Albay	275,296	925	0.00	27,665	3,273	11.83
Camarines Norte	122,785	2,051	0.00	14,894	4,542	30.50
Camarines Sur	348,936	1,685	0.00	43,855	8,161	18.61
Catanduanes	49,528	3,465	0.00	5,395	2,090	38.74
Masbate	159,670	41	0.00	22,495	4,813	21.40
Sorsogon	157,019	14,975	0.00	18,065	4,590	25.41
City of Naga	47,465	22	0.00	4,242	652	15.37
**Region 6**	**1,592,066**	**16,847**	**0.00**	**147,934**	**19,196**	**12.98**
Aklan	126,123	9,843	0.00	11,281	1,308	11.59
Antique	116,765	254	0.00	11,899	1,790	15.04
Capiz	162,833	3,491	0.00	13,872	2,940	21.19
Guimaras	38,389	234	0.00	3,361	898	26.72
Iloilo	408,763	1,170	0.00	35,981	3,858	10.72
Negros Occidental	509,071	1,855	0.00	52,445	6,017	11.47
City of Bacolod	129,594	0	0.00	10,361	2,385	23.02
City of Iloilo	100,528	0	0.00	8,734	0	0.00
**Region 7**	**1,724,303**	**18,434**	**0.00**	**167,016**	**34,810**	**20.84**
Bohol	269,090	47	0.00	26,210	5,399	20.60
Cebu	696,388	242	0.00	72,875	11,619	15.94
Negros Oriental	288,752	4,219	0.00	28,980	2,651	9.15
Siquijor	20,611	8,014	0.00	1,671	553	33.09
City of Cebu	229,267	4,353	0.00	19,466	9,861	50.66
City of Lapu-Lapu	126,623	1,029	0.00	10,833	1,284	11.85
City of Mandaue	93,572	530	0.00	6,981	3,443	49.32
**Region 8**	**857,807**	**3,278**	**0.00**	**93,713**	**8,181**	**8.73**
Biliran	33,145	146	0.00	3,378	1,698	50.27
Eastern Samar	88,096	31	0.00	9,594	1,010	10.53
Leyte	289,364	211	0.00	31,504	487	1.55
Northern Samar	115,475	2,179	0.00	14,053	893	6.35
Southern Leyte	81,557	65	0.00	7,368	1,588	21.55
Samar	146,964	645	0.00	16,946	1,233	7.28
Ormoc City	48,456	0	0.00	5,344	1,047	19.59
City of Tacloban	54,750	1	0.00	5,526	225	4.07

Table 2.E.3.3 Soil Transmitted Helminthiasis Prevention and Control
Women of reproductive age (WRA) 20-49 years old, and pregnant women who completed 2 doses of deworming tablets
Philippines, 2024

Area	Eligible Population (Female 20-49 years old)	WRA who completed 2 doses of deworming tablets		Eligible Population (0-11 months)	Pregnant Women who completed 1 dose of deworming tablets	
		Number	%		Number	%
**Region 9**	**776,442**	**3,154**	**0.00**	**85,348**	**11,316**	**13.26**
Zamboanga del Norte	199,460	1,359	0.00	24,977	3,563	14.27
Zamboanga del Sur	205,382	295	0.00	23,905	1,099	4.60
Zamboanga Sibugay	130,429	91	0.00	13,903	1,236	8.89
City of Isabela	26,408	91	0.00	3,209	598	18.64
City of Zamboanga	214,763	1,318	0.00	19,354	4,820	24.90
**Region 10**	**1,040,683**	**19,154**	**0.00**	**110,290**	**24,963**	**22.63**
Bukidnon	313,728	1,663	0.00	33,913	8,639	25.47
Camiguin	17,328	2,632	0.00	1,727	670	38.80
Lanao del Norte	146,350	313	0.00	17,881	2,380	13.31
Misamis Occidental	122,134	116	0.00	12,342	515	4.17
Misamis Oriental	191,643	5	0.00	22,184	1,263	5.69
City of Cagayan De Oro	171,664	0	0.00	14,503	7,910	54.54
City of Iligan	77,836	14,425	0.00	7,740	3,586	46.33
**Region 11**	**1,113,470**	**11,912**	**0.00**	**107,934**	**32,425**	**30.04**
Davao de Oro	153,666	2,165	0.00	15,434	6,565	42.54
Davao del Norte	240,919	1,872	0.00	23,685	8,412	35.52
Davao Oriental	110,768	1,641	0.00	12,025	4,560	37.92
Davao del Sur	141,222	782	0.00	13,178	2,216	16.82
Davao Occidental	57,960	1,582	0.00	6,606	2,875	43.52
City of Davao	408,935	3,870	0.00	37,006	7,797	21.07
**Region 12**	**926,063**	**66,754**	**0.00**	**95,296**	**30,246**	**31.74**
Cotabato	263,563	400	0.00	25,696	4,647	18.08
Sarangani	113,665	3,570	0.00	13,501	7,888	58.43
South Cotabato	208,030	4,176	0.00	21,842	6,557	30.02
Sultan Kudarat	179,001	58,421	0.00	19,613	10,186	51.93
City of General Santos	161,804	187	0.00	14,644	968	6.61
**Caraga**	**551,853**	**3,398**	**0.00**	**55,371**	**3,891**	**7.03**
Agusan del Norte	75,701	25	0.00	7,331	624	8.51
Agusan del Sur	140,411	0	0.00	16,180	921	5.69
Surigao del Norte	108,067	3,325	0.00	10,168	840	8.26
Surigao del Sur	124,786	44	0.00	12,834	1,463	11.40
Dinagat Islands	23,694	4	0.00	2,117	43	2.03
City of Butuan	79,194	0	0.00	6,741	0	0.00
**BARMM**	**1,013,582**	**10,252**	**0.00**	**134,585**	**7,970**	**5.92**
Basilan	83,032	245	0.00	11,653	154	1.32
Lanao del Sur	241,739	7,684	0.00	33,339	1,240	3.72
Maguindanao del Norte	127,233	610	0.00	18,923	796	4.21
Maguindanao del Sur	143,347	804	0.00	23,214	2,916	12.56
Sulu	205,763	909	0.00	21,381	2,117	9.90
Tawi-Tawi	89,512	0	0.00	12,602	0	0.00
SGA	46,079	13	0.00	6,305	118	1.87
City of Cotabato	76,877	0	0.00	7,168	747	10.42

Legend:
0 = No Case
* = No Report

Table 2.E.4.1 HIV-AIDS/STI Prevention and Control
Pregnant Women Screened and Positive with Syphilis and Screened for HIV
Philippines, 2024

Area	Eligible Population	Screened for Syphilis	%	Positive for Syphilis	%	Screened for HIV	%
**Philippines**	**2,200,865**	**662,514**	**30.10**	**4,940**	**0.75**	**627,502**	**28.51**
**N C R**	**263,248**	**126,138**	**47.92**	**419**	**0.33**	**127,878**	**48.58**
City of Malabon	7,447	2,148	28.84	14	0.65	2,152	28.90
City of Navotas	5,263	3,482	66.16	12	0.34	3,459	65.72
City of Valenzuela	13,601	6,201	45.59	33	0.53	6,314	46.42
City of Caloocan	33,499	17,430	52.03	44	0.25	16,941	50.57
City of Marikina	8,399	2,006	23.88	2	0.10	1,535	18.28
City of Pasig	17,856	9,201	51.53	33	0.36	9,112	51.03
Pateros	1,120	277	24.73	7	2.53	356	31.79
City of Taguig	19,226	9,539	49.62	64	0.67	9,861	51.29
Quezon City	59,607	33,444	56.11	50	0.15	34,185	57.35
City of Makati	10,991	1,497	13.62	5	0.33	1,916	17.43
City of Mandaluyong	7,989	5,643	70.63	2	0.04	5,677	71.06
City of San Juan	2,173	742	34.15	0	0.00	741	34.10
City of Manila	34,268	17,612	51.39	80	0.45	17,644	51.49
City of Las Piñas	11,346	3,844	33.88	10	0.26	4,606	40.60
City of Muntinlupa	9,949	3,224	32.41	9	0.28	3,543	35.61
City of Parañaque	13,385	4,935	36.87	24	0.49	4,972	37.15
Pasay City	7,129	4,913	68.92	30	0.61	4,864	68.23
**C A R**	**31,490**	**22,836**	**72.52**	**52**	**0.23**	**12,472**	**39.61**
Abra	3,639	1,655	45.48	4	0.24	1,575	43.28
Apayao	2,222	3,030	136.36	31	1.02	1,219	54.86
Benguet	8,900	7,054	79.26	1	0.01	3,520	39.55
Ifugao	4,145	2,141	51.65	5	0.23	1,424	34.35
Kalinga	4,582	1,663	36.29	1	0.06	1,113	24.29
Mountain Province	2,615	4,444	169.94	2	0.05	1,018	38.93
City of Baguio	5,387	2,849	52.89	8	0.28	2,603	48.32
**Region 1**	**96,024**	**34,549**	**35.98**	**75**	**0.22**	**25,356**	**26.41** *
Ilocos Norte	8,809	5,553	63.04	2	0.04	4,906	55.69
Ilocos Sur	9,834	6,783	68.97	2	0.03	4,809	48.90
La Union	13,436	5,940	44.21	11	0.19	5,528	41.14
Pangasinan	60,591	15,517	25.61	57	0.37	9,466	15.62
City of Dagupan	3,354	756	22.54	3	0.40	647	19.29
**Region 2**	**64,034**	**15,652**	**24.44**	**85**	**0.54**	**12,411**	**19.38**
Batanes	241	91	37.76	1	1.10	79	32.78
Cagayan	20,723	2,562	12.36	16	0.62	2,856	13.78
Isabela	27,362	9,110	33.29	48	0.53	6,136	22.43
Nueva Vizcaya	8,880	2,299	25.89	12	0.52	1,859	20.93
Quirino	3,865	1,398	36.17	7	0.50	1,299	33.61
City of Santiago	2,963	192	6.48	1	0.52	182	6.14
**Region 3**	**235,313**	**83,980**	**35.69**	**459**	**0.55**	**91,138**	**38.73**
Aurora	4,578	1,995	43.58	6	0.30	2,164	47.27
Bataan	17,556	8,268	47.10	42	0.51	8,851	50.42
Bulacan	68,115	37,451	54.98	90	0.24	37,893	55.63
Nueva Ecija	43,626	9,510	21.80	93	0.98	11,303	25.91
Pampanga	46,146	13,375	28.98	155	1.16	15,392	33.36
Tarlac	29,655	7,519	25.35	50	0.66	8,281	27.92
Zambales	11,796	3,221	27.31	16	0.50	4,683	39.70
City of Angeles	9,232	1,211	13.12	1	0.08	1,266	13.71
City of Olongapo	4,609	1,430	31.03	6	0.42	1,305	28.31

Table 2.E.4.1 HIV-AIDS/STI Prevention and Control
Pregnant Women Screened and Positive with Syphilis and Screened for HIV
Philippines, 2024

Area	Eligible Population	Screened for Syphilis	%	Positive for Syphilis	%	Screened for HIV	%
**Region 4A**	**310,150**	**94,909**	**30.60**	**1,228**	**1.29**	**90,433**	**29.16**
Batangas	53,484	11,222	20.98	83	0.74	9,542	17.84
Cavite	78,589	24,015	30.56	215	0.90	22,589	28.74
Laguna	61,967	24,618	39.73	515	2.09	22,535	36.37
Quezon	40,954	9,432	23.03	150	1.59	9,052	22.10
Rizal	68,903	23,467	34.06	243	1.04	25,019	36.31
City of Lucena	6,253	2,155	34.46	22	1.02	1,696	27.12
**Region 4B**	**66,508**	**19,348**	**29.09**	**262**	**1.35**	**15,873**	**23.87** *
Marinduque	4,124	1,358	32.93	3	0.22	1,020	24.73
Occidental Mindoro	11,748	2,738	23.31	41	1.50	620	5.28
Oriental Mindoro	19,007	4,534	23.85	37	0.82	3,068	16.14
Palawan	20,503	6,226	30.37	86	1.38	6,983	34.06
Romblon	5,488	2,006	36.55	12	0.60	1,384	25.22
City of Puerto Princesa	5,638	2,486	44.09	83	3.34	2,798	49.63
**Region 5**	**136,611**	**24,017**	**17.58**	**197**	**0.82**	**28,875**	**21.14**
Albay	27,665	4,069	14.71	33	0.81	4,876	17.63
Camarines Norte	14,894	1,464	9.83	38	2.60	1,293	8.68
Camarines Sur	43,855	6,283	14.33	10	0.16	8,269	18.86
Catanduanes	5,395	2,390	44.30	5	0.21	2,464	45.67
Masbate	22,495	5,163	22.95	55	1.07	5,560	24.72
Sorsogon	18,065	3,905	21.62	53	1.36	4,804	26.59
City of Naga	4,242	743	17.52	3	0.40	1,609	37.93
**Region 6**	**147,934**	**47,850**	**32.35**	**445**	**0.93**	**34,431**	**23.27** *
Aklan	11,281	3,803	33.71	22	0.58	2,201	19.51
Antique	11,899	3,684	30.96	71	1.93	2,628	22.09
Capiz	13,872	3,660	26.38	48	1.31	2,169	15.64
Guimaras	3,361	1,936	57.60	8	0.41	1,781	52.99
Iloilo	35,981	13,639	37.91	96	0.70	6,758	18.78
Negros Occidental	52,445	14,391	27.44	104	0.72	13,132	25.04
City of Bacolod	10,361	3,815	36.82	80	2.10	3,178	30.67
City of Iloilo	8,734	2,922	33.46	16	0.55	2,584	29.59
**Region 7**	**167,016**	**68,606**	**41.08**	**624**	**0.91**	**69,511**	**41.62**
Bohol	26,210	10,558	40.28	83	0.79	8,141	31.06
Cebu	72,875	17,896	24.56	342	1.91	21,220	29.12
Negros Oriental	28,980	5,689	19.63	26	0.46	4,914	16.96
Siquijor	1,671	762	45.60	2	0.26	807	48.29
City of Cebu	19,466	9,085	46.67	3	0.03	9,454	48.57
City of Lapu-Lapu	10,833	5,269	48.64	156	2.96	5,525	51.00
City of Mandaue	6,981	19,347	277.14	12	0.06	19,450	278.61
**Region 8**	**93,713**	**16,716**	**17.84**	**101**	**0.60**	**20,123**	**21.47**
Biliran	3,378	1,586	46.95	12	0.76	1,720	50.92
Eastern Samar	9,594	2,520	26.27	9	0.36	2,741	28.57
Leyte	31,504	1,836	5.83	8	0.44	3,447	10.94
Northern Samar	14,053	3,903	27.77	32	0.82	4,152	29.55
Southern Leyte	7,368	337	4.57	1	0.30	952	12.92
Samar	16,946	1,935	11.42	22	1.14	2,340	13.81
Ormoc City	5,344	1,818	34.02	16	0.88	2,045	38.27
City of Tacloban	5,526	2,781	50.33	1	0.04	2,726	49.33

Table 2.E.4.1 HIV-AIDS/STI Prevention and Control

Pregnant Women Screened and Positive with Syphilis and Screened for HIV

Philippines, 2024

Area	Eligible Population	Screened for Syphilis	%	Positive for Syphilis	%	Screened for HIV	%
**Region 9**	**85,348**	**4,927**	**5.77**	**17**	**0.35**	**4,136**	**4.85**
Zamboanga del Norte	24,977	788	3.15	2	0.25	771	3.09
Zamboanga del Sur	23,905	889	3.72	8	0.90	263	1.10
Zamboanga Sibugay	13,903	1,380	9.93	4	0.29	1,616	11.62
City of Isabela	3,209	615	19.16	2	0.33	615	19.16
City of Zamboanga	19,354	1,255	6.48	1	0.08	871	4.50
**Region 10**	**110,290**	**28,872**	**26.18**	**257**	**0.89**	**26,705**	**24.21**
Bukidnon	33,913	6,563	19.35	67	1.02	5,827	17.18
Camiguin	1,727	1,025	59.35	0	0.00	1,049	60.74
Lanao del Norte	17,881	2,203	12.32	10	0.45	3,641	20.36
Misamis Occidental	12,342	1,258	10.19	0	0.00	4,163	33.73
Misamis Oriental	22,184	4,547	20.50	11	0.24	5,027	22.66
City of Cagayan De Oro	14,503	12,323	84.97	169	1.37	4,380	30.20
City of Iligan	7,740	953	12.31	0	0.00	2,618	33.82
**Region 11**	**107,934**	**31,432**	**29.12**	**134**	**0.43**	**30,088**	**27.88**
Davao de Oro	15,434	1,095	7.09	22	2.01	2,072	13.42
Davao del Norte	23,685	6,134	25.90	15	0.24	6,762	28.55
Davao Oriental	12,025	773	6.43	6	0.78	1,253	10.42
Davao del Sur	13,178	2,506	19.02	7	0.28	3,586	27.21
Davao Occidental	6,606	1,204	18.23	20	1.66	1,763	26.69
City of Davao	37,006	19,720	53.29	64	0.32	14,652	39.59
**Region 12**	**95,296**	**26,069**	**27.36**	**500**	**1.92**	**18,782**	**19.71**
Cotabato	25,696	3,891	15.14	70	1.80	3,529	13.73
Sarangani	13,501	5,472	40.53	95	1.74	2,497	18.49
South Cotabato	21,842	8,353	38.24	51	0.61	8,063	36.92
Sultan Kudarat	19,613	5,446	27.77	187	3.43	1,884	9.61
City of General Santos	14,644	2,907	19.85	97	3.34	2,809	19.18
**Caraga**	**55,371**	**8,669**	**15.66**	**38**	**0.44**	**10,563**	**19.08**
Agusan del Norte	7,331	1,104	15.06	8	0.72	1,076	14.68
Agusan del Sur	16,180	1,911	11.81	8	0.42	2,863	17.69
Surigao del Norte	10,168	1,762	17.33	14	0.79	1,652	16.25
Surigao del Sur	12,834	497	3.87	4	0.80	1,105	8.61
Dinagat Islands	2,117	931	43.98	3	0.32	905	42.75
City of Butuan	6,741	2,464	36.55	1	0.04	2,962	43.94
**BARMM**	**134,585**	**7,944**	**5.90**	**47**	**0.59**	**8,727**	**6.48**
Basilan	11,653	118	1.01	0	0.00	421	3.61
Lanao del Sur	33,339	3,574	10.72	1	0.03	3,556	10.67
Maguindanao del Norte	18,923	135	0.71	1	0.74	330	1.74
Maguindanao del Sur	23,214	1,070	4.61	21	1.96	782	3.37
Sulu	21,381	28	0.13	0	0.00	30	0.14
Tawi-Tawi	12,602	148	1.17	3	2.03	590	4.68
SGA	6,305	8	0.13	1	12.50	6	0.10
City of Cotabato	7,168	2,863	39.94	20	0.70	3,012	42.02

Legend:
0 = No Case
* = No Report

Table 2.E.5.1 Leprosy Prevention and Control
Prevalence Rate and Case Detection Rate
Philippines, 2024

Area	Population	Leprosy Cases undergoing Treatment			Prevalence Rate	Newly Detected Case			Case Detection Rate
		Male	Female	Total		Male	Female	Total	
**Philippines**	**112,079,845**	**845**	**427**	**1,272**	**0.11**	**786**	**524**	**1,310**	**1.17**
**N C R**	**13,902,112**	**7**	**3**	**10**	**0.01**	**1**	**2**	**3**	**0.02**
City of Malabon	393,282	0	0	0	0.00	0	0	0	0.00
City of Navotas	257,469	0	0	0	0.00	0	0	0	0.00
City of Valenzuela	738,827	0	0	0	0.00	0	0	0	0.00
City of Caloocan	1,724,390	0	0	0	0.00	0	0	0	0.00
City of Marikina	468,372	0	0	0	0.00	0	0	0	0.00
City of Pasig	833,749	6	3	9	0.11	0	1	1	0.12
Pateros	66,567	1	0	1	0.15	1	0	1	1.50
City of Taguig	928,943	0	0	0	0.00	0	1	1	0.11
Quezon City	3,052,364	0	0	0	0.00	0	0	0	0.00
City of Makati	640,885	0	0	0	0.00	0	0	0	0.00
City of Mandaluyong	438,436	0	0	0	0.00	0	0	0	0.00
City of San Juan	127,586	0	0	0	0.00	0	0	0	0.00
City of Manila	1,891,095	0	0	0	0.00	0	0	0	0.00
City of Las Piñas	623,636	0	0	0	0.00	0	0	0	0.00
City of Muntinlupa	557,576	0	0	0	0.00	0	0	0	0.00
City of Parañaque	710,582	0	0	0	0.00	0	0	0	0.00
Pasay City	448,353	0	0	0	0.00	0	0	0	0.00
**C A R**	**1,857,123**	**13**	**7**	**20**	**0.11**	**12**	**2**	**14**	**0.75**
Abra	251,241	1	4	5	0.20	1	1	2	0.80
Apayao	127,176	2	1	3	0.24	1	0	1	0.79
Benguet	477,423	0	0	0	0.00	0	0	0	0.00
Ifugao	217,579	3	0	3	0.14	4	0	4	1.84
Kalinga	240,365	0	0	0	0.00	0	0	0	0.00
Mountain Province	162,512	0	0	0	0.00	0	0	0	0.00
City of Baguio	380,827	7	2	9	0.24	6	1	7	1.84
**Region 1**	**5,371,956**	**94**	**41**	**135**	**0.25**	**79**	**33**	**112**	**2.08**
Ilocos Norte	610,577	12	2	14	0.23	12	2	14	2.29
Ilocos Sur	706,868	9	7	16	0.23	9	4	13	1.84
La Union	829,158	11	6	17	0.21	13	6	19	2.29
Pangasinan	3,045,191	58	26	84	0.28	43	21	64	2.10
City of Dagupan	180,162	4	0	4	0.22	2	0	2	1.11
**Region 2**	**3,760,607**	**30**	**11**	**41**	**0.11**	**29**	**11**	**40**	**1.06**
Batanes	19,266	0	0	0	0.00	0	0	0	0.00
Cagayan	1,283,068	12	3	15	0.12	11	4	15	1.17
Isabela	1,573,655	16	6	22	0.14	15	7	22	1.40
Nueva Vizcaya	517,600	1	1	2	0.04	1	0	1	0.19
Quirino	212,120	0	1	1	0.05	1	0	1	0.47
City of Santiago	154,898	1	0	1	0.06	1	0	1	0.65
**Region 3**	**12,781,127**	**78**	**38**	**116**	**0.09**	**42**	**19**	**61**	**0.48**
Aurora	243,753	2	0	2	0.08	1	0	1	0.41
Bataan	882,586	0	0	0	0.00	0	0	0	0.00
Bulacan	3,815,209	4	2	6	0.02	4	1	5	0.13
Nueva Ecija	2,356,971	20	7	27	0.11	10	3	13	0.55
Pampanga	2,532,962	5	1	6	0.02	2	1	3	0.12
Tarlac	1,537,751	39	22	61	0.40	22	12	34	2.21
Zambales	667,072	3	2	5	0.07	2	1	3	0.45
City of Angeles	479,199	3	4	7	0.15	1	1	2	0.42
City of Olongapo	265,624	2	0	2	0.08	0	0	0	0.00

Table 2.E.5.1 Leprosy Prevention and Control
Prevalence Rate and Case Detection Rate
Philippines, 2024

Area	Population	Leprosy Cases undergoing Treatment			Prevalence Rate	Newly Detected Case			Case Detection Rate
		Male	Female	Total		Male	Female	Total	
**Region 4A**	**16,675,819**	**15**	**3**	**18**	**0.01**	**24**	**11**	**35**	**0.21**
Batangas	2,976,731	3	1	4	0.01	2	0	2	0.07
Cavite	4,482,531	2	2	4	0.01	6	2	8	0.18
Laguna	3,480,921	5	0	5	0.01	4	4	8	0.23
Quezon	1,996,436	5	0	5	0.03	8	1	9	0.45
Rizal	3,450,627	0	0	0	0.00	2	3	5	0.14
City of Lucena	288,573	0	0	0	0.00	2	1	3	1.04
**Region 4B**	**3,311,838**	**55**	**16**	**71**	**0.21**	**26**	**12**	**38**	**1.15**
Marinduque	240,500	18	6	24	1.00	6	2	8	3.33
Occidental Mindoro	543,612	18	3	21	0.39	4	1	5	0.92
Oriental Mindoro	926,271	4	2	6	0.06	4	2	6	0.65
Palawan	971,373	7	5	12	0.12	10	6	16	1.65
Romblon	313,035	0	0	0	0.00	0	1	1	0.32
City of Puerto Princesa	317,047	8	0	8	0.25	2	0	2	0.63
**Region 5**	**6,285,893**	**10**	**7**	**17**	**0.03**	**15**	**9**	**24**	**0.38**
Albay	1,421,135	2	1	3	0.02	2	2	4	0.28
Camarines Norte	660,386	0	2	2	0.03	2	2	4	0.61
Camarines Sur	1,923,038	2	1	3	0.02	2	3	5	0.26
Catanduanes	277,230	1	0	1	0.04	1	0	1	0.36
Masbate	932,021	2	3	5	0.05	5	2	7	0.75
Sorsogon	853,928	3	0	3	0.04	2	0	2	0.23
City of Naga	218,155	0	0	0	0.00	1	0	1	0.46
**Region 6**	**8,026,781**	**20**	**8**	**28**	**0.03**	**26**	**14**	**40**	**0.50**
Aklan	625,070	3	1	4	0.06	4	1	5	0.80
Antique	614,750	0	0	0	0.00	1	2	3	0.49
Capiz	813,912	1	0	1	0.01	2	0	2	0.25
Guimaras	191,037	0	0	0	0.00	0	0	0	0.00
Iloilo	2,065,439	5	2	7	0.03	6	2	8	0.39
Negros Occidental	2,657,997	11	5	16	0.06	12	9	21	0.79
City of Bacolod	598,149	0	0	0	0.00	1	0	1	0.17
City of Iloilo	460,427	0	0	0	0.00	0	0	0	0.00
**Region 7**	**8,352,783**	**71**	**14**	**85**	**0.10**	**54**	**15**	**69**	**0.83**
Bohol	1,415,451	5	3	8	0.06	20	6	26	1.84
Cebu	3,443,332	29	4	33	0.10	23	5	28	0.81
Negros Oriental	1,478,907	0	0	0	0.00	0	0	0	0.00
Siquijor	103,397	1	0	1	0.10	0	0	0	0.00
City of Cebu	1,003,708	8	3	11	0.11	4	3	7	0.70
City of Lapu-Lapu	525,400	28	4	32	0.61	7	1	8	1.52
City of Mandaue	382,588	0	0	0	0.00	0	0	0	0.00
**Region 8**	**4,627,073**	**117**	**34**	**151**	**0.33**	**111**	**37**	**148**	**3.20**
Biliran	183,266	6	3	9	0.49	4	2	6	3.27
Eastern Samar	483,811	11	1	12	0.25	11	1	12	2.48
Leyte	1,572,543	31	11	42	0.27	38	13	51	3.24
Northern Samar	648,722	30	9	39	0.60	20	12	32	4.93
Southern Leyte	430,474	7	2	9	0.21	5	2	7	1.63
Samar	807,949	24	6	30	0.37	20	6	26	3.22
Ormoc City	239,823	1	1	2	0.08	3	0	3	1.25
City of Tacloban	260,485	7	1	8	0.31	10	1	11	4.22

Table 2.E.5.1 Leprosy Prevention and Control
Prevalence Rate and Case Detection Rate
Philippines, 2024

Area	Population	Leprosy Cases undergoing Treatment			Prevalence Rate	Newly Detected Case			Case Detection Rate
		Male	Female	Total		Male	Female	Total	
**Region 9**	**3,982,251**	**134**	**169**	**303**	**0.76**	**173**	**278**	**451**	**11.33**
Zamboanga del Norte	1,072,509	57	97	154	1.44	104	164	268	24.99
Zamboanga del Sur	1,080,585	73	59	132	1.22	63	100	163	15.08
Zamboanga Sibugay	686,729	3	12	15	0.22	3	13	16	2.33
City of Isabela	131,555	0	0	0	0.00	2	0	2	1.52
City of Zamboanga	1,010,873	1	1	2	0.02	1	1	2	0.20
**Region 10**	**5,232,243**	**78**	**26**	**104**	**0.20**	**85**	**21**	**106**	**2.03**
Bukidnon	1,627,773	12	2	14	0.09	20	6	26	1.60
Camiguin	93,920	0	0	0	0.00	0	0	0	0.00
Lanao del Norte	745,269	0	4	4	0.05	3	0	3	0.40
Misamis Occidental	630,533	30	4	34	0.54	16	1	17	2.70
Misamis Oriental	993,490	8	6	14	0.14	10	5	15	1.51
City of Cagayan De Oro	766,153	17	5	22	0.29	21	6	27	3.52
City of Iligan	375,105	11	5	16	0.43	15	3	18	4.80
**Region 11**	**5,421,241**	**22**	**6**	**28**	**0.05**	**19**	**11**	**30**	**0.55**
Davao de Oro	799,461	0	0	0	0.00	0	0	0	0.00
Davao del Norte	1,166,096	12	2	14	0.12	7	2	9	0.77
Davao Oriental	594,939	5	1	6	0.10	1	2	3	0.50
Davao del Sur	700,668	3	2	5	0.07	1	3	4	0.57
Davao Occidental	318,092	0	0	0	0.00	5	3	8	2.51
City of Davao	1,841,985	2	1	3	0.02	5	1	6	0.33
**Region 12**	**4,555,599**	**57**	**21**	**78**	**0.17**	**51**	**26**	**77**	**1.69**
Cotabato	1,329,326	22	6	28	0.21	8	4	12	0.90
Sarangani	586,956	2	1	3	0.05	6	1	7	1.19
South Cotabato	1,016,509	6	5	11	0.11	5	4	9	0.89
Sultan Kudarat	889,774	10	3	13	0.15	2	2	4	0.45
City of General Santos	733,034	17	6	23	0.31	30	15	45	6.14
**Caraga**	**2,880,174**	**32**	**10**	**42**	**0.15**	**25**	**12**	**37**	**1.28**
Agusan del Norte	396,858	0	0	0	0.00	2	2	4	1.01
Agusan del Sur	763,159	10	1	11	0.14	6	2	8	1.05
Surigao del Norte	543,833	6	1	7	0.13	5	2	7	1.29
Surigao del Sur	664,065	7	3	10	0.15	2	1	3	0.45
Dinagat Islands	128,190	0	1	1	0.08	1	1	2	1.56
City of Butuan	384,069	9	4	13	0.34	9	4	13	3.38
**BARMM**	**5,055,225**	**12**	**13**	**25**	**0.05**	**14**	**11**	**25**	**0.49**
Basilan	430,622	3	5	8	0.19	4	3	7	1.63
Lanao del Sur	1,206,544	0	1	1	0.01	0	1	1	0.08
Maguindanao del Norte	646,802	1	0	1	0.02	0	0	0	0.00
Maguindanao del Sur	759,130	1	0	1	0.01	0	0	0	0.00
Sulu	1,005,907	7	6	13	0.13	10	6	16	1.59
Tawi-Tawi	441,708	0	0	0	0.00	0	0	0	0.00
SGA	226,137	0	1	1	0.04	0	1	1	0.44
City of Cotabato	338,375	0	0	0	0.00	0	0	0	0.00

Legend:
0 = No Case
* = No Report

2.E.6.1 Rabies
Animal Bites and Deaths Due to Rabies
Philippines, 2024

Area	Animal Bites			Death due to rabies			Rate
	Male	Female	Total	Male	Female	Total	
**Philippines**	**1,111,338**	**1,345,084**	**2,456,422**	**210**	**89**	**299**	**0.01**
**N C R**	**202,748**	**259,114**	**461,862**	**9**	**6**	**15**	**0.00**
City of Malabon	8,659	10,204	18,863	1	0	1	0.01
City of Navotas	4,514	5,047	9,561	1	0	1	0.01
City of Valenzuela	9,232	11,754	20,986	0	0	0	0.00
City of Caloocan	16,730	20,685	37,415	3	2	5	0.01
City of Marikina	4,013	5,067	9,080	0	0	0	0.00
City of Pasig	25,752	34,323	60,075	0	1	1	0.00
Pateros	1,160	1,550	2,710	0	0	0	0.00
City of Taguig	54,971	71,043	126,014	0	0	0	0.00
Quezon City	37,906	47,704	85,610	0	2	2	0.00
City of Makati	4,570	6,243	10,813	0	0	0	0.00
City of Mandaluyong	7,086	9,586	16,672	0	0	0	0.00
City of San Juan	3,729	5,014	8,743	0	0	0	0.00
City of Manila	6,911	8,611	15,522	1	1	2	0.01
City of Las Piñas	4,584	5,899	10,483	1	0	1	0.01
City of Muntinlupa	4,957	6,334	11,291	1	0	1	0.01
City of Parañaque	4,259	5,609	9,868	1	0	1	0.01
Pasay City	3,715	4,441	8,156	0	0	0	0.00
**C A R**	**29,580**	**32,532**	**62,112**	**1**	**1**	**2**	**0.00**
Abra	3,568	3,991	7,559	1	0	1	0.01
Apayao	2,737	2,731	5,468	0	0	0	0.00
Benguet	3,766	3,919	7,685	0	0	0	0.00
Ifugao	3,016	2,764	5,780	0	0	0	0.00
Kalinga	2,566	2,288	4,854	0	1	1	0.02
Mountain Province	1,805	1,637	3,442	0	0	0	0.00
City of Baguio	12,122	15,202	27,324	0	0	0	0.00
**Region 1**	**79,650**	**98,147**	**177,797**	**15**	**4**	**19**	**0.01**
Ilocos Norte	9,822	10,142	19,964	2	0	2	0.01
Ilocos Sur	12,638	16,061	28,699	1	0	1	0.00
La Union	15,805	22,413	38,218	1	1	2	0.01
Pangasinan	36,170	43,112	79,282	11	3	14	0.02
City of Dagupan	5,215	6,419	11,634	0	0	0	0.00
**Region 2**	**59,937**	**69,289**	**129,226**	**13**	**0**	**13**	**0.01**
Batanes	11	17	28	0	0	0	0.00
Cagayan	6,967	7,248	14,215	0	0	0	0.00
Isabela	34,959	40,741	75,700	13	0	13	0.02
Nueva Vizcaya	12,548	15,359	27,907	0	0	0	0.00
Quirino	3,379	3,405	6,784	0	0	0	0.00
City of Santiago	2,073	2,519	4,592	0	0	0	0.00
**Region 3**	**112,459**	**138,678**	**251,137**	**29**	**10**	**39**	**0.02**
Aurora	6,887	8,200	15,087	1	0	1	0.01
Bataan	11,770	14,683	26,453	3	0	3	0.01
Bulacan	25,987	32,046	58,033	9	4	13	0.02
Nueva Ecija	28,437	35,832	64,269	7	2	9	0.01
Pampanga	9,142	10,832	19,974	1	3	4	0.02
Tarlac	14,715	17,213	31,928	4	1	5	0.02
Zambales	6,849	8,391	15,240	4	0	4	0.03
City of Angeles	5,290	7,110	12,400	0	0	0	0.00
City of Olongapo	3,382	4,371	7,753	0	0	0	0.00

2.E.6.1 Rabies

Animal Bites and Deaths Due to Rabies

Philippines, 2024

Area	Animal Bites			Death due to rabies			Rate
	Male	Female	Total	Male	Female	Total	
**Region 4A**	**141,920**	**177,103**	**319,023**	**18**	**9**	**27**	**0.01**
Batangas	17,472	22,283	39,755	3	1	4	0.01
Cavite	27,418	33,393	60,811	9	3	12	0.02
Laguna	24,454	30,355	54,809	2	1	3	0.01
Quezon	22,099	28,624	50,723	2	3	5	0.01
Rizal	49,183	60,879	110,062	2	0	2	0.00
City of Lucena	1,294	1,569	2,863	0	1	1	0.03
**Region 4B**	**21,844**	**24,868**	**46,712**	**13**	**16**	**29**	**0.06**
Marinduque	3,471	4,507	7,978	1	0	1	0.01
Occidental Mindoro	4,841	5,123	9,964	1	1	2	0.02
Oriental Mindoro	3,868	4,553	8,421	0	5	5	0.06
Palawan	4,274	4,598	8,872	10	8	18	0.20
Romblon	1,883	2,231	4,114	1	2	3	0.07
City of Puerto Princesa	3,507	3,856	7,363	0	0	0	0.00
**Region 5**	**28,134**	**34,592**	**62,726**	**7**	**1**	**8**	**0.01**
Albay	3,852	4,491	8,343	0	0	0	0.00
Camarines Norte	563	736	1,299	0	0	0	0.00
Camarines Sur	4,316	5,387	9,703	1	0	1	0.01
Catanduanes	4,325	5,488	9,813	0	0	0	0.00
Masbate	2,253	2,632	4,885	0	1	1	0.02
Sorsogon	8,947	11,210	20,157	6	0	6	0.03
City of Naga	3,878	4,648	8,526	0	0	0	0.00
**Region 6**	**63,185**	**74,469**	**137,654**	**17**	**6**	**23**	**0.02**
Aklan	7,383	8,469	15,852	2	0	2	0.01
Antique	1,372	1,685	3,057	2	0	2	0.07
Capiz	9,147	11,441	20,588	4	2	6	0.03
Guimaras	3,259	3,783	7,042	0	1	1	0.01
Iloilo	11,859	13,922	25,781	3	2	5	0.02
Negros Occidental	24,095	27,728	51,823	4	1	5	0.01
City of Bacolod	5,125	6,324	11,449	1	0	1	0.01
City of Iloilo	945	1,117	2,062	1	0	1	0.05
**Region 7**	**98,228**	**116,023**	**214,251**	**5**	**4**	**9**	**0.00**
Bohol	11,209	13,660	24,869	0	1	1	0.00
Cebu	40,996	47,840	88,836	3	1	4	0.00
Negros Oriental	8,620	10,232	18,852	0	0	0	0.00
Siquijor	1,360	1,769	3,129	0	0	0	0.00
City of Cebu	14,058	15,845	29,903	1	2	3	0.01
City of Lapu-Lapu	12,870	15,932	28,802	0	0	0	0.00
City of Mandaue	9,115	10,745	19,860	1	0	1	0.01
**Region 8**	**27,085**	**32,249**	**59,334**	**6**	**2**	**8**	**0.01**
Biliran	2,426	2,813	5,239	0	0	0	0.00
Eastern Samar	794	1,123	1,917	1	1	2	0.10
Leyte	4,828	5,709	10,537	1	0	1	0.01
Northern Samar	430	474	904	0	0	0	0.00
Southern Leyte	4,986	5,854	10,840	0	0	0	0.00
Samar	6,987	8,429	15,416	1	0	1	0.01
Ormoc City	3,943	4,596	8,539	0	0	0	0.00
City of Tacloban	2,691	3,251	5,942	3	1	4	0.07

2.E.6.1 Rabies

Animal Bites and Deaths Due to Rabies
Philippines, 2024

Area	Animal Bites			Death due to rabies			Rate
	Male	Female	Total	Male	Female	Total	
**Region 9**	**28,915**	**35,253**	**64,168**	**6**	**2**	**8**	**0.01**
Zamboanga del Norte	3,552	4,014	7,566	2	0	2	0.03
Zamboanga del Sur	7,557	8,964	16,521	1	0	1	0.01
Zamboanga Sibugay	8,046	9,454	17,500	0	0	0	0.00
City of Isabela	468	541	1,009	0	0	0	0.00
City of Zamboanga	9,292	12,280	21,572	3	2	5	0.02
**Region 10**	**54,306**	**62,387**	**116,693**	**9**	**4**	**13**	**0.01**
Bukidnon	11,832	13,301	25,133	2	0	2	0.01
Camiguin	1,588	1,860	3,448	0	0	0	0.00
Lanao del Norte	6,094	4,668	10,762	0	0	0	0.00
Misamis Occidental	5,208	5,824	11,032	3	0	3	0.03
Misamis Oriental	12,053	14,034	26,087	1	1	2	0.01
City of Cagayan De Oro	9,113	12,061	21,174	2	3	5	0.02
City of Iligan	8,418	10,639	19,057	1	0	1	0.01
**Region 11**	**89,227**	**104,973**	**194,200**	**25**	**11**	**36**	**0.02**
Davao de Oro	10,281	12,214	22,495	5	2	7	0.03
Davao del Norte	25,760	28,661	54,421	9	4	13	0.02
Davao Oriental	6,866	8,614	15,480	3	1	4	0.03
Davao del Sur	7,289	8,792	16,081	3	2	5	0.03
Davao Occidental	2,256	2,532	4,788	2	2	4	0.08
City of Davao	36,775	44,160	80,935	3	0	3	0.00
**Region 12**	**39,223**	**45,470**	**84,693**	**29**	**10**	**39**	**0.05**
Cotabato	6,624	7,499	14,123	6	2	8	0.06
Sarangani	3,374	3,749	7,123	6	2	8	0.11
South Cotabato	13,988	16,610	30,598	6	0	6	0.02
Sultan Kudarat	10,285	11,554	21,839	7	2	9	0.04
City of General Santos	4,952	6,058	11,010	4	4	8	0.07
**Caraga**	**30,710**	**35,099**	**65,809**	**4**	**2**	**6**	**0.01**
Agusan del Norte	504	564	1,068	0	0	0	0.00
Agusan del Sur	8,632	9,710	18,342	4	2	6	0.03
Surigao del Norte	8,712	10,034	18,746	0	0	0	0.00
Surigao del Sur	7,979	9,128	17,107	0	0	0	0.00
Dinagat Islands	647	818	1,465	0	0	0	0.00
City of Butuan	4,236	4,845	9,081	0	0	0	0.00
**BARMM**	**4,187**	**4,838**	**9,025**	**4**	**1**	**5**	**0.06**
Basilan	858	885	1,743	1	0	1	0.06
Lanao del Sur	468	472	940	0	0	0	0.00
Maguindanao del Norte	197	221	418	0	0	0	0.00
Maguindanao del Sur	528	636	1,164	0	0	0	0.00
Sulu	44	95	139	0	0	0	0.00
Tawi-Tawi	434	403	837	0	0	0	0.00
SGA	2	4	6	0	0	0	0.00
City of Cotabato	1,656	2,122	3,778	3	1	4	0.11

Legend:
0 = No Case
* = No Report

Table 2.E.7.1 Tuberculosis Prevention and Control
Case Notification Rate (All forms and DTRB)
Philippines, 2024

PSGC	Area	Total Population	Notified TB, All Forms (New and Relapse)			Case Notification Rate (All Forms)	Registered Bacteriologically Confirmed Drug Resistant			Case Notification Rate (DRTB)
			Male	Female	Total		Male	Female	Total	
1300000000	Philippines	112,079,845	347,071	197,877	544,948	486	7,137	3,074	10,211	9.11
1300000000	N C R	13,902,112	60,784	39,540	100,324	722	1,493	720	2,213	15.92
1380100000	City of Caloocan	1,724,390	7,397	4,702	12,099	702	175	78	253	14.67
1380200000	City of Las Piñas	623,636	2,189	1,341	3,530	566	42	28	70	11.22
1380300000	City of Makati	297,966	1,911	1,558	3,469	1,164	33	26	59	19.80
1380400000	City of Malabon	393,282	2,477	1,491	3,968	1,009	52	33	85	21.61
1380500000	City of Mandaluyong	438,436	2,068	1,262	3,330	760	32	15	47	10.72
1380600000	City of Manila	1,891,095	8,692	5,764	14,456	765	314	143	457	24.20
1380700000	City of Marikina	468,372	2,143	1,384	3,527	753	56	26	82	17.51
1380800000	City of Muntinlupa	557,576	2,258	1,026	3,284	589	85	19	104	18.65
1380900000	City of Navotas	257,469	1,606	941	2,547	989	38	21	59	22.92
1381000000	City of Parañaque	710,582	2,573	1,557	4,130	581	38	21	59	8.30
1381100000	Pasay City	448,353	2,130	1,599	3,729	832	25	15	40	8.92
1381200000	City of Pasig	833,749	3,140	2,134	5,274	633	49	20	69	8.28
1381300000	Quezon City	3,052,364	13,689	9,014	22,703	744	367	208	575	18.84
1381400000	City of San Juan	127,586	528	402	930	729	18	10	28	21.95
1381500000	City of Taguig	1,271,862	4,613	3,101	7,714	607	114	31	145	11.40
1381600000	City of Valenzuela	738,827	3,055	2,019	5,074	687	50	25	75	10.15
1381701000	Pateros	66,567	315	245	560	841	5	1	6	9.01
1400000000	C A R	1,857,123	3,933	1,958	5,891	317	72	11	83	4.47
1430300000	City of Baguio	380,827	1,023	527	1,550	407	26	4	30	7.88
1400100000	Abra	251,241	804	417	1,221	486	11	3	14	5.57
1400101000	Bangued	50,483	245	139	384	761	3	0	3	5.94
1400102000	Boliney	4,551	12	3	15	330	0	0	0	0.00
1400103000	Bucay	17,953	54	10	64	356	3	0	3	16.71
1400104000	Bucloc	2,395	15	9	24	1,002	0	0	0	0.00
1400105000	Daguioman	2,019	5	3	8	396	0	0	0	0.00
1400106000	Danglas	4,074	12	9	21	515	1	0	1	24.55
1400107000	Dolores	11,513	33	19	52	452	0	0	0	0.00
1400108000	La Paz	16,528	50	23	73	442	0	0	0	0.00
1400109000	Lacub	3,646	12	13	25	686	0	0	0	0.00
1400110000	Lagangilang	14,914	50	25	75	503	0	0	0	0.00
1400111000	Lagayan	4,488	8	7	15	334	0	0	0	0.00
1400112000	Langiden	3,576	8	3	11	308	0	0	0	0.00
1400113000	Licuan-Baay	4,566	9	3	12	263	0	0	0	0.00
1400114000	Luba	6,518	9	7	16	245	0	0	0	0.00
1400115000	Malibcong	4,027	19	5	24	596	0	0	0	0.00
1400116000	Manabo	11,611	31	18	49	422	0	0	0	0.00
1400117000	Peñarrubia	6,954	15	3	18	259	1	0	1	14.38
1400118000	Pidigan	12,475	20	8	28	224	0	0	0	0.00
1400119000	Pilar	10,146	23	20	43	424	0	0	0	0.00
1400120000	Sallapadan	6,389	36	12	48	751	0	1	1	15.65
1400121000	San Isidro	4,745	15	4	19	400	0	0	0	0.00
1400122000	San Juan	10,689	34	14	48	449	2	0	2	18.71
1400123000	San Quintin	5,705	8	4	12	210	0	1	1	17.53
1400124000	Tayum	14,871	25	19	44	296	0	0	0	0.00
1400125000	Tineg	5,056	16	13	29	574	0	0	0	0.00
1400126000	Tubo	5,674	18	9	27	476	0	1	1	17.62
1400127000	Villaviciosa	5,675	22	15	37	652	1	0	1	17.62
1401100000	Benguet	477,423	516	193	709	149	12	0	12	2.51
1401101000	Atok	19,840	8	1	9	45	0	0	0	0.00
1401103000	Bakun	15,046	2	4	6	40	0	0	0	0.00
1401104000	Bokod	14,841	5	0	5	34	0	0	0	0.00
1401105000	Buguias	47,075	15	7	22	47	0	0	0	0.00
1401106000	Itogon	63,091	107	28	135	214	1	0	1	1.59
1401107000	Kabayan	16,114	18	7	25	155	0	0	0	0.00
1401108000	Kapangan	19,298	5	4	9	47	0	0	0	0.00
1401109000	Kibungan	17,872	7	1	8	45	0	0	0	0.00
1401110000	La Trinidad	144,035	238	106	344	239	8	0	8	5.55
1401111000	Mankayan	39,058	30	4	34	87	0	0	0	0.00
1401112000	Sablan	11,632	11	4	15	129	0	0	0	0.00
1401113000	Tuba	49,506	52	17	69	139	3	0	3	6.06
1401114000	Tublay	20,015	18	10	28	140	0	0	0	0.00
1402700000	Ifugao	217,579	268	125	393	181	4	1	5	2.30
1402701000	Banaue	21,574	24	15	39	181	0	0	0	0.00
1402702000	Hungduan	9,246	9	3	12	130	1	0	1	10.82
1402703000	Kiangan	18,538	22	11	33	178	1	0	1	5.39
1402704000	Lagawe	19,712	21	18	39	198	0	0	0	0.00
1402705000	Lamut	27,681	70	27	97	350	1	1	2	7.23
1402706000	Mayoyao	15,698	12	4	16	102	0	0	0	0.00
1402707000	Alfonso Lista	35,812	54	17	71	198	1	0	1	2.79
1402708000	Aguinaldo	22,570	23	10	33	146	0	0	0	0.00
1402709000	Hingyon	10,150	21	11	32	315	0	0	0	0.00
1402710000	Tinoc	19,804	9	1	10	50	0	0	0	0.00
1402711000	Asipulo	16,794	3	8	11	66	0	0	0	0.00
1403200000	Kalinga	240,365	633	277	910	379	8	2	10	4.16
1403201000	Balbalan	12,947	24	7	31	239	0	0	0	0.00
1403206000	Lubuagan	9,688	22	4	26	268	0	0	0	0.00
1403208000	Pasil	10,615	26	10	36	339	0	0	0	0.00
1403209000	Pinukpuk	35,493	68	31	99	279	2	0	2	5.63
1403211000	Rizal	20,518	33	12	45	219	3	0	3	14.62
1403213000	City of Tabuk	128,827	416	194	610	474	3	2	5	3.88
1403214000	Tanudan	8,855	17	8	25	282	0	0	0	0.00
1403215000	Tinglayan	13,422	27	11	38	283	0	0	0	0.00
1404400000	Mountain Province	162,512	229	99	328	202	4	0	4	2.46
1404401000	Barlig	4,796	4	4	8	167	0	0	0	0.00
1404402000	Bauko	33,722	40	8	48	142	2	0	2	5.93
1404403000	Besao	6,875	22	4	26	378	0	0	0	0.00
1404404000	Bontoc	24,316	37	16	53	218	0	0	0	0.00
1404405000	Natonin	10,516	14	16	30	285	0	0	0	0.00
1404406000	Paracelis	33,096	53	30	83	251	0	0	0	0.00
1404407000	Sabangan	9,623	16	2	18	187	0	0	0	0.00
1404408000	Sadanga	8,496	13	9	22	259	0	0	0	0.00
1404409000	Sagada	11,562	13	4	17	147	2	0	2	17.30
1404410000	Tadian	19,510	17	6	23	118	0	0	0	0.00
1408100000	Apayao	127,176	460	320	780	613	7	1	8	6.29
1408101000	Calanasan	12,762	27	15	42	329	1	0	1	7.84
1408102000	Conner	28,795	63	46	109	379	3	0	3	10.42
1408103000	Flora	18,246	20	14	34	186	1	0	1	5.48
1408104000	Kabugao	16,664	39	18	57	342	0	0	0	0.00
1408105000	Luna	21,632	284	193	477	2,205	2	1	3	13.87
1408106000	Pudtol	15,732	13	7	20	127	0	0	0	0.00
1408107000	Santa Marcela	13,345	14	27	41	307	0	0	0	0.00
100000000	Region 1	5,371,956	14,487	7,999	22,486	419	288	92	380	7.07
102800000	Ilocos Norte	610,577	1,047	626	1,673	274	10	1	11	1.80
102801000	Adams	2,253	3	0	3	133	0	0	0	0.00
102802000	Bacarra	33,496	45	36	81	242	0	0	0	0.00
102803000	Badoc	32,532	46	36	82	252	1	0	1	3.07
102804000	Bangui	15,020	33	15	48	320	1	0	1	6.66
102805000	City of Batac	55,537	123	68	191	344	1	0	1	1.80
102806000	Burgos	10,759	28	18	46	428	0	0	0	0.00
102807000	Carasi	1,607	2	2	4	249	0	1	1	62.23
102808000	Currimao	12,215	38	26	64	524	1	0	1	8.19
102809000	Dingras	40,236	64	37	101	251	0	0	0	0.00
102810000	Dumalneg	3,098	14	12	26	839	0	0	0	0.00
102811000	Banna	19,297	27	20	47	244	0	0	0	0.00
102812000	City of Laoag	111,975	193	97	290	259	0	0	0	0.00
102813000	Marcos	18,036	24	13	37	205	0	0	0	0.00
102814000	Nueva Era	12,056	16	17	33	274	0	0	0	0.00
102815000	Pagudpud	25,256	43	40	83	329	1	0	1	3.96
102816000	Paoay	25,001	28	18	46	184	0	0	0	0.00
102817000	Pasuquin	29,739	23	8	31	104	1	0	1	3.36
102818000	Piddig	22,482	48	14	62	276	0	0	0	0.00
102819000	Pinili	17,626	18	9	27	153	0	0	0	0.00

Table 2.E.7.1 Tuberculosis Prevention and Control
Case Notification Rate (All forms and DTRB)
Philippines, 2024

PSGC	Area	Total Population	Notified TB, All Forms (New and Relapse)			Case Notification Rate (All Forms)	Registered Bacteriologically Confirmed Drug Resistant			Case Notification Rate (DRTB)
			Male	Female	Total		Male	Female	Total	
102820000	San Nicolas	38,977	60	22	82	210	2	0	2	5.13
102821000	Sarrat	25,186	56	35	91	361	1	0	1	3.97
102822000	Solsona	24,854	74	50	124	499	1	0	1	4.02
102823000	Vintar	33,339	41	33	74	222	0	0	0	0.00
**102900000**	**Ilocos Sur**	**706,868**	**1,908**	**1,168**	**3,076**	**435**	**38**	**5**	**43**	**6.08**
102901000	Alilem	7,361	26	13	39	530	0	0	0	0.00
102902000	Banayoyo	7,931	12	4	16	202	0	0	0	0.00
102903000	Bantay	37,157	290	184	474	1,276	5	1	6	16.15
102904000	Burgos	12,793	50	47	97	758	2	0	2	15.63
102905000	Cabugao	39,044	52	27	79	202	1	0	1	2.56
**102906000**	**City of Candon**	**61,438**	**206**	**114**	**320**	**521**	**1**	**0**	**1**	**1.63**
102907000	Caoayan	19,574	57	45	102	521	0	0	0	0.00
102908000	Cervantes	19,933	29	11	40	201	0	0	0	0.00
102909000	Galimuyod	10,244	19	8	27	264	1	1	2	19.52
102910000	Gregorio del Pilar	4,472	8	6	14	313	0	0	0	0.00
102911000	Lidlidda	4,705	9	1	10	213	0	0	0	0.00
102912000	Magsingal	31,400	96	64	160	510	2	0	2	6.37
102913000	Nagbukel	5,465	19	6	25	457	0	0	0	0.00
102914000	Narvacan	46,271	86	54	140	303	0	0	0	0.00
102915000	Quirino	9,315	11	7	18	193	0	0	0	0.00
102916000	Salcedo	11,110	31	19	50	450	0	0	0	0.00
102917000	San Emilio	7,211	13	5	18	250	0	0	0	0.00
102918000	San Esteban	8,381	28	13	41	489	0	0	0	0.00
102919000	San Ildefonso	8,190	17	15	32	391	1	0	1	12.21
102920000	San Juan	26,674	53	41	94	352	1	0	1	3.75
102921000	San Vicente	13,118	29	11	40	305	1	0	1	7.62
102922000	Santa	14,992	36	22	58	387	0	0	0	0.00
102923000	Santa Catalina	14,493	56	37	93	642	0	0	0	0.00
102924000	Santa Cruz	41,366	65	36	101	244	1	0	1	2.42
102925000	Santa Lucia	25,966	65	61	126	485	1	0	1	3.85
102926000	Santa Maria	30,013	64	29	93	310	0	0	0	0.00
102927000	Santiago	19,473	54	33	87	447	1	0	1	5.14
102928000	Santo Domingo	29,041	38	20	58	200	11	3	14	48.21
102929000	Sigay	2,552	8	8	16	627	0	0	0	0.00
102930000	Sinait	25,998	47	21	68	262	3	0	3	11.54
102931000	Sugpon	4,931	7	7	14	284	0	0	0	0.00
102932000	Suyo	10,766	37	25	62	576	0	0	0	0.00
102933000	Tagudin	41,544	118	72	190	457	1	0	1	2.41
**102934000**	**City of Vigan**	**53,946**	**172**	**102**	**274**	**508**	**5**	**0**	**5**	**9.27**
**103300000**	**La Union**	**829,158**	**2,401**	**1,509**	**3,910**	**472**	**32**	**12**	**44**	**5.31**
103301000	Agoo	66,518	286	201	487	732	3	2	5	7.52
103302000	Aringay	51,144	95	65	160	313	1	0	1	1.96
103303000	Bacnotan	44,596	107	70	177	397	2	1	3	6.73
103304000	Bagulin	14,790	36	14	50	338	1	0	1	6.76
103305000	Balaoan	40,352	105	69	174	431	0	0	0	0.00
103306000	Bangar	38,044	84	59	143	376	0	0	0	0.00
103307000	Bauang	79,360	165	87	252	318	3	0	3	3.78
103308000	Burgos	9,010	9	10	19	211	0	0	0	0.00
103309000	Caba	23,205	55	21	76	328	0	0	0	0.00
103310000	Luna	37,318	188	105	293	785	3	0	3	8.04
103311000	Naguilian	52,385	190	139	329	628	2	0	2	3.82
103312000	Pugo	19,353	45	27	72	372	0	0	0	0.00
103313000	Rosario	60,981	92	54	146	239	4	2	6	9.84
**103314000**	**City of San Fernando**	**127,860**	**553**	**333**	**886**	**693**	**9**	**6**	**15**	**11.73**
103315000	San Gabriel	19,209	51	40	91	474	1	0	1	5.21
103316000	San Juan	40,571	108	71	179	441	1	1	2	4.93
103317000	Santo Tomas	41,149	65	25	90	219	1	0	1	2.43
103318000	Santol	14,289	32	23	55	385	1	0	1	7.00
103319000	Sudipen	17,187	45	28	73	425	0	0	0	0.00
103320000	Tubao	31,837	90	68	158	496	0	0	0	0.00
**105500000**	**Pangasinan**	**3,225,353**	**9,131**	**4,696**	**13,827**	**429**	**208**	**74**	**282**	**8.74**
105501000	Agno	30,247	61	38	99	327	1	0	1	3.31
105502000	Aguilar	46,478	104	52	156	336	4	0	4	8.61
**105503000**	**City of Alaminos**	**101,965**	**566**	**353**	**919**	**901**	**12**	**7**	**19**	**18.63**
105504000	Alcala	48,912	96	37	133	272	0	1	1	2.04
105505000	Anda	42,381	83	54	137	323	2	1	3	7.08
105506000	Asingan	57,913	162	54	216	373	3	1	4	6.91
105507000	Balungao	30,028	72	40	112	373	2	0	2	6.66
105508000	Bani	53,412	141	74	215	403	3	0	3	5.62
105509000	Basista	38,865	108	84	192	494	4	1	5	12.87
105510000	Bautista	35,734	111	69	180	504	3	0	3	8.40
105511000	Bayambang	131,926	296	142	438	332	11	4	15	11.37
105512000	Binalonan	56,383	144	74	218	387	2	1	3	5.32
105513000	Binmaley	88,903	165	65	230	259	4	5	9	10.12
105514000	Bolinao	86,937	163	82	245	282	8	2	10	11.50
105515000	Bugallon	77,380	156	67	223	288	4	0	4	5.17
105516000	Burgos	23,814	34	14	48	202	0	0	0	0.00
105517000	Calasiao	103,324	234	90	324	314	10	5	15	14.52
**105518000**	**City of Dagupan**	**180,162**	**1,020**	**548**	**1,568**	**870**	**22**	**8**	**30**	**16.65**
105519000	Dasol	31,747	121	58	179	564	1	0	1	3.15
105520000	Infanta	27,115	61	36	97	358	0	0	0	0.00
105521000	Labrador	27,110	45	24	69	255	2	1	3	11.07
105522000	Lingayen	109,735	354	182	536	488	4	2	6	5.47
105523000	Mabini	26,967	61	36	97	360	0	0	0	0.00
105524000	Malasiqui	146,736	356	197	553	377	3	3	6	4.09
105525000	Manaoag	77,487	162	63	225	290	4	3	7	9.03
105526000	Mangaldan	116,133	235	103	338	291	13	6	19	16.36
105527000	Mangatarem	79,475	226	116	342	430	4	1	5	6.29
105528000	Mapandan	38,859	106	33	139	358	4	0	4	10.29
105529000	Natividad	25,772	70	45	115	446	3	1	4	15.52
105530000	Pozorrubio	75,133	139	69	208	277	0	0	0	0.00
105531000	Rosales	67,006	245	136	381	569	5	2	7	10.45
**105532000**	**City of San Carlos**	**211,102**	**681**	**347**	**1,028**	**487**	**23**	**9**	**32**	**15.16**
105533000	San Fabian	89,142	195	87	282	316	5	0	5	5.61
105534000	San Jacinto	45,532	84	41	125	275	0	0	0	0.00
105535000	San Manuel	55,155	113	47	160	290	1	0	1	1.81
105536000	San Nicolas	39,955	78	45	123	308	2	0	2	5.01
105537000	San Quintin	34,231	121	61	182	532	1	0	1	2.92
105538000	Santa Barbara	94,910	205	90	295	311	5	0	5	5.27
105539000	Santa Maria	34,313	87	58	145	423	3	0	3	8.74
105540000	Santo Tomas	14,878	53	16	69	464	1	0	1	6.72
105541000	Sison	52,510	118	61	179	341	3	0	3	5.71
105542000	Sual	40,247	49	32	81	201	5	0	5	12.42
105543000	Tayug	45,984	221	126	347	755	1	1	2	4.35
105544000	Umingan	77,635	219	117	336	433	6	2	8	10.30
105545000	Urbiztondo	57,648	92	51	143	248	5	3	8	13.88
**105546000**	**City of Urdaneta**	**148,239**	**756**	**379**	**1,135**	**766**	**5**	**2**	**7**	**4.72**
105547000	Villasis	65,705	112	81	193	294	2	1	3	4.57
105548000	Laoac	34,128	50	22	72	211	2	1	3	8.79
**200000000**	**Region 2**	**3,760,607**	**10,425**	**6,436**	**16,861**	**448**	**138**	**38**	**176**	**4.68**
**203135000**	**City of Santiago**	**154,898**	**926**	**453**	**1,379**	**890**	**10**	**3**	**13**	**8.39**
**200900000**	**Batanes**	**19,266**	**35**	**13**	**48**	**249**	**1**	**0**	**1**	**5.19**
200901000	Basco	9,955	13	8	21	211	0	0	0	0.00
200902000	Itbayat	3,128	8	1	9	288	0	0	0	0.00
200903000	Ivana	1,407	3	1	4	284	1	0	1	71.07
200904000	Mahatao	1,703	4	0	4	235	0	0	0	0.00
200905000	Sabtang	1,696	4	3	7	413	0	0	0	0.00
200906000	Uyugan	1,377	3	0	3	218	0	0	0	0.00
**201500000**	**Cagayan**	**1,283,068**	**3,000**	**1,782**	**4,782**	**373**	**47**	**8**	**55**	**4.29**
201501000	Abulug	34,924	53	42	95	272	1	0	1	2.86
201502000	Alcala	41,365	51	22	73	176	0	0	0	0.00
201503000	Allacapan	35,604	43	35	78	219	2	0	2	5.62
201504000	Amulung	50,664	76	52	128	253	1	0	1	1.97
201505000	Aparri	70,715	196	118	314	444	4	1	5	7.07
201506000	Baggao	88,156	133	69	202	229	1	0	1	1.13

Table 2.E.7.1 Tuberculosis Prevention and Control
Case Notification Rate (All forms and DTRB)
Philippines, 2024

PSGC	Area	Total Population	Notified TB, All Forms (New and Relapse)			Case Notification Rate (All Forms)	Registered Bacteriologically Confirmed Drug Resistant			Case Notification Rate (DRTB)
			Male	Female	Total		Male	Female	Total	
201507000	Ballesteros	34,786	125	101	226	650	1	0	1	2.87
201508000	Buguey	32,342	85	44	129	399	2	0	2	6.18
201509000	Calayan	17,855	18	20	38	213	0	0	0	0.00
201510000	Camalaniugan	25,256	78	59	137	542	3	0	3	11.88
201511000	Claveria	31,900	82	38	120	376	1	0	1	3.13
201512000	Enrile	36,717	63	23	86	234	1	0	1	2.72
201513000	Gattaran	59,070	116	85	201	340	0	0	0	0.00
201514000	Gonzaga	42,819	126	100	226	528	1	0	1	2.34
201515000	Iguig	30,183	50	21	71	235	3	0	3	9.94
201516000	Lal-Lo	49,113	102	65	167	340	4	0	4	8.14
201517000	Lasam	41,282	53	36	89	216	1	1	2	4.84
201518000	Pamplona	25,021	62	43	105	420	0	1	1	4.00
201519000	Peñablanca	52,057	76	28	104	200	1	0	1	1.92
201520000	Piat	24,815	62	26	88	355	2	1	3	12.09
201521000	Rizal	19,078	31	16	47	246	0	0	0	0.00
201522000	Sanchez-Mira	26,164	63	44	107	409	0	0	0	0.00
201523000	Santa Ana	37,399	74	54	128	342	5	1	6	16.04
201524000	Santa Praxedes	4,434	9	3	12	271	0	0	0	0.00
201525000	Santa Teresita	19,724	64	45	109	553	0	0	0	0.00
201526000	Santo Niño	28,581	43	42	85	297	0	0	0	0.00
201527000	Solana	88,900	221	135	356	400	0	1	1	1.12
201528000	Tuao	64,248	168	70	238	370	4	0	4	6.23
**201529000**	**Tuguegarao City**	**169,896**	**677**	**346**	**1,023**	**602**	**9**	**2**	**11**	**6.47**
**203100000**	**Isabela**	**1,728,553**	**5,987**	**3,783**	**9,770**	**565**	**81**	**26**	**107**	**6.19**
203101000	Alicia	74,991	206	131	337	449	1	0	1	1.33
203102000	Angadanan	44,992	137	59	196	436	2	1	3	6.67
203103000	Aurora	36,623	128	113	241	658	1	0	1	2.73
203104000	Benito Soliven	30,070	122	79	201	668	0	1	1	3.33
203105000	Burgos	26,182	103	76	179	684	2	1	3	11.46
203106000	Cabagan	55,146	263	263	526	954	0	0	0	0.00
203107000	Cabatuan	40,021	108	66	174	435	1	0	1	2.50
**203108000**	**City of Cauayan**	**146,468**	**550**	**298**	**848**	**579**	**6**	**2**	**8**	**5.46**
203109000	Cordon	48,142	124	97	221	459	0	0	0	0.00
203110000	Dinapigue	6,065	22	13	35	577	0	0	0	0.00
203111000	Divilacan	5,851	4	3	7	120	3	0	3	51.27
203112000	Echague	89,416	231	96	327	366	4	1	5	5.59
203113000	Gamu	30,951	94	84	178	575	1	3	4	12.92
**203114000**	**City of Ilagan**	**161,602**	**682**	**502**	**1,184**	**733**	**14**	**3**	**17**	**10.52**
203115000	Jones	45,687	169	150	319	698	1	0	1	2.19
203116000	Luna	20,702	41	21	62	299	0	0	0	0.00
203117000	Maconacon	3,977	9	1	10	251	0	0	0	0.00
203118000	Delfin Albano	29,949	119	51	170	568	14	6	20	66.78
203119000	Mallig	32,919	129	69	198	601	0	0	0	0.00
203120000	Naguilian	33,852	129	102	231	682	0	0	0	0.00
203121000	Palanan	18,117	43	25	68	375	1	0	1	5.52
203122000	Quezon	27,634	56	41	97	351	0	1	1	3.62
203123000	Quirino	25,026	75	61	136	543	1	0	1	4.00
203124000	Ramon	58,285	136	59	195	335	3	0	3	5.15
203125000	Reina Mercedes	28,190	119	59	178	631	3	0	3	10.64
203126000	Roxas	67,522	241	141	382	566	2	0	2	2.96
203127000	San Agustin	22,128	51	32	83	375	0	0	0	0.00
203128000	San Guillermo	21,394	57	31	88	411	1	0	1	4.67
203129000	San Isidro	27,272	90	34	124	455	1	0	1	3.67
203130000	San Manuel	34,799	116	85	201	578	0	0	0	0.00
203131000	San Mariano	62,147	228	180	408	657	1	2	3	4.83
203132000	San Mateo	67,048	117	61	178	265	3	1	4	5.97
203133000	San Pablo	26,702	44	37	81	303	2	0	2	7.49
203134000	Santa Maria	26,411	118	54	172	651	0	0	0	0.00
203136000	Santo Tomas	24,562	43	38	81	330	0	0	0	0.00
203137000	Tumauini	72,812	157	118	275	378	3	1	4	5.49
**205000000**	**Nueva Vizcaya**	**517,600**	**902**	**550**	**1,452**	**281**	**7**	**2**	**9**	**1.74**
205001000	Ambaguio	16,593	8	3	11	66	0	0	0	0.00
205002000	Aritao	43,480	79	45	124	285	0	0	0	0.00
205003000	Bagabag	38,868	75	35	110	283	0	0	0	0.00
205004000	Bambang	58,236	118	68	186	319	2	0	2	3.43
205005000	Bayombong	70,955	160	107	267	376	2	1	3	4.23
205006000	Diadi	19,770	85	70	155	784	0	0	0	0.00
205007000	Dupax del Norte	34,599	55	46	101	292	0	0	0	0.00
205008000	Dupax del Sur	21,722	23	12	35	161	0	0	0	0.00
205009000	Kasibu	44,384	35	12	47	106	0	0	0	0.00
205010000	Kayapa	27,394	19	10	29	106	0	0	0	0.00
205011000	Quezon	25,395	23	18	41	161	0	0	0	0.00
205012000	Santa Fe	19,040	32	22	54	284	0	0	0	0.00
205013000	Solano	67,514	153	82	235	348	2	1	3	4.44
205014000	Villaverde	20,950	27	14	41	196	1	0	1	4.77
205015000	Alfonso Castaneda	8,700	10	6	16	184	0	0	0	0.00
**205700000**	**Quirino**	**212,120**	**501**	**308**	**809**	**381**	**2**	**2**	**4**	**1.89**
205701000	Aglipay	31,604	37	57	94	297	0	0	0	0.00
205702000	Cabarroguis	35,132	104	52	156	444	1	0	1	2.85
205703000	Diffun	58,733	181	118	299	509	1	0	1	1.70
205704000	Maddela	42,184	88	29	117	277	0	1	1	2.37
205705000	Saguday	17,668	64	38	102	577	0	1	1	5.66
205706000	Nagtipunan	26,799	27	14	41	153	0	0	0	0.00
**300000000**	**Region 3**	**12,781,127**	**40,007**	**22,988**	**62,995**	**493**	**1,046**	**431**	**1,477**	**11.56**
**330100000**	**City of Angeles**	**479,199**	**1,380**	**829**	**2,209**	**461**	**35**	**10**	**45**	**9.39**
**331400000**	**City of Olongapo**	**265,624**	**1,205**	**709**	**1,914**	**721**	**22**	**7**	**29**	**10.92**
**300800000**	**Bataan**	**882,586**	**3,250**	**1,894**	**5,144**	**583**	**60**	**22**	**82**	**9.29**
300801000	Abucay	44,232	122	80	202	457	3	0	3	6.78
300802000	Bagac	32,570	89	50	139	427	0	0	0	0.00
**300803000**	**City of Balanga**	**107,639**	**518**	**361**	**879**	**817**	**21**	**6**	**27**	**25.08**
300804000	Dinalupihan	121,880	499	292	791	649	6	4	10	8.20
300805000	Hermosa	79,846	300	166	466	584	4	3	7	8.77
300806000	Limay	81,645	186	108	294	360	2	1	3	3.67
300807000	Mariveles	156,144	678	410	1,088	697	2	1	3	1.92
300808000	Morong	36,982	208	112	320	865	5	0	5	13.52
300809000	Orani	72,593	250	129	379	522	10	4	14	19.29
300810000	Orion	62,240	197	76	273	439	3	2	5	8.03
300811000	Pilar	47,303	101	69	170	359	1	0	1	2.11
300812000	Samal	39,512	102	41	143	362	3	1	4	10.12
**301400000**	**Bulacan**	**3,815,209**	**13,195**	**8,104**	**21,299**	**558**	**489**	**194**	**683**	**17.90**
301401000	Angat	68,114	198	141	339	498	5	1	6	8.81
301402000	Balagtas	78,741	449	317	766	973	7	6	13	16.51
**301403000**	**City of Baliwag**	**172,399**	**602**	**333**	**935**	**542**	**21**	**10**	**31**	**17.98**
301404000	Bocaue	146,415	887	554	1,441	984	12	5	17	11.61
301405000	Bulacan	81,825	261	144	405	495	18	3	21	25.66
301406000	Bustos	79,602	243	123	366	460	11	3	14	17.59
301407000	Calumpit	120,913	290	170	460	380	7	3	10	8.27
301408000	Guiguinto	116,210	394	201	595	512	10	6	16	13.77
301409000	Hagonoy	136,230	465	298	763	560	7	2	9	6.61
**301410000**	**City of Malolos**	**264,943**	**841**	**482**	**1,323**	**499**	**25**	**12**	**37**	**13.97**
301411000	Marilao	264,425	1,003	675	1,678	635	20	6	26	9.83
**301412000**	**City of Meycauayan**	**233,210**	**964**	**581**	**1,545**	**662**	**24**	**6**	**30**	**12.86**
301413000	Norzagaray	141,309	307	188	495	350	10	7	17	12.03
301414000	Obando	60,750	230	194	424	698	8	1	9	14.81
301415000	Pandi	160,651	348	233	581	362	15	5	20	12.45
301416000	Paombong	56,606	132	69	201	355	4	1	5	8.83
301417000	Plaridel	117,905	346	206	552	468	10	5	15	12.72
301418000	Pulilan	111,906	252	149	401	358	3	1	4	3.57
301419000	San Ildefonso	118,744	297	143	440	371	7	2	9	7.58
**301420000**	**City of San Jose Del Monte**	**672,212**	**2,901**	**1,892**	**4,793**	**713**	**219**	**88**	**307**	**45.67**
301421000	San Miguel	177,088	375	191	566	320	17	5	22	12.42
301422000	San Rafael	105,165	209	119	328	312	2	1	3	2.85
301423000	Santa Maria	299,855	1,126	656	1,782	594	26	14	40	13.34
301424000	Doña Remedios Trinidad	29,991	75	45	120	400	1	1	2	6.67
**304900000**	**Nueva Ecija**	**2,356,971**	**6,166**	**3,331**	**9,497**	**403**	**160**	**56**	**216**	**9.16**

Table 2.E.7.1 Tuberculosis Prevention and Control
Case Notification Rate (All forms and DTRB)
Philippines, 2024

PSGC	Area	Total Population	Notified TB, All Forms (New and Relapse)			Case Notification Rate (All Forms)	Registered Bacteriologically Confirmed Drug Resistant			Case Notification Rate (DRTB)
			Male	Female	Total		Male	Female	Total	
304901000	Aliaga	71,566	116	46	162	226	2	1	3	4.19
304902000	Bongabon	68,241	120	69	189	277	0	2	2	2.93
**304903000**	**City of Cabanatuan**	**336,524**	**1,298**	**690**	**1,988**	**591**	**17**	**6**	**23**	**6.83**
304904000	Cabiao	88,657	241	121	362	408	9	3	12	13.54
304905000	Carranglan	42,918	80	55	135	315	0	0	0	0.00
304906000	Cuyapo	68,072	204	113	317	466	4	0	4	5.88
304907000	Gabaldon	40,377	82	55	137	339	2	0	2	4.95
**304908000**	**City of Gapan**	**127,162**	**435**	**221**	**656**	**516**	**39**	**6**	**45**	**35.39**
304909000	General Mamerto Natividad	44,866	59	31	90	201	1	1	2	4.46
304910000	General Tinio	57,692	86	81	167	289	2	3	5	8.67
304911000	Guimba	128,164	233	107	340	265	4	0	4	3.12
304912000	Jaen	81,323	115	68	183	225	3	1	4	4.92
304913000	Laur	38,851	64	52	116	299	1	0	1	2.57
304914000	Licab	29,463	32	11	43	146	0	0	0	0.00
304915000	Llanera	42,474	53	25	78	184	1	1	2	4.71
304916000	Lupao	46,336	59	30	89	192	3	1	4	8.63
**304917000**	**Science City of Muñoz**	**84,501**	**197**	**91**	**288**	**341**	**2**	**2**	**4**	**4.73**
304918000	Nampicuan	14,471	38	27	65	449	0	0	0	0.00
**304919000**	**City of Palayan**	**46,912**	**142**	**57**	**199**	**424**	**1**	**1**	**2**	**4.26**
304920000	Pantabangan	32,139	74	48	122	380	0	0	0	0.00
304921000	Peñaranda	32,961	53	28	81	246	4	0	4	12.14
304922000	Quezon	42,486	80	41	121	285	0	0	0	0.00
304923000	Rizal	70,212	143	61	204	291	2	1	3	4.27
304924000	San Antonio	85,460	177	118	295	345	1	1	2	2.34
304925000	San Isidro	55,180	124	59	183	332	2	0	2	3.62
**304926000**	**San Jose City**	**155,121**	**600**	**361**	**961**	**620**	**6**	**0**	**6**	**3.87**
304927000	San Leonardo	70,533	209	105	314	445	3	3	6	8.51
304928000	Santa Rosa	78,124	210	101	311	398	4	0	4	5.12
304929000	Santo Domingo	61,553	249	174	423	687	27	19	46	74.73
304930000	Talavera	134,816	330	147	477	354	18	4	22	16.32
304931000	Talugtug	25,238	91	50	141	559	2	0	2	7.92
304932000	Zaragoza	54,578	172	88	260	476	0	0	0	0.00
**305400000**	**Pampanga**	**2,532,962**	**7,167**	**4,049**	**11,216**	**443**	**145**	**67**	**212**	**8.37**
305402000	Apalit	121,185	356	177	533	440	6	4	10	8.25
305403000	Arayat	150,912	539	278	817	541	8	1	9	5.96
305404000	Bacolor	50,063	88	48	136	272	1	0	1	2.00
305405000	Candaba	124,508	193	93	286	230	5	2	7	5.62
305406000	Floridablanca	140,395	300	201	501	357	6	6	12	8.55
305407000	Guagua	132,049	533	241	774	586	12	1	13	9.84
305408000	Lubao	180,143	599	323	922	512	9	6	15	8.33
**305409000**	**Mabalacat City**	**305,559**	**756**	**506**	**1,262**	**413**	**12**	**7**	**19**	**6.22**
305410000	Macabebe	80,609	320	177	497	617	7	1	8	9.92
305411000	Magalang	129,253	249	143	392	303	5	4	9	6.96
305412000	Masantol	59,939	96	52	148	247	1	1	2	3.34
305413000	Mexico	180,494	577	339	916	508	5	8	13	7.20
305414000	Minalin	49,793	144	75	219	440	3	2	5	10.04
305415000	Porac	148,252	421	242	663	447	9	3	12	8.09
**305416000**	**City of San Fernando**	**370,274**	**1,324**	**813**	**2,137**	**577**	**33**	**12**	**45**	**12.15**
305417000	San Luis	61,026	191	97	288	472	1	1	2	3.28
305418000	San Simon	61,685	100	49	149	242	4	1	5	8.11
305419000	Santa Ana	63,341	88	55	143	226	6	3	9	14.21
305420000	Santa Rita	48,939	91	39	130	266	4	2	6	12.26
305421000	Sto. Tomas	44,204	105	51	156	353	1	1	2	4.52
305422000	Sasmuan	30,339	97	50	147	485	7	1	8	26.37
**306900000**	**Tarlac**	**1,537,751**	**4,394**	**2,247**	**6,641**	**432**	**62**	**42**	**104**	**6.76**
306901000	Anao	12,208	24	15	39	319	0	0	0	0.00
306902000	Bamban	80,799	147	75	222	275	6	4	10	12.38
306903000	Camiling	87,320	422	241	663	759	5	4	9	10.31
306904000	Capas	163,189	351	181	532	326	3	2	5	3.06
306905000	Concepcion	176,117	544	271	815	463	8	10	18	10.22
306906000	Gerona	94,486	219	106	325	344	4	1	5	5.29
306907000	La Paz	69,184	133	52	185	267	3	0	3	4.34
306908000	Mayantoc	32,598	70	26	96	295	0	1	1	3.07
306909000	Moncada	62,819	154	86	240	382	3	2	5	7.96
306910000	Paniqui	103,004	371	231	602	584	4	1	5	4.85
306911000	Pura	25,807	56	39	95	368	1	0	1	3.87
306912000	Ramos	22,879	65	31	96	420	1	1	2	8.74
306913000	San Clemente	13,182	29	6	35	266	0	0	0	0.00
306914000	San Manuel	28,390	59	21	80	282	1	0	1	3.52
306915000	Santa Ignacia	51,627	132	58	190	368	1	1	2	3.87
**306916000**	**City of Tarlac**	**401,828**	**1,447**	**725**	**2,172**	**541**	**21**	**15**	**36**	**8.96**
306917000	Victoria	69,372	108	51	159	229	1	0	1	1.44
306918000	San Jose	42,942	63	32	95	221	0	0	0	0.00
**307100000**	**Zambales**	**667,072**	**2,464**	**1,418**	**3,882**	**582**	**58**	**30**	**88**	**13.19**
307101000	Botolan	68,610	213	147	360	525	2	3	5	7.29
307102000	Cabangan	28,607	77	40	117	409	0	0	0	0.00
307103000	Candelaria	31,016	131	97	228	735	0	1	1	3.22
307104000	Castillejos	70,048	232	113	345	493	6	0	6	8.57
307105000	Iba	57,377	206	101	307	535	4	1	5	8.71
307106000	Masinloc	56,684	224	100	324	572	7	2	9	15.88
307108000	Palauig	40,847	135	66	201	492	4	5	9	22.03
307109000	San Antonio	37,728	110	70	180	477	4	2	6	15.90
307110000	San Felipe	25,234	89	44	133	527	2	2	4	15.85
307111000	San Marcelino	38,001	121	100	221	582	8	2	10	26.32
307112000	San Narciso	30,764	88	49	137	445	2	0	2	6.50
307113000	Santa Cruz	65,757	222	132	354	538	7	2	9	13.69
307114000	Subic	116,399	616	359	975	838	12	10	22	18.90
**307700000**	**Aurora**	**243,753**	**786**	**407**	**1,193**	**489**	**15**	**3**	**18**	**7.38**
307701000	Baler	45,577	128	65	193	423	3	3	6	13.16
307702000	Casiguran	27,288	73	22	95	348	2	0	2	7.33
307703000	Dilasag	17,797	44	26	70	393	1	0	1	5.62
307704000	Dinalungan	13,014	19	13	32	246	0	0	0	0.00
307705000	Dingalan	29,065	189	108	297	1,022	2	0	2	6.88
307706000	Dipaculao	34,173	101	51	152	445	2	0	2	5.85
307707000	Maria Aurora	45,975	144	75	219	476	2	0	2	4.35
307708000	San Luis	30,864	88	47	135	437	3	0	3	9.72
**400000000**	**Region 4A**	**16,675,819**	**55,392**	**34,286**	**89,678**	**538**	**1,080**	**524**	**1,604**	**9.62**
**431200000**	**City of Lucena**	**288,573**	**1,083**	**652**	**1,735**	**601**	**20**	**18**	**38**	**13.17**
**401000000**	**Batangas**	**2,976,731**	**12,656**	**7,853**	**20,509**	**689**	**160**	**52**	**212**	**7.12**
401001000	Agoncillo	39,787	157	143	300	754	1	0	1	2.51
401002000	Alitagtag	26,821	94	57	151	563	4	0	4	14.91
401003000	Balayan	96,637	807	400	1,207	1,249	12	1	13	13.45
401004000	Balete	24,817	52	47	99	399	1	2	3	12.09
**401005000**	**Batangas City**	**356,240**	**1,589**	**823**	**2,412**	**677**	**9**	**1**	**10**	**2.81**
401006000	Bauan	91,488	625	494	1,119	1,223	7	4	11	12.02
**401007000**	**City of Calaca**	**89,378**	**334**	**271**	**605**	**677**	**5**	**5**	**10**	**11.19**
401008000	Calatagan	59,978	196	99	295	492	2	0	2	3.33
401009000	Cuenca	36,423	151	87	238	653	4	0	4	10.98
401010000	Ibaan	59,159	290	167	457	772	6	1	7	11.83
401011000	Laurel	44,570	135	62	197	442	6	1	7	15.71
401012000	Lemery	94,417	609	385	994	1,053	2	3	5	5.30
401013000	Lian	57,640	210	103	313	543	6	0	6	10.41
**401014000**	**City of Lipa**	**388,640**	**1,625**	**949**	**2,574**	**662**	**17**	**6**	**23**	**5.92**
401015000	Lobo	40,737	165	101	266	653	2	0	2	4.91
401016000	Mabini	50,858	129	83	212	417	4	0	4	7.87
401017000	Malvar	66,879	344	188	532	795	5	0	5	7.48
401018000	Mataasnakahoy	30,868	104	67	171	554	1	1	2	6.48
401019000	Nasugbu	141,110	668	381	1,049	743	6	3	9	6.38
401020000	Padre Garcia	54,097	163	99	262	484	5	2	7	12.94
401021000	Rosario	131,823	399	274	673	511	8	1	9	6.83
401022000	San Jose	81,945	454	344	798	974	3	3	6	7.32
401023000	San Juan	117,631	481	266	747	635	4	0	4	3.40
401024000	San Luis	36,174	119	89	208	575	2	2	4	11.06
401025000	San Nicolas	23,909	93	54	147	615	1	0	1	4.18
401026000	San Pascual	69,544	285	184	469	674	1	3	4	5.75

Table 2.E.7.1 Tuberculosis Prevention and Control
Case Notification Rate (All forms and DTRB)
Philippines, 2024

PSGC	Area	Total Population	Notified TB, All Forms (New and Relapse)			Case Notification Rate (All Forms)	Registered Bacteriologically Confirmed Drug Resistant			Case Notification Rate (DRTB)
			Male	Female	Total		Male	Female	Total	
401027000	Santa Teresita	21,559	128	113	241	1,118	2	0	2	9.28
**401028000**	**City of Sto. Tomas**	**227,734**	**712**	**447**	**1,159**	**509**	**6**	**5**	**11**	**4.83**
401029000	Taal	62,067	284	207	491	791	9	2	11	17.72
401030000	Talisay	47,013	119	70	189	402	3	1	4	8.51
**401031000**	**City of Tanauan**	**200,385**	**757**	**568**	**1,325**	**661**	**14**	**4**	**18**	**8.98**
401032000	Taysan	40,630	158	115	273	672	0	0	0	0.00
401033000	Tingloy	19,215	41	26	67	349	1	1	2	10.41
401034000	Tuy	46,558	179	90	269	578	1	0	1	2.15
**402100000**	**Cavite**	**4,482,531**	**12,711**	**7,803**	**20,514**	**458**	**247**	**123**	**370**	**8.25**
402101000	Alfonso	59,558	127	87	214	359	1	1	2	3.36
402102000	Amadeo	42,552	81	46	127	298	0	0	0	0.00
**402103000**	**City of Bacoor**	**687,235**	**1,556**	**929**	**2,485**	**362**	**21**	**14**	**35**	**5.09**
402104000	Carmona	110,465	314	181	495	448	2	3	5	4.53
**402105000**	**City of Cavite**	**100,674**	**388**	**205**	**593**	**589**	**6**	**0**	**6**	**5.96**
**402106000**	**City of Dasmariñas**	**722,708**	**2,075**	**1,296**	**3,371**	**466**	**47**	**31**	**78**	**10.79**
402107000	General Emilio Aguinaldo	24,293	32	20	52	214	0	0	0	0.00
**402108000**	**City of General Trias**	**467,318**	**1,484**	**917**	**2,401**	**514**	**22**	**18**	**40**	**8.56**
**402109000**	**City of Imus**	**507,781**	**1,203**	**740**	**1,943**	**383**	**28**	**10**	**38**	**7.48**
402110000	Indang	68,943	184	77	261	379	4	1	5	7.25
402111000	Kawit	109,913	368	263	631	574	11	3	14	12.74
402112000	Magallanes	24,607	33	12	45	183	0	0	0	0.00
402113000	Maragondon	41,393	122	68	190	459	2	1	3	7.25
402114000	Mendez	35,037	83	59	142	405	2	0	2	5.71
402115000	Naic	168,387	482	286	768	456	13	3	16	9.50
402116000	Noveleta	50,503	189	133	322	638	2	2	4	7.92
402117000	Rosario	115,218	525	309	834	724	10	2	12	10.42
402118000	Silang	306,522	842	505	1,347	439	19	11	30	9.79
**402119000**	**City of Tagaytay**	**87,208**	**332**	**213**	**545**	**625**	**4**	**4**	**8**	**9.17**
402120000	Tanza	326,720	772	509	1,281	392	15	8	23	7.04
402121000	Ternate	25,478	60	29	89	349	3	0	3	11.77
**402122000**	**City of Trece Martires**	**221,291**	**909**	**620**	**1,529**	**691**	**17**	**5**	**22**	**9.94**
402123000	Gen. Mariano Alvarez	178,727	550	299	849	475	18	6	24	13.43
**403400000**	**Laguna**	**3,480,921**	**11,630**	**7,178**	**18,808**	**540**	**220**	**99**	**319**	**9.16**
403401000	Alaminos	53,328	228	145	373	699	4	1	5	9.38
403402000	Bay	69,508	318	287	605	870	4	0	4	5.75
**403403000**	**City of Biñan**	**423,072**	**1,434**	**947**	**2,381**	**563**	**25**	**16**	**41**	**9.69**
**403404000**	**City of Cabuyao**	**368,772**	**966**	**603**	**1,569**	**425**	**24**	**10**	**34**	**9.22**
**403405000**	**City of Calamba**	**561,764**	**1,805**	**1,042**	**2,847**	**507**	**44**	**17**	**61**	**10.86**
403406000	Calauan	89,693	232	140	372	415	0	1	1	1.11
403407000	Cavinti	24,218	32	17	49	202	1	0	1	4.13
403408000	Famy	17,237	40	35	75	435	0	1	1	5.80
403409000	Kalayaan	25,469	84	56	140	550	3	0	3	11.78
403410000	Liliw	39,505	99	78	177	448	4	2	6	15.19
403411000	Los Baños	117,775	417	238	655	556	5	0	5	4.25
403412000	Luisiana	20,944	101	50	151	721	0	0	0	0.00
403413000	Lumban	32,645	95	65	160	490	4	1	5	15.32
403414000	Mabitac	21,658	71	39	110	508	0	0	0	0.00
403415000	Magdalena	28,084	74	40	114	406	2	0	2	7.12
403416000	Majayjay	28,076	110	51	161	573	0	0	0	0.00
403417000	Nagcarlan	64,928	384	249	633	975	0	1	1	1.54
403418000	Paete	25,191	69	37	106	421	1	2	3	11.91
403419000	Pagsanjan	45,019	104	65	169	375	3	0	3	6.66
403420000	Pakil	23,859	151	92	243	1,018	3	2	5	20.96
403421000	Pangil	25,642	73	62	135	526	6	0	6	23.40
403422000	Pila	55,585	194	135	329	592	3	4	7	12.59
403423000	Rizal	18,366	48	24	72	392	1	0	1	5.44
**403424000**	**City of San Pablo**	**291,359**	**1,208**	**727**	**1,935**	**664**	**22**	**10**	**32**	**10.98**
**403425000**	**City of San Pedro**	**334,748**	**1,263**	**761**	**2,024**	**605**	**22**	**14**	**36**	**10.75**
403426000	Santa Cruz	126,465	557	330	887	701	18	13	31	24.51
403427000	Santa Maria	34,827	87	43	130	373	0	0	0	0.00
**403428000**	**City of Santa Rosa**	**428,568**	**1,164**	**658**	**1,822**	**425**	**19**	**2**	**21**	**4.90**
403429000	Siniloan	40,293	167	125	292	725	1	1	2	4.96
403430000	Victoria	44,323	55	37	92	208	1	1	2	4.51
**405600000**	**Quezon**	**1,996,436**	**6,067**	**3,773**	**9,840**	**493**	**133**	**63**	**196**	**9.82**
405601000	Agdangan	12,817	33	25	58	453	5	2	7	54.61
405602000	Alabat	15,942	82	47	129	809	0	1	1	6.27
405603000	Atimonan	64,848	279	183	462	712	0	1	1	1.54
405605000	Buenavista	31,648	116	49	165	521	1	0	1	3.16
405606000	Burdeos	25,033	64	45	109	435	1	0	1	3.99
405607000	Calauag	72,186	302	144	446	618	4	2	6	8.31
405608000	Candelaria	143,154	761	561	1,322	923	4	5	9	6.29
405610000	Catanauan	75,219	199	91	290	386	2	1	3	3.99
405615000	Dolores	33,111	66	31	97	293	1	0	1	3.02
405616000	General Luna	25,269	54	38	92	364	0	0	0	0.00
405617000	General Nakar	35,508	63	29	92	259	0	0	0	0.00
405618000	Guinayangan	44,438	115	64	179	403	0	0	0	0.00
405619000	Gumaca	72,672	349	225	574	790	3	2	5	6.88
405620000	Infanta	78,522	226	215	441	562	3	0	3	3.82
405621000	Jomalig	7,916	11	5	16	202	0	1	1	12.63
405622000	Lopez	95,779	463	250	713	744	8	2	10	10.44
405623000	Lucban	53,838	155	140	295	548	2	3	5	9.29
405625000	Macalelon	27,582	41	26	67	243	17	10	27	97.89
405627000	Mauban	73,131	224	180	404	552	6	1	7	9.57
405628000	Mulanay	57,330	124	72	196	342	2	2	4	6.98
405629000	Padre Burgos	23,511	50	16	66	281	1	1	2	8.51
405630000	Pagbilao	80,057	212	101	313	391	2	2	4	5.00
405631000	Panukulan	16,714	25	17	42	251	1	0	1	5.98
405632000	Patnanungan	15,611	10	17	27	173	0	4	4	25.62
405633000	Perez	12,834	32	20	52	405	1	0	1	7.79
405634000	Pitogo	22,798	56	21	77	338	27	6	33	144.75
405635000	Plaridel	10,139	19	10	29	286	0	1	1	9.86
405636000	Polillo	32,514	121	46	167	514	1	1	2	6.15
405637000	Quezon	15,886	37	20	57	359	2	0	2	12.59
405638000	Real	39,999	77	32	109	273	1	0	1	2.50
405639000	Sampaloc	13,631	37	12	49	359	4	1	5	36.68
405640000	San Andres	38,023	66	22	88	231	0	1	1	2.63
405641000	San Antonio	36,571	76	27	103	282	3	1	4	10.94
405642000	San Francisco	63,995	154	82	236	369	0	2	2	3.13
405644000	San Narciso	52,865	153	95	248	469	2	1	3	5.67
405645000	Sariaya	169,358	428	313	741	438	14	4	18	10.63
405646000	Tagkawayan	54,733	222	160	382	698	5	0	5	9.14
**405647000**	**City of Tayabas**	**116,109**	**266**	**160**	**426**	**367**	**2**	**2**	**4**	**3.45**
405648000	Tiaong	109,686	239	168	407	371	7	3	10	9.12
405649000	Unisan	25,459	60	14	74	291	1	0	1	3.93
**405800000**	**Rizal**	**3,450,627**	**11,245**	**7,027**	**18,272**	**530**	**300**	**169**	**469**	**13.59**
405801000	Angono	134,881	402	281	683	506	11	6	17	12.60
**405802000**	**City of Antipolo**	**928,349**	**3,289**	**2,099**	**5,388**	**580**	**65**	**40**	**105**	**11.31**
405803000	Baras	91,010	212	126	338	371	3	6	9	9.89
405804000	Binangonan	321,774	840	515	1,355	421	34	16	50	15.54
405805000	Cainta	388,368	1,038	504	1,542	397	28	11	39	10.04
405806000	Cardona	50,868	151	82	233	458	5	2	7	13.76
405807000	Jala-Jala	34,512	115	81	196	568	5	0	5	14.49
405808000	Rodriguez	459,145	1,775	1,259	3,034	661	68	33	101	22.00
405809000	Morong	73,083	603	348	951	1,301	10	3	13	17.79
405810000	Pililla	74,256	198	104	302	407	5	2	7	9.43
405811000	San Mateo	281,208	852	553	1,405	500	24	19	43	15.29
405812000	Tanay	144,810	511	296	807	557	7	7	14	9.67
405813000	Taytay	401,564	1,018	628	1,646	410	32	23	55	13.70
405814000	Teresa	66,799	241	151	392	587	3	1	4	5.99
**1700000000**	**Region 4B**	**3,311,838**	**10,032**	**5,680**	**15,712**	**474**	**196**	**85**	**281**	**8.48**
**1731500000**	**City of Puerto Princesa**	**317,047**	**1,246**	**692**	**1,938**	**611**	**20**	**14**	**34**	**10.72**
**1704000000**	**Marinduque**	**240,500**	**713**	**472**	**1,185**	**493**	**18**	**4**	**22**	**9.15**
1704001000	Boac	57,574	154	119	273	474	6	0	6	10.42
1704002000	Buenavista	26,800	92	68	160	597	1	0	1	3.73
1704003000	Gasan	36,241	119	85	204	563	2	2	4	11.04

Table 2.E.7.1 Tuberculosis Prevention and Control
Case Notification Rate (All forms and DTRB)
Philippines, 2024

PSGC	Area	Total Population	Notified TB, All Forms (New and Relapse)			Case Notification Rate (All Forms)	Registered Bacteriologically Confirmed Drug Resistant			Case Notification Rate (DRTB)
			Male	Female	Total		Male	Female	Total	
1704004000	Mogpog	34,538	100	53	153	443	5	1	6	17.37
1704005000	Santa Cruz	54,692	146	92	238	435	3	1	4	7.31
1704006000	Torrijos	30,655	102	55	157	512	1	0	1	3.26
**1705100000**	**Occidental Mindoro**	**543,612**	**1,617**	**882**	**2,499**	**460**	**44**	**18**	**62**	**11.41**
1705101000	Abra De Ilog	36,683	138	98	236	643	5	2	7	19.08
1705102000	Calintaan	30,977	68	47	115	371	0	0	0	0.00
1705103000	Looc	7,802	7	7	14	179	0	0	0	0.00
1705104000	Lubang	17,437	42	6	48	275	0	0	0	0.00
1705105000	Magsaysay	41,334	75	72	147	356	0	0	0	0.00
1705106000	Mamburao	49,930	193	100	293	587	8	2	10	20.03
1705107000	Paluan	19,092	94	68	162	849	3	3	6	31.43
1705108000	Rizal	41,831	87	41	128	306	1	0	1	2.39
1705109000	Sablayan	97,284	361	139	500	514	16	4	20	20.56
1705110000	San Jose	157,260	447	243	690	439	9	7	16	10.17
1705111000	Santa Cruz	43,982	105	61	166	377	2	0	2	4.55
**1705200000**	**Oriental Mindoro**	**926,271**	**3,185**	**1,727**	**4,912**	**530**	**48**	**29**	**77**	**8.31**
1705201000	Baco	40,647	183	105	288	709	1	1	2	4.92
1705202000	Bansud	43,855	139	68	207	472	1	2	3	6.84
1705203000	Bongabong	79,318	262	146	408	514	11	2	13	16.39
1705204000	Bulalacao	45,712	142	99	241	527	2	2	4	8.75
**1705205000**	**City of Calapan**	**148,561**	**744**	**381**	**1,125**	**757**	**7**	**7**	**14**	**9.42**
1705206000	Gloria	51,173	139	102	241	471	3	4	7	13.68
1705207000	Mansalay	61,456	159	81	240	391	3	1	4	6.51
1705208000	Naujan	110,027	253	122	375	341	5	4	9	8.18
1705209000	Pinamalayan	90,953	344	172	516	567	9	3	12	13.19
1705210000	Pola	35,480	87	41	128	361	0	0	0	0.00
1705211000	Puerto Galera	43,157	117	55	172	399	1	0	1	2.32
1705212000	Roxas	61,100	238	129	367	601	2	1	3	4.91
1705213000	San Teodoro	19,458	53	37	90	463	2	1	3	15.42
1705214000	Socorro	42,092	144	82	226	537	0	1	1	2.38
1705215000	Victoria	53,282	181	107	288	541	1	0	1	1.88
**1705300000**	**Palawan**	**971,373**	**2,196**	**1,181**	**3,377**	**348**	**42**	**15**	**57**	**5.87**
1705301000	Aborlan	40,321	67	33	100	248	0	1	1	2.48
1705302000	Agutaya	12,946	28	10	38	294	0	0	0	0.00
1705303000	Araceli	14,583	60	40	100	686	1	0	1	6.86
1705304000	Balabac	44,784	28	26	54	121	0	2	2	4.47
1705305000	Bataraza	89,110	216	97	313	351	1	0	1	1.12
1705306000	Brooke'S Point	77,457	429	202	631	815	6	3	9	11.62
1705307000	Busuanga	26,395	43	37	80	303	2	1	3	11.37
1705308000	Cagayancillo	6,884	17	13	30	436	0	0	0	0.00
1705309000	Coron	66,745	154	95	249	373	6	3	9	13.48
1705310000	Cuyo	23,675	42	29	71	300	0	0	0	0.00
1705311000	Dumaran	24,169	42	29	71	294	0	0	0	0.00
1705312000	El Nido	52,461	106	67	173	330	4	2	6	11.44
1705313000	Linapacan	16,851	16	10	26	154	1	0	1	5.93
1705314000	Magsaysay	12,609	26	12	38	301	0	0	0	0.00
1705315000	Narra	81,293	154	72	226	278	6	1	7	8.61
1705317000	Quezon	67,203	105	35	140	208	2	0	2	2.98
1705318000	Roxas	71,769	143	58	201	280	2	0	2	2.79
1705319000	San Vicente	34,707	69	34	103	297	0	0	0	0.00
1705320000	Taytay	86,215	155	60	215	249	5	2	7	8.12
1705321000	Kalayaan	193	*	*	*	0	0	0	0	0.00
1705322000	Culion	23,376	64	32	96	411	2	0	2	8.56
1705323000	Rizal	58,536	172	135	307	524	2	0	2	3.42
1705324000	Sofronio Española	39,091	60	55	115	294	2	0	2	5.12
**1705900000**	**Romblon**	**313,035**	**1,075**	**726**	**1,801**	**575**	**24**	**5**	**29**	**9.26**
1705901000	Alcantara	17,171	57	38	95	553	0	0	0	0.00
1705902000	Banton	5,737	25	45	70	1,220	0	2	2	34.86
1705903000	Cajidiocan	23,779	49	21	70	294	1	0	1	4.21
1705904000	Calatrava	11,600	21	19	40	345	0	0	0	0.00
1705905000	Concepcion	3,561	22	19	41	1,151	0	0	0	0.00
1705906000	Corcuera	10,112	32	31	63	623	0	0	0	0.00
1705907000	Looc	21,863	89	47	136	622	1	0	1	4.57
1705908000	Magdiwang	15,772	84	60	144	913	4	0	4	25.36
1705909000	Odiongan	49,662	166	76	242	487	6	0	6	12.08
1705910000	Romblon	40,919	182	129	311	760	5	1	6	14.66
1705911000	San Agustin	24,607	64	41	105	427	0	1	1	4.06
1705912000	San Andres	16,310	41	24	65	399	1	0	1	6.13
1705913000	San Fernando	24,908	118	76	194	779	1	0	1	4.01
1705914000	San Jose	12,189	41	19	60	492	1	0	1	8.20
1705915000	Santa Fe	17,829	40	31	71	398	2	0	2	11.22
1705916000	Ferrol	8,006	23	34	57	712	2	0	2	24.98
1705917000	Santa Maria	9,010	21	16	37	411	0	1	1	11.10
**500000000**	**Region 5**	**6,285,893**	**20,064**	**10,915**	**30,979**	**493**	**522**	**231**	**753**	**11.98**
500500000	**Albay**	1,421,135	5,243	2,955	8,198	577	**120**	**51**	171	12.03
500501000	Bacacay	74,698	182	110	292	391	4	1	5	6.69
500502000	Camalig	73,670	193	119	312	424	3	2	5	6.79
500503000	Daraga	138,544	777	444	1,221	881	24	11	35	25.26
500504000	Guinobatan	88,095	127	59	186	211	2	2	4	4.54
500505000	Jovellar	18,032	28	27	55	305	0	0	0	0.00
**500506000**	**City of Legazpi**	**217,901**	**1,400**	**820**	**2,220**	**1,019**	**19**	**13**	**32**	**14.69**
500507000	Libon	77,553	154	80	234	302	2	0	2	2.58
**500508000**	**City of Ligao**	**122,274**	**421**	**255**	**676**	**553**	**11**	**3**	**14**	**11.45**
500509000	Malilipot	42,448	147	80	227	535	7	3	10	23.56
500510000	Malinao	49,030	138	69	207	422	2	0	2	4.08
500511000	Manito	27,213	98	56	154	566	1	1	2	7.35
500512000	Oas	67,321	115	56	171	254	3	0	3	4.46
500513000	Pio Duran	50,907	84	51	135	265	1	2	3	5.89
500514000	Polangui	92,597	169	95	264	285	7	1	8	8.64
500515000	Rapu-Rapu	36,948	53	28	81	219	2	1	3	8.12
500516000	Santo Domingo	38,368	143	71	214	558	4	2	6	15.64
**500517000**	**City of Tabaco**	**146,228**	**817**	**427**	**1,244**	**851**	**19**	**7**	**26**	**17.78**
500518000	Tiwi	59,308	197	108	305	514	9	2	11	18.55
501600000	Camarines Norte	660,386	2,057	857	2,914	441	**78**	**26**	104	15.75
501601000	Basud	47,105	84	39	123	261	3	1	4	8.49
501602000	Capalonga	37,655	69	28	97	258	2	0	2	5.31
501603000	Daet	116,864	515	277	792	678	20	6	26	22.25
501604000	San Lorenzo Ruiz	16,441	30	5	35	213	1	1	2	12.16
501605000	Jose Panganiban	67,139	279	78	357	532	11	7	18	26.81
501606000	Labo	115,060	239	86	325	282	12	2	14	12.17
501607000	Mercedes	58,010	195	112	307	529	11	5	16	27.58
501608000	Paracale	63,975	305	84	389	608	8	2	10	15.63
501609000	San Vicente	13,063	29	11	40	306	1	0	1	7.66
501610000	Santa Elena	45,671	90	30	120	263	2	0	2	4.38
501611000	Talisay	28,034	66	30	96	342	4	0	4	14.27
501612000	Vinzons	51,369	156	77	233	454	3	2	5	9.73
501700000	Camarines Sur	2,141,193	6,721	3,939	10,660	498	**144**	**81**	225	10.51
501701000	Baao	64,044	153	98	251	392	2	2	4	6.25
501702000	Balatan	32,529	103	29	132	406	0	0	0	0.00
501703000	Bato	54,084	82	42	124	229	2	0	2	3.70
501704000	Bombon	18,735	49	21	70	374	0	0	0	0.00
501705000	Buhi	84,302	206	92	298	353	3	1	4	4.74
501706000	Bula	76,008	95	63	158	208	1	0	1	1.32
501707000	Cabusao	19,736	66	36	102	517	0	0	0	0.00
501708000	Calabanga	92,221	253	138	391	424	2	2	4	4.34
501709000	Camaligan	25,812	68	58	126	488	2	0	2	7.75
501710000	Canaman	37,128	78	36	114	307	2	2	4	10.77
501711000	Caramoan	52,674	125	75	200	380	2	2	4	7.59
501712000	Del Gallego	27,022	79	31	110	407	3	0	3	11.10
501713000	Gainza	11,904	38	21	59	496	2	2	4	33.60
501714000	Garchitorena	30,876	87	58	145	470	1	1	2	6.48
501715000	Goa	75,192	262	154	416	553	5	2	7	9.31
**501716000**	**City of Iriga**	**117,400**	**728**	**422**	**1,150**	**980**	**3**	**3**	**6**	**5.11**
501717000	Lagonoy	58,552	165	90	255	436	3	1	4	6.83
501718000	Libmanan	115,932	271	167	438	378	5	3	8	6.90

Table 2.E.7.1 Tuberculosis Prevention and Control
Case Notification Rate (All forms and DTRB)
Philippines, 2024

PSGC	Area	Total Population	Notified TB, All Forms (New and Relapse)			Case Notification Rate (All Forms)	Registered Bacteriologically Confirmed Drug Resistant			Case Notification Rate (DRTB)
			Male	Female	Total		Male	Female	Total	
501719000	Lupi	34,258	84	42	126	368	2	1	3	8.76
501720000	Magarao	27,382	70	48	118	431	0	0	0	0.00
501721000	Milaor	35,269	82	42	124	352	1	1	2	5.67
501722000	Minalabac	35,269	155	83	238	424	2	0	2	3.56
501723000	Nabua	87,448	279	117	396	453	7	3	10	11.44
**501724000**	**City of Naga**	**218,155**	**1,512**	**1,058**	**2,570**	**1,178**	**55**	**37**	**92**	**42.17**
501725000	Ocampo	53,093	103	50	153	288	1	1	2	3.77
501726000	Pamplona	40,842	105	61	166	406	2	1	3	7.35
501727000	Pasacao	55,892	80	41	121	216	4	3	7	12.52
501728000	Pili	104,330	235	120	355	340	2	3	5	4.79
501729000	Presentacion	23,076	77	53	130	563	0	0	0	0.00
501730000	Ragay	61,562	176	83	259	421	2	1	3	4.87
501731000	Sagñay	38,067	104	72	176	462	3	0	3	7.88
501732000	San Fernando	39,976	98	51	149	373	2	0	2	5.00
501733000	San Jose	45,461	147	86	233	513	3	3	6	13.20
501734000	Sipocot	69,699	119	58	177	254	3	1	4	5.74
501735000	Siruma	20,054	43	23	66	329	0	0	0	0.00
501736000	Tigaon	63,169	144	88	232	367	6	2	8	12.66
501737000	Tinambac	73,202	200	132	332	454	11	3	14	19.13
**502000000**	**Catanduanes**	**277,230**	**891**	**479**	**1,370**	**494**	**23**	**7**	**30**	**10.82**
502001000	Bagamanoc	11,087	26	11	37	334	1	0	1	9.02
502002000	Baras	13,492	34	14	48	356	0	0	0	0.00
502003000	Bato	22,006	32	15	47	214	3	1	4	18.18
502004000	Caramoran	33,162	47	26	73	220	2	0	2	6.03
502005000	Gigmoto	9,046	13	3	16	177	0	0	0	0.00
502006000	Pandan	21,873	49	30	79	361	1	0	1	4.57
502007000	Panganiban	9,756	15	8	23	236	0	0	0	0.00
502008000	San Andres	39,274	91	27	118	300	0	1	1	2.55
502009000	San Miguel	15,869	31	17	48	302	2	1	3	18.90
502010000	Viga	23,121	45	20	65	281	1	0	1	4.33
502011000	Virac	78,544	508	308	816	1,039	13	4	17	21.64
**504100000**	**Masbate**	**932,021**	**2,717**	**1,381**	**4,098**	**440**	**43**	**22**	**65**	**6.97**
504101000	Aroroy	90,859	348	146	494	544	6	4	10	11.01
504102000	Baleno	29,451	89	34	123	418	2	1	3	10.19
504103000	Balud	40,731	77	50	127	312	0	2	2	4.91
504104000	Batuan	14,636	24	19	43	294	2	2	4	27.33
504105000	Cataingan	51,219	106	35	141	275	4	1	5	9.76
504106000	Cawayan	71,508	146	81	227	317	2	1	3	4.20
504107000	Claveria	42,736	65	36	101	236	1	1	2	4.68
504108000	Dimasalang	25,233	54	33	87	345	0	0	0	0.00
504109000	Esperanza	17,537	52	24	76	433	0	0	0	0.00
504110000	Mandaon	45,460	142	96	238	524	4	2	6	13.20
**504111000**	**City of Masbate**	**108,996**	**659**	**322**	**981**	**900**	**4**	**2**	**6**	**5.50**
504112000	Milagros	60,073	163	90	253	421	1	0	1	1.66
504113000	Mobo	42,264	58	32	90	213	1	2	3	7.10
504114000	Monreal	25,409	80	39	119	468	2	0	2	7.87
504115000	Palanas	27,936	66	29	95	340	3	0	3	10.74
504116000	Pio V. Corpuz	24,066	46	26	72	299	0	0	0	0.00
504117000	Placer	58,858	192	134	326	554	3	1	4	6.80
504118000	San Fernando	21,601	74	28	102	472	2	1	3	13.89
504119000	San Jacinto	30,073	76	33	109	362	3	0	3	9.98
504120000	San Pascual	45,043	59	26	85	189	1	1	2	4.44
504121000	Uson	58,332	141	68	209	358	2	1	3	5.14
**506200000**	**Sorsogon**	**853,928**	**2,435**	**1,304**	**3,739**	**438**	**114**	**44**	**158**	**18.50**
506202000	Barcelona	20,987	78	39	117	557	1	0	1	4.76
506203000	Bulan	109,010	245	111	356	327	3	3	6	5.50
506204000	Bulusan	23,932	76	24	100	418	1	0	1	4.18
506205000	Casiguran	37,224	98	41	139	373	6	1	7	18.81
506206000	Castilla	63,091	119	66	185	293	4	1	5	7.93
506207000	Donsol	51,687	84	40	124	240	4	4	8	15.48
506208000	Gubat	60,478	223	106	329	544	4	3	7	11.57
506209000	Irosin	62,285	190	87	277	445	4	1	5	8.03
506210000	Juban	36,323	93	50	143	394	60	26	86	236.76
506211000	Magallanes	38,638	93	43	136	352	3	1	4	10.35
506212000	Matnog	43,285	93	49	142	328	5	1	6	13.86
506213000	Pilar	78,655	142	80	222	282	4	0	4	5.09
506214000	Prieto Diaz	22,794	80	60	140	614	1	1	2	8.77
506215000	Santa Magdalena	17,120	35	15	50	292	1	0	1	5.84
**506216000**	**City of Sorsogon**	**188,419**	**786**	**493**	**1,279**	**679**	**13**	**2**	**15**	**7.96**
**600000000**	**Region 6**	**8,026,781**	**30,837**	**16,077**	**46,914**	**983**	**576**	**217**	**793**	**16.62**
**1830200000**	**City of Bacolod**	**598,149**	**3,751**	**2,258**	**6,009**	**1,005**	**80**	**32**	**112**	**18.72**
**631000000**	**City of Iloilo**	**460,427**	**2,948**	**1,730**	**4,678**	**1,016**	**46**	**16**	**62**	**13.47**
**600400000**	**Aklan**	**625,070**	**1,742**	**903**	**2,645**	**423**	**15**	**11**	**26**	**4.16**
600401000	Altavas	25,717	99	43	142	552	0	2	2	7.78
600402000	Balete	30,949	32	11	43	139	1	0	1	3.23
600403000	Banga	40,364	67	28	95	235	0	0	0	0.00
600404000	Batan	33,484	45	17	62	185	0	1	1	2.99
600405000	Buruanga	19,357	35	22	57	294	0	0	0	0.00
600406000	Ibajay	52,892	153	93	246	465	2	1	3	5.67
600407000	Kalibo	91,349	562	343	905	991	4	2	6	6.57
600408000	Lezo	15,640	57	28	85	543	0	0	0	0.00
600409000	Libacao	28,790	50	30	80	278	1	0	1	3.47
600410000	Madalag	18,949	24	2	26	137	1	0	1	5.28
600411000	Makato	30,068	51	22	73	243	0	1	1	3.33
600412000	Malay	61,791	229	87	316	511	2	2	4	6.47
600413000	Malinao	24,529	61	25	86	351	2	0	2	8.15
600414000	Nabas	41,560	34	26	60	144	0	1	1	2.41
600415000	New Washington	49,257	124	41	165	335	1	1	2	4.06
600416000	Numancia	36,211	97	70	167	461	0	0	0	0.00
600417000	Tangalan	24,163	22	15	37	153	1	0	1	4.14
**600600000**	**Antique**	**614,750**	**2,346**	**1,388**	**3,734**	**607**	**121**	**49**	**170**	**27.65**
600601000	Anini-Y	22,018	49	19	68	309	1	0	1	4.54
600602000	Barbaza	23,359	59	48	107	458	1	0	1	4.28
600603000	Belison	14,129	53	35	88	623	0	0	0	0.00
600604000	Bugasong	34,676	137	73	210	606	2	0	2	5.77
600605000	Caluya	40,211	78	40	118	293	1	2	3	7.46
600606000	Culasi	44,494	179	128	307	690	3	0	3	6.74
600607000	Tobias Fornier	33,816	80	47	127	376	0	0	0	0.00
600608000	Hamtic	52,685	192	100	292	554	2	0	2	3.80
600609000	Laua-An	26,584	134	79	213	801	1	0	1	3.76
600610000	Libertad	17,507	66	48	114	651	1	0	1	5.71
600611000	Pandan	35,965	125	57	182	506	0	2	2	5.56
600612000	Patnongon	38,330	124	77	201	524	4	0	4	10.44
600613000	San Jose	65,417	528	339	867	1,325	95	43	138	210.95
600614000	San Remigio	34,094	106	65	171	502	4	0	4	11.73
600615000	Sebaste	18,817	72	40	112	595	3	0	3	15.94
600616000	Sibalom	63,972	211	115	326	510	3	2	5	7.82
600617000	Tibiao	28,702	91	39	130	453	0	0	0	0.00
600618000	Valderrama	19,974	62	39	101	506	0	0	0	0.00
**601900000**	**Capiz**	**813,912**	**2,646**	**1,335**	**3,981**	**489**	**49**	**18**	**67**	**8.23**
601901000	Cuartero	27,993	54	26	80	286	0	0	0	0.00
601902000	Dao	33,846	82	30	112	331	0	0	0	0.00
601903000	Dumalag	30,099	67	30	97	322	0	0	0	0.00
601904000	Dumarao	49,809	146	72	218	438	1	0	1	2.01
601905000	Ivisan	32,405	42	18	60	185	1	1	2	6.17
601906000	Jamindan	38,931	76	39	115	295	3	0	3	7.71
601907000	Ma-Ayon	41,605	89	38	127	305	3	0	3	7.21
601908000	Mambusao	40,759	65	23	88	216	3	0	3	7.36
601909000	Panay	48,924	95	50	145	296	4	0	4	8.18
601910000	Panitan	42,694	92	33	125	293	4	3	7	16.40
601911000	Pilar	47,926	126	68	194	405	4	2	6	12.52
601912000	Pontevedra	49,950	86	30	116	232	0	0	0	0.00
601913000	President Roxas	31,650	77	45	122	385	2	3	5	15.80
**601914000**	**City of Roxas**	**183,670**	**1,297**	**728**	**2,025**	**1,103**	**19**	**8**	**27**	**14.70**
601915000	Sapi-An	27,281	47	17	64	235	4	1	5	18.33

Table 2.E.7.1 Tuberculosis Prevention and Control
Case Notification Rate (All forms and DTRB)
Philippines, 2024

PSGC	Area	Total Population	Notified TB, All Forms (New and Relapse)			Case Notification Rate (All Forms)	Registered Bacteriologically Confirmed Drug Resistant			Case Notification Rate (DRTB)
			Male	Female	Total		Male	Female	Total	
601916000	Sigma	31,901	105	50	155	486	0	0	0	0.00
601917000	Tapaz	54,469	100	38	138	253	1	0	1	1.84
**603000000**	**Iloilo**	**2,065,439**	**6,332**	**3,156**	**9,488**	**459**	**81**	**33**	**114**	**5.52**
603001000	Ajuy	54,470	106	54	160	294	4	1	5	9.18
603002000	Alimodian	39,852	115	70	185	464	0	0	0	0.00
603003000	Anilao	30,661	72	29	101	329	1	1	2	6.52
603004000	Badiangan	27,056	63	20	83	307	2	0	2	7.39
603005000	Balasan	35,087	143	58	201	573	0	0	0	0.00
603006000	Banate	33,493	134	79	213	636	5	1	6	17.91
603007000	Barotac Nuevo	58,266	257	153	410	704	3	4	7	12.01
603008000	Barotac Viejo	49,601	201	93	294	593	0	0	0	0.00
603009000	Batad	22,228	52	31	83	373	0	0	0	0.00
603010000	Bingawan	16,385	47	16	63	385	0	0	0	0.00
603012000	Cabatuan	61,257	178	99	277	452	3	0	3	4.90
603013000	Calinog	63,594	140	56	196	308	1	0	1	1.57
603014000	Carles	72,985	183	96	279	382	1	1	2	2.74
603015000	Concepcion	46,060	111	48	159	345	1	0	1	2.17
603016000	Dingle	45,975	150	69	219	476	1	0	1	2.18
603017000	Dueñas	34,598	128	58	186	538	1	0	1	2.89
603018000	Dumangas	73,926	263	130	393	532	8	2	10	13.53
603019000	Estancia	53,872	139	57	196	364	2	0	2	3.71
603020000	Guimbal	35,022	100	51	151	431	1	0	1	2.86
603021000	Igbaras	32,197	94	48	142	441	0	0	0	0.00
603023000	Janiuay	66,895	231	82	313	468	4	1	5	7.47
603025000	Lambunao	81,799	193	88	281	344	6	0	6	7.34
603026000	Leganes	35,185	118	50	168	477	2	0	2	5.68
603027000	Lemery	31,572	126	78	204	646	1	0	1	3.17
603028000	Leon	52,184	138	87	225	431	0	2	2	3.83
603029000	Maasin	38,484	86	57	143	372	0	3	3	7.80
603030000	Miagao	68,115	146	84	230	338	2	3	5	7.34
603031000	Mina	24,042	100	60	160	666	1	0	1	4.16
603032000	New Lucena	24,322	74	21	95	391	0	1	1	4.11
603034000	Oton	99,308	233	114	347	349	2	0	2	2.01
**603035000**	**City of Passi**	**90,193**	**360**	**218**	**578**	**641**	**5**	**1**	**6**	**6.65**
603036000	Pavia	71,701	210	124	334	466	2	1	3	4.18
603037000	Pototan	79,014	493	158	651	824	6	1	7	8.86
603038000	San Dionisio	39,327	104	57	161	409	2	2	4	10.17
603039000	San Enrique	36,956	82	45	127	344	3	1	4	10.82
603040000	San Joaquin	52,617	191	140	331	629	3	1	4	7.60
603041000	San Miguel	30,314	67	27	94	310	0	0	0	0.00
603042000	San Rafael	18,086	62	21	83	459	1	1	2	11.06
603043000	Santa Barbara	67,885	173	91	264	389	1	1	2	2.95
603044000	Sara	55,161	156	87	243	441	2	0	2	3.63
603045000	Tigbauan	65,331	154	74	228	349	1	1	2	3.06
603046000	Tubungan	23,021	43	26	69	300	1	0	1	4.34
603047000	Zarraga	27,342	116	52	168	614	2	3	5	18.29
**1804500000**	**Negros Occidental**	**2,657,997**	**10,547**	**4,991**	**15,538**	**585**	**178**	**52**	**230**	**8.65**
**1804502000**	**City of Bago**	**191,210**	**1,074**	**468**	**1,542**	**806**	**17**	**1**	**18**	**9.41**
1804503000	Binalbagan	73,026	234	133	367	503	6	1	7	9.59
**1804504000**	**City of Cadiz**	**160,081**	**640**	**296**	**936**	**585**	**13**	**4**	**17**	**10.62**
1804505000	Calatrava	84,302	247	93	340	403	2	1	3	3.56
1804506000	Candoni	23,978	63	43	106	442	0	0	0	0.00
1804507000	Cauayan	110,893	290	110	400	361	1	3	4	3.61
1804508000	Enrique B. Magalona	64,290	157	90	247	384	2	1	3	4.67
**1804509000**	**City of Escalante**	**98,260**	**241**	**129**	**370**	**377**	**3**	**1**	**4**	**4.07**
**1804510000**	**City of Himamaylan**	**116,955**	**409**	**185**	**594**	**508**	**13**	**4**	**17**	**14.54**
1804511000	Hinigaran	88,909	567	276	843	948	5	1	6	6.75
1804512000	Hinoba-an	62,405	166	89	255	409	3	1	4	6.41
1804513000	Ilog	60,504	187	89	276	456	1	0	1	1.65
1804514000	Isabela	64,571	317	185	502	777	6	0	6	9.29
**1804515000**	**City of Kabankalan**	**205,627**	**635**	**287**	**922**	**448**	**4**	**2**	**6**	**2.92**
**1804516000**	**City of La Carlota**	**66,664**	**389**	**187**	**576**	**864**	**4**	**1**	**5**	**7.50**
1804517000	La Castellana	81,156	337	176	513	632	5	2	7	8.63
1804518000	Manapla	55,083	176	57	233	423	5	0	5	9.08
1804519000	Moises Padilla	43,805	198	91	289	660	2	0	2	4.57
1804520000	Murcia	91,449	435	214	649	710	1	2	3	3.28
1804521000	Pontevedra	54,502	243	137	380	697	5	3	8	14.68
1804522000	Pulupandan	30,117	164	72	236	784	2	2	4	13.28
**1804523000**	**City of Sagay**	**152,073**	**573**	**200**	**773**	**508**	**7**	**2**	**9**	**5.92**
**1804524000**	**City of San Carlos**	**135,958**	**396**	**212**	**608**	**447**	**5**	**2**	**7**	**5.15**
1804525000	San Enrique	24,177	152	84	236	976	3	1	4	16.54
**1804526000**	**City of Silay**	**131,377**	**843**	**403**	**1,246**	**948**	**13**	**5**	**18**	**13.70**
**1804527000**	**City of Sipalay**	**73,021**	**228**	**101**	**329**	**451**	**1**	**0**	**1**	**1.37**
**1804528000**	**City of Talisay**	**110,333**	**432**	**224**	**656**	**595**	**33**	**8**	**41**	**37.16**
1804529000	Toboso	44,835	93	46	139	310	1	0	1	2.23
1804530000	Valladolid	39,996	150	74	224	560	3	1	4	10.00
**1804531000**	**City of Victorias**	**90,101**	**454**	**210**	**664**	**737**	**12**	**3**	**15**	**16.65**
1804532000	Salvador Benedicto	28,339	57	30	87	307	0	0	0	0.00
**607900000**	**Guimaras**	**191,037**	**525**	**316**	**841**	**440**	**6**	**6**	**12**	**6.28**
607901000	Buenavista	53,005	121	65	186	351	4	2	6	11.32
607902000	Jordan	40,903	126	79	205	501	1	2	3	7.33
607903000	Nueva Valencia	43,220	145	72	217	502	1	2	3	6.94
607904000	San Lorenzo	30,327	74	58	132	435	0	0	0	0.00
607905000	Sibunag	23,582	59	42	101	428	0	0	0	0.00
**700000000**	**Region 7**	**8,352,783**	**23,563**	**11,995**	**35,558**	**525**	**319**	**118**	**437**	**6.45**
**730600000**	**City of Cebu**	**1,003,708**	**4,663**	**2,578**	**7,241**	**721**	**89**	**34**	**123**	**12.25**
**731100000**	**City of Lapu-Lapu**	**525,400**	**2,117**	**1,144**	**3,261**	**621**	**24**	**9**	**33**	**6.28**
**731300000**	**City of Mandaue**	**382,588**	**1,437**	**805**	**2,242**	**586**	**17**	**6**	**23**	**6.01**
**701200000**	**Bohol**	**1,415,451**	**2,542**	**1,274**	**3,816**	**270**	**25**	**14**	**39**	**2.76**
701201000	Alburquerque	11,246	13	7	20	178	0	0	0	0.00
701202000	Alicia	24,803	54	18	72	290	1	0	1	4.03
701203000	Anda	17,780	35	14	49	276	0	0	0	0.00
701204000	Antequera	14,990	14	2	16	107	0	0	0	0.00
701205000	Baclayon	22,462	35	23	58	258	0	0	0	0.00
701206000	Balilihan	18,694	18	8	26	139	1	0	1	5.35
701207000	Batuan	13,871	23	20	43	310	0	0	0	0.00
701208000	Bilar	18,539	20	9	29	156	1	0	1	5.39
701209000	Buenavista	30,178	39	25	64	212	2	0	2	6.63
701210000	Calape	33,094	43	24	67	202	0	0	0	0.00
701211000	Candijay	30,137	68	40	108	358	1	0	1	3.32
701212000	Carmen	50,853	59	24	83	163	0	0	0	0.00
701213000	Catigbian	23,945	35	21	56	234	0	0	0	0.00
701214000	Clarin	21,249	32	12	44	207	1	0	1	4.71
701215000	Corella	9,480	7	4	11	116	0	0	0	0.00
701216000	Cortes	18,418	21	6	27	147	1	1	2	10.86
701217000	Dagohoy	20,463	12	10	22	108	0	0	0	0.00
701218000	Danao	20,891	27	15	42	201	0	0	0	0.00
701219000	Dauis	53,938	66	34	100	185	2	0	2	3.71
701220000	Dimiao	14,889	21	9	30	201	0	0	0	0.00
701221000	Duero	18,861	34	13	47	249	0	1	1	5.30
701222000	Garcia Hernandez	24,430	39	13	52	213	0	1	1	4.09
701223000	Guindulman	34,105	41	31	72	211	0	0	0	0.00
701224000	Inabanga	48,720	92	35	127	261	1	0	1	2.05
701225000	Jagna	35,836	53	24	77	215	2	0	2	5.58
701226000	Getafe	34,749	59	21	80	230	1	1	2	5.76
701227000	Lila	12,240	12	12	24	196	0	0	0	0.00
701228000	Loay	17,855	22	15	37	207	0	0	0	0.00
701229000	Loboc	17,418	28	8	36	207	1	0	1	5.74
701230000	Loon	44,224	66	32	98	222	1	0	1	2.26
701231000	Mabini	28,734	41	24	65	226	0	1	1	3.48
701232000	Maribojoc	22,178	26	13	39	176	0	0	0	0.00
701233000	Panglao	41,282	70	34	104	252	0	0	0	0.00
701234000	Pilar	29,420	45	18	63	214	0	0	0	0.00
701235000	President Carlos P. Garcia	23,794	35	18	53	223	0	0	0	0.00
701236000	Sagbayan	24,603	29	9	38	154	1	0	1	4.06

Table 2.E.7.1 Tuberculosis Prevention and Control
Case Notification Rate (All forms and DTRB)
Philippines, 2024

PSGC	Area	Total Population	Notified TB, All Forms (New and Relapse)			Case Notification Rate (All Forms)	Registered Bacteriologically Confirmed Drug Resistant			Case Notification Rate (DRTB)
			Male	Female	Total		Male	Female	Total	
701237000	San Isidro	9,909	9	1	10	101	0	0	0	0.00
701238000	San Miguel	26,122	33	12	45	172	0	0	0	0.00
701239000	Sevilla	11,376	19	6	25	220	0	0	0	0.00
701240000	Sierra Bullones	26,368	46	23	69	262	0	0	0	0.00
701241000	Sikatuna	6,906	11	1	12	174	0	0	0	0.00
**701242000**	**City of Tagbilaran**	**107,452**	**480**	**295**	**775**	**721**	**1**	**3**	**4**	**3.72**
701243000	Talibon	73,854	156	82	238	322	2	1	3	4.06
701244000	Trinidad	36,051	65	24	89	247	0	1	1	2.77
701245000	Tubigon	47,932	65	32	97	202	2	1	3	6.26
701246000	Ubay	85,545	244	114	358	418	2	2	4	4.68
701247000	Valencia	28,398	35	17	52	183	0	0	0	0.00
701248000	Bien Unido	27,169	45	22	67	247	1	1	2	7.36
**702200000**	**Cebu**	**3,443,332**	**8,461**	**3,930**	**12,391**	**360**	**124**	**44**	**168**	**4.88**
702201000	Alcantara	17,530	34	10	44	251	0	0	0	0.00
702202000	Alcoy	19,809	25	14	39	197	1	0	1	5.05
702203000	Alegria	26,267	36	11	47	179	2	0	2	7.61
702204000	Aloguinsan	35,607	56	27	83	233	3	0	3	8.43
702205000	Argao	79,225	150	54	204	257	3	0	3	3.79
702206000	Asturias	56,863	87	33	120	211	3	0	3	5.28
702207000	Badian	44,517	102	38	140	314	0	0	0	0.00
702208000	Balamban	98,559	183	94	277	281	2	1	3	3.04
702209000	Bantayan	87,992	288	154	442	502	7	2	9	10.23
702210000	Barili	83,013	119	60	179	216	1	1	2	2.41
**702211000**	**City of Bogo**	**91,677**	**346**	**155**	**501**	**546**	**6**	**6**	**12**	**13.09**
702212000	Boljoon	17,534	40	12	52	297	0	0	0	0.00
702213000	Borbon	39,174	85	28	113	288	1	0	1	2.55
**702214000**	**City of Carcar**	**142,947**	**568**	**218**	**786**	**550**	**0**	**2**	**2**	**1.40**
702215000	Carmen	60,068	158	79	237	395	2	1	3	4.99
702216000	Catmon	34,266	33	9	42	123	1	0	1	2.92
702218000	Compostela	58,678	167	80	247	421	2	1	3	5.11
702219000	Consolacion	155,292	367	183	550	354	6	3	9	5.80
702220000	Cordova	73,740	177	92	269	365	1	1	2	2.71
702221000	Daanbantayan	96,132	269	105	374	389	6	3	9	9.36
702222000	Dalaguete	76,580	86	35	121	158	1	0	1	1.31
**702223000**	**Danao City**	**163,632**	**389**	**236**	**625**	**382**	**6**	**3**	**9**	**5.50**
702224000	Dumanjug	59,711	117	48	165	276	1	0	1	1.67
702225000	Ginatilan	16,906	18	6	24	142	1	0	1	5.92
702227000	Liloan	159,441	286	169	455	285	5	1	6	3.76
702228000	Madridejos	43,470	92	41	133	306	2	3	5	11.50
702229000	Malabuyoc	20,160	47	28	75	372	0	0	0	0.00
702231000	Medellin	61,703	131	58	189	306	0	1	1	1.62
702232000	Minglanilla	157,197	404	194	598	380	8	1	9	5.73
702233000	Moalboal	37,881	105	50	155	409	0	0	0	0.00
**702234000**	**City of Naga**	**139,911**	**278**	**111**	**389**	**278**	**1**	**2**	**3**	**2.14**
702235000	Oslob	29,393	68	48	116	395	1	0	1	3.40
702236000	Pilar	12,506	32	11	43	344	0	0	0	0.00
702237000	Pinamungajan	77,427	123	35	158	204	2	0	2	2.58
702238000	Poro	26,307	53	20	73	277	4	2	6	22.81
702239000	Ronda	21,032	56	17	73	347	2	0	2	9.51
702240000	Samboan	20,413	28	11	39	191	0	0	0	0.00
702241000	San Fernando	75,942	180	61	241	317	0	0	0	0.00
702242000	San Francisco	61,783	133	62	195	316	3	1	4	6.47
702243000	San Remigio	67,642	161	89	250	370	5	0	5	7.39
702244000	Santa Fe	35,850	113	62	175	488	3	1	4	11.16
702245000	Santander	18,715	38	18	56	299	1	0	1	5.34
702246000	Sibonga	55,416	126	57	183	330	5	1	6	10.83
702247000	Sogod	40,157	175	93	268	667	3	0	3	7.47
702248000	Tabogon	42,438	68	17	85	200	4	1	5	11.78
702249000	Tabuelan	29,832	47	20	67	225	0	0	0	0.00
**702250000**	**City of Talisay**	**274,161**	**939**	**461**	**1,400**	**511**	**13**	**5**	**18**	**6.57**
**702251000**	**City of Toledo**	**218,576**	**723**	**348**	**1,071**	**490**	**0**	**1**	**1**	**0.46**
702252000	Tuburan	68,956	138	65	203	294	5	0	5	7.25
702253000	Tudela	11,304	17	3	20	177	1	0	1	8.85
**1804600000**	**Negros Oriental**	**1,478,907**	**4,203**	**2,197**	**6,400**	**433**	**40**	**11**	**51**	**3.45**
1804601000	Amlan	26,372	58	17	75	284	0	0	0	0.00
1804602000	Ayungon	47,458	123	57	180	379	2	1	3	6.32
1804603000	Bacong	42,303	100	54	154	364	0	0	0	0.00
**1804604000**	**City of Bais**	**87,010**	**276**	**138**	**414**	**476**	**5**	**3**	**8**	**9.19**
1804605000	Basay	29,831	80	43	123	412	2	1	3	10.06
**1804606000**	**City of Bayawan**	**127,915**	**410**	**195**	**605**	**473**	**3**	**0**	**3**	**2.35**
1804607000	Bindoy	41,356	74	38	112	271	0	0	0	0.00
**1804608000**	**City of Canlaon**	**60,658**	**227**	**130**	**357**	**589**	**2**	**0**	**2**	**3.30**
1804609000	Dauin	30,135	67	42	109	362	1	1	2	6.64
**1804610000**	**City of Dumaguete**	**137,821**	**932**	**584**	**1,516**	**1,100**	**11**	**3**	**14**	**10.16**
**1804611000**	**City of Guihulngan**	**106,387**	**201**	**93**	**294**	**276**	**2**	**0**	**2**	**1.88**
1804612000	Jimalalud	33,207	25	8	33	99	1	1	2	6.02
1804613000	La Libertad	42,283	46	29	75	177	0	0	0	0.00
1804614000	Mabinay	86,258	257	152	409	474	1	0	1	1.16
1804615000	Manjuyod	46,028	56	27	83	180	0	0	0	0.00
1804616000	Pamplona	41,808	94	49	143	342	2	0	2	4.78
1804617000	San Jose	22,680	44	21	65	287	1	0	1	4.41
1804618000	Santa Catalina	81,179	126	68	194	239	1	1	2	2.46
1804619000	Siaton	86,699	171	89	260	300	1	0	1	1.15
1804620000	Sibulan	66,960	177	90	267	399	0	0	0	0.00
**1804621000**	**City of Tanjay**	**84,139**	**250**	**78**	**328**	**390**	**3**	**0**	**3**	**3.57**
1804622000	Tayasan	38,657	69	31	100	259	1	0	1	2.59
1804623000	Valencia	39,723	56	16	72	181	0	0	0	0.00
1804624000	Vallehermoso	41,855	141	82	223	533	1	0	1	2.39
1804625000	Zamboanguita	30,185	143	66	209	692	0	0	0	0.00
**1806100000**	**Siquijor**	**103,397**	**140**	**67**	**207**	**200**	**0**	**0**	**0**	**0.00**
1806101000	Enrique Villanueva	6,790	12	9	21	309	0	0	0	0.00
1806102000	Larena	14,454	20	8	28	194	0	0	0	0.00
1806103000	Lazi	22,488	45	17	62	276	0	0	0	0.00
1806104000	Maria	14,385	20	14	34	236	0	0	0	0.00
1806105000	San Juan	16,365	23	10	33	202	0	0	0	0.00
1806106000	Siquijor	28,915	20	9	29	100	0	0	0	0.00
**800000000**	**Region 8**	**4,627,073**	**10,741**	**5,649**	**16,390**	**354**	**309**	**122**	**431**	**9.31**
**803738000**	**Ormoc City**	**239,823**	**556**	**291**	**847**	**353**	**9**	**2**	**11**	**4.59**
**831600000**	**City of Tacloban**	**260,485**	**1,435**	**925**	**2,360**	**906**	**10**	**4**	**14**	**5.37**
**802600000**	**Eastern Samar**	**483,811**	**1,109**	**623**	**1,732**	**358**	**47**	**27**	**74**	**15.30**
802601000	Arteche	16,690	28	24	52	312	2	1	3	17.97
802602000	Balangiga	14,593	27	17	44	302	0	0	0	0.00
802603000	Balangkayan	10,228	28	11	39	381	0	0	0	0.00
**802604000**	**City of Borongan**	**73,107**	**285**	**170**	**455**	**622**	**3**	**1**	**4**	**5.47**
802605000	Can-Avid	22,269	31	19	50	225	1	0	1	4.49
802606000	Dolores	46,901	104	57	161	343	0	0	0	0.00
802607000	General Macarthur	14,489	24	23	47	324	0	0	0	0.00
802608000	Giporlos	13,183	22	11	33	250	3	0	3	22.76
802609000	Guiuan	54,042	152	81	233	431	11	5	16	29.61
802610000	Hernani	8,531	26	11	37	434	0	0	0	0.00
802611000	Jipapad	8,463	11	6	17	201	0	0	0	0.00
802612000	Lawaan	13,064	18	11	29	222	0	0	0	0.00
802613000	Llorente	21,483	31	16	47	219	0	2	2	9.31
802614000	Maslog	5,570	5	1	6	108	0	0	0	0.00
802615000	Maydolong	15,734	21	22	43	273	16	17	33	209.74
802616000	Mercedes	6,112	11	2	13	213	1	0	1	16.36
802617000	Oras	37,625	87	43	130	346	5	1	6	15.95
802618000	Quinapondan	14,509	29	11	40	276	0	0	0	0.00
802619000	Salcedo	22,142	37	10	47	212	2	0	2	9.03
802620000	San Julian	14,808	26	16	42	284	1	0	1	6.75
802621000	San Policarpo	15,412	18	8	26	169	2	0	2	12.98
802622000	Sulat	15,763	57	37	94	596	0	0	0	0.00
802623000	Taft	19,093	31	16	47	246	0	0	0	0.00
**803700000**	**Leyte**	**1,812,366**	**3,707**	**1,834**	**5,541**	**306**	**146**	**53**	**199**	**10.98**
803701000	Abuyog	61,250	185	53	238	389	5	2	7	11.43

Table 2.E.7.1 Tuberculosis Prevention and Control
Case Notification Rate (All forms and DTRB)
Philippines, 2024

PSGC	Area	Total Population	Notified TB, All Forms (New and Relapse)			Case Notification Rate (All Forms)	Registered Bacteriologically Confirmed Drug Resistant			Case Notification Rate (DRTB)
			Male	Female	Total		Male	Female	Total	
803702000	Alangalang	57,358	156	85	241	420	2	0	2	3.49
803703000	Albuera	49,244	120	47	167	339	1	0	1	2.03
803705000	Babatngon	30,135	51	29	80	265	0	0	0	0.00
803706000	Barugo	35,099	88	50	138	393	0	1	1	2.85
803707000	Bato	39,544	65	39	104	263	3	3	6	15.17
**803708000**	**City of Baybay**	**112,822**	**314**	**186**	**500**	**443**	**6**	**3**	**9**	**7.98**
803710000	Burauen	53,150	102	46	148	278	0	0	0	0.00
803713000	Calubian	31,649	70	43	113	357	1	2	3	9.48
803714000	Capoocan	34,511	42	24	66	191	1	0	1	2.90
803715000	Carigara	55,901	142	81	223	399	5	1	6	10.73
803717000	Dagami	36,455	42	16	58	159	2	1	3	8.23
803718000	Dulag	49,412	204	116	320	648	63	13	76	153.81
803719000	Hilongos	65,523	130	64	194	296	0	0	0	0.00
803720000	Hindang	20,871	30	15	45	216	1	0	1	4.79
803721000	Inopacan	21,449	41	25	66	308	0	0	0	0.00
803722000	Isabel	47,792	95	32	127	266	3	0	3	6.28
803723000	Jaro	44,965	71	30	101	225	0	0	0	0.00
803724000	Javier	26,879	43	17	60	223	0	0	0	0.00
803725000	Julita	15,789	40	13	53	336	1	0	1	6.33
803726000	Kananga	62,078	115	49	164	264	1	0	1	1.61
803728000	La Paz	19,175	42	18	60	313	0	0	0	0.00
803729000	Leyte	41,680	49	25	74	178	0	0	0	0.00
803730000	Macarthur	21,721	43	24	67	308	32	20	52	239.40
803731000	Mahaplag	27,892	55	24	79	283	0	0	0	0.00
803733000	Matag-Ob	17,696	35	18	53	300	0	0	0	0.00
803734000	Matalom	32,648	40	28	68	208	1	0	1	3.06
803735000	Mayorga	18,652	34	9	43	231	0	0	0	0.00
803736000	Merida	32,054	43	17	60	187	0	0	0	0.00
803739000	Palo	79,960	147	67	214	268	2	1	3	3.75
803740000	Palompon	58,340	95	39	134	230	2	1	3	5.14
803741000	Pastrana	19,784	21	16	37	187	0	0	0	0.00
803742000	San Isidro	30,808	25	14	39	127	0	0	0	0.00
803743000	San Miguel	20,320	48	15	63	310	1	0	1	4.92
803744000	Santa Fe	22,798	53	25	78	342	1	2	3	13.16
803745000	Tabango	34,157	47	27	74	217	1	0	1	2.93
803746000	Tabontabon	12,048	16	21	37	307	0	0	0	0.00
803748000	Tanauan	58,709	94	49	143	244	1	0	1	1.70
803749000	Tolosa	21,318	52	21	73	342	1	0	1	4.69
803750000	Tunga	7,798	12	6	18	231	0	1	1	12.82
803751000	Villaba	43,109	54	20	74	172	0	0	0	0.00
**804800000**	**Northern Samar**	**648,722**	**1,400**	**807**	**2,207**	**340**	**23**	**5**	**28**	**4.32**
804801000	Allen	25,911	45	12	57	220	2	0	2	7.72
804802000	Biri	11,659	29	7	36	309	0	0	0	0.00
804803000	Bobon	26,122	57	37	94	360	2	0	2	7.66
804804000	Capul	12,402	18	12	30	242	0	0	0	0.00
804805000	Catarman	99,620	456	277	733	736	2	1	3	3.01
804806000	Catubig	32,546	38	21	59	181	2	0	2	6.15
804807000	Gamay	23,768	55	45	100	421	0	0	0	0.00
804808000	Laoang	60,625	129	70	199	328	0	2	2	3.30
804809000	Lapinig	12,097	19	14	33	273	1	0	1	8.27
804810000	Las Navas	36,667	28	16	44	120	0	0	0	0.00
804811000	Lavezares	30,092	73	39	112	372	0	0	0	0.00
804812000	Mapanas	14,743	20	19	39	265	0	0	0	0.00
804813000	Mondragon	42,875	79	40	119	278	1	0	1	2.33
804814000	Palapag	34,596	59	36	95	275	1	1	2	5.78
804815000	Pambujan	35,689	68	28	96	269	1	0	1	2.80
804816000	Rosario	11,258	19	11	30	266	2	0	2	17.77
804817000	San Antonio	8,882	11	13	24	270	1	0	1	11.26
804818000	San Isidro	28,324	60	25	85	300	2	0	2	7.06
804819000	San Jose	17,709	28	12	40	226	2	1	3	16.94
804820000	San Roque	30,800	38	34	72	234	1	0	1	3.25
804821000	San Vicente	6,928	14	10	24	346	0	0	0	0.00
804822000	Silvino Lobos	15,100	13	7	20	132	0	0	0	0.00
804823000	Victoria	15,573	26	14	40	257	2	0	2	12.84
804824000	Lope De Vega	14,736	18	8	26	176	1	0	1	6.79
**806000000**	**Samar**	**807,949**	**1,745**	**890**	**2,635**	**326**	**46**	**18**	**64**	**7.92**
806001000	Almagro	9,273	19	10	29	313	0	0	0	0.00
806002000	Basey	56,752	87	45	132	233	4	1	5	8.81
**806003000**	**City of Calbayog**	**189,532**	**649**	**341**	**990**	**522**	**8**	**5**	**13**	**6.86**
806004000	Calbiga	23,585	30	26	56	237	1	2	3	12.72
**806005000**	**City of Catbalogan**	**111,927**	**348**	**190**	**538**	**481**	**3**	**2**	**5**	**4.47**
806006000	Daram	42,516	59	24	83	195	1	0	1	2.35
806007000	Gandara	35,242	43	26	69	196	1	1	2	5.68
806008000	Hinabangan	14,002	10	4	14	100	0	0	0	0.00
806009000	Jiabong	19,638	33	11	44	224	2	0	2	10.18
806010000	Marabut	17,852	51	24	75	420	0	1	1	5.60
806011000	Matuguinao	7,605	6	8	14	184	1	0	1	13.15
806012000	Motiong	15,798	13	7	20	127	1	0	1	6.33
806013000	Pinabacdao	18,321	19	12	31	169	0	0	0	0.00
806014000	San Jose De Buan	8,049	7	2	9	112	0	0	0	0.00
806015000	San Sebastian	8,717	8	8	16	184	0	0	0	0.00
806016000	Santa Margarita	27,467	48	11	59	215	18	3	21	76.46
806017000	Santa Rita	43,112	56	27	83	193	2	0	2	4.64
806018000	Santo Niño	12,519	25	12	37	296	0	0	0	0.00
806019000	Talalora	8,028	13	9	22	274	0	1	1	12.46
806020000	Tarangnan	26,269	67	22	89	339	0	0	0	0.00
806021000	Villareal	27,571	28	13	41	149	0	0	0	0.00
806022000	Paranas	33,297	45	18	63	189	1	1	2	6.01
806023000	Zumarraga	16,446	22	8	30	182	1	0	1	6.08
806024000	Tagapul-An	8,824	12	5	17	193	1	0	1	11.33
806025000	San Jorge	17,648	31	14	45	255	1	0	1	5.67
806026000	Pagsanghan	7,959	16	13	29	364	0	1	1	12.56
**806400000**	**Southern Leyte**	**430,474**	**885**	**385**	**1,270**	**295**	**31**	**12**	**43**	**9.99**
806401000	Anahawan	8,429	21	8	29	344	2	0	2	23.73
806402000	Bontoc	29,811	63	22	85	285	0	1	1	3.35
806403000	Hinunangan	29,149	66	29	95	326	0	0	0	0.00
806404000	Hinundayan	12,398	33	12	45	363	1	0	1	8.07
806405000	Libagon	15,244	41	12	53	348	1	0	1	6.56
806406000	Liloan	24,800	46	16	62	250	3	0	3	12.10
**806407000**	**City of Maasin**	**87,646**	**203**	**105**	**308**	**351**	**4**	**1**	**5**	**5.70**
806408000	Macrohon	26,580	46	29	75	282	2	0	2	7.52
806409000	Malitbog	23,256	27	15	42	181	0	0	0	0.00
806410000	Padre Burgos	11,159	16	8	24	215	0	0	0	0.00
806411000	Pintuyan	10,202	23	12	35	343	1	0	1	9.80
806412000	Saint Bernard	28,440	75	29	104	366	1	1	2	7.03
806413000	San Francisco	13,436	28	14	42	313	2	0	2	14.89
806414000	San Juan	14,912	50	15	65	436	0	1	1	6.71
806415000	San Ricardo	10,500	33	15	48	457	0	0	0	0.00
806416000	Silago	13,120	19	10	29	221	12	7	19	144.82
806417000	Sogod	48,211	60	24	84	174	2	1	3	6.22
806418000	Tomas Oppus	16,990	23	4	27	159	0	0	0	0.00
806419000	Limasawa	6,191	12	6	18	291	0	0	0	0.00
**807800000**	**Biliran**	**183,266**	**460**	**185**	**645**	**352**	**6**	**3**	**9**	**4.91**
807801000	Almeria	18,024	42	21	63	350	1	0	1	5.55
807802000	Biliran	18,095	50	18	68	376	0	1	1	5.53
807803000	Cabucgayan	21,755	36	9	45	207	2	0	2	9.19
807804000	Caibiran	25,180	51	22	73	290	0	0	0	0.00
807805000	Culaba	13,216	13	4	17	129	0	0	0	0.00
807806000	Kawayan	20,477	47	19	66	322	1	0	1	4.88
807807000	Maripipi	6,373	25	10	35	549	0	0	0	0.00
807808000	Naval	60,146	196	82	278	462	2	2	4	6.65
**900000000**	**Region 9**	**3,982,251**	**10,576**	**4,899**	**15,475**	**389**	**192**	**74**	**266**	**6.68**
**931700000**	**City of Zamboanga**	**1,010,873**	**3,904**	**1,992**	**5,896**	**583**	**41**	**12**	**53**	**5.24**
**990101000**	**City of Isabela**	**131,555**	**427**	**283**	**710**	**540**	**3**	**5**	**8**	**6.08**
**907200000**	**Zamboanga del Norte**	**1,072,509**	**2,285**	**971**	**3,256**	**304**	**81**	**29**	**110**	**10.26**

Table 2.E.7.1 Tuberculosis Prevention and Control
Case Notification Rate (All forms and DTRB)
Philippines, 2024

PSGC	Area	Total Population	Notified TB, All Forms (New and Relapse)			Case Notification Rate (All Forms)	Registered Bacteriologically Confirmed Drug Resistant			Case Notification Rate (DRTB)
			Male	Female	Total		Male	Female	Total	
**907201000**	**City of Dapitan**	**86,210**	**184**	**59**	**243**	**282**	**7**	**1**	**8**	**9.28**
**907202000**	**City of Dipolog**	**141,011**	**700**	**392**	**1,092**	**774**	**4**	**2**	**6**	**4.25**
907203000	Katipunan	44,796	75	33	108	241	0	0	0	0.00
907204000	La Libertad	8,119	22	10	32	394	0	1	1	12.32
907205000	Labason	45,973	81	30	111	241	63	21	84	182.72
907206000	Liloy	43,023	100	34	134	311	0	0	0	0.00
907207000	Manukan	37,130	64	11	75	202	0	0	0	0.00
907208000	Mutia	11,731	16	7	23	196	0	0	0	0.00
907209000	Piñan	20,363	35	10	45	221	0	0	0	0.00
907210000	Polanco	42,747	107	29	136	318	0	0	0	0.00
907211000	Pres. Manuel A. Roxas	39,458	54	23	77	195	1	0	1	2.53
907212000	Rizal	15,052	40	20	60	399	0	0	0	0.00
907213000	Salug	33,282	49	19	68	204	1	1	2	6.01
907214000	Sergio Osmeña Sr.	32,995	49	10	59	179	0	0	0	0.00
907215000	Siayan	37,874	34	14	48	127	0	1	1	2.64
907216000	Sibuco	37,427	66	31	97	259	0	1	1	2.67
907217000	Sibutad	17,456	43	9	52	298	0	0	0	0.00
907218000	Sindangan	107,702	226	97	323	300	1	1	2	1.86
907219000	Siocon	50,285	47	30	77	153	0	0	0	0.00
907220000	Sirawai	32,532	39	15	54	166	1	0	1	3.07
907221000	Tampilisan	25,248	34	9	43	170	1	0	1	3.96
907222000	Jose Dalman	30,062	55	13	68	226	1	0	1	3.33
907223000	Gutalac	37,482	61	20	81	216	1	0	1	2.67
907224000	Baliguian	23,837	18	5	23	96	0	0	0	0.00
907225000	Godod	17,810	24	13	37	208	0	0	0	0.00
907226000	Bacungan	28,622	36	14	50	175	0	0	0	0.00
907227000	Kalawit	24,282	26	14	40	165	0	0	0	0.00
**907300000**	**Zamboanga del Sur**	**1,080,585**	**2,388**	**1,021**	**3,409**	**315**	**36**	**18**	**54**	**5.00**
907302000	Aurora	54,491	75	23	98	180	0	0	0	0.00
907303000	Bayog	34,664	61	26	87	251	3	1	4	11.54
907305000	Dimataling	32,483	58	25	83	256	0	0	0	0.00
907306000	Dinas	37,201	55	17	72	194	1	0	1	2.69
907307000	Dumalinao	33,666	74	29	103	306	3	0	3	8.91
907308000	Dumingag	50,342	85	60	145	288	0	0	0	0.00
907311000	Kumalarang	30,476	59	25	84	276	0	0	0	0.00
907312000	Labangan	46,376	77	32	109	235	1	0	1	2.16
907313000	Lapuyan	27,839	84	48	132	474	3	2	5	17.96
907315000	Mahayag	48,942	120	45	165	337	1	2	3	6.13
907317000	Margosatubig	39,686	95	48	143	360	7	5	12	30.24
907318000	Midsalip	34,489	44	19	63	183	0	0	0	0.00
907319000	Molave	54,757	137	43	180	329	0	1	1	1.83
**907322000**	**City of Pagadian**	**219,983**	**751**	**312**	**1,063**	**483**	**6**	**1**	**7**	**3.18**
907323000	Ramon Magsaysay	27,427	73	34	107	390	0	1	1	3.65
907324000	San Miguel	20,446	27	13	40	196	0	0	0	0.00
907325000	San Pablo	27,343	31	14	45	165	1	1	2	7.31
907327000	Tabina	26,340	36	18	54	205	0	1	1	3.80
907328000	Tambulig	38,480	85	48	133	346	1	0	1	2.60
907330000	Tukuran	43,640	96	41	137	314	3	2	5	11.46
907333000	Lakewood	21,806	39	14	53	243	0	0	0	0.00
907337000	Josefina	12,413	19	4	23	185	1	0	1	8.06
907338000	Pitogo	28,586	49	19	68	238	1	0	1	3.50
907340000	Sominot	19,177	19	7	26	136	0	0	0	0.00
907341000	Vincenzo A. Sagun	25,043	42	15	57	228	0	1	1	3.99
907343000	Guipos	22,337	61	19	80	358	4	0	4	17.91
907344000	Tigbao	22,152	36	23	59	266	0	0	0	0.00
**908300000**	**Zamboanga Sibugay**	**686,729**	**1,572**	**632**	**2,204**	**321**	**31**	**10**	**41**	**5.97**
908301000	Alicia	40,798	69	30	99	243	0	1	1	2.45
908302000	Buug	38,678	81	62	143	370	1	0	1	2.59
908303000	Diplahan	32,840	75	20	95	289	2	2	4	12.18
908304000	Imelda	26,901	54	22	76	283	1	0	1	3.72
908305000	Ipil	92,486	501	177	678	733	14	4	18	19.46
908306000	Kabasalan	47,825	96	25	121	253	0	0	0	0.00
908307000	Mabuhay	37,702	30	5	35	93	0	0	0	0.00
908308000	Malangas	32,576	116	48	164	503	2	1	3	9.21
908309000	Naga	43,124	68	34	102	237	2	0	2	4.64
908310000	Olutanga	38,938	32	13	45	116	0	0	0	0.00
908311000	Payao	35,700	60	29	89	249	0	0	0	0.00
908312000	Roseller Lim	44,802	115	43	158	353	0	1	1	2.23
908313000	Siay	41,854	106	57	163	389	2	0	2	4.78
908314000	Talusan	28,105	29	12	41	146	1	0	1	3.56
908315000	Titay	55,873	75	32	107	192	2	0	2	3.58
908316000	Tungawan	48,527	65	23	88	181	4	1	5	10.30
**1000000000**	**Region 10**	**5,232,243**	**12,774**	**7,010**	**19,784**	**378**	**249**	**102**	**351**	**6.71**
**1030500000**	**City of Cagayan De Oro**	**766,153**	**3,090**	**2,065**	**5,155**	**673**	**32**	**21**	**53**	**6.92**
**1030900000**	**City of Iligan**	**375,105**	**1,882**	**1,052**	**2,934**	**782**	**18**	**8**	**26**	**6.93**
**1001300000**	**Bukidnon**	**1,627,773**	**2,000**	**811**	**2,811**	**173**	**39**	**18**	**57**	**3.50**
1001301000	Baungon	38,697	33	13	46	119	0	0	0	0.00
1001302000	Damulog	40,688	33	14	47	116	23	12	35	86.02
1001303000	Dangcagan	27,630	23	9	32	116	1	0	1	3.62
1001304000	Don Carlos	73,689	102	42	144	195	0	0	0	0.00
1001305000	Impasug-ong	56,720	28	12	40	71	0	0	0	0.00
1001306000	Kadingilan	35,937	29	13	42	117	0	0	0	0.00
1001307000	Kalilangan	46,661	58	22	80	171	0	1	1	2.14
1001308000	Kibawe	44,490	101	31	132	297	2	0	2	4.50
1001309000	Kitaotao	56,381	34	12	46	82	1	0	1	1.77
1001310000	Lantapan	69,567	34	6	40	58	0	0	0	0.00
1001311000	Libona	51,998	48	15	63	121	0	0	0	0.00
**1001312000**	**City of Malaybalay**	**201,389**	**318**	**119**	**437**	**217**	**3**	**3**	**6**	**2.98**
1001313000	Malitbog	28,430	75	43	118	415	1	0	1	3.52
1001314000	Manolo Fortich	119,801	92	38	130	109	1	1	2	1.67
1001315000	Maramag	115,228	133	46	179	155	2	0	2	1.74
1001316000	Pangantucan	60,891	70	27	97	159	0	0	0	0.00
1001317000	Quezon	116,059	162	73	235	202	0	0	0	0.00
1001318000	San Fernando	66,593	58	17	75	113	0	0	0	0.00
1001319000	Sumilao	30,968	33	11	44	142	0	0	0	0.00
1001320000	Talakag	79,169	53	42	95	120	0	1	1	1.26
**1001321000**	**City of Valencia**	**228,222**	**442**	**194**	**636**	**279**	**4**	**0**	**4**	**1.75**
1001322000	Cabanglasan	38,565	41	12	53	137	1	0	1	2.59
**1001800000**	**Camiguin**	**93,920**	**156**	**53**	**209**	**223**	**62**	**16**	**78**	**83.05**
1001801000	Catarman	17,575	28	16	44	250	2	0	2	11.38
1001802000	Guinsiliban	6,734	14	4	18	267	0	0	0	0.00
1001803000	Mahinog	14,678	27	9	36	245	0	0	0	0.00
1001804000	Mambajao	41,811	85	23	108	258	59	16	75	179.38
1001805000	Sagay	13,122	2	1	3	23	1	0	1	7.62
**1003500000**	**Lanao del Norte**	**745,269**	**1,711**	**929**	**2,640**	**354**	**34**	**20**	**54**	**7.25**
1003501000	Bacolod	24,629	100	62	162	658	2	0	2	8.12
1003502000	Baloi	70,638	72	63	135	191	0	0	0	0.00
1003503000	Baroy	24,915	136	29	165	662	2	0	2	8.03
1003505000	Kapatagan	65,430	230	73	303	463	4	2	6	9.17
1003506000	Sultan Naga Dimaporo	64,642	84	74	158	244	1	4	5	7.73
1003507000	Kauswagan	25,326	50	22	72	284	5	0	5	19.74
1003508000	Kolambugan	28,909	77	39	116	401	1	2	3	10.38
1003509000	Lala	77,067	287	154	441	572	3	1	4	5.19
1003510000	Linamon	21,705	71	36	107	493	2	0	2	9.21
1003511000	Magsaysay	20,576	58	30	88	428	0	0	0	0.00
1003512000	Maigo	24,237	52	31	83	342	1	0	1	4.13
1003513000	Matungao	15,668	39	52	91	581	1	2	3	19.15
1003514000	Munai	35,410	25	26	51	144	0	0	0	0.00
1003515000	Nunungan	18,904	16	10	26	138	1	0	1	5.29
1003516000	Pantao Ragat	30,271	8	8	16	53	0	0	0	0.00
1003517000	Poona Piagapo	29,852	16	9	25	84	0	0	0	0.00
1003518000	Salvador	32,928	65	38	103	313	0	2	2	6.07
1003519000	Sapad	23,896	51	30	81	339	0	1	1	4.18
1003520000	Tagoloan	15,387	19	11	30	195	1	0	1	6.50
1003521000	Tangcal	16,752	18	7	25	149	0	0	0	0.00

Table 2.E.7.1 Tuberculosis Prevention and Control
Case Notification Rate (All forms and DTRB)
Philippines, 2024

PSGC	Area	Total Population	Notified TB, All Forms (New and Relapse)			Case Notification Rate (All Forms)	Registered Bacteriologically Confirmed Drug Resistant			Case Notification Rate (DRTB)
			Male	Female	Total		Male	Female	Total	
1003522000	Tubod	51,418	214	98	312	607	10	6	16	31.12
1003523000	Pantar	26,709	23	27	50	187	0	0	0	0.00
1004200000	Misamis Occidental	630,533	1,643	804	2,447	388	25	9	34	5.39
1004201000	Aloran	27,934	48	18	66	236	1	0	1	3.58
1004202000	Baliangao	18,475	34	10	44	238	0	0	0	0.00
1004203000	Bonifacio	35,597	52	19	71	199	3	0	3	8.43
1004204000	Calamba	23,689	55	30	85	359	0	0	0	0.00
1004205000	Clarin	39,828	92	45	137	344	1	0	1	2.51
1004206000	Concepcion	9,324	9	2	11	118	0	0	0	0.00
1004207000	Jimenez	28,962	104	46	150	518	1	0	1	3.45
1004208000	Lopez Jaena	25,751	43	17	60	233	1	0	1	3.88
1004209000	City of Oroquieta	73,554	146	63	209	284	4	2	6	8.16
1004210000	City of Ozamiz	145,762	684	408	1,092	749	12	6	18	12.35
1004211000	Panaon	10,797	20	4	24	222	0	0	0	0.00
1004212000	Plaridel	40,267	96	31	127	315	0	1	1	2.48
1004213000	Sapang Dalaga	20,540	44	16	60	292	1	0	1	4.87
1004214000	Sinacaban	19,689	30	11	41	208	0	0	0	0.00
1004215000	City of Tangub	71,301	145	72	217	304	0	0	0	0.00
1004216000	Tudela	28,931	35	10	45	156	1	0	1	3.46
1004217000	Don Victoriano Chiongbian	10,132	6	2	8	79	0	0	0	0.00
1004300000	Misamis Oriental	993,490	2,292	1,296	3,588	361	39	10	49	4.93
1004301000	Alubijid	33,258	59	21	80	241	1	0	1	3.01
1004302000	Balingasag	77,362	239	100	339	438	3	2	5	6.46
1004303000	Balingoan	11,298	19	13	32	283	1	0	1	8.85
1004304000	Binuangan	7,495	15	7	22	294	0	0	0	0.00
1004306000	Claveria	55,124	58	29	87	158	0	1	1	1.81
1004307000	City of El Salvador	61,032	129	64	193	316	4	1	5	8.19
1004308000	City of Gingoog	142,822	441	256	697	488	8	2	10	7.00
1004309000	Gitagum	18,092	34	10	44	243	0	0	0	0.00
1004310000	Initao	34,376	103	40	143	416	1	0	1	2.91
1004311000	Jasaan	59,149	35	12	47	79	0	0	0	0.00
1004312000	Kinoguitan	14,329	19	4	23	161	0	0	0	0.00
1004313000	Lagonglong	25,264	46	19	65	257	1	0	1	3.96
1004314000	Laguindingan	26,880	105	61	166	618	3	0	3	11.16
1004315000	Libertad	13,136	35	24	59	449	1	0	1	7.61
1004316000	Lugait	20,909	74	52	126	603	0	0	0	0.00
1004317000	Magsaysay	38,520	51	33	84	218	2	0	2	5.19
1004318000	Manticao	30,014	80	38	118	393	2	0	2	6.66
1004319000	Medina	37,044	252	179	431	1,163	1	0	1	2.70
1004320000	Naawan	23,088	52	42	94	407	0	1	1	4.33
1004321000	Opol	69,357	118	82	200	288	3	0	3	4.33
1004322000	Salay	30,899	50	36	86	278	4	2	6	19.42
1004323000	Sugbongcogon	10,037	8	5	13	130	0	0	0	0.00
1004324000	Tagoloan	84,946	156	91	247	291	2	0	2	2.35
1004325000	Talisayan	26,289	50	31	81	308	1	0	1	3.80
1004326000	Villanueva	42,770	64	47	111	260	1	1	2	4.68
1100000000	Region 11	5,421,241	14,010	6,633	20,643	381	211	73	284	5.24
1130700000	City of Davao	1,841,985	5,199	2,559	7,758	421	68	24	92	4.99
1102300000	Davao del Norte	1,166,096	3,107	1,435	4,542	390	75	20	95	8.15
1102301000	Asuncion	64,047	143	84	227	354	4	1	5	7.81
1102303000	Carmen	86,016	183	83	266	309	3	0	3	3.49
1102305000	Kapalong	84,060	265	182	447	532	1	1	2	2.38
1102314000	New Corella	59,623	162	78	240	403	1	0	1	1.68
1102315000	City of Panabo	217,245	598	261	859	395	11	5	16	7.36
1102317000	Island Garden City of Samal	120,329	228	113	341	283	2	2	4	3.32
1102318000	Santo Tomas	133,260	333	141	474	356	2	1	3	2.25
1102319000	City of Tagum	307,856	792	392	1,184	385	30	8	38	12.34
1102322000	Talaingod	29,120	37	29	66	227	1	0	1	3.43
1102323000	Braulio E. Dujali	36,573	292	31	323	883	20	1	21	57.42
1102324000	San Isidro	27,967	74	41	115	411	0	1	1	3.58
1102400000	Davao del Sur	700,668	1,915	902	2,817	402	36	16	52	7.42
1102401000	Bansalan	64,283	190	95	285	443	1	0	1	1.56
1102403000	City of Digos	195,611	715	360	1,075	550	21	11	32	16.36
1102404000	Hagonoy	58,024	113	59	172	296	1	1	2	3.45
1102406000	Kiblawan	49,934	79	29	108	216	0	0	0	0.00
1102407000	Magsaysay	57,562	127	37	164	285	0	1	1	1.74
1102408000	Malalag	41,322	118	53	171	414	1	0	1	2.42
1102410000	Matanao	61,748	116	61	177	287	2	0	2	3.24
1102411000	Padada	29,989	137	64	201	670	3	1	4	13.34
1102412000	Santa Cruz	106,705	246	114	360	337	1	2	3	2.81
1102414000	Sulop	35,490	74	30	104	293	6	0	6	16.91
1102500000	Davao Oriental	594,939	1,274	605	1,879	316	13	2	15	2.52
1102501000	Baganga	60,104	145	76	221	368	4	0	4	6.66
1102502000	Banaybanay	45,697	116	55	171	374	1	0	1	2.19
1102503000	Boston	14,986	28	12	40	267	0	0	0	0.00
1102504000	Caraga	40,292	68	21	89	221	1	0	1	2.48
1102505000	Cateel	45,225	129	86	215	475	2	0	2	4.42
1102506000	Governor Generoso	61,611	108	57	165	268	1	1	2	3.25
1102507000	Lupon	68,890	99	43	142	206	0	0	0	0.00
1102508000	Manay	40,514	46	14	60	148	0	0	0	0.00
1102509000	City of Mati	155,397	419	195	614	395	2	1	3	1.93
1102510000	San Isidro	34,085	45	21	66	194	1	0	1	2.93
1102511000	Tarragona	28,138	71	25	96	341	1	0	1	3.55
1108200000	Davao de Oro	799,461	2,024	870	2,894	362	16	10	26	3.25
1108201000	Compostela	93,726	300	148	448	478	3	2	5	5.33
1108202000	Laak	83,512	120	56	176	211	0	0	0	0.00
1108203000	Mabini	45,317	88	35	123	271	2	3	5	11.03
1108204000	Maco	85,999	193	75	268	312	2	0	2	2.33
1108205000	Maragusan	67,620	60	29	89	132	0	0	0	0.00
1108206000	Mawab	41,111	152	72	224	545	0	1	1	2.43
1108207000	Monkayo	97,944	308	114	422	431	4	1	5	5.10
1108208000	Montevista	48,149	164	61	225	467	3	1	4	8.31
1108209000	Nabunturan	87,371	401	201	602	689	1	1	2	2.29
1108210000	New Bataan	52,982	107	36	143	270	1	1	2	3.77
1108211000	Pantukan	95,730	131	43	174	182	0	0	0	0.00
1108600000	Davao Occidental	318,092	491	262	753	237	3	1	4	1.26
1108601000	Don Marcelino	45,540	77	27	104	228	0	0	0	0.00
1108602000	Jose Abad Santos	73,448	114	77	191	260	0	0	0	0.00
1108603000	Malita	118,206	164	95	259	219	2	1	3	2.54
1108604000	Santa Maria	58,281	96	42	138	237	1	0	1	1.72
1108605000	Sarangani	22,617	40	21	61	270	0	0	0	0.00
1200000000	Region 12	4,555,599	13,710	6,525	20,235	444	131	57	188	4.13
1230800000	City of General Santos	733,034	2,989	1,562	4,551	621	35	9	44	6.00
1204700000	Cotabato	1,329,326	3,887	1,907	5,794	436	32	18	50	3.76
1204701000	Alamada	72,792	99	42	141	194	1	1	2	2.75
1204702000	Carmen	85,680	121	67	188	219	0	0	0	0.00
1204703000	Kabacan	80,320	191	92	283	352	2	1	3	3.74
1204704000	City of Kidapawan	167,674	1,003	563	1,566	934	9	3	12	7.16
1204705000	Libungan	58,721	151	61	212	361	1	0	1	1.70
1204706000	Magpet	56,152	92	38	130	232	0	0	0	0.00
1204707000	Makilala	91,500	200	115	315	344	2	0	2	2.19
1204708000	Matalam	84,521	175	76	251	297	3	2	5	5.92
1204709000	Midsayap	120,582	657	292	949	787	2	1	3	2.49
1204710000	M'Lang	100,702	263	103	366	363	6	1	7	6.95
1204711000	Pigkawayan	54,585	146	64	210	385	1	3	4	7.33
1204712000	Pikit	70,716	203	120	323	457	1	3	4	5.66
1204713000	President Roxas	53,882	157	85	242	449	2	0	2	3.71
1204714000	Tulunan	62,874	103	48	151	240	1	2	3	4.77
1204715000	Antipas	27,699	62	33	95	343	0	1	1	3.61
1204716000	Banisilan	49,548	76	40	116	234	0	0	0	0.00
1204717000	Aleosan	38,936	92	26	118	303	0	0	0	0.00
1204718000	Arakan	52,442	96	42	138	263	1	0	1	1.91
1206300000	South Cotabato	1,016,509	3,298	1,385	4,683	461	29	15	44	4.33
1206302000	Banga	91,952	249	71	320	348	2	2	4	4.35
1206306000	City of Koronadal	203,757	999	388	1,387	681	7	3	10	4.91

Table 2.E.7.1 Tuberculosis Prevention and Control
Case Notification Rate (All forms and DTRB)
Philippines, 2024

PSGC	Area	Total Population	Notified TB, All Forms (New and Relapse)			Case Notification Rate (All Forms)	Registered Bacteriologically Confirmed Drug Resistant			Case Notification Rate (DRTB)
			Male	Female	Total		Male	Female	Total	
1206311000	Norala	47,986	231	89	320	667	3	0	3	6.25
1206312000	Polomolok	180,777	573	250	823	455	1	4	5	2.77
1206313000	Surallah	92,986	247	99	346	372	8	2	10	10.75
1206314000	Tampakan	42,612	133	57	190	446	0	1	1	2.35
1206315000	Tantangan	47,184	158	69	227	481	0	0	0	0.00
1206316000	T'Boli	106,857	176	86	262	245	1	0	1	0.94
1206317000	Tupi	77,093	253	123	376	488	2	2	4	5.19
1206318000	Santo Niño	40,978	117	55	172	420	2	0	2	4.88
1206319000	Lake Sebu	84,327	162	98	260	308	3	1	4	4.74
**1206500000**	**Sultan Kudarat**	**889,774**	**2,338**	**1,064**	**3,402**	**382**	**29**	**10**	**39**	**4.38**
1206501000	Bagumbayan	73,436	177	83	260	354	1	2	3	4.09
1206502000	Columbio	35,113	64	32	96	273	1	0	1	2.85
1206503000	Esperanza	77,290	177	73	250	323	0	1	1	1.29
1206504000	Isulan	102,333	332	142	474	463	7	0	7	6.84
1206505000	Kalamansig	53,436	130	79	209	391	1	2	3	5.61
1206506000	Lebak	94,522	243	137	380	402	0	1	1	1.06
1206507000	Lutayan	67,640	97	46	143	211	1	0	1	1.48
1206508000	Lambayong	82,962	186	97	283	341	2	1	3	3.62
1206509000	Palimbang	96,193	142	59	201	209	3	0	3	3.12
1206510000	President Quirino	43,261	98	45	143	331	3	0	3	6.93
**1206511000**	**City of Tacurong**	**113,626**	**640**	**243**	**883**	**777**	**10**	**3**	**13**	**11.44**
1206512000	Sen. Ninoy Aquino	49,962	52	28	80	160	0	0	0	0.00
**1208000000**	**Sarangani**	**586,956**	**1,198**	**607**	**1,805**	**308**	**6**	**5**	**11**	**1.87**
1208001000	Alabel	91,708	188	63	251	274	0	0	0	0.00
1208002000	Glan	115,320	226	120	346	300	0	2	2	1.73
1208003000	Kiamba	70,188	132	77	209	298	3	1	4	5.70
1208004000	Maasim	69,243	133	68	201	290	1	0	1	1.44
1208005000	Maitum	47,031	108	67	175	372	0	0	0	0.00
1208006000	Malapatan	83,629	204	115	319	381	0	2	2	2.39
1208007000	Malungon	109,837	207	97	304	277	2	0	2	1.82
**1600000000**	**Caraga**	**2,880,174**	**8,143**	**4,130**	**12,273**	**426**	**166**	**71**	**237**	**8.23**
**1630400000**	**City of Butuan**	**384,069**	**1,177**	**625**	**1,802**	**469**	**8**	**6**	**14**	**3.65**
**1600200000**	**Agusan del Norte**	**396,858**	**917**	**431**	**1,348**	**340**	**21**	**5**	**26**	**6.55**
1600201000	Buenavista	70,747	133	53	186	263	6	0	6	8.48
**1600203000**	**City of Cabadbaran**	**82,438**	**287**	**153**	**440**	**534**	**2**	**0**	**2**	**2.43**
1600204000	Carmen	24,186	61	30	91	376	1	1	2	8.27
1600205000	Jabonga	25,155	47	20	67	266	2	1	3	11.93
1600206000	Kitcharao	21,488	52	32	84	391	3	1	4	18.62
1600207000	Las Nieves	31,037	60	17	77	248	0	0	0	0.00
1600208000	Magallanes	22,968	34	14	48	209	0	0	0	0.00
1600209000	Nasipit	45,072	81	38	119	264	3	0	3	6.66
1600210000	Santiago	29,605	51	22	73	247	1	1	2	6.76
1600211000	Tubay	26,656	71	38	109	409	3	1	4	15.01
1600212000	Remedios T. Romualdez	17,506	40	14	54	308	0	0	0	0.00
**1600300000**	**Agusan del Sur**	**763,159**	**1,866**	**807**	**2,673**	**350**	**33**	**15**	**48**	**6.29**
**1600301000**	**City of Bayugan**	**113,241**	**244**	**132**	**376**	**332**	**0**	**3**	**3**	**2.65**
1600302000	Bunawan	49,536	250	75	325	656	0	0	0	0.00
1600303000	Esperanza	60,339	90	51	141	234	2	0	2	3.31
1600304000	La Paz	31,132	51	29	80	257	4	6	10	32.12
1600305000	Loreto	44,922	156	54	210	467	3	1	4	8.90
1600306000	Prosperidad	91,000	242	148	390	429	5	0	5	5.49
1600307000	Rosario	51,762	142	56	198	383	8	1	9	17.39
1600308000	San Francisco	84,344	196	43	239	283	8	1	9	10.67
1600309000	San Luis	35,805	72	41	113	316	1	1	2	5.59
1600310000	Santa Josefa	27,377	58	24	82	300	1	0	1	3.65
1600311000	Talacogon	41,675	86	31	117	281	0	0	0	0.00
1600312000	Trento	56,690	134	50	184	325	1	1	2	3.53
1600313000	Veruela	40,689	92	41	133	327	0	0	0	0.00
1600314000	Sibagat	34,647	53	32	85	245	0	1	1	2.89
**1606700000**	**Surigao del Norte**	**543,833**	**1,830**	**991**	**2,821**	**519**	**43**	**12**	**55**	**10.11**
1606701000	Alegria	16,339	45	19	64	392	0	0	0	0.00
1606702000	Bacuag	14,970	42	30	72	481	2	0	2	13.36
1606704000	Burgos	4,190	15	13	28	668	1	0	1	23.87
1606706000	Claver	37,425	112	50	162	433	0	0	0	0.00
1606707000	Dapa	29,573	92	59	151	511	1	0	1	3.38
1606708000	Del Carmen	20,328	44	22	66	325	2	0	2	9.84
1606710000	General Luna	23,418	57	25	82	350	4	1	5	21.35
1606711000	Gigaquit	22,083	35	28	63	285	0	1	1	4.53
1606714000	Mainit	28,316	82	43	125	441	1	1	2	7.06
1606715000	Malimono	18,857	44	28	72	382	2	0	2	10.61
1606716000	Pilar	10,378	28	5	33	318	1	1	2	19.27
1606717000	Placer	29,795	57	24	81	272	2	0	2	6.71
1606718000	San Benito	5,729	17	8	25	436	0	1	1	17.46
1606719000	San Francisco	15,347	39	11	50	326	1	0	1	6.52
1606720000	San Isidro	8,583	9	9	18	210	0	0	0	0.00
1606721000	Santa Monica	9,423	30	15	45	478	1	0	1	10.61
1606722000	Sison	14,424	25	22	47	326	0	0	0	0.00
1606723000	Socorro	27,253	23	11	34	125	0	0	0	0.00
**1606724000**	**City of Surigao**	**174,810**	**939**	**523**	**1,462**	**836**	**23**	**7**	**30**	**17.16**
1606725000	Tagana-An	17,510	33	10	43	246	2	0	2	11.42
1606727000	Tubod	15,082	62	36	98	650	0	0	0	0.00
**1606800000**	**Surigao del Sur**	**664,065**	**2,100**	**1,133**	**3,233**	**487**	**54**	**32**	**86**	**12.95**
1606801000	Barobo	55,488	135	59	194	350	5	5	10	18.02
1606802000	Bayabas	9,360	24	16	40	427	0	0	0	0.00
**1606803000**	**City of Bislig**	**102,442**	**499**	**334**	**833**	**813**	**7**	**7**	**14**	**13.67**
1606804000	Cagwait	22,409	63	22	85	379	0	1	1	4.46
1606805000	Cantilan	34,876	104	35	139	399	3	2	5	14.34
1606806000	Carmen	11,910	22	13	35	294	11	2	13	109.15
1606807000	Carrascal	25,523	79	35	114	447	2	1	3	11.75
1606808000	Cortes	18,369	46	24	70	381	1	1	2	10.89
1606809000	Hinatuan	45,131	93	56	149	330	6	3	9	19.94
1606810000	Lanuza	13,706	51	20	71	518	1	0	1	7.30
1606811000	Lianga	35,115	77	32	109	310	4	1	5	14.24
1606812000	Lingig	35,817	105	58	163	455	2	2	4	11.17
1606813000	Madrid	16,668	57	21	78	468	2	1	3	18.00
1606814000	Marihatag	20,502	43	17	60	293	0	1	1	4.88
1606815000	San Agustin	23,471	33	23	56	239	1	0	1	4.26
1606816000	San Miguel	43,838	152	64	216	493	1	1	2	4.56
1606817000	Tagbina	42,617	96	51	147	345	2	2	4	9.39
1606818000	Tago	40,933	83	50	133	325	1	2	3	7.33
**1606819000**	**City of Tandag**	**65,890**	**338**	**203**	**541**	**821**	**5**	**0**	**5**	**7.59**
**1608500000**	**Dinagat Islands**	**128,190**	**253**	**143**	**396**	**309**	**7**	**1**	**8**	**6.24**
1608501000	Basilisa	36,911	56	31	87	236	1	0	1	2.71
1608502000	Cagdianao	18,388	42	28	70	381	2	1	3	16.31
1608503000	Dinagat	10,621	38	16	54	508	1	0	1	9.42
1608504000	Libjo	18,062	22	16	38	210	0	0	0	0.00
1608505000	Loreto	9,690	8	11	19	196	0	0	0	0.00
1608506000	San Jose	26,398	68	39	107	405	2	0	2	7.58
1608507000	Tubajon	8,120	19	2	21	259	1	0	1	12.32
**1900000000**	**BARMM**	**5,055,225**	**7,593**	**5,157**	**12,750**	**252**	**149**	**108**	**257**	**5.08**
**1900700000**	**Basilan**	**430,622**	**677**	**428**	**1,105**	**257**	**6**	**5**	**11**	**2.55**
**1900702000**	**City of Lamitan**	**100,900**	**230**	**122**	**352**	**349**	**3**	**3**	**6**	**5.95**
1900703000	Lantawan	31,063	49	31	80	258	1	0	1	3.22
1900704000	Maluso	46,828	89	65	154	329	1	0	1	2.14
1900705000	Sumisip	48,637	76	60	136	280	0	1	1	2.06
1900706000	Tipo-Tipo	26,312	52	35	87	331	0	0	0	0.00
1900707000	Tuburan	24,823	19	11	30	121	0	0	0	0.00
1900708000	Akbar	23,098	13	10	23	100	0	0	0	0.00
1900709000	Al-Barka	23,736	25	18	43	181	0	1	1	4.21
1900710000	Hadji Mohammad Ajul	24,631	36	12	48	195	1	0	1	4.06
1900711000	Ungkaya Pukan	24,373	53	36	89	365	0	0	0	0.00
1900712000	Hadji Muhtamad	26,867	14	11	25	93	0	0	0	0.00
1900713000	Tabuan-Lasa	29,354	21	17	38	129	0	0	0	0.00
**1903600000**	**Lanao del Sur**	**1,206,544**	**1,341**	**1,179**	**2,520**	**209**	**59**	**52**	**111**	**9.20**
1903601000	Bacolod-Kalawi	23,129	12	9	21	91	1	1	2	8.65

Table 2.E.7.1 Tuberculosis Prevention and Control
Case Notification Rate (All forms and DTRB)
Philippines, 2024

PSGC	Area	Total Population	Notified TB, All Forms (New and Relapse)			Case Notification Rate (All Forms)	Registered Bacteriologically Confirmed Drug Resistant			Case Notification Rate (DRTB)
			Male	Female	Total		Male	Female	Total	
1903602000	Balabagan	29,874	56	42	98	328	0	1	1	3.35
1903603000	Balindong	32,812	34	18	52	158	2	4	6	18.29
1903604000	Bayang	28,023	9	1	10	36	0	0	0	0.00
1903605000	Binidayan	25,965	16	11	27	104	0	0	0	0.00
1903606000	Bubong	26,574	19	38	57	215	1	2	3	11.29
1903607000	Butig	23,008	11	12	23	100	1	0	1	4.35
1903609000	Ganassi	30,802	16	12	28	91	0	3	3	9.74
1903610000	Kapai	20,582	7	9	16	78	0	0	0	0.00
1903611000	Lumba-Bayabao	45,915	22	23	45	98	0	0	0	0.00
1903612000	Lumbatan	22,780	23	18	41	180	0	1	1	4.39
1903613000	Madalum	26,478	12	11	23	87	0	0	0	0.00
1903614000	Madamba	22,050	13	12	25	113	1	1	2	9.07
1903615000	Malabang	49,331	98	102	200	405	1	3	4	8.11
1903616000	Marantao	38,682	61	51	112	290	1	0	1	2.59
**1903617000**	**City of Marawi**	**212,743**	**469**	**431**	**900**	**423**	**10**	**10**	**20**	**9.40**
1903618000	Masiu	33,620	30	6	36	107	0	0	0	0.00
1903619000	Mulondo	19,983	10	20	30	150	0	1	1	5.00
1903620000	Pagayawan	15,057	4	9	13	86	0	0	0	0.00
1903621000	Piagapo	30,244	34	36	70	231	1	1	2	6.61
1903622000	Poona Bayabao	31,255	12	13	25	80	0	0	0	0.00
1903623000	Pualas	14,526	12	7	19	131	0	0	0	0.00
1903624000	Ditsaan-Ramain	24,592	19	20	39	159	2	4	6	24.40
1903625000	Saguiaran	27,002	43	36	79	293	1	2	3	11.11
1903626000	Tamparan	32,105	15	19	34	106	0	0	0	0.00
1903627000	Taraka	27,184	33	25	58	213	0	0	0	0.00
1903628000	Tubaran	16,896	9	3	12	71	0	0	0	0.00
1903629000	Tugaya	24,783	16	12	28	113	1	0	1	4.04
1903630000	Wao	52,560	51	25	76	145	30	14	44	83.71
1903631000	Marogong	30,151	24	25	49	163	0	2	2	6.63
1903632000	Calanogas	14,985	17	19	36	240	1	0	1	6.67
1903633000	Buadiposo-Buntong	18,050	19	11	30	166	1	0	1	5.54
1903634000	Maguing	30,462	9	13	22	72	0	0	0	0.00
1903635000	Picong	18,911	47	47	94	497	3	2	5	26.44
1903636000	Lumbayanague	19,091	9	10	19	100	0	0	0	0.00
1903637000	Amai Manabilang	12,538	3	1	4	32	0	0	0	0.00
1903638000	Tagoloan Ii	12,603	2	0	2	16	0	0	0	0.00
1903639000	Kapatagan	20,550	27	15	42	204	1	0	1	4.87
1903640000	Sultan Dumalondong	12,517	9	3	12	96	0	0	0	0.00
1903641000	Lumbaca-Unayan	8,131	9	4	13	160	0	0	0	0.00
**1906600000**	**Sulu**	**1,005,907**	**972**	**668**	**1,640**	**163**	**17**	**11**	**28**	**2.78**
1906601000	Indanan	93,841	83	62	145	155	2	1	3	3.20
1906602000	Jolo	140,679	395	271	666	473	6	4	10	7.11
1906603000	Kalingalan Caluang	39,806	30	12	42	106	1	1	2	5.02
1906604000	Luuk	37,916	32	18	50	132	0	1	1	2.64
1906605000	Maimbung	59,834	45	31	76	127	0	0	0	0.00
1906606000	Hadji Panglima Tahil	7,906	8	2	10	126	1	0	1	12.65
1906607000	Old Panamao	49,852	25	20	45	90	1	0	1	2.01
1906608000	Pangutaran	36,992	32	23	55	149	0	1	1	2.70
1906609000	Parang	71,545	59	38	97	136	0	2	2	2.80
1906610000	Pata	24,736	6	5	11	44	0	0	0	0.00
1906611000	Patikul	79,708	101	66	167	210	4	1	5	6.27
1906612000	Siasi	81,689	42	18	60	73	0	0	0	0.00
1906613000	Talipao	100,088	29	9	38	38	1	0	1	1.00
1906614000	Tapul	20,799	1	3	4	19	0	0	0	0.00
1906615000	Tongkil	35,616	33	32	65	183	0	0	0	0.00
1906616000	Panglima Estino	34,265	14	16	30	88	1	0	1	2.92
1906617000	Lugus	29,100	4	7	11	38	0	0	0	0.00
1906618000	Pandami	33,199	15	15	30	90	0	0	0	0.00
1906619000	Omar	28,336	18	20	38	134	0	0	0	0.00
**1907000000**	**Tawi-Tawi**	**441,708**	**477**	**323**	**800**	**181**	**1**	**0**	**1**	**0.23**
1907001000	Panglima Sugala	48,055	29	26	55	114	0	0	0	0.00
1907002000	Bongao	117,175	235	167	402	343	0	0	0	0.00
1907003000	Mapun	30,259	29	11	40	132	0	0	0	0.00
1907004000	Simunul	34,245	26	22	48	140	0	0	0	0.00
1907005000	Sitangkai	37,455	33	15	48	128	0	0	0	0.00
1907006000	South Ubian	29,583	20	15	35	118	0	0	0	0.00
1907007000	Tandubas	34,317	16	5	21	61	0	0	0	0.00
1907008000	Turtle Islands	5,683	8	2	10	176	0	0	0	0.00
1907009000	Languyan	37,101	17	10	27	73	1	0	1	2.70
1907010000	Sapa-Sapa	33,592	20	17	37	110	0	0	0	0.00
1907011000	Sibutu	34,243	44	33	77	225	0	0	0	0.00
**1908700000**	**Maguindanao del Norte**	**985,177**	**3,231**	**1,964**	**5,195**	**527**	**47**	**28**	**75**	**7.61**
1908701000	Barira	37,722	28	15	43	114	0	0	0	0.00
1908702000	Buldon	40,797	38	28	66	162	0	1	1	2.45
**1908703000**	**City of Cotabato**	**338,375**	**2,305**	**1,417**	**3,722**	**1,100**	**34**	**21**	**55**	**16.25**
1908704000	Datu Blah T. Sinsuat	28,451	15	18	33	116	0	0	0	0.00
1908705000	Datu Odin Sinsuat	122,480	246	139	385	314	5	1	6	4.90
1908706000	Kabuntalan	25,734	24	19	43	167	0	0	0	0.00
1908707000	Matanog	37,934	37	22	59	156	1	0	1	2.64
1908708000	Northern Kabuntalan	26,807	16	6	22	82	1	0	1	3.73
1908709000	Parang	107,532	133	70	203	189	3	2	5	4.65
1908710000	Sultan Kudarat	111,569	144	86	230	206	1	2	3	2.69
1908711000	Sultan Mastura	28,448	59	29	88	309	1	0	1	3.52
1908712000	Talitay	17,702	14	8	22	124	0	0	0	0.00
1908713000	Upi	61,626	172	107	279	453	1	1	2	3.25
**1908800000**	**Maguindanao del Sur**	**759,130**	**895**	**595**	**1,490**	**196**	**19**	**12**	**31**	**4.08**
1908801000	Ampatuan	30,242	48	20	68	225	0	0	0	0.00
1908802000	Buluan	61,773	103	56	159	257	0	0	0	0.00
1908803000	Datu Abdullah Sangki	31,592	29	21	50	158	1	0	1	3.17
1908804000	Datu Anggal Midtimbang	28,980	19	12	31	107	0	0	0	0.00
1908805000	Datu Hoffer Ampatuan	27,290	20	15	35	128	0	0	0	0.00
1908806000	Datu Paglas	37,124	31	25	56	151	0	0	0	0.00
1908807000	Datu Piang	30,044	41	34	75	250	0	1	1	3.33
1908808000	Datu Salibo	19,470	6	11	17	87	1	0	1	5.14
1908809000	Datu Saudi Ampatuan	33,759	30	24	54	160	1	2	3	8.89
1908810000	Datu Unsay	13,234	17	19	36	272	0	0	0	0.00
1908811000	Gen. S.K. Pendatun	34,896	53	33	86	246	1	1	2	5.73
1908812000	Guindulungan	25,487	21	19	40	157	0	0	0	0.00
1908813000	Mamasapano	28,568	28	19	47	165	2	1	3	10.50
1908814000	Mangudadatu	27,095	20	16	36	133	0	0	0	0.00
1908815000	Pagagawan	38,624	78	44	122	316	1	1	2	5.18
1908816000	Pagalungan	49,449	34	33	67	135	1	0	1	2.02
1908817000	Paglat	19,753	39	17	56	284	0	0	0	0.00
1908818000	Pandag	27,198	23	5	28	103	0	0	0	0.00
1908819000	Rajah Buayan	28,535	18	11	29	102	0	0	0	0.00
1908820000	Shariff Aguak	35,924	48	27	75	209	7	6	13	36.19
1908821000	Shariff Saydona Mustapha	25,288	25	13	38	150	1	0	1	3.95
1908822000	South Upi	44,798	72	58	130	290	1	0	1	2.23
1908823000	Sultan Sa Barongis	25,273	42	22	64	253	0	0	0	0.00
1908824000	Talayan	34,734	50	41	91	262	2	0	2	5.76

The provinces under the Special Geographic Area (SGA) are not yet included in the 2024 TB report, as they are still counted under Cotabato City in the 2024 reporting year. This is due to the administrative transition of SGA not yet being reflected in the current Integrated Tuberculosis Information System (ITIS) dataset.

Legend:
0 = No Case
* = No Report

Table 2.E.7.2 Tuberculosis Prevention and Control
Treatment Success Rate (TSR), DSTB-all forms and MDRTB
Philippines, 2024

PSGC	Area	TB all forms cured and Completely Treated			Registered TB all forms Registered during Specified Period			Treatment Success Rate	Registered Bacteriology Confirmed Drug Resistant			Registered Bacteriologically Confirmed Drug Resistant Specified			Treatment Success Rate
		Male	Female	Total	Male	Female	Total		Male	Female	Total	Male	Female	Total	
1300000000	Philippines	257,683	141,881	399,564	277,293	150,941	428,215	93.31	4,131	1,721	5,852	5,424	2,308	7,732	75.69
1300000000	N C R	41,125	25,087	66,212	44,570	26,782	71,352	92.80	894	395	1,289	1,278	563	1,841	70.02
1380100000	City of Caloocan	4,309	2,697	7,006	4,685	2,882	7,567	92.59	101	47	148	147	67	214	69.16
1380200000	City of Las Piñas	1,209	699	1,908	1,330	761	2,091	91.25	21	16	37	40	22	62	59.68
1380300000	City of Makati	1,572	1,029	2,601	1,665	1,095	2,760	94.24	31	2	33	42	5	47	70.21
1380400000	City of Malabon	1,630	1,016	2,646	1,795	1,120	2,915	90.77	47	20	67	66	26	92	72.83
1380500000	City of Mandaluyong	1,506	951	2,457	1,678	1,023	2,701	90.97	24	11	35	30	15	45	77.78
1380600000	City of Manila	6,754	4,202	10,956	7,341	4,453	11,794	92.89	140	68	208	247	119	366	56.83
1380700000	City of Marikina	1,080	634	1,714	1,200	677	1,877	91.32	25	16	41	33	21	54	75.93
1380800000	City of Muntinlupa	2,137	761	2,898	2,338	843	3,181	91.10	71	5	76	90	8	98	77.55
1380900000	City of Navotas	922	479	1,401	1,014	513	1,527	91.75	37	6	43	52	12	64	67.19
1381000000	City of Parañaque	1,726	1,024	2,750	1,833	1,066	2,899	94.86	29	6	35	39	12	51	68.63
1381100000	Pasay City	1,517	876	2,393	1,646	964	2,610	91.69	22	11	33	31	14	45	73.33
1381200000	City of Pasig	2,173	1,442	3,615	2,477	1,610	4,087	88.45	46	21	67	60	30	90	74.44
1381300000	Quezon City	8,742	5,542	14,284	9,364	5,886	15,250	93.67	172	112	284	234	148	382	74.35
1381400000	City of San Juan	299	168	467	324	189	513	91.03	4	6	10	9	9	18	55.56
1381500000	City of Taguig	3,263	2,085	5,348	3,406	2,126	5,532	96.67	90	21	111	108	25	133	83.46
1381600000	City of Valenzuela	2,052	1,347	3,399	2,222	1,434	3,656	92.97	34	27	61	50	30	80	76.25
1381701000	Pateros	234	135	369	252	140	392	94.13	0	0	0	0	0	0	0.0
1400000000	C A R	2,302	1,198	3,500	2,495	1,283	3,778	92.64	27	6	33	35	6	41	80.49
1430300000	City of Baguio	402	222	624	451	247	698	89.40	7	0	7	9	0	9	77.78
1400100000	Abra	542	298	840	591	318	909	92.41	9	2	11	11	2	13	84.62
1400101000	Bangued	116	66	182	125	73	198	91.92	9	2	11	11	2	13	84.62
1400102000	Boliney	3	2	5	3	2	5	100.00	0	0	0	0	0	0	0.0
1400103000	Bucay	45	17	62	53	21	74	83.78	0	0	0	0	0	0	0.0
1400104000	Bucloc	10	11	21	10	11	21	100.00	0	0	0	0	0	0	0.0
1400105000	Daguioman	6	5	11	6	5	11	100.00	0	0	0	0	0	0	0.0
1400106000	Danglas	14	12	26	16	12	28	92.86	0	0	0	0	0	0	0.0
1400107000	Dolores	19	10	29	21	10	31	93.55	0	0	0	0	0	0	0.0
1400108000	La Paz	47	16	63	53	17	70	90.00	0	0	0	0	0	0	0.0
1400109000	Lacub	6	6	12	6	6	12	100.00	0	0	0	0	0	0	0.0
1400110000	Lagangilang	44	23	67	48	24	72	93.06	0	0	0	0	0	0	0.0
1400111000	Lagayan	4	8	12	4	8	12	100.00	0	0	0	0	0	0	0.0
1400112000	Langiden	18	6	24	21	7	28	85.71	0	0	0	0	0	0	0.0
1400113000	Licuan-Baay	9	6	15	10	7	17	88.24	0	0	0	0	0	0	0.0
1400114000	Luba	15	4	19	15	4	19	100.00	0	0	0	0	0	0	0.0
1400115000	Malibcong	5	2	7	9	3	12	58.33	0	0	0	0	0	0	0.0
1400116000	Manabo	39	19	58	39	19	58	100.00	0	0	0	0	0	0	0.0
1400117000	Peñarrubia	12	1	13	12	1	13	100.00	0	0	0	0	0	0	0.0
1400118000	Pidigan	9	7	16	10	7	17	94.12	0	0	0	0	0	0	0.0
1400119000	Pilar	11	5	16	12	5	17	94.12	0	0	0	0	0	0	0.0
1400120000	Sallapadan	8	3	11	9	3	12	91.67	0	0	0	0	0	0	0.0
1400121000	San Isidro	9	0	9	9	1	10	90.00	0	0	0	0	0	0	0.0
1400122000	San Juan	20	21	41	21	22	43	95.35	0	0	0	0	0	0	0.0
1400123000	San Quintin	9	3	12	9	3	12	100.00	0	0	0	0	0	0	0.0
1400124000	Tayum	30	17	47	33	18	51	92.16	0	0	0	0	0	0	0.0
1400125000	Tineg	11	6	17	12	6	18	94.44	0	0	0	0	0	0	0.0
1400126000	Tubo	11	7	18	11	8	19	94.74	0	0	0	0	0	0	0.0
1400127000	Villaviciosa	12	15	27	14	15	29	93.10	0	0	0	0	0	0	0.0
1401100000	Benguet	332	111	443	360	118	478	92.68	0	0	0	0	0	0	0.0
1401101000	Atok	7	5	12	9	5	14	85.71	0	0	0	0	0	0	0.0
1401103000	Bakun	4	1	5	5	1	6	83.33	0	0	0	0	0	0	0.0
1401104000	Bokod	6	2	8	6	2	8	100.00	0	0	0	0	0	0	0.0
1401105000	Buguias	11	3	14	11	4	15	93.33	0	0	0	0	0	0	0.0
1401106000	Itogon	107	19	126	115	20	135	93.33	0	0	0	0	0	0	0.0
1401107000	Kabayan	3	5	8	4	6	10	80.00	0	0	0	0	0	0	0.0
1401108000	Kapangan	12	3	15	13	3	16	93.75	0	0	0	0	0	0	0.0
1401109000	Kibungan	8	4	12	8	4	12	100.00	0	0	0	0	0	0	0.0
1401110000	La Trinidad	87	28	115	94	31	125	92.00	0	0	0	0	0	0	0.0
1401111000	Mankayan	28	12	40	30	12	42	95.24	0	0	0	0	0	0	0.0
1401112000	Sablan	12	8	20	14	8	22	90.91	0	0	0	0	0	0	0.0
1401113000	Tuba	35	17	52	37	18	55	94.55	0	0	0	0	0	0	0.0
1401114000	Tublay	12	4	16	14	4	18	88.89	0	0	0	0	0	0	0.0
1402700000	Ifugao	216	132	348	223	132	355	98.03	2	1	3	3	1	4	75.00
1402701000	Banaue	25	13	38	26	13	39	97.44	0	0	0	0	0	0	0.0
1402702000	Hungduan	9	1	10	10	1	11	90.91	0	0	0	0	0	0	0.0
1402703000	Kiangan	15	8	23	16	8	24	95.83	0	0	0	0	0	0	0.0
1402704000	Lagawe	20	12	32	21	12	33	96.97	0	0	0	0	0	0	0.0
1402705000	Lamut	29	19	48	30	19	49	97.96	2	1	3	3	1	4	75.00
1402706000	Mayoyao	21	12	33	21	12	33	100.00	0	0	0	0	0	0	0.0
1402707000	Alfonso Lista	48	33	81	49	33	82	98.78	0	0	0	0	0	0	0.0
1402708000	Aguinaldo	24	18	42	24	18	42	100.00	0	0	0	0	0	0	0.0
1402709000	Hingyon	17	5	22	18	5	23	95.65	0	0	0	0	0	0	0.0
1402710000	Tinoc	3	4	7	3	4	7	100.00	0	0	0	0	0	0	0.0
1402711000	Asipulo	5	7	12	5	7	12	100.00	0	0	0	0	0	0	0.0
1403200000	Kalinga	389	193	582	411	203	614	94.79	3	1	4	4	1	5	80.00
1403201000	Balbalan	16	10	26	16	10	26	100.00	0	0	0	0	0	0	0.0
1403206000	Lubuagan	17	7	24	17	7	24	100.00	0	0	0	0	0	0	0.0
1403208000	Pasil	24	7	31	24	7	31	100.00	0	0	0	0	0	0	0.0
1403209000	Pinukpuk	67	33	100	71	35	106	94.34	0	0	0	0	0	0	0.0
1403211000	Rizal	24	12	36	26	13	39	92.31	0	0	0	0	0	0	0.0
1403213000	City of Tabuk	214	114	328	228	119	347	94.52	3	1	4	4	1	5	80.00
1403214000	Tanudan	5	3	8	5	3	8	100.00	0	0	0	0	0	0	0.0
1403215000	Tinglayan	22	7	29	24	9	33	87.88	0	0	0	0	0	0	0.0
1404400000	Mountain Province	181	99	280	194	106	300	93.33	1	0	1	2	0	2	50.0
1404401000	Barlig	2	0	2	2	0	2	100.00	0	0	0	0	0	0	0.0
1404402000	Bauko	29	16	45	31	19	50	90.00	0	0	0	0	0	0	0.0
1404403000	Besao	9	5	14	9	6	15	93.33	0	0	0	0	0	0	0.0
1404404000	Bontoc	42	13	55	44	13	57	96.49	1	0	1	2	0	2	50.0
1404405000	Natonin	10	4	14	10	4	14	100.00	0	0	0	0	0	0	0.0
1404406000	Paracelis	48	38	86	53	39	92	93.48	0	0	0	0	0	0	0.0
1404407000	Sabangan	4	4	8	4	5	9	88.89	0	0	0	0	0	0	0.0
1404408000	Sadanga	6	3	9	8	3	11	81.82	0	0	0	0	0	0	0.0
1404409000	Sagada	11	4	15	12	5	17	88.24	0	0	0	0	0	0	0.0
1404410000	Tadian	20	12	32	21	12	33	96.97	0	0	0	0	0	0	0.0
1408100000	Apayao	240	143	383	265	159	424	90.33	5	2	7	6	2	8	87.50
1408101000	Calanasan	11	1	12	12	1	13	92.31	0	0	0	0	0	0	0.0
1408102000	Conner	60	34	94	63	37	100	94.00	0	0	0	0	0	0	0.0
1408103000	Flora	16	14	30	17	16	33	90.91	0	0	0	0	0	0	0.0
1408104000	Kabugao	26	11	37	29	12	41	90.24	0	0	0	0	0	0	0.0
1408105000	Luna	84	53	137	99	62	161	85.09	0	0	0	0	0	0	0.0
1408106000	Pudtol	25	9	34	25	9	34	100.00	5	2	7	6	2	8	87.50
1408107000	Santa Marcela	18	21	39	20	22	42	92.86	0	0	0	0	0	0	0.0
100000000	Region 1	11,019	6,071	17,090	11,563	6,285	17,848	95.75	198	90	288	244	101	345	83.48
102800000	Ilocos Norte	774	421	1,195	849	447	1,296	92.21	6	4	10	7	5	12	83.33
102801000	Adams	2	0	2	2	0	2	100.00	0	0	0	0	0	0	0.0
102802000	Bacarra	29	14	43	33	14	47	91.49	1	1	2	2	1	3	66.67
102803000	Badoc	37	19	56	40	23	63	88.89	0	0	0	0	0	0	0.0
102804000	Bangui	25	16	41	27	16	43	95.35	0	0	0	0	0	0	0.0
102805000	City of Batac	60	29	89	67	32	99	89.90	3	1	4	3	2	5	80.00
102806000	Burgos	12	6	18	12	7	19	94.74	0	2	2	0	2	2	100.0
102807000	Carasi	2	2	4	2	2	4	100.00	0	0	0	0	0	0	0.0
102808000	Currimao	25	20	45	26	20	46	97.83	0	0	0	0	0	0	0.0
102809000	Dingras	53	18	71	53	18	71	100.00	2	0	2	2	0	2	100.0

Table 2.E.7.2 Tuberculosis Prevention and Control
Treatment Success Rate (TSR), DSTB-all forms and MDRTB
Philippines, 2024

PSGC	Area	TB all forms cured and Completely Treated			Registered TB all forms Registered during Specified Period			Treatment Success Rate	Registered Bacteriology Confirmed Drug Resistant			Registered Bacteriologically Confirmed Drug Resistant Specified Period			Treatment Success Rate
		Male	Female	Total	Male	Female	Total		Male	Female	Total	Male	Female	Total	
102810000	Dumalneg	6	6	12	6	6	12	100.00	0	0	0	0	0	0	0.0
102811000	Banna	24	21	45	26	22	48	93.75	0	0	0	0	0	0	0.0
**102812000**	**City of Laoag**	**134**	**80**	**214**	**154**	**88**	**242**	**88.43**	**0**	**0**	**0**	**0**	**0**	**0**	**0.0**
102813000	Marcos	32	14	46	33	14	47	97.87	0	0	0	0	0	0	0.0
102814000	Nueva Era	11	12	23	12	12	24	95.83	0	0	0	0	0	0	0.0
102815000	Pagudpud	51	22	73	56	24	80	91.25	0	0	0	0	0	0	0.0
102816000	Paoay	11	6	17	16	6	22	77.27	0	0	0	0	0	0	0.0
102817000	Pasuquin	37	10	47	41	10	51	92.16	0	0	0	0	0	0	0.0
102818000	Piddig	25	16	41	27	16	43	95.35	0	0	0	0	0	0	0.0
102819000	Pinili	13	7	20	16	8	24	83.33	0	0	0	0	0	0	0.0
102820000	San Nicolas	50	22	72	57	28	85	84.71	0	0	0	0	0	0	0.0
102821000	Sarrat	41	18	59	42	18	60	98.33	0	0	0	0	0	0	0.0
102822000	Solsona	46	35	81	47	35	82	98.78	0	0	0	0	0	0	0.0
102823000	Vintar	48	28	76	54	28	82	92.68	0	0	0	0	0	0	0.0
**102900000**	**Ilocos Sur**	**1,379**	**727**	**2,106**	**1,423**	**756**	**2,179**	**96.65**	**16**	**8**	**24**	**22**	**11**	**33**	**72.73**
102901000	Alilem	12	13	25	12	13	25	100.00	0	0	0	0	0	0	0.0
102902000	Banayoyo	14	8	22	15	9	24	91.67	0	0	0	0	0	0	0.0
102903000	Bantay	233	144	377	236	147	383	98.43	6	1	7	7	2	9	77.78
102904000	Burgos	45	22	67	47	23	70	95.71	0	0	0	0	0	0	0.0
102905000	Cabugao	40	11	51	42	13	55	92.73	0	0	0	0	0	0	0.0
**102906000**	**City of Candon**	**91**	**29**	**120**	**92**	**29**	**121**	**99.17**	**0**	**0**	**0**	**0**	**0**	**0**	**0.0**
102907000	Caoayan	75	46	121	75	46	121	100.00	0	0	0	0	0	0	0.0
102908000	Cervantes	27	10	37	27	10	37	100.00	0	0	0	0	0	0	0.0
102909000	Galimuyod	16	5	21	17	5	22	95.45	0	0	0	0	0	0	0.0
102910000	Gregorio del Pilar	4	0	4	4	0	4	100.00	0	0	0	0	0	0	0.0
102911000	Lidlidda	6	2	8	6	2	8	100.00	0	0	0	0	0	0	0.0
102912000	Magsingal	38	27	65	39	29	68	95.59	0	0	0	0	0	0	0.0
102913000	Nagbukel	14	16	30	14	16	30	100.00	0	0	0	0	0	0	0.0
102914000	Narvacan	87	31	118	95	37	132	89.39	0	0	0	0	0	0	0.0
102915000	Quirino	8	1	9	8	1	9	100.00	0	0	0	0	0	0	0.0
102916000	Salcedo	20	10	30	21	10	31	96.77	0	0	0	0	0	0	0.0
102917000	San Emilio	10	6	16	10	6	16	100.00	0	0	0	0	0	0	0.0
102918000	San Esteban	14	6	20	14	6	20	100.00	0	0	0	0	0	0	0.0
102919000	San Ildefonso	32	26	58	32	26	58	100.00	0	0	0	0	0	0	0.0
102920000	San Juan	38	26	64	38	26	64	100.00	0	0	0	0	0	0	0.0
102921000	San Vicente	26	4	30	28	6	34	88.24	0	0	0	0	0	0	0.0
102922000	Santa	38	14	52	38	14	52	100.00	0	0	0	0	0	0	0.0
102923000	Santa Catalina	24	23	47	24	23	47	100.00	0	0	0	0	0	0	0.0
102924000	Santa Cruz	62	28	90	63	33	96	93.75	6	4	10	10	4	14	71.43
102925000	Santa Lucia	42	26	68	47	26	73	93.15	0	0	0	0	0	0	0.0
102926000	Santa Maria	33	8	41	33	8	41	100.00	0	0	0	0	0	0	0.0
102927000	Santiago	38	21	59	38	21	59	100.00	0	0	0	0	0	0	0.0
102928000	Santo Domingo	35	22	57	38	25	63	90.48	0	0	0	0	0	0	0.0
102929000	Sigay	1	2	3	2	2	4	75.00	0	0	0	0	0	0	0.0
102930000	Sinait	40	12	52	46	14	60	86.67	0	0	0	0	0	0	0.0
102931000	Sugpon	6	3	9	6	3	9	100.00	0	0	0	0	0	0	0.0
102932000	Suyo	13	6	19	15	6	21	90.48	0	0	0	0	0	0	0.0
102933000	Tagudin	90	67	157	94	68	162	96.91	0	0	0	0	0	0	0.0
**102934000**	**City of Vigan**	**107**	**52**	**159**	**107**	**53**	**160**	**99.38**	**4**	**3**	**7**	**5**	**5**	**10**	**70.00**
**103300000**	**La Union**	**1,970**	**1,161**	**3,131**	**2,122**	**1,209**	**3,331**	**94.00**	**26**	**11**	**37**	**38**	**12**	**50**	**74.00**
103301000	Agoo	272	152	424	290	158	448	94.64	11	3	14	12	3	15	93.33
103302000	Aringay	83	32	115	84	32	116	99.14	0	0	0	0	0	0	0.0
103303000	Bacnotan	92	50	142	95	53	148	95.95	0	0	0	1	0	1	0.0
103304000	Bagulin	16	9	25	16	9	25	100.00	0	0	0	0	0	0	0.0
103305000	Balaoan	76	48	124	81	50	131	94.66	0	0	0	0	0	0	0.0
103306000	Bangar	79	47	126	86	47	133	94.74	0	0	0	0	0	0	0.0
103307000	Bauang	144	73	217	155	76	231	93.94	0	0	0	0	0	0	0.0
103308000	Burgos	39	29	68	39	30	69	98.55	0	0	0	0	0	0	0.0
103309000	Caba	47	14	61	50	16	66	92.42	0	0	0	0	0	0	0.0
103310000	Luna	103	64	167	103	64	167	100.00	0	0	0	0	0	0	0.0
103311000	Naguilian	132	118	250	151	118	269	92.94	0	0	0	0	0	0	0.0
103312000	Pugo	51	35	86	52	35	87	98.85	0	0	0	0	0	0	0.0
103313000	Rosario	98	47	145	103	47	150	96.67	0	0	0	0	0	0	0.0
**103314000**	**City of San Fernando**	**395**	**249**	**644**	**457**	**275**	**732**	**87.98**	**15**	**8**	**23**	**25**	**9**	**34**	**67.65**
103315000	San Gabriel	66	42	108	66	42	108	100.00	0	0	0	0	0	0	0.0
103316000	San Juan	53	22	75	57	24	81	92.59	0	0	0	0	0	0	0.0
103317000	Santo Tomas	60	22	82	60	22	82	100.00	0	0	0	0	0	0	0.0
103318000	Santol	41	32	73	47	34	81	90.12	0	0	0	0	0	0	0.0
103319000	Sudipen	45	20	65	50	21	71	91.55	0	0	0	0	0	0	0.0
103320000	Tubao	78	56	134	80	56	136	98.53	0	0	0	0	0	0	0.0
**105500000**	**Pangasinan**	**6,896**	**3,762**	**10,658**	**7,169**	**3,873**	**11,042**	**96.52**	**150**	**67**	**217**	**177**	**73**	**250**	**86.80**
105501000	Agno	39	18	57	40	19	59	96.61	0	0	0	0	0	0	0.0
105502000	Aguilar	124	66	190	134	72	206	92.23	0	0	0	0	0	0	0.0
**105503000**	**City of Alaminos**	**235**	**125**	**360**	**235**	**125**	**360**	**100.00**	**17**	**6**	**23**	**18**	**6**	**24**	**95.83**
105504000	Alcala	76	31	107	76	31	107	100.00	0	0	0	0	0	0	0.0
105505000	Anda	71	34	105	72	34	106	99.06	0	0	0	0	0	0	0.0
105506000	Asingan	117	59	176	117	59	176	100.00	0	0	0	0	0	0	0.0
105507000	Balungao	91	37	128	94	37	131	97.71	0	0	0	0	0	0	0.0
105508000	Bani	89	42	131	89	42	131	100.00	0	0	0	0	0	0	0.0
105509000	Basista	117	86	203	121	86	207	98.07	0	0	0	0	0	0	0.0
105510000	Bautista	86	48	134	87	48	135	99.26	0	0	0	0	0	0	0.0
105511000	Bayambang	246	163	409	267	170	437	93.59	0	0	0	0	0	0	0.0
105512000	Binalonan	131	65	196	131	65	196	100.00	0	0	0	0	0	0	0.0
105513000	Binmaley	128	71	199	136	77	213	93.43	0	0	0	0	0	0	0.0
105514000	Bolinao	145	78	223	145	80	225	99.11	0	0	0	0	0	0	0.0
105515000	Bugallon	142	80	222	148	82	230	96.52	0	0	0	0	1	1	0.0
105516000	Burgos	44	21	65	44	21	65	100.00	0	0	0	0	0	0	0.0
105517000	Calasiao	179	94	273	201	99	300	91.00	0	0	0	0	0	0	0.0
**105518000**	**City of Dagupan**	**580**	**302**	**882**	**609**	**323**	**932**	**94.64**	**40**	**15**	**55**	**47**	**19**	**66**	**83.33**
105519000	Dasol	108	59	167	108	59	167	100.00	0	0	0	0	0	0	0.0
105520000	Infanta	42	22	64	42	22	64	100.00	0	0	0	0	0	0	0.0
105521000	Labrador	35	21	56	36	21	57	98.25	0	0	0	0	0	0	0.0
105522000	Lingayen	233	129	362	233	129	362	100.00	0	0	0	0	0	0	0.0
105523000	Mabini	56	37	93	56	37	93	100.00	0	0	0	0	0	0	0.0
105524000	Malasiqui	349	197	546	374	202	576	94.79	1	0	1	1	0	1	100.0
105525000	Manaoag	146	58	204	151	59	210	97.14	0	0	0	0	0	0	0.0
105526000	Mangaldan	189	90	279	196	91	287	97.21	0	0	0	0	0	0	0.0
105527000	Mangatarem	173	131	304	174	131	305	99.67	0	0	0	0	0	0	0.0
105528000	Mapandan	64	46	110	67	46	113	97.35	0	0	0	0	0	0	0.0
105529000	Natividad	73	42	115	75	42	117	98.29	0	0	0	0	0	0	0.0
105530000	Pozorrubio	126	60	186	136	63	199	93.47	0	0	0	0	0	0	0.0
105531000	Rosales	265	154	419	269	157	426	98.36	0	0	0	0	0	0	0.0
**105532000**	**City of San Carlos**	**367**	**242**	**609**	**394**	**252**	**646**	**94.27**	**65**	**29**	**94**	**77**	**29**	**106**	**88.68**
105533000	San Fabian	184	81	265	189	82	271	97.79	0	0	0	0	0	0	0.0
105534000	San Jacinto	73	30	103	77	32	109	94.50	0	0	0	0	0	0	0.0
105535000	San Manuel	91	59	150	91	59	150	100.00	0	0	0	0	0	0	0.0
105536000	San Nicolas	43	38	81	43	39	82	98.78	0	0	0	0	0	0	0.0
105537000	San Quintin	138	80	218	138	80	218	100.00	0	0	0	0	0	0	0.0
105538000	Santa Barbara	100	56	156	104	57	161	96.89	0	0	0	0	0	0	0.0
105539000	Santa Maria	70	35	105	79	39	118	88.98	0	0	0	0	0	0	0.0
105540000	Santo Tomas	35	19	54	35	19	54	100.00	0	0	0	0	0	0	0.0
105541000	Sison	71	26	97	76	27	103	94.17	0	0	0	0	0	0	0.0
105542000	Sual	53	30	83	54	30	84	98.81	0	0	0	0	0	0	0.0
105543000	Tayug	177	82	259	184	90	274	94.53	0	0	0	0	0	0	0.0
105544000	Umingan	302	182	484	308	187	495	97.78	0	0	0	0	0	0	0.0
105545000	Urbiztondo	125	68	193	127	68	195	98.97	0	0	0	0	0	0	0.0

Table 2.E.7.2 Tuberculosis Prevention and Control
Treatment Success Rate (TSR), DSTB-all forms and MDRTB
Philippines, 2024

PSGC	Area	TB all forms cured and Completely Treated			Registered TB all forms Registered during Specified Period			Treatment Success Rate	Registered Bacteriology Confirmed Drug Resistant			Registered Bacteriologically Confirmed Drug Resistant Specified Period			Treatment Success Rate
		Male	Female	Total	Male	Female	Total		Male	Female	Total	Male	Female	Total	
105546000	**City of Urdaneta**	335	139	474	364	153	517	91.68	27	17	44	34	18	52	84.62
105547000	Villasis	181	102	283	191	103	294	96.26	0	0	0	0	0	0	0.0
105548000	Laoac	52	27	79	52	27	79	100.00	0	0	0	0	0	0	0.0
200000000	**Region 2**	8,609	5,330	13,939	9,061	5,540	14,601	95.47	68	27	95	80	38	118	80.51
203135000	**City of Santiago**	541	314	855	550	316	866	98.73	18	14	32	22	18	40	80.00
200900000	**Batanes**	13	12	25	15	13	28	89.29	0	0	0	0	0	0	0.0
200901000	Basco	7	7	14	8	7	15	93.33	0	0	0	0	0	0	0.0
200902000	Itbayat	2	0	2	2	0	2	100.00	0	0	0	0	0	0	0.0
200903000	Ivana	3	0	3	3	1	4	75.00	0	0	0	0	0	0	0.0
200904000	Mahatao	0	0	0	0	0	0	0.00	0	0	0	0	0	0	0.00
200905000	Sabtang	1	4	5	1	4	5	100.00	0	0	0	0	0	0	0.0
200906000	Uyugan	0	1	1	1	1	2	50.00	0	0	0	0	0	0	0.0
201500000	**Cagayan**	2,872	1,724	4,596	3,001	1,785	4,786	96.03	36	9	45	42	14	56	80.36
201501000	Abulug	74	43	117	75	44	119	98.32	0	0	0	0	0	0	0.0
201502000	Alcala	54	36	90	61	38	99	90.91	0	0	0	0	0	0	0.0
201503000	Allacapan	45	17	62	46	20	66	93.94	0	0	0	0	0	0	0.0
201504000	Amulung	50	27	77	52	27	79	97.47	0	0	0	0	0	0	0.0
201505000	Aparri	210	119	329	213	119	332	99.10	3	2	5	5	2	7	71.43
201506000	Baggao	156	85	241	160	85	245	98.37	0	0	0	0	0	0	0.0
201507000	Ballesteros	98	71	169	100	73	173	97.69	0	0	0	0	0	0	0.0
201508000	Buguey	46	19	65	46	20	66	98.48	0	0	0	0	0	0	0.0
201509000	Calayan	26	17	43	26	17	43	100.00	0	0	0	0	0	0	0.0
201510000	Camalaniugan	58	39	97	59	40	99	97.98	0	0	0	0	0	0	0.0
201511000	Claveria	100	59	159	102	61	163	97.55	0	0	0	0	0	0	0.0
201512000	Enrile	78	25	103	80	26	106	97.17	0	0	0	0	0	0	0.0
201513000	Gattaran	169	131	300	184	136	320	93.75	0	0	0	0	0	0	0.0
201514000	Gonzaga	146	90	236	149	91	240	98.33	0	0	0	0	0	0	0.0
201515000	Iguig	77	57	134	90	71	161	83.23	0	0	0	0	0	0	0.0
201516000	Lal-Lo	90	49	139	100	50	150	92.67	0	0	0	0	0	0	0.0
201517000	Lasam	56	26	82	57	28	85	96.47	0	0	0	0	0	0	0.0
201518000	Pamplona	72	57	129	83	60	143	90.21	0	0	0	0	0	0	0.0
201519000	Peñablanca	70	46	116	71	46	117	99.15	0	0	0	0	0	0	0.0
201520000	Piat	60	23	83	64	25	89	93.26	0	0	0	0	0	0	0.0
201521000	Rizal	79	92	171	82	93	175	97.71	0	0	0	0	0	0	0.0
201522000	Sanchez-Mira	77	76	153	78	77	155	98.71	0	0	0	0	0	0	0.0
201523000	Santa Ana	87	42	129	92	47	139	92.81	0	0	0	0	0	0	0.0
201524000	Santa Praxedes	15	5	20	16	5	21	95.24	0	0	0	0	0	0	0.0
201525000	Santa Teresita	36	26	62	39	27	66	93.94	0	0	0	0	0	0	0.0
201526000	Santo Niño	47	27	74	49	29	78	94.87	0	0	0	0	0	0	0.0
201527000	Solana	247	146	393	255	148	403	97.52	0	0	0	0	0	0	0.0
201528000	Tuao	156	64	220	156	64	220	100.00	0	0	0	0	0	0	0.0
201529000	**Tuguegarao City**	393	210	603	416	218	634	95.11	33	7	40	37	12	49	81.63
203100000	**Isabela**	4,583	2,916	7,499	4,816	3,015	7,831	95.76	18	14	32	22	18	40	80.00
203101000	Alicia	170	96	266	172	97	269	98.88	0	0	0	0	0	0	0.0
203102000	Angadanan	117	61	178	117	61	178	100.00	0	0	0	0	0	0	0.0
203103000	Aurora	116	84	200	116	84	200	100.00	0	0	0	0	0	0	0.0
203104000	Benito Soliven	49	38	87	54	39	93	93.55	0	0	0	0	0	0	0.0
203105000	Burgos	82	51	133	92	54	146	91.10	0	0	0	0	0	0	0.0
203106000	Cabagan	258	244	502	274	249	523	95.98	0	0	0	0	0	0	0.0
203107000	Cabatuan	129	73	202	129	73	202	100.00	0	0	0	0	0	0	0.0
203108000	**City of Cauayan**	272	148	420	305	165	470	89.36	0	0	0	0	0	0	0.0
203109000	Cordon	113	46	159	119	49	168	94.64	0	0	0	0	0	0	0.0
203110000	Dinapigue	23	14	37	24	14	38	97.37	0	0	0	0	0	0	0.0
203111000	Divilacan	6	5	11	8	6	14	78.57	0	0	0	0	0	0	0.0
203112000	Echague	187	84	271	195	85	280	96.79	0	0	0	0	0	0	0.0
203113000	Gamu	86	56	142	101	60	161	88.20	0	0	0	0	0	0	0.0
203114000	**City of Ilagan**	450	285	735	478	297	775	94.84	0	0	0	0	0	0	0.0
203115000	Jones	85	52	137	112	67	179	76.54	0	0	0	0	0	0	0.0
203116000	Luna	51	26	77	52	26	78	98.72	0	0	0	0	0	0	0.0
203117000	Maconacon	4	2	6	5	2	7	85.71	0	0	0	0	0	0	0.0
203118000	Delfin Albano	84	64	148	86	65	151	98.01	0	0	0	0	0	0	0.0
203119000	Mallig	174	122	296	176	123	299	99.00	0	0	0	0	0	0	0.0
203120000	Naguilian	109	89	198	109	89	198	100.00	0	0	0	0	0	0	0.0
203121000	Palanan	42	19	61	42	19	61	100.00	0	0	0	0	0	0	0.0
203122000	Quezon	39	16	55	39	16	55	100.00	0	0	0	0	0	0	0.0
203123000	Quirino	58	47	105	58	47	105	100.00	0	0	0	0	0	0	0.0
203124000	Ramon	103	48	151	116	50	166	90.96	0	0	0	0	0	0	0.0
203125000	Reina Mercedes	98	77	175	98	77	175	100.00	0	0	0	0	0	0	0.0
203126000	Roxas	170	98	268	184	113	297	90.24	0	0	0	0	0	0	0.0
203127000	San Agustin	38	36	74	51	40	91	81.32	0	0	0	0	0	0	0.0
203128000	San Guillermo	43	32	75	44	32	76	98.68	0	0	0	0	0	0	0.0
203129000	San Isidro	68	24	92	68	24	92	100.00	0	0	0	0	0	0	0.0
203130000	San Manuel	113	57	170	118	60	178	95.51	0	0	0	0	0	0	0.0
203131000	San Mariano	251	209	460	251	210	461	99.78	0	0	0	0	0	0	0.0
203132000	San Mateo	75	47	122	81	49	130	93.85	0	0	0	0	0	0	0.0
203133000	San Pablo	65	53	118	66	54	120	98.33	0	0	0	0	0	0	0.0
203134000	Santa Maria	107	68	175	107	68	175	100.00	0	0	0	0	0	0	0.0
203136000	Santo Tomas	55	35	90	59	37	96	93.75	0	0	0	0	0	0	0.0
203137000	Tumauini	152	96	248	160	98	258	96.12	0	0	0	0	0	0	0.0
205000000	**Nueva Vizcaya**	704	366	1,070	775	409	1,184	90.37	9	2	11	11	3	14	78.57
205001000	Ambaguio	8	7	15	11	12	23	65.22	0	0	0	0	0	0	0.0
205002000	Aritao	69	22	91	79	29	108	84.26	0	0	0	0	0	0	0.0
205003000	Bagabag	58	24	82	60	27	87	94.25	0	0	0	0	0	0	0.0
205004000	Bambang	83	50	133	112	58	170	78.24	0	0	0	0	0	0	0.0
205005000	Bayombong	146	82	228	151	88	239	95.40	9	2	11	11	3	14	78.57
205006000	Diadi	34	18	52	36	18	54	96.30	0	0	0	0	0	0	0.0
205007000	Dupax del Norte	47	25	72	50	26	76	94.74	0	0	0	0	0	0	0.0
205008000	Dupax del Sur	14	21	35	18	23	41	85.37	0	0	0	0	0	0	0.0
205009000	Kasibu	31	16	47	34	18	52	90.38	0	0	0	0	0	0	0.0
205010000	Kayapa	19	7	26	21	8	29	89.66	0	0	0	0	0	0	0.0
205011000	Quezon	30	14	44	35	16	51	86.27	0	0	0	0	0	0	0.0
205012000	Santa Fe	21	14	35	22	16	38	92.11	0	0	0	0	0	0	0.0
205013000	Solano	115	54	169	117	56	173	97.69	0	0	0	0	0	0	0.0
205014000	Villaverde	20	10	30	20	12	32	93.75	0	0	0	0	0	0	0.0
205015000	Alfonso Castaneda	9	2	11	9	2	11	100.00	0	0	0	0	0	0	0.0
205700000	**Quirino**	437	312	749	454	318	772	97.02	5	2	7	5	3	8	87.50
205701000	Aglipay	56	26	82	57	26	83	98.80	0	0	0	0	0	0	0.0
205702000	Cabarroguis	119	90	209	131	94	225	92.89	5	2	7	5	3	8	87.50
205703000	Diffun	107	82	189	109	82	191	98.95	0	0	0	0	0	0	0.0
205704000	Maddela	63	49	112	64	51	115	97.39	0	0	0	0	0	0	0.0
205705000	Saguday	59	49	108	59	49	108	100.00	0	0	0	0	0	0	0.0
205706000	Nagtipunan	33	16	49	34	16	50	98.00	0	0	0	0	0	0	0.0
300000000	**Region 3**	29,222	16,648	45,870	31,213	17,539	48,752	94.09	570	237	807	746	316	1,062	75.99
330100000	**City of Angeles**	828	421	1,249	895	456	1,351	92.45	26	10	36	32	11	43	83.72
331400000	**City of Olongapo**	700	421	1,121	800	454	1,254	89.39	12	10	22	25	16	41	53.66
300800000	**Bataan**	2,624	1,441	4,065	2,748	1,491	4,239	95.90	35	15	50	55	24	79	63.29
300801000	Abucay	81	57	138	81	57	138	100.00	0	0	0	0	0	0	0.0
300802000	Bagac	64	24	88	70	25	95	92.63	0	0	0	0	0	0	0.0
300803000	**City of Balanga**	275	142	417	297	149	446	93.50	24	13	37	41	16	57	64.91
300804000	Dinalupihan	372	217	589	401	238	639	92.18	11	2	13	14	8	22	59.09
300805000	Hermosa	248	120	368	248	120	368	100.00	0	0	0	0	0	0	0.0
300806000	Limay	224	137	361	241	140	381	94.75	0	0	0	0	0	0	0.0
300807000	Mariveles	591	345	936	626	355	981	95.41	0	0	0	0	0	0	0.0
300808000	Morong	171	77	248	174	78	252	98.41	0	0	0	0	0	0	0.0
300809000	Orani	201	82	283	204	84	288	98.26	0	0	0	0	0	0	0.0
300810000	Orion	212	110	322	213	114	327	98.47	0	0	0	0	0	0	0.0

Table 2.E.7.2 Tuberculosis Prevention and Control
Treatment Success Rate (TSR), DSTB-all forms and MDRTB
Philippines, 2024

PSGC	Area	TB all forms cured and Completely Treated			Registered TB all forms Registered during Specified Period			Treatment Success Rate	Registered Bacteriology Confirmed Drug Resistant			Registered Bacteriologically Confirmed Drug Resistant Specified Period			Treatment Success Rate
		Male	Female	Total	Male	Female	Total		Male	Female	Total	Male	Female	Total	
300811000	Pilar	98	66	164	105	66	171	95.91	0	0	0	0	0	0	0.0
300812000	Samal	87	64	151	88	65	153	98.69	0	0	0	0	0	0	0.0
**301400000**	**Bulacan**	**9,758**	**5,888**	**15,646**	**10,431**	**6,170**	**16,601**	**94.25**	**199**	**77**	**276**	**265**	**105**	**370**	**74.59**
301401000	Angat	163	61	224	167	61	228	98.25	0	0	0	0	0	0	0.0
301402000	Balagtas	266	186	452	274	189	463	97.62	0	0	0	0	0	0	0.0
**301403000**	**City of Baliwag**	**400**	**206**	**606**	**408**	**210**	**618**	**98.06**	**20**	**11**	**31**	**22**	**13**	**35**	**88.57**
301404000	Bocaue	387	216	603	460	256	716	84.22	0	0	0	0	0	0	0.0
301405000	Bulacan	206	116	322	224	119	343	93.88	0	0	0	0	0	0	0.0
301406000	Bustos	186	124	310	188	125	313	99.04	0	0	0	0	0	0	0.0
301407000	Calumpit	176	87	263	187	94	281	93.59	0	0	0	0	0	0	0.0
301408000	Guiguinto	269	147	416	271	149	420	99.05	0	0	0	0	0	0	0.0
301409000	Hagonoy	382	219	601	407	223	630	95.40	13	4	17	16	5	21	80.95
**301410000**	**City of Malolos**	**557**	**319**	**876**	**612**	**337**	**949**	**92.31**	**52**	**12**	**64**	**71**	**24**	**95**	**67.37**
301411000	Marilao	845	568	1,413	903	597	1,500	94.20	0	0	0	0	0	0	0.0
**301412000**	**City of Meycauayan**	**582**	**352**	**934**	**636**	**379**	**1,015**	**92.02**	**0**	**0**	**0**	**0**	**0**	**0**	**0.0**
301413000	Norzagaray	246	155	401	285	171	456	87.94	0	0	0	0	0	0	0.0
301414000	Obando	200	148	348	218	156	374	93.05	0	0	0	0	0	0	0.0
301415000	Pandi	274	174	448	310	184	494	90.69	0	0	0	0	0	0	0.0
301416000	Paombong	88	45	133	101	52	153	86.93	0	0	0	0	0	0	0.0
301417000	Plaridel	266	146	412	300	153	453	90.95	0	0	0	0	0	0	0.0
301418000	Pulilan	224	136	360	248	148	396	90.91	0	0	0	0	0	0	0.0
301419000	San Ildefonso	252	118	370	261	121	382	96.86	0	0	0	0	0	0	0.0
**301420000**	**City of San Jose Del Monte**	**2,233**	**1,467**	**3,700**	**2,271**	**1,481**	**3,752**	**98.61**	**41**	**22**	**63**	**58**	**26**	**84**	**75.00**
301421000	San Miguel	330	171	501	357	178	535	93.64	7	4	11	10	4	14	78.57
301422000	San Rafael	232	115	347	251	122	373	93.03	0	0	0	0	0	0	0.0
301423000	Santa Maria	915	579	1,494	1,007	627	1,634	91.43	66	24	90	88	33	121	74.38
301424000	Doña Remedios Trinidad	79	33	112	85	38	123	91.06	0	0	0	0	0	0	0.0
**304900000**	**Nueva Ecija**	**4,722**	**2,700**	**7,422**	**4,985**	**2,811**	**7,796**	**95.20**	**75**	**33**	**108**	**94**	**45**	**139**	**77.70**
304901000	Aliaga	119	91	210	137	104	241	87.14	0	0	0	0	0	0	0.0
304902000	Bongabon	110	59	169	112	59	171	98.83	0	0	0	0	0	0	0.0
**304903000**	**City of Cabanatuan**	**832**	**483**	**1,315**	**848**	**494**	**1,342**	**97.99**	**31**	**13**	**44**	**33**	**21**	**54**	**81.48**
304904000	Cabiao	138	98	236	139	100	239	98.74	0	0	0	0	0	0	0.0
304905000	Carranglan	80	50	130	85	54	139	93.53	0	0	0	0	0	0	0.0
304906000	Cuyapo	243	123	366	254	127	381	96.06	0	0	0	0	0	0	0.0
304907000	Gabaldon	85	40	125	89	45	134	93.28	0	0	0	0	0	0	0.0
**304908000**	**City of Gapan**	**329**	**151**	**480**	**334**	**151**	**485**	**98.97**	**0**	**0**	**0**	**0**	**0**	**0**	**0.0**
304909000	General Mamerto Natividad	63	34	97	69	38	107	90.65	0	0	0	0	0	0	0.0
304910000	General Tinio	108	79	187	119	89	208	89.90	0	0	0	0	0	0	0.0
304911000	Guimba	302	192	494	306	193	499	99.00	7	4	11	10	4	14	78.57
304912000	Jaen	108	61	169	109	63	172	98.26	0	0	0	0	0	0	0.0
304913000	Laur	82	49	131	86	49	135	97.04	0	0	0	0	0	0	0.0
304914000	Licab	28	11	39	33	11	44	88.64	0	0	0	0	0	0	0.0
304915000	Llanera	51	22	73	53	22	75	97.33	0	0	0	0	0	0	0.0
304916000	Lupao	66	23	89	81	26	107	83.18	0	0	0	0	0	0	0.0
**304917000**	**Science City of Muñoz**	**114**	**79**	**193**	**126**	**84**	**210**	**91.90**	**0**	**0**	**0**	**0**	**0**	**0**	**0.0**
304918000	Nampicuan	52	38	90	53	39	92	97.83	0	0	0	0	0	0	0.0
**304919000**	**City of Palayan**	**123**	**78**	**201**	**130**	**83**	**213**	**94.37**	**0**	**0**	**0**	**0**	**0**	**0**	**0.0**
304920000	Pantabangan	50	34	84	51	35	86	97.67	0	0	0	0	0	0	0.0
304921000	Peñaranda	71	33	104	72	33	105	99.05	0	0	0	0	0	0	0.0
304922000	Quezon	67	28	95	73	29	102	93.14	0	0	0	0	0	0	0.0
304923000	Rizal	130	76	206	131	76	207	99.52	0	0	0	0	0	0	0.0
304924000	San Antonio	145	67	212	148	68	216	98.15	20	14	34	27	18	45	75.56
304925000	San Isidro	72	30	102	77	31	108	94.44	0	0	0	0	0	0	0.0
**304926000**	**San Jose City**	**290**	**176**	**466**	**346**	**187**	**533**	**87.43**	**17**	**2**	**19**	**24**	**2**	**26**	**73.08**
304927000	San Leonardo	112	62	174	125	67	192	90.63	0	0	0	0	0	0	0.0
304928000	Santa Rosa	177	130	307	183	131	314	97.77	0	0	0	0	0	0	0.0
304929000	Santo Domingo	152	90	242	154	94	248	97.58	0	0	0	0	0	0	0.0
304930000	Talavera	260	131	391	290	146	436	89.68	0	0	0	0	0	0	0.0
304931000	Talugtug	77	51	128	77	52	129	99.22	0	0	0	0	0	0	0.0
304932000	Zaragoza	86	31	117	95	31	126	92.86	0	0	0	0	0	0	0.0
**305400000**	**Pampanga**	**4,954**	**2,695**	**7,649**	**5,338**	**2,862**	**8,200**	**93.28**	**128**	**59**	**187**	**153**	**67**	**220**	**85.00**
305402000	Apalit	248	136	384	263	141	404	95.05	0	0	0	0	0	0	0.0
305403000	Arayat	243	154	397	273	162	435	91.26	16	8	24	19	8	27	88.89
305404000	Bacolor	81	37	118	85	38	123	95.93	0	0	0	0	0	0	0.0
305405000	Candaba	183	103	286	195	106	301	95.02	0	0	0	0	0	0	0.0
305406000	Floridablanca	299	171	470	301	172	473	99.37	7	5	12	7	7	14	85.71
305407000	Guagua	287	156	443	308	166	474	93.46	0	0	0	0	0	0	0.0
305408000	Lubao	320	182	502	338	194	532	94.36	22	8	30	27	10	37	81.08
**305409000**	**Mabalacat City**	**607**	**402**	**1,009**	**675**	**440**	**1,115**	**90.49**	**24**	**5**	**29**	**29**	**6**	**35**	**82.86**
305410000	Macabebe	201	110	311	223	121	344	90.41	11	6	17	13	6	19	89.47
305411000	Magalang	251	108	359	293	119	412	87.14	0	0	0	0	0	0	0.0
305412000	Masantol	80	36	116	90	38	128	90.63	0	0	0	0	0	0	0.0
305413000	Mexico	337	169	506	360	180	540	93.70	0	0	0	0	0	0	0.0
305414000	Minalin	128	56	184	134	59	193	95.34	0	0	0	0	0	0	0.0
305415000	Porac	313	176	489	346	187	533	91.74	0	0	0	0	0	0	0.0
**305416000**	**City of San Fernando**	**854**	**445**	**1,299**	**898**	**466**	**1,364**	**95.23**	**48**	**27**	**75**	**58**	**30**	**88**	**85.23**
305417000	San Luis	100	43	143	104	47	151	94.70	0	0	0	0	0	0	0.0
305418000	San Simon	65	30	95	73	38	111	85.59	0	0	0	0	0	0	0.0
305419000	Santa Ana	97	44	141	107	46	153	92.16	0	0	0	0	0	0	0.0
305420000	Santa Rita	99	63	162	100	66	166	97.59	0	0	0	0	0	0	0.0
305421000	Sto. Tomas	79	35	114	83	35	118	96.61	0	0	0	0	0	0	0.0
305422000	Sasmuan	82	39	121	89	41	130	93.08	0	0	0	0	0	0	0.0
**306900000**	**Tarlac**	**3,218**	**1,538**	**4,756**	**3,318**	**1,585**	**4,903**	**97.00**	**49**	**18**	**67**	**60**	**29**	**89**	**75.28**
306901000	Anao	35	12	47	35	12	47	100.00	0	0	0	0	0	0	0.0
306902000	Bamban	131	64	195	146	74	220	88.64	0	0	0	0	0	0	0.0
306903000	Camiling	190	73	263	191	74	265	99.25	3	0	3	4	1	5	60.0
306904000	Capas	314	157	471	320	159	479	98.33	0	0	0	0	0	0	0.0
306905000	Concepcion	280	151	431	290	154	444	97.07	12	5	17	15	7	22	77.27
306906000	Gerona	203	100	303	204	102	306	99.02	0	0	0	0	0	0	0.0
306907000	La Paz	157	72	229	169	74	243	94.24	0	0	0	0	0	0	0.0
306908000	Mayantoc	60	34	94	62	34	96	97.92	0	0	0	0	0	0	0.0
306909000	Moncada	135	80	215	135	80	215	100.00	3	1	4	4	1	5	80.00
306910000	Paniqui	244	120	364	244	121	365	99.73	10	5	15	11	6	17	88.24
306911000	Pura	52	21	73	52	21	73	100.00	0	0	0	0	0	0	0.0
306912000	Ramos	55	28	83	55	28	83	100.00	0	0	0	0	0	0	0.0
306913000	San Clemente	13	6	19	13	7	20	95.00	0	0	0	0	0	0	0.0
306914000	San Manuel	50	24	74	50	24	74	100.00	0	0	0	0	0	0	0.0
306915000	Santa Ignacia	89	47	136	89	48	137	99.27	0	0	0	0	0	0	0.0
**306916000**	**City of Tarlac**	**1,043**	**463**	**1,506**	**1,096**	**486**	**1,582**	**95.20**	**21**	**7**	**28**	**26**	**14**	**40**	**70.00**
306917000	Victoria	105	57	162	105	57	162	100.00	0	0	0	0	0	0	0.0
306918000	San Jose	62	29	91	62	30	92	98.91	0	0	0	0	0	0	0.0
**307100000**	**Zambales**	**1,758**	**1,202**	**2,960**	**2,004**	**1,365**	**3,369**	**87.86**	**34**	**12**	**46**	**44**	**15**	**59**	**77.97**
307101000	Botolan	173	147	320	195	162	357	89.64	0	0	0	0	0	0	0.0
307102000	Cabangan	49	29	78	49	30	79	98.73	0	0	0	0	0	0	0.0
307103000	Candelaria	88	58	146	91	59	150	97.33	0	0	0	0	0	0	0.0
307104000	Castillejos	143	119	262	155	133	288	90.97	0	0	0	0	0	0	0.0
307105000	Iba	147	85	232	174	104	278	83.45	17	7	24	22	10	32	75.00
307106000	Masinloc	204	119	323	206	119	325	99.38	0	0	0	0	0	0	0.0
307108000	Palauig	101	64	165	114	74	188	87.77	0	0	0	0	0	0	0.0
307109000	San Antonio	135	110	245	135	110	245	100.00	0	0	0	0	0	0	0.0
307110000	San Felipe	91	42	133	95	47	142	93.66	0	0	0	0	0	0	0.0
307111000	San Marcelino	76	56	132	130	89	219	60.27	0	0	0	0	0	0	0.0
307112000	San Narciso	75	35	110	76	38	114	96.49	0	0	0	0	0	0	0.0
307113000	Santa Cruz	185	124	309	198	130	328	94.21	0	0	0	0	0	0	0.0
307114000	Subic	291	214	505	386	270	656	76.98	17	5	22	22	5	27	81.48
**307700000**	**Aurora**	**660**	**342**	**1,002**	**694**	**345**	**1,039**	**96.44**	**12**	**3**	**15**	**18**	**4**	**22**	**68.18**

Table 2.E.7.2 Tuberculosis Prevention and Control
Treatment Success Rate (TSR), DSTB-all forms and MDRTB
Philippines, 2024

PSGC	Area	TB all forms cured and Completely Treated			Registered TB all forms Registered during Specified Period			Treatment Success Rate	Registered Bacteriology Confirmed Drug Resistant			Registered Bacteriologically Confirmed Drug Resistant Specified Period			Treatment Success Rate
		Male	Female	Total	Male	Female	Total		Male	Female	Total	Male	Female	Total	
307701000	Baler	159	63	222	168	63	231	96.10	0	0	0	0	0	0	0.0
307702000	Casiguran	78	30	108	81	31	112	96.43	0	0	0	0	0	0	0.0
307703000	Dilasag	44	19	63	45	19	64	98.44	0	0	0	0	0	0	0.0
307704000	Dinalungan	31	18	49	32	18	50	98.00	0	0	0	0	0	0	0.0
307705000	Dingalan	86	51	137	89	52	141	97.16	0	0	0	0	0	0	0.0
307706000	Dipaculao	59	27	86	65	27	92	93.48	0	0	0	0	0	0	0.0
307707000	Maria Aurora	143	93	236	150	94	244	96.72	12	3	15	18	4	22	68.18
307708000	San Luis	60	41	101	64	41	105	96.19	0	0	0	0	0	0	0.0
**400000000**	**Region 4A**	**39,440**	**23,516**	**62,956**	**42,197**	**24,811**	**67,008**	**93.95**	**720**	**327**	**1,047**	**938**	**450**	**1,388**	**75.43**
**431200000**	**City of Lucena**	**700**	**388**	**1,088**	**774**	**438**	**1,212**	**89.77**	**52**	**23**	**75**	**64**	**26**	**90**	**83.33**
**401000000**	**Batangas**	**6,774**	**4,329**	**11,103**	**7,180**	**4,491**	**11,671**	**95.13**	**103**	**40**	**143**	**132**	**53**	**185**	**77.30**
401001000	Agoncillo	102	80	182	103	80	183	99.45	0	0	0	0	0	0	0.0
401002000	Alitagtag	88	85	173	92	85	177	97.74	0	0	0	0	0	0	0.0
401003000	Balayan	238	122	360	253	132	385	93.51	0	0	0	0	0	0	0.0
401004000	Balete	25	37	62	27	39	66	93.94	0	0	0	0	0	0	0.0
**401005000**	**Batangas City**	**823**	**366**	**1,189**	**933**	**402**	**1,335**	**89.06**	**26**	**10**	**36**	**43**	**16**	**59**	**61.02**
401006000	Bauan	277	172	449	277	172	449	100.00	0	0	0	0	0	0	0.0
**401007000**	**City of Calaca**	**320**	**266**	**586**	**322**	**266**	**588**	**99.66**	**0**	**0**	**0**	**0**	**0**	**0**	**0.0**
401008000	Calatagan	102	87	189	102	87	189	100.00	0	0	0	0	0	0	0.0
401009000	Cuenca	126	106	232	137	112	249	93.17	0	0	0	0	0	0	0.0
401010000	Ibaan	193	163	356	193	163	356	100.00	0	0	0	0	0	0	0.0
401011000	Laurel	66	43	109	78	48	126	86.51	0	0	0	0	0	0	0.0
401012000	Lemery	184	125	309	184	125	309	100.00	0	0	0	0	0	0	0.0
401013000	Lian	128	53	181	149	60	209	86.60	0	0	0	0	0	0	0.0
**401014000**	**City of Lipa**	**701**	**404**	**1,105**	**754**	**427**	**1,181**	**93.56**	**33**	**13**	**46**	**37**	**19**	**56**	**82.14**
401015000	Lobo	94	51	145	99	51	150	96.67	0	0	0	0	0	0	0.0
401016000	Mabini	114	71	185	116	72	188	98.40	0	0	0	0	0	0	0.0
401017000	Malvar	130	65	195	130	65	195	100.00	0	0	0	0	0	0	0.0
401018000	Mataasnakahoy	69	72	141	70	72	142	99.30	0	0	0	0	0	0	0.0
401019000	Nasugbu	349	201	550	351	201	552	99.64	21	5	26	24	5	29	89.66
401020000	Padre Garcia	96	56	152	103	60	163	93.25	0	0	0	0	0	0	0.0
401021000	Rosario	252	165	417	272	174	446	93.50	0	0	0	0	0	0	0.0
401022000	San Jose	303	202	505	312	209	521	96.93	0	0	0	0	0	0	0.0
401023000	San Juan	392	296	688	417	303	720	95.56	6	3	9	6	3	9	100.0
401024000	San Luis	98	76	174	98	76	174	100.00	0	0	0	0	0	0	0.0
401025000	San Nicolas	54	35	89	54	35	89	100.00	0	0	0	0	0	0	0.0
401026000	San Pascual	190	129	319	192	132	324	98.46	0	0	0	0	0	0	0.0
401027000	Santa Teresita	61	38	99	62	38	100	99.00	0	0	0	0	0	0	0.0
**401028000**	**City of Sto. Tomas**	**281**	**186**	**467**	**310**	**200**	**510**	**91.57**	**0**	**0**	**0**	**0**	**0**	**0**	**0.0**
401029000	Taal	212	132	344	217	137	354	97.18	17	9	26	22	10	32	81.25
401030000	Talisay	104	62	166	112	67	179	92.74	0	0	0	0	0	0	0.0
**401031000**	**City of Tanauan**	**344**	**220**	**564**	**372**	**233**	**605**	**93.22**	**0**	**0**	**0**	**0**	**0**	**0**	**0.0**
401032000	Taysan	121	88	209	140	88	228	91.67	0	0	0	0	0	0	0.0
401033000	Tingloy	40	19	59	41	20	61	96.72	0	0	0	0	0	0	0.0
401034000	Tuy	97	56	153	108	60	168	91.07	0	0	0	0	0	0	0.0
**402100000**	**Cavite**	**10,255**	**6,259**	**16,514**	**10,981**	**6,571**	**17,552**	**94.09**	**171**	**78**	**249**	**220**	**108**	**328**	**75.91**
402101000	Alfonso	105	41	146	107	42	149	97.99	0	0	0	0	0	0	0.0
402102000	Amadeo	45	27	72	53	28	81	88.89	0	0	0	0	0	0	0.0
**402103000**	**City of Bacoor**	**1,193**	**725**	**1,918**	**1,296**	**777**	**2,073**	**92.52**	**17**	**7**	**24**	**26**	**12**	**38**	**63.16**
402104000	Carmona	254	152	406	262	156	418	97.13	0	0	0	0	0	0	0.0
**402105000**	**City of Cavite**	**340**	**205**	**545**	**369**	**217**	**586**	**93.00**	**0**	**0**	**0**	**0**	**0**	**0**	**0.0**
**402106000**	**City of Dasmariñas**	**1,813**	**1,219**	**3,032**	**1,964**	**1,298**	**3,262**	**92.95**	**42**	**16**	**58**	**50**	**24**	**74**	**78.38**
402107000	General Emilio Aguinaldo	42	20	62	45	20	65	95.38	0	0	0	0	0	0	0.0
**402108000**	**City of General Trias**	**963**	**604**	**1,567**	**995**	**620**	**1,615**	**97.03**	**0**	**0**	**0**	**0**	**0**	**0**	**0.0**
**402109000**	**City of Imus**	**861**	**526**	**1,387**	**971**	**573**	**1,544**	**89.83**	**31**	**19**	**50**	**41**	**25**	**66**	**75.76**
402110000	Indang	137	68	205	141	70	211	97.16	0	0	0	0	0	0	0.0
402111000	Kawit	339	186	525	357	193	550	95.45	0	0	0	0	0	0	0.0
402112000	Magallanes	43	7	50	48	8	56	89.29	0	0	0	0	0	0	0.0
402113000	Maragondon	78	47	125	83	49	132	94.70	0	0	0	0	0	0	0.0
402114000	Mendez	56	19	75	61	22	83	90.36	0	0	0	0	0	0	0.0
402115000	Naic	444	233	677	491	246	737	91.86	0	0	0	0	0	0	0.0
402116000	Noveleta	143	79	222	154	89	243	91.36	0	0	0	0	0	0	0.0
402117000	Rosario	376	205	581	390	210	600	96.83	0	0	0	0	0	0	0.0
402118000	Silang	614	333	947	647	343	990	95.66	0	0	0	0	0	0	0.0
**402119000**	**City of Tagaytay**	**243**	**142**	**385**	**244**	**142**	**386**	**99.74**	**16**	**6**	**22**	**19**	**8**	**27**	**81.48**
402120000	Tanza	794	476	1,270	852	503	1,355	93.73	0	0	0	0	0	0	0.0
402121000	Ternate	45	24	69	51	25	76	90.79	0	0	0	0	0	0	0.0
**402122000**	**City of Trece Martires**	**832**	**611**	**1,443**	**842**	**616**	**1,458**	**98.97**	**52**	**24**	**76**	**68**	**28**	**96**	**79.17**
402123000	Gen. Mariano Alvarez	495	310	805	558	324	882	91.27	13	6	19	16	11	27	70.37
**403400000**	**Laguna**	**8,678**	**4,841**	**13,519**	**9,060**	**5,026**	**14,086**	**95.97**	**167**	**70**	**237**	**214**	**99**	**313**	**75.72**
403401000	Alaminos	146	90	236	161	96	257	91.83	0	0	0	0	0	0	0.0
403402000	Bay	145	85	230	149	85	234	98.29	0	0	0	0	0	0	0.0
**403403000**	**City of Biñan**	**1,140**	**620**	**1,760**	**1,144**	**623**	**1,767**	**99.60**	**0**	**0**	**0**	**0**	**0**	**0**	**0.0**
**403404000**	**City of Cabuyao**	**741**	**422**	**1,163**	**788**	**437**	**1,225**	**94.94**	**0**	**0**	**0**	**0**	**0**	**0**	**0.0**
**403405000**	**City of Calamba**	**1,473**	**803**	**2,276**	**1,490**	**808**	**2,298**	**99.04**	**61**	**19**	**80**	**72**	**30**	**102**	**78.43**
403406000	Calauan	211	97	308	212	98	310	99.35	0	0	0	0	0	0	0.0
403407000	Cavinti	34	30	64	37	30	67	95.52	0	0	0	0	0	0	0.0
403408000	Famy	56	26	82	60	30	90	91.11	0	0	0	0	0	0	0.0
403409000	Kalayaan	91	52	143	103	53	156	91.67	0	0	0	0	0	0	0.0
403410000	Liliw	110	78	188	122	79	201	93.53	0	0	0	0	0	0	0.0
403411000	Los Baños	252	168	420	285	192	477	88.05	18	5	23	23	9	32	71.88
403412000	Luisiana	36	24	60	42	27	69	86.96	0	0	0	0	0	0	0.0
403413000	Lumban	135	89	224	135	89	224	100.00	0	0	0	0	0	0	0.0
403414000	Mabitac	66	52	118	66	52	118	100.00	0	0	0	0	0	0	0.0
403415000	Magdalena	76	29	105	80	33	113	92.92	0	0	0	0	0	0	0.0
403416000	Majayjay	64	35	99	69	37	106	93.40	0	0	0	1	0	1	0.0
403417000	Nagcarlan	182	125	307	191	130	321	95.64	0	0	0	0	0	0	0.0
403418000	Paete	66	50	116	69	52	121	95.87	0	0	0	0	0	0	0.0
403419000	Pagsanjan	196	120	316	207	124	331	95.47	25	10	35	39	18	57	61.4
403420000	Pakil	40	16	56	43	16	59	94.92	0	0	0	0	0	0	0.0
403421000	Pangil	78	41	119	86	42	128	92.97	9	3	12	11	5	16	75.00
403422000	Pila	185	81	266	195	85	280	95.00	0	0	0	0	0	0	0.0
403423000	Rizal	21	16	37	24	17	41	90.24	0	0	0	0	0	0	0.0
**403424000**	**City of San Pablo**	**704**	**433**	**1,137**	**783**	**488**	**1,271**	**89.46**	**0**	**0**	**0**	**0**	**0**	**0**	**0.0**
**403425000**	**City of San Pedro**	**813**	**376**	**1,189**	**825**	**387**	**1,212**	**98.10**	**0**	**0**	**0**	**0**	**0**	**0**	**0.0**
403426000	Santa Cruz	355	190	545	373	195	568	95.95	0	0	0	0	0	0	0.0
403427000	Santa Maria	114	66	180	118	67	185	97.30	0	0	0	0	0	0	0.0
**403428000**	**City of Santa Rosa**	**865**	**466**	**1,331**	**915**	**487**	**1,402**	**94.94**	**54**	**33**	**87**	**68**	**37**	**105**	**82.86**
403429000	Siniloan	198	121	319	202	126	328	97.26	0	0	0	0	0	0	0.0
403430000	Victoria	85	40	125	86	41	127	98.43	0	0	0	0	0	0	0.0
**405600000**	**Quezon**	**4,513**	**2,480**	**6,993**	**4,977**	**2,687**	**7,664**	**91.24**	**58**	**27**	**85**	**75**	**36**	**111**	**76.58**
405601000	Agdangan	27	22	49	27	22	49	100.00	0	0	0	0	0	0	0.0
405602000	Alabat	63	51	114	74	55	129	88.37	0	0	0	0	0	0	0.0
405603000	Atimonan	157	59	216	162	61	223	96.86	0	0	0	0	0	0	0.0
405605000	Buenavista	105	46	151	105	46	151	100.00	0	0	0	0	0	0	0.0
405606000	Burdeos	56	33	89	64	37	101	88.12	0	0	0	0	0	0	0.0
405607000	Calauag	150	73	223	235	129	364	61.26	0	0	0	0	0	0	0.0
405608000	Candelaria	425	252	677	430	253	683	99.12	0	0	0	0	0	0	0.0
405610000	Catanauan	139	76	215	142	76	218	98.62	9	2	11	12	3	15	73.33
405615000	Dolores	58	30	88	79	36	115	76.52	0	0	0	0	0	0	0.0
405616000	General Luna	79	44	123	84	47	131	93.89	0	0	0	0	0	0	0.0
405617000	General Nakar	79	42	121	90	47	137	88.32	0	0	0	0	0	0	0.0
405618000	Guinayangan	91	64	155	103	67	170	91.18	0	0	0	0	0	0	0.0
405619000	Gumaca	168	97	265	210	109	319	83.07	39	11	50	47	15	62	80.65
405620000	Infanta	142	75	217	157	80	237	91.56	5	10	15	8	14	22	68.18
405621000	Jomalig	12	9	21	22	10	32	65.63	0	0	0	0	0	0	0.0

Table 2.E.7.2 Tuberculosis Prevention and Control
Treatment Success Rate (TSR), DSTB-all forms and MDRTB
Philippines, 2024

PSGC	Area	TB all forms cured and Completely Treated			Registered TB all forms Registered during Specified Period			Treatment Success Rate	Registered Bacteriology Confirmed Drug Resistant			Registered Bacteriologically Confirmed Drug Resistant Specified Period			Treatment Success Rate
		Male	Female	Total	Male	Female	Total		Male	Female	Total	Male	Female	Total	
405622000	Lopez	186	75	261	196	79	275	94.91	0	0	0	0	0	0	0.0
405623000	Lucban	98	53	151	98	54	152	99.34	0	0	0	0	0	0	0.0
405625000	Macalelon	48	22	70	51	23	74	94.59	0	0	0	0	0	0	0.0
405627000	Mauban	188	105	293	203	109	312	93.91	5	4	9	8	4	12	75.00
405628000	Mulanay	92	52	144	127	76	203	70.94	0	0	0	0	0	0	0.0
405629000	Padre Burgos	45	20	65	49	22	71	91.55	0	0	0	0	0	0	0.0
405630000	Pagbilao	216	91	307	226	92	318	96.54	0	0	0	0	0	0	0.0
405631000	Panukulan	51	39	90	53	43	96	93.75	0	0	0	0	0	0	0.0
405632000	Patnanungan	13	12	25	15	15	30	83.33	0	0	0	0	0	0	0.0
405633000	Perez	43	17	60	45	19	64	93.75	0	0	0	0	0	0	0.0
405634000	Pitogo	59	34	93	60	34	94	98.94	0	0	0	0	0	0	0.0
405635000	Plaridel	37	21	58	41	22	63	92.06	0	0	0	0	0	0	0.0
405636000	Polillo	63	43	106	72	48	120	88.33	0	0	0	0	0	0	0.0
405637000	Quezon	48	27	75	58	31	89	84.27	0	0	0	0	0	0	0.0
405638000	Real	86	54	140	93	62	155	90.32	0	0	0	0	0	0	0.0
405639000	Sampaloc	25	11	36	26	11	37	97.30	0	0	0	0	0	0	0.0
405640000	San Andres	83	29	112	102	36	138	81.16	0	0	0	0	0	0	0.0
405641000	San Antonio	75	42	117	80	43	123	95.12	0	0	0	0	0	0	0.0
405642000	San Francisco	116	84	200	118	85	203	98.52	0	0	0	0	0	0	0.0
405644000	San Narciso	171	81	252	179	84	263	95.82	0	0	0	0	0	0	0.0
405645000	Sariaya	344	232	576	357	235	592	97.30	0	0	0	0	0	0	0.0
405646000	Tagkawayan	131	78	209	153	88	241	86.72	0	0	0	0	0	0	0.0
**405647000**	**City of Tayabas**	**245**	**131**	**376**	**270**	**140**	**410**	**91.71**	**0**	**0**	**0**	**0**	**0**	**0**	**0.0**
405648000	Tiaong	264	137	401	286	144	430	93.26	0	0	0	0	0	0	0.0
405649000	Unisan	35	17	52	35	17	52	100.00	0	0	0	0	0	0	0.0
**405800000**	**Rizal**	**8,520**	**5,219**	**13,739**	**9,225**	**5,598**	**14,823**	**92.69**	**169**	**89**	**258**	**233**	**128**	**361**	**71.47**
405801000	Angono	321	160	481	362	187	549	87.61	0	0	0	0	0	0	0.0
**405802000**	**City of Antipolo**	**2,190**	**1,401**	**3,591**	**2,416**	**1,536**	**3,952**	**90.87**	**49**	**29**	**78**	**66**	**46**	**112**	**69.64**
405803000	Baras	241	111	352	246	112	358	98.32	0	0	0	0	0	0	0.0
405804000	Binangonan	581	368	949	624	395	1,019	93.13	0	0	0	0	0	0	0.0
405805000	Cainta	902	430	1,332	911	435	1,346	98.96	34	21	55	54	31	85	64.71
405806000	Cardona	127	101	228	129	101	230	99.13	0	0	0	0	0	0	0.0
405807000	Jala-Jala	71	40	111	82	43	125	88.80	0	0	0	0	0	0	0.0
405808000	Rodriguez	1,466	946	2,412	1,633	1,035	2,668	90.40	40	19	59	52	21	73	80.82
405809000	Morong	197	135	332	203	137	340	97.65	0	0	0	0	0	0	0.0
405810000	Pililla	169	90	259	172	90	262	98.85	0	0	0	0	0	0	0.0
405811000	San Mateo	783	577	1,360	883	630	1,513	89.89	21	6	27	28	11	39	69.23
405812000	Tanay	502	321	823	530	340	870	94.60	25	14	39	33	19	52	75.00
405813000	Taytay	724	415	1,139	783	430	1,213	93.90	0	0	0	0	0	0	0.0
405814000	Teresa	246	124	370	251	127	378	97.88	0	0	0	0	0	0	0.0
**1700000000**	**Region 4B**	**8,916**	**4,958**	**13,874**	**9,695**	**5,322**	**15,017**	**92.39**	**185**	**72**	**257**	**211**	**86**	**297**	**86.53**
**1731500000**	**City of Puerto Princesa**	**988**	**445**	**1,433**	**1,046**	**467**	**1,513**	**94.71**	**35**	**10**	**45**	**39**	**13**	**52**	**86.54**
**1704000000**	**Marinduque**	**834**	**514**	**1,348**	**965**	**574**	**1,539**	**87.59**	**14**	**1**	**15**	**16**	**2**	**18**	**83.33**
1704001000	Boac	194	120	314	230	128	358	87.71	14	1	15	16	2	18	83.33
1704002000	Buenavista	137	114	251	162	133	295	85.08	0	0	0	0	0	0	0.0
1704003000	Gasan	165	96	261	174	100	274	95.26	0	0	0	0	0	0	0.0
1704004000	Mogpog	124	80	204	142	94	236	86.44	0	0	0	0	0	0	0.0
1704005000	Santa Cruz	130	57	187	156	63	219	85.39	0	0	0	0	0	0	0.0
1704006000	Torrijos	84	47	131	101	56	157	83.44	0	0	0	0	0	0	0.0
**1705100000**	**Occidental Mindoro**	**1,628**	**945**	**2,573**	**1,760**	**1,003**	**2,763**	**93.12**	**39**	**19**	**58**	**46**	**22**	**68**	**85.29**
1705101000	Abra De Ilog	133	100	233	144	107	251	92.83	0	0	0	0	0	0	0.0
1705102000	Calintaan	88	54	142	91	58	149	95.30	0	0	0	0	0	0	0.0
1705103000	Looc	12	3	15	15	3	18	83.33	0	0	0	0	0	0	0.0
1705104000	Lubang	37	36	73	49	39	88	82.95	0	0	0	0	0	0	0.0
1705105000	Magsaysay	84	65	149	94	76	170	87.65	0	0	0	0	0	0	0.0
1705106000	Mamburao	211	135	346	249	147	396	87.37	39	19	58	46	22	68	85.29
1705107000	Paluan	90	63	153	95	66	161	95.03	0	0	0	0	0	0	0.0
1705108000	Rizal	96	48	144	101	48	149	96.64	0	0	0	0	0	0	0.0
1705109000	Sablayan	353	123	476	376	133	509	93.52	0	0	0	0	0	0	0.0
1705110000	San Jose	417	240	657	431	243	674	97.48	0	0	0	0	0	0	0.0
1705111000	Santa Cruz	107	78	185	115	83	198	93.43	0	0	0	0	0	0	0.0
**1705200000**	**Oriental Mindoro**	**2,814**	**1,551**	**4,365**	**2,989**	**1,650**	**4,639**	**94.09**	**59**	**22**	**81**	**62**	**24**	**86**	**94.19**
1705201000	Baco	143	132	275	146	134	280	98.21	0	0	0	0	0	0	0.0
1705202000	Bansud	166	71	237	167	72	239	99.16	0	0	0	0	0	0	0.0
1705203000	Bongabong	213	122	335	263	152	415	80.72	0	0	0	0	0	0	0.0
1705204000	Bulalacao	105	65	170	113	70	183	92.90	0	0	0	0	0	0	0.0
**1705205000**	**City of Calapan**	**460**	**226**	**686**	**470**	**234**	**704**	**97.44**	**27**	**13**	**40**	**28**	**13**	**41**	**97.56**
1705206000	Gloria	165	97	262	172	99	271	96.68	0	0	0	0	0	0	0.0
1705207000	Mansalay	136	93	229	149	101	250	91.60	0	0	0	0	0	0	0.0
1705208000	Naujan	291	172	463	304	181	485	95.46	0	0	0	0	0	0	0.0
1705209000	Pinamalayan	337	154	491	347	159	506	97.04	0	0	0	0	0	0	0.0
1705210000	Pola	89	42	131	89	42	131	100.00	0	0	0	0	0	0	0.0
1705211000	Puerto Galera	99	46	145	103	47	150	96.67	0	0	0	0	0	0	0.0
1705212000	Roxas	243	100	343	254	105	359	95.54	32	9	41	33	11	44	93.18
1705213000	San Teodoro	60	31	91	65	33	98	92.86	0	0	0	0	0	0	0.0
1705214000	Socorro	112	66	178	123	71	194	91.75	0	0	0	0	0	0	0.0
1705215000	Victoria	195	134	329	224	150	374	87.97	0	0	0	1	0	1	0.0
**1705300000**	**Palawan**	**1,750**	**1,031**	**2,781**	**1,964**	**1,115**	**3,079**	**90.32**	**27**	**13**	**40**	**36**	**17**	**53**	**75.47**
1705301000	Aborlan	76	39	115	80	39	119	96.64	0	0	0	0	0	0	0.0
1705302000	Agutaya	22	21	43	24	23	47	91.49	0	0	0	0	0	0	0.0
1705303000	Araceli	72	49	121	76	50	126	96.03	0	0	0	0	0	0	0.0
1705304000	Balabac	36	24	60	41	28	69	86.96	0	0	0	0	0	0	0.0
1705305000	Bataraza	172	125	297	185	126	311	95.50	0	0	0	0	0	0	0.0
1705306000	Brooke'S Point	239	79	318	251	83	334	95.21	5	0	5	6	1	7	71.43
1705307000	Busuanga	38	31	69	43	33	76	90.79	0	0	0	0	0	0	0.0
1705308000	Cagayancillo	10	7	17	11	8	19	89.47	0	0	0	0	0	0	0.0
1705309000	Coron	128	83	211	150	94	244	86.48	9	8	17	16	11	27	62.96
1705310000	Cuyo	33	10	43	38	10	48	89.58	0	0	0	0	0	0	0.0
1705311000	Dumaran	31	33	64	34	38	72	88.89	0	0	0	0	0	0	0.0
1705312000	El Nido	90	54	144	109	57	166	86.75	0	0	0	0	0	0	0.0
1705313000	Linapacan	14	10	24	14	10	24	100.00	0	0	0	0	0	0	0.0
1705314000	Magsaysay	14	15	29	18	19	37	78.38	0	0	0	0	0	0	0.0
1705315000	Narra	196	94	290	212	101	313	92.65	4	1	5	4	1	5	100.0
1705317000	Quezon	87	56	143	96	63	159	89.94	0	0	0	0	0	0	0.0
1705318000	Roxas	112	62	174	143	73	216	80.56	0	0	0	0	0	0	0.0
1705319000	San Vicente	51	49	100	61	54	115	86.96	0	0	0	0	0	0	0.0
1705320000	Taytay	140	58	198	157	65	222	89.19	9	4	13	10	4	14	92.86
1705321000	Kalayaan	0	0	0	0	0	0	0.00	0	0	0	0	0	0	0.00
1705322000	Culion	73	55	128	87	59	146	87.67	0	0	0	0	0	0	0.0
1705323000	Rizal	66	44	110	70	45	115	95.65	0	0	0	0	0	0	0.0
1705324000	Sofronio Española	50	33	83	64	37	101	82.18	0	0	0	0	0	0	0.0
**1705900000**	**Romblon**	**902**	**472**	**1,374**	**971**	**513**	**1,484**	**92.59**	**11**	**7**	**18**	**12**	**8**	**20**	**90.00**
1705901000	Alcantara	51	27	78	61	35	96	81.25	0	0	0	0	0	0	0.0
1705902000	Banton	11	8	19	12	8	20	95.00	0	0	0	0	0	0	0.0
1705903000	Cajidiocan	71	39	110	71	39	110	100.00	0	0	0	0	0	0	0.0
1705904000	Calatrava	21	8	29	25	10	35	82.86	0	0	0	0	0	0	0.0
1705905000	Concepcion	22	11	33	26	11	37	89.19	0	0	0	0	0	0	0.0
1705906000	Corcuera	30	18	48	33	18	51	94.12	0	0	0	0	0	0	0.0
1705907000	Looc	63	20	83	71	21	92	90.22	0	0	0	0	0	0	0.0
1705908000	Magdiwang	50	37	87	58	43	101	86.14	0	0	0	0	0	0	0.0
1705909000	Odiongan	138	56	194	144	56	200	97.00	11	7	18	12	8	20	90.00
1705910000	Romblon	156	85	241	161	91	252	95.63	0	0	0	0	0	0	0.0
1705911000	San Agustin	78	46	124	84	47	131	94.66	0	0	0	0	0	0	0.0
1705912000	San Andres	43	29	72	43	32	75	96.00	0	0	0	0	0	0	0.0
1705913000	San Fernando	76	42	118	85	50	135	87.41	0	0	0	0	0	0	0.0
1705914000	San Jose	25	10	35	26	11	37	94.59	0	0	0	0	0	0	0.0

Table 2.E.7.2 Tuberculosis Prevention and Control
Treatment Success Rate (TSR), DSTB-all forms and MDRTB
Philippines, 2024

PSGC	Area	TB all forms cured and Completely Treated			Registered TB all forms Registered during Specified Period			Treatment Success Rate	Registered Bacteriology Confirmed Drug Resistant			Registered Bacteriologically Confirmed Drug Resistant Specified Period			Treatment Success Rate
		Male	Female	Total	Male	Female	Total		Male	Female	Total	Male	Female	Total	
1705915000	Santa Fe	36	14	50	37	18	55	90.91	0	0	0	0	0	0	0.0
1705916000	Ferrol	11	13	24	13	14	27	88.89	0	0	0	0	0	0	0.0
1705917000	Santa Maria	20	9	29	21	9	30	96.67	0	0	0	0	0	0	0.0
**500000000**	**Region 5**	**15,704**	**8,548**	**24,252**	**17,230**	**9,204**	**26,434**	**91.75**	**262**	**129**	**391**	**336**	**168**	**504**	**77.58**
500500000	**Albay**	3,628	1,920	5,548	4,104	2,143	6,247	88.81	74	43	117	97	57	154	75.97
500501000	Bacacay	173	121	294	188	125	313	93.93	0	0	0	2	0	2	0.0
500502000	Camalig	180	108	288	180	108	288	100.00	0	0	0	0	0	0	0.0
500503000	Daraga	401	209	610	412	213	625	97.60	40	31	71	52	39	91	78.02
500504000	Guinobatan	158	98	256	163	99	262	97.71	0	0	0	1	0	1	0.0
500505000	Jovellar	30	15	45	30	15	45	100.00	0	0	0	0	0	0	0.0
**500506000**	**City of Legazpi**	**598**	**301**	**899**	**887**	**451**	**1,338**	**67.19**	**0**	**0**	**0**	**5**	**3**	**8**	**0.0**
500507000	Libon	212	96	308	223	102	325	94.77	0	0	0	0	0	0	0.0
**500508000**	**City of Ligao**	**282**	**141**	**423**	**366**	**184**	**550**	**76.91**	**34**	**12**	**46**	**36**	**13**	**49**	**93.88**
500509000	Malilipot	141	102	243	145	103	248	97.98	0	0	0	0	0	0	0.0
500510000	Malinao	138	66	204	145	67	212	96.23	0	0	0	0	0	0	0.0
500511000	Manito	86	49	135	91	50	141	95.74	0	0	0	0	0	0	0.0
500512000	Oas	126	67	193	133	68	201	96.02	0	0	0	0	0	0	0.0
500513000	Pio Duran	114	76	190	123	79	202	94.06	0	0	0	0	0	0	0.0
500514000	Polangui	199	87	286	203	88	291	98.28	0	0	0	0	0	0	0.0
500515000	Rapu-Rapu	47	20	67	50	20	70	95.71	0	0	0	1	2	3	0.0
500516000	Santo Domingo	115	56	171	118	57	175	97.71	0	0	0	0	0	0	0.0
**500517000**	**City of Tabaco**	**418**	**196**	**614**	**437**	**201**	**638**	**96.24**	**0**	**0**	**0**	**0**	**0**	**0**	**0.0**
500518000	Tiwi	210	112	322	210	113	323	99.69	0	0	0	0	0	0	0.0
501600000	Camarines Norte	1,596	699	2,295	1,737	745	2,482	92.47	52	11	63	59	14	73	86.30
501601000	Basud	83	47	130	86	47	133	97.74	52	11	63	59	14	73	86.30
501602000	Capalonga	49	22	71	55	26	81	87.65	0	0	0	0	0	0	0.0
501603000	Daet	232	150	382	258	164	422	90.52	0	0	0	0	0	0	0.0
501604000	San Lorenzo Ruiz	29	17	46	29	17	46	100.00	0	0	0	0	0	0	0.0
501605000	Jose Panganiban	251	86	337	269	93	362	93.09	0	0	0	0	0	0	0.0
501606000	Labo	214	89	303	237	90	327	92.66	0	0	0	0	0	0	0.0
501607000	Mercedes	152	76	228	165	81	246	92.68	0	0	0	0	0	0	0.0
501608000	Paracale	300	74	374	325	80	405	92.35	0	0	0	0	0	0	0.0
501609000	San Vicente	15	3	18	16	4	20	90.00	0	0	0	0	0	0	0.0
501610000	Santa Elena	92	48	140	102	48	150	93.33	0	0	0	0	0	0	0.0
501611000	Talisay	58	21	79	63	23	86	91.86	0	0	0	0	0	0	0.0
501612000	Vinzons	121	66	187	132	72	204	91.67	0	0	0	0	0	0	0.0
501700000	Camarines Sur	5,372	3,080	8,452	5,887	3,307	9,194	91.93	73	48	121	91	57	148	81.76
501701000	Baao	195	100	295	205	108	313	94.25	0	0	0	0	0	0	0.0
501702000	Balatan	102	57	159	106	58	164	96.95	0	0	0	0	0	0	0.0
501703000	Bato	67	38	105	79	44	123	85.37	0	0	0	0	0	0	0.0
501704000	Bombon	44	37	81	47	39	86	94.19	0	0	0	0	0	0	0.0
501705000	Buhi	201	125	326	237	140	377	86.47	1	0	1	1	0	1	100.0
501706000	Bula	112	57	169	125	61	186	90.86	0	1	1	0	1	1	100.0
501707000	Cabusao	63	45	108	70	48	118	91.53	18	6	24	20	8	28	85.71
501708000	Calabanga	213	154	367	251	168	419	87.59	0	0	0	1	0	1	0.0
501709000	Camaligan	76	51	127	77	51	128	99.22	0	0	0	0	0	0	0.0
501710000	Canaman	59	38	97	76	44	120	80.83	0	0	0	0	0	0	0.0
501711000	Caramoan	137	78	215	145	83	228	94.30	0	0	0	1	0	1	0.0
501712000	Del Gallego	66	25	91	74	31	105	86.67	1	0	1	1	0	1	100.0
501713000	Gainza	48	48	96	51	53	104	92.31	1	0	1	1	0	1	100.0
501714000	Garchitorena	64	49	113	69	50	119	94.96	0	0	0	0	0	0	0.0
501715000	Goa	271	152	423	292	172	464	91.16	0	0	0	0	0	0	0.0
**501716000**	**City of Iriga**	**298**	**169**	**467**	**318**	**177**	**495**	**94.34**	**0**	**0**	**0**	**0**	**0**	**0**	**0.0**
501717000	Lagonoy	186	111	297	192	113	305	97.38	0	0	0	0	0	0	0.0
501718000	Libmanan	262	167	429	273	168	441	97.28	0	0	0	0	0	0	0.0
501719000	Lupi	74	52	126	81	54	135	93.33	0	0	0	0	0	0	0.0
501720000	Magarao	81	30	111	86	33	119	93.28	0	0	0	0	0	0	0.0
501721000	Milaor	98	52	150	104	56	160	93.75	0	0	0	0	0	0	0.0
501722000	Minalabac	153	55	208	167	56	223	93.27	0	0	0	0	0	0	0.0
501723000	Nabua	154	88	242	167	99	266	90.98	0	0	0	0	0	0	0.0
**501724000**	**City of Naga**	**610**	**342**	**952**	**658**	**358**	**1,016**	**93.70**	**52**	**40**	**92**	**66**	**47**	**113**	**81.42**
501725000	Ocampo	111	65	176	132	75	207	85.02	0	0	0	0	0	0	0.0
501726000	Pamplona	79	46	125	86	47	133	93.98	0	0	0	0	0	0	0.0
501727000	Pasacao	120	65	185	134	76	210	88.10	0	0	0	0	0	0	0.0
501728000	Pili	273	137	410	302	146	448	91.52	0	0	0	0	0	0	0.0
501729000	Presentacion	82	48	130	99	55	154	84.42	0	0	0	0	0	0	0.0
501730000	Ragay	125	73	198	140	79	219	90.41	0	0	0	0	0	0	0.0
501731000	Sagñay	110	68	178	124	76	200	89.00	0	0	0	0	0	0	0.0
501732000	San Fernando	119	71	190	131	75	206	92.23	0	0	0	0	0	0	0.0
501733000	San Jose	150	92	242	167	100	267	90.64	0	1	1	0	1	1	100.0
501734000	Sipocot	158	77	235	182	86	268	87.69	0	0	0	0	0	0	0.0
501735000	Siruma	52	21	73	53	22	75	97.33	0	0	0	0	0	0	0.0
501736000	Tigaon	171	82	253	177	87	264	95.83	0	0	0	0	0	0	0.0
501737000	Tinambac	188	115	303	210	119	329	92.10	0	0	0	0	0	0	0.0
**502000000**	**Catanduanes**	**673**	**385**	**1,058**	**733**	**412**	**1,145**	**92.40**	**7**	**6**	**13**	**9**	**10**	**19**	**68.42**
502001000	Bagamanoc	24	7	31	24	8	32	96.88	0	0	0	0	0	0	0.0
502002000	Baras	40	18	58	42	19	61	95.08	7	6	13	9	10	19	68.42
502003000	Bato	37	29	66	41	32	73	90.41	0	0	0	0	0	0	0.0
502004000	Caramoran	72	39	111	74	40	114	97.37	0	0	0	0	0	0	0.0
502005000	Gigmoto	20	4	24	20	4	24	100.00	0	0	0	0	0	0	0.0
502006000	Pandan	71	43	114	79	44	123	92.68	0	0	0	0	0	0	0.0
502007000	Panganiban	15	7	22	15	7	22	100.00	0	0	0	0	0	0	0.0
502008000	San Andres	89	66	155	97	71	168	92.26	0	0	0	0	0	0	0.0
502009000	San Miguel	44	19	63	46	21	67	94.03	0	0	0	0	0	0	0.0
502010000	Viga	66	37	103	68	38	106	97.17	0	0	0	0	0	0	0.0
502011000	Virac	195	116	311	227	128	355	87.61	0	0	0	0	0	0	0.0
**504100000**	**Masbate**	**2,316**	**1,317**	**3,633**	**2,486**	**1,388**	**3,874**	**93.78**	**21**	**9**	**30**	**31**	**14**	**45**	**66.67**
504101000	Aroroy	343	165	508	374	176	550	92.36	0	0	0	0	0	0	0.0
504102000	Baleno	58	30	88	61	31	92	95.65	0	0	0	0	0	0	0.0
504103000	Balud	57	28	85	70	43	113	75.22	0	0	0	0	0	0	0.0
504104000	Batuan	26	16	42	31	17	48	87.50	0	0	0	0	0	0	0.0
504105000	Cataingan	90	30	120	106	38	144	83.33	0	0	0	0	0	0	0.0
504106000	Cawayan	150	90	240	154	91	245	97.96	0	0	0	0	0	0	0.0
504107000	Claveria	71	30	101	77	32	109	92.66	0	0	0	0	0	0	0.0
504108000	Dimasalang	58	50	108	62	50	112	96.43	0	0	0	0	0	0	0.0
504109000	Esperanza	44	13	57	50	13	63	90.48	0	0	0	0	0	0	0.0
504110000	Mandaon	154	115	269	154	115	269	100.00	0	0	0	0	0	0	0.0
**504111000**	**City of Masbate**	**340**	**176**	**516**	**344**	**178**	**522**	**98.85**	**21**	**9**	**30**	**31**	**14**	**45**	**66.67**
504112000	Milagros	190	114	304	209	123	332	91.57	0	0	0	0	0	0	0.0
504113000	Mobo	51	35	86	51	35	86	100.00	0	0	0	0	0	0	0.0
504114000	Monreal	79	47	126	80	48	128	98.44	0	0	0	0	0	0	0.0
504115000	Palanas	81	58	139	81	58	139	100.00	0	0	0	0	0	0	0.0
504116000	Pio V. Corpuz	60	41	101	61	41	102	99.02	0	0	0	0	0	0	0.0
504117000	Placer	133	95	228	144	101	245	93.06	0	0	0	0	0	0	0.0
504118000	San Fernando	66	26	92	71	26	97	94.85	0	0	0	0	0	0	0.0
504119000	San Jacinto	53	30	83	57	32	89	93.26	0	0	0	0	0	0	0.0
504120000	San Pascual	94	49	143	106	53	159	89.94	0	0	0	0	0	0	0.0
504121000	Uson	118	79	197	143	87	230	85.65	0	0	0	0	0	0	0.0
**506200000**	**Sorsogon**	**2,119**	**1,147**	**3,266**	**2,283**	**1,209**	**3,492**	**93.53**	**35**	**12**	**47**	**49**	**16**	**65**	**72.31**
506202000	Barcelona	56	37	93	61	41	102	91.18	0	0	0	0	0	0	0.0
506203000	Bulan	309	164	473	316	167	483	97.93	0	0	0	1	0	1	0.0
506204000	Bulusan	52	36	88	61	39	100	88.00	0	0	0	0	0	0	0.0
506205000	Casiguran	87	40	127	96	44	140	90.71	0	0	0	0	0	0	0.0
506206000	Castilla	116	39	155	124	41	165	93.94	1	0	1	2	0	2	50.0
506207000	Donsol	95	54	149	107	63	170	87.65	0	0	0	0	0	0	0.0
506208000	Gubat	179	82	261	213	90	303	86.14	0	0	0	0	0	0	0.0

Table 2.E.7.2 Tuberculosis Prevention and Control
Treatment Success Rate (TSR), DSTB-all forms and MDRTB
Philippines, 2024

PSGC	Area	TB all forms cured and Completely Treated			Registered TB all forms Registered during Specified Period			Treatment Success Rate	Registered Bacteriology Confirmed Drug Resistant			Registered Bacteriologically Confirmed Drug Resistant Specified			Treatment Success Rate
		Male	Female	Total	Male	Female	Total		Male	Female	Total	Male	Female	Total	
506209000	Irosin	214	98	312	219	102	321	97.20	0	0	0	0	0	0	0.0
506210000	Juban	83	48	131	87	49	136	96.32	0	0	0	0	0	0	0.0
506211000	Magallanes	69	50	119	71	51	122	97.54	0	0	0	0	0	0	0.0
506212000	Matnog	95	59	154	103	65	168	91.67	0	0	0	0	1	1	0.0
506213000	Pilar	146	78	224	159	83	242	92.56	0	0	0	0	0	0	0.0
506214000	Prieto Diaz	64	52	116	72	55	127	91.34	0	0	0	0	0	0	0.0
506215000	Santa Magdalena	39	17	56	42	17	59	94.92	0	0	0	1	0	1	0.0
**506216000**	**City of Sorsogon**	**515**	**293**	**808**	**552**	**302**	**854**	**94.61**	**34**	**12**	**46**	**45**	**15**	**60**	**76.67**
**600000000**	**Region 6**	**24,643**	**12,133**	**36,776**	**25,850**	**12,659**	**38,509**	**95.50**	**331**	**106**	**437**	**402**	**135**	**537**	**81.38**
**1830200000**	**City of Bacolod**	**2,153**	**1,101**	**3,254**	**2,314**	**1,179**	**3,493**	**93.16**	**42**	**12**	**54**	**58**	**17**	**75**	**72.00**
**631000000**	**City of Iloilo**	**1,779**	**908**	**2,687**	**1,900**	**977**	**2,877**	**93.40**	**45**	**13**	**58**	**55**	**18**	**73**	**79.45**
**600400000**	**Aklan**	**1,554**	**761**	**2,315**	**1,645**	**808**	**2,453**	**94.37**	**20**	**7**	**27**	**28**	**11**	**39**	**69.23**
600401000	Altavas	62	35	97	70	40	110	88.18	0	1	1	0	1	1	100.0
600402000	Balete	47	27	74	49	30	79	93.67	0	0	0	0	0	0	0.0
600403000	Banga	69	26	95	73	27	100	95.00	0	0	0	0	0	0	0.0
600404000	Batan	43	15	58	43	15	58	100.00	0	0	0	0	0	0	0.0
600405000	Buruanga	51	28	79	52	30	82	96.34	0	1	1	0	1	1	100.0
600406000	Ibajay	122	42	164	125	43	168	97.62	1	0	1	1	0	1	100.0
600407000	Kalibo	395	189	584	438	216	654	89.30	11	3	14	18	5	23	60.87
600408000	Lezo	28	17	45	28	17	45	100.00	1	0	1	1	0	1	100.0
600409000	Libacao	41	19	60	41	19	60	100.00	0	0	0	0	0	0	0.0
600410000	Madalag	37	33	70	38	33	71	98.59	0	0	0	0	0	0	0.0
600411000	Makato	82	39	121	82	39	121	100.00	0	0	0	0	0	0	0.0
600412000	Malay	150	75	225	165	78	243	92.59	0	0	0	0	0	0	0.0
600413000	Malinao	48	19	67	51	20	71	94.37	0	0	0	0	1	1	0.0
600414000	Nabas	96	57	153	99	59	158	96.84	6	1	7	7	1	8	87.50
600415000	New Washington	141	54	195	144	55	199	97.99	1	1	2	1	2	3	66.67
600416000	Numancia	112	73	185	115	73	188	98.40	0	0	0	0	0	0	0.0
600417000	Tangalan	30	13	43	32	14	46	93.48	0	0	0	0	0	0	0.0
**600600000**	**Antique**	**2,020**	**995**	**3,015**	**2,146**	**1,035**	**3,181**	**94.78**	**21**	**9**	**30**	**25**	**13**	**38**	**78.95**
600601000	Anini-Y	58	21	79	60	21	81	97.53	0	0	0	0	0	0	0.0
600602000	Barbaza	63	37	100	63	37	100	100.00	0	0	0	0	0	0	0.0
600603000	Belison	43	19	62	51	22	73	84.93	0	0	0	0	0	0	0.0
600604000	Bugasong	113	58	171	113	59	172	99.42	0	1	1	0	1	1	100.0
600605000	Caluya	92	44	136	100	47	147	92.52	0	0	0	0	0	0	0.0
600606000	Culasi	156	92	248	174	100	274	90.51	0	1	1	1	1	2	50.0
600607000	Tobias Fornier	89	37	126	105	40	145	86.90	0	0	0	0	0	0	0.0
600608000	Hamtic	143	94	237	152	97	249	95.18	1	1	2	1	1	2	100.0
600609000	Laua-An	145	66	211	157	68	225	93.78	0	0	0	1	0	1	0.0
600610000	Libertad	76	33	109	79	33	112	97.32	0	0	0	0	0	0	0.0
600611000	Pandan	99	41	140	101	42	143	97.90	0	0	0	0	1	1	0.0
600612000	Patnongon	114	60	174	116	61	177	98.31	0	0	0	0	0	0	0.0
600613000	San Jose	287	109	396	298	112	410	96.59	18	4	22	19	5	24	91.67
600614000	San Remigio	123	74	197	125	74	199	98.99	0	0	0	0	0	0	0.0
600615000	Sebaste	67	34	101	72	36	108	93.52	1	2	3	2	3	5	60.0
600616000	Sibalom	206	98	304	228	103	331	91.84	0	0	0	0	1	1	0.0
600617000	Tibiao	77	39	116	77	39	116	100.00	0	0	0	0	0	0	0.0
600618000	Valderrama	69	39	108	75	44	119	90.76	1	0	1	1	0	1	100.0
**601900000**	**Capiz**	**2,166**	**1,142**	**3,308**	**2,273**	**1,193**	**3,466**	**95.44**	**38**	**14**	**52**	**42**	**17**	**59**	**88.14**
601901000	Cuartero	45	12	57	51	12	63	90.48	0	0	0	0	0	0	0.0
601902000	Dao	71	38	109	79	39	118	92.37	1	0	1	1	0	1	100.0
601903000	Dumalag	69	23	92	70	23	93	98.92	0	0	0	0	0	0	0.0
601904000	Dumarao	144	95	239	149	96	245	97.55	1	0	1	1	0	1	100.0
601905000	Ivisan	49	30	79	49	30	79	100.00	0	0	0	0	0	0	0.0
601906000	Jamindan	75	23	98	77	23	100	98.00	0	0	0	0	0	0	0.0
601907000	Ma-Ayon	86	44	130	92	44	136	95.59	0	1	1	0	1	1	100.0
601908000	Mambusao	73	21	94	76	22	98	95.92	1	0	1	1	0	1	100.0
601909000	Panay	73	37	110	76	37	113	97.35	2	1	3	2	1	3	100.0
601910000	Panitan	59	19	78	61	19	80	97.50	0	0	0	0	0	0	0.0
601911000	Pilar	132	82	214	132	82	214	100.00	0	1	1	0	1	1	100.0
601912000	Pontevedra	91	39	130	92	39	131	99.24	0	0	0	1	0	1	0.0
601913000	President Roxas	75	28	103	75	29	104	99.04	0	0	0	0	0	0	0.0
**601914000**	**City of Roxas**	**868**	**524**	**1,392**	**927**	**564**	**1,491**	**93.36**	**32**	**11**	**43**	**35**	**14**	**49**	**87.76**
601915000	Sapi-An	83	31	114	83	31	114	100.00	0	0	0	0	0	0	0.0
601916000	Sigma	83	56	139	92	61	153	90.85	1	0	1	1	0	1	100.0
601917000	Tapaz	90	40	130	92	42	134	97.01	0	0	0	0	0	0	0.0
**603000000**	**Iloilo**	**5,400**	**2,560**	**7,960**	**5,610**	**2,647**	**8,257**	**96.40**	**40**	**11**	**51**	**46**	**11**	**57**	**89.47**
603001000	Ajuy	112	55	167	112	55	167	100.00	0	1	1	0	1	1	100.0
603002000	Alimodian	130	63	193	137	64	201	96.02	1	0	1	1	0	1	100.0
603003000	Anilao	71	27	98	73	27	100	98.00	0	0	0	0	0	0	0.0
603004000	Badiangan	54	17	71	57	18	75	94.67	0	0	0	0	0	0	0.0
603005000	Balasan	127	67	194	136	68	204	95.10	12	2	14	16	2	18	77.78
603006000	Banate	105	50	155	108	53	161	96.27	0	0	0	0	0	0	0.0
603007000	Barotac Nuevo	187	96	283	194	98	292	96.92	0	0	0	0	0	0	0.0
603008000	Barotac Viejo	217	95	312	217	95	312	100.00	0	0	0	0	0	0	0.0
603009000	Batad	76	29	105	76	30	106	99.06	0	0	0	0	0	0	0.0
603010000	Bingawan	43	14	57	44	14	58	98.28	0	0	0	1	0	1	0.0
603012000	Cabatuan	179	87	266	179	87	266	100.00	1	0	1	1	0	1	100.0
603013000	Calinog	128	92	220	129	93	222	99.10	0	0	0	0	0	0	0.0
603014000	Carles	150	62	212	153	65	218	97.25	0	0	0	0	0	0	0.0
603015000	Concepcion	121	44	165	132	48	180	91.67	1	0	1	1	0	1	100.0
603016000	Dingle	111	59	170	111	59	170	100.00	0	0	0	0	0	0	0.0
603017000	Dueñas	109	47	156	110	47	157	99.36	0	1	1	0	1	1	100.0
603018000	Dumangas	210	74	284	217	79	296	95.95	0	0	0	0	0	0	0.0
603019000	Estancia	120	57	177	141	63	204	86.76	1	0	1	1	0	1	100.0
603020000	Guimbal	108	69	177	109	69	178	99.44	8	1	9	9	1	10	90.00
603021000	Igbaras	75	24	99	79	27	106	93.40	0	0	0	0	0	0	0.0
603023000	Janiuay	134	68	202	147	72	219	92.24	2	0	2	2	0	2	100.0
603025000	Lambunao	175	70	245	179	74	253	96.84	0	0	0	0	0	0	0.0
603026000	Leganes	95	36	131	97	36	133	98.50	0	0	0	0	0	0	0.0
603027000	Lemery	107	54	161	107	54	161	100.00	0	0	0	0	0	0	0.0
603028000	Leon	121	69	190	129	75	204	93.14	0	0	0	0	0	0	0.0
603029000	Maasin	89	55	144	90	57	147	97.96	0	0	0	0	0	0	0.0
603030000	Miagao	145	66	211	145	66	211	100.00	2	0	2	2	0	2	100.0
603031000	Mina	64	41	105	67	41	108	97.22	0	0	0	0	0	0	0.0
603032000	New Lucena	42	26	68	43	27	70	97.14	0	0	0	0	0	0	0.0
603034000	Oton	238	106	344	238	106	344	100.00	0	1	1	0	1	1	100.0
**603035000**	**City of Passi**	**335**	**146**	**481**	**360**	**155**	**515**	**93.40**	**8**	**5**	**13**	**8**	**5**	**13**	**100.0**
603036000	Pavia	164	96	260	164	96	260	100.00	1	0	1	1	0	1	100.0
603037000	Pototan	289	112	401	307	118	425	94.35	0	0	0	0	0	0	0.0
603038000	San Dionisio	111	45	156	117	48	165	94.55	0	0	0	0	0	0	0.0
603039000	San Enrique	113	36	149	113	36	149	100.00	0	0	0	0	0	0	0.0
603040000	San Joaquin	142	79	221	159	84	243	90.95	0	0	0	0	0	0	0.0
603041000	San Miguel	58	30	88	60	32	92	95.65	1	0	1	1	0	1	100.0
603042000	San Rafael	36	18	54	38	19	57	94.74	0	0	0	0	0	0	0.0
603043000	Santa Barbara	148	90	238	164	95	259	91.89	0	0	0	0	0	0	0.0
603044000	Sara	120	66	186	122	68	190	97.89	1	0	1	1	0	1	100.0
603045000	Tigbauan	133	86	219	133	87	220	99.55	0	0	0	0	1	1	0.0
603046000	Tubungan	28	12	40	29	13	42	95.24	1	0	1	1	0	1	100.0
603047000	Zarraga	80	25	105	88	29	117	89.74	0	0	0	0	0	0	0.0
**1804500000**	**Negros Occidental**	**9,169**	**4,489**	**13,658**	**9,545**	**4,633**	**14,178**	**96.33**	**125**	**39**	**164**	**148**	**47**	**195**	**84.10**
**1804502000**	**City of Bago**	**981**	**463**	**1,444**	**994**	**466**	**1,460**	**98.90**	**1**	**1**	**2**	**3**	**1**	**4**	**50.0**
1804503000	Binalbagan	266	127	393	293	131	424	92.69	0	1	1	0	1	1	100.0
**1804504000**	**City of Cadiz**	**591**	**266**	**857**	**627**	**275**	**902**	**95.01**	**18**	**3**	**21**	**23**	**5**	**28**	**75.00**
1804505000	Calatrava	228	92	320	232	97	329	97.26	1	0	1	1	0	1	100.0
1804506000	Candoni	64	36	100	65	36	101	99.01	0	0	0	0	0	0	0.0

Table 2.E.7.2 Tuberculosis Prevention and Control
Treatment Success Rate (TSR), DSTB-all forms and MDRTB
Philippines, 2024

PSGC	Area	TB all forms cured and Completely Treated			Registered TB all forms Registered during Specified Period			Treatment Success Rate	Registered Bacteriology Confirmed Drug Resistant			Registered Bacteriologically Confirmed Drug Resistant Specified Period			Treatment Success Rate
		Male	Female	Total	Male	Female	Total		Male	Female	Total	Male	Female	Total	
1804507000	Cauayan	322	176	498	324	177	501	99.40	2	0	2	2	0	2	100.0
1804508000	Enrique B. Magalona	198	80	278	207	83	290	95.86	0	0	0	0	0	0	0.0
1804509000	City of Escalante	250	103	353	252	103	355	99.44	0	0	0	0	0	0	0.0
1804510000	City of Himamaylan	405	236	641	416	239	655	97.86	3	0	3	3	0	3	100.0
1804511000	Hinigaran	327	184	511	339	189	528	96.78	1	2	3	1	2	3	100.0
1804512000	Hinoba-an	174	73	247	188	83	271	91.14	0	0	0	0	0	0	0.0
1804513000	Ilog	137	71	208	148	75	223	93.27	1	0	1	1	0	1	100.0
1804514000	Isabela	259	152	411	261	153	414	99.28	0	0	0	0	0	0	0.0
1804515000	City of Kabankalan	406	204	610	431	211	642	95.02	21	5	26	26	6	32	81.25
1804516000	City of La Carlota	304	148	452	326	158	484	93.39	35	11	46	37	12	49	93.88
1804517000	La Castellana	278	167	445	286	169	455	97.80	2	0	2	2	0	2	100.0
1804518000	Manapla	142	58	200	162	64	226	88.50	1	0	1	3	0	3	33.33
1804519000	Moises Padilla	202	109	311	204	109	313	99.36	0	0	0	0	0	0	0.0
1804520000	Murcia	373	202	575	387	214	601	95.67	1	0	1	1	0	1	100.0
1804521000	Pontevedra	257	106	363	267	108	375	96.80	0	0	0	2	0	2	0.0
1804522000	Pulupandan	170	83	253	178	84	262	96.56	0	1	1	0	2	2	50.0
1804523000	City of Sagay	538	220	758	552	228	780	97.18	2	2	4	2	2	4	100.0
1804524000	City of San Carlos	359	149	508	389	158	547	92.87	12	3	15	15	4	19	78.95
1804525000	San Enrique	108	69	177	117	73	190	93.16	0	0	0	0	0	0	0.0
1804526000	City of Silay	505	273	778	513	274	787	98.86	18	9	27	20	10	30	90.00
1804527000	City of Sipalay	225	86	311	229	93	322	96.58	0	0	0	0	0	0	0.0
1804528000	City of Talisay	354	178	532	377	188	565	94.16	3	0	3	3	0	3	100.0
1804529000	Toboso	119	66	185	130	73	203	91.13	1	0	1	1	1	2	50.0
1804530000	Valladolid	151	71	222	160	75	235	94.47	2	1	3	2	1	3	100.0
1804531000	City of Victorias	427	208	635	440	214	654	97.09	0	0	0	0	0	0	0.0
1804532000	Salvador Benedicto	49	33	82	51	33	84	97.62	0	0	0	0	0	0	0.0
607900000	Guimaras	402	177	579	417	187	604	95.86	0	1	1	0	1	1	100.0
607901000	Buenavista	106	44	150	111	48	159	94.34	0	0	0	0	0	0	0.0
607902000	Jordan	81	35	116	84	38	122	95.08	0	0	0	0	0	0	0.0
607903000	Nueva Valencia	131	60	191	133	60	193	98.96	0	1	1	0	1	1	100.0
607904000	San Lorenzo	50	15	65	52	17	69	94.20	0	0	0	0	0	0	0.0
607905000	Sibunag	34	23	57	37	24	61	93.44	0	0	0	0	0	0	0.0
700000000	Region 7	17,362	8,478	25,840	19,472	9,397	28,869	89.51	190	64	254	270	102	372	68.28
730600000	City of Cebu	3,090	1,452	4,542	3,876	1,824	5,700	79.68	77	16	93	113	41	154	60.39
731100000	City of Lapu-Lapu	1,361	653	2,014	1,503	702	2,205	91.34	0	0	0	0	0	0	0.0
731300000	City of Mandaue	932	418	1,350	1,108	488	1,596	84.59	48	28	76	70	38	108	70.37
701200000	Bohol	2,469	1,261	3,730	2,674	1,356	4,030	92.56	37	10	47	52	11	63	74.60
701201000	Alburquerque	23	10	33	25	11	36	91.67	0	0	0	0	0	0	0.0
701202000	Alicia	58	36	94	68	36	104	90.38	0	0	0	0	0	0	0.0
701203000	Anda	61	29	90	63	32	95	94.74	0	0	0	0	0	0	0.0
701204000	Antequera	23	10	33	26	12	38	86.84	0	0	0	0	0	0	0.0
701205000	Baclayon	37	16	53	39	17	56	94.64	0	0	0	0	0	0	0.0
701206000	Balilihan	33	14	47	37	15	52	90.38	0	0	0	0	0	0	0.0
701207000	Batuan	22	17	39	22	18	40	97.50	0	0	0	0	0	0	0.0
701208000	Bilar	34	8	42	35	8	43	97.67	0	0	0	0	0	0	0.0
701209000	Buenavista	38	24	62	39	26	65	95.38	0	0	0	0	0	0	0.0
701210000	Calape	38	19	57	49	20	69	82.61	0	0	0	0	0	0	0.0
701211000	Candijay	75	43	118	86	47	133	88.72	0	0	0	0	0	0	0.0
701212000	Carmen	58	29	87	68	32	100	87.00	0	0	0	0	0	0	0.0
701213000	Catigbian	42	16	58	42	16	58	100.00	0	0	0	0	0	0	0.0
701214000	Clarin	24	9	33	27	10	37	89.19	0	0	0	0	0	0	0.0
701215000	Corella	6	3	9	8	3	11	81.82	0	0	0	0	0	0	0.0
701216000	Cortes	15	9	24	15	11	26	92.31	0	0	0	0	0	0	0.0
701217000	Dagohoy	24	10	34	24	11	35	97.14	0	0	0	0	0	0	0.0
701218000	Danao	37	22	59	40	22	62	95.16	0	0	0	0	0	0	0.0
701219000	Dauis	92	37	129	102	40	142	90.85	0	0	0	0	0	0	0.0
701220000	Dimiao	22	13	35	22	13	35	100.00	0	0	0	0	0	0	0.0
701221000	Duero	33	11	44	34	14	48	91.67	0	0	0	0	0	0	0.0
701222000	Garcia Hernandez	25	15	40	30	16	46	86.96	0	0	0	0	0	0	0.0
701223000	Guindulman	41	30	71	41	31	72	98.61	0	0	0	0	0	0	0.0
701224000	Inabanga	75	69	144	79	70	149	96.64	0	0	0	0	0	0	0.0
701225000	Jagna	40	22	62	44	29	73	84.93	0	0	0	0	0	0	0.0
701226000	Getafe	52	20	72	57	23	80	90.00	0	0	0	0	0	0	0.0
701227000	Lila	22	8	30	25	10	35	85.71	0	0	0	0	0	0	0.0
701228000	Loay	15	11	26	16	12	28	92.86	0	0	0	0	0	0	0.0
701229000	Loboc	18	5	23	18	5	23	100.00	0	0	0	0	0	0	0.0
701230000	Loon	48	24	72	56	27	83	86.75	0	0	0	0	0	0	0.0
701231000	Mabini	61	27	88	64	29	93	94.62	0	0	0	0	0	0	0.0
701232000	Maribojoc	30	13	43	30	13	43	100.00	0	0	0	0	0	0	0.0
701233000	Panglao	65	29	94	67	30	97	96.91	0	0	0	0	0	0	0.0
701234000	Pilar	43	23	66	46	26	72	91.67	0	0	0	0	0	0	0.0
701235000	President Carlos P. Garcia	39	19	58	44	21	65	89.23	0	0	0	0	0	0	0.0
701236000	Sagbayan	34	30	64	40	31	71	90.14	0	0	0	0	0	0	0.0
701237000	San Isidro	16	13	29	16	14	30	96.67	0	0	0	0	0	0	0.0
701238000	San Miguel	47	17	64	49	20	69	92.75	0	0	0	0	0	0	0.0
701239000	Sevilla	13	10	23	14	10	24	95.83	0	0	0	0	0	0	0.0
701240000	Sierra Bullones	64	35	99	68	38	106	93.40	0	0	0	0	0	0	0.0
701241000	Sikatuna	9	12	21	9	12	21	100.00	0	0	0	0	0	0	0.0
701242000	City of Tagbilaran	354	155	509	398	176	574	88.68	25	6	31	35	6	41	75.61
701243000	Talibon	165	90	255	175	92	267	95.51	0	0	0	0	0	0	0.0
701244000	Trinidad	47	25	72	50	26	76	94.74	0	0	0	0	0	0	0.0
701245000	Tubigon	75	37	112	78	38	116	96.55	0	0	0	0	0	0	0.0
701246000	Ubay	205	106	311	215	110	325	95.69	12	4	16	17	5	22	72.73
701247000	Valencia	21	11	32	23	12	35	91.43	0	0	0	0	0	0	0.0
701248000	Bien Unido	50	20	70	51	21	72	97.22	0	0	0	0	0	0	0.0
702200000	Cebu	6,291	3,232	9,523	6,816	3,453	10,269	92.74	0	0	0	0	0	0	0.0
702201000	Alcantara	34	17	51	39	21	60	85.00	0	0	0	0	0	0	0.0
702202000	Alcoy	26	11	37	29	12	41	90.24	0	0	0	0	0	0	0.0
702203000	Alegria	22	12	34	26	13	39	87.18	0	0	0	0	0	0	0.0
702204000	Aloguinsan	49	18	67	51	21	72	93.06	0	0	0	0	0	0	0.0
702205000	Argao	104	56	160	107	59	166	96.39	0	0	0	0	0	0	0.0
702206000	Asturias	77	35	112	81	37	118	94.92	0	0	0	0	0	0	0.0
702207000	Badian	110	56	166	114	61	175	94.86	0	0	0	0	0	0	0.0
702208000	Balamban	167	77	244	176	81	257	94.94	0	0	0	0	0	0	0.0
702209000	Bantayan	198	124	322	208	131	339	94.99	0	0	0	0	0	0	0.0
702210000	Barili	118	69	187	124	71	195	95.90	0	0	0	0	0	0	0.0
702211000	City of Bogo	178	77	255	186	80	266	95.86	0	0	0	0	0	0	0.0
702212000	Boljoon	34	10	44	36	15	51	86.27	0	0	0	0	0	0	0.0
702213000	Borbon	76	53	129	80	53	133	96.99	0	0	0	0	0	0	0.0
702214000	City of Carcar	222	86	308	241	93	334	92.22	0	0	0	0	0	0	0.0
702215000	Carmen	73	35	108	86	42	128	84.38	0	0	0	0	0	0	0.0
702216000	Catmon	41	17	58	44	19	63	92.06	0	0	0	0	0	0	0.0
702218000	Compostela	114	55	169	122	59	181	93.37	0	0	0	0	0	0	0.0
702219000	Consolacion	317	172	489	323	172	495	98.79	0	0	0	0	0	0	0.0
702220000	Cordova	182	73	255	185	76	261	97.70	0	0	0	0	0	0	0.0
702221000	Daanbantayan	179	88	267	189	90	279	95.70	0	0	0	0	0	0	0.0
702222000	Dalaguete	97	38	135	108	42	150	90.00	0	0	0	0	0	0	0.0
702223000	Danao City	374	211	585	398	229	627	93.30	0	0	0	0	0	0	0.0
702224000	Dumanjug	112	65	177	122	71	193	91.71	0	0	0	0	0	0	0.0
702225000	Ginatilan	28	7	35	33	8	41	85.37	0	0	0	0	0	0	0.0
702227000	Liloan	216	113	329	224	119	343	95.92	0	0	0	0	0	0	0.0
702228000	Madridejos	78	35	113	85	35	120	94.17	0	0	0	0	0	0	0.0
702229000	Malabuyoc	18	7	25	20	7	27	92.59	0	0	0	0	0	0	0.0
702231000	Medellin	100	45	145	114	51	165	87.88	0	0	0	0	0	0	0.0
702232000	Minglanilla	318	160	478	333	164	497	96.18	0	0	0	0	0	0	0.0
702233000	Moalboal	84	46	130	87	51	138	94.20	0	0	0	0	0	0	0.0

Table 2.E.7.2 Tuberculosis Prevention and Control
Treatment Success Rate (TSR), DSTB-all forms and MDRTB
Philippines, 2024

PSGC	Area	TB all forms cured and Completely Treated			Registered TB all forms Registered during Specified Period			Treatment Success Rate	Registered Bacteriology Confirmed Drug Resistant			Registered Bacteriologically Confirmed Drug Resistant Specified Period			Treatment Success Rate
		Male	Female	Total	Male	Female	Total		Male	Female	Total	Male	Female	Total	
**702234000**	**City of Naga**	**191**	**112**	**303**	**253**	**129**	**382**	**79.32**	**0**	**0**	**0**	**0**	**0**	**0**	**0.0**
702235000	Oslob	62	37	99	75	44	119	83.19	0	0	0	0	0	0	0.0
702236000	Pilar	30	11	41	32	14	46	89.13	0	0	0	0	0	0	0.0
702237000	Pinamungajan	140	52	192	153	55	208	92.31	0	0	0	0	0	0	0.0
702238000	Poro	54	19	73	56	21	77	94.81	0	0	0	0	0	0	0.0
702239000	Ronda	46	29	75	50	29	79	94.94	0	0	0	0	0	0	0.0
702240000	Samboan	41	27	68	43	33	76	89.47	0	0	0	0	0	0	0.0
702241000	San Fernando	123	54	177	136	58	194	91.24	0	0	0	0	0	0	0.0
702242000	San Francisco	131	57	188	150	63	213	88.26	0	0	0	0	0	0	0.0
702243000	San Remigio	115	45	160	119	46	165	96.97	0	0	0	0	0	0	0.0
702244000	Santa Fe	75	51	126	85	52	137	91.97	0	0	0	0	0	0	0.0
702245000	Santander	40	82	122	45	87	132	92.42	0	0	0	0	0	0	0.0
702246000	Sibonga	55	23	78	60	26	86	90.70	0	0	0	0	0	0	0.0
702247000	Sogod	80	40	120	91	41	132	90.91	0	0	0	0	0	0	0.0
702248000	Tabogon	104	50	154	109	51	160	96.25	0	0	0	0	0	0	0.0
702249000	Tabuelan	53	27	80	60	28	88	90.91	0	0	0	0	0	0	0.0
**702250000**	**City of Talisay**	**633**	**397**	**1,030**	**717**	**432**	**1,149**	**89.64**	**0**	**0**	**0**	**0**	**0**	**0**	**0.0**
**702251000**	**City of Toledo**	**422**	**172**	**594**	**454**	**180**	**634**	**93.69**	**0**	**0**	**0**	**0**	**0**	**0**	**0.0**
702252000	Tuburan	121	69	190	125	71	196	96.94	0	0	0	0	0	0	0.0
702253000	Tudela	29	10	39	32	10	42	92.86	0	0	0	0	0	0	0.0
**1804600000**	**Negros Oriental**	**3,055**	**1,399**	**4,454**	**3,323**	**1,505**	**4,828**	**92.25**	**28**	**10**	**38**	**35**	**12**	**47**	**80.85**
1804601000	Amlan	49	12	61	57	15	72	84.72	0	0	0	0	0	0	0.0
1804602000	Ayungon	131	63	194	141	66	207	93.72	0	0	0	0	0	0	0.0
1804603000	Bacong	71	31	102	79	33	112	91.07	0	0	0	0	0	0	0.0
**1804604000**	**City of Bais**	**179**	**101**	**280**	**189**	**103**	**292**	**95.89**	**0**	**0**	**0**	**0**	**0**	**0**	**0.0**
1804605000	Basay	77	25	102	87	33	120	85.00	0	0	0	0	0	0	0.0
**1804606000**	**City of Bayawan**	**227**	**114**	**341**	**263**	**125**	**388**	**87.89**	**0**	**0**	**0**	**0**	**0**	**0**	**0.0**
1804607000	Bindoy	72	32	104	78	34	112	92.86	0	0	0	0	0	0	0.0
**1804608000**	**City of Canlaon**	**156**	**108**	**264**	**168**	**114**	**282**	**93.62**	**0**	**0**	**0**	**0**	**0**	**0**	**0.0**
1804609000	Dauin	45	26	71	51	29	80	88.75	0	0	0	0	0	0	0.0
**1804610000**	**City of Dumaguete**	**386**	**165**	**551**	**425**	**176**	**601**	**91.68**	**28**	**10**	**38**	**35**	**12**	**47**	**80.85**
**1804611000**	**City of Guihulngan**	**203**	**83**	**286**	**215**	**88**	**303**	**94.39**	**0**	**0**	**0**	**0**	**0**	**0**	**0.0**
1804612000	Jimalalud	36	7	43	40	8	48	89.58	0	0	0	0	0	0	0.0
1804613000	La Libertad	71	32	103	73	33	106	97.17	0	0	0	0	0	0	0.0
1804614000	Mabinay	216	73	289	251	88	339	85.25	0	0	0	0	0	0	0.0
1804615000	Manjuyod	54	26	80	56	28	84	95.24	0	0	0	0	0	0	0.0
1804616000	Pamplona	98	67	165	106	74	180	91.67	0	0	0	0	0	0	0.0
1804617000	San Jose	39	25	64	46	29	75	85.33	0	0	0	0	0	0	0.0
1804618000	Santa Catalina	166	73	239	175	80	255	93.73	0	0	0	0	0	0	0.0
1804619000	Siaton	193	63	256	198	65	263	97.34	0	0	0	0	0	0	0.0
1804620000	Sibulan	139	61	200	157	65	222	90.09	0	0	0	0	0	0	0.0
**1804621000**	**City of Tanjay**	**180**	**64**	**244**	**183**	**65**	**248**	**98.39**	**0**	**0**	**0**	**0**	**0**	**0**	**0.0**
1804622000	Tayasan	59	37	96	62	39	101	95.05	0	0	0	0	0	0	0.0
1804623000	Valencia	44	22	66	46	23	69	95.65	0	0	0	0	0	0	0.0
1804624000	Vallehermoso	99	59	158	107	61	168	94.05	0	0	0	0	0	0	0.0
1804625000	Zamboanguita	65	30	95	70	31	101	94.06	0	0	0	0	0	0	0.0
**1806100000**	**Siquijor**	**164**	**63**	**227**	**172**	**69**	**241**	**94.19**	**0**	**0**	**0**	**0**	**0**	**0**	**0.0**
1806101000	Enrique Villanueva	15	4	19	15	4	19	100.00	0	0	0	0	0	0	0.0
1806102000	Larena	14	5	19	15	6	21	90.48	0	0	0	0	0	0	0.0
1806103000	Lazi	33	10	43	35	11	46	93.48	0	0	0	0	0	0	0.0
1806104000	Maria	21	8	29	22	8	30	96.67	0	0	0	0	0	0	0.0
1806105000	San Juan	27	17	44	28	18	46	95.65	0	0	0	0	0	0	0.0
1806106000	Siquijor	54	19	73	57	22	79	92.41	0	0	0	0	0	0	0.0
**800000000**	**Region 8**	**8,431**	**4,466**	**12,897**	**9,186**	**4,825**	**14,011**	**92.05**	**155**	**41**	**196**	**184**	**57**	**241**	**81.33**
**803738000**	**Ormoc City**	**428**	**237**	**665**	**477**	**262**	**739**	**89.99**	**0**	**0**	**0**	**0**	**0**	**0**	**0.0**
**831600000**	**City of Tacloban**	**867**	**538**	**1,405**	**968**	**593**	**1,561**	**90.01**	**0**	**0**	**0**	**0**	**0**	**0**	**0.0**
**802600000**	**Eastern Samar**	**759**	**415**	**1,174**	**815**	**442**	**1,257**	**93.40**	**23**	**5**	**28**	**28**	**7**	**35**	**80.00**
802601000	Arteche	31	19	50	36	19	55	90.91	0	0	0	1	0	1	0.0
802602000	Balangiga	31	3	34	39	5	44	77.27	0	0	0	0	0	0	0.0
802603000	Balangkayan	11	12	23	12	12	24	95.83	0	0	0	0	0	0	0.0
**802604000**	**City of Borongan**	**140**	**79**	**219**	**142**	**79**	**221**	**99.10**	**23**	**5**	**28**	**26**	**7**	**33**	**84.85**
802605000	Can-Avid	41	16	57	42	17	59	96.61	0	0	0	0	0	0	0.0
802606000	Dolores	65	28	93	67	33	100	93.00	0	0	0	0	0	0	0.0
802607000	General Macarthur	16	11	27	21	11	32	84.38	0	0	0	0	0	0	0.0
802608000	Giporlos	9	7	16	10	11	21	76.19	0	0	0	0	0	0	0.0
802609000	Guiuan	66	25	91	75	31	106	85.85	0	0	0	0	0	0	0.0
802610000	Hernani	18	13	31	21	13	34	91.18	0	0	0	0	0	0	0.0
802611000	Jipapad	14	13	27	14	14	28	96.43	0	0	0	0	0	0	0.0
802612000	Lawaan	29	9	38	30	10	40	95.00	0	0	0	1	0	1	0.0
802613000	Llorente	27	13	40	31	13	44	90.91	0	0	0	0	0	0	0.0
802614000	Maslog	4	2	6	4	2	6	100.00	0	0	0	0	0	0	0.0
802615000	Maydolong	26	12	38	26	12	38	100.00	0	0	0	0	0	0	0.0
802616000	Mercedes	7	4	11	9	5	14	78.57	0	0	0	0	0	0	0.0
802617000	Oras	56	41	97	56	42	98	98.98	0	0	0	0	0	0	0.0
802618000	Quinapondan	17	8	25	21	9	30	83.33	0	0	0	0	0	0	0.0
802619000	Salcedo	28	21	49	30	21	51	96.08	0	0	0	0	0	0	0.0
802620000	San Julian	34	18	52	38	20	58	89.66	0	0	0	0	0	0	0.0
802621000	San Policarpo	20	11	31	22	11	33	93.94	0	0	0	0	0	0	0.0
802622000	Sulat	43	28	71	43	30	73	97.26	0	0	0	0	0	0	0.0
802623000	Taft	26	22	48	26	22	48	100.00	0	0	0	0	0	0	0.0
**803700000**	**Leyte**	**3,084**	**1,481**	**4,565**	**3,357**	**1,603**	**4,960**	**92.04**	**74**	**24**	**98**	**83**	**32**	**115**	**85.22**
803701000	Abuyog	170	72	242	186	81	267	90.64	0	0	0	0	0	0	0.0
803702000	Alangalang	161	88	249	161	88	249	100.00	0	0	0	0	0	0	0.0
803703000	Albuera	106	58	164	108	58	166	98.80	0	0	0	0	0	0	0.0
803705000	Babatngon	64	28	92	67	28	95	96.84	0	0	0	0	0	0	0.0
803706000	Barugo	80	28	108	80	28	108	100.00	0	0	0	0	0	0	0.0
803707000	Bato	58	24	82	59	24	83	98.80	0	0	0	0	0	0	0.0
**803708000**	**City of Baybay**	**179**	**84**	**263**	**211**	**102**	**313**	**84.03**	**0**	**0**	**0**	**0**	**0**	**0**	**0.0**
803710000	Burauen	57	24	81	65	29	94	86.17	0	0	0	0	0	0	0.0
803713000	Calubian	54	28	82	59	31	90	91.11	0	0	0	0	0	0	0.0
803714000	Capoocan	38	23	61	47	27	74	82.43	0	0	0	0	0	0	0.0
803715000	Carigara	99	33	132	109	37	146	90.41	0	0	0	0	0	0	0.0
803717000	Dagami	45	33	78	45	33	78	100.00	0	0	0	0	0	0	0.0
803718000	Dulag	67	46	113	71	48	119	94.96	0	0	0	0	0	0	0.0
803719000	Hilongos	99	39	138	102	41	143	96.50	0	0	0	0	0	0	0.0
803720000	Hindang	37	17	54	38	17	55	98.18	0	0	0	0	0	0	0.0
803721000	Inopacan	32	12	44	35	13	48	91.67	0	0	0	0	0	0	0.0
803722000	Isabel	87	22	109	94	23	117	93.16	0	0	0	0	0	0	0.0
803723000	Jaro	93	50	143	94	50	144	99.31	0	0	0	0	0	0	0.0
803724000	Javier	48	31	79	53	36	89	88.76	0	0	0	0	0	0	0.0
803725000	Julita	39	22	61	40	23	63	96.83	0	0	0	0	0	0	0.0
803726000	Kananga	100	39	139	122	42	164	84.76	0	0	0	0	0	0	0.0
803728000	La Paz	36	11	47	40	12	52	90.38	0	0	0	0	0	0	0.0
803729000	Leyte	55	20	75	64	23	87	86.21	0	0	0	0	0	0	0.0
803730000	Macarthur	39	17	56	46	17	63	88.89	0	0	0	0	0	0	0.0
803731000	Mahaplag	56	29	85	59	31	90	94.44	0	0	0	0	0	0	0.0
803733000	Matag-Ob	35	29	64	39	31	70	91.43	0	0	0	0	0	0	0.0
803734000	Matalom	61	46	107	67	50	117	91.45	0	0	0	0	0	0	0.0
803735000	Mayorga	34	20	54	34	20	54	100.00	0	0	0	0	0	0	0.0
803736000	Merida	41	10	51	41	12	53	96.23	0	0	0	0	0	0	0.0
803739000	Palo	146	47	193	146	47	193	100.00	74	24	98	83	32	115	85.22
803740000	Palompon	90	47	137	107	54	161	85.09	0	0	0	0	0	0	0.0
803741000	Pastrana	25	11	36	28	13	41	87.80	0	0	0	0	0	0	0.0
803742000	San Isidro	31	12	43	33	13	46	93.48	0	0	0	0	0	0	0.0
803743000	San Miguel	53	12	65	59	13	72	90.28	0	0	0	0	0	0	0.0
803744000	Santa Fe	46	27	73	47	28	75	97.33	0	0	0	0	0	0	0.0

Table 2.E.7.2 Tuberculosis Prevention and Control
Treatment Success Rate (TSR), DSTB-all forms and MDRTB
Philippines, 2024

PSGC	Area	TB all forms cured and Completely Treated			Registered TB all forms Registered during Specified Period			Treatment Success Rate	Registered Bacteriology Confirmed Drug Resistant			Registered Bacteriologically Confirmed Drug Resistant Specified Period			Treatment Success Rate
		Male	Female	Total	Male	Female	Total		Male	Female	Total	Male	Female	Total	
803745000	Tabango	47	27	74	51	28	79	93.67	0	0	0	0	0	0	0.0
803746000	Tabontabon	17	8	25	20	9	29	86.21	0	0	0	0	0	0	0.0
803748000	Tanauan	37	17	54	39	17	56	96.43	0	0	0	0	0	0	0.0
803749000	Tolosa	39	14	53	44	17	61	86.89	0	0	0	0	0	0	0.0
803750000	Tunga	14	12	26	14	12	26	100.00	0	0	0	0	0	0	0.0
803751000	Villaba	41	27	68	56	35	91	74.73	0	0	0	0	0	0	0.0
**804800000**	**Northern Samar**	**1,213**	**670**	**1,883**	**1,312**	**714**	**2,026**	**92.94**	**18**	**2**	**20**	**22**	**3**	**25**	**80.00**
804801000	Allen	57	22	79	65	24	89	88.76	0	0	0	0	0	0	0.0
804802000	Biri	18	8	26	18	9	27	96.30	0	0	0	0	0	0	0.0
804803000	Bobon	62	39	101	65	40	105	96.19	0	0	0	0	0	0	0.0
804804000	Capul	30	7	37	31	9	40	92.50	0	0	0	0	0	0	0.0
804805000	Catarman	277	164	441	302	176	478	92.26	18	2	20	22	3	25	80.00
804806000	Catubig	47	26	73	55	28	83	87.95	0	0	0	0	0	0	0.0
804807000	Gamay	37	35	72	47	38	85	84.71	0	0	0	0	0	0	0.0
804808000	Laoang	140	79	219	147	85	232	94.40	0	0	0	0	0	0	0.0
804809000	Lapinig	22	11	33	22	12	34	97.06	0	0	0	0	0	0	0.0
804810000	Las Navas	10	7	17	16	9	25	68.00	0	0	0	0	0	0	0.0
804811000	Lavezares	79	42	121	84	43	127	95.28	0	0	0	0	0	0	0.0
804812000	Mapanas	29	14	43	29	14	43	100.00	0	0	0	0	0	0	0.0
804813000	Mondragon	72	37	109	72	37	109	100.00	0	0	0	0	0	0	0.0
804814000	Palapag	50	25	75	55	27	82	91.46	0	0	0	0	0	0	0.0
804815000	Pambujan	32	25	57	38	28	66	86.36	0	0	0	0	0	0	0.0
804816000	Rosario	21	8	29	22	8	30	96.67	0	0	0	0	0	0	0.0
804817000	San Antonio	18	10	28	18	10	28	100.00	0	0	0	0	0	0	0.0
804818000	San Isidro	48	15	63	54	17	71	88.73	0	0	0	0	0	0	0.0
804819000	San Jose	24	14	38	25	15	40	95.00	0	0	0	0	0	0	0.0
804820000	San Roque	70	45	115	71	46	117	98.29	0	0	0	0	0	0	0.0
804821000	San Vicente	12	10	22	12	10	22	100.00	0	0	0	0	0	0	0.0
804822000	Silvino Lobos	12	11	23	15	11	26	88.46	0	0	0	0	0	0	0.0
804823000	Victoria	32	10	42	34	11	45	93.33	0	0	0	0	0	0	0.0
804824000	Lope De Vega	14	6	20	15	7	22	90.91	0	0	0	0	0	0	0.0
**806000000**	**Samar**	**1,216**	**660**	**1,876**	**1,336**	**719**	**2,055**	**91.29**	**15**	**4**	**19**	**22**	**7**	**29**	**65.52**
806001000	Almagro	11	4	15	12	4	16	93.75	0	0	0	0	0	0	0.0
806002000	Basey	66	32	98	70	34	104	94.23	0	0	0	0	0	0	0.0
**806003000**	**City of Calbayog**	**331**	**190**	**521**	**382**	**217**	**599**	**86.98**	**15**	**4**	**19**	**22**	**7**	**29**	**65.52**
806004000	Calbiga	16	7	23	17	7	24	95.83	0	0	0	0	0	0	0.0
**806005000**	**City of Catbalogan**	**217**	**116**	**333**	**236**	**123**	**359**	**92.76**	**0**	**0**	**0**	**0**	**0**	**0**	**0.0**
806006000	Daram	62	24	86	69	28	97	88.66	0	0	0	0	0	0	0.0
806007000	Gandara	50	35	85	57	36	93	91.40	0	0	0	0	0	0	0.0
806008000	Hinabangan	11	11	22	12	12	24	91.67	0	0	0	0	0	0	0.0
806009000	Jiabong	29	5	34	29	5	34	100.00	0	0	0	0	0	0	0.0
806010000	Marabut	40	20	60	42	24	66	90.91	0	0	0	0	0	0	0.0
806011000	Matuguinao	10	6	16	10	6	16	100.00	0	0	0	0	0	0	0.0
806012000	Motiong	12	11	23	13	12	25	92.00	0	0	0	0	0	0	0.0
806013000	Pinabacdao	15	13	28	16	13	29	96.55	0	0	0	0	0	0	0.0
806014000	San Jose De Buan	4	5	9	8	6	14	64.29	0	0	0	0	0	0	0.0
806015000	San Sebastian	14	6	20	15	7	22	90.91	0	0	0	0	0	0	0.0
806016000	Santa Margarita	29	8	37	33	10	43	86.05	0	0	0	0	0	0	0.0
806017000	Santa Rita	60	25	85	66	27	93	91.40	0	0	0	0	0	0	0.0
806018000	Santo Niño	30	8	38	35	10	45	84.44	0	0	0	0	0	0	0.0
806019000	Talalora	13	6	19	13	7	20	95.00	0	0	0	0	0	0	0.0
806020000	Tarangnan	58	41	99	58	41	99	100.00	0	0	0	0	0	0	0.0
806021000	Villareal	41	27	68	42	27	69	98.55	0	0	0	0	0	0	0.0
806022000	Paranas	23	15	38	25	16	41	92.68	0	0	0	0	0	0	0.0
806023000	Zumarraga	19	15	34	19	15	34	100.00	0	0	0	0	0	0	0.0
806024000	Tagapul-An	6	7	13	8	8	16	81.25	0	0	0	0	0	0	0.0
806025000	San Jorge	28	19	47	28	19	47	100.00	0	0	0	0	0	0	0.0
806026000	Pagsanghan	21	4	25	21	5	26	96.15	0	0	0	0	0	0	0.0
**806400000**	**Southern Leyte**	**857**	**472**	**1,329**	**928**	**507**	**1,435**	**92.61**	**25**	**6**	**31**	**29**	**8**	**37**	**83.78**
806401000	Anahawan	17	10	27	20	11	31	87.10	0	0	0	0	0	0	0.0
806402000	Bontoc	52	23	75	52	23	75	100.00	0	0	0	0	0	0	0.0
806403000	Hinunangan	69	36	105	71	36	107	98.13	0	0	0	0	0	0	0.0
806404000	Hinundayan	38	11	49	43	14	57	85.96	0	0	0	0	0	0	0.0
806405000	Libagon	29	10	39	31	12	43	90.70	0	0	0	0	0	0	0.0
806406000	Liloan	50	32	82	51	32	83	98.80	0	0	0	0	0	0	0.0
**806407000**	**City of Maasin**	**216**	**148**	**364**	**224**	**157**	**381**	**95.54**	**25**	**6**	**31**	**29**	**8**	**37**	**83.78**
806408000	Macrohon	49	29	78	52	31	83	93.98	0	0	0	0	0	0	0.0
806409000	Malitbog	37	17	54	37	17	54	100.00	0	0	0	0	0	0	0.0
806410000	Padre Burgos	24	11	35	26	12	38	92.11	0	0	0	0	0	0	0.0
806411000	Pintuyan	20	17	37	22	17	39	94.87	0	0	0	0	0	0	0.0
806412000	Saint Bernard	83	36	119	96	39	135	88.15	0	0	0	0	0	0	0.0
806413000	San Francisco	28	12	40	29	12	41	97.56	0	0	0	0	0	0	0.0
806414000	San Juan	32	19	51	35	20	55	92.73	0	0	0	0	0	0	0.0
806415000	San Ricardo	24	14	38	25	15	40	95.00	0	0	0	0	0	0	0.0
806416000	Silago	0	0	0	20	12	32	0.00	0	0	0	0	0	0	0.0
806417000	Sogod	74	29	103	76	29	105	98.10	0	0	0	0	0	0	0.0
806418000	Tomas Oppus	10	9	19	12	9	21	90.48	0	0	0	0	0	0	0.0
806419000	Limasawa	5	9	14	6	9	15	93.33	0	0	0	0	0	0	0.0
**807800000**	**Biliran**	**435**	**230**	**665**	**470**	**247**	**717**	**92.75**	**0**	**0**	**0**	**0**	**0**	**0**	**0.0**
807801000	Almeria	43	27	70	45	27	72	97.22	0	0	0	0	0	0	0.0
[truncated: 1,441,144 more chars]
