# Supplementary material for: Neglected Tropical Diseases Elimination in the Philippines: Challenges and Gaps
Source: Trop Med Infect Dis. 2026 Apr 17;11(4):106. doi: 10.3390/tropicalmed11040106 (PMC13120366; doi:10.3390/tropicalmed11040106)
Supplement: Supplementary file 1 [file Supplementary File S3. FHSIS 2021.pdf › Supplementary File S3. FHSIS 2021.pdf]

2021 ANNUAL REPORT

FHSIS

FIELD HEALTH SERVICES INFORMATION SYSTEM

MONITORING AND EVALUATION DIVISION
EPIDEMIOLOGY BUREAU
DEPARTMENT OF HEALTH
SAN LAZARO COMPOUND, RIZAL AVENUE,
STA. CRUZ, MANILA

Republic of the Philippines
Department of Health

BEVERLY LORRAINE C. HO, MD, MPH
OIC - Undersecretary of Health, Public Health Services Team

GLORIA NENITA V. VELASCO, MD, DipEpi, MScPH
Director III, Epidemiology Bureau

VITO G. ROQUE JR., MD, PHSAE
Chief, Monitoring and Evaluation Division

FHSIS UNIT STAFF

MARIEL A. DEJESA, MD, MPM
Medical Officer IV

Jose M. Hernaez
Information Systems Analyst III
Joel V. Cantero
Computer Programmer II
Hernan T. Tayer
Computer Programmer II
Julius T. Castro
Computer Programmer II
Lester C. Recio
Health Program Officer II

Denisse Lou M. Adriano
Senior Health Program Officer
Allan P. Ignacio
Statistician II
Dexter Jay B. Flores
Health Program Officer II
Karla Mae C. Ramirez
Health Program Officer II

FOREWORD

The Department of Health (DOH) presents the 2021 Field Health Services Information System (FHSIS) Annual Report. This report reflects the data on the extent of coverage of essential public health services offered by the field health care facilities. It also accounts selected demographic, mortality and natality data, and morbidity data captured in public health facilities throughout the year.

Relevant FHSIS data is essential to guide local health officers and coordinators in managing service delivery in their respective locality, and in monitoring and evaluating the effectiveness of interventions. It also serves as a supplement to DOH data in crafting policies/guidelines, prioritization and allocation of resources, and improvement of systems towards Universal Health Care. Over the years, the FHSIS reports have been of great value for the health sector, academe, researchers and other organizations.

We are grateful for the hard work and contribution to this report of our health workers and staff from the barangay, municipal, city and provincial offices, our partners from the Local Government Units, Centers for Health Development, Disease Prevention and Control Bureau, and the Epidemiology Bureau. It is hoped that the translation of this publication in creating and updating policies and evidence-informed interventions will improve health outcomes and provide better health services advantageous to all Filipinos.

BEVERLY LORRAINE C. HO, MD, MPH
OIC - Undersecretary of Health
Public Health Services Team

TABLE OF CONTENTS

Field Health Services Information System
2021 Annual Report

CHAPTER 1 – ACCOMPLISHMENT OF PUBLIC HEALTH PROGRAMS

**1.A. Family Planning Services**	
Highlights	
1. Number of New Acceptors 2020 vs. 2021	**3**
2. Number of Current Users 2020 vs. 2021	**3**
3. Number of Drop-outs 2020 vs. 2021	**4**
4. Proportion of Women of Reproductive Age with Unmet Need	**4**
Summary Tables (by Method, Region, Province/City, Age Group)	
1. Number of New Acceptors – Table 1.A.1	**5**
2. Number of Other Acceptors – Table 1.A.2	**25**
3. Number of Drop-outs – Table 1.A.3	**45**
4. Number of Current Users End – Table 1.A.4	**65**
5. Number of Unmet Needs – Table 1.A.5	**85**
Figures and Graphs	
1. Percentage Distribution of New Acceptors (10-49 years old) – Figure 1.A.1	**91**
2. Percentage Distribution of Other Acceptors (10-49 years old) – Figure 1.A.2	**91**
3. Percentage Distribution of Drop-outs (10-49 years old) – Figure 1.A.3	**92**
4. Percentage Distribution of Current Users End (10-49 years old) – Figure 1.A.4	**92**
5. Percentage Distribution of Unmet Needs by Region – Figure 1.A.5	**93**
**1.B. Maternal Care and Services**	
Highlights	
1. Proportion of Women with at least 4 or more Prenatal Check-ups by Region, 2020 vs. 2021	**95**
2. Proportion of Women 10-49 years old who gave birth with at least 4 Prenatal Check-ups by Region	**95**
3. Proportion of Pregnant Women for the 1st, 2nd and more times given Tetanus Diphtheria Vaccine by Region	**95**
4. Proportion of Pregnant Women given Tetanus Diphtheria by Age Group	**96**
5. Proportion of Pregnant and Postpartum Women who completed doses of Iron and Folic Acid (FA) 2020 vs. 2021	**96**
6. Proportion of Deliveries by Skilled Health Professionals (2020 vs. 2021)	**96**
7. Percentage Distribution of Deliveries attended by Skilled Health Professionals	**96**
8. Proportion of Deliveries Attended by Skilled Health Professionals by Region	**97**
9. Proportion of Facility Based Deliveries by Region	**97**
10. Proportion of Livebirths by Weight (2020 vs. 2021)	**97**
11. Proportion of Postpartum Women with at least 2 Postpartum Check-ups by Age group (2020 vs. 2021)	**98**
12. Proportion of Postpartum Women together with their Newborn who completed at least 2 Postpartum check-ups by Region	**98**
13. Proportion of Postpartum Women who Completed Iron with Folic Acid (FA) and Vitamin A Supplementation (2020 vs. 2021)	**98**
1.B.1 Prenatal Care	
Summary Tables (by Region, Province/City and Age Group)	
1. Number and Proportion of Women who gave birth with at least 4 or more Prenatal Check-ups - Table 1.B.1.1	**99**
2. Number and Proportion of Pregnant Women seen according to their Nutritional Status - Table 1.B.1.2	**104**
3. Number and Proportion of Pregnant Women for the 1st time given at least 2 doses of Tetanus Diphtheria (Td) vaccination – Table 1.B.1.3	**108**
4. Number and Proportion of Preganant Women for the 2nd or more times given at least 3 doses of Td vaccination (Td2 Plus) – Table 1.B.1.4	**113**
5. Number and Proportion of Pregnant Women who completed Iron with Folic Acid Supplementation – Table	**118**
6. Number and Proportion of Pregnant Women who completed doses of Calcium Carbonate Supplementation – Table 1.B.1.6	**123**
7. Number and Proportion of Pregnant Women given Iodine capsules – Table 1.B.1.7	**128**
8. Number and Proportion of Pregnant Women given 1 dose of deworming tablet – Table 1.B.1.8	**133**
9. Number and Proportion of Pregnant Women screened for Syphilis – Table 1.B.1.9	**138**
10. Number and Proportion of Pregnant Women tested positive for Syphilis – Table 1.B.1.10	**143**
11. Number and Proportion of Pregnant Women screened for Hepatitis B -Table 1.B.1.11	**148**

12.	Number and Proportion of Pregnant Women tested positive for Hepatitis B – Table 1.B.1.12	**153**
13.	Number and Proportion of Pregnant Women screened for HIV – Table 1.B.1.13	**158**
14.	Number and Proportion of Pregnant Women tested for Complete Blood Count (CBC) or Hemoglobin (Hgb) & Hemotocrit (Hct) Count - Table 1.B.1.14	**163**
15.	Number and Proportion of Pregnant Women tested for Complete Blood Count or Hgb & Hct count diagnosed with Anemia – Table 1.B.1.15	**168**
16.	Number and Proportion of Pregnant Women screened for Gestational Diabetes – Table 1.B.1.16	**173**
17.	Number and Proportion of Pregnant Women tested positive for Gestational Diabetes – Table 1.B.1.17	**178**
Figures and Graphs		
1.	Women 10-14 years old who gave birth with at least 4 Prenatal Check-ups – Figure 1.B.1.1	**183**
2.	Women 15-19 years old who gave birth with at least 4 Prenatal Check-ups – Figure 1.B.1.2	**183**
3.	Women 20-49 years old who gave birth with at least 4 Prenatal Check-ups – Figure 1.B.1.3	**184**
4.	Women 10-49 years old who gave birth with at least 4 Prenatal Check-ups – Figure 1.B.1.4	**184**
5.	Percentage Distribution of Pregnant Women seen during the 1st trimester with Normal BMI – Figure 1.B.1.5	**185**
6.	Percentage Distribution of Pregnant Women seen during the 1st trimester with Low BMI – Figure 1.B.1.6	**185**
7.	Percentage Distribution of Pregnant Women seen during the 1st trimester with High BMI – Figure 1.B.1.7	**186**
8.	Percentage Distribution of Women (10-14 years old) Pregnant for the 1st time given at least 2 doses of Tetanus Diphtheria (Td) vaccination – Figure 1.B.1.8	**186**
9.	Percentage Distribution of Women (15-19 years old) pregnant for the 1st time given at least 2 doses of Td vaccination – Figure 1.B.1.9	**187**
10.	Percentage Distribution of Women (20-49 years old) pregnant for the 1st time given at least 2 doses of Td vaccination – Figure 1.B.1.10	**187**
11.	Percentage Distribution of Women Pregnant for the 1st time given at least 2 doses of Td vaccination BY Age Group – Figure 1.B.1.11	**188**
12.	Percentage Distribution of Women (10-14 years old) Pregnant for the 2nd time or more given at least 3 doses of Td vaccination – Figure 1.B.1.12	**188**
13.	Percentage Distribution of Women (15-19 years old) Pregnant for the 2nd time or more given at least 3 doses of Td vaccination – Figure 1.B.1.13	**189**
14.	Percentage Distribution of Women (20-49 years old) Pregnant for the 2nd time or more given at least 3 doses of Td vaccination – Figure 1.B.1.14	**189**
15.	Percentage Distribution of Women (10-49 years old) Pregnant for the 2nd time or more given at least 3 doses of Td vaccination by Age Group – Figure 1.B.1.15	**190**
16.	Percentage Distribution of Pregnant Women (10-14 years old) who completed Iron with Folic Acid – Figure 1.B.1.16	**190**
17.	Percentage Distribution of Pregnant Women (15-19 years old) who completed Iron with Folic Acid – Figure 1.B.1.17	**191**
18.	Percentage Distribution of Pregnant Women (20-49 years old) who completed Iron with Folic Acid – Figure 1.B.1.18	**191**
19.	Percentage Distribution of Pregnant Women (10-49 years old) who completed Iron with Folic Acid – Figure 1.B.1.19	**192**
20.	Percentage Distribution of Pregnant Women (10-14 years old) who completed Calcium Carbonate – Figure 1.B.1.20	**192**
21.	Percentage Distribution of Pregnant Women (15-19 years old) who completed Calcium Carbonate – Figure 1.B.1.21	**193**
22.	Percentage Distribution of Pregnant Women (20-49 years old) who completed Calcium Carbonate – Figure 1.B.1.22	**193**
23.	Percentage Distribution of Pregnant Women (10-49 years old) who completed Calcium Carbonate – Figure 1.B.1.23	**194**
24.	Percentage Distribution of Pregnant Women (10-49 years old) who completed Calcium Carbonate by Age Group – Figure 1.B.1.24	**194**
25.	Percentage Distribution of Pregnant Women (20-49 years old) who completed Iodine – Figure 1.B.1.25	**195**
26.	Percentage Distribution of Pregnant Women (10-49 years old) who completed Iodine – Figure 1.B.1.26	**195**
27.	Percentage Distribution of Pregnant Women (10-49 years old) who completed Iodine by Age Group – Figure 1.B.1.27	**196**
28.	Percentage Distribution of Pregnant Women (10-14 years old) given 1 dose of Deworming Tablet – Figure 1.B.1.28	**196**
29.	Percentage Distribution of Pregnant Women (15-19 years old) given one 1 of Deworming Tablet – Figure	**197**

30.	Percentage Distribution of Pregnant Women (20-49 years old) given 1 dose of Deworming Tablet – Figure 1.B.1.30	**197**
31.	Percentage Distribution of Pregnant Women (10-49 years old) given 1 dose of Deworming Tablet – Figure 1.B.1.31	**198**
32.	Percentage Distribution of Pregnant Women (10-49 years old) given 1 dose of Deworming Tablet by Age Group – Figure 1.B.1.32	**198**
33.	Percentage Distribution of Pregnant Women (10-14 years old) Screened for Syphilis – Figure 1.B.1.33	**199**
34.	Percentage Distribution of Pregnant Women (15-19 years old) Screened for Syphilis – Figure 1.B.1.34	**199**
35.	Percentage Distribution of Pregnant Women (20-49 years old) Screened for Syphilis – Figure 1.B.1.35	**200**
36.	Percentage Distribution of Pregnant Women (10-49 years old) Screened for Syphilis – Figure 1.B.1.36	**200**
37.	Percentage Distribution of Pregnant Women (10-49 years old) Screened for Syphilis by Age Group – Figure 1.B.1.37	**201**
38.	Percentage Distribution of Pregnant Women (10-14 years old) tested positive for Syphilis – Figure 1.B.1.38	**201**
39.	Percentage Distribution of Pregnant Women (15-19 years old) tested positive for Syphilis – Figure 1.B.1.39	**202**
40.	Percentage Distribution of Pregnant Women (20-49 years old) tested positive for Syphilis – Figure 1.B.1.40	**202**
41.	Percentage Distribution of Pregnant Women (10-49 years old) tested positive for Syphilis – Figure 1.B.1.41	**203**
42.	Percentage Distribution of Pregnant Women (10-49 years old) tested positive for Syphilis by Age Group – Figure 1.B.1.42	**203**
43.	Percentage Distribution of Pregnant Women (10-14 years old) screened for Hepatitis B – Figure 1.B.1.43	**204**
44.	Percentage Distribution of Pregnant Women (15-19 years old) screened for Hepatitis B – Figure 1.B.1.44	**204**
45.	Percentage Distribution of Pregnant Women (20-49 years old) screened for Hepatitis B – Figure 1.B.1.45	**205**
46.	Percentage Distribution of Pregnant Women (10-49 years old) screened for Hepatitis B – Figure 1.B.1.46	**205**
47.	Percentage Distribution of Pregnant Women (10-49 years old) screened for Hepatitis B by Age Group – Figure 1.B.1.47	**206**
48.	Percentage Distribution of Pregnant Women (10-14 years old) tested positive for Hepatitis B – Figure 1.B.1.48	**206**
49.	Percentage Distribution of Pregnant Women (15-19 years old) tested positive for Hepatitis B – Figure 1.B.1.49	**207**
50.	Percentage Distribution of Pregnant Women (20-49 years old) tested positive for Hepatitis B – Figure 1.B.1.50	**207**
51.	Percentage Distribution of Pregnant Women (10-49 years old) tested positive for Hepatitis B – Figure 1.B.1.51	**208**
52.	Percentage Distribution of Pregnant Women (10-49 years old) tested positive for Hepatitis B by Age Group – Figure 1.B.1.52	**208**
53.	Percentage Distribution of Pregnant Women (10-14 years old) screened for HIV – Figure 1.B.1.53	**209**
54.	Percentage Distribution of Pregnant Women (15-19 years old) screened for HIV – Figure 1.B.1.54	**209**
55.	Percentage Distribution of Pregnant Women (20-49 years old) screened for HIV – Figure 1.B.1.55	**210**
56.	Percentage Distribution of Pregnant Women (10-49 years old) screened for HIV – Figure 1.B.1.56	**210**
57.	Percentage Distribution of Pregnant Women (10-49 years old) screened for HIV by Age Group – Figure 1.B.1.57	**211**
58.	Percentage Distribution of Pregnant Women (10-14 years old) tested for Complete Blood Count (CBC) or Hemoglobin (Hgb) & Hemotocrit (Hct) Count – Figure 1.B.1.58	**211**
59.	Percentage Distribution of Pregnant Women (15-19 years old) tested for Complete Blood Count (CBC) or Hemoglobin (Hgb) & Hemotocrit (Hct) Count – Figure 1.B.1.59	**212**
60.	Percentage Distribution of Pregnant Women (20-49 years old) tested for Complete Blood Count (CBC) or Hemoglobin (Hgb) & Hemotocrit (Hct) Count – Figure 1.B.1.60	**212**
61.	Percentage Distribution of Pregnant Women (10-49 years old) tested for Complete Blood Count (CBC) or Hemoglobin (Hgb) & Hemotocrit (Hct) Count – Figure 1.B.1.61	**213**
62.	Percentage Distribution of Pregnant Women (10-49 years old) tested for Complete Blood Count (CBC) or Hemoglobin (Hgb) & Hemotocrit (Hct) Count by Age Group – Figure 1.B.1.62	**213**
63.	Percentage Distribution of Pregnant Women (10-14 years old) tested for Complete Blood Count (CBC) or Hemoglobin (Hgb) & Hemotocrit (Hct) Diagnosed with Anemia – Figure 1.B.1.63	**214**
64.	Percentage Distribution of Pregnant Women (15-19 years old) tested for Complete Blood Count (CBC) or Hemoglobin (Hgb) & Hemotocrit (Hct) Diagnosed with Anemia – Figure 1.B.1.64	**214**
65.	Percentage Distribution of Pregnant Women (20-49 years old) tested for Complete Blood Count (CBC) or Hemoglobin (Hgb) & Hemotocrit (Hct) Diagnosed with Anemia – Figure 1.B.1.65	**215**
66.	Percentage Distribution of Pregnant Women (10-49 years old) tested for Complete Blood Count (CBC) or Hemoglobin (Hgb) & Hemotocrit (Hct) Diagnosed with Anemia – Figure 1.B.1.66	**215**

67. Percentage Distribution of Pregnant Women (10-49 years old) tested for Complete Blood Count (CBC) or Hemoglobin (Hgb) & Hemotocrit (Hct) Diagnosed with Anemia by Age Group – Figure 1.B.1.67	216
68. Percentage Distribution of Pregnant Women (10-14 years old) screened for Gestational Diabetes – Figure 1.B.1.68	216
69. Percentage Distribution of Pregnant Women (15-19 years old) screened for Gestational Diabetes – Figure 1.B.1.69	217
70. Percentage Distribution of Pregnant Women (20-49 years old) screened for Gestational Diabetes – Figure 1.B.1.70	217
71. Percentage Distribution of Pregnant Women (10-49 years old) screened for Gestational Diabetes – Figure 1.B.1.71	218
72. Percentage Distribution of Pregnant Women (10-49 years old) screened for Gestational Diabetes by Age Group – Figure 1.B.1.72	218
73. Percentage Distribution of Pregnant Women (10-14 years old) tested positive for Gestational Diabetes – Figure 1.B.1.73	219
74. Percentage Distribution of Pregnant Women (15-19 years old) tested positive for Gestational Diabetes – Figure 1.B.1.74	219
75. Percentage Distribution of Pregnant Women (20-49 years old) tested positive for Gestational Diabetes – Figure 1.B.1.75	220
76. Percentage Distribution of Pregnant Women (10-49 years old) tested positive for Gestational Diabetes – Figure 1.B.1.76	220
77. Percentage Distribution of Pregnant Women tested positive for Gestational Diabetes by Age group – Figure 1.B.1.77	221
1.B.2 Intrapartum Care and Delivery Outcome	
Summary Tables (by Region, Province/City and Age Group)	
1. Total Number of Women who Delivered a Live Baby or Stillbirth/Fetal Death, Deliveries Attended by Skilled Health Professionals and Facility Based Delivery – Table 1.B.2.1	222
2. Number and Proportion of Delivery by Type (Vaginal and Cesarean) – Table 1.B.2.2	225
3. Number and Proportion of Pregnancy by Outcome (Full Term and Pre-term) – Table 1.B.2.3	230
4. Number and Proportion of Pregnancy by Outcome (Fetal Deaths & Abortion) – Table 1.B.2.4	235
5. Number and Proportion of Live Births by Birth Weight – Table 1.B.2.5	240
Figures and Graphs	
1. Percentage of deliveries attended by Skilled Health Personnel – Figure 1.B.2.1	245
2. Percentage of deliveries attended by Physician – Figure 1.B.2.2	245
3. Percentage of deliveries attended by Nurse – Figure 1.B.2.3	246
4. Percentage deliveries attended by Midwife – Figure 1.B.2.4	246
5. Facility Based Delivery - Figure 1.B.2.5	247
6. Facility Based Delivery by Ownership (Public and Private) – Figure 1.B.2.6	247
7. Facility Based Delivery by Ownership (Public) - Figure 1.B.2.7	248
8. Facility Based Delivery by Ownership (Private) - Figure 1.B.2.8	248
9. Percentage of Deliveries by Type (Vaginal) – Figure 1.B.2.9	249
10. Percentage of Deliveries by Type (Cesarean Section) – Figure 1.B.2.10	249
11. Percentage of Pregnancy Outcome (Full Term) – Figure 1.B.2.11	250
12. Percentage of Pregnancy Outcome (Preterm) – Figure 1.B.2.12	250
13. Percentage of Pregnancy Outcome (Fetal Death) – Figure 1.B.2.13	251
14. Percentage Distribution of Normal Birth Weight – Figure 1.B.2.14	251
15. Percentage Distribution of Low Birth Weight – Figure 1.B.2.15	252
16. Percentage Distribution of Unknown Birth Weight – Figure 1.B.2.16	252
1.B.3 Postpartum and Newborn Care	
Summary Tables (by Region, Province/City and Age Group)	
1. Number and Proportion of Postpartum Women together with their Newborn who completed at least 2 Postpartum Check-ups – Table 1.B.3.1	253
2. Number and Proportion of Postpartum Women who completed Iron with Folic Acid – Table 1.B.3.2	257
3. Number and Proportion of Postpartum Women who completed Vitamin A supplementation – Table 1.B.3.3	262
Figures and Graphs	
1. Postpartum Women together with their Newborn who completed at least 2 Postpartum Check-ups - Figure 1.B.3.1	267
2. Percentage of Postpartum Women together with their Newborn who completed at least 2 Postpartum Check-ups by Age Group - Figure 1.B.3.2	267
3. Postpartum Women who completed Iron with Folic Acid – Figure 1.B.3.3	268
4. Postpartum Women (10-14 years old) who completed Iron with Folic Acid – Figure 1.B.3.4	268

5.	Postpartum Women (15-19 years old) who completed Iron with Folic Acid – Figure 1.B.3.5	**269**
6.	Postpartum Women (20-49 years old) who completed Iron with Folic Acid – Figure 1.B.3.6	**269**
7.	Postpartum Women who completed Vitamin A Supplementation – Figure 1.B.3.7	**270**
8.	Postpartum Women (10-14 years old) who completed Vitamin A Supplementation – Figure 1.B.3.8	**270**
9.	Postpartum Women (15-19 years old) who completed Vitamin A Supplementation – Figure 1.B.3.9	**271**
10.	Postpartum Women (20-49 years old) who completed Vitamin A Supplementation – Figure 1.B.3.10	**271**
**1.C Child Care and Services**		
1.C.1 Immunization Services for Infants and Children		
Highlights		
1.	Proportion of Fully Immunized Children (FIC), 2017-2021	**273**
2.	Proportion of Completely Immunized Children (CIC), 2017-2021	**273**
3.	Proportion of Fully Immunized Children (FIC) and Completely Immunized Children (CIC) by Region	**273**
4.	Proportion of Children Protected at Birth (CPAB), 2017-2021	**274**
5.	Proportion of Newborns/Infants Vaccinated with BCG and Hepatitis B1 Antigens, 2017-2021	**274**
6.	Proportion of Newborns/Infants Vaccinated with BCG and Hepatitis B1 Antigens by Region	**274**
7.	Proportion of Infants Given 3 Doses of DPT-HiB-HepB Antigens, 2017-2021	**274**
8.	Proportion of Infants Given 3 Doses of DPT-HiB-HepB Antigens by Region	**275**
9.	Proportion of Infants Given 3 Doses of Oral Polio Vaccine (OPV), 2017-2021	**275**
10.	Proportion of Infants Given 3 Doses of Oral Polio Vaccine (OPV) by Region	**275**
11.	Proportion of Infants Vaccinated with Inactivated Polio Vaccine (IPV), 2019-2021	**275**
12.	Proportion of Infants Vaccinated with Inactivated Polio Vaccine (IPV) by Region	**276**
13.	Proportion of Infants Given 3 Doses of Pneumococcal Conjugate Vaccine (PCV), 2017-2021	**276**
14.	Proportion of Infants Given 3 Doses of Pneumococcal Conjugate Vaccine (PCV) by Region	**276**
15.	Proportion of Infants Vaccinated with 2 Doses of Measles Containing Vaccine (MCV), 2017-2021	**276**
16.	Proportion of Infants Vaccinated with 2 Doses of Measles Containing Vaccine (MCV) by Region	**277**
17.	Proportion of Newborns who were Initiated on Breastfeeding Immediately After Birth	**277**
18.	Proportion of Infants Exclusively Breastfed until 5th month and 29 days, 2017-2021	**277**
19.	Proportion of Infants Exclusively Breastfed until 5th month and 29 days by Region	**278**
20.	Proportion of Infants Born Preterm or with Low Birth Weight Given Iron Supplements	**278**
21.	Proportion of Sick Infants and Children Given Vitamin A, 2017-2021	**278**
22.	Proportion of Sick Infants and Children Given Vitamin A by Region	**279**
23.	Proportion of Children (0-59 months) with Diarrhea Given ORS and ORS with Zinc, 2017-2021	**279**
24.	Proportion of Children (0-59 months) with Diarrhea Given ORS and ORS with Zinc by Region	**279**
25.	Proportion of Children (0-59 months) with Pneumonia who Received Treatment, 2017-2021	**279**
26.	Proportion of Children (0-59 months) with Pneumonia who Received Treatment by Region	**280**
Summary Tables (by Region, Province/City and Age Group)		
1.	Number and Proportion of Children Protected at Birth (CPAB), Newborn / Infants Vaccinated with BCG and Hepatitis B antigen – Table 1.C.1.1	**281**
2.	Number and Proportion of Infants who completed 3 doses of DPT-HiB-HepB antigen – Table 1.C.1.2	**285**
3.	Number and Proportion of Infants who completed 3 doses of Oral Polio Vaccine (OPV) and Inactive Polio Vaccine (IPV) – Table 1.C.1.3	**289**
4.	Number and Proportion of Infants who completed 3 doses of Pneumococcal Conjugate Vaccine (PCV) – Table 1.C.1.4	**292**
5.	Number and Proportion of Children vaccinated with 2 doses of Measles Containing Vaccine (MCV) – Table 1.C.1.5	**296**
6.	Number and Proportion of Fully Immunized Children (FIC) and Completely Immunized Children (CIC) – Table 1.C.1.6	**301**
Figures and Graphs		
1.	Percentage Distribution of Child Protected at Birth – Figure 1.C.1.1	**306**
2.	Percentage Distribution of Newborn/Infants given BCG – Figure 1.C.1.2	**306**
3.	Percentage Distribution of Newborn given Hepatitis B1 Vaccine within 24 hours After Birth – Figure 1.C.1.3	**307**
4.	Percentage Distribution of Infants vaccinated with DPT-HiB-HepB 1– Figure 1.C.1.4	**307**
5.	Percentage Distribution of Infants vaccinated with DPT-HiB-HepB 2 – Figure 1.C.1.5	**308**
6.	Percentage Distribution of Infants vaccinated with DPT-HiB-HepB 3 – Figure 1.C.1.6	**308**
7.	Percentage Distribution of Infants who completed 3 doses of DPT-HiB-HepB antigen – Figure 1.C.1.7	**309**
8.	Percentage Distribution of Infants given Oral Polio Vaccine (OPV) 1 – Figure 1.C.1.8	**309**
9.	Percentage Distribution of Infants given Oral Polio Vaccine (OPV) 2 – Figure 1.C.1.9	**310**

10.	Percentage Distribution of Infants given Oral Polio Vaccine (OPV) 3 – Figure 1.C.1.10	**310**
11.	Percentage Distribution of Infants given Inactivated Polio Vaccine (IPV) – Figure 1.C.1.11	**311**
12.	Percentage Distribution of Infants who completed 3 doses of Oral Polio Vaccine (OPV) – Figure 1.C.1.12	**311**
13.	Percentage Distribution of Infants given Pneumococcal Conjugate Vaccine (PCV) 1 – Figure 1.C.1.13	**312**
14.	Percentage Distribution of Infants given Pneumococcal Conjugate Vaccine (PCV) 2 – Figure 1.C.1.14	**312**
15.	Percentage Distribution of Infants given Pneumococcal Conjugate Vaccine (PCV) 3 – Figure 1.C.1.15	**313**
16.	Percentage Distribution of Infants who completed 3 doses of Pneumococcal Conjugate Vaccine (PCV) – Figure 1.C.1.16	**313**
17.	Percentage Distribution of Children vaccinated with Measles Containing Vaccine (MCV) 1 – Figure 1.C.1.17	**314**
18.	Percentage Distribution of Children vaccinated with Measles Containing Vaccine (MCV) 2 – Figure 1.C.1.18	**314**
19.	Percentage Distribution of Children vaccinated with 2 dose of Measles Containing Vaccine (MCV) – Figure 1.C.1.19	**315**
20.	Percentage Distribution of Fully Immunized Children (FIC) - Figure 1.C.1.20	**315**
21.	Percentage Distribution of Completely Immunized Children (CIC) – Figure 1.C.1.21	**316**
1.C.2 Nutrition Services for Infants and Children		
Summary Tables (by Region, Province/City and Age Group)		
1.	Number and Proportion of Newborns who were Initiated on Breastfeeding immediately after birth lasting for at least 90 minutes, infants Exclusively Breastfed until 5th month and 29 days, and infants who Continued Breastfeeding and were Introduced to Complementary Feeding beginning at 6 months of age – Table 1.C.2.1	**317**
2.	Number and Proportion of Infants Born Preterm or with Low Birth Weight Given Iron Supplements and Infants/Children 6-11 and 12-59 months old who completed Vitamin A Supplementation – Table 1.C.2.2	**321**
3.	Number and Proportion of Infants 6-11 months old and children 12-23 months old who completed Micronutrient Powder (MNP) Supplementation – Table 1.C.2.3	**325**
4.	Number and Proportion of Children 0-59 months old whose Nutritional Status are Normal, Stunted and Overweight/Obese – Table 1.C.2.4	**330**
5.	Number and Proportion of Children 0-59 months old whose Nutritional Status are Wasted-MAM, Wasted-SAM and Wasted – Table 1.C.2.5	**334**
Figures and Graphs		
1.	Percentage Distribution of Newborns initiated on breastfeeding immediately after birth– Figure 1.C.2.1	**338**
2.	Percentage Distribution of Infants Exclusively Breastfed until 5th month and 29 days – Figure 1.C.2.2	**338**
3.	Percentage Distribution of Infants who continued Breastfeeding and were introduced to Complementary Feeding – Figure 1.C.2.3	**339**
4.	Percentage Distribution of Infants Born Preterm or with Low Birth Weight Given Iron supplementation – Figure 1.C.2.4	**339**
5.	Percentage Distribution of Infants/Children (6-11 months old) who completed Vitamin A supplementation – Figure 1.C.2.5	**340**
6.	Percentage Distribution of Infants/Children (12-59 months old) who completed Vitamin A supplementation – Figure 1.C.2.6	**340**
7.	Percentage Distribution of Infants 6-11 months old who completed Micronutrient Powder (MNP) supplementation by Region – Figure 1.C.2.7	**341**
8.	Percentage Distribution of Children 12-23 months old who completed Micronutrient Powder (MNP) supplementation by Region – Figure 1.C.2.8	**341**
9.	Percentage of Infants 6-11 months old and Children 12-23 months old who completed Micronutrient Powder (MNP) supplementation – Figure 1.C.2.9	**342**
10.	Percentage Distribution of Children 0-59 months old whose Nutritional Status is Normal – Figure 1.C.2.10	**342**
11.	Percentage Distribution of Children 0-59 months old whose Nutritional Status is Stunted – Figure 1.C.2.11	**343**
12.	Percentage Distribution of Children 0-59 months old whose Nutritional Status is Overweight/Obese – Figure 1.C.2.12	**343**
13.	Percentage Distribution of Children 0-59 months old whose Nutritional Status is Wasted – Figure 1.C.2.13	**344**
14.	Percentage Distribution of Children 0-59 months old according to their Nutritional status – Figure 1.C.2.14	**344**
1.C.3 Deworming Services for Children and Adolescents		
Summary Tables (by Region, Province/City and Age Group)		
1.	Number and Proportion of Children/Adolescents who completed 2 doses of Deworming Tablet – Table 1.C.3.1	**345**
Figures and Graphs		
1.	Percentage Distribution of Children 1-4 years old given 2 doses of Deworming Drugs – Figure 1.C.3.1	**348**
2.	Percentage Distribution of Children 5-9 years old given 2 doses of Deworming Drugs – Figure 1.C.3.2	**348**
3.	Percentage Distribution of Children 10-19 years old given 2 doses of Deworming Drugs – Figure 1.C.3.3	**349**

1.C.4 Management of Sick Infants and Children		
Summary Tables (by Region, Province/City and Age Group)		
1.	Number and Proportion of High Risk Infants and Children with Measles and/or Persistent Diarrhea who received Vitamin A capsule aside from routine supplementation – Table 1.C.4.1	**350**
2.	Number and Proportion of Diarrhea Cases Seen (0-59 months old) who received Oral Rehydration Salt Solution (ORS) and ORS with Zinc drops or syrup and Pneumonia cases seen and received treatment – Table 1.C.4.2	**354**
Figures and Graphs		
1.	Percentage Distribution of Sick Infants aged 6-11 months Vitamin A 100,000 IU – Figure 1.C.4.1	**357**
2.	Percentage Distribution of Sick Infants aged 12-59 months Vitamin A 200,000 IU – Figure 1.C.4.2	**357**
3.	Percentage Distribution of Children (0-59 months old) with Diarrhea given ORS – Figure 1.C.4.3	**358**
4.	Percentage Distribution of Children (0-59 months old) with Diarrhea given ORS with Zinc – Figure 1.C.4.4	**358**
5.	Percentage Distribution of Children (0-59 months old) who are treated with Pneumonia – Figure 1.C.4.5	**359**
**1.D Oral Health Care and Services**		
Highlights		
1.	Proportion of Children 12-59 months old who are Orally Fit upon examination or after rehabilitation	**361**
2.	Proportion of Basic Oral Health Care Received by Age Group	**361**
3.	Proportion of Pregnant Women who received Basic Oral Health Care by Age Group	**361**
Summary Tables (by Region, Province/City and Age Group)		
1.	Number and Proportion of Children 12-59 months old who are orally fit upon oral examination or after oral rehabilitation – Table 1.D.1	**362**
2.	Number and Proportion of Children 5 years old and above with new cases of Decayed-Missing Filled Teeth (DMFT) – Table 1.D.2	**368**
3.	Number and Proportion of Infants 0-11 months old who received BOHC – Table 1.D.3	**373**
4.	Number and Proportion of Children 1-4 years old who received BOHC – Table 1.D.4	**378**
5.	Number and Proportion of Children 5-9 years old who received BOHC – Table 1.D.5	**384**
6.	Number and Proportion of Adolescents 10-14 years old who received BOHC – Table 1.D.6	**390**
7.	Number and Proportion of Adolescents 15-19 years old who received BOHC – Table 1.D.7	**395**
8.	Number and Proportion of Adolescents 20-59 years old who received BOHC – Table 1.D.8	**400**
9.	Number and Proportion of Senior Citizen 60 years old and above who received BOHC – Table 1.D.9	**405**
10.	Number and Proportion of Pregnan Women (10-14, 15-19, and 20-49 years old) who received BOHC – Table 1.D.10	**410**
Figures and Graphs		
1.	Percentage Distribution of Children 12-59 months old who are orally fit upon oral examination by Region – Figure 1.D.1	**416**
2.	Percentage Distribution of Children 12-59 months old who are orally fit upon oral examination by Sex – Figure 1.D.2	**416**
3.	Percentage Distribution of Children 5 years old and above with cases of Decayed, Missing Filled Teeth (DMFT) by Region – Figure 1.D.3	**417**
4.	Percentage Distribution of Children 5 years old and above with cases of DMFT by Sex– Figure 1.D.4	**417**
5.	Percentage Distribution of Infants 0-11 months old who received Basic Oral Health Care (BOHC) by Region – Figure 1.D.5	**418**
6.	Percentage Distribution of Infants 0-11 months old who received BOHC by Sex – Figure 1.D.6	**418**
7.	Percentage Distribution of Children 1-4 years old who received BOHC by Region – Figure 1.D.7	**419**
8.	Percentage Distribution of Children 1-4 years old who received BOHC by Sex – Figure 1.D.8	**419**
9.	Percentage Distribution of Children 5-9 years old who received BOHC by Region – Figure 1.D.9	**420**
10.	Percentage Distribution of Children 5-9 years old who received BOHC by Sex – Figure 1.D.10	**420**
11.	Percentage Distribution of Adolescent 10-14 years old who received BOHC by Region – Figure 1.D.11	**421**
12.	Percentage Distribution of Adolescent 10-14 years old who received BOHC by Sex – Figure 1.D.12	**421**
13.	Percentage Distribution of Adolescent 15-19 years old who received BOHC by Region – Figure 1.D.13	**422**
14.	Percentage Distribution of Adolescent 15-19 years old who received BOHC by Sex – Figure 1.D.14	**422**
15.	Percentage Distribution of Adult 20-59 years old who received BOHC by Region - Figure 1.D.15	**423**
16.	Percentage Distribution of Adult 20-59 years old who received BOHC by Sex – Figure 1.D.16	**423**
17.	Percentage Distribution of Senior Citizen 60 years old and above who received BOHC by Region – Figure 1.D.17	**424**
18.	Percentage Distribution of Senior Citizen 60 years old and above who received BOHC by Sex – Figure 1.D.18	**424**
19.	Percentage Distribution of Pregnan Women 10-14 years old who received BOHC – Figure 1.D.19	**425**
20.	Percentage Distribution of Pregnan Women 15-19 years old who received BOHC – Figure 1.D.20	**425**
21.	Percentage Distribution of Pregnan Women 20-49 years old who received BOHC – Figure 1.D.21	**426**

1.E Infectious Disease Prevention and Control Services	
1.E.1 Filariasis Prevention and Control	
Summary Tables (by Region, Province/City and Age Group)	
1. Number and Proportion of Positive Cases Found, Case Detection Rate, Lymphatic Filariasis Cases and Clinical Rate - Table 1.E.1	**428**
Figures and Graphs	
1. Filariasis Case Detection Rate – Figure 1.E.1.1	**434**
2. Lymphatic Filariasis Clinical Rate – Figure 1.E.1.2	**434**
3. Filariasis Case Detection Rate by Year – Figure 1.E.1.3	**435**
4. Lymphatic Filariasis Clinical Rate by Year – Figure 1.E.1.4	**435**
1.E.2 Schistosomiasis Prevention and Control	
Summary Tables (by Region, Province/City and Age Group)	
1. Number and Proportion of Suspected Schistosomiasis Cases Seen, Acute Clinically Diagnosed Cases Seen and Acute Confirmed Cases – Table 1.E.2.1	**436**
2. Number and Proportion of Chronic Clinically Diagnosed Cases Seen and Confirmed Chronic Cases – Table 1.E.2.2	**442**
3. Number and Proportion of Chronic Clinically Diagnosed Cases Treated in the Health Facility and Confirmed Chronic Cases referred to a Hospital Facility - Table 1.E.2.3	**448**
Figures and Graphs	
1. Proportion of Suspected Cases Seen – Figure 1.E.2.1	**454**
2. Proportion of Acute Clinically Diagnosed Cases Seen – Figure 1.E.2.2	**454**
3. Proportion of Acute Confirmed Cases – Figure 1.E.2.3	**455**
4. Proportion of Chronic Clinically Diagnosed Cases Seen – Figure 1.E.2.4	**455**
5. Proportion of Chronic Clinically Diagnosed Confirmed Cases – Figure 1.E.2.5	**456**
6. Proportion of Chronic Clinically Diagnosed Cases Treated in the Health Facility – Figure 1.E.2.6	**456**
7. Proportion of Confirmed Chronic Cases Referred to Hospital Facility – Figure 1.E.2.7	**457**
1.E.3 Soil Transmitted Helminthiasis Prevention and Control	
Summary Tables (by Region, Province/City and Age Group)	
1. Number and Proportion of PSAC and SAC who completed 2 doses of Deworming Tablets - Table 1.E.3.1	**458**
2. Number and Proportion of Adolescents 10-19 years old and WRA 20-49 years old who completed 2 doses of Deworming Tablet – Table 1.E.3.2	**462**
3. Number and Proportion of Pregnant Women who completed 1 dose of Deworming Tablet – Table 1.E.3.3	**468**
Figures and Graphs	
1. Proportion of PSAC (1-4 years old) who completed 2 doses of Deworming Tablet – Figure 1.E.3.1	**474**
2. Proportion of SAC (5-9 years old) who completed 2 doses of Deworming Tablet – Figure 1.E.3.2	**474**
3. Proportion of Adolescents (10-19 years old) who completed 2 doses of Deworming Tablet – Figure 1.E.3.3	**475**
4. Proportion of WRA (20-49 years old) who completed 2 doses of Deworming Tablet – Figure 1.E.3.4	**475**
5. Proportion of Pregnant Women who completed 1 dose of Deworming Tablet – Figure 1.E.3.5	**476**
1.E.4 HIV-AIDS/STI Prevention and Control	
Summary Tables (by Region, Province/City and Age Group)	
1. Number and Proportion of Pregnant Women Screened for Syphilis, Positive for Syphilis and Screened for HIV – Table 1.E.4	**477**
Figures and Graphs	
1. Proportion of Pregnant Women Screened for Syphilis - Figure 1.E.4.1	**483**
2. Proportion of Pregnant Women Tested Positive for Syphilis – Figure 1.E.4.2	**483**
3. Proportion of Pregnant Women Tested for HIV – Figure 1.E.4.3	**484**
1.E.5 Malaria Prevention and Control	
Summary Tables (by Region, Province/City and Age Group)	
1. Number and Rates of Probable/Clinically and Confirmed Malaria Cases (<5 years old and >=5 years old) – Table 1.E.5.1	**485**
2. Number and Rates of Laboratory Confirmed Malaria Deaths (<5 years old and >=5 years old) – Table 1.E.5.2	**490**
3. Number of Confirmed Malaria Cases and Annual Parasite Incidence – Table 1.E.5.3	**495**
Figures and Graphs	
1. Morbidity Rate of <5 years old Probable/Clinically and Confirmed Malaria – Figure 1.E.5.1	**500**
2. Morbidity Rate of >=5 years old Probable/Clinically and Confirmed Malaria – Figure 1.E.5.2	**500**
3. Morbidity Rate of <5 years old Probable/Clinically and Confirmed Malaria, 2019-2021 – Figure 1.E.5.3	**501**
4. Morbidity Rate of >=5 years old Probable/Clinically and Confirmed Malaria, 2019-2021 – Figure 1.E.5.4	**501**

5. Mortality Rate of <5 years old Laboratory Confirmed Malaria Cases – Figure 1.E.5.5	**502**
6. Mortality Rate of >= 5 years old Laboratory Confirmed Malaria Cases – Figure 1.E.5.6	**502**
7. Malaria Annual Parasite Incidence – Figure 1.E.5.7	**503**
8. Malaria Annual Parasite Incidence, 2019-2021 – Figure 1.E.5.8	**503**
1.E.6 Leprosy Prevention and Control	
Summary Tables (by Region, Province/City and Age Group)	
1. Leprosy Cases Undergoing Treatment, Prevalence Rate and Case Detection Rate – Table 1.E.6	**504**
Figures and Graphs	
1. Leprosy Prevalence Rate– Figure 1.E.6.1	**509**
2. Leprosy Case Detection Rate – Figure 1.E.6.2	**509**
3. Leprosy Annual Case Detection Rate, 2019-2021 – Figure 1.E.6.3	**510**
4. Leprosy Annual Prevalence Rate, 2019-2021 – Figure 1.E.6.4	**510**
1.E.7 Rabies	
Summary Tables (by Region, Province/City and Age Group)	
1. Number and Proportion of Deaths due to Rabies – Table 1.E.7	**511**
Figures and Graphs	
1. Proportion of Deaths due to Rabies – Figure 1.E.7.1	**516**
2. Proportion of Deaths due to Rabies, 2019-2021 – Figure 1.E.7.2	**516**
1.E.8 Tuberculosis Prevention and Control	
Summary Tables (by Region, Province/City and Age Group)	
1. Case Notification Rate (CNR), all forms – Table 1.E.8.1	**517**
2. Case Notification Rate (DRTB) – Table 1.E.8.2	**522**
3. Treatment Success Rate (TSR), DSTB-all forms – Table 1.E.8.3	**527**
4. Treatment Success Rate (TSR), MDRTB – Table 1.E.8.4	**532**
Figures and Graphs	
1. Case Notification Rate (CNR), all forms – Figure 1.E.8.1	**537**
2. Case Notification Rate (DRTB) – Figure 1.E.8.2	**537**
3. Treatment Success Rate (TSR), DSTB-all forms – Figure 1.E.8.3	**538**
4. Treatment Success Rate (TSR), MDRTB – Figure 1.E.8.4	**538**
**1.F Non-Communicable Disease Prevention and Control Services**	
1.F.1 Lifestyle Related Diseases (Risk Assessment Using PhilPEN protocol)	
Highlights	
1. Proportion of Adults 20 years old and above who were risk-assessed as Current Smokers, Binge Drinkers and Overweight/Obese	**540**
2. Proportion of Adults 20 years old and above who were risk-assessed as Current Smokers, Binge Drinkers and Overweight/Obese by Region	**540**
3. Number of Women 20 years old and above who were screened for Cervical Cancer and Breast Mass	**540**
4. Proportion of Adults 20 years old and above newly-identified with Hypertensive and Type II Diabetes Mellitus	**541**
5. Number of Senior Citizens 60 years old and above who were Screened and Diagnosed with Eye Problems	**541**
6. Proportion of Senior Citizens 60 years old and above who received 1 dose of Pneumococcal Polysaccharide Vaccine (PPV) and Influenza Vaccine	**541**
Summary Tables (by Region, Province/City and Age Group)	
1. Number and Proportion of Adults Age 20 years old and above who were Risk-assessed using the PhilPEN Protocol – Table 1.F.1.1	**542**
2. Number and Proportion of Adults 20 years old and above who are Current Smokers, Binge Drinkers and Overweight/Obese based on the PhilPEN Protocol – Table 1.F.1.2	**547**
1.F.2 Cancer Prevention and Control	
Summary Tables (by Region, Province/City and Age Group)	
1. Number and Proportion of Women 20 years old and above Screened for Cervical and Breast Cancer – Table 1.F.2	**551**
1.F.3 Cardiovascular Disease and Diabetes Mellitus Prevention and Control	
Summary Tables (by Region, Province/City and Age Group)	
1. Number and Proportion of Newly-identified Hypertensive and with Type 2 Diabetes Mellitus among 20 years old and above adults – Table 1.F.3	**556**
1.F.4 Blindness Prevention Program	
Summary Tables (by Region, Province/City and Age Group)	
1. Number and Proportion of Senior Citizens 60 years old and above Screened for Visual Acuity and Diagnosed with Eye Disease/s – Table 1.F.4	**561**

1.F.5 Immunization for Senior Citizens		
Summary Tables (by Region, Province/City and Age Group)		
1.	Number and Proportion of Senior Citizens 60 years old and above who received 1 dose of Pneumococcal Polysaccharide Vaccine (PPV) and 1 dose of Influenza Vaccine – Table 1.F.5	**566**
Figures and Graphs		
1.	Percentage Distribution of Adults aged 20 years old and above who were Risk-assessed using the PhilPEN Protocol – Figure 1.F.1	**571**
2.	Percentage Distribution of Adults aged 20 years old and above who are Current Smokers – Figure 1.F.2	**571**
3.	Percentage Distribution of Adults aged 20 years old and above who are Binge Drinkers – Figure 1.F.3	**572**
4.	Percentage Distribution of Adults aged 20 years old and above who are Overweight or Obese – Figure 1.F.4	**572**
5.	Percentage Distribution of Adults aged 20 years old and above who are Current Smokers, Binge Drinkers and Overweight or Obese – Figure 1.F.5	**573**
6.	Percentage Distribution of Women 20 years old and above Screened for Breast Mass – Figure 1.F.6	**573**
7.	Percentage Distribution of Women 20 years old and above with Suspicious Breast Mass – Figure 1.F.7	**574**
8.	Percentage Distribution of Women 20 years old and above Screened for Cervical Cancer, Found Positive or Suspected for Cervical Cancer, Screened for Breast Mass, and Women with Suspicious Breast Mass – Figure 1.F.8	**574**
9.	Percentage Distribution of Newly-identified 20 years old and above adults with Hypertension – Figure 1.F.9	**575**
10.	Percentage Distribution of Newly-identified 20 years old and above adults with Hypertension by Sex – Figure 1.F.10	**575**
11.	Percentage Distribution of Newly-identified 20 years old and above adults with Type 2 Diabetes Mellitus – Figure 1.F.11	**576**
12.	Percentage Distribution of Newly-identified 20 years old and above adults with Type 2 Diabetes Mellitus by Sex – Figure 1.F.12	**576**
13.	Percentage Distribution of Senior Citizens (60 years old and above) Screened for Visual Acuity – Figure 1.F.13	**577**
14.	Percentage Distribution of Senior Citizens (60 years old and above) Screened for Visual Acuity and Diagnosed with Eye Diseases – Figure 1.F.14	**577**
15.	Percentage Distribution of Senior Citizens (60 years old and above) Diagnosed with Eye Diseases – Figure 1.F.15	**578**
16.	Percentage Distribution of Senior Citizens 60 years old and above who received 1 dose of Pneumococcal Polysaccharide Vaccine (PPV) – Figure 1.F.5.16	**578**
17.	Percentage Distribution of Senior Citizens 60 years old and above who received 1 dose of Influenza Vaccine – Figure 1.F.17	**579**
18.	Percentage Distribution of Senior Citizens 60 years old and above who received 1 dose of PPV and 1 dose of Influenza Vaccine – Figure 1.F.18	**579**
**1.G Environmental Health and Sanitation Services**		
Highlights		
1.	Proportion of Households with Access to Basic Safe Water Supply and Basic Sanitation Facility, 2017-2021	**581**
2.	Proportion of Households with Access to Basic Safe Water Supply by Source Type	**581**
3.	Proportion of Households using Safely Managed Drinking Water Services and Sanitation Services, 2019-2021	**581**
4.	Proportion of Industrial Establishments with Sanitary Permits	**582**
5.	Proportion of Barangay Declared Zero Open Defecation (ZOD) Area	**582**
Summary Tables (by Region, Province/City and Age Group)		
1.	Number and Proportion of Households with Access to Basic Safe Water Supply (Level I, II, III) and using Safely Managed Drinking-Water Services – Table 1.G.1	**583**
2.	Number and Proportion of Households with Basic Sanitation Facility and using Safely Managed Sanitation Service – Table 1.G.2	**588**
3.	Number and Proportion of Industrial Establishments, Industrial Establishments Issued with Sanitary Permit, Total Number of Barangay and Number of Barangays Certified as Zero Open Defecation (ZOD) – Table 1.G.3	**593**
Figures and Graphs		
1.	Percentage of Households with access to Basic Safe Water Supply (Level 1) – Figure 1.G.1	**599**
2.	Percentage of Households with access to Basic Safe Water Supply (Level 2) – Figure 1.G.2	**599**
3.	Percentage of Households with access to Basic Safe Water Supply (Level 3) – Figure 1.G.3	**600**
4.	Percentage of Households with access to Basic Safe Water Supply (Levels 1, 2, 3) – Figure 1.G.4	**600**
5.	Percentage of Households using Safely Manage Water Services – Figure 1.G.5	**601**
6.	Percentage of Households with Basic Sanitation Facility (Septic Tank) – Figure 1.G.6	**601**
7.	Percentage of Households with Basic Sanitation Facility (Community Sewerage System) – Figure 1.G.7	**602**
8.	Percentage of Households with Basic Sanitation Facility (Ventilated Improve Pit latrine) – Figure 1.G.8	**602**

9.	Percentage of Households using Safely Manage Sanitation Service – Figure 1.G.9	**603**
10.	Percentage of Industrial Establishment with Sanitary Permit – Figure 1.G.10	**603**
11.	Percentage of Barangay Certified as ZOD Area – Figure 1.G.11	**604**

CHAPTER 2 – MORBIDITY, MORTALITY AND NATALITY

**2.A Morbidity**		
Highlights		
1.	Top 10 Leading Causes of Morbidity	**607**
**2.B Mortality**		
Highlights		
1.	Mortality Rate, 2017-2021	**607**
2.	Proportion of Deaths by Sex	**607**
3.	Maternal Mortality Ratio, 2017-2021	**608**
4.	Maternal Mortality Ratio by Region	**608**
5.	Under Five Mortality Rate, 2017-2021	**608**
6.	Under Five Mortality Rate by Region	**608**
7.	Infant Mortality Rate, 2017-2021	**609**
8.	Infant Mortality Rate by Region	**609**
9.	Neonatal Mortality Rate, 2019-2021	**609**
10.	Neonatal Mortality Rate by Region	**609**
11.	Perinatal Mortality Rate, 2017-2021	**610**
12.	Perinatal Mortality Rate by Region	**610**
**2.C Natality**		
Highlights		
1.	Crude Birth Rate, 2017-2021	**610**
2.	Proportion of Live Births by Sex	**611**
3.	Adolescent Birth Rate, 2019-2021	**611**
**2.A Morbidity**		
Summary Tables		
2.A.1 Top Ten Leading Causes of Morbidity		
1.	Top Ten Leading Causes of Morbidity (Philippines) – Table 2.A.1	**612**
2.A.2 Top Ten Leading Causes of Morbidity by Gender		
1.	Top Ten Leading Causes of Morbidity by Gender (Male) – Table 2.A.2.1	**613**
2.	Top Ten Leading Causes of Morbidity by Gender (Female) – Table 2.A.2.2	**613**
2.A.3 Top Ten Leading Causes of Morbidity by Region		
1.	Nation Capital Region (NCR) – Table 2.A.3.1	**614**
2.	Cordillera Administrative Region (CAR) – Table 2.A.3.2	**614**
3.	Region 1 – Table 2.A.3.3	**614**
4.	Region 2 – Table 2.A.3.4	**615**
5.	Region 3 – Table 2.A.3.5	**615**
6.	Region 4A – Table 2.A.3.6	**615**
7.	Region 4B – Table 2.A.3.7	**616**
8.	Region 5 – Table 2.A.3.8	**616**
9.	Region 6 – Table 2.A.3.9	**616**
10.	Region 7 – Table 2.A.3.10	**617**
11.	Region 8 – Table 2.A.3.11	**617**
12.	Region 9 – Table 2.A.3.12	**617**
13.	Region 10 – Table 2.A.3.13	**618**
14.	Region 11 – Table 2.A.3.14	**618**
15.	Region 12 – Table 2.A.3.15	**618**
16.	Bangsamoro Autonomous Region in Muslim Mindanao (BARMM) – Table 2.A.3.16	**619**
17.	Caraga – Table 2.A.3.17	**619**

2.A.4 Morbidity Rate (by Type of Disease, Sex and Age Group)	
1. Acute Bloody Diarrhea – Table 2.A.4.1	**620**
2. Acute Febrile Illness - Table 2.A.4.2	**626**
3. Acute Flaccid Paralysis – Table 2.A.4.3	**632**
4. Acute Hemorrhagic Fever – Table 2.A.4.4	**638**
5. Acute Lower Respiratory Tract Infection – Table 2.A.4.5	**644**
6. Animal Bites – Table 2.A.4.6	**650**
7. Acute Respiratory Infection (less than 5 years old) – Table 2.A.4.7	**656**
8. Acute Respiratory Infection (more than 5 years old) – Table 2.A.4.8	**660**
9. Acute Watery Diarrhea – Table 2.A.4.9	**666**
10. Bronchitis – Table 2.A.4.10	**672**
11. Cholera – Table 2.A.4.11	**678**
12. Chronic Obstructive Pulmonary Disease – Table 2.A.4.12	**684**
13. Diphtheria – Table 2.A.4.13	**690**
14. Diseases of the Heart – Table 2.A.4.14	**696**
15. Filariasis – Table 2.A.4.15	**702**
16. Fever of Unknown Origin – Table 2.A.4.16	**708**
17. Genital Ulcer – Table 2.A.4.17	**714**
18. Gonorrhea – Table 2.A.4.18	**720**
19. Hypertension – Table 2.A.4.19	**726**
20. Influenza-Like Illness – Table 2.A.4.20	**732**
21. Influenza – Table 2.A.4.21	**738**
22. Leprosy – Table 2.A.4.22	**744**
23. Leptospirosis – Table 2.A.4.23	**750**
24. Malaria – Table 2.A.4.24	**756**
25. Measles – Table 2.A.4.25	**762**
26. Meningococcal Infection – Table 2.A.4.26	**768**
27. Neonatal Tetanus – Table 2.A.4.27	**774**
28. Non-Neonatal Tetanus – Table 2.A.4.28	**779**
29. Pneumonia – Table 2.A.4.29	**785**
30. Rabies (Human) – Table 2.A.4.30	**791**
31. Paralytic Shellfish Poisoning – Table 2.A.4.31	**797**
32. Schistosomiasis – Table 2.A.4.32	**803**
33. Skin Diseases – Table 2.A.4.33	**809**
34. Syphilis – Table 2.A.4.34	**815**
35. Tuberculosis (All Forms) – Table 2.A.4.35	**821**
36. Typhoid and Paratyphoid Fever – Table 2.A.4.36	**827**
37. Urethral Discharge – Table 2.A.4.37	**833**
38. Urinary Tract Infection – Table 2.A.4.38	**839**
39. Viral Encephalitis – Table 2.A.4.39	**845**
40. Viral Hepatitis – Table 2.A.4.40	**851**
41. Viral Meningitis – Table 2.A.4.41	**857**
42. Whooping Cough – Table 2.A.4.42	**863**
Figures and Graphs	
1. Acute Bloody Diarrhea, 2017-2021 – Figure 2.A.3.1	**869**
2. Acute Bloody Diarrhea, 2021 – Figure 2.A.3.2	**869**
3. Acute Febrille Illness, 2017-2021– Figure 2.A.3.3	**870**
4. Acute Febrille Illness, 2021 – Figure 2.A.3.4	**870**
5. Acute Flaccid Paralysis, 2017-2021– Figure 2.A.3.5	**871**
6. Acute Flaccid Paralysis, 2021 – Figure 2.A.3.6	**871**
7. Acute Hemorrhagic Fever, 2017-2021– Figure 2.A.3.7	**872**
8. Acute Hemorrhagic Fever, 2021 – Figure 2.A.3.8	**872**
9. Acute Lower Respiratory Tract Infection, 2019-2021 – Figure 2.A.3.9	**873**
10. Acute Lower Respiratory Tract Infection, 2021 – Figure 2.A.3.10	**873**
11. Animal Bites, 2019-2021 – Figure 2.A.3.11	**874**
12. Animal Bites, 2021 – Figure 2.A.3.12	**874**
13. Acute Respiratory Infection (<5 years old), 2019-2021 – Figure 2.A.3.13	**875**
14. Acute Respiratory Infection (<5 years old), 2021 – Figure 2.A.3.14	**875**

15.	Acute Respiratory Infection (>5 years old), 2019-2021 – Figure 2.A.3.15	**876**
16.	Acute Respiratory Infection (>5 years old), 2021 – Figure 2.A.3.16	**876**
17.	Acute Watery Diarrhea, 2017-2021 – Figure 2.A.3.17	**877**
18.	Acute Watery Diarrhea, 2021 – Figure 2.A.3.18	**877**
19.	Bronchitis, 2017-2021 – Figure 2.A.3.19	**878**
20.	Bronchitis, 2021 – Figure 2.A.3.20	**878**
21.	Cholera, 2017-2021 – Figure 2.A.3.21	**879**
22.	Cholera, 2021– Figure 2.A.3.22	**879**
23.	Chronic Obstructive Pulmonary Disease (COPD), 2019-2021– Figure 2.A.3.23	**880**
24.	Chronic Obstructive Pulmonary Disease, 2021 – Figure 2.A.3.24	**880**
25.	Diphtheria, 2017-2021 – Figure 2.A.3.25	**881**
26.	Diptheria, 2021– Figure 2.A.3.26	**881**
27.	Diseases Of The Heart, 2017-2021 – Figure 2.A.3.27	**882**
28.	Diseases Of The Heart, 2021 – Figure 2.A.3.28	**882**
29.	Filariasis, 2017-2021 – Figure 2.A.3.29	**883**
30.	Filariasis, 2021 – Figure 2.A.3.30	**883**
31.	Fever of Unknown Origin, 2019-2021 – Figure 2.A.3.31	**884**
32.	Fever of Unknown Origin, 2021 – Figure 2.A.3.32	**884**
33.	Genital Ulcer, 2019-2021 – Figure 2.A.3.33	**885**
34.	Genital Ulcer, 2021 – Figure 2.A.3.34	**885**
35.	Gonorrhea, 2019-2021 – Figure 2.A.3.35	**886**
36.	Gonorrhea, 2021 – Figure 2.A.3.36	**886**
37.	Hypertension, 2017-2021 – Figure 2.A.3.37	**887**
38.	Hypertension, 2021 – Figure 2.A.3.38	**887**
39.	Influenza-Like Illness, 2019-2021 – Figure 2.A.3.39	**888**
40.	Influenza-Like Illness, 2021 – Figure 2.A.3.40	**888**
41.	Influenza, 2017-2021 – Figure 2.A.3.41	**889**
42.	Influenza, 2021 – Figure 2.A.3.42	**889**
43.	Leprosy, 2017-2021 – Figure 2.A.3.43	**890**
44.	Leprosy, 2021 – Figure 2.A.3.44	**890**
45.	Leptospirosis, 2017-2021 – Figure 2.A.3.45	**891**
46.	Leptospirosis, 2021 – Figure 2.A.3.46	**891**
47.	Malaria, 2017-2021 – Figure 2.A.3.47	**892**
48.	Malaria, 2021 – Figure 2.A.3.48	**892**
49.	Measles, 2017-2021 – Figure 2.A.3.49	**893**
50.	Measles, 2021 – Figure 2.A.3.50	**893**
51.	Meningococcemia, 2017-2021 – Figure 2.A.3.51	**894**
52.	Meningococcemia, 2021 – Figure 2.A.3.52	**894**
53.	Neonatal Tetanus, 2017-2021 – Figure 2.A.3.53	**895**
54.	Neonatal Tetanus, 2021 – Figure 2.A.3.54	**895**
55.	Non-Neonatal Tetanus, 2017-2021 – Figure 2.A.3.55	**896**
56.	Non-Neonatal Tetanus, 2021 – Figure 2.A.3.56	**896**
57.	Pneumonia, 2019-2021 – Figure 2.A.3.57	**897**
58.	Pneumonia, 2021 – Figure 2.A.3.58	**897**
59.	Rabies (Human), 2017-2021 – Figure 2.A.3.59	**898**
60.	Rabies (Human), 2021 – Figure 2.A.3.60	**898**
61.	Paralytic Shellfish Poisoning, 2017-2021 – Figure 2.A.3.61	**899**
62.	Paralytic Shellfish Poisoning, 2021 – Figure 2.A.3.62	**899**
63.	Schistosomiasis, 2017-2021 – Figure 2.A.3.63	**900**
64.	Schistosomiasis, 2021 – Figure 2.A.3.64	**900**
65.	Skin Disease, 2019-2021 – Figure 2.A.3.65	**901**
66.	Skin Disease, 2021 – Figure 2.A.3.66	**901**
67.	Syphilis, 2019-2021 – Figure 2.A.3.67	**902**
68.	Syphilis, 2021 – Figure 2.A.3.68	**902**
69.	Tuberculosis All Forms, 2017-2021 – Figure 2.A.3.69	**903**
70.	Tuberculosis All Forms, 2021 – Figure 2.A.3.70	**903**

71. Typhoid and Paratyphoid Fever, 2017-2021 – Figure 2.A.3.71	**904**
72. Typhoid and Paratyphoid Fever, 2021 – Figure 2.A.3.72	**904**
73. Urethral Discharge, 2019-2021 – Figure 2.A.3.73	**905**
74. Urethral Discharge, 2021 – Figure 2.A.3.74	**905**
75. Urinary Tract Infection, 2017-2021 – Figure 2.A.3.75	**906**
76. Urinary Tract Infection, 2021 – Figure 2.A.3.76	**906**
77. Viral Encephalitis, 2017-2021 – Figure 2.A.3.77	**907**
78. Viral Encephalitis, 2021 – Figure 2.A.3.78	**907**
79. Viral Hepatitis, 2017-2021 – Figure 2.A.3.79	**908**
80. Viral Hepatitis, 2021 – Figure 2.A.3.80	**908**
81. Viral Meningitis, 2017-2021 – Figure 2.A.3.81	**909**
82. Viral Meningitis, 2021 – Figure 2.A.3.82	**909**
83. Whooping Cough, 2017-2021 – Figure 2.A.3.83	**910**
84. Whooping Cough, 2021 – Figure 2.A.3.84	**910**
**2.B Mortality**	
Summary Tables	
1. Total Number of Deaths, Maternal Deaths and Under Five Years Old Deaths – Table 2.B.1	**911**
2. Total Number of Infant Deaths, Neonatal Deaths and Perinatal Deaths – Table 2.B.2	**915**
Figure and Graphs	
1. Mortality Rate – Figure 2.B.1	**919**
2. Maternal Mortality Ratio (MMR) – Figure 2.B.2	**919**
3. Under Five Mortality Rate (UFMR) – Figure 2.B.3	**920**
4. Infant Mortality Rate (IMR) – Figure 2.B.4	**920**
5. Neonatal Mortality Rate (NMR) – Figure 2.B.5	**921**
6. Fetal Mortality Rate – Figure 2.B.6	**921**
7. Early Neonatal Mortality Rate – Figure 2.B.7	**922**
8. Perinatal Mortality Rate (PMR) – Figure 2.B.8	**922**
**2.C Natality**	
Summary Tables	
1. Number of Live Births, Crude Birth Rate and Adolescent Birth Rate – Table 2.C	**923**
Figure and Graphs	
1. Crude Birth Rate (CBR) – Figure 2.C.1	**927**
2. Adolescent Birth Rate – Figure 2.C.2	**927**

CHAPTER 3 – DEMOGRAPHIC DATA

**3.A Demographic Data**	
Highlights	
1. Projected Population and Number of Households	**930**
2. Number and Ratio of Barangay	**930**
3. Number and Ratio of Health Facilities	**931**
4. Number of LGU and DOH Hired Public Health Workers	**931**
Summary Tables	
1. Projected Population and Number of Households – Table 3.A.1	**932**
2. Number and Ratio of Barangays, Health Centers and Barangay Health Stations – Table 3.A.2	**937**
3. Number and Ratio of Physicians, Dentists and Public Health Nurses – Table 3.A.3	**941**
4. Number and Ratio of Midwives, Nutritionists and Medical Technologists – Table 3.A.4	**944**
5. Number and Ratio of Sanitary Engineers, Sanitary Inspectors and Active BHWs – Table 3.A.5	**947**
Figure and Graphs	
1. Number of Physicians per 20,000 Population – Figure 3.A.1	**950**
2. Number of Dentists per 50,000 Population – Figure 3.A.2	**950**
3. Number of Public Health Nurses per 10,000 Population – Figure 3.A.3	**951**
4. Number of Rural Health Midwives per 5,000 Population – Figure 3.A.4	**951**
5. Number of Nutritionists per 20,000 Population – Figure 3.A.5	**952**
6. Number of Medical Technologists per 50,000 Population – Figure 3.A.6	**952**
7. Number Sanitary Engineers – Figure 3.A.7	**953**
8. Number of Sanitary Inspectors per 20,000 Population – Figure 3.A.8	**953**
9. Number of BHWs per 20 Households – Figure 3.A.9	**954**

FIELD HEALTH SERVICE
INFORMATION SYSTEM

CHAPTER 1

Annual Report 2021

FAMILY PLANNING SERVICES

FHSIS ANNUAL REPORT 2021

1.A. Family Planning (FP)

In 2021, NFP LAM was the FP method with the highest number (340,641) of New Acceptors followed by Pills-COC (147,171) and Injectables (132,582). Compared to the previous year, there was a decrease in the number of New Acceptors of FP methods except for Pills-POP which had a 17% increase and NFP-CMM which had a 88% increase. The method with the highest decrease in New Acceptors was MSTR/NSV (64%) followed by FSTR/BTL (50%).

Table 1.A.1 Number of New Acceptors
by Type of Family Planning Method
Philippines, 2020 vs. 2021

Method	New Acceptors		% Change
	2020	2021	
CONDOM	41,523	28,338	-32%
FSTR/BTL	30,344	15,317	-50%
IMPLANTS	36,501	45,069	23%
INJECTABLES	170,066	132,582	-22%
IUD-INTERVAL	17,830	11,708	-34%
IUD-POSTPARTUM	22,633	11,573	-49%
MSTR/NSV	1,223	442	-64%
NFP-BBT	1,727	1,045	-39%
NFP-CMM	2,464	4,628	88%
NFP-LAM	399,835	340,641	-15%
NFP-SDM	9,805	8,155	-17%
NFP-STM	745	1,350	81%
PILLS-COC	185,971	147,171	-21%
PILLS-POP	57,408	67,406	17%

The FP method with the highest number of Current Users was Pills-COC (2,660,391) followed by Injectables (1,531,638) and FSTR/BTL (783,073). A decrease in the Current Users of Pills -POP (40%) , NFP-BBT (17%), FSTR/BTL (5%) and NFP-LAM (4%) was observed compared to the previous year.

Table 1.A.2 Number of Current Users
by Type of Family Planning Method
Philippines, 2020 vs. 2021

Method	Current Users		% Change
	2020	2021	
CONDOM	371,545	372,201	0%
FSTR/BTL	827,672	783,073	-5%
IMPLANTS	434,815	493,679	14%
INJECTABLES	1,483,691	1,531,638	3%
IUD-INTERVAL	411,911	426,502	4%
IUD-POSTPARTUM	121,232	135,261	12%
MSTR/NSV	9,605	11,577	21%
NFP-BBT	5,125	4,254	-17%
NFP-CMM	44,034	50,418	14%
NFP-LAM	629,318	606,265	-4%
NFP-SDM	92,447	114,500	24%
NFP-STM	3,425	4,519	32%
PILLS-COC	2,247,194	2,660,391	18%
PILLS-POP	684,645	408,534	-40%

For the number of Drop Outs by FP method, NFP-LAM had the highest with 619,483 followed by Pills-COC (315,081) and Injectables (286,399). There was a decrease in Drop Outs for majority of FP methods in 2021 except for NFP-BBT with a 52% increase, Implants with 31% increase and NFP-STM with 3% increase.

Table 1.A.3 Number of Drop-outs
by Type of Family Planning Method
Philippines, 2020 vs. 2021

Method	Drop Outs		% Change
	2020	2021	
CONDOM	127,256	62,360	-51%
FSTR/BTL	94,836	49,564	-48%
IMPLANTS	80,159	105,383	31%
INJECTABLES	403,332	286,399	-29%
IUD-INTERVAL	66,656	36,216	-46%
IUD-POSTPARTUM	22,295	13,167	-41%
MSTR/NSV	2,568	1,004	-61%
NFP-BBT	1,594	2,423	52%
NFP-CMM	34,011	7,847	-77%
NFP-LAM	786,674	619,483	-21%
NFP-SDM	27,362	18,209	-33%
NFP-STM	1,295	1,329	3%
PILLS-COC	451,212	315,081	-30%
PILLS-POP	115,904	101,743	-12%

In 2021, the proportion of Women of Reproductive Age with Unmet Need for Modern FP in the Philippines was 3.26%. The region with the highest unmet need was Region 11 with 21.35% followed by Region 8 (14.93%) and Region 6 (8.12%). Compared to 2020, half of the Regions had an increase in Modern FP Unmet Need. A significant increase was noted for Regions 11 and 8.

Figure 1A.1 Proportion of Women of Reproductive Age with Unmet Need for Modern Family Planning by Region Philippines, 2021

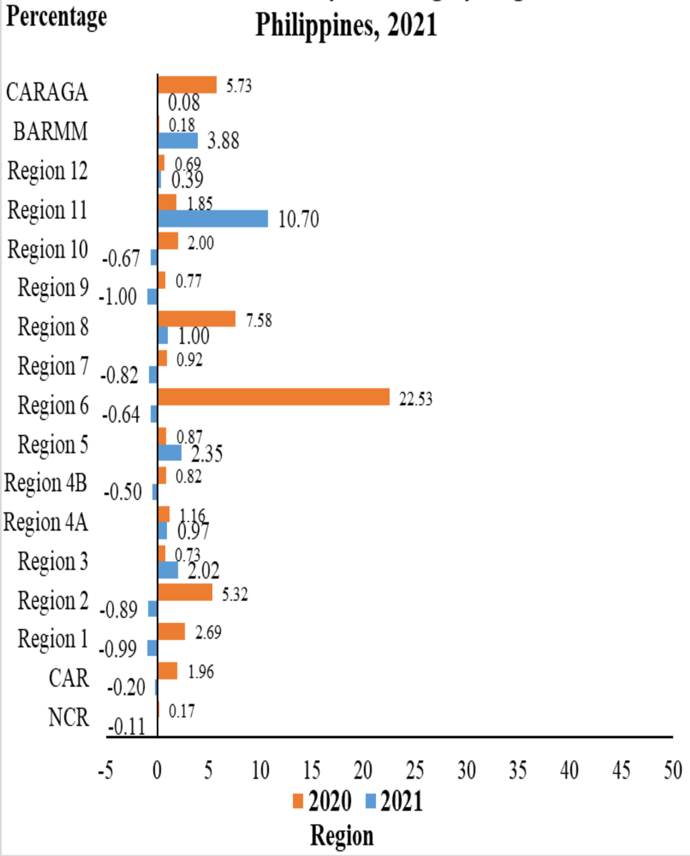

Table 1.A.1 - MODERN METHOD OF FAMILY PLANNING

New Acceptors

Philippines, 2021

Area	Total Current Users	FSTR/BTL Age group 10-14	FSTR/BTL Age group 15-19	FSTR/BTL Age group 20-49	Total	MSTR/NSV Age group 10-14	MSTR/NSV Age group 15-19	MSTR/NSV Age group 20-49	Total
**PHILIPPINES**	**7,279,694**	**2**	**147**	**15,168**	**15,317**	**0**	**56**	**386**	**442**
**N C R**	**726,121**	**0**	**6**	**1,406**	**1,412**	**0**	**1**	**24**	**25**
Malabon	32,409	0	0	8	8	0	0	0	0
Navotas	23,787	0	0	5	5	0	0	0	0
Valenzuela City	39,524	0	0	111	111	0	0	0	0
Caloocan City	67,903	0	0	22	22	0	0	0	0
Marikina City	15,458	0	0	20	20	0	0	0	0
Pasig City	37,512	0	0	109	109	0	0	0	0
Pateros	2,543	0	0	17	17	0	0	0	0
Taguig	57,590	0	0	1	1	0	1	19	20
Quezon City	259,021	0	0	203	203	0	0	0	0
Makati City	12,799	0	0	165	165	0	0	1	1
Mandaluyong City	16,826	0	6	81	87	0	0	3	3
San Juan	5,760	0	0	9	9	0	0	0	0
Manila City	31,616	0	0	315	315	0	0	0	0
Las Piñas City	22,298	0	0	60	60	0	0	0	0
Muntinlupa City	40,077	0	0	86	86	0	0	0	0
Parañaque City	33,986	0	0	135	135	0	0	1	1
Pasay City	27,012	0	0	59	59	0	0	0	0
**C A R**	**131,969**	**0**	**0**	**130**	**130**	**0**	**0**	**0**	**0**
Abra	16,967	0	0	23	23	0	0	0	0
Apayao	12,113	0	0	1	1	0	0	0	0
Benguet	30,721	0	0	59	59	0	0	0	0
Ifugao	19,056	0	0	0	0	0	0	0	0
Kalinga	18,965	0	0	39	39	0	0	0	0
Mt. Province	12,528	0	0	4	4	0	0	0	0
Baguio City	21,619	0	0	4	4	0	0	0	0
**Region 1**	**466,771**	**0**	**0**	**228**	**228**	**0**	**0**	**1**	**1**
Ilocos Norte	41,297	0	0	0	0	0	0	0	0
Ilocos Sur	58,069	0	0	114	114	0	0	0	0
La Union	59,945	0	0	51	51	0	0	0	0
Pangasinan	233,753	0	0	40	40	0	0	1	1
Alaminos City	10,247	0	0	9	9	0	0	0	0
Candon City	7,036	0	0	2	2	0	0	0	0
Dagupan City	6,893	0	0	0	0	0	0	0	0
Laoag City	8,101	0	0	0	0	0	0	0	0
San Carlos City	11,818	0	0	0	0	0	0	0	0
San Fernando City	12,806	0	0	12	12	0	0	0	0
Urdaneta City	12,828	0	0	0	0	0	0	0	0
Vigan City	3,978	0	0	0	0	0	0	0	0
**Region 2**	**314,925**	**0**	**4**	**163**	**167**	**0**	**0**	**0**	**0**
Batanes	938	0	0	0	0	0	0	0	0
Cagayan	87,099	0	0	20	20	0	0	0	0
Isabela	108,463	0	0	35	35	0	0	0	0
Nueva Vizcaya	45,287	0	3	77	80	0	0	0	0
Quirino	19,270	0	0	5	5	0	0	0	0
Cauayan City	9,889	0	0	3	3	0	0	0	0
Ilagan City	15,732	0	1	16	17	0	0	0	0
Santiago City	13,933	0	0	1	1	0	0	0	0
Tuguegarao City	14,314	0	0	6	6	0	0	0	0
**Region 3**	**794,245**	**0**	**11**	**6,147**	**6,158**	**0**	**34**	**195**	**229**
Aurora	21,216	0	0	5	5	0	0	0	0
Bataan	50,598	0	0	50	50	0	0	2	2

Table 1.A.1 - MODERN METHOD OF FAMILY PLANNING

New Acceptors

Philippines, 2021

Area	Total Current Users	FSTR/BTL Age group 10-14	15-19	20-49	Total	MSTR/NSV Age group 10-14	15-19	20-49	Total
Bulacan	148,620	0	1	3,159	3,160	0	0	1	1
Nueva Ecija	128,909	0	0	178	178	0	2	3	5
Pampanga	109,928	0	7	2,160	2,167	0	27	176	203
Tarlac	80,640	0	0	29	29	0	0	0	0
Zambales	42,194	0	0	180	180	0	0	0	0
Angeles City	23,390	0	0	92	92	0	0	0	0
Balanga City	4,908	0	0	3	3	0	0	0	0
Cabanatuan City	19,136	0	0	7	7	0	0	0	0
City of San Fernando	9,567	0	3	167	170	0	5	13	18
Gapan City	7,511	0	0	2	2	0	0	0	0
Mabalacat City	22,703	0	0	27	27	0	0	0	0
Malolos City	7,548	0	0	0	0	0	0	0	0
Meycauayan	7,990	0	0	4	4	0	0	0	0
Olongapo	10,438	0	0	28	28	0	0	0	0
Palayan City	3,016	0	0	1	1	0	0	0	0
San Jose City	12,975	0	0	11	11	0	0	0	0
San Jose del Monte City	57,952	0	0	10	10	0	0	0	0
Science City of Munoz	6,335	0	0	0	0	0	0	0	0
Tarlac City	18,671	0	0	34	34	0	0	0	0
**Region 4A**	**502,769**	**0**	**1**	**665**	**666**	**0**	**0**	**14**	**14**
Batangas	69,044	0	0	88	88	0	0	0	0
Cavite	50,364	0	0	74	74	0	0	1	1
Laguna	39,915	0	1	64	65	0	0	0	0
Quezon	65,304	0	0	33	33	0	0	0	0
Rizal	46,762	0	0	34	34	0	0	0	0
Antipolo City	6,069	0	0	203	203	0	0	0	0
Bacoor City	10,158	0	0	14	14	0	0	0	0
Batangas City	15,493	0	0	0	0	0	0	0	0
Biñan City	42,782	0	0	88	88	0	0	3	3
Cabuyao City	6,608	0	0	4	4	0	0	0	0
Calamba City	22,351	0	0	36	36	0	0	3	3
Cavite City	4,279	0	0	2	2	0	0	0	0
Dasmariñas City	37,251	0	0	4	4	0	0	0	0
General Trias City	5,008	0	0	0	0	0	0	0	0
Imus City	6,503	0	0	4	4	0	0	0	0
Lipa City	5,955	0	0	5	5	0	0	0	0
Lucena City	5,207	0	0	1	1	0	0	0	0
San Pablo City	5,471	0	0	3	3	0	0	0	0
San Pedro City	10,235	0	0	3	3	0	0	0	0
Santa Rosa City	23,903	0	0	5	5	0	0	7	7
Tagaytay City	8,089	0	0	0	0	0	0	0	0
Tanauan City	4,819	0	0	0	0	0	0	0	0
Tayabas City	7,446	0	0	0	0	0	0	0	0
Trece Martires City	3,753	0	0	0	0	0	0	0	0
**Region 4B**	**262,639**	**0**	**15**	**215**	**230**	**0**	**0**	**2**	**2**
Marinduque	13,771	0	15	17	32	0	0	1	1
Mindoro Occidental	45,760	0	0	49	49	0	0	0	0
Mindoro Oriental	64,019	0	0	61	61	0	0	1	1
Palawan	85,631	0	0	32	32	0	0	0	0
Romblon	22,521	0	0	27	27	0	0	0	0
Puerto Princesa City	30,937	0	0	29	29	0	0	0	0
**Region 5**	**467,956**	**0**	**9**	**2,108**	**2,117**	**0**	**0**	**8**	**8**
Albay	86,082	0	0	33	33	0	0	0	0
Camarines Norte	42,496	0	0	97	97	0	0	0	0
Camarines Sur	106,478	0	0	0	0	0	0	1	1
Catanduanes	25,339	0	0	2	2	0	0	0	0
Masbate	82,338	0	0	36	36	0	0	0	0
Sorsogon	63,538	0	9	1,871	1,880	0	0	7	7
Iriga City	12,388	0	0	1	1	0	0	0	0

Table 1.A.1 - MODERN METHOD OF FAMILY PLANNING

New Acceptors

Philippines, 2021

Area	Total Current Users	FSTR/BTL Age group 10-14	15-19	20-49	Total	MSTR/NSV Age group 10-14	15-19	20-49	Total
Legaspi City	23,098	0	0	11	11	0	0	0	0
Naga City	26,199	0	0	57	57	0	0	0	0
**Region 6**	**597,683**	**0**	**0**	**366**	**366**	**0**	**0**	**15**	**15**
Aklan	44,142	0	0	37	37	0	0	0	0
Antique	46,065	0	0	43	43	0	0	0	0
Capiz	74,407	0	0	34	34	0	0	6	6
Guimaras	15,917	0	0	7	7	0	0	0	0
Iloilo	153,978	0	0	12	12	0	0	0	0
Negros Occidental	190,516	0	0	203	203	0	0	3	3
Bacolod City	26,414	0	0	23	23	0	0	0	0
Iloilo City	46,244	0	0	7	7	0	0	6	6
**Region 7**	**402,202**	**0**	**0**	**397**	**397**	**0**	**0**	**19**	**19**
Bohol	80,267	0	0	70	70	0	0	0	0
Cebu	142,224	0	0	21	21	0	0	8	8
Negros Oriental	81,104	0	0	76	76	0	0	0	0
Siquijor	8,593	0	0	67	67	0	0	0	0
Cebu City	33,223	0	0	103	103	0	0	7	7
Lapu-Lapu City	35,312	0	0	41	41	0	0	4	4
Mandaue City	21,479	0	0	19	19	0	0	0	0
**Region 8**	**342,050**	**0**	**0**	**503**	**503**	**0**	**0**	**16**	**16**
Biliran	11,102	0	0	5	5	0	0	0	0
Eastern Samar	38,666	0	0	54	54	0	0	9	9
Northern Leyte	113,942	0	0	335	335	0	0	6	6
Northern Samar	46,536	0	0	21	21	0	0	0	0
Southern Leyte	24,868	0	0	4	4	0	0	0	0
Western Samar	26,826	0	0	19	19	0	0	0	0
Calbayog City	8,836	0	0	25	25	0	0	0	0
Maasin City	5,096	0	0	19	19	0	0	0	0
Ormoc City	20,009	0	0	6	6	0	0	1	1
Tacloban City	46,169	0	0	15	15	0	0	0	0
**Region 9**	**314,587**	**0**	**0**	**741**	**741**	**0**	**0**	**0**	**0**
Zamboanga del Norte	98,783	0	0	4	4	0	0	0	0
Zamboanga del Sur	65,366	0	0	16	16	0	0	0	0
Zamboanga Sibugay	42,607	0	0	18	18	0	0	0	0
Dapitan City	7,733	0	0	699	699	0	0	0	0
Dipolog City	16,393	0	0	0	0	0	0	0	0
Isabela City	9,369	0	0	0	0	0	0	0	0
Pagadian City	19,991	0	0	4	4	0	0	0	0
Zamboanga City	54,345	0	0	0	0	0	0	0	0
**Region 10**	**469,808**	**2**	**9**	**306**	**317**	**0**	**20**	**51**	**71**
Bukidnon	110,248	0	0	51	51	0	0	0	0
Camiguin	6,471	0	0	3	3	0	0	0	0
Lanao del Norte	60,033	0	3	33	36	0	0	0	0
Misamis Occidental	25,727	0	1	15	16	0	0	6	6
Misamis Oriental	79,462	0	0	16	16	0	0	0	0
Cagayan de Oro City	60,104	0	1	120	121	0	0	0	0
El Salvador City	6,228	0	0	5	5	0	0	0	0
Gingoog City	29,566	2	3	12	17	0	0	7	7
Iligan City	27,468	0	0	23	23	0	0	0	0
Malaybalay City	19,334	0	0	8	8	0	0	0	0
Oroquieta City	6,837	0	0	0	0	0	0	0	0
Ozamis City	13,612	0	0	0	0	0	20	38	58
Tangub City	3,838	0	0	4	4	0	0	0	0
Valencia City	20,880	0	1	16	17	0	0	0	0

Table 1.A.1 - MODERN METHOD OF FAMILY PLANNING

New Acceptors

Philippines, 2021

Area	Total Current Users	FSTR/BTL Age group 10-14	FSTR/BTL Age group 15-19	FSTR/BTL Age group 20-49	Total	MSTR/NSV Age group 10-14	MSTR/NSV Age group 15-19	MSTR/NSV Age group 20-49	Total
**Region 11**	**501,561**	**0**	**44**	**836**	**880**	**0**	**1**	**18**	**19**
Davao de Oro	83,015	0	2	35	37	0	0	2	2
Davao del Norte	100,273	0	2	183	185	0	1	2	3
Davao Oriental	51,379	0	0	104	104	0	0	1	1
Davao del Sur	65,425	0	5	80	85	0	0	1	1
Davao Occidental	31,994	0	0	6	6	0	0	0	0
Davao City	169,475	0	35	428	463	0	0	12	12
**Region 12**	**468,656**	**0**	**15**	**522**	**537**	**0**	**0**	**19**	**19**
North Cotabato	136,386	0	2	292	294	0	0	9	9
Sarangani	65,258	0	10	49	59	0	0	3	3
South Cotabato	91,960	0	3	126	129	0	0	6	6
Sultan Kudarat	93,390	0	0	21	21	0	0	0	0
					0				0
Cotabato City	30,632	0	0	30	30	0	0	0	0
Gen. Santos City	51,030	0	0	4	4	0	0	1	1
**BARMM**	**266,572**	**0**	**32**	**243**	**275**	**0**	**0**	**0**	**0**
Basilan	10,532	0	0	3	3	0	0	0	0
Lanao del Sur	40,004	0	0	31	31	0	0	0	0
Maguindanao	89,662	0	5	99	104	0	0	0	0
Sulu	88,420	0	27	104	131	0	0	0	0
Tawi-Tawi	27,695	0	0	1	1	0	0	0	0
					0				0
Lamitan City	4,938	0	0	0	0	0	0	0	0
Marawi City	5,321	0	0	5	5	0	0	0	0
**CARAGA**	**249,180**	**0**	**1**	**192**	**193**	**0**	**0**	**4**	**4**
Agusan del Norte	37,360	0	0	19	19	0	0	0	0
Agusan del Sur	76,525	0	1	14	15	0	0	0	0
Surigao del Norte	30,743	0	0	77	77	0	0	4	4
Surigao del Sur	42,013	0	0	1	1	0	0	0	0
Province of Dinagat	10,218	0	0	1	1	0	0	0	0
Bislig City	9,004	0	0	0	0	0	0	0	0
Butuan City	30,541	0	0	32	32	0	0	0	0
Surigao City	12,776	0	0	48	48	0	0	0	0

Table 1.A.1 - MODERN METHOD OF FAMILY PLANNING

New Acceptors

Philippines, 2021

Area	CONDOM Age group			Total	IUD-INTERVAL Age group			Total	IUD-POSTPARTUM Age group			Total
	10-14	15-19	20-49		10-14	15-19	20-49		10-14	15-19	20-49	
**PHILIPPINES**	**73**	**2,500**	**25,765**	**28,338**	**13**	**731**	**10,964**	**11,708**	**56**	**1,529**	**9,988**	**11,573**
**N C R**	**31**	**535**	**4,607**	**5,173**	**0**	**61**	**365**	**426**	**25**	**592**	**2,638**	**3,255**
Malabon	0	15	125	140	0	0	1	1	0	0	11	11
Navotas	0	52	204	256	0	0	1	1	0	0	0	0
Valenzuela City	0	0	143	143	0	0	10	10	0	0	10	10
Caloocan City	0	33	208	241	0	0	2	2	0	13	37	50
Marikina City	0	4	35	39	0	1	2	3	11	149	633	793
Pasig City	0	10	153	163	0	1	40	41	6	141	528	675
Pateros	0	0	18	18	0	0	5	5	0	0	0	0
Taguig	30	104	1,071	1,205	0	1	6	7	0	4	75	79
Quezon City	1	117	1,170	1,288	0	22	74	96	3	176	575	754
Makati City	0	1	262	263	0	3	95	98	0	24	201	225
Mandaluyong City	0	74	222	296	0	4	20	24	3	35	197	235
San Juan	0	0	2	2	0	0	20	20	0	0	0	0
Manila City	0	52	385	437	0	9	42	51	1	11	61	73
Las Piñas City	0	9	176	185	0	5	22	27	1	36	227	264
Muntinlupa City	0	10	166	176	0	0	8	8	0	0	0	0
Parañaque City	0	3	84	87	0	15	17	32	0	3	20	23
Pasay City	0	51	183	234	0	0	0	0	0	0	63	63
**C A R**	**0**	**16**	**165**	**181**	**0**	**5**	**47**	**52**	**0**	**1**	**22**	**23**
Abra	0	1	22	23	0	1	1	2	0	0	0	0
Apayao	0	5	1	6	0	0	2	2	0	0	4	4
Benguet	0	3	59	62	0	1	22	23	0	0	1	1
Ifugao	0	0	2	2	0	0	0	0	0	0	0	0
Kalinga	0	0	3	3	0	0	5	5	0	1	6	7
Mt. Province	0	0	10	10	0	3	4	7	0	0	1	1
Baguio City	0	7	68	75	0	0	13	13	0	0	10	10
**Region 1**	**1**	**87**	**892**	**980**	**0**	**44**	**482**	**526**	**0**	**5**	**34**	**39**
Ilocos Norte	0	0	60	60	0	1	11	12	0	1	2	3
Ilocos Sur	0	35	183	218	0	0	8	8	0	1	22	23
La Union	0	0	21	21	0	4	17	21	0	3	8	11
Pangasinan	1	42	430	473	0	31	222	253	0	0	0	0
Alaminos City	0	0	0	0	0	8	204	212	0	0	0	0
Candon City	0	0	136	136	0	0	2	2	0	0	0	0
Dagupan City	0	4	8	12	0	0	1	1	0	0	0	0
Laoag City	0	0	0	0	0	0	0	0	0	0	0	0
San Carlos City	0	1	20	21	0	0	7	7	0	0	0	0
San Fernando City	0	5	31	36	0	0	8	8	0	0	2	2
Urdaneta City	0	0	3	3	0	0	2	2	0	0	0	0
Vigan City	0	0	0	0	0	0	0	0	0	0	0	0
**Region 2**	**0**	**20**	**185**	**205**	**0**	**19**	**157**	**176**	**0**	**35**	**125**	**160**
Batanes	0	0	0	0	0	0	0	0	0	0	0	0
Cagayan	0	0	15	15	0	15	51	66	0	10	38	48
Isabela	0	5	104	109	0	4	84	88	0	16	60	76
Nueva Vizcaya	0	0	31	31	0	0	5	5	0	3	2	5
Quirino	0	12	28	40	0	0	2	2	0	2	2	4
Cauayan City	0	0	1	1	0	0	0	0	0	0	1	1
Ilagan City	0	0	5	5	0	0	1	1	0	0	3	3
Santiago City	0	3	0	3	0	0	2	2	0	0	0	0
Tuguegarao City	0	0	1	1	0	0	12	12	0	4	19	23
**Region 3**	**1**	**269**	**3,390**	**3,660**	**0**	**35**	**498**	**533**	**2**	**179**	**2,251**	**2,432**
Aurora	0	0	7	7	0	1	1	2	0	0	0	0
Bataan	1	17	162	180	0	0	7	7	0	4	10	14

Table 1.A.1 - MODERN METHOD OF FAMILY PLANNING

New Acceptors

Philippines, 2021

Area	CONDOM			Total	IUD-INTERVAL			Total	IUD-POSTPARTUM			Total
	Age group				Age group				Age group			
	10-14	15-19	20-49		10-14	15-19	20-49		10-14	15-19	20-49	
Bulacan	0	23	211	234	0	0	185	185	0	0	345	345
Nueva Ecija	0	21	117	138	0	10	48	58	2	105	401	508
Pampanga	0	22	606	628	0	19	144	163	0	52	1,356	1,408
Tarlac	0	53	832	885	0	1	18	19	0	0	11	11
Zambales	0	2	69	71	0	1	13	14	0	0	24	24
Angeles City	0	0	46	46	0	0	0	0	0	2	4	6
Balanga City	0	0	4	4	0	0	3	3	0	0	1	1
Cabanatuan City	0	4	51	55	0	0	2	2	0	1	4	5
City of San Fernando	0	12	237	249	0	2	10	12	0	5	21	26
Gapan City	0	0	17	17	0	0	0	0	0	0	2	2
Mabalacat City	0	9	113	122	0	0	3	3	0	1	1	2
Malolos City	0	0	45	45	0	0	1	1	0	0	0	0
Meycauayan	0	11	74	85	0	0	3	3	0	0	0	0
Olongapo	0	0	42	42	0	1	18	19	0	8	61	69
Palayan City	0	0	0	0	0	0	0	0	0	0	0	0
San Jose City	0	4	30	34	0	0	1	1	0	1	9	10
San Jose del Monte City	0	39	572	611	0	0	0	0	0	0	0	0
Science City of Munoz	0	0	2	2	0	0	0	0	0	0	0	0
Tarlac City	0	52	153	205	0	0	41	41	0	0	1	1
**Region 4A**	**0**	**527**	**2,567**	**3,094**	**0**	**14**	**311**	**325**	**10**	**24**	**355**	**389**
Batangas	0	92	326	418	0	0	26	26	0	1	20	21
Cavite	0	2	24	26	0	1	5	6	0	0	2	2
Laguna	0	0	32	32	0	3	77	80	1	3	14	18
Quezon	0	5	52	57	0	3	38	41	0	3	9	12
Rizal	0	4	66	70	0	7	14	21	0	17	31	48
Antipolo City	0	0	15	15	0	0	103	103	0	0	258	258
Bacoor City	0	0	6	6	0	0	4	4	0	0	5	5
Batangas City	0	2	25	27	0	0	0	0	0	0	0	0
Biñan City	0	318	1,498	1,816	0	0	32	32	0	0	1	1
Cabuyao City	0	21	102	123	0	0	1	1	0	0	0	0
Calamba City	0	4	49	53	0	0	5	5	0	0	2	2
Cavite City	0	0	0	0	0	0	0	0	0	0	0	0
Dasmariñas City	0	15	41	56	0	0	0	0	0	0	0	0
General Trias City	0	0	0	0	0	0	0	0	0	0	0	0
Imus City	0	0	7	7	0	0	1	1	0	0	0	0
Lipa City	0	0	3	3	0	0	2	2	0	0	0	0
Lucena City	0	0	1	1	0	0	0	0	0	0	0	0
San Pablo City	0	0	2	2	0	0	0	0	0	0	1	1
San Pedro City	0	4	7	11	0	0	1	1	0	0	0	0
Santa Rosa City	0	59	306	365	0	0	1	1	9	0	11	20
Tagaytay City	0	0	0	0	0	0	0	0	0	0	1	1
Tanauan City	0	0	1	1	0	0	1	1	0	0	0	0
Tayabas City	0	0	0	0	0	0	0	0	0	0	0	0
Trece Martires City	0	1	4	5	0	0	0	0	0	0	0	0
**Region 4B**	**9**	**12**	**215**	**236**	**0**	**4**	**86**	**90**	**0**	**11**	**125**	**136**
Marinduque	0	4	9	13	0	0	2	2	0	0	0	0
Mindoro Occidental	0	0	17	17	0	3	0	3	0	0	0	0
Mindoro Oriental	0	7	75	82	0	0	64	64	0	11	114	125
Palawan	9	1	49	59	0	1	18	19	0	0	3	3
Romblon	0	0	25	25	0	0	2	2	0	0	1	1
Puerto Princesa City	0	0	40	40	0	0	0	0	0	0	7	7
**Region 5**	**0**	**62**	**2,935**	**2,997**	**0**	**33**	**321**	**354**	**0**	**19**	**104**	**123**
Albay	0	15	484	499	0	0	50	50	0	2	26	28
Camarines Norte	0	7	182	189	0	22	71	93	0	1	7	8
Camarines Sur	0	19	340	359	0	6	26	32	0	10	35	45
Catanduanes	0	0	6	6	0	0	1	1	0	0	2	2
Masbate	0	9	92	101	0	0	30	30	0	2	6	8
Sorsogon	0	5	1,155	1,160	0	0	121	121	0	0	10	10
Iriga City	0	4	71	75	0	2	5	7	0	1	2	3

Table 1.A.1 - MODERN METHOD OF FAMILY PLANNING

New Acceptors

Philippines, 2021

Area	CONDOM			Total	IUD-INTERVAL			Total	IUD-POSTPARTUM			Total
	Age group				Age group				Age group			
	10-14	15-19	20-49		10-14	15-19	20-49		10-14	15-19	20-49	
Legaspi City	0	0	22	22	0	0	0	0	0	0	4	4
Naga City	0	3	583	586	0	3	17	20	0	3	12	15
**Region 6**	**0**	**52**	**764**	**816**	**0**	**48**	**589**	**637**	**2**	**50**	**330**	**382**
Aklan	0	7	91	98	0	0	5	5	0	0	1	1
Antique	0	3	72	75	0	0	8	8	0	0	5	5
Capiz	0	4	80	84	0	0	35	35	0	0	9	9
Guimaras	0	0	9	9	0	0	2	2	0	0	0	0
Iloilo	0	10	199	209	0	23	134	157	0	5	82	87
Negros Occidental	0	24	251	275	0	24	260	284	2	43	204	249
Bacolod City	0	2	32	34	0	1	80	81	0	1	18	19
Iloilo City	0	2	30	32	0	0	65	65	0	1	11	12
**Region 7**	**2**	**129**	**2,489**	**2,620**	**3**	**111**	**4,930**	**5,044**	**3**	**117**	**894**	**1,014**
Bohol	0	18	324	342	1	31	340	372	0	76	406	482
Cebu	1	43	773	817	0	47	587	634	0	13	313	326
Negros Oriental	1	6	841	848	0	7	3,658	3,665	0	12	58	70
Siquijor	0	0	16	16	0	0	5	5	0	0	0	0
Cebu City	0	9	211	220	0	13	243	256	0	10	69	79
Lapu-Lapu City	0	48	284	332	2	11	71	84	3	5	20	28
Mandaue City	0	5	40	45	0	2	26	28	0	1	28	29
**Region 8**	**1**	**30**	**467**	**498**	**0**	**16**	**324**	**340**	**0**	**38**	**187**	**225**
Biliran	0	2	9	11	0	0	3	3	0	0	1	1
Eastern Samar	1	5	46	52	0	1	6	7	0	0	1	1
Northern Leyte	0	4	118	122	0	3	213	216	0	3	51	54
Northern Samar	0	6	53	59	0	4	10	14	0	4	6	10
Southern Leyte	0	0	17	17	0	0	19	19	0	3	8	11
Western Samar	0	0	5	5	0	0	1	1	0	3	33	36
Calbayog City	0	3	44	47	0	0	4	4	0	0	0	0
Maasin City	0	0	19	19	0	5	35	40	0	4	44	48
Ormoc City	0	1	5	6	0	2	3	5	0	10	3	13
Tacloban City	0	9	151	160	0	1	30	31	0	11	40	51
**Region 9**	**3**	**24**	**393**	**420**	**0**	**30**	**227**	**257**	**0**	**68**	**179**	**247**
Zamboanga del Norte	3	9	188	200	0	4	110	114	0	5	18	23
Zamboanga del Sur	0	2	38	40	0	13	52	65	0	52	73	125
Zamboanga Sibugay	0	10	92	102	0	11	38	49	0	5	16	21
Dapitan City	0	0	2	2	0	0	1	1	0	0	0	0
Dipolog City	0	0	0	0	0	0	1	1	0	1	50	51
Isabela City	0	0	3	3	0	0	1	1	0	0	0	0
Pagadian City	0	0	5	5	0	0	6	6	0	5	16	21
Zamboanga City	0	3	65	68	0	2	18	20	0	0	6	6
**Region 10**	**13**	**119**	**990**	**1,122**	**0**	**107**	**551**	**658**	**3**	**170**	**1,030**	**1,203**
Bukidnon	0	17	108	125	0	30	193	223	1	7	129	137
Camiguin	0	0	1	1	0	0	0	0	0	1	1	2
Lanao del Norte	0	11	286	297	0	8	43	51	0	31	224	255
Misamis Occidental	0	12	149	161	0	4	5	9	0	0	1	1
Misamis Oriental	0	14	70	84	0	14	76	90	0	15	66	81
Cagayan de Oro City	0	20	118	138	0	21	113	134	1	74	517	592
El Salvador City	0	0	0	0	0	2	11	13	1	9	6	16
Gingoog City	13	9	58	80	0	6	7	13	0	5	9	14
Iligan City	0	15	124	139	0	18	65	83	0	22	74	96
Malaybalay City	0	1	9	10	0	2	2	4	0	4	0	4
Oroquieta City	0	0	0	0	0	0	0	0	0	0	0	0
Ozamis City	0	3	28	31	0	0	3	3	0	0	0	0
Tangub City	0	0	2	2	0	0	0	0	0	0	0	0
Valencia City	0	17	37	54	0	2	33	35	0	2	3	5

Table 1.A.1 - MODERN METHOD OF FAMILY PLANNING

New Acceptors

Philippines, 2021

Area	CONDOM			Total	IUD-INTERVAL			Total	IUD-POSTPARTUM			Total
	Age group				Age group				Age group			
	10-14	15-19	20-49		10-14	15-19	20-49		10-14	15-19	20-49	
**Region 11**	**12**	**232**	**1,644**	**1,888**	**7**	**60**	**555**	**622**	**4**	**52**	**574**	**630**
Davao de Oro	5	14	101	120	0	8	28	36	2	4	8	14
Davao del Norte	0	15	158	173	0	9	89	98	1	16	90	107
Davao Oriental	0	5	74	79	0	1	42	43	0	1	9	10
Davao del Sur	0	51	99	150	6	22	88	116	1	11	39	51
Davao Occidental	0	0	13	13	0	6	7	13	0	1	4	5
Davao City	7	147	1,199	1,353	1	14	301	316	0	19	424	443
**Region 12**	**0**	**102**	**1,396**	**1,498**	**0**	**50**	**786**	**836**	**0**	**49**	**427**	**476**
North Cotabato	0	41	759	800	0	27	547	574	0	10	118	128
Sarangani	0	24	74	98	0	2	19	21	0	4	5	9
South Cotabato	0	21	269	290	0	17	100	117	0	7	70	77
Sultan Kudarat	0	5	54	59	0	0	24	24	0	15	28	43
				0				0				0
Cotabato City	0	2	138	140	0	0	30	30	0	8	150	158
Gen. Santos City	0	9	102	111	0	4	66	70	0	5	56	61
**BARMM**	**0**	**142**	**1,656**	**1,798**	**1**	**13**	**166**	**180**	**3**	**32**	**281**	**316**
Basilan	0	14	62	76	0	0	1	1	0	0	0	0
Lanao del Sur	0	12	778	790	0	2	3	5	0	1	0	1
Maguindanao	0	47	632	679	0	0	74	74	0	17	239	256
Sulu	0	0	125	125	0	0	62	62	3	0	4	7
Tawi-Tawi	0	0	40	40	0	0	0	0	0	0	0	0
				0				0				0
Lamitan City	0	0	1	1	1	11	26	38	0	13	38	51
Marawi City	0	69	18	87	0	0	0	0	0	1	0	1
**CARAGA**	**0**	**142**	**1,010**	**1,152**	**2**	**81**	**569**	**652**	**4**	**87**	**432**	**523**
Agusan del Norte	0	25	156	181	0	6	50	56	1	15	55	71
Agusan del Sur	0	28	122	150	1	48	323	372	1	41	82	124
Surigao del Norte	0	54	415	469	0	17	88	105	0	5	115	120
Surigao del Sur	0	10	50	60	1	3	28	32	2	2	27	31
Province of Dinagat	0	6	33	39	0	2	35	37	0	0	10	10
Bislig City	0	4	17	21	0	2	1	3	0	2	5	7
Butuan City	0	11	183	194	0	0	3	3	0	19	60	79
Surigao City	0	4	34	38	0	3	41	44	0	3	78	81

Table 1.A.1 - MODERN METHOD OF FAMILY PLANNING

New Acceptors

Philippines, 2021

Area	PILLS-POP			Total	PILLS-COC			Total	INJECTABLES			Total
	Age group				Age group				Age group			
	10-14	15-19	20-49		10-14	15-19	20-49		10-14	15-19	20-49	
**PHILIPPINES**	**139**	**10,081**	**57,186**	**67,406**	**114**	**9,753**	**137,304**	**147,171**	**144**	**15,109**	**117,329**	**132,582**
**N C R**	**6**	**1,495**	**9,626**	**11,127**	**11**	**828**	**11,572**	**12,411**	**9**	**2,305**	**18,691**	**21,005**
Malabon	0	52	325	377	0	27	347	374	0	94	444	538
Navotas	0	64	164	228	0	75	312	387	0	132	346	478
Valenzuela City	0	73	740	813	0	48	1,770	1,818	1	146	1,619	1,766
Caloocan City	1	145	669	815	0	129	953	1,082	0	384	1,644	2,028
Marikina City	0	43	296	339	0	12	266	278	1	109	989	1,099
Pasig City	0	58	444	502	0	87	718	805	1	134	1,812	1,947
Pateros	0	1	17	18	0	1	68	69	0	1	98	99
Taguig	0	112	982	1,094	9	57	871	937	0	134	1,618	1,752
Quezon City	1	321	1,738	2,060	1	129	2,140	2,270	4	431	2,714	3,149
Makati City	0	27	705	732	0	10	654	664	0	63	1,363	1,426
Mandaluyong City	0	79	291	370	0	40	436	476	0	105	540	645
San Juan	0	0	4	4	0	0	99	99	0	0	88	88
Manila City	4	340	1,715	2,059	1	89	789	879	0	154	1,317	1,471
Las Piñas City	0	77	728	805	0	42	861	903	2	206	1,724	1,932
Muntinlupa City	0	45	372	417	0	45	617	662	0	119	1,135	1,254
Parañaque City	0	33	293	326	0	37	428	465	0	93	822	915
Pasay City	0	25	143	168	0	0	243	243	0	0	418	418
**C A R**	**2**	**103**	**733**	**838**	**0**	**89**	**716**	**805**	**3**	**149**	**1,182**	**1,334**
Abra	0	17	88	105	0	50	162	212	0	31	166	197
Apayao	2	9	56	67	0	7	48	55	2	21	53	76
Benguet	0	32	136	168	0	16	233	249	0	36	299	335
Ifugao	0	3	17	20	0	0	13	13	0	2	15	17
Kalinga	0	18	32	50	0	6	78	84	0	4	60	64
Mt. Province	0	2	18	20	0	5	38	43	0	17	45	62
Baguio City	0	22	386	408	0	5	144	149	1	38	544	583
**Region 1**	**0**	**85**	**1,798**	**1,883**	**6**	**496**	**5,444**	**5,946**	**2**	**382**	**3,684**	**4,068**
Ilocos Norte	0	12	419	431	0	1	395	396	0	3	128	131
Ilocos Sur	0	50	1,198	1,248	6	174	1,099	1,279	0	111	864	975
La Union	0	11	84	95	0	20	206	226	1	30	220	251
Pangasinan	0	0	0	0	0	266	2,977	3,243	1	160	1,918	2,079
Alaminos City	0	0	0	0	0	1	106	107	0	2	53	55
Candon City	0	0	4	4	0	0	8	8	0	0	8	8
Dagupan City	0	1	11	12	0	7	76	83	0	42	201	243
Laoag City	0	0	8	8	0	0	164	164	0	0	11	11
San Carlos City	0	0	0	0	0	12	239	251	0	22	173	195
San Fernando City	0	11	74	85	0	7	92	99	0	9	66	75
Urdaneta City	0	0	0	0	0	8	82	90	0	3	42	45
Vigan City	0	0	0	0	0	0	0	0	0	0	0	0
**Region 2**	**7**	**243**	**1,329**	**1,579**	**3**	**279**	**2,831**	**3,113**	**2**	**327**	**1,332**	**1,661**
Batanes	0	0	0	0	0	0	0	0	0	0	1	1
Cagayan	0	49	302	351	0	34	433	467	0	64	380	444
Isabela	5	126	638	769	2	151	1,952	2,105	1	166	561	728
Nueva Vizcaya	1	16	58	75	1	45	183	229	0	25	99	124
Quirino	0	10	36	46	0	23	78	101	0	16	88	104
Cauayan City	0	2	12	14	0	13	28	41	0	14	24	38
Ilagan City	1	15	97	113	0	3	50	53	1	24	62	87
Santiago City	0	0	0	0	0	6	17	23	0	11	24	35
Tuguegarao City	0	25	186	211	0	4	90	94	0	7	93	100
**Region 3**	**2**	**984**	**5,183**	**6,169**	**2**	**1,415**	**12,202**	**13,619**	**18**	**2,540**	**16,872**	**19,430**
Aurora	0	5	31	36	0	1	42	43	0	5	33	38
Bataan	2	162	496	660	1	64	672	737	4	276	1,479	1,759

Table 1.A.1 - MODERN METHOD OF FAMILY PLANNING

New Acceptors

Philippines, 2021

Area	PILLS-POP			Total	PILLS-COC			Total	INJECTABLES			Total
	Age group				Age group				Age group			
	10-14	15-19	20-49		10-14	15-19	20-49		10-14	15-19	20-49	
Bulacan	0	92	837	929	0	198	2,024	2,222	0	419	2,873	3,292
Nueva Ecija	0	152	650	802	0	151	1,074	1,225	1	316	1,567	1,884
Pampanga	0	65	590	655	0	140	1,078	1,218	3	144	1,790	1,937
Tarlac	0	40	328	368	1	152	2,063	2,216	4	164	1,942	2,110
Zambales	0	24	160	184	0	29	378	407	2	194	982	1,178
Angeles City	0	33	139	172	0	57	543	600	0	89	669	758
Balanga City	0	3	14	17	0	7	32	39	3	24	102	129
Cabanatuan City	0	98	298	396	0	52	426	478	0	71	425	496
City of San Fernando	0	24	156	180	0	69	846	915	0	48	297	345
Gapan City	0	5	69	74	0	12	124	136	0	22	231	253
Mabalacat City	0	14	26	40	0	38	374	412	0	106	740	846
Malolos City	0	0	0	0	0	1	90	91	0	4	142	146
Meycauayan	0	31	134	165	0	48	262	310	0	30	254	284
Olongapo	0	11	86	97	0	21	235	256	1	76	575	652
Palayan City	0	0	2	2	0	1	12	13	0	7	19	26
San Jose City	0	36	87	123	0	39	268	307	0	93	396	489
San Jose del Monte City	0	55	630	685	0	105	1,081	1,186	0	188	1,597	1,785
Science City of Munoz	0	0	8	8	0	0	5	5	0	6	39	45
Tarlac City	0	134	442	576	0	230	573	803	0	258	720	978
**Region 4A**	**11**	**576**	**576**	**1,163**	**4**	**660**	**5,593**	**6,257**	**10**	**821**	**7,126**	**7,957**
Batangas	1	143	143	287	1	75	1,008	1,084	7	117	906	1,030
Cavite	0	16	16	32	0	33	288	321	0	57	566	623
Laguna	0	20	20	40	0	17	197	214	0	27	351	378
Quezon	0	32	32	64	2	45	347	394	2	104	635	741
Rizal	0	41	41	82	1	43	437	481	1	102	778	881
Antipolo City	0	0	0	0	0	0	7	7	0	0	397	397
Bacoor City	0	3	3	6	0	7	85	92	0	23	166	189
Batangas City	0	7	7	14	0	6	186	192	0	14	106	120
Biñan City	0	200	200	400	0	261	1,528	1,789	0	190	1,412	1,602
Cabuyao City	0	29	29	58	0	45	267	312	0	33	261	294
Calamba City	1	19	19	39	0	10	234	244	0	12	279	291
Cavite City	0	0	0	0	0	2	11	13	0	2	13	15
Dasmariñas City	0	8	8	16	0	25	195	220	0	24	271	295
General Trias City	0	0	0	0	0	0	0	0	0	0	0	0
Imus City	0	3	3	6	0	6	40	46	0	7	78	85
Lipa City	0	3	3	6	0	0	34	34	0	1	55	56
Lucena City	0	0	0	0	0	7	36	43	0	26	150	176
San Pablo City	0	2	2	4	0	6	41	47	0	9	55	64
San Pedro City	0	26	26	52	0	4	109	113	0	3	56	59
Santa Rosa City	9	22	22	53	0	66	502	568	0	54	480	534
Tagaytay City	0	0	0	0	0	0	8	8	0	0	12	12
Tanauan City	0	2	2	4	0	0	3	3	0	7	37	44
Tayabas City	0	0	0	0	0	0	0	0	0	0	0	0
Trece Martires City	0	0	0	0	0	2	30	32	0	9	62	71
**Region 4B**	**0**	**51**	**592**	**643**	**7**	**133**	**1,491**	**1,631**	**2**	**306**	**2,423**	**2,731**
Marinduque	0	9	179	188	0	49	140	189	0	19	126	145
Mindoro Occidental	0	4	27	31	1	20	84	105	0	32	126	158
Mindoro Oriental	0	4	221	225	0	9	301	310	0	38	392	430
Palawan	0	30	125	155	0	53	616	669	1	204	862	1,067
Romblon	0	4	40	44	6	2	119	127	1	13	131	145
Puerto Princesa City	0	0	0	0	0	0	231	231	0	0	786	786
**Region 5**	**1**	**257**	**1,961**	**2,219**	**3**	**513**	**19,878**	**20,394**	**4**	**424**	**7,169**	**7,597**
Albay	0	51	520	571	1	73	1,408	1,482	0	81	1,246	1,327
Camarines Norte	0	45	274	319	1	35	1,095	1,131	1	55	422	478
Camarines Sur	0	106	680	786	0	115	2,118	2,233	1	106	1,215	1,322
Catanduanes	0	0	3	3	0	2	23	25	0	0	49	49
Masbate	1	23	178	202	1	68	976	1,045	2	64	714	780
Sorsogon	0	14	68	82	0	199	12,816	13,015	0	102	2,944	3,046
Iriga City	0	8	92	100	0	15	235	250	0	3	104	107

Table 1.A.1 - MODERN METHOD OF FAMILY PLANNING

New Acceptors

Philippines, 2021

Area	PILLS-POP			Total	PILLS-COC			Total	INJECTABLES			Total
	Age group				Age group				Age group			
	10-14	15-19	20-49		10-14	15-19	20-49		10-14	15-19	20-49	
Legaspi City	0	4	64	68	0	1	51	52	0	1	29	30
Naga City	0	6	82	88	0	5	1,156	1,161	0	12	446	458
**Region 6**	**6**	**523**	**2,642**	**3,171**	**3**	**733**	**8,052**	**8,788**	**8**	**884**	**8,203**	**9,095**
Aklan	2	30	232	264	0	42	534	576	1	59	625	685
Antique	0	39	250	289	0	19	527	546	0	103	916	1,019
Capiz	0	28	164	192	2	57	691	750	0	75	973	1,048
Guimaras	0	6	34	40	0	13	139	152	0	13	65	78
Iloilo	0	69	410	479	1	121	1,405	1,527	2	126	1,578	1,706
Negros Occidental	4	334	1,332	1,670	0	391	3,187	3,578	5	375	2,901	3,281
Bacolod City	0	8	182	190	0	30	232	262	0	66	690	756
Iloilo City	0	9	38	47	0	60	1,337	1,397	0	67	455	522
**Region 7**	**7**	**603**	**4,251**	**4,861**	**7**	**547**	**9,407**	**9,961**	**5**	**1,314**	**11,492**	**12,811**
Bohol	0	62	418	480	1	50	1,242	1,293	0	120	1,076	1,196
Cebu	4	233	1,419	1,656	1	207	2,956	3,164	1	442	4,020	4,463
Negros Oriental	2	80	426	508	2	114	2,742	2,858	0	296	2,150	2,446
Siquijor	0	11	102	113	0	2	54	56	1	13	131	145
Cebu City	1	121	1,003	1,125	3	109	1,647	1,759	3	319	2,544	2,866
Lapu-Lapu City	0	71	639	710	0	51	506	557	0	84	1,089	1,173
Mandaue City	0	25	244	269	0	14	260	274	0	40	482	522
**Region 8**	**4**	**146**	**1,089**	**1,239**	**3**	**245**	**4,347**	**4,595**	**5**	**257**	**2,613**	**2,875**
Biliran	0	10	30	40	0	10	76	86	0	14	89	103
Eastern Samar	1	14	124	139	2	66	1,631	1,699	5	41	373	419
Northern Leyte	0	18	255	273	0	49	996	1,045	0	53	704	757
Northern Samar	2	37	182	221	0	49	567	616	0	46	490	536
Southern Leyte	0	7	26	33	0	4	106	110	0	11	87	98
Western Samar	1	15	203	219	1	12	364	377	0	26	248	274
Calbayog City	0	5	43	48	0	29	290	319	0	24	253	277
Maasin City	0	4	30	34	0	2	115	117	0	3	79	82
Ormoc City	0	20	64	84	0	21	44	65	0	26	45	71
Tacloban City	0	16	132	148	0	3	158	161	0	13	245	258
**Region 9**	**0**	**143**	**640**	**783**	**3**	**326**	**3,914**	**4,243**	**6**	**428**	**3,227**	**3,661**
Zamboanga del Norte	0	29	171	200	1	64	619	684	1	59	445	505
Zamboanga del Sur	0	24	124	148	0	32	1,666	1,698	1	38	678	717
Zamboanga Sibugay	0	24	69	93	2	74	360	436	2	104	504	610
Dapitan City	0	3	3	6	0	0	3	3	0	7	24	31
Dipolog City	0	0	2	2	0	5	92	97	0	0	2	2
Isabela City	0	5	41	46	0	16	110	126	0	33	292	325
Pagadian City	0	1	30	31	0	5	94	99	0	8	137	145
Zamboanga City	0	57	200	257	0	130	970	1,100	2	179	1,145	1,326
**Region 10**	**4**	**402**	**1,979**	**2,385**	**5**	**670**	**8,612**	**9,287**	**5**	**535**	**4,743**	**5,283**
Bukidnon	2	162	416	580	0	148	790	938	3	99	548	650
Camiguin	0	0	4	4	0	0	6	6	0	0	18	18
Lanao del Norte	0	36	322	358	0	53	674	727	0	48	677	725
Misamis Occidental	0	4	97	101	0	17	267	284	1	13	168	182
Misamis Oriental	0	34	189	223	1	29	227	257	1	39	218	258
Cagayan de Oro City	1	20	222	243	0	43	357	400	0	82	493	575
El Salvador City	0	7	22	29	0	2	15	17	0	13	40	53
Gingoog City	0	12	28	40	0	12	19	31	0	3	56	59
Iligan City	1	50	274	325	1	50	491	542	0	46	382	428
Malaybalay City	0	19	45	64	0	24	147	171	0	28	71	99
Oroquieta City	0	1	11	12	0	1	11	12	0	0	10	10
Ozamis City	0	33	259	292	0	256	5,305	5,561	0	151	1,922	2,073
Tangub City	0	2	10	12	0	7	71	78	0	1	34	35
Valencia City	0	22	80	102	3	28	232	263	0	12	106	118

Table 1.A.1 - MODERN METHOD OF FAMILY PLANNING

New Acceptors

Philippines, 2021

Area	PILLS-POP			Total	PILLS-COC			Total	INJECTABLES			Total
	Age group				Age group				Age group			
	10-14	15-19	20-49		10-14	15-19	20-49		10-14	15-19	20-49	
**Region 11**	**68**	**1,185**	**4,955**	**6,208**	**36**	**691**	**11,972**	**12,699**	**40**	**1,120**	**5,163**	**6,323**
Davao de Oro	6	239	732	977	2	60	2,824	2,886	3	134	502	639
Davao del Norte	11	202	957	1,170	12	96	939	1,047	6	178	1,066	1,250
Davao Oriental	11	41	298	350	11	45	505	561	10	35	375	420
Davao del Sur	17	258	612	887	1	151	4,860	5,012	13	306	764	1,083
Davao Occidental	0	18	40	58	2	44	124	170	1	133	232	366
Davao City	23	427	2,316	2,766	8	295	2,720	3,023	7	334	2,224	2,565
**Region 12**	**10**	**2,358**	**15,019**	**17,387**	**9**	**1,105**	**8,418**	**9,532**	**11**	**1,818**	**9,813**	**11,642**
North Cotabato	8	1,832	13,036	14,876	8	492	3,925	4,425	7	744	4,080	4,831
Sarangani	0	167	305	472	0	147	471	618	0	146	423	569
South Cotabato	2	227	864	1,093	1	130	1,216	1,347	2	399	1,744	2,145
Sultan Kudarat	0	25	100	125	0	181	806	987	0	176	648	824
				0				0				0
Cotabato City	0	19	264	283	0	44	1,213	1,257	0	137	1,720	1,857
Gen. Santos City	0	88	450	538	0	111	787	898	2	216	1,198	1,416
**BARMM**	**1**	**294**	**2,731**	**3,026**	**6**	**553**	**9,466**	**10,025**	**10**	**974**	**10,889**	**11,873**
Basilan	0	33	153	186	0	55	442	497	1	93	529	623
Lanao del Sur	0	28	430	458	0	12	718	730	0	36	776	812
Maguindanao	1	134	972	1,107	0	295	2,625	2,920	0	606	3,099	3,705
Sulu	0	62	981	1,043	1	98	4,802	4,901	6	141	5,595	5,742
Tawi-Tawi	0	30	123	153	5	72	667	744	0	36	546	582
				0				0				0
Lamitan City	0	6	28	34	0	15	114	129	3	60	258	321
Marawi City	0	1	44	45	0	6	98	104	0	2	86	88
**CARAGA**	**10**	**633**	**2,082**	**2,725**	**6**	**470**	**13,389**	**13,865**	**4**	**525**	**2,707**	**3,236**
Agusan del Norte	4	250	678	932	3	55	417	475	2	101	455	558
Agusan del Sur	0	141	398	539	1	68	748	817	0	107	587	694
Surigao del Norte	0	67	331	398	0	162	10,986	11,148	1	152	654	807
Surigao del Sur	0	66	174	240	2	45	239	286	0	29	185	214
Province of Dinagat	0	19	94	113	0	17	140	157	0	19	166	185
Bislig City	0	10	28	38	0	15	50	65	0	9	31	40
Butuan City	6	63	301	370	0	63	472	535	1	71	439	511
Surigao City	0	17	78	95	0	45	337	382	0	37	190	227

Table 1.A.1 - MODERN METHOD OF FAMILY PLANNING

New Acceptors

Philippines, 2021

Area	IMPLANTS			Total	NFP-CMM			Total	NFP-BBT			Total
	Age group				Age group				Age group			
	10-14	15-19	20-49		10-14	15-19	20-49		10-14	15-19	20-49	
**PHILIPPINES**	**154**	**6,007**	**38,908**	**45,069**	**9**	**84**	**4,535**	**4,628**	**22**	**135**	**888**	**1,045**
**N C R**	**56**	**1,279**	**6,217**	**7,552**	**0**	**1**	**68**	**69**	**0**	**0**	**3**	**3**
Malabon	0	18	114	132	0	0	0	0	0	0	0	0
Navotas	2	39	55	96	0	0	0	0	0	0	0	0
Valenzuela City	2	77	510	589	0	0	40	40	0	0	0	0
Caloocan City	0	60	279	339	0	0	0	0	0	0	0	0
Marikina City	4	57	369	430	0	0	1	1	0	0	0	0
Pasig City	6	96	669	771	0	0	0	0	0	0	0	0
Pateros	0	1	7	8	0	0	0	0	0	0	0	0
Taguig	1	8	255	264	0	0	0	0	0	0	0	0
Quezon City	7	222	952	1,181	0	0	0	0	0	0	3	3
Makati City	1	6	256	263	0	0	0	0	0	0	0	0
Mandaluyong City	0	10	266	276	0	1	27	28	0	0	0	0
San Juan	0	0	15	15	0	0	0	0	0	0	0	0
Manila City	32	596	1,839	2,467	0	0	0	0	0	0	0	0
Las Piñas City	1	74	257	332	0	0	0	0	0	0	0	0
Muntinlupa City	0	12	241	253	0	0	0	0	0	0	0	0
Parañaque City	0	3	91	94	0	0	0	0	0	0	0	0
Pasay City	0	0	42	42	0	0	0	0	0	0	0	0
**C A R**	**0**	**132**	**534**	**666**	**0**	**0**	**108**	**108**	**0**	**2**	**77**	**79**
Abra	0	8	45	53	0	0	105	105	0	0	77	77
Apayao	0	9	19	28	0	0	0	0	0	0	0	0
Benguet	0	6	53	59	0	0	0	0	0	2	0	2
Ifugao	0	1	3	4	0	0	0	0	0	0	0	0
Kalinga	0	7	20	27	0	0	0	0	0	0	0	0
Mt. Province	0	0	16	16	0	0	3	3	0	0	0	0
Baguio City	0	101	378	479	0	0	0	0	0	0	0	0
**Region 1**	**0**	**110**	**1,210**	**1,320**	**0**	**0**	**85**	**85**	**0**	**6**	**64**	**70**
Ilocos Norte	0	3	129	132	0	0	1	1	0	0	20	20
Ilocos Sur	0	2	57	59	0	0	14	14	0	3	5	8
La Union	0	25	139	164	0	0	70	70	0	0	0	0
Pangasinan	0	65	696	761	0	0	0	0	0	3	37	40
Alaminos City	0	2	38	40	0	0	0	0	0	0	0	0
Candon City	0	0	0	0	0	0	0	0	0	0	0	0
Dagupan City	0	2	33	35	0	0	0	0	0	0	2	2
Laoag City	0	0	1	1	0	0	0	0	0	0	0	0
San Carlos City	0	2	23	25	0	0	0	0	0	0	0	0
San Fernando City	0	9	91	100	0	0	0	0	0	0	0	0
Urdaneta City	0	0	3	3	0	0	0	0	0	0	0	0
Vigan City	0	0	0	0	0	0	0	0	0	0	0	0
**Region 2**	**0**	**91**	**344**	**435**	**0**	**0**	**0**	**0**	**12**	**21**	**48**	**81**
Batanes	0	0	0	0	0	0	0	0	0	0	0	0
Cagayan	0	15	82	97	0	0	0	0	0	1	15	16
Isabela	0	51	179	230	0	0	0	0	0	6	19	25
Nueva Vizcaya	0	19	68	87	0	0	0	0	12	14	14	40
Quirino	0	1	3	4	0	0	0	0	0	0	0	0
Cauayan City	0	2	1	3	0	0	0	0	0	0	0	0
Ilagan City	0	0	3	3	0	0	0	0	0	0	0	0
Santiago City	0	3	2	5	0	0	0	0	0	0	0	0
Tuguegarao City	0	0	6	6	0	0	0	0	0	0	0	0
**Region 3**	**16**	**437**	**3,091**	**3,544**	**0**	**19**	**151**	**170**	**0**	**5**	**19**	**24**
Aurora	0	1	3	4	0	0	0	0	0	0	2	2
Bataan	12	107	287	406	0	0	9	9	0	1	4	5

Table 1.A.1 - MODERN METHOD OF FAMILY PLANNING

New Acceptors

Philippines, 2021

Area	IMPLANTS			Total	NFP-CMM			Total	NFP-BBT			Total
	Age group				Age group				Age group			
	10-14	15-19	20-49		10-14	15-19	20-49		10-14	15-19	20-49	
Bulacan	0	34	561	595	0	0	6	6	0	0	0	0
Nueva Ecija	1	145	547	693	0	1	1	2	0	0	0	0
Pampanga	0	22	743	765	0	17	127	144	0	2	6	8
Tarlac	0	6	143	149	0	0	0	0	0	0	0	0
Zambales	1	31	194	226	0	0	0	0	0	0	0	0
Angeles City	0	20	206	226	0	0	0	0	0	0	0	0
Balanga City	0	4	9	13	0	0	0	0	0	0	0	0
Cabanatuan City	0	0	7	7	0	0	0	0	0	0	0	0
City of San Fernando	0	0	16	16	0	0	1	1	0	0	4	4
Gapan City	0	2	10	12	0	0	1	1	0	0	0	0
Mabalacat City	0	1	6	7	0	0	0	0	0	0	0	0
Malolos City	0	0	2	2	0	0	0	0	0	0	0	0
Meycauayan	0	5	8	13	0	1	6	7	0	0	0	0
Olongapo	2	38	114	154	0	0	0	0	0	0	0	0
Palayan City	0	0	0	0	0	0	0	0	0	0	0	0
San Jose City	0	0	10	10	0	0	0	0	0	0	0	0
San Jose del Monte City	0	19	175	194	0	0	0	0	0	0	0	0
Science City of Munoz	0	0	0	0	0	0	0	0	0	0	0	0
Tarlac City	0	2	50	52	0	0	0	0	0	2	3	5
**Region 4A**	**10**	**100**	**852**	**962**	**9**	**0**	**37**	**46**	**9**	**7**	**19**	**35**
Batangas	1	2	29	32	0	0	0	0	0	0	0	0
Cavite	0	5	78	83	0	0	8	8	0	0	0	0
Laguna	0	8	126	134	0	0	10	10	0	0	0	0
Quezon	0	4	31	35	0	0	0	0	0	0	2	2
Rizal	0	38	139	177	0	0	19	19	0	6	4	10
Antipolo City	0	0	32	32	0	0	0	0	0	0	13	13
Bacoor City	0	2	6	8	0	0	0	0	0	0	0	0
Batangas City	0	0	3	3	0	0	0	0	0	0	0	0
Biñan City	0	20	128	148	0	0	0	0	0	0	0	0
Cabuyao City	0	0	12	12	0	0	0	0	0	1	0	1
Calamba City	0	13	166	179	0	0	0	0	0	0	0	0
Cavite City	0	0	0	0	0	0	0	0	0	0	0	0
Dasmariñas City	0	2	33	35	0	0	0	0	0	0	0	0
General Trias City	0	0	0	0	0	0	0	0	0	0	0	0
Imus City	0	0	1	1	0	0	0	0	0	0	0	0
Lipa City	0	0	0	0	0	0	0	0	0	0	0	0
Lucena City	0	0	0	0	0	0	0	0	0	0	0	0
San Pablo City	0	2	1	3	0	0	0	0	0	0	0	0
San Pedro City	0	1	3	4	0	0	0	0	0	0	0	0
Santa Rosa City	9	2	63	74	9	0	0	9	9	0	0	9
Tagaytay City	0	1	0	1	0	0	0	0	0	0	0	0
Tanauan City	0	0	0	0	0	0	0	0	0	0	0	0
Tayabas City	0	0	0	0	0	0	0	0	0	0	0	0
Trece Martires City	0	0	1	1	0	0	0	0	0	0	0	0
**Region 4B**	**1**	**50**	**770**	**821**	**0**	**0**	**42**	**42**	**0**	**1**	**1**	**2**
Marinduque	0	7	78	85	0	0	0	0	0	0	0	0
Mindoro Occidental	1	0	19	20	0	0	4	4	0	0	0	0
Mindoro Oriental	0	9	113	122	0	0	1	1	0	0	1	1
Palawan	0	29	486	515	0	0	23	23	0	0	0	0
Romblon	0	5	61	66	0	0	14	14	0	1	0	1
Puerto Princesa City	0	0	13	13	0	0	0	0	0	0	0	0
**Region 5**	**2**	**186**	**2,668**	**2,856**	**0**	**33**	**3,482**	**3,515**	**0**	**8**	**43**	**51**
Albay	0	42	455	497	0	1	33	34	0	2	22	24
Camarines Norte	0	12	79	91	0	0	25	25	0	0	2	2
Camarines Sur	1	83	905	989	0	1	121	122	0	6	11	17
Catanduanes	0	1	8	9	0	0	0	0	0	0	0	0
Masbate	1	29	332	362	0	10	151	161	0	0	0	0
Sorsogon	0	9	723	732	0	21	3,151	3,172	0	0	4	4
Iriga City	0	10	99	109	0	0	0	0	0	0	0	0

Table 1.A.1 - MODERN METHOD OF FAMILY PLANNING

New Acceptors

Philippines, 2021

Area	IMPLANTS			Total	NFP-CMM			Total	NFP-BBT			Total
	Age group				Age group				Age group			
	10-14	15-19	20-49		10-14	15-19	20-49		10-14	15-19	20-49	
Legaspi City	0	0	11	11	0	0	0	0	0	0	0	0
Naga City	0	0	56	56	0	0	1	1	0	0	4	4
**Region 6**	**6**	**259**	**2,163**	**2,428**	**0**	**6**	**165**	**171**	**0**	**10**	**32**	**42**
Aklan	2	7	146	155	0	0	3	3	0	0	2	2
Antique	0	25	121	146	0	5	151	156	0	0	5	5
Capiz	0	10	153	163	0	1	1	2	0	1	1	2
Guimaras	0	2	9	11	0	0	0	0	0	0	0	0
Iloilo	0	66	535	601	0	0	10	10	0	0	1	1
Negros Occidental	2	122	809	933	0	0	0	0	0	3	8	11
Bacolod City	2	26	265	293	0	0	0	0	0	6	15	21
Iloilo City	0	1	125	126	0	0	0	0	0	0	0	0
**Region 7**	**2**	**299**	**3,497**	**3,798**	**0**	**0**	**1**	**1**	**0**	**25**	**105**	**130**
Bohol	0	56	349	405	0	0	0	0	0	0	4	4
Cebu	0	143	1,511	1,654	0	0	0	0	0	22	90	112
Negros Oriental	1	27	584	612	0	0	1	1	0	3	11	14
Siquijor	0	2	17	19	0	0	0	0	0	0	0	0
Cebu City	0	46	600	646	0	0	0	0	0	0	0	0
Lapu-Lapu City	0	11	212	223	0	0	0	0	0	0	0	0
Mandaue City	1	14	224	239	0	0	0	0	0	0	0	0
**Region 8**	**0**	**136**	**1,381**	**1,517**	**0**	**1**	**12**	**13**	**0**	**3**	**82**	**85**
Biliran	0	5	18	23	0	0	6	6	0	0	0	0
Eastern Samar	0	8	104	112	0	0	4	4	0	0	0	0
Northern Leyte	0	18	659	677	0	0	0	0	0	3	54	57
Northern Samar	0	35	191	226	0	0	0	0	0	0	0	0
Southern Leyte	0	9	77	86	0	0	0	0	0	0	0	0
Western Samar	0	14	128	142	0	1	2	3	0	0	19	19
Calbayog City	0	4	52	56	0	0	0	0	0	0	0	0
Maasin City	0	6	38	44	0	0	0	0	0	0	0	0
Ormoc City	0	12	11	23	0	0	0	0	0	0	9	9
Tacloban City	0	25	103	128	0	0	0	0	0	0	0	0
**Region 9**	**6**	**358**	**1,647**	**2,011**	**0**	**0**	**57**	**57**	**0**	**1**	**1**	**2**
Zamboanga del Norte	3	111	417	531	0	0	54	54	0	0	0	0
Zamboanga del Sur	2	108	375	485	0	0	0	0	0	1	1	2
Zamboanga Sibugay	1	101	373	475	0	0	3	3	0	0	0	0
Dapitan City	0	0	0	0	0	0	0	0	0	0	0	0
Dipolog City	0	1	123	124	0	0	0	0	0	0	0	0
Isabela City	0	7	81	88	0	0	0	0	0	0	0	0
Pagadian City	0	0	5	5	0	0	0	0	0	0	0	0
Zamboanga City	0	30	273	303	0	0	0	0	0	0	0	0
**Region 10**	**5**	**339**	**2,613**	**2,957**	**0**	**3**	**158**	**161**	**0**	**14**	**316**	**330**
Bukidnon	1	155	800	956	0	0	2	2	0	9	304	313
Camiguin	0	0	0	0	0	0	0	0	0	0	0	0
Lanao del Norte	0	34	323	357	0	0	0	0	0	0	0	0
Misamis Occidental	0	1	28	29	0	0	4	4	0	0	0	0
Misamis Oriental	0	18	107	125	0	0	8	8	0	0	0	0
Cagayan de Oro City	0	50	411	461	0	0	0	0	0	0	0	0
El Salvador City	0	8	43	51	0	0	0	0	0	0	0	0
Gingoog City	0	8	44	52	0	0	4	4	0	4	9	13
Iligan City	0	7	54	61	0	0	0	0	0	0	0	0
Malaybalay City	0	16	30	46	0	1	2	3	0	0	0	0
Oroquieta City	0	0	1	1	0	0	0	0	0	0	0	0
Ozamis City	0	38	753	791	0	2	135	137	0	1	3	4
Tangub City	0	0	0	0	0	0	0	0	0	0	0	0
Valencia City	4	4	19	27	0	0	3	3	0	0	0	0

Table 1.A.1 - MODERN METHOD OF FAMILY PLANNING

New Acceptors

Philippines, 2021

Area	IMPLANTS			Total	NFP-CMM			Total	NFP-BBT			Total
	Age group				Age group				Age group			
	10-14	15-19	20-49		10-14	15-19	20-49		10-14	15-19	20-49	
**Region 11**	**28**	**470**	**2,628**	**3,126**	**0**	**14**	**123**	**137**	**0**	**2**	**14**	**16**
Davao de Oro	4	58	188	250	0	3	19	22	0	0	0	0
Davao del Norte	4	89	539	632	0	1	14	15	0	0	4	4
Davao Oriental	1	4	81	86	0	4	55	59	0	2	4	6
Davao del Sur	2	68	244	314	0	1	2	3	0	0	0	0
Davao Occidental	10	30	65	105	0	0	0	0	0	0	0	0
Davao City	7	221	1,511	1,739	0	5	33	38	0	0	6	6
**Region 12**	**7**	**872**	**3,419**	**4,298**	**0**	**0**	**14**	**14**	**1**	**24**	**35**	**60**
North Cotabato	4	232	1,225	1,461	0	0	7	7	0	4	18	22
Sarangani	0	90	389	479	0	0	0	0	0	0	0	0
South Cotabato	3	279	701	983	0	0	0	0	1	8	7	16
Sultan Kudarat	0	145	278	423	0	0	0	0	0	0	0	0
				0				0				0
Cotabato City	0	17	371	388	0	0	7	7	0	4	10	14
Gen. Santos City	0	109	455	564	0	0	0	0	0	8	0	8
**BARMM**	**9**	**536**	**3,997**	**4,542**	**0**	**0**	**4**	**4**	**0**	**4**	**7**	**11**
Basilan	1	40	226	267	0	0	0	0	0	0	0	0
Lanao del Sur	1	5	181	187	0	0	1	1	0	0	0	0
Maguindanao	0	252	985	1,237	0	0	1	1	0	0	2	2
Sulu	7	185	2,048	2,240	0	0	0	0	0	0	0	0
Tawi-Tawi	0	29	445	474	0	0	0	0	0	0	0	0
				0				0				0
Lamitan City	0	25	64	89	0	0	2	2	0	4	5	9
Marawi City	0	0	48	48	0	0	0	0	0	0	0	0
**CARAGA**	**6**	**353**	**1,877**	**2,236**	**0**	**7**	**28**	**35**	**0**	**2**	**22**	**24**
Agusan del Norte	1	62	220	283	0	0	0	0	0	0	0	0
Agusan del Sur	0	106	387	493	0	2	2	4	0	1	2	3
Surigao del Norte	2	57	697	756	0	4	25	29	0	1	15	16
Surigao del Sur	0	43	174	217	0	0	0	0	0	0	5	5
Province of Dinagat	3	25	171	199	0	0	0	0	0	0	0	0
Bislig City	0	4	15	19	0	1	1	2	0	0	0	0
Butuan City	0	28	107	135	0	0	0	0	0	0	0	0
Surigao City	0	28	106	134	0	0	0	0	0	0	0	0

Table 1.A.1 - MODERN METHOD OF FAMILY PLANNING

New Acceptors

Philippines, 2021

Area	NFP-STM			Total	NFP-SDM			Total	NFP-LAM			Total	Total New Acceptors
	Age group				Age group				Age group				
	10-14	15-19	20-49		10-14	15-19	20-49		10-14	15-19	20-49		
**PHILIPPINES**	14	133	1,203	1,350	13	382	7,760	8,155	805	49,798	290,038	340,641	815,425
**N C R**	0	0	0	0	1	0	36	37	56	6,438	55,081	61,575	124,070
Malabon	0	0	0	0	0	0	0	0	0	122	511	633	2,214
Navotas	0	0	0	0	0	0	8	8	1	118	833	952	2,411
Valenzuela City	0	0	0	0	0	0	5	5	0	49	2,739	2,788	8,093
Caloocan City	0	0	0	0	0	0	1	1	3	796	3,289	4,088	8,668
Marikina City	0	0	0	0	0	0	0	0	1	40	383	424	3,426
Pasig City	0	0	0	0	0	0	0	0	1	149	2,885	3,035	8,048
Pateros	0	0	0	0	0	0	0	0	0	14	716	730	964
Taguig	0	0	0	0	1	0	4	5	10	812	8,699	9,521	14,885
Quezon City	0	0	0	0	0	0	14	14	24	1,345	7,745	9,114	20,132
Makati City	0	0	0	0	0	0	0	0	0	136	3,488	3,624	7,461
Mandaluyong City	0	0	0	0	0	0	4	4	0	286	2,769	3,055	5,499
San Juan	0	0	0	0	0	0	0	0	0	15	478	493	730
Manila City	0	0	0	0	0	0	0	0	8	930	7,323	8,261	16,013
Las Piñas City	0	0	0	0	0	0	0	0	1	294	3,357	3,652	8,160
Muntinlupa City	0	0	0	0	0	0	0	0	0	274	2,952	3,226	6,082
Parañaque City	0	0	0	0	0	0	0	0	7	958	6,163	7,128	9,206
Pasay City	0	0	0	0	0	0	0	0	0	100	751	851	2,078
**C A R**	0	0	0	0	0	5	32	37	18	840	3,738	4,596	8,849
Abra	0	0	0	0	0	3	5	8	8	188	989	1,185	1,990
Apayao	0	0	0	0	0	0	0	0	4	114	345	463	702
Benguet	0	0	0	0	0	0	8	8	1	145	847	993	1,959
Ifugao	0	0	0	0	0	0	3	3	2	151	482	635	694
Kalinga	0	0	0	0	0	2	9	11	2	155	600	757	1,047
Mt. Province	0	0	0	0	0	0	5	5	1	78	339	418	589
Baguio City	0	0	0	0	0	0	2	2	0	9	136	145	1,868
**Region 1**	0	16	17	33	0	29	214	243	20	2,043	17,133	19,196	34,618
Ilocos Norte	0	0	0	0	0	0	3	3	1	87	1,415	1,503	2,692
Ilocos Sur	0	5	0	5	0	10	113	123	3	293	3,404	3,700	7,774
La Union	0	0	0	0	0	0	4	4	3	383	1,521	1,907	2,821
Pangasinan	0	11	17	28	0	19	73	92	7	1,027	6,994	8,028	15,038
Alaminos City	0	0	0	0	0	0	0	0	2	33	1,089	1,124	1,547
Candon City	0	0	0	0	0	0	0	0	0	0	136	136	296
Dagupan City	0	0	0	0	0	0	5	5	4	106	740	850	1,243
Laoag City	0	0	0	0	0	0	0	0	0	19	1,265	1,284	1,468
San Carlos City	0	0	0	0	0	0	0	0	0	40	176	216	715
San Fernando City	0	0	0	0	0	0	16	16	0	10	123	133	566
Urdaneta City	0	0	0	0	0	0	0	0	0	35	204	239	382
Vigan City	0	0	0	0	0	0	0	0	0	10	66	76	76
**Region 2**	0	0	4	4	0	2	12	14	36	1,904	6,721	8,661	16,256
Batanes	0	0	0	0	0	0	0	0	2	20	74	96	97
Cagayan	0	0	4	4	0	0	0	0	1	488	2,251	2,740	4,268
Isabela	0	0	0	0	0	2	11	13	8	472	1,470	1,950	6,128
Nueva Vizcaya	0	0	0	0	0	0	1	1	17	418	1,162	1,597	2,274
Quirino	0	0	0	0	0	0	0	0	0	64	190	254	560
Cauayan City	0	0	0	0	0	0	0	0	3	124	398	525	626
Ilagan City	0	0	0	0	0	0	0	0	2	86	410	498	780
Santiago City	0	0	0	0	0	0	0	0	3	220	521	744	813
Tuguegarao City	0	0	0	0	0	0	0	0	0	12	245	257	710
**Region 3**	0	0	13	13	1	73	615	689	26	3,806	27,044	30,876	87,546
Aurora	0	0	0	0	0	0	0	0	1	153	336	490	627
Bataan	0	0	0	0	0	0	0	0	6	444	2,423	2,873	6,702

Table 1.A.1 - MODERN METHOD OF FAMILY PLANNING

New Acceptors

Philippines, 2021

Area	NFP-STM Age group 10-14	NFP-STM 15-19	NFP-STM 20-49	Total	NFP-SDM Age group 10-14	NFP-SDM 15-19	NFP-SDM 20-49	Total	NFP-LAM Age group 10-14	NFP-LAM 15-19	NFP-LAM 20-49	Total	Total New Acceptors
Bulacan	0	0	0	0	0	0	13	13	0	554	4,560	5,114	16,096
Nueva Ecija	0	0	0	0	0	1	1	2	5	449	2,361	2,815	8,310
Pampanga	0	0	13	13	1	72	589	662	0	209	3,347	3,556	13,527
Tarlac	0	0	0	0	0	0	12	12	1	361	4,304	4,666	10,465
Zambales	0	0	0	0	0	0	0	0	1	176	870	1,047	3,331
Angeles City	0	0	0	0	0	0	0	0	0	97	1,055	1,152	3,052
Balanga City	0	0	0	0	0	0	0	0	4	69	159	232	441
Cabanatuan City	0	0	0	0	0	0	0	0	2	277	1,098	1,377	2,823
City of San Fernando	0	0	0	0	0	0	0	0	0	57	698	755	2,691
Gapan City	0	0	0	0	0	0	0	0	0	61	461	522	1,019
Mabalacat City	0	0	0	0	0	0	0	0	0	217	1,389	1,606	3,065
Malolos City	0	0	0	0	0	0	0	0	0	1	5	6	291
Meycauayan	0	0	0	0	0	0	0	0	0	77	862	939	1,810
Olongapo	0	0	0	0	0	0	0	0	1	66	509	576	1,893
Palayan City	0	0	0	0	0	0	0	0	0	4	48	52	94
San Jose City	0	0	0	0	0	0	0	0	5	218	773	996	1,981
San Jose del Monte City	0	0	0	0	0	0	0	0	0	0	0	0	4,471
Science City of Munoz	0	0	0	0	0	0	0	0	0	0	0	0	60
Tarlac City	0	0	0	0	0	0	0	0	0	316	1,786	2,102	4,797
**Region 4A**	**9**	**0**	**89**	**98**	**0**	**66**	**86**	**152**	**98**	**1,429**	**10,767**	**12,294**	**33,452**
Batangas	0	0	0	0	0	65	67	132	72	147	1,148	1,367	4,485
Cavite	0	0	0	0	0	1	1	2	1	63	920	984	2,162
Laguna	0	0	0	0	0	0	4	4	1	82	350	433	1,408
Quezon	0	0	0	0	0	0	4	4	3	132	692	827	2,210
Rizal	0	0	0	0	0	0	0	0	4	220	1,165	1,389	3,212
Antipolo City	0	0	89	89	0	0	1	1	0	0	534	534	1,652
Bacoor City	0	0	0	0	0	0	0	0	0	54	704	758	1,082
Batangas City	0	0	0	0	0	0	5	5	0	0	3	3	364
Biñan City	0	0	0	0	0	0	4	4	0	241	1,201	1,442	7,325
Cabuyao City	0	0	0	0	0	0	0	0	0	103	1,149	1,252	2,057
Calamba City	0	0	0	0	0	0	0	0	2	57	429	488	1,340
Cavite City	0	0	0	0	0	0	0	0	0	0	7	7	37
Dasmariñas City	0	0	0	0	0	0	0	0	0	75	718	793	1,419
General Trias City	0	0	0	0	0	0	0	0	0	0	0	0	0
Imus City	0	0	0	0	0	0	0	0	0	7	87	94	244
Lipa City	0	0	0	0	0	0	0	0	0	0	0	0	106
Lucena City	0	0	0	0	0	0	0	0	0	0	5	5	226
San Pablo City	0	0	0	0	0	0	0	0	3	45	222	270	394
San Pedro City	0	0	0	0	0	0	0	0	1	37	312	350	593
Santa Rosa City	9	0	0	9	0	0	0	0	9	96	910	1,015	2,669
Tagaytay City	0	0	0	0	0	0	0	0	1	21	81	103	125
Tanauan City	0	0	0	0	0	0	0	0	0	5	32	37	90
Tayabas City	0	0	0	0	0	0	0	0	1	42	89	132	132
Trece Martires City	0	0	0	0	0	0	0	0	0	2	9	11	120
**Region 4B**	**0**	**0**	**1**	**1**	**3**	**0**	**1,108**	**1,111**	**29**	**1,491**	**6,405**	**7,925**	**15,601**
Marinduque	0	0	0	0	0	0	0	0	0	57	597	654	1,309
Mindoro Occidental	0	0	0	0	0	0	1,073	1,073	13	766	1,300	2,079	3,539
Mindoro Oriental	0	0	1	1	0	0	1	1	0	56	774	830	2,254
Palawan	0	0	0	0	0	0	9	9	8	454	1,666	2,128	4,679
Romblon	0	0	0	0	3	0	10	13	8	158	1,746	1,912	2,377
Puerto Princesa City	0	0	0	0	0	0	15	15	0	0	322	322	1,443
**Region 5**	**0**	**12**	**893**	**905**	**0**	**37**	**3,429**	**3,466**	**39**	**3,588**	**17,593**	**21,220**	**67,822**
Albay	0	0	0	0	0	6	546	552	2	370	3,667	4,039	9,136
Camarines Norte	0	8	768	776	0	14	515	529	4	418	1,661	2,083	5,821
Camarines Sur	0	4	114	118	0	5	156	161	17	1,038	4,670	5,725	11,910
Catanduanes	0	0	0	0	0	0	9	9	0	53	210	263	369
Masbate	0	0	3	3	0	6	668	674	12	1,023	3,018	4,053	7,455
Sorsogon	0	0	4	4	0	6	1,377	1,383	4	621	2,619	3,244	27,860
Iriga City	0	0	0	0	0	0	38	38	0	44	323	367	1,057

Table 1.A.1 - MODERN METHOD OF FAMILY PLANNING

New Acceptors

Philippines, 2021

Area	NFP-STM			Total	NFP-SDM			Total	NFP-LAM			Total	Total New Acceptors
	Age group				Age group				Age group				
	10-14	15-19	20-49		10-14	15-19	20-49		10-14	15-19	20-49		
Legaspi City	0	0	0	0	0	0	2	2	0	5	104	109	309
Naga City	0	0	4	4	0	0	118	118	0	16	1,321	1,337	3,905
**Region 6**	**0**	**0**	**1**	**1**	**0**	**5**	**87**	**92**	**55**	**3,302**	**21,294**	**24,651**	**50,655**
Aklan	0	0	1	1	0	2	3	5	8	188	1,909	2,105	3,937
Antique	0	0	0	0	0	0	4	4	1	162	1,507	1,670	3,966
Capiz	0	0	0	0	0	0	0	0	0	106	1,385	1,491	3,816
Guimaras	0	0	0	0	0	0	0	0	0	114	539	653	952
Iloilo	0	0	0	0	0	0	44	44	21	632	5,958	6,611	11,444
Negros Occidental	0	0	0	0	0	3	33	36	25	2,008	9,194	11,227	21,750
Bacolod City	0	0	0	0	0	0	0	0	0	92	770	862	2,541
Iloilo City	0	0	0	0	0	0	3	3	0	0	32	32	2,249
**Region 7**	**0**	**0**	**0**	**0**	**0**	**1**	**201**	**202**	**61**	**4,814**	**33,650**	**38,525**	**79,383**
Bohol	0	0	0	0	0	0	9	9	2	406	2,175	2,583	7,236
Cebu	0	0	0	0	0	1	162	163	17	1,675	13,072	14,764	27,782
Negros Oriental	0	0	0	0	0	0	0	0	27	1,130	5,370	6,527	17,625
Siquijor	0	0	0	0	0	0	30	30	0	55	191	246	697
Cebu City	0	0	0	0	0	0	0	0	15	1,234	9,336	10,585	17,646
Lapu-Lapu City	0	0	0	0	0	0	0	0	0	311	3,269	3,580	6,732
Mandaue City	0	0	0	0	0	0	0	0	0	3	237	240	1,665
**Region 8**	**0**	**83**	**151**	**234**	**0**	**5**	**73**	**78**	**21**	**1,305**	**8,109**	**9,435**	**21,653**
Biliran	0	0	0	0	0	0	4	4	4	92	373	469	751
Eastern Samar	0	4	1	5	0	5	27	32	4	153	1,079	1,236	3,769
Northern Leyte	0	0	0	0	0	0	0	0	0	231	2,020	2,251	5,793
Northern Samar	0	0	0	0	0	0	6	6	3	183	1,555	1,741	3,450
Southern Leyte	0	0	0	0	0	0	0	0	0	107	499	606	984
Western Samar	0	0	0	0	0	0	0	0	8	101	473	582	1,677
Calbayog City	0	0	0	0	0	0	0	0	0	98	700	798	1,574
Maasin City	0	0	0	0	0	0	27	27	2	12	128	142	572
Ormoc City	0	79	150	229	0	0	9	9	0	235	548	783	1,304
Tacloban City	0	0	0	0	0	0	0	0	0	93	734	827	1,779
**Region 9**	**0**	**0**	**17**	**17**	**0**	**1**	**82**	**83**	**30**	**2,793**	**7,910**	**10,733**	**23,255**
Zamboanga del Norte	0	0	6	6	0	1	73	74	8	872	1,914	2,794	5,189
Zamboanga del Sur	0	0	0	0	0	0	1	1	6	323	549	878	4,175
Zamboanga Sibugay	0	0	0	0	0	0	8	8	2	406	989	1,397	3,212
Dapitan City	0	0	0	0	0	0	0	0	1	93	194	288	1,030
Dipolog City	0	0	11	11	0	0	0	0	2	158	475	635	923
Isabela City	0	0	0	0	0	0	0	0	0	132	531	663	1,252
Pagadian City	0	0	0	0	0	0	0	0	0	29	247	276	592
Zamboanga City	0	0	0	0	0	0	0	0	11	780	3,011	3,802	6,882
**Region 10**	**0**	**0**	**9**	**9**	**3**	**131**	**1,148**	**1,282**	**79**	**4,024**	**17,597**	**21,700**	**46,765**
Bukidnon	0	0	4	4	2	59	120	181	21	1,243	2,679	3,943	8,103
Camiguin	0	0	0	0	0	0	0	0	2	36	206	244	278
Lanao del Norte	0	0	5	5	0	0	49	49	2	311	2,642	2,955	5,815
Misamis Occidental	0	0	0	0	0	0	3	3	4	124	1,112	1,240	2,036
Misamis Oriental	0	0	0	0	0	2	47	49	12	320	1,494	1,826	3,017
Cagayan de Oro City	0	0	0	0	0	0	0	0	13	643	4,341	4,997	7,661
El Salvador City	0	0	0	0	0	0	0	0	0	64	168	232	416
Gingoog City	0	0	0	0	1	19	49	69	4	166	305	475	874
Iligan City	0	0	0	0	0	5	16	21	6	358	1,572	1,936	3,654
Malaybalay City	0	0	0	0	0	1	13	14	3	265	591	859	1,282
Oroquieta City	0	0	0	0	0	0	0	0	0	4	19	23	58
Ozamis City	0	0	0	0	0	25	851	876	1	251	1,877	2,129	11,955
Tangub City	0	0	0	0	0	20	0	20	4	11	60	75	226
Valencia City	0	0	0	0	0	0	0	0	7	228	531	766	1,390

Table 1.A.1 - MODERN METHOD OF FAMILY PLANNING

New Acceptors

Philippines, 2021

Area	NFP-STM			Total	NFP-SDM			Total	NFP-LAM			Total	Total New Acceptors
	Age group				Age group				Age group				
	10-14	15-19	20-49		10-14	15-19	20-49		10-14	15-19	20-49		
**Region 11**	**0**	**5**	**4**	**9**	**1**	**5**	**115**	**121**	**108**	**2,120**	**10,819**	**13,047**	**45,725**
Davao de Oro	0	0	0	0	0	0	4	4	10	123	617	750	5,737
Davao del Norte	0	1	0	1	0	0	29	29	16	331	1,958	2,305	7,019
Davao Oriental	0	3	2	5	0	2	5	7	26	516	1,362	1,904	3,635
Davao del Sur	0	0	0	0	0	0	7	7	15	118	561	694	8,403
Davao Occidental	0	0	0	0	0	0	25	25	4	205	230	439	1,200
Davao City	0	1	2	3	1	3	45	49	37	827	6,091	6,955	19,731
**Region 12**	**5**	**16**	**2**	**23**	**0**	**0**	**19**	**19**	**55**	**5,214**	**19,043**	**24,312**	**70,653**
North Cotabato	0	0	0	0	0	0	4	4	20	1,366	6,057	7,443	34,874
Sarangani	0	0	0	0	0	0	3	3	0	1,166	2,183	3,349	5,680
South Cotabato	0	0	0	0	0	0	12	12	25	1,332	4,188	5,545	11,760
Sultan Kudarat	0	0	0	0	0	0	0	0	0	502	1,396	1,898	4,404
				0				0				0	
Cotabato City	0	1	2	3	0	0	0	0	2	408	2,952	3,362	7,529
Gen. Santos City	5	15	0	20	0	0	0	0	8	440	2,267	2,715	6,406
**BARMM**	**0**	**0**	**0**	**0**	**0**	**2**	**35**	**37**	**37**	**2,475**	**20,229**	**22,741**	**54,828**
Basilan	0	0	0	0	0	0	0	0	2	128	692	822	2,475
Lanao del Sur	0	0	0	0	0	2	19	21	0	330	7,371	7,701	10,737
Maguindanao	0	0	0	0	0	0	12	12	8	1,289	6,499	7,796	17,893
Sulu	0	0	0	0	0	0	3	3	20	394	2,858	3,272	17,526
Tawi-Tawi	0	0	0	0	0	0	1	1	3	211	1,805	2,019	4,014
				0				0				0	
Lamitan City	0	0	0	0	0	0	0	0	4	98	369	471	1,145
Marawi City	0	0	0	0	0	0	0	0	0	25	635	660	1,038
**CARAGA**	**0**	**1**	**2**	**3**	**4**	**20**	**468**	**492**	**37**	**2,212**	**6,905**	**9,154**	**34,294**
Agusan del Norte	0	0	0	0	0	3	2	5	2	229	644	875	3,455
Agusan del Sur	0	1	2	3	0	13	84	97	6	553	1,219	1,778	5,089
Surigao del Norte	0	0	0	0	0	4	299	303	2	224	1,110	1,336	15,568
Surigao del Sur	0	0	0	0	0	0	1	1	12	362	1,190	1,564	2,651
Province of Dinagat	0	0	0	0	4	0	6	10	0	74	457	531	1,282
Bislig City	0	0	0	0	0	0	0	0	12	240	424	676	871
Butuan City	0	0	0	0	0	0	1	1	3	426	1,115	1,544	3,404
Surigao City	0	0	0	0	0	0	75	75	0	104	746	850	1,974

Table 1.A.2 - MODERN METHOD OF FAMILY PLANNING

Other Acceptors

Philippines, 2021

Area	Total Current Users	FSTR/BTL Age group 10-14	FSTR/BTL Age group 15-19	FSTR/BTL Age group 20-49	Total	MSTR/NSV Age group 10-14	MSTR/NSV Age group 15-19	MSTR/NSV Age group 20-49	Total
**PHILIPPINES**	**7,279,694**	**5**	**423**	**34,891**	**35,319**	**0**	**15**	**652**	**667**
**N C R**	**726,121**	**0**	**1**	**4,619**	**4,620**	**0**	**0**	**25**	**25**
Malabon	32,409	0	0	90	90	0	0	1	1
Navotas	23,787	0	0	181	181	0	0	0	0
Valenzuela City	39,524	0	0	832	832	0	0	17	17
Caloocan City	67,903	0	0	390	390	0	0	3	3
Marikina City	15,458	0	0	36	36	0	0	0	0
Pasig City	37,512	0	0	267	267	0	0	0	0
Pateros	2,543	0	0	2	2	0	0	0	0
Taguig	57,590	0	0	370	370	0	0	0	0
Quezon City	259,021	0	0	1,662	1,662	0	0	3	3
Makati City	12,799	0	0	38	38	0	0	1	1
Mandaluyong City	16,826	0	0	14	14	0	0	0	0
San Juan	5,760	0	0	0	0	0	0	0	0
Manila City	31,616	0	0	359	359	0	0	0	0
Las Piñas City	22,298	0	0	168	168	0	0	0	0
Muntinlupa City	40,077	0	0	48	48	0	0	0	0
Parañaque City	33,986	0	1	64	65	0	0	0	0
Pasay City	27,012	0	0	98	98	0	0	0	0
**C A R**	**131,969**	**3**	**9**	**2,433**	**2,445**	**0**	**0**	**8**	**8**
Abra	16,967	3	1	99	103	0	0	0	0
Apayao	12,113	0	0	606	606	0	0	5	5
Benguet	30,721	0	0	519	519	0	0	0	0
Ifugao	19,056	0	0	226	226	0	0	1	1
Kalinga	18,965	0	0	106	106	0	0	0	0
Mt. Province	12,528	0	8	566	574	0	0	2	2
Baguio City	21,619	0	0	311	311	0	0	0	0
**Region 1**	**466,771**	**0**	**8**	**1,384**	**1,392**	**0**	**0**	**8**	**8**
Ilocos Norte	41,297	0	0	163	163	0	0	0	0
Ilocos Sur	58,069	0	0	58	58	0	0	0	0
La Union	59,945	0	5	310	315	0	0	0	0
Pangasinan	233,753	0	3	693	696	0	0	8	8
Alaminos City	10,247	0	0	6	6	0	0	0	0
Candon City	7,036	0	0	0	0	0	0	0	0
Dagupan City	6,893	0	0	1	1	0	0	0	0
Laoag City	8,101	0	0	2	2	0	0	0	0
San Carlos City	11,818	0	0	79	79	0	0	0	0
San Fernando City	12,806	0	0	23	23	0	0	0	0
Urdaneta City	12,828	0	0	0	0	0	0	0	0
Vigan City	3,978	0	0	49	49	0	0	0	0
**Region 2**	**314,925**	**0**	**10**	**1,670**	**1,680**	**0**	**0**	**7**	**7**
Batanes	938	0	0	12	12	0	0	0	0
Cagayan	87,099	0	0	156	156	0	0	0	0
Isabela	108,463	0	4	373	377	0	0	3	3
Nueva Vizcaya	45,287	0	6	954	960	0	0	4	4
Quirino	19,270	0	0	59	59	0	0	0	0
Cauayan City	9,889	0	0	63	63	0	0	0	0
Ilagan City	15,732	0	0	21	21	0	0	0	0
Santiago City	13,933	0	0	22	22	0	0	0	0
Tuguegarao City	14,314	0	0	10	10	0	0	0	0
**Region 3**	**794,245**	**0**	**79**	**10,533**	**10,612**	**0**	**4**	**146**	**150**
Aurora	21,216	0	0	46	46	0	0	4	4
Bataan	50,598	0	0	618	618	0	0	0	0

Table 1.A.2 - MODERN METHOD OF FAMILY PLANNING

Other Acceptors

Philippines, 2021

Area	Total Current Users	FSTR/BTL Age group 10-14	15-19	20-49	Total	MSTR/NSV Age group 10-14	15-19	20-49	Total
Bulacan	148,620	0	0	596	596	0	0	1	1
Nueva Ecija	128,909	0	3	2,138	2,141	0	0	7	7
Pampanga	109,928	0	67	5,410	5,477	0	4	43	47
Tarlac	80,640	0	1	316	317	0	0	1	1
Zambales	42,194	0	0	279	279	0	0	85	85
Angeles City	23,390	0	0	225	225	0	0	5	5
Balanga City	4,908	0	0	12	12	0	0	0	0
Cabanatuan City	19,136	0	1	257	258	0	0	0	0
City of San Fernando	9,567	0	0	6	6	0	0	0	0
Gapan City	7,511	0	0	12	12	0	0	0	0
Mabalacat City	22,703	0	0	1	1	0	0	0	0
Malolos City	7,548	0	0	130	130	0	0	0	0
Meycauayan	7,990	0	0	3	3	0	0	0	0
Olongapo	10,438	0	0	87	87	0	0	0	0
Palayan City	3,016	0	0	5	5	0	0	0	0
San Jose City	12,975	0	0	7	7	0	0	0	0
San Jose del Monte City	57,952	0	0	12	12	0	0	0	0
Science City of Munoz	6,335	0	0	6	6	0	0	0	0
Tarlac City	18,671	0	7	367	374	0	0	0	0
**Region 4A**	**502,769**	**0**	**2**	**875**	**877**	**0**	**0**	**2**	**2**
Batangas	69,044	0	1	177	178	0	0	0	0
Cavite	50,364	0	0	49	49	0	0	0	0
Laguna	39,915	0	0	71	71	0	0	0	0
Quezon	65,304	0	1	200	201	0	0	1	1
Rizal	46,762	0	0	102	102	0	0	0	0
Antipolo City	6,069	0	0	5	5	0	0	0	0
Bacoor City	10,158	0	0	1	1	0	0	0	0
Batangas City	15,493	0	0	7	7	0	0	0	0
Biñan City	42,782	0	0	6	6	0	0	0	0
Cabuyao City	6,608	0	0	0	0	0	0	0	0
Calamba City	22,351	0	0	140	140	0	0	0	0
Cavite City	4,279	0	0	2	2	0	0	0	0
Dasmariñas City	37,251	0	0	0	0	0	0	0	0
General Trias City	5,008	0	0	18	18	0	0	0	0
Imus City	6,503	0	0	4	4	0	0	0	0
Lipa City	5,955	0	0	3	3	0	0	0	0
Lucena City	5,207	0	0	22	22	0	0	0	0
San Pablo City	5,471	0	0	12	12	0	0	0	0
San Pedro City	10,235	0	0	7	7	0	0	0	0
Santa Rosa City	23,903	0	0	14	14	0	0	0	0
Tagaytay City	8,089	0	0	20	20	0	0	0	0
Tanauan City	4,819	0	0	2	2	0	0	1	1
Tayabas City	7,446	0	0	10	10	0	0	0	0
Trece Martires City	3,753	0	0	3	3	0	0	0	0
**Region 4B**	**262,639**	**1**	**2**	**1,173**	**1,176**	**0**	**0**	**22**	**22**
Marinduque	13,771	0	0	111	111	0	0	12	12
Mindoro Occidental	45,760	0	0	95	95	0	0	0	0
Mindoro Oriental	64,019	0	1	565	566	0	0	2	2
Palawan	85,631	1	1	210	212	0	0	8	8
Romblon	22,521	0	0	50	50	0	0	0	0
Puerto Princesa City	30,937	0	0	142	142	0	0	0	0
**Region 5**	**467,956**	**0**	**225**	**2,483**	**2,708**	**0**	**0**	**166**	**166**
Albay	86,082	0	225	1,494	1,719	0	0	67	67
Camarines Norte	42,496	0	0	77	77	0	0	0	0
Camarines Sur	106,478	0	0	135	135	0	0	83	83
Catanduanes	25,339	0	0	150	150	0	0	8	8
Masbate	82,338	0	0	249	249	0	0	7	7
Sorsogon	63,538	0	0	338	338	0	0	1	1
Iriga City	12,388	0	0	1	1	0	0	0	0

Table 1.A.2 - MODERN METHOD OF FAMILY PLANNING

Other Acceptors

Philippines, 2021

Area	Total Current Users	FSTR/BTL Age group 10-14	15-19	20-49	Total	MSTR/NSV Age group 10-14	15-19	20-49	Total
Legaspi City	23,098	0	0	4	4	0	0	0	0
Naga City	26,199	0	0	35	35	0	0	0	0
**Region 6**	**597,683**	**0**	**8**	**1,587**	**1,595**	**0**	**0**	**53**	**53**
Aklan	44,142	0	0	39	39	0	0	0	0
Antique	46,065	0	0	270	270	0	0	0	0
Capiz	74,407	0	0	252	252	0	0	41	41
Guimaras	15,917	0	2	76	78	0	0	0	0
Iloilo	153,978	0	0	95	95	0	0	3	3
Negros Occidental	190,516	0	2	315	317	0	0	9	9
Bacolod City	26,414	0	0	231	231	0	0	0	0
Iloilo City	46,244	0	4	309	313	0	0	0	0
**Region 7**	**402,202**	**0**	**2**	**490**	**492**	**0**	**0**	**20**	**20**
Bohol	80,267	0	2	52	54	0	0	0	0
Cebu	142,224	0	0	178	178	0	0	10	10
Negros Oriental	81,104	0	0	99	99	0	0	0	0
Siquijor	8,593	0	0	7	7	0	0	0	0
Cebu City	33,223	0	0	105	105	0	0	9	9
Lapu-Lapu City	35,312	0	0	47	47	0	0	1	1
Mandaue City	21,479	0	0	2	2	0	0	0	0
**Region 8**	**342,050**	**0**	**0**	**725**	**725**	**0**	**0**	**52**	**52**
Biliran	11,102	0	0	30	30	0	0	3	3
Eastern Samar	38,666	0	0	85	85	0	0	10	10
Northern Leyte	113,942	0	0	137	137	0	0	14	14
Northern Samar	46,536	0	0	45	45	0	0	0	0
Southern Leyte	24,868	0	0	134	134	0	0	17	17
Western Samar	26,826	0	0	86	86	0	0	2	2
Calbayog City	8,836	0	0	50	50	0	0	0	0
Maasin City	5,096	0	0	18	18	0	0	0	0
Ormoc City	20,009	0	0	138	138	0	0	6	6
Tacloban City	46,169	0	0	2	2	0	0	0	0
**Region 9**	**314,587**	**0**	**2**	**130**	**132**	**0**	**2**	**6**	**8**
Zamboanga del Norte	98,783	0	0	55	55	0	0	1	1
Zamboanga del Sur	65,366	0	2	59	61	0	2	5	7
Zamboanga Sibugay	42,607	0	0	4	4	0	0	0	0
Dapitan City	7,733	0	0	0	0	0	0	0	0
Dipolog City	16,393	0	0	9	9	0	0	0	0
Isabela City	9,369	0	0	0	0	0	0	0	0
Pagadian City	19,991	0	0	3	3	0	0	0	0
Zamboanga City	54,345	0	0	0	0	0	0	0	0
**Region 10**	**469,808**	**1**	**21**	**1,127**	**1,149**	**0**	**6**	**51**	**57**
Bukidnon	110,248	0	0	403	403	0	0	15	15
Camiguin	6,471	0	0	19	19	0	0	0	0
Lanao del Norte	60,033	0	0	28	28	0	0	0	0
Misamis Occidental	25,727	0	0	58	58	0	0	5	5
Misamis Oriental	79,462	0	0	32	32	0	0	0	0
Cagayan de Oro City	60,104	0	7	171	178	0	6	19	25
El Salvador City	6,228	0	0	13	13	0	0	0	0
Gingoog City	29,566	0	8	109	117	0	0	5	5
Iligan City	27,468	0	0	27	27	0	0	0	0
Malaybalay City	19,334	0	0	205	205	0	0	5	5
Oroquieta City	6,837	0	0	1	1	0	0	0	0
Ozamis City	13,612	0	0	5	5	0	0	1	1
Tangub City	3,838	0	4	4	8	0	0	0	0
Valencia City	20,880	1	2	52	55	0	0	1	1

Table 1.A.2 - MODERN METHOD OF FAMILY PLANNING

Other Acceptors

Philippines, 2021

Area	Total Current Users	FSTR/BTL Age group 10-14	15-19	20-49	Total	MSTR/NSV Age group 10-14	15-19	20-49	Total
**Region 11**	**501,561**	**0**	**40**	**1,493**	**1,533**	**0**	**1**	**47**	**48**
Davao de Oro	83,015	0	3	214	217	0	0	0	0
Davao del Norte	100,273	0	1	264	265	0	0	7	7
Davao Oriental	51,379	0	13	316	329	0	0	12	12
Davao del Sur	65,425	0	1	99	100	0	0	3	3
Davao Occidental	31,994	0	0	66	66	0	0	0	0
Davao City	169,475	0	22	534	556	0	1	25	26
**Region 12**	**468,656**	**0**	**11**	**1,896**	**1,907**	**0**	**2**	**17**	**19**
North Cotabato	136,386	0	10	745	755	0	1	5	6
Sarangani	65,258	0	1	192	193	0	1	4	5
South Cotabato	91,960	0	0	414	414	0	0	8	8
Sultan Kudarat	93,390	0	0	222	222	0	0	0	0
					0				0
Cotabato City	30,632	0	0	255	255	0	0	0	0
Gen. Santos City	51,030	0	0	68	68	0	0	0	0
**BARMM**	**266,572**	**0**	**0**	**619**	**619**	**0**	**0**	**1**	**1**
Basilan	10,532	0	0	20	20	0	0	0	0
Lanao del Sur	40,004	0	0	84	84	0	0	0	0
Maguindanao	89,662	0	0	178	178	0	0	1	1
Sulu	88,420	0	0	9	9	0	0	0	0
Tawi-Tawi	27,695	0	0	4	4	0	0	0	0
					0				0
Lamitan City	4,938	0	0	317	317	0	0	0	0
Marawi City	5,321	0	0	7	7	0	0	0	0
**CARAGA**	**249,180**	**0**	**3**	**1,654**	**1,657**	**0**	**0**	**21**	**21**
Agusan del Norte	37,360	0	1	181	182	0	0	0	0
Agusan del Sur	76,525	0	2	980	982	0	0	16	16
Surigao del Norte	30,743	0	0	94	94	0	0	2	2
Surigao del Sur	42,013	0	0	49	49	0	0	3	3
Province of Dinagat	10,218	0	0	8	8	0	0	0	0
Bislig City	9,004	0	0	143	143	0	0	0	0
Butuan City	30,541	0	0	66	66	0	0	0	0
Surigao City	12,776	0	0	133	133	0	0	0	0

Table 1.A.2 - MODERN METHOD OF FAMILY PLANNING

Other Acceptors

Philippines, 2021

Area	CONDOM			Total	IUD-INTERVAL			Total	IUD-POSTPARTUM			Total
	Age group				Age group				Age group			
	10-14	15-19	20-49		10-14	15-19	20-49		10-14	15-19	20-49	
**PHILIPPINES**	**54**	**2,557**	**45,667**	**48,278**	**21**	**1,099**	**26,743**	**27,863**	**15**	**892**	**12,149**	**13,056**
**N C R**	**8**	**656**	**14,969**	**15,633**	**1**	**78**	**1,384**	**1,463**	**1**	**147**	**2,701**	**2,849**
Malabon	0	11	290	301	0	2	49	51	0	1	12	13
Navotas	3	75	146	224	0	4	17	21	0	0	0	0
Valenzuela City	0	7	399	406	0	0	113	113	0	0	93	93
Caloocan City	0	28	380	408	1	13	170	184	0	15	188	203
Marikina City	0	0	34	34	0	1	3	4	0	4	70	74
Pasig City	0	3	131	134	0	1	54	55	0	13	185	198
Pateros	0	0	70	70	0	0	5	5	0	0	0	0
Taguig	1	6	152	159	0	18	225	243	1	5	58	64
Quezon City	4	383	12,512	12,899	0	31	592	623	0	105	1,612	1,717
Makati City	0	1	104	105	0	2	25	27	0	1	14	15
Mandaluyong City	0	11	75	86	0	0	5	5	0	1	6	7
San Juan	0	0	4	4	0	0	0	0	0	0	0	0
Manila City	0	25	241	266	0	5	63	68	0	0	60	60
Las Piñas City	0	5	125	130	0	1	14	15	0	1	88	89
Muntinlupa City	0	0	8	8	0	0	1	1	0	0	5	5
Parañaque City	0	15	15	30	0	0	30	30	0	1	15	16
Pasay City	0	86	283	369	0	0	18	18	0	0	295	295
**C A R**	**1**	**32**	**1,275**	**1,308**	**0**	**31**	**1,059**	**1,090**	**1**	**21**	**550**	**572**
Abra	0	4	33	37	0	0	9	9	0	5	20	25
Apayao	0	1	128	129	0	7	591	598	0	8	343	351
Benguet	0	8	414	422	0	8	192	200	0	1	48	49
Ifugao	0	2	169	171	0	2	31	33	0	1	20	21
Kalinga	0	4	99	103	0	2	92	94	0	2	30	32
Mt. Province	1	8	186	195	0	8	55	63	1	4	64	69
Baguio City	0	5	246	251	0	4	89	93	0	0	25	25
**Region 1**	**0**	**30**	**1,646**	**1,676**	**7**	**48**	**838**	**893**	**0**	**6**	**64**	**70**
Ilocos Norte	0	1	247	248	0	2	23	25	0	0	6	6
Ilocos Sur	0	1	116	117	0	0	7	7	0	0	2	2
La Union	0	5	119	124	1	6	107	114	0	6	53	59
Pangasinan	0	18	992	1,010	6	32	663	701	0	0	0	0
Alaminos City	0	0	1	1	0	1	5	6	0	0	0	0
Candon City	0	0	0	0	0	0	0	0	0	0	0	0
Dagupan City	0	1	17	18	0	0	1	1	0	0	0	0
Laoag City	0	0	67	67	0	0	0	0	0	0	0	0
San Carlos City	0	3	26	29	0	7	19	26	0	0	0	0
San Fernando City	0	0	35	35	0	0	9	9	0	0	3	3
Urdaneta City	0	0	0	0	0	0	4	4	0	0	0	0
Vigan City	0	1	26	27	0	0	0	0	0	0	0	0
**Region 2**	**0**	**21**	**835**	**856**	**4**	**58**	**899**	**961**	**0**	**38**	**224**	**262**
Batanes	0	0	9	9	0	0	0	0	0	0	0	0
Cagayan	0	0	76	76	0	19	231	250	0	15	76	91
Isabela	0	7	294	301	0	8	127	135	0	12	62	74
Nueva Vizcaya	0	9	380	389	4	29	459	492	0	4	65	69
Quirino	0	1	20	21	0	0	30	30	0	0	1	1
Cauayan City	0	0	8	8	0	0	18	18	0	0	0	0
Ilagan City	0	0	11	11	0	0	8	8	0	0	0	0
Santiago City	0	4	36	40	0	2	7	9	0	0	0	0
Tuguegarao City	0	0	1	1	0	0	19	19	0	7	20	27
**Region 3**	**4**	**291**	**5,025**	**5,320**	**1**	**46**	**1,177**	**1,224**	**0**	**43**	**642**	**685**
Aurora	1	14	192	207	0	1	19	20	0	0	6	6
Bataan	0	4	175	179	0	0	4	4	0	0	3	3

Table 1.A.2 - MODERN METHOD OF FAMILY PLANNING

Other Acceptors

Philippines, 2021

Area	CONDOM			Total	IUD-INTERVAL			Total	IUD-POSTPARTUM			Total
	Age group				Age group				Age group			
	10-14	15-19	20-49		10-14	15-19	20-49		10-14	15-19	20-49	
Bulacan	0	20	849	869	0	0	74	74	0	22	53	75
Nueva Ecija	0	47	1,388	1,435	0	2	201	203	0	3	39	42
Pampanga	1	45	1,023	1,069	0	35	606	641	0	15	392	407
Tarlac	0	65	520	585	0	1	27	28	0	0	7	7
Zambales	0	13	202	215	0	4	29	33	0	1	18	19
Angeles City	0	0	25	25	0	0	4	4	0	0	8	8
Balanga City	0	0	14	14	0	0	2	2	0	0	0	0
Cabanatuan City	0	1	87	88	0	2	25	27	0	0	3	3
City of San Fernando	2	2	7	11	0	1	0	1	0	0	0	0
Gapan City	0	0	4	4	0	0	1	1	0	0	0	0
Mabalacat City	0	3	79	82	0	0	20	20	0	0	0	0
Malolos City	0	0	52	52	0	0	19	19	0	0	0	0
Meycauayan	0	7	45	52	0	0	1	1	0	0	0	0
Olongapo	0	0	22	22	0	0	0	0	0	1	13	14
Palayan City	0	1	7	8	0	0	5	5	0	0	0	0
San Jose City	0	1	30	31	0	0	0	0	0	1	1	2
San Jose del Monte City	0	28	211	239	0	0	13	13	0	0	0	0
Science City of Munoz	0	0	2	2	0	0	1	1	0	0	0	0
Tarlac City	0	40	91	131	1	0	126	127	0	0	99	99
**Region 4A**	**9**	**174**	**1,348**	**1,531**	**0**	**33**	**425**	**458**	**5**	**33**	**390**	**428**
Batangas	0	18	150	168	0	0	24	24	0	0	17	17
Cavite	0	3	129	132	0	2	31	33	1	1	0	2
Laguna	0	5	67	72	0	4	91	95	0	1	10	11
Quezon	0	5	190	195	0	1	151	152	2	1	44	47
Rizal	0	18	50	68	0	24	65	89	1	8	168	177
Antipolo City	0	0	198	198	0	0	8	8	0	0	0	0
Bacoor City	0	0	7	7	0	0	2	2	0	0	0	0
Batangas City	0	0	24	24	0	0	3	3	1	19	107	127
Biñan City	0	96	282	378	0	0	4	4	0	0	0	0
Cabuyao City	0	2	17	19	0	0	0	0	0	0	0	0
Calamba City	0	1	38	39	0	0	26	26	0	0	38	38
Cavite City	0	1	7	8	0	0	0	0	0	0	0	0
Dasmariñas City	0	6	27	33	0	0	1	1	0	0	0	0
General Trias City	0	1	19	20	0	0	2	2	0	1	3	4
Imus City	0	1	7	8	0	0	0	0	0	0	0	0
Lipa City	0	0	0	0	0	0	0	0	0	0	0	0
Lucena City	0	2	15	17	0	0	0	0	0	0	1	1
San Pablo City	0	2	7	9	0	1	5	6	0	0	0	0
San Pedro City	0	0	7	7	0	0	1	1	0	1	0	1
Santa Rosa City	9	12	77	98	0	0	0	0	0	0	1	1
Tagaytay City	0	0	6	6	0	0	6	6	0	0	1	1
Tanauan City	0	0	0	0	0	0	0	0	0	1	0	1
Tayabas City	0	1	14	15	0	1	3	4	0	0	0	0
Trece Martires City	0	0	10	10	0	0	2	2	0	0	0	0
**Region 4B**	**22**	**55**	**698**	**775**	**0**	**10**	**271**	**281**	**2**	**6**	**55**	**63**
Marinduque	0	0	26	26	0	0	18	18	0	0	3	3
Mindoro Occidental	0	3	58	61	0	1	29	30	0	0	0	0
Mindoro Oriental	0	5	70	75	0	7	109	116	0	1	17	18
Palawan	22	47	346	415	0	2	108	110	2	5	6	13
Romblon	0	0	23	23	0	0	7	7	0	0	7	7
Puerto Princesa City	0	0	175	175	0	0	0	0	0	0	22	22
**Region 5**	**4**	**549**	**3,442**	**3,995**	**0**	**43**	**1,025**	**1,068**	**0**	**9**	**527**	**536**
Albay	0	486	725	1,211	0	36	521	557	0	1	279	280
Camarines Norte	0	8	448	456	0	3	84	87	0	1	12	13
Camarines Sur	3	13	854	870	0	1	37	38	0	6	127	133
Catanduanes	0	0	114	114	0	0	2	2	0	0	39	39
Masbate	1	29	632	662	0	2	288	290	0	1	60	61
Sorsogon	0	10	393	403	0	0	81	81	0	0	6	6
Iriga City	0	2	20	22	0	1	5	6	0	0	1	1

Table 1.A.2 - MODERN METHOD OF FAMILY PLANNING

Other Acceptors

Philippines, 2021

Area	CONDOM			Total	IUD-INTERVAL			Total	IUD-POSTPARTUM			Total
	Age group				Age group				Age group			
	10-14	15-19	20-49		10-14	15-19	20-49		10-14	15-19	20-49	
Legaspi City	0	1	10	11	0	0	0	0	0	0	0	0
Naga City	0	0	246	246	0	0	7	7	0	0	3	3
**Region 6**	**0**	**75**	**2,192**	**2,267**	**3**	**70**	**1,182**	**1,255**	**1**	**35**	**547**	**583**
Aklan	0	1	99	100	0	0	8	8	0	0	2	2
Antique	0	3	152	155	0	2	77	79	0	0	7	7
Capiz	0	4	139	143	0	7	81	88	0	0	14	14
Guimaras	0	4	62	66	0	0	19	19	0	1	4	5
Iloilo	0	20	492	512	2	20	199	221	1	16	129	146
Negros Occidental	0	15	583	598	1	18	398	417	0	9	235	244
Bacolod City	0	4	34	38	0	4	187	191	0	5	29	34
Iloilo City	0	24	631	655	0	19	213	232	0	4	127	131
**Region 7**	**0**	**73**	**2,112**	**2,185**	**0**	**89**	**3,196**	**3,285**	**0**	**124**	**1,261**	**1,385**
Bohol	0	18	565	583	0	34	757	791	0	47	508	555
Cebu	0	23	745	768	0	36	1,504	1,540	0	61	556	617
Negros Oriental	0	13	453	466	0	4	570	574	0	2	40	42
Siquijor	0	6	61	67	0	1	90	91	0	0	2	2
Cebu City	0	4	97	101	0	14	233	247	0	14	138	152
Lapu-Lapu City	0	9	173	182	0	0	39	39	0	0	11	11
Mandaue City	0	0	18	18	0	0	3	3	0	0	6	6
**Region 8**	**0**	**37**	**963**	**1,000**	**1**	**35**	**849**	**885**	**0**	**33**	**472**	**505**
Biliran	0	0	62	62	0	0	23	23	0	0	3	3
Eastern Samar	0	2	154	156	1	2	14	17	0	5	12	17
Northern Leyte	0	1	64	65	0	9	177	186	0	20	134	154
Northern Samar	0	5	151	156	0	5	74	79	0	0	21	21
Southern Leyte	0	6	185	191	0	14	321	335	0	5	183	188
Western Samar	0	0	16	16	0	0	59	59	0	3	93	96
Calbayog City	0	0	33	33	0	0	2	2	0	0	1	1
Maasin City	0	0	17	17	0	0	27	27	0	0	2	2
Ormoc City	0	23	269	292	0	5	152	157	0	0	22	22
Tacloban City	0	0	12	12	0	0	0	0	0	0	1	1
**Region 9**	**0**	**50**	**1,503**	**1,553**	**0**	**131**	**5,705**	**5,836**	**1**	**99**	**1,062**	**1,162**
Zamboanga del Norte	0	18	517	535	0	12	283	295	0	3	96	99
Zamboanga del Sur	0	11	321	332	0	33	552	585	0	55	570	625
Zamboanga Sibugay	0	8	286	294	0	83	4,597	4,680	1	38	328	367
Dapitan City	0	2	26	28	0	0	4	4	0	0	2	2
Dipolog City	0	9	180	189	0	2	220	222	0	0	11	11
Isabela City	0	0	6	6	0	0	0	0	0	0	0	0
Pagadian City	0	2	119	121	0	0	31	31	0	3	47	50
Zamboanga City	0	0	48	48	0	1	18	19	0	0	8	8
**Region 10**	**0**	**94**	**2,058**	**2,152**	**0**	**160**	**1,923**	**2,083**	**0**	**57**	**566**	**623**
Bukidnon	0	24	610	634	0	101	1,166	1,267	0	22	200	222
Camiguin	0	2	7	9	0	0	7	7	0	0	0	0
Lanao del Norte	0	28	432	460	0	1	71	72	0	0	0	0
Misamis Occidental	0	1	278	279	0	0	75	75	0	0	1	1
Misamis Oriental	0	2	100	102	0	8	123	131	0	6	47	53
Cagayan de Oro City	0	0	18	18	0	0	58	58	0	11	214	225
El Salvador City	0	0	13	13	0	7	36	43	0	5	27	32
Gingoog City	0	15	144	159	0	2	43	45	0	3	14	17
Iligan City	0	6	87	93	0	5	79	84	0	7	41	48
Malaybalay City	0	3	258	261	0	35	148	183	0	0	1	1
Oroquieta City	0	0	1	1	0	0	1	1	0	0	0	0
Ozamis City	0	0	19	19	0	0	0	0	0	0	0	0
Tangub City	0	0	6	6	0	0	3	3	0	0	0	0
Valencia City	0	13	85	98	0	1	113	114	0	3	21	24

Table 1.A.2 - MODERN METHOD OF FAMILY PLANNING

Other Acceptors

Philippines, 2021

Area	CONDOM			Total	IUD-INTERVAL			Total	IUD-POSTPARTUM			Total
	Age group				Age group				Age group			
	10-14	15-19	20-49		10-14	15-19	20-49		10-14	15-19	20-49	
**Region 11**	**3**	**183**	**1,872**	**2,058**	**0**	**90**	**1,170**	**1,260**	**0**	**40**	**463**	**503**
Davao de Oro	0	2	207	209	0	10	240	250	0	0	29	29
Davao del Norte	0	19	337	356	0	12	181	193	0	8	110	118
Davao Oriental	0	53	414	467	0	7	189	196	0	2	26	28
Davao del Sur	0	11	168	179	0	10	154	164	0	2	40	42
Davao Occidental	0	1	34	35	0	14	94	108	0	0	6	6
Davao City	3	97	712	812	0	37	312	349	0	28	252	280
**Region 12**	**0**	**82**	**1,869**	**1,951**	**3**	**66**	**1,814**	**1,883**	**1**	**82**	**1,108**	**1,191**
North Cotabato	0	14	571	585	0	22	1,079	1,101	0	21	262	283
Sarangani	0	20	285	305	0	9	130	139	0	1	52	53
South Cotabato	0	13	385	398	0	8	333	341	0	8	291	299
Sultan Kudarat	0	24	294	318	0	10	84	94	0	7	149	156
				0				0				0
Cotabato City	0	5	221	226	3	12	128	143	1	43	323	367
Gen. Santos City	0	6	113	119	0	5	60	65	0	2	31	33
**BARMM**	**3**	**49**	**1,353**	**1,405**	**1**	**41**	**330**	**372**	**1**	**70**	**607**	**678**
Basilan	0	1	58	59	0	0	3	3	0	0	1	1
Lanao del Sur	0	9	682	691	0	0	4	4	0	0	12	12
Maguindanao	3	34	479	516	0	5	79	84	0	4	58	62
Sulu	0	2	32	34	0	0	6	6	0	0	5	5
Tawi-Tawi	0	0	12	12	0	0	0	0	0	0	0	0
				0				0				0
Lamitan City	0	0	29	29	1	36	238	275	1	66	531	598
Marawi City	0	3	61	64	0	0	0	0	0	0	0	0
**CARAGA**	**0**	**106**	**2,507**	**2,613**	**0**	**70**	**3,496**	**3,566**	**2**	**49**	**910**	**961**
Agusan del Norte	0	14	353	367	0	21	216	237	0	8	85	93
Agusan del Sur	0	53	958	1,011	0	25	2,631	2,656	1	22	527	550
Surigao del Norte	0	6	224	230	0	3	250	253	1	2	48	51
Surigao del Sur	0	7	326	333	0	13	175	188	0	3	90	93
Province of Dinagat	0	1	93	94	0	1	63	64	0	0	7	7
Bislig City	0	4	127	131	0	3	56	59	0	1	39	40
Butuan City	0	21	392	413	0	3	82	85	0	2	64	66
Surigao City	0	0	34	34	0	1	23	24	0	11	50	61

Table 1.A.2 - MODERN METHOD OF FAMILY PLANNING

Other Acceptors

Philippines, 2021

Area	PILLS-POP			Total	PILLS-COC			Total	INJECTABLES			Total
	Age group				Age group				Age group			
	10-14	15-19	20-49		10-14	15-19	20-49		10-14	15-19	20-49	
**PHILIPPINES**	**150**	**6,086**	**66,890**	**73,126**	**101**	**13,685**	**297,458**	**311,244**	**169**	**13,134**	**195,860**	**209,163**
**N C R**	**5**	**835**	**11,899**	**12,739**	**5**	**1,297**	**42,617**	**43,919**	**17**	**1,949**	**40,330**	**42,296**
Malabon	0	46	459	505	0	74	1,543	1,617	1	76	1,107	1,184
Navotas	0	30	196	226	0	96	644	740	0	135	814	949
Valenzuela City	0	40	885	925	0	124	5,661	5,785	1	140	3,224	3,365
Caloocan City	0	103	831	934	0	240	2,903	3,143	1	239	9,619	9,859
Marikina City	0	10	182	192	0	16	456	472	0	34	778	812
Pasig City	3	39	301	343	2	85	727	814	5	112	1,422	1,539
Pateros	0	0	5	5	0	2	68	70	0	3	50	53
Taguig	0	14	285	299	0	32	724	756	0	38	1,084	1,122
Quezon City	2	405	6,744	7,151	1	471	25,637	26,109	8	707	16,453	17,168
Makati City	0	3	237	240	0	10	357	367	0	8	679	687
Mandaluyong City	0	34	213	247	0	31	474	505	0	48	203	251
San Juan	0	0	0	0	0	0	54	54	0	0	126	126
Manila City	0	60	880	940	2	64	916	982	1	101	1,310	1,412
Las Piñas City	0	35	231	266	0	32	760	792	0	90	927	1,017
Muntinlupa City	0	4	26	30	0	7	580	587	0	12	540	552
Parañaque City	0	12	118	130	0	13	392	405	0	206	1,039	1,245
Pasay City	0	0	306	306	0	0	721	721	0	0	955	955
**C A R**	**0**	**301**	**4,048**	**4,349**	**2**	**557**	**26,086**	**26,645**	**6**	**481**	**7,447**	**7,934**
Abra	0	25	233	258	0	39	554	593	5	75	474	554
Apayao	0	103	1,677	1,780	0	261	16,072	16,333	0	125	1,042	1,167
Benguet	0	41	589	630	0	54	1,681	1,735	0	60	1,352	1,412
Ifugao	0	49	409	458	2	58	1,662	1,722	0	57	1,204	1,261
Kalinga	0	45	269	314	0	49	1,805	1,854	0	32	786	818
Mt. Province	0	17	178	195	0	71	3,261	3,332	1	77	811	889
Baguio City	0	21	693	714	0	25	1,051	1,076	0	55	1,778	1,833
**Region 1**	**1**	**65**	**994**	**1,060**	**5**	**476**	**10,577**	**11,058**	**0**	**356**	**5,438**	**5,794**
Ilocos Norte	0	8	275	283	0	12	1,171	1,183	0	10	414	424
Ilocos Sur	0	10	437	447	5	25	1,328	1,358	0	14	412	426
La Union	1	39	203	243	0	46	1,088	1,134	0	95	956	1,051
Pangasinan	0	0	0	0	0	305	6,277	6,582	0	181	3,222	3,403
Alaminos City	0	0	0	0	0	2	79	81	0	3	39	42
Candon City	0	0	0	0	0	0	0	0	0	0	0	0
Dagupan City	0	0	4	4	0	0	73	73	0	6	58	64
Laoag City	0	0	0	0	0	0	27	27	0	0	0	0
San Carlos City	0	0	0	0	0	67	355	422	0	29	188	217
San Fernando City	0	8	75	83	0	9	52	61	0	12	97	109
Urdaneta City	0	0	0	0	0	10	125	135	0	5	25	30
Vigan City	0	0	0	0	0	0	2	2	0	1	27	28
**Region 2**	**1**	**375**	**4,176**	**4,552**	**3**	**744**	**11,702**	**12,449**	**6**	**512**	**7,748**	**8,266**
Batanes	0	0	7	7	0	1	61	62	0	14	154	168
Cagayan	1	82	734	817	0	76	2,497	2,573	3	85	1,407	1,495
Isabela	0	71	654	725	0	133	3,830	3,963	3	213	3,406	3,622
Nueva Vizcaya	0	171	2,352	2,523	3	420	3,562	3,985	0	108	1,680	1,788
Quirino	0	19	154	173	0	43	382	425	0	23	266	289
Cauayan City	0	14	86	100	0	20	368	388	0	14	179	193
Ilagan City	0	7	99	106	0	8	224	232	0	14	131	145
Santiago City	0	0	0	0	0	39	655	694	0	39	464	503
Tuguegarao City	0	11	90	101	0	4	123	127	0	2	61	63
**Region 3**	**4**	**502**	**7,577**	**8,083**	**9**	**1,180**	**21,044**	**22,233**	**9**	**1,557**	**21,455**	**23,021**
Aurora	0	26	512	538	0	62	1,227	1,289	0	64	734	798
Bataan	1	17	341	359	0	56	882	938	1	83	1,802	1,886

Table 1.A.2 - MODERN METHOD OF FAMILY PLANNING

Other Acceptors

Philippines, 2021

Area	PILLS-POP			Total	PILLS-COC			Total	INJECTABLES			Total
	Age group				Age group				Age group			
	10-14	15-19	20-49		10-14	15-19	20-49		10-14	15-19	20-49	
Bulacan	1	83	1,038	1,122	1	157	4,208	4,366	1	238	4,289	4,528
Nueva Ecija	0	63	1,881	1,944	1	154	5,086	5,241	0	169	3,437	3,606
Pampanga	0	66	2,287	2,353	2	257	3,513	3,772	1	308	3,402	3,711
Tarlac	0	57	391	448	4	98	2,607	2,709	4	173	2,043	2,220
Zambales	1	18	281	300	0	45	832	877	2	124	1,980	2,106
Angeles City	0	3	33	36	0	13	188	201	0	10	173	183
Balanga City	0	0	31	31	0	13	146	159	0	28	404	432
Cabanatuan City	1	27	173	201	0	26	274	300	0	12	292	304
City of San Fernando	0	2	23	25	1	19	128	148	0	3	30	33
Gapan City	0	0	10	10	0	0	42	42	0	1	83	84
Mabalacat City	0	3	24	27	0	10	220	230	0	20	369	389
Malolos City	0	0	0	0	0	11	314	325	0	15	131	146
Meycauayan	0	21	70	91	0	18	126	144	0	14	179	193
Olongapo	0	5	76	81	0	14	339	353	0	36	651	687
Palayan City	0	0	0	0	0	1	63	64	0	4	47	51
San Jose City	0	8	67	75	0	23	183	206	0	38	368	406
San Jose del Monte City	0	17	131	148	0	56	383	439	0	64	641	705
Science City of Munoz	0	1	13	14	0	4	55	59	0	7	60	67
Tarlac City	0	85	195	280	0	143	228	371	0	146	340	486
**Region 4A**	**1**	**229**	**229**	**459**	**19**	**516**	**7,512**	**8,047**	**59**	**503**	**7,936**	**8,498**
Batangas	0	26	26	52	10	40	622	672	48	19	303	370
Cavite	0	4	4	8	0	42	706	748	0	48	920	968
Laguna	0	6	6	12	0	39	572	611	2	19	507	528
Quezon	0	21	21	42	0	65	2,147	2,212	0	71	2,058	2,129
Rizal	0	15	15	30	0	47	753	800	0	112	1,179	1,291
Antipolo City	0	0	0	0	0	0	31	31	0	0	529	529
Bacoor City	0	1	1	2	0	3	70	73	0	17	142	159
Batangas City	0	4	4	8	0	2	238	240	0	8	168	176
Biñan City	0	95	95	190	0	169	594	763	0	97	422	519
Cabuyao City	0	10	10	20	0	13	124	137	0	12	65	77
Calamba City	0	14	14	28	0	35	512	547	0	9	210	219
Cavite City	0	0	0	0	0	3	35	38	0	4	20	24
Dasmariñas City	0	9	9	18	0	16	163	179	0	14	264	278
General Trias City	1	2	2	5	0	3	134	137	0	5	185	190
Imus City	0	0	0	0	0	1	61	62	0	2	113	115
Lipa City	0	0	0	0	0	0	1	1	0	1	7	8
Lucena City	0	1	1	2	0	9	141	150	0	8	206	214
San Pablo City	0	1	1	2	0	8	105	113	0	12	74	86
San Pedro City	0	14	14	28	0	7	123	130	0	8	102	110
Santa Rosa City	0	1	1	2	9	8	155	172	9	18	85	112
Tagaytay City	0	0	0	0	0	0	50	50	0	0	96	96
Tanauan City	0	0	0	0	0	0	11	11	0	0	19	19
Tayabas City	0	5	5	10	0	2	90	92	0	14	165	179
Trece Martires City	0	0	0	0	0	4	74	78	0	5	97	102
**Region 4B**	**94**	**150**	**1,664**	**1,908**	**10**	**325**	**10,925**	**11,260**	**7**	**484**	**10,368**	**10,859**
Marinduque	0	0	46	46	0	1	833	834	0	0	137	137
Mindoro Occidental	0	22	335	357	0	93	2,256	2,349	0	115	1,811	1,926
Mindoro Oriental	1	35	247	283	0	41	946	987	0	57	704	761
Palawan	93	85	958	1,136	10	185	4,209	4,404	7	301	4,845	5,153
Romblon	0	8	78	86	0	5	626	631	0	11	479	490
Puerto Princesa City	0	0	0	0	0	0	2,055	2,055	0	0	2,392	2,392
**Region 5**	**0**	**313**	**4,229**	**4,542**	**3**	**1,604**	**33,150**	**34,757**	**6**	**813**	**10,284**	**11,103**
Albay	0	23	408	431	0	963	13,944	14,907	1	345	1,129	1,475
Camarines Norte	0	77	722	799	0	114	2,598	2,712	0	88	1,421	1,509
Camarines Sur	0	91	1,454	1,545	2	189	6,253	6,444	4	106	2,565	2,675
Catanduanes	0	4	349	353	0	13	273	286	0	26	559	585
Masbate	0	68	699	767	1	221	5,853	6,075	1	124	2,150	2,275
Sorsogon	0	47	421	468	0	100	3,235	3,335	0	117	2,239	2,356
Iriga City	0	2	47	49	0	2	150	152	0	0	64	64

Table 1.A.2 - MODERN METHOD OF FAMILY PLANNING

Other Acceptors

Philippines, 2021

Area	PILLS-POP			Total	PILLS-COC			Total	INJECTABLES			Total
	Age group				Age group				Age group			
	10-14	15-19	20-49		10-14	15-19	20-49		10-14	15-19	20-49	
Legaspi City	0	1	22	23	0	1	49	50	0	6	22	28
Naga City	0	0	107	107	0	1	795	796	0	1	135	136
**Region 6**	**1**	**258**	**4,164**	**4,423**	**0**	**622**	**14,701**	**15,323**	**5**	**953**	**9,318**	**10,276**
Aklan	0	5	187	192	0	14	596	610	0	22	458	480
Antique	0	19	372	391	0	62	1,222	1,284	0	80	1,593	1,673
Capiz	0	5	297	302	0	42	1,156	1,198	0	31	825	856
Guimaras	0	7	84	91	0	17	526	543	0	20	175	195
Iloilo	1	24	525	550	0	72	2,953	3,025	1	47	1,287	1,335
Negros Occidental	0	182	2,557	2,739	0	274	5,531	5,805	4	657	3,882	4,543
Bacolod City	0	1	63	64	0	30	257	287	0	56	534	590
Iloilo City	0	15	79	94	0	111	2,460	2,571	0	40	564	604
**Region 7**	**3**	**272**	**3,081**	**3,356**	**1**	**408**	**12,418**	**12,827**	**3**	**526**	**12,321**	**12,850**
Bohol	3	23	353	379	0	52	2,143	2,195	2	66	1,842	1,910
Cebu	0	94	1,257	1,351	1	112	4,007	4,120	0	104	3,420	3,524
Negros Oriental	0	78	623	701	0	156	3,995	4,151	1	197	4,193	4,391
Siquijor	0	8	128	136	0	13	345	358	0	22	430	452
Cebu City	0	32	366	398	0	52	1,516	1,568	0	103	1,958	2,061
Lapu-Lapu City	0	33	304	337	0	21	310	331	0	30	363	393
Mandaue City	0	4	50	54	0	2	102	104	0	4	115	119
**Region 8**	**0**	**166**	**2,485**	**2,651**	**1**	**314**	**9,108**	**9,423**	**0**	**354**	**5,627**	**5,981**
Biliran	0	13	123	136	0	32	742	774	0	30	342	372
Eastern Samar	0	14	103	117	1	21	616	638	0	18	482	500
Northern Leyte	0	6	185	191	0	34	1,126	1,160	0	46	699	745
Northern Samar	0	17	259	276	0	64	1,641	1,705	0	79	1,076	1,155
Southern Leyte	0	14	257	271	0	32	1,360	1,392	0	40	622	662
Western Samar	0	22	351	373	0	17	375	392	0	21	576	597
Calbayog City	0	12	662	674	0	18	865	883	0	18	491	509
Maasin City	0	1	10	11	0	2	85	87	0	3	59	62
Ormoc City	0	65	502	567	0	91	2,245	2,336	0	93	1,233	1,326
Tacloban City	0	2	33	35	0	3	53	56	0	6	47	53
**Region 9**	**3**	**279**	**2,775**	**3,057**	**4**	**790**	**14,894**	**15,688**	**2**	**636**	**9,622**	**10,260**
Zamboanga del Norte	1	68	427	496	1	237	3,849	4,087	1	200	2,515	2,716
Zamboanga del Sur	0	49	320	369	1	208	4,441	4,650	0	120	1,900	2,020
Zamboanga Sibugay	0	27	542	569	1	136	3,433	3,570	0	139	2,179	2,318
Dapitan City	0	3	42	45	0	12	257	269	0	11	195	206
Dipolog City	2	25	219	246	1	60	1,094	1,155	1	35	691	727
Isabela City	0	11	64	75	0	19	229	248	0	20	247	267
Pagadian City	0	71	698	769	0	20	224	244	0	15	569	584
Zamboanga City	0	25	463	488	0	98	1,367	1,465	0	96	1,326	1,422
**Region 10**	**6**	**395**	**2,870**	**3,271**	**20**	**970**	**12,615**	**13,605**	**16**	**633**	**6,613**	**7,262**
Bukidnon	3	210	1,251	1,464	2	548	5,763	6,313	4	310	2,839	3,153
Camiguin	0	1	9	10	0	8	101	109	0	2	61	63
Lanao del Norte	1	29	373	403	0	45	1,174	1,219	0	67	840	907
Misamis Occidental	0	5	202	207	0	8	421	429	0	10	228	238
Misamis Oriental	0	18	197	215	1	34	609	644	0	17	413	430
Cagayan de Oro City	0	8	41	49	0	10	281	291	0	8	143	151
El Salvador City	0	3	29	32	0	6	79	85	0	14	151	165
Gingoog City	0	14	123	137	1	23	329	353	0	28	261	289
Iligan City	1	15	192	208	0	31	641	672	0	21	515	536
Malaybalay City	1	34	221	256	0	155	2,347	2,502	0	67	629	696
Oroquieta City	0	0	5	5	0	26	7	33	0	0	16	16
Ozamis City	0	3	19	22	0	6	101	107	2	5	75	82
Tangub City	0	16	16	32	0	2	82	84	0	32	35	67
Valencia City	0	39	192	231	16	68	680	764	10	52	407	469

Table 1.A.2 - MODERN METHOD OF FAMILY PLANNING

Other Acceptors

Philippines, 2021

Area	PILLS-POP			Total	PILLS-COC			Total	INJECTABLES			Total
	Age group				Age group				Age group			
	10-14	15-19	20-49		10-14	15-19	20-49		10-14	15-19	20-49	
**Region 11**	**9**	**496**	**4,213**	**4,718**	**4**	**1,079**	**17,981**	**19,064**	**18**	**690**	**6,509**	**7,217**
Davao de Oro	1	86	798	885	0	186	2,213	2,399	5	72	1,025	1,102
Davao del Norte	0	70	721	791	0	111	2,105	2,216	2	98	1,183	1,283
Davao Oriental	2	87	607	696	3	228	2,681	2,912	2	99	1,198	1,299
Davao del Sur	6	48	566	620	0	144	5,987	6,131	0	99	1,026	1,125
Davao Occidental	0	7	72	79	0	73	877	950	0	95	646	741
Davao City	0	198	1,449	1,647	1	337	4,118	4,456	9	227	1,431	1,667
**Region 12**	**7**	**745**	**6,394**	**7,146**	**6**	**1,456**	**25,141**	**26,603**	**2**	**1,572**	**16,054**	**17,628**
North Cotabato	7	322	3,231	3,560	0	450	9,779	10,229	0	488	5,145	5,633
Sarangani	0	169	828	997	0	231	3,871	4,102	0	159	1,589	1,748
South Cotabato	0	108	1,248	1,356	4	326	5,291	5,621	0	349	3,492	3,841
Sultan Kudarat	0	20	261	281	0	212	3,069	3,281	0	220	1,990	2,210
				0				0				0
Cotabato City	0	39	399	438	1	101	1,717	1,819	1	204	2,079	2,284
Gen. Santos City	0	87	427	514	1	136	1,414	1,551	1	152	1,759	1,912
**BARMM**	**4**	**328**	**2,331**	**2,663**	**5**	**503**	**9,107**	**9,615**	**8**	**681**	**11,420**	**12,109**
Basilan	0	24	153	177	0	48	509	557	0	43	571	614
Lanao del Sur	0	26	758	784	1	17	1,144	1,162	0	24	1,232	1,256
Maguindanao	0	224	677	901	2	179	3,085	3,266	2	295	4,151	4,448
Sulu	0	20	395	415	1	72	2,078	2,151	0	91	3,183	3,274
Tawi-Tawi	1	5	107	113	0	45	915	960	0	26	682	708
				0				0				0
Lamitan City	3	27	179	209	1	141	1,314	1,456	6	202	1,534	1,742
Marawi City	0	2	62	64	0	1	62	63	0	0	67	67
**CARAGA**	**11**	**377**	**3,761**	**4,149**	**4**	**844**	**17,880**	**18,728**	**5**	**434**	**7,370**	**7,809**
Agusan del Norte	2	84	742	828	1	76	2,406	2,483	1	96	1,201	1,298
Agusan del Sur	1	109	1,170	1,280	2	441	7,084	7,527	3	184	2,777	2,964
Surigao del Norte	2	29	437	468	0	32	3,109	3,141	0	22	765	787
Surigao del Sur	0	48	688	736	0	96	2,336	2,432	1	44	973	1,018
Province of Dinagat	0	8	76	84	1	14	381	396	0	9	300	309
Bislig City	0	17	186	203	0	17	479	496	0	15	294	309
Butuan City	6	73	398	477	0	162	1,790	1,952	0	56	831	887
Surigao City	0	9	64	73	0	6	295	301	0	8	229	237

Table 1.A.2 - MODERN METHOD OF FAMILY PLANNING
Other Acceptors
Philippines, 2021

Area	IMPLANTS			Total	NFP-CMM			Total	NFP-BBT			Total
	Age group				Age group				Age group			
	10-14	15-19	20-49		10-14	15-19	20-49		10-14	15-19	20-49	
**PHILIPPINES**	**429**	**5,074**	**79,103**	**84,606**	**120**	**421**	**7,376**	**7,917**	**14**	**38**	**911**	**963**
**N C R**	**1**	**584**	**9,022**	**9,607**	**0**	**0**	**21**	**21**	**0**	**0**	**0**	**0**
Malabon	0	24	259	283	0	0	0	0	0	0	0	0
Navotas	0	34	122	156	0	0	1	1	0	0	0	0
Valenzuela City	0	96	1,724	1,820	0	0	6	6	0	0	0	0
Caloocan City	1	64	1,022	1,087	0	0	0	0	0	0	0	0
Marikina City	0	14	198	212	0	0	8	8	0	0	0	0
Pasig City	0	20	566	586	0	0	0	0	0	0	0	0
Pateros	0	0	9	9	0	0	0	0	0	0	0	0
Taguig	0	35	205	240	0	0	0	0	0	0	0	0
Quezon City	0	209	3,073	3,282	0	0	0	0	0	0	0	0
Makati City	0	7	60	67	0	0	0	0	0	0	0	0
Mandaluyong City	0	0	31	31	0	0	6	6	0	0	0	0
San Juan	0	0	4	4	0	0	0	0	0	0	0	0
Manila City	0	58	1,142	1,200	0	0	0	0	0	0	0	0
Las Piñas City	0	23	317	340	0	0	0	0	0	0	0	0
Muntinlupa City	0	0	19	19	0	0	0	0	0	0	0	0
Parañaque City	0	0	155	155	0	0	0	0	0	0	0	0
Pasay City	0	0	116	116	0	0	0	0	0	0	0	0
**C A R**	**2**	**137**	**2,690**	**2,829**	**0**	**128**	**762**	**890**	**5**	**1**	**25**	**31**
Abra	0	19	184	203	0	0	3	3	0	1	6	7
Apayao	1	20	185	206	0	0	2	2	5	0	0	5
Benguet	0	6	357	363	0	0	14	14	0	0	2	2
Ifugao	0	33	303	336	0	13	133	146	0	0	0	0
Kalinga	0	12	353	365	0	115	538	653	0	0	2	2
Mt. Province	1	18	334	353	0	0	72	72	0	0	9	9
Baguio City	0	29	974	1,003	0	0	0	0	0	0	6	6
**Region 1**	**0**	**156**	**2,082**	**2,238**	**0**	**12**	**261**	**273**	**0**	**0**	**358**	**358**
Ilocos Norte	0	2	36	38	0	2	29	31	0	0	0	0
Ilocos Sur	0	4	22	26	0	0	18	18	0	0	3	3
La Union	0	30	377	407	0	0	94	94	0	0	1	1
Pangasinan	0	89	1,442	1,531	0	0	0	0	0	0	195	195
Alaminos City	0	1	22	23	0	0	0	0	0	0	0	0
Candon City	0	0	0	0	0	0	0	0	0	0	0	0
Dagupan City	0	0	29	29	0	0	0	0	0	0	0	0
Laoag City	0	0	0	0	0	0	0	0	0	0	0	0
San Carlos City	0	15	37	52	0	0	0	0	0	0	0	0
San Fernando City	0	12	115	127	0	0	0	0	0	0	0	0
Urdaneta City	0	0	2	2	0	0	0	0	0	0	0	0
Vigan City	0	3	0	3	0	10	120	130	0	0	159	159
**Region 2**	**2**	**108**	**2,226**	**2,336**	**0**	**0**	**91**	**91**	**9**	**9**	**16**	**34**
Batanes	0	0	0	0	0	0	27	27	0	0	0	0
Cagayan	0	29	458	487	0	0	17	17	0	0	0	0
Isabela	0	58	1,186	1,244	0	0	1	1	0	0	9	9
Nueva Vizcaya	2	18	453	473	0	0	42	42	9	9	7	25
Quirino	0	0	23	23	0	0	4	4	0	0	0	0
Cauayan City	0	1	37	38	0	0	0	0	0	0	0	0
Ilagan City	0	0	4	4	0	0	0	0	0	0	0	0
Santiago City	0	1	55	56	0	0	0	0	0	0	0	0
Tuguegarao City	0	1	10	11	0	0	0	0	0	0	0	0
**Region 3**	**1**	**179**	**4,537**	**4,717**	**0**	**19**	**119**	**138**	**0**	**11**	**78**	**89**
Aurora	0	6	241	247	0	0	40	40	0	0	8	8
Bataan	0	38	451	489	0	0	0	0	0	0	0	0

Table 1.A.2 - MODERN METHOD OF FAMILY PLANNING

Other Acceptors

Philippines, 2021

Area	IMPLANTS			Total	NFP-CMM			Total	NFP-BBT			Total
	Age group				Age group				Age group			
	10-14	15-19	20-49		10-14	15-19	20-49		10-14	15-19	20-49	
Bulacan	0	19	557	576	0	0	0	0	0	0	0	0
Nueva Ecija	0	41	1,224	1,265	0	13	4	17	0	1	2	3
Pampanga	0	54	1,017	1,071	0	6	49	55	0	10	67	77
Tarlac	1	3	185	189	0	0	0	0	0	0	0	0
Zambales	0	9	419	428	0	0	0	0	0	0	1	1
Angeles City	0	1	87	88	0	0	0	0	0	0	0	0
Balanga City	0	4	54	58	0	0	0	0	0	0	0	0
Cabanatuan City	0	1	25	26	0	0	0	0	0	0	0	0
City of San Fernando	0	0	0	0	0	0	0	0	0	0	0	0
Gapan City	0	0	6	6	0	0	0	0	0	0	0	0
Mabalacat City	0	0	2	2	0	0	0	0	0	0	0	0
Malolos City	0	0	84	84	0	0	0	0	0	0	0	0
Meycauayan	0	0	36	36	0	0	26	26	0	0	0	0
Olongapo	0	0	19	19	0	0	0	0	0	0	0	0
Palayan City	0	0	0	0	0	0	0	0	0	0	0	0
San Jose City	0	1	31	32	0	0	0	0	0	0	0	0
San Jose del Monte City	0	2	81	83	0	0	0	0	0	0	0	0
Science City of Munoz	0	0	6	6	0	0	0	0	0	0	0	0
Tarlac City	0	0	12	12	0	0	0	0	0	0	0	0
**Region 4A**	**1**	**72**	**1,453**	**1,526**	**0**	**0**	**99**	**99**	**0**	**0**	**9**	**9**
Batangas	1	2	47	50	0	0	0	0	0	0	2	2
Cavite	0	7	92	99	0	0	4	4	0	0	0	0
Laguna	0	8	165	173	0	0	8	8	0	0	0	0
Quezon	0	9	144	153	0	0	1	1	0	0	2	2
Rizal	0	23	284	307	0	0	0	0	0	0	0	0
Antipolo City	0	0	51	51	0	0	86	86	0	0	0	0
Bacoor City	0	0	2	2	0	0	0	0	0	0	0	0
Batangas City	0	2	29	31	0	0	0	0	0	0	0	0
Biñan City	0	7	60	67	0	0	0	0	0	0	0	0
Cabuyao City	0	1	19	20	0	0	0	0	0	0	0	0
Calamba City	0	10	246	256	0	0	0	0	0	0	0	0
Cavite City	0	2	4	6	0	0	0	0	0	0	0	0
Dasmariñas City	0	0	63	63	0	0	0	0	0	0	0	0
General Trias City	0	0	11	11	0	0	0	0	0	0	1	1
Imus City	0	0	1	1	0	0	0	0	0	0	0	0
Lipa City	0	0	2	2	0	0	0	0	0	0	0	0
Lucena City	0	0	1	1	0	0	0	0	0	0	0	0
San Pablo City	0	1	2	3	0	0	0	0	0	0	1	1
San Pedro City	0	0	11	11	0	0	0	0	0	0	0	0
Santa Rosa City	0	0	144	144	0	0	0	0	0	0	0	0
Tagaytay City	0	0	68	68	0	0	0	0	0	0	0	0
Tanauan City	0	0	4	4	0	0	0	0	0	0	3	3
Tayabas City	0	0	1	1	0	0	0	0	0	0	0	0
Trece Martires City	0	0	2	2	0	0	0	0	0	0	0	0
**Region 4B**	**2**	**161**	**3,493**	**3,656**	**102**	**110**	**194**	**406**	**0**	**1**	**9**	**10**
Marinduque	0	0	94	94	0	0	1	1	0	0	0	0
Mindoro Occidental	1	23	248	272	0	11	153	164	0	0	0	0
Mindoro Oriental	0	17	379	396	0	0	0	0	0	0	0	0
Palawan	1	118	2,390	2,509	101	98	12	211	0	1	7	8
Romblon	0	3	57	60	1	1	28	30	0	0	2	2
Puerto Princesa City	0	0	325	325	0	0	0	0	0	0	0	0
**Region 5**	**1**	**282**	**6,021**	**6,304**	**16**	**70**	**3,208**	**3,294**	**0**	**3**	**144**	**147**
Albay	0	100	1,742	1,842	0	1	141	142	0	0	35	35
Camarines Norte	0	20	415	435	0	1	107	108	0	0	13	13
Camarines Sur	0	76	1,828	1,904	0	5	255	260	0	1	52	53
Catanduanes	1	13	271	285	0	8	142	150	0	0	0	0
Masbate	0	42	850	892	16	30	1,975	2,021	0	1	37	38
Sorsogon	0	29	778	807	0	25	587	612	0	1	7	8
Iriga City	0	2	96	98	0	0	0	0	0	0	0	0

Table 1.A.2 - MODERN METHOD OF FAMILY PLANNING

Other Acceptors

Philippines, 2021

Area	IMPLANTS Age group 10-14	15-19	20-49	Total	NFP-CMM Age group 10-14	15-19	20-49	Total	NFP-BBT Age group 10-14	15-19	20-49	Total
Legaspi City	0	0	9	9	0	0	0	0	0	0	0	0
Naga City	0	0	32	32	0	0	1	1	0	0	0	0
**Region 6**	**1**	**285**	**3,229**	**3,515**	**0**	**15**	**390**	**405**	**0**	**0**	**20**	**20**
Aklan	0	3	105	108	0	0	1	1	0	0	0	0
Antique	0	22	589	611	0	15	388	403	0	0	6	6
Capiz	0	6	212	218	0	0	0	0	0	0	1	1
Guimaras	0	9	32	41	0	0	0	0	0	0	0	0
Iloilo	0	58	652	710	0	0	0	0	0	0	3	3
Negros Occidental	0	128	976	1,104	0	0	1	1	0	0	6	6
Bacolod City	1	45	459	505	0	0	0	0	0	0	0	0
Iloilo City	0	14	204	218	0	0	0	0	0	0	4	4
**Region 7**	**1**	**200**	**5,602**	**5,803**	**0**	**1**	**10**	**11**	**0**	**0**	**19**	**19**
Bohol	0	19	1,121	1,140	0	0	0	0	0	0	1	1
Cebu	0	95	2,736	2,831	0	0	0	0	0	0	0	0
Negros Oriental	1	46	553	600	0	1	8	9	0	0	18	18
Siquijor	0	1	65	66	0	0	0	0	0	0	0	0
Cebu City	0	25	618	643	0	0	2	2	0	0	0	0
Lapu-Lapu City	0	1	94	95	0	0	0	0	0	0	0	0
Mandaue City	0	13	415	428	0	0	0	0	0	0	0	0
**Region 8**	**0**	**102**	**2,735**	**2,837**	**0**	**0**	**194**	**194**	**0**	**1**	**59**	**60**
Biliran	0	7	177	184	0	0	0	0	0	1	1	2
Eastern Samar	0	1	208	209	0	0	16	16	0	0	0	0
Northern Leyte	0	13	493	506	0	0	0	0	0	0	4	4
Northern Samar	0	19	404	423	0	0	2	2	0	0	0	0
Southern Leyte	0	17	265	282	0	0	2	2	0	0	6	6
Western Samar	0	11	294	305	0	0	174	174	0	0	44	44
Calbayog City	0	2	170	172	0	0	0	0	0	0	0	0
Maasin City	0	1	34	35	0	0	0	0	0	0	0	0
Ormoc City	0	22	657	679	0	0	0	0	0	0	4	4
Tacloban City	0	9	33	42	0	0	0	0	0	0	0	0
**Region 9**	**396**	**569**	**8,750**	**9,715**	**0**	**0**	**0**	**0**	**0**	**1**	**18**	**19**
Zamboanga del Norte	1	122	2,202	2,325	0	0	0	0	0	0	5	5
Zamboanga del Sur	2	247	3,849	4,098	0	0	0	0	0	0	1	1
Zamboanga Sibugay	392	127	1,454	1,973	0	0	0	0	0	1	12	13
Dapitan City	0	2	41	43	0	0	0	0	0	0	0	0
Dipolog City	0	27	287	314	0	0	0	0	0	0	0	0
Isabela City	0	13	119	132	0	0	0	0	0	0	0	0
Pagadian City	0	5	118	123	0	0	0	0	0	0	0	0
Zamboanga City	1	26	680	707	0	0	0	0	0	0	0	0
**Region 10**	**8**	**450**	**4,990**	**5,448**	**1**	**53**	**527**	**581**	**0**	**5**	**56**	**61**
Bukidnon	2	337	2,889	3,228	1	18	279	298	0	0	2	2
Camiguin	0	0	6	6	0	0	0	0	0	0	0	0
Lanao del Norte	0	9	507	516	0	0	0	0	0	0	0	0
Misamis Occidental	0	0	35	35	0	0	0	0	0	0	0	0
Misamis Oriental	0	18	274	292	0	2	38	40	0	0	8	8
Cagayan de Oro City	0	8	131	139	0	0	0	0	0	0	0	0
El Salvador City	0	11	124	135	0	0	0	0	0	0	0	0
Gingoog City	0	0	17	17	0	15	94	109	0	0	4	4
Iligan City	0	3	129	132	0	0	55	55	0	0	0	0
Malaybalay City	1	57	728	786	0	0	3	3	0	0	4	4
Oroquieta City	0	0	0	0	0	0	0	0	0	0	0	0
Ozamis City	0	0	32	32	0	0	0	0	0	0	1	1
Tangub City	0	0	6	6	0	0	0	0	0	0	0	0
Valencia City	5	7	112	124	0	18	58	76	0	5	37	42

Table 1.A.2 - MODERN METHOD OF FAMILY PLANNING

Other Acceptors

Philippines, 2021

Area	IMPLANTS Age group			Total	NFP-CMM Age group			Total	NFP-BBT Age group			Total
	10-14	15-19	20-49		10-14	15-19	20-49		10-14	15-19	20-49	
**Region 11**	**4**	**347**	**3,330**	**3,681**	**1**	**4**	**375**	**380**	**0**	**0**	**5**	**5**
Davao de Oro	2	70	670	742	0	2	41	43	0	0	1	1
Davao del Norte	1	65	642	708	0	0	0	0	0	0	0	0
Davao Oriental	0	21	398	419	0	1	222	223	0	0	2	2
Davao del Sur	0	36	395	431	0	0	13	13	0	0	0	0
Davao Occidental	0	31	257	288	0	0	0	0	0	0	0	0
Davao City	1	124	968	1,093	1	1	99	101	0	0	2	2
**Region 12**	**2**	**824**	**8,707**	**9,533**	**0**	**1**	**127**	**128**	**0**	**0**	**70**	**70**
North Cotabato	0	192	2,648	2,840	0	1	105	106	0	0	68	68
Sarangani	0	110	1,280	1,390	0	0	16	16	0	0	0	0
South Cotabato	1	313	2,482	2,796	0	0	0	0	0	0	0	0
Sultan Kudarat	0	102	1,213	1,315	0	0	6	6	0	0	2	2
				0				0				0
Cotabato City	0	49	468	517	0	0	0	0	0	0	0	0
Gen. Santos City	1	58	616	675	0	0	0	0	0	0	0	0
**BARMM**	**3**	**288**	**4,495**	**4,786**	**0**	**0**	**6**	**6**	**0**	**0**	**6**	**6**
Basilan	0	29	215	244	0	0	1	1	0	0	0	0
Lanao del Sur	0	9	311	320	0	0	1	1	0	0	0	0
Maguindanao	1	67	964	1,032	0	0	2	2	0	0	0	0
Sulu	0	68	1,754	1,822	0	0	0	0	0	0	0	0
Tawi-Tawi	0	7	524	531	0	0	0	0	0	0	0	0
				0				0				0
Lamitan City	2	107	700	809	0	0	2	2	0	0	6	6
Marawi City	0	1	27	28	0	0	0	0	0	0	0	0
**CARAGA**	**4**	**330**	**5,741**	**6,075**	**0**	**8**	**992**	**1,000**	**0**	**6**	**19**	**25**
Agusan del Norte	0	73	799	872	0	0	1	1	0	0	3	3
Agusan del Sur	0	146	2,295	2,441	0	8	955	963	0	6	10	16
Surigao del Norte	3	16	456	475	0	0	0	0	0	0	4	4
Surigao del Sur	1	54	927	982	0	0	22	22	0	0	1	1
Province of Dinagat	0	9	300	309	0	0	0	0	0	0	0	0
Bislig City	0	8	135	143	0	0	14	14	0	0	1	1
Butuan City	0	20	728	748	0	0	0	0	0	0	0	0
Surigao City	0	4	101	105	0	0	0	0	0	0	0	0

Table 1.A.2 - MODERN METHOD OF FAMILY PLANNING

Other Acceptors

Philippines, 2021

Area	NFP-STM			Total	NFP-SDM			Total	NFP-LAM			Total	Total Other Acceptors
	Age group				Age group				Age group				
	10-14	15-19	20-49		10-14	15-19	20-49		10-14	15-19	20-49		
**PHILIPPINES**	**12**	**23**	**1,044**	**1,079**	**15**	**572**	**15,073**	**15,660**	**356**	**15,313**	**283,291**	**298,960**	**1,127,901**
**N C R**	**0**	**0**	**0**	**0**	**2**	**3**	**113**	**118**	**24**	**1,727**	**30,180**	**31,931**	**165,221**
Malabon	0	0	0	0	0	0	0	0	0	44	823	867	4,912
Navotas	0	0	0	0	0	0	0	0	3	109	1,196	1,308	3,806
Valenzuela City	0	0	0	0	0	0	11	11	0	128	1,880	2,008	15,381
Caloocan City	0	0	0	0	0	3	8	11	0	231	3,025	3,256	19,478
Marikina City	0	0	0	0	0	0	0	0	0	7	135	142	1,986
Pasig City	0	0	0	0	0	0	0	0	1	13	367	381	4,317
Pateros	0	0	0	0	0	0	0	0	0	0	52	52	266
Taguig	0	0	0	0	2	0	0	2	0	24	378	402	3,657
Quezon City	0	0	0	0	0	0	24	24	3	698	16,680	17,381	88,019
Makati City	0	0	0	0	0	0	0	0	0	0	74	74	1,621
Mandaluyong City	0	0	0	0	0	0	0	0	3	22	77	102	1,254
San Juan	0	0	0	0	0	0	0	0	0	0	52	52	240
Manila City	0	0	0	0	0	0	65	65	9	191	1,065	1,265	6,617
Las Piñas City	0	0	0	0	0	0	0	0	0	7	182	189	3,006
Muntinlupa City	0	0	0	0	0	0	0	0	0	10	10	20	1,270
Parañaque City	0	0	0	0	0	0	5	5	5	93	353	451	2,532
Pasay City	0	0	0	0	0	0	0	0	0	150	3,831	3,981	6,859
**C A R**	**0**	**0**	**8**	**8**	**0**	**16**	**1,533**	**1,549**	**5**	**382**	**9,078**	**9,465**	**59,123**
Abra	0	0	0	0	0	3	72	75	4	80	740	824	2,691
Apayao	0	0	1	1	0	0	2	2	0	70	1,287	1,357	22,542
Benguet	0	0	0	0	0	4	560	564	0	88	2,107	2,195	8,105
Ifugao	0	0	2	2	0	5	582	587	0	60	1,782	1,842	6,806
Kalinga	0	0	1	1	0	0	64	64	0	23	1,669	1,692	6,098
Mt. Province	0	0	4	4	0	4	241	245	1	56	1,175	1,232	7,234
Baguio City	0	0	0	0	0	0	12	12	0	5	318	323	5,647
**Region 1**	**0**	**0**	**185**	**185**	**0**	**17**	**615**	**632**	**2**	**636**	**16,842**	**17,480**	**43,117**
Ilocos Norte	0	0	0	0	0	0	1	1	0	8	901	909	3,311
Ilocos Sur	0	0	0	0	0	7	216	223	0	34	1,107	1,141	3,826
La Union	0	0	0	0	0	2	95	97	1	197	3,462	3,660	7,299
Pangasinan	0	0	10	10	0	7	284	291	1	281	9,606	9,888	24,315
Alaminos City	0	0	0	0	0	0	0	0	0	9	296	305	464
Candon City	0	0	0	0	0	0	0	0	0	0	0	0	0
Dagupan City	0	0	0	0	0	0	0	0	0	0	0	0	190
Laoag City	0	0	0	0	0	0	0	0	0	0	0	0	96
San Carlos City	0	0	0	0	0	0	0	0	0	35	989	1,024	1,849
San Fernando City	0	0	0	0	0	1	18	19	0	9	131	140	609
Urdaneta City	0	0	0	0	0	0	0	0	0	34	136	170	341
Vigan City	0	0	175	175	0	0	1	1	0	29	214	243	817
**Region 2**	**0**	**4**	**26**	**30**	**0**	**16**	**236**	**252**	**7**	**963**	**13,255**	**14,225**	**46,001**
Batanes	0	0	0	0	0	0	2	2	0	5	154	159	446
Cagayan	0	0	0	0	0	0	1	1	3	114	2,703	2,820	8,783
Isabela	0	0	0	0	0	0	8	8	0	168	3,742	3,910	14,372
Nueva Vizcaya	0	4	26	30	0	16	218	234	3	565	2,737	3,305	14,319
Quirino	0	0	0	0	0	0	6	6	1	22	502	525	1,556
Cauayan City	0	0	0	0	0	0	0	0	0	27	804	831	1,639
Ilagan City	0	0	0	0	0	0	0	0	0	8	328	336	863
Santiago City	0	0	0	0	0	0	0	0	0	49	2,151	2,200	3,524
Tuguegarao City	0	0	0	0	0	0	1	1	0	5	134	139	499
**Region 3**	**0**	**3**	**410**	**413**	**0**	**0**	**720**	**720**	**4**	**1,057**	**15,577**	**16,638**	**94,043**
Aurora	0	0	0	0	0	0	1	1	1	70	1,455	1,526	4,730
Bataan	0	0	0	0	0	0	0	0	0	34	834	868	5,344

Table 1.A.2 - MODERN METHOD OF FAMILY PLANNING

Other Acceptors

Philippines, 2021

Area	NFP-STM			Total	NFP-SDM			Total	NFP-LAM			Total	Total Other Acceptors
	Age group				Age group				Age group				
	10-14	15-19	20-49		10-14	15-19	20-49		10-14	15-19	20-49		
Bulacan	0	0	0	0	0	0	328	328	0	123	2,611	2,734	15,269
Nueva Ecija	0	0	0	0	0	0	5	5	1	167	2,683	2,851	18,760
Pampanga	0	3	410	413	0	0	382	382	2	134	1,691	1,827	21,302
Tarlac	0	0	0	0	0	0	3	3	0	93	1,333	1,426	7,933
Zambales	0	0	0	0	0	0	0	0	0	148	1,041	1,189	5,532
Angeles City	0	0	0	0	0	0	0	0	0	23	60	83	858
Balanga City	0	0	0	0	0	0	1	1	0	17	382	399	1,108
Cabanatuan City	0	0	0	0	0	0	0	0	0	29	1,328	1,357	2,564
City of San Fernando	0	0	0	0	0	0	0	0	0	8	42	50	274
Gapan City	0	0	0	0	0	0	0	0	0	11	93	104	263
Mabalacat City	0	0	0	0	0	0	0	0	0	70	554	624	1,375
Malolos City	0	0	0	0	0	0	0	0	0	1	67	68	824
Meycauayan	0	0	0	0	0	0	0	0	0	7	169	176	722
Olongapo	0	0	0	0	0	0	0	0	0	6	251	257	1,520
Palayan City	0	0	0	0	0	0	0	0	0	11	44	55	188
San Jose City	0	0	0	0	0	0	0	0	0	39	584	623	1,382
San Jose del Monte City	0	0	0	0	0	0	0	0	0	0	0	0	1,639
Science City of Munoz	0	0	0	0	0	0	0	0	0	0	0	0	155
Tarlac City	0	0	0	0	0	0	0	0	0	66	355	421	2,301
**Region 4A**	**0**	**0**	**3**	**3**	**0**	**48**	**360**	**408**	**9**	**405**	**7,880**	**8,294**	**30,639**
Batangas	0	0	0	0	0	47	333	380	7	41	1,744	1,792	3,705
Cavite	0	0	3	3	0	1	13	14	0	25	599	624	2,684
Laguna	0	0	0	0	0	0	8	8	0	45	491	536	2,125
Quezon	0	0	0	0	0	0	2	2	1	44	947	992	6,129
Rizal	0	0	0	0	0	0	1	1	0	54	1,207	1,261	4,126
Antipolo City	0	0	0	0	0	0	0	0	0	0	457	457	1,365
Bacoor City	0	0	0	0	0	0	0	0	0	0	84	84	330
Batangas City	0	0	0	0	0	0	3	3	0	5	266	271	890
Biñan City	0	0	0	0	0	0	0	0	0	103	219	322	2,249
Cabuyao City	0	0	0	0	0	0	0	0	0	18	69	87	360
Calamba City	0	0	0	0	0	0	0	0	1	17	295	313	1,606
Cavite City	0	0	0	0	0	0	0	0	0	0	20	20	98
Dasmariñas City	0	0	0	0	0	0	0	0	0	14	290	304	876
General Trias City	0	0	0	0	0	0	0	0	0	2	154	156	544
Imus City	0	0	0	0	0	0	0	0	0	0	55	55	245
Lipa City	0	0	0	0	0	0	0	0	0	0	0	0	14
Lucena City	0	0	0	0	0	0	0	0	0	2	61	63	470
San Pablo City	0	0	0	0	0	0	0	0	0	9	142	151	383
San Pedro City	0	0	0	0	0	0	0	0	0	12	134	146	441
Santa Rosa City	0	0	0	0	0	0	0	0	0	3	73	76	619
Tagaytay City	0	0	0	0	0	0	0	0	0	0	178	178	425
Tanauan City	0	0	0	0	0	0	0	0	0	1	77	78	119
Tayabas City	0	0	0	0	0	0	0	0	0	10	312	322	633
Trece Martires City	0	0	0	0	0	0	0	0	0	0	6	6	203
**Region 4B**	**12**	**0**	**6**	**18**	**3**	**30**	**290**	**323**	**149**	**645**	**14,934**	**15,728**	**46,485**
Marinduque	0	0	0	0	0	0	3	3	0	4	303	307	1,592
Mindoro Occidental	0	0	0	0	0	0	8	8	1	225	5,061	5,287	10,549
Mindoro Oriental	0	0	0	0	0	0	6	6	0	39	2,415	2,454	5,664
Palawan	12	0	6	18	3	30	123	156	147	345	5,507	5,999	20,352
Romblon	0	0	0	0	0	0	122	122	1	32	993	1,026	2,534
Puerto Princesa City	0	0	0	0	0	0	28	28	0	0	655	655	5,794
**Region 5**	**0**	**5**	**256**	**261**	**1**	**128**	**5,456**	**5,585**	**19**	**1,197**	**30,280**	**31,496**	**105,962**
Albay	0	0	14	14	1	7	1,424	1,432	4	144	2,746	2,894	27,006
Camarines Norte	0	0	3	3	0	5	269	274	0	188	4,328	4,516	11,002
Camarines Sur	0	4	142	146	0	35	840	875	7	312	9,532	9,851	25,012
Catanduanes	0	0	0	0	0	0	214	214	3	44	745	792	2,978
Masbate	0	1	9	10	0	75	2,041	2,116	4	319	6,128	6,451	21,914
Sorsogon	0	0	88	88	0	3	528	531	1	185	6,269	6,455	15,489
Iriga City	0	0	0	0	0	3	111	114	0	5	271	276	783

Table 1.A.2 - MODERN METHOD OF FAMILY PLANNING

Other Acceptors

Philippines, 2021

Area	NFP-STM			Total	NFP-SDM			Total	NFP-LAM			Total	Total Other Acceptors
	Age group				Age group				Age group				
	10-14	15-19	20-49		10-14	15-19	20-49		10-14	15-19	20-49		
Legaspi City	0	0	0	0	0	0	0	0	0	0	17	17	142
Naga City	0	0	0	0	0	0	29	29	0	0	244	244	1,636
**Region 6**	**0**	**0**	**1**	**1**	**7**	**13**	**575**	**595**	**7**	**735**	**18,183**	**18,925**	**59,236**
Aklan	0	0	0	0	0	0	16	16	0	31	1,056	1,087	2,643
Antique	0	0	0	0	0	0	30	30	2	54	1,748	1,804	6,713
Capiz	0	0	0	0	0	0	1	1	1	29	591	621	3,735
Guimaras	0	0	0	0	0	0	14	14	2	46	1,288	1,336	2,388
Iloilo	0	0	0	0	0	3	275	278	0	115	3,378	3,493	10,371
Negros Occidental	0	0	1	1	7	8	231	246	2	427	9,516	9,945	25,975
Bacolod City	0	0	0	0	0	0	0	0	0	29	477	506	2,446
Iloilo City	0	0	0	0	0	2	8	10	0	4	129	133	4,965
**Region 7**	**0**	**2**	**0**	**2**	**0**	**3**	**70**	**73**	**1**	**632**	**19,637**	**20,270**	**62,578**
Bohol	0	0	0	0	0	0	5	5	0	64	2,678	2,742	10,355
Cebu	0	0	0	0	0	0	22	22	0	182	5,417	5,599	20,560
Negros Oriental	0	2	0	2	0	0	9	9	1	199	6,210	6,410	17,472
Siquijor	0	0	0	0	0	3	34	37	0	7	344	351	1,567
Cebu City	0	0	0	0	0	0	0	0	0	95	3,417	3,512	8,798
Lapu-Lapu City	0	0	0	0	0	0	0	0	0	85	1,426	1,511	2,947
Mandaue City	0	0	0	0	0	0	0	0	0	0	145	145	879
**Region 8**	**0**	**5**	**61**	**66**	**0**	**2**	**652**	**654**	**4**	**499**	**9,504**	**10,007**	**35,040**
Biliran	0	0	50	50	0	0	38	38	1	47	684	732	2,409
Eastern Samar	0	5	11	16	0	0	325	325	1	35	608	644	2,750
Northern Leyte	0	0	0	0	0	0	9	9	0	84	1,800	1,884	5,055
Northern Samar	0	0	0	0	0	0	49	49	0	121	1,810	1,931	5,842
Southern Leyte	0	0	0	0	0	2	33	35	0	55	1,152	1,207	4,722
Western Samar	0	0	0	0	0	0	0	0	1	42	827	870	3,014
Calbayog City	0	0	0	0	0	0	0	0	1	6	237	244	2,568
Maasin City	0	0	0	0	0	0	27	27	0	3	55	58	344
Ormoc City	0	0	0	0	0	0	171	171	0	89	2,025	2,114	7,812
Tacloban City	0	0	0	0	0	0	0	0	0	17	306	323	524
**Region 9**	**0**	**0**	**0**	**0**	**2**	**49**	**457**	**508**	**5**	**1,370**	**17,694**	**19,069**	**67,007**
Zamboanga del Norte	0	0	0	0	2	49	448	499	3	376	5,167	5,546	16,659
Zamboanga del Sur	0	0	0	0	0	0	0	0	1	267	2,253	2,521	15,269
Zamboanga Sibugay	0	0	0	0	0	0	6	6	0	133	2,339	2,472	16,266
Dapitan City	0	0	0	0	0	0	0	0	0	24	522	546	1,143
Dipolog City	0	0	0	0	0	0	3	3	0	74	1,347	1,421	4,297
Isabela City	0	0	0	0	0	0	0	0	0	12	310	322	1,050
Pagadian City	0	0	0	0	0	0	0	0	0	38	1,186	1,224	3,149
Zamboanga City	0	0	0	0	0	0	0	0	1	446	4,570	5,017	9,174
**Region 10**	**0**	**4**	**43**	**47**	**0**	**182**	**2,063**	**2,245**	**40**	**1,131**	**16,289**	**17,460**	**56,044**
Bukidnon	0	0	30	30	0	44	544	588	6	539	5,057	5,602	23,219
Camiguin	0	0	0	0	0	0	0	0	0	8	447	455	678
Lanao del Norte	0	0	0	0	0	0	45	45	2	87	1,859	1,948	5,598
Misamis Occidental	0	0	0	0	0	1	76	77	0	14	594	608	2,012
Misamis Oriental	0	0	0	0	0	1	67	68	3	63	1,282	1,348	3,363
Cagayan de Oro City	0	0	0	0	0	0	0	0	0	17	379	396	1,530
El Salvador City	0	0	0	0	0	0	4	4	0	9	307	316	838
Gingoog City	0	4	12	16	0	35	164	199	0	91	1,061	1,152	2,619
Iligan City	0	0	0	0	0	9	222	231	0	73	1,672	1,745	3,831
Malaybalay City	0	0	0	0	0	44	814	858	1	75	1,597	1,673	7,433
Oroquieta City	0	0	0	0	0	0	0	0	0	4	93	97	154
Ozamis City	0	0	0	0	0	0	5	5	0	22	484	506	780
Tangub City	0	0	0	0	0	40	53	93	0	4	78	82	381
Valencia City	0	0	1	1	0	8	69	77	28	125	1,379	1,532	3,608

Table 1.A.2 - MODERN METHOD OF FAMILY PLANNING

Other Acceptors

Philippines, 2021

Area	NFP-STM			Total	NFP-SDM			Total	NFP-LAM			Total	Total Other Acceptors
	Age group				Age group				Age group				
	10-14	15-19	20-49		10-14	15-19	20-49		10-14	15-19	20-49		
**Region 11**	**0**	**0**	**35**	**35**	**0**	**8**	**228**	**236**	**35**	**859**	**7,970**	**8,864**	**49,602**
Davao de Oro	0	0	0	0	0	0	10	10	3	31	351	385	6,272
Davao del Norte	0	0	1	1	0	1	6	7	1	109	1,404	1,514	7,459
Davao Oriental	0	0	1	1	0	6	164	170	3	278	3,104	3,385	10,139
Davao del Sur	0	0	10	10	0	1	6	7	0	23	203	226	9,051
Davao Occidental	0	0	0	0	0	0	13	13	0	36	309	345	2,631
Davao City	0	0	23	23	0	0	29	29	28	382	2,599	3,009	14,050
**Region 12**	**0**	**0**	**6**	**6**	**0**	**10**	**167**	**177**	**7**	**1,675**	**20,875**	**22,557**	**90,799**
North Cotabato	0	0	0	0	0	0	55	55	0	473	5,740	6,213	31,434
Sarangani	0	0	0	0	0	2	33	35	0	460	4,621	5,081	14,064
South Cotabato	0	0	6	6	0	8	36	44	7	355	4,197	4,559	19,683
Sultan Kudarat	0	0	0	0	0	0	36	36	0	133	3,484	3,617	11,538
				0				0				0	
Cotabato City	0	0	0	0	0	0	5	5	0	77	1,007	1,084	7,138
Gen. Santos City	0	0	0	0	0	0	2	2	0	177	1,826	2,003	6,942
**BARMM**	**0**	**0**	**0**	**0**	**0**	**4**	**60**	**64**	**26**	**640**	**20,300**	**20,966**	**53,290**
Basilan	0	0	0	0	0	0	0	0	0	22	481	503	2,179
Lanao del Sur	0	0	0	0	0	0	25	25	0	111	5,239	5,350	9,689
Maguindanao	0	0	0	0	0	4	27	31	10	308	7,949	8,267	18,788
Sulu	0	0	0	0	0	0	3	3	0	51	3,514	3,565	11,284
Tawi-Tawi	0	0	0	0	0	0	3	3	13	54	2,214	2,281	4,612
				0				0				0	
Lamitan City	0	0	0	0	0	0	2	2	3	79	425	507	5,952
Marawi City	0	0	0	0	0	0	0	0	0	15	478	493	786
**CARAGA**	**0**	**0**	**4**	**4**	**0**	**43**	**1,478**	**1,521**	**12**	**760**	**14,813**	**15,585**	**63,714**
Agusan del Norte	0	0	0	0	0	0	9	9	0	76	1,351	1,427	7,800
Agusan del Sur	0	0	4	4	0	41	1,016	1,057	6	330	5,163	5,499	26,966
Surigao del Norte	0	0	0	0	0	1	352	353	0	38	918	956	6,814
Surigao del Sur	0	0	0	0	0	1	50	51	6	136	3,061	3,203	9,111
Province of Dinagat	0	0	0	0	0	0	14	14	0	8	233	241	1,526
Bislig City	0	0	0	0	0	0	13	13	0	26	933	959	2,511
Butuan City	0	0	0	0	0	0	17	17	0	129	2,935	3,064	7,775
Surigao City	0	0	0	0	0	0	7	7	0	17	219	236	1,211

Table 1.A.3 - MODERN METHOD OF FAMILY PLANNING

Drop Outs

Philippines, 2021

Area	Total Current Users	FSTR/BTL			Total	MSTR/NSV			Total
		Age group				Age group			
		10-14	15-19	20-49		10-14	15-19	20-49	
**PHILIPPINES**	**7,279,694**	**4**	**581**	**48,979**	**49,564**	**0**	**7**	**997**	**1,004**
**N C R**	**726,121**	**0**	**1**	**4,333**	**4,334**	**0**	**0**	**38**	**38**
Malabon	32,409	0	0	124	124	0	0	0	0
Navotas	23,787	0	1	99	100	0	0	0	0
Valenzuela City	39,524	0	0	433	433	0	0	12	12
Caloocan City	67,903	0	0	1,635	1,635	0	0	4	4
Marikina City	15,458	0	0	51	51	0	0	1	1
Pasig City	37,512	0	0	53	53	0	0	3	3
Pateros	2,543	0	0	47	47	0	0	0	0
Taguig	57,590	0	0	71	71	0	0	0	0
Quezon City	259,021	0	0	1,193	1,193	0	0	8	8
Makati City	12,799	0	0	1	1	0	0	0	0
Mandaluyong City	16,826	0	0	122	122	0	0	6	6
San Juan	5,760	0	0	4	4	0	0	0	0
Manila City	31,616	0	0	88	88	0	0	1	1
Las Piñas City	22,298	0	0	245	245	0	0	0	0
Muntinlupa City	40,077	0	0	27	27	0	0	0	0
Parañaque City	33,986	0	0	10	10	0	0	0	0
Pasay City	27,012	0	0	130	130	0	0	3	3
**C A R**	**131,969**	**3**	**9**	**2,292**	**2,304**	**0**	**0**	**29**	**29**
Abra	16,967	3	1	553	557	0	0	4	4
Apayao	12,113	0	0	317	317	0	0	10	10
Benguet	30,721	0	0	549	549	0	0	4	4
Ifugao	19,056	0	0	150	150	0	0	10	10
Kalinga	18,965	0	0	391	391	0	0	0	0
Mt. Province	12,528	0	8	237	245	0	0	1	1
Baguio City	21,619	0	0	95	95	0	0	0	0
**Region 1**	**466,771**	**0**	**9**	**3,960**	**3,969**	**0**	**0**	**18**	**18**
Ilocos Norte	41,297	0	0	913	913	0	0	0	0
Ilocos Sur	58,069	0	0	594	594	0	0	0	0
La Union	59,945	0	5	512	517	0	0	5	5
Pangasinan	233,753	0	4	1,501	1,505	0	0	13	13
Alaminos City	10,247	0	0	12	12	0	0	0	0
Candon City	7,036	0	0	11	11	0	0	0	0
Dagupan City	6,893	0	0	12	12	0	0	0	0
Laoag City	8,101	0	0	111	111	0	0	0	0
San Carlos City	11,818	0	0	139	139	0	0	0	0
San Fernando City	12,806	0	0	36	36	0	0	0	0
Urdaneta City	12,828	0	0	40	40	0	0	0	0
Vigan City	3,978	0	0	79	79	0	0	0	0
**Region 2**	**314,925**	**0**	**16**	**2,472**	**2,488**	**0**	**0**	**22**	**22**
Batanes	938	0	0	16	16	0	0	0	0
Cagayan	87,099	0	0	259	259	0	0	2	2
Isabela	108,463	0	0	800	800	0	0	4	4
Nueva Vizcaya	45,287	0	10	969	979	0	0	15	15
Quirino	19,270	0	0	103	103	0	0	0	0
Cauayan City	9,889	0	6	78	84	0	0	0	0
Ilagan City	15,732	0	0	66	66	0	0	0	0
Santiago City	13,933	0	0	128	128	0	0	0	0
Tuguegarao City	14,314	0	0	53	53	0	0	1	1
**Region 3**	**794,245**	**0**	**253**	**12,137**	**12,390**	**0**	**4**	**31**	**35**
Aurora	21,216	0	0	42	42	0	0	0	0
Bataan	50,598	0	0	93	93	0	0	0	0
Bulacan	148,620	0	3	1,344	1,347	0	0	0	0
Nueva Ecija	128,909	0	153	1,971	2,124	0	2	11	13

Table 1.A.3 - MODERN METHOD OF FAMILY PLANNING

Drop Outs

Philippines, 2021

Area	Total Current Users	FSTR/BTL			Total	MSTR/NSV			Total
		Age group				Age group			
		10-14	15-19	20-49		10-14	15-19	20-49	
Pampanga	109,928	0	94	6,579	6,673	0	2	11	13
Tarlac	80,640	0	0	314	314	0	0	0	0
Zambales	42,194	0	0	193	193	0	0	1	1
Angeles City	23,390	0	0	76	76	0	0	5	5
Balanga City	4,908	0	0	68	68	0	0	2	2
Cabanatuan City	19,136	0	3	291	294	0	0	0	0
City of San Fernando	9,567	0	0	94	94	0	0	0	0
Gapan City	7,511	0	0	28	28	0	0	0	0
Mabalacat City	22,703	0	0	254	254	0	0	1	1
Malolos City	7,548	0	0	61	61	0	0	0	0
Meycauayan	7,990	0	0	25	25	0	0	0	0
Olongapo	10,438	0	0	7	7	0	0	0	0
Palayan City	3,016	0	0	14	14	0	0	0	0
San Jose City	12,975	0	0	0	0	0	0	0	0
San Jose del Monte City	57,952	0	0	620	620	0	0	0	0
Science City of Munoz	6,335	0	0	23	23	0	0	0	0
Tarlac City	18,671	0	0	40	40	0	0	0	0
**Region 4A**	**502,769**	**0**	**9**	**1,314**	**1,323**	**0**	**0**	**17**	**17**
Batangas	69,044	0	1	162	163	0	0	0	0
Cavite	50,364	0	0	90	90	0	0	7	7
Laguna	39,915	0	8	150	158	0	0	3	3
Quezon	65,304	0	0	170	170	0	0	2	2
Rizal	46,762	0	0	121	121	0	0	1	1
Antipolo City	6,069	0	0	0	0	0	0	0	0
Bacoor City	10,158	0	0	43	43	0	0	0	0
Batangas City	15,493	0	0	68	68	0	0	0	0
Biñan City	42,782	0	0	3	3	0	0	0	0
Cabuyao City	6,608	0	0	26	26	0	0	0	0
Calamba City	22,351	0	0	233	233	0	0	1	1
Cavite City	4,279	0	0	29	29	0	0	0	0
Dasmariñas City	37,251	0	0	96	96	0	0	0	0
General Trias City	5,008	0	0	48	48	0	0	0	0
Imus City	6,503	0	0	8	8	0	0	0	0
Lipa City	5,955	0	0	3	3	0	0	0	0
Lucena City	5,207	0	0	5	5	0	0	0	0
San Pablo City	5,471	0	0	14	14	0	0	1	1
San Pedro City	10,235	0	0	0	0	0	0	0	0
Santa Rosa City	23,903	0	0	0	0	0	0	0	0
Tagaytay City	8,089	0	0	18	18	0	0	2	2
Tanauan City	4,819	0	0	7	7	0	0	0	0
Tayabas City	7,446	0	0	19	19	0	0	0	0
Trece Martires City	3,753	0	0	1	1	0	0	0	0
**Region 4B**	**262,639**	**1**	**16**	**2,754**	**2,771**	**0**	**0**	**10**	**10**
Marinduque	13,771	0	15	223	238	0	0	3	3
Mindoro Occidental	45,760	0	0	161	161	0	0	0	0
Mindoro Oriental	64,019	0	1	1,809	1,810	0	0	1	1
Palawan	85,631	1	0	223	224	0	0	4	4
Romblon	22,521	0	0	169	169	0	0	1	1
Puerto Princesa City	30,937	0	0	169	169	0	0	1	1
**Region 5**	**467,956**	**0**	**24**	**3,442**	**3,466**	**0**	**0**	**128**	**128**
Albay	86,082	0	12	374	386	0	0	9	9
Camarines Norte	42,496	0	1	389	390	0	0	6	6
Camarines Sur	106,478	0	1	867	868	0	0	87	87
Catanduanes	25,339	0	0	56	56	0	0	8	8
Masbate	82,338	0	1	323	324	0	0	3	3
Sorsogon	63,538	0	9	1,327	1,336	0	0	8	8
Iriga City	12,388	0	0	63	63	0	0	5	5
Legaspi City	23,098	0	0	10	10	0	0	0	0
Naga City	26,199	0	0	33	33	0	0	2	2

Table 1.A.3 - MODERN METHOD OF FAMILY PLANNING

Drop Outs

Philippines, 2021

Area	Total Current Users	FSTR/BTL Age group 10-14	15-19	20-49	Total	MSTR/NSV Age group 10-14	15-19	20-49	Total
**Region 6**	**597,683**	**0**	**13**	**2,261**	**2,274**	**0**	**0**	**112**	**112**
Aklan	44,142	0	0	114	114	0	0	11	11
Antique	46,065	0	0	483	483	0	0	11	11
Capiz	74,407	0	0	131	131	0	0	15	15
Guimaras	15,917	0	1	64	65	0	0	1	1
Iloilo	153,978	0	0	394	394	0	0	9	9
Negros Occidental	190,516	0	7	651	658	0	0	45	45
Bacolod City	26,414	0	0	127	127	0	0	9	9
Iloilo City	46,244	0	5	297	302	0	0	11	11
**Region 7**	**402,202**	**0**	**0**	**2,280**	**2,280**	**0**	**0**	**126**	**126**
Bohol	80,267	0	0	1,020	1,020	0	0	44	44
Cebu	142,224	0	0	197	197	0	0	23	23
Negros Oriental	81,104	0	0	150	150	0	0	23	23
Siquijor	8,593	0	0	67	67	0	0	0	0
Cebu City	33,223	0	0	314	314	0	0	36	36
Lapu-Lapu City	35,312	0	0	530	530	0	0	0	0
Mandaue City	21,479	0	0	2	2	0	0	0	0
**Region 8**	**342,050**	**0**	**0**	**1,357**	**1,357**	**0**	**0**	**113**	**113**
Biliran	11,102	0	0	49	49	0	0	41	41
Eastern Samar	38,666	0	0	165	165	0	0	9	9
Northern Leyte	113,942	0	0	152	152	0	0	6	6
Northern Samar	46,536	0	0	96	96	0	0	11	11
Southern Leyte	24,868	0	0	391	391	0	0	34	34
Western Samar	26,826	0	0	73	73	0	0	2	2
Calbayog City	8,836	0	0	156	156	0	0	0	0
Maasin City	5,096	0	0	72	72	0	0	0	0
Ormoc City	20,009	0	0	181	181	0	0	10	10
Tacloban City	46,169	0	0	22	22	0	0	0	0
**Region 9**	**314,587**	**0**	**2**	**955**	**957**	**0**	**0**	**1**	**1**
Zamboanga del Norte	98,783	0	0	302	302	0	0	0	0
Zamboanga del Sur	65,366	0	2	206	208	0	0	0	0
Zamboanga Sibugay	42,607	0	0	100	100	0	0	1	1
Dapitan City	7,733	0	0	46	46	0	0	0	0
Dipolog City	16,393	0	0	174	174	0	0	0	0
Isabela City	9,369	0	0	27	27	0	0	0	0
Pagadian City	19,991	0	0	19	19	0	0	0	0
Zamboanga City	54,345	0	0	81	81	0	0	0	0
**Region 10**	**469,808**	**0**	**17**	**1,453**	**1,470**	**0**	**0**	**67**	**67**
Bukidnon	110,248	0	10	415	425	0	0	32	32
Camiguin	6,471	0	0	18	18	0	0	0	0
Lanao del Norte	60,033	0	0	157	157	0	0	0	0
Misamis Occidental	25,727	0	0	40	40	0	0	0	0
Misamis Oriental	79,462	0	0	112	112	0	0	1	1
Cagayan de Oro City	60,104	0	0	77	77	0	0	0	0
El Salvador City	6,228	0	0	8	8	0	0	0	0
Gingoog City	29,566	0	7	87	94	0	0	0	0
Iligan City	27,468	0	0	81	81	0	0	4	4
Malaybalay City	19,334	0	0	334	334	0	0	20	20
Oroquieta City	6,837	0	0	2	2	0	0	0	0
Ozamis City	13,612	0	0	8	8	0	0	1	1
Tangub City	3,838	0	0	33	33	0	0	0	0
Valencia City	20,880	0	0	81	81	0	0	9	9

Table 1.A.3 - MODERN METHOD OF FAMILY PLANNING

Drop Outs

Philippines, 2021

Area	Total Current Users	FSTR/BTL Age group			Total	MSTR/NSV Age group			Total
		10-14	15-19	20-49		10-14	15-19	20-49	
**Region 11**	**501,561**	**0**	**34**	**2,411**	**2,445**	**0**	**2**	**114**	**116**
Davao de Oro	83,015	0	0	551	551	0	0	18	18
Davao del Norte	100,273	0	1	503	504	0	1	22	23
Davao Oriental	51,379	0	3	394	397	0	0	22	22
Davao del Sur	65,425	0	2	234	236	0	0	9	9
Davao Occidental	31,994	0	0	176	176	0	0	1	1
Davao City	169,475	0	28	553	581	0	1	42	43
**Region 12**	**468,656**	**0**	**177**	**3,282**	**3,459**	**0**	**0**	**105**	**105**
North Cotabato	136,386	0	14	1,050	1,064	0	0	36	36
Sarangani	65,258	0	156	355	511	0	0	3	3
South Cotabato	91,960	0	6	1,063	1,069	0	0	56	56
Sultan Kudarat	93,390	0	1	448	449	0	0	9	9
					0				0
Cotabato City	30,632	0	0	232	232	0	0	0	0
Gen. Santos City	51,030	0	0	134	134	0	0	1	1
**BARMM**	**266,572**	**0**	**0**	**917**	**917**	**0**	**0**	**21**	**21**
Basilan	10,532	0	0	16	16	0	0	0	0
Lanao del Sur	40,004	0	0	158	158	0	0	3	3
Maguindanao	89,662	0	0	555	555	0	0	16	16
Sulu	88,420	0	0	85	85	0	0	2	2
Tawi-Tawi	27,695	0	0	64	64	0	0	0	0
					0				0
Lamitan City	4,938	0	0	31	31	0	0	0	0
Marawi City	5,321	0	0	8	8	0	0	0	0
**CARAGA**	**249,180**	**0**	**1**	**1,359**	**1,360**	**0**	**1**	**45**	**46**
Agusan del Norte	37,360	0	0	161	161	0	0	3	3
Agusan del Sur	76,525	0	0	362	362	0	1	19	20
Surigao del Norte	30,743	0	0	272	272	0	0	17	17
Surigao del Sur	42,013	0	0	186	186	0	0	4	4
Province of Dinagat	10,218	0	1	178	179	0	0	2	2
Bislig City	9,004	0	0	39	39	0	0	0	0
Butuan City	30,541	0	0	98	98	0	0	0	0
Surigao City	12,776	0	0	63	63	0	0	0	0

Table 1.A.3 - MODERN METHOD OF FAMILY PLANNING

Drop Outs

Philippines, 2021

Area	CONDOM			Total	IUD-INTERVAL			Total	IUD-POSTPARTUM			Total
	Age group				Age group				Age group			
	10-14	15-19	20-49		10-14	15-19	20-49		10-14	15-19	20-49	
**PHILIPPINES**	**115**	**3,018**	**59,227**	**62,360**	**24**	**1,466**	**34,726**	**36,216**	**18**	**1,171**	**11,978**	**13,167**
**N C R**	**55**	**883**	**18,407**	**19,345**	**7**	**160**	**4,150**	**4,317**	**7**	**260**	**1,853**	**2,120**
Malabon	1	23	396	420	0	6	79	85	0	0	1	1
Navotas	3	110	283	396	0	5	43	48	0	0	0	0
Valenzuela City	0	3	461	464	0	15	195	210	0	3	116	119
Caloocan City	0	62	1,325	1,387	3	91	1,779	1,873	4	112	377	493
Marikina City	0	0	184	184	0	0	7	7	0	0	22	22
Pasig City	0	11	228	239	0	8	138	146	0	59	341	400
Pateros	0	1	83	84	0	0	38	38	0	0	0	0
Taguig	31	17	439	487	0	0	64	64	0	13	87	100
Quezon City	5	380	12,643	13,028	0	22	1,156	1,178	1	67	642	710
Makati City	0	2	120	122	0	0	5	5	0	0	2	2
Mandaluyong City	0	27	267	294	0	1	31	32	0	0	4	4
San Juan	0	0	49	49	0	0	18	18	0	0	0	0
Manila City	0	94	614	708	0	3	140	143	0	3	51	54
Las Piñas City	0	23	754	777	0	9	134	143	0	2	118	120
Muntinlupa City	0	4	115	119	0	0	176	176	0	0	0	0
Parañaque City	15	9	182	206	4	0	18	22	2	1	19	22
Pasay City	0	117	264	381	0	0	129	129	0	0	73	73
**C A R**	**1**	**43**	**1,389**	**1,433**	**1**	**22**	**1,060**	**1,083**	**1**	**46**	**506**	**553**
Abra	0	2	64	66	0	1	16	17	0	0	2	2
Apayao	0	9	120	129	0	2	373	375	0	11	318	329
Benguet	0	14	377	391	1	3	337	341	0	4	43	47
Ifugao	0	4	177	181	0	2	41	43	0	1	10	11
Kalinga	0	3	160	163	0	1	100	101	0	0	5	5
Mt. Province	1	7	181	189	0	9	104	113	1	29	75	105
Baguio City	0	4	310	314	0	4	89	93	0	1	53	54
**Region 1**	**0**	**44**	**1,809**	**1,853**	**1**	**56**	**1,146**	**1,203**	**1**	**4**	**75**	**80**
Ilocos Norte	0	2	44	46	0	2	25	27	0	0	3	3
Ilocos Sur	0	5	128	133	0	0	28	28	0	0	14	14
La Union	0	3	135	138	1	9	123	133	1	3	40	44
Pangasinan	0	24	1,063	1,087	0	28	567	595	0	0	0	0
Alaminos City	0	0	2	2	0	9	349	358	0	0	0	0
Candon City	0	0	40	40	0	0	0	0	0	0	3	3
Dagupan City	0	6	35	41	0	2	5	7	0	0	4	4
Laoag City	0	0	100	100	0	0	6	6	0	0	0	0
San Carlos City	0	2	46	48	0	5	34	39	0	0	0	0
San Fernando City	0	1	45	46	0	1	7	8	0	1	11	12
Urdaneta City	0	0	168	168	0	0	0	0	0	0	0	0
Vigan City	0	1	3	4	0	0	2	2	0	0	0	0
**Region 2**	**1**	**56**	**799**	**856**	**5**	**64**	**1,064**	**1,133**	**1**	**48**	**315**	**364**
Batanes	0	0	6	6	0	0	2	2	0	0	0	0
Cagayan	0	2	100	102	0	15	333	348	0	18	82	100
Isabela	0	16	261	277	0	11	266	277	1	19	114	134
Nueva Vizcaya	1	20	316	337	4	27	291	322	0	4	95	99
Quirino	0	15	44	59	0	3	27	30	0	2	5	7
Cauayan City	0	1	25	26	0	0	30	30	0	1	0	1
Ilagan City	0	0	15	15	0	0	19	19	0	0	2	2
Santiago City	0	2	26	28	1	7	59	67	0	0	0	0
Tuguegarao City	0	0	6	6	0	1	37	38	0	4	17	21
**Region 3**	**2**	**404**	**5,994**	**6,400**	**0**	**110**	**2,059**	**2,169**	**0**	**50**	**516**	**566**
Aurora	1	2	60	63	0	1	16	17	0	1	14	15
Bataan	0	7	250	257	0	2	39	41	0	0	8	8
Bulacan	0	8	335	343	0	2	128	130	0	20	126	146
Nueva Ecija	0	41	613	654	0	32	530	562	0	19	173	192

Table 1.A.3 - MODERN METHOD OF FAMILY PLANNING

Drop Outs

Philippines, 2021

Area	CONDOM			Total	IUD-INTERVAL			Total	IUD-POSTPARTUM			Total
	Age group				Age group				Age group			
	10-14	15-19	20-49		10-14	15-19	20-49		10-14	15-19	20-49	
Pampanga	1	115	1,575	1,691	0	46	764	810	0	4	143	147
Tarlac	0	58	344	402	0	1	70	71	0	0	1	1
Zambales	0	5	311	316	0	3	48	51	0	0	8	8
Angeles City	0	0	83	83	0	0	4	4	0	0	20	20
Balanga City	0	0	15	15	0	0	12	12	0	0	0	0
Cabanatuan City	0	7	74	81	0	1	22	23	0	4	2	6
City of San Fernando	0	0	70	70	0	0	1	1	0	0	1	1
Gapan City	0	0	4	4	0	1	7	8	0	0	0	0
Mabalacat City	0	21	260	281	0	1	61	62	0	0	0	0
Malolos City	0	6	61	67	0	0	13	13	0	0	0	0
Meycauayan	0	12	68	80	0	0	0	0	0	0	0	0
Olongapo	0	0	44	44	0	0	0	0	0	0	7	7
Palayan City	0	0	1	1	0	0	20	20	0	0	0	0
San Jose City	0	8	28	36	0	0	13	13	0	2	0	2
San Jose del Monte City	0	96	1,686	1,782	0	16	185	201	0	0	0	0
Science City of Munoz	0	0	5	5	0	2	19	21	0	0	0	0
Tarlac City	0	18	107	125	0	2	107	109	0	0	13	13
**Region 4A**	**0**	**246**	**2,047**	**2,293**	**1**	**30**	**1,028**	**1,059**	**2**	**40**	**876**	**918**
Batangas	0	3	74	77	0	0	49	49	0	0	3	3
Cavite	0	0	90	90	0	1	65	66	0	1	1	2
Laguna	0	14	108	122	1	16	164	181	0	11	133	144
Quezon	0	9	183	192	0	8	295	303	2	0	15	17
Rizal	0	10	226	236	0	1	40	41	0	15	32	47
Antipolo City	0	0	27	27	0	0	6	6	0	0	149	149
Bacoor City	0	0	20	20	0	0	13	13	0	0	3	3
Batangas City	0	1	54	55	0	4	13	17	0	12	41	53
Biñan City	0	119	697	816	0	0	32	32	0	0	0	0
Cabuyao City	0	1	77	78	0	0	1	1	0	0	2	2
Calamba City	0	0	110	110	0	0	318	318	0	0	466	466
Cavite City	0	0	1	1	0	0	0	0	0	0	0	0
Dasmariñas City	0	10	80	90	0	0	4	4	0	0	12	12
General Trias City	0	0	19	19	0	0	1	1	0	0	0	0
Imus City	0	2	50	52	0	0	1	1	0	0	0	0
Lipa City	0	0	4	4	0	0	3	3	0	0	0	0
Lucena City	0	0	11	11	0	0	1	1	0	0	0	0
San Pablo City	0	1	8	9	0	0	17	17	0	1	2	3
San Pedro City	0	1	29	30	0	0	0	0	0	0	0	0
Santa Rosa City	0	75	132	207	0	0	0	0	0	0	0	0
Tagaytay City	0	0	2	2	0	0	0	0	0	0	10	10
Tanauan City	0	0	7	7	0	0	2	2	0	0	7	7
Tayabas City	0	0	9	9	0	0	3	3	0	0	0	0
Trece Martires City	0	0	29	29	0	0	0	0	0	0	0	0
**Region 4B**	**1**	**27**	**1,075**	**1,103**	**0**	**1**	**475**	**476**	**1**	**7**	**108**	**116**
Marinduque	0	4	63	67	0	0	81	81	0	0	3	3
Mindoro Occidental	0	3	226	229	0	0	97	97	0	2	3	5
Mindoro Oriental	0	5	107	112	0	1	125	126	0	2	30	32
Palawan	1	15	417	433	0	0	142	142	1	3	11	15
Romblon	0	0	85	85	0	0	30	30	0	0	27	27
Puerto Princesa City	0	0	177	177	0	0	0	0	0	0	34	34
**Region 5**	**0**	**101**	**3,530**	**3,631**	**0**	**24**	**951**	**975**	**0**	**13**	**293**	**306**
Albay	0	35	496	531	0	6	116	122	0	3	46	49
Camarines Norte	0	15	427	442	0	3	182	185	0	0	8	8
Camarines Sur	0	23	1,090	1,113	0	6	254	260	0	7	70	77
Catanduanes	0	17	67	84	0	5	54	59	0	0	5	5
Masbate	0	0	3	3	0	3	175	178	0	3	145	148
Sorsogon	0	9	959	968	0	1	105	106	0	0	16	16
Iriga City	0	0	80	80	0	0	50	50	0	0	3	3
Legaspi City	0	1	45	46	0	0	0	0	0	0	0	0
Naga City	0	1	363	364	0	0	15	15	0	0	0	0

Table 1.A.3 - MODERN METHOD OF FAMILY PLANNING

Drop Outs

Philippines, 2021

Area	CONDOM			Total	IUD-INTERVAL			Total	IUD-POSTPARTUM			Total
	Age group				Age group				Age group			
	10-14	15-19	20-49		10-14	15-19	20-49		10-14	15-19	20-49	
**Region 6**	**0**	**109**	**2,814**	**2,923**	**1**	**95**	**1,926**	**2,022**	**0**	**38**	**496**	**534**
Aklan	0	28	287	315	0	0	17	17	0	0	6	6
Antique	0	12	246	258	0	3	110	113	0	0	37	37
Capiz	0	12	321	333	0	2	254	256	0	0	3	3
Guimaras	0	4	50	54	0	1	35	36	0	0	8	8
Iloilo	0	3	887	890	0	17	458	475	0	8	56	64
Negros Occidental	0	18	700	718	1	26	915	942	0	20	314	334
Bacolod City	0	13	85	98	0	8	65	73	0	1	29	30
Iloilo City	0	19	238	257	0	38	72	110	0	9	43	52
**Region 7**	**0**	**106**	**3,139**	**3,245**	**0**	**75**	**3,014**	**3,089**	**0**	**143**	**1,540**	**1,683**
Bohol	0	13	952	965	0	24	1,325	1,349	0	91	906	997
Cebu	0	30	772	802	0	24	609	633	0	43	284	327
Negros Oriental	0	17	400	417	0	6	340	346	0	1	33	34
Siquijor	0	1	74	75	0	0	41	41	0	1	55	56
Cebu City	0	15	198	213	0	19	468	487	0	7	88	95
Lapu-Lapu City	0	30	675	705	0	2	226	228	0	0	174	174
Mandaue City	0	0	68	68	0	0	5	5	0	0	0	0
**Region 8**	**0**	**83**	**1,494**	**1,577**	**0**	**53**	**1,673**	**1,726**	**0**	**51**	**589**	**640**
Biliran	0	11	118	129	0	1	42	43	0	0	9	9
Eastern Samar	0	2	168	170	0	1	36	37	0	2	18	20
Northern Leyte	0	1	114	115	0	31	520	551	0	36	286	322
Northern Samar	0	3	106	109	0	2	84	86	0	1	21	22
Southern Leyte	0	10	267	277	0	7	401	408	0	7	159	166
Western Samar	0	30	49	79	0	0	11	11	0	3	28	31
Calbayog City	0	0	153	153	0	3	49	52	0	0	7	7
Maasin City	0	0	30	30	0	0	157	157	0	0	6	6
Ormoc City	0	22	288	310	0	8	258	266	0	2	23	25
Tacloban City	0	4	201	205	0	0	115	115	0	0	32	32
**Region 9**	**3**	**194**	**2,206**	**2,403**	**0**	**85**	**1,743**	**1,828**	**0**	**66**	**412**	**478**
Zamboanga del Norte	3	101	847	951	0	10	466	476	0	4	45	49
Zamboanga del Sur	0	49	541	590	0	58	720	778	0	51	220	271
Zamboanga Sibugay	0	29	401	430	0	14	272	286	0	3	21	24
Dapitan City	0	2	82	84	0	0	6	6	0	0	8	8
Dipolog City	0	13	198	211	0	1	99	100	0	1	36	37
Isabela City	0	0	5	5	0	0	0	0	0	0	0	0
Pagadian City	0	0	61	61	0	2	144	146	0	7	68	75
Zamboanga City	0	0	71	71	0	0	36	36	0	0	14	14
**Region 10**	**0**	**69**	**2,466**	**2,535**	**0**	**215**	**2,539**	**2,754**	**2**	**150**	**571**	**723**
Bukidnon	0	11	628	639	0	48	1,025	1,073	0	14	267	281
Camiguin	0	0	23	23	0	0	9	9	0	0	2	2
Lanao del Norte	0	30	352	382	0	6	175	181	0	1	20	21
Misamis Occidental	0	5	189	194	0	1	118	119	0	0	3	3
Misamis Oriental	0	4	151	155	0	15	249	264	1	12	18	31
Cagayan de Oro City	0	3	284	287	0	81	289	370	1	100	189	290
El Salvador City	0	1	5	6	0	15	131	146	0	1	5	6
Gingoog City	0	7	97	104	0	5	52	57	0	10	12	22
Iligan City	0	3	215	218	0	28	154	182	0	7	8	15
Malaybalay City	0	2	427	429	0	3	236	239	0	5	26	31
Oroquieta City	0	0	0	0	0	0	0	0	0	0	0	0
Ozamis City	0	1	14	15	0	0	8	8	0	0	0	0
Tangub City	0	0	3	3	0	0	4	4	0	0	2	2
Valencia City	0	2	78	80	0	13	89	102	0	0	19	19

Table 1.A.3 - MODERN METHOD OF FAMILY PLANNING

Drop Outs

Philippines, 2021

Area	CONDOM			Total	IUD-INTERVAL			Total	IUD-POSTPARTUM			Total
	Age group				Age group				Age group			
	10-14	15-19	20-49		10-14	15-19	20-49		10-14	15-19	20-49	
**Region 11**	**51**	**288**	**2,484**	**2,823**	**3**	**81**	**2,687**	**2,771**	**1**	**50**	**441**	**492**
Davao de Oro	2	11	363	376	0	6	530	536	0	2	15	17
Davao del Norte	0	11	472	483	0	12	466	478	0	3	94	97
Davao Oriental	0	18	246	264	0	6	378	384	0	3	12	15
Davao del Sur	0	6	250	256	3	11	402	416	1	4	73	78
Davao Occidental	0	4	71	75	0	3	124	127	0	12	57	69
Davao City	49	238	1,082	1,369	0	43	787	830	0	26	190	216
**Region 12**	**0**	**161**	**3,119**	**3,280**	**3**	**155**	**4,201**	**4,359**	**1**	**128**	**1,612**	**1,741**
North Cotabato	0	58	1,029	1,087	0	81	2,183	2,264	0	21	422	443
Sarangani	0	26	314	340	0	19	201	220	0	5	23	28
South Cotabato	0	32	970	1,002	0	30	1,295	1,325	0	22	570	592
Sultan Kudarat	0	19	253	272	0	3	285	288	0	24	162	186
				0				0				0
Cotabato City	0	13	397	410	3	18	150	171	1	51	368	420
Gen. Santos City	0	13	156	169	0	4	87	91	0	5	67	72
**BARMM**	**1**	**98**	**4,318**	**4,417**	**1**	**38**	**1,509**	**1,548**	**0**	**18**	**217**	**235**
Basilan	0	34	143	177	0	1	42	43	0	0	2	2
Lanao del Sur	1	18	2,614	2,633	0	0	116	116	0	1	6	7
Maguindanao	0	31	1,045	1,076	1	37	1,232	1,270	0	12	159	171
Sulu	0	15	294	309	0	0	92	92	0	0	20	20
Tawi-Tawi	0	0	78	78	0	0	0	0	0	0	0	0
				0				0				0
Lamitan City	0	0	1	1	0	0	27	27	0	5	30	35
Marawi City	0	0	143	143	0	0	0	0	0	0	0	0
**CARAGA**	**0**	**106**	**2,137**	**2,243**	**1**	**202**	**3,501**	**3,704**	**1**	**59**	**1,558**	**1,618**
Agusan del Norte	0	19	356	375	0	16	697	713	0	12	55	67
Agusan del Sur	0	60	794	854	1	151	1,541	1,693	0	21	1,283	1,304
Surigao del Norte	0	3	278	281	0	9	474	483	0	1	31	32
Surigao del Sur	0	11	322	333	0	17	421	438	1	17	104	122
Province of Dinagat	0	1	135	136	0	2	133	135	0	0	2	2
Bislig City	0	7	122	129	0	5	96	101	0	4	27	31
Butuan City	0	3	80	83	0	2	105	107	0	2	24	26
Surigao City	0	2	50	52	0	0	34	34	0	2	32	34

Table 1.A.3 - MODERN METHOD OF FAMILY PLANNING

Drop Outs

Philippines, 2021

Area	PILLS-POP			Total	PILLS-COC			Total	INJECTABLES			Total
	Age group				Age group				Age group			
	10-14	15-19	20-49		10-14	15-19	20-49		10-14	15-19	20-49	
**PHILIPPINES**	**101**	**8,078**	**93,564**	**101,743**	**248**	**17,040**	**297,793**	**315,081**	**226**	**20,200**	**265,973**	**286,399**
**N C R**	**17**	**1,886**	**15,377**	**17,280**	**93**	**1,781**	**46,011**	**47,885**	**71**	**3,540**	**54,295**	**57,906**
Malabon	0	50	571	621	0	81	1,072	1,153	0	326	3,085	3,411
Navotas	0	121	383	504	0	210	800	1,010	2	253	1,127	1,382
Valenzuela City	0	99	1,740	1,839	0	103	7,392	7,495	1	325	6,034	6,360
Caloocan City	0	107	889	996	0	263	2,996	3,259	1	624	7,746	8,371
Marikina City	0	30	187	217	0	22	896	918	1	113	1,417	1,531
Pasig City	0	68	334	402	0	132	1,076	1,208	0	161	1,573	1,734
Pateros	0	2	5	7	0	1	113	114	0	8	95	103
Taguig	0	52	662	714	0	42	1,787	1,829	0	59	2,918	2,977
Quezon City	0	670	6,049	6,719	1	503	22,841	23,345	2	744	18,751	19,497
Makati City	0	34	300	334	0	18	462	480	0	70	1,161	1,231
Mandaluyong City	0	64	545	609	0	53	463	516	0	71	581	652
San Juan	0	26	11	37	0	17	158	175	0	0	122	122
Manila City	4	390	2,392	2,786	41	177	2,309	2,527	3	268	3,734	4,005
Las Piñas City	2	104	805	911	1	109	2,299	2,409	1	242	2,322	2,565
Muntinlupa City	0	26	224	250	0	34	694	728	1	51	2,307	2,359
Parañaque City	11	25	118	154	50	16	399	465	59	225	965	1,249
Pasay City	0	18	162	180	0	0	254	254	0	0	357	357
**C A R**	**1**	**291**	**11,189**	**11,481**	**1**	**321**	**9,041**	**9,363**	**2**	**440**	**7,299**	**7,741**
Abra	0	24	209	233	0	21	626	647	0	24	329	353
Apayao	1	87	5,167	5,255	0	67	2,376	2,443	0	73	817	890
Benguet	0	59	497	556	0	69	1,552	1,621	0	100	1,362	1,462
Ifugao	0	26	578	604	0	42	1,271	1,313	0	28	1,068	1,096
Kalinga	0	35	1,219	1,254	0	55	901	956	0	22	693	715
Mt. Province	0	35	2,673	2,708	1	45	878	924	1	82	763	846
Baguio City	0	25	846	871	0	22	1,437	1,459	1	111	2,267	2,379
**Region 1**	**0**	**73**	**1,267**	**1,340**	**6**	**618**	**11,386**	**12,010**	**1**	**637**	**7,498**	**8,136**
Ilocos Norte	0	7	144	151	0	5	1,771	1,776	0	13	349	362
Ilocos Sur	0	17	808	825	5	69	775	849	0	64	666	730
La Union	0	36	138	174	0	87	1,220	1,307	0	161	1,275	1,436
Pangasinan	0	0	0	0	1	397	6,325	6,723	1	342	4,112	4,455
Alaminos City	0	0	0	0	0	0	116	116	0	2	73	75
Candon City	0	0	0	0	0	0	71	71	0	0	53	53
Dagupan City	0	0	19	19	0	2	198	200	0	7	131	138
Laoag City	0	0	26	26	0	0	18	18	0	0	10	10
San Carlos City	0	0	0	0	0	35	742	777	0	24	534	558
San Fernando City	0	13	118	131	0	4	66	70	0	14	133	147
Urdaneta City	0	0	0	0	0	15	53	68	0	10	73	83
Vigan City	0	0	14	14	0	4	31	35	0	0	89	89
**Region 2**	**3**	**480**	**3,071**	**3,554**	**6**	**709**	**13,112**	**13,827**	**6**	**665**	**9,384**	**10,055**
Batanes	0	0	11	11	0	2	57	59	1	12	152	165
Cagayan	0	95	502	597	1	117	2,248	2,366	2	93	1,475	1,570
Isabela	1	152	1,065	1,218	3	225	4,212	4,440	1	343	4,108	4,452
Nueva Vizcaya	2	159	1,197	1,358	2	257	3,815	4,074	2	86	1,766	1,854
Quirino	0	25	108	133	0	26	381	407	0	40	414	454
Cauayan City	0	12	33	45	0	20	413	433	0	17	223	240
Ilagan City	0	24	110	134	0	2	611	613	0	9	269	278
Santiago City	0	0	0	0	0	50	1,278	1,328	0	60	839	899
Tuguegarao City	0	13	45	58	0	10	97	107	0	5	138	143
**Region 3**	**4**	**675**	**5,944**	**6,623**	**9**	**1,623**	**29,200**	**30,832**	**11**	**2,380**	**27,675**	**30,066**
Aurora	0	23	365	388	0	30	640	670	0	27	405	432
Bataan	1	63	337	401	0	79	1,276	1,355	2	223	2,515	2,740
Bulacan	1	75	1,042	1,118	1	252	5,235	5,488	1	420	5,571	5,992
Nueva Ecija	0	145	1,179	1,324	0	138	4,353	4,491	0	278	3,649	3,927

Table 1.A.3 - MODERN METHOD OF FAMILY PLANNING

Drop Outs

Philippines, 2021

Area	PILLS-POP Age group 10-14	PILLS-POP Age group 15-19	PILLS-POP Age group 20-49	Total	PILLS-COC Age group 10-14	PILLS-COC Age group 15-19	PILLS-COC Age group 20-49	Total	INJECTABLES Age group 10-14	INJECTABLES Age group 15-19	INJECTABLES Age group 20-49	Total
Pampanga	0	75	1,236	1,311	7	249	7,263	7,519	4	238	3,881	4,123
Tarlac	0	107	489	596	1	181	1,487	1,669	0	158	1,479	1,637
Zambales	1	28	263	292	0	68	1,223	1,291	2	161	2,420	2,583
Angeles City	0	6	76	82	0	67	612	679	0	56	542	598
Balanga City	0	0	9	9	0	14	198	212	1	35	596	632
Cabanatuan City	1	31	119	151	0	46	850	896	0	53	348	401
City of San Fernando	0	18	129	147	0	30	155	185	0	20	129	149
Gapan City	0	5	24	29	0	17	208	225	0	21	195	216
Mabalacat City	0	17	134	151	0	28	886	914	0	101	1,142	1,243
Malolos City	0	0	0	0	0	12	230	242	0	10	185	195
Meycauayan	0	9	53	62	0	39	401	440	0	47	485	532
Olongapo	0	9	125	134	0	27	583	610	1	81	1,132	1,214
Palayan City	0	0	19	19	0	2	126	128	0	3	59	62
San Jose City	0	12	123	135	0	25	628	653	0	36	641	677
San Jose del Monte City	0	18	77	95	0	229	2,242	2,471	0	345	1,807	2,152
Science City of Munoz	0	2	15	17	0	4	153	157	0	2	45	47
Tarlac City	0	32	130	162	0	86	451	537	0	65	449	514
**Region 4A**	**0**	**351**	**351**	**702**	**7**	**668**	**10,180**	**10,855**	**61**	**731**	**10,926**	**11,718**
Batangas	0	24	24	48	6	25	1,039	1,070	49	11	446	506
Cavite	0	12	12	24	0	35	676	711	3	98	1,222	1,323
Laguna	0	21	21	42	0	40	927	967	0	37	359	396
Quezon	0	45	45	90	1	96	2,179	2,276	1	146	2,612	2,759
Rizal	0	34	34	68	0	115	1,148	1,263	8	146	1,902	2,056
Antipolo City	0	0	0	0	0	0	35	35	0	0	362	362
Bacoor City	0	3	3	6	0	2	161	163	0	19	299	318
Batangas City	0	5	5	10	0	13	319	332	0	20	195	215
Biñan City	0	133	133	266	0	185	670	855	0	116	642	758
Cabuyao City	0	19	19	38	0	31	320	351	0	33	162	195
Calamba City	0	21	21	42	0	17	1,109	1,126	0	10	742	752
Cavite City	0	0	0	0	0	3	24	27	0	5	33	38
Dasmariñas City	0	10	10	20	0	18	358	376	0	12	384	396
General Trias City	0	8	8	16	0	1	128	129	0	10	401	411
Imus City	0	4	4	8	0	3	189	192	0	6	260	266
Lipa City	0	1	1	2	0	0	13	13	0	0	10	10
Lucena City	0	0	0	0	0	5	105	110	0	12	183	195
San Pablo City	0	1	1	2	0	10	150	160	0	6	157	163
San Pedro City	0	1	1	2	0	7	127	134	0	2	75	77
Santa Rosa City	0	0	0	0	0	59	188	247	0	28	129	157
Tagaytay City	0	0	0	0	0	0	84	84	0	0	38	38
Tanauan City	0	3	3	6	0	0	33	33	0	0	37	37
Tayabas City	0	4	4	8	0	3	156	159	0	10	229	239
Trece Martires City	0	2	2	4	0	0	42	42	0	4	47	51
**Region 4B**	**2**	**70**	**1,483**	**1,555**	**8**	**283**	**8,234**	**8,525**	**3**	**565**	**10,586**	**11,154**
Marinduque	0	0	164	164	0	45	212	257	0	6	252	258
Mindoro Occidental	0	11	85	96	0	68	1,916	1,984	0	99	1,955	2,054
Mindoro Oriental	0	5	328	333	1	12	704	717	0	30	635	665
Palawan	2	47	794	843	7	148	3,334	3,489	3	422	5,594	6,019
Romblon	0	7	112	119	0	10	442	452	0	8	480	488
Puerto Princesa City	0	0	0	0	0	0	1,626	1,626	0	0	1,670	1,670
**Region 5**	**0**	**350**	**10,597**	**10,947**	**5**	**1,139**	**26,098**	**27,242**	**5**	**1,148**	**19,966**	**21,119**
Albay	0	97	7,930	8,027	2	230	2,529	2,761	0	148	3,115	3,263
Camarines Norte	0	64	494	558	0	133	2,362	2,495	0	153	3,118	3,271
Camarines Sur	0	107	1,265	1,372	1	248	5,876	6,125	1	252	6,103	6,356
Catanduanes	0	2	42	44	1	34	282	317	0	256	456	712
Masbate	0	51	432	483	1	240	4,511	4,752	4	169	2,495	2,668
Sorsogon	0	23	291	314	0	248	9,137	9,385	0	165	4,106	4,271
Iriga City	0	2	27	29	0	4	229	233	0	3	235	238
Legaspi City	0	4	73	77	0	0	139	139	0	2	41	43
Naga City	0	0	43	43	0	2	1,033	1,035	0	0	297	297

Table 1.A.3 - MODERN METHOD OF FAMILY PLANNING

Drop Outs

Philippines, 2021

Area	PILLS-POP Age group 10-14	PILLS-POP 15-19	PILLS-POP 20-49	Total	PILLS-COC Age group 10-14	PILLS-COC 15-19	PILLS-COC 20-49	Total	INJECTABLES Age group 10-14	INJECTABLES 15-19	INJECTABLES 20-49	Total
**Region 6**	**5**	**382**	**2,950**	**3,337**	**2**	**1,447**	**15,569**	**17,018**	**3**	**1,435**	**13,321**	**14,759**
Aklan	0	16	218	234	0	41	831	872	0	47	790	837
Antique	0	31	353	384	0	84	1,301	1,385	1	194	1,672	1,867
Capiz	0	23	174	197	0	44	803	847	0	70	861	931
Guimaras	0	14	59	73	0	13	381	394	0	11	221	232
Iloilo	0	28	879	907	1	121	2,292	2,414	1	88	1,691	1,780
Negros Occidental	5	239	1,083	1,327	1	929	8,359	9,289	1	846	6,544	7,391
Bacolod City	0	5	78	83	0	44	720	764	0	82	1,082	1,164
Iloilo City	0	26	106	132	0	171	882	1,053	0	97	460	557
**Region 7**	**3**	**324**	**3,084**	**3,411**	**4**	**642**	**15,976**	**16,622**	**9**	**947**	**16,220**	**17,176**
Bohol	0	37	399	436	0	63	2,639	2,702	0	74	2,227	2,301
Cebu	0	106	806	912	1	156	3,562	3,719	2	241	3,631	3,874
Negros Oriental	1	41	365	407	0	145	3,958	4,103	1	264	4,944	5,209
Siquijor	0	10	129	139	0	10	407	417	1	13	367	381
Cebu City	2	93	810	905	3	224	4,163	4,390	3	303	3,801	4,107
Lapu-Lapu City	0	36	551	587	0	44	1,044	1,088	2	51	1,021	1,074
Mandaue City	0	1	24	25	0	0	203	203	0	1	229	230
**Region 8**	**2**	**214**	**2,439**	**2,655**	**0**	**547**	**9,840**	**10,387**	**0**	**562**	**7,142**	**7,704**
Biliran	0	17	93	110	0	29	486	515	0	42	539	581
Eastern Samar	2	18	102	122	0	22	801	823	0	40	471	511
Northern Leyte	0	10	102	112	0	58	1,362	1,420	0	25	667	692
Northern Samar	0	16	165	181	0	86	1,465	1,551	0	69	1,069	1,138
Southern Leyte	0	24	175	199	0	84	1,002	1,086	0	56	678	734
Western Samar	0	41	270	311	0	142	763	905	0	152	826	978
Calbayog City	0	33	901	934	0	37	1,032	1,069	0	44	793	837
Maasin City	0	2	48	50	0	1	213	214	0	1	82	83
Ormoc City	0	49	276	325	0	78	1,885	1,963	0	101	1,331	1,432
Tacloban City	0	4	307	311	0	10	831	841	0	32	686	718
**Region 9**	**2**	**209**	**1,322**	**1,533**	**3**	**981**	**15,625**	**16,609**	**2**	**957**	**11,700**	**12,659**
Zamboanga del Norte	0	72	444	516	2	298	3,862	4,162	1	211	2,443	2,655
Zamboanga del Sur	0	49	274	323	0	221	3,526	3,747	0	124	2,199	2,323
Zamboanga Sibugay	0	9	135	144	1	167	3,320	3,488	0	206	3,067	3,273
Dapitan City	0	2	33	35	0	1	297	298	0	17	179	196
Dipolog City	2	28	90	120	0	72	996	1,068	0	22	665	687
Isabela City	0	7	62	69	0	11	237	248	0	34	413	447
Pagadian City	0	2	26	28	0	31	589	620	0	33	478	511
Zamboanga City	0	40	258	298	0	180	2,798	2,978	1	310	2,256	2,567
**Region 10**	**6**	**374**	**3,888**	**4,268**	**14**	**1,352**	**16,083**	**17,449**	**11**	**929**	**10,434**	**11,374**
Bukidnon	3	202	995	1,200	5	476	6,146	6,627	7	494	4,647	5,148
Camiguin	0	1	18	19	0	6	98	104	0	3	46	49
Lanao del Norte	0	20	452	472	7	422	788	1,217	0	71	757	828
Misamis Occidental	0	13	75	88	0	1	246	247	0	11	266	277
Misamis Oriental	2	61	1,036	1,099	2	66	3,013	3,081	1	76	1,389	1,466
Cagayan de Oro City	0	11	133	144	0	39	546	585	1	58	548	607
El Salvador City	0	4	49	53	0	7	144	151	1	7	173	181
Gingoog City	0	1	793	794	0	58	1,283	1,341	0	25	207	232
Iligan City	0	14	117	131	0	82	591	673	1	49	768	818
Malaybalay City	1	14	73	88	0	122	2,441	2,563	0	98	1,092	1,190
Oroquieta City	0	0	0	0	0	0	16	16	0	0	1	1
Ozamis City	0	2	14	16	0	5	65	70	0	5	108	113
Tangub City	0	0	1	1	0	0	68	68	0	0	13	13
Valencia City	0	31	132	163	0	68	638	706	0	32	419	451

Table 1.A.3 - MODERN METHOD OF FAMILY PLANNING

Drop Outs

Philippines, 2021

Area	PILLS-POP			Total	PILLS-COC			Total	INJECTABLES			Total
	Age group				Age group				Age group			
	10-14	15-19	20-49		10-14	15-19	20-49		10-14	15-19	20-49	
**Region 11**	**20**	**631**	**5,200**	**5,851**	**67**	**1,320**	**14,498**	**15,885**	**23**	**1,067**	**9,573**	**10,663**
Davao de Oro	4	130	736	870	1	79	2,995	3,075	4	119	1,672	1,795
Davao del Norte	1	73	618	692	2	151	2,968	3,121	2	143	1,766	1,911
Davao Oriental	2	60	879	941	50	456	1,781	2,287	1	97	881	979
Davao del Sur	2	90	396	488	1	119	1,398	1,518	2	161	1,347	1,510
Davao Occidental	0	12	68	80	2	47	801	850	2	98	1,110	1,210
Davao City	11	266	2,503	2,780	11	468	4,555	5,034	12	449	2,797	3,258
**Region 12**	**18**	**819**	**5,917**	**6,754**	**7**	**1,931**	**25,863**	**27,801**	**6**	**2,662**	**22,190**	**24,858**
North Cotabato	16	397	3,111	3,524	2	629	10,784	11,415	2	848	6,401	7,251
Sarangani	0	134	338	472	0	370	2,523	2,893	0	302	2,395	2,697
South Cotabato	2	122	1,151	1,275	4	452	6,165	6,621	2	679	6,393	7,074
Sultan Kudarat	0	23	380	403	0	188	2,815	3,003	0	294	2,120	2,414
				0				0				0
Cotabato City	0	38	541	579	1	92	1,993	2,086	0	236	2,761	2,997
Gen. Santos City	0	105	396	501	0	200	1,583	1,783	2	303	2,120	2,425
**BARMM**	**1**	**481**	**4,447**	**4,929**	**9**	**807**	**19,565**	**20,381**	**6**	**939**	**20,725**	**21,670**
Basilan	0	50	259	309	0	89	896	985	0	97	882	979
Lanao del Sur	0	87	1,277	1,364	1	73	6,412	6,486	0	91	5,546	5,637
Maguindanao	0	245	918	1,163	7	460	4,840	5,307	5	518	5,806	6,329
Sulu	0	78	1,760	1,838	1	112	5,723	5,836	0	150	6,745	6,895
Tawi-Tawi	0	13	135	148	0	43	1,312	1,355	0	24	1,397	1,421
				0				0				0
Lamitan City	1	7	39	47	0	30	239	269	1	59	220	280
Marawi City	0	1	59	60	0	0	143	143	0	0	129	129
**CARAGA**	**17**	**468**	**15,038**	**15,523**	**7**	**871**	**11,512**	**12,390**	**6**	**596**	**7,039**	**7,641**
Agusan del Norte	1	119	698	818	3	156	1,785	1,944	0	116	1,132	1,248
Agusan del Sur	0	167	2,408	2,575	1	473	4,013	4,487	4	207	1,911	2,122
Surigao del Norte	1	32	11,025	11,058	0	49	1,901	1,950	0	49	1,270	1,319
Surigao del Sur	1	71	426	498	3	101	2,066	2,170	2	81	1,364	1,447
Province of Dinagat	0	13	89	102	0	17	404	421	0	30	269	299
Bislig City	0	1	55	56	0	20	444	464	0	29	316	345
Butuan City	14	49	273	336	0	40	702	742	0	52	549	601
Surigao City	0	16	64	80	0	15	197	212	0	32	228	260

Table 1.A.3 - MODERN METHOD OF FAMILY PLANNING
Drop Outs
Philippines, 2021

Area	IMPLANTS Age group			Total	NFP-CMM Age group			Total	NFP-BBT Age group			Total
	10-14	15-19	20-49		10-14	15-19	20-49		10-14	15-19	20-49	
**PHILIPPINES**	69	6,263	99,051	105,383	4	247	7,596	7,847	27	188	2,208	2,423
**N C R**	32	1,922	14,729	16,683	0	3	233	236	0	67	292	359
Malabon	9	54	714	777	0	0	0	0	0	0	0	0
Navotas	1	52	181	234	0	0	0	0	0	0	0	0
Valenzuela City	7	58	1,661	1,726	0	0	72	72	0	0	0	0
Caloocan City	5	187	1,787	1,979	0	0	0	0	0	0	0	0
Marikina City	0	2	367	369	0	0	11	11	0	0	0	0
Pasig City	2	31	547	580	0	0	0	0	0	0	0	0
Pateros	0	0	8	8	0	0	0	0	0	0	0	0
Taguig	0	54	468	522	0	0	0	0	0	0	0	0
Quezon City	1	162	1,908	2,071	0	0	0	0	0	0	3	3
Makati City	0	8	147	155	0	0	0	0	0	0	0	0
Mandaluyong City	1	20	135	156	0	3	20	23	0	0	0	0
San Juan	0	0	2	2	0	0	0	0	0	0	0	0
Manila City	4	1,199	5,018	6,221	0	0	0	0	0	67	288	355
Las Piñas City	2	92	1,329	1,423	0	0	47	47	0	0	0	0
Muntinlupa City	0	0	113	113	0	0	0	0	0	0	0	0
Parañaque City	0	3	173	176	0	0	15	15	0	0	1	1
Pasay City	0	0	171	171	0	0	68	68	0	0	0	0
**C A R**	3	145	2,556	2,704	0	18	430	448	5	3	48	56
Abra	0	15	350	365	0	0	2	2	0	1	6	7
Apayao	1	47	210	258	0	0	3	3	5	0	0	5
Benguet	0	16	514	530	0	0	13	13	0	2	2	4
Ifugao	1	9	296	306	0	12	98	110	0	0	0	0
Kalinga	0	8	231	239	0	6	252	258	0	0	0	0
Mt. Province	1	18	127	146	0	0	59	59	0	0	23	23
Baguio City	0	32	828	860	0	0	3	3	0	0	17	17
**Region 1**	0	126	1,962	2,088	0	0	129	129	0	3	234	237
Ilocos Norte	0	1	80	81	0	0	20	20	0	0	5	5
Ilocos Sur	0	1	85	86	0	0	25	25	0	0	1	1
La Union	0	41	297	338	0	0	56	56	0	0	0	0
Pangasinan	0	51	1,110	1,161	0	0	0	0	0	1	228	229
Alaminos City	0	1	42	43	0	0	0	0	0	0	0	0
Candon City	0	0	0	0	0	0	11	11	0	0	0	0
Dagupan City	0	3	51	54	0	0	0	0	0	0	0	0
Laoag City	0	0	0	0	0	0	0	0	0	0	0	0
San Carlos City	0	13	182	195	0	0	0	0	0	0	0	0
San Fernando City	0	11	91	102	0	0	0	0	0	0	0	0
Urdaneta City	0	3	19	22	0	0	0	0	0	0	0	0
Vigan City	0	1	5	6	0	0	17	17	0	2	0	2
**Region 2**	3	150	3,175	3,328	1	28	76	105	21	23	23	67
Batanes	0	0	2	2	0	1	13	14	0	0	0	0
Cagayan	1	14	482	497	0	0	53	53	0	0	0	0
Isabela	0	74	1,828	1,902	0	0	0	0	0	0	0	0
Nueva Vizcaya	2	42	544	588	1	27	7	35	21	23	23	67
Quirino	0	1	59	60	0	0	3	3	0	0	0	0
Cauayan City	0	7	29	36	0	0	0	0	0	0	0	0
Ilagan City	0	1	98	99	0	0	0	0	0	0	0	0
Santiago City	0	8	84	92	0	0	0	0	0	0	0	0
Tuguegarao City	0	3	49	52	0	0	0	0	0	0	0	0
**Region 3**	1	347	6,551	6,899	0	10	218	228	0	10	137	147
Aurora	0	6	149	155	0	2	109	111	0	0	1	1
Bataan	0	13	452	465	0	0	44	44	0	0	71	71
Bulacan	0	37	1,122	1,159	0	0	0	0	0	0	0	0
Nueva Ecija	0	50	1,298	1,348	0	3	7	10	0	1	2	3

Table 1.A.3 - MODERN METHOD OF FAMILY PLANNING

Drop Outs

Philippines, 2021

Area	IMPLANTS Age group 10-14	15-19	20-49	Total	NFP-CMM Age group 10-14	15-19	20-49	Total	NFP-BBT Age group 10-14	15-19	20-49	Total
Pampanga	1	104	1,440	1,545	0	5	42	47	0	6	60	66
Tarlac	0	23	228	251	0	0	0	0	0	0	0	0
Zambales	0	46	521	567	0	0	2	2	0	0	0	0
Angeles City	0	3	142	145	0	0	0	0	0	0	0	0
Balanga City	0	8	117	125	0	0	0	0	0	0	0	0
Cabanatuan City	0	5	88	93	0	0	0	0	0	0	0	0
City of San Fernando	0	0	7	7	0	0	0	0	0	0	0	0
Gapan City	0	22	89	111	0	0	0	0	0	0	0	0
Mabalacat City	0	0	80	80	0	0	0	0	0	0	0	0
Malolos City	0	0	23	23	0	0	0	0	0	0	0	0
Meycauayan	0	4	55	59	0	0	14	14	0	0	0	0
Olongapo	0	1	56	57	0	0	0	0	0	0	0	0
Palayan City	0	0	0	0	0	0	0	0	0	0	0	0
San Jose City	0	1	42	43	0	0	0	0	0	0	0	0
San Jose del Monte City	0	24	594	618	0	0	0	0	0	0	0	0
Science City of Munoz	0	0	0	0	0	0	0	0	0	0	0	0
Tarlac City	0	0	48	48	0	0	0	0	0	3	3	6
**Region 4A**	**3**	**116**	**2,977**	**3,096**	**0**	**0**	**62**	**62**	**0**	**1**	**10**	**11**
Batangas	2	0	34	36	0	0	0	0	0	0	4	4
Cavite	0	8	375	383	0	0	15	15	0	0	0	0
Laguna	1	11	278	290	0	0	13	13	0	1	1	2
Quezon	0	9	286	295	0	0	2	2	0	0	2	2
Rizal	0	45	552	597	0	0	0	0	0	0	0	0
Antipolo City	0	0	30	30	0	0	0	0	0	0	0	0
Bacoor City	0	5	134	139	0	0	0	0	0	0	0	0
Batangas City	0	9	5	14	0	0	0	0	0	0	0	0
Biñan City	0	0	22	22	0	0	0	0	0	0	0	0
Cabuyao City	0	1	66	67	0	0	0	0	0	0	0	0
Calamba City	0	10	286	296	0	0	0	0	0	0	0	0
Cavite City	0	0	54	54	0	0	0	0	0	0	0	0
Dasmariñas City	0	9	525	534	0	0	0	0	0	0	1	1
General Trias City	0	2	92	94	0	0	0	0	0	0	0	0
Imus City	0	1	43	44	0	0	0	0	0	0	0	0
Lipa City	0	1	4	5	0	0	0	0	0	0	0	0
Lucena City	0	0	1	1	0	0	0	0	0	0	0	0
San Pablo City	0	1	29	30	0	0	0	0	0	0	0	0
San Pedro City	0	0	50	50	0	0	0	0	0	0	0	0
Santa Rosa City	0	3	31	34	0	0	0	0	0	0	0	0
Tagaytay City	0	0	50	50	0	0	0	0	0	0	0	0
Tanauan City	0	0	8	8	0	0	0	0	0	0	2	2
Tayabas City	0	0	12	12	0	0	32	32	0	0	0	0
Trece Martires City	0	1	10	11	0	0	0	0	0	0	0	0
**Region 4B**	**0**	**68**	**2,991**	**3,059**	**0**	**12**	**581**	**593**	**0**	**0**	**14**	**14**
Marinduque	0	0	75	75	0	0	114	114	0	0	0	0
Mindoro Occidental	0	3	81	84	0	11	218	229	0	0	0	0
Mindoro Oriental	0	5	288	293	0	0	0	0	0	0	0	0
Palawan	0	53	1,956	2,009	0	0	176	176	0	0	10	10
Romblon	0	7	248	255	0	1	73	74	0	0	4	4
Puerto Princesa City	0	0	343	343	0	0	0	0	0	0	0	0
**Region 5**	**1**	**246**	**5,984**	**6,231**	**2**	**86**	**3,936**	**4,024**	**0**	**39**	**1,082**	**1,121**
Albay	1	32	403	436	0	22	821	843	0	27	934	961
Camarines Norte	0	16	278	294	0	5	18	23	0	6	11	17
Camarines Sur	0	84	1,929	2,013	0	3	188	191	0	5	64	69
Catanduanes	0	10	239	249	0	13	200	213	0	0	10	10
Masbate	0	84	2,095	2,179	2	24	731	757	0	1	27	28
Sorsogon	0	16	929	945	0	19	1,919	1,938	0	0	3	3
Iriga City	0	0	53	53	0	0	59	59	0	0	32	32
Legaspi City	0	4	11	15	0	0	0	0	0	0	0	0
Naga City	0	0	47	47	0	0	0	0	0	0	1	1

Table 1.A.3 - MODERN METHOD OF FAMILY PLANNING

Drop Outs

Philippines, 2021

Area	IMPLANTS			Total	NFP-CMM			Total	NFP-BBT			Total
	Age group				Age group				Age group			
	10-14	15-19	20-49		10-14	15-19	20-49		10-14	15-19	20-49	
**Region 6**	**4**	**361**	**8,399**	**8,764**	**0**	**7**	**148**	**155**	**0**	**8**	**62**	**70**
Aklan	0	28	745	773	0	0	3	3	0	0	0	0
Antique	0	61	882	943	0	7	118	125	0	0	5	5
Capiz	0	12	479	491	0	0	27	27	0	0	1	1
Guimaras	0	9	223	232	0	0	0	0	0	0	0	0
Iloilo	2	70	1,813	1,885	0	0	0	0	0	0	34	34
Negros Occidental	2	117	2,607	2,726	0	0	0	0	0	8	19	27
Bacolod City	0	38	1,467	1,505	0	0	0	0	0	0	3	3
Iloilo City	0	26	183	209	0	0	0	0	0	0	0	0
**Region 7**	**0**	**325**	**9,253**	**9,578**	**0**	**0**	**114**	**114**	**0**	**3**	**15**	**18**
Bohol	0	52	1,935	1,987	0	0	4	4	0	0	1	1
Cebu	0	115	3,269	3,384	0	0	0	0	0	0	0	0
Negros Oriental	0	36	480	516	0	0	110	110	0	3	14	17
Siquijor	0	3	27	30	0	0	0	0	0	0	0	0
Cebu City	0	112	2,840	2,952	0	0	0	0	0	0	0	0
Lapu-Lapu City	0	1	514	515	0	0	0	0	0	0	0	0
Mandaue City	0	6	188	194	0	0	0	0	0	0	0	0
**Region 8**	**0**	**210**	**4,472**	**4,682**	**0**	**0**	**210**	**210**	**0**	**0**	**71**	**71**
Biliran	0	13	121	134	0	0	57	57	0	0	0	0
Eastern Samar	0	10	168	178	0	0	4	4	0	0	0	0
Northern Leyte	0	10	504	514	0	0	0	0	0	0	49	49
Northern Samar	0	36	1,243	1,279	0	0	0	0	0	0	0	0
Southern Leyte	0	29	528	557	0	0	12	12	0	0	13	13
Western Samar	0	68	267	335	0	0	111	111	0	0	1	1
Calbayog City	0	9	303	312	0	0	0	0	0	0	0	0
Maasin City	0	1	92	93	0	0	3	3	0	0	1	1
Ormoc City	0	29	960	989	0	0	0	0	0	0	7	7
Tacloban City	0	5	286	291	0	0	23	23	0	0	0	0
**Region 9**	**3**	**350**	**8,259**	**8,612**	**0**	**0**	**14**	**14**	**0**	**0**	**2**	**2**
Zamboanga del Norte	1	78	1,275	1,354	0	0	14	14	0	0	1	1
Zamboanga del Sur	1	109	2,973	3,083	0	0	0	0	0	0	0	0
Zamboanga Sibugay	1	124	3,157	3,282	0	0	0	0	0	0	1	1
Dapitan City	0	4	121	125	0	0	0	0	0	0	0	0
Dipolog City	0	16	88	104	0	0	0	0	0	0	0	0
Isabela City	0	2	93	95	0	0	0	0	0	0	0	0
Pagadian City	0	0	42	42	0	0	0	0	0	0	0	0
Zamboanga City	0	17	510	527	0	0	0	0	0	0	0	0
**Region 10**	**1**	**235**	**4,378**	**4,614**	**1**	**11**	**302**	**314**	**0**	**19**	**62**	**81**
Bukidnon	0	153	2,353	2,506	1	7	187	195	0	3	9	12
Camiguin	0	0	11	11	0	0	0	0	0	0	0	0
Lanao del Norte	0	20	229	249	0	0	0	0	0	0	5	5
Misamis Occidental	0	1	52	53	0	0	0	0	0	0	0	0
Misamis Oriental	0	10	426	436	0	4	76	80	0	0	1	1
Cagayan de Oro City	0	21	341	362	0	0	0	0	0	0	0	0
El Salvador City	0	1	24	25	0	0	0	0	0	0	0	0
Gingoog City	0	6	104	110	0	0	1	1	0	10	12	22
Iligan City	0	0	236	236	0	0	10	10	0	0	0	0
Malaybalay City	1	18	451	470	0	0	0	0	0	5	15	20
Oroquieta City	0	0	0	0	0	0	0	0	0	0	0	0
Ozamis City	0	0	39	39	0	0	2	2	0	0	3	3
Tangub City	0	0	2	2	0	0	0	0	0	0	0	0
Valencia City	0	5	110	115	0	0	26	26	0	1	17	18

Table 1.A.3 - MODERN METHOD OF FAMILY PLANNING

Drop Outs

Philippines, 2021

Area	IMPLANTS			Total	NFP-CMM			Total	NFP-BBT			Total
	Age group				Age group				Age group			
	10-14	15-19	20-49		10-14	15-19	20-49		10-14	15-19	20-49	
**Region 11**	**6**	**365**	**5,671**	**6,042**	**0**	**59**	**578**	**637**	**1**	**0**	**74**	**75**
Davao de Oro	0	52	1,312	1,364	0	1	102	103	0	0	3	3
Davao del Norte	0	64	798	862	0	0	21	21	0	0	0	0
Davao Oriental	1	38	779	818	0	5	180	185	0	0	2	2
Davao del Sur	4	52	544	600	0	53	166	219	1	0	9	10
Davao Occidental	1	39	447	487	0	0	2	2	0	0	4	4
Davao City	0	120	1,791	1,911	0	0	107	107	0	0	56	56
**Region 12**	**9**	**775**	**8,054**	**8,838**	**0**	**11**	**222**	**233**	**0**	**4**	**43**	**47**
North Cotabato	2	273	3,466	3,741	0	10	151	161	0	0	32	32
Sarangani	0	65	499	564	0	1	19	20	0	0	1	1
South Cotabato	6	235	1,802	2,043	0	0	42	42	0	0	0	0
Sultan Kudarat	0	108	1,033	1,141	0	0	2	2	0	0	0	0
				0				0				0
Cotabato City	0	41	512	553	0	0	7	7	0	4	10	14
Gen. Santos City	1	53	742	796	0	0	1	1	0	0	0	0
**BARMM**	**1**	**259**	**4,643**	**4,903**	**0**	**0**	**13**	**13**	**0**	**0**	**2**	**2**
Basilan	0	42	434	476	0	0	1	1	0	0	0	0
Lanao del Sur	0	4	702	706	0	0	1	1	0	0	2	2
Maguindanao	1	155	1,389	1,545	0	0	0	0	0	0	0	0
Sulu	0	39	1,249	1,288	0	0	8	8	0	0	0	0
Tawi-Tawi	0	6	666	672	0	0	3	3	0	0	0	0
				0				0				0
Lamitan City	0	13	169	182	0	0	0	0	0	0	0	0
Marawi City	0	0	34	34	0	0	0	0	0	0	0	0
**CARAGA**	**2**	**263**	**4,997**	**5,262**	**0**	**2**	**330**	**332**	**0**	**8**	**37**	**45**
Agusan del Norte	1	53	709	763	0	0	37	37	0	3	5	8
Agusan del Sur	1	106	1,955	2,062	0	2	117	119	0	5	20	25
Surigao del Norte	0	11	410	421	0	0	155	155	0	0	3	3
Surigao del Sur	0	30	648	678	0	0	3	3	0	0	9	9
Province of Dinagat	0	24	472	496	0	0	0	0	0	0	0	0
Bislig City	0	14	122	136	0	0	18	18	0	0	0	0
Butuan City	0	20	559	579	0	0	0	0	0	0	0	0
Surigao City	0	5	122	127	0	0	0	0	0	0	0	0

Table 1.A.3 - MODERN METHOD OF FAMILY PLANNING

Drop Outs

Philippines, 2021

Area	NFP-STM			Total	NFP-SDM			Total	NFP-LAM			Total	Total Drop-outs Acceptors
	Age group				Age group				Age group				
	10-14	15-19	20-49		10-14	15-19	20-49		10-14	15-19	20-49		
**PHILIPPINES**	0	39	1,290	1,329	12	660	17,537	18,209	888	58,167	560,428	619,483	1,620,208
**N C R**	0	0	8	8	2	7	309	318	263	7,559	83,498	91,320	262,149
Malabon	0	0	0	0	0	0	0	0	1	135	1,128	1,264	7,856
Navotas	0	0	0	0	0	0	1	1	3	366	2,537	2,906	6,581
Valenzuela City	0	0	0	0	0	7	4	11	2	91	4,242	4,335	23,076
Caloocan City	0	0	0	0	0	0	25	25	8	1,065	6,798	7,871	27,893
Marikina City	0	0	0	0	0	0	4	4	0	49	486	535	3,850
Pasig City	0	0	0	0	0	0	0	0	1	146	2,585	2,732	7,497
Pateros	0	0	0	0	0	0	0	0	0	11	687	698	1,099
Taguig	0	0	0	0	2	0	0	2	6	593	7,717	8,316	15,082
Quezon City	0	0	0	0	0	0	135	135	9	2,197	24,225	26,431	94,318
Makati City	0	0	0	0	0	0	0	0	0	101	3,445	3,546	5,876
Mandaluyong City	0	0	1	1	0	0	0	0	3	340	3,317	3,660	6,075
San Juan	0	0	0	0	0	0	0	0	0	5	522	527	934
Manila City	0	0	0	0	0	0	66	66	18	1,033	8,753	9,804	26,758
Las Piñas City	0	0	0	0	0	0	7	7	4	393	4,057	4,454	13,101
Muntinlupa City	0	0	0	0	0	0	0	0	0	213	2,605	2,818	6,590
Parañaque City	0	0	0	0	0	0	1	1	208	797	5,159	6,164	8,485
Pasay City	0	0	7	7	0	0	66	66	0	24	5,235	5,259	7,078
**C A R**	0	0	7	7	0	18	798	816	19	1,054	12,942	14,015	52,033
Abra	0	0	0	0	0	5	38	43	5	192	1,793	1,990	4,286
Apayao	0	0	1	1	0	0	16	16	5	149	1,295	1,449	11,480
Benguet	0	0	0	0	0	2	161	163	1	244	3,127	3,372	9,053
Ifugao	0	0	2	2	0	9	309	318	5	146	2,258	2,409	6,553
Kalinga	0	0	0	0	0	0	78	78	1	162	2,644	2,807	6,967
Mt. Province	0	0	4	4	0	2	163	165	2	154	1,452	1,608	7,136
Baguio City	0	0	0	0	0	0	33	33	0	7	373	380	6,558
**Region 1**	0	1	30	31	2	21	787	810	18	2,537	32,790	35,345	67,249
Ilocos Norte	0	0	0	0	0	0	108	108	0	80	2,335	2,415	5,907
Ilocos Sur	0	1	0	1	2	8	133	143	3	219	3,584	3,806	7,235
La Union	0	0	3	3	0	2	102	104	3	618	5,381	6,002	10,257
Pangasinan	0	0	0	0	0	10	388	398	5	1,262	16,682	17,949	34,115
Alaminos City	0	0	0	0	0	0	0	0	2	33	785	820	1,426
Candon City	0	0	3	3	0	0	0	0	0	0	134	134	326
Dagupan City	0	0	0	0	0	0	0	0	1	138	736	875	1,350
Laoag City	0	0	0	0	0	0	0	0	0	20	1,121	1,141	1,412
San Carlos City	0	0	0	0	0	0	0	0	0	77	1,371	1,448	3,204
San Fernando City	0	0	0	0	0	1	44	45	0	31	326	357	954
Urdaneta City	0	0	0	0	0	0	0	0	4	33	224	261	642
Vigan City	0	0	24	24	0	0	12	12	0	26	111	137	421
**Region 2**	0	3	27	30	0	55	293	348	47	2,517	19,999	22,563	58,740
Batanes	0	0	0	0	0	0	11	11	2	18	215	235	521
Cagayan	0	0	0	0	0	0	0	0	5	452	5,016	5,473	11,367
Isabela	0	0	0	0	0	0	9	9	8	637	5,240	5,885	19,398
Nueva Vizcaya	0	3	27	30	0	55	272	327	24	871	4,026	4,921	15,006
Quirino	0	0	0	0	0	0	1	1	0	72	716	788	2,045
Cauayan City	0	0	0	0	0	0	0	0	5	134	1,103	1,242	2,137
Ilagan City	0	0	0	0	0	0	0	0	0	58	611	669	1,895
Santiago City	0	0	0	0	0	0	0	0	3	254	2,713	2,970	5,512
Tuguegarao City	0	0	0	0	0	0	0	0	0	21	359	380	859
**Region 3**	0	3	476	479	1	75	1,339	1,415	15	4,461	41,440	45,916	144,165
Aurora	0	0	0	0	0	0	1	1	0	228	1,722	1,950	3,845
Bataan	0	0	0	0	0	0	0	0	5	438	3,127	3,570	9,045
Bulacan	0	0	0	0	0	0	120	120	0	593	7,012	7,605	23,448
Nueva Ecija	0	0	0	0	0	0	12	12	2	554	5,195	5,751	20,411

Table 1.A.3 - MODERN METHOD OF FAMILY PLANNING

Drop Outs

Philippines, 2021

Area	NFP-STM Age group 10-14	NFP-STM 15-19	NFP-STM 20-49	Total	NFP-SDM Age group 10-14	NFP-SDM 15-19	NFP-SDM 20-49	Total	NFP-LAM Age group 10-14	NFP-LAM 15-19	NFP-LAM 20-49	Total	Total Drop-outs Acceptors
Pampanga	0	3	463	466	1	75	1,193	1,269	1	347	4,540	4,888	30,568
Tarlac	0	0	1	1	0	0	3	3	0	382	5,094	5,476	10,421
Zambales	0	0	0	0	0	0	1	1	1	242	2,009	2,252	7,557
Angeles City	0	0	0	0	0	0	5	5	0	159	1,291	1,450	3,147
Balanga City	0	0	0	0	0	0	3	3	3	120	633	756	1,834
Cabanatuan City	0	0	0	0	0	0	0	0	3	319	2,239	2,561	4,506
City of San Fernando	0	0	3	3	0	0	0	0	0	62	521	583	1,240
Gapan City	0	0	0	0	0	0	0	0	0	60	539	599	1,220
Mabalacat City	0	0	0	0	0	0	1	1	0	303	2,391	2,694	5,681
Malolos City	0	0	0	0	0	0	0	0	0	4	70	74	675
Meycauayan	0	0	9	9	0	0	0	0	0	88	838	926	2,147
Olongapo	0	0	0	0	0	0	0	0	0	78	821	899	2,972
Palayan City	0	0	0	0	0	0	0	0	0	19	171	190	434
San Jose City	0	0	0	0	0	0	0	0	0	254	1,584	1,838	3,397
San Jose del Monte City	0	0	0	0	0	0	0	0	0	0	0	0	7,939
Science City of Munoz	0	0	0	0	0	0	0	0	0	0	0	0	270
Tarlac City	0	0	0	0	0	0	0	0	0	211	1,643	1,854	3,408
**Region 4A**	**0**	**0**	**2**	**2**	**0**	**1**	**149**	**150**	**115**	**1,483**	**17,926**	**19,524**	**51,730**
Batangas	0	0	0	0	0	0	76	76	51	146	2,887	3,084	5,116
Cavite	0	0	0	0	0	0	8	8	1	68	1,605	1,674	4,393
Laguna	0	0	0	0	0	0	13	13	3	111	886	1,000	3,331
Quezon	0	0	0	0	0	1	6	7	53	141	1,713	1,907	8,022
Rizal	0	0	0	0	0	0	1	1	2	264	2,525	2,791	7,222
Antipolo City	0	0	0	0	0	0	0	0	0	0	429	429	1,038
Bacoor City	0	0	0	0	0	0	0	0	0	57	866	923	1,628
Batangas City	0	0	0	0	0	0	3	3	0	5	309	314	1,081
Biñan City	0	0	0	0	0	0	13	13	0	214	981	1,195	3,960
Cabuyao City	0	0	0	0	0	0	0	0	0	89	1,088	1,177	1,935
Calamba City	0	0	0	0	0	0	28	28	2	60	851	913	4,285
Cavite City	0	0	0	0	0	0	0	0	1	2	31	34	183
Dasmariñas City	0	0	0	0	0	0	1	1	0	63	844	907	2,437
General Trias City	0	0	0	0	0	0	0	0	0	24	281	305	1,023
Imus City	0	0	0	0	0	0	0	0	0	10	131	141	712
Lipa City	0	0	0	0	0	0	0	0	0	0	0	0	40
Lucena City	0	0	0	0	0	0	0	0	0	0	40	40	363
San Pablo City	0	0	0	0	0	0	0	0	1	60	460	521	920
San Pedro City	0	0	0	0	0	0	0	0	0	54	449	503	796
Santa Rosa City	0	0	0	0	0	0	0	0	0	62	808	870	1,515
Tagaytay City	0	0	0	0	0	0	0	0	0	0	266	266	470
Tanauan City	0	0	0	0	0	0	0	0	0	5	30	35	144
Tayabas City	0	0	2	2	0	0	0	0	1	44	425	470	953
Trece Martires City	0	0	0	0	0	0	0	0	0	4	21	25	163
**Region 4B**	**0**	**0**	**23**	**23**	**0**	**8**	**365**	**373**	**59**	**1,825**	**21,242**	**23,126**	**52,898**
Marinduque	0	0	0	0	0	0	13	13	1	25	1,037	1,063	2,336
Mindoro Occidental	0	0	0	0	0	0	24	24	20	934	6,520	7,474	12,437
Mindoro Oriental	0	0	0	0	0	0	0	0	0	73	1,994	2,067	6,156
Palawan	0	0	23	23	0	8	121	129	34	675	7,576	8,285	21,801
Romblon	0	0	0	0	0	0	175	175	4	118	3,107	3,229	5,108
Puerto Princesa City	0	0	0	0	0	0	32	32	0	0	1,008	1,008	5,060
**Region 5**	**0**	**15**	**568**	**583**	**0**	**221**	**6,403**	**6,624**	**47**	**5,197**	**48,903**	**54,147**	**140,544**
Albay	0	8	224	232	0	21	283	304	3	391	6,297	6,691	24,615
Camarines Norte	0	0	3	3	0	2	274	276	2	679	5,957	6,638	14,606
Camarines Sur	0	7	246	253	0	22	1,603	1,625	22	1,383	15,057	16,462	36,871
Catanduanes	0	0	17	17	0	92	233	325	3	658	1,331	1,992	4,091
Masbate	0	0	9	9	0	68	2,502	2,570	11	1,182	9,055	10,248	24,350
Sorsogon	0	0	50	50	0	14	1,312	1,326	6	866	9,275	10,147	30,813
Iriga City	0	0	19	19	0	2	154	156	0	36	681	717	1,737
Legaspi City	0	0	0	0	0	0	0	0	0	2	167	169	499
Naga City	0	0	0	0	0	0	42	42	0	0	1,083	1,083	2,962

Table 1.A.3 - MODERN METHOD OF FAMILY PLANNING

Drop Outs

Philippines, 2021

Area	NFP-STM			Total	NFP-SDM			Total	NFP-LAM			Total	Total Drop-outs Acceptors
	Age group				Age group				Age group				
	10-14	15-19	20-49		10-14	15-19	20-49		10-14	15-19	20-49		
**Region 6**	**0**	**1**	**3**	**4**	**7**	**23**	**519**	**549**	**29**	**3,611**	**39,310**	**42,950**	**95,471**
Aklan	0	0	1	1	0	0	25	25	1	186	2,962	3,149	6,357
Antique	0	0	0	0	0	0	44	44	8	254	3,209	3,471	9,126
Capiz	0	0	0	0	0	0	12	12	0	102	2,112	2,214	5,458
Guimaras	0	0	0	0	0	0	1	1	3	147	1,703	1,853	2,949
Iloilo	0	0	1	1	0	5	139	144	5	581	8,460	9,046	18,043
Negros Occidental	0	1	1	2	7	16	281	304	12	2,154	19,155	21,321	45,084
Bacolod City	0	0	0	0	0	0	0	0	0	161	1,468	1,629	5,485
Iloilo City	0	0	0	0	0	2	17	19	0	26	241	267	2,969
**Region 7**	**0**	**0**	**0**	**0**	**0**	**5**	**179**	**184**	**37**	**4,071**	**45,692**	**49,800**	**107,326**
Bohol	0	0	0	0	0	0	72	72	2	347	5,222	5,571	17,449
Cebu	0	0	0	0	0	0	34	34	8	964	12,213	13,185	27,090
Negros Oriental	0	0	0	0	0	0	16	16	14	1,110	10,425	11,549	22,897
Siquijor	0	0	0	0	0	5	57	62	0	40	582	622	1,890
Cebu City	0	0	0	0	0	0	0	0	12	1,265	12,011	13,288	26,787
Lapu-Lapu City	0	0	0	0	0	0	0	0	1	344	4,907	5,252	10,153
Mandaue City	0	0	0	0	0	0	0	0	0	1	332	333	1,060
**Region 8**	**0**	**4**	**2**	**6**	**0**	**4**	**391**	**395**	**14**	**1,758**	**15,665**	**17,437**	**48,960**
Biliran	0	0	0	0	0	0	14	14	3	111	954	1,068	2,750
Eastern Samar	0	4	0	4	0	0	97	97	9	119	1,115	1,243	3,383
Northern Leyte	0	0	0	0	0	0	7	7	0	146	2,346	2,492	6,432
Northern Samar	0	0	0	0	0	0	41	41	1	170	2,699	2,870	7,384
Southern Leyte	0	0	0	0	0	2	70	72	0	166	1,875	2,041	5,990
Western Samar	0	0	0	0	0	0	0	0	1	495	1,463	1,959	4,796
Calbayog City	0	0	0	0	0	0	0	0	0	87	1,056	1,143	4,663
Maasin City	0	0	0	0	0	0	4	4	0	10	211	221	934
Ormoc City	0	0	0	0	0	2	143	145	0	301	2,677	2,978	8,631
Tacloban City	0	0	2	2	0	0	15	15	0	153	1,269	1,422	3,997
**Region 9**	**0**	**0**	**0**	**0**	**0**	**19**	**282**	**301**	**29**	**3,447**	**24,816**	**28,292**	**73,689**
Zamboanga del Norte	0	0	0	0	0	19	236	255	8	1,124	7,452	8,584	19,319
Zamboanga del Sur	0	0	0	0	0	0	1	1	4	417	2,800	3,221	14,545
Zamboanga Sibugay	0	0	0	0	0	0	27	27	3	454	3,769	4,226	15,282
Dapitan City	0	0	0	0	0	0	1	1	2	107	775	884	1,683
Dipolog City	0	0	0	0	0	0	17	17	2	247	2,057	2,306	4,824
Isabela City	0	0	0	0	0	0	0	0	0	85	699	784	1,675
Pagadian City	0	0	0	0	0	0	0	0	0	90	1,224	1,314	2,816
Zamboanga City	0	0	0	0	0	0	0	0	10	923	6,040	6,973	13,545
**Region 10**	**0**	**1**	**13**	**14**	**0**	**130**	**2,231**	**2,361**	**39**	**4,299**	**30,578**	**34,916**	**82,940**
Bukidnon	0	1	5	6	0	37	444	481	15	1,427	7,190	8,632	27,257
Camiguin	0	0	0	0	0	0	2	2	0	36	736	772	1,009
Lanao del Norte	0	0	0	0	0	0	3	3	5	507	4,306	4,818	8,333
Misamis Occidental	0	0	0	0	0	0	4	4	0	69	1,217	1,286	2,311
Misamis Oriental	0	0	1	1	0	7	386	393	5	369	2,657	3,031	10,151
Cagayan de Oro City	0	0	7	7	0	0	0	0	7	527	4,107	4,641	7,370
El Salvador City	0	0	0	0	0	0	7	7	0	69	485	554	1,137
Gingoog City	0	0	0	0	0	40	164	204	4	170	1,218	1,392	4,373
Iligan City	0	0	0	0	0	5	217	222	2	377	3,322	3,701	6,291
Malaybalay City	0	0	0	0	0	41	917	958	1	409	2,649	3,059	9,401
Oroquieta City	0	0	0	0	0	0	6	6	0	1	86	87	112
Ozamis City	0	0	0	0	0	0	2	2	0	54	536	590	867
Tangub City	0	0	0	0	0	0	75	75	0	16	98	114	315
Valencia City	0	0	0	0	0	0	4	4	0	268	1,971	2,239	4,013

Table 1.A.3 - MODERN METHOD OF FAMILY PLANNING

Drop Outs

Philippines, 2021

Area	NFP-STM			Total	NFP-SDM			Total	NFP-LAM			Total	Total Drop-outs Acceptors
	Age group				Age group				Age group				
	10-14	15-19	20-49		10-14	15-19	20-49		10-14	15-19	20-49		
**Region 11**	**0**	**0**	**55**	**55**	**0**	**10**	**429**	**439**	**79**	**2,255**	**17,953**	**20,287**	**68,581**
Davao de Oro	0	0	0	0	0	0	10	10	3	95	978	1,076	9,794
Davao del Norte	0	0	1	1	0	0	21	21	8	323	3,148	3,479	11,693
Davao Oriental	0	0	10	10	0	10	264	274	28	658	4,317	5,003	11,581
Davao del Sur	0	0	0	0	0	0	16	16	2	130	713	845	6,201
Davao Occidental	0	0	3	3	0	0	18	18	1	184	596	781	3,883
Davao City	0	0	41	41	0	0	100	100	37	865	8,201	9,103	25,429
**Region 12**	**0**	**4**	**50**	**54**	**0**	**5**	**302**	**307**	**22**	**6,040**	**38,907**	**44,969**	**126,805**
North Cotabato	0	3	42	45	0	0	92	92	12	1,470	10,715	12,197	43,352
Sarangani	0	0	0	0	0	0	12	12	0	1,453	5,944	7,397	15,158
South Cotabato	0	0	6	6	0	5	145	150	8	1,372	9,290	10,670	31,925
Sultan Kudarat	0	0	0	0	0	0	42	42	0	721	4,806	5,527	13,736
				0				0				0	
Cotabato City	0	1	2	3	0	0	11	11	2	382	4,021	4,405	11,888
Gen. Santos City	0	0	0	0	0	0	0	0	0	642	4,131	4,773	10,746
**BARMM**	**0**	**0**	**2**	**2**	**0**	**0**	**225**	**225**	**17**	**3,153**	**46,205**	**49,375**	**108,638**
Basilan	0	0	0	0	0	0	0	0	0	190	1,469	1,659	4,647
Lanao del Sur	0	0	2	2	0	0	188	188	3	513	15,493	16,009	33,312
Maguindanao	0	0	0	0	0	0	31	31	11	1,737	15,348	17,096	34,559
Sulu	0	0	0	0	0	0	0	0	0	395	8,158	8,553	24,926
Tawi-Tawi	0	0	0	0	0	0	4	4	2	223	4,194	4,419	8,164
				0				0				0	
Lamitan City	0	0	0	0	0	0	1	1	1	72	458	531	1,404
Marawi City	0	0	0	0	0	0	1	1	0	23	1,085	1,108	1,626
**CARAGA**	**0**	**7**	**24**	**31**	**0**	**58**	**2,536**	**2,594**	**39**	**2,900**	**22,562**	**25,501**	**78,290**
Agusan del Norte	0	0	13	13	0	0	182	182	2	287	2,099	2,388	8,720
Agusan del Sur	0	7	11	18	0	53	1,494	1,547	10	919	7,181	8,110	25,298
Surigao del Norte	0	0	0	0	0	5	710	715	2	218	2,395	2,615	19,321
Surigao del Sur	0	0	0	0	0	0	32	32	17	530	4,397	4,944	10,864
Province of Dinagat	0	0	0	0	0	0	21	21	0	93	770	863	2,656
Bislig City	0	0	0	0	0	0	69	69	6	149	1,253	1,408	2,796
Butuan City	0	0	0	0	0	0	16	16	2	613	3,555	4,170	6,758
Surigao City	0	0	0	0	0	0	12	12	0	91	912	1,003	1,877

Table 1.A.4 - MODERN METHOD OF FAMILY PLANNING

Current User (Ending)

Philippines, 2021

Area	Total Current Users	FSTR/BTL Age group 10-14	FSTR/BTL Age group 15-19	FSTR/BTL Age group 20-49	Total	MSTR/NSV Age group 10-14	MSTR/NSV Age group 15-19	MSTR/NSV Age group 20-49	Total
**PHILIPPINES**	**7,602,812**	**3**	**1,533**	**778,491**	**783,073**	**0**	**94**	**11,483**	**11,577**
**N C R**	**753,263**	**0**	**263**	**85,153**	**88,462**	**0**	**7**	**658**	**665**
Malabon	31,679	0	0	3,046	3,046	0	0	4	4
Navotas	23,423	0	1	614	615	0	4	4	8
Valenzuela City	39,922	0	0	6,832	6,832	0	0	73	73
Caloocan City	68,156	0	1	8,788	8,789	0	0	12	12
Marikina City	17,020	0	0	2,223	2,223	0	0	194	194
Pasig City	42,380	0	1	3,671	3,672	0	0	17	17
Pateros	2,674	0	0	332	332	0	0	4	4
Taguig	61,050	0	0	3,241	3,241	0	1	26	27
Quezon City	272,854	0	67	35,350	35,417	0	0	217	217
Makati City	16,005	0	0	1,469	1,469	0	0	10	10
Mandaluyong City	17,504	0	7	1,940	1,947	0	0	18	18
San Juan	5,796	0	0	262	262	0	0	10	10
Manila City	27,488	0	1	4,917	4,918	0	0	13	13
Las Piñas City	20,363	0	0	3,582	3,582	0	0	10	10
Muntinlupa City	40,839	0	1	3,253	3,254	0	1	12	13
Parañaque City	37,239	0	184	2,488	2,672	0	1	30	31
Pasay City	28,871	0	0	6,191	6,191	0	0	4	4
**C A R**	**147,908**	**0**	**0**	**30,908**	**30,908**	**0**	**0**	**132**	**132**
Abra	17,362	0	0	3,819	3,819	0	0	5	5
Apayao	23,877	0	0	2,218	2,218	0	0	3	3
Benguet	31,732	0	0	7,965	7,965	0	0	15	15
Ifugao	20,003	0	0	2,856	2,856	0	0	92	92
Kalinga	19,143	0	0	3,229	3,229	0	0	5	5
Mt. Province	13,215	0	0	5,004	5,004	0	0	4	4
Baguio City	22,576	0	0	5,817	5,817	0	0	8	8
**Region 1**	**477,257**	**0**	**42**	**70,157**	**70,199**	**0**	**0**	**102**	**102**
Ilocos Norte	41,393	0	9	7,789	7,798	0	0	16	16
Ilocos Sur	62,434	0	10	8,632	8,642	0	0	5	5
La Union	59,808	0	7	10,722	10,729	0	0	16	16
Pangasinan	238,991	0	16	30,685	30,701	0	0	39	39
Alaminos City	10,832	0	0	1,098	1,098	0	0	0	0
Candon City	7,006	0	0	885	885	0	0	0	0
Dagupan City	6,976	0	0	929	929	0	0	2	2
Laoag City	8,253	0	0	1,826	1,826	0	0	0	0
San Carlos City	11,178	0	0	2,427	2,427	0	0	0	0
San Fernando City	13,027	0	0	2,357	2,357	0	0	23	23
Urdaneta City	12,909	0	0	1,624	1,624	0	0	1	1
Vigan City	4,450	0	0	1,183	1,183	0	0	0	0
**Region 2**	**318,442**	**0**	**30**	**45,479**	**45,509**	**0**	**0**	**94**	**94**
Batanes	960	0	0	170	170	0	0	0	0
Cagayan	88,783	0	20	11,464	11,484	0	0	26	26
Isabela	109,565	0	7	16,345	16,352	0	0	21	21
Nueva Vizcaya	46,874	0	2	8,260	8,262	0	0	22	22
Quirino	19,341	0	0	2,431	2,431	0	0	7	7
Cauayan City	10,017	0	0	1,721	1,721	0	0	0	0
Ilagan City	15,480	0	1	1,337	1,338	0	0	0	0
Santiago City	12,758	0	0	1,378	1,378	0	0	0	0
Tuguegarao City	14,664	0	0	2,373	2,373	0	0	18	18
**Region 3**	**831,669**	**0**	**57**	**154,999**	**155,056**	**0**	**34**	**833**	**867**
Aurora	22,728	0	3	1,987	1,990	0	0	14	14
Bataan	53,599	0	7	6,988	6,995	0	0	8	8

Table 1.A.4 - MODERN METHOD OF FAMILY PLANNING

Current User (Ending)

Philippines, 2021

Area	Total Current Users	FSTR/BTL Age group			Total	MSTR/NSV Age group			Total
		10-14	15-19	20-49		10-14	15-19	20-49	
Bulacan	156,537	0	4	29,375	29,379	0	0	375	375
Nueva Ecija	135,568	0	5	27,358	27,363	0	0	1	1
Pampanga	114,189	0	2	39,117	39,119	0	29	223	252
Tarlac	88,617	0	2	11,774	11,776	0	0	8	8
Zambales	43,500	0	2	7,750	7,752	0	0	93	93
Angeles City	24,153	0	0	5,705	5,705	0	0	72	72
Balanga City	4,623	0	0	872	872	0	0	1	1
Cabanatuan City	20,017	0	6	3,852	3,858	0	0	0	0
City of San Fernando	11,292	0	16	1,755	1,771	0	5	15	20
Gapan City	7,573	0	0	1,806	1,806	0	0	0	0
Mabalacat City	21,462	0	0	2,703	2,703	0	0	0	0
Malolos City	7,988	0	0	1,352	1,352	0	0	6	6
Meycauayan	8,375	0	1	363	364	0	0	5	5
Olongapo	10,879	0	2	2,071	2,073	0	0	3	3
Palayan City	2,864	0	0	542	542	0	0	0	0
San Jose City	12,941	0	0	808	808	0	0	2	2
San Jose del Monte City	56,123	0	0	5,560	5,560	0	0	5	5
Science City of Munoz	6,280	0	0	1,257	1,257	0	0	1	1
Tarlac City	22,361	0	7	2,004	2,011	0	0	1	1
**Region 4A**	**515,130**	**0**	**52**	**64,936**	**64,988**	**0**	**0**	**170**	**170**
Batangas	72,118	0	3	12,213	12,216	0	0	17	17
Cavite	50,817	0	1	8,779	8,780	0	0	27	27
Laguna	40,117	0	2	5,705	5,707	0	0	22	22
Quezon	65,621	0	2	6,504	6,506	0	0	6	6
Rizal	46,878	0	0	5,783	5,783	0	0	17	17
Antipolo City	8,048	0	0	1,165	1,165	0	0	8	8
Bacoor City	9,942	0	0	1,630	1,630	0	0	2	2
Batangas City	15,666	0	0	2,320	2,320	0	0	3	3
Biñan City	48,396	0	3	1,147	1,150	0	0	3	3
Cabuyao City	7,090	0	0	694	694	0	0	1	1
Calamba City	21,012	0	2	3,062	3,064	0	0	4	4
Cavite City	4,231	0	0	1,286	1,286	0	0	2	2
Dasmariñas City	37,109	0	3	4,200	4,203	0	0	11	11
General Trias City	4,529	0	0	1,223	1,223	0	0	0	0
Imus City	6,280	0	0	894	894	0	0	2	2
Lipa City	6,035	0	0	1,223	1,223	0	0	0	0
Lucena City	5,540	0	0	219	219	0	0	1	1
San Pablo City	5,328	0	0	966	966	0	0	0	0
San Pedro City	10,473	0	5	681	686	0	0	15	15
Santa Rosa City	25,676	0	4	1,087	1,091	0	0	11	11
Tagaytay City	8,169	0	0	1,109	1,109	0	0	11	11
Tanauan City	4,884	0	0	986	986	0	0	2	2
Tayabas City	7,258	0	26	1,297	1,323	0	0	4	4
Trece Martires City	3,913	0	1	763	764	0	0	1	1
**Region 4B**	**271,827**	**0**	**56**	**23,520**	**23,576**	**0**	**0**	**1,369**	**1,369**
Marinduque	14,336	0	0	1,835	1,835	0	0	19	19
Mindoro Occidental	47,411	0	0	2,387	2,387	0	0	9	9
Mindoro Oriental	65,781	0	1	4,983	4,984	0	0	1,281	1,281
Palawan	88,861	0	55	7,969	8,024	0	0	4	4
Romblon	22,324	0	0	3,597	3,597	0	0	45	45
Puerto Princesa City	33,114	0	0	2,749	2,749	0	0	11	11
**Region 5**	**501,196**	**0**	**244**	**31,172**	**31,416**	**0**	**0**	**384**	**384**
Albay	97,609	0	213	5,920	6,133	0	0	86	86
Camarines Norte	44,713	0	8	1,767	1,775	0	0	7	7
Camarines Sur	106,529	0	18	5,421	5,439	0	0	20	20
Catanduanes	24,595	0	0	3,460	3,460	0	0	98	98
Masbate	87,357	0	0	2,877	2,877	0	0	36	36
Sorsogon	76,074	0	5	7,044	7,049	0	0	72	72
Iriga City	12,491	0	0	564	564	0	0	7	7

Table 1.A.4 - MODERN METHOD OF FAMILY PLANNING
Current User (Ending)
Philippines, 2021

Area	Total Current Users	FSTR/BTL Age group 10-14	15-19	20-49	Total	MSTR/NSV Age group 10-14	15-19	20-49	Total
Legaspi City	23,050	0	0	1,859	1,859	0	0	0	0
Naga City	28,778	0	0	2,260	2,260	0	0	58	58
**Region 6**	**612,103**	**0**	**5**	**47,595**	**47,600**	**0**	**0**	**1,993**	**1,993**
Aklan	44,365	0	1	3,652	3,653	0	0	3	3
Antique	47,618	0	0	7,002	7,002	0	0	84	84
Capiz	76,500	0	0	4,423	4,423	0	0	365	365
Guimaras	16,308	0	1	1,301	1,302	0	0	6	6
Iloilo	157,750	0	0	12,620	12,620	0	0	300	300
Negros Occidental	193,157	0	2	11,714	11,716	0	0	1,140	1,140
Bacolod City	25,916	0	0	1,858	1,858	0	0	83	83
Iloilo City	50,489	0	1	5,025	5,026	0	0	12	12
**Region 7**	**436,837**	**0**	**7**	**28,886**	**28,893**	**0**	**0**	**1,292**	**1,292**
Bohol	80,409	0	7	9,820	9,827	0	0	221	221
Cebu	163,476	0	0	11,356	11,356	0	0	414	414
Negros Oriental	93,304	0	0	2,974	2,974	0	0	264	264
Siquijor	8,967	0	0	486	486	0	0	1	1
Cebu City	32,880	0	0	1,803	1,803	0	0	340	340
Lapu-Lapu City	34,838	0	0	2,168	2,168	0	0	51	51
Mandaue City	22,963	0	0	279	279	0	0	1	1
**Region 8**	**349,783**	**0**	**0**	**29,934**	**29,934**	**0**	**0**	**457**	**457**
Biliran	11,512	0	0	1,188	1,188	0	0	18	18
Eastern Samar	41,802	0	0	5,156	5,156	0	0	189	189
Northern Leyte	118,358	0	0	9,669	9,669	0	0	92	92
Northern Samar	48,444	0	0	1,934	1,934	0	0	77	77
Southern Leyte	24,584	0	0	3,044	3,044	0	0	30	30
Western Samar	26,721	0	0	1,559	1,559	0	0	6	6
Calbayog City	8,315	0	0	1,215	1,215	0	0	8	8
Maasin City	5,078	0	0	625	625	0	0	4	4
Ormoc City	20,494	0	0	1,410	1,410	0	0	12	12
Tacloban City	44,475	0	0	4,134	4,134	0	0	21	21
**Region 9**	**331,160**	**0**	**6**	**14,582**	**14,588**	**0**	**2**	**207**	**209**
Zamboanga del Norte	101,312	0	0	4,383	4,383	0	0	93	93
Zamboanga del Sur	70,265	0	0	1,869	1,869	0	2	37	39
Zamboanga Sibugay	46,803	0	4	1,953	1,957	0	0	16	16
Dapitan City	8,223	0	0	1,397	1,397	0	0	12	12
Dipolog City	16,789	0	0	765	765	0	0	2	2
Isabela City	9,996	0	2	853	855	0	0	4	4
Pagadian City	20,916	0	0	1,139	1,139	0	0	43	43
Zamboanga City	56,856	0	0	2,223	2,223	0	0	0	0
**Region 10**	**489,677**	**3**	**134**	**31,968**	**32,105**	**0**	**28**	**846**	**874**
Bukidnon	114,313	0	19	6,782	6,801	0	0	69	69
Camiguin	6,418	0	0	1,098	1,098	0	0	6	6
Lanao del Norte	63,113	0	5	1,170	1,175	0	0	8	8
Misamis Occidental	27,464	0	7	1,462	1,469	0	0	20	20
Misamis Oriental	75,691	0	1	6,276	6,277	0	0	56	56
Cagayan de Oro City	61,925	0	13	4,730	4,743	0	6	478	484
El Salvador City	6,345	0	0	392	392	0	0	0	0
Gingoog City	28,686	2	54	2,466	2,522	0	2	44	46
Iligan City	28,662	0	3	1,754	1,757	0	0	20	20
Malaybalay City	18,648	0	0	1,498	1,498	0	0	20	20
Oroquieta City	6,937	0	0	797	797	0	0	2	2
Ozamis City	25,480	0	24	923	947	0	20	50	70
Tangub City	4,130	0	4	309	313	0	0	0	0
Valencia City	21,865	1	4	2,311	2,316	0	0	73	73

Table 1.A.4 - MODERN METHOD OF FAMILY PLANNING

Current User (Ending)

Philippines, 2021

Area	Total Current Users	FSTR/BTL Age group			Total	MSTR/NSV Age group			Total
		10-14	15-19	20-49		10-14	15-19	20-49	
**Region 11**	**528,307**	**0**	**325**	**46,123**	**46,448**	**0**	**15**	**1,810**	**1,825**
Davao de Oro	85,230	0	61	7,599	7,660	0	0	163	163
Davao del Norte	103,058	0	95	11,381	11,476	0	0	598	598
Davao Oriental	53,572	0	14	5,524	5,538	0	0	101	101
Davao del Sur	76,678	0	11	4,932	4,943	0	11	72	83
Davao Occidental	31,942	0	53	4,287	4,340	0	1	65	66
Davao City	177,827	0	91	12,400	12,491	0	3	811	814
**Region 12**	**503,303**	**0**	**269**	**45,477**	**45,746**	**0**	**7**	**916**	**923**
North Cotabato	159,342	0	14	10,381	10,395	0	1	130	131
Sarangani	69,844	0	159	4,208	4,367	0	2	100	102
South Cotabato	91,478	0	31	9,990	10,021	0	4	423	427
Sultan Kudarat	95,596	0	3	9,435	9,438	0	0	165	165
Cotabato City	33,411	0	0	2,438	2,438	0	0	0	0
Gen. Santos City	53,632	0	62	9,025	9,087	0	0	98	98
**B.A.R.M.M.**	**266,052**	**0**	**33**	**7,806**	**7,839**	**0**	**0**	**19**	**19**
Basilan	10,539	0	0	347	347	0	0	0	0
Lanao del Sur	27,118	0	0	1,333	1,333	0	0	9	9
Maguindanao	91,784	0	6	3,018	3,024	0	0	14	14
Sulu	92,304	0	27	1,594	1,621	0	0	-4	-4
Tawi-Tawi	28,157	0	0	601	601	0	0	0	0
Lamitan City	10,631	0	0	577	577	0	0	0	0
Marawi City	5,519	0	0	336	336	0	0	0	0
**CARAGA**	**268,898**	**0**	**10**	**19,796**	**19,806**	**0**	**1**	**201**	**202**
Agusan del Norte	39,895	0	3	3,224	3,227	0	0	8	8
Agusan del Sur	83,282	0	3	6,713	6,716	0	0	59	59
Surigao del Norte	33,804	0	2	1,955	1,957	0	0	26	26
Surigao del Sur	42,911	0	1	3,008	3,009	0	0	62	62
Province of Dinagat	10,370	0	0	383	383	0	0	2	2
Bislig City	9,590	0	0	789	789	0	0	5	5
Butuan City	34,962	0	1	2,645	2,646	0	0	18	18
Surigao City	14,084	0	0	1,079	1,079	0	1	21	22

Table 1.A.4 - MODERN METHOD OF FAMILY PLANNING

Current User (Ending)

Philippines, 2021

Area	CONDOM			Total	IUD-INTERVAL			Total	IUD-POSTPARTUM			Total
	Age group				Age group				Age group			
	10-14	15-19	20-49		10-14	15-19	20-49		10-14	15-19	20-49	
**PHILIPPINES**	**127**	**13,245**	**358,829**	**372,201**	**91**	**8,614**	**417,797**	**426,502**	**213**	**7,951**	**127,097**	**135,261**
**N C R**	**24**	**3,538**	**73,557**	**77,119**	**5**	**932**	**42,567**	**43,504**	**72**	**2,566**	**21,856**	**24,494**
Malabon	1	905	1,979	2,885	0	137	1,637	1,774	0	96	222	318
Navotas	1	246	1,723	1,970	0	5	416	421	0	4	47	51
Valenzuela City	0	5	863	868	0	1	389	390	0	29	665	694
Caloocan City	0	298	6,400	6,698	2	171	3,734	3,907	1	59	531	591
Marikina City	0	5	520	525	0	46	1,151	1,197	21	409	1,854	2,284
Pasig City	8	117	2,035	2,160	0	20	2,025	2,045	11	335	1,190	1,536
Pateros	0	0	182	182	0	0	332	332	0	0	0	0
Taguig	7	261	3,492	3,760	1	43	1,808	1,852	1	20	255	276
Quezon City	7	1,066	48,375	49,448	0	346	19,779	20,125	31	962	13,990	14,983
Makati City	0	23	860	883	0	15	583	598	0	32	357	389
Mandaluyong City	0	76	1,105	1,181	0	5	527	532	3	36	238	277
San Juan	0	0	115	115	0	0	1,360	1,360	0	0	0	0
Manila City	0	30	449	479	0	15	642	657	1	59	693	753
Las Piñas City	0	15	803	818	0	8	457	465	2	35	222	259
Muntinlupa City	0	60	1,119	1,179	1	68	3,061	3,130	0	7	57	64
Parañaque City	0	195	1,903	2,098	1	52	1,108	1,161	1	483	651	1,135
Pasay City	0	236	1,634	1,870	0	0	3,558	3,558	0	0	884	884
**C A R**	**0**	**66**	**5,432**	**5,498**	**0**	**38**	**6,351**	**6,389**	**0**	**17**	**6,162**	**6,179**
Abra	0	6	312	318	0	0	59	59	0	5	42	47
Apayao	0	3	145	148	0	7	573	580	0	1	44	45
Benguet	0	16	2,509	2,525	0	19	2,506	2,525	0	0	471	471
Ifugao	0	7	559	566	0	3	1,004	1,007	0	1	17	18
Kalinga	0	4	600	604	0	4	778	782	0	5	61	66
Mt. Province	0	4	528	532	0	5	471	476	0	5	51	56
Baguio City	0	26	779	805	0	0	960	960	0	0	5,476	5,476
**Region 1**	**1**	**509**	**20,289**	**20,799**	**6**	**400**	**10,558**	**10,964**	**0**	**35**	**559**	**594**
Ilocos Norte	0	16	1,707	1,723	0	16	516	532	0	7	128	135
Ilocos Sur	0	45	1,481	1,526	0	1	528	529	0	2	106	108
La Union	0	14	1,439	1,453	0	39	1,498	1,537	0	16	117	133
Pangasinan	1	366	13,364	13,731	6	295	7,072	7,373	0	0	0	0
Alaminos City	0	4	273	277	0	0	0	0	0	0	0	0
Candon City	0	0	1,060	1,060	0	0	2	2	0	0	133	133
Dagupan City	0	7	161	168	0	4	73	77	0	4	66	70
Laoag City	0	0	0	0	0	0	64	64	0	0	0	0
San Carlos City	0	2	367	369	0	9	380	389	0	0	0	0
San Fernando City	0	6	280	286	0	13	162	175	0	6	9	15
Urdaneta City	0	49	131	180	0	23	262	285	0	0	0	0
Vigan City	0	0	26	26	0	0	1	1	0	0	0	0
**Region 2**	**4**	**143**	**3,603**	**3,750**	**2**	**413**	**17,685**	**18,100**	**1**	**242**	**1,236**	**1,479**
Batanes	0	0	19	19	0	0	11	11	0	0	0	0
Cagayan	1	13	328	342	2	131	8,660	8,793	0	75	223	298
Isabela	2	48	1,314	1,364	0	113	3,188	3,301	1	89	464	554
Nueva Vizcaya	0	2	1,305	1,307	0	6	983	989	0	5	47	52
Quirino	0	17	267	284	0	0	733	733	0	0	2	2
Cauayan City	0	0	65	65	0	3	282	285	0	3	24	27
Ilagan City	1	54	107	162	0	27	895	922	0	4	128	132
Santiago City	0	5	64	69	0	15	338	353	0	0	0	0
Tuguegarao City	0	4	134	138	0	118	2,595	2,713	0	66	348	414
**Region 3**	**13**	**1,135**	**35,009**	**36,157**	**1**	**273**	**12,203**	**12,477**	**22**	**1,068**	**9,254**	**10,344**
Aurora	1	31	1,194	1,226	0	8	286	294	0	2	62	64
Bataan	7	93	2,453	2,553	0	29	506	535	1	15	485	501

Table 1.A.4 - MODERN METHOD OF FAMILY PLANNING

Current User (Ending)

Philippines, 2021

Area	CONDOM			Total	IUD-INTERVAL			Total	IUD-POSTPARTUM			Total
	Age group				Age group				Age group			
	10-14	15-19	20-49		10-14	15-19	20-49		10-14	15-19	20-49	
Bulacan	0	87	3,646	3,733	0	25	3,618	3,643	0	31	1,603	1,634
Nueva Ecija	0	168	3,434	3,602	0	50	2,205	2,255	21	903	2,568	3,492
Pampanga	0	82	4,239	4,321	0	16	706	722	0	79	3,722	3,801
Tarlac	0	144	4,004	4,148	0	62	934	996	0	5	234	239
Zambales	0	26	1,005	1,031	0	4	589	593	0	7	196	203
Angeles City	0	16	589	605	0	1	275	276	0	2	48	50
Balanga City	0	2	102	104	0	0	32	32	0	0	1	1
Cabanatuan City	0	17	446	463	0	6	111	117	0	2	37	39
City of San Fernando	5	56	666	727	0	6	22	28	0	6	52	58
Gapan City	0	12	130	142	0	0	35	35	0	1	5	6
Mabalacat City	0	37	2,051	2,088	0	6	248	254	0	2	2	4
Malolos City	0	7	423	430	0	1	80	81	0	0	0	0
Meycauayan	0	43	434	477	0	0	19	19	0	1	26	27
Olongapo	0	1	643	644	0	1	68	69	0	10	94	104
Palayan City	0	1	23	24	0	1	166	167	0	0	0	0
San Jose City	0	6	208	214	0	1	109	110	0	2	16	18
San Jose del Monte City	0	95	8,340	8,435	0	4	1,667	1,671	0	0	0	0
Science City of Munoz	0	0	83	83	0	0	102	102	0	0	0	0
Tarlac City	0	211	896	1,107	1	52	425	478	0	0	103	103
**Region 4A**	**27**	**2,728**	**31,670**	**34,425**	**8**	**296**	**21,096**	**21,400**	**21**	**304**	**5,936**	**6,261**
Batangas	7	132	4,235	4,374	6	18	2,195	2,219	0	17	668	685
Cavite	6	17	1,763	1,786	1	25	2,096	2,122	2	17	131	150
Laguna	0	100	3,187	3,287	1	67	3,331	3,399	5	38	956	999
Quezon	0	24	1,745	1,769	0	31	6,030	6,061	0	7	168	175
Rizal	0	44	1,910	1,954	0	44	1,462	1,506	1	27	366	394
Antipolo City	0	0	588	588	0	0	344	344	0	0	114	114
Bacoor City	0	1	253	254	0	11	253	264	0	0	43	43
Batangas City	0	7	1,032	1,039	0	3	626	629	4	141	1,843	1,988
Biñan City	0	1,860	7,854	9,714	0	9	672	681	0	5	111	116
Cabuyao City	0	25	230	255	0	0	182	182	0	0	104	104
Calamba City	2	71	1,063	1,136	0	25	640	665	0	24	510	534
Cavite City	0	1	55	56	0	3	74	77	0	0	11	11
Dasmariñas City	0	81	1,836	1,917	0	41	1,406	1,447	0	9	358	367
General Trias City	0	2	53	55	0	1	87	88	0	1	12	13
Imus City	0	1	298	299	0	1	35	36	0	1	27	28
Lipa City	0	0	405	405	0	1	227	228	0	0	48	48
Lucena City	0	3	100	103	0	0	14	14	0	0	2	2
San Pablo City	0	7	108	115	0	5	378	383	0	4	67	71
San Pedro City	0	14	370	384	0	7	285	292	0	5	100	105
Santa Rosa City	9	326	3,757	4,092	0	1	205	206	9	1	82	92
Tagaytay City	3	7	152	162	0	0	8	8	0	0	132	132
Tanauan City	0	2	336	338	0	0	199	199	0	7	83	90
Tayabas City	0	1	295	296	0	1	158	159	0	0	0	0
Trece Martires City	0	2	45	47	0	2	189	191	0	0	0	0
**Region 4B**	**30**	**57**	**6,592**	**6,679**	**0**	**45**	**6,459**	**6,504**	**1**	**22**	**4,342**	**4,365**
Marinduque	0	1	291	292	0	0	393	393	0	0	0	0
Mindoro Occidental	0	6	1,030	1,036	0	11	1,343	1,354	0	2	14	16
Mindoro Oriental	0	14	1,618	1,632	0	25	2,682	2,707	0	18	3,155	3,173
Palawan	30	36	2,116	2,182	0	3	1,072	1,075	1	2	13	16
Romblon	0	0	574	574	0	6	969	975	0	0	805	805
Puerto Princesa City	0	0	963	963	0	0	0	0	0	0	355	355
**Region 5**	**14**	**873**	**30,583**	**31,470**	**0**	**144**	**7,503**	**7,647**	**0**	**66**	**995**	**1,061**
Albay	0	552	8,619	9,171	0	30	1,159	1,189	0	7	352	359
Camarines Norte	10	56	2,294	2,360	0	88	1,632	1,720	0	27	49	76
Camarines Sur	3	62	4,682	4,747	0	10	1,345	1,355	0	23	226	249
Catanduanes	0	5	577	582	0	0	58	58	0	0	66	66
Masbate	1	82	2,983	3,066	0	5	2,019	2,024	0	3	155	158
Sorsogon	0	30	3,120	3,150	0	1	447	448	0	1	40	41
Iriga City	0	11	638	649	0	3	385	388	0	1	2	3

Table 1.A.4 - MODERN METHOD OF FAMILY PLANNING

Current User (Ending)

Philippines, 2021

Area	CONDOM			Total	IUD-INTERVAL			Total	IUD-POSTPARTUM			Total
	Age group				Age group				Age group			
	10-14	15-19	20-49		10-14	15-19	20-49		10-14	15-19	20-49	
Legaspi City	0	63	2,075	2,138	0	2	104	106	0	0	69	69
Naga City	0	12	5,595	5,607	0	5	354	359	0	4	36	40
**Region 6**	**2**	**368**	**30,363**	**30,733**	**8**	**474**	**35,473**	**35,955**	**7**	**334**	**10,412**	**10,753**
Aklan	0	54	2,753	2,807	0	3	699	702	0	0	12	12
Antique	1	17	1,495	1,513	0	5	686	691	0	0	28	28
Capiz	0	37	3,918	3,955	0	38	7,520	7,558	0	2	51	53
Guimaras	0	5	953	958	0	0	338	338	0	1	7	8
Iloilo	1	81	8,491	8,573	5	130	8,527	8,662	2	35	2,612	2,649
Negros Occidental	0	126	7,171	7,297	2	199	12,895	13,096	2	240	5,231	5,473
Bacolod City	0	1	1,416	1,417	1	34	3,019	3,054	3	27	1,978	2,008
Iloilo City	0	47	4,166	4,213	0	65	1,789	1,854	0	29	493	522
**Region 7**	**2**	**333**	**22,350**	**22,685**	**5**	**735**	**62,078**	**62,818**	**3**	**430**	**6,805**	**7,238**
Bohol	0	62	5,161	5,223	2	216	12,988	13,206	0	185	2,595	2,780
Cebu	1	118	8,338	8,457	1	302	26,839	27,142	0	133	2,765	2,898
Negros Oriental	1	38	3,696	3,735	0	51	11,711	11,762	0	19	342	361
Siquijor	0	7	407	414	0	4	517	521	0	1	99	100
Cebu City	0	20	921	941	0	119	3,499	3,618	0	68	693	761
Lapu-Lapu City	0	83	1,908	1,991	2	41	4,133	4,176	3	23	273	299
Mandaue City	0	5	1,919	1,924	0	2	2,391	2,393	0	1	38	39
**Region 8**	**1**	**316**	**13,966**	**14,283**	**2**	**260**	**24,887**	**25,149**	**1**	**353**	**4,165**	**4,519**
Biliran	0	2	363	365	1	14	575	590	0	3	44	47
Eastern Samar	1	68	3,640	3,709	1	4	390	395	0	8	116	124
Northern Leyte	0	55	3,768	3,823	0	113	12,144	12,257	0	117	1,494	1,611
Northern Samar	0	31	1,324	1,355	0	18	2,043	2,061	0	21	425	446
Southern Leyte	0	16	851	867	0	22	3,215	3,237	0	14	364	378
Western Samar	0	-27	440	413	0	22	708	730	1	59	574	634
Calbayog City	0	5	278	283	0	-2	84	82	0	0	-4	-4
Maasin City	0	0	155	155	0	6	1,355	1,361	0	6	54	60
Ormoc City	0	11	932	943	0	11	1,457	1,468	0	12	34	46
Tacloban City	0	155	2,215	2,370	0	52	2,916	2,968	0	113	1,064	1,177
**Region 9**	**1**	**145**	**11,547**	**11,693**	**0**	**314**	**19,290**	**19,604**	**47**	**505**	**19,886**	**20,438**
Zamboanga del Norte	1	32	4,373	4,406	0	11	943	954	20	67	8,352	8,439
Zamboanga del Sur	0	51	1,394	1,445	0	105	7,416	7,521	5	253	2,572	2,830
Zamboanga Sibugay	0	43	1,687	1,730	0	172	8,595	8,767	2	86	703	791
Dapitan City	0	2	586	588	0	0	1	1	0	0	840	840
Dipolog City	0	2	1,449	1,451	0	1	122	123	20	51	1,753	1,824
Isabela City	0	5	406	411	0	0	153	153	0	0	2	2
Pagadian City	0	7	1,196	1,203	0	15	2,060	2,075	0	46	1,066	1,112
Zamboanga City	0	3	456	459	0	10	0	10	0	2	4,598	4,600
**Region 10**	**25**	**742**	**20,008**	**20,775**	**20**	**2,466**	**54,505**	**56,991**	**9**	**788**	**11,522**	**12,319**
Bukidnon	0	72	3,023	3,095	0	529	14,238	14,767	4	129	1,107	1,240
Camiguin	0	4	259	263	0	1	738	739	0	2	37	39
Lanao del Norte	0	230	4,153	4,383	2	88	3,797	3,887	0	36	749	785
Misamis Occidental	11	110	1,899	2,020	0	27	2,191	2,218	0	11	133	144
Misamis Oriental	0	83	3,065	3,148	18	1,124	13,436	14,578	-1	67	1,540	1,606
Cagayan de Oro City	0	48	2,336	2,384	0	270	8,480	8,750	1	319	6,986	7,306
El Salvador City	0	1	103	104	0	35	1,862	1,897	2	62	195	259
Gingoog City	14	97	1,469	1,580	0	173	2,554	2,727	0	50	347	397
Iligan City	0	49	1,626	1,675	0	134	3,067	3,201	3	107	374	484
Malaybalay City	0	3	638	641	0	45	877	922	0	-1	-23	-24
Oroquieta City	0	0	194	194	0	0	631	631	0	0	0	0
Ozamis City	0	16	719	735	0	2	630	632	0	0	0	0
Tangub City	0	0	82	82	0	0	63	63	0	0	8	8
Valencia City	0	29	442	471	0	38	1,941	1,979	0	6	69	75

Table 1.A.4 - MODERN METHOD OF FAMILY PLANNING

Current User (Ending)

Philippines, 2021

Area	CONDOM			Total	IUD-INTERVAL			Total	IUD-POSTPARTUM			Total
	Age group				Age group				Age group			
	10-14	15-19	20-49		10-14	15-19	20-49		10-14	15-19	20-49	
**Region 11**	**-22**	**964**	**20,010**	**20,952**	**20**	**654**	**40,490**	**41,164**	**15**	**255**	**5,873**	**6,143**
Davao de Oro	5	110	3,151	3,266	1	83	7,174	7,258	4	31	482	517
Davao del Norte	1	165	4,252	4,418	4	148	6,354	6,506	3	81	2,457	2,541
Davao Oriental	0	53	1,154	1,207	0	9	3,248	3,257	0	5	315	320
Davao del Sur	2	119	1,795	1,916	7	165	8,612	8,784	1	35	480	516
Davao Occidental	1	20	371	392	1	62	2,114	2,177	0	9	154	163
Davao City	-31	497	9,287	9,753	7	187	12,988	13,182	7	94	1,985	2,086
**Region 12**	**0**	**627**	**15,435**	**16,062**	**10**	**574**	**27,025**	**27,609**	**2**	**457**	**12,196**	**12,655**
North Cotabato	0	167	4,194	4,361	10	185	12,069	12,264	0	103	1,443	1,546
Sarangani	0	124	2,203	2,327	0	61	2,540	2,601	0	12	159	171
South Cotabato	0	79	2,861	2,940	0	144	5,318	5,462	0	105	2,117	2,222
Sultan Kudarat	0	131	2,085	2,216	0	116	4,678	4,794	0	101	4,070	4,171
Cotabato City	0	57	2,135	2,192	0	30	903	933	2	121	1,190	1,313
Gen. Santos City	0	69	1,957	2,026	0	38	1,517	1,555	0	15	3,217	3,232
**B.A.R.M.M.**	**5**	**359**	**7,179**	**7,543**	**1**	**117**	**3,225**	**3,343**	**6**	**169**	**1,987**	**2,162**
Basilan	0	6	277	283	0	-1	52	51	0	0	25	25
Lanao del Sur	-1	43	2,102	2,144	0	24	760	784	0	1	199	200
Maguindanao	6	130	2,951	3,087	-1	23	1,644	1,666	0	32	544	576
Sulu	0	22	724	746	0	0	308	308	3	0	157	160
Tawi-Tawi	0	0	332	332	0	0	0	0	0	0	0	0
Lamitan City	0	0	47	47	2	71	442	515	3	135	1,062	1,200
Marawi City	0	158	746	904	0	0	19	19	0	1	0	1
**CARAGA**	**0**	**342**	**11,236**	**11,578**	**3**	**479**	**26,402**	**26,884**	**6**	**340**	**3,911**	**4,257**
Agusan del Norte	0	52	1,586	1,638	0	184	4,931	5,115	1	62	1,199	1,262
Agusan del Sur	0	76	3,511	3,587	1	154	9,105	9,260	2	57	1,073	1,132
Surigao del Norte	0	79	1,294	1,373	1	29	2,146	2,176	1	9	153	163
Surigao del Sur	0	56	1,902	1,958	1	44	4,252	4,297	2	35	512	549
Province of Dinagat	0	13	761	774	0	16	1,005	1,021	0	0	24	24
Bislig City	0	10	626	636	0	18	1,173	1,191	0	9	143	152
Butuan City	0	47	1,411	1,458	0	22	1,758	1,780	0	151	663	814
Surigao City	0	9	145	154	0	12	2,032	2,044	0	17	144	161

Table 1.A.4 - MODERN METHOD OF FAMILY PLANNING

Current User (Ending)

Philippines, 2021

Area	PILLS-POP Age group			Total	PILLS-COC Age group			Total	INJECTABLES Age group			Total
	10-14	15-19	20-49		10-14	15-19	20-49		10-14	15-19	20-49	
**PHILIPPINES**	**728**	**31,098**	**376,708**	**408,534**	**421**	**74,762**	**2,585,208**	**2,660,391**	**926**	**75,346**	**1,455,366**	**1,531,638**
**N C R**	**93**	**5,211**	**34,597**	**39,901**	**35**	**7,029**	**157,990**	**165,054**	**107**	**12,383**	**147,129**	**159,619**
Malabon	0	1,102	2,164	3,266	0	1,306	6,628	7,934	4	1,508	4,878	6,390
Navotas	6	100	1,235	1,341	0	590	6,274	6,864	0	1,579	7,714	9,293
Valenzuela City	0	49	227	276	0	342	8,981	9,323	1	423	5,692	6,116
Caloocan City	2	314	1,968	2,284	4	632	9,339	9,975	2	1,190	21,822	23,014
Marikina City	0	86	767	853	0	43	2,379	2,422	6	407	4,183	4,596
Pasig City	3	196	1,397	1,596	3	329	8,359	8,691	8	928	13,476	14,412
Pateros	0	2	34	36	0	3	822	825	0	3	749	752
Taguig	15	969	3,523	4,507	11	787	11,402	12,200	0	603	13,856	14,459
Quezon City	61	1,432	14,434	15,927	10	1,514	69,171	70,695	14	2,404	34,297	36,715
Makati City	0	50	1,276	1,326	0	51	2,083	2,134	0	130	4,325	4,455
Mandaluyong City	0	92	1,164	1,256	0	64	3,235	3,299	0	178	3,633	3,811
San Juan	0	0	0	0	0	3	1,906	1,909	0	0	1,694	1,694
Manila City	0	83	727	810	1	120	1,392	1,513	17	269	3,403	3,689
Las Piñas City	0	95	1,154	1,249	1	120	4,022	4,143	2	333	4,074	4,409
Muntinlupa City	6	223	901	1,130	5	607	11,290	11,902	6	1,373	8,123	9,502
Parañaque City	0	354	2,625	2,979	0	515	5,717	6,232	47	1,055	7,904	9,006
Pasay City	0	64	1,001	1,065	0	3	4,990	4,993	0	0	7,306	7,306
**C A R**	**4**	**500**	**13,417**	**13,921**	**7**	**977**	**37,467**	**38,451**	**19**	**824**	**20,380**	**21,223**
Abra	2	101	837	940	5	420	6,343	6,768	15	230	1,953	2,198
Apayao	1	78	1,917	1,996	0	218	14,461	14,679	2	135	2,223	2,360
Benguet	0	100	969	1,069	0	95	8,241	8,336	1	129	4,823	4,953
Ifugao	1	59	3,814	3,874	2	70	2,262	2,334	0	104	3,089	3,193
Kalinga	0	102	4,736	4,838	0	64	1,551	1,615	0	35	3,323	3,358
Mt. Province	0	8	234	242	0	84	2,694	2,778	1	74	1,630	1,705
Baguio City	0	52	910	962	0	26	1,915	1,941	0	117	3,339	3,456
**Region 1**	**2**	**303**	**5,420**	**5,725**	**9**	**4,948**	**195,322**	**200,279**	**5**	**3,504**	**94,518**	**98,027**
Ilocos Norte	1	72	1,873	1,946	1	171	18,309	18,481	0	114	5,728	5,842
Ilocos Sur	0	130	2,780	2,910	6	437	24,361	24,804	0	296	10,773	11,069
La Union	1	84	674	759	0	196	22,395	22,591	1	449	14,745	15,195
Pangasinan	0	0	0	0	2	2,363	101,721	104,086	4	1,952	49,433	51,389
Alaminos City	0	0	0	0	0	1,270	2,741	4,011	0	393	1,410	1,803
Candon City	0	0	4	4	0	0	2,426	2,426	0	0	2,038	2,038
Dagupan City	0	3	44	47	0	37	2,536	2,573	0	107	1,713	1,820
Laoag City	0	0	0	0	0	0	2,843	2,843	0	0	2,060	2,060
San Carlos City	0	0	0	0	0	57	4,644	4,701	0	49	2,200	2,249
San Fernando City	0	14	45	59	0	120	6,739	6,859	0	12	1,769	1,781
Urdaneta City	0	0	0	0	0	292	5,051	5,343	0	131	2,161	2,292
Vigan City	0	0	0	0	0	5	1,556	1,561	0	1	488	489
**Region 2**	**6**	**2,290**	**12,647**	**14,943**	**9**	**3,724**	**149,907**	**153,640**	**37**	**2,579**	**49,812**	**52,428**
Batanes	0	1	1	2	0	2	183	185	0	17	325	342
Cagayan	1	410	2,529	2,940	3	467	45,673	46,143	4	348	11,302	11,654
Isabela	4	404	3,272	3,680	3	801	56,679	57,483	5	821	16,851	17,677
Nueva Vizcaya	0	164	2,705	2,869	2	589	20,162	20,753	0	335	6,214	6,549
Quirino	0	45	292	337	1	363	9,147	9,511	27	144	4,358	4,529
Cauayan City	0	25	179	204	0	104	5,301	5,405	0	46	1,441	1,487
Ilagan City	1	1,131	2,868	4,000	0	1,147	1,840	2,987	1	669	4,390	5,060
Santiago City	0	0	0	0	0	100	4,777	4,877	0	152	3,655	3,807
Tuguegarao City	0	110	801	911	0	151	6,145	6,296	0	47	1,276	1,323
**Region 3**	**4**	**2,362**	**20,940**	**23,306**	**17**	**9,391**	**278,141**	**287,549**	**36**	**11,870**	**204,309**	**216,215**
Aurora	0	55	1,088	1,143	0	145	10,482	10,627	0	154	3,335	3,489
Bataan	2	209	1,474	1,685	5	527	12,952	13,484	7	1,447	18,308	19,762

Table 1.A.4 - MODERN METHOD OF FAMILY PLANNING

Current User (Ending)

Philippines, 2021

Area	PILLS-POP			Total	PILLS-COC			Total	INJECTABLES			Total
	Age group				Age group				Age group			
	10-14	15-19	20-49		10-14	15-19	20-49		10-14	15-19	20-49	
Bulacan	0	271	3,017	3,288	0	1,612	54,430	56,042	9	2,302	37,982	40,293
Nueva Ecija	0	515	3,978	4,493	2	1,939	51,460	53,401	1	1,636	28,425	30,062
Pampanga	1	204	3,434	3,639	2	755	28,497	29,254	2	991	22,407	23,400
Tarlac	0	174	2,483	2,657	7	920	34,326	35,253	9	769	24,948	25,726
Zambales	0	84	821	905	0	331	12,660	12,991	5	790	13,181	13,976
Angeles City	0	61	347	408	0	347	8,139	8,486	0	374	5,058	5,432
Balanga City	0	3	37	40	0	73	1,418	1,491	2	134	1,465	1,601
Cabanatuan City	1	168	739	908	0	223	9,330	9,553	0	197	3,019	3,216
City of San Fernando	0	91	823	914	1	191	3,206	3,398	0	191	2,086	2,277
Gapan City	0	20	177	197	0	103	2,365	2,468	0	87	1,782	1,869
Mabalacat City	0	21	123	144	0	197	5,559	5,756	0	350	6,682	7,032
Malolos City	0	0	0	0	0	19	3,268	3,287	0	25	2,402	2,427
Meycauayan	0	48	210	258	0	275	2,774	3,049	0	118	2,159	2,277
Olongapo	0	29	223	252	0	108	2,530	2,638	1	172	2,494	2,667
Palayan City	0	2	3	5	0	26	1,534	1,560	0	42	491	533
San Jose City	0	49	185	234	0	161	5,405	5,566	0	296	4,378	4,674
San Jose del Monte City	0	64	802	866	0	477	17,480	17,957	0	805	17,143	17,948
Science City of Munoz	0	3	39	42	0	61	3,106	3,167	0	42	1,559	1,601
Tarlac City	0	291	937	1,228	0	901	7,220	8,121	0	948	5,005	5,953
**Region 4A**	**137**	**2,461**	**2,461**	**5,059**	**77**	**6,146**	**174,924**	**181,147**	**299**	**5,935**	**117,849**	**124,083**
Batangas	119	304	304	727	42	294	25,246	25,582	286	361	11,210	11,857
Cavite	2	79	79	160	2	335	16,959	17,296	0	547	13,712	14,259
Laguna	1	101	101	203	14	322	13,348	13,684	5	206	6,294	6,505
Quezon	0	331	331	662	2	597	25,167	25,766	4	651	18,280	18,935
Rizal	0	124	124	248	1	384	16,350	16,735	-7	672	14,625	15,290
Antipolo City	0	0	0	0	0	0	6	6	0	0	3,506	3,506
Bacoor City	0	32	32	64	0	53	2,106	2,159	0	169	3,108	3,277
Batangas City	0	54	54	108	0	43	6,252	6,295	0	91	2,459	2,550
Biñan City	0	847	847	1,694	0	1,613	15,785	17,398	0	1,345	10,897	12,242
Cabuyao City	0	42	42	84	0	35	3,159	3,194	0	57	1,475	1,532
Calamba City	5	90	90	185	6	262	7,940	8,208	2	199	4,232	4,433
Cavite City	0	1	1	2	0	37	1,166	1,203	0	72	859	931
Dasmariñas City	0	84	84	168	0	257	9,437	9,694	0	388	7,724	8,112
General Trias City	1	9	9	19	1	17	1,182	1,200	0	37	1,240	1,277
Imus City	0	7	7	14	0	44	2,322	2,366	0	93	1,900	1,993
Lipa City	0	8	8	16	0	15	2,928	2,943	0	8	940	948
Lucena City	0	4	4	8	0	156	2,020	2,176	0	207	2,637	2,844
San Pablo City	0	8	8	16	0	58	1,969	2,027	0	66	1,171	1,237
San Pedro City	0	172	172	344	0	233	4,883	5,116	0	101	1,466	1,567
Santa Rosa City	9	120	120	249	9	1,324	9,311	10,644	9	461	3,068	3,538
Tagaytay City	0	0	0	0	0	2	3,552	3,554	0	2	2,170	2,172
Tanauan City	0	26	26	52	0	22	1,417	1,439	0	41	1,545	1,586
Tayabas City	0	17	17	34	0	7	1,674	1,681	0	58	1,836	1,894
Trece Martires City	0	1	1	2	0	36	745	781	0	103	1,495	1,598
**Region 4B**	**93**	**266**	**10,480**	**10,839**	**15**	**793**	**99,936**	**100,744**	**6**	**891**	**56,280**	**57,177**
Marinduque	0	42	300	342	0	26	6,544	6,570	0	45	2,242	2,287
Mindoro Occidental	0	54	978	1,032	4	305	15,455	15,764	0	318	8,494	8,812
Mindoro Oriental	1	55	7,341	7,397	0	278	25,233	25,511	0	210	10,434	10,644
Palawan	92	95	1,425	1,612	4	159	33,332	33,495	5	277	18,367	18,649
Romblon	0	20	436	456	7	25	6,608	6,640	1	41	3,460	3,502
Puerto Princesa City	0	0	0	0	0	0	12,764	12,764	0	0	13,283	13,283
**Region 5**	**2**	**908**	**12,210**	**13,120**	**7**	**3,876**	**199,447**	**203,330**	**9**	**2,862**	**78,378**	**81,249**
Albay	0	179	2,405	2,584	1	1,084	38,568	39,653	1	686	18,863	19,550
Camarines Norte	0	135	1,269	1,404	2	516	21,403	21,921	1	446	6,380	6,827
Camarines Sur	1	251	3,243	3,495	2	608	39,418	40,028	6	464	15,915	16,385
Catanduanes	0	6	439	445	0	151	4,589	4,740	0	210	4,660	4,870
Masbate	1	161	1,681	1,843	2	824	38,445	39,271	1	419	10,869	11,289
Sorsogon	0	93	1,273	1,366	0	435	30,634	31,069	0	443	12,216	12,659
Iriga City	0	21	327	348	0	63	3,923	3,986	0	62	1,532	1,594

Table 1.A.4 - MODERN METHOD OF FAMILY PLANNING

Current User (Ending)

Philippines, 2021

Area	PILLS-POP			Total	PILLS-COC			Total	INJECTABLES			Total
	Age group				Age group				Age group			
	10-14	15-19	20-49		10-14	15-19	20-49		10-14	15-19	20-49	
Legaspi City	0	29	908	937	0	159	11,091	11,250	0	96	4,939	5,035
Naga City	0	33	665	698	0	36	11,376	11,412	0	36	3,004	3,040
**Region 6**	**136**	**1,085**	**12,971**	**14,192**	**25**	**4,884**	**259,499**	**264,408**	**18**	**4,014**	**116,374**	**120,406**
Aklan	2	100	1,889	1,991	2	294	16,050	16,346	2	553	12,069	12,624
Antique	0	66	838	904	0	217	14,790	15,007	0	354	12,532	12,886
Capiz	0	52	950	1,002	10	389	34,828	35,227	0	472	18,140	18,612
Guimaras	0	11	254	265	0	83	9,701	9,784	0	59	2,103	2,162
Iloilo	1	129	3,018	3,148	10	600	68,096	68,706	6	660	28,947	29,613
Negros Occidental	5	661	5,232	5,898	3	2,807	80,927	83,737	10	1,547	31,565	33,122
Bacolod City	128	28	508	664	0	170	5,880	6,050	0	137	4,632	4,769
Iloilo City	0	38	282	320	0	324	29,227	29,551	0	232	6,386	6,618
**Region 7**	**11**	**1,203**	**13,926**	**15,140**	**5**	**2,312**	**130,785**	**133,102**	**14**	**3,336**	**84,630**	**87,980**
Bohol	6	130	1,393	1,529	2	221	24,456	24,679	3	417	11,087	11,507
Cebu	4	405	4,288	4,697	1	602	48,654	49,257	1	916	27,768	28,685
Negros Oriental	1	205	1,417	1,623	2	753	35,347	36,102	3	1,062	23,231	24,296
Siquijor	0	40	392	432	0	40	3,371	3,411	0	86	2,423	2,509
Cebu City	0	159	1,414	1,573	0	522	6,016	6,538	2	522	5,752	6,276
Lapu-Lapu City	0	236	4,706	4,942	0	158	4,995	5,153	5	290	8,239	8,534
Mandaue City	0	28	316	344	0	16	7,946	7,962	0	43	6,130	6,173
**Region 8**	**3**	**808**	**14,498**	**15,309**	**8**	**2,336**	**126,847**	**129,191**	**9**	**2,098**	**56,993**	**59,100**
Biliran	0	46	658	704	0	94	4,789	4,883	0	81	1,409	1,490
Eastern Samar	0	55	2,177	2,232	3	335	14,430	14,768	5	271	6,267	6,543
Northern Leyte	0	176	5,559	5,735	0	683	44,061	44,744	1	594	17,807	18,402
Northern Samar	2	101	1,688	1,791	3	243	17,841	18,087	3	163	8,826	8,992
Southern Leyte	0	19	355	374	0	80	9,789	9,869	0	121	2,901	3,022
Western Samar	1	-4	284	281	2	122	10,038	10,162	0	107	5,640	5,747
Calbayog City	0	18	769	787	0	38	3,318	3,356	0	37	1,484	1,521
Maasin City	0	5	190	195	0	6	1,495	1,501	0	6	434	440
Ormoc City	0	89	595	684	0	164	9,114	9,278	0	121	1,898	2,019
Tacloban City	0	303	2,223	2,526	0	571	11,972	12,543	0	597	10,327	10,924
**Region 9**	**13**	**776**	**107,177**	**107,966**	**12**	**2,280**	**36,786**	**39,078**	**13**	**1,708**	**57,330**	**59,051**
Zamboanga del Norte	2	121	40,327	40,450	0	385	6,337	6,722	3	452	19,607	20,062
Zamboanga del Sur	0	221	27,960	28,181	2	551	6,461	7,014	1	509	8,542	9,052
Zamboanga Sibugay	0	185	2,295	2,480	9	556	13,925	14,490	7	502	7,907	8,416
Dapitan City	0	12	3,676	3,688	0	23	6	29	0	14	695	709
Dipolog City	10	30	7,709	7,749	1	13	571	585	1	18	1,398	1,417
Isabela City	0	85	200	285	0	49	2,553	2,602	0	79	2,083	2,162
Pagadian City	0	80	991	1,071	0	477	6,700	7,177	0	73	3,426	3,499
Zamboanga City	1	42	24,019	24,062	0	226	233	459	1	61	13,672	13,734
**Region 10**	**32**	**2,097**	**17,993**	**20,122**	**40**	**5,752**	**177,716**	**183,508**	**44**	**3,485**	**63,151**	**66,680**
Bukidnon	6	590	3,233	3,829	3	1,916	46,227	48,146	9	737	10,549	11,295
Camiguin	0	2	35	37	2	48	2,668	2,718	0	10	686	696
Lanao del Norte	21	519	4,421	4,961	9	592	23,956	24,557	15	971	12,337	13,323
Misamis Occidental	2	107	1,418	1,527	2	287	11,377	11,666	3	63	2,917	2,983
Misamis Oriental	-2	105	1,830	1,933	2	486	25,258	25,746	0	164	9,013	9,177
Cagayan de Oro City	1	90	1,390	1,481	0	407	15,750	16,157	0	441	9,955	10,396
El Salvador City	0	29	174	203	0	52	1,454	1,506	0	72	1,019	1,091
Gingoog City	1	258	1,427	1,686	1	514	8,000	8,515	0	218	3,044	3,262
Iligan City	3	92	804	899	2	356	9,202	9,560	5	339	5,642	5,986
Malaybalay City	0	54	247	301	0	200	9,473	9,673	0	74	919	993
Oroquieta City	0	57	675	732	0	109	2,143	2,252	0	20	1,189	1,209
Ozamis City	0	75	580	655	0	480	10,541	11,021	2	290	3,773	4,065
Tangub City	0	25	1,318	1,343	0	12	1,177	1,189	0	33	56	89
Valencia City	0	94	441	535	19	293	10,490	10,802	10	53	2,052	2,115

Table 1.A.4 - MODERN METHOD OF FAMILY PLANNING

Current User (Ending)

Philippines, 2021

Area	PILLS-POP			Total	PILLS-COC			Total	INJECTABLES			Total
	Age group				Age group				Age group			
	10-14	15-19	20-49		10-14	15-19	20-49		10-14	15-19	20-49	
**Region 11**	**143**	**3,412**	**26,952**	**30,507**	**121**	**8,056**	**215,649**	**223,826**	**266**	**6,213**	**76,505**	**82,984**
Davao de Oro	21	634	3,297	3,952	20	2,118	41,623	43,761	20	889	10,123	11,032
Davao del Norte	25	692	7,150	7,867	71	1,019	43,417	44,507	36	901	13,293	14,230
Davao Oriental	14	159	2,058	2,231	10	544	24,893	25,447	14	254	6,294	6,562
Davao del Sur	39	515	3,496	4,050	10	1,145	36,899	38,054	163	1,210	11,585	12,958
Davao Occidental	1	91	542	634	9	771	11,259	12,039	14	1,051	7,123	8,188
Davao City	43	1,321	10,409	11,773	1	2,459	57,558	60,018	19	1,908	28,087	30,014
**Region 12**	**9**	**4,929**	**49,397**	**54,335**	**10**	**6,466**	**164,218**	**170,694**	**12**	**7,678**	**96,766**	**104,456**
North Cotabato	8	2,375	18,110	20,493	7	1,935	58,356	60,298	6	1,893	28,924	30,823
Sarangani	0	790	2,989	3,779	0	1,727	29,332	31,059	0	1,345	12,011	13,356
South Cotabato	1	466	3,271	3,738	2	1,138	36,359	37,499	4	1,281	15,270	16,555
Sultan Kudarat	0	719	16,981	17,700	0	1,205	21,321	22,526	0	2,303	18,508	20,811
Cotabato City	0	92	1,066	1,158	0	278	11,444	11,722	1	500	10,106	10,607
Gen. Santos City	0	487	6,980	7,467	1	183	7,406	7,590	1	356	11,947	12,304
**B.A.R.M.M.**	**30**	**1,087**	**9,184**	**10,301**	**16**	**3,082**	**73,912**	**77,010**	**20**	**4,058**	**97,264**	**101,342**
Basilan	21	180	388	589	0	269	3,364	3,633	3	399	3,152	3,554
Lanao del Sur	4	75	1,544	1,623	0	84	6,578	6,662	0	163	6,439	6,602
Maguindanao	2	402	2,995	3,399	2	1,088	24,752	25,842	1	1,854	32,557	34,412
Sulu	0	337	3,454	3,791	2	1,273	25,066	26,341	8	1,181	41,810	42,999
Tawi-Tawi	1	22	95	118	10	86	10,757	10,853	0	51	9,553	9,604
Lamitan City	2	50	315	367	2	254	2,320	2,576	8	384	2,825	3,217
Marawi City	0	21	393	414	0	28	1,075	1,103	0	26	928	954
**CARAGA**	**10**	**1,400**	**12,438**	**13,848**	**8**	**2,710**	**106,662**	**109,380**	**12**	**1,908**	**37,698**	**39,618**
Agusan del Norte	6	337	2,478	2,821	2	316	14,211	14,529	4	293	5,742	6,039
Agusan del Sur	3	362	2,521	2,886	4	770	34,324	35,098	2	558	10,960	11,520
Surigao del Norte	1	127	2,963	3,091	0	267	13,778	14,045	5	215	5,051	5,271
Surigao del Sur	0	223	2,161	2,384	1	463	18,511	18,975	0	264	4,318	4,582
Province of Dinagat	0	51	262	313	1	85	3,641	3,727	0	64	1,550	1,614
Bislig City	0	50	427	477	0	122	3,656	3,778	0	48	763	811
Butuan City	0	223	1,498	1,721	0	565	13,674	14,239	1	408	6,140	6,549
Surigao City	0	27	128	155	0	122	4,867	4,989	0	58	3,174	3,232

Table 1.A.4 - MODERN METHOD OF FAMILY PLANNING

Current User (Ending)

Philippines, 2021

Area	IMPLANTS			Total	NFP-CMM			Total	NFP-BBT			Total
	Age group				Age group				Age group			
	10-14	15-19	20-49		10-14	15-19	20-49		10-14	15-19	20-49	
**PHILIPPINES**	**832**	**28,927**	**463,920**	**493,679**	**129**	**918**	**49,371**	**50,418**	**51**	**322**	**3,881**	**4,254**
**N C R**	**212**	**7,916**	**60,368**	**68,496**	**0**	**2**	**413**	**415**	**2**	**53**	**343**	**398**
Malabon	0	528	1,992	2,520	0	0	0	0	0	0	0	0
Navotas	1	108	974	1,083	0	0	1	1	0	0	0	0
Valenzuela City	52	1,656	10,093	11,801	0	0	1	1	0	0	0	0
Caloocan City	7	583	6,443	7,033	0	0	11	11	0	0	0	0
Marikina City	5	179	1,831	2,015	0	2	1	3	0	0	0	0
Pasig City	6	300	3,565	3,871	0	0	11	11	0	0	0	0
Pateros	0	1	54	55	0	0	0	0	0	0	0	0
Taguig	2	19	2,617	2,638	0	0	0	0	0	0	0	0
Quezon City	46	1,774	13,840	15,660	0	0	13	13	0	0	0	0
Makati City	1	83	1,232	1,316	0	0	0	0	0	0	0	0
Mandaluyong City	0	37	1,147	1,184	0	0	318	318	0	0	0	0
San Juan	0	0	95	95	0	0	0	0	0	0	0	0
Manila City	87	1,936	9,011	11,034	0	0	0	0	2	53	343	398
Las Piñas City	2	312	2,769	3,083	0	0	0	0	0	0	0	0
Muntinlupa City	1	99	1,625	1,725	0	0	0	0	0	0	0	0
Parañaque City	2	301	1,714	2,017	0	0	0	0	0	0	0	0
Pasay City	0	0	1,366	1,366	0	0	57	57	0	0	0	0
**C A R**	**4**	**350**	**7,875**	**8,229**	**0**	**111**	**2,630**	**2,741**	**0**	**4**	**181**	**185**
Abra	3	62	949	1,014	0	0	178	178	0	0	135	135
Apayao	0	20	644	664	0	0	2	2	0	0	0	0
Benguet	0	18	992	1,010	0	0	7	7	0	0	0	0
Ifugao	0	38	1,272	1,310	0	1	740	741	0	2	7	9
Kalinga	0	33	1,042	1,075	0	110	1,568	1,678	0	2	34	36
Mt. Province	0	14	558	572	0	0	135	135	0	0	5	5
Baguio City	1	165	2,418	2,584	0	0	0	0	0	0	0	0
**Region 1**	**0**	**989**	**15,009**	**15,998**	**0**	**13**	**1,839**	**1,852**	**0**	**17**	**312**	**329**
Ilocos Norte	0	22	603	625	0	3	373	376	0	1	22	23
Ilocos Sur	0	20	467	487	0	0	679	679	0	5	28	33
La Union	0	128	1,508	1,636	0	0	320	320	0	0	16	16
Pangasinan	0	539	10,373	10,912	0	0	4	4	0	11	18	29
Alaminos City	0	190	285	475	0	0	0	0	0	0	0	0
Candon City	0	0	4	4	0	0	44	44	0	0	24	24
Dagupan City	0	21	500	521	0	0	0	0	0	0	4	4
Laoag City	0	0	4	4	0	0	0	0	0	0	0	0
San Carlos City	0	12	384	396	0	0	0	0	0	0	0	0
San Fernando City	0	27	501	528	0	0	0	0	0	0	0	0
Urdaneta City	0	28	377	405	0	0	0	0	0	0	0	0
Vigan City	0	2	3	5	0	10	419	429	0	0	200	200
**Region 2**	**3**	**477**	**9,827**	**10,307**	**0**	**0**	**216**	**216**	**0**	**8**	**52**	**60**
Batanes	0	0	3	3	0	0	78	78	0	0	0	0
Cagayan	3	107	2,574	2,684	0	0	24	24	0	1	16	17
Isabela	0	250	4,448	4,698	0	0	6	6	0	6	35	41
Nueva Vizcaya	0	63	1,481	1,544	0	0	84	84	0	1	1	2
Quirino	0	0	363	363	0	0	24	24	0	0	0	0
Cauayan City	0	5	182	187	0	0	0	0	0	0	0	0
Ilagan City	0	29	221	250	0	0	0	0	0	0	0	0
Santiago City	0	14	426	440	0	0	0	0	0	0	0	0
Tuguegarao City	0	9	129	138	0	0	0	0	0	0	0	0
**Region 3**	**29**	**1,947**	**33,459**	**35,435**	**0**	**40**	**1,082**	**1,122**	**0**	**19**	**81**	**100**
Aurora	1	22	1,112	1,135	0	5	717	722	0	0	10	10
Bataan	12	264	3,290	3,566	0	0	39	39	0	1	9	10

Table 1.A.4 - MODERN METHOD OF FAMILY PLANNING

Current User (Ending)

Philippines, 2021

Area	IMPLANTS Age group			Total	NFP-CMM Age group			Total	NFP-BBT Age group			Total
	10-14	15-19	20-49		10-14	15-19	20-49		10-14	15-19	20-49	
Bulacan	0	209	6,923	7,132	0	0	9	9	0	0	4	4
Nueva Ecija	9	732	5,503	6,244	0	14	4	18	0	0	0	0
Pampanga	0	119	3,463	3,582	0	20	265	285	0	7	23	30
Tarlac	1	54	1,549	1,604	0	0	0	0	0	0	0	0
Zambales	1	111	2,375	2,487	0	0	28	28	0	1	1	2
Angeles City	0	75	1,445	1,520	0	0	0	0	0	2	1	3
Balanga City	0	25	126	151	0	0	0	0	0	0	0	0
Cabanatuan City	0	8	259	267	0	0	0	0	0	0	0	0
City of San Fernando	0	2	261	263	0	0	1	1	0	6	30	36
Gapan City	0	36	342	378	0	0	1	1	0	0	0	0
Mabalacat City	0	13	460	473	0	0	0	0	0	0	0	0
Malolos City	0	3	390	393	0	0	0	0	0	0	0	0
Meycauayan	0	26	359	385	0	1	18	19	0	0	0	0
Olongapo	5	150	1,133	1,288	0	0	0	0	0	0	0	0
Palayan City	0	0	0	0	0	0	0	0	0	0	0	0
San Jose City	0	16	273	289	0	0	0	0	0	0	0	0
San Jose del Monte City	0	53	3,628	3,681	0	0	0	0	0	0	0	0
Science City of Munoz	0	0	27	27	0	0	0	0	0	0	0	0
Tarlac City	0	29	541	570	0	0	0	0	0	2	3	5
**Region 4A**	**42**	**1,155**	**26,568**	**27,765**	**9**	**14**	**5,948**	**5,971**	**9**	**13**	**161**	**183**
Batangas	28	12	763	803	0	0	4,731	4,731	0	0	-2	-2
Cavite	2	104	3,322	3,428	0	0	10	10	0	0	0	0
Laguna	1	149	3,085	3,235	0	3	328	331	0	0	0	0
Quezon	0	36	1,577	1,613	0	0	46	46	0	0	101	101
Rizal	1	111	1,953	2,065	0	0	32	32	0	8	6	14
Antipolo City	0	0	237	237	0	0	150	150	0	0	14	14
Bacoor City	0	51	613	664	0	0	0	0	0	0	0	0
Batangas City	0	19	233	252	0	0	0	0	0	0	0	0
Biñan City	0	48	493	541	0	0	0	0	0	0	0	0
Cabuyao City	0	4	229	233	0	0	0	0	0	1	0	1
Calamba City	1	84	1,588	1,673	0	0	10	10	0	0	0	0
Cavite City	0	18	502	520	0	1	0	1	0	3	0	3
Dasmariñas City	0	429	7,875	8,304	0	0	18	18	0	0	19	19
General Trias City	0	9	408	417	0	0	0	0	0	0	2	2
Imus City	0	12	338	350	0	0	0	0	0	0	0	0
Lipa City	0	3	197	200	0	0	0	0	0	0	11	11
Lucena City	0	0	45	45	0	0	0	0	0	0	0	0
San Pablo City	0	11	105	116	0	0	0	0	0	1	1	2
San Pedro City	0	22	636	658	0	10	91	101	0	0	8	8
Santa Rosa City	9	25	1,231	1,265	9	0	0	9	9	0	0	9
Tagaytay City	0	1	468	469	0	0	0	0	0	0	0	0
Tanauan City	0	0	46	46	0	0	0	0	0	0	1	1
Tayabas City	0	0	155	155	0	0	532	532	0	0	0	0
Trece Martires City	0	7	469	476	0	0	0	0	0	0	0	0
**Region 4B**	**3**	**340**	**21,811**	**22,154**	**102**	**146**	**5,082**	**5,330**	**0**	**3**	**108**	**111**
Marinduque	0	17	755	772	0	0	78	78	0	0	1	1
Mindoro Occidental	2	89	2,096	2,187	0	41	2,169	2,210	0	1	74	75
Mindoro Oriental	0	78	2,554	2,632	0	0	321	321	0	0	16	16
Palawan	1	126	13,383	13,510	101	105	509	715	0	1	11	12
Romblon	0	30	1,197	1,227	1	0	1,993	1,994	0	1	6	7
Puerto Princesa City	0	0	1,826	1,826	0	0	12	12	0	0	0	0
**Region 5**	**6**	**1,062**	**28,282**	**29,350**	**17**	**196**	**17,127**	**17,340**	**0**	**9**	**540**	**549**
Albay	3	233	4,436	4,672	0	10	1,574	1,584	0	2	82	84
Camarines Norte	0	63	1,471	1,534	0	1	149	150	0	0	16	16
Camarines Sur	1	374	9,890	10,265	3	16	2,679	2,698	0	5	245	250
Catanduanes	1	63	2,002	2,066	0	17	1,641	1,658	0	0	0	0
Masbate	1	193	5,288	5,482	14	47	4,608	4,669	0	1	15	16
Sorsogon	0	76	3,104	3,180	0	104	6,110	6,214	0	1	11	12
Iriga City	0	27	799	826	0	1	255	256	0	0	131	131

Table 1.A.4 - MODERN METHOD OF FAMILY PLANNING

Current User (Ending)

Philippines, 2021

Area	IMPLANTS			Total	NFP-CMM			Total	NFP-BBT			Total
	Age group				Age group				Age group			
	10-14	15-19	20-49		10-14	15-19	20-49		10-14	15-19	20-49	
Legaspi City	0	27	610	637	0	0	44	44	0	0	0	0
Naga City	0	6	682	688	0	0	67	67	0	0	40	40
**Region 6**	**8**	**1,109**	**27,025**	**28,142**	**0**	**75**	**4,555**	**4,630**	**40**	**13**	**188**	**241**
Aklan	2	21	1,513	1,536	0	1	63	64	0	0	2	2
Antique	0	61	1,851	1,912	0	72	4,282	4,354	0	0	7	7
Capiz	0	48	2,172	2,220	0	1	94	95	0	1	1	2
Guimaras	0	10	220	230	0	0	0	0	0	0	6	6
Iloilo	1	221	7,895	8,117	0	0	50	50	0	2	84	86
Negros Occidental	2	578	10,200	10,780	0	0	36	36	0	4	54	58
Bacolod City	3	96	1,294	1,393	0	0	29	29	40	6	29	75
Iloilo City	0	74	1,880	1,954	0	1	1	2	0	0	5	5
**Region 7**	**5**	**1,556**	**39,323**	**40,884**	**0**	**1**	**246**	**247**	**0**	**22**	**151**	**173**
Bohol	0	402	7,476	7,878	0	0	4	4	0	0	6	6
Cebu	0	692	20,675	21,367	0	0	16	16	0	22	91	113
Negros Oriental	4	111	2,967	3,082	0	1	224	225	0	0	54	54
Siquijor	0	16	368	384	0	0	0	0	0	0	0	0
Cebu City	0	262	3,152	3,414	0	0	2	2	0	0	0	0
Lapu-Lapu City	0	52	1,235	1,287	0	0	0	0	0	0	0	0
Mandaue City	1	21	3,450	3,472	0	0	0	0	0	0	0	0
**Region 8**	**1**	**810**	**29,172**	**29,983**	**0**	**6**	**1,777**	**1,783**	**0**	**9**	**310**	**319**
Biliran	0	56	848	904	0	0	76	76	0	1	1	2
Eastern Samar	0	50	2,032	2,082	0	0	95	95	0	3	16	19
Northern Leyte	1	232	10,968	11,201	0	5	184	189	0	5	170	175
Northern Samar	0	107	4,127	4,234	0	0	114	114	0	0	0	0
Southern Leyte	0	38	1,595	1,633	0	0	103	103	0	0	6	6
Western Samar	0	19	3,609	3,628	0	1	1,102	1,103	0	0	62	62
Calbayog City	0	4	558	562	0	0	0	0	0	0	0	0
Maasin City	0	9	330	339	0	0	100	100	0	0	45	45
Ormoc City	0	53	1,721	1,774	0	0	3	3	0	0	10	10
Tacloban City	0	242	3,384	3,626	0	0	0	0	0	0	0	0
**Region 9**	**400**	**1,513**	**28,719**	**30,632**	**0**	**3**	**123**	**126**	**0**	**2**	**25**	**27**
Zamboanga del Norte	3	262	8,111	8,376	0	0	112	112	0	0	5	5
Zamboanga del Sur	4	644	9,029	9,677	0	0	0	0	0	1	2	3
Zamboanga Sibugay	393	455	4,694	5,542	0	0	7	7	0	1	17	18
Dapitan City	0	2	348	350	0	0	0	0	0	0	0	0
Dipolog City	0	24	894	918	0	0	0	0	0	0	0	0
Isabela City	0	57	2,090	2,147	0	0	0	0	0	0	0	0
Pagadian City	0	19	962	981	0	0	0	0	0	0	0	0
Zamboanga City	0	50	2,591	2,641	0	3	4	7	0	0	1	1
**Region 10**	**19**	**1,736**	**26,874**	**28,629**	**0**	**212**	**3,991**	**4,203**	**0**	**73**	**808**	**881**
Bukidnon	6	948	10,351	11,305	0	64	941	1,005	0	7	338	345
Camiguin	0	6	147	153	0	0	0	0	0	0	0	0
Lanao del Norte	2	138	2,937	3,077	0	0	77	77	0	0	9	9
Misamis Occidental	0	2	392	394	0	22	48	70	0	45	48	93
Misamis Oriental	0	113	2,643	2,756	0	8	1,500	1,508	0	0	102	102
Cagayan de Oro City	0	134	3,299	3,433	0	0	0	0	0	3	72	75
El Salvador City	1	49	443	493	0	0	0	0	0	0	0	0
Gingoog City	0	105	1,091	1,196	0	96	827	923	0	13	119	132
Iligan City	1	53	1,389	1,443	0	0	124	124	0	0	1	1
Malaybalay City	0	99	1,714	1,813	0	1	5	6	0	0	1	1
Oroquieta City	0	10	194	204	0	0	0	0	0	0	0	0
Ozamis City	0	73	1,474	1,547	0	2	258	260	0	1	3	4
Tangub City	0	0	4	4	0	0	0	0	0	0	0	0
Valencia City	9	6	796	811	0	19	211	230	0	4	115	119

Table 1.A.4 - MODERN METHOD OF FAMILY PLANNING

Current User (Ending)

Philippines, 2021

Area	IMPLANTS			Total	NFP-CMM			Total	NFP-BBT			Total
	Age group				Age group				Age group			
	10-14	15-19	20-49		10-14	15-19	20-49		10-14	15-19	20-49	
**Region 11**	**67**	**2,406**	**35,324**	**37,797**	**1**	**84**	**2,295**	**2,380**	**-1**	**4**	**317**	**320**
Davao de Oro	13	463	5,278	5,754	0	12	448	460	0	0	41	41
Davao del Norte	23	438	6,114	6,575	0	28	176	204	0	0	99	99
Davao Oriental	0	100	2,946	3,046	0	7	812	819	0	2	24	26
Davao del Sur	4	268	3,542	3,814	0	20	292	312	-1	0	-7	-8
Davao Occidental	9	283	2,570	2,862	0	0	1	1	0	0	-3	-3
Davao City	18	854	14,874	15,746	1	17	566	584	0	2	163	165
**Region 12**	**6**	**2,977**	**34,193**	**37,176**	**0**	**0**	**588**	**588**	**1**	**59**	**121**	**181**
North Cotabato	3	634	10,669	11,306	0	-4	355	351	0	4	85	89
Sarangani	0	361	4,380	4,741	0	4	61	65	0	5	15	20
South Cotabato	2	937	6,291	7,230	0	0	19	19	1	15	19	35
Sultan Kudarat	0	752	7,723	8,475	0	0	81	81	0	0	2	2
Cotabato City	0	88	1,435	1,523	0	0	56	56	0	0	0	0
Gen. Santos City	1	205	3,695	3,901	0	0	16	16	0	35	0	35
**B.A.R.M.M.**	**17**	**1,435**	**21,978**	**23,430**	**0**	**0**	**16**	**16**	**0**	**4**	**13**	**17**
Basilan	1	129	1,207	1,337	0	0	4	4	0	0	0	0
Lanao del Sur	2	18	1,038	1,058	0	0	4	4	0	0	0	0
Maguindanao	2	571	5,472	6,045	0	0	9	9	0	0	2	2
Sulu	10	471	9,973	10,454	0	0	-8	-8	0	0	0	0
Tawi-Tawi	0	35	2,833	2,868	0	0	3	3	0	0	0	0
Lamitan City	2	205	1,128	1,335	0	0	4	4	0	4	11	15
Marawi City	0	6	327	333	0	0	0	0	0	0	0	0
**CARAGA**	**10**	**1,149**	**18,113**	**19,272**	**0**	**15**	**1,443**	**1,458**	**0**	**10**	**170**	**180**
Agusan del Norte	0	199	2,682	2,881	0	0	39	39	0	0	3	3
Agusan del Sur	0	298	5,659	5,957	0	8	1,009	1,017	0	8	8	16
Surigao del Norte	6	164	2,280	2,450	0	4	152	156	0	2	36	38
Surigao del Sur	1	148	2,548	2,697	0	0	121	121	0	0	122	122
Province of Dinagat	3	75	1,530	1,608	0	0	0	0	0	0	0	0
Bislig City	0	29	436	465	0	3	120	123	0	0	1	1
Butuan City	0	193	2,193	2,386	0	0	2	2	0	0	0	0
Surigao City	0	43	785	828	0	0	0	0	0	0	0	0

Table 1.A.4 - MODERN METHOD OF FAMILY PLANNING

Current User (Ending)

Philippines, 2021

Area	NFP-STM			Total	NFP-SDM			Total	NFP-LAM			Total
	Age group				Age group				Age group			
	10-14	15-19	20-49		10-14	15-19	20-49		10-14	15-19	20-49	
**PHILIPPINES**	**26**	**207**	**4,286**	**4,519**	**31**	**1,924**	**112,545**	**114,500**	**1,218**	**58,737**	**546,310**	**606,265**
**N C R**	**0**	**7**	**32**	**39**	**1**	**3**	**1,740**	**1,744**	**102**	**9,144**	**74,107**	**83,353**
Malabon	0	0	0	0	0	0	4	4	2	942	2,594	3,538
Navotas	0	0	0	0	0	0	10	10	1	350	1,415	1,766
Valenzuela City	0	0	0	0	0	0	16	16	0	176	3,356	3,532
Caloocan City	0	0	0	0	0	3	104	107	3	636	5,096	5,735
Marikina City	0	0	0	0	0	0	2	2	1	163	542	706
Pasig City	0	0	0	0	0	0	18	18	5	232	4,114	4,351
Pateros	0	0	0	0	0	0	0	0	0	7	149	156
Taguig	0	0	0	0	1	0	7	8	13	1,356	16,713	18,082
Quezon City	0	0	0	0	0	0	1,480	1,480	36	1,544	10,594	12,174
Makati City	0	0	0	0	0	0	0	0	0	153	3,272	3,425
Mandaluyong City	0	0	32	32	0	0	4	4	2	149	3,494	3,645
San Juan	0	0	0	0	0	0	10	10	0	10	331	341
Manila City	0	0	0	0	0	0	0	0	0	347	2,877	3,224
Las Piñas City	0	0	0	0	0	0	24	24	1	179	2,141	2,321
Muntinlupa City	0	0	0	0	0	0	0	0	1	1,377	7,562	8,940
Parañaque City	0	7	0	7	0	0	61	61	37	1,297	8,506	9,840
Pasay City	0	0	0	0	0	0	0	0	0	226	1,351	1,577
**C A R**	**0**	**0**	**69**	**69**	**0**	**23**	**4,884**	**4,907**	**92**	**926**	**8,058**	**9,076**
Abra	0	0	24	24	0	8	212	220	69	314	1,254	1,637
Apayao	0	0	0	0	0	0	7	7	1	117	1,057	1,175
Benguet	0	0	0	0	0	4	1,199	1,203	0	116	1,537	1,653
Ifugao	0	0	9	9	0	7	2,208	2,215	0	185	1,594	1,779
Kalinga	0	0	36	36	0	2	426	428	21	133	1,239	1,393
Mt. Province	0	0	0	0	0	2	729	731	1	51	923	975
Baguio City	0	0	0	0	0	0	103	103	0	10	454	464
**Region 1**	**0**	**15**	**369**	**384**	**1**	**138**	**6,335**	**6,474**	**33**	**3,689**	**41,809**	**45,531**
Ilocos Norte	0	0	64	64	0	39	1,058	1,097	1	172	2,562	2,735
Ilocos Sur	0	4	53	57	1	64	3,301	3,366	4	456	7,759	8,219
La Union	0	0	7	7	0	2	776	778	3	391	4,244	4,638
Pangasinan	0	11	27	38	0	30	596	626	22	1,952	18,089	20,063
Alaminos City	0	0	0	0	0	0	0	0	0	265	2,903	3,168
Candon City	0	0	31	31	0	0	28	28	0	0	327	327
Dagupan City	0	0	0	0	0	0	40	40	3	31	691	725
Laoag City	0	0	0	0	0	0	0	0	0	42	1,414	1,456
San Carlos City	0	0	0	0	0	0	0	0	0	32	615	647
San Fernando City	0	0	0	0	0	3	483	486	0	31	427	458
Urdaneta City	0	0	0	0	0	0	52	52	0	287	2,440	2,727
Vigan City	0	0	187	187	0	0	1	1	0	30	338	368
**Region 2**	**0**	**2**	**114**	**116**	**0**	**4**	**482**	**486**	**28**	**1,966**	**15,320**	**17,314**
Batanes	0	0	0	0	0	0	23	23	0	15	112	127
Cagayan	0	0	6	6	0	0	143	143	12	615	3,602	4,229
Isabela	0	0	2	2	0	2	31	33	6	437	3,910	4,353
Nueva Vizcaya	0	2	106	108	0	1	236	237	6	278	3,812	4,096
Quirino	0	0	0	0	0	0	27	27	1	160	932	1,093
Cauayan City	0	0	0	0	0	0	2	2	1	91	542	634
Ilagan City	0	0	0	0	0	0	0	0	2	83	544	629
Santiago City	0	0	0	0	0	0	0	0	0	276	1,558	1,834
Tuguegarao City	0	0	0	0	0	1	20	21	0	11	308	319
**Region 3**	**0**	**0**	**12**	**12**	**0**	**14**	**3,928**	**3,942**	**30**	**4,568**	**44,489**	**49,087**
Aurora	0	0	0	0	0	1	74	75	3	233	1,703	1,939
Bataan	0	0	0	0	0	1	120	121	8	483	3,849	4,340

Table 1.A.4 - MODERN METHOD OF FAMILY PLANNING

Current User (Ending)

Philippines, 2021

Area	NFP-STM Age group 10-14	NFP-STM Age group 15-19	NFP-STM Age group 20-49	Total	NFP-SDM Age group 10-14	NFP-SDM Age group 15-19	NFP-SDM Age group 20-49	Total	NFP-LAM Age group 10-14	NFP-LAM Age group 15-19	NFP-LAM Age group 20-49	Total
Bulacan	0	0	0	0	0	3	2,890	2,893	0	586	7,526	8,112
Nueva Ecija	0	0	0	0	0	1	9	10	4	582	4,041	4,627
Pampanga	0	0	7	7	0	4	593	597	1	263	4,916	5,180
Tarlac	0	0	0	0	0	0	21	21	1	289	5,899	6,189
Zambales	0	0	5	5	0	0	44	44	2	447	2,941	3,390
Angeles City	0	0	0	0	0	0	74	74	1	168	1,353	1,522
Balanga City	0	0	0	0	0	0	19	19	2	44	265	311
Cabanatuan City	0	0	0	0	0	0	0	0	0	192	1,404	1,596
City of San Fernando	0	0	0	0	0	4	79	83	1	170	1,545	1,716
Gapan City	0	0	0	0	0	0	0	0	0	88	583	671
Mabalacat City	0	0	0	0	0	0	5	5	0	177	2,826	3,003
Malolos City	0	0	0	0	0	0	0	0	0	0	12	12
Meycauayan	0	0	0	0	0	0	0	0	1	166	1,328	1,495
Olongapo	0	0	0	0	0	0	0	0	1	75	1,065	1,141
Palayan City	0	0	0	0	0	0	0	0	0	8	25	33
San Jose City	0	0	0	0	0	0	0	0	5	139	882	1,026
San Jose del Monte City	0	0	0	0	0	0	0	0	0	0	0	0
Science City of Munoz	0	0	0	0	0	0	0	0	0	0	0	0
Tarlac City	0	0	0	0	0	0	0	0	0	458	2,326	2,784
**Region 4A**	**9**	**1**	**282**	**292**	**6**	**327**	**6,968**	**7,301**	**100**	**3,346**	**32,639**	**36,085**
Batangas	0	0	0	0	6	122	5,145	5,273	80	215	3,341	3,636
Cavite	0	0	150	150	0	9	180	189	1	136	2,323	2,460
Laguna	0	0	10	10	0	9	1,080	1,089	0	174	1,472	1,646
Quezon	0	0	1	1	0	10	191	201	0	270	3,509	3,779
Rizal	0	0	2	2	0	0	13	13	4	277	2,544	2,825
Antipolo City	0	0	103	103	0	0	25	25	0	0	1,788	1,788
Bacoor City	0	0	0	0	0	0	0	0	1	119	1,465	1,585
Batangas City	0	0	0	0	0	1	75	76	0	8	398	406
Biñan City	0	0	0	0	0	0	14	14	0	647	4,196	4,843
Cabuyao City	0	0	0	0	0	0	0	0	0	72	738	810
Calamba City	0	0	0	0	0	0	45	45	0	73	982	1,055
Cavite City	0	0	0	0	0	7	2	9	0	8	122	130
Dasmariñas City	0	1	0	1	0	123	115	238	0	177	2,433	2,610
General Trias City	0	0	0	0	0	0	0	0	0	4	231	235
Imus City	0	0	0	0	0	0	0	0	0	13	285	298
Lipa City	0	0	0	0	0	0	2	2	0	0	11	11
Lucena City	0	0	0	0	0	0	0	0	0	11	117	128
San Pablo City	0	0	0	0	0	0	0	0	2	47	346	395
San Pedro City	0	0	0	0	0	2	44	46	1	85	1,065	1,151
Santa Rosa City	9	0	0	9	0	0	11	11	9	876	3,565	4,450
Tagaytay City	0	0	0	0	0	44	0	44	1	21	486	508
Tanauan City	0	0	0	0	0	0	0	0	1	3	141	145
Tayabas City	0	0	16	16	0	0	26	26	0	104	1,034	1,138
Trece Martires City	0	0	0	0	0	0	0	0	0	6	47	53
**Region 4B**	**12**	**0**	**41**	**53**	**6**	**30**	**12,522**	**12,558**	**213**	**1,239**	**18,916**	**20,368**
Marinduque	0	0	0	0	0	0	49	49	0	132	1,566	1,698
Mindoro Occidental	0	0	4	4	0	2	8,185	8,187	28	576	3,734	4,338
Mindoro Oriental	0	0	1	1	0	0	17	17	0	173	5,292	5,465
Palawan	12	0	35	47	3	25	3,414	3,442	179	220	5,679	6,078
Romblon	0	0	1	1	3	3	710	716	6	138	1,641	1,785
Puerto Princesa City	0	0	0	0	0	0	147	147	0	0	1,004	1,004
**Region 5**	**0**	**23**	**2,001**	**2,024**	**2**	**633**	**38,845**	**39,480**	**41**	**3,786**	**38,949**	**42,776**
Albay	0	0	41	41	1	30	5,738	5,769	8	496	6,230	6,734
Camarines Norte	0	8	772	780	0	37	1,835	1,872	3	386	3,882	4,271
Camarines Sur	0	14	815	829	0	103	11,363	11,466	18	983	8,302	9,303
Catanduanes	0	0	17	17	0	0	2,312	2,312	0	190	4,033	4,223
Masbate	0	1	8	9	0	404	8,836	9,240	11	1,050	6,316	7,377
Sorsogon	0	0	47	47	1	30	4,828	4,859	1	463	5,444	5,908
Iriga City	0	0	210	210	0	28	2,400	2,428	0	118	983	1,101

Table 1.A.4 - MODERN METHOD OF FAMILY PLANNING

Current User (Ending)

Philippines, 2021

Area	NFP-STM			Total	NFP-SDM			Total	NFP-LAM			Total
	Age group				Age group				Age group			
	10-14	15-19	20-49		10-14	15-19	20-49		10-14	15-19	20-49	
Legaspi City	0	0	54	54	0	0	90	90	0	69	762	831
Naga City	0	0	37	37	0	1	1,443	1,444	0	31	2,997	3,028
**Region 6**	**0**	**3**	**86**	**89**	**2**	**72**	**6,798**	**6,872**	**56**	**3,750**	**42,283**	**46,089**
Aklan	0	3	23	26	0	2	269	271	9	330	3,989	4,328
Antique	0	0	10	10	0	0	307	307	2	173	2,738	2,913
Capiz	0	0	0	0	0	0	6	6	1	141	2,840	2,982
Guimaras	0	0	0	0	0	0	35	35	0	86	1,128	1,214
Iloilo	0	0	29	29	2	9	2,594	2,605	18	880	11,694	12,592
Negros Occidental	0	0	24	24	0	61	3,360	3,421	24	2,105	15,230	17,359
Bacolod City	0	0	0	0	0	0	38	38	2	14	4,462	4,478
Iloilo City	0	0	0	0	0	0	189	189	0	21	202	223
**Region 7**	**0**	**2**	**17**	**19**	**0**	**3**	**1,852**	**1,855**	**50**	**3,969**	**30,492**	**34,511**
Bohol	0	0	0	0	0	0	606	606	2	408	2,533	2,943
Cebu	0	0	0	0	0	1	512	513	20	1,324	7,217	8,561
Negros Oriental	0	2	17	19	0	2	246	248	18	934	7,607	8,559
Siquijor	0	0	0	0	0	0	488	488	0	47	174	221
Cebu City	0	0	0	0	0	0	0	0	10	868	6,736	7,614
Lapu-Lapu City	0	0	0	0	0	0	0	0	0	386	5,851	6,237
Mandaue City	0	0	0	0	0	0	0	0	0	2	374	376
**Region 8**	**0**	**84**	**560**	**644**	**0**	**49**	**5,007**	**5,056**	**17**	**2,584**	**31,455**	**34,056**
Biliran	0	0	50	50	0	0	113	113	2	97	983	1,082
Eastern Samar	0	5	358	363	0	24	3,288	3,312	-3	267	2,551	2,815
Northern Leyte	0	0	1	1	0	5	155	160	0	815	9,484	10,299
Northern Samar	0	0	1	1	0	4	341	345	3	407	8,597	9,007
Southern Leyte	0	0	0	0	0	2	138	140	0	241	1,640	1,881
Western Samar	0	0	0	0	0	0	2	2	12	-17	2,399	2,394
Calbayog City	0	0	0	0	0	0	0	0	1	58	446	505
Maasin City	0	0	0	0	0	0	100	100	2	8	143	153
Ormoc City	0	79	150	229	0	14	869	883	0	203	1,532	1,735
Tacloban City	0	0	0	0	0	0	1	1	0	505	3,680	4,185
**Region 9**	**0**	**0**	**18**	**18**	**2**	**47**	**1,860**	**1,909**	**26**	**2,030**	**23,765**	**25,821**
Zamboanga del Norte	0	0	6	6	2	47	1,681	1,730	13	400	5,161	5,574
Zamboanga del Sur	0	0	0	0	0	0	0	0	4	461	2,169	2,634
Zamboanga Sibugay	0	0	0	0	0	0	120	120	0	548	1,921	2,469
Dapitan City	0	0	0	0	0	0	10	10	0	33	566	599
Dipolog City	0	0	11	11	0	0	11	11	2	14	1,917	1,933
Isabela City	0	0	0	0	0	0	38	38	0	122	1,215	1,337
Pagadian City	0	0	0	0	0	0	0	0	0	16	2,600	2,616
Zamboanga City	0	0	1	1	0	0	0	0	7	436	8,216	8,659
**Region 10**	**0**	**45**	**415**	**460**	**6**	**442**	**13,651**	**14,099**	**125**	**5,125**	**42,781**	**48,031**
Bukidnon	0	0	154	154	2	131	2,549	2,682	26	1,404	8,150	9,580
Camiguin	0	0	1	1	0	0	79	79	3	30	556	589
Lanao del Norte	0	0	5	5	0	1	254	255	1	450	6,160	6,611
Misamis Occidental	0	37	52	89	0	18	512	530	7	265	3,969	4,241
Misamis Oriental	0	0	96	96	0	33	1,795	1,828	16	493	6,371	6,880
Cagayan de Oro City	0	0	0	0	0	0	56	56	10	630	6,020	6,660
El Salvador City	0	0	0	0	0	0	28	28	0	62	310	372
Gingoog City	0	8	104	112	4	65	2,334	2,403	5	544	2,636	3,185
Iligan City	0	0	2	2	0	50	962	1,012	11	383	2,104	2,498
Malaybalay City	0	0	0	0	0	26	2,091	2,117	3	84	600	687
Oroquieta City	0	0	0	0	0	0	259	259	0	74	583	657
Ozamis City	0	0	0	0	0	49	1,691	1,740	2	412	3,390	3,804
Tangub City	0	0	0	0	0	60	498	558	5	1	475	481
Valencia City	0	0	1	1	0	9	543	552	36	293	1,457	1,786

Table 1.A.4 - MODERN METHOD OF FAMILY PLANNING

Current User (Ending)

Philippines, 2021

Area	NFP-STM			Total	NFP-SDM			Total	NFP-LAM			Total
	Age group				Age group				Age group			
	10-14	15-19	20-49		10-14	15-19	20-49		10-14	15-19	20-49	
**Region 11**	**0**	**10**	**198**	**208**	**1**	**37**	**2,548**	**2,586**	**152**	**3,241**	**27,774**	**31,167**
Davao de Oro	0	0	3	3	0	1	222	223	16	229	895	1,140
Davao del Norte	0	1	52	53	0	3	231	234	38	576	3,136	3,750
Davao Oriental	0	3	0	3	0	10	1,396	1,406	16	520	3,073	3,609
Davao del Sur	0	0	13	13	0	2	124	126	14	116	987	1,117
Davao Occidental	0	0	-3	-3	0	16	296	312	5	242	527	774
Davao City	0	6	133	139	1	5	279	285	63	1,558	19,156	20,777
**Region 12**	**5**	**13**	**16**	**34**	**0**	**11**	**681**	**692**	**57**	**4,644**	**27,451**	**32,152**
North Cotabato	0	-2	-21	-23	0	0	158	158	13	1,119	6,018	7,150
Sarangani	0	0	32	32	0	2	83	85	0	1,357	5,782	7,139
South Cotabato	0	0	5	5	0	9	248	257	36	1,000	4,032	5,068
Sultan Kudarat	0	0	0	0	0	0	125	125	0	776	4,316	5,092
Cotabato City	0	0	0	0	0	0	65	65	0	197	1,207	1,404
Gen. Santos City	5	15	0	20	0	0	2	2	8	195	6,096	6,299
**B.A.R.M.M.**	**0**	**0**	**2**	**2**	**0**	**6**	**243**	**249**	**62**	**2,629**	**30,088**	**32,779**
Basilan	0	0	1	1	0	0	21	21	4	88	602	694
Lanao del Sur	0	0	-1	-1	0	2	34	36	-3	364	6,303	6,664
Maguindanao	0	0	0	0	0	4	106	110	18	1,346	12,234	13,598
Sulu	0	0	2	2	0	0	15	15	21	499	5,359	5,879
Tawi-Tawi	0	0	0	0	0	0	56	56	15	58	3,649	3,722
Lamitan City	0	0	0	0	0	0	2	2	7	160	609	776
Marawi City	0	0	0	0	0	0	9	9	0	114	1,332	1,446
**CARAGA**	**0**	**2**	**54**	**56**	**4**	**85**	**4,201**	**4,290**	**34**	**2,101**	**15,934**	**18,069**
Agusan del Norte	0	0	0	0	0	3	177	180	1	255	1,897	2,153
Agusan del Sur	0	0	1	1	0	50	1,359	1,409	12	585	4,027	4,624
Surigao del Norte	0	0	9	9	0	7	1,257	1,264	2	167	1,616	1,785
Surigao del Sur	0	1	44	45	0	14	788	802	10	367	2,931	3,308
Province of Dinagat	0	0	0	0	4	1	244	249	0	74	581	655
Bislig City	0	0	0	0	0	10	235	245	6	152	759	917
Butuan City	0	0	0	0	0	0	24	24	3	408	2,914	3,325
Surigao City	0	1	0	1	0	0	117	117	0	93	1,209	1,302

Table 1.A.5 - MODERN FAMILY PLANNING

No. and proportion of WRA 15-49 yrs old with unmet needs

Philippines, 2021

Area	Estimated No. of WRA (TP x GR Factor)	Total No. of WRA (15-49 yrs old) with Unmet Needs	%
**PHILIPPINES**	**28,354,144**	**930,909**	**3.28**
**N C R**	**3,873,231**	**5,787**	**0.15**
Malabon	**109,940**	0	**0.00**
Navotas	**75,033**	288	**0.38**
Valenzuela City	**186,610**	972	**0.52**
Caloocan City	**476,429**	0	**0.00**
Marikina City	**135,576**	957	**0.71**
Pasig City	**227,179**	1,117	**0.49**
Pateros	**19,202**	0	**0.00**
Taguig	**242,103**	*	**0.00**
Quezon City	**883,127**	0	**0.00**
Makati City	**175,236**	2,266	**1.29**
Mandaluyong City	**116,185**	106	**0.09**
San Juan	**36,748**	*	**0.00**
Manila City	**535,435**	*	**0.00**
Las Piñas City	**177,132**	*	**0.00**
Muntinlupa City	**151,746**	*	**0.00**
Parañaque City	**200,266**	0	**0.00**
Pasay City	**125,284**	81	**0.06**
**C A R**	**476,905**	**7,379**	**1.55**
Abra	**61,877**	4,152	**6.71**
Apayao	**30,855**	95	**0.31**
Benguet	**134,227**	1,216	**0.91**
Ifugao	**53,037**	0	**0.00**
Kalinga	**54,478**	69	**0.13**
Mt. Province	**38,547**	172	**0.45**
Baguio City	**103,884**	1,675	**1.61**
**Region 1**	**1,320,697**	**473**	**0.04**
Ilocos Norte	**128,356**	345	**0.27**
Ilocos Sur	**152,408**	*	**0.00**
La Union	**181,055**	*	**0.00**
Pangasinan	**614,906**	*	**0.00**
Alaminos City	**23,234**	*	**0.00**
Candon City	**16,067**	*	**0.00**
Dagupan City	**44,356**	*	**0.00**
Laoag City	**29,597**	128	**0.43**
San Carlos City	**48,834**	*	**0.00**

Table 1.A.5 - MODERN FAMILY PLANNING
No. and proportion of WRA 15-49 yrs old with unmet needs
Philippines, 2021

Area	Estimated No. of WRA (TP x GR Factor)	Total No. of WRA (15-49 yrs old) with Unmet Needs	%
San Fernando City	**33,175**	*	**0.00**
Urdaneta City	**34,431**	*	**0.00**
Vigan City	**14,278**	*	**0.00**
**Region 2**	**924,169**	**5,363**	**0.58**
Batanes	**4,071**	0	**0.00**
Cagayan	**280,475**	32	**0.01**
Isabela	**317,749**	3,495	**1.10**
Nueva Vizcaya	**121,776**	299	**0.25**
Quirino	**48,900**	123	**0.25**
Cauayan City	**34,765**	626	**1.80**
Ilagan City	**39,079**	273	**0.70**
Santiago City	**36,192**	259	**0.72**
Tuguegarao City	**41,162**	256	**0.62**
**Region 3**	**3,279,355**	**71,413**	**2.18**
Aurora	**57,076**	120	**0.21**
Bataan	**194,565**	1,868	**0.96**
Bulacan	**677,725**	0	**0.00**
Nueva Ecija	**422,093**	11,268	**2.67**
Pampanga	**494,589**	2,864	**0.58**
Tarlac	**281,905**	531	**0.19**
Zambales	**167,126**	380	**0.23**
Angeles City	**124,088**	191	**0.15**
Balanga City	**28,124**	158	**0.56**
Cabanatuan City	**86,389**	768	**0.89**
City of San Fernando	**92,442**	106	**0.11**
Gapan City	**31,530**	0	**0.00**
Mabalacat City	**75,605**	59	**0.08**
Malolos City	**75,700**	1,071	**1.41**
Meycauayan	**62,789**	0	**0.00**
Olongapo	**65,916**	0	**0.00**
Palayan City	**11,729**	32	**0.27**
San Jose City	**39,943**	0	**0.00**
San Jose del Monte City	**172,397**	51,425	**29.83**
Science City of Munoz	**23,289**	0	**0.00**
Tarlac City	**94,335**	572	**0.61**
**Region 4A**	**4,401,403**	**98,549**	**2.24**
Batangas	**567,066**	16,615	**2.93**
Cavite	**444,446**	3,065	**0.69**
Laguna	**306,859**	32,481	**10.58**
Quezon	**467,832**	11,555	**2.47**
Rizal	**645,898**	1,602	**0.25**

Table 1.A.5 - MODERN FAMILY PLANNING
No. and proportion of WRA 15-49 yrs old with unmet needs
Philippines, 2021

Area	Estimated No. of WRA (TP x GR Factor)	Total No. of WRA (15-49 yrs old) with Unmet Needs	%
Antipolo City	**237,903**	0	**0.00**
Bacoor City	**194,720**	325	**0.17**
Batangas City	**100,639**	445	**0.44**
Biñan City	**102,900**	1,244	**1.21**
Cabuyao City	**95,395**	632	**0.66**
Calamba City	**140,427**	687	**0.49**
Cavite City	**33,327**	*	**0.00**
Dasmariñas City	**213,664**	55	**0.03**
General Trias City	**101,899**	304	**0.30**
Imus City	**130,913**	1,345	**1.03**
Lipa City	**101,406**	25,867	**25.51**
Lucena City	**70,897**	131	**0.18**
San Pablo City	**82,214**	132	**0.16**
San Pedro City	**100,666**	78	**0.08**
Santa Rosa City	**109,307**	148	**0.14**
Tagaytay City	**23,077**	7	**0.03**
Tanauan City	**52,893**	1,831	**3.46**
Tayabas City	**26,571**	0	**0.00**
Trece Martires City	**50,484**	0	**0.00**
**Region 4B**	**771,870**	**3,110**	**0.40**
Marinduque	**55,443**	144	**0.26**
Mindoro Occidental	**121,881**	0	**0.00**
Mindoro Oriental	**220,321**	1	**0.00**
Palawan	**234,662**	1,810	**0.77**
Romblon	**69,091**	1,155	**1.67**
Puerto Princesa City	**70,472**	0	**0.00**
**Region 5**	**1,482,490**	**42,876**	**2.89**
Albay	**294,861**	1,125	**0.38**
Camarines Norte	**151,506**	54	**0.04**
Camarines Sur	**419,760**	610	**0.15**
Catanduanes	**64,004**	25,072	**39.17**
Masbate	**220,059**	12,555	**5.71**
Sorsogon	**201,903**	1,102	**0.55**
Iriga City	**28,521**	0	**0.00**
Legaspi City	**51,855**	2,358	**4.55**
Naga City	**50,021**	0	**0.00**
**Region 6**	**1,984,482**	**161,163**	**8.12**
Aklan	**153,288**	38,878	**25.36**
Antique	**144,334**	24,929	**17.27**
Capiz	**206,662**	1,355	**0.66**

Table 1.A.5 - MODERN FAMILY PLANNING
No. and proportion of WRA 15-49 yrs old with unmet needs
Philippines, 2021

Area	Estimated No. of WRA (TP x GR Factor)	Total No. of WRA (15-49 yrs old) with Unmet Needs	%
Guimaras	**46,793**	1,562	**3.34**
Iloilo	**522,575**	11,603	**2.22**
Negros Occidental	**644,843**	68,431	**10.61**
Bacolod City	**145,086**	14,405	**9.93**
Iloilo City	**120,901**	0	**0.00**
**Region 7**	**2,036,382**	**3,286**	**0.16**
Bohol	**323,146**	0	**0.00**
Cebu	**850,626**	0	**0.00**
Negros Oriental	**348,519**	0	**0.00**
Siquijor	**23,986**	0	**0.00**
Cebu City	**267,020**	2,395	**0.90**
Lapu-Lapu City	**118,120**	891	**0.75**
Mandaue City	**104,965**	0	**0.00**
**Region 8**	**1,147,726**	**171,389**	**14.93**
Biliran	**42,954**	1,716	**3.99**
Eastern Samar	**119,489**	46,534	**38.94**
Northern Leyte	**400,786**	50,791	**12.67**
Northern Samar	**160,535**	35,828	**22.32**
Southern Leyte	**85,357**	560	**0.66**
Western Samar	**149,478**	29,917	**20.01**
Calbayog City	**46,052**	3,215	**6.98**
Maasin City	**21,722**	746	**3.43**
Ormoc City	**57,086**	2,078	**3.64**
Tacloban City	**64,267**	4	**0.01**
**Region 9**	**962,241**	**0**	**0.00**
Zamboanga del Norte	**203,562**	*	**0.00**
Zamboanga del Sur	**216,523**	*	**0.00**
Zamboanga Sibugay	**170,121**	*	**0.00**
Dapitan City	**21,020**	*	**0.00**
Dipolog City	**33,351**	*	**0.00**
Isabela City	**34,650**	*	**0.00**
Pagadian City	**53,104**	*	**0.00**
Zamboanga City	**229,910**	*	**0.00**
**Region 10**	**1,282,897**	**8,328**	**0.65**
Bukidnon	**283,051**	236	**0.08**
Camiguin	**21,928**	0	**0.00**
Lanao del Norte	**189,688**	0	**0.00**

Table 1.A.5 - MODERN FAMILY PLANNING
No. and proportion of WRA 15-49 yrs old with unmet needs
Philippines, 2021

Area	Estimated No. of WRA (TP x GR Factor)	Total No. of WRA (15-49 yrs old) with Unmet Needs	%
Misamis Occidental	**82,898**	1,211	**1.46**
Misamis Oriental	**200,700**	0	**0.00**
Cagayan de Oro City	**190,094**	339	**0.18**
El Salvador City	**14,119**	0	**0.00**
Gingoog City	**35,056**	6,414	**18.30**
Iligan City	**96,077**	54	**0.06**
Malaybalay City	**47,179**	0	**0.00**
Oroquieta City	**17,960**	0	**0.00**
Ozamis City	**36,003**	0	**0.00**
Tangub City	**15,998**	0	**0.00**
Valencia City	**52,146**	74	**0.14**
**Region 11**	**1,356,597**	**289,657**	**21.35**
Davao de Oro	**192,069**	45,833	**23.86**
Davao del Norte	**268,636**	64,661	**24.07**
Davao Oriental	**143,503**	67,236	**46.85**
Davao del Sur	**186,349**	60,521	**32.48**
Davao Occidental	**84,990**	8,829	**10.39**
Davao City	**481,050**	42,577	**8.85**
**Region 12**	**1,282,761**	**12,085**	**0.94**
North Cotabato	**391,579**	171	**0.04**
Sarangani	**148,152**	10,731	**7.24**
South Cotabato	**263,438**	633	**0.24**
Sultan Kudarat	**222,932**	0	**0.00**
Cotabato City	**85,558**	211	**0.25**
Gen. Santos City	**171,102**	339	**0.20**
**BARMM**	**1,098,663**	**9,210**	**0.84**
Basilan	**78,086**	1,661	**2.13**
Lanao del Sur	**248,333**	1,108	**0.45**
Maguindanao	**342,940**		**0.00**
Sulu	**240,891**		**0.00**
Tawi-Tawi	**107,522**	1,806	**1.68**
Lamitan City	**21,487**		**0.00**
Marawi City	**59,404**	4,635	**7.80**

Table 1.A.5 - MODERN FAMILY PLANNING

No. and proportion of WRA 15-49 yrs old with unmet needs

Philippines, 2021

Area	Estimated No. of WRA (TP x GR Factor)	Total No. of WRA (15-49 yrs old) with Unmet Needs	%
CARAGA	672,275	40,841	6.08
Agusan del Norte	**94,557**	0	**0.00**
Agusan del Sur	**178,045**	0	**0.00**
Surigao del Norte	**88,665**	1,120	**1.26**
Surigao del Sur	**125,012**	1,551	**1.24**
Province of Dinagat	**31,055**	0	**0.00**
Bislig City	**23,745**	6,733	**28.36**
Butuan City	**89,902**	3,763	**4.19**
Surigao City	**41,294**	27,674	**67.02**

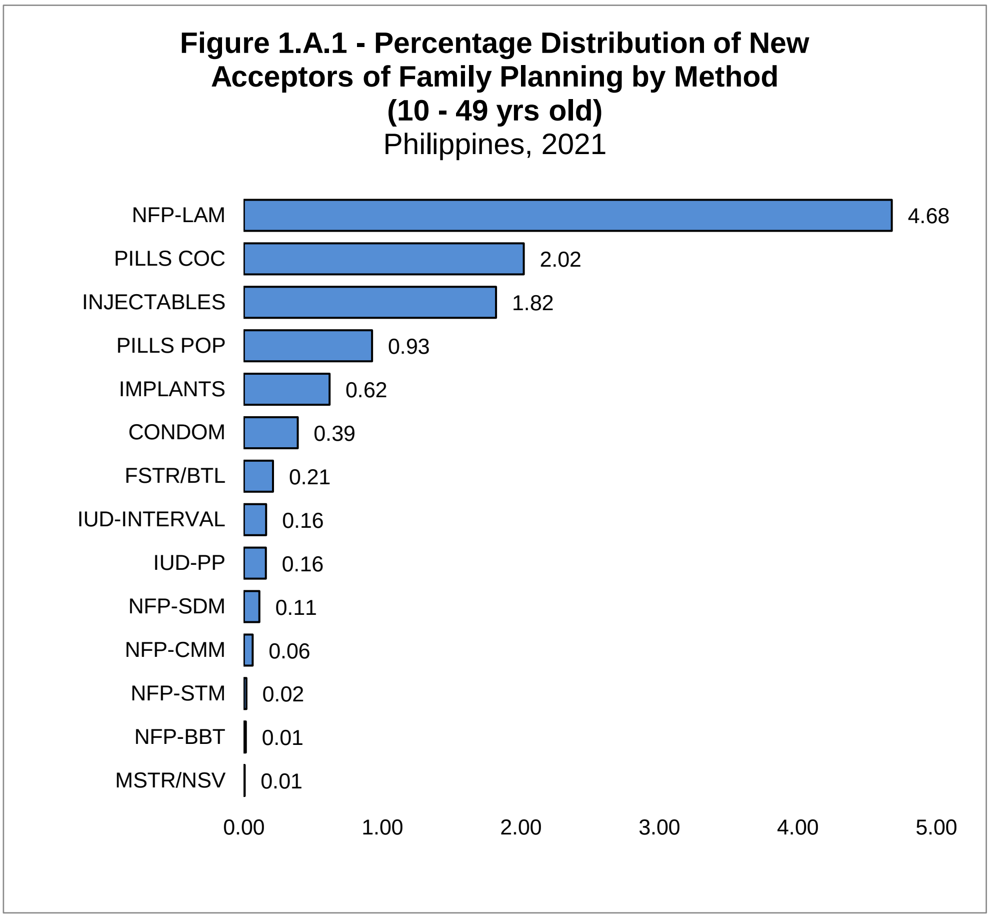
Figure 1.A.1 - Percentage Distribution of New Acceptors of Family Planning by Method
(10 - 49 yrs old)
Philippines, 2021
NFP-LAM
4.68
PILLS COC
2.02
INJECTABLES
1.82
PILLS POP
0.93
IMPLANTS
0.62
CONDOM
0.39
FSTR/BTL
0.21
IUD-INTERVAL
0.16
IUD-PP
0.16
NFP-SDM
0.11
NFP-CMM
0.06
NFP-STM
0.02
NFP-BBT
0.01
MSTR/NSV
0.01
0.00
1.00
2.00
3.00
4.00
5.00

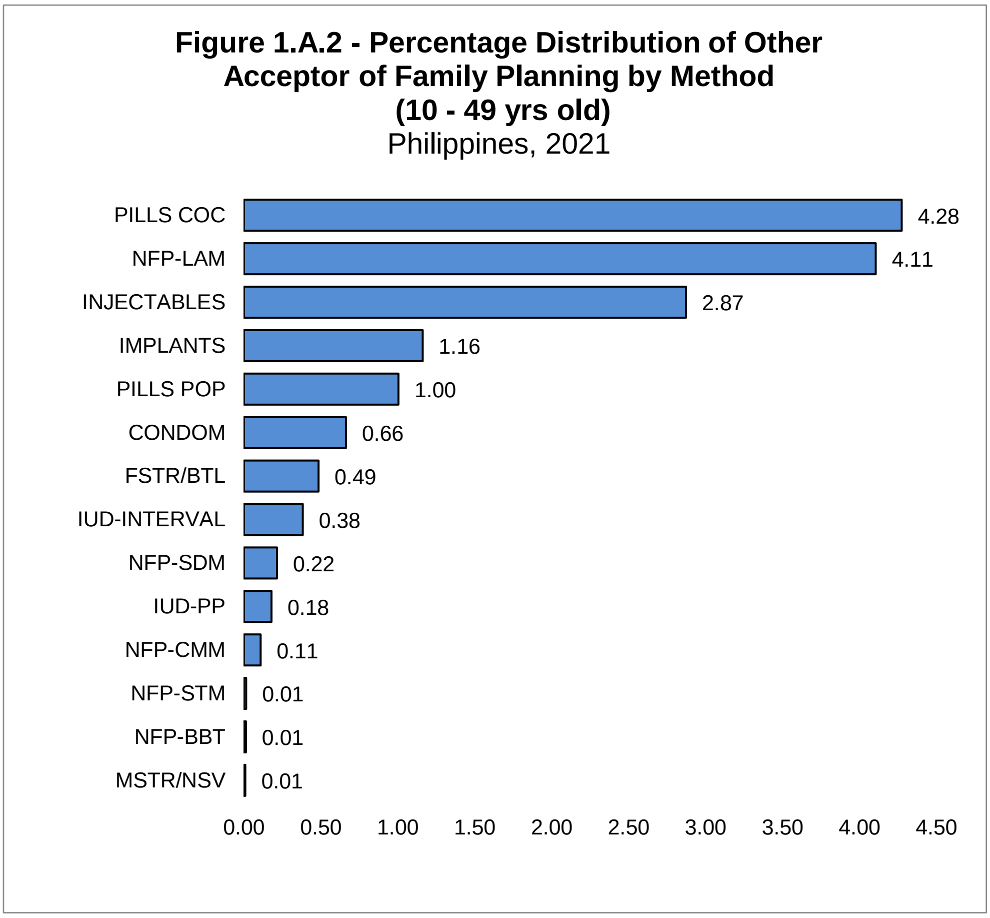
Figure 1.A.2 - Percentage Distribution of Other Acceptor of Family Planning by Method
(10 - 49 yrs old)
Philippines, 2021
PILLS COC
4.28
NFP-LAM
4.11
INJECTABLES
2.87
IMPLANTS
1.16
PILLS POP
1.00
CONDOM
0.66
FSTR/BTL
0.49
IUD-INTERVAL
0.38
NFP-SDM
0.22
IUD-PP
0.18
NFP-CMM
0.11
NFP-STM
0.01
NFP-BBT
0.01
MSTR/NSV
0.01
0.00
0.50
1.00
1.50
2.00
2.50
3.00
3.50
4.00
4.50

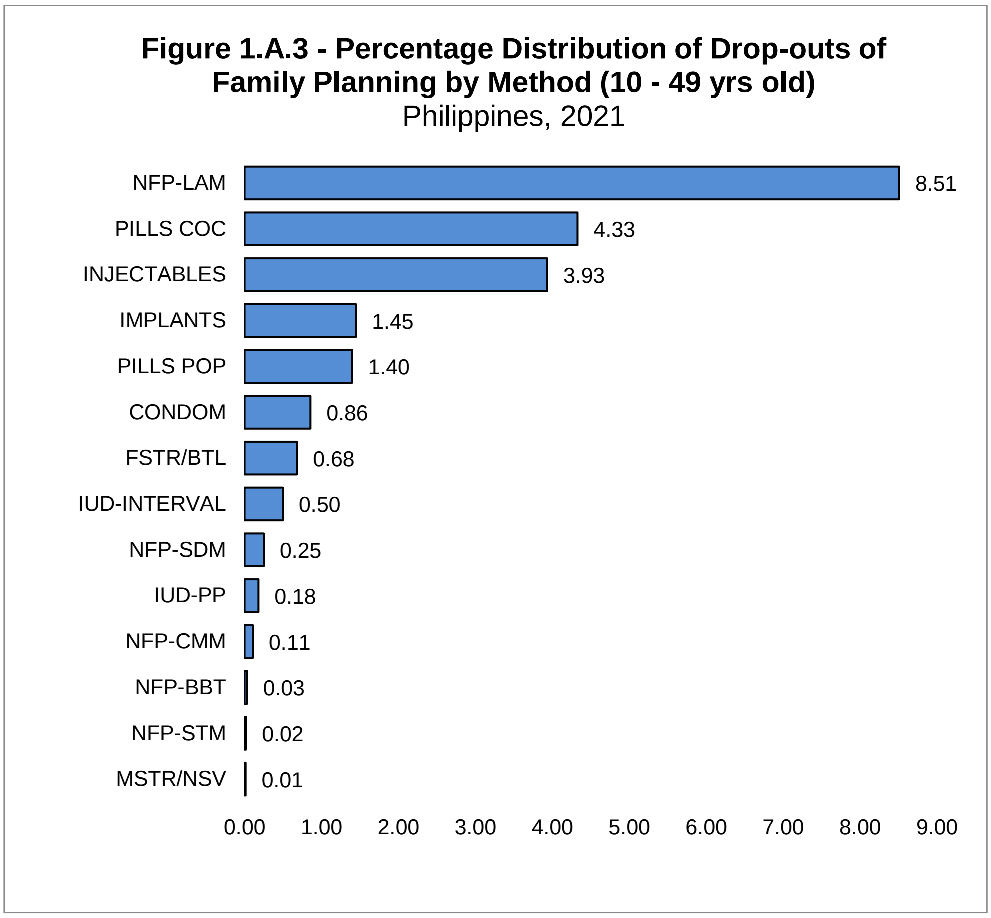

Figure 1.A.3 - Percentage Distribution of Drop-outs of Family Planning by Method (10 - 49 yrs old)
Philippines, 2021
NFP-LAM
8.51
PILLS COC
4.33
INJECTABLES
3.93
IMPLANTS
1.45
PILLS POP
1.40
CONDOM
0.86
FSTR/BTL
0.68
IUD-INTERVAL
0.50
NFP-SDM
0.25
IUD-PP
0.18
NFP-CMM
0.11
NFP-BBT
0.03
NFP-STM
0.02
MSTR/NSV
0.01
0.00
1.00
2.00
3.00
4.00
5.00
6.00
7.00
8.00
9.00

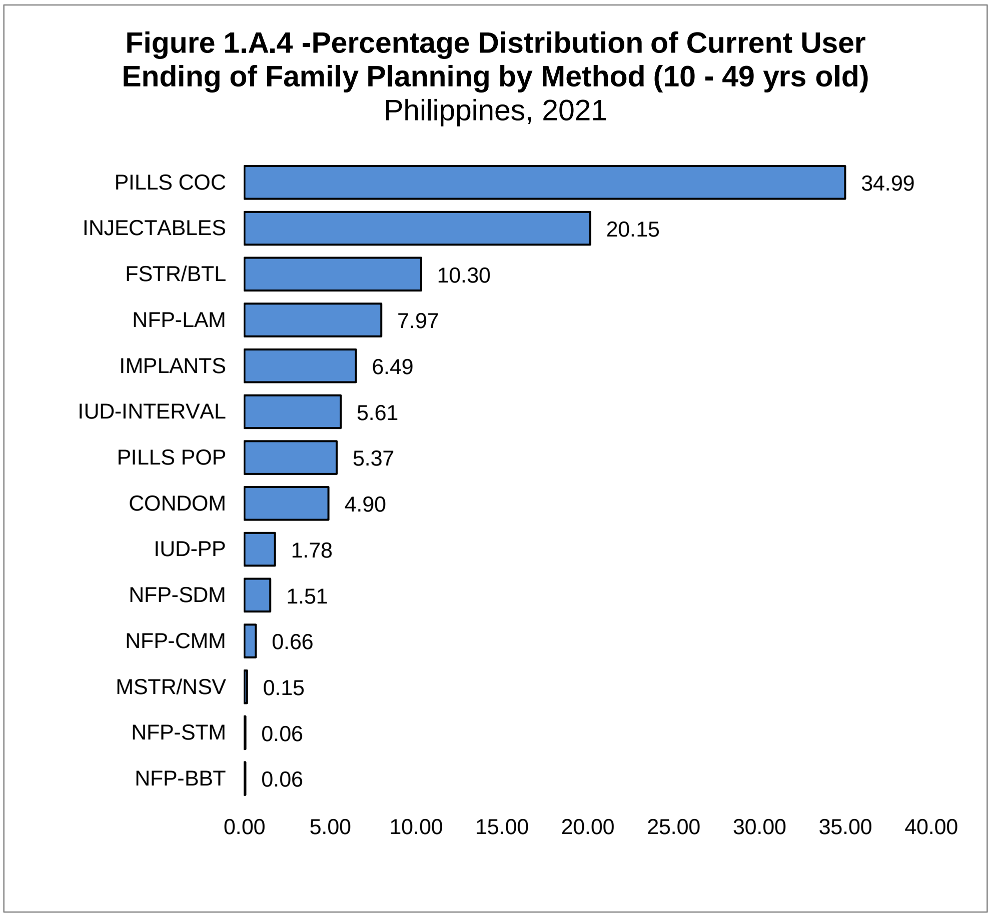

Figure 1.A.4 -Percentage Distribution of Current User Ending of Family Planning by Method (10 - 49 yrs old)
Philippines, 2021
PILLS COC
34.99
INJECTABLES
20.15
FSTR/BTL
10.30
NFP-LAM
7.97
IMPLANTS
6.49
IUD-INTERVAL
5.61
PILLS POP
5.37
CONDOM
4.90
IUD-PP
1.78
NFP-SDM
1.51
NFP-CMM
0.66
MSTR/NSV
0.15
NFP-STM
0.06
NFP-BBT
0.06
0.00
5.00
10.00
15.00
20.00
25.00
30.00
35.00
40.00

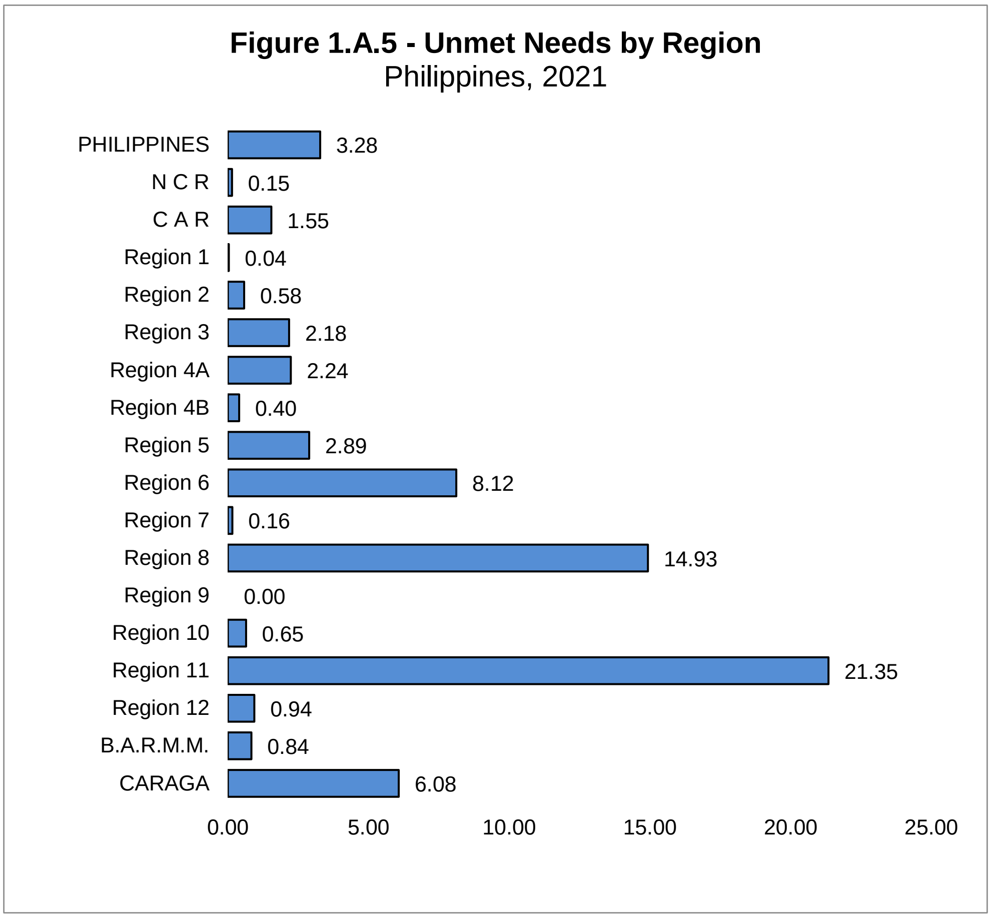
Figure 1.A.5 - Unmet Needs by Region
Philippines, 2021
PHILIPPINES 3.28
N C R 0.15
C A R 1.55
Region 1 0.04
Region 2 0.58
Region 3 2.18
Region 4A 2.24
Region 4B 0.40
Region 5 2.89
Region 6 8.12
Region 7 0.16
Region 8 14.93
Region 9 0.00
Region 10 0.65
Region 11 21.35
Region 12 0.94
B.A.R.M.M. 0.84
CARAGA 6.08
0.00
5.00
10.00
15.00
20.00
25.00

MATERNAL CARE AND SERVICES

FHSIS ANNUAL REPORT 2021

1.B. Maternal Care and Services

- **Prenatal Care**

The proportion of pregnant women with at least 4 or more prenatal check-ups in 2021 was 85.28% which was 53% higher than 2020. The regions with the highest increase in the proportion of pregnant women with at least 4 or more prenatal check-ups were Region 4A at 125%, Region 1 at 77% and Region 7 at 69% *(Table 1.B.1)*.

Out of the pregnant women who had 4ANC in 2021, 2,073 (0.17%) were 10 – 14 years old, while 120,966 (75.46%) were 15 – 19 years old and 945,880 (75.46%) were 20-49 years old.

Table 1.B.1 Proportion of Pregnant Women with at least 4 or more Prenatal Check-ups by Region Philippines, 2020 vs 2021

	2020	2021	% change
PHILIPPINES	55.76	85.28	53%
N C R	59.51	79.41	33%
C A R	53.89	70.34	31%
Region 1	67.17	118.90	77%
Region 2	61.5	94.31	53%
Region 3	64.8	102.79	59%
Region 4A	31.79	71.39	125%
Region 4B	45.87	75.63	65%
Region 5	52.91	69.91	32%
Region 6	52.63	79.52	51%
Region 7	57.08	96.27	69%
Region 8	49.14	79.37	62%
Region 9	60.54	81.41	34%
Region 10	73.83	98.76	34%
Region 11	59.75	78.41	31%
Region 12	65.85	90.30	37%
BARMM	67.6	95.56	41%
CARAGA	55.82	75.29	35%

In 2021, the top 3 regions with the highest proportion of pregnant women (10-49 years old) who have at least 4 prenatal check-ups were from Region 1 at 118.90%, followed by Region 3 at 102.79% and Region 10 at 98.76%. Meanwhile, the lowest proportion of pregnant women who have had at least 4 prenatal check-ups were Region 5 and CAR at 69.91% and 70.34%, respectively.

Figure 1.B.1 Proportion of Women 10-49 years old who gave birth with atleast 4 Prenatal check-up by Region Philippines, 2021

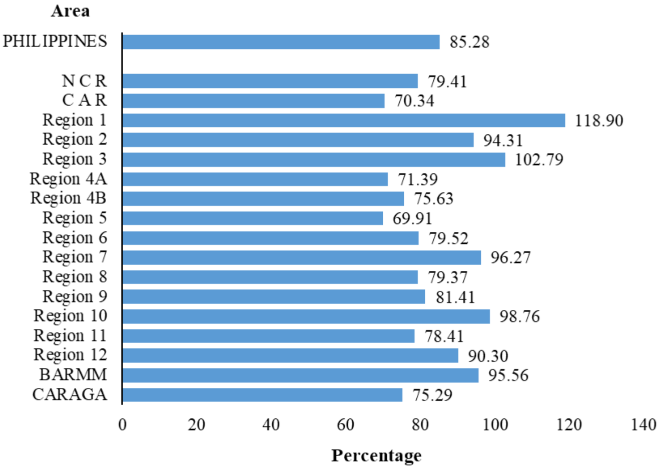

In 2021, 63.55% of the pregnant women seen in the government health centers in the country were given sufficient doses of Tetanus Diphtheria (Td) vaccine. Of this, 530,038 (24.91%) of pregnant women were vaccinated with Td (2 doses) during their first pregnancy and 822,287 (38.64%) were vaccinated with Td (at least 3 doses) during their 2nd and succeeding pregnancies.

Figure 1.B.2 Proportion of Pregnant Women for the 1st, 2nd and more times given Tetanus Diphtheria Vaccine by Region Philippines, 2021

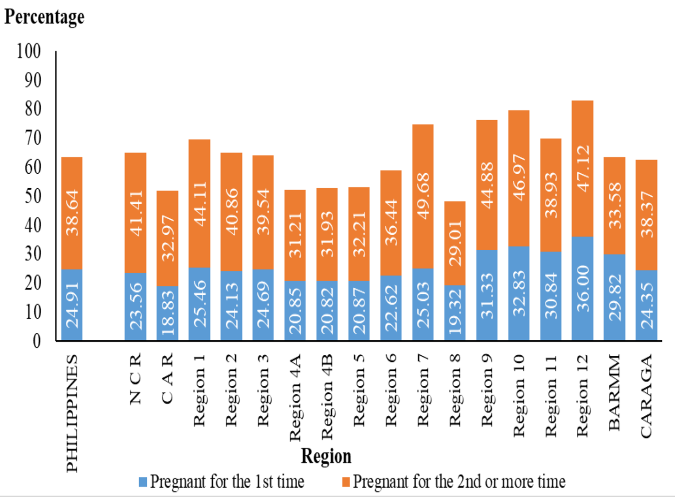

In the 20-49 years old age group, 19.97% were given 2 doses of Tetanus Diphtheria Vaccine on their 1st pregnancy and 35.38% were given at least 3 doses of Tetanus Diphtheria Vaccine on their 2nd and succeeding pregnancies.

Figure 1.B.3 Proportion of Pregnant Women given Tetanus Diphtheria by Age Group Philippines, 2021

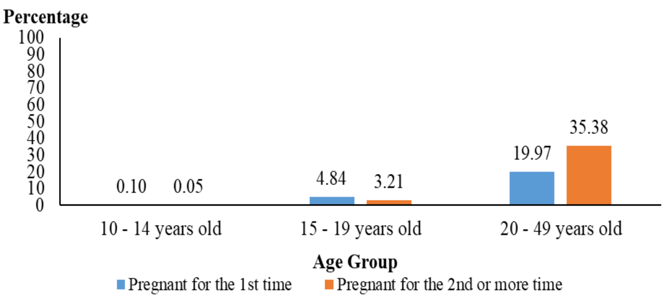

Figure 1.B.4 illustrates a slight decrease both in the proportion of pregnant women who completed doses of Iron and Folic Acid from 50.93% in 2020 to 48.79% in 2021 and on the proportion of postpartum women who completed Iron and Folic Acid supplementation from 52.62% in 2020 to 49.95% in 2021.

Figure 1.B.4 Proportion of Pregnant and Postpartum Women who completed doses of Iron and Folic Acid (FA) Philippines, 2020 vs. 2021

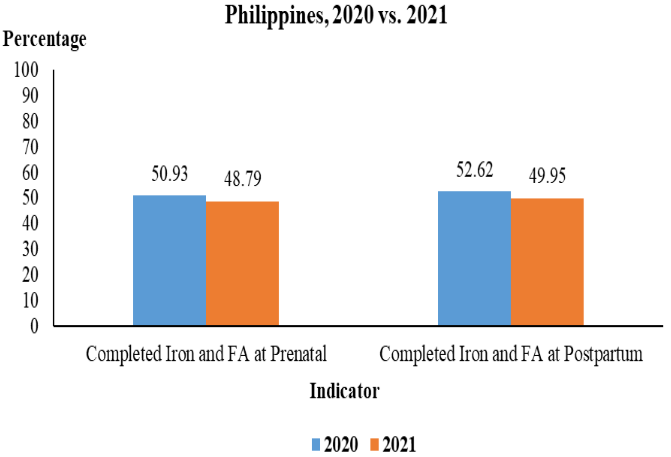

- **Intrapartum Care**

The proportion of deliveries by Physicians had a slight decrease from 56.25% in 2020 to 52.64% in 2021. For the deliveries by Nurses and Midwives, there was a slight increase from 1.44% in 2020 to 1.47% in 2021 for deliveries by Nurses and 32.46% in 2020 to 38.35% in 2021 for deliveries by Midwives.

Figure 1.B.5 Proportion of Deliveries by Skilled Health Professionals Philippines, 2020 vs 2021

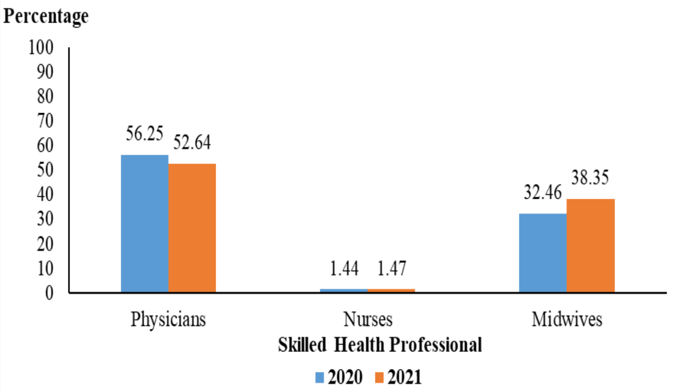

Of the total deliveries in 2021, 1,253,409 (92.46%) were delivered by Skilled Health Professionals; 659,775 (52.64%) were attended by Physicians while 18,386 (1.47%) were delivered by Nurses and 480,698 (38.35%) were delivered by Midwives.

Figure 1.B.6 Percentage Distribution of Deliveries Attended by Skilled Health Professionals Philippines, 2021

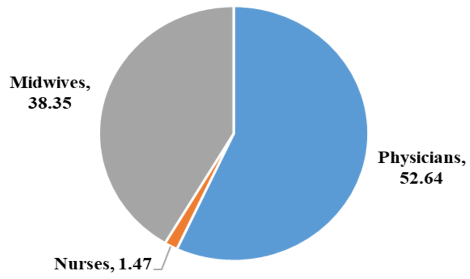

The regions with the highest percentage of deliveries attended by skilled health professional were Region 1 with 48,640 (99.75%) deliveries followed by Region 2 with 39,421 (97.55%). The regions with the lowest percentage of deliveries by skilled health professional was seen in BARMM and Region 4B with 45,153 (72.14%) and 34,222 (83.49%) deliveries, respectively.

Figure 1.B.7 Proportion of Deliveries Attended by Skilled Health Personnel by Region Philippines, 2021

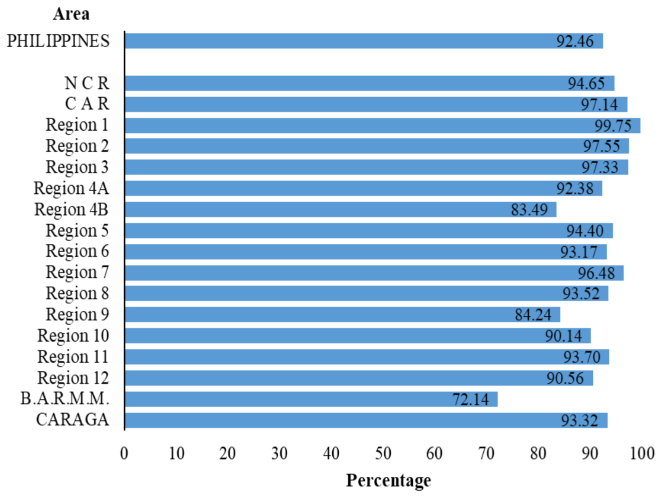

A slight increase in Facility Based Deliveries was noted from 2020 at 89.41% to 90.48% in 2021. Region 1 had the highest births in health facilities with 99.59% followed by Region 2 96.68% and Region 3 with 95.27%. The region with the lowest recorded facility based delivery was BARMM with 59.70%.

Figure 1.B.8 Proportion of Facility Based Deliveries by Region Philippines, 2021

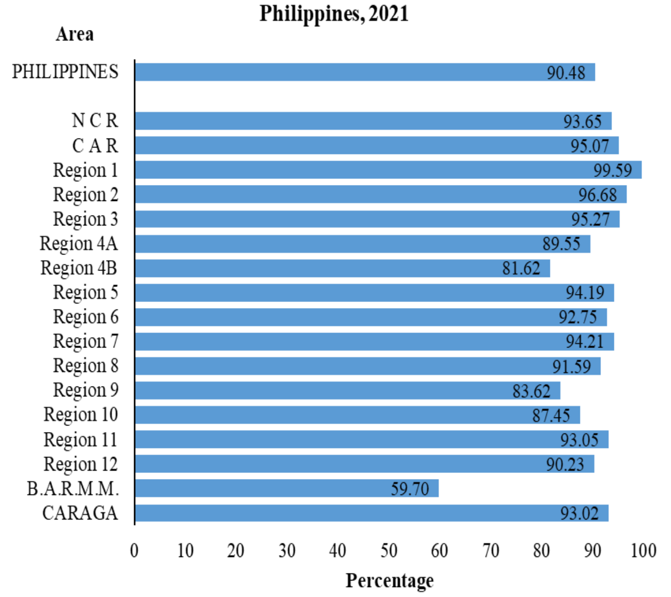

In 2021, 91.90% were classified as having normal birthweight or with birth weight ≥ 2,500 grams. Figure 1.B.9 shows a slight increase in the percentage of neonates with normal birth weight from 2020 to 2021. Neonates with low birth weight (<2,500 grams) had a slight decrease from 2020 at 6.17% to 5.53% in 2021. Births with no reported weight increased from 2.31% in 2020 to 2.58% in 2021.

Figure 1.B.9 Proportion of Livebirths by Weight Philippines 2020 vs. 2021

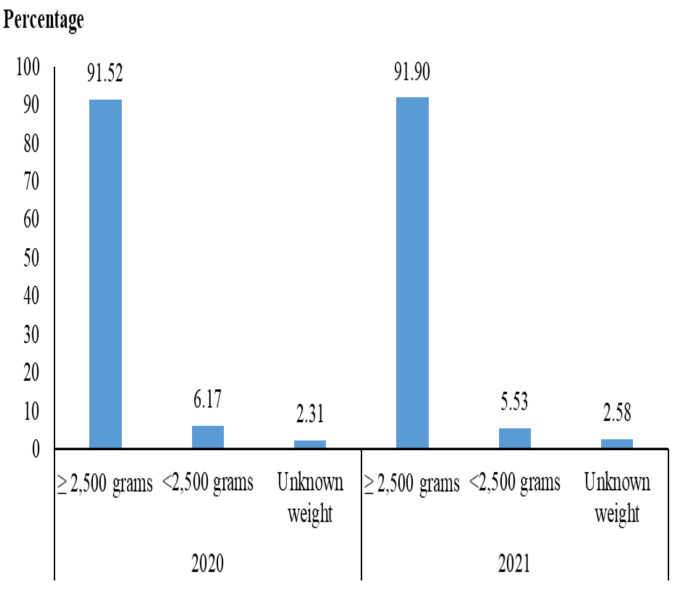

- **Postpartum Care**

Among women aged 10-49 years old who gave birth in 2021, a total of 90.86% completed two postpartum check-ups together with their newborn. The majority of the postpartum women belonged to the 20-49 age group at 81.19% followed by the 15-19 age group at 9.51%.

There was an increase in the proportion of postpartum women with at least 2 postpartum check-ups from 2020 to 2021. The highest increase was seen in the 20-49 years old age group from 51.09% in 2020 to 81.19% in 2021.

Figure 1.B.10 Proportion of Postpartum Women with at least 2 Postpartum Check-ups by Age Group Philippines, 2020 vs. 2021

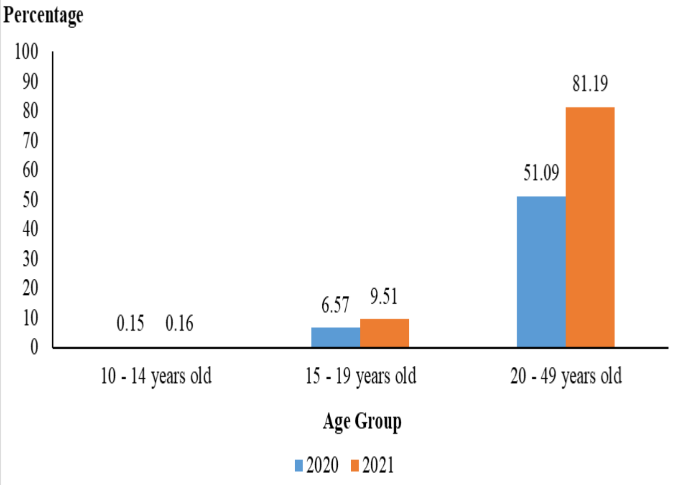

In 2021, the regions with the highest proportion of women with 2 postpartum check-ups were seen in Region 1 at 111.81%, followed by Region 3 at 105.49% and Region 2 at 98.40%. The regions with the least proportion of women with 2 postpartum check-ups were recorded in CAR at 76.72%, followed by CARAGA at 78.76% and Region 5 at 81.70%.

Figure 1.B.11 Proportion of Postpatrum Women together with their Newborn who completed at least 2 Postpartum check-ups by Region Philippines, 2021

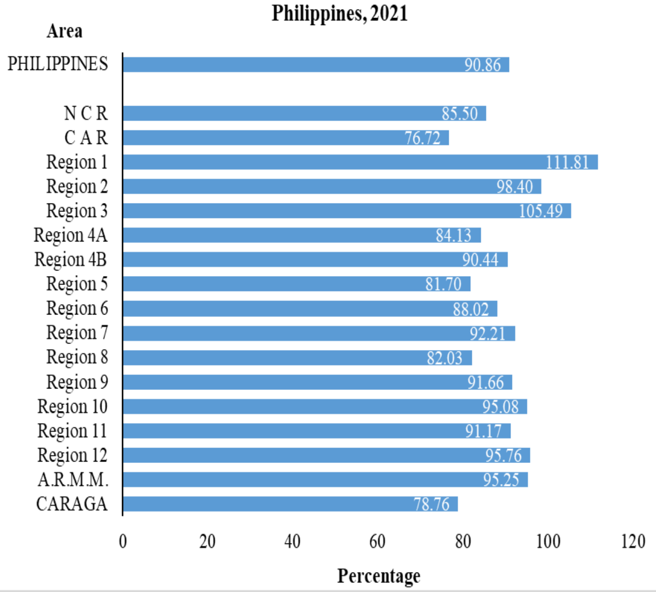

For the proportion of postpartum women who completed Iron with Folic Acid and Vitamin A supplementation, there was a slight decrease from 52.62% in 2020 to 49.95% in 2021 for Iron with Folic Acid Supplementation and 52.62% in 2020 to 50.93% in 2021 for Vitamin A supplementation.

Figure 1.B.12 Proportion of Postpartum Women who Completed Iron with Folic Acid (FA) and Vitamin A Supplementation Philippines, 2020 vs. 2021

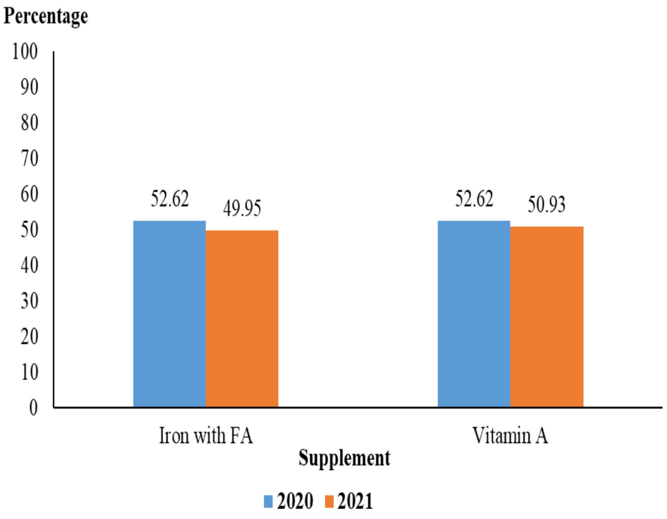

Table 1.B.1.1 - Prenatal Care

Number and Proportion of Women who gave birth with at least 4 or more Prenatal Check-ups by Region, Province/City and Age Group

Philippines, 2021

Area	Total Deliveries	Women who gave birth with at least 4 prenatal check-ups							
		Age Group						Total	%
		10-14 yrs old		15-19 yrs old		20-49 yrs old			
		No.	%	No.	%	No.	%		
**PHILIPPINES**	**1,253,409**	**2,073**	**0.17**	**120,966**	**9.65**	**945,880**	**75.46**	**1,068,923**	**85.28**
**N C R**	**147,582**	**146**	**0.10**	**11,378**	**7.71**	**105,675**	**71.60**	**117,199**	**79.41**
Malabon	5,166	12	0.23	528	10.22	3,246	62.83	3,786	73.29
Navotas	3,245	5	0.15	506	15.59	2,734	84.25	3,245	100.00
Valenzuela City	6,550	7	0.11	632	9.65	5,693	86.92	6,332	96.67
Caloocan City	14,253	22	0.15	1,910	13.40	12,321	86.44	14,253	100.00
Marikina City	7,079	4	0.06	280	3.96	3,115	44.00	3,399	48.02
Pasig City	11,767	6	0.05	684	5.81	7,329	62.28	8,019	68.15
Pateros	714	3	0.42	46	6.44	244	34.17	293	41.04
Taguig	8,868	11	0.12	716	8.07	6,017	67.85	6,744	76.05
Quezon City	44,126	24	0.05	2,429	5.50	34,314	77.76	36,767	83.32
Makati City	5,726	6	0.10	226	3.95	2,584	45.13	2,816	49.18
Mandaluyong City	3,436	1	0.03	272	7.92	3,036	88.36	3,309	96.30
San Juan	1,470	2	0.14	35	2.38	874	59.46	911	61.97
Manila City	13,248	19	0.14	1,335	10.08	7,503	56.63	8,857	66.86
Las Piñas City	5,520	0	0.00	442	8.01	4,041	73.21	4,483	81.21
Muntinlupa City	6,733	13	0.19	535	7.95	6,185	91.86	6,733	100.00
Parañaque City	5,487	8	0.15	483	8.80	2,637	48.06	3,128	57.01
Pasay City	4,194	3	0.07	319	7.61	3,802	90.65	4,124	98.33
**C A R**	**22,444**	**13**	**0.06**	**1,277**	**5.69**	**14,498**	**64.60**	**15,788**	**70.34**
Abra	2,679	0	0.00	173	6.46	1,561	58.27	1,734	64.73
Apayao	1,702	6	0.35	159	9.34	936	54.99	1,101	64.69
Benguet	4,616	0	0.00	225	4.87	3,912	84.75	4,137	89.62
Ifugao	2,796	3	0.11	142	5.08	1,777	63.56	1,922	68.74
Kalinga	2,381	2	0.08	315	13.23	2,002	84.08	2,319	97.40
Mt. Province	2,461	1	0.04	119	4.84	1,433	58.23	1,553	63.10
Baguio City	5,809	1	0.02	144	2.48	2,877	49.53	3,022	52.02
**Region 1**	**48,764**	**46**	**0.09**	**4,927**	**10.10**	**53,006**	**108.70**	**57,979**	**118.90**
Ilocos Norte	4,382	5	0.11	254	5.80	3,649	83.27	3,908	89.18
Ilocos Sur	4,215	7	0.17	421	9.99	6,464	153.36	6,892	163.51
La Union	4,241	4	0.09	614	14.48	5,561	131.12	6,179	145.70
Pangasinan	14,420	15	0.10	2,665	18.48	26,517	183.89	29,197	202.48
Alaminos City	1,250	0	0.00	97	7.76	1,242	99.36	1,339	107.12
Candon City	1,075	0	0.00	1	0.09	1,064	98.98	1,065	99.07
Dagupan City	8,147	1	0.01	337	4.14	1,700	20.87	2,038	25.02
Laoag City	1,965	0	0.00	35	1.78	1,909	97.15	1,944	98.93
San Carlos City	2,280	4	0.18	173	7.59	1,861	81.62	2,038	89.39
San Fernando City	3,530	5	0.14	89	2.52	1,063	30.11	1,157	32.78
Urdaneta City	1,843	3	0.16	169	9.17	1,480	80.30	1,652	89.64
Vigan City	1,416	2	0.14	72	5.08	496	35.03	570	40.25

Table 1.B.1.1 - Prenatal Care

Number and Proportion of Women who gave birth with at least 4 or more Prenatal Check-ups by Region, Province/City and Age Group

Philippines, 2021

Area	Total Deliveries	Women who gave birth with at least 4 prenatal check-ups							
		Age Group						Total	%
		10-14 yrs old		15-19 yrs old		20-49 yrs old			
		No.	%	No.	%	No.	%		
**Region 2**	**40,412**	**82**	**0.20**	**4,424**	**10.95**	**33,607**	**83.16**	**38,113**	**94.31**
Batanes	269	1	0.37	8	2.97	127	47.21	136	50.56
Cagayan	9,734	22	0.23	1,116	11.46	7,895	81.11	9,033	92.80
Isabela	12,520	33	0.26	1,561	12.47	11,689	93.36	13,283	106.09
Nueva Vizcaya	6,736	10	0.15	783	11.62	5,424	80.52	6,217	92.30
Quirino	2,634	1	0.04	270	10.25	2,611	99.13	2,882	109.42
Cauayan City	1,645	4	0.24	216	13.13	1,298	78.91	1,518	92.28
Ilagan City	2,646	8	0.30	173	6.54	1,511	57.11	1,692	63.95
Santiago City	2,861	2	0.07	268	9.37	2,521	88.12	2,791	97.55
Tuguegarao City	1,367	1	0.07	29	2.12	531	38.84	561	41.04
**Region 3**	**117,383**	**217**	**0.18**	**15,397**	**13.12**	**105,047**	**89.49**	**120,661**	**102.79**
Aurora	3,008	2	0.07	307	10.21	2,393	79.55	2,702	89.83
Bataan	5,148	28	0.54	988	19.19	6,673	129.62	7,689	149.36
Bulacan	22,840	52	0.23	3,031	13.27	20,608	90.23	23,691	103.73
Nueva Ecija	9,489	26	0.27	1,522	16.04	9,886	104.18	11,434	120.50
Pampanga	9,794	28	0.29	1,739	17.76	11,540	117.83	13,307	135.87
Tarlac	11,344	7	0.06	1,086	9.57	9,665	85.20	10,758	94.83
Zambales	7,601	5	0.07	490	6.45	3,280	43.15	3,775	49.66
Angeles City	7,027	3	0.04	476	6.77	2,953	42.02	3,432	48.84
Balanga City	1,290	4	0.31	133	10.31	738	57.21	875	67.83
Cabanatuan City	4,236	7	0.17	604	14.26	3,466	81.82	4,077	96.25
City of San Fernando	4,596	10	0.22	423	9.20	2,516	54.74	2,949	64.16
Gapan City	966	1	0.10	125	12.94	805	83.33	931	96.38
Mabalacat City	4,337	6	0.14	746	17.20	4,453	102.67	5,205	120.01
Malolos City	3,489	6	0.17	226	6.48	1,815	52.02	2,047	58.67
Meycauayan	2,351	3	0.13	477	20.29	3,201	136.15	3,681	156.57
Olongapo	3,425	7	0.20	252	7.36	1,879	54.86	2,138	62.42
Palayan City	77	0	0.00	64	83.12	313	406.49	377	489.61
San Jose City	1,940	4	0.21	327	16.86	1,750	90.21	2,081	107.27
San Jose del Monte City	6,642	10	0.15	1,194	17.98	11,040	166.21	12,244	184.34
Science City of Munoz	105	4	3.81	108	102.86	667	635.24	779	741.90
Tarlac City	7,678	4	0.05	1,079	14.05	5,406	70.41	6,489	84.51
**Region 4A**	**169,896**	**314**	**0.18**	**12,869**	**7.57**	**108,108**	**63.63**	**121,291**	**71.39**
Batangas	21,266	32	0.15	1,539	7.24	17,606	82.79	19,177	90.18
Cavite	16,518	24	0.15	908	5.50	7,336	44.41	8,268	50.05
Laguna	11,693	45	0.38	1,236	10.57	7,783	66.56	9,064	77.52
Quezon	18,491	49	0.26	1,557	8.42	11,251	60.85	12,857	69.53
Rizal	21,048	35	0.17	1,825	8.67	12,030	57.16	13,890	65.99
Antipolo City	11,094	12	0.11	923	8.32	10,245	92.35	11,180	100.78
Bacoor City	6,756	1	0.01	119	1.76	1,021	15.11	1,141	16.89
Batangas City	3,484	9	0.26	300	8.61	2,994	85.94	3,303	94.80
Biñan City	7,973	3	0.04	898	11.26	5,329	66.84	6,230	78.14
Cabuyao City	3,188	2	0.06	378	11.86	3,411	106.99	3,791	118.91

Table 1.B.1.1 - Prenatal Care

Number and Proportion of Women who gave birth with at least 4 or more Prenatal Check-ups by Region, Province/City and Age Group

Philippines, 2021

Area	Total Deliveries	Women who gave birth with at least 4 prenatal check-ups							
		Age Group						Total	%
		10-14 yrs old		15-19 yrs old		20-49 yrs old			
		No.	%	No.	%	No.	%		
Calamba City	8,294	10	0.12	711	8.57	6,715	80.96	7,436	89.66
Cavite City	659	1	0.15	31	4.70	124	18.82	156	23.67
Dasmariñas City	6,573	12	0.18	409	6.22	4,143	63.03	4,564	69.44
General Trias City	729	15	2.06	172	23.59	1,804	247.46	1,991	273.11
Imus City	4,356	2	0.05	79	1.81	703	16.14	784	18.00
Lipa City	4,231	9	0.21	135	3.19	1,656	39.14	1,800	42.54
Lucena City	2,473	2	0.08	203	8.21	1,284	51.92	1,489	60.21
San Pablo City	4,772	14	0.29	395	8.28	2,285	47.88	2,694	56.45
San Pedro City	1,302	1	0.08	206	15.82	1,241	95.31	1,448	111.21
Santa Rosa City	7,494	18	0.24	528	7.05	4,922	65.68	5,468	72.97
Tagaytay City	1,189	2	0.17	56	4.71	1,078	90.66	1,136	95.54
Tanauan City	2,707	2	0.07	89	3.29	1,753	64.76	1,844	68.12
Tayabas City	1,345	14	1.04	143	10.63	1,124	83.57	1,281	95.24
Trece Martires City	2,261	0	0.00	29	1.28	270	11.94	299	13.22
**Region 4B**	**40,990**	**60**	**0.15**	**3,795**	**9.26**	**27,147**	**66.23**	**31,002**	**75.63**
Marinduque	2,785	1	0.04	183	6.57	2,026	72.75	2,210	79.35
Mindoro Occidental	7,794	13	0.17	964	12.37	5,892	75.60	6,869	88.13
Mindoro Oriental	11,017	7	0.06	844	7.66	7,575	68.76	8,426	76.48
Palawan	12,500	36	0.29	1,539	12.31	9,179	73.43	10,754	86.03
Romblon	3,895	3	0.08	265	6.80	2,475	63.54	2,743	70.42
Puerto Princesa City	2,999	0	0.00	0	0.00	0	0.00	0	0.00
**Region 5**	**78,718**	**55**	**0.07**	**5,171**	**6.57**	**49,809**	**63.28**	**55,035**	**69.91**
Albay	9,202	5	0.05	600	6.52	8,175	88.84	8,780	95.41
Camarines Norte	9,661	5	0.05	884	9.15	6,454	66.80	7,343	76.01
Camarines Sur	15,499	16	0.10	809	5.22	9,710	62.65	10,535	67.97
Catanduanes	4,006	1	0.02	248	6.19	2,450	61.16	2,699	67.37
Masbate	12,154	18	0.15	1,393	11.46	8,087	66.54	9,498	78.15
Sorsogon	10,424	3	0.03	760	7.29	8,190	78.57	8,953	85.89
Iriga City	1,629	0	0.00	111	6.81	1,056	64.83	1,167	71.64
Legaspi City	4,511	3	0.07	159	3.52	3,906	86.59	4,068	90.18
Naga City	11,632	4	0.03	207	1.78	1,781	15.31	1,992	17.13
**Region 6**	**82,095**	**110**	**0.13**	**7,297**	**8.89**	**57,878**	**70.50**	**65,285**	**79.52**
Aklan	5,568	7	0.13	410	7.36	4,433	79.62	4,850	87.10
Antique	7,312	4	0.05	433	5.92	4,729	64.67	5,166	70.65
Capiz	7,458	8	0.11	517	6.93	5,221	70.01	5,746	77.04
Guimaras	2,319	3	0.13	173	7.46	2,005	86.46	2,181	94.05
Iloilo	19,237	35	0.18	1,634	8.49	14,158	73.60	15,827	82.27
Negros Occidental	31,802	47	0.15	3,341	10.51	21,376	67.22	24,764	77.87
Bacolod City	3,858	1	0.03	371	9.62	2,577	66.80	2,949	76.44
Iloilo City	4,541	5	0.11	418	9.21	3,379	74.41	3,802	83.73
**Region 7**	**92,739**	**106**	**0.11**	**9,591**	**10.34**	**79,585**	**85.82**	**89,282**	**96.27**
Bohol	17,266	10	0.06	1,303	7.55	12,472	72.23	13,785	79.84
Cebu	27,663	34	0.12	3,827	13.83	30,863	111.57	34,724	125.53

Table 1.B.1.1 - Prenatal Care

Number and Proportion of Women who gave birth with at least 4 or more Prenatal Check-ups by Region, Province/City and Age Group

Philippines, 2021

Area	Total Deliveries	Women who gave birth with at least 4 prenatal check-ups							
		Age Group						Total	%
		10-14 yrs old		15-19 yrs old		20-49 yrs old			
		No.	%	No.	%	No.	%		
Negros Oriental	18,889	30	0.16	1,747	9.25	10,939	57.91	12,716	67.32
Siquijor	1,138	0	0.00	83	7.29	754	66.26	837	73.55
Cebu City	16,354	27	0.17	1,555	9.51	12,151	74.30	13,733	83.97
Lapu-Lapu City	7,391	2	0.03	527	7.13	7,243	98.00	7,772	105.15
Mandaue City	4,038	3	0.07	549	13.60	5,163	127.86	5,715	141.53
**Region 8**	**52,130**	**96**	**0.18**	**4,356**	**8.36**	**36,923**	**70.83**	**41,375**	**79.37**
Biliran	2,621	3	0.11	211	8.05	1,498	57.15	1,712	65.32
Eastern Samar	6,181	7	0.11	550	8.90	4,931	79.78	5,488	88.79
Northern Leyte	14,081	16	0.11	1,184	8.41	11,172	79.34	12,372	87.86
Northern Samar	8,565	3	0.04	499	5.83	4,931	57.57	5,433	63.43
Southern Leyte	2,521	4	0.16	330	13.09	2,185	86.67	2,519	99.92
Western Samar	6,475	56	0.86	899	13.88	5,412	83.58	6,367	98.33
Calbayog City	1,938	0	0.00	139	7.17	1,228	63.36	1,367	70.54
Maasin City	2,252	0	0.00	42	1.87	646	28.69	688	30.55
Ormoc City	4,748	0	0.00	316	6.66	2,373	49.98	2,689	56.63
Tacloban City	2,748	7	0.25	186	6.77	2,547	92.69	2,740	99.71
**Region 9**	**46,771**	**32**	**0.07**	**4,595**	**9.82**	**33,443**	**71.50**	**38,074**	**81.41**
Zamboanga del Norte	9,489	6	0.06	1,082	11.40	6,894	72.65	7,982	84.12
Zamboanga del Sur	6,651	9	0.14	528	7.94	3,497	52.58	4,037	60.70
Zamboanga Sibugay	6,965	8	0.11	621	8.92	4,296	61.68	4,923	70.68
Dapitan City	1,598	3	0.19	66	4.13	1,381	86.42	1,450	90.74
Dipolog City	2,277	1	0.04	242	10.63	1,728	75.89	1,973	86.65
Isabela City	1,629	3	0.18	222	13.63	1,294	79.44	1,517	93.12
Pagadian City	2,298	0	0.00	223	9.70	1,371	59.66	1,594	69.36
Zamboanga City	15,864	2	0.01	1,611	10.16	12,982	81.83	14,598	92.02
**Region 10**	**71,984**	**236**	**0.33**	**10,620**	**14.75**	**60,234**	**83.68**	**71,090**	**98.76**
Bukidnon	19,679	110	0.56	3,353	17.04	13,711	69.67	17,174	87.27
Camiguin	1,100	0	0.00	49	4.45	508	46.18	557	50.64
Lanao del Norte	8,578	8	0.09	739	8.62	8,059	93.95	8,806	102.66
Misamis Occidental	3,779	8	0.21	345	9.13	3,441	91.06	3,794	100.40
Misamis Oriental	8,821	14	0.16	1,688	19.14	9,752	110.55	11,454	129.85
Cagayan de Oro City	11,751	39	0.33	1,671	14.22	10,656	90.68	12,366	105.23
El Salvador City	249	0	0.00	60	24.10	356	142.97	416	167.07
Gingoog City	2,893	31	1.07	808	27.93	1,904	65.81	2,743	94.82
Iligan City	6,035	14	0.23	677	11.22	4,507	74.68	5,198	86.13
Malaybalay City	2,899	6	0.21	367	12.66	2,072	71.47	2,445	84.34
Oroquieta City	744	0	0.00	38	5.11	490	65.86	528	70.97
Ozamis City	1,944	2	0.10	203	10.44	1,705	87.71	1,910	98.25
Tangub City	1,173	0	0.00	241	20.55	909	77.49	1,150	98.04
Valencia City	2,339	4	0.17	381	16.29	2,164	92.52	2,549	108.98

Table 1.B.1.1 - Prenatal Care

Number and Proportion of Women who gave birth with at least 4 or more Prenatal Check-ups by Region, Province/City and Age Group

Philippines, 2021

Area	Total Deliveries	Women who gave birth with at least 4 prenatal check-ups							
		Age Group						Total	%
		10-14 yrs old		15-19 yrs old		20-49 yrs old			
		No.	%	No.	%	No.	%		
**Region 11**	**76,188**	**304**	**0.40**	**7,915**	**10.39**	**51,522**	**67.62**	**59,741**	**78.41**
Davao de Oro	11,332	57	0.50	1,663	14.68	8,447	74.54	10,167	89.72
Davao del Norte	17,041	88	0.52	1,784	10.47	11,747	68.93	13,619	79.92
Davao Oriental	8,236	31	0.38	970	11.78	5,333	64.75	6,334	76.91
Davao del Sur	8,170	32	0.39	781	9.56	4,367	53.45	5,180	63.40
Davao Occidental	4,276	33	0.77	522	12.21	1,247	29.16	1,802	42.14
Davao City	27,133	63	0.23	2,195	8.09	20,381	75.12	22,639	83.44
**Region 12**	**65,637**	**168**	**0.26**	**8,810**	**13.42**	**50,293**	**76.62**	**59,271**	**90.30**
North Cotabato	18,335	32	0.17	2,346	12.80	13,995	76.33	16,373	89.30
Sarangani	9,714	45	0.46	1,772	18.24	7,254	74.68	9,071	93.38
South Cotabato	16,353	40	0.24	2,079	12.71	12,600	77.05	14,719	90.01
Sultan Kudarat	12,030	44	0.37	1,659	13.79	9,233	76.75	10,936	90.91
Cotabato City	3,298	2	0.06	255	7.73	2,513	76.20	2,770	83.99
Gen. Santos City	5,907	5	0.08	699	11.83	4,698	79.53	5,402	91.45
**BARMM**	**62,595**	**46**	**0.07**	**5,713**	**9.13**	**54,059**	**86.36**	**59,818**	**95.56**
Basilan	3,253	7	0.22	243	7.47	1,823	56.04	2,073	63.73
Lanao del Sur	17,519	4	0.02	684	3.90	15,779	90.07	16,467	94.00
Maguindanao	20,188	28	0.14	2,513	12.45	18,836	93.30	21,377	105.89
Sulu	8,891	0	0.00	0	0.00	8,033	90.35	8,033	90.35
Tawi-Tawi	5,681	1	0.02	285	5.02	4,395	77.36	4,681	82.40
Lamitan City	1,226	5	0.41	195	15.91	776	63.30	976	79.61
Marawi City	5,837	1	0.02	1,793	30.72	4,417	75.67	6,211	106.41
**CARAGA**	**37,081**	**42**	**0.11**	**2,831**	**7.63**	**25,046**	**67.54**	**27,919**	**75.29**
Agusan del Norte	3,599	2	0.06	289	8.03	2,365	65.71	2,656	73.80
Agusan del Sur	11,882	18	0.15	982	8.26	7,347	61.83	8,347	70.25
Surigao del Norte	3,409	3	0.09	231	6.78	2,562	75.15	2,796	82.02
Surigao del Sur	6,454	6	0.09	472	7.31	4,154	64.36	4,632	71.77
Province of Dinagat	1,020	1	0.10	76	7.45	730	71.57	807	79.12
Bislig City	1,294	3	0.23	122	9.43	1,054	81.45	1,179	91.11
Butuan City	5,267	9	0.17	398	7.56	4,860	92.27	5,267	100.00
Surigao City	4,156	0	0.00	261	6.28	1,974	47.50	2,235	53.78

Note: Put asterisk (*) for No Report and Zero (0) for No Case

Table 1.B.1.2 - Prenatal Care

Number and Proportion of Pregnant Women Seen According to their Nutritional Status by Region, Province/City and Age Group
Philippines, 2021

Area	Eligible Pop. (Under 1)	Pregnant women seen during the 1st trimester according to their Body Mass Index (BMI)														
		Normal BMI					Low BMI					High BMI				
		Age Group			Total	%	Age Group			Total	%	Age Group			Total	%
		10-14	15-19	20-49			10-14	15-19	20-49			10-14	15-19	20-49		
**PHILIPPINES**	**2,128,168**	**2,309**	**105,558**	**689,069**	**796,936**	**37.45**	**506**	**11,494**	**36,337**	**48,337**	**2.27**	**233**	**7,212**	**80,885**	**88,330**	**4.15**
**N C R**	**237,921**	**127**	**6,749**	**59,788**	**66,664**	**28.02**	**54**	**1,359**	**3,649**	**5,062**	**2.13**	**10**	**802**	**10,402**	**11,214**	**4.71**
Malabon	6,757	14	404	2,122	2,540	37.59	14	236	428	678	10.03	0	100	1,034	1,134	16.78
Navotas	4,607	5	522	2,121	2,648	57.48	0	24	43	67	1.45	0	14	123	137	2.97
Valenzuela City	11,465	8	380	3,325	3,713	32.39	2	83	311	396	3.45	0	79	1,081	1,160	10.12
Caloocan City	29,270	10	909	5,084	6,003	20.51	9	161	425	595	2.03	0	80	898	978	3.34
Marikina City	8,330	0	76	475	551	6.61	1	27	68	96	1.15	0	28	369	397	4.77
Pasig City	13,954	3	226	1,782	2,011	14.41	4	75	209	288	2.06	2	54	953	1,009	7.23
Pateros	1,181	1	57	327	385	32.60	0	3	3	6	0.51	0	0	5	5	0.42
Taguig	14,874	8	429	3,491	3,928	26.41	6	122	419	547	3.68	0	90	1,021	1,111	7.47
Quezon City	54,246	32	1,335	19,956	21,323	39.31	6	197	537	740	1.36	2	52	815	869	1.60
Makati City	10,765	3	51	772	826	7.67	2	24	132	158	1.47	3	24	544	571	5.30
Mandaluyong City	7,137	1	153	1,566	1,720	24.10	0	6	34	40	0.56	0	8	125	133	1.86
San Juan	2,254	2	43	814	859	38.11	0	1	10	11	0.49	0	1	59	60	2.66
Manila City	32,887	15	832	4,183	5,030	15.29	3	112	325	440	1.34	1	110	963	1,074	3.27
Las Piñas City	10,879	2	276	2,478	2,756	25.33	1	79	187	267	2.45	1	68	1,000	1,069	9.83
Muntinlupa City	9,317	6	370	6,777	7,153	76.77	6	133	286	425	4.56	1	32	623	656	7.04
Parañaque City	12,298	15	455	2,843	3,313	26.94	0	26	122	148	1.20	0	13	243	256	2.08
Pasay City	7,700	2	231	1,672	1,905	24.74	0	50	110	160	2.08	0	49	546	595	7.73
**C A R**	**35,143**	**35**	**1,622**	**13,360**	**15,017**	**42.73**	**6**	**95**	**378**	**479**	**1.36**	**3**	**105**	**2,007**	**2,115**	**6.02**
Abra	4,287	4	257	1,425	1,686	39.33	0	18	57	75	1.75	0	7	138	145	3.38
Apayao	2,476	5	200	1,082	1,287	51.98	2	27	61	90	3.63	0	15	175	190	7.67
Benguet	9,140	12	441	4,408	4,861	53.18	1	8	59	68	0.74	0	28	582	610	6.67
Ifugao	4,439	9	254	2,158	2,421	54.54	0	7	44	51	1.15	1	12	194	207	4.66
Kalinga	4,660	2	212	1,796	2,010	43.13	3	20	93	116	2.49	1	14	254	269	5.77
Mt. Province	3,066	2	129	1,233	1,364	44.49	0	7	27	34	1.11	1	18	407	426	13.89
Baguio City	7,075	1	129	1,258	1,388	19.62	0	8	37	45	0.64	0	11	257	268	3.79
**Region 1**	**97,198**	**33**	**3,483**	**35,461**	**38,977**	**40.10**	**6**	**274**	**1,103**	**1,383**	**1.42**	**0**	**155**	**1,764**	**1,919**	**1.97**
Ilocos Norte	7,986	6	252	2,909	3,167	39.66	0	16	114	130	1.63	0	8	61	69	0.86
Ilocos Sur	9,197	6	349	4,790	5,145	55.94	0	17	79	96	1.04	0	8	203	211	2.29
La Union	11,429	6	564	4,401	4,971	43.49	1	51	151	203	1.78	0	22	242	264	2.31
Pangasinan	50,454	10	1,799	16,729	18,538	36.74	0	148	657	805	1.60	0	100	1,074	1,174	2.33
Alaminos City	1,905	0	82	893	975	51.18	0	0	0	0	0.00	0	0	0	0	0.00
Candon City	976	0	1	575	576	59.02	0	0	0	0	0.00	0	0	6	6	0.61
Dagupan City	3,636	1	126	677	804	22.11	0	2	13	15	0.41	0	0	8	8	0.22
Laoag City	1,845	0	35	1,909	1,944	105.37	0	2	2	4	0.22	0	0	0	0	0.00
San Carlos City	3,999	0	78	678	756	18.90	0	15	61	76	1.90	0	6	54	60	1.50
San Fernando City	2,092	1	78	675	754	36.04	0	0	4	4	0.19	0	1	6	7	0.33
Urdaneta City	2,819	1	98	996	1,095	38.84	0	10	22	32	1.14	0	6	81	87	3.09
Vigan City	860	2	21	229	252	29.30	5	13	0	18	2.09	0	4	29	33	3.84
**Region 2**	**69,228**	**89**	**3,817**	**25,087**	**28,993**	**41.88**	**16**	**303**	**904**	**1,223**	**1.77**	**5**	**245**	**2,770**	**3,020**	**4.36**
Batanes	351	1	7	89	97	27.64	0	4	5	9	2.56	0	1	75	76	21.65
Cagayan	20,563	12	1,036	6,472	7,520	36.57	3	71	299	373	1.81	3	65	529	597	2.90
Isabela	23,543	62	1,262	8,204	9,528	40.47	9	130	343	482	2.05	1	93	1,105	1,199	5.09
Nueva Vizcaya	9,555	5	630	3,997	4,632	48.48	2	39	108	149	1.56	0	36	572	608	6.36
Quirino	4,038	2	262	1,956	2,220	54.98	1	28	68	97	2.40	0	21	147	168	4.16
Cauayan City	2,576	1	180	955	1,136	44.10	0	9	30	39	1.51	0	2	44	46	1.79
Ilagan City	2,901	3	146	953	1,102	37.99	0	17	26	43	1.48	1	9	74	84	2.90
Santiago City	2,683	3	244	2,048	2,295	85.54	1	4	15	20	0.75	0	10	104	114	4.25
Tuguegarao City	3,018	0	50	413	463	15.34	0	1	10	11	0.36	0	8	120	128	4.24
**Region 3**	**220,202**	**295**	**12,749**	**76,676**	**89,720**	**40.74**	**42**	**991**	**3,022**	**4,055**	**1.84**	**18**	**559**	**6,068**	**6,645**	**3.02**
Aurora	4,764	7	364	2,134	2,505	52.58	1	40	99	140	2.94	0	6	96	102	2.14
Bataan	13,814	35	824	4,064	4,923	35.64	2	121	265	388	2.81	0	55	786	841	6.09
Bulacan	43,722	52	2,723	17,638	20,413	46.69	17	265	985	1,267	2.90	3	151	1,553	1,707	3.90
Nueva Ecija	28,915	23	1,309	7,821	9,153	31.65	10	188	602	800	2.77	5	77	553	635	2.20
Pampanga	31,503	30	1,336	8,083	9,449	29.99	3	78	182	263	0.83	3	44	653	700	2.22
Tarlac	19,565	5	1,028	8,153	9,186	46.95	1	56	203	260	1.33	1	59	651	711	3.63
Zambales	12,551	46	465	2,500	3,011	23.99	4	31	51	86	0.69	0	13	194	207	1.65
Angeles City	7,905	6	409	2,376	2,791	35.31	0	10	25	35	0.44	0	17	188	205	2.59
Balanga City	1,996	0	114	555	669	33.52	3	21	45	69	3.46	0	11	167	178	8.92
Cabanatuan City	5,921	4	257	1,364	1,625	27.44	0	5	35	40	0.68	0	20	208	228	3.85
City of San Fernando	5,890	21	352	2,077	2,450	41.60	0	30	183	213	3.62	1	5	55	61	1.04
Gapan City	2,159	2	123	585	710	32.89	0	0	0	0	0.00	0	0	0	0	0.00
Mabalacat City	4,811	9	300	2,021	2,330	48.43	0	32	68	100	2.08	1	31	241	273	5.67

Table 1.B.1.2 - Prenatal Care

Number and Proportion of Pregnant Women Seen According to their Nutritional Status by Region, Province/City and Age Group
Philippines, 2021

Area	Eligible Pop. (Under 1)	Pregnant women seen during the 1st trimester according to their Body Mass Index (BMI)														
		Normal BMI					Low BMI					High BMI				
		Age Group			Total	%	Age Group			Total	%	Age Group			Total	%
		10-14	15-19	20-49			10-14	15-19	20-49			10-14	15-19	20-49		
Malolos City	4,885	10	246	1,666	1,922	39.34	1	25	55	81	1.66	4	22	214	240	4.91
Meycauayan	4,050	2	466	3,143	3,611	89.16	0	15	20	35	0.86	0	8	34	42	1.04
Olongapo	4,952	2	174	964	1,140	23.02	0	48	138	186	3.76	0	29	291	320	6.46
Palayan City	805	0	47	233	280	34.78	0	3	8	11	1.37	0	0	41	41	5.09
San Jose City	2,736	4	247	1,383	1,634	59.72	0	11	33	44	1.61	0	3	61	64	2.34
San Jose del Monte City	11,120	15	620	4,805	5,440	48.92	0	11	20	31	0.28	0	8	75	83	0.75
Science City of Munoz	1,594	4	78	378	460	28.86	0	0	0	0	0.00	0	0	0	0	0.00
Tarlac City	6,544	18	1,267	4,733	6,018	91.96	0	1	5	6	0.09	0	0	7	7	0.11
**Region 4A**	**298,398**	**268**	**10,651**	**73,113**	**84,032**	**28.16**	**121**	**1,658**	**6,505**	**8,284**	**2.78**	**23**	**865**	**8,038**	**8,926**	**2.99**
Batangas	38,848	20	1,111	11,288	12,419	31.97	21	208	804	1,033	2.66	1	83	555	639	1.64
Cavite	27,948	19	761	5,591	6,371	22.80	10	111	597	718	2.57	1	76	817	894	3.20
Laguna	19,727	31	886	5,009	5,926	30.04	7	158	630	795	4.03	2	67	718	787	3.99
Quezon	37,644	66	1,762	9,727	11,555	30.70	17	225	916	1,158	3.08	4	105	1,178	1,287	3.42
Rizal	44,826	24	1,484	7,857	9,365	20.89	12	312	1,263	1,587	3.54	1	149	631	781	1.74
Antipolo City	16,510	5	765	4,634	5,404	32.73	0	53	349	402	2.43	0	26	164	190	1.15
Bacoor City	12,252	0	118	775	893	7.29	0	14	42	56	0.46	0	10	88	98	0.80
Batangas City	6,895	1	135	883	1,019	14.78	2	35	112	149	2.16	0	30	342	372	5.40
Biñan City	6,607	28	805	4,684	5,517	83.50	32	29	66	127	1.92	0	6	70	76	1.15
Cabuyao City	6,128	2	333	2,682	3,017	49.23	0	50	110	160	2.61	0	27	342	369	6.02
Calamba City	9,025	9	429	3,672	4,110	45.54	7	132	337	476	5.27	1	58	921	980	10.86
Cavite City	2,098	0	25	115	140	6.67	1	9	11	21	1.00	0	10	39	49	2.34
Dasmariñas City	13,442	11	297	2,997	3,305	24.59	2	44	213	259	1.93	1	19	241	261	1.94
General Trias City	6,410	13	144	1,120	1,277	19.92	1	10	84	95	1.48	0	10	101	111	1.73
Imus City	8,235	0	130	703	833	10.12	0	29	85	114	1.38	0	23	199	222	2.70
Lipa City	6,954	2	155	1,935	2,092	30.08	0	17	117	134	1.93	0	3	47	50	0.72
Lucena City	5,704	5	329	1,527	1,861	32.63	1	35	108	144	2.52	0	7	80	87	1.53
San Pablo City	5,277	2	96	628	726	13.76	0	26	69	95	1.80	2	18	184	204	3.87
San Pedro City	6,465	7	222	1,168	1,397	21.61	1	39	155	195	3.02	0	11	121	132	2.04
Santa Rosa City	7,023	17	344	2,718	3,079	43.84	4	87	263	354	5.04	8	97	771	876	12.47
Tagaytay City	1,451	1	39	752	792	54.58	0	0	19	19	1.31	0	0	3	3	0.21
Tanauan City	3,620	2	76	1,332	1,410	38.95	0	14	65	79	2.18	0	7	79	86	2.38
Tayabas City	2,134	2	132	1,000	1,134	53.14	2	7	57	66	3.09	0	8	114	122	5.72
Trece Martires City	3,175	1	73	316	390	12.28	1	14	33	48	1.51	2	15	233	250	7.87
**Region 4B**	**72,035**	**38**	**2,821**	**17,805**	**20,664**	**28.69**	**7**	**329**	**1,071**	**1,407**	**1.95**	**4**	**99**	**1,688**	**1,791**	**2.49**
Marinduque	4,906	2	166	1,482	1,650	33.63	1	16	100	117	2.38	0	11	199	210	4.28
Mindoro Occidental	12,449	3	569	3,388	3,960	31.81	1	53	142	196	1.57	1	17	376	394	3.16
Mindoro Oriental	20,011	8	773	5,508	6,289	31.43	1	49	150	200	1.00	0	12	163	175	0.87
Palawan	21,676	18	1,054	5,562	6,634	30.61	3	163	464	630	2.91	2	46	720	768	3.54
Romblon	6,482	7	259	1,865	2,131	32.88	1	48	215	264	4.07	1	13	230	244	3.76
Puerto Princesa City	6,511	0	0	0	0	0.00	0	0	0	0	0.00	0	0	0	0	0.00
**Region 5**	**137,350**	**59**	**6,873**	**43,857**	**50,789**	**36.98**	**16**	**757**	**2,854**	**3,627**	**2.64**	**8**	**287**	**4,752**	**5,047**	**3.67**
Albay	23,903	8	751	9,086	9,845	41.19	2	71	444	517	2.16	1	23	562	586	2.45
Camarines Norte	14,340	10	827	4,668	5,505	38.39	2	154	396	552	3.85	5	64	842	911	6.35
Camarines Sur	39,151	16	1,148	10,132	11,296	28.85	6	179	782	967	2.47	1	68	1,178	1,247	3.19
Catanduanes	6,381	1	155	1,712	1,868	29.27	0	40	239	279	4.37	0	18	313	331	5.19
Masbate	22,565	11	1,155	6,194	7,360	32.62	3	98	317	418	1.85	0	42	604	646	2.86
Sorsogon	19,470	5	694	6,145	6,844	35.15	2	133	538	673	3.46	1	57	1,074	1,132	5.81
Iriga City	2,665	0	116	961	1,077	40.41	1	14	25	40	1.50	0	5	81	86	3.23
Legaspi City	4,205	2	1,879	3,835	5,716	135.93	0	57	90	147	3.50	0	4	27	31	0.74
Naga City	4,670	6	148	1,124	1,278	27.37	0	11	23	34	0.73	0	6	71	77	1.65
**Region 6**	**146,545**	**105**	**5,662**	**41,988**	**47,755**	**32.59**	**24**	**620**	**2,148**	**2,792**	**1.91**	**6**	**308**	**4,125**	**4,439**	**3.03**
Aklan	11,231	4	303	3,169	3,476	30.95	3	33	165	201	1.79	0	17	487	504	4.49
Antique	12,985	8	452	4,029	4,489	34.57	1	39	178	218	1.68	0	21	370	391	3.01
Capiz	13,984	12	427	3,355	3,794	27.13	0	21	144	165	1.18	2	15	255	272	1.95
Guimaras	3,085	3	132	1,436	1,571	50.92	0	15	71	86	2.79	0	18	326	344	11.15
Iloilo	36,414	35	1,340	11,895	13,270	36.44	5	119	397	521	1.43	1	50	547	598	1.64
Negros Occidental	49,314	33	2,604	15,398	18,035	36.57	14	341	1,009	1,364	2.77	3	159	1,639	1,801	3.65
Bacolod City	11,102	2	127	831	960	8.65	1	36	103	140	1.26	0	21	336	357	3.22
Iloilo City	8,430	8	277	1,875	2,160	25.62	0	16	81	97	1.15	0	7	165	172	2.04
**Region 7**	**162,860**	**112**	**6,580**	**48,970**	**55,662**	**34.18**	**21**	**616**	**2,137**	**2,774**	**1.70**	**12**	**625**	**7,908**	**8,545**	**5.25**
Bohol	27,637	6	610	6,539	7,155	25.89	3	18	56	77	0.28	0	0	155	155	0.56
Cebu	67,046	34	2,525	18,116	20,675	30.84	6	267	1,102	1,375	2.05	5	264	3,097	3,366	5.02
Negros Oriental	27,922	40	1,485	7,720	9,245	33.11	7	140	427	574	2.06	2	126	1,506	1,634	5.85
Siquijor	1,636	1	78	473	552	33.74	0	12	45	57	3.48	1	11	141	153	9.35
Cebu City	21,037	23	1,064	6,716	7,803	37.09	3	155	302	460	2.19	4	188	2,392	2,584	12.28
Lapu-Lapu City	9,310	4	449	5,168	5,621	60.38	2	9	83	94	1.01	0	23	468	491	5.27
Mandaue City	8,272	4	369	4,238	4,611	55.74	0	15	122	137	1.66	0	13	149	162	1.96

Table 1.B.1.2 - Prenatal Care

Number and Proportion of Pregnant Women Seen According to their Nutritional Status by Region, Province/City and Age Group
Philippines, 2021

Area	Eligible Pop. (Under 1)	Pregnant women seen during the 1st trimester according to their Body Mass Index (BMI)														
		Normal BMI					Low BMI					High BMI				
		Age Group			Total	%	Age Group			Total	%	Age Group			Total	%
		10-14	15-19	20-49			10-14	15-19	20-49			10-14	15-19	20-49		
**Region 8**	**104,016**	**89**	**4,301**	**31,313**	**35,703**	**34.32**	**13**	**389**	**1,846**	**2,248**	**2.16**	**2**	**240**	**2,762**	**3,004**	**2.89**
Biliran	3,856	4	217	1,344	1,565	40.59	1	29	96	126	3.27	0	32	324	356	9.23
Eastern Samar	11,635	14	559	3,796	4,369	37.55	0	18	65	83	0.71	0	37	437	474	4.07
Northern Leyte	35,052	33	1,557	11,583	13,173	37.58	3	73	392	468	1.34	1	64	629	694	1.98
Northern Samar	15,655	9	494	3,680	4,183	26.72	0	32	149	181	1.16	0	21	186	207	1.32
Southern Leyte	6,556	2	199	1,899	2,100	32.03	1	24	59	84	1.28	0	19	292	311	4.74
Western Samar	14,515	19	543	3,650	4,212	29.02	7	73	463	543	3.74	0	23	257	280	1.93
Calbayog City	4,474	0	137	785	922	20.61	0	79	364	443	9.90	0	1	108	109	2.44
Maasin City	1,661	0	25	424	449	27.03	0	10	42	52	3.13	0	5	155	160	9.63
Ormoc City	4,989	4	320	1,882	2,206	44.22	1	35	76	112	2.24	1	35	361	397	7.96
Tacloban City	5,623	4	250	2,270	2,524	44.89	0	16	140	156	2.77	0	3	13	16	0.28
**Region 9**	**79,552**	**107**	**5,842**	**32,455**	**38,404**	**48.28**	**21**	**402**	**1,117**	**1,540**	**1.94**	**1**	**180**	**1,786**	**1,967**	**2.47**
Zamboanga del Norte	17,075	39	1,286	6,330	7,655	44.83	7	79	202	288	1.69	1	22	215	238	1.39
Zamboanga del Sur	17,506	24	1,122	5,834	6,980	39.87	5	128	285	418	2.39	0	35	476	511	2.92
Zamboanga Sibugay	14,992	21	920	4,751	5,692	37.97	3	69	146	218	1.45	0	35	311	346	2.31
Dapitan City	1,755	0	44	1,442	1,486	84.67	2	5	6	13	0.74	0	5	29	34	1.94
Dipolog City	2,794	5	280	1,722	2,007	71.83	3	15	66	84	3.01	0	2	137	139	4.97
Isabela City	2,544	5	240	1,300	1,545	60.73	0	19	63	82	3.22	0	1	46	47	1.85
Pagadian City	4,290	0	292	1,454	1,746	40.70	0	23	75	98	2.28	0	13	55	68	1.59
Zamboanga City	18,596	13	1,658	9,622	11,293	60.73	1	64	274	339	1.82	0	67	517	584	3.14
**Region 10**	**100,690**	**209**	**8,182**	**43,423**	**51,814**	**51.46**	**39**	**888**	**2,689**	**3,616**	**3.59**	**49**	**832**	**6,969**	**7,850**	**7.80**
Bukidnon	23,307	88	2,827	9,807	12,722	54.58	11	391	1,109	1,511	6.48	16	276	2,990	3,282	14.08
Camiguin	1,854	1	56	429	486	26.21	0	0	1	1	0.05	0	0	11	11	0.59
Lanao del Norte	14,955	9	687	6,962	7,658	51.21	0	46	318	364	2.43	0	7	91	98	0.66
Misamis Occidental	6,413	9	301	3,012	3,322	51.80	9	27	77	113	1.76	9	18	212	239	3.73
Misamis Oriental	15,058	9	1,542	7,715	9,266	61.54	3	94	250	347	2.30	0	24	308	332	2.20
Cagayan de Oro City	14,263	14	678	5,430	6,122	42.92	6	150	508	664	4.66	0	178	1,827	2,005	14.06
El Salvador City	1,061	1	62	244	307	28.93	0	2	6	8	0.75	0	6	31	37	3.49
Gingoog City	2,627	31	534	1,570	2,135	81.27	9	103	280	392	14.92	21	209	569	799	30.41
Iligan City	7,575	11	421	2,395	2,827	37.32	0	31	65	96	1.27	1	28	282	311	4.11
Malaybalay City	3,882	3	285	1,292	1,580	40.70	0	15	32	47	1.21	0	15	176	191	4.92
Oroquieta City	1,389	1	34	353	388	27.93	1	0	7	8	0.58	0	0	13	13	0.94
Ozamis City	2,782	4	252	1,672	1,928	69.30	0	0	4	4	0.14	0	2	33	35	1.26
Tangub City	1,234	21	63	476	560	45.38	0	0	0	0	0.00	0	0	0	0	0.00
Valencia City	4,290	7	440	2,066	2,513	58.58	0	29	32	61	1.42	2	69	426	497	11.59
**Region 11**	**107,847**	**416**	**8,709**	**41,739**	**50,864**	**47.16**	**79**	**1,152**	**2,881**	**4,112**	**3.81**	**65**	**1,073**	**11,026**	**12,164**	**11.28**
Davao de Oro	15,526	78	1,593	5,936	7,607	49.00	16	262	500	778	5.01	14	286	3,269	3,569	22.99
Davao del Norte	21,184	85	1,951	10,603	12,639	59.66	13	300	747	1,060	5.00	11	191	2,644	2,846	13.43
Davao Oriental	13,125	50	1,007	4,220	5,277	40.21	4	103	280	387	2.95	7	138	1,343	1,488	11.34
Davao del Sur	14,355	49	908	3,903	4,860	33.86	7	143	400	550	3.83	8	191	1,319	1,518	10.57
Davao Occidental	6,598	40	652	1,373	2,065	31.30	8	86	123	217	3.29	8	61	356	425	6.44
Davao City	37,059	114	2,598	15,704	18,416	49.69	31	258	831	1,120	3.02	17	206	2,095	2,318	6.25
**Region 12**	**104,693**	**189**	**9,575**	**44,784**	**54,548**	**52.10**	**25**	**856**	**1,833**	**2,714**	**2.59**	**17**	**513**	**5,454**	**5,984**	**5.72**
North Cotabato	33,933	37	2,699	13,088	15,824	46.63	9	183	412	604	1.78	3	116	1,080	1,199	3.53
Sarangani	12,904	40	1,760	6,716	8,516	66.00	3	155	300	458	3.55	8	151	1,055	1,214	9.41
South Cotabato	21,039	45	2,179	10,523	12,747	60.59	7	212	442	661	3.14	1	112	1,581	1,694	8.05
Sultan Kudarat	17,331	60	1,685	7,632	9,377	54.11	1	115	315	431	2.49	4	61	895	960	5.54
Cotabato City	5,831	0	235	2,020	2,255	38.67	1	12	46	59	1.01	0	12	122	134	2.30
Gen. Santos City	13,655	7	1,017	4,805	5,829	42.69	4	179	318	501	3.67	1	61	721	783	5.73
**BARMM**	**94,148**	**34**	**3,843**	**33,967**	**37,844**	**40.20**	**10**	**562**	**1,437**	**2,009**	**2.13**	**9**	**140**	**1,377**	**1,526**	**1.62**
Basilan	7,672	3	253	1,355	1,611	21.00	1	30	91	122	1.59	0	3	53	56	0.73
Lanao del Sur	21,391	7	693	13,915	14,615	68.32	0	8	44	52	0.24	0	1	45	46	0.22
Maguindanao	31,650	19	1,505	12,372	13,896	43.91	8	137	303	448	1.42	9	70	813	892	2.82
Sulu	16,891	0	0	0	0	0.00	0	0	0	0	0.00	0	0	0	0	0.00
Tawi-Tawi	9,319	0	271	3,260	3,531	37.89	1	33	107	141	1.51	0	12	200	212	2.27
Lamitan City	2,112	4	137	378	519	24.57	0	11	18	29	1.37	0	11	48	59	2.79
Marawi City	5,113	1	984	2,687	3,672	71.82	0	343	874	1,217	23.80	0	43	218	261	5.10

Table 1.B.1.2 - Prenatal Care

Number and Proportion of Pregnant Women Seen According to their Nutritional Status by Region, Province/City and Age Group
Philippines, 2021

Area	Eligible Pop. (Under 1)	Pregnant women seen during the 1st trimester according to their Body Mass Index (BMI)														
		Normal BMI					Low BMI					High BMI				
		Age Group			Total	%	Age Group			Total	%	Age Group			Total	%
		10-14	15-19	20-49			10-14	15-19	20-49			10-14	15-19	20-49		
**CARAGA**	**60,342**	**104**	**4,099**	**25,283**	**29,486**	**48.86**	**6**	**243**	**763**	**1,012**	**1.68**	**1**	**184**	**1,989**	**2,174**	**3.60**
Agusan del Norte	8,116	28	633	3,255	3,916	48.25	0	21	81	102	1.26	0	26	216	242	2.98
Agusan del Sur	17,658	32	1,408	8,705	10,145	57.45	1	54	184	239	1.35	1	78	457	536	3.04
Surigao del Norte	7,195	9	448	2,902	3,359	46.69	0	31	68	99	1.38	0	10	173	183	2.54
Surigao del Sur	11,532	15	599	3,763	4,377	37.96	3	50	161	214	1.86	0	20	493	513	4.45
Province of Dinagat	2,574	1	131	962	1,094	42.50	0	4	35	39	1.52	0	3	66	69	2.68
Bislig City	2,185	4	135	824	963	44.07	0	19	13	32	1.46	0	5	128	133	6.09
Butuan City	7,732	8	490	3,332	3,830	49.53	2	28	116	146	1.89	0	17	195	212	2.74
Surigao City	3,350	7	255	1,540	1,802	53.79	0	36	105	141	4.21	0	25	261	286	8.54

Note: Put asterisk (*) for No Report and Zero (0) for No Case

Table 1.B.1.3 - Prenatal Care

Number and proportion of pregnant women given Tetanus Diptheria (Td) Vaccination

Philippines, 2021

Area	Eligible Pop. (Under 1)	Women pregnant for the 1st time given at least two (2) doses of Td Vaccine							
		Age Group						Total	%
		10-14 yrs old		15-19 yrs old		20-49 yrs old			
		No.	%	No.	%	No.	%		
**PHILIPPINES**	**2,128,168**	**2,095**	**0.10**	**102,979**	**4.84**	**424,964**	**19.97**	**530,038**	**24.91**
**N C R**	**237,921**	**138**	**0.06**	**8,232**	**3.46**	**47,678**	**20.04**	**56,048**	**23.56**
Malabon	6,757	16	0.24	426	6.30	1,704	25.22	2,146	31.76
Navotas	4,607	2	0.04	277	6.01	1,190	25.83	1,469	31.89
Valenzuela City	11,465	6	0.05	377	3.29	4,403	38.40	4,786	41.74
Caloocan City	29,270	14	0.05	1,341	4.58	9,451	32.29	10,806	36.92
Marikina City	8,330	6	0.07	236	2.83	1,242	14.91	1,484	17.82
Pasig City	13,954	2	0.01	417	2.99	3,264	23.39	3,683	26.39
Pateros	1,181	0	0.00	31	2.62	159	13.46	190	16.09
Taguig	14,874	7	0.05	498	3.35	1,859	12.50	2,364	15.89
Quezon City	54,246	44	0.08	1,915	3.53	10,026	18.48	11,985	22.09
Makati City	10,765	0	0.00	67	0.62	1,224	11.37	1,291	11.99
Mandaluyong City	7,137	4	0.06	159	2.23	1,215	17.02	1,378	19.31
San Juan	2,254	1	0.04	18	0.80	365	16.19	384	17.04
Manila City	32,887	10	0.03	1,293	3.93	4,710	14.32	6,013	18.28
Las Piñas City	10,879	3	0.03	265	2.44	2,195	20.18	2,463	22.64
Muntinlupa City	9,317	7	0.08	367	3.94	2,159	23.17	2,533	27.19
Parañaque City	12,298	8	0.07	301	2.45	1,311	10.66	1,620	13.17
Pasay City	7,700	8	0.10	244	3.17	1,201	15.60	1,453	18.87
**C A R**	**35,143**	**20**	**0.06**	**1,070**	**3.04**	**5,529**	**15.73**	**6,619**	**18.83**
Abra	4,287	1	0.02	153	3.57	612	14.28	766	17.87
Apayao	2,476	6	0.24	158	6.38	354	14.30	518	20.92
Benguet	9,140	1	0.01	202	2.21	1,556	17.02	1,759	19.25
Ifugao	4,439	4	0.09	148	3.33	913	20.57	1,065	23.99
Kalinga	4,660	3	0.06	211	4.53	755	16.20	969	20.79
Mt. Province	3,066	1	0.03	85	2.77	441	14.38	527	17.19
Baguio City	7,075	4	0.06	113	1.60	898	12.69	1,015	14.35
**Region 1**	**97,198**	**32**	**0.03**	**3,650**	**3.76**	**21,060**	**21.67**	**24,742**	**25.46**
Ilocos Norte	7,986	0	0.00	200	2.50	1,680	21.04	1,880	23.54
Ilocos Sur	9,197	3	0.03	246	2.67	2,337	25.41	2,586	28.12
La Union	11,429	5	0.04	532	4.65	2,733	23.91	3,270	28.61
Pangasinan	50,454	12	0.02	1,967	3.90	10,103	20.02	12,082	23.95
Alaminos City	1,905	0	0.00	64	3.36	546	28.66	610	32.02
Candon City	976	0	0.00	1	0.10	544	55.74	545	55.84
Dagupan City	3,636	1	0.03	168	4.62	449	12.35	618	17.00
Laoag City	1,845	0	0.00	3	0.16	535	29.00	538	29.16
San Carlos City	3,999	4	0.10	177	4.43	664	16.60	845	21.13
San Fernando City	2,092	3	0.14	101	4.83	422	20.17	526	25.14
Urdaneta City	2,819	2	0.07	137	4.86	852	30.22	991	35.15

Table 1.B.1.3 - Prenatal Care

Number and proportion of pregnant women given Tetanus Diptheria (Td) Vaccination
Philippines, 2021

Area	Eligible Pop. (Under 1)	Women pregnant for the 1st time given at least two (2) doses of Td Vaccine							
		Age Group						Total	%
		10-14 yrs old		15-19 yrs old		20-49 yrs old			
		No.	%	No.	%	No.	%		
Vigan City	860	2	0.23	54	6.28	195	22.67	251	29.19
**Region 2**	**69,228**	**59**	**0.09**	**3,429**	**4.95**	**13,215**	**19.09**	**16,703**	**24.13**
Batanes	351	2	0.57	7	1.99	72	20.51	81	23.08
Cagayan	20,563	10	0.05	965	4.69	3,344	16.26	4,319	21.00
Isabela	23,543	31	0.13	1,218	5.17	4,540	19.28	5,789	24.59
Nueva Vizcaya	9,555	5	0.05	536	5.61	1,919	20.08	2,460	25.75
Quirino	4,038	0	0.00	217	5.37	1,066	26.40	1,283	31.77
Cauayan City	2,576	3	0.12	137	5.32	609	23.64	749	29.08
Ilagan City	2,901	3	0.10	102	3.52	438	15.10	543	18.72
Santiago City	2,683	4	0.15	199	7.42	881	32.84	1,084	40.40
Tuguegarao City	3,018	1	0.03	48	1.59	346	11.46	395	13.09
**Region 3**	**220,202**	**170**	**0.08**	**10,223**	**4.64**	**43,969**	**19.97**	**54,362**	**24.69**
Aurora	4,764	0	0.00	256	5.37	853	17.91	1,109	23.28
Bataan	13,814	22	0.16	665	4.81	1,989	14.40	2,676	19.37
Bulacan	43,722	29	0.07	2,099	4.80	8,457	19.34	10,585	24.21
Nueva Ecija	28,915	24	0.08	1,112	3.85	3,904	13.50	5,040	17.43
Pampanga	31,503	24	0.08	1,067	3.39	5,237	16.62	6,328	20.09
Tarlac	19,565	7	0.04	1,065	5.44	5,576	28.50	6,648	33.98
Zambales	12,551	6	0.05	361	2.88	1,230	9.80	1,597	12.72
Angeles City	7,905	0	0.00	171	2.16	1,139	14.41	1,310	16.57
Balanga City	1,996	3	0.15	114	5.71	287	14.38	404	20.24
Cabanatuan City	5,921	3	0.05	309	5.22	973	16.43	1,285	21.70
City of San Fernando	5,890	7	0.12	205	3.48	1,069	18.15	1,281	21.75
Gapan City	2,159	0	0.00	65	3.01	308	14.27	373	17.28
Mabalacat City	4,811	7	0.15	379	7.88	1,381	28.71	1,767	36.73
Malolos City	4,885	4	0.08	89	1.82	671	13.74	764	15.64
Meycauayan	4,050	2	0.05	341	8.42	1,830	45.19	2,173	53.65
Olongapo	4,952	5	0.10	148	2.99	820	16.56	973	19.65
Palayan City	805	0	0.00	22	2.73	105	13.04	127	15.78
San Jose City	2,736	6	0.22	241	8.81	853	31.18	1,100	40.20
San Jose del Monte City	11,120	16	0.14	760	6.83	4,863	43.73	5,639	50.71
Science City of Munoz	1,594	2	0.13	77	4.83	221	13.86	300	18.82
Tarlac City	6,544	3	0.05	677	10.35	2,203	33.66	2,883	44.06
**Region 4A**	**298,398**	**202**	**0.07**	**10,310**	**3.46**	**51,692**	**17.32**	**62,204**	**20.85**
Batangas	38,848	15	0.04	1,073	2.76	7,410	19.07	8,498	21.88
Cavite	27,948	18	0.06	843	3.02	3,780	13.53	4,641	16.61
Laguna	19,727	30	0.15	958	4.86	3,581	18.15	4,569	23.16
Quezon	37,644	48	0.13	1,686	4.48	6,880	18.28	8,614	22.88
Rizal	44,826	20	0.04	1,165	2.60	3,994	8.91	5,179	11.55
Antipolo City	16,510	0	0.00	689	4.17	5,129	31.07	5,818	35.24
Bacoor City	12,252	1	0.01	147	1.20	747	6.10	895	7.30
Batangas City	6,895	3	0.04	236	3.42	1,460	21.17	1,699	24.64

Table 1.B.1.3 - Prenatal Care

Number and proportion of pregnant women given Tetanus Diptheria (Td) Vaccination

Philippines, 2021

Area	Eligible Pop. (Under 1)	Women pregnant for the 1st time given at least two (2) doses of Td Vaccine							
		Age Group						Total	%
		10-14 yrs old		15-19 yrs old		20-49 yrs old			
		No.	%	No.	%	No.	%		
Biñan City	6,607	1	0.02	884	13.38	3,610	54.64	4,495	68.03
Cabuyao City	6,128	3	0.05	333	5.43	1,588	25.91	1,924	31.40
Calamba City	9,025	11	0.12	359	3.98	2,089	23.15	2,459	27.25
Cavite City	2,098	0	0.00	32	1.53	112	5.34	144	6.86
Dasmariñas City	13,442	8	0.06	267	1.99	1,983	14.75	2,258	16.80
General Trias City	6,410	3	0.05	156	2.43	983	15.34	1,142	17.82
Imus City	8,235	0	0.00	107	1.30	492	5.97	599	7.27
Lipa City	6,954	2	0.03	170	2.44	1,065	15.31	1,237	17.79
Lucena City	5,704	2	0.04	262	4.59	1,279	22.42	1,543	27.05
San Pablo City	5,277	3	0.06	254	4.81	691	13.09	948	17.96
San Pedro City	6,465	9	0.14	106	1.64	582	9.00	697	10.78
Santa Rosa City	7,023	16	0.23	354	5.04	2,365	33.68	2,735	38.94
Tagaytay City	1,451	1	0.07	29	2.00	500	34.46	530	36.53
Tanauan City	3,620	3	0.08	53	1.46	876	24.20	932	25.75
Tayabas City	2,134	4	0.19	91	4.26	309	14.48	404	18.93
Trece Martires City	3,175	1	0.03	56	1.76	187	5.89	244	7.69
**Region 4B**	**72,035**	**55**	**0.08**	**3,181**	**4.42**	**11,761**	**16.33**	**14,997**	**20.82**
Marinduque	4,906	2	0.04	185	3.77	751	15.31	938	19.12
Mindoro Occidental	12,449	15	0.12	842	6.76	2,162	17.37	3,019	24.25
Mindoro Oriental	20,011	9	0.04	809	4.04	3,405	17.02	4,223	21.10
Palawan	21,676	22	0.10	1,109	5.12	4,325	19.95	5,456	25.17
Romblon	6,482	7	0.11	236	3.64	1,118	17.25	1,361	21.00
Puerto Princesa City	6,511	0	0.00	0	0.00	0	0.00	0	0.00
**Region 5**	**137,350**	**49**	**0.04**	**5,415**	**3.94**	**23,198**	**16.89**	**28,662**	**20.87**
Albay	23,903	9	0.04	644	2.69	4,535	18.97	5,188	21.70
Camarines Norte	14,340	9	0.06	836	5.83	2,322	16.19	3,167	22.09
Camarines Sur	39,151	10	0.03	1,148	2.93	6,032	15.41	7,190	18.36
Catanduanes	6,381	4	0.06	230	3.60	822	12.88	1,056	16.55
Masbate	22,565	13	0.06	1,555	6.89	4,354	19.30	5,922	26.24
Sorsogon	19,470	3	0.02	685	3.52	2,801	14.39	3,489	17.92
Iriga City	2,665	1	0.04	50	1.88	350	13.13	401	15.05
Legaspi City	4,205	0	0.00	126	3.00	1,150	27.35	1,276	30.34
Naga City	4,670	0	0.00	141	3.02	832	17.82	973	20.84
**Region 6**	**146,545**	**96**	**0.07**	**6,354**	**4.34**	**26,698**	**18.22**	**33,148**	**22.62**
Aklan	11,231	7	0.06	333	2.97	2,150	19.14	2,490	22.17
Antique	12,985	5	0.04	449	3.46	2,121	16.33	2,575	19.83
Capiz	13,984	10	0.07	421	3.01	2,132	15.25	2,563	18.33
Guimaras	3,085	2	0.06	124	4.02	761	24.67	887	28.75
Iloilo	36,414	19	0.05	1,293	3.55	7,576	20.81	8,888	24.41
Negros Occidental	49,314	39	0.08	3,082	6.25	9,358	18.98	12,479	25.31
Bacolod City	11,102	5	0.05	331	2.98	1,379	12.42	1,715	15.45
Iloilo City	8,430	9	0.11	321	3.81	1,221	14.48	1,551	18.40

Table 1.B.1.3 - Prenatal Care

Number and proportion of pregnant women given Tetanus Diptheria (Td) Vaccination

Philippines, 2021

Area	Eligible Pop. (Under 1)	Women pregnant for the 1st time given at least two (2) doses of Td Vaccine							
		Age Group						Total	%
		10-14 yrs old		15-19 yrs old		20-49 yrs old			
		No.	%	No.	%	No.	%		
**Region 7**	**162,860**	**134**	**0.08**	**8,493**	**5.21**	**32,136**	**19.73**	**40,763**	**25.03**
Bohol	27,637	8	0.03	1,084	3.92	6,225	22.52	7,317	26.48
Cebu	67,046	57	0.09	3,525	5.26	11,390	16.99	14,972	22.33
Negros Oriental	27,922	47	0.17	1,831	6.56	5,041	18.05	6,919	24.78
Siquijor	1,636	0	0.00	86	5.26	396	24.21	482	29.46
Cebu City	21,037	15	0.07	1,222	5.81	4,115	19.56	5,352	25.44
Lapu-Lapu City	9,310	1	0.01	390	4.19	2,768	29.73	3,159	33.93
Mandaue City	8,272	6	0.07	355	4.29	2,201	26.61	2,562	30.97
**Region 8**	**104,016**	**71**	**0.07**	**3,693**	**3.55**	**16,328**	**15.70**	**20,092**	**19.32**
Biliran	3,856	2	0.05	197	5.11	660	17.12	859	22.28
Eastern Samar	11,635	4	0.03	441	3.79	1,749	15.03	2,194	18.86
Northern Leyte	35,052	26	0.07	1,075	3.07	4,908	14.00	6,009	17.14
Northern Samar	15,655	7	0.04	487	3.11	2,625	16.77	3,119	19.92
Southern Leyte	6,556	5	0.08	234	3.57	1,148	17.51	1,387	21.16
Western Samar	14,515	15	0.10	512	3.53	1,794	12.36	2,321	15.99
Calbayog City	4,474	0	0.00	176	3.93	775	17.32	951	21.26
Maasin City	1,661	0	0.00	48	2.89	381	22.94	429	25.83
Ormoc City	4,989	7	0.14	326	6.53	858	17.20	1,191	23.87
Tacloban City	5,623	5	0.09	197	3.50	1,430	25.43	1,632	29.02
**Region 9**	**79,552**	**78**	**0.10**	**5,772**	**7.26**	**19,074**	**23.98**	**24,924**	**31.33**
Zamboanga del Norte	17,075	30	0.18	1,524	8.93	4,939	28.93	6,493	38.03
Zamboanga del Sur	17,506	19	0.11	1,206	6.89	3,435	19.62	4,660	26.62
Zamboanga Sibugay	14,992	8	0.05	991	6.61	2,529	16.87	3,528	23.53
Dapitan City	1,755	0	0.00	43	2.45	1,036	59.03	1,079	61.48
Dipolog City	2,794	4	0.14	196	7.02	1,041	37.26	1,241	44.42
Isabela City	2,544	0	0.00	174	6.84	767	30.15	941	36.99
Pagadian City	4,290	0	0.00	260	6.06	1,299	30.28	1,559	36.34
Zamboanga City	18,596	17	0.09	1,378	7.41	4,028	21.66	5,423	29.16
**Region 10**	**100,690**	**178**	**0.18**	**7,837**	**7.78**	**25,042**	**24.87**	**33,057**	**32.83**
Bukidnon	23,307	89	0.38	2,497	10.71	4,861	20.86	7,447	31.95
Camiguin	1,854	1	0.05	55	2.97	190	10.25	246	13.27
Lanao del Norte	14,955	17	0.11	678	4.53	5,545	37.08	6,240	41.73
Misamis Occidental	6,413	3	0.05	252	3.93	1,481	23.09	1,736	27.07
Misamis Oriental	15,058	16	0.11	1,269	8.43	3,968	26.35	5,253	34.89
Cagayan de Oro City	14,263	15	0.11	1,379	9.67	3,573	25.05	4,967	34.82
El Salvador City	1,061	1	0.09	87	8.20	161	15.17	249	23.47
Gingoog City	2,627	12	0.46	273	10.39	752	28.63	1,037	39.47
Iligan City	7,575	7	0.09	451	5.95	1,490	19.67	1,948	25.72
Malaybalay City	3,882	5	0.13	292	7.52	738	19.01	1,035	26.66

Table 1.B.1.3 - Prenatal Care

Number and proportion of pregnant women given Tetanus Diptheria (Td) Vaccination

Philippines, 2021

Area	Eligible Pop. (Under 1)	Women pregnant for the 1st time given at least two (2) doses of Td Vaccine							
		Age Group						Total	%
		10-14 yrs old		15-19 yrs old		20-49 yrs old			
		No.	%	No.	%	No.	%		
Oroquieta City	1,389	1	0.07	29	2.09	316	22.75	346	24.91
Ozamis City	2,782	3	0.11	233	8.38	570	20.49	806	28.97
Tangub City	1,234	2	0.16	80	6.48	553	44.81	635	51.46
Valencia City	4,290	6	0.14	262	6.11	844	19.67	1,112	25.92
**Region 11**	**107,847**	**495**	**0.46**	**8,891**	**8.24**	**23,874**	**22.14**	**33,260**	**30.84**
Davao de Oro	15,526	89	0.57	1,546	9.96	2,846	18.33	4,481	28.86
Davao del Norte	21,184	117	0.55	1,869	8.82	5,246	24.76	7,232	34.14
Davao Oriental	13,125	47	0.36	1,002	7.63	2,095	15.96	3,144	23.95
Davao del Sur	14,355	65	0.45	1,176	8.19	2,129	14.83	3,370	23.48
Davao Occidental	6,598	62	0.94	957	14.50	826	12.52	1,845	27.96
Davao City	37,059	115	0.31	2,341	6.32	10,732	28.96	13,188	35.59
**Region 12**	**104,693**	**194**	**0.19**	**9,018**	**8.61**	**28,478**	**27.20**	**37,690**	**36.00**
North Cotabato	33,933	41	0.12	2,433	7.17	8,815	25.98	11,289	33.27
Sarangani	12,904	44	0.34	1,657	12.84	3,814	29.56	5,515	42.74
South Cotabato	21,039	30	0.14	1,970	9.36	6,378	30.32	8,378	39.82
Sultan Kudarat	17,331	54	0.31	1,586	9.15	4,396	25.36	6,036	34.83
Cotabato City	5,831	0	0.00	226	3.88	2,017	34.59	2,243	38.47
Gen. Santos City	13,655	25	0.18	1,146	8.39	3,058	22.39	4,229	30.97
**BARMM**	**94,148**	**54**	**0.06**	**3,882**	**4.12**	**24,136**	**25.64**	**28,072**	**29.82**
Basilan	7,672	2	0.03	205	2.67	1,162	15.15	1,369	17.84
Lanao del Sur	21,391	6	0.03	511	2.39	8,422	39.37	8,939	41.79
Maguindanao	31,650	37	0.12	1,891	5.97	8,005	25.29	9,933	31.38
Sulu	16,891	0	0.00	0	0.00	0	0.00	0	0.00
Tawi-Tawi	9,319	2	0.02	383	4.11	3,217	34.52	3,602	38.65
Lamitan City	2,112	6	0.28	191	9.04	372	17.61	569	26.94
Marawi City	5,113	1	0.02	701	13.71	2,958	57.85	3,660	71.58
**CARAGA**	**60,342**	**70**	**0.12**	**3,529**	**5.85**	**11,096**	**18.39**	**14,695**	**24.35**
Agusan del Norte	8,116	13	0.16	462	5.69	1,567	19.31	2,042	25.16
Agusan del Sur	17,658	28	0.16	1,157	6.55	3,179	18.00	4,364	24.71
Surigao del Norte	7,195	7	0.10	320	4.45	1,325	18.42	1,652	22.96
Surigao del Sur	11,532	7	0.06	666	5.78	2,177	18.88	2,850	24.71
Province of Dinagat	2,574	0	0.00	91	3.54	372	14.45	463	17.99
Bislig City	2,185	4	0.18	116	5.31	304	13.91	424	19.41
Butuan City	7,732	9	0.12	518	6.70	1,552	20.07	2,079	26.89
Surigao City	3,350	2	0.06	199	5.94	620	18.51	821	24.51

Note: Put asterisk (*) for No Report and Zero (0) for No Case

Table 1.B.1.4 - Prenatal Care

Number and proportion of pregnant women given Tetanus Diptheria (Td) Vaccination
Philippines, 2021

Area	Eligible Pop. (Under 1)	Women pregnant for the 2nd or more times given at least three (3) doses of Td Vaccine							
		Age Group						Total	%
		10-14 yrs old		15-19 yrs old		20-49 yrs old			
		No.	%	No.	%	No.	%		
**PHILIPPINES**	**2,128,168**	**1,027**	**0.05**	**68,342**	**3.21**	**752,918**	**35.38**	**822,287**	**38.64**
**N C R**	**237,921**	**83**	**0.03**	**6,746**	**2.84**	**91,699**	**38.54**	**98,528**	**41.41**
Malabon	6,757	2	0.03	190	2.81	2,718	40.22	2,910	43.07
Navotas	4,607	3	0.07	301	6.53	1,911	41.48	2,215	48.08
Valenzuela City	11,465	3	0.03	234	2.04	2,936	25.61	3,173	27.68
Caloocan City	29,270	3	0.01	857	2.93	9,967	34.05	10,827	36.99
Marikina City	8,330	1	0.01	86	1.03	2,020	24.25	2,107	25.29
Pasig City	13,954	2	0.01	491	3.52	7,110	50.95	7,603	54.49
Pateros	1,181	0	0.00	13	1.10	255	21.59	268	22.69
Taguig	14,874	6	0.04	616	4.14	5,336	35.87	5,958	40.06
Quezon City	54,246	24	0.04	1,237	2.28	32,233	59.42	33,494	61.74
Makati City	10,765	2	0.02	151	1.40	3,207	29.79	3,360	31.21
Mandaluyong City	7,137	3	0.04	202	2.83	2,319	32.49	2,524	35.36
San Juan	2,254	0	0.00	6	0.27	429	19.03	435	19.30
Manila City	32,887	6	0.02	1,208	3.67	8,148	24.78	9,362	28.47
Las Piñas City	10,879	4	0.04	274	2.52	3,655	33.60	3,933	36.15
Muntinlupa City	9,317	1	0.01	221	2.37	3,366	36.13	3,588	38.51
Parañaque City	12,298	15	0.12	349	2.84	2,858	23.24	3,222	26.20
Pasay City	7,700	8	0.10	310	4.03	3,231	41.96	3,549	46.09
**C A R**	**35,143**	**9**	**0.03**	**762**	**2.17**	**10,817**	**30.78**	**11,588**	**32.97**
Abra	4,287	0	0.00	138	3.22	1,175	27.41	1,313	30.63
Apayao	2,476	2	0.08	66	2.67	796	32.15	864	34.89
Benguet	9,140	0	0.00	99	1.08	2,785	30.47	2,884	31.55
Ifugao	4,439	3	0.07	155	3.49	1,600	36.04	1,758	39.60
Kalinga	4,660	4	0.09	182	3.91	2,027	43.50	2,213	47.49
Mt. Province	3,066	0	0.00	70	2.28	1,150	37.51	1,220	39.79
Baguio City	7,075	0	0.00	52	0.73	1,284	18.15	1,336	18.88
**Region 1**	**97,198**	**14**	**0.01**	**2,545**	**2.62**	**40,318**	**41.48**	**42,877**	**44.11**
Ilocos Norte	7,986	1	0.01	134	1.68	2,236	28.00	2,371	29.69
Ilocos Sur	9,197	1	0.01	128	1.39	4,028	43.80	4,157	45.20
La Union	11,429	2	0.02	299	2.62	4,151	36.32	4,452	38.95
Pangasinan	50,454	6	0.01	1,515	3.00	21,829	43.27	23,350	46.28
Alaminos City	1,905	0	0.00	46	2.41	942	49.45	988	51.86
Candon City	976	0	0.00	0	0.00	590	60.45	590	60.45
Dagupan City	3,636	0	0.00	109	3.00	1,093	30.06	1,202	33.06
Laoag City	1,845	0	0.00	22	1.19	1,625	88.08	1,647	89.27
San Carlos City	3,999	0	0.00	27	0.68	1,235	30.88	1,262	31.56
San Fernando City	2,092	4	0.19	77	3.68	1,013	48.42	1,094	52.29
Urdaneta City	2,819	0	0.00	130	4.61	1,217	43.17	1,347	47.78
Vigan City	860	0	0.00	58	6.74	359	41.74	417	48.49

Table 1.B.1.4 - Prenatal Care

Number and proportion of pregnant women given Tetanus Diptheria (Td) Vaccination
Philippines, 2021

Area	Eligible Pop. (Under 1)	Women pregnant for the 2nd or more times given at least three (3) doses of Td Vaccine							
		Age Group						Total	%
		10-14 yrs old		15-19 yrs old		20-49 yrs old			
		No.	%	No.	%	No.	%		
**Region 2**	**69,228**	**47**	**0.07**	**2,362**	**3.41**	**25,875**	**37.38**	**28,284**	**40.86**
Batanes	351	0	0.00	5	1.42	88	25.07	93	26.50
Cagayan	20,563	6	0.03	457	2.22	6,193	30.12	6,656	32.37
Isabela	23,543	25	0.11	895	3.80	8,761	37.21	9,681	41.12
Nueva Vizcaya	9,555	5	0.05	377	3.95	3,628	37.97	4,010	41.97
Quirino	4,038	1	0.02	134	3.32	1,695	41.98	1,830	45.32
Cauayan City	2,576	3	0.12	160	6.21	1,060	41.15	1,223	47.48
Ilagan City	2,901	1	0.03	76	2.62	1,309	45.12	1,386	47.78
Santiago City	2,683	4	0.15	225	8.39	2,501	93.22	2,730	101.75
Tuguegarao City	3,018	2	0.07	33	1.09	640	21.21	675	22.37
**Region 3**	**220,202**	**225**	**0.10**	**8,041**	**3.65**	**78,803**	**35.79**	**87,069**	**39.54**
Aurora	4,764	0	0.00	73	1.53	1,640	34.42	1,713	35.96
Bataan	13,814	5	0.04	424	3.07	5,175	37.46	5,604	40.57
Bulacan	43,722	16	0.04	1,573	3.60	15,218	34.81	16,807	38.44
Nueva Ecija	28,915	18	0.06	921	3.19	7,938	27.45	8,877	30.70
Pampanga	31,503	6	0.02	922	2.93	8,237	26.15	9,165	29.09
Tarlac	19,565	2	0.01	804	4.11	9,035	46.18	9,841	50.30
Zambales	12,551	15	0.12	202	1.61	2,524	20.11	2,741	21.84
Angeles City	7,905	1	0.01	291	3.68	2,306	29.17	2,598	32.87
Balanga City	1,996	0	0.00	60	3.01	650	32.57	710	35.57
Cabanatuan City	5,921	1	0.02	111	1.87	1,960	33.10	2,072	34.99
City of San Fernando	5,890	4	0.07	217	3.68	1,612	27.37	1,833	31.12
Gapan City	2,159	0	0.00	62	2.87	724	33.53	786	36.41
Mabalacat City	4,811	3	0.06	457	9.50	2,428	50.47	2,888	60.03
Malolos City	4,885	2	0.04	136	2.78	1,578	32.30	1,716	35.13
Meycauayan	4,050	0	0.00	243	6.00	2,083	51.43	2,326	57.43
Olongapo	4,952	0	0.00	89	1.80	1,756	35.46	1,845	37.26
Palayan City	805	0	0.00	12	1.49	169	20.99	181	22.48
San Jose City	2,736	4	0.15	182	6.65	1,331	48.65	1,517	55.45
San Jose del Monte City	11,120	5	0.04	669	6.02	8,490	76.35	9,164	82.41
Science City of Munoz	1,594	4	0.25	93	5.83	656	41.15	753	47.24
Tarlac City	6,544	139	2.12	500	7.64	3,293	50.32	3,932	60.09
**Region 4A**	**298,398**	**107**	**0.04**	**7,636**	**2.56**	**85,392**	**28.62**	**93,135**	**31.21**
Batangas	38,848	10	0.03	718	1.85	13,343	34.35	14,071	36.22
Cavite	27,948	17	0.06	750	2.68	7,729	27.65	8,496	30.40
Laguna	19,727	15	0.08	643	3.26	5,545	28.11	6,203	31.44
Quezon	37,644	20	0.05	880	2.34	10,730	28.50	11,630	30.89
Rizal	44,826	20	0.04	1,082	2.41	8,376	18.69	9,478	21.14
Antipolo City	16,510	0	0.00	719	4.35	7,411	44.89	8,130	49.24
Bacoor City	12,252	0	0.00	134	1.09	1,144	9.34	1,278	10.43
Batangas City	6,895	0	0.00	46	0.67	1,657	24.03	1,703	24.70
Biñan City	6,607	1	0.02	747	11.31	3,925	59.41	4,673	70.73
Cabuyao City	6,128	2	0.03	250	4.08	2,725	44.47	2,977	48.58

Table 1.B.1.4 - Prenatal Care

Number and proportion of pregnant women given Tetanus Diptheria (Td) Vaccination
Philippines, 2021

Area	Eligible Pop. (Under 1)	Women pregnant for the 2nd or more times given at least three (3) doses of Td Vaccine							
		Age Group						Total	%
		10-14 yrs old		15-19 yrs old		20-49 yrs old			
		No.	%	No.	%	No.	%		
Calamba City	9,025	2	0.02	195	2.16	3,742	41.46	3,939	43.65
Cavite City	2,098	0	0.00	26	1.24	201	9.58	227	10.82
Dasmariñas City	13,442	5	0.04	209	1.55	2,747	20.44	2,961	22.03
General Trias City	6,410	4	0.06	117	1.83	1,647	25.69	1,768	27.58
Imus City	8,235	0	0.00	76	0.92	741	9.00	817	9.92
Lipa City	6,954	2	0.03	150	2.16	2,611	37.55	2,763	39.73
Lucena City	5,704	0	0.00	175	3.07	1,596	27.98	1,771	31.05
San Pablo City	5,277	1	0.02	231	4.38	1,823	34.55	2,055	38.94
San Pedro City	6,465	0	0.00	128	1.98	1,109	17.15	1,237	19.13
Santa Rosa City	7,023	5	0.07	214	3.05	2,986	42.52	3,205	45.64
Tagaytay City	1,451	1	0.07	22	1.52	797	54.93	820	56.51
Tanauan City	3,620	2	0.06	43	1.19	1,538	42.49	1,583	43.73
Tayabas City	2,134	0	0.00	53	2.48	800	37.49	853	39.97
Trece Martires City	3,175	0	0.00	28	0.88	469	14.77	497	15.65
**Region 4B**	**72,035**	**21**	**0.03**	**1,723**	**2.39**	**21,255**	**29.51**	**22,999**	**31.93**
									**0.00**
Marinduque	4,906	0	0.00	38	0.77	1,481	30.19	1,519	30.96
Mindoro Occidental	12,449	2	0.02	484	3.89	4,951	39.77	5,437	43.67
Mindoro Oriental	20,011	4	0.02	567	2.83	6,331	31.64	6,902	34.49
Palawan	21,676	11	0.05	485	2.24	6,042	27.87	6,538	30.16
Romblon	6,482	4	0.06	149	2.30	2,450	37.80	2,603	40.16
Puerto Princesa City	6,511	0	0.00	0	0.00	0	0.00	0	0.00
**Region 5**	**137,350**	**12**	**0.01**	**2,613**	**1.90**	**41,618**	**30.30**	**44,243**	**32.21**
Albay	23,903	0	0.00	266	1.11	7,900	33.05	8,166	34.16
Camarines Norte	14,340	1	0.01	314	2.19	5,404	37.68	5,719	39.88
Camarines Sur	39,151	3	0.01	395	1.01	8,519	21.76	8,917	22.78
Catanduanes	6,381	3	0.05	214	3.35	1,396	21.88	1,613	25.28
Masbate	22,565	5	0.02	881	3.90	7,997	35.44	8,883	39.37
Sorsogon	19,470	0	0.00	395	2.03	6,715	34.49	7,110	36.52
Iriga City	2,665	0	0.00	21	0.79	528	19.81	549	20.60
Legaspi City	4,205	0	0.00	29	0.69	1,688	40.14	1,717	40.83
Naga City	4,670	0	0.00	98	2.10	1,471	31.50	1,569	33.60
**Region 6**	**146,545**	**46**	**0.03**	**4,366**	**2.98**	**48,984**	**33.43**	**53,396**	**36.44**
Aklan	11,231	2	0.02	175	1.56	3,566	31.75	3,743	33.33
Antique	12,985	4	0.03	298	2.29	3,983	30.67	4,285	33.00
Capiz	13,984	1	0.01	296	2.12	4,119	29.46	4,416	31.58
Guimaras	3,085	2	0.06	136	4.41	1,715	55.59	1,853	60.06
Iloilo	36,414	9	0.02	1,013	2.78	12,435	34.15	13,457	36.96
Negros Occidental	49,314	20	0.04	2,110	4.28	19,228	38.99	21,358	43.31
Bacolod City	11,102	4	0.04	177	1.59	2,145	19.32	2,326	20.95
Iloilo City	8,430	4	0.05	161	1.91	1,793	21.27	1,958	23.23

Table 1.B.1.4 - Prenatal Care

Number and proportion of pregnant women given Tetanus Diptheria (Td) Vaccination
Philippines, 2021

Area	Eligible Pop. (Under 1)	Women pregnant for the 2nd or more times given at least three (3) doses of Td Vaccine							
		Age Group						Total	%
		10-14 yrs old		15-19 yrs old		20-49 yrs old			
		No.	%	No.	%	No.	%		
**Region 7**	**162,860**	**68**	**0.04**	**7,551**	**4.64**	**73,289**	**45.00**	**80,908**	**49.68**
Bohol	27,637	13	0.05	1,633	5.91	11,962	43.28	13,608	49.24
Cebu	67,046	12	0.02	2,412	3.60	27,106	40.43	29,530	44.04
Negros Oriental	27,922	30	0.11	1,453	5.20	11,813	42.31	13,296	47.62
Siquijor	1,636	0	0.00	95	5.81	756	46.21	851	52.02
Cebu City	21,037	11	0.05	1,283	6.10	11,795	56.07	13,089	62.22
Lapu-Lapu City	9,310	0	0.00	309	3.32	5,645	60.63	5,954	63.95
Mandaue City	8,272	2	0.02	366	4.42	4,212	50.92	4,580	55.37
**Region 8**	**104,016**	**18**	**0.02**	**1,826**	**1.76**	**28,336**	**27.24**	**30,180**	**29.01**
Biliran	3,856	2	0.05	113	2.93	1,500	38.90	1,615	41.88
Eastern Samar	11,635	2	0.02	220	1.89	3,211	27.60	3,433	29.51
Northern Leyte	35,052	9	0.03	773	2.21	10,537	30.06	11,319	32.29
Northern Samar	15,655	0	0.00	174	1.11	3,123	19.95	3,297	21.06
Southern Leyte	6,556	1	0.02	48	0.73	1,566	23.89	1,615	24.63
Western Samar	14,515	2	0.01	187	1.29	3,281	22.60	3,470	23.91
Calbayog City	4,474	1	0.02	104	2.32	1,141	25.50	1,246	27.85
Maasin City	1,661	0	0.00	23	1.38	345	20.77	368	22.16
Ormoc City	4,989	1	0.02	108	2.16	1,686	33.79	1,795	35.98
Tacloban City	5,623	0	0.00	76	1.35	1,946	34.61	2,022	35.96
**Region 9**	**79,552**	**39**	**0.05**	**3,547**	**4.46**	**32,114**	**40.37**	**35,700**	**44.88**
Zamboanga del Norte	17,075	2	0.01	521	3.05	6,315	36.98	6,838	40.05
Zamboanga del Sur	17,506	7	0.04	393	2.24	4,610	26.33	5,010	28.62
Zamboanga Sibugay	14,992	10	0.07	700	4.67	5,177	34.53	5,887	39.27
Dapitan City	1,755	0	0.00	19	1.08	973	55.44	992	56.52
Dipolog City	2,794	5	0.18	196	7.02	1,520	54.40	1,721	61.60
Isabela City	2,544	0	0.00	55	2.16	619	24.33	674	26.49
Pagadian City	4,290	0	0.00	352	8.21	1,912	44.57	2,264	52.77
Zamboanga City	18,596	15	0.08	1,311	7.05	10,988	59.09	12,314	66.22
**Region 10**	**100,690**	**67**	**0.07**	**5,167**	**5.13**	**42,062**	**41.77**	**47,296**	**46.97**
Bukidnon	23,307	30	0.13	1,618	6.94	9,670	41.49	11,318	48.56
Camiguin	1,854	0	0.00	14	0.76	527	28.43	541	29.18
Lanao del Norte	14,955	3	0.02	422	2.82	5,693	38.07	6,118	40.91
Misamis Occidental	6,413	1	0.02	192	2.99	2,200	34.31	2,393	37.31
Misamis Oriental	15,058	10	0.07	766	5.09	5,863	38.94	6,639	44.09
Cagayan de Oro City	14,263	10	0.07	1,001	7.02	7,872	55.19	8,883	62.28
El Salvador City	1,061	1	0.09	34	3.20	391	36.85	426	40.15
Gingoog City	2,627	1	0.04	139	5.29	995	37.88	1,135	43.21
Iligan City	7,575	5	0.07	305	4.03	2,911	38.43	3,221	42.52
Malaybalay City	3,882	3	0.08	170	4.38	1,589	40.93	1,762	45.39
Oroquieta City	1,389	0	0.00	19	1.37	506	36.43	525	37.80
Ozamis City	2,782	0	0.00	77	2.77	1,416	50.90	1,493	53.67

Table 1.B.1.4 - Prenatal Care

Number and proportion of pregnant women given Tetanus Diptheria (Td) Vaccination

Philippines, 2021

Area	Eligible Pop. (Under 1)	Women pregnant for the 2nd or more times given at least three (3) doses of Td Vaccine							
		Age Group						Total	%
		10-14 yrs old		15-19 yrs old		20-49 yrs old			
		No.	%	No.	%	No.	%		
Tangub City	1,234	0	0.00	45	3.65	234	18.96	279	22.61
Valencia City	4,290	3	0.07	365	8.51	2,195	51.17	2,563	59.74
**Region 11**	**107,847**	**121**	**0.11**	**2,911**	**2.70**	**38,953**	**36.12**	**41,985**	**38.93**
Davao de Oro	15,526	20	0.13	535	3.45	6,762	43.55	7,317	47.13
Davao del Norte	21,184	27	0.13	551	2.60	8,826	41.66	9,404	44.39
Davao Oriental	13,125	7	0.05	353	2.69	4,696	35.78	5,056	38.52
Davao del Sur	14,355	6	0.04	356	2.48	5,278	36.77	5,640	39.29
Davao Occidental	6,598	3	0.05	216	3.27	2,240	33.95	2,459	37.27
Davao City	37,059	58	0.16	900	2.43	11,151	30.09	12,109	32.67
**Region 12**	**104,693**	**110**	**0.11**	**6,415**	**6.13**	**42,809**	**40.89**	**49,334**	**47.12**
North Cotabato	33,933	26	0.08	1,762	5.19	11,683	34.43	13,471	39.70
Sarangani	12,904	9	0.07	785	6.08	5,367	41.59	6,161	47.74
South Cotabato	21,039	22	0.10	1,570	7.46	10,602	50.39	12,194	57.96
Sultan Kudarat	17,331	34	0.20	1,245	7.18	8,081	46.63	9,360	54.01
Cotabato City	5,831	0	0.00	127	2.18	1,621	27.80	1,748	29.98
Gen. Santos City	13,655	19	0.14	926	6.78	5,455	39.95	6,400	46.87
**BARMM**	**94,148**	**26**	**0.03**	**2,545**	**2.70**	**29,041**	**30.85**	**31,612**	**33.58**
Basilan	7,672	0	0.00	137	1.79	1,246	16.24	1,383	18.03
Lanao del Sur	21,391	3	0.01	214	1.00	9,850	46.05	10,067	47.06
Maguindanao	31,650	20	0.06	779	2.46	11,858	37.47	12,657	39.99
Sulu	16,891	0	0.00	0	0.00	0	0.00	0	0.00
Tawi-Tawi	9,319	2	0.02	281	3.02	3,912	41.98	4,195	45.02
Lamitan City	2,112	1	0.05	71	3.36	409	19.37	481	22.77
Marawi City	5,113	0	0.00	1,063	20.79	1,766	34.54	2,829	55.33
**CARAGA**	**60,342**	**14**	**0.02**	**1,586**	**2.63**	**21,553**	**35.72**	**23,153**	**38.37**
Agusan del Norte	8,116	4	0.05	211	2.60	2,497	30.77	2,712	33.42
Agusan del Sur	17,658	5	0.03	472	2.67	6,703	37.96	7,180	40.66
Surigao del Norte	7,195	1	0.01	132	1.83	2,453	34.09	2,586	35.94
Surigao del Sur	11,532	3	0.03	435	3.77	4,261	36.95	4,699	40.75
Province of Dinagat	2,574	0	0.00	26	1.01	552	21.45	578	22.46
Bislig City	2,185	0	0.00	69	3.16	807	36.93	876	40.09
Butuan City	7,732	0	0.00	130	1.68	2,957	38.24	3,087	39.92
Surigao City	3,350	1	0.03	111	3.31	1,323	39.49	1,435	42.84

Note: Put asterisk (*) for No Report and Zero (0) for No Case

Table 1.B.1.5 - Prenatal Care

Number of Pregnant Women who Completed Iron with Folic Acid by Region
Philippines, 2021

Area	Eligible Pop. (Under 1)	Iron with Folic Acid Supplementation							
		Age Group						Total	%
		10-14 yrs old		15-19 yrs old		20-49 yrs old			
		No.	%	No.	%	No.	%		
**PHILIPPINES**	**2,128,168**	**2,189**	**0.10**	**120,395**	**5.66**	**915,662**	**43.03**	**1,038,246**	**48.79**
**N C R**	**237,921**	**203**	**0.09**	**12,377**	**5.20**	**99,678**	**41.90**	**112,258**	**47.18**
Malabon	6,757	28	0.41	890	13.17	4,622	68.40	5,540	81.99
Navotas	4,607	10	0.22	597	12.96	2,738	59.43	3,345	72.61
Valenzuela City	11,465	4	0.03	494	4.31	5,039	43.95	5,537	48.29
Caloocan City	29,270	14	0.05	1,642	5.61	16,353	55.87	18,009	61.53
Marikina City	8,330	4	0.05	273	3.28	2,778	33.35	3,055	36.67
Pasig City	13,954	12	0.09	806	5.78	7,273	52.12	8,091	57.98
Pateros	1,181	4	0.34	135	11.43	676	57.24	815	69.01
Taguig	14,874	17	0.11	1,239	8.33	9,467	63.65	10,723	72.09
Quezon City	54,246	21	0.04	997	1.84	10,224	18.85	11,242	20.72
Makati City	10,765	4	0.04	145	1.35	3,038	28.22	3,187	29.61
Mandaluyong City	7,137	5	0.07	258	3.61	2,603	36.47	2,866	40.16
San Juan	2,254	1	0.04	36	1.60	954	42.32	991	43.97
Manila City	32,887	33	0.10	2,403	7.31	12,975	39.45	15,411	46.86
Las Piñas City	10,879	1	0.01	412	3.79	3,806	34.98	4,219	38.78
Muntinlupa City	9,317	13	0.14	706	7.58	7,268	78.01	7,987	85.73
Parañaque City	12,298	30	0.24	849	6.90	5,274	42.89	6,153	50.03
Pasay City	7,700	2	0.03	495	6.43	4,590	59.61	5,087	66.06
**C A R**	**35,143**	**17**	**0.05**	**1,300**	**3.70**	**15,367**	**43.73**	**16,684**	**47.47**
Abra	4,287	1	0.02	197	4.60	1,568	36.58	1,766	41.19
Apayao	2,476	5	0.20	128	5.17	1,042	42.08	1,175	47.46
Benguet	9,140	2	0.02	331	3.62	4,830	52.84	5,163	56.49
Ifugao	4,439	4	0.09	173	3.90	2,336	52.62	2,513	56.61
Kalinga	4,660	2	0.04	223	4.79	2,288	49.10	2,513	53.93
Mt. Province	3,066	1	0.03	122	3.98	1,480	48.27	1,603	52.28
Baguio City	7,075	2	0.03	126	1.78	1,823	25.77	1,951	27.58
**Region 1**	**97,198**	**36**	**0.04**	**5,202**	**5.35**	**49,841**	**51.28**	**55,079**	**56.67**
Ilocos Norte	7,986	2	0.03	531	6.65	3,389	42.44	3,922	49.11
Ilocos Sur	9,197	13	0.14	412	4.48	4,895	53.22	5,320	57.84
La Union	11,429	2	0.02	618	5.41	5,731	50.14	6,351	55.57
Pangasinan	50,454	9	0.02	2,635	5.22	25,738	51.01	28,382	56.25
Alaminos City	1,905	0	0.00	79	4.15	1,151	60.42	1,230	64.57
Candon City	976	0	0.00	1	0.10	1,057	108.30	1,058	108.40
Dagupan City	3,636	2	0.06	340	9.35	1,708	46.97	2,050	56.38
Laoag City	1,845	0	0.00	35	1.90	1,909	103.47	1,944	105.37
San Carlos City	3,999	2	0.05	243	6.08	1,528	38.21	1,773	44.34
San Fernando City	2,092	3	0.14	98	4.68	1,022	48.85	1,123	53.68
Urdaneta City	2,819	2	0.07	146	5.18	1,233	43.74	1,381	48.99
Vigan City	860	1	0.12	64	7.44	480	55.81	545	63.37
**Region 2**	**69,228**	**56**	**0.08**	**4,152**	**6.00**	**31,482**	**45.48**	**35,690**	**51.55**
Batanes	351	0	0.00	9	2.56	124	35.33	133	37.89
Cagayan	20,563	6	0.03	917	4.46	6,965	33.87	7,888	38.36
Isabela	23,543	26	0.11	1,500	6.37	11,302	48.01	12,828	54.49
Nueva Vizcaya	9,555	9	0.09	759	7.94	5,275	55.21	6,043	63.24

Table 1.B.1.5 - Prenatal Care

Number of Pregnant Women who Completed Iron with Folic Acid by Region
Philippines, 2021

Area	Eligible Pop. (Under 1)	Iron with Folic Acid Supplementation							
		Age Group						Total	%
		10-14 yrs old		15-19 yrs old		20-49 yrs old			
		No.	%	No.	%	No.	%		
Quirino	4,038	2	0.05	251	6.22	2,410	59.68	2,663	65.95
Cauayan City	2,576	5	0.19	218	8.46	1,334	51.79	1,557	60.44
Ilagan City	2,901	5	0.17	170	5.86	1,448	49.91	1,623	55.95
Santiago City	2,683	3	0.11	265	9.88	1,900	70.82	2,168	80.81
Tuguegarao City	3,018	0	0.00	63	2.09	724	23.99	787	26.08
**Region 3**	**220,202**	**219**	**0.10**	**14,998**	**6.81**	**102,888**	**46.72**	**118,105**	**53.63**
Aurora	4,764	2	0.04	266	5.58	2,108	44.25	2,376	49.87
Bataan	13,814	15	0.11	920	6.66	6,063	43.89	6,998	50.66
Bulacan	43,722	33	0.08	2,863	6.55	19,448	44.48	22,344	51.10
Nueva Ecija	28,915	25	0.09	1,407	4.87	9,341	32.31	10,773	37.26
Pampanga	31,503	25	0.08	1,607	5.10	10,693	33.94	12,325	39.12
Tarlac	19,565	20	0.10	1,425	7.28	12,564	64.22	14,009	71.60
Zambales	12,551	35	0.28	471	3.75	2,937	23.40	3,443	27.43
Angeles City	7,905	2	0.03	544	6.88	3,061	38.72	3,607	45.63
Balanga City	1,996	4	0.20	148	7.41	839	42.03	991	49.65
Cabanatuan City	5,921	5	0.08	534	9.02	3,262	55.09	3,801	64.20
City of San Fernando	5,890	9	0.15	364	6.18	2,394	40.65	2,767	46.98
Gapan City	2,159	5	0.23	121	5.60	848	39.28	974	45.11
Mabalacat City	4,811	2	0.04	721	14.99	3,780	78.57	4,503	93.60
Malolos City	4,885	5	0.10	248	5.08	1,902	38.94	2,155	44.11
Meycauayan	4,050	2	0.05	439	10.84	2,763	68.22	3,204	79.11
Olongapo	4,952	7	0.14	280	5.65	2,149	43.40	2,436	49.19
Palayan City	805	0	0.00	53	6.58	206	25.59	259	32.17
San Jose City	2,736	2	0.07	290	10.60	1,853	67.73	2,145	78.40
San Jose del Monte City	11,120	17	0.15	1,181	10.62	10,904	98.06	12,102	108.83
Science City of Munoz	1,594	1	0.06	71	4.45	493	30.93	565	35.45
Tarlac City	6,544	3	0.05	1,045	15.97	5,280	80.68	6,328	96.70
**Region 4A**	**298,398**	**189**	**0.06**	**13,031**	**4.37**	**107,316**	**35.96**	**120,536**	**40.39**
Batangas	38,848	13	0.03	1,385	3.57	16,779	43.19	18,177	46.79
Cavite	27,948	8	0.03	670	2.40	6,493	23.23	7,171	25.66
Laguna	19,727	27	0.14	1,158	5.87	7,775	39.41	8,960	45.42
Quezon	37,644	34	0.09	1,464	3.89	10,644	28.28	12,142	32.25
Rizal	44,826	50	0.11	1,869	4.17	11,340	25.30	13,259	29.58
Antipolo City	16,510	1	0.01	872	5.28	10,344	62.65	11,217	67.94
Bacoor City	12,252	3	0.02	138	1.13	1,101	8.99	1,242	10.14
Batangas City	6,895	6	0.09	293	4.25	3,018	43.77	3,317	48.11
Biñan City	6,607	1	0.02	952	14.41	5,299	80.20	6,252	94.63
Cabuyao City	6,128	3	0.05	472	7.70	3,471	56.64	3,946	64.39
Calamba City	9,025	6	0.07	1,312	14.54	7,683	85.13	9,001	99.73
Cavite City	2,098	0	0.00	33	1.57	114	5.43	147	7.01
Dasmariñas City	13,442	3	0.02	308	2.29	3,398	25.28	3,709	27.59
General Trias City	6,410	2	0.03	110	1.72	1,299	20.27	1,411	22.01
Imus City	8,235	0	0.00	71	0.86	745	9.05	816	9.91
Lipa City	6,954	4	0.06	185	2.66	2,645	38.04	2,834	40.75
Lucena City	5,704	5	0.09	324	5.68	1,919	33.64	2,248	39.41
San Pablo City	5,277	5	0.09	326	6.18	2,039	38.64	2,370	44.91
San Pedro City	6,465	0	0.00	201	3.11	1,569	24.27	1,770	27.38
Santa Rosa City	7,023	13	0.19	577	8.22	5,102	72.65	5,692	81.05
Tagaytay City	1,451	0	0.00	35	2.41	1,090	75.12	1,125	77.53
Tanauan City	3,620	2	0.06	56	1.55	1,684	46.52	1,742	48.12
Tayabas City	2,134	2	0.09	141	6.61	1,186	55.58	1,329	62.28
Trece Martires City	3,175	1	0.03	79	2.49	579	18.24	659	20.76

Table 1.B.1.5 - Prenatal Care

Number of Pregnant Women who Completed Iron with Folic Acid by Region
Philippines, 2021

Area	Eligible Pop. (Under 1)	Iron with Folic Acid Supplementation							
		Age Group						Total	%
		10-14 yrs old		15-19 yrs old		20-49 yrs old			
		No.	%	No.	%	No.	%		
**Region 4B**	**72,035**	**27**	**0.04**	**3,190**	**4.43**	**25,601**	**35.54**	**28,818**	**40.01**
Marinduque	4,906	0	0.00	178	3.63	1,998	40.73	2,176	44.35
Mindoro Occidental	12,449	12	0.10	890	7.15	5,774	46.38	6,676	53.63
Mindoro Oriental	20,011	2	0.01	740	3.70	6,691	33.44	7,433	37.14
Palawan	21,676	11	0.05	1,165	5.37	8,919	41.15	10,095	46.57
Romblon	6,482	2	0.03	217	3.35	2,219	34.23	2,438	37.61
Puerto Princesa City	6,511	0	0.00	0	0.00	0	0.00	0	0.00
**Region 5**	**137,350**	**49**	**0.04**	**5,527**	**4.02**	**53,987**	**39.31**	**59,563**	**43.37**
Albay	23,903	10	0.04	740	3.10	11,268	47.14	12,018	50.28
Camarines Norte	14,340	4	0.03	881	6.14	6,188	43.15	7,073	49.32
Camarines Sur	39,151	5	0.01	1,116	2.85	12,276	31.36	13,397	34.22
Catanduanes	6,381	1	0.02	218	3.42	2,151	33.71	2,370	37.14
Masbate	22,565	24	0.11	1,352	5.99	8,499	37.66	9,875	43.76
Sorsogon	19,470	4	0.02	712	3.66	8,054	41.37	8,770	45.04
Iriga City	2,665	0	0.00	93	3.49	978	36.70	1,071	40.19
Legaspi City	4,205	1	0.02	185	4.40	2,679	63.71	2,865	68.13
Naga City	4,670	0	0.00	230	4.93	1,894	40.56	2,124	45.48
**Region 6**	**146,545**	**95**	**0.06**	**6,969**	**4.76**	**60,029**	**40.96**	**67,093**	**45.78**
Aklan	11,231	9	0.08	431	3.84	5,997	53.40	6,437	57.31
Antique	12,985	5	0.04	394	3.03	4,408	33.95	4,807	37.02
Capiz	13,984	2	0.01	332	2.37	4,170	29.82	4,504	32.21
Guimaras	3,085	1	0.03	163	5.28	1,971	63.89	2,135	69.21
Iloilo	36,414	23	0.06	1,477	4.06	15,474	42.49	16,974	46.61
Negros Occidental	49,314	40	0.08	3,413	6.92	22,559	45.75	26,012	52.75
Bacolod City	11,102	3	0.03	339	3.05	2,235	20.13	2,577	23.21
Iloilo City	8,430	12	0.14	420	4.98	3,215	38.14	3,647	43.26
**Region 7**	**162,860**	**99**	**0.06**	**8,851**	**5.43**	**73,126**	**44.90**	**82,076**	**50.40**
Bohol	27,637	11	0.04	1,527	5.53	12,150	43.96	13,688	49.53
Cebu	67,046	27	0.04	3,503	5.22	29,408	43.86	32,938	49.13
Negros Oriental	27,922	25	0.09	1,679	6.01	10,565	37.84	12,269	43.94
Siquijor	1,636	0	0.00	87	5.32	833	50.92	920	56.23
Cebu City	21,037	26	0.12	1,242	5.90	9,480	45.06	10,748	51.09
Lapu-Lapu City	9,310	3	0.03	500	5.37	6,988	75.06	7,491	80.46
Mandaue City	8,272	7	0.08	313	3.78	3,702	44.75	4,022	48.62
**Region 8**	**104,016**	**97**	**0.09**	**4,343**	**4.18**	**38,727**	**37.23**	**43,167**	**41.50**
Biliran	3,856	2	0.05	165	4.28	1,513	39.24	1,680	43.57
Eastern Samar	11,635	4	0.03	486	4.18	4,399	37.81	4,889	42.02
Northern Leyte	35,052	39	0.11	1,559	4.45	14,592	41.63	16,190	46.19
Northern Samar	15,655	1	0.01	545	3.48	4,853	31.00	5,399	34.49
Southern Leyte	6,556	5	0.08	227	3.46	2,522	38.47	2,754	42.01
Western Samar	14,515	43	0.30	673	4.64	4,741	32.66	5,457	37.60
Calbayog City	4,474	0	0.00	96	2.15	687	15.36	783	17.50
Maasin City	1,661	0	0.00	49	2.95	585	35.22	634	38.17
Ormoc City	4,989	2	0.04	287	5.75	2,153	43.15	2,442	48.95

Table 1.B.1.5 - Prenatal Care

Number of Pregnant Women who Completed Iron with Folic Acid by Region
Philippines, 2021

Area	Eligible Pop. (Under 1)	Iron with Folic Acid Supplementation							
		Age Group						Total	%
		10-14 yrs old		15-19 yrs old		20-49 yrs old			
		No.	%	No.	%	No.	%		
Tacloban City	5,623	1	0.02	256	4.55	2,682	47.70	2,939	52.27
**Region 9**	**79,552**	**70**	**0.09**	**5,738**	**7.21**	**36,573**	**45.97**	**42,381**	**53.27**
Zamboanga del Norte	17,075	19	0.11	1,319	7.72	7,956	46.59	9,294	54.43
Zamboanga del Sur	17,506	9	0.05	1,036	5.92	5,528	31.58	6,573	37.55
Zamboanga Sibugay	14,992	5	0.03	744	4.96	4,573	30.50	5,322	35.50
Dapitan City	1,755	1	0.06	54	3.08	1,479	84.27	1,534	87.41
Dipolog City	2,794	10	0.36	248	8.88	1,836	65.71	2,094	74.95
Isabela City	2,544	4	0.16	205	8.06	1,216	47.80	1,425	56.01
Pagadian City	4,290	0	0.00	229	5.34	1,464	34.13	1,693	39.46
Zamboanga City	18,596	22	0.12	1,903	10.23	12,521	67.33	14,446	77.68
**Region 10**	**100,690**	**189**	**0.19**	**9,671**	**9.60**	**56,942**	**56.55**	**66,802**	**66.34**
Bukidnon	23,307	80	0.34	3,144	13.49	13,495	57.90	16,719	71.73
Camiguin	1,854	1	0.05	70	3.78	706	38.08	777	41.91
Lanao del Norte	14,955	7	0.05	625	4.18	7,574	50.65	8,206	54.87
Misamis Occidental	6,413	2	0.03	324	5.05	3,190	49.74	3,516	54.83
Misamis Oriental	15,058	23	0.15	1,558	10.35	9,289	61.69	10,870	72.19
Cagayan de Oro City	14,263	17	0.12	1,435	10.06	8,996	63.07	10,448	73.25
El Salvador City	1,061	1	0.09	78	7.35	442	41.66	521	49.10
Gingoog City	2,627	30	1.14	590	22.46	1,907	72.59	2,527	96.19
Iligan City	7,575	12	0.16	590	7.79	3,973	52.45	4,575	60.40
Malaybalay City	3,882	6	0.15	366	9.43	2,073	53.40	2,445	62.98
Oroquieta City	1,389	0	0.00	50	3.60	493	35.49	543	39.09
Ozamis City	2,782	4	0.14	259	9.31	1,870	67.22	2,133	76.67
Tangub City	1,234	0	0.00	82	6.65	597	48.38	679	55.02
Valencia City	4,290	6	0.14	500	11.66	2,337	54.48	2,843	66.27
**Region 11**	**107,847**	**574**	**0.53**	**8,958**	**8.31**	**55,756**	**51.70**	**65,288**	**60.54**
Davao de Oro	15,526	92	0.59	1,679	10.81	8,238	53.06	10,009	64.47
Davao del Norte	21,184	122	0.58	2,014	9.51	12,668	59.80	14,804	69.88
Davao Oriental	13,125	49	0.37	1,092	8.32	5,998	45.70	7,139	54.39
Davao del Sur	14,355	79	0.55	1,013	7.06	5,322	37.07	6,414	44.68
Davao Occidental	6,598	35	0.53	545	8.26	1,337	20.26	1,917	29.05
Davao City	37,059	197	0.53	2,615	7.06	22,193	59.89	25,005	67.47
**Region 12**	**104,693**	**166**	**0.16**	**9,089**	**8.68**	**49,399**	**47.18**	**58,654**	**56.02**
North Cotabato	33,933	32	0.09	2,400	7.07	13,997	41.25	16,429	48.42
Sarangani	12,904	37	0.29	2,013	15.60	7,712	59.76	9,762	75.65
South Cotabato	21,039	31	0.15	2,126	10.11	12,435	59.10	14,592	69.36
Sultan Kudarat	17,331	47	0.27	1,539	8.88	8,928	51.51	10,514	60.67
Cotabato City	5,831	0	0.00	184	3.16	1,643	28.18	1,827	31.33
Gen. Santos City	13,655	19	0.14	827	6.06	4,684	34.30	5,530	40.50
**B.A.R.M.M.**	**94,148**	**45**	**0.05**	**3,304**	**3.51**	**31,703**	**33.67**	**35,052**	**37.23**
Basilan	7,672	1	0.01	173	2.25	1,266	16.50	1,440	18.77
Lanao del Sur	21,391	2	0.01	368	1.72	10,841	50.68	11,211	52.41
Maguindanao	31,650	36	0.11	1,648	5.21	13,131	41.49	14,815	46.81
Sulu	16,891	0	0.00	0	0.00	0	0.00	0	0.00
Tawi-Tawi	9,319	1	0.01	215	2.31	3,168	34.00	3,384	36.31

Table 1.B.1.5 - Prenatal Care

Number of Pregnant Women who Completed Iron with Folic Acid by Region
Philippines, 2021

Area	Eligible Pop. (Under 1)	Iron with Folic Acid Supplementation							
		Age Group						Total	%
		10-14 yrs old		15-19 yrs old		20-49 yrs old			
		No.	%	No.	%	No.	%		
Lamitan City	2,112	4	0.19	140	6.63	534	25.28	678	32.10
Marawi City	5,113	1	0.02	760	14.86	2,763	54.04	3,524	68.92
**CARAGA**	**60,342**	**58**	**0.10**	**3,695**	**6.12**	**27,247**	**45.15**	**31,000**	**51.37**
Agusan del Norte	8,116	4	0.05	558	6.88	3,181	39.19	3,743	46.12
Agusan del Sur	17,658	24	0.14	1,233	6.98	8,996	50.95	10,253	58.06
Surigao del Norte	7,195	6	0.08	344	4.78	2,780	38.64	3,130	43.50
Surigao del Sur	11,532	8	0.07	459	3.98	3,921	34.00	4,388	38.05
Province of Dinagat	2,574	1	0.04	104	4.04	907	35.24	1,012	39.32
Bislig City	2,185	10	0.46	115	5.26	988	45.22	1,113	50.94
Butuan City	7,732	3	0.04	667	8.63	4,602	59.52	5,272	68.18
Surigao City	3,350	2	0.06	215	6.42	1,872	55.88	2,089	62.36

Note: Put asterisk (*) for No Report and Zero (0) for No Case

Table 1.B.1.6 - Prenatal Care

Number of Pregnant Women who Completed Calcium Carbonate Supplementation
Philippines, 2021

Area	Eligible Pop. (Under 1)	Calcium carbonate supplementation							
		Age Group						Total	%
		10-14 yrs old		15-19 yrs old		20-49 yrs old			
		No.	%	No.	%	No.	%		
**PHILIPPINES**	**2,128,168**	**1,290**	**0.06**	**76,373**	**3.59**	**587,852**	**27.62**	**665,515**	**31.27**
**N C R**	**237,921**	**113**	**0.05**	**6,334**	**2.66**	**54,578**	**22.94**	**61,025**	**25.65**
Malabon	6,757	14	0.21	584	8.64	3,670	54.31	4,268	63.16
Navotas	4,607	6	0.13	389	8.44	2,169	47.08	2,564	55.65
Valenzuela City	11,465	2	0.02	213	1.86	1,751	15.27	1,966	17.15
Caloocan City	29,270	18	0.06	1,266	4.33	13,091	44.72	14,375	49.11
Marikina City	8,330	4	0.05	231	2.77	1,813	21.76	2,048	24.59
Pasig City	13,954	3	0.02	323	2.31	4,771	34.19	5,097	36.53
Pateros	1,181	1	0.08	34	2.88	94	7.96	129	10.92
Taguig	14,874	10	0.07	920	6.19	7,334	49.31	8,264	55.56
Quezon City	54,246	1	0.00	267	0.49	2,410	4.44	2,678	4.94
Makati City	10,765	2	0.02	137	1.27	3,526	32.75	3,665	34.05
Mandaluyong City	7,137	3	0.04	45	0.63	570	7.99	618	8.66
San Juan	2,254	3	0.13	34	1.51	669	29.68	706	31.32
Manila City	32,887	36	0.11	1,153	3.51	5,680	17.27	6,869	20.89
Las Piñas City	10,879	0	0.00	29	0.27	302	2.78	331	3.04
Muntinlupa City	9,317	0	0.00	75	0.80	703	7.55	778	8.35
Parañaque City	12,298	7	0.06	169	1.37	1,526	12.41	1,702	13.84
Pasay City	7,700	3	0.04	465	6.04	4,499	58.43	4,967	64.51
**C A R**	**35,143**	**12**	**0.03**	**1,939**	**5.52**	**11,848**	**33.71**	**13,799**	**39.27**
Abra	4,287	1	0.02	159	3.71	1,196	27.90	1,356	31.63
Apayao	2,476	6	0.24	116	4.68	938	37.88	1,060	42.81
Benguet	9,140	3	0.03	225	2.46	3,989	43.64	4,217	46.14
Ifugao	4,439	1	0.02	79	1.78	957	21.56	1,037	23.36
Kalinga	4,660	1	0.02	1,267	27.19	3,429	73.58	4,697	100.79
Mt. Province	3,066	0	0.00	38	1.24	706	23.03	744	24.27
Baguio City	7,075	0	0.00	55	0.78	633	8.95	688	9.72
**Region 1**	**97,198**	**37**	**0.04**	**4,381**	**4.51**	**46,852**	**48.20**	**51,270**	**52.75**
Ilocos Norte	7,986	3	0.04	238	2.98	3,266	40.90	3,507	43.91
Ilocos Sur	9,197	12	0.13	383	4.16	5,479	59.57	5,874	63.87
La Union	11,429	4	0.03	582	5.09	5,424	47.46	6,010	52.59
Pangasinan	50,454	10	0.02	2,332	4.62	23,284	46.15	25,626	50.79
Alaminos City	1,905	0	0.00	64	3.36	1,024	53.75	1,088	57.11
Candon City	976	0	0.00	26	2.66	1,000	102.46	1,026	105.12
Dagupan City	3,636	2	0.06	277	7.62	1,424	39.16	1,703	46.84
Laoag City	1,845	0	0.00	35	1.90	1,909	103.47	1,944	105.37
San Carlos City	3,999	0	0.00	121	3.03	1,187	29.68	1,308	32.71
San Fernando City	2,092	3	0.14	101	4.83	992	47.42	1,096	52.39
Urdaneta City	2,819	2	0.07	166	5.89	1,447	51.33	1,615	57.29
Vigan City	860	1	0.12	56	6.51	416	48.37	473	55.00
**Region 2**	**69,228**	**57**	**0.08**	**3,299**	**4.77**	**26,907**	**38.87**	**30,263**	**43.71**
Batanes	351	0	0.00	2	0.57	57	16.24	59	16.81
Cagayan	20,563	6	0.03	576	2.80	4,670	22.71	5,252	25.54

Table 1.B.1.6 - Prenatal Care

Number of Pregnant Women who Completed Calcium Carbonate Supplementation
Philippines, 2021

Area	Eligible Pop. (Under 1)	Calcium carbonate supplementation							
		Age Group						Total	%
		10-14 yrs old		15-19 yrs old		20-49 yrs old			
		No.	%	No.	%	No.	%		
Isabela	23,543	27	0.11	1,158	4.92	9,415	39.99	10,600	45.02
Nueva Vizcaya	9,555	11	0.12	744	7.79	5,102	53.40	5,857	61.30
Quirino	4,038	1	0.02	244	6.04	2,432	60.23	2,677	66.30
Cauayan City	2,576	3	0.12	123	4.77	827	32.10	953	37.00
Ilagan City	2,901	6	0.21	156	5.38	1,387	47.81	1,549	53.40
Santiago City	2,683	3	0.11	242	9.02	2,418	90.12	2,663	99.25
Tuguegarao City	3,018	0	0.00	54	1.79	599	19.85	653	21.64
**Region 3**	**220,202**	**128**	**0.06**	**9,887**	**4.49**	**67,625**	**30.71**	**77,640**	**35.26**
Aurora	4,764	1	0.02	104	2.18	755	15.85	860	18.05
Bataan	13,814	9	0.07	481	3.48	3,246	23.50	3,736	27.05
Bulacan	43,722	23	0.05	2,014	4.61	13,687	31.30	15,724	35.96
Nueva Ecija	28,915	22	0.08	1,038	3.59	7,004	24.22	8,064	27.89
Pampanga	31,503	25	0.08	1,074	3.41	7,267	23.07	8,366	26.56
Tarlac	19,565	7	0.04	898	4.59	8,147	41.64	9,052	46.27
Zambales	12,551	0	0.00	149	1.19	958	7.63	1,107	8.82
Angeles City	7,905	2	0.03	282	3.57	1,658	20.97	1,942	24.57
Balanga City	1,996	4	0.20	90	4.51	459	23.00	553	27.71
Cabanatuan City	5,921	3	0.05	301	5.08	1,887	31.87	2,191	37.00
City of San Fernando	5,890	4	0.07	244	4.14	1,586	26.93	1,834	31.14
Gapan City	2,159	3	0.14	104	4.82	647	29.97	754	34.92
Mabalacat City	4,811	2	0.04	403	8.38	2,344	48.72	2,749	57.14
Malolos City	4,885	2	0.04	121	2.48	1,002	20.51	1,125	23.03
Meycauayan	4,050	1	0.02	308	7.60	1,873	46.25	2,182	53.88
Olongapo	4,952	1	0.02	109	2.20	812	16.40	922	18.62
Palayan City	805	0	0.00	60	7.45	251	31.18	311	38.63
San Jose City	2,736	2	0.07	126	4.61	865	31.62	993	36.29
San Jose del Monte City	11,120	15	0.13	1,055	9.49	8,747	78.66	9,817	88.28
Science City of Munoz	1,594	0	0.00	66	4.14	413	25.91	479	30.05
Tarlac City	6,544	2	0.03	860	13.14	4,017	61.38	4,879	74.56
**Region 4A**	**298,398**	**172**	**0.06**	**8,734**	**2.93**	**67,209**	**22.52**	**76,115**	**25.51**
Batangas	38,848	36	0.09	624	1.61	7,855	20.22	8,515	21.92
Cavite	27,948	5	0.02	382	1.37	3,704	13.25	4,091	14.64
Laguna	19,727	25	0.13	718	3.64	4,867	24.67	5,610	28.44
Quezon	37,644	10	0.03	584	1.55	4,394	11.67	4,988	13.25
Rizal	44,826	24	0.05	1,184	2.64	7,374	16.45	8,582	19.15
Antipolo City	16,510	5	0.03	871	5.28	7,034	42.60	7,910	47.91
Bacoor City	12,252	0	0.00	64	0.52	519	4.24	583	4.76
Batangas City	6,895	4	0.06	264	3.83	2,491	36.13	2,759	40.01
Biñan City	6,607	2	0.03	819	12.40	4,556	68.96	5,377	81.38
Cabuyao City	6,128	1	0.02	333	5.43	2,677	43.68	3,011	49.14
Calamba City	9,025	7	0.08	1,318	14.60	5,946	65.88	7,271	80.57
Cavite City	2,098	0	0.00	16	0.76	66	3.15	82	3.91
Dasmariñas City	13,442	3	0.02	201	1.50	2,182	16.23	2,386	17.75
General Trias City	6,410	31	0.48	38	0.59	503	7.85	572	8.92
Imus City	8,235	0	0.00	39	0.47	479	5.82	518	6.29
Lipa City	6,954	1	0.01	89	1.28	1,460	21.00	1,550	22.29
Lucena City	5,704	3	0.05	198	3.47	1,240	21.74	1,441	25.26
San Pablo City	5,277	5	0.09	225	4.26	1,458	27.63	1,688	31.99
San Pedro City	6,465	1	0.02	137	2.12	1,125	17.40	1,263	19.54
Santa Rosa City	7,023	7	0.10	467	6.65	4,445	63.29	4,919	70.04

Table 1.B.1.6 - Prenatal Care

Number of Pregnant Women who Completed Calcium Carbonate Supplementation
Philippines, 2021

Area	Eligible Pop. (Under 1)	Calcium carbonate supplementation							
		Age Group						Total	%
		10-14 yrs old		15-19 yrs old		20-49 yrs old			
		No.	%	No.	%	No.	%		
Tagaytay City	1,451	0	0.00	33	2.27	1,075	74.09	1,108	76.36
Tanauan City	3,620	2	0.06	53	1.46	1,083	29.92	1,138	31.44
Tayabas City	2,134	0	0.00	40	1.87	348	16.31	388	18.18
Trece Martires City	3,175	0	0.00	37	1.17	328	10.33	365	11.50
**Region 4B**	**72,035**	**23**	**0.03**	**1,513**	**2.10**	**12,422**	**17.24**	**13,958**	**19.38**
Marinduque	4,906	0	0.00	121	2.47	1,571	32.02	1,692	34.49
Mindoro Occidental	12,449	4	0.03	458	3.68	3,072	24.68	3,534	28.39
Mindoro Oriental	20,011	3	0.01	380	1.90	3,337	16.68	3,720	18.59
Palawan	21,676	5	0.02	437	2.02	3,073	14.18	3,515	16.22
Romblon	6,482	11	0.17	117	1.80	1,369	21.12	1,497	23.09
Puerto Princesa City	6,511	0	0.00	0	0.00	0	0.00	0	0.00
**Region 5**	**137,350**	**35**	**0.03**	**3,414**	**2.49**	**35,770**	**26.04**	**39,219**	**28.55**
Albay	23,903	14	0.06	652	2.73	9,755	40.81	10,421	43.60
Camarines Norte	14,340	2	0.01	471	3.28	3,486	24.31	3,959	27.61
Camarines Sur	39,151	4	0.01	772	1.97	8,479	21.66	9,255	23.64
Catanduanes	6,381	0	0.00	105	1.65	1,146	17.96	1,251	19.61
Masbate	22,565	11	0.05	698	3.09	4,609	20.43	5,318	23.57
Sorsogon	19,470	3	0.02	472	2.42	5,115	26.27	5,590	28.71
Iriga City	2,665	0	0.00	60	2.25	476	17.86	536	20.11
Legaspi City	4,205	1	0.02	145	3.45	2,154	51.22	2,300	54.70
Naga City	4,670	0	0.00	39	0.84	550	11.78	589	12.61
**Region 6**	**146,545**	**44**	**0.03**	**3,141**	**2.14**	**28,582**	**19.50**	**31,767**	**21.68**
Aklan	11,231	5	0.04	216	1.92	2,600	23.15	2,821	25.12
Antique	12,985	0	0.00	72	0.55	917	7.06	989	7.62
Capiz	13,984	1	0.01	158	1.13	1,815	12.98	1,974	14.12
Guimaras	3,085	0	0.00	112	3.63	1,254	40.65	1,366	44.28
Iloilo	36,414	15	0.04	873	2.40	8,995	24.70	9,883	27.14
Negros Occidental	49,314	15	0.03	1,354	2.75	9,980	20.24	11,349	23.01
Bacolod City	11,102	0	0.00	95	0.86	897	8.08	992	8.94
Iloilo City	8,430	8	0.09	261	3.10	2,124	25.20	2,393	28.39
**Region 7**	**162,860**	**44**	**0.03**	**5,086**	**3.12**	**46,197**	**28.37**	**51,327**	**31.52**
Bohol	27,637	0	0.00	425	1.54	5,855	21.19	6,280	22.72
Cebu	67,046	12	0.02	1,742	2.60	15,094	22.51	16,848	25.13
Negros Oriental	27,922	4	0.01	468	1.68	3,060	10.96	3,532	12.65
Siquijor	1,636	0	0.00	45	2.75	480	29.34	525	32.09
Cebu City	21,037	19	0.09	1,639	7.79	12,130	57.66	13,788	65.54
Lapu-Lapu City	9,310	3	0.03	406	4.36	5,937	63.77	6,346	68.16
Mandaue City	8,272	6	0.07	361	4.36	3,641	44.02	4,008	48.45
**Region 8**	**104,016**	**67**	**0.06**	**2,925**	**2.81**	**26,393**	**25.37**	**29,385**	**28.25**
Biliran	3,856	5	0.13	125	3.24	1,145	29.69	1,275	33.07
Eastern Samar	11,635	2	0.02	412	3.54	3,458	29.72	3,872	33.28
Northern Leyte	35,052	17	0.05	978	2.79	9,527	27.18	10,522	30.02
Northern Samar	15,655	0	0.00	360	2.30	3,259	20.82	3,619	23.12

Table 1.B.1.6 - Prenatal Care

Number of Pregnant Women who Completed Calcium Carbonate Supplementation
Philippines, 2021

Area	Eligible Pop. (Under 1)	Calcium carbonate supplementation							
		Age Group						Total	%
		10-14 yrs old		15-19 yrs old		20-49 yrs old			
		No.	%	No.	%	No.	%		
Southern Leyte	6,556	4	0.06	229	3.49	2,358	35.97	2,591	39.52
Western Samar	14,515	36	0.25	495	3.41	3,194	22.00	3,725	25.66
Calbayog City	4,474	0	0.00	22	0.49	191	4.27	213	4.76
Maasin City	1,661	0	0.00	39	2.35	537	32.33	576	34.68
Ormoc City	4,989	2	0.04	96	1.92	1,000	20.04	1,098	22.01
Tacloban City	5,623	1	0.02	169	3.01	1,724	30.66	1,894	33.68
**Region 9**	**79,552**	**44**	**0.06**	**2,318**	**2.91**	**15,625**	**19.64**	**17,987**	**22.61**
Zamboanga del Norte	17,075	13	0.08	855	5.01	5,430	31.80	6,298	36.88
Zamboanga del Sur	17,506	10	0.06	632	3.61	3,429	19.59	4,071	23.25
Zamboanga Sibugay	14,992	4	0.03	391	2.61	2,688	17.93	3,083	20.56
Dapitan City	1,755	0	0.00	17	0.97	587	33.45	604	34.42
Dipolog City	2,794	4	0.14	133	4.76	943	33.75	1,080	38.65
Isabela City	2,544	0	0.00	20	0.79	202	7.94	222	8.73
Pagadian City	4,290	0	0.00	41	0.96	437	10.19	478	11.14
Zamboanga City	18,596	13	0.07	229	1.23	1,909	10.27	2,151	11.57
**Region 10**	**100,690**	**125**	**0.12**	**7,719**	**7.67**	**48,118**	**47.79**	**55,962**	**55.58**
Bukidnon	23,307	48	0.21	2,508	10.76	11,106	47.65	13,662	58.62
Camiguin	1,854	1	0.05	52	2.80	525	28.32	578	31.18
Lanao del Norte	14,955	6	0.04	677	4.53	7,313	48.90	7,996	53.47
Misamis Occidental	6,413	2	0.03	240	3.74	2,770	43.19	3,012	46.97
Misamis Oriental	15,058	22	0.15	1,431	9.50	8,417	55.90	9,870	65.55
Cagayan de Oro City	14,263	10	0.07	1,133	7.94	9,124	63.97	10,267	71.98
El Salvador City	1,061	0	0.00	82	7.73	449	42.32	531	50.05
Gingoog City	2,627	20	0.76	510	19.41	1,350	51.39	1,880	71.56
Iligan City	7,575	7	0.09	522	6.89	3,256	42.98	3,785	49.97
Malaybalay City	3,882	1	0.03	51	1.31	364	9.38	416	10.72
Oroquieta City	1,389	0	0.00	20	1.44	324	23.33	344	24.77
Ozamis City	2,782	4	0.14	195	7.01	1,438	51.69	1,637	58.84
Tangub City	1,234	0	0.00	82	6.65	588	47.65	670	54.29
Valencia City	4,290	4	0.09	216	5.03	1,094	25.50	1,314	30.63
**Region 11**	**107,847**	**203**	**0.19**	**6,098**	**5.65**	**32,102**	**29.77**	**38,403**	**35.61**
Davao de Oro	15,526	51	**0.33**	1,293	**8.33**	4,994	**32.17**	**6,338**	**40.82**
Davao del Norte	21,184	48	**0.23**	1,533	**7.24**	7,913	**37.35**	**9,494**	**44.82**
Davao Oriental	13,125	28	**0.21**	629	**4.79**	2,893	**22.04**	**3,550**	**27.05**
Davao del Sur	14,355	24	**0.17**	553	**3.85**	2,239	**15.60**	**2,816**	**19.62**
Davao Occidental	6,598	9	**0.14**	141	**2.14**	260	**3.94**	**410**	**6.21**
Davao City	37,059	43	**0.12**	1,949	**5.26**	13,803	**37.25**	**15,795**	**42.62**
**Region 12**	**104,693**	**111**	**0.11**	**5,645**	**5.39**	**31,402**	**29.99**	**37,158**	**35.49**
North Cotabato	33,933	28	**0.08**	1,603	**4.72**	9,259	**27.29**	**10,890**	**32.09**
Sarangani	12,904	22	**0.17**	1,163	**9.01**	4,326	**33.52**	**5,511**	**42.71**
South Cotabato	21,039	10	**0.05**	954	**4.53**	6,770	**32.18**	**7,734**	**36.76**
Sultan Kudarat	17,331	38	**0.22**	1,146	**6.61**	6,751	**38.95**	**7,935**	**45.79**
Cotabato City	5,831	0	**0.00**	58	**0.99**	704	**12.07**	**762**	**13.07**
Gen. Santos City	13,655	13	**0.10**	721	**5.28**	3,592	**26.31**	**4,326**	**31.68**

Table 1.B.1.6 - Prenatal Care

Number of Pregnant Women who Completed Calcium Carbonate Supplementation
Philippines, 2021

Area	Eligible Pop. (Under 1)	Calcium carbonate supplementation							
		Age Group						Total	%
		10-14 yrs old		15-19 yrs old		20-49 yrs old			
		No.	%	No.	%	No.	%		
**BARMM**	**94,148**	**38**	**0.04**	**1,610**	**1.71**	**17,435**	**18.52**	**19,083**	**20.27**
Basilan	7,672	0	**0.00**	71	**0.93**	385	**5.02**	**456**	**5.94**
Lanao del Sur	21,391	1	**0.00**	275	**1.29**	8,017	**37.48**	**8,293**	**38.77**
Maguindanao	31,650	35	**0.11**	893	**2.82**	6,923	**21.87**	**7,851**	**24.81**
Sulu	16,891	0	**0.00**	0	**0.00**	0	**0.00**	**0**	**0.00**
Tawi-Tawi	9,319	0	**0.00**	52	**0.56**	542	**5.82**	**594**	**6.37**
Lamitan City	2,112	1	**0.05**	47	**2.23**	230	**10.89**	**278**	**13.16**
Marawi City	5,113	1	**0.02**	272	**5.32**	1,338	**26.17**	**1,611**	**31.51**
**CARAGA**	**60,342**	**37**	**0.06**	**2,330**	**3.86**	**18,787**	**31.13**	**21,154**	**35.06**
Agusan del Norte	8,116	0	**0.00**	0	**0.00**	0	**0.00**	**0**	**0.00**
Agusan del Sur	17,658	18	**0.10**	893	**5.06**	7,067	**40.02**	**7,978**	**45.18**
Surigao del Norte	7,195	7	**0.10**	283	**3.93**	2,375	**33.01**	**2,665**	**37.04**
Surigao del Sur	11,532	2	**0.02**	334	**2.90**	2,874	**24.92**	**3,210**	**27.84**
Province of Dinagat	2,574	0	**0.00**	82	**3.19**	710	**27.58**	**792**	**30.77**
Bislig City	2,185	5	**0.23**	120	**5.49**	964	**44.12**	**1,089**	**49.84**
Butuan City	7,732	3	**0.04**	430	**5.56**	3,113	**40.26**	**3,546**	**45.86**
Surigao City	3,350	2	**0.06**	188	**5.61**	1,684	**50.27**	**1,874**	**55.94**

Note: Put asterisk (*) for No Report and Zero (0) for No Case

Table 1.B.1.7 - Prenatal Care

Number of Pregnant Women who Completed Iodine
Philippines, 2021

Area	Eligible Pop. (Under 1)	Iodine Capsule							
		Age Group						Total	%
		10-14 yrs old		15-19 yrs old		20-49 yrs old			
		No.	%	No.	%	No.	%		
**PHILIPPINES**	**2,128,168**	**385**	**0.018**	**14,813**	**0.70**	**93,165**	**4.38**	**108,363**	**5.09**
**N C R**	**237,921**	**5**	**0.002**	**89**	**0.04**	**531**	**0.22**	**625**	**0.26**
Malabon	6,757	0	0.000	0	0.00	0	0.00	0	0.00
Navotas	4,607	2	0.043	31	0.67	83	1.80	116	2.52
Valenzuela City	11,465	0	0.000	18	0.16	133	1.16	151	1.32
Caloocan City	29,270	0	0.000	4	0.01	81	0.28	85	0.29
Marikina City	8,330	0	0.000	0	0.00	0	0.00	0	0.00
Pasig City	13,954	0	0.000	0	0.00	0	0.00	0	0.00
Pateros	1,181	0	0.000	0	0.00	0	0.00	0	0.00
Taguig	14,874	1	0.007	20	0.13	142	0.95	163	1.10
Quezon City	54,246	0	0.000	0	0.00	0	0.00	0	0.00
Makati City	10,765	0	0.000	0	0.00	0	0.00	0	0.00
Mandaluyong City	7,137	2	0.028	16	0.22	66	0.92	84	1.18
San Juan	2,254	0	0.000	0	0.00	0	0.00	0	0.00
Manila City	32,887	0	0.000	0	0.00	0	0.00	0	0.00
Las Piñas City	10,879	0	0.000	0	0.00	5	0.05	5	0.05
Muntinlupa City	9,317	0	0.000	0	0.00	0	0.00	0	0.00
Parañaque City	12,298	0	0.000	0	0.00	21	0.17	21	0.17
Pasay City	7,700	0	0.000	0	0.00	0	0.00	0	0.00
**C A R**	**35,143**	**11**	**0.031**	**754**	**2.15**	**7,634**	**21.72**	**8,399**	**23.90**
Abra	4,287	1	0.023	165	3.85	1,040	24.26	1,206	28.13
Apayao	2,476	1	0.040	50	2.02	320	12.92	371	14.98
Benguet	9,140	5	0.055	211	2.31	2,580	28.23	2,796	30.59
Ifugao	4,439	2	0.045	125	2.82	1,159	26.11	1,286	28.97
Kalinga	4,660	1	0.021	99	2.12	875	18.78	975	20.92
Mt. Province	3,066	0	0.000	44	1.44	444	14.48	488	15.92
Baguio City	7,075	1	0.014	60	0.85	1,216	17.19	1,277	18.05
**Region 1**	**97,198**	**5**	**0.005**	**1,080**	**1.11**	**9,361**	**9.63**	**10,446**	**10.75**
Ilocos Norte	7,986	1	0.013	58	0.73	826	10.34	885	11.08
Ilocos Sur	9,197	0	0.000	80	0.87	1,165	12.67	1,245	13.54
La Union	11,429	1	0.009	176	1.54	1,098	9.61	1,275	11.16
Pangasinan	50,454	1	0.002	633	1.25	5,454	10.81	6,088	12.07
Alaminos City	1,905	0	0.000	0	0.00	28	1.47	28	1.47
Candon City	976	0	0.000	1	0.10	26	2.66	27	2.77
Dagupan City	3,636	1	0.028	53	1.46	236	6.49	290	7.98
Laoag City	1,845	0	0.000	0	0.00	0	0.00	0	0.00
San Carlos City	3,999	0	0.000	16	0.40	112	2.80	128	3.20
San Fernando City	2,092	1	0.048	15	0.72	114	5.45	130	6.21
Urdaneta City	2,819	0	0.000	48	1.70	302	10.71	350	12.42
Vigan City	860	0	0.000	0	0.00	0	0.00	0	0.00
**Region 2**	**69,228**	**2**	**0.003**	**91**	**0.13**	**775**	**1.12**	**868**	**1.25**
Batanes	351	0	0.000	0	0.00	0	0.00	0	0.00

Table 1.B.1.7 - Prenatal Care
Number of Pregnant Women who Completed Iodine
Philippines, 2021

Area	Eligible Pop. (Under 1)	Iodine Capsule							
		Age Group						Total	%
		10-14 yrs old		15-19 yrs old		20-49 yrs old			
		No.	%	No.	%	No.	%		
Cagayan	20,563	0	0.000	2	0.01	46	0.22	48	0.23
Isabela	23,543	1	0.004	25	0.11	248	1.05	274	1.16
Nueva Vizcaya	9,555	0	0.000	23	0.24	102	1.07	125	1.31
Quirino	4,038	0	0.000	0	0.00	0	0.00	0	0.00
Cauayan City	2,576	0	0.000	0	0.00	0	0.00	0	0.00
Ilagan City	2,901	1	0.034	41	1.41	379	13.06	421	14.51
Santiago City	2,683	0	0.000	0	0.00	0	0.00	0	0.00
Tuguegarao City	3,018	0	0.000	0	0.00	0	0.00	0	0.00
**Region 3**	**220,202**	**1**	**0.000**	**353**	**0.16**	**3,483**	**1.58**	**3,837**	**1.74**
Aurora	4,764	0	0.000	2	0.04	19	0.40	21	0.44
Bataan	13,814	0	0.000	5	0.04	71	0.51	76	0.55
Bulacan	43,722	0	0.000	158	0.36	1,048	2.40	1,206	2.76
Nueva Ecija	28,915	0	0.000	31	0.11	775	2.68	806	2.79
Pampanga	31,503	0	0.000	16	0.05	110	0.35	126	0.40
Tarlac	19,565	0	0.000	99	0.51	1,010	5.16	1,109	5.67
Zambales	12,551	0	0.000	0	0.00	4	0.03	4	0.03
Angeles City	7,905	0	0.000	4	0.05	17	0.22	21	0.27
Balanga City	1,996	0	0.000	0	0.00	0	0.00	0	0.00
Cabanatuan City	5,921	0	0.000	0	0.00	0	0.00	0	0.00
City of San Fernando	5,890	1	0.017	16	0.27	89	1.51	106	1.80
Gapan City	2,159	0	0.000	0	0.00	2	0.09	2	0.09
Mabalacat City	4,811	0	0.000	0	0.00	45	0.94	45	0.94
Malolos City	4,885	0	0.000	0	0.00	0	0.00	0	0.00
Meycauayan	4,050	0	0.000	20	0.49	175	4.32	195	4.81
Olongapo	4,952	0	0.000	2	0.04	36	0.73	38	0.77
Palayan City	805	0	0.000	0	0.00	0	0.00	0	0.00
San Jose City	2,736	0	0.000	0	0.00	0	0.00	0	0.00
San Jose del Monte City	11,120	0	0.000	0	0.00	80	0.72	80	0.72
Science City of Munoz	1,594	0	0.000	0	0.00	2	0.13	2	0.13
Tarlac City	6,544	0	0.000	0	0.00	0	0.00	0	0.00
**Region 4A**	**298,398**	**18**	**0.006**	**949**	**0.32**	**5,808**	**1.95**	**6,775**	**2.27**
Batangas	38,848	0	0.000	62	0.16	230	0.59	292	0.75
Cavite	27,948	2	0.007	28	0.10	218	0.78	248	0.89
Laguna	19,727	2	0.010	22	0.11	150	0.76	174	0.88
Quezon	37,644	3	0.008	74	0.20	427	1.13	504	1.34
Rizal	44,826	10	0.022	125	0.28	601	1.34	736	1.64
Antipolo City	16,510	0	0.000	0	0.00	0	0.00	0	0.00
Bacoor City	12,252	0	0.000	4	0.03	42	0.34	46	0.38
Batangas City	6,895	0	0.000	1	0.01	8	0.12	9	0.13
Biñan City	6,607	0	0.000	512	7.75	3,089	46.75	3,601	54.50
Cabuyao City	6,128	0	0.000	6	0.10	75	1.22	81	1.32
Calamba City	9,025	0	0.000	29	0.32	237	2.63	266	2.95
Cavite City	2,098	1	0.048	1	0.05	5	0.24	7	0.33
Dasmariñas City	13,442	0	0.000	15	0.11	170	1.26	185	1.38
General Trias City	6,410	0	0.000	0	0.00	0	0.00	0	0.00
Imus City	8,235	0	0.000	0	0.00	2	0.02	2	0.02
Lipa City	6,954	0	0.000	4	0.06	141	2.03	145	2.09
Lucena City	5,704	0	0.000	30	0.53	197	3.45	227	3.98
San Pablo City	5,277	0	0.000	0	0.00	0	0.00	0	0.00

Table 1.B.1.7 - Prenatal Care

Number of Pregnant Women who Completed Iodine
Philippines, 2021

Area	Eligible Pop. (Under 1)	Iodine Capsule							
		Age Group						Total	%
		10-14 yrs old		15-19 yrs old		20-49 yrs old			
		No.	%	No.	%	No.	%		
San Pedro City	6,465	0	0.000	8	0.12	63	0.97	71	1.10
Santa Rosa City	7,023	0	0.000	5	0.07	62	0.88	67	0.95
Tagaytay City	1,451	0	0.000	19	1.31	40	2.76	59	4.07
Tanauan City	3,620	0	0.000	2	0.06	45	1.24	47	1.30
Tayabas City	2,134	0	0.000	2	0.09	6	0.28	8	0.37
Trece Martires City	3,175	0	0.000	0	0.00	0	0.00	0	0.00
**Region 4B**	**72,035**	**5**	**0.007**	**24**	**0.03**	**397**	**0.55**	**426**	**0.59**
Marinduque	4,906	0	0.000	0	0.00	13	0.26	13	0.26
Mindoro Occidental	12,449	0	0.000	2	0.02	17	0.14	19	0.15
Mindoro Oriental	20,011	5	0.025	3	0.01	167	0.83	175	0.87
Palawan	21,676	0	0.000	18	0.08	131	0.60	149	0.69
Romblon	6,482	0	0.000	1	0.02	69	1.06	70	1.08
Puerto Princesa City	6,511	0	0.000	0	0.00	0	0.00	0	0.00
**Region 5**	**137,350**	**0**	**0.000**	**70**	**0.05**	**841**	**0.61**	**911**	**0.66**
Albay	23,903	0	0.000	19	0.08	300	1.26	319	1.33
Camarines Norte	14,340	0	0.000	19	0.13	133	0.93	152	1.06
Camarines Sur	39,151	0	0.000	17	0.04	207	0.53	224	0.57
Catanduanes	6,381	0	0.000	1	0.02	57	0.89	58	0.91
Masbate	22,565	0	0.000	5	0.02	50	0.22	55	0.24
Sorsogon	19,470	0	0.000	4	0.02	33	0.17	37	0.19
Iriga City	2,665	0	0.000	0	0.00	0	0.00	0	0.00
Legaspi City	4,205	0	0.000	2	0.05	20	0.48	22	0.52
Naga City	4,670	0	0.000	3	0.06	41	0.88	44	0.94
**Region 6**	**146,545**	**26**	**0.018**	**1,026**	**0.70**	**9,089**	**6.20**	**10,141**	**6.92**
Aklan	11,231	3	0.027	17	0.15	200	1.78	220	1.96
Antique	12,985	1	0.008	85	0.65	881	6.78	967	7.45
Capiz	13,984	2	0.014	24	0.17	386	2.76	412	2.95
Guimaras	3,085	0	0.000	0	0.00	0	0.00	0	0.00
Iloilo	36,414	11	0.030	364	1.00	4,065	11.16	4,440	12.19
Negros Occidental	49,314	9	0.018	452	0.92	2,804	5.69	3,265	6.62
Bacolod City	11,102	0	0.000	37	0.33	278	2.50	315	2.84
Iloilo City	8,430	0	0.000	47	0.56	475	5.63	522	6.19
**Region 7**	**162,860**	**5**	**0.003**	**582**	**0.36**	**3,911**	**2.40**	**4,498**	**2.76**
Bohol	27,637	0	0.000	51	0.18	219	0.79	270	0.98
Cebu	67,046	0	0.000	65	0.10	564	0.84	629	0.94
Negros Oriental	27,922	5	0.018	338	1.21	2,374	8.50	2,717	9.73
Siquijor	1,636	0	0.000	1	0.06	3	0.18	4	0.24
Cebu City	21,037	0	0.000	21	0.10	124	0.59	145	0.69
Lapu-Lapu City	9,310	0	0.000	28	0.30	264	2.84	292	3.14
Mandaue City	8,272	0	0.000	78	0.94	363	4.39	441	5.33
**Region 8**	**104,016**	**3**	**0.003**	**129**	**0.12**	**1,110**	**1.07**	**1,242**	**1.19**
Biliran	3,856	0	0.000	10	0.26	30	0.78	40	1.04

Table 1.B.1.7 - Prenatal Care

Number of Pregnant Women who Completed Iodine
Philippines, 2021

Area	Eligible Pop. (Under 1)	Iodine Capsule							
		Age Group						Total	%
		10-14 yrs old		15-19 yrs old		20-49 yrs old			
		No.	%	No.	%	No.	%		
Eastern Samar	11,635	0	0.000	11	0.09	87	0.75	98	0.84
Northern Leyte	35,052	0	0.000	34	0.10	379	1.08	413	1.18
Northern Samar	15,655	0	0.000	39	0.25	292	1.87	331	2.11
Southern Leyte	6,556	2	0.031	5	0.08	26	0.40	33	0.50
Western Samar	14,515	1	0.007	20	0.14	175	1.21	196	1.35
Calbayog City	4,474	0	0.000	0	0.00	24	0.54	24	0.54
Maasin City	1,661	0	0.000	0	0.00	7	0.42	7	0.42
Ormoc City	4,989	0	0.000	0	0.00	0	0.00	0	0.00
Tacloban City	5,623	0	0.000	10	0.18	90	1.60	100	1.78
**Region 9**	**79,552**	**23**	**0.029**	**946**	**1.19**	**5,329**	**6.70**	**6,298**	**7.92**
Zamboanga del Norte	17,075	18	0.105	674	3.95	3,287	19.25	3,979	23.30
Zamboanga del Sur	17,506	0	0.000	3	0.02	42	0.24	45	0.26
Zamboanga Sibugay	14,992	0	0.000	4	0.03	23	0.15	27	0.18
Dapitan City	1,755	0	0.000	38	2.17	398	22.68	436	24.84
Dipolog City	2,794	5	0.179	204	7.30	1,493	53.44	1,702	60.92
Isabela City	2,544	0	0.000	0	0.00	0	0.00	0	0.00
Pagadian City	4,290	0	0.000	0	0.00	0	0.00	0	0.00
Zamboanga City	18,596	0	0.000	23	0.12	86	0.46	109	0.59
**Region 10**	**100,690**	**30**	**0.030**	**1,832**	**1.82**	**10,757**	**10.68**	**12,619**	**12.53**
Bukidnon	23,307	24	0.103	747	3.21	3,171	13.61	3,942	16.91
Camiguin	1,854	0	0.000	0	0.00	0	0.00	0	0.00
Lanao del Norte	14,955	1	0.007	401	2.68	3,877	25.92	4,279	28.61
Misamis Occidental	6,413	1	0.016	73	1.14	630	9.82	704	10.98
Misamis Oriental	15,058	0	0.000	158	1.05	846	5.62	1,004	6.67
Cagayan de Oro City	14,263	0	0.000	9	0.06	128	0.90	137	0.96
El Salvador City	1,061	1	0.094	85	8.01	438	41.28	524	49.39
Gingoog City	2,627	0	0.000	21	0.80	54	2.06	75	2.85
Iligan City	7,575	0	0.000	0	0.00	0	0.00	0	0.00
Malaybalay City	3,882	1	0.026	30	0.77	141	3.63	172	4.43
Oroquieta City	1,389	0	0.000	13	0.94	146	10.51	159	11.45
Ozamis City	2,782	1	0.036	110	3.95	644	23.15	755	27.14
Tangub City	1,234	0	0.000	1	0.08	6	0.49	7	0.57
Valencia City	4,290	1	0.023	184	4.29	676	15.76	861	20.07
**Region 11**	**107,847**	**147**	**0.136**	**3,315**	**3.07**	**16,824**	**15.60**	**20,286**	**18.81**
Davao de Oro	15,526	32	0.206	838	5.40	3,793	24.43	4,663	30.03
Davao del Norte	21,184	25	0.118	608	2.87	3,510	16.57	4,143	19.56
Davao Oriental	13,125	0	0.000	30	0.23	192	1.46	222	1.69
Davao del Sur	14,355	19	0.132	194	1.35	844	5.88	1,057	7.36
Davao Occidental	6,598	33	0.500	607	9.20	1,520	23.04	2,160	32.74
Davao City	37,059	38	0.103	1,038	2.80	6,965	18.79	8,041	21.70
**Region 12**	**104,693**	**104**	**0.099**	**3,498**	**3.34**	**16,753**	**16.00**	**20,355**	**19.44**
North Cotabato	33,933	12	0.035	718	2.12	3,806	11.22	4,536	13.37
Sarangani	12,904	31	0.240	918	7.11	3,518	27.26	4,467	34.62
South Cotabato	21,039	8	0.038	520	2.47	2,372	11.27	2,900	13.78

Table 1.B.1.7 - Prenatal Care

Number of Pregnant Women who Completed Iodine
Philippines, 2021

Area	Eligible Pop. (Under 1)	Iodine Capsule							
		Age Group						Total	%
		10-14 yrs old		15-19 yrs old		20-49 yrs old			
		No.	%	No.	%	No.	%		
Sultan Kudarat	17,331	42	0.242	951	5.49	4,655	26.86	5,648	32.59
Cotabato City	5,831	0	0.000	70	1.20	640	10.98	710	12.18
Gen. Santos City	13,655	11	0.081	321	2.35	1,762	12.90	2,094	15.34
**BARMM**	**94,148**	**0**	**0.000**	**19**	**0.02**	**220**	**0.23**	**239**	**0.25**
Basilan	7,672	0	0.000	6	0.08	24	0.31	30	0.39
Lanao del Sur	21,391	0	0.000	5	0.02	150	0.70	155	0.72
Maguindanao	31,650	0	0.000	1	0.00	4	0.01	5	0.02
Sulu	16,891	0	0.000	0	0.00	0	0.00	0	0.00
Tawi-Tawi	9,319	0	0.000	0	0.00	5	0.05	5	0.05
Lamitan City	2,112	0	0.000	7	0.33	37	1.75	44	2.08
Marawi City	5,113	0	0.000	0	0.00	0	0.00	0	0.00
**CARAGA**	**60,342**	**0**	**0.000**	**56**	**0.09**	**342**	**0.57**	**398**	**0.66**
Agusan del Norte	8,116	0	0.000	10	0.12	100	1.23	110	1.36
Agusan del Sur	17,658	0	0.000	33	0.19	148	0.84	181	1.03
Surigao del Norte	7,195	0	0.000	0	0.00	0	0.00	0	0.00
Surigao del Sur	11,532	0	0.000	11	0.10	73	0.63	84	0.73
Province of Dinagat	2,574	0	0.000	1	0.04	19	0.74	20	0.78
Bislig City	2,185	0	0.000	0	0.00	0	0.00	0	0.00
Butuan City	7,732	0	0.000	1	0.01	2	0.03	3	0.04
Surigao City	3,350	0	0.000	0	0.00	0	0.00	0	0.00

Note: Put asterisk (*) for No Report and Zero (0) for No Case

Table 1.B.1.8 - Prenatal Care

Number and Proportion of Pregnant Women given one (1) dose of Deworming Tablet by Region
Philippines, 2021

Area	Eligible Pop. (Under 1)	Pregnant women given one (1) dose of Deworming Tablet							
		Age Group						Total	%
		10-14 yrs old		15-19 yrs old		20-49 yrs old			
		No.	%	No.	%	No.	%		
**PHILIPPINES**	**2,128,168**	**629**	**0.030**	**32,672**	**1.54**	**226,033**	**10.62**	**259,334**	**12.19**
**N C R**	**237,921**	**39**	**0.016**	**2,760**	**1.16**	**25,866**	**10.87**	**28,665**	**12.05**
Malabon	6,757	0	0.000	0	0.00	0	0.00	0	0.00
Navotas	4,607	0	0.000	7	0.15	14	0.30	21	0.46
Valenzuela City	11,465	0	0.000	12	0.10	131	1.14	143	1.25
Caloocan City	29,270	0	0.000	0	0.00	0	0.00	0	0.00
Marikina City	8,330	1	0.012	98	1.18	499	5.99	598	7.18
Pasig City	13,954	0	0.000	2	0.01	9	0.06	11	0.08
Pateros	1,181	0	0.000	58	4.91	248	21.00	306	25.91
Taguig	14,874	2	0.013	96	0.65	985	6.62	1,083	7.28
Quezon City	54,246	34	0.063	2,127	3.92	22,512	41.50	24,673	45.48
Makati City	10,765	0	0.000	113	1.05	231	2.15	344	3.20
Mandaluyong City	7,137	0	0.000	0	0.00	0	0.00	0	0.00
San Juan	2,254	1	0.044	8	0.35	114	5.06	123	5.46
Manila City	32,887	0	0.000	91	0.28	236	0.72	327	0.99
Las Piñas City	10,879	0	0.000	0	0.00	0	0.00	0	0.00
Muntinlupa City	9,317	0	0.000	0	0.00	0	0.00	0	0.00
Parañaque City	12,298	1	0.008	57	0.46	310	2.52	368	2.99
Pasay City	7,700	0	0.000	91	1.18	577	7.49	668	8.68
**C A R**	**35,143**	**12**	**0.034**	**608**	**1.73**	**7,312**	**20.81**	**7,932**	**22.57**
Abra	4,287	0	0.000	77	1.80	522	12.18	599	13.97
Apayao	2,476	6	0.242	141	5.69	1,022	41.28	1,169	47.21
Benguet	9,140	1	0.011	95	1.04	1,497	16.38	1,593	17.43
Ifugao	4,439	3	0.068	90	2.03	1,397	31.47	1,490	33.57
Kalinga	4,660	2	0.043	158	3.39	1,757	37.70	1,917	41.14
Mt. Province	3,066	0	0.000	4	0.13	59	1.92	63	2.05
Baguio City	7,075	0	0.000	43	0.61	1,058	14.95	1,101	15.56
**Region 1**	**97,198**	**2**	**0.002**	**862**	**0.89**	**8,794**	**9.05**	**9,658**	**9.94**
Ilocos Norte	7,986	0	0.000	74	0.93	885	11.08	959	12.01
Ilocos Sur	9,197	0	0.000	6	0.07	150	1.63	156	1.70
La Union	11,429	1	0.009	192	1.68	1,580	13.82	1,773	15.51
Pangasinan	50,454	0	0.000	473	0.94	4,838	9.59	5,311	10.53
Alaminos City	1,905	0	0.000	0	0.00	10	0.52	10	0.52
Candon City	976	0	0.000	0	0.00	215	22.03	215	22.03
Dagupan City	3,636	0	0.000	1	0.03	66	1.82	67	1.84
Laoag City	1,845	0	0.000	0	0.00	0	0.00	0	0.00
San Carlos City	3,999	0	0.000	15	0.38	146	3.65	161	4.03
San Fernando City	2,092	0	0.000	14	0.67	154	7.36	168	8.03
Urdaneta City	2,819	1	0.035	87	3.09	750	26.61	838	29.73
Vigan City	860	0	0.000	0	0.00	0	0.00	0	0.00

Table 1.B.1.8 - Prenatal Care

Number and Proportion of Pregnant Women given one (1) dose of Deworming Tablet by Region
Philippines, 2021

Area	Eligible Pop. (Under 1)	Pregnant women given one (1) dose of Deworming Tablet							
		Age Group						Total	%
		10-14 yrs old		15-19 yrs old		20-49 yrs old			
		No.	%	No.	%	No.	%		
**Region 2**	**69,228**	**21**	**0.030**	**670**	**0.97**	**4,732**	**6.84**	**5,423**	**7.83**
Batanes	351	0	0.000	1	0.28	17	4.84	18	5.13
Cagayan	20,563	10	0.049	203	0.99	1,425	6.93	1,638	7.97
Isabela	23,543	1	0.004	169	0.72	982	4.17	1,152	4.89
Nueva Vizcaya	9,555	9	0.094	155	1.62	999	10.46	1,163	12.17
Quirino	4,038	0	0.000	0	0.00	0	0.00	0	0.00
Cauayan City	2,576	0	0.000	41	1.59	303	11.76	344	13.35
Ilagan City	2,901	1	0.034	48	1.65	425	14.65	474	16.34
Santiago City	2,683	0	0.000	9	0.34	24	0.89	33	1.23
Tuguegarao City	3,018	0	0.000	44	1.46	557	18.46	601	19.91
**Region 3**	**220,202**	**22**	**0.010**	**746**	**0.34**	**5,801**	**2.63**	**6,569**	**2.98**
Aurora	4,764	1	0.021	26	0.55	147	3.09	174	3.65
Bataan	13,814	3	0.022	11	0.08	131	0.95	145	1.05
Bulacan	43,722	1	0.002	67	0.15	418	0.96	486	1.11
Nueva Ecija	28,915	0	0.000	36	0.12	162	0.56	198	0.68
Pampanga	31,503	0	0.000	8	0.03	266	0.84	274	0.87
Tarlac	19,565	6	0.031	270	1.38	2,747	14.04	3,023	15.45
Zambales	12,551	5	0.040	69	0.55	378	3.01	452	3.60
Angeles City	7,905	0	0.000	0	0.00	0	0.00	0	0.00
Balanga City	1,996	0	0.000	0	0.00	0	0.00	0	0.00
Cabanatuan City	5,921	0	0.000	0	0.00	0	0.00	0	0.00
City of San Fernando	5,890	0	0.000	26	0.44	153	2.60	179	3.04
Gapan City	2,159	0	0.000	9	0.42	51	2.36	60	2.78
Mabalacat City	4,811	0	0.000	27	0.56	195	4.05	222	4.61
Malolos City	4,885	0	0.000	0	0.00	0	0.00	0	0.00
Meycauayan	4,050	0	0.000	31	0.77	124	3.06	155	3.83
Olongapo	4,952	3	0.061	65	1.31	445	8.99	513	10.36
Palayan City	805	0	0.000	2	0.25	5	0.62	7	0.87
San Jose City	2,736	0	0.000	0	0.00	0	0.00	0	0.00
San Jose del Monte City	11,120	0	0.000	0	0.00	0	0.00	0	0.00
Science City of Munoz	1,594	3	0.188	93	5.83	565	35.45	661	41.47
Tarlac City	6,544	0	0.000	6	0.09	14	0.21	20	0.31
**Region 4A**	**298,398**	**12**	**0.004**	**1,167**	**0.39**	**8,070**	**2.70**	**9,249**	**3.10**
Batangas	38,848	0	0.000	3	0.01	101	0.26	104	0.27
Cavite	27,948	2	0.007	46	0.16	295	1.06	343	1.23
Laguna	19,727	4	0.020	158	0.80	939	4.76	1,101	5.58
Quezon	37,644	1	0.003	138	0.37	706	1.88	845	2.24
Rizal	44,826	1	0.002	299	0.67	1,557	3.47	1,857	4.14
Antipolo City	16,510	0	0.000	4	0.02	10	0.06	14	0.08
Bacoor City	12,252	0	0.000	12	0.10	82	0.67	94	0.77
Batangas City	6,895	2	0.029	0	0.00	0	0.00	2	0.03
Biñan City	6,607	0	0.000	298	4.51	2,421	36.64	2,719	41.15
Cabuyao City	6,128	0	0.000	22	0.36	104	1.70	126	2.06
Calamba City	9,025	0	0.000	11	0.12	286	3.17	297	3.29
Cavite City	2,098	0	0.000	0	0.00	0	0.00	0	0.00
Dasmariñas City	13,442	1	0.007	16	0.12	281	2.09	298	2.22

Table 1.B.1.8 - Prenatal Care

Number and Proportion of Pregnant Women given one (1) dose of Deworming Tablet by Region
Philippines, 2021

Area	Eligible Pop. (Under 1)	Pregnant women given one (1) dose of Deworming Tablet							
		Age Group						Total	%
		10-14 yrs old		15-19 yrs old		20-49 yrs old			
		No.	%	No.	%	No.	%		
General Trias City	6,410	0	0.000	8	0.12	146	2.28	154	2.40
Imus City	8,235	0	0.000	14	0.17	84	1.02	98	1.19
Lipa City	6,954	0	0.000	0	0.00	1	0.01	1	0.01
Lucena City	5,704	0	0.000	0	0.00	34	0.60	34	0.60
San Pablo City	5,277	0	0.000	1	0.02	7	0.13	8	0.15
San Pedro City	6,465	0	0.000	23	0.36	112	1.73	135	2.09
Santa Rosa City	7,023	0	0.000	74	1.05	550	7.83	624	8.89
Tagaytay City	1,451	0	0.000	0	0.00	13	0.90	13	0.90
Tanauan City	3,620	0	0.000	11	0.30	87	2.40	98	2.71
Tayabas City	2,134	0	0.000	0	0.00	0	0.00	0	0.00
Trece Martires City	3,175	1	0.031	29	0.91	254	8.00	284	8.94
**Region 4B**	**72,035**	**10**	**0.014**	**599**	**0.83**	**4,692**	**6.51**	**5,301**	**7.36**
Marinduque	4,906	0	0.000	40	0.82	490	9.99	530	10.80
Mindoro Occidental	12,449	2	0.016	84	0.67	500	4.02	586	4.71
Mindoro Oriental	20,011	0	0.000	185	0.92	1,681	8.40	1,866	9.32
Palawan	21,676	8	0.037	259	1.19	1,768	8.16	2,035	9.39
Romblon	6,482	0	0.000	31	0.48	253	3.90	284	4.38
Puerto Princesa City	6,511	0	0.000	0	0.00	0	0.00	0	0.00
**Region 5**	**137,350**	**34**	**0.025**	**2,713**	**1.98**	**24,165**	**17.59**	**26,912**	**19.59**
Albay	23,903	6	0.025	221	0.92	3,454	14.45	3,681	15.40
Camarines Norte	14,340	7	0.049	578	4.03	4,256	29.68	4,841	33.76
Camarines Sur	39,151	5	0.013	611	1.56	5,947	15.19	6,563	16.76
Catanduanes	6,381	2	0.031	140	2.19	1,140	17.87	1,282	20.09
Masbate	22,565	11	0.049	752	3.33	4,664	20.67	5,427	24.05
Sorsogon	19,470	2	0.010	293	1.50	3,363	17.27	3,658	18.79
Iriga City	2,665	1	0.038	69	2.59	832	31.22	902	33.85
Legaspi City	4,205	0	0.000	0	0.00	41	0.98	41	0.98
Naga City	4,670	0	0.000	49	1.05	468	10.02	517	11.07
**Region 6**	**146,545**	**27**	**0.018**	**1,600**	**1.09**	**13,312**	**9.08**	**14,939**	**10.19**
Aklan	11,231	0	0.000	148	1.32	1,416	12.61	1,564	13.93
Antique	12,985	1	0.008	153	1.18	1,375	10.59	1,529	11.78
Capiz	13,984	3	0.021	169	1.21	1,837	13.14	2,009	14.37
Guimaras	3,085	1	0.032	81	2.63	884	28.65	966	31.31
Iloilo	36,414	6	0.016	235	0.65	2,375	6.52	2,616	7.18
Negros Occidental	49,314	16	0.032	808	1.64	5,385	10.92	6,209	12.59
Bacolod City	11,102	0	0.000	1	0.01	17	0.15	18	0.16
Iloilo City	8,430	0	0.000	5	0.06	23	0.27	28	0.33
**Region 7**	**162,860**	**44**	**0.027**	**3,953**	**2.43**	**31,590**	**19.40**	**35,587**	**21.85**
Bohol	27,637	4	0.014	461	1.67	3,996	14.46	4,461	16.14
Cebu	67,046	23	0.034	1,478	2.20	11,614	17.32	13,115	19.56
Negros Oriental	27,922	2	0.007	359	1.29	2,322	8.32	2,683	9.61
Siquijor	1,636	0	0.000	61	3.73	587	35.88	648	39.61

Table 1.B.1.8 - Prenatal Care

Number and Proportion of Pregnant Women given one (1) dose of Deworming Tablet by Region
Philippines, 2021

Area	Eligible Pop. (Under 1)	Pregnant women given one (1) dose of Deworming Tablet							
		Age Group						Total	%
		10-14 yrs old		15-19 yrs old		20-49 yrs old			
		No.	%	No.	%	No.	%		
Cebu City	21,037	14	0.067	1,270	6.04	9,286	44.14	10,570	50.24
Lapu-Lapu City	9,310	0	0.000	93	1.00	1,441	15.48	1,534	16.48
Mandaue City	8,272	1	0.012	231	2.79	2,344	28.34	2,576	31.14
**Region 8**	**104,016**	**14**	**0.013**	**1,362**	**1.31**	**10,051**	**9.66**	**11,427**	**10.99**
Biliran	3,856	4	0.104	153	3.97	1,352	35.06	1,509	39.13
Eastern Samar	11,635	3	0.026	278	2.39	1,977	16.99	2,258	19.41
Northern Leyte	35,052	6	0.017	194	0.55	1,505	4.29	1,705	4.86
Northern Samar	15,655	0	0.000	177	1.13	1,303	8.32	1,480	9.45
Southern Leyte	6,556	1	0.015	105	1.60	753	11.49	859	13.10
Western Samar	14,515	0	0.000	165	1.14	1,368	9.42	1,533	10.56
Calbayog City	4,474	0	0.000	38	0.85	222	4.96	260	5.81
Maasin City	1,661	0	0.000	24	1.44	295	17.76	319	19.21
Ormoc City	4,989	0	0.000	228	4.57	1,258	25.22	1,486	29.79
Tacloban City	5,623	0	0.000	0	0.00	18	0.32	18	0.32
**Region 9**	**79,552**	**10**	**0.013**	**1,350**	**1.70**	**8,635**	**10.85**	**9,995**	**12.56**
Zamboanga del Norte	17,075	2	0.012	330	1.93	1,650	9.66	1,982	11.61
Zamboanga del Sur	17,506	3	0.017	151	0.86	827	4.72	981	5.60
Zamboanga Sibugay	14,992	1	0.007	270	1.80	1,412	9.42	1,683	11.23
Dapitan City	1,755	2	0.114	48	2.74	1,420	80.91	1,470	83.76
Dipolog City	2,794	0	0.000	30	1.07	148	5.30	178	6.37
Isabela City	2,544	0	0.000	75	2.95	310	12.19	385	15.13
Pagadian City	4,290	0	0.000	2	0.05	10	0.23	12	0.28
Zamboanga City	18,596	2	0.011	444	2.39	2,858	15.37	3,304	17.77
**Region 10**	**100,690**	**62**	**0.062**	**2,642**	**2.62**	**13,130**	**13.04**	**15,834**	**15.73**
Bukidnon	23,307	46	0.197	1,524	6.54	6,329	27.15	7,899	33.89
Camiguin	1,854	0	0.000	3	0.16	88	4.75	91	4.91
Lanao del Norte	14,955	1	0.007	41	0.27	454	3.04	496	3.32
Misamis Occidental	6,413	0	0.000	25	0.39	277	4.32	302	4.71
Misamis Oriental	15,058	1	0.007	182	1.21	1,026	6.81	1,209	8.03
Cagayan de Oro City	14,263	0	0.000	9	0.06	87	0.61	96	0.67
El Salvador City	1,061	0	0.000	0	0.00	0	0.00	0	0.00
Gingoog City	2,627	3	0.114	16	0.61	35	1.33	54	2.06
Iligan City	7,575	7	0.092	381	5.03	2,331	30.77	2,719	35.89
Malaybalay City	3,882	0	0.000	27	0.70	205	5.28	232	5.98
Oroquieta City	1,389	0	0.000	0	0.00	0	0.00	0	0.00
Ozamis City	2,782	0	0.000	78	2.80	453	16.28	531	19.09
Tangub City	1,234	0	0.000	0	0.00	0	0.00	0	0.00
Valencia City	4,290	4	0.093	356	8.30	1,845	43.01	2,205	51.40
**Region 11**	**107,847**	**213**	**0.198**	**4,648**	**4.31**	**24,416**	**22.64**	**29,277**	**27.15**
Davao de Oro	15,526	52	0.335	1,182	7.61	5,542	35.69	6,776	43.64
Davao del Norte	21,184	34	0.160	1,103	5.21	6,164	29.10	7,301	34.46
Davao Oriental	13,125	21	0.160	598	4.56	3,167	24.13	3,786	28.85
Davao del Sur	14,355	23	0.160	238	1.66	1,131	7.88	1,392	9.70
Davao Occidental	6,598	50	0.758	550	8.34	1,406	21.31	2,006	30.40
Davao City	37,059	33	0.089	977	2.64	7,006	18.90	8,016	21.63

Table 1.B.1.8 - Prenatal Care

Number and Proportion of Pregnant Women given one (1) dose of Deworming Tablet by Region
Philippines, 2021

Area	Eligible Pop. (Under 1)	Pregnant women given one (1) dose of Deworming Tablet							
		Age Group						Total	%
		10-14 yrs old		15-19 yrs old		20-49 yrs old			
		No.	%	No.	%	No.	%		
**Region 12**	**104,693**	**95**	**0.091**	**5,264**	**5.03**	**24,519**	**23.42**	**29,878**	**28.54**
North Cotabato	33,933	4	0.012	680	2.00	3,273	9.65	3,957	11.66
Sarangani	12,904	42	0.325	1,881	14.58	7,341	56.89	9,264	71.79
South Cotabato	21,039	11	0.052	919	4.37	4,849	23.05	5,779	27.47
Sultan Kudarat	17,331	30	0.173	1,389	8.01	6,754	38.97	8,173	47.16
Cotabato City	5,831	0	0.000	115	1.97	778	13.34	893	15.31
Gen. Santos City	13,655	8	0.059	280	2.05	1,524	11.16	1,812	13.27
**BARMM**	**94,148**	**5**	**0.005**	**1,333**	**1.42**	**8,709**	**9.25**	**10,047**	**10.67**
Basilan	7,672	0	0.000	54	0.70	364	4.74	418	5.45
Lanao del Sur	21,391	2	0.009	101	0.47	3,395	15.87	3,498	16.35
Maguindanao	31,650	1	0.003	74	0.23	1,236	3.91	1,311	4.14
Sulu	16,891	0	0.000	0	0.00	0	0.00	0	0.00
Tawi-Tawi	9,319	2	0.021	138	1.48	1,244	13.35	1,384	14.85
Lamitan City	2,112	0	0.000	17	0.80	40	1.89	57	2.70
Marawi City	5,113	0	0.000	949	18.56	2,430	47.53	3,379	66.09
**CARAGA**	**60,342**	**7**	**0.012**	**395**	**0.65**	**2,239**	**3.71**	**2,641**	**4.38**
Agusan del Norte	8,116	0	0.000	4	0.05	16	0.20	20	0.25
Agusan del Sur	17,658	0	0.000	100	0.57	463	2.62	563	3.19
Surigao del Norte	7,195	0	0.000	1	0.01	41	0.57	42	0.58
Surigao del Sur	11,532	5	0.043	196	1.70	1,223	10.61	1,424	12.35
Province of Dinagat	2,574	2	0.078	17	0.66	104	4.04	123	4.78
Bislig City	2,185	0	0.000	17	0.78	60	2.75	77	3.52
Butuan City	7,732	0	0.000	4	0.05	36	0.47	40	0.52
Surigao City	3,350	0	0.000	56	1.67	296	8.84	352	10.51

Note: Put asterisk (*) for No Report and Zero (0) for No Case

Table 1.B.1.9 - Prenatal Care

Number and Proportion of Pregnant Women screened for Syphillis by Region

Philippines, 2021

Area	Eligible Pop. (Under 1)	Screened for Syphillis							
		Age Group						Total	%
		10-14 yrs old		15-19 yrs old		20-49 yrs old			
		No.	%	No.	%	No.	%		
**PHILIPPINES**	**2,128,168**	**933**	**0.04**	**49,350**	**2.32**	**378,010**	**17.76**	**428,293**	**20.12**
**N C R**	**237,921**	**195**	**0.08**	**10,404**	**4.37**	**83,998**	**35.30**	**94,597**	**39.76**
Malabon	6,757	18	0.27	522	7.73	2,572	38.06	3,112	46.06
Navotas	4,607	7	0.15	570	12.37	2,469	53.59	3,046	66.12
Valenzuela City	11,465	14	0.12	582	5.08	5,531	48.24	6,127	53.44
Caloocan City	29,270	9	0.03	1,029	3.52	7,618	26.03	8,656	29.57
								0	
Marikina City	8,330	3	0.04	171	2.05	1,508	18.10	1,682	20.19
Pasig City	13,954	7	0.05	758	5.43	7,592	54.41	8,357	59.89
Pateros	1,181	2	0.17	97	8.21	598	50.64	697	59.02
Taguig	14,874	15	0.10	856	5.76	5,878	39.52	6,749	45.37
Quezon City	54,246	45	0.08	2,195	4.05	21,592	39.80	23,832	43.93
Makati City	10,765	0	0.00	68	0.63	2,877	26.73	2,945	27.36
Mandaluyong City	7,137	6	0.08	200	2.80	2,271	31.82	2,477	34.71
San Juan	2,254	1	0.04	33	1.46	749	33.23	783	34.74
Manila City	32,887	39	0.12	1,937	5.89	11,413	34.70	13,389	40.71
Las Piñas City	10,879	2	0.02	197	1.81	2,001	18.39	2,200	20.22
Muntinlupa City	9,317	8	0.09	298	3.20	2,222	23.85	2,528	27.13
Parañaque City	12,298	12	0.10	504	4.10	3,829	31.14	4,345	35.33
Pasay City	7,700	7	0.09	387	5.03	3,278	42.57	3,672	47.69
**C A R**	**35,143**	**51**	**0.15**	**1,482**	**4.22**	**12,843**	**36.54**	**14,376**	**40.91**
Abra	4,287	0	0.00	46	1.07	271	6.32	317	7.39
Apayao	2,476	8	0.32	118	4.77	822	33.20	948	38.29
Benguet	9,140	12	0.13	566	6.19	5,872	64.25	6,450	70.57
Ifugao	4,439	0	0.00	123	2.77	1,196	26.94	1,319	29.71
Kalinga	4,660	2	0.04	140	3.00	1,133	24.31	1,275	27.36
Mt. Province	3,066	3	0.10	75	2.45	770	25.11	848	27.66
Baguio City	7,075	26	0.37	414	5.85	2,779	39.28	3,219	45.50
**Region 1**	**97,198**	**21**	**0.02**	**2,070**	**2.13**	**24,696**	**25.41**	**26,787**	**27.56**
Ilocos Norte	7,986	4	0.05	202	2.53	2,465	30.87	2,671	33.45
Ilocos Sur	9,197	6	0.07	364	3.96	4,497	48.90	4,867	52.92
La Union	11,429	2	0.02	366	3.20	3,296	28.84	3,664	32.06
Pangasinan	50,454	6	0.01	754	1.49	8,623	17.09	9,383	18.60
Alaminos City	1,905	0	0.00	59	3.10	759	39.84	818	42.94
Candon City	976	0	0.00	1	0.10	575	58.91	576	59.02
Dagupan City	3,636	0	0.00	93	2.56	690	18.98	783	21.53
Laoag City	1,845	0	0.00	35	1.90	1,909	103.47	1,944	105.37
San Carlos City	3,999	1	0.03	33	0.83	372	9.30	406	10.15
San Fernando City	2,092	0	0.00	36	1.72	452	21.61	488	23.33
Urdaneta City	2,819	2	0.07	118	4.19	920	32.64	1,040	36.89
Vigan City	860	0	0.00	9	1.05	138	16.05	147	17.09
**Region 2**	**69,228**	**34**	**0.05**	**1,365**	**1.97**	**8,931**	**12.90**	**10,330**	**14.92**
Batanes	351	1	0.28	7	1.99	155	44.16	163	46.44
Cagayan	20,563	2	0.01	130	0.63	812	3.95	944	4.59
Isabela	23,543	18	0.08	581	2.47	3,400	14.44	3,999	16.99
Nueva Vizcaya	9,555	3	0.03	280	2.93	1,788	18.71	2,071	21.67
Quirino	4,038	3	0.07	107	2.65	854	21.15	964	23.87

Table 1.B.1.9 - Prenatal Care

Number and Proportion of Pregnant Women screened for Syphillis by Region
Philippines, 2021

Area	Eligible Pop. (Under 1)	Screened for Syphillis							
		Age Group						Total	%
		10-14 yrs old		15-19 yrs old		20-49 yrs old			
		No.	%	No.	%	No.	%		
Cauayan City	2,576	1	0.04	81	3.14	443	17.20	525	20.38
Ilagan City	2,901	6	0.21	132	4.55	1,087	37.47	1,225	42.23
Santiago City	2,683	0	0.00	47	1.75	344	12.82	391	14.57
Tuguegarao City	3,018	0	0.00	0	0.00	48	1.59	48	1.59
**Region 3**	**220,202**	**196**	**0.09**	**8,552**	**3.88**	**57,337**	**26.04**	**66,085**	**30.01**
Aurora	4,764	5	0.10	203	4.26	1,318	27.67	1,526	32.03
Bataan	13,814	20	0.14	654	4.73	4,320	31.27	4,994	36.15
Bulacan	43,722	35	0.08	1,802	4.12	11,532	26.38	13,369	30.58
Nueva Ecija	28,915	22	0.08	760	2.63	4,438	15.35	5,220	18.05
Pampanga	31,503	23	0.07	809	2.57	5,359	17.01	6,191	19.65
Tarlac	19,565	11	0.06	692	3.54	4,974	25.42	5,677	29.02
Zambales	12,551	6	0.05	304	2.42	2,230	17.77	2,540	20.24
Angeles City	7,905	1	0.01	128	1.62	857	10.84	986	12.47
Balanga City	1,996	2	0.10	135	6.76	843	42.23	980	49.10
Cabanatuan City	5,921	4	0.07	283	4.78	1,413	23.86	1,700	28.71
City of San Fernando	5,890	9	0.15	220	3.74	1,659	28.17	1,888	32.05
Gapan City	2,159	0	0.00	75	3.47	386	17.88	461	21.35
Mabalacat City	4,811	5	0.10	339	7.05	2,002	41.61	2,346	48.76
Malolos City	4,885	3	0.06	146	2.99	870	17.81	1,019	20.86
Meycauayan	4,050	5	0.12	237	5.85	1,263	31.19	1,505	37.16
Olongapo	4,952	2	0.04	143	2.89	991	20.01	1,136	22.94
Palayan City	805	0	0.00	22	2.73	122	15.16	144	17.89
San Jose City	2,736	6	0.22	152	5.56	598	21.86	756	27.63
San Jose del Monte City	11,120	35	0.31	901	8.10	9,419	84.70	10,355	93.12
Science City of Munoz	1,594	0	0.00	76	4.77	342	21.46	418	26.22
Tarlac City	6,544	2	0.03	471	7.20	2,401	36.69	2,874	43.92
**Region 4A**	**298,398**	**83**	**0.03**	**6,050**	**2.03**	**45,138**	**15.13**	**51,271**	**17.18**
Batangas	38,848	2	0.01	237	0.61	2,573	6.62	2,812	7.24
Cavite	27,948	7	0.03	457	1.64	4,058	14.52	4,522	16.18
Laguna	19,727	10	0.05	468	2.37	3,228	16.36	3,706	18.79
Quezon	37,644	19	0.05	597	1.59	3,827	10.17	4,443	11.80
Rizal	44,826	24	0.05	1,580	3.52	9,170	20.46	10,774	24.04
Antipolo City	16,510	0	0.00	234	1.42	1,002	6.07	1,236	7.49
Bacoor City	12,252	1	0.01	100	0.82	759	6.19	860	7.02
Batangas City	6,895	0	0.00	108	1.57	1,056	15.32	1,164	16.88
Biñan City	6,607	0	0.00	581	8.79	3,559	53.87	4,140	62.66
Cabuyao City	6,128	0	0.00	87	1.42	1,783	29.10	1,870	30.52
Calamba City	9,025	4	0.04	161	1.78	1,800	19.94	1,965	21.77
Cavite City	2,098	1	0.05	100	4.77	372	17.73	473	22.55
Dasmariñas City	13,442	0	0.00	286	2.13	2,969	22.09	3,255	24.22
General Trias City	6,410	0	0.00	38	0.59	608	9.49	646	10.08
Imus City	8,235	0	0.00	55	0.67	374	4.54	429	5.21
Lipa City	6,954	1	0.01	64	0.92	888	12.77	953	13.70
Lucena City	5,704	3	0.05	306	5.36	1,201	21.06	1,510	26.47
San Pablo City	5,277	0	0.00	24	0.45	125	2.37	149	2.82
San Pedro City	6,465	1	0.02	163	2.52	1,101	17.03	1,265	19.57
Santa Rosa City	7,023	7	0.10	233	3.32	2,277	32.42	2,517	35.84
Tagaytay City	1,451	0	0.00	38	2.62	794	54.72	832	57.34
Tanauan City	3,620	2	0.06	69	1.91	1,143	31.57	1,214	33.54
Tayabas City	2,134	0	0.00	4	0.19	7	0.33	11	0.52
Trece Martires City	3,175	1	0.03	60	1.89	464	14.61	525	16.54
**Region 4B**	**72,035**	**13**	**0.02**	**907**	**1.26**	**7,450**	**10.34**	**8,370**	**11.62**
Marinduque	4,906	0	0.00	41	0.84	620	12.64	661	13.47

Table 1.B.1.9 - Prenatal Care

Number and Proportion of Pregnant Women screened for Syphillis by Region
Philippines, 2021

Area	Eligible Pop. (Under 1)	Screened for Syphillis							
		Age Group						Total	%
		10-14 yrs old		15-19 yrs old		20-49 yrs old			
		No.	%	No.	%	No.	%		
Mindoro Occidental	12,449	5	0.04	229	1.84	1,706	13.70	1,940	15.58
Mindoro Oriental	20,011	1	0.00	230	1.15	2,224	11.11	2,455	12.27
Palawan	21,676	5	0.02	246	1.13	1,631	7.52	1,882	8.68
Romblon	6,482	2	0.03	161	2.48	1,269	19.58	1,432	22.09
Puerto Princesa City	6,511	0	0.00	0	0.00	0	0.00	0	0.00
**Region 5**	**137,350**	**22**	**0.02**	**1,783**	**1.30**	**15,387**	**11.20**	**17,192**	**12.52**
Albay	23,903	1	0.00	297	1.24	4,317	18.06	4,615	19.31
Camarines Norte	14,340	3	0.02	260	1.81	1,603	11.18	1,866	13.01
Camarines Sur	39,151	2	0.01	119	0.30	1,626	4.15	1,747	4.46
Catanduanes	6,381	10	0.16	134	2.10	658	10.31	802	12.57
Masbate	22,565	5	0.02	532	2.36	2,939	13.02	3,476	15.40
Sorsogon	19,470	0	0.00	161	0.83	1,506	7.73	1,667	8.56
Iriga City	2,665	0	0.00	51	1.91	566	21.24	617	23.15
Legaspi City	4,205	1	0.02	90	2.14	972	23.12	1,063	25.28
Naga City	4,670	0	0.00	139	2.98	1,200	25.70	1,339	28.67
**Region 6**	**146,545**	**62**	**0.04**	**3,573**	**2.44**	**30,787**	**21.01**	**34,422**	**23.49**
Aklan	11,231	13	0.12	319	2.84	3,047	27.13	3,379	30.09
Antique	12,985	5	0.04	225	1.73	2,113	16.27	2,343	18.04
Capiz	13,984	4	0.03	190	1.36	1,951	13.95	2,145	15.34
Guimaras	3,085	4	0.13	135	4.38	1,541	49.95	1,680	54.46
Iloilo	36,414	18	0.05	1,258	3.45	11,902	32.69	13,178	36.19
Negros Occidental	49,314	7	0.01	686	1.39	5,037	10.21	5,730	11.62
Bacolod City	11,102	4	0.04	463	4.17	3,117	28.08	3,584	32.28
Iloilo City	8,430	7	0.08	297	3.52	2,079	24.66	2,383	28.27
**Region 7**	**162,860**	**40**	**0.02**	**3,357**	**2.06**	**27,669**	**16.99**	**31,066**	**19.08**
Bohol	27,637	0	0.00	305	1.10	4,583	16.58	4,888	17.69
Cebu	67,046	13	0.02	900	1.34	6,186	9.23	7,099	10.59
Negros Oriental	27,922	1	0.00	166	0.59	1,234	4.42	1,401	5.02
Siquijor	1,636	1	0.06	67	4.10	523	31.97	591	36.12
Cebu City	21,037	17	0.08	1,331	6.33	9,282	44.12	10,630	50.53
Lapu-Lapu City	9,310	4	0.04	341	3.66	3,705	39.80	4,050	43.50
Mandaue City	8,272	4	0.05	247	2.99	2,156	26.06	2,407	29.10
**Region 8**	**104,016**	**15**	**0.01**	**1,093**	**1.05**	**9,346**	**8.99**	**10,454**	**10.05**
Biliran	3,856	3	0.08	158	4.10	1,074	27.85	1,235	32.03
Eastern Samar	11,635	0	0.00	121	1.04	817	7.02	938	8.06
Northern Leyte	35,052	3	0.01	158	0.45	1,473	4.20	1,634	4.66
Northern Samar	15,655	0	0.00	240	1.53	1,930	12.33	2,170	13.86
Southern Leyte	6,556	1	0.02	40	0.61	324	4.94	365	5.57
Western Samar	14,515	1	0.01	95	0.65	602	4.15	698	4.81
Calbayog City	4,474	0	0.00	22	0.49	192	4.29	214	4.78
Maasin City	1,661	0	0.00	1	0.06	4	0.24	5	0.30
Ormoc City	4,989	5	0.10	49	0.98	374	7.50	428	8.58
Tacloban City	5,623	2	0.04	209	3.72	2,556	45.46	2,767	49.21
**Region 9**	**79,552**	**8**	**0.01**	**573**	**0.72**	**3,107**	**3.91**	**3,688**	**4.64**
Zamboanga del Norte	17,075	3	0.02	88	0.52	374	2.19	465	2.72
Zamboanga del Sur	17,506	1	0.01	147	0.84	620	3.54	768	4.39

Table 1.B.1.9 - Prenatal Care

Number and Proportion of Pregnant Women screened for Syphillis by Region
Philippines, 2021

Area	Eligible Pop. (Under 1)	Screened for Syphillis							
		Age Group						Total	%
		10-14 yrs old		15-19 yrs old		20-49 yrs old			
		No.	%	No.	%	No.	%		
Zamboanga Sibugay	14,992	3	0.02	272	1.81	1,741	11.61	2,016	13.45
Dapitan City	1,755	1	0.06	15	0.85	90	5.13	106	6.04
Dipolog City	2,794	0	0.00	0	0.00	5	0.18	5	0.18
Isabela City	2,544	0	0.00	0	0.00	0	0.00	0	0.00
Pagadian City	4,290	0	0.00	28	0.65	156	3.64	184	4.29
Zamboanga City	18,596	0	0.00	23	0.12	121	0.65	144	0.77
**Region 10**	**100,690**	**26**	**0.03**	**1,667**	**1.66**	**10,548**	**10.48**	**12,241**	**12.16**
Bukidnon	23,307	6	0.03	165	0.71	687	2.95	858	3.68
Camiguin	1,854	1	0.05	23	1.24	245	13.21	269	14.51
Lanao del Norte	14,955	4	0.03	71	0.47	543	3.63	618	4.13
Misamis Occidental	6,413	0	0.00	16	0.25	132	2.06	148	2.31
Misamis Oriental	15,058	6	0.04	259	1.72	1,656	11.00	1,921	12.76
Cagayan de Oro City	14,263	7	0.05	733	5.14	4,793	33.60	5,533	38.79
El Salvador City	1,061	0	0.00	8	0.75	27	2.54	35	3.30
Gingoog City	2,627	0	0.00	14	0.53	77	2.93	91	3.46
Iligan City	7,575	0	0.00	38	0.50	330	4.36	368	4.86
Malaybalay City	3,882	1	0.03	144	3.71	719	18.52	864	22.26
Oroquieta City	1,389	0	0.00	1	0.07	18	1.30	19	1.37
Ozamis City	2,782	1	0.04	156	5.61	1,105	39.72	1,262	45.36
Tangub City	1,234	0	0.00	0	0.00	0	0.00	0	0.00
Valencia City	4,290	0	0.00	39	0.91	216	5.03	255	5.94
**Region 11**	**107,847**	**85**	**0.08**	**2,316**	**2.15**	**16,143**	**14.97**	**18,544**	**17.19**
Davao de Oro	15,526	0	0.00	15	0.10	126	0.81	141	0.91
Davao del Norte	21,184	19	0.09	561	2.65	3,948	18.64	4,528	21.37
Davao Oriental	13,125	0	0.00	18	0.14	109	0.83	127	0.97
Davao del Sur	14,355	4	0.03	121	0.84	493	3.43	618	4.31
Davao Occidental	6,598	6	0.09	46	0.70	103	1.56	155	2.35
Davao City	37,059	56	0.15	1,555	4.20	11,364	30.66	12,975	35.01
**Region 12**	**104,693**	**58**	**0.06**	**2,986**	**2.85**	**16,727**	**15.98**	**19,771**	**18.88**
North Cotabato	33,933	4	0.01	266	0.78	1,556	4.59	1,826	5.38
Sarangani	12,904	9	0.07	526	4.08	2,379	18.44	2,914	22.58
South Cotabato	21,039	9	0.04	817	3.88	4,851	23.06	5,677	26.98
Sultan Kudarat	17,331	24	0.14	947	5.46	5,146	29.69	6,117	35.30
Cotabato City	5,831	0	0.00	25	0.43	361	6.19	386	6.62
Gen. Santos City	13,655	12	0.09	405	2.97	2,434	17.82	2,851	20.88
**BARMM**	**94,148**	**0**	**0.00**	**10**	**0.01**	**331**	**0.35**	**341**	**0.36**
Basilan	7,672	0	0.00	4	0.05	8	0.10	12	0.16
Lanao del Sur	21,391	0	0.00	0	0.00	253	1.18	253	1.18
Maguindanao	31,650	0	0.00	6	0.02	70	0.22	76	0.24
Sulu	16,891	0	0.00	0	0.00	0	0.00	0	0.00
Tawi-Tawi	9,319	0	0.00	0	0.00	0	0.00	0	0.00
Lamitan City	2,112	0	0.00	0	0.00	0	0.00	0	0.00
Marawi City	5,113	0	0.00	0	0.00	0	0.00	0	0.00

Table 1.B.1.9 - Prenatal Care

Number and Proportion of Pregnant Women screened for Syphillis by Region
Philippines, 2021

Area	Eligible Pop. (Under 1)	Screened for Syphillis							
		Age Group						Total	%
		10-14 yrs old		15-19 yrs old		20-49 yrs old			
		No.	%	No.	%	No.	%		
**CARAGA**	**60,342**	**24**	**0.04**	**1,162**	**1.93**	**7,572**	**12.55**	**8,758**	**14.51**
Agusan del Norte	8,116	3	0.04	101	1.24	812	10.00	916	11.29
Agusan del Sur	17,658	6	0.03	395	2.24	1,854	10.50	2,255	12.77
Surigao del Norte	7,195	0	0.00	79	1.10	521	7.24	600	8.34
Surigao del Sur	11,532	0	0.00	47	0.41	448	3.88	495	4.29
Province of Dinagat	2,574	5	0.19	61	2.37	543	21.10	609	23.66
Bislig City	2,185	1	0.05	8	0.37	30	1.37	39	1.78
Butuan City	7,732	2	0.03	341	4.41	2,400	31.04	2,743	35.48
Surigao City	3,350	7	0.21	130	3.88	964	28.78	1,101	32.87

Note: Put asterisk (*) for No Report and Zero (0) for No Case

Table 1.B.1.10 - Prenatal Care

Number and proportion of pregnant women tested positive for Syphillis by Region
Philippines, 2021

Area	Total No. of Pregnant Women Screened for Syphillis	Tested Positive for Syphillis							
		Age Group						Total	%
		10-14 yrs old		15-19 yrs old		20-49 yrs old			
		No.	%	No.	%	No.	%		
**PHILIPPINES**	**428,293**	**54**	**0.01**	**1,030**	**0.24**	**6,604**	**1.54**	**7,688**	**1.80**
**N C R**	**94,597**	**1**	**0.00**	**127**	**0.13**	**827**	**0.87**	**955**	**1.01**
Malabon	3,112	0	0.00	8	0.26	10	0.32	18	0.58
Navotas	3,046	0	0.00	14	0.46	61	2.00	75	2.46
Valenzuela City	6,127	0	0.00	17	0.28	183	2.99	200	3.26
Caloocan City	8,656	0	0.00	4	0.05	16	0.18	20	0.23
								0	
Marikina City	1,682	0	0.00	3	0.18	10	0.59	13	0.77
Pasig City	8,357	0	0.00	1	0.01	11	0.13	12	0.14
Pateros	697	0	0.00	1	0.14	0	0.00	1	0.14
Taguig	6,749	1	0.01	27	0.40	190	2.82	218	3.23
Quezon City	23,832	0	0.00	14	0.06	51	0.21	65	0.27
Makati City	2,945	0	0.00	0	0.00	15	0.51	15	0.51
Mandaluyong City	2,477	0	0.00	0	0.00	3	0.12	3	0.12
San Juan	783	0	0.00	0	0.00	1	0.13	1	0.13
Manila City	13,389	0	0.00	17	0.13	41	0.31	58	0.43
Las Piñas City	2,200	0	0.00	2	0.09	16	0.73	18	0.82
Muntinlupa City	2,528	0	0.00	1	0.04	9	0.36	10	0.40
Parañaque City	4,345	0	0.00	10	0.23	198	4.56	208	4.79
Pasay City	3,672	0	0.00	8	0.22	12	0.33	20	0.54
**C A R**	**14,376**	**0**	**0.00**	**5**	**0.03**	**43**	**0.30**	**48**	**0.33**
Abra	317	0	0.00	1	0.32	7	2.21	8	2.52
Apayao	948	0	0.00	0	0.00	5	0.53	5	0.53
Benguet	6,450	0	0.00	1	0.02	9	0.14	10	0.16
Ifugao	1,319	0	0.00	0	0.00	1	0.08	1	0.08
Kalinga	1,275	0	0.00	1	0.08	4	0.31	5	0.39
Mt. Province	848	0	0.00	1	0.12	0	0.00	1	0.12
Baguio City	3,219	0	0.00	1	0.03	17	0.53	18	0.56
**Region 1**	**26,787**	**1**	**0.00**	**9**	**0.03**	**90**	**0.34**	**100**	**0.37**
Ilocos Norte	2,671	0	0.00	0	0.00	6	0.22	6	0.22
Ilocos Sur	4,867	0	0.00	0	0.00	2	0.04	2	0.04
La Union	3,664	0	0.00	2	0.05	38	1.04	40	1.09
Pangasinan	9,383	1	0.01	7	0.07	42	0.45	50	0.53
Alaminos City	818	0	0.00	0	0.00	0	0.00	0	0.00
Candon City	576	0	0.00	0	0.00	0	0.00	0	0.00
Dagupan City	783	0	0.00	0	0.00	2	0.26	2	0.26
Laoag City	1,944	0	0.00	0	0.00	0	0.00	0	0.00
San Carlos City	406	0	0.00	0	0.00	0	0.00	0	0.00
San Fernando City	488	0	0.00	0	0.00	0	0.00	0	0.00
Urdaneta City	1,040	0	0.00	0	0.00	0	0.00	0	0.00
Vigan City	147	0	0.00	0	0.00	0	0.00	0	0.00
**Region 2**	**10,330**	**1**	**0.01**	**17**	**0.16**	**146**	**1.41**	**164**	**1.59**
Batanes	163	0	0.00	0	0.00	0	0.00	0	0.00
Cagayan	944	0	0.00	3	0.32	18	1.91	21	2.22

Table 1.B.1.10 - Prenatal Care

Number and proportion of pregnant women tested positive for Syphillis by Region
Philippines, 2021

Area	Total No. of Pregnant Women Screened for Syphillis	Tested Positive for Syphillis							
		Age Group						Total	%
		10-14 yrs old		15-19 yrs old		20-49 yrs old			
		No.	%	No.	%	No.	%		
Isabela	3,999	0	0.00	4	0.10	33	0.83	37	0.93
Nueva Vizcaya	2,071	1	0.05	3	0.14	28	1.35	32	1.55
Quirino	964	0	0.00	5	0.52	55	5.71	60	6.22
Cauayan City	525	0	0.00	0	0.00	0	0.00	0	0.00
Ilagan City	1,225	0	0.00	0	0.00	6	0.49	6	0.49
Santiago City	391	0	0.00	2	0.51	3	0.77	5	1.28
Tuguegarao City	48	0	0.00	0	0.00	3	6.25	3	6.25
**Region 3**	**66,085**	**4**	**0.01**	**161**	**0.24**	**776**	**1.17**	**941**	**1.42**
Aurora	1,526	0	0.00	0	0.00	0	0.00	0	0.00
Bataan	4,994	0	0.00	5	0.10	38	0.76	43	0.86
Bulacan	13,369	0	0.00	60	0.45	256	1.91	316	2.36
Nueva Ecija	5,220	0	0.00	7	0.13	66	1.26	73	1.40
Pampanga	6,191	0	0.00	7	0.11	21	0.34	28	0.45
Tarlac	5,677	0	0.00	8	0.14	35	0.62	43	0.76
Zambales	2,540	0	0.00	4	0.16	15	0.59	19	0.75
Angeles City	986	4	0.41	26	2.64	146	14.81	176	17.85
Balanga City	980	0	0.00	0	0.00	10	1.02	10	1.02
Cabanatuan City	1,700	0	0.00	2	0.12	9	0.53	11	0.65
City of San Fernando	1,888	0	0.00	3	0.16	6	0.32	9	0.48
Gapan City	461	0	0.00	0	0.00	1	0.22	1	0.22
Mabalacat City	2,346	0	0.00	23	0.98	98	4.18	121	5.16
Malolos City	1,019	0	0.00	0	0.00	6	0.59	6	0.59
Meycauayan	1,505	0	0.00	1	0.07	0	0.00	1	0.07
Olongapo	1,136	0	0.00	10	0.88	43	3.79	53	4.67
Palayan City	144	0	0.00	0	0.00	0	0.00	0	0.00
San Jose City	756	0	0.00	0	0.00	0	0.00	0	0.00
San Jose del Monte City	10,355	0	0.00	5	0.05	21	0.20	26	0.25
Science City of Munoz	418	0	0.00	0	0.00	2	0.48	2	0.48
Tarlac City	2,874	0	0.00	0	0.00	3	0.10	3	0.10
**Region 4A**	**51,271**	**23**	**0.04**	**151**	**0.29**	**1,335**	**2.60**	**1,509**	**2.94**
Batangas	2,812	0	0.00	3	0.11	38	1.35	41	1.46
Cavite	4,522	4	0.09	8	0.18	156	3.45	168	3.72
Laguna	3,706	1	0.03	11	0.30	65	1.75	77	2.08
Quezon	4,443	2	0.05	20	0.45	114	2.57	136	3.06
Rizal	10,774	0	0.00	30	0.28	195	1.81	225	2.09
Antipolo City	1,236	0	0.00	5	0.40	5	0.40	10	0.81
Bacoor City	860	0	0.00	2	0.23	9	1.05	11	1.28
Batangas City	1,164	0	0.00	0	0.00	6	0.52	6	0.52
Biñan City	4,140	0	0.00	20	0.48	297	7.17	317	7.66
Cabuyao City	1,870	0	0.00	0	0.00	0	0.00	0	0.00
Calamba City	1,965	7	0.36	4	0.20	55	2.80	66	3.36
Cavite City	473	2	0.42	4	0.85	15	3.17	21	4.44
Dasmariñas City	3,255	0	0.00	5	0.15	94	2.89	99	3.04
General Trias City	646	0	0.00	0	0.00	13	2.01	13	2.01
Imus City	429	7	1.63	2	0.47	36	8.39	45	10.49
Lipa City	953	0	0.00	0	0.00	5	0.52	5	0.52
Lucena City	1,510	0	0.00	10	0.66	38	2.52	48	3.18
San Pablo City	149	0	0.00	1	0.67	1	0.67	2	1.34
San Pedro City	1,265	0	0.00	7	0.55	37	2.92	44	3.48
Santa Rosa City	2,517	0	0.00	17	0.68	124	4.93	141	5.60

Table 1.B.1.10 - Prenatal Care

Number and proportion of pregnant women tested positive for Syphillis by Region

Philippines, 2021

Area	Total No. of Pregnant Women Screened for Syphillis	Tested Positive for Syphillis							
		Age Group						Total	%
		10-14 yrs old		15-19 yrs old		20-49 yrs old			
		No.	%	No.	%	No.	%		
Tagaytay City	832	0	0.00	0	0.00	11	1.32	11	1.32
Tanauan City	1,214	0	0.00	0	0.00	8	0.66	8	0.66
Tayabas City	11	0	0.00	1	9.09	0	0.00	1	9.09
Trece Martires City	525	0	0.00	1	0.19	13	2.48	14	2.67
**Region 4B**	**8,370**	**0**	**0.00**	**45**	**0.54**	**289**	**3.45**	**334**	**3.99**
Marinduque	661	0	0.00	0	0.00	12	1.82	12	1.82
Mindoro Occidental	1,940	0	0.00	3	0.15	1	0.05	4	0.21
Mindoro Oriental	2,455	0	0.00	25	1.02	166	6.76	191	7.78
Palawan	1,882	0	0.00	13	0.69	64	3.40	77	4.09
Romblon	1,432	0	0.00	4	0.28	46	3.21	50	3.49
Puerto Princesa City	0	0	0.00	0	0.00	0	0.00	0	0.00
**Region 5**	**17,192**	**3**	**0.02**	**27**	**0.16**	**297**	**1.73**	**327**	**1.90**
Albay	4,615	0	0.00	3	0.07	83	1.80	86	1.86
Camarines Norte	1,866	0	0.00	4	0.21	42	2.25	46	2.47
Camarines Sur	1,747	0	0.00	2	0.11	16	0.92	18	1.03
Catanduanes	802	0	0.00	1	0.12	9	1.12	10	1.25
Masbate	3,476	3	0.09	8	0.23	77	2.22	88	2.53
Sorsogon	1,667	0	0.00	7	0.42	47	2.82	54	3.24
Iriga City	617	0	0.00	0	0.00	9	1.46	9	1.46
Legaspi City	1,063	0	0.00	0	0.00	10	0.94	10	0.94
Naga City	1,339	0	0.00	2	0.15	4	0.30	6	0.45
**Region 6**	**34,422**	**10**	**0.03**	**71**	**0.21**	**620**	**1.80**	**701**	**2.04**
Aklan	3,379	0	0.00	8	0.24	45	1.33	53	1.57
Antique	2,343	4	0.17	5	0.21	61	2.60	70	2.99
Capiz	2,145	0	0.00	4	0.19	33	1.54	37	1.72
Guimaras	1,680	0	0.00	2	0.12	8	0.48	10	0.60
Iloilo	13,178	1	0.01	18	0.14	247	1.87	266	2.02
Negros Occidental	5,730	0	0.00	18	0.31	83	1.45	101	1.76
Bacolod City	3,584	5	0.14	9	0.25	86	2.40	100	2.79
Iloilo City	2,383	0	0.00	7	0.29	57	2.39	64	2.69
**Region 7**	**31,066**	**5**	**0.02**	**224**	**0.72**	**1,016**	**3.27**	**1,245**	**4.01**
Bohol	4,888	1	0.02	58	1.19	109	2.23	168	3.44
Cebu	7,099	2	0.03	62	0.87	477	6.72	541	7.62
Negros Oriental	1,401	0	0.00	10	0.71	32	2.28	42	3.00
Siquijor	591	0	0.00	0	0.00	2	0.34	2	0.34
Cebu City	10,630	1	0.01	83	0.78	287	2.70	371	3.49
Lapu-Lapu City	4,050	0	0.00	0	0.00	0	0.00	0	0.00
Mandaue City	2,407	1	0.04	11	0.46	109	4.53	121	5.03
**Region 8**	**10,454**	**2**	**0.02**	**43**	**0.41**	**296**	**2.83**	**341**	**3.26**
Biliran	1,235	0	0.00	2	0.16	12	0.97	14	1.13
Eastern Samar	938	0	0.00	8	0.85	13	1.39	21	2.24
Northern Leyte	1,634	0	0.00	3	0.18	36	2.20	39	2.39
Northern Samar	2,170	0	0.00	1	0.05	25	1.15	26	1.20

Table 1.B.1.10 - Prenatal Care

Number and proportion of pregnant women tested positive for Syphillis by Region

Philippines, 2021

Area	Total No. of Pregnant Women Screened for Syphillis	Tested Positive for Syphillis							
		Age Group						Total	%
		10-14 yrs old		15-19 yrs old		20-49 yrs old			
		No.	%	No.	%	No.	%		
Southern Leyte	365	0	0.00	0	0.00	19	5.21	19	5.21
Western Samar	698	2	0.29	24	3.44	144	20.63	170	24.36
Calbayog City	214	0	0.00	0	0.00	0	0.00	0	0.00
Maasin City	5	0	0.00	1	20.00	4	80.00	5	100.00
Ormoc City	428	0	0.00	0	0.00	12	2.80	12	2.80
Tacloban City	2,767	0	0.00	4	0.14	31	1.12	35	1.26
**Region 9**	**3,688**	**0**	**0.00**	**7**	**0.19**	**50**	**1.36**	**57**	**1.55**
Zamboanga del Norte	465	0	0.00	0	0.00	9	1.94	9	1.94
Zamboanga del Sur	768	0	0.00	0	0.00	12	1.56	12	1.56
Zamboanga Sibugay	2,016	0	0.00	7	0.35	26	1.29	33	1.64
Dapitan City	106	0	0.00	0	0.00	0	0.00	0	0.00
Dipolog City	5	0	0.00	0	0.00	2	40.00	2	40.00
Isabela City	0	0	0.00	0	0.00	0	0.00	0	0.00
Pagadian City	184	0	0.00	0	0.00	0	0.00	0	0.00
Zamboanga City	144	0	0.00	0	0.00	1	0.69	1	0.69
**Region 10**	**12,241**	**3**	**0.02**	**53**	**0.43**	**197**	**1.61**	**253**	**2.07**
Bukidnon	858	0	0.00	7	0.82	9	1.05	16	1.86
Camiguin	269	0	0.00	1	0.37	19	7.06	20	7.43
Lanao del Norte	618	0	0.00	0	0.00	1	0.16	1	0.16
Misamis Occidental	148	0	0.00	2	1.35	10	6.76	12	8.11
Misamis Oriental	1,921	0	0.00	0	0.00	4	0.21	4	0.21
Cagayan de Oro City	5,533	2	0.04	33	0.60	112	2.02	147	2.66
El Salvador City	35	0	0.00	0	0.00	0	0.00	0	0.00
Gingoog City	91	0	0.00	0	0.00	0	0.00	0	0.00
Iligan City	368	0	0.00	0	0.00	13	3.53	13	3.53
Malaybalay City	864	1	0.12	9	1.04	27	3.13	37	4.28
Oroquieta City	19	0	0.00	1	5.26	0	0.00	1	5.26
Ozamis City	1,262	0	0.00	0	0.00	0	0.00	0	0.00
Tangub City	0	0	0.00	0	0.00	0	0.00	0	0.00
Valencia City	255	0	0.00	0	0.00	2	0.78	2	0.78
**Region 11**	**18,544**	**0**	**0.00**	**11**	**0.06**	**39**	**0.21**	**50**	**0.27**
Davao de Oro	141	0	0.00	0	0.00	0	0.00	0	0.00
Davao del Norte	4,528	0	0.00	0	0.00	5	0.11	5	0.11
Davao Oriental	127	0	0.00	6	4.72	20	15.75	26	20.47
Davao del Sur	618	0	0.00	2	0.32	1	0.16	3	0.49
Davao Occidental	155	0	0.00	0	0.00	4	2.58	4	2.58
Davao City	12,975	0	0.00	3	0.02	9	0.07	12	0.09
**Region 12**	**19,771**	**1**	**0.01**	**45**	**0.23**	**350**	**1.77**	**396**	**2.00**
North Cotabato	1,826	0	0.00	1	0.05	66	3.61	67	3.67
Sarangani	2,914	0	0.00	17	0.58	114	3.91	131	4.50
South Cotabato	5,677	0	0.00	9	0.16	19	0.33	28	0.49
Sultan Kudarat	6,117	0	0.00	3	0.05	65	1.06	68	1.11
Cotabato City	386	0	0.00	0	0.00	0	0.00	0	0.00
Gen. Santos City	2,851	1	0.04	15	0.53	86	3.02	102	3.58

Table 1.B.1.10 - Prenatal Care

Number and proportion of pregnant women tested positive for Syphillis by Region
Philippines, 2021

Area	Total No. of Pregnant Women Screened for Syphillis	Tested Positive for Syphillis							
		Age Group						Total	%
		10-14 yrs old		15-19 yrs old		20-49 yrs old			
		No.	%	No.	%	No.	%		
**BARMM**	**341**	**0**	**0.00**	**2**	**0.59**	**6**	**1.76**	**8**	**2.35**
Basilan	12	0	0.00	0	0.00	4	33.33	4	33.33
Lanao del Sur	253	0	0.00	0	0.00	0	0.00	0	0.00
Maguindanao	76	0	0.00	2	2.63	2	2.63	4	5.26
Sulu	0	0	0.00	0	0.00	0	0.00	0	0.00
Tawi-Tawi	0	0	0.00	0	0.00	0	0.00	0	0.00
Lamitan City	0	0	0.00	0	0.00	0	0.00	0	0.00
Marawi City	0	0	0.00	0	0.00	0	0.00	0	0.00
**CARAGA**	**8,758**	**0**	**0.00**	**32**	**0.37**	**227**	**2.59**	**259**	**2.96**
Agusan del Norte	916	0	0.00	12	1.31	113	12.34	125	13.65
Agusan del Sur	2,255	0	0.00	9	0.40	27	1.20	36	1.60
Surigao del Norte	600	0	0.00	0	0.00	15	2.50	15	2.50
Surigao del Sur	495	0	0.00	4	0.81	30	6.06	34	6.87
Province of Dinagat	609	0	0.00	0	0.00	12	1.97	12	1.97
Bislig City	39	0	0.00	0	0.00	0	0.00	0	0.00
Butuan City	2,743	0	0.00	7	0.26	27	0.98	34	1.24
Surigao City	1,101	0	0.00	0	0.00	3	0.27	3	0.27

Note: Put asterisk (*) for No Report and Zero (0) for No Case

Table 1.B.1.11 - Prenatal Care

Number and proportion of pregnant women screened for Hepatitis B by Region
Philippines, 2021

Area	Eligible Pop. (Under 1)	Pregnant Women Screened for Hepatitis B							
		Age Group						Total	%
		10-14 yrs old		15-19 yrs old		20-49 yrs old			
		No.	%	No.	%	No.	%		
**PHILIPPINES**	**2,128,168**	**1,065**	**0.05**	**58,706**	**2.76**	**442,151**	**20.78**	**501,922**	**23.58**
**N C R**	**237,921**	**191**	**0.08**	**9,628**	**4.05**	**76,404**	**32.11**	**86,223**	**36.24**
Malabon	6,757	18	0.27	550	8.14	2,666	39.46	3,234	47.86
Navotas	4,607	7	0.15	545	11.83	2,478	53.79	3,030	65.77
Valenzuela City	11,465	12	0.10	488	4.26	4,806	41.92	5,306	46.28
Caloocan City	29,270	11	0.04	1,010	3.45	7,629	26.06	8,650	29.55
Marikina City	8,330	4	0.05	162	1.94	1,398	16.78	1,564	18.78
Pasig City	13,954	7	0.05	792	5.68	7,788	55.81	8,587	61.54
Pateros	1,181	2	0.17	103	8.72	632	53.51	737	62.40
Taguig	14,874	15	0.10	868	5.84	5,793	38.95	6,676	44.88
Quezon City	54,246	36	0.07	1,658	3.06	17,347	31.98	19,041	35.10
Makati City	10,765	15	0.14	339	3.15	3,181	29.55	3,535	32.84
Mandaluyong City	7,137	7	0.10	182	2.55	2,198	30.80	2,387	33.45
San Juan	2,254	1	0.04	33	1.46	742	32.92	776	34.43
Manila City	32,887	31	0.09	1,694	5.15	10,162	30.90	11,887	36.14
Las Piñas City	10,879	3	0.03	221	2.03	2,083	19.15	2,307	21.21
Muntinlupa City	9,317	8	0.09	261	2.80	1,941	20.83	2,210	23.72
Parañaque City	12,298	12	0.10	514	4.18	3,820	31.06	4,346	35.34
Pasay City	7,700	2	0.03	208	2.70	1,740	22.60	1,950	25.32
**C A R**	**35,143**	**28**	**0.08**	**1,320**	**3.76**	**12,646**	**35.98**	**13,994**	**39.82**
Abra	4,287	0	0.00	28	0.65	241	5.62	269	6.27
Apayao	2,476	7	0.28	145	5.86	784	31.66	936	37.80
Benguet	9,140	12	0.13	580	6.35	6,130	67.07	6,722	73.54
Ifugao	4,439	1	0.02	128	2.88	1,271	28.63	1,400	31.54
Kalinga	4,660	2	0.04	107	2.30	706	15.15	815	17.49
Mt. Province	3,066	2	0.07	97	3.16	838	27.33	937	30.56
Baguio City	7,075	4	0.06	235	3.32	2,676	37.82	2,915	41.20
**Region 1**	**97,198**	**23**	**0.02**	**2,355**	**2.42**	**26,338**	**27.10**	**28,716**	**29.54**
Ilocos Norte	7,986	4	0.05	213	2.67	2,628	32.91	2,845	35.62
Ilocos Sur	9,197	8	0.09	385	4.19	4,664	50.71	5,057	54.99
La Union	11,429	2	0.02	386	3.38	3,281	28.71	3,669	32.10
Pangasinan	50,454	7	0.01	925	1.83	9,870	19.56	10,802	21.41
Alaminos City	1,905	0	0.00	67	3.52	794	41.68	861	45.20
Candon City	976	0	0.00	1	0.10	575	58.91	576	59.02
Dagupan City	3,636	0	0.00	106	2.92	716	19.69	822	22.61
Laoag City	1,845	0	0.00	35	1.90	1,909	103.47	1,944	105.37
San Carlos City	3,999	1	0.03	33	0.83	347	8.68	381	9.53
San Fernando City	2,092	0	0.00	38	1.82	517	24.71	555	26.53
Urdaneta City	2,819	1	0.04	118	4.19	914	32.42	1,033	36.64
Vigan City	860	0	0.00	48	5.58	123	14.30	171	19.88
**Region 2**	**69,228**	**44**	**0.06**	**1,768**	**2.55**	**11,936**	**17.24**	**13,748**	**19.86**
Batanes	351	1	0.28	7	1.99	149	42.45	157	44.73
Cagayan	20,563	3	0.01	110	0.53	842	4.09	955	4.64
Isabela	23,543	21	0.09	719	3.05	4,555	19.35	5,295	22.49
Nueva Vizcaya	9,555	7	0.07	468	4.90	2,927	30.63	3,402	35.60
Quirino	4,038	5	0.12	145	3.59	1,066	26.40	1,216	30.11

Table 1.B.1.11 - Prenatal Care

Number and proportion of pregnant women screened for Hepatitis B by Region

Philippines, 2021

Area	Eligible Pop. (Under 1)	Pregnant Women Screened for Hepatitis B							
		Age Group						Total	%
		10-14 yrs old		15-19 yrs old		20-49 yrs old			
		No.	%	No.	%	No.	%		
Cauayan City	2,576	1	0.04	118	4.58	736	28.57	855	33.19
Ilagan City	2,901	6	0.21	139	4.79	1,053	36.30	1,198	41.30
Santiago City	2,683	0	0.00	61	2.27	578	21.54	639	23.82
Tuguegarao City	3,018	0	0.00	1	0.03	30	0.99	31	1.03
**Region 3**	**220,202**	**181**	**0.08**	**7,965**	**3.62**	**53,235**	**24.18**	**61,381**	**27.87**
Aurora	4,764	1	0.02	226	4.74	1,411	29.62	1,638	34.38
Bataan	13,814	23	0.17	689	4.99	4,497	32.55	5,209	37.71
Bulacan	43,722	39	0.09	1,762	4.03	11,372	26.01	13,173	30.13
Nueva Ecija	28,915	17	0.06	697	2.41	4,011	13.87	4,725	16.34
Pampanga	31,503	20	0.06	698	2.22	4,180	13.27	4,898	15.55
Tarlac	19,565	6	0.03	506	2.59	3,435	17.56	3,947	20.17
Zambales	12,551	0	0.00	268	2.14	1,748	13.93	2,016	16.06
Angeles City	7,905	1	0.01	77	0.97	538	6.81	616	7.79
Balanga City	1,996	1	0.05	107	5.36	708	35.47	816	40.88
Cabanatuan City	5,921	5	0.08	161	2.72	850	14.36	1,016	17.16
City of San Fernando	5,890	9	0.15	230	3.90	1,703	28.91	1,942	32.97
Gapan City	2,159	3	0.14	89	4.12	457	21.17	549	25.43
Mabalacat City	4,811	5	0.10	349	7.25	2,137	44.42	2,491	51.78
Malolos City	4,885	2	0.04	67	1.37	496	10.15	565	11.57
Meycauayan	4,050	5	0.12	257	6.35	1,534	37.88	1,796	44.35
Olongapo	4,952	2	0.04	140	2.83	973	19.65	1,115	22.52
Palayan City	805	0	0.00	22	2.73	122	15.16	144	17.89
San Jose City	2,736	4	0.15	117	4.28	433	15.83	554	20.25
San Jose del Monte City	11,120	35	0.31	900	8.09	9,420	84.71	10,355	93.12
Science City of Munoz	1,594	0	0.00	37	2.32	172	10.79	209	13.11
Tarlac City	6,544	3	0.05	566	8.65	3,038	46.42	3,607	55.12
**Region 4A**	**298,398**	**120**	**0.04**	**8,444**	**2.83**	**62,641**	**20.99**	**71,205**	**23.86**
Batangas	38,848	10	0.03	503	1.29	6,515	16.77	7,028	18.09
Cavite	27,948	8	0.03	562	2.01	4,871	17.43	5,441	19.47
Laguna	19,727	11	0.06	703	3.56	4,490	22.76	5,204	26.38
Quezon	37,644	28	0.07	1,109	2.95	7,257	19.28	8,394	22.30
Rizal	44,826	26	0.06	1,489	3.32	8,704	19.42	10,219	22.80
Antipolo City	16,510	4	0.02	675	4.09	1,824	11.05	2,503	15.16
Bacoor City	12,252	1	0.01	115	0.94	966	7.88	1,082	8.83
Batangas City	6,895	0	0.00	107	1.55	1,046	15.17	1,153	16.72
Biñan City	6,607	0	0.00	708	10.72	4,707	71.24	5,415	81.96
Cabuyao City	6,128	0	0.00	304	4.96	3,215	52.46	3,519	57.42
Calamba City	9,025	7	0.08	235	2.60	2,793	30.95	3,035	33.63
Cavite City	2,098	4	0.19	49	2.34	185	8.82	238	11.34
Dasmariñas City	13,442	0	0.00	300	2.23	3,176	23.63	3,476	25.86
General Trias City	6,410	2	0.03	81	1.26	679	10.59	762	11.89
Imus City	8,235	1	0.01	92	1.12	678	8.23	771	9.36
Lipa City	6,954	1	0.01	166	2.39	1,749	25.15	1,916	27.55
Lucena City	5,704	3	0.05	339	5.94	1,442	25.28	1,784	31.28
San Pablo City	5,277	0	0.00	57	1.08	380	7.20	437	8.28
San Pedro City	6,465	1	0.02	193	2.99	1,212	18.75	1,406	21.75
Santa Rosa City	7,023	6	0.09	357	5.08	3,091	44.01	3,454	49.18
Tagaytay City	1,451	0	0.00	38	2.62	811	55.89	849	58.51
Tanauan City	3,620	2	0.06	66	1.82	1,312	36.24	1,380	38.12
Tayabas City	2,134	4	0.19	147	6.89	1,108	51.92	1,259	59.00
Trece Martires City	3,175	1	0.03	49	1.54	430	13.54	480	15.12

Table 1.B.1.11 - Prenatal Care

Number and proportion of pregnant women screened for Hepatitis B by Region

Philippines, 2021

Area	Eligible Pop. (Under 1)	Pregnant Women Screened for Hepatitis B							
		Age Group						Total	%
		10-14 yrs old		15-19 yrs old		20-49 yrs old			
		No.	%	No.	%	No.	%		
**Region 4B**	**72,035**	**17**	**0.02**	**1,158**	**1.61**	**9,607**	**13.34**	**10,782**	**14.97**
Marinduque	4,906	0	0.00	69	1.41	939	19.14	1,008	20.55
Mindoro Occidental	12,449	6	0.05	324	2.60	2,480	19.92	2,810	22.57
Mindoro Oriental	20,011	5	0.02	321	1.60	2,975	14.87	3,301	16.50
Palawan	21,676	4	0.02	256	1.18	1,653	7.63	1,913	8.83
Romblon	6,482	2	0.03	188	2.90	1,560	24.07	1,750	27.00
Puerto Princesa City	6,511	0	0.00	0	0.00	0	0.00	0	0.00
**Region 5**	**137,350**	**14**	**0.01**	**1,886**	**1.37**	**17,223**	**12.54**	**19,123**	**13.92**
Albay	23,903	1	0.00	347	1.45	4,876	20.40	5,224	21.85
Camarines Norte	14,340	5	0.03	287	2.00	1,695	11.82	1,987	13.86
Camarines Sur	39,151	2	0.01	153	0.39	2,001	5.11	2,156	5.51
Catanduanes	6,381	2	0.03	126	1.97	854	13.38	982	15.39
Masbate	22,565	3	0.01	501	2.22	2,850	12.63	3,354	14.86
Sorsogon	19,470	0	0.00	307	1.58	2,807	14.42	3,114	15.99
Iriga City	2,665	0	0.00	21	0.79	227	8.52	248	9.31
Legaspi City	4,205	1	0.02	108	2.57	1,579	37.55	1,688	40.14
Naga City	4,670	0	0.00	36	0.77	334	7.15	370	7.92
**Region 6**	**146,545**	**65**	**0.04**	**5,154**	**3.52**	**41,816**	**28.53**	**47,035**	**32.10**
Aklan	11,231	8	0.07	288	2.56	3,313	29.50	3,609	32.13
Antique	12,985	4	0.03	306	2.36	2,495	19.21	2,805	21.60
Capiz	13,984	7	0.05	282	2.02	3,087	22.08	3,376	24.14
Guimaras	3,085	4	0.13	131	4.25	1,595	51.70	1,730	56.08
Iloilo	36,414	22	0.06	1,421	3.90	13,530	37.16	14,973	41.12
Negros Occidental	49,314	10	0.02	1,911	3.88	12,171	24.68	14,092	28.58
Bacolod City	11,102	3	0.03	493	4.44	3,396	30.59	3,892	35.06
Iloilo City	8,430	7	0.08	322	3.82	2,229	26.44	2,558	30.34
**Region 7**	**162,860**	**55**	**0.03**	**4,251**	**2.61**	**36,136**	**22.19**	**40,442**	**24.83**
Bohol	27,637	3	0.01	427	1.55	6,420	23.23	6,850	24.79
Cebu	67,046	16	0.02	1,158	1.73	8,426	12.57	9,600	14.32
Negros Oriental	27,922	9	0.03	646	2.31	4,663	16.70	5,318	19.05
Siquijor	1,636	1	0.06	50	3.06	404	24.69	455	27.81
Cebu City	21,037	14	0.07	1,204	5.72	8,447	40.15	9,665	45.94
Lapu-Lapu City	9,310	4	0.04	432	4.64	4,874	52.35	5,310	57.04
Mandaue City	8,272	8	0.10	334	4.04	2,902	35.08	3,244	39.22
**Region 8**	**104,016**	**31**	**0.03**	**2,148**	**2.07**	**19,201**	**18.46**	**21,380**	**20.55**
Biliran	3,856	2	0.05	197	5.11	1,347	34.93	1,546	40.09
Eastern Samar	11,635	4	0.03	173	1.49	1,363	11.71	1,540	13.24
Northern Leyte	35,052	11	0.03	667	1.90	6,166	17.59	6,844	19.53
Northern Samar	15,655	0	0.00	326	2.08	3,272	20.90	3,598	22.98
Southern Leyte	6,556	3	0.05	199	3.04	1,994	30.41	2,196	33.50
Western Samar	14,515	3	0.02	206	1.42	1,132	7.80	1,341	9.24
Calbayog City	4,474	0	0.00	30	0.67	229	5.12	259	5.79
Maasin City	1,661	0	0.00	29	1.75	423	25.47	452	27.21
Ormoc City	4,989	0	0.00	93	1.86	600	12.03	693	13.89
Tacloban City	5,623	8	0.14	228	4.05	2,675	47.57	2,911	51.77

Table 1.B.1.11 - Prenatal Care

Number and proportion of pregnant women screened for Hepatitis B by Region
Philippines, 2021

Area	Eligible Pop. (Under 1)	Pregnant Women Screened for Hepatitis B							
		Age Group						Total	%
		10-14 yrs old		15-19 yrs old		20-49 yrs old			
		No.	%	No.	%	No.	%		
**Region 9**	**79,552**	**4**	**0.01**	**391**	**0.49**	**1,957**	**2.46**	**2,352**	**2.96**
Zamboanga del Norte	17,075	3	0.02	126	0.74	638	3.74	767	4.49
Zamboanga del Sur	17,506	0	0.00	86	0.49	377	2.15	463	2.64
Zamboanga Sibugay	14,992	0	0.00	70	0.47	422	2.81	492	3.28
Dapitan City	1,755	1	0.06	15	0.85	90	5.13	106	6.04
Dipolog City	2,794	0	0.00	0	0.00	10	0.36	10	0.36
Isabela City	2,544	0	0.00	13	0.51	59	2.32	72	2.83
Pagadian City	4,290	0	0.00	7	0.16	41	0.96	48	1.12
Zamboanga City	18,596	0	0.00	74	0.40	320	1.72	394	2.12
**Region 10**	**100,690**	**58**	**0.06**	**3,812**	**3.79**	**21,206**	**21.06**	**25,076**	**24.90**
Bukidnon	23,307	8	0.03	516	2.21	2,323	9.97	2,847	12.22
Camiguin	1,854	0	0.00	27	1.46	340	18.34	367	19.80
Lanao del Norte	14,955	9	0.06	107	0.72	503	3.36	619	4.14
Misamis Occidental	6,413	0	0.00	28	0.44	217	3.38	245	3.82
Misamis Oriental	15,058	13	0.09	1,081	7.18	5,440	36.13	6,534	43.39
Cagayan de Oro City	14,263	8	0.06	1,127	7.90	7,392	51.83	8,527	59.78
El Salvador City	1,061	1	0.09	57	5.37	314	29.59	372	35.06
Gingoog City	2,627	13	0.49	275	10.47	1,022	38.90	1,310	49.87
Iligan City	7,575	1	0.01	119	1.57	822	10.85	942	12.44
Malaybalay City	3,882	3	0.08	242	6.23	1,267	32.64	1,512	38.95
Oroquieta City	1,389	0	0.00	1	0.07	22	1.58	23	1.66
Ozamis City	2,782	2	0.07	159	5.72	1,159	41.66	1,320	47.45
Tangub City	1,234	0	0.00	0	0.00	4	0.32	4	0.32
Valencia City	4,290	0	0.00	73	1.70	381	8.88	454	10.58
**Region 11**	**107,847**	**135**	**0.13**	**2,790**	**2.59**	**18,786**	**17.42**	**21,711**	**20.13**
Davao de Oro	15,526	1	0.01	43	0.28	309	1.99	353	2.27
Davao del Norte	21,184	34	0.16	709	3.35	4,609	21.76	5,352	25.26
Davao Oriental	13,125	0	0.00	25	0.19	115	0.88	140	1.07
Davao del Sur	14,355	18	0.13	234	1.63	1,256	8.75	1,508	10.51
Davao Occidental	6,598	0	0.00	11	0.17	43	0.65	54	0.82
Davao City	37,059	82	0.22	1,768	4.77	12,454	33.61	14,304	38.60
**Region 12**	**104,693**	**63**	**0.06**	**4,026**	**3.85**	**22,042**	**21.05**	**26,131**	**24.96**
North Cotabato	33,933	8	0.02	752	2.22	4,278	12.61	5,038	14.85
Sarangani	12,904	13	0.10	914	7.08	3,760	29.14	4,687	36.32
South Cotabato	21,039	10	0.05	924	4.39	5,750	27.33	6,684	31.77
Sultan Kudarat	17,331	24	0.14	914	5.27	4,809	27.75	5,747	33.16
Cotabato City	5,831	0	0.00	42	0.72	516	8.85	558	9.57
Gen. Santos City	13,655	8	0.06	480	3.52	2,929	21.45	3,417	25.02
**BARMM**	**94,148**	**0**	**0.00**	**19**	**0.02**	**578**	**0.61**	**597**	**0.63**
Basilan	7,672	0	0.00	0	0.00	0	0.00	0	0.00
Lanao del Sur	21,391	0	0.00	15	0.07	541	2.53	556	2.60
Maguindanao	31,650	0	0.00	3	0.01	32	0.10	35	0.11
Sulu	16,891	0	0.00	0	0.00	0	0.00	0	0.00
Tawi-Tawi	9,319	0	0.00	0	0.00	0	0.00	0	0.00
Lamitan City	2,112	0	0.00	1	0.05	5	0.24	6	0.28
Marawi City	5,113	0	0.00	0	0.00	0	0.00	0	0.00

Table 1.B.1.11 - Prenatal Care

Number and proportion of pregnant women screened for Hepatitis B by Region
Philippines, 2021

Area	Eligible Pop. (Under 1)	Pregnant Women Screened for Hepatitis B							
		Age Group						Total	%
		10-14 yrs old		15-19 yrs old		20-49 yrs old			
		No.	%	No.	%	No.	%		
**CARAGA**	**60,342**	**36**	**0.06**	**1,591**	**2.64**	**10,399**	**17.23**	**12,026**	**19.93**
Agusan del Norte	8,116	9	0.11	238	2.93	1,488	18.33	1,735	21.38
Agusan del Sur	17,658	5	0.03	347	1.97	1,653	9.36	2,005	11.35
Surigao del Norte	7,195	1	0.01	157	2.18	1,307	18.17	1,465	20.36
Surigao del Sur	11,532	8	0.07	149	1.29	1,061	9.20	1,218	10.56
Province of Dinagat	2,574	5	0.19	61	2.37	562	21.83	628	24.40
Bislig City	2,185	0	0.00	0	0.00	3	0.14	3	0.14
Butuan City	7,732	1	0.01	407	5.26	2,836	36.68	3,244	41.96
Surigao City	3,350	7	0.21	232	6.93	1,489	44.45	1,728	51.58

Note: Put asterisk (*) for No Report and Zero (0) for No Case

Table 1.B.1.12 - Prenatal Care

Number and proportion of pregnant women tested positive for Hepatitis B by Region
Philippines, 2021

Area	Total No. of Pregnant Women Screened for Hepatitis B	Pregnant Women Tested Positive for Hepatitis B							
		Age Group						Total	%
		10-14 yrs old		15-19 yrs old		20-49 yrs old			
		No.	%	No.	%	No.	%		
**PHILIPPINES**	**501,922**	**48**	**0.01**	**1,393**	**0.28**	**10,305**	**2.05**	**11,746**	**2.34**
**N C R**	**86,223**	**3**	**0.00**	**163**	**0.19**	**1,636**	**1.90**	**1,802**	**2.09**
Malabon	3,234	0	0.00	2	0.06	26	0.80	28	0.87
Navotas	3,030	0	0.00	3	0.10	26	0.86	29	0.96
Valenzuela City	5,306	0	0.00	24	0.45	310	5.84	334	6.29
Caloocan City	8,650	0	0.00	5	0.06	72	0.83	77	0.89
Marikina City	1,564	0	0.00	0	0.00	18	1.15	18	1.15
Pasig City	8,587	0	0.00	4	0.05	49	0.57	53	0.62
Pateros	737	0	0.00	2	0.27	9	1.22	11	1.49
Taguig	6,676	3	0.04	56	0.84	413	6.19	472	7.07
Quezon City	19,041	0	0.00	8	0.04	165	0.87	173	0.91
Makati City	3,535	0	0.00	3	0.08	74	2.09	77	2.18
Mandaluyong City	2,387	0	0.00	0	0.00	13	0.54	13	0.54
San Juan	776	0	0.00	0	0.00	0	0.00	0	0.00
Manila City	11,887	0	0.00	19	0.16	122	1.03	141	1.19
Las Piñas City	2,307	0	0.00	10	0.43	62	2.69	72	3.12
Muntinlupa City	2,210	0	0.00	0	0.00	1	0.05	1	0.05
Parañaque City	4,346	0	0.00	24	0.55	241	5.55	265	6.10
Pasay City	1,950	0	0.00	3	0.15	35	1.79	38	1.95
**C A R**	**13,994**	**0**	**0.00**	**21**	**0.15**	**198**	**1.41**	**219**	**1.56**
Abra	269	0	0.00	0	0.00	2	0.74	2	0.74
Apayao	936	0	0.00	2	0.21	16	1.71	18	1.92
Benguet	6,722	0	0.00	7	0.10	72	1.07	79	1.18
Ifugao	1,400	0	0.00	1	0.07	24	1.71	25	1.79
Kalinga	815	0	0.00	2	0.25	8	0.98	10	1.23
Mt. Province	937	0	0.00	8	0.85	49	5.23	57	6.08
Baguio City	2,915	0	0.00	1	0.03	27	0.93	28	0.96
**Region 1**	**28,716**	**0**	**0.00**	**20**	**0.07**	**239**	**0.83**	**259**	**0.90**
Ilocos Norte	2,845	0	0.00	2	0.07	20	0.70	22	0.77
Ilocos Sur	5,057	0	0.00	0	0.00	23	0.45	23	0.45
La Union	3,669	0	0.00	0	0.00	37	1.01	37	1.01
Pangasinan	10,802	0	0.00	17	0.16	139	1.29	156	1.44
Alaminos City	861	0	0.00	0	0.00	0	0.00	0	0.00
Candon City	576	0	0.00	0	0.00	0	0.00	0	0.00
Dagupan City	822	0	0.00	1	0.12	18	2.19	19	2.31
Laoag City	1,944	0	0.00	0	0.00	0	0.00	0	0.00
San Carlos City	381	0	0.00	0	0.00	1	0.26	1	0.26
San Fernando City	555	0	0.00	0	0.00	0	0.00	0	0.00
Urdaneta City	1,033	0	0.00	0	0.00	1	0.10	1	0.10
Vigan City	171	0	0.00	0	0.00	0	0.00	0	0.00
**Region 2**	**13,748**	**0**	**0.00**	**35**	**0.25**	**231**	**1.68**	**266**	**1.93**
Batanes	157	0	0.00	0	0.00	0	0.00	0	0.00
Cagayan	955	0	0.00	4	0.42	17	1.78	21	2.20
Isabela	5,295	0	0.00	12	0.23	88	1.66	100	1.89
Nueva Vizcaya	3,402	0	0.00	13	0.38	75	2.20	88	2.59

Table 1.B.1.12 - Prenatal Care

Number and proportion of pregnant women tested positive for Hepatitis B by Region
Philippines, 2021

Area	Total No. of Pregnant Women Screened for Hepatitis B	Pregnant Women Tested Positive for Hepatitis B							
		Age Group						Total	%
		10-14 yrs old		15-19 yrs old		20-49 yrs old			
		No.	%	No.	%	No.	%		
Quirino	1,216	0	0.00	4	0.33	39	3.21	43	3.54
Cauayan City	855	0	0.00	0	0.00	1	0.12	1	0.12
Ilagan City	1,198	0	0.00	0	0.00	1	0.08	1	0.08
Santiago City	639	0	0.00	2	0.31	8	1.25	10	1.56
Tuguegarao City	31	0	0.00	0	0.00	2	6.45	2	6.45
**Region 3**	**61,381**	**2**	**0.00**	**85**	**0.14**	**644**	**1.05**	**731**	**1.19**
Aurora	1,638	0	0.00	4	0.24	22	1.34	26	1.59
Bataan	5,209	0	0.00	13	0.25	94	1.80	107	2.05
Bulacan	13,173	2	0.02	13	0.10	112	0.85	127	0.96
Nueva Ecija	4,725	0	0.00	12	0.25	145	3.07	157	3.32
Pampanga	4,898	0	0.00	6	0.12	29	0.59	35	0.71
Tarlac	3,947	0	0.00	5	0.13	54	1.37	59	1.49
Zambales	2,016	0	0.00	1	0.05	17	0.84	18	0.89
Angeles City	616	0	0.00	1	0.16	6	0.97	7	1.14
Balanga City	816	0	0.00	1	0.12	3	0.37	4	0.49
Cabanatuan City	1,016	0	0.00	3	0.30	7	0.69	10	0.98
City of San Fernando	1,942	0	0.00	3	0.15	25	1.29	28	1.44
Gapan City	549	0	0.00	0	0.00	0	0.00	0	0.00
Mabalacat City	2,491	0	0.00	3	0.12	27	1.08	30	1.20
Malolos City	565	0	0.00	0	0.00	3	0.53	3	0.53
Meycauayan	1,796	0	0.00	1	0.06	5	0.28	6	0.33
Olongapo	1,115	0	0.00	13	1.17	56	5.02	69	6.19
Palayan City	144	0	0.00	0	0.00	0	0.00	0	0.00
San Jose City	554	0	0.00	0	0.00	0	0.00	0	0.00
San Jose del Monte City	10,355	0	0.00	6	0.06	30	0.29	36	0.35
Science City of Munoz	209	0	0.00	0	0.00	3	1.44	3	1.44
Tarlac City	3,607	0	0.00	0	0.00	6	0.17	6	0.17
**Region 4A**	**71,205**	**19**	**0.03**	**271**	**0.38**	**1,938**	**2.72**	**2,228**	**3.13**
Batangas	7,028	0	0.00	8	0.11	69	0.98	77	1.10
Cavite	5,441	0	0.00	14	0.26	171	3.14	185	3.40
Laguna	5,204	0	0.00	19	0.37	114	2.19	133	2.56
Quezon	8,394	3	0.04	42	0.50	343	4.09	388	4.62
Rizal	10,219	0	0.00	31	0.30	185	1.81	216	2.11
Antipolo City	2,503	4	0.16	51	2.04	263	10.51	318	12.70
Bacoor City	1,082	0	0.00	2	0.18	16	1.48	18	1.66
Batangas City	1,153	0	0.00	6	0.52	27	2.34	33	2.86
Biñan City	5,415	0	0.00	12	0.22	74	1.37	86	1.59
Cabuyao City	3,519	0	0.00	7	0.20	38	1.08	45	1.28
Calamba City	3,035	0	0.00	7	0.23	108	3.56	115	3.79
Cavite City	238	1	0.42	0	0.00	3	1.26	4	1.68
Dasmariñas City	3,476	0	0.00	5	0.14	94	2.70	99	2.85
General Trias City	762	0	0.00	3	0.39	22	2.89	25	3.28
Imus City	771	1	0.13	4	0.52	22	2.85	27	3.50
Lipa City	1,916	0	0.00	5	0.26	38	1.98	43	2.24
Lucena City	1,784	0	0.00	6	0.34	74	4.15	80	4.48
San Pablo City	437	0	0.00	2	0.46	14	3.20	16	3.66
San Pedro City	1,406	0	0.00	10	0.71	41	2.92	51	3.63
Santa Rosa City	3,454	0	0.00	23	0.67	179	5.18	202	5.85
Tagaytay City	849	0	0.00	3	0.35	14	1.65	17	2.00
Tanauan City	1,380	1	0.07	1	0.07	5	0.36	7	0.51
Tayabas City	1,259	0	0.00	1	0.08	12	0.95	13	1.03
Trece Martires City	480	9	1.88	9	1.88	12	2.50	30	6.25

Table 1.B.1.12 - Prenatal Care

Number and proportion of pregnant women tested positive for Hepatitis B by Region

Philippines, 2021

Area	Total No. of Pregnant Women Screened for Hepatitis B	Pregnant Women Tested Positive for Hepatitis B							
		Age Group						Total	%
		10-14 yrs old		15-19 yrs old		20-49 yrs old			
		No.	%	No.	%	No.	%		
**Region 4B**	**10,782**	**1**	**0.01**	**46**	**0.43**	**362**	**3.36**	**409**	**3.79**
Marinduque	1,008	0	0.00	2	0.20	9	0.89	11	1.09
Mindoro Occidental	2,810	0	0.00	7	0.25	60	2.14	67	2.38
Mindoro Oriental	3,301	0	0.00	30	0.91	190	5.76	220	6.66
Palawan	1,913	1	0.05	6	0.31	69	3.61	76	3.97
Romblon	1,750	0	0.00	1	0.06	34	1.94	35	2.00
Puerto Princesa City	0	0	0.00	0	0.00	0	0.00	0	0.00
**Region 5**	**19,123**	**1**	**0.01**	**51**	**0.27**	**470**	**2.46**	**522**	**2.73**
Albay	5,224	0	0.00	4	0.08	94	1.80	98	1.88
Camarines Norte	1,987	1	0.05	8	0.40	58	2.92	67	3.37
Camarines Sur	2,156	0	0.00	3	0.14	35	1.62	38	1.76
Catanduanes	982	0	0.00	8	0.81	49	4.99	57	5.80
Masbate	3,354	0	0.00	11	0.33	69	2.06	80	2.39
Sorsogon	3,114	0	0.00	13	0.42	131	4.21	144	4.62
Iriga City	248	0	0.00	0	0.00	2	0.81	2	0.81
Legaspi City	1,688	0	0.00	2	0.12	19	1.13	21	1.24
Naga City	370	0	0.00	2	0.54	13	3.51	15	4.05
**Region 6**	**47,035**	**3**	**0.01**	**125**	**0.27**	**1,036**	**2.20**	**1,164**	**2.47**
Aklan	3,609	1	0.03	9	0.25	58	1.61	68	1.88
Antique	2,805	0	0.00	6	0.21	96	3.42	102	3.64
Capiz	3,376	1	0.03	5	0.15	93	2.75	99	2.93
Guimaras	1,730	0	0.00	2	0.12	26	1.50	28	1.62
Iloilo	14,973	1	0.01	44	0.29	279	1.86	324	2.16
Negros Occidental	14,092	0	0.00	51	0.36	358	2.54	409	2.90
Bacolod City	3,892	0	0.00	4	0.10	80	2.06	84	2.16
Iloilo City	2,558	0	0.00	4	0.16	46	1.80	50	1.95
**Region 7**	**40,442**	**3**	**0.01**	**170**	**0.42**	**938**	**2.32**	**1,111**	**2.75**
Bohol	6,850	0	0.00	16	0.23	166	2.42	182	2.66
Cebu	9,600	2	0.02	50	0.52	422	4.40	474	4.94
Negros Oriental	5,318	0	0.00	20	0.38	97	1.82	117	2.20
Siquijor	455	0	0.00	0	0.00	6	1.32	6	1.32
Cebu City	9,665	1	0.01	67	0.69	165	1.71	233	2.41
Lapu-Lapu City	5,310	0	0.00	1	0.02	12	0.23	13	0.24
Mandaue City	3,244	0	0.00	16	0.49	70	2.16	86	2.65
**Region 8**	**21,380**	**2**	**0.01**	**67**	**0.31**	**685**	**3.20**	**754**	**3.53**
Biliran	1,546	0	0.00	5	0.32	21	1.36	26	1.68
Eastern Samar	1,540	0	0.00	5	0.32	48	3.12	53	3.44
Northern Leyte	6,844	0	0.00	31	0.45	322	4.70	353	5.16
Northern Samar	3,598	0	0.00	7	0.19	61	1.70	68	1.89
Southern Leyte	2,196	0	0.00	2	0.09	61	2.78	63	2.87
Western Samar	1,341	2	0.15	4	0.30	61	4.55	67	5.00
Calbayog City	259	0	0.00	0	0.00	2	0.77	2	0.77
Maasin City	452	0	0.00	3	0.66	26	5.75	29	6.42
Ormoc City	693	0	0.00	3	0.43	14	2.02	17	2.45

Table 1.B.1.12 - Prenatal Care

Number and proportion of pregnant women tested positive for Hepatitis B by Region

Philippines, 2021

Area	Total No. of Pregnant Women Screened for Hepatitis B	Pregnant Women Tested Positive for Hepatitis B							
		Age Group						Total	%
		10-14 yrs old		15-19 yrs old		20-49 yrs old			
		No.	%	No.	%	No.	%		
Tacloban City	2,911	0	0.00	7	0.24	69	2.37	76	2.61
**Region 9**	**2,352**	**0**	**0.00**	**20**	**0.85**	**64**	**2.72**	**84**	**3.57**
Zamboanga del Norte	767	0	0.00	8	1.04	14	1.83	22	2.87
Zamboanga del Sur	463	0	0.00	5	1.08	21	4.54	26	5.62
Zamboanga Sibugay	492	0	0.00	4	0.81	12	2.44	16	3.25
Dapitan City	106	0	0.00	1	0.94	4	3.77	5	4.72
Dipolog City	10	0	0.00	0	0.00	0	0.00	0	0.00
Isabela City	72	0	0.00	0	0.00	1	1.39	1	1.39
Pagadian City	48	0	0.00	0	0.00	0	0.00	0	0.00
Zamboanga City	394	0	0.00	2	0.51	12	3.05	14	3.55
**Region 10**	**25,076**	**10**	**0.04**	**150**	**0.60**	**699**	**2.79**	**859**	**3.43**
Bukidnon	2,847	8	0.28	14	0.49	69	2.42	91	3.20
Camiguin	367	0	0.00	0	0.00	9	2.45	9	2.45
Lanao del Norte	619	2	0.32	5	0.81	12	1.94	19	3.07
Misamis Occidental	245	0	0.00	1	0.41	5	2.04	6	2.45
Misamis Oriental	6,534	0	0.00	9	0.14	86	1.32	95	1.45
Cagayan de Oro City	8,527	0	0.00	110	1.29	399	4.68	509	5.97
El Salvador City	372	0	0.00	1	0.27	0	0.00	1	0.27
Gingoog City	1,310	0	0.00	2	0.15	36	2.75	38	2.90
Iligan City	942	0	0.00	0	0.00	20	2.12	20	2.12
Malaybalay City	1,512	0	0.00	8	0.53	44	2.91	52	3.44
Oroquieta City	23	0	0.00	0	0.00	2	8.70	2	8.70
Ozamis City	1,320	0	0.00	0	0.00	6	0.45	6	0.45
Tangub City	4	0	0.00	0	0.00	0	0.00	0	0.00
Valencia City	454	0	0.00	0	0.00	11	2.42	11	2.42
**Region 11**	**21,711**	**4**	**0.02**	**25**	**0.12**	**196**	**0.90**	**225**	**1.04**
Davao de Oro	353	0	0.00	0	0.00	10	2.83	10	2.83
Davao del Norte	5,352	1	0.02	3	0.06	45	0.84	49	0.92
Davao Oriental	140	0	0.00	0	0.00	0	0.00	0	0.00
Davao del Sur	1,508	1	0.07	8	0.53	26	1.72	35	2.32
Davao Occidental	54	0	0.00	0	0.00	2	3.70	2	3.70
Davao City	14,304	2	0.01	14	0.10	113	0.79	129	0.90
**Region 12**	**26,131**	**0**	**0.00**	**100**	**0.38**	**657**	**2.51**	**757**	**2.90**
North Cotabato	5,038	0	0.00	22	0.44	184	3.65	206	4.09
Sarangani	4,687	0	0.00	37	0.79	165	3.52	202	4.31
South Cotabato	6,684	0	0.00	8	0.12	106	1.59	114	1.71
Sultan Kudarat	5,747	0	0.00	12	0.21	102	1.77	114	1.98
Cotabato City	558	0	0.00	0	0.00	3	0.54	3	0.54
Gen. Santos City	3,417	0	0.00	21	0.61	97	2.84	118	3.45
**BARMM**	**597**	**0**	**0.00**	**0**	**0.00**	**5**	**0.84**	**5**	**0.84**
Basilan	0	0	0.00	0	0.00	0	0.00	0	0.00
Lanao del Sur	556	0	0.00	0	0.00	1	0.18	1	0.18
Maguindanao	35	0	0.00	0	0.00	4	11.43	4	11.43
Sulu	0	0	0.00	0	0.00	0	0.00	0	0.00
Tawi-Tawi	0	0	0.00	0	0.00	0	0.00	0	0.00

Table 1.B.1.12 - Prenatal Care

Number and proportion of pregnant women tested positive for Hepatitis B by Region
Philippines, 2021

Area	Total No. of Pregnant Women Screened for Hepatitis B	Pregnant Women Tested Positive for Hepatitis B							
		Age Group						Total	%
		10-14 yrs old		15-19 yrs old		20-49 yrs old			
		No.	%	No.	%	No.	%		
Lamitan City	6	0	0.00	0	0.00	0	0.00	0	0.00
Marawi City	0	0	0.00	0	0.00	0	0.00	0	0.00
**CARAGA**	**12,026**	**0**	**0.00**	**44**	**0.37**	**307**	**2.55**	**351**	**2.92**
Agusan del Norte	1,735	0	0.00	14	0.81	90	5.19	104	5.99
Agusan del Sur	2,005	0	0.00	11	0.55	53	2.64	64	3.19
Surigao del Norte	1,465	0	0.00	5	0.34	57	3.89	62	4.23
Surigao del Sur	1,218	0	0.00	4	0.33	25	2.05	29	2.38
Province of Dinagat	628	0	0.00	4	0.64	31	4.94	35	5.57
Bislig City	3	0	0.00	0	0.00	0	0.00	0	0.00
Butuan City	3,244	0	0.00	6	0.18	37	1.14	43	1.33
Surigao City	1,728	0	0.00	0	0.00	14	0.81	14	0.81

Note: Put asterisk (*) for No Report and Zero (0) for No Case

Table 1.B.1.13 - Prenatal Care

Number and proportion of pregnant women screened for HIV
Philippines, 2021

Area	Eligible Pop. (Under 1)	Pregnant Women Screened for HIV							
		Age Group						Total	%
		10-14 yrs old		15-19 yrs old		20-49 yrs old			
		No.	%	No.	%	No.	%		
**PHILIPPINES**	**2,128,168**	**1,031**	**0.05**	**37,298**	**1.75**	**281,963**	**13.25**	**320,292**	**15.05**
**N C R**	**237,921**	**184**	**0.08**	**10,361**	**4.35**	**81,692**	**34.34**	**92,237**	**38.77**
Malabon	6,757	18	0.27	518	7.67	2,500	37.00	3,036	44.93
Navotas	4,607	5	0.11	515	11.18	2,259	49.03	2,779	60.32
Valenzuela City	11,465	13	0.11	617	5.38	6,131	53.48	6,761	58.97
Caloocan City	29,270	7	0.02	987	3.37	7,160	24.46	8,154	27.86
Marikina City	8,330	1	0.01	92	1.10	826	9.92	919	11.03
Pasig City	13,954	7	0.05	757	5.42	7,613	54.56	8,377	60.03
Pateros	1,181	0	0.00	27	2.29	219	18.54	246	20.83
Taguig	14,874	10	0.07	892	6.00	5,810	39.06	6,712	45.13
Quezon City	54,246	43	0.08	2,139	3.94	21,359	39.37	23,541	43.40
Makati City	10,765	1	0.01	176	1.63	2,226	20.68	2,403	22.32
Mandaluyong City	7,137	8	0.11	190	2.66	2,236	31.33	2,434	34.10
San Juan	2,254	1	0.04	33	1.46	758	33.63	792	35.14
Manila City	32,887	45	0.14	2,143	6.52	12,250	37.25	14,438	43.90
Las Piñas City	10,879	1	0.01	91	0.84	999	9.18	1,091	10.03
Muntinlupa City	9,317	7	0.08	258	2.77	2,114	22.69	2,379	25.53
Parañaque City	12,298	10	0.08	542	4.41	4,057	32.99	4,609	37.48
Pasay City	7,700	7	0.09	384	4.99	3,175	41.23	3,566	46.31
**C A R**	**35,143**	**12**	**0.03**	**795**	**2.26**	**7,633**	**21.72**	**8,440**	**24.02**
Abra	4,287	0	0.00	35	0.82	224	5.23	259	6.04
Apayao	2,476	0	0.00	30	1.21	107	4.32	137	5.53
Benguet	9,140	7	0.08	409	4.47	3,955	43.27	4,371	47.82
Ifugao	4,439	0	0.00	81	1.82	905	20.39	986	22.21
Kalinga	4,660	1	0.02	27	0.58	132	2.83	160	3.43
Mt. Province	3,066	0	0.00	11	0.36	173	5.64	184	6.00
Baguio City	7,075	4	0.06	202	2.86	2,137	30.20	2,343	33.12
**Region 1**	**97,198**	**8**	**0.01**	**1,235**	**1.27**	**14,891**	**15.32**	**16,134**	**16.60**
Ilocos Norte	7,986	1	0.01	70	0.88	1,047	13.11	1,118	14.00
Ilocos Sur	9,197	4	0.04	209	2.27	2,971	32.30	3,184	34.62
La Union	11,429	1	0.01	234	2.05	2,098	18.36	2,333	20.41
Pangasinan	50,454	1	0.00	368	0.73	4,318	8.56	4,687	9.29
Alaminos City	1,905	0	0.00	59	3.10	763	40.05	822	43.15
Candon City	976	0	0.00	1	0.10	126	12.91	127	13.01
Dagupan City	3,636	0	0.00	95	2.61	624	17.16	719	19.77
Laoag City	1,845	0	0.00	30	1.63	1,435	77.78	1,465	79.40
San Carlos City	3,999	0	0.00	17	0.43	144	3.60	161	4.03
San Fernando City	2,092	0	0.00	35	1.67	461	22.04	496	23.71
Urdaneta City	2,819	1	0.04	117	4.15	899	31.89	1,017	36.08
Vigan City	860	0	0.00	0	0.00	5	0.58	5	0.58

Table 1.B.1.13 - Prenatal Care

Number and proportion of pregnant women screened for HIV
Philippines, 2021

Area	Eligible Pop. (Under 1)	Pregnant Women Screened for HIV							
		Age Group						Total	%
		10-14 yrs old		15-19 yrs old		20-49 yrs old			
		No.	%	No.	%	No.	%		
**Region 2**	**69,228**	**16**	**0.02**	**655**	**0.95**	**4,788**	**6.92**	**5,459**	**7.89**
Batanes	351	0	0.00	4	1.14	74	21.08	78	22.22
Cagayan	20,563	2	0.01	72	0.35	446	2.17	520	2.53
Isabela	23,543	12	0.05	195	0.83	1,308	5.56	1,515	6.44
Nueva Vizcaya	9,555	0	0.00	149	1.56	1,046	10.95	1,195	12.51
Quirino	4,038	2	0.05	105	2.60	877	21.72	984	24.37
Cauayan City	2,576	0	0.00	43	1.67	245	9.51	288	11.18
Ilagan City	2,901	0	0.00	26	0.90	211	7.27	237	8.17
Santiago City	2,683	0	0.00	61	2.27	574	21.39	635	23.67
Tuguegarao City	3,018	0	0.00	0	0.00	7	0.23	7	0.23
**Region 3**	**220,202**	**167**	**0.08**	**6,937**	**3.15**	**46,805**	**21.26**	**53,909**	**24.48**
Aurora	4,764	5	0.10	206	4.32	1,172	24.60	1,383	29.03
Bataan	13,814	19	0.14	576	4.17	3,706	26.83	4,301	31.14
Bulacan	43,722	31	0.07	1,400	3.20	9,191	21.02	10,622	24.29
Nueva Ecija	28,915	13	0.04	531	1.84	3,253	11.25	3,797	13.13
Pampanga	31,503	23	0.07	695	2.21	4,339	13.77	5,057	16.05
Tarlac	19,565	3	0.02	383	1.96	2,655	13.57	3,041	15.54
Zambales	12,551	4	0.03	278	2.21	2,009	16.01	2,291	18.25
Angeles City	7,905	1	0.01	122	1.54	826	10.45	949	12.01
Balanga City	1,996	1	0.05	150	7.52	864	43.29	1,015	50.85
Cabanatuan City	5,921	4	0.07	284	4.80	1,431	24.17	1,719	29.03
City of San Fernando	5,890	9	0.15	225	3.82	1,644	27.91	1,878	31.88
Gapan City	2,159	0	0.00	81	3.75	405	18.76	486	22.51
Mabalacat City	4,811	5	0.10	280	5.82	1,572	32.68	1,857	38.60
Malolos City	4,885	3	0.06	149	3.05	898	18.38	1,050	21.49
Meycauayan	4,050	5	0.12	209	5.16	1,241	30.64	1,455	35.93
Olongapo	4,952	0	0.00	81	1.64	661	13.35	742	14.98
Palayan City	805	0	0.00	13	1.61	120	14.91	133	16.52
San Jose City	2,736	5	0.18	107	3.91	332	12.13	444	16.23
San Jose del Monte City	11,120	35	0.31	873	7.85	9,225	82.96	10,133	91.12
Science City of Munoz	1,594	0	0.00	75	4.71	334	20.95	409	25.66
Tarlac City	6,544	1	0.02	219	3.35	927	14.17	1,147	17.53
**Region 4A**	**298,398**	**92**	**0.03**	**4,857**	**1.63**	**34,491**	**11.56**	**39,440**	**13.22**
Batangas	38,848	1	0.00	162	0.42	2,759	7.10	2,922	7.52
Cavite	27,948	2	0.01	278	0.99	2,698	9.65	2,978	10.66
Laguna	19,727	5	0.03	316	1.60	2,376	12.04	2,697	13.67
Quezon	37,644	20	0.05	426	1.13	2,711	7.20	3,157	8.39
Rizal	44,826	30	0.07	1,424	3.18	8,256	18.42	9,710	21.66
Antipolo City	16,510	4	0.02	538	3.26	1,968	11.92	2,510	15.20
Bacoor City	12,252	0	0.00	106	0.87	757	6.18	863	7.04
Batangas City	6,895	0	0.00	27	0.39	191	2.77	218	3.16
Biñan City	6,607	0	0.00	602	9.11	3,391	51.32	3,993	60.44
Cabuyao City	6,128	0	0.00	64	1.04	548	8.94	612	9.99
Calamba City	9,025	3	0.03	57	0.63	680	7.53	740	8.20
Cavite City	2,098	0	0.00	50	2.38	187	8.91	237	11.30
Dasmariñas City	13,442	0	0.00	246	1.83	2,683	19.96	2,929	21.79

Table 1.B.1.13 - Prenatal Care

Number and proportion of pregnant women screened for HIV
Philippines, 2021

Area	Eligible Pop. (Under 1)	Pregnant Women Screened for HIV							
		Age Group						Total	%
		10-14 yrs old		15-19 yrs old		20-49 yrs old			
		No.	%	No.	%	No.	%		
General Trias City	6,410	0	0.00	36	0.56	443	6.91	479	7.47
Imus City	8,235	0	0.00	69	0.84	569	6.91	638	7.75
Lipa City	6,954	0	0.00	23	0.33	461	6.63	484	6.96
Lucena City	5,704	0	0.00	60	1.05	353	6.19	413	7.24
San Pablo City	5,277	0	0.00	0	0.00	17	0.32	17	0.32
San Pedro City	6,465	0	0.00	82	1.27	627	9.70	709	10.97
Santa Rosa City	7,023	16	0.23	213	3.03	1,959	27.89	2,188	31.15
Tagaytay City	1,451	0	0.00	22	1.52	388	26.74	410	28.26
Tanauan City	3,620	2	0.06	39	1.08	397	10.97	438	12.10
Tayabas City	2,134	0	0.00	3	0.14	3	0.14	6	0.28
Trece Martires City	3,175	9	0.28	14	0.44	69	2.17	92	2.90
**Region 4B**	**72,035**	**8**	**0.01**	**560**	**0.78**	**4,779**	**6.63**	**5,347**	**7.42**
Marinduque	4,906	0	0.00	25	0.51	382	7.79	407	8.30
Mindoro Occidental	12,449	0	0.00	32	0.26	357	2.87	389	3.12
Mindoro Oriental	20,011	2	0.01	167	0.83	1,615	8.07	1,784	8.92
Palawan	21,676	6	0.03	225	1.04	1,488	6.86	1,719	7.93
Romblon	6,482	0	0.00	111	1.71	937	14.46	1,048	16.17
Puerto Princesa City	6,511	0	0.00	0	0.00	0	0.00	0	0.00
**Region 5**	**137,350**	**7**	**0.01**	**985**	**0.72**	**9,287**	**6.76**	**10,279**	**7.48**
Albay	23,903	0	0.00	187	0.78	2,749	11.50	2,936	12.28
Camarines Norte	14,340	0	0.00	41	0.29	243	1.69	284	1.98
Camarines Sur	39,151	3	0.01	95	0.24	1,286	3.28	1,384	3.54
Catanduanes	6,381	0	0.00	61	0.96	298	4.67	359	5.63
Masbate	22,565	2	0.01	202	0.90	1,094	4.85	1,298	5.75
Sorsogon	19,470	0	0.00	69	0.35	633	3.25	702	3.61
Iriga City	2,665	0	0.00	44	1.65	384	14.41	428	16.06
Legaspi City	4,205	2	0.05	100	2.38	1,038	24.68	1,140	27.11
Naga City	4,670	0	0.00	186	3.98	1,562	33.45	1,748	37.43
**Region 6**	**146,545**	**298**	**0.20**	**1,795**	**1.22**	**13,977**	**9.54**	**16,070**	**10.97**
Aklan	11,231	5	0.04	143	1.27	1,886	16.79	2,034	18.11
Antique	12,985	1	0.01	139	1.07	1,016	7.82	1,156	8.90
Capiz	13,984	0	0.00	34	0.24	366	2.62	400	2.86
Guimaras	3,085	0	0.00	48	1.56	540	17.50	588	19.06
Iloilo	36,414	3	0.01	373	1.02	3,343	9.18	3,719	10.21
Negros Occidental	49,314	4	0.01	490	0.99	2,835	5.75	3,329	6.75
Bacolod City	11,102	277	2.50	312	2.81	2,237	20.15	2,826	25.45
Iloilo City	8,430	8	0.09	256	3.04	1,754	20.81	2,018	23.94
**Region 7**	**162,860**	**55**	**0.03**	**3,837**	**2.36**	**29,232**	**17.95**	**33,124**	**20.34**
Bohol	27,637	0	0.00	242	0.88	1,647	5.96	1,889	6.84
Cebu	67,046	26	0.04	1,402	2.09	9,680	14.44	11,108	16.57
Negros Oriental	27,922	1	0.00	74	0.27	349	1.25	424	1.52
Siquijor	1,636	1	0.06	82	5.01	601	36.74	684	41.81
Cebu City	21,037	16	0.08	1,221	5.80	8,665	41.19	9,902	47.07
Lapu-Lapu City	9,310	4	0.04	441	4.74	4,868	52.29	5,313	57.07
Mandaue City	8,272	7	0.08	375	4.53	3,422	41.37	3,804	45.99

Table 1.B.1.13 - Prenatal Care

Number and proportion of pregnant women screened for HIV
Philippines, 2021

Area	Eligible Pop. (Under 1)	Pregnant Women Screened for HIV							
		Age Group						Total	%
		10-14 yrs old		15-19 yrs old		20-49 yrs old			
		No.	%	No.	%	No.	%		
**Region 8**	**104,016**	**12**	**0.01**	**945**	**0.91**	**7,246**	**6.97**	**8,203**	**7.89**
Biliran	3,856	2	0.05	175	4.54	1,246	32.31	1,423	36.90
Eastern Samar	11,635	0	0.00	92	0.79	704	6.05	796	6.84
Northern Leyte	35,052	1	0.00	108	0.31	842	2.40	951	2.71
Northern Samar	15,655	0	0.00	230	1.47	1,857	11.86	2,087	13.33
Southern Leyte	6,556	3	0.05	43	0.66	360	5.49	406	6.19
Western Samar	14,515	3	0.02	79	0.54	554	3.82	636	4.38
Calbayog City	4,474	0	0.00	2	0.04	13	0.29	15	0.34
Maasin City	1,661	0	0.00	0	0.00	2	0.12	2	0.12
Ormoc City	4,989	2	0.04	109	2.18	719	14.41	830	16.64
Tacloban City	5,623	1	0.02	107	1.90	949	16.88	1,057	18.80
**Region 9**	**79,552**	**4**	**0.01**	**155**	**0.19**	**772**	**0.97**	**931**	**1.17**
Zamboanga del Norte	17,075	3	0.02	49	0.29	281	1.65	333	1.95
Zamboanga del Sur	17,506	0	0.00	24	0.14	99	0.57	123	0.70
Zamboanga Sibugay	14,992	0	0.00	33	0.22	163	1.09	196	1.31
Dapitan City	1,755	1	0.06	15	0.85	90	5.13	106	6.04
Dipolog City	2,794	0	0.00	0	0.00	5	0.18	5	0.18
Isabela City	2,544	0	0.00	6	0.24	4	0.16	10	0.39
Pagadian City	4,290	0	0.00	0	0.00	0	0.00	0	0.00
Zamboanga City	18,596	0	0.00	28	0.15	130	0.70	158	0.85
**Region 10**	**100,690**	**84**	**0.08**	**991**	**0.98**	**5,021**	**4.99**	**6,096**	**6.05**
Bukidnon	23,307	62	0.27	151	0.65	464	1.99	677	2.90
Camiguin	1,854	0	0.00	15	0.81	162	8.74	177	9.55
Lanao del Norte	14,955	5	0.03	58	0.39	382	2.55	445	2.98
Misamis Occidental	6,413	0	0.00	13	0.20	117	1.82	130	2.03
Misamis Oriental	15,058	4	0.03	163	1.08	713	4.74	880	5.84
Cagayan de Oro City	14,263	1	0.01	248	1.74	1,207	8.46	1,456	10.21
El Salvador City	1,061	0	0.00	10	0.94	38	3.58	48	4.52
Gingoog City	2,627	10	0.38	75	2.85	234	8.91	319	12.14
Iligan City	7,575	0	0.00	6	0.08	89	1.17	95	1.25
Malaybalay City	3,882	1	0.03	54	1.39	237	6.11	292	7.52
Oroquieta City	1,389	0	0.00	0	0.00	20	1.44	20	1.44
Ozamis City	2,782	1	0.04	141	5.07	1,036	37.24	1,178	42.34
Tangub City	1,234	0	0.00	0	0.00	2	0.16	2	0.16
Valencia City	4,290	0	0.00	57	1.33	320	7.46	377	8.79
**Region 11**	**107,847**	**48**	**0.04**	**1,034**	**0.96**	**7,578**	**7.03**	**8,660**	**8.03**
Davao de Oro	15,526	0	0.00	8	0.05	43	0.28	51	0.33
Davao del Norte	21,184	6	0.03	236	1.11	1,875	8.85	2,117	9.99
Davao Oriental	13,125	8	0.06	35	0.27	103	0.78	146	1.11
Davao del Sur	14,355	2	0.01	87	0.61	389	2.71	478	3.33
Davao Occidental	6,598	3	0.05	34	0.52	100	1.52	137	2.08
Davao City	37,059	29	0.08	634	1.71	5,068	13.68	5,731	15.46

Table 1.B.1.13 - Prenatal Care

Number and proportion of pregnant women screened for HIV
Philippines, 2021

Area	Eligible Pop. (Under 1)	Pregnant Women Screened for HIV							
		Age Group						Total	%
		10-14 yrs old		15-19 yrs old		20-49 yrs old			
		No.	%	No.	%	No.	%		
**Region 12**	**104,693**	**21**	**0.02**	**1,435**	**1.37**	**8,287**	**7.92**	**9,743**	**9.31**
North Cotabato	33,933	3	0.01	194	0.57	1,040	3.06	1,237	3.65
Sarangani	12,904	4	0.03	204	1.58	970	7.52	1,178	9.13
South Cotabato	21,039	9	0.04	697	3.31	4,260	20.25	4,966	23.60
Sultan Kudarat	17,331	3	0.02	119	0.69	488	2.82	610	3.52
Cotabato City	5,831	0	0.00	23	0.39	226	3.88	249	4.27
Gen. Santos City	13,655	2	0.01	198	1.45	1,303	9.54	1,503	11.01
**BARMM**	**94,148**	**0**	**0.00**	**0**	**0.00**	**145**	**0.15**	**145**	**0.15**
Basilan	7,672	0	0.00	0	0.00	0	0.00	0	0.00
Lanao del Sur	21,391	0	0.00	0	0.00	135	0.63	135	0.63
Maguindanao	31,650	0	0.00	0	0.00	10	0.03	10	0.03
Sulu	16,891	0	0.00	0	0.00	0	0.00	0	0.00
Tawi-Tawi	9,319	0	0.00	0	0.00	0	0.00	0	0.00
Lamitan City	2,112	0	0.00	0	0.00	0	0.00	0	0.00
Marawi City	5,113	0	0.00	0	0.00	0	0.00	0	0.00
**CARAGA**	**60,342**	**15**	**0.02**	**721**	**1.19**	**5,339**	**8.85**	**6,075**	**10.07**
Agusan del Norte	8,116	2	0.02	50	0.62	435	5.36	487	6.00
Agusan del Sur	17,658	1	0.01	137	0.78	739	4.19	877	4.97
Surigao del Norte	7,195	0	0.00	40	0.56	299	4.16	339	4.71
Surigao del Sur	11,532	0	0.00	33	0.29	202	1.75	235	2.04
Province of Dinagat	2,574	4	0.16	48	1.86	518	20.12	570	22.14
Bislig City	2,185	0	0.00	0	0.00	2	0.09	2	0.09
Butuan City	7,732	1	0.01	267	3.45	2,082	26.93	2,350	30.39
Surigao City	3,350	7	0.21	146	4.36	1,062	31.70	1,215	36.27

Note: Put asterisk (*) for No Report and Zero (0) for No Case

Table 1.B.1.14 - Prenatal Care

Number and proportion of pregnant women tested for CBC or Hemoglobin and Hematocrit Count by Region
Philippines, 2021

Area	Eligible Pop. (Under 1)	Tested for CBC/Hgb and Hct							
		Age Group						Total	%
		10-14 yrs old		15-19 yrs old		20-49 yrs old			
		No.	%	No.	%	No.	%		
**PHILIPPINES**	**2,128,168**	**1,762**	**0.08**	**82,965**	**3.90**	**590,299**	**27.74**	**675,026**	**31.72**
**N C R**	**237,921**	**182**	**0.08**	**10,287**	**4.32**	**82,960**	**34.87**	**93,429**	**39.27**
Malabon	6,757	10	0.15	486	7.19	2,420	35.81	2,916	43.16
Navotas	4,607	5	0.11	528	11.46	2,286	49.62	2,819	61.19
Valenzuela City	11,465	12	0.10	577	5.03	5,265	45.92	5,854	51.06
Caloocan City	29,270	5	0.02	795	2.72	7,071	24.16	7,871	26.89
Marikina City	8,330	4	0.05	162	1.94	1,449	17.39	1,615	19.39
Pasig City	13,954	8	0.06	841	6.03	8,132	58.28	8,981	64.36
Pateros	1,181	3	0.25	120	10.16	677	57.32	800	67.74
Taguig	14,874	19	0.13	985	6.62	6,614	44.47	7,618	51.22
Quezon City	54,246	48	0.09	2,074	3.82	20,604	37.98	22,726	41.89
Makati City	10,765	3	0.03	243	2.26	3,835	35.62	4,081	37.91
Mandaluyong City	7,137	9	0.13	206	2.89	2,303	32.27	2,518	35.28
San Juan	2,254	0	0.00	34	1.51	710	31.50	744	33.01
Manila City	32,887	25	0.08	1,652	5.02	9,190	27.94	10,867	33.04
Las Piñas City	10,879	3	0.03	237	2.18	2,187	20.10	2,427	22.31
Muntinlupa City	9,317	7	0.08	349	3.75	2,514	26.98	2,870	30.80
Parañaque City	12,298	15	0.12	545	4.43	4,027	32.75	4,587	37.30
Pasay City	7,700	6	0.08	453	5.88	3,676	47.74	4,135	53.70
**C A R**	**35,143**	**46**	**0.13**	**1,578**	**4.49**	**13,455**	**38.29**	**15,079**	**42.91**
Abra	4,287	18	0.42	244	5.69	554	12.92	816	19.03
Apayao	2,476	7	0.28	173	6.99	1,032	41.68	1,212	48.95
Benguet	9,140	11	0.12	468	5.12	4,595	50.27	5,074	55.51
Ifugao	4,439	1	0.02	161	3.63	1,790	40.32	1,952	43.97
Kalinga	4,660	3	0.06	194	4.16	1,560	33.48	1,757	37.70
Mt. Province	3,066	3	0.10	98	3.20	1,032	33.66	1,133	36.95
Baguio City	7,075	3	0.04	240	3.39	2,892	40.88	3,135	44.31
**Region 1**	**97,198**	**25**	**0.03**	**3,079**	**3.17**	**33,859**	**34.84**	**36,963**	**38.03**
Ilocos Norte	7,986	3	0.04	235	2.94	2,818	35.29	3,056	38.27
Ilocos Sur	9,197	7	0.08	410	4.46	4,858	52.82	5,275	57.36
La Union	11,429	3	0.03	524	4.58	4,013	35.11	4,540	39.72
Pangasinan	50,454	9	0.02	1,495	2.96	15,700	31.12	17,204	34.10
Alaminos City	1,905	0	0.00	45	2.36	729	38.27	774	40.63
Candon City	976	0	0.00	1	0.10	850	87.09	851	87.19
Dagupan City	3,636	0	0.00	104	2.86	705	19.39	809	22.25
Laoag City	1,845	0	0.00	35	1.90	1,909	103.47	1,944	105.37
San Carlos City	3,999	1	0.03	45	1.13	488	12.20	534	13.35
San Fernando City	2,092	0	0.00	45	2.15	630	30.11	675	32.27
Urdaneta City	2,819	2	0.07	126	4.47	1,024	36.32	1,152	40.87
Vigan City	860	0	0.00	14	1.63	135	15.70	149	17.33
**Region 2**	**69,228**	**63**	**0.09**	**3,162**	**4.57**	**21,094**	**30.47**	**24,319**	**35.13**
Batanes	351	1	0.28	8	2.28	134	38.18	143	40.74
Cagayan	20,563	21	0.10	898	4.37	5,303	25.79	6,222	30.26
Isabela	23,543	22	0.09	988	4.20	6,718	28.54	7,728	32.83
Nueva Vizcaya	9,555	5	0.05	482	5.04	3,442	36.02	3,929	41.12
Quirino	4,038	9	0.22	378	9.36	2,396	59.34	2,783	68.92

Table 1.B.1.14 - Prenatal Care

Number and proportion of pregnant women tested for CBC or Hemoglobin and Hematocrit Count by Region
Philippines, 2021

Area	Eligible Pop. (Under 1)	Tested for CBC/Hgb and Hct							
		Age Group						Total	%
		10-14 yrs old		15-19 yrs old		20-49 yrs old			
		No.	%	No.	%	No.	%		
Cauayan City	2,576	0	0.00	164	6.37	917	35.60	1,081	41.96
Ilagan City	2,901	5	0.17	147	5.07	1,219	42.02	1,371	47.26
Santiago City	2,683	0	0.00	65	2.42	632	23.56	697	25.98
Tuguegarao City	3,018	0	0.00	32	1.06	333	11.03	365	12.09
**Region 3**	**220,202**	**184**	**0.08**	**8,248**	**3.75**	**55,390**	**25.15**	**63,822**	**28.98**
Aurora	4,764	6	0.13	114	2.39	767	16.10	887	18.62
Bataan	13,814	23	0.17	785	5.68	5,053	36.58	5,861	42.43
Bulacan	43,722	31	0.07	1,815	4.15	11,734	26.84	13,580	31.06
Nueva Ecija	28,915	23	0.08	722	2.50	4,359	15.08	5,104	17.65
Pampanga	31,503	16	0.05	494	1.57	2,757	8.75	3,267	10.37
Tarlac	19,565	10	0.05	792	4.05	5,972	30.52	6,774	34.62
Zambales	12,551	5	0.04	264	2.10	1,782	14.20	2,051	16.34
Angeles City	7,905	1	0.01	172	2.18	831	10.51	1,004	12.70
Balanga City	1,996	4	0.20	157	7.87	962	48.20	1,123	56.26
Cabanatuan City	5,921	3	0.05	173	2.92	967	16.33	1,143	19.30
City of San Fernando	5,890	8	0.14	218	3.70	1,742	29.58	1,968	33.41
Gapan City	2,159	2	0.09	111	5.14	572	26.49	685	31.73
Mabalacat City	4,811	2	0.04	77	1.60	990	20.58	1,069	22.22
Malolos City	4,885	1	0.02	146	2.99	784	16.05	931	19.06
Meycauayan	4,050	3	0.07	278	6.86	1,504	37.14	1,785	44.07
Olongapo	4,952	3	0.06	137	2.77	1,085	21.91	1,225	24.74
Palayan City	805	0	0.00	20	2.48	122	15.16	142	17.64
San Jose City	2,736	5	0.18	164	5.99	610	22.30	779	28.47
San Jose del Monte City	11,120	35	0.31	972	8.74	9,586	86.21	10,593	95.26
Science City of Munoz	1,594	0	0.00	72	4.52	317	19.89	389	24.40
Tarlac City	6,544	3	0.05	565	8.63	2,894	44.22	3,462	52.90
**Region 4A**	**298,398**	**131**	**0.04**	**9,448**	**3.17**	**69,085**	**23.15**	**78,664**	**26.36**
Batangas	38,848	4	0.01	562	1.45	6,970	17.94	7,536	19.40
Cavite	27,948	9	0.03	565	2.02	5,007	17.92	5,581	19.97
Laguna	19,727	9	0.05	778	3.94	5,011	25.40	5,798	29.39
Quezon	37,644	41	0.11	1,434	3.81	9,344	24.82	10,819	28.74
Rizal	44,826	30	0.07	1,542	3.44	8,699	19.41	10,271	22.91
Antipolo City	16,510	2	0.01	780	4.72	2,828	17.13	3,610	21.87
Bacoor City	12,252	1	0.01	146	1.19	999	8.15	1,146	9.35
Batangas City	6,895	0	0.00	107	1.55	1,100	15.95	1,207	17.51
Biñan City	6,607	2	0.03	797	12.06	4,786	72.44	5,585	84.53
Cabuyao City	6,128	3	0.05	322	5.25	3,390	55.32	3,715	60.62
Calamba City	9,025	8	0.09	293	3.25	3,143	34.83	3,444	38.16
Cavite City	2,098	0	0.00	48	2.29	191	9.10	239	11.39
Dasmariñas City	13,442	3	0.02	339	2.52	3,252	24.19	3,594	26.74
General Trias City	6,410	0	0.00	68	1.06	671	10.47	739	11.53
Imus City	8,235	0	0.00	93	1.13	794	9.64	887	10.77
Lipa City	6,954	1	0.01	188	2.70	2,214	31.84	2,403	34.56
Lucena City	5,704	3	0.05	387	6.78	1,567	27.47	1,957	34.31
San Pablo City	5,277	0	0.00	67	1.27	457	8.66	524	9.93
San Pedro City	6,465	1	0.02	218	3.37	1,303	20.15	1,522	23.54
Santa Rosa City	7,023	7	0.10	405	5.77	3,608	51.37	4,020	57.24
Tagaytay City	1,451	0	0.00	37	2.55	817	56.31	854	58.86
Tanauan City	3,620	2	0.06	69	1.91	1,306	36.08	1,377	38.04
Tayabas City	2,134	4	0.19	144	6.75	1,167	54.69	1,315	61.62
Trece Martires City	3,175	1	0.03	59	1.86	461	14.52	521	16.41

Table 1.B.1.14 - Prenatal Care

Number and proportion of pregnant women tested for CBC or Hemoglobin and Hematocrit Count by Region
Philippines, 2021

Area	Eligible Pop. (Under 1)	Tested for CBC/Hgb and Hct							
		Age Group						Total	%
		10-14 yrs old		15-19 yrs old		20-49 yrs old			
		No.	%	No.	%	No.	%		
**Region 4B**	**72,035**	**23**	**0.03**	**1,583**	**2.20**	**12,433**	**17.26**	**14,039**	**19.49**
Marinduque	4,906	0	0.00	71	1.45	903	18.41	974	19.85
Mindoro Occidental	12,449	8	0.06	352	2.83	2,674	21.48	3,034	24.37
Mindoro Oriental	20,011	3	0.01	470	2.35	4,276	21.37	4,749	23.73
Palawan	21,676	10	0.05	483	2.23	2,850	13.15	3,343	15.42
Romblon	6,482	2	0.03	207	3.19	1,730	26.69	1,939	29.91
Puerto Princesa City	6,511	0	0.00	0	0.00	0	0.00	0	0.00
**Region 5**	**137,350**	**25**	**0.02**	**2,810**	**2.05**	**28,125**	**20.48**	**30,960**	**22.54**
Albay	23,903	4	0.02	496	2.08	7,136	29.85	7,636	31.95
Camarines Norte	14,340	3	0.02	491	3.42	3,158	22.02	3,652	25.47
Camarines Sur	39,151	3	0.01	225	0.57	2,821	7.21	3,049	7.79
Catanduanes	6,381	2	0.03	171	2.68	1,280	20.06	1,453	22.77
Masbate	22,565	5	0.02	594	2.63	3,619	16.04	4,218	18.69
Sorsogon	19,470	7	0.04	669	3.44	6,237	32.03	6,913	35.51
Iriga City	2,665	0	0.00	13	0.49	77	2.89	90	3.38
Legaspi City	4,205	1	0.02	146	3.47	3,736	88.85	3,883	92.34
Naga City	4,670	0	0.00	5	0.11	61	1.31	66	1.41
**Region 6**	**146,545**	**91**	**0.06**	**5,987**	**4.09**	**46,982**	**32.06**	**53,060**	**36.21**
Aklan	11,231	8	0.07	330	2.94	3,770	33.57	4,108	36.58
Antique	12,985	8	0.06	377	2.90	3,016	23.23	3,401	26.19
Capiz	13,984	8	0.06	272	1.95	3,263	23.33	3,543	25.34
Guimaras	3,085	4	0.13	142	4.60	1,719	55.72	1,865	60.45
Iloilo	36,414	25	0.07	1,464	4.02	14,069	38.64	15,558	42.73
Negros Occidental	49,314	18	0.04	2,511	5.09	15,271	30.97	17,800	36.10
Bacolod City	11,102	11	0.10	550	4.95	3,452	31.09	4,013	36.15
Iloilo City	8,430	9	0.11	341	4.05	2,422	28.73	2,772	32.88
**Region 7**	**162,860**	**68**	**0.04**	**5,237**	**3.22**	**43,454**	**26.68**	**48,759**	**29.94**
Bohol	27,637	5	0.02	718	2.60	7,836	28.35	8,559	30.97
Cebu	67,046	23	0.03	1,341	2.00	10,595	15.80	11,959	17.84
Negros Oriental	27,922	13	0.05	1,020	3.65	7,063	25.30	8,096	29.00
Siquijor	1,636	0	0.00	55	3.36	505	30.87	560	34.23
Cebu City	21,037	17	0.08	1,263	6.00	8,880	42.21	10,160	48.30
Lapu-Lapu City	9,310	4	0.04	470	5.05	5,256	56.46	5,730	61.55
Mandaue City	8,272	6	0.07	370	4.47	3,319	40.12	3,695	44.67
**Region 8**	**104,016**	**60**	**0.06**	**3,057**	**2.94**	**26,454**	**25.43**	**29,571**	**28.43**
Biliran	3,856	2	0.05	201	5.21	1,477	38.30	1,680	43.57
Eastern Samar	11,635	5	0.04	254	2.18	1,891	16.25	2,150	18.48
Northern Leyte	35,052	15	0.04	1,031	2.94	9,147	26.10	10,193	29.08
Northern Samar	15,655	3	0.02	524	3.35	4,754	30.37	5,281	33.73
Southern Leyte	6,556	3	0.05	238	3.63	2,415	36.84	2,656	40.51
Western Samar	14,515	27	0.19	306	2.11	1,930	13.30	2,263	15.59
Calbayog City	4,474	0	0.00	22	0.49	244	5.45	266	5.95
Maasin City	1,661	0	0.00	42	2.53	610	36.72	652	39.25
Ormoc City	4,989	3	0.06	218	4.37	1,327	26.60	1,548	31.03
Tacloban City	5,623	2	0.04	221	3.93	2,659	47.29	2,882	51.25

Table 1.B.1.14 - Prenatal Care

Number and proportion of pregnant women tested for CBC or Hemoglobin and Hematocrit Count by Region
Philippines, 2021

Area	Eligible Pop. (Under 1)	Tested for CBC/Hgb and Hct							
		Age Group						Total	%
		10-14 yrs old		15-19 yrs old		20-49 yrs old			
		No.	%	No.	%	No.	%		
**Region 9**	**79,552**	**30**	**0.04**	**1,478**	**1.86**	**8,536**	**10.73**	**10,044**	**12.63**
Zamboanga del Norte	17,075	7	0.04	433	2.54	2,189	12.82	2,629	15.40
Zamboanga del Sur	17,506	4	0.02	280	1.60	1,575	9.00	1,859	10.62
Zamboanga Sibugay	14,992	5	0.03	364	2.43	2,255	15.04	2,624	17.50
Dapitan City	1,755	1	0.06	10	0.57	83	4.73	94	5.36
Dipolog City	2,794	3	0.11	4	0.14	20	0.72	27	0.97
Isabela City	2,544	0	0.00	24	0.94	174	6.84	198	7.78
Pagadian City	4,290	0	0.00	173	4.03	1,250	29.14	1,423	33.17
Zamboanga City	18,596	10	0.05	190	1.02	990	5.32	1,190	6.40
**Region 10**	**100,690**	**160**	**0.16**	**6,303**	**6.26**	**32,121**	**31.90**	**38,584**	**38.32**
Bukidnon	23,307	80	0.34	2,126	9.12	8,096	34.74	10,302	44.20
Camiguin	1,854	0	0.00	33	1.78	418	22.55	451	24.33
Lanao del Norte	14,955	23	0.15	205	1.37	876	5.86	1,104	7.38
Misamis Occidental	6,413	0	0.00	148	2.31	1,743	27.18	1,891	29.49
Misamis Oriental	15,058	12	0.08	1,094	7.27	6,097	40.49	7,203	47.84
Cagayan de Oro City	14,263	18	0.13	1,056	7.40	6,261	43.90	7,335	51.43
El Salvador City	1,061	2	0.19	91	8.58	424	39.96	517	48.73
Gingoog City	2,627	11	0.42	339	12.90	1,218	46.36	1,568	59.69
Iligan City	7,575	3	0.04	229	3.02	1,612	21.28	1,844	24.34
Malaybalay City	3,882	3	0.08	295	7.60	1,476	38.02	1,774	45.70
Oroquieta City	1,389	0	0.00	12	0.86	144	10.37	156	11.23
Ozamis City	2,782	3	0.11	241	8.66	1,600	57.51	1,844	66.28
Tangub City	1,234	0	0.00	3	0.24	8	0.65	11	0.89
Valencia City	4,290	5	0.12	431	10.05	2,148	50.07	2,584	60.23
**Region 11**	**107,847**	**467**	**0.43**	**9,467**	**8.78**	**51,991**	**48.21**	**61,925**	**57.42**
Davao de Oro	15,526	99	0.64	1,898	12.22	8,879	57.19	10,876	70.05
Davao del Norte	21,184	111	0.52	2,280	10.76	12,908	60.93	15,299	72.22
Davao Oriental	13,125	36	0.27	1,033	7.87	5,253	40.02	6,322	48.17
Davao del Sur	14,355	67	0.47	1,099	7.66	5,465	38.07	6,631	46.19
Davao Occidental	6,598	30	0.45	318	4.82	819	12.41	1,167	17.69
Davao City	37,059	124	0.33	2,839	7.66	18,667	50.37	21,630	58.37
**Region 12**	**104,693**	**116**	**0.11**	**6,969**	**6.66**	**35,767**	**34.16**	**42,852**	**40.93**
North Cotabato	33,933	26	0.08	1,936	5.71	10,401	30.65	12,363	36.43
Sarangani	12,904	36	0.28	1,576	12.21	6,280	48.67	7,892	61.16
South Cotabato	21,039	18	0.09	1,571	7.47	8,878	42.20	10,467	49.75
Sultan Kudarat	17,331	29	0.17	1,078	6.22	6,086	35.12	7,193	41.50
Cotabato City	5,831	0	0.00	89	1.53	773	13.26	862	14.78
Gen. Santos City	13,655	7	0.05	719	5.27	3,349	24.53	4,075	29.84
**BARMM**	**94,148**	**6**	**0.01**	**396**	**0.42**	**3,675**	**3.90**	**4,077**	**4.33**
Basilan	7,672	0	0.00	6	0.08	18	0.23	24	0.31
Lanao del Sur	21,391	1	0.00	145	0.68	2,051	9.59	2,197	10.27
Maguindanao	31,650	4	0.01	140	0.44	839	2.65	983	3.11
Sulu	16,891	0	0.00	0	0.00	0	0.00	0	0.00
Tawi-Tawi	9,319	0	0.00	1	0.01	1	0.01	2	0.02
Lamitan City	2,112	1	0.05	60	2.84	148	7.01	209	9.90
Marawi City	5,113	0	0.00	44	0.86	618	12.09	662	12.95

Table 1.B.1.14 - Prenatal Care

Number and proportion of pregnant women tested for CBC or Hemoglobin and Hematocrit Count by Region
Philippines, 2021

Area	Eligible Pop. (Under 1)	Tested for CBC/Hgb and Hct							
		Age Group						Total	%
		10-14 yrs old		15-19 yrs old		20-49 yrs old			
		No.	%	No.	%	No.	%		
**CARAGA**	**60,342**	**85**	**0.14**	**3,876**	**6.42**	**24,918**	**41.29**	**28,879**	**47.86**
Agusan del Norte	8,116	8	0.10	495	6.10	3,270	40.29	3,773	46.49
Agusan del Sur	17,658	23	0.13	1,164	6.59	7,059	39.98	8,246	46.70
Surigao del Norte	7,195	8	0.11	375	5.21	2,617	36.37	3,000	41.70
Surigao del Sur	11,532	23	0.20	742	6.43	5,020	43.53	5,785	50.16
Province of Dinagat	2,574	5	0.19	93	3.61	781	30.34	879	34.15
Bislig City	2,185	3	0.14	138	6.32	1,051	48.10	1,192	54.55
Butuan City	7,732	9	0.12	608	7.86	3,391	43.86	4,008	51.84
Surigao City	3,350	6	0.18	261	7.79	1,729	51.61	1,996	59.58

Note: Put asterisk (*) for No Report and Zero (0) for No Case

Table 1.B.1.15 - Prenatal Care

Number and proportion of pregnant women tested for Complete Blood Count or Hemoglobin and Hematocrit Count diagnosed with anemia by Region

Philippines, 2021

Area	Total No. tested for CBC or Hgb or Hct count	Tested for CBC/Hgb and Hct diagnosed with anemia							
		Age Group						Total	%
		10-14 yrs old		15-19 yrs old		20-49 yrs old			
		No.	%	No.	%	No.	%		
**PHILIPPINES**	**675,026**	**365**	**0.05**	**13,104**	**1.94**	**70,247**	**10.41**	**83,716**	**12.40**
**N C R**	**93,429**	**19**	**0.02**	**1,348**	**1.44**	**8,093**	**8.66**	**9,460**	**10.13**
Malabon	2,916	2	0.07	52	1.78	282	9.67	336	11.52
Navotas	2,819	0	0.00	55	1.95	261	9.26	316	11.21
Valenzuela City	5,854	0	0.00	46	0.79	442	7.55	488	8.34
Caloocan City	7,871	1	0.01	139	1.77	956	12.15	1,096	13.92
Marikina City	1,615	1	0.06	25	1.55	150	9.29	176	10.90
Pasig City	8,981	1	0.01	202	2.25	1,036	11.54	1,239	13.80
Pateros	800	0	0.00	10	1.25	36	4.50	46	5.75
Taguig	7,618	1	0.01	180	2.36	937	12.30	1,118	14.68
Quezon City	22,726	10	0.04	251	1.10	1,703	7.49	1,964	8.64
Makati City	4,081	0	0.00	21	0.51	439	10.76	460	11.27
Mandaluyong City	2,518	0	0.00	26	1.03	192	7.63	218	8.66
San Juan	744	0	0.00	0	0.00	0	0.00	0	0.00
Manila City	10,867	1	0.01	188	1.73	735	6.76	924	8.50
Las Piñas City	2,427	0	0.00	37	1.52	263	10.84	300	12.36
Muntinlupa City	2,870	0	0.00	0	0.00	0	0.00	0	0.00
Parañaque City	4,587	1	0.02	36	0.78	176	3.84	213	4.64
Pasay City	4,135	1	0.02	80	1.93	485	11.73	566	13.69
**C A R**	**15,079**	**3**	**0.02**	**126**	**0.84**	**1,076**	**7.14**	**1,205**	**7.99**
Abra	816	0	0.00	1	0.12	2	0.25	3	0.37
Apayao	1,212	1	0.08	5	0.41	60	4.95	66	5.45
Benguet	5,074	0	0.00	16	0.32	147	2.90	163	3.21
Ifugao	1,952	1	0.05	27	1.38	182	9.32	210	10.76
Kalinga	1,757	1	0.06	59	3.36	474	26.98	534	30.39
Mt. Province	1,133	0	0.00	6	0.53	95	8.38	101	8.91
Baguio City	3,135	0	0.00	12	0.38	116	3.70	128	4.08
**Region 1**	**36,963**	**1**	**0.00**	**269**	**0.73**	**2,170**	**5.87**	**2,440**	**6.60**
Ilocos Norte	3,056	0	0.00	1	0.03	31	1.01	32	1.05
Ilocos Sur	5,275	0	0.00	26	0.49	318	6.03	344	6.52
La Union	4,540	0	0.00	37	0.81	149	3.28	186	4.10
Pangasinan	17,204	0	0.00	131	0.76	1,092	6.35	1,223	7.11
Alaminos City	774	0	0.00	0	0.00	0	0.00	0	0.00
Candon City	851	0	0.00	0	0.00	23	2.70	23	2.70
Dagupan City	809	0	0.00	69	8.53	498	61.56	567	70.09
Laoag City	1,944	0	0.00	0	0.00	0	0.00	0	0.00
San Carlos City	534	1	0.19	3	0.56	39	7.30	43	8.05
San Fernando City	675	0	0.00	0	0.00	14	2.07	14	2.07
Urdaneta City	1,152	0	0.00	0	0.00	0	0.00	0	0.00
Vigan City	149	0	0.00	2	1.34	6	4.03	8	5.37
**Region 2**	**24,319**	**9**	**0.04**	**449**	**1.85**	**2,429**	**9.99**	**2,887**	**11.87**
Batanes	143	0	0.00	0	0.00	2	1.40	2	1.40
Cagayan	6,222	4	0.06	139	2.23	829	13.32	972	15.62
Isabela	7,728	1	0.01	162	2.10	702	9.08	865	11.19

Table 1.B.1.15 - Prenatal Care

Number and proportion of pregnant women tested for Complete Blood Count or Hemoglobin and Hematocrit Count diagnosed with anemia by Region

Philippines, 2021

Area	Total No. tested for CBC or Hgb or Hct count	Tested for CBC/Hgb and Hct diagnosed with anemia							
		Age Group						Total	%
		10-14 yrs old		15-19 yrs old		20-49 yrs old			
		No.	%	No.	%	No.	%		
Nueva Vizcaya	3,929	1	0.03	60	1.53	380	9.67	441	11.22
Quirino	2,783	3	0.11	42	1.51	145	5.21	190	6.83
Cauayan City	1,081	0	0.00	11	1.02	35	3.24	46	4.26
Ilagan City	1,371	0	0.00	20	1.46	183	13.35	203	14.81
Santiago City	697	0	0.00	10	1.43	105	15.06	115	16.50
Tuguegarao City	365	0	0.00	5	1.37	48	13.15	53	14.52
**Region 3**	**63,822**	**17**	**0.03**	**756**	**1.18**	**4,126**	**6.46**	**4,899**	**7.68**
Aurora	887	0	0.00	7	0.79	31	3.49	38	4.28
Bataan	5,861	2	0.03	101	1.72	636	10.85	739	12.61
Bulacan	13,580	3	0.02	150	1.10	686	5.05	839	6.18
Nueva Ecija	5,104	1	0.02	114	2.23	737	14.44	852	16.69
Pampanga	3,267	0	0.00	27	0.83	119	3.64	146	4.47
Tarlac	6,774	2	0.03	58	0.86	196	2.89	256	3.78
Zambales	2,051	1	0.05	8	0.39	48	2.34	57	2.78
Angeles City	1,004	0	0.00	7	0.70	50	4.98	57	5.68
Balanga City	1,123	2	0.18	25	2.23	198	17.63	225	20.04
Cabanatuan City	1,143	0	0.00	15	1.31	47	4.11	62	5.42
City of San Fernando	1,968	2	0.10	38	1.93	258	13.11	298	15.14
Gapan City	685	0	0.00	3	0.44	17	2.48	20	2.92
Mabalacat City	1,069	0	0.00	36	3.37	406	37.98	442	41.35
Malolos City	931	0	0.00	4	0.43	12	1.29	16	1.72
Meycauayan	1,785	0	0.00	5	0.28	30	1.68	35	1.96
Olongapo	1,225	0	0.00	13	1.06	46	3.76	59	4.82
Palayan City	142	0	0.00	0	0.00	6	4.23	6	4.23
San Jose City	779	0	0.00	0	0.00	0	0.00	0	0.00
San Jose del Monte City	10,593	2	0.02	91	0.86	446	4.21	539	5.09
Science City of Munoz	389	0	0.00	0	0.00	0	0.00	0	0.00
Tarlac City	3,462	2	0.06	54	1.56	157	4.53	213	6.15
**Region 4A**	**78,664**	**45**	**0.06**	**1,161**	**1.48**	**6,599**	**8.39**	**7,805**	**9.92**
Batangas	7,536	0	0.00	50	0.66	416	5.52	466	6.18
Cavite	5,581	2	0.04	91	1.63	605	10.84	698	12.51
Laguna	5,798	1	0.02	138	2.38	642	11.07	781	13.47
Quezon	10,819	1	0.01	193	1.78	1,037	9.58	1,231	11.38
Rizal	10,271	3	0.03	137	1.33	686	6.68	826	8.04
Antipolo City	3,610	5	0.14	64	1.77	186	5.15	255	7.06
Bacoor City	1,146	1	0.09	10	0.87	74	6.46	85	7.42
Batangas City	1,207	0	0.00	19	1.57	148	12.26	167	13.84
Biñan City	5,585	0	0.00	55	0.98	308	5.51	363	6.50
Cabuyao City	3,715	0	0.00	41	1.10	105	2.83	146	3.93
Calamba City	3,444	3	0.09	63	1.83	753	21.86	819	23.78
Cavite City	239	1	0.42	7	2.93	10	4.18	18	7.53
Dasmariñas City	3,594	25	0.70	46	1.28	347	9.65	418	11.63
General Trias City	739	0	0.00	7	0.95	103	13.94	110	14.88
Imus City	887	0	0.00	10	1.13	89	10.03	99	11.16
Lipa City	2,403	1	0.04	30	1.25	218	9.07	249	10.36
Lucena City	1,957	1	0.05	73	3.73	298	15.23	372	19.01
San Pablo City	524	0	0.00	13	2.48	60	11.45	73	13.93
San Pedro City	1,522	0	0.00	23	1.51	86	5.65	109	7.16
Santa Rosa City	4,020	1	0.02	84	2.09	357	8.88	442	11.00
Tagaytay City	854	0	0.00	0	0.00	14	1.64	14	1.64
Tanauan City	1,377	0	0.00	2	0.15	40	2.90	42	3.05

Table 1.B.1.15 - Prenatal Care

Number and proportion of pregnant women tested for Complete Blood Count or Hemoglobin and Hematocrit Count diagnosed with anemia by Region

Philippines, 2021

Area	Total No. tested for CBC or Hgb or Hct count	Tested for CBC/Hgb and Hct diagnosed with anemia							
		Age Group						Total	%
		10-14 yrs old		15-19 yrs old		20-49 yrs old			
		No.	%	No.	%	No.	%		
Tayabas City	1,315	0	0.00	1	0.08	8	0.61	9	0.68
Trece Martires City	521	0	0.00	4	0.77	9	1.73	13	2.50
**Region 4B**	**14,039**	**5**	**0.04**	**155**	**1.10**	**868**	**6.18**	**1,028**	**7.32**
Marinduque	974	0	0.00	6	0.62	21	2.16	27	2.77
Mindoro Occidental	3,034	0	0.00	50	1.65	231	7.61	281	9.26
Mindoro Oriental	4,749	0	0.00	24	0.51	205	4.32	229	4.82
Palawan	3,343	5	0.15	65	1.94	336	10.05	406	12.14
Romblon	1,939	0	0.00	10	0.52	75	3.87	85	4.38
Puerto Princesa City	0	0	0.00	0	0.00	0	0.00	0	0.00
**Region 5**	**30,960**	**4**	**0.01**	**363**	**1.17**	**2,539**	**8.20**	**2,906**	**9.39**
Albay	7,636	1	0.01	41	0.54	516	6.76	558	7.31
Camarines Norte	3,652	0	0.00	136	3.72	698	19.11	834	22.84
Camarines Sur	3,049	0	0.00	18	0.59	178	5.84	196	6.43
Catanduanes	1,453	0	0.00	22	1.51	184	12.66	206	14.18
Masbate	4,218	2	0.05	65	1.54	331	7.85	398	9.44
Sorsogon	6,913	1	0.01	69	1.00	489	7.07	559	8.09
Iriga City	90	0	0.00	2	2.22	5	5.56	7	7.78
Legaspi City	3,883	0	0.00	8	0.21	128	3.30	136	3.50
Naga City	66	0	0.00	2	3.03	10	15.15	12	18.18
**Region 6**	**53,060**	**6**	**0.01**	**530**	**1.00**	**3,869**	**7.29**	**4,405**	**8.30**
Aklan	4,108	0	0.00	44	1.07	292	7.11	336	8.18
Antique	3,401	3	0.09	19	0.56	182	5.35	204	6.00
Capiz	3,543	0	0.00	23	0.65	315	8.89	338	9.54
Guimaras	1,865	1	0.05	17	0.91	152	8.15	170	9.12
Iloilo	15,558	0	0.00	79	0.51	1,113	7.15	1,192	7.66
Negros Occidental	17,800	2	0.01	260	1.46	1,258	7.07	1,520	8.54
Bacolod City	4,013	0	0.00	61	1.52	403	10.04	464	11.56
Iloilo City	2,772	0	0.00	27	0.97	154	5.56	181	6.53
**Region 7**	**48,759**	**11**	**0.02**	**704**	**1.44**	**5,473**	**11.22**	**6,188**	**12.69**
Bohol	8,559	3	0.04	204	2.38	2,321	27.12	2,528	29.54
Cebu	11,959	3	0.03	133	1.11	1,072	8.96	1,208	10.10
Negros Oriental	8,096	1	0.01	139	1.72	756	9.34	896	11.07
Siquijor	560	0	0.00	13	2.32	62	11.07	75	13.39
Cebu City	10,160	3	0.03	118	1.16	674	6.63	795	7.82
Lapu-Lapu City	5,730	0	0.00	33	0.58	175	3.05	208	3.63
Mandaue City	3,695	1	0.03	64	1.73	413	11.18	478	12.94
**Region 8**	**29,571**	**4**	**0.01**	**301**	**1.02**	**1,899**	**6.42**	**2,204**	**7.45**
Biliran	1,680	0	0.00	45	2.68	262	15.60	307	18.27
Eastern Samar	2,150	0	0.00	16	0.74	156	7.26	172	8.00
Northern Leyte	10,193	1	0.01	72	0.71	548	5.38	621	6.09
Northern Samar	5,281	0	0.00	35	0.66	246	4.66	281	5.32
Southern Leyte	2,656	0	0.00	21	0.79	115	4.33	136	5.12
Western Samar	2,263	3	0.13	63	2.78	278	12.28	344	15.20

Table 1.B.1.15 - Prenatal Care

Number and proportion of pregnant women tested for Complete Blood Count or Hemoglobin and Hematocrit Count diagnosed with anemia by Region

Philippines, 2021

Area	Total No. tested for CBC or Hgb or Hct count	Tested for CBC/Hgb and Hct diagnosed with anemia							
		Age Group						Total	%
		10-14 yrs old		15-19 yrs old		20-49 yrs old			
		No.	%	No.	%	No.	%		
Calbayog City	266	0	0.00	3	1.13	17	6.39	20	7.52
Maasin City	652	0	0.00	12	1.84	104	15.95	116	17.79
Ormoc City	1,548	0	0.00	30	1.94	111	7.17	141	9.11
Tacloban City	2,882	0	0.00	4	0.14	62	2.15	66	2.29
**Region 9**	**10,044**	**6**	**0.06**	**180**	**1.79**	**937**	**9.33**	**1,123**	**11.18**
Zamboanga del Norte	2,629	2	0.08	29	1.10	157	5.97	188	7.15
Zamboanga del Sur	1,859	3	0.16	61	3.28	251	13.50	315	16.94
Zamboanga Sibugay	2,624	0	0.00	32	1.22	236	8.99	268	10.21
Dapitan City	94	0	0.00	7	7.45	37	39.36	44	46.81
Dipolog City	27	1	3.70	0	0.00	0	0.00	1	3.70
Isabela City	198	0	0.00	15	7.58	45	22.73	60	30.30
Pagadian City	1,423	0	0.00	30	2.11	133	9.35	163	11.45
Zamboanga City	1,190	0	0.00	6	0.50	78	6.55	84	7.06
**Region 10**	**38,584**	**27**	**0.07**	**1,407**	**3.65**	**5,793**	**15.01**	**7,227**	**18.73**
Bukidnon	10,302	11	0.11	514	4.99	1,855	18.01	2,380	23.10
Camiguin	451	0	0.00	2	0.44	13	2.88	15	3.33
Lanao del Norte	1,104	2	0.18	24	2.17	148	13.41	174	15.76
Misamis Occidental	1,891	0	0.00	16	0.85	106	5.61	122	6.45
Misamis Oriental	7,203	1	0.01	186	2.58	595	8.26	782	10.86
Cagayan de Oro City	7,335	1	0.01	352	4.80	1,960	26.72	2,313	31.53
El Salvador City	517	0	0.00	10	1.93	47	9.09	57	11.03
Gingoog City	1,568	9	0.57	126	8.04	382	24.36	517	32.97
Iligan City	1,844	0	0.00	28	1.52	99	5.37	127	6.89
Malaybalay City	1,774	0	0.00	24	1.35	128	7.22	152	8.57
Oroquieta City	156	0	0.00	0	0.00	2	1.28	2	1.28
Ozamis City	1,844	1	0.05	9	0.49	63	3.42	73	3.96
Tangub City	11	0	0.00	0	0.00	0	0.00	0	0.00
Valencia City	2,584	2	0.08	116	4.49	395	15.29	513	19.85
**Region 11**	**61,925**	**178**	**0.29**	**3,413**	**5.51**	**15,629**	**25.24**	**19,220**	**31.04**
Davao de Oro	10,876	42	0.39	809	7.44	3,155	29.01	4,006	36.83
Davao del Norte	15,299	35	0.23	745	4.87	3,255	21.28	4,035	26.37
Davao Oriental	6,322	11	0.17	358	5.66	1,555	24.60	1,924	30.43
Davao del Sur	6,631	20	0.30	405	6.11	1,925	29.03	2,350	35.44
Davao Occidental	1,167	9	0.77	117	10.03	320	27.42	446	38.22
Davao City	21,630	61	0.28	979	4.53	5,419	25.05	6,459	29.86
**Region 12**	**42,852**	**12**	**0.03**	**1,004**	**2.34**	**4,173**	**9.74**	**5,189**	**12.11**
North Cotabato	12,363	1	0.01	349	2.82	1,619	13.10	1,969	15.93
Sarangani	7,892	7	0.09	307	3.89	976	12.37	1,290	16.35
South Cotabato	10,467	1	0.01	139	1.33	653	6.24	793	7.58
Sultan Kudarat	7,193	0	0.00	76	1.06	303	4.21	379	5.27
Cotabato City	862	0	0.00	9	1.04	51	5.92	60	6.96
Gen. Santos City	4,075	3	0.07	124	3.04	571	14.01	698	17.13

Table 1.B.1.15 - Prenatal Care

Number and proportion of pregnant women tested for Complete Blood Count or Hemoglobin and Hematocrit Count diagnosed with anemia by Region

Philippines, 2021

Area	Total No. tested for CBC or Hgb or Hct count	Tested for CBC/Hgb and Hct diagnosed with anemia							
		Age Group						Total	%
		10-14 yrs old		15-19 yrs old		20-49 yrs old			
		No.	%	No.	%	No.	%		
**BARMM**	**4,077**	**0**	**0.00**	**42**	**1.03**	**140**	**3.43**	**182**	**4.46**
Basilan	24	0	0.00	4	16.67	3	12.50	7	29.17
Lanao del Sur	2,197	0	0.00	10	0.46	24	1.09	34	1.55
Maguindanao	983	0	0.00	27	2.75	111	11.29	138	14.04
Sulu	0	0	0.00	0	0.00	0	0.00	0	0.00
Tawi-Tawi	2	0	0.00	0	0.00	0	0.00	0	0.00
Lamitan City	209	0	0.00	1	0.48	2	0.96	3	1.44
Marawi City	662	0	0.00	0	0.00	0	0.00	0	0.00
**CARAGA**	**28,879**	**18**	**0.06**	**896**	**3.10**	**4,434**	**15.35**	**5,348**	**18.52**
Agusan del Norte	3,773	2	0.05	83	2.20	400	10.60	485	12.85
Agusan del Sur	8,246	7	0.08	335	4.06	1,366	16.57	1,708	20.71
Surigao del Norte	3,000	1	0.03	63	2.10	307	10.23	371	12.37
Surigao del Sur	5,785	2	0.03	155	2.68	1,047	18.10	1,204	20.81
Province of Dinagat	879	2	0.23	15	1.71	131	14.90	148	16.84
Bislig City	1,192	1	0.08	54	4.53	314	26.34	369	30.96
Butuan City	4,008	3	0.07	124	3.09	577	14.40	704	17.56
Surigao City	1,996	0	0.00	67	3.36	292	14.63	359	17.99

Note: Put asterisk (*) for No Report and Zero (0) for No Case

Tabe 1.B.1.16 - Prenatal Care

Number and proportion of pregnant women screened for Gestational Diabetes by Region
Philippines, 2021

Area	Eligible Pop. (Under 1)	Screened for Gestational Diabetes							
		Age Group						Total	%
		10-14 yrs old		15-19 yrs old		20-49 yrs old			
		No.	%	No.	%	No.	%		
PHILIPPINES	2,128,168	448	0.021	22,453	1.06	197,218	9.27	220,119	10.34
N C R	237,921	77	0.032	4,122	1.73	49,000	20.60	53,199	22.36
Malabon	6,757	0	0.000	60	0.89	494	7.31	554	8.20
Navotas	4,607	1	0.022	183	3.97	869	18.86	1,053	22.86
Valenzuela City	11,465	1	0.009	70	0.61	504	4.40	575	5.02
Caloocan City	29,270	1	0.003	276	0.94	2,712	9.27	2,989	10.21
Marikina City	8,330	2	0.024	86	1.03	811	9.74	899	10.79
Pasig City	13,954	4	0.029	417	2.99	5,680	40.71	6,101	43.72
Pateros	1,181	2	0.169	34	2.88	287	24.30	323	27.35
Taguig	14,874	3	0.020	159	1.07	1,547	10.40	1,709	11.49
Quezon City	54,246	26	0.048	1,001	1.85	21,733	40.06	22,760	41.96
Makati City	10,765	0	0.000	63	0.59	1,200	11.15	1,263	11.73
Mandaluyong City	7,137	2	0.028	76	1.06	876	12.27	954	13.37
San Juan	2,254	1	0.044	22	0.98	564	25.02	587	26.04
Manila City	32,887	17	0.052	965	2.93	6,009	18.27	6,991	21.26
Las Piñas City	10,879	0	0.000	70	0.64	657	6.04	727	6.68
Muntinlupa City	9,317	1	0.011	71	0.76	484	5.19	556	5.97
Parañaque City	12,298	12	0.098	346	2.81	2,450	19.92	2,808	22.83
Pasay City	7,700	4	0.052	223	2.90	2,123	27.57	2,350	30.52
C A R	35,143	11	0.031	527	1.50	5,033	14.32	5,571	15.85
Abra	4,287	0	0.000	21	0.49	163	3.80	184	4.29
Apayao	2,476	1	0.040	87	3.51	440	17.77	528	21.32
Benguet	9,140	6	0.066	142	1.55	1,387	15.18	1,535	16.79
Ifugao	4,439	0	0.000	15	0.34	269	6.06	284	6.40
Kalinga	4,660	0	0.000	18	0.39	100	2.15	118	2.53
Mt. Province	3,066	0	0.000	26	0.85	295	9.62	321	10.47
Baguio City	7,075	4	0.057	218	3.08	2,379	33.63	2,601	36.76
Region 1	97,198	12	0.012	1,234	1.27	14,507	14.93	15,753	16.21
Ilocos Norte	7,986	1	0.013	86	1.08	1,305	16.34	1,392	17.43
Ilocos Sur	9,197	4	0.043	276	3.00	2,840	30.88	3,120	33.92
La Union	11,429	1	0.009	142	1.24	1,210	10.59	1,353	11.84
Pangasinan	50,454	4	0.008	589	1.17	6,037	11.97	6,630	13.14
Alaminos City	1,905	0	0.000	48	2.52	671	35.22	719	37.74
Candon City	976	0	0.000	1	0.10	438	44.88	439	44.98
Dagupan City	3,636	0	0.000	4	0.11	14	0.39	18	0.50
Laoag City	1,845	0	0.000	10	0.54	972	52.68	982	53.22
San Carlos City	3,999	1	0.025	14	0.35	165	4.13	180	4.50
San Fernando City	2,092	0	0.000	38	1.82	543	25.96	581	27.77
Urdaneta City	2,819	1	0.035	26	0.92	312	11.07	339	12.03
Vigan City	860	0	0.000	0	0.00	0	0.00	0	0.00
Region 2	69,228	17	0.025	643	0.93	4,638	6.70	5,298	7.65
Batanes	351	0	0.000	1	0.28	65	18.52	66	18.80
Cagayan	20,563	3	0.015	97	0.47	470	2.29	570	2.77
Isabela	23,543	11	0.047	268	1.14	2,047	8.69	2,326	9.88
Nueva Vizcaya	9,555	1	0.010	133	1.39	889	9.30	1,023	10.71

Tabe 1.B.1.16 - Prenatal Care

Number and proportion of pregnant women screened for Gestational Diabetes by Region

Philippines, 2021

Area	Eligible Pop. (Under 1)	Screened for Gestational Diabetes							
		Age Group						Total	%
		10-14 yrs old		15-19 yrs old		20-49 yrs old			
		No.	%	No.	%	No.	%		
Quirino	4,038	2	0.050	37	0.92	346	8.57	385	9.53
Cauayan City	2,576	0	0.000	55	2.14	339	13.16	394	15.30
Ilagan City	2,901	0	0.000	28	0.97	253	8.72	281	9.69
Santiago City	2,683	0	0.000	24	0.89	226	8.42	250	9.32
Tuguegarao City	3,018	0	0.000	0	0.00	3	0.10	3	0.10
**Region 3**	**220,202**	**91**	**0.041**	**2,929**	**1.33**	**21,684**	**9.85**	**24,704**	**11.22**
Aurora	4,764	1	0.021	38	0.80	189	3.97	228	4.79
Bataan	13,814	15	0.109	249	1.80	1,669	12.08	1,933	13.99
Bulacan	43,722	11	0.025	647	1.48	4,243	9.70	4,901	11.21
Nueva Ecija	28,915	11	0.038	303	1.05	1,720	5.95	2,034	7.03
Pampanga	31,503	5	0.016	155	0.49	1,156	3.67	1,316	4.18
Tarlac	19,565	6	0.031	282	1.44	1,779	9.09	2,067	10.56
Zambales	12,551	0	0.000	48	0.38	357	2.84	405	3.23
Angeles City	7,905	0	0.000	8	0.10	193	2.44	201	2.54
Balanga City	1,996	1	0.050	8	0.40	148	7.41	157	7.87
Cabanatuan City	5,921	0	0.000	51	0.86	317	5.35	368	6.22
City of San Fernando	5,890	10	0.170	211	3.58	1,651	28.03	1,872	31.78
Gapan City	2,159	0	0.000	7	0.32	64	2.96	71	3.29
Mabalacat City	4,811	0	0.000	4	0.08	175	3.64	179	3.72
Malolos City	4,885	0	0.000	0	0.00	21	0.43	21	0.43
Meycauayan	4,050	0	0.000	43	1.06	316	7.80	359	8.86
Olongapo	4,952	2	0.040	70	1.41	532	10.74	604	12.20
Palayan City	805	0	0.000	0	0.00	0	0.00	0	0.00
San Jose City	2,736	0	0.000	0	0.00	0	0.00	0	0.00
San Jose del Monte City	11,120	26	0.234	604	5.43	6,322	56.85	6,952	62.52
Science City of Munoz	1,594	0	0.000	0	0.00	0	0.00	0	0.00
Tarlac City	6,544	3	0.046	201	3.07	832	12.71	1,036	15.83
**Region 4A**	**298,398**	**42**	**0.014**	**2,355**	**0.79**	**21,917**	**7.34**	**24,314**	**8.15**
Batangas	38,848	1	0.003	151	0.39	2,904	7.48	3,056	7.87
Cavite	27,948	3	0.011	151	0.54	1,600	5.72	1,754	6.28
Laguna	19,727	1	0.005	177	0.90	1,331	6.75	1,509	7.65
Quezon	37,644	4	0.011	138	0.37	1,126	2.99	1,268	3.37
Rizal	44,826	13	0.029	772	1.72	4,767	10.63	5,552	12.39
Antipolo City	16,510	0	0.000	32	0.19	145	0.88	177	1.07
Bacoor City	12,252	0	0.000	26	0.21	325	2.65	351	2.86
Batangas City	6,895	0	0.000	50	0.73	361	5.24	411	5.96
Biñan City	6,607	0	0.000	325	4.92	2,576	38.99	2,901	43.91
Cabuyao City	6,128	0	0.000	78	1.27	1,660	27.09	1,738	28.36
Calamba City	9,025	6	0.066	66	0.73	994	11.01	1,066	11.81
Cavite City	2,098	5	0.238	1	0.05	2	0.10	8	0.38
Dasmariñas City	13,442	2	0.015	61	0.45	759	5.65	822	6.12
General Trias City	6,410	0	0.000	23	0.36	369	5.76	392	6.12
Imus City	8,235	0	0.000	15	0.18	158	1.92	173	2.10
Lipa City	6,954	2	0.029	4	0.06	65	0.93	71	1.02
Lucena City	5,704	0	0.000	19	0.33	176	3.09	195	3.42
San Pablo City	5,277	0	0.000	17	0.32	143	2.71	160	3.03
San Pedro City	6,465	0	0.000	36	0.56	216	3.34	252	3.90
Santa Rosa City	7,023	5	0.071	180	2.56	1,506	21.44	1,691	24.08
Tagaytay City	1,451	0	0.000	28	1.93	551	37.97	579	39.90
Tanauan City	3,620	0	0.000	3	0.08	160	4.42	163	4.50
Tayabas City	2,134	0	0.000	0	0.00	1	0.05	1	0.05
Trece Martires City	3,175	0	0.000	2	0.06	22	0.69	24	0.76

Tabe 1.B.1.16 - Prenatal Care

Number and proportion of pregnant women screened for Gestational Diabetes by Region
Philippines, 2021

Area	Eligible Pop. (Under 1)	Screened for Gestational Diabetes							
		Age Group						Total	%
		10-14 yrs old		15-19 yrs old		20-49 yrs old			
		No.	%	No.	%	No.	%		
**Region 4B**	**72,035**	**1**	**0.001**	**323**	**0.45**	**2,484**	**3.45**	**2,808**	**3.90**
Marinduque	4,906	0	0.000	29	0.59	404	8.23	433	8.83
Mindoro Occidental	12,449	0	0.000	30	0.24	239	1.92	269	2.16
Mindoro Oriental	20,011	0	0.000	43	0.21	366	1.83	409	2.04
Palawan	21,676	1	0.005	126	0.58	840	3.88	967	4.46
Romblon	6,482	0	0.000	95	1.47	635	9.80	730	11.26
Puerto Princesa City	6,511	0	0.000	0	0.00	0	0.00	0	0.00
**Region 5**	**137,350**	**4**	**0.003**	**671**	**0.49**	**7,270**	**5.29**	**7,945**	**5.78**
Albay	23,903	0	0.000	143	0.60	2,028	8.48	2,171	9.08
Camarines Norte	14,340	0	0.000	49	0.34	282	1.97	331	2.31
Camarines Sur	39,151	2	0.005	154	0.39	1,642	4.19	1,798	4.59
Catanduanes	6,381	0	0.000	2	0.03	81	1.27	83	1.30
Masbate	22,565	1	0.004	150	0.66	1,128	5.00	1,279	5.67
Sorsogon	19,470	1	0.005	110	0.56	1,187	6.10	1,298	6.67
Iriga City	2,665	0	0.000	11	0.41	35	1.31	46	1.73
Legaspi City	4,205	0	0.000	50	1.19	792	18.83	842	20.02
Naga City	4,670	0	0.000	2	0.04	95	2.03	97	2.08
**Region 6**	**146,545**	**37**	**0.025**	**3,098**	**2.11**	**25,763**	**17.58**	**28,898**	**19.72**
Aklan	11,231	4	0.036	201	1.79	2,298	20.46	2,503	22.29
Antique	12,985	2	0.015	86	0.66	814	6.27	902	6.95
Capiz	13,984	2	0.014	149	1.07	1,549	11.08	1,700	12.16
Guimaras	3,085	3	0.097	111	3.60	1,263	40.94	1,377	44.64
Iloilo	36,414	17	0.047	1,013	2.78	9,546	26.22	10,576	29.04
Negros Occidental	49,314	4	0.008	1,041	2.11	6,517	13.22	7,562	15.33
Bacolod City	11,102	2	0.018	352	3.17	2,487	22.40	2,841	25.59
Iloilo City	8,430	3	0.036	145	1.72	1,289	15.29	1,437	17.05
**Region 7**	**162,860**	**17**	**0.010**	**1,271**	**0.78**	**10,603**	**6.51**	**11,891**	**7.30**
Bohol	27,637	0	0.000	102	0.37	1,223	4.43	1,325	4.79
Cebu	67,046	9	0.013	448	0.67	2,855	4.26	3,312	4.94
Negros Oriental	27,922	1	0.004	81	0.29	489	1.75	571	2.04
Siquijor	1,636	0	0.000	6	0.37	55	3.36	61	3.73
Cebu City	21,037	7	0.033	482	2.29	3,950	18.78	4,439	21.10
Lapu-Lapu City	9,310	0	0.000	85	0.91	1,465	15.74	1,550	16.65
Mandaue City	8,272	0	0.000	67	0.81	566	6.84	633	7.65
**Region 8**	**104,016**	**5**	**0.005**	**529**	**0.51**	**4,435**	**4.26**	**4,969**	**4.78**
Biliran	3,856	0	0.000	27	0.70	187	4.85	214	5.55
Eastern Samar	11,635	0	0.000	52	0.45	513	4.41	565	4.86
Northern Leyte	35,052	1	0.003	77	0.22	588	1.68	666	1.90
Northern Samar	15,655	0	0.000	161	1.03	1,164	7.44	1,325	8.46
Southern Leyte	6,556	1	0.015	80	1.22	622	9.49	703	10.72
Western Samar	14,515	1	0.007	54	0.37	287	1.98	342	2.36
Calbayog City	4,474	0	0.000	3	0.07	56	1.25	59	1.32
Maasin City	1,661	0	0.000	1	0.06	7	0.42	8	0.48
Ormoc City	4,989	0	0.000	28	0.56	220	4.41	248	4.97

Tabe 1.B.1.16 - Prenatal Care

Number and proportion of pregnant women screened for Gestational Diabetes by Region
Philippines, 2021

Area	Eligible Pop. (Under 1)	Screened for Gestational Diabetes							
		Age Group						Total	%
		10-14 yrs old		15-19 yrs old		20-49 yrs old			
		No.	%	No.	%	No.	%		
Tacloban City	5,623	2	0.036	46	0.82	791	14.07	839	14.92
**Region 9**	**79,552**	**6**	**0.008**	**219**	**0.28**	**1,435**	**1.80**	**1,660**	**2.09**
Zamboanga del Norte	17,075	4	0.023	116	0.68	707	4.14	827	4.84
Zamboanga del Sur	17,506	2	0.011	42	0.24	224	1.28	268	1.53
Zamboanga Sibugay	14,992	0	0.000	45	0.30	375	2.50	420	2.80
Dapitan City	1,755	0	0.000	1	0.06	6	0.34	7	0.40
Dipolog City	2,794	0	0.000	0	0.00	6	0.21	6	0.21
Isabela City	2,544	0	0.000	4	0.16	51	2.00	55	2.16
Pagadian City	4,290	0	0.000	0	0.00	0	0.00	0	0.00
Zamboanga City	18,596	0	0.000	11	0.06	66	0.35	77	0.41
**Region 10**	**100,690**	**16**	**0.016**	**1,279**	**1.27**	**7,389**	**7.34**	**8,684**	**8.62**
Bukidnon	23,307	4	0.017	258	1.11	878	3.77	1,140	4.89
Camiguin	1,854	1	0.054	20	1.08	253	13.65	274	14.78
Lanao del Norte	14,955	0	0.000	40	0.27	338	2.26	378	2.53
Misamis Occidental	6,413	0	0.000	16	0.25	172	2.68	188	2.93
Misamis Oriental	15,058	5	0.033	293	1.95	1,500	9.96	1,798	11.94
Cagayan de Oro City	14,263	2	0.014	418	2.93	2,649	18.57	3,069	21.52
El Salvador City	1,061	1	0.094	4	0.38	20	1.89	25	2.36
Gingoog City	2,627	2	0.076	59	2.25	267	10.16	328	12.49
Iligan City	7,575	1	0.013	27	0.36	261	3.45	289	3.82
Malaybalay City	3,882	0	0.000	32	0.82	183	4.71	215	5.54
Oroquieta City	1,389	0	0.000	0	0.00	1	0.07	1	0.07
Ozamis City	2,782	0	0.000	111	3.99	842	30.27	953	34.26
Tangub City	1,234	0	0.000	0	0.00	4	0.32	4	0.32
Valencia City	4,290	0	0.000	1	0.02	21	0.49	22	0.51
**Region 11**	**107,847**	**72**	**0.067**	**1,027**	**0.95**	**8,216**	**7.62**	**9,315**	**8.64**
Davao de Oro	15,526	0	0.000	26	0.17	208	1.34	234	1.51
Davao del Norte	21,184	4	0.019	67	0.32	1,050	4.96	1,121	5.29
Davao Oriental	13,125	1	0.008	37	0.28	186	1.42	224	1.71
Davao del Sur	14,355	4	0.028	53	0.37	263	1.83	320	2.23
Davao Occidental	6,598	6	0.091	42	0.64	106	1.61	154	2.33
Davao City	37,059	57	0.154	802	2.16	6,403	17.28	7,262	19.60
**Region 12**	**104,693**	**28**	**0.027**	**1,481**	**1.41**	**7,593**	**7.25**	**9,102**	**8.69**
North Cotabato	33,933	5	0.015	363	1.07	1,830	5.39	2,198	6.48
Sarangani	12,904	15	0.116	473	3.67	2,207	17.10	2,695	20.88
South Cotabato	21,039	1	0.005	209	0.99	1,102	5.24	1,312	6.24
Sultan Kudarat	17,331	6	0.035	259	1.49	1,421	8.20	1,686	9.73
Cotabato City	5,831	0	0.000	24	0.41	224	3.84	248	4.25
Gen. Santos City	13,655	1	0.007	153	1.12	809	5.92	963	7.05
**BARMM**	**94,148**	**3**	**0.003**	**297**	**0.32**	**1,911**	**2.03**	**2,211**	**2.35**
Basilan	7,672	0	0.000	0	0.00	0	0.00	0	0.00
Lanao del Sur	21,391	0	0.000	28	0.13	874	4.09	902	4.22
Maguindanao	31,650	3	0.009	56	0.18	373	1.18	432	1.36
Sulu	16,891	0	0.000	0	0.00	0	0.00	0	0.00
Tawi-Tawi	9,319	0	0.000	0	0.00	0	0.00	0	0.00

Tabe 1.B.1.16 - Prenatal Care

Number and proportion of pregnant women screened for Gestational Diabetes by Region
Philippines, 2021

Area	Eligible Pop. (Under 1)	Screened for Gestational Diabetes							
		Age Group						Total	%
		10-14 yrs old		15-19 yrs old		20-49 yrs old			
		No.	%	No.	%	No.	%		
Lamitan City	2,112	0	0.000	0	0.00	4	0.19	4	0.19
Marawi City	5,113	0	0.000	213	4.17	660	12.91	873	17.07
**CARAGA**	**60,342**	**9**	**0.015**	**448**	**0.74**	**3,340**	**5.54**	**3,797**	**6.29**
Agusan del Norte	8,116	0	0.000	44	0.54	363	4.47	407	5.01
Agusan del Sur	17,658	3	0.017	159	0.90	885	5.01	1,047	5.93
Surigao del Norte	7,195	1	0.014	47	0.65	300	4.17	348	4.84
Surigao del Sur	11,532	1	0.009	51	0.44	479	4.15	531	4.60
Province of Dinagat	2,574	0	0.000	9	0.35	158	6.14	167	6.49
Bislig City	2,185	0	0.000	4	0.18	30	1.37	34	1.56
Butuan City	7,732	0	0.000	68	0.88	604	7.81	672	8.69
Surigao City	3,350	4	0.119	66	1.97	521	15.55	591	17.64

Note: Put asterisk (*) for No Report and Zero (0) for No Case

Table 1.B.1.17 - Prenatal Care

Number and proportion of pregnant women tested positive for Gestational Diabetes by Region
Philippines, 2021

Area	Total No. of Pregnant Women Screened for Gestational Diabetes	Tested positive for Gestational Diabetes							
		Age Group						Total	%
		10-14 yrs old		15-19 yrs old		20-49 yrs old			
		No.	%	No.	%	No.	%		
**PHILIPPINES**	**220,119**	**33**	**0.015**	**437**	**0.20**	**5,271**	**2.39**	**5,741**	**2.61**
**N C R**	**53,199**	**1**	**0.002**	**120**	**0.23**	**1,380**	**2.59**	**1,501**	**2.82**
Malabon	554	0	0.000	0	0.00	38	6.86	38	6.86
Navotas	1,053	0	0.000	27	2.56	68	6.46	95	9.02
Valenzuela City	575	0	0.000	2	0.35	44	7.65	46	8.00
Caloocan City	2,989	0	0.000	3	0.10	52	1.74	55	1.84
Marikina City	899	0	0.000	0	0.00	16	1.78	16	1.78
Pasig City	6,101	0	0.000	38	0.62	356	5.84	394	6.46
Pateros	323	0	0.000	0	0.00	1	0.31	1	0.31
Taguig	1,709	1	0.059	9	0.53	106	6.20	116	6.79
Quezon City	22,760	0	0.000	15	0.07	349	1.53	364	1.60
Makati City	1,263	0	0.000	0	0.00	129	10.21	129	10.21
Mandaluyong City	954	0	0.000	1	0.10	9	0.94	10	1.05
San Juan	587	0	0.000	0	0.00	0	0.00	0	0.00
Manila City	6,991	0	0.000	22	0.31	139	1.99	161	2.30
Las Piñas City	727	0	0.000	0	0.00	34	4.68	34	4.68
Muntinlupa City	556	0	0.000	0	0.00	8	1.44	8	1.44
Parañaque City	2,808	0	0.000	2	0.07	9	0.32	11	0.39
Pasay City	2,350	0	0.000	1	0.04	22	0.94	23	0.98
**C A R**	**5,571**	**0**	**0.000**	**3**	**0.05**	**35**	**0.63**	**38**	**0.68**
Abra	184	0	0.000	0	0.00	0	0.00	0	0.00
Apayao	528	0	0.000	0	0.00	6	1.14	6	1.14
Benguet	1,535	0	0.000	0	0.00	11	0.72	11	0.72
Ifugao	284	0	0.000	0	0.00	1	0.35	1	0.35
Kalinga	118	0	0.000	3	2.54	7	5.93	10	8.47
Mt. Province	321	0	0.000	0	0.00	5	1.56	5	1.56
Baguio City	2,601	0	0.000	0	0.00	5	0.19	5	0.19
**Region 1**	**15,753**	**0**	**0.000**	**3**	**0.02**	**103**	**0.65**	**106**	**0.67**
Ilocos Norte	1,392	0	0.000	1	0.07	8	0.57	9	0.65
Ilocos Sur	3,120	0	0.000	0	0.00	13	0.42	13	0.42
La Union	1,353	0	0.000	0	0.00	11	0.81	11	0.81
Pangasinan	6,630	0	0.000	2	0.03	62	0.94	64	0.97
Alaminos City	719	0	0.000	0	0.00	2	0.28	2	0.28
Candon City	439	0	0.000	0	0.00	5	1.14	5	1.14
Dagupan City	18	0	0.000	0	0.00	0	0.00	0	0.00
Laoag City	982	0	0.000	0	0.00	0	0.00	0	0.00
San Carlos City	180	0	0.000	0	0.00	0	0.00	0	0.00
San Fernando City	581	0	0.000	0	0.00	2	0.34	2	0.34
Urdaneta City	339	0	0.000	0	0.00	0	0.00	0	0.00
Vigan City	0	0	0.000	0	0.00	0	0.00	0	0.00
**Region 2**	**5,298**	**0**	**0.000**	**8**	**0.15**	**103**	**1.94**	**111**	**2.10**
Batanes	66	0	0.000	0	0.00	2	3.03	2	3.03
Cagayan	570	0	0.000	2	0.35	18	3.16	20	3.51
Isabela	2,326	0	0.000	4	0.17	38	1.63	42	1.81
Nueva Vizcaya	1,023	0	0.000	2	0.20	37	3.62	39	3.81

Table 1.B.1.17 - Prenatal Care

Number and proportion of pregnant women tested positive for Gestational Diabetes by Region
Philippines, 2021

Area	Total No. of Pregnant Women Screened for Gestational Diabetes	Tested positive for Gestational Diabetes							
		Age Group						Total	%
		10-14 yrs old		15-19 yrs old		20-49 yrs old			
		No.	%	No.	%	No.	%		
Quirino	385	0	0.000	0	0.00	2	0.52	2	0.52
Cauayan City	394	0	0.000	0	0.00	0	0.00	0	0.00
Ilagan City	281	0	0.000	0	0.00	5	1.78	5	1.78
Santiago City	250	0	0.000	0	0.00	1	0.40	1	0.40
Tuguegarao City	3	0	0.000	0	0.00	0	0.00	0	0.00
**Region 3**	**24,704**	**4**	**0.016**	**18**	**0.07**	**409**	**1.66**	**431**	**1.74**
Aurora	228	0	0.000	0	0.00	2	0.88	2	0.88
Bataan	1,933	0	0.000	2	0.10	55	2.85	57	2.95
Bulacan	4,901	0	0.000	0	0.00	60	1.22	60	1.22
Nueva Ecija	2,034	0	0.000	3	0.15	23	1.13	26	1.28
Pampanga	1,316	0	0.000	0	0.00	26	1.98	26	1.98
Tarlac	2,067	0	0.000	2	0.10	52	2.52	54	2.61
Zambales	405	0	0.000	0	0.00	0	0.00	0	0.00
Angeles City	201	0	0.000	0	0.00	1	0.50	1	0.50
Balanga City	157	0	0.000	0	0.00	45	28.66	45	28.66
Cabanatuan City	368	0	0.000	0	0.00	0	0.00	0	0.00
City of San Fernando	1,872	3	0.160	6	0.32	95	5.07	104	5.56
Gapan City	71	1	1.408	1	1.41	2	2.82	4	5.63
Mabalacat City	179	0	0.000	0	0.00	1	0.56	1	0.56
Malolos City	21	0	0.000	0	0.00	1	4.76	1	4.76
Meycauayan	359	0	0.000	0	0.00	7	1.95	7	1.95
Olongapo	604	0	0.000	1	0.17	27	4.47	28	4.64
Palayan City	0	0	0.000	0	0.00	0	0.00	0	0.00
San Jose City	0	0	0.000	0	0.00	0	0.00	0	0.00
San Jose del Monte City	6,952	0	0.000	0	0.00	5	0.07	5	0.07
Science City of Munoz	0	0	0.000	0	0.00	0	0.00	0	0.00
Tarlac City	1,036	0	0.000	3	0.29	7	0.68	10	0.97
**Region 4A**	**24,314**	**15**	**0.062**	**39**	**0.16**	**628**	**2.58**	**682**	**2.80**
Batangas	3,056	0	0.000	2	0.07	27	0.88	29	0.95
Cavite	1,754	1	0.057	6	0.34	85	4.85	92	5.25
Laguna	1,509	12	0.795	8	0.53	60	3.98	80	5.30
Quezon	1,268	0	0.000	1	0.08	103	8.12	104	8.20
Rizal	5,552	0	0.000	15	0.27	114	2.05	129	2.32
Antipolo City	177	0	0.000	0	0.00	3	1.69	3	1.69
Bacoor City	351	0	0.000	0	0.00	11	3.13	11	3.13
Batangas City	411	2	0.487	0	0.00	28	6.81	30	7.30
Biñan City	2,901	0	0.000	0	0.00	2	0.07	2	0.07
Cabuyao City	1,738	0	0.000	0	0.00	18	1.04	18	1.04
Calamba City	1,066	0	0.000	0	0.00	52	4.88	52	4.88
Cavite City	8	0	0.000	0	0.00	0	0.00	0	0.00
Dasmariñas City	822	0	0.000	4	0.49	13	1.58	17	2.07
General Trias City	392	0	0.000	0	0.00	14	3.57	14	3.57
Imus City	173	0	0.000	0	0.00	9	5.20	9	5.20
Lipa City	71	0	0.000	0	0.00	3	4.23	3	4.23
Lucena City	195	0	0.000	1	0.51	10	5.13	11	5.64
San Pablo City	160	0	0.000	0	0.00	3	1.88	3	1.88
San Pedro City	252	0	0.000	0	0.00	11	4.37	11	4.37
Santa Rosa City	1,691	0	0.000	2	0.12	52	3.08	54	3.19
Tagaytay City	579	0	0.000	0	0.00	1	0.17	1	0.17
Tanauan City	163	0	0.000	0	0.00	9	5.52	9	5.52
Tayabas City	1	0	0.000	0	0.00	0	0.00	0	0.00
Trece Martires City	24	0	0.000	0	0.00	0	0.00	0	0.00

Table 1.B.1.17 - Prenatal Care

Number and proportion of pregnant women tested positive for Gestational Diabetes by Region
Philippines, 2021

Area	Total No. of Pregnant Women Screened for Gestational Diabetes	Tested positive for Gestational Diabetes							
		Age Group						Total	%
		10-14 yrs old		15-19 yrs old		20-49 yrs old			
		No.	%	No.	%	No.	%		
**Region 4B**	**2,808**	**0**	**0.000**	**22**	**0.78**	**78**	**2.78**	**100**	**3.56**
Marinduque	433	0	0.000	1	0.23	5	1.15	6	1.39
Mindoro Occidental	269	0	0.000	0	0.00	4	1.49	4	1.49
Mindoro Oriental	409	0	0.000	11	2.69	11	2.69	22	5.38
Palawan	967	0	0.000	9	0.93	40	4.14	49	5.07
Romblon	730	0	0.000	1	0.14	18	2.47	19	2.60
Puerto Princesa City	0	0	0.000	0	0.00	0	0.00	0	0.00
**Region 5**	**7,945**	**0**	**0.000**	**11**	**0.14**	**319**	**4.02**	**330**	**4.15**
Albay	2,171	0	0.000	4	0.18	122	5.62	126	5.80
Camarines Norte	331	0	0.000	2	0.60	24	7.25	26	7.85
Camarines Sur	1,798	0	0.000	0	0.00	43	2.39	43	2.39
Catanduanes	83	0	0.000	0	0.00	2	2.41	2	2.41
Masbate	1,279	0	0.000	1	0.08	27	2.11	28	2.19
Sorsogon	1,298	0	0.000	3	0.23	76	5.86	79	6.09
Iriga City	46	0	0.000	0	0.00	0	0.00	0	0.00
Legaspi City	842	0	0.000	1	0.12	25	2.97	26	3.09
Naga City	97	0	0.000	0	0.00	0	0.00	0	0.00
**Region 6**	**28,898**	**0**	**0.000**	**39**	**0.13**	**458**	**1.58**	**497**	**1.72**
Aklan	2,503	0	0.000	2	0.08	32	1.28	34	1.36
Antique	902	0	0.000	1	0.11	38	4.21	39	4.32
Capiz	1,700	0	0.000	2	0.12	29	1.71	31	1.82
Guimaras	1,377	0	0.000	0	0.00	9	0.65	9	0.65
Iloilo	10,576	0	0.000	7	0.07	104	0.98	111	1.05
Negros Occidental	7,562	0	0.000	19	0.25	119	1.57	138	1.82
Bacolod City	2,841	0	0.000	4	0.14	69	2.43	73	2.57
Iloilo City	1,437	0	0.000	4	0.28	58	4.04	62	4.31
**Region 7**	**11,891**	**0**	**0.000**	**62**	**0.52**	**648**	**5.45**	**710**	**5.97**
Bohol	1,325	0	0.000	18	1.36	279	21.06	297	22.42
Cebu	3,312	0	0.000	8	0.24	68	2.05	76	2.29
Negros Oriental	571	0	0.000	4	0.70	22	3.85	26	4.55
Siquijor	61	0	0.000	0	0.00	4	6.56	4	6.56
Cebu City	4,439	0	0.000	2	0.05	24	0.54	26	0.59
Lapu-Lapu City	1,550	0	0.000	0	0.00	12	0.77	12	0.77
Mandaue City	633	0	0.000	30	4.74	239	37.76	269	42.50
**Region 8**	**4,969**	**0**	**0.000**	**23**	**0.46**	**234**	**4.71**	**257**	**5.17**
Biliran	214	0	0.000	2	0.93	18	8.41	20	9.35
Eastern Samar	565	0	0.000	1	0.18	8	1.42	9	1.59
Northern Leyte	666	0	0.000	3	0.45	39	5.86	42	6.31
Northern Samar	1,325	0	0.000	2	0.15	40	3.02	42	3.17
Southern Leyte	703	0	0.000	2	0.28	13	1.85	15	2.13
Western Samar	342	0	0.000	12	3.51	85	24.85	97	28.36
Calbayog City	59	0	0.000	0	0.00	5	8.47	5	8.47
Maasin City	8	0	0.000	1	12.50	2	25.00	3	37.50
Ormoc City	248	0	0.000	0	0.00	11	4.44	11	4.44

Table 1.B.1.17 - Prenatal Care

Number and proportion of pregnant women tested positive for Gestational Diabetes by Region
Philippines, 2021

Area	Total No. of Pregnant Women Screened for Gestational Diabetes	Tested positive for Gestational Diabetes							
		Age Group						Total	%
		10-14 yrs old		15-19 yrs old		20-49 yrs old			
		No.	%	No.	%	No.	%		
Tacloban City	839	0	0.000	0	0.00	13	1.55	13	1.55
**Region 9**	**1,660**	**0**	**0.000**	**8**	**0.48**	**36**	**2.17**	**44**	**2.65**
Zamboanga del Norte	827	0	0.000	0	0.00	6	0.73	6	0.73
Zamboanga del Sur	268	0	0.000	1	0.37	14	5.22	15	5.60
Zamboanga Sibugay	420	0	0.000	0	0.00	3	0.71	3	0.71
Dapitan City	7	0	0.000	0	0.00	0	0.00	0	0.00
Dipolog City	6	0	0.000	0	0.00	0	0.00	0	0.00
Isabela City	55	0	0.000	0	0.00	2	3.64	2	3.64
Pagadian City	0	0	0.000	0	0.00	0	0.00	0	0.00
Zamboanga City	77	0	0.000	7	9.09	11	14.29	18	23.38
**Region 10**	**8,684**	**0**	**0.000**	**23**	**0.26**	**282**	**3.25**	**305**	**3.51**
Bukidnon	1,140	0	0.000	9	0.79	49	4.30	58	5.09
Camiguin	274	0	0.000	0	0.00	0	0.00	0	0.00
Lanao del Norte	378	0	0.000	0	0.00	42	11.11	42	11.11
Misamis Occidental	188	0	0.000	1	0.53	24	12.77	25	13.30
Misamis Oriental	1,798	0	0.000	4	0.22	41	2.28	45	2.50
Cagayan de Oro City	3,069	0	0.000	8	0.26	87	2.83	95	3.10
El Salvador City	25	0	0.000	0	0.00	5	20.00	5	20.00
Gingoog City	328	0	0.000	0	0.00	3	0.91	3	0.91
Iligan City	289	0	0.000	0	0.00	13	4.50	13	4.50
Malaybalay City	215	0	0.000	1	0.47	10	4.65	11	5.12
Oroquieta City	1	0	0.000	0	0.00	1	100.00	1	100.00
Ozamis City	953	0	0.000	0	0.00	5	0.52	5	0.52
Tangub City	4	0	0.000	0	0.00	0	0.00	0	0.00
Valencia City	22	0	0.000	0	0.00	2	9.09	2	9.09
**Region 11**	**9,315**	**12**	**0.129**	**6**	**0.06**	**132**	**1.42**	**150**	**1.61**
Davao de Oro	234	0	0.000	3	1.28	24	10.26	27	11.54
Davao del Norte	1,121	1	0.089	0	0.00	38	3.39	39	3.48
Davao Oriental	224	0	0.000	1	0.45	1	0.45	2	0.89
Davao del Sur	320	1	0.313	0	0.00	9	2.81	10	3.13
Davao Occidental	154	0	0.000	1	0.65	4	2.60	5	3.25
Davao City	7,262	10	0.138	1	0.01	56	0.77	67	0.92
**Region 12**	**9,102**	**1**	**0.011**	**21**	**0.23**	**215**	**2.36**	**237**	**2.60**
North Cotabato	2,198	1	0.045	8	0.36	88	4.00	97	4.41
Sarangani	2,695	0	0.000	11	0.41	61	2.26	72	2.67
South Cotabato	1,312	0	0.000	1	0.08	27	2.06	28	2.13
Sultan Kudarat	1,686	0	0.000	1	0.06	22	1.30	23	1.36
Cotabato City	248	0	0.000	0	0.00	2	0.81	2	0.81
Gen. Santos City	963	0	0.000	0	0.00	15	1.56	15	1.56
**BARMM**	**2,211**	**0**	**0.000**	**5**	**0.23**	**12**	**0.54**	**17**	**0.77**
Basilan	0	0	0.000	0	0.00	0	0.00	0	0.00
Lanao del Sur	902	0	0.000	0	0.00	3	0.33	3	0.33
Maguindanao	432	0	0.000	5	1.16	9	2.08	14	3.24
Sulu	0	0	0.000	0	0.00	0	0.00	0	0.00
Tawi-Tawi	0	0	0.000	0	0.00	0	0.00	0	0.00

Table 1.B.1.17 - Prenatal Care

Number and proportion of pregnant women tested positive for Gestational Diabetes by Region
Philippines, 2021

Area	Total No. of Pregnant Women Screened for Gestational Diabetes	Tested positive for Gestational Diabetes							
		Age Group						Total	%
		10-14 yrs old		15-19 yrs old		20-49 yrs old			
		No.	%	No.	%	No.	%		
Lamitan City	4	0	0.000	0	0.00	0	0.00	0	0.00
Marawi City	873	0	0.000	0	0.00	0	0.00	0	0.00
**CARAGA**	**3,797**	**0**	**0.000**	**26**	**0.68**	**199**	**5.24**	**225**	**5.93**
Agusan del Norte	407	0	0.000	1	0.25	12	2.95	13	3.19
Agusan del Sur	1,047	0	0.000	11	1.05	99	9.46	110	10.51
Surigao del Norte	348	0	0.000	1	0.29	11	3.16	12	3.45
Surigao del Sur	531	0	0.000	3	0.56	43	8.10	46	8.66
Province of Dinagat	167	0	0.000	0	0.00	11	6.59	11	6.59
Bislig City	34	0	0.000	2	5.88	4	11.76	6	17.65
Butuan City	672	0	0.000	8	1.19	14	2.08	22	3.27
Surigao City	591	0	0.000	0	0.00	5	0.85	5	0.85

Note: Put asterisk (*) for No Report and Zero (0) for No Case

Figure 1.B.1.1 - Women 10-14 years old who gave bith with at least 4 Prenatal check-up by Region
Philippines, 2021

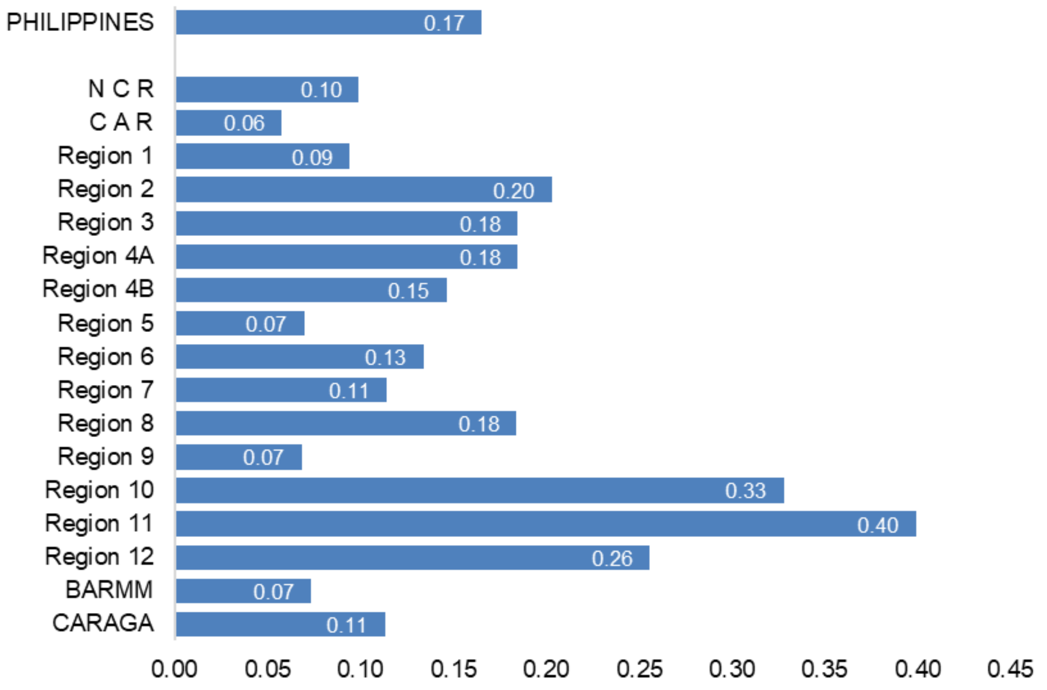

Figure 1.B.1.2 - Women 15-19 years old who gave birth with at least 4 Prenatal check-up by Region
Philippines, 2021

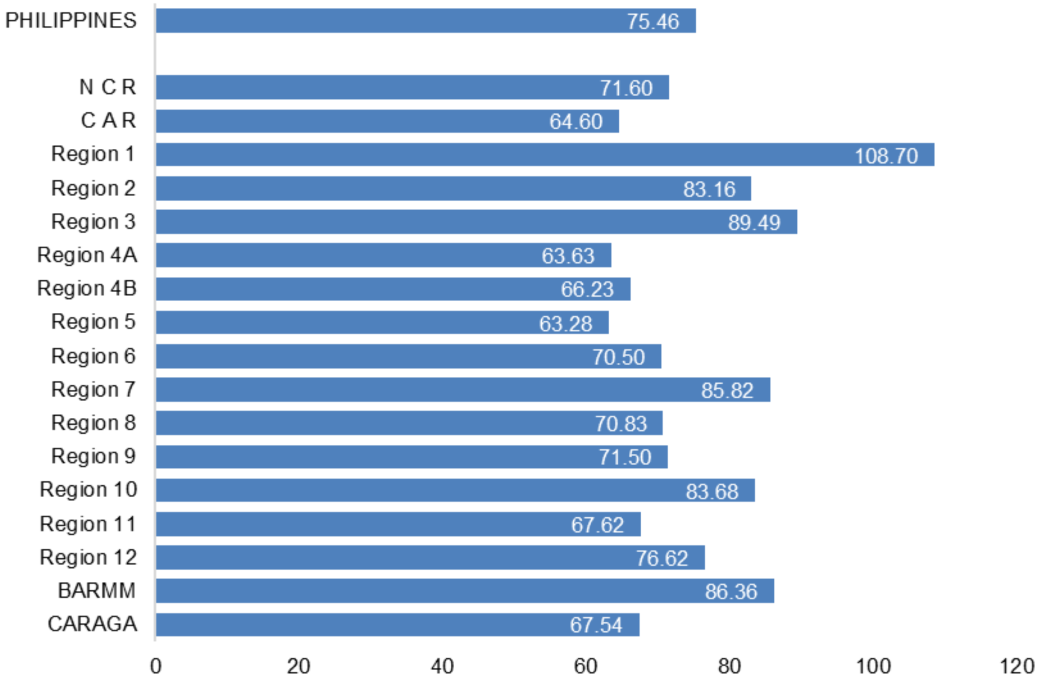

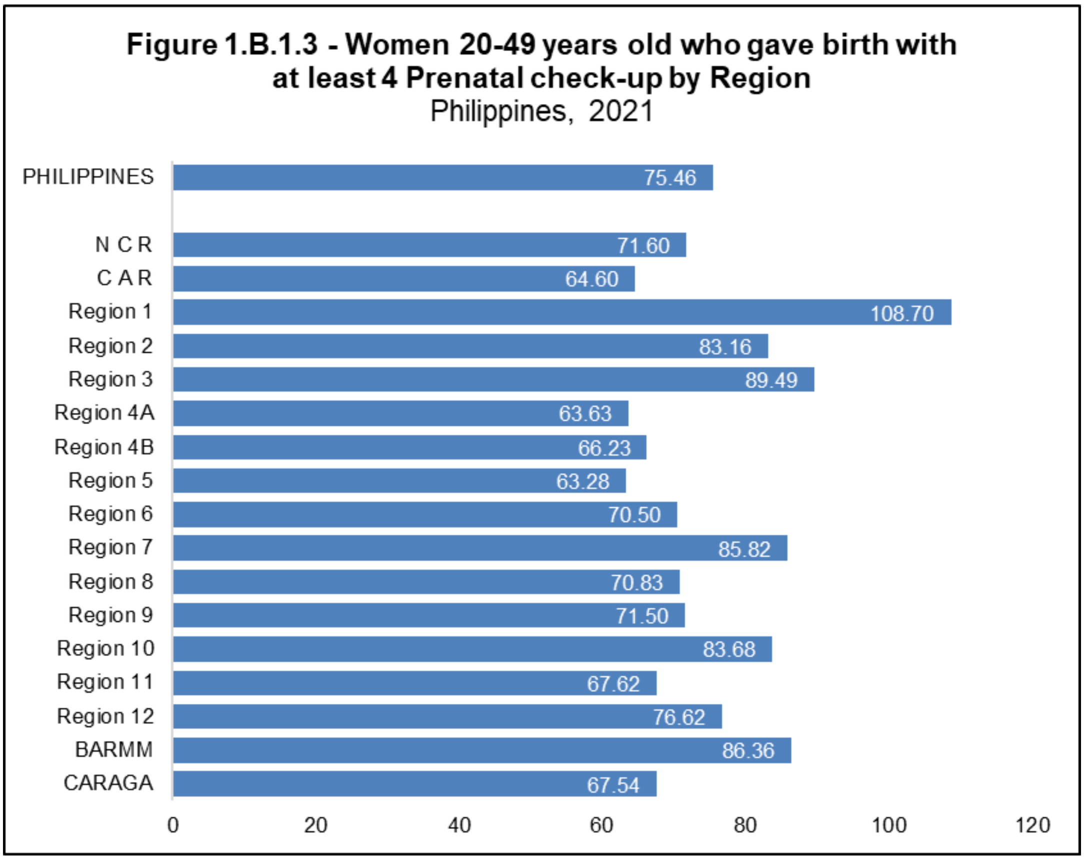
Figure 1.B.1.3 - Women 20-49 years old who gave birth with at least 4 Prenatal check-up by Region
Philippines, 2021
PHILIPPINES 75.46
N C R 71.60
C A R 64.60
Region 1 108.70
Region 2 83.16
Region 3 89.49
Region 4A 63.63
Region 4B 66.23
Region 5 63.28
Region 6 70.50
Region 7 85.82
Region 8 70.83
Region 9 71.50
Region 10 83.68
Region 11 67.62
Region 12 76.62
BARMM 86.36
CARAGA 67.54
0 20 40 60 80 100 120

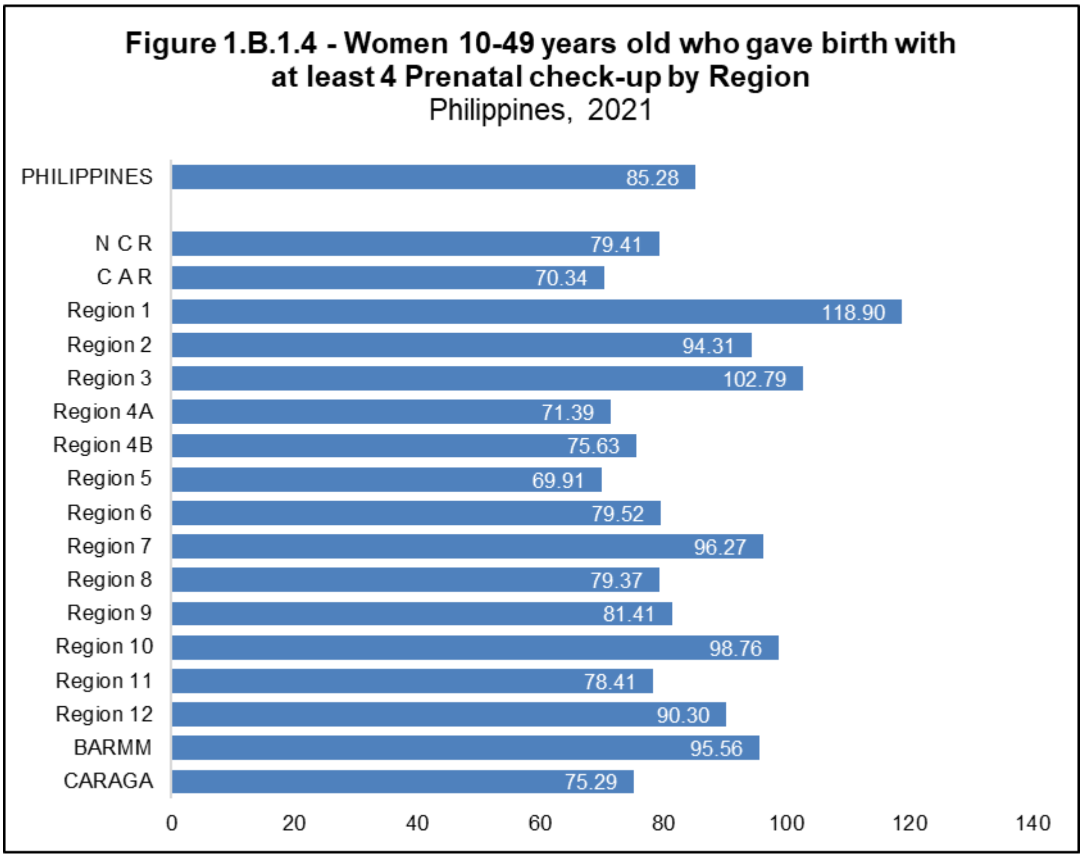
Figure 1.B.1.4 - Women 10-49 years old who gave birth with at least 4 Prenatal check-up by Region
Philippines, 2021
PHILIPPINES 85.28
N C R 79.41
C A R 70.34
Region 1 118.90
Region 2 94.31
Region 3 102.79
Region 4A 71.39
Region 4B 75.63
Region 5 69.91
Region 6 79.52
Region 7 96.27
Region 8 79.37
Region 9 81.41
Region 10 98.76
Region 11 78.41
Region 12 90.30
BARMM 95.56
CARAGA 75.29
0 20 40 60 80 100 120 140

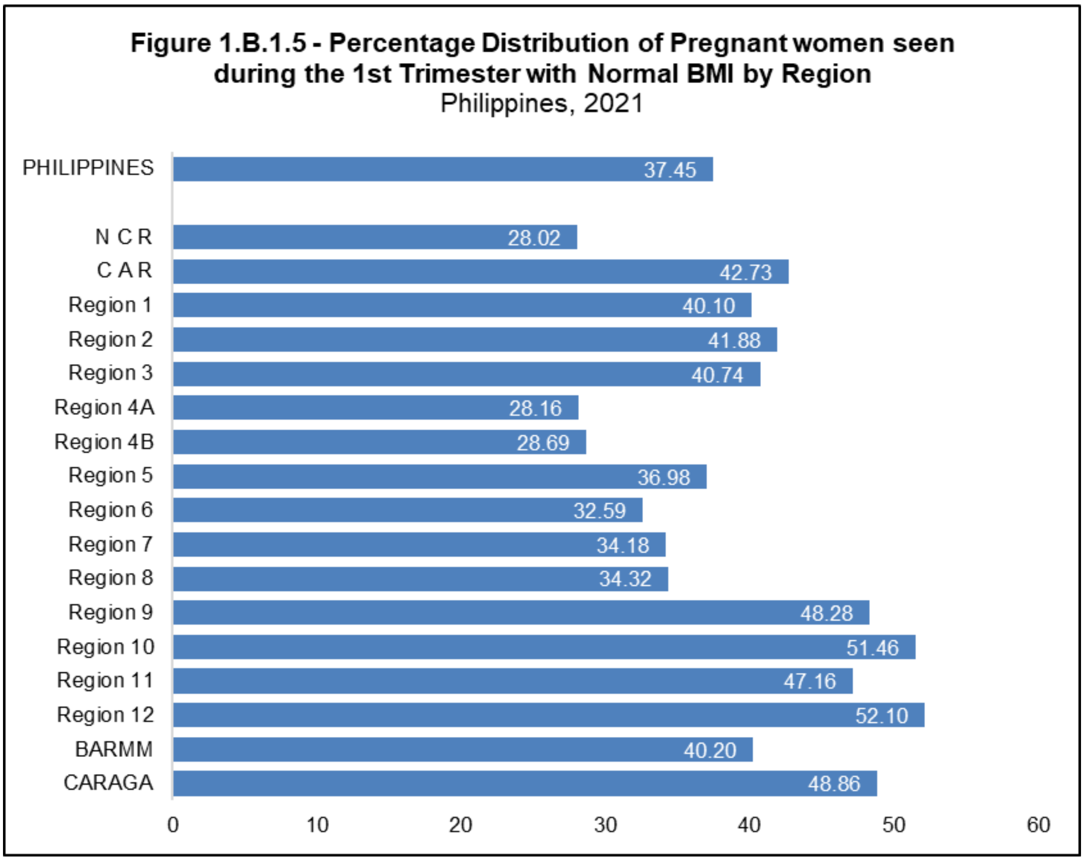
Figure 1.B.1.5 - Percentage Distribution of Pregnant women seen during the 1st Trimester with Normal BMI by Region
Philippines, 2021
PHILIPPINES
37.45
N C R
28.02
C A R
42.73
Region 1
40.10
Region 2
41.88
Region 3
40.74
Region 4A
28.16
Region 4B
28.69
Region 5
36.98
Region 6
32.59
Region 7
34.18
Region 8
34.32
Region 9
48.28
Region 10
51.46
Region 11
47.16
Region 12
52.10
BARMM
40.20
CARAGA
48.86
0
10
20
30
40
50
60

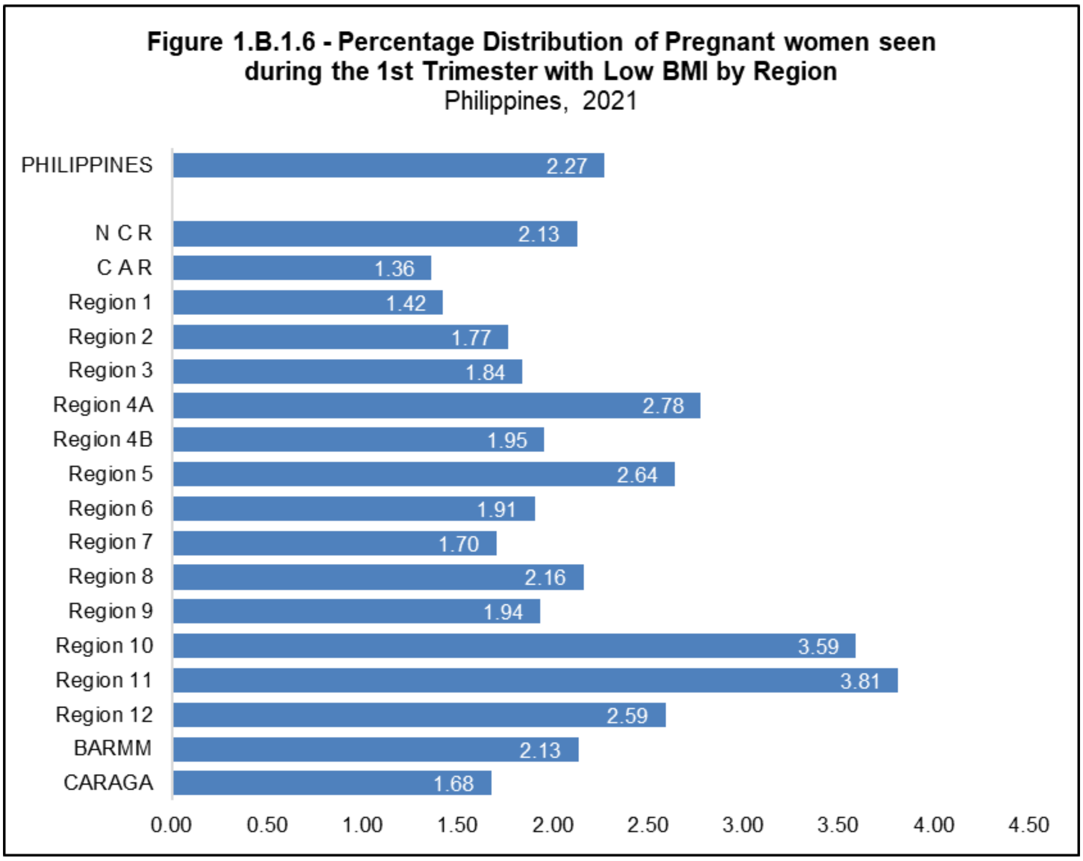
Figure 1.B.1.6 - Percentage Distribution of Pregnant women seen during the 1st Trimester with Low BMI by Region
Philippines, 2021
PHILIPPINES
2.27
N C R
2.13
C A R
1.36
Region 1
1.42
Region 2
1.77
Region 3
1.84
Region 4A
2.78
Region 4B
1.95
Region 5
2.64
Region 6
1.91
Region 7
1.70
Region 8
2.16
Region 9
1.94
Region 10
3.59
Region 11
3.81
Region 12
2.59
BARMM
2.13
CARAGA
1.68
0.00
0.50
1.00
1.50
2.00
2.50
3.00
3.50
4.00
4.50

Figure 1.B.1.7 - Percentage Distribution of Pregnant women seen during the 1st Trimester with High BMI by Region
Philippines, 2021

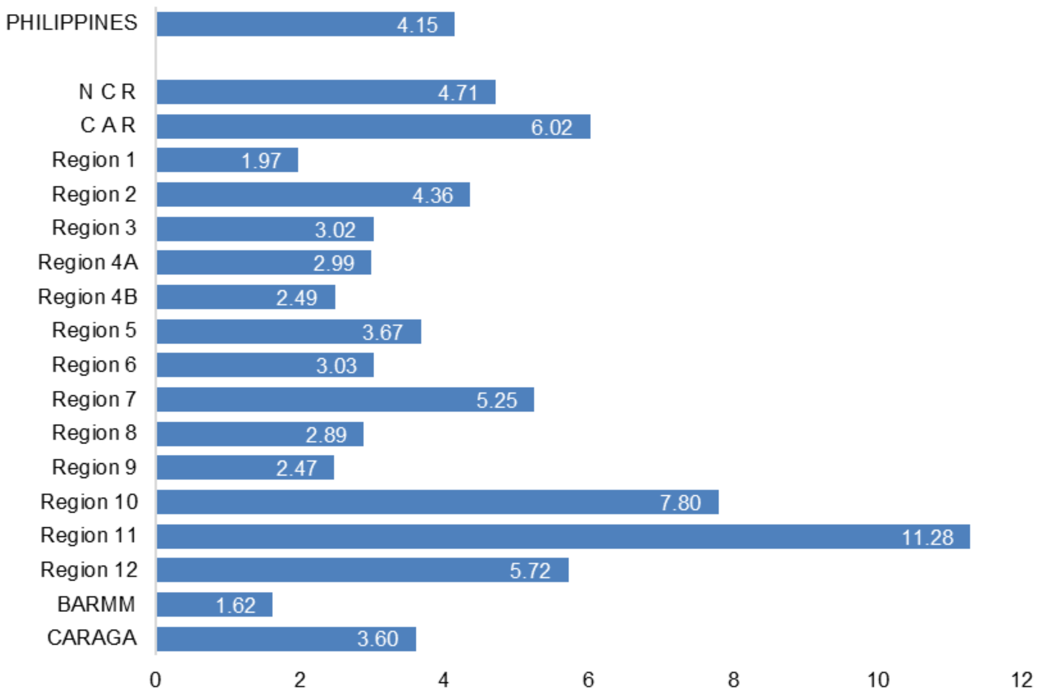

Figure 1.B.1.8 - Percentage Distribution of Women (10-14 yr. old) pregnant for the 1st time given at least 2 doses of Tetanus Diptheria (Td) Vaccination by Region
Philippines, 2021

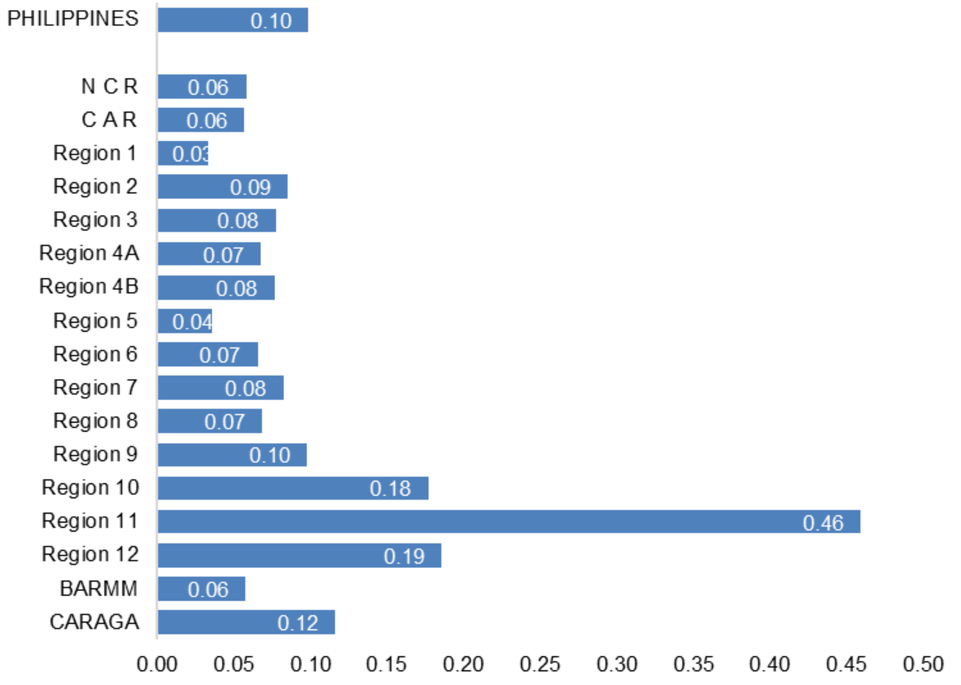

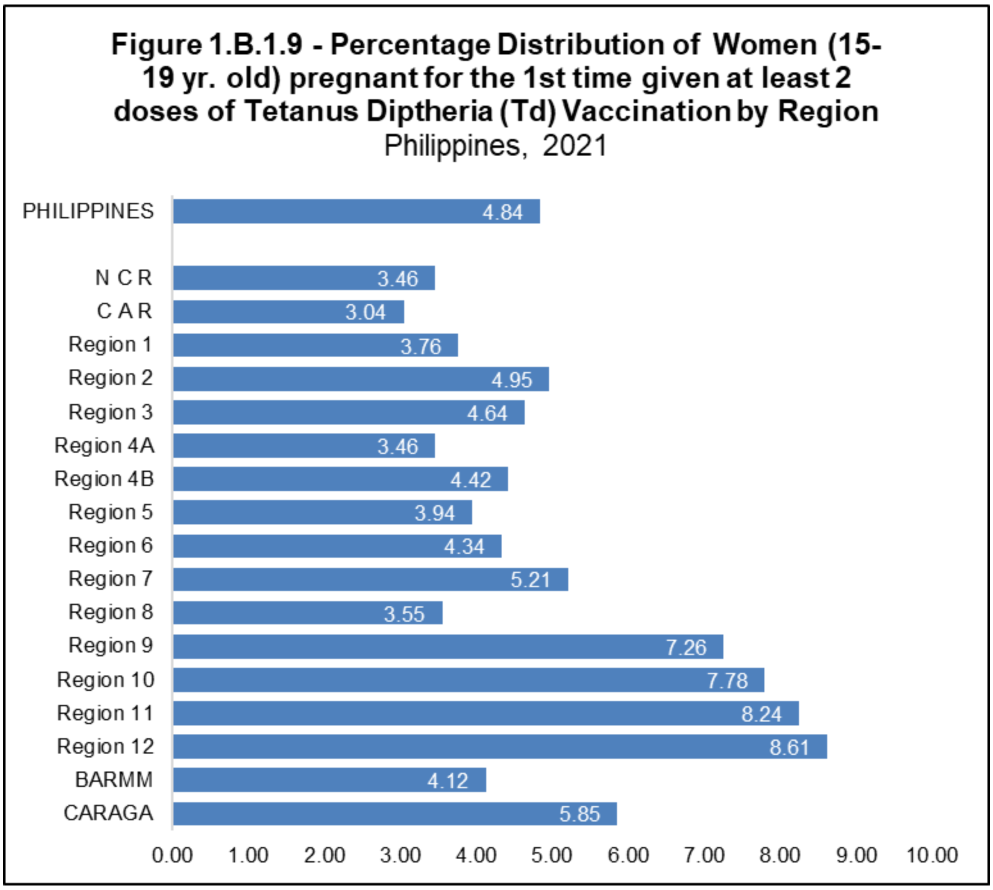

Figure 1.B.1.9 - Percentage Distribution of Women (15-19 yr. old) pregnant for the 1st time given at least 2 doses of Tetanus Diptheria (Td) Vaccination by Region
Philippines, 2021
PHILIPPINES 4.84
N C R 3.46
C A R 3.04
Region 1 3.76
Region 2 4.95
Region 3 4.64
Region 4A 3.46
Region 4B 4.42
Region 5 3.94
Region 6 4.34
Region 7 5.21
Region 8 3.55
Region 9 7.26
Region 10 7.78
Region 11 8.24
Region 12 8.61
BARMM 4.12
CARAGA 5.85
0.00 1.00 2.00 3.00 4.00 5.00 6.00 7.00 8.00 9.00 10.00

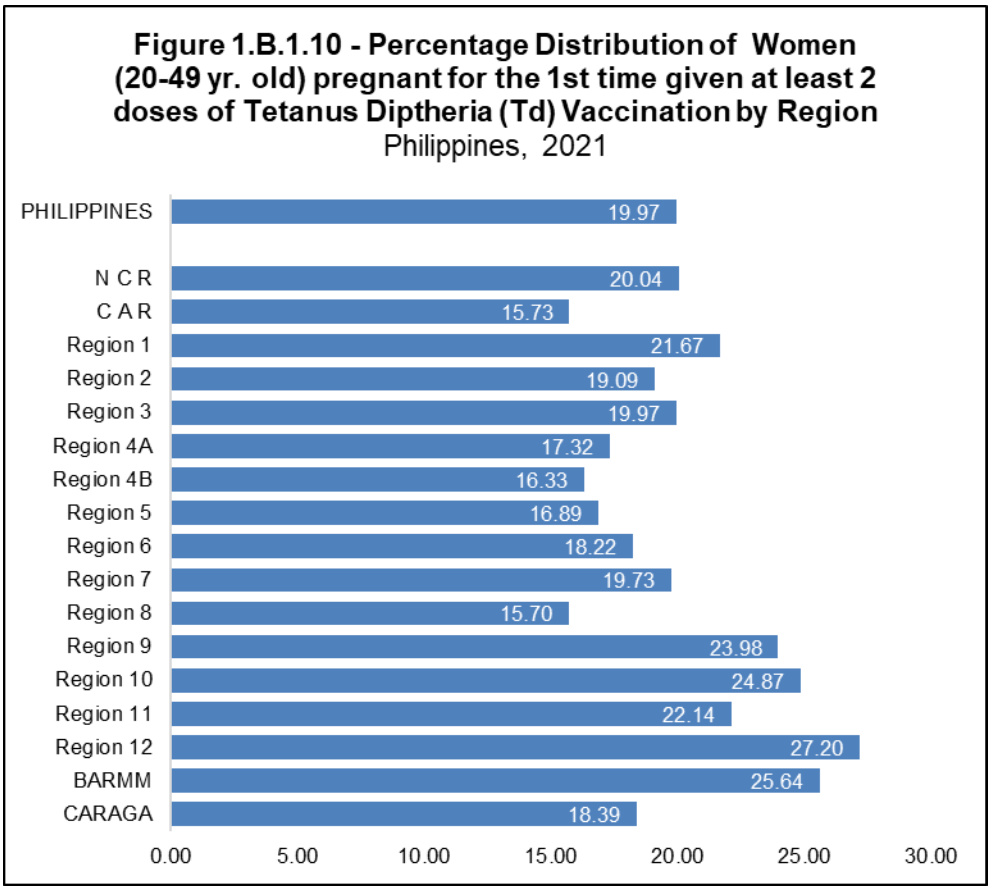

Figure 1.B.1.10 - Percentage Distribution of Women (20-49 yr. old) pregnant for the 1st time given at least 2 doses of Tetanus Diptheria (Td) Vaccination by Region
Philippines, 2021
PHILIPPINES 19.97
N C R 20.04
C A R 15.73
Region 1 21.67
Region 2 19.09
Region 3 19.97
Region 4A 17.32
Region 4B 16.33
Region 5 16.89
Region 6 18.22
Region 7 19.73
Region 8 15.70
Region 9 23.98
Region 10 24.87
Region 11 22.14
Region 12 27.20
BARMM 25.64
CARAGA 18.39
0.00 5.00 10.00 15.00 20.00 25.00 30.00

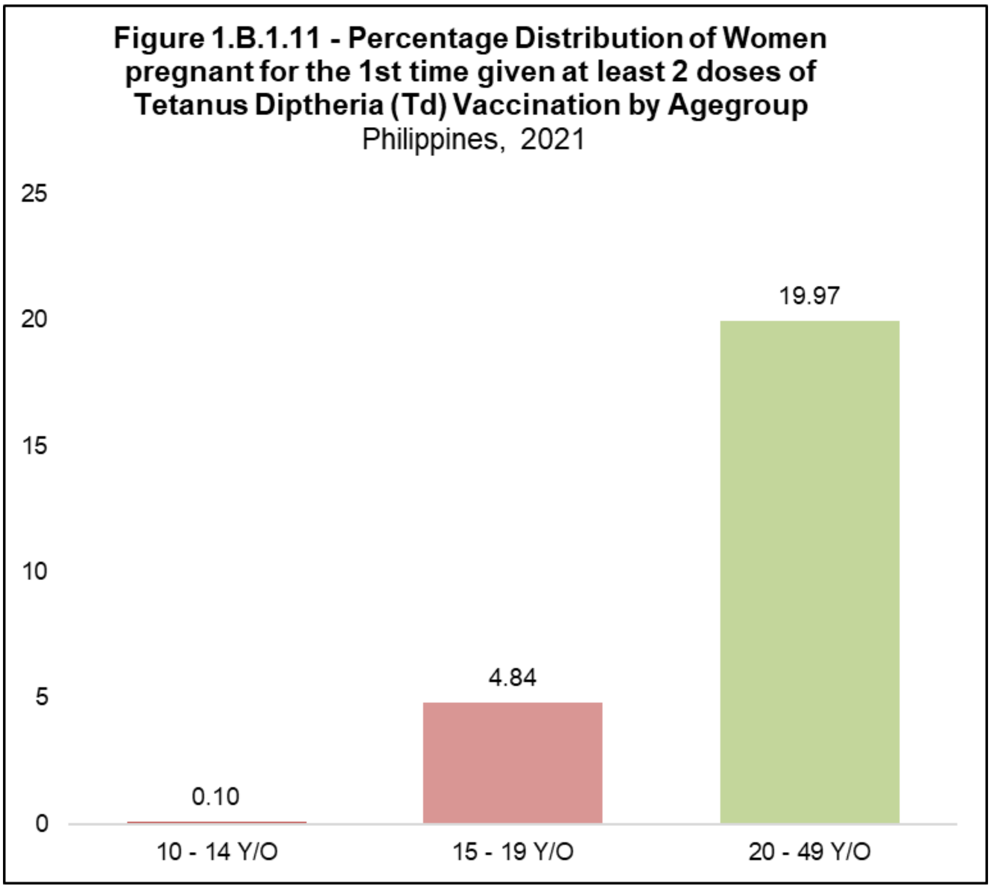

Figure 1.B.1.11 - Percentage Distribution of Women pregnant for the 1st time given at least 2 doses of Tetanus Diptheria (Td) Vaccination by Agegroup
Philippines, 2021
25
20
15
10
5
0
0.10
4.84
19.97
10 - 14 Y/O
15 - 19 Y/O
20 - 49 Y/O

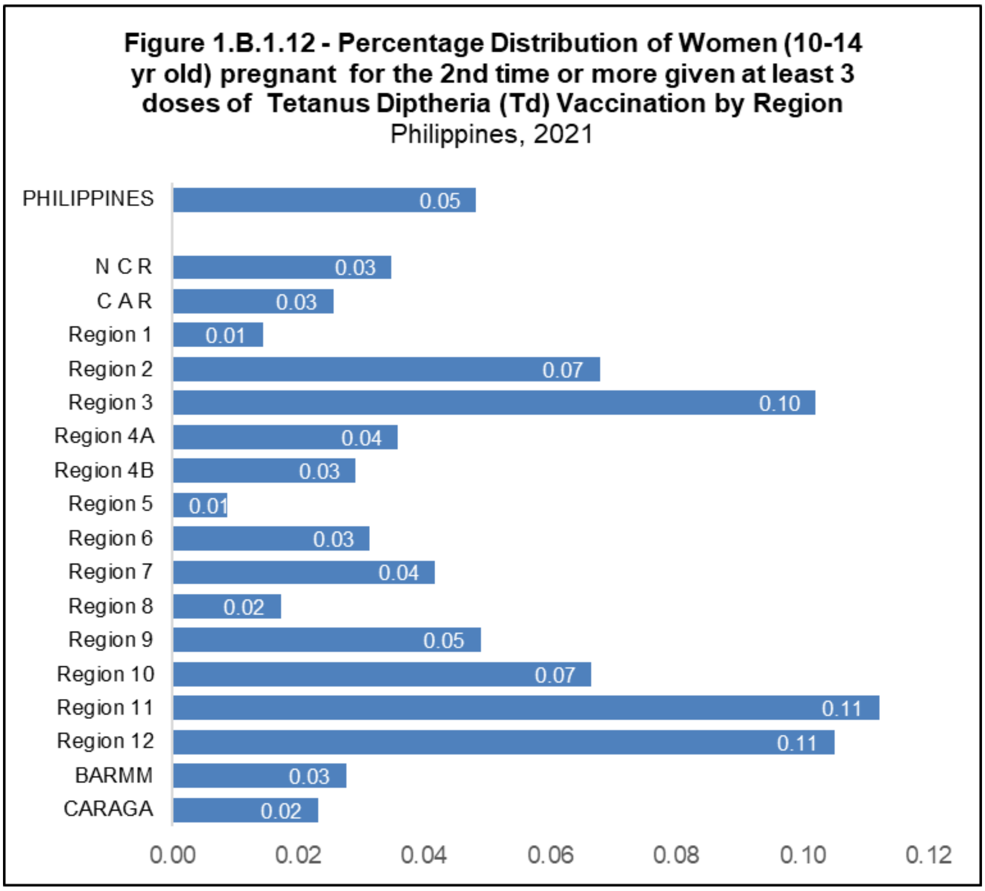

Figure 1.B.1.12 - Percentage Distribution of Women (10-14 yr old) pregnant for the 2nd time or more given at least 3 doses of Tetanus Diptheria (Td) Vaccination by Region
Philippines, 2021
PHILIPPINES 0.05
N C R 0.03
C A R 0.03
Region 1 0.01
Region 2 0.07
Region 3 0.10
Region 4A 0.04
Region 4B 0.03
Region 5 0.01
Region 6 0.03
Region 7 0.04
Region 8 0.02
Region 9 0.05
Region 10 0.07
Region 11 0.11
Region 12 0.11
BARMM 0.03
CARAGA 0.02
0.00
0.02
0.04
0.06
0.08
0.10
0.12

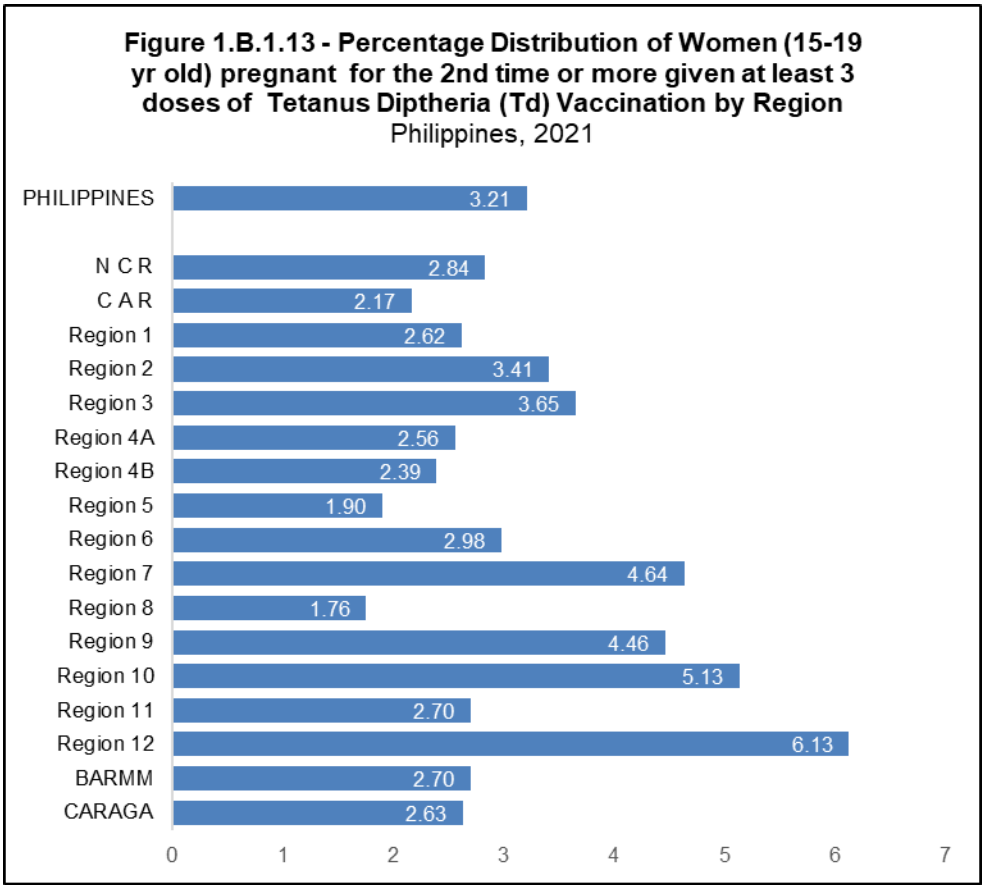
Figure 1.B.1.13 - Percentage Distribution of Women (15-19 yr old) pregnant for the 2nd time or more given at least 3 doses of Tetanus Diptheria (Td) Vaccination by Region
Philippines, 2021
PHILIPPINES 3.21
N C R 2.84
C A R 2.17
Region 1 2.62
Region 2 3.41
Region 3 3.65
Region 4A 2.56
Region 4B 2.39
Region 5 1.90
Region 6 2.98
Region 7 4.64
Region 8 1.76
Region 9 4.46
Region 10 5.13
Region 11 2.70
Region 12 6.13
BARMM 2.70
CARAGA 2.63
0 1 2 3 4 5 6 7

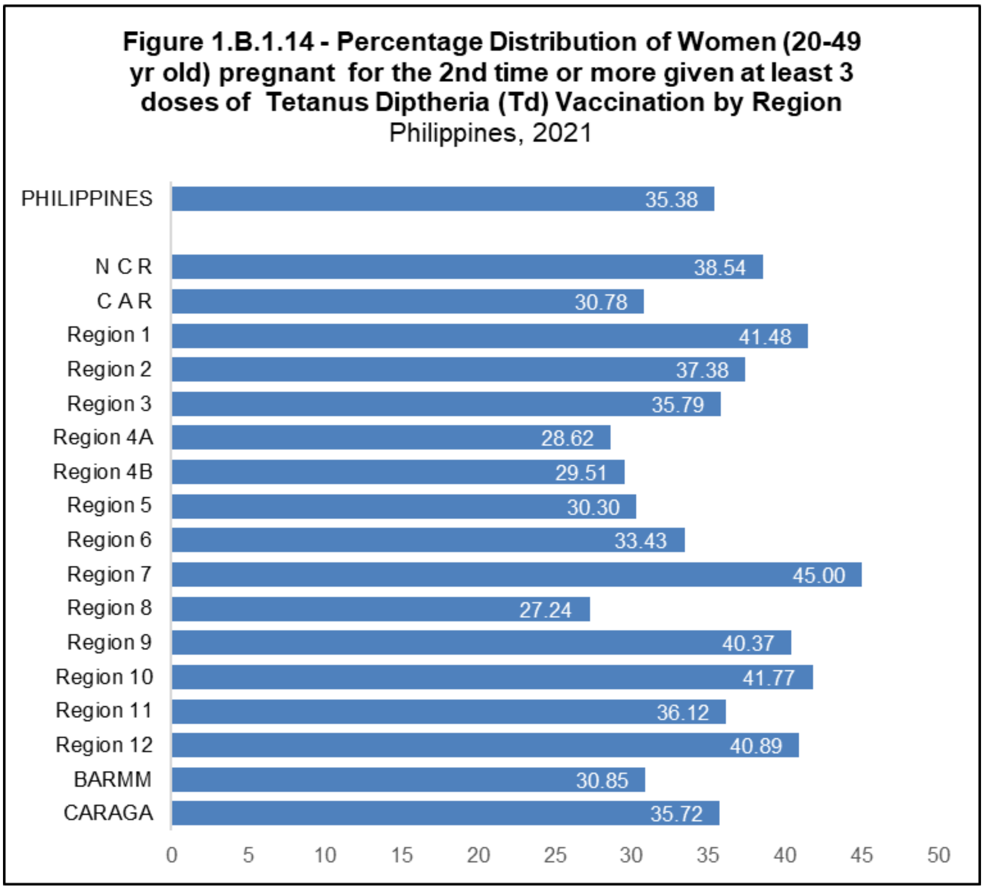
Figure 1.B.1.14 - Percentage Distribution of Women (20-49 yr old) pregnant for the 2nd time or more given at least 3 doses of Tetanus Diptheria (Td) Vaccination by Region
Philippines, 2021
PHILIPPINES 35.38
N C R 38.54
C A R 30.78
Region 1 41.48
Region 2 37.38
Region 3 35.79
Region 4A 28.62
Region 4B 29.51
Region 5 30.30
Region 6 33.43
Region 7 45.00
Region 8 27.24
Region 9 40.37
Region 10 41.77
Region 11 36.12
Region 12 40.89
BARMM 30.85
CARAGA 35.72
0 5 10 15 20 25 30 35 40 45 50

Figure 1.B.1.15- Percentage Distribution of Women (10-49 yrs.old) pregnant for the 2nd time or more given Tetanus Diptheria (Td) Vaccination by Agegroup
Philippines, 2021

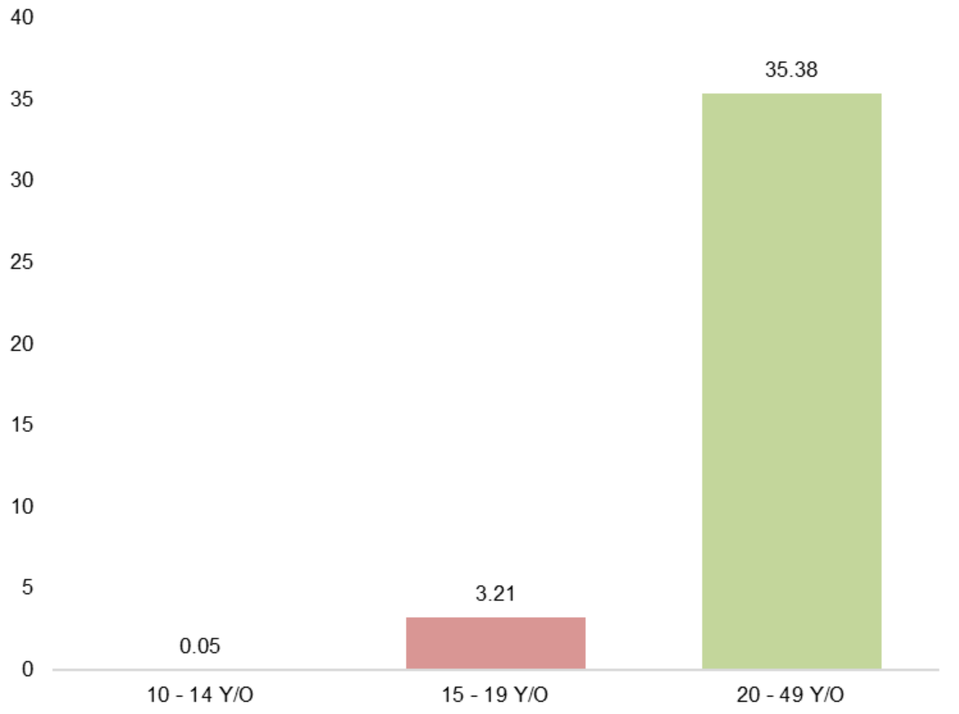

Figure 1.B.1.16 - Percentage Distribution of Pregnant Women (10 - 14 yrs. old) who completed Iron with Folic Acid by Region
Philippines, 2021

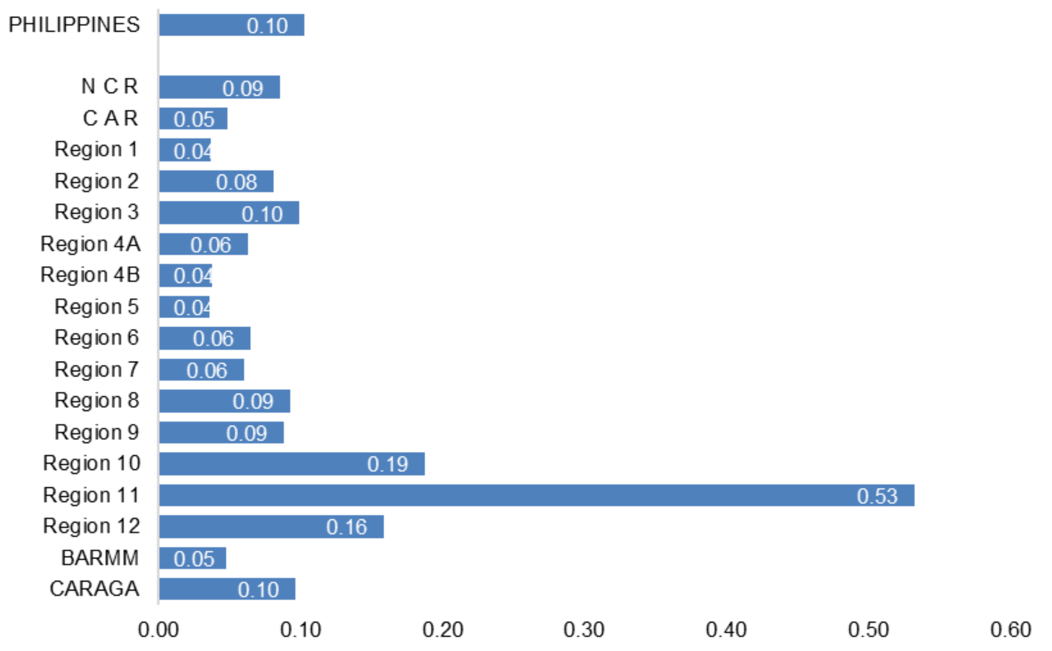

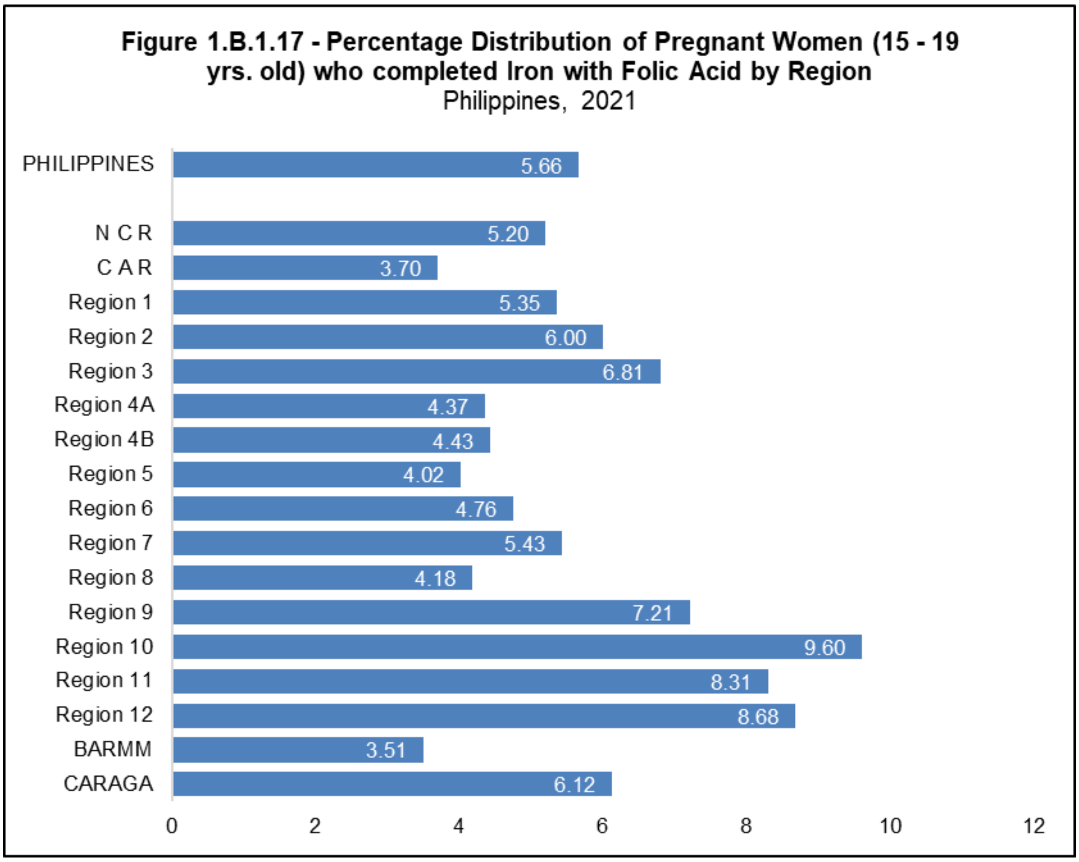
Figure 1.B.1.17 - Percentage Distribution of Pregnant Women (15 - 19 yrs. old) who completed Iron with Folic Acid by Region
Philippines, 2021
PHILIPPINES
5.66
N C R
5.20
C A R
3.70
Region 1
5.35
Region 2
6.00
Region 3
6.81
Region 4A
4.37
Region 4B
4.43
Region 5
4.02
Region 6
4.76
Region 7
5.43
Region 8
4.18
Region 9
7.21
Region 10
9.60
Region 11
8.31
Region 12
8.68
BARMM
3.51
CARAGA
6.12
0
2
4
6
8
10
12

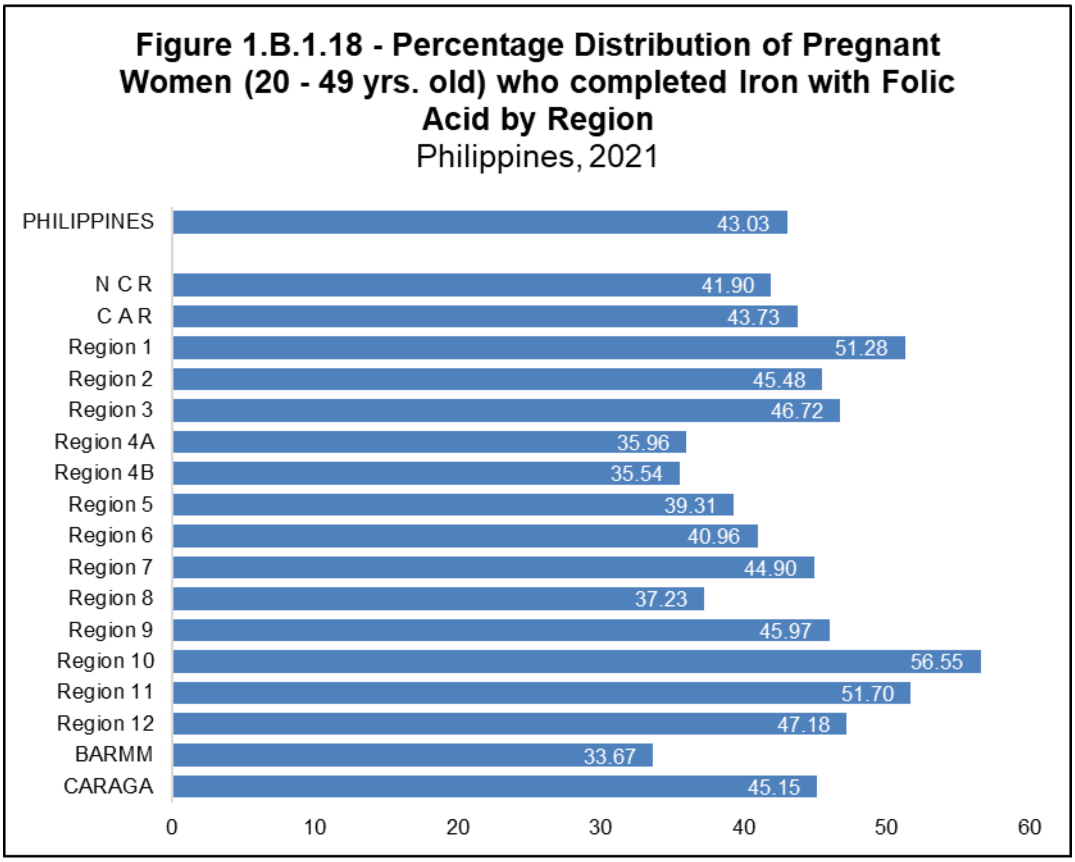
Figure 1.B.1.18 - Percentage Distribution of Pregnant Women (20 - 49 yrs. old) who completed Iron with Folic Acid by Region
Philippines, 2021
PHILIPPINES
43.03
N C R
41.90
C A R
43.73
Region 1
51.28
Region 2
45.48
Region 3
46.72
Region 4A
35.96
Region 4B
35.54
Region 5
39.31
Region 6
40.96
Region 7
44.90
Region 8
37.23
Region 9
45.97
Region 10
56.55
Region 11
51.70
Region 12
47.18
BARMM
33.67
CARAGA
45.15
0
10
20
30
40
50
60

Figure 1.B.1.19 - Percentage Distribution of Pregnant Women (10 - 49 yrs. old) who completed Iron with Folic Acid by Region
Philippines, 2021

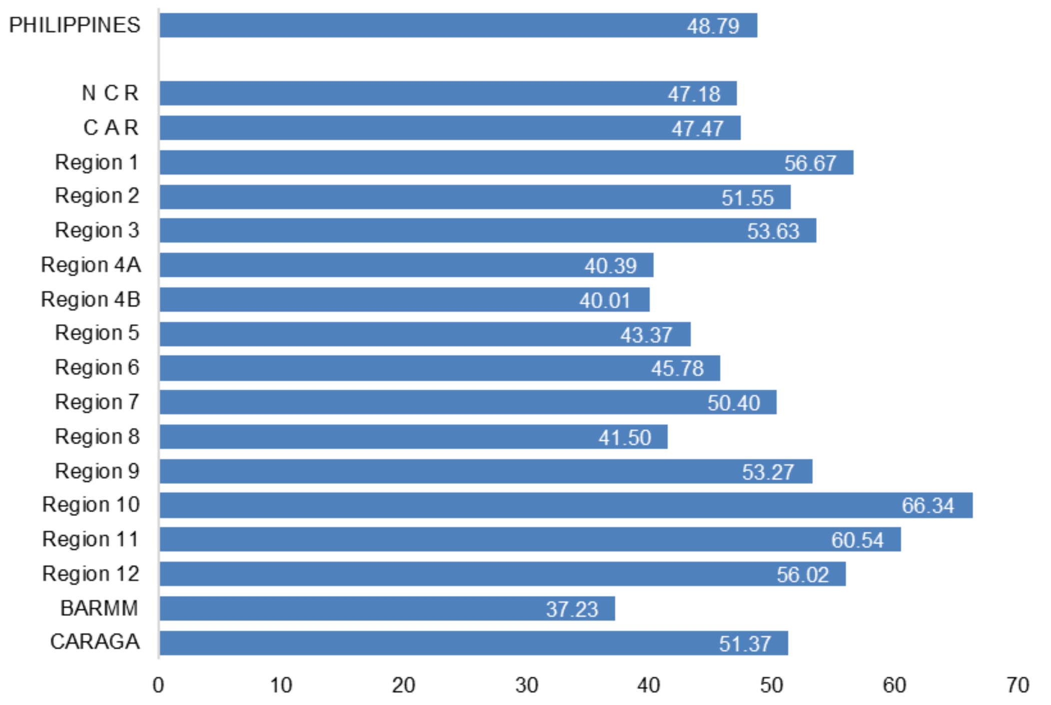

Figure 1.B.1.20 - Percentage Distribution of Pregnant Women (10-14 yrs. old) who completed Calcium Carbonate Supplementation by Region
Philippines, 2021

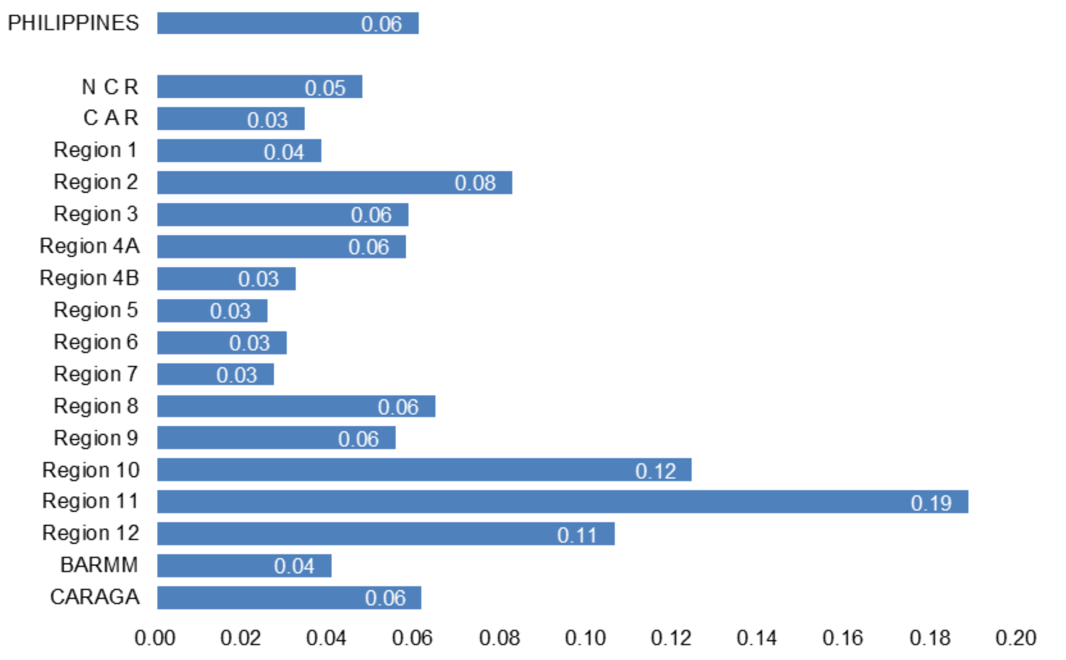

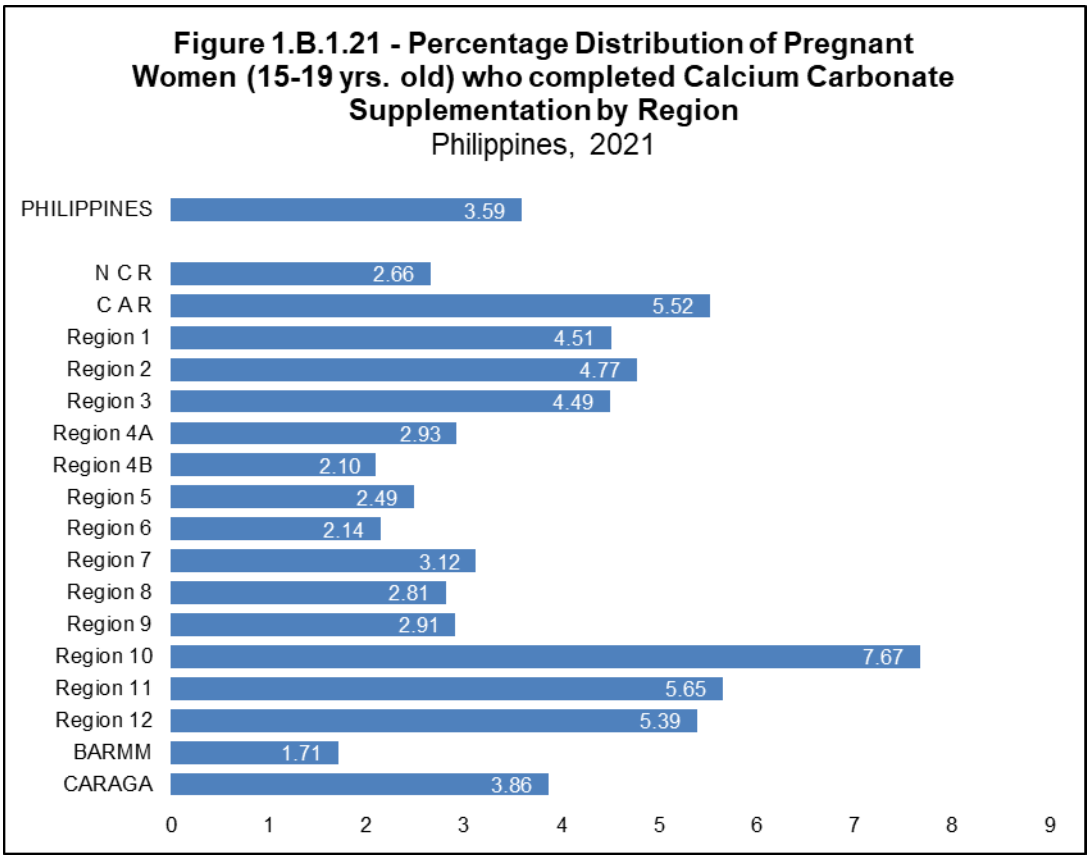
Figure 1.B.1.21 - Percentage Distribution of Pregnant Women (15-19 yrs. old) who completed Calcium Carbonate Supplementation by Region
Philippines, 2021
PHILIPPINES 3.59
N C R 2.66
C A R 5.52
Region 1 4.51
Region 2 4.77
Region 3 4.49
Region 4A 2.93
Region 4B 2.10
Region 5 2.49
Region 6 2.14
Region 7 3.12
Region 8 2.81
Region 9 2.91
Region 10 7.67
Region 11 5.65
Region 12 5.39
BARMM 1.71
CARAGA 3.86
0 1 2 3 4 5 6 7 8 9

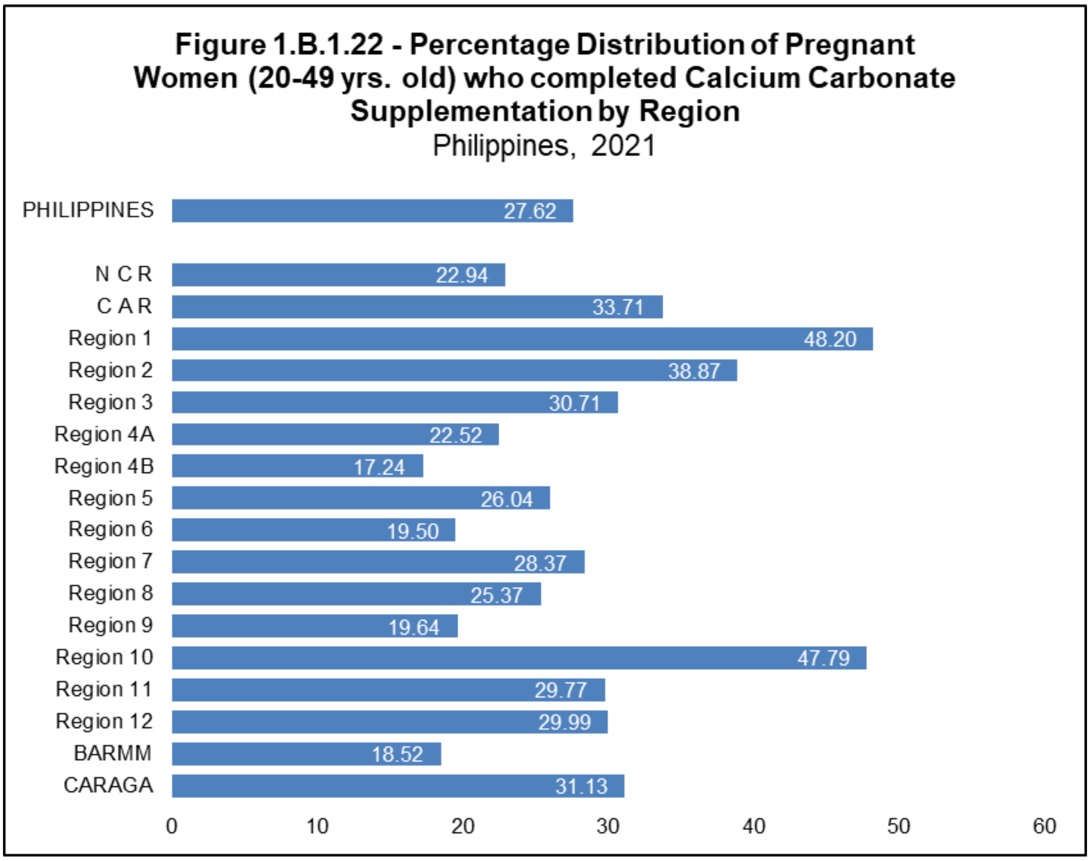
Figure 1.B.1.22 - Percentage Distribution of Pregnant Women (20-49 yrs. old) who completed Calcium Carbonate Supplementation by Region
Philippines, 2021
PHILIPPINES 27.62
N C R 22.94
C A R 33.71
Region 1 48.20
Region 2 38.87
Region 3 30.71
Region 4A 22.52
Region 4B 17.24
Region 5 26.04
Region 6 19.50
Region 7 28.37
Region 8 25.37
Region 9 19.64
Region 10 47.79
Region 11 29.77
Region 12 29.99
BARMM 18.52
CARAGA 31.13
0 10 20 30 40 50 60

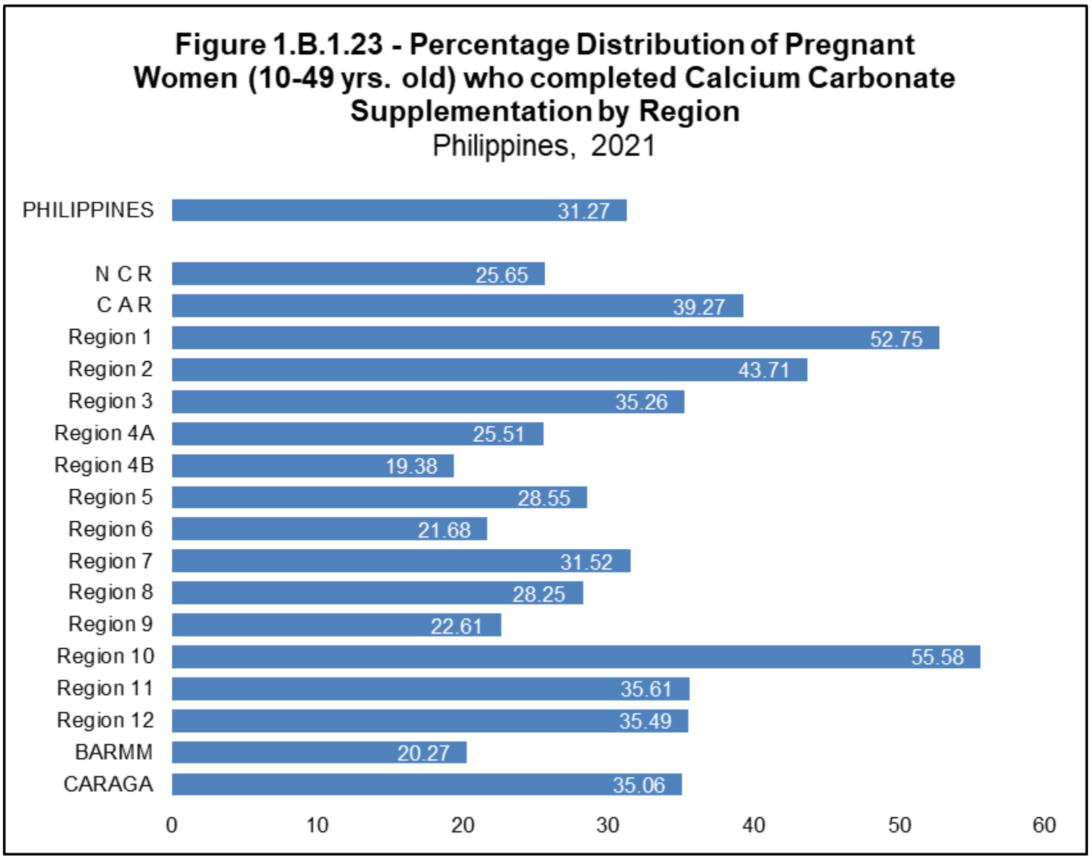

Figure 1.B.1.23 - Percentage Distribution of Pregnant Women (10-49 yrs. old) who completed Calcium Carbonate Supplementation by Region
Philippines, 2021
PHILIPPINES
31.27
N C R
25.65
C A R
39.27
Region 1
52.75
Region 2
43.71
Region 3
35.26
Region 4A
25.51
Region 4B
19.38
Region 5
28.55
Region 6
21.68
Region 7
31.52
Region 8
28.25
Region 9
22.61
Region 10
55.58
Region 11
35.61
Region 12
35.49
BARMM
20.27
CARAGA
35.06
0
10
20
30
40
50
60

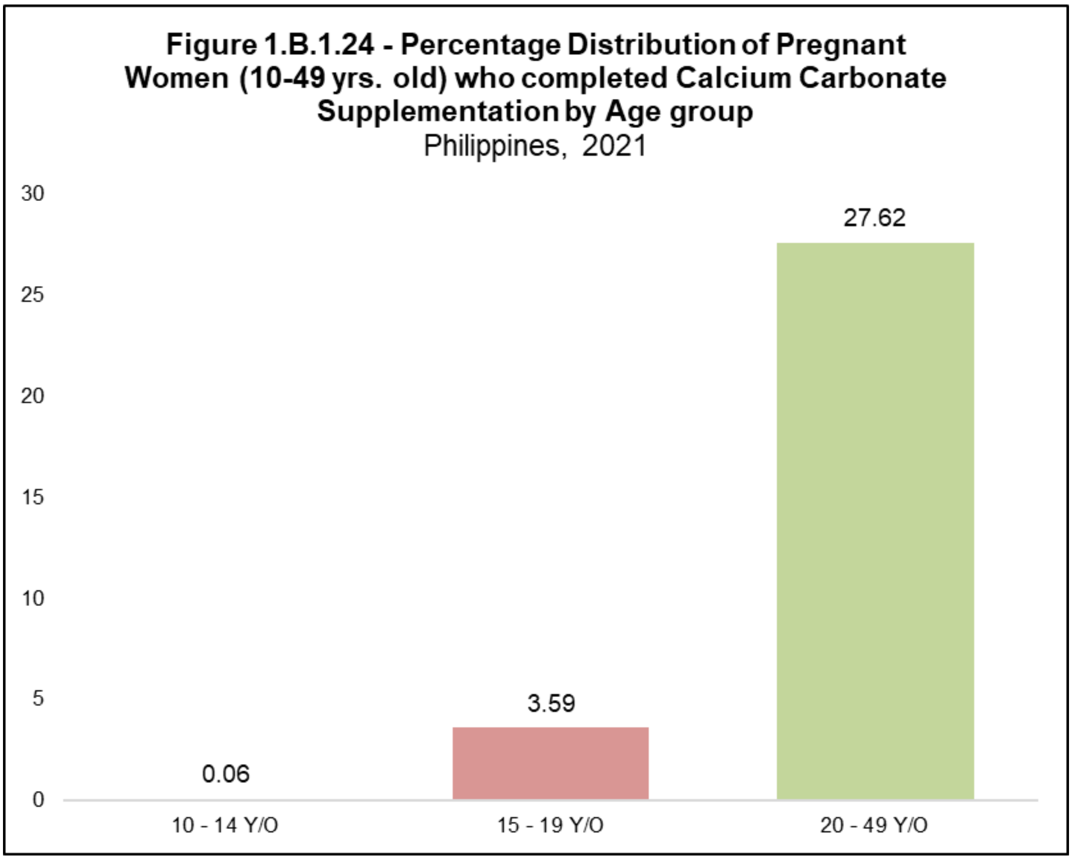

Figure 1.B.1.24 - Percentage Distribution of Pregnant Women (10-49 yrs. old) who completed Calcium Carbonate Supplementation by Age group
Philippines, 2021
30
25
20
15
10
5
0
0.06
3.59
27.62
10 - 14 Y/O
15 - 19 Y/O
20 - 49 Y/O

Figure 1.B.1.25 - Percentage Distribution of Pregnant Women (20-49 yrs. old) who completed Iodine by Region
Philippines, 2021

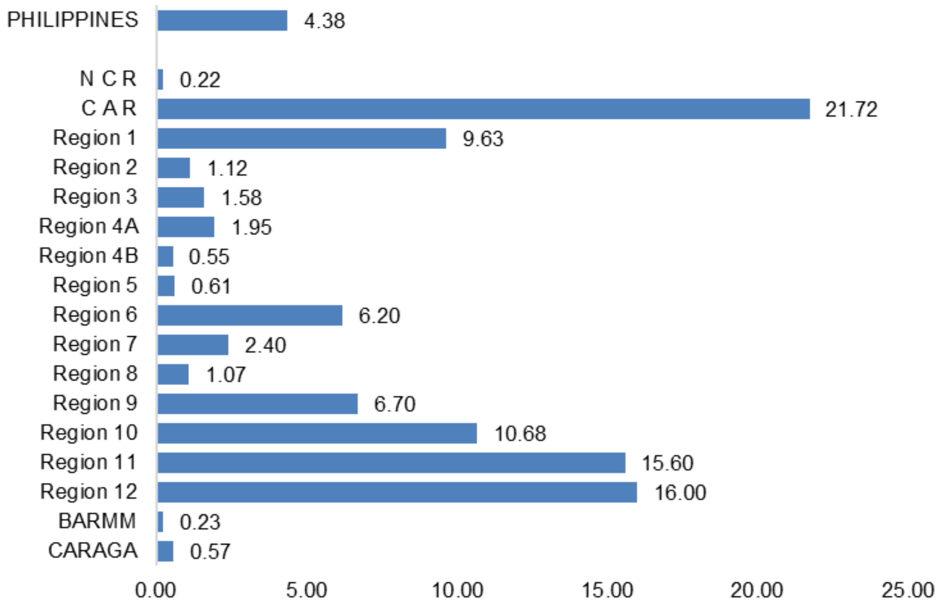

Figure 1.B.1.26 - Percentage Distribution of Pregnant Women (10-49 yrs. old) who completed Iodine by Region
Philippines, 2021

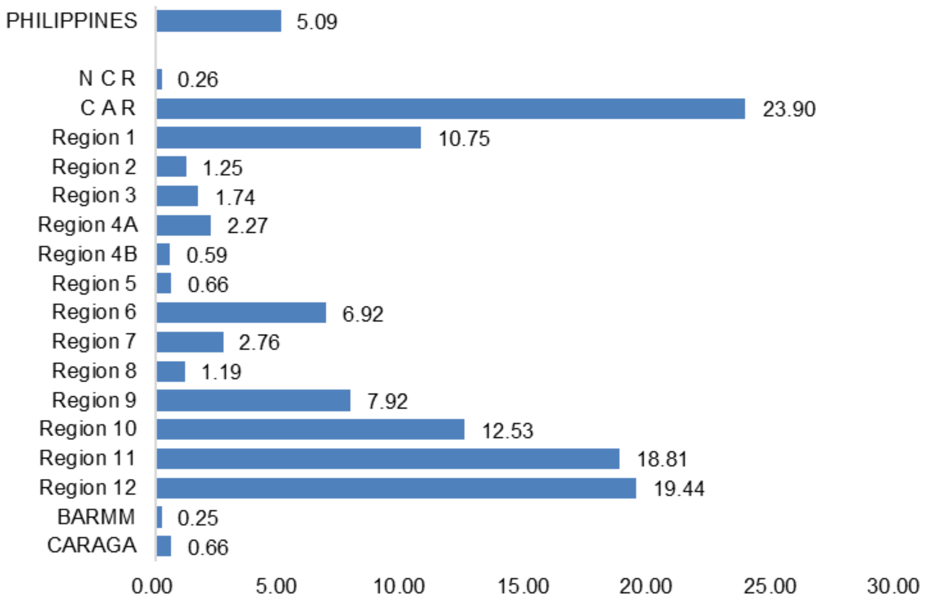

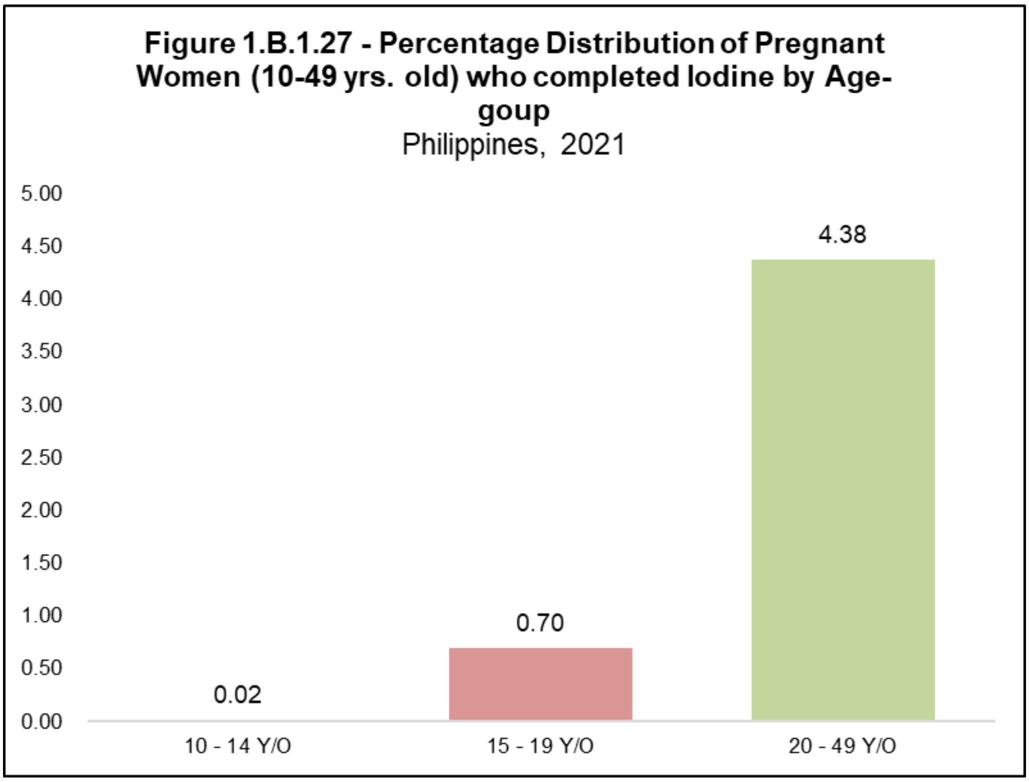
Figure 1.B.1.27 - Percentage Distribution of Pregnant Women (10-49 yrs. old) who completed Iodine by Age-goup
Philippines, 2021
5.00
4.50
4.00
3.50
3.00
2.50
2.00
1.50
1.00
0.50
0.00
0.02
0.70
4.38
10 - 14 Y/O
15 - 19 Y/O
20 - 49 Y/O

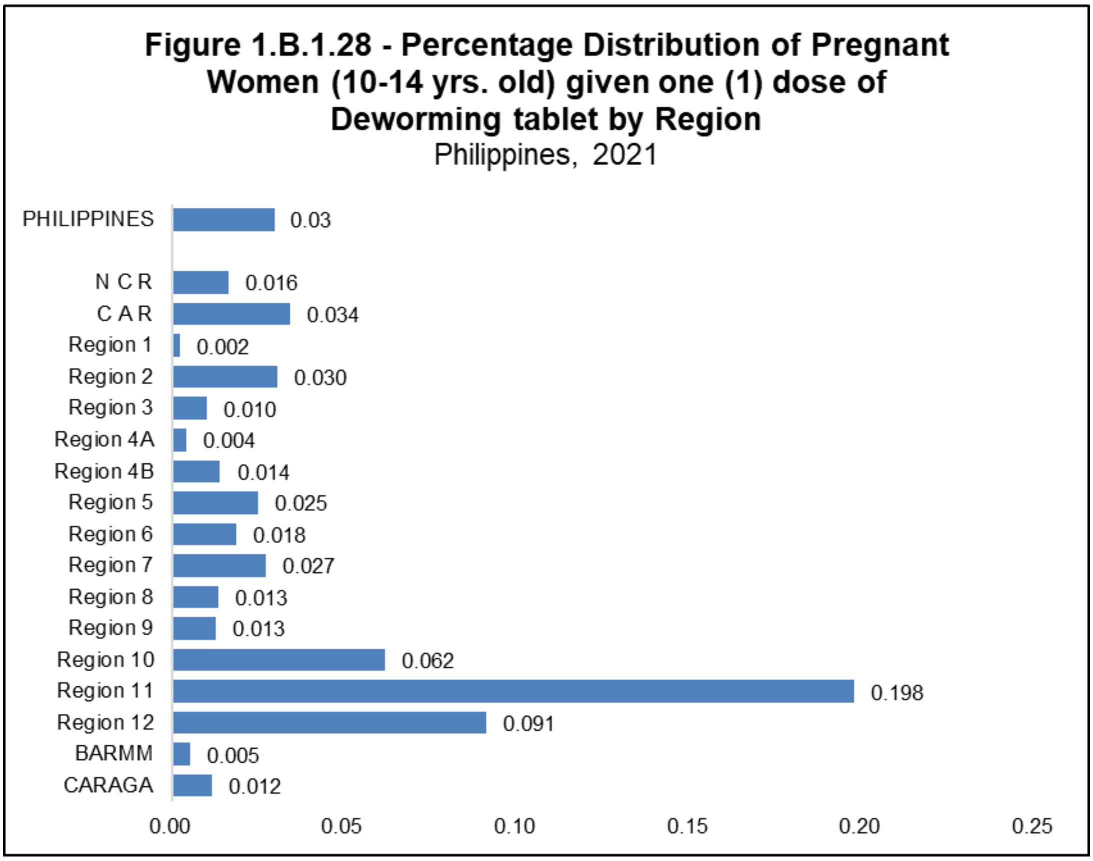
Figure 1.B.1.28 - Percentage Distribution of Pregnant Women (10-14 yrs. old) given one (1) dose of Deworming tablet by Region
Philippines, 2021
PHILIPPINES 0.03
N C R 0.016
C A R 0.034
Region 1 0.002
Region 2 0.030
Region 3 0.010
Region 4A 0.004
Region 4B 0.014
Region 5 0.025
Region 6 0.018
Region 7 0.027
Region 8 0.013
Region 9 0.013
Region 10 0.062
Region 11 0.198
Region 12 0.091
BARMM 0.005
CARAGA 0.012
0.00
0.05
0.10
0.15
0.20
0.25

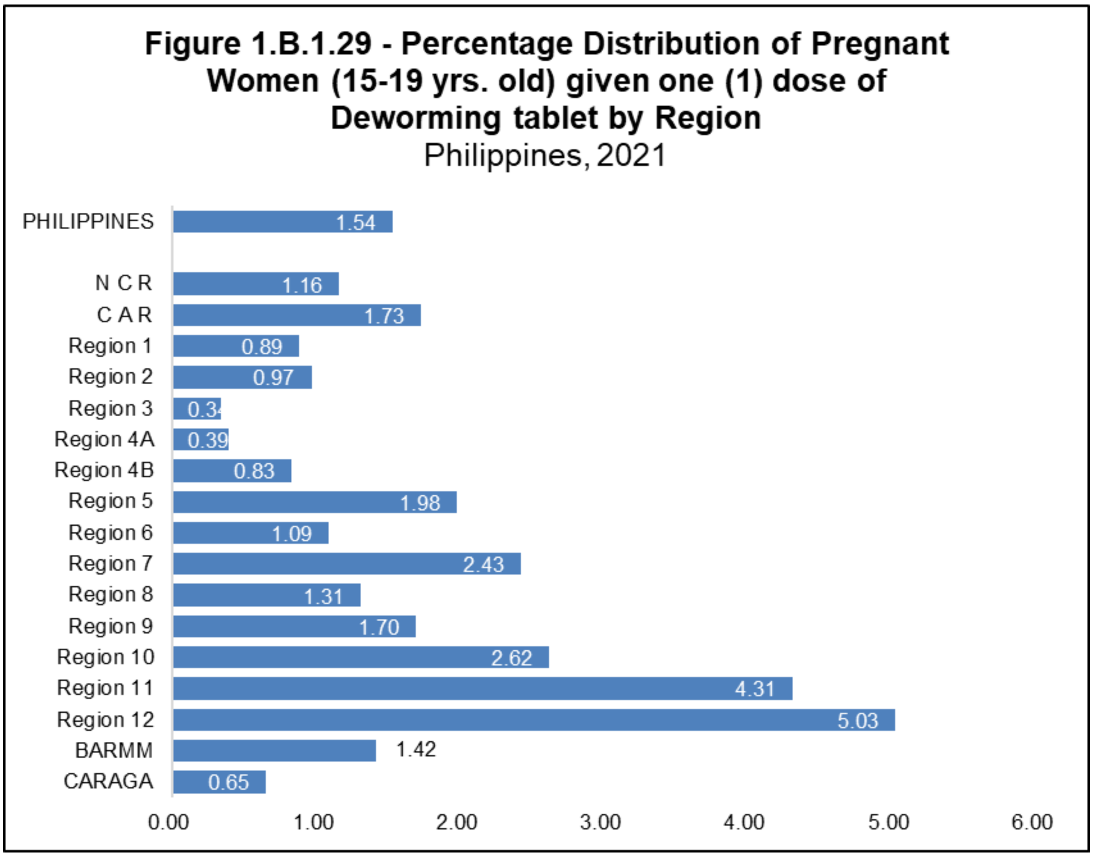
Figure 1.B.1.29 - Percentage Distribution of Pregnant Women (15-19 yrs. old) given one (1) dose of Deworming tablet by Region
Philippines, 2021
PHILIPPINES 1.54
N C R 1.16
C A R 1.73
Region 1 0.89
Region 2 0.97
Region 3 0.3
Region 4A 0.39
Region 4B 0.83
Region 5 1.98
Region 6 1.09
Region 7 2.43
Region 8 1.31
Region 9 1.70
Region 10 2.62
Region 11 4.31
Region 12 5.03
BARMM 1.42
CARAGA 0.65
0.00 1.00 2.00 3.00 4.00 5.00 6.00

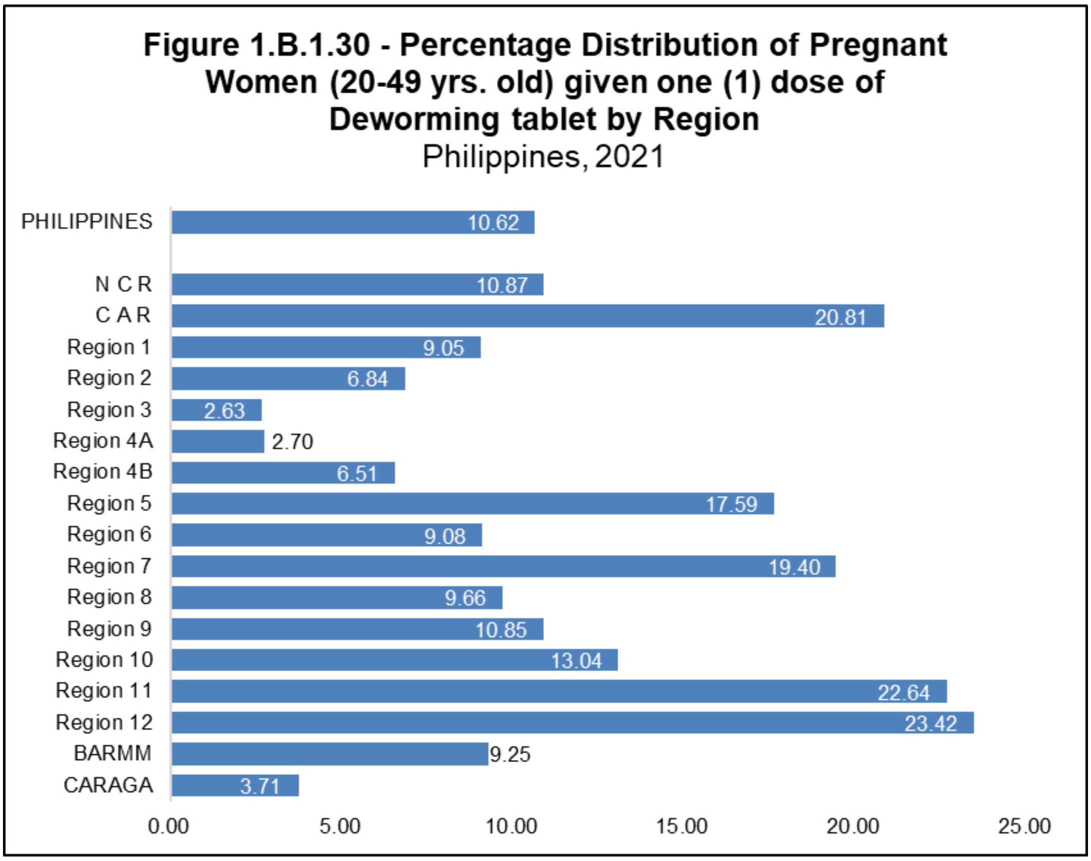
Figure 1.B.1.30 - Percentage Distribution of Pregnant Women (20-49 yrs. old) given one (1) dose of Deworming tablet by Region
Philippines, 2021
PHILIPPINES 10.62
N C R 10.87
C A R 20.81
Region 1 9.05
Region 2 6.84
Region 3 2.63
Region 4A 2.70
Region 4B 6.51
Region 5 17.59
Region 6 9.08
Region 7 19.40
Region 8 9.66
Region 9 10.85
Region 10 13.04
Region 11 22.64
Region 12 23.42
BARMM 9.25
CARAGA 3.71
0.00 5.00 10.00 15.00 20.00 25.00

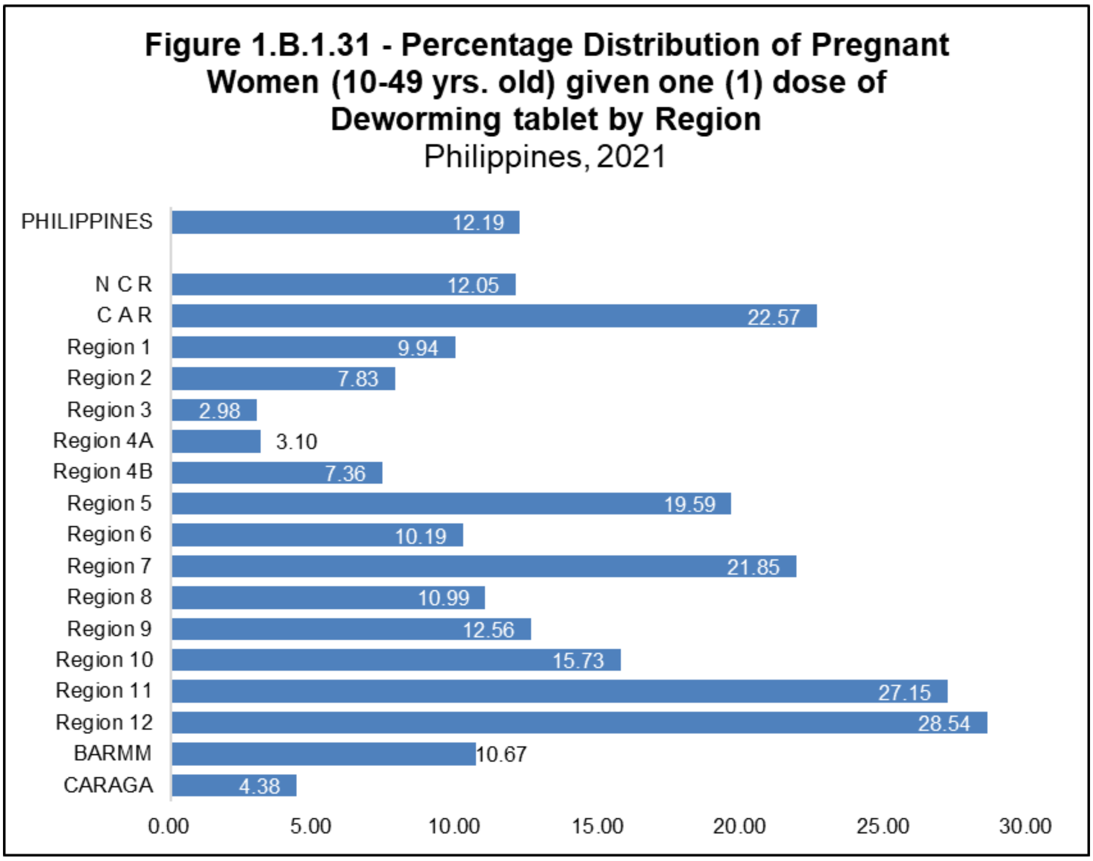
Figure 1.B.1.31 - Percentage Distribution of Pregnant Women (10-49 yrs. old) given one (1) dose of Deworming tablet by Region
Philippines, 2021
PHILIPPINES 12.19
N C R 12.05
C A R 22.57
Region 1 9.94
Region 2 7.83
Region 3 2.98
Region 4A 3.10
Region 4B 7.36
Region 5 19.59
Region 6 10.19
Region 7 21.85
Region 8 10.99
Region 9 12.56
Region 10 15.73
Region 11 27.15
Region 12 28.54
BARMM 10.67
CARAGA 4.38
0.00
5.00
10.00
15.00
20.00
25.00
30.00

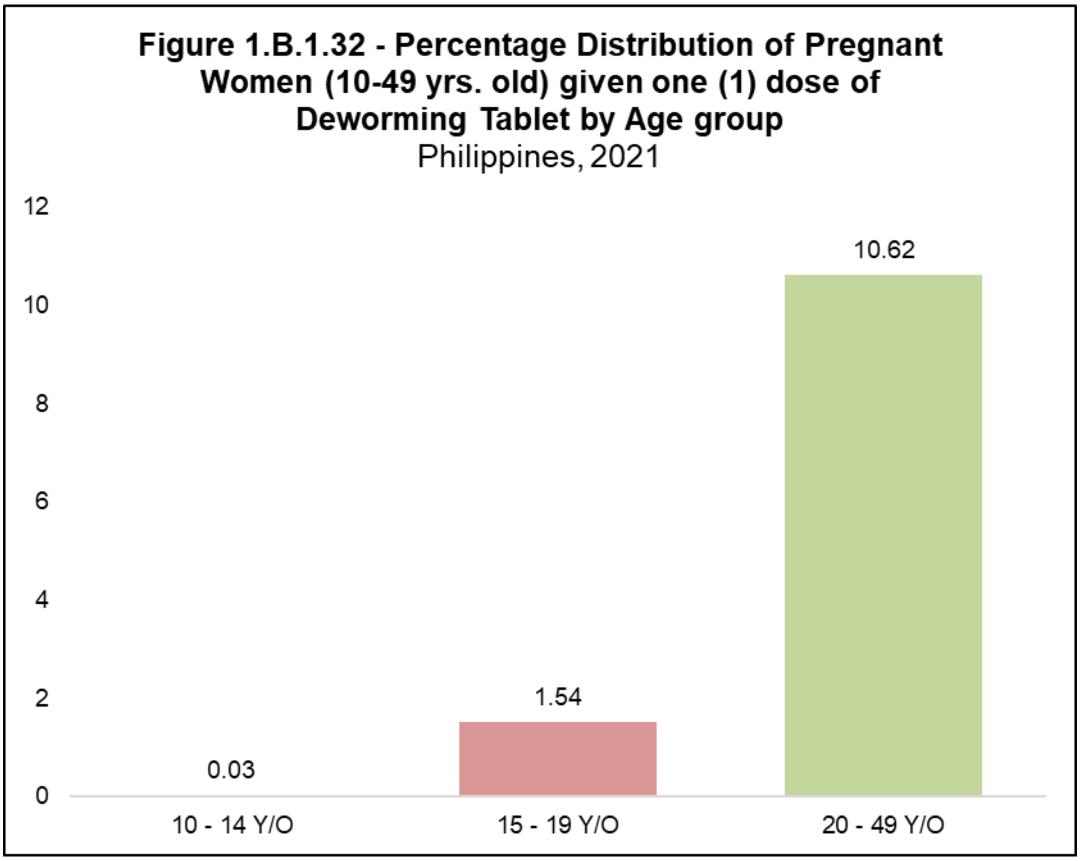
Figure 1.B.1.32 - Percentage Distribution of Pregnant Women (10-49 yrs. old) given one (1) dose of Deworming Tablet by Age group
Philippines, 2021
12
10
8
6
4
2
0
0.03
1.54
10.62
10 - 14 Y/O
15 - 19 Y/O
20 - 49 Y/O

Figure 1.B.1.33 - Percentage Distribution of Pregnant Women (10-14 yrs. old) screened for Syphillis by Region
Philippines, 2021

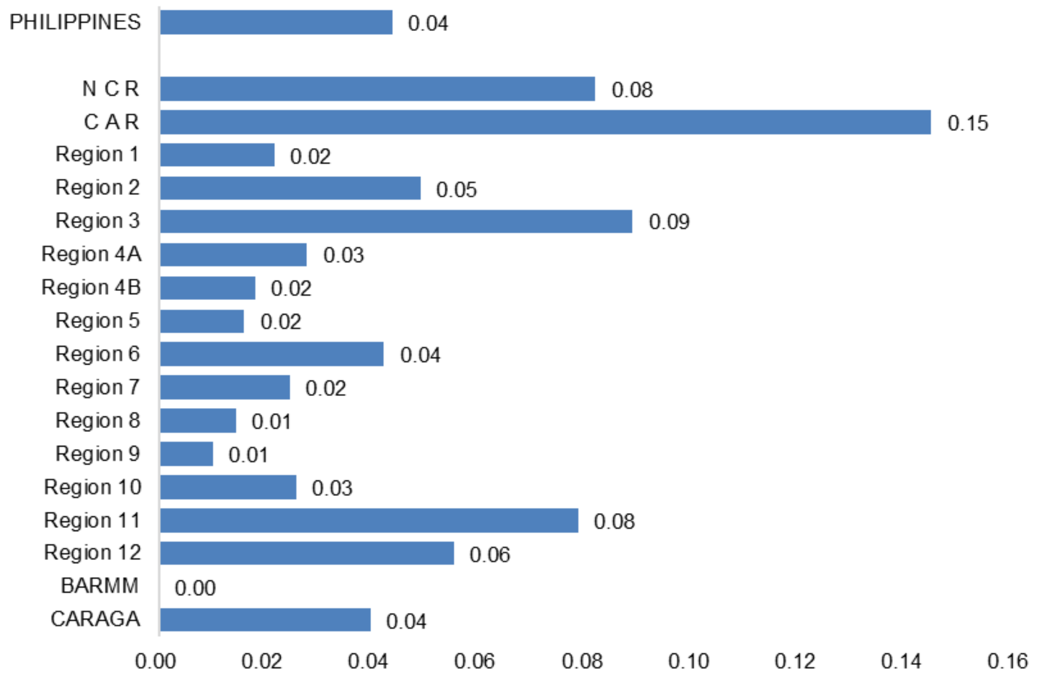

Figure 1.B.1.34 - Percentage Distribution of Pregnant Women (15-19 yrs. old) screened for Syphillis by Region
Philippines, 2021

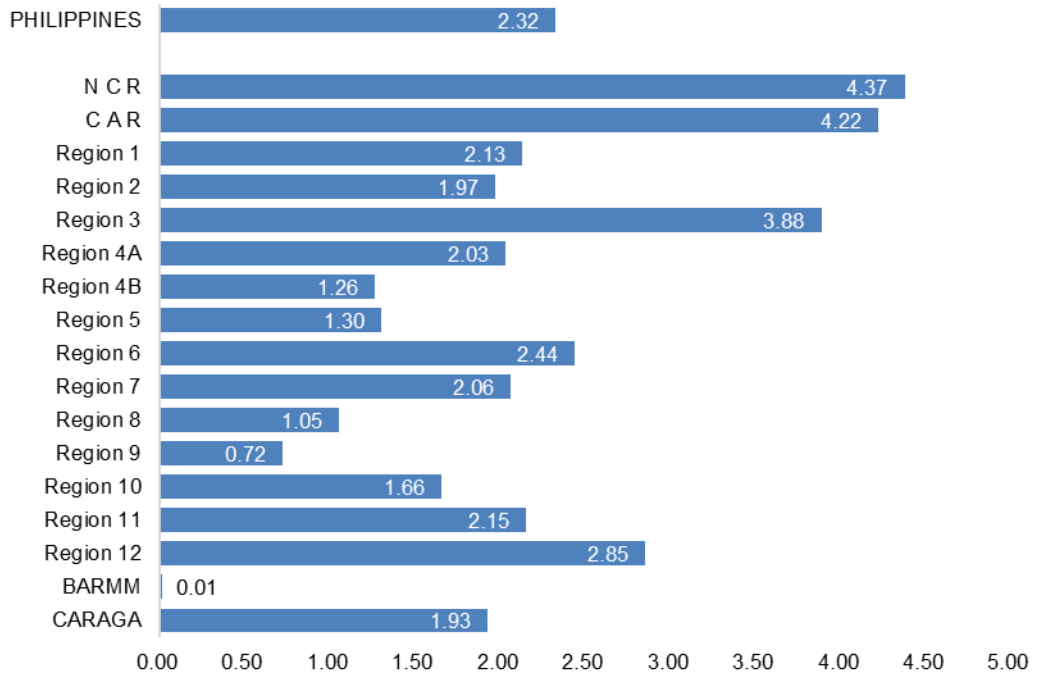

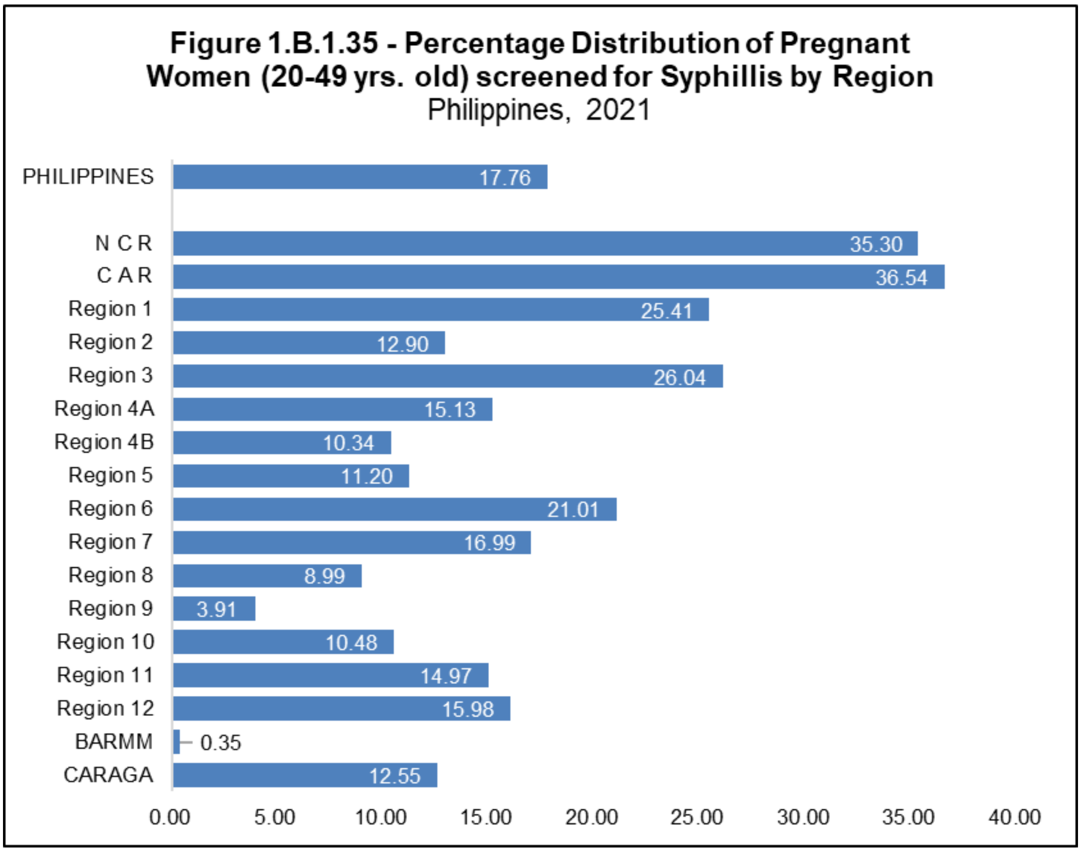
Figure 1.B.1.35 - Percentage Distribution of Pregnant Women (20-49 yrs. old) screened for Syphillis by Region
Philippines, 2021
PHILIPPINES
N C R
C A R
Region 1
Region 2
Region 3
Region 4A
Region 4B
Region 5
Region 6
Region 7
Region 8
Region 9
Region 10
Region 11
Region 12
BARMM
CARAGA
17.76
35.30
36.54
25.41
12.90
26.04
15.13
10.34
11.20
21.01
16.99
8.99
3.91
10.48
14.97
15.98
0.35
12.55
0.00
5.00
10.00
15.00
20.00
25.00
30.00
35.00
40.00

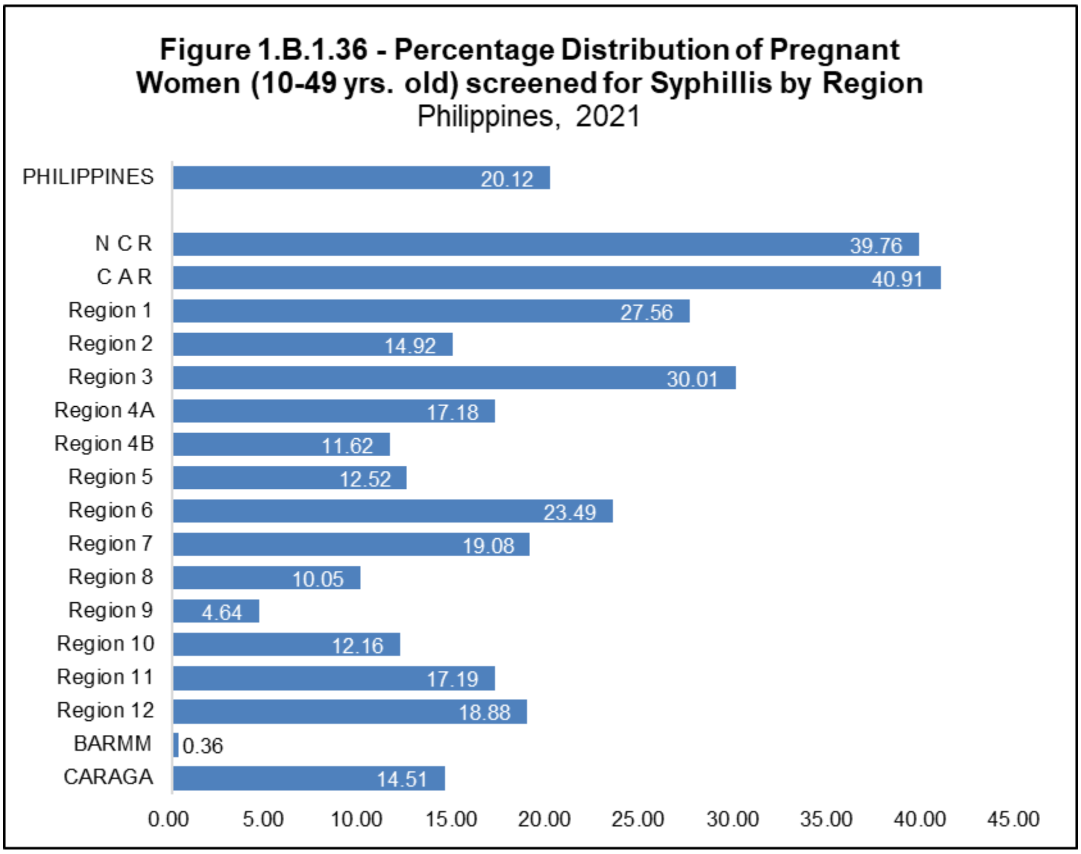
Figure 1.B.1.36 - Percentage Distribution of Pregnant Women (10-49 yrs. old) screened for Syphillis by Region
Philippines, 2021
PHILIPPINES
N C R
C A R
Region 1
Region 2
Region 3
Region 4A
Region 4B
Region 5
Region 6
Region 7
Region 8
Region 9
Region 10
Region 11
Region 12
BARMM
CARAGA
20.12
39.76
40.91
27.56
14.92
30.01
17.18
11.62
12.52
23.49
19.08
10.05
4.64
12.16
17.19
18.88
0.36
14.51
0.00
5.00
10.00
15.00
20.00
25.00
30.00
35.00
40.00
45.00

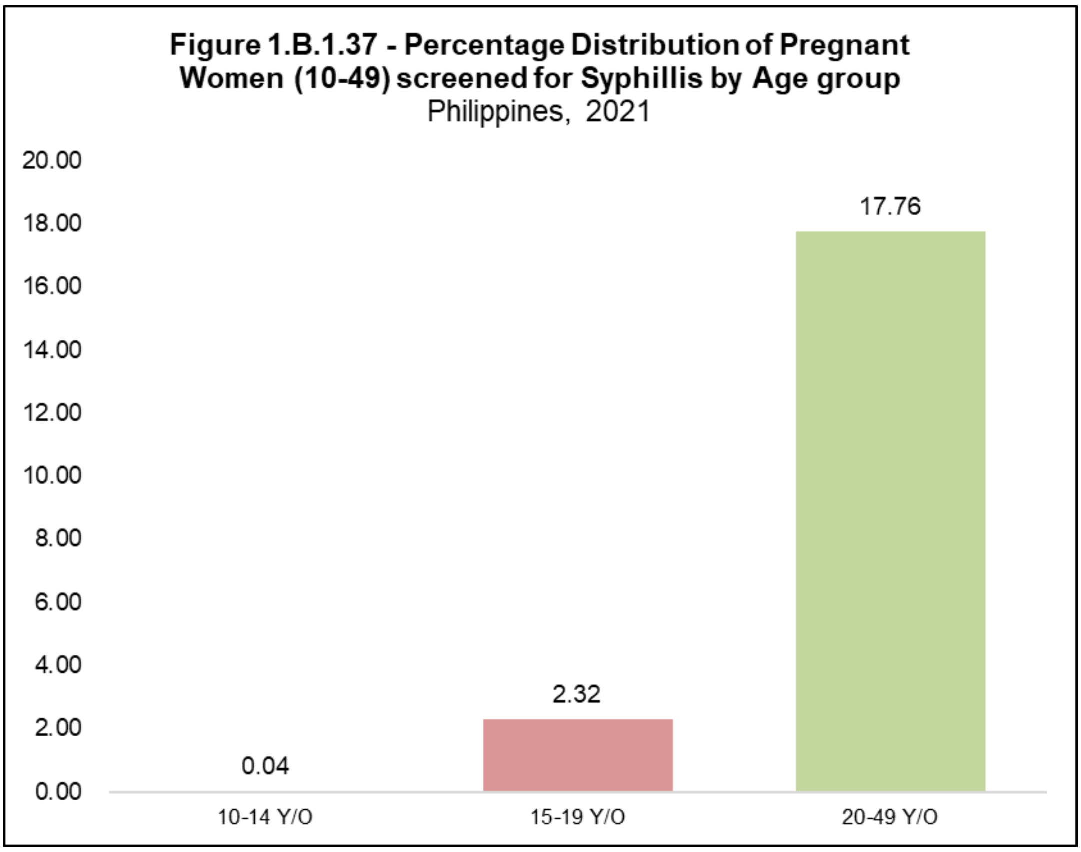
Figure 1.B.1.37 - Percentage Distribution of Pregnant Women (10-49) screened for Syphillis by Age group
Philippines, 2021
20.00
18.00
16.00
14.00
12.00
10.00
8.00
6.00
4.00
2.00
0.00
17.76
2.32
0.04
10-14 Y/O
15-19 Y/O
20-49 Y/O

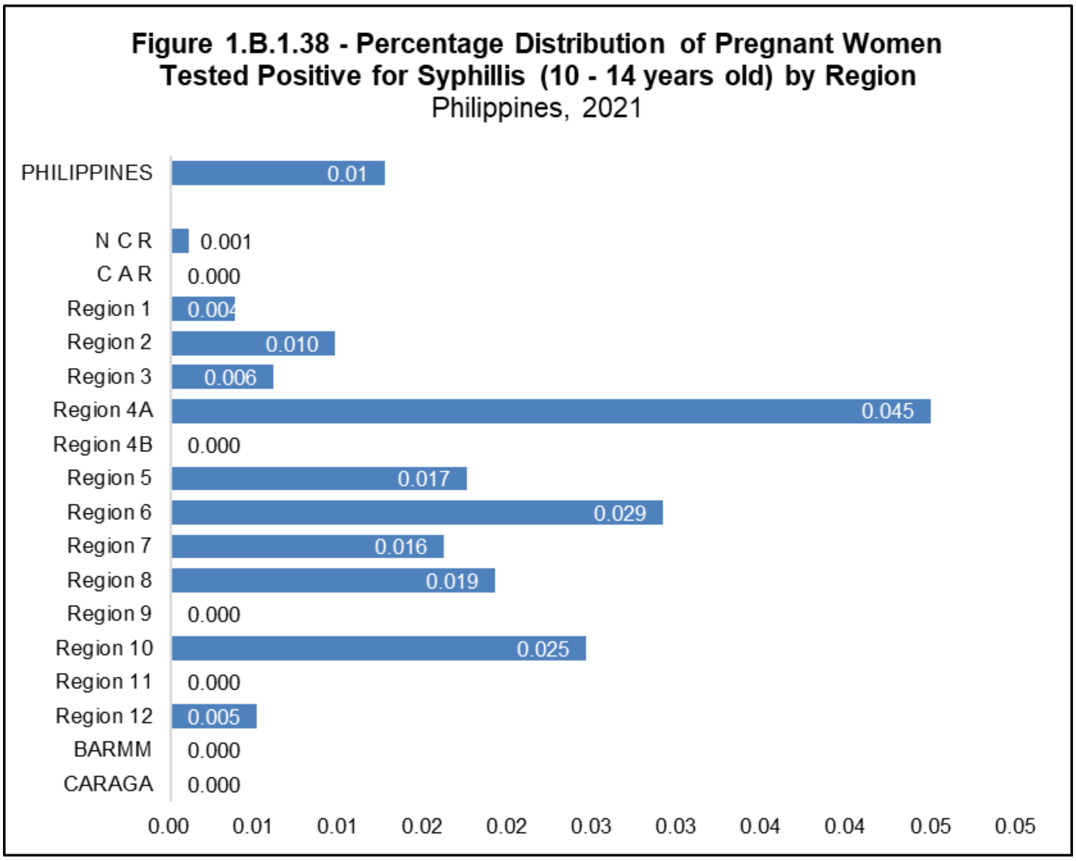
Figure 1.B.1.38 - Percentage Distribution of Pregnant Women Tested Positive for Syphillis (10 - 14 years old) by Region
Philippines, 2021
PHILIPPINES 0.01
N C R 0.001
C A R 0.000
Region 1 0.004
Region 2 0.010
Region 3 0.006
Region 4A 0.045
Region 4B 0.000
Region 5 0.017
Region 6 0.029
Region 7 0.016
Region 8 0.019
Region 9 0.000
Region 10 0.025
Region 11 0.000
Region 12 0.005
BARMM 0.000
CARAGA 0.000
0.00 0.01 0.01 0.02 0.02 0.03 0.03 0.04 0.04 0.05 0.05

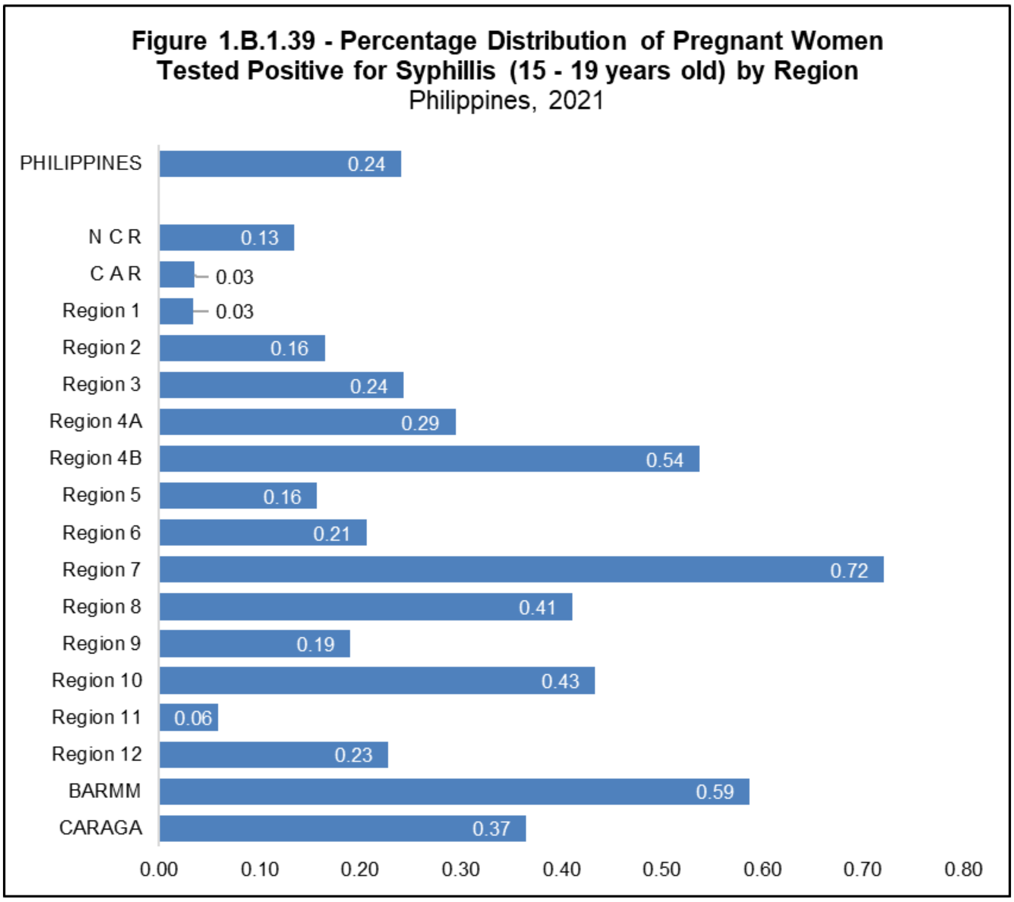
Figure 1.B.1.39 - Percentage Distribution of Pregnant Women
Tested Positive for Syphillis (15 - 19 years old) by Region
Philippines, 2021
PHILIPPINES
0.24
N C R
0.13
C A R
0.03
Region 1
0.03
Region 2
0.16
Region 3
0.24
Region 4A
0.29
Region 4B
0.54
Region 5
0.16
Region 6
0.21
Region 7
0.72
Region 8
0.41
Region 9
0.19
Region 10
0.43
Region 11
0.06
Region 12
0.23
BARMM
0.59
CARAGA
0.37
0.00
0.10
0.20
0.30
0.40
0.50
0.60
0.70
0.80

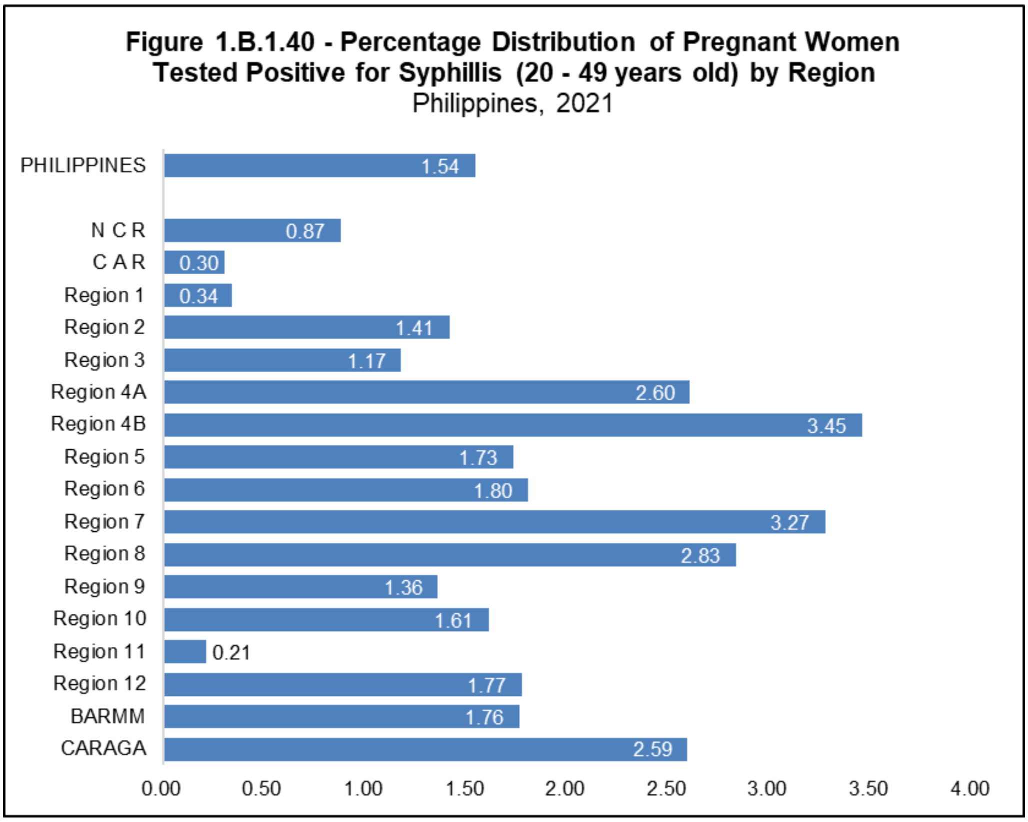
Figure 1.B.1.40 - Percentage Distribution of Pregnant Women
Tested Positive for Syphillis (20 - 49 years old) by Region
Philippines, 2021
PHILIPPINES
1.54
N C R
0.87
C A R
0.30
Region 1
0.34
Region 2
1.41
Region 3
1.17
Region 4A
2.60
Region 4B
3.45
Region 5
1.73
Region 6
1.80
Region 7
3.27
Region 8
2.83
Region 9
1.36
Region 10
1.61
Region 11
0.21
Region 12
1.77
BARMM
1.76
CARAGA
2.59
0.00
0.50
1.00
1.50
2.00
2.50
3.00
3.50
4.00

Figure 1.B.1.41 - Percentage Distribution of Pregnant Women tested positive for Syphilis (10-49 years old) by Region
Philippines, 2021

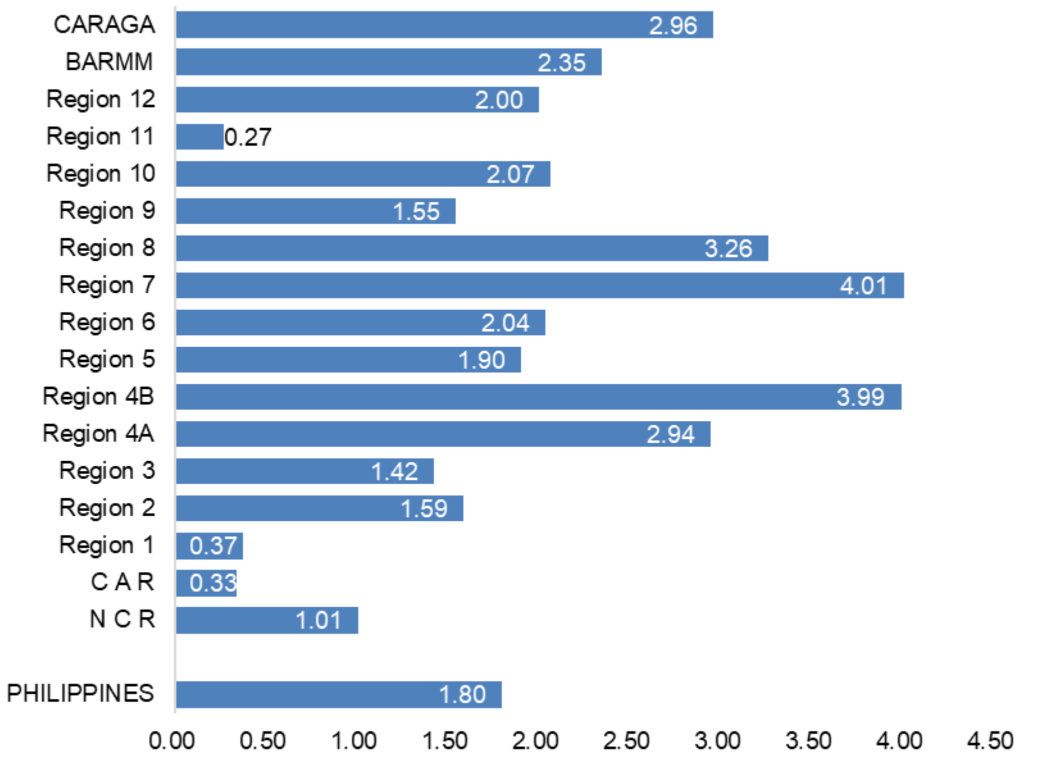

Figure 1.B.1.42 - Percentage Distribution of Pregnant Women (10-49 years old) Tested Positive for Syphillis by Age group
Philippines, 2021

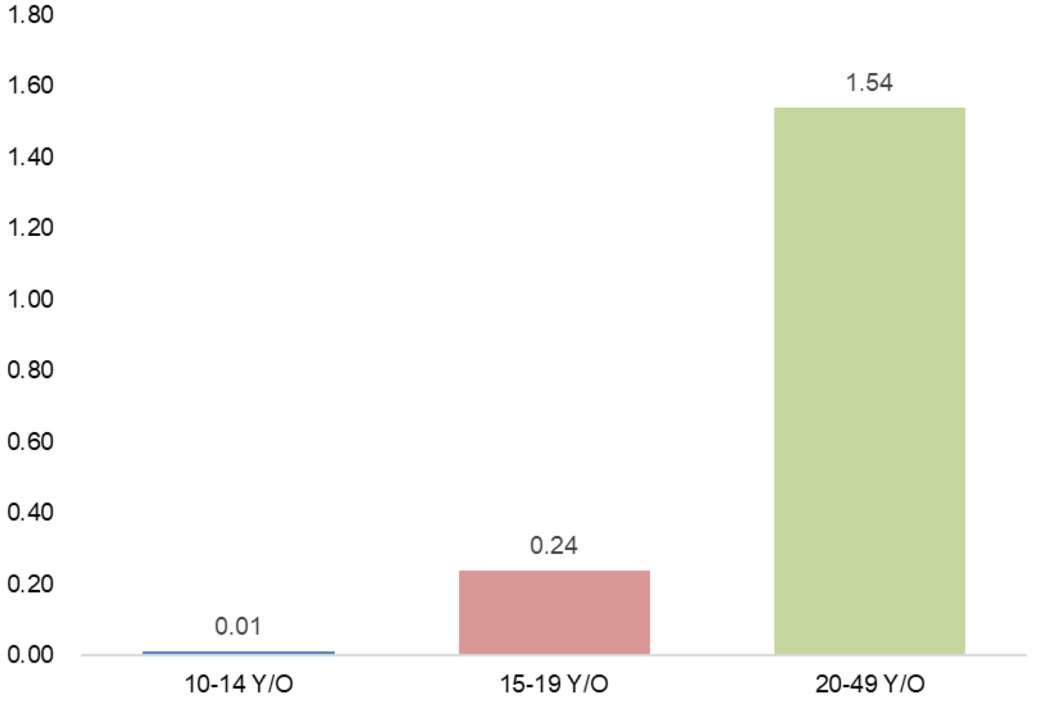

Figure 1.B.1.43 - Percentage Distribution of Pregnant Women (10-14 years old) screened for Hepatitis B by Region
Philippines, 2021

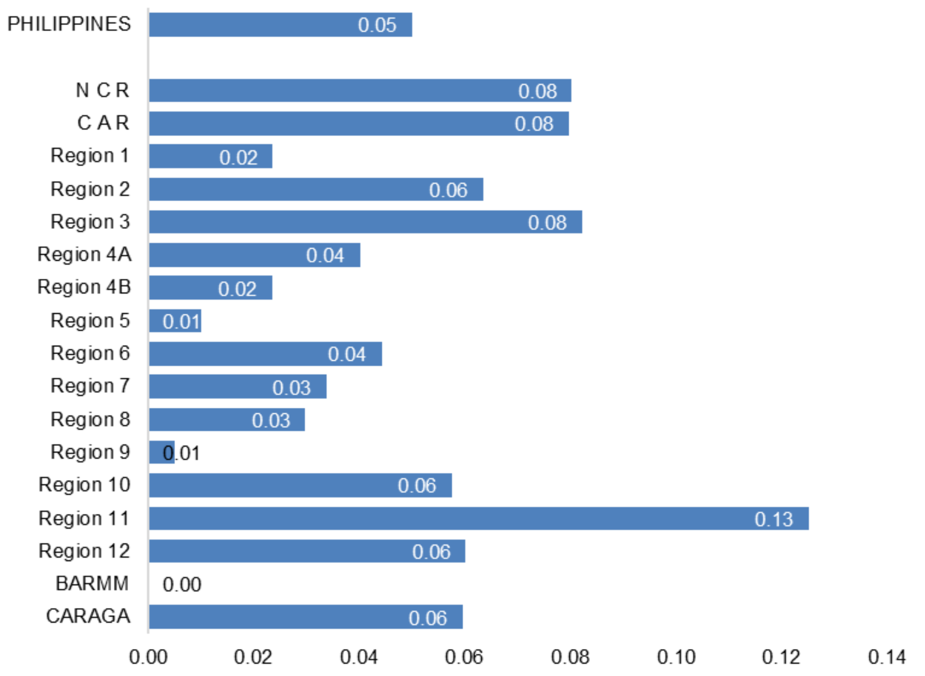

Figure 1.B.1.44 - Percentage Distribution of Pregnant Women (15-19 years old) screened for Hepatitis B by Region
Philippines, 2021

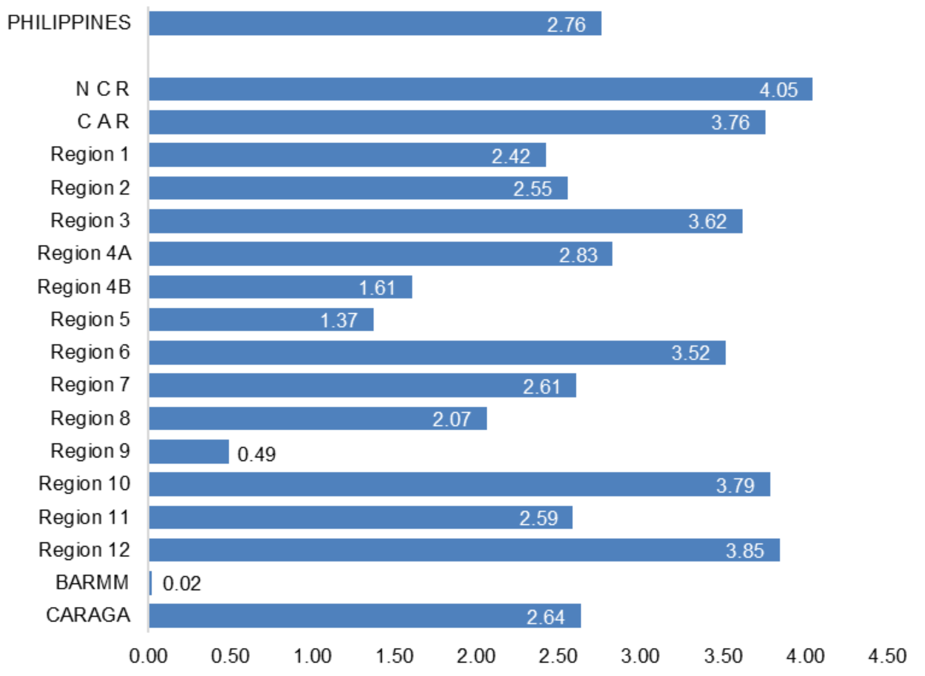

Figure 1.B.1.45 - Percentage Distribution of Pregnant Women (20-49 years old) screened for Hepatitis B by Region
Philippines, 2021

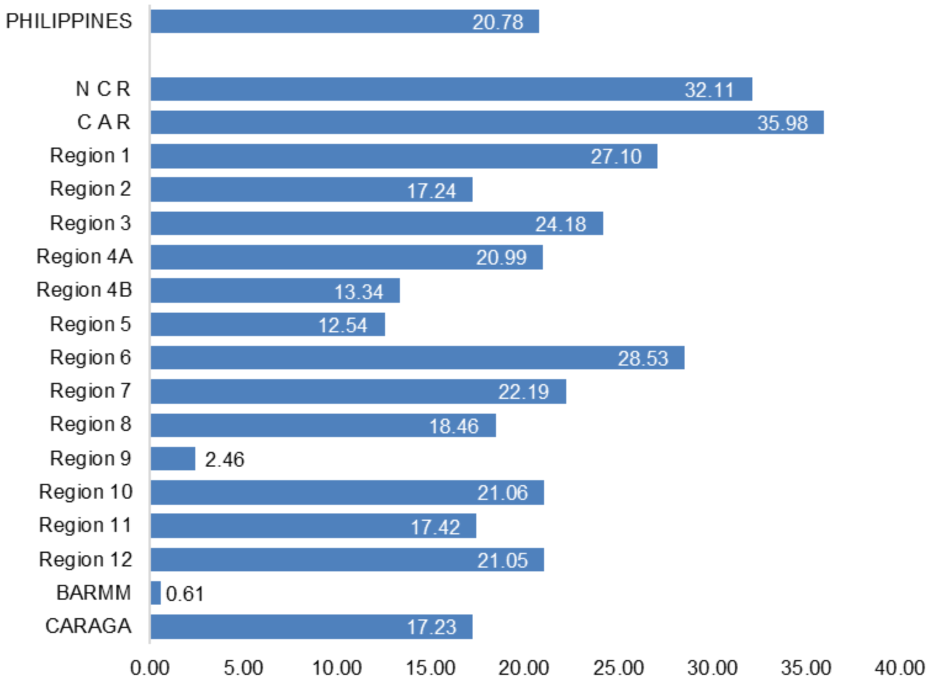

Figure 1.B.1.46 - Percentage Distribution of Pregnant Women (10 - 49 years old) screened for Hepatitis B by Region
Philippines, 2021

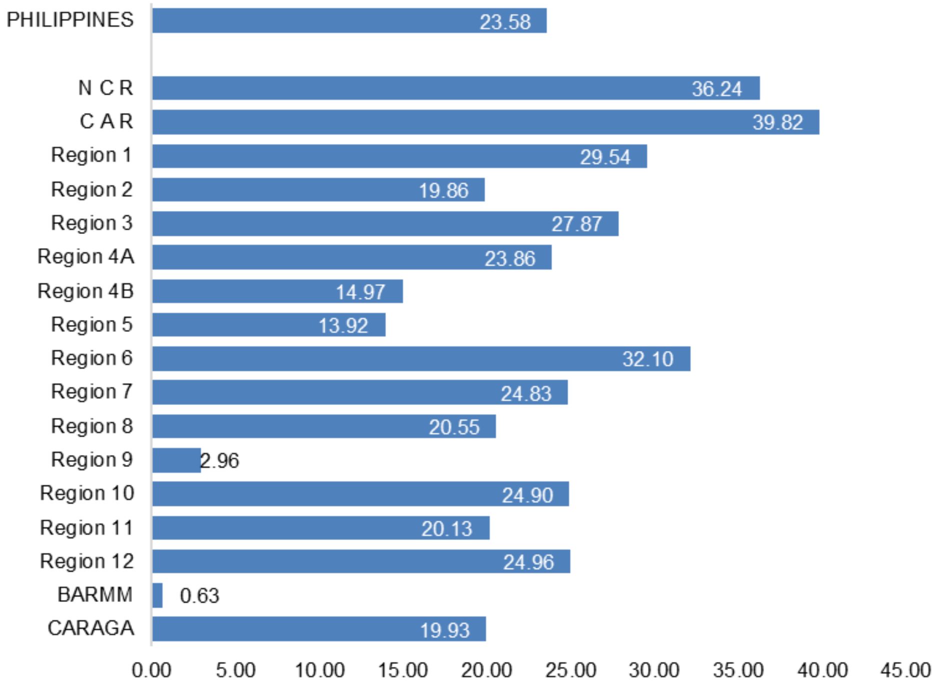

Figure 1.B.1.47 - Percentage Distribution of Pregnant Women (10-49 years old) screened for Hepatitis B by Age group
Philippines, 2021

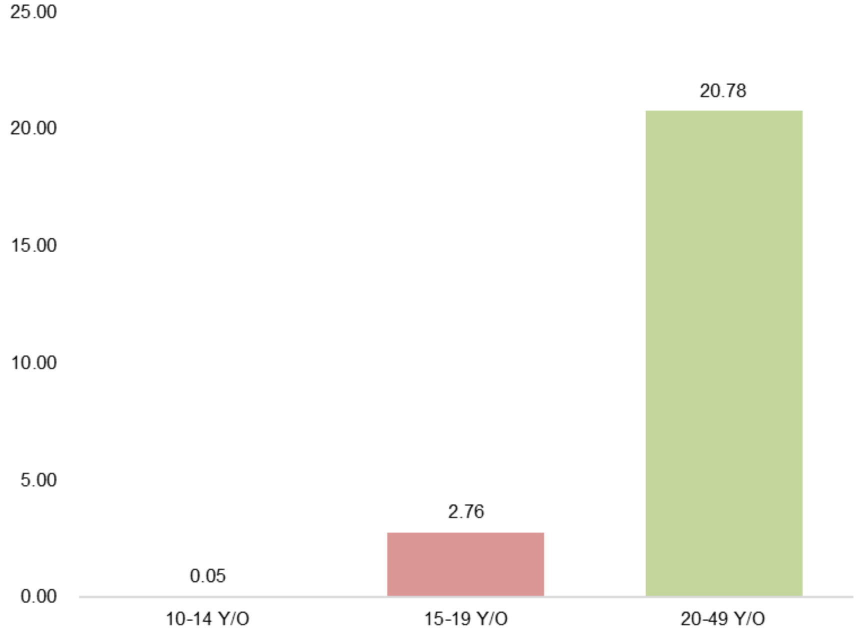

Figure 1.B.1.48 - Percentage Distribution of Pregnant Women (10-14 years old) Tested Positive for Hepatitis B by Region
Philippines, 2021

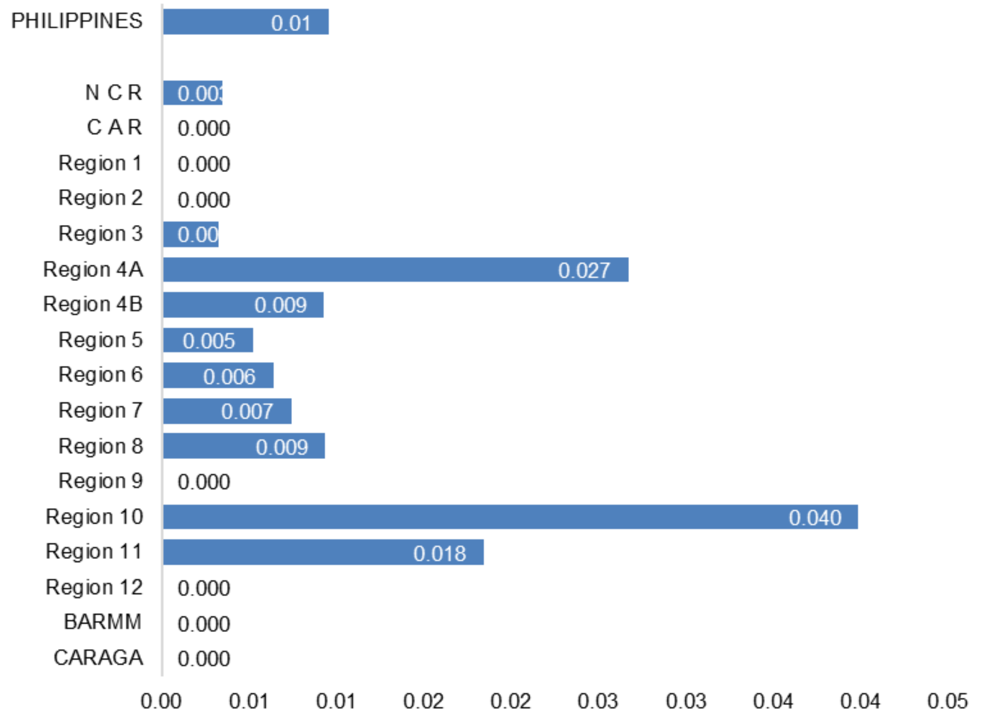

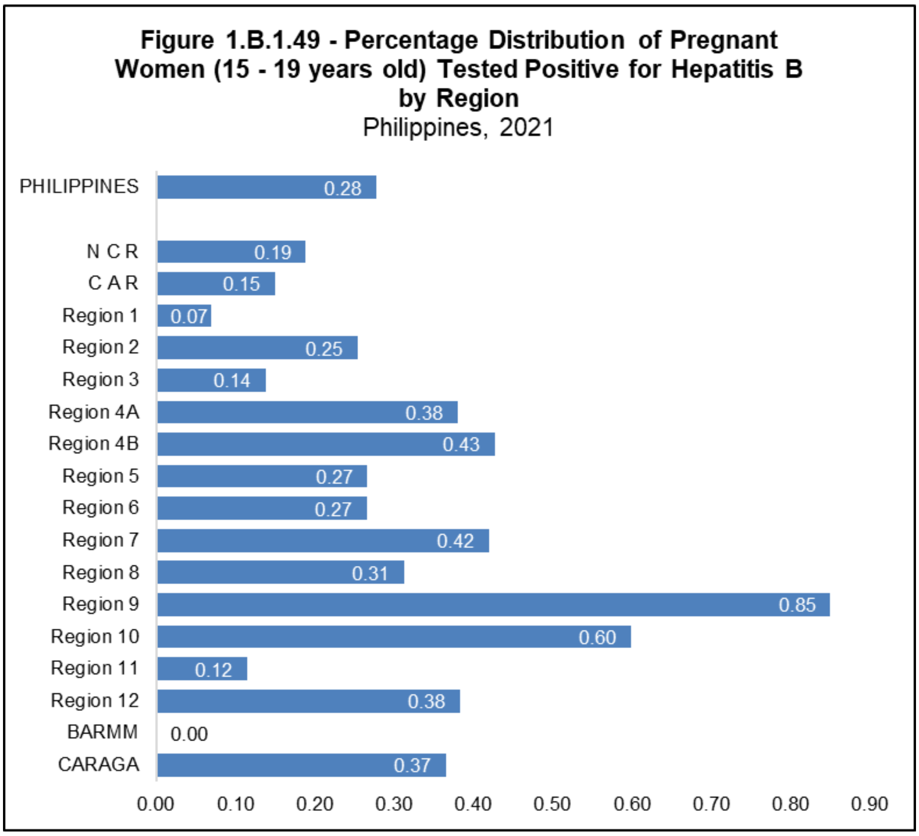
Figure 1.B.1.49 - Percentage Distribution of Pregnant Women (15 - 19 years old) Tested Positive for Hepatitis B by Region
Philippines, 2021
PHILIPPINES 0.28
N C R 0.19
C A R 0.15
Region 1 0.07
Region 2 0.25
Region 3 0.14
Region 4A 0.38
Region 4B 0.43
Region 5 0.27
Region 6 0.27
Region 7 0.42
Region 8 0.31
Region 9 0.85
Region 10 0.60
Region 11 0.12
Region 12 0.38
BARMM 0.00
CARAGA 0.37
0.00 0.10 0.20 0.30 0.40 0.50 0.60 0.70 0.80 0.90

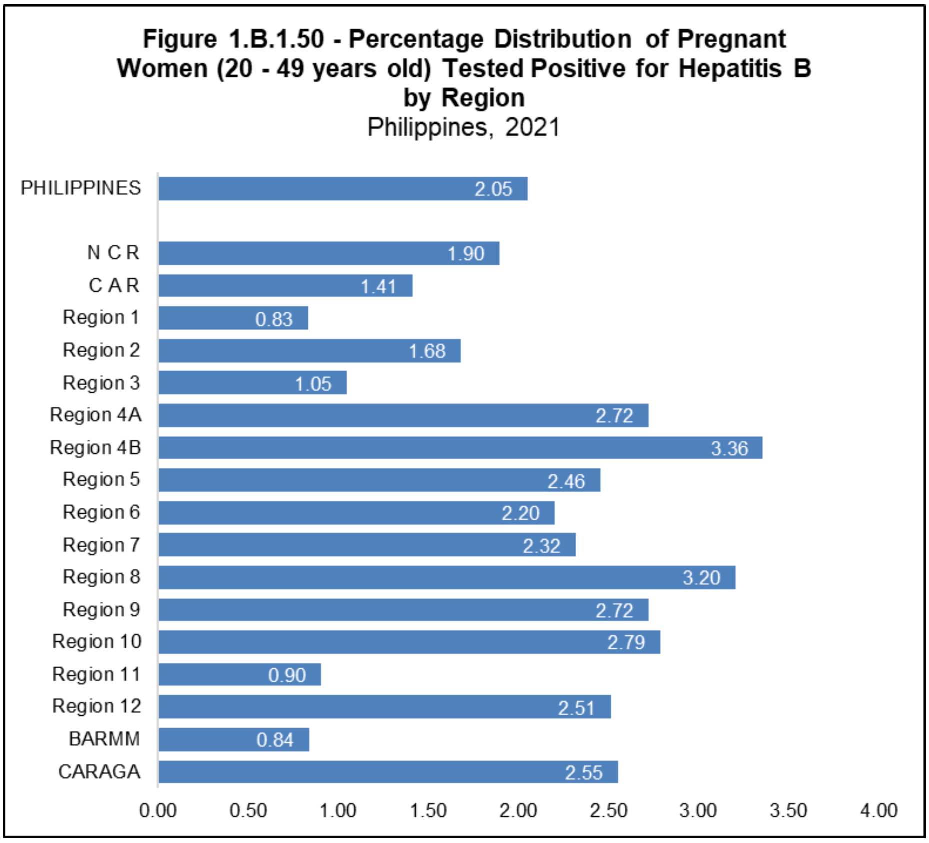
Figure 1.B.1.50 - Percentage Distribution of Pregnant Women (20 - 49 years old) Tested Positive for Hepatitis B by Region
Philippines, 2021
PHILIPPINES 2.05
N C R 1.90
C A R 1.41
Region 1 0.83
Region 2 1.68
Region 3 1.05
Region 4A 2.72
Region 4B 3.36
Region 5 2.46
Region 6 2.20
Region 7 2.32
Region 8 3.20
Region 9 2.72
Region 10 2.79
Region 11 0.90
Region 12 2.51
BARMM 0.84
CARAGA 2.55
0.00 0.50 1.00 1.50 2.00 2.50 3.00 3.50 4.00

Figure 1.B.1.51 - Percentage Distribution of Pregnant Women (10 - 49 years old) Tested Positive for Hepatitis B by Region
Philippines, 2021

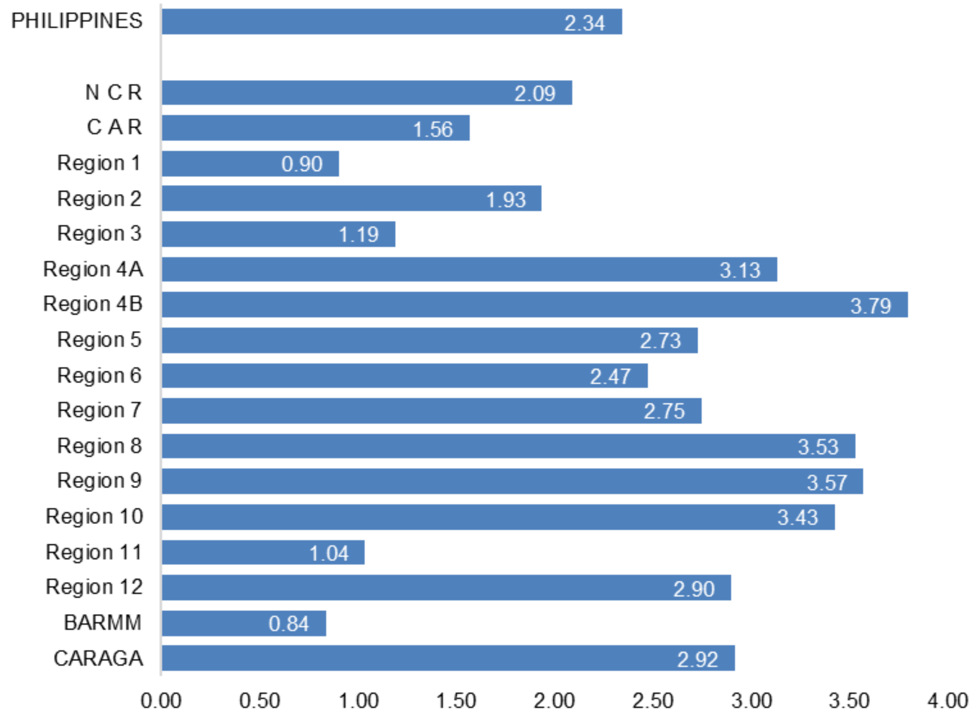

Figure 1.B.1.52 - Percentage Distribution of Pregnant Women Tested Positive for Hepatitis B by Age group
Philippines, 2021

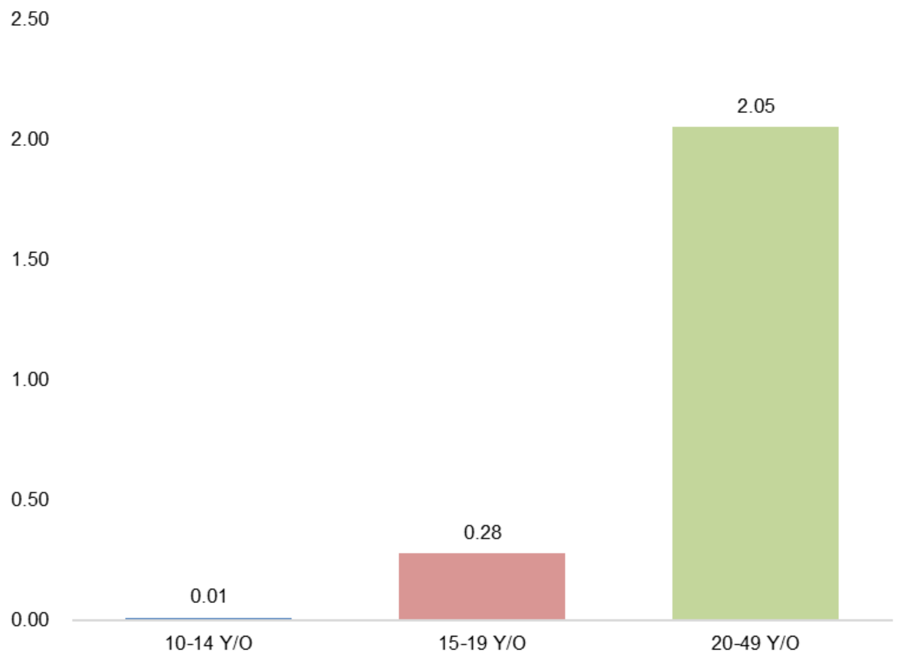

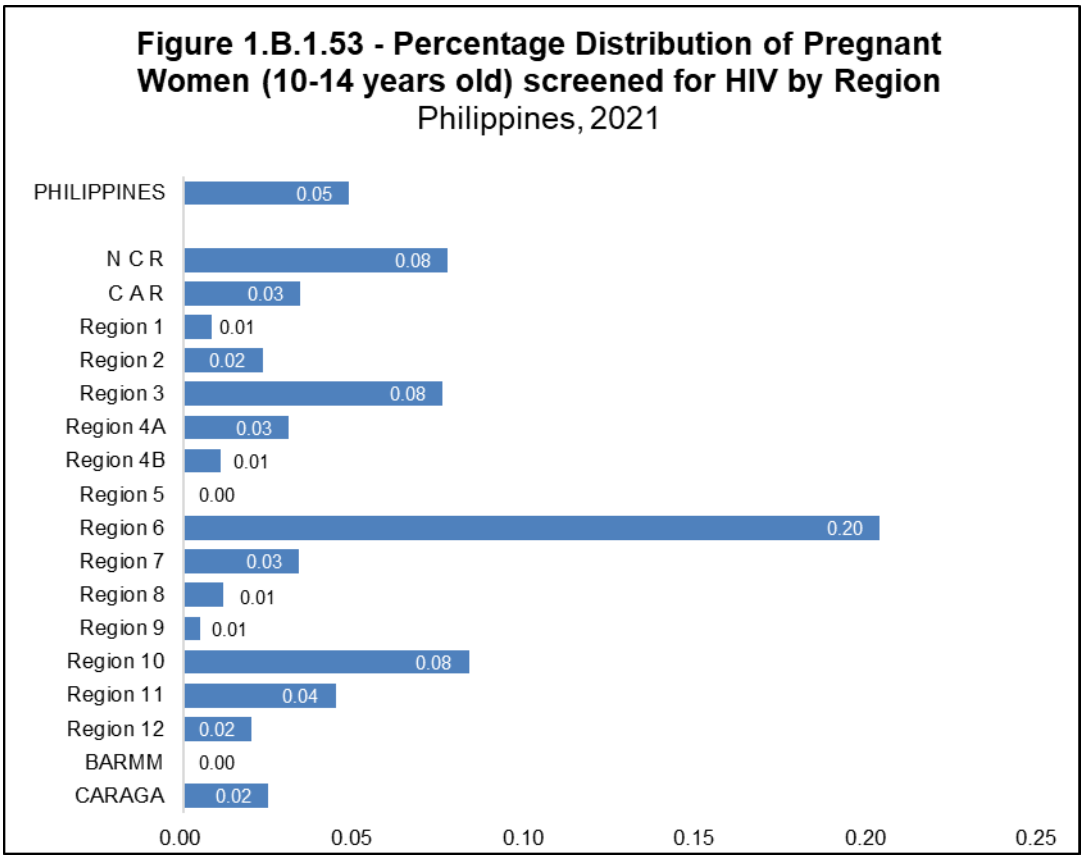
Figure 1.B.1.53 - Percentage Distribution of Pregnant Women (10-14 years old) screened for HIV by Region
Philippines, 2021
PHILIPPINES
0.05
N C R
0.08
C A R
0.03
Region 1
0.01
Region 2
0.02
Region 3
0.08
Region 4A
0.03
Region 4B
0.01
Region 5
0.00
Region 6
0.20
Region 7
0.03
Region 8
0.01
Region 9
0.01
Region 10
0.08
Region 11
0.04
Region 12
0.02
BARMM
0.00
CARAGA
0.02
0.00
0.05
0.10
0.15
0.20
0.25

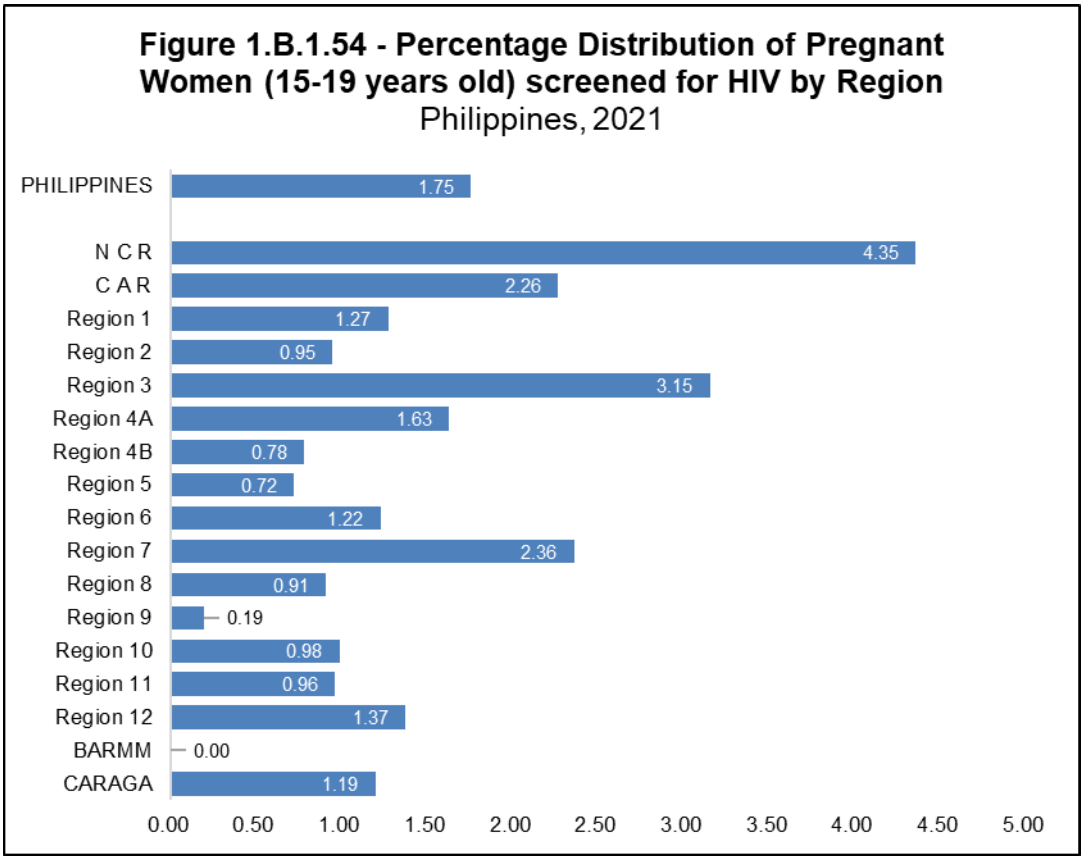
Figure 1.B.1.54 - Percentage Distribution of Pregnant Women (15-19 years old) screened for HIV by Region
Philippines, 2021
PHILIPPINES
1.75
N C R
4.35
C A R
2.26
Region 1
1.27
Region 2
0.95
Region 3
3.15
Region 4A
1.63
Region 4B
0.78
Region 5
0.72
Region 6
1.22
Region 7
2.36
Region 8
0.91
Region 9
0.19
Region 10
0.98
Region 11
0.96
Region 12
1.37
BARMM
0.00
CARAGA
1.19
0.00
0.50
1.00
1.50
2.00
2.50
3.00
3.50
4.00
4.50
5.00

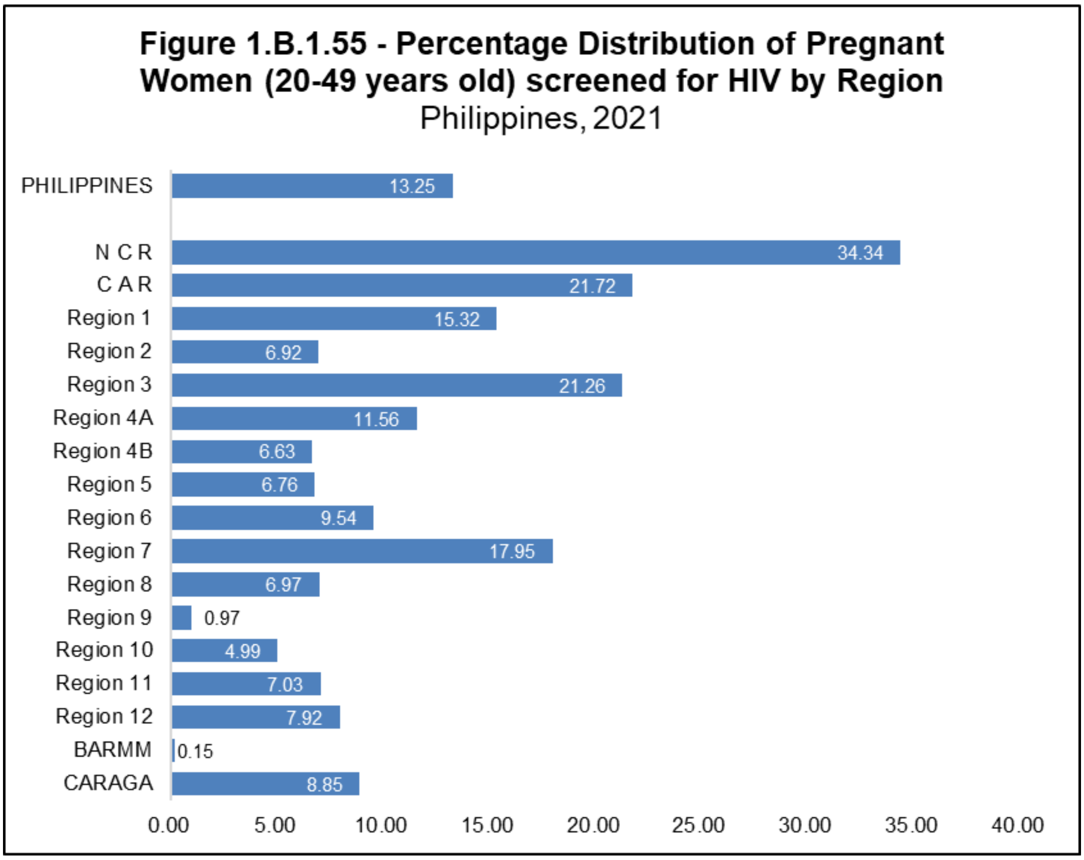

Figure 1.B.1.55 - Percentage Distribution of Pregnant Women (20-49 years old) screened for HIV by Region
Philippines, 2021
PHILIPPINES
13.25
N C R
34.34
C A R
21.72
Region 1
15.32
Region 2
6.92
Region 3
21.26
Region 4A
11.56
Region 4B
6.63
Region 5
6.76
Region 6
9.54
Region 7
17.95
Region 8
6.97
Region 9
0.97
Region 10
4.99
Region 11
7.03
Region 12
7.92
BARMM
0.15
CARAGA
8.85
0.00
5.00
10.00
15.00
20.00
25.00
30.00
35.00
40.00

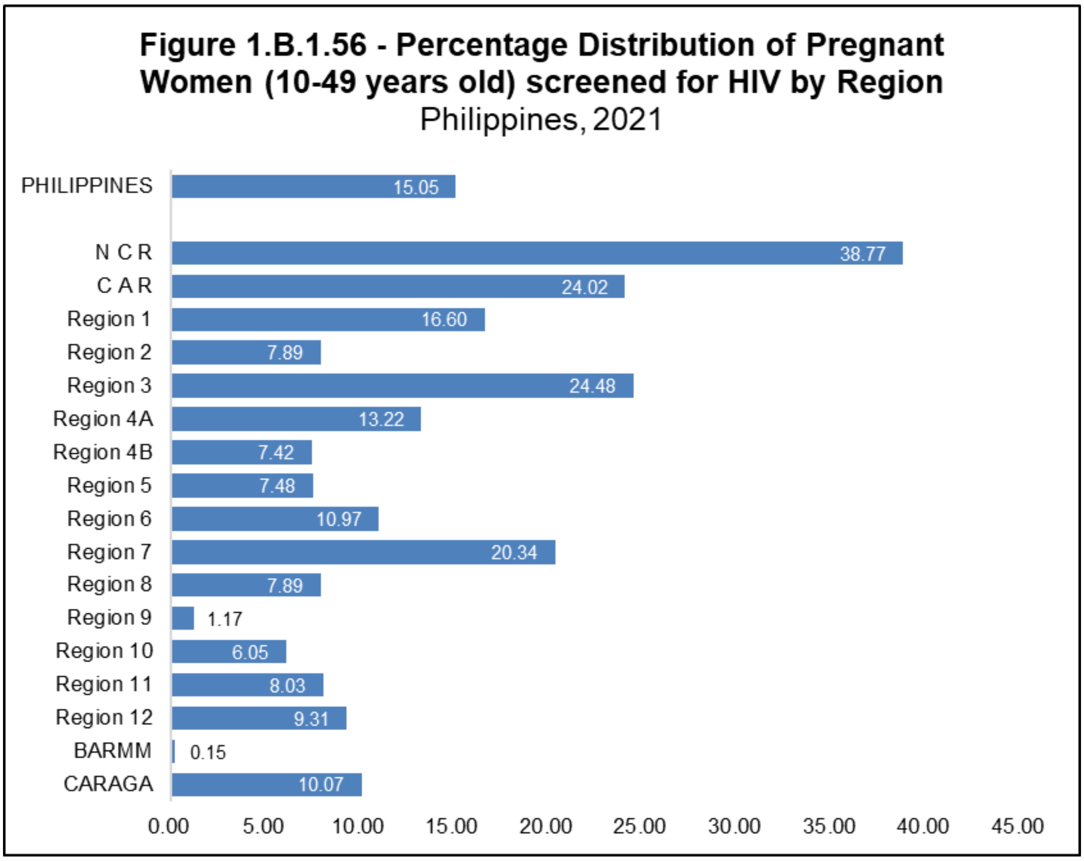

Figure 1.B.1.56 - Percentage Distribution of Pregnant Women (10-49 years old) screened for HIV by Region
Philippines, 2021
PHILIPPINES
15.05
N C R
38.77
C A R
24.02
Region 1
16.60
Region 2
7.89
Region 3
24.48
Region 4A
13.22
Region 4B
7.42
Region 5
7.48
Region 6
10.97
Region 7
20.34
Region 8
7.89
Region 9
1.17
Region 10
6.05
Region 11
8.03
Region 12
9.31
BARMM
0.15
CARAGA
10.07
0.00
5.00
10.00
15.00
20.00
25.00
30.00
35.00
40.00
45.00

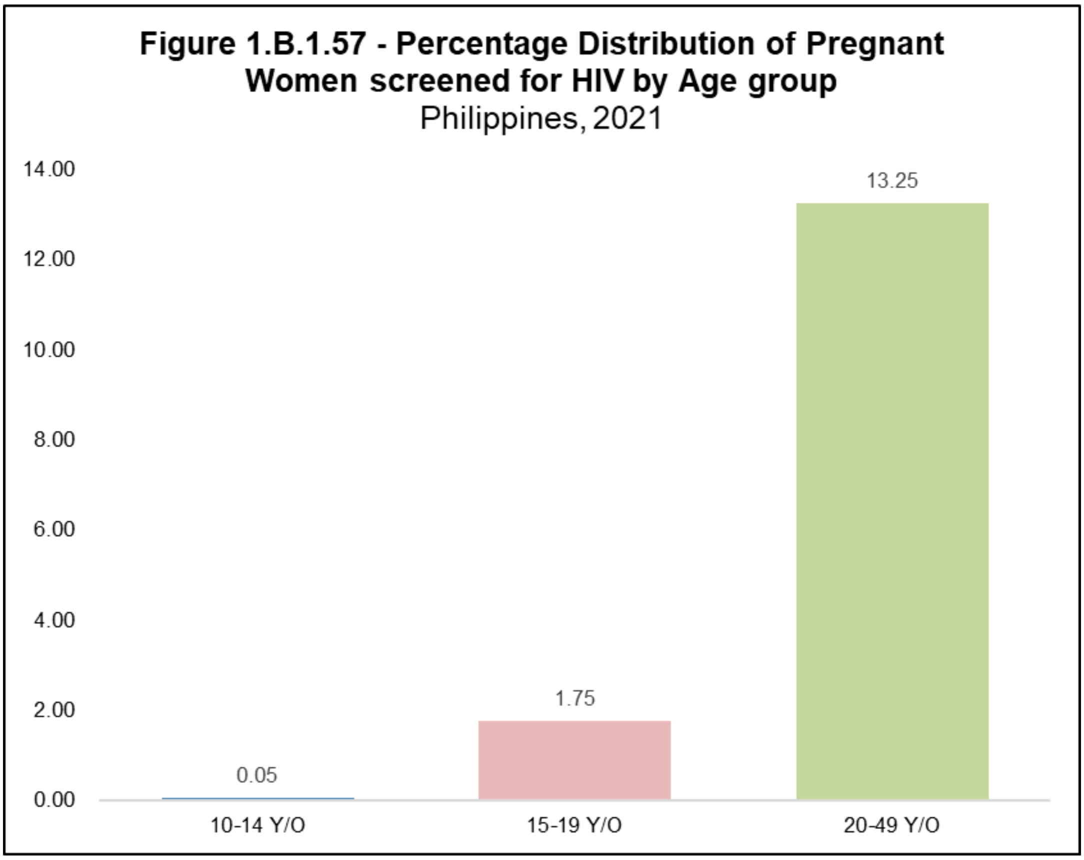
Figure 1.B.1.57 - Percentage Distribution of Pregnant Women screened for HIV by Age group
Philippines, 2021
14.00
12.00
10.00
8.00
6.00
4.00
2.00
0.00
0.05
1.75
13.25
10-14 Y/O
15-19 Y/O
20-49 Y/O

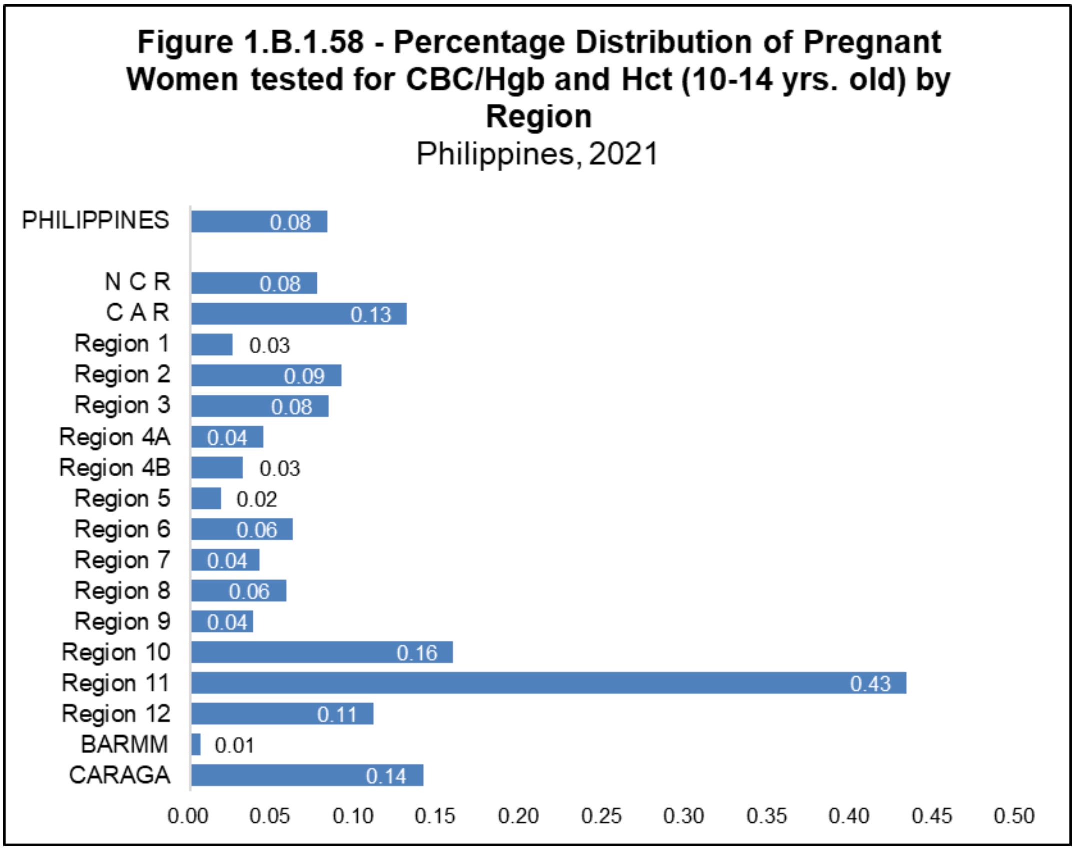
Figure 1.B.1.58 - Percentage Distribution of Pregnant Women tested for CBC/Hgb and Hct (10-14 yrs. old) by Region
Philippines, 2021
PHILIPPINES 0.08
N C R 0.08
C A R 0.13
Region 1 0.03
Region 2 0.09
Region 3 0.08
Region 4A 0.04
Region 4B 0.03
Region 5 0.02
Region 6 0.06
Region 7 0.04
Region 8 0.06
Region 9 0.04
Region 10 0.16
Region 11 0.43
Region 12 0.11
BARMM 0.01
CARAGA 0.14
0.00
0.05
0.10
0.15
0.20
0.25
0.30
0.35
0.40
0.45
0.50

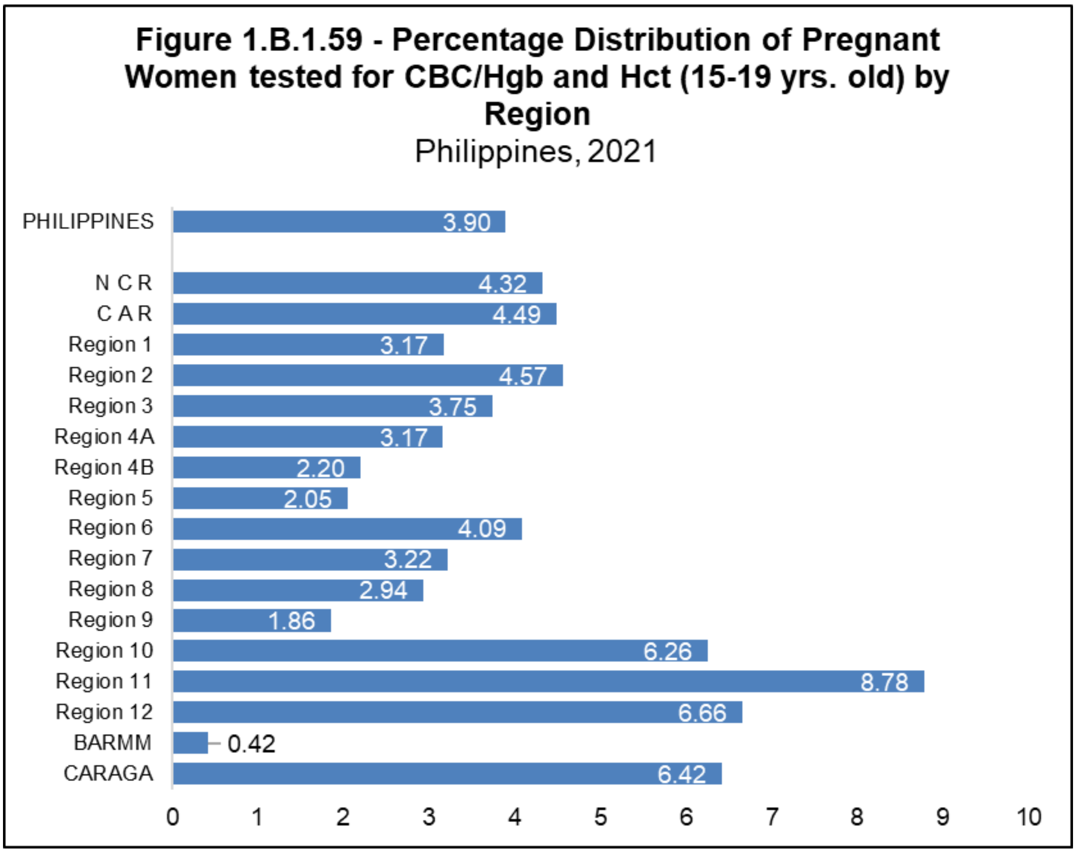
Figure 1.B.1.59 - Percentage Distribution of Pregnant Women tested for CBC/Hgb and Hct (15-19 yrs. old) by Region
Philippines, 2021
PHILIPPINES
3.90
N C R
4.32
C A R
4.49
Region 1
3.17
Region 2
4.57
Region 3
3.75
Region 4A
3.17
Region 4B
2.20
Region 5
2.05
Region 6
4.09
Region 7
3.22
Region 8
2.94
Region 9
1.86
Region 10
6.26
Region 11
8.78
Region 12
6.66
BARMM
0.42
CARAGA
6.42
0 1 2 3 4 5 6 7 8 9 10

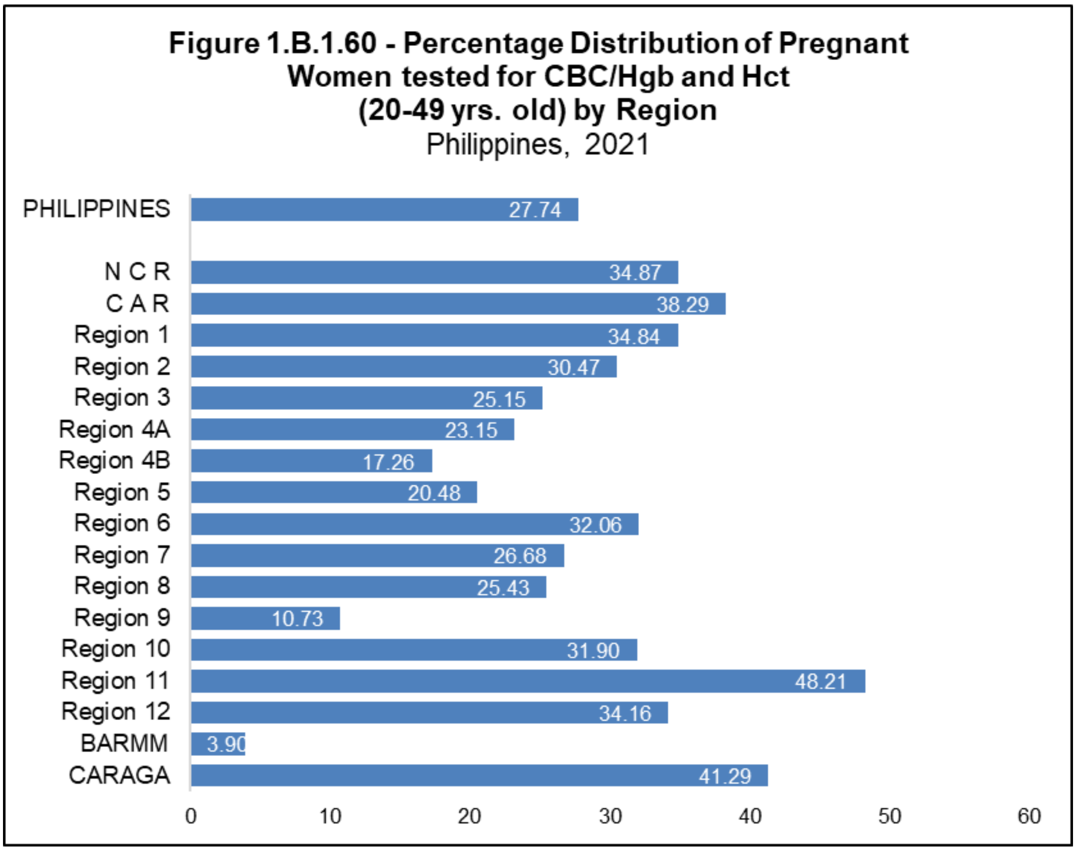
Figure 1.B.1.60 - Percentage Distribution of Pregnant Women tested for CBC/Hgb and Hct (20-49 yrs. old) by Region
Philippines, 2021
PHILIPPINES
27.74
N C R
34.87
C A R
38.29
Region 1
34.84
Region 2
30.47
Region 3
25.15
Region 4A
23.15
Region 4B
17.26
Region 5
20.48
Region 6
32.06
Region 7
26.68
Region 8
25.43
Region 9
10.73
Region 10
31.90
Region 11
48.21
Region 12
34.16
BARMM
3.90
CARAGA
41.29
0 10 20 30 40 50 60

Figure 1.B.1.61 - Percentage Distribution of Pregnant Women tested for CBC/Hgb and Hct (10-49 yrs. old) by Region
Philippines, 2021

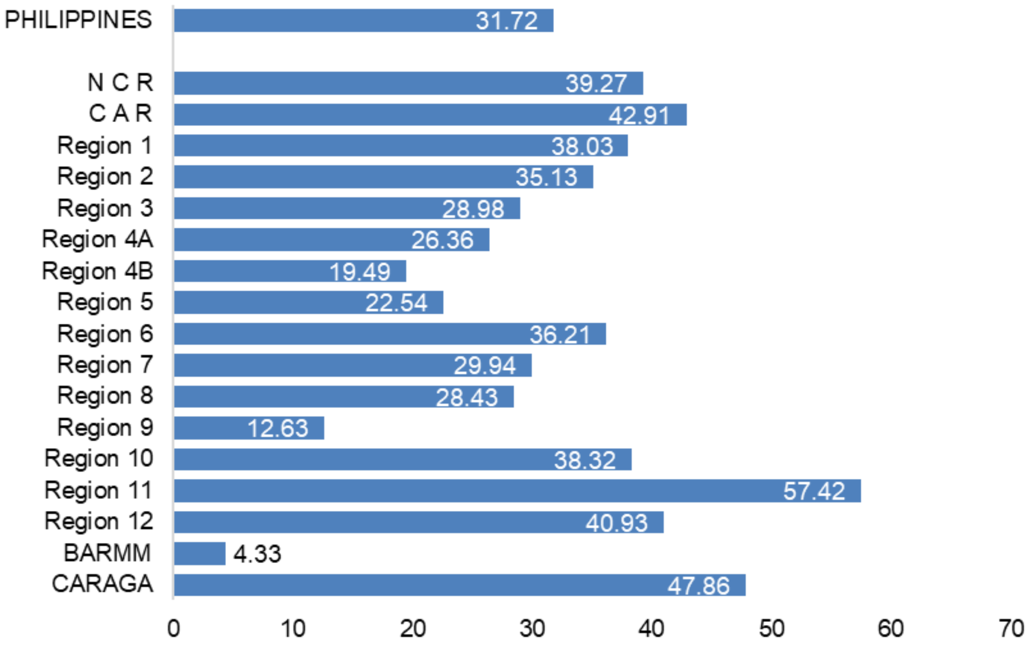

Figure 1.B.1.62 - Percentage Distribution of Pregnant Women tested for CBC/Hgb and Hct by Age group
Philippines, 2021

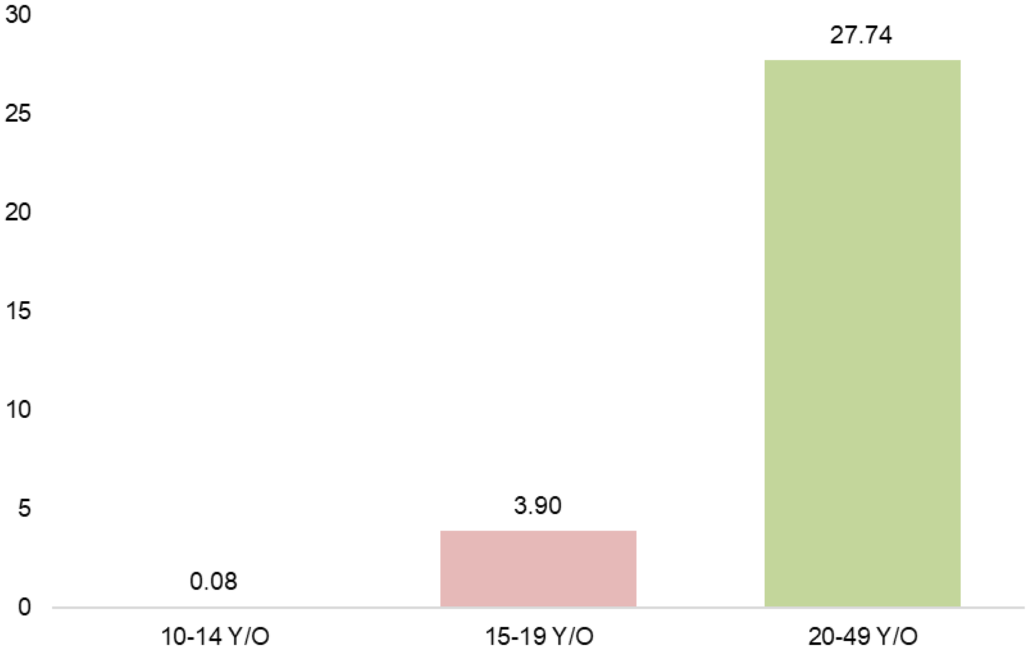

Figure 1.B.1.63 - Percentage Distribution of Pregnant Women tested for CBC/Hgb and Hct diagnosed with anemia (10-14 yrs. old) by Region
Philippines, 2021

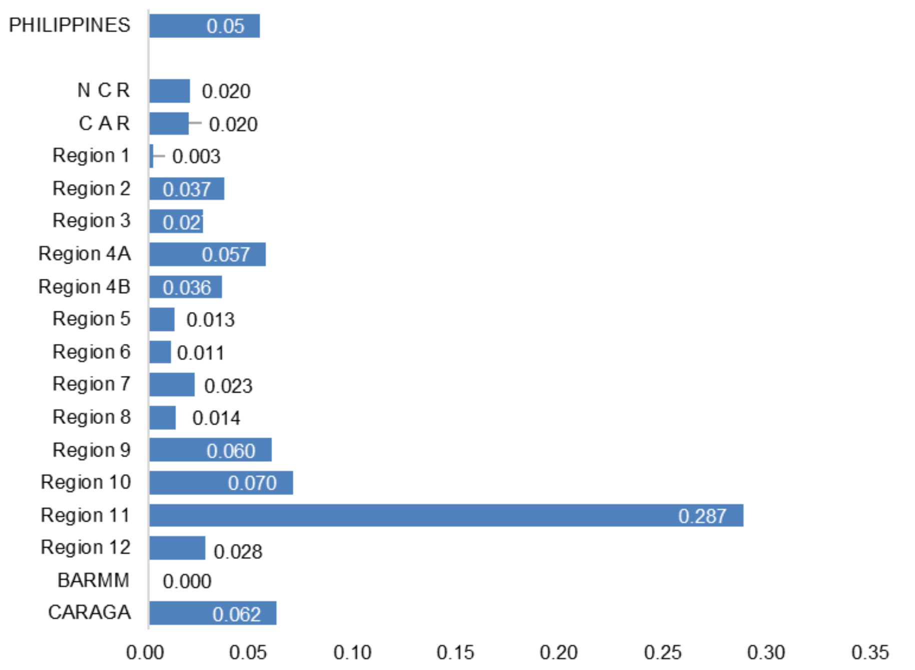

Figure 1.B.1.64 - Percentage Distribution of Pregnant Women tested for CBC/Hgb and Hct diagnosed with anemia (15-19 yrs. old) by Region
Philippines, 2021

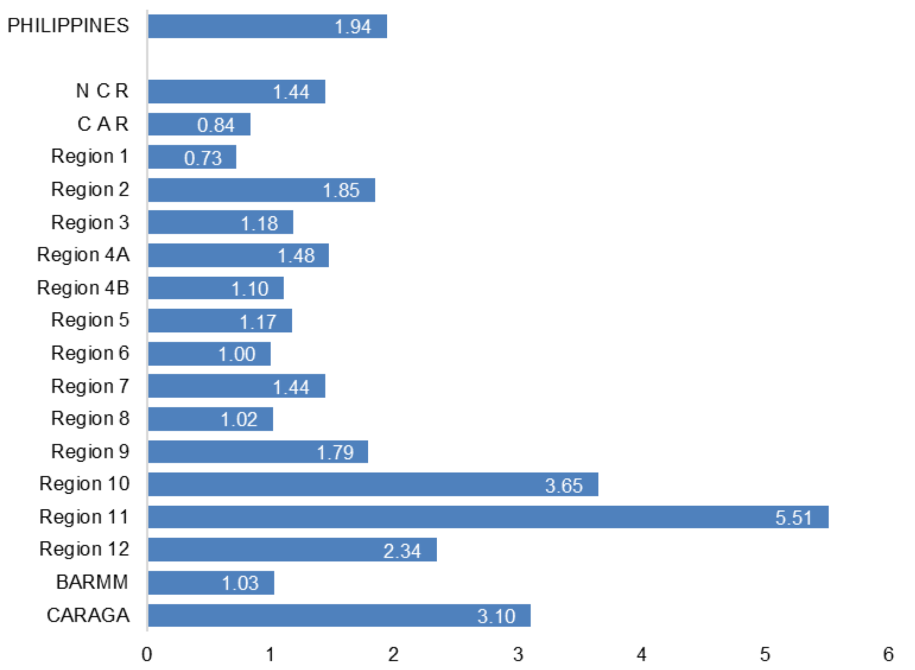

Figure 1.B.1.65 - Percentage Distribution of Pregnant Women tested for CBC/Hgb and Hct diagnosed with anemia (20-49 yrs. old) by Region
Philippines, 2021

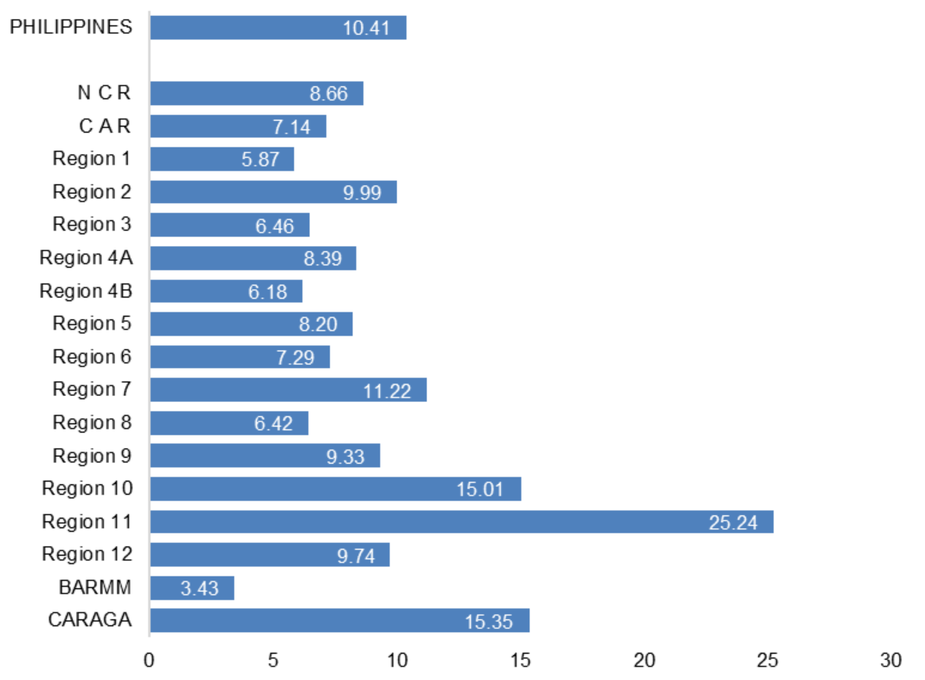

Figure 1.B.1.66 - Percentage Distribution of Pregnant Women tested for CBC/Hgb and Hct diagnosed with anemia (10-49 yrs. old) by Region
Philippines, 2021

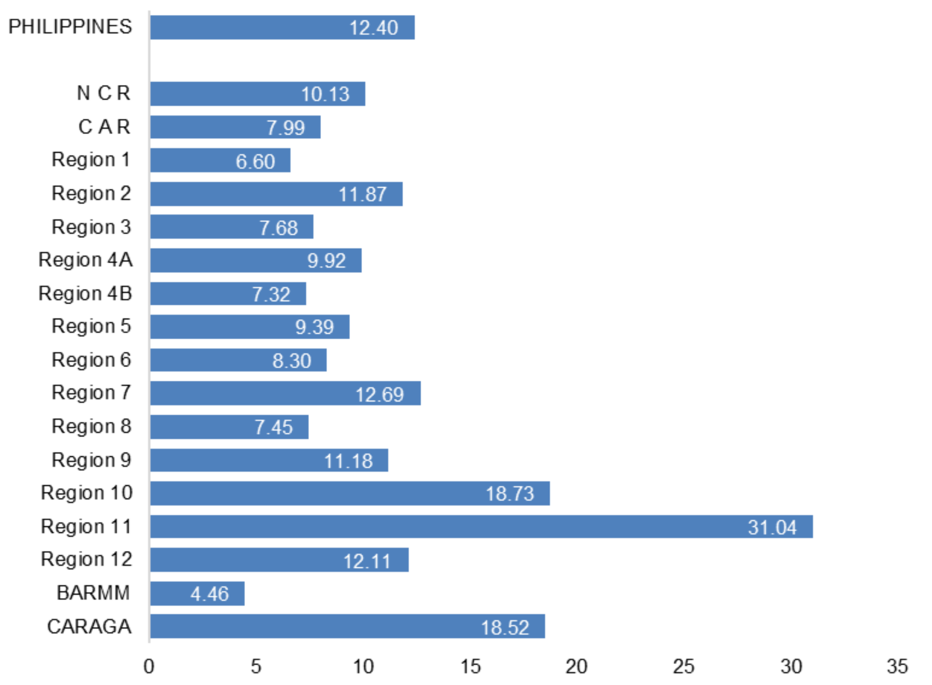

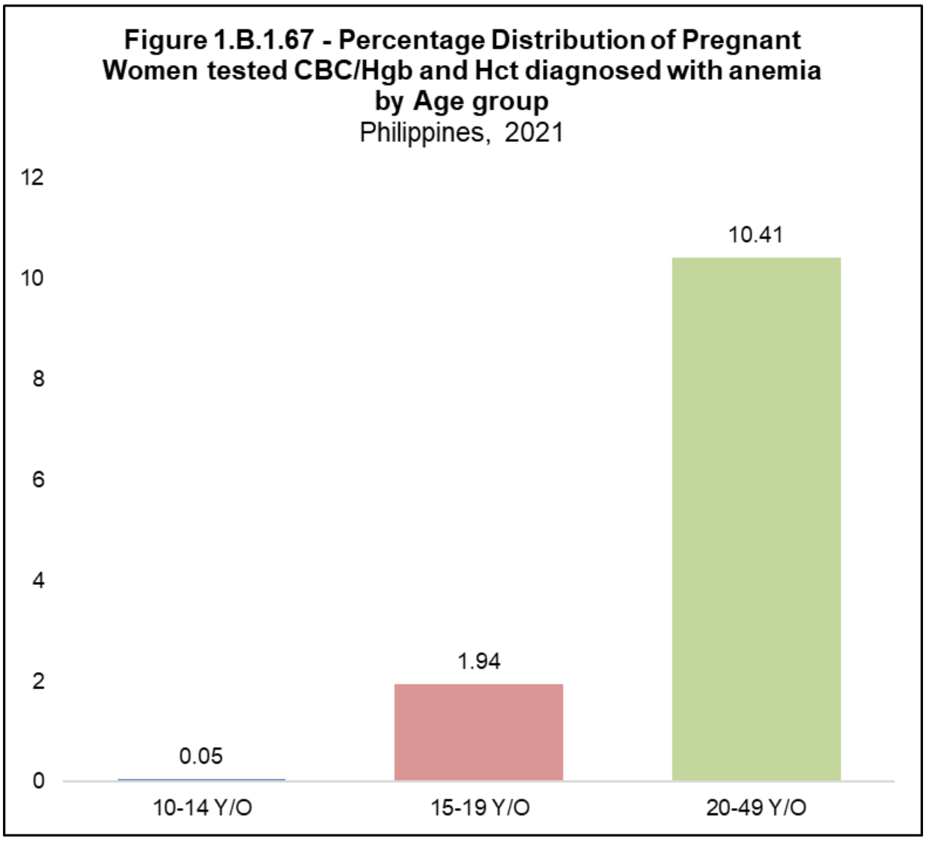
Figure 1.B.1.67 - Percentage Distribution of Pregnant Women tested CBC/Hgb and Hct diagnosed with anemia by Age group
Philippines, 2021
12
10
8
6
4
2
0
0.05
1.94
10.41
10-14 Y/O
15-19 Y/O
20-49 Y/O

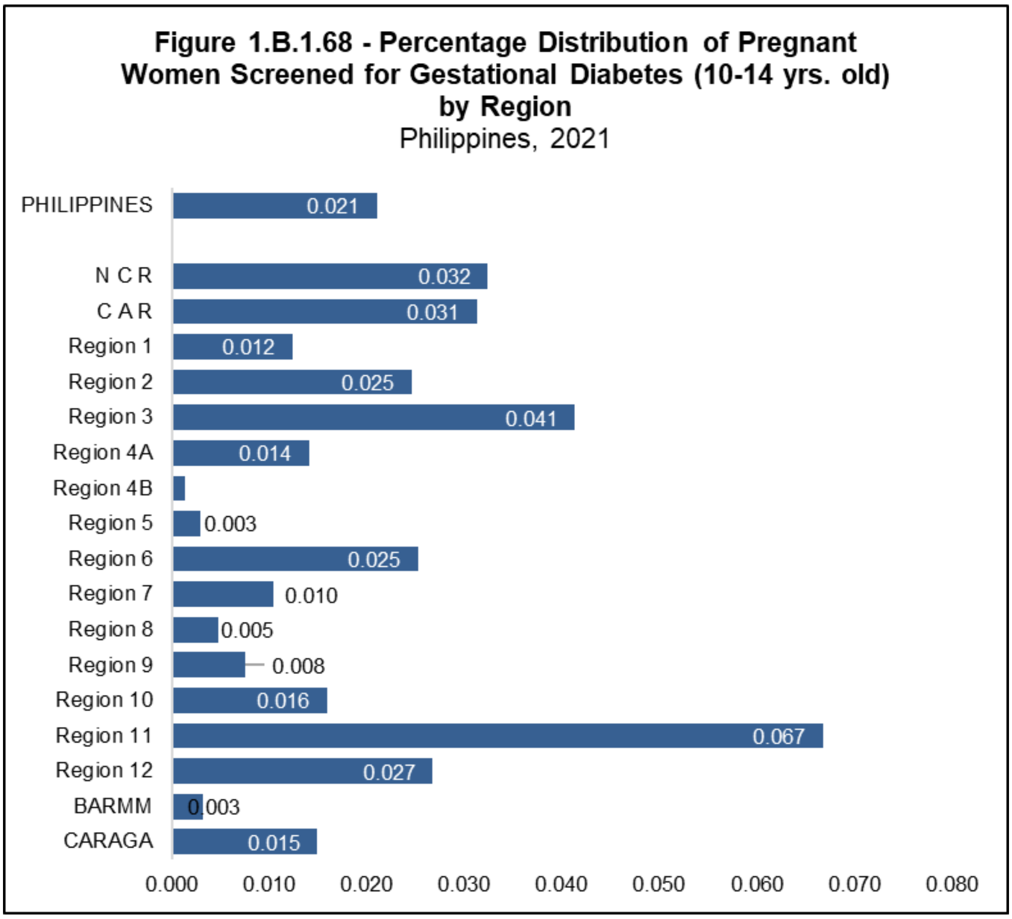
Figure 1.B.1.68 - Percentage Distribution of Pregnant Women Screened for Gestational Diabetes (10-14 yrs. old) by Region
Philippines, 2021
PHILIPPINES 0.021
N C R 0.032
C A R 0.031
Region 1 0.012
Region 2 0.025
Region 3 0.041
Region 4A 0.014
Region 4B
Region 5 0.003
Region 6 0.025
Region 7 0.010
Region 8 0.005
Region 9 0.008
Region 10 0.016
Region 11 0.067
Region 12 0.027
BARMM 0.003
CARAGA 0.015
0.000
0.010
0.020
0.030
0.040
0.050
0.060
0.070
0.080

Figure 1.B.1.69 - Percentage Distribution of Pregnant Women Screened for Gestational Diabetes (15-19 yrs. old) by Region
Philippines, 2021

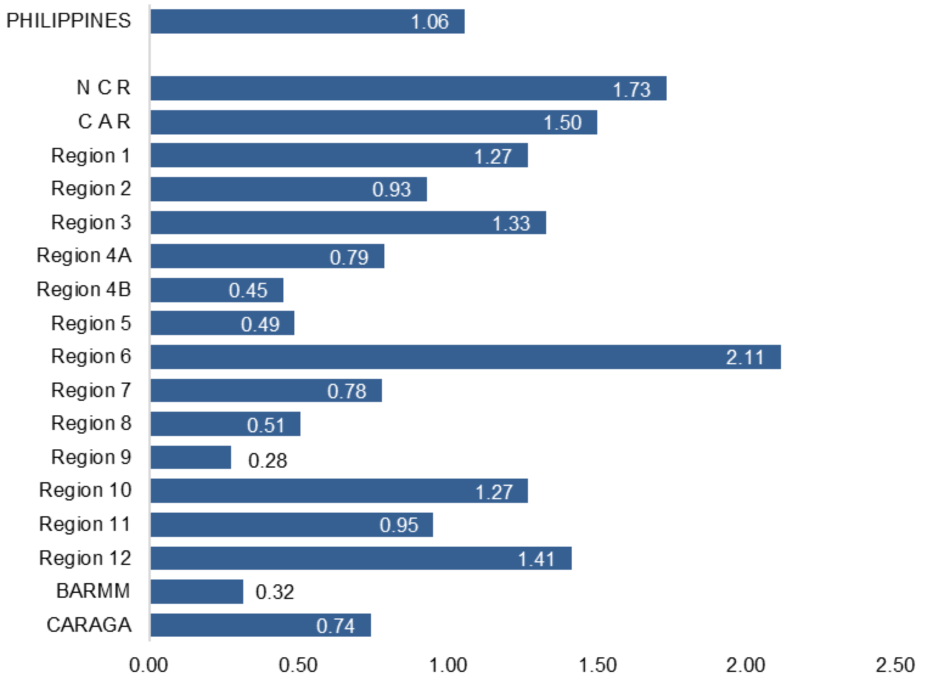

Figure 1.B.1.70 - Percentage Distribution of Pregnant Women Screened for Gestational Diabetes (20-49 yrs. old) by Region
Philippines, 2021

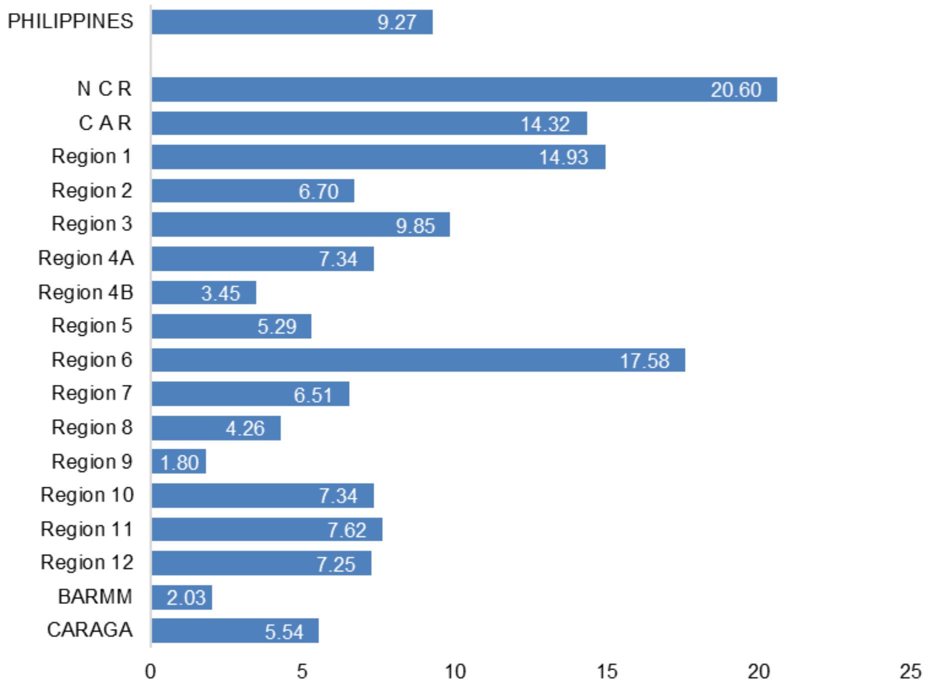

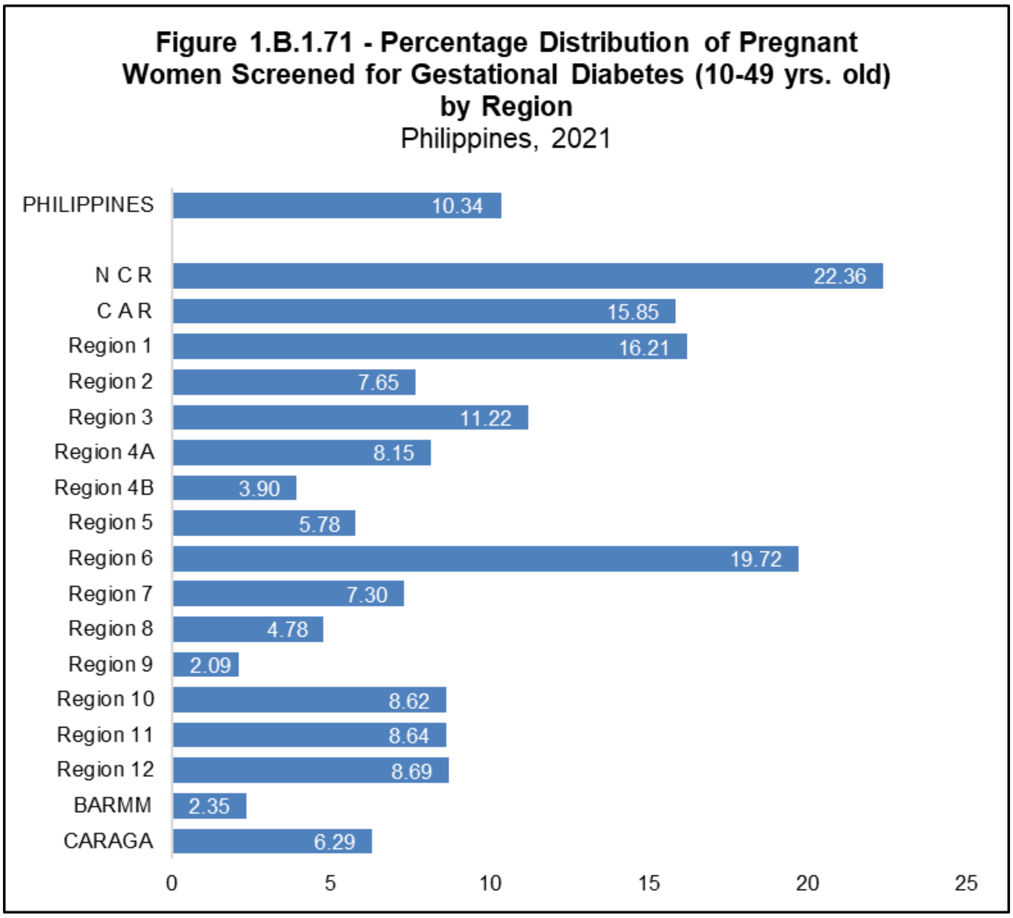
Figure 1.B.1.71 - Percentage Distribution of Pregnant Women Screened for Gestational Diabetes (10-49 yrs. old) by Region
Philippines, 2021
PHILIPPINES 10.34
N C R 22.36
C A R 15.85
Region 1 16.21
Region 2 7.65
Region 3 11.22
Region 4A 8.15
Region 4B 3.90
Region 5 5.78
Region 6 19.72
Region 7 7.30
Region 8 4.78
Region 9 2.09
Region 10 8.62
Region 11 8.64
Region 12 8.69
BARMM 2.35
CARAGA 6.29
0
5
10
15
20
25

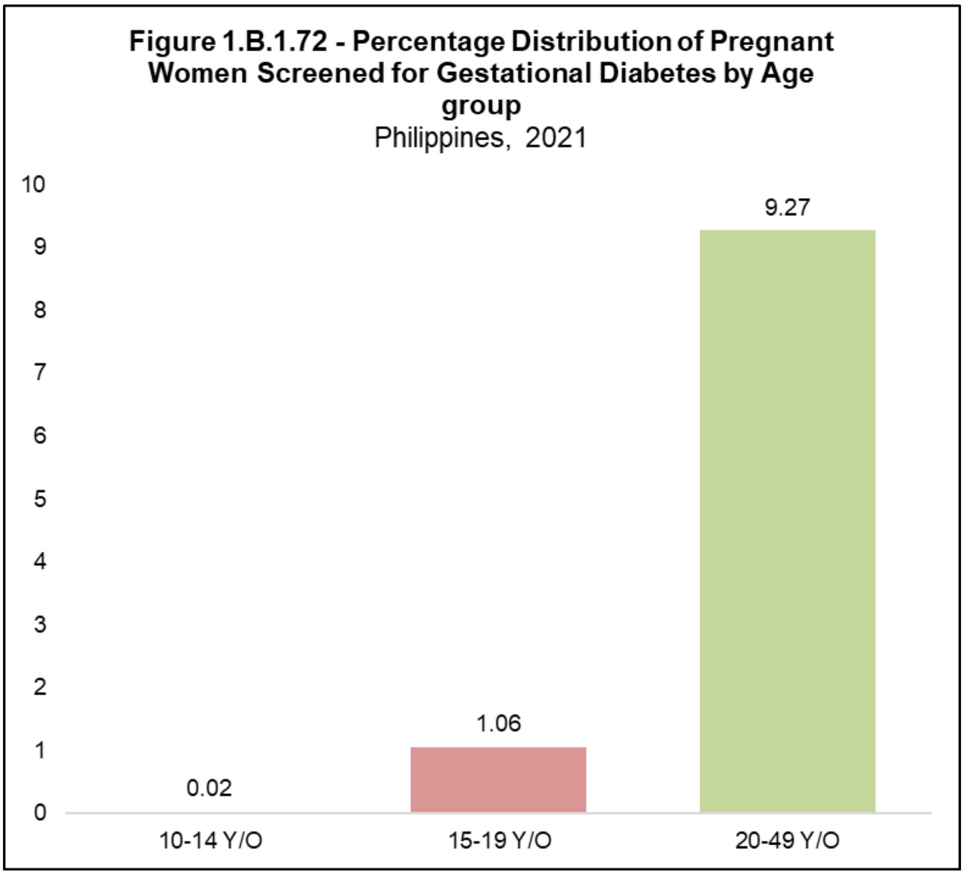
Figure 1.B.1.72 - Percentage Distribution of Pregnant Women Screened for Gestational Diabetes by Age group
Philippines, 2021
10
9
8
7
6
5
4
3
2
1
0
0.02
1.06
9.27
10-14 Y/O
15-19 Y/O
20-49 Y/O

Figure 1.B.1.73 - Percentage Distribution of Pregnant Women Tested Positive for Gestational Diabetes (10-14 yrs. old) by Region
Philippines, 2021

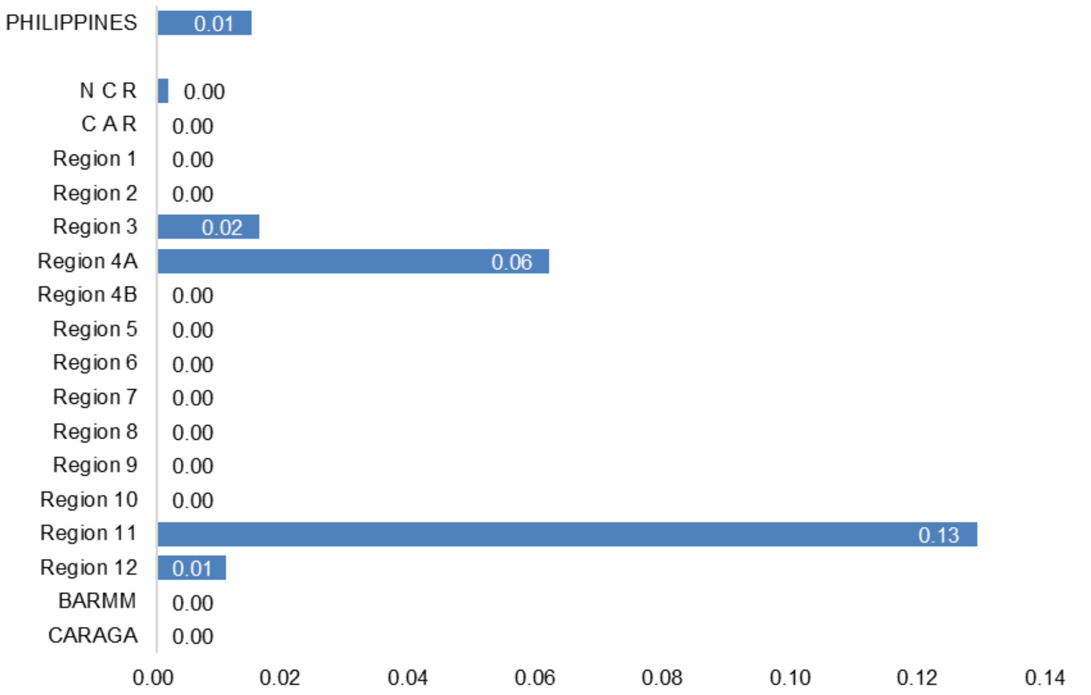

Figure 1.B.1.74 - Percentage Distribution of Pregnant Women Tested Positive for Gestational Diabetes (15-19 yrs. old) by Region
Philippines, 2021

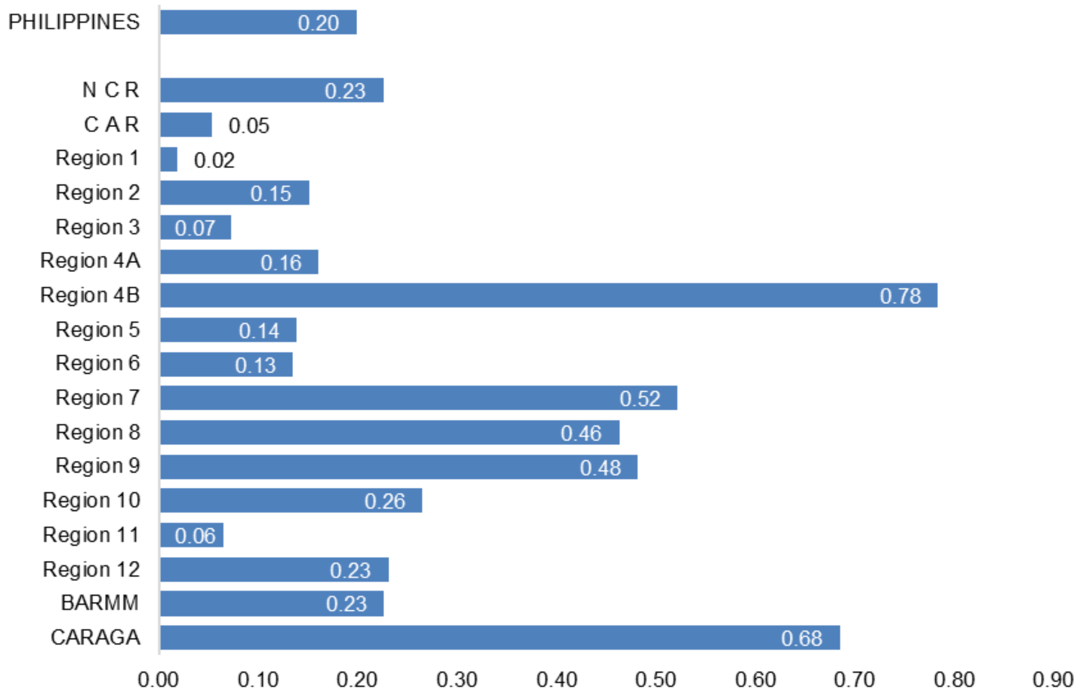

Figure 1.B.1.75 - Percentage Distribution of Pregnant Women Tested Positive for Gestational Diabetes (20-49 yrs. old) by Region
Philippines, 2021

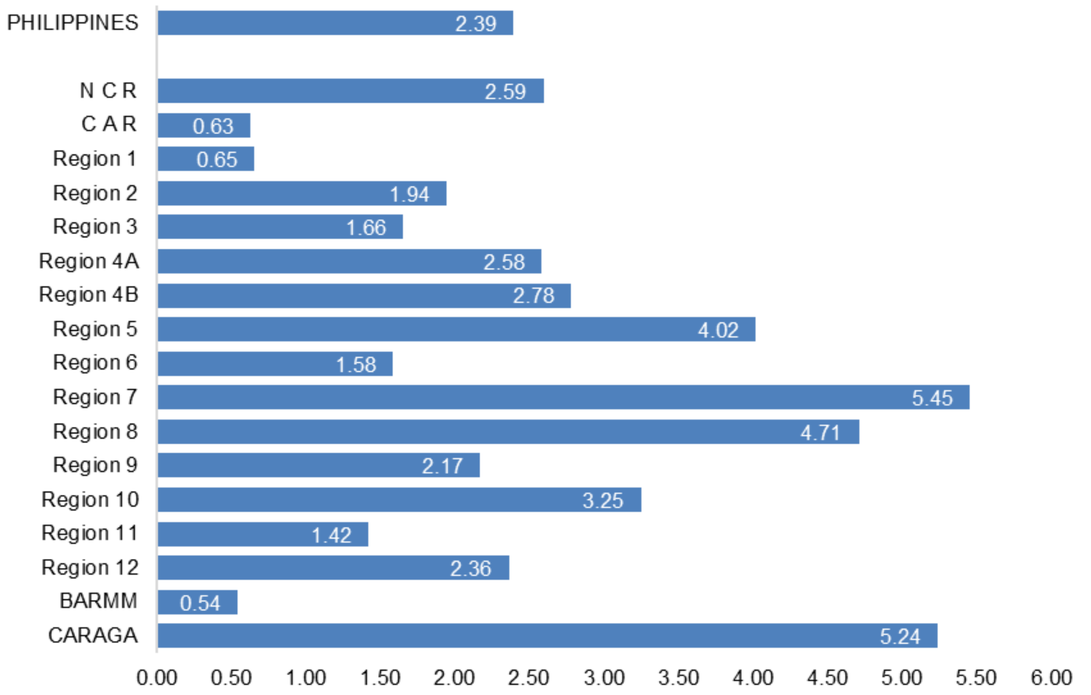

Figure 1.B.1.76 - Percentage Distribution of Pregnant Women Tested Positive for Gestational Diabetes (10-49 yrs. old) by Region
Philippines, 2021

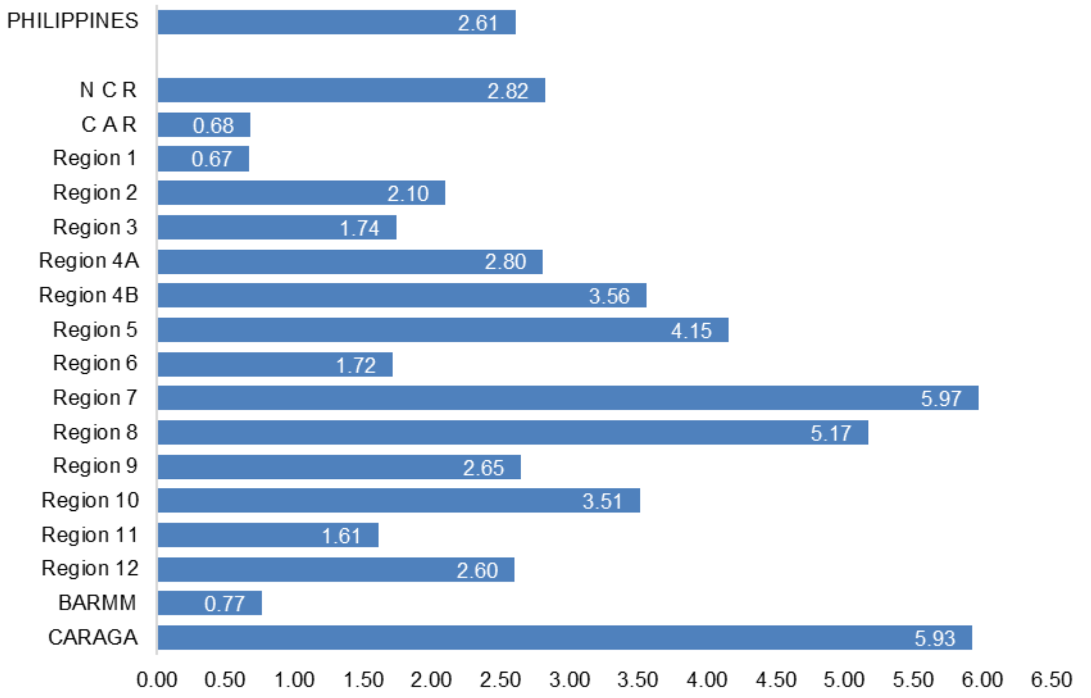

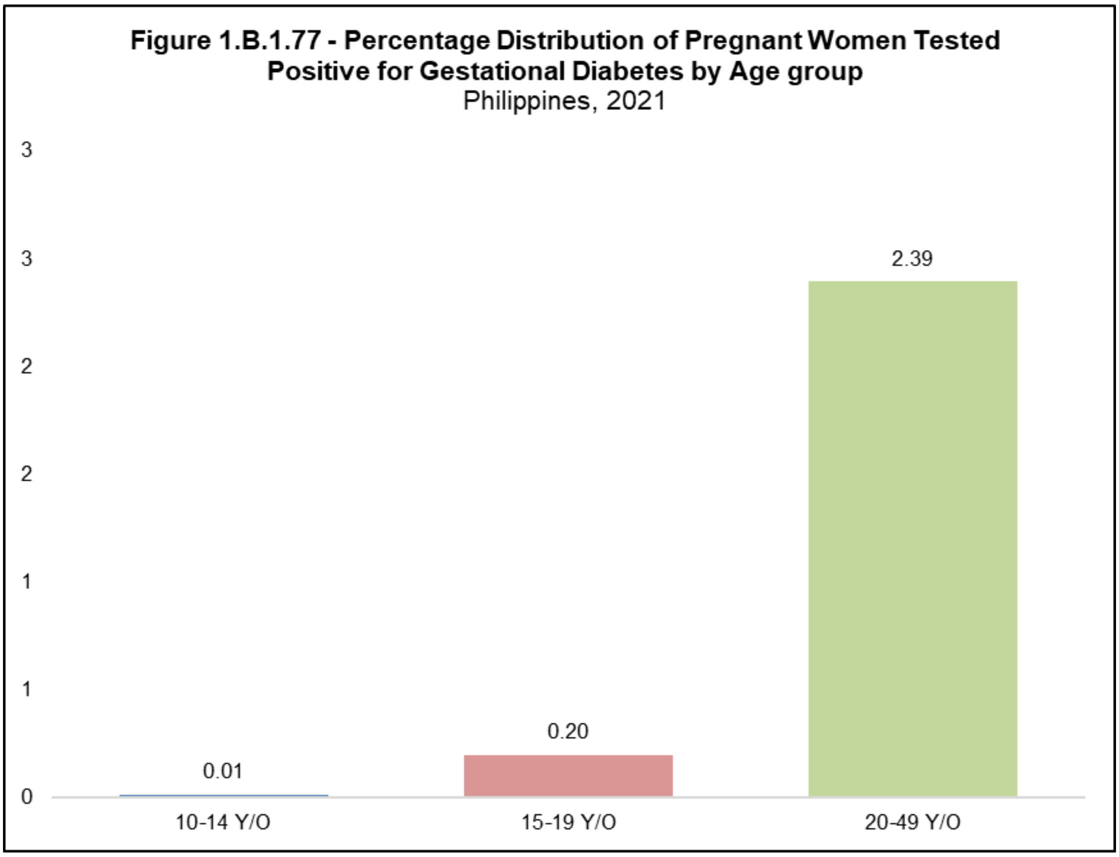

Figure 1.B.1.77 - Percentage Distribution of Pregnant Women Tested Positive for Gestational Diabetes by Age group
Philippines, 2021
3
3
2
2
1
1
0
2.39
0.20
0.01
10-14 Y/O
15-19 Y/O
20-49 Y/O

Table 1.B.2.1 - Intrapartum Care and Delivery Outcome

Total Number of women who delivered a live baby or stillbirth/fetal death, deliveries attended by skilled health professionals & deliveries in health facilities

Philippines, 2021

Area	Total Deliveries				Skilled Health Professional								Facility Based Delivery					
	Total Number of women who delivered				Deliveries attended by skilled health professional								Type of Health Facility					
	Age Group (in Years)			Total	MD	%	Nurses	%	Midwives	%	Total	%	Public	%	Private	%	Total	%
	10-14	15-19	20-49															
**PHILIPPINES**	**2,299**	**125,598**	**1,125,512**	**1,253,409**	**659,775**	**52.64**	**18,386**	**1.47**	**480,698**	**38.35**	**1,158,859**	**92.46**	**718,327**	**57.31**	**415,735**	**33.17**	**1,134,062**	**90.48**
**N C R**	**288**	**12,198**	**135,096**	**147,582**	**91,053**	**61.70**	**536**	**0.36**	**48,090**	**32.59**	**139,679**	**94.65**	**70,653**	**47.87**	**67,563**	**45.78**	**138,216**	**93.65**
Malabon	10	508	4,648	5,166	2,580	49.94	0	0.00	2,378	46.03	4,958	95.97	2,400	46.46	2,522	48.82	4,922	95.28
Navotas	2	322	2,921	3,245	1,607	49.52	0	0.00	1,635	50.39	3,242	99.91	1,707	52.60	1,521	46.87	3,228	99.48
Valenzuela City	6	544	6,000	6,550	3,292	50.26	12	0.18	3,214	49.07	6,518	99.51	4,178	63.79	2,245	34.27	6,423	98.06
Caloocan City	9	772	13,472	14,253	4,877	34.22	13	0.09	9,363	65.69	14,253	100.00	3,354	23.53	10,864	76.22	14,218	99.75
Marikina City	12	502	6,565	7,079	3,944	55.71	199	2.81	2,728	38.54	6,871	97.06	1,697	23.97	5,108	72.16	6,805	96.13
Pasig City	16	798	10,953	11,767	9,020	76.66	20	0.17	2,043	17.36	11,083	94.19	5,610	47.68	5,366	45.60	10,976	93.28
Pateros	0	42	672	714	191	26.75	0	0.00	517	72.41	708	99.16	124	17.37	549	76.89	673	94.26
Taguig	28	737	8,103	8,868	4,312	48.62	162	1.83	3,967	44.73	8,441	95.18	5,223	58.90	3,196	36.04	8,419	94.94
Quezon City	69	3,484	40,573	44,126	29,041	65.81	22	0.05	11,019	24.97	40,082	90.84	18,180	41.20	21,076	47.76	39,256	88.96
Makati City	7	216	5,503	5,726	4,902	85.61	2	0.03	714	12.47	5,618	98.11	2,690	46.98	2,923	51.05	5,613	98.03
Mandaluyong City	4	293	3,139	3,436	2,513	73.14	0	0.00	921	26.80	3,434	99.94	2,985	86.87	441	12.83	3,426	99.71
San Juan	0	54	1,416	1,470	1,410	95.92	46	3.13	12	0.82	1,468	99.86	563	38.30	901	61.29	1,464	99.59
Manila City	15	1,633	11,600	13,248	10,494	79.21	1	0.01	1,972	14.89	12,467	94.10	10,605	80.05	2,117	15.98	12,722	96.03
Las Piñas City	3	516	5,001	5,520	1,551	28.10	39	0.71	3,561	64.51	5,151	93.32	3,209	58.13	1,960	35.51	5,169	93.64
Muntinlupa City	5	877	5,851	6,733	4,219	62.66	5	0.07	1,642	24.39	5,866	87.12	2,007	29.81	3,600	53.47	5,607	83.28
Parañaque City	102	607	4,778	5,487	3,617	65.92	13	0.24	1,725	31.44	5,355	97.59	2,920	53.22	2,211	40.30	5,131	93.51
Pasay City	0	293	3,901	4,194	3,483	83.05	2	0.05	679	16.19	4,164	99.28	3,201	76.32	963	22.96	4,164	99.28
**C A R**	**18**	**1,677**	**20,749**	**22,444**	**18,418**	**82.06**	**508**	**2.26**	**2,877**	**12.82**	**21,803**	**97.14**	**17,094**	**76.16**	**4,244**	**18.91**	**21,338**	**95.07**
Abra	0	297	2,382	2,679	2,304	86.00	92	3.43	208	7.76	2,604	97.20	2,533	94.55	5	0.19	2,538	94.74
Apayao	6	205	1,491	1,702	1,429	83.96	46	2.70	89	5.23	1,564	91.89	1,502	88.25	48	2.82	1,550	91.07
Benguet	0	261	4,355	4,616	4,114	89.12	37	0.80	242	5.24	4,393	95.17	3,107	67.31	1,209	26.19	4,316	93.50
Ifugao	2	242	2,552	2,796	1,735	62.05	112	4.01	862	30.83	2,709	96.89	2,322	83.05	275	9.84	2,597	92.88
Kalinga	0	166	2,215	2,381	1,147	48.17	100	4.20	1,119	47.00	2,366	99.37	1,482	62.24	840	35.28	2,322	97.52
Mt. Province	3	186	2,272	2,461	1,988	80.78	121	4.92	282	11.46	2,391	97.16	2,148	87.28	95	3.86	2,243	91.14
Baguio City	7	320	5,482	5,809	5,701	98.14	0	0.00	75	1.29	5,776	99.43	4,000	68.86	1,772	30.50	5,772	99.36
**Region 1**	**40**	**3,830**	**44,894**	**48,764**	**38,055**	**78.04**	**142**	**0.29**	**10,443**	**21.42**	**48,640**	**99.75**	**35,348**	**72.49**	**13,218**	**27.11**	**48,566**	**99.59**
Ilocos Norte	5	348	4,029	4,382	4,031	91.99	8	0.18	342	7.80	4,381	99.98	3,731	85.14	645	14.72	4,376	99.86
Ilocos Sur	6	279	3,930	4,215	4,034	95.71	5	0.12	173	4.10	4,212	99.93	2,348	55.71	1,857	44.06	4,205	99.76
La Union	0	410	3,831	4,241	3,337	78.68	4	0.09	865	20.40	4,206	99.17	3,961	93.40	228	5.38	4,189	98.77
Pangasinan	3	1,153	13,264	14,420	8,932	61.94	55	0.38	5,403	37.47	14,390	99.79	11,956	82.91	2,420	16.78	14,376	99.69
Alaminos City	0	42	1,208	1,250	1,250	100.00	0	0.00	0	0.00	1,250	100.00	1,020	81.60	230	18.40	1,250	100.00
Candon City	0	1	1,074	1,075	1,052	97.86	4	0.37	19	1.77	1,075	100.00	111	10.33	964	89.67	1,075	100.00
Dagupan City	16	733	7,398	8,147	5,866	72.00	30	0.37	2,224	27.30	8,120	99.67	3,875	47.56	4,218	51.77	8,093	99.34
Laoag City	0	35	1,930	1,965	1,965	100.00	0	0.00	0	0.00	1,965	100.00	1,803	91.76	162	8.24	1,965	100.00
San Carlos City	2	201	2,077	2,280	1,579	69.25	11	0.48	662	29.04	2,252	98.77	976	42.81	1,272	55.79	2,248	98.60
San Fernando City	5	318	3,207	3,530	3,442	97.51	25	0.71	63	1.78	3,530	100.00	3,130	88.67	400	11.33	3,530	100.00
Urdaneta City	0	180	1,663	1,843	1,151	62.45	0	0.00	692	37.55	1,843	100.00	1,192	64.68	651	35.32	1,843	100.00
Vigan City	3	130	1,283	1,416	1,416	100.00	0	0.00	0	0.00	1,416	100.00	1,245	87.92	171	12.08	1,416	100.00
**Region 2**	**74**	**4,401**	**35,937**	**40,412**	**26,916**	**66.60**	**214**	**0.53**	**12,291**	**30.41**	**39,421**	**97.55**	**27,565**	**68.21**	**11,504**	**28.47**	**39,069**	**96.68**
Batanes	2	24	243	269	265	98.51	0	0.00	4	1.49	269	100.00	266	98.88	0	0.00	266	98.88
Cagayan	11	1,070	8,653	9,734	5,685	58.40	100	1.03	3,689	37.90	9,474	97.33	7,996	82.15	1,318	13.54	9,314	95.69
Isabela	36	1,444	11,040	12,520	7,402	59.12	85	0.68	4,737	37.84	12,224	97.64	6,005	47.96	6,094	48.67	12,099	96.64
Nueva Vizcaya	8	815	5,913	6,736	5,580	82.84	25	0.37	811	12.04	6,416	95.25	6,180	91.75	197	2.92	6,377	94.67
Quirino	5	318	2,311	2,634	2,268	86.10	0	0.00	304	11.54	2,572	97.65	2,472	93.85	93	3.53	2,565	97.38
Cauayan City	6	200	1,439	1,645	1,122	68.21	0	0.00	498	30.27	1,620	98.48	1,033	62.80	573	34.83	1,606	97.63
Ilagan City	1	194	2,451	2,646	2,618	98.94	0	0.00	7	0.26	2,625	99.21	2,053	77.59	572	21.62	2,625	99.21
Santiago City	3	259	2,599	2,861	1,038	36.28	1	0.03	1,822	63.68	2,861	100.00	767	26.81	2,091	73.09	2,858	99.90
Tuguegarao City	2	77	1,288	1,367	938	68.62	3	0.22	419	30.65	1,360	99.49	793	58.01	566	41.40	1,359	99.41
**Region 3**	**152**	**11,361**	**105,870**	**117,383**	**67,880**	**57.83**	**533**	**0.45**	**45,834**	**39.05**	**114,247**	**97.33**	**54,241**	**46.21**	**57,591**	**49.06**	**111,832**	**95.27**
Aurora	2	336	2,670	3,008	2,130	70.81	13	0.43	630	20.94	2,773	92.19	2,400	79.79	355	11.80	2,755	91.59
Bataan	14	533	4,601	5,148	3,456	67.13	15	0.29	1,507	29.27	4,978	96.70	3,594	69.81	1,286	24.98	4,880	94.79
Bulacan	23	1,694	21,123	22,840	8,324	36.44	83	0.36	13,481	59.02	21,888	95.83	3,936	17.23	16,828	73.68	20,764	90.91
Nueva Ecija	23	706	8,760	9,489	5,034	53.05	17	0.18	3,742	39.44	8,793	92.67	2,758	29.07	5,583	58.84	8,341	87.90
Pampanga	7	1,008	8,779	9,794	7,689	78.51	62	0.63	1,893	19.33	9,644	98.47	6,223	63.54	3,352	34.23	9,575	97.76
Tarlac	8	1,061	10,275	11,344	6,601	58.19	142	1.25	4,494	39.62	11,237	99.06	8,146	71.81	3,011	26.54	11,157	98.35
Zambales	11	926	6,664	7,601	5,569	73.27	6	0.08	2,026	26.65	7,601	100.00	7,255	95.45	346	4.55	7,601	100.00
Angeles City	2	595	6,430	7,027	3,479	49.51	2	0.03	3,408	48.50	6,889	98.04	1,112	15.82	5,717	81.36	6,829	97.18
Balanga City	9	175	1,106	1,290	1,238	95.97	0	0.00	40	3.10	1,278	99.07	938	72.71	340	26.36	1,278	99.07
Cabanatuan City	5	460	3,771	4,236	3,527	83.26	1	0.02	582	13.74	4,110	97.03	2,987	70.51	1,084	25.59	4,071	96.10
City of San Fernando	9	487	4,100	4,596	3,488	75.89	0	0.00	977	21.26	4,465	97.15	3,251	70.74	1,172	25.50	4,423	96.24
Gapan City	1	112	853	966	578	59.83	0	0.00	388	40.17	966	100.00	505	52.28	461	47.72	966	100.00
Mabalacat City	4	407	3,926	4,337	2,405	55.45	1	0.02	1,958	45.15	4,364	100.62	1,368	31.54	2,985	68.83	4,353	100.37
Malolos City	0	79	3,410	3,489	2,699	77.36	9	0.26	744	21.32	3,452	98.94	25	0.72	3,265	93.58	3,290	94.30
Meycauayan	1	127	2,223	2,351	303	12.89	0	0.00	2,023	86.05	2,326	98.94	292	12.42	1,983	84.35	2,275	96.77
Olongapo	4	354	3,067	3,425	2,252	65.75	9	0.26	928	27.09	3,189	93.11	1,583	46.22	1,448	42.28	3,031	88.50
Palayan City	0	6	71	77	0	0.00	6	7.79	62	80.52	68	88.31	66	85.71	0	0.00	66	85.71
San Jose City	8	249	1,683	1,940	744	38.35	136	7.01	1,033	53.25	1,913	98.61	1,273	65.62	632	32.58	1,905	98.20
San Jose del Monte City	12	820	5,810	6,642	3,930	59.17	0	0.00	2,705	40.73	6,635	99.89	2,841	42.77	3,792	57.09	6,633	99.86
Science City of Munoz	1	18	86	105	0	0.00	0	0.00	68	64.76	68	64.76	2	1.90	62	59.05	64	60.95
Tarlac City	8	1,208	6,462	7,678	4,434	57.75	31	0.40	3,145	40.96	7,610	99.11	3,686	48.01	3,889	50.65	7,575	98.66

Table 1.B.2.1 - Intrapartum Care and Delivery Outcome

Total Number of women who delivered a live baby or stillbirth/fetal death, deliveries attended by skilled health professionals & deliveries in health facilities

Philippines, 2021

Area	Total Deliveries				Skilled Health Professional								Facility Based Delivery					
	Total Number of women who delivered				Deliveries attended by skilled health professional								Type of Health Facility					
	Age Group (in Years)			Total	MD	%	Nurses	%	Midwives	%	Total	%	Public	%	Private	%	Total	%
	10-14	15-19	20-49															
**Region 4A**	**248**	**13,587**	**156,061**	**169,896**	**80,897**	**47.62**	**2,887**	**1.70**	**73,169**	**43.07**	**156,953**	**92.38**	**67,901**	**39.97**	**84,235**	**49.58**	**152,136**	**89.55**
Batangas	20	1,509	19,737	21,266	14,776	69.48	80	0.38	4,585	21.56	19,441	91.42	5,656	26.60	12,748	59.95	18,404	86.54
Cavite	51	1,160	15,307	16,518	6,018	36.43	75	0.45	9,520	57.63	15,613	94.52	3,621	21.92	11,420	69.14	15,041	91.06
Laguna	21	1,245	10,427	11,693	7,157	61.21	62	0.53	3,668	31.37	10,887	93.11	7,845	67.09	2,919	24.96	10,764	92.06
Quezon	31	2,095	16,365	18,491	7,503	40.58	400	2.16	7,664	41.45	15,567	84.19	10,955	59.25	4,363	23.60	15,318	82.84
Rizal	41	2,233	18,774	21,048	11,281	53.60	540	2.57	7,898	37.52	19,719	93.69	11,011	52.31	7,446	35.38	18,457	87.69
Antipolo City	22	160	10,912	11,094	6,122	55.18	81	0.73	3,350	30.20	9,553	86.11	6,594	59.44	2,240	20.19	8,834	79.63
Bacoor City	3	410	6,343	6,756	2,324	34.40	44	0.65	3,568	52.81	5,936	87.86	1,198	17.73	4,697	69.52	5,895	87.26
Batangas City	6	343	3,135	3,484	2,670	76.64	0	0.00	498	14.29	3,168	90.93	1,567	44.98	1,588	45.58	3,155	90.56
Biñan City	1	911	7,061	7,973	1,979	24.82	104	1.30	5,698	71.47	7,781	97.59	1,928	24.18	5,684	71.29	7,612	95.47
Cabuyao City	0	158	3,030	3,188	423	13.27	0	0.00	2,765	86.73	3,188	100.00	369	11.57	2,777	87.11	3,146	98.68
Calamba City	16	680	7,598	8,294	3,440	41.48	46	0.55	4,523	54.53	8,009	96.56	5,064	61.06	2,927	35.29	7,991	96.35
Cavite City	0	38	621	659	414	62.82	21	3.19	217	32.93	652	98.94	57	8.65	592	89.83	649	98.48
Dasmariñas City	9	465	6,099	6,573	1,931	29.38	345	5.25	4,336	65.97	6,612	100.59	2,122	32.28	4,577	69.63	6,699	101.92
General Trias City	0	80	649	729	193	26.47	0	0.00	486	66.67	679	93.14	74	10.15	528	72.43	602	82.58
Imus City	4	151	4,201	4,356	2,239	51.40	38	0.87	2,016	46.28	4,293	98.55	879	20.18	3,250	74.61	4,129	94.79
Lipa City	4	259	3,968	4,231	2,304	54.46	18	0.43	1,261	29.80	3,583	84.68	1,488	35.17	1,835	43.37	3,323	78.54
Lucena City	1	273	2,199	2,473	1,108	44.80	14	0.57	1,220	49.33	2,342	94.70	1,487	60.13	887	35.87	2,374	96.00
San Pablo City	8	443	4,321	4,772	2,360	49.46	943	19.76	1,449	30.36	4,752	99.58	364	7.63	4,271	89.50	4,635	97.13
San Pedro City	0	98	1,204	1,302	505	38.79	10	0.77	762	58.53	1,277	98.08	611	46.93	611	46.93	1,222	93.86
Santa Rosa City	6	521	6,967	7,494	3,203	42.74	8	0.11	3,571	47.65	6,782	90.50	2,860	38.16	4,020	53.64	6,880	91.81
Tagaytay City	1	49	1,139	1,189	520	43.73	1	0.08	665	55.93	1,186	99.75	22	1.85	1,158	97.39	1,180	99.24
Tanauan City	0	89	2,618	2,707	1,376	50.83	47	1.74	1,020	37.68	2,443	90.25	921	34.02	1,445	53.38	2,366	87.40
Tayabas City	2	67	1,276	1,345	237	17.62	0	0.00	1,096	81.49	1,333	99.11	791	58.81	543	40.37	1,334	99.18
Trece Martires City	1	150	2,110	2,261	814	36.00	10	0.44	1,333	58.96	2,157	95.40	417	18.44	1,709	75.59	2,126	94.03
**Region 4B**	**53**	**4,161**	**36,776**	**40,990**	**22,158**	**54.06**	**759**	**1.85**	**11,305**	**27.58**	**34,222**	**83.49**	**27,471**	**67.02**	**5,987**	**14.61**	**33,458**	**81.62**
Marinduque	3	246	2,536	2,785	2,393	85.92	37	1.33	296	10.63	2,726	97.88	2,715	97.49	2	0.07	2,717	97.56
Mindoro Occidental	11	1,054	6,729	7,794	2,768	35.51	55	0.71	2,668	34.23	5,491	70.45	4,461	57.24	953	12.23	5,414	69.46
Mindoro Oriental	3	472	10,542	11,017	8,263	75.00	106	0.96	1,253	11.37	9,622	87.34	7,265	65.94	2,124	19.28	9,389	85.22
Palawan	24	1,739	10,737	12,500	5,000	40.00	495	3.96	4,159	33.27	9,654	77.23	8,088	64.70	1,158	9.26	9,246	73.97
Romblon	9	356	3,530	3,895	2,578	66.19	31	0.80	1,204	30.91	3,813	97.89	3,610	92.68	179	4.60	3,789	97.28
Puerto Princesa City	3	294	2,702	2,999	1,156	38.55	35	1.17	1,725	57.52	2,916	97.23	1,332	44.41	1,571	52.38	2,903	96.80
**Region 5**	**63**	**7,421**	**71,234**	**78,718**	**30,447**	**38.68**	**5,116**	**6.50**	**38,744**	**49.22**	**74,307**	**94.40**	**47,089**	**59.82**	**27,055**	**34.37**	**74,144**	**94.19**
Albay	5	578	8,619	9,202	2,731	29.68	452	4.91	5,702	61.96	8,885	96.56	4,153	45.13	4,723	51.33	8,876	96.46
Camarines Norte	2	1,165	8,494	9,661	1,855	19.20	2,916	30.18	4,412	45.67	9,183	95.05	6,710	69.45	2,471	25.58	9,181	95.03
Camarines Sur	1	1,251	14,247	15,499	2,727	17.59	634	4.09	10,075	65.00	13,436	86.69	4,723	30.47	8,671	55.95	13,394	86.42
Catanduanes	6	387	3,613	4,006	3,211	80.15	152	3.79	527	13.16	3,890	97.10	3,467	86.55	407	10.16	3,874	96.70
Masbate	18	1,718	10,418	12,154	1,842	15.16	468	3.85	8,812	72.50	11,122	91.51	10,327	84.97	724	5.96	11,051	90.92
Sorsogon	8	880	9,536	10,424	6,443	61.81	387	3.71	3,457	33.16	10,287	98.69	7,979	76.54	2,286	21.93	10,265	98.47
Iriga City	2	101	1,526	1,629	426	26.15	62	3.81	1,129	69.31	1,617	99.26	154	9.45	1,462	89.75	1,616	99.20
Legaspi City	4	188	4,319	4,511	1,525	33.81	45	1.00	2,926	64.86	4,496	99.67	1,513	33.54	2,983	66.13	4,496	99.67
Naga City	17	1,153	10,462	11,632	9,687	83.28	0	0.00	1,704	14.65	11,391	97.93	8,063	69.32	3,328	28.61	11,391	97.93
**Region 6**	**111**	**8,646**	**73,338**	**82,095**	**43,522**	**53.01**	**611**	**0.74**	**32,359**	**39.42**	**76,492**	**93.17**	**61,771**	**75.24**	**14,373**	**17.51**	**76,144**	**92.75**
Aklan	14	447	5,107	5,568	3,842	69.00	65	1.17	1,072	19.25	4,979	89.42	4,335	77.86	501	9.00	4,836	86.85
Antique	6	614	6,692	7,312	4,024	55.03	59	0.81	2,612	35.72	6,695	91.56	6,210	84.93	441	6.03	6,651	90.96
Capiz	7	676	6,775	7,458	3,584	48.06	5	0.07	3,350	44.92	6,939	93.04	3,418	45.83	3,521	47.21	6,939	93.04
Guimaras	3	191	2,125	2,319	2,131	91.89	2	0.09	168	7.24	2,301	99.22	2,217	95.60	75	3.23	2,292	98.84
Iloilo	20	1,815	17,402	19,237	14,461	75.17	260	1.35	3,970	20.64	18,691	97.16	17,550	91.23	1,085	5.64	18,635	96.87
Negros Occidental	58	4,168	27,576	31,802	10,180	32.01	171	0.54	18,529	58.26	28,880	90.81	23,611	74.24	5,210	16.38	28,821	90.63
Bacolod City	2	291	3,565	3,858	2,563	66.43	8	0.21	950	24.62	3,521	91.26	1,777	46.06	1,720	44.58	3,497	90.64
Iloilo City	1	444	4,096	4,541	2,737	60.27	41	0.90	1,708	37.61	4,486	98.79	2,653	58.42	1,820	40.08	4,473	98.50
**Region 7**	**92**	**7,067**	**85,580**	**92,739**	**46,722**	**50.38**	**443**	**0.48**	**42,310**	**45.62**	**89,475**	**96.48**	**57,025**	**61.49**	**30,345**	**32.72**	**87,370**	**94.21**
Bohol	8	993	16,265	17,266	11,302	65.46	97	0.56	5,657	32.76	17,056	98.78	13,924	80.64	3,274	18.96	17,198	99.61
Cebu	15	1,499	26,149	27,663	7,581	27.40	147	0.53	18,530	66.98	26,258	94.92	15,948	57.65	10,205	36.89	26,153	94.54
Negros Oriental	37	2,225	16,627	18,889	10,151	53.74	90	0.48	6,206	32.86	16,447	87.07	13,364	70.75	2,979	15.77	16,343	86.52
Siquijor	0	107	1,031	1,138	955	83.92	0	0.00	224	19.68	1,179	103.60	1,070	94.02	67	5.89	1,137	99.91
Cebu City	24	1,544	14,786	16,354	11,510	70.38	54	0.33	6,657	40.71	18,221	111.42	8,827	53.97	6,652	40.68	15,479	94.65
Lapu-Lapu City	8	406	6,977	7,391	2,010	27.20	54	0.73	4,171	56.43	6,235	84.36	2,198	29.74	4,816	65.16	7,014	94.90
Mandaue City	0	293	3,745	4,038	3,213	79.57	1	0.02	865	21.42	4,079	101.02	1,694	41.95	2,352	58.25	4,046	100.20
**Region 8**	**151**	**5,260**	**46,719**	**52,130**	**27,062**	**51.91**	**862**	**1.65**	**20,829**	**39.96**	**48,753**	**93.52**	**33,144**	**63.58**	**14,600**	**28.01**	**47,744**	**91.59**
Biliran	5	346	2,270	2,621	1,159	44.22	24	0.92	1,348	51.43	2,531	96.57	2,588	98.74	0	0.00	2,588	98.74
Eastern Samar	12	659	5,510	6,181	4,437	71.78	52	0.84	1,289	20.85	5,778	93.48	3,203	51.82	2,552	41.29	5,755	93.11
Northern Leyte	74	1,351	12,656	14,081	5,246	37.26	161	1.14	8,475	60.19	13,882	98.59	8,029	57.02	5,211	37.01	13,240	94.03
Northern Samar	32	923	7,610	8,565	4,745	55.40	55	0.64	2,449	28.59	7,249	84.64	6,919	80.78	319	3.72	7,238	84.51
Southern Leyte	4	286	2,231	2,521	2,068	82.03	5	0.20	438	17.37	2,511	99.60	2,059	81.67	397	15.75	2,456	97.42
Western Samar	15	683	5,777	6,475	2,796	43.18	303	4.68	2,692	41.58	5,791	89.44	4,468	69.00	995	15.37	5,463	84.37
Calbayog City	1	211	1,726	1,938	999	51.55	0	0.00	317	16.36	1,316	67.91	1,039	53.61	276	14.24	1,315	67.85
Maasin City	0	212	2,040	2,252	1,727	76.69	44	1.95	477	21.18	2,248	99.82	1,738	77.18	508	22.56	2,246	99.73
Ormoc City	7	411	4,330	4,748	1,971	41.51	194	4.09	2,540	53.50	4,705	99.09	853	17.97	3,848	81.04	4,701	99.01
Tacloban City	1	178	2,569	2,748	1,914	69.65	24	0.87	804	29.26	2,742	99.78	2,248	81.80	494	17.98	2,742	99.78

Table 1.B.2.1 - Intrapartum Care and Delivery Outcome

Total Number of women who delivered a live baby or stillbirth/fetal death, deliveries attended by skilled health professionals & deliveries in health facilities
Philippines, 2021

Area	Total Deliveries				Skilled Health Professional								Facility Based Delivery					
	Total Number of women who delivered				Deliveries attended by skilled health professional								Type of Health Facility					
	Age Group (in Years)			Total	MD	%	Nurses	%	Midwives	%	Total	%	Public	%	Private	%	Total	%
	10-14	15-19	20-49															
**Region 9**	**60**	**5,819**	**40,892**	**46,771**	**21,332**	**45.61**	**929**	**1.99**	**17,140**	**36.65**	**39,401**	**84.24**	**34,496**	**73.76**	**4,616**	**9.87**	**39,112**	**83.62**
Zamboanga del Norte	12	1,396	8,081	9,489	2,819	29.71	128	1.35	5,201	54.81	8,148	85.87	8,097	85.33	17	0.18	8,114	85.51
Zamboanga del Sur	10	910	5,731	6,651	811	12.19	160	2.41	4,358	65.52	5,329	80.12	3,902	58.67	1,367	20.55	5,269	79.22
Zamboanga Sibugay	10	951	6,004	6,965	1,710	24.55	134	1.92	4,031	57.88	5,875	84.35	5,623	80.73	200	2.87	5,823	83.60
Dapitan City	4	86	1,508	1,598	1,541	96.43	24	1.50	25	1.56	1,590	99.50	1,573	98.44	17	1.06	1,590	99.50
Dipolog City	2	274	2,001	2,277	2,074	91.08	2	0.09	49	2.15	2,125	93.32	2,084	91.52	37	1.62	2,121	93.15
Isabela City	3	221	1,405	1,629	826	50.71	22	1.35	74	4.54	922	56.60	788	48.37	134	8.23	922	56.60
Pagadian City	1	218	2,079	2,298	1,135	49.39	0	0.00	1,019	44.34	2,154	93.73	1,135	49.39	1,019	44.34	2,154	93.73
Zamboanga City	18	1,763	14,083	15,864	10,416	65.66	459	2.89	2,383	15.02	13,258	83.57	11,294	71.19	1,825	11.50	13,119	82.70
**Region 10**	**296**	**9,994**	**61,694**	**71,984**	**38,924**	**54.07**	**713**	**0.99**	**25,247**	**35.07**	**64,884**	**90.14**	**51,504**	**71.55**	**11,443**	**15.90**	**62,947**	**87.45**
Bukidnon	92	3,710	15,877	19,679	9,399	47.76	235	1.19	8,079	41.05	17,713	90.01	14,159	71.95	2,500	12.70	16,659	84.65
Camiguin	2	93	1,005	1,100	840	76.36	10	0.91	249	22.64	1,099	99.91	965	87.73	129	11.73	1,094	99.45
Lanao del Norte	5	718	7,855	8,578	3,783	44.10	46	0.54	2,344	27.33	6,173	71.96	5,589	65.16	307	3.58	5,896	68.73
Misamis Occidental	2	301	3,476	3,779	2,485	65.76	65	1.72	1,031	27.28	3,581	94.76	3,095	81.90	342	9.05	3,437	90.95
Misamis Oriental	9	1,396	7,416	8,821	5,131	58.17	136	1.54	3,253	36.88	8,520	96.59	7,307	82.84	1,101	12.48	8,408	95.32
Cagayan de Oro City	26	1,433	10,292	11,751	6,315	53.74	33	0.28	5,258	44.75	11,606	98.77	10,380	88.33	1,096	9.33	11,476	97.66
El Salvador City	0	25	224	249	242	97.19	0	0.00	0	0.00	242	97.19	242	97.19	0	0.00	242	97.19
Gingoog City	57	509	2,327	2,893	1,569	54.23	127	4.39	885	30.59	2,581	89.22	1,824	63.05	647	22.36	2,471	85.41
Iligan City	6	557	5,472	6,035	5,204	86.23	6	0.10	490	8.12	5,700	94.45	3,502	58.03	2,084	34.53	5,586	92.56
Malaybalay City	4	434	2,461	2,899	1,012	34.91	16	0.55	1,442	49.74	2,470	85.20	1,096	37.81	1,361	46.95	2,457	84.75
Oroquieta City	2	97	645	744	615	82.66	0	0.00	100	13.44	715	96.10	614	82.53	129	17.34	743	99.87
Ozamis City	2	206	1,736	1,944	1,417	72.89	0	0.00	493	25.36	1,910	98.25	1,359	69.91	549	28.24	1,908	98.15
Tangub City	80	191	902	1,173	577	49.19	38	3.24	177	15.09	792	67.52	744	63.43	16	1.36	760	64.79
Valencia City	9	324	2,006	2,339	335	14.32	1	0.04	1,446	61.82	1,782	76.19	628	26.85	1,182	50.53	1,810	77.38
**Region 11**	**390**	**10,860**	**64,938**	**76,188**	**44,196**	**58.01**	**250**	**0.33**	**26,939**	**35.36**	**71,385**	**93.70**	**40,299**	**52.89**	**30,596**	**40.16**	**70,895**	**93.05**
Davao de Oro	71	1,909	9,352	11,332	7,704	67.98	49	0.43	3,360	29.65	11,113	98.07	8,520	75.19	2,628	23.19	11,148	98.38
Davao del Norte	111	2,390	14,540	17,041	10,277	60.31	59	0.35	6,078	35.67	16,414	96.32	9,401	55.17	6,872	40.33	16,273	95.49
Davao Oriental	43	1,298	6,895	8,236	6,302	76.52	75	0.91	1,409	17.11	7,786	94.54	7,586	92.11	121	1.47	7,707	93.58
Davao del Sur	37	1,275	6,858	8,170	4,428	54.20	42	0.51	3,250	39.78	7,720	94.49	1,811	22.17	5,858	71.70	7,669	93.87
Davao Occidental	56	1,154	3,066	4,276	1,796	42.00	8	0.19	949	22.19	2,753	64.38	1,816	42.47	896	20.95	2,712	63.42
Davao City	72	2,834	24,227	27,133	13,689	50.45	17	0.06	11,893	43.83	25,599	94.35	11,165	41.15	14,221	52.41	25,386	93.56
**Region 12**	**181**	**9,428**	**56,028**	**65,637**	**30,997**	**47.22**	**508**	**0.77**	**27,934**	**42.56**	**59,439**	**90.56**	**38,880**	**59.23**	**20,346**	**31.00**	**59,226**	**90.23**
North Cotabato	39	2,631	15,665	18,335	10,022	54.66	153	0.83	5,382	29.35	15,557	84.85	9,799	53.44	5,719	31.19	15,518	84.64
Sarangani	37	1,857	7,820	9,714	3,301	33.98	61	0.63	5,471	56.32	8,833	90.93	6,403	65.92	2,402	24.73	8,805	90.64
South Cotabato	42	2,387	13,924	16,353	10,273	62.82	54	0.33	4,998	30.56	15,325	93.71	11,037	67.49	4,245	25.96	15,282	93.45
Sultan Kudarat	57	1,806	10,167	12,030	4,937	41.04	40	0.33	6,160	51.21	11,137	92.58	8,488	70.56	2,581	21.45	11,069	92.01
Cotabato City	2	279	3,017	3,298	1,610	48.82	0	0.00	1,286	38.99	2,896	87.81	1,712	51.91	1,182	35.84	2,894	87.75
Gen. Santos City	4	468	5,435	5,907	854	14.46	200	3.39	4,637	78.50	5,691	96.34	1,441	24.39	4,217	71.39	5,658	95.78
**BARMM**	**27**	**5,917**	**56,651**	**62,595**	**10,727**	**17.14**	**2,945**	**4.70**	**31,481**	**50.29**	**45,153**	**72.14**	**30,401**	**48.57**	**6,970**	**11.14**	**37,371**	**59.70**
Basilan	7	384	2,862	3,253	430	13.22	409	12.57	944	29.02	1,783	54.81	1,051	32.31	57	1.75	1,108	34.06
Lanao del Sur	4	795	16,720	17,519	5,361	30.60	603	3.44	9,533	54.42	15,497	88.46	11,528	65.80	3,330	19.01	14,858	84.81
Maguindanao	12	1,952	18,224	20,188	905	4.48	1,047	5.19	12,181	60.34	14,133	70.01	6,118	30.31	2,199	10.89	8,317	41.20
Sulu	1	350	8,540	8,891	219	2.46	98	1.10	3,126	35.16	3,443	38.72	3,262	36.69	52	0.58	3,314	37.27
Tawi-Tawi	2	495	5,184	5,681	378	6.65	456	8.03	3,868	68.09	4,702	82.77	4,251	74.83	18	0.32	4,269	75.15
							0		0									
Lamitan City	1	247	978	1,226	57	4.65	9	0.73	1,004	81.89	1,070	87.28	983	80.18	19	1.55	1,002	81.73
Marawi City	0	1,694	4,143	5,837	3,377	57.86	323	5.53	825	14.13	4,525	77.52	3,208	54.96	1,295	22.19	4,503	77.15
**CARAGA**	**55**	**3,971**	**33,055**	**37,081**	**20,469**	**55.20**	**430**	**1.16**	**13,706**	**36.96**	**34,605**	**93.32**	**23,445**	**63.23**	**11,049**	**29.80**	**34,494**	**93.02**
Agusan del Norte	2	393	3,204	3,599	1,787	49.65	2	0.06	1,578	43.85	3,367	93.55	1,378	38.29	1,921	53.38	3,299	91.66
Agusan del Sur	15	1,200	10,667	11,882	5,883	49.51	67	0.56	4,851	40.83	10,801	90.90	6,507	54.76	4,236	35.65	10,743	90.41
Surigao del Norte	3	346	3,060	3,409	1,159	34.00	269	7.89	1,759	51.60	3,187	93.49	3,150	92.40	18	0.53	3,168	92.93
Surigao del Sur	15	775	5,664	6,454	4,704	72.89	36	0.56	1,126	17.45	5,866	90.89	5,763	89.29	95	1.47	5,858	90.77
Province of Dinagat	2	123	895	1,020	645	63.24	47	4.61	273	26.76	965	94.61	956	93.73	0	0.00	956	93.73
Bislig City	9	150	1,135	1,294	1,259	97.30	0	0.00	14	1.08	1,273	98.38	1,133	87.56	139	10.74	1,272	98.30
Butuan City	9	398	4,860	5,267	1,426	27.07	9	0.17	3,587	68.10	5,022	95.35	784	14.89	4,292	81.49	5,076	96.37
Surigao City	0	586	3,570	4,156	3,606	86.77	0	0.00	518	12.46	4,124	99.23	3,774	90.81	348	8.37	4,122	99.18

Note: Put asterisk (*) for No Report and Zero (0) for No Case

Table 1.B.2.2 - Intrapartum Care and Delivery Outcome
Number and proportion of delivey by type and age group
Philippines, 2021

Area	Total number of Deliveries	Delivery by Type									
		Vaginal					Cesarean Section				
		Age Group in Year			Total	%	Age Group in Year			Total	%
		10-14	15-19	20-49			10-14	15-19	20-49		
**PHILIPPINES**	**1,253,409**	**2,066**	**111,299**	**957,702**	**1,071,067**	**85.45**	**961**	**9,786**	**126,101**	**136,848**	**10.92**
**N C R**	**147,582**	**250**	**9,817**	**102,643**	**112,710**	**76.37**	**131**	**794**	**9,616**	**10,541**	**7.14**
Malabon	5,166	10	470	4,118	4,598	89.01	0	38	528	566	10.96
Navotas	3,245	2	281	2,588	2,871	88.47	0	32	341	373	11.49
Valenzuela City	6,550	4	460	5,392	5,856	89.40	0	75	660	735	11.22
Caloocan City	14,253	11	967	12,560	13,538	94.98	0	48	667	715	5.02
Marikina City	7,079	0	0	0	0	0.00	0	0	0	0	0.00
Pasig City	11,767	5	531	5,898	6,434	54.68	0	47	877	924	7.85
Pateros	714	0	40	618	658	92.16	0	2	54	56	7.84
Taguig	8,868	35	728	7,562	8,325	93.88	124	115	585	824	9.29
Quezon City	44,126	65	3,357	38,456	41,878	94.91	2	98	2,273	2,373	5.38
Makati City	5,726	0	0	0	0	0.00	0	0	0	0	0.00
Mandaluyong City	3,436	3	261	2,859	3,123	90.89	1	32	280	313	9.11
San Juan	1,470	0	11	234	245	16.67	0	1	106	107	7.28
Manila City	13,248	10	1,404	10,225	11,639	87.85	4	198	1,349	1,551	11.71
Las Piñas City	5,520	3	475	4,566	5,044	91.38	0	40	435	475	8.61
Muntinlupa City	6,733	0	0	0	0	0.00	0	0	0	0	0.00
Parañaque City	5,487	102	582	3,846	4,530	82.56	0	25	932	957	17.44
Pasay City	4,194	0	250	3,721	3,971	94.68	0	43	529	572	13.64
**C A R**	**22,444**	**17**	**1,389**	**15,086**	**16,492**	**73.48**	**1**	**288**	**5,663**	**5,952**	**26.52**
Abra	2,679	0	213	1,584	1,797	67.08	0	84	798	882	32.92
Apayao	1,702	6	194	1,224	1,424	83.67	0	11	267	278	16.33
Benguet	4,616	0	227	3,439	3,666	79.42	0	34	916	950	20.58
Ifugao	2,796	1	214	2,081	2,296	82.12	1	28	471	500	17.88
Kalinga	2,381	0	159	1,953	2,112	88.70	0	7	262	269	11.30
Mt. Province	2,461	3	157	1,608	1,768	71.84	0	29	664	693	28.16
Baguio City	5,809	7	225	3,197	3,429	59.03	0	95	2,285	2,380	40.97
**Region 1**	**48,764**	**39**	**3,571**	**39,007**	**42,617**	**87.39**	**1**	**259**	**5,887**	**6,147**	**12.61**
Ilocos Norte	4,382	5	338	3,839	4,182	95.44	0	10	190	200	4.56
Ilocos Sur	4,215	5	214	2,948	3,167	75.14	1	65	982	1,048	24.86
La Union	4,241	0	403	3,720	4,123	97.22	0	7	111	118	2.78
Pangasinan	14,420	3	1,100	12,620	13,723	95.17	0	53	644	697	4.83
Alaminos City	1,250	0	37	1,065	1,102	88.16	0	5	143	148	11.84
Candon City	1,075	0	1	908	909	84.56	0	0	166	166	15.44
Dagupan City	8,147	16	692	4,970	5,678	69.69	0	41	2,428	2,469	30.31
Laoag City	1,965	0	35	1,855	1,890	96.18	0	0	75	75	3.82
San Carlos City	2,280	2	183	1,668	1,853	81.27	0	18	409	427	18.73
San Fernando City	3,530	5	298	2,779	3,082	87.31	0	20	428	448	12.69
Urdaneta City	1,843	0	166	1,497	1,663	90.23	0	14	166	180	9.77
Vigan City	1,416	3	104	1,138	1,245	87.92	0	26	145	171	12.08
**Region 2**	**40,412**	**59**	**3,803**	**28,909**	**32,771**	**81.09**	**15**	**583**	**7,043**	**7,641**	**18.91**
Batanes	269	2	23	187	212	78.81	0	1	56	57	21.19
Cagayan	9,734	11	1,022	7,855	8,888	91.31	0	48	798	846	8.69
Isabela	12,520	26	1,204	8,385	9,615	76.80	10	236	2,659	2,905	23.20
Nueva Vizcaya	6,736	5	662	4,405	5,072	75.30	3	144	1,517	1,664	24.70
Quirino	2,634	5	271	1,744	2,020	76.69	0	48	566	614	23.31

Table 1.B.2.2 - Intrapartum Care and Delivery Outcome

Number and proportion of delivey by type and age group

Philippines, 2021

Area	Delivery by Type										
	Total number of Deliveries	Vaginal					Cesarean Section				
		Age Group in Year			Total	%	Age Group in Year			Total	%
		10-14	15-19	20-49			10-14	15-19	20-49		
Cauayan City	1,645	6	158	1,110	1,274	77.45	0	42	329	371	22.55
Ilagan City	2,646	1	170	2,061	2,232	84.35	0	23	391	414	15.65
Santiago City	2,861	2	229	2,197	2,428	84.87	1	30	402	433	15.13
Tuguegarao City	1,367	1	64	965	1,030	75.35	1	11	325	337	24.65
**Region 3**	**117,383**	**120**	**8,688**	**78,507**	**87,315**	**74.38**	**25**	**1,844**	**21,350**	**23,219**	**19.78**
Aurora	3,008	2	305	2,175	2,482	82.51	0	32	490	522	17.35
Bataan	5,148	16	496	4,005	4,517	87.74	0	38	593	631	12.26
Bulacan	22,840	13	1,348	15,271	16,632	72.82	5	141	3,837	3,983	17.44
Nueva Ecija	9,489	20	515	4,402	4,937	52.03	4	372	4,033	4,409	46.46
Pampanga	9,794	6	772	7,213	7,991	81.59	1	180	1,622	1,803	18.41
Tarlac	11,344	6	905	9,024	9,935	87.58	2	156	1,251	1,409	12.42
Zambales	7,601	14	612	4,545	5,171	68.03	4	241	2,185	2,430	31.97
Angeles City	7,027	1	466	6,074	6,541	93.08	1	133	472	606	8.62
Balanga City	1,290	8	129	666	803	62.25	1	46	440	487	37.75
Cabanatuan City	4,236	3	390	3,184	3,577	84.44	2	70	587	659	15.56
City of San Fernando	4,596	1	31	886	918	19.97	0	1	1	2	0.04
Gapan City	966	1	71	564	636	65.84	0	37	293	330	34.16
Mabalacat City	4,337	2	332	3,014	3,348	77.20	0	1	67	68	1.57
Malolos City	3,489	0	57	1,299	1,356	38.87	0	18	2,115	2,133	61.13
Meycauayan	2,351	1	120	2,107	2,228	94.77	0	7	116	123	5.23
Olongapo	3,425	4	355	3,006	3,365	98.25	0	0	60	60	1.75
Palayan City	77	0	6	71	77	100.00	0	0	0	0	0.00
San Jose City	1,940	4	177	1,222	1,403	72.32	4	71	462	537	27.68
San Jose del Monte City	6,642	12	755	5,028	5,795	87.25	0	25	822	847	12.75
Science City of Munoz	105	5	15	85	105	100.00	0	0	0	0	0.00
Tarlac City	7,678	1	831	4,666	5,498	71.61	1	275	1,904	2,180	28.39
**Region 4A**	**169,896**	**132**	**11,682**	**130,096**	**141,910**	**83.53**	**29**	**1,378**	**23,894**	**25,301**	**14.89**
Batangas	21,266	14	1,188	14,312	15,514	72.95	5	235	5,265	5,505	25.89
Cavite	16,518	14	974	13,187	14,175	85.82	0	93	1,883	1,976	11.96
Laguna	11,693	19	1,076	8,816	9,911	84.76	2	98	1,682	1,782	15.24
Quezon	18,491	22	1,993	14,737	16,752	90.60	9	116	1,436	1,561	8.44
Rizal	21,048	21	1,816	15,810	17,647	83.84	0	198	3,182	3,380	16.06
Antipolo City	11,094	0	364	7,996	8,360	75.36	2	91	845	938	8.46
Bacoor City	6,756	2	302	5,383	5,687	84.18	1	29	979	1,009	14.93
Batangas City	3,484	5	308	2,270	2,583	74.14	1	38	855	894	25.66
Biñan City	7,973	0	652	6,776	7,428	93.16	1	125	419	545	6.84
Cabuyao City	3,188	0	168	2,800	2,968	93.10	0	7	213	220	6.90
Calamba City	8,294	14	628	6,375	7,017	84.60	2	50	1,225	1,277	15.40
Cavite City	659	0	35	546	581	88.16	0	2	73	75	11.38
Dasmariñas City	6,573	3	365	5,691	6,059	92.18	0	34	627	661	10.06
General Trias City	729	0	71	644	715	98.08	0	0	48	48	6.58
Imus City	4,356	3	133	3,380	3,516	80.72	0	16	798	814	18.69
Lipa City	4,231	1	209	2,762	2,972	70.24	2	36	1,102	1,140	26.94
Lucena City	2,473	1	239	1,902	2,142	86.62	0	19	309	328	13.26
San Pablo City	4,772	4	368	3,249	3,621	75.88	4	76	1,071	1,151	24.12
San Pedro City	1,302	0	61	1,078	1,139	87.48	0	12	151	163	12.52
Santa Rosa City	7,494	6	431	6,320	6,757	90.17	0	83	654	737	9.83
Tagaytay City	1,189	1	35	929	965	81.16	0	8	214	222	18.67
Tanauan City	2,707	0	60	2,161	2,221	82.05	0	6	465	471	17.40
Tayabas City	1,345	2	67	1,092	1,161	86.32	0	0	184	184	13.68
Trece Martires City	2,261	0	139	1,880	2,019	89.30	0	6	214	220	9.73
**Region 4B**	**40,990**	**52**	**3,957**	**33,394**	**37,403**	**91.25**	**0**	**166**	**3,297**	**3,463**	**8.45**
Marinduque	2,785	2	215	2,151	2,368	85.03	0	20	397	417	14.97

Table 1.B.2.2 - Intrapartum Care and Delivery Outcome

Number and proportion of delivey by type and age group

Philippines, 2021

Area	Total number of Deliveries	Delivery by Type									
		Vaginal					Cesarean Section				
		Age Group in Year			Total	%	Age Group in Year			Total	%
		10-14	15-19	20-49			10-14	15-19	20-49		
Mindoro Occidental	7,794	10	996	6,449	7,455	95.65	0	15	319	334	4.29
Mindoro Oriental	11,017	3	470	8,800	9,273	84.17	0	65	1,679	1,744	15.83
Palawan	12,500	25	1,643	10,199	11,867	94.94	0	51	473	524	4.19
Romblon	3,895	9	331	3,282	3,622	92.99	0	8	255	263	6.75
Puerto Princesa City	2,999	3	302	2,513	2,818	93.96	0	7	174	181	6.04
**Region 5**	**78,718**	**54**	**6,418**	**61,079**	**67,551**	**85.81**	**18**	**963**	**10,186**	**11,167**	**14.19**
Albay	9,202	5	576	8,405	8,986	97.65	0	0	216	216	2.35
Camarines Norte	9,661	2	1,065	7,462	8,529	88.28	0	78	1,054	1,132	11.72
Camarines Sur	15,499	1	1,250	14,152	15,403	99.38	0	1	95	96	0.62
Catanduanes	4,006	15	334	2,881	3,230	80.63	0	40	736	776	19.37
Masbate	12,154	18	1,715	10,356	12,089	99.47	0	4	61	65	0.53
Sorsogon	10,424	7	821	8,296	9,124	87.53	1	58	1,241	1,300	12.47
Iriga City	1,629	2	94	1,208	1,304	80.05	0	5	320	325	19.95
Legaspi City	4,511	3	170	3,932	4,105	91.00	1	17	388	406	9.00
Naga City	11,632	1	393	4,387	4,781	41.10	16	760	6,075	6,851	58.90
**Region 6**	**82,095**	**107**	**8,274**	**67,127**	**75,508**	**91.98**	**2**	**362**	**6,223**	**6,587**	**8.02**
Aklan	5,568	13	421	4,697	5,131	92.15	0	14	423	437	7.85
Antique	7,312	6	601	6,095	6,702	91.66	0	13	597	610	8.34
Capiz	7,458	6	657	6,245	6,908	92.63	1	19	530	550	7.37
Guimaras	2,319	3	186	1,922	2,111	91.03	0	4	204	208	8.97
Iloilo	19,237	19	1,708	16,149	17,876	92.93	1	107	1,253	1,361	7.07
Negros Occidental	31,802	57	4,000	25,355	29,412	92.48	0	173	2,217	2,390	7.52
Bacolod City	3,858	2	277	3,018	3,297	85.46	0	11	550	561	14.54
Iloilo City	4,541	1	424	3,646	4,071	89.65	0	21	449	470	10.35
**Region 7**	**92,739**	**296**	**6,788**	**79,374**	**86,458**	**93.23**	**8**	**287**	**6,133**	**6,428**	**6.93**
Bohol	17,266	8	976	15,490	16,474	95.41	0	21	771	792	4.59
Cebu	27,663	15	1,448	24,664	26,127	94.45	0	50	1,486	1,536	5.55
Negros Oriental	18,889	37	2,166	15,358	17,561	92.97	0	59	1,269	1,328	7.03
Siquijor	1,138	2	111	922	1,035	90.95	0	4	99	103	9.05
Cebu City	16,354	226	1,475	13,618	15,319	93.67	8	66	1,108	1,182	7.23
Lapu-Lapu City	7,391	8	340	6,395	6,743	91.23	0	66	582	648	8.77
Mandaue City	4,038	0	272	2,927	3,199	79.22	0	21	818	839	20.78
**Region 8**	**52,130**	**107**	**4,570**	**40,172**	**44,849**	**86.03**	**652**	**323**	**4,140**	**5,115**	**9.81**
Biliran	2,621	6	335	2,083	2,424	92.48	0	9	171	180	6.87
Eastern Samar	6,181	13	593	4,750	5,356	86.65	0	51	775	826	13.36
Northern Leyte	14,081	41	933	9,814	10,788	76.61	642	36	469	1,147	8.15
Northern Samar	8,565	23	842	7,137	8,002	93.43	9	59	495	563	6.57
Southern Leyte	2,521	4	279	2,079	2,362	93.69	0	4	151	155	6.15
Western Samar	6,475	12	676	5,304	5,992	92.54	0	64	419	483	7.46
Calbayog City	1,938	1	204	1,587	1,792	92.47	0	7	139	146	7.53
Maasin City	2,252	0	173	1,438	1,611	71.54	0	39	602	641	28.46
Ormoc City	4,748	6	377	3,647	4,030	84.88	1	34	683	718	15.12
Tacloban City	2,748	1	158	2,333	2,492	90.68	0	20	236	256	9.32
**Region 9**	**46,771**	**58**	**5,602**	**39,387**	**45,047**	**96.31**	**4**	**134**	**1,302**	**1,440**	**3.08**
Zamboanga del Norte	9,489	12	1,388	8,015	9,415	99.22	0	8	66	74	0.78

Table 1.B.2.2 - Intrapartum Care and Delivery Outcome

Number and proportion of delivey by type and age group
Philippines, 2021

Area	Total number of Deliveries	Delivery by Type									
		Vaginal					Cesarean Section				
		Age Group in Year			Total	%	Age Group in Year			Total	%
		10-14	15-19	20-49			10-14	15-19	20-49		
Zamboanga del Sur	6,651	11	896	5,688	6,595	99.16	0	2	54	56	0.84
Zamboanga Sibugay	6,965	10	932	5,800	6,742	96.80	0	19	204	223	3.20
Dapitan City	1,598	0	78	1,485	1,563	97.81	3	8	23	34	2.13
Dipolog City	2,277	5	236	1,750	1,991	87.44	0	5	155	160	7.03
Isabela City	1,629	3	198	1,274	1,475	90.55	0	8	106	114	7.00
Pagadian City	2,298	0	188	1,946	2,134	92.86	0	7	40	47	2.05
Zamboanga City	15,864	17	1,686	13,429	15,132	95.39	1	77	654	732	4.61
**Region 10**	**71,984**	**217**	**9,403**	**57,096**	**66,716**	**92.68**	**13**	**377**	**4,134**	**4,524**	**6.28**
Bukidnon	19,679	88	3,516	14,109	17,713	90.01	4	149	1,346	1,499	7.62
Camiguin	1,100	2	89	888	979	89.00	0	3	118	121	11.00
Lanao del Norte	8,578	6	664	7,565	8,235	96.00	0	28	204	232	2.70
Misamis Occidental	3,779	2	271	3,254	3,527	93.33	0	12	240	252	6.67
Misamis Oriental	8,821	9	1,244	7,176	8,429	95.56	0	18	208	226	2.56
Cagayan de Oro City	11,751	35	1,364	9,555	10,954	93.22	0	39	758	797	6.78
El Salvador City	249	0	25	224	249	100.00	0	0	0	0	0.00
Gingoog City	2,893	53	516	2,046	2,615	90.39	7	41	230	278	9.61
Iligan City	6,035	6	535	5,128	5,669	93.94	0	22	344	366	6.06
Malaybalay City	2,899	4	411	2,201	2,616	90.24	0	22	261	283	9.76
Oroquieta City	744	2	90	577	669	89.92	0	7	68	75	10.08
Ozamis City	1,944	1	191	1,529	1,721	88.53	1	17	205	223	11.47
Tangub City	1,173	1	176	961	1,138	97.02	0	6	29	35	2.98
Valencia City	2,339	8	311	1,883	2,202	94.14	1	13	123	137	5.86
**Region 11**	**76,188**	**311**	**9,285**	**53,682**	**63,278**	**83.06**	**48**	**802**	**8,758**	**9,608**	**12.61**
Davao de Oro	11,332	67	1,766	8,181	10,014	88.37	5	135	1,215	1,355	11.96
Davao del Norte	17,041	72	2,219	12,614	14,905	87.47	14	143	2,030	2,187	12.83
Davao Oriental	8,236	38	1,240	6,042	7,320	88.88	3	54	569	626	7.60
Davao del Sur	8,170	37	1,130	5,826	6,993	85.59	6	76	795	877	10.73
Davao Occidental	4,276	34	722	1,798	2,554	59.73	3	22	180	205	4.79
Davao City	27,133	63	2,208	19,221	21,492	79.21	17	372	3,969	4,358	16.06
**Region 12**	**65,637**	**169**	**8,873**	**50,779**	**59,821**	**91.14**	**13**	**537**	**5,266**	**5,816**	**8.86**
North Cotabato	18,335	35	2,402	14,135	16,572	90.38	4	224	1,535	1,763	9.62
Sarangani	9,714	35	1,815	7,496	9,346	96.21	2	42	324	368	3.79
South Cotabato	16,353	41	2,268	12,307	14,616	89.38	1	108	1,628	1,737	10.62
Sultan Kudarat	12,030	52	1,722	9,002	10,776	89.58	5	84	1,165	1,254	10.42
Cotabato City	3,298	2	254	2,668	2,924	88.66	0	19	355	374	11.34
Gen. Santos City	5,907	4	412	5,171	5,587	94.58	1	60	259	320	5.42
**BARMM**	**62,595**	**23**	**5,351**	**50,185**	**55,559**	**88.76**	**1**	**565**	**1,482**	**2,048**	**3.27**
Basilan	3,253	6	354	2,805	3,165	97.29	0	4	72	76	2.34
Lanao del Sur	17,519	2	731	16,292	17,025	97.18	0	15	459	474	2.71
Maguindanao	20,188	11	1,922	17,944	19,877	98.46	1	30	280	311	1.54
Sulu	8,891	1	271	3,738	4,010	45.10	0	0	3	3	0.03
Tawi-Tawi	5,681	2	364	5,177	5,543	97.57	0	6	54	60	1.06
Lamitan City	1,226	1	243	967	1,211	98.78	0	4	11	15	1.22
Marawi City	5,837	0	1,466	3,262	4,728	81.00	0	506	603	1,109	19.00

Table 1.B.2.2 - Intrapartum Care and Delivery Outcome

Number and proportion of delivey by type and age group
Philippines, 2021

Area	Total number of Deliveries	Delivery by Type									
		Vaginal					Cesarean Section				
		Age Group in Year			Total	%	Age Group in Year			Total	%
		10-14	15-19	20-49			10-14	15-19	20-49		
**CARAGA**	**37,081**	**55**	**3,828**	**31,179**	**35,062**	**94.56**	**0**	**124**	**1,727**	**1,851**	**4.99**
Agusan del Norte	3,599	2	391	3,189	3,582	99.53	0	0	17	17	0.47
Agusan del Sur	11,882	15	1,165	9,885	11,065	93.12	0	19	798	817	6.88
Surigao del Norte	3,409	3	343	3,063	3,409	100.00	0	0	0	0	0.00
Surigao del Sur	6,454	15	743	5,596	6,354	98.45	0	7	93	100	1.55
Province of Dinagat	1,020	2	124	894	1,020	100.00	0	0	0	0	0.00
Bislig City	1,294	9	146	1,023	1,178	91.04	0	4	112	116	8.96
Butuan City	5,267	9	393	4,043	4,445	84.39	0	31	623	654	12.42
Surigao City	4,156	0	523	3,486	4,009	96.46	0	63	84	147	3.54

Note: Put asterisk (*) for No Report and Zero (0) for No Case

Vaginal - deliveries by NSD, Vaccum and Forcep

Deliveries should be reported by place of occurrence.

Table 1.B.2.3 - Intrapartum Care and Delivery Outcome

A. Number and Proportion of Pregnancy Outcome (Full term and Pre-term)
Philippines, 2021

Area	Total number of Deliveries	Full term (37-42 weeks AOG)					Pre-term (22-36 weeks AOG)				
		Age Group in Year			Total	%	Age Group in Year			Total	%
		10-14	15-19	20-49			10-14	15-19	20-49		
**PHILIPPINES**	**1,253,409**	**2,925**	**116,351**	**1,056,311**	**1,175,587**	**93.79**	**616**	**2,476**	**14,487**	**17,579**	**1.40**
**N C R**	**147,582**	**153**	**9,635**	**104,651**	**114,439**	**77.54**	**49**	**106**	**595**	**750**	**0.51**
Malabon	5,166	10	502	4,628	5,140	99.50	0	4	22	26	0.50
Navotas	3,245	2	303	2,920	3,225	99.38	0	1	13	14	0.43
Valenzuela City	6,550	4	399	4,674	5,077	77.51	0	13	51	64	0.98
Caloocan City	14,253	9	1,004	13,209	14,222	99.78	0	9	17	26	0.18
Marikina City	7,079	0	0	0	0	0.00	0	0	0	0	0.00
Pasig City	11,767	9	557	6,751	7,317	62.18	0	6	45	51	0.43
Pateros	714	0	42	668	710	99.44	0	0	3	3	0.42
Taguig	8,868	40	684	6,797	7,521	84.81	49	33	163	245	2.76
Quezon City	44,126	56	3,416	40,229	43,701	99.04	0	7	62	69	0.16
Makati City	5,726	0	0	0	0	0.00	0	0	0	0	0.00
Mandaluyong City	3,436	4	289	3,107	3,400	98.95	0	4	14	18	0.52
San Juan	1,470	0	47	1,311	1,358	92.38	0	4	106	110	7.48
Manila City	13,248	16	1,592	11,531	13,139	99.18	0	17	42	59	0.45
Las Piñas City	5,520	3	515	4,994	5,512	99.86	0	0	3	3	0.05
Muntinlupa City	6,733	0	0	0	0	0.00	0	0	0	0	0.00
Parañaque City	5,487	0	0	0	0	0.00	0	0	0	0	0.00
Pasay City	4,194	0	285	3,832	4,117	98.16	0	8	54	62	1.48
**C A R**	**22,444**	**16**	**1,546**	**19,917**	**21,479**	**95.70**	**2**	**118**	**643**	**763**	**3.40**
Abra	2,679	0	233	2,198	2,431	90.74	0	63	171	234	8.73
Apayao	1,702	6	200	1,462	1,668	98.00	0	5	20	25	1.47
Benguet	4,616	0	242	4,253	4,495	97.38	0	13	87	100	2.17
Ifugao	2,796	2	236	2,494	2,732	97.71	0	6	36	42	1.50
Kalinga	2,381	0	160	2,190	2,350	98.70	0	4	12	16	0.67
Mt. Province	2,461	3	179	2,173	2,355	95.69	0	3	58	61	2.48
Baguio City	5,809	5	296	5,147	5,448	93.79	2	24	259	285	4.91
**Region 1**	**48,764**	**39**	**3,763**	**44,617**	**48,419**	**99.29**	**5**	**79**	**267**	**351**	**0.72**
Ilocos Norte	4,382	4	329	3,954	4,287	97.83	3	19	75	97	2.21
Ilocos Sur	4,215	4	280	3,896	4,180	99.17	2	12	29	43	1.02
La Union	4,241	0	403	3,805	4,208	99.22	0	6	25	31	0.73
Pangasinan	14,420	3	1,128	13,199	14,330	99.38	0	25	65	90	0.62
Alaminos City	1,250	0	42	1,208	1,250	100.00	0	0	0	0	0.00
Candon City	1,075	0	1	1,073	1,074	99.91	0	0	1	1	0.09
Dagupan City	8,147	16	726	7,387	8,129	99.78	0	7	11	18	0.22
Laoag City	1,965	0	35	1,930	1,965	100.00	0	0	0	0	0.00
San Carlos City	2,280	2	198	2,058	2,258	99.04	0	3	19	22	0.96
San Fernando City	3,530	5	313	3,174	3,492	98.92	0	5	27	32	0.91
Urdaneta City	1,843	0	179	1,654	1,833	99.46	0	1	9	10	0.54
Vigan City	1,416	5	129	1,279	1,413	99.79	0	1	6	7	0.49
**Region 2**	**40,412**	**68**	**4,255**	**35,163**	**39,486**	**97.71**	**4**	**110**	**480**	**594**	**1.47**
Batanes	269	2	24	234	260	96.65	0	0	5	5	1.86
Cagayan	9,734	8	1,032	8,465	9,505	97.65	1	30	129	160	1.64
Isabela	12,520	33	1,413	10,817	12,263	97.95	3	47	125	175	1.40
Nueva Vizcaya	6,736	8	781	5,739	6,528	96.91	0	16	126	142	2.11

Table 1.B.2.3 - Intrapartum Care and Delivery Outcome

A. Number and Proportion of Pregnancy Outcome (Full term and Pre-term)
Philippines, 2021

Area	Total number of Deliveries	Full term (37-42 weeks AOG)					Pre-term (22-36 weeks AOG)				
		Age Group in Year			Total	%	Age Group in Year			Total	%
		10-14	15-19	20-49			10-14	15-19	20-49		
Quirino	2,634	5	290	2,257	2,552	96.89	0	10	52	62	2.35
Cauayan City	1,645	6	192	1,417	1,615	98.18	0	4	9	13	0.79
Ilagan City	2,646	1	190	2,440	2,631	99.43	0	0	9	9	0.34
Santiago City	2,861	3	258	2,585	2,846	99.48	0	1	14	15	0.52
Tuguegarao City	1,367	2	75	1,209	1,286	94.07	0	2	11	13	0.95
**Region 3**	**117,383**	**135**	**10,090**	**97,291**	**107,516**	**91.59**	**2**	**139**	**671**	**812**	**0.69**
Aurora	3,008	3	307	2,589	2,899	96.38	0	22	53	75	2.49
Bataan	5,148	15	511	4,515	5,041	97.92	1	9	65	75	1.46
Bulacan	22,840	17	1,587	19,629	21,233	92.96	0	10	113	123	0.54
Nueva Ecija	9,489	24	585	8,083	8,692	91.60	0	5	16	21	0.22
Pampanga	9,794	7	954	8,760	9,721	99.25	0	6	56	62	0.63
Tarlac	11,344	8	1,060	10,244	11,312	99.72	0	1	29	30	0.26
Zambales	7,601	12	842	6,540	7,394	97.28	0	10	134	144	1.89
Angeles City	7,027	2	556	6,380	6,938	98.73	0	39	57	96	1.37
Balanga City	1,290	9	166	1,051	1,226	95.04	0	8	39	47	3.64
Cabanatuan City	4,236	5	454	3,724	4,183	98.75	0	5	18	23	0.54
City of San Fernando	4,596	1	31	882	914	19.89	0	0	0	0	0.00
Gapan City	966	2	108	853	963	99.69	0	0	2	2	0.21
Mabalacat City	4,337	2	134	1,285	1,421	32.76	0	1	19	20	0.46
Malolos City	3,489	0	64	3,419	3,483	99.83	0	5	1	6	0.17
Meycauayan	2,351	1	126	2,224	2,351	100.00	0	0	0	0	0.00
Olongapo	3,425	4	354	3,052	3,410	99.56	0	1	5	6	0.18
Palayan City	77	0	6	71	77	100.00	0	0	0	0	0.00
San Jose City	1,940	7	246	1,673	1,926	99.28	1	2	5	8	0.41
San Jose del Monte City	6,642	12	821	5,806	6,639	99.95	0	0	0	0	0.00
Science City of Munoz	105	0	14	90	104	99.05	0	0	1	1	0.95
Tarlac City	7,678	4	1,164	6,421	7,589	98.84	0	15	58	73	0.95
**Region 4A**	**169,896**	**135**	**12,227**	**149,334**	**161,696**	**95.17**	**9**	**228**	**1,102**	**1,339**	**0.79**
Batangas	21,266	14	1,296	18,857	20,167	94.83	1	25	122	148	0.70
Cavite	16,518	10	1,007	14,756	15,773	95.49	1	23	130	154	0.93
Laguna	11,693	20	1,129	10,420	11,569	98.94	1	23	98	122	1.04
Quezon	18,491	21	1,842	15,578	17,441	94.32	1	42	151	194	1.05
Rizal	21,048	19	1,899	18,273	20,191	95.93	1	29	255	285	1.35
Antipolo City	11,094	2	422	7,812	8,236	74.24	0	0	16	16	0.14
Bacoor City	6,756	2	294	5,845	6,141	90.90	0	3	15	18	0.27
Batangas City	3,484	5	335	3,035	3,375	96.87	1	7	37	45	1.29
Biñan City	7,973	1	760	7,169	7,930	99.46	1	23	7	31	0.39
Cabuyao City	3,188	0	178	3,004	3,182	99.81	0	3	3	6	0.19
Calamba City	8,294	16	656	7,545	8,217	99.07	0	16	68	84	1.01
Cavite City	659	0	34	616	650	98.63	0	1	0	1	0.15
Dasmariñas City	6,573	1	388	6,228	6,617	100.67	0	6	24	30	0.46
General Trias City	729	0	67	700	767	105.21	0	1	0	1	0.14
Imus City	4,356	3	127	4,108	4,238	97.29	0	0	23	23	0.53
Lipa City	4,231	2	214	3,776	3,992	94.35	1	5	28	34	0.80
Lucena City	2,473	1	239	2,103	2,343	94.74	0	2	40	42	1.70
San Pablo City	4,772	8	443	4,321	4,772	100.00	0	0	0	0	0.00
San Pedro City	1,302	2	98	1,189	1,289	99.00	0	2	13	15	1.15
Santa Rosa City	7,494	5	504	6,941	7,450	99.41	1	9	23	33	0.44
Tagaytay City	1,189	1	36	1,167	1,204	101.26	0	0	1	1	0.08
Tanauan City	2,707	0	54	2,592	2,646	97.75	0	1	11	12	0.44
Tayabas City	1,345	2	66	1,255	1,323	98.36	0	1	9	10	0.74
Trece Martires City	2,261	0	139	2,044	2,183	96.55	0	6	28	34	1.50

Table 1.B.2.3 - Intrapartum Care and Delivery Outcome

A. Number and Proportion of Pregnancy Outcome (Full term and Pre-term)
Philippines, 2021

Area	Total number of Deliveries	Full term (37-42 weeks AOG)					Pre-term (22-36 weeks AOG)				
		Age Group in Year			Total	%	Age Group in Year			Total	%
		10-14	15-19	20-49			10-14	15-19	20-49		
**Region 4B**	**40,990**	**46**	**3,939**	**36,072**	**40,057**	**97.72**	**3**	**70**	**267**	**340**	**0.83**
Marinduque	2,785	1	225	2,500	2,726	97.88	0	6	25	31	1.11
Mindoro Occidental	7,794	9	999	6,598	7,606	97.59	1	15	45	61	0.78
Mindoro Oriental	11,017	2	472	10,405	10,879	98.75	1	5	62	68	0.62
Palawan	12,500	23	1,621	10,475	12,119	96.95	1	33	77	111	0.89
Romblon	3,895	8	327	3,465	3,800	97.56	0	6	36	42	1.08
Puerto Princesa City	2,999	3	295	2,629	2,927	97.60	0	5	22	27	0.90
**Region 5**	**78,718**	**55**	**6,999**	**69,080**	**76,134**	**96.72**	**5**	**365**	**1,600**	**1,970**	**2.50**
Albay	9,202	5	568	8,585	9,158	99.52	0	5	14	19	0.21
Camarines Norte	9,661	2	989	7,390	8,381	86.75	0	188	985	1,173	12.14
Camarines Sur	15,499	0	1,226	14,113	15,339	98.97	1	13	65	79	0.51
Catanduanes	4,006	6	356	3,520	3,882	96.90	0	19	49	68	1.70
Masbate	12,154	17	1,675	10,201	11,893	97.85	1	23	79	103	0.85
Sorsogon	10,424	3	757	9,074	9,834	94.34	2	108	343	453	4.35
Iriga City	1,629	2	100	1,479	1,581	97.05	0	1	23	24	1.47
Legaspi City	4,511	4	177	4,291	4,472	99.14	0	7	22	29	0.64
Naga City	11,632	16	1,151	10,427	11,594	99.67	1	1	20	22	0.19
**Region 6**	**82,095**	**112**	**8,396**	**72,019**	**80,527**	**98.09**	**5**	**172**	**885**	**1,062**	**1.29**
Aklan	5,568	14	435	5,035	5,484	98.49	0	12	46	58	1.04
Antique	7,312	15	567	6,426	7,008	95.84	0	13	175	188	2.57
Capiz	7,458	6	676	6,750	7,432	99.65	1	4	30	35	0.47
Guimaras	2,319	2	180	2,095	2,277	98.19	1	11	33	45	1.94
Iloilo	19,237	20	1,787	17,185	18,992	98.73	0	25	158	183	0.95
Negros Occidental	31,802	53	4,027	26,939	31,019	97.54	3	99	381	483	1.52
Bacolod City	3,858	1	285	3,522	3,808	98.70	0	3	32	35	0.91
Iloilo City	4,541	1	439	4,067	4,507	99.25	0	5	30	35	0.77
**Region 7**	**92,739**	**294**	**7,065**	**84,047**	**91,406**	**98.56**	**5**	**378**	**4,953**	**5,336**	**5.75**
Bohol	17,266	7	986	16,153	17,146	99.30	1	207	4,053	4,261	24.68
Cebu	27,663	14	1,467	25,963	27,444	99.21	1	25	147	173	0.63
Negros Oriental	18,889	36	2,144	16,226	18,406	97.44	1	71	294	366	1.94
Siquijor	1,138	2	113	1,022	1,137	99.91	0	2	5	7	0.62
Cebu City	16,354	228	1,706	14,266	16,200	99.06	1	34	162	197	1.20
Lapu-Lapu City	7,391	7	375	6,789	7,171	97.02	1	31	144	176	2.38
Mandaue City	4,038	0	274	3,628	3,902	96.63	0	8	148	156	3.86
**Region 8**	**52,130**	**982**	**4,502**	**42,017**	**47,501**	**91.12**	**502**	**96**	**620**	**1,218**	**2.34**
Biliran	2,621	5	339	2,221	2,565	97.86	1	4	32	37	1.41
Eastern Samar	6,181	12	621	5,326	5,959	96.41	0	13	54	67	1.08
Northern Leyte	14,081	923	796	8,689	10,408	73.92	490	7	66	563	4.00
Northern Samar	8,565	23	844	7,388	8,255	96.38	6	42	262	310	3.62
Southern Leyte	2,521	3	283	2,186	2,472	98.06	0	0	6	6	0.24
Western Samar	6,475	10	629	5,718	6,357	98.18	3	10	50	63	0.97
Calbayog City	1,938	1	210	1,713	1,924	99.28	0	1	6	7	0.36
Maasin City	2,252	0	211	2,020	2,231	99.07	0	1	7	8	0.36
Ormoc City	4,748	5	398	4,208	4,611	97.11	2	13	122	137	2.89

Table 1.B.2.3 - Intrapartum Care and Delivery Outcome

A. Number and Proportion of Pregnancy Outcome (Full term and Pre-term)
Philippines, 2021

Area	Total number of Deliveries	Full term (37-42 weeks AOG)					Pre-term (22-36 weeks AOG)				
		Age Group in Year			Total	%	Age Group in Year			Total	%
		10-14	15-19	20-49			10-14	15-19	20-49		
Tacloban City	2,748	0	171	2,548	2,719	98.94	0	5	15	20	0.73
**Region 9**	**46,771**	**57**	**5,632**	**40,317**	**46,006**	**98.36**	**3**	**80**	**192**	**275**	**0.59**
Zamboanga del Norte	9,489	11	1,356	7,991	9,358	98.62	1	25	51	77	0.81
Zamboanga del Sur	6,651	9	898	5,694	6,601	99.25	1	6	20	27	0.41
Zamboanga Sibugay	6,965	10	927	5,921	6,858	98.46	0	17	37	54	0.78
Dapitan City	1,598	3	79	1,491	1,573	98.44	0	7	14	21	1.31
Dipolog City	2,277	4	244	1,881	2,129	93.50	0	1	12	13	0.57
Isabela City	1,629	3	202	1,371	1,576	96.75	0	0	5	5	0.31
Pagadian City	2,298	0	194	1,986	2,180	94.87	0	0	0	0	0.00
Zamboanga City	15,864	17	1,732	13,982	15,731	99.16	1	24	53	78	0.49
**Region 10**	**71,984**	**236**	**9,559**	**61,066**	**70,861**	**98.44**	**11**	**168**	**644**	**823**	**1.14**
Bukidnon	19,679	85	3,636	15,540	19,261	97.88	8	63	204	275	1.40
Camiguin	1,100	2	92	998	1,092	99.27	0	1	7	8	0.73
Lanao del Norte	8,578	6	709	7,648	8,363	97.49	0	8	33	41	0.48
Misamis Occidental	3,779	2	289	3,442	3,733	98.78	0	8	38	46	1.22
Misamis Oriental	8,821	8	1,212	7,511	8,731	98.98	0	25	85	110	1.25
Cagayan de Oro City	11,751	59	1,360	10,228	11,647	99.11	0	24	83	107	0.91
El Salvador City	249	0	25	224	249	100.00	0	0	1	1	0.40
Gingoog City	2,893	54	468	2,299	2,821	97.51	0	7	65	72	2.49
Iligan City	6,035	6	550	5,435	5,991	99.27	0	7	37	44	0.73
Malaybalay City	2,899	3	424	2,424	2,851	98.34	1	8	40	49	1.69
Oroquieta City	744	2	96	638	736	98.92	0	0	8	8	1.08
Ozamis City	1,944	2	202	1,715	1,919	98.71	0	4	21	25	1.29
Tangub City	1,173	0	179	973	1,152	98.21	1	7	14	22	1.88
Valencia City	2,339	7	317	1,991	2,315	98.97	1	6	8	15	0.64
**Region 11**	**76,188**	**346**	**10,295**	**62,884**	**73,525**	**96.50**	**8**	**141**	**599**	**748**	**0.98**
Davao de Oro	11,332	70	1,864	9,214	11,148	98.38	2	32	89	123	1.09
Davao del Norte	17,041	83	2,320	14,241	16,644	97.67	4	42	162	208	1.22
Davao Oriental	8,236	39	1,191	6,237	7,467	90.66	0	19	54	73	0.89
Davao del Sur	8,170	36	1,239	6,686	7,961	97.44	2	28	90	120	1.47
Davao Occidental	4,276	49	992	2,677	3,718	86.95	0	3	30	33	0.77
Davao City	27,133	69	2,689	23,829	26,587	97.99	0	17	174	191	0.70
**Region 12**	**65,637**	**177**	**9,163**	**55,188**	**64,528**	**98.31**	**3**	**113**	**494**	**610**	**0.93**
North Cotabato	18,335	38	2,549	15,458	18,045	98.42	1	31	153	185	1.01
Sarangani	9,714	36	1,820	7,704	9,560	98.41	0	24	61	85	0.88
South Cotabato	16,353	41	2,321	13,647	16,009	97.90	1	23	104	128	0.78
Sultan Kudarat	12,030	57	1,764	9,954	11,775	97.88	0	29	129	158	1.31
Cotabato City	3,298	2	272	3,000	3,274	99.27	0	0	18	18	0.55
Gen. Santos City	5,907	3	437	5,425	5,865	99.29	1	6	29	36	0.61
**BARMM**	**62,595**	**19**	**5,468**	**50,035**	**55,522**	**88.70**	**0**	**39**	**234**	**273**	**0.44**
Basilan	3,253	3	260	2,147	2,410	74.09	0	4	43	47	1.44
Lanao del Sur	17,519	2	738	16,755	17,495	99.86	0	9	44	53	0.30
Maguindanao	20,188	8	1,399	13,402	14,809	73.36	0	5	39	44	0.22
Sulu	8,891	2	528	8,077	8,607	96.81	0	3	63	66	0.74
Tawi-Tawi	5,681	2	348	4,866	5,216	91.81	0	6	8	14	0.25

Table 1.B.2.3 - Intrapartum Care and Delivery Outcome

A. Number and Proportion of Pregnancy Outcome (Full term and Pre-term)
Philippines, 2021

Area	Total number of Deliveries	Full term (37-42 weeks AOG)					Pre-term (22-36 weeks AOG)				
		Age Group in Year			Total	%	Age Group in Year			Total	%
		10-14	15-19	20-49			10-14	15-19	20-49		
Lamitan City	1,226	1	245	967	1,213	98.94	0	2	1	3	0.24
Marawi City	5,837	1	1,950	3,821	5,772	98.89	0	10	36	46	0.79
**CARAGA**	**37,081**	**55**	**3,817**	**32,613**	**36,485**	**98.39**	**0**	**74**	**241**	**315**	**0.85**
Agusan del Norte	3,599	2	393	3,186	3,581	99.50	0	1	14	15	0.42
Agusan del Sur	11,882	15	1,172	10,591	11,778	99.12	0	14	31	45	0.38
Surigao del Norte	3,409	3	338	2,985	3,326	97.57	0	6	24	30	0.88
Surigao del Sur	6,454	15	742	5,558	6,315	97.85	0	14	56	70	1.08
Province of Dinagat	1,020	2	123	880	1,005	98.53	0	0	5	5	0.49
Bislig City	1,294	9	143	1,111	1,263	97.60	0	5	13	18	1.39
Butuan City	5,267	9	378	4,764	5,151	97.80	0	12	68	80	1.52
Surigao City	4,156	0	528	3,538	4,066	97.83	0	22	30	52	1.25

Note: Put asterisk (*) for No Report and Zero (0) for No Case

Table 1.B.2.4 - Intrapartum Care and Delivery Outcome

B. Number and Proportion of Pregnancy Outcome (Fetal Deaths and Abortion)

Philippines, 2021

Area	Total number of Deliveries	Fetal Deaths					Abortion (Counts Only)			
		Age Group			Total	%	Age Group			Total
		10-14	15-19	20-49			10-14	15-19	20-49	
**PHILIPPINES**	**1,253,409**	**1,287**	**857**	**7,061**	**9,205**	**0.73**	**48**	**688**	**5,898**	**6,634**
**N C R**	**147,582**	**1**	**50**	**1,593**	**1,644**	**1.11**	**0**	**22**	**184**	**206**
Malabon	5,166	0	0	7	7	0.14	0	4	26	30
Navotas	3,245	0	0	3	3	0.09	0	1	2	3
Valenzuela City	6,550	0	3	45	48	0.73	0	7	67	74
Caloocan City	14,253	0	0	0	0	0.00	0	0	2	2
Marikina City	7,079	1	4	56	61	0.86	0	0	0	0
Pasig City	11,767	0	6	106	112	0.95	0	0	4	4
Pateros	714	0	0	6	6	0.84	0	1	3	4
Taguig	8,868	0	0	110	110	1.24	0	7	41	48
Quezon City	44,126	0	32	431	463	1.05	0	0	29	29
Makati City	5,726	0	0	56	56	0.98	0	0	0	0
Mandaluyong City	3,436	0	0	25	25	0.73	0	1	5	6
San Juan	1,470	0	0	14	14	0.95	0	0	0	0
Manila City	13,248	0	0	529	529	3.99	0	1	3	4
Las Piñas City	5,520	0	0	59	59	1.07	0	0	2	2
Muntinlupa City	6,733	0	0	58	58	0.86	0	0	0	0
Parañaque City	5,487	0	5	74	79	1.44	0	0	0	0
Pasay City	4,194	0	0	14	14	0.33	0	0	0	0
**C A R**	**22,444**	**0**	**13**	**189**	**202**	**0.90**	**0**	**29**	**693**	**722**
Abra	2,679	0	1	13	14	0.52	0	0	5	5
Apayao	1,702	0	0	9	9	0.53	0	5	23	28
Benguet	4,616	0	6	15	21	0.45	0	11	169	180
Ifugao	2,796	0	0	22	22	0.79	0	1	54	55
Kalinga	2,381	0	2	13	15	0.63	0	1	12	13
Mt. Province	2,461	0	4	41	45	1.83	0	3	91	94
Baguio City	5,809	0	0	76	76	1.31	0	8	339	347
**Region 1**	**48,764**	**0**	**20**	**172**	**192**	**0.39**	**0**	**11**	**114**	**125**
Ilocos Norte	4,382	0	6	22	28	0.64	0	2	22	24
Ilocos Sur	4,215	0	1	4	5	0.12	0	0	6	6
La Union	4,241	0	5	7	12	0.28	0	6	45	51
Pangasinan	14,420	0	2	21	23	0.16	0	1	36	37
Alaminos City	1,250	0	0	0	0	0.00	0	0	0	0
Candon City	1,075	0	0	0	0	0.00	0	0	0	0
Dagupan City	8,147	0	5	92	97	1.19	0	0	0	0
Laoag City	1,965	0	0	0	0	0.00	0	0	2	2
San Carlos City	2,280	0	1	19	20	0.88	0	0	0	0
San Fernando City	3,530	0	0	5	5	0.14	0	2	3	5
Urdaneta City	1,843	0	0	2	2	0.11	0	0	0	0
Vigan City	1,416	0	0	0	0	0.00	0	0	0	0

Table 1.B.2.4 - Intrapartum Care and Delivery Outcome
B. Number and Proportion of Pregnancy Outcome (Fetal Deaths and Abortion)
Philippines, 2021

Area	Total number of Deliveries	Fetal Deaths					Abortion (Counts Only)			
		Age Group			Total	%	Age Group			Total
		10-14	15-19	20-49			10-14	15-19	20-49	
**Region 2**	**40,412**	**3**	**40**	**227**	**270**	**0.67**	**3**	**35**	**522**	**560**
Batanes	269	0	0	6	6	2.23	0	0	14	14
Cagayan	9,734	3	9	57	69	0.71	0	5	126	131
Isabela	12,520	0	13	69	82	0.65	2	11	107	120
Nueva Vizcaya	6,736	0	9	55	64	0.95	0	18	232	250
Quirino	2,634	0	4	16	20	0.76	1	0	24	25
Cauayan City	1,645	0	5	12	17	1.03	0	1	10	11
Ilagan City	2,646	0	0	6	6	0.23	0	0	1	1
Santiago City	2,861	0	0	0	0	0.00	0	0	0	0
Tuguegarao City	1,367	0	0	6	6	0.44	0	0	8	8
**Region 3**	**117,383**	**2**	**28**	**251**	**281**	**0.24**	**0**	**17**	**196**	**213**
Aurora	3,008	0	3	33	36	1.20	0	1	20	21
Bataan	5,148	1	6	25	32	0.62	0	8	109	117
Bulacan	22,840	0	2	28	30	0.13	0	0	7	7
Nueva Ecija	9,489	1	5	12	18	0.19	0	0	5	5
Pampanga	9,794	0	1	10	11	0.11	0	0	12	12
Tarlac	11,344	0	0	2	2	0.02	0	2	9	11
Zambales	7,601	0	6	57	63	0.83	0	2	5	7
Angeles City	7,027	0	1	2	3	0.04	0	0	0	0
Balanga City	1,290	0	1	16	17	1.32	0	3	17	20
Cabanatuan City	4,236	0	1	29	30	0.71	0	1	1	2
City of San Fernando	4,596	0	0	0	0	0.00	0	0	0	0
Gapan City	966	0	1	0	1	0.10	0	0	0	0
Mabalacat City	4,337	0	0	3	3	0.07	0	0	2	2
Malolos City	3,489	0	0	0	0	0.00	0	0	3	3
Meycauayan	2,351	0	0	0	0	0.00	0	0	0	0
Olongapo	3,425	0	0	9	9	0.26	0	0	1	1
Palayan City	77	0	0	0	0	0.00	0	0	0	0
San Jose City	1,940	0	0	6	6	0.31	0	0	3	3
San Jose del Monte City	6,642	0	0	4	4	0.06	0	0	0	0
Science City of Munoz	105	0	0	0	0	0.00	0	0	2	2
Tarlac City	7,678	0	1	15	16	0.21	0	0	0	0
**Region 4A**	**169,896**	**6**	**43**	**374**	**423**	**0.25**	**5**	**35**	**379**	**419**
Batangas	21,266	1	5	86	92	0.43	2	1	34	37
Cavite	16,518	1	6	35	42	0.25	1	7	73	81
Laguna	11,693	0	1	10	11	0.09	0	4	16	20
Quezon	18,491	0	20	101	121	0.65	0	13	126	139
Rizal	21,048	0	0	42	42	0.20	0	0	18	18
						0.00				
Antipolo City	11,094	0	0	0	0	0.00	0	0	0	0
Bacoor City	6,756	0	0	1	1	0.01	0	0	1	1
Batangas City	3,484	0	1	18	19	0.55	0	0	13	13

Table 1.B.2.4 - Intrapartum Care and Delivery Outcome

B. Number and Proportion of Pregnancy Outcome (Fetal Deaths and Abortion)
Philippines, 2021

Area	Total number of Deliveries	Fetal Deaths					Abortion (Counts Only)			
		Age Group			Total	%	Age Group			Total
		10-14	15-19	20-49			10-14	15-19	20-49	
Biñan City	7,973	0	0	13	13	0.16	0	1	0	1
Cabuyao City	3,188	0	0	0	0	0.00	0	0	0	0
Calamba City	8,294	0	2	9	11	0.13	0	3	45	48
Cavite City	659	3	0	0	3	0.46	0	0	2	2
Dasmariñas City	6,573	0	1	2	3	0.05	0	0	8	8
General Trias City	729	0	0	1	1	0.14	0	0	3	3
Imus City	4,356	0	0	0	0	0.00	0	0	0	0
Lipa City	4,231	0	2	21	23	0.54	1	1	22	24
Lucena City	2,473	0	2	7	9	0.36	0	1	5	6
San Pablo City	4,772	0	0	0	0	0.00	0	0	0	0
San Pedro City	1,302	0	0	1	1	0.08	0	0	0	0
Santa Rosa City	7,494	1	2	8	11	0.15	0	2	6	8
Tagaytay City	1,189	0	0	0	0	0.00	0	0	0	0
Tanauan City	2,707	0	0	5	5	0.18	0	0	5	5
Tayabas City	1,345	0	0	8	8	0.59	0	1	0	1
Trece Martires City	2,261	0	1	6	7	0.31	1	1	2	4
**Region 4B**	**40,990**	**0**	**43**	**380**	**423**	**1.03**	**0**	**30**	**186**	**216**
Marinduque	2,785	0	1	27	28	1.01	0	2	9	11
Mindoro Occidental	7,794	0	10	85	95	1.22	0	4	41	45
Mindoro Oriental	11,017	0	0	70	70	0.64	0	3	7	10
Palawan	12,500	0	27	117	144	1.15	0	17	94	111
Romblon	3,895	0	2	39	41	1.05	0	4	35	39
Puerto Princesa City	2,999	0	3	42	45	1.50	0	0	0	0
**Region 5**	**78,718**	**1**	**70**	**545**	**616**	**0.78**	**1**	**69**	**659**	**729**
Albay	9,202	0	0	25	25	0.27	0	7	67	74
Camarines Norte	9,661	0	13	94	107	1.11	0	5	43	48
Camarines Sur	15,499	0	11	71	82	0.53	0	10	107	117
Catanduanes	4,006	0	10	46	56	1.40	0	4	23	27
Masbate	12,154	0	17	142	159	1.31	0	16	108	124
Sorsogon	10,424	1	18	118	137	1.31	1	21	254	276
Iriga City	1,629	0	0	24	24	1.47	0	0	4	4
Legaspi City	4,511	0	0	10	10	0.22	0	3	52	55
Naga City	11,632	0	1	15	16	0.14	0	3	1	4
**Region 6**	**82,095**	**3**	**68**	**489**	**560**	**0.68**	**1**	**55**	**463**	**519**
Aklan	5,568	0	3	23	26	0.47	0	3	32	35
Antique	7,312	3	12	100	115	1.57	0	5	38	43
Capiz	7,458	0	1	13	14	0.19	0	4	16	20
Guimaras	2,319	0	3	19	22	0.95	0	3	39	42
Iloilo	19,237	0	3	59	62	0.32	0	4	76	80
Negros Occidental	31,802	0	43	243	286	0.90	1	35	237	273
Bacolod City	3,858	0	1	11	12	0.31	0	0	10	10
Iloilo City	4,541	0	2	21	23	0.51	0	1	15	16

Table 1.B.2.4 - Intrapartum Care and Delivery Outcome

B. Number and Proportion of Pregnancy Outcome (Fetal Deaths and Abortion)
Philippines, 2021

Area	Total number of Deliveries	Fetal Deaths					Abortion (Counts Only)			
		Age Group			Total	%	Age Group			Total
		10-14	15-19	20-49			10-14	15-19	20-49	
**Region 7**	**92,739**	**0**	**32**	**379**	**411**	**0.44**	**2**	**8**	**349**	**359**
Bohol	17,266	0	7	39	46	0.27	0	0	44	44
Cebu	27,663	0	8	72	80	0.29	0	4	15	19
Negros Oriental	18,889	0	10	107	117	0.62	1	0	157	158
Siquijor	1,138	0	0	41	41	3.60	1	0	61	62
Cebu City	16,354	0	2	14	16	0.10	0	0	8	8
Lapu-Lapu City	7,391	0	0	74	74	1.00	0	3	58	61
Mandaue City	4,038	0	5	32	37	0.92	0	1	6	7
**Region 8**	**52,130**	**1,260**	**55**	**390**	**1,705**	**3.27**	**16**	**19**	**274**	**309**
Biliran	2,621	1	15	36	52	1.98	1	3	55	59
Eastern Samar	6,181	0	5	42	47	0.76	0	2	29	31
Northern Leyte	14,081	1,255	5	80	1,340	9.52	15	6	125	146
Northern Samar	8,565	4	24	101	129	1.51	0	3	42	45
Southern Leyte	2,521	0	1	9	10	0.40	0	1	9	10
Western Samar	6,475	0	1	14	15	0.23	0	2	9	11
Calbayog City	1,938	0	3	10	13	0.67	0	2	0	2
Maasin City	2,252	0	0	48	48	2.13	0	0	5	5
Ormoc City	4,748	0	0	39	39	0.82	0	0	0	0
Tacloban City	2,748	0	1	11	12	0.44	0	0	0	0
**Region 9**	**46,771**	**0**	**33**	**177**	**210**	**0.45**	**0**	**47**	**225**	**272**
Zamboanga del Norte	9,489	0	14	41	55	0.58	0	18	74	92
Zamboanga del Sur	6,651	0	3	24	27	0.41	0	8	31	39
Zamboanga Sibugay	6,965	0	7	46	53	0.76	0	10	79	89
Dapitan City	1,598	0	5	25	30	1.88	0	6	7	13
Dipolog City	2,277	0	1	7	8	0.35	0	1	7	8
Isabela City	1,629	0	1	5	6	0.37	0	0	4	4
Pagadian City	2,298	0	1	2	3	0.13	0	0	0	0
Zamboanga City	15,864	0	1	27	28	0.18	0	4	23	27
**Region 10**	**71,984**	**1**	**57**	**389**	**447**	**0.62**	**2**	**41**	**296**	**339**
Bukidnon	19,679	0	34	145	179	0.91	0	24	157	181
Camiguin	1,100	0	0	21	21	1.91	0	0	0	0
Lanao del Norte	8,578	0	3	26	29	0.34	1	0	10	11
Misamis Occidental	3,779	0	1	4	5	0.13	0	1	5	6
Misamis Oriental	8,821	0	3	32	35	0.40	0	1	15	16
Cagayan de Oro City	11,751	0	1	33	34	0.29	1	5	54	60
El Salvador City	249	0	0	0	0	0.00	0	0	0	0
Gingoog City	2,893	0	9	36	45	1.56	0	4	13	17
Iligan City	6,035	0	1	56	57	0.94	0	2	31	33
Malaybalay City	2,899	0	3	16	19	0.66	0	4	8	12

Table 1.B.2.4 - Intrapartum Care and Delivery Outcome

B. Number and Proportion of Pregnancy Outcome (Fetal Deaths and Abortion)
Philippines, 2021

Area	Total number of Deliveries	Fetal Deaths					Abortion (Counts Only)			
		Age Group			Total	%	Age Group			Total
		10-14	15-19	20-49			10-14	15-19	20-49	
Oroquieta City	744	0	0	1	1	0.13	0	0	2	2
Ozamis City	1,944	0	1	12	13	0.67	0	0	1	1
Tangub City	1,173	0	0	0	0	0.00	0	0	0	0
Valencia City	2,339	1	1	7	9	0.38	0	0	0	0
**Region 11**	**76,188**	**6**	**122**	**467**	**595**	**0.78**	**11**	**155**	**742**	**908**
Davao de Oro	11,332	0	24	87	111	0.98	2	33	107	142
Davao del Norte	17,041	1	27	133	161	0.94	2	53	229	284
Davao Oriental	8,236	0	15	64	79	0.96	0	9	75	84
Davao del Sur	8,170	1	14	82	97	1.19	4	22	96	122
Davao Occidental	4,276	3	36	57	96	2.25	0	22	77	99
Davao City	27,133	1	6	44	51	0.19	3	16	158	177
**Region 12**	**65,637**	**1**	**77**	**324**	**402**	**0.61**	**3**	**81**	**325**	**409**
North Cotabato	18,335	0	27	78	105	0.57	1	12	87	100
Sarangani	9,714	1	13	55	69	0.71	0	28	62	90
South Cotabato	16,353	0	16	103	119	0.73	1	17	101	119
Sultan Kudarat	12,030	0	18	79	97	0.81	1	24	73	98
Cotabato City	3,298	0	1	5	6	0.18	0	0	0	0
Gen. Santos City	5,907	0	2	4	6	0.10	0	0	2	2
**BARMM**	**62,595**	**2**	**70**	**492**	**564**	**0.90**	**2**	**20**	**105**	**127**
Basilan	3,253	2	39	312	353	10.85	1	7	12	20
Lanao del Sur	17,519	0	1	27	28	0.16	0	1	30	31
Maguindanao	20,188	0	5	54	59	0.29	1	2	24	27
Sulu	8,891	0	18	89	107	1.20	0	5	26	31
Tawi-Tawi	5,681	0	2	8	10	0.18	0	0	7	7
Lamitan City	1,226	0	0	0	0	0.00	0	0	0	0
Marawi City	5,837	0	5	2	7	0.12	0	5	6	11
**CARAGA**	**37,081**	**1**	**36**	**223**	**260**	**0.70**	**2**	**14**	**186**	**202**
Agusan del Norte	3,599	0	0	4	4	0.11	0	0	12	12
Agusan del Sur	11,882	0	3	56	59	0.50	2	4	45	51
Surigao del Norte	3,409	0	2	51	53	1.55	0	1	21	22
Surigao del Sur	6,454	0	19	50	69	1.07	0	4	66	70
Province of Dinagat	1,020	0	0	10	10	0.98	0	2	26	28
Bislig City	1,294	0	2	11	13	1.00	0	3	13	16
Butuan City	5,267	1	10	38	49	0.93	0	0	3	3
Surigao City	4,156	0	0	3	3	0.07	0	0	0	0

Note: Put asterisk (*) for No Report and Zero (0) for No Case

Table 1.B.2.5 - Intrapartum Care and Delivery Outcome
Number and Proportion of Livebirths by Birth Weight
Philippines, 2021

Area	Total No. of Livebirths	NBW (≥ 2500 grams)		LBW (< 2500)		UBW	
		No.	%	No.	%	No.	%
**PHILIPPINES**	**1,247,069**	**1,146,009**	**91.90**	**68,902**	**5.53**	**32,158**	**2.58**
**N C R**	**147,024**	**132,388**	**90.05**	**13,325**	**9.06**	**1,311**	**0.89**
Malabon	5,158	4,838	93.80	166	3.22	154	2.99
Navotas	3,241	3,161	97.53	76	2.34	4	0.12
Valenzuela City	6,520	6,122	93.90	269	4.13	129	1.98
Caloocan City	14,402	13,280	92.21	1,113	7.73	9	0.06
Marikina City	7,010	6,305	89.94	705	10.06	0	0.00
Pasig City	11,761	9,958	84.67	1,768	15.03	35	0.30
Pateros	714	707	99.02	7	0.98	0	0.00
Taguig	8,594	7,539	87.72	641	7.46	414	4.82
Quezon City	43,663	38,046	87.14	5,534	12.67	83	0.19
Makati City	5,691	5,056	88.84	576	10.12	59	1.04
Mandaluyong City	3,418	3,310	96.84	107	3.13	1	0.03
San Juan	1,468	1,308	89.10	155	10.56	5	0.34
Manila City	13,237	12,693	95.89	413	3.12	131	0.99
Las Piñas City	5,515	5,291	95.94	62	1.12	162	2.94
Muntinlupa City	6,733	5,895	87.55	838	12.45	0	0.00
Parañaque City	5,720	4,970	86.89	625	10.93	125	2.19
Pasay City	4,179	3,909	93.54	270	6.46	0	0.00
**C A R**	**22,381**	**20,556**	**91.85**	**1,764**	**7.88**	**61**	**0.27**
Abra	2,701	2,387	88.37	281	10.40	33	1.22
Apayao	1,701	1,565	92.00	130	7.64	6	0.35
Benguet	4,617	4,288	92.87	314	6.80	15	0.32
Ifugao	2,776	2,690	96.90	82	2.95	4	0.14
Kalinga	2,373	2,299	96.88	72	3.03	2	0.08
Mt. Province	2,434	2,271	93.30	163	6.70	0	0.00
Baguio City	5,779	5,056	87.49	722	12.49	1	0.02
**Region 1**	**48,712**	**46,058**	**94.55**	**2,401**	**4.93**	**253**	**0.52**
Ilocos Norte	4,378	4,230	96.62	144	3.29	4	0.09
Ilocos Sur	4,223	4,106	97.23	115	2.72	2	0.05
La Union	4,236	4,060	95.85	173	4.08	3	0.07
Pangasinan	14,410	14,249	98.88	148	1.03	13	0.09
Alaminos City	1,255	1,254	99.92	1	0.08	0	0.00
Candon City	1,075	1,073	99.81	2	0.19	0	0.00
Dagupan City	8,100	6,741	83.22	1,241	15.32	118	1.46
Laoag City	1,965	1,965	100.00	0	0.00	0	0.00
San Carlos City	2,267	2,226	98.19	39	1.72	2	0.09
San Fernando City	3,538	3,088	87.28	339	9.58	111	3.14
Urdaneta City	1,845	1,819	98.59	26	1.41	0	0.00

Table 1.B.2.5 - Intrapartum Care and Delivery Outcome

Number and Proportion of Livebirths by Birth Weight
Philippines, 2021

Area	Total No. of Livebirths	NBW (≥ 2500 grams)		LBW (< 2500)		UBW	
		No.	%	No.	%	No.	%
Vigan City	1,420	1,247	87.82	173	12.18	0	0.00
**Region 2**	**40,270**	**38,625**	**95.92**	**1,573**	**3.91**	**72**	**0.18**
Batanes	267	235	88.01	32	11.99	0	0.00
Cagayan	9,697	9,432	97.27	236	2.43	29	0.30
Isabela	12,459	11,987	96.21	437	3.51	35	0.28
Nueva Vizcaya	6,692	6,023	90.00	666	9.95	3	0.04
Quirino	2,624	2,497	95.16	122	4.65	5	0.19
Cauayan City	1,642	1,615	98.36	27	1.64	0	0.00
Ilagan City	2,640	2,629	99.58	11	0.42	0	0.00
Santiago City	2,883	2,851	98.89	32	1.11	0	0.00
Tuguegarao City	1,366	1,356	99.27	10	0.73	0	0.00
**Region 3**	**116,676**	**109,413**	**93.78**	**6,568**	**5.63**	**695**	**0.60**
Aurora	2,993	2,709	90.51	256	8.55	28	0.94
Bataan	5,144	4,760	92.53	348	6.77	36	0.70
Bulacan	22,189	20,457	92.19	1,618	7.29	114	0.51
Nueva Ecija	9,275	8,978	96.80	105	1.13	192	2.07
Pampanga	9,787	9,315	95.18	353	3.61	119	1.22
Tarlac	11,347	11,238	99.04	89	0.78	20	0.18
Zambales	7,560	6,777	89.64	678	8.97	105	1.39
Angeles City	7,279	6,479	89.01	791	10.87	9	0.12
Balanga City	1,283	1,192	92.91	91	7.09	0	0.00
Cabanatuan City	4,225	4,113	97.35	100	2.37	12	0.28
City of San Fernando	4,596	3,877	84.36	719	15.64	0	0.00
Gapan City	967	965	99.79	2	0.21	0	0.00
Mabalacat City	4,326	3,618	83.63	703	16.25	5	0.12
Malolos City	3,489	3,476	99.63	13	0.37	0	0.00
Meycauayan	2,351	2,335	99.32	1	0.04	15	0.64
Olongapo	3,418	3,126	91.46	261	7.64	31	0.91
Palayan City	77	77	100.00	0	0.00	0	0.00
San Jose City	1,940	1,876	96.70	55	2.84	9	0.46
San Jose del Monte City	6,631	6,261	94.42	370	5.58	0	0.00
Science City of Munoz	106	101	95.28	5	4.72	0	0.00
Tarlac City	7,693	7,683	99.87	10	0.13	0	0.00
**Region 4A**	**168,998**	**155,074**	**91.76**	**8,350**	**4.94**	**5,574**	**3.30**
Batangas	20,740	19,994	96.40	479	2.31	267	1.29
Cavite	16,556	15,367	92.82	731	4.42	458	2.77
Laguna	11,691	10,409	89.03	892	7.63	390	3.34
Quezon	17,992	16,507	91.75	799	4.44	686	3.81
Rizal	20,786	18,789	90.39	979	4.71	1,018	4.90
Antipolo City	11,983	10,292	85.89	1,337	11.16	354	2.95
Bacoor City	6,509	5,526	84.90	315	4.84	668	10.26
Batangas City	3,501	3,236	92.43	256	7.31	9	0.26

Table 1.B.2.5 - Intrapartum Care and Delivery Outcome

Number and Proportion of Livebirths by Birth Weight
Philippines, 2021

Area	Total No. of Livebirths	NBW (≥ 2500 grams)		LBW (< 2500)		UBW	
		No.	%	No.	%	No.	%
Biñan City	7,961	7,718	96.95	145	1.82	98	1.23
Cabuyao City	3,188	3,178	99.69	10	0.31	0	0.00
Calamba City	8,301	7,528	90.69	375	4.52	398	4.79
Cavite City	655	634	96.79	21	3.21	0	0.00
Dasmariñas City	6,527	6,196	94.93	286	4.38	45	0.69
General Trias City	729	673	92.32	23	3.16	33	4.53
Imus City	4,347	4,004	92.11	191	4.39	152	3.50
Lipa City	4,093	3,892	95.09	110	2.69	91	2.22
Lucena City	2,427	2,223	91.59	158	6.51	46	1.90
San Pablo City	4,772	4,039	84.64	428	8.97	305	6.39
San Pedro City	1,304	1,072	82.21	141	10.81	91	6.98
Santa Rosa City	7,483	6,785	90.67	385	5.14	313	4.18
Tagaytay City	1,189	1,182	99.41	3	0.25	4	0.34
Tanauan City	2,675	2,457	91.85	120	4.49	98	3.66
Tayabas City	1,336	1,300	97.31	36	2.69	0	0.00
Trece Martires City	2,253	2,073	92.01	130	5.77	50	2.22
**Region 4B**	**41,216**	**37,076**	**89.96**	**2,078**	**5.04**	**2,062**	**5.00**
Marinduque	2,765	2,639	95.44	115	4.16	11	0.40
Mindoro Occidental	7,731	6,876	88.94	326	4.22	529	6.84
Mindoro Oriental	11,156	9,695	86.90	765	6.86	696	6.24
Palawan	12,572	11,426	90.88	496	3.95	650	5.17
Romblon	3,925	3,531	89.96	230	5.86	164	4.18
Puerto Princesa City	3,067	2,909	94.85	146	4.76	12	0.39
**Region 5**	**78,461**	**69,208**	**88.21**	**7,030**	**8.96**	**2,223**	**2.83**
Albay	9,197	8,687	94.45	379	4.12	131	1.42
Camarines Norte	9,555	8,292	86.78	1,222	12.79	41	0.43
Camarines Sur	15,437	13,108	84.91	876	5.67	1,453	9.41
Catanduanes	3,972	3,495	87.99	462	11.63	15	0.38
Masbate	12,054	10,800	89.60	695	5.77	559	4.64
Sorsogon	10,324	9,311	90.19	995	9.64	18	0.17
Iriga City	1,612	1,434	88.96	178	11.04	0	0.00
Legaspi City	4,506	4,390	97.43	111	2.46	5	0.11
Naga City	11,804	9,691	82.10	2,112	17.89	1	0.01
**Region 6**	**81,900**	**75,048**	**91.63**	**5,702**	**6.96**	**1,150**	**1.40**
Aklan	5,536	5,133	92.72	300	5.42	103	1.86
Antique	7,250	6,063	83.63	1,073	14.80	114	1.57
Capiz	7,494	7,067	94.30	308	4.11	119	1.59
Guimaras	2,322	2,110	90.87	212	9.13	0	0.00
Iloilo	19,202	17,705	92.20	1,384	7.21	113	0.59
Negros Occidental	31,682	29,214	92.21	1,952	6.16	516	1.63
Bacolod City	3,872	3,375	87.16	329	8.50	168	4.34
Iloilo City	4,542	4,381	96.46	144	3.17	17	0.37

Table 1.B.2.5 - Intrapartum Care and Delivery Outcome

Number and Proportion of Livebirths by Birth Weight
Philippines, 2021

Area	Total No. of Livebirths	NBW (≥ 2500 grams)		LBW (< 2500)		UBW	
		No.	%	No.	%	No.	%
**Region 7**	**92,365**	**84,827**	**91.84**	**5,869**	**6.35**	**1,669**	**1.81**
Bohol	17,238	15,543	90.17	1,695	9.83	0	0.00
Cebu	27,617	26,201	94.87	1,069	3.87	347	1.26
Negros Oriental	18,802	16,327	86.84	1,175	6.25	1,300	6.91
Siquijor	1,174	1,027	87.48	147	12.52	0	0.00
Cebu City	16,209	15,475	95.47	716	4.42	18	0.11
Lapu-Lapu City	7,256	6,702	92.36	551	7.59	3	0.04
Mandaue City	4,069	3,552	87.29	516	12.68	1	0.02
**Region 8**	**53,194**	**48,026**	**90.28**	**3,077**	**5.78**	**2,091**	**3.93**
Biliran	2,613	2,359	90.28	248	9.49	6	0.23
Eastern Samar	6,130	5,722	93.34	183	2.99	225	3.67
Northern Leyte	15,328	13,943	90.96	405	2.64	980	6.39
Northern Samar	8,498	7,378	86.82	608	7.15	512	6.02
Southern Leyte	2,480	2,258	91.05	222	8.95	0	0.00
Western Samar	6,501	6,099	93.82	273	4.20	129	1.98
Calbayog City	1,929	1,615	83.72	75	3.89	239	12.39
Maasin City	2,213	1,869	84.46	344	15.54	0	0.00
Ormoc City	4,761	4,073	85.55	688	14.45	0	0.00
Tacloban City	2,741	2,710	98.87	31	1.13	0	0.00
**Region 9**	**46,201**	**43,358**	**93.85**	**1,101**	**2.38**	**1,742**	**3.77**
Zamboanga del Norte	9,468	8,288	87.54	283	2.99	897	9.47
Zamboanga del Sur	6,641	5,852	88.12	183	2.76	606	9.13
Zamboanga Sibugay	6,936	6,477	93.38	346	4.99	113	1.63
Dapitan City	1,595	1,564	98.06	31	1.94	0	0.00
Dipolog City	1,964	1,915	97.51	42	2.14	7	0.36
Isabela City	1,586	1,472	92.81	4	0.25	110	6.94
Pagadian City	2,182	2,147	98.40	35	1.60	0	0.00
Zamboanga City	15,829	15,643	98.82	177	1.12	9	0.06
**Region 10**	**70,977**	**66,379**	**93.52**	**2,212**	**3.12**	**2,386**	**3.36**
Bukidnon	18,729	17,526	93.58	912	4.87	291	1.55
Camiguin	1,084	1,045	96.40	39	3.60	0	0.00
Lanao del Norte	8,583	7,413	86.37	106	1.23	1,064	12.40
Misamis Occidental	3,866	3,838	99.28	19	0.49	9	0.23
Misamis Oriental	8,817	7,551	85.64	472	5.35	794	9.01
Cagayan de Oro City	11,740	11,405	97.15	240	2.04	95	0.81
El Salvador City	250	245	98.00	1	0.40	4	1.60
Gingoog City	2,848	2,826	99.23	16	0.56	6	0.21
Iligan City	5,977	5,765	96.45	162	2.71	50	0.84
Malaybalay City	2,900	2,785	96.03	85	2.93	30	1.03

Table 1.B.2.5 - Intrapartum Care and Delivery Outcome
Number and Proportion of Livebirths by Birth Weight
Philippines, 2021

Area	Total No. of Livebirths	NBW (≥ 2500 grams)		LBW (< 2500)		UBW	
		No.	%	No.	%	No.	%
Oroquieta City	743	684	92.06	59	7.94	0	0.00
Ozamis City	1,933	1,912	98.91	21	1.09	0	0.00
Tangub City	1,173	1,109	94.54	34	2.90	30	2.56
Valencia City	2,334	2,275	97.47	46	1.97	13	0.56
**Region 11**	**76,593**	**70,197**	**91.65**	**3,673**	**4.80**	**2,723**	**3.56**
Davao de Oro	11,442	10,717	93.66	623	5.44	102	0.89
Davao del Norte	17,083	15,852	92.79	859	5.03	372	2.18
Davao Oriental	8,309	7,821	94.13	387	4.66	101	1.22
Davao del Sur	8,254	7,570	91.71	432	5.23	252	3.05
Davao Occidental	4,197	3,066	73.05	159	3.79	972	23.16
Davao City	27,308	25,171	92.17	1,213	4.44	924	3.38
**Region 12**	**65,475**	**61,619**	**94.11**	**1,661**	**2.54**	**2,195**	**3.35**
North Cotabato	18,326	16,657	90.89	512	2.79	1,157	6.31
Sarangani	9,677	9,229	95.37	168	1.74	280	2.89
South Cotabato	16,306	15,687	96.20	396	2.43	223	1.37
Sultan Kudarat	11,962	11,245	94.01	389	3.25	328	2.74
Cotabato City	3,299	3,128	94.82	38	1.15	133	4.03
Gen. Santos City	5,905	5,673	96.07	158	2.68	74	1.25
**BARMM**	**59,731**	**53,839**	**90.14**	**1,006**	**1.68**	**4,886**	**8.18**
Basilan	3,206	2,394	74.67	152	4.74	660	20.59
Lanao del Sur	17,544	16,521	94.17	53	0.30	970	5.53
Maguindanao	20,142	18,531	92.00	257	1.28	1,354	6.72
Sulu	7,079	6,580	92.95	76	1.07	423	5.98
Tawi-Tawi	5,659	5,402	95.46	65	1.15	192	3.39
Lamitan City	1,362	1,342	98.53	19	1.40	1	0.07
Marawi City	4,739	3,069	64.76	384	8.10	1,286	27.14
**CARAGA**	**36,895**	**34,318**	**93.02**	**1,512**	**4.10**	**1,065**	**2.89**
Agusan del Norte	3,603	3,261	90.51	186	5.16	156	4.33
Agusan del Sur	11,850	11,374	95.98	236	1.99	240	2.03
Surigao del Norte	3,361	2,960	88.07	217	6.46	184	5.47
Surigao del Sur	6,394	5,679	88.82	325	5.08	390	6.10
Province of Dinagat	1,015	939	92.51	63	6.21	13	1.28
Bislig City	1,288	1,273	98.84	13	1.01	2	0.16
Butuan City	5,228	5,011	95.85	161	3.08	56	1.07
Surigao City	4,156	3,821	91.94	311	7.48	24	0.58

Note: Put asterisk (*) for No Report and Zero (0) for No Case
Live births should be reported by place of occurrence.

NBW - Normal Birth Weight, LBW - Low Birth Weight
UBW - Unknown Birth Weight

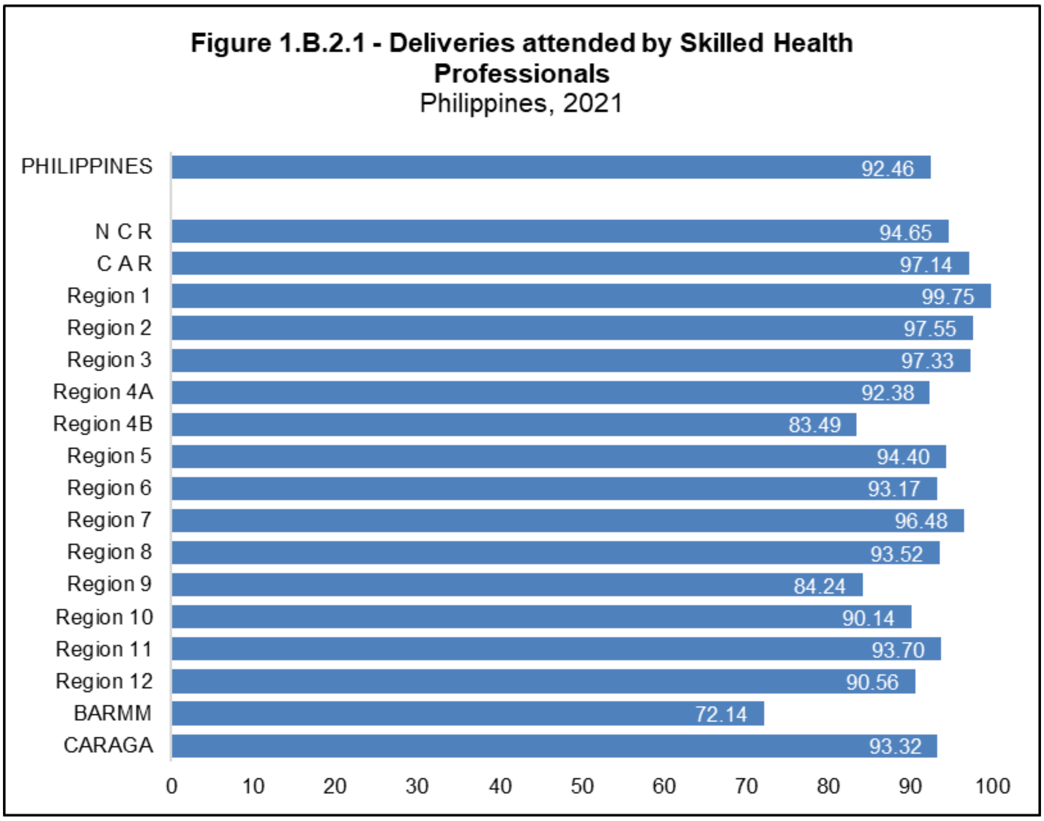
Figure 1.B.2.1 - Deliveries attended by Skilled Health Professionals
Philippines, 2021
PHILIPPINES
N C R
C A R
Region 1
Region 2
Region 3
Region 4A
Region 4B
Region 5
Region 6
Region 7
Region 8
Region 9
Region 10
Region 11
Region 12
BARMM
CARAGA
92.46
94.65
97.14
99.75
97.55
97.33
92.38
83.49
94.40
93.17
96.48
93.52
84.24
90.14
93.70
90.56
72.14
93.32
0
10
20
30
40
50
60
70
80
90
100

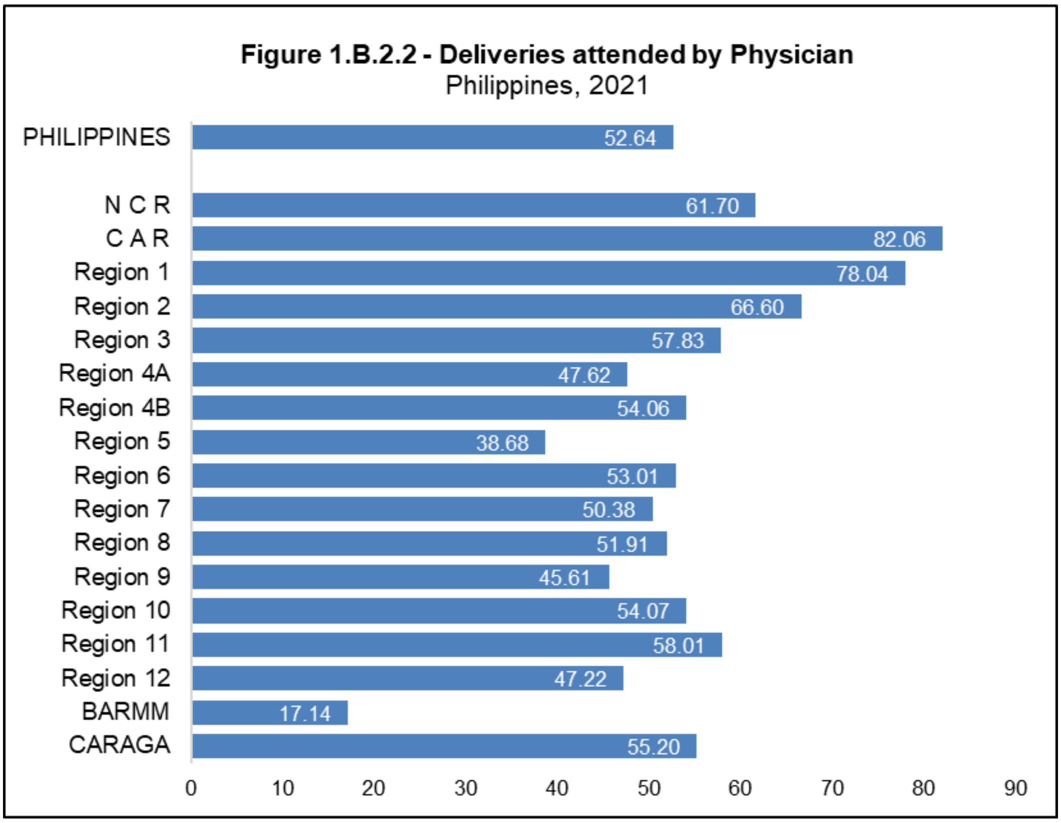
Figure 1.B.2.2 - Deliveries attended by Physician
Philippines, 2021
PHILIPPINES
N C R
C A R
Region 1
Region 2
Region 3
Region 4A
Region 4B
Region 5
Region 6
Region 7
Region 8
Region 9
Region 10
Region 11
Region 12
BARMM
CARAGA
52.64
61.70
82.06
78.04
66.60
57.83
47.62
54.06
38.68
53.01
50.38
51.91
45.61
54.07
58.01
47.22
17.14
55.20
0
10
20
30
40
50
60
70
80
90

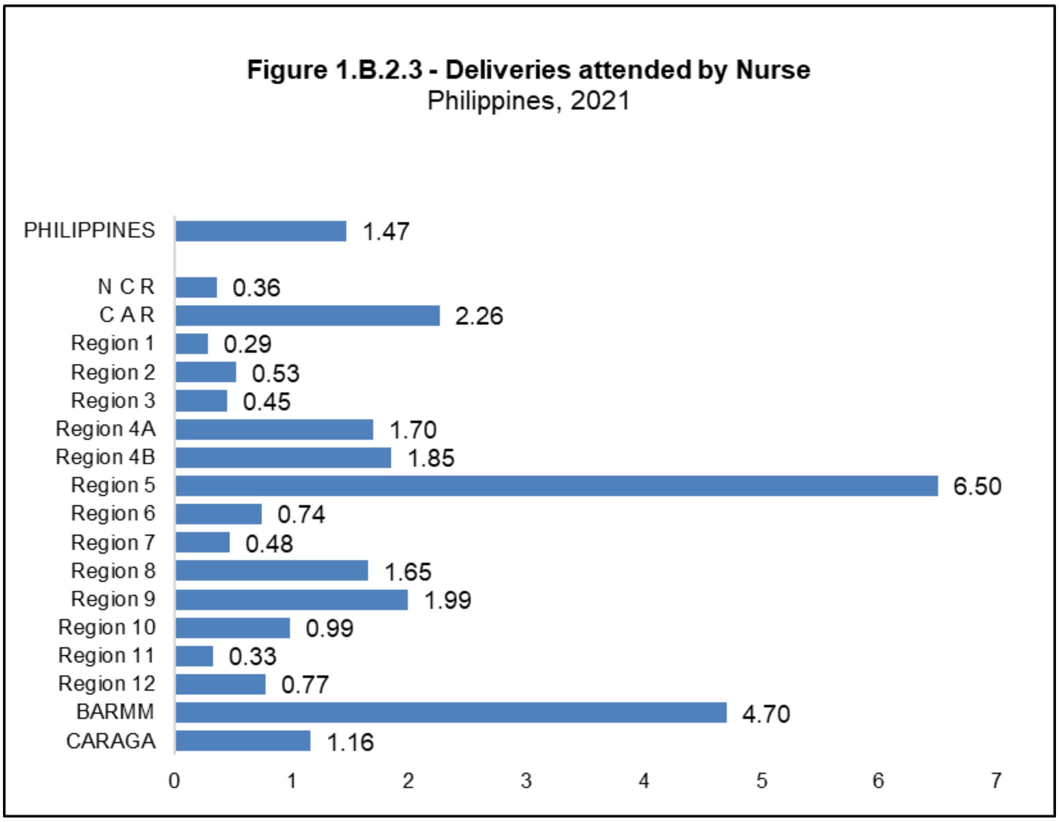
Figure 1.B.2.3 - Deliveries attended by Nurse
Philippines, 2021
PHILIPPINES 1.47
N C R 0.36
C A R 2.26
Region 1 0.29
Region 2 0.53
Region 3 0.45
Region 4A 1.70
Region 4B 1.85
Region 5 6.50
Region 6 0.74
Region 7 0.48
Region 8 1.65
Region 9 1.99
Region 10 0.99
Region 11 0.33
Region 12 0.77
BARMM 4.70
CARAGA 1.16
0 1 2 3 4 5 6 7

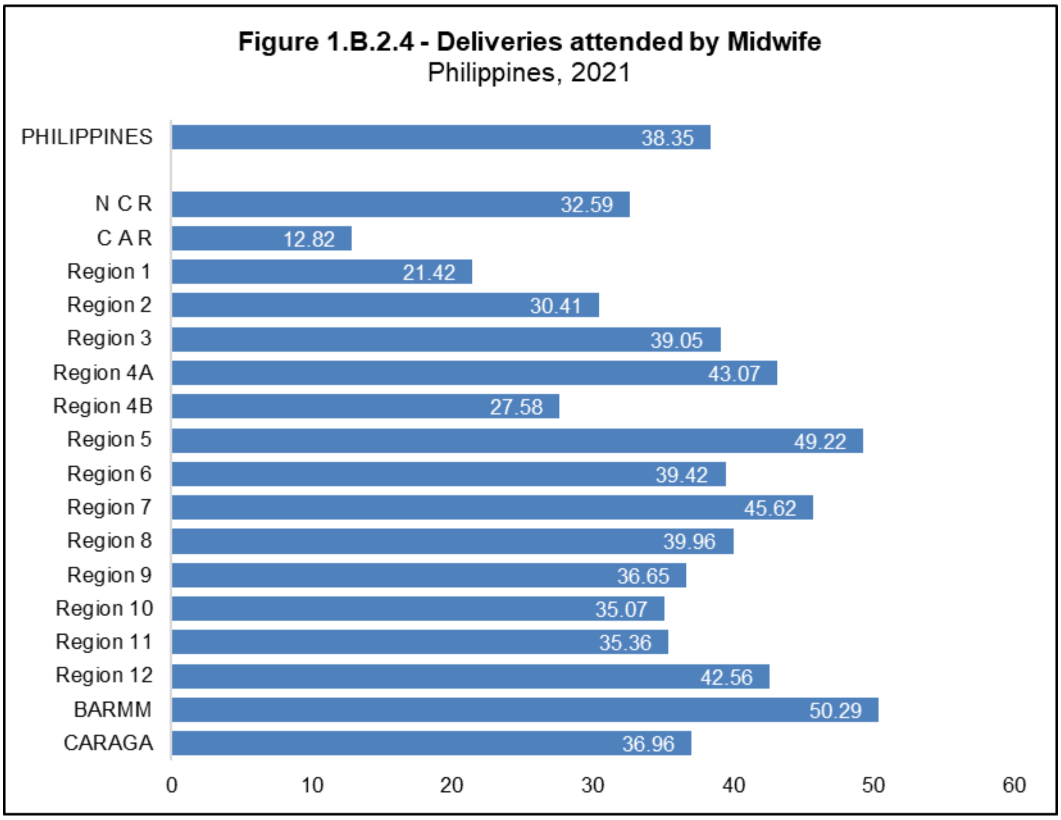
Figure 1.B.2.4 - Deliveries attended by Midwife
Philippines, 2021
PHILIPPINES 38.35
N C R 32.59
C A R 12.82
Region 1 21.42
Region 2 30.41
Region 3 39.05
Region 4A 43.07
Region 4B 27.58
Region 5 49.22
Region 6 39.42
Region 7 45.62
Region 8 39.96
Region 9 36.65
Region 10 35.07
Region 11 35.36
Region 12 42.56
BARMM 50.29
CARAGA 36.96
0 10 20 30 40 50 60

Figure 1.B.2.5 - Facility Based Delivery
Philippines, 2021

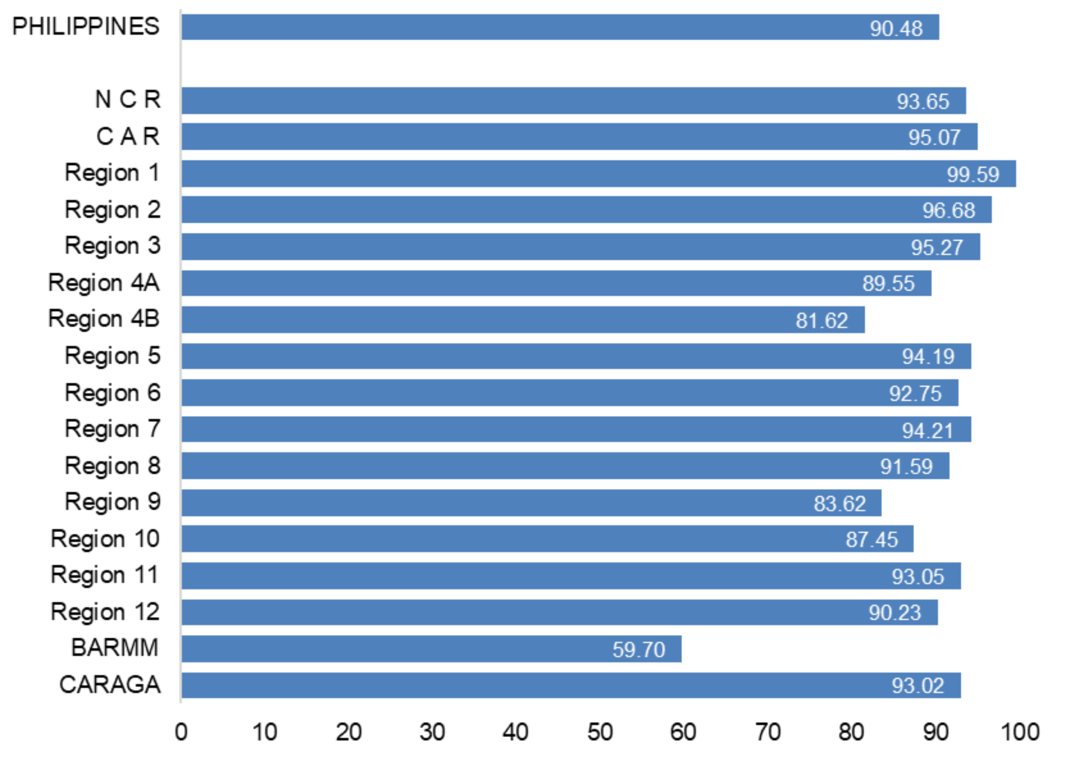

Figure 1.B.2.6 - Facility Based Deliver by Ownership (Public and Private)
Philippines, 2021

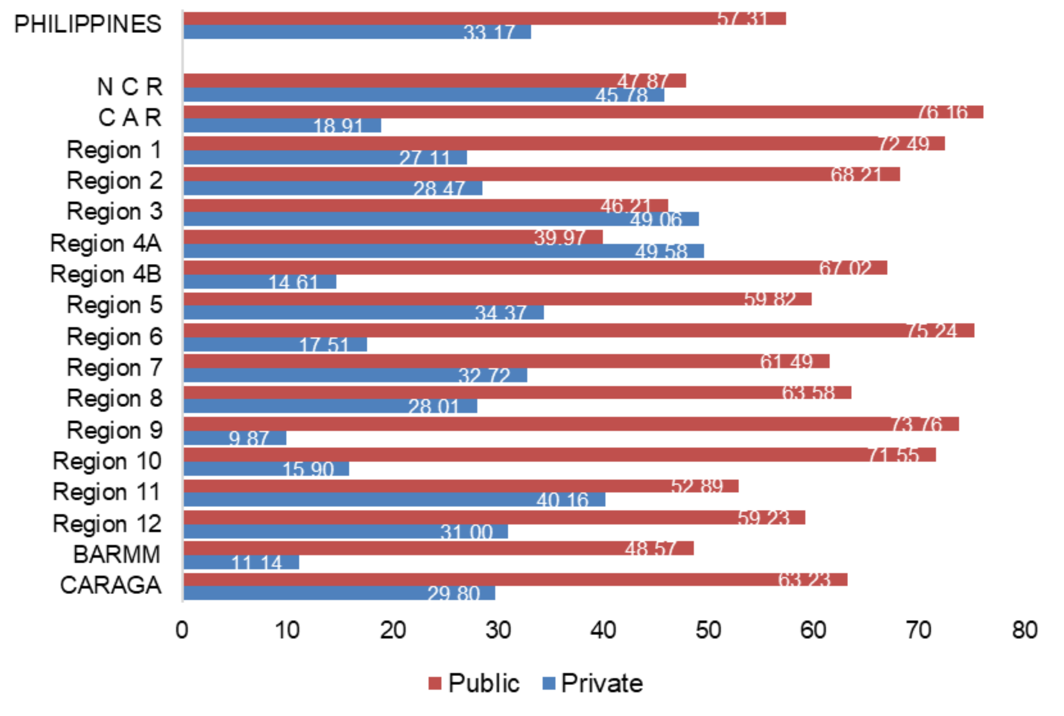

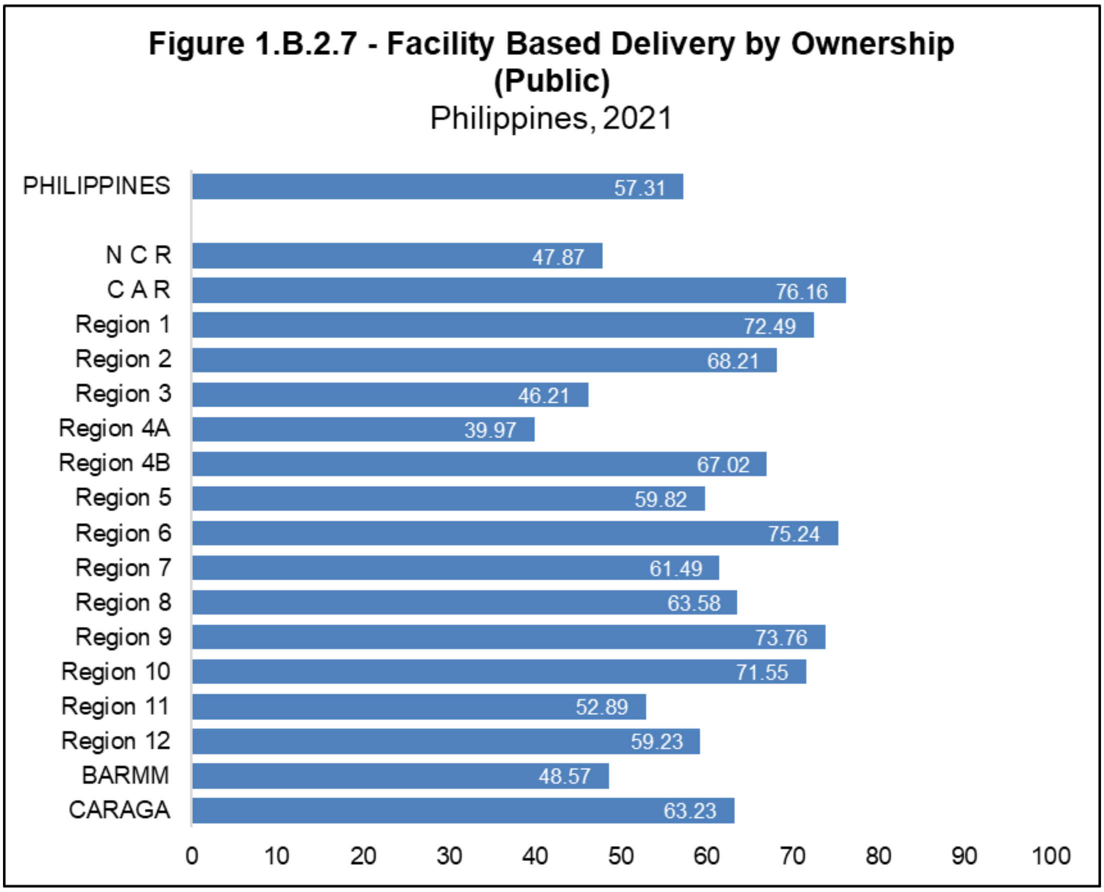
Figure 1.B.2.7 - Facility Based Delivery by Ownership (Public)
Philippines, 2021
PHILIPPINES
N C R
C A R
Region 1
Region 2
Region 3
Region 4A
Region 4B
Region 5
Region 6
Region 7
Region 8
Region 9
Region 10
Region 11
Region 12
BARMM
CARAGA
57.31
47.87
76.16
72.49
68.21
46.21
39.97
67.02
59.82
75.24
61.49
63.58
73.76
71.55
52.89
59.23
48.57
63.23
0
10
20
30
40
50
60
70
80
90
100

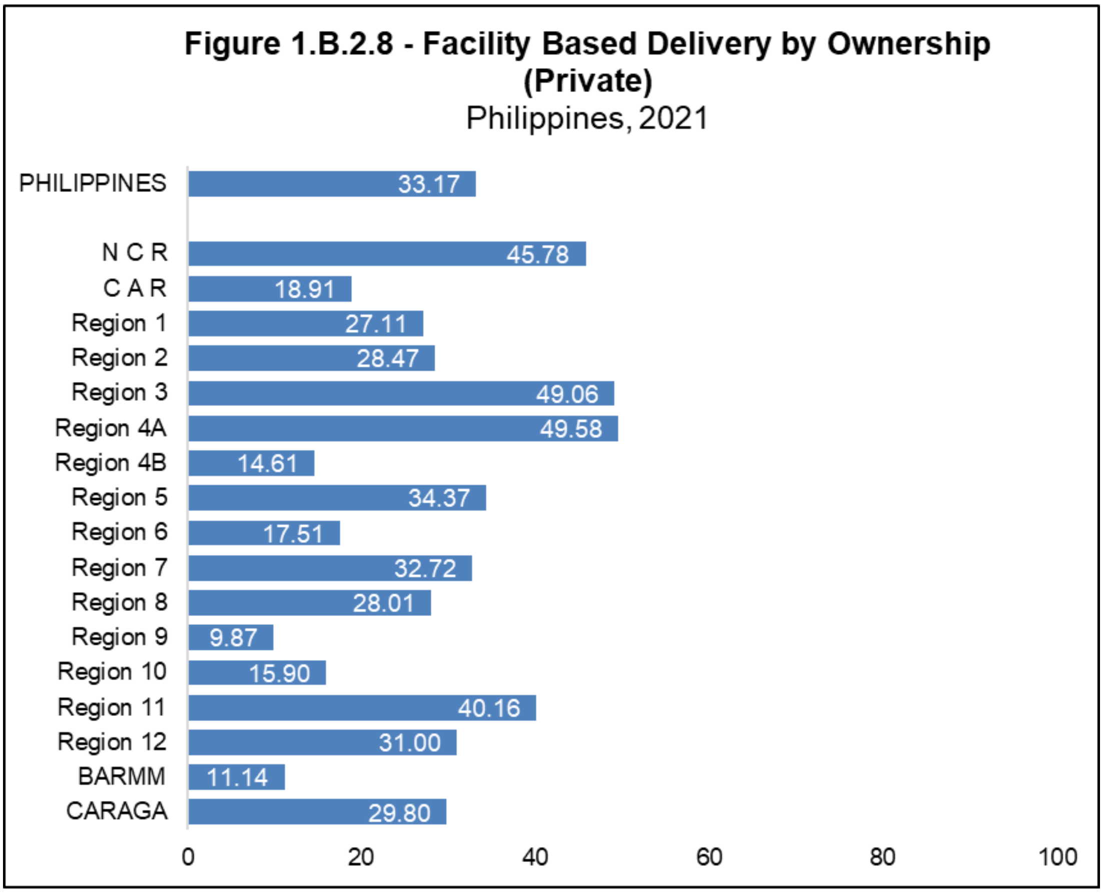
Figure 1.B.2.8 - Facility Based Delivery by Ownership (Private)
Philippines, 2021
PHILIPPINES
N C R
C A R
Region 1
Region 2
Region 3
Region 4A
Region 4B
Region 5
Region 6
Region 7
Region 8
Region 9
Region 10
Region 11
Region 12
BARMM
CARAGA
33.17
45.78
18.91
27.11
28.47
49.06
49.58
14.61
34.37
17.51
32.72
28.01
9.87
15.90
40.16
31.00
11.14
29.80
0
20
40
60
80
100

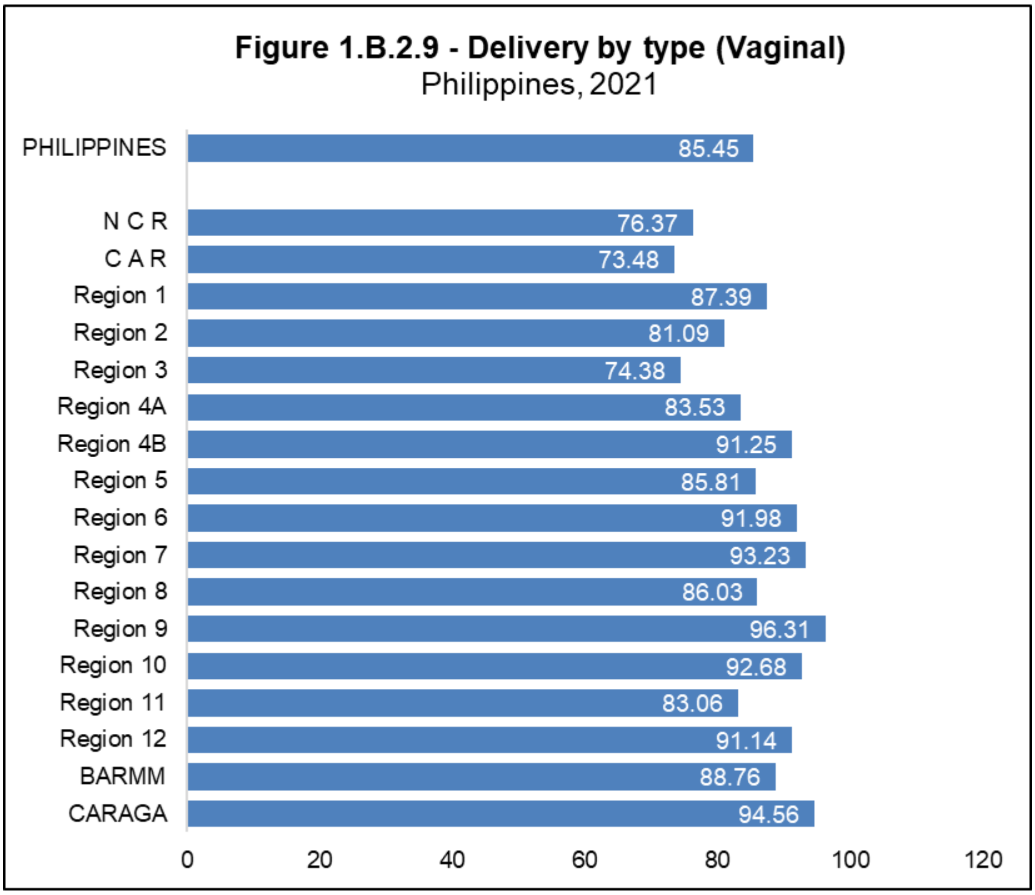
Figure 1.B.2.9 - Delivery by type (Vaginal)
Philippines, 2021
PHILIPPINES
85.45
N C R
76.37
C A R
73.48
Region 1
87.39
Region 2
81.09
Region 3
74.38
Region 4A
83.53
Region 4B
91.25
Region 5
85.81
Region 6
91.98
Region 7
93.23
Region 8
86.03
Region 9
96.31
Region 10
92.68
Region 11
83.06
Region 12
91.14
BARMM
88.76
CARAGA
94.56
0
20
40
60
80
100
120

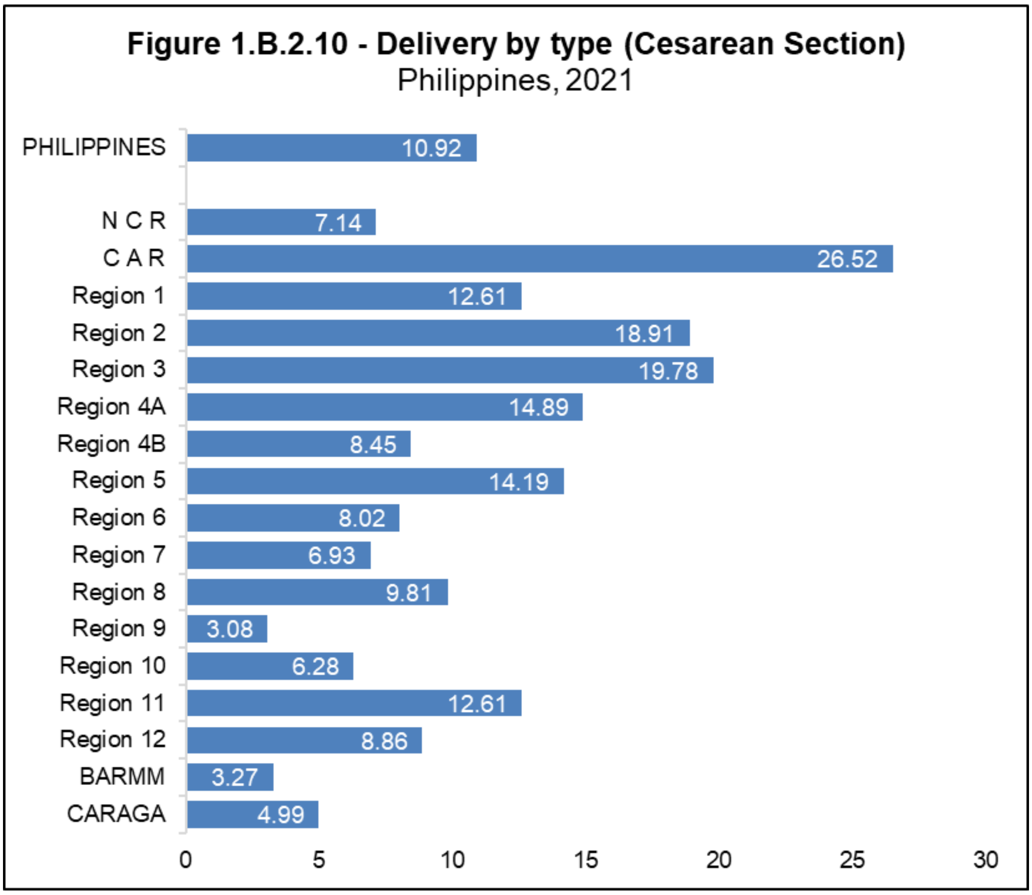
Figure 1.B.2.10 - Delivery by type (Cesarean Section)
Philippines, 2021
PHILIPPINES
10.92
N C R
7.14
C A R
26.52
Region 1
12.61
Region 2
18.91
Region 3
19.78
Region 4A
14.89
Region 4B
8.45
Region 5
14.19
Region 6
8.02
Region 7
6.93
Region 8
9.81
Region 9
3.08
Region 10
6.28
Region 11
12.61
Region 12
8.86
BARMM
3.27
CARAGA
4.99
0
5
10
15
20
25
30

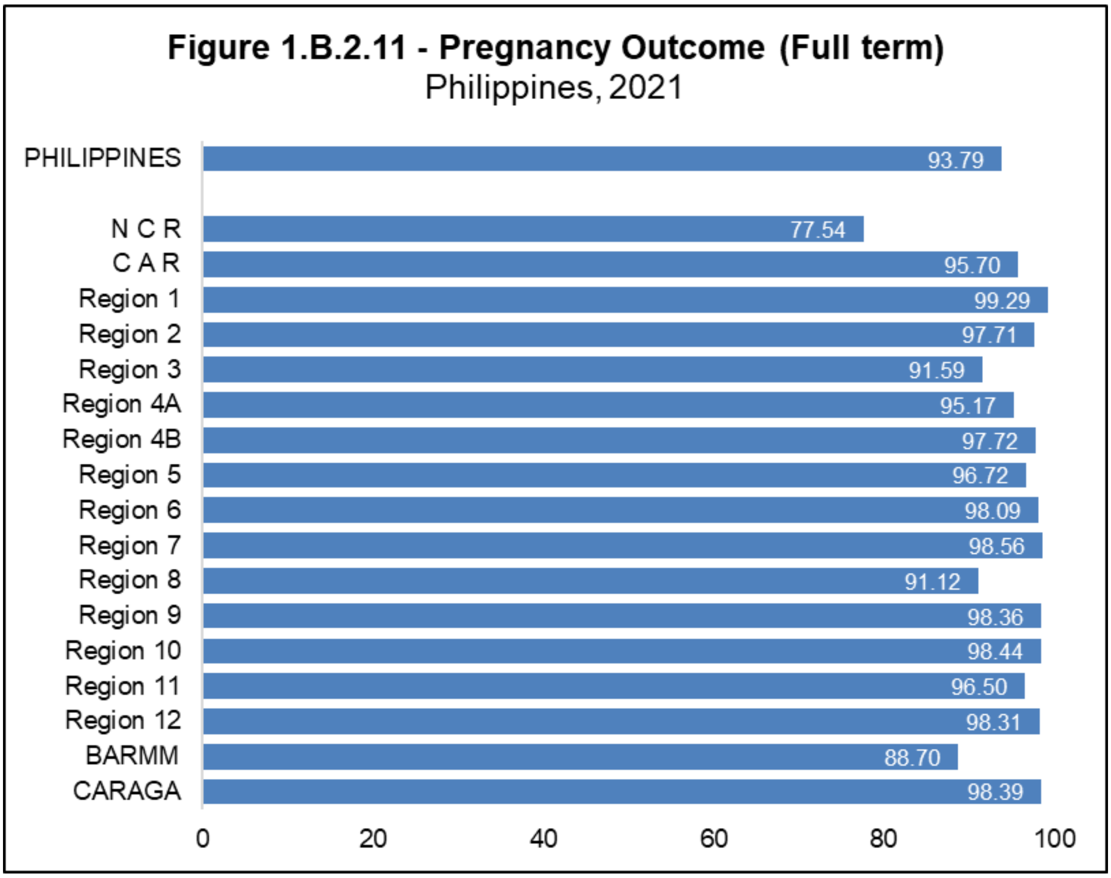
Figure 1.B.2.11 - Pregnancy Outcome (Full term)
Philippines, 2021
PHILIPPINES
93.79
N C R
77.54
C A R
95.70
Region 1
99.29
Region 2
97.71
Region 3
91.59
Region 4A
95.17
Region 4B
97.72
Region 5
96.72
Region 6
98.09
Region 7
98.56
Region 8
91.12
Region 9
98.36
Region 10
98.44
Region 11
96.50
Region 12
98.31
BARMM
88.70
CARAGA
98.39
0
20
40
60
80
100

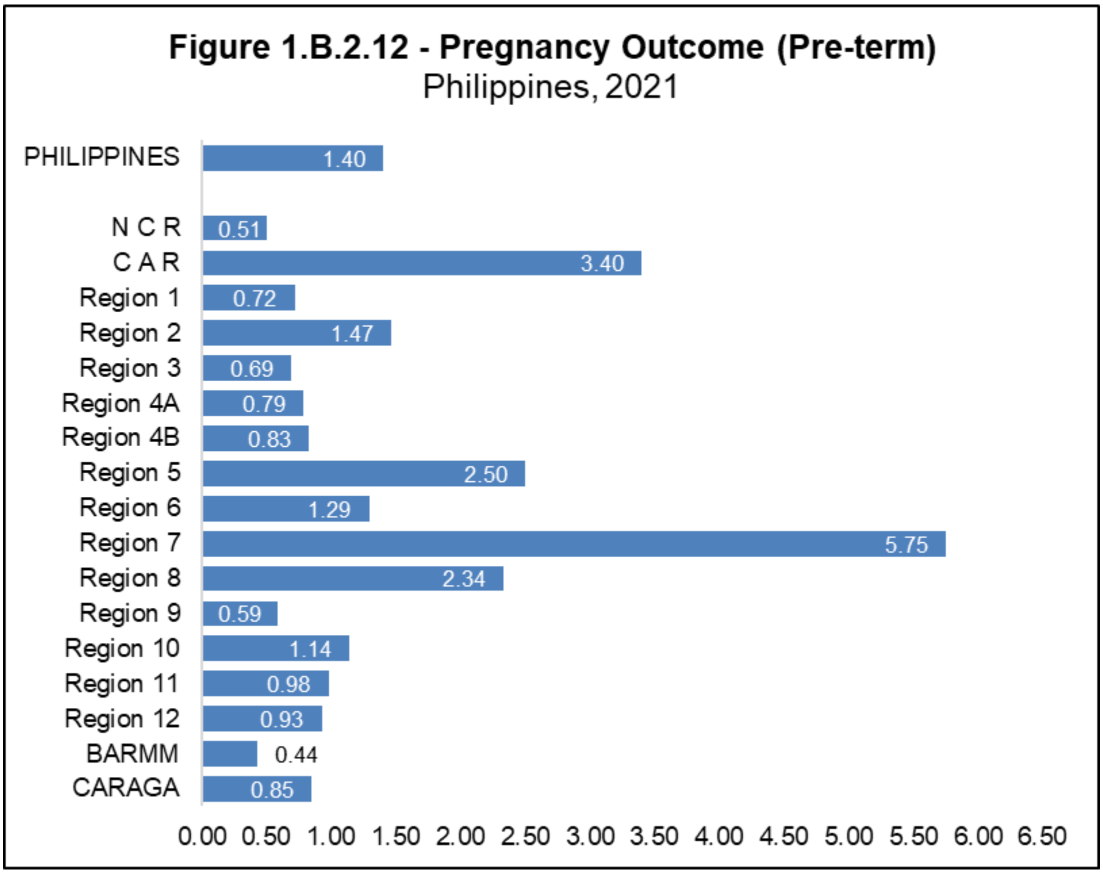
Figure 1.B.2.12 - Pregnancy Outcome (Pre-term)
Philippines, 2021
PHILIPPINES
1.40
N C R
0.51
C A R
3.40
Region 1
0.72
Region 2
1.47
Region 3
0.69
Region 4A
0.79
Region 4B
0.83
Region 5
2.50
Region 6
1.29
Region 7
5.75
Region 8
2.34
Region 9
0.59
Region 10
1.14
Region 11
0.98
Region 12
0.93
BARMM
0.44
CARAGA
0.85
0.00
0.50
1.00
1.50
2.00
2.50
3.00
3.50
4.00
4.50
5.00
5.50
6.00
6.50

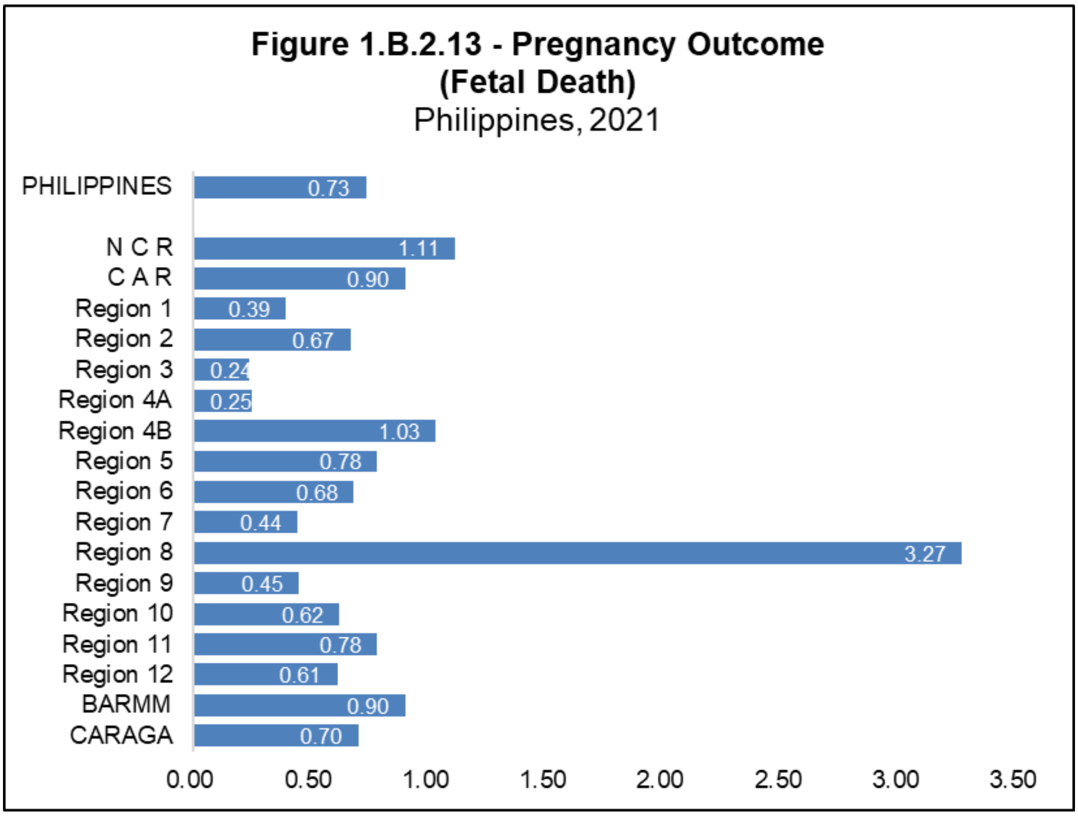

Figure 1.B.2.13 - Pregnancy Outcome
(Fetal Death)
Philippines, 2021
PHILIPPINES 0.73
N C R 1.11
C A R 0.90
Region 1 0.39
Region 2 0.67
Region 3 0.24
Region 4A 0.25
Region 4B 1.03
Region 5 0.78
Region 6 0.68
Region 7 0.44
Region 8 3.27
Region 9 0.45
Region 10 0.62
Region 11 0.78
Region 12 0.61
BARMM 0.90
CARAGA 0.70
0.00 0.50 1.00 1.50 2.00 2.50 3.00 3.50

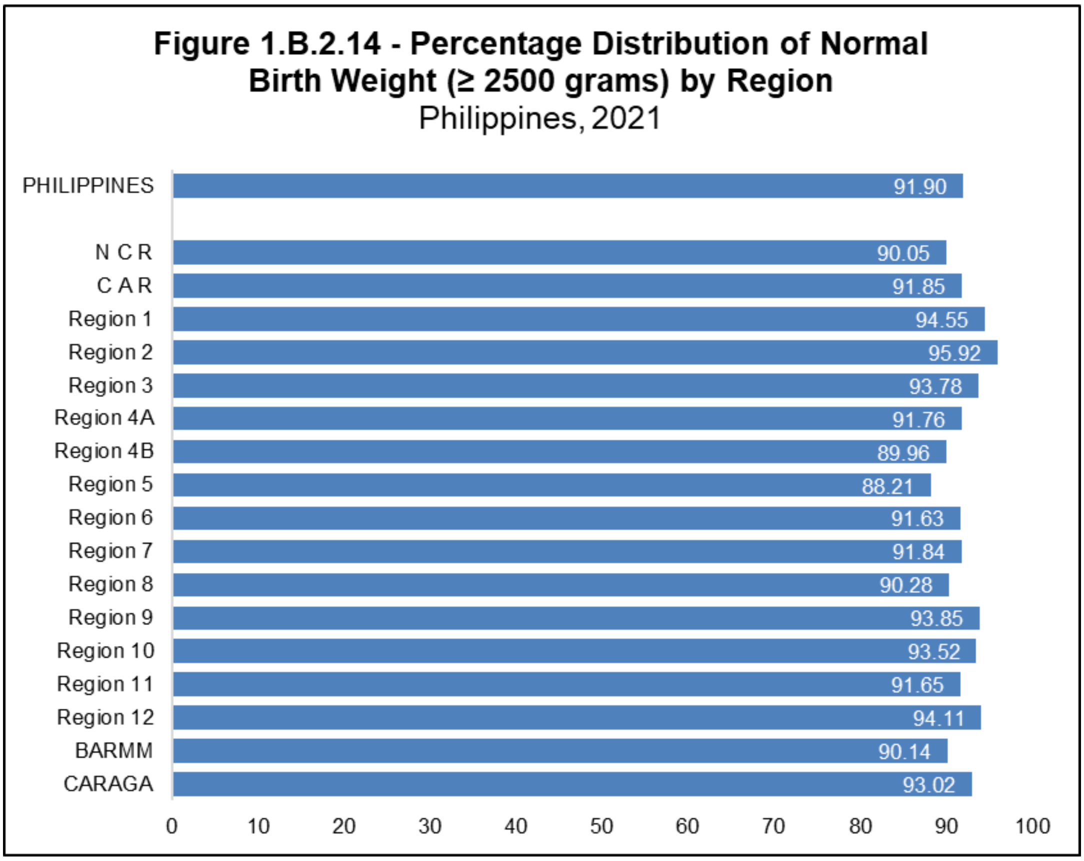

Figure 1.B.2.14 - Percentage Distribution of Normal
Birth Weight (≥ 2500 grams) by Region
Philippines, 2021
PHILIPPINES 91.90
N C R 90.05
C A R 91.85
Region 1 94.55
Region 2 95.92
Region 3 93.78
Region 4A 91.76
Region 4B 89.96
Region 5 88.21
Region 6 91.63
Region 7 91.84
Region 8 90.28
Region 9 93.85
Region 10 93.52
Region 11 91.65
Region 12 94.11
BARMM 90.14
CARAGA 93.02
0 10 20 30 40 50 60 70 80 90 100

Figure 1.B.2.15 - Percentage Distribution Low Birth Weight (< 2500) by Region
Philippines, 2021

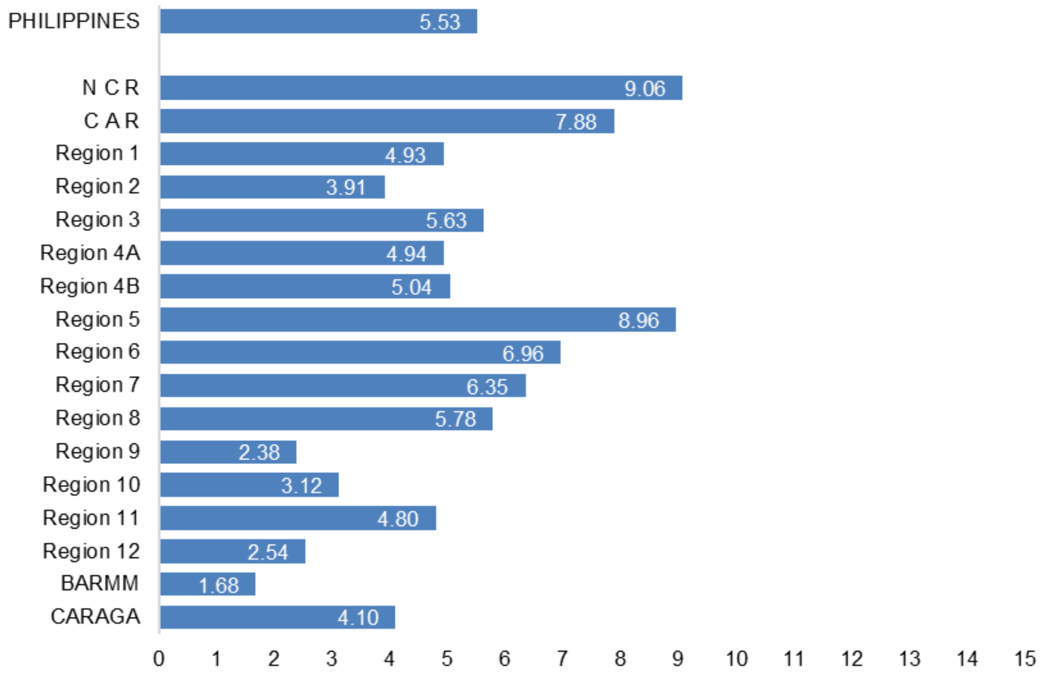

Figure 1.B.2.16 - Percentage Distribution of Unknown Birth Weight by Region
Philippines, 2021

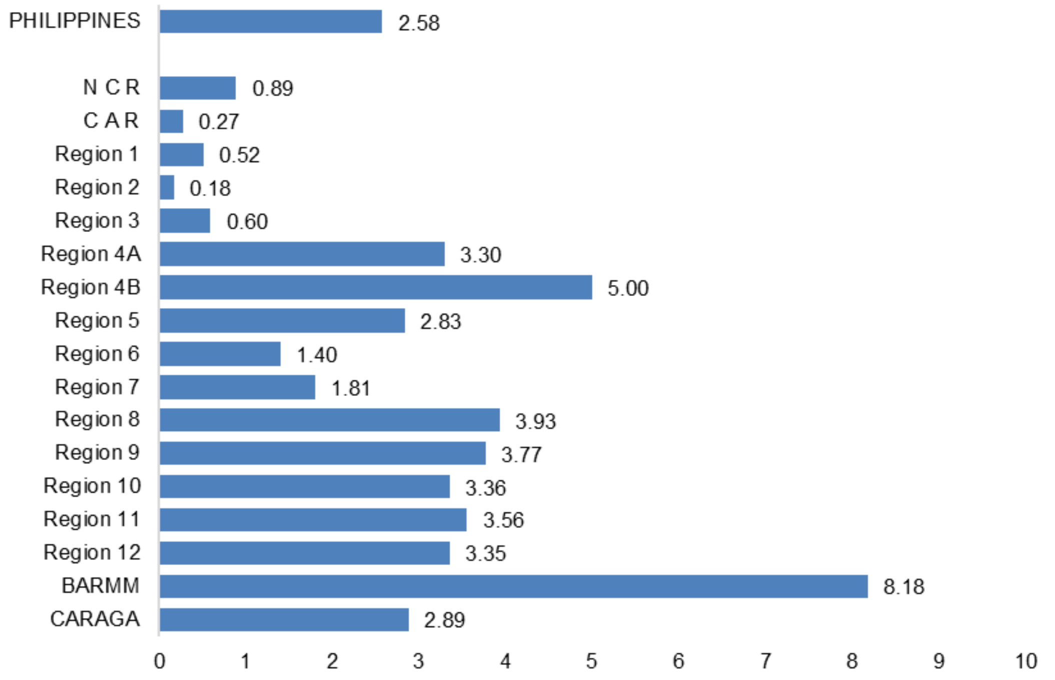

Table 1.B.3.1 - Postpartum and Newborn Care

Number and proportion of postpartum women together with their newborn who completed at least 2 postpartum check-ups
Philippines, 2021

Area	Total Deliveries	Completed at least 2 postpartum check-ups							
		Age Group in Year						Total	%
		10 - 14		15-19		20-49			
		No.	%	No.	%	No.	%		
**PHILIPPINES**	**1,253,409**	**2,055**	**0.16**	**119,164**	**9.51**	**1,017,612**	**81.19**	**1,138,831**	**90.86**
**N C R**	**147,582**	**153**	**0.10**	**10,407**	**7.05**	**115,621**	**78.34**	**126,181**	**85.50**
Malabon	5,166	10	0.19	490	9.49	4,632	89.66	5,132	99.34
Navotas	3,245	3	0.09	310	9.55	2,618	80.68	2,931	90.32
Valenzuela City	6,550	4	0.06	476	7.27	5,277	80.56	5,757	87.89
Caloocan City	14,253	13	0.09	1,285	9.02	12,955	90.89	14,253	100.00
Marikina City	7,079	3	0.04	163	2.30	2,391	33.78	2,557	36.12
Pasig City	11,767	8	0.07	594	5.05	7,107	60.40	7,709	65.51
Pateros	714	0	0.00	42	5.88	671	93.98	713	99.86
Taguig	8,868	5	0.06	768	8.66	7,915	89.25	8,688	97.97
Quezon City	44,126	28	0.06	1,998	4.53	33,808	76.62	35,834	81.21
Makati City	5,726	30	0.52	229	4.00	3,348	58.47	3,607	62.99
Mandaluyong City	3,436	4	0.12	275	8.00	3,074	89.46	3,353	97.58
San Juan	1,470	1	0.07	33	2.24	854	58.10	888	60.41
Manila City	13,248	13	0.10	1,701	12.84	11,534	87.06	13,248	100.00
Las Piñas City	5,520	3	0.05	521	9.44	4,826	87.43	5,350	96.92
Muntinlupa City	6,733	10	0.15	552	8.20	6,171	91.65	6,733	100.00
Parañaque City	5,487	18	0.33	682	12.43	4,672	85.15	5,372	97.90
Pasay City	4,194	0	0.00	288	6.87	3,768	89.84	4,056	96.71
**C A R**	**22,444**	**21**	**0.09**	**1,973**	**8.79**	**15,226**	**67.84**	**17,220**	**76.72**
Abra	2,679	0	0.00	267	9.97	1,899	70.88	2,166	80.85
Apayao	1,702	13	0.76	174	10.22	973	57.17	1,160	68.16
Benguet	4,616	0	0.00	245	5.31	4,002	86.70	4,247	92.01
Ifugao	2,796	2	0.07	211	7.55	1,758	62.88	1,971	70.49
Kalinga	2,381	1	0.04	220	9.24	2,133	89.58	2,354	98.87
Mt. Province	2,461	2	0.08	144	5.85	1,745	70.91	1,891	76.84
Baguio City	5,809	3	0.05	712	12.26	2,716	46.76	3,431	59.06
**Region 1**	**48,764**	**30**	**0.06**	**4,299**	**8.82**	**50,192**	**102.93**	**54,521**	**111.81**
Ilocos Norte	4,382	5	0.11	335	7.64	3,999	91.26	4,339	99.02
Ilocos Sur	4,215	7	0.17	380	9.02	5,705	135.35	6,092	144.53
La Union	4,241	0	0.00	384	9.05	3,548	83.66	3,932	92.71
Pangasinan	14,420	5	0.03	2,097	14.54	23,614	163.76	25,716	178.34
Alaminos City	1,250	0	0.00	43	3.44	1,205	96.40	1,248	99.84
Candon City	1,075	0	0.00	1	0.09	1,074	99.91	1,075	100.00
Dagupan City	8,147	5	0.06	270	3.31	1,957	24.02	2,232	27.40
Laoag City	1,965	0	0.00	35	1.78	1,930	98.22	1,965	100.00
San Carlos City	2,280	1	0.04	195	8.55	2,012	88.25	2,208	96.84
San Fernando City	3,530	5	0.14	318	9.01	3,207	90.85	3,530	100.00
Urdaneta City	1,843	0	0.00	167	9.06	1,448	78.57	1,615	87.63
Vigan City	1,416	2	0.14	74	5.23	493	34.82	569	40.18
**Region 2**	**40,412**	**67**	**0.17**	**4,421**	**10.94**	**35,279**	**87.30**	**39,767**	**98.40**
Batanes	269	2	0.74	20	7.43	248	92.19	270	100.37
Cagayan	9,734	12	0.12	1,208	12.41	9,309	95.63	10,529	108.17
Isabela	12,520	32	0.26	1,504	12.01	11,516	91.98	13,052	104.25
Nueva Vizcaya	6,736	6	0.09	669	9.93	5,164	76.66	5,839	86.68
Quirino	2,634	3	0.11	266	10.10	2,599	98.67	2,868	108.88
Cauayan City	1,645	6	0.36	194	11.79	1,429	86.87	1,629	99.03
Ilagan City	2,646	1	0.04	230	8.69	1,335	50.45	1,566	59.18

Table 1.B.3.1 - Postpartum and Newborn Care

Number and proportion of postpartum women together with their newborn who completed at least 2 postpartum check-ups
Philippines, 2021

Area	Total Deliveries	Completed at least 2 postpartum check-ups							
		Age Group in Year						Total	%
		10 - 14		15-19		20-49			
		No.	%	No.	%	No.	%		
Santiago City	2,861	3	0.10	259	9.05	2,421	84.62	2,683	93.78
Tuguegarao City	1,367	2	0.15	71	5.19	1,258	92.03	1,331	97.37
	**0**								
**Region 3**	**117,383**	**250**	**0.21**	**13,458**	**11.47**	**110,120**	**93.81**	**123,828**	**105.49**
Aurora	3,008	2	0.07	276	9.18	2,125	70.64	2,403	79.89
Bataan	5,148	28	0.54	873	16.96	6,899	134.01	7,800	151.52
Bulacan	22,840	28	0.12	2,721	11.91	24,430	106.96	27,179	119.00
Nueva Ecija	9,489	30	0.32	1,163	12.26	8,622	90.86	9,815	103.44
Pampanga	9,794	42	0.43	1,459	14.90	12,362	126.22	13,863	141.55
Tarlac	11,344	7	0.06	1,047	9.23	10,179	89.73	11,233	99.02
Zambales	7,601	5	0.07	441	5.80	3,880	51.05	4,326	56.91
Angeles City	7,027	2	0.03	387	5.51	2,365	33.66	2,754	39.19
Balanga City	1,290	8	0.62	163	12.64	1,074	83.26	1,245	96.51
Cabanatuan City	4,236	5	0.12	466	11.00	3,715	87.70	4,186	98.82
City of San Fernando	4,596	8	0.17	362	7.88	2,876	62.58	3,246	70.63
Gapan City	966	1	0.10	100	10.35	859	88.92	960	99.38
Mabalacat City	4,337	50	1.15	660	15.22	4,337	100.00	5,047	116.37
Malolos City	3,489	3	0.09	197	5.65	1,886	54.06	2,086	59.79
Meycauayan	2,351	0	0.00	285	12.12	2,372	100.89	2,657	113.02
Olongapo	3,425	13	0.38	239	6.98	2,320	67.74	2,572	75.09
Palayan City	77	0	0.00	26	33.77	227	294.81	253	328.57
San Jose City	1,940	5	0.26	247	12.73	1,633	84.18	1,885	97.16
San Jose del Monte City	6,642	5	0.08	939	14.14	11,852	178.44	12,796	192.65
Science City of Munoz	105	2	1.90	95	90.48	674	641.90	771	734.29
Tarlac City	7,678	6	0.08	1,312	17.09	5,433	70.76	6,751	87.93
**Region 4A**	**169,896**	**152**	**0.09**	**12,112**	**7.13**	**130,674**	**76.91**	**142,938**	**84.13**
Batangas	21,266	18	0.08	1,466	6.89	19,900	93.58	21,384	100.55
Cavite	16,518	11	0.07	914	5.53	11,992	72.60	12,917	78.20
Laguna	11,693	14	0.12	971	8.30	7,972	68.18	8,957	76.60
Quezon	18,491	18	0.10	1,810	9.79	14,197	76.78	16,025	86.66
Rizal	21,048	23	0.11	1,684	8.00	14,414	68.48	16,121	76.59
Antipolo City	11,094	3	0.03	509	4.59	9,582	86.37	10,094	90.99
Bacoor City	6,756	1	0.01	198	2.93	3,120	46.18	3,319	49.13
Batangas City	3,484	6	0.17	347	9.96	3,174	91.10	3,527	101.23
Biñan City	7,973	0	0.00	882	11.06	5,708	71.59	6,590	82.65
Cabuyao City	3,188	0	0.00	291	9.13	3,850	120.77	4,141	129.89
Calamba City	8,294	12	0.14	620	7.48	6,935	83.61	7,567	91.23
Cavite City	659	0	0.00	25	3.79	220	33.38	245	37.18
Dasmariñas City	6,573	1	0.02	393	5.98	5,752	87.51	6,146	93.50
General Trias City	729	16	2.19	155	21.26	1,501	205.90	1,672	229.36
Imus City	4,356	2	0.05	110	2.53	1,971	45.25	2,083	47.82
Lipa City	4,231	4	0.09	262	6.19	3,732	88.21	3,998	94.49
Lucena City	2,473	1	0.04	231	9.34	1,971	79.70	2,203	89.08
San Pablo City	4,772	8	0.17	347	7.27	2,481	51.99	2,836	59.43
San Pedro City	1,302	2	0.15	162	12.44	2,027	155.68	2,191	168.28
Santa Rosa City	7,494	7	0.09	402	5.36	4,400	58.71	4,809	64.17
Tagaytay City	1,189	1	0.08	28	2.35	1,154	97.06	1,183	99.50
Tanauan City	2,707	1	0.04	46	1.70	1,771	65.42	1,818	67.16
Tayabas City	1,345	3	0.22	138	10.26	1,103	82.01	1,244	92.49
Trece Martires City	2,261	0	0.00	121	5.35	1,747	77.27	1,868	82.62
**Region 4B**	**40,990**	**53**	**0.13**	**4,173**	**10.18**	**32,845**	**80.13**	**37,071**	**90.44**
Marinduque	2,785	1	0.04	229	8.22	2,511	90.16	2,741	98.42
Mindoro Occidental	7,794	11	0.14	1,106	14.19	6,909	88.65	8,026	102.98
Mindoro Oriental	11,017	12	0.11	727	6.60	7,172	65.10	7,911	71.81
Palawan	12,500	19	0.15	1,530	12.24	10,459	83.67	12,008	96.06
Romblon	3,895	9	0.23	346	8.88	3,414	87.65	3,769	96.77

Table 1.B.3.1 - Postpartum and Newborn Care

Number and proportion of postpartum women together with their newborn who completed at least 2 postpartum check-ups
Philippines, 2021

Area	Total Deliveries	Completed at least 2 postpartum check-ups							
		Age Group in Year						Total	%
		10 - 14		15-19		20-49			
		No.	%	No.	%	No.	%		
Puerto Princesa City	2,999	1	0.03	235	7.84	2,380	79.36	2,616	87.23
**Region 5**	**78,718**	**55**	**0.07**	**5,742**	**7.29**	**58,517**	**74.34**	**64,314**	**81.70**
Albay	9,202	7	0.08	577	6.27	8,496	92.33	9,080	98.67
Camarines Norte	9,661	5	0.05	781	8.08	6,627	68.60	7,413	76.73
Camarines Sur	15,499	10	0.06	1,321	8.52	14,620	94.33	15,951	102.92
Catanduanes	4,006	6	0.15	338	8.44	3,240	80.88	3,584	89.47
Masbate	12,154	18	0.15	1,500	12.34	9,410	77.42	10,928	89.91
Sorsogon	10,424	5	0.05	862	8.27	9,028	86.61	9,895	94.93
Iriga City	1,629	0	0.00	67	4.11	915	56.17	982	60.28
Legaspi City	4,511	2	0.04	131	2.90	4,014	88.98	4,147	91.93
Naga City	11,632	2	0.02	165	1.42	2,167	18.63	2,334	20.07
**Region 6**	**82,095**	**117**	**0.14**	**7,864**	**9.58**	**64,280**	**78.30**	**72,261**	**88.02**
Aklan	5,568	7	0.13	406	7.29	4,631	83.17	5,044	90.59
Antique	7,312	6	0.08	531	7.26	5,816	79.54	6,353	86.88
Capiz	7,458	5	0.07	515	6.91	4,698	62.99	5,218	69.97
Guimaras	2,319	3	0.13	172	7.42	2,086	89.95	2,261	97.50
Iloilo	19,237	21	0.11	1,673	8.70	15,430	80.21	17,124	89.02
Negros Occidental	31,802	63	0.20	3,875	12.18	25,476	80.11	29,414	92.49
Bacolod City	3,858	5	0.13	252	6.53	2,129	55.18	2,386	61.85
Iloilo City	4,541	7	0.15	440	9.69	4,014	88.39	4,461	98.24
**Region 7**	**92,739**	**90**	**0.10**	**7,682**	**8.28**	**77,745**	**83.83**	**85,517**	**92.21**
Bohol	17,266	12	0.07	1,283	7.43	13,019	75.40	14,314	82.90
Cebu	27,663	35	0.13	3,002	10.85	32,545	117.65	35,582	128.63
Negros Oriental	18,889	32	0.17	1,726	9.14	13,003	68.84	14,761	78.15
Siquijor	1,138	1	0.09	123	10.81	1,008	88.58	1,132	99.47
Cebu City	16,354	10	0.06	802	4.90	7,378	45.11	8,190	50.08
Lapu-Lapu City	7,391	0	0.00	413	5.59	6,902	93.38	7,315	98.97
Mandaue City	4,038	0	0.00	333	8.25	3,890	96.33	4,223	104.58
**Region 8**	**52,130**	**186**	**0.36**	**4,290**	**8.23**	**38,284**	**73.44**	**42,760**	**82.03**
Biliran	2,621	5	0.19	302	11.52	2,103	80.24	2,410	91.95
Eastern Samar	6,181	11	0.18	651	10.53	5,301	85.76	5,963	96.47
Northern Leyte	14,081	42	0.30	942	6.69	10,334	73.39	11,318	80.38
Northern Samar	8,565	103	1.20	679	7.93	5,768	67.34	6,550	76.47
Southern Leyte	2,521	5	0.20	343	13.61	2,158	85.60	2,506	99.40
Western Samar	6,475	13	0.20	620	9.58	5,110	78.92	5,743	88.69
Calbayog City	1,938	2	0.10	171	8.82	1,436	74.10	1,609	83.02
Maasin City	2,252	2	0.09	65	2.89	707	31.39	774	34.37
Ormoc City	4,748	2	0.04	355	7.48	2,875	60.55	3,232	68.07
Tacloban City	2,748	1	0.04	162	5.90	2,492	90.68	2,655	96.62
**Region 9**	**46,771**	**59**	**0.13**	**5,134**	**10.98**	**37,676**	**80.55**	**42,869**	**91.66**
Zamboanga del Norte	9,489	9	0.09	1,135	11.96	7,118	75.01	8,262	87.07
Zamboanga del Sur	6,651	13	0.20	738	11.10	4,518	67.93	5,269	79.22
Zamboanga Sibugay	6,965	10	0.14	861	12.36	5,569	79.96	6,440	92.46
Dapitan City	1,598	3	0.19	86	5.38	1,508	94.37	1,597	99.94
Dipolog City	2,277	3	0.13	238	10.45	1,886	82.83	2,127	93.41
Isabela City	1,629	5	0.31	198	12.15	1,335	81.95	1,538	94.41
Pagadian City	2,298	0	0.00	189	8.22	1,944	84.60	2,133	92.82

Table 1.B.3.1 - Postpartum and Newborn Care

Number and proportion of postpartum women together with their newborn who completed at least 2 postpartum check-ups
Philippines, 2021

Area	Total Deliveries	Completed at least 2 postpartum check-ups							
		Age Group in Year						Total	%
		10 - 14		15-19		20-49			
		No.	%	No.	%	No.	%		
Zamboanga City	15,864	16	0.10	1,689	10.65	13,798	86.98	15,503	97.72
**Region 10**	**71,984**	**283**	**0.39**	**9,645**	**13.40**	**58,516**	**81.29**	**68,444**	**95.08**
Bukidnon	19,679	88	0.45	3,323	16.89	13,872	70.49	17,283	87.82
Camiguin	1,100	2	0.18	92	8.36	968	88.00	1,062	96.55
Lanao del Norte	8,578	5	0.06	597	6.96	7,285	84.93	7,887	91.94
Misamis Occidental	3,779	1	0.03	272	7.20	3,380	89.44	3,653	96.67
Misamis Oriental	8,821	75	0.85	1,485	16.83	8,848	100.31	10,408	117.99
Cagayan de Oro City	11,751	38	0.32	1,454	12.37	9,869	83.98	11,361	96.68
El Salvador City	249	1	0.40	96	38.55	593	238.15	690	277.11
Gingoog City	2,893	48	1.66	510	17.63	1,910	66.02	2,468	85.31
Iligan City	6,035	6	0.10	539	8.93	3,965	65.70	4,510	74.73
Malaybalay City	2,899	4	0.14	434	14.97	2,461	84.89	2,899	100.00
Oroquieta City	744	2	0.27	97	13.04	644	86.56	743	99.87
Ozamis City	1,944	2	0.10	202	10.39	1,696	87.24	1,900	97.74
Tangub City	1,173	3	0.26	182	15.52	984	83.89	1,169	99.66
Valencia City	2,339	8	0.34	362	15.48	2,041	87.26	2,411	103.08
**Region 11**	**76,188**	**292**	**0.38**	**9,786**	**12.84**	**59,383**	**77.94**	**69,461**	**91.17**
Davao de Oro	11,332	65	0.57	1,876	16.55	9,059	79.94	11,000	97.07
Davao del Norte	17,041	60	0.35	2,113	12.40	12,697	74.51	14,870	87.26
Davao Oriental	8,236	30	0.36	1,242	15.08	6,528	79.26	7,800	94.71
Davao del Sur	8,170	38	0.47	1,169	14.31	6,290	76.99	7,497	91.76
Davao Occidental	4,276	38	0.89	807	18.87	2,046	47.85	2,891	67.61
Davao City	27,133	61	0.22	2,579	9.51	22,763	83.89	25,403	93.62
**Region 12**	**65,637**	**171**	**0.26**	**9,144**	**13.93**	**53,537**	**81.57**	**62,852**	**95.76**
North Cotabato	18,335	36	0.20	2,565	13.99	14,990	81.76	17,591	95.94
Sarangani	9,714	35	0.36	1,819	18.73	7,629	78.54	9,483	97.62
South Cotabato	16,353	39	0.24	2,313	14.14	13,601	83.17	15,953	97.55
Sultan Kudarat	12,030	52	0.43	1,676	13.93	9,575	79.59	11,303	93.96
Cotabato City	3,298	2	0.06	250	7.58	2,683	81.35	2,935	88.99
Gen. Santos City	5,907	7	0.12	521	8.82	5,059	85.64	5,587	94.58
**BARMM**	**62,595**	**28**	**0.04**	**5,873**	**9.38**	**53,721**	**85.82**	**59,622**	**95.25**
Basilan	3,253	5	0.15	292	8.98	2,204	67.75	2,501	76.88
Lanao del Sur	17,519	2	0.01	736	4.20	16,132	92.08	16,870	96.30
Maguindanao	20,188	17	0.08	2,204	10.92	18,760	92.93	20,981	103.93
Sulu	8,891	0	0.00	290	3.26	7,107	79.93	7,397	83.20
Tawi-Tawi	5,681	3	0.05	336	5.91	5,096	89.70	5,435	95.67
Lamitan City	1,226	1	0.08	247	20.15	978	79.77	1,226	100.00
Marawi City	5,837	0	0.00	1,768	30.29	3,444	59.00	5,212	89.29
**CARAGA**	**37,081**	**48**	**0.13**	**3,161**	**8.52**	**25,996**	**70.11**	**29,205**	**78.76**
Agusan del Norte	3,599	1	0.03	309	8.59	2,440	67.80	2,750	76.41
Agusan del Sur	11,882	15	0.13	991	8.34	7,432	62.55	8,438	71.01
Surigao del Norte	3,409	2	0.06	203	5.95	2,391	70.14	2,596	76.15
Surigao del Sur	6,454	10	0.15	555	8.60	4,707	72.93	5,272	81.69
Province of Dinagat	1,020	2	0.20	116	11.37	866	84.90	984	96.47
Bislig City	1,294	9	0.70	145	11.21	1,128	87.17	1,282	99.07
Butuan City	5,267	9	0.17	398	7.56	4,860	92.27	5,267	100.00
Surigao City	4,156	0	0.00	444	10.68	2,172	52.26	2,616	62.95

Note: Put asterisk (*) for No Report and Zero (0) for No Case

Table 1.B.3.2 - Postpartum and Newborn Care

Number and Proportion of Postpartum Qomen who completed Iron with Folic Acid Supplementation
Philippines, 2021

Area	Eligible Pop. (Under 1)	Iron with Folic Acid Supplementation							
		Age Group in Year						Total	%
		10-14		15-19		20-49			
		No.	%	No.	%	No.	%		
**PHILIPPINES**	**2,128,168**	**1,826**	**0.09**	**109,673**	**5.15**	**951,552**	**44.71**	**1,063,051**	**49.95**
**N C R**	**237,921**	**113**	**0.05**	**9,534**	**4.01**	**98,345**	**41.34**	**107,992**	**45.39**
Malabon	6,757	10	0.15	508	7.52	4,686	69.35	5,204	77.02
Navotas	4,607	2	0.04	315	6.84	2,676	58.09	2,993	64.97
Valenzuela City	11,465	9	0.08	489	4.27	5,625	49.06	6,123	53.41
Caloocan City	29,270	13	0.04	1,361	4.65	15,714	53.69	17,088	58.38
Marikina City	8,330	3	0.04	209	2.51	2,870	34.45	3,082	37.00
Pasig City	13,954	3	0.02	594	4.26	5,856	41.97	6,453	46.24
Pateros	1,181	0	0.00	33	2.79	610	51.65	643	54.45
Taguig	14,874	9	0.06	799	5.37	8,001	53.79	8,809	59.22
Quezon City	54,246	10	0.02	732	1.35	9,388	17.31	10,130	18.67
Makati City	10,765	0	0.00	152	1.41	3,989	37.06	4,141	38.47
Mandaluyong City	7,137	4	0.06	266	3.73	2,922	40.94	3,192	44.72
San Juan	2,254	1	0.04	26	1.15	826	36.65	853	37.84
Manila City	32,887	25	0.08	2,066	6.28	13,776	41.89	15,867	48.25
Las Piñas City	10,879	3	0.03	555	5.10	4,993	45.90	5,551	51.02
Muntinlupa City	9,317	10	0.11	559	6.00	7,514	80.65	8,083	86.76
Parañaque City	12,298	11	0.09	577	4.69	5,098	41.45	5,686	46.24
Pasay City	7,700	0	0.00	293	3.81	3,801	49.36	4,094	53.17
**C A R**	**35,143**	**17**	**0.05**	**1,350**	**3.84**	**17,875**	**50.86**	**19,242**	**54.75**
Abra	4,287	1	0.02	261	6.09	1,771	41.31	2,033	47.42
Apayao	2,476	13	0.53	148	5.98	1,266	51.13	1,427	57.63
Benguet	9,140	0	0.00	318	3.48	5,881	64.34	6,199	67.82
Ifugao	4,439	2	0.05	158	3.56	1,951	43.95	2,111	47.56
Kalinga	4,660	0	0.00	225	4.83	2,590	55.58	2,815	60.41
Mt. Province	3,066	0	0.00	136	4.44	1,471	47.98	1,607	52.41
Baguio City	7,075	1	0.01	104	1.47	2,945	41.63	3,050	43.11
**Region 1**	**97,198**	**34**	**0.03**	**4,294**	**4.42**	**49,871**	**51.31**	**54,199**	**55.76**
Ilocos Norte	7,986	2	0.03	300	3.76	3,786	47.41	4,088	51.19
Ilocos Sur	9,197	5	0.05	326	3.54	5,531	60.14	5,862	63.74
La Union	11,429	8	0.07	703	6.15	5,763	50.42	6,474	56.65
Pangasinan	50,454	10	0.02	2,112	4.19	24,043	47.65	26,165	51.86
Alaminos City	1,905	0	0.00	38	1.99	1,197	62.83	1,235	64.83
Candon City	976	0	0.00	1	0.10	1,074	110.04	1,075	110.14
Dagupan City	3,636	4	0.11	292	8.03	1,886	51.87	2,182	60.01
Laoag City	1,845	0	0.00	35	1.90	1,930	104.61	1,965	106.50
San Carlos City	3,999	3	0.08	178	4.45	1,886	47.16	2,067	51.69
San Fernando City	2,092	0	0.00	68	3.25	839	40.11	907	43.36
Urdaneta City	2,819	0	0.00	167	5.92	1,448	51.37	1,615	57.29
Vigan City	860	2	0.23	74	8.60	488	56.74	564	65.58
**Region 2**	**69,228**	**47**	**0.07**	**3,853**	**5.57**	**32,414**	**46.82**	**36,314**	**52.46**
Batanes	351	2	0.57	14	3.99	211	60.11	227	64.67
Cagayan	20,563	6	0.03	995	4.84	8,227	40.01	9,228	44.88
Isabela	23,543	28	0.12	1,427	6.06	10,686	45.39	12,141	51.57
Nueva Vizcaya	9,555	3	0.03	568	5.94	4,636	48.52	5,207	54.50

Table 1.B.3.2 - Postpartum and Newborn Care

Number and Proportion of Postpartum Qomen who completed Iron with Folic Acid Supplementation
Philippines, 2021

Area	Eligible Pop. (Under 1)	Iron with Folic Acid Supplementation							
		Age Group in Year						Total	%
		10-14		15-19		20-49			
		No.	%	No.	%	No.	%		
Quirino	4,038	2	0.05	242	5.99	2,502	61.96	2,746	68.00
Cauayan City	2,576	3	0.12	208	8.07	1,361	52.83	1,572	61.02
Ilagan City	2,901	1	0.03	109	3.76	1,209	41.68	1,319	45.47
Santiago City	2,683	1	0.04	215	8.01	2,386	88.93	2,602	96.98
Tuguegarao City	3,018	1	0.03	75	2.49	1,196	39.63	1,272	42.15
**Region 3**	**220,202**	**187**	**0.08**	**13,761**	**6.25**	**107,995**	**49.04**	**121,943**	**55.38**
Aurora	4,764	4	0.08	261	5.48	2,117	44.44	2,382	50.00
Bataan	13,814	17	0.12	915	6.62	7,337	53.11	8,269	59.86
Bulacan	43,722	23	0.05	2,738	6.26	23,088	52.81	25,849	59.12
Nueva Ecija	28,915	26	0.09	1,158	4.00	8,827	30.53	10,011	34.62
Pampanga	31,503	17	0.05	1,321	4.19	12,328	39.13	13,666	43.38
Tarlac	19,565	20	0.10	1,253	6.40	11,837	60.50	13,110	67.01
Zambales	12,551	4	0.03	317	2.53	2,734	21.78	3,055	24.34
Angeles City	7,905	4	0.05	370	4.68	2,595	32.83	2,969	37.56
Balanga City	1,996	6	0.30	172	8.62	1,070	53.61	1,248	62.53
Cabanatuan City	5,921	3	0.05	415	7.01	3,457	58.39	3,875	65.45
City of San Fernando	5,890	4	0.07	350	5.94	2,616	44.41	2,970	50.42
Gapan City	2,159	1	0.05	103	4.77	872	40.39	976	45.21
Mabalacat City	4,811	28	0.58	513	10.66	4,161	86.49	4,702	97.73
Malolos City	4,885	1	0.02	198	4.05	1,844	37.75	2,043	41.82
Meycauayan	4,050	0	0.00	228	5.63	1,927	47.58	2,155	53.21
Olongapo	4,952	13	0.26	202	4.08	1,934	39.05	2,149	43.40
Palayan City	805	0	0.00	25	3.11	229	28.45	254	31.55
San Jose City	2,736	5	0.18	224	8.19	1,709	62.46	1,938	70.83
San Jose del Monte City	11,120	4	0.04	1,605	14.43	11,343	102.01	12,952	116.47
Science City of Munoz	1,594	4	0.25	86	5.40	694	43.54	784	49.18
Tarlac City	6,544	3	0.05	1,307	19.97	5,276	80.62	6,586	100.64
**Region 4A**	**298,398**	**129**	**0.04**	**11,175**	**3.74**	**122,337**	**41.00**	**133,641**	**44.79**
Batangas	38,848	19	0.05	1,364	3.51	19,633	50.54	21,016	54.10
Cavite	27,948	6	0.02	811	2.90	10,531	37.68	11,348	40.60
Laguna	19,727	14	0.07	975	4.94	7,852	39.80	8,841	44.82
Quezon	37,644	19	0.05	1,455	3.87	12,530	33.29	14,004	37.20
Rizal	44,826	23	0.05	1,608	3.59	14,313	31.93	15,944	35.57
Antipolo City	16,510	3	0.02	568	3.44	7,287	44.14	7,858	47.60
Bacoor City	12,252	2	0.02	176	1.44	2,905	23.71	3,083	25.16
Batangas City	6,895	2	0.03	284	4.12	3,284	47.63	3,570	51.78
Biñan City	6,607	0	0.00	885	13.39	5,690	86.12	6,575	99.52
Cabuyao City	6,128	0	0.00	294	4.80	3,849	62.81	4,143	67.61
Calamba City	9,025	10	0.11	632	7.00	6,953	77.04	7,595	84.16
Cavite City	2,098	1	0.05	12	0.57	219	10.44	232	11.06
Dasmariñas City	13,442	0	0.00	345	2.57	5,215	38.80	5,560	41.36
General Trias City	6,410	0	0.00	132	2.06	1,478	23.06	1,610	25.12
Imus City	8,235	4	0.05	71	0.86	1,643	19.95	1,718	20.86
Lipa City	6,954	9	0.13	210	3.02	3,417	49.14	3,636	52.29
Lucena City	5,704	0	0.00	209	3.66	1,804	31.63	2,013	35.29
San Pablo City	5,277	6	0.11	289	5.48	2,261	42.85	2,556	48.44
San Pedro City	6,465	1	0.02	147	2.27	1,807	27.95	1,955	30.24
Santa Rosa City	7,023	7	0.10	396	5.64	4,337	61.75	4,740	67.49
Tagaytay City	1,451	0	0.00	23	1.59	1,162	80.08	1,185	81.67
Tanauan City	3,620	0	0.00	49	1.35	1,715	47.38	1,764	48.73
Tayabas City	2,134	3	0.14	138	6.47	1,092	51.17	1,233	57.78
Trece Martires City	3,175	0	0.00	102	3.21	1,360	42.83	1,462	46.05

Table 1.B.3.2 - Postpartum and Newborn Care

Number and Proportion of Postpartum Qomen who completed Iron with Folic Acid Supplementation
Philippines, 2021

Area	Eligible Pop. (Under 1)	Iron with Folic Acid Supplementation							
		Age Group in Year						Total	%
		10-14		15-19		20-49			
		No.	%	No.	%	No.	%		
**Region 4B**	**72,035**	**51**	**0.07**	**3,682**	**5.11**	**30,255**	**42.00**	**33,988**	**47.18**
Marinduque	4,906	0	0.00	189	3.85	2,302	46.92	2,491	50.77
Mindoro Occidental	12,449	16	0.13	1,055	8.47	7,053	56.66	8,124	65.26
Mindoro Oriental	20,011	8	0.04	572	2.86	5,999	29.98	6,579	32.88
Palawan	21,676	19	0.09	1,388	6.40	10,039	46.31	11,446	52.80
Romblon	6,482	7	0.11	309	4.77	3,094	47.73	3,410	52.61
Puerto Princesa City	6,511	1	0.02	169	2.60	1,768	27.15	1,938	29.77
**Region 5**	**137,350**	**44**	**0.03**	**5,326**	**3.88**	**53,691**	**39.09**	**59,061**	**43.00**
Albay	23,903	8	0.03	535	2.24	10,275	42.99	10,818	45.26
Camarines Norte	14,340	4	0.03	803	5.60	6,413	44.72	7,220	50.35
Camarines Sur	39,151	13	0.03	1,069	2.73	12,998	33.20	14,080	35.96
Catanduanes	6,381	1	0.02	366	5.74	2,585	40.51	2,952	46.26
Masbate	22,565	12	0.05	1,555	6.89	8,228	36.46	9,795	43.41
Sorsogon	19,470	4	0.02	706	3.63	8,359	42.93	9,069	46.58
Iriga City	2,665	1	0.04	51	1.91	830	31.14	882	33.10
Legaspi City	4,205	1	0.02	94	2.24	2,197	52.25	2,292	54.51
Naga City	4,670	0	0.00	147	3.15	1,806	38.67	1,953	41.82
**Region 6**	**146,545**	**107**	**0.07**	**7,148**	**4.88**	**61,681**	**42.09**	**68,936**	**47.04**
Aklan	11,231	7	0.06	398	3.54	4,875	43.41	5,280	47.01
Antique	12,985	5	0.04	494	3.80	5,507	42.41	6,006	46.25
Capiz	13,984	4	0.03	443	3.17	4,279	30.60	4,726	33.80
Guimaras	3,085	1	0.03	110	3.57	1,957	63.44	2,068	67.03
Iloilo	36,414	14	0.04	1,483	4.07	15,511	42.60	17,008	46.71
Negros Occidental	49,314	66	0.13	3,624	7.35	24,445	49.57	28,135	57.05
Bacolod City	11,102	5	0.05	184	1.66	1,472	13.26	1,661	14.96
Iloilo City	8,430	5	0.06	412	4.89	3,635	43.12	4,052	48.07
**Region 7**	**162,860**	**71**	**0.04**	**6,488**	**3.98**	**72,173**	**44.32**	**78,732**	**48.34**
Bohol	27,637	11	0.04	914	3.31	9,955	36.02	10,880	39.37
Cebu	67,046	26	0.04	2,278	3.40	29,546	44.07	31,850	47.50
Negros Oriental	27,922	22	0.08	1,544	5.53	11,891	42.59	13,457	48.19
Siquijor	1,636	2	0.12	270	16.50	2,071	126.59	2,343	143.22
Cebu City	21,037	9	0.04	809	3.85	7,881	37.46	8,699	41.35
Lapu-Lapu City	9,310	1	0.01	363	3.90	7,050	75.73	7,414	79.63
Mandaue City	8,272	0	0.00	310	3.75	3,779	45.68	4,089	49.43
**Region 8**	**104,016**	**145**	**0.14**	**4,177**	**4.02**	**40,195**	**38.64**	**44,517**	**42.80**
Biliran	3,856	3	0.08	256	6.64	1,985	51.48	2,244	58.20
Eastern Samar	11,635	5	0.04	551	4.74	4,560	39.19	5,116	43.97
Northern Leyte	35,052	10	0.03	1,164	3.32	13,697	39.08	14,871	42.43
Northern Samar	15,655	104	0.66	605	3.86	5,328	34.03	6,037	38.56
Southern Leyte	6,556	5	0.08	342	5.22	2,818	42.98	3,165	48.28
Western Samar	14,515	13	0.09	640	4.41	5,046	34.76	5,699	39.26
Calbayog City	4,474	0	0.00	129	2.88	1,142	25.53	1,271	28.41
Maasin City	1,661	1	0.06	54	3.25	652	39.25	707	42.56
Ormoc City	4,989	3	0.06	293	5.87	2,636	52.84	2,932	58.77

Table 1.B.3.2 - Postpartum and Newborn Care

Number and Proportion of Postpartum Qomen who completed Iron with Folic Acid Supplementation
Philippines, 2021

Area	Eligible Pop. (Under 1)	Iron with Folic Acid Supplementation							
		Age Group in Year						Total	%
		10-14		15-19		20-49			
		No.	%	No.	%	No.	%		
Tacloban City	5,623	1	0.02	143	2.54	2,331	41.45	2,475	44.02
**Region 9**	**79,552**	**62**	**0.08**	**5,472**	**6.88**	**39,797**	**50.03**	**45,331**	**56.98**
Zamboanga del Norte	17,075	12	0.07	1,219	7.14	7,876	46.13	9,107	53.34
Zamboanga del Sur	17,506	18	0.10	1,017	5.81	6,419	36.67	7,454	42.58
Zamboanga Sibugay	14,992	7	0.05	839	5.60	5,200	34.69	6,046	40.33
Dapitan City	1,755	3	0.17	86	4.90	1,504	85.70	1,593	90.77
Dipolog City	2,794	3	0.11	221	7.91	1,780	63.71	2,004	71.73
Isabela City	2,544	4	0.16	213	8.37	1,317	51.77	1,534	60.30
Pagadian City	4,290	0	0.00	195	4.55	1,943	45.29	2,138	49.84
Zamboanga City	18,596	15	0.08	1,682	9.04	13,758	73.98	15,455	83.11
**Region 10**	**100,690**	**154**	**0.15**	**8,439**	**8.38**	**53,721**	**53.35**	**62,314**	**61.89**
Bukidnon	23,307	71	0.30	3,058	13.12	13,588	58.30	16,717	71.73
Camiguin	1,854	2	0.11	90	4.85	962	51.89	1,054	56.85
Lanao del Norte	14,955	4	0.03	518	3.46	6,632	44.35	7,154	47.84
Misamis Occidental	6,413	0	0.00	254	3.96	2,972	46.34	3,226	50.30
Misamis Oriental	15,058	15	0.10	993	6.59	6,893	45.78	7,901	52.47
Cagayan de Oro City	14,263	27	0.19	1,300	9.11	8,752	61.36	10,079	70.67
El Salvador City	1,061	1	0.09	100	9.43	577	54.38	678	63.90
Gingoog City	2,627	21	0.80	353	13.44	1,597	60.79	1,971	75.03
Iligan City	7,575	4	0.05	526	6.94	3,988	52.65	4,518	59.64
Malaybalay City	3,882	4	0.10	434	11.18	2,461	63.40	2,899	74.68
Oroquieta City	1,389	0	0.00	83	5.98	587	42.26	670	48.24
Ozamis City	2,782	2	0.07	203	7.30	1,705	61.29	1,910	68.66
Tangub City	1,234	1	0.08	94	7.62	790	64.02	885	71.72
Valencia City	4,290	2	0.05	433	10.09	2,217	51.68	2,652	61.82
**Region 11**	**107,847**	**424**	**0.39**	**9,466**	**8.78**	**56,235**	**52.14**	**66,125**	**61.31**
Davao de Oro	15,526	103	0.66	1,755	11.30	8,697	56.02	10,555	67.98
Davao del Norte	21,184	99	0.47	2,421	11.43	14,184	66.96	16,704	78.85
Davao Oriental	13,125	47	0.36	1,110	8.46	6,060	46.17	7,217	54.99
Davao del Sur	14,355	40	0.28	1,035	7.21	5,071	35.33	6,146	42.81
Davao Occidental	6,598	54	0.82	780	11.82	1,954	29.62	2,788	42.26
Davao City	37,059	81	0.22	2,365	6.38	20,269	54.69	22,715	61.29
**Region 12**	**104,693**	**156**	**0.15**	**8,108**	**7.74**	**47,841**	**45.70**	**56,105**	**53.59**
North Cotabato	33,933	33	0.10	2,154	6.35	13,137	38.71	15,324	45.16
Sarangani	12,904	35	0.27	1,679	13.01	7,381	57.20	9,095	70.48
South Cotabato	21,039	33	0.16	2,181	10.37	12,908	61.35	15,122	71.88
Sultan Kudarat	17,331	48	0.28	1,539	8.88	8,928	51.51	10,515	60.67
Cotabato City	5,831	0	0.00	163	2.80	1,851	31.74	2,014	34.54
Gen. Santos City	13,655	7	0.05	392	2.87	3,636	26.63	4,035	29.55
**BARMM**	**94,148**	**30**	**0.03**	**3,585**	**3.81**	**39,342**	**41.79**	**42,957**	**45.63**
Basilan	7,672	3	0.04	200	2.61	2,591	33.77	2,794	36.42
Lanao del Sur	21,391	1	0.00	336	1.57	9,832	45.96	10,169	47.54
Maguindanao	31,650	21	0.07	1,624	5.13	15,176	47.95	16,821	53.15
Sulu	16,891	2	0.01	367	2.17	4,304	25.48	4,673	27.67
Tawi-Tawi	9,319	0	0.00	196	2.10	3,405	36.54	3,601	38.64

Table 1.B.3.2 - Postpartum and Newborn Care

Number and Proportion of Postpartum Qomen who completed Iron with Folic Acid Supplementation
Philippines, 2021

Area	Eligible Pop. (Under 1)	Iron with Folic Acid Supplementation							
		Age Group in Year						Total	%
		10-14		15-19		20-49			
		No.	%	No.	%	No.	%		
Lamitan City	2,112	3	0.14	250	11.84	1,074	50.85	1,327	62.83
Marawi City	5,113	0	0.00	612	11.97	2,960	57.89	3,572	69.86
**CARAGA**	**60,342**	**55**	**0.09**	**3,815**	**6.32**	**27,784**	**46.04**	**31,654**	**52.46**
Agusan del Norte	8,116	9	0.11	509	6.27	3,328	41.01	3,846	47.39
Agusan del Sur	17,658	14	0.08	1,222	6.92	8,877	50.27	10,113	57.27
Surigao del Norte	7,195	4	0.06	320	4.45	2,833	39.37	3,157	43.88
Surigao del Sur	11,532	11	0.10	449	3.89	4,173	36.19	4,633	40.18
Province of Dinagat	2,574	2	0.08	140	5.44	1,050	40.79	1,192	46.31
Bislig City	2,185	4	0.18	112	5.13	1,032	47.23	1,148	52.54
Butuan City	7,732	11	0.14	662	8.56	4,428	57.27	5,101	65.97
Surigao City	3,350	0	0.00	401	11.97	2,063	61.58	2,464	73.55

Note: Put asterisk (*) for No Report and Zero (0) for No Case

Table 1.B.3.3 - Postpartum and Newborn Care

Number and Proportion of Postpartum Women who completed Vitamin A Suplementation
Philippines, 2021

Area	Eligible Pop. (Under 1)	Vitamin A Suplementation							
		Age Group in Year						Total	%
		10-14		15-19		20-49			
		No.	%	No.	%	No.	%		
**PHILIPPINES**	**2,128,168**	**2,004**	**0.09**	**113,113**	**5.32**	**968,803**	**45.52**	**1,083,920**	**50.93**
**N C R**	**237,921**	**122**	**0.05**	**10,432**	**4.38**	**122,798**	**51.61**	**133,352**	**56.05**
Malabon	6,757	10	0.15	466	6.90	4,412	65.30	4,888	72.34
Navotas	4,607	0	0.00	304	6.60	2,615	56.76	2,919	63.36
Valenzuela City	11,465	6	0.05	385	3.36	5,421	47.28	5,812	50.69
Caloocan City	29,270	12	0.04	1,197	4.09	15,160	51.79	16,369	55.92
Marikina City	8,330	3	0.04	215	2.58	2,279	27.36	2,497	29.98
Pasig City	13,954	2	0.01	582	4.17	5,649	40.48	6,233	44.67
Pateros	1,181	0	0.00	42	3.56	672	56.90	714	60.46
Taguig	14,874	8	0.05	808	5.43	8,463	56.90	9,279	62.38
Quezon City	54,246	30	0.06	2,207	4.07	36,610	67.49	38,847	71.61
Makati City	10,765	4	0.04	186	1.73	3,606	33.50	3,796	35.26
Mandaluyong City	7,137	3	0.04	221	3.10	2,635	36.92	2,859	40.06
San Juan	2,254	1	0.04	34	1.51	849	37.67	884	39.22
Manila City	32,887	14	0.04	1,752	5.33	12,435	37.81	14,201	43.18
Las Piñas City	10,879	3	0.03	494	4.54	5,172	47.54	5,669	52.11
Muntinlupa City	9,317	10	0.11	559	6.00	7,434	79.79	8,003	85.90
Parañaque City	12,298	16	0.13	703	5.72	5,704	46.38	6,423	52.23
Pasay City	7,700	0	0.00	277	3.60	3,682	47.82	3,959	51.42
**C A R**	**35,143**	**21**	**0.06**	**1,454**	**4.14**	**16,588**	**47.20**	**18,063**	**51.40**
Abra	4,287	2	0.05	267	6.23	1,803	42.06	2,072	48.33
Apayao	2,476	14	0.57	204	8.24	1,368	55.25	1,586	64.05
Benguet	9,140	1	0.01	259	2.83	4,227	46.25	4,487	49.09
Ifugao	4,439	2	0.05	254	5.72	2,232	50.28	2,488	56.05
Kalinga	4,660	0	0.00	219	4.70	2,490	53.43	2,709	58.13
Mt. Province	3,066	2	0.07	135	4.40	1,507	49.15	1,644	53.62
Baguio City	7,075	0	0.00	116	1.64	2,961	41.85	3,077	43.49
**Region 1**	**97,198**	**37**	**0.04**	**4,194**	**4.31**	**47,505**	**48.87**	**51,736**	**53.23**
Ilocos Norte	7,986	3	0.04	336	4.21	3,825	47.90	4,164	52.14
Ilocos Sur	9,197	7	0.08	350	3.81	5,542	60.26	5,899	64.14
La Union	11,429	8	0.07	689	6.03	5,579	48.81	6,276	54.91
Pangasinan	50,454	7	0.01	1,853	3.67	20,542	40.71	22,402	44.40
Alaminos City	1,905	0	0.00	34	1.78	1,096	57.53	1,130	59.32
Candon City	976	0	0.00	1	0.10	1,016	104.10	1,017	104.20
Dagupan City	3,636	5	0.14	242	6.66	1,931	53.11	2,178	59.90
Laoag City	1,845	0	0.00	49	2.66	1,628	88.24	1,677	90.89
San Carlos City	3,999	1	0.03	170	4.25	1,888	47.21	2,059	51.49
San Fernando City	2,092	4	0.19	234	11.19	2,538	121.32	2,776	132.70
Urdaneta City	2,819	0	0.00	167	5.92	1,448	51.37	1,615	57.29
Vigan City	860	2	0.23	69	8.02	472	54.88	543	63.14
**Region 2**	**69,228**	**63**	**0.09**	**4,197**	**6.06**	**34,481**	**49.81**	**38,741**	**55.96**
Batanes	351	2	0.57	20	5.70	246	70.09	268	76.35
Cagayan	20,563	11	0.05	1,146	5.57	8,555	41.60	9,712	47.23
Isabela	23,543	32	0.14	1,502	6.38	11,707	49.73	13,241	56.24
Nueva Vizcaya	9,555	6	0.06	623	6.52	4,979	52.11	5,608	58.69

Table 1.B.3.3 - Postpartum and Newborn Care

Number and Proportion of Postpartum Women who completed Vitamin A Suplementation
Philippines, 2021

Area	Eligible Pop. (Under 1)	Vitamin A Suplementation							
		Age Group in Year						Total	%
		10-14		15-19		20-49			
		No.	%	No.	%	No.	%		
Quirino	4,038	3	0.07	255	6.32	2,555	63.27	2,813	69.66
Cauayan City	2,576	6	0.23	200	7.76	1,437	55.78	1,643	63.78
Ilagan City	2,901	1	0.03	162	5.58	1,426	49.16	1,589	54.77
Santiago City	2,683	1	0.04	215	8.01	2,334	86.99	2,550	95.04
Tuguegarao City	3,018	1	0.03	74	2.45	1,242	41.15	1,317	43.64
**Region 3**	**220,202**	**217**	**0.10**	**11,070**	**5.03**	**94,122**	**42.74**	**105,409**	**47.87**
Aurora	4,764	2	0.04	242	5.08	2,047	42.97	2,291	48.09
Bataan	13,814	33	0.24	811	5.87	6,665	48.25	7,509	54.36
Bulacan	43,722	26	0.06	2,230	5.10	19,348	44.25	21,604	49.41
Nueva Ecija	28,915	30	0.10	704	2.43	5,931	20.51	6,665	23.05
Pampanga	31,503	14	0.04	1,156	3.67	10,671	33.87	11,841	37.59
Tarlac	19,565	17	0.09	1,159	5.92	10,697	54.67	11,873	60.68
Zambales	12,551	2	0.02	255	2.03	2,307	18.38	2,564	20.43
Angeles City	7,905	2	0.03	263	3.33	1,789	22.63	2,054	25.98
Balanga City	1,996	6	0.30	176	8.82	1,051	52.66	1,233	61.77
Cabanatuan City	5,921	6	0.10	447	7.55	3,513	59.33	3,966	66.98
City of San Fernando	5,890	7	0.12	326	5.53	2,577	43.75	2,910	49.41
Gapan City	2,159	0	0.00	50	2.32	302	13.99	352	16.30
Mabalacat City	4,811	32	0.67	512	10.64	3,618	75.20	4,162	86.51
Malolos City	4,885	3	0.06	184	3.77	1,723	35.27	1,910	39.10
Meycauayan	4,050	0	0.00	156	3.85	1,271	31.38	1,427	35.23
Olongapo	4,952	13	0.26	191	3.86	1,740	35.14	1,944	39.26
Palayan City	805	0	0.00	24	2.98	229	28.45	253	31.43
San Jose City	2,736	16	0.58	245	8.95	1,674	61.18	1,935	70.72
San Jose del Monte City	11,120	5	0.04	949	8.53	11,768	105.83	12,722	114.41
Science City of Munoz	1,594	1	0.06	82	5.14	639	40.09	722	45.29
Tarlac City	6,544	2	0.03	908	13.88	4,562	69.71	5,472	83.62
**Region 4A**	**298,398**	**135**	**0.05**	**11,071**	**3.71**	**115,781**	**38.80**	**126,987**	**42.56**
Batangas	38,848	13	0.03	1,014	2.61	12,809	32.97	13,836	35.62
Cavite	27,948	12	0.04	920	3.29	11,913	42.63	12,845	45.96
Laguna	19,727	12	0.06	926	4.69	7,585	38.45	8,523	43.20
Quezon	37,644	16	0.04	1,770	4.70	13,857	36.81	15,643	41.56
Rizal	44,826	20	0.04	1,561	3.48	13,956	31.13	15,537	34.66
Antipolo City	16,510	5	0.03	553	3.35	6,909	41.85	7,467	45.23
Bacoor City	12,252	4	0.03	184	1.50	2,918	23.82	3,106	25.35
Batangas City	6,895	6	0.09	345	5.00	3,189	46.25	3,540	51.34
Biñan City	6,607	0	0.00	606	9.17	4,507	68.22	5,113	77.39
Cabuyao City	6,128	0	0.00	293	4.78	3,847	62.78	4,140	67.56
Calamba City	9,025	15	0.17	627	6.95	6,864	76.06	7,506	83.17
Cavite City	2,098	1	0.05	27	1.29	253	12.06	281	13.39
Dasmariñas City	13,442	2	0.01	388	2.89	5,505	40.95	5,895	43.86
General Trias City	6,410	1	0.02	126	1.97	1,470	22.93	1,597	24.91
Imus City	8,235	1	0.01	90	1.09	1,675	20.34	1,766	21.45
Lipa City	6,954	4	0.06	201	2.89	2,979	42.84	3,184	45.79
Lucena City	5,704	1	0.02	215	3.77	2,020	35.41	2,236	39.20
San Pablo City	5,277	8	0.15	347	6.58	2,487	47.13	2,842	53.86
San Pedro City	6,465	2	0.03	171	2.65	1,863	28.82	2,036	31.49
Santa Rosa City	7,023	7	0.10	370	5.27	4,147	59.05	4,524	64.42
Tagaytay City	1,451	1	0.07	28	1.93	1,151	79.32	1,180	81.32
Tanauan City	3,620	0	0.00	51	1.41	1,701	46.99	1,752	48.40
Tayabas City	2,134	3	0.14	140	6.56	1,077	50.47	1,220	57.17
Trece Martires City	3,175	1	0.03	118	3.72	1,099	34.61	1,218	38.36

Table 1.B.3.3 - Postpartum and Newborn Care

Number and Proportion of Postpartum Women who completed Vitamin A Suplementation
Philippines, 2021

Area	Eligible Pop. (Under 1)	Vitamin A Suplementation							
		Age Group in Year						Total	%
		10-14		15-19		20-49			
		No.	%	No.	%	No.	%		
**Region 4B**	**72,035**	**91**	**0.13**	**3,582**	**4.97**	**28,439**	**39.48**	**32,112**	**44.58**
Marinduque	4,906	1	0.02	232	4.73	2,525	51.47	2,758	56.22
Mindoro Occidental	12,449	26	0.21	809	6.50	5,011	40.25	5,846	46.96
Mindoro Oriental	20,011	19	0.09	511	2.55	5,714	28.55	6,244	31.20
Palawan	21,676	25	0.12	1,450	6.69	9,765	45.05	11,240	51.85
Romblon	6,482	18	0.28	333	5.14	3,192	49.24	3,543	54.66
Puerto Princesa City	6,511	2	0.03	247	3.79	2,232	34.28	2,481	38.10
**Region 5**	**137,350**	**83**	**0.06**	**5,636**	**4.10**	**55,288**	**40.25**	**61,007**	**44.42**
Albay	23,903	13	0.05	645	2.70	9,601	40.17	10,259	42.92
Camarines Norte	14,340	5	0.03	779	5.43	6,399	44.62	7,183	50.09
Camarines Sur	39,151	10	0.03	1,027	2.62	11,182	28.56	12,219	31.21
Catanduanes	6,381	10	0.16	356	5.58	2,874	45.04	3,240	50.78
Masbate	22,565	37	0.16	1,592	7.06	9,417	41.73	11,046	48.95
Sorsogon	19,470	5	0.03	869	4.46	9,059	46.53	9,933	51.02
Iriga City	2,665	0	0.00	66	2.48	950	35.65	1,016	38.12
Legaspi City	4,205	3	0.07	168	4.00	4,159	98.91	4,330	102.97
Naga City	4,670	0	0.00	134	2.87	1,647	35.27	1,781	38.14
**Region 6**	**146,545**	**107**	**0.07**	**7,777**	**5.31**	**65,098**	**44.42**	**72,982**	**49.80**
Aklan	11,231	7	0.06	389	3.46	4,741	42.21	5,137	45.74
Antique	12,985	5	0.04	537	4.14	5,877	45.26	6,419	49.43
Capiz	13,984	5	0.04	449	3.21	4,633	33.13	5,087	36.38
Guimaras	3,085	3	0.10	176	5.71	2,104	68.20	2,283	74.00
Iloilo	36,414	20	0.05	1,746	4.79	16,740	45.97	18,506	50.82
Negros Occidental	49,314	62	0.13	3,862	7.83	25,375	51.46	29,299	59.41
Bacolod City	11,102	4	0.04	193	1.74	1,644	14.81	1,841	16.58
Iloilo City	8,430	1	0.01	425	5.04	3,984	47.26	4,410	52.31
**Region 7**	**162,860**	**78**	**0.05**	**6,532**	**4.01**	**67,703**	**41.57**	**74,313**	**45.63**
Bohol	27,637	12	0.04	906	3.28	9,995	36.17	10,913	39.49
Cebu	67,046	21	0.03	2,200	3.28	25,561	38.12	27,782	41.44
Negros Oriental	27,922	34	0.12	1,818	6.51	13,327	47.73	15,179	54.36
Siquijor	1,636	1	0.06	119	7.27	1,001	61.19	1,121	68.52
Cebu City	21,037	10	0.05	776	3.69	7,483	35.57	8,269	39.31
Lapu-Lapu City	9,310	0	0.00	391	4.20	6,561	70.47	6,952	74.67
Mandaue City	8,272	0	0.00	322	3.89	3,775	45.64	4,097	49.53
**Region 8**	**104,016**	**159**	**0.15**	**4,382**	**4.21**	**40,355**	**38.80**	**44,896**	**43.16**
Biliran	3,856	5	0.13	294	7.62	2,108	54.67	2,407	62.42
Eastern Samar	11,635	12	0.10	584	5.02	4,802	41.27	5,398	46.39
Northern Leyte	35,052	9	0.03	1,209	3.45	13,871	39.57	15,089	43.05
Northern Samar	15,655	104	0.66	643	4.11	5,743	36.68	6,490	41.46
Southern Leyte	6,556	5	0.08	354	5.40	2,884	43.99	3,243	49.47
Western Samar	14,515	21	0.14	659	4.54	5,131	35.35	5,811	40.03
Calbayog City	4,474	0	0.00	180	4.02	1,437	32.12	1,617	36.14
Maasin City	1,661	1	0.06	61	3.67	675	40.64	737	44.37
Ormoc City	4,989	2	0.04	356	7.14	2,876	57.65	3,234	64.82

Table 1.B.3.3 - Postpartum and Newborn Care

Number and Proportion of Postpartum Women who completed Vitamin A Suplementation
Philippines, 2021

Area	Eligible Pop. (Under 1)	Vitamin A Suplementation							
		Age Group in Year						Total	%
		10-14		15-19		20-49			
		No.	%	No.	%	No.	%		
Tacloban City	5,623	0	0.00	42	0.75	828	14.73	870	15.47
**Region 9**	**79,552**	**96**	**0.12**	**5,662**	**7.12**	**39,532**	**49.69**	**45,290**	**56.93**
Zamboanga del Norte	17,075	12	0.07	1,287	7.54	7,671	44.93	8,970	52.53
Zamboanga del Sur	17,506	45	0.26	1,099	6.28	6,459	36.90	7,603	43.43
Zamboanga Sibugay	14,992	13	0.09	886	5.91	5,496	36.66	6,395	42.66
Dapitan City	1,755	3	0.17	86	4.90	1,504	85.70	1,593	90.77
Dipolog City	2,794	3	0.11	246	8.80	1,859	66.54	2,108	75.45
Isabela City	2,544	3	0.12	176	6.92	1,127	44.30	1,306	51.34
Pagadian City	4,290	0	0.00	166	3.87	1,583	36.90	1,749	40.77
Zamboanga City	18,596	17	0.09	1,716	9.23	13,833	74.39	15,566	83.71
**Region 10**	**100,690**	**170**	**0.17**	**8,202**	**8.15**	**51,989**	**51.63**	**60,361**	**59.95**
Bukidnon	23,307	83	0.36	3,062	13.14	13,069	56.07	16,214	69.57
Camiguin	1,854	2	0.11	92	4.96	978	52.75	1,072	57.82
Lanao del Norte	14,955	5	0.03	542	3.62	6,731	45.01	7,278	48.67
Misamis Occidental	6,413	1	0.02	222	3.46	2,920	45.53	3,143	49.01
Misamis Oriental	15,058	12	0.08	877	5.82	5,811	38.59	6,700	44.49
Cagayan de Oro City	14,263	27	0.19	1,410	9.89	9,920	69.55	11,357	79.63
El Salvador City	1,061	1	0.09	100	9.43	585	55.14	686	64.66
Gingoog City	2,627	17	0.65	122	4.64	557	21.20	696	26.49
Iligan City	7,575	6	0.08	546	7.21	3,904	51.54	4,456	58.83
Malaybalay City	3,882	4	0.10	434	11.18	2,456	63.27	2,894	74.55
Oroquieta City	1,389	2	0.14	97	6.98	645	46.44	744	53.56
Ozamis City	2,782	2	0.07	203	7.30	1,705	61.29	1,910	68.66
Tangub City	1,234	0	0.00	57	4.62	644	52.19	701	56.81
Valencia City	4,290	8	0.19	438	10.21	2,064	48.11	2,510	58.51
**Region 11**	**107,847**	**353**	**0.33**	**10,430**	**9.67**	**61,926**	**57.42**	**72,709**	**67.42**
Davao de Oro	15,526	77	0.50	1,859	11.97	9,089	58.54	11,025	71.01
Davao del Norte	21,184	69	0.33	2,313	10.92	13,802	65.15	16,184	76.40
Davao Oriental	13,125	36	0.27	1,302	9.92	6,721	51.21	8,059	61.40
Davao del Sur	14,355	46	0.32	1,244	8.67	6,665	46.43	7,955	55.42
Davao Occidental	6,598	45	0.68	1,014	15.37	2,617	39.66	3,676	55.71
Davao City	37,059	80	0.22	2,698	7.28	23,032	62.15	25,810	69.65
**Region 12**	**104,693**	**167**	**0.16**	**8,368**	**7.99**	**49,251**	**47.04**	**57,786**	**55.20**
North Cotabato	33,933	35	0.10	2,145	6.32	12,915	38.06	15,095	44.48
Sarangani	12,904	35	0.27	1,772	13.73	7,570	58.66	9,377	72.67
South Cotabato	21,039	39	0.19	2,253	10.71	13,466	64.00	15,758	74.90
Sultan Kudarat	17,331	51	0.29	1,651	9.53	9,525	54.96	11,227	64.78
Cotabato City	5,831	2	0.03	253	4.34	2,672	45.82	2,927	50.20
Gen. Santos City	13,655	5	0.04	294	2.15	3,103	22.72	3,402	24.91
**BARMM**	**94,148**	**26**	**0.03**	**6,150**	**6.53**	**49,880**	**52.98**	**56,056**	**59.54**
Basilan	7,672	6	0.08	305	3.98	2,415	31.48	2,726	35.53
Lanao del Sur	21,391	1	0.00	635	2.97	12,822	59.94	13,458	62.91
Maguindanao	31,650	14	0.04	2,291	7.24	19,957	63.06	22,262	70.34
Sulu	16,891	2	0.01	431	2.55	5,785	34.25	6,218	36.81
Tawi-Tawi	9,319	1	0.01	322	3.46	4,740	50.86	5,063	54.33

Table 1.B.3.3 - Postpartum and Newborn Care

Number and Proportion of Postpartum Women who completed Vitamin A Suplementation
Philippines, 2021

Area	Eligible Pop. (Under 1)	Vitamin A Suplementation							
		Age Group in Year						Total	%
		10-14		15-19		20-49			
		No.	%	No.	%	No.	%		
Lamitan City	2,112	2	0.09	255	12.07	1,084	51.33	1,341	63.49
Marawi City	5,113	0	0.00	1,911	37.38	3,077	60.18	4,988	97.56
**CARAGA**	**60,342**	**79**	**0.13**	**3,974**	**6.59**	**28,067**	**46.51**	**32,120**	**53.23**
Agusan del Norte	8,116	12	0.15	489	6.03	3,315	40.85	3,816	47.02
Agusan del Sur	17,658	19	0.11	1,179	6.68	8,243	46.68	9,441	53.47
Surigao del Norte	7,195	6	0.08	232	3.22	2,116	29.41	2,354	32.72
Surigao del Sur	11,532	24	0.21	776	6.73	5,687	49.31	6,487	56.25
Province of Dinagat	2,574	3	0.12	125	4.86	992	38.54	1,120	43.51
Bislig City	2,185	9	0.41	148	6.77	1,142	52.27	1,299	59.45
Butuan City	7,732	6	0.08	694	8.98	4,963	64.19	5,663	73.24
Surigao City	3,350	0	0.00	331	9.88	1,609	48.03	1,940	57.91

Note: Put asterisk (*) for No Report and Zero (0) for No Case

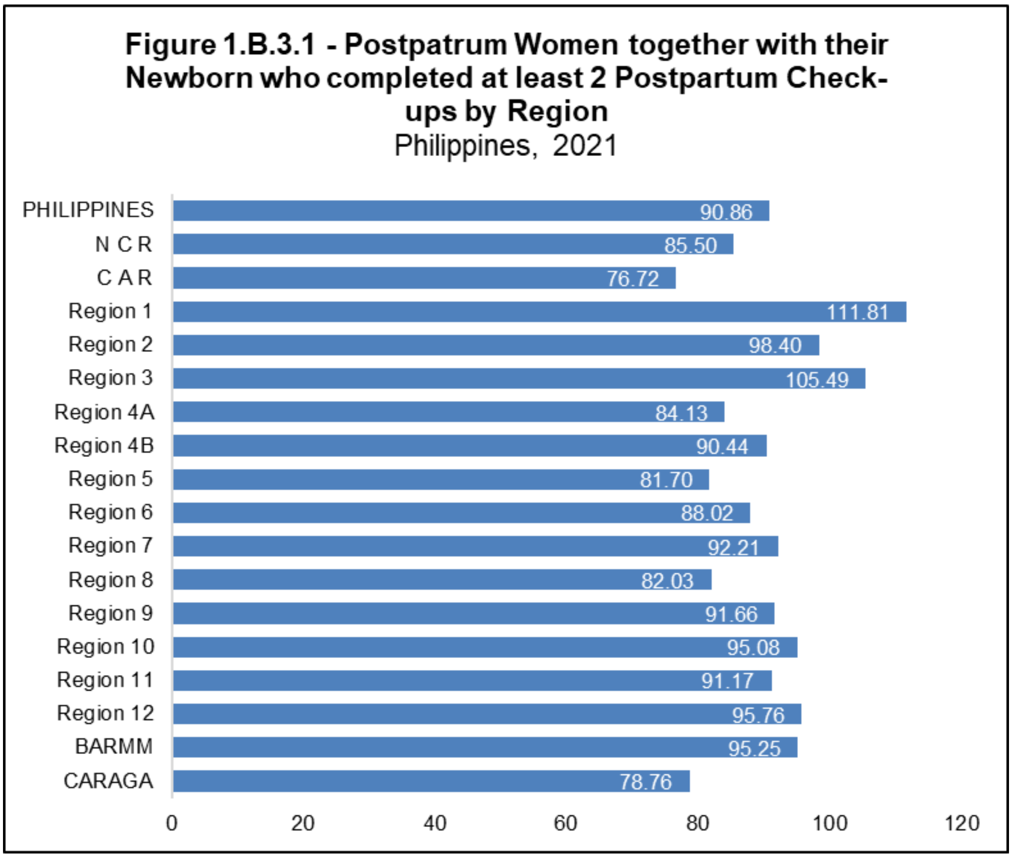
Figure 1.B.3.1 - Postpatrum Women together with their Newborn who completed at least 2 Postpartum Check-ups by Region
Philippines, 2021
PHILIPPINES
N C R
C A R
Region 1
Region 2
Region 3
Region 4A
Region 4B
Region 5
Region 6
Region 7
Region 8
Region 9
Region 10
Region 11
Region 12
BARMM
CARAGA
90.86
85.50
76.72
111.81
98.40
105.49
84.13
90.44
81.70
88.02
92.21
82.03
91.66
95.08
91.17
95.76
95.25
78.76
0
20
40
60
80
100
120

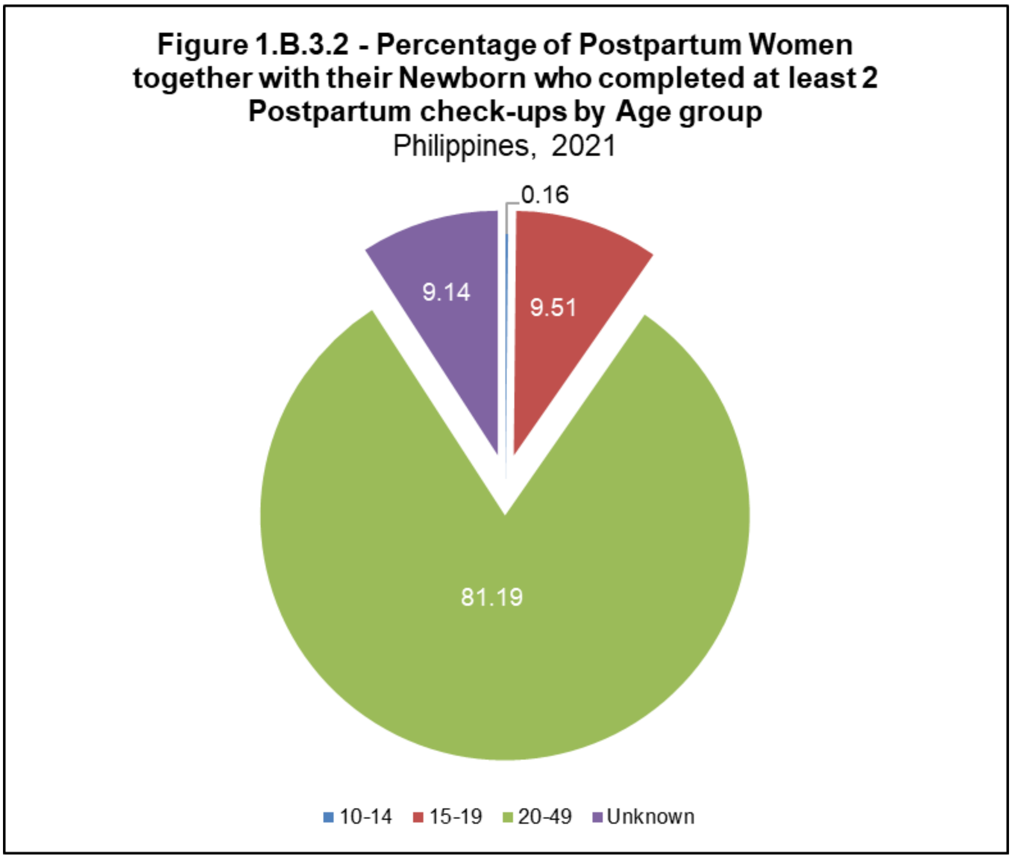
Figure 1.B.3.2 - Percentage of Postpartum Women together with their Newborn who completed at least 2 Postpartum check-ups by Age group
Philippines, 2021
0.16
9.14
9.51
81.19
10-14
15-19
20-49
Unknown

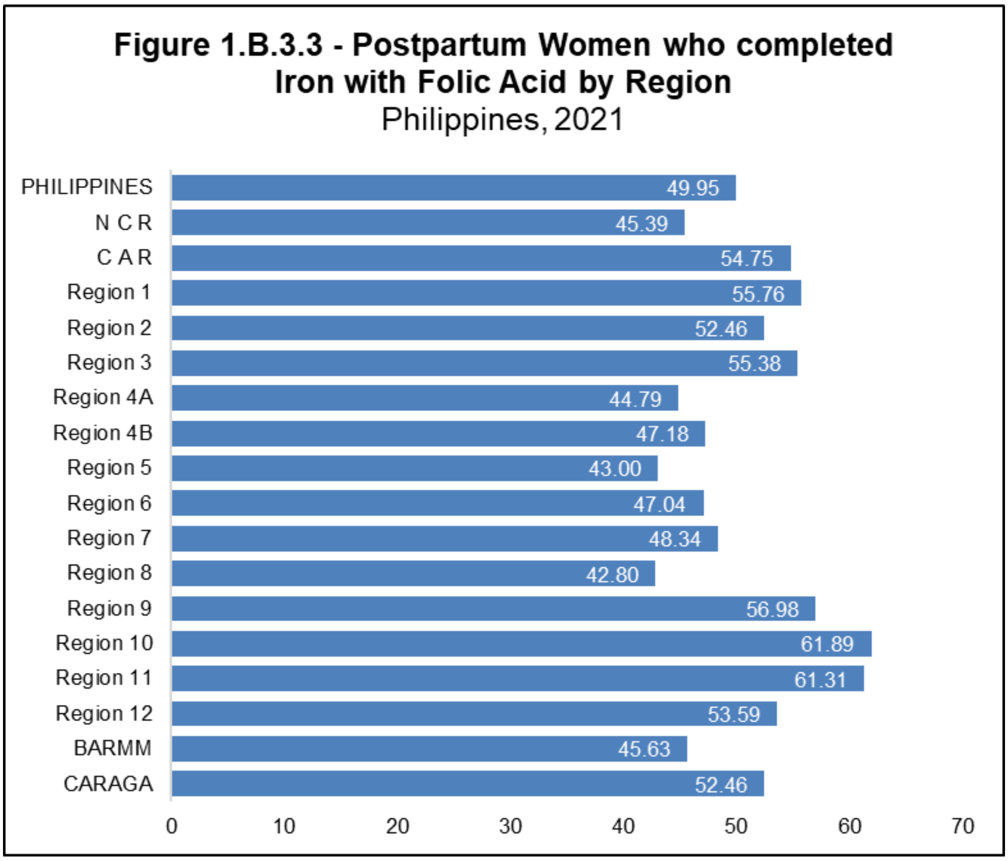
Figure 1.B.3.3 - Postpartum Women who completed Iron with Folic Acid by Region
Philippines, 2021
PHILIPPINES 49.95
N C R 45.39
C A R 54.75
Region 1 55.76
Region 2 52.46
Region 3 55.38
Region 4A 44.79
Region 4B 47.18
Region 5 43.00
Region 6 47.04
Region 7 48.34
Region 8 42.80
Region 9 56.98
Region 10 61.89
Region 11 61.31
Region 12 53.59
BARMM 45.63
CARAGA 52.46
0 10 20 30 40 50 60 70

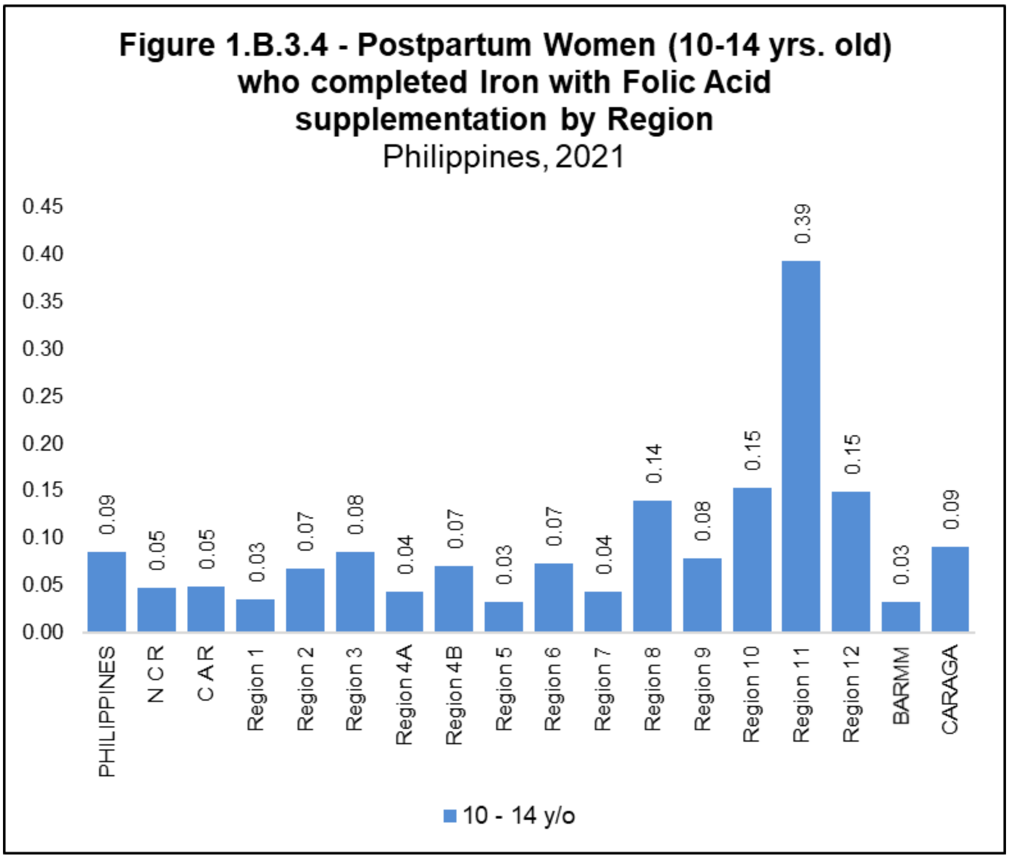
Figure 1.B.3.4 - Postpartum Women (10-14 yrs. old) who completed Iron with Folic Acid supplementation by Region
Philippines, 2021
0.45 0.40 0.35 0.30 0.25 0.20 0.15 0.10 0.05 0.00
PHILIPPINES 0.09
N C R 0.05
C A R 0.05
Region 1 0.03
Region 2 0.07
Region 3 0.08
Region 4A 0.04
Region 4B 0.07
Region 5 0.03
Region 6 0.07
Region 7 0.04
Region 8 0.14
Region 9 0.08
Region 10 0.15
Region 11 0.39
Region 12 0.15
BARMM 0.03
CARAGA 0.09
10 - 14 y/o

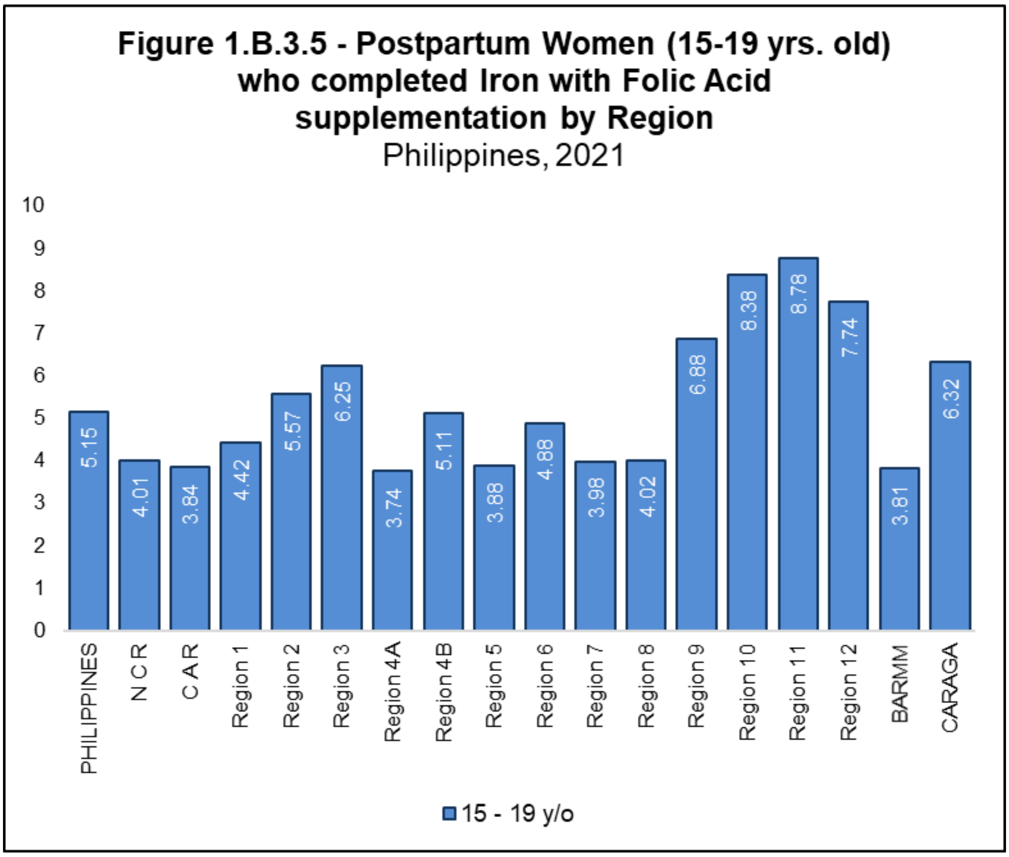
Figure 1.B.3.5 - Postpartum Women (15-19 yrs. old) who completed Iron with Folic Acid supplementation by Region
Philippines, 2021
10
9
8
7
6
5
4
3
2
1
0
5.15
4.01
3.84
4.42
5.57
6.25
3.74
5.11
3.88
4.88
3.98
4.02
6.88
8.38
8.78
7.74
3.81
6.32
PHILIPPINES
N C R
C A R
Region 1
Region 2
Region 3
Region 4A
Region 4B
Region 5
Region 6
Region 7
Region 8
Region 9
Region 10
Region 11
Region 12
BARMM
CARAGA
15 - 19 y/o

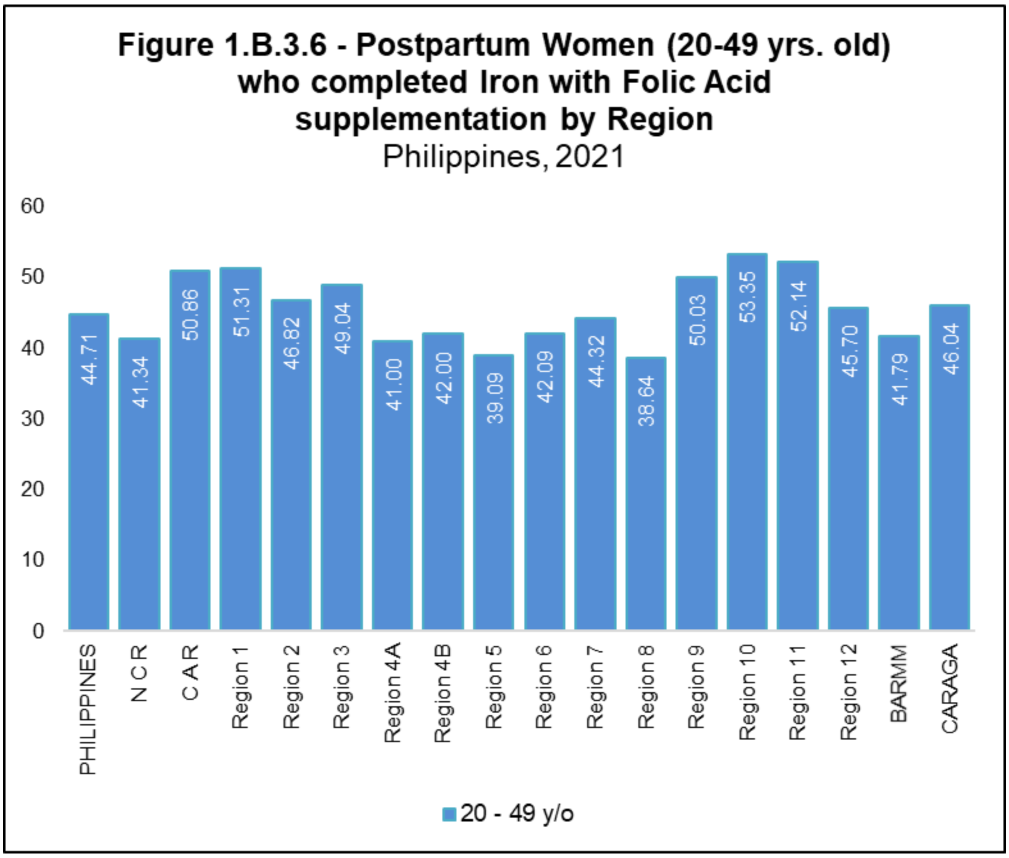
Figure 1.B.3.6 - Postpartum Women (20-49 yrs. old) who completed Iron with Folic Acid supplementation by Region
Philippines, 2021
60
50
40
30
20
10
0
44.71
41.34
50.86
51.31
46.82
49.04
41.00
42.00
39.09
42.09
44.32
38.64
50.03
53.35
52.14
45.70
41.79
46.04
PHILIPPINES
N C R
C A R
Region 1
Region 2
Region 3
Region 4A
Region 4B
Region 5
Region 6
Region 7
Region 8
Region 9
Region 10
Region 11
Region 12
BARMM
CARAGA
20 - 49 y/o

Figure 1.B.3.7 - Postpartum Women who completed Vitamin A supplementation by Region
Philippines, 2021

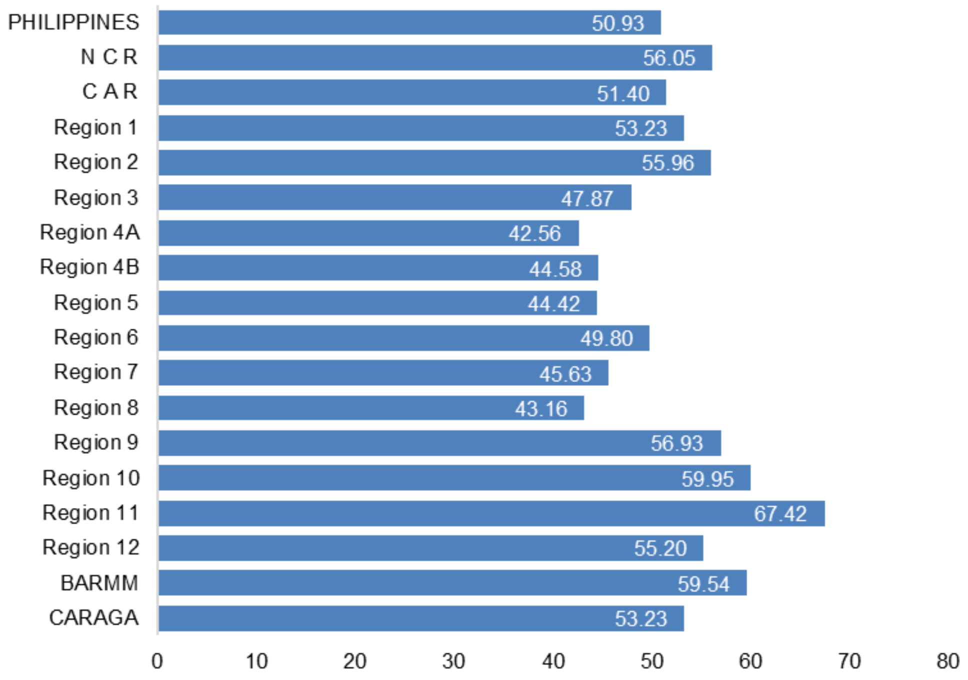

Figure 1.B.3.8 - Postpartum Women (10-14 yrs. old) who completed Vitamin A supplementation by Region
Philippines, 2021

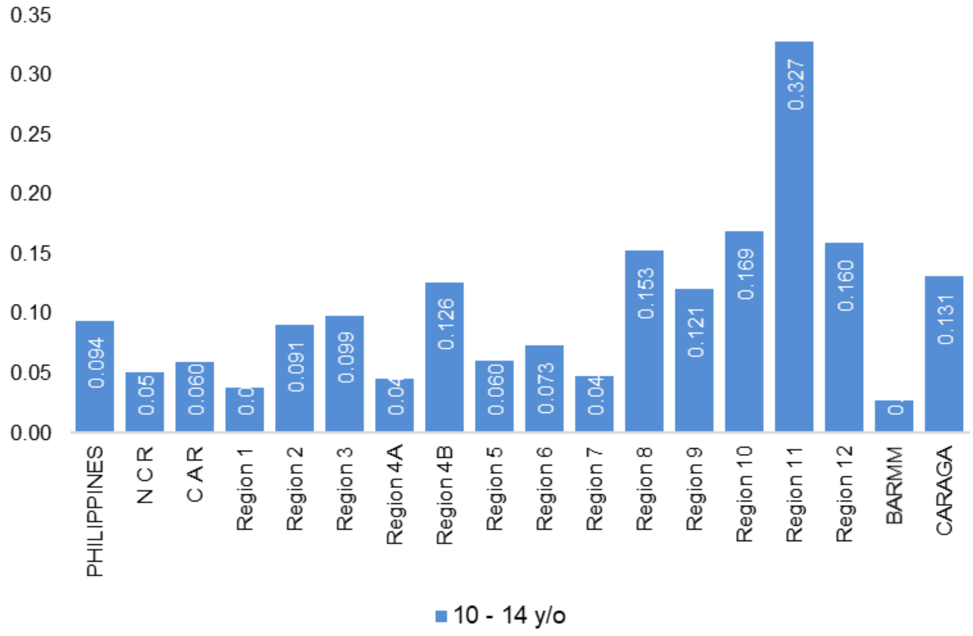

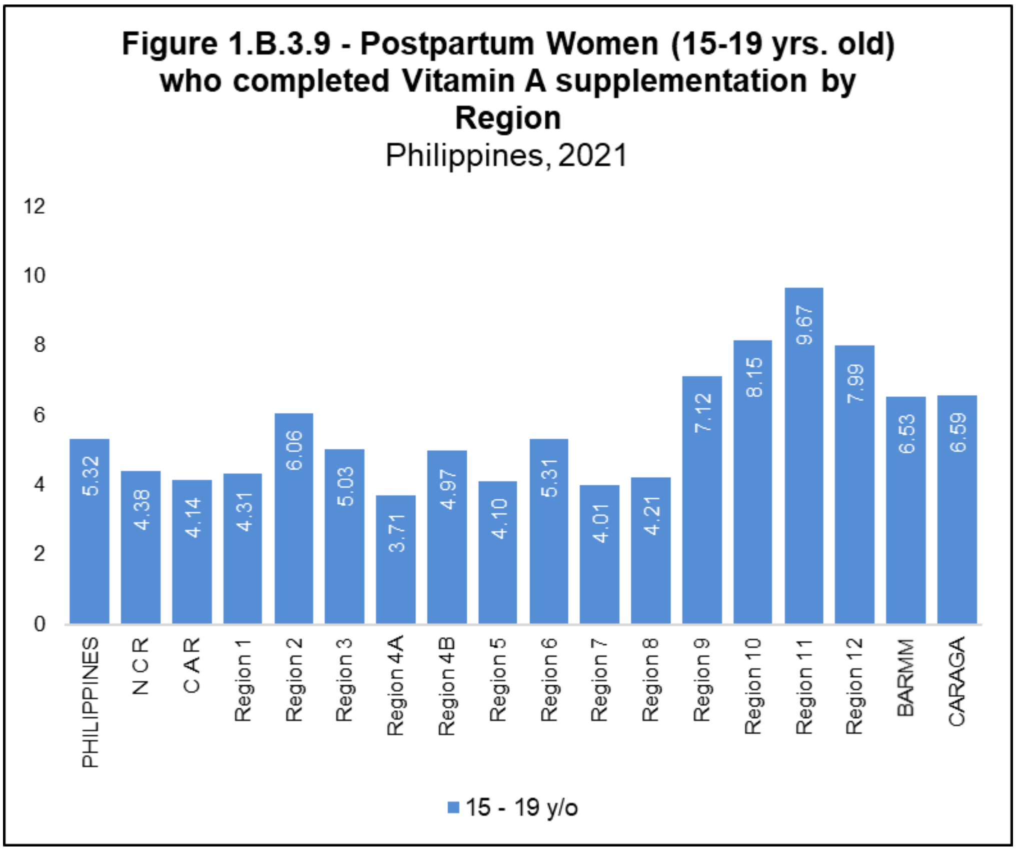
Figure 1.B.3.9 - Postpartum Women (15-19 yrs. old) who completed Vitamin A supplementation by Region
Philippines, 2021
12
10
8
6
4
2
0
5.32
4.38
4.14
4.31
6.06
5.03
3.71
4.97
4.10
5.31
4.01
4.21
7.12
8.15
9.67
7.99
6.53
6.59
PHILIPPINES
NCR
CAR
Region 1
Region 2
Region 3
Region 4A
Region 4B
Region 5
Region 6
Region 7
Region 8
Region 9
Region 10
Region 11
Region 12
BARMM
CARAGA
15 - 19 y/o

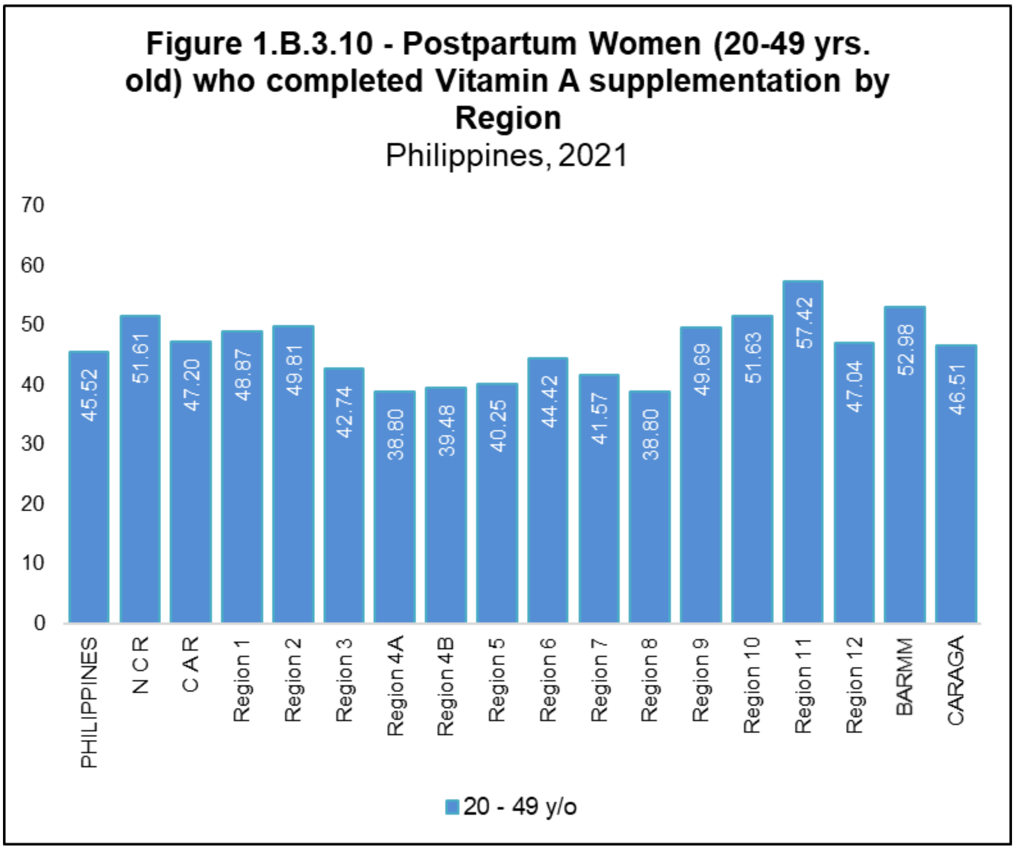
Figure 1.B.3.10 - Postpartum Women (20-49 yrs. old) who completed Vitamin A supplementation by Region
Philippines, 2021
70
60
50
40
30
20
10
0
45.52
51.61
47.20
48.87
49.81
42.74
38.80
39.48
40.25
44.42
41.57
38.80
49.69
51.63
57.42
47.04
52.98
46.51
PHILIPPINES
NCR
CAR
Region 1
Region 2
Region 3
Region 4A
Region 4B
Region 5
Region 6
Region 7
Region 8
Region 9
Region 10
Region 11
Region 12
BARMM
CARAGA
20 - 49 y/o

CHILD CARE AND SERVICES

FHSIS ANNUAL REPORT 2021

1.C. Child Care and Services

- **Immunization Services for Infants and Children**

A Fully Immunized Child (FIC) is an infant who received one dose of BCG, three doses each of OPV, DPT, and Hepatitis B vaccines, and two doses of Measles Containing Vaccine by 12 months of age. In the Philippines, there were 1,337,743 (62.86%) FIC in 2021. This was 32.40% below the national program target of 95%. Figure 1.C.1 shows that in 5 years, the annual proportion of fully immunized children has not achieved the desired immunization program coverage and it has gradually declined from 2017 to 2021, noting a slight increase in the year 2019.

Figure 1.C.1 Proportion of Fully Immunized Children Philippines, 2017 - 2021

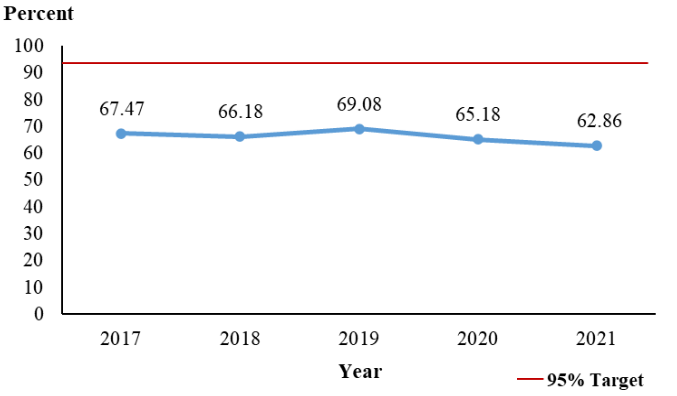

There was a decrease in the proportion of Completely Immunized Children (CIC) or children 13-23 months who were completely immunized outside the recommended vaccination schedule, from 14.62% in 2020 to 10.73% in 2021.

Figure 1.C.2 Proportion of Completely Immunized Children Philippines, 2017 - 2021

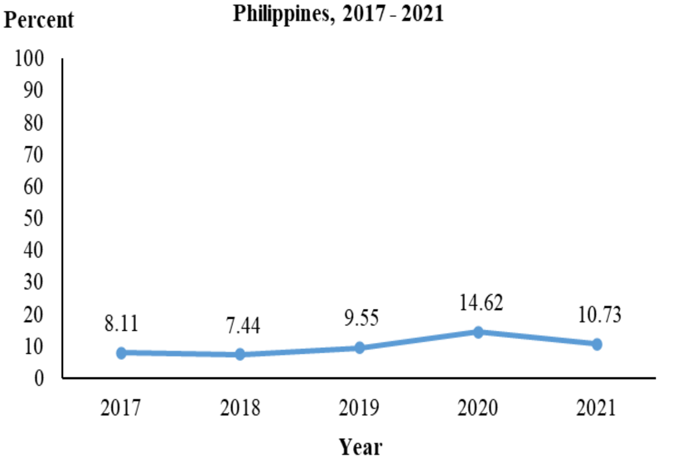

Figure 1.C.3 shows that all regions have FIC percentages of more than 50%. The top 3 regions with the highest proportion of FIC were Region 1 at 77.36%, Region 10 at 76.51% and CARAGA at 73.88% while Region 8 has the lowest proportion at 52.76%. For CIC, NCR recorded the highest proportion at 21.75% followed by Region 7 at 15.30%. Region 2 has the lowest proportion at 3.12%.

Figure 1.C.3 Proportion Fully Immunized Children (FIC) and Completely Immunized Children (CIC) by Region Philippines, 2021

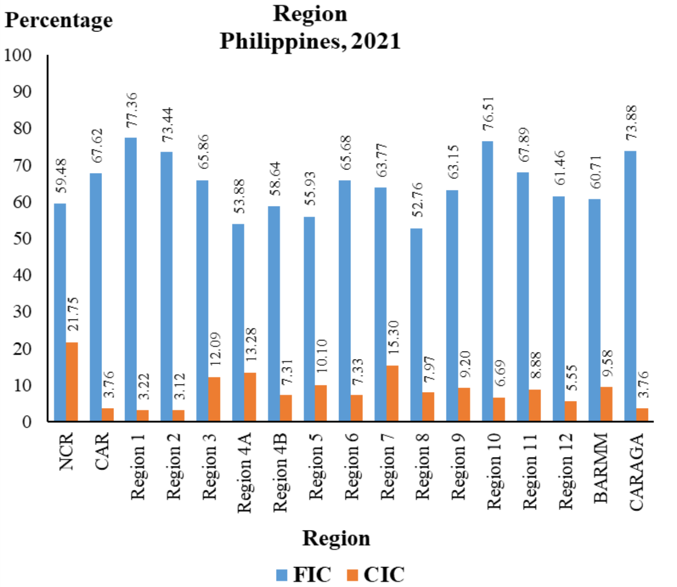

A continuous decrease was observed from 2017 (82.42%) to 2021 (53.79%) in the proportion of Children Protected at Birth (CPAB). The CPAB refers to newborns whose mothers have received the recommended dosage of Tetanus Toxoid or Tetanus Diphtheria Toxoid vaccinations.

Figure 1.C.4 Proportion of Children Protected at Birth Philippines, 2017 - 2021

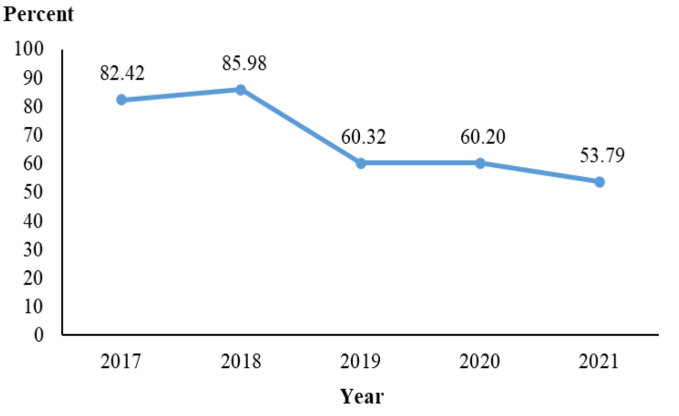

The proportion of newborn/infants vaccinated with BCG and Hepatitis B1 antigens registered the lowest in 2021 with 58.91% and 48.97% respectively in the 5-year period. A gradual decrease since 2019 was observed for both indicators.

Figure 1.C.5 Proportion of Newborns/Infants Vaccinated with BCG and Hepatitis B1* Antigens Philippines, 2017 - 2021

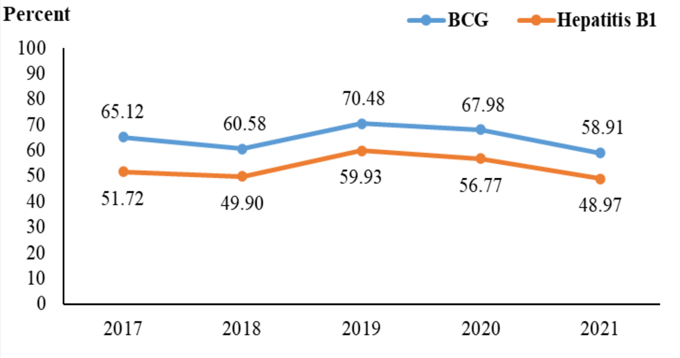

*Given within 24hrs. after birth

The region with the highest proportion of newborn/infants given BCG and Hepatitis B1 vaccine was recorded in NCR at 74.42% and 66.99% respectively, followed by Region 11 (73.64%, 62.80%). However, Region 5 had the lowest proportion of BCG vaccination at 46.57% while Region 4B registered lowest for Hepatitis B1 vaccination at 36.73%.

Figure 1.C.6 Proportion of Newborns/Infants Vaccinated with BCG and Hepatitis B1* Antigens by Region Philippines, 2021

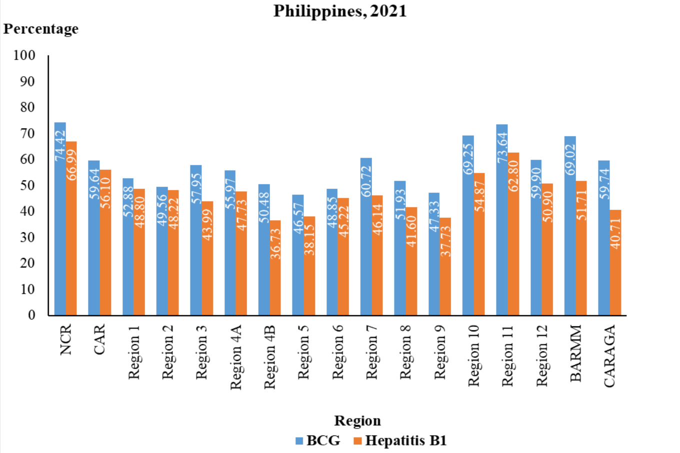

Diphtheria, Pertussis, Tetanus, (DPT), Hepatitis B (HepB), and *Haemophilus influenza type B* (Hib) vaccines are part of the Expanded Program on Immunization in the Philippines. The proportion of infants vaccinated with all 3 doses is adjacent to one another for all years as presented in Figure 1.C.7. However, a decrease was noted for all 3 doses in 2021 after an increase in the past 2 years.

Figure 1.C.7 Proportion of Infants Given 3 Doses of DPT-HiB-HepB Antigens Philippines, 2017 - 2021

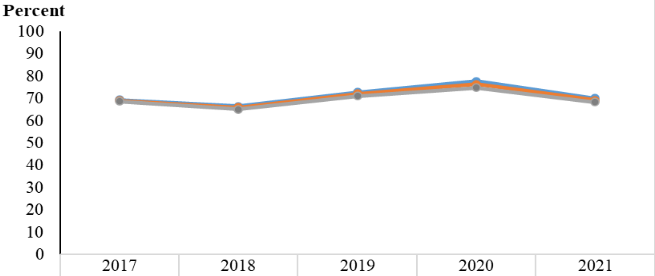

	2017	2018	2019	2020	2021
Dose 1	69.29	66.47	72.83	77.77	70.09
Dose 2	69.09	65.90	72.10	76.32	69.27
Dose 3	68.65	65.10	71.08	74.80	68.34

Year

In 2021, the region with the highest proportion of infants vaccinated for all 3 doses of DPT-Hib-HepB was Region 10 (79.78%-79.02%-76.64%) followed by Region 3 (76.79%-76.60%-76.55%). Region 8 registered the lowest for the 3 doses of DPT-HiB-HepB coverage.

Figure 1.C.8 Proportion of Infants Given 3 Doses of DPT-HiB-HepB Antigens by Region Philippines, 2021

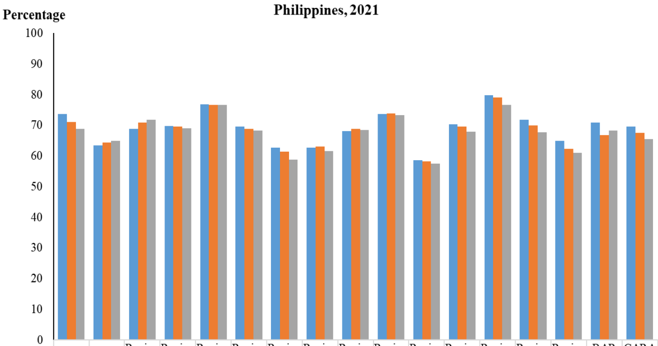

	NCR	CAR	Region 1	Region 2	Region 3	Region 4A	Region 4B	Region 5	Region 6	Region 7	Region 8	Region 9	Region 10	Region 11	Region 12	BARMM	CARAGA
Dose 1	73.70	63.51	68.90	69.70	76.79	69.58	62.61	62.64	68.05	73.69	58.63	70.30	79.78	71.76	64.87	70.77	69.48
Dose 2	71.07	64.33	70.85	69.55	76.60	68.79	61.46	63.04	68.86	73.80	58.22	69.50	79.02	69.84	62.39	66.69	67.60
Dose 3	68.89	64.88	71.84	69.02	76.55	68.23	58.75	61.55	68.48	73.27	57.39	67.93	76.64	67.66	60.93	68.31	65.39

Region

As shown in Figure 1.C.9, the proportion of infants vaccinated with OPV 1, 2, and 3 had a relative decline from 2019 to 2021. It was also observed that the coverage rates decreased for each subsequent dose.

Figure 1.C.9 Proportion of Infants Given 3 Doses of Oral Polio Vaccine Philippines, 2017 - 2021

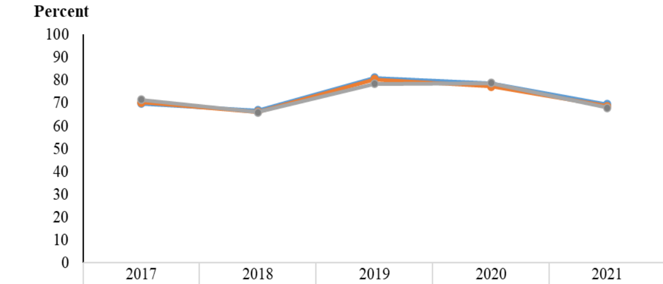

	2017	2018	2019	2020	2021
Dose 1	69.89	66.90	81.13	78.55	69.53
Dose 2	70.33	66.22	80.43	77.28	68.71
Dose 3	71.50	66.02	78.43	78.78	68.05

Year

The region with the highest proportion of infants given OPV 1, 2, and 3 was recorded in Region 10 with 80.89%, 77.32%, and 76.28%, respectively. This figure also shows that all regions had a proportion of more than 50% for infants given OPV 1, 2, and 3.

Figure 1.C.10 Proportion of Infants Given 3 Doses of Oral Polio Vaccine by Region Philippines, 2021

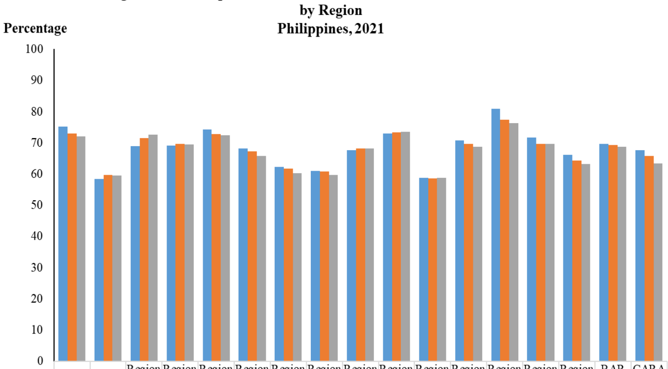

	NCR	CAR	Region 1	Region 2	Region 3	Region 4A	Region 4B	Region 5	Region 6	Region 7	Region 8	Region 9	Region 10	Region 11	Region 12	BARMM	CARAGA
Dose 1	75.10	58.39	68.92	69.18	74.22	68.23	62.24	61.05	67.57	72.95	58.81	70.82	80.89	71.70	66.23	69.69	67.58
Dose 2	72.95	59.61	71.51	69.67	72.81	67.24	61.66	60.77	68.19	73.36	58.62	69.60	77.32	69.73	64.39	69.23	65.81
Dose 3	71.96	59.48	72.53	69.44	72.33	65.82	60.19	59.60	68.23	73.55	58.75	68.80	76.28	69.63	63.22	68.69	63.42

Region

As shown in Figure 1.C.11, the proportion of infants vaccinated with Inactivated Polio Vaccine (IPV) decreased in 2021 (67.18) subsequent to the increase in 2020.

Figure 1.C.11 Proportion of Infants Vaccinated with Inactivated Polio Vaccine Philippines, 2019 - 2021

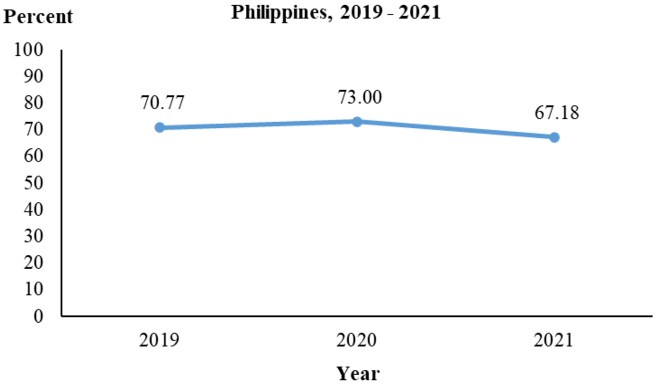

The region with the highest proportion of infants given one dose of IPV was recorded in Region 3 at 75.21%, followed by Region 10 at 74.35%, then Region 7 at 73.56%. The rest of the regions have recorded at least 50% coverage for infants vaccinated with IPV.

Figure 1.C.12 Proportion of Infants Vaccinated with Inactivated Polio Vaccine by Region
Philippines, 2021

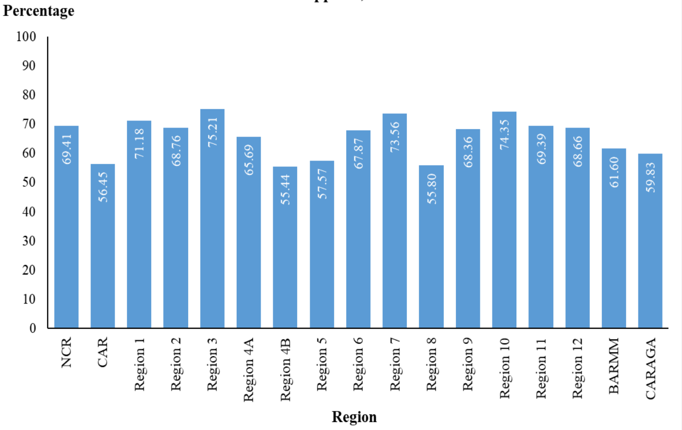

The proportion of infants who completed 3 doses of Pneumococcal Conjugate Vaccine (PCV) steadily increased from 2017 to 2020, but decreased in 2021. It was also observed that the coverage rates decreased for each subsequent dose in 5 years.

Figure 1.C.13 Proportion of Infants Given 3 Doses of Pneumococcal Conjugate Vaccine
Philippines, 2017 - 2021

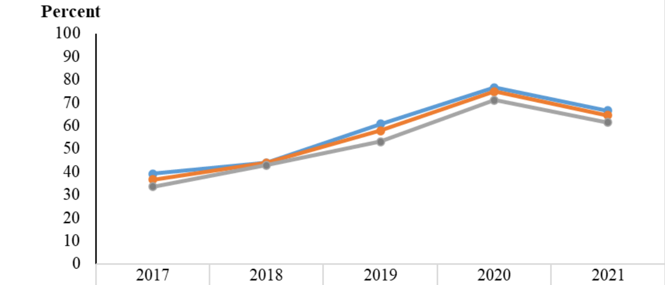

	2017	2018	2019	2020	2021
Dose 1	39.15	43.79	60.75	76.42	66.38
Dose 2	36.51	43.79	57.85	74.73	64.32
Dose 3	33.44	42.77	52.96	70.97	61.28

Year

In 2021, the region with the highest proportion of infants vaccinated with PCV1 was Region 10 (72.67%) while Region 1 registered the highest for PCV 2 (69.96%) and PCV 3 (70.68%). Region 4B had the least coverage for PCV 1 (52.71%), PCV 2 (49.00%) and PCV 3 (44.74%) vaccination.

Figure 1.C.14 Proportion of Infants Given 3 Doses of Pneumococcal Conjugate Vaccine by Region
Philippines, 2021

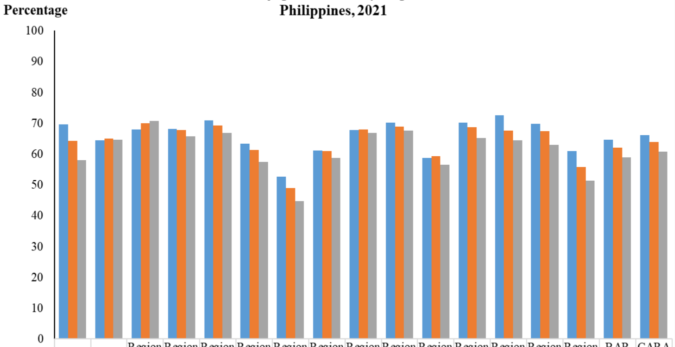

	NCR	CAR	Region 1	Region 2	Region 3	Region 4A	Region 4B	Region 5	Region 6	Region 7	Region 8	Region 9	Region 10	Region 11	Region 12	BARMM	CARAGA
Dose 1	69.71	64.50	68.03	68.22	70.89	63.43	52.71	61.22	67.71	70.10	58.83	70.11	72.67	69.78	60.93	64.68	66.06
Dose 2	64.32	64.96	69.96	67.77	69.22	61.29	49.00	60.91	67.91	68.89	59.25	68.67	67.56	67.35	55.76	62.02	63.83
Dose 3	57.95	64.56	70.68	65.74	66.94	57.53	44.74	58.74	66.81	67.56	56.51	65.18	64.40	63.01	51.44	58.95	60.73

Region

The proportion of infants vaccinated with 2 doses of Measles Containing Vaccine (MCV) decreased in 2021, subsequent to the increase since 2019. It was also observed that the coverage gap between 2 doses decreased over time in the 5-year period.

Figure 1.C.15 Proportion of Infants Vaccinated with 2 Doses of Measles Containing Vaccine (MCV)
Philippines, 2017 - 2021

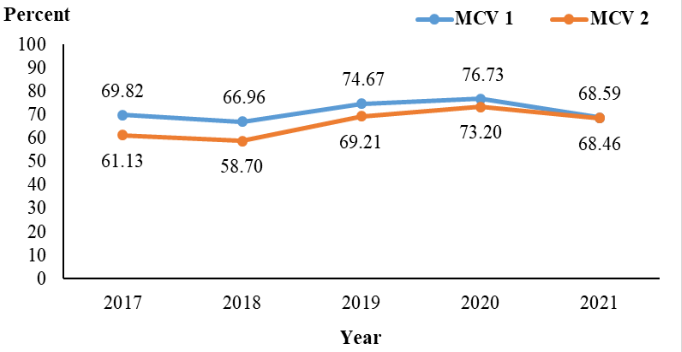

Region 10 had the highest proportion of infants vaccinated with MCV1 at 78.76% followed by Region 1 at 75.93%. For MCV2 however, Region 1 registered highest at 80.59% followed by Region 10 at 78.64%. In both doses, a coverage of more than 50% was observed for all regions.

Figure 1.C.16 Proportion of Infants Vaccinated with 2 Doses of Measles Containing Vaccine (MCV) by Region Philippines, 2021

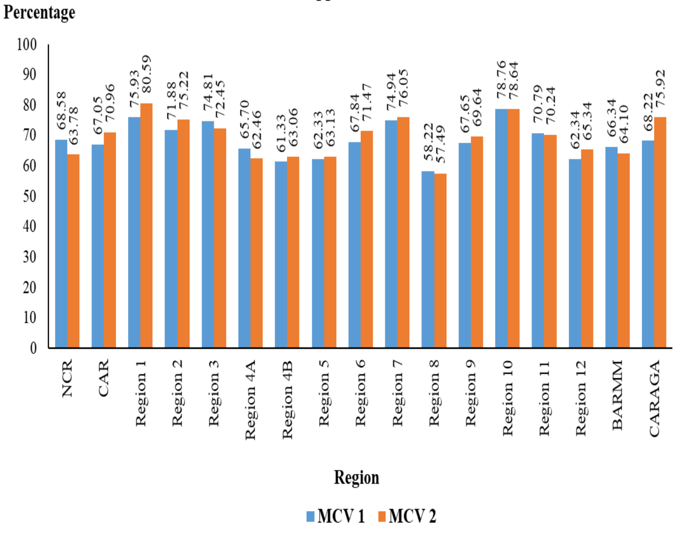

- **Nutrition Services for Infants and Children**

In the Philippines, the number of newborns initiated on breastfeeding immediately after birth reached 1,019,937 or 82.39%. The regions with the highest proportion of newborns initiated on breastfeeding immediately after birth were Region 1 at 99.83%, followed by Region 7 at 94.99%, and Region 12 at 93.46%. Among the regions, BARMM has the lowest proportion at 63.99%, followed by NCR at 67.69%.

Figure 1.C.17 Proportion of Newborns Who Were Initiated on Breastfeeding Immediately After Birth by Region Philippines, 2021

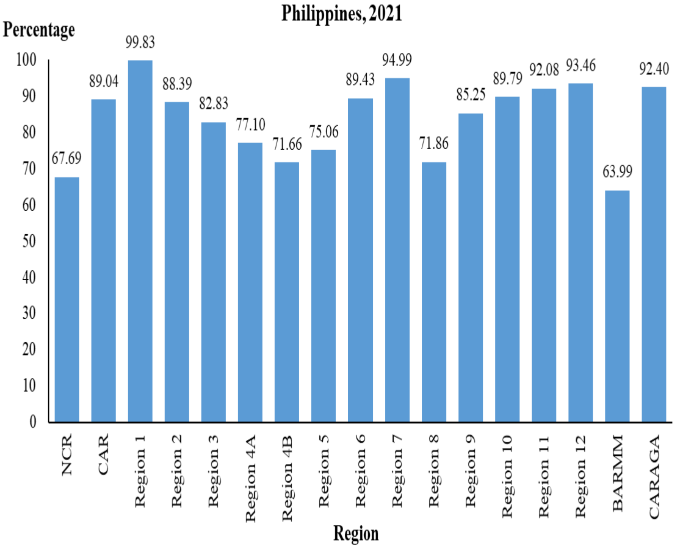

In 2021, the proportion of infants who exclusively breastfed until the 5th month and 29 days after birth was at 49.77%. A decreasing trend was observed since 2019.

Figure 1.C.18 Proportion of Infants Exclusively Breastfed until 5th month and 29 days Philippines, 2017 - 2021

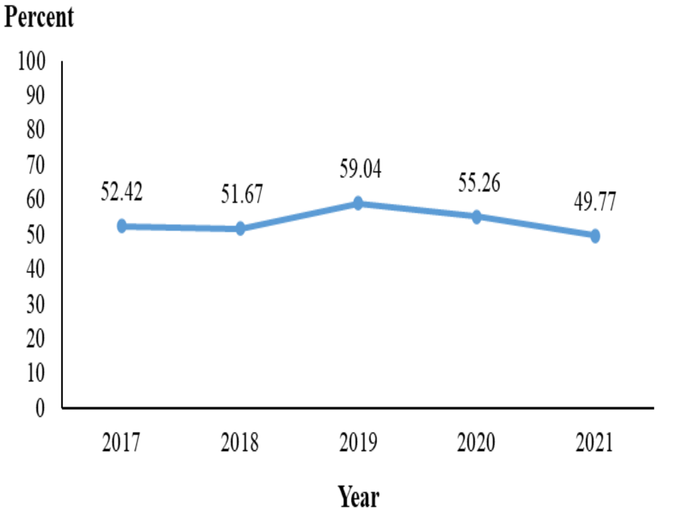

The top 3 regions with the highest proportion of infants who exclusively breastfed within the first 6 months after birth were Region 10 at 70.32%, Region 1 at 65.71% and Region 12 at 63.27%. It was also observed that half of the regions did not reach the national target of at least 50%.

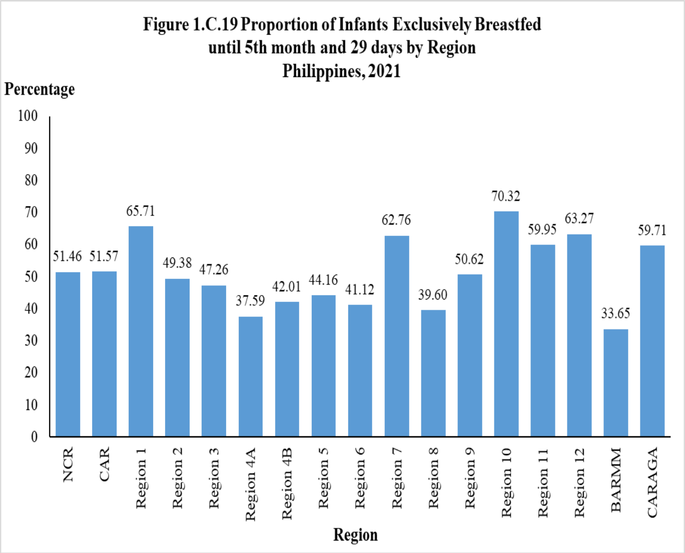

For the proportion of infants born preterm or with low birth weight (LBW) who were given iron supplementation, the regions with the highest proportion were BARMM at 79.03%, followed by Region 10 at 63.90% and Region 12 at 55.30%. Meanwhile, Region 7 registered the lowest at 1.95%

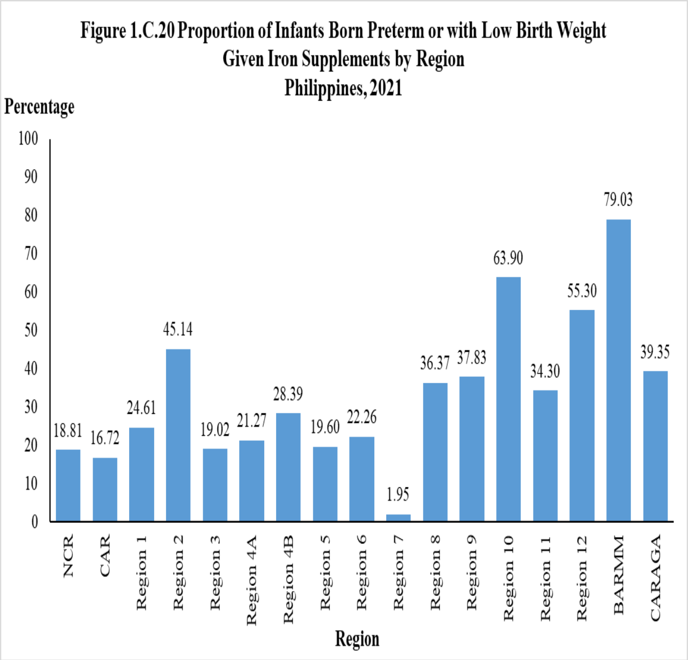

- **Management of Sick Infants and Children**

Figure 1.C.21 shows a fluctuating trend from 2017 (52.12%) to 2021 (57.10%) in the proportion of sick infants aged 6-11 months who were given Vitamin A. Although, 2021 registered the highest coverage in the 5-year period. However, for the sick children aged 12-59 months who were given Vitamin A in health facilities, a decrease was noted in 2021 after a continuous increase in the previous 4 years.

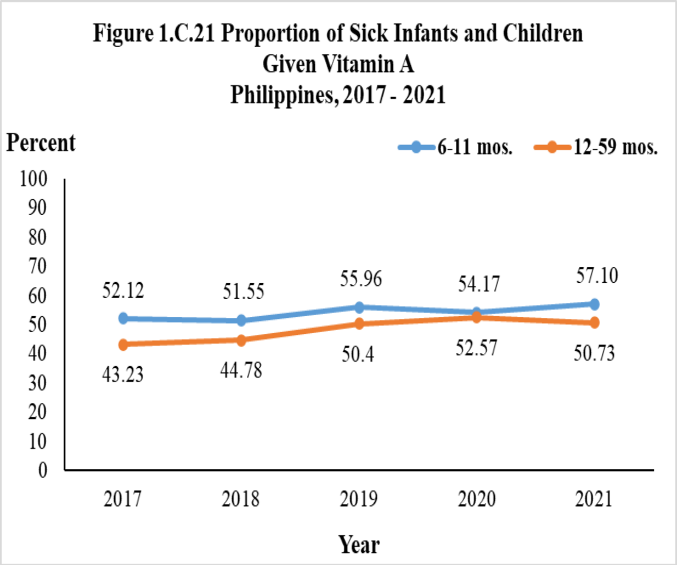

The region with the highest percentage of sick infants aged 6-11 months who were given Vitamin A supplementation was Region 1 at 86.77%, while Region 11 registered highest (91.04%) for the coverage of sick children aged 12-59 months given Vitamin A supplementation. Considering the program target of 100% for both indicators, it should be noted that 14 regions (82%) were below 75% coverage for both the 6-11 months and 12-59 months' age group.

Figure 1.C.22 Proportion of Sick Infants and Children Given Vitamin A by Region
Philippines, 2021

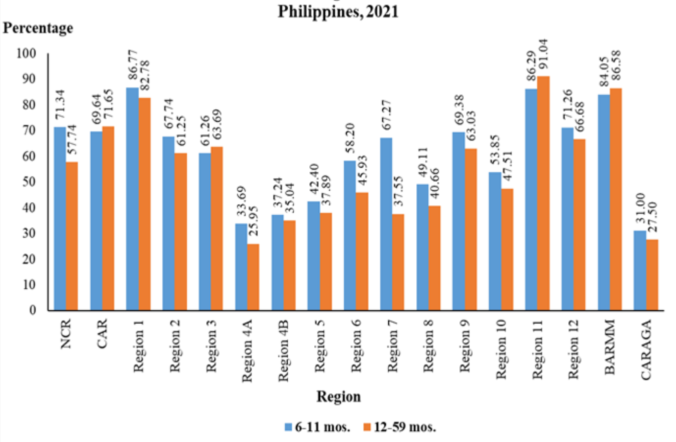

Figure 1.C.23 shows a steady decrease in the proportion of children aged 0-59 months with diarrhea who were given Oral Rehydration Salt Solution (ORS) alone from 2017 to 2020, followed by a slight increase in 2021. Conversely, for the proportion of children aged 0-59 months given ORS with Zinc drops/syrup, an increase was noted from 2018 at 47.28% to 58.79% in 2021.

Figure 1.C.23 Proportion of Children (0-59 mos.) with Diarrhea Given ORS and ORS with Zinc
Philippines, 2017 - 2021

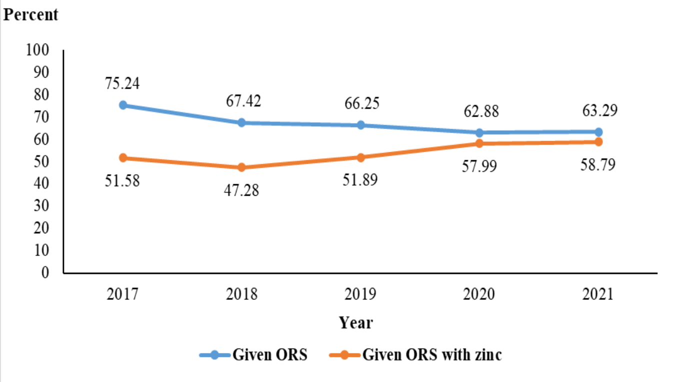

The regions with the highest percentage of children aged 0-59 months old with diarrhea given ORS were Region 10 at 92.18%, followed by Region 4A at 87.78% then Region 8 at 87.71%. For the same population given ORS and Zinc, Region 2 has reported the highest coverage which accounted to 82.66% while the lowest was registered in Region 12 at 34.48%.

Figure 1.C.24 Proportion of Children (0-59 mos.) with Diarrhea Given ORS and ORS with Zinc by Region
Philippines, 2021

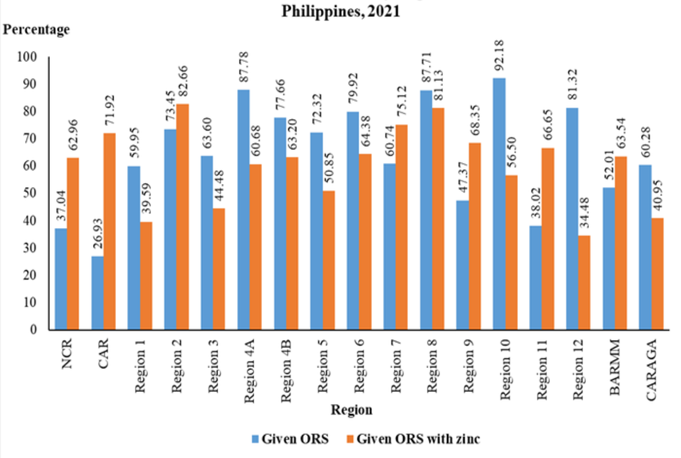

Figure 1.C.25 shows that in 2021, the proportion of children aged 0-59 months with Pneumonia who received treatment was at 96.29%. From 2018 to 2021, a steady coverage at 96% was noted. Further, the coverage for the 5-year period remained at more than 95%.

Figure 1.C.25 Proportion of Children (0-59 mos.) with Pneumonia who Received Treatment
Philippines, 2017 - 2021

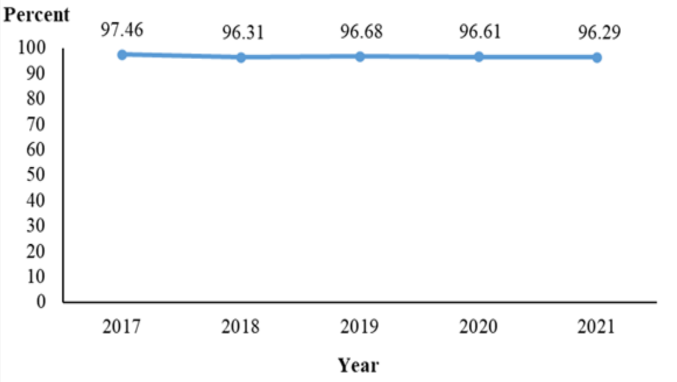

The Region with the highest reported proportion of children 0-59 months old with Pneumonia who were treated was Region 2 at 98.59%. Moreover, all regions registered a coverage of at least 90% which was close to the program target of 100%.

Figure 1.C.26 Proportion of Children (0-59 mos.) with Pneumonia who Received Treatment by Region Philippines, 2021

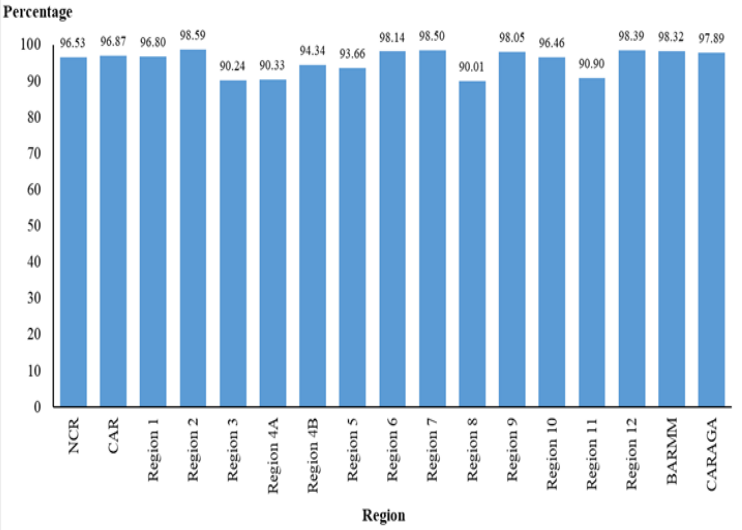

Table 1.C.1.1 - Immunization Services for Infants and Children

Number and Proportion of Children Protected at Birth (CPAB), Newborn/Infants Given BCG & Hepatitis B1 Vaccines
Philippines, 2021

Area	Eligible Pop (Under 1 y.o.)	Child Protected at Birth (CPAB)				BCG				Hepa B1 (w/in 24 Hrs.) after birth			
		Male	Female	Total	%	Male	Female	Total	%	Male	Female	Total	%
**PHILIPPINES**	**2,128,168**	**575,503**	**569,142**	**1,144,645**	**53.79**	**637,424**	**616,263**	**1,253,687**	**58.91**	**527,919**	**514,346**	**1,042,265**	**48.97**
**N C R**	**237,921**	**70,996**	**72,532**	**143,528**	**60.33**	**89,054**	**88,014**	**177,068**	**74.42**	**80,228**	**79,160**	**159,388**	**66.99**
Malabon	6,757	2670	2761	5,431	80.38	2,492	2,534	5,026	74.38	2,547	2,352	4,899	72.50
Navotas	4,607	1566	1536	3,102	67.33	1,717	1,671	3,388	73.54	1,655	1,614	3,269	70.96
Valenzuela City	11,465	2465	2632	5,097	44.46	2,159	2,213	4,372	38.13	1,195	1,251	2,446	21.33
Caloocan City	29,270	6949	7783	14,732	50.33	9,706	9,495	19,201	65.60	8,098	7,854	15,952	54.50
Marikina City	8,330	2595	2313	4,908	58.92	4,071	3,870	7,941	95.33	3,817	3,773	7,590	91.12
Pasig City	13,954	2854	2692	5,546	39.74	5,203	5,006	10,209	73.16	3,646	3,382	7,028	50.37
Pateros	1,181	368	344	712	60.29	391	368	759	64.27	376	350	726	61.47
Taguig	14,874	2665	2866	5,531	37.19	3,266	3,307	6,573	44.19	2,638	2,652	5,290	35.57
Quezon City	54,246	23386	23591	46,977	86.60	24,558	24,540	49,098	90.51	22,940	23,062	46,002	84.80
Makati City	10,765	2385	2237	4,622	42.94	3,620	3,261	6,881	63.92	3,509	3,176	6,685	62.10
Mandaluyong City	7,137	831	926	1,757	24.62	1,618	1,623	3,241	45.41	1,388	1,254	2,642	37.02
San Juan	2,254	667	697	1,364	60.51	404	380	784	34.78	403	374	777	34.47
Manila City	32,887	11031	12050	23,081	70.18	16,105	16,781	32,886	100.00	16,067	16,819	32,886	100.00
Las Piñas City	10,879	2474	2523	4,997	45.93	3,231	3,138	6,369	58.54	2,512	2,398	4,910	45.13
Muntinlupa City	9,317	2894	2832	5,726	61.46	3,298	3,121	6,419	68.90	2,840	2,693	5,533	59.39
Parañaque City	12,298	3164	2738	5,902	47.99	3,754	3,300	7,054	57.36	3,334	2,956	6,290	51.15
Pasay City	7,700	2032	2011	4,043	52.51	3,461	3,406	6,867	89.18	3,263	3,200	6,463	83.94
**C A R**	**35,143**	**8,604**	**8,640**	**17,244**	**49.07**	**10,510**	**10,450**	**20,960**	**59.64**	**9,845**	**9,870**	**19,715**	**56.10**
Abra	4,287	815	975	1,790	41.75	912	1,240	2,152	50.20	868	1,195	2,063	48.12
Apayao	2,476	678	731	1,409	56.91	487	520	1,007	40.67	410	463	873	35.26
Benguet	9,140	2,395	2,380	4,775	52.24	2,332	2,156	4,488	49.10	2,261	2,086	4,347	47.56
Ifugao	4,439	1,018	973	1,991	44.85	1,457	1,363	2,820	63.53	1,214	1,117	2,331	52.51
Kalinga	4,660	1,507	1,377	2,884	61.89	1,170	1,179	2,349	50.41	1,018	1,041	2,059	44.18
Mt. Province	3,066	892	872	1,764	57.53	1,170	1,227	2,397	78.18	1,141	1,267	2,408	78.54
Baguio City	7,075	1,299	1,332	2,631	37.19	2,982	2,765	5,747	81.23	2,933	2,701	5,634	79.63
**Region 1**	**97,198**	**27,744**	**26,545**	**54,289**	**55.85**	**26,313**	**25,087**	**51,400**	**52.88**	**24,216**	**23,215**	**47,431**	**48.80**
Ilocos Norte	7,986	2,184	2,153	4,337	54.31	2,216	2,179	4,395	55.03	2,225	2,178	4,403	55.13
Ilocos Sur	9,197	3,554	3,487	7,041	76.56	3,659	3,556	7,215	78.45	3,638	3,542	7,180	78.07
La Union	11,429	3,207	3,109	6,316	55.26	3,247	3,120	6,367	55.71	3,238	3,147	6,385	55.87
Pangasinan	50,454	12,638	12,000	24,638	48.83	10,452	9,975	20,427	40.49	8,594	8,271	16,865	33.43
Alaminos City	1,905	685	570	1,255	65.88	685	570	1,255	65.88	685	570	1,255	65.88
Candon City	976	554	521	1,075	110.14	554	521	1,075	110.14	554	521	1,075	110.14
Dagupan City	3,636	959	964	1,923	52.89	1,266	1,221	2,487	68.40	1,211	1,198	2,409	66.25
Laoag City	1,845	960	1,005	1,965	106.50	960	1,005	1,965	106.50	960	1,005	1,965	106.50
San Carlos City	3,999	1,116	975	2,091	52.29	1,401	1,231	2,632	65.82	1,312	1,146	2,458	61.47
San Fernando City	2,092	633	566	1,199	57.31	633	566	1,199	57.31	633	566	1,199	57.31
Urdaneta City	2,819	791	752	1,543	54.74	791	752	1,543	54.74	791	752	1,543	54.74
Vigan City	860	463	443	906	105.35	449	391	840	97.67	375	319	694	80.70
**Region 2**	**69,228**	**20,894**	**20,149**	**41,043**	**59.29**	**17,640**	**16,666**	**34,306**	**49.56**	**17,233**	**16,147**	**33,380**	**48.22**
Batanes	351	125	141	266	75.78	121	115	236	67.24	125	142	267	76.07
Cagayan	20,563	5,212	4,941	10,153	49.38	4,066	3,816	7,882	38.33	3,924	3,643	7,567	36.80
Isabela	23,543	6,923	6,665	13,588	57.72	6,381	6,126	12,507	53.12	6,284	5,985	12,269	52.11
Nueva Vizcaya	9,555	2,780	2,796	5,576	58.36	1,326	1,287	2,613	27.35	1,229	1,146	2,375	24.86
Quirino	4,038	1,454	1,462	2,916	72.21	1,465	1,470	2,935	72.68	1,429	1,450	2,879	71.30
Cauayan City	2,576	824	790	1,614	62.66	847	798	1,645	63.86	858	775	1,633	63.39
Ilagan City	2,901	1,492	1,389	2,881	99.31	1,396	1,246	2,642	91.07	1,395	1,245	2,640	91.00
Santiago City	2,683	1,393	1,290	2,683	100.00	1,393	1,290	2,683	100.00	1,393	1,290	2,683	100.00
Tuguegarao City	3,018	691	675	1,366	45.26	645	518	1,163	38.54	596	471	1,067	35.35
**Region 3**	**220,202**	**59,764**	**59,266**	**119,030**	**54.05**	**64,394**	**63,211**	**127,605**	**57.95**	**48,534**	**48,323**	**96,857**	**43.99**
Aurora	4,764	1,147	1,078	2,225	46.70	1,559	1,408	2,967	62.28	1,330	1,232	2,562	53.78
Bataan	13,814	4,068	3,875	7,943	57.50	1,961	1,882	3,843	27.82	1,802	1,715	3,517	25.46
Bulacan	43,722	11,043	10,678	21,721	49.68	16,109	15,384	31,493	72.03	12,221	12,303	24,524	56.09
Nueva Ecija	28,915	5,802	5,600	11,402	39.43	4,417	4,318	8,735	30.21	2,452	2,353	4,805	16.62

Table 1.C.1.1 - Immunization Services for Infants and Children
Number and Proportion of Children Protected at Birth (CPAB), Newborn/Infants Given BCG & Hepatitis B1 Vaccines
Philippines, 2021

Area	Eligible Pop (Under 1 y.o.)	Child Protected at Birth (CPAB)				BCG				Hepa B1 (w/in 24 Hrs.) after birth			
		Male	Female	Total	%	Male	Female	Total	%	Male	Female	Total	%
Pampanga	31,503	9,956	9,845	19,801	62.85	12,126	12,434	24,560	77.96	11,481	11,785	23,266	73.85
Tarlac	19,565	5,660	5,702	11,362	58.07	4,995	5,020	10,015	51.19	3,362	3,539	6,901	35.27
Zambales	12,551	2,132	2,205	4,337	34.56	2,301	2,242	4,543	36.20	789	815	1,604	12.78
Angeles City	7,905	1,759	1,902	3,661	46.31	2,278	2,428	4,706	59.53	802	836	1,638	20.72
Balanga City	1,996	623	603	1,226	61.42	2,574	2,319	4,893	245.14	2,348	2,060	4,408	220.84
Cabanatuan City	5,921	2,282	2,262	4,544	76.74	2,263	2,198	4,461	75.34	1,858	1,810	3,668	61.95
City of San Fernando	5,890	1,444	1,365	2,809	47.69	1,002	948	1,950	33.11	1,009	967	1,976	33.55
Gapan City	2,159	587	567	1,154	53.45	609	616	1,225	56.74	288	275	563	26.08
Mabalacat City	4,811	1,955	1,906	3,861	80.25	1,363	1,310	2,673	55.56	1,460	1,371	2,831	58.84
Malolos City	4,885	809	780	1,589	32.53	2,147	1,991	4,138	84.71	1,557	1,398	2,955	60.49
Meycauayan	4,050	1,509	1,684	3,193	78.84	1,659	1,772	3,431	84.72	1,341	1,525	2,866	70.77
Olongapo	4,952	1,398	1,465	2,863	57.82	556	529	1,085	21.91	252	268	520	10.50
Palayan City	805	244	219	463	57.52	131	124	255	31.68	48	31	79	9.81
San Jose City	2,736	946	828	1,774	64.84	1,008	862	1,870	68.35	930	801	1,731	63.27
San Jose del Monte City	11,120	3,349	3,518	6,867	61.75	3,103	3,202	6,305	56.70	1,588	1,624	3,212	28.88
Science City of Munoz	1,594	528	556	1,084	68.01	258	271	529	33.19	13	19	32	2.01
Tarlac City	6,544	2,523	2,628	5,151	78.71	1,975	1,953	3,928	60.02	1,603	1,596	3,199	48.88
**Region 4A**	**298,398**	**69,807**	**67,422**	**137,229**	**45.99**	**85,637**	**81,377**	**167,014**	**55.97**	**73,084**	**69,340**	**142,424**	**47.73**
Batangas	38,848	10,270	9,858	20,128	51.81	11,344	10,707	22,051	56.76	10,531	9,951	20,482	52.72
Cavite	27,948	6,844	6,613	13,457	48.15	7,920	7,638	15,558	55.67	7,258	6,980	14,238	50.94
Laguna	19,727	2,992	2,871	5,863	29.72	3,749	3,488	7,237	36.69	2,456	2,312	4,768	24.17
Quezon	37,644	7,659	7,422	15,081	40.06	9,011	8,618	17,629	46.83	7,130	6,898	14,028	37.26
Rizal	44,826	8,600	8,324	16,924	37.75	11,198	10,600	21,798	48.63	8,345	7,884	16,229	36.20
Antipolo City	16,510	3,176	3,166	6,342	38.41	4,875	4,502	9,377	56.80	2,981	2,892	5,873	35.57
Bacoor City	12,252	2,490	2,323	4,813	39.28	3,374	3,040	6,414	52.35	2,538	2,307	4,845	39.54
Batangas City	6,895	1,688	1,631	3,319	48.14	2,160	2,118	4,278	62.04	2,106	2,048	4,154	60.25
Biñan City	6,607	3,251	3,232	6,483	98.12	3,474	3,484	6,958	105.31	3,207	3,195	6,402	96.90
Cabuyao City	6,128	2,255	2,379	4,634	75.62	2,460	2,459	4,919	80.27	1,972	2,018	3,990	65.11
Calamba City	9,025	4,033	4,032	8,065	89.36	4,030	4,012	8,042	89.11	3,875	3,897	7,772	86.12
Cavite City	2,098	142	142	284	13.54	228	211	439	20.92	189	174	363	17.30
Dasmariñas City	13,442	2,623	2,422	5,045	37.53	3,522	3,237	6,759	50.28	3,852	3,033	6,885	51.22
General Trias City	6,410	1,043	1,065	2,108	32.89	1,729	1,699	3,428	53.48	1,600	1,552	3,152	49.17
Imus City	8,235	1,342	1,288	2,630	31.94	2,229	2,094	4,323	52.50	1,967	1,959	3,926	47.67
Lipa City	6,954	1,987	1,855	3,842	55.25	2,163	2,083	4,246	61.06	1,666	1,609	3,275	47.10
Lucena City	5,704	1,100	1,058	2,158	37.83	1,234	1,173	2,407	42.20	1,184	1,134	2,318	40.64
San Pablo City	5,277	1,415	1,366	2,781	52.70	1,941	1,903	3,844	72.84	1,862	1,840	3,702	70.15
San Pedro City	6,465	1,157	1,103	2,260	34.96	1,743	1,697	3,440	53.21	1,566	1,438	3,004	46.47
Santa Rosa City	7,023	2,633	2,370	5,003	71.24	2,876	2,594	5,470	77.89	2,713	2,441	5,154	73.39
Tagaytay City	1,451	632	568	1,200	82.70	628	566	1,194	82.29	623	566	1,189	81.94
Tanauan City	3,620	848	816	1,664	45.97	1,606	1,477	3,083	85.17	1,371	1,283	2,654	73.31
Tayabas City	2,134	722	612	1,334	62.51	836	690	1,526	71.51	833	691	1,524	71.42
Trece Martires City	3,175	905	906	1,811	57.04	1,307	1,287	2,594	81.70	1,259	1,238	2,497	78.65
**Region 4B**	**72,035**	**17,374**	**16,443**	**33,817**	**46.95**	**18,559**	**17,804**	**36,363**	**50.48**	**13,556**	**12,906**	**26,462**	**36.73**
Marinduque	4,906	1,369	1,273	2,642	53.85	1,372	1,275	2,647	53.95	1,339	1,263	2,602	53.04
Mindoro Occidental	12,449	3,508	3,299	6,807	54.68	4,042	3,763	7,805	62.70	2,877	2,651	5,528	44.41
Mindoro Oriental	20,011	3,949	3,775	7,724	38.60	4,445	4,048	8,493	42.44	3,238	3,007	6,245	31.21
Palawan	21,676	5,415	5,140	10,555	48.69	5,534	5,637	11,171	51.54	3,615	3,610	7,225	33.33
Romblon	6,482	1,880	1,812	3,692	56.96	1,778	1,709	3,487	53.80	1,795	1,764	3,559	54.91
Puerto Princesa City	6,511	1,253	1,144	2,397	36.81	1,388	1,372	2,760	42.39	692	611	1,303	20.01
**Region 5**	**137,350**	**29,052**	**28,697**	**57,749**	**42.05**	**32,687**	**31,271**	**63,958**	**46.57**	**26,522**	**25,876**	**52,398**	**38.15**
Albay	23,903	5,303	5,158	10,461	43.76	4,805	4,620	9,425	39.43	4,166	4,066	8,232	34.44
Camarines Norte	14,340	3,381	3,411	6,792	47.36	4,275	3,954	8,229	57.38	4,097	3,769	7,866	54.85
Camarines Sur	39,151	5,175	5,090	10,265	26.22	7,501	7,300	14,801	37.80	5,205	5,017	10,222	26.11
Catanduanes	6,381	1,634	1,585	3,219	50.45	1,610	1,587	3,197	50.10	1,574	1,560	3,134	49.11
Masbate	22,565	5,815	6,010	11,825	52.40	7,039	6,723	13,762	60.99	4,347	4,649	8,996	39.87
Sorsogon	19,470	4,840	4,669	9,509	48.84	4,920	4,718	9,638	49.50	4,847	4,703	9,550	49.05
Iriga City	2,665	797	758	1,555	58.35	365	353	718	26.94	196	196	392	14.71
Legaspi City	4,205	1,167	1,091	2,258	53.70	1,093	1,004	2,097	49.87	1,062	964	2,026	48.18
Naga City	4,670	940	925	1,865	39.94	1,079	1,012	2,091	44.78	1,028	952	1,980	42.40

Table 1.C.1.1 - Immunization Services for Infants and Children

Number and Proportion of Children Protected at Birth (CPAB), Newborn/Infants Given BCG & Hepatitis B1 Vaccines
Philippines, 2021

Area	Eligible Pop (Under 1 y.o.)	Child Protected at Birth (CPAB)				BCG				Hepa B1 (w/in 24 Hrs.) after birth			
		Male	Female	Total	%	Male	Female	Total	%	Male	Female	Total	%
**Region 6**	**146,545**	**38,290**	**37,009**	**75,299**	**51.38**	**36,670**	**34,914**	**71,584**	**48.85**	**33,886**	**32,382**	**66,268**	**45.22**
Aklan	11,231	2,559	2,573	5,132	45.69	2,059	1,871	3,930	34.99	1,357	1,266	2,623	23.35
Antique	12,985	3,148	3,001	6,149	47.35	3,452	3,271	6,723	51.78	2,980	2,951	5,931	45.68
Capiz	13,984	2,929	2,769	5,698	40.75	3,670	3,451	7,121	50.92	3,448	3,236	6,684	47.80
Guimaras	3,085	1,175	1,083	2,258	73.19	607	577	1,184	38.38	643	591	1,234	40.00
Iloilo	36,414	9,581	9,023	18,604	51.09	8,303	8,067	16,370	44.96	9,360	8,570	17,930	49.24
Negros Occidental	49,314	14,355	14,382	28,737	58.27	16,609	15,885	32,494	65.89	14,711	14,480	29,191	59.19
Bacolod City	11,102	2,362	2,181	4,543	40.92	1,014	930	1,944	17.51	957	878	1,835	16.53
Iloilo City	8,430	2,181	1,997	4,178	49.56	956	862	1,818	21.57	430	410	840	9.96
**Region 7**	**162,860**	**42,485**	**42,089**	**84,574**	**51.93**	**50,609**	**48,272**	**98,881**	**60.72**	**38,335**	**36,816**	**75,151**	**46.14**
Bohol	27,637	6,813	6,701	13,514	48.90	7,240	6,854	14,094	51.00	7,852	7,497	15,349	55.54
Cebu	67,046	15,205	14,806	30,011	44.76	18,964	17,636	36,600	54.59	12,095	11,526	23,621	35.23
Negros Oriental	27,922	7,045	7,467	14,512	51.97	10,099	10,402	20,501	73.42	5,924	6,090	12,014	43.03
Siquijor	1,636	540	529	1,069	65.34	611	547	1,158	70.78	589	544	1,133	69.25
Cebu City	21,037	7,918	7,607	15,525	73.80	7,823	7,207	15,030	71.45	7,618	7,109	14,727	70.01
Lapu-Lapu City	9,310	3,834	3,809	7,643	82.09	3,925	3,855	7,780	83.57	3,559	3,415	6,974	74.91
Mandaue City	8,272	1,130	1,170	2,300	27.80	1,947	1,771	3,718	44.95	698	635	1,333	16.11
**Region 8**	**104,016**	**22,826**	**22,588**	**45,414**	**43.66**	**27,502**	**26,511**	**54,013**	**51.93**	**21,993**	**21,275**	**43,268**	**41.60**
Biliran	3,856	1,328	1,239	2,567	66.57	1,337	1,251	2,588	67.12	1,358	1,268	2,626	68.10
Eastern Samar	11,635	3,155	3,168	6,323	54.34	3,166	3,059	6,225	53.50	2,120	2,005	4,125	35.45
Northern Leyte	35,052	8,096	7,719	15,815	45.12	8,883	8,355	17,238	49.18	7,839	7,304	15,143	43.20
Northern Samar	15,655	2,781	2,858	5,639	36.02	4,721	4,545	9,266	59.19	3,160	3,038	6,198	39.59
Southern Leyte	6,556	1,652	1,585	3,237	49.37	1,638	1,571	3,209	48.95	1,453	1,415	2,868	43.75
Western Samar	14,515	2,153	1,982	4,135	28.49	3,493	3,343	6,836	47.10	2,631	2,604	5,235	36.07
Calbayog City	4,474	663	686	1,349	30.15	1,212	1,120	2,332	52.12	562	492	1,054	23.56
Maasin City	1,661	287	352	639	38.47	320	335	655	39.43	117	126	243	14.63
Ormoc City	4,989	1,536	1,529	3,065	61.44	1,532	1,449	2,981	59.75	1,524	1,500	3,024	60.61
Tacloban City	5,623	1,175	1,470	2,645	47.04	1,200	1,483	2,683	47.71	1,229	1,523	2,752	48.94
**Region 9**	**79,552**	**21,957**	**22,381**	**44,338**	**55.73**	**18,920**	**18,732**	**37,652**	**47.33**	**14,820**	**15,193**	**30,013**	**37.73**
Zamboanga del Norte	17,075	4,392	4,338	8,730	51.13	4,905	4,751	9,656	56.55	3,775	3,733	7,508	43.97
Zamboanga del Sur	17,506	3,055	3,259	6,314	36.07	2,896	3,048	5,944	33.95	2,234	2,724	4,958	28.32
Zamboanga Sibugay	14,992	3,202	3,287	6,489	43.28	3,139	3,221	6,360	42.42	2,908	3,000	5,908	39.41
Dapitan City	1,755	757	837	1,594	90.83	750	821	1,571	89.52	744	810	1,554	88.55
Dipolog City	2,794	1,082	958	2,040	73.01	1,020	930	1,950	69.79	982	893	1,875	67.11
Isabela City	2,544	656	618	1,274	50.08	823	788	1,611	63.33	525	472	997	39.19
Pagadian City	4,290	1,238	1,040	2,278	53.10	1,237	1,026	2,263	52.75	1,202	1,011	2,213	51.59
Zamboanga City	18,596	7,575	8,044	15,619	83.99	4,150	4,147	8,297	44.62	2,450	2,550	5,000	26.89
**Region 10**	**100,690**	**32,960**	**33,441**	**66,401**	**65.95**	**35,246**	**34,480**	**69,726**	**69.25**	**27,292**	**27,959**	**55,251**	**54.87**
Bukidnon	23,307	8,168	8,208	16,376	70.26	9,343	8,176	17,519	75.17	6,842	6,927	13,769	59.08
Camiguin	1,854	560	507	1,067	57.55	532	430	962	51.89	553	493	1,046	56.42
Lanao del Norte	14,955	3,516	3,807	7,323	48.97	3,884	4,014	7,898	52.81	2,716	2,764	5,480	36.64
Misamis Occidental	6,413	1,758	1,921	3,679	57.37	1,872	1,996	3,868	60.31	1,662	1,778	3,440	53.64
Misamis Oriental	15,058	5,676	5,809	11,485	76.27	5,686	5,875	11,561	76.78	5,355	5,451	10,806	71.76
Cagayan de Oro City	14,263	5,820	5,606	11,426	80.11	5,370	5,423	10,793	75.67	3,770	3,839	7,609	53.35
El Salvador City	1,061	132	118	250	23.56	401	345	746	70.31	358	328	686	64.66
Gingoog City	2,627	1,129	1,390	2,519	95.89	1,094	1,377	2,471	94.06	1,121	1,294	2,415	91.93
Iligan City	7,575	2,047	1,939	3,986	52.62	2,148	2,044	4,192	55.34	1,800	1,745	3,545	46.80
Malaybalay City	3,882	1,332	1,231	2,563	66.02	1,469	1,324	2,793	71.95	274	237	511	13.16
Oroquieta City	1,389	360	383	743	53.49	335	359	694	49.96	294	304	598	43.05
Ozamis City	2,782	930	961	1,891	67.97	929	965	1,894	68.08	929	965	1,894	68.08
Tangub City	1,234	461	424	885	71.72	428	398	826	66.94	242	450	692	56.08
Valencia City	4,290	1,071	1,137	2,208	51.47	1,755	1,754	3,509	81.79	1,376	1,384	2,760	64.34
**Region 11**	**107,847**	**37,438**	**34,877**	**72,315**	**67.05**	**41,496**	**37,924**	**79,420**	**73.64**	**35,096**	**32,628**	**67,724**	**62.80**
Davao de Oro	15,526	5,759	5,205	10,964	70.62	6,532	5,641	12,173	78.40	5,376	4,689	10,065	64.83
Davao del Norte	21,184	8,722	8,004	16,726	78.96	10,165	9,018	19,183	90.55	9,028	8,288	17,316	81.74
Davao Oriental	13,125	3,932	3,762	7,694	58.62	5,042	4,276	9,318	70.99	3,719	3,611	7,330	55.85

Table 1.C.1.1 - Immunization Services for Infants and Children

Number and Proportion of Children Protected at Birth (CPAB), Newborn/Infants Given BCG & Hepatitis B1 Vaccines
Philippines, 2021

Area	Eligible Pop (Under 1 y.o.)	Child Protected at Birth (CPAB)				BCG				Hepa B1 (w/in 24 Hrs.) after birth			
		Male	Female	Total	%	Male	Female	Total	%	Male	Female	Total	%
Davao del Sur	14,355	4,224	3,935	8,159	56.84	4,105	3,906	8,011	55.81	4,036	3,773	7,809	54.40
Davao Occidental	6,598	1,377	1,324	2,701	40.94	1,480	1,395	2,875	43.57	461	423	884	13.40
Davao City	37,059	13,424	12,647	26,071	70.35	14,172	13,688	27,860	75.18	12,476	11,844	24,320	65.63
**Region 12**	**104,693**	**30,481**	**29,713**	**60,194**	**57.50**	**31,942**	**30,770**	**62,712**	**59.90**	**27,099**	**26,189**	**53,288**	**50.90**
North Cotabato	33,933	8,289	8,530	16,819	49.57	8,594	8,612	17,206	50.71	6,818	6,896	13,714	40.41
Sarangani	12,904	4,380	4,139	8,519	66.02	4,877	4,615	9,492	73.56	4,288	4,101	8,389	65.01
South Cotabato	21,039	8,044	7,861	15,905	75.60	8,063	7,677	15,740	74.81	7,594	7,212	14,806	70.37
Sultan Kudarat	17,331	5,951	5,411	11,362	65.56	6,156	5,707	11,863	68.45	5,569	5,123	10,692	61.69
Cotabato City	5,831	1,455	1,548	3,003	51.50	1,695	1,769	3,464	59.41	900	973	1,873	32.12
Gen. Santos City	13,655	2,362	2,224	4,586	33.58	2,557	2,390	4,947	36.23	1,930	1,884	3,814	27.93
**BARMM**	**94,148**	**28,753**	**30,613**	**59,366**	**63.06**	**31,914**	**33,063**	**64,977**	**69.02**	**23,886**	**24,797**	**48,683**	**51.71**
Basilan	7,672	1,079	1,093	2,172	28.31	1,526	1,554	3,080	40.15	706	741	1,447	18.86
Lanao del Sur	21,391	8,385	9,025	17,410	81.39	8,521	9,077	17,598	82.27	7,749	8,285	16,034	74.96
Maguindanao	31,650	9,990	10,426	20,416	64.51	11,441	11,610	23,051	72.83	7,302	7,459	14,761	46.64
Sulu	16,891	4,187	4,470	8,657	51.25	4,947	5,336	10,283	60.88	3,435	3,696	7,131	42.22
Tawi-Tawi	9,319	2,738	2,780	5,518	59.21	2,895	2,932	5,827	62.53	2,453	2,478	4,931	52.91
Lamitan City	2,112	531	559	1,090	51.61	566	549	1,115	52.79	652	537	1,189	56.30
Marawi City	5,113	1,843	2,260	4,103	80.25	2,018	2,005	4,023	78.68	1,589	1,601	3,190	62.39
**CARAGA**	**60,342**	**16,078**	**16,737**	**32,815**	**54.38**	**18,331**	**17,717**	**36,048**	**59.74**	**12,294**	**12,270**	**24,564**	**40.71**
Agusan del Norte	8,116	1,893	1,873	3,766	46.40	2,370	2,306	4,676	57.61	836	906	1,742	21.46
Agusan del Sur	17,658	4,872	5,109	9,981	56.52	6,041	5,943	11,984	67.87	3,987	3,916	7,903	44.76
Surigao del Norte	7,195	1,769	1,793	3,562	49.51	2,165	2,078	4,243	58.97	1,345	1,469	2,814	39.11
Surigao del Sur	11,532	2,944	3,024	5,968	51.75	3,053	2,973	6,026	52.25	2,627	2,610	5,237	45.41
Province of Dinagat	2,574	468	530	998	38.77	542	533	1,075	41.76	381	412	793	30.81
Bislig City	2,185	681	607	1,288	58.95	700	621	1,321	60.46	670	599	1,269	58.08
Butuan City	7,732	2,524	2,835	5,359	69.31	2,623	2,494	5,117	66.18	2,128	2,045	4,173	53.97
Surigao City	3,350	927	966	1,893	56.51	837	769	1,606	47.94	320	313	633	18.90

Note: Put asterisk (*) for No Report and Zero (0) for No Case

Table 1.C.1.2 - Immunization Services for Infants

Number and Proportion of Infants who completed 3 doses of DPT-HiB-HepB antigen

Philippines, 2021

Area	Eligible Pop (Under 1 y.o.)	DPT-HiB-HepB 1				DPT-HiB-HepB 2				DPT-HiB-HepB 3			
		Male	Female	Total	%	Male	Female	Total	%	Male	Female	Total	%
**PHILIPPINES**	**2,128,168**	**763,301**	**728,298**	**1,491,599**	**70.09**	**753,496**	**720,727**	**1,474,223**	**69.27**	**742,358**	**712,092**	**1,454,450**	**68.34**
**N C R**	**237,921**	**88,832**	**86,514**	**175,346**	**73.70**	**85,149**	**83,931**	**169,080**	**71.07**	**82,351**	**81,562**	**163,913**	**68.89**
Malabon	6,757	3018	2953	5,971	88.37	2,770	2,718	5,488	81.22	2,590	2,495	5,085	75.26
Navotas	4,607	2189	2050	4,239	92.01	2,079	2,028	4,107	89.15	1,988	1,975	3,963	86.02
Valenzuela City	11,465	4323	4101	8,424	73.48	4,450	4,223	8,673	75.65	4,470	4,224	8,694	75.83
Caloocan City	29,270	9698	9465	19,163	65.47	8,773	8,279	17,052	58.26	7,568	7,307	14,875	50.82
Marikina City	8,330	3319	2959	6,278	75.37	3,282	2,808	6,090	73.11	3,116	2,777	5,893	70.74
Pasig City	13,954	4647	4327	8,974	64.31	4,589	4,291	8,880	63.64	4,397	4,224	8,621	61.78
Pateros	1,181	511	484	995	84.25	529	505	1,034	87.55	540	525	1,065	90.18
Taguig	14,874	5182	4831	10,013	67.32	5,271	5,002	10,273	69.07	5,116	4,803	9,919	66.69
Quezon City	54,246	23367	23120	46,487	85.70	22,204	22,043	44,247	81.57	21,880	22,108	43,988	81.09
Makati City	10,765	3134	2719	5,853	54.37	3,074	2,743	5,817	54.04	3,141	2,801	5,942	55.20
Mandaluyong City	7,137	2545	2490	5,035	70.55	2,459	2,365	4,824	67.59	2,381	2,504	4,885	68.45
San Juan	2,254	450	436	886	39.31	450	449	899	39.88	509	469	978	43.39
Manila City	32,887	11436	12488	23,924	72.75	10,796	12,222	23,018	69.99	10,450	11,692	22,142	67.33
Las Piñas City	10,879	3934	3770	7,704	70.82	3,761	3,653	7,414	68.15	3,694	3,529	7,223	66.39
Muntinlupa City	9,317	3624	3367	6,991	75.03	3,633	3,420	7,053	75.70	3,703	3,495	7,198	77.26
Parañaque City	12,298	4106	3743	7,849	63.82	3,911	3,528	7,439	60.49	3,777	3,486	7,263	59.06
Pasay City	7,700	3349	3211	6,560	85.19	3,118	3,654	6,772	87.95	3,031	3,148	6,179	80.25
**C A R**	**35,143**	**11,543**	**10,778**	**22,321**	**63.51**	**11,597**	**11,011**	**22,608**	**64.33**	**11,696**	**11,104**	**22,800**	**64.88**
Abra	4,287	1,255	1,252	2,507	58.48	1,284	1,269	2,553	59.55	1,357	1,317	2,674	62.37
Apayao	2,476	868	795	1,663	67.16	831	807	1,638	66.16	806	823	1,629	65.79
Benguet	9,140	2,882	2,655	5,537	60.58	2,941	2,720	5,661	61.94	2,993	2,787	5,780	63.24
Ifugao	4,439	1,626	1,386	3,012	67.85	1,631	1,456	3,087	69.54	1,603	1,405	3,008	67.76
Kalinga	4,660	1,749	1,659	3,408	73.13	1,766	1,683	3,449	74.01	1,794	1,711	3,505	75.21
Mt. Province	3,066	1,031	981	2,012	65.62	1,061	973	2,034	66.34	1,066	1,004	2,070	67.51
Baguio City	7,075	2,132	2,050	4,182	59.11	2,083	2,103	4,186	59.17	2,077	2,057	4,134	58.43
**Region 1**	**97,198**	**34,669**	**32,302**	**66,971**	**68.90**	**35,304**	**33,564**	**68,868**	**70.85**	**35,824**	**34,007**	**69,831**	**71.84**
Ilocos Norte	7,986	2,543	2,457	5,000	62.61	2,530	2,486	5,016	62.81	2,608	2,436	5,044	63.16
Ilocos Sur	9,197	3,502	3,347	6,849	74.47	3,654	3,417	7,071	76.88	3,522	3,343	6,865	74.64
La Union	11,429	3,942	3,559	7,501	65.63	4,118	3,893	8,011	70.09	4,115	3,901	8,016	70.14
Pangasinan	50,454	17,814	16,557	34,371	68.12	18,036	17,255	35,291	69.95	18,611	17,570	36,181	71.71
Alaminos City	1,905	723	656	1,379	72.39	741	623	1,364	71.60	708	660	1,368	71.81
Candon City	976	451	445	896	91.80	439	428	867	88.83	416	395	811	83.09
Dagupan City	3,636	1,231	1,184	2,415	66.42	1,288	1,261	2,549	70.10	1,299	1,315	2,614	71.89
Laoag City	1,845	965	987	1,952	105.80	966	989	1,955	105.96	964	999	1,963	106.40
San Carlos City	3,999	1,532	1,357	2,889	72.24	1,495	1,331	2,826	70.67	1,456	1,311	2,767	69.19
San Fernando City	2,092	672	582	1,254	59.94	680	614	1,294	61.85	687	673	1,360	65.01
Urdaneta City	2,819	979	901	1,880	66.69	1,050	972	2,022	71.73	995	991	1,986	70.45
Vigan City	860	315	270	585	68.02	307	295	602	70.00	443	413	856	99.53
**Region 2**	**69,228**	**24,540**	**23,709**	**48,249**	**69.70**	**24,730**	**23,421**	**48,151**	**69.55**	**24,585**	**23,195**	**47,780**	**69.02**
Batanes	351	134	114	248	70.66	133	118	251	71.51	142	106	248	70.66
Cagayan	20,563	6,756	6,174	12,930	62.88	6,818	6,248	13,066	63.54	6,614	6,151	12,765	62.08
Isabela	23,543	8,077	8,383	16,460	69.91	8,102	7,886	15,988	67.91	8,127	7,716	15,843	67.29
Nueva Vizcaya	9,555	3,425	3,229	6,654	69.64	3,415	3,203	6,618	69.26	3,330	3,048	6,378	66.75
Quirino	4,038	1,590	1,539	3,129	77.49	1,638	1,555	3,193	79.07	1,628	1,593	3,221	79.77
Cauayan City	2,576	871	836	1,707	66.27	903	917	1,820	70.65	1,004	985	1,989	77.21
Ilagan City	2,901	1,299	1,261	2,560	88.25	1,302	1,245	2,547	87.80	1,275	1,290	2,565	88.42
Santiago City	2,683	1,340	1,234	2,574	95.94	1,368	1,286	2,654	98.92	1,338	1,315	2,653	98.88
Tuguegarao City	3,018	1,048	939	1,987	65.84	1,051	963	2,014	66.73	1,127	991	2,118	70.18
**Region 3**	**220,202**	**86,042**	**83,060**	**169,102**	**76.79**	**85,614**	**83,052**	**168,666**	**76.60**	**85,740**	**82,831**	**168,571**	**76.55**
Aurora	4,764	1,822	1,666	3,488	73.22	1,840	1,695	3,535	74.20	1,850	1,712	3,562	74.77
Bataan	13,814	5,749	5,104	10,853	78.57	5,759	5,157	10,916	79.02	5,679	5,227	10,906	78.95
Bulacan	43,722	16,952	17,324	34,276	78.40	17,158	17,394	34,552	79.03	16,628	16,865	33,493	76.60
Nueva Ecija	28,915	7,532	7,240	14,772	51.09	7,730	7,409	15,139	52.36	8,021	7,373	15,394	53.24

Table 1.C.1.2 - Immunization Services for Infants

Number and Proportion of Infants who completed 3 doses of DPT-HiB-HepB antigen
Philippines, 2021

Area	Eligible Pop (Under 1 y.o.)	DPT-HiB-HepB 1				DPT-HiB-HepB 2				DPT-HiB-HepB 3			
		Male	Female	Total	%	Male	Female	Total	%	Male	Female	Total	%
Pampanga	31,503	13,094	12,826	25,920	82.28	12,776	12,493	25,269	80.21	12,810	12,630	25,440	80.75
Tarlac	19,565	8,784	8,215	16,999	86.88	8,810	8,363	17,173	87.77	8,929	8,507	17,436	89.12
Zambales	12,551	3,772	3,439	7,211	57.45	3,604	3,386	6,990	55.69	3,430	3,226	6,656	53.03
Angeles City	7,905	3,628	3,598	7,226	91.41	3,618	3,608	7,226	91.41	3,601	3,562	7,163	90.61
Balanga City	1,996	730	683	1,413	70.79	714	684	1,398	70.04	710	699	1,409	70.59
Cabanatuan City	5,921	2,441	2,371	4,812	81.27	2,423	2,307	4,730	79.89	2,429	2,343	4,772	80.59
City of San Fernando	5,890	2,502	2,322	4,824	81.90	2,455	2,258	4,713	80.02	2,482	2,160	4,642	78.81
Gapan City	2,159	912	856	1,768	81.89	905	844	1,749	81.01	894	846	1,740	80.59
Mabalacat City	4,811	2,139	2,017	4,156	86.39	2,075	2,012	4,087	84.95	2,122	2,086	4,208	87.47
Malolos City	4,885	1,582	1,473	3,055	62.54	1,516	1,493	3,009	61.60	1,559	1,520	3,079	63.03
Meycauayan	4,050	1,725	1,657	3,382	83.51	1,675	1,696	3,371	83.23	1,680	1,690	3,370	83.21
Olongapo	4,952	1,590	1,575	3,165	63.91	1,628	1,536	3,164	63.89	1,656	1,537	3,193	64.48
Palayan City	805	324	290	614	76.27	335	293	628	78.01	312	300	612	76.02
San Jose City	2,736	1,234	1,154	2,388	87.28	1,178	1,132	2,310	84.43	1,180	1,125	2,305	84.25
San Jose del Monte City	11,120	5,800	5,732	11,532	103.71	5,764	5,705	11,469	103.14	5,875	5,739	11,614	104.44
Science City of Munoz	1,594	576	544	1,120	70.26	617	581	1,198	75.16	623	594	1,217	76.35
Tarlac City	6,544	3,154	2,974	6,128	93.64	3,034	3,006	6,040	92.30	3,270	3,090	6,360	97.19
**Region 4A**	**298,398**	**107,505**	**100,135**	**207,640**	**69.58**	**105,410**	**99,845**	**205,255**	**68.79**	**104,998**	**98,587**	**203,585**	**68.23**
Batangas	38,848	14,100	12,785	26,885	69.21	13,775	12,847	26,622	68.53	13,466	12,557	26,023	66.99
Cavite	27,948	9,986	9,270	19,256	68.90	9,844	9,237	19,081	68.27	9,681	9,081	18,762	67.13
Laguna	19,727	6,795	6,127	12,922	65.50	6,590	6,262	12,852	65.15	6,469	6,114	12,583	63.79
Quezon	37,644	12,095	11,195	23,290	61.87	11,985	11,388	23,373	62.09	11,797	11,240	23,037	61.20
Rizal	44,826	12,805	12,108	24,913	55.58	12,700	11,852	24,552	54.77	13,499	11,831	25,330	56.51
Antipolo City	16,510	7,753	7,200	14,953	90.57	7,468	7,050	14,518	87.93	7,050	6,615	13,665	82.77
Bacoor City	12,252	4,056	3,846	7,902	64.50	4,008	3,580	7,588	61.93	3,742	3,529	7,271	59.35
Batangas City	6,895	2,297	2,425	4,722	68.48	2,238	2,276	4,514	65.47	2,213	2,180	4,393	63.71
Biñan City	6,607	3,729	3,552	7,281	110.20	3,549	3,620	7,169	108.51	3,626	3,680	7,306	110.58
Cabuyao City	6,128	2,913	2,748	5,661	92.38	2,894	2,778	5,672	92.56	2,987	2,875	5,862	95.66
Calamba City	9,025	4,259	4,088	8,347	92.49	4,273	4,104	8,377	92.82	4,164	4,033	8,197	90.83
Cavite City	2,098	275	225	500	23.83	282	283	565	26.93	284	273	557	26.55
Dasmariñas City	13,442	4,454	4,092	8,546	63.58	4,216	4,038	8,254	61.40	4,249	4,076	8,325	61.93
General Trias City	6,410	2,522	2,467	4,989	77.83	2,517	2,367	4,884	76.19	2,539	2,349	4,888	76.26
Imus City	8,235	2,811	2,556	5,367	65.17	2,663	2,609	5,272	64.02	2,686	2,635	5,321	64.61
Lipa City	6,954	2,853	2,671	5,524	79.44	2,767	2,683	5,450	78.37	2,758	2,620	5,378	77.34
Lucena City	5,704	2,145	1,966	4,111	72.07	2,020	1,934	3,954	69.32	1,972	1,905	3,877	67.97
San Pablo City	5,277	1,892	1,839	3,731	70.70	1,915	1,798	3,713	70.36	1,973	1,861	3,834	72.65
San Pedro City	6,465	1,997	1,933	3,930	60.79	2,036	1,897	3,933	60.84	1,976	1,791	3,767	58.27
Santa Rosa City	7,023	3,558	3,129	6,687	95.22	3,535	3,298	6,833	97.29	3,691	3,399	7,090	100.95
Tagaytay City	1,451	597	579	1,176	81.05	611	576	1,187	81.81	616	623	1,239	85.39
Tanauan City	3,620	1,330	1,198	2,528	69.83	1,244	1,223	2,467	68.15	1,264	1,164	2,428	67.07
Tayabas City	2,134	800	726	1,526	71.51	830	756	1,586	74.32	839	731	1,570	73.57
Trece Martires City	3,175	1,483	1,410	2,893	91.12	1,450	1,389	2,839	89.42	1,457	1,425	2,882	90.77
**Region 4B**	**72,035**	**23,153**	**21,947**	**45,100**	**62.61**	**22,627**	**21,649**	**44,276**	**61.46**	**22,018**	**20,305**	**42,323**	**58.75**
Marinduque	4,906	1,532	1,364	2,896	59.03	1,533	1,421	2,954	60.21	1,532	1,396	2,928	59.68
Mindoro Occidental	12,449	4,587	4,249	8,836	70.98	4,461	4,152	8,613	69.19	4,168	3,843	8,011	64.35
Mindoro Oriental	20,011	5,970	5,643	11,613	58.03	5,912	5,723	11,635	58.14	5,705	5,105	10,810	54.02
Palawan	21,676	6,595	6,438	13,033	60.13	6,400	6,311	12,711	58.64	6,263	6,004	12,267	56.59
Romblon	6,482	2,050	1,960	4,010	61.86	2,098	1,962	4,060	62.63	2,120	1,901	4,021	62.03
Puerto Princesa City	6,511	2,419	2,293	4,712	72.37	2,223	2,080	4,303	66.09	2,230	2,056	4,286	65.83
**Region 5**	**137,350**	**44,479**	**41,559**	**86,038**	**62.64**	**45,047**	**41,534**	**86,581**	**63.04**	**43,582**	**40,955**	**84,537**	**61.55**
Albay	23,903	7,765	7,292	15,057	62.99	8,033	7,334	15,367	64.29	7,606	7,250	14,856	62.15
Camarines Norte	14,340	5,003	4,531	9,534	66.49	4,813	4,458	9,271	64.65	4,645	4,319	8,964	62.51
Camarines Sur	39,151	12,818	11,940	24,758	63.24	12,824	11,910	24,734	63.18	12,382	11,717	24,099	61.55
Catanduanes	6,381	1,904	1,831	3,735	58.53	2,003	1,857	3,860	60.49	1,908	1,775	3,683	57.72
Masbate	22,565	7,368	6,941	14,309	63.41	7,317	6,895	14,212	62.98	7,106	6,600	13,706	60.74
Sorsogon	19,470	5,715	5,342	11,057	56.79	5,982	5,489	11,471	58.92	6,019	5,531	11,550	59.32
Iriga City	2,665	767	689	1,456	54.63	746	710	1,456	54.63	722	667	1,389	52.12
Legaspi City	4,205	1,497	1,492	2,989	71.08	1,688	1,390	3,078	73.20	1,557	1,597	3,154	75.01
Naga City	4,670	1,642	1,501	3,143	67.30	1,641	1,491	3,132	67.07	1,637	1,499	3,136	67.15

Table 1.C.1.2 - Immunization Services for Infants

Number and Proportion of Infants who completed 3 doses of DPT-HiB-HepB antigen
Philippines, 2021

Area	Eligible Pop (Under 1 y.o.)	DPT-HiB-HepB 1				DPT-HiB-HepB 2				DPT-HiB-HepB 3			
		Male	Female	Total	%	Male	Female	Total	%	Male	Female	Total	%
**Region 6**	**146,545**	**51,495**	**48,233**	**99,728**	**68.05**	**51,600**	**49,310**	**100,910**	**68.86**	**51,532**	**48,818**	**100,350**	**68.48**
Aklan	11,231	3,713	3,484	7,197	64.08	3,729	3,482	7,211	64.21	3,777	3,523	7,300	65.00
Antique	12,985	4,140	3,800	7,940	61.15	4,139	3,851	7,990	61.53	4,121	3,820	7,941	61.16
Capiz	13,984	4,695	4,552	9,247	66.13	4,795	4,711	9,506	67.98	4,697	4,663	9,360	66.93
Guimaras	3,085	1,193	1,171	2,364	76.63	1,224	1,203	2,427	78.67	1,202	1,151	2,353	76.27
Iloilo	36,414	12,113	11,208	23,321	64.04	12,511	12,013	24,524	67.35	12,581	11,725	24,306	66.75
Negros Occidental	49,314	18,849	17,857	36,706	74.43	18,664	18,017	36,681	74.38	18,565	17,911	36,476	73.97
Bacolod City	11,102	3,987	3,645	7,632	68.74	3,808	3,459	7,267	65.46	3,780	3,403	7,183	64.70
Iloilo City	8,430	2,805	2,516	5,321	63.12	2,730	2,574	5,304	62.92	2,809	2,622	5,431	64.42
**Region 7**	**162,860**	**61,162**	**58,847**	**120,009**	**73.69**	**61,496**	**58,699**	**120,195**	**73.80**	**61,066**	**58,261**	**119,327**	**73.27**
Bohol	27,637	8,763	8,558	17,321	62.67	9,025	8,605	17,630	63.79	8,762	8,471	17,233	62.35
Cebu	67,046	25,423	24,193	49,616	74.00	25,690	23,998	49,688	74.11	25,909	24,133	50,042	74.64
Negros Oriental	27,922	11,122	11,236	22,358	80.07	10,624	10,957	21,581	77.29	10,121	10,249	20,370	72.95
Siquijor	1,636	636	556	1,192	72.86	645	616	1,261	77.08	675	602	1,277	78.06
Cebu City	21,037	7,922	7,245	15,167	72.10	7,870	7,167	15,037	71.48	8,001	7,391	15,392	73.17
Lapu-Lapu City	9,310	4,472	4,381	8,853	95.09	4,550	4,403	8,953	96.17	4,465	4,442	8,907	95.67
Mandaue City	8,272	2,824	2,678	5,502	66.51	3,092	2,953	6,045	73.08	3,133	2,973	6,106	73.82
**Region 8**	**104,016**	**31,304**	**29,681**	**60,985**	**58.63**	**31,095**	**29,466**	**60,561**	**58.22**	**30,659**	**29,031**	**59,690**	**57.39**
Biliran	3,856	1,308	1,232	2,540	65.87	1,300	1,203	2,503	64.91	1,303	1,215	2,518	65.30
Eastern Samar	11,635	3,359	3,238	6,597	56.70	3,460	3,186	6,646	57.12	3,336	3,106	6,442	55.37
Northern Leyte	35,052	10,005	9,203	19,208	54.80	10,026	9,254	19,280	55.00	9,829	9,118	18,947	54.05
Northern Samar	15,655	4,755	4,624	9,379	59.91	4,670	4,501	9,171	58.58	4,571	4,451	9,022	57.63
Southern Leyte	6,556	1,931	1,722	3,653	55.72	1,906	1,823	3,729	56.88	1,906	1,770	3,676	56.07
Western Samar	14,515	4,354	4,239	8,593	59.20	4,233	4,125	8,358	57.58	4,173	3,972	8,145	56.11
Calbayog City	4,474	1,457	1,410	2,867	64.08	1,403	1,347	2,750	61.47	1,351	1,285	2,636	58.92
Maasin City	1,661	442	457	899	54.12	445	453	898	54.06	473	427	900	54.18
Ormoc City	4,989	1,779	1,682	3,461	69.37	1,715	1,649	3,364	67.43	1,673	1,617	3,290	65.95
Tacloban City	5,623	1,914	1,874	3,788	67.37	1,937	1,925	3,862	68.68	2,044	2,070	4,114	73.16
**Region 9**	**79,552**	**28,747**	**27,177**	**55,924**	**70.30**	**28,300**	**26,986**	**55,286**	**69.50**	**27,716**	**26,326**	**54,042**	**67.93**
Zamboanga del Norte	17,075	6,392	5,816	12,208	71.50	6,445	6,178	12,623	73.93	6,241	5,952	12,193	71.41
Zamboanga del Sur	17,506	5,454	5,165	10,619	60.66	5,344	5,179	10,523	60.11	5,124	4,794	9,918	56.65
Zamboanga Sibugay	14,992	4,295	4,303	8,598	57.35	4,267	4,215	8,482	56.58	4,211	4,086	8,297	55.34
Dapitan City	1,755	779	829	1,608	91.62	777	816	1,593	90.77	767	813	1,580	90.03
Dipolog City	2,794	1,097	1,056	2,153	77.06	1,079	1,005	2,084	74.59	1,053	1,020	2,073	74.19
Isabela City	2,544	973	895	1,868	73.43	1,020	886	1,906	74.92	990	903	1,893	74.41
Pagadian City	4,290	1,755	1,418	3,173	73.96	1,723	1,411	3,134	73.05	1,768	1,422	3,190	74.36
Zamboanga City	18,596	8,002	7,695	15,697	84.41	7,645	7,296	14,941	80.35	7,562	7,336	14,898	80.11
**Region 10**	**100,690**	**40,739**	**39,589**	**80,328**	**79.78**	**39,963**	**39,606**	**79,569**	**79.02**	**38,828**	**38,338**	**77,166**	**76.64**
Bukidnon	23,307	10,167	9,820	19,987	85.76	9,496	9,166	18,662	80.07	9,127	8,779	17,906	76.83
Camiguin	1,854	591	545	1,136	61.27	603	521	1,124	60.63	621	511	1,132	61.06
Lanao del Norte	14,955	4,790	4,768	9,558	63.91	4,733	5,699	10,432	69.76	4,685	4,729	9,414	62.95
Misamis Occidental	6,413	2,632	2,418	5,050	78.75	2,532	2,458	4,990	77.81	2,473	2,404	4,877	76.05
Misamis Oriental	15,058	6,819	6,926	13,745	91.28	6,947	6,983	13,930	92.51	6,771	7,218	13,989	92.90
Cagayan de Oro City	14,263	5,351	5,176	10,527	73.81	5,423	4,999	10,422	73.07	5,303	5,114	10,417	73.04
El Salvador City	1,061	442	405	847	79.83	438	420	858	80.87	409	395	804	75.78
Gingoog City	2,627	1,397	1,530	2,927	111.42	1,441	1,547	2,988	113.74	1,395	1,524	2,919	111.12
Iligan City	7,575	2,671	2,466	5,137	67.82	2,637	2,391	5,028	66.38	2,636	2,453	5,089	67.18
Malaybalay City	3,882	1,884	1,635	3,519	90.65	1,852	1,606	3,458	89.08	1,727	1,575	3,302	85.06
Oroquieta City	1,389	477	471	948	68.25	482	485	967	69.62	471	454	925	66.59
Ozamis City	2,782	1,029	1,026	2,055	73.87	985	1,002	1,987	71.42	991	954	1,945	69.91
Tangub City	1,234	496	438	934	75.69	511	458	969	78.53	518	513	1,031	83.55
Valencia City	4,290	1,993	1,965	3,958	92.26	1,883	1,871	3,754	87.51	1,701	1,715	3,416	79.63
**Region 11**	107,847	39,513	37,878	77,391	71.76	38,710	36,610	75,320	69.84	37,498	35,471	72,969	67.66
Davao de Oro	15,526	6,176	5,670	11,846	76.30	6,045	5,449	11,494	74.03	5,791	5,202	10,993	70.80
Davao del Norte	21,184	8,712	8,643	17,355	81.93	8,706	8,364	17,070	80.58	8,336	8,075	16,411	77.47
Davao Oriental	13,125	4,460	4,286	8,746	66.64	4,464	4,157	8,621	65.68	4,142	3,971	8,113	61.81

Table 1.C.1.2 - Immunization Services for Infants

Number and Proportion of Infants who completed 3 doses of DPT-HiB-HepB antigen
Philippines, 2021

Area	Eligible Pop (Under 1 y.o.)	DPT-HiB-HepB 1				DPT-HiB-HepB 2				DPT-HiB-HepB 3			
		Male	Female	Total	%	Male	Female	Total	%	Male	Female	Total	%
Davao del Sur	14,355	4,774	4,514	9,288	64.70	4,778	4,505	9,283	64.67	4,675	4,269	8,944	62.31
Davao Occidental	6,598	1,570	1,567	3,137	47.54	1,535	1,436	2,971	45.03	1,450	1,356	2,806	42.53
Davao City	37,059	13,821	13,198	27,019	72.91	13,182	12,699	25,881	69.84	13,104	12,598	25,702	69.35
**Region 12**	**104,693**	**35,129**	**32,782**	**67,911**	**64.87**	**33,690**	**31,625**	**65,315**	**62.39**	**32,539**	**31,255**	**63,794**	**60.93**
North Cotabato	33,933	9,547	9,013	18,560	54.70	8,685	8,299	16,984	50.05	8,260	7,969	16,229	47.83
Sarangani	12,904	4,868	4,545	9,413	72.95	4,712	4,329	9,041	70.06	4,641	4,432	9,073	70.31
South Cotabato	21,039	8,175	7,611	15,786	75.03	7,971	7,327	15,298	72.71	7,743	7,459	15,202	72.26
Sultan Kudarat	17,331	6,401	5,782	12,183	70.30	6,279	5,791	12,070	69.64	6,076	5,815	11,891	68.61
Cotabato City	5,831	1,854	1,806	3,660	62.77	1,818	1,781	3,599	61.72	1,766	1,764	3,530	60.54
Gen. Santos City	13,655	4,284	4,025	8,309	60.85	4,225	4,098	8,323	60.95	4,053	3,816	7,869	57.63
**BARMM**	**94,148**	**32,935**	**33,697**	**66,632**	**70.77**	**32,343**	**30,445**	**62,788**	**66.69**	**31,581**	**32,736**	**64,317**	**68.31**
Basilan	7,672	1,692	1,692	3,384	44.11	1,589	1,556	3,145	40.99	1,369	1,427	2,796	36.44
Lanao del Sur	21,391	8,417	8,814	17,231	80.55	8,204	8,873	17,077	79.83	8,015	8,780	16,795	78.51
Maguindanao	31,650	12,299	12,245	24,544	77.55	12,242	9,143	21,385	67.57	12,080	11,886	23,966	75.72
Sulu	16,891	4,940	5,220	10,160	60.15	4,696	5,014	9,710	57.49	4,562	4,757	9,319	55.17
Tawi-Tawi	9,319	2,933	3,057	5,990	64.28	2,936	3,198	6,134	65.82	2,893	3,129	6,022	64.62
Lamitan City	2,112	612	614	1,226	58.05	580	584	1,164	55.11	572	573	1,145	54.21
Marawi City	5,113	2,042	2,055	4,097	80.13	2,096	2,077	4,173	81.62	2,090	2,184	4,274	83.59
**CARAGA**	**60,342**	**21,514**	**20,410**	**41,924**	**69.48**	**20,821**	**19,973**	**40,794**	**67.60**	**20,145**	**19,310**	**39,455**	**65.39**
Agusan del Norte	8,116	2,843	2,633	5,476	67.47	2,793	2,567	5,360	66.04	2,621	2,513	5,134	63.26
Agusan del Sur	17,658	6,423	6,222	12,645	71.61	6,092	6,048	12,140	68.75	5,917	5,760	11,677	66.13
Surigao del Norte	7,195	2,569	2,389	4,958	68.91	2,589	2,419	5,008	69.60	2,429	2,397	4,826	67.07
Surigao del Sur	11,532	4,034	3,822	7,856	68.12	3,885	3,682	7,567	65.62	3,658	3,471	7,129	61.82
Province of Dinagat	2,574	659	651	1,310	50.89	697	657	1,354	52.60	710	671	1,381	53.65
Bislig City	2,185	744	673	1,417	64.85	696	705	1,401	64.12	736	661	1,397	63.94
Butuan City	7,732	2,866	2,744	5,610	72.56	2,738	2,622	5,360	69.32	2,726	2,556	5,282	68.31
Surigao City	3,350	1,376	1,276	2,652	79.16	1,331	1,273	2,604	77.73	1,348	1,281	2,629	78.48

Note: Put asterisk (*) for No Report and Zero (0) for No Case

Table 1.C.1.3 - Immunization Services for Infants and Children

Number and Proportion of Infants who completed 3 doses of Oral Polio Vaccine (OPV) and Inactivated Polio Vaccine (IPV)
Philippines, 2021

Area	Eligible Pop (Under 1 y.o.)	OPV 1				OPV 2				OPV 3				IPV			
		Male	Female	Total	%	Male	Female	Total	%	Male	Female	Total	%	Male	Female	Total	%
**PHILIPPINES**	**2,128,168**	**759,302**	**720,332**	**1,479,634**	**69.53**	**745,874**	**716,339**	**1,462,213**	**68.71**	**736,846**	**711,371**	**1,448,217**	**68.05**	**726,275**	**703,463**	**1,429,738**	**67.18**
**N C R**	**237,921**	**90,691**	**87,986**	**178,677**	**75.10**	**87,418**	**86,146**	**173,564**	**72.95**	**85,689**	**85,517**	**171,206**	**71.96**	**83,311**	**81,829**	**165,140**	**69.41**
Malabon	6,757	3,029	2,906	5,935	87.83	2,743	2,690	5,433	80.41	2,550	2,519	5,069	75.02	2,607	2,534	5,141	76.08
Navotas	4,607	2,174	2,038	4,212	91.43	2,044	2,019	4,063	88.19	1,984	2,143	4,127	89.58	2,017	1,991	4,008	87.00
Valenzuela City	11,465	4,322	4,112	8,434	73.56	4,458	4,148	8,606	75.06	4,414	4,255	8,669	75.61	4,945	4,753	9,698	84.59
Caloocan City	29,270	10,434	9,875	20,309	69.39	9,805	9,466	19,271	65.84	9,260	8,852	18,112	61.88	8,975	8,577	17,552	59.97
Marikina City	8,330	3,345	2,958	6,303	75.67	3,260	2,802	6,062	72.77	3,118	2,765	5,883	70.62	3,051	2,701	5,752	69.05
Pasig City	13,954	4,725	4,319	9,044	64.81	4,596	4,324	8,920	63.92	4,415	4,220	8,635	61.88	4,471	4,210	8,681	62.21
Pateros	1,181	511	484	995	84.25	529	505	1,034	87.55	540	525	1,065	90.18	536	524	1,060	89.75
Taguig	14,874	5,412	5,245	10,657	71.65	5,494	5,318	10,812	72.69	5,502	5,364	10,866	73.05	5,336	4,936	10,272	69.06
Quezon City	54,246	24,266	23,914	48,180	88.82	23,455	23,415	46,870	86.40	23,304	23,183	46,487	85.70	20,836	20,638	41,474	76.46
Makati City	10,765	3,107	2,721	5,828	54.14	3,111	2,752	5,863	54.46	3,126	2,808	5,934	55.12	2,825	2,597	5,422	50.37
Mandaluyong City	7,137	2,543	2,487	5,030	70.48	2,447	2,364	4,811	67.41	2,394	2,493	4,887	68.47	2,544	2,663	5,207	72.96
San Juan	2,254	420	408	828	36.73	429	432	861	38.20	471	438	909	40.33	471	442	913	40.51
Manila City	32,887	11,478	12,542	24,020	73.04	10,797	12,056	22,853	69.49	10,440	11,685	22,125	67.28	10,299	11,463	21,762	66.17
Las Piñas City	10,879	3,914	3,727	7,641	70.24	3,763	3,662	7,425	68.25	3,681	3,500	7,181	66.01	3,617	3,429	7,046	64.77
Muntinlupa City	9,317	3,568	3,345	6,913	74.20	3,579	3,385	6,964	74.75	3,656	3,487	7,143	76.67	3,564	3,401	6,965	74.76
Parañaque City	12,298	4,125	3,727	7,852	63.85	3,882	3,614	7,496	60.95	3,793	3,487	7,280	59.20	4,414	3,954	8,368	68.04
Pasay City	7,700	3,318	3,178	6,496	84.36	3,026	3,194	6,220	80.78	3,041	3,793	6,834	88.75	2,803	3,016	5,819	75.57
**C A R**	**35,143**	**10,546**	**9,974**	**20,520**	**58.39**	**10,733**	**10,216**	**20,949**	**59.61**	**10,680**	**10,223**	**20,903**	**59.48**	**10,136**	**9,701**	**19,837**	**56.45**
Abra	4,287	1,253	1,248	2,501	58.34	1,288	1,274	2,562	59.76	1,359	1,334	2,693	62.82	1,358	1,336	2,694	62.84
Apayao	2,476	833	794	1,627	65.71	792	801	1,593	64.34	739	736	1,475	59.57	760	756	1,516	61.23
Benguet	9,140	2,873	2,674	5,547	60.69	2,978	2,725	5,703	62.40	3,020	2,851	5,871	64.23	2,892	2,705	5,597	61.24
Ifugao	4,439	1,561	1,365	2,926	65.92	1,608	1,454	3,062	68.98	1,568	1,401	2,969	66.88	1,368	1,256	2,624	59.11
Kalinga	4,660	877	871	1,748	37.51	969	876	1,845	39.59	880	869	1,749	37.53	675	666	1,341	28.78
Mt. Province	3,066	1,020	985	2,005	65.39	1,051	987	2,038	66.47	1,039	969	2,008	65.49	1,053	964	2,017	65.79
Baguio City	7,075	2,129	2,037	4,166	58.88	2,047	2,099	4,146	58.60	2,075	2,063	4,138	58.49	2,030	2,018	4,048	57.22
**Region 1**	**97,198**	**34,693**	**32,298**	**66,991**	**68.92**	**35,703**	**33,804**	**69,507**	**71.51**	**36,175**	**34,320**	**70,495**	**72.53**	**35,422**	**33,759**	**69,181**	**71.18**
Ilocos Norte	7,986	2,538	2,463	5,001	62.62	2,531	2,483	5,014	62.78	2,604	2,458	5,062	63.39	2,600	2,462	5,062	63.39
Ilocos Sur	9,197	3,515	3,331	6,846	74.44	3,634	3,397	7,031	76.45	3,523	3,334	6,857	74.56	3,412	3,244	6,656	72.37
La Union	11,429	3,987	3,571	7,558	66.13	4,227	4,001	8,228	71.99	4,148	3,905	8,053	70.46	4,073	3,853	7,926	69.35
Pangasinan	50,454	17,803	16,556	34,359	68.10	18,326	17,376	35,702	70.76	18,825	17,800	36,625	72.59	18,356	17,489	35,845	71.04
Alaminos City	1,905	712	647	1,359	71.34	748	608	1,356	71.18	720	671	1,391	73.02	646	589	1,235	64.83
Candon City	976	451	445	896	91.80	439	428	867	88.83	416	395	811	83.09	416	395	811	83.09
Dagupan City	3,636	1,227	1,194	2,421	66.58	1,289	1,286	2,575	70.82	1,373	1,367	2,740	75.36	1,373	1,367	2,740	75.36
Laoag City	1,845	965	987	1,952	105.80	966	989	1,955	105.96	964	999	1,963	106.40	964	999	1,963	106.40
San Carlos City	3,999	1,527	1,355	2,882	72.07	1,496	1,345	2,841	71.04	1,461	1,324	2,785	69.64	1,448	1,296	2,744	68.62
San Fernando City	2,092	667	584	1,251	59.80	686	625	1,311	62.67	693	660	1,353	64.67	686	658	1,344	64.24
Urdaneta City	2,819	983	897	1,880	66.69	1,050	972	2,022	71.73	995	991	1,986	70.45	995	991	1,986	70.45
Vigan City	860	318	268	586	68.14	311	294	605	70.35	453	416	869	101.05	453	416	869	101.05
**Region 2**	**69,228**	**24,814**	**23,078**	**47,892**	**69.18**	**24,553**	**23,680**	**48,233**	**69.67**	**24,797**	**23,277**	**48,074**	**69.44**	**24,191**	**23,409**	**47,600**	**68.76**
Batanes	351	133	114	247	70.37	133	117	250	71.23	141	106	247	70.37	141	106	247	70.37
Cagayan	20,563	6,674	6,173	12,847	62.48	6,770	6,592	13,362	64.98	6,725	6,190	12,915	62.81	6,549	6,104	12,653	61.53
Isabela	23,543	8,119	7,790	15,909	67.57	8,166	7,913	16,079	68.30	8,209	7,817	16,026	68.07	8,020	8,158	16,178	68.72
Nueva Vizcaya	9,555	3,669	3,093	6,762	70.77	3,158	2,975	6,133	64.19	3,045	2,881	5,926	62.02	2,977	2,837	5,814	60.85
Quirino	4,038	1,593	1,628	3,221	79.77	1,636	1,558	3,194	79.10	1,716	1,549	3,265	80.86	1,649	1,495	3,144	77.86
Cauayan City	2,576	969	885	1,854	71.97	1,089	1,077	2,166	84.08	1,173	1,058	2,231	86.61	1,155	1,007	2,162	83.93
Ilagan City	2,901	1,256	1,226	2,482	85.56	1,197	1,186	2,383	82.14	1,336	1,359	2,695	92.90	1,249	1,404	2,653	91.45
Santiago City	2,683	1,346	1,230	2,576	96.01	1,381	1,297	2,678	99.81	1,338	1,328	2,666	99.37	1,340	1,324	2,664	99.29
Tuguegarao City	3,018	1,055	939	1,994	66.07	1,023	965	1,988	65.87	1,114	989	2,103	69.68	1,111	974	2,085	69.09
**Region 3**	**220,202**	**83,297**	**80,130**	**163,427**	**74.22**	**81,521**	**78,799**	**160,320**	**72.81**	**80,999**	**78,278**	**159,277**	**72.33**	**84,518**	**81,095**	**165,613**	**75.21**
Aurora	4,764	1,669	1,493	3,162	66.37	1,654	1,464	3,118	65.45	1,583	1,458	3,041	63.83	1,692	1,542	3,234	67.88
Bataan	13,814	5,621	4,970	10,591	76.67	5,520	4,921	10,441	75.58	5,308	4,854	10,162	73.56	5,602	5,096	10,698	77.44
Bulacan	43,722	16,443	16,524	32,967	75.40	16,075	16,323	32,398	74.10	15,398	15,600	30,998	70.90	15,807	15,840	31,647	72.38
Nueva Ecija	28,915	7,933	7,715	15,648	54.12	8,000	7,524	15,524	53.69	8,347	7,753	16,100	55.68	8,824	8,165	16,989	58.75
Pampanga	31,503	12,684	12,502	25,186	79.95	12,372	12,107	24,479	77.70	12,232	12,028	24,260	77.01	12,556	12,190	24,746	78.55
Tarlac	19,565	8,620	8,111	16,731	85.51	8,605	8,208	16,813	85.93	8,764	8,259	17,023	87.01	8,742	8,208	16,950	86.63
Zambales	12,551	3,677	3,339	7,016	55.90	3,448	3,281	6,729	53.61	3,388	3,196	6,584	52.46	3,420	3,238	6,658	53.05
Angeles City	7,905	3,167	3,029	6,196	78.38	3,051	3,074	6,125	77.48	2,870	2,968	5,838	73.85	3,321	3,340	6,661	84.26
Balanga City	1,996	724	680	1,404	70.34	722	682	1,404	70.34	713	692	1,405	70.39	712	692	1,404	70.34
Cabanatuan City	5,921	2,235	2,143	4,378	73.94	2,115	2,035	4,150	70.09	2,094	2,006	4,100	69.25	2,335	2,282	4,617	77.98
City of San Fernando	5,890	2,392	2,207	4,599	78.08	2,285	2,073	4,358	73.99	2,150	1,849	3,999	67.89	2,486	2,174	4,660	79.12
Gapan City	2,159	938	897	1,835	84.99	827	769	1,596	73.92	841	762	1,603	74.25	822	732	1,554	71.98
Mabalacat City	4,811	2,050	1,912	3,962	82.35	1,944	1,899	3,843	79.88	1,988	1,945	3,933	81.75	2,103	2,041	4,144	86.14
Malolos City	4,885	1,532	1,427	2,959	60.57	1,473	1,434	2,907	59.51	1,490	1,475	2,965	60.70	1,558	1,527	3,085	63.15
Meycauayan	4,050	1,526	1,486	3,012	74.37	1,581	1,505	3,086	76.20	1,460	1,497	2,957	73.01	1,821	1,867	3,688	91.06
Olongapo	4,952	1,462	1,458	2,920	58.97	1,460	1,405	2,865	57.86	1,514	1,434	2,948	59.53	1,734	1,608	3,342	67.49
Palayan City	805	323	285	608	75.53	343	304	647	80.37	348	291	639	79.38	348	291	639	79.38
San Jose City	2,736	1,142	1,090	2,232	81.58	1,081	1,047	2,128	77.78	1,349	1,325	2,674	97.73	1,108	1,026	2,134	78.00
San Jose del Monte City	11,120	5,596	5,500	11,096	99.78	5,482	5,393	10,875	97.80	5,573	5,439	11,012	99.03	5,769	5,630	11,399	102.51
Science City of Munoz	1,594	557	533	1,090	68.38	591	555	1,146	71.89	603	584	1,187	74.47	587	570	1,157	72.58
Tarlac City	6,544	3,006	2,829	5,835	89.17	2,892	2,796	5,688	86.92	2,986	2,863	5,849	89.38	3,171	3,036	6,207	94.85

Table 1.C.1.3 - Immunization Services for Infants and Children

Number and Proportion of Infants who completed 3 doses of Oral Polio Vaccine (OPV) and Inactivated Polio Vaccine (IPV)
Philippines, 2021

Area	Eligible Pop (Under 1 y.o.)	OPV 1				OPV 2				OPV 3				IPV			
		Male	Female	Total	%	Male	Female	Total	%	Male	Female	Total	%	Male	Female	Total	%
**Region 4A**	**298,398**	**105,561**	**98,027**	**203,588**	**68.23**	**103,062**	**97,592**	**200,654**	**67.24**	**100,591**	**95,828**	**196,419**	**65.82**	**100,695**	**95,316**	**196,011**	**65.69**
Batangas	38,848	13,805	12,668	26,473	68.15	13,723	12,807	26,530	68.29	13,325	12,460	25,785	66.37	13,063	12,205	25,268	65.04
Cavite	27,948	9,945	9,247	19,192	68.67	9,862	9,220	19,082	68.28	9,628	9,069	18,697	66.90	9,736	9,151	18,887	67.58
Laguna	19,727	6,686	6,025	12,711	64.43	6,492	6,215	12,707	64.41	6,411	6,054	12,465	63.19	6,400	6,058	12,458	63.15
Quezon	37,644	11,241	10,549	21,790	57.88	10,821	10,489	21,310	56.61	10,505	10,030	20,535	54.55	10,655	10,175	20,830	55.33
Rizal	44,826	12,869	11,902	24,771	55.26	12,351	11,664	24,015	53.57	11,936	11,544	23,480	52.38	11,612	11,023	22,635	50.50
Antipolo City	16,510	7,863	7,275	15,138	91.69	7,401	6,993	14,394	87.18	7,015	6,504	13,519	81.88	7,176	6,621	13,797	83.57
Bacoor City	12,252	3,997	3,527	7,524	61.41	3,915	3,522	7,437	60.70	3,686	3,486	7,172	58.54	3,682	3,464	7,146	58.33
Batangas City	6,895	2,298	2,269	4,567	66.24	2,235	2,263	4,498	65.24	2,205	2,181	4,386	63.61	2,197	2,172	4,369	63.36
Biñan City	6,607	3,716	3,534	7,250	109.73	3,961	3,593	7,554	114.33	3,616	3,582	7,198	108.95	3,613	3,591	7,204	109.04
Cabuyao City	6,128	2,930	2,796	5,726	93.44	2,905	2,772	5,677	92.64	2,978	2,835	5,813	94.86	3,016	2,856	5,872	95.82
Calamba City	9,025	4,020	3,872	7,892	87.45	3,968	3,740	7,708	85.41	3,814	3,754	7,568	83.86	3,859	3,715	7,574	83.92
Cavite City	2,098	270	225	495	23.59	285	287	572	27.26	284	270	554	26.41	293	307	600	28.60
Dasmariñas City	13,442	4,361	4,021	8,382	62.36	4,012	3,861	7,873	58.57	4,063	3,866	7,929	58.99	4,149	3,996	8,145	60.59
General Trias City	6,410	2,516	2,477	4,993	77.89	2,495	2,367	4,862	75.85	2,443	2,353	4,796	74.82	2,407	2,318	4,725	73.71
Imus City	8,235	2,730	2,533	5,263	63.91	2,638	2,664	5,302	64.38	2,670	2,583	5,253	63.79	2,706	2,606	5,312	64.51
Lipa City	6,954	2,812	2,567	5,379	77.35	2,655	2,569	5,224	75.12	2,591	2,503	5,094	73.25	2,610	2,445	5,055	72.69
Lucena City	5,704	2,071	1,883	3,954	69.32	1,943	1,854	3,797	66.57	1,902	1,853	3,755	65.83	1,992	1,939	3,931	68.92
San Pablo City	5,277	1,888	1,812	3,700	70.12	1,885	1,815	3,700	70.12	1,907	1,831	3,738	70.84	2,005	1,924	3,929	74.46
San Pedro City	6,465	1,923	1,859	3,782	58.50	1,959	1,820	3,779	58.45	1,857	1,727	3,584	55.44	1,899	1,737	3,636	56.24
Santa Rosa City	7,023	3,500	3,102	6,602	94.01	3,506	3,216	6,722	95.71	3,677	3,429	7,106	101.18	3,470	3,091	6,561	93.42
Tagaytay City	1,451	597	584	1,181	81.39	617	584	1,201	82.77	608	628	1,236	85.18	565	558	1,123	77.39
Tanauan City	3,620	1,243	1,141	2,384	65.86	1,170	1,139	2,309	63.78	1,174	1,117	2,291	63.29	1,296	1,241	2,537	70.08
Tayabas City	2,134	808	725	1,533	71.84	814	740	1,554	72.82	826	736	1,562	73.20	832	735	1,567	73.43
Trece Martires City	3,175	1,472	1,434	2,906	91.53	1,449	1,398	2,847	89.67	1,470	1,433	2,903	91.43	1,462	1,388	2,850	89.76
**Region 4B**	**72,035**	**23,085**	**21,752**	**44,837**	**62.24**	**22,734**	**21,681**	**44,415**	**61.66**	**22,261**	**21,098**	**43,359**	**60.19**	**20,617**	**19,317**	**39,934**	**55.44**
Marinduque	4,906	1,524	1,364	2,888	58.87	1,534	1,455	2,989	60.93	1,539	1,409	2,948	60.09	1,526	1,375	2,901	59.13
Mindoro Occidental	12,449	4,619	4,191	8,810	70.77	4,533	4,178	8,711	69.97	4,421	4,128	8,549	68.67	4,166	3,841	8,007	64.32
Mindoro Oriental	20,011	5,824	5,572	11,396	56.95	5,772	5,574	11,346	56.70	5,627	5,376	11,003	54.98	5,070	4,865	9,935	49.65
Palawan	21,676	6,667	6,472	13,139	60.62	6,610	6,482	13,092	60.40	6,454	6,271	12,725	58.71	5,769	5,487	11,256	51.93
Romblon	6,482	2,044	1,931	3,975	61.32	2,096	1,942	4,038	62.30	2,116	1,912	4,028	62.14	2,104	1,970	4,074	62.85
Puerto Princesa City	6,511	2,407	2,222	4,629	71.10	2,189	2,050	4,239	65.11	2,104	2,002	4,106	63.06	1,982	1,779	3,761	57.76
**Region 5**	**137,350**	**43,298**	**40,559**	**83,857**	**61.05**	**42,937**	**40,536**	**83,473**	**60.77**	**41,349**	**40,514**	**81,863**	**59.60**	**40,735**	**38,334**	**79,069**	**57.57**
Albay	23,903	7,685	7,186	14,871	62.21	7,935	7,368	15,303	64.02	7,652	7,303	14,955	62.57	7,594	7,203	14,797	61.90
Camarines Norte	14,340	4,955	4,926	9,881	68.91	4,856	4,894	9,750	67.99	5,237	5,344	10,581	73.79	3,997	3,840	7,837	54.65
Camarines Sur	39,151	12,526	11,539	24,065	61.47	12,260	11,457	23,717	60.58	11,495	10,950	22,445	57.33	11,688	11,024	22,712	58.01
Catanduanes	6,381	1,853	1,822	3,675	57.59	1,968	1,839	3,807	59.66	1,808	1,776	3,584	56.17	1,853	1,816	3,669	57.50
Masbate	22,565	6,775	6,439	13,214	58.56	6,381	6,048	12,429	55.08	5,486	6,363	11,849	52.51	5,982	5,515	11,497	50.95
Sorsogon	19,470	5,587	5,206	10,793	55.43	5,695	5,295	10,990	56.45	5,777	5,263	11,040	56.70	5,909	5,357	11,266	57.86
Iriga City	2,665	765	677	1,442	54.11	743	713	1,456	54.63	745	673	1,418	53.21	737	751	1,488	55.83
Legaspi City	4,205	1,564	1,339	2,903	69.04	1,531	1,509	3,040	72.29	1,619	1,440	3,059	72.75	1,377	1,390	2,767	65.80
Naga City	4,670	1,588	1,425	3,013	64.52	1,568	1,413	2,981	63.83	1,530	1,402	2,932	62.78	1,598	1,438	3,036	65.01
**Region 6**	**146,545**	**51,120**	**47,899**	**99,019**	**67.57**	**51,258**	**48,667**	**99,925**	**68.19**	**51,325**	**48,656**	**99,981**	**68.23**	**51,028**	**48,426**	**99,454**	**67.87**
Aklan	11,231	3,648	3,440	7,088	63.11	3,826	3,543	7,369	65.61	3,758	3,441	7,199	64.10	3,749	3,431	7,180	63.93
Antique	12,985	4,052	3,730	7,782	59.93	4,025	3,736	7,761	59.77	4,069	3,783	7,852	60.47	3,892	3,646	7,538	58.05
Capiz	13,984	4,687	4,548	9,235	66.04	4,651	4,547	9,198	65.78	4,516	4,505	9,021	64.51	4,628	4,620	9,248	66.13
Guimaras	3,085	1,196	1,170	2,366	76.69	1,232	1,207	2,439	79.06	1,203	1,154	2,357	76.40	1,196	1,156	2,352	76.24
Iloilo	36,414	12,169	11,337	23,506	64.55	12,630	11,920	24,550	67.42	12,668	12,007	24,675	67.76	12,647	11,937	24,584	67.51
Negros Occidental	49,314	18,721	17,668	36,389	73.79	18,328	17,675	36,003	73.01	18,516	17,772	36,288	73.59	18,339	17,663	36,002	73.01
Bacolod City	11,102	3,843	3,499	7,342	66.13	3,830	3,465	7,295	65.71	3,783	3,375	7,158	64.47	3,767	3,344	7,111	64.05
Iloilo City	8,430	2,804	2,507	5,311	63.00	2,736	2,574	5,310	62.99	2,812	2,619	5,431	64.42	2,810	2,629	5,439	64.52
**Region 7**	**162,860**	**60,841**	**57,965**	**118,806**	**72.95**	**61,128**	**58,346**	**119,474**	**73.36**	**61,296**	**58,484**	**119,780**	**73.55**	**61,192**	**58,612**	**119,804**	**73.56**
Bohol	27,637	8,819	8,660	17,479	63.24	9,187	8,817	18,004	65.14	9,350	9,100	18,450	66.76	9,285	9,074	18,359	66.43
Cebu	67,046	25,352	23,521	48,873	72.89	25,694	23,904	49,598	73.98	25,669	23,959	49,628	74.02	25,883	24,289	50,172	74.83
Negros Oriental	27,922	10,765	10,777	21,542	77.15	10,163	10,479	20,642	73.93	9,992	10,050	20,042	71.78	9,763	9,867	19,630	70.30
Siquijor	1,636	601	527	1,128	68.95	617	571	1,188	72.62	576	513	1,089	66.56	646	582	1,228	75.06
Cebu City	21,037	7,886	7,216	15,102	71.79	7,795	7,130	14,925	70.95	7,929	7,306	15,235	72.42	7,906	7,302	15,208	72.29
Lapu-Lapu City	9,310	4,602	4,552	9,154	98.32	4,592	4,498	9,090	97.64	4,644	4,609	9,253	99.39	4,629	4,587	9,216	98.99
Mandaue City	8,272	2,816	2,712	5,528	66.83	3,080	2,947	6,027	72.86	3,136	2,947	6,083	73.54	3,080	2,911	5,991	72.43
**Region 8**	**104,016**	**31,384**	**29,784**	**61,168**	**58.81**	**31,265**	**29,712**	**60,977**	**58.62**	**30,983**	**30,124**	**61,107**	**58.75**	**29,690**	**28,350**	**58,040**	**55.80**
Biliran	3,856	1,280	1,201	2,481	64.34	1,258	1,191	2,449	63.51	1,278	1,190	2,468	64.00	1,290	1,199	2,489	64.55
Eastern Samar	11,635	3,393	3,281	6,674	57.36	3,576	3,258	6,834	58.74	3,516	3,270	6,786	58.32	3,289	3,151	6,440	55.35
Northern Leyte	35,052	10,584	9,653	20,237	57.73	10,379	9,627	20,006	57.08	10,235	10,240	20,475	58.41	10,050	9,259	19,309	55.09
Northern Samar	15,655	4,592	4,543	9,135	58.35	4,608	4,451	9,059	57.87	4,389	4,353	8,742	55.84	3,859	3,890	7,749	49.50
Southern Leyte	6,556	1,912	1,742	3,654	55.74	1,874	1,821	3,695	56.36	1,864	1,766	3,630	55.37	1,801	1,727	3,528	53.81
Western Samar	14,515	4,213	4,053	8,266	56.95	4,239	4,142	8,381	57.74	4,260	4,044	8,304	57.21	4,021	3,913	7,934	54.66
Calbayog City	4,474	1,331	1,313	2,644	59.10	1,261	1,208	2,469	55.19	1,246	1,166	2,412	53.91	1,249	1,172	2,421	54.11
Maasin City	1,661	432	457	889	53.52	439	444	883	53.16	467	438	905	54.49	461	434	895	53.88
Ormoc City	4,989	1,753	1,666	3,419	68.53	1,708	1,653	3,361	67.37	1,686	1,613	3,299	66.13	1,697	1,605	3,302	66.19
Tacloban City	5,623	1,894	1,875	3,769	67.03	1,923	1,917	3,840	68.29	2,042	2,044	4,086	72.67	1,973	2,000	3,973	70.66

Table 1.C.1.3 - Immunization Services for Infants and Children

Number and Proportion of Infants who completed 3 doses of Oral Polio Vaccine (OPV) and Inactivated Polio Vaccine (IPV)
Philippines, 2021

Area	Eligible Pop (Under 1 y.o.)	OPV 1				OPV 2				OPV 3				IPV			
		Male	Female	Total	%	Male	Female	Total	%	Male	Female	Total	%	Male	Female	Total	%
**Region 9**	**79,552**	**28,782**	**27,557**	**56,339**	**70.82**	**28,286**	**27,083**	**55,369**	**69.60**	**27,985**	**26,746**	**54,731**	**68.80**	**27,908**	**26,472**	**54,380**	**68.36**
Zamboanga del Norte	17,075	6,405	6,091	12,496	73.18	6,328	6,073	12,401	72.63	6,291	5,985	12,276	71.89	6,191	5,845	12,036	70.49
Zamboanga del Sur	17,506	5,525	5,392	10,917	62.36	5,393	5,323	10,716	61.21	5,255	5,088	10,343	59.08	5,270	5,052	10,322	58.96
Zamboanga Sibugay	14,992	4,341	4,279	8,620	57.50	4,311	4,237	8,548	57.02	4,281	4,169	8,450	56.36	4,142	4,077	8,219	54.82
Dapitan City	1,755	777	833	1,610	91.74	770	832	1,602	91.28	763	827	1,590	90.60	766	823	1,589	90.54
Dipolog City	2,794	1,076	1,032	2,108	75.45	1,081	1,022	2,103	75.27	1,051	1,011	2,062	73.80	1,055	1,035	2,090	74.80
Isabela City	2,544	976	893	1,869	73.47	1,018	892	1,910	75.08	1,000	937	1,937	76.14	981	923	1,904	74.84
Pagadian City	4,290	1,739	1,419	3,158	73.61	1,722	1,409	3,131	72.98	1,787	1,420	3,207	74.76	1,879	1,368	3,247	75.69
Zamboanga City	18,596	7,943	7,618	15,561	83.68	7,663	7,295	14,958	80.44	7,557	7,309	14,866	79.94	7,624	7,349	14,973	80.52
**Region 10**	**100,690**	**42,182**	**39,271**	**81,453**	**80.89**	**39,609**	**38,241**	**77,850**	**77.32**	**38,890**	**37,912**	**76,802**	**76.28**	**37,880**	**36,984**	**74,864**	**74.35**
Bukidnon	23,307	11,695	9,771	21,466	92.10	9,475	8,998	18,473	79.26	8,951	8,528	17,479	74.99	8,334	8,054	16,388	70.31
Camiguin	1,854	587	552	1,139	61.43	599	518	1,117	60.25	623	512	1,135	61.22	611	521	1,132	61.06
Lanao del Norte	14,955	4,757	4,678	9,435	63.09	4,691	4,847	9,538	63.78	4,751	4,828	9,579	64.05	4,701	4,719	9,420	62.99
Misamis Occidental	6,413	2,522	2,498	5,020	78.28	2,450	2,460	4,910	76.56	2,458	2,434	4,892	76.28	2,381	2,427	4,808	74.97
Misamis Oriental	15,058	6,818	6,763	13,581	90.19	6,857	6,746	13,603	90.34	6,799	6,838	13,637	90.56	6,752	6,919	13,671	90.79
Cagayan de Oro City	14,263	5,402	5,122	10,524	73.79	5,362	4,939	10,301	72.22	5,259	5,070	10,329	72.42	5,299	5,012	10,311	72.29
El Salvador City	1,061	436	383	819	77.19	426	416	842	79.36	418	389	807	76.06	405	381	786	74.08
Gingoog City	2,627	1,443	1,546	2,989	113.78	1,419	1,528	2,947	112.18	1,432	1,555	2,987	113.70	1,386	1,448	2,834	107.88
Iligan City	7,575	2,659	2,395	5,054	66.72	2,608	2,381	4,989	65.86	2,674	2,490	5,164	68.17	2,627	2,398	5,025	66.34
Malaybalay City	3,882	1,875	1,638	3,513	90.49	1,826	1,561	3,387	87.25	1,726	1,547	3,273	84.31	1,631	1,439	3,070	79.08
Oroquieta City	1,389	473	448	921	66.31	481	482	963	69.33	473	461	934	67.24	521	502	1,023	73.65
Ozamis City	2,782	981	963	1,944	69.88	967	984	1,951	70.13	1,049	973	2,022	72.68	1,052	979	2,031	73.01
Tangub City	1,234	499	441	940	76.18	510	475	985	79.82	519	498	1,017	82.41	475	407	882	71.47
Valencia City	4,290	2,035	2,073	4,108	95.76	1,938	1,906	3,844	89.60	1,758	1,789	3,547	82.68	1,705	1,778	3,483	81.19
**Region 11**	**107,847**	**39,611**	**37,718**	**77,329**	**71.70**	**38,685**	**36,521**	**75,206**	**69.73**	**38,669**	**36,425**	**75,094**	**69.63**	**38,471**	**36,366**	**74,837**	**69.39**
Davao de Oro	15,526	6,263	5,528	11,791	75.94	6,196	5,590	11,786	75.91	6,263	5,578	11,841	76.27	6,101	5,443	11,544	74.35
Davao del Norte	21,184	8,804	8,592	17,396	82.12	8,661	8,313	16,974	80.13	8,548	8,212	16,760	79.12	8,390	8,067	16,457	77.69
Davao del Sur	13,125	4,366	4,217	8,583	65.39	4,301	3,995	8,296	63.21	4,199	4,012	8,211	62.56	4,135	4,062	8,197	62.45
Davao Oriental	14,355	4,805	4,565	9,370	65.27	4,838	4,545	9,383	65.36	4,786	4,447	9,233	64.32	4,884	4,501	9,385	65.38
Davao Occidental	6,598	1,452	1,439	2,891	43.82	1,439	1,315	2,754	41.74	1,480	1,352	2,832	42.92	1,477	1,369	2,846	43.13
Davao City	37,059	13,921	13,377	27,298	73.66	13,250	12,763	26,013	70.19	13,393	12,824	26,217	70.74	13,484	12,924	26,408	71.26
**Region 12**	**104,693**	**35,941**	**33,394**	**69,335**	**66.23**	**34,702**	**32,709**	**67,411**	**64.39**	**33,801**	**32,386**	**66,187**	**63.22**	**33,585**	**38,293**	**71,878**	**68.66**
North Cotabato	33,933	10,132	9,448	19,580	57.70	9,421	9,054	18,475	54.45	9,257	8,824	18,081	53.28	9,307	8,764	18,071	53.25
Sarangani	12,904	4,860	4,548	9,408	72.91	4,855	4,447	9,302	72.09	4,747	4,488	9,235	71.57	4,849	4,553	9,402	72.86
South Cotabato	21,039	8,169	7,652	15,821	75.20	7,981	7,542	15,523	73.78	7,912	7,561	15,473	73.54	8,017	7,688	15,705	74.65
Sultan Kudarat	17,331	6,446	5,831	12,277	70.84	6,300	5,869	12,169	70.22	6,112	5,869	11,981	69.13	5,803	11,840	17,643	101.80
Cotabato City	5,831	1,858	1,791	3,649	62.58	1,831	1,770	3,601	61.76	1,800	1,785	3,585	61.48	1,786	1,762	3,548	60.85
Gen. Santos City	13,655	4,476	4,124	8,600	62.98	4,314	4,027	8,341	61.08	3,973	3,859	7,832	57.36	3,823	3,686	7,509	54.99
**BARMM**	**94,148**	**32,491**	**33,123**	**65,614**	**69.69**	**32,018**	**33,157**	**65,175**	**69.23**	**31,815**	**32,855**	**64,670**	**68.69**	**28,363**	**29,632**	**57,995**	**61.60**
Basilan	7,672	1,640	1,496	3,136	40.88	1,482	1,434	2,916	38.01	1,292	1,286	2,578	33.60	934	971	1,905	24.83
Lanao del Sur	21,391	8,557	8,964	17,521	81.91	8,443	9,098	17,541	82.00	8,489	9,153	17,642	82.47	8,347	8,995	17,342	81.07
Maguindanao	31,650	12,152	12,158	24,310	76.81	12,165	12,083	24,248	76.61	12,021	11,841	23,862	75.39	11,339	11,264	22,603	71.42
Sulu	16,891	4,660	4,939	9,599	56.83	4,412	4,855	9,267	54.86	4,515	4,790	9,305	55.09	3,348	3,683	7,031	41.63
Tawi-Tawi	9,319	2,994	3,126	6,120	65.67	2,944	3,130	6,074	65.18	2,899	3,129	6,028	64.69	2,880	3,115	5,995	64.33
Lamitan City	2,112	587	557	1,144	54.17	549	544	1,093	51.75	533	519	1,052	49.81	535	543	1,078	51.04
Marawi City	5,113	1,901	1,883	3,784	74.01	2,023	2,013	4,036	78.94	2,066	2,137	4,203	82.20	980	1,061	2,041	39.92
**CARAGA**	**60,342**	**20,965**	**19,817**	**40,782**	**67.58**	**20,262**	**19,449**	**39,711**	**65.81**	**19,541**	**18,728**	**38,269**	**63.42**	**18,533**	**17,568**	**36,101**	**59.83**
Agusan del Norte	8,116	2,753	2,544	5,297	65.27	2,708	2,463	5,171	63.71	2,548	2,414	4,962	61.14	2,559	2,399	4,958	61.09
Agusan del Sur	17,658	6,214	6,086	12,300	69.66	5,957	5,887	11,844	67.07	5,765	5,536	11,301	64.00	5,003	4,928	9,931	56.24
Surigao del Norte	7,195	2,529	2,367	4,896	68.05	2,544	2,375	4,919	68.37	2,470	2,437	4,907	68.20	2,441	2,286	4,727	65.70
Surigao del Sur	11,532	4,047	3,747	7,794	67.59	3,801	3,666	7,467	64.75	3,512	3,344	6,856	59.45	3,173	2,941	6,114	53.02
Province of Dinagat	2,574	657	644	1,301	50.54	656	661	1,317	51.17	668	658	1,326	51.52	682	643	1,325	51.48
Bislig City	2,185	732	659	1,391	63.66	700	710	1,410	64.53	741	680	1,421	65.03	703	658	1,361	62.29
Butuan City	7,732	2,665	2,488	5,153	66.65	2,564	2,444	5,008	64.77	2,501	2,367	4,868	62.96	2,694	2,485	5,179	66.98
Surigao City	3,350	1,368	1,282	2,650	79.10	1,332	1,243	2,575	76.87	1,336	1,292	2,628	78.45	1,278	1,228	2,506	74.81

Table 1.C.1.4 - Immunization Services for Infants and Children

Number and Proportion of Infants who completed 3 doses of Pneumococcal Conjugate Vaccine (PCV)
Philippines, 2021

Area	Eligible Pop (Under 1 y.o.)	PCV 1				PCV 2				PCV 3			
		Male	Female	Total	%	Male	Female	Total	%	Male	Female	Total	%
**PHILIPPINES**	**2,128,168**	**724,599**	**688,052**	**1,412,651**	**66.38**	**697,936**	**671,005**	**1,368,941**	**64.32**	**663,564**	**640,559**	**1,304,123**	**61.28**
**N C R**	**237,921**	**84,035**	**81,827**	**165,862**	**69.71**	**76,901**	**76,118**	**153,019**	**64.32**	**68,937**	**68,932**	**137,869**	**57.95**
Malabon	6,757	3,497	3,255	6,752	99.93	2,884	2,895	5,779	85.53	2,078	2,054	4,132	61.15
Navotas	4,607	2,112	2,052	4,164	90.38	2,092	2,060	4,152	90.12	2,147	2,076	4,223	91.66
Valenzuela City	11,465	3,989	3,825	7,814	68.16	3,738	3,704	7,442	64.91	3,467	3,602	7,069	61.66
Caloocan City	29,270	9,670	8,840	18,510	63.24	7,474	7,317	14,791	50.53	5,807	5,523	11,330	38.71
Marikina City	8,330	3,261	2,926	6,187	74.27	3,192	2,774	5,966	71.62	3,045	2,683	5,728	68.76
Pasig City	13,954	4,237	3,903	8,140	58.33	4,086	3,711	7,797	55.88	3,524	3,447	6,971	49.96
Pateros	1,181	575	518	1,093	92.55	558	593	1,151	97.46	592	588	1,180	99.92
Taguig	14,874	5,112	4,879	9,991	67.17	4,874	4,644	9,518	63.99	4,867	4,624	9,491	63.81
Quezon City	54,246	21,198	21,397	42,595	78.52	19,347	19,521	38,868	71.65	16,834	17,183	34,017	62.71
Makati City	10,765	2,906	2,499	5,405	50.21	2,828	2,449	5,277	49.02	2,527	2,349	4,876	45.29
Mandaluyong City	7,137	2,273	2,258	4,531	63.49	2,228	2,158	4,386	61.45	2,197	2,306	4,503	63.09
San Juan	2,254	497	468	965	42.81	482	466	948	42.06	500	470	970	43.03
Manila City	32,887	10,634	11,804	22,438	68.23	9,759	10,964	20,723	63.01	8,425	9,483	17,908	54.45
Las Piñas City	10,879	3,789	3,548	7,337	67.44	3,514	3,467	6,981	64.17	3,390	3,231	6,621	60.86
Muntinlupa City	9,317	3,143	2,953	6,096	65.43	3,188	3,019	6,207	66.62	2,945	2,872	5,817	62.43
Parañaque City	12,298	4,170	3,777	7,947	64.62	4,009	3,634	7,643	62.15	3,815	3,485	7,300	59.36
Pasay City	7,700	2,972	2,925	5,897	76.58	2,648	2,742	5,390	70.00	2,777	2,956	5,733	74.45
**C A R**	**35,143**	**11,705**	**10,963**	**22,668**	**64.50**	**11,677**	**11,153**	**22,830**	**64.96**	**11,554**	**11,135**	**22,689**	**64.56**
Abra	4,287	1,262	1,254	2,516	58.69	1,280	1,274	2,554	59.58	1,359	1,315	2,674	62.37
Apayao	2,476	849	801	1,650	66.64	835	825	1,660	67.04	795	795	1,590	64.22
Benguet	9,140	2,955	2,716	5,671	62.05	2,992	2,788	5,780	63.24	2,896	2,811	5,707	62.44
Ifugao	4,439	1,589	1,386	2,975	67.02	1,617	1,454	3,071	69.18	1,548	1,403	2,951	66.48
Kalinga	4,660	1,864	1,736	3,600	77.25	1,840	1,737	3,577	76.76	1,805	1,748	3,553	76.24
Mt. Province	3,066	1,031	960	1,991	64.94	1,074	1,004	2,078	67.78	1,069	967	2,036	66.41
Baguio City	7,075	2,155	2,110	4,265	60.28	2,039	2,071	4,110	58.09	2,082	2,096	4,178	59.05
**Region 1**	**97,198**	**34,121**	**32,005**	**66,126**	**68.03**	**34,774**	**33,226**	**68,000**	**69.96**	**35,180**	**33,515**	**68,695**	**70.68**
Ilocos Norte	7,986	2,546	2,457	5,003	62.65	2,530	2,496	5,026	62.94	2,607	2,459	5,066	63.44
Ilocos Sur	9,197	3,542	3,344	6,886	74.87	3,624	3,428	7,052	76.68	3,519	3,352	6,871	74.71
La Union	11,429	3,943	3,542	7,485	65.49	4,087	3,815	7,902	69.14	4,096	3,890	7,986	69.87
Pangasinan	50,454	17,419	16,397	33,816	67.02	17,868	17,223	35,091	69.55	18,366	17,421	35,787	70.93
Alaminos City	1,905	718	652	1,370	71.92	722	627	1,349	70.81	655	646	1,301	68.29
Candon City	976	445	442	887	90.88	435	408	843	86.37	441	387	828	84.84
Dagupan City	3,636	1,076	1,087	2,163	59.49	1,061	1,061	2,122	58.36	1,070	1,062	2,132	58.64
Laoag City	1,845	965	987	1,952	105.80	966	989	1,955	105.96	964	999	1,963	106.40
San Carlos City	3,999	1,502	1,344	2,846	71.17	1,433	1,287	2,720	68.02	1,349	1,240	2,589	64.74
San Fernando City	2,092	671	581	1,252	59.85	686	626	1,312	62.72	672	653	1,325	63.34
Urdaneta City	2,819	979	901	1,880	66.69	1,050	972	2,022	71.73	995	991	1,986	70.45
Vigan City	860	315	271	586	68.14	312	294	606	70.47	446	415	861	100.12
**Region 2**	**69,228**	**24,354**	**22,874**	**47,228**	**68.22**	**24,057**	**22,862**	**46,919**	**67.77**	**23,398**	**22,115**	**45,513**	**65.74**
Batanes	351	135	114	249	70.94	132	118	250	71.23	144	106	250	71.23
Cagayan	20,563	6,686	6,066	12,752	62.01	6,524	6,047	12,571	61.13	6,302	5,803	12,105	58.87
Isabela	23,543	7,926	7,623	15,549	66.05	7,989	7,712	15,701	66.69	7,527	7,383	14,910	63.33
Nueva Vizcaya	9,555	3,290	3,063	6,353	66.49	3,129	2,907	6,036	63.17	3,067	2,700	5,767	60.36
Quirino	4,038	1,623	1,661	3,284	81.33	1,610	1,587	3,197	79.17	1,609	1,538	3,147	77.93
Cauayan City	2,576	1,032	945	1,977	76.75	1,039	1,002	2,041	79.23	1,112	1,012	2,124	82.45
Ilagan City	2,901	1,290	1,239	2,529	87.18	1,275	1,273	2,548	87.83	1,283	1,337	2,620	90.31
Santiago City	2,683	1,326	1,228	2,554	95.19	1,326	1,259	2,585	96.35	1,267	1,288	2,555	95.23
Tuguegarao City	3,018	1,046	935	1,981	65.64	1,033	957	1,990	65.94	1,087	948	2,035	67.43
**Region 3**	**220,202**	**79,586**	**76,511**	**156,097**	**70.89**	**76,669**	**75,755**	**152,424**	**69.22**	**74,743**	**72,669**	**147,412**	**66.94**
Aurora	4,764	1,820	1,660	3,480	73.05	1,825	1,689	3,514	73.76	1,825	1,697	3,522	73.93
Bataan	13,814	5,699	5,031	10,730	77.67	5,724	5,112	10,836	78.44	5,601	5,101	10,702	77.47
Bulacan	43,722	14,413	14,761	29,174	66.73	13,884	13,724	27,608	63.14	12,882	12,975	25,857	59.14

Table 1.C.1.4 - Immunization Services for Infants and Children

Number and Proportion of Infants who completed 3 doses of Pneumococcal Conjugate Vaccine (PCV)
Philippines, 2021

Area	Eligible Pop (Under 1 y.o.)	PCV 1				PCV 2				PCV 3			
		Male	Female	Total	%	Male	Female	Total	%	Male	Female	Total	%
Nueva Ecija	28,915	6,801	6,506	13,307	46.02	5,827	7,559	13,386	46.29	5,518	5,127	10,645	36.81
Pampanga	31,503	12,415	12,014	24,429	77.54	11,876	11,587	23,463	74.48	11,311	11,695	23,006	73.03
Tarlac	19,565	8,063	7,538	15,601	79.74	7,815	7,480	15,295	78.18	7,863	7,450	15,313	78.27
Zambales	12,551	3,863	3,521	7,384	58.83	3,695	3,428	7,123	56.75	3,464	3,291	6,755	53.82
Angeles City	7,905	3,278	3,197	6,475	81.91	3,338	3,159	6,497	82.19	3,263	3,151	6,414	81.14
Balanga City	1,996	733	681	1,414	70.84	718	683	1,401	70.19	711	696	1,407	70.49
Cabanatuan City	5,921	2,471	2,370	4,841	81.76	2,435	2,329	4,764	80.46	2,445	2,334	4,779	80.71
City of San Fernando	5,890	2,360	2,143	4,503	76.45	2,261	2,047	4,308	73.14	2,200	1,945	4,145	70.37
Gapan City	2,159	856	816	1,672	77.44	786	736	1,522	70.50	752	722	1,474	68.27
Mabalacat City	4,811	2,073	1,921	3,994	83.02	1,945	1,933	3,878	80.61	1,991	1,913	3,904	81.15
Malolos City	4,885	1,591	1,497	3,088	63.21	1,545	1,491	3,036	62.15	1,568	1,541	3,109	63.64
Meycauayan	4,050	1,733	1,700	3,433	84.77	1,697	1,698	3,395	83.83	1,713	1,741	3,454	85.28
Olongapo	4,952	1,515	1,516	3,031	61.21	1,509	1,469	2,978	60.14	1,575	1,485	3,060	61.79
Palayan City	805	327	301	628	78.01	345	292	637	79.13	319	299	618	76.77
San Jose City	2,736	890	826	1,716	62.72	868	859	1,727	63.12	846	802	1,648	60.23
San Jose del Monte City	11,120	5,762	5,707	11,469	103.14	5,732	5,714	11,446	102.93	5,907	5,777	11,684	105.07
Science City of Munoz	1,594	567	535	1,102	69.13	590	559	1,149	72.08	603	593	1,196	75.03
Tarlac City	6,544	2,356	2,270	4,626	70.69	2,254	2,207	4,461	68.17	2,386	2,334	4,720	72.13
**Region 4A**	**298,398**	**97,971**	**91,304**	**189,275**	**63.43**	**94,074**	**88,819**	**182,893**	**61.29**	**87,859**	**83,805**	**171,664**	**57.53**
Batangas	38,848	12,476	11,589	24,065	61.95	11,808	11,012	22,820	58.74	10,858	9,958	20,816	53.58
Cavite	27,948	8,735	8,072	16,807	60.14	7,972	7,602	15,574	55.72	7,248	6,990	14,238	50.94
Laguna	19,727	6,663	5,967	12,630	64.02	6,277	5,958	12,235	62.02	5,842	5,432	11,274	57.15
Quezon	37,644	10,171	9,637	19,808	52.62	9,463	9,150	18,613	49.44	8,848	8,580	17,428	46.30
Rizal	44,826	12,723	11,839	24,562	54.79	13,044	11,539	24,583	54.84	11,858	11,297	23,155	51.66
Antipolo City	16,510	7,569	6,814	14,383	87.12	7,287	6,950	14,237	86.23	6,416	6,313	12,729	77.10
Bacoor City	12,252	3,622	3,710	7,332	59.84	3,229	3,009	6,238	50.91	2,678	2,529	5,207	42.50
Batangas City	6,895	1,591	1,496	3,087	44.77	1,496	1,439	2,935	42.57	1,398	1,359	2,757	39.99
Biñan City	6,607	2,894	2,759	5,653	85.56	2,809	2,816	5,625	85.14	2,849	2,905	5,754	87.09
Cabuyao City	6,128	2,888	2,741	5,629	91.86	2,929	2,759	5,688	92.82	3,388	2,888	6,276	102.42
Calamba City	9,025	4,282	4,123	8,405	93.13	4,007	3,875	7,882	87.34	3,417	3,215	6,632	73.48
Cavite City	2,098	227	192	419	19.97	227	234	461	21.97	262	257	519	24.74
Dasmariñas City	13,442	4,325	4,011	8,336	62.01	4,252	4,097	8,349	62.11	4,123	3,951	8,074	60.07
General Trias City	6,410	2,003	1,915	3,918	61.12	1,910	1,850	3,760	58.66	1,823	1,706	3,529	55.05
Imus City	8,235	2,712	2,591	5,303	64.40	2,680	2,762	5,442	66.08	2,543	2,618	5,161	62.67
Lipa City	6,954	2,750	2,530	5,280	75.93	2,665	2,412	5,077	73.01	2,466	2,274	4,740	68.16
Lucena City	5,704	1,837	1,729	3,566	62.52	1,622	1,681	3,303	57.91	1,547	1,482	3,029	53.10
San Pablo City	5,277	1,095	987	2,082	39.45	1,089	1,048	2,137	40.50	1,173	1,119	2,292	43.43
San Pedro City	6,465	1,999	1,916	3,915	60.56	2,014	1,864	3,878	59.98	1,887	1,781	3,668	56.74
Santa Rosa City	7,023	3,308	2,929	6,237	88.81	3,332	2,965	6,297	89.66	3,308	3,409	6,717	95.64
Tagaytay City	1,451	609	588	1,197	82.49	610	583	1,193	82.22	625	617	1,242	85.60
Tanauan City	3,620	1,216	1,070	2,286	63.15	1,086	1,113	2,199	60.75	1,065	1,013	2,078	57.40
Tayabas City	2,134	764	690	1,454	68.13	813	716	1,529	71.65	769	678	1,447	67.81
Trece Martires City	3,175	1,512	1,409	2,921	92.00	1,453	1,385	2,838	89.39	1,468	1,434	2,902	91.40
**Region 4B**	**72,035**	**19,493**	**18,476**	**37,969**	**52.71**	**18,086**	**17,210**	**35,296**	**49.00**	**16,603**	**15,629**	**32,232**	**44.74**
Marinduque	4,906	1,520	1,343	2,863	58.36	1,509	1,408	2,917	59.46	1,541	1,366	2,907	59.25
Mindoro Occidental	12,449	3,597	3,255	6,852	55.04	3,332	3,002	6,334	50.88	3,046	2,761	5,807	46.65
Mindoro Oriental	20,011	5,316	5,003	10,319	51.57	4,973	4,853	9,826	49.10	4,582	4,419	9,001	44.98
Palawan	21,676	5,739	5,778	11,517	53.13	5,265	5,193	10,458	48.25	4,832	4,663	9,495	43.80
Romblon	6,482	1,955	1,824	3,779	58.30	1,991	1,811	3,802	58.65	1,908	1,774	3,682	56.80
Puerto Princesa City	6,511	1,366	1,273	2,639	40.53	1,016	943	1,959	30.09	694	646	1,340	20.58
**Region 5**	**137,350**	**43,539**	**40,549**	**84,088**	**61.22**	**43,218**	**40,442**	**83,660**	**60.91**	**41,454**	**39,222**	**80,676**	**58.74**
Albay	23,903	7,778	7,314	15,092	63.14	8,016	7,424	15,440	64.59	7,462	7,163	14,625	61.18
Camarines Norte	14,340	4,461	4,139	8,600	59.97	4,233	3,861	8,094	56.44	3,891	3,776	7,667	53.47
Camarines Sur	39,151	12,837	11,911	24,748	63.21	12,415	11,773	24,188	61.78	11,975	11,241	23,216	59.30
Catanduanes	6,381	1,929	1,833	3,762	58.96	2,045	1,911	3,956	62.00	1,883	1,849	3,732	58.49
Masbate	22,565	6,814	6,429	13,243	58.69	6,656	6,289	12,945	57.37	6,603	6,218	12,821	56.82
Sorsogon	19,470	5,766	5,354	11,120	57.11	6,048	5,569	11,617	59.67	5,980	5,483	11,463	58.88
Iriga City	2,665	749	682	1,431	53.70	748	692	1,440	54.03	728	638	1,366	51.26
Legaspi City	4,205	1,538	1,319	2,857	67.94	1,426	1,428	2,854	67.87	1,345	1,406	2,751	65.42
Naga City	4,670	1,667	1,568	3,235	69.27	1,631	1,495	3,126	66.94	1,587	1,448	3,035	64.99

Table 1.C.1.4 - Immunization Services for Infants and Children

Number and Proportion of Infants who completed 3 doses of Pneumococcal Conjugate Vaccine (PCV)
Philippines, 2021

Area	Eligible Pop (Under 1 y.o.)	PCV 1				PCV 2				PCV 3			
		Male	Female	Total	%	Male	Female	Total	%	Male	Female	Total	%
**Region 6**	**146,545**	**51,286**	**47,945**	**99,231**	**67.71**	**51,074**	**48,439**	**99,513**	**67.91**	**50,022**	**47,882**	**97,904**	**66.81**
Aklan	11,231	3,695	3,460	7,155	63.71	3,737	3,510	7,247	64.53	3,783	3,482	7,265	64.69
Antique	12,985	4,147	3,780	7,927	61.05	4,112	3,833	7,945	61.19	4,112	3,814	7,926	61.04
Capiz	13,984	4,704	4,607	9,311	66.58	4,813	4,728	9,541	68.23	4,645	4,684	9,329	66.71
Guimaras	3,085	1,194	1,169	2,363	76.60	1,222	1,209	2,431	78.80	1,192	1,144	2,336	75.72
Iloilo	36,414	12,122	11,140	23,262	63.88	12,328	11,616	23,944	65.75	11,991	11,471	23,462	64.43
Negros Occidental	49,314	18,596	17,674	36,270	73.55	18,437	17,635	36,072	73.15	18,059	17,565	35,624	72.24
Bacolod City	11,102	4,034	3,595	7,629	68.72	3,686	3,341	7,027	63.29	3,462	3,106	6,568	59.16
Iloilo City	8,430	2,794	2,520	5,314	63.04	2,739	2,567	5,306	62.94	2,778	2,616	5,394	63.99
**Region 7**	**162,860**	**58,506**	**55,658**	**114,164**	**70.10**	**57,292**	**54,899**	**112,191**	**68.89**	**56,068**	**53,966**	**110,034**	**67.56**
Bohol	27,637	8,806	8,703	17,509	63.35	8,836	8,729	17,565	63.56	9,033	8,701	17,734	64.17
Cebu	67,046	24,554	22,809	47,363	70.64	24,037	22,444	46,481	69.33	23,634	22,442	46,076	68.72
Negros Oriental	27,922	9,708	9,687	19,395	69.46	9,137	9,385	18,522	66.33	8,402	8,919	17,321	62.03
Siquijor	1,636	628	551	1,179	72.07	635	610	1,245	76.10	659	586	1,245	76.10
Cebu City	21,037	7,896	7,171	15,067	71.62	7,749	7,072	14,821	70.45	7,574	6,950	14,524	69.04
Lapu-Lapu City	9,310	4,153	4,106	8,259	88.71	4,081	4,018	8,099	86.99	4,031	3,775	7,806	83.85
Mandaue City	8,272	2,761	2,631	5,392	65.18	2,817	2,641	5,458	65.98	2,735	2,593	5,328	64.41
**Region 8**	**104,016**	**31,560**	**29,636**	**61,196**	**58.83**	**31,921**	**29,710**	**61,631**	**59.25**	**30,133**	**28,650**	**58,783**	**56.51**
Biliran	3,856	1,325	1,223	2,548	66.08	1,285	1,216	2,501	64.86	1,277	1,188	2,465	63.93
Eastern Samar	11,635	3,336	3,239	6,575	56.51	3,459	3,165	6,624	56.93	3,295	3,012	6,307	54.21
Northern Leyte	35,052	10,744	9,826	20,570	58.68	11,326	9,791	21,117	60.24	10,124	9,544	19,668	56.11
Northern Samar	15,655	4,680	4,510	9,190	58.70	4,750	4,534	9,284	59.30	4,557	4,428	8,985	57.39
Southern Leyte	6,556	1,766	1,557	3,323	50.69	1,742	1,704	3,446	52.56	1,761	1,646	3,407	51.97
Western Samar	14,515	4,286	4,072	8,358	57.58	4,044	4,046	8,090	55.74	3,946	3,745	7,691	52.99
Calbayog City	4,474	1,403	1,269	2,672	59.72	1,350	1,329	2,679	59.88	1,185	1,108	2,293	51.25
Maasin City	1,661	442	461	903	54.36	434	442	876	52.74	465	429	894	53.82
Ormoc City	4,989	1,649	1,587	3,236	64.86	1,611	1,534	3,145	63.04	1,475	1,496	2,971	59.55
Tacloban City	5,623	1,929	1,892	3,821	67.95	1,920	1,949	3,869	68.81	2,048	2,054	4,102	72.95
**Region 9**	**79,552**	**28,709**	**27,066**	**55,775**	**70.11**	**27,856**	**26,772**	**54,628**	**68.67**	**26,578**	**25,273**	**51,851**	**65.18**
Zamboanga del Norte	17,075	6,483	6,149	12,632	73.98	6,412	6,214	12,626	73.94	6,074	5,957	12,031	70.46
Zamboanga del Sur	17,506	5,427	5,086	10,513	60.05	5,210	5,061	10,271	58.67	4,843	4,512	9,355	53.44
Zamboanga Sibugay	14,992	4,354	4,162	8,516	56.80	4,205	4,117	8,322	55.51	4,075	3,966	8,041	53.64
Dapitan City	1,755	770	835	1,605	91.45	764	832	1,596	90.94	762	829	1,591	90.66
Dipolog City	2,794	1,076	1,039	2,115	75.70	1,080	1,030	2,110	75.52	1,035	980	2,015	72.12
Isabela City	2,544	922	838	1,760	69.18	941	839	1,780	69.97	918	867	1,785	70.17
Pagadian City	4,290	1,766	1,385	3,151	73.45	1,718	1,412	3,130	72.96	1,706	1,323	3,029	70.61
Zamboanga City	18,596	7,911	7,572	15,483	83.26	7,526	7,267	14,793	79.55	7,165	6,839	14,004	75.31
**Region 10**	**100,690**	**37,285**	**35,886**	**73,171**	**72.67**	**34,620**	**33,403**	**68,023**	**67.56**	**32,604**	**32,244**	**64,848**	**64.40**
Bukidnon	23,307	9,368	8,892	18,260	78.35	8,564	8,197	16,761	71.91	7,657	7,559	15,216	65.29
Camiguin	1,854	592	534	1,126	60.73	578	509	1,087	58.63	593	474	1,067	57.55
Lanao del Norte	14,955	4,712	4,703	9,415	62.96	4,644	4,695	9,339	62.45	4,557	4,662	9,219	61.64
Misamis Occidental	6,413	2,310	2,246	4,556	71.04	2,257	2,265	4,522	70.51	2,173	2,181	4,354	67.89
Misamis Oriental	15,058	6,206	6,102	12,308	81.74	5,679	5,454	11,133	73.93	5,327	5,335	10,662	70.81
Cagayan de Oro City	14,263	4,885	4,636	9,521	66.75	4,611	4,288	8,899	62.39	4,397	4,281	8,678	60.84
El Salvador City	1,061	431	393	824	77.66	437	402	839	79.08	395	365	760	71.63
Gingoog City	2,627	1,396	1,503	2,899	110.35	1,388	1,482	2,870	109.25	1,364	1,516	2,880	109.63
Iligan City	7,575	1,824	1,625	3,449	45.53	1,454	1,313	2,767	36.53	1,429	1,307	2,736	36.12
Malaybalay City	3,882	1,795	1,563	3,358	86.50	1,653	1,417	3,070	79.08	1,561	1,456	3,017	77.72
Oroquieta City	1,389	413	412	825	59.40	316	341	657	47.30	265	264	529	38.08
Ozamis City	2,782	1,020	992	2,012	72.32	978	991	1,969	70.78	984	973	1,957	70.35
Tangub City	1,234	474	451	925	74.96	491	471	962	77.96	503	475	978	79.25
Valencia City	4,290	1,859	1,834	3,693	86.08	1,570	1,578	3,148	73.38	1,399	1,396	2,795	65.15

Table 1.C.1.4 - Immunization Services for Infants and Children

Number and Proportion of Infants who completed 3 doses of Pneumococcal Conjugate Vaccine (PCV)
Philippines, 2021

Area	Eligible Pop (Under 1 y.o.)	PCV 1				PCV 2				PCV 3			
		Male	Female	Total	%	Male	Female	Total	%	Male	Female	Total	%
**Region 11**	**107,847**	**38,855**	**36,403**	**75,258**	**69.78**	**37,270**	**35,364**	**72,634**	**67.35**	**34,761**	**33,191**	**67,952**	**63.01**
Davao de Oro	15,526	6,180	5,570	11,750	75.68	6,081	5,564	11,645	75.00	5,640	5,037	10,677	68.77
Davao del Norte	21,184	8,695	8,256	16,951	80.02	8,618	8,136	16,754	79.09	8,223	7,932	16,155	76.26
Davao del Sur	13,125	4,404	4,188	8,592	65.46	4,192	4,002	8,194	62.43	3,783	3,675	7,458	56.82
Davao Oriental	14,355	4,833	4,501	9,334	65.02	4,772	4,452	9,224	64.26	4,555	4,276	8,831	61.52
Davao Occidental	6,598	1,578	1,433	3,011	45.64	1,419	1,341	2,760	41.83	1,282	1,207	2,489	37.72
Davao City	37,059	13,165	12,455	25,620	69.13	12,188	11,869	24,057	64.92	11,278	11,064	22,342	60.29
**Region 12**	**104,693**	**33,007**	**30,782**	**63,789**	**60.93**	**30,044**	**28,334**	**58,378**	**55.76**	**27,430**	**26,427**	**53,857**	**51.44**
North Cotabato	33,933	8,913	8,457	17,370	51.19	7,335	7,085	14,420	42.50	6,092	5,987	12,079	35.60
Sarangani	12,904	4,603	4,257	8,860	68.66	4,304	3,926	8,230	63.78	3,907	3,740	7,647	59.26
South Cotabato	21,039	7,557	7,115	14,672	69.74	7,106	6,661	13,767	65.44	6,738	6,471	13,209	62.78
Sultan Kudarat	17,331	6,258	5,591	11,849	68.37	6,052	5,651	11,703	67.53	5,773	5,499	11,272	65.04
Cotabato City	5,831	1,738	1,651	3,389	58.12	1,670	1,670	3,340	57.28	1,620	1,517	3,137	53.80
Gen. Santos City	13,655	3,938	3,711	7,649	56.02	3,577	3,341	6,918	50.66	3,300	3,213	6,513	47.70
**BARMM**	**94,148**	**30,004**	**30,891**	**60,895**	**64.68**	**28,601**	**29,787**	**58,388**	**62.02**	**27,563**	**27,933**	**55,496**	**58.95**
Basilan	7,672	1,561	1,549	3,110	40.54	1,396	1,448	2,844	37.07	1,255	1,296	2,551	33.25
Lanao del Sur	21,391	8,513	8,955	17,468	81.66	8,338	9,014	17,352	81.12	8,248	8,222	16,470	76.99
Maguindanao	31,650	9,472	9,409	18,881	59.66	8,694	8,467	17,161	54.22	7,916	7,838	15,754	49.78
Sulu	16,891	4,858	5,182	10,040	59.44	4,654	4,991	9,645	57.10	4,654	4,857	9,511	56.31
Tawi-Tawi	9,319	2,947	3,073	6,020	64.60	2,869	3,153	6,022	64.62	2,852	3,063	5,915	63.47
Lamitan City	2,112	601	595	1,196	56.63	555	535	1,090	51.61	530	517	1,047	49.57
Marawi City	5,113	2,052	2,128	4,180	81.75	2,095	2,179	4,274	83.59	2,108	2,140	4,248	83.08
**CARAGA**	**60,342**	**20,583**	**19,276**	**39,859**	**66.06**	**19,802**	**18,712**	**38,514**	**63.83**	**18,677**	**17,971**	**36,648**	**60.73**
Agusan del Norte	8,116	2,803	2,620	5,423	66.82	2,763	2,510	5,273	64.97	2,550	2,469	5,019	61.84
Agusan del Sur	17,658	6,078	5,934	12,012	68.03	5,774	5,635	11,409	64.61	5,615	5,491	11,106	62.90
Surigao del Norte	7,195	2,544	2,343	4,887	67.92	2,585	2,413	4,998	69.46	2,367	2,330	4,697	65.28
Surigao del Sur	11,532	4,048	3,804	7,852	68.09	3,847	3,629	7,476	64.83	3,628	3,364	6,992	60.63
Province of Dinagat	2,574	652	624	1,276	49.57	692	672	1,364	52.99	681	684	1,365	53.03
Bislig City	2,185	696	645	1,341	61.37	679	654	1,333	61.01	622	648	1,270	58.12
Butuan City	7,732	2,532	2,226	4,758	61.54	2,276	2,132	4,408	57.01	2,061	1,973	4,034	52.17
Surigao City	3,350	1,230	1,080	2,310	68.96	1,186	1,067	2,253	67.25	1,153	1,012	2,165	64.63

Table 1.C.1.5 - Immunization Services for Infants and Children

Number and Proportion of Children vaccinated with 2 doses of Measles Containing Vaccine (MCV)
Philippines, 2021

Area	Eligible Pop (Under 1 y.o.)	MCV 1				MCV 2			
		Male	Female	Total	%	Male	Female	Total	%
**PHILIPPINES**	**2,128,168**	**752,031**	**709,203**	**1,461,234**	**68.66**	**745,190**	**713,225**	**1,458,415**	**68.53**
**N C R**	**237,921**	**82,856**	**80,304**	**163,160**	**68.58**	**76,205**	**75,538**	**151,743**	**63.78**
Malabon	6,757	2,438	2,282	4,720	69.85	2,460	2,479	4,939	73.09
Navotas	4,607	2,060	1,999	4,059	88.11	2,121	2,056	4,177	90.67
Valenzuela City	11,465	4,175	3,986	8,161	71.18	4,080	3,795	7,875	68.69
Caloocan City	29,270	9,751	9,059	18,810	64.26	8,394	8,051	16,445	56.18
Marikina City	8,330	2,696	2,525	5,221	62.68	2,365	2,209	4,574	54.91
Pasig City	13,954	4,270	4,088	8,358	59.90	3,663	3,562	7,225	51.78
Pateros	1,181	469	504	973	82.39	453	498	951	80.52
Taguig	14,874	4,894	4,827	9,721	65.36	4,665	4,406	9,071	60.99
Quezon City	54,246	22,478	22,059	44,537	82.10	20,647	20,839	41,486	76.48
Makati City	10,765	3,262	3,014	6,276	58.30	2,632	2,451	5,083	47.22
Mandaluyong City	7,137	2,139	2,109	4,248	59.52	1,989	1,993	3,982	55.79
San Juan	2,254	471	503	974	43.21	528	534	1,062	47.12
Manila City	32,887	9,373	9,903	19,276	58.61	8,156	9,165	17,321	52.67
Las Piñas City	10,879	3,884	3,732	7,616	70.01	4,649	4,548	9,197	84.54
Muntinlupa City	9,317	3,948	3,599	7,547	81.00	3,349	3,127	6,476	69.51
Parañaque City	12,298	3,428	3,133	6,561	53.35	2,970	2,864	5,834	47.44
Pasay City	7,700	3,120	2,982	6,102	79.25	3,084	2,961	6,045	78.51
**C A R**	**35,143**	**12,193**	**11,371**	**23,564**	**67.05**	**12,911**	**12,028**	**24,939**	**70.96**
Abra	4,287	1,328	1,271	2,599	60.63	1,424	1,392	2,816	65.69
Apayao	2,476	896	816	1,712	69.14	877	837	1,714	69.22
Benguet	9,140	3,216	3,005	6,221	68.06	3,326	3,059	6,385	69.86
Ifugao	4,439	1,593	1,407	3,000	67.58	1,601	1,488	3,089	69.59
Kalinga	4,660	1,977	1,842	3,819	81.95	2,125	1,872	3,997	85.77
Mt. Province	3,066	1,041	951	1,992	64.97	1,097	1,007	2,104	68.62
Baguio City	7,075	2,142	2,079	4,221	59.66	2,461	2,373	4,834	68.33
**Region 1**	**97,198**	**38,174**	**35,631**	**73,805**	**75.93**	**40,358**	**37,971**	**78,329**	**80.59**
Ilocos Norte	7,986	2,800	2,623	5,423	67.91	2,830	2,764	5,594	70.05
Ilocos Sur	9,197	3,971	3,779	7,750	84.27	4,186	3,994	8,180	88.94
La Union	11,429	4,349	4,094	8,443	73.87	4,559	4,264	8,823	77.20
Pangasinan	50,454	19,883	18,448	38,331	75.97	21,334	19,858	41,192	81.64
Alaminos City	1,905	776	692	1,468	77.06	815	719	1,534	80.52
Candon City	976	497	449	946	96.93	496	455	951	97.44
Dagupan City	3,636	1,264	1,123	2,387	65.65	1,381	1,257	2,638	72.55
Laoag City	1,845	986	1,010	1,996	108.18	987	1,023	2,010	108.94
San Carlos City	3,999	1,670	1,483	3,153	78.84	1,565	1,468	3,033	75.84
San Fernando City	2,092	686	653	1,339	64.01	719	684	1,403	67.07
Urdaneta City	2,819	972	1,004	1,976	70.10	1,028	1,073	2,101	74.53
Vigan City	860	320	273	593	68.95	458	412	870	101.16

Table 1.C.1.5 - Immunization Services for Infants and Children

Number and Proportion of Children vaccinated with 2 doses of Measles Containing Vaccine (MCV)
Philippines, 2021

Area	Eligible Pop (Under 1 y.o.)	MCV 1				MCV 2			
		Male	Female	Total	%	Male	Female	Total	%
**Region 2**	**69,228**	**25,764**	**24,000**	**49,764**	**71.88**	**26,799**	**25,271**	**52,070**	**75.22**
Batanes	351	108	105	213	60.68	128	114	242	68.95
Cagayan	20,563	7,101	6,388	13,489	65.60	7,222	6,529	13,751	66.87
Isabela	23,543	8,470	8,057	16,527	70.20	8,789	8,539	17,328	73.60
Nueva Vizcaya	9,555	3,419	3,168	6,587	68.94	3,782	3,474	7,256	75.94
Quirino	4,038	1,739	1,696	3,435	85.07	1,927	1,894	3,821	94.63
Cauayan City	2,576	1,138	971	2,109	81.87	1,050	1,005	2,055	79.77
Ilagan City	2,901	1,322	1,326	2,648	91.28	1,411	1,398	2,809	96.83
Santiago City	2,683	1,372	1,311	2,683	100.00	1,389	1,294	2,683	100.00
Tuguegarao City	3,018	1,095	978	2,073	68.69	1,101	1,024	2,125	70.41
**Region 3**	**220,202**	**84,360**	**80,368**	**164,728**	**74.81**	**81,251**	**78,284**	**159,535**	**72.45**
Aurora	4,764	1,712	1,610	3,322	69.73	1,826	1,784	3,610	75.78
Bataan	13,814	5,665	5,262	10,927	79.10	4,958	4,734	9,692	70.16
Bulacan	43,722	15,369	15,485	30,854	70.57	15,093	15,312	30,405	69.54
Nueva Ecija	28,915	8,629	7,976	16,605	57.43	8,382	7,823	16,205	56.04
Pampanga	31,503	13,272	12,883	26,155	83.02	12,552	12,186	24,738	78.53
Tarlac	19,565	8,995	8,444	17,439	89.13	9,346	8,799	18,145	92.74
Zambales	12,551	3,619	3,313	6,932	55.23	3,187	3,056	6,243	49.74
Angeles City	7,905	3,505	3,236	6,741	85.28	3,486	3,235	6,721	85.02
Balanga City	1,996	730	701	1,431	71.69	726	662	1,388	69.54
Cabanatuan City	5,921	2,469	2,224	4,693	79.26	2,444	2,293	4,737	80.00
City of San Fernando	5,890	2,482	2,217	4,699	79.78	2,139	1,915	4,054	68.83
Gapan City	2,159	792	818	1,610	74.57	731	781	1,512	70.03
Mabalacat City	4,811	2,170	2,082	4,252	88.38	2,192	2,219	4,411	91.69
Malolos City	4,885	1,564	1,369	2,933	60.04	1,278	1,155	2,433	49.81
Meycauayan	4,050	1,680	1,663	3,343	82.54	1,609	1,597	3,206	79.16
Olongapo	4,952	1,485	1,365	2,850	57.55	1,271	1,164	2,435	49.17
Palayan City	805	329	298	627	77.89	307	289	596	74.04
San Jose City	2,736	1,339	1,240	2,579	94.26	1,340	1,206	2,546	93.06
San Jose del Monte City	11,120	4,836	4,634	9,470	85.16	4,547	4,382	8,929	80.30
Science City of Munoz	1,594	606	574	1,180	74.03	611	627	1,238	77.67
Tarlac City	6,544	3,112	2,974	6,086	93.00	3,226	3,065	6,291	96.13
**Region 4A**	**298,398**	**101,931**	**94,126**	**196,057**	**65.70**	**96,281**	**90,087**	**186,368**	**62.46**
Batangas	38,848	14,095	12,734	26,829	69.06	13,729	12,568	26,297	67.69
Cavite	27,948	9,949	9,414	19,363	69.28	9,808	9,041	18,849	67.44
Laguna	19,727	6,457	5,956	12,413	62.92	6,002	5,561	11,563	58.62
Quezon	37,644	11,339	10,501	21,840	58.02	10,792	10,054	20,846	55.38
Rizal	44,826	11,674	10,752	22,426	50.03	10,449	9,861	20,310	45.31
Antipolo City	16,510	6,373	5,830	12,203	73.91	5,669	5,504	11,173	67.67
Bacoor City	12,252	3,399	3,153	6,552	53.48	3,076	2,853	5,929	48.39
Batangas City	6,895	2,144	2,035	4,179	60.61	2,087	1,905	3,992	57.90
Biñan City	6,607	3,582	3,526	7,108	107.58	3,586	3,468	7,054	106.77
Cabuyao City	6,128	2,921	2,762	5,683	92.74	2,841	2,728	5,569	90.88
Calamba City	9,025	3,899	3,574	7,473	82.80	3,710	3,437	7,147	79.19
Cavite City	2,098	237	205	442	21.07	193	170	363	17.30
Dasmariñas City	13,442	4,445	4,073	8,518	63.37	4,076	3,714	7,790	57.95

Table 1.C.1.5 - Immunization Services for Infants and Children

Number and Proportion of Children vaccinated with 2 doses of Measles Containing Vaccine (MCV)
Philippines, 2021

Area	Eligible Pop (Under 1 y.o.)	MCV 1				MCV 2			
		Male	Female	Total	%	Male	Female	Total	%
General Trias City	6,410	2,578	2,441	5,019	78.30	2,378	2,293	4,671	72.87
Imus City	8,235	2,778	2,636	5,414	65.74	2,612	2,484	5,096	61.88
Lipa City	6,954	2,657	2,422	5,079	73.04	2,317	2,181	4,498	64.68
Lucena City	5,704	1,963	1,912	3,875	67.93	1,931	1,844	3,775	66.18
San Pablo City	5,277	2,059	1,791	3,850	72.96	2,052	1,876	3,928	74.44
San Pedro City	6,465	1,783	1,586	3,369	52.11	1,533	1,405	2,938	45.44
Santa Rosa City	7,023	3,371	2,979	6,350	90.42	3,496	3,451	6,947	98.92
Tagaytay City	1,451	712	684	1,396	96.21	788	736	1,524	105.03
Tanauan City	3,620	1,212	1,069	2,281	63.01	1,135	994	2,129	58.81
Tayabas City	2,134	845	802	1,647	77.18	797	797	1,594	74.70
Trece Martires City	3,175	1,459	1,289	2,748	86.55	1,224	1,162	2,386	75.15
**Region 4B**	**72,035**	**22,910**	**21,266**	**44,176**	**61.33**	**23,345**	**22,082**	**45,427**	**63.06**
Marinduque	4,906	1,635	1,374	3,009	61.33	1,725	1,532	3,257	66.39
Mindoro Occidental	12,449	4,266	3,927	8,193	65.81	4,388	4,136	8,524	68.47
Mindoro Oriental	20,011	5,965	5,529	11,494	57.44	6,090	5,680	11,770	58.82
Palawan	21,676	6,544	6,262	12,806	59.08	6,671	6,488	13,159	60.71
Romblon	6,482	2,287	2,130	4,417	68.14	2,410	2,225	4,635	71.51
Puerto Princesa City	6,511	2,213	2,044	4,257	65.38	2,061	2,021	4,082	62.69
**Region 5**	**137,350**	**44,495**	**41,115**	**85,610**	**62.33**	**44,713**	**42,002**	**86,715**	**63.13**
Albay	23,903	7,906	7,332	15,238	63.75	8,442	7,987	16,429	68.73
Camarines Norte	14,340	4,813	4,413	9,226	64.34	4,859	4,520	9,379	65.40
Camarines Sur	39,151	12,610	11,693	24,303	62.08	12,114	11,311	23,425	59.83
Catanduanes	6,381	1,955	1,853	3,808	59.68	2,066	1,913	3,979	62.36
Masbate	22,565	7,068	6,665	13,733	60.86	6,957	6,636	13,593	60.24
Sorsogon	19,470	6,196	5,636	11,832	60.77	6,150	5,812	11,962	61.44
Iriga City	2,665	766	687	1,453	54.52	871	762	1,633	61.28
Legaspi City	4,205	1,626	1,422	3,048	72.49	1,600	1,583	3,183	75.70
Naga City	4,670	1,555	1,414	2,969	63.58	1,654	1,478	3,132	67.07
**Region 6**	**146,545**	**51,584**	**47,826**	**99,410**	**67.84**	**53,974**	**50,761**	**104,735**	**71.47**
Aklan	11,231	3,844	3,524	7,368	65.60	3,927	3,691	7,618	67.83
Antique	12,985	3,925	3,605	7,530	57.99	4,284	4,007	8,291	63.85
Capiz	13,984	4,542	4,383	8,925	63.82	4,582	4,468	9,050	64.72
Guimaras	3,085	1,205	1,182	2,387	77.37	1,347	1,288	2,635	85.41
Iloilo	36,414	12,618	11,484	24,102	66.19	13,306	12,471	25,777	70.79
Negros Occidental	49,314	18,774	17,418	36,192	73.39	19,262	18,108	37,370	75.78
Bacolod City	11,102	3,632	3,307	6,939	62.50	3,917	3,502	7,419	66.83
Iloilo City	8,430	3,044	2,923	5,967	70.78	3,349	3,226	6,575	78.00
**Region 7**	**162,860**	**62,335**	**59,714**	**122,049**	**74.94**	**62,659**	**61,195**	**123,854**	**76.05**
Bohol	27,637	9,345	9,188	18,533	67.06	9,167	9,232	18,399	66.57
Cebu	67,046	26,682	24,773	51,455	76.75	26,831	25,477	52,308	78.02
Negros Oriental	27,922	9,901	10,203	20,104	72.00	9,253	9,596	18,849	67.51
Siquijor	1,636	642	620	1,262	77.14	688	627	1,315	80.38

Table 1.C.1.5 - Immunization Services for Infants and Children

Number and Proportion of Children vaccinated with 2 doses of Measles Containing Vaccine (MCV)
Philippines, 2021

Area	Eligible Pop (Under 1 y.o.)	MCV 1				MCV 2			
		Male	Female	Total	%	Male	Female	Total	%
Cebu City	21,037	7,312	6,662	13,974	66.43	8,192	7,855	16,047	76.28
Lapu-Lapu City	9,310	4,930	4,845	9,775	104.99	4,941	4,881	9,822	105.50
Mandaue City	8,272	3,523	3,423	6,946	83.97	3,587	3,527	7,114	86.00
**Region 8**	**104,016**	**31,902**	**28,659**	**60,561**	**58.22**	**30,778**	**29,017**	**59,795**	**57.49**
Biliran	3,856	1,351	1,172	2,523	65.43	1,382	1,245	2,627	68.13
Eastern Samar	11,635	3,181	2,984	6,165	52.99	3,448	3,143	6,591	56.65
Northern Leyte	35,052	11,656	9,770	21,426	61.13	10,334	9,591	19,925	56.84
Northern Samar	15,655	4,507	4,164	8,671	55.39	4,342	4,146	8,488	54.22
Southern Leyte	6,556	1,932	1,792	3,724	56.80	2,023	1,814	3,837	58.53
Western Samar	14,515	4,200	3,877	8,077	55.65	4,152	4,051	8,203	56.51
Calbayog City	4,474	1,100	1,047	2,147	47.99	1,053	976	2,029	45.35
Maasin City	1,661	523	475	998	60.08	569	545	1,114	67.07
Ormoc City	4,989	1,759	1,632	3,391	67.97	1,762	1,691	3,453	69.21
Tacloban City	5,623	1,693	1,746	3,439	61.16	1,713	1,815	3,528	62.74
**Region 9**	**79,552**	**27,672**	**26,144**	**53,816**	**67.65**	**28,309**	**27,094**	**55,403**	**69.64**
Zamboanga del Norte	17,075	6,274	6,042	12,316	72.13	6,677	6,435	13,112	76.79
Zamboanga del Sur	17,506	5,111	4,979	10,090	57.64	5,225	5,191	10,416	59.50
Zamboanga Sibugay	14,992	4,379	4,188	8,567	57.14	4,420	4,298	8,718	58.15
Dapitan City	1,755	771	831	1,602	91.28	761	829	1,590	90.60
Dipolog City	2,794	1,073	1,043	2,116	75.73	1,067	1,051	2,118	75.81
Isabela City	2,544	953	863	1,816	71.38	1,058	1,022	2,080	81.76
Pagadian City	4,290	1,694	1,252	2,946	68.67	1,649	1,217	2,866	66.81
Zamboanga City	18,596	7,417	6,946	14,363	77.24	7,452	7,051	14,503	77.99
**Region 10**	**100,690**	**40,688**	**38,618**	**79,306**	**78.76**	**40,503**	**38,680**	**79,183**	**78.64**
Bukidnon	23,307	9,389	8,576	17,965	77.08	8,792	8,428	17,220	73.88
Camiguin	1,854	627	582	1,209	65.21	611	575	1,186	63.97
Lanao del Norte	14,955	4,616	4,592	9,208	61.57	4,771	4,735	9,506	63.56
Misamis Occidental	6,413	2,637	2,499	5,136	80.09	2,542	2,362	4,904	76.47
Misamis Oriental	15,058	7,226	7,169	14,395	95.60	7,043	6,856	13,899	92.30
Cagayan de Oro City	14,263	5,713	5,412	11,125	78.00	6,122	5,725	11,847	83.06
El Salvador City	1,061	428	375	803	75.68	422	406	828	78.04
Gingoog City	2,627	1,535	1,603	3,138	119.45	1,431	1,514	2,945	112.11
Iligan City	7,575	2,796	2,481	5,277	69.66	2,948	2,654	5,602	73.95
Malaybalay City	3,882	1,808	1,545	3,353	86.37	1,740	1,571	3,311	85.29
Oroquieta City	1,389	519	375	894	64.36	577	515	1,092	78.62
Ozamis City	2,782	1,160	1,078	2,238	80.45	1,345	1,122	2,467	88.68
Tangub City	1,234	516	522	1,038	84.12	468	476	944	76.50
Valencia City	4,290	1,718	1,809	3,527	82.21	1,691	1,741	3,432	80.00
**Region 11**	**107,847**	**39,534**	**36,808**	**76,342**	**70.79**	**38,979**	**36,768**	**75,747**	**70.24**
Davao de Oro	15,526	5,749	5,162	10,911	70.28	5,812	5,339	11,151	71.82
Davao del Norte	21,184	8,899	8,483	17,382	82.05	9,385	8,943	18,328	86.52
Davao Oriental	13,125	4,382	4,083	8,465	64.50	4,391	4,144	8,535	65.03
Davao del Sur	14,355	5,593	4,894	10,487	73.05	4,897	4,567	9,464	65.93
Davao Occidental	6,598	1,396	1,388	2,784	42.19	1,340	1,340	2,680	40.62
Davao City	37,059	13,515	12,798	26,313	71.00	13,154	12,435	25,589	69.05

Table 1.C.1.5 - Immunization Services for Infants and Children

Number and Proportion of Children vaccinated with 2 doses of Measles Containing Vaccine (MCV)
Philippines, 2021

Area	Eligible Pop (Under 1 y.o.)	MCV 1				MCV 2			
		Male	Female	Total	%	Male	Female	Total	%
**Region 12**	**104,693**	**33,663**	**31,598**	**65,261**	**62.34**	**35,093**	**33,314**	**68,407**	**65.34**
North Cotabato	33,933	8,998	8,489	17,487	51.53	9,346	8,674	18,020	53.10
Sarangani	12,904	4,694	4,434	9,128	70.74	5,055	4,820	9,875	76.53
South Cotabato	21,039	7,948	7,531	15,479	73.57	8,248	8,118	16,366	77.79
Sultan Kudarat	17,331	6,130	5,601	11,731	67.69	6,595	6,075	12,670	73.11
Cotabato City	5,831	1,704	1,632	3,336	57.21	1,885	1,785	3,670	62.94
Gen. Santos City	13,655	4,189	3,911	8,100	59.32	3,964	3,842	7,806	57.17
**BARMM**	**94,148**	**30,650**	**31,812**	**62,462**	**66.34**	**29,685**	**30,667**	**60,352**	**64.10**
Basilan	7,672	1,127	1,181	2,308	30.08	1,003	1,037	2,040	26.59
Lanao del Sur	21,391	8,219	8,879	17,098	79.93	8,535	9,090	17,625	82.39
Maguindanao	31,650	11,634	11,663	23,297	73.61	11,204	10,982	22,186	70.10
Sulu	16,891	4,147	4,495	8,642	51.16	4,063	4,348	8,411	49.80
Tawi-Tawi	9,319	2,831	3,017	5,848	62.75	2,655	2,969	5,624	60.35
Lamitan City	2,112	505	476	981	46.45	472	492	964	45.64
Marawi City	5,113	2,187	2,101	4,288	83.86	1,753	1,749	3,502	68.49
**CARAGA**	**60,342**	**21,320**	**19,843**	**41,163**	**68.22**	**23,347**	**22,466**	**45,813**	**75.92**
Agusan del Norte	8,116	2,717	2,581	5,298	65.28	3,046	2,850	5,896	72.65
Agusan del Sur	17,658	6,001	5,565	11,566	65.50	6,891	6,783	13,674	77.44
Surigao del Norte	7,195	2,730	2,524	5,254	73.02	2,874	2,786	5,660	78.67
Surigao del Sur	11,532	3,780	3,474	7,254	62.90	3,803	3,598	7,401	64.18
Province of Dinagat	2,574	774	684	1,458	56.64	817	760	1,577	61.27
Bislig City	2,185	766	710	1,476	67.55	786	728	1,514	69.29
Butuan City	7,732	3,206	3,056	6,262	80.99	3,673	3,629	7,302	94.44
Surigao City	3,350	1,346	1,249	2,595	77.46	1,457	1,332	2,789	83.25

Table 1.C.1.6 - Immunization Services for Infants and Children

Number and Proportion of Fully Immunized Children & Completely Immunized Children
Philippines, 2021

Area	Eligible Pop (Under 1 y.o.)	FIC				CIC			
		Male	Female	Total	%	Male	Female	Total	%
**PHILIPPINES**	**2,128,168**	**682,772**	**654,971**	**1,337,743**	**62.86**	**116,132**	**112,298**	**228,430**	**10.73**
**N C R**	**237,921**	**70,884**	**70,639**	**141,523**	**59.48**	**25,804**	**25,953**	**51,757**	**21.75**
Malabon	6,757	2,552	2,593	5,145	76.14	2,498	2,530	5,028	74.41
Navotas	4,607	1,911	1,840	3,751	81.42	231	211	442	9.59
Valenzuela City	11,465	3,472	3,212	6,684	58.30	1,184	1,108	2,292	19.99
Caloocan City	29,270	7,008	6,739	13,747	46.97	2,630	2,506	5,136	17.55
Marikina City	8,330	2,147	2,027	4,174	50.11	281	292	573	6.88
Pasig City	13,954	3,984	3,853	7,837	56.16	1,094	1,034	2,128	15.25
Pateros	1,181	453	494	947	80.19	0	2	2	0.17
Taguig	14,874	4,500	4,183	8,683	58.38	1,896	1,804	3,700	24.88
Quezon City	54,246	18,877	19,153	38,030	70.11	8,583	8,817	17,400	32.08
Makati City	10,765	3,305	3,092	6,397	59.42	240	235	475	4.41
Mandaluyong City	7,137	1,819	1,851	3,670	51.42	181	157	338	4.74
San Juan	2,254	459	480	939	41.66	77	62	139	6.17
Manila City	32,887	7,139	8,190	15,329	46.61	2,997	3,413	6,410	19.49
Las Piñas City	10,879	3,364	3,272	6,636	61.00	1,223	1,227	2,450	22.52
Muntinlupa City	9,317	3,802	3,441	7,243	77.74	1,560	1,543	3,103	33.30
Parañaque City	12,298	3,202	3,070	6,272	51.00	543	484	1,027	8.35
Pasay City	7,700	2,890	3,149	6,039	78.43	586	528	1,114	14.47
**C A R**	**35,143**	**12,292**	**11,470**	**23,762**	**67.62**	**705**	**616**	**1,321**	**3.76**
Abra	4,287	1,404	1,375	2,779	64.82	14	31	45	1.05
Apayao	2,476	819	791	1,610	65.02	17	13	30	1.21
Benguet	9,140	3,177	2,918	6,095	66.68	171	161	332	3.63
Ifugao	4,439	1,528	1,412	2,940	66.23	81	65	146	3.29
Kalinga	4,660	2,095	1,842	3,937	84.48	37	28	65	1.39
Mt. Province	3,066	1,070	988	2,058	67.12	25	16	41	1.34
Baguio City	7,075	2,199	2,144	4,343	61.39	360	302	662	9.36
**Region 1**	**97,198**	**38,702**	**36,487**	**75,189**	**77.36**	**1,649**	**1,481**	**3,130**	**3.22**
Ilocos Norte	7,986	2,830	2,763	5,593	70.04	0	1	1	0.01
Ilocos Sur	9,197	4,170	3,972	8,142	88.53	16	22	38	0.41
La Union	11,429	4,400	4,131	8,531	74.64	152	130	282	2.47
Pangasinan	50,454	20,386	19,041	39,427	78.14	948	817	1,765	3.50
Alaminos City	1,905	800	689	1,489	78.16	15	30	45	2.36
Candon City	976	496	455	951	97.44	0	0	0	0.00
Dagupan City	3,636	892	810	1,702	46.81	489	447	936	25.74
Laoag City	1,845	987	1,023	2,010	108.94	0	0	0	0.00
San Carlos City	3,999	1,536	1,434	2,970	74.27	29	34	63	1.58
San Fernando City	2,092	719	684	1,403	67.07	0	0	0	0.00
Urdaneta City	2,819	1,028	1,073	2,101	74.53	0	0	0	0.00
Vigan City	860	458	412	870	101.16	0	0	0	0.00

Table 1.C.1.6 - Immunization Services for Infants and Children

Number and Proportion of Fully Immunized Children & Completely Immunized Children
Philippines, 2021

Area	Eligible Pop (Under 1 y.o.)	FIC				CIC			
		Male	Female	Total	%	Male	Female	Total	%
**Region 2**	**69,228**	**26,158**	**24,684**	**50,842**	**73.44**	**1,140**	**1,020**	**2,160**	**3.12**
Batanes	351	128	114	242	68.95	0	0	0	0.00
Cagayan	20,563	7,068	6,326	13,394	65.14	408	347	755	3.67
Isabela	23,543	8,653	8,470	17,123	72.73	382	315	697	2.96
Nueva Vizcaya	9,555	3,645	3,354	6,999	73.25	154	162	316	3.31
Quirino	4,038	1,925	1,890	3,815	94.48	13	19	32	0.79
Cauayan City	2,576	912	823	1,735	67.35	76	87	163	6.33
Ilagan City	2,901	1,406	1,390	2,796	96.38	22	23	45	1.55
Santiago City	2,683	1,389	1,294	2,683	100.00	4	4	8	0.30
Tuguegarao City	3,018	1,032	1,023	2,055	68.09	81	63	144	4.77
**Region 3**	**220,202**	**74,001**	**71,020**	**145,021**	**65.86**	**13,535**	**13,084**	**26,619**	**12.09**
Aurora	4,764	1,826	1,781	3,607	75.71	186	121	307	6.44
Bataan	13,814	4,998	4,837	9,835	71.20	1,077	982	2,059	14.91
Bulacan	43,722	12,785	12,642	25,427	58.16	3,167	3,462	6,629	15.16
Nueva Ecija	28,915	7,059	6,613	13,672	47.28	1,543	1,436	2,979	10.30
Pampanga	31,503	11,830	11,348	23,178	73.57	1,548	1,518	3,066	9.73
Tarlac	19,565	8,663	8,160	16,823	85.99	740	676	1,416	7.24
Zambales	12,551	2,601	2,478	5,079	40.47	833	768	1,601	12.76
Angeles City	7,905	3,290	3,090	6,380	80.71	421	397	818	10.35
Balanga City	1,996	719	649	1,368	68.54	111	128	239	11.97
Cabanatuan City	5,921	2,056	1,954	4,010	67.73	383	344	727	12.28
City of San Fernando	5,890	1,972	1,794	3,766	63.94	480	406	886	15.04
Gapan City	2,159	706	746	1,452	67.25	84	80	164	7.60
Mabalacat City	4,811	2,040	2,030	4,070	84.60	122	139	261	5.43
Malolos City	4,885	1,269	1,134	2,403	49.19	315	264	579	11.85
Meycauayan	4,050	1,601	1,571	3,172	78.32	547	540	1,087	26.84
Olongapo	4,952	1,039	984	2,023	40.85	359	294	653	13.19
Palayan City	805	252	234	486	60.37	84	75	159	19.75
San Jose City	2,736	1,190	1,086	2,276	83.19	137	109	246	8.99
San Jose del Monte City	11,120	4,482	4,410	8,892	79.96	888	850	1,738	15.63
Science City of Munoz	1,594	570	579	1,149	72.08	44	45	89	5.58
Tarlac City	6,544	3,053	2,900	5,953	90.97	466	450	916	14.00
**Region 4A**	**298,398**	**83,000**	**77,784**	**160,784**	**53.88**	**20,405**	**19,225**	**39,630**	**13.28**
Batangas	38,848	11,916	10,792	22,708	58.45	3,769	3,438	7,207	18.55
Cavite	27,948	8,598	8,023	16,621	59.47	1,202	1,068	2,270	8.12
Laguna	19,727	4,826	4,494	9,320	47.24	1,313	1,198	2,511	12.73
Quezon	37,644	8,737	8,480	17,217	45.74	2,234	2,057	4,291	11.40
Rizal	44,826	9,028	8,425	17,453	38.93	2,263	2,267	4,530	10.11
Antipolo City	16,510	4,771	4,618	9,389	56.87	1,394	1,468	2,862	17.33
Bacoor City	12,252	2,445	2,212	4,657	38.01	565	524	1,089	8.89
Batangas City	6,895	1,866	1,696	3,562	51.66	216	206	422	6.12
Biñan City	6,607	3,541	3,473	7,014	106.16	909	863	1,772	26.82
Cabuyao City	6,128	2,718	2,616	5,334	87.04	800	839	1,639	26.75
Calamba City	9,025	3,309	3,042	6,351	70.37	468	471	939	10.40
Cavite City	2,098	179	157	336	16.02	3	1	4	0.19
Dasmariñas City	13,442	3,741	3,534	7,275	54.12	861	739	1,600	11.90

Table 1.C.1.6 - Immunization Services for Infants and Children

Number and Proportion of Fully Immunized Children & Completely Immunized Children
Philippines, 2021

Area	Eligible Pop (Under 1 y.o.)	FIC				CIC			
		Male	Female	Total	%	Male	Female	Total	%
General Trias City	6,410	1,849	1,817	3,666	57.19	539	475	1,014	15.82
Imus City	8,235	2,124	2,057	4,181	50.77	500	445	945	11.48
Lipa City	6,954	2,016	1,799	3,815	54.86	666	642	1,308	18.81
Lucena City	5,704	1,572	1,498	3,070	53.82	731	663	1,394	24.44
San Pablo City	5,277	1,679	1,592	3,271	61.99	366	324	690	13.08
San Pedro City	6,465	1,337	1,278	2,615	40.45	289	247	536	8.29
Santa Rosa City	7,023	3,392	3,097	6,489	92.40	532	525	1,057	15.05
Tagaytay City	1,451	788	736	1,524	105.03	1	1	2	0.14
Tanauan City	3,620	938	801	1,739	48.04	369	331	700	19.34
Tayabas City	2,134	626	624	1,250	58.58	163	158	321	15.04
Trece Martires City	3,175	1,004	923	1,927	60.69	252	275	527	16.60
**Region 4B**	**72,035**	**21,695**	**20,544**	**42,239**	**58.64**	**2,725**	**2,538**	**5,263**	**7.31**
Marinduque	4,906	1,630	1,446	3,076	62.70	99	74	173	3.53
Mindoro Occidental	12,449	3,885	3,595	7,480	60.09	497	477	974	7.82
Mindoro Oriental	20,011	6,075	5,612	11,687	58.40	805	721	1,526	7.63
Palawan	21,676	5,952	5,960	11,912	54.95	745	665	1,410	6.50
Romblon	6,482	2,327	2,152	4,479	69.10	90	84	174	2.68
Puerto Princesa City	6,511	1,826	1,779	3,605	55.37	489	517	1,006	15.45
**Region 5**	**137,350**	**39,189**	**37,637**	**76,826**	**55.93**	**7,142**	**6,732**	**13,874**	**10.10**
Albay	23,903	7,343	7,131	14,474	60.55	1,273	1,225	2,498	10.45
Camarines Norte	14,340	4,342	4,066	8,408	58.63	559	564	1,123	7.83
Camarines Sur	39,151	9,972	9,590	19,562	49.97	2,422	2,180	4,602	11.75
Catanduanes	6,381	1,976	1,813	3,789	59.38	199	155	354	5.55
Masbate	22,565	6,052	6,207	12,259	54.33	1,509	1,477	2,986	13.23
Sorsogon	19,470	5,655	5,329	10,984	56.41	587	575	1,162	5.97
Iriga City	2,665	817	702	1,519	57.00	94	101	195	7.32
Legaspi City	4,205	1,460	1,372	2,832	67.35	298	280	578	13.75
Naga City	4,670	1,572	1,427	2,999	64.22	201	175	376	8.05
**Region 6**	**146,545**	**49,589**	**46,663**	**96,252**	**65.68**	**5,528**	**5,214**	**10,742**	**7.33**
Aklan	11,231	3,668	3,427	7,095	63.17	643	620	1,263	11.25
Antique	12,985	4,118	3,862	7,980	61.46	180	173	353	2.72
Capiz	13,984	3,974	3,798	7,772	55.58	758	755	1,513	10.82
Guimaras	3,085	1,229	1,197	2,426	78.64	112	92	204	6.61
Iloilo	36,414	13,205	12,378	25,583	70.26	121	100	221	0.61
Negros Occidental	49,314	17,359	16,327	33,686	68.31	2,496	2,336	4,832	9.80
Bacolod City	11,102	2,770	2,530	5,300	47.74	1,135	1,057	2,192	19.74
Iloilo City	8,430	3,266	3,144	6,410	76.04	83	81	164	1.95
**Region 7**	**162,860**	**52,930**	**50,929**	**103,859**	**63.77**	**12,576**	**12,339**	**24,915**	**15.30**
Bohol	27,637	6,851	6,569	13,420	48.56	2,804	2,519	5,323	19.26
Cebu	67,046	22,418	21,223	43,641	65.09	4,917	4,686	9,603	14.32
Negros Oriental	27,922	7,686	7,820	15,506	55.53	2,204	2,473	4,677	16.75
Siquijor	1,636	650	586	1,236	75.55	38	41	79	4.83

Table 1.C.1.6 - Immunization Services for Infants and Children

Number and Proportion of Fully Immunized Children & Completely Immunized Children
Philippines, 2021

Area	Eligible Pop (Under 1 y.o.)	FIC				CIC			
		Male	Female	Total	%	Male	Female	Total	%
Cebu City	21,037	7,052	6,684	13,736	65.29	1,247	1,256	2,503	11.90
Lapu-Lapu City	9,310	4,466	4,389	8,855	95.11	450	464	914	9.82
Mandaue City	8,272	3,807	3,658	7,465	90.24	916	900	1,816	21.95
**Region 8**	**104,016**	**28,271**	**26,604**	**54,875**	**52.76**	**4,237**	**4,054**	**8,291**	**7.97**
Biliran	3,856	1,268	1,135	2,403	62.32	135	127	262	6.79
Eastern Samar	11,635	3,199	2,907	6,106	52.48	668	632	1,300	11.17
Northern Leyte	35,052	9,963	9,241	19,204	54.79	1,109	969	2,078	5.93
Northern Samar	15,655	3,631	3,381	7,012	44.79	1,032	1,102	2,134	13.63
Southern Leyte	6,556	1,915	1,778	3,693	56.33	110	108	218	3.33
Western Samar	14,515	3,663	3,562	7,225	49.78	657	669	1,326	9.14
Calbayog City	4,474	929	853	1,782	39.83	126	92	218	4.87
Maasin City	1,661	520	498	1,018	61.29	63	51	114	6.86
Ormoc City	4,989	1,505	1,465	2,970	59.53	262	227	489	9.80
Tacloban City	5,623	1,678	1,784	3,462	61.57	75	77	152	2.70
**Region 9**	**79,552**	**25,294**	**24,942**	**50,236**	**63.15**	**3,660**	**3,660**	**7,320**	**9.20**
Zamboanga del Norte	17,075	6,118	5,941	12,059	70.62	813	818	1,631	9.55
Zamboanga del Sur	17,506	4,129	4,977	9,106	52.02	877	938	1,815	10.37
Zamboanga Sibugay	14,992	3,827	3,698	7,525	50.19	610	599	1,209	8.06
Dapitan City	1,755	761	829	1,590	90.60	22	28	50	2.85
Dipolog City	2,794	988	954	1,942	69.51	141	134	275	9.84
Isabela City	2,544	954	852	1,806	70.99	183	196	379	14.90
Pagadian City	4,290	1,596	1,145	2,741	63.89	66	102	168	3.92
Zamboanga City	18,596	6,921	6,546	13,467	72.42	948	845	1,793	9.64
**Region 10**	**100,690**	**39,263**	**37,770**	**77,033**	**76.51**	**3,404**	**3,328**	**6,732**	**6.69**
Bukidnon	23,307	8,530	8,239	16,769	71.95	833	826	1,659	7.12
Camiguin	1,854	567	535	1,102	59.44	55	56	111	5.99
Lanao del Norte	14,955	4,588	4,497	9,085	60.75	319	360	679	4.54
Misamis Occidental	6,413	2,620	2,435	5,055	78.82	339	346	685	10.68
Misamis Oriental	15,058	6,880	6,809	13,689	90.91	451	435	886	5.88
Cagayan de Oro City	14,263	5,917	5,608	11,525	80.80	548	481	1,029	7.21
El Salvador City	1,061	362	347	709	66.82	60	59	119	11.22
Gingoog City	2,627	1,349	1,461	2,810	106.97	227	279	506	19.26
Iligan City	7,575	2,804	2,528	5,332	70.39	100	71	171	2.26
Malaybalay City	3,882	1,740	1,571	3,311	85.29	263	249	512	13.19
Oroquieta City	1,389	574	514	1,088	78.33	2	1	3	0.22
Ozamis City	2,782	1,313	1,148	2,461	88.46	38	10	48	1.73
Tangub City	1,234	427	450	877	71.07	11	9	20	1.62
Valencia City	4,290	1,592	1,628	3,220	75.06	158	146	304	7.09
**Region 11**	**107,847**	**37,739**	**35,480**	**73,219**	**67.89**	**4,972**	**4,604**	**9,576**	**8.88**
Davao de Oro	15,526	5,689	5,220	10,909	70.26	103	73	176	1.13
Davao del Norte	21,184	9,012	8,684	17,696	83.53	1,033	965	1,998	9.43
Davao del Sur	13,125	4,562	4,249	8,811	67.13	613	564	1,177	8.97
Davao Oriental	14,355	4,526	4,150	8,676	60.44	674	638	1,312	9.14
Davao Occidental	6,598	1,208	1,127	2,335	35.39	102	125	227	3.44
Davao City	37,059	12,742	12,050	24,792	66.90	2,447	2,239	4,686	12.64

Table 1.C.1.6 - Immunization Services for Infants and Children

Number and Proportion of Fully Immunized Children & Completely Immunized Children
Philippines, 2021

Area	Eligible Pop (Under 1 y.o.)	FIC				CIC			
		Male	Female	Total	%	Male	Female	Total	%
**Region 12**	**104,693**	**32,947**	**31,398**	**64,345**	**61.46**	**3,018**	**2,797**	**5,815**	**5.55**
North Cotabato	33,933	8,435	7,863	16,298	48.03	1,193	1,141	2,334	6.88
Sarangani	12,904	4,897	4,741	9,638	74.69	218	189	407	3.15
South Cotabato	21,039	7,745	7,579	15,324	72.84	614	603	1,217	5.78
Sultan Kudarat	17,331	6,364	5,889	12,253	70.70	304	237	541	3.12
Cotabato City	5,831	1,833	1,735	3,568	61.19	52	50	102	1.75
Gen. Santos City	13,655	3,673	3,591	7,264	53.20	637	577	1,214	8.89
**BARMM**	**94,148**	**28,150**	**29,006**	**57,156**	**60.71**	**4,475**	**4,540**	**9,015**	**9.58**
Basilan	7,672	940	961	1,901	24.78	311	347	658	8.58
Lanao del Sur	21,391	8,326	8,875	17,201	80.41	237	227	464	2.17
Maguindanao	31,650	9,961	9,855	19,816	62.61	1,620	1,637	3,257	10.29
Sulu	16,891	3,447	3,667	7,114	42.12	1,330	1,306	2,636	15.61
Tawi-Tawi	9,319	2,848	3,115	5,963	63.99	233	284	517	5.55
Lamitan City	2,112	422	418	840	39.77	51	79	130	6.16
Marawi City	5,113	2,206	2,115	4,321	84.51	693	660	1,353	26.46
**CARAGA**	**60,342**	**22,668**	**21,914**	**44,582**	**73.88**	**1,157**	**1,113**	**2,270**	**3.76**
Agusan del Norte	8,116	2,897	2,678	5,575	68.69	172	162	334	4.12
Agusan del Sur	17,658	6,885	6,859	13,744	77.83	168	161	329	1.86
Surigao del Norte	7,195	2,721	2,631	5,352	74.38	176	182	358	4.98
Surigao del Sur	11,532	3,582	3,400	6,982	60.54	471	454	925	8.02
Province of Dinagat	2,574	731	731	1,462	56.80	64	55	119	4.62
Bislig City	2,185	796	723	1,519	69.52	32	27	59	2.70
Butuan City	7,732	3,637	3,585	7,222	93.40	36	44	80	1.03
Surigao City	3,350	1,419	1,307	2,726	81.37	38	28	66	1.97

Note: Put asterisk (*) for No Report and Zero (0) for No Case

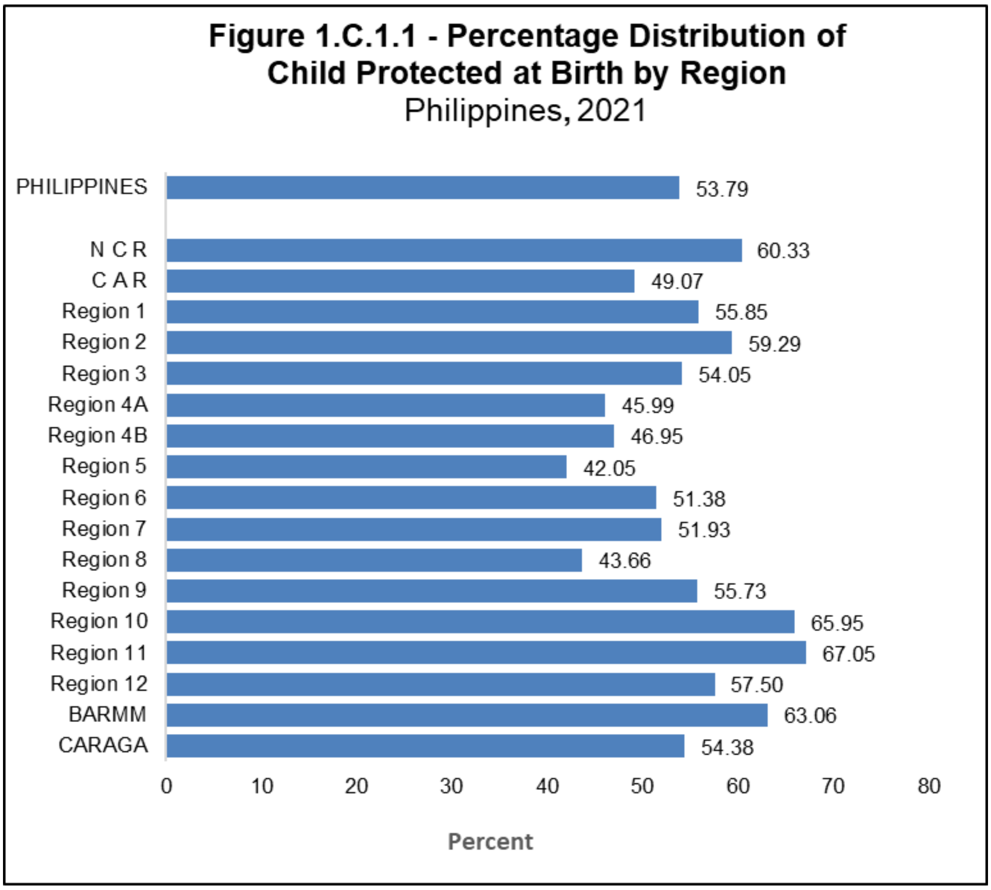
Figure 1.C.1.1 - Percentage Distribution of
Child Protected at Birth by Region
Philippines, 2021
PHILIPPINES 53.79
N C R 60.33
C A R 49.07
Region 1 55.85
Region 2 59.29
Region 3 54.05
Region 4A 45.99
Region 4B 46.95
Region 5 42.05
Region 6 51.38
Region 7 51.93
Region 8 43.66
Region 9 55.73
Region 10 65.95
Region 11 67.05
Region 12 57.50
BARMM 63.06
CARAGA 54.38
0 10 20 30 40 50 60 70 80
Percent

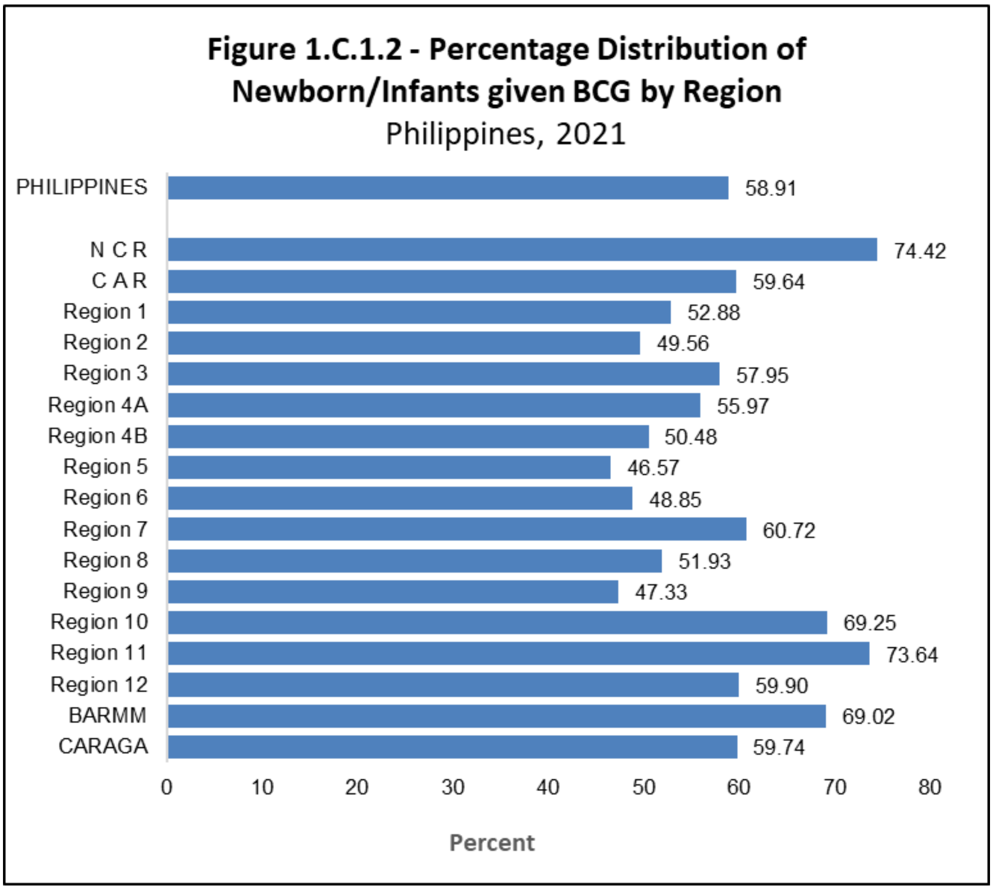
Figure 1.C.1.2 - Percentage Distribution of
Newborn/Infants given BCG by Region
Philippines, 2021
PHILIPPINES 58.91
N C R 74.42
C A R 59.64
Region 1 52.88
Region 2 49.56
Region 3 57.95
Region 4A 55.97
Region 4B 50.48
Region 5 46.57
Region 6 48.85
Region 7 60.72
Region 8 51.93
Region 9 47.33
Region 10 69.25
Region 11 73.64
Region 12 59.90
BARMM 69.02
CARAGA 59.74
0 10 20 30 40 50 60 70 80
Percent

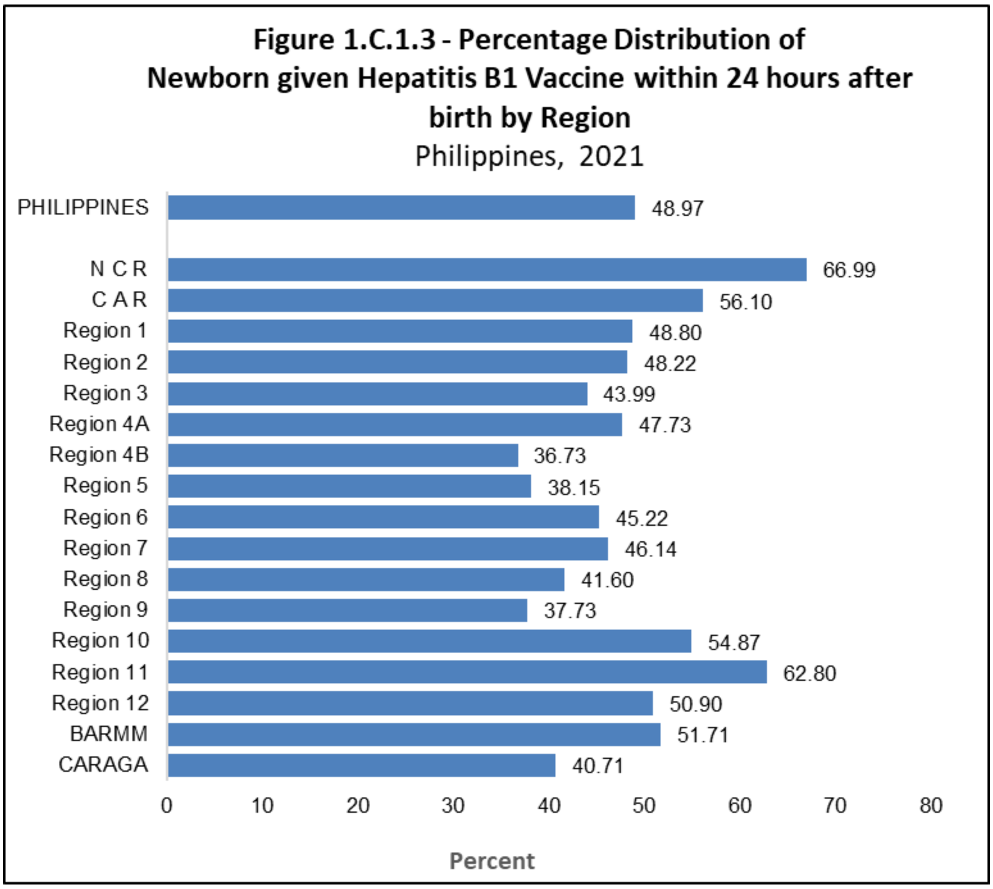

Figure 1.C.1.3 - Percentage Distribution of
Newborn given Hepatitis B1 Vaccine within 24 hours after
birth by Region
Philippines, 2021
PHILIPPINES 48.97
N C R 66.99
C A R 56.10
Region 1 48.80
Region 2 48.22
Region 3 43.99
Region 4A 47.73
Region 4B 36.73
Region 5 38.15
Region 6 45.22
Region 7 46.14
Region 8 41.60
Region 9 37.73
Region 10 54.87
Region 11 62.80
Region 12 50.90
BARMM 51.71
CARAGA 40.71
0 10 20 30 40 50 60 70 80
Percent

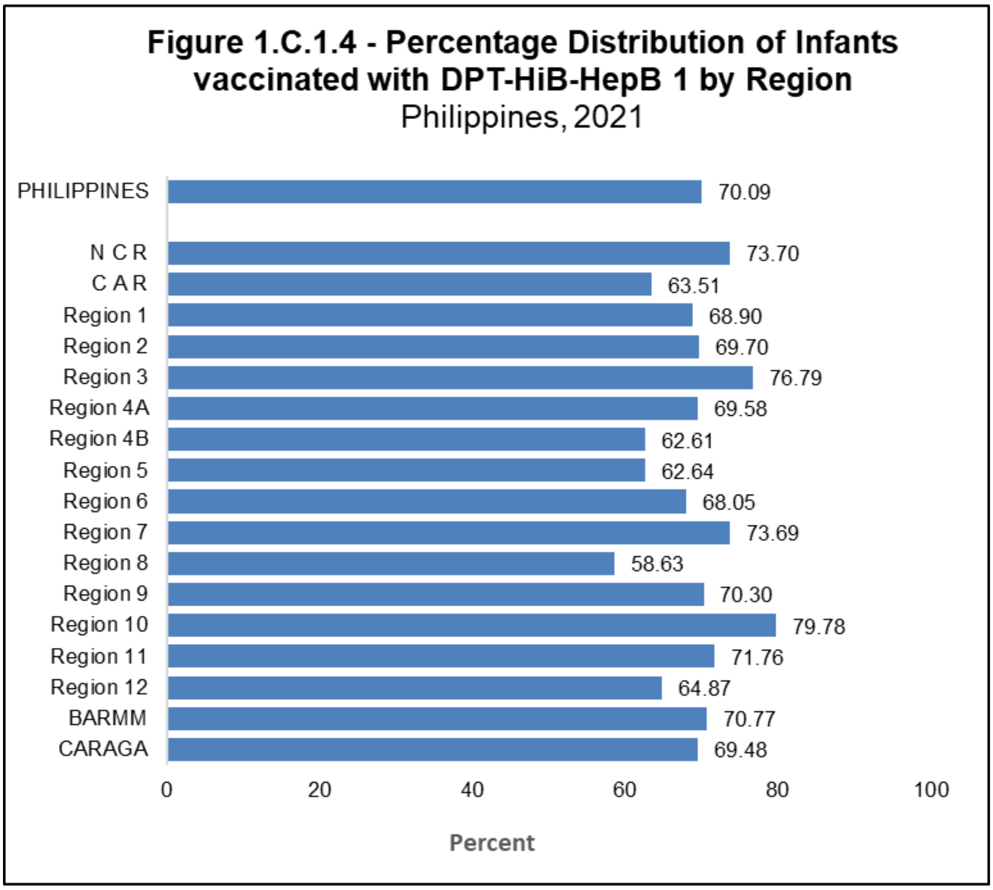

Figure 1.C.1.4 - Percentage Distribution of Infants
vaccinated with DPT-HiB-HepB 1 by Region
Philippines, 2021
PHILIPPINES 70.09
N C R 73.70
C A R 63.51
Region 1 68.90
Region 2 69.70
Region 3 76.79
Region 4A 69.58
Region 4B 62.61
Region 5 62.64
Region 6 68.05
Region 7 73.69
Region 8 58.63
Region 9 70.30
Region 10 79.78
Region 11 71.76
Region 12 64.87
BARMM 70.77
CARAGA 69.48
0 20 40 60 80 100
Percent

Figure 1.C.1.5 - Percentage Distribution of Infants vaccinated with DPT-HiB-HepB 2 by Region
Philippines, 2021

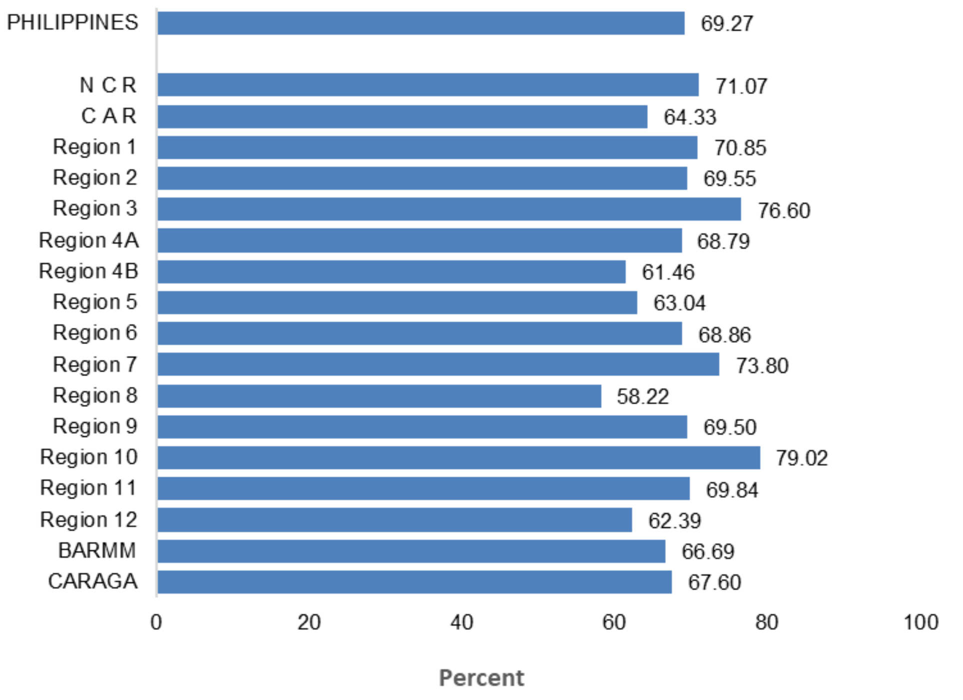

Figure 1.C.1.6 - Percentage Distribution of Infants vaccinated with DPT-HiB-HepB 3 by Region
Philippines, 2021

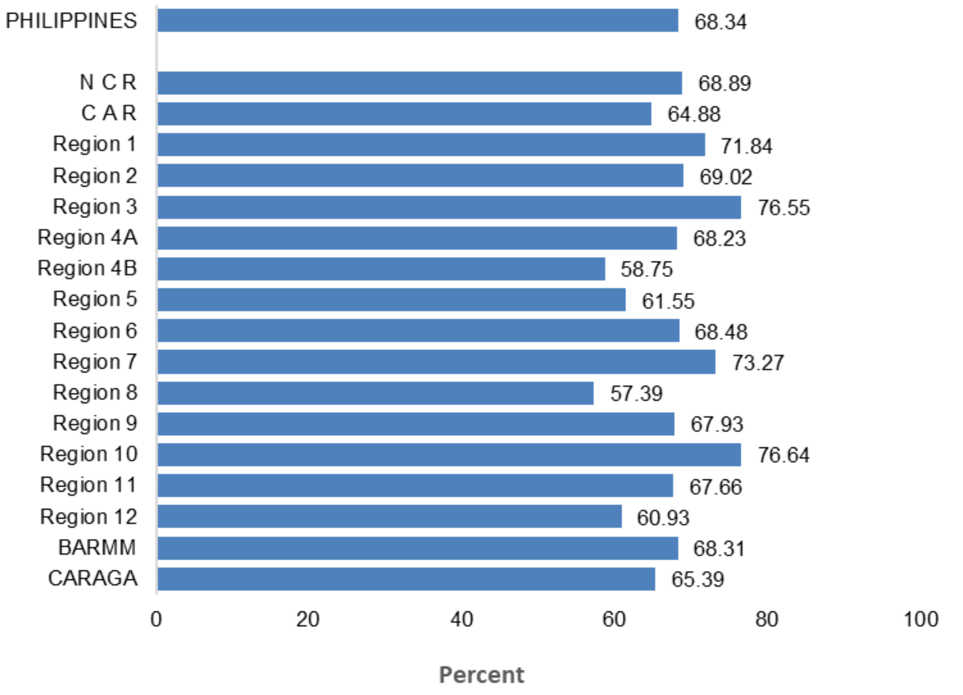

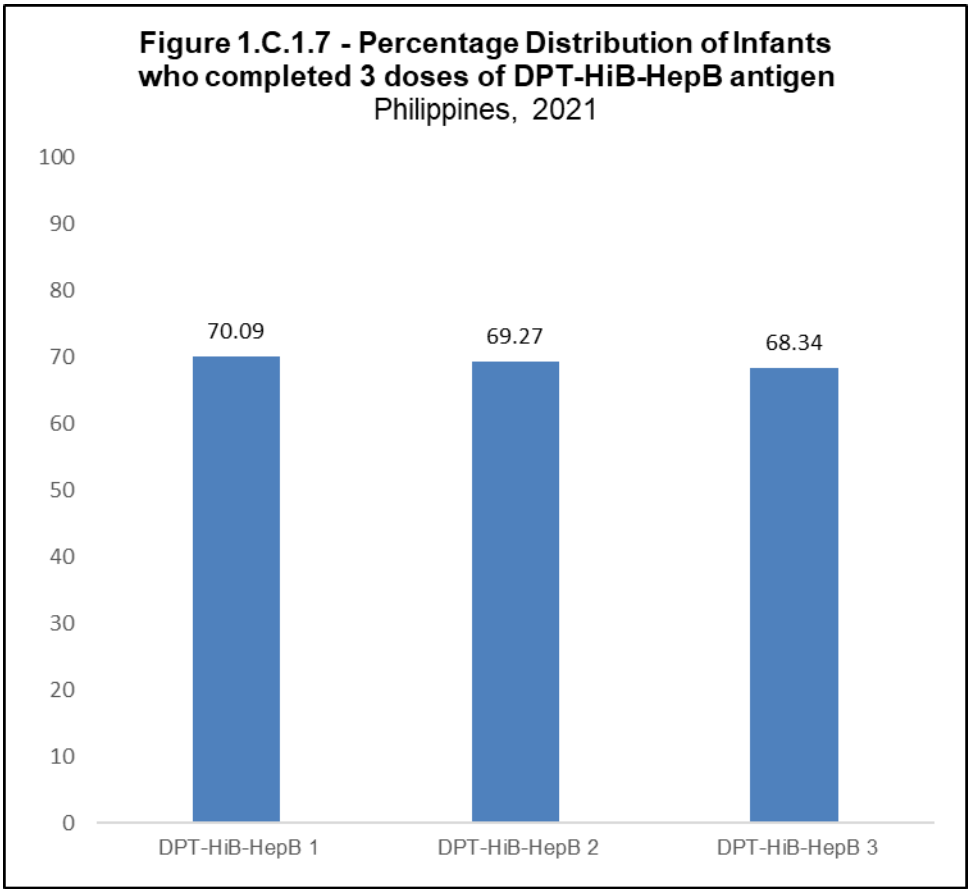
Figure 1.C.1.7 - Percentage Distribution of Infants who completed 3 doses of DPT-HiB-HepB antigen
Philippines, 2021
100
90
80
70
60
50
40
30
20
10
0
70.09
69.27
68.34
DPT-HiB-HepB 1
DPT-HiB-HepB 2
DPT-HiB-HepB 3

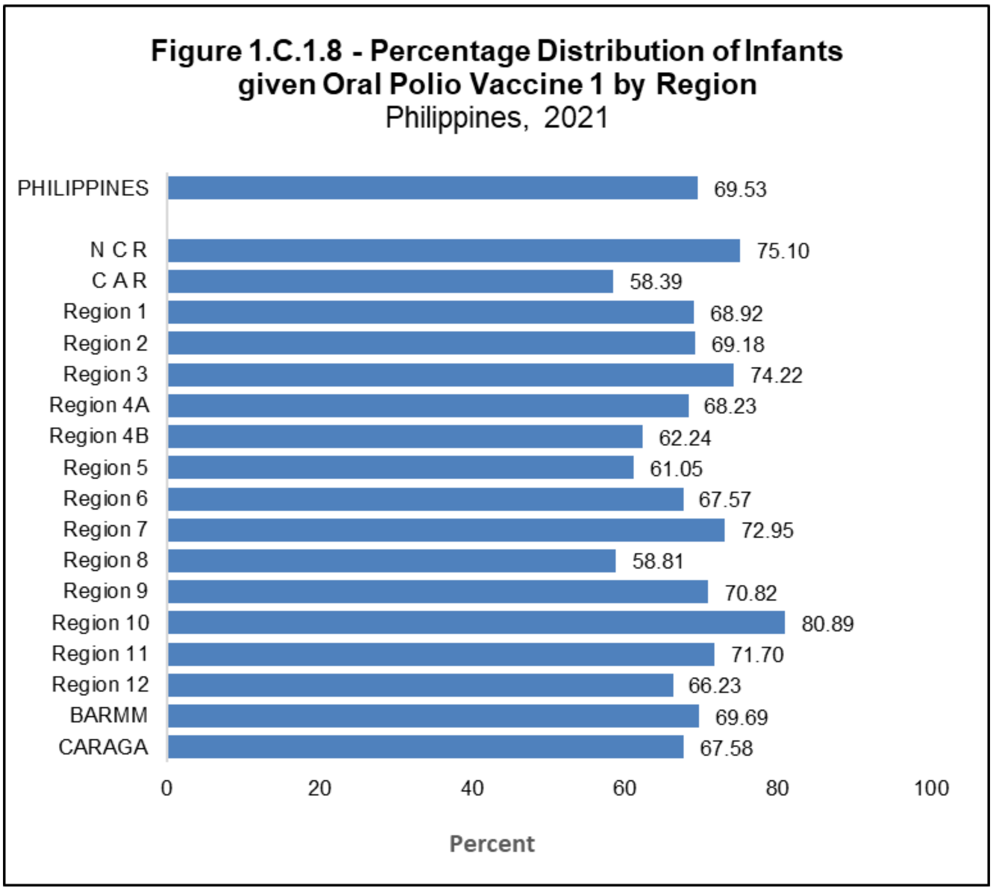
Figure 1.C.1.8 - Percentage Distribution of Infants given Oral Polio Vaccine 1 by Region
Philippines, 2021
PHILIPPINES 69.53
N C R 75.10
C A R 58.39
Region 1 68.92
Region 2 69.18
Region 3 74.22
Region 4A 68.23
Region 4B 62.24
Region 5 61.05
Region 6 67.57
Region 7 72.95
Region 8 58.81
Region 9 70.82
Region 10 80.89
Region 11 71.70
Region 12 66.23
BARMM 69.69
CARAGA 67.58
0
20
40
60
80
100
Percent

Figure 1.C.1.9 - Percentage Distribution of Infants given Oral Polio Vaccine 2 by Region
Philippines, 2021

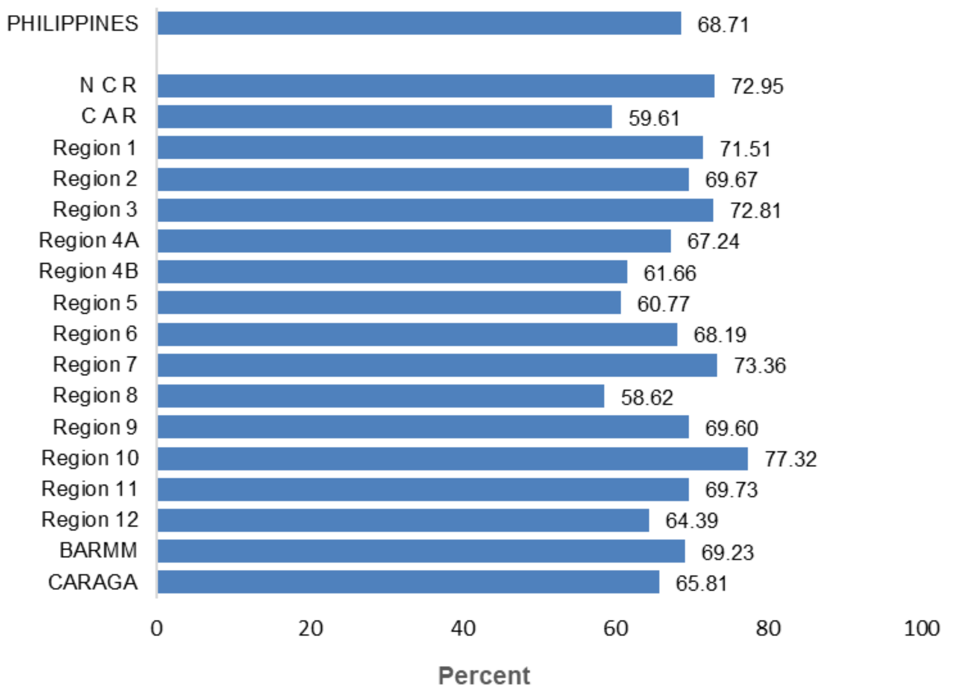

Figure 1.C.1.10 - Percentage Distribution of Infants given Oral Polio Vaccine 3 by Region
Philippines, 2021

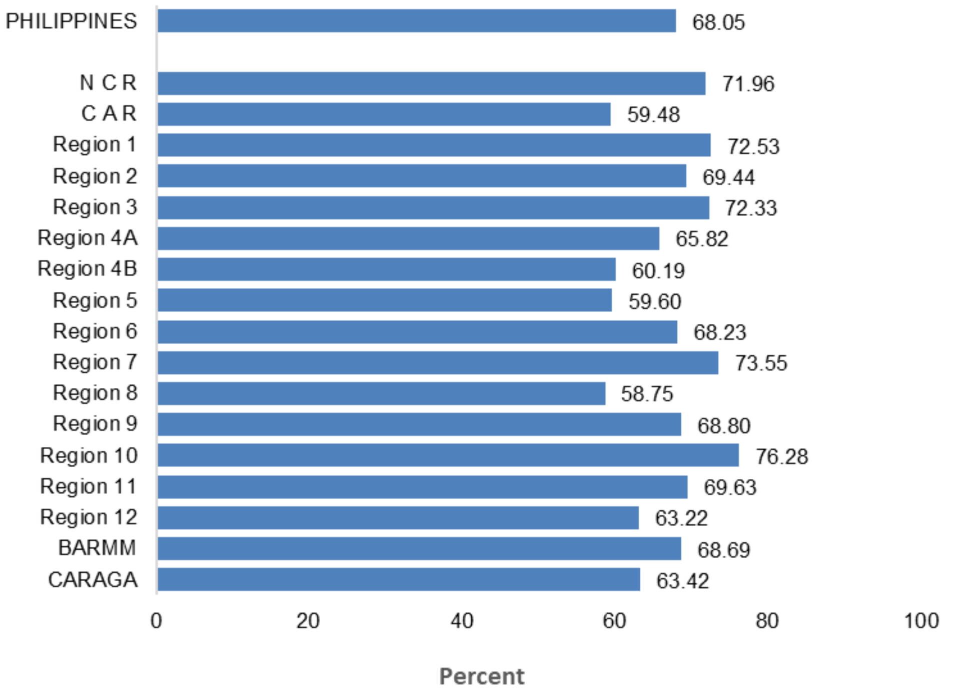

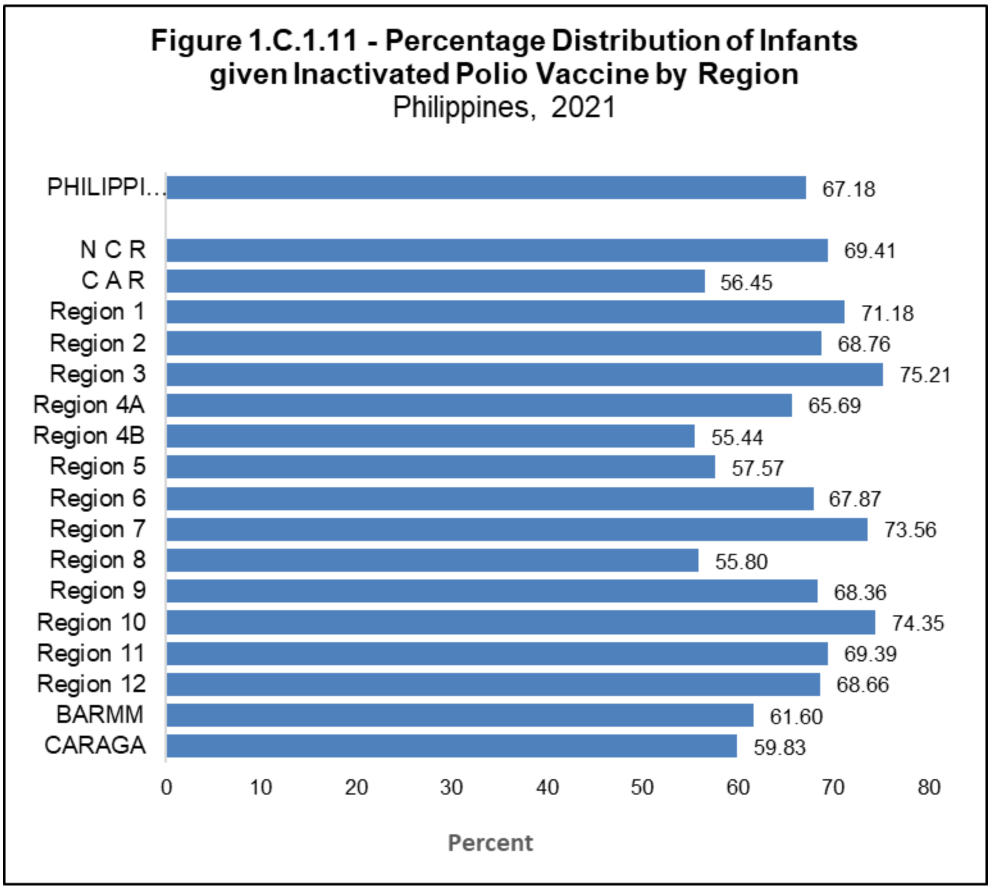
Figure 1.C.1.11 - Percentage Distribution of Infants given Inactivated Polio Vaccine by Region
Philippines, 2021
PHILIPPI...
N C R
C A R
Region 1
Region 2
Region 3
Region 4A
Region 4B
Region 5
Region 6
Region 7
Region 8
Region 9
Region 10
Region 11
Region 12
BARMM
CARAGA
67.18
69.41
56.45
71.18
68.76
75.21
65.69
55.44
57.57
67.87
73.56
55.80
68.36
74.35
69.39
68.66
61.60
59.83
0
10
20
30
40
50
60
70
80
Percent

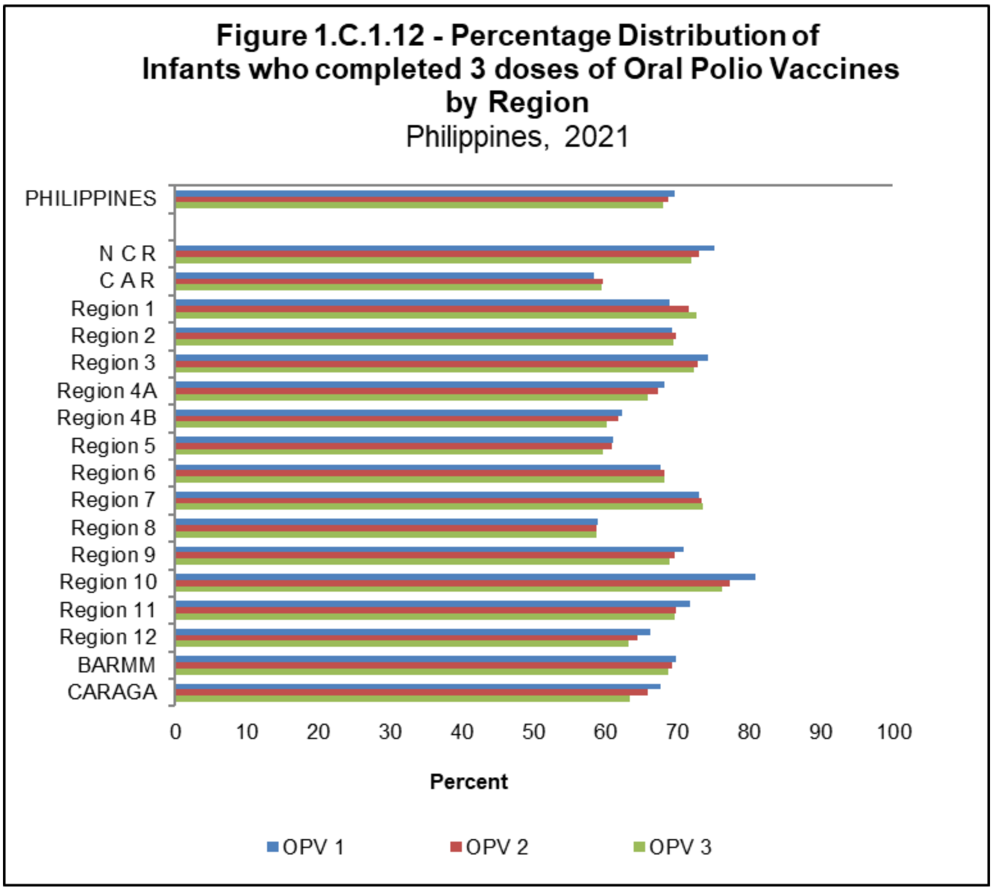
Figure 1.C.1.12 - Percentage Distribution of Infants who completed 3 doses of Oral Polio Vaccines by Region
Philippines, 2021
PHILIPPINES
N C R
C A R
Region 1
Region 2
Region 3
Region 4A
Region 4B
Region 5
Region 6
Region 7
Region 8
Region 9
Region 10
Region 11
Region 12
BARMM
CARAGA
0
10
20
30
40
50
60
70
80
90
100
Percent
OPV 1
OPV 2
OPV 3

Figure 1.C.1.13 - Percentage Distribution of Infants vaccinated with Pneumococcal Conjugate Vaccine 1 by Region
Philippines, 2021

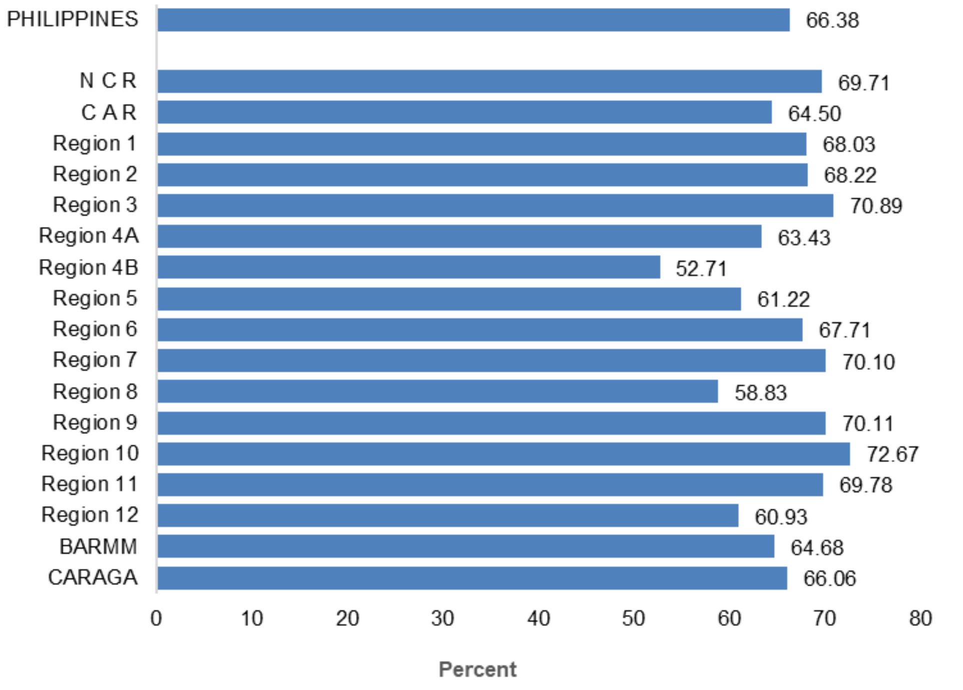

Figure 1.C.1.14 - Percentage Distribution of infants vaccinated with Pneumococcal Conjugate Vaccine 2 by Region
Philippines, 2021

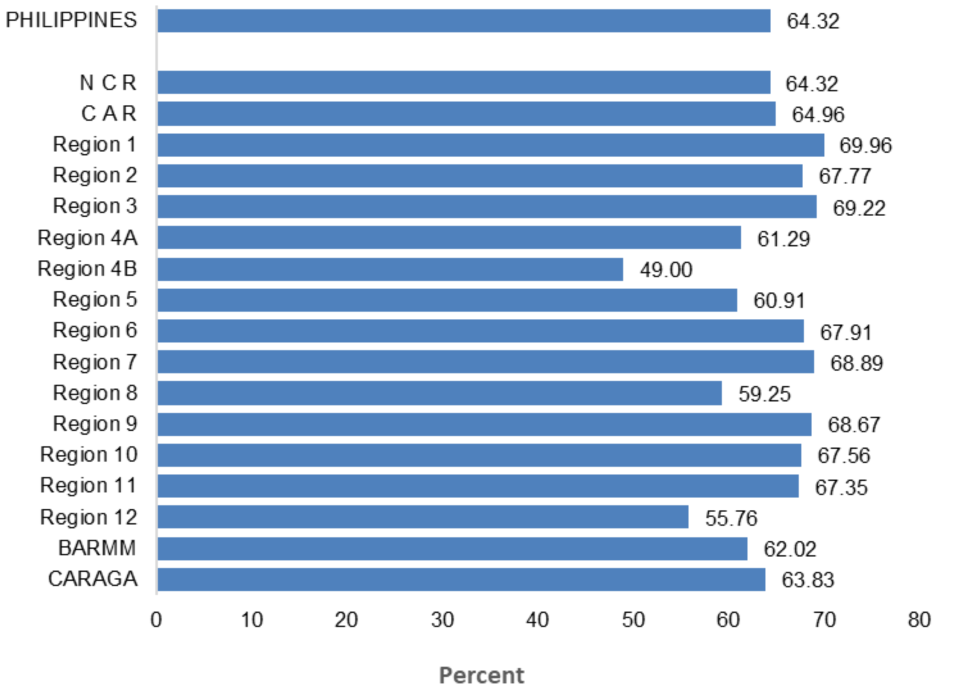

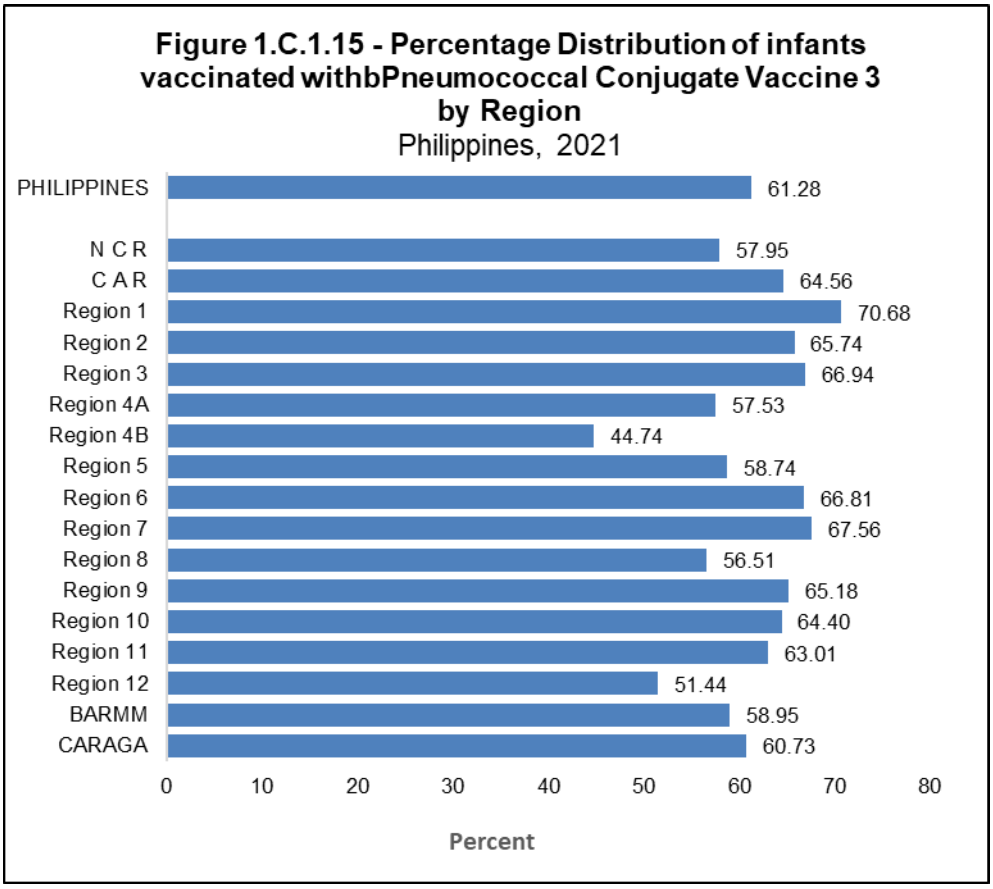
Figure 1.C.1.15 - Percentage Distribution of infants vaccinated withbPneumococcal Conjugate Vaccine 3 by Region
Philippines, 2021
PHILIPPINES 61.28
N C R 57.95
C A R 64.56
Region 1 70.68
Region 2 65.74
Region 3 66.94
Region 4A 57.53
Region 4B 44.74
Region 5 58.74
Region 6 66.81
Region 7 67.56
Region 8 56.51
Region 9 65.18
Region 10 64.40
Region 11 63.01
Region 12 51.44
BARMM 58.95
CARAGA 60.73
0 10 20 30 40 50 60 70 80
Percent

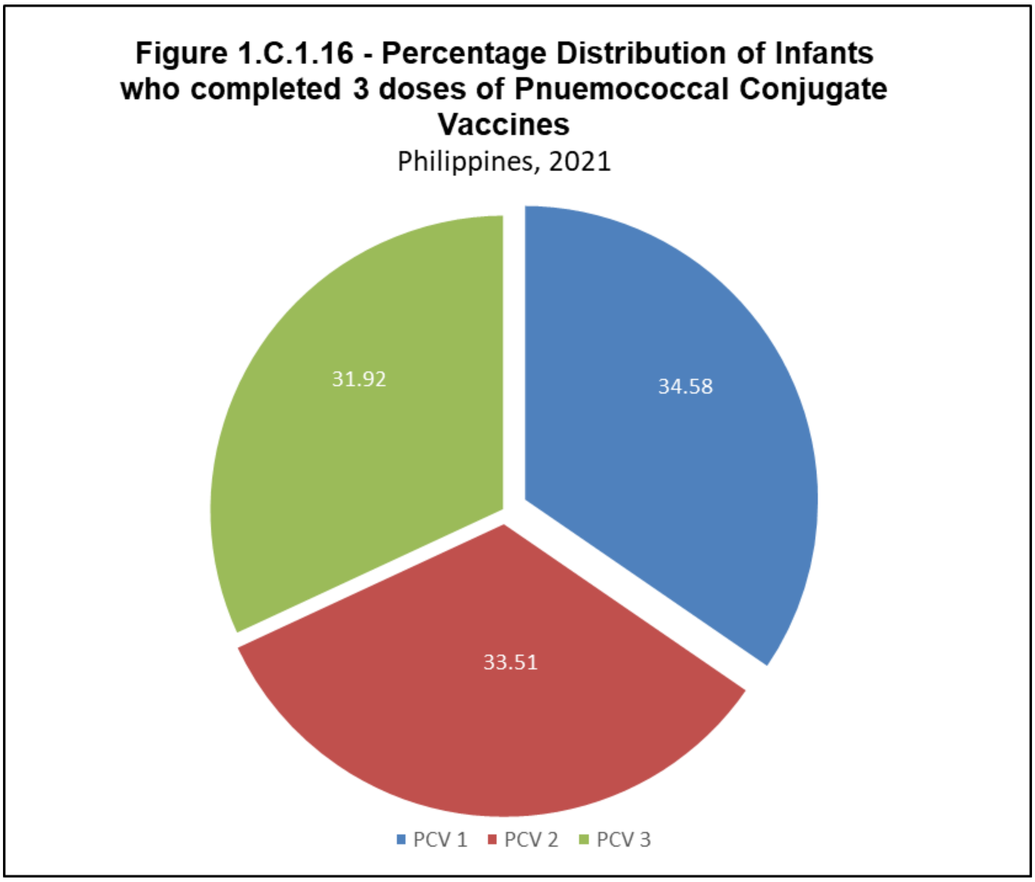
Figure 1.C.1.16 - Percentage Distribution of Infants who completed 3 doses of Pnuemococcal Conjugate Vaccines
Philippines, 2021
34.58
33.51
31.92
PCV 1
PCV 2
PCV 3

Figure 1.C.1.17 - Percentage Distribution of Children vaccinated with Measles Containing Vaccine 1 by Region
Philippines, 2021

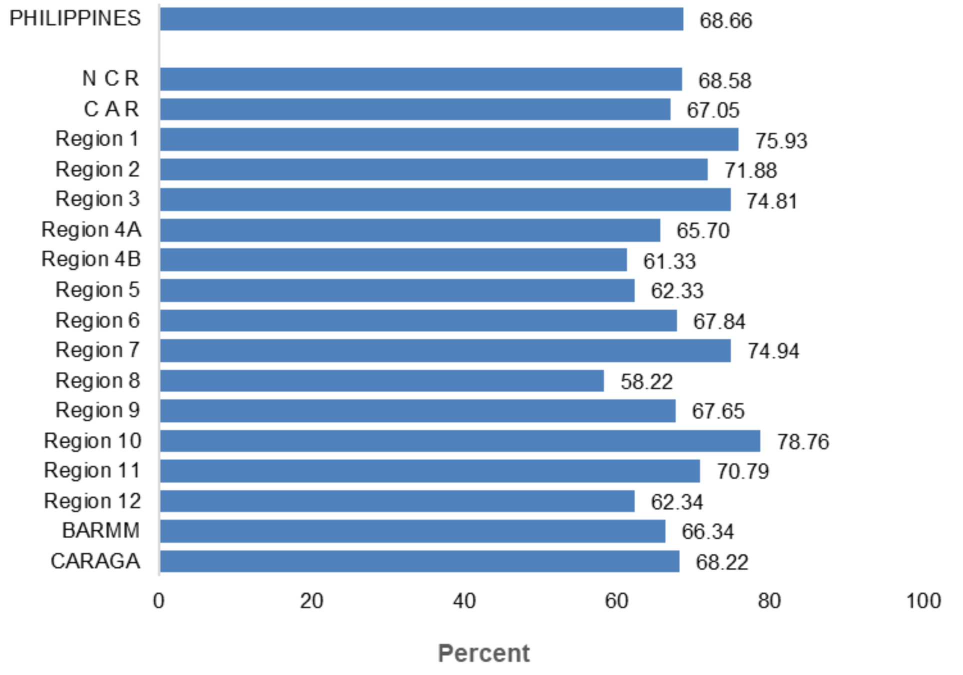

Figure 1.C.1.18 - Percentage Distribution of Children vaccinated with Measles Containing Vaccine 2 by Region
Philippines, 2021

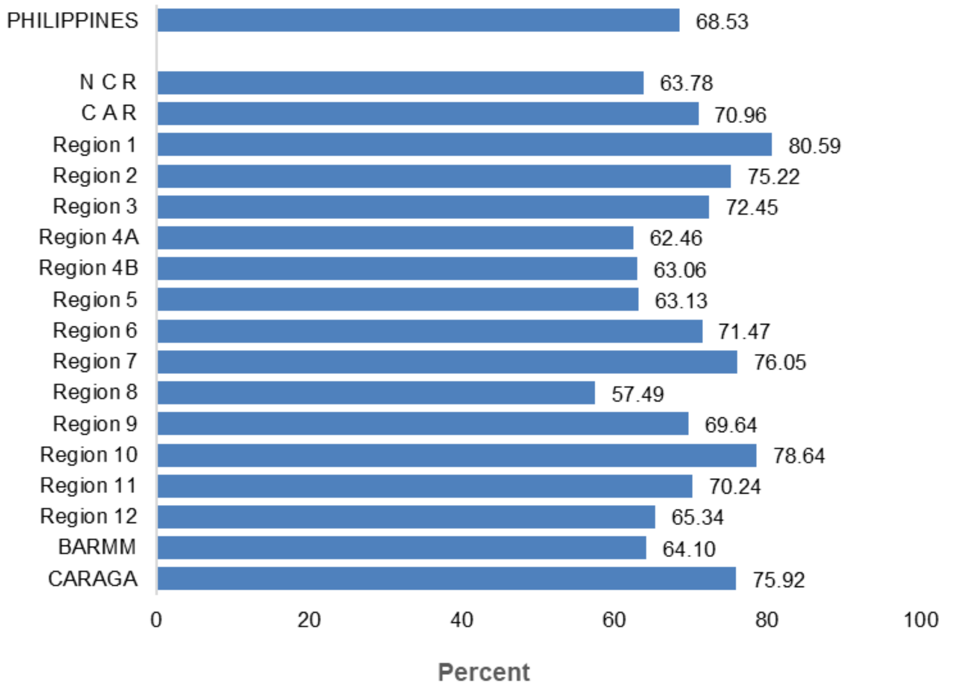

Figure 1.C.1.19 - Percentage Distribution of Children vaccinated with 2 doses of Measles Contatining Vaccine
Philippines, 2021

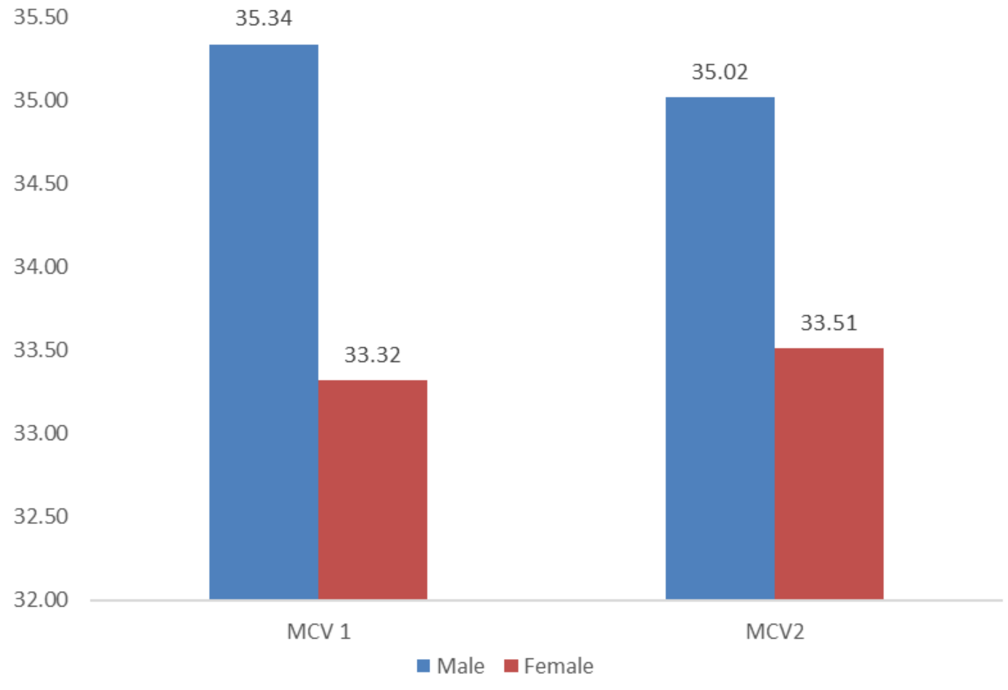

Figure 1.C.1.20 - Percentage Distribution of Fully Immunized Children by Region
Philippines, 2021

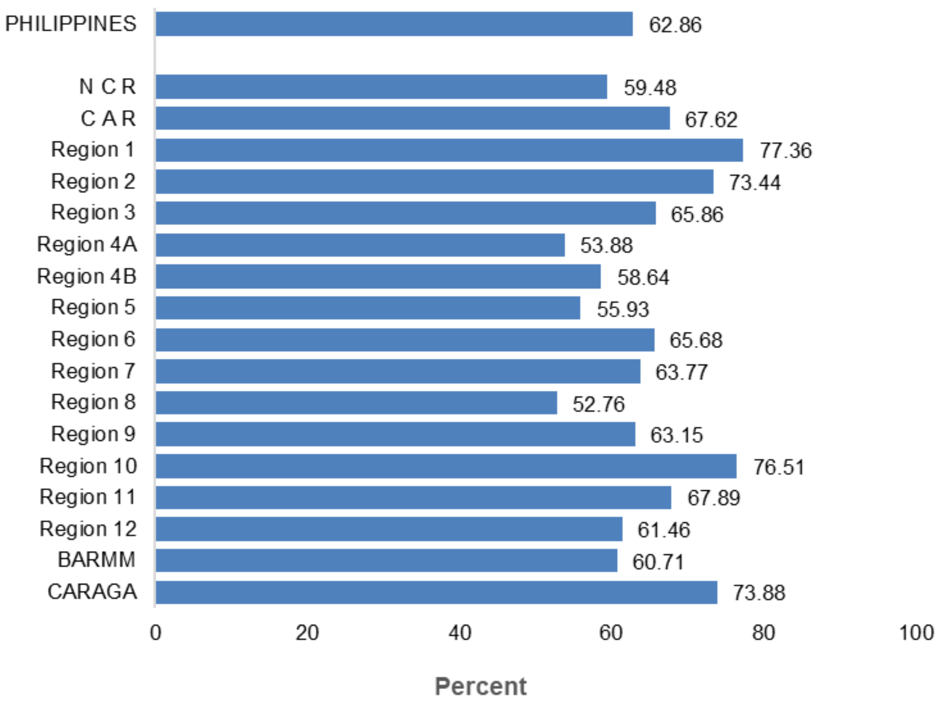

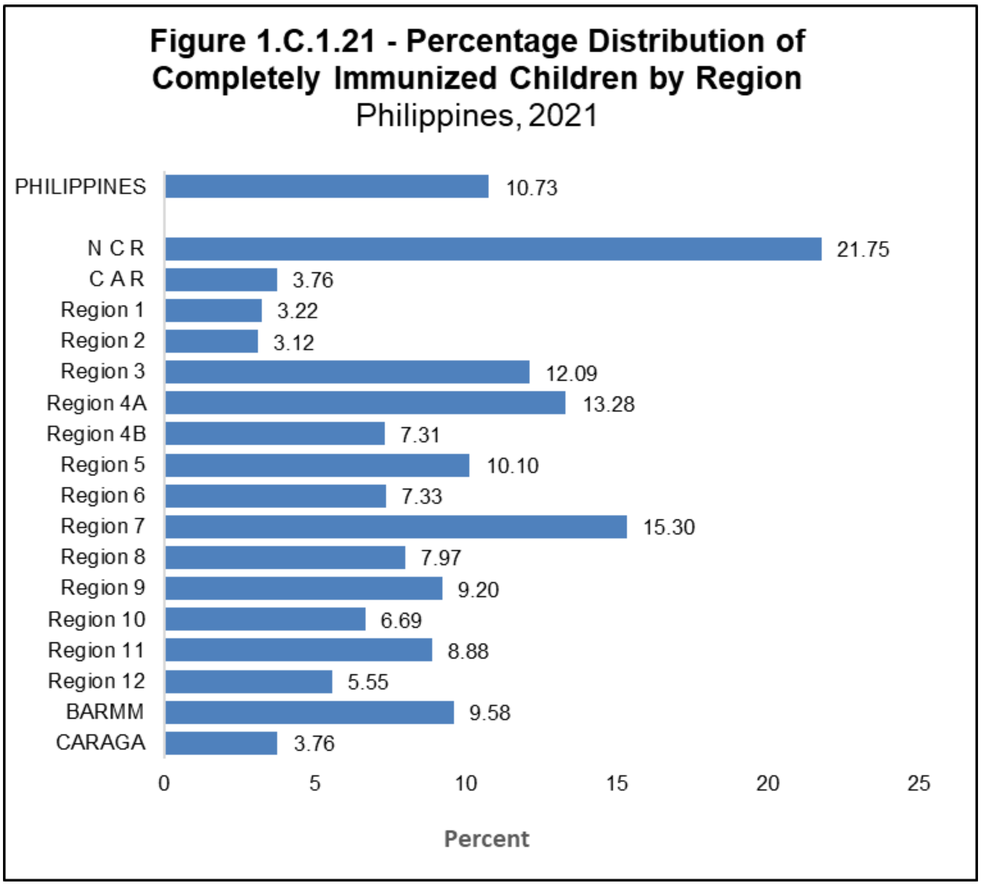

Figure 1.C.1.21 - Percentage Distribution of Completely Immunized Children by Region
Philippines, 2021
PHILIPPINES
10.73
N C R
21.75
C A R
3.76
Region 1
3.22
Region 2
3.12
Region 3
12.09
Region 4A
13.28
Region 4B
7.31
Region 5
10.10
Region 6
7.33
Region 7
15.30
Region 8
7.97
Region 9
9.20
Region 10
6.69
Region 11
8.88
Region 12
5.55
BARMM
9.58
CARAGA
3.76
0
5
10
15
20
25
Percent

Table 1.C.2.1 - Nutrition Services for Infants and Children

Numbers and Proportion of newborns initiated on breastfeeding immediately after birth lasting for at least 90 minutes, infants exclusively breastfed until 5th month and 29 days, and infants who continued breastfeeding and were introduced to complementary feeding beginning at 6 months of age

Philippines, 2021

Area	Live births	Newborns initiated on breastfeeding immediately after birth				Eligible Pop (Under 1 y.o.)	Infants Exclusively Breastfed until 5th month and 29 days				E.Pop (6-11 Mos.)	Infants who continued breastfeeding and were introduced to complementary feeding			
		Male	Female	Total	%		Male	Female	Total	%		Male	Female	Total	%
**PHILIPPINES**	**1,237,902**	**505,275**	**514,662**	**1,019,937**	**82.39**	**2,128,168**	**530,397**	**528,701**	**1,059,098**	**49.77**	**1,064,115**	**486,797**	**482,495**	**969,292**	**91.09**
**N C R**	**147,024**	**48,677**	**50,849**	**99,526**	**67.69**	**237,921**	**60,761**	**61,662**	**122,423**	**51.46**	**118,939**	**56,392**	**57,366**	**113,758**	**95.64**
Malabon	5,158	1,699	2,127	3,826	74.18	6,757	2,471	2,841	5,312	78.61	3,375	2,177	2,556	4,733	140.24
Navotas	3,241	1,539	1,531	3,070	94.72	4,607	2,204	2,155	4,359	94.62	2,305	1,890	1,925	3,815	165.51
Valenzuela City	6,520	1,590	1,364	2,954	45.31	11,465	2,874	2,738	5,612	48.95	5,733	1,967	1,723	3,690	64.36
Caloocan City	14,402	1,673	2,002	3,675	25.52	29,270	4,101	4,016	8,117	27.73	14,629	4,324	4,219	8,543	58.40
Marikina City	7,010	852	1,075	1,927	27.49	8,330	1,528	1,513	3,041	36.51	4,162	1,624	1,529	3,153	75.76
Pasig City	11,761	2,205	2,200	4,405	37.45	13,954	2,291	2,199	4,490	32.18	6,981	2,134	2,122	4,256	60.97
Pateros	714	358	338	696	97.48	1,181	328	379	707	59.86	587	312	354	666	113.46
Taguig	8,594	3,939	4,554	8,493	98.82	14,874	6,537	5,953	12,490	83.97	7,430	5,229	5,345	10,574	142.31
Quezon City	43,663	14,511	15,893	30,404	69.63	54,246	17,124	18,353	35,477	65.40	27,121	16,210	17,208	33,418	123.22
Makati City	5,691	1,262	1,150	2,412	42.38	10,765	1,232	1,050	2,282	21.20	5,376	1,191	1,012	2,203	40.98
Mandaluyong City	3,418	1,108	1,116	2,224	65.07	7,137	1,477	1,406	2,883	40.40	3,565	1,339	1,295	2,634	73.88
San Juan	1,468	404	453	857	58.38	2,254	269	293	562	24.93	1,129	229	270	499	44.20
Manila City	13,237	6,810	6,427	13,237	100.00	32,887	6,731	7,527	14,258	43.35	16,442	6,263	6,964	13,227	80.45
Las Piñas City	5,515	2,561	2,632	5,193	94.16	10,879	3,130	3,086	6,216	57.14	5,440	3,107	3,024	6,131	112.70
Muntinlupa City	6,733	3,241	3,347	6,588	97.85	9,317	2,996	2,854	5,850	62.79	4,661	3,228	3,009	6,237	133.81
Parañaque City	5,720	2,893	2,578	5,471	95.65	12,298	3,637	3,478	7,115	57.85	6,153	3,393	3,076	6,469	105.14
Pasay City	4,179	2,032	2,062	4,094	97.97	7,700	1,831	1,821	3,652	47.43	3,850	1,775	1,735	3,510	91.17
**C A R**	**22,381**	**10,165**	**9,763**	**19,928**	**89.04**	**35,143**	**9,175**	**8,947**	**18,122**	**51.57**	**17,594**	**7,995**	**7,867**	**15,862**	**90.16**
Abra	2,701	823	749	1,572	58.20	4,287	941	913	1,854	43.25	2,146	868	871	1,739	81.03
Apayao	1,701	595	677	1,272	74.78	2,476	697	685	1,382	55.82	1,235	621	610	1,231	99.68
Benguet	4,617	2,392	2,137	4,529	98.09	9,140	2,496	2,391	4,887	53.47	4,568	2,174	2,125	4,299	94.11
Ifugao	2,776	1,097	1,085	2,182	78.60	4,439	979	977	1,956	44.06	2,219	936	941	1,877	84.59
Kalinga	2,373	1,115	1,165	2,280	96.08	4,660	1,596	1,605	3,201	68.69	2,335	1,114	1,076	2,190	93.79
Mt. Province	2,434	1,183	1,210	2,393	98.32	3,066	900	839	1,739	56.72	1,539	769	753	1,522	98.90
Baguio City	5,779	2,960	2,740	5,700	98.63	7,075	1,566	1,537	3,103	43.86	3,552	1,513	1,491	3,004	84.57
**Region 1**	**48,712**	**24,738**	**23,893**	**48,631**	**99.83**	**97,198**	**32,719**	**31,150**	**63,869**	**65.71**	**48,596**	**30,858**	**29,743**	**60,601**	**124.70**
Ilocos Norte	4,378	2,170	2,208	4,378	100.00	7,986	2,368	2,185	4,553	57.01	4,000	2,150	2,061	4,211	105.28
Ilocos Sur	4,223	2,051	2,091	4,142	98.08	9,197	3,755	3,655	7,410	80.57	4,600	3,573	3,579	7,152	155.48
La Union	4,236	2,149	2,087	4,236	100.00	11,429	3,610	3,553	7,163	62.67	5,714	3,438	3,404	6,842	119.74
Pangasinan	14,410	7,226	7,184	14,410	100.00	50,454	17,284	16,476	33,760	66.91	25,208	15,777	15,225	31,002	122.98
Alaminos City	1,255	686	569	1,255	100.00	1,905	769	654	1,423	74.70	952	758	572	1,330	139.71
Candon City	1,075	554	521	1,075	100.00	976	330	294	624	63.93	485	446	339	785	161.86
Dagupan City	8,100	4,184	3,916	8,100	100.00	3,636	663	620	1,283	35.29	1,820	729	712	1,441	79.18
Laoag City	1,965	960	1,005	1,965	100.00	1,845	949	977	1,926	104.39	917	948	981	1,929	210.36
San Carlos City	2,267	1,216	1,051	2,267	100.00	3,999	1,136	1,052	2,188	54.71	2,003	1,126	1,050	2,176	108.64
San Fernando City	3,538	1,820	1,718	3,538	100.00	2,092	574	537	1,111	53.11	1,048	540	512	1,052	100.38
Urdaneta City	1,845	939	906	1,845	100.00	2,819	713	702	1,415	50.20	1,417	828	814	1,642	115.88
Vigan City	1,420	783	637	1,420	100.00	860	568	445	1,013	117.79	432	545	494	1,039	240.51
**Region 2**	**40,270**	**18,050**	**17,543**	**35,593**	**88.39**	**69,228**	**17,153**	**17,031**	**34,184**	**49.38**	**34,606**	**16,104**	**15,860**	**31,964**	**92.37**
Batanes	267	124	142	266	99.63	351	89	94	183	52.14	179	88	91	179	100.00
Cagayan	9,697	4,288	4,303	8,591	88.59	20,563	5,534	5,227	10,761	52.33	10,261	5,225	4,933	10,158	99.00
Isabela	12,459	5,253	5,219	10,472	84.05	23,543	4,664	4,629	9,293	39.47	11,782	4,757	4,802	9,559	81.13
Nueva Vizcaya	6,692	2,642	2,760	5,402	80.72	9,555	2,850	2,945	5,795	60.65	4,779	2,415	2,305	4,720	98.77
Quirino	2,624	1,149	1,193	2,342	89.25	4,038	946	876	1,822	45.12	2,017	924	836	1,760	87.26
Cauayan City	1,642	833	798	1,631	99.33	2,576	722	743	1,465	56.87	1,287	633	654	1,287	100.00
Ilagan City	2,640	1,561	1,079	2,640	100.00	2,901	934	1,004	1,938	66.80	1,449	652	797	1,449	100.00
Santiago City	2,883	1,493	1,390	2,883	100.00	2,683	587	752	1,339	49.91	1,339	587	752	1,339	100.00
Tuguegarao City	1,366	707	659	1,366	100.00	3,018	827	761	1,588	52.62	1,513	823	690	1,513	100.00
**Region 3**	**116,580**	**47,050**	**49,519**	**96,569**	**82.83**	**220,202**	**52,125**	**51,949**	**104,074**	**47.26**	**110,063**	**46,450**	**45,414**	**91,864**	**83.46**
Aurora	2,993	1,046	973	2,019	67.46	4,764	1,360	1,380	2,740	57.51	2,383	1,293	1,288	2,581	108.31
Bataan	5,144	3,714	3,572	7,286	141.64	13,814	4,561	4,468	9,029	65.36	6,909	4,052	4,050	8,102	117.27
Bulacan	22,651	7,949	8,494	16,443	72.59	43,722	6,591	6,853	13,444	30.75	21,847	6,768	6,282	13,050	59.73
Nueva Ecija	9,008	2,811	2,853	5,664	62.88	28,915	5,640	5,301	10,941	37.84	14,444	4,089	3,884	7,973	55.20
Pampanga	9,788	4,823	5,069	9,892	101.06	31,503	5,140	5,152	10,292	32.67	15,744	4,653	4,735	9,388	59.63
Tarlac	11,347	4,767	5,092	9,859	86.89	19,565	7,112	6,860	13,972	71.41	9,780	5,476	5,243	10,719	109.60
Zambales	7,560	3,083	3,274	6,357	84.09	12,551	2,152	2,317	4,469	35.61	6,281	2,233	2,308	4,541	72.30
Angeles City	6,987	1,780	1,905	3,685	52.74	7,905	1,722	1,918	3,640	46.05	3,951	1,369	1,493	2,862	72.44
Balanga City	1,283	640	613	1,253	97.66	1,996	601	552	1,153	57.77	999	587	531	1,118	111.91
Cabanatuan City	4,225	2,114	2,097	4,211	99.67	5,921	1,399	1,378	2,777	46.90	2,954	1,661	1,602	3,263	110.46
City of San Fernando	4,596	483	463	946	20.58	5,890	1,820	1,718	3,538	60.07	2,945	1,527	1,454	2,981	101.22
Gapan City	967	429	450	879	90.90	2,159	574	607	1,181	54.70	1,081	565	592	1,157	107.03

Table 1.C.2.1 - Nutrition Services for Infants and Children

Numbers and Proportion of newborns initiated on breastfeeding immediately after birth lasting for at least 90 minutes, infants exclusively breastfed until 5th month and 29 days, and infants who continued breastfeeding and were introduced to complementary feeding beginning at 6 months of age

Philippines, 2021

Area	Live births	Newborns initiated on breastfeeding immediately after birth				Eligible Pop (Under 1 y.o.)	Infants Exclusively Breastfed until 5th month and 29 days				E.Pop (6-11 Mos.)	Infants who continued breastfeeding and were introduced to complementary feeding			
		Male	Female	Total	%		Male	Female	Total	%		Male	Female	Total	%
Mabalacat City	4,326	965	1,142	2,107	48.71	4,811	1,152	1,073	2,225	46.25	2,410	993	950	1,943	80.62
Malolos City	3,489	775	812	1,587	45.49	4,885	701	684	1,385	28.35	2,439	650	665	1,315	53.92
Meycauayan	2,351	1,326	1,511	2,837	120.67	4,050	1,572	1,554	3,126	77.19	2,025	1,437	1,483	2,920	144.20
Olongapo	3,418	1,130	1,279	2,409	70.48	4,952	638	705	1,343	27.12	2,474	315	310	625	25.26
Palayan City	77	44	29	73	94.81	805	206	218	424	52.67	400	177	199	376	94.00
San Jose City	1,940	993	922	1,915	98.71	2,736	1,347	1,250	2,597	94.92	1,370	1,047	978	2,025	147.81
San Jose del Monte City	6,631	5,433	5,722	11,155	168.23	11,120	4,817	4,847	9,664	86.91	5,562	4,052	4,073	8,125	146.08
Science City of Munoz	106	60	73	133	125.47	1,594	571	596	1,167	73.21	796	469	490	959	120.48
Tarlac City	7,693	2,685	3,174	5,859	76.16	6,544	2,449	2,518	4,967	75.90	3,269	3,037	2,804	5,841	178.68
**Region 4A**	**168,998**	**64,357**	**65,943**	**130,300**	**77.10**	**298,398**	**56,164**	**56,000**	**112,164**	**37.59**	**149,202**	**54,051**	**53,955**	**108,006**	**72.39**
Batangas	20,093	9,703	9,661	19,364	96.37	38,848	8,598	8,215	16,813	43.28	19,436	8,813	8,380	17,193	88.46
Cavite	10,280	6,237	6,411	12,648	123.04	27,948	4,669	4,652	9,321	33.35	13,969	4,290	4,321	8,611	61.64
Laguna	14,770	3,423	3,534	6,957	47.10	19,727	3,898	3,831	7,729	39.18	9,870	3,854	3,732	7,586	76.86
Quezon	18,194	6,817	7,262	14,079	77.38	37,644	7,022	7,158	14,180	37.67	18,813	6,630	6,888	13,518	71.85
Rizal	17,448	8,780	8,920	17,700	101.44	44,826	6,595	6,609	13,204	29.46	22,421	5,883	6,012	11,895	53.05
Antipolo City	9,300	690	774	1,464	15.74	16,510	2,194	2,131	4,325	26.20	8,257	1,765	1,828	3,593	43.51
Bacoor City	2,763	1,821	1,889	3,710	134.27	12,252	1,528	1,559	3,087	25.20	6,123	1,372	1,452	2,824	46.12
Batangas City	4,260	1,684	1,653	3,337	78.33	6,895	552	584	1,136	16.48	3,445	887	951	1,838	53.35
Biñan City	10,059	3,424	3,389	6,813	67.73	6,607	3,067	3,045	6,112	92.51	3,303	2,793	2,853	5,646	170.94
Cabuyao City	4,028	1,681	1,817	3,498	86.84	6,128	1,804	1,929	3,733	60.92	3,064	1,604	1,686	3,290	107.38
Calamba City	10,488	4,125	4,150	8,275	78.90	9,025	3,704	3,466	7,170	79.45	4,511	3,306	3,225	6,531	144.78
Cavite City	346	84	85	169	48.84	2,098	78	67	145	6.91	1,046	88	83	171	16.35
Dasmariñas City	6,907	3,061	3,124	6,185	89.55	13,442	3,048	3,075	6,123	45.55	6,717	2,860	2,867	5,727	85.26
General Trias City	4,528	864	937	1,801	39.77	6,410	559	605	1,164	18.16	3,205	647	655	1,302	40.62
Imus City	3,980	1,524	1,491	3,015	75.75	8,235	899	977	1,876	22.78	4,117	685	753	1,438	34.93
Lipa City	2,776	1,530	1,446	2,976	107.20	6,954	415	401	816	11.73	3,473	1,071	928	1,999	57.56
Lucena City	1,713	1,126	1,107	2,233	130.36	5,704	980	1,040	2,020	35.41	2,855	993	1,037	2,030	71.10
San Pablo City	6,029	1,246	1,360	2,606	43.22	5,277	971	905	1,876	35.55	2,646	820	754	1,574	59.49
San Pedro City	1,648	1,155	1,311	2,466	149.64	6,465	1,082	1,283	2,365	36.58	3,232	942	998	1,940	60.02
Santa Rosa City	9,454	2,220	2,571	4,791	50.68	7,023	2,144	2,146	4,290	61.09	3,511	1,999	1,913	3,912	111.42
Tagaytay City	815	509	499	1,008	123.68	1,451	603	611	1,214	83.67	726	585	592	1,177	162.12
Tanauan City	4,597	929	869	1,798	39.11	3,620	337	327	664	18.34	1,811	763	710	1,473	81.34
Tayabas City	1,688	717	626	1,343	79.56	2,134	759	732	1,491	69.87	1,064	745	712	1,457	136.94
Trece Martires City	2,834	1,007	1,057	2,064	72.83	3,175	658	652	1,310	41.26	1,587	656	625	1,281	80.72
**Region 4B**	**41,216**	**14,773**	**14,763**	**29,536**	**71.66**	**72,035**	**14,967**	**15,296**	**30,263**	**42.01**	**36,035**	**7,083**	**6,663**	**13,746**	**38.15**
Marinduque	2,765	1,357	1,293	2,650	95.84	4,906	1,224	1,159	2,383	48.57	2,464	79	61	140	5.68
Mindoro Occidental	7,731	3,358	3,233	6,591	85.25	12,449	3,938	3,599	7,537	60.54	6,230	3,749	3,364	7,113	114.17
Mindoro Oriental	11,156	1,627	1,727	3,354	30.06	20,011	1,763	2,530	4,293	21.45	10,004	603	590	1,193	11.93
Palawan	12,572	5,215	5,346	10,561	84.00	21,676	5,450	5,435	10,885	50.22	10,843	1,279	1,296	2,575	23.75
Romblon	3,925	1,844	1,818	3,662	93.30	6,482	1,882	1,850	3,732	57.57	3,236	337	338	675	20.86
Puerto Princesa City	3,067	1,372	1,346	2,718	88.62	6,511	710	723	1,433	22.01	3,258	1,036	1,014	2,050	62.92
**Region 5**	**78,461**	**29,271**	**29,622**	**58,893**	**75.06**	**137,350**	**30,484**	**30,168**	**60,652**	**44.16**	**68,682**	**30,189**	**30,226**	**60,415**	**87.96**
Albay	9,197	3,971	3,975	7,946	86.40	23,903	4,768	4,699	9,467	39.61	11,965	4,692	4,711	9,403	78.59
Camarines Norte	9,555	4,235	4,565	8,800	92.10	14,340	3,735	3,531	7,266	50.67	7,162	3,915	3,828	7,743	108.11
Camarines Sur	15,437	6,770	6,582	13,352	86.49	39,151	9,369	9,651	19,020	48.58	19,610	8,680	8,850	17,530	89.39
Catanduanes	3,972	1,345	1,343	2,688	67.67	6,381	1,438	1,434	2,872	45.01	3,178	1,359	1,380	2,739	86.19
Masbate	12,054	5,247	5,219	10,466	86.83	22,565	5,002	4,855	9,857	43.68	11,277	5,689	5,647	11,336	100.52
Sorsogon	10,324	4,748	5,137	9,885	95.75	19,470	3,121	3,058	6,179	31.74	9,726	2,933	2,859	5,792	59.55
Iriga City	1,612	796	768	1,564	97.02	2,665	678	635	1,313	49.27	1,328	641	664	1,305	98.27
Legaspi City	4,506	1,080	1,044	2,124	47.14	4,205	1,052	1,040	2,092	49.75	2,101	1,075	1,147	2,222	105.76
Naga City	11,804	1,079	989	2,068	17.52	4,670	1,321	1,265	2,586	55.37	2,335	1,205	1,140	2,345	100.43
**Region 6**	**81,900**	**36,943**	**36,304**	**73,247**	**89.43**	**146,545**	**30,342**	**29,920**	**60,262**	**41.12**	**73,273**	**29,289**	**28,755**	**58,044**	**79.22**
Aklan	5,536	2,538	2,550	**5,088**	**91.91**	11,231	2,992	2,930	5,922	52.73	5,611	2,864	2,826	5,690	101.41
Antique	7,250	3,397	3,177	**6,574**	**90.68**	12,985	2,595	2,609	5,204	40.08	6,486	2,407	2,391	4,798	73.97
Capiz	7,494	3,757	3,539	**7,296**	**97.36**	13,984	3,157	3,223	6,380	45.62	6,992	3,035	3,083	6,118	87.50
Guimaras	2,322	1,164	1,063	**2,227**	**95.91**	3,085	998	989	1,987	64.41	1,549	962	991	1,953	126.08
Iloilo	19,202	8,421	8,529	**16,950**	**88.27**	36,414	8,807	8,328	17,135	47.06	18,209	8,206	7,817	16,023	87.99
Negros Occidental	31,682	14,371	14,630	**29,001**	**91.54**	49,314	9,212	9,350	18,562	37.64	24,667	9,027	8,949	17,976	72.87
Bacolod City	3,872	1,243	876	**2,119**	**54.73**	11,102	954	872	1,826	16.45	5,544	1,144	1,051	2,195	39.59
Iloilo City	4,542	2,052	1,940	**3,992**	**87.89**	8,430	1,627	1,619	3,246	38.51	4,215	1,644	1,647	3,291	78.08
**Region 7**	**92,365**	**43,998**	**43,738**	**87,736**	**94.99**	**162,860**	**51,377**	**50,831**	**102,208**	**62.76**	**81,443**	**46,210**	**45,311**	**91,521**	**112.37**
Bohol	17,238	8,511	8,086	16,597	96.28	27,637	7,657	7,442	15,099	54.63	13,837	6,027	5,509	11,536	83.37
Cebu	27,617	12,849	12,531	25,380	91.90	67,046	21,714	20,941	42,655	63.62	33,510	20,110	19,195	39,305	117.29
Negros Oriental	18,802	8,258	8,592	16,850	89.62	27,922	9,079	9,831	18,910	67.72	13,961	9,064	9,887	18,951	135.74
Siquijor	1,174	415	387	802	68.31	1,636	643	561	1,204	73.59	817	625	541	1,166	142.72

Table 1.C.2.1 - Nutrition Services for Infants and Children

Numbers and Proportion of newborns initiated on breastfeeding immediately after birth lasting for at least 90 minutes, infants exclusively breastfed until 5th month and 29 days, and infants who continued breastfeeding and were introduced to complementary feeding beginning at 6 months of age

Philippines, 2021

Area	Live births	Newborns initiated on breastfeeding immediately after birth				Eligible Pop (Under 1 y.o.)	Infants Exclusively Breastfed until 5th month and 29 days				E.Pop (6-11 Mos.)	Infants who continued breastfeeding and were introduced to complementary feeding			
		Male	Female	Total	%		Male	Female	Total	%		Male	Female	Total	%
Cebu City	16,209	7,917	7,771	15,688	96.79	21,037	6,237	6,129	12,366	58.78	10,527	4,574	4,292	8,866	84.22
Lapu-Lapu City	7,256	3,750	3,749	7,499	103.35	9,310	3,564	3,543	7,107	76.34	4,657	3,380	3,403	6,783	145.65
Mandaue City	4,069	2,298	2,622	4,920	120.91	8,272	2,483	2,384	4,867	58.84	4,134	2,430	2,484	4,914	118.87
**Region 8**	**53,342**	**18,916**	**19,416**	**38,332**	**71.86**	**104,016**	**21,124**	**20,068**	**41,192**	**39.60**	**52,044**	**17,080**	**16,588**	**33,668**	**64.69**
Biliran	2,613	1,220	996	2,216	84.81	3,856	981	616	1,597	41.42	1,915	816	438	1,254	65.48
Eastern Samar	6,130	2,606	2,900	5,506	89.82	11,635	2,309	2,403	4,712	40.50	5,849	1,784	1,855	3,639	62.22
Northern Leyte	15,328	4,708	4,536	9,244	60.31	35,052	5,839	5,516	11,355	32.39	17,534	4,630	4,651	9,281	52.93
Northern Samar	8,498	2,659	2,907	5,566	65.50	15,655	3,159	3,070	6,229	39.79	7,822	2,793	2,725	5,518	70.54
Southern Leyte	2,628	1,132	1,024	2,156	82.04	6,556	1,567	1,584	3,151	48.06	3,280	1,234	1,191	2,425	73.93
Western Samar	6,501	2,868	2,885	5,753	88.49	14,515	2,948	2,727	5,675	39.10	7,272	2,673	2,616	5,289	72.73
	-	0	0				0	0				0	0		
Calbayog City	1,929	588	723	1,311	67.96	4,474	614	656	1,270	28.39	2,234	560	588	1,148	51.39
Maasin City	2,213	306	341	647	29.24	1,661	408	383	791	47.62	833	341	308	649	77.91
Ormoc City	4,761	1,690	1,592	3,282	68.94	4,989	1,492	1,434	2,926	58.65	2,498	1,246	1,219	2,465	98.68
Tacloban City	2,741	1,139	1,512	2,651	96.72	5,623	1,807	1,679	3,486	62.00	2,807	1,003	997	2,000	71.25
**Region 9**	**48,052**	**19,167**	**21,798**	**40,965**	**85.25**	**79,552**	**19,829**	**20,442**	**40,271**	**50.62**	**39,757**	**18,120**	**18,645**	**36,765**	**92.47**
Zamboanga del Norte	10,809	3,862	3,934	7,796	72.13	17,075	4,053	4,185	8,238	48.25	8,519	3,658	3,729	7,387	86.71
Zamboanga del Sur	6,563	2,348	3,070	5,418	82.55	17,506	3,802	3,791	7,593	43.37	8,750	3,559	3,527	7,086	80.98
Zamboanga Sibugay	6,888	2,716	3,107	5,823	84.54	14,992	2,679	2,852	5,531	36.89	7,499	2,539	2,620	5,159	68.80
Dapitan City	1,595	741	808	1,549	97.12	1,755	303	313	616	35.10	883	294	318	612	69.31
Dipolog City	2,325	1,069	1,004	2,073	89.16	2,794	1,118	1,031	2,149	76.91	1,396	1,020	1,125	2,145	153.65
Isabela City	1,688	526	454	980	58.06	2,544	586	602	1,188	46.70	1,268	530	586	1,116	88.01
Pagadian City	2,355	893	1,477	2,370	100.64	4,290	645	706	1,351	31.49	2,147	541	610	1,151	53.61
Zamboanga City	15,829	7,012	7,944	14,956	94.48	18,596	6,643	6,962	13,605	73.16	9,295	5,979	6,130	12,109	130.27
**Region 10**	**70,977**	**30,482**	**33,248**	**63,730**	**89.79**	**100,690**	**35,215**	**35,595**	**70,810**	**70.32**	**50,346**	**33,063**	**32,805**	**65,868**	**130.83**
Bukidnon	18,729	8,098	9,333	17,431	93.07	23,307	7,771	7,882	15,653	67.16	11,655	8,357	7,807	16,164	138.69
Camiguin	1,084	523	475	998	92.07	1,854	543	479	1,022	55.12	930	666	588	1,254	134.84
Lanao del Norte	8,583	2,894	3,417	6,311	73.53	14,955	4,268	4,666	8,934	59.74	7,486	3,707	3,989	7,696	102.81
Misamis Occidental	3,866	1,516	1,715	3,231	83.57	6,413	2,316	2,276	4,592	71.60	3,193	1,981	1,928	3,909	122.42
Misamis Oriental	8,817	3,519	3,963	7,482	84.86	15,058	4,695	4,696	9,391	62.37	7,526	4,199	4,199	8,398	111.59
Cagayan de Oro City	11,740	6,184	6,410	12,594	107.27	14,263	6,916	6,638	13,554	95.03	7,126	6,488	6,483	12,971	182.02
El Salvador City	250	132	118	250	100.00	1,061	323	355	678	63.90	529	319	330	649	122.68
Gingoog City	2,848	1,005	1,231	2,236	78.51	2,627	1,245	1,498	2,743	104.42	1,319	1,187	1,339	2,526	191.51
Iligan City	5,977	2,248	2,144	4,392	73.48	7,575	2,369	2,149	4,518	59.64	3,785	1,672	1,503	3,175	83.88
Malaybalay City	2,900	1,520	1,380	2,900	100.00	3,882	1,675	1,634	3,309	85.24	1,945	1,561	1,497	3,058	157.22
Oroquieta City	743	354	389	743	100.00	1,389	267	312	579	41.68	693	234	278	512	73.88
Ozamis City	1,933	850	1,003	1,853	95.86	2,782	1,209	1,305	2,514	90.37	1,390	1,211	1,307	2,518	181.15
Tangub City	1,173	597	566	1,163	99.15	1,234	264	268	532	43.11	621	250	247	497	80.03
Valencia City	2,334	1,042	1,104	2,146	91.95	4,290	1,354	1,437	2,791	65.06	2,148	1,231	1,310	2,541	118.30
**Region 11**	**76,595**	**36,444**	**34,088**	**70,532**	**92.08**	**107,847**	**32,995**	**31,657**	**64,652**	**59.95**	**53,895**	**35,233**	**33,381**	**68,614**	**127.31**
Davao de Oro	11,442	5,819	5,288	11,107	97.07	15,526	5,195	4,750	9,945	64.05	7,770	5,379	4,972	10,351	133.22
Davao del Norte	17,083	8,660	7,995	16,655	97.49	21,184	7,015	6,781	13,796	65.12	10,575	7,482	7,152	14,634	138.38
Davao del Sur	8,309	3,879	3,805	7,684	92.48	13,125	4,092	3,881	7,973	60.75	6,555	4,195	3,919	8,114	123.78
Davao Oriental	8,254	3,983	3,724	7,707	93.37	14,355	4,022	3,719	7,741	53.93	7,173	4,722	4,357	9,079	126.57
Davao Occidental	4,199	1,030	938	1,968	46.87	6,598	1,512	1,383	2,895	43.88	3,294	1,534	1,405	2,939	89.22
Davao City	27,308	13,073	12,338	25,411	93.05	37,059	11,159	11,143	22,302	60.18	18,528	11,921	11,576	23,497	126.82
**Region 12**	**65,475**	**30,396**	**30,798**	**61,194**	**93.46**	**104,693**	**33,013**	**33,228**	**66,241**	**63.27**	**52,360**	**29,847**	**29,718**	**59,565**	**113.76**
North Cotabato	18,326	8,196	8,750	16,946	92.47	33,933	9,084	9,540	18,624	54.88	16,966	7,922	8,011	15,933	93.91
Sarangani	9,677	4,738	4,710	9,448	97.63	12,904	5,267	5,190	10,457	81.04	6,452	4,718	4,787	9,505	147.32
South Cotabato	16,306	7,845	7,799	15,644	95.94	21,039	7,088	7,132	14,220	67.59	10,523	6,123	6,188	12,311	116.99
Sultan Kudarat	11,962	5,921	5,463	11,384	95.17	17,331	7,141	6,710	13,851	79.92	8,677	7,141	6,710	13,851	159.63
Cotabato City	3,299	1,562	1,656	3,218	97.54	5,831	1,561	1,509	3,070	52.65	2,917	1,453	1,463	2,916	99.97
Gen. Santos City	5,905	2,134	2,420	4,554	77.12	13,655	2,872	3,147	6,019	44.08	6,825	2,490	2,559	5,049	73.98
**BARMM**	**48,659**	**14,867**	**16,268**	**31,135**	**63.99**	**94,148**	**15,129**	**16,549**	**31,678**	**33.65**	**47,089**	**13,100**	**14,639**	**27,739**	**58.91**
Basilan	2,250	928	996	1,924	85.51	7,672	669	681	1,350	17.60	3,842	787	829	1,616	42.06
Lanao del Sur	13,376	2,013	2,264	4,277	31.98	21,391	1,893	2,234	4,127	19.29	10,681	1,719	2,069	3,788	35.46
Maguindanao	20,142	7,652	8,282	15,934	79.11	31,650	8,595	9,010	17,605	55.62	15,840	7,295	7,851	15,146	95.62
Sulu	0	0	0	0	0.00	16,891	0	0	0	0.00	8,442	0	0	0	0.00
Tawi-Tawi	5,720	2,151	2,508	4,659	81.45	9,319	2,023	2,524	4,547	48.79	4,664	1,609	2,061	3,670	78.69
Lamitan City	1,362	638	623	1,261	92.58	2,112	455	527	982	46.50	1,059	343	391	734	69.31
Marawi City	5,809	1,485	1,595	3,080	53.02	5,113	1,494	1,573	3,067	59.98	2,561	1,347	1,438	2,785	108.75

Table 1.C.2.1 - Nutrition Services for Infants and Children

Numbers and Proportion of newborns initiated on breastfeeding immediately after birth lasting for at least 90 minutes, infants exclusively breastfed until 5th month and 29 days, and infants who continued breastfeeding and were introduced to complementary feeding beginning at 6 months of age

Philippines, 2021

Area	Live births	Newborns initiated on breastfeeding immediately after birth				Eligible Pop (Under 1 y.o.)	Infants Exclusively Breastfed until 5th month and 29 days				E.Pop (6-11 Mos.)	Infants who continued breastfeeding and were introduced to complementary feeding			
		Male	Female	Total	%		Male	Female	Total	%		Male	Female	Total	%
**CARAGA**	**36,895**	**16,981**	**17,109**	**34,090**	**92.40**	**60,342**	**17,825**	**18,208**	**36,033**	**59.71**	**30,191**	**15,733**	**15,559**	**31,292**	**103.65**
Agusan del Norte	3,603	1,790	1,661	3,451	95.78	8,116	2,432	2,442	4,874	60.05	4,069	1,907	1,923	3,830	94.13
Agusan del Sur	11,850	4,980	5,230	10,210	86.16	17,658	5,989	6,340	12,329	69.82	8,826	5,112	5,436	10,548	119.51
Surigao del Norte	3,361	1,511	1,667	3,178	94.56	7,195	1,781	1,833	3,614	50.23	3,603	1,461	1,502	2,963	82.24
Surigao del Sur	6,394	3,078	3,029	6,107	95.51	11,532	3,213	3,169	6,382	55.34	5,763	3,112	3,057	6,169	107.04
Province of Dinagat	1,015	444	501	945	93.10	2,574	389	463	852	33.10	1,292	336	439	775	59.98
Bislig City	1,288	674	612	1,286	99.84	2,185	569	598	1,167	53.41	1,094	511	535	1,046	95.61
Butuan City	5,228	2,395	2,366	4,761	91.07	7,732	2,412	2,339	4,751	61.45	3,862	2,323	1,714	4,037	104.53
Surigao City	4,156	2,109	2,043	4,152	99.90	3,350	1,040	1,024	2,064	61.61	1,682	971	953	1,924	114.39

Note: Put asterisk (*) for No Report and Zero (0) for No Case

Table 1.C.2.2- Nutrition Services for Infants and Children

Number of Infants born preterm or with low birth weight given iron supplements and infants 6-11 mos. old and children 12-59 mos. old given Vitamin A supplementation
Philippines, 2021

Area	Total No. of live births with low birth weight	Infants preterm or with LBW (< 2500 grams) given Iron				Eligible Pop (6-11 months)	Infants 6-11 mos. old Given Vitamin A				Eligible Pop (12-59 months)	Children 12-59 mos. Old Given Vitamin A			
		Male	Female	Total	%		Male	Female	Total	%		Male	Female	Total	%
**PHILIPPINES**	**115,933**	**9,074**	**9,830**	**18,904**	**16.31**	**1,064,115**	**619,516**	**606,548**	**1,226,064**	**115.22**	**8,962,535**	**2,614,414**	**2,559,339**	**5,173,753**	**57.73**
**NCR**	**13,325**	**1,169**	**1,338**	**2,507**	**18.81**	**118,939**	**57,937**	**59,750**	**117,687**	**98.95**	**955,786**	**381,744**	**389,974**	**771,718**	**80.74**
Malabon	166	40	27	67	40.36	3,375	1,591	1,783	3,374	99.97	27,129	9,582	10,798	20,380	75.12
Navotas	76	44	31	75	98.68	2,305	1,186	1,117	2,303	99.91	18,518	5,600	5,199	10,799	58.32
Valenzuela City	269	104	165	269	100.00	5,733	2,727	3,001	5,728	99.91	46,052	15,602	14,177	29,779	64.66
Caloocan City	1,113	1	0	1	0.09	14,629	7,432	7,193	14,625	99.97	117,560	55,503	54,248	109,751	93.36
Marikina City	705	96	100	196	27.80	4,162	2,168	1,995	4,163	100.02	33,452	5,278	5,482	10,760	32.17
Pasig City	1,768	12	16	28	1.58	6,981	3,587	3,388	6,975	99.91	56,059	19,858	19,318	39,176	69.88
Pateros	7	4	3	7	100.00	587	296	293	589	100.34	4,739	1,499	1,458	2,957	62.40
Taguig	641	275	366	641	100.00	7,430	3,954	3,481	7,435	100.07	59,742	29,606	30,138	59,744	100.00
Quezon City	5,534	103	106	209	3.78	27,121	13,042	14,075	27,117	99.99	217,929	94,812	98,063	192,875	88.50
Makati City	576	54	54	108	18.75	5,376	2,344	1,983	4,327	80.49	43,242	13,470	12,768	26,238	60.68
Mandaluyong City	107	16	22	38	35.51	3,565	1,810	1,757	3,567	100.06	28,674	6,280	6,609	12,889	44.95
San Juan	155	12	18	30	19.35	1,129	521	609	1,130	100.09	9,067	2,165	2,517	4,682	51.64
Manila City	413	192	212	404	97.82	16,442	7,211	9,232	16,443	100.01	132,127	61,341	68,506	129,847	98.27
Las Piñas City	62	31	31	62	100.00	5,440	2,784	2,655	5,439	99.98	43,706	10,863	11,151	22,014	50.37
Muntinlupa City	838	39	45	84	10.02	4,661	2,302	2,358	4,660	99.98	37,449	18,392	18,860	37,252	99.47
Parañaque City	625	66	81	147	23.52	6,153	3,129	2,834	5,963	96.91	49,421	16,668	16,675	33,343	67.47
Pasay City	270	80	61	141	52.22	3,850	1,853	1,996	3,849	99.97	30,920	15,225	14,007	29,232	94.54
**C A R**	**1,764**	**118**	**177**	**295**	**16.72**	**17,594**	**8,749**	**8,420**	**17,169**	**97.58**	**147,119**	**44,689**	**40,607**	**85,296**	**57.98**
Abra	281	34	69	103	36.65	2,146	950	1,019	1,969	91.75	19,047	5,019	4,708	9,727	51.07
Apayao	130	16	28	44	33.85	1,235	646	582	1,228	99.43	10,999	4,655	3,991	8,646	78.61
Benguet	314	8	11	19	6.05	4,568	2,382	2,146	4,528	99.12	36,250	9,348	8,632	17,980	49.60
Ifugao	82	6	4	10	12.20	2,219	1,076	993	2,069	93.24	19,270	5,516	4,926	10,442	54.19
Kalinga	72	15	21	36	50.00	2,335	1,169	1,165	2,334	99.96	19,904	5,186	3,991	9,177	46.11
Mt. Province	163	18	24	42	25.77	1,539	780	751	1,531	99.48	13,600	5,159	4,527	9,686	71.22
Baguio City	722	21	20	41	5.68	3,552	1,746	1,764	3,510	98.82	28,049	9,806	9,832	19,638	70.01
**Region 1**	**2,401**	**264**	**327**	**591**	**24.61**	**48,596**	**31,376**	**29,494**	**60,870**	**125.26**	**407,388**	**121,461**	**110,144**	**231,605**	**56.85**
Ilocos Norte	144	42	74	116	80.56	4,000	3,174	2,996	6,170	154.25	32,506	12,912	12,120	25,032	77.01
Ilocos Sur	115	48	41	89	77.39	4,600	4,652	4,534	9,186	199.70	40,200	19,546	18,446	37,992	94.51
La Union	173	54	52	106	61.27	5,714	5,861	5,802	11,663	204.11	48,309	31,100	28,956	60,056	124.32
Pangasinan	148	82	105	187	126.35	25,208	12,184	11,142	23,326	92.53	210,411	38,233	33,244	71,477	33.97
Alaminos City	1	1	0	1	100.00	952	574	439	1,013	106.41	7,947	506	443	949	11.94
Candon City	2	0	0	0	0.00	485	661	548	1,209	249.28	4,234	965	1,243	2,208	52.15
Dagupan City	1,241	19	38	57	4.59	1,820	701	697	1,398	76.81	15,175	4,715	4,314	9,029	59.50
Laoag City	0	0	0	0	0.00	917	837	758	1,595	173.94	7,498	3,462	2,021	5,483	73.13
San Carlos City	39	7	5	12	30.77	2,003	735	664	1,399	69.85	16,715	426	356	782	4.68
San Fernando City	339	5	9	14	4.13	1,048	955	892	1,847	176.24	8,849	7,281	6,702	13,983	158.02
Urdaneta City	26	3	3	6	23.08	1,417	547	582	1,129	79.68	11,778	670	815	1,485	12.61
Vigan City	173	3	0	3	1.73	432	495	440	935	216.44	3,766	1,645	1,484	3,129	83.09
**Region 2**	**1,573**	**337**	**373**	**710**	**45.14**	**34,606**	**16,708**	**16,666**	**33,374**	**96.44**	**290,609**	**95,682**	**93,949**	**189,631**	**65.25**
Batanes	32	3	2	5	15.63	179	82	88	170	94.97	1,575	68	230	298	18.92
Cagayan	236	76	88	164	69.49	10,261	5,053	4,944	9,997	97.43	86,326	26,004	23,773	49,777	57.66
Isabela	437	99	122	221	50.57	11,782	5,662	5,698	11,360	96.42	98,455	40,026	38,064	78,090	79.32
Nueva Vizcaya	666	91	95	186	27.93	4,779	2,101	2,223	4,324	90.48	40,263	14,315	16,520	30,835	76.58
Quirino	122	39	38	77	63.11	2,017	1,019	917	1,936	95.98	17,233	3,904	3,720	7,624	44.24
Cauayan City	27	12	11	23	85.19	1,287	674	613	1,287	100.00	10,769	1,661	1,468	3,129	29.06
Ilagan City	11	6	5	11	100.00	1,449	656	792	1,448	99.93	12,101	1,945	2,734	4,679	38.67
Santiago City	32	8	11	19	59.38	1,339	638	701	1,339	100.00	11,213	2,012	1,845	3,857	34.40
Tuguegarao City	10	3	1	4	40.00	1,513	823	690	1,513	100.00	12,674	5,747	5,595	11,342	89.49
**Region 3**	**6,568**	**583**	**666**	**1,249**	**19.02**	**110,063**	**66,231**	**65,034**	**131,265**	**119.26**	**913,592**	**223,518**	**219,205**	**442,723**	**48.46**
Aurora	256	5	7	12	4.69	2,383	1,919	1,897	3,816	160.13	20,457	7,402	7,079	14,481	70.79
Bataan	348	64	65	129	37.07	6,909	5,297	5,065	10,362	149.98	54,805	26,200	24,776	50,976	93.01
Bulacan	1,618	164	142	306	18.91	21,847	9,052	9,112	18,164	83.14	181,335	19,925	19,808	39,733	21.91
Nueva Ecija	105	112	92	204	194.29	14,444	5,131	4,976	10,107	69.97	122,063	22,678	22,992	45,670	37.42
Pampanga	353	33	34	67	18.98	15,744	7,746	7,589	15,335	97.40	131,439	24,661	23,691	48,352	36.79
Tarlac	89	8	4	12	13.48	9,780	5,411	5,744	11,155	114.06	80,858	11,620	16,259	27,879	34.48
Zambales	678	64	69	133	19.62	6,281	3,637	3,626	7,263	115.63	50,969	17,563	16,689	34,252	67.20
Angeles City	791	14	16	30	3.79	3,951	2,783	2,638	5,421	137.21	32,975	3,774	3,924	7,698	23.34
Balanga City	91	15	20	35	38.46	999	499	504	1,003	100.40	7,919	4,025	3,647	7,672	96.88
Cabanatuan City	100	23	31	54	54.00	2,954	2,648	2,431	5,079	171.94	24,979	3,615	3,245	6,860	27.46
City of San Fernando	719	15	21	36	5.01	2,945	2,311	2,061	4,372	148.46	24,564	7,908	6,358	14,266	58.08
Gapan City	2	2	0	2	100.00	1,081	623	605	1,228	113.60	9,116	4,644	4,333	8,977	98.48
Mabalacat City	703	27	38	65	9.25	2,410	3,441	3,499	6,940	287.97	20,093	13,084	12,627	25,711	127.96

Table 1.C.2.2- Nutrition Services for Infants and Children

Number of Infants born preterm or with low birth weight given iron supplements and infants 6-11 mos. old and children 12-59 mos. old given Vitamin A supplementation
Philippines, 2021

Area	Total No. of live births with low birth weight	Infants preterm or with LBW (< 2500 grams) given Iron				Eligible Pop (6-11 months)	Infants 6-11 mos. old Given Vitamin A				Eligible Pop (12-59 months)	Children 12-59 mos. Old Given Vitamin A			
		Male	Female	Total	%		Male	Female	Total	%		Male	Female	Total	%
Malolos City	13	11	11	22	169.23	2,439	1,277	1,281	2,558	104.88	20,252	10,714	10,325	21,039	103.89
Meycauayan	1	1	0	1	100.00	2,025	1,544	1,526	3,070	151.60	16,798	1,516	1,552	3,068	18.26
Olongapo	261	10	103	113	43.30	2,474	1,806	1,662	3,468	140.18	20,104	4,576	4,307	8,883	44.19
Palayan City	0	0	0	0	0.00	400	257	285	542	135.50	3,393	1,771	1,820	3,591	105.84
San Jose City	55	13	12	25	45.45	1,370	1,500	1,373	2,873	209.71	11,553	12,863	11,656	24,519	212.23
San Jose del Monte City	370	0	0	0	0.00	5,562	6,019	5,834	11,853	213.11	46,129	15,286	14,919	30,205	65.48
Science City of Munoz	5	0	0	0	0.00	796	394	389	783	98.37	6,737	2,906	2,836	5,742	85.23
Tarlac City	10	2	1	3	30.00	3,269	2,936	2,937	5,873	179.66	27,054	6,787	6,362	13,149	48.60
**Region 4A**	**8,350**	**847**	**929**	**1,776**	**21.27**	**149,202**	**87,540**	**84,125**	**171,665**	**115.06**	**1,233,724**	**251,234**	**243,424**	**494,658**	**40.09**
Batangas	479	145	172	317	66.18	19,436	7,986	7,786	15,772	81.15	163,720	38,831	36,822	75,653	46.21
Cavite	731	59	71	130	17.78	13,969	8,494	7,652	16,146	115.58	114,923	25,013	22,882	47,895	41.68
Laguna	892	49	42	91	10.20	9,870	6,281	6,010	12,291	124.53	80,579	19,819	18,860	38,679	48.00
Quezon	799	178	181	359	44.93	18,813	10,386	9,622	20,008	106.35	159,600	39,377	41,947	81,324	50.95
Rizal	979	121	161	282	28.80	22,421	9,499	9,528	19,027	84.86	181,961	15,097	14,486	29,583	16.26
Antipolo City	1,337	1	10	11	0.82	8,257	5,417	5,156	10,573	128.05	67,021	10,790	10,158	20,948	31.26
Bacoor City	315	4	3	7	2.22	6,123	3,246	3,115	6,361	103.89	50,346	4,874	4,726	9,600	19.07
Batangas City	256	11	18	29	11.33	3,445	2,234	2,212	4,446	129.06	29,056	3,846	3,591	7,437	25.60
Biñan City	145	54	44	98	67.59	3,303	3,968	3,804	7,772	235.30	27,018	17,968	17,138	35,106	129.94
Cabuyao City	10	27	10	37	370.00	3,064	2,409	2,521	4,930	160.90	25,045	4,296	4,289	8,585	34.28
Calamba City	375	37	33	70	18.67	4,511	4,315	4,210	8,525	188.98	36,868	12,305	11,498	23,803	64.56
Cavite City	21	3	1	4	19.05	1,046	237	201	438	41.87	8,610	38	28	66	0.77
Dasmariñas City	286	31	26	57	19.93	6,717	4,869	4,657	9,526	141.82	55,249	13,436	12,797	26,233	47.48
General Trias City	23	2	4	6	26.09	3,205	1,571	1,675	3,246	101.28	26,348	2,392	2,496	4,888	18.55
Imus City	191	9	22	31	16.23	4,117	2,519	2,410	4,929	119.72	33,852	6,365	6,167	12,532	37.02
Lipa City	110	3	4	7	6.36	3,473	517	528	1,045	30.09	29,275	1,025	949	1,974	6.74
Lucena City	158	23	23	46	29.11	2,855	2,669	2,631	5,300	185.64	24,184	7,220	7,520	14,740	60.95
San Pablo City	428	12	10	22	5.14	2,646	1,788	1,585	3,373	127.48	21,585	5,018	4,744	9,762	45.23
San Pedro City	141	8	4	12	8.51	3,232	1,332	1,405	2,737	84.68	26,430	1,618	1,570	3,188	12.06
Santa Rosa City	385	9	22	31	8.05	3,511	3,895	3,554	7,449	212.16	28,699	6,139	5,554	11,693	40.74
Tagaytay City	3	19	19	38	1266.67	726	675	697	1,372	188.98	5,966	2,346	2,255	4,601	77.12
Tanauan City	120	9	5	14	11.67	1,811	1,183	1,197	2,380	131.42	15,272	3,340	3,218	6,558	42.94
Tayabas City	36	26	33	59	163.89	1,064	695	593	1,288	121.05	9,064	6,043	5,753	11,796	130.14
Trece Martires City	130	7	11	18	13.85	1,587	1,355	1,376	2,731	172.09	13,053	4,038	3,976	8,014	61.40
**Region 4B**	**2,078**	**276**	**314**	**590**	**28.39**	**36,035**	**17,311**	**16,770**	**34,081**	**94.58**	**301,672**	**92,594**	**87,084**	**179,678**	**59.56**
Marinduque	115	41	45	86	74.78	2,464	1,850	1,618	3,468	140.75	22,017	7,868	6,922	14,790	67.18
Mindoro Occidental	326	15	26	41	12.58	6,230	4,079	4,024	8,103	130.06	50,503	29,480	27,962	57,442	113.74
Mindoro Oriental	765	48	40	88	11.50	10,004	2,219	2,008	4,227	42.25	83,982	11,921	11,194	23,115	27.52
Palawan	496	76	97	173	34.88	10,843	6,600	6,656	13,256	122.25	89,558	34,916	33,018	67,934	75.85
Romblon	230	60	71	131	56.96	3,236	1,647	1,559	3,206	99.07	28,715	6,360	6,176	12,536	43.66
Puerto Princesa City	146	36	35	71	48.63	3,258	916	905	1,821	55.89	26,897	2,049	1,812	3,861	14.35
**Region 5**	**7,029**	**654**	**724**	**1,378**	**19.60**	**68,682**	**35,529**	**34,273**	**69,802**	**101.63**	**591,236**	**165,140**	**161,493**	**326,633**	**55.25**
Albay	379	93	132	225	59.37	11,965	6,086	5,870	11,956	99.92	103,949	38,665	36,364	75,029	72.18
Camarines Norte	1,222	72	112	184	15.06	7,162	3,476	3,372	6,848	95.62	60,107	27,457	25,418	52,875	87.97
Camarines Sur	875	60	86	146	16.69	19,610	9,068	8,628	17,696	90.24	167,647	26,739	25,436	52,175	31.12
Catanduanes	462	98	133	231	50.00	3,178	1,921	1,908	3,829	120.48	26,943	6,764	7,020	13,784	51.16
Masbate	695	193	137	330	47.48	11,277	4,606	4,477	9,083	80.54	99,576	22,435	22,744	45,179	45.37
Sorsogon	995	107	89	196	19.70	9,726	5,433	5,368	10,801	111.05	83,359	21,842	20,585	42,427	50.90
Iriga City	178	16	26	42	23.60	1,328	620	658	1,278	96.23	11,391	386	384	770	6.76
Legaspi City	111	11	4	15	13.51	2,101	2,634	2,366	5,000	237.98	18,283	15,000	17,753	32,753	179.14
Naga City	2,112	4	5	9	0.43	2,335	1,685	1,626	3,311	141.80	19,981	5,852	5,789	11,641	58.26
**Region 6**	**5,702**	**582**	**687**	**1,269**	**22.26**	**73,273**	**39,502**	**37,676**	**77,178**	**105.33**	**623,781**	**198,556**	**191,677**	**390,233**	**62.56**
Aklan	300	43	36	79	26.33	5,611	3,865	3,562	7,427	132.36	48,127	8,136	10,824	18,960	39.40
Antique	1,073	52	51	103	9.60	6,486	3,854	3,620	7,474	115.23	54,526	10,329	9,558	19,887	36.47
Capiz	308	38	43	81	26.30	6,992	5,193	5,370	10,563	151.07	60,055	19,018	18,397	37,415	62.30
Guimaras	212	47	36	83	39.15	1,549	1,119	1,062	2,181	140.80	14,503	5,899	5,679	11,578	79.83
Iloilo	1,384	108	149	257	18.57	18,209	6,252	5,744	11,996	65.88	153,119	69,721	65,605	135,326	88.38
Negros Occidental	1,952	216	276	492	25.20	24,667	14,098	13,562	27,660	112.13	210,638	67,522	64,295	131,817	62.58
Bacolod City	329	66	86	152	46.20	5,544	2,539	2,316	4,855	87.57	47,393	4,601	4,469	9,070	19.14
Iloilo City	144	12	10	22	15.28	4,215	2,582	2,440	5,022	119.15	35,420	13,330	12,850	26,180	73.91
**Region 7**	**51,289**	**492**	**507**	**999**	**1.95**	**81,443**	**50,435**	**49,628**	**100,063**	**122.86**	**650,762**	**146,555**	**149,269**	**295,824**	**45.46**
Bohol	17,238	46	42	88	0.51	13,837	6,466	6,366	12,832	92.74	116,463	22,812	22,862	45,674	39.22
Cebu	1,069	122	139	261	24.42	33,510	21,502	20,071	41,573	124.06	261,301	60,137	57,500	117,637	45.02
Negros Oriental	1,175	134	158	292	24.85	13,961	6,178	7,097	13,275	95.09	114,898	27,697	35,000	62,697	54.57
Siquijor	1,101	1	1	2	0.18	817	694	622	1,316	161.08	7,543	1,717	1,692	3,409	45.19
Cebu City	16,209	114	108	222	1.37	10,527	7,835	7,757	15,592	148.11	82,030	23,963	22,581	46,544	56.74
Lapu-Lapu City	7,319	47	41	88	1.20	4,657	5,189	5,073	10,262	220.36	36,283	7,361	6,896	14,257	39.29

Table 1.C.2.2- Nutrition Services for Infants and Children

Number of Infants born preterm or with low birth weight given iron supplements and infants 6-11 mos. old and children 12-59 mos. old given Vitamin A supplementation
Philippines, 2021

Area	Total No. of live births with low birth weight	Infants preterm or with LBW (< 2500 grams) given Iron				Eligible Pop (6-11 months)	Infants 6-11 mos. old Given Vitamin A				Eligible Pop (12-59 months)	Children 12-59 mos. Old Given Vitamin A			
		Male	Female	Total	%		Male	Female	Total	%		Male	Female	Total	%
Mandaue City	7,178	28	18	46	0.64	4,134	2,571	2,642	5,213	126.10	32,244	2,868	2,738	5,606	17.39
**Region 8**	**3,077**	**555**	**564**	**1,119**	**36.37**	**52,044**	**25,816**	**25,537**	**51,353**	**98.67**	**439,152**	**118,384**	**113,606**	**231,990**	**52.83**
Biliran	248	103	88	191	77.02	1,915	1,094	821	1,915	100.00	16,889	2,680	2,455	5,135	30.40
Eastern Samar	183	31	24	55	30.05	5,849	2,810	2,978	5,788	98.96	47,349	10,845	12,257	23,102	48.79
Northern Leyte	405	73	74	147	36.30	17,534	8,712	8,569	17,281	98.56	145,057	39,132	36,977	76,109	52.47
Northern Samar	608	96	91	187	30.76	7,822	3,910	3,862	7,772	99.36	68,641	21,603	21,300	42,903	62.50
Southern Leyte	222	38	35	73	32.88	3,280	1,494	1,581	3,075	93.75	29,248	8,737	7,747	16,484	56.36
Western Samar	273	47	46	93	34.07	7,272	3,588	3,670	7,258	99.81	61,611	13,212	12,473	25,685	41.69
Calbayog City	75	17	12	29	38.67	2,234	1,159	1,075	2,234	100.00	18,987	3,250	3,122	6,372	33.56
Maasin City	344	55	60	115	33.43	833	442	390	832	99.88	7,450	2,880	2,512	5,392	72.38
Ormoc City	688	92	116	208	30.23	2,498	1,219	1,243	2,462	98.56	20,661	5,090	4,740	9,830	47.58
Tacloban City	31	3	18	21	67.74	2,807	1,388	1,348	2,736	97.47	23,259	10,955	10,023	20,978	90.19
**Region 9**	**1,134**	**173**	**256**	**429**	**37.83**	**39,757**	**23,064**	**23,265**	**46,329**	**116.53**	**337,340**	**66,108**	**67,274**	**133,382**	**39.54**
Zamboanga del Norte	283	73	70	143	50.53	8,519	5,318	5,324	10,642	124.92	74,039	16,048	16,695	32,743	44.22
Zamboanga del Sur	183	43	86	129	70.49	8,750	4,897	4,795	9,692	110.77	71,567	16,935	16,618	33,553	46.88
Zamboanga Sibugay	346	24	40	64	18.50	7,499	4,322	4,876	9,198	122.66	66,255	16,435	16,877	33,312	50.28
Dapitan City	31	12	17	29	93.55	883	362	441	803	90.94	7,646	207	251	458	5.99
Dipolog City	38	2	7	9	23.68	1,396	1,166	1,064	2,230	159.74	12,129	2,600	2,767	5,367	44.25
Isabela City	4	1	1	2	50.00	1,268	547	592	1,139	89.83	12,150	394	373	767	6.31
Pagadian City	72	3	4	7	9.72	2,147	1,167	1,190	2,357	109.78	17,557	1,219	1,560	2,779	15.83
Zamboanga City	177	15	31	46	25.99	9,295	5,285	4,983	10,268	110.47	75,997	12,270	12,133	24,403	32.11
**Region 10**	**3,801**	**1,290**	**1,139**	**2,429**	**63.90**	**50,346**	**38,311**	**37,120**	**75,431**	**149.83**	**431,863**	**222,906**	**214,118**	**437,024**	**101.20**
Bukidnon	912	250	217	467	51.21	11,655	7,594	7,114	14,708	126.19	98,908	61,193	57,732	118,925	120.24
Camiguin	41	18	23	41	100.00	930	418	366	784	84.30	7,997	3,594	3,406	7,000	87.53
Lanao del Norte	615	261	353	614	99.84	7,486	6,303	6,525	12,828	171.36	67,455	31,914	30,870	62,784	93.08
Misamis Occidental	607	10	7	17	2.80	3,193	1,512	1,683	3,195	100.06	27,457	13,036	14,284	27,320	99.50
Misamis Oriental	408	42	48	90	22.06	7,526	4,635	4,496	9,131	121.33	62,945	34,221	33,020	67,241	106.83
Cagayan de Oro City	534	232	302	534	100.00	7,126	11,167	10,484	21,651	303.83	59,625	51,916	48,069	99,985	167.69
El Salvador City	11	5	6	11	100.00	529	75	94	169	31.95	4,426	110	99	209	4.72
Gingoog City	572	438	134	572	100.00	1,319	123	161	284	21.53	11,000	492	643	1,135	10.32
Iligan City	47	17	30	47	100.00	3,785	2,479	2,395	4,874	128.77	34,174	10,280	10,358	20,638	60.39
Malaybalay City	23	2	7	9	39.13	1,945	464	348	812	41.75	16,486	0	0	0	0.00
Oroquieta City	0	0	0	0	0.00	693	649	610	1,259	181.67	5,950	3,141	2,793	5,934	99.73
Ozamis City	10	3	7	10	100.00	1,390	800	722	1,522	109.50	11,924	3,747	3,853	7,600	63.74
Tangub City	11	4	3	7	63.64	621	160	188	348	56.04	5,299	2	1	3	0.06
Valencia City	10	8	2	10	100.00	2,148	1,932	1,934	3,866	179.98	18,217	9,260	8,990	18,250	100.18
**Region 11**	**3,673**	**624**	**636**	**1,260**	**34.30**	**53,895**	**41,412**	**39,671**	**81,083**	**150.45**	**450,626**	**220,924**	**209,993**	**430,917**	**95.63**
Davao de Oro	623	145	164	309	49.60	7,770	6,088	5,774	11,862	152.66	68,026	42,057	39,322	81,379	119.63
Davao del Norte	859	174	167	341	39.70	10,575	8,248	7,892	16,140	152.62	87,061	52,849	50,678	103,527	118.91
Davao del Sur	387	60	58	118	30.49	6,555	4,847	4,435	9,282	141.60	56,666	24,323	23,215	47,538	83.89
Davao Oriental	432	51	46	97	22.45	7,173	4,936	4,539	9,475	132.09	58,178	22,240	21,049	43,289	74.41
Davao Occidental	159	7	6	13	8.18	3,294	2,177	2,028	4,205	127.66	30,486	11,476	10,942	22,418	73.54
Davao City	1,213	187	195	382	31.49	18,528	15,116	15,003	30,119	162.56	150,209	67,979	64,787	132,766	88.39
**Region 12**	**1,651**	**461**	**452**	**913**	**55.30**	**52,360**	**36,308**	**34,904**	**71,212**	**136.00**	**437,961**	**122,468**	**118,976**	**241,444**	**55.13**
North Cotabato	512	64	88	152	29.69	16,966	8,047	7,840	15,887	93.64	140,154	43,410	41,629	85,039	60.68
Sarangani	168	56	48	104	61.90	6,452	7,186	6,894	14,080	218.23	54,636	22,245	22,015	44,260	81.01
South Cotabato	386	163	160	323	83.68	10,523	7,991	7,877	15,868	150.79	84,890	30,492	29,692	60,184	70.90
Sultan Kudarat	389	116	102	218	56.04	8,677	7,647	7,211	14,858	171.23	74,810	19,327	19,296	38,623	51.63
Cotabato City	38	1	1	2	5.26	2,917	1,449	1,451	2,900	99.42	28,340	1,718	1,714	3,432	12.11
Gen. Santos City	158	61	53	114	72.15	6,825	3,988	3,631	7,619	111.63	55,131	5,276	4,630	9,906	17.97
**BARMM**	**1,006**	**351**	**444**	**795**	**79.03**	**47,089**	**21,393**	**22,630**	**44,023**	**93.49**	**490,094**	**49,943**	**57,164**	**107,107**	**21.85**
Basilan	152	81	95	176	115.79	3,842	884	897	1,781	46.36	36,154	2,194	2,101	4,295	11.88
Lanao del Sur	53	2	0	2	3.77	10,681	1,522	1,668	3,190	29.87	111,572	4,991	5,129	10,120	9.07
Maguindanao	257	11	46	57	22.18	15,840	9,635	9,756	19,391	122.42	151,474	7,830	8,246	16,076	10.61
Sulu	76	207	233	440	578.95	8,442	4,679	5,025	9,704	114.95	109,019	22,730	27,180	49,910	45.78
Tawi-Tawi	65	6	8	14	21.54	4,664	2,633	3,244	5,877	126.01	45,239	7,598	9,659	17,257	38.15
Lamitan City	19	4	2	6	31.58	1,059	162	166	328	30.97	9,948	355	361	716	7.20
Marawi City	384	40	60	100	26.04	2,561	1,878	1,874	3,752	146.51	26,688	4,245	4,488	8,733	32.72

Table 1.C.2.2- Nutrition Services for Infants and Children

Number of Infants born preterm or with low birth weight given iron supplements and infants 6-11 mos. old and children 12-59 mos. old given Vitamin A supplementation
Philippines, 2021

Area	Total No. of live births with low birth weight	Infants preterm or with LBW (< 2500 grams) given Iron				Eligible Pop (6-11 months)	Infants 6-11 mos. old Given Vitamin A				Eligible Pop (12-59 months)	Children 12-59 mos. Old Given Vitamin A			
		Male	Female	Total	%		Male	Female	Total	%		Male	Female	Total	%
**CARAGA**	**1,512**	**298**	**297**	**595**	**39.35**	**30,191**	**21,894**	**21,585**	**43,479**	**144.01**	**259,830**	**92,508**	**91,382**	**183,890**	**70.77**
Agusan del Norte	186	19	20	39	20.97	4,069	2,486	2,546	5,032	123.67	34,174	10,652	11,276	21,928	64.17
Agusan del Sur	236	95	77	172	72.88	8,826	5,215	5,080	10,295	116.64	77,089	29,070	28,021	57,091	74.06
Surigao del Norte	217	53	71	124	57.14	3,603	1,899	1,876	3,775	104.77	31,994	9,233	9,270	18,503	57.83
Surigao del Sur	325	50	57	107	32.92	5,763	3,298	3,324	6,622	114.91	48,690	20,088	20,443	40,531	83.24
Province of Dinagat	63	11	9	20	31.75	1,292	662	739	1,401	108.44	11,239	4,619	4,568	9,187	81.74
Bislig City	13	2	2	4	30.77	1,094	520	530	1,050	95.98	9,250	4,758	4,485	9,243	99.92
Butuan City	161	22	17	39	24.22	3,862	6,613	6,350	12,963	335.66	32,495	12,555	11,957	24,512	75.43
Surigao City	311	46	44	90	28.94	1,682	1,201	1,140	2,341	139.18	14,899	1,533	1,362	2,895	19.43

Note: Put asterisk (*) for No Report and Zero (0) for No Case

Table 1.C.2.3 - Nutrition Services for Infants and Children

Number and Proportion of Infants 6-11 mos. old and Children 12-23 mos. old who completed Micronutrient Powder Supplementation
Philippines, 2021

Area	Eligible Pop (6-11 mos.)	Infants 6-11 mos. old who completed MNP supplementation				Eligible Pop (12-23 mos.)	Children 12-23 mos. old who completed MNP supplementation			
		Male	Female	Total	%		Male	Female	Total	%
**PHILIPPINES**	**1,064,115**	**67,809**	**66,125**	**133,934**	**12.59**	**2,143,492**	**97,158**	**99,073**	**196,231**	**9.15**
**N C R**	**118,939**	**16,582**	**16,680**	**33,262**	**27.97**	**235,286**	**27,973**	**30,012**	**57,985**	**24.64**
Malabon	3,375	1631	1743	3,374	99.97	6,679	3,200	3,479	6,679	100.00
Navotas	2,305	340	371	711	30.85	4,557	259	279	538	11.81
Valenzuela City	5,733	115	145	260	4.54	11,336	76	1,148	1,224	10.80
Caloocan City	14,629	1194	1042	2,236	15.28	28,942	1,008	1,016	2,024	6.99
Marikina City	4,162	173	172	345	8.29	8,236	0	0	0	0.00
Pasig City	6,981	0	0	0	0.00	13,798	0	0	0	0.00
Pateros	587	23	27	50	8.52	1,167	24	18	42	3.60
Taguig	7,430	2690	2589	5,279	71.05	14,709	4,569	4,746	9,315	63.33
Quezon City	27,121	269	304	573	2.11	53,648	181	201	382	0.71
Makati City	5,376	1869	1698	3,567	66.35	10,644	1,563	1,319	2,882	27.08
Mandaluyong City	3,565	172	184	356	9.99	7,058	92	89	181	2.56
San Juan	1,129	548	582	1,130	100.09	2,232	967	1,265	2,232	100.00
Manila City	16,442	3208	3502	6,710	40.81	32,528	7,161	7,567	14,728	45.28
Las Piñas City	5,440	43	52	95	1.75	10,762	12	26	38	0.35
Muntinlupa City	4,661	2096	2218	4,314	92.56	9,218	4,553	4,667	9,220	100.02
Parañaque City	6,153	206	207	413	6.71	12,164	405	481	886	7.28
Pasay City	3,850	2005	1844	3,849	99.97	7,608	3,903	3,711	7,614	100.08
**C A R**	**17,594**	**550**	**565**	**1,115**	**6.34**	**35,902**	**447**	**464**	**911**	**2.54**
Abra	2,146	130	116	246	11.46	4,409	41	54	95	2.15
Apayao	1,235	1	3	4	0.32	2,507	0	0	0	0.00
Benguet	4,568	285	315	600	13.13	9,269	146	149	295	3.18
Ifugao	2,219	8	9	17	0.77	4,739	5	3	8	0.17
Kalinga	2,335	108	107	215	9.21	4,753	154	150	304	6.40
Mt. Province	1,539	4	4	8	0.52	3,061	0	0	0	0.00
Baguio City	3,552	14	11	25	0.70	7,164	101	108	209	2.92
**Region 1**	**48,596**	**6,421**	**6,145**	**12,566**	**25.86**	**97,412**	**7,728**	**7,400**	**15,128**	**15.53**
Ilocos Norte	4,000	350	353	703	17.58	7,702	607	599	1,206	15.66
Ilocos Sur	4,600	1124	1132	2256	49.04	9,500	1757	1812	3,569	37.57
La Union	5,714	83	88	171	2.99	11,425	373	393	766	6.70
Pangasinan	25,208	3056	2846	5902	23.41	50,627	3149	2866	6,015	11.88
Alaminos City	952	393	345	738	77.52	1,910	390	383	773	40.47
Candon City	485	522	460	982	202.47	997	541	495	1,036	103.91
Dagupan City	1,820	709	704	1413	77.64	3,649	580	581	1,161	31.82
Laoag City	917	0	0	0	0.00	1,771	0	0	0	0.00
San Carlos City	2,003	58	59	117	5.84	4,018	87	89	176	4.38
San Fernando City	1,048	6	18	24	2.29	2,090	150	100	250	11.96
Urdaneta City	1,417	119	139	258	18.21	2,833	94	82	176	6.21
Vigan City	432	1	1	2	0.46	890	0	0	0	0.00
**Region 2**	**34,606**	**2,851**	**2,534**	**5,385**	**15.56**	**70,015**	**3,301**	**3,179**	**6,480**	**9.26**
Batanes	179	10	6	16	8.94	353	0	0	0	0.00
Cagayan	10,261	551	499	1050	10.23	20,956	917	896	1,813	8.65
Isabela	11,782	924	831	1755	14.90	23,494	762	775	1,537	6.54
Nueva Vizcaya	4,779	449	344	793	16.59	9,727	611	502	1,113	11.44
Quirino	2,017	201	176	377	18.69	4,264	138	137	275	6.45

Table 1.C.2.3 - Nutrition Services for Infants and Children

Number and Proportion of Infants 6-11 mos. old and Children 12-23 mos. old who completed Micronutrient Powder Supplementation
Philippines, 2021

Area	Eligible Pop (6-11 mos.)	Infants 6-11 mos. old who completed MNP supplementation				Eligible Pop (12-23 mos.)	Children 12-23 mos. old who completed MNP supplementation			
		Male	Female	Total	%		Male	Female	Total	%
Cauayan City	1,287	295	254	549	42.66	2,576	301	267	568	22.05
Ilagan City	1,449	56	54	110	7.59	2,893	33	39	72	2.49
Santiago City	1,339	195	205	400	29.87	2,677	204	232	436	16.29
Tuguegarao City	1,513	170	165	335	22.14	3,075	335	331	666	21.66
**Region 3**	**110,063**	**6,742**	**6,444**	**13,186**	**11.98**	**218,435**	**4,173**	**3,987**	**8,160**	**3.74**
Aurora	2,383	49	56	105	4.41	4,937	49	43	92	1.86
Bataan	6,909	546	535	1,081	15.65	13,339	1,129	1,044	2,173	16.29
Bulacan	21,847	369	433	802	3.67	43,256	156	182	338	0.78
Nueva Ecija	14,444	4,066	3,784	7,850	54.35	29,077	640	640	1,280	4.40
Pampanga	15,744	123	105	228	1.45	31,109	102	90	192	0.62
Tarlac	9,780	545	491	1,036	10.59	19,345	741	668	1,409	7.28
Zambales	6,281	4	0	4	0.06	12,519	0	0	0	0.00
Angeles City	3,951	265	267	532	13.46	7,806	445	444	889	11.39
Balanga City	999	38	33	71	7.11	1,927	52	45	97	5.03
Cabanatuan City	2,954	16	16	32	1.08	5,950	13	27	40	0.67
City of San Fernando	2,945	134	139	273	9.27	5,818	3	3	6	0.10
Gapan City	1,081	33	35	68	6.29	2,172	22	24	46	2.12
Mabalacat City	2,410	222	209	431	17.88	4,760	296	256	552	11.60
Malolos City	2,439	0	0	0	0.00	4,835	0	0	0	0.00
Meycauayan	2,025	74	74	148	7.31	4,010	80	79	159	3.97
Olongapo	2,474	7	2	9	0.36	4,942	0	0	0	0.00
Palayan City	400	0	0	0	0.00	805	0	0	0	0.00
San Jose City	1,370	6	4	10	0.73	2,750	11	6	17	0.62
San Jose del Monte City	5,562	131	151	282	5.07	11,003	304	297	601	5.46
Science City of Munoz	796	0	0	0	0.00	1,600	0	0	0	0.00
Tarlac City	3,269	114	110	224	6.85	6,475	130	139	269	4.15
**Region 4A**	**149,202**	**9,846**	**9,440**	**19,286**	**12.93**	**296,631**	**19,439**	**19,558**	**38,997**	**13.15**
Batangas	19,436	247	201	448	2.31	39,103	1,342	1,544	2,886	7.38
Cavite	13,969	933	934	1,867	13.37	27,486	354	331	685	2.49
Laguna	9,870	708	685	1,393	14.11	19,483	527	581	1,108	5.69
Quezon	18,813	511	469	980	5.21	38,303	422	351	773	2.02
Rizal	22,421	830	686	1,516	6.76	44,156	658	708	1,366	3.09
Antipolo City	8,257	195	247	442	5.35	16,264	160	200	360	2.21
Bacoor City	6,123	407	380	787	12.85	12,040	342	348	690	5.73
Batangas City	3,445	47	30	77	2.24	6,936	3	2	5	0.07
Biñan City	3,303	3,682	3,563	7,245	219.35	6,533	12,957	13,046	26,003	398.03
Cabuyao City	3,064	203	170	373	12.17	6,058	205	195	400	6.60
Calamba City	4,511	362	338	700	15.52	8,919	286	251	537	6.02
Cavite City	1,046	6	0	6	0.57	2,061	0	0	0	0.00
Dasmariñas City	6,717	592	560	1,152	17.15	13,205	607	535	1,142	8.65
General Trias City	3,205	63	37	100	3.12	6,294	17	19	36	0.57
Imus City	4,117	118	160	278	6.75	8,087	165	142	307	3.80
Lipa City	3,473	3	0	3	0.09	6,989	0	0	0	0.00
Lucena City	2,855	26	27	53	1.86	5,799	29	30	59	1.02
San Pablo City	2,646	5	7	12	0.45	5,215	0	0	0	0.00
San Pedro City	3,232	431	472	903	27.94	6,390	375	347	722	11.30
Santa Rosa City	3,511	274	294	568	16.18	6,936	681	626	1,307	18.84
Tagaytay City	726	161	137	298	41.05	1,429	265	259	524	36.67
Tanauan City	1,811	8	10	18	0.99	3,647	42	41	83	2.28
Tayabas City	1,064	30	31	61	5.73	2,176	0	0	0	0.00
Trece Martires City	1,587	4	2	6	0.38	3,122	2	2	4	0.13

Table 1.C.2.3 - Nutrition Services for Infants and Children

Number and Proportion of Infants 6-11 mos. old and Children 12-23 mos. old who completed Micronutrient Powder Supplementation
Philippines, 2021

Area	Eligible Pop (6-11 mos.)	Infants 6-11 mos. old who completed MNP supplementation				Eligible Pop (12-23 mos.)	Children 12-23 mos. old who completed MNP supplementation			
		Male	Female	Total	%		Male	Female	Total	%
**Region 4B**	**36,035**	**1,047**	**1,006**	**2,053**	**5.70**	**72,046**	**1,714**	**1,877**	**3,591**	**4.98**
Marinduque	2,464	148	136	284	11.53	4,977	313	348	661	13.28
Mindoro Occidental	6,230	229	241	470	7.54	12,541	0	0	0	0.00
Mindoro Oriental	10,004	115	102	217	2.17	19,773	358	395	753	3.81
Palawan	10,843	449	432	881	8.13	21,633	497	530	1,027	4.75
Romblon	3,236	73	68	141	4.36	6,626	514	579	1,093	16.50
Puerto Princesa City	3,258	33	27	60	1.84	6,496	32	25	57	0.88
**Region 5**	**68,682**	**4,065**	**3,777**	**7,842**	**11.42**	**140,333**	**5,307**	**5,095**	**10,402**	**7.41**
Albay	11,965	372	387	759	6.34	24,549	1,471	1,482	2,953	12.03
Camarines Norte	7,162	167	150	317	4.43	14,005	79	75	154	1.10
Camarines Sur	19,610	208	182	390	1.99	39,844	219	222	441	1.11
Catanduanes	3,178	5	5	10	0.31	6,554	60	32	92	1.40
Masbate	11,277	712	485	1,197	10.61	23,811	444	430	874	3.67
Sorsogon	9,726	2,235	2,226	4,461	45.87	19,791	2,241	2,179	4,420	22.33
Iriga City	1,328	39	47	86	6.48	2,708	91	99	190	7.02
Legaspi City	2,101	50	100	150	7.14	4,320	407	324	731	16.92
Naga City	2,335	277	195	472	20.21	4,751	295	252	547	11.51
**Region 6**	**73,273**	**5,922**	**5,899**	**11,821**	**16.13**	**147,788**	**6,903**	**7,094**	**13,997**	**9.47**
Aklan	5,611	274	252	526	9.37	11,516	373	340	713	6.19
Antique	6,486	41	16	57	0.88	13,016	65	47	112	0.86
Capiz	6,992	182	211	393	5.62	14,260	72	89	161	1.13
Guimaras	1,549	0	0	0	0.00	3,339	0	0	0	0.00
Iloilo	18,209	3,557	3,561	7,118	39.09	36,386	4,349	4,491	8,840	24.30
Negros Occidental	24,667	1,854	1,856	3,710	15.04	49,672	1,638	1,585	3,223	6.49
Bacolod City	5,544	14	3	17	0.31	11,178	406	542	948	8.48
Iloilo City	4,215	0	0	0	0.00	8,421	0	0	0	0.00
**Region 7**	**81,443**	**2,765**	**2,818**	**5,583**	**6.86**	**159,302**	**3,966**	**4,406**	**8,372**	**5.26**
Bohol	13,837	343	322	665	4.81	27,664	1,068	1,013	2,081	7.52
Cebu	33,510	671	684	1,355	4.04	64,764	819	743	1,562	2.41
Negros Oriental	13,961	63	113	176	1.26	27,803	622	1,081	1,703	6.13
Siquijor	817	107	0	107	13.10	1,760	0	0	0	0.00
Cebu City	10,527	94	71	165	1.57	20,330	204	227	431	2.12
Lapu-Lapu City	4,657	957	1,019	1,976	42.43	8,992	900	913	1,813	20.16
Mandaue City	4,134	530	609	1,139	27.55	7,989	353	429	782	9.79
**Region 8**	**52,044**	**2,610**	**2,478**	**5,088**	**9.78**	**103,914**	**5,703**	**4,988**	**10,691**	**10.29**
Biliran	1,915	260	201	461	24.07	4,073	253	173	426	10.46
Eastern Samar	5,849	59	107	166	2.84	11,260	693	209	902	8.01
Northern Leyte	17,534	496	455	951	5.42	34,369	606	643	1,249	3.63
Northern Samar	7,822	956	987	1,943	24.84	16,000	1,223	1,070	2,293	14.33
Southern Leyte	3,280	8	12	20	0.61	6,927	13	9	22	0.32
Western Samar	7,272	494	402	896	12.32	14,607	380	353	733	5.02
Calbayog City	2,234	240	205	445	19.92	4,501	1,755	1,741	3,496	77.67
Maasin City	833	31	41	72	8.64	1,764	511	488	999	56.63
Ormoc City	2,498	52	57	109	4.36	4,894	187	213	400	8.17
Tacloban City	2,807	14	11	25	0.89	5,519	82	89	171	3.10

Table 1.C.2.3 - Nutrition Services for Infants and Children

Number and Proportion of Infants 6-11 mos. old and Children 12-23 mos. old who completed Micronutrient Powder Supplementation
Philippines, 2021

Area	Eligible Pop (6-11 mos.)	Infants 6-11 mos. old who completed MNP supplementation				Eligible Pop (12-23 mos.)	Children 12-23 mos. old who completed MNP supplementation			
		Male	Female	Total	%		Male	Female	Total	%
**Region 9**	**39,757**	**1,085**	**1,063**	**2,148**	**5.40**	**79,833**	**2,212**	**2,382**	**4,594**	**5.75**
Zamboanga del Norte	8,519	475	499	974	11.43	17,325	732	840	1,572	9.07
Zamboanga del Sur	8,750	168	159	327	3.74	17,144	117	145	262	1.53
Zamboanga Sibugay	7,499	70	77	147	1.96	15,468	80	167	247	1.60
Dapitan City	883	0	0	0	0.00	1,785	0	0	0	0.00
Dipolog City	1,396	79	82	161	11.53	2,839	66	52	118	4.16
Isabela City	1,268	3	3	6	0.47	2,864	0	0	0	0.00
Pagadian City	2,147	0	0	0	0.00	4,207	0	0	0	0.00
Zamboanga City	9,295	290	243	533	5.73	18,201	1,217	1,178	2,395	13.16
**Region 10**	**50,346**	**2,605**	**2,645**	**5,250**	**10.43**	**103,468**	**5,354**	**5,808**	**11,162**	**10.79**
Bukidnon	11,655	958	956	1,914	16.42	23,967	738	753	1,491	6.22
Camiguin	930	0	0	0	0.00	1,872	103	96	199	10.63
Lanao del Norte	7,486	706	702	1,408	18.81	15,733	1,452	1,548	3,000	19.07
Misamis Occidental	3,193	375	403	778	24.37	6,565	1,033	1,106	2,139	32.58
Misamis Oriental	7,526	136	148	284	3.77	15,235	545	650	1,195	7.84
Cagayan de Oro City	7,126	260	265	525	7.37	14,435	278	277	555	3.84
El Salvador City	529	0	0	0	0.00	1,072	0	0	0	0.00
Gingoog City	1,319	71	80	151	11.45	2,663	1,089	1,292	2,381	89.41
Iligan City	3,785	5	2	7	0.18	7,969	0	0	0	0.00
Malaybalay City	1,945	6	3	9	0.46	4,002	0	0	0	0.00
Oroquieta City	693	17	22	39	5.63	1,422	5	6	11	0.77
Ozamis City	1,390	39	38	77	5.54	2,853	84	76	160	5.61
Tangub City	621	0	0	0	0.00	1,267	0	0	0	0.00
Valencia City	2,148	32	26	58	2.70	4,413	27	4	31	0.70
**Region 11**	**53,895**	**549**	**482**	**1,031**	**1.91**	**108,712**	**912**	**817**	**1,729**	**1.59**
Davao de Oro	7,770	70	11	81	1.04	16,159	66	9	75	0.46
Davao del Norte	10,575	414	409	823	7.78	21,193	18	23	41	0.19
Davao del Sur	6,555	34	33	67	1.02	13,347	354	322	676	5.06
Davao Oriental	7,173	1	4	5	0.07	14,220	415	370	785	5.52
Davao Occidental	3,294	6	1	7	0.21	7,091	0	0	0	0.00
Davao City	18,528	24	24	48	0.26	36,702	59	93	152	0.41
**Region 12**	**52,360**	**2,209**	**2,257**	**4,466**	**8.53**	**105,179**	**1,242**	**1,240**	**2,482**	**2.36**
North Cotabato	16,966	653	717	1,370	8.07	33,693	350	411	761	2.26
Sarangani	6,452	508	498	1,006	15.59	13,289	367	304	671	5.05
South Cotabato	10,523	736	719	1,455	13.83	20,753	382	372	754	3.63
Sultan Kudarat	8,677	179	201	380	4.38	17,783	95	96	191	1.07
Cotabato City	2,917	0	0	0	0.00	6,182	0	0	0	0.00
Gen. Santos City	6,825	133	122	255	3.74	13,479	48	57	105	0.78
**BARMM**	**47,089**	**136**	**129**	**265**	**0.56**	**107,360**	**77**	**81**	**158**	**0.15**
Basilan	3,842	7	10	17	0.44	7,995	67	74	141	1.76
Lanao del Sur	10,681	6	9	15	0.14	25,061	1	0	1	0.00
Maguindanao	15,840	27	15	42	0.27	34,778	9	7	16	0.05
Sulu	8,442	0	0	0	0.00	21,517	0	0	0	0.00
Tawi-Tawi	4,664	0	0	0	0.00	9,815	0	0	0	0.00
Lamitan City	1,059	1	3	4	0.38	2,202	0	0	0	0.00
Marawi City	2,561	95	92	187	7.30	5,992	0	0	0	0.00

Table 1.C.2.3 - Nutrition Services for Infants and Children

Number and Proportion of Infants 6-11 mos. old and Children 12-23 mos. old who completed Micronutrient Powder Supplementation
Philippines, 2021

Area	Eligible Pop (6-11 mos.)	Infants 6-11 mos. old who completed MNP supplementation				Eligible Pop (12-23 mos.)	Children 12-23 mos. old who completed MNP supplementation			
		Male	Female	Total	%		Male	Female	Total	%
**CARAGA**	**30,191**	**1,824**	**1,763**	**3,587**	**11.88**	**61,876**	**707**	**685**	**1,392**	**2.25**
Agusan del Norte	4,069	203	63	266	6.54	8,207	10	7	17	0.21
Agusan del Sur	8,826	1,149	1,266	2,415	27.36	18,477	257	264	521	2.82
Surigao del Norte	3,603	0	0	0	0.00	7,589	0	0	0	0.00
Surigao del Sur	5,763	56	45	101	1.75	11,424	133	117	250	2.19
Province of Dinagat	1,292	42	10	52	4.02	2,662	59	47	106	3.98
Bislig City	1,094	105	127	232	21.21	2,168	67	72	139	6.41
Butuan City	3,862	65	73	138	3.57	7,811	21	20	41	0.52
Surigao City	1,682	204	179	383	22.77	3,538	160	158	318	8.99

Table 1.C.2.4 - Nutrition Services for Infants and Children

Number and Proportion of children 0-59 mos. old whose nutritional status are normal, stunted and overweight/obese

Philippines, 2021

Area	Eligible Pop (0-59 mos)	Normal				Stunted				Overweight/obese			
		Male	Female	Total	%	Male	Female	Total	%	Male	Female	Total	%
**PHILIPPINES**	**11,090,559**	**2,475,179**	**2,338,028**	**4,813,207**	**43.40**	**208,887**	**175,791**	**384,678**	**3.47**	**79,075**	**68,823**	**147,898**	**1.33**
**N C R**	**1,193,686**	**187,703**	**177,299**	**365,002**	**30.58**	**5,769**	**4,356**	**10,125**	**0.85**	**1,970**	**1,709**	**3,679**	**0.31**
Malabon	33,881	9,578	9,380	18,958	55.95	740	558	1,298	3.83	100	85	185	0.55
Navotas	23,124	*	*	0	0.00	*	*	0	0.00	*	*	0	0.00
Valenzuela City	57,511	*	*	0	0.00	*	*	0	0.00	*	*	0	0.00
Caloocan City	146,825	46,975	44,749	91,724	62.47	1,208	794	2,002	1.36	635	648	1,283	0.87
Marikina City	41,782	*	*	0	0.00	*	*	0	0.00	*	*	0	0.00
Pasig City	70,013	*	*	0	0.00	*	*	0	0.00	*	*	0	0.00
Pateros	5,919	*	*	0	0.00	*	*	0	0.00	*	*	0	0.00
Taguig	74,613	*	*	0	0.00	*	*	0	0.00	*	*	0	0.00
Quezon City	272,173	*	*	0	0.00	*	*	0	0.00	*	*	0	0.00
Makati City	54,005	12,333	10,919	23,252	43.06	165	140	305	0.56	275	172	447	0.83
Mandaluyong City	35,804	14,657	13,900	28,557	79.76	82	63	145	0.40	16	11	27	0.08
San Juan	11,333	4,207	3,884	8,091	71.39	64	52	116	1.02	73	55	128	1.13
Manila City	165,015	67,847	64,372	132,219	80.13	2,167	1,722	3,889	2.36	400	351	751	0.46
Las Piñas City	54,587	*	*	0	0.00	*	*	0	0.00	*	*	0	0.00
Muntinlupa City	46,765	*	*	0	0.00	*	*	0	0.00	*	*	0	0.00
Parañaque City	61,719	19,273	17,978	37,251	60.36	768	598	1,366	2.21	261	188	449	0.73
Pasay City	38,617	12,833	12,117	24,950	64.61	575	429	1,004	2.60	210	199	409	1.06
**C A R**	**182,278**	**63,684**	**58,222**	**121,906**	**66.88**	**6,125**	**4,313**	**10,438**	**5.73**	**1,496**	**1,039**	**2,535**	**1.39**
Abra	23,348	2,210	1,954	4,164	17.83	420	341	761	3.26	115	82	197	0.84
Apayao	13,463	892	874	1,766	13.12	71	64	135	1.00	60	49	109	0.81
Benguet	45,396	18,149	16,596	34,745	76.54	880	829	1,709	3.76	493	328	821	1.81
Ifugao	23,716	8,879	8,558	17,437	73.52	1,488	378	1,866	7.87	122	87	209	0.88
Kalinga	24,564	11,273	10,582	21,855	88.97	1,154	897	2,051	8.35	432	268	700	2.85
Mt. Province	16,656	9,266	7,388	16,654	99.99	1,911	1,615	3,526	21.17	186	171	357	2.14
Baguio City	35,135	13,015	12,270	25,285	71.97	201	189	390	1.11	88	54	142	0.40
**Region 1**	**504,596**	**258,170**	**240,711**	**498,881**	**98.87**	**9,424**	**7,834**	**17,258**	**3.42**	**6,552**	**5,088**	**11,640**	**2.31**
Ilocos Norte	40,486	31,491	30,485	61,976	153.08	1,721	1,472	3,193	7.89	1,353	1,162	2,515	6.21
Ilocos Sur	49,428	18,815	17,483	36,298	73.44	1,417	1,122	2,539	5.14	981	812	1,793	3.63
La Union	59,720	56,114	52,528	108,642	181.92	2,485	2,230	4,715	7.90	1,507	1,100	2,607	4.37
Pangasinan	260,849	113,137	104,137	217,274	83.29	2,718	2,053	4,771	1.83	1,638	1,296	2,934	1.12
Alaminos City	9,857	4,074	3,770	7,844	79.58	61	63	124	1.26	81	61	142	1.44
Candon City	5,208	2,264	2,031	4,295	82.47	102	90	192	3.69	83	66	149	2.86
Dagupan City	18,811	2,291	2,236	4,527	24.07	160	143	303	1.61	78	56	134	0.71
Laoag City	9,337	5,129	5,027	10,156	108.77	57	54	111	1.19	94	2	96	1.03
San Carlos City	20,720	7,046	6,723	13,769	66.45	325	258	583	2.81	325	253	578	2.79
San Fernando City	10,944	9,488	8,742	18,230	166.58	276	244	520	4.75	326	218	544	4.97
Urdaneta City	14,606	6,288	5,732	12,020	82.29	82	87	169	1.16	81	61	142	0.97
Vigan City	4,630	2,033	1,817	3,850	83.15	20	18	38	0.82	5	1	6	0.13
**Region 2**	**359,821**	**108,201**	**103,295**	**211,496**	**58.78**	**9,022**	**8,007**	**17,029**	**4.73**	**4,195**	**3,477**	**7,672**	**2.13**
Batanes	1,924	516	526	1,042	54.16	31	21	52	2.70	57	40	97	5.04
Cagayan	106,898	33,847	33,691	67,538	63.18	2,596	2,214	4,810	4.50	1,044	904	1,948	1.82
Isabela	121,957	41,578	38,782	80,360	65.89	4,901	3,963	8,864	7.27	2,561	2,036	4,597	3.77
Nueva Vizcaya	49,835	1,202	1,180	2,382	4.78	762	1,126	1,888	3.79	191	234	425	0.85
Quirino	21,281	9,082	8,474	17,556	82.50	436	406	842	3.96	163	126	289	1.36
Cauayan City	13,344	5,445	5,206	10,651	79.82	69	77	146	1.09	44	42	86	0.64
Ilagan City	15,003	6,299	5,840	12,139	80.91	202	182	384	2.56	92	64	156	1.04
Santiago City	13,893	7,189	6,603	13,792	99.27	25	18	43	0.31	17	6	23	0.17
Tuguegarao City	15,686	3,043	2,993	6,036	38.48	0	0	0	0.00	26	25	51	0.33
**Region 3**	**1,133,743**	**280,850**	**269,405**	**550,255**	**48.53**	**22,272**	**20,072**	**42,344**	**3.73**	**14,748**	**13,545**	**28,293**	**2.50**
Aurora	25,223	6,892	6,006	12,898	51.14	747	704	1,451	5.75	239	181	420	1.67
Bataan	68,608	31222	30096	61,318	89.37	938	882	1,820	2.65	895	701	1,596	2.33
Bulacan	225,061	7,978	5,794	13,772	6.12	96	55	151	0.07	212	149	361	0.16
Nueva Ecija	150,975	9,960	9,153	19,113	12.66	1344	1344	2,688	1.78	1427	1356	2,783	1.84

Table 1.C.2.4 - Nutrition Services for Infants and Children

Number and Proportion of children 0-59 mos. old whose nutritional status are normal, stunted and overweight/obese

Philippines, 2021

Area	Eligible Pop (0-59 mos)	Normal				Stunted				Overweight/obese			
		Male	Female	Total	%	Male	Female	Total	%	Male	Female	Total	%
Pampanga	162,929	72667	68652	141,319	86.74	7484	7490	14,974	9.19	5313	5330	10,643	6.53
Tarlac	100,395	6,424	6,483	12,907	12.86	122	103	225	0.22	233	152	385	0.38
Zambales	63,515	4,323	4,051	8,374	13.18	649	596	1,245	1.96	271	234	505	0.80
Angeles City	40,879	16597	16553	33,150	81.09	0	0	0	0.00	505	440	945	2.31
Balanga City	9,918	4,289	4,072	8,361	84.30	163	172	335	3.38	202	161	363	3.66
Cabanatuan City	30,901	14,776	17,523	32,299	104.52	153	158	311	1.01	146	145	291	0.94
City of San Fernando	30,451	0	0	0	0.00	0	0	0	0.00	0	0	0	0.00
Gapan City	11,278	5277	4950	10,227	90.68	245	198	443	3.93	208	137	345	3.06
Mabalacat City	24,906	0	0	0	0.00	0	0	0	0.00	0	0	0	0.00
Malolos City	25,137	38	55	93	0.37	0	1	1	0.00	0	1	1	0.00
Meycauayan	20,848	3,004	2,926	5,930	28.44	19	14	33	0.16	24	17	41	0.20
Olongapo	25,051	2,243	2,174	4,417	17.63	38	26	64	0.26	50	44	94	0.38
Palayan City	4,199	1616	1440	3,056	72.78	229	174	403	9.60	118	68	186	4.43
San Jose City	14,290	6,298	6,440	12,738	89.14	247	159	406	2.84	34	21	55	0.38
San Jose del Monte City	57,244	48705	46712	95,417	166.68	7127	5846	12,973	22.66	4380	3895	8,275	14.46
Science City of Munoz	8,334	3458	3174	6,632	79.58	366	338	704	8.45	54	65	119	1.43
Tarlac City	33,601	35083	33151	68,234	203.07	2305	1812	4,117	12.25	437	448	885	2.63
**Region 4A**	**1,532,116**	**163,319**	**153,274**	**316,593**	**20.66**	**11,475**	**9,040**	**20,515**	**1.34**	**4,868**	**3,934**	**8,802**	**0.57**
Batangas	202,551	38,659	37,348	76,007	37.52	1,898	1,413	3,311	1.63	1,582	1,131	2,713	1.34
Cavite	142,880	4,005	2,853	6,858	4.80	1,207	805	2,012	1.41	575	350	925	0.65
Laguna	100,287	27,938	25,105	53,043	52.89	1,409	1,156	2,565	2.56	699	713	1,412	1.41
Quezon	197,226	28,627	26,961	55,588	28.18	3,782	3,184	6,966	3.53	1,381	1,207	2,588	1.31
Rizal	226,799	4,074	3,976	8,050	3.55	25	22	47	0.02	129	122	251	0.11
Antipolo City	83,535	*	1	1	0.00	*	*	0	0.00	*	*	0	0.00
Bacoor City	62,603	*	*	0	0.00	*	*	0	0.00	*	*	0	0.00
Batangas City	35,956	*	*	0	0.00	*	*	0	0.00	*	*	0	0.00
Biñan City	33,629	12,609	13,164	25,773	76.64	4	*	4	0.01	25	23	48	0.14
Cabuyao City	31,176	*	*	0	0.00	*	*	0	0.00	*	*	0	0.00
Calamba City	45,889	28,197	25,883	54,080	117.85	1,604	1,245	2,849	6.21	164	138	302	0.66
Cavite City	10,713	*	*	0	0.00	*	*	0	0.00	*	*	0	0.00
Dasmariñas City	68,688	258	214	472	0.69	*	9	9	0.01	3	*	3	0.00
General Trias City	32,758	*	*	0	0.00	*	*	0	0.00	*	*	0	0.00
Imus City	42,085	*	*	0	0.00	*	*	0	0.00	*	*	0	0.00
Lipa City	36,217	*	*	0	0.00	*	*	0	0.00	*	*	0	0.00
Lucena City	29,892	3,832	3,490	7,322	24.49	424	319	743	2.49	245	206	451	1.51
San Pablo City	26,871	*	*	0	0.00	*	*	0	0.00	*	*	0	0.00
San Pedro City	32,895	*	*	0	0.00	*	*	0	0.00	*	*	0	0.00
Santa Rosa City	35,720	8,753	8,297	17,050	47.73	247	166	413	1.16	2	2	4	0.01
Tagaytay City	7,416	*	*	0	0.00	*	*	0	0.00	*	*	0	0.00
Tanauan City	18,893	6,367	5,982	12,349	65.36	*	*	0	0.00	63	42	105	0.56
Tayabas City	11,206	*	*	0	0.00	875	721	1,596	14.24	*	*	0	0.00
Trece Martires City	16,231	*	*	0	0.00	*	*	0	0.00	*	*	0	0.00
**Region 4B**	**373,725**	**69,522**	**67,693**	**137,215**	**36.72**	**8,263**	**7,126**	**15,389**	**4.12**	**1,255**	**1,070**	**2,325**	**0.62**
Marinduque	26,946	5,270	4,719	9,989	37.07	603	540	1,143	4.24	111	109	220	0.82
Mindoro Occidental	62,954	25,861	24,449	50,310	79.92	4,636	3,795	8,431	13.39	523	455	978	1.55
Mindoro Oriental	103,990	*	*	0	0.00	*	*	0	0.00	*	*	0	0.00
Palawan	111,234	23,905	23,371	47,276	42.50	2,122	2,033	4,155	3.74	414	319	733	0.66
Romblon	35,194	13,561	14,231	27,792	78.97	831	704	1,535	4.36	174	176	350	0.99
Puerto Princesa City	33,407	925	923	1,848	5.53	71	54	125	0.37	33	11	44	0.13
**Region 5**	**728,621**	**233,091**	**197,127**	**430,218**	**59.05**	**30,476**	**25,095**	**55,571**	**7.63**	**7,882**	**6,480**	**14,362**	**1.97**
Albay	127,867	75,721	70,380	146,101	114.26	7,220	6,120	13,340	10.43	2,015	1,637	3,652	2.86
Camarines Norte	74,442	110	83	193	0.26	2,915	2,301	5,216	7.01	626	434	1,060	1.42
Camarines Sur	206,826	30,366	29,895	60,261	29.14	8,035	6,785	14,820	7.17	1,770	1,804	3,574	1.73
Catanduanes	33,331	4,650	4,622	9,272	27.82	1,051	922	1,973	5.92	137	88	225	0.68
Masbate	122,161	54,974	31,918	86,892	71.13	4,282	3,680	7,962	6.52	935	671	1,606	1.31
Sorsogon	102,811	36,284	33,933	70,217	68.30	5,698	4,451	10,149	9.87	1,955	1,500	3,455	3.36
Iriga City	14,056	6,975	4,991	11,966	85.13	705	336	1,041	7.41	46	42	88	0.63
Legaspi City	22,479	9,436	8,612	18,048	80.29	187	186	373	1.66	251	201	452	2.01
Naga City	24,648	14,575	12,693	27,268	110.63	383	314	697	2.83	147	103	250	1.01

Table 1.C.2.4 - Nutrition Services for Infants and Children

Number and Proportion of children 0-59 mos. old whose nutritional status are normal, stunted and overweight/obese

Philippines, 2021

Area	Eligible Pop (0-59 mos)	Normal				Stunted				Overweight/obese			
		Male	Female	Total	%	Male	Female	Total	%	Male	Female	Total	%
**Region 6**	**770,257**	**260,290**	**246,260**	**506,550**	**65.76**	**24,064**	**21,404**	**45,468**	**5.90**	**11,021**	**11,720**	**22,741**	**2.95**
Aklan	59,345	19,085	17,826	36,911	62.20	2,383	1,963	4,346	7.32	362	312	674	1.14
Antique	67,488	16,880	15,887	32,767	48.55	1,810	1,547	3,357	4.97	414	345	759	1.12
Capiz	74,045	30,544	28,722	59,266	80.04	2,076	1,562	3,638	4.91	1,222	1,071	2,293	3.10
Guimaras	17,593	7,670	7,320	14,990	85.20	457	414	871	4.95	267	223	490	2.79
Iloilo	189,498	65,516	61,378	126,894	66.96	10,097	9,391	19,488	10.28	5,016	4,365	9,381	4.95
Negros Occidental	259,955	75,061	74,629	149,690	57.58	5,150	4,522	9,672	3.72	1,389	1,118	2,507	0.96
Bacolod City	58,489	27,887	23,871	51,758	88.49	1,643	1,674	3,317	5.67	1,810	3,786	5,596	9.57
Iloilo City	43,844	17,647	16,627	34,274	78.17	448	331	779	1.78	541	500	1,041	2.37
**Region 7**	**813,613**	**45,217**	**50,897**	**96,114**	**11.81**	**4,618**	**4,069**	**8,687**	**1.07**	**1,021**	**969**	**1,990**	**0.24**
Bohol	144,101	0	0	0	0.00	0	0	0	0.00	0	0	0	0.00
Cebu	328,344	12,558	12,073	24,631	7.50	156	80	236	0.07	152	117	269	0.08
Negros Oriental	142,799	32,659	38,824	71,483	50.06	4,462	3,989	8,451	5.92	869	852	1,721	1.21
Siquijor	9,187	0	0	0	0.00	0	0	0	0.00	0	0	0	0.00
Cebu City	103,071	0	0	0	0.00	0	0	0	0.00	0	0	0	0.00
Lapu-Lapu City	45,594	0	0	0	0.00	0	0	0	0.00	0	0	0	0.00
Mandaue City	40,517	0	0	0	0.00	0	0	0	0.00	0	0	0	0.00
**Region 8**	**543,128**	**155,812**	**145,675**	**301,487**	**55.51**	**31,140**	**24,530**	**55,670**	**10.25**	**9,544**	**8,028**	**17,572**	**3.24**
Biliran	20,744	7,943	7,274	15,217	73.36	1,181	919	2,100	10.12	342	297	639	3.08
Eastern Samar	58,978	13,867	12,956	26,823	45.48	2,520	2,030	4,550	7.71	970	848	1,818	3.08
Northern Leyte	180,081	50,903	47,291	98,194	54.53	9,946	8,000	17,946	9.97	3,566	3,020	6,586	3.66
Northern Samar	84,271	25,274	23,558	48,832	57.95	5,508	4,333	9,841	11.68	1,358	1,141	2,499	2.97
Southern Leyte	35,819	11,069	10,318	21,387	59.71	2,150	1,246	3,396	9.48	897	613	1,510	4.22
Western Samar	76,123	34,043	32,431	66,474	87.32	7,793	6,431	14,224	18.69	1,688	1,528	3,216	4.22
Calbayog City	23,454	0	0	0	0.00	202	144	346	1.48	81	73	154	0.66
Maasin City	9,115	2,916	2,618	5,534	60.71	182	130	312	3.42	62	47	109	1.20
Ormoc City	25,653	9,797	9,229	19,026	74.17	1,649	1,292	2,941	11.46	580	460	1,040	4.05
Tacloban City	28,890	0	0	0	0.00	9	5	14	0.05	0	1	1	0.00
**Region 9**	**416,892**	**48,497**	**46,775**	**95,272**	**22.85**	**5,744**	**5,663**	**11,407**	**2.74**	**1,517**	**1,279**	**2,796**	**0.67**
Zamboanga del Norte	91,102	38,518	36,921	75,439	82.81	3,679	3,243	6,922	7.60	974	836	1,810	1.99
Zamboanga del Sur	89,100	3,064	3,007	6,071	6.81	572	501	1,073	1.20	126	98	224	0.25
Zamboanga Sibugay	81,223	4,223	4,102	8,325	10.25	1,438	1,868	3,306	4.07	315	257	572	0.70
Dapitan City	9,407	0	0	0	0.00	0	0	0	0.00	0	0	0	0.00
Dipolog City	14,921	0	0	0	0.00	0	0	0	0.00	0	0	0	0.00
Isabela City	14,692	167	161	328	2.23	48	41	89	0.61	101	87	188	1.28
Pagadian City	21,848	0	0	0	0.00	0	0	0	0.00	0	0	0	0.00
Zamboanga City	94,599	2,525	2,584	5,109	5.40	7	10	17	0.02	1	1	2	0.00
**Region 10**	**532,602**	**191,512**	**187,006**	**378,518**	**71.07**	**11,739**	**9,579**	**21,318**	**4.00**	**2,987**	**2,712**	**5,699**	**1.07**
Bukidnon	122,218	56,850	53,318	110,168	90.14	5,684	4,494	10,178	8.33	1,121	908	2,029	1.66
Camiguin	9,860	3,209	3,447	6,656	67.51	27	18	45	0.46	34	37	71	0.72
Lanao del Norte	82,428	31,720	31,699	63,419	76.94	3,109	2,629	5,738	6.96	1,054	1,050	2,104	2.55
Misamis Occidental	33,861	11,909	11,199	23,108	68.24	868	752	1,620	4.78	145	175	320	0.95
Misamis Oriental	78,018	8	2	10	0.01	45	38	83	0.11	3	3	6	0.01
Cagayan de Oro City	73,888	34,126	36,887	71,013	96.11	261	231	492	0.67	10	9	19	0.03
El Salvador City	5,487	2,602	2,407	5,009	91.29	24	25	49	0.89	5	4	9	0.16
Gingoog City	13,629	0	0	0	0.00	0	0	0	0.00	0	0	0	0.00
Iligan City	41,744	18,101	16,808	34,909	83.63	767	604	1,371	3.28	329	334	663	1.59
Malaybalay City	20,378	8,672	8,069	16,741	82.15	637	531	1,168	5.73	206	142	348	1.71
Oroquieta City	7,336	2,086	2,029	4,115	56.09	150	94	244	3.33	54	37	91	1.24
Ozamis City	14,710	10,145	9,987	20,132	136.86	11	9	20	0.14	13	10	23	0.16
Tangub City	6,530	0	0	0	0.00	0	0	0	0.00	0	0	0	0.00
Valencia City	22,515	12,084	11,154	23,238	103.21	156	154	310	1.38	13	3	16	0.07

Table 1.C.2.4 - Nutrition Services for Infants and Children

Number and Proportion of children 0-59 mos. old whose nutritional status are normal, stunted and overweight/obese

Philippines, 2021

Area	Eligible Pop (0-59 mos)	Normal				Stunted				Overweight/obese			
		Male	Female	Total	%	Male	Female	Total	%	Male	Female	Total	%
**Region 11**	**558,480**	**240,745**	**227,112**	**467,857**	**83.77**	**17,498**	**14,345**	**31,843**	**5.70**	**5,177**	**3,915**	**9,092**	**1.63**
Davao de Oro	83,550	34,135	31,564	65,699	78.63	2,464	2,105	4,569	5.47	636	451	1,087	1.30
Davao del Norte	108,231	51,343	48,068	99,411	91.85	2,487	2,027	4,514	4.17	927	723	1,650	1.52
Davao del Sur	69,787	28,089	26,156	54,245	77.73	2,540	1,918	4,458	6.39	855	628	1,483	2.13
Davao Oriental	72,554	26,768	25,074	51,842	71.45	3,082	2,321	5,403	7.45	906	695	1,601	2.21
Davao Occidental	37,089	13,013	12,382	25,395	68.47	2,345	1,905	4,250	11.46	410	358	768	2.07
Davao City	187,269	87,397	83,868	171,265	91.45	4,580	4,069	8,649	4.62	1,443	1,060	2,503	1.34
**Region 12**	**542,638**	**66,725**	**62,978**	**129,703**	**23.90**	**3,527**	**3,546**	**7,073**	**1.30**	**1,888**	**1,444**	**3,332**	**0.61**
North Cotabato	174,075	0	0	0	0.00	0	0	0	0.00	0	0	0	0.00
Sarangani	67,551	29,987	28,759	58,746	86.97	2,073	2,098	4,171	6.17	414	293	707	1.05
South Cotabato	105,905	0	0	0	0.00	0	0	0	0.00	0	0	0	0.00
Sultan Kudarat	92,144	0	0	0	0.00	0	0	0	0.00	626	548	1,174	1.27
Cotabato City	34,179	6,762	6,443	13,205	38.63	351	279	630	1.84	159	142	301	0.88
Gen. Santos City	68,784	29,976	27,776	57,752	83.96	1,103	1,169	2,272	3.30	689	461	1,150	1.67
**BARMM**	**584,203**	**12,280**	**12,836**	**25,116**	**4.30**	**1,179**	**1,095**	**2,274**	**0.39**	**387**	**361**	**748**	**0.13**
Basilan	43,840	0	0	0	0.00	0	0	0	0.00	0	0	0	0.00
Lanao del Sur	132,904	0	0	0	0.00	0	0	0	0.00	0	0	0	0.00
Maguindanao	183,130	0	0	0	0.00	0	0	0	0.00	0	0	0	0.00
Sulu	125,905	0	0	0	0.00	0	0	0	0.00	0	0	0	0.00
Tawi-Tawi	54,559	12,136	12,696	24,832	45.51	1,172	1,091	2,263	4.15	385	359	744	1.36
Lamitan City	12,064	144	140	284	2.35	7	4	11	0.09	2	2	4	0.03
Marawi City	31,801	0	0	0	0.00	0	0	0	0.00	0	0	0	0.00
**CARAGA**	**320,160**	**89,561**	**91,463**	**181,024**	**56.54**	**6,552**	**5,717**	**12,269**	**3.83**	**2,567**	**2,053**	**4,620**	**1.44**
Agusan del Norte	42,294	20,997	20,854	41,851	98.95	999	980	1,979	4.68	544	442	986	2.33
Agusan del Sur	94,737	3,359	3,890	7,249	7.65	548	562	1,110	1.17	76	72	148	0.16
Surigao del Norte	39,189	20,045	21,459	41,504	105.91	2,007	1,824	3,831	9.78	916	754	1,670	4.26
Surigao del Sur	60,208	21,625	20,698	42,323	70.29	2,005	1,616	3,621	6.01	475	415	890	1.48
Province of Dinagat	13,825	3,405	5,230	8,635	62.46	290	206	496	3.59	295	174	469	3.39
Bislig City	11,435	291	323	614	5.37	28	42	70	0.61	14	10	24	0.21
Butuan City	40,214	13,473	13,337	26,810	66.67	258	134	392	0.97	24	20	44	0.11
Surigao City	18,258	6,366	5,672	12,038	65.93	417	353	770	4.22	223	166	389	2.13

Note: Put asterisk (*) for No Report and Zero (0) for No Case

Table 1.C.2.5 - Nutrition Services for Infants and Children

Number and Proportion of children 0-59 mos. old whose nutritional status are Wasted-MAM, Wasted-SAM and Wasted
Philippines, 2021

Area	Eligible Pop (0-59 mos)	Wasted-MAM				Wasted-SAM				Wasted			
		Male	Female	Total	%	Male	Female	Total	%	Male	Female	Total	%
**PHILIPPINES**	**11,090,559**	**38,492**	**31,800**	**70,292**	**0.63**	**12,971**	**10,735**	**23,706**	**0.21**	**51,463**	**42,535**	**93,998**	**0.85**
**N C R**	**1,193,686**	**1,474**	**1,145**	**2,619**	**0.22**	**533**	**422**	**955**	**0.08**	**2,007**	**1,567**	**3,574**	**0.30**
Malabon	33,881	195	165	360	1.06	68	35	103	0.30	263	200	463	1.37
Navotas	23,124	*	*	0	0.00	*	*	0	0.00	0	0	0	0.00
Valenzuela City	57,511	*	*	0	0.00	*	*	0	0.00	0	0	0	0.00
Caloocan City	146,825	265	194	459	0.31	99	92	191	0.13	364	286	650	0.44
Marikina City	41,782	*	*	0	0.00	*	*	0	0.00	0	0	0	0.00
Pasig City	70,013	*	*	0	0.00	*	*	0	0.00	0	0	0	0.00
Pateros	5,919	*	*	0	0.00	*	*	0	0.00	0	0	0	0.00
Taguig	74,613	*	*	0	0.00	*	*	0	0.00	0	0	0	0.00
Quezon City	272,173	*	*	0	0.00	*	*	0	0.00	0	0	0	0.00
										0	0		
Makati City	54,005	26	26	52	0.10	7	4	11	0.02	33	30	63	0.12
Mandaluyong City	35,804	45	31	76	0.21	15	16	31	0.09	60	47	107	0.30
San Juan	11,333	17	21	38	0.34	0	0	0	0.00	17	21	38	0.34
Manila City	165,015	701	521	1,222	0.74	307	249	556	0.34	1,008	770	1,778	1.08
Las Piñas City	54,587	*	*	0	0.00	*	*	0	0.00	0	0	0	0.00
Muntinlupa City	46,765	*	*	0	0.00	*	*	0	0.00	0	0	0	0.00
Parañaque City	61,719	140	113	253	0.41	17	14	31	0.05	157	127	284	0.46
Pasay City	38,617	85	74	159	0.41	20	12	32	0.08	105	86	191	0.49
**C A R**	**182,278**	**578**	**483**	**1,061**	**0.58**	**183**	**150**	**333**	**0.18**	**761**	**633**	**1,394**	**0.76**
Abra	23,348	91	62	153	0.66	32	23	55	0.24	123	85	208	0.89
Apayao	13,463	66	50	116	0.86	27	29	56	0.42	93	79	172	1.28
Benguet	45,396	92	57	149	0.33	28	25	53	0.12	120	82	202	0.44
Ifugao	23,716	44	36	80	0.34	18	12	30	0.13	62	48	110	0.46
Kalinga	24,564	214	196	410	1.67	64	45	109	0.44	278	241	519	2.11
Mt. Province	16,656	50	65	115	0.69	5	9	14	0.08	55	74	129	0.77
Baguio City	35,135	21	17	38	0.11	9	7	16	0.05	30	24	54	0.15
**Region 1**	**504,596**	**2,528**	**2,088**	**4,616**	**0.91**	**672**	**512**	**1,184**	**0.23**	**3,200**	**2,600**	**5,800**	**1.15**
Ilocos Norte	40,486	114	106	220	0.54	10	11	21	0.05	124	117	241	0.60
Ilocos Sur	49,428	264	211	475	0.96	0	0	0	0.00	264	211	475	0.96
La Union	59,720	460	367	827	1.38	20	9	29	0.05	480	376	856	1.43
Pangasinan	260,849	1,332	1,050	2,382	0.91	491	377	868	0.33	1,823	1,427	3,250	1.25
Alaminos City	9,857	16	12	28	0.28	7	9	16	0.16	23	21	44	0.45
Candon City	5,208	14	14	28	0.54	0	0	0	0.00	14	14	28	0.54
Dagupan City	18,811	50	54	104	0.55	2	4	6	0.03	52	58	110	0.58
Laoag City	9,337	0	0	0	0.00	8	9	17	0.18	8	9	17	0.18
San Carlos City	20,720	152	152	304	1.47	103	66	169	0.82	255	218	473	2.28
San Fernando City	10,944	94	94	188	1.72	14	14	28	0.26	108	108	216	1.97
Urdaneta City	14,606	29	27	56	0.38	17	13	30	0.21	46	40	86	0.59
Vigan City	4,630	3	1	4	0.09	0	0	0	0.00	3	1	4	0.09
**Region 2**	**359,821**	**2,888**	**2,516**	**5,404**	**1.50**	**697**	**664**	**1,361**	**0.38**	**3,585**	**3,180**	**6,765**	**1.88**
Batanes	1,924	12	9	21	1.09	2	2	4	0.21	14	11	25	1.30
Cagayan	106,898	1,607	1,437	3,044	2.85	152	170	322	0.30	1,759	1,607	3,366	3.15
Isabela	121,957	1,152	946	2,098	1.72	492	452	944	0.77	1,644	1,398	3,042	2.49
Nueva Vizcaya	49,835	8	9	17	0.03	0	0	0	0.00	8	9	17	0.03
Quirino	21,281	28	35	63	0.30	14	11	25	0.12	42	46	88	0.41
Cauayan City	13,344	48	56	104	0.78	28	22	50	0.37	76	78	154	1.15
Ilagan City	15,003	33	24	57	0.38	9	7	16	0.11	42	31	73	0.49
Santiago City	13,893	0	0	0	0.00	0	0	0	0.00	0	0	0	0.00
Tuguegarao City	15,686	0	0	0	0.00	0	0	0	0.00	0	0	0	0.00
**Region 3**	**1,133,743**	**5,817**	**4,661**	**10,478**	**0.92**	**3,307**	**2,683**	**5,990**	**0.53**	**9,124**	**7,344**	**16,468**	**1.45**
Aurora	25,223	113	100	213	0.84	17	18	35	0.14	130	118	248	0.98
Bataan	68,608	520	361	881	1.28	163	157	320	0.47	683	518	1,201	1.75
Bulacan	225,061	0	0	0	0.00	0	0	0	0.00	0	0	0	0.00
Nueva Ecija	150,975	0	0	0	0.00	0	0	0	0.00	0	0	0	0.00
Pampanga	162,929	2086	1732	3,818	2.34	1373	1118	2,491	1.53	3,459	2,850	6,309	3.87

Table 1.C.2.5 - Nutrition Services for Infants and Children

Number and Proportion of children 0-59 mos. old whose nutritional status are Wasted-MAM, Wasted-SAM and Wasted
Philippines, 2021

Area	Eligible Pop (0-59 mos)	Wasted-MAM				Wasted-SAM				Wasted			
		Male	Female	Total	%	Male	Female	Total	%	Male	Female	Total	%
Tarlac	100,395	17	30	47	0.05	41	14	55	0.05	58	44	102	0.10
Zambales	63,515	216	189	405	0.64	46	40	86	0.14	262	229	491	0.77
Angeles City	40,879	0	0	0	0.00	0	0	0	0.00	0	0	0	0.00
Balanga City	9,918	72	61	133	1.34	50	60	110	1.11	122	121	243	2.45
Cabanatuan City	30,901	77	81	158	0.51	0	0	0	0.00	77	81	158	0.51
City of San Fernando	30,451	0	0	0	0.00	0	0	0	0.00	0	0	0	0.00
Gapan City	11,278	90	71	161	1.43	38	59	97	0.86	128	130	258	2.29
Mabalacat City	24,906	0	0	0	0.00	0	0	0	0.00	0	0	0	0.00
Malolos City	25,137	0	0	0	0.00	0	0	0	0.00	0	0	0	0.00
Meycauayan	20,848	16	13	29	0.14	0	0	0	0.00	16	13	29	0.14
Olongapo	25,051	0	0	0	0.00	0	0	0	0.00	0	0	0	0.00
Palayan City	4,199	0	0	0	0.00	0	0	0	0.00	0	0	0	0.00
San Jose City	14,290	155	150	305	2.13	0	0	0	0.00	155	150	305	2.13
San Jose del Monte City	57,244	1785	1377	3,162	5.52	1230	909	2,139	3.74	3,015	2,286	5,301	9.26
Science City of Munoz	8,334	114	93	207	2.48	27	54	81	0.97	141	147	288	3.46
Tarlac City	33,601	556	403	959	2.85	322	254	576	1.71	878	657	1,535	4.57
**Region 4A**	**1,532,116**	**1,343**	**1,190**	**2,533**	**0.17**	**163**	**149**	**312**	**0.02**	**1,506**	**1,339**	**2,845**	**0.19**
Batangas	202,551	206	288	494	0.24	14	4	18	0.01	220	292	512	0.25
Cavite	142,880	525	370	895	0.63	92	80	172	0.12	617	450	1,067	0.75
Laguna	100,287	106	96	202	0.20	4	5	9	0.01	110	101	211	0.21
Quezon	197,226	259	221	480	0.24	25	28	53	0.03	284	249	533	0.27
Rizal	226,799	26	32	58	0.03	22	28	50	0.02	48	60	108	0.05
Antipolo City	83,535	*	*	0	0.00	*	*	0	0.00	0	0	0	0.00
Bacoor City	62,603	*	*	0	0.00	*	*	0	0.00	0	0	0	0.00
Batangas City	35,956	*	*	0	0.00	*	*	0	0.00	0	0	0	0.00
Biñan City	33,629	*	*	0	0.00	*	*	0	0.00	0	0	0	0.00
Cabuyao City	31,176	*	*	0	0.00	*	*	0	0.00	0	0	0	0.00
Calamba City	45,889	49	32	81	0.18	1	4	5	0.01	50	36	86	0.19
Cavite City	10,713	*	*	0	0.00	*	*	0	0.00	0	0	0	0.00
Dasmariñas City	68,688	6	*	6	0.01	*	*	0	0.00	6	0	6	0.01
General Trias City	32,758	*	*	0	0.00	*	*	0	0.00	0	0	0	0.00
Imus City	42,085	*	*	0	0.00	*	*	0	0.00	0	0	0	0.00
Lipa City	36,217	*	*	0	0.00	*	*	0	0.00	0	0	0	0.00
Lucena City	29,892	20	*	20	0.07	5	*	5	0.02	25	0	25	0.08
San Pablo City	26,871	*	*	0	0.00	*	*	0	0.00	0	0	0	0.00
San Pedro City	32,895	*	*	0	0.00	*	*	0	0.00	0	0	0	0.00
Santa Rosa City	35,720	51	61	112	0.31	*	*	0	0.00	51	61	112	0.31
Tagaytay City	7,416	*	*	0	0.00	*	*	0	0.00	0	0	0	0.00
Tanauan City	18,893	*	*	0	0.00	*	*	0	0.00	0	0	0	0.00
Tayabas City	11,206	95	90	185	1.65	*	*	0	0.00	95	90	185	1.65
Trece Martires City	16,231	*	*	0	0.00	*	*	0	0.00	0	0	0	0.00
**Region 4B**	**373,725**	**1,723**	**1,409**	**3,132**	**0.84**	**298**	**219**	**517**	**0.14**	**2,021**	**1,628**	**3,649**	**0.98**
Marinduque	26,946	240	175	415	1.54	43	26	69	0.26	283	201	484	1.80
Mindoro Occidental	62,954	737	555	1,292	2.05	225	156	381	0.61	962	711	1,673	2.66
Mindoro Oriental	103,990	*	*	0	0.00	*	*	0	0.00	0	0	0	0.00
Palawan	111,234	601	568	1,169	1.05	7	3	10	0.01	608	571	1,179	1.06
Romblon	35,194	134	99	233	0.66	22	28	50	0.14	156	127	283	0.80
Puerto Princesa City	33,407	11	12	23	0.07	1	6	7	0.02	12	18	30	0.09
**Region 5**	**728,621**	**4,594**	**3,928**	**8,522**	**1.17**	**901**	**725**	**1,626**	**0.22**	**5,495**	**4,653**	**10,148**	**1.39**
Albay	127,867	746	679	1,425	1.11	124	161	285	0.22	870	840	1,710	1.34
Camarines Norte	74,442	722	749	1,471	1.98	0	0	0	0.00	722	749	1,471	1.98
Camarines Sur	206,826	812	695	1,507	0.73	211	119	330	0.16	1,023	814	1,837	0.89
Catanduanes	33,331	242	182	424	1.27	56	33	89	0.27	298	215	513	1.54
Masbate	122,161	873	750	1,623	1.33	255	204	459	0.38	1,128	954	2,082	1.70
Sorsogon	102,811	701	610	1,311	1.28	200	126	326	0.32	901	736	1,637	1.59
Iriga City	14,056	307	72	379	2.70	2	2	4	0.03	309	74	383	2.72
Legaspi City	22,479	96	80	176	0.78	45	28	73	0.32	141	108	249	1.11
Naga City	24,648	95	111	206	0.84	8	52	60	0.24	103	163	266	1.08
**Region 6**	**770,257**	**3,691**	**3,321**	**7,012**	**0.91**	**1,478**	**1,123**	**2,601**	**0.34**	**5,169**	**4,444**	**9,613**	**1.25**
Aklan	59,345	682	646	1,328	2.24	200	128	328	0.55	882	774	1,656	2.79
Antique	67,488	97	94	191	0.28	46	34	80	0.12	143	128	271	0.40
Capiz	74,045	572	445	1,017	1.37	212	151	363	0.49	784	596	1,380	1.86
Guimaras	17,593	82	84	166	0.94	25	12	37	0.21	107	96	203	1.15
Iloilo	189,498	0	0	0	0.00	0	0	0	0.00	0	0	0	0.00
Negros Occidental	259,955	1,460	1,258	2,718	1.05	769	604	1,373	0.53	2,229	1,862	4,091	1.57
Bacolod City	58,489	625	641	1,266	2.16	148	135	283	0.48	773	776	1,549	2.65
Iloilo City	43,844	173	153	326	0.74	78	59	137	0.31	251	212	463	1.06

Table 1.C.2.5 - Nutrition Services for Infants and Children

Number and Proportion of children 0-59 mos. old whose nutritional status are Wasted-MAM, Wasted-SAM and Wasted
Philippines, 2021

Area	Eligible Pop (0-59 mos)	Wasted-MAM				Wasted-SAM				Wasted			
		Male	Female	Total	%	Male	Female	Total	%	Male	Female	Total	%
**Region 7**	**813,613**	**24**	**11**	**35**	**0.00**	**2**	**0**	**2**	**0.00**	**26**	**11**	**37**	**0.00**
Bohol	144,101	0	0	0	0.00	0	0	0	0.00	0	0	0	0.00
Cebu	328,344	24	11	35	0.01	2	0	2	0.00	26	11	37	0.01
Negros Oriental	142,799	0	0	0	0.00	0	0	0	0.00	0	0	0	0.00
Siquijor	9,187	0	0	0	0.00	0	0	0	0.00	0	0	0	0.00
Cebu City	103,071	0	0	0	0.00	0	0	0	0.00	0	0	0	0.00
Lapu-Lapu City	45,594	0	0	0	0.00	0	0	0	0.00	0	0	0	0.00
Mandaue City	40,517	0	0	0	0.00	0	0	0	0.00	0	0	0	0.00
**Region 8**	**543,128**	**4,720**	**3,536**	**8,256**	**1.52**	**1,932**	**1,523**	**3,455**	**0.64**	**6,652**	**5,059**	**11,711**	**2.16**
Biliran	20,744	106	90	196	0.94	75	51	126	0.61	181	141	322	1.55
Eastern Samar	58,978	0	0	0	0.00	160	127	287	0.49	160	127	287	0.49
Northern Leyte	180,081	1,814	1,427	3,241	1.80	927	694	1,621	0.90	2,741	2,121	4,862	2.70
Northern Samar	84,271	845	596	1,441	1.71	299	258	557	0.66	1,144	854	1,998	2.37
Southern Leyte	35,819	309	245	554	1.55	133	97	230	0.64	442	342	784	2.19
Western Samar	76,123	1,093	789	1,882	2.47	193	171	364	0.48	1,286	960	2,246	2.95
Calbayog City	23,454	104	54	158	0.67	0	0	0	0.00	104	54	158	0.67
Maasin City	9,115	80	50	130	1.43	0	0	0	0.00	80	50	130	1.43
Ormoc City	25,653	369	285	654	2.55	145	125	270	1.05	514	410	924	3.60
Tacloban City	28,890	0	0	0	0.00	0	0	0	0.00	0	0	0	0.00
**Region 9**	**416,892**	**1,500**	**1,253**	**2,753**	**0.66**	**499**	**461**	**960**	**0.23**	**1,999**	**1,714**	**3,713**	**0.89**
Zamboanga del Norte	91,102	818	770	1,588	1.74	313	293	606	0.67	1,131	1,063	2,194	2.41
Zamboanga del Sur	89,100	671	476	1,147	1.29	181	164	345	0.39	852	640	1,492	1.67
Zamboanga Sibugay	81,223	2	2	4	0.00	0	0	0	0.00	2	2	4	0.00
Dapitan City	9,407	9	5	14	0.15	5	4	9	0.10	14	9	23	0.24
Dipolog City	14,921			0	0.00			0	0.00	0	0	0	0.00
Isabela City	14,692	0	0	0	0.00	0	0	0	0.00	0	0	0	0.00
Pagadian City	21,848	0	0	0	0.00	0	0	0	0.00	0	0	0	0.00
Zamboanga City	94,599	0	0	0	0.00	0	0	0	0.00	0	0	0	0.00
**Region 10**	**532,602**	**2,608**	**2,139**	**4,747**	**0.89**	**844**	**708**	**1,552**	**0.29**	**3,452**	**2,847**	**6,299**	**1.18**
Bukidnon	122,218	1,049	865	1,914	1.57	302	245	547	0.45	1,351	1,110	2,461	2.01
Camiguin	9,860	13	6	19	0.19	4	4	8	0.08	17	10	27	0.27
Lanao del Norte	82,428	706	557	1,263	1.53	307	227	534	0.65	1,013	784	1,797	2.18
Misamis Occidental	33,861	246	183	429	1.27	72	76	148	0.44	318	259	577	1.70
Misamis Oriental	78,018	0	0	0	0.00	0	0	0	0.00	0	0	0	0.00
Cagayan de Oro City	73,888	65	47	112	0.15	48	29	77	0.10	113	76	189	0.26
El Salvador City	5,487	3	6	9	0.16	1	1	2	0.04	4	7	11	0.20
Gingoog City	13,629	0	0	0	0.00	0	0	0	0.00	0	0	0	0.00
Iligan City	41,744	262	270	532	1.27	40	53	93	0.22	302	323	625	1.50
Malaybalay City	20,378	211	149	360	1.77	60	65	125	0.61	271	214	485	2.38
Oroquieta City	7,336	8	2	10	0.14	0	0	0	0.00	8	2	10	0.14
Ozamis City	14,710	18	22	40	0.27	0	0	0	0.00	18	22	40	0.27
Tangub City	6,530	0	0	0	0.00	0	0	0	0.00	0	0	0	0.00
Valencia City	22,515	27	32	59	0.26	10	8	18	0.08	37	40	77	0.34
**Region 11**	**558,480**	**2,702**	**2,205**	**4,907**	**0.88**	**829**	**890**	**1,719**	**0.31**	**3,531**	**3,095**	**6,626**	**1.19**
Davao de Oro	83,550	305	261	566	0.68	89	68	157	0.19	394	329	723	0.87
Davao del Norte	108,231	454	323	777	0.72	61	60	121	0.11	515	383	898	0.83
Davao del Sur	69,787	579	488	1,067	1.53	201	160	361	0.52	780	648	1,428	2.05
Davao Oriental	72,554	473	343	816	1.12	203	144	347	0.48	676	487	1,163	1.60
Davao Occidental	37,089	500	427	927	2.50	198	379	577	1.56	698	806	1,504	4.06
Davao City	187,269	391	363	754	0.40	77	79	156	0.08	468	442	910	0.49
**Region 12**	**542,638**	**568**	**572**	**1,140**	**0.21**	**90**	**87**	**177**	**0.03**	**658**	**659**	**1,317**	**0.24**
North Cotabato	174,075	0	0	0	0.00	0	0	0	0.00	0	0	0	0.00
Sarangani	67,551	338	359	697	1.03	49	52	101	0.15	387	411	798	1.18
South Cotabato	105,905	0	0	0	0.00	0	0	0	0.00	0	0	0	0.00
Sultan Kudarat	92,144	169	169	338	0.37	26	24	50	0.05	195	193	388	0.42
Cotabato City	34,179	50	28	78	0.23	15	10	25	0.07	65	38	103	0.30
Gen. Santos City	68,784	11	16	27	0.04	0	1	1	0.00	11	17	28	0.04

Table 1.C.2.5 - Nutrition Services for Infants and Children

Number and Proportion of children 0-59 mos. old whose nutritional status are Wasted-MAM, Wasted-SAM and Wasted

Philippines, 2021

Area	Eligible Pop (0-59 mos)	Wasted-MAM				Wasted-SAM				Wasted			
		Male	Female	Total	%	Male	Female	Total	%	Male	Female	Total	%
**BARMM**	**584,203**	**255**	**227**	**482**	**0.08**	**129**	**117**	**246**	**0.04**	**384**	**344**	**728**	**0.12**
Basilan	43,840	0	0	0	0.00	0	0	0	0.00	0	0	0	0.00
Lanao del Sur	132,904	0	0	0	0.00	0	0	0	0.00	0	0	0	0.00
Maguindanao	183,130	0	0	0	0.00	0	0	0	0.00	0	0	0	0.00
Sulu	125,905	0	0	0	0.00	0	0	0	0.00	0	0	0	0.00
Tawi-Tawi	54,559	244	223	467	0.86	121	113	234	0.43	365	336	701	1.28
Lamitan City	12,064	11	4	15	0.12	8	4	12	0.10	19	8	27	0.22
Marawi City	31,801	0	0	0	0.00	0	0	0	0.00	0	0	0	0.00
**CARAGA**	**320,160**	**1,479**	**1,116**	**2,595**	**0.81**	**414**	**302**	**716**	**0.22**	**1,893**	**1,418**	**3,311**	**1.03**
Agusan del Norte	42,294	226	215	441	1.04	64	53	117	0.28	290	268	558	1.32
Agusan del Sur	94,737	42	34	76	0.08	12	15	27	0.03	54	49	103	0.11
Surigao del Norte	39,189	470	421	891	2.27	117	93	210	0.54	587	514	1,101	2.81
Surigao del Sur	60,208	35	23	58	0.10	10	13	23	0.04	45	36	81	0.13
Province of Dinagat	13,825	470	256	726	5.25	149	88	237	1.71	619	344	963	6.97
Bislig City	11,435	2	2	4	0.03	2	3	5	0.04	4	5	9	0.08
Butuan City	40,214	37	23	60	0.15	3	5	8	0.02	40	28	68	0.17
Surigao City	18,258	197	142	339	1.86	57	32	89	0.49	254	174	428	2.34

Note: Put asterisk (*) for No Report and Zero (0) for No Case

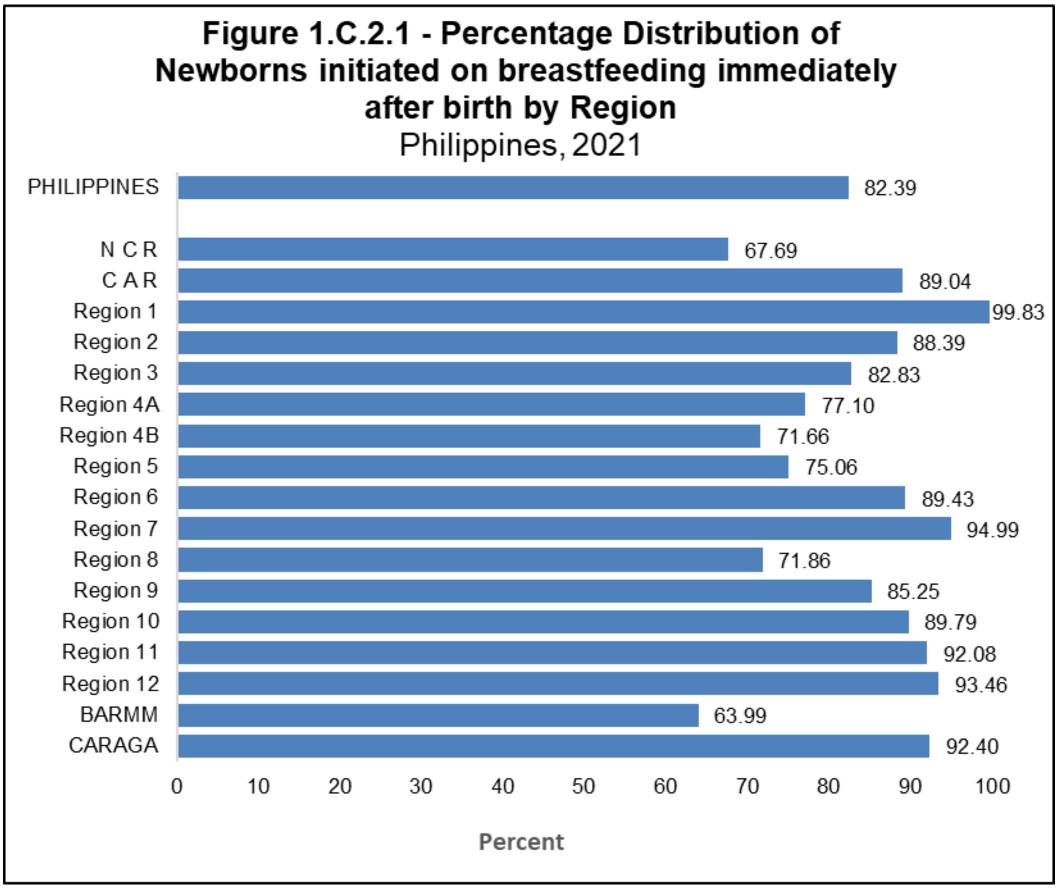

Figure 1.C.2.1 - Percentage Distribution of Newborns initiated on breastfeeding immediately after birth by Region
Philippines, 2021
PHILIPPINES 82.39
N C R 67.69
C A R 89.04
Region 1 99.83
Region 2 88.39
Region 3 82.83
Region 4A 77.10
Region 4B 71.66
Region 5 75.06
Region 6 89.43
Region 7 94.99
Region 8 71.86
Region 9 85.25
Region 10 89.79
Region 11 92.08
Region 12 93.46
BARMM 63.99
CARAGA 92.40
0 10 20 30 40 50 60 70 80 90 100
Percent

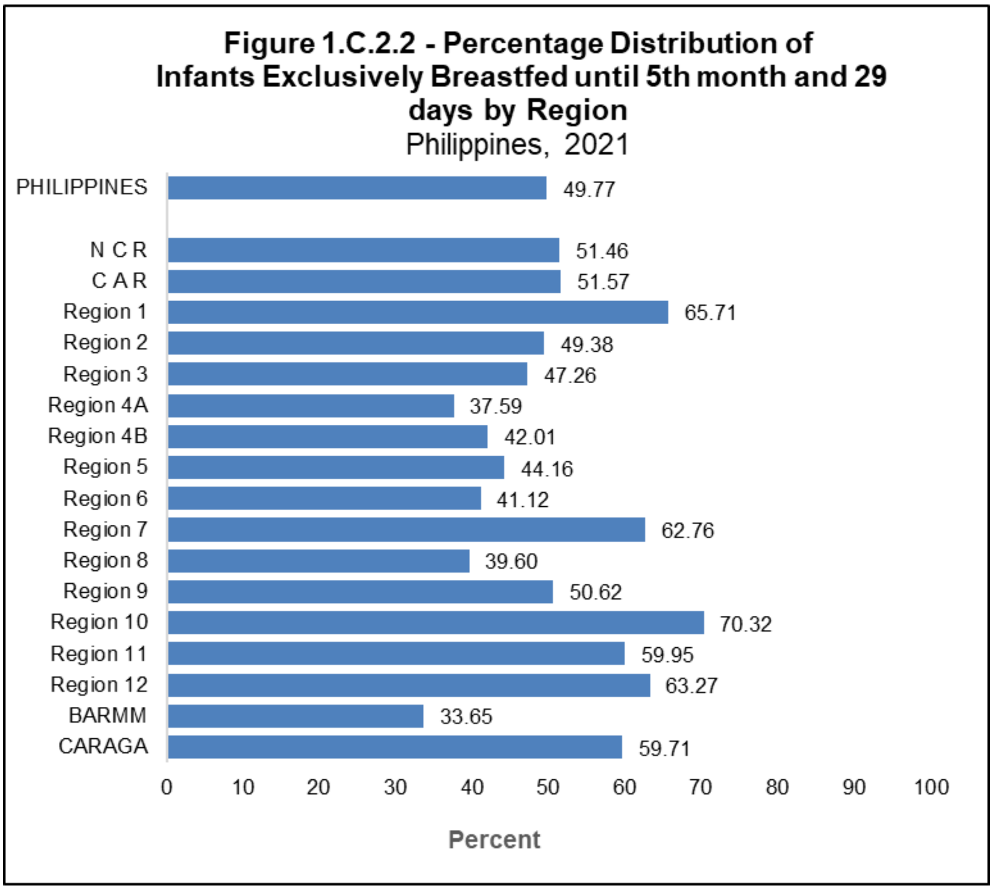

Figure 1.C.2.2 - Percentage Distribution of Infants Exclusively Breastfed until 5th month and 29 days by Region
Philippines, 2021
PHILIPPINES 49.77
N C R 51.46
C A R 51.57
Region 1 65.71
Region 2 49.38
Region 3 47.26
Region 4A 37.59
Region 4B 42.01
Region 5 44.16
Region 6 41.12
Region 7 62.76
Region 8 39.60
Region 9 50.62
Region 10 70.32
Region 11 59.95
Region 12 63.27
BARMM 33.65
CARAGA 59.71
0 10 20 30 40 50 60 70 80 90 100
Percent

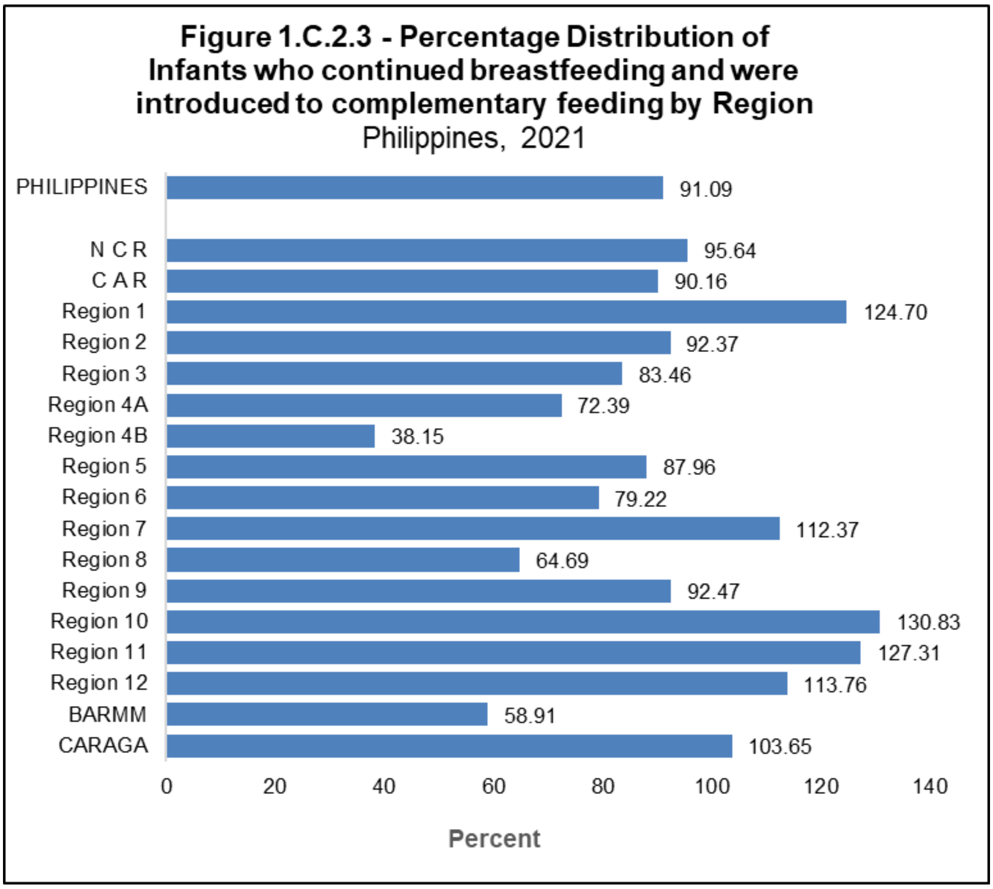
Figure 1.C.2.3 - Percentage Distribution of Infants who continued breastfeeding and were introduced to complementary feeding by Region
Philippines, 2021
PHILIPPINES
91.09
N C R
95.64
C A R
90.16
Region 1
124.70
Region 2
92.37
Region 3
83.46
Region 4A
72.39
Region 4B
38.15
Region 5
87.96
Region 6
79.22
Region 7
112.37
Region 8
64.69
Region 9
92.47
Region 10
130.83
Region 11
127.31
Region 12
113.76
BARMM
58.91
CARAGA
103.65
0
20
40
60
80
100
120
140
Percent

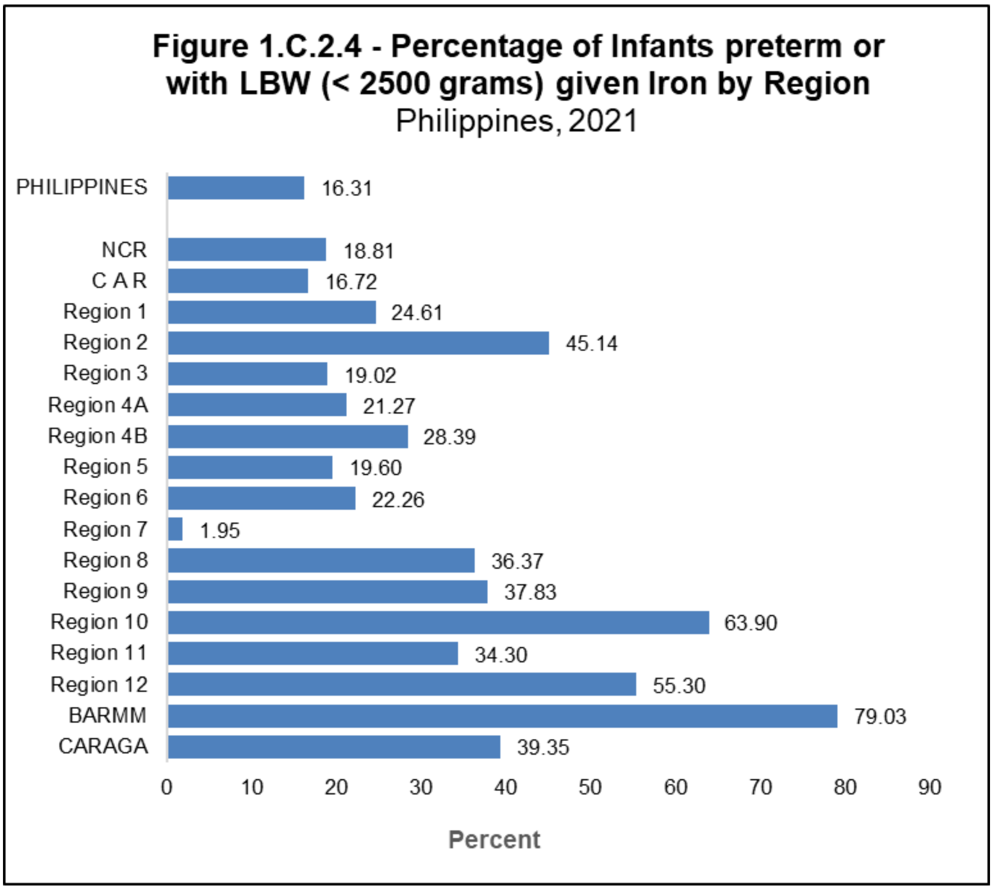
Figure 1.C.2.4 - Percentage of Infants preterm or with LBW (< 2500 grams) given Iron by Region
Philippines, 2021
PHILIPPINES
16.31
NCR
18.81
C A R
16.72
Region 1
24.61
Region 2
45.14
Region 3
19.02
Region 4A
21.27
Region 4B
28.39
Region 5
19.60
Region 6
22.26
Region 7
1.95
Region 8
36.37
Region 9
37.83
Region 10
63.90
Region 11
34.30
Region 12
55.30
BARMM
79.03
CARAGA
39.35
0
10
20
30
40
50
60
70
80
90
Percent

Figure 1.C.2.5 - Percentage Distribution of Infants 6-11 mos. old Given Vitamin A by Region
Philippines, 2021

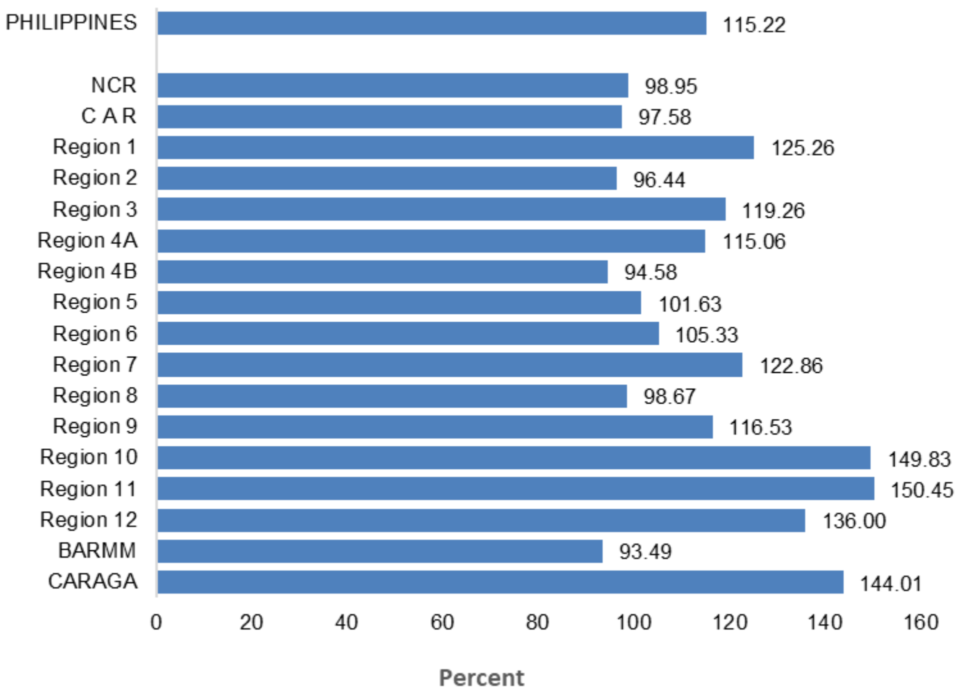

Figure 1.C.2.6 - Percentage Distribution of Children 12-59 mos. old Given Vitamin A by Region
Philippines, 2021

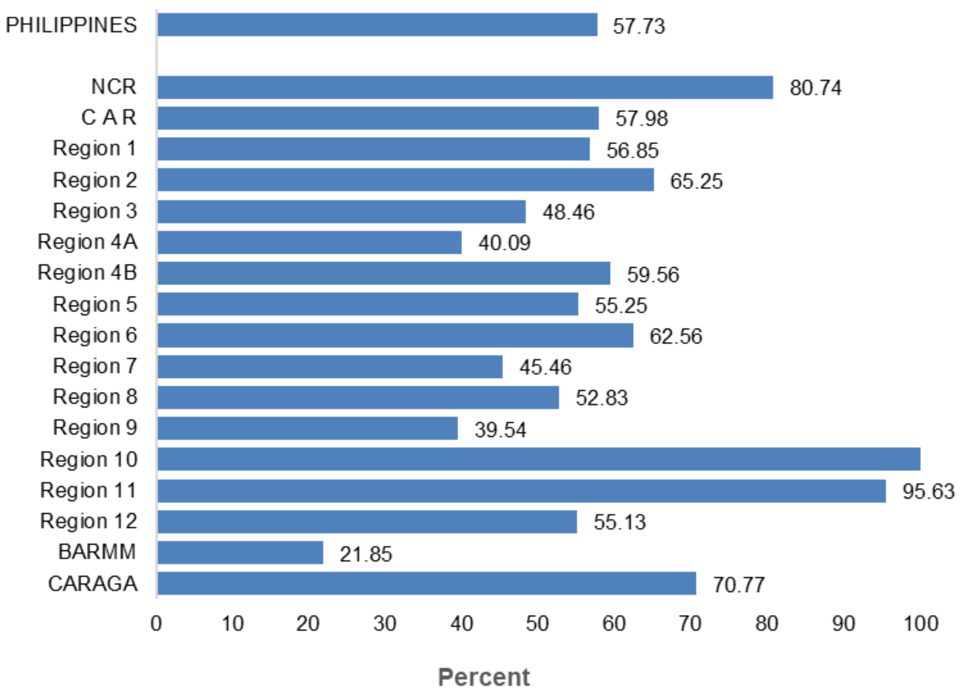

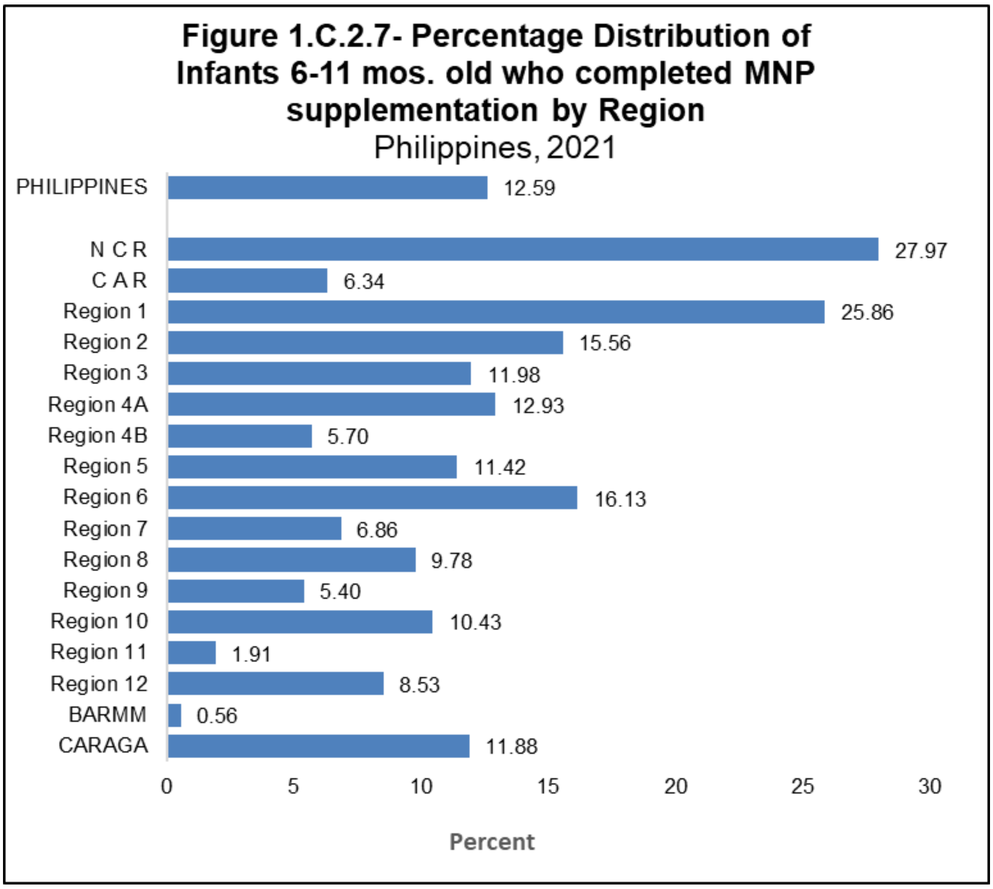
Figure 1.C.2.7- Percentage Distribution of Infants 6-11 mos. old who completed MNP supplementation by Region
Philippines, 2021
PHILIPPINES
12.59
N C R
27.97
C A R
6.34
Region 1
25.86
Region 2
15.56
Region 3
11.98
Region 4A
12.93
Region 4B
5.70
Region 5
11.42
Region 6
16.13
Region 7
6.86
Region 8
9.78
Region 9
5.40
Region 10
10.43
Region 11
1.91
Region 12
8.53
BARMM
0.56
CARAGA
11.88
0
5
10
15
20
25
30
Percent

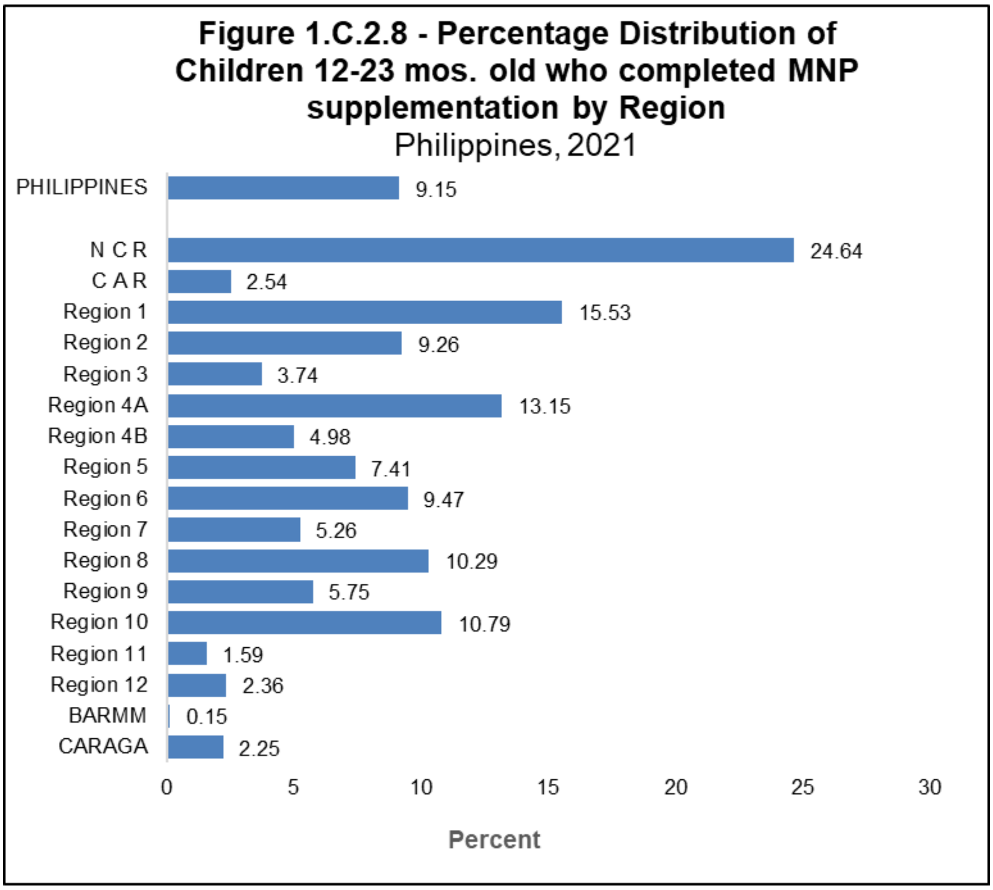
Figure 1.C.2.8 - Percentage Distribution of Children 12-23 mos. old who completed MNP supplementation by Region
Philippines, 2021
PHILIPPINES
9.15
N C R
24.64
C A R
2.54
Region 1
15.53
Region 2
9.26
Region 3
3.74
Region 4A
13.15
Region 4B
4.98
Region 5
7.41
Region 6
9.47
Region 7
5.26
Region 8
10.29
Region 9
5.75
Region 10
10.79
Region 11
1.59
Region 12
2.36
BARMM
0.15
CARAGA
2.25
0
5
10
15
20
25
30
Percent

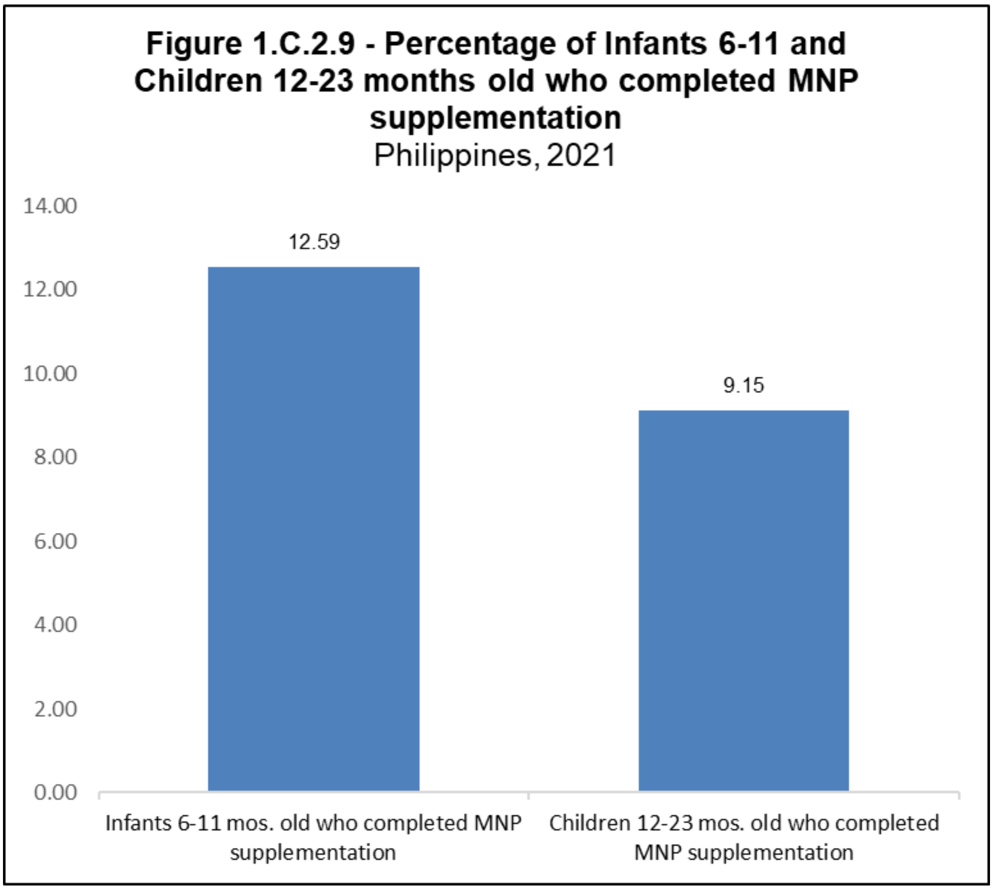
Figure 1.C.2.9 - Percentage of Infants 6-11 and Children 12-23 months old who completed MNP supplementation
Philippines, 2021
14.00
12.00
10.00
8.00
6.00
4.00
2.00
0.00
12.59
9.15
Infants 6-11 mos. old who completed MNP supplementation
Children 12-23 mos. old who completed MNP supplementation

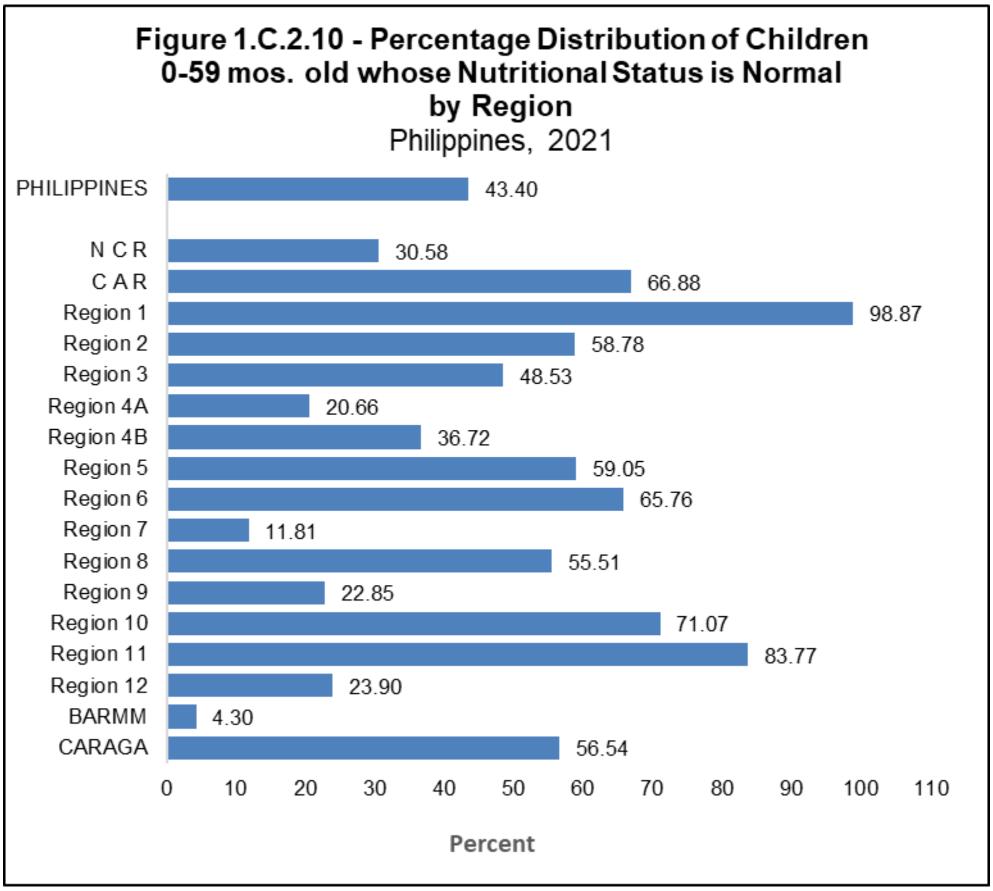
Figure 1.C.2.10 - Percentage Distribution of Children 0-59 mos. old whose Nutritional Status is Normal by Region
Philippines, 2021
PHILIPPINES 43.40
N C R 30.58
C A R 66.88
Region 1 98.87
Region 2 58.78
Region 3 48.53
Region 4A 20.66
Region 4B 36.72
Region 5 59.05
Region 6 65.76
Region 7 11.81
Region 8 55.51
Region 9 22.85
Region 10 71.07
Region 11 83.77
Region 12 23.90
BARMM 4.30
CARAGA 56.54
0 10 20 30 40 50 60 70 80 90 100 110
Percent

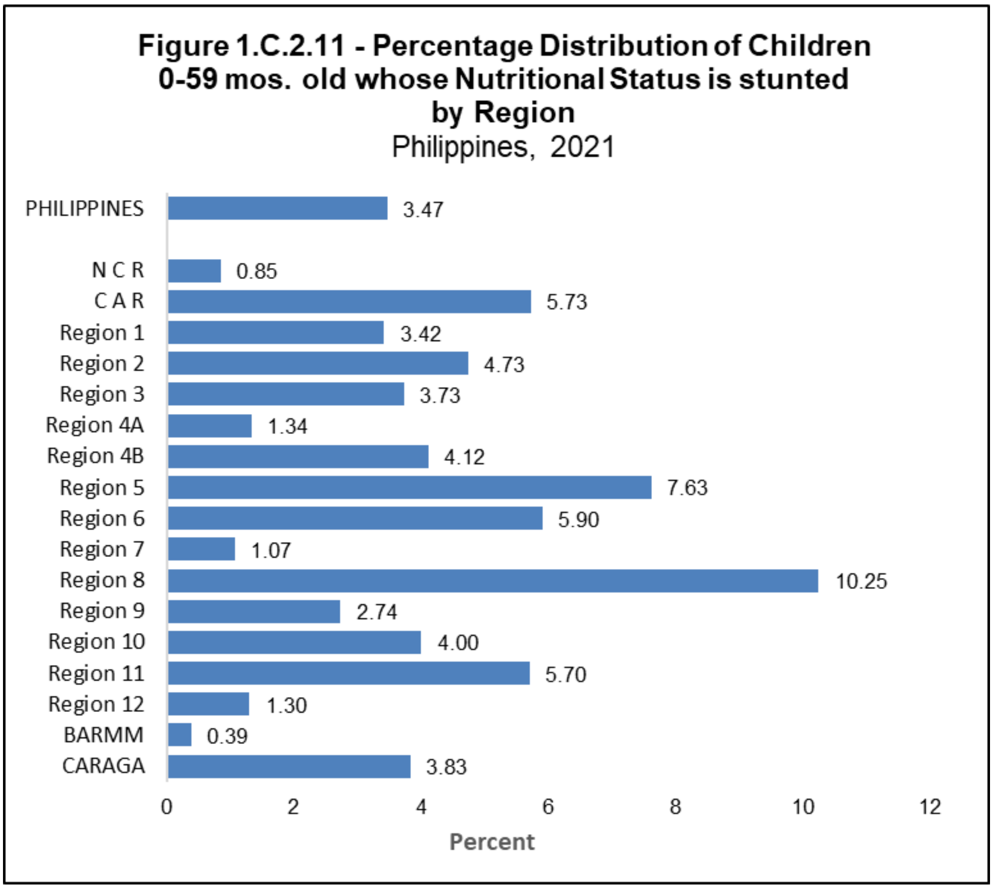
Figure 1.C.2.11 - Percentage Distribution of Children
0-59 mos. old whose Nutritional Status is stunted
by Region
Philippines, 2021
PHILIPPINES
3.47
N C R
0.85
C A R
5.73
Region 1
3.42
Region 2
4.73
Region 3
3.73
Region 4A
1.34
Region 4B
4.12
Region 5
7.63
Region 6
5.90
Region 7
1.07
Region 8
10.25
Region 9
2.74
Region 10
4.00
Region 11
5.70
Region 12
1.30
BARMM
0.39
CARAGA
3.83
0
2
4
6
8
10
12
Percent

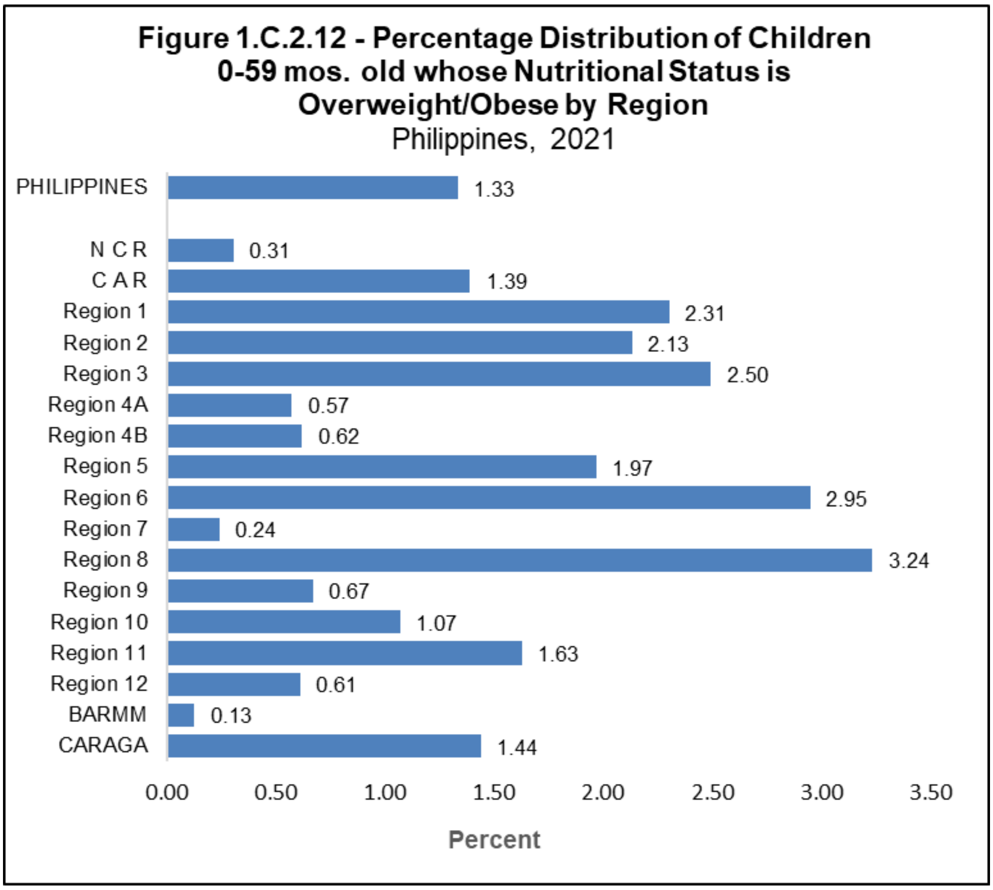
Figure 1.C.2.12 - Percentage Distribution of Children
0-59 mos. old whose Nutritional Status is
Overweight/Obese by Region
Philippines, 2021
PHILIPPINES
1.33
N C R
0.31
C A R
1.39
Region 1
2.31
Region 2
2.13
Region 3
2.50
Region 4A
0.57
Region 4B
0.62
Region 5
1.97
Region 6
2.95
Region 7
0.24
Region 8
3.24
Region 9
0.67
Region 10
1.07
Region 11
1.63
Region 12
0.61
BARMM
0.13
CARAGA
1.44
0.00
0.50
1.00
1.50
2.00
2.50
3.00
3.50
Percent

Figure 1.C.2.13 - Percentage Distribution of Children 0-59 mos. old whose Nutritional Status is Wasted by Region
Philippines, 2021

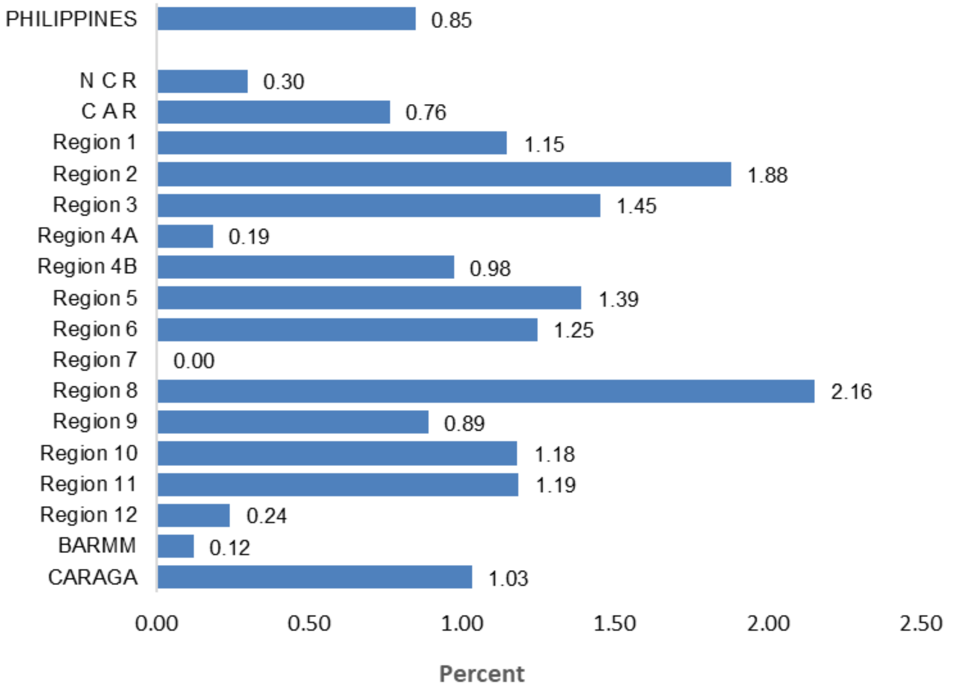

Figure 1.C.2.14 - Percentage of Children 0-59 mos. old according to their Nutritional Status
Philippines, 2021

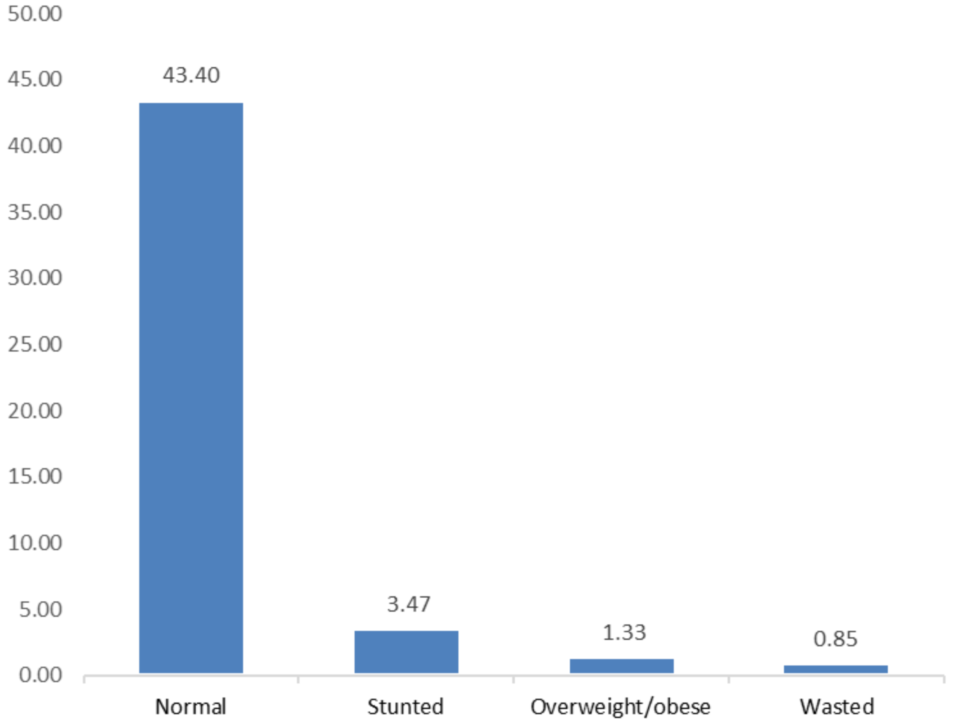

Table 1.C.3.1 - Deworming Services for Children and Adolescents

Number and Proportion of Children/Adolescents who completed 2 doses of deworming tablet
Philippines, 2021

Area	Eligible Pop (1-4 years old)	No. of children 1-4 y/o given 2 doses of deworming drugs				Eligible Pop (5-9 years old)	No. of Children 5-9 y/o given 2 doses of deworming drugs				Eligible Pop (10-19 years old)	No. of adolescents 10-19 y/o given 2 doses of deworming drugs			
		Male	Female	Total	%		Male	Female	Total	%		Male	Female	Total	%
**PHILIPPINES**	**8,962,535**	**2,308,035**	**2,262,753**	**4,570,788**	**51.00**	**11,114,941**	**1,971,685**	**1,935,944**	**3,907,629**	**35.16**	**21,185,431**	**2,861,120**	**2,811,597**	**5,672,717**	**26.78**
**N C R**	**955,786**	**267,903**	**257,000**	**524,903**	**54.92**	**1,227,409**	**153,314**	**142,312**	**295,626**	**24.09**	**2,439,351**	**139,228**	**129,054**	**268,282**	**11.00**
Malabon	27,129	2,726	3,994	6,720	24.77	34,839	689	1,749	2,438	7.00	69,242	392	830	1,222	1.76
Navotas	18,518	1,512	1,447	2,959	15.98	23,779	793	833	1,626	6.84	47,258	608	637	1,245	2.63
Valenzuela City	46,052	8,208	8,416	16,624	36.10	59,139	6,649	6,968	13,617	23.03	117,528	8,668	8,545	17,213	14.65
Caloocan City	117,560	53,993	51,562	105,555	89.79	150,966	33,001	32,367	65,368	43.30	300,055	27,807	23,550	51,357	17.12
Marikina City	33,452	2,591	2,639	5,230	15.63	42,965	1,133	974	2,107	4.90	85,386	662	692	1,354	1.59
Pasig City	56,059	1,268	1,265	2,533	4.52	71,991	587	528	1,115	1.55	143,078	267	278	545	0.38
Pateros	4,739	1,579	1,529	3,108	65.58	6,082	713	595	1,308	21.51	12,093	308	294	602	4.98
Taguig	59,742	31,958	27,786	59,744	100.00	76,721	28,153	27,889	56,042	73.05	152,478	38,291	37,534	75,825	49.73
Quezon City	217,929	61,252	63,859	125,111	57.41	279,868	33,019	22,313	55,332	19.77	556,187	22,652	15,146	37,798	6.80
Makati City	43,242	11,728	10,806	22,534	52.11	55,531	5,657	5,179	10,836	19.51	110,363	3,179	3,510	6,689	6.06
Mandaluyong City	28,674	2,529	2,555	5,084	17.73	36,818	18	19	37	0.10	73,172	13	16	29	0.04
San Juan	9,067	2,157	2,142	4,299	47.41	11,646	746	739	1,485	12.75	23,144	571	620	1,191	5.15
Manila City	132,127	27,104	31,268	58,372	44.18	169,678	15,469	17,471	32,940	19.41	337,210	11,276	12,127	23,403	6.94
Las Piñas City	43,706	4,638	4,724	9,362	21.42	56,130	714	768	1,482	2.64	111,554	422	505	927	0.83
Muntinlupa City	37,449	19,089	18,200	37,289	99.57	48,089	17,735	17,234	34,969	72.72	95,569	18,807	20,743	39,550	41.38
Parañaque City	49,421	14,653	14,808	29,461	59.61	63,463	2,695	2,759	5,454	8.59	126,128	1,795	1,669	3,464	2.75
Pasay City	30,920	20,918	10,000	30,918	99.99	39,704	5,543	3,927	9,470	23.85	78,906	3,510	2,358	5,868	7.44
**C A R**	**147,119**	**40,650**	**37,392**	**78,042**	**53.05**	**180,536**	**18,280**	**17,886**	**36,166**	**20.03**	**348,366**	**32,292**	**32,093**	**64,385**	**18.48**
Abra	19,047	5,781	5,448	11,229	58.95	23,644	1,176	981	2,157	9.12	46,837	1,773	1,626	3,399	7.26
Apayao	10,999	3,472	3,342	6,814	61.95	13,279	1,434	1,335	2,769	20.85	24,903	2,272	2,087	4,359	17.50
Benguet	36,250	9,054	8,256	17,310	47.75	44,397	1,419	1,910	3,329	7.50	88,250	4,125	3,703	7,828	8.87
Ifugao	19,270	7,709	7,204	14,913	77.39	23,425	6,766	6,558	13,324	56.88	43,086	12,437	11,764	24,201	56.17
Kalinga	19,904	4,795	4,917	9,712	48.79	24,419	4,690	4,730	9,420	38.58	45,205	8,643	9,814	18,457	40.83
Mt. Province	13,600	5,260	4,813	10,073	74.07	17,011	1,238	1,084	2,322	13.65	31,777	1,651	1,546	3,197	10.06
Baguio City	28,049	4,579	3,412	7,991	28.49	34,361	1,557	1,288	2,845	8.28	68,308	1,391	1,553	2,944	4.31
**Region 1**	**407,388**	**101,624**	**97,079**	**198,703**	**48.77**	**519,205**	**82,746**	**77,624**	**160,370**	**30.89**	**1,000,199**	**132,256**	**128,733**	**260,989**	**26.09**
Ilocos Norte	32,506	11,382	11,283	22,665	69.73	41,488	1,055	1,136	2,191	5.28	86,044	350	162	512	0.60
Ilocos Sur	40,200	14,703	13,030	27,733	68.99	52,091	16,862	15,693	32,555	62.50	105,704	27,821	26,711	54,532	51.59
La Union	48,309	29,311	27,104	56,415	116.78	61,959	38,994	35,143	74,137	119.65	126,165	68,452	66,121	134,573	106.66
Pangasinan	210,411	37,051	36,978	74,029	35.18	266,888	18,078	18,456	36,534	13.69	496,491	19,818	21,309	41,127	8.28
Alaminos City	7,947	2,496	2,420	4,916	61.86	10,081	0	0	0	0.00	18,758	0	0	0	0.00
Candon City	4,234	0	0	0	0.00	5,495	0	0	0	0.00	11,138	0	0	0	0.00
Dagupan City	15,175	1,937	1,882	3,819	25.17	19,252	2,045	2,008	4,053	21.05	35,815	3,629	3,528	7,157	19.98
Laoag City	7,498	0	0	0	0.00	9,568	0	0	0	0.00	19,837	0	0	0	0.00
San Carlos City	16,715	1,248	1,150	2,398	14.35	21,198	258	226	484	2.28	39,428	145	135	280	0.71
San Fernando City	8,849	3,489	3,227	6,716	75.90	11,355	5,454	4,962	10,416	91.73	23,116	12,039	10,764	22,803	98.65
Urdaneta City	11,778	7	5	12	0.10	14,947	0	0	0	0.00	27,801	2	3	5	0.02
Vigan City	3,766	0	0	0	0.00	4,883	0	0	0	0.00	9,902	0	0	0	0.00
**Region 2**	**290,609**	**84,143**	**85,019**	**169,162**	**58.21**	**365,588**	**38,787**	**38,963**	**77,750**	**21.27**	**686,897**	**55,511**	**54,087**	**109,598**	**15.96**
Batanes	1,575	733	673	1,406	89.27	1,917	308	113	421	21.96	3,397	202	380	582	17.13
Cagayan	86,326	30,339	29,347	59,686	69.14	108,797	12,979	12,069	25,048	23.02	206,766	20,149	19,006	39,155	18.94
Isabela	98,455	21,864	25,153	47,017	47.75	124,710	9,729	10,611	20,340	16.31	234,016	12,496	13,078	25,574	10.93
Nueva Vizcaya	40,263	7,356	7,069	14,425	35.83	49,489	1,611	1,973	3,584	7.24	93,116	5,253	4,895	10,148	10.90
Quirino	17,233	5,556	5,541	11,097	64.39	21,527	1,018	1,494	2,512	11.67	38,203	675	608	1,283	3.36
Cauayan City	10,769	735	670	1,405	13.05	13,647	401	331	732	5.36	25,611	183	144	327	1.28
Ilagan City	12,101	6,265	5,836	12,101	100.00	15,334	5,837	5,513	11,350	74.02	28,781	5,383	5,219	10,602	36.84
Santiago City	11,213	5,842	5,371	11,213	100.00	14,203	5,719	5,705	11,424	80.43	26,656	9,944	9,545	19,489	73.11
Tuguegarao City	12,674	5,453	5,359	10,812	85.31	15,964	1,185	1,154	2,339	14.65	30,351	1,226	1,212	2,438	8.03
**Region 3**	**913,592**	**218,816**	**225,789**	**444,605**	**48.67**	**1,160,338**	**191,738**	**188,764**	**380,502**	**32.79**	**2,319,836**	**265,215**	**263,798**	**529,013**	**22.80**
Aurora	20,457	9,539	8,945	18,484	90.36	24,680	9,600	9,127	18,727	75.88	45,730	15,191	14,722	29,913	65.41
Bataan	54,805	23,391	22,561	45,952	83.85	69,326	10,089	9,574	19,663	28.36	136,603	9,433	9,108	18,541	13.57
Bulacan	181,335	25,851	31,528	57,379	31.64	232,817	35,400	39,793	75,193	32.30	469,928	59,059	70,237	129,296	27.51
Nueva Ecija	122,063	13,749	13,076	26,825	21.98	155,359	9,816	9,142	18,958	12.20	304,433	10,306	10,081	20,387	6.70
Pampanga	131,439	14,518	14,518	29,036	22.09	167,494	9,162	9,198	18,360	10.96	343,154	13,530	13,819	27,349	7.97
Tarlac	80,858	14,904	20,604	35,508	43.91	102,418	14,027	14,165	28,192	27.53	205,964	17,862	18,369	36,231	17.59
Zambales	50,969	13,203	12,817	26,020	51.05	62,560	12,848	13,429	26,277	42.00	120,477	18,597	19,286	37,883	31.44
Angeles City	32,975	2,223	2,231	4,454	13.51	42,024	1,496	1,360	2,856	6.80	86,098	723	771	1,494	1.74
Balanga City	7,919	3,952	3,522	7,474	94.38	10,023	3,644	3,284	6,928	69.12	19,742	4,015	3,856	7,871	39.87
Cabanatuan City	24,979	16,621	15,392	32,013	128.16	31,794	12,047	11,640	23,687	74.50	62,305	12,126	11,686	23,812	38.22
City of San Fernando	24,564	5,119	5,143	10,262	41.78	31,308	3,549	3,251	6,800	21.72	64,139	3,602	3,050	6,652	10.37
Gapan City	9,116	4,815	4,848	9,663	106.00	11,603	2,255	0	2,255	19.43	22,741	0	0	0	0.00
Mabalacat City	20,093	13,667	14,233	27,900	138.85	25,604	16,680	15,819	32,499	126.93	52,457	23,485	23,262	46,747	89.11
Malolos City	20,252	9,187	8,635	17,822	88.00	26,003	10,504	9,694	20,198	77.68	52,491	16,753	16,282	33,035	62.93
Meycauayan	16,798	5,472	5,237	10,709	63.75	21,567	7,435	7,791	15,226	70.60	43,535	18,248	8,052	26,300	60.41
Olongapo	20,104	2,362	2,371	4,733	23.54	24,676	1,905	1,804	3,709	15.03	47,520	2,201	2,162	4,363	9.18

Table 1.C.3.1 - Deworming Services for Children and Adolescents

Number and Proportion of Children/Adolescents who completed 2 doses of deworming tablet
Philippines, 2021

Area	Eligible Pop (1-4 years old)	No. of children 1-4 y/o given 2 doses of deworming drugs				Eligible Pop (5-9 years old)	No. of Children 5-9 y/o given 2 doses of deworming drugs				Eligible Pop (10-19 years old)	No. of adolescents 10-19 y/o given 2 doses of deworming drugs			
		Male	Female	Total	%		Male	Female	Total	%		Male	Female	Total	%
Palayan City	3,393	2,737	2,614	5,351	157.71	4,317	1,757	1,651	3,408	78.94	8,461	1,917	1,926	3,843	45.42
San Jose City	11,553	4,209	4,026	8,235	71.28	14,699	5,413	5,002	10,415	70.86	28,808	8,460	8,078	16,538	57.41
San Jose del Monte City	46,129	19,378	20,402	39,780	86.24	59,224	14,384	13,671	28,055	47.37	119,541	16,986	16,629	33,615	28.12
Science City of Munoz	6,737	6,000	5,549	11,549	171.43	8,572	112	126	238	2.78	16,797	138	126	264	1.57
Tarlac City	27,054	7,919	7,537	15,456	57.13	34,270	9,615	9,243	18,858	55.03	68,912	12,583	12,296	24,879	36.10
**Region 4A**	**1,233,724**	**210,073**	**201,913**	**411,986**	**33.39**	**1,546,430**	**184,424**	**177,844**	**362,268**	**23.43**	**3,060,405**	**261,488**	**250,526**	**512,014**	**16.73**
Batangas	163,720	40,626	38,148	78,774	48.12	201,384	42,285	40,408	82,693	41.06	400,219	68,357	64,333	132,690	33.15
Cavite	114,923	23,443	20,188	43,631	37.97	145,884	23,262	20,041	43,303	29.68	295,548	31,201	28,088	59,289	20.06
Laguna	80,579	13,740	13,026	26,766	33.22	101,215	12,375	11,895	24,270	23.98	200,053	17,494	16,996	34,490	17.24
Quezon	159,600	33,313	30,966	64,279	40.28	200,093	27,860	26,896	54,756	27.37	380,891	45,274	44,032	89,306	23.45
Rizal	181,961	17,410	16,620	34,030	18.70	227,987	14,841	14,930	29,771	13.06	449,952	11,676	11,966	23,642	5.25
Antipolo City	67,021	1,429	3,109	4,538	6.77	83,973	169	192	361	0.43	165,728	0	0	0	0.00
Bacoor City	50,346	4,082	4,207	8,289	16.46	63,916	768	1,025	1,793	2.81	129,491	382	364	746	0.58
Batangas City	29,056	1,362	1,292	2,654	9.13	35,741	712	734	1,446	4.05	71,027	698	727	1,425	2.01
Biñan City	27,018	22,069	22,959	45,028	166.66	33,941	22,191	22,027	44,218	130.28	67,073	37,014	35,787	72,801	108.54
Cabuyao City	25,045	1,642	1,695	3,337	13.32	31,467	2,365	2,381	4,746	15.08	62,187	1,902	2,029	3,931	6.32
Calamba City	36,868	5,271	4,965	10,236	27.76	46,330	4,934	4,949	9,883	21.33	91,534	5,068	4,854	9,922	10.84
Cavite City	8,610	9	5	14	0.16	10,936	0	0	0	0.00	22,165	0	0	0	0.00
Dasmariñas City	55,249	9,649	9,984	19,633	35.54	70,129	4,678	5,017	9,695	13.82	142,083	4,128	3,724	7,852	5.53
General Trias City	26,348	1,472	1,559	3,031	11.50	33,449	806	749	1,555	4.65	67,767	827	786	1,613	2.38
Imus City	33,852	5,482	5,226	10,708	31.63	42,970	4,627	4,477	9,104	21.19	87,053	4,825	4,756	9,581	11.01
Lipa City	29,275	386	373	759	2.59	36,012	335	296	631	1.75	71,567	409	402	811	1.13
Lucena City	24,184	5,804	5,880	11,684	48.31	30,324	2,217	2,467	4,684	15.45	57,725	4,030	4,398	8,428	14.60
San Pablo City	21,585	438	429	867	4.02	27,112	436	437	873	3.22	53,595	462	461	923	1.72
San Pedro City	26,430	1,477	1,387	2,864	10.84	33,210	344	324	668	2.01	65,622	275	284	559	0.85
Santa Rosa City	28,699	6,631	5,909	12,540	43.69	36,058	2,319	2,051	4,370	12.12	71,250	1,197	1,211	2,408	3.38
Tagaytay City	5,966	5,422	5,460	10,882	182.40	7,579	6,366	6,535	12,901	170.22	15,345	11,969	12,087	24,056	156.77
Tanauan City	15,272	1,806	1,733	3,539	23.17	18,782	1,322	1,027	2,349	12.51	37,326	1,323	1,242	2,565	6.87
Tayabas City	9,064	4,216	4,101	8,317	91.76	11,366	5,924	5,710	11,634	102.36	21,633	9,161	8,618	17,779	82.18
Trece Martires City	13,053	2,894	2,692	5,586	42.79	16,572	3,288	3,276	6,564	39.61	33,571	3,816	3,381	7,197	21.44
**Region 4B**	**301,672**	**82,416**	**79,429**	**161,845**	**53.65**	**373,153**	**87,760**	**83,307**	**171,067**	**45.84**	**669,357**	**133,692**	**124,691**	**258,383**	**38.60**
Marinduque	22,017	6,916	6,261	13,177	59.85	27,446	7,844	7,397	15,241	55.53	49,106	12,059	11,919	23,978	48.83
Mindoro Occidental	50,503	26,482	25,898	52,380	103.72	62,379	30,338	28,195	58,533	93.83	110,453	43,979	41,544	85,523	77.43
Mindoro Oriental	83,982	10,925	10,511	21,436	25.52	104,890	11,308	10,303	21,611	20.60	187,150	20,671	19,521	40,192	21.48
Palawan	89,558	28,384	26,902	55,286	61.73	110,257	27,539	26,715	54,254	49.21	199,843	43,892	39,438	83,330	41.70
Romblon	28,715	4,815	4,766	9,581	33.37	35,073	4,225	4,407	8,632	24.61	62,787	5,721	5,303	11,024	17.56
Puerto Princesa City	26,897	4,894	5,091	9,985	37.12	33,108	6,506	6,290	12,796	38.65	60,018	7,370	6,966	14,336	23.89
**Region 5**	**591,236**	**172,986**	**166,897**	**339,883**	**57.49**	**708,265**	**221,796**	**212,468**	**434,264**	**61.31**	**1,301,201**	**370,687**	**359,126**	**729,813**	**56.09**
Albay	103,949	35,994	36,795	72,789	70.02	128,265	44,275	43,902	88,177	68.75	243,398	71,583	74,489	146,072	60.01
Camarines Norte	60,107	27,611	26,043	53,654	89.26	70,858	35,060	34,547	69,607	98.23	128,854	63,662	59,746	123,408	95.77
Camarines Sur	167,647	49,087	46,834	95,921	57.22	201,887	65,540	61,688	127,228	63.02	370,218	105,175	101,590	206,765	55.85
Catanduanes	26,943	5,552	5,022	10,574	39.25	32,009	8,419	7,791	16,210	50.64	57,554	15,626	14,356	29,982	52.09
Masbate	99,576	19,045	17,242	36,287	36.44	115,770	26,906	24,968	51,874	44.81	208,571	44,670	42,040	86,710	41.57
Sorsogon	83,359	20,476	19,858	40,334	48.39	99,151	26,128	24,317	50,445	50.88	180,540	46,437	43,321	89,758	49.72
Iriga City	11,391	134	146	280	2.46	13,716	119	141	260	1.90	25,153	124	151	275	1.09
Legaspi City	18,283	7,882	7,743	15,625	85.46	22,552	7,043	6,801	13,844	61.39	42,796	12,315	12,224	24,539	57.34
Naga City	19,981	7,205	7,214	14,419	72.16	24,057	8,306	8,313	16,619	69.08	44,117	11,095	11,209	22,304	50.56
**Region 6**	**623,781**	**219,110**	**211,558**	**430,668**	**69.04**	**778,724**	**180,430**	**174,429**	**354,859**	**45.57**	**1,501,799**	**283,173**	**275,966**	**559,139**	**37.23**
Aklan	48,127	17,047	15,206	32,253	67.02	60,517	18,708	17,437	36,145	59.73	114,161	32,661	30,719	63,380	55.52
Antique	54,526	19,461	18,283	37,744	69.22	68,116	23,758	23,103	46,861	68.80	122,311	42,137	41,510	83,647	68.39
Capiz	60,055	26,975	26,278	53,253	88.67	73,943	25,376	24,782	50,158	67.83	149,074	37,438	36,965	74,403	49.91
Guimaras	14,503	6,246	5,738	11,984	82.63	17,631	8,750	8,260	17,010	96.48	33,938	15,611	15,107	30,718	90.51
Iloilo	153,119	70,345	67,989	138,334	90.34	192,523	64,677	61,630	126,307	65.61	379,792	107,079	103,303	210,382	55.39
Negros Occidental	210,638	51,414	51,180	102,594	48.71	262,407	26,678	25,909	52,587	20.04	501,764	35,596	35,193	70,789	14.11
Bacolod City	47,393	15,555	15,392	30,947	65.30	59,048	12,483	13,308	25,791	43.68	112,898	12,651	13,169	25,820	22.87
Iloilo City	35,420	12,067	11,492	23,559	66.51	44,539	0	0	0	0.00	87,861	0	0	0	0.00
**Region 7**	**650,762**	**92,836**	**96,404**	**189,240**	**29.08**	**814,009**	**45,904**	**44,296**	**90,200**	**11.08**	**1,540,685**	**42,063**	**36,330**	**78,393**	**5.09**
Bohol	116,463	12,520	11,239	23,759	20.40	146,004	1,314	1,198	2,512	1.72	272,060	170	127	297	0.11
Cebu	261,301	36,311	36,919	73,230	28.03	327,647	27,172	24,956	52,128	15.91	620,513	31,707	28,664	60,371	9.73
Negros Oriental	114,898	25,091	29,734	54,825	47.72	141,917	11,043	11,876	22,919	16.15	272,255	6,912	4,138	11,050	4.06
Siquijor	7,543	0	0	0	0.00	9,649	0	0	0	0.00	18,340	0	0	0	0.00
Cebu City	82,030	12,781	12,389	25,170	30.68	102,864	3,656	3,551	7,207	7.01	194,786	1,912	1,926	3,838	1.97
Lapu-Lapu City	36,283	5,898	5,901	11,799	32.52	45,499	2,577	2,535	5,112	11.24	86,164	1,249	1,335	2,584	3.00
Mandaue City	32,244	235	222	457	1.42	40,429	142	180	322	0.80	76,567	113	140	253	0.33
**Region 8**	**439,152**	**74,221**	**73,235**	**147,456**	**33.58**	**528,354**	**27,765**	**28,395**	**56,160**	**10.63**	**984,689**	**37,326**	**39,670**	**76,996**	**7.82**
Biliran	16,889	2,114	2,057	4,171	24.70	19,923	1,282	1,209	2,491	12.50	36,252	1,462	1,328	2,790	7.70
Eastern Samar	47,349	7,491	7,626	15,117	31.93	57,439	2,007	2,108	4,115	7.16	105,850	2,363	2,839	5,202	4.91

Table 1.C.3.1 - Deworming Services for Children and Adolescents

Number and Proportion of Children/Adolescents who completed 2 doses of deworming tablet
Philippines, 2021

Area	Eligible Pop (1-4 years old)	No. of children 1-4 y/o given 2 doses of deworming drugs				Eligible Pop (5-9 years old)	No. of Children 5-9 y/o given 2 doses of deworming drugs				Eligible Pop (10-19 years old)	No. of adolescents 10-19 y/o given 2 doses of deworming drugs			
		Male	Female	Total	%		Male	Female	Total	%		Male	Female	Total	%
Northern Leyte	145,057	27,371	25,941	53,312	36.75	175,854	5,019	5,176	10,195	5.80	331,194	4,378	4,717	9,095	2.75
Northern Samar	68,641	8,690	9,053	17,743	25.85	80,867	6,414	6,743	13,157	16.27	147,616	10,240	11,235	21,475	14.55
Southern Leyte	29,248	9,060	8,813	17,873	61.11	36,601	5,643	6,007	11,650	31.83	68,560	8,584	8,632	17,216	25.11
Western Samar	61,611	6,415	7,211	13,626	22.12	72,696	2,133	2,282	4,415	6.07	135,680	2,898	3,266	6,164	4.54
Calbayog City	18,987	1,234	1,272	2,506	13.20	22,414	1,600	1,534	3,134	13.98	41,821	2,934	3,024	5,958	14.25
Maasin City	7,450	2,347	2,117	4,464	59.92	9,317	1,989	1,886	3,875	41.59	17,442	3,496	3,625	7,121	40.83
Ormoc City	20,661	9,193	8,554	17,747	85.90	25,050	1,631	1,403	3,034	12.11	47,163	830	865	1,695	3.59
Tacloban City	23,259	306	591	897	3.86	28,193	47	47	94	0.33	53,111	141	139	280	0.53
**Region 9**	**337,340**	**110,300**	**113,164**	**223,464**	**66.24**	**410,204**	**147,449**	**155,536**	**302,985**	**73.86**	**776,000**	**249,232**	**262,659**	**511,891**	**65.97**
Zamboanga del Norte	74,039	38,461	44,191	82,652	111.63	89,183	49,539	58,915	108,454	121.61	168,195	90,226	106,088	196,314	116.72
Zamboanga del Sur	71,567	31,923	24,458	56,381	78.78	88,185	40,219	34,074	74,293	84.25	170,085	73,596	60,712	134,308	78.97
Zamboanga Sibugay	66,255	149	119	268	0.40	79,431	189	147	336	0.42	143,447	332	318	650	0.45
Dapitan City	7,646	465	529	994	13.00	9,213	576	671	1,247	13.54	17,368	2,016	2,427	4,443	25.58
Dipolog City	12,129	10,120	14,434	24,554	202.44	14,609	14,452	19,645	34,097	233.40	27,552	26,829	35,442	62,271	226.01
Isabela City	12,150	2,783	2,690	5,473	45.05	14,307	3,434	3,208	6,642	46.42	27,047	0	0	0	0.00
Pagadian City	17,557	6,945	7,080	14,025	79.88	21,631	10,989	11,776	22,765	105.24	41,716	13,430	13,568	26,998	64.72
Zamboanga City	75,997	19,454	19,663	39,117	51.47	93,645	28,051	27,100	55,151	58.89	180,590	42,803	44,104	86,907	48.12
**Region 10**	**431,863**	**195,696**	**194,097**	**389,793**	**90.26**	**525,500**	**251,261**	**254,878**	**506,139**	**96.32**	**996,834**	**403,637**	**408,776**	**812,413**	**81.50**
Bukidnon	98,908	48,364	44,966	93,330	94.36	119,217	63,457	60,585	124,042	104.05	224,694	96,652	98,741	195,393	86.96
Camiguin	7,997	2,676	2,938	5,614	70.20	9,584	5,197	3,340	8,537	89.08	17,955	9,197	5,976	15,173	84.51
Lanao del Norte	67,455	37,860	39,832	77,692	115.18	81,571	49,683	57,456	107,139	131.34	153,610	81,820	88,027	169,847	110.57
Misamis Occidental	27,457	10,746	11,412	22,158	80.70	34,210	13,979	14,844	28,823	84.25	65,844	24,810	26,345	51,155	77.69
Misamis Oriental	62,945	39,266	37,170	76,436	121.43	77,039	50,658	48,884	99,542	129.21	147,116	82,398	78,804	161,202	109.57
Cagayan de Oro City	59,625	21,766	22,881	44,647	74.88	72,981	26,144	27,414	53,558	73.39	139,341	42,757	44,784	87,541	62.83
El Salvador City	4,426	1,803	2,203	4,006	90.51	5,423	2,573	3,144	5,717	105.42	10,347	4,052	4,953	9,005	87.03
Gingoog City	11,000	2,542	2,664	5,206	47.33	13,462	3,478	3,515	6,993	51.95	25,695	5,689	5,636	11,325	44.07
Iligan City	34,174	12,373	12,276	24,649	72.13	41,309	11,667	12,090	23,757	57.51	77,805	17,963	18,847	36,810	47.31
Malaybalay City	16,486	4,057	3,946	8,003	48.54	19,872	4,057	3,946	8,003	40.27	37,460	7,325	6,812	14,137	37.74
Oroquieta City	5,950	3,997	4,072	8,069	135.61	7,411	5,917	5,885	11,802	159.25	14,269	9,265	9,519	18,784	131.64
Ozamis City	11,924	810	971	1,781	14.94	14,856	1,265	1,307	2,572	17.31	28,599	2,340	2,327	4,667	16.32
Tangub City	5,299	1,934	1,873	3,807	71.84	6,603	3,448	3,163	6,611	100.12	12,705	5,383	5,175	10,558	83.10
Valencia City	18,217	7,502	6,893	14,395	79.02	21,962	9,738	9,305	19,043	86.71	41,394	13,986	12,830	26,816	64.78
**Region 11**	**450,626**	**172,602**	**165,869**	**338,471**	**75.11**	**560,189**	**79,431**	**82,156**	**161,587**	**28.85**	**1,044,422**	**68,849**	**65,865**	**134,714**	**12.90**
Davao de Oro	68,026	31,145	28,721	59,866	88.00	82,832	15,122	13,891	29,013	35.03	151,938	15,918	14,556	30,474	20.06
Davao del Norte	87,061	37,866	35,734	73,600	84.54	109,027	16,224	15,393	31,617	29.00	203,114	20,412	20,158	40,570	19.97
Davao del Sur	56,666	16,191	15,449	31,640	55.84	68,323	10,570	10,244	20,814	30.46	124,927	11,410	10,951	22,361	17.90
Davao Oriental	58,178	15,591	14,583	30,174	51.86	73,008	6,021	5,607	11,628	15.93	138,407	5,272	4,846	10,118	7.31
Davao Occidental	30,486	1,958	1,950	3,908	12.82	38,544	1,238	1,128	2,366	6.14	68,741	1,424	1,811	3,235	4.71
Davao City	150,209	69,851	69,432	139,283	92.73	188,455	30,256	35,893	66,149	35.10	357,295	14,413	13,543	27,956	7.82
**Region 12**	**437,961**	**150,583**	**144,760**	**295,343**	**67.44**	**540,004**	**128,418**	**125,479**	**253,897**	**47.02**	**1,005,492**	**185,423**	**181,295**	**366,718**	**36.47**
North Cotabato	140,154	42,872	40,295	83,167	59.34	172,865	14,865	15,146	30,011	17.36	316,814	17,355	18,096	35,451	11.19
Sarangani	54,636	20,133	19,940	40,073	73.35	66,597	20,621	20,075	40,696	61.11	125,028	32,152	31,452	63,604	50.87
South Cotabato	84,890	34,199	32,638	66,837	78.73	104,142	31,712	30,981	62,693	60.20	196,587	44,232	43,089	87,321	44.42
Sultan Kudarat	74,810	26,072	26,269	52,341	69.97	93,721	35,835	35,188	71,023	75.78	174,233	60,236	57,869	118,105	67.79
Cotabato City	28,340	3,978	4,165	8,143	28.73	35,041	3,682	3,918	7,600	21.69	65,157	3,737	4,476	8,213	12.60
Gen. Santos City	55,131	23,329	21,453	44,782	81.23	67,638	21,703	20,171	41,874	61.91	127,673	27,711	26,313	54,024	42.31
**BARMM**	**490,094**	**14,126**	**14,637**	**28,763**	**5.87**	**568,489**	**14,799**	**15,709**	**30,508**	**5.37**	**951,682**	**20,147**	**22,945**	**43,092**	**4.53**
Basilan	36,154	1,976	2,175	4,151	11.48	41,761	2,197	2,361	4,558	10.91	70,058	3,598	4,643	8,241	11.76
Lanao del Sur	111,572	0	0	0	0.00	128,178	0	0	0	0.00	208,241	0	0	0	0.00
Maguindanao	151,474	969	1,035	2,004	1.32	178,701	626	628	1,254	0.70	303,512	471	527	998	0.33
Sulu	109,019	0	0	0	0.00	124,395	0	0	0	0.00	208,089	0	0	0	0.00
Tawi-Tawi	45,239	7,834	7,929	15,763	34.84	53,302	8,226	8,770	16,996	31.89	92,701	11,089	12,409	23,498	25.35
Lamitan City	9,948	317	306	623	6.26	11,485	318	333	651	5.67	19,277	282	339	621	3.22
Marawi City	26,688	3,030	3,192	6,222	23.31	30,667	3,432	3,617	7,049	22.99	49,804	4,707	5,027	9,734	19.54
**CARAGA**	**259,830**	**99,950**	**98,511**	**198,461**	**76.38**	**308,544**	**117,383**	**115,898**	**233,281**	**75.61**	**558,216**	**180,901**	**175,983**	**356,884**	**63.93**
Agusan del Norte	34,174	7,645	7,942	15,587	45.61	40,788	11,015	11,092	22,107	54.20	75,504	18,741	19,787	38,528	51.03
Agusan del Sur	77,089	36,103	35,018	71,121	92.26	87,967	31,019	30,902	61,921	70.39	151,458	37,909	34,939	72,848	48.10
Surigao del Norte	31,994	8,844	8,716	17,560	54.89	39,306	12,821	12,834	25,655	65.27	73,752	22,326	21,514	43,840	59.44
Surigao del Sur	48,690	20,034	19,020	39,054	80.21	58,174	26,340	24,897	51,237	88.08	105,529	44,648	42,310	86,958	82.40
Province of Dinagat	11,239	5,934	5,934	11,868	105.60	14,189	6,584	6,677	13,261	93.46	25,792	12,379	11,803	24,182	93.76
Bislig City	9,250	4,087	4,065	8,152	88.13	11,046	5,391	5,488	10,879	98.49	20,050	8,481	8,076	16,557	82.58
Butuan City	32,495	15,200	15,914	31,114	95.75	38,773	20,970	20,989	41,959	108.22	71,788	31,773	32,983	64,756	90.20
Surigao City	14,899	2,103	1,902	4,005	26.88	18,301	3,243	3,019	6,262	34.22	34,343	4,644	4,571	9,215	26.83

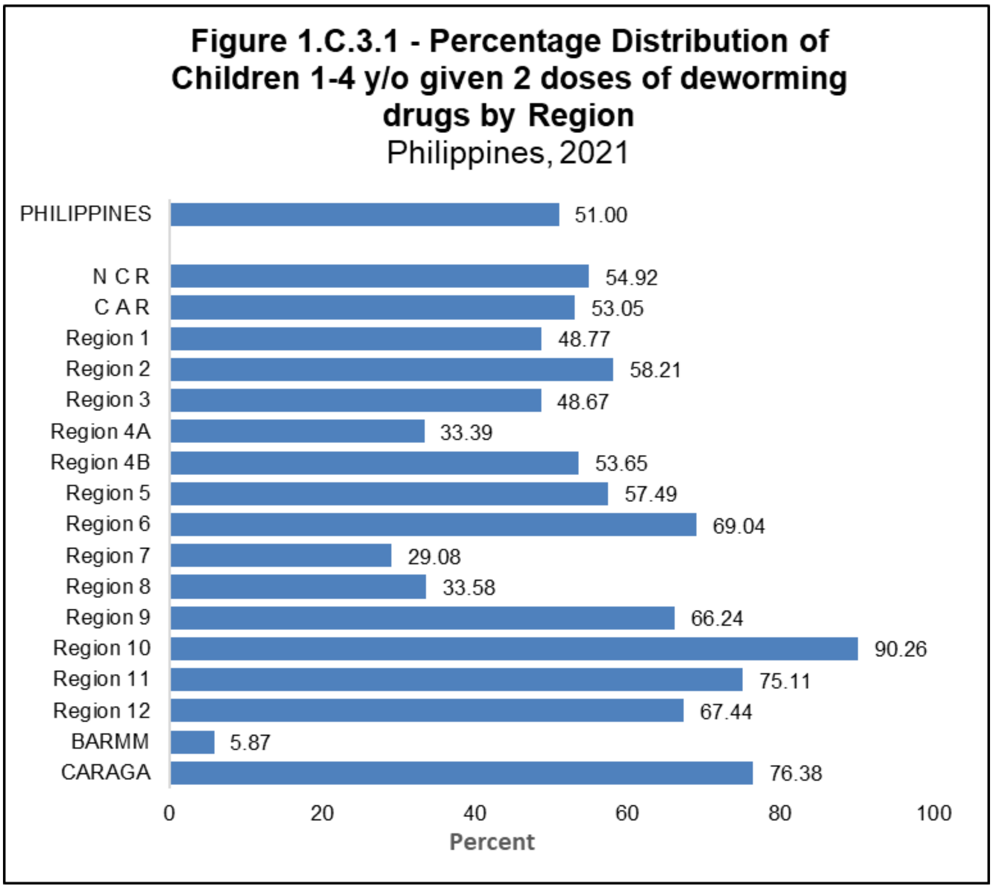
Figure 1.C.3.1 - Percentage Distribution of Children 1-4 y/o given 2 doses of deworming drugs by Region
Philippines, 2021
PHILIPPINES 51.00
N C R 54.92
C A R 53.05
Region 1 48.77
Region 2 58.21
Region 3 48.67
Region 4A 33.39
Region 4B 53.65
Region 5 57.49
Region 6 69.04
Region 7 29.08
Region 8 33.58
Region 9 66.24
Region 10 90.26
Region 11 75.11
Region 12 67.44
BARMM 5.87
CARAGA 76.38
0 20 40 60 80 100
Percent

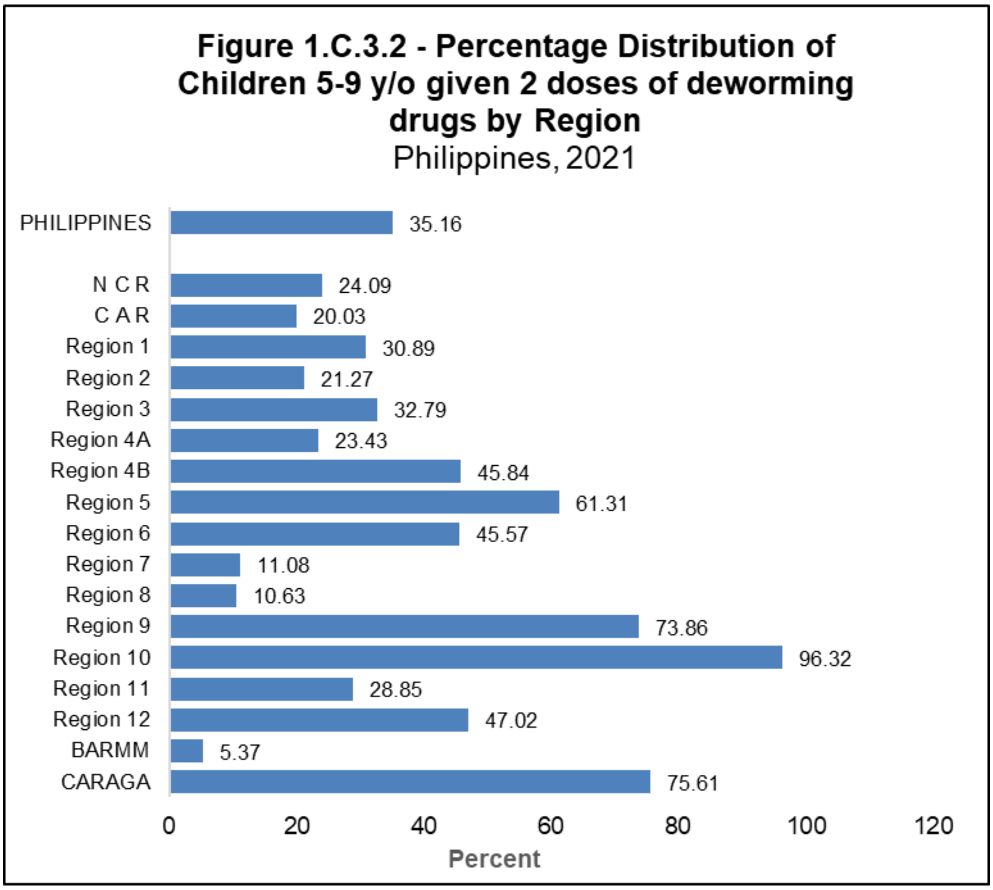
Figure 1.C.3.2 - Percentage Distribution of Children 5-9 y/o given 2 doses of deworming drugs by Region
Philippines, 2021
PHILIPPINES 35.16
N C R 24.09
C A R 20.03
Region 1 30.89
Region 2 21.27
Region 3 32.79
Region 4A 23.43
Region 4B 45.84
Region 5 61.31
Region 6 45.57
Region 7 11.08
Region 8 10.63
Region 9 73.86
Region 10 96.32
Region 11 28.85
Region 12 47.02
BARMM 5.37
CARAGA 75.61
0 20 40 60 80 100 120
Percent

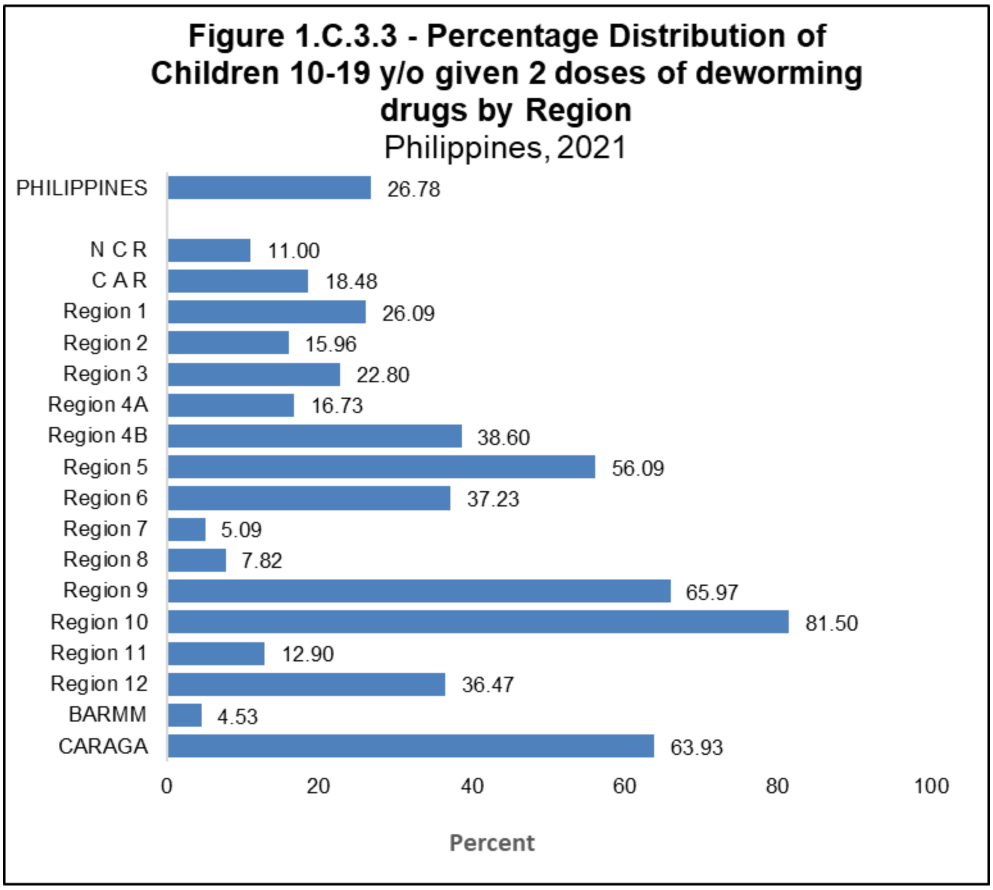

Figure 1.C.3.3 - Percentage Distribution of Children 10-19 y/o given 2 doses of deworming drugs by Region
Philippines, 2021
PHILIPPINES
26.78
N C R
11.00
C A R
18.48
Region 1
26.09
Region 2
15.96
Region 3
22.80
Region 4A
16.73
Region 4B
38.60
Region 5
56.09
Region 6
37.23
Region 7
5.09
Region 8
7.82
Region 9
65.97
Region 10
81.50
Region 11
12.90
Region 12
36.47
BARMM
4.53
CARAGA
63.93
0
20
40
60
80
100
Percent

Table 1.C.4.1 - Management of Sick Infants and Children

Number of high risk infants and children with measles and/or persistent diarrhea who received Vitamin A capsule aside from routine supplementation
Philippines, 2021

Area	Sick Infants aged 6-11 mos.							Sick Children aged 12-59 mos.						
	Seen			given Vitamin A 100,000 IU				Seen			given Vitamin A 200,000 IU			
	Male	Female	Total	Male	Female	Total	%	Male	Female	Total	Male	Female	Total	%
**PHILIPPINES**	**60,612**	**59,147**	**119,759**	**34,214**	**34,169**	**68,383**	**57.10**	**121,881**	**119,010**	**240,891**	**62,201**	**60,000**	**122,201**	**50.73**
**N C R**	**5,161**	**4,908**	**10,069**	**3,451**	**3,732**	**7,183**	**71.34**	**10,498**	**10,912**	**21,410**	**5,968**	**6,395**	**12,363**	**57.74**
Malabon	156	137	293	6	10	16	5.46	260	292	552	19	27	46	8.33
Navotas	398	382	780	382	377	759	97.31	1,050	985	2,035	829	783	1,612	79.21
Valenzuela City	139	139	278	61	102	163	58.63	399	415	814	195	133	328	40.29
Caloocan City	71	81	152	71	81	152	100.00	415	437	852	415	437	852	100.00
Marikina City	8	4	12	8	4	12	100.00	7	8	15	7	7	14	93.33
Pasig City	0	0	0	0	0	0	0.00	0	0	0	0	0	0	0.00
Pateros	6	9	15	0	2	2	13.33	39	37	76	3	5	8	10.53
Taguig	1,606	1,133	2,739	547	496	1,043	38.08	4,382	4,389	8,771	1,227	1,256	2,483	28.31
Quezon City	109	116	225	109	116	225	100.00	151	168	319	151	168	319	100.00
							0.00							0.00
Makati City	45	41	86	40	41	81	94.19	77	56	133	71	51	122	91.73
Mandaluyong City	3	3	6	3	3	6	100.00	4	2	6	4	2	6	100.00
San Juan	2	2	4	2	2	4	100.00	4	3	7	4	3	7	100.00
Manila City	2,488	2,721	5,209	2,136	2,389	4,525	86.87	3,316	3,732	7,048	2,787	3,255	6,042	85.73
							0.00							0.00
Las Piñas City	0	0	0	0	0	0	0.00	0	0	0	0	0	0	0.00
Muntinlupa City	43	48	91	26	30	56	61.54	123	143	266	78	98	176	66.17
Parañaque City	87	92	179	60	79	139	77.65	271	245	516	178	170	348	67.44
Pasay City	0	0	0	0	0	0	0.00	0	0	0	0	0	0	0.00
**C A R**	**303**	**280**	**583**	**220**	**186**	**406**	**69.64**	**583**	**581**	**1,164**	**417**	**417**	**834**	**71.65**
Abra	106	91	197	65	44	109	55.33	226	236	462	152	152	304	65.80
Apayao	27	34	61	16	12	28	45.90	48	56	104	32	44	76	73.08
Benguet	31	31	62	31	31	62	100.00	66	58	124	66	58	124	100.00
Ifugao	102	87	189	79	67	146	77.25	157	138	295	103	81	184	62.37
Kalinga	12	8	20	12	8	20	100.00	26	31	57	22	28	50	87.72
Mt. Province	5	6	11	1	3	4	36.36	16	6	22	0	0	0	0.00
Baguio City	20	23	43	16	21	37	86.05	44	56	100	42	54	96	96.00
**Region 1**	**2,374**	**2,140**	**4,514**	**2,065**	**1,852**	**3,917**	**86.77**	**4,560**	**4,263**	**8,823**	**3,787**	**3,517**	**7,304**	**82.78**
Ilocos Norte	59	58	117	42	41	83	70.94	314	290	604	198	188	386	63.91
Ilocos Sur	408	363	771	303	275	578	74.97	764	737	1501	442	424	866	57.69
La Union	134	112	246	76	67	143	58.13	360	311	671	155	126	281	41.88
Pangasinan	1542	1370	2912	1454	1286	2740	94.09	2722	2544	5266	2722	2544	5,266	100.00
Alaminos City	77	64	141	77	64	141	100.00	78	70	148	78	70	148	100.00
Candon City	34	39	73	34	39	73	100.00	55	47	102	55	47	102	100.00
Dagupan City	0	1	1	0	1	1	100.00	0	2	2	0	2	2	100.00
Laoag City	0	0	0	0	0	0	0.00	0	0	0	0	0	0	0.00
San Carlos City	65	69	134	65	69	134	100.00	119	97	216	119	97	216	100.00
San Fernando City	41	54	95	0	0	0	0.00	130	146	276	0	0	0	0.00
Urdaneta City	14	10	24	14	10	24	100.00	18	19	37	18	19	37	100.00
Vigan City	0	0	0	0	0	0	0.00	0	0	0	0	0	0	0.00
**Region 2**	**2,648**	**2,609**	**5,257**	**1,795**	**1,766**	**3,561**	**67.74**	**4,962**	**4,600**	**9,562**	**3,024**	**2,833**	**5,857**	**61.25**
Batanes	0	0	0	0	0	0	0.00	0	0	0	0	0	0	0.00
Cagayan	267	269	536	171	217	388	72.39	722	580	1302	322	258	580	44.55
Isabela	1529	1493	3022	839	804	1643	54.37	2677	2638	5315	1280	1308	2,588	48.69
Nueva Vizcaya	165	181	346	123	112	235	67.92	229	205	434	149	140	289	66.59
Quirino	172	188	360	148	155	303	84.17	341	334	675	281	284	565	83.70
Cauayan City	462	441	903	462	441	903	100.00	870	739	1609	870	739	1,609	100.00
Ilagan City	53	36	89	52	36	88	98.88	120	102	222	119	102	221	99.55
Santiago City	0	0	0	0	0	0	0.00	0	0	0	0	0	0	0.00
Tuguegarao City	0	1	1	0	1	1	100.00	3	2	5	3	2	5	100.00

Table 1.C.4.1 - Management of Sick Infants and Children

Number of high risk infants and children with measles and/or persistent diarrhea who received Vitamin A capsule aside from routine supplementation
Philippines, 2021

Area	Sick Infants aged 6-11 mos.							Sick Children aged 12-59 mos.						
	Seen			given Vitamin A 100,000 IU				Seen			given Vitamin A 200,000 IU			
	Male	Female	Total	Male	Female	Total	%	Male	Female	Total	Male	Female	Total	%
**Region 3**	**7,470**	**7,061**	**14,531**	**4,586**	**4,316**	**8,902**	**61.26**	**17,886**	**15,118**	**33,004**	**11,605**	**9,416**	**21,021**	**63.69**
Aurora	152	169	321	66	66	132	41.12	282	284	566	83	104	187	33.04
Bataan	588	489	1077	177	125	302	28.04	1418	1396	2814	331	306	637	22.64
Bulacan	1539	1513	3052	1021	1036	2057	67.40	4,938	4539	9477	3153	3080	6,233	65.77
Nueva Ecija	863	867	1730	414	424	838	48.44	3005	2788	5793	2430	2303	4,733	81.70
Pampanga	175	188	363	156	164	320	88.15	238	220	458	209	196	405	88.43
Tarlac	1135	1117	2252	860	814	1674	74.33	1762	1729	3491	1107	1095	2,202	63.08
Zambales	465	457	922	196	203	399	43.28	812	822	1634	385	407	792	48.47
Angeles City	281	308	589	227	247	474	80.48	452	537	989	350	407	757	76.54
Balanga City	4	3	7	2	1	3	42.86	7	2	9	1	0	1	11.11
Cabanatuan City	5	5	10	5	5	10	100.00	9	11	20	9	11	20	100.00
City of San Fernando	228	222	450	165	177	342	76.00	329	334	663	277	279	556	83.86
Gapan City	87	85	172	21	28	49	28.49	109	124	233	29	36	65	27.90
Mabalacat City	291	29	320	281	15	296	92.50	2016	28	2044	1993	11	2,004	98.04
Malolos City	19	15	34	3	7	10	29.41	37	32	69	9	7	16	23.19
Meycauayan	1184	1098	2282	602	590	1192	52.23	1789	1593	3382	696	641	1,337	39.53
Olongapo	7	5	12	6	5	11	91.67	18	19	37	17	16	33	89.19
Palayan City	5	4	9	5	4	9	100.00	5	3	8	5	3	8	100.00
San Jose City	15	23	38	15	23	38	100.00	60	49	109	58	46	104	95.41
San Jose del Monte City	25	26	51	25	26	51	100.00	52	64	116	52	64	116	100.00
Science City of Munoz	0	0	0	0	0	0	0.00	0	0	0	0	0	0	0.00
Tarlac City	402	438	840	339	356	695	82.74	548	544	1092	411	404	815	74.63
**Region 4A**	**9,921**	**9,711**	**19,632**	**3,167**	**3,448**	**6,615**	**33.69**	**20,027**	**19,947**	**39,974**	**5,006**	**5,368**	**10,374**	**25.95**
Batangas	253	230	483	74	79	153	31.68	563	432	995	145	83	228	22.91
Cavite	1920	1949	3869	296	320	616	15.92	3527	3753	7280	321	326	647	8.89
Laguna	434	422	856	88	106	194	22.66	847	788	1635	64	64	128	7.83
Quezon	1766	1609	3375	378	406	784	23.23	2998	3447	6445	624	1132	1,756	27.25
Rizal	782	707	1489	240	236	476	31.97	1869	1825	3694	365	328	693	18.76
Antipolo City	712	811	1523	562	714	1276	83.78	1508	1541	3049	803	778	1,581	51.85
Bacoor City	620	666	1286	207	223	430	33.44	1723	1577	3300	288	290	578	17.52
Batangas City	35	30	65	2	7	9	13.85	36	30	66	4	6	10	15.15
Biñan City	236	264	500	214	234	448	89.60	304	331	635	249	275	524	82.52
Cabuyao City	58	72	130	36	36	72	55.38	287	251	538	189	180	369	68.59
Calamba City	817	845	1662	336	355	691	41.58	2067	1966	4033	563	527	1,090	27.03
Cavite City	9	8	17	1	0	1	5.88	12	23	35	0	0	0	0.00
Dasmariñas City	573	537	1110	274	263	537	48.38	577	512	1089	292	286	578	53.08
General Trias City	192	181	373	20	11	31	8.31	346	343	689	9	9	18	2.61
Imus City	698	574	1272	201	203	404	31.76	1861	1621	3482	699	648	1,347	38.68
Lipa City	3	2	5	2	2	4	80.00	2	4	6	2	4	6	100.00
Lucena City	459	466	925	190	205	395	42.70	872	927	1799	293	336	629	34.96
San Pablo City	4	4	8	1	0	1	12.50	7	5	12	0	0	0	0.00
San Pedro City	5	10	15	3	2	5	33.33	3	0	3	0	0	0	0.00
Santa Rosa City	143	131	274	27	32	59	21.53	277	229	506	51	54	105	20.75
Tagaytay City	108	102	210	14	13	27	12.86	264	271	535	34	34	68	12.71
Tanauan City	10	7	17	0	0	0	0.00	30	13	43	0	0	0	0.00
Tayabas City	71	67	138	1	1	2	1.45	15	12	27	11	8	19	70.37
Trece Martires City	13	17	30	0	0	0	0.00	32	46	78	0	0	0	0.00
**Region 4B**	**988**	**881**	**1,869**	**374**	**322**	**696**	**37.24**	**2,704**	**2,570**	**5,274**	**903**	**945**	**1,848**	**35.04**
Marinduque	88	60	148	85	59	144	97.30	157	155	312	136	138	274	87.82
Mindoro Occidental	471	412	883	89	73	162	18.35	1,200	1,089	2,289	165	145	310	13.54
Mindoro Oriental	72	83	155	27	20	47	30.32	171	166	337	55	61	116	34.42
Palawan	185	177	362	139	143	282	77.90	702	772	1,474	522	581	1,103	74.83
Romblon	137	117	254	32	26	58	22.83	392	334	726	17	13	30	4.13
Puerto Princesa City	35	32	67	2	1	3	4.48	82	54	136	8	7	15	11.03
**Region 5**	**5,090**	**4,958**	**10,048**	**2,135**	**2,125**	**4,260**	**42.40**	**10,842**	**10,849**	**21,691**	**4,132**	**4,087**	**8,219**	**37.89**
Albay	1460	1327	2787	554	496	1050	37.67	3411	3640	7051	1168	1154	2,322	32.93
Camarines Norte	632	537	1169	326	271	597	51.07	1278	1195	2473	916	847	1,763	71.29

Table 1.C.4.1 - Management of Sick Infants and Children

Number of high risk infants and children with measles and/or persistent diarrhea who received Vitamin A capsule aside from routine supplementation
Philippines, 2021

Area	Sick Infants aged 6-11 mos.							Sick Children aged 12-59 mos.						
	Seen			given Vitamin A 100,000 IU				Seen			given Vitamin A 200,000 IU			
	Male	Female	Total	Male	Female	Total	%	Male	Female	Total	Male	Female	Total	%
Camarines Sur	436	478	914	170	182	352	38.51	1293	1299	2592	356	340	696	26.85
Catanduanes	65	66	131	12	8	20	15.27	336	285	621	61	59	120	19.32
Masbate	671	662	1333	324	329	653	48.99	879	841	1720	384	436	820	47.67
Sorsogon	1231	1288	2519	484	462	946	37.55	2558	2554	5112	794	780	1,574	30.79
Iriga City	79	122	201	76	113	189	94.03	202	203	405	181	182	363	89.63
Legaspi City	455	424	879	144	214	358	40.73	800	756	1556	223	234	457	29.37
Naga City	61	54	115	45	50	95	82.61	85	76	161	49	55	104	64.60
**Region 6**	**2,911**	**2,824**	**5,735**	**1,687**	**1,651**	**3,338**	**58.20**	**6,170**	**5,881**	**12,051**	**2,785**	**2,750**	**5,535**	**45.93**
Aklan	504	509	1,013	370	373	743	73.35	1,006	965	1,971	702	703	1,405	71.28
Antique	390	501	891	150	171	321	36.03	784	802	1,586	297	295	592	37.33
Capiz	39	63	102	30	46	76	74.51	71	64	135	45	49	94	69.63
Guimaras	13	7	20	10	3	13	65.00	11	13	24	1	4	5	20.83
Iloilo	956	734	1,690	482	392	874	51.72	2,177	1,971	4,148	672	620	1,292	31.15
Negros Occidental	888	889	1,777	568	600	1,168	65.73	1,849	1,815	3,664	951	979	1,930	52.67
Bacolod City	58	51	109	49	38	87	79.82	64	58	122	51	38	89	72.95
Iloilo City	63	70	133	28	28	56	42.11	208	193	401	66	62	128	31.92
**Region 7**	**1,010**	**1,022**	**2,032**	**700**	**667**	**1,367**	**67.27**	**2,094**	**2,061**	**4,155**	**753**	**807**	**1,560**	**37.55**
Bohol	168	160	328	88	72	160	48.78	536	589	1125	177	201	378	33.60
Cebu	501	548	1049	431	422	853	81.32	739	711	1450	260	298	558	38.48
Negros Oriental	36	41	77	32	35	67	87.01	90	95	185	85	89	174	94.05
Siquijor	69	55	124	0	0	0	0.00	305	287	592	0	0	0	0.00
Cebu City	133	118	251	74	68	142	56.57	263	217	480	118	104	222	46.25
Lapu-Lapu City	69	63	132	69	63	132	100.00	103	95	198	103	95	198	100.00
Mandaue City	34	37	71	6	7	13	18.31	58	67	125	10	20	30	24.00
**Region 8**	**4,237**	**4,328**	**8,565**	**1,956**	**2,250**	**4,206**	**49.11**	**8,655**	**8,830**	**17,485**	**3,592**	**3,518**	**7,110**	**40.66**
Biliran	189	549	738	124	476	600	81.30	354	863	1217	115	114	229	18.82
Eastern Samar	567	487	1054	117	112	229	21.73	1173	1176	2349	306	325	631	26.86
Northern Leyte	666	605	1271	364	351	715	56.25	1114	1039	2153	444	431	875	40.64
Northern Samar	1544	1501	3045	745	751	1496	49.13	3445	3211	6656	1624	1559	3,183	47.82
Southern Leyte	87	75	162	22	33	55	33.95	177	193	370	63	63	126	34.05
Western Samar	1052	969	2021	534	475	1009	49.93	2037	2004	4041	913	899	1,812	44.84
Calbayog City	1	1	2	1	1	2	100.00	0	0	0	0	0	0	0.00
Maasin City	39	43	82	16	8	24	29.27	20	23	43	6	3	9	20.93
Ormoc City	0	0	0	0	0	0	0.00	1	2	3	1	2	3	100.00
Tacloban City	92	98	190	33	43	76	40.00	334	319	653	120	122	242	37.06
**Region 9**	**3,841**	**3,684**	**7,525**	**2,703**	**2,518**	**5,221**	**69.38**	**6,720**	**6,725**	**13,445**	**4,229**	**4,246**	**8,475**	**63.03**
Zamboanga del Norte	2036	1995	4031	1490	1459	2949	73.16	2988	3005	5993	1896	1893	3,789	63.22
Zamboanga del Sur	541	532	1073	439	373	812	75.68	1219	1223	2442	828	838	1,666	68.22
Zamboanga Sibugay	569	551	1120	289	257	546	48.75	1324	1301	2625	766	773	1,539	58.63
Dapitan City	54	53	107	22	26	48	44.86	63	64	127	33	44	77	60.63
Dipolog City	42	29	71	34	22	56	78.87	62	48	110	46	36	82	74.55
Isabela City	78	68	146	60	58	118	80.82	140	148	288	80	89	169	58.68
Pagadian City	18	14	32	18	14	32	100.00	25	21	46	25	21	46	100.00
Zamboanga City	503	442	945	351	309	660	69.84	899	915	1,814	555	552	1,107	61.03
**Region 10**	**5,537**	**5,812**	**11,349**	**2,978**	**3,134**	**6,112**	**53.85**	**9,460**	**9,728**	**19,188**	**4,480**	**4,637**	**9,117**	**47.51**
Bukidnon	1239	1442	2681	590	682	1272	47.44	2329	2412	4741	1018	1046	2,064	43.54
Camiguin	0	0	0	0	0	0	0.00	42	54	96	6	12	18	18.75
Lanao del Norte	1215	1350	2565	937	978	1915	74.66	1721	1682	3403	1187	1165	2,352	69.12
Misamis Occidental	411	358	769	183	208	391	50.85	395	459	854	271	355	626	73.30
Misamis Oriental	1408	1329	2737	535	497	1032	37.71	2479	2613	5092	556	531	1,087	21.35
Cagayan de Oro City	155	174	329	112	107	219	66.57	214	218	432	80	98	178	41.20

Table 1.C.4.1 - Management of Sick Infants and Children

Number of high risk infants and children with measles and/or persistent diarrhea who received Vitamin A capsule aside from routine supplementation
Philippines, 2021

Area	Sick Infants aged 6-11 mos.							Sick Children aged 12-59 mos.						
	Seen			given Vitamin A 100,000 IU				Seen			given Vitamin A 200,000 IU			
	Male	Female	Total	Male	Female	Total	%	Male	Female	Total	Male	Female	Total	%
El Salvador City	0	0	0	0	0	0	0.00	0	0	0	0	0	0	0.00
Gingoog City	211	249	460	90	112	202	43.91	526	555	1081	274	359	633	58.56
Iligan City	486	494	980	387	396	783	79.90	953	950	1903	775	771	1,546	81.24
Malaybalay City	271	278	549	96	103	199	36.25	398	380	778	45	47	92	11.83
Oroquieta City	0	0	0	0	0	0	0.00	0	0	0	0	0	0	0.00
Ozamis City	1	0	1	0	0	0	0.00	1	0	1	0	0	0	0.00
Tangub City	0	0	0	0	0	0	0.00	1	1	2	1	1	2	100.00
Valencia City	140	138	278	48	51	99	35.61	401	404	805	267	252	519	64.47
**Region 11**	**1,135**	**959**	**2,094**	**991**	**816**	**1,807**	**86.29**	**1,684**	**1,487**	**3,171**	**1,535**	**1,352**	**2,887**	**91.04**
Davao de Oro	24	23	47	15	15	30	63.83	62	48	110	34	28	62	56.36
Davao del Norte	84	69	153	79	64	143	93.46	168	142	310	239	204	443	142.90
Davao del Sur	57	32	89	48	27	75	84.27	68	46	114	60	39	99	86.84
Davao Oriental	496	402	898	482	394	876	97.55	730	642	1372	690	609	1,299	94.68
Davao Occidental	43	44	87	51	45	96	110.34	79	71	150	34	35	69	46.00
Davao City	431	389	820	316	271	587	71.59	577	538	1115	478	437	915	82.06
**Region 12**	**4,223**	**4,148**	**8,371**	**3,028**	**2,937**	**5,965**	**71.26**	**7,172**	**7,172**	**14,344**	**4,751**	**4,814**	**9,565**	**66.68**
North Cotabato	1914	1997	3911	1070	1087	2157	55.15	2998	3020	6018	1371	1438	2,809	46.68
Sarangani	1785	1667	3452	1508	1448	2956	85.63	3145	3199	6344	2754	2810	5,564	87.70
South Cotabato	275	226	501	236	191	427	85.23	624	605	1229	308	293	601	48.90
Sultan Kudarat	96	81	177	89	73	162	91.53	121	114	235	120	110	230	97.87
Cotabato City	0	0	0	0	0	0	0.00	0	0	0	0	0	0	0.00
Gen. Santos City	153	177	330	125	138	263	79.70	284	234	518	198	163	361	69.69
**BARMM**	**2,236**	**2,430**	**4,666**	**1,895**	**2,027**	**3,922**	**84.05**	**4,735**	**4,898**	**9,633**	**4,399**	**3,941**	**8,340**	**86.58**
Basilan	852	915	1767	839	896	1735	98.19	1875	1810	3685	2384	1772	4,156	112.78
Lanao del Sur	163	172	335	111	109	220	65.67	294	348	642	204	240	444	69.16
Maguindanao	0	0	0	0	0	0	0.00	0	0	0	0	0	0	0.00
Sulu	381	561	942	270	394	664	70.49	920	1185	2105	679	827	1,506	71.54
Tawi-Tawi	247	247	494	85	95	180	36.44	643	608	1251	134	155	289	23.10
Lamitan City	14	9	23	11	7	18	78.26	26	16	42	21	16	37	88.10
Marawi City	579	526	1105	579	526	1105	100.00	977	931	1908	977	931	1,908	100.00
**CARAGA**	**1,527**	**1,392**	**2,919**	**483**	**422**	**905**	**31.00**	**3,129**	**3,388**	**6,517**	**835**	**957**	**1,792**	**27.50**
Agusan del Norte	184	155	339	58	56	114	33.63	410	424	834	132	128	260	31.18
Agusan del Sur	511	475	986	162	164	326	33.06	1,006	1,199	2,205	278	395	673	30.52
Surigao del Norte	504	478	982	74	62	136	13.85	1,181	1,242	2,423	125	138	263	10.85
Surigao del Sur	36	21	57	35	21	56	98.25	56	63	119	52	59	111	93.28
Province of Dinagat	31	37	68	2	4	6	8.82	66	62	128	18	9	27	21.09
Bislig City	17	15	32	17	15	32	100.00	40	31	71	38	29	67	94.37
Butuan City	114	83	197	49	36	85	43.15	166	159	325	85	89	174	53.54
Surigao City	130	128	258	86	64	150	58.14	204	208	412	107	110	217	52.67

Note: Put asterisk (*) for No Report and Zero (0) for No Case

Table 1.C.4.2 - Management of Sick Infants and Children

Number and proportion of diarrhea cases 0-59 mos. old who received Oral Rehydration Salt (ORS) solution and ORS with Zinc drops/syrup and Pneumonia cases among children 0-59 mos. old who received treatment

Philippines, 2021

Area	Children 0-59 mos. old with Diarrhea											Children 0-59 mos. old with Pneumonia						
	Seen			Given ORS				Given ORS w/ zinc				Seen			Received treatment			
	Male	Female	Total	Male	Female	Total	%	Male	Female	Total	%	Male	Female	Total	Male	Female	Total	%
**PHILIPPINES**	**38,552**	**36,967**	**75,519**	**24,290**	**23,503**	**47,793**	**63.29**	**22,869**	**21,529**	**44,398**	**58.79**	**23,793**	**22,240**	**46,033**	**22,884**	**21,440**	**44,324**	**96.29**
**N C R**	**3,604**	**3,356**	**6,960**	**1,349**	**1,229**	**2,578**	**37.04**	**2,255**	**2,127**	**4,382**	**62.96**	**1,621**	**1,610**	**3,231**	**1,552**	**1,567**	**3,119**	**96.53**
Malabon	65	68	133	14	5	19	14.29	51	63	114	85.71	35	37	72	19	35	54	75.00
Navotas	356	275	631	147	85	232	36.77	209	190	399	63.23	50	34	84	28	12	40	47.62
Valenzuela City	238	211	449	214	172	386	85.97	24	39	63	14.03	140	78	218	114	64	178	81.65
Caloocan City	13	13	26	0	0	0	0.00	13	13	26	100.00	0	1	1	0	1	1	100.00
Marikina City	22	18	40	13	11	24	60.00	9	7	16	40.00	2	1	3	2	0	2	66.67
Pasig City	155	118	273	99	71	170	62.27	56	47	103	37.73	30	15	45	30	15	45	100.00
Pateros	11	11	22	9	7	16	72.73	2	4	6	27.27	9	10	19	7	6	13	68.42
Taguig	646	590	1,236	300	281	581	47.01	346	309	655	52.99	16	24	40	13	24	37	92.50
Quezon City	146	124	270	102	78	180	66.67	44	46	90	33.33	122	85	207	122	85	207	100.00
Makati City	110	60	170	8	6	14	8.24	102	54	156	91.76	37	30	67	37	30	67	100.00
Mandaluyong City	43	40	83	18	24	42	50.60	25	16	41	49.40	39	35	74	39	35	74	100.00
San Juan	2	2	4	2	2	4	100.00	0	0	0	0.00	0	1	1	0	1	1	100.00
Manila City	1,181	1,314	2,495	324	403	727	29.14	857	911	1,768	70.86	963	1,046	2,009	963	1,046	2,009	100.00
Las Piñas City	209	218	427	0	0	0	0.00	209	218	427	100.00	27	35	62	27	35	62	100.00
Muntinlupa City	183	134	317	31	26	57	17.98	152	108	260	82.02	110	102	212	110	102	212	100.00
Parañaque City	90	79	169	68	58	126	74.56	22	21	43	25.44	5	8	13	5	8	13	100.00
Pasay City	134	81	215	0	0	0	0.00	134	81	215	100.00	36	68	104	36	68	104	100.00
**C A R**	**1,834**	**1,474**	**3,308**	**486**	**405**	**891**	**26.93**	**1,336**	**1,043**	**2,379**	**71.92**	**772**	**666**	**1,438**	**742**	**651**	**1,393**	**96.87**
Abra	222	191	413	48	50	98	23.73	169	130	299	72.40	79	61	140	77	61	138	98.57
Apayao	74	90	164	31	37	68	41.46	43	53	96	58.54	43	32	75	43	32	75	100.00
Benguet	292	219	511	68	59	127	24.85	224	160	384	75.15	107	89	196	92	81	173	88.27
Ifugao	421	293	714	165	103	268	37.54	252	186	438	61.34	108	105	213	108	105	213	100.00
Kalinga	602	474	1,076	57	58	115	10.69	544	406	950	88.29	212	176	388	204	173	377	97.16
Mt. Province	193	189	382	113	96	209	54.71	78	92	170	44.50	142	101	243	137	100	237	97.53
Baguio City	30	18	48	4	2	6	12.50	26	16	42	87.50	81	102	183	81	99	180	98.36
**Region 1**	**1,742**	**1,729**	**3,471**	**1,025**	**1,056**	**2,081**	**59.95**	**711**	**663**	**1,374**	**39.59**	**708**	**605**	**1,313**	**687**	**584**	**1,271**	**96.80**
Ilocos Norte	57	59	116	6	5	11	9.48	45	46	91	78.45	14	3	17	14	3	17	100.00
Ilocos Sur	283	250	533	24	23	47	8.82	259	225	484	90.81	126	121	247	105	100	205	83.00
La Union	158	172	330	32	31	63	19.09	126	141	267	80.91	111	73	184	111	73	184	100.00
Pangasinan	854	933	1,787	699	818	1,517	84.89	155	115	270	15.11	363	328	691	363	328	691	100.00
Alaminos City	70	53	123	58	43	101	82.11	12	10	22	17.89	18	14	32	18	14	32	100.00
Candon City	42	34	76	42	34	76	100.00	0	0	0	0.00	21	16	37	21	16	37	100.00
Dagupan City	17	20	37	0	0	0	0.00	17	20	37	100.00	12	11	23	12	11	23	100.00
Laoag City	0	0	0	0	0	0	0.00	0	0	0	0.00	0	0	0	0	0	0	0.00
San Carlos City	244	191	435	164	102	266	61.15	80	89	169	38.85	29	21	50	29	21	50	100.00
San Fernando City	0	0	0	0	0	0	0.00	0	0	0	0.00	11	9	20	11	9	20	100.00
Urdaneta City	15	10	25	0	0	0	0.00	15	10	25	100.00	0	0	0	0	0	0	0.00
Vigan City	2	7	9	0	0	0	0.00	2	7	9	100.00	3	9	12	3	9	12	100.00
**Region 2**	**1,821**	**1,644**	**3,465**	**1,327**	**1,218**	**2,545**	**73.45**	**1,492**	**1,372**	**2,864**	**82.66**	**1,103**	**949**	**2,052**	**1,082**	**941**	**2,023**	**98.59**
Batanes	5	1	6	1	1	2	33.33	3	1	4	66.67	8	7	15	5	5	10	66.67
Cagayan	259	251	510	225	220	445	87.25	162	167	329	64.51	83	56	139	74	56	130	93.53
Isabela	951	887	1,838	878	803	1,681	91.46	821	754	1,575	85.69	983	843	1,826	974	841	1,815	99.40
Nueva Vizcaya	268	195	463	171	140	311	67.17	176	153	329	71.06	10	20	30	10	16	26	86.67
Quirino	21	20	41	21	20	41	100.00	14	13	27	65.85	2	5	7	2	5	7	100.00
Cauayan City	276	253	529	1	3	4	0.76	275	250	525	99.24	1	3	4	1	3	4	100.00
Ilagan City	29	27	56	29	27	56	100.00	29	26	55	98.21	14	11	25	14	11	25	100.00
Santiago City	0	0	0	0	0	0	0.00	0	0	0	0.00	0	0	0	0	0	0	0.00
Tuguegarao City	12	10	22	1	4	5	22.73	12	8	20	90.91	2	4	6	2	4	6	100.00
**Region 3**	**6,853**	**6,627**	**13,480**	**4,297**	**4,276**	**8,573**	**63.60**	**3,089**	**2,907**	**5,996**	**44.48**	**652**	**619**	**1,271**	**579**	**568**	**1,147**	**90.24**
Aurora	38	30	68	25	20	45	66.18	23	19	42	61.76	31	27	58	29	26	55	94.83
Bataan	781	645	1,426	286	229	515	36.12	531	481	1,012	70.97	143	136	279	139	136	275	98.57
Bulacan	1,223	1,284	2,507	923	1,017	1,940	77.38	667	610	1,277	50.94	89	83	172	86	83	169	98.26
Nueva Ecija	720	665	1,385	273	206	479	34.58	237	218	455	32.85	67	57	124	47	41	88	70.97
Pampanga	260	255	515	218	212	430	83.50	40	34	74	14.37	101	88	189	96	82	178	94.18
Tarlac	1,366	1,349	2,715	1,251	1,255	2,506	92.30	116	95	211	7.77	59	66	125	58	66	124	99.20
Zambales	166	154	320	115	122	237	74.06	92	96	188	58.75	9	10	19	3	7	10	52.63
Angeles City	198	174	372	139	125	264	70.97	156	146	302	81.18	9	9	18	9	9	18	100.00
Balanga City	15	19	34	2	1	3	8.82	13	18	31	91.18	1	0	1	1	0	1	100.00
Cabanatuan City	217	232	449	210	225	435	96.88	9	16	25	5.57	0	3	3	0	3	3	100.00
City of San Fernando	52	50	102	47	43	90	88.24	8	9	17	16.67	4	5	9	4	5	9	100.00
Gapan City	183	173	356	73	71	144	40.45	109	102	211	59.27	20	15	35	20	15	35	100.00
Mabalacat City	22	14	36	7	7	14	38.89	16	8	24	66.67	5	2	7	5	2	7	100.00
Malolos City	14	19	33	9	13	22	66.67	9	13	22	66.67	0	0	0	0	0	0	0.00
Meycauayan	474	486	960	296	303	599	62.40	221	233	454	47.29	6	5	11	6	5	11	100.00
Olongapo	30	16	46	9	5	14	30.43	20	13	33	71.74	0	1	1	1	0	1	100.00
Palayan City	8	6	14	8	6	14	100.00	2	6	8	57.14	1	4	5	1	4	5	100.00
San Jose City	21	15	36	7	6	13	36.11	14	11	25	69.44	8	7	15	8	6	14	93.33
San Jose del Monte City	418	419	837	86	82	168	20.07	338	342	680	81.24	0	2	2	0	2	2	100.00
Science City of Munoz	86	63	149	11	11	22	14.77	77	53	130	87.25	20	19	39	20	19	39	100.00
Tarlac City	561	559	1,120	302	317	619	55.27	391	384	775	69.20	79	80	159	46	57	103	64.78

Table 1.C.4.2 - Management of Sick Infants and Children

Number and proportion of diarrhea cases 0-59 mos. old who received Oral Rehydration Salt (ORS) solution and ORS with Zinc drops/syrup and Pneumonia cases among children 0-59 mos. old who received treatment

Philippines, 2021

Area	Children 0-59 mos. old with Diarrhea											Children 0-59 mos. old with Pneumonia					
	Seen			Given ORS				Given ORS w/ zinc				Seen			Received treatment		
	Male	Female	Total	Male	Female	Total	%	Male	Female	Total	%	Male	Female	Total	Male	Female	Total	%
**Region 4A**	**2,771**	**2,648**	**5,419**	**2,451**	**2,306**	**4,757**	**87.78**	**1,714**	**1,574**	**3,288**	**60.68**	**836**	**860**	**1,696**	**756**	**776**	**1,532**	**90.33**
Batangas	125	121	246	69	66	135	54.88	64	59	123	50.00	45	45	90	45	44	89	98.89
Cavite	333	318	651	233	203	436	66.97	147	126	273	41.94	31	24	55	26	18	44	80.00
Laguna	145	116	261	135	103	238	91.19	85	69	154	59.00	5	6	11	3	4	7	63.64
Quezon	482	442	924	438	393	831	89.94	307	254	561	60.71	438	484	922	405	434	839	91.00
Rizal	421	403	824	399	372	771	93.57	332	333	665	80.70	83	76	159	78	71	149	93.71
Antipolo City	157	179	336	157	177	334	99.40	164	148	312	92.86	77	101	178	77	101	178	100.00
Bacoor City	104	112	216	87	91	178	82.41	78	81	159	73.61	2	2	4	2	2	4	100.00
Batangas City	62	37	99	46	35	81	81.82	49	19	68	68.69	0	0	0	0	0	0	0.00
Biñan City	244	242	486	244	242	486	100.00	125	119	244	50.21	0	0	0	0	0	0	0.00
Cabuyao City	109	124	233	94	116	210	90.13	70	87	157	67.38	0	0	0	0	0	0	0.00
Calamba City	175	184	359	164	174	338	94.15	81	84	165	45.96	10	5	15	6	4	10	66.67
Cavite City	2	5	7	2	4	6	85.71	0	0	0	0.00	0	0	0	0	0	0	0.00
Dasmariñas City	82	83	165	67	68	135	81.82	53	54	107	64.85	27	17	44	27	16	43	97.73
General Trias City	10	6	16	9	3	12	75.00	1	1	2	12.50	32	33	65	32	33	65	100.00
Imus City	70	55	125	68	48	116	92.80	2	6	8	6.40	37	19	56	7	3	10	17.86
Lipa City	2	1	3	2	1	3	100.00	0	1	1	33.33	0	1	1	0	1	1	100.00
Lucena City	183	158	341	175	150	325	95.31	133	116	249	73.02	21	26	47	21	25	46	97.87
San Pablo City	0	1	1	0	1	1	100.00	0	0	0	0.00	0	0	0	0	0	0	0.00
San Pedro City	1	0	1	1	0	1	100.00	0	0	0	0.00	0	0	0	0	0	0	0.00
Santa Rosa City	38	46	84	36	45	81	96.43	9	6	15	17.86	3	2	5	3	2	5	100.00
Tagaytay City	1	0	1	0	0	0	0.00	1	0	1	100.00	0	0	0	0	0	0	0.00
Tanauan City	3	1	4	3	1	4	100.00	1	0	1	25.00	0	0	0	0	0	0	0.00
Tayabas City	20	11	31	20	10	30	96.77	10	8	18	58.06	25	19	44	24	18	42	95.45
Trece Martires City	2	3	5	2	3	5	100.00	2	3	5	100.00	0	0	0	0	0	0	0.00
**Region 4B**	**669**	**611**	**1,280**	**520**	**474**	**994**	**77.66**	**433**	**376**	**809**	**63.20**	**199**	**172**	**371**	**189**	**161**	**350**	**94.34**
Marinduque	56	31	87	54	30	84	96.55	48	27	75	86.21	61	37	98	61	37	98	100.00
Mindoro Occidental	209	204	413	102	104	206	49.88	128	116	244	59.08	8	7	15	8	6	14	93.33
Mindoro Oriental	41	45	86	31	37	68	79.07	33	33	66	76.74	22	25	47	14	17	31	65.96
Palawan	292	258	550	284	246	530	96.36	212	195	407	74.00	71	83	154	71	82	153	99.35
Romblon	43	30	73	27	23	50	68.49	11	2	13	17.81	36	20	56	34	19	53	94.64
Puerto Princesa City	28	43	71	22	34	56	78.87	1	3	4	5.63	1	0	1	1	0	1	100.00
**Region 5**	**1,978**	**1,888**	**3,866**	**1,433**	**1,363**	**2,796**	**72.32**	**1,042**	**924**	**1,966**	**50.85**	**643**	**555**	**1,198**	**612**	**510**	**1,122**	**93.66**
Albay	567	532	1,099	476	437	913	83.08	332	278	610	55.51	145	144	289	136	127	263	91.00
Camarines Norte	289	241	530	259	223	482	90.94	204	159	363	68.49	90	69	159	87	65	152	95.60
Camarines Sur	512	488	1,000	263	255	518	51.80	240	223	463	46.30	218	193	411	213	188	401	97.57
Catanduanes	30	30	60	27	31	58	96.67	1	4	5	8.33	57	45	102	55	44	99	97.06
Masbate	293	334	627	213	243	456	72.73	144	161	305	48.64	71	53	124	68	51	119	95.97
Sorsogon	169	139	308	119	91	210	68.18	81	59	140	45.45	45	26	71	39	23	62	87.32
Iriga City	54	62	116	18	24	42	36.21	34	37	71	61.21	4	10	14	1	0	1	7.14
Legaspi City	45	43	88	39	40	79	89.77	6	3	9	10.23	3	5	8	3	2	5	62.50
Naga City	19	19	38	19	19	38	100.00	0	0	0	0.00	10	10	20	10	10	20	100.00
**Region 6**	**1,277**	**1,258**	**2,535**	**1,023**	**1,003**	**2,026**	**79.92**	**815**	**817**	**1,632**	**64.38**	**2,004**	**1,967**	**3,971**	**1,965**	**1,932**	**3,897**	**98.14**
Aklan	140	148	288	115	123	238	82.64	37	44	81	28.13	94	92	186	93	92	185	99.46
Antique	133	147	280	67	72	139	49.64	39	44	83	29.64	486	570	1,056	478	568	1,046	99.05
Capiz	35	56	91	13	15	28	30.77	34	54	88	96.70	266	247	513	265	247	512	99.81
Guimaras	1	1	2	0	0	0	0.00	1	1	2	100.00	2	2	4	1	2	3	75.00
Iloilo	178	175	353	158	156	314	88.95	92	105	197	55.81	357	361	718	357	352	709	98.75
Negros Occidental	725	670	1,395	618	585	1,203	86.24	579	540	1,119	80.22	630	564	1,194	619	547	1,166	97.65
Bacolod City	50	42	92	39	35	74	80.43	27	26	53	57.61	13	5	18	4	4	8	44.44
Iloilo City	15	19	34	13	17	30	88.24	6	3	9	26.47	156	126	282	148	120	268	95.04
**Region 7**	**2,314**	**2,304**	**4,618**	**1,454**	**1,351**	**2,805**	**60.74**	**1,742**	**1,727**	**3,469**	**75.12**	**3,672**	**3,546**	**7,218**	**3,618**	**3,492**	**7,110**	**98.50**
Bohol	1,151	1,100	2,251	1,122	1,080	2,202	97.82	794	711	1,505	66.86	946	957	1,903	939	955	1,894	99.53
Cebu	337	291	628	200	161	361	57.48	226	197	423	67.36	753	682	1,435	735	650	1,385	96.52
Negros Oriental	473	599	1,072	0	0	0	0.00	458	585	1,043	97.29	1,562	1,529	3,091	1,548	1,511	3,059	98.96
Siquijor	32	38	70	12	9	21	30.00	19	29	48	68.57	41	37	78	37	36	73	93.59
Cebu City	115	93	208	76	59	135	64.90	82	62	144	69.23	215	196	411	204	195	399	97.08
Lapu-Lapu City	203	176	379	41	36	77	20.32	162	140	302	79.68	146	135	281	146	135	281	100.00
Mandaue City	3	7	10	3	6	9	90.00	1	3	4	40.00	9	10	19	9	10	19	100.00
**Region 8**	**1,600**	**1,394**	**2,994**	**1,391**	**1,235**	**2,626**	**87.71**	**1,297**	**1,132**	**2,429**	**81.13**	**2,094**	**1,670**	**3,764**	**1,872**	**1,516**	**3,388**	**90.01**
Biliran	89	66	155	44	29	73	47.10	72	55	127	81.94	96	75	171	87	73	160	93.57
Eastern Samar	118	138	256	92	122	214	83.59	86	93	179	69.92	468	451	919	318	342	660	71.82
Northern Leyte	266	244	510	224	220	444	87.06	164	174	338	66.27	334	282	616	323	269	592	96.10
Northern Samar	601	460	1,061	593	433	1,026	96.70	537	415	952	89.73	261	183	444	251	179	430	96.85
Southern Leyte	52	38	90	9	13	22	24.44	43	24	67	74.44	78	50	128	78	50	128	100.00
Western Samar	386	378	764	359	361	720	94.24	343	325	668	87.43	668	468	1,136	626	442	1,068	94.01
Calbayog City	2	2	4	2	2	4	100.00	2	2	4	100.00	2	0	2	2	0	2	100.00
Maasin City	38	17	55	35	16	51	92.73	19	11	30	54.55	5	2	7	5	2	7	100.00
Ormoc City	19	15	34	8	6	14	41.18	11	9	20	58.82	95	75	170	95	75	170	100.00
Tacloban City	29	36	65	25	33	58	89.23	20	24	44	67.69	87	84	171	87	84	171	100.00

Table 1.C.4.2 - Management of Sick Infants and Children

Number and proportion of diarrhea cases 0-59 mos. old who received Oral Rehydration Salt (ORS) solution and ORS with Zinc drops/syrup and Pneumonia cases among children 0-59 mos. old who received treatment

Philippines, 2021

Area	Children 0-59 mos. old with Diarrhea											Children 0-59 mos. old with Pneumonia					
	Seen			Given ORS				Given ORS w/ zinc				Seen			Received treatment		
	Male	Female	Total	Male	Female	Total	%	Male	Female	Total	%	Male	Female	Total	Male	Female	Total	%
**Region 9**	**2,101**	**2,032**	**4,133**	**982**	**976**	**1,958**	**47.37**	**1,454**	**1,371**	**2,825**	**68.35**	**1,279**	**1,239**	**2,518**	**1,257**	**1,212**	**2,469**	**98.05**
Zamboanga del Norte	891	866	1,757	415	441	856	48.72	461	417	878	49.97	777	757	1,534	763	744	1,507	98.24
Zamboanga del Sur	298	279	577	268	251	519	89.95	265	246	511	88.56	311	278	589	306	271	577	97.96
Zamboanga Sibugay	170	191	361	51	63	114	31.58	114	121	235	65.10	78	97	175	76	91	167	95.43
Dapitan City	31	31	62	0	0	0	0.00	31	31	62	100.00	4	1	5	4	1	5	100.00
Dipolog City	24	19	43	14	12	26	60.47	14	7	21	48.84	12	19	31	12	19	31	100.00
Isabela City	265	242	507	187	176	363	71.60	196	179	375	73.96	71	70	141	71	70	141	100.00
Pagadian City	0	0	0	0	0	0	0.00	0	0	0	0.00	0	0	0	0	0	0	0.00
Zamboanga City	422	404	826	47	33	80	9.69	373	370	743	89.95	26	17	43	25	16	41	95.35
**Region 10**	**2,134**	**2,213**	**4,347**	**1,954**	**2,053**	**4,007**	**92.18**	**1,198**	**1,258**	**2,456**	**56.50**	**2,444**	**2,475**	**4,919**	**2,357**	**2,388**	**4,745**	**96.46**
Bukidnon	487	489	976	480	469	949	97.23	405	409	814	83.40	735	757	1,492	670	716	1,386	92.90
Camiguin	5	7	12	2	5	7	58.33	3	2	5	41.67	11	10	21	11	8	19	90.48
Lanao del Norte	314	332	646	290	330	620	95.98	177	219	396	61.30	228	225	453	224	224	448	98.90
Misamis Occidental	87	99	186	87	98	185	99.46	79	95	174	93.55	98	131	229	97	124	221	96.51
Misamis Oriental	352	328	680	299	287	586	86.18	302	295	597	87.79	285	295	580	279	286	565	97.41
Cagayan de Oro City	121	121	242	115	113	228	94.21	63	62	125	51.65	131	125	256	131	122	253	98.83
El Salvador City	1	7	8	1	7	8	100.00	0	2	2	25.00	0	0	0	0	0	0	0.00
Gingoog City	30	45	75	28	44	72	96.00	12	25	37	49.33	34	44	78	24	31	55	70.51
Iligan City	144	144	288	130	135	265	92.01	73	61	134	46.53	70	66	136	69	64	133	97.79
Malaybalay City	99	102	201	31	27	58	28.86	63	70	133	66.17	102	122	224	102	121	223	99.55
Oroquieta City	0	0	0	0	0	0	0.00	0	0	0	0.00	0	0	0	0	0	0	0.00
Ozamis City	441	498	939	441	498	939	100.00	0	0	0	0.00	677	626	1,303	677	626	1,303	100.00
Tangub City	0	0	0	0	0	0	0.00	0	0	0	0.00	0	0	0	0	0	0	0.00
Valencia City	53	41	94	50	40	90	95.74	21	18	39	41.49	73	74	147	73	66	139	94.56
**Region 11**	**1,043**	**927**	**1,970**	**398**	**351**	**749**	**38.02**	**708**	**605**	**1,313**	**66.65**	**931**	**806**	**1,737**	**856**	**723**	**1,579**	**90.90**
Davao de Oro	80	90	170	47	48	95	55.88	43	36	79	46.47	93	81	174	87	79	166	95.40
Davao del Norte	296	277	573	91	83	174	30.37	213	185	398	69.46	119	101	220	119	100	219	99.55
Davao del Sur	112	89	201	21	13	34	16.92	84	68	152	75.62	119	109	228	109	102	211	92.54
Davao Oriental	147	136	283	37	42	79	27.92	132	128	260	91.87	252	217	469	230	175	405	86.35
Davao Occidental	68	37	105	66	39	105	100.00	46	30	76	72.38	26	15	41	18	10	28	68.29
Davao City	340	298	638	136	126	262	41.07	190	158	348	54.55	322	283	605	293	257	550	90.91
**Region 12**	**2,045**	**2,007**	**4,052**	**1,688**	**1,607**	**3,295**	**81.32**	**698**	**699**	**1,397**	**34.48**	**2,553**	**2,300**	**4,853**	**2,520**	**2,255**	**4,775**	**98.39**
North Cotabato	648	680	1,328	546	545	1,091	82.15	235	271	506	38.10	812	795	1,607	793	767	1,560	97.08
Sarangani	486	445	931	474	436	910	97.74	0	0	0	0.00	330	284	614	323	278	601	97.88
South Cotabato	392	400	792	283	248	531	67.05	260	256	516	65.15	788	703	1,491	782	693	1,475	98.93
Sultan Kudarat	359	336	695	242	244	486	69.93	117	92	209	30.07	228	218	446	228	217	445	99.78
Cotabato City	24	28	52	23	26	49	94.23	1	2	3	5.77	318	246	564	318	246	564	100.00
Gen. Santos City	136	118	254	120	108	228	89.76	85	78	163	64.17	77	54	131	76	54	130	99.24
**BARMM**	**4,083**	**4,234**	**8,317**	**2,114**	**2,212**	**4,326**	**52.01**	**2,589**	**2,696**	**5,285**	**63.54**	**1,800**	**1,830**	**3,630**	**1,768**	**1,801**	**3,569**	**98.32**
Basilan	516	556	1,072	397	415	812	75.75	344	382	726	67.72	250	257	507	239	246	485	95.66
Lanao del Sur	336	375	711	309	332	641	90.15	178	182	360	50.63	83	80	163	78	75	153	93.87
Maguindanao	2,395	2,416	4,811	624	642	1,266	26.31	1,771	1,774	3,545	73.69	1,252	1,249	2,501	1,236	1,236	2,472	98.84
Sulu	326	409	735	279	352	631	85.85	235	296	531	72.24	73	105	178	73	105	178	100.00
Tawi-Tawi	0	0	0	0	0	0	0.00	0	0	0	0.00	0	0	0	0	0	0	0.00
Lamitan City	15	23	38	10	16	26	68.42	9	10	19	50.00	14	1	15	14	1	15	100.00
Marawi City	495	455	950	495	455	950	100.00	52	52	104	10.95	128	138	266	128	138	266	100.00
**CARAGA**	**683**	**621**	**1,304**	**398**	**388**	**786**	**60.28**	**296**	**238**	**534**	**40.95**	**482**	**371**	**853**	**472**	**363**	**835**	**97.89**
Agusan del Norte	61	45	106	46	35	81	76.42	29	21	50	47.17	9	5	14	1	1	2	14.29
Agusan del Sur	241	200	441	119	113	232	52.61	122	90	212	48.07	38	15	53	38	15	53	100.00
Surigao del Norte	128	125	253	78	89	167	66.01	50	34	84	33.20	130	109	239	130	109	239	100.00
Surigao del Sur	103	99	202	48	46	94	46.53	52	51	103	50.99	134	107	241	133	106	239	99.17
Province of Dinagat	23	34	57	6	10	16	28.07	17	19	36	63.16	2	4	6	2	4	6	100.00
Bislig City	46	42	88	43	39	82	93.18	3	3	6	6.82	127	95	222	126	92	218	98.20
Butuan City	33	31	64	19	23	42	65.63	14	8	22	34.38	0	0	0	0	0	0	0.00
Surigao City	48	45	93	39	33	72	77.42	9	12	21	22.58	42	36	78	42	36	78	100.00

Note: Put asterisk (*) for No Report and Zero (0) for No Case

Figure 1.C.4.1 - Percentage Distribution of Sick Infants aged 6-11 mos. given Vitamin A 100,000 IU by Region
Philippines, 2021

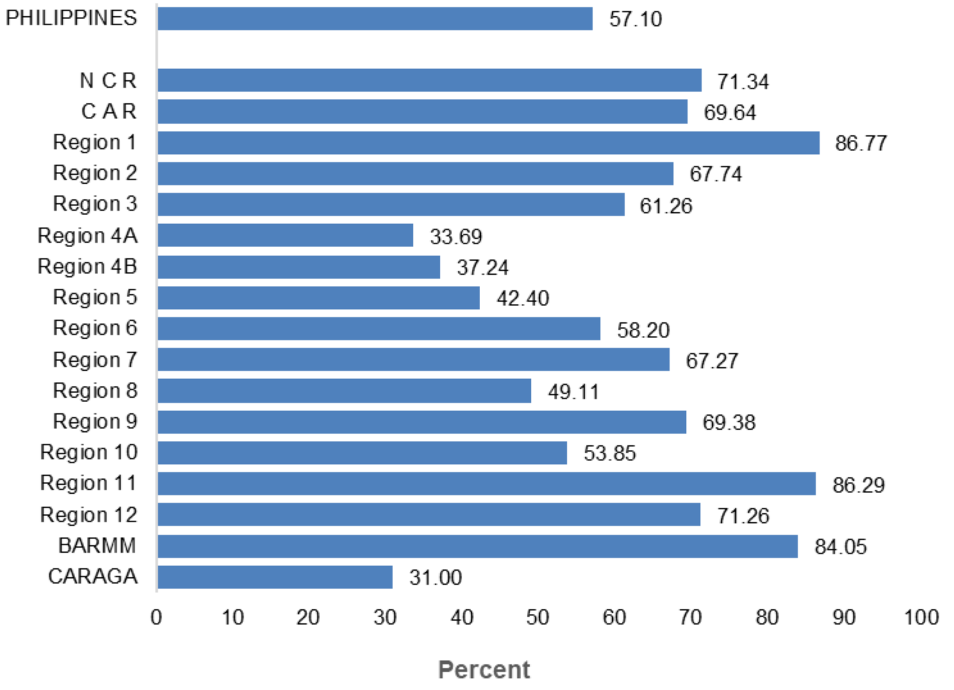

Figure 1.C.4.2 - Percentage Distribution of Sick Children aged 12-59 mos. given Vitamin A 200,000 IU by Region
Philippines, 2021

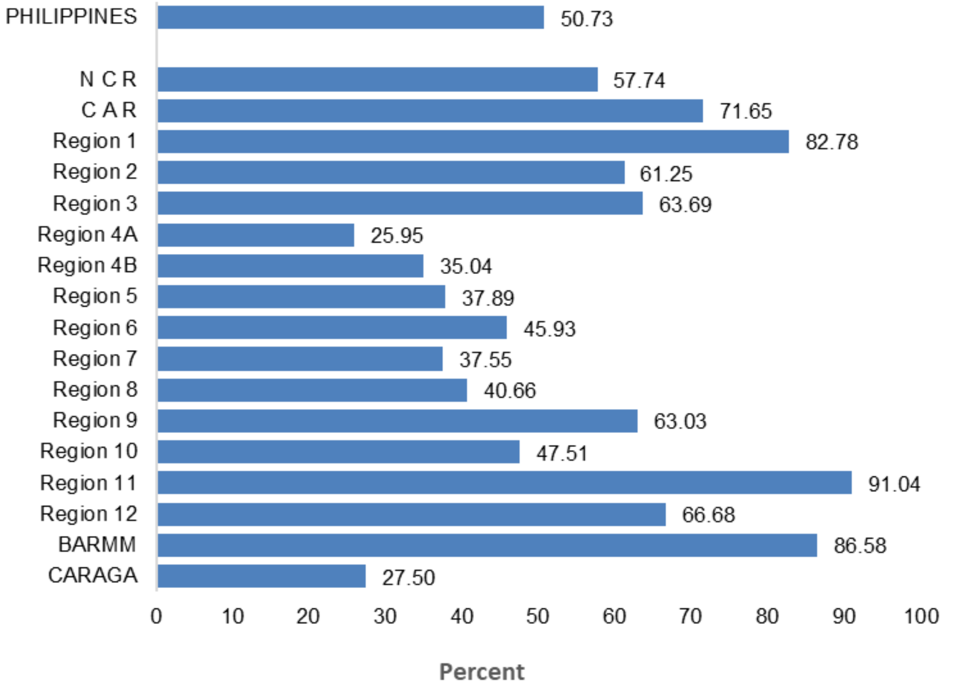

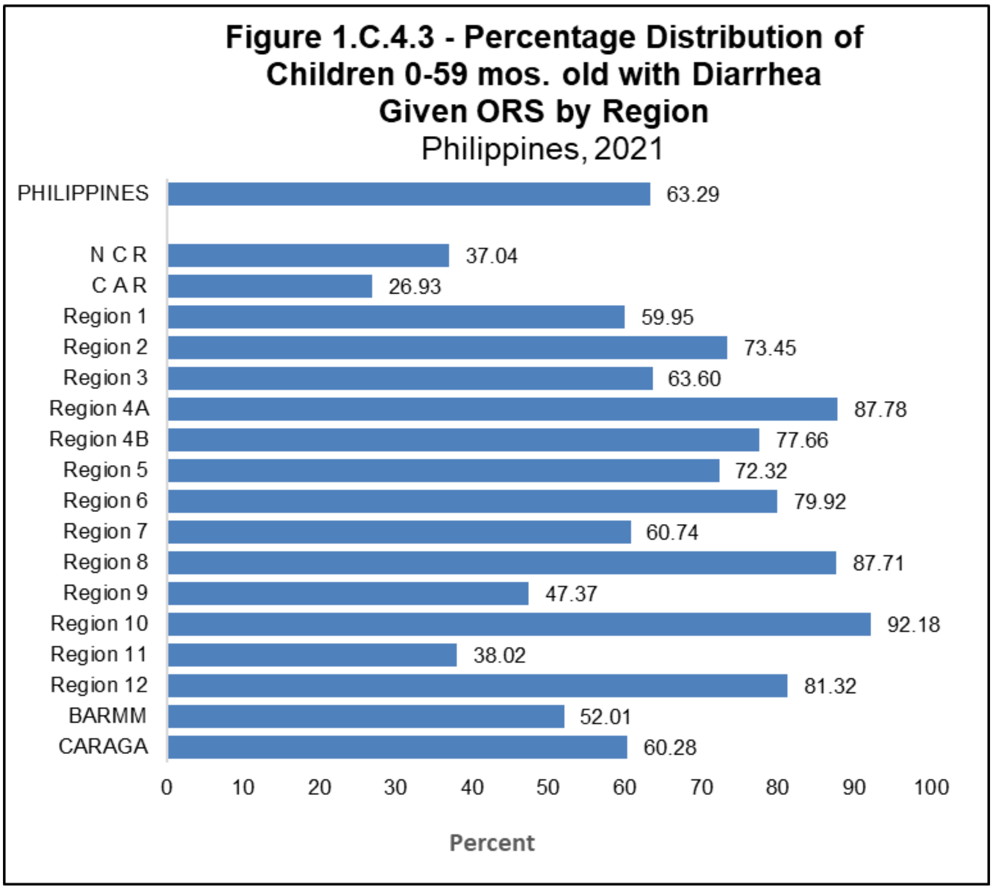
Figure 1.C.4.3 - Percentage Distribution of Children 0-59 mos. old with Diarrhea Given ORS by Region
Philippines, 2021
PHILIPPINES
N C R
C A R
Region 1
Region 2
Region 3
Region 4A
Region 4B
Region 5
Region 6
Region 7
Region 8
Region 9
Region 10
Region 11
Region 12
BARMM
CARAGA
63.29
37.04
26.93
59.95
73.45
63.60
87.78
77.66
72.32
79.92
60.74
87.71
47.37
92.18
38.02
81.32
52.01
60.28
0
10
20
30
40
50
60
70
80
90
100
Percent

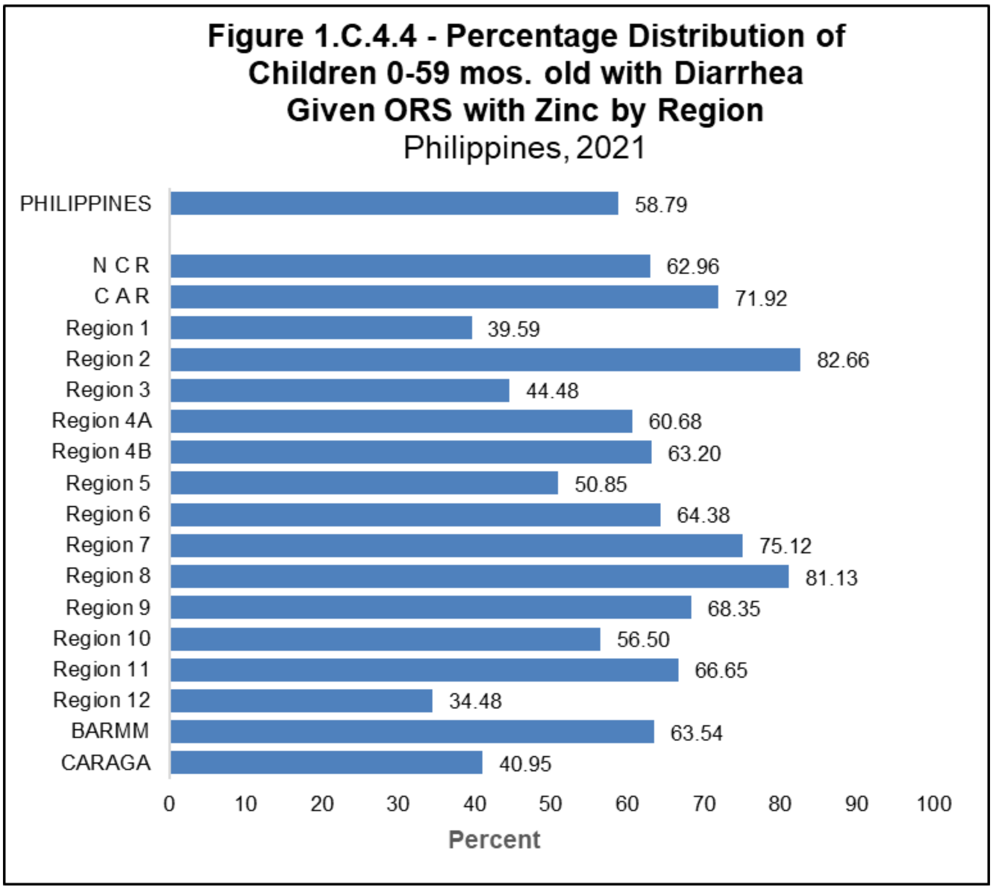
Figure 1.C.4.4 - Percentage Distribution of Children 0-59 mos. old with Diarrhea Given ORS with Zinc by Region
Philippines, 2021
PHILIPPINES
N C R
C A R
Region 1
Region 2
Region 3
Region 4A
Region 4B
Region 5
Region 6
Region 7
Region 8
Region 9
Region 10
Region 11
Region 12
BARMM
CARAGA
58.79
62.96
71.92
39.59
82.66
44.48
60.68
63.20
50.85
64.38
75.12
81.13
68.35
56.50
66.65
34.48
63.54
40.95
0
10
20
30
40
50
60
70
80
90
100
Percent

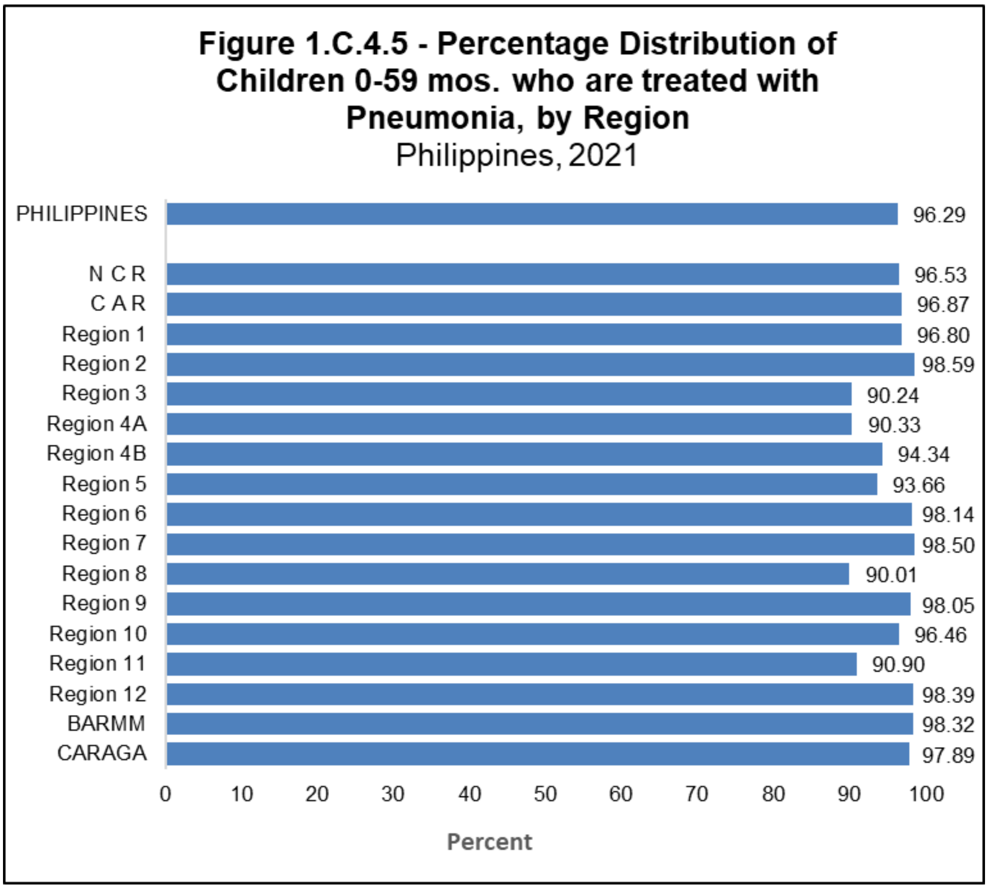
Figure 1.C.4.5 - Percentage Distribution of Children 0-59 mos. who are treated with Pneumonia, by Region
Philippines, 2021
PHILIPPINES
96.29
N C R
96.53
C A R
96.87
Region 1
96.80
Region 2
98.59
Region 3
90.24
Region 4A
90.33
Region 4B
94.34
Region 5
93.66
Region 6
98.14
Region 7
98.50
Region 8
90.01
Region 9
98.05
Region 10
96.46
Region 11
90.90
Region 12
98.39
BARMM
98.32
CARAGA
97.89
0
10
20
30
40
50
60
70
80
90
100
Percent

ORAL HEALTH CARE AND SERVICES

FHSIS ANNUAL REPORT 2021

1.D. Oral Health Care and Services

In 2021, a total of 339,117 or 18.92% children 12-59 months old were orally fit upon oral examination or after oral rehabilitation among the service target (20%) of the total estimated number of 12-59 months old children. Among the regions that reported, the highest proportion with orally fit children was NCR with 44.62% followed by Region 5 with 31.95%.

Figure 1.D.1 Proportion of Children 12-59 Months Old Who Are Orally Fit Upon Examination or After Rehabilitation by Region Philippines, 2021

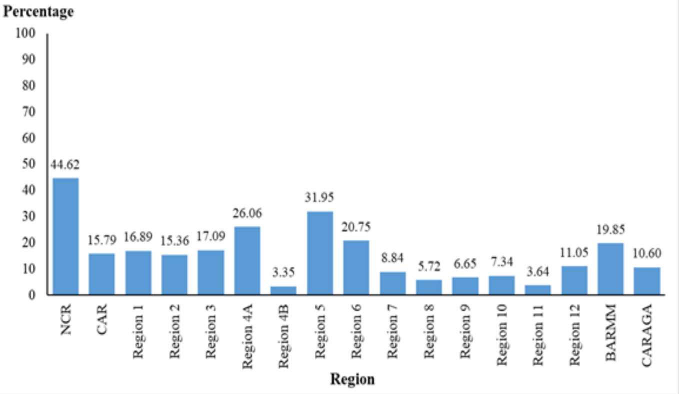

Basic Oral Health Care (BOHC) can be rendered to all age groups where a 30% service target among the eligible population concerned is observed. For 2021, the BOHC coverage for age groups of less than one year old and 1 to 4 years old was 28.62%, which is highest among all age groups. However, for the 20 to 49 years old, only 5.01% received BOHC.

Figure 1.D.2 Proportion of Basic Oral Health Care Received by Age Group Philippines, 2021

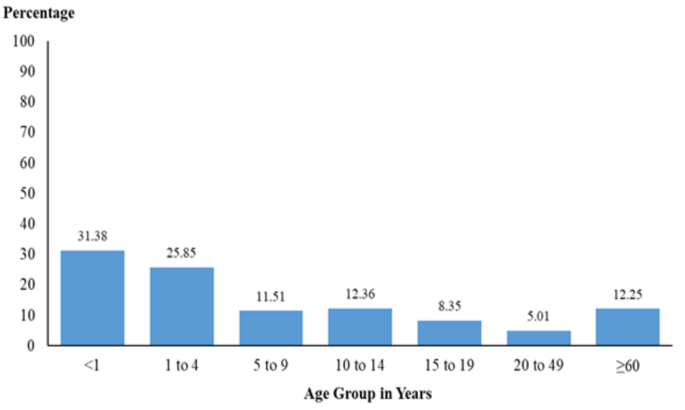

For the percentage distribution of Pregnant Women who received Basic Oral Health Care, the 20-49 years old age group availed most of the services at 10.39%.

Figure 1.D.3 Proportion of Pregnant Women Who Received Basic Oral Health Care by Age Group Philippines, 2021

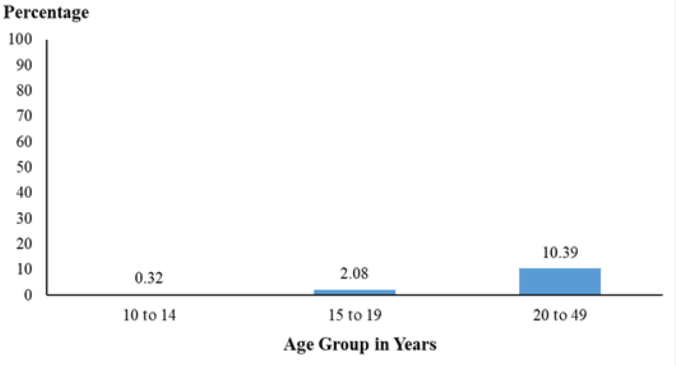

Table 1.D.1 - Oral Health Care

Number and proportion of children 12-59 months old who are orally fit upon oral examination or after oral rehabilitation

Philippines, 2021

Area	Eligible Population	Orally fit		Total	%
		Male	Female		
PHILIPPINES	1,792,507	166,386	172,731	339,117	18.92
N C R	191,157	41,964	43,323	85,287	44.62
Malabon	5,426	145	152	297	5.47
Navotas	3,704	245	287	532	14.36
Valenzuela City	9,210	1,467	1,538	3,005	32.63
Caloocan City	23,512	4,933	5,244	10,177	43.28
Marikina City	6,690	104	103	207	3.09
Pasig City	11,212	3,225	3,374	6,599	58.86
Pateros	948	13	13	26	2.74
Taguig	11,948	505	499	1,004	8.40
Quezon City	43,586	13,596	13,732	27,328	62.70
Makati City	8,648	1,595	1,780	3,375	39.02
Mandaluyong City	5,735	1,971	1,958	3,929	68.51
San Juan	1,813	474	500	974	53.71
Manila City	26,425	7,907	8,255	16,162	61.16
Las Piñas City	8,741	2,447	2,426	4,873	55.75
Muntinlupa City	7,490	1,228	1,244	2,472	33.00
Parañaque City	9,884	2,087	2,203	4,290	43.40
Pasay City	6,184	22	15	37	0.60
C A R	29,424	2,353	2,293	4,646	15.79
Abra	3,809	76	83	159	4.17
Apayao	2,200	267	263	530	24.09
Benguet	7,250	1,526	1,530	3,056	42.15
Ifugao	3,854	81	48	129	3.35
Kalinga	3,981	67	80	147	3.69
Mt. Province	2,720	95	74	169	6.21
Baguio City	5,610	241	215	456	8.13
Region 1	81,478	6,636	7,127	13,763	16.89
Ilocos Norte	6,501	597	619	1,216	18.70
Ilocos Sur	8,040	956	1,045	2,001	24.89
La Union	9,662	2,726	2,559	5,285	54.70
Pangasinan	42,082	1,711	2,220	3,931	9.34
Alaminos City	1,589	220	250	470	29.57
Candon City	847	78	72	150	17.71
Dagupan City	3,035	82	72	154	5.07

Table 1.D.1 - Oral Health Care

Number and proportion of children 12-59 months old who are orally fit upon oral examination or after oral rehabilitation

Philippines, 2021

Area	Eligible Population	Orally fit		Total	%
		Male	Female		
Laoag City	1,500	0	0	0	0.00
San Carlos City	3,343	121	120	241	7.21
San Fernando City	1,770	27	21	48	2.71
Urdaneta City	2,356	6	5	11	0.47
Vigan City	753	112	144	256	33.99
**Region 2**	58,122	4,284	4,643	8,927	15.36
Batanes	315	134	112	246	78.10
Cagayan	17,265	650	683	1,333	7.72
Isabela	19,691	787	819	1,606	8.16
Nueva Vizcaya	8,053	1,020	1,208	2,228	27.67
Quirino	3,447	54	70	124	3.60
Cauayan City	2,154	194	260	454	21.08
Ilagan City	2,420	1,191	1,223	2,414	99.74
Santiago City	2,243	254	268	522	23.28
Tuguegarao City	2,535	0	0	0	0.00
**Region 3**	182,718	15,280	15,952	31,232	17.09
Aurora	4,091	236	250	486	11.88
Bataan	10,961	339	326	665	6.07
Bulacan	36,267	826	840	1,666	4.59
Nueva Ecija	24,413	5,344	5,455	10,799	44.24
Pampanga	26,288	1,693	1,719	3,412	12.98
Tarlac	16,172	2,568	2,642	5,210	32.22
Zambales	10,194	757	835	1,592	15.62
Angeles City	6,595	0	0	0	0.00
Balanga City	1,584	223	257	480	30.31
Cabanatuan City	4,996	223	264	487	9.75
City of San Fernando	4,913	36	28	64	1.30
Gapan City	1,823	19	26	45	2.47
Mabalacat City	4,019	1,433	1,629	3,062	76.20
Malolos City	4,050	196	196	392	9.68
Meycauayan	3,360	461	485	946	28.16
Olongapo	4,021	0	0	0	0.00
Palayan City	679	16	14	30	4.42
San Jose City	2,311	351	384	735	31.81
San Jose del Monte City	9,226	119	159	278	3.01
Science City of Munoz	1,347	53	75	128	9.50
Tarlac City	5,411	387	368	755	13.95
**Region 4A**	246,745	32,296	32,005	64,301	26.06
Batangas	32,744	2,116	2,188	4,304	13.14

Table 1.D.1 - Oral Health Care

Number and proportion of children 12-59 months old who are orally fit upon oral examination or after oral rehabilitation

Philippines, 2021

Area	Eligible Population	Orally fit		Total	%
		Male	Female		
Cavite	22,985	84	102	186	0.81
Laguna	16,116	9,333	9,500	18,833	116.86
Quezon	31,920	2,057	1,951	4,008	12.56
Rizal	36,392	1,367	1,427	2,794	7.68
Antipolo City	13,404	14,002	13,468	27,470	204.94
Bacoor City	10,069	4	5	9	0.09
Batangas City	5,811	160	142	302	5.20
Biñan City	5,404	208	189	397	7.35
Cabuyao City	5,009	98	126	224	4.47
Calamba City	7,374	757	685	1,442	19.56
Cavite City	1,722	1	0	1	0.06
Dasmariñas City	11,050	0	0	0	0.00
General Trias City	5,270	0	0	0	0.00
Imus City	6,770	169	176	345	5.10
Lipa City	5,855	38	38	76	1.30
Lucena City	4,837	4	16	20	0.41
San Pablo City	4,317	97	120	217	5.03
San Pedro City	5,286	298	325	623	11.79
Santa Rosa City	5,740	560	618	1,178	20.52
Tagaytay City	1,193	4	9	13	1.09
Tanauan City	3,054	232	275	507	16.60
Tayabas City	1,813	707	645	1,352	74.58
Trece Martires City	2,611	0	0	0	0.00
**Region 4B**	60,334	1,014	1,006	2,020	3.35
Marinduque	4,403	238	215	453	10.29
Mindoro Occidental	10,101	0	0	0	0.00
Mindoro Oriental	16,796	417	460	877	5.22
Palawan	17,912	251	235	486	2.71
Romblon	5,743	108	96	204	3.55
Puerto Princesa City	5,379	0	0	0	0.00
**Region 5**	118,247	18,783	18,996	37,779	31.95
Albay	20,790	3,925	3,983	7,908	38.04
Camarines Norte	12,021	2,181	2,152	4,333	36.04
Camarines Sur	33,529	3,676	3,340	7,016	20.92
Catanduanes	5,389	1,506	1,520	3,026	56.16
Masbate	19,915	2,256	2,595	4,851	24.36
Sorsogon	16,672	3,944	3,992	7,936	47.60
Iriga City	2,278	329	378	707	31.03
Legaspi City	3,657	691	747	1,438	39.33
Naga City	3,996	275	289	564	14.11

Table 1.D.1 - Oral Health Care

Number and proportion of children 12-59 months old who are orally fit upon oral examination or after oral rehabilitation

Philippines, 2021

Area	Eligible Population	Orally fit		Total	%
		Male	Female		
**Region 6**	124,756	13,283	12,601	25,884	20.75
Aklan	9,625	350	407	757	7.86
Antique	10,905	166	180	346	3.17
Capiz	12,011	1,248	1,355	2,603	21.67
Guimaras	2,901	0	0	0	0.00
Iloilo	30,624	9,114	9,685	18,799	61.39
Negros Occidental	42,128	425	358	783	1.86
Bacolod City	9,479	143	173	316	3.33
Iloilo City	7,084	1,837	443	2,280	32.19
**Region 7**	130,152	5,557	5,945	11,502	8.84
Bohol	23,293	326	320	646	2.77
Cebu	52,260	4,747	5,090	9,837	18.82
Negros Oriental	22,980	316	372	688	2.99
Siquijor	1,509	14	11	25	1.66
Cebu City	16,406	126	126	252	1.54
Lapu-Lapu City	7,257	24	23	47	0.65
Mandaue City	6,449	4	3	7	0.11
**Region 8**	87,830	2,569	2,457	5,026	5.72
Biliran	3,378	91	65	156	4.62
Eastern Samar	9,470	283	268	551	5.82
Northern Leyte	29,011	799	788	1,587	5.47
Northern Samar	13,728	224	274	498	3.63
Southern Leyte	5,850	37	44	81	1.38
Western Samar	12,322	814	751	1,565	12.70
Calbayog City	3,797	30	47	77	2.03
Maasin City	1,490	71	74	145	9.73
Ormoc City	4,132	220	146	366	8.86
Tacloban City	4,652	0	0	0	0.00
**Region 9**	67,468	2,088	2,400	4,488	6.65
Zamboanga del Norte	14,808	514	582	1,096	7.40
Zamboanga del Sur	14,313	1,201	1,369	2,570	17.96
Zamboanga Sibugay	13,251	174	237	411	3.10
Dapitan City	1,529	171	173	344	22.50
Dipolog City	2,426	1	3	4	0.16
Isabela City	2,430	2	0	2	0.08

Table 1.D.1 - Oral Health Care

Number and proportion of children 12-59 months old who are orally fit upon oral examination or after oral rehabilitation

Philippines, 2021

Area	Eligible Population	Orally fit		Total	%
		Male	Female		
Pagadian City	3,511	25	36	61	1.74
Zamboanga City	15,199	0	0	0	0.00
**Region 10**	86,373	2,368	3,969	6,337	7.34
Bukidnon	19,782	389	513	902	4.56
Camiguin	1,599	4	4	8	0.50
Lanao del Norte	13,491	85	97	182	1.35
Misamis Occidental	5,491	799	2,150	2,949	53.70
Misamis Oriental	12,589	477	529	1,006	7.99
Cagayan de Oro City	11,925	0	0	0	0.00
El Salvador City	885	4	7	11	1.24
Gingoog City	2,200	143	159	302	13.73
Iligan City	6,835	13	9	22	0.32
Malaybalay City	3,297	309	342	651	19.74
Oroquieta City	1,190	0	0	0	0.00
Ozamis City	2,385	5	4	9	0.38
Tangub City	1,060	68	70	138	13.02
Valencia City	3,643	72	85	157	4.31
**Region 11**	90,125	1,405	1,875	3,280	3.64
Davao de Oro	13,605	324	480	804	5.91
Davao del Norte	17,412	305	282	587	3.37
Davao Oriental	11,333	296	442	738	6.51
Davao del Sur	11,636	140	201	341	2.93
Davao Occidental	6,097	90	132	222	3.64
Davao City	30,042	250	338	588	1.96
**Region 12**	87,592	4,642	5,036	9,678	11.05
North Cotabato	28,031	1,873	1,900	3,773	13.46
Sarangani	10,927	865	919	1,784	16.33
South Cotabato	16,978	320	364	684	4.03
Sultan Kudarat	14,962	1,371	1,635	3,006	20.09
Cotabato City	5,668	75	79	154	2.72
Gen. Santos City	11,026	138	139	277	2.51
**BARMM**	98,019	9,406	10,052	19,458	19.85
Basilan	7,231	62	67	129	1.78
Lanao del Sur	22,314	464	644	1,108	4.97
Maguindanao	30,295	4,269	4,627	8,896	29.36
Sulu	21,804	4,494	4,572	9,066	41.58

Table 1.D.1 - Oral Health Care

Number and proportion of children 12-59 months old who are orally fit upon oral examination or after oral rehabilitation

Philippines, 2021

Area	Eligible Population	Orally fit		Total	%
		Male	Female		
Tawi-Tawi	9,048	0	0	0	0.00
Lamitan City	1,990	0	0	0	0.00
Marawi City	5,338	117	142	259	4.85
**CARAGA**	51,966	2,458	3,051	5,509	10.60
Agusan del Norte	6,835	308	699	1,007	14.73
Agusan del Sur	15,418	297	414	711	4.61
Surigao del Norte	6,399	278	314	592	9.25
Surigao del Sur	9,738	607	635	1,242	12.75
Province of Dinagat	2,248	178	197	375	16.68
Bislig City	1,850	29	19	48	2.59
Butuan City	6,499	452	511	963	14.82
Surigao City	2,980	309	262	571	19.16

Note: Put asterisk (*) for No Report and Zero (0) for No Case

Table 1.D.2 - Oral Health Care

Number and Proportion of Children 5 years old and above with Cases of Decayed, Missing, Filled Teeth (DMFT)
Philippines, 2021

Area	Total Examined	Cases of 5 y/o and above with DMFT		Total with DMFT	%
		Male	Female		
**PHILIPPINES**	**1,287,967**	**418,937**	**659,667**	**1,078,604**	**83.74**
**N C R**	**406,560**	**145,262**	**261,298**	**406,560**	**100.00**
Malabon	4,946	2,185	2,761	4,946	100.00
Navotas	605	295	310	605	100.00
Valenzuela City	0	0	0	0	0.00
Caloocan City	57,510	22,499	35,011	57,510	100.00
Marikina City	0	0	0	0	0.00
Pasig City	46,859	18,003	28,856	46,859	100.00
Pateros	94	46	48	94	100.00
Taguig	2,753	1,147	1,606	2,753	100.00
Quezon City	29,617	5,600	24,017	29,617	100.00
Makati City	5,773	1,978	3,795	5,773	100.00
Mandaluyong City	6,466	1,726	4,740	6,466	100.00
San Juan	1,175	490	685	1,175	100.00
Manila City	26,554	7,920	18,634	26,554	100.00
Las Piñas City	0	0	0	0	0.00
Muntinlupa City	221,513	82,110	139,403	221,513	100.00
Parañaque City	1,271	648	623	1,271	100.00
Pasay City	1,424	615	809	1,424	100.00
**C A R**	**43,957**	**7,531**	**14,794**	**22,325**	**50.79**
Abra	1,588	489	699	1,188	74.81
Apayao	1,004	399	489	888	88.45
Benguet	11,259	2,524	4,998	7,522	66.81
Ifugao	2,741	788	1,543	2,331	85.04
Kalinga	462	78	275	353	76.41
Mt. Province	3,342	286	491	777	23.25
Baguio City	23,561	2,967	6,299	9,266	39.33
**Region 1**	**159,197**	**29,301**	**38,132**	**67,433**	**42.36**
Ilocos Norte	12,531	568	743	1,311	10.46
Ilocos Sur	47,971	2,756	3,582	6,338	13.21
La Union	34,106	801	926	1,727	5.06
Pangasinan	48,609	22,285	28,344	50,629	104.16
Alaminos City	5,235	2,255	2,980	5,235	100.00
Candon City	2,321	34	45	79	3.40
Dagupan City	2,158	273	1,069	1,342	62.19
Laoag City	458	0	0	0	0.00
San Carlos City	299	144	155	299	100.00
San Fernando City	2,400	5	1	6	0.25
Urdaneta City	25	11	14	25	100.00
Vigan City	3,084	169	273	442	14.33

Table 1.D.2 - Oral Health Care

Number and Proportion of Children 5 years old and above with Cases of Decayed, Missing, Filled Teeth (DMFT)
Philippines, 2021

Area	Total Examined	Cases of 5 y/o and above with DMFT		Total with DMFT	%
		Male	Female		
**Region 2**	**67,573**	**26,114**	**41,459**	**67,573**	**100.00**
Batanes	0	0	0	0	0.00
Cagayan	1,902	567	1,335	1,902	100.00
Isabela	24,358	9,876	14,482	24,358	100.00
Nueva Vizcaya	26,233	9,060	17,173	26,233	100.00
Quirino	183	80	103	183	100.00
Cauayan City	733	280	453	733	100.00
Ilagan City	13,781	6,089	7,692	13,781	100.00
Santiago City	383	162	221	383	100.00
Tuguegarao City	0	0	0	0	0.00
**Region 3**	**63,434**	**26,503**	**35,178**	**61,681**	**97.24**
Aurora	1,373	606	767	1,373	100.00
Bataan	964	377	587	964	100.00
Bulacan	4,111	1,209	2,902	4,111	100.00
Nueva Ecija	10,803	5,256	5,547	10,803	100.00
Pampanga	3,212	1,393	1,431	2,824	87.92
Tarlac	15,325	6,301	9,024	15,325	100.00
Zambales	3,406	1,224	1,859	3,083	90.52
Angeles City	0	0	0	0	0.00
Balanga City	1	1	0	1	100.00
Cabanatuan City	313	147	166	313	100.00
City of San Fernando	232	67	165	232	100.00
Gapan City	102	45	57	102	100.00
Mabalacat City	13,125	5,754	6,889	12,643	96.33
Malolos City	4	4	0	4	100.00
Meycauayan	372	203	169	372	100.00
Olongapo	0	0	0	0	0.00
Palayan City	334	161	173	334	100.00
San Jose City	1,442	540	902	1,442	100.00
San Jose del Monte City	417	55	55	110	26.38
Science City of Munoz	1,783	442	1,154	1,596	89.51
Tarlac City	6,115	2,718	3,331	6,049	98.92
**Region 4A**	**123,101**	**53,650**	**67,095**	**120,745**	**98.09**
Batangas	79,038	37,108	41,209	78,317	99.09
Cavite	5,257	2,184	3,071	5,255	99.96
Laguna	5,242	2,215	3,027	5,242	100.00
Quezon	4,204	948	2,617	3,565	84.80
Rizal	7,366	2,705	4,661	7,366	100.00
Antipolo City	0	0	0	0	0.00
Bacoor City	1,943	648	1,295	1,943	100.00
Batangas City	2,256	983	1,258	2,241	99.34
Biñan City	2,234	949	1,285	2,234	100.00
Cabuyao City	1,620	657	963	1,620	100.00

Table 1.D.2 - Oral Health Care

Number and Proportion of Children 5 years old and above with Cases of Decayed, Missing, Filled Teeth (DMFT)
Philippines, 2021

Area	Total Examined	Cases of 5 y/o and above with DMFT		Total with DMFT	%
		Male	Female		
Calamba City	2,415	983	1,432	2,415	100.00
Cavite City	0	0	0	0	0.00
Dasmariñas City	0	0	0	0	0.00
General Trias City	0	0	0	0	0.00
Imus City	2,150	594	1,556	2,150	100.00
Lipa City	105	58	47	105	100.00
Lucena City	18	5	5	10	55.56
San Pablo City	1,896	830	1,066	1,896	100.00
San Pedro City	1,733	695	1,038	1,733	100.00
Santa Rosa City	36	16	20	36	100.00
Tagaytay City	440	146	260	406	92.27
Tanauan City	1,975	372	666	1,038	52.56
Tayabas City	0	0	0	0	0.00
Trece Martires City	3,173	1,554	1,619	3,173	100.00
**Region 4B**	**3,611**	**1,304**	**2,219**	**3,523**	**97.56**
Marinduque	706	217	453	670	94.90
Mindoro Occidental	0	0	0	0	0.00
Mindoro Oriental	0	0	0	0	0.00
Palawan	1,505	563	942	1,505	100.00
Romblon	1,400	524	824	1,348	96.29
Puerto Princesa City	0	0	0	0	0.00
**Region 5**	**114,971**	**23,040**	**39,856**	**62,896**	**54.71**
Albay	16,896	3,050	7,429	10,479	62.02
Camarines Norte	11,086	2,842	4,449	7,291	65.77
Camarines Sur	54,965	7,185	13,651	20,836	37.91
Catanduanes	4,017	1,639	2,248	3,887	96.76
Masbate	16,784	5,290	7,709	12,999	77.45
Sorsogon	3,878	1,210	1,482	2,692	69.42
Iriga City	2,437	792	1,519	2,311	94.83
Legaspi City	1,762	729	1,033	1,762	100.00
Naga City	3,146	303	336	639	20.31
**Region 6**	**121,682**	**29,287**	**47,753**	**77,040**	**63.31**
Aklan	2,808	863	1,621	2,484	88.46
Antique	8,439	2,073	2,746	4,819	57.10
Capiz	9,261	2,678	4,015	6,693	72.27
Guimaras	30	7	7	14	46.67
Iloilo	75,720	21,423	33,700	55,123	72.80
Negros Occidental	8,770	260	842	1,102	12.57
Bacolod City	11,303	1,119	1,868	2,987	26.43
Iloilo City	5,351	864	2,954	3,818	71.35

Table 1.D.2 - Oral Health Care

Number and Proportion of Children 5 years old and above with Cases of Decayed, Missing, Filled Teeth (DMFT)
Philippines, 2021

Area	Total Examined	Cases of 5 y/o and above with DMFT		Total with DMFT	%
		Male	Female		
**Region 7**	**30,157**	**12,011**	**19,170**	**31,181**	**103.40**
Bohol	0	654	1,054	1,708	0.00
Cebu	23,680	9,615	14,065	23,680	100.00
Negros Oriental	698	34	40	74	10.60
Siquijor	266	92	161	253	95.11
Cebu City	5,340	1,567	3,773	5,340	100.00
Lapu-Lapu City	47	0	0	0	0.00
Mandaue City	126	49	77	126	100.00
**Region 8**	**27,292**	**4,133**	**7,487**	**11,620**	**42.58**
Biliran	11,714	186	239	425	3.63
Eastern Samar	54	13	9	22	40.74
Northern Leyte	9,592	3,049	6,106	9,155	95.44
Northern Samar	4,296	528	669	1,197	27.86
Southern Leyte	0	0	0	0	0.00
Western Samar	593	53	49	102	17.20
Calbayog City	406	183	223	406	100.00
Maasin City	345	111	183	294	85.22
Ormoc City	0	0	0	0	0.00
Tacloban City	292	10	9	19	6.51
**Region 9**	**0**	**6,114**	**7,790**	**13,904**	**0.00**
Zamboanga del Norte	0	161	235	396	0.00
Zamboanga del Sur	0	2,798	3,156	5,954	0.00
Zamboanga Sibugay	0	2,380	3,304	5,684	0.00
Dapitan City	0	151	208	359	0.00
Dipolog City	0	592	848	1,440	0.00
Isabela City	0	32	39	71	0.00
Pagadian City	0	0	0	0	0.00
Zamboanga City	0	0	0	0	0.00
**Region 10**	**35,777**	**11,661**	**22,056**	**33,717**	**94.24**
Bukidnon	5,892	2,242	3,650	5,892	100.00
Camiguin	2,099	20	19	39	1.86
Lanao del Norte	3,840	1,083	2,757	3,840	100.00
Misamis Occidental	5,406	1,942	3,464	5,406	100.00
Misamis Oriental	1,297	456	841	1,297	100.00
Cagayan de Oro City	0	0	0	0	0.00
El Salvador City	0	0	0	0	0.00
Gingoog City	257	137	120	257	100.00
Iligan City	9	4	5	9	100.00
Malaybalay City	276	119	157	276	100.00
Oroquieta City	0	0	0	0	0.00
Ozamis City	3	1	2	3	100.00

Table 1.D.2 - Oral Health Care

Number and Proportion of Children 5 years old and above with Cases of Decayed, Missing, Filled Teeth (DMFT)
Philippines, 2021

Area	Total Examined	Cases of 5 y/o and above with DMFT		Total with DMFT	%
		Male	Female		
Tangub City	300	142	158	300	100.00
Valencia City	16,398	5,515	10,883	16,398	100.00
**Region 11**	**1,878**	**816**	**877**	**1,693**	**90.15**
Davao de Oro	504	223	230	453	89.88
Davao del Norte	376	158	169	327	86.97
Davao Oriental	269	106	139	245	91.08
Davao del Sur	196	78	89	167	85.20
Davao Occidental	97	39	48	87	89.69
Davao City	436	212	202	414	94.95
**Region 12**	**43,339**	**16,758**	**22,532**	**39,290**	**90.66**
North Cotabato	10,914	3,931	6,983	10,914	100.00
Sarangani	4,012	1,393	1,302	2,695	67.17
South Cotabato	1,233	296	937	1,233	100.00
Sultan Kudarat	13,037	5,758	7,279	13,037	100.00
Cotabato City	13,752	5,246	5,774	11,020	80.13
Gen. Santos City	391	134	257	391	100.00
**BARMM**	**25,932**	**18,554**	**19,363**	**37,917**	**146.22**
Basilan	92	46	46	92	100.00
Lanao del Sur	2,540	1,916	2,401	4,317	169.96
Maguindanao	23,252	11,566	11,686	23,252	100.00
Sulu	0	4,977	5,176	10,153	0.00
Tawi-Tawi	0	0	0	0	0.00
Lamitan City	0	0	0	0	0.00
Marawi City	48	49	54	103	214.58
**CARAGA**	**19,506**	**6,898**	**12,608**	**19,506**	**100.00**
Agusan del Norte	3,225	1,222	2,003	3,225	100.00
Agusan del Sur	4,251	1,727	2,524	4,251	100.00
Surigao del Norte	632	259	373	632	100.00
Surigao del Sur	7,663	1,969	5,694	7,663	100.00
Province of Dinagat	237	118	119	237	100.00
Bislig City	36	19	17	36	100.00
Butuan City	1,336	640	696	1,336	100.00
Surigao City	2,126	944	1,182	2,126	100.00

Note: Put asterisk (*) for No Report and Zero (0) for No Case

Table 1.D.3 - Oral Health Care

Number and proportion of infants 0-11 months old who received Basic Oral Health Care (BOHC)

Philippines, 2021

Area	Infants 0-11 months old who received BOHC				
	Eligible Population	Male	Female	Total	%
**PHILIPPINES**	**638,450**	**99,328**	**101,009**	**200,337**	**31.38**
**N C R**	**71,376**	**27,745**	**27,894**	**55,639**	**77.95**
Malabon	2,027	257	263	520	25.65
Navotas	1,382	345	432	777	56.22
Valenzuela City	3,440	1,685	1,753	3,438	99.96
Caloocan City	8,781	4,210	4,569	8,779	99.98
Marikina City	2,499	92	69	161	6.44
Pasig City	4,186	2,216	1,970	4,186	100.00
Pateros	354	176	178	354	99.92
Taguig	4,462	236	221	457	10.24
Quezon City	16,274	8,347	7,925	16,272	99.99
Makati City	3,230	860	788	1,648	51.03
Mandaluyong City	2,141	1,096	1,045	2,141	100.00
San Juan	676	354	323	677	100.12
Manila City	9,866	4,761	5,105	9,866	100.00
Las Piñas City	3,264	891	896	1,787	54.75
Muntinlupa City	2,795	1,318	1,479	2,797	100.07
Parañaque City	3,689	901	878	1,779	48.22
Pasay City	2,310	0	0	0	0.00
**C A R**	**10,543**	**1,716**	**1,521**	**3,237**	**30.70**
Abra	1,286	333	265	598	46.50
Apayao	743	84	77	161	21.67
Benguet	2,742	737	740	1,477	53.87
Ifugao	1,332	28	24	52	3.90
Kalinga	1,398	36	29	65	4.65
Mt. Province	920	76	58	134	14.57
Baguio City	2,123	422	328	750	35.34
**Region 1**	**29,159**	**4,989**	**5,207**	**10,196**	**34.97**
Ilocos Norte	2,396	356	339	695	29.01
Ilocos Sur	2,759	506	540	1,046	37.91
La Union	3,429	1,106	1,122	2,228	64.98
Pangasinan	15,136	1,128	1,377	2,505	16.55
Alaminos City	572	182	215	397	69.47
Candon City	293	27	26	53	18.10
Dagupan City	1,091	203	191	394	36.12
Laoag City	554	0	0	0	0.00
San Carlos City	1,200	151	171	322	26.84
San Fernando City	628	30	22	52	8.29
Urdaneta City	846	0	0	0	0.00
Vigan City	258	1,300	1,204	2,504	970.54

Table 1.D.3 - Oral Health Care

Number and proportion of infants 0-11 months old who received Basic Oral Health Care (BOHC)

Philippines, 2021

Area	Infants 0-11 months old who received BOHC				
	Eligible Population	Male	Female	Total	%
**Region 2**	**20,768**	**3,823**	**4,287**	**8,110**	**39.05**
Batanes	105	0	0	0	0.00
Cagayan	6,169	228	194	422	6.84
Isabela	7,063	1,394	1,706	3,100	43.89
Nueva Vizcaya	2,867	1,219	1,299	2,518	87.84
Quirino	1,211	24	24	48	3.96
Cauayan City	773	147	204	351	45.42
Ilagan City	870	420	446	866	99.51
Santiago City	805	391	414	805	100.01
Tuguegarao City	905	0	0	0	0.00
**Region 3**	**66,061**	**10,846**	**11,466**	**22,312**	**33.78**
Aurora	1,429	232	279	511	35.75
Bataan	4,144	57	36	93	2.24
Bulacan	13,117	1,018	913	1,931	14.72
Nueva Ecija	8,675	1,032	1,111	2,143	24.70
Pampanga	9,451	1,197	1,029	2,226	23.55
Tarlac	5,870	1,438	1,562	3,000	51.11
Zambales	3,765	520	482	1,002	26.61
Angeles City	2,372	0	0	0	0.00
Balanga City	599	91	123	214	35.74
Cabanatuan City	1,776	21	23	44	2.48
City of San Fernando	1,767	41	51	92	5.21
Gapan City	648	209	226	435	67.16
Mabalacat City	1,443	454	505	959	66.44
Malolos City	1,466	398	473	871	59.43
Meycauayan	1,215	2	2	4	0.33
Olongapo	1,486	0	0	0	0.00
Palayan City	242	0	0	0	0.00
San Jose City	821	810	909	1,719	209.43
San Jose del Monte City	3,336	3,014	3,397	6,411	192.18
Science City of Munoz	478	3	8	11	2.30
Tarlac City	1,963	309	337	646	32.91
**Region 4A**	**89,519**	**8,369**	**8,211**	**16,580**	**18.52**
Batangas	11,654	1,409	1,413	2,822	24.21
Cavite	8,384	42	39	81	0.97
Laguna	5,918	113	100	213	3.60
Quezon	11,293	1,416	1,389	2,805	24.84
Rizal	13,448	3,981	3,865	7,846	58.34
Antipolo City	4,953	0	0	0	0.00
Bacoor City	3,676	0	0	0	0.00
Batangas City	2,069	571	586	1,157	55.93
Biñan City	1,982	40	40	80	4.04
Cabuyao City	1,838	37	37	74	4.03

Table 1.D.3 - Oral Health Care

Number and proportion of infants 0-11 months old who received Basic Oral Health Care (BOHC)

Philippines, 2021

Area	Infants 0-11 months old who received BOHC				
	Eligible Population	Male	Female	Total	%
Calamba City	2,708	55	54	109	4.03
Cavite City	629	0	0	0	0.00
Dasmariñas City	4,033	0	0	0	0.00
General Trias City	1,923	0	0	0	0.00
Imus City	2,471	364	397	761	30.80
Lipa City	2,086	2	0	2	0.10
Lucena City	1,711	8	5	13	0.76
San Pablo City	1,583	32	32	64	4.04
San Pedro City	1,940	39	39	78	4.02
Santa Rosa City	2,107	0	0	0	0.00
Tagaytay City	435	0	0	0	0.00
Tanauan City	1,086	51	30	81	7.46
Tayabas City	640	209	185	394	61.54
Trece Martires City	953	0	0	0	0.00
**Region 4B**	**21,611**	**1,134**	**1,052**	**2,186**	**10.12**
Marinduque	1,472	151	138	289	19.64
Mindoro Occidental	3,735	312	299	611	16.36
Mindoro Oriental	6,003	448	403	851	14.18
Palawan	6,503	128	136	264	4.06
Romblon	1,945	95	76	171	8.79
Puerto Princesa City	1,953	0	0	0	0.00
**Region 5**	**41,205**	**5,839**	**5,153**	**10,992**	**26.68**
Albay	7,171	459	467	926	12.91
Camarines Norte	4,302	1,987	1,386	3,373	78.41
Camarines Sur	11,745	1,101	1,067	2,168	18.46
Catanduanes	1,914	422	371	793	41.43
Masbate	6,770	839	884	1,723	25.45
Sorsogon	5,841	650	537	1,187	20.32
Iriga City	800	242	303	545	68.17
Legaspi City	1,262	12	20	32	2.54
Naga City	1,401	127	118	245	17.49
**Region 6**	**43,964**	**7,131**	**7,253**	**14,384**	**32.72**
Aklan	3,369	320	269	589	17.48
Antique	3,896	273	297	570	14.63
Capiz	4,195	820	853	1,673	39.88
Guimaras	926	286	273	559	60.40
Iloilo	10,924	3,974	4,191	8,165	74.74
Negros Occidental	14,794	94	94	188	1.27
Bacolod City	3,331	2	4	6	0.18
Iloilo City	2,529	1,362	1,272	2,634	104.15

Table 1.D.3 - Oral Health Care

Number and proportion of infants 0-11 months old who received Basic Oral Health Care (BOHC)

Philippines, 2021

Area	Infants 0-11 months old who received BOHC				
	Eligible Population	Male	Female	Total	%
**Region 7**	**48,858**	**6,163**	**6,244**	**12,407**	**25.39**
Bohol	8,291	218	227	445	5.37
Cebu	20,114	4,201	4,309	8,510	42.31
Negros Oriental	8,377	654	630	1,284	15.33
Siquijor	491	3	5	8	1.63
Cebu City	6,311	203	265	468	7.42
Lapu-Lapu City	2,793	0	0	0	0.00
Mandaue City	2,482	884	808	1,692	68.18
**Region 8**	**31,205**	**1,958**	**2,038**	**3,996**	**12.81**
Biliran	1,157	231	214	445	38.47
Eastern Samar	3,491	216	253	469	13.44
Northern Leyte	10,516	282	284	566	5.38
Northern Samar	4,697	51	64	115	2.45
Southern Leyte	1,967	0	0	0	0.00
Western Samar	4,355	187	212	399	9.16
Calbayog City	1,342	23	37	60	4.47
Maasin City	498	4	7	11	2.21
Ormoc City	1,497	896	900	1,796	120.00
Tacloban City	1,687	68	67	135	8.00
**Region 9**	**23,866**	**1,191**	**1,234**	**2,425**	**10.16**
Zamboanga del Norte	5,123	180	187	367	7.16
Zamboanga del Sur	5,252	734	802	1,536	29.25
Zamboanga Sibugay	4,498	277	245	522	11.61
Dapitan City	527	0	0	0	0.00
Dipolog City	838	0	0	0	0.00
Isabela City	763	0	0	0	0.00
Pagadian City	1,287	0	0	0	0.00
Zamboanga City	5,579	0	0	0	0.00
**Region 10**	**30,207**	**2,417**	**2,770**	**5,187**	**17.17**
Bukidnon	6,992	305	424	729	10.43
Camiguin	556	0	0	0	0.00
Lanao del Norte	4,487	5	16	21	0.47
Misamis Occidental	1,924	607	780	1,387	72.09
Misamis Oriental	4,517	497	427	924	20.45
Cagayan de Oro City	4,279	35	41	76	1.78
El Salvador City	318	46	14	60	18.85
Gingoog City	788	57	185	242	30.71
Iligan City	2,273	93	111	204	8.98
Malaybalay City	1,165	583	579	1,162	99.78
Oroquieta City	417	0	0	0	0.00
Ozamis City	835	0	0	0	0.00

Table 1.D.3 - Oral Health Care

Number and proportion of infants 0-11 months old who received Basic Oral Health Care (BOHC)

Philippines, 2021

Area	Infants 0-11 months old who received BOHC				
	Eligible Population	Male	Female	Total	%
Tangub City	370	123	125	248	66.99
Valencia City	1,287	66	68	134	10.41
**Region 11**	**32,354**	**515**	**740**	**1,255**	**3.88**
Davao de Oro	4,658	71	105	176	3.78
Davao del Norte	6,355	103	133	236	3.71
Davao Oriental	3,938	65	93	158	4.01
Davao del Sur	4,307	74	130	204	4.74
Davao Occidental	1,979	33	41	74	3.74
Davao City	11,118	169	238	407	3.66
**Region 12**	**31,408**	**8,767**	**8,828**	**17,595**	**56.02**
North Cotabato	10,180	3,588	3,278	6,866	67.45
Sarangani	3,871	669	659	1,328	34.30
South Cotabato	6,312	438	438	876	13.88
Sultan Kudarat	5,199	894	1,003	1,897	36.49
Cotabato City	1,749	666	779	1,445	82.60
Gen. Santos City	4,097	2,512	2,671	5,183	126.52
**BARMM**	**28,244**	**3,357**	**3,614**	**6,971**	**24.68**
Basilan	2,302	14	9	23	1.00
Lanao del Sur	6,417	373	495	868	13.53
Maguindanao	9,495	2,881	3,006	5,887	62.00
Sulu	5,067	19	20	39	0.77
Tawi-Tawi	2,796	0	0	0	0.00
Lamitan City	634	0	0	0	0.00
Marawi City	1,534	70	84	154	10.04
**CARAGA**	**18,103**	**3,368**	**3,497**	**6,865**	**37.92**
Agusan del Norte	2,435	694	865	1,559	64.03
Agusan del Sur	5,297	60	75	135	2.55
Surigao del Norte	2,159	417	419	836	38.73
Surigao del Sur	3,460	481	516	997	28.82
Province of Dinagat	772	317	305	622	80.55
Bislig City	656	119	84	203	30.97
Butuan City	2,320	749	759	1,508	65.01
Surigao City	1,005	531	474	1,005	100.00

Note: Put asterisk (*) for No Report and Zero (0) for No Case

Table 1.D.4 - Oral Health Care

Number and proportion of children 1-4 years old who received Basic Oral Health Care (BOHC)

Philippines, 2021

Area	Infants 1-4 years old who received BOHC				
	Eligible Population	Male	Female	Total	%
**PHILIPPINES**	**2,688,761**	**338,526**	**356,567**	**695,093**	**25.85**
**N C R**	**286,736**	**68,157**	**69,519**	**137,676**	**48.01**
Malabon	8,139	695	711	1,406	17.28
Navotas	5,555	493	571	1,064	19.15
Valenzuela City	13,816	2,394	2,567	4,961	35.91
Caloocan City	35,268	9,004	9,646	18,650	52.88
Marikina City	10,036	199	201	400	3.99
Pasig City	16,818	7,861	8,957	16,818	100.00
Pateros	1,422	694	612	1,306	91.86
Taguig	17,923	1,496	1,396	2,892	16.14
Quezon City	65,379	13,711	13,846	27,557	42.15
Makati City	12,973	2,008	1,872	3,880	29.91
Mandaluyong City	8,602	2,864	2,891	5,755	66.90
San Juan	2,720	840	758	1,598	58.75
Manila City	39,638	11,589	11,820	23,409	59.06
Las Piñas City	13,112	4,985	5,041	10,026	76.47
Muntinlupa City	11,235	5,865	5,369	11,234	99.99
Parañaque City	14,826	3,371	3,172	6,543	44.13
Pasay City	9,276	88	89	177	1.91
**C A R**	**44,136**	**4,192**	**3,966**	**8,158**	**18.48**
Abra	5,714	585	579	1,164	20.37
Apayao	3,300	548	526	1,074	32.55
Benguet	10,875	2,173	2,003	4,176	38.40
Ifugao	5,781	217	198	415	7.18
Kalinga	5,971	167	155	322	5.39
Mt. Province	4,080	54	51	105	2.57
Baguio City	8,415	448	454	902	10.72
**Region 1**	**122,216**	**14,225**	**25,184**	**39,409**	**32.25**
Ilocos Norte	9,752	1,368	1,443	2,811	28.83
Ilocos Sur	12,060	2,134	2,304	4,438	36.80
La Union	14,493	5,171	15,175	20,346	140.39
Pangasinan	63,123	3,432	4,001	7,433	11.78
Alaminos City	2,384	223	295	518	21.73

Table 1.D.4 - Oral Health Care

Number and proportion of children 1-4 years old who received Basic Oral Health Care (BOHC)
Philippines, 2021

Area	Infants 1-4 years old who received BOHC				
	Eligible Population	Male	Female	Total	%
Candon City	1,270	264	299	563	44.32
Dagupan City	4,553	123	113	236	5.18
Laoag City	2,249	0	0	0	0.00
San Carlos City	5,015	615	636	1,251	24.95
San Fernando City	2,655	496	457	953	35.90
Urdaneta City	3,533	6	5	11	0.31
Vigan City	1,130	393	456	849	75.15
**Region 2**	**87,183**	**10,227**	**10,917**	**21,144**	**24.25**
Batanes	473	156	163	319	67.51
Cagayan	25,898	1,335	1,373	2,708	10.46
Isabela	29,537	2,444	2,588	5,032	17.04
Nueva Vizcaya	12,079	2,161	2,714	4,875	40.36
Quirino	5,170	229	271	500	9.67
Cauayan City	3,231	687	791	1,478	45.75
Ilagan City	3,630	1,697	1,535	3,232	89.03
Santiago City	3,364	1,517	1,482	2,999	89.15
Tuguegarao City	3,802	1	0	1	0.03
**Region 3**	**274,078**	**33,833**	**33,052**	**66,885**	**24.40**
Aurora	6,137	564	602	1,166	19.00
Bataan	16,442	512	529	1,041	6.33
Bulacan	54,401	2,356	2,352	4,708	8.65
Nueva Ecija	36,619	4,519	4,458	8,977	24.51
Pampanga	39,432	3,812	3,506	7,318	18.56
Tarlac	24,257	6,931	7,040	13,971	57.59
Zambales	15,291	1,478	1,447	2,925	19.13
Angeles City	9,893	243	243	486	4.91
Balanga City	2,376	653	813	1,466	61.71
Cabanatuan City	7,494	1,298	1,382	2,680	35.76
City of San Fernando	7,369	10	17	27	0.37
Gapan City	2,735	42	53	95	3.47
Mabalacat City	6,028	5,080	3,958	9,038	149.94
Malolos City	6,076	336	331	667	10.98
Meycauayan	5,039	512	495	1,007	19.98
Olongapo	6,031	0	0	0	0.00
Palayan City	1,018	25	42	67	6.58
San Jose City	3,466	1,161	1,245	2,406	69.42
San Jose del Monte City	13,839	3,301	3,555	6,856	49.54
Science City of Munoz	2,021	307	294	601	29.74
Tarlac City	8,116	693	690	1,383	17.04

Table 1.D.4 - Oral Health Care

Number and proportion of children 1-4 years old who received Basic Oral Health Care (BOHC)

Philippines, 2021

Area	Infants 1-4 years old who received BOHC				
	Eligible Population	Male	Female	Total	%
**Region 4A**	**370,117**	**76,817**	**78,506**	**155,323**	**41.97**
Batangas	49,116	6,303	6,018	12,321	25.09
Cavite	34,477	847	837	1,684	4.88
Laguna	24,174	7,653	8,028	15,681	64.87
Quezon	47,880	6,237	6,291	12,528	26.17
Rizal	54,588	1,970	1,986	3,956	7.25
Antipolo City	20,106	13,223	12,221	25,444	126.55
Bacoor City	15,104	20	13	33	0.22
Batangas City	8,717	790	715	1,505	17.27
Biñan City	8,105	4,781	4,438	9,219	113.74
Cabuyao City	7,514	3,829	4,062	7,891	105.02
Calamba City	11,060	21,115	22,397	43,512	393.40
Cavite City	2,583	4	6	10	0.39
Dasmariñas City	16,575	0	0	0	0.00
General Trias City	7,904	0	0	0	0.00
Imus City	10,156	212	204	416	4.10
Lipa City	8,783	41	65	106	1.21
Lucena City	7,255	17	19	36	0.50
San Pablo City	6,476	1,366	1,654	3,020	46.64
San Pedro City	7,929	2,495	2,889	5,384	67.90
Santa Rosa City	8,610	5,423	6,058	11,481	133.35
Tagaytay City	1,790	38	40	78	4.36
Tanauan City	4,582	267	339	606	13.23
Tayabas City	2,719	124	164	288	10.59
Trece Martires City	3,916	62	62	124	3.17
**Region 4B**	**90,502**	**3,021**	**2,985**	**6,006**	**6.64**
Marinduque	6,605	600	547	1,147	17.37
Mindoro Occidental	15,151	290	263	553	3.65
Mindoro Oriental	25,195	1,670	1,708	3,378	13.41
Palawan	26,867	264	274	538	2.00
Romblon	8,615	197	193	390	4.53
Puerto Princesa City	8,069	0	0	0	0.00
**Region 5**	**177,371**	**40,284**	**40,142**	**80,426**	**45.34**
Albay	31,185	7,445	8,138	15,583	49.97
Camarines Norte	18,032	4,171	3,705	7,876	43.68
Camarines Sur	50,294	8,788	8,685	17,473	34.74
Catanduanes	8,083	4,678	4,511	9,189	113.68

Table 1.D.4 - Oral Health Care

Number and proportion of children 1-4 years old who received Basic Oral Health Care (BOHC)

Philippines, 2021

Area	Infants 1-4 years old who received BOHC				
	Eligible Population	Male	Female	Total	%
Masbate	29,873	5,234	5,374	10,608	35.51
Sorsogon	25,008	6,570	6,306	12,876	51.49
Iriga City	3,417	920	1,060	1,980	57.94
Legaspi City	5,485	1,975	1,836	3,811	69.48
Naga City	5,994	503	527	1,030	17.18
**Region 6**	**187,134**	**26,748**	**26,709**	**53,457**	**28.57**
Aklan	14,438	819	943	1,762	12.20
Antique	16,358	605	605	1,210	7.40
Capiz	18,017	2,737	2,912	5,649	31.35
Guimaras	4,351	1,977	1,921	3,898	89.59
Iloilo	45,936	15,927	16,628	32,555	70.87
Negros Occidental	63,191	1,601	1,592	3,193	5.05
Bacolod City	14,218	286	313	599	4.21
Iloilo City	10,626	2,796	1,795	4,591	43.21
**Region 7**	**195,229**	**11,437**	**11,961**	**23,398**	**11.98**
Bohol	34,939	0	0	0	0.00
Cebu	78,390	9,216	9,580	18,796	23.98
Negros Oriental	34,469	359	403	762	2.21
Siquijor	2,263	52	57	109	4.82
Cebu City	24,609	1,376	1,404	2,780	11.30
Lapu-Lapu City	10,885	320	374	694	6.38
Mandaue City	9,673	114	143	257	2.66
**Region 8**	**131,746**	**5,156**	**5,369**	**10,525**	**7.99**
Biliran	5,067	106	97	203	4.01
Eastern Samar	14,205	778	914	1,692	11.91
Northern Leyte	43,517	1,140	1,193	2,333	5.36
Northern Samar	20,592	708	814	1,522	7.39
Southern Leyte	8,774	0	0	0	0.00
Western Samar	18,483	648	583	1,231	6.66
Calbayog City	5,696	32	51	83	1.46
Maasin City	2,235	235	228	463	20.72
Ormoc City	6,198	1,497	1,465	2,962	47.79
Tacloban City	6,978	12	24	36	0.52

Table 1.D.4 - Oral Health Care

Number and proportion of children 1-4 years old who received Basic Oral Health Care (BOHC)
Philippines, 2021

Area	Infants 1-4 years old who received BOHC				
	Eligible Population	Male	Female	Total	%
**Region 9**	**101,202**	**3,695**	**4,398**	**8,093**	**8.00**
Zamboanga del Norte	22,212	447	540	987	4.44
Zamboanga del Sur	21,470	2,556	2,988	5,544	25.82
Zamboanga Sibugay	19,877	475	548	1,023	5.15
Dapitan City	2,294	115	173	288	12.56
Dipolog City	3,639	51	77	128	3.52
Isabela City	3,645	0	0	0	0.00
Pagadian City	5,267	51	72	123	2.34
Zamboanga City	22,799	0	0	0	0.00
**Region 10**	**129,559**	**9,125**	**10,142**	**19,267**	**14.87**
Bukidnon	29,672	1,090	1,287	2,377	8.01
Camiguin	2,399	6	9	15	0.63
Lanao del Norte	20,237	120	141	261	1.29
Misamis Occidental	8,237	2,565	3,017	5,582	67.77
Misamis Oriental	18,884	2,299	2,382	4,681	24.79
Cagayan de Oro City	17,888	218	263	481	2.69
El Salvador City	1,328	31	49	80	6.03
Gingoog City	3,300	1,439	1,584	3,023	91.61
Iligan City	10,252	217	252	469	4.57
Malaybalay City	4,946	278	276	554	11.20
Oroquieta City	1,785	0	0	0	0.00
Ozamis City	3,577	35	37	72	2.01
Tangub City	1,590	265	297	562	35.35
Valencia City	5,465	562	548	1,110	20.31
**Region 11**	**135,188**	**1,086**	**1,231**	**2,317**	**1.71**
Davao de Oro	20,408	192	207	399	1.96
Davao del Norte	26,118	232	296	528	2.02
Davao Oriental	17,000	158	175	333	1.96
Davao del Sur	17,453	103	154	257	1.47
Davao Occidental	9,146	54	84	138	1.51
Davao City	45,063	347	315	662	1.47
**Region 12**	**131,388**	**11,443**	**12,163**	**23,606**	**17.97**
North Cotabato	42,046	3,960	3,979	7,939	18.88
Sarangani	16,391	2,580	2,676	5,256	32.07
South Cotabato	25,467	1,016	1,118	2,134	8.38

Table 1.D.4 - Oral Health Care

Number and proportion of children 1-4 years old who received Basic Oral Health Care (BOHC)

Philippines, 2021

Area	Infants 1-4 years old who received BOHC				
	Eligible Population	Male	Female	Total	%
Sultan Kudarat	22,443	1,371	1,785	3,156	14.06
Cotabato City	8,502	934	958	1,892	22.25
Gen. Santos City	16,539	1,582	1,647	3,229	19.52
**BARMM**	**147,028**	**11,712**	**12,460**	**24,172**	**16.44**
Basilan	10,846	72	76	148	1.36
Lanao del Sur	33,472	1,564	1,818	3,382	10.10
Maguindanao	45,442	4,865	5,033	9,898	21.78
Sulu	32,706	4,731	4,976	9,707	29.68
Tawi-Tawi	13,572	0	0	0	0.00
Lamitan City	2,984	0	0	0	0.00
Marawi City	8,006	480	557	1,037	12.95
**CARAGA**	**77,949**	**7,368**	**7,863**	**15,231**	**19.54**
Agusan del Norte	10,252	1,360	1,671	3,031	29.56
Agusan del Sur	23,127	691	776	1,467	6.34
Surigao del Norte	9,598	665	645	1,310	13.65
Surigao del Sur	14,607	1,153	1,217	2,370	16.23
Province of Dinagat	3,372	1,390	1,428	2,818	83.58
Bislig City	2,775	327	337	664	23.93
Butuan City	9,749	1,145	1,229	2,374	24.35
Surigao City	4,470	637	560	1,197	26.78

Note: Put asterisk (*) for No Report and Zero (0) for No Case

Table 1.D.5 - Oral Health Care

Number and proportion of children 5-9 years old who received Basic Oral Health Care (BOHC)

Philippines, 2021

Area	Children 5-9 years old who received BOHC				
	Eligible Population	Male	Female	Total	%
**PHILIPPINES**	**3,334,482**	**185,077**	**198,637**	**383,714**	**11.51**
**N C R**	**368,223**	**29,352**	**32,259**	**61,611**	**16.73**
Malabon	10,452	1,154	1,186	2,340	22.39
Navotas	7,134	363	430	793	11.12
Valenzuela City	17,742	2,920	3,216	6,136	34.59
Caloocan City	45,290	4,970	5,864	10,834	23.92
Marikina City	12,890	42	27	69	0.54
Pasig City	21,597	9,997	11,005	21,002	97.24
Pateros	1,825	193	154	347	19.02
Taguig	23,016	813	978	1,791	7.78
Quezon City	83,960	12	16	28	0.03
Makati City	16,659	249	353	602	3.61
Mandaluyong City	11,045	530	610	1,140	10.32
San Juan	3,494	264	253	517	14.80
Manila City	50,903	2,161	2,377	4,538	8.91
Las Piñas City	16,839	219	201	420	2.49
Muntinlupa City	14,427	4,131	4,265	8,396	58.20
Parañaque City	19,039	1,157	1,122	2,279	11.97
Pasay City	11,911	177	202	379	3.18
**C A R**	**54,161**	**4,999**	**5,398**	**10,397**	**19.20**
Abra	7,093	205	238	443	6.25
Apayao	3,984	561	577	1,138	28.57
Benguet	13,319	2,245	2,307	4,552	34.18
Ifugao	7,028	365	427	792	11.27
Kalinga	7,326	111	133	244	3.33
Mt. Province	5,103	143	132	275	5.39
Baguio City	10,308	1,369	1,584	2,953	28.65
**Region 1**	**155,762**	**14,243**	**15,707**	**29,950**	**19.23**
Ilocos Norte	12,446	1,160	1,313	2,473	19.87
Ilocos Sur	15,627	2,540	2,688	5,228	33.45
La Union	18,588	2,998	3,092	6,090	32.76
Pangasinan	80,066	6,080	7,055	13,135	16.41
Alaminos City	3,024	550	600	1,150	38.03

Table 1.D.5 - Oral Health Care

Number and proportion of children 5-9 years old who received Basic Oral Health Care (BOHC)
Philippines, 2021

Area	Children 5-9 years old who received BOHC				
	Eligible Population	Male	Female	Total	%
Candon City	1,649	77	69	146	8.86
Dagupan City	5,776	59	73	132	2.29
Laoag City	2,870	0	0	0	0.00
San Carlos City	6,359	312	330	642	10.10
San Fernando City	3,407	112	166	278	8.16
Urdaneta City	4,484	11	14	25	0.56
Vigan City	1,465	344	307	651	44.44
**Region 2**	**109,676**	**8,128**	**8,742**	**16,870**	**15.38**
Batanes	575	114	133	247	42.95
Cagayan	32,639	903	1,003	1,906	5.84
Isabela	37,413	1,612	1,923	3,535	9.45
Nueva Vizcaya	14,847	2,152	2,564	4,716	31.76
Quirino	6,458	203	289	492	7.62
Cauayan City	4,094	98	157	255	6.23
Ilagan City	4,600	1,794	1,514	3,308	71.91
Santiago City	4,261	1,252	1,159	2,411	56.58
Tuguegarao City	4,789	0	0	0	0.00
**Region 3**	**348,101**	**15,788**	**16,501**	**32,289**	**9.28**
Aurora	7,404	451	484	935	12.63
Bataan	20,798	137	157	294	1.41
Bulacan	69,845	745	782	1,527	2.19
Nueva Ecija	46,608	2,824	3,366	6,190	13.28
Pampanga	50,248	775	821	1,596	3.18
Tarlac	30,725	3,089	3,288	6,377	20.75
Zambales	18,768	1,153	1,085	2,238	11.92
Angeles City	12,607	199	231	430	3.41
Balanga City	3,007	158	144	302	10.04
Cabanatuan City	9,538	519	630	1,149	12.05
City of San Fernando	9,392	2	3	5	0.05
Gapan City	3,481	0	0	0	0.00
Mabalacat City	7,681	3,137	2,759	5,896	76.76
Malolos City	7,801	114	127	241	3.09
Meycauayan	6,470	317	311	628	9.71
Olongapo	7,403	0	0	0	0.00
Palayan City	1,295	75	60	135	10.42
San Jose City	4,410	566	527	1,093	24.79
San Jose del Monte City	17,767	792	946	1,738	9.78
Science City of Munoz	2,572	179	257	436	16.95
Tarlac City	10,281	556	523	1,079	10.50

Table 1.D.5 - Oral Health Care

Number and proportion of children 5-9 years old who received Basic Oral Health Care (BOHC)
Philippines, 2021

Area	Children 5-9 years old who received BOHC				
	Eligible Population	Male	Female	Total	%
**Region 4A**	**463,929**	**19,601**	**19,813**	**39,414**	**8.50**
Batangas	60,415	7,342	6,701	14,043	23.24
Cavite	43,765	569	573	1,142	2.61
Laguna	30,365	2,242	2,467	4,709	15.51
Quezon	60,028	3,993	4,157	8,150	13.58
Rizal	68,396	1,544	1,618	3,162	4.62
Antipolo City	25,192	0	0	0	0.00
Bacoor City	19,175	105	158	263	1.37
Batangas City	10,722	302	335	637	5.94
Biñan City	10,182	615	645	1,260	12.37
Cabuyao City	9,440	389	460	849	8.99
Calamba City	13,899	643	722	1,365	9.82
Cavite City	3,281	11	21	32	0.98
Dasmariñas City	21,039	0	0	0	0.00
General Trias City	10,035	0	0	0	0.00
Imus City	12,891	113	133	246	1.91
Lipa City	10,804	102	78	180	1.67
Lucena City	9,097	25	24	49	0.54
San Pablo City	8,134	916	942	1,858	22.84
San Pedro City	9,963	382	449	831	8.34
Santa Rosa City	10,817	17	24	41	0.38
Tagaytay City	2,274	30	38	68	2.99
Tanauan City	5,635	96	106	202	3.58
Tayabas City	3,410	48	43	91	2.67
Trece Martires City	4,972	117	119	236	4.75
**Region 4B**	**111,946**	**2,564**	**2,758**	**5,322**	**4.75**
Marinduque	8,234	391	385	776	9.42
Mindoro Occidental	18,714	241	302	543	2.90
Mindoro Oriental	31,467	1,170	1,259	2,429	7.72
Palawan	33,077	366	444	810	2.45
Romblon	10,522	396	368	764	7.26
Puerto Princesa City	9,932	0	0	0	0.00
**Region 5**	**212,480**	**21,872**	**22,410**	**44,282**	**20.84**
Albay	38,480	4,172	4,510	8,682	22.56
Camarines Norte	21,257	3,237	2,676	5,913	27.82
Camarines Sur	60,566	5,469	5,678	11,147	18.40
Catanduanes	9,603	1,394	1,395	2,789	29.04

Table 1.D.5 - Oral Health Care

Number and proportion of children 5-9 years old who received Basic Oral Health Care (BOHC)
Philippines, 2021

Area	Children 5-9 years old who received BOHC				
	Eligible Population	Male	Female	Total	%
Masbate	34,731	3,345	3,641	6,986	20.11
Sorsogon	29,745	2,531	2,727	5,258	17.68
Iriga City	4,115	272	271	543	13.20
Legaspi City	6,766	1,075	1,098	2,173	32.12
Naga City	7,217	377	414	791	10.96
**Region 6**	**233,617**	**13,294**	**14,309**	**27,603**	**11.82**
Aklan	18,155	390	527	917	5.05
Antique	20,435	596	683	1,279	6.26
Capiz	22,183	725	721	1,446	6.52
Guimaras	5,289	180	130	310	5.86
Iloilo	57,757	8,489	9,274	17,763	30.75
Negros Occidental	78,722	752	816	1,568	1.99
Bacolod City	17,714	762	838	1,600	9.03
Iloilo City	13,362	1,400	1,320	2,720	20.36
**Region 7**	**244,203**	**9,745**	**10,567**	**20,312**	**8.32**
Bohol	43,801	683	737	1,420	3.24
Cebu	98,294	7,227	7,997	15,224	15.49
Negros Oriental	42,575	250	316	566	1.33
Siquijor	2,895	104	106	210	7.25
Cebu City	30,859	902	841	1,743	5.65
Lapu-Lapu City	13,650	465	427	892	6.53
Mandaue City	12,129	114	143	257	2.12
**Region 8**	**158,506**	**4,798**	**5,178**	**9,976**	**6.29**
Biliran	5,977	129	134	263	4.40
Eastern Samar	17,232	611	788	1,399	8.12
Northern Leyte	52,756	947	955	1,902	3.61
Northern Samar	24,260	879	1,015	1,894	7.81
Southern Leyte	10,980	0	0	0	0.00
Western Samar	21,809	634	685	1,319	6.05
Calbayog City	6,724	64	103	167	2.48
Maasin City	2,795	46	36	82	2.93
Ormoc City	7,515	1,466	1,450	2,916	38.80
Tacloban City	8,458	22	12	34	0.40

Table 1.D.5 - Oral Health Care

Number and proportion of children 5-9 years old who received Basic Oral Health Care (BOHC)
Philippines, 2021

Area	Children 5-9 years old who received BOHC				
	Eligible Population	Male	Female	Total	%
**Region 9**	**123,061**	**3,971**	**4,758**	**8,729**	**7.09**
Zamboanga del Norte	26,755	240	300	540	2.02
Zamboanga del Sur	26,456	2,664	3,067	5,731	21.66
Zamboanga Sibugay	23,829	568	563	1,131	4.75
Dapitan City	2,764	162	219	381	13.78
Dipolog City	4,383	47	50	97	2.21
Isabela City	4,292	10	19	29	0.68
Pagadian City	6,489	280	540	820	12.64
Zamboanga City	28,094	0	0	0	0.00
**Region 10**	**157,650**	**8,352**	**9,179**	**17,531**	**11.12**
Bukidnon	35,765	981	1,108	2,089	5.84
Camiguin	2,875	16	23	39	1.36
Lanao del Norte	24,471	226	242	468	1.91
Misamis Occidental	10,263	3,515	4,031	7,546	73.53
Misamis Oriental	23,112	1,419	1,393	2,812	12.17
Cagayan de Oro City	21,894	154	220	374	1.71
El Salvador City	1,627	11	11	22	1.35
Gingoog City	4,039	495	469	964	23.87
Iligan City	12,393	418	515	933	7.53
Malaybalay City	5,962	311	356	667	11.19
Oroquieta City	2,223	0	0	0	0.00
Ozamis City	4,457	0	0	0	0.00
Tangub City	1,981	374	346	720	36.35
Valencia City	6,589	432	465	897	13.61
**Region 11**	**168,057**	**1,250**	**1,677**	**2,927**	**1.74**
Davao de Oro	24,850	262	350	612	2.46
Davao del Norte	32,708	300	352	652	1.99
Davao Oriental	20,497	232	290	522	2.55
Davao del Sur	21,902	99	128	227	1.04
Davao Occidental	11,563	54	88	142	1.23
Davao City	56,537	303	469	772	1.37
**Region 12**	**162,001**	**8,373**	**9,545**	**17,918**	**11.06**
North Cotabato	51,860	3,932	4,398	8,330	16.06
Sarangani	19,979	998	1,275	2,273	11.38
South Cotabato	31,243	570	692	1,262	4.04

Table 1.D.5 - Oral Health Care

Number and proportion of children 5-9 years old who received Basic Oral Health Care (BOHC)
Philippines, 2021

Area	Children 5-9 years old who received BOHC				
	Eligible Population	Male	Female	Total	%
Sultan Kudarat	28,116	1,499	1,877	3,376	12.01
Cotabato City	10,512	1,010	905	1,915	18.22
Gen. Santos City	20,291	364	398	762	3.76
**BARMM**	**170,547**	**11,160**	**11,390**	**22,550**	**13.22**
Basilan	12,528	79	91	170	1.36
Lanao del Sur	38,453	1,295	1,441	2,736	7.12
Maguindanao	53,610	4,701	4,944	9,645	17.99
Sulu	37,319	4,498	4,371	8,869	23.77
Tawi-Tawi	15,991	0	0	0	0.00
Lamitan City	3,446	0	0	0	0.00
Marawi City	9,200	587	543	1,130	12.28
**CARAGA**	**92,563**	**7,587**	**8,446**	**16,033**	**17.32**
Agusan del Norte	12,236	1,345	1,698	3,043	24.87
Agusan del Sur	26,390	1,282	1,497	2,779	10.53
Surigao del Norte	11,792	437	542	979	8.30
Surigao del Sur	17,452	1,348	1,453	2,801	16.05
Province of Dinagat	4,257	1,277	1,301	2,578	60.56
Bislig City	3,314	119	98	217	6.55
Butuan City	11,632	1,062	1,142	2,204	18.95
Surigao City	5,490	717	715	1,432	26.08

Note: Put asterisk (*) for No Report and Zero (0) for No Case

Table 1.D.6 - Oral Health Care

Number and proportion of adolescents 10-14 years who received Basic Oral Health Care (BOHC)
Philippines, 2021

Area	Adolescents 10-14 years old who received BOHC				
	Eligible Population	Male	Female	Total	%
**PHILIPPINES**	**3,251,253**	**186,176**	**215,711**	**401,887**	**12.36**
**N C R**	**375,489**	**18,974**	**20,472**	**39,446**	**10.51**
Malabon	10,658	215	252	467	4.38
Navotas	7,274	235	288	523	7.19
Valenzuela City	18,091	0	0	0	0.00
Caloocan City	46,189	3,833	4,345	8,178	17.71
Marikina City	13,143	0	1	1	0.01
Pasig City	22,023	7,999	8,768	16,767	76.14
Pateros	1,861	35	53	88	4.73
Taguig	23,470	339	320	659	2.81
Quezon City	85,616	83	93	176	0.21
Makati City	16,988	199	218	417	2.45
Mandaluyong City	11,264	334	327	661	5.87
San Juan	3,563	218	194	412	11.56
Manila City	51,907	1,749	1,868	3,617	6.97
Las Piñas City	17,172	59	54	113	0.66
Muntinlupa City	14,711	3,176	3,181	6,357	43.21
Parañaque City	19,415	252	222	474	2.44
Pasay City	12,146	248	288	536	4.41
**C A R**	**52,409**	**3,785**	**4,686**	**8,471**	**16.16**
Abra	7,062	108	265	373	5.28
Apayao	3,828	241	227	468	12.23
Benguet	12,871	2,092	2,140	4,232	32.88
Ifugao	6,693	142	281	423	6.32
Kalinga	7,031	194	276	470	6.69
Mt. Province	4,961	110	179	289	5.83
Baguio City	9,962	898	1,318	2,216	22.24
**Region 1**	**155,497**	**7,721**	**8,859**	**16,580**	**10.66**
Ilocos Norte	12,608	295	316	611	4.85
Ilocos Sur	15,919	1,871	2,093	3,964	24.90
La Union	18,742	1,211	1,682	2,893	15.44
Pangasinan	79,267	3,704	4,043	7,747	9.77
Alaminos City	2,996	240	265	505	16.86
Candon City	1,678	101	75	176	10.49
Dagupan City	5,718	39	62	101	1.77
Laoag City	2,909	0	0	0	0.00
San Carlos City	6,298	113	134	247	3.92
San Fernando City	3,434	26	43	69	2.01
Urdaneta City	4,439	21	39	60	1.35

Table 1.D.6 - Oral Health Care

Number and proportion of adolescents 10-14 years who received Basic Oral Health Care (BOHC)
Philippines, 2021

Area	Adolescents 10-14 years old who received BOHC				
	Eligible Population	Male	Female	Total	%
Vigan City	1,491	100	107	207	13.88
**Region 2**	**107,306**	**6,075**	**7,266**	**13,341**	**12.43**
Batanes	586	82	117	199	33.95
Cagayan	31,839	422	603	1,025	3.22
Isabela	36,673	1,833	2,386	4,219	11.50
Nueva Vizcaya	14,548	1,140	1,498	2,638	18.13
Quirino	6,285	23	49	72	1.15
Cauayan City	4,014	37	51	88	2.19
Ilagan City	4,511	1,580	1,573	3,153	69.90
Santiago City	4,177	958	989	1,947	46.61
Tuguegarao City	4,673	0	0	0	0.00
**Region 3**	**346,583**	**6,075**	**7,361**	**13,436**	**3.88**
Aurora	6,980	219	240	459	6.58
Bataan	20,573	96	105	201	0.98
Bulacan	69,551	560	707	1,267	1.82
Nueva Ecija	46,366	1,300	1,760	3,060	6.60
Pampanga	50,960	504	719	1,223	2.40
Tarlac	30,485	549	625	1,174	3.85
Zambales	18,148	792	864	1,656	9.12
Angeles City	12,786	27	31	58	0.45
Balanga City	2,974	20	35	55	1.85
Cabanatuan City	9,488	12	35	47	0.50
City of San Fernando	9,524	1	1	2	0.02
Gapan City	3,463	0	0	0	0.00
Mabalacat City	7,790	393	425	818	10.50
Malolos City	7,768	72	54	126	1.62
Meycauayan	6,444	90	89	179	2.78
Olongapo	7,157	0	0	0	0.00
Palayan City	1,289	48	64	112	8.69
San Jose City	4,387	256	332	588	13.40
San Jose del Monte City	17,692	548	758	1,306	7.38
Science City of Munoz	2,557	47	78	125	4.89
Tarlac City	10,202	541	439	980	9.61
**Region 4A**	**460,270**	**89,147**	**103,566**	**192,713**	**41.87**
Batangas	59,717	7,123	6,674	13,797	23.10
Cavite	44,000	574	731	1,305	2.97
Laguna	30,248	9,879	12,474	22,353	73.90
Quezon	58,667	1,228	1,507	2,735	4.66
Rizal	67,472	2,151	2,502	4,653	6.90
Antipolo City	24,851	0	0	0	0.00
Bacoor City	19,276	114	151	265	1.37
Batangas City	10,598	180	251	431	4.07

Table 1.D.6 - Oral Health Care

Number and proportion of adolescents 10-14 years who received Basic Oral Health Care (BOHC)
Philippines, 2021

Area	Adolescents 10-14 years old who received BOHC				
	Eligible Population	Male	Female	Total	%
Biñan City	10,143	16,029	19,203	35,232	347.36
Cabuyao City	9,403	1,818	3,084	4,902	52.13
Calamba City	13,843	29,419	33,044	62,463	451.23
Cavite City	3,299	17	25	42	1.27
Dasmariñas City	21,152	0	0	0	0.00
General Trias City	10,088	0	0	0	0.00
Imus City	12,958	90	170	260	2.01
Lipa City	10,678	89	71	160	1.50
Lucena City	8,891	4	1	5	0.06
San Pablo City	8,105	3,052	4,017	7,069	87.22
San Pedro City	9,922	3,425	4,473	7,898	79.60
Santa Rosa City	10,774	13,435	14,709	28,144	261.22
Tagaytay City	2,285	10	16	26	1.14
Tanauan City	5,570	56	89	145	2.60
Tayabas City	3,332	66	74	140	4.20
Trece Martires City	4,998	388	300	688	13.77
**Region 4B**	**106,950**	**2,126**	**2,899**	**5,025**	**4.70**
Marinduque	7,924	184	222	406	5.12
Mindoro Occidental	17,968	137	146	283	1.58
Mindoro Oriental	30,112	1,360	1,971	3,331	11.06
Palawan	31,348	288	390	678	2.16
Romblon	10,186	157	170	327	3.21
Puerto Princesa City	9,413	0	0	0	0.00
**Region 5**	**202,778**	**6,041**	**7,428**	**13,469**	**6.64**
Albay	37,401	951	1,047	1,998	5.34
Camarines Norte	19,961	1,405	1,844	3,249	16.28
Camarines Sur	58,035	871	1,203	2,074	3.57
Catanduanes	9,142	183	219	402	4.40
Masbate	32,158	1,926	2,108	4,034	12.54
Sorsogon	28,647	477	686	1,163	4.06
Iriga City	3,943	70	118	188	4.77
Legaspi City	6,578	6	4	10	0.15
Naga City	6,915	152	199	351	5.08
**Region 6**	**227,310**	**7,478**	**8,794**	**16,272**	**7.16**
Aklan	17,403	160	235	395	2.27
Antique	19,989	764	907	1,671	8.36
Capiz	21,759	495	627	1,122	5.16
Guimaras	5,075	24	28	52	1.02
Iloilo	56,676	4,482	5,081	9,563	16.87
Negros Occidental	76,162	611	817	1,428	1.87
Bacolod City	17,137	827	999	1,826	10.66
Iloilo City	13,111	115	100	215	1.64

Table 1.D.6 - Oral Health Care

Number and proportion of adolescents 10-14 years who received Basic Oral Health Care (BOHC)
Philippines, 2021

Area	Adolescents 10-14 years old who received BOHC				
	Eligible Population	Male	Female	Total	%
**Region 7**	**237,698**	**5,027**	**6,003**	**11,030**	**4.64**
Bohol	43,487	336	332	668	1.54
Cebu	95,202	3,405	4,360	7,765	8.16
Negros Oriental	41,250	192	288	480	1.16
Siquijor	2,905	61	89	150	5.16
Cebu City	29,885	127	28	155	0.52
Lapu-Lapu City	13,221	906	906	1,812	13.71
Mandaue City	11,748	0	0	0	0.00
**Region 8**	**151,043**	**2,812**	**3,888**	**6,700**	**4.44**
Biliran	5,553	69	106	175	3.15
Eastern Samar	16,446	340	499	839	5.10
Northern Leyte	50,135	1,037	1,628	2,665	5.32
Northern Samar	22,943	504	767	1,271	5.54
Southern Leyte	10,681	0	0	0	0.00
Western Samar	20,934	515	437	952	4.55
Calbayog City	6,449	116	194	310	4.81
Maasin City	2,718	35	31	66	2.43
Ormoc City	7,143	183	198	381	5.33
Tacloban City	8,041	13	28	41	0.51
**Region 9**	**117,303**	**2,203**	**2,846**	**5,049**	**4.30**
Zamboanga del Norte	25,420	50	54	104	0.41
Zamboanga del Sur	25,397	872	878	1,750	6.89
Zamboanga Sibugay	22,373	366	540	906	4.05
Dapitan City	2,624	79	72	151	5.76
Dipolog City	4,164	80	110	190	4.56
Isabela City	4,129	100	186	286	6.93
Pagadian City	6,229	656	1,006	1,662	26.68
Zamboanga City	26,968	0	0	0	0.00
**Region 10**	**150,772**	**8,374**	**9,532**	**17,906**	**11.88**
Bukidnon	33,636	784	1,065	1,849	5.50
Camiguin	2,785	70	117	187	6.71
Lanao del Norte	23,252	135	160	295	1.27
Misamis Occidental	10,114	4,365	4,843	9,208	91.04
Misamis Oriental	22,296	1,039	1,038	2,077	9.32
Cagayan de Oro City	21,114	30	33	63	0.30
El Salvador City	1,568	4	13	17	1.08
Gingoog City	3,893	272	320	592	15.21
Iligan City	11,776	254	351	605	5.14
Malaybalay City	5,607	257	349	606	10.81

Table 1.D.6 - Oral Health Care

Number and proportion of adolescents 10-14 years who received Basic Oral Health Care (BOHC)
Philippines, 2021

Area	Adolescents 10-14 years old who received BOHC				
	Eligible Population	Male	Female	Total	%
Oroquieta City	2,192	0	0	0	0.00
Ozamis City	4,391	0	0	0	0.00
Tangub City	1,950	383	334	717	36.77
Valencia City	6,197	781	909	1,690	27.27
**Region 11**	**162,704**	**571**	**814**	**1,385**	**0.85**
Davao de Oro	23,765	121	178	299	1.26
Davao del Norte	31,595	133	175	308	0.97
Davao Oriental	19,510	94	143	237	1.21
Davao del Sur	21,449	75	110	185	0.86
Davao Occidental	11,011	32	57	89	0.81
Davao City	55,374	116	151	267	0.48
**Region 12**	**155,296**	**5,151**	**6,213**	**11,364**	**7.32**
North Cotabato	49,109	2,355	2,855	5,210	10.61
Sarangani	19,118	0	0	0	0.00
South Cotabato	30,150	350	475	825	2.74
Sultan Kudarat	27,009	629	843	1,472	5.45
Cotabato City	10,329	1,759	1,960	3,719	36.00
Gen. Santos City	19,580	58	80	138	0.70
**BARMM**	**154,513**	**11,261**	**11,334**	**22,595**	**14.62**
Basilan	11,260	46	59	105	0.93
Lanao del Sur	33,753	952	997	1,949	5.77
Maguindanao	48,597	4,527	4,588	9,115	18.76
Sulu	34,766	5,462	5,379	10,841	31.18
Tawi-Tawi	14,968	0	0	0	0.00
Lamitan City	3,099	0	0	0	0.00
Marawi City	8,070	274	311	585	7.25
**CARAGA**	**87,334**	**3,355**	**3,750**	**7,105**	**8.14**
Agusan del Norte	11,510	495	561	1,056	9.17
Agusan del Sur	24,034	219	279	498	2.07
Surigao del Norte	11,664	205	270	475	4.07
Surigao del Sur	16,440	807	889	1,696	10.32
Province of Dinagat	4,185	864	871	1,735	41.46
Bislig City	3,124	24	21	45	1.44
Butuan City	10,945	504	671	1,175	10.74
Surigao City	5,433	237	188	425	7.82

Note: Put asterisk (*) for No Report and Zero (0) for No Case

Table 1.D.7 - Oral Health Care

Number and proportion of adolescents 15- 19 years old who received Basic Oral Health Care (BOHC)

Philippines, 2021

Area	Adolescents 15-19 years old who received BOHC				
	Eligible Population	Male	Female	Total	%
**PHILIPPINES**	**3,104,307**	**115,902**	**143,295**	**259,197**	**8.35**
**N C R**	**356,312**	**19,386**	**24,154**	**43,540**	**12.22**
Malabon	10,114	289	315	604	5.97
Navotas	6,903	233	285	518	7.50
Valenzuela City	17,166	1,899	1,967	3,866	22.52
Caloocan City	43,829	4,177	5,658	9,835	22.44
Marikina City	12,473	0	0	0	0.00
Pasig City	20,900	7,469	9,831	17,300	82.78
Pateros	1,766	18	44	62	3.51
Taguig	22,272	257	343	600	2.69
Quezon City	81,239	108	191	299	0.37
Makati City	16,120	238	367	605	3.75
Mandaluyong City	10,689	198	237	435	4.07
San Juan	3,380	111	140	251	7.43
Manila City	49,257	1,671	2,054	3,725	7.56
Las Piñas City	16,294	54	88	142	0.87
Muntinlupa City	13,959	2,106	2,028	4,134	29.61
Parañaque City	18,423	153	150	303	1.64
Pasay City	11,528	405	456	861	7.47
**C A R**	**52,107**	**4,108**	**6,521**	**10,629**	**20.40**
Abra	6,992	515	562	1,077	15.40
Apayao	3,641	123	230	353	9.70
Benguet	13,608	2,214	3,248	5,462	40.14
Ifugao	6,234	310	804	1,114	17.87
Kalinga	6,530	226	370	596	9.13
Mt. Province	4,572	128	242	370	8.09
Baguio City	10,530	592	1,065	1,657	15.74
**Region 1**	**144,561**	**10,152**	**11,958**	**22,110**	**15.29**
Ilocos Norte	13,199	320	359	679	5.14
Ilocos Sur	15,786	1,957	2,205	4,162	26.37
La Union	19,110	1,290	1,703	2,993	15.66
Pangasinan	69,683	5,626	6,563	12,189	17.49
Alaminos City	2,633	500	625	1,125	42.73
Candon City	1,665	136	135	271	16.28
Dagupan City	5,027	42	58	100	1.99
Laoag City	3,043	0	0	0	0.00
San Carlos City	5,534	144	148	292	5.28
San Fernando City	3,501	28	35	63	1.80
Urdaneta City	3,902	0	0	0	0.00
Vigan City	1,478	109	127	236	15.97

Table 1.D.7 - Oral Health Care

Number and proportion of adolescents 15- 19 years old who received Basic Oral Health Care (BOHC)

Philippines, 2021

Area	Adolescents 15-19 years old who received BOHC				
	Eligible Population	Male	Female	Total	%
**Region 2**	**98,742**	**5,135**	**6,876**	**12,011**	**12.16**
Batanes	432	84	109	193	44.68
Cagayan	30,190	560	812	1,372	4.54
Isabela	33,517	1,278	1,674	2,952	8.81
Nueva Vizcaya	13,387	890	1,530	2,420	18.08
Quirino	5,175	16	56	72	1.39
Cauayan City	3,668	49	83	132	3.60
Ilagan City	4,122	1,346	1,618	2,964	71.91
Santiago City	3,819	912	994	1,906	49.91
Tuguegarao City	4,432	0	0	0	0.00
**Region 3**	**349,374**	**5,412**	**6,575**	**11,987**	**3.43**
Aurora	6,737	289	289	578	8.58
Bataan	20,406	105	144	249	1.22
Bulacan	71,433	570	850	1,420	1.99
Nueva Ecija	44,969	0	0	0	0.00
Pampanga	51,989	204	509	713	1.37
Tarlac	31,299	1,202	1,285	2,487	7.95
Zambales	17,995	551	713	1,264	7.02
Angeles City	13,044	21	23	44	0.34
Balanga City	2,950	28	51	79	2.68
Cabanatuan City	9,203	26	53	79	0.86
City of San Fernando	9,718	0	0	0	0.00
Gapan City	3,359	0	0	0	0.00
Mabalacat City	7,947	378	383	761	9.58
Malolos City	7,979	68	90	158	1.98
Meycauayan	6,617	75	92	167	2.52
Olongapo	7,099	0	0	0	0.00
Palayan City	1,250	9	14	23	1.84
San Jose City	4,256	655	805	1,460	34.30
San Jose del Monte City	18,171	331	394	725	3.99
Science City of Munoz	2,481	51	72	123	4.96
Tarlac City	10,473	849	808	1,657	15.82
**Region 4A**	**457,837**	**20,935**	**24,403**	**45,338**	**9.90**
Batangas	60,342	8,049	8,373	16,422	27.21
Cavite	44,670	892	1,167	2,059	4.61
Laguna	29,763	2,969	3,669	6,638	22.30
Quezon	55,601	1,703	2,225	3,928	7.06
Rizal	67,513	3,053	3,580	6,633	9.82
Antipolo City	24,867	0	0	0	0.00
Bacoor City	19,568	155	260	415	2.12
Batangas City	10,709	173	447	620	5.79
Biñan City	9,980	571	747	1,318	13.21
Cabuyao City	9,252	235	360	595	6.43

Table 1.D.7 - Oral Health Care

Number and proportion of adolescents 15- 19 years old who received Basic Oral Health Care (BOHC)

Philippines, 2021

Area	Adolescents 15-19 years old who received BOHC				
	Eligible Population	Male	Female	Total	%
Calamba City	13,621	911	1,341	2,252	16.53
Cavite City	3,350	19	42	61	1.82
Dasmariñas City	21,473	0	0	0	0.00
General Trias City	10,241	0	0	0	0.00
Imus City	13,155	152	253	405	3.08
Lipa City	10,790	92	107	199	1.84
Lucena City	8,426	9	10	19	0.23
San Pablo City	7,975	85	167	252	3.16
San Pedro City	9,763	1,044	782	1,826	18.70
Santa Rosa City	10,601	34	64	98	0.92
Tagaytay City	2,319	7	20	27	1.16
Tanauan City	5,627	144	149	293	5.21
Tayabas City	3,158	204	276	480	15.20
Trece Martires City	5,074	434	364	798	15.73
**Region 4B**	**93,865**	**873**	**1,459**	**2,332**	**2.48**
Marinduque	6,810	198	308	506	7.43
Mindoro Occidental	15,167	86	174	260	1.71
Mindoro Oriental	26,033	0	9	9	0.03
Palawan	28,611	416	717	1,133	3.96
Romblon	8,652	173	251	424	4.90
Puerto Princesa City	8,593	0	0	0	0.00
**Region 5**	**187,578**	**5,449**	**7,387**	**12,836**	**6.84**
Albay	35,619	1,038	1,198	2,236	6.28
Camarines Norte	18,701	1,350	1,872	3,222	17.23
Camarines Sur	53,034	529	854	1,383	2.61
Catanduanes	8,121	178	183	361	4.45
Masbate	30,410	1,457	1,739	3,196	10.51
Sorsogon	25,507	549	1,048	1,597	6.26
Iriga City	3,602	124	188	312	8.66
Legaspi City	6,265	59	82	141	2.25
Naga City	6,320	165	223	388	6.14
**Region 6**	**223,218**	**5,559**	**7,427**	**12,986**	**5.82**
Aklan	16,848	294	389	683	4.05
Antique	16,694	0	0	0	0.00
Capiz	22,962	622	819	1,441	6.28
Guimaras	5,108	17	15	32	0.63
Iloilo	57,254	3,021	3,977	6,998	12.22
Negros Occidental	74,367	639	970	1,609	2.16
Bacolod City	16,735	904	1,180	2,084	12.45
Iloilo City	13,249	62	77	139	1.05

Table 1.D.7 - Oral Health Care

Number and proportion of adolescents 15- 19 years old who received Basic Oral Health Care (BOHC)

Philippines, 2021

Area	Adolescents 15-19 years old who received BOHC				
	Eligible Population	Male	Female	Total	%
**Region 7**	**224,496**	**4,325**	**6,231**	**10,556**	**4.70**
Bohol	38,123	290	397	687	1.80
Cebu	90,938	2,764	3,985	6,749	7.42
Negros Oriental	40,433	275	641	916	2.27
Siquijor	2,599	42	74	116	4.46
Cebu City	28,551	148	364	512	1.79
Lapu-Lapu City	12,630	806	770	1,576	12.48
Mandaue City	11,222	0	0	0	0.00
**Region 8**	**144,344**	**1,489**	**2,175**	**3,664**	**2.54**
Biliran	5,321	60	56	116	2.18
Eastern Samar	15,299	258	432	690	4.51
Northern Leyte	49,226	0	0	0	0.00
Northern Samar	21,343	395	650	1,045	4.90
Southern Leyte	9,880	0	0	0	0.00
Western Samar	19,765	262	469	731	3.70
Calbayog City	6,092	58	91	149	2.45
Maasin City	2,515	27	40	67	2.66
Ormoc City	7,009	429	437	866	12.36
Tacloban City	7,896	0	0	0	0.00
**Region 9**	**115,499**	**1,683**	**2,474**	**4,157**	**3.60**
Zamboanga del Norte	25,047	19	31	50	0.20
Zamboanga del Sur	25,627	926	1,378	2,304	8.99
Zamboanga Sibugay	20,654	286	468	754	3.65
Dapitan City	2,586	82	117	199	7.70
Dipolog City	4,101	50	82	132	3.22
Isabela City	3,987	320	398	718	18.01
Pagadian City	6,286	0	0	0	0.00
Zamboanga City	27,212	0	0	0	0.00
**Region 10**	**148,272**	**9,323**	**11,692**	**21,015**	**14.17**
Bukidnon	33,762	1,039	1,487	2,526	7.48
Camiguin	2,602	30	37	67	2.58
Lanao del Norte	22,833	97	137	234	1.02
Misamis Occidental	9,644	4,607	5,228	9,835	101.98
Misamis Oriental	21,841	1,074	1,085	2,159	9.89
Cagayan de Oro City	20,687	0	0	0	0.00
El Salvador City	1,536	1	9	10	0.65
Gingoog City	3,815	442	469	911	23.88
Iligan City	11,566	214	327	541	4.68
Malaybalay City	5,629	365	553	918	16.31
Oroquieta City	2,089	0	0	0	0.00
Ozamis City	4,188	3	2	5	0.12

Table 1.D.7 - Oral Health Care

Number and proportion of adolescents 15- 19 years old who received Basic Oral Health Care (BOHC)

Philippines, 2021

Area	Adolescents 15-19 years old who received BOHC				
	Eligible Population	Male	Female	Total	%
Tangub City	1,860	422	689	1,111	59.73
Valencia City	6,220	1,029	1,669	2,698	43.38
**Region 11**	**150,623**	**659**	**748**	**1,407**	**0.93**
Davao de Oro	21,816	125	114	239	1.10
Davao del Norte	29,336	163	160	323	1.10
Davao Oriental	17,971	110	111	221	1.23
Davao del Sur	20,072	58	100	158	0.79
Davao Occidental	9,611	35	46	81	0.84
Davao City	51,818	168	217	385	0.74
**Region 12**	**146,348**	**6,972**	**8,931**	**15,903**	**10.87**
North Cotabato	45,933	2,350	2,845	5,195	11.31
Sarangani	18,393	0	0	0	0.00
South Cotabato	28,824	642	1,043	1,685	5.85
Sultan Kudarat	25,259	1,294	1,788	3,082	12.20
Cotabato City	9,218	2,547	2,778	5,325	57.77
Gen. Santos City	18,721	139	477	616	3.29
**BARMM**	**131,008**	**11,153**	**10,477**	**21,630**	**16.51**
Basilan	9,761	41	57	98	1.00
Lanao del Sur	28,716	648	796	1,444	5.03
Maguindanao	42,466	4,289	4,251	8,540	20.11
Sulu	27,671	6,022	5,263	11,285	40.78
Tawi-Tawi	12,841	0	0	0	0.00
Lamitan City	2,685	0	0	0	0.00
Marawi City	6,868	153	110	263	3.83
**CARAGA**	**80,124**	**3,289**	**3,807**	**7,096**	**8.86**
Agusan del Norte	11,140	249	327	576	5.17
Agusan del Sur	21,398	242	267	509	2.38
Surigao del Norte	10,464	125	194	319	3.05
Surigao del Sur	15,218	857	951	1,808	11.88
Province of Dinagat	3,550	980	1,011	1,991	56.09
Bislig City	2,890	32	35	67	2.32
Butuan City	10,594	526	670	1,196	11.29
Surigao City	4,872	278	352	630	12.93

Note: Put asterisk (*) for No Report and Zero (0) for No Case

Table 1.D.8 - Oral Health Care

Number and proportion of adolescents 20 -59 years old who received Basic Oral Health Care (BOHC)
Philippines, 2021

Area	Adults 20-59 years old who received BOHC				
	Eligible Population	Male	Female	Total	%
**PHILIPPINES**	**17,083,404**	**333,936**	**521,973**	**855,909**	**5.01**
**N C R**	**2,346,732**	**56,618**	**90,374**	**146,992**	**6.26**
Malabon	66,612	362	483	845	1.27
Navotas	45,462	300	354	654	1.44
Valenzuela City	113,065	2,394	2,567	4,961	4.39
Caloocan City	288,662	8,341	12,434	20,775	7.20
Marikina City	82,142	8	16	24	0.03
Pasig City	137,644	31,513	54,617	86,130	62.57
Pateros	11,635	153	247	400	3.44
Taguig	146,686	909	1,707	2,616	1.78
Quezon City	535,074	387	726	1,113	0.21
Makati City	106,173	1,995	2,635	4,630	4.36
Mandaluyong City	70,395	509	638	1,147	1.63
San Juan	22,266	482	878	1,360	6.11
Manila City	324,413	2,942	4,646	7,588	2.34
Las Piñas City	107,319	123	481	604	0.56
Muntinlupa City	91,941	5,035	6,113	11,148	12.13
Parañaque City	121,338	240	407	647	0.53
Pasay City	75,906	925	1,425	2,350	3.10
**C A R**	**286,862**	**14,750**	**24,573**	**39,323**	**13.71**
Abra	38,299	661	987	1,648	4.30
Apayao	18,835	315	771	1,086	5.77
Benguet	79,942	10,662	14,495	25,157	31.47
Ifugao	31,697	582	1,671	2,253	7.11
Kalinga	32,861	348	975	1,323	4.03
Mt. Province	23,354	303	630	933	4.00
Baguio City	61,874	1,879	5,044	6,923	11.19
**Region 1**	**813,888**	**34,748**	**47,196**	**81,944**	**10.07**
Ilocos Norte	81,016	1,779	2,178	3,957	4.88
Ilocos Sur	95,793	9,052	10,446	19,498	20.35
La Union	112,881	3,113	7,522	10,635	9.42
Pangasinan	374,009	18,005	23,884	41,889	11.20
Alaminos City	14,132	1,345	1,111	2,456	17.38
Candon City	10,096	342	413	755	7.48
Dagupan City	26,979	173	323	496	1.84
Laoag City	18,682	0	0	0	0.00
San Carlos City	29,706	274	443	717	2.41
San Fernando City	20,682	344	414	758	3.67
Urdaneta City	20,943	56	96	152	0.73
Vigan City	8,970	265	366	631	7.03

Table 1.D.8 - Oral Health Care

Number and proportion of adolescents 20 -59 years old who received Basic Oral Health Care (BOHC)
Philippines, 2021

Area	Adults 20-59 years old who received BOHC				
	Eligible Population	Male	Female	Total	%
**Region 2**	**574,595**	**14,155**	**25,315**	**39,470**	**6.87**
Batanes	2,618	251	381	632	24.14
Cagayan	174,253	1,468	2,761	4,229	2.43
Isabela	198,293	2,447	4,663	7,110	3.59
Nueva Vizcaya	74,851	3,541	7,604	11,145	14.89
Quirino	30,335	217	693	910	3.00
Cauayan City	21,698	134	328	462	2.13
Ilagan City	24,385	2,889	5,078	7,967	32.67
Santiago City	22,587	3,208	3,807	7,015	31.06
Tuguegarao City	25,576	0	0	0	0.00
**Region 3**	**2,010,897**	**20,655**	**28,940**	**49,595**	**2.47**
Aurora	34,457	857	854	1,711	4.97
Bataan	119,196	362	703	1,065	0.89
Bulacan	413,300	1,860	3,276	5,136	1.24
Nueva Ecija	261,645	2,106	3,288	5,394	2.06
Pampanga	300,522	1,653	946	2,599	0.86
Tarlac	176,181	2,942	3,688	6,630	3.76
Zambales	102,374	1,328	1,663	2,991	2.92
Angeles City	75,401	81	87	168	0.22
Balanga City	17,229	219	1,063	1,282	7.44
Cabanatuan City	53,551	19	55	74	0.14
City of San Fernando	56,173	2	1	3	0.01
Gapan City	19,544	46	39	85	0.43
Mabalacat City	45,940	2,616	2,499	5,115	11.13
Malolos City	46,161	225	310	535	1.16
Meycauayan	38,290	154	190	344	0.90
Olongapo	40,378	0	0	0	0.00
Palayan City	7,272	24	26	50	0.69
San Jose City	24,758	1,320	1,943	3,263	13.18
San Jose del Monte City	105,135	2,498	5,393	7,891	7.51
Science City of Munoz	14,438	220	725	945	6.55
Tarlac City	58,953	2,123	2,191	4,314	7.32
**Region 4A**	**2,639,107**	**79,273**	**115,307**	**194,580**	**7.37**
Batangas	341,644	27,970	33,268	61,238	17.92
Cavite	265,181	3,321	5,522	8,843	3.33
Laguna	184,211	9,792	13,916	23,708	12.87
Quezon	282,702	3,387	6,327	9,714	3.44
Rizal	385,728	5,510	8,511	14,021	3.63
Antipolo City	142,076	7,751	10,010	17,761	12.50
Bacoor City	116,179	680	1,296	1,976	1.70
Batangas City	60,634	1,161	4,297	5,458	9.00
Biñan City	61,768	2,900	4,114	7,014	11.36
Cabuyao City	57,265	1,656	2,372	4,028	7.03

Table 1.D.8 - Oral Health Care

Number and proportion of adolescents 20 -59 years old who received Basic Oral Health Care (BOHC)
Philippines, 2021

Area	Adults 20-59 years old who received BOHC				
	Eligible Population	Male	Female	Total	%
Calamba City	84,298	6,149	9,385	15,534	18.43
Cavite City	19,888	86	301	387	1.95
Dasmariñas City	127,479	0	0	0	0.00
General Trias City	60,799	0	0	0	0.00
Imus City	78,109	663	1,639	2,302	2.95
Lipa City	61,094	151	223	374	0.61
Lucena City	42,846	20	35	55	0.13
San Pablo City	49,349	1,590	4,501	6,091	12.34
San Pedro City	60,429	4,013	6,522	10,535	17.43
Santa Rosa City	65,616	214	269	483	0.74
Tagaytay City	13,769	96	190	286	2.08
Tanauan City	31,866	1,207	1,429	2,636	8.27
Tayabas City	16,057	403	791	1,194	7.44
Trece Martires City	30,121	553	389	942	3.13
**Region 4B**	**458,311**	**4,348**	**7,552**	**11,900**	**2.60**
Marinduque	33,636	407	756	1,163	3.46
Mindoro Occidental	73,111	399	826	1,225	1.68
Mindoro Oriental	131,027	2,188	3,928	6,116	4.67
Palawan	137,493	1,034	1,485	2,519	1.83
Romblon	41,751	320	557	877	2.10
Puerto Princesa City	41,292	0	0	0	0.00
**Region 5**	**875,505**	**14,418**	**29,193**	**43,611**	**4.98**
Albay	175,694	3,665	4,903	8,568	4.88
Camarines Norte	90,518	2,450	3,876	6,326	6.99
Camarines Sur	247,136	1,335	7,924	9,259	3.75
Catanduanes	38,257	1,259	1,743	3,002	7.85
Masbate	127,281	3,326	5,316	8,642	6.79
Sorsogon	119,480	1,675	4,120	5,795	4.85
Iriga City	16,792	329	704	1,033	6.15
Legaspi City	30,899	98	153	251	0.81
Naga City	29,450	281	454	735	2.50
**Region 6**	**1,222,957**	**16,493**	**27,598**	**44,091**	**3.61**
Aklan	93,989	518	1,035	1,553	1.65
Antique	89,547	1,492	1,766	3,258	3.64
Capiz	127,385	941	1,248	2,189	1.72
Guimaras	29,347	46	80	126	0.43
Iloilo	322,573	8,979	16,118	25,097	7.78
Negros Occidental	396,319	1,727	3,416	5,143	1.30
Bacolod City	89,170	2,596	3,592	6,188	6.94
Iloilo City	74,627	194	343	537	0.72

Table 1.D.8 - Oral Health Care

Number and proportion of adolescents 20 -59 years old who received Basic Oral Health Care (BOHC)
Philippines, 2021

Area	Adults 20-59 years old who received BOHC				
	Eligible Population	Male	Female	Total	%
**Region 7**	**1,234,501**	**9,434**	**18,310**	**27,744**	**2.25**
Bohol	198,925	370	849	1,219	0.61
Cebu	511,764	6,601	11,734	18,335	3.58
Negros Oriental	213,649	966	2,129	3,095	1.45
Siquijor	15,296	111	207	318	2.08
Cebu City	160,653	699	2,592	3,291	2.05
Lapu-Lapu City	71,065	591	616	1,207	1.70
Mandaue City	63,149	96	183	279	0.44
**Region 8**	**690,066**	**4,187**	**7,486**	**11,673**	**1.69**
Biliran	25,906	146	211	357	1.38
Eastern Samar	72,272	578	980	1,558	2.16
Northern Leyte	242,754	1,484	3,024	4,508	1.86
Northern Samar	93,040	500	1,057	1,557	1.67
Southern Leyte	52,830	137	301	438	0.83
Western Samar	88,912	305	389	694	0.78
Calbayog City	27,399	443	577	1,020	3.72
Maasin City	13,447	14	17	31	0.23
Ormoc City	34,580	537	740	1,277	3.69
Tacloban City	38,928	43	190	233	0.60
**Region 9**	**568,332**	**5,925**	**9,442**	**15,367**	**2.70**
Zamboanga del Norte	122,676	52	307	359	0.29
Zamboanga del Sur	127,148	1,984	2,677	4,661	3.67
Zamboanga Sibugay	100,126	1,045	1,893	2,938	2.93
Dapitan City	12,665	108	156	264	2.08
Dipolog City	20,096	332	543	875	4.35
Isabela City	19,426	1,276	1,692	2,968	15.28
Pagadian City	31,186	1,128	2,174	3,302	10.59
Zamboanga City	135,009	0	0	0	0.00
**Region 10**	**775,646**	**23,528**	**32,163**	**55,691**	**7.18**
Bukidnon	173,077	1,376	2,392	3,768	2.18
Camiguin	13,614	218	259	477	3.50
Lanao del Norte	109,209	160	219	379	0.35
Misamis Occidental	51,509	15,961	19,542	35,503	68.93
Misamis Oriental	122,579	747	1,750	2,497	2.04
Cagayan de Oro City	116,103	0	0	0	0.00
El Salvador City	8,624	28	138	166	1.92
Gingoog City	21,409	496	453	949	4.43
Iligan City	55,316	757	1,955	2,712	4.90
Malaybalay City	28,850	549	1,094	1,643	5.70
Oroquieta City	11,160	16	17	33	0.30
Ozamis City	22,372	0	0	0	0.00

Table 1.D.8 - Oral Health Care

Number and proportion of adolescents 20 -59 years old who received Basic Oral Health Care (BOHC)
Philippines, 2021

Area	Adults 20-59 years old who received BOHC				
	Eligible Population	Male	Female	Total	%
Tangub City	9,940	1,278	1,867	3,145	31.64
Valencia City	31,885	1,942	2,477	4,419	13.86
**Region 11**	**824,881**	**692**	**1,120**	**1,812**	**0.22**
Davao de Oro	119,904	100	143	243	0.20
Davao del Norte	165,217	150	222	372	0.23
Davao Oriental	87,805	102	196	298	0.34
Davao del Sur	111,894	122	140	262	0.23
Davao Occidental	51,216	32	50	82	0.16
Davao City	288,845	186	369	555	0.19
**Region 12**	**759,797**	**12,511**	**30,971**	**43,482**	**5.72**
North Cotabato	231,850	3,970	14,545	18,515	7.99
Sarangani	87,634	1,139	2,562	3,701	4.22
South Cotabato	157,865	1,807	3,232	5,039	3.19
Sultan Kudarat	132,539	2,031	3,352	5,383	4.06
Cotabato City	47,384	2,280	2,313	4,593	9.69
Gen. Santos City	102,526	1,284	4,967	6,251	6.10
**BARMM**	**593,892**	**16,291**	**16,829**	**33,120**	**5.58**
Basilan	42,020	50	73	123	0.29
Lanao del Sur	130,654	1,064	1,896	2,960	2.27
Maguindanao	189,487	8,380	8,904	17,284	9.12
Sulu	129,695	6,539	5,482	12,021	9.27
Tawi-Tawi	59,222	0	0	0	0.00
Lamitan City	11,561	0	0	0	0.00
Marawi City	31,254	258	474	732	2.34
**CARAGA**	**407,437**	**5,910**	**9,604**	**15,514**	**3.81**
Agusan del Norte	57,191	1,426	2,613	4,039	7.06
Agusan del Sur	106,516	686	583	1,269	1.19
Surigao del Norte	54,095	343	740	1,083	2.00
Surigao del Sur	76,385	1,847	3,173	5,020	6.57
Province of Dinagat	19,170	243	441	684	3.57
Bislig City	14,508	90	119	209	1.44
Butuan City	54,378	963	1,526	2,489	4.58
Surigao City	25,194	312	409	721	2.86

Note: Put asterisk (*) for No Report and Zero (0) for No Case

Table 1.D.9 - Oral Health Care

Number and proportion of senior citizens 60 years old and above who received Basic Oral Health Care (BOHC)
Philippines, 2021

Area	Senior citizens 60 years old and above who received BOHC				
	Eligible Population	Male	Female	Total	%
**PHILIPPINES**	**2,958,918**	**193,670**	**168,797**	**362,467**	**12.25**
**N C R**	**385,012**	**23,496**	**30,442**	**53,938**	**14.01**
Malabon	10,929	165	189	354	3.24
Navotas	7,460	101	150	251	3.36
Valenzuela City	18,548	2,920	3,216	6,136	33.08
Caloocan City	47,360	2,111	2,266	4,377	9.24
Marikina City	13,477	1	2	3	0.02
Pasig City	22,583	9,580	12,691	22,271	98.62
Pateros	1,909	22	26	48	2.51
Taguig	24,066	139	191	330	1.37
Quezon City	87,786	1,016	1,446	2,462	2.80
Makati City	17,419	271	297	568	3.26
Mandaluyong City	11,549	174	213	387	3.35
San Juan	3,653	414	780	1,194	32.69
Manila City	53,224	2,568	3,147	5,715	10.74
Las Piñas City	17,607	109	148	257	1.46
Muntinlupa City	15,084	3,602	5,347	8,949	59.33
Parañaque City	19,907	132	178	310	1.56
Pasay City	12,452	171	155	326	2.62
**C A R**	**46,599**	**4,228**	**5,632**	**9,860**	**21.16**
Abra	8,099	361	481	842	10.40
Apayao	3,093	124	147	271	8.76
Benguet	11,820	2,976	4,074	7,050	59.64
Ifugao	4,849	177	304	481	9.92
Kalinga	5,357	72	86	158	2.95
Mt. Province	4,235	141	141	282	6.66
Baguio City	9,147	377	399	776	8.48
**Region 1**	**172,887**	**9,089**	**11,052**	**20,141**	**11.65**
Ilocos Norte	18,956	727	817	1,544	8.15
Ilocos Sur	22,258	2,082	2,251	4,333	19.47
La Union	24,556	1,074	1,398	2,472	10.07
Pangasinan	75,332	3,868	4,865	8,733	11.59
Alaminos City	2,847	580	700	1,280	44.96
Candon City	2,346	158	213	371	15.81
Dagupan City	5,435	32	37	69	1.27
Laoag City	4,373	0	0	0	0.00
San Carlos City	5,984	169	211	380	6.35
San Fernando City	4,499	327	489	816	18.14
Urdaneta City	4,219	7	12	19	0.45

Table 1.D.9 - Oral Health Care

Number and proportion of senior citizens 60 years old and above who received Basic Oral Health Care (BOHC)
Philippines, 2021

Area	Senior citizens 60 years old and above who received BOHC				
	Eligible Population	Male	Female	Total	%
Vigan City	2,084	65	59	124	5.95
**Region 2**	**109,828**	**7,836**	**10,281**	**18,117**	**16.50**
Batanes	615	45	69	114	18.54
Cagayan	35,475	299	449	748	2.11
Isabela	36,718	1,287	1,589	2,876	7.83
Nueva Vizcaya	13,894	1,320	1,932	3,252	23.41
Quirino	5,206	54	91	145	2.79
Cauayan City	4,018	323	437	760	18.92
Ilagan City	4,514	1,857	2,286	4,143	91.78
Santiago City	4,181	2,651	3,428	6,079	145.38
Tuguegarao City	5,206	0	0	0	0.00
**Region 3**	**359,077**	**8,886**	**10,228**	**19,114**	**5.32**
Aurora	5,807	226	263	489	8.42
Bataan	21,100	37	56	93	0.44
Bulacan	71,112	471	669	1,140	1.60
Nueva Ecija	49,150	668	864	1,532	3.12
Pampanga	52,504	609	498	1,107	2.11
Tarlac	33,088	1,482	1,555	3,037	9.18
Zambales	18,714	429	433	862	4.61
Angeles City	13,176	31	23	54	0.41
Balanga City	3,050	181	237	418	13.71
Cabanatuan City	10,060	2	9	11	0.11
City of San Fernando	9,815	0	0	0	0.00
Gapan City	3,672	0	0	0	0.00
Mabalacat City	8,028	865	882	1,747	21.76
Malolos City	7,944	51	55	106	1.33
Meycauayan	6,589	114	102	216	3.28
Olongapo	7,382	0	0	0	0.00
Palayan City	1,367	4	3	7	0.51
San Jose City	4,651	288	357	645	13.87
San Jose del Monte City	18,090	2,254	2,818	5,072	28.04
Science City of Munoz	2,711	127	318	445	16.42
Tarlac City	11,071	1,047	1,086	2,133	19.27
**Region 4A**	**427,537**	**99,428**	**50,535**	**149,963**	**35.08**
Batangas	60,330	7,001	6,939	13,940	23.11
Cavite	40,921	758	744	1,502	3.67
Laguna	28,863	10,068	7,805	17,873	61.92
Quezon	50,877	1,097	1,317	2,414	4.74
Rizal	58,916	1,889	2,254	4,143	7.03
Antipolo City	21,701	1,768	2,277	4,045	18.64
Bacoor City	17,927	145	202	347	1.94
Batangas City	10,706	154	247	401	3.75

Table 1.D.9 - Oral Health Care

Number and proportion of senior citizens 60 years old and above who received Basic Oral Health Care (BOHC)
Philippines, 2021

Area	Senior citizens 60 years old and above who received BOHC				
	Eligible Population	Male	Female	Total	%
Biñan City	9,678	47,935	6,010	53,945	557.40
Cabuyao City	8,973	3,181	2,801	5,982	66.66
Calamba City	13,207	17,511	8,148	25,659	194.28
Cavite City	3,067	11	29	40	1.30
Dasmariñas City	19,672	0	0	0	0.00
General Trias City	9,381	0	0	0	0.00
Imus City	12,053	146	259	405	3.36
Lipa City	10,787	99	121	220	2.04
Lucena City	7,712	3	0	3	0.04
San Pablo City	7,731	609	1,713	2,322	30.03
San Pedro City	9,468	1,766	2,514	4,280	45.21
Santa Rosa City	10,281	4,514	6,218	10,732	104.39
Tagaytay City	2,123	10	9	19	0.90
Tanauan City	5,626	351	496	847	15.06
Tayabas City	2,890	108	107	215	7.44
Trece Martires City	4,648	304	325	629	13.53
**Region 4B**	**80,480**	**1,269**	**1,880**	**3,149**	**3.91**
Marinduque	8,045	222	275	497	6.18
Mindoro Occidental	12,150	341	635	976	8.03
Mindoro Oriental	23,557	417	584	1,001	4.25
Palawan	21,169	155	231	386	1.82
Romblon	9,203	134	155	289	3.14
Puerto Princesa City	6,356	0	0	0	0.00
**Region 5**	**160,964**	**3,917**	**5,285**	**9,202**	**5.72**
Albay	34,267	767	926	1,693	4.94
Camarines Norte	16,057	553	792	1,345	8.38
Camarines Sur	44,387	271	566	837	1.89
Catanduanes	7,388	543	626	1,169	15.82
Masbate	22,262	1,222	1,531	2,753	12.37
Sorsogon	22,272	434	661	1,095	4.92
Iriga City	3,015	52	85	137	4.54
Legaspi City	6,027	15	23	38	0.63
Naga City	5,289	60	75	135	2.55
**Region 6**	**252,968**	**6,797**	**10,017**	**16,814**	**6.65**
Aklan	19,085	92	145	237	1.24
Antique	18,416	802	870	1,672	9.08
Capiz	27,087	490	679	1,169	4.32
Guimaras	6,006	4	9	13	0.22
Iloilo	69,771	4,003	5,471	9,474	13.58
Negros Occidental	78,744	979	2,250	3,229	4.10
Bacolod City	17,716	372	491	863	4.87
Iloilo City	16,143	55	102	157	0.97

Table 1.D.9 - Oral Health Care

Number and proportion of senior citizens 60 years old and above who received Basic Oral Health Care (BOHC)
Philippines, 2021

Area	Senior citizens 60 years old and above who received BOHC				
	Eligible Population	Male	Female	Total	%
**Region 7**	**231,440**	**3,927**	**4,616**	**8,543**	**3.69**
Bohol	44,657	109	183	292	0.65
Cebu	88,219	2,797	3,473	6,270	7.11
Negros Oriental	43,817	170	218	388	0.89
Siquijor	3,923	61	89	150	3.82
Cebu City	27,691	127	28	155	0.56
Lapu-Lapu City	12,249	663	625	1,288	10.51
Mandaue City	10,885	0	0	0	0.00
**Region 8**	**134,192**	**1,692**	**1,944**	**3,636**	**2.71**
Biliran	5,236	149	221	370	7.07
Eastern Samar	14,433	463	433	896	6.21
Northern Leyte	48,021	282	408	690	1.44
Northern Samar	15,688	136	196	332	2.12
Southern Leyte	12,291	0	0	0	0.00
Western Samar	15,941	190	164	354	2.22
Calbayog City	4,913	183	218	401	8.16
Maasin City	3,130	2	2	4	0.13
Ormoc City	6,840	272	290	562	8.22
Tacloban City	7,699	15	12	27	0.35
**Region 9**	**93,599**	**1,274**	**1,610**	**2,884**	**3.08**
Zamboanga del Norte	22,221	50	116	166	0.75
Zamboanga del Sur	20,664	860	1,005	1,865	9.03
Zamboanga Sibugay	14,903	256	350	606	4.07
Dapitan City	2,294	16	22	38	1.66
Dipolog City	3,640	19	36	55	1.51
Isabela City	2,875	27	29	56	1.95
Pagadian City	5,067	46	52	98	1.93
Zamboanga City	21,934	0	0	0	0.00
**Region 10**	**130,253**	**5,362**	**6,425**	**11,787**	**9.05**
Bukidnon	26,150	359	512	871	3.33
Camiguin	3,100	18	31	49	1.58
Lanao del Norte	16,296	39	41	80	0.49
Misamis Occidental	11,056	2,461	3,053	5,514	49.88
Misamis Oriental	21,391	323	525	848	3.96
Cagayan de Oro City	20,258	0	0	0	0.00
El Salvador City	1,505	97	205	302	20.07
Gingoog City	3,737	696	693	1,389	37.17
Iligan City	8,254	86	149	235	2.85
Malaybalay City	4,359	614	646	1,260	28.90

Table 1.D.9 - Oral Health Care

Number and proportion of senior citizens 60 years old and above who received Basic Oral Health Care (BOHC)
Philippines, 2021

Area	Senior citizens 60 years old and above who received BOHC				
	Eligible Population	Male	Female	Total	%
Oroquieta City	2,395	1	0	1	0.04
Ozamis City	4,802	0	0	0	0.00
Tangub City	2,134	229	255	484	22.68
Valencia City	4,818	439	315	754	15.65
**Region 11**	**137,572**	**183**	**247**	**430**	**0.31**
Davao de Oro	18,818	17	32	49	0.26
Davao del Norte	27,698	38	54	92	0.33
Davao Oriental	15,640	54	54	108	0.69
Davao del Sur	18,731	22	33	55	0.29
Davao Occidental	8,338	8	16	24	0.29
Davao City	48,346	44	58	102	0.21
**Region 12**	**109,528**	**4,900**	**6,363**	**11,263**	**10.28**
North Cotabato	34,944	1,742	2,038	3,780	10.82
Sarangani	11,838	900	1,061	1,961	16.57
South Cotabato	23,519	281	442	723	3.07
Sultan Kudarat	18,269	879	1,068	1,947	10.66
Cotabato City	5,686	827	1,028	1,855	32.63
Gen. Santos City	15,273	271	726	997	6.53
**BARMM**	**55,826**	**9,562**	**9,651**	**19,213**	**34.42**
Basilan	4,327	30	56	86	1.99
Lanao del Sur	11,335	342	363	705	6.22
Maguindanao	17,689	2,848	3,123	5,971	33.76
Sulu	12,656	6,302	6,076	12,378	97.80
Tawi-Tawi	5,918	0	0	0	0.00
Lamitan City	1,192	0	0	0	0.00
Marawi City	2,709	40	33	73	2.69
**CARAGA**	**71,155**	**1,824**	**2,589**	**4,413**	**6.20**
Agusan del Norte	10,118	274	440	714	7.06
Agusan del Sur	15,542	95	124	219	1.41
Surigao del Norte	10,229	156	269	425	4.15
Surigao del Sur	14,129	628	862	1,490	10.55
Province of Dinagat	4,071	210	311	521	12.80
Bislig City	2,684	29	26	55	2.05
Butuan City	9,618	322	412	734	7.63
Surigao City	4,765	110	145	255	5.35

Note: Put asterisk (*) for No Report and Zero (0) for No Case

Table 1.D.10 - Oral Health Care

Number and proportion of pregnant women 10-14, 15-19, 20-49 years old who received
Basic Oral Health Care (BOHC)
Philippines, 2021

Area	Eligible Pop.	Pregnant Women 10-14 y/o received BOHC		Pregnant Women 15-19 y/o received BOHC		Pregnant Women 20-49 y/o received BOHC	
		No.	%	No.	%	No.	%
**PHILIPPINES**	**2,128,168**	**6,826**	**0.32**	**44,243**	**2.08**	**221,011**	**10.39**
**N C R**	**237,921**	**330**	**0.14**	**8,749**	**3.68**	**44,852**	**18.85**
Malabon	6,757	4	0.06	80	1.18	452	6.69
Navotas	4,607	45	0.98	117	2.54	423	9.18
Valenzuela City	11,465	5	0.04	345	3.01	2,215	19.32
Caloocan City	29,270	88	0.30	1,974	6.74	4,744	16.21
Marikina City	8,330	24	0.29	54	0.65	108	1.30
Pasig City	13,954	27	0.19	1,820	13.04	10,248	73.44
Pateros	1,181	0	0.00	16	1.35	159	13.46
Taguig	14,874	7	0.05	148	1.00	539	3.62
Quezon City	54,246	6	0.01	264	0.49	2,286	4.21
Makati City	10,765	13	0.12	433	4.02	3,632	33.74
Mandaluyong City	7,137	18	0.25	344	4.82	2,363	33.11
San Juan	2,254	0	0.00	31	1.38	341	15.13
Manila City	32,887	30	0.09	1,768	5.38	9,662	29.38
Las Piñas City	10,879	2	0.02	112	1.03	593	5.45
Muntinlupa City	9,317	37	0.40	636	6.83	3,513	37.71
Parañaque City	12,298	8	0.07	201	1.63	1,645	13.38
Pasay City	7,700	16	0.21	406	5.27	1,929	25.05
**C A R**	**35,143**	**28**	**0.08**	**611**	**1.74**	**5,210**	**14.83**
Abra	4,287	6	0.14	106	2.47	651	15.19
Apayao	2,476	0	0.00	77	3.11	366	14.78
Benguet	9,140	14	0.15	166	1.82	1,656	18.12
Ifugao	4,439	1	0.02	21	0.47	313	7.05
Kalinga	4,660	0	0.00	19	0.41	198	4.25
Mt. Province	3,066	4	0.13	7	0.23	118	3.85
Baguio City	7,075	3	0.04	215	3.04	1,908	26.97
**Region 1**	**97,198**	**106**	**0.11**	**2,201**	**2.26**	**15,796**	**16.25**
Ilocos Norte	7,986	2	0.03	123	1.54	944	11.82
Ilocos Sur	9,197	10	0.11	338	3.68	2,318	25.20
La Union	11,429	64	0.56	576	5.04	2,497	21.85
Pangasinan	50,454	25	0.05	794	1.57	6,457	12.80
Alaminos City	1,905	0	0.00	0	0.00	831	43.62
Candon City	976	0	0.00	16	1.64	139	14.24
Dagupan City	3,636	0	0.00	122	3.36	508	13.97
Laoag City	1,845	0	0.00	12	0.65	446	24.17

Table 1.D.10 - Oral Health Care

Number and proportion of pregnant women 10-14, 15-19, 20-49 years old who received
Basic Oral Health Care (BOHC)
Philippines, 2021

Area	Eligible Pop.	Pregnant Women 10-14 y/o received BOHC		Pregnant Women 15-19 y/o received BOHC		Pregnant Women 20-49 y/o received BOHC	
		No.	%	No.	%	No.	%
San Carlos City	3,999	0	0.00	69	1.73	736	18.40
San Fernando City	2,092	2	0.10	39	1.86	243	11.62
Urdaneta City	2,819	0	0.00	0	0.00	0	0.00
Vigan City	860	3	0.35	112	13.02	677	78.72
**Region 2**	**69,228**	**68**	**0.10**	**1,892**	**2.73**	**11,623**	**16.79**
Batanes	351	0	0.00	2	0.57	94	26.78
Cagayan	20,563	20	0.10	448	2.18	1,589	7.73
Isabela	23,543	2	0.01	206	0.87	1,804	7.66
Nueva Vizcaya	9,555	20	0.21	469	4.91	3,155	33.02
Quirino	4,038	0	0.00	143	3.54	488	12.09
Cauayan City	2,576	5	0.19	95	3.69	413	16.03
Ilagan City	2,901	17	0.59	218	7.51	1,652	56.95
Santiago City	2,683	4	0.15	311	11.59	2,428	90.50
Tuguegarao City	3,018	0	0.00	0	0.00	0	0.00
**Region 3**	**220,202**	**338**	**0.15**	**4,054**	**1.84**	**20,616**	**9.36**
Aurora	4,764	10	0.21	222	4.66	809	16.98
Bataan	13,814	1	0.01	4	0.03	21	0.15
Bulacan	43,722	108	0.25	669	1.53	2,932	6.71
Nueva Ecija	28,915	0	0.00	0	0.00	1,615	5.59
Pampanga	31,503	9	0.03	162	0.51	725	2.30
Tarlac	19,565	85	0.43	604	3.09	2,602	13.30
Zambales	12,551	37	0.29	350	2.79	1,422	11.33
Angeles City	7,905	0	0.00	61	0.77	232	2.93
Balanga City	1,996	8	0.40	164	8.22	853	42.74
Cabanatuan City	5,921	0	0.00	5	0.08	37	0.62
City of San Fernando	5,890	0	0.00	0	0.00	0	0.00
Gapan City	2,159	0	0.00	1	0.05	7	0.32
Mabalacat City	4,811	1	0.02	467	9.71	1,533	31.86
Malolos City	4,885	5	0.10	61	1.25	383	7.84
Meycauayan	4,050	9	0.22	106	2.62	411	10.15
Olongapo	4,952	0	0.00	4	0.08	31	0.63
Palayan City	805	0	0.00	0	0.00	1	0.12
San Jose City	2,736	8	0.29	250	9.14	531	19.41
San Jose del Monte City	11,120	5	0.04	766	6.89	5,221	46.95
Science City of Munoz	1,594	0	0.00	9	0.56	57	3.58
Tarlac City	6,544	52	0.79	149	2.28	1,193	18.23
**Region 4A**	**298,398**	**2,013**	**0.67**	**3,557**	**1.19**	**29,460**	**9.87**
Batangas	38,848	197	0.51	704	1.81	6,093	15.68
Cavite	27,948	35	0.13	12	0.04	344	1.23
Laguna	19,727	0	0.00	269	1.36	2,986	15.14

Table 1.D.10 - Oral Health Care

Number and proportion of pregnant women 10-14, 15-19, 20-49 years old who received Basic Oral Health Care (BOHC)

Philippines, 2021

Area	Eligible Pop.	Pregnant Women 10-14 y/o received BOHC		Pregnant Women 15-19 y/o received BOHC		Pregnant Women 20-49 y/o received BOHC	
		No.	%	No.	%	No.	%
Quezon	37,644	839	2.23	1,161	3.08	5,050	13.42
Rizal	44,826	245	0.55	1,000	2.23	2,745	6.12
Antipolo City	16,510	0	0.00	0	0.00	5,807	35.17
Bacoor City	12,252	1	0.01	90	0.73	256	2.09
Batangas City	6,895	343	4.97	17	0.25	49	0.71
Biñan City	6,607	0	0.00	63	0.95	813	12.31
Cabuyao City	6,128	0	0.00	27	0.44	492	8.03
Calamba City	9,025	0	0.00	36	0.40	668	7.40
Cavite City	2,098	50	2.38	0	0.00	0	0.00
Dasmariñas City	13,442	6	0.04	0	0.00	0	0.00
General Trias City	6,410	0	0.00	0	0.00	0	0.00
Imus City	8,235	57	0.69	16	0.19	121	1.47
Lipa City	6,954	0	0.00	3	0.04	71	1.02
Lucena City	5,704	0	0.00	0	0.00	39	0.68
San Pablo City	5,277	0	0.00	47	0.89	613	11.62
San Pedro City	6,465	0	0.00	80	1.24	958	14.82
Santa Rosa City	7,023	0	0.00	17	0.24	157	2.24
Tagaytay City	1,451	2	0.14	1	0.07	1	0.07
Tanauan City	3,620	1	0.03	8	0.22	333	9.20
Tayabas City	2,134	237	11.11	0	0.00	1,475	69.12
Trece Martires City	3,175	0	0.00	6	0.19	389	12.25
**Region 4B**	**72,035**	**1,736**	**2.41**	**397**	**0.55**	**1,703**	**2.36**
Marinduque	4,906	1	0.02	59	1.20	526	10.72
Mindoro Occidental	12,449	0	0.00	0	0.00	0	0.00
Mindoro Oriental	20,011	1,718	8.59	5	0.02	22	0.11
Palawan	21,676	13	0.06	278	1.28	710	3.28
Romblon	6,482	4	0.06	55	0.85	445	6.87
Puerto Princesa City	6,511	0	0.00	0	0.00	0	0.00
**Region 5**	**137,350**	**529**	**0.39**	**5,212**	**3.79**	**10,418**	**7.59**
Albay	23,903	0	0.00	102	0.43	1,808	7.56
Camarines Norte	14,340	64	0.45	527	3.68	1,769	12.34
Camarines Sur	39,151	190	0.49	1,511	3.86	1,725	4.41
Catanduanes	6,381	6	0.09	120	1.88	686	10.75
Masbate	22,565	67	0.30	2,217	9.82	361	1.60
Sorsogon	19,470	199	1.02	620	3.18	3,514	18.05
Iriga City	2,665	0	0.00	30	1.13	193	7.24
Legaspi City	4,205	1	0.02	65	1.55	136	3.23
Naga City	4,670	2	0.04	20	0.43	226	4.84

Table 1.D.10 - Oral Health Care

Number and proportion of pregnant women 10-14, 15-19, 20-49 years old who received Basic Oral Health Care (BOHC)

Philippines, 2021

Area	Eligible Pop.	Pregnant Women 10-14 y/o received BOHC		Pregnant Women 15-19 y/o received BOHC		Pregnant Women 20-49 y/o received BOHC	
		No.	%	No.	%	No.	%
**Region 6**	**146,545**	**112**	**0.08**	**2,016**	**1.38**	**13,310**	**9.08**
Aklan	11,231	4	0.04	95	0.85	562	5.00
Antique	12,985	1	0.01	0	0.00	1,504	11.58
Capiz	13,984	19	0.14	265	1.90	1,244	8.90
Guimaras	3,085	0	0.00	5	0.16	55	1.78
Iloilo	36,414	18	0.05	548	1.50	4,481	12.31
Negros Occidental	49,314	26	0.05	787	1.60	3,132	6.35
Bacolod City	11,102	1	0.01	72	0.65	705	6.35
Iloilo City	8,430	43	0.51	244	2.89	1,627	19.30
**Region 7**	**162,860**	**113**	**0.07**	**2,166**	**1.33**	**8,255**	**5.07**
Bohol	27,637	0	0.00	126	0.46	246	0.89
Cebu	67,046	27	0.04	973	1.45	6,113	9.12
Negros Oriental	27,922	8	0.03	310	1.11	1,245	4.46
Siquijor	1,636	0	0.00	0	0.00	36	2.20
Cebu City	21,037	78	0.37	698	3.32	402	1.91
Lapu-Lapu City	9,310	0	0.00	11	0.12	16	0.17
Mandaue City	8,272	0	0.00	48	0.58	197	2.38
**Region 8**	**104,016**	**116**	**0.11**	**400**	**0.38**	**6,053**	**5.82**
Biliran	3,856	1	0.03	28	0.73	112	2.90
Eastern Samar	11,635	12	0.10	82	0.70	916	7.87
Northern Leyte	35,052	0	0.00	165	0.47	1,112	3.17
Northern Samar	15,655	0	0.00	0	0.00	662	4.23
Southern Leyte	6,556	0	0.00	0	0.00	0	0.00
Western Samar	14,515	60	0.41	73	0.50	93	0.64
Calbayog City	4,474	41	0.92	42	0.94	32	0.72
Maasin City	1,661	0	0.00	6	0.36	70	4.21
Ormoc City	4,989	0	0.00	0	0.00	2,943	58.99
Tacloban City	5,623	2	0.04	4	0.07	113	2.01
**Region 9**	**79,552**	**63**	**0.08**	**1,350**	**1.70**	**5,428**	**6.82**
Zamboanga del Norte	17,075	1	0.01	483	2.83	2,796	16.37
Zamboanga del Sur	17,506	18	0.10	469	2.68	1,738	9.93
Zamboanga Sibugay	14,992	1	0.01	75	0.50	449	2.99
Dapitan City	1,755	25	1.42	112	6.38	165	9.40
Dipolog City	2,794	0	0.00	0	0.00	14	0.50
Isabela City	2,544	0	0.00	15	0.59	48	1.89
Pagadian City	4,290	18	0.42	196	4.57	218	5.08
Zamboanga City	18,596	0	0.00	0	0.00	0	0.00

Table 1.D.10 - Oral Health Care

Number and proportion of pregnant women 10-14, 15-19, 20-49 years old who received
Basic Oral Health Care (BOHC)
Philippines, 2021

Area	Eligible Pop.	Pregnant Women 10-14 y/o received BOHC		Pregnant Women 15-19 y/o received BOHC		Pregnant Women 20-49 y/o received BOHC	
		No.	%	No.	%	No.	%
**Region 10**	**100,690**	**171**	**0.17**	**3,391**	**3.37**	**9,793**	**9.73**
Bukidnon	23,307	7	0.03	478	2.05	2,088	8.96
Camiguin	1,854	0	0.00	17	0.92	40	2.16
Lanao del Norte	14,955	2	0.01	268	1.79	315	2.11
Misamis Occidental	6,413	0	0.00	937	14.61	1,822	28.41
Misamis Oriental	15,058	91	0.60	476	3.16	1,553	10.31
Cagayan de Oro City	14,263	0	0.00	0	0.00	0	0.00
El Salvador City	1,061	1	0.09	19	1.79	126	11.88
Gingoog City	2,627	26	0.99	525	19.98	652	24.82
Iligan City	7,575	1	0.01	21	0.28	134	1.77
Malaybalay City	3,882	9	0.23	168	4.33	524	13.50
Oroquieta City	1,389	0	0.00	6	0.43	36	2.59
Ozamis City	2,782	0	0.00	0	0.00	0	0.00
Tangub City	1,234	34	2.76	39	3.16	425	34.44
Valencia City	4,290	0	0.00	437	10.19	2,078	48.44
**Region 11**	**107,847**	**56**	**0.05**	**860**	**0.80**	**3,951**	**3.66**
Davao de Oro	15,526	5	0.03	145	0.93	685	4.41
Davao del Norte	21,184	8	0.04	176	0.83	808	3.81
Davao Oriental	13,125	5	0.04	128	0.98	513	3.91
Davao del Sur	14,355	8	0.06	120	0.84	343	2.39
Davao Occidental	6,598	6	0.09	35	0.53	145	2.20
Davao City	37,059	24	0.06	256	0.69	1,457	3.93
**Region 12**	**104,693**	**552**	**0.53**	**4,424**	**4.23**	**18,488**	**17.66**
North Cotabato	33,933	265	0.78	1,244	3.67	4,960	14.62
Sarangani	12,904	0	0.00	0	0.00	2,091	16.20
South Cotabato	21,039	8	0.04	438	2.08	2,378	11.30
Sultan Kudarat	17,331	13	0.08	532	3.07	3,246	18.73
Cotabato City	5,831	222	3.81	1,398	23.98	1,895	32.50
Gen. Santos City	13,655	44	0.32	812	5.95	3,918	28.69
**BARMM**	**94,148**	**354**	**0.38**	**1,143**	**1.21**	**5,570**	**5.92**
Basilan	7,672	2	0.03	13	0.17	72	0.94
Lanao del Sur	21,391	272	1.27	296	1.38	1,298	6.07
Maguindanao	31,650	63	0.20	666	2.10	3,833	12.11
Sulu	16,891	17	0.10	41	0.24	199	1.18
Tawi-Tawi	9,319	0	0.00	0	0.00	0	0.00
Lamitan City	2,112	0	0.00	0	0.00	0	0.00
Marawi City	5,113	0	0.00	127	2.48	168	3.29

Table 1.D.10 - Oral Health Care

Number and proportion of pregnant women 10-14, 15-19, 20-49 years old who received
Basic Oral Health Care (BOHC)
Philippines, 2021

Area	Eligible Pop.	Pregnant Women 10-14 y/o received BOHC		Pregnant Women 15-19 y/o received BOHC		Pregnant Women 20-49 y/o received BOHC	
		No.	%	No.	%	No.	%
**CARAGA**	**60,342**	**141**	**0.23**	**1,820**	**3.02**	**10,485**	**17.38**
Agusan del Norte	8,116	28	0.34	199	2.45	831	10.24
Agusan del Sur	17,658	3	0.02	326	1.85	1,390	7.87
Surigao del Norte	7,195	3	0.04	140	1.95	1,183	16.44
Surigao del Sur	11,532	33	0.29	559	4.85	3,091	26.80
Province of Dinagat	2,574	60	2.33	78	3.03	1,051	40.83
Bislig City	2,185	5	0.23	76	3.48	458	20.96
Butuan City	7,732	0	0.00	210	2.72	1,284	16.61
Surigao City	3,350	9	0.27	232	6.93	1,197	35.73

Note: Put asterisk (*) for No Report and Zero (0) for No Case

Figure 1.D.1 - Percentage Distribution of Children aged 12-59 mos. old who were Orally Fit upon Oral Examination by Region
Philippines, 2021

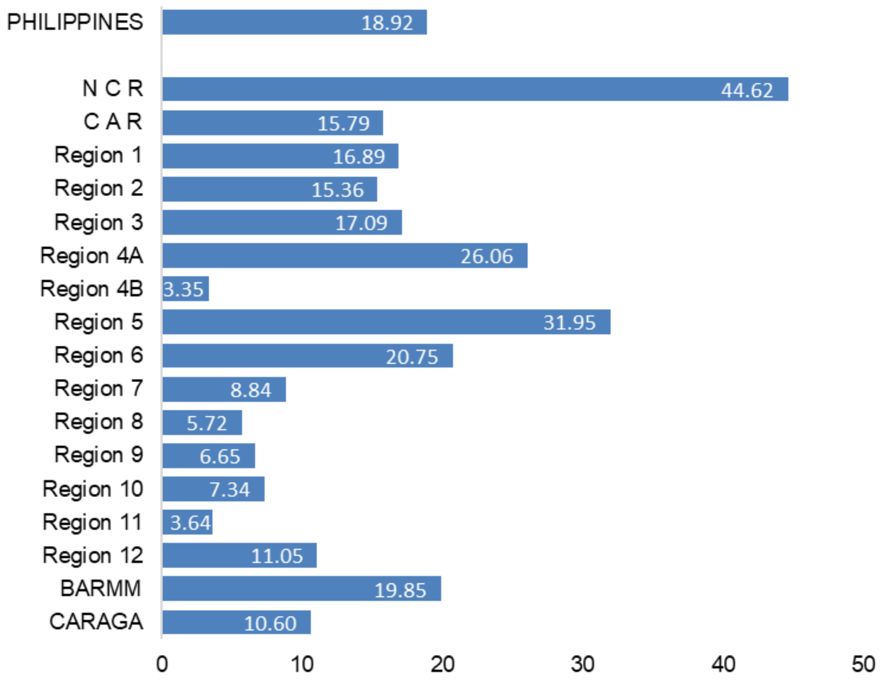

Figure 1.D.2 - Percentage Distribution of Children aged 12-59 months old who were Orally Fit upon Oral Examination or after Oral ehabilitation by Sex and Region
Philippines, 2021

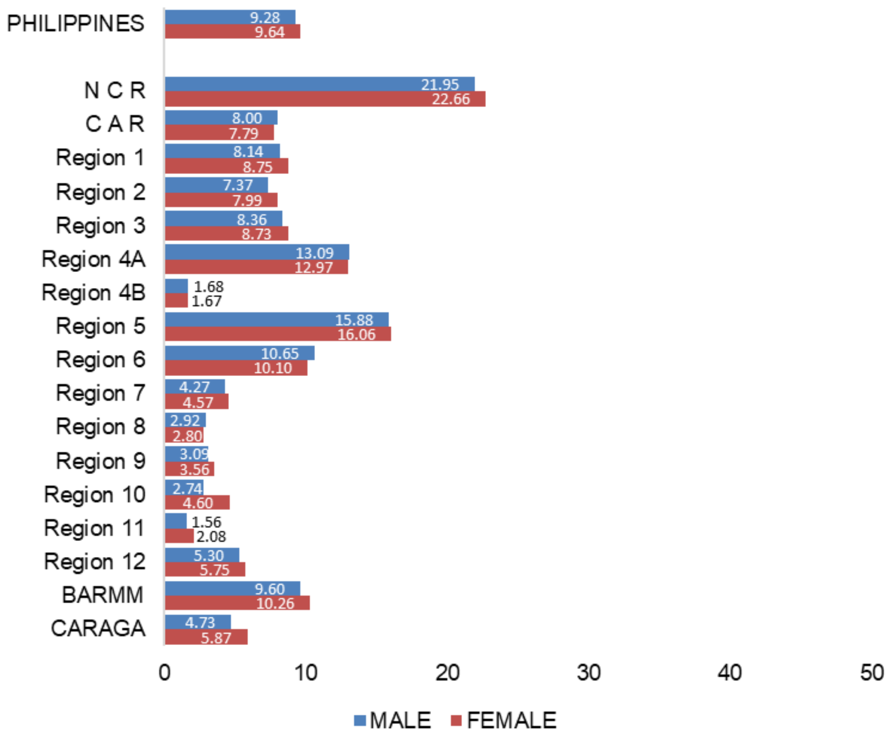

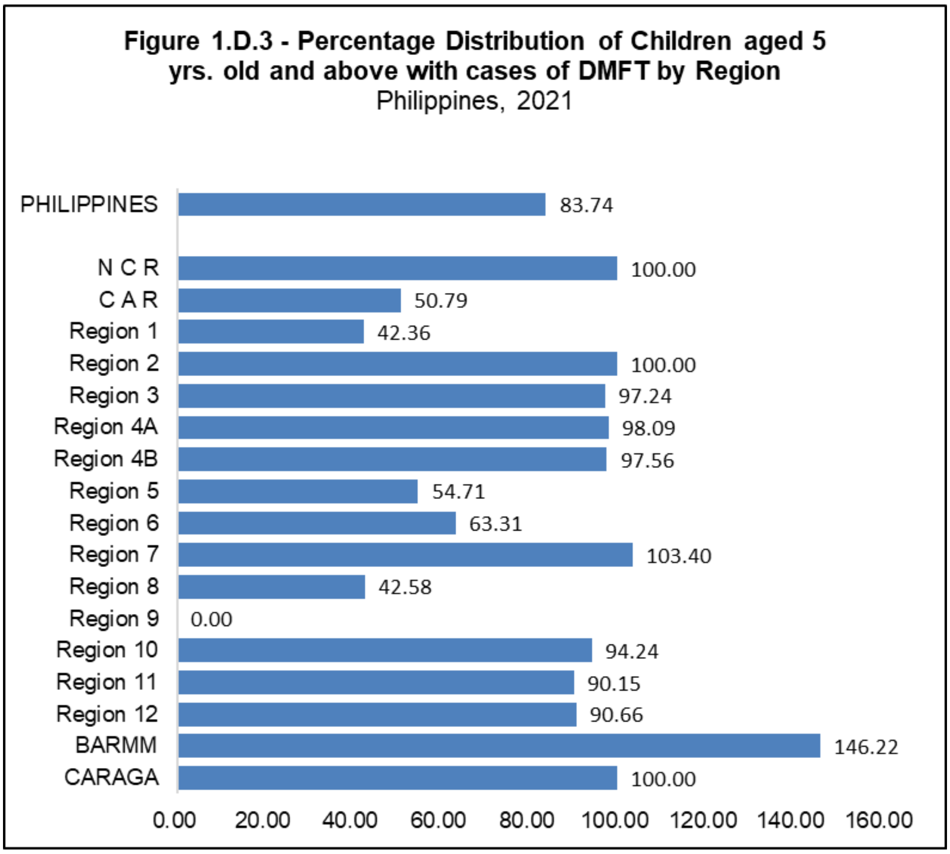
Figure 1.D.3 - Percentage Distribution of Children aged 5 yrs. old and above with cases of DMFT by Region
Philippines, 2021
PHILIPPINES
83.74
N C R
100.00
C A R
50.79
Region 1
42.36
Region 2
100.00
Region 3
97.24
Region 4A
98.09
Region 4B
97.56
Region 5
54.71
Region 6
63.31
Region 7
103.40
Region 8
42.58
Region 9
0.00
Region 10
94.24
Region 11
90.15
Region 12
90.66
BARMM
146.22
CARAGA
100.00
0.00 20.00 40.00 60.00 80.00 100.00 120.00 140.00 160.00

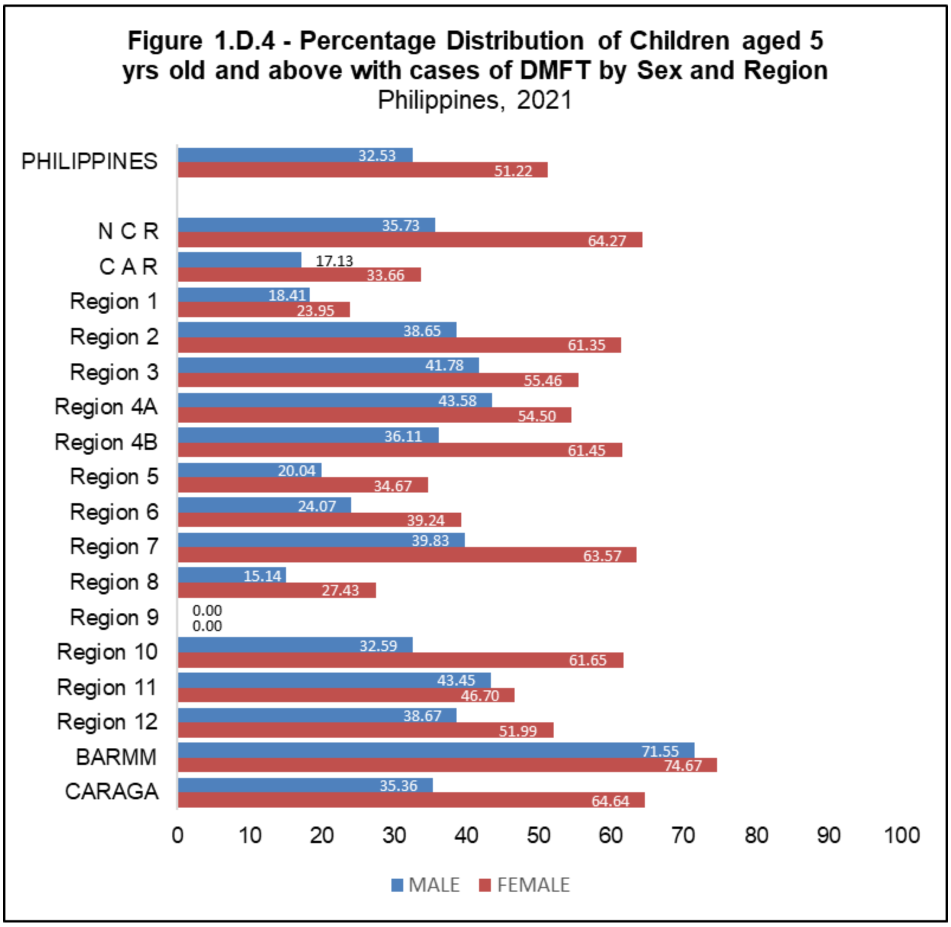
Figure 1.D.4 - Percentage Distribution of Children aged 5 yrs old and above with cases of DMFT by Sex and Region
Philippines, 2021
PHILIPPINES
32.53
51.22
N C R
35.73
64.27
C A R
17.13
33.66
Region 1
18.41
23.95
Region 2
38.65
61.35
Region 3
41.78
55.46
Region 4A
43.58
54.50
Region 4B
36.11
61.45
Region 5
20.04
34.67
Region 6
24.07
39.24
Region 7
39.83
63.57
Region 8
15.14
27.43
Region 9
0.00
0.00
Region 10
32.59
61.65
Region 11
43.45
46.70
Region 12
38.67
51.99
BARMM
71.55
74.67
CARAGA
35.36
64.64
0 10 20 30 40 50 60 70 80 90 100
MALE
FEMALE

Figure 1.D.5 - Percentage Distribution of Infants aged 0-11 mos. old who received BOHC by Region
Philippines, 2021

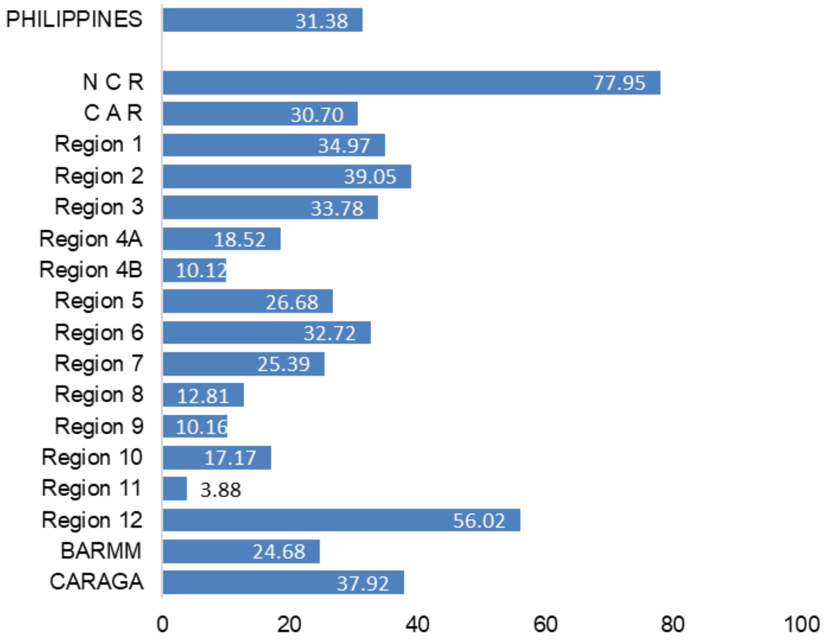

Figure 1.D.6 - Percentage Distribution of Infants 0-11 mos. old who received BOHC by Sex and Region
Philippines, 2021

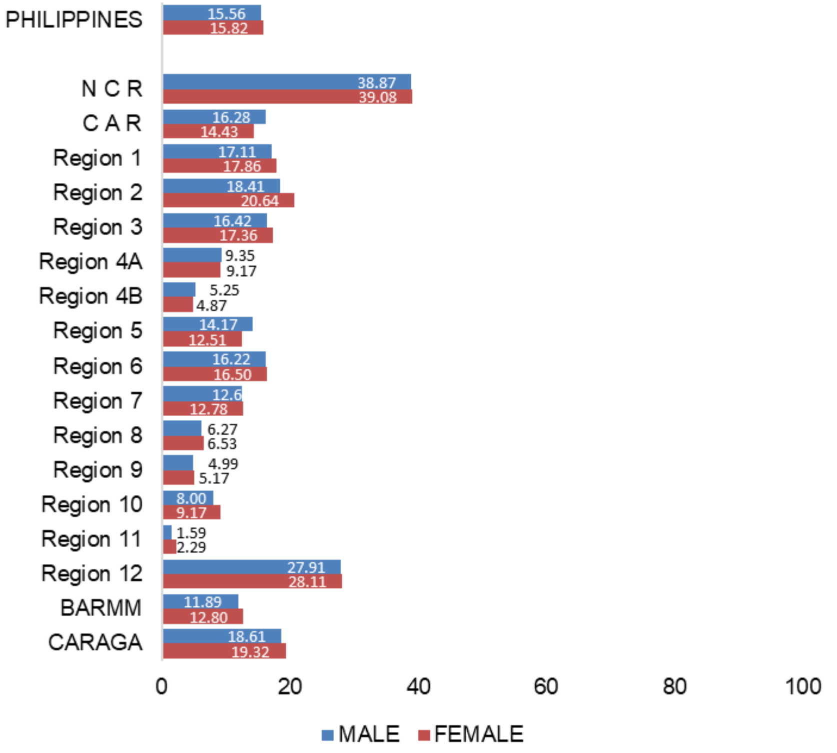

Figure 1.D.7 - Percentage Distribution of Children aged 1-4 yrs. old who received BOHC by Region
Philippinesl, 2021

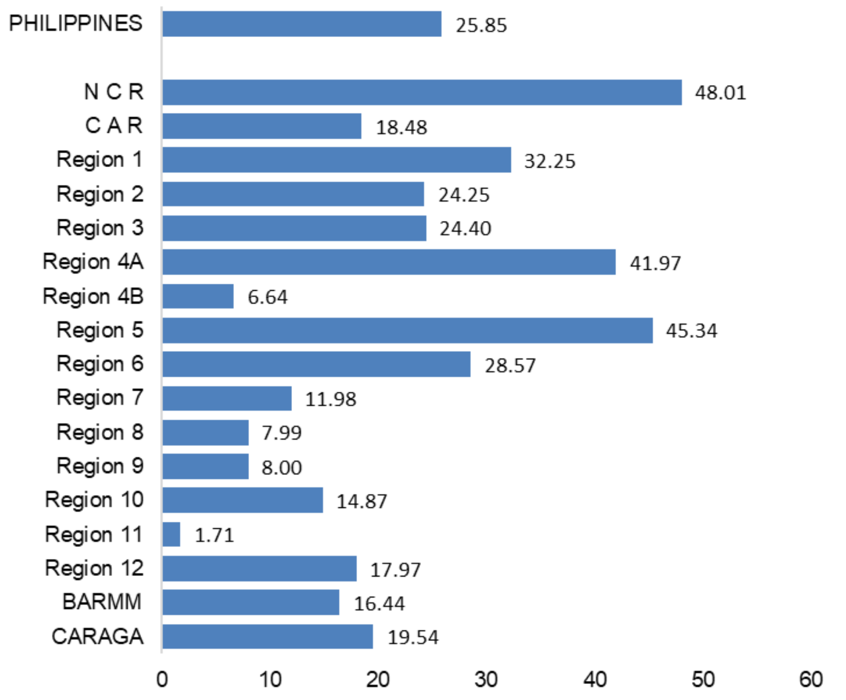

Figure 1.D.8 - Percentage Distribution of Children aged 1-4 yrs. old who received BOHC by Sex and Region
Philippines, 2021

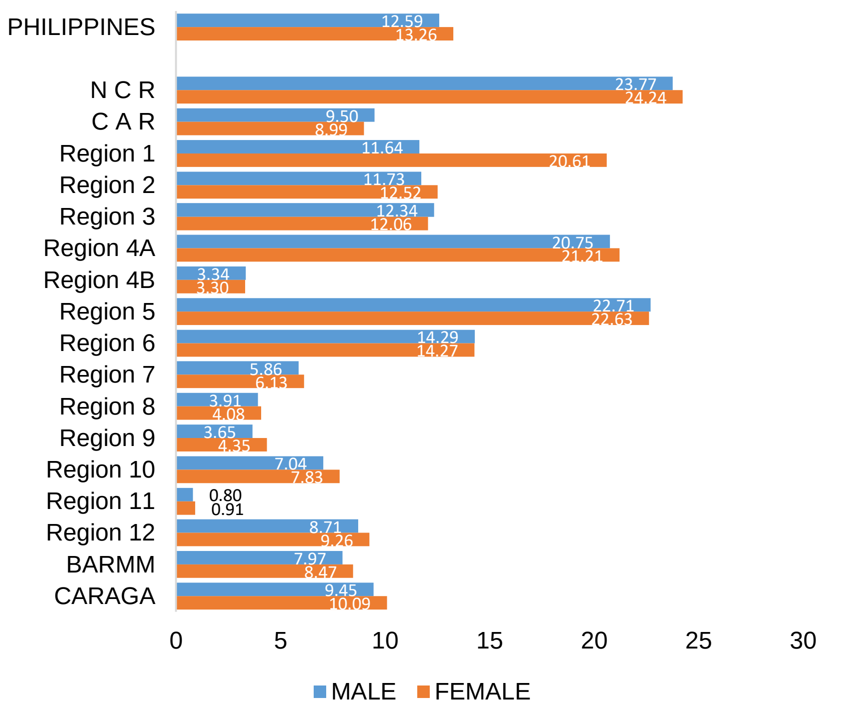

Figure 1.D.9 - Percentage Distribution of Children aged 5-9 yrs. old who received BOHC by Region
Phillippines, 2021

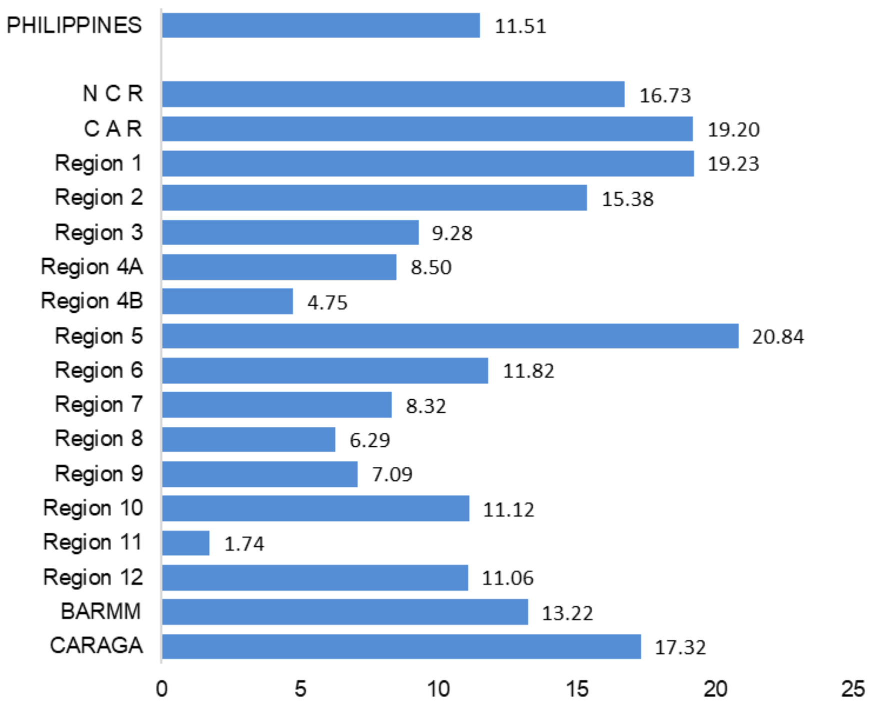

Figure 1.D.10 - Percentage Distribution of Children aged 5-9 yrs. old who received BOHC by Sex and Region
Philippines, 2021

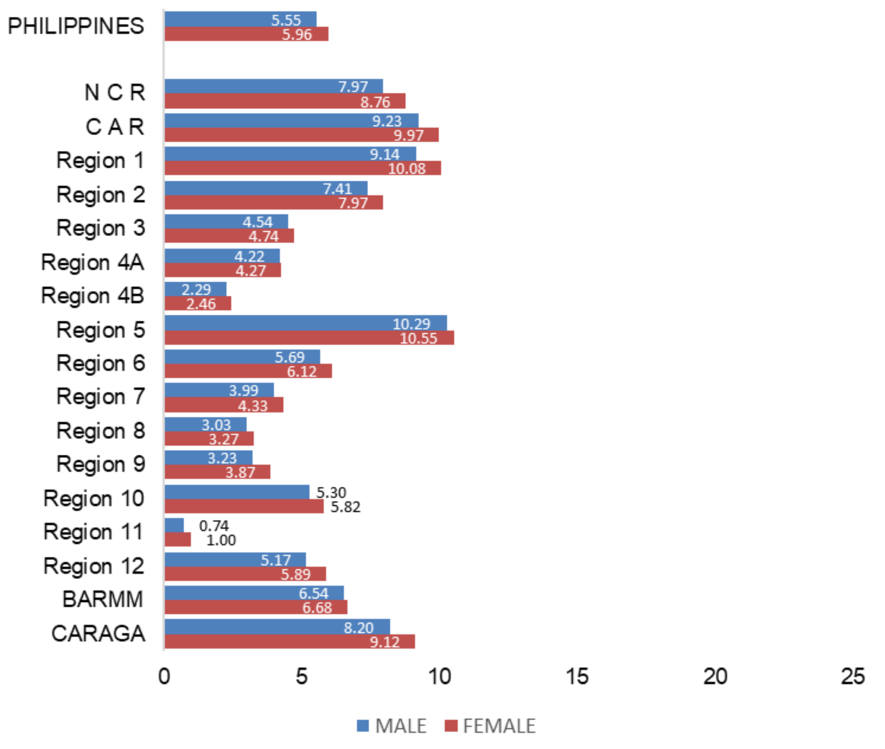

Figure 1.D.11 - Percentage Distribution of Adolescent aged 10-14 years old who received BOHC by Region
Philippines, 2021

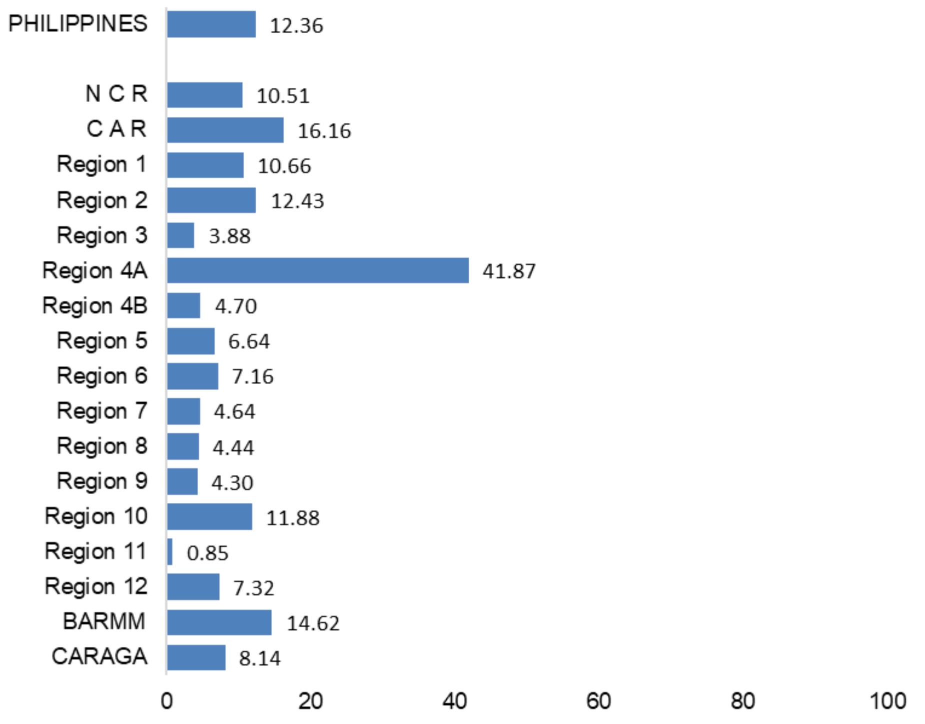

Figure 1.D.12 - Percentage Distribution of Adolescent aged 10-14 years old who received BOHC by Sex and Region
Philippines, 2021

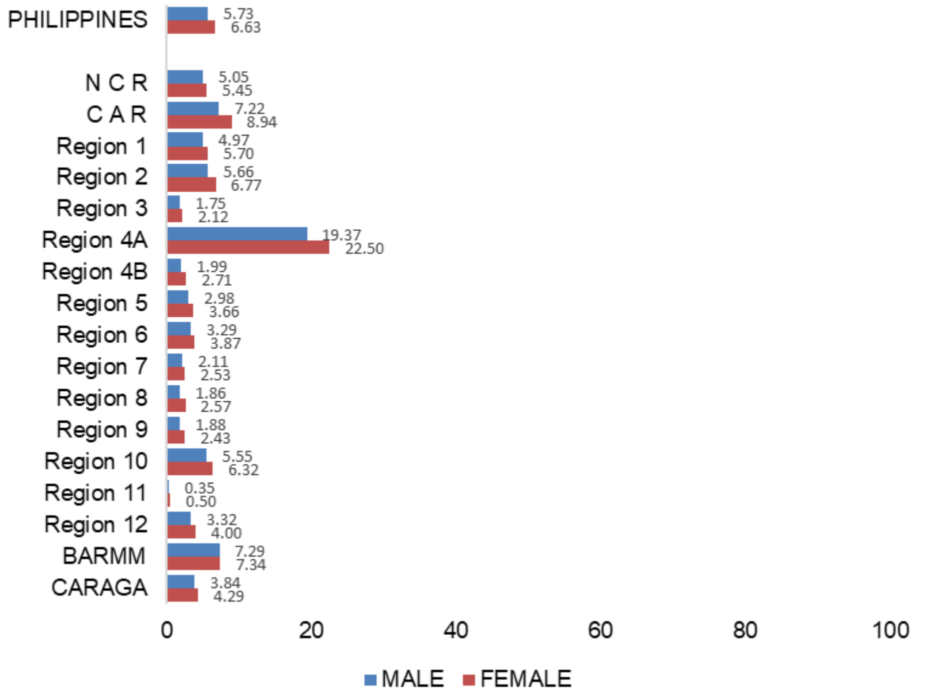

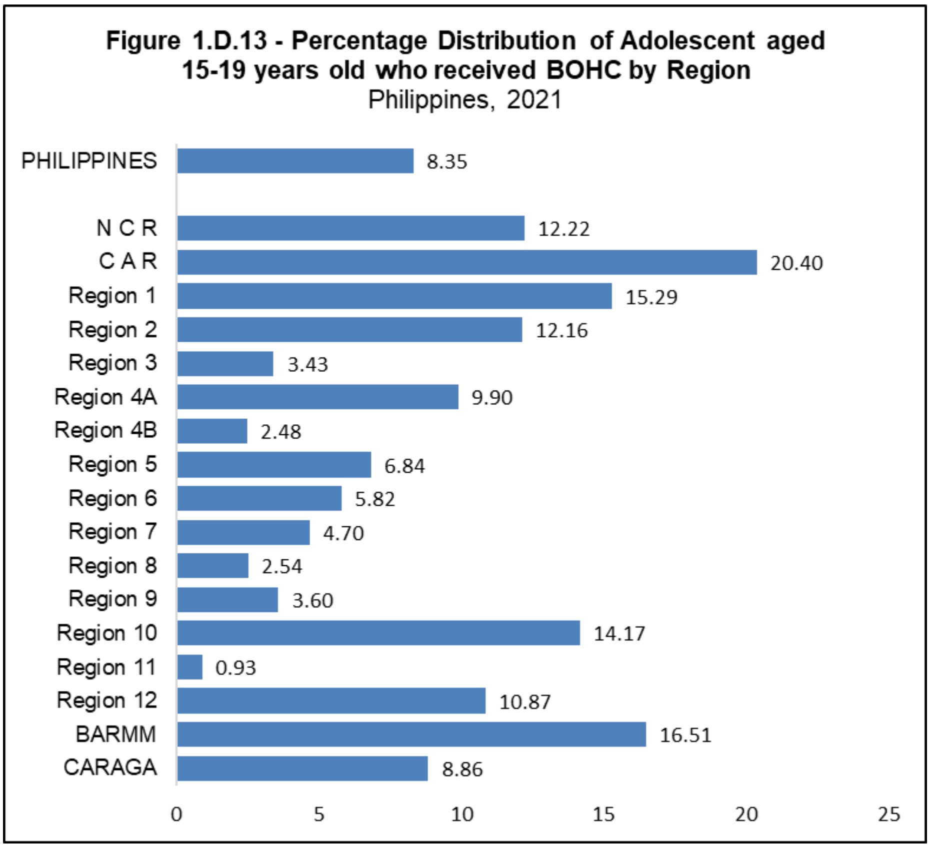
Figure 1.D.13 - Percentage Distribution of Adolescent aged 15-19 years old who received BOHC by Region
Philippines, 2021
PHILIPPINES 8.35
N C R 12.22
C A R 20.40
Region 1 15.29
Region 2 12.16
Region 3 3.43
Region 4A 9.90
Region 4B 2.48
Region 5 6.84
Region 6 5.82
Region 7 4.70
Region 8 2.54
Region 9 3.60
Region 10 14.17
Region 11 0.93
Region 12 10.87
BARMM 16.51
CARAGA 8.86
0 5 10 15 20 25

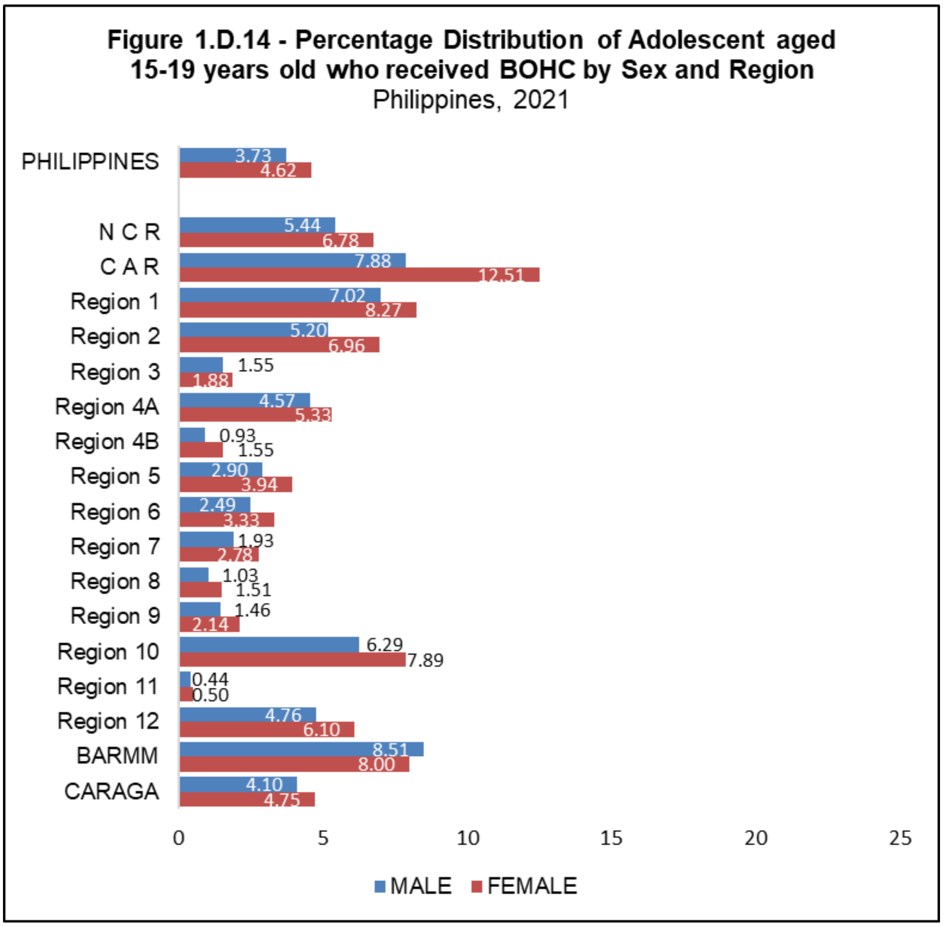
Figure 1.D.14 - Percentage Distribution of Adolescent aged 15-19 years old who received BOHC by Sex and Region
Philippines, 2021
PHILIPPINES 3.73 4.62
N C R 5.44 6.78
C A R 7.88 12.51
Region 1 7.02 8.27
Region 2 5.20 6.96
Region 3 1.55 1.88
Region 4A 4.57 5.33
Region 4B 0.93 1.55
Region 5 2.90 3.94
Region 6 2.49 3.33
Region 7 1.93 2.78
Region 8 1.03 1.51
Region 9 1.46 2.14
Region 10 6.29 7.89
Region 11 0.44 0.50
Region 12 4.76 6.10
BARMM 8.51 8.00
CARAGA 4.10 4.75
0 5 10 15 20 25
MALE FEMALE

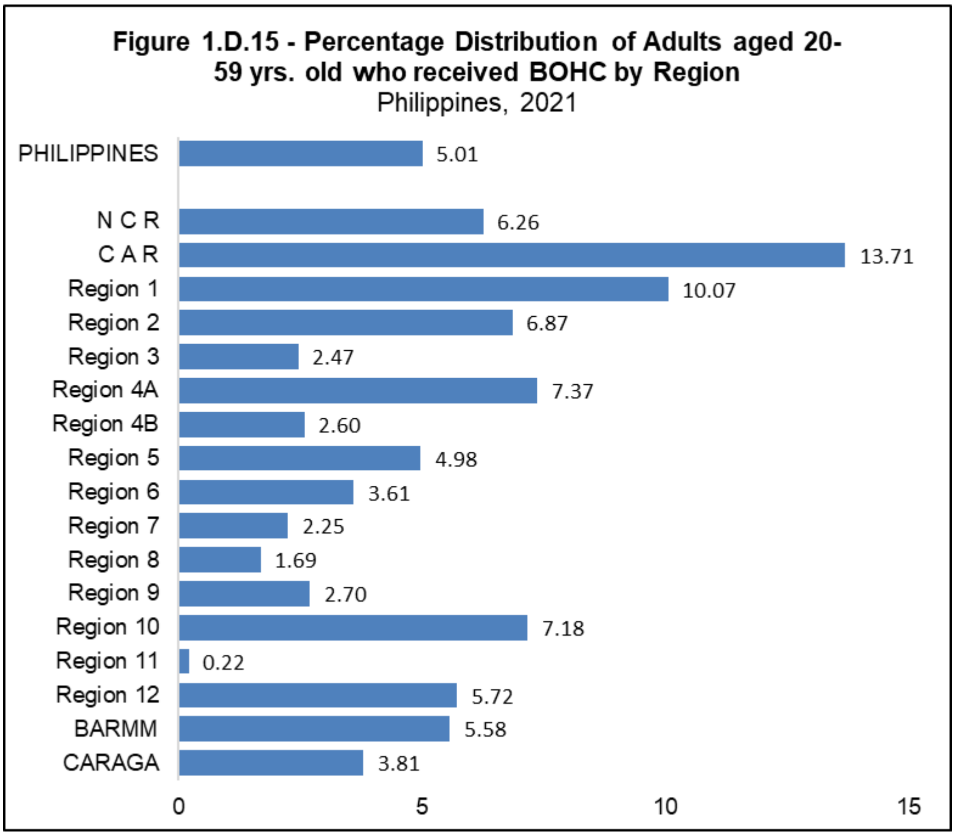
Figure 1.D.15 - Percentage Distribution of Adults aged 20-59 yrs. old who received BOHC by Region
Philippines, 2021
PHILIPPINES 5.01
N C R 6.26
C A R 13.71
Region 1 10.07
Region 2 6.87
Region 3 2.47
Region 4A 7.37
Region 4B 2.60
Region 5 4.98
Region 6 3.61
Region 7 2.25
Region 8 1.69
Region 9 2.70
Region 10 7.18
Region 11 0.22
Region 12 5.72
BARMM 5.58
CARAGA 3.81
0
5
10
15

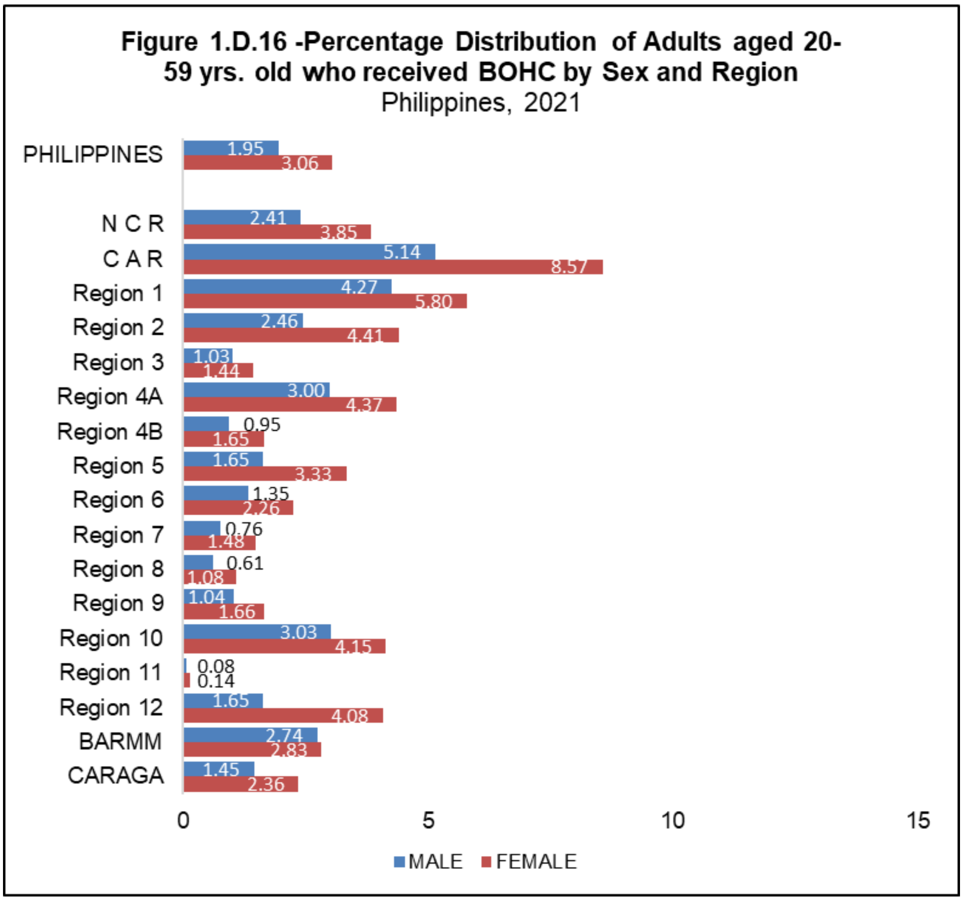
Figure 1.D.16 -Percentage Distribution of Adults aged 20-59 yrs. old who received BOHC by Sex and Region
Philippines, 2021
PHILIPPINES 1.95 3.06
N C R 2.41 3.85
C A R 5.14 8.57
Region 1 4.27 5.80
Region 2 2.46 4.41
Region 3 1.03 1.44
Region 4A 3.00 4.37
Region 4B 0.95 1.65
Region 5 1.65 3.33
Region 6 1.35 2.26
Region 7 0.76 1.48
Region 8 0.61 1.08
Region 9 1.04 1.66
Region 10 3.03 4.15
Region 11 0.08 0.14
Region 12 1.65 4.08
BARMM 2.74 2.83
CARAGA 1.45 2.36
0
5
10
15
MALE
FEMALE

Figure 1.D.17 - Percentage Distribution of Senior Citizens aged 60 yrs. old and above who received BOHC
Philippines, 2021

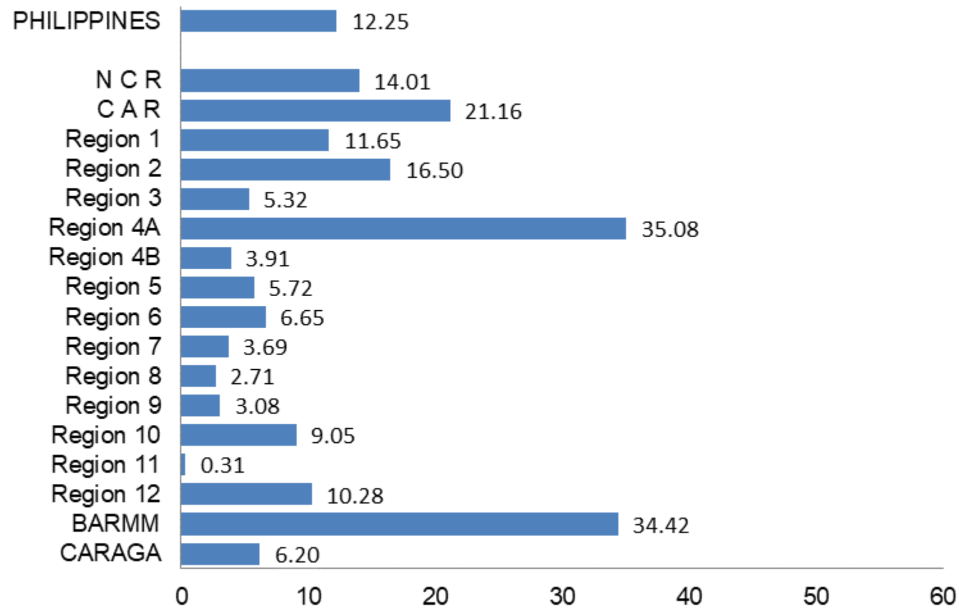

Figure 1.D.18 - Percentage Distribution of Senior Citizens aged 60 yrs. old and above who received BOHC by Sex and Region
Philippines, 2021

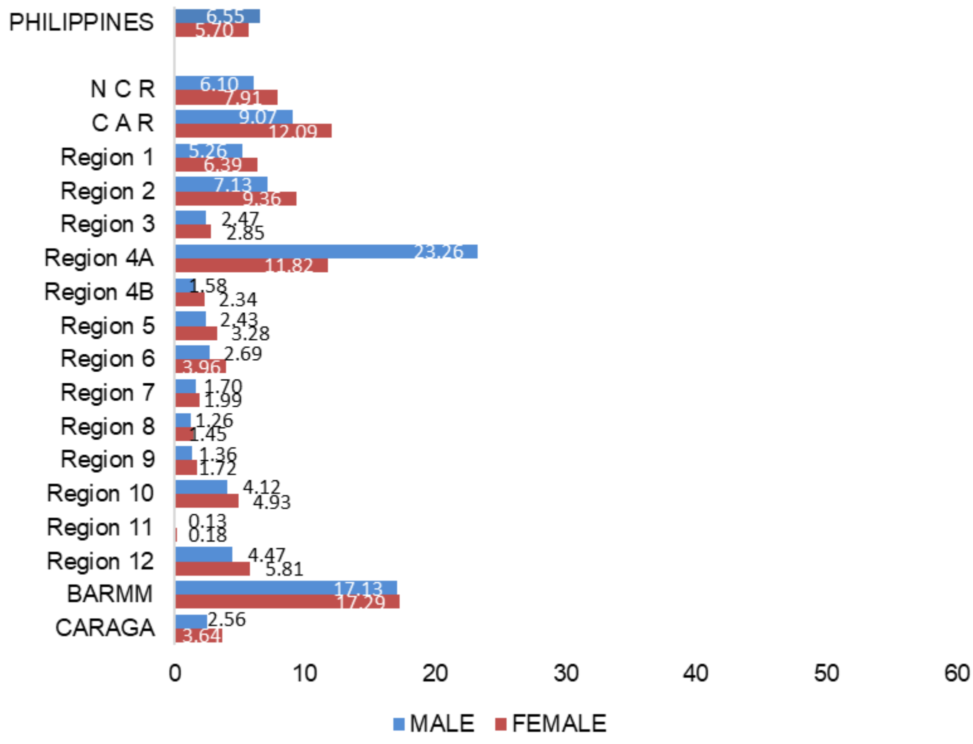

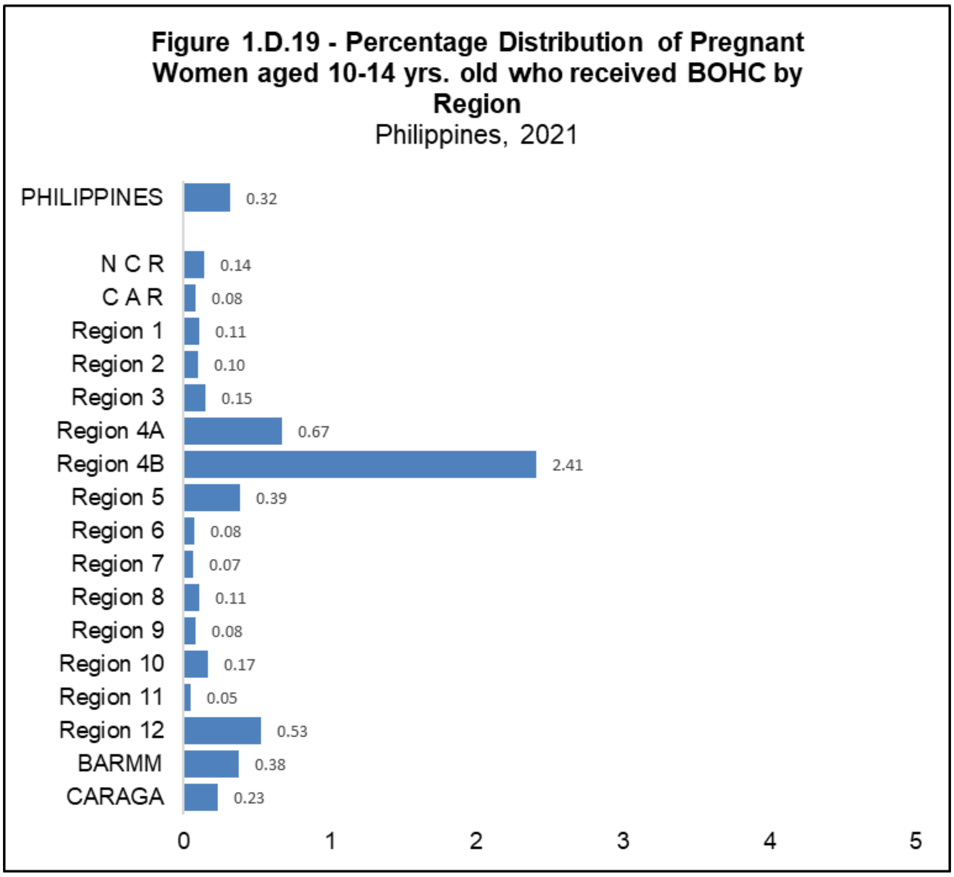

Figure 1.D.19 - Percentage Distribution of Pregnant Women aged 10-14 yrs. old who received BOHC by Region
Philippines, 2021
PHILIPPINES
N C R
C A R
Region 1
Region 2
Region 3
Region 4A
Region 4B
Region 5
Region 6
Region 7
Region 8
Region 9
Region 10
Region 11
Region 12
BARMM
CARAGA
0.32
0.14
0.08
0.11
0.10
0.15
0.67
2.41
0.39
0.08
0.07
0.11
0.08
0.17
0.05
0.53
0.38
0.23
0
1
2
3
4
5

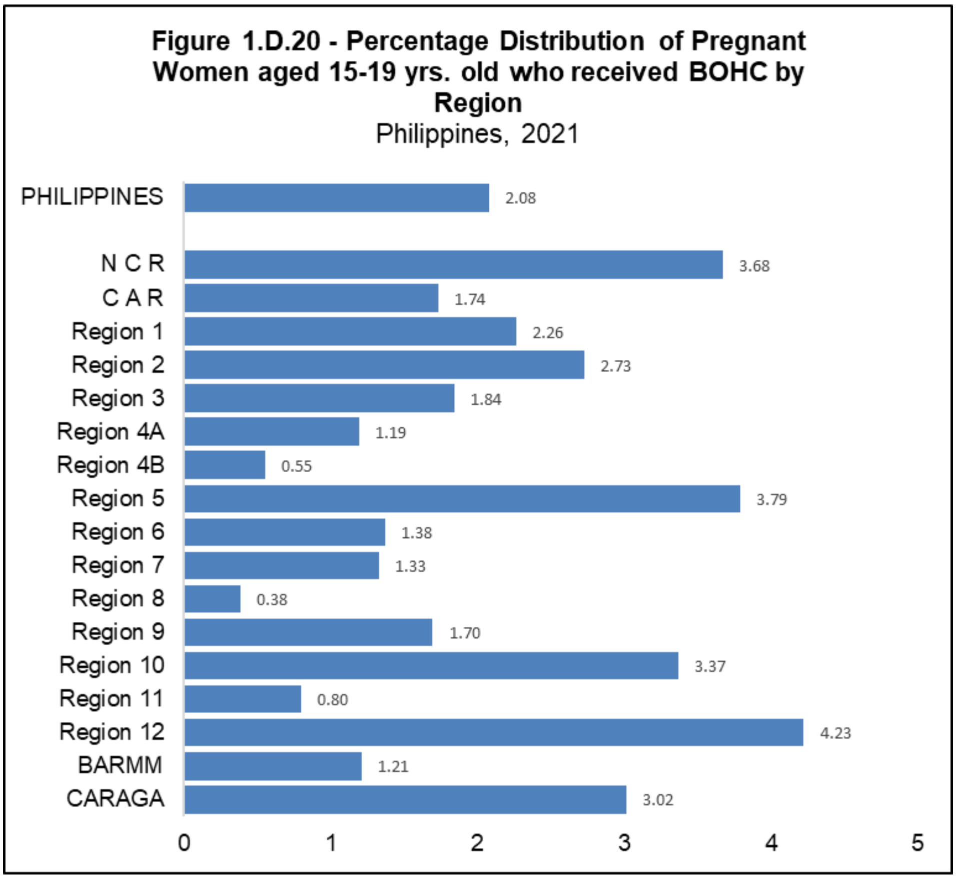

Figure 1.D.20 - Percentage Distribution of Pregnant Women aged 15-19 yrs. old who received BOHC by Region
Philippines, 2021
PHILIPPINES
N C R
C A R
Region 1
Region 2
Region 3
Region 4A
Region 4B
Region 5
Region 6
Region 7
Region 8
Region 9
Region 10
Region 11
Region 12
BARMM
CARAGA
2.08
3.68
1.74
2.26
2.73
1.84
1.19
0.55
3.79
1.38
1.33
0.38
1.70
3.37
0.80
4.23
1.21
3.02
0
1
2
3
4
5

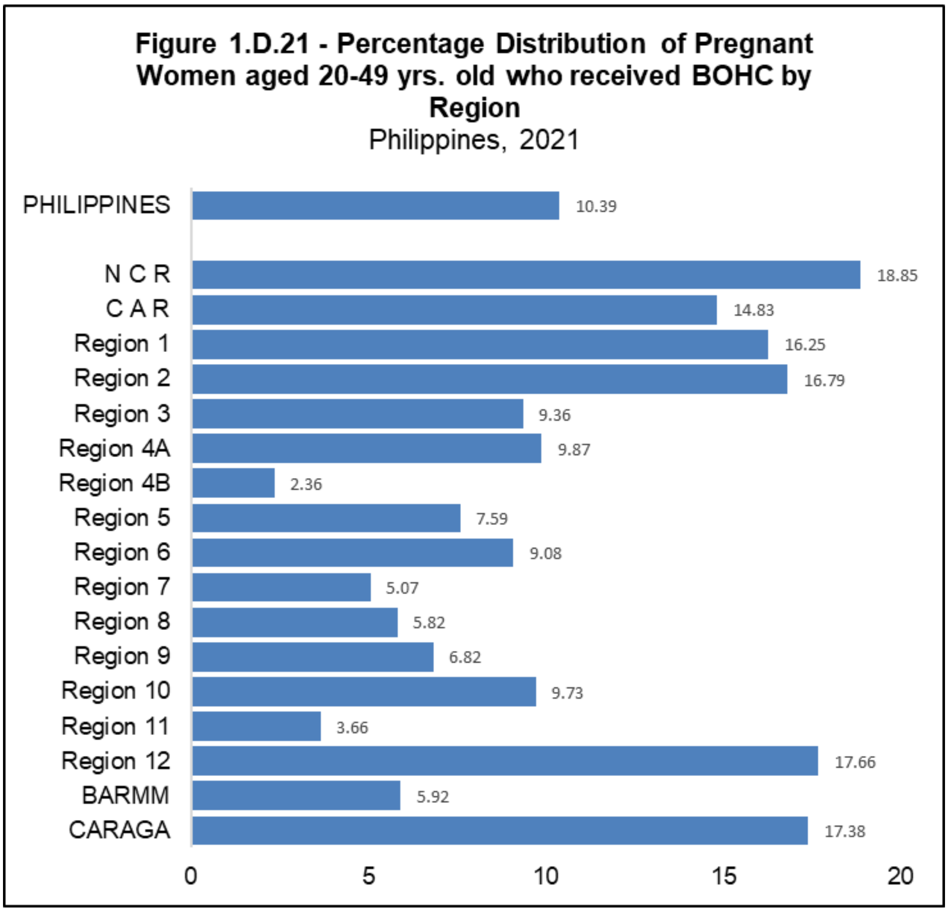

Figure 1.D.21 - Percentage Distribution of Pregnant Women aged 20-49 yrs. old who received BOHC by Region
Philippines, 2021
PHILIPPINES
10.39
N C R
18.85
C A R
14.83
Region 1
16.25
Region 2
16.79
Region 3
9.36
Region 4A
9.87
Region 4B
2.36
Region 5
7.59
Region 6
9.08
Region 7
5.07
Region 8
5.82
Region 9
6.82
Region 10
9.73
Region 11
3.66
Region 12
17.66
BARMM
5.92
CARAGA
17.38
0
5
10
15
20

INFECTIOUS DISEASE PREVENTION AND CONTROL SERVICES

FHSIS ANNUAL REPORT 2021

Table 1.E.1 - Filariasis Prevention and Control

Case Detection Rate (CDR) and Clinical Rate of Lymphatic Filariasis
Philippines, 2021

Area	Total Examined for lymphatic filariasis	Number positive cases found				Lymphatic filariasis cases examined with disabilities/deformities			
		Male	Female	Total	Case Detection Rate	Male	Female	Total	Clinical Rate
**PHILIPPINES**	**10,777**	**132**	**113**	**245**	**2.27**	**22**	**17**	**39**	**15.92**
**N C R**	**0**	**0**	**0**	**0**	**0.00**	**0**	**0**	**0**	**0.00**
Malabon City	0	0	0	0	0.00	0	0	0	0.00
Navotas City	0	0	0	0	0.00	0	0	0	0.00
Valenzuela City	0	0	0	0	0.00	0	0	0	0.00
Caloocan City	0	0	0	0	0.00	0	0	0	0.00
Marikina City	0	0	0	0	0.00	0	0	0	0.00
Pasig City	0	0	0	0	0.00	0	0	0	0.00
Pateros	0	0	0	0	0.00	0	0	0	0.00
Taguig City	0	0	0	0	0.00	0	0	0	0.00
Quezon City	0	0	0	0	0.00	0	0	0	0.00
Makati City	0	0	0	0	0.00	0	0	0	0.00
Mandaluyong City	0	0	0	0	0.00	0	0	0	0.00
San Juan City	0	0	0	0	0.00	0	0	0	0.00
Manila City	0	0	0	0	0.00	0	0	0	0.00
Las Piñas City	0	0	0	0	0.00	0	0	0	0.00
Muntinlupa City	0	0	0	0	0.00	0	0	0	0.00
Parañaque City	0	0	0	0	0.00	0	0	0	0.00
Pasay City	0	0	0	0	0.00	0	0	0	0.00
**C A R**	**0**	**0**	**0**	**0**	**0.00**	**0**	**0**	**0**	**0.00**
Abra	0	0	0	0	0.00	0	0	0	0.00
Apayao	0	0	0	0	0.00	0	0	0	0.00
Benguet	0	0	0	0	0.00	0	0	0	0.00
Ifugao	0	0	0	0	0.00	0	0	0	0.00
Kalinga	0	0	0	0	0.00	0	0	0	0.00
Mt. Province	0	0	0	0	0.00	0	0	0	0.00
Baguio City	0	0	0	0	0.00	0	0	0	0.00
**Region 1**	**0**	**0**	**0**	**0**	**0.00**	**0**	**0**	**0**	**0.00**
Ilocos Norte	0	0	0	0	0.00	0	0	0	0.00
Ilocos Sur	0	0	0	0	0.00	0	0	0	0.00
La Union	0	0	0	0	0.00	0	0	0	0.00
Pangasinan	0	0	0	0	0.00	0	0	0	0.00
Alaminos City	0	0	0	0	0.00	0	0	0	0.00
Candon City	0	0	0	0	0.00	0	0	0	0.00

Table 1.E.1 - Filariasis Prevention and Control

Case Detection Rate (CDR) and Clinical Rate of Lymphatic Filariasis

Philippines, 2021

Area	Total Examined for lymphatic filariasis	Number positive cases found				Lymphatic filariasis cases examined with disabilities/deformities			
		Male	Female	Total	Case Detection Rate	Male	Female	Total	Clinical Rate
Dagupan City	0	0	0	0	0.00	0	0	0	0.00
Laoag City	0	0	0	0	0.00	0	0	0	0.00
San Carlos City	0	0	0	0	0.00	0	0	0	0.00
San Fernando City	0	0	0	0	0.00	0	0	0	0.00
Urdaneta City	0	0	0	0	0.00	0	0	0	0.00
Vigan City	0	0	0	0	0.00	0	0	0	0.00
**Region 2**	**0**	**0**	**0**	**0**	**0.00**	**0**	**0**	**0**	**0.00**
Batanes	0	0	0	0	0.00	0	0	0	0.00
Cagayan	0	0	0	0	0.00	0	0	0	0.00
Isabela	0	0	0	0	0.00	0	0	0	0.00
Nueva Vizcaya	0	0	0	0	0.00	0	0	0	0.00
Quirino	0	0	0	0	0.00	0	0	0	0.00
Cauayan City	0	0	0	0	0.00	0	0	0	0.00
Ilagan City	0	0	0	0	0.00	0	0	0	0.00
Santiago City	0	0	0	0	0.00	0	0	0	0.00
Tuguegarao City	0	0	0	0	0.00	0	0	0	0.00
**Region 3**	**0**	**0**	**0**	**0**	**0.00**	**0**	**0**	**0**	**0.00**
Aurora	0	0	0	0	0.00	0	0	0	0.00
Bataan	0	0	0	0	0.00	0	0	0	0.00
Bulacan	0	0	0	0	0.00	0	0	0	0.00
Nueva Ecija	0	0	0	0	0.00	0	0	0	0.00
Pampanga	0	0	0	0	0.00	0	0	0	0.00
Tarlac	0	0	0	0	0.00	0	0	0	0.00
Zambales	0	0	0	0	0.00	0	0	0	0.00
Angeles City	0	0	0	0	0.00	0	0	0	0.00
Balanga City	0	0	0	0	0.00	0	0	0	0.00
Cabanatuan City	0	0	0	0	0.00	0	0	0	0.00
City of San Fernando	0	0	0	0	0.00	0	0	0	0.00
Gapan City	0	0	0	0	0.00	0	0	0	0.00
Mabalacat City	0	0	0	0	0.00	0	0	0	0.00
Malolos City	0	0	0	0	0.00	0	0	0	0.00
Meycauayan City	0	0	0	0	0.00	0	0	0	0.00
Olongapo City	0	0	0	0	0.00	0	0	0	0.00
Palayan City	0	0	0	0	0.00	0	0	0	0.00
San Jose City	0	0	0	0	0.00	0	0	0	0.00
San Jose del Monte City	0	0	0	0	0.00	0	0	0	0.00
Science City of Munoz	0	0	0	0	0.00	0	0	0	0.00
Tarlac City	0	0	0	0	0.00	0	0	0	0.00

Table 1.E.1 - Filariasis Prevention and Control
Case Detection Rate (CDR) and Clinical Rate of Lymphatic Filariasis
Philippines, 2021

Area	Total Examined for lymphatic filariasis	Number positive cases found				Lymphatic filariasis cases examined with disabilities/deformities			
		Male	Female	Total	Case Detection Rate	Male	Female	Total	Clinical Rate
**Region 4A**	**3**	**0**	**0**	**0**	**0.00**	**0**	**0**	**0**	**0.00**
Batangas	1	0	0	0	0.00	0	0	0	0.00
Cavite	0	0	0	0	0.00	0	0	0	0.00
Laguna	0	0	0	0	0.00	0	0	0	0.00
Quezon	2	0	0	0	0.00	0	0	0	0.00
Rizal	0	0	0	0	0.00	0	0	0	0.00
Antipolo City	0	0	0	0	0.00	0	0	0	0.00
Bacoor City	0	0	0	0	0.00	0	0	0	0.00
Batangas City	0	0	0	0	0.00	0	0	0	0.00
Biñan City	0	0	0	0	0.00	0	0	0	0.00
Cabuyao City	0	0	0	0	0.00	0	0	0	0.00
Calamba City	0	0	0	0	0.00	0	0	0	0.00
Cavite City	0	0	0	0	0.00	0	0	0	0.00
Dasmariñas City	0	0	0	0	0.00	0	0	0	0.00
General Trias City	0	0	0	0	0.00	0	0	0	0.00
Imus City	0	0	0	0	0.00	0	0	0	0.00
Lipa City	0	0	0	0	0.00	0	0	0	0.00
Lucena City	0	0	0	0	0.00	0	0	0	0.00
San Pablo City	0	0	0	0	0.00	0	0	0	0.00
San Pedro City	0	0	0	0	0.00	0	0	0	0.00
Santa Rosa City	0	0	0	0	0.00	0	0	0	0.00
Tagaytay City	0	0	0	0	0.00	0	0	0	0.00
Tanauan City	0	0	0	0	0.00	0	0	0	0.00
Tayabas City	0	0	0	0	0.00	0	0	0	0.00
Trece Martires City	0	0	0	0	0.00	0	0	0	0.00
**Region 4B**	**2,400**	**56**	**54**	**110**	**4.58**	**20**	**17**	**37**	**33.64**
Marinduque	0	0	0	0	0.00	0	0	0	0.00
Mindoro Occidental	0	0	0	0	0.00	0	0	0	0.00
Mindoro Oriental	2,400	56	54	110	4.58	20	17	37	33.64
Palawan	0	0	0	0	0.00	0	0	0	0.00
Romblon	0	0	0	0	0.00	0	0	0	0.00
Puerto Princesa City	0	0	0	0	0.00	0	0	0	0.00
**Region 5**	**0**	**0**	**0**	**0**	**0.00**	**0**	**0**	**0**	**0.00**
Albay	0	0	0	0	0.00	0	0	0	0.00
Camarines Norte	0	0	0	0	0.00	0	0	0	0.00
Camarines Sur	0	0	0	0	0.00	0	0	0	0.00
Catanduanes	0	0	0	0	0.00	0	0	0	0.00
Masbate	0	0	0	0	0.00	0	0	0	0.00

Table 1.E.1 - Filariasis Prevention and Control

Case Detection Rate (CDR) and Clinical Rate of Lymphatic Filariasis
Philippines, 2021

Area	Total Examined for lymphatic filariasis	Number positive cases found				Lymphatic filariasis cases examined with disabilities/deformities			
		Male	Female	Total	Case Detection Rate	Male	Female	Total	Clinical Rate
Sorsogon	0	0	0	0	0.00	0	0	0	0.00
Iriga City	0	0	0	0	0.00	0	0	0	0.00
Legaspi City	0	0	0	0	0.00	0	0	0	0.00
Naga City	0	0	0	0	0.00	0	0	0	0.00
**Region 6**	**536**	**3**	**0**	**3**	**0.56**	**0**	**0**	**0**	**0.00**
Aklan	0	0	0	0	0.00	0	0	0	0.00
Antique	536	3	0	3	0.56	0	0	0	0.00
Capiz	0	0	0	0	0.00	0	0	0	0.00
Guimaras	0	0	0	0	0.00	0	0	0	0.00
Iloilo	0	0	0	0	0.00	0	0	0	0.00
Negros Occidental	0	0	0	0	0.00	0	0	0	0.00
Bacolod City	0	0	0	0	0.00	0	0	0	0.00
Iloilo City	0	0	0	0	0.00	0	0	0	0.00
**Region 7**	**0**	**0**	**0**	**0**	**0.00**	**0**	**0**	**0**	**0.00**
Bohol	0	0	0	0	0.00	0	0	0	0.00
Cebu	0	0	0	0	0.00	0	0	0	0.00
Negros Oriental	0	0	0	0	0.00	0	0	0	0.00
Siquijor	0	0	0	0	0.00	0	0	0	0.00
Cebu City	0	0	0	0	0.00	0	0	0	0.00
Lapu-Lapu City	0	0	0	0	0.00	0	0	0	0.00
Mandaue City	0	0	0	0	0.00	0	0	0	0.00
**Region 8**	**7,835**	**72**	**54**	**126**	**1.61**	**2**	**0**	**2**	**1.59**
Biliran	1,372	1	0	1	0.07	0	0	0	0.00
Eastern Samar	1,284	15	2	17	1.32	0	0	0	0.00
Northern Leyte	950	21	15	36	3.79	1	0	1	2.78
Northern Samar	2,450	20	21	41	1.67	0	0	0	0.00
Southern Leyte	648	3	2	5	0.77	1	0	1	20.00
Western Samar	853	10	10	20	2.34	0	0	0	0.00
Calbayog City	278	2	4	6	2.16	0	0	0	0.00
Maasin City	0	0	0	0	0.00	0	0	0	0.00
Ormoc City	0	0	0	0	0.00	0	0	0	0.00
Tacloban City	0	0	0	0	0.00	0	0	0	0.00

Table 1.E.1 - Filariasis Prevention and Control

Case Detection Rate (CDR) and Clinical Rate of Lymphatic Filariasis
Philippines, 2021

Area	Total Examined for lymphatic filariasis	Number positive cases found				Lymphatic filariasis cases examined with disabilities/deformities			
		Male	Female	Total	Case Detection Rate	Male	Female	Total	Clinical Rate
**Region 9**	**0**	**0**	**0**	**0**	**0.00**	**0**	**0**	**0**	**0.00**
Zamboanga del Norte	0	0	0	0	0.00	0	0	0	0.00
Zamboanga del Sur	0	0	0	0	0.00	0	0	0	0.00
Zamboanga Sibugay	0	0	0	0	0.00	0	0	0	0.00
Dapitan City	0	0	0	0	0.00	0	0	0	0.00
Dipolog City	0	0	0	0	0.00	0	0	0	0.00
Isabela City	0	0	0	0	0.00	0	0	0	0.00
Pagadian City	0	0	0	0	0.00	0	0	0	0.00
Zamboanga City	0	0	0	0	0.00	0	0	0	0.00
**Region 10**	**0**	**0**	**0**	**0**	**0.00**	**0**	**0**	**0**	**0.00**
Bukidnon	0	0	0	0	0.00	0	0	0	0.00
Camiguin	0	0	0	0	0.00	0	0	0	0.00
Lanao del Norte	0	0	0	0	0.00	0	0	0	0.00
Misamis Occidental	0	0	0	0	0.00	0	0	0	0.00
Misamis Oriental	0	0	0	0	0.00	0	0	0	0.00
Cagayan de Oro City	0	0	0	0	0.00	0	0	0	0.00
El Salvador City	0	0	0	0	0.00	0	0	0	0.00
Gingoog City	0	0	0	0	0.00	0	0	0	0.00
Iligan City	0	0	0	0	0.00	0	0	0	0.00
Malaybalay City	0	0	0	0	0.00	0	0	0	0.00
Oroquieta City	0	0	0	0	0.00	0	0	0	0.00
Ozamis City	0	0	0	0	0.00	0	0	0	0.00
Tangub City	0	0	0	0	0.00	0	0	0	0.00
Valencia City	0	0	0	0	0.00	0	0	0	0.00
**Region 11**	**0**	**0**	**0**	**0**	**0.00**	**0**	**0**	**0**	**0.00**
Davao de Oro	0	0	0	0	0.00	0	0	0	0.00
Davao del Norte	0	0	0	0	0.00	0	0	0	0.00
Davao del Sur	0	0	0	0	0.00	0	0	0	0.00
Davao Oriental	0	0	0	0	0.00	0	0	0	0.00
Davao Occidental	0	0	0	0	0.00	0	0	0	0.00
Davao City	0	0	0	0	0.00	0	0	0	0.00
**Region 12**	**0**	**1**	**3**	**4**	**0.00**	**0**	**0**	**0**	**0.00**
North Cotabato	0	0	0	0	0.00	0	0	0	0.00
Sarangani	0	0	0	0	0.00	0	0	0	0.00
South Cotabato	0	0	0	0	0.00	0	0	0	0.00
Sultan Kudarat	0	1	3	4	0.00	0	0	0	0.00

Table 1.E.1 - Filariasis Prevention and Control

Case Detection Rate (CDR) and Clinical Rate of Lymphatic Filariasis
Philippines, 2021

Area	Total Examined for lymphatic filariasis	Number positive cases found				Lymphatic filariasis cases examined with disabilities/deformities			
		Male	Female	Total	Case Detection Rate	Male	Female	Total	Clinical Rate
Cotabato City	0	0	0	0	0.00	0	0	0	0.00
Gen. Santos City	0	0	0	0	0.00	0	0	0	0.00
**BARMM**	**0**	**0**	**0**	**0**	**0.00**	**0**	**0**	**0**	**0.00**
Basilan	0	0	0	0	0.00	0	0	0	0.00
Lanao del Sur	0	0	0	0	0.00	0	0	0	0.00
Maguindanao	*	*	*	0	0.00	*	*		0.00
Sulu	*	*	*	0	0.00	*	*		0.00
Tawi-Tawi	*	*	*	0	0.00	*	*		0.00
Lamitan City	*	*	*	0	0.00	*	*		0.00
Marawi City	*	*	*	0	0.00	*	*		0.00
**CARAGA**	**3**	**0**	**2**	**2**	**66.67**	**0**	**0**	**0**	**0.00**
Agusan del Norte	0	0	0	0	0.00	0	0	0	0.00
Agusan del Sur	3	0	2	2	66.67	0	0	0	0.00
Surigao del Norte	0	0	0	0	0.00	0	0	0	0.00
Surigao del Sur	0	0	0	0	0.00	0	0	0	0.00
Province of Dinagat	0	0	0	0	0.00	0	0	0	0.00
Bislig City	0	0	0	0	0.00	0	0	0	0.00
Butuan City	0	0	0	0	0.00	0	0	0	0.00
Surigao City	0	0	0	0	0.00	0	0	0	0.00

Legend:
0 = No Case
** = No Report*

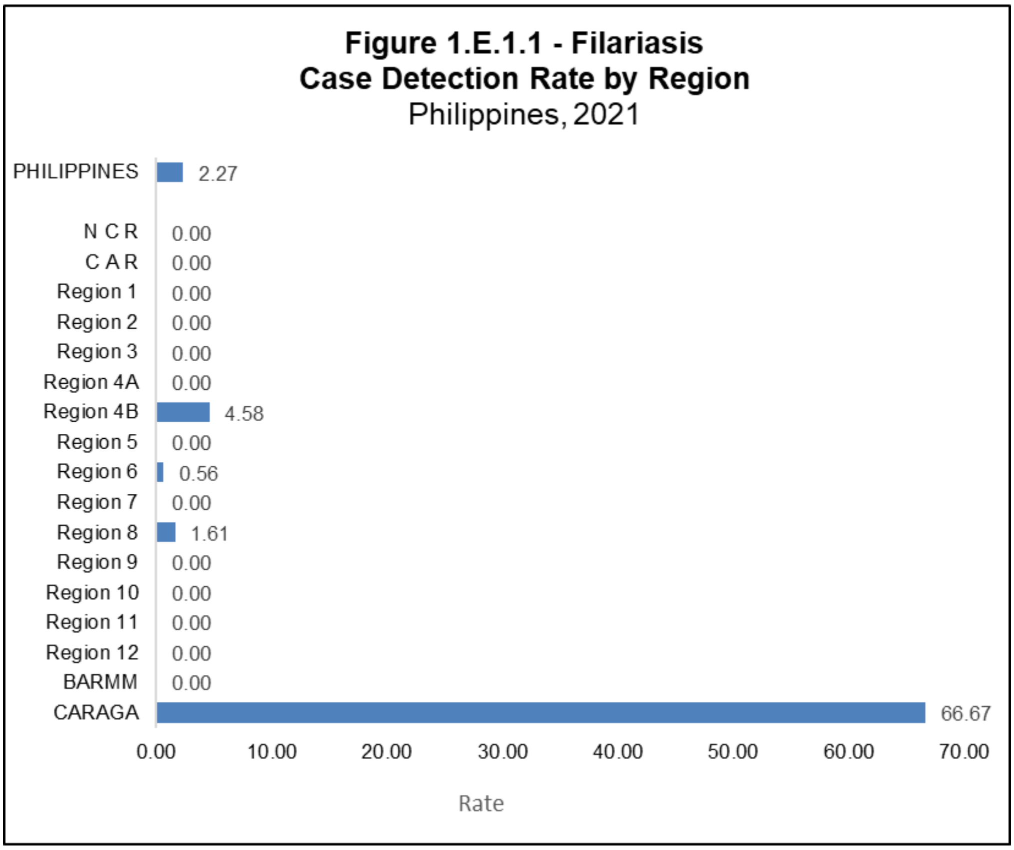
Figure 1.E.1.1 - Filariasis
Case Detection Rate by Region
Philippines, 2021
PHILIPPINES 2.27
N C R 0.00
C A R 0.00
Region 1 0.00
Region 2 0.00
Region 3 0.00
Region 4A 0.00
Region 4B 4.58
Region 5 0.00
Region 6 0.56
Region 7 0.00
Region 8 1.61
Region 9 0.00
Region 10 0.00
Region 11 0.00
Region 12 0.00
BARMM 0.00
CARAGA 66.67
0.00 10.00 20.00 30.00 40.00 50.00 60.00 70.00
Rate

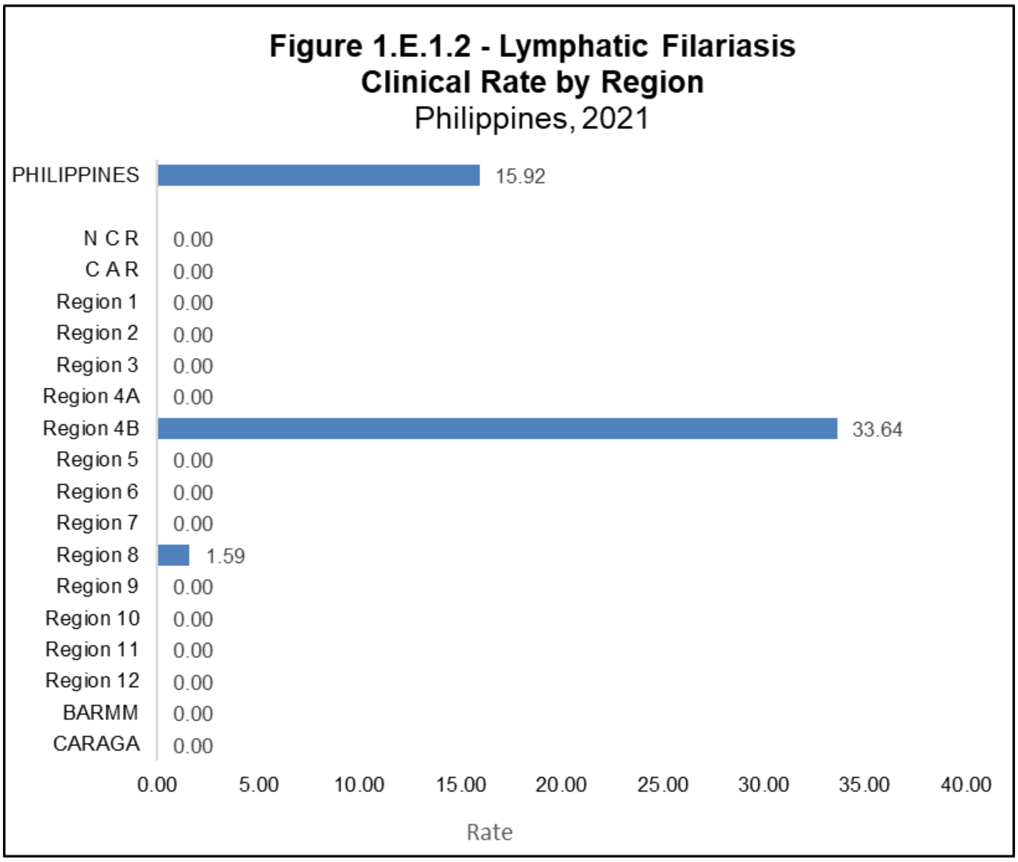
Figure 1.E.1.2 - Lymphatic Filariasis
Clinical Rate by Region
Philippines, 2021
PHILIPPINES 15.92
N C R 0.00
C A R 0.00
Region 1 0.00
Region 2 0.00
Region 3 0.00
Region 4A 0.00
Region 4B 33.64
Region 5 0.00
Region 6 0.00
Region 7 0.00
Region 8 1.59
Region 9 0.00
Region 10 0.00
Region 11 0.00
Region 12 0.00
BARMM 0.00
CARAGA 0.00
0.00 5.00 10.00 15.00 20.00 25.00 30.00 35.00 40.00
Rate

Figure 1.E.1.3 - Filariasis
Case Detection Rate by Year
Philippines, 2017-2021

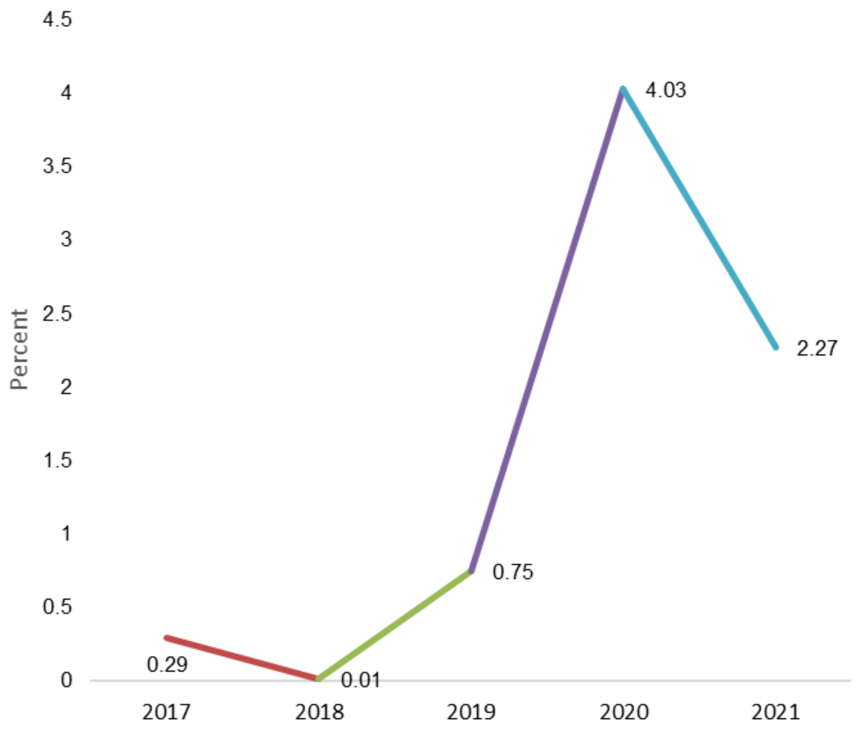

Figure 1.E.1.4 - Lymphatic Filariasis
Clinical Rate by Year
Philippines, 2017-2021

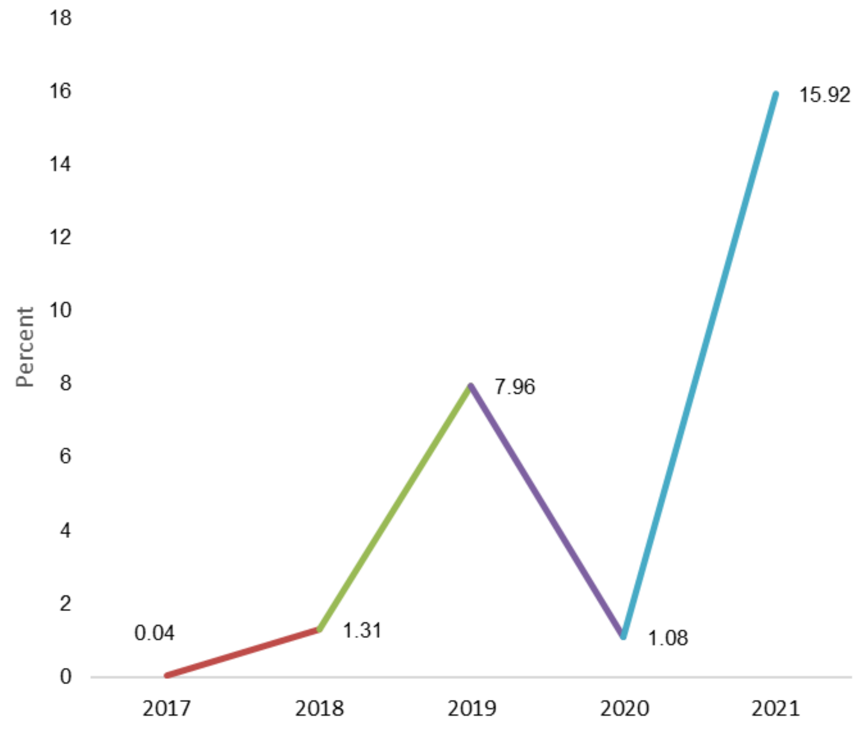

Table 1.E.2.1 - Schistosomiasis Prevention and Control

Number and Proportion of Suspected Schistosomiasis cases seen, Acute clinically diagnosed cases seen and Acute Confirmed Cases by Stool Examination

Philippines, 2021

Area	Total Patients seen	Number of Suspected Cases				No. of Acute Clinically Diagnosed Cases				No. of Acute Cases Confirmed Positive by Stool Examination			
		Male	Female	Total	%	Male	Female	Total	%	Male	Female	Total	%
**PHILIPPINES**	**61,334**	**7,778**	**6,416**	**14,194**	**23.14**	**368**	**232**	**600**	**4.23**	**168**	**112**	**280**	**46.67**
**N C R**	**0**	**0**	**0**	**0**	**0.00**	**0**	**0**	**0**	**0.00**	**0**	**0**	**0**	**0.00**
Malabon City	0	0	0	0	0.00	0	0	0	0.00	0	0	0	0.00
Navotas City	0	0	0	0	0.00	0	0	0	0.00	0	0	0	0.00
Valenzuela City	0	0	0	0	0.00	0	0	0	0.00	0	0	0	0.00
Caloocan City	0	0	0	0	0.00	0	0	0	0.00	0	0	0	0.00
Marikina City	0	0	0	0	0.00	0	0	0	0.00	0	0	0	0.00
Pasig City	0	0	0	0	0.00	0	0	0	0.00	0	0	0	0.00
Pateros	0	0	0	0	0.00	0	0	0	0.00	0	0	0	0.00
Taguig City	0	0	0	0	0.00	0	0	0	0.00	0	0		0.00
Quezon City	0	0	0	0	0.00	0	0	0	0.00	0	0	0	0.00
Makati City	0	0	0	0	0.00	0	0	0	0.00	0	0	0	0.00
Mandaluyong City	0	0	0	0	0.00	0	0	0	0.00	0	0	0	0.00
San Juan City	0	0	0	0	0.00	0	0	0	0.00	0	0	0	0.00
Manila City	0	0	0	0	0.00	0	0	0	0.00	0	0	0	0.00
Las Piñas City	0	0	0	0	0.00	0	0	0	0.00	0	0	0	0.00
Muntinlupa City	0	0	0	0	0.00	0	0	0	0.00	0	0	0	0.00
Parañaque City	0	0	0	0	0.00	0	0	0	0.00	0	0	0	0.00
Pasay City	0	0	0	0	0.00	0	0	0	0.00	0	0	0	0.00
**C A R**	**0**	**0**	**0**	**0**	**0.00**	**0**	**0**	**0**	**0.00**	**0**	**0**	**0**	**0.00**
Abra	0	0	0	0	0.00	0	0	0	0.00	0	0	0	0.00
Apayao	0	0	0	0	0.00	0	0	0	0.00	0	0	0	0.00
Benguet	0	0	0	0	0.00	0	0	0	0.00	0	0	0	0.00
Ifugao	0	0	0	0	0.00	0	0	0	0.00	0	0	0	0.00
Kalinga	0	0	0	0	0.00	0	0	0	0.00	0	0	0	0.00
Mt. Province	0	0	0	0	0.00	0	0	0	0.00	0	0	0	0.00
Baguio City	0	0	0	0	0.00	0	0	0	0.00	0	0	0	0.00
**Region 1**	**0**	**0**	**0**	**0**	**0.00**	**0**	**0**	**0**	**0.00**	**0**	**0**	**0**	**0.00**
Ilocos Norte	0	0	0	0	0.00	0	0	0	0.00	0	0	0	0.00
Ilocos Sur	0	0	0	0	0.00	0	0	0	0.00	0	0	0	0.00
La Union	0	0	0	0	0.00	0	0	0	0.00	0	0	0	0.00
Pangasinan	0	0	0	0	0.00	0	0	0	0.00	0	0	0	0.00
Alaminos City	0	0	0	0	0.00	0	0	0	0.00	0	0	0	0.00
Candon City	0	0	0	0	0.00	0	0	0	0.00	0	0	0	0.00
Dagupan City	0	0	0	0	0.00	0	0	0	0.00	0	0	0	0.00
Laoag City	0	0	0	0	0.00	0	0	0	0.00	0	0	0	0.00

Table 1.E.2.1 - Schistosomiasis Prevention and Control

Number and Proportion of Suspected Schistosomiasis cases seen, Acute clinically diagnosed cases seen and Acute Confirmed Cases by Stool Examination

Philippines, 2021

Area	Total Patients seen	Number of Suspected Cases				No. of Acute Clinically Diagnosed Cases				No. of Acute Cases Confirmed Positive by Stool Examination			
		Male	Female	Total	%	Male	Female	Total	%	Male	Female	Total	%
San Carlos City	0	0	0	0	0.00	0	0	0	0.00	0	0	0	0.00
San Fernando City	0	0	0	0	0.00	0	0	0	0.00	0	0	0	0.00
Urdaneta City	0	0	0	0	0.00	0	0	0	0.00	0	0	0	0.00
Vigan City	0	0	0	0	0.00	0	0	0	0.00	0	0	0	0.00
**Region 2**	**0**	**0**	**0**	**0**	**0.00**	**0**	**0**	**0**	**0.00**	**0**	**0**	**0**	**0.00**
Batanes	0	0	0	0	0.00	0	0	0	0.00	0	0	0	0.00
Cagayan	0	0	0	0	0.00	0	0	0	0.00	0	0	0	0.00
Isabela	0	0	0	0	0.00	0	0	0	0.00	0	0	0	0.00
Nueva Vizcaya	0	0	0	0	0.00	0	0	0	0.00	0	0	0	0.00
Quirino	0	0	0	0	0.00	0	0	0	0.00	0	0	0	0.00
Cauayan City	0	0	0	0	0.00	0	0	0	0.00	0	0	0	0.00
Ilagan City	0	0	0	0	0.00	0	0	0	0.00	0	0	0	0.00
Santiago City	0	0	0	0	0.00	0	0	0	0.00	0	0	0	0.00
Tuguegarao City	0	0	0	0	0.00	0	0	0	0.00	0	0	0	0.00
**Region 3**	**0**	**0**	**0**	**0**	**0.00**	**0**	**0**	**0**	**0.00**	**0**	**0**	**0**	**0.00**
Aurora	0	0	0	0	0.00	0	0	0	0.00	0	0	0	0.00
Bataan	0	0	0	0	0.00	0	0	0	0.00	0	0	0	0.00
Bulacan	0	0	0	0	0.00	0	0	0	0.00	0	0	0	0.00
Nueva Ecija	0	0	0	0	0.00	0	0	0	0.00	0	0	0	0.00
Pampanga	0	0	0	0	0.00	0	0	0	0.00	0	0	0	0.00
Tarlac	0	0	0	0	0.00	0	0	0	0.00	0	0	0	0.00
Zambales	0	0	0	0	0.00	0	0	0	0.00	0	0	0	0.00
Angeles City	0	0	0	0	0.00	0	0	0	0.00	0	0	0	0.00
Balanga City	0	0	0	0	0.00	0	0	0	0.00	0	0	0	0.00
Cabanatuan City	0	0	0	0	0.00	0	0	0	0.00	0	0	0	0.00
City of San Fernando	0	0	0	0	0.00	0	0	0	0.00	0	0	0	0.00
Gapan City	0	0	0	0	0.00	0	0	0	0.00	0	0	0	0.00
Mabalacat City	0	0	0	0	0.00	0	0	0	0.00	0	0	0	0.00
Malolos City	0	0	0	0	0.00	0	0	0	0.00	0	0	0	0.00
Meycauayan City	0	0	0	0	0.00	0	0	0	0.00	0	0	0	0.00
Olongapo City	0	0	0	0	0.00	0	0	0	0.00	0	0	0	0.00
Palayan City	0	0	0	0	0.00	0	0	0	0.00	0	0	0	0.00
San Jose City	0	0	0	0	0.00	0	0	0	0.00	0	0	0	0.00
San Jose del Monte City	0	0	0	0	0.00	0	0	0	0.00	0	0	0	0.00
Science City of Munoz	0	0	0	0	0.00	0	0	0	0.00	0	0	0	0.00
Tarlac City	0	0	0	0	0.00	0	0	0	0.00	0	0	0	0.00
**Region 4A**	**280**	**60**	**23**	**83**	**29.64**	**1**	**0**	**1**	**1.20**	**0**	**0**	**0**	**0.00**
Batangas	31	20	11	31	100.00	0	0	0	0.00	0	0	0	0.00
Cavite	9	6	3	9	100.00	0	0	0	0.00	0	0	0	0.00

Table 1.E.2.1 - Schistosomiasis Prevention and Control

Number and Proportion of Suspected Schistosomiasis cases seen, Acute clinically diagnosed cases seen and Acute Confirmed Cases by Stool Examination

Philippines, 2021

Area	Total Patients seen	Number of Suspected Cases				No. of Acute Clinically Diagnosed Cases				No. of Acute Cases Confirmed Positive by Stool Examination			
		Male	Female	Total	%	Male	Female	Total	%	Male	Female	Total	%
Laguna	0	0	0	0	0.00	0	0	0	0.00	0	0	0	0.00
Quezon	33	21	9	30	90.91	0	0	0	0.00	0	0	0	0.00
Rizal	2	2	0	2	100.00	0	0	0	0.00	0	0	0	0.00
Antipolo City	0	0	0	0	0.00	0	0	0	0.00	0	0	0	0.00
Bacoor City	2	0	0	0	0.00	0	0	0	0.00	0	0	0	0.00
Batangas City	0	0	0	0	0.00	0	0	0	0.00	0	0	0	0.00
Biñan City	0	0	0	0	0.00	0	0	0	0.00	0	0	0	0.00
Cabuyao City	0	0	0	0	0.00	0	0	0	0.00	0	0	0	0.00
Calamba City	0	0	0	0	0.00	0	0	0	0.00	0	0	0	0.00
Cavite City	0	0	0	0	0.00	0	0	0	0.00	0	0	0	0.00
Dasmariñas City	15	11	0	11	73.33	1	0	1	9.09	0	0	0	0.00
General Trias City	3	0	0	0	0.00	0	0	0	0.00	0	0	0	0.00
Imus City	0	0	0	0	0.00	0	0	0	0.00	0	0	0	0.00
Lipa City	0	0	0	0	0.00	0	0	0	0.00	0	0	0	0.00
Lucena City	185	0	0	0	0.00	0	0	0	0.00	0	0	0	0.00
San Pablo City	0	0	0	0	0.00	0	0	0	0.00	0	0	0	0.00
San Pedro City	0	0	0	0	0.00	0	0	0	0.00	0	0	0	0.00
Santa Rosa City	0	0	0	0	0.00	0	0	0	0.00	0	0	0	0.00
Tagaytay City	0	0	0	0	0.00	0	0	0	0.00	0	0	0	0.00
Tanauan City	0	0	0	0	0.00	0	0	0	0.00	0	0	0	0.00
Tayabas City	0	0	0	0	0.00	0	0	0	0.00	0	0	0	0.00
Trece Martires City	0	0	0	0	0.00	0	0	0	0.00	0	0	0	0.00
**Region 4B**	**0**	**0**	**0**	**0**	**0.00**	**0**	**0**	**0**	**0.00**	**0**	**0**	**0**	**0.00**
Marinduque	0	0	0	0	0.00	0	0	0	0.00	0	0	0	0.00
Mindoro Occidental	0	0	0	0	0.00	0	0	0	0.00	0	0	0	0.00
Mindoro Oriental	0	0	0	0	0.00	0	0	0	0.00	0	0	0	0.00
Palawan	0	0	0	0	0.00	0	0	0	0.00	0	0	0	0.00
Romblon	0	0	0	0	0.00	0	0	0	0.00	0	0	0	0.00
Puerto Princesa City	0	0	0	0	0.00	0	0	0	0.00	0	0	0	0.00
**Region 5**	**29**	**23**	**6**	**29**	**100.00**	**7**	**2**	**9**	**31.03**	**2**	**0**	**2**	**22.22**
Albay	3	2	1	3	100.00	1	1	2	66.67	0	0	0	0.00
Camarines Norte	0	0	0	0	0.00	0	0	0	0.00	0	0	0	0.00
Camarines Sur	0	0	0	0	0.00	0	0	0	0.00	0	0	0	0.00
Catanduanes	0	0	0	0	0.00	0	0	0	0.00	0	0	0	0.00
Masbate	0	0	0	0	0.00	0	0	0	0.00	0	0	0	0.00
Sorsogon	26	21	5	26	100.00	6	1	7	26.92	2	0	2	28.57
Iriga City	0	0	0	0	0.00	0	0	0	0.00	0	0	0	0.00
Legaspi City	0	0	0	0	0.00	0	0	0	0.00	0	0	0	0.00
Naga City	0	0	0	0	0.00	0	0	0	0.00	0	0	0	0.00

Table 1.E.2.1 - Schistosomiasis Prevention and Control

Number and Proportion of Suspected Schistosomiasis cases seen, Acute clinically diagnosed cases seen and Acute Confirmed Cases by Stool Examination

Philippines, 2021

Area	Total Patients seen	Number of Suspected Cases				No. of Acute Clinically Diagnosed Cases				No. of Acute Cases Confirmed Positive by Stool Examination			
		Male	Female	Total	%	Male	Female	Total	%	Male	Female	Total	%
**Region 6**	**0**	**0**	**0**	**0**	**0.00**	**0**	**0**	**0**	**0.00**	**0**	**0**	**0**	**0.00**
Aklan	0	0	0	0	0.00	0	0	0	0.00	0	0	0	0.00
Antique	0	0	0	0	0.00	0	0	0	0.00	0	0	0	0.00
Capiz	0	0	0	0	0.00	0	0	0	0.00	0	0	0	0.00
Guimaras	0	0	0	0	0.00	0	0	0	0.00	0	0	0	0.00
Iloilo	0	0	0	0	0.00	0	0	0	0.00	0	0	0	0.00
Negros Occidental	0	0	0	0	0.00	0	0	0	0.00	0	0	0	0.00
Bacolod City	0	0	0	0	0.00	0	0	0	0.00	0	0	0	0.00
Bago City	0	0	0	0	0.00	0	0	0	0.00	0	0	0	0.00
**Region 7**	**0**	**0**	**0**	**0**	**0.00**	**0**	**0**	**0**	**0.00**	**0**	**0**	**0**	**0.00**
Bohol	0	0	0	0	0.00	0	0	0	0.00	0	0	0	0.00
Cebu	0	0	0	0	0.00	0	0	0	0.00	0	0	0	0.00
Negros Oriental	0	0	0	0	0.00	0	0	0	0.00	0	0	0	0.00
Siquijor	0	0	0	0	0.00	0	0	0	0.00	0	0	0	0.00
Cebu City	0	0	0	0	0.00	0	0	0	0.00	0	0	0	0.00
Lapu-Lapu City	0	0	0	0	0.00	0	0	0	0.00	0	0	0	0.00
Mandaue City	0	0	0	0	0.00	0	0	0	0.00	0	0	0	0.00
**Region 8**	**0**	**0**	**0**	**0**	**0.00**	**0**	**0**	**0**	**0.00**	**0**	**0**	**0**	**0.00**
Biliran	0	0	0	0	0.00	0	0	0	0.00	0	0	0	0.00
Eastern Samar	0	0	0	0	0.00	0	0	0	0.00	0	0	0	0.00
Northern Leyte	0	0	0	0	0.00	0	0	0	0.00	0	0	0	0.00
Northern Samar	0	0	0	0	0.00	0	0	0	0.00	0	0	0	0.00
Southern Leyte	0	0	0	0	0.00	0	0	0	0.00	0	0	0	0.00
Western Samar	0	0	0	0	0.00	0	0	0	0.00	0	0	0	0.00
Calbayog City	0	0	0	0	0.00	0	0	0	0.00	0	0	0	0.00
Maasin City	0	0	0	0	0.00	0	0	0	0.00	0	0	0	0.00
Ormoc City	0	0	0	0	0.00	0	0	0	0.00	0	0	0	0.00
Tacloban City	0	0	0	0	0.00	0	0	0	0.00	0	0	0	0.00
**Region 9**	**0**	**0**	**0**	**0**	**0.00**	**0**	**0**	**0**	**0.00**	**0**	**0**	**0**	**0.00**
Zamboanga del Norte	0	0	0	0	0.00	0	0	0	0.00	0	0	0	0.00
Zamboanga del Sur	0	0	0	0	0.00	0	0	0	0.00	0	0	0	0.00
Zamboanga Sibugay	0	0	0	0	0.00	0	0	0	0.00	0	0	0	0.00
Dapitan City	0	0	0	0	0.00	0	0	0	0.00	0	0	0	0.00
Dipolog City	0	0	0	0	0.00	0	0	0	0.00	0	0	0	0.00
Isabela City	0	0	0	0	0.00	0	0	0	0.00	0	0	0	0.00
Pagadian City	0	0	0	0	0.00	0	0	0	0.00	0	0	0	0.00
Zamboanga City	0	0	0	0	0.00	0	0	0	0.00	0	0	0	0.00

Table 1.E.2.1 - Schistosomiasis Prevention and Control

Number and Proportion of Suspected Schistosomiasis cases seen, Acute clinically diagnosed cases seen and Acute Confirmed Cases by Stool Examination

Philippines, 2021

Area	Total Patients seen	Number of Suspected Cases				No. of Acute Clinically Diagnosed Cases				No. of Acute Cases Confirmed Positive by Stool Examination			
		Male	Female	Total	%	Male	Female	Total	%	Male	Female	Total	%
**Region 10**	**844**	**409**	**224**	**633**	**75.00**	**227**	**140**	**367**	**57.98**	**99**	**54**	**153**	**41.69**
Bukidnon	49	30	15	45	91.84	8	5	13	28.89	4	1	5	38.46
Camiguin	0	0	0	0	0.00	0	0	0	0.00	0	0	0	0.00
Lanao del Norte	55	38	17	55	100.00	10	3	13	23.64	10	3	13	100.00
Misamis Occidental	0	0	0	0	0.00	0	0	0	0.00	0	0	0	0.00
Misamis Oriental	0	0	0	0	0.00	0	0	0	0.00	0	0	0	0.00
Cagayan de Oro City	0	0	0	0	0.00	0	0	0	0.00	0	0	0	0.00
El Salvador City	0	0	0	0	0.00	0	0	0	0.00	0	0	0	0.00
Gingoog City	0	0	0	0	0.00	0	0	0	0.00	0	0	0	0.00
Iligan City	0	0	0	0	0.00	0	0	0	0.00	0	0	0	0.00
Malaybalay City	122	66	56	122	100.00	66	56	122	100.00	4	6	10	8.20
Oroquieta City	0	0	0	0	0.00	0	0	0	0.00	0	0	0	0.00
Ozamis City	10	5	5	10	100.00	2	1	3	30.00	0	0	0	0.00
Tangub City	15	14	1	15	100.00	0	0	0	0.00	0	0	0	0.00
Valencia City	593	256	130	386	65.09	141	75	216	55.96	81	44	125	57.87
**Region 11**	**5,767**	**342**	**334**	**676**	**11.72**	**36**	**17**	**53**	**7.84**	**10**	**6**	**16**	**30.19**
Davao de Oro	708	309	318	627	88.56	4	3	7	1.12	4	3	7	100.00
Davao del Norte	421	33	16	49	11.64	32	14	46	93.88	6	3	9	19.57
Davao del Sur	0	0	0	0	0.00	0	0	0	0.00	0	0	0	0.00
Davao Oriental	4,638	0	0	0	0.00	0	0	0	0.00	0	0	0	0.00
Davao Occidental	0	0	0	0	0.00	0	0	0	0.00	0	0	0	0.00
Davao City	0	0	0	0	0.00	0	0	0	0.00	0	0	0	0.00
**Region 12**	**150**	**32**	**13**	**45**	**30.00**	**25**	**11**	**36**	**80.00**	**17**	**9**	**26**	**72.22**
North Cotabato	128	29	12	41	32.03	22	10	32	78.05	17	9	26	81.25
Sarangani	0	0	0	0	0.00	0	0	0	0.00	0	0	0	0.00
South Cotabato	3	1	1	2	66.67	1	1	2	100.00	0	0	0	0.00
Sultan Kudarat	0	0	0	0	0.00	0	0	0	0.00	0	0	0	0.00
Cotabato City	0	0	0	0	0.00	0	0	0	0.00	0	0	0	0.00
Gen. Santos City	19	2	0	2	10.53	2	0	2	100.00	0	0	0	0.00
**BARMM**	**13**	**8**	**5**	**13**	**100.00**	**8**	**5**	**13**	**100.00**	**0**	**0**	**0**	**0.00**
Basilan	0	0	0	0	0.00	0	0	0	0.00	0	0	0	0.00
Lanao del Sur	0	0	0	0	0.00	0	0	0	0.00	0	0	0	0.00
Maguindanao	13	8	5	13	100.00	8	5	13	100.00	0	0	0	0.00
Sulu	0	0	0	0	0.00	0	0	0	0.00	0	0	0	0.00
Tawi-Tawi	0	0	0	0	0.00	0	0	0	0.00	0	0	0	0.00

Table 1.E.2.1 - Schistosomiasis Prevention and Control

Number and Proportion of Suspected Schistosomiasis cases seen, Acute clinically diagnosed cases seen and Acute Confirmed Cases by Stool Examination

Philippines, 2021

Area	Total Patients seen	Number of Suspected Cases				No. of Acute Clinically Diagnosed Cases				No. of Acute Cases Confirmed Positive by Stool Examination			
		Male	Female	Total	%	Male	Female	Total	%	Male	Female	Total	%
Lamitan City	0	0	0	0	0.00	0	0	0	0.00	0	0	0	0.00
Marawi City	0	0	0	0	0.00	0	0	0	0.00	0	0	0	0.00
**CARAGA**	**54,251**	**6,904**	**5,811**	**12,715**	**23.44**	**64**	**57**	**121**	**0.95**	**40**	**43**	**83**	**68.60**
Agusan del Norte	5,589	826	659	1,485	26.57	6	5	11	0.74	2	1	3	27.27
Agusan del Sur	47,062	6,049	5,142	11,191	23.78	38	45	83	0.74	23	36	59	71.08
Surigao del Norte	262	26	9	35	13.36	17	6	23	65.71	14	6	20	86.96
Surigao del Sur	1	0	0	0	0.00	0	1	1	0.00	0	0	0	0.00
Province of Dinagat	0	0	0	0	0.00	0	0	0	0.00	0	0	0	0.00
Bislig City	0	0	0	0	0.00	0	0	0	0.00	0	0	0	0.00
Butuan City	1,337	3	1	4	0.30	3	0	3	75.00	1	0	1	33.33
Surigao City	0	0	0	0	0.00	0	0	0	0.00	0	0	0	0.00

Legend:

0 = No Case

** = No Report*

Table 1.E.2.2 - Schistosomiasis Prevention and Control

Number and Proportion of Chronic Clinically Diagnosed Cases and Chronic Cllinically diagnosed cases confirmed by stool examination
Philippines, 2021

Area	No. of Clinically Diagnosed Chronic Cases				No. of chronic clinically diagnoses cases confirmed by stool examination (KKT) or rectal biopsy			
	Male	Female	Total	%	Male	Female	Total	%
**PHILIPPINES**	**294**	**222**	**516**	**3.64**	**188**	**168**	**356**	**68.99**
**N C R**	**0**	**0**	**0**	**0.00**	**0**	**0**	**0**	**0.00**
Malabon City	0	0	0	0.00	0	0	0	0.00
Navotas City	0	0	0	0.00	0	0	0	0.00
Valenzuela City	0	0	0	0.00	0	0	0	0.00
Caloocan City	0	0	0	0.00	0	0	0	0.00
Marikina City	0	0	0	0.00	0	0	0	0.00
Pasig City	0	0	0	0.00	0	0	0	0.00
Pateros	0	0	0	0.00	0	0	0	0.00
Taguig City	0	0	0	0.00	0	0	0	0.00
Quezon City	0	0	0	0.00	0	0	0	0.00
Makati City	0	0	0	0.00	0	0	0	0.00
Mandaluyong City	0	0	0	0.00	0	0	0	0.00
San Juan City	0	0	0	0.00	0	0	0	0.00
Manila City	0	0	0	0.00	0	0	0	0.00
Las Piñas City	0	0	0	0.00	0	0	0	0.00
Muntinlupa City	0	0	0	0.00	0	0	0	0.00
Parañaque City	0	0	0	0.00	0	0	0	0.00
Pasay City	0	0	0	0.00	0	0	0	0.00
**C A R**	**0**	**0**	**0**	**0.00**	**0**	**0**	**0**	**0.00**
Abra	0	0	0	0.00	0	0	0	0.00
Apayao	0	0	0	0.00	0	0	0	0.00
Benguet	0	0	0	0.00	0	0	0	0.00
Ifugao	0	0	0	0.00	0	0	0	0.00
Kalinga	0	0	0	0.00	0	0	0	0.00
Mt. Province	0	0	0	0.00	0	0	0	0.00
Baguio City	0	0	0	0.00	0	0	0	0.00
**Region 1**	**0**	**0**	**0**	**0.00**	**0**	**0**	**0**	**0.00**
Ilocos Norte	0	0	0	0.00	0	0	0	0.00
Ilocos Sur	0	0	0	0.00	0	0	0	0.00
La Union	0	0	0	0.00	0	0	0	0.00
Pangasinan	0	0	0	0.00	0	0	0	0.00

Table 1.E.2.2 - Schistosomiasis Prevention and Control

Number and Proportion of Chronic Clinically Diagnosed Cases and Chronic Cllinically diagnosed cases confirmed by stool examination

Philippines, 2021

Area	No. of Clinically Diagnosed Chronic Cases				No. of chronic clinically diagnoses cases confirmed by stool examination (KKT) or rectal biopsy			
	Male	Female	Total	%	Male	Female	Total	%
Alaminos City	0	0	0	0.00	0	0	0	0.00
Candon City	0	0	0	0.00	0	0	0	0.00
Dagupan City	0	0	0	0.00	0	0	0	0.00
Laoag City	0	0	0	0.00	0	0	0	0.00
San Carlos City	0	0	0	0.00	0	0	0	0.00
San Fernando City	0	0	0	0.00	0	0	0	0.00
Urdaneta City	0	0	0	0.00	0	0	0	0.00
Vigan City	0	0	0	0.00	0	0	0	0.00
**Region 2**	**0**	**0**	**0**	**0.00**	**0**	**0**	**0**	**0.00**
Batanes	0	0	0	0.00	0	0	0	0.00
Cagayan	0	0	0	0.00	0	0	0	0.00
Isabela	0	0	0	0.00	0	0	0	0.00
Nueva Vizcaya	0	0	0	0.00	0	0	0	0.00
Quirino	0	0	0	0.00	0	0	0	0.00
Cauayan City	0	0	0	0.00	0	0	0	0.00
Ilagan City	0	0	0	0.00	0	0	0	0.00
Santiago City	0	0	0	0.00	0	0	0	0.00
Tuguegarao City	0	0	0	0.00	0	0	0	0.00
**Region 3**	**0**	**0**	**0**	**0.00**	**0**	**0**	**0**	**0.00**
Aurora	0	0	0	0.00	0	0	0	0.00
Bataan	0	0	0	0.00	0	0	0	0.00
Bulacan	0	0	0	0.00	0	0	0	0.00
Nueva Ecija	0	0	0	0.00	0	0	0	0.00
Pampanga	0	0	0	0.00	0	0	0	0.00
Tarlac	0	0	0	0.00	0	0	0	0.00
Zambales	0	0	0	0.00	0	0	0	0.00
Angeles City	0	0	0	0.00	0	0	0	0.00
Balanga City	0	0	0	0.00	0	0	0	0.00
Cabanatuan City	0	0	0	0.00	0	0	0	0.00
City of San Fernando	0	0	0	0.00	0	0	0	0.00
Gapan City	0	0	0	0.00	0	0	0	0.00
Mabalacat City	0	0	0	0.00	0	0	0	0.00
Malolos City	0	0	0	0.00	0	0	0	0.00
Meycauayan City	0	0	0	0.00	0	0	0	0.00
Olongapo City	0	0	0	0.00	0	0	0	0.00
Palayan City	0	0	0	0.00	0	0	0	0.00
San Jose City	0	0	0	0.00	0	0	0	0.00
San Jose del Monte City	0	0	0	0.00	0	0	0	0.00

Table 1.E.2.2 - Schistosomiasis Prevention and Control

Number and Proportion of Chronic Clinically Diagnosed Cases and Chronic Cllinically diagnosed cases confirmed by stool examination

Philippines, 2021

Area	No. of Clinically Diagnosed Chronic Cases				No. of chronic clinically diagnoses cases confirmed by stool examination (KKT) or rectal biopsy			
	Male	Female	Total	%	Male	Female	Total	%
Science City of Munoz	0	0	0	0.00	0	0	0	0.00
Tarlac City	0	0	0	0.00	0	0	0	0.00
**Region 4A**	**0**	**0**	**0**	**0.00**	**0**	**0**	**0**	**0.00**
Batangas	0	0	0	0.00	0	0	0	0.00
Cavite	0	0	0	0.00	0	0	0	0.00
Laguna	0	0	0	0.00	0	0	0	0.00
Quezon	0	0	0	0.00	0	0	0	0.00
Rizal	0	0	0	0.00	0	0	0	0.00
Antipolo City	0	0	0	0.00	0	0	0	0.00
Bacoor City	0	0	0	0.00	0	0	0	0.00
Batangas City	0	0	0	0.00	0	0	0	0.00
Biñan City	0	0	0	0.00	0	0	0	0.00
Cabuyao City	0	0	0	0.00	0	0	0	0.00
Calamba City	0	0	0	0.00	0	0	0	0.00
Cavite City	0	0	0	0.00	0	0	0	0.00
Dasmariñas City	0	0	0	0.00	0	0	0	0.00
General Trias City	0	0	0	0.00	0	0	0	0.00
Imus City	0	0	0	0.00	0	0	0	0.00
Lipa City	0	0	0	0.00	0	0	0	0.00
Lucena City	0	0	0	0.00	0	0	0	0.00
San Pablo City	0	0	0	0.00	0	0	0	0.00
San Pedro City	0	0	0	0.00	0	0	0	0.00
Santa Rosa City	0	0	0	0.00	0	0	0	0.00
Tagaytay City	0	0	0	0.00	0	0	0	0.00
Tanauan City	0	0	0	0.00	0	0	0	0.00
Tayabas City	0	0	0	0.00	0	0	0	0.00
Trece Martires City	0	0	0	0.00	0	0	0	0.00
**Region 4B**	**0**	**0**	**0**	**0.00**	**0**	**0**	**0**	**0.00**
Marinduque	0	0	0	0.00	0	0	0	0.00
Mindoro Occidental	0	0	0	0.00	0	0	0	0.00
Mindoro Oriental	0	0	0	0.00	0	0	0	0.00
Palawan	0	0	0	0.00	0	0	0	0.00
Romblon	0	0	0	0.00	0	0	0	0.00
Puerto Princesa City	0	0	0	0.00	0	0	0	0.00

Table 1.E.2.2 - Schistosomiasis Prevention and Control

Number and Proportion of Chronic Clinically Diagnosed Cases and Chronic Cllinically diagnosed cases confirmed by stool examination
Philippines, 2021

Area	No. of Clinically Diagnosed Chronic Cases				No. of chronic clinically diagnoses cases confirmed by stool examination (KKT) or rectal biopsy			
	Male	Female	Total	%	Male	Female	Total	%
**Region 5**	**15**	**4**	**19**	**65.52**	**4**	**1**	**5**	**26.32**
Albay	0	0	0	0.00	0	0	0	0.00
Camarines Norte	0	0	0	0.00	0	0	0	0.00
Camarines Sur	0	0	0	0.00	0	0	0	0.00
Catanduanes	0	0	0	0.00	0	0	0	0.00
Masbate	0	0	0	0.00	0	0	0	0.00
Sorsogon	15	4	19	73.08	4	1	5	26.32
Iriga City	0	0	0	0.00	0	0	0	0.00
Legaspi City	0	0	0	0.00	0	0	0	0.00
Naga City	0	0	0	0.00	0	0	0	0.00
**Region 6**	**0**	**0**	**0**	**0.00**	**0**	**0**	**0**	**0.00**
Aklan	0	0	0	0.00	0	0	0	0.00
Antique	0	0	0	0.00	0	0	0	0.00
Capiz	0	0	0	0.00	0	0	0	0.00
Guimaras	0	0	0	0.00	0	0	0	0.00
Iloilo	0	0	0	0.00	0	0	0	0.00
Negros Occidental	0	0	0	0.00	0	0	0	0.00
Bacolod City	0	0	0	0.00	0	0	0	0.00
Bago City	0	0	0	0.00	0	0	0	0.00
**Region 7**	**0**	**0**	**0**	**0.00**	**0**	**0**	**0**	**0.00**
Bohol	0	0	0	0.00	0	0	0	0.00
Cebu	0	0	0	0.00	0	0	0	0.00
Negros Oriental	0	0	0	0.00	0	0	0	0.00
Siquijor	0	0	0	0.00	0	0	0	0.00
				0.00				
Cebu City	0	0	0	0.00	0	0	0	0.00
Lapu-Lapu City	0	0	0	0.00	0	0	0	0.00
Mandaue City	0	0	0	0.00	0	0	0	0.00
**Region 8**	**0**	**0**	**0**	**0.00**	**0**	**0**	**0**	**0.00**
Biliran	0	0	0	0.00	0	0	0	0.00
Eastern Samar	0	0	0	0.00	0	0	0	0.00
Northern Leyte	0	0	0	0.00	0	0	0	0.00
Northern Samar	0	0	0	0.00	0	0	0	0.00
Southern Leyte	0	0	0	0.00	0	0	0	0.00

Table 1.E.2.2 - Schistosomiasis Prevention and Control

Number and Proportion of Chronic Clinically Diagnosed Cases and Chronic Cllinically diagnosed cases confirmed by stool examination

Philippines, 2021

Area	No. of Clinically Diagnosed Chronic Cases				No. of chronic clinically diagnoses cases confirmed by stool examination (KKT) or rectal biopsy			
	Male	Female	Total	%	Male	Female	Total	%
Western Samar	0	0	0	0.00	0	0	0	0.00
Calbayog City	0	0	0	0.00	0	0	0	0.00
Maasin City	0	0	0	0.00	0	0	0	0.00
Ormoc City	0	0	0	0.00	0	0	0	0.00
Tacloban City	0	0	0	0.00	0	0	0	0.00
**Region 9**	**0**	**0**	**0**	**0.00**	**0**	**0**	**0**	**0.00**
Zamboanga del Norte	0	0	0	0.00	0	0	0	0.00
Zamboanga del Sur	0	0	0	0.00	0	0	0	0.00
Zamboanga Sibugay	0	0	0	0.00	0	0	0	0.00
Dapitan City	0	0	0	0.00	0	0	0	0.00
Dipolog City	0	0	0	0.00	0	0	0	0.00
Isabela City	0	0	0	0.00	0	0	0	0.00
Pagadian City	0	0	0	0.00	0	0	0	0.00
Zamboanga City	0	0	0	0.00	0	0	0	0.00
**Region 10**	**234**	**177**	**411**	**64.93**	**163**	**151**	**314**	**76.40**
Bukidnon	7	5	12	26.67	5	5	10	83.33
Camiguin	0	0	0	0.00	0	0	0	0.00
Lanao del Norte	0	0	0	0.00	0	0	0	0.00
Misamis Occidental	0	0	0	0.00	0	0	0	0.00
Misamis Oriental	0	0	0	0.00	0	0	0	0.00
Cagayan de Oro City	0	0	0	0.00	0	0	0	0.00
El Salvador City	0	0	0	0.00	0	0	0	0.00
Gingoog City	0	0	0	0.00	0	0	0	0.00
Iligan City	0	0	0	0.00	0	0	0	0.00
Malaybalay City	0	0	0	0.00	0	0	0	0.00
Oroquieta City	0	0	0	0.00	0	0	0	0.00
Ozamis City	3	4	7	70.00	3	4	7	100.00
Tangub City	14	1	15	100.00	14	1	15	100.00
Valencia City	210	167	377	97.67	141	141	282	74.80
**Region 11**	**4**	**3**	**7**	**1.04**	**4**	**3**	**7**	**100.00**
Davao de Oro	0	0	0	0.00	0	0	0	0.00
Davao del Norte	4	3	7	14.29	4	3	7	100.00
Davao del Sur	0	0	0	0.00	0	0	0	0.00
Davao Oriental	0	0	0	0.00	0	0	0	0.00

Table 1.E.2.2 - Schistosomiasis Prevention and Control

Number and Proportion of Chronic Clinically Diagnosed Cases and Chronic Cllinically diagnosed cases confirmed by stool examination
Philippines, 2021

Area	No. of Clinically Diagnosed Chronic Cases				No. of chronic clinically diagnoses cases confirmed by stool examination (KKT) or rectal biopsy			
	Male	Female	Total	%	Male	Female	Total	%
Davao Occidental	0	0	0	0.00	0	0	0	0.00
							0	0.00
Davao City	0	0	0	0.00	0	0	0	0.00
**Region 12**	**6**	**1**	**7**	**15.56**	**5**	**1**	**6**	**85.71**
North Cotabato	4	1	5	12.20	4	1	5	100.00
Sarangani	0	0	0	0.00	0	0	0	0.00
South Cotabato	0	0	0	0.00	0	0	0	0.00
Sultan Kudarat	0	0	0	0.00	0	0	0	0.00
Cotabato City	0	0	0	0.00	0	0	0	0.00
Gen. Santos City	2	0	2	100.00	1	0	1	50.00
**BARMM**	**0**	**0**	**0**	**0.00**	**0**	**0**	**0**	**0.00**
							0	0.00
Basilan	0	0	0	0.00	0	0	0	0.00
Lanao del Sur	0	0	0	0.00	0	0	0	0.00
Maguindanao	0	0	0	0.00	0	0	0	0.00
Sulu	0	0	0	0.00	0	0	0	0.00
Tawi-Tawi	0	0	0	0.00	0	0	0	0.00
Lamitan City	0	0	0	0.00	0	0	0	0.00
Marawi City	0	0	0	0.00	0	0	0	0.00
**CARAGA**	**35**	**37**	**72**	**0.57**	**12**	**12**	**24**	**33.33**
Agusan del Norte	3	2	5	0.3	3	1	4	80.00
Agusan del Sur	25	14	39	0.35	4	0	4	10.26
Surigao del Norte	6	2	8	22.86	5	1	6	75.00
Surigao del Sur	0	0	0	0.00	0	0	0	0.00
Province of Dinagat	0	0	0	0.00	0	0	0	0.00
Bislig City	0	0	0	0.00	0	0	0	0.00
Butuan City	1	19	20	500.00	0	10	10	50.00
Surigao City	0	0	0	0.00	0	0	0	0.00

Legend:
0 = No Case
** = No Report*

Table 1.E.2.3 - Schistosomiasis Prevention and Control

Number and Proportion of Chronic Clinically Diagnosed cases treated in the Health Facility and Confirmed Chronic Cases referred to a Hospital Facility
Philippines, 2021

Area	No. of Chronic Clinically Diagnosed cases treated in the Health Facility				No. of Confirmed Chronic Cases referred to Hospital Facility			
	Male	Female	Total	%	Male	Female	Total	%
**PHILIPPINES**	**339**	**171**	**510**	**80.19**	**7**	**2**	**9**	**2.53**
**N C R**	**0**	**0**	**0**	**0.00**	**0**	**0**	**0**	**0.00**
Malabon City	0	0	0	0.00	0	0	0	0.00
Navotas City	0	0	0	0.00	0	0	0	0.00
Valenzuela City	0	0	0	0.00	0	0	0	0.00
Caloocan City	0	0	0	0.00	0	0	0	0.00
Marikina City	0	0	0	0.00	0	0	0	0.00
Pasig City	0	0	0	0.00	0	0	0	0.00
Pateros	0	0	0	0.00	0	0	0	0.00
Taguig City	0	0	0	0.00	0	0	0	0.00
Quezon City	0	0	0	0.00	0	0	0	0.00
Makati City	0	0	0	0.00	0	0	0	0.00
Mandaluyong City	0	0	0	0.00	0	0	0	0.00
San Juan City	0	0	0	0.00	0	0	0	0.00
Manila City	0	0	0	0.00	0	0	0	0.00
Las Piñas City	0	0	0	0.00	0	0	0	0.00
Muntinlupa City	0	0	0	0.00	0	0	0	0.00
Parañaque City	0	0	0	0.00	0	0	0	0.00
Pasay City	0	0	0	0.00	0	0	0	0.00
**C A R**	**0**	**0**	**0**	**0.00**	**0**	**0**	**0**	**0.00**
Abra	0	0	0	0.00	0	0	0	0.00
Apayao	0	0	0	0.00	0	0	0	0.00
Benguet	0	0	0	0.00	0	0	0	0.00
Ifugao	0	0	0	0.00	0	0	0	0.00
Kalinga	0	0	0	0.00	0	0	0	0.00
Mt. Province	0	0	0	0.00	0	0	0	0.00
Baguio City	0	0	0	0.00	0	0	0	0.00
**Region 1**	**0**	**0**	**0**	**0.00**	**0**	**0**	**0**	**0.00**
Ilocos Norte	0	0	0	0.00	0	0	0	0.00
Ilocos Sur	0	0	0	0.00	0	0	0	0.00
La Union	0	0	0	0.00	0	0	0	0.00
Pangasinan	0	0	0	0.00	0	0	0	0.00
Alaminos City	0	0	0	0.00	0	0	0	0.00
Candon City	0	0	0	0.00	0	0	0	0.00
Dagupan City	0	0	0	0.00	0	0	0	0.00
Laoag City	0	0	0	0.00	0	0	0	0.00
San Carlos City	0	0	0	0.00	0	0	0	0.00

Table 1.E.2.3 - Schistosomiasis Prevention and Control

Number and Proportion of Chronic Clinically Diagnosed cases treated in the Health Facility and Confirmed Chronic Cases referred to a Hospital Facility
Philippines, 2021

Area	No. of Chronic Clinically Diagnosed cases treated in the Health Facility				No. of Confirmed Chronic Cases referred to Hospital Facility			
	Male	Female	Total	%	Male	Female	Total	%
San Fernando City	0	0	0	0.00	0	0	0	0.00
Urdaneta City	0	0	0	0.00	0	0	0	0.00
Vigan City	0	0	0	0.00	0	0	0	0.00
**Region 2**	**0**	**0**	**0**	**0.00**	**0**	**0**	**0**	**0.00**
Batanes	0	0	0	0.00	0	0	0	0.00
Cagayan	0	0	0	0.00	0	0	0	0.00
Isabela	0	0	0	0.00	0	0	0	0.00
Nueva Vizcaya	0	0	0	0.00	0	0	0	0.00
Quirino	0	0	0	0.00	0	0	0	0.00
Cauayan City	0	0	0	0.00	0	0	0	0.00
Ilagan City	0	0	0	0.00	0	0	0	0.00
Santiago City	0	0	0	0.00	0	0	0	0.00
Tuguegarao City	0	0	0	0.00	0	0	0	0.00
**Region 3**	**0**	**0**	**0**	**0.00**	**0**	**0**	**0**	**0.00**
Aurora	0	0	0	0.00	0	0	0	0.00
Bataan	0	0	0	0.00	0	0	0	0.00
Bulacan	0	0	0	0.00	0	0	0	0.00
Nueva Ecija	0	0	0	0.00	0	0	0	0.00
Pampanga	0	0	0	0.00	0	0	0	0.00
Tarlac	0	0	0	0.00	0	0	0	0.00
Zambales	0	0	0	0.00	0	0	0	0.00
Angeles City	0	0	0	0.00	0	0	0	0.00
Balanga City	0	0	0	0.00	0	0	0	0.00
Cabanatuan City	0	0	0	0.00	0	0	0	0.00
City of San Fernando	0	0	0	0.00	0	0	0	0.00
Gapan City	0	0	0	0.00	0	0	0	0.00
Mabalacat City	0	0	0	0.00	0	0	0	0.00
Malolos City	0	0	0	0.00	0	0	0	0.00
Meycauayan City	0	0	0	0.00	0	0	0	0.00
Olongapo City	0	0	0	0.00	0	0	0	0.00
Palayan City	0	0	0	0.00	0	0	0	0.00
San Jose City	0	0	0	0.00	0	0	0	0.00
San Jose del Monte City	0	0	0	0.00	0	0	0	0.00
Science City of Munoz	0	0	0	0.00	0	0	0	0.00
Tarlac City	0	0	0	0.00	0	0	0	0.00
**Region 4A**	**0**	**0**	**0**	**0.00**	**0**	**0**	**0**	**0.00**
Batangas	0	0	0	0.00	0	0	0	0.00
Cavite	0	0	0	0.00	0	0	0	0.00
Laguna	0	0	0	0.00	0	0	0	0.00
Quezon	0	0	0	0.00	0	0	0	0.00

Table 1.E.2.3 - Schistosomiasis Prevention and Control

Number and Proportion of Chronic Clinically Diagnosed cases treated in the Health Facility and Confirmed Chronic Cases referred to a Hospital Facility
Philippines, 2021

Area	No. of Chronic Clinically Diagnosed cases treated in the Health Facility				No. of Confirmed Chronic Cases referred to Hospital Facility			
	Male	Female	Total	%	Male	Female	Total	%
Rizal	0	0	0	0.00	0	0	0	0.00
Antipolo City	0	0	0	0.00	0	0	0	0.00
Bacoor City	0	0	0	0.00	0	0	0	0.00
Batangas City	0	0	0	0.00	0	0	0	0.00
Biñan City	0	0	0	0.00	0	0	0	0.00
Cabuyao City	0	0	0	0.00	0	0	0	0.00
Calamba City	0	0	0	0.00	0	0	0	0.00
Cavite City	0	0	0	0.00	0	0	0	0.00
Dasmariñas City	0	0	0	0.00	0	0	0	0.00
General Trias City	0	0	0	0.00	0	0	0	0.00
Imus City	0	0	0	0.00	0	0	0	0.00
Lipa City	0	0	0	0.00	0	0	0	0.00
Lucena City	0	0	0	0.00	0	0	0	0.00
San Pablo City	0	0	0	0.00	0	0	0	0.00
San Pedro City	0	0	0	0.00	0	0	0	0.00
Santa Rosa City	0	0	0	0.00	0	0	0	0.00
Tagaytay City	0	0	0	0.00	0	0	0	0.00
Tanauan City	0	0	0	0.00	0	0	0	0.00
Tayabas City	0	0	0	0.00	0	0	0	0.00
Trece Martires City	0	0	0	0.00	0	0	0	0.00
**Region 4B**	**0**	**0**	**0**	**0.00**	**0**	**0**	**0**	**0.00**
Marinduque	0	0	0	0.00	0	0	0	0.00
Mindoro Occidental	0	0	0	0.00	0	0	0	0.00
Mindoro Oriental	0	0	0	0.00	0	0	0	0.00
Palawan	0	0	0	0.00	0	0	0	0.00
Romblon	0	0	0	0.00	0	0	0	0.00
Puerto Princesa City	0	0	0	0.00	0	0	0	0.00
**Region 5**	**0**	**0**	**0**	**0.00**	**0**	**0**	**0**	**0.00**
Albay	0	0	0	0.00	0	0	0	0.00
Camarines Norte	0	0	0	0.00	0	0	0	0.00
Camarines Sur	0	0	0	0.00	0	0	0	0.00
Catanduanes	0	0	0	0.00	0	0	0	0.00
Masbate	0	0	0	0.00	0	0	0	0.00
Sorsogon	0	0	0	0.00	0	0	0	0.00
Iriga City	0	0	0	0.00	0	0	0	0.00
Legaspi City	0	0	0	0.00	0	0	0	0.00
Naga City	0	0	0	0.00	0	0	0	0.00
**Region 6**	**0**	**0**	**0**	**0.00**	**0**	**0**	**0**	**0.00**
Aklan	0	0	0	0.00	0	0	0	0.00

Table 1.E.2.3 - Schistosomiasis Prevention and Control

Number and Proportion of Chronic Clinically Diagnosed cases treated in the Health Facility and Confirmed Chronic Cases referred to a Hospital Facility

Philippines, 2021

Area	No. of Chronic Clinically Diagnosed cases treated in the Health Facility				No. of Confirmed Chronic Cases referred to Hospital Facility			
	Male	Female	Total	%	Male	Female	Total	%
Antique	0	0	0	0.00	0	0	0	0.00
Capiz	0	0	0	0.00	0	0	0	0.00
Guimaras	0	0	0	0.00	0	0	0	0.00
Iloilo	0	0	0	0.00	0	0	0	0.00
Negros Occidental	0	0	0	0.00	0	0	0	0.00
Bacolod City	0	0	0	0.00	0	0	0	0.00
Bago City	0	0	0	0.00	0	0	0	0.00
**Region 7**	**0**	**0**	**0**	**0.00**	**0**	**0**	**0**	**0.00**
Bohol	0	0	0	0.00	0	0	0	0.00
Cebu	0	0	0	0.00	0	0	0	0.00
Negros Oriental	0	0	0	0.00	0	0	0	0.00
Siquijor	0	0	0	0.00	0	0	0	0.00
Cebu City	0	0	0	0.00	0	0	0	0.00
Lapu-Lapu City	0	0	0	0.00	0	0	0	0.00
Mandaue City	0	0	0	0.00	0	0	0	0.00
**Region 8**	**0**	**0**	**0**	**0.00**	**0**	**0**	**0**	**0.00**
Biliran	0	0	0	0.00	0	0	0	0.00
Eastern Samar	0	0	0	0.00	0	0	0	0.00
Northern Leyte	0	0	0	0.00	0	0	0	0.00
Northern Samar	0	0	0	0.00	0	0	0	0.00
Southern Leyte	0	0	0	0.00	0	0	0	0.00
Western Samar	0	0	0	0.00	0	0	0	0.00
Calbayog City	0	0	0	0.00	0	0	0	0.00
Maasin City	0	0	0	0.00	0	0	0	0.00
Ormoc City	0	0	0	0.00	0	0	0	0.00
Tacloban City	0	0	0	0.00	0	0	0	0.00
**Region 9**	**0**	**0**	**0**	**0.00**	**0**	**0**	**0**	**0.00**
Zamboanga del Norte	0	0	0	0.00	0	0	0	0.00
Zamboanga del Sur	0	0	0	0.00	0	0	0	0.00
Zamboanga Sibugay	0	0	0	0.00	0	0	0	0.00
Dapitan City	0	0	0	0.00	0	0	0	0.00
Dipolog City	0	0	0	0.00	0	0	0	0.00
Isabela City	0	0	0	0.00	0	0	0	0.00
Pagadian City	0	0	0	0.00	0	0	0	0.00
Zamboanga City	0	0	0	0.00	0	0	0	0.00

Table 1.E.2.3 - Schistosomiasis Prevention and Control

Number and Proportion of Chronic Clinically Diagnosed cases treated in the Health Facility and Confirmed Chronic Cases referred to a Hospital Facility
Philippines, 2021

Area	No. of Chronic Clinically Diagnosed cases treated in the Health Facility				No. of Confirmed Chronic Cases referred to Hospital Facility			
	Male	Female	Total	%	Male	Female	Total	%
**Region 10**	**283**	**143**	**426**	**91.22**	**2**	**0**	**2**	**0.64**
Bukidnon	8	7	15	100.00	2	0	2	20.00
Camiguin	0	0	0	0.00	0	0	0	0.00
Lanao del Norte	0	0	0	0.00	0	0	0	0.00
Misamis Occidental	0	0	0	0.00	0	0	0	0.00
Misamis Oriental	0	0	0	0.00	0	0	0	0.00
Cagayan de Oro City	0	0	0	0.00	0	0	0	0.00
El Salvador City	0	0	0	0.00	0	0	0	0.00
Gingoog City	0	0	0	0.00	0	0	0	0.00
Iligan City	0	0	0	0.00	0	0	0	0.00
Malaybalay City	0	0	0	0.00	0	0	0	0.00
Oroquieta City	0	0	0	0.00	0	0	0	0.00
Ozamis City	5	5	10	142.86	0	0	0	0.00
Tangub City	14	1	15	100.00	0	0	0	0.00
Valencia City	256	130	386	94.84	0	0	0	0.00
**Region 11**	**8**	**5**	**13**	**56.52**	**0**	**1**	**1**	**14.29**
Davao de Oro	0	0	0	0.00	0	0	0	0.00
Davao del Norte	8	5	13	81.25	0	1	1	14.29
Davao del Sur	0	0	0	0.00	0	0	0	0.00
Davao Oriental	0	0	0	0.00	0	0	0	0.00
Davao Occidental	0	0	0	0.00	0	0	0	0.00
Davao City	0	0	0	0.00	0	0	0	0.00
**Region 12**	**14**	**6**	**20**	**62.50**	**3**	**0**	**3**	**50.00**
North Cotabato	13	6	19	61.29	3	0	3	60.00
Sarangani	0	0	0	0.00	0	0	0	0.00
South Cotabato	0	0	0	0.00	0	0	0	0.00
Sultan Kudarat	0	0	0	0.00	0	0	0	0.00
Cotabato City	0	0	0	0.00	0	0	0	0.00
Gen. Santos City	1	0	1	100.00	0	0	0	0.00
**BARMM**	**8**	**4**	**12**	**0.00**	**0**	**0**	**0**	**0.00**
Basilan	0	0	0	0.00	0	0	0	0.00
Lanao del Sur	0	0	0	0.00	0	0	0	0.00
Maguindanao	8	4	12	0.00	0	0	0	0.00
Sulu	0	0	0	0.00	0	0	0	0.00
Tawi-Tawi	0	0	0	0.00	0	0	0	0.00
Lamitan City	0	0	0	0.00	0	0	0	0.00
Marawi City	0	0	0	0.00	0	0	0	0.00

Table 1.E.2.3 - Schistosomiasis Prevention and Control

Number and Proportion of Chronic Clinically Diagnosed cases treated in the Health Facility and Confirmed Chronic Cases referred to a Hospital Facility
Philippines, 2021

Area	No. of Chronic Clinically Diagnosed cases treated in the Health Facility				No. of Confirmed Chronic Cases referred to Hospital Facility			
	Male	Female	Total	%	Male	Female	Total	%
**CARAGA**	**26**	**13**	**39**	**36.45**	**2**	**1**	**3**	**12.50**
Agusan del Norte	3	2	5	71.43	1	0	1	25.00
Agusan del Sur	17	11	28	44.44	1	0	1	25.00
Surigao del Norte	6	0	6	23.08	0	1	1	16.67
Surigao del Sur	0	0	0	0.00	0	0	0	0.00
Province of Dinagat	0	0	0	0.00	0	0	0	0.00
Bislig City	0	0	0	0.00	0	0	0	0.00
Butuan City	0	0	0	0.00	0	0	0	0.00
Surigao City	0	0	0	0.00	0	0	0	0.00

Legend:
0 = No Case
** = No Report*

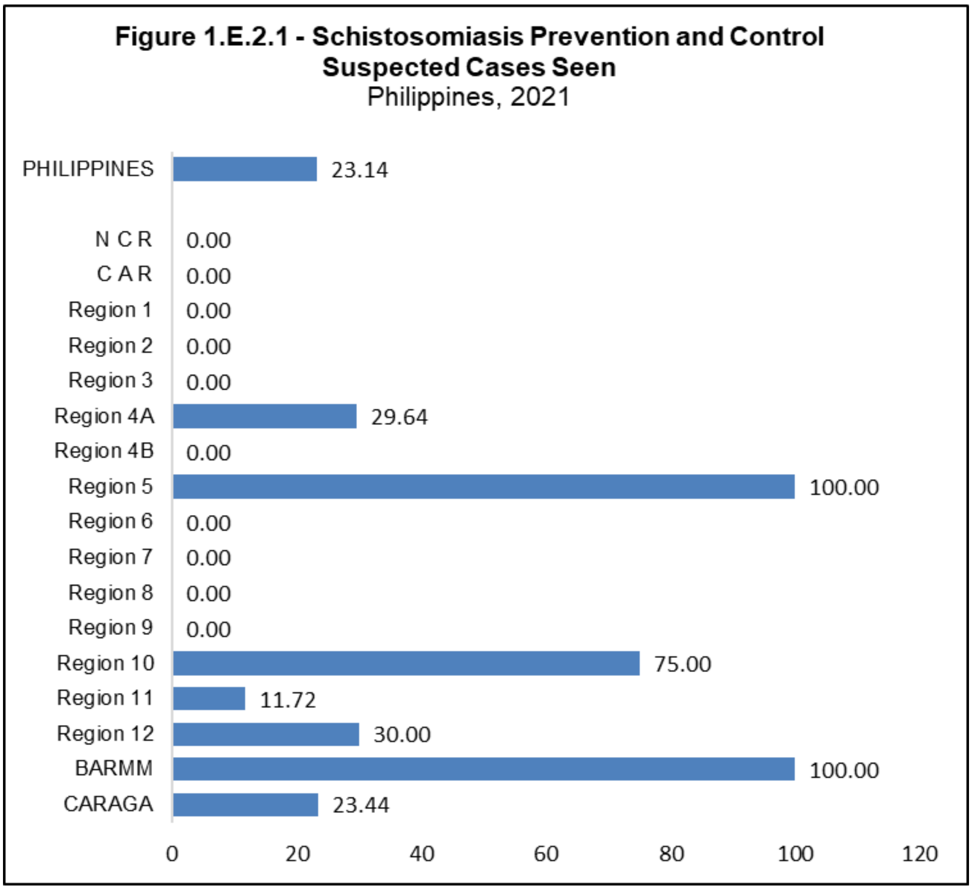

Figure 1.E.2.1 - Schistosomiasis Prevention and Control
Suspected Cases Seen
Philippines, 2021
PHILIPPINES
23.14
N C R
0.00
C A R
0.00
Region 1
0.00
Region 2
0.00
Region 3
0.00
Region 4A
29.64
Region 4B
0.00
Region 5
100.00
Region 6
0.00
Region 7
0.00
Region 8
0.00
Region 9
0.00
Region 10
75.00
Region 11
11.72
Region 12
30.00
BARMM
100.00
CARAGA
23.44
0
20
40
60
80
100
120

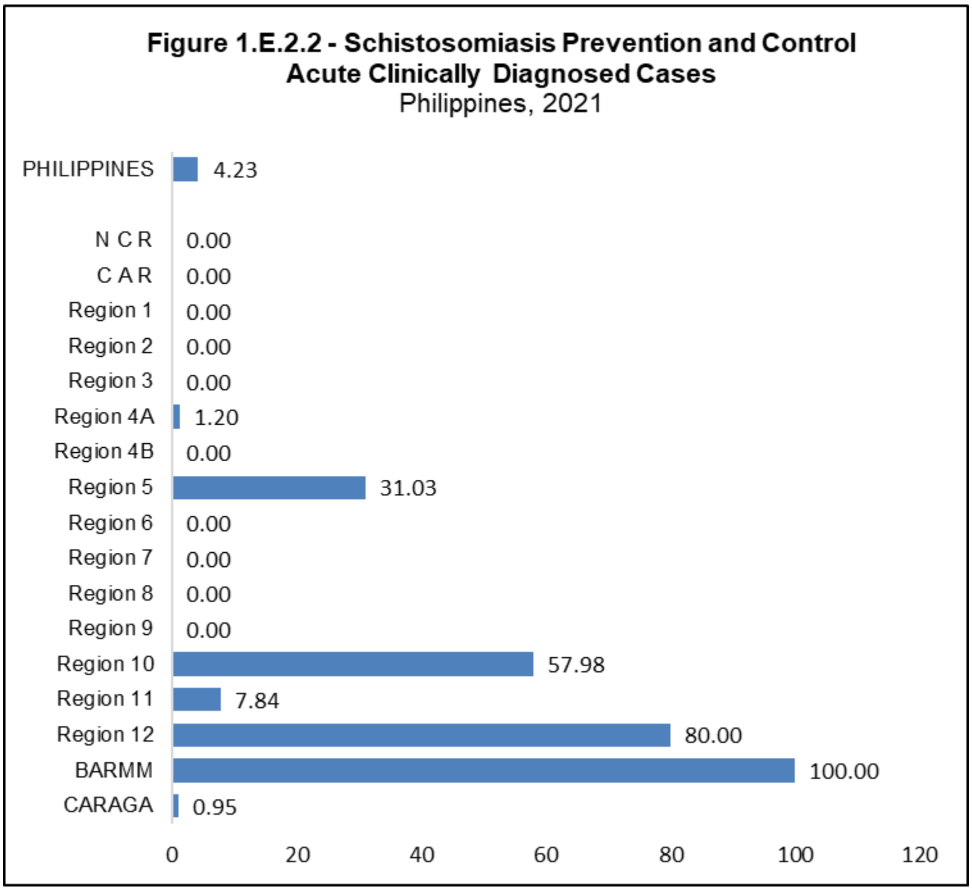

Figure 1.E.2.2 - Schistosomiasis Prevention and Control
Acute Clinically Diagnosed Cases
Philippines, 2021
PHILIPPINES
4.23
N C R
0.00
C A R
0.00
Region 1
0.00
Region 2
0.00
Region 3
0.00
Region 4A
1.20
Region 4B
0.00
Region 5
31.03
Region 6
0.00
Region 7
0.00
Region 8
0.00
Region 9
0.00
Region 10
57.98
Region 11
7.84
Region 12
80.00
BARMM
100.00
CARAGA
0.95
0
20
40
60
80
100
120

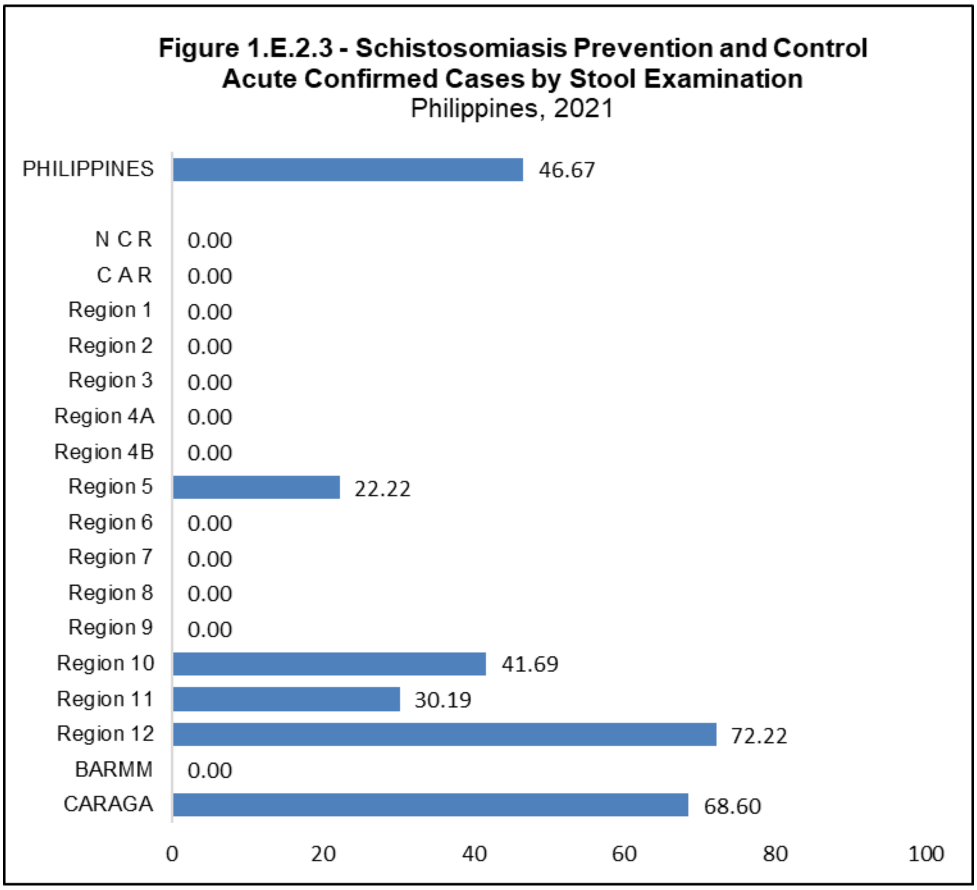

Figure 1.E.2.3 - Schistosomiasis Prevention and Control
Acute Confirmed Cases by Stool Examination
Philippines, 2021
PHILIPPINES
46.67
N C R
0.00
C A R
0.00
Region 1
0.00
Region 2
0.00
Region 3
0.00
Region 4A
0.00
Region 4B
0.00
Region 5
22.22
Region 6
0.00
Region 7
0.00
Region 8
0.00
Region 9
0.00
Region 10
41.69
Region 11
30.19
Region 12
72.22
BARMM
0.00
CARAGA
68.60
0
20
40
60
80
100

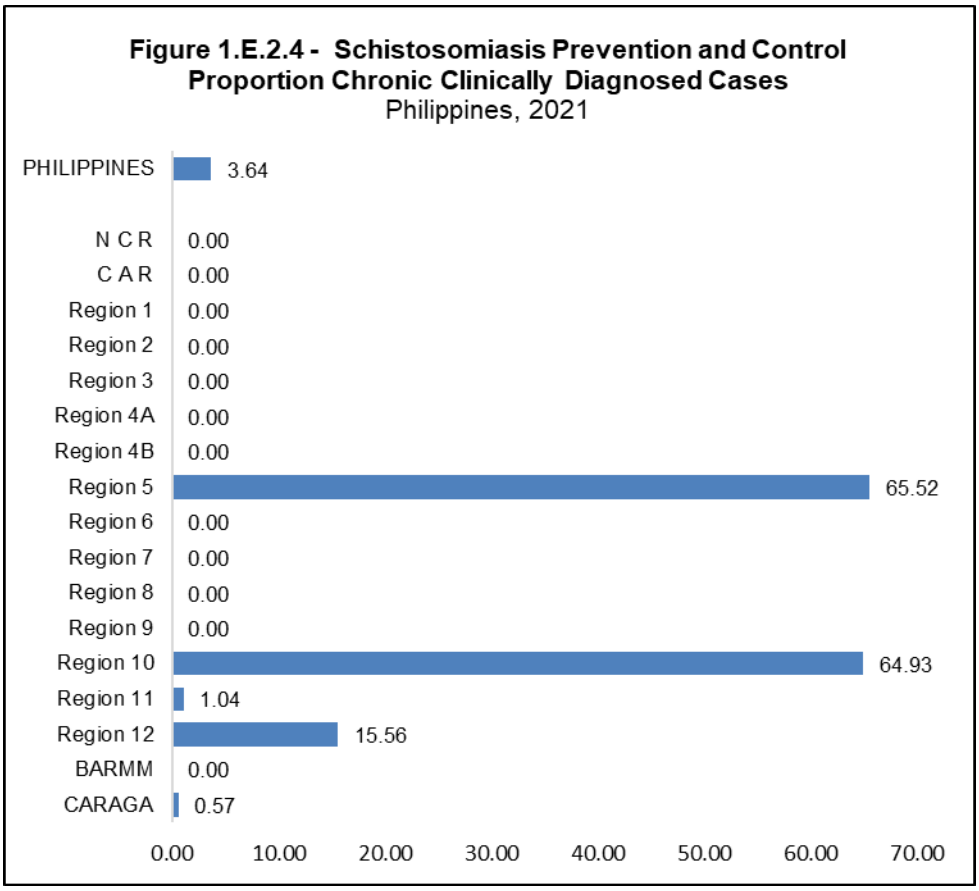

Figure 1.E.2.4 - Schistosomiasis Prevention and Control
Proportion Chronic Clinically Diagnosed Cases
Philippines, 2021
PHILIPPINES
3.64
N C R
0.00
C A R
0.00
Region 1
0.00
Region 2
0.00
Region 3
0.00
Region 4A
0.00
Region 4B
0.00
Region 5
65.52
Region 6
0.00
Region 7
0.00
Region 8
0.00
Region 9
0.00
Region 10
64.93
Region 11
1.04
Region 12
15.56
BARMM
0.00
CARAGA
0.57
0.00
10.00
20.00
30.00
40.00
50.00
60.00
70.00

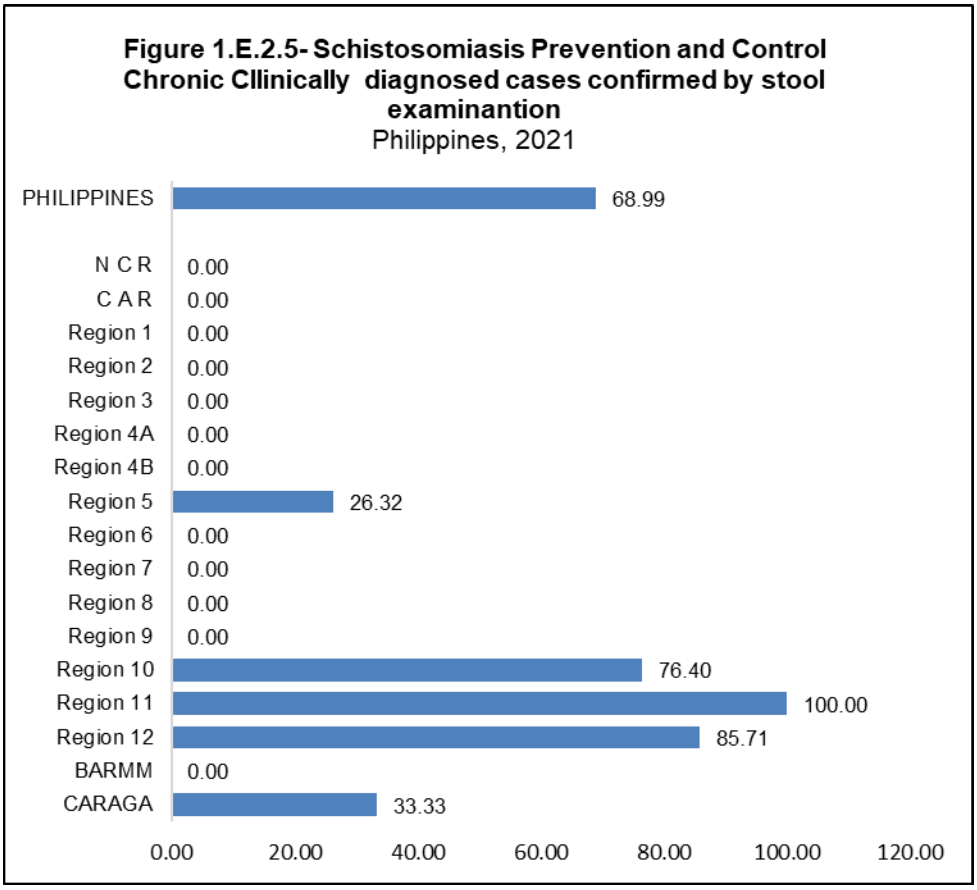

Figure 1.E.2.5- Schistosomiasis Prevention and Control
Chronic Cllinically diagnosed cases confirmed by stool examinantion
Philippines, 2021
PHILIPPINES
68.99
N C R
C A R
Region 1
Region 2
Region 3
Region 4A
Region 4B
Region 5
Region 6
Region 7
Region 8
Region 9
Region 10
Region 11
Region 12
BARMM
CARAGA
0.00
26.32
76.40
100.00
85.71
33.33
0.00
20.00
40.00
60.00
80.00
100.00
120.00

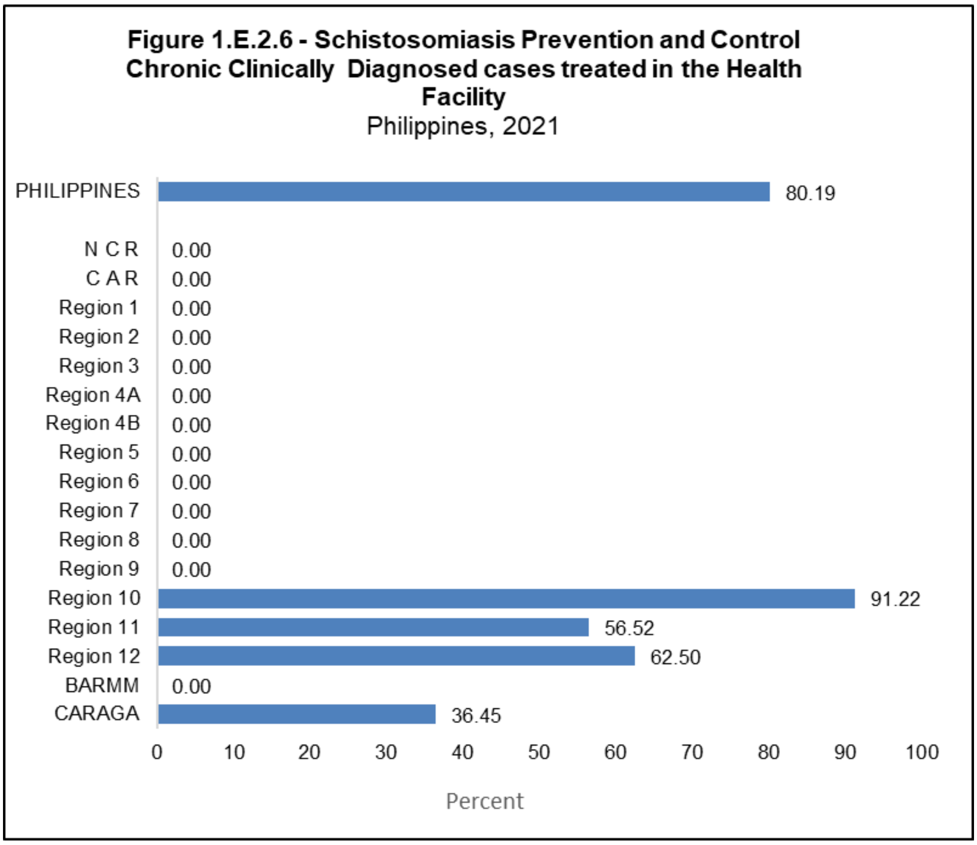

Figure 1.E.2.6 - Schistosomiasis Prevention and Control
Chronic Clinically Diagnosed cases treated in the Health Facility
Philippines, 2021
PHILIPPINES
80.19
N C R
C A R
Region 1
Region 2
Region 3
Region 4A
Region 4B
Region 5
Region 6
Region 7
Region 8
Region 9
Region 10
Region 11
Region 12
BARMM
CARAGA
0.00
91.22
56.52
62.50
36.45
0
10
20
30
40
50
60
70
80
90
100
Percent

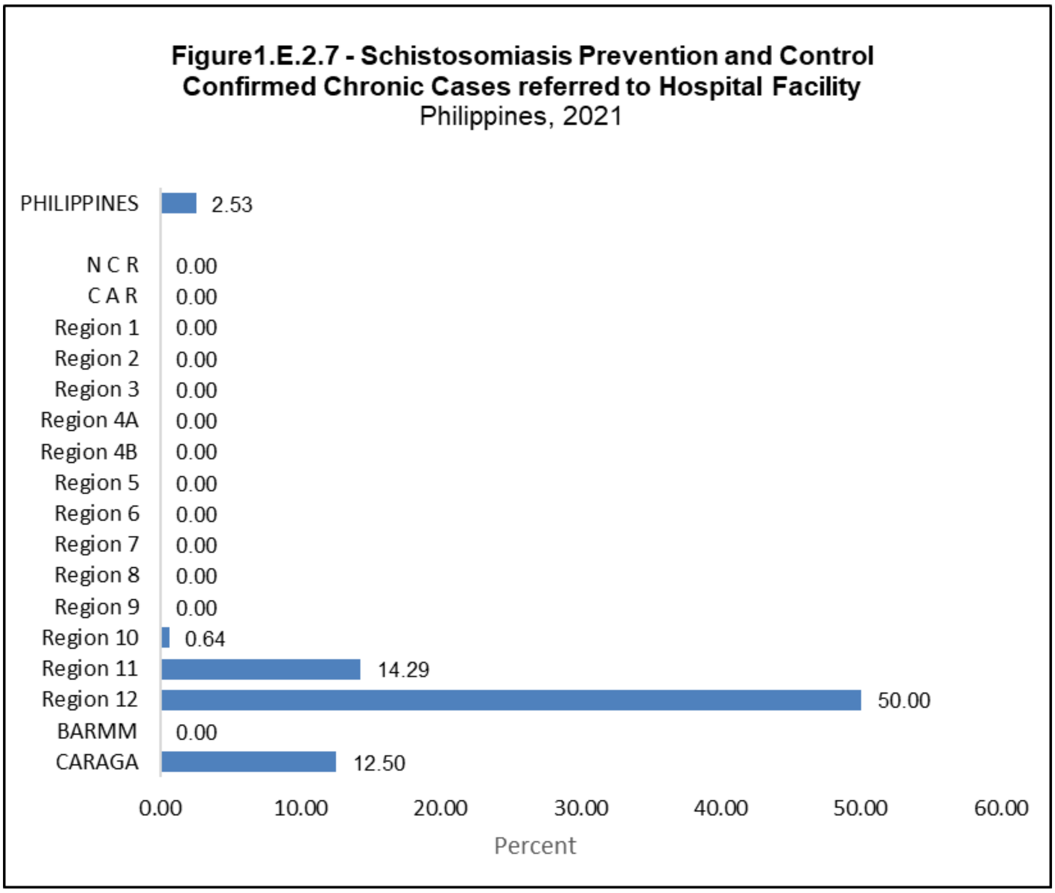

Figure1.E.2.7 - Schistosomiasis Prevention and Control
Confirmed Chronic Cases referred to Hospital Facility
Philippines, 2021
PHILIPPINES
2.53
N C R
C A R
Region 1
Region 2
Region 3
Region 4A
Region 4B
Region 5
Region 6
Region 7
Region 8
Region 9
Region 10
Region 11
Region 12
BARMM
CARAGA
0.00
0.64
14.29
50.00
12.50
0.00
10.00
20.00
30.00
40.00
50.00
60.00
Percent

Table 1.E.3.1 - Soil Transmitted Helminthiasis Prevention and Control

Number and Proportion of PSAC [1](1-4) and SAC [2](5-9) years old who completed 2 doses of deworming tablets

Philippines, 2021

Area	Elig. Pop	Number PSAC [1] (1-4 yrs. Old) who completed 2 doses of deworming tablets				Elig. Pop	Number SAC [2] (5-9 yrs. Old) who completed 2 doses of deworming tablets			
		Male	Female	Total	%		Male	Female	Total	%
**PHILIPPINES**	**8,962,535**	**2,260,392**	**2,227,299**	**4,487,691**	**50.07**	**11,114,941**	**2,024,658**	**1,866,992**	**3,891,650**	**35.01**
**NCR**	**955,786**	**267,903**	**257,000**	**524,903**	**54.92**	**1,227,409**	**153,314**	**142,312**	**295,626**	**24.09**
Malabon City	27,129	2,726	3,994	6,720	24.77	34,839	689	1,749	2,438	7.00
Navotas City	18,518	1,512	1,447	2,959	15.98	23,779	793	833	1,626	6.84
Valenzuela City	46,052	8,208	8,416	16,624	36.10	59,139	6,649	6,968	13,617	23.03
Caloocan City	117,560	53,993	51,562	105,555	89.79	150,966	33,001	32,367	65,368	43.30
Marikina City	33,452	2,591	2,639	5,230	15.63	42,965	1,133	974	2,107	4.90
Pasig City	56,059	1,268	1,265	2,533	4.52	71,991	587	528	1,115	1.55
Pateros	4,739	1,579	1,529	3,108	65.58	6,082	713	595	1,308	21.51
Taguig City	59,742	31,958	27,786	59,744	100.00	76,721	28,153	27,889	56,042	73.05
Quezon City	217,929	61,252	63,859	125,111	57.41	279,868	33,019	22,313	55,332	19.77
Makati City	43,242	11,728	10,806	22,534	52.11	55,531	5,657	5,179	10,836	19.51
Mandaluyong City	28,674	2,529	2,555	5,084	17.73	36,818	18	19	37	0.10
San Juan City	9,067	2,157	2,142	4,299	47.41	11,646	746	739	1,485	12.75
Manila City	132,127	27,104	31,268	58,372	44.18	169,678	15,469	17,471	32,940	19.41
Las Piñas City	43,706	4,638	4,724	9,362	21.42	56,130	714	768	1,482	2.64
Muntinlupa City	37,449	19,089	18,200	37,289	99.57	48,089	17,735	17,234	34,969	72.72
Parañaque City	49,421	14,653	14,808	29,461	59.61	63,463	2,695	2,759	5,454	8.59
Pasay City	30,920	20,918	10,000	30,918	99.99	39,704	5,543	3,927	9,470	23.85
**CAR**	**147,119**	**40,650**	**37,392**	**78,042**	**53.05**	**180,536**	**18,280**	**17,886**	**36,166**	**20.03**
Abra	19,047	5,781	5,448	11,229	58.95	23,644	1,176	981	2,157	11.32
Apayao	10,999	3,472	3,342	6,814	61.95	13,279	1,434	1,335	2,769	25.18
Benguet	36,250	9,054	8,256	17,310	47.75	44,397	1,419	1,910	3,329	9.18
Ifugao	19,270	7,709	7,204	14,913	77.39	23,425	6,766	6,558	13,324	69.14
Kalinga	19,904	4,795	4,917	9,712	48.79	24,419	4,690	4,730	9,420	47.33
Mt. Province	13,600	5,260	4,813	10,073	74.07	17,011	1,238	1,084	2,322	17.07
Baguio City	28,049	4,579	3,412	7,991	28.49	34,361	1,557	1,288	2,845	10.14
**Region 1**	**407,388**	**101,624**	**97,079**	**198,703**	**48.77**	**519,205**	**82,746**	**77,624**	**160,370**	**30.89**
Ilocos Norte	32,506	11,382	11,283	22,665	69.73	41,488	1,055	1,136	2,191	5.28
Ilocos Sur	40,200	14,703	13,030	27,733	68.99	52,091	16,862	15,693	32,555	62.50
La Union	48,309	29,311	27,104	56,415	116.78	61,959	38,994	35,143	74,137	119.65
Pangasinan	210,411	37,051	36,978	74,029	35.18	266,888	18,078	18,456	36,534	13.69
Alaminos City	7,947	2,496	2,420	4,916	61.86	10,081	0	0	0	0.00
Candon City	4,234	0	0	0	0.00	5,495	0	0	0	0.00
Dagupan City	15,175	1,937	1,882	3,819	25.17	19,252	2,045	2,008	4,053	21.05
Laoag City	7,498	0	0	0	0.00	9,568	0	0	0	0.00
San Carlos City	16,715	1,248	1,150	2,398	14.35	21,198	258	226	484	2.28
San Fernando City	8,849	3,489	3,227	6,716	75.90	11,355	5,454	4,962	10,416	91.73
Urdaneta City	11,778	7	5	12	0.10	14,947	0	0	0	0.00
Vigan City	3,766	0	0	0	0.00	4,883	0	0	0	0.00
**Region 2**	**290,609**	**84,143**	**85,019**	**169,162**	**58.21**	**365,588**	**38,787**	**38,963**	**77,750**	**21.27**
Batanes	1,575	733	673	1,406	89.27	1,917	308	113	421	21.96
Cagayan	86,326	30,339	29,347	59,686	69.14	108,797	12,979	12,069	25,048	23.02
Isabela	98,455	21,864	25,153	47,017	47.75	124,710	9,729	10,611	20,340	16.31
Nueva Vizcaya	40,263	7,356	7,069	14,425	35.83	49,489	1,611	1,973	3,584	7.24
Quirino	17,233	5,556	5,541	11,097	64.39	21,527	1,018	1,494	2,512	11.67
Cauayan City	10,769	735	670	1,405	13.05	13,647	401	331	732	5.36
Ilagan City	12,101	6,265	5,836	12,101	100.00	15,334	5,837	5,513	11,350	74.02
Santiago City	11,213	5,842	5,371	11,213	100.00	14,203	5,719	5,705	11,424	80.43
Tuguegarao City	12,674	5,453	5,359	10,812	85.31	15,964	1,185	1,154	2,339	14.65

Table 1.E.3.1 - Soil Transmitted Helminthiasis Prevention and Control

Number and Proportion of PSAC [1](1-4) and SAC [2](5-9) years old who completed 2 doses of deworming tablets
Philippines, 2021

Area	Elig. Pop	Number PSAC [1] (1-4 yrs. Old) who completed 2 doses of deworming tablets				Elig. Pop	Number SAC [2] (5-9 yrs. Old) who completed 2 doses of deworming tablets			
		Male	Female	Total	%		Male	Female	Total	%
**Region 3**	**913,592**	**218,816**	**225,789**	**444,605**	**48.67**	**1,160,338**	**191,738**	**188,764**	**380,502**	**32.79**
Aurora	20,457	9,539	8,945	18,484	90.36	24,680	9,600	9,127	18,727	75.88
Bataan	54,805	23,391	22,561	45,952	83.85	69,326	10,089	9,574	19,663	28.36
Bulacan	181,335	25,851	31,528	57,379	31.64	232,817	35,400	39,793	75,193	32.30
Nueva Ecija	122,063	13,749	13,076	26,825	21.98	155,359	9,816	9,142	18,958	12.20
Pampanga	131,439	14,518	14,518	29,036	22.09	167,494	9,162	9,198	18,360	10.96
Tarlac	80,858	14,904	20,604	35,508	43.91	102,418	14,027	14,165	28,192	27.53
Zambales	50,969	13,203	12,817	26,020	51.05	62,560	12,848	13,429	26,277	42.00
Angeles City	32,975	2,223	2,231	4,454	13.51	42,024	1,496	1,360	2,856	6.80
Balanga City	7,919	3,952	3,522	7,474	94.38	10,023	3,644	3,284	6,928	69.12
Cabanatuan City	24,979	16,621	15,392	32,013	128.16	31,794	12,047	11,640	23,687	74.50
City of San Fernando	24,564	5,119	5,143	10,262	41.78	31,308	3,549	3,251	6,800	21.72
Gapan City	9,116	4,815	4,848	9,663	106.00	11,603	2,255	0	2,255	19.43
Mabalacat City	20,093	13,667	14,233	27,900	138.85	25,604	16,680	15,819	32,499	126.93
Malolos City	20,252	9,187	8,635	17,822	88.00	26,003	10,504	9,694	20,198	77.68
Meycauayan City	16,798	5,472	5,237	10,709	63.75	21,567	7,435	7,791	15,226	70.60
Olongapo City	20,104	2,362	2,371	4,733	23.54	24,676	1,905	1,804	3,709	15.03
Palayan City	3,393	2,737	2,614	5,351	157.71	4,317	1,757	1,651	3,408	78.94
San Jose City	11,553	4,209	4,026	8,235	71.28	14,699	5,413	5,002	10,415	70.86
San Jose del Monte City	46,129	19,378	20,402	39,780	86.24	59,224	14,384	13,671	28,055	47.37
Science City of Munoz	6,737	6,000	5,549	11,549	171.43	8,572	112	126	238	2.78
Tarlac City	27,054	7,919	7,537	15,456	57.13	34,270	9,615	9,243	18,858	55.03
**Region 4A**	**1,233,724**	**210,073**	**201,913**	**411,986**	**33.39**	**1,546,430**	**184,424**	**177,844**	**362,268**	**23.43**
Batangas	163,720	40,626	38,148	78,774	48.12	201,384	42,285	40,408	82,693	41.06
Cavite	114,923	23,443	20,188	43,631	37.97	145,884	23,262	20,041	43,303	29.68
Laguna	80,579	13,740	13,026	26,766	33.22	101,215	12,375	11,895	24,270	23.98
Quezon	159,600	33,313	30,966	64,279	40.28	200,093	27,860	26,896	54,756	27.37
Rizal	181,961	17,410	16,620	34,030	18.70	227,987	14,841	14,930	29,771	13.06
Antipolo City	67,021	1,429	3,109	4,538	6.77	83,973	169	192	361	0.43
Bacoor City	50,346	4,082	4,207	8,289	16.46	63,916	768	1,025	1,793	2.81
Batangas City	29,056	1,362	1,292	2,654	9.13	35,741	712	734	1,446	4.05
Biñan City	27,018	22,069	22,959	45,028	166.66	33,941	22,191	22,027	44,218	130.28
Cabuyao City	25,045	1,642	1,695	3,337	13.32	31,467	2,365	2,381	4,746	15.08
Calamba City	36,868	5,271	4,965	10,236	27.76	46,330	4,934	4,949	9,883	21.33
Cavite City	8,610	9	5	14	0.16	10,936	0	0	0	0.00
Dasmariñas City	55,249	9,649	9,984	19,633	35.54	70,129	4,678	5,017	9,695	13.82
General Trias City	26,348	1,472	1,559	3,031	11.50	33,449	806	749	1,555	4.65
Imus City	33,852	5,482	5,226	10,708	31.63	42,970	4,627	4,477	9,104	21.19
Lipa City	29,275	386	373	759	2.59	36,012	335	296	631	1.75
Lucena City	24,184	5,804	5,880	11,684	48.31	30,324	2,217	2,467	4,684	15.45
San Pablo City	21,585	438	429	867	4.02	27,112	436	437	873	3.22
San Pedro City	26,430	1,477	1,387	2,864	10.84	33,210	344	324	668	2.01
Santa Rosa City	28,699	6,631	5,909	12,540	43.69	36,058	2,319	2,051	4,370	12.12
Tagaytay City	5,966	5,422	5,460	10,882	182.40	7,579	6,366	6,535	12,901	170.22
Tanauan City	15,272	1,806	1,733	3,539	23.17	18,782	1,322	1,027	2,349	12.51
Tayabas City	9,064	4,216	4,101	8,317	91.76	11,366	5,924	5,710	11,634	102.36
Trece Martires City	13,053	2,894	2,692	5,586	42.79	16,572	3,288	3,276	6,564	39.61
**Region 4B**	**301,672**	**82,416**	**79,429**	**161,845**	**53.65**	**373,153**	**87,760**	**83,307**	**171,067**	**45.84**
Marinduque	22,017	6,916	6,261	13,177	59.85	27,446	7,844	7,397	15,241	55.53
Mindoro Occidental	50,503	26,482	25,898	52,380	103.72	62,379	30,338	28,195	58,533	93.83
Mindoro Oriental	83,982	10,925	10,511	21,436	25.52	104,890	11,308	10,303	21,611	20.60
Palawan	89,558	28,384	26,902	55,286	61.73	110,257	27,539	26,715	54,254	49.21
Romblon	28,715	4,815	4,766	9,581	33.37	35,073	4,225	4,407	8,632	24.61
Puerto Princesa City	26,897	4,894	5,091	9,985	37.12	33,108	6,506	6,290	12,796	38.65

Table 1.E.3.1 - Soil Transmitted Helminthiasis Prevention and Control

Number and Proportion of PSAC [1](1-4) and SAC [2](5-9) years old who completed 2 doses of deworming tablets
Philippines, 2021

Area	Elig. Pop	Number PSAC [1] (1-4 yrs. Old) who completed 2 doses of deworming tablets				Elig. Pop	Number SAC [2] (5-9 yrs. Old) who completed 2 doses of deworming tablets			
		Male	Female	Total	%		Male	Female	Total	%
**Region 5**	**591,236**	**172,986**	**166,897**	**339,883**	**57.49**	**708,265**	**221,796**	**212,468**	**434,264**	**61.31**
Albay	103,949	35,994	36,795	72,789	70.02	128,265	44,275	43,902	88,177	68.75
Camarines Norte	60,107	27,611	26,043	53,654	89.26	70,858	35,060	34,547	69,607	98.23
Camarines Sur	167,647	49,087	46,834	95,921	57.22	201,887	65,540	61,688	127,228	63.02
Catanduanes	26,943	5,552	5,022	10,574	39.25	32,009	8,419	7,791	16,210	50.64
Masbate	99,576	19,045	17,242	36,287	36.44	115,770	26,906	24,968	51,874	44.81
Sorsogon	83,359	20,476	19,858	40,334	48.39	99,151	26,128	24,317	50,445	50.88
Iriga City	11,391	134	146	280	2.46	13,716	119	141	260	1.90
Legaspi City	18,283	7,882	7,743	15,625	85.46	22,552	7,043	6,801	13,844	61.39
Naga City	19,981	7,205	7,214	14,419	72.16	24,057	8,306	8,313	16,619	69.08
**Region 6**	**623,781**	**219,110**	**211,558**	**430,668**	**69.04**	**778,724**	**180,430**	**174,429**	**354,859**	**45.57**
Aklan	48,127	17,047	15,206	32,253	67.02	60,517	18,708	17,437	36,145	59.73
Antique	54,526	19,461	18,283	37,744	69.22	68,116	23,758	23,103	46,861	68.80
Capiz	60,055	26,975	26,278	53,253	88.67	73,943	25,376	24,782	50,158	67.83
Guimaras	14,503	6,246	5,738	11,984	82.63	17,631	8,750	8,260	17,010	96.48
Iloilo	153,119	70,345	67,989	138,334	90.34	192,523	64,677	61,630	126,307	65.61
Negros Occidental	210,638	51,414	51,180	102,594	48.71	262,407	26,678	25,909	52,587	20.04
Bacolod City	47,393	15,555	15,392	30,947	65.30	59,048	12,483	13,308	25,791	43.68
Iloilo City	35,420	12,067	11,492	23,559	66.51	44,539	0	0	0	0.00
**Region 7**	**650,762**	**92,836**	**96,404**	**189,240**	**29.08**	**814,009**	**45,904**	**44,296**	**90,200**	**11.08**
Bohol	116,463	12,520	11,239	23,759	20.40	146,004	1,314	1,198	2,512	1.72
Cebu	261,301	36,311	36,919	73,230	28.03	327,647	27,172	24,956	52,128	15.91
Negros Oriental	114,898	25,091	29,734	54,825	47.72	141,917	11,043	11,876	22,919	16.15
Siquijor	7,543	0	0	0	0.00	9,649	0	0	0	0.00
Cebu City	82,030	12,781	12,389	25,170	30.68	102,864	3,656	3,551	7,207	7.01
Lapu-Lapu City	36,283	5,898	5,901	11,799	32.52	45,499	2,577	2,535	5,112	11.24
Mandaue City	32,244	235	222	457	1.42	40,429	142	180	322	0.80
**Region 8**	**439,152**	**71,195**	**70,398**	**141,593**	**32.24**	**528,354**	**26,732**	**26,348**	**53,080**	**10.05**
Biliran	16,889	2,114	2,057	4,171	24.70	19,923	1,282	1,209	2,491	12.50
Eastern Samar	47,349	7,491	7,626	15,117	31.93	57,439	2,007	2,108	4,115	7.16
Northern Leyte	145,057	26,363	24,812	51,175	35.28	175,854	4,993	5,151	10,144	5.77
Northern Samar	68,641	8,690	9,053	17,743	25.85	80,867	6,414	6,743	13,157	16.27
Southern Leyte	29,248	7,888	7,951	15,839	54.15	36,601	4,761	4,105	8,866	24.22
Western Samar	61,611	5,569	6,365	11,934	19.37	72,696	2,008	2,162	4,170	5.74
Calbayog City	18,987	1,234	1,272	2,506	13.20	22,414	1,600	1,534	3,134	13.98
Maasin City	7,450	2,347	2,117	4,464	59.92	9,317	1,989	1,886	3,875	41.59
Ormoc City	20,661	9,193	8,554	17,747	85.90	25,050	1,631	1,403	3,034	12.11
Tacloban City	23,259	306	591	897	3.86	28,193	47	47	94	0.33
**Region 9**	**337,340**	**124,327**	**137,001**	**261,328**	**77.47**	**410,204**	**247,384**	**132,701**	**380,085**	**92.66**
Zamboanga del Norte	74,039	39,226	45,556	84,782	114.51	89,183	50,658	60,430	111,088	124.56
Zamboanga del Sur	71,567	25,998	27,164	53,162	74.28	88,185	14,845	9,762	24,607	27.90
Zamboanga Sibugay	66,255	24,478	25,042	49,520	74.74	79,431	132,555	8,286	140,841	177.31
Dapitan City	7,646	465	529	994	13.00	9,213	576	671	1,247	13.54
Dipolog City	12,129	10,120	14,434	24,554	202.44	14,609	14,452	19,645	34,097	233.40
Isabela City	12,150	2,783	2,690	5,473	45.05	14,307	3,434	3,208	6,642	46.42
Pagadian City	17,557	1,803	1,923	3,726	21.22	21,631	2,813	3,599	6,412	29.64
Zamboanga City	75,997	19,454	19,663	39,117	51.47	93,645	28,051	27,100	55,151	58.89

Table 1.E.3.1 - Soil Transmitted Helminthiasis Prevention and Control

Number and Proportion of PSAC [1](1-4) and SAC [2](5-9) years old who completed 2 doses of deworming tablets
Philippines, 2021

Area	Elig. Pop	Number PSAC [1] (1-4 yrs. Old) who completed 2 doses of deworming tablets				Elig. Pop	Number SAC [2] (5-9 yrs. Old) who completed 2 doses of deworming tablets			
		Male	Female	Total	%		Male	Female	Total	%
**Region 10**	**431,863**	**185,046**	**183,596**	**368,642**	**85.36**	**525,500**	**234,830**	**241,444**	**476,274**	**90.63**
Bukidnon	98,908	39,911	36,766	76,677	77.52	119,217	51,432	49,333	100,765	84.52
Camiguin	7,997	1,043	1,182	2,225	27.82	9,584	1,712	1,974	3,686	38.46
Lanao del Norte	67,455	37,860	39,832	77,692	115.18	81,571	49,683	57,456	107,139	131.34
Misamis Occidental	27,457	10,746	11,412	22,158	80.70	34,210	13,979	14,844	28,823	84.25
Misamis Oriental	62,945	39,266	37,170	76,436	121.43	77,039	50,658	48,884	99,542	129.21
Cagayan de Oro City	59,625	21,766	22,881	44,647	74.88	72,981	26,144	27,414	53,558	73.39
El Salvador City	4,426	1,803	2,203	4,006	90.51	5,423	2,573	3,144	5,717	105.42
Gingoog City	11,000	2,542	2,664	5,206	47.33	13,462	3,478	3,515	6,993	51.95
Iligan City	34,174	12,373	12,276	24,649	72.13	41,309	11,667	12,090	23,757	57.51
Malaybalay City	16,486	4,057	3,946	8,003	48.54	19,872	4,057	3,946	8,003	40.27
Oroquieta City	5,950	3,997	4,072	8,069	135.61	7,411	5,917	5,885	11,802	159.25
Ozamis City	11,924	810	971	1,781	14.94	14,856	1,265	1,307	2,572	17.31
Tangub City	5,299	1,370	1,328	2,698	50.92	6,603	2,527	2,347	4,874	73.81
Valencia City	18,217	7,502	6,893	14,395	79.02	21,962	9,738	9,305	19,043	86.71
**Region 11**	**450,626**	**138,515**	**134,351**	**272,866**	**60.55**	**560,189**	**64,520**	**66,978**	**131,498**	**23.47**
Davao de Oro	68,026	10,077	9,788	19,865	29.20	82,832	3,537	3,334	6,871	8.30
Davao del Norte	87,061	33,225	31,349	64,574	74.17	109,027	14,963	14,131	29,094	26.69
Davao del Sur	56,666	14,453	13,490	27,943	49.31	68,323	10,398	8,046	18,444	27.00
Davao Oriental	58,178	15,591	14,583	30,174	51.86	73,008	6,021	5,607	11,628	15.93
Davao Occidental	30,486	1,958	1,950	3,908	12.82	38,544	1,591	1,475	3,066	7.95
Davao City	150,209	63,211	63,191	126,402	84.15	188,455	28,010	34,385	62,395	33.11
**Region 12**	**437,961**	**150,583**	**144,760**	**295,343**	**67.44**	**540,004**	**128,418**	**125,479**	**253,897**	**47.02**
North Cotabato	140,154	42,872	40,295	83,167	59.34	172,865	14,865	15,146	30,011	17.36
Sarangani	54,636	20,133	19,940	40,073	73.35	66,597	20,621	20,075	40,696	61.11
South Cotabato	84,890	34,199	32,638	66,837	78.73	104,142	31,712	30,981	62,693	60.20
Sultan Kudarat	74,810	26,072	26,269	52,341	69.97	93,721	35,835	35,188	71,023	75.78
Cotabato City	28,340	3,978	4,165	8,143	28.73	35,041	3,682	3,918	7,600	21.69
Gen. Santos City	55,131	23,329	21,453	44,782	81.23	67,638	21,703	20,171	41,874	61.91
**BARMM**	**490,094**	**219**	**202**	**421**	**0.09**	**568,489**	**212**	**251**	**463**	**0.08**
Basilan	36,154	0	0	0	0.00	41,761	0	0	0	0.00
Lanao del Sur	111,572	0	0	0	0.00	128,178	0	0	0	0.00
Maguindanao	151,474	0	0	0	0.00	178,701	0	0	0	0.00
Sulu	109,019	0	0	0	0.00	124,395	0	0	0	0.00
Tawi-Tawi	45,239	0	0	0	0.00	53,302	0	0	0	0.00
Lamitan City	9,948	219	202	421	4.23	11,485	212	251	463	4.03
Marawi City	26,688	0	0	0	0.00	30,667	0	0	0	0.00
**CARAGA**	**259,830**	**99,950**	**98,511**	**198,461**	**76.38**	**308,544**	**117,383**	**115,898**	**233,281**	**75.61**
Agusan del Norte	34,174	7,645	7,942	15,587	45.61	40,788	11,015	11,092	22,107	54.20
Agusan del Sur	77,089	36,103	35,018	71,121	92.26	87,967	31,019	30,902	61,921	70.39
Surigao del Norte	31,994	8,844	8,716	17,560	54.89	39,306	12,821	12,834	25,655	65.27
Surigao del Sur	48,690	20,034	19,020	39,054	80.21	58,174	26,340	24,897	51,237	88.08
Province of Dinagat	11,239	5,934	5,934	11,868	105.60	14,189	6,584	6,677	13,261	93.46
Bislig City	9,250	4,087	4,065	8,152	88.13	11,046	5,391	5,488	10,879	98.49
Butuan City	32,495	15,200	15,914	31,114	95.75	38,773	20,970	20,989	41,959	108.22
Surigao City	14,899	2,103	1,902	4,005	26.88	18,301	3,243	3,019	6,262	34.22

Legend:
0 = No Case
** = No Report*

PSAC [1] - Pre School Age Children
SAC [2] - School Age Children

Table 1.E.3.2 - Soil Transmitted Helminthiasis Prevention and Control

Number and Proportion of 10-19 and Women 20-49 years old who completed 2 doses of deworming tablets
Philippines, 2021

Area	Elig. Pop.	No. of Adolecents (10-19 yrs. Old) who completed 2 doses of deworming tablets				Elig. Pop.	No of WRA (20 - 49 yrs. Old) who completed 2 doses of deworming tablets	%
		Male	Female	Total	%			
**PHILIPPINES**	**21,185,431**	**2,929,150**	**2,773,349**	**5,702,499**	**26.92**	**23,288,684**	**230,913**	**0.99**
**N C R**	**2,439,351**	**139,228**	**129,054**	**268,282**	**11.00**	**3,279,826**	**27,183**	**0.83**
Malabon City	69,242	392	830	1,222	1.76	93,097	0	0.00
Navotas City	47,258	608	637	1,245	2.63	63,539	0	0.00
Valenzuela City	117,528	8,668	8,545	17,213	14.65	158,022	148	0.09
Caloocan City	300,055	27,807	23,550	51,357	17.12	403,445	19,095	4.73
Marikina City	85,386	662	692	1,354	1.59	114,803	871	0.76
Pasig City	143,078	267	278	545	0.38	192,373	0	0.00
Pateros	12,093	308	294	602	4.98	16,261	22	0.14
Taguig City	152,478	38,291	37,534	75,825	49.73	205,013	0	0.00
Quezon City	556,187	22,652	15,146	37,798	6.80	747,828	2,999	0.40
Makati City	110,363	3,179	3,510	6,689	6.06	148,386	2,716	1.83
Mandaluyong City	73,172	13	16	29	0.04	98,383	0	0.00
San Juan City	23,144	571	620	1,191	5.15	31,117	0	0.00
Manila City	337,210	11,276	12,127	23,403	6.94	453,402	0	0.00
Las Piñas City	111,554	422	505	927	0.83	149,990	0	0.00
Muntinlupa City	95,569	18,807	20,743	39,550	41.38	128,499	0	0.00
Parañaque City	126,128	1,795	1,669	3,464	2.75	169,584	1,233	0.73
Pasay City	78,906	3,510	2,358	5,868	7.44	106,084	99	0.09
**C A R**	**348,366**	**32,292**	**32,093**	**64,385**	**18.48**	**391,512**	**0**	**0.00**
Abra	46,837	1,773	1,626	3,399	7.26	50,658	0	0.00
Apayao	24,903	2,272	2,087	4,359	17.50	24,986	0	0.00
Benguet	88,250	4,125	3,703	7,828	8.87	111,448	0	0.000
Ifugao	43,086	12,437	11,764	24,201	56.17	42,890	0	0.00
Kalinga	45,205	8,643	9,814	18,457	40.83	44,092	0	0.00
Mt. Province	31,777	1,651	1,546	3,197	10.06	31,179	0	0.00
Baguio City	68,308	1,391	1,553	2,944	4.31	86,259	0	0.00
**Region 1**	**1,000,199**	**132,256**	**128,733**	**260,989**	**26.09**	**1,088,313**	**6,861**	**0.63**
Ilocos Norte	86,044	350	162	512	0.60	107,240	129	0.12
Ilocos Sur	105,704	27,821	26,711	54,532	51.59	127,024	567	0.45
La Union	126,165	68,452	66,121	134,573	106.66	150,231	6,139	4.09
Pangasinan	496,491	19,818	21,309	41,127	8.28	502,895	0	0.00
Alaminos City	18,758	0	0	0	0.00	19,002	0	0.00
Candon City	11,138	0	0	0	0.00	13,392	0	0.00
Dagupan City	35,815	3,629	3,528	7,157	19.98	36,274	26	0.07
Laoag City	19,837	0	0	0	0.00	24,728	0	0.00
San Carlos City	39,428	145	135	280	0.71	39,940	0	0.00

Table 1.E.3.2 - Soil Transmitted Helminthiasis Prevention and Control

Number and Proportion of 10-19 and Women 20-49 years old who completed 2 doses of deworming tablets
Philippines, 2021

Area	Elig. Pop.	No. of Adolecents (10-19 yrs. Old) who completed 2 doses of deworming tablets				Elig. Pop.	No of WRA (20 - 49 yrs. Old) who completed 2 doses of deworming tablets	%
		Male	Female	Total	%			
San Fernando City	23,116	12,039	10,764	22,803	98.65	27,528	0	0.00
Urdaneta City	27,801	2	3	5	0.02	28,156	0	0.00
Vigan City	9,902	0	0	0	0.00	11,903	0	0.00
**Region 2**	**686,897**	**55,511**	**54,087**	**109,598**	**15.96**	**764,506**	**2,376**	**0.31**
Batanes	3,397	202	380	582	17.13	3,363	2	0.06
Cagayan	206,766	20,149	19,006	39,155	18.94	231,800	1,218	0.53
Isabela	234,016	12,496	13,078	25,574	10.93	263,654	266	0.10
Nueva Vizcaya	93,116	5,253	4,895	10,148	10.90	99,787	292	0.29
Quirino	38,203	675	608	1,283	3.36	40,572	127	0.31
Cauayan City	25,611	183	144	327	1.28	28,848	285	0.99
Ilagan City	28,781	5,383	5,219	10,602	36.84	32,427	186	0.57
Santiago City	26,656	9,944	9,545	19,489	73.11	30,033	0	0.00
Tuguegarao City	30,351	1,226	1,212	2,438	8.03	34,022	0	0.00
**Region 3**	**2,319,836**	**265,219**	**263,705**	**528,924**	**22.80**	**2,712,593**	**1,546**	**0.06**
Aurora	45,730	15,191	14,722	29,913	65.41	46,225	0	0.00
Bataan	136,603	9,433	9,108	18,541	13.57	161,670	145	0.09
Bulacan	469,928	59,059	70,237	129,296	27.51	561,429	0	0.00
Nueva Ecija	304,433	10,306	10,081	20,387	6.70	349,365	162	0.05
Pampanga	343,154	13,530	13,819	27,349	7.97	409,967	0	0.00
Tarlac	205,964	17,862	18,369	36,231	17.59	231,384	0	0.00
Zambales	120,477	18,597	19,286	37,883	31.44	137,993	88	0.06
Angeles City	86,098	723	771	1,494	1.74	102,857	0	0.00
Balanga City	19,742	4,019	3,763	7,782	39.42	23,366	0	0.00
Cabanatuan City	62,305	12,126	11,686	23,812	38.22	71,508	0	0.00
City of San Fernando	64,139	3,602	3,050	6,652	10.37	76,624	72	0.09
Gapan City	22,741	0	0	0	0.00	26,098	0	0.00
Mabalacat City	52,457	23,485	23,262	46,747	89.11	62,668	0	0.00
Malolos City	52,491	16,753	16,282	33,035	62.93	62,709	0	0.00
Meycauayan City	43,535	18,248	8,052	26,300	60.41	52,013	35	0.07
Olongapo City	47,520	2,201	2,162	4,363	9.18	54,426	1,044	1.92
Palayan City	8,461	1,917	1,926	3,843	45.42	9,710	0	0.00
San Jose City	28,808	8,460	8,078	16,538	57.41	33,060	0	0.00
San Jose del Monte City	119,541	16,986	16,629	33,615	28.12	142,818	0	0.00
Science City of Munoz	16,797	138	126	264	1.57	19,278	0	0.00
Tarlac City	68,912	12,583	12,296	24,879	36.10	77,425	0	0.00
**Region 4A**	**3,060,405**	**261,488**	**250,526**	**512,014**	**16.73**	**3,654,153**	**9,956**	**0.27**
Batangas	400,219	68,357	64,333	132,690	33.15	468,815	4,717	1.01
Cavite	295,548	31,201	28,088	59,289	20.06	371,290	1	0.00
Laguna	200,053	17,494	16,996	34,490	17.24	258,162	293	0.11
Quezon	380,891	45,274	44,032	89,306	23.45	378,040	661	0.17

Table 1.E.3.2 - Soil Transmitted Helminthiasis Prevention and Control

Number and Proportion of 10-19 and Women 20-49 years old who completed 2 doses of deworming tablets
Philippines, 2021

Area	Elig. Pop.	No. of Adolecents (10-19 yrs. Old) who completed 2 doses of deworming tablets				Elig. Pop.	No of WRA (20 - 49 yrs. Old) who completed 2 doses of deworming tablets	%
		Male	Female	Total	%			
Rizal	449,952	11,676	11,966	23,642	5.25	535,409	502	0.09
Antipolo City	165,728	0	0	0	0.00	197,209	0	0.00
Bacoor City	129,491	382	364	746	0.58	162,671	0	0.00
Batangas City	71,027	698	727	1,425	2.01	83,205	0	0.00
Biñan City	67,073	37,014	35,787	72,801	108.54	86,566	498	0.58
Cabuyao City	62,187	1,902	2,029	3,931	6.32	80,254	4	0.00
Calamba City	91,534	5,068	4,854	9,922	10.84	118,134	0	0.00
Cavite City	22,165	0	0	0	0.00	27,847	0	0.00
Dasmariñas City	142,083	4,128	3,724	7,852	5.53	178,488	106	0.06
General Trias City	67,767	827	786	1,613	2.38	85,125	0	0.00
Imus City	87,053	4,825	4,756	9,581	11.01	109,365	341	0.31
Lipa City	71,567	409	402	811	1.13	83,834	0	0.00
Lucena City	57,725	4,030	4,398	8,428	14.60	57,291	2,779	4.85
San Pablo City	53,595	462	461	923	1.72	69,156	5	0.01
San Pedro City	65,622	275	284	559	0.85	84,688	0	0.00
Santa Rosa City	71,250	1,197	1,211	2,408	3.38	91,954	48	0.05
Tagaytay City	15,345	11,969	12,087	24,056	156.77	19,278	0	0.00
Tanauan City	37,326	1,323	1,242	2,565	6.87	43,728	1	0.00
Tayabas City	21,633	9,161	8,618	17,779	82.18	21,470	0	0.00
Trece Martires City	33,571	3,816	3,381	7,197	21.44	42,174	0	0.00
**Region 4B**	**669,357**	**133,692**	**124,691**	**258,383**	**38.60**	**619,712**	**106**	**0.02**
Marinduque	49,106	12,059	11,919	23,978	48.83	44,553	0	0.00
Mindoro Occidental	110,453	43,979	41,544	85,523	77.43	97,805	0	0.00
Mindoro Oriental	187,150	20,671	19,521	40,192	21.48	177,987	62	0.03
Palawan	199,843	43,892	39,438	83,330	41.70	187,782	44	0.02
Romblon	62,787	5,721	5,303	11,024	17.56	55,189	0	0.00
Puerto Princesa City	60,018	7,370	6,966	14,336	23.89	56,396	0	0.00
**Region 5**	**1,301,201**	**370,687**	**359,126**	**729,813**	**56.09**	**1,180,092**	**51,303**	**4.35**
Albay	243,398	71,583	74,489	146,072	60.01	237,558	3,235	1.36
Camarines Norte	128,854	63,662	59,746	123,408	95.77	120,910	5,137	4.25
Camarines Sur	370,218	105,175	101,590	206,765	55.85	334,438	15,481	4.63
Catanduanes	57,554	15,626	14,356	29,982	52.09	51,068	169	0.33
Masbate	208,571	44,670	42,040	86,710	41.57	171,326	532	0.31
Sorsogon	180,540	46,437	43,321	89,758	49.72	160,434	26,472	16.50
Iriga City	25,153	124	151	275	1.09	22,728	86	0.38
Legaspi City	42,796	12,315	12,224	24,539	57.34	41,772	191	0.46
Naga City	44,117	11,095	11,209	22,304	50.56	39,858	0	0.00
**Region 6**	**1,501,799**	**283,173**	**275,966**	**559,139**	**37.23**	**1,622,936**	**17,725**	**1.09**
Aklan	114,161	32,661	30,719	63,380	55.52	125,722	3,875	3.08

Table 1.E.3.2 - Soil Transmitted Helminthiasis Prevention and Control

Number and Proportion of 10-19 and Women 20-49 years old who completed 2 doses of deworming tablets
Philippines, 2021

Area	Elig. Pop.	No. of Adolecents (10-19 yrs. Old) who completed 2 doses of deworming tablets				Elig. Pop.	No of WRA (20 - 49 yrs. Old) who completed 2 doses of deworming tablets	%
		Male	Female	Total	%			
Antique	122,311	42,137	41,510	83,647	68.39	117,667	306	0.26
Capiz	149,074	37,438	36,965	74,403	49.91	168,944	1,841	1.09
Guimaras	33,938	15,611	15,107	30,718	90.51	38,554	906	2.35
Iloilo	379,792	107,079	103,303	210,382	55.39	429,819	3,446	0.80
Negros Occidental	501,764	35,596	35,193	70,789	14.11	524,729	6,145	1.17
Bacolod City	112,898	12,651	13,169	25,820	22.87	118,063	1,206	1.02
Iloilo City	87,861	0	0	0	0.00	99,438	0	0.00
**Region 7**	**1,540,685**	**42,063**	**36,330**	**78,393**	**5.09**	**1,671,549**	**503**	**0.03**
Bohol	272,060	170	127	297	0.11	262,405	0	0.00
Cebu	620,513	31,707	28,664	60,371	9.73	702,262	0	0.00
Negros Oriental	272,255	6,912	4,138	11,050	4.06	282,382	131	0.05
Siquijor	18,340	0	0	0	0.00	19,863	0	0.00
Cebu City	194,786	1,912	1,926	3,838	1.97	220,460	0	0.00
Lapu-Lapu City	86,164	1,249	1,335	2,584	3.00	97,519	372	0.38
Mandaue City	76,567	113	140	253	0.33	86,658	0	0.00
**Region 8**	**984,689**	**35,362**	**38,003**	**73,365**	**7.45**	**915,751**	**1,426**	**0.16**
Biliran	36,252	1,462	1,328	2,790	7.70	34,610	0	0.00
Eastern Samar	105,850	2,363	2,839	5,202	4.91	95,075	0	0.00
Northern Leyte	331,194	3,908	4,234	8,142	2.46	321,254	0	0.00
Northern Samar	147,616	10,240	11,235	21,475	14.55	126,246	85	0.07
Southern Leyte	68,560	7,090	7,448	14,538	21.20	69,508	1,316	1.89
Western Samar	135,680	2,898	3,266	6,164	4.54	117,796	0	0.00
Calbayog City	41,821	2,934	3,024	5,958	14.25	36,303	0	0.00
Maasin City	17,442	3,496	3,625	7,121	40.83	17,688	0	0.00
Ormoc City	47,163	830	865	1,695	3.59	45,754	25	0.05
Tacloban City	53,111	141	139	280	0.53	51,517	0	0.00
**Region 9**	**776,000**	**338,367**	**252,191**	**590,558**	**76.10**	**772,969**	**6,751**	**0.87**
Zamboanga del Norte	168,195	90,226	106,088	196,314	116.72	163,031	2,009	1.23
Zamboanga del Sur	170,085	26,028	17,639	43,667	25.67	174,294	2,146	1.23
Zamboanga Sibugay	143,447	128,206	24,020	152,226	106.12	136,337	92	0.07
Dapitan City	17,368	2,219	2,729	4,948	28.49	16,831	1,218	7.24
Dipolog City	27,552	26,829	35,442	62,271	226.01	26,704	162	0.61
Isabela City	27,047	0	0	0	0.00	27,947	365	1.31
Pagadian City	41,716	22,056	22,169	44,225	106.01	42,750	28	0.07
Zamboanga City	180,590	42,803	44,104	86,907	48.12	185,075	731	0.39

Table 1.E.3.2 - Soil Transmitted Helminthiasis Prevention and Control

Number and Proportion of 10-19 and Women 20-49 years old who completed 2 doses of deworming tablets
Philippines, 2021

Area	Elig. Pop.	No. of Adolecents (10-19 yrs. Old) who completed 2 doses of deworming tablets				Elig. Pop.	No of WRA (20 - 49 yrs. Old) who completed 2 doses of deworming tablets	%
		Male	Female	Total	%			
**Region 10**	**996,834**	**396,140**	**396,680**	**792,820**	**79.53**	**1,040,047**	**11,115**	**1.07**
Bukidnon	224,694	90,681	88,074	178,755	79.55	228,256	709	0.31
Camiguin	17,955	9,197	5,976	15,173	84.51	17,713	0	0.00
Lanao del Norte	153,610	81,820	88,027	169,847	110.57	151,679	54	0.04
Misamis Occidental	65,844	24,810	26,345	51,155	77.69	67,238	328	0.49
Misamis Oriental	147,116	82,398	78,804	161,202	109.57	164,897	473	0.29
Cagayan de Oro City	139,341	42,757	44,784	87,541	62.83	156,183	0	0.00
El Salvador City	10,347	4,052	4,953	9,005	87.03	11,599	0	0.00
Gingoog City	25,695	5,689	5,636	11,325	44.07	28,802	506	1.76
Iligan City	77,805	17,963	18,847	36,810	47.31	76,830	9,045	11.77
Malaybalay City	37,460	7,325	6,812	14,137	37.74	38,048	0	0.00
Oroquieta City	14,269	9,265	9,519	18,784	131.64	14,569	0	0.00
Ozamis City	28,599	2,340	2,327	4,667	16.32	29,204	0	0.00
Tangub City	12,705	3,857	3,746	7,603	59.84	12,978	0	0.00
Valencia City	41,394	13,986	12,830	26,816	64.78	42,051	0	0.00
**Region 11**	**1,044,422**	**77,144**	**74,687**	**151,831**	**14.54**	**1,109,682**	**19,692**	**1.77**
Davao de Oro	151,938	18,450	16,718	35,168	23.15	157,287	2,719	1.73
Davao del Norte	203,114	30,051	29,721	59,772	29.43	221,456	5,425	2.45
Davao del Sur	124,927	8,790	8,311	17,101	13.69	114,875	1,541	1.34
Davao Oriental	138,407	5,272	4,846	10,118	7.31	152,724	4,625	3.03
Davao Occidental	68,741	2,094	2,369	4,463	6.49	69,099	401	0.58
Davao City	357,295	12,487	12,722	25,209	7.06	394,241	4,981	1.26
**Region 12**	**1,005,492**	**185,423**	**181,295**	**366,718**	**36.47**	**1,042,194**	**74,370**	**7.14**
North Cotabato	316,814	17,355	18,096	35,451	11.19	315,764	374	0.12
Sarangani	125,028	32,152	31,452	63,604	50.87	118,405	4,636	3.92
South Cotabato	196,587	44,232	43,089	87,321	44.42	216,149	1,704	0.79
Sultan Kudarat	174,233	60,236	57,869	118,105	67.79	181,697	67,307	37.04
Cotabato City	65,157	3,737	4,476	8,213	12.60	69,796	0	0.00
Gen. Santos City	127,673	27,711	26,313	54,024	42.31	140,383	349	0.25
**BARMM**	**951,682**	**204**	**199**	**403**	**0.04**	**880,454**	**0**	**0.00**
Basilan	70,058	0	0	0	0.00	62,018	0	0.00
Lanao del Sur	208,241	0	0	0	0.00	199,424	0	0.00
Maguindanao	303,512	0	0	0	0.00	274,082	0	0.00
Sulu	208,089	0	0	0	0.00	194,030	0	0.00
Tawi-Tawi	92,701	0	0	0	0.00	86,141	0	0.00
Lamitan City	19,277	204	199	403	2.09	17,062	0	0.00
Marawi City	49,804	0	0	0	0.00	47,697	0	0.00

Table 1.E.3.2 - Soil Transmitted Helminthiasis Prevention and Control

Number and Proportion of 10-19 and Women 20-49 years old who completed 2 doses of deworming tablets
Philippines, 2021

Area	Elig. Pop.	No. of Adolecents (10-19 yrs. Old) who completed 2 doses of deworming tablets				Elig. Pop.	No of WRA (20 - 49 yrs. Old) who completed 2 doses of deworming tablets	%
		Male	Female	Total	%			
**CARAGA**	**558,216**	**180,901**	**175,983**	**356,884**	**63.93**	**542,395**	**0**	**0.00**
Agusan del Norte	75,504	18,741	19,787	38,528	51.03	76,287	0	0.00
Agusan del Sur	151,458	37,909	34,939	72,848	48.10	143,087	0	0.00
Surigao del Norte	73,752	22,326	21,514	43,840	59.44	71,950	0	0.00
Surigao del Sur	105,529	44,648	42,310	86,958	82.40	100,560	0	0.00
Province of Dinagat	25,792	12,379	11,803	24,182	93.76	25,372	0	0.00
								0.00
Bislig City	20,050	8,481	8,076	16,557	82.58	19,099	0	0.00
Butuan City	71,788	31,773	32,983	64,756	90.20	72,532	0	0.00
Surigao City	34,343	4,644	4,571	9,215	26.83	33,508	0	0.00

Legend:
0 = No Case
** = No Report*

PSAC [1] - Pre School Age Children
SAC [2] - School Age Children

Table 1.E.3.3 - Soil Transmitted Helminthiasis Prevention and Control

Number and Proportion of Pregnant Women who completed 1 dose of deworming tablet
Philippines, 2021

Area	Elig. Pop	Pregnant Women who completed 1 dose of deworming tablet	
		No.	%
**PHILIPPINES**	**2,128,168**	**259,334**	**12.19**
**N C R**	**237,921**	**28,665**	**12.05**
Malabon City	6,757	0	0.00
Navotas City	4,607	21	0.46
Valenzuela City	11,465	143	1.25
Caloocan City	29,270	0	0.00
Marikina City	8,330	598	7.18
Pasig City	13,954	11	0.08
Pateros	1,181	306	25.91
Taguig City	14,874	1,083	7.28
Quezon City	54,246	24,673	45.48
Makati City	10,765	344	3.20
Mandaluyong City	7,137	0	0.00
San Juan City	2,254	123	5.46
Manila City	32,887	327	0.99
Las Piñas City	10,879	0	0.00
Muntinlupa City	9,317	0	0.00
Parañaque City	12,298	368	2.99
Pasay City	7,700	668	8.68
**C A R**	**35,143**	**7,932**	**22.57**
Abra	4,287	599	13.97
Apayao	2,476	1,169	47.21
Benguet	9,140	1,593	17.43
Ifugao	4,439	1,490	33.57
Kalinga	4,660	1,917	41.14
Mt. Province	3,066	63	2.05
Baguio City	7,075	1,101	15.56
**Region 1**	**97,198**	**9,658**	**9.94**
Ilocos Norte	7,986	959	12.01
Ilocos Sur	9,197	156	1.70
La Union	11,429	1,773	15.51
Pangasinan	50,454	5,311	10.53

Table 1.E.3.3 - Soil Transmitted Helminthiasis Prevention and Control

Number and Proportion of Pregnant Women who completed 1 dose of deworming tablet
Philippines, 2021

Area	Elig. Pop	Pregnant Women who completed 1 dose of deworming tablet	
		No.	%
Alaminos City	1,905	10	0.52
Candon City	976	215	22.03
Dagupan City	3,636	67	1.84
Laoag City	1,845	0	0.00
San Carlos City	3,999	161	4.03
San Fernando City	2,092	168	8.03
Urdaneta City	2,819	838	29.73
Vigan City	860	0	0.00
**Region 2**	**69,228**	**5,423**	**7.83**
Batanes	351	18	5.13
Cagayan	20,563	1,638	7.97
Isabela	23,543	1,152	4.89
Nueva Vizcaya	9,555	1,163	12.17
Quirino	4,038	0	8.52
Cauayan City	2,576	344	0.00
Ilagan City	2,901	474	16.34
Santiago City	2,683	33	1.23
Tuguegarao City	3,018	601	19.91
**Region 3**	**220,202**	**6,569**	**2.98**
Aurora	4,764	174	3.65
Bataan	13,814	145	1.05
Bulacan	43,722	486	1.11
Nueva Ecija	28,915	198	0.68
Pampanga	31,503	274	0.87
Tarlac	19,565	3,023	15.45
Zambales	12,551	452	3.60
Angeles City	7,905	0	0.00
Balanga City	1,996	0	0.00
Cabanatuan City	5,921	0	0.00
City of San Fernando	5,890	179	3.04
Gapan City	2,159	60	2.78
Mabalacat City	4,811	222	4.61
Malolos City	4,885	0	0.00
Meycauayan City	4,050	155	3.83
Olongapo City	4,952	513	10.36
Palayan City	805	7	0.87
San Jose City	2,736	0	0.00

Table 1.E.3.3 - Soil Transmitted Helminthiasis Prevention and Control

Number and Proportion of Pregnant Women who completed 1 dose of deworming tablet
Philippines, 2021

Area	Elig. Pop	Pregnant Women who completed 1 dose of deworming tablet	
		No.	%
San Jose del Monte City	11,120	0	0.00
Science City of Munoz	1,594	661	41.47
Tarlac City	6,544	20	0.31
**Region 4A**	**298,398**	**9,249**	**3.10**
Batangas	38,848	104	0.27
Cavite	27,948	343	1.23
Laguna	19,727	1,101	5.58
Quezon	37,644	845	2.24
Rizal	44,826	1,857	4.14
Antipolo City	16,510	14	0.08
Bacoor City	12,252	94	0.77
Batangas City	6,895	2	0.03
Biñan City	6,607	2,719	41.15
Cabuyao City	6,128	126	2.06
Calamba City	9,025	297	3.29
Cavite City	2,098	0	0.00
Dasmariñas City	13,442	298	2.22
General Trias City	6,410	154	2.40
Imus City	8,235	98	1.19
Lipa City	6,954	1	0.01
Lucena City	5,704	34	0.60
San Pablo City	5,277	8	0.15
San Pedro City	6,465	135	2.09
Santa Rosa City	7,023	624	8.89
Tagaytay City	1,451	13	0.90
Tanauan City	3,620	98	2.71
Tayabas City	2,134	0	0.00
Trece Martires City	3,175	284	8.94
**Region 4B**	**72,035**	**5,301**	**7.36**
Marinduque	4,906	530	10.80
Mindoro Occidental	12,449	586	4.71
Mindoro Oriental	20,011	1,866	9.32
Palawan	21,676	2,035	9.39
Romblon	6,482	284	4.38
Puerto Princesa City	6,511	0	0.00

Table 1.E.3.3 - Soil Transmitted Helminthiasis Prevention and Control

Number and Proportion of Pregnant Women who completed 1 dose of deworming tablet

Philippines, 2021

Area	Elig. Pop	Pregnant Women who completed 1 dose of deworming tablet	
		No.	%
**Region 5**	**137,350**	**26,912**	**19.59**
Albay	23,903	3,681	15.40
Camarines Norte	14,340	4,841	33.76
Camarines Sur	39,151	6,563	16.76
Catanduanes	6,381	1,282	20.09
Masbate	22,565	5,427	24.05
Sorsogon	19,470	3,658	18.79
Iriga City	2,665	902	33.85
Legaspi City	4,205	41	0.98
Naga City	4,670	517	11.07
**Region 6**	**146,545**	**14,939**	**10.19**
Aklan	11,231	1,564	13.93
Antique	12,985	1,529	11.78
Capiz	13,984	2,009	14.37
Guimaras	3,085	966	31.31
Iloilo	36,414	2,616	7.18
Negros Occidental	49,314	6,209	12.59
Bacolod City	11,102	18	0.16
Iloilo City	8,430	28	0.33
**Region 7**	**162,860**	**35,587**	**21.85**
Bohol	27,637	4,461	16.14
Cebu	67,046	13,115	19.56
Negros Oriental	27,922	2,683	9.61
Siquijor	1,636	648	39.61
Cebu City	21,037	10,570	50.24
Lapu-Lapu City	9,310	1,534	16.48
Mandaue City	8,272	2,576	31.14
**Region 8**	**104,016**	**11,427**	**10.99**
Biliran	3,856	1,509	39.13
Eastern Samar	11,635	2,258	19.41
Northern Leyte	35,052	1,705	4.86
Northern Samar	15,655	1,480	9.45
Southern Leyte	6,556	859	13.10

Table 1.E.3.3 - Soil Transmitted Helminthiasis Prevention and Control

Number and Proportion of Pregnant Women who completed 1 dose of deworming tablet
Philippines, 2021

Area	Elig. Pop	Pregnant Women who completed 1 dose of deworming tablet	
		No.	%
Western Samar	14,515	1,533	10.56
Calbayog City	4,474	260	5.81
Maasin City	1,661	319	19.21
Ormoc City	4,989	1,486	29.79
Tacloban City	5,623	18	0.32
**Region 9**	**79,552**	**9,995**	**12.56**
Zamboanga del Norte	17,075	1,982	11.61
Zamboanga del Sur	17,506	981	5.60
Zamboanga Sibugay	14,992	1,683	11.23
Dapitan City	1,755	1,470	83.76
Dipolog City	2,794	178	6.37
Isabela City	2,544	385	15.13
Pagadian City	4,290	12	0.28
Zamboanga City	18,596	3,304	17.77
**Region 10**	**100,690**	**15,834**	**15.73**
Bukidnon	23,307	7,899	33.89
Camiguin	1,854	91	4.91
Lanao del Norte	14,955	496	3.32
Misamis Occidental	6,413	302	4.71
Misamis Oriental	15,058	1,209	8.03
Cagayan de Oro City	14,263	96	0.67
El Salvador City	1,061	0	0.00
Gingoog City	2,627	54	2.06
Iligan City	7,575	2,719	35.89
Malaybalay City	3,882	232	5.98
Oroquieta City	1,389	0	0.00
Ozamis City	2,782	531	19.09
Tangub City	1,234	0	0.00
Valencia City	4,290	2,205	51.40
**Region 11**	**107,847**	**29,277**	**27.15**
Davao de Oro	15,526	6,776	43.64
Davao del Norte	21,184	7,301	34.46
Davao del Sur	13,125	3,786	28.85
Davao Oriental	14,355	1,392	9.70

Table 1.E.3.3 - Soil Transmitted Helminthiasis Prevention and Control

Number and Proportion of Pregnant Women who completed 1 dose of deworming tablet
Philippines, 2021

Area	Elig. Pop	Pregnant Women who completed 1 dose of deworming tablet	
		No.	%
Davao Occidental	6,598	2,006	30.40
Davao City	37,059	8,016	21.63
**Region 12**	**104,693**	**29,878**	**28.54**
North Cotabato	33,933	3,957	11.66
Sarangani	12,904	9,264	71.79
South Cotabato	21,039	5,779	27.47
Sultan Kudarat	17,331	8,173	47.16
Cotabato City	5,831	893	15.31
Gen. Santos City	13,655	1,812	13.27
**BARMM**	**94,148**	**10,047**	**10.67**
Basilan	7,672	418	5.45
Lanao del Sur	21,391	3,498	16.35
Maguindanao	31,650	1,311	4.14
Sulu	16,891	0	0.00
Tawi-Tawi	9,319	1,384	14.85
Lamitan City	2,112	57	2.70
Marawi City	5,113	3,379	66.09
**CARAGA**	**60,342**	**2,641**	**4.38**
Agusan del Norte	8,116	20	0.25
Agusan del Sur	17,658	563	3.19
Surigao del Norte	7,195	42	0.58
Surigao del Sur	11,532	1,424	12.35
Province of Dinagat	2,574	123	4.78
Bislig City	2,185	77	3.52
Butuan City	7,732	40	0.52
Surigao City	3,350	352	10.51

Legend:
0 = No Case
** = No Report*

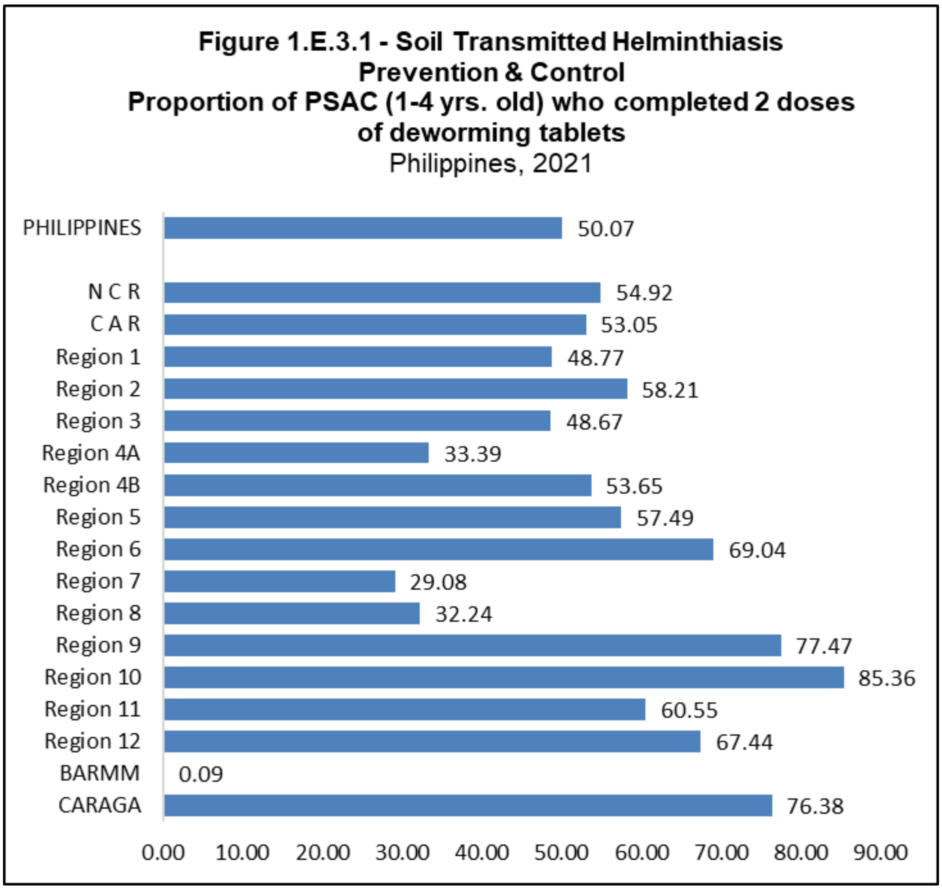
Figure 1.E.3.1 - Soil Transmitted Helminthiasis
Prevention & Control
Proportion of PSAC (1-4 yrs. old) who completed 2 doses of deworming tablets
Philippines, 2021
PHILIPPINES 50.07
N C R 54.92
C A R 53.05
Region 1 48.77
Region 2 58.21
Region 3 48.67
Region 4A 33.39
Region 4B 53.65
Region 5 57.49
Region 6 69.04
Region 7 29.08
Region 8 32.24
Region 9 77.47
Region 10 85.36
Region 11 60.55
Region 12 67.44
BARMM 0.09
CARAGA 76.38
0.00 10.00 20.00 30.00 40.00 50.00 60.00 70.00 80.00 90.00

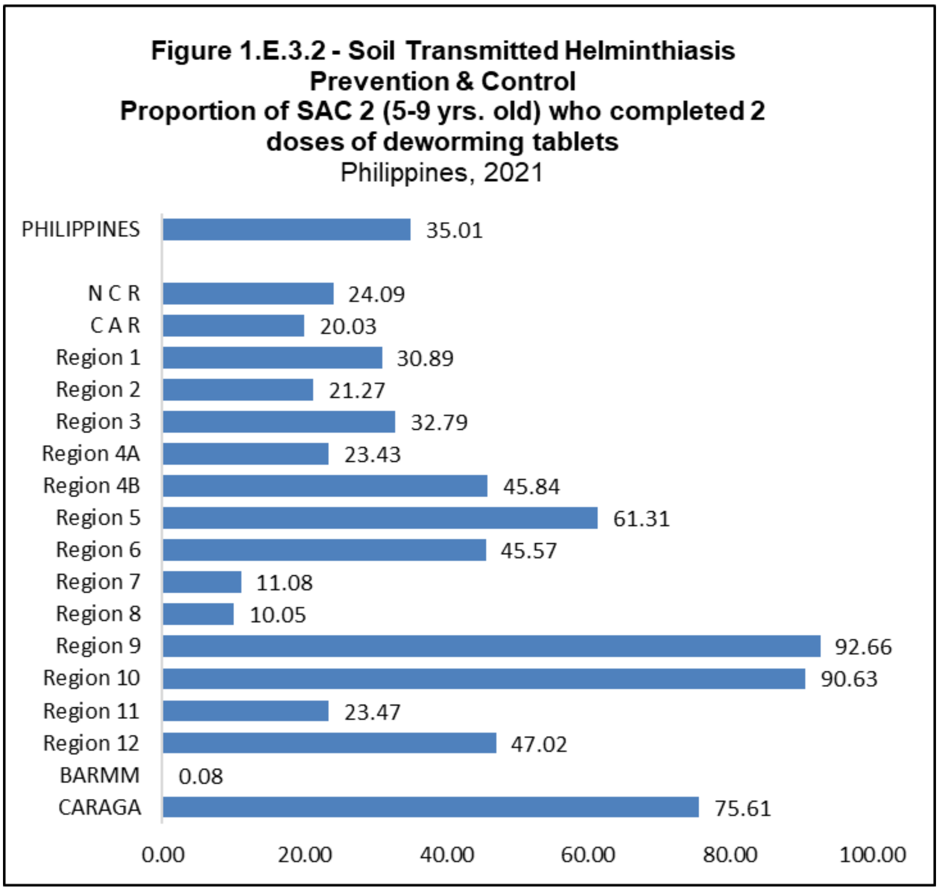
Figure 1.E.3.2 - Soil Transmitted Helminthiasis
Prevention & Control
Proportion of SAC 2 (5-9 yrs. old) who completed 2 doses of deworming tablets
Philippines, 2021
PHILIPPINES 35.01
N C R 24.09
C A R 20.03
Region 1 30.89
Region 2 21.27
Region 3 32.79
Region 4A 23.43
Region 4B 45.84
Region 5 61.31
Region 6 45.57
Region 7 11.08
Region 8 10.05
Region 9 92.66
Region 10 90.63
Region 11 23.47
Region 12 47.02
BARMM 0.08
CARAGA 75.61
0.00 20.00 40.00 60.00 80.00 100.00

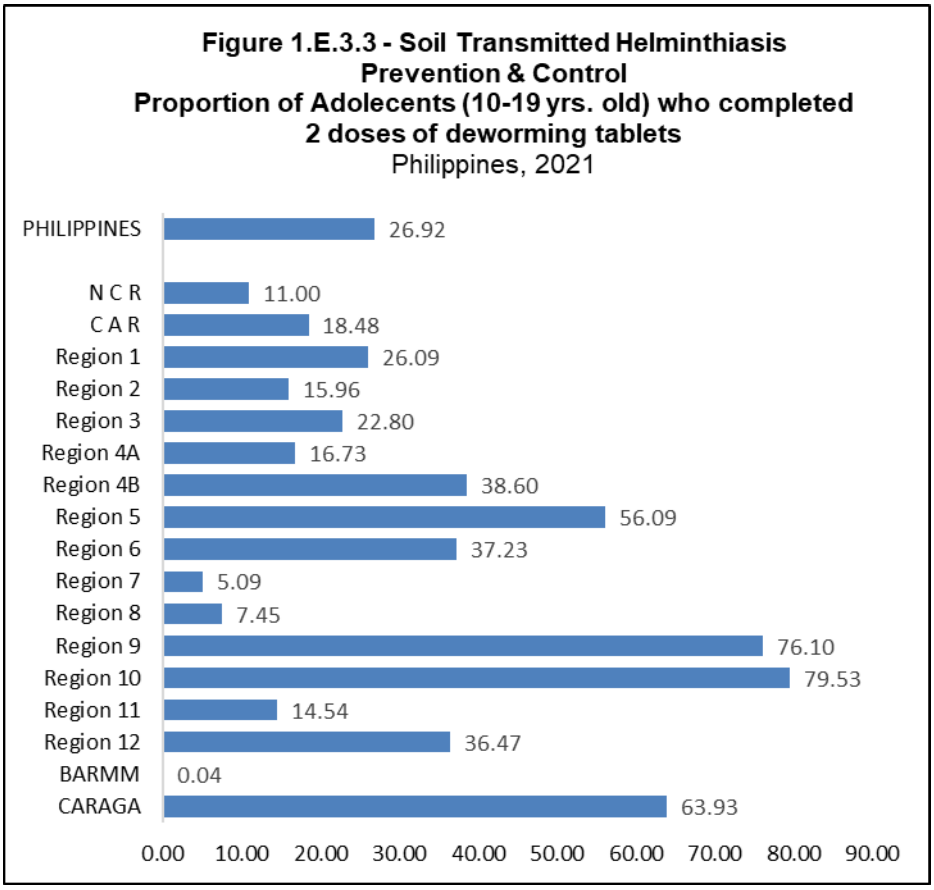

Figure 1.E.3.3 - Soil Transmitted Helminthiasis
Prevention & Control
Proportion of Adolecents (10-19 yrs. old) who completed
2 doses of deworming tablets
Philippines, 2021
PHILIPPINES 26.92
N C R 11.00
C A R 18.48
Region 1 26.09
Region 2 15.96
Region 3 22.80
Region 4A 16.73
Region 4B 38.60
Region 5 56.09
Region 6 37.23
Region 7 5.09
Region 8 7.45
Region 9 76.10
Region 10 79.53
Region 11 14.54
Region 12 36.47
BARMM 0.04
CARAGA 63.93
0.00 10.00 20.00 30.00 40.00 50.00 60.00 70.00 80.00 90.00

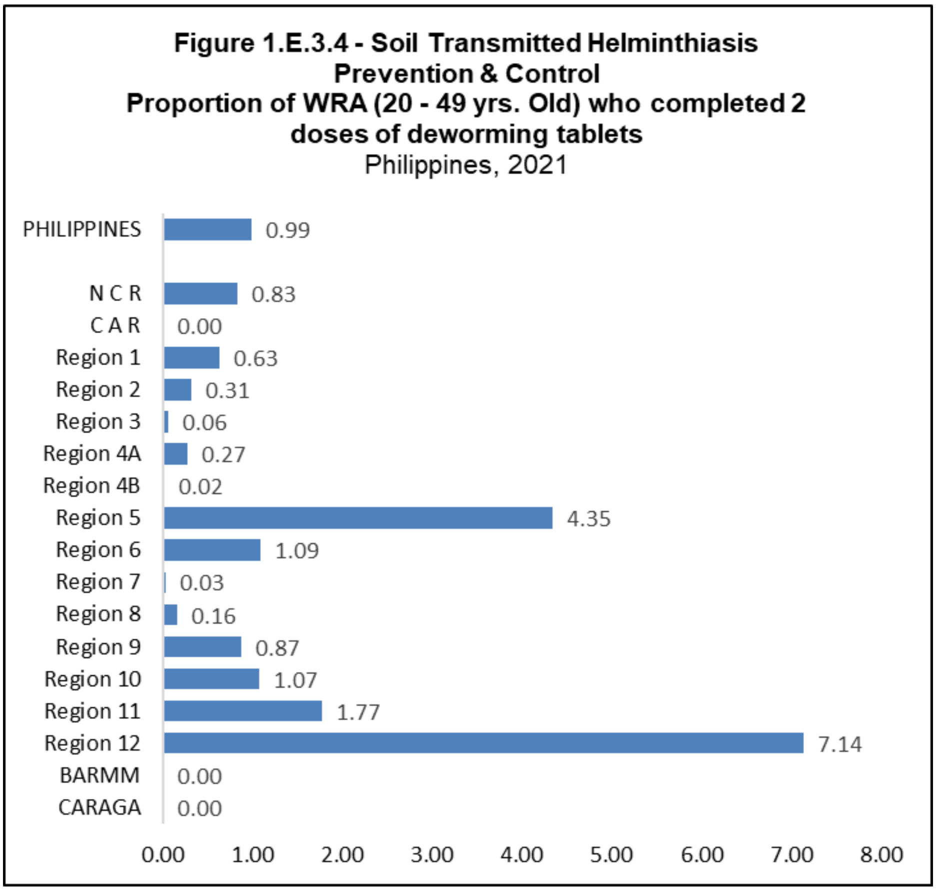

Figure 1.E.3.4 - Soil Transmitted Helminthiasis
Prevention & Control
Proportion of WRA (20 - 49 yrs. Old) who completed 2
doses of deworming tablets
Philippines, 2021
PHILIPPINES 0.99
N C R 0.83
C A R 0.00
Region 1 0.63
Region 2 0.31
Region 3 0.06
Region 4A 0.27
Region 4B 0.02
Region 5 4.35
Region 6 1.09
Region 7 0.03
Region 8 0.16
Region 9 0.87
Region 10 1.07
Region 11 1.77
Region 12 7.14
BARMM 0.00
CARAGA 0.00
0.00 1.00 2.00 3.00 4.00 5.00 6.00 7.00 8.00

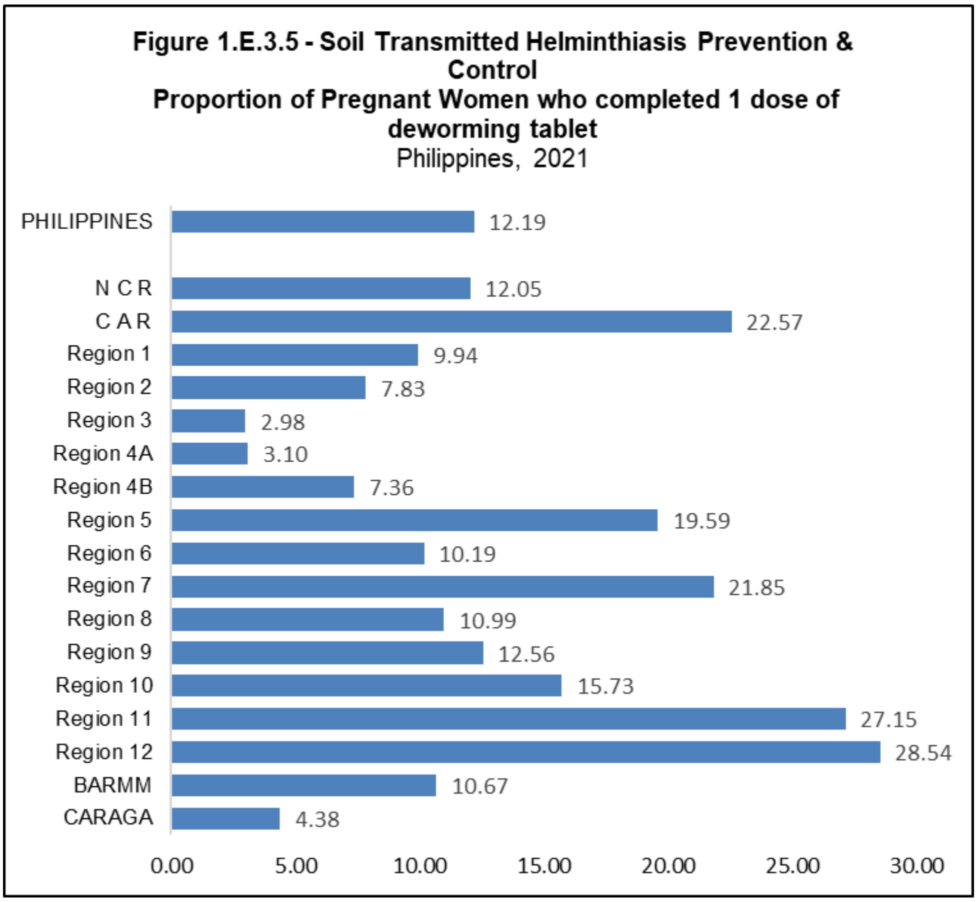
Figure 1.E.3.5 - Soil Transmitted Helminthiasis Prevention & Control
Proportion of Pregnant Women who completed 1 dose of deworming tablet
Philippines, 2021
PHILIPPINES
12.19
N C R
12.05
C A R
22.57
Region 1
9.94
Region 2
7.83
Region 3
2.98
Region 4A
3.10
Region 4B
7.36
Region 5
19.59
Region 6
10.19
Region 7
21.85
Region 8
10.99
Region 9
12.56
Region 10
15.73
Region 11
27.15
Region 12
28.54
BARMM
10.67
CARAGA
4.38
0.00
5.00
10.00
15.00
20.00
25.00
30.00

Table 1.E.4 - HIV-AIDS/STI Prevention and Control Services

Proportion of Pregnant Women Screened for HIV, Screened and Positive for Syphilis
Philippines, 2021

Area	Eligible Pop.	Pregnant Women					
		Screened for Syphilis	%	Positive for Sphilis	%	Screened for HIV	%
**PHILIPPINES**	**2,128,168**	**427,244**	**20.08**	**7,659**	**1.79**	**319,675**	**15.02**
**NCR**	**237,921**	**94,597**	**39.76**	**955**	**1.01**	**92,237**	**38.77**
Malabon City	6,757	3,112	46.06	18	0.58	3,036	44.93
Navotas City	4,607	3,046	66.12	75	2.46	2,779	60.32
Valenzuela City	11,465	6,127	53.44	200	3.26	6,761	58.97
Caloocan City	29,270	8,656	29.57	20	0.23	8,154	27.86
Marikina City	8,330	1,682	20.19	13	0.77	919	11.03
Pasig City	13,954	8,357	59.89	12	0.14	8,377	60.03
Pateros	1,181	697	59.02	1	0.14	246	20.83
Taguig City	14,874	6,749	45.37	218	3.23	6,712	45.13
Quezon City	54,246	23,832	43.93	65	0.27	23,541	43.40
Makati City	10,765	2,945	27.36	15	0.51	2,403	22.32
Mandaluyong City	7,137	2,477	34.71	3	0.12	2,434	34.10
San Juan City	2,254	783	34.74	1	0.13	792	35.14
Manila City	32,887	13,389	40.71	58	0.43	14,438	43.90
Las Piñas City	10,879	2,200	20.22	18	0.82	1,091	10.03
Muntinlupa City	9,317	2,528	27.13	10	0.40	2,379	25.53
Parañaque City	12,298	4,345	35.33	208	4.79	4,609	37.48
Pasay City	7,700	3,672	47.69	20	0.54	3,566	46.31
**CAR**	**35,143**	**14,376**	**40.91**	**48**	**0.33**	**8,440**	**24.02**
Abra	4,287	317	7.39	8	2.52	259	6.04
Apayao	2,476	948	38.29	5	0.53	137	5.53
Benguet	9,140	6,450	70.57	10	0.16	4,371	47.82
Ifugao	4,439	1,319	29.71	1	0.08	986	22.21
Kalinga	4,660	1,275	27.36	5	0.39	160	3.43
Mt. Province	3,066	848	27.66	1	0.12	184	6.00
Baguio City	7,075	3,219	45.50	18	0.56	2,343	33.12
**Region 1**	**97,198**	**26,787**	**27.56**	**100**	**0.37**	**16,134**	**16.60**
Ilocos Norte	7,986	2,671	33.45	6	0.22	1,256	15.73
Ilocos Sur	9,197	4,867	52.92	2	0.04	3,184	34.62
La Union	11,429	3,664	32.06	40	1.09	2,333	20.41
Pangasinan	50,454	9,383	18.60	50	0.53	4,687	9.29
Alaminos City	1,905	818	42.94	0	0.00	822	43.15
Candon City	976	576	59.02	0	0.00	127	13.01
Dagupan City	3,636	783	21.53	2	0.26	719	19.77

Table 1.E.4 - HIV-AIDS/STI Prevention and Control Services

Proportion of Pregnant Women Screened for HIV, Screened and Positive for Syphilis
Philippines, 2021

Area	Eligible Pop.	Pregnant Women					
		Screened for Syphilis	%	Positive for Sphilis	%	Screened for HIV	%
Laoag City	1,845	1,944	105.37	0	0.00	1,327	71.92
San Carlos City	3,999	406	10.15	0	0.00	161	4.03
San Fernando City	2,092	488	23.33	0	0.00	496	23.71
Urdaneta City	2,819	1,040	36.89	0	0.00	1,017	36.08
Vigan City	860	147	17.09	0	0.00	5	0.58
**Region 2**	**69,228**	**10,330**	**14.92**	**164**	**1.59**	**5,459**	**7.89**
Batanes	351	163	46.44	0	0.00	78	22.22
Cagayan	20,563	944	4.59	21	2.22	520	2.53
Isabela	23,543	3,999	16.99	37	0.93	1,515	6.44
Nueva Vizcaya	9,555	2,071	21.67	32	1.55	1,195	12.51
Quirino	4,038	964	23.87	60	6.22	984	24.37
Cauayan City	2,576	525	20.38	0	0.00	288	11.18
Ilagan City	2,901	1,225	42.23	6	0.49	237	8.17
Santiago City	2,683	391	14.57	5	1.28	635	23.67
Tuguegarao City	3,018	48	1.59	3	6.25	7	0.23
**Region 3**	**220,202**	**66,085**	**30.01**	**941**	**1.42**	**53,909**	**24.48**
Aurora	4,764	1,526	32.03	0	0.00	1,383	29.03
Bataan	13,814	4,994	36.15	43	0.86	4,301	31.14
Bulacan	43,722	13,369	30.58	316	2.36	10,622	24.29
Nueva Ecija	28,915	5,220	18.05	73	1.40	3,797	13.13
Pampanga	31,503	6,191	19.65	28	0.45	5,057	16.05
Tarlac	19,565	5,677	29.02	43	0.76	3,041	15.54
Zambales	12,551	2,540	20.24	19	0.75	2,291	18.25
Angeles City	7,905	986	12.47	176	17.85	949	12.01
Balanga City	1,996	980	49.10	10	1.02	1,015	50.85
Cabanatuan City	5,921	1,700	28.71	11	0.65	1,719	29.03
City of San Fernando	5,890	1,888	32.05	9	0.48	1,878	31.88
Gapan City	2,159	461	21.35	1	0.22	486	22.51
Mabalacat City	4,811	2,346	48.76	121	5.16	1,857	38.60
Malolos City	4,885	1,019	20.86	6	0.59	1,050	21.49
Meycauayan City	4,050	1,505	37.16	1	0.07	1,455	35.93
Olongapo City	4,952	1,136	22.94	53	4.67	742	14.98
Palayan City	805	144	17.89	0	0.00	133	16.52
San Jose City	2,736	756	27.63	0	0.00	444	16.23
San Jose del Monte City	11,120	10,355	93.12	26	0.25	10,133	91.12
Science City of Munoz	1,594	418	26.22	2	0.48	409	25.66
Tarlac City	6,544	2,874	43.92	3	0.10	1,147	17.53

Table 1.E.4 - HIV-AIDS/STI Prevention and Control Services

Proportion of Pregnant Women Screened for HIV, Screened and Positive for Syphilis
Philippines, 2021

Area	Eligible Pop.	Pregnant Women					
		Screened for Syphilis	%	Positive for Sphilis	%	Screened for HIV	%
**Region 4A**	**298,398**	**49,252**	**16.51**	**1,461**	**2.97**	**39,440**	**13.22**
Batangas	38,848	2,812	7.24	41	1.46	2,922	7.52
Cavite	27,948	4,503	16.11	168	3.73	2,978	10.66
Laguna	19,727	3,706	18.79	77	2.08	2,697	13.67
Quezon	37,644	4,443	11.80	136	3.06	3,157	8.39
Rizal	44,826	9,080	20.26	188	2.07	9,710	21.66
Antipolo City	16,510	1,236	7.49	10	0.81	2,510	15.20
Bacoor City	12,252	860	7.02	11	1.28	863	7.04
Batangas City	6,895	1,164	16.88	6	0.52	218	3.16
Biñan City	6,607	4,140	62.66	317	7.66	3,993	60.44
Cabuyao City	6,128	1,870	30.52	0	0.00	612	9.99
Calamba City	9,025	1,965	21.77	66	3.36	740	8.20
Cavite City	2,098	237	11.30	10	4.22	237	11.30
Dasmariñas City	13,442	3,185	23.69	99	3.11	2,929	21.79
General Trias City	6,410	646	10.08	13	2.01	479	7.47
Imus City	8,235	429	5.21	45	10.49	638	7.75
Lipa City	6,954	953	13.70	5	0.52	484	6.96
Lucena City	5,704	1,510	26.47	48	3.18	413	7.24
San Pablo City	5,277	149	2.82	2	1.34	17	0.32
San Pedro City	6,465	1,265	19.57	44	3.48	709	10.97
Santa Rosa City	7,023	2,517	35.84	141	5.60	2,188	31.15
Tagaytay City	1,451	832	57.34	11	1.32	410	28.26
Tanauan City	3,620	1,214	33.54	8	0.66	438	12.10
Tayabas City	2,134	11	0.52	1	9.09	6	0.28
Trece Martires City	3,175	525	16.54	14	2.67	92	2.90
**Region 4B**	**72,035**	**8,302**	**11.52**	**334**	**4.02**	**5,281**	**7.33**
Marinduque	4,906	661	13.47	12	1.82	407	8.30
Mindoro Occidental	12,449	1,932	15.52	4	0.21	386	3.10
Mindoro Oriental	20,011	2,455	12.27	191	7.78	1,784	8.92
Palawan	21,676	1,882	8.68	77	4.09	1,716	7.92
Romblon	6,482	1,372	21.17	50	3.64	988	15.24
Puerto Princesa City	6,511	0	0.00	0	0.00	0	0.00
**Region 5**	**137,350**	**17,192**	**12.52**	**327**	**1.90**	**10,279**	**7.48**
Albay	23,903	4,615	19.31	86	1.86	2,936	12.28
Camarines Norte	14,340	1,866	13.01	46	2.47	284	1.98
Camarines Sur	39,151	1,747	4.46	18	1.03	1,384	3.54
Catanduanes	6,381	802	12.57	10	1.25	359	5.63
Masbate	22,565	3,476	15.40	88	2.53	1,298	5.75
Sorsogon	19,470	1,667	8.56	54	3.24	702	3.61

Table 1.E.4 - HIV-AIDS/STI Prevention and Control Services

Proportion of Pregnant Women Screened for HIV, Screened and Positive for Syphilis
Philippines, 2021

Area	Eligible Pop.	Pregnant Women					
		Screened for Syphilis	%	Positive for Sphilis	%	Screened for HIV	%
Iriga City	2,665	617	23.15	9	1.46	428	16.06
Legaspi City	4,205	1,063	25.28	10	0.94	1,140	27.11
Naga City	4,670	1,339	28.67	6	0.45	1,748	37.43
**Region 6**	**146,545**	**34,422**	**23.49**	**701**	**2.04**	**16,070**	**10.97**
Aklan	11,231	3,379	30.09	53	1.57	2,034	18.11
Antique	12,985	2,343	18.04	70	2.99	1,156	8.90
Capiz	13,984	2,145	15.34	37	1.72	400	2.86
Guimaras	3,085	1,680	54.46	10	0.60	588	19.06
Iloilo	36,414	13,178	36.19	266	2.02	3,719	10.21
Negros Occidental	49,314	5,730	11.62	101	1.76	3,329	6.75
Bacolod City	11,102	3,584	32.28	100	2.79	2,826	25.45
Iloilo City	8,430	2,383	28.27	64	2.69	2,018	23.94
**Region 7**	**162,860**	**31,066**	**19.08**	**1,219**	**3.92**	**33,124**	**20.34**
Bohol	27,637	4,888	17.69	168	3.44	1,889	6.84
Cebu	67,046	7,099	10.59	529	7.45	11,108	16.57
Negros Oriental	27,922	1,401	5.02	37	2.64	424	1.52
Siquijor	1,636	591	36.12	2	0.34	684	41.81
Cebu City	21,037	10,630	50.53	362	3.41	9,902	47.07
Lapu-Lapu City	9,310	4,050	43.50	0	0.00	5,313	57.07
Mandaue City	8,272	2,407	29.10	121	5.03	3,804	45.99
**Region 8**	**104,016**	**12,485**	**12.00**	**388**	**3.11**	**8,203**	**7.89**
Biliran	3,856	1,235	32.03	14	1.13	1,423	36.90
Eastern Samar	11,635	938	8.06	21	2.24	796	6.84
Northern Leyte	35,052	3,665	10.46	86	2.35	951	2.71
Northern Samar	15,655	2,170	13.86	26	1.20	2,087	13.33
Southern Leyte	6,556	365	5.57	19	5.21	406	6.19
Western Samar	14,515	698	4.81	170	24.36	636	4.38
Calbayog City	4,474	214	4.78	0	0.00	15	0.34
Maasin City	1,661	5	0.30	5	100.00	2	0.12
Ormoc City	4,989	428	8.58	12	2.80	830	16.64
Tacloban City	5,623	2,767	49.21	35	1.26	1,057	18.80
**Region 9**	**79,552**	**3,156**	**3.97**	**45**	**1.43**	**747**	**0.94**
Zamboanga del Norte	17,075	465	2.72	0	0.00	333	1.95
Zamboanga del Sur	17,506	247	1.41	9	3.64	3	0.02
Zamboanga Sibugay	14,992	2,016	13.45	33	1.64	160	1.07

Table 1.E.4 - HIV-AIDS/STI Prevention and Control Services

Proportion of Pregnant Women Screened for HIV, Screened and Positive for Syphilis
Philippines, 2021

Area	Eligible Pop.	Pregnant Women					
		Screened for Syphilis	%	Positive for Sphilis	%	Screened for HIV	%
Dapitan City	1,755	106	6.04	0	0.00	85	4.84
Dipolog City	2,794	5	0.18	2	40.00	3	0.11
Isabela City	2,544	0	0.00	0	0.00	10	0.39
Pagadian City	4,290	173	4.03	1	0.58	0	0.00
Zamboanga City	18,596	144	0.77	0	0.00	153	0.82
**Region 10**	**100,690**	**11,760**	**11.68**	**257**	**2.19**	**5,840**	**5.80**
Bukidnon	23,307	859	3.69	16	1.86	644	2.76
Camiguin	1,854	269	14.51	20	7.43	177	9.55
Lanao del Norte	14,955	618	4.13	1	0.16	445	2.98
Misamis Occidental	6,413	148	2.31	12	8.11	130	2.03
Misamis Oriental	15,058	1,437	9.54	4	0.28	657	4.36
Cagayan de Oro City	14,263	5,533	38.79	147	2.66	1,456	10.21
El Salvador City	1,061	35	3.30	0	0.00	48	4.52
Gingoog City	2,627	91	3.46	0	0.00	319	12.14
Iligan City	7,575	368	4.86	13	3.53	95	1.25
Malaybalay City	3,882	864	22.26	37	4.28	292	7.52
Oroquieta City	1,389	19	1.37	1	5.26	20	1.44
Ozamis City	2,782	1,262	45.36	4	0.32	1,178	42.34
Tangub City	1,234	2	0.16	0	0.00	2	0.16
Valencia City	4,290	255	5.94	2	0.78	377	8.79
**Region 11**	**107,847**	**19,028**	**17.64**	**62**	**0.33**	**8,660**	**8.03**
Davao de Oro	15,526	143	0.92	0	0.00	51	0.33
Davao del Norte	21,184	4,560	21.53	13	0.29	2,117	9.99
Davao del Sur	13,125	128	0.98	26	20.31	146	1.11
Davao Oriental	14,355	618	4.31	3	0.49	478	3.33
Davao Occidental	6,598	159	2.41	3	1.89	137	2.08
Davao City	37,059	13,420	36.21	17	0.13	5,731	15.46
**Region 12**	**104,693**	**19,648**	**18.77**	**398**	**2.03**	**9,777**	**9.34**
North Cotabato	33,933	1,826	5.38	67	3.67	1,237	3.65
Sarangani	12,904	2,914	22.58	131	4.50	1,178	9.13
South Cotabato	21,039	5,737	27.27	28	0.49	5,053	24.02
Sultan Kudarat	17,331	6,117	35.30	68	1.11	610	3.52
Cotabato City	5,831	203	3.48	2	0.99	196	3.36
Gen. Santos City	13,655	2,851	20.88	102	3.58	1,503	11.01

Table 1.E.4 - HIV-AIDS/STI Prevention and Control Services

Proportion of Pregnant Women Screened for HIV, Screened and Positive for Syphilis
Philippines, 2021

Area	Eligible Pop.	Pregnant Women					
		Screened for Syphilis	%	Positive for Sphilis	%	Screened for HIV	%
**BARMM**	**94,148**	**0**	**0.00**	**0**	**0.00**	**0**	**0.00**
Basilan	7,672	0	0.00	0	0.00	0	0.00
Lanao del Sur	21,391	0	0.00	0	0.00	0	0.00
Maguindanao	31,650	0	0.00	0	0.00	0	0.00
Sulu	16,891	0	0.00	0	0.00	0	0.00
Tawi-Tawi	9,319	0	0.00	0	0.00	0	0.00
Lamitan City	2,112	0	0.00	0	0.00	0	0.00
Marawi City	5,113	0	0.00	0	0.00	0	0.00
**CARAGA**	**60,342**	**8,758**	**14.51**	**259**	**2.96**	**6,075**	**10.07**
Agusan del Norte	8,116	916	11.29	125	13.65	487	6.00
Agusan del Sur	17,658	2,255	12.77	36	1.60	877	4.97
Surigao del Norte	7,195	600	8.34	15	2.50	339	4.71
Surigao del Sur	11,532	495	4.29	34	6.87	235	2.04
Province of Dinagat	2,574	609	23.66	12	1.97	570	22.14
Bislig City	2,185	39	1.78	0	0.00	2	0.09
Butuan City	7,732	2,743	35.48	34	1.24	2,350	30.39
Surigao City	3,350	1,101	32.87	3	0.27	1,215	36.27

Legend:
0 = No Case
** = No Report*

Figure 1.E.4.1 - HIV-AIDS/STI Prevention and Control Services
Pregnant Women Screen for Syphillis
Philippines, 2021

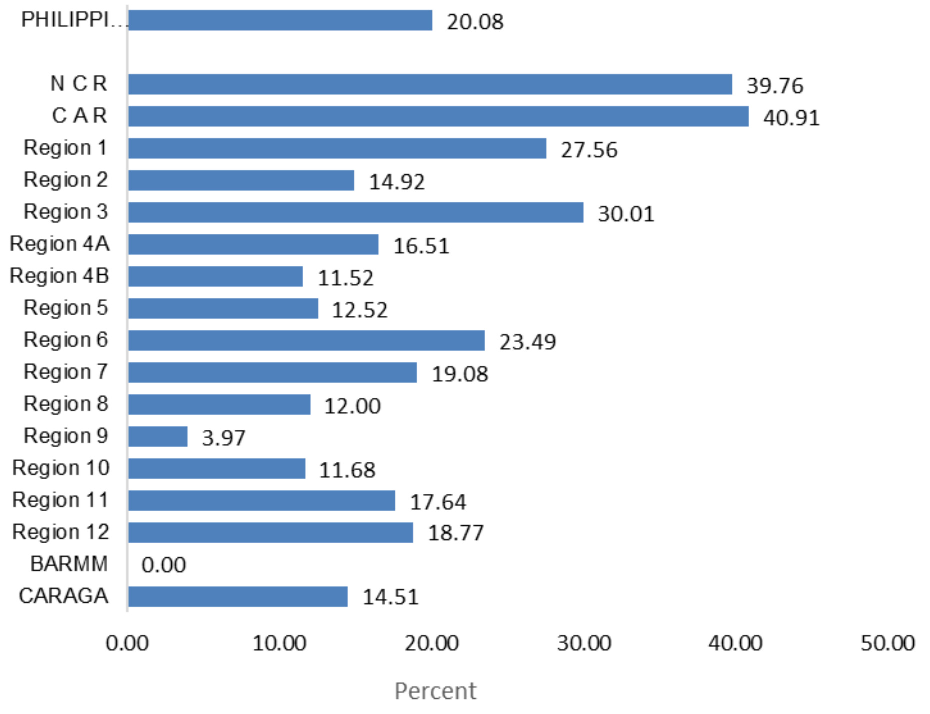

Figure 1.E.4.2 - HIV-AIDS/STI Prevention and Control Services
Pregnant Women Positive for Syphillis
Philippines, 2021

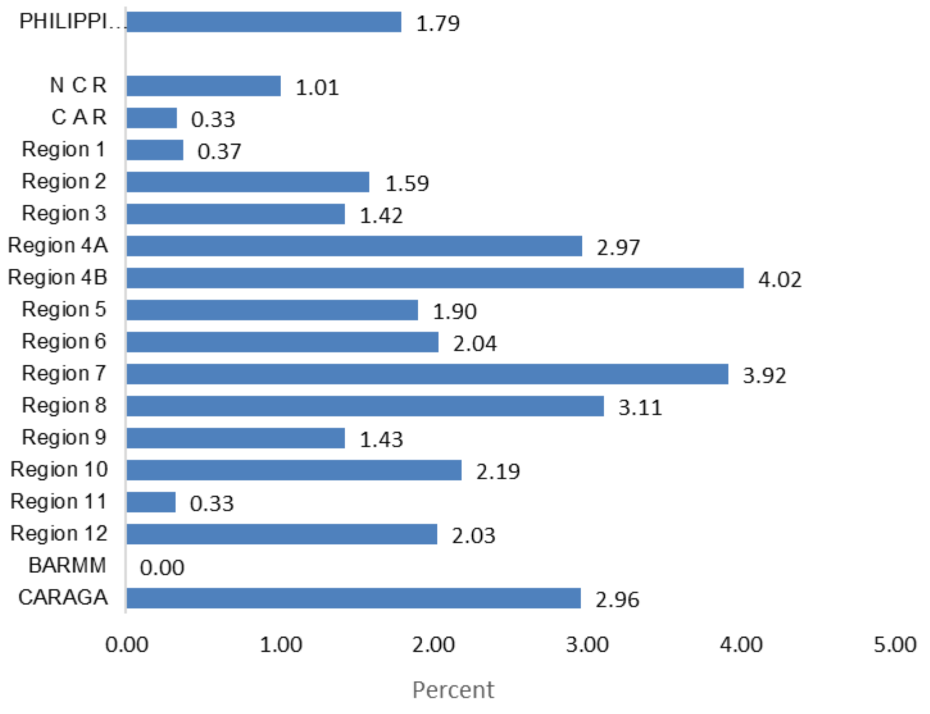

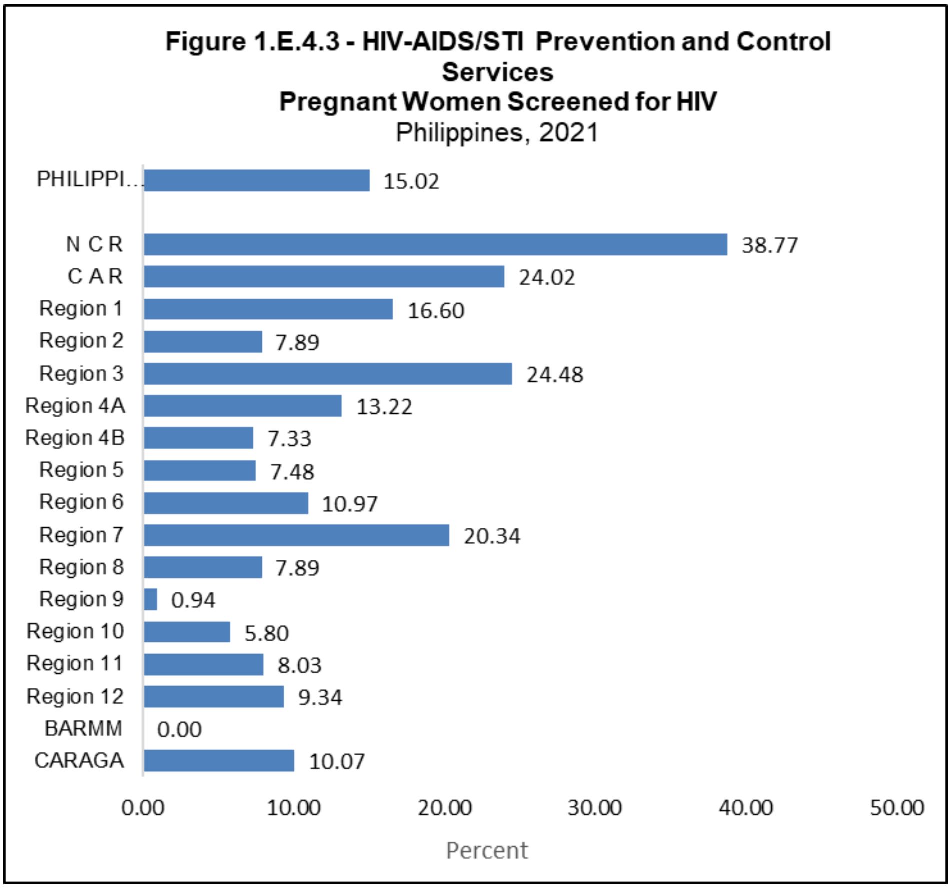
Figure 1.E.4.3 - HIV-AIDS/STI Prevention and Control Services
Pregnant Women Screened for HIV
Philippines, 2021
PHILIPPI...
15.02
N C R
38.77
C A R
24.02
Region 1
16.60
Region 2
7.89
Region 3
24.48
Region 4A
13.22
Region 4B
7.33
Region 5
7.48
Region 6
10.97
Region 7
20.34
Region 8
7.89
Region 9
0.94
Region 10
5.80
Region 11
8.03
Region 12
9.34
BARMM
0.00
CARAGA
10.07
0.00
10.00
20.00
30.00
40.00
50.00
Percent

Table 1.E.5.1 - Malaria

Probable/Clinically and Confirmed Malaria Cases Disaggregated by Age Group and Sex
Philippines, 2021

Area	Population	Probable/Clinically and Confirmed Malaria Cases							
		<5yrs Old				>=5 Yrs Old			
		Male	Female	Total	Morbidity Rate	Male	Female	Total	Morbidity Rate
**PHILIPPINES**	**110,198,654**	**30**	**13**	**43**	**0.04**	**1,570**	**1,099**	**2,669**	**2.42**
**N C R**	**13,966,223**	**0**	**0**	**0**	**0.00**	**0**	**0**	**0**	**0.00**
Malabon City	396,434	0	0	0	0.00	0	0	0	0.00
Navotas City	270,559	0	0	0	0.00	0	0	0	0.00
Valenzuela City	672,888	0	0	0	0.00	0	0	0	0.00
Caloocan City	1,717,928	0	0	0	0.00	0	0	0	0.00
Marikina City	488,857	0	0	0	0.00	0	0	0	0.00
Pasig City	819,171	0	0	0	0.00	0	0	0	0.00
Pateros	69,239	0	0	0	0.00	0	0	0	0.00
Taguig City	872,980	0	0	0	0.00	0	0	0	0.00
Quezon City	3,184,407	0	0	0	0.00	0	0	0	0.00
Makati City	631,867	0	0	0	0.00	0	0	0	0.00
Mandaluyong City	418,942	0	0	0	0.00	0	0	0	0.00
San Juan City	132,512	0	0	0	0.00	0	0	0	0.00
Manila City	1,930,696	0	0	0	0.00	0	0	0	0.00
Las Piñas City	638,694	0	0	0	0.00	0	0	0	0.00
Muntinlupa City	547,173	0	0	0	0.00	0	0	0	0.00
Parañaque City	722,126	0	0	0	0.00	0	0	0	0.00
Pasay City	451,750	0	0	0	0.00	0	0	0	0.00
**C A R**	**1,822,719**	**0**	**0**	**0**	**0.00**	**0**	**0**	**0**	**0.00**
Abra	248,468	0	0	0	0.00	0	0	0	0.00
Apayao	124,728	0	0	0	0.00	0	0	0	0.00
Benguet	483,934	0	0	0	0.00	0	0	0	0.00
Ifugao	212,063	0	0	0	0.00	0	0	0	0.00
Kalinga	221,563	0	0	0	0.00	0	0	0	0.00
Mt. Province	157,413	0	0	0	0.00	0	0	0	0.00
Baguio City	374,550	0	0	0	0.00	0	0	0	0.00
**Region 1**	**5,313,223**	**0**	**0**	**0**	**0.00**	**0**	**0**	**0**	**0.00**
Ilocos Norte	501,276	0	0	0	0.00	0	0	0	0.00
Ilocos Sur	600,674	0	0	0	0.00	0	0	0	0.00
La Union	705,988	0	0	0	0.00	0	0	0	0.00
Pangasinan	2,522,027	0	0	0	0.00	0	0	0	0.00
Alaminos City	95,291	0	0	0	0.00	0	0	0	0.00
Candon City	63,312	0	0	0	0.00	0	0	0	0.00
Dagupan City	181,929	0	0	0	0.00	0	0	0	0.00
Laoag City	115,582	0	0	0	0.00	0	0	0	0.00
San Carlos City	200,310	0	0	0	0.00	0	0	0	0.00
San Fernando City	129,350	0	0	0	0.00	0	0	0	0.00
Urdaneta City	141,215	0	0	0	0.00	0	0	0	0.00
Vigan City	56,269	0	0	0	0.00	0	0	0	0.00

Table 1.E.5.1 - Malaria

Probable/Clinically and Confirmed Malaria Cases Disaggregated by Age Group and Sex
Philippines, 2021

Area	Population	Probable/Clinically and Confirmed Malaria Cases							
		<5yrs Old				>=5 Yrs Old			
		Male	Female	Total	Morbidity Rate	Male	Female	Total	Morbidity Rate
**Region 2**	**3,693,664**	**0**	**0**	**0**	**0.00**	**0**	**0**	**0**	**0.00**
Batanes	18,013	0	0	0	0.00	0	0	0	0.00
Cagayan	1,121,561	0	0	0	0.00	0	0	0	0.00
Isabela	1,264,006	0	0	0	0.00	0	0	0	0.00
Nueva Vizcaya	488,238	0	0	0	0.00	0	0	0	0.00
Quirino	199,473	0	0	0	0.00	0	0	0	0.00
Cauayan City	138,317	0	0	0	0.00	0	0	0	0.00
Ilagan City	155,452	0	0	0	0.00	0	0	0	0.00
Santiago City	143,982	0	0	0	0.00	0	0	0	0.00
Tuguegarao City	164,622	0	0	0	0.00	0	0	0	0.00
**Region 3**	**12,513,847**	**0**	**0**	**0**	**0.00**	**0**	**0**	**0**	**0.00**
Aurora	229,841	0	0	0	0.00	0	0	0	0.00
Bataan	742,197	0	0	0	0.00	0	0	0	0.00
Bulacan	2,542,502	0	0	0	0.00	0	0	0	0.00
Nueva Ecija	1,646,738	0	0	0	0.00	0	0	0	0.00
Pampanga	1,850,369	0	0	0	0.00	0	0	0	0.00
Tarlac	1,106,339	0	0	0	0.00	0	0	0	0.00
Zambales	650,180	0	0	0	0.00	0	0	0	0.00
Angeles City	464,253	0	0	0	0.00	0	0	0	0.00
Balanga City	107,277	0	0	0	0.00	0	0	0	0.00
Cabanatuan City	337,037	0	0	0	0.00	0	0	0	0.00
City of San Fernando	345,857	0	0	0	0.00	0	0	0	0.00
Gapan City	123,009	0	0	0	0.00	0	0	0	0.00
Mabalacat City	282,858	0	0	0	0.00	0	0	0	0.00
Malolos City	283,977	0	0	0	0.00	0	0	0	0.00
Meycauayan City	235,549	0	0	0	0.00	0	0	0	0.00
Olongapo City	256,443	0	0	0	0.00	0	0	0	0.00
Palayan City	45,768	0	0	0	0.00	0	0	0	0.00
San Jose City	155,831	0	0	0	0.00	0	0	0	0.00
San Jose del Monte City	646,756	0	0	0	0.00	0	0	0	0.00
Science City of Munoz	90,867	0	0	0	0.00	0	0	0	0.00
Tarlac City	370,199	0	0	0	0.00	0	0	0	0.00
**Region 4A**	**16,361,107**	**0**	**0**	**0**	**0.00**	**0**	**0**	**0**	**0.00**
Batangas	2,144,028	0	0	0	0.00	0	0	0	0.00
Cavite	1,604,676	0	0	0	0.00	0	0	0	0.00
Laguna	1,111,798	0	0	0	0.00	0	0	0	0.00
Quezon	1,890,167	0	0	0	0.00	0	0	0	0.00
Rizal	2,386,862	0	0	0	0.00	0	0	0	0.00
Antipolo City	879,159	0	0	0	0.00	0	0	0	0.00
Bacoor City	703,029	0	0	0	0.00	0	0	0	0.00
Batangas City	380,515	0	0	0	0.00	0	0	0	0.00
Biñan City	372,804	0	0	0	0.00	0	0	0	0.00
Cabuyao City	345,623	0	0	0	0.00	0	0	0	0.00
Calamba City	508,770	0	0	0	0.00	0	0	0	0.00
Cavite City	120,342	0	0	0	0.00	0	0	0	0.00

Table 1.E.5.1 - Malaria

Probable/Clinically and Confirmed Malaria Cases Disaggregated by Age Group and Sex
Philippines, 2021

Area	Population	Probable/Clinically and Confirmed Malaria Cases							
		<5yrs Old				>=5 Yrs Old			
		Male	Female	Total	Morbidity Rate	Male	Female	Total	Morbidity Rate
Dasmariñas City	771,409	0	0	0	0.00	0	0	0	0.00
General Trias City	367,906	0	0	0	0.00	0	0	0	0.00
Imus City	472,646	0	0	0	0.00	0	0	0	0.00
Lipa City	383,404	0	0	0	0.00	0	0	0	0.00
Lucena City	286,460	0	0	0	0.00	0	0	0	0.00
San Pablo City	297,847	0	0	0	0.00	0	0	0	0.00
San Pedro City	364,721	0	0	0	0.00	0	0	0	0.00
Santa Rosa City	396,019	0	0	0	0.00	0	0	0	0.00
Tagaytay City	83,320	0	0	0	0.00	0	0	0	0.00
Tanauan City	199,978	0	0	0	0.00	0	0	0	0.00
Tayabas City	107,355	0	0	0	0.00	0	0	0	0.00
Trece Martires City	182,269	0	0	0	0.00	0	0	0	0.00
**Region 4B**	**3,212,211**	**19**	**12**	**31**	**0.97**	**1,519**	**1,078**	**2,597**	**80.85**
Marinduque	242,420	0	0	0	0.00	0	0	0	0.00
Mindoro Occidental	519,990	0	0	0	0.00	1	0	1	0.19
Mindoro Oriental	911,307	0	0	0	0.00	1	29	30	3.29
Palawan	950,209	19	12	31	3.26	1,517	1,049	2,566	270.05
Romblon	302,917	0	0	0	0.00	0	0	0	0.00
Puerto Princesa City	285,368	0	0	0	0.00	0	0	0	0.00
**Region 5**	**6,192,924**	**0**	**0**	**0**	**0.00**	**1**	**0**	**1**	**0.02**
Albay	1,199,362	0	0	0	0.00	0	0	0	0.00
Camarines Norte	629,402	0	0	0	0.00	0	0	0	0.00
Camarines Sur	1,750,680	0	0	0	0.00	0	0	0	0.00
Catanduanes	275,043	0	0	0	0.00	0	0	0	0.00
Masbate	944,945	0	0	0	0.00	0	0	0	0.00
Sorsogon	855,000	0	0	0	0.00	1	0	1	0.12
Iriga City	118,952	0	0	0	0.00	0	0	0	0.00
Legaspi City	210,918	0	0	0	0.00	0	0	0	0.00
Naga City	208,622	0	0	0	0.00	0	0	0	0.00
**Region 6**	**7,970,551**	**0**	**0**	**0**	**0.00**	**0**	**0**	**0**	**0.00**
Aklan	610,948	0	0	0	0.00	0	0	0	0.00
Antique	617,788	0	0	0	0.00	0	0	0	0.00
Capiz	811,948	0	0	0	0.00	0	0	0	0.00
Guimaras	187,011	0	0	0	0.00	0	0	0	0.00
Iloilo	2,069,653	0	0	0	0.00	0	0	0	0.00
Negros Occidental	2,607,671	0	0	0	0.00	0	0	0	0.00
Bacolod City	586,715	0	0	0	0.00	0	0	0	0.00
Iloilo City	478,817	0	0	0	0.00	0	0	0	0.00
**Region 7**	**8,054,772**	**0**	**0**	**0**	**0.00**	**0**	**0**	**0**	**0.00**
Bohol	1,374,101	0	0	0	0.00	0	0	0	0.00
Cebu	3,276,440	0	0	0	0.00	0	0	0	0.00
Negros Oriental	1,415,196	0	0	0	0.00	0	0	0	0.00

Table 1.E.5.1 - Malaria

Probable/Clinically and Confirmed Malaria Cases Disaggregated by Age Group and Sex
Philippines, 2021

Area	Population	Probable/Clinically and Confirmed Malaria Cases							
		<5yrs Old				>=5 Yrs Old			
		Male	Female	Total	Morbidity Rate	Male	Female	Total	Morbidity Rate
Siquijor	101,227	0	0	0	0.00	0	0	0	0.00
Cebu City	1,028,542	0	0	0	0.00	0	0	0	0.00
Lapu-Lapu City	454,972	0	0	0	0.00	0	0	0	0.00
Mandaue City	404,294	0	0	0	0.00	0	0	0	0.00
**Region 8**	**4,803,767**	**0**	**0**	**0**	**0.00**	**0**	**0**	**0**	**0.00**
Biliran	180,717	0	0	0	0.00	0	0	0	0.00
Eastern Samar	511,290	0	0	0	0.00	0	0	0	0.00
Northern Leyte	1,656,424	0	0	0	0.00	0	0	0	0.00
Northern Samar	675,196	0	0	0	0.00	0	0	0	0.00
Southern Leyte	358,038	0	0	0	0.00	0	0	0	0.00
Western Samar	634,040	0	0	0	0.00	0	0	0	0.00
Calbayog City	195,379	0	0	0	0.00	0	0	0	0.00
Maasin City	91,120	0	0	0	0.00	0	0	0	0.00
Ormoc City	235,937	0	0	0	0.00	0	0	0	0.00
Tacloban City	265,626	0	0	0	0.00	0	0	0	0.00
**Region 9**	**3,809,540**	**0**	**0**	**0**	**0.00**	**0**	**0**	**0**	**0.00**
Zamboanga del Norte	831,480	0	0	0	0.00	0	0	0	0.00
Zamboanga del Sur	840,050	0	0	0	0.00	0	0	0	0.00
Zamboanga Sibugay	687,546	0	0	0	0.00	0	0	0	0.00
Dapitan City	85,853	0	0	0	0.00	0	0	0	0.00
Dipolog City	136,208	0	0	0	0.00	0	0	0	0.00
Isabela City	130,380	0	0	0	0.00	0	0	0	0.00
Pagadian City	206,034	0	0	0	0.00	0	0	0	0.00
Zamboanga City	891,989	0	0	0	0.00	0	0	0	0.00
**Region 10**	**5,074,563**	**0**	**0**	**0**	**0.00**	**0**	**0**	**0**	**0.00**
Bukidnon	1,130,220	0	0	0	0.00	0	0	0	0.00
Camiguin	93,118	0	0	0	0.00	0	0	0	0.00
Lanao del Norte	735,928	0	0	0	0.00	0	0	0	0.00
Misamis Occidental	287,446	0	0	0	0.00	0	0	0	0.00
Misamis Oriental	837,081	0	0	0	0.00	0	0	0	0.00
Cagayan de Oro City	740,740	0	0	0	0.00	0	0	0	0.00
El Salvador City	55,016	0	0	0	0.00	0	0	0	0.00
Gingoog City	136,593	0	0	0	0.00	0	0	0	0.00
Iligan City	372,772	0	0	0	0.00	0	0	0	0.00
Malaybalay City	188,395	0	0	0	0.00	0	0	0	0.00
Oroquieta City	74,210	0	0	0	0.00	0	0	0	0.00
Ozamis City	148,747	0	0	0	0.00	0	0	0	0.00
Tangub City	66,085	0	0	0	0.00	0	0	0	0.00
Valencia City	208,212	0	0	0	0.00	0	0	0	0.00
**Region 11**	**5,371,254**	**0**	**0**	**0**	**0.00**	**0**	**0**	**0**	**0.00**
Davao de Oro	780,742	0	0	0	0.00	0	0	0	0.00

Table 1.E.5.1 - Malaria

Probable/Clinically and Confirmed Malaria Cases Disaggregated by Age Group and Sex
Philippines, 2021

Area	Population	Probable/Clinically and Confirmed Malaria Cases							
		<5yrs Old				>=5 Yrs Old			
		Male	Female	Total	Morbidity Rate	Male	Female	Total	Morbidity Rate
Davao del Norte	1,063,436	0	0	0	0.00	0	0	0	0.00
Davao del Sur	607,848	0	0	0	0.00	0	0	0	0.00
Davao Oriental	719,362	0	0	0	0.00	0	0	0	0.00
Davao Occidental	342,874	0	0	0	0.00	0	0	0	0.00
Davao City	1,856,992	0	0	0	0.00	0	0	0	0.00
**Region 12**	**4,985,878**	**11**	**1**	**12**	**0.24**	**50**	**21**	**71**	**1.42**
North Cotabato	1,553,081	0	0	0	0.00	1	1	2	0.13
Sarangani	590,746	0	0	0	0.00	0	0	0	0.00
South Cotabato	1,011,240	0	0	0	0.00	0	0	0	0.00
Sultan Kudarat	862,787	11	1	12	1.39	39	20	59	6.84
Cotabato City	311,264	0	0	0	0.00	0	0	0	0.00
Gen. Santos City	656,760	0	0	0	0.00	10	0	10	1.52
**BARMM**	**4,270,193**	**0**	**0**	**0**	**0.00**	**0**	**0**	**0**	**0.00**
Basilan	310,153	0	0	0	0.00	0	0	0	0.00
Lanao del Sur	942,654	0	0	0	0.00	0	0	0	0.00
Maguindanao	1,355,941	0	0	0	0.00	0	0	0	0.00
Sulu	932,940	0	0	0	0.00	0	0	0	0.00
Tawi-Tawi	417,702	0	0	0	0.00	0	0	0	0.00
Lamitan City	85,335	0	0	0	0.00	0	0	0	0.00
Marawi City	225,468	0	0	0	0.00	0	0	0	0.00
**CARAGA**	**2,782,218**	**0**	**0**	**0**	**0.00**	**0**	**0**	**0**	**0.00**
Agusan del Norte	382,946	0	0	0	0.00	0	0	0	0.00
Agusan del Sur	741,035	0	0	0	0.00	0	0	0	0.00
Surigao del Norte	366,639	0	0	0	0.00	0	0	0	0.00
Surigao del Sur	525,627	0	0	0	0.00	0	0	0	0.00
Province of Dinagat	131,274	0	0	0	0.00	0	0	0	0.00
Bislig City	99,839	0	0	0	0.00	0	0	0	0.00
Butuan City	364,103	0	0	0	0.00	0	0	0	0.00
Surigao City	170,755	0	0	0	0.00	0	0	0	0.00

Legend:
0 = No Case
** = No Report*

Table 1.E.5.2 - Malaria

Age<5 and Age >=5 yrs. old Laboratory Confirmed Malaria Deaths disaggregated by Age Group and Sex
Philippines, 2021

Area	Population	Laboratory Confirmed Malaria Deaths							
		<5yrs Old				>=5 Yrs Old			
		Male	Female	Total	Mortality Rate	Male	Female	Total	Mortality Rate
**PHILIPPINES**	**110,198,654**	**0**	**0**	**0**	**0.00**	**0**	**3**	**3**	**0.00**
**N C R**	**13,966,223**	**0**	**0**	**0**	**0.00**	**0**	**0**	**0**	**0.00**
Malabon City	396,434	0	0	0	0.00	0	0	0	0.00
Navotas City	270,559	0	0	0	0.00	0	0	0	0.00
Valenzuela City	672,888	0	0	0	0.00	0	0	0	0.00
Caloocan City	1,717,928	0	0	0	0.00	0	0	0	0.00
Marikina City	488,857	0	0	0	0.00	0	0	0	0.00
Pasig City	819,171	0	0	0	0.00	0	0	0	0.00
Pateros	69,239	0	0	0	0.00	0	0	0	0.00
Taguig City	872,980	0	0	0	0.00	0	0	0	0.00
Quezon City	3,184,407	0	0	0	0.00	0	0	0	0.00
Makati City	631,867	0	0	0	0.00	0	0	0	0.00
Mandaluyong City	418,942	0	0	0	0.00	0	0	0	0.00
San Juan City	132,512	0	0	0	0.00	0	0	0	0.00
Manila City	1,930,696	0	0	0	0.00	0	0	0	0.00
Las Piñas City	638,694	0	0	0	0.00	0	0	0	0.00
Muntinlupa City	547,173	0	0	0	0.00	0	0	0	0.00
Parañaque City	722,126	0	0	0	0.00	0	0	0	0.00
Pasay City	451,750	0	0	0	0.00	0	0	0	0.00
**C A R**	**1,822,719**	**0**	**0**	**0**	**0.00**	**0**	**0**	**0**	**0.00**
Abra	248,468	0	0	0	0.00	0	0	0	0.00
Apayao	124,728	0	0	0	0.00	0	0	0	0.00
Benguet	483,934	0	0	0	0.00	0	0	0	0.00
Ifugao	212,063	0	0	0	0.00	0	0	0	0.00
Kalinga	221,563	0	0	0	0.00	0	0	0	0.00
Mt. Province	157,413	0	0	0	0.00	0	0	0	0.00
Baguio City	374,550	0	0	0	0.00	0	0	0	0.00
**Region 1**	**5,313,223**	**0**	**0**	**0**	**0.00**	**0**	**0**	**0**	**0.00**
Ilocos Norte	501,276	0	0	0	0.00	0	0	0	0.00
Ilocos Sur	600,674	0	0	0	0.00	0	0	0	0.00
La Union	705,988	0	0	0	0.00	0	0	0	0.00
Pangasinan	2,522,027	0	0	0	0.00	0	0	0	0.00
Alaminos City	95,291	0	0	0	0.00	0	0	0	0.00
Candon City	63,312	0	0	0	0.00	0	0	0	0.00
Dagupan City	181,929	0	0	0	0.00	0	0	0	0.00
Laoag City	115,582	0	0	0	0.00	0	0	0	0.00
San Carlos City	200,310	0	0	0	0.00	0	0	0	0.00
San Fernando City	129,350	0	0	0	0.00	0	0	0	0.00
Urdaneta City	141,215	0	0	0	0.00	0	0	0	0.00
Vigan City	56,269	0	0	0	0.00	0	0	0	0.00

Table 1.E.5.2 - Malaria

Age<5 and Age >=5 yrs. old Laboratory Confirmed Malaria Deaths disaggregated by Age Group and Sex
Philippines, 2021

Area	Population	Laboratory Confirmed Malaria Deaths							
		<5yrs Old				>=5 Yrs Old			
		Male	Female	Total	Mortality Rate	Male	Female	Total	Mortality Rate
**Region 2**	**3,693,664**	**0**	**0**	**0**	**0.00**	**0**	**0**	**0**	**0.00**
Batanes	18,013	0	0	0	0.00	0	0	0	0.00
Cagayan	1,121,561	0	0	0	0.00	0	0	0	0.00
Isabela	1,264,006	0	0	0	0.00	0	0	0	0.00
Nueva Vizcaya	488,238	0	0	0	0.00	0	0	0	0.00
Quirino	199,473	0	0	0	0.00	0	0	0	0.00
Cauayan City	138,317	0	0	0	0.00	0	0	0	0.00
Ilagan City	155,452	0	0	0	0.00	0	0	0	0.00
Santiago City	143,982	0	0	0	0.00	0	0	0	0.00
Tuguegarao City	164,622	0	0	0	0.00	0	0	0	0.00
**Region 3**	**12,513,847**	**0**	**0**	**0**	**0.00**	**0**	**0**	**0**	**0.00**
Aurora	229,841	0	0	0	0.00	0	0	0	0.00
Bataan	742,197	0	0	0	0.00	0	0	0	0.00
Bulacan	2,542,502	0	0	0	0.00	0	0	0	0.00
Nueva Ecija	1,646,738	0	0	0	0.00	0	0	0	0.00
Pampanga	1,850,369	0	0	0	0.00	0	0	0	0.00
Tarlac	1,106,339	0	0	0	0.00	0	0	0	0.00
Zambales	650,180	0	0	0	0.00	0	0	0	0.00
		0	0	0	0.00	0	0	0	0.00
Angeles City	464,253	0	0	0	0.00	0	0	0	0.00
Balanga City	107,277	0	0	0	0.00	0	0	0	0.00
Cabanatuan City	337,037	0	0	0	0.00	0	0	0	0.00
City of San Fernando	345,857	0	0	0	0.00	0	0	0	0.00
Gapan City	123,009	0	0	0	0.00	0	0	0	0.00
Mabalacat City	282,858	0	0	0	0.00	0	0	0	0.00
Malolos City	283,977	0	0	0	0.00	0	0	0	0.00
Meycauayan City	235,549	0	0	0	0.00	0	0	0	0.00
Olongapo City	256,443	0	0	0	0.00	0	0	0	0.00
Palayan City	45,768	0	0	0	0.00	0	0	0	0.00
San Jose City	155,831	0	0	0	0.00	0	0	0	0.00
San Jose del Monte City	646,756	0	0	0	0.00	0	0	0	0.00
Science City of Munoz	90,867	0	0	0	0.00	0	0	0	0.00
Tarlac City	370,199	0	0	0	0.00	0	0	0	0.00
**Region 4A**	**16,361,107**	**0**	**0**	**0**	**0.00**	**0**	**0**	**0**	**0.00**
Batangas	2,144,028	0	0	0	0.00	0	0	0	0.00
Cavite	1,604,676	0	0	0	0.00	0	0	0	0.00
Laguna	1,111,798	0	0	0	0.00	0	0	0	0.00
Quezon	1,890,167	0	0	0	0.00	0	0	0	0.00
Rizal	2,386,862	0	0	0	0.00	0	0	0	0.00
Antipolo City	879,159	0	0	0	0.00	0	0	0	0.00
Bacoor City	703,029	0	0	0	0.00	0	0	0	0.00
Batangas City	380,515	0	0	0	0.00	0	0	0	0.00
Biñan City	372,804	0	0	0	0.00	0	0	0	0.00
Cabuyao City	345,623	0	0	0	0.00	0	0	0	0.00
Calamba City	508,770	0	0	0	0.00	0	0	0	0.00
Cavite City	120,342	0	0	0	0.00	0	0	0	0.00

Table 1.E.5.2 - Malaria

Age<5 and Age >=5 yrs. old Laboratory Confirmed Malaria Deaths disaggregated by Age Group and Sex
Philippines, 2021

Area	Population	Laboratory Confirmed Malaria Deaths							
		<5yrs Old				>=5 Yrs Old			
		Male	Female	Total	Mortality Rate	Male	Female	Total	Mortality Rate
Dasmariñas City	771,409	0	0	0	0.00	0	0	0	0.00
General Trias City	367,906	0	0	0	0.00	0	0	0	0.00
Imus City	472,646	0	0	0	0.00	0	0	0	0.00
Lipa City	383,404	0	0	0	0.00	0	0	0	0.00
Lucena City	286,460	0	0	0	0.00	0	0	0	0.00
San Pablo City	297,847	0	0	0	0.00	0	0	0	0.00
San Pedro City	364,721	0	0	0	0.00	0	0	0	0.00
Santa Rosa City	396,019	0	0	0	0.00	0	0	0	0.00
Tagaytay City	83,320	0	0	0	0.00	0	0	0	0.00
Tanauan City	199,978	0	0	0	0.00	0	0	0	0.00
Tayabas City	107,355	0	0	0	0.00	0	0	0	0.00
Trece Martires City	182,269	0	0	0	0.00	0	0	0	0.00
**Region 4B**	**3,212,211**	**0**	**0**	**0**	**0.00**	**0**	**2**	**2**	**0.06**
Marinduque	242,420	0	0	0	0.00	0	0	0	0.00
Mindoro Occidental	519,990	0	0	0	0.00	0	0	0	0.00
Mindoro Oriental	911,307	0	0	0	0.00	0	0	0	0.00
Palawan	950,209	0	0	0	0.00	0	2	2	0.21
Romblon	302,917	0	0	0	0.00	0	0	0	0.00
Puerto Princesa City	285,368	0	0	0	0.00	0	0	0	0.00
**Region 5**	**6,192,924**	**0**	**0**	**0**	**0.00**	**0**	**0**	**0**	**0.00**
Albay	1,199,362	0	0	0	0.00	0	0	0	0.00
Camarines Norte	629,402	0	0	0	0.00	0	0	0	0.00
Camarines Sur	1,750,680	0	0	0	0.00	0	0	0	0.00
Catanduanes	275,043	0	0	0	0.00	0	0	0	0.00
Masbate	944,945	0	0	0	0.00	0	0	0	0.00
Sorsogon	855,000	0	0	0	0.00	0	0	0	0.00
Iriga City	118,952	0	0	0	0.00	0	0	0	0.00
Legaspi City	210,918	0	0	0	0.00	0	0	0	0.00
Naga City	208,622	0	0	0	0.00	0	0	0	0.00
**Region 6**	**7,970,551**	**0**	**0**	**0**	**0.00**	**0**	**0**	**0**	**0.00**
Aklan	610,948	0	0	0	0.00	0	0	0	0.00
Antique	617,788	0	0	0	0.00	0	0	0	0.00
Capiz	811,948	0	0	0	0.00	0	0	0	0.00
Guimaras	187,011	0	0	0	0.00	0	0	0	0.00
Iloilo	2,069,653	0	0	0	0.00	0	0	0	0.00
Negros Occidental	2,607,671	0	0	0	0.00	0	0	0	0.00
Bacolod City	586,715	0	0	0	0.00	0	0	0	0.00
Iloilo City	478,817	0	0	0	0.00	0	0	0	0.00
**Region 7**	**8,054,772**	**0**	**0**	**0**	**0.00**	**0**	**0**	**0**	**0.00**
Bohol	1,374,101	0	0	0	0.00	0	0	0	0.00
Cebu	3,276,440	0	0	0	0.00	0	0	0	0.00
Negros Oriental	1,415,196	0	0	0	0.00	0	0	0	0.00

Table 1.E.5.2 - Malaria

Age<5 and Age >=5 yrs. old Laboratory Confirmed Malaria Deaths disaggregated by Age Group and Sex
Philippines, 2021

Area	Population	Laboratory Confirmed Malaria Deaths							
		<5yrs Old				>=5 Yrs Old			
		Male	Female	Total	Mortality Rate	Male	Female	Total	Mortality Rate
Siquijor	101,227	0	0	0	0.00	0	0	0	0.00
Cebu City	1,028,542	0	0	0	0.00	0	0	0	0.00
Lapu-Lapu City	454,972	0	0	0	0.00	0	0	0	0.00
Mandaue City	404,294	0	0	0	0.00	0	0	0	0.00
**Region 8**	**4,803,767**	**0**	**0**	**0**	**0.00**	**0**	**0**	**0**	**0.00**
Biliran	180,717	0	0	0	0.00	0	0	0	0.00
Eastern Samar	511,290	0	0	0	0.00	0	0	0	0.00
Northern Leyte	1,656,424	0	0	0	0.00	0	0	0	0.00
Northern Samar	675,196	0	0	0	0.00	0	0	0	0.00
Southern Leyte	358,038	0	0	0	0.00	0	0	0	0.00
Western Samar	634,040	0	0	0	0.00	0	0	0	0.00
Calbayog City	195,379	0	0	0	0.00	0	0	0	0.00
Maasin City	91,120	0	0	0	0.00	0	0	0	0.00
Ormoc City	235,937	0	0	0	0.00	0	0	0	0.00
Tacloban City	265,626	0	0	0	0.00	0	0	0	0.00
**Region 9**	**3,809,540**	**0**	**0**	**0**	**0.00**	**0**	**0**	**0**	**0.00**
Zamboanga del Norte	831,480	0	0	0	0.00	0	0	0	0.00
Zamboanga del Sur	840,050	0	0	0	0.00	0	0	0	0.00
Zamboanga Sibugay	687,546	0	0	0	0.00	0	0	0	0.00
Dapitan City	85,853	0	0	0	0.00	0	0	0	0.00
Dipolog City	136,208	0	0	0	0.00	0	0	0	0.00
Isabela City	130,380	0	0	0	0.00	0	0	0	0.00
Pagadian City	206,034	0	0	0	0.00	0	0	0	0.00
Zamboanga City	891,989	0	0	0	0.00	0	0	0	0.00
**Region 10**	**5,074,563**	**0**	**0**	**0**	**0.00**	**0**	**0**	**0**	**0.00**
Bukidnon	1,130,220	0	0	0	0.00	0	0	0	0.00
Camiguin	93,118	0	0	0	0.00	0	0	0	0.00
Lanao del Norte	735,928	0	0	0	0.00	0	0	0	0.00
Misamis Occidental	342,462	0	0	0	0.00	0	0	0	0.00
Misamis Oriental	782,065	0	0	0	0.00	0	0	0	0.00
Cagayan de Oro City	740,740	0	0	0	0.00	0	0	0	0.00
El Salvador City	55,016	0	0	0	0.00	0	0	0	0.00
Gingoog City	136,593	0	0	0	0.00	0	0	0	0.00
Iligan City	372,772	0	0	0	0.00	0	0	0	0.00
Malaybalay City	188,395	0	0	0	0.00	0	0	0	0.00
Oroquieta City	74,210	0	0	0	0.00	0	0	0	0.00
Ozamis City	148,747	0	0	0	0.00	0	0	0	0.00
Tangub City	66,085	0	0	0	0.00	0	0	0	0.00
Valencia City	208,212	0	0	0	0.00	0	0	0	0.00
**Region 11**	**5,371,254**	**0**	**0**	**0**	**0.00**	**0**	**0**	**0**	**0.00**
Davao de Oro	780,742	0	0	0	0.00	0	0	0	0.00

Table 1.E.5.2 - Malaria

Age<5 and Age >=5 yrs. old Laboratory Confirmed Malaria Deaths disaggregated by Age Group and Sex
Philippines, 2021

Area	Population	Laboratory Confirmed Malaria Deaths							
		<5yrs Old				>=5 Yrs Old			
		Male	Female	Total	Mortality Rate	Male	Female	Total	Mortality Rate
Davao del Norte	1,063,436	0	0	0	0.00	0	0	0	0.00
Davao del Sur	607,848	0	0	0	0.00	0	0	0	0.00
Davao Oriental	719,362	0	0	0	0.00	0	0	0	0.00
Davao Occidental	342,874	0	0	0	0.00	0	0	0	0.00
Davao City	1,856,992	0	0	0	0.00	0	0	0	0.00
**Region 12**	**4,985,878**	**0**	**0**	**0**	**0.00**	**0**	**1**	**1**	**0.02**
North Cotabato	1,553,081	0	0	0	0.00	0	0	0	0.00
Sarangani	590,746	0	0	0	0.00	0	0	0	0.00
South Cotabato	1,011,240	0	0	0	0.00	0	0	0	0.00
Sultan Kudarat	862,787	0	0	0	0.00	0	1	1	0.12
Cotabato City	311,264	0	0	0	0.00	0	0	0	0.00
Gen. Santos City	656,760	0	0	0	0.00	0	0	0	0.00
**BARMM**	**4,270,193**	**0**	**0**	**0**	**0.00**	**0**	**0**	**0**	**0.00**
Basilan	310,153	0	0	0	0.00	0	0	0	0.00
Lanao del Sur	942,654	0	0	0	0.00	0	0	0	0.00
Maguindanao	1,355,941	0	0	0	0.00	0	0	0	0.00
Sulu	932,940	0	0	0	0.00	0	0	0	0.00
Tawi-Tawi	417,702	0	0	0	0.00	0	0	0	0.00
Lamitan City	85,335	0	0	0	0.00	0	0	0	0.00
Marawi City	225,468	0	0	0	0.00	0	0	0	0.00
**CARAGA**	**2,782,218**	**0**	**0**	**0**	**0.00**	**0**	**0**	**0**	**0.00**
Agusan del Norte	382,946	0	0	0	0.00	0	0	0	0.00
Agusan del Sur	741,035	0	0	0	0.00	0	0	0	0.00
Surigao del Norte	366,639	0	0	0	0.00	0	0	0	0.00
Surigao del Sur	525,627	0	0	0	0.00	0	0	0	0.00
Province of Dinagat	131,274	0	0	0	0.00	0	0	0	0.00
Bislig City	99,839	0	0	0	0.00	0	0	0	0.00
Butuan City	364,103	0	0	0	0.00	0	0	0	0.00
Surigao City	170,755	0	0	0	0.00	0	0	0	0.00

Legend:
0 = No Case
** = No Report*

Table 1.E.5.3 - Malaria

Number of confirmed Malaria Cases and Annual Parasite Incidence
Philippines, 2021

Area	Population at Risk	Confirmed Malaria Cases			Annual Parasite Incidence
		Male	Female	Total	
**PHILIPPINES**	**228,750**	**103**	**40**	**143**	**0.63**
**NCR**	**0**	**0**	**0**	**0**	**0.00**
Malabon City	0	0	0	0	0.00
Navotas City	0	0	0	0	0.00
Valenzuela City	0	0	0	0	0.00
Caloocan City	0	0	0	0	0.00
Marikina City	0	0	0	0	0.00
Pasig City	0	0	0	0	0.00
Pateros	0	0	0	0	0.00
Taguig City	0	0	0	0	0.00
Quezon City	0	0	0	0	0.00
Makati City	0	0	0	0	0.00
Mandaluyong City	0	0	0	0	0.00
San Juan City	0	0	0	0	0.00
Manila City	0	0	0	0	0.00
Las Piñas City	0	0	0	0	0.00
Muntinlupa City	0	0	0	0	0.00
Parañaque City	0	0	0	0	0.00
Pasay City	0	0	0	0	0.00
**CAR**	**0**	**0**	**0**	**0**	**0.00**
Abra	0	0	0	0	0.00
Apayao	0	0	0	0	0.00
Benguet	0	0	0	0	0.00
Ifugao	0	0	0	0	0.00
Kalinga	0	0	0	0	0.00
Mt. Province	0	0	0	0	0.00
Baguio City	0	0	0	0	0.00
**Region 1**	**0**	**0**	**0**	**0**	**0.00**
Ilocos Norte	0	0	0	0	0.00
Ilocos Sur	0	0	0	0	0.00
La Union	0	0	0	0	0.00
Pangasinan	0	0	0	0	0.00
Alaminos City	0	0	0	0	0.00
Candon City	0	0	0	0	0.00
Dagupan City	0	0	0	0	0.00
Laoag City	0	0	0	0	0.00
San Carlos City	0	0	0	0	0.00
San Fernando City	0	0	0	0	0.00
Urdaneta City	0	0	0	0	0.00
Vigan City	0	0	0	0	0.00

Table 1.E.5.3 - Malaria

Number of confirmed Malaria Cases and Annual Parasite Incidence
Philippines, 2021

Area	Population at Risk	Confirmed Malaria Cases			Annual Parasite Incidence
		Male	Female	Total	
**Region 2**	**0**	**0**	**0**	**0**	**0.00**
Batanes	0	0	0	0	0.00
Cagayan	0	0	0	0	0.00
Isabela	0	0	0	0	0.00
Nueva Vizcaya	0	0	0	0	0.00
Quirino	0	0	0	0	0.00
Cauayan City	0	0	0	0	0.00
Ilagan City	0	0	0	0	0.00
Santiago City	0	0	0	0	0.00
Tuguegarao City	0	0	0	0	0.00
**Region 3**	**0**	**0**	**0**	**0**	**0.00**
Aurora	0	0	0	0	0.00
Bataan	0	0	0	0	0.00
Bulacan	0	0	0	0	0.00
Nueva Ecija	0	0	0	0	0.00
Pampanga	0	0	0	0	0.00
Tarlac	0	0	0	0	0.00
Zambales	0	0	0	0	0.00
Angeles City	0	0	0	0	0.00
Balanga City	0	0	0	0	0.00
Cabanatuan City	0	0	0	0	0.00
City of San Fernando	0	0	0	0	0.00
Gapan City	0	0	0	0	0.00
Mabalacat City	0	0	0	0	0.00
Malolos City	0	0	0	0	0.00
Meycauayan City	0	0	0	0	0.00
Olongapo City	0	0	0	0	0.00
Palayan City	0	0	0	0	0.00
San Jose City	0	0	0	0	0.00
San Jose del Monte City	0	0	0	0	0.00
Science City of Munoz	0	0	0	0	0.00
Tarlac City	0	0	0	0	0.00
**Region 4A**	**0**	**0**	**0**	**0**	**0.00**
Batangas	0	0	0	0	0.00
Cavite	0	0	0	0	0.00
Laguna	0	0	0	0	0.00
Quezon	0	0	0	0	0.00
Rizal	0	0	0	0	0.00
Antipolo City	0	0	0	0	0.00
Bacoor City	0	0	0	0	0.00
Batangas City	0	0	0	0	0.00
Biñan City	0	0	0	0	0.00
Cabuyao City	0	0	0	0	0.00

Table 1.E.5.3 - Malaria

Number of confirmed Malaria Cases and Annual Parasite Incidence
Philippines, 2021

Area	Population at Risk	Confirmed Malaria Cases			Annual Parasite Incidence
		Male	Female	Total	
Calamba City	0	0	0	0	0.00
Cavite City	0	0	0	0	0.00
Dasmariñas City	0	0	0	0	0.00
General Trias City	0	0	0	0	0.00
Imus City	0	0	0	0	0.00
Lipa City	0	0	0	0	0.00
Lucena City	0	0	0	0	0.00
San Pablo City	0	0	0	0	0.00
San Pedro City	0	0	0	0	0.00
Santa Rosa City	0	0	0	0	0.00
Tagaytay City	0	0	0	0	0.00
Tanauan City	0	0	0	0	0.00
Tayabas City	0	0	0	0	0.00
Trece Martires City	0	0	0	0	0.00
**Region 4B**	**81,857**	**43**	**20**	**63**	**0.77**
Marinduque	0	0	0	0	0.00
Mindoro Occidental	0	0	0	0	0.00
Mindoro Oriental	0	0	0	0	0.00
Palawan	81,857	43	20	63	0.77
Romblon	0	0	0	0	0.00
Puerto Princesa City	0	0	0	0	0.00
**Region 5**	**0**	**0**	**0**	**0**	**0.00**
Albay	0	0	0	0	0.00
Camarines Norte	0	0	0	0	0.00
Camarines Sur	0	0	0	0	0.00
Catanduanes	0	0	0	0	0.00
Masbate	0	0	0	0	0.00
Sorsogon	0	0	0	0	0.00
Iriga City	0	0	0	0	0.00
Legaspi City	0	0	0	0	0.00
Naga City	0	0	0	0	0.00
**Region 6**	**0**	**0**	**0**	**0**	**0.00**
Aklan	0	0	0	0	0.00
Antique	0	0	0	0	0.00
Capiz	0	0	0	0	0.00
Guimaras	0	0	0	0	0.00
Iloilo	0	0	0	0	0.00
Negros Occidental	0	0	0	0	0.00
Bacolod City	0	0	0	0	0.00
Iloilo City	0	0	0	0	0.00

Table 1.E.5.3 - Malaria

Number of confirmed Malaria Cases and Annual Parasite Incidence
Philippines, 2021

Area	Population at Risk	Confirmed Malaria Cases			Annual Parasite Incidence
		Male	Female	Total	
**Region 7**	**0**	**0**	**0**	**0**	**0.00**
Bohol	0	0	0	0	0.00
Cebu	0	0	0	0	0.00
Negros Oriental	0	0	0	0	0.00
Siquijor	0	0	0	0	0.00
Cebu City	0	0	0	0	0.00
Lapu-Lapu City	0	0	0	0	0.00
Mandaue City	0	0	0	0	0.00
**Region 8**	**0**	**0**	**0**	**0**	**0.00**
Biliran	0	0	0	0	0.00
Eastern Samar	0	0	0	0	0.00
Northern Leyte	0	0	0	0	0.00
Northern Samar	0	0	0	0	0.00
Southern Leyte	0	0	0	0	0.00
Western Samar	0	0	0	0	0.00
Calbayog City	0	0	0	0	0.00
Maasin City	0	0	0	0	0.00
Ormoc City	0	0	0	0	0.00
Tacloban City	0	0	0	0	0.00
**Region 9**	**0**	**0**	**0**	**0**	**0.00**
Zamboanga del Norte	0	0	0	0	0.00
Zamboanga del Sur	0	0	0	0	0.00
Zamboanga Sibugay	0	0	0	0	0.00
Dapitan City	0	0	0	0	0.00
Dipolog City	0	0	0	0	0.00
Isabela City	0	0	0	0	0.00
Pagadian City	0	0	0	0	0.00
Zamboanga City	0	0	0	0	0.00
**Region 10**	**0**	**0**	**0**	**0**	**0.00**
Bukidnon	0	0	0	0	0.00
Camiguin	0	0	0	0	0.00
Lanao del Norte	0	0	0	0	0.00
Misamis Occidental	0	0	0	0	0.00
Misamis Oriental	0	0	0	0	0.00
Cagayan de Oro City	0	0	0	0	0.00
El Salvador City	0	0	0	0	0.00
Gingoog City	0	0	0	0	0.00
Iligan City	0	0	0	0	0.00
Malaybalay City	0	0	0	0	0.00
Oroquieta City	0	0	0	0	0.00
Ozamis City	0	0	0	0	0.00

Table 1.E.5.3 - Malaria

Number of confirmed Malaria Cases and Annual Parasite Incidence
Philippines, 2021

Area	Population at Risk	Confirmed Malaria Cases			Annual Parasite Incidence
		Male	Female	Total	
Tangub City	0	0	0	0	0.00
Valencia City	0	0	0	0	0.00
**Region 11**	**0**	**0**	**0**	**0**	**0.00**
Davao de Oro	0	0	0	0	0.00
Davao del Norte	0	0	0	0	0.00
Davao del Sur	0	0	0	0	0.00
Davao Oriental	0	0	0	0	0.00
Davao Occidental	0	0	0	0	0.00
Davao City	0	0	0	0	0.00
**Region 12**	**146,893**	**60**	**20**	**80**	**0.54**
North Cotabato	0	0	0	0	0.00
Sarangani	0	0	0	0	0.00
South Cotabato	0	0	0	0	0.00
Sultan Kudarat	146,893	50	20	70	0.48
Cotabato City	0	0	0	0	0.00
Gen. Santos City	0	10	0	10	0.00
**BARMM**	**0**	**0**	**0**	**0**	**0.00**
Basilan	0	0	0	0	0.00
Lanao del Sur	0	0	0	0	0.00
Maguindanao	0	0	0	0	0.00
Sulu	0	0	0	0	0.00
Tawi-Tawi	0	0	0	0	0.00
Lamitan City	0	0	0	0	0.00
Marawi City	0	0	0	0	0.00
**CARAGA**	**0**	**0**	**0**	**0**	**0.00**
Agusan del Norte	0	0	0	0	0.00
Agusan del Sur	0	0	0	0	0.00
Surigao del Norte	0	0	0	0	0.00
Surigao del Sur	0	0	0	0	0.00
Province of Dinagat	0	0	0	0	0.00
Bislig City	0	0	0	0	0.00
Butuan City	0	0	0	0	0.00
Surigao City	0	0	0	0	0.00

Legend:
0 = No Case
** = No Report*

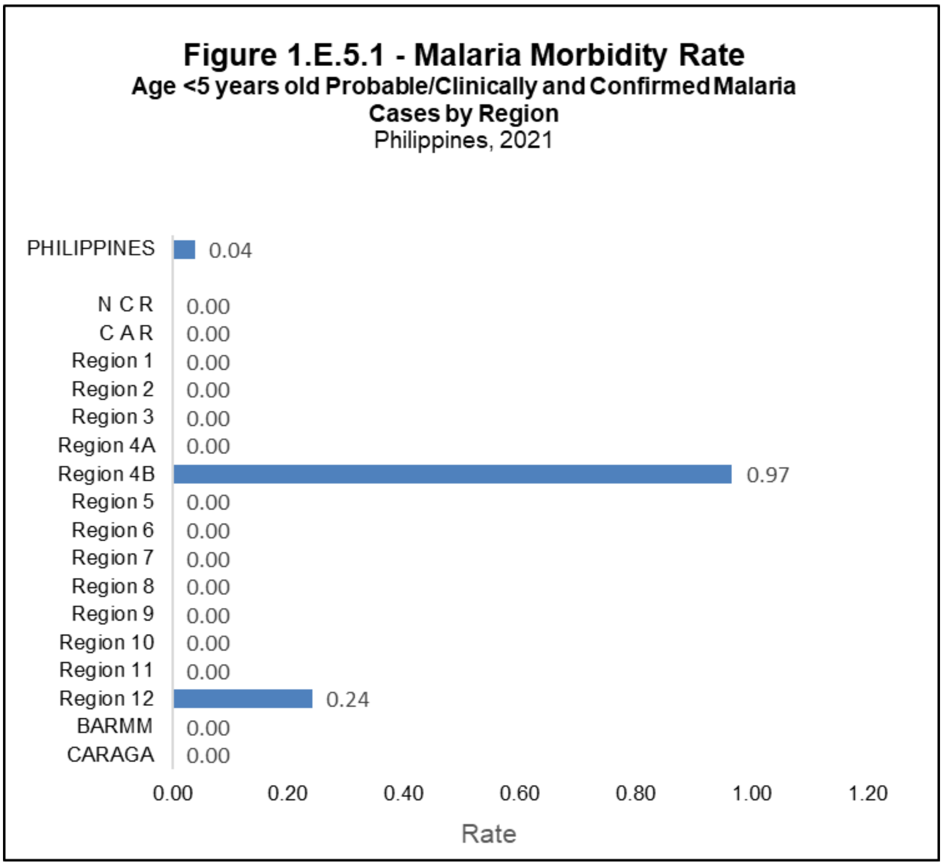

Figure 1.E.5.1 - Malaria Morbidity Rate
Age <5 years old Probable/Clinically and Confirmed Malaria Cases by Region
Philippines, 2021
PHILIPPINES
0.04
N C R
C A R
Region 1
Region 2
Region 3
Region 4A
Region 4B
Region 5
Region 6
Region 7
Region 8
Region 9
Region 10
Region 11
Region 12
BARMM
CARAGA
0.00
0.97
0.24
0.00
0.20
0.40
0.60
0.80
1.00
1.20
Rate

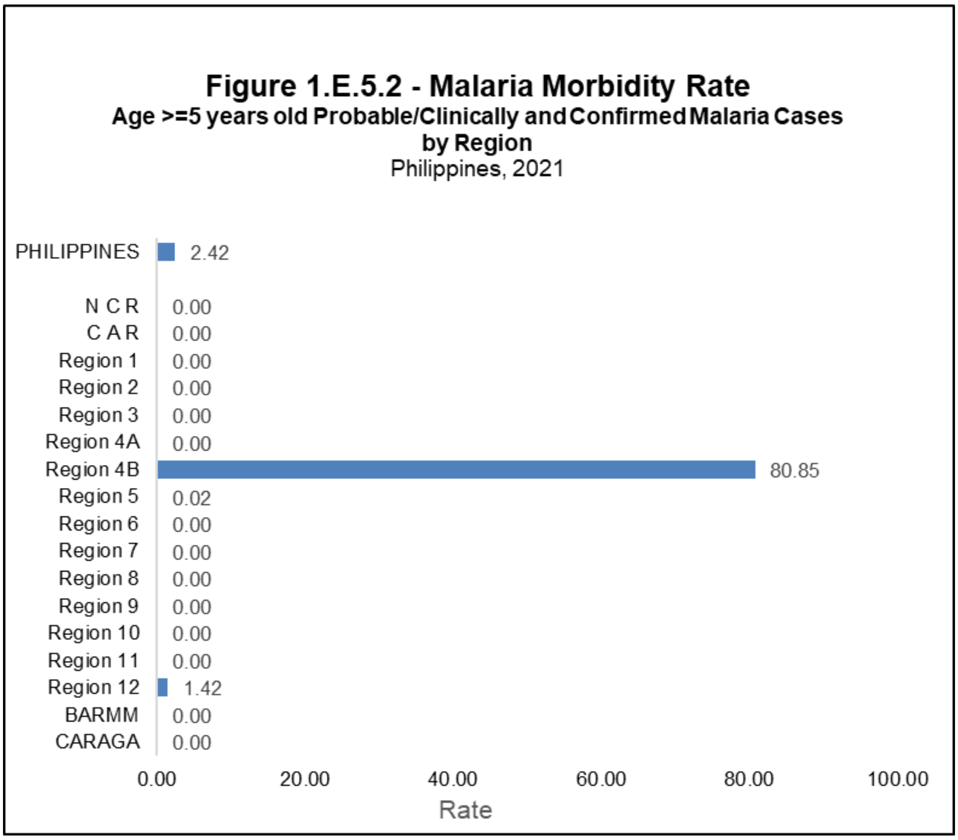

Figure 1.E.5.2 - Malaria Morbidity Rate
Age >=5 years old Probable/Clinically and Confirmed Malaria Cases by Region
Philippines, 2021
PHILIPPINES
2.42
N C R
C A R
Region 1
Region 2
Region 3
Region 4A
Region 4B
Region 5
Region 6
Region 7
Region 8
Region 9
Region 10
Region 11
Region 12
BARMM
CARAGA
0.00
80.85
0.02
1.42
0.00
20.00
40.00
60.00
80.00
100.00
Rate

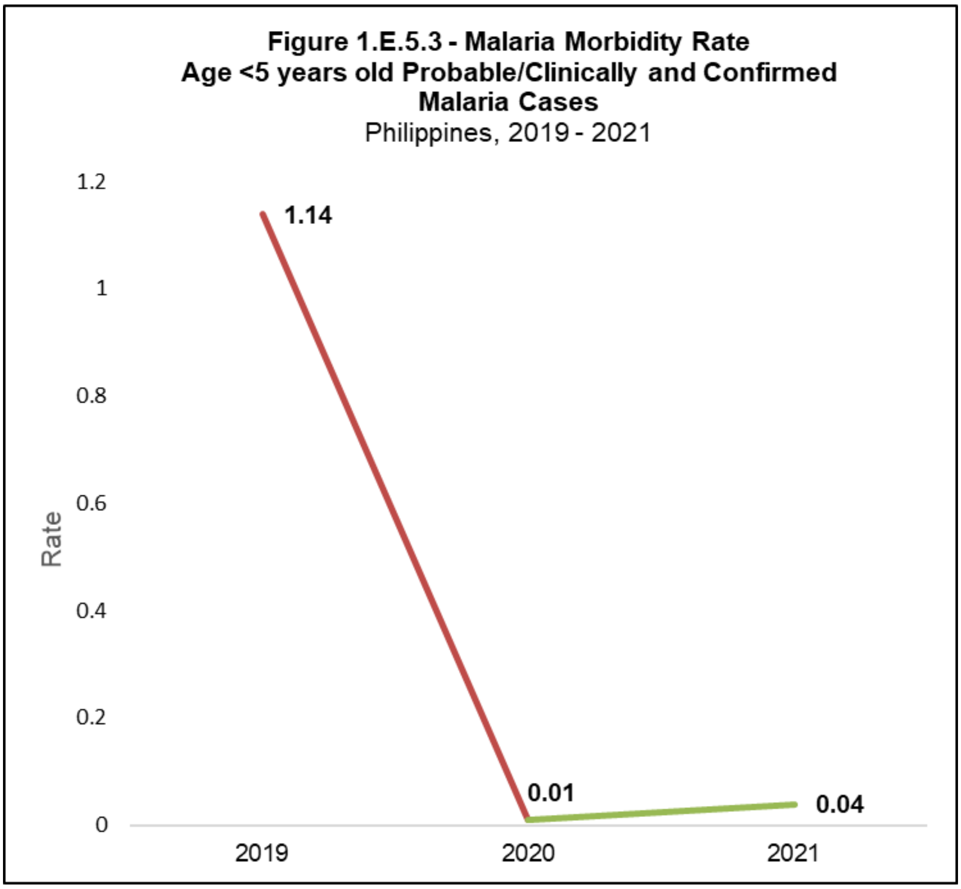

Figure 1.E.5.3 - Malaria Morbidity Rate
Age <5 years old Probable/Clinically and Confirmed Malaria Cases
Philippines, 2019 - 2021
Rate
1.2
1
0.8
0.6
0.4
0.2
0
1.14
0.01
0.04
2019
2020
2021

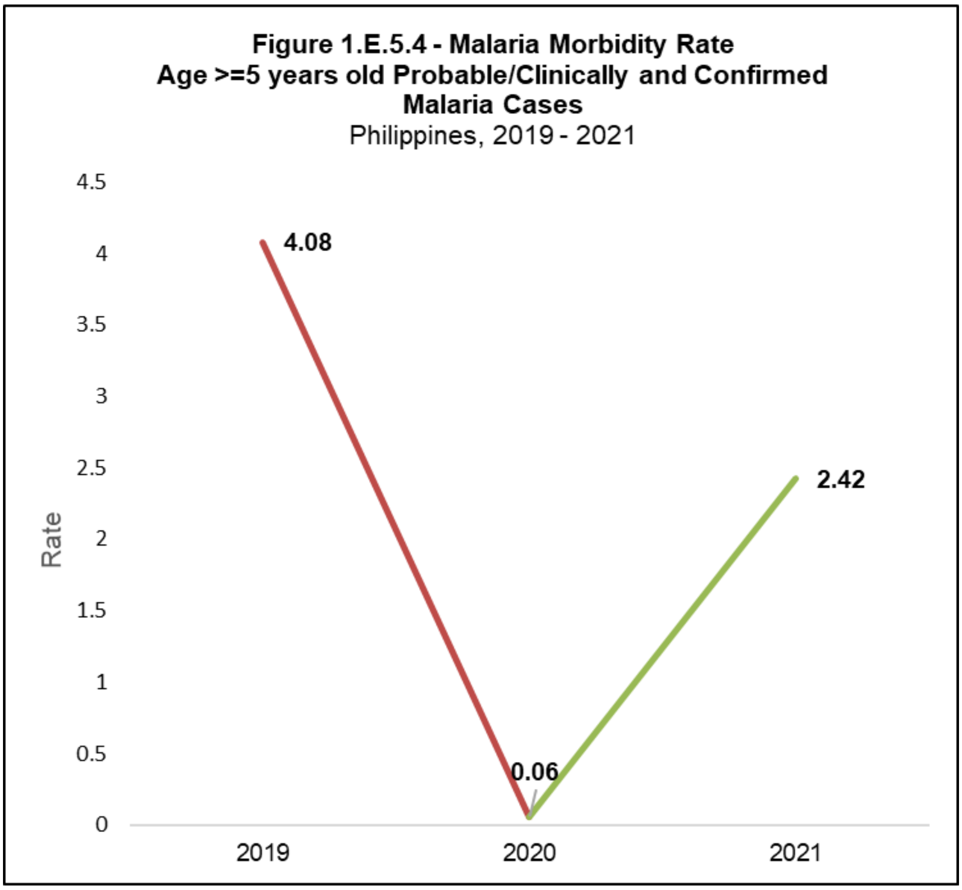

Figure 1.E.5.4 - Malaria Morbidity Rate
Age >=5 years old Probable/Clinically and Confirmed Malaria Cases
Philippines, 2019 - 2021
Rate
4.5
4
3.5
3
2.5
2
1.5
1
0.5
0
4.08
0.06
2.42
2019
2020
2021

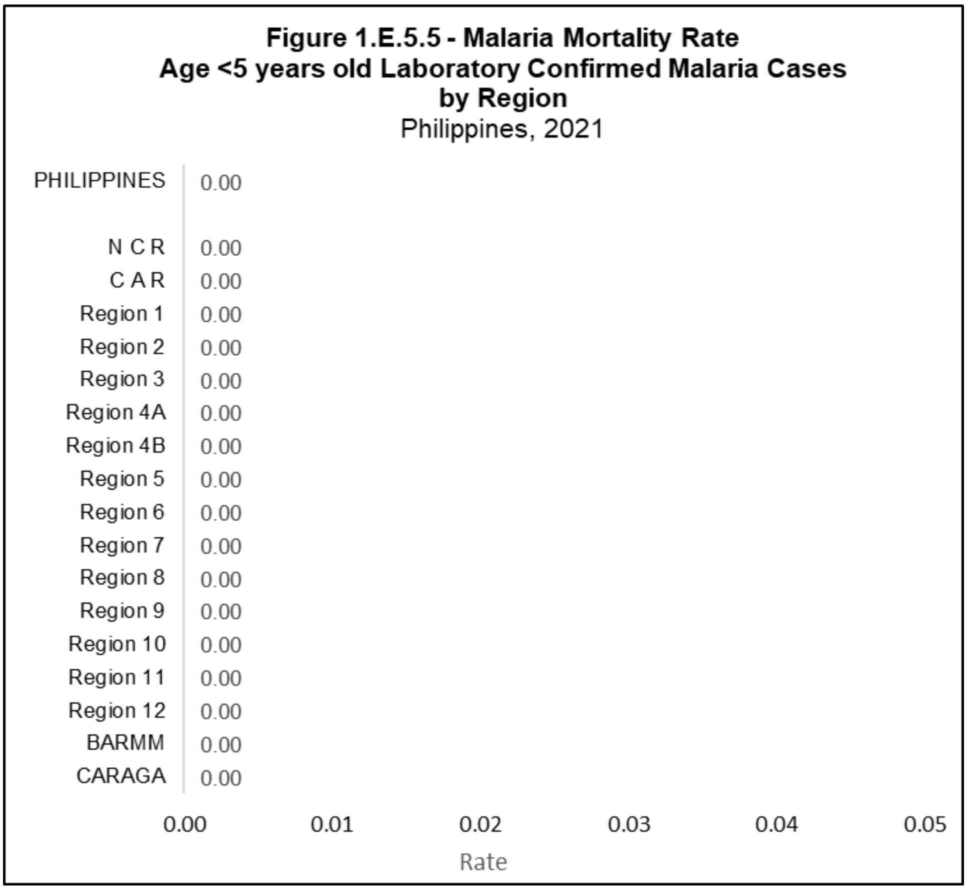
Figure 1.E.5.5 - Malaria Mortality Rate
Age <5 years old Laboratory Confirmed Malaria Cases
by Region
Philippines, 2021
PHILIPPINES 0.00
N C R 0.00
C A R 0.00
Region 1 0.00
Region 2 0.00
Region 3 0.00
Region 4A 0.00
Region 4B 0.00
Region 5 0.00
Region 6 0.00
Region 7 0.00
Region 8 0.00
Region 9 0.00
Region 10 0.00
Region 11 0.00
Region 12 0.00
BARMM 0.00
CARAGA 0.00
0.00 0.01 0.02 0.03 0.04 0.05
Rate

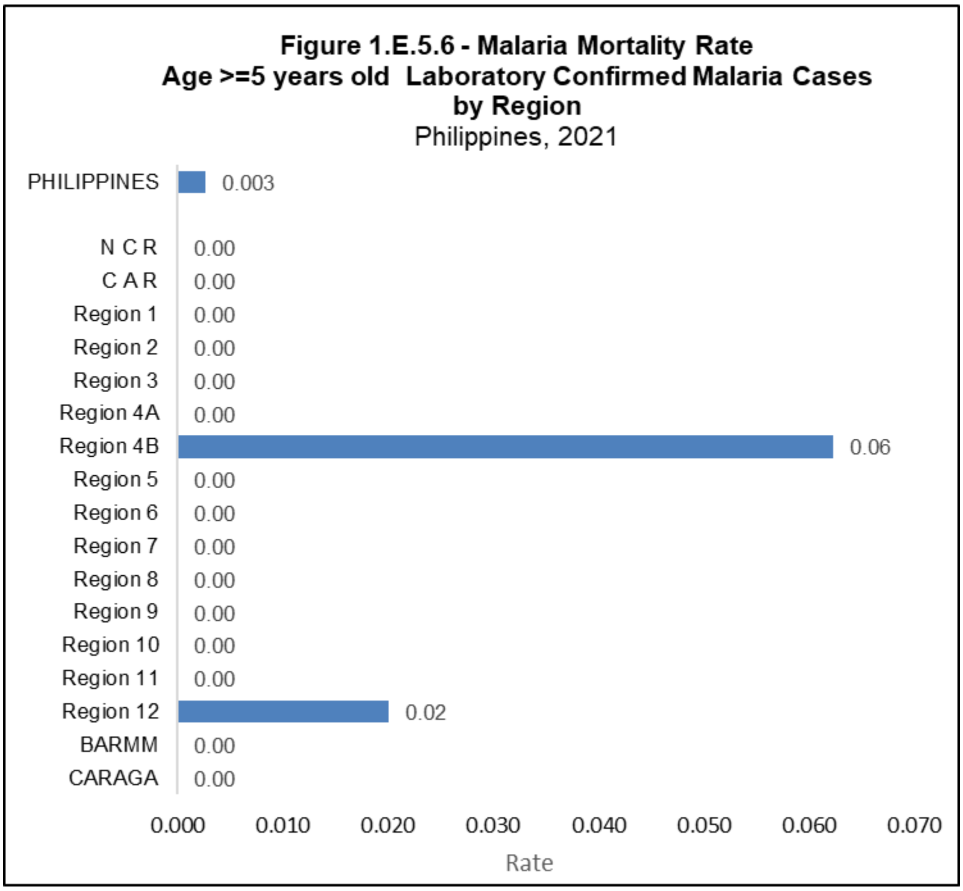
Figure 1.E.5.6 - Malaria Mortality Rate
Age >=5 years old Laboratory Confirmed Malaria Cases
by Region
Philippines, 2021
PHILIPPINES 0.003
N C R 0.00
C A R 0.00
Region 1 0.00
Region 2 0.00
Region 3 0.00
Region 4A 0.00
Region 4B 0.06
Region 5 0.00
Region 6 0.00
Region 7 0.00
Region 8 0.00
Region 9 0.00
Region 10 0.00
Region 11 0.00
Region 12 0.02
BARMM 0.00
CARAGA 0.00
0.000 0.010 0.020 0.030 0.040 0.050 0.060 0.070
Rate

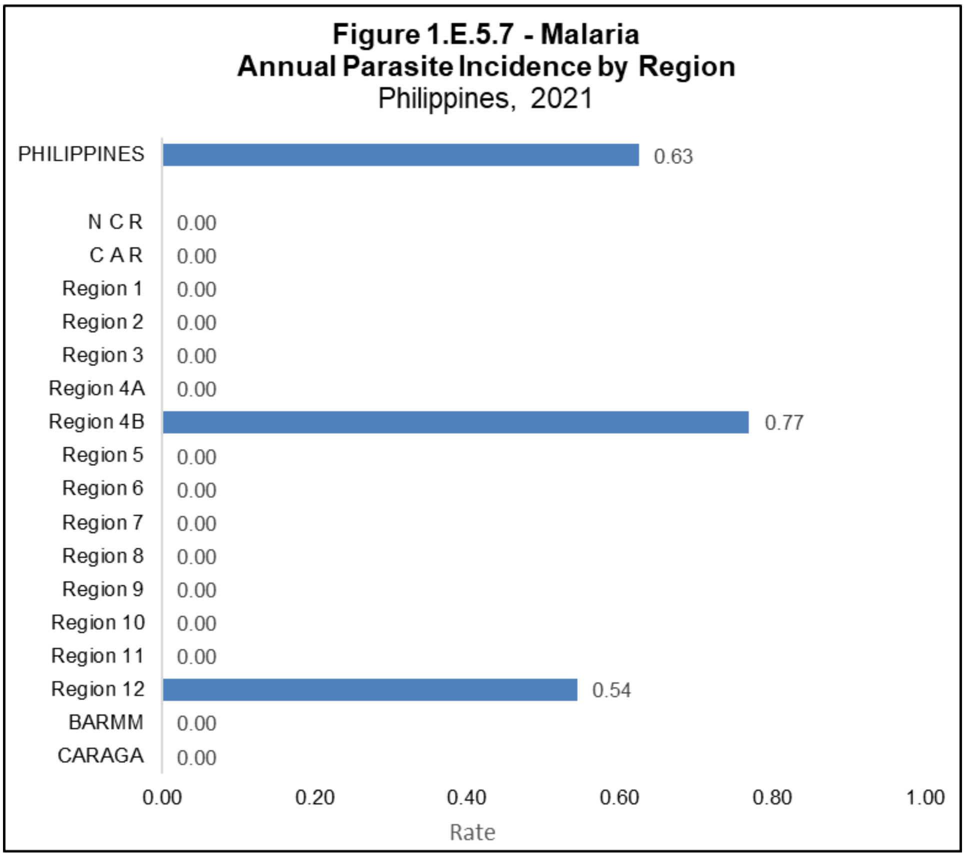

Figure 1.E.5.7 - Malaria
Annual Parasite Incidence by Region
Philippines, 2021
PHILIPPINES
0.63
N C R
C A R
Region 1
Region 2
Region 3
Region 4A
Region 4B
0.77
Region 5
Region 6
Region 7
Region 8
Region 9
Region 10
Region 11
Region 12
0.54
BARMM
CARAGA
0.00
0.00
0.20
0.40
0.60
0.80
1.00
Rate

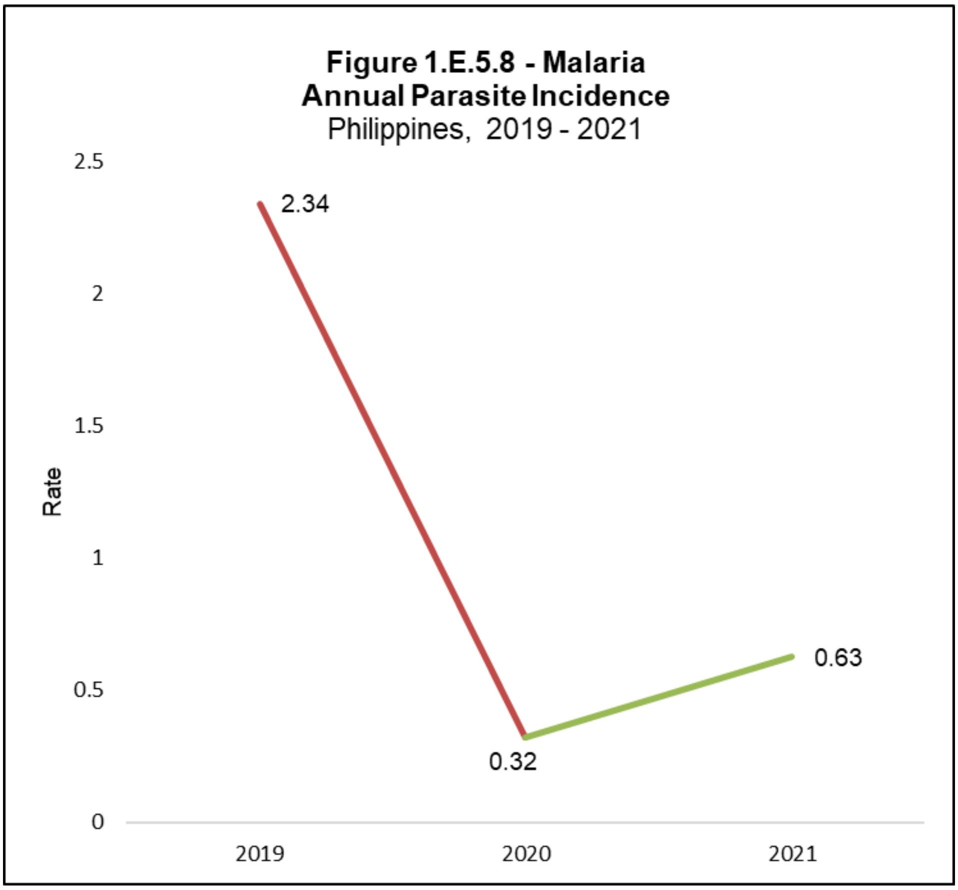

Figure 1.E.5.8 - Malaria
Annual Parasite Incidence
Philippines, 2019 - 2021
Rate
2.5
2
1.5
1
0.5
0
2.34
0.32
0.63
2019
2020
2021

Table 1.E.6 - Leprosy

Prevalence and Detection Rate

Philippines, 2021

Area	Population	Leprosy Cases undergoing Treatment				Newly Detected Case			
		Male	Female	Total	Prevalence Rate	Male	Female	Total	Case Detection Rate
**PHILIPPINES**	**110,198,654**	**309**	**97**	**406**	**0.04**	**393**	**130**	**523**	**0.47**
**N C R**	**13,966,223**	**4**	**4**	**8**	**0.01**	**1**	**2**	**3**	**0.02**
Malabon City	396,434	0	0	0	0.00	0	0	0	0.00
Navotas City	270,559	0	0	0	0.00	0	0	0	0.00
Valenzuela City	672,888	1	1	2	0.03	1	1	2	0.30
Caloocan City	1,717,928	0	0	0	0.00	0	0	0	0.00
Marikina City	488,857	0	0	0	0.00	0	0	0	0.00
Pasig City	819,171	1	0	1	0.01	0	0	0	0.00
Pateros	69,239	0	0	0	0.00	0	0	0	0.00
Taguig City	872,980	2	0	2	0.02	0	0	0	0.00
Quezon City	3,184,407	0	0	0	0.00	0	0	0	0.00
Makati City	631,867	0	3	3	0.05	0	1	1	0.16
Mandaluyong City	418,942	0	0	0	0.00	0	0	0	0.00
San Juan City	132,512	0	0	0	0.00	0	0	0	0.00
Manila City	1,930,696	0	0	0	0.00	0	0	0	0.00
Las Piñas City	638,694	0	0	0	0.00	0	0	0	0.00
Muntinlupa City	547,173	0	0	0	0.00	0	0	0	0.00
Parañaque City	722,126	0	0	0	0.00	0	0	0	0.00
Pasay City	451,750	0	0	0	0.00	0	0	0	0.00
**C A R**	**1,822,719**	**11**	**5**	**16**	**0.09**	**5**	**2**	**7**	**0.38**
Abra	248,468	7	2	9	0.36	2	0	2	0.80
Apayao	124,728	0	0	0	0.00	0	0	0	0.00
Benguet	483,934	0	1	1	0.02	1	0	1	0.21
Ifugao	212,063	2	0	2	0.09	1	0	1	0.47
Kalinga	221,563	0	0	0	0.00	0	0	0	0.00
Mt. Province	157,413	0	1	1	0.06	0	0	0	0.00
Baguio City	374,550	2	1	3	0.08	1	2	3	0.80
**Region 1**	**5,313,223**	**44**	**22**	**66**	**0.12**	**45**	**17**	**62**	**1.17**
Ilocos Norte	501,276	0	0	0	0.00	8	1	9	1.80
Ilocos Sur	600,674	7	1	8	0.13	6	1	7	1.17
La Union	705,988	2	3	5	0.07	4	1	5	0.71
Pangasinan	2,522,027	24	12	36	0.14	21	12	33	1.31
Alaminos City	95,291	0	0	0	0.00	0	0	0	0.00
Candon City	63,312	4	0	4	0.63	0	0	0	0.00
Dagupan City	181,929	0	0	0	0.00	1	0	1	0.55
Laoag City	115,582	0	0	0	0.00	0	0	0	0.00
San Carlos City	200,310	2	1	3	0.15	3	0	3	1.50
San Fernando City	129,350	1	1	2	0.15	1	1	2	1.55
Urdaneta City	141,215	4	4	8	0.57	1	1	2	1.42
Vigan City	56,269	0	0	0	0.00	0	0	0	0.00

Table 1.E.6 - Leprosy
Prevalence and Detection Rate
Philippines, 2021

Area	Population	Leprosy Cases undergoing Treatment				Newly Detected Case			
		Male	Female	Total	Prevalence Rate	Male	Female	Total	Case Detection Rate
**Region 2**	**3,693,664**	**10**	**2**	**12**	**0.03**	**11**	**2**	**13**	**0.35**
Batanes	18,013	0	0	0	0.00	1	0	1	5.55
Cagayan	1,121,561	1	1	2	0.02	0	1	1	0.09
Isabela	1,264,006	3	0	3	0.02	7	0	7	0.55
Nueva Vizcaya	488,238	0	0	0	0.00	1	0	1	0.20
Quirino	199,473	2	0	2	0.10	1	0	1	0.50
Cauayan City	138,317	3	1	4	0.29	1	1	2	1.45
Ilagan City	155,452	0	0	0	0.00	0	0	0	0.00
Santiago City	143,982	1	0	1	0.07	0	0	0	0.00
Tuguegarao City	164,622	0	0	0	0.00	0	0	0	0.00
**Region 3**	**12,513,847**	**5**	**0**	**5**	**0.00**	**11**	**2**	**13**	**0.10**
Aurora	229,841	0	0	0	0.00	0	0	0	0.00
Bataan	742,197	0	0	0	0.00	0	1	1	0.13
Bulacan	2,542,502	1	0	1	0.00	2	1	3	0.12
Nueva Ecija	1,646,738	0	0	0	0.00	0	0	0	0.00
Pampanga	1,850,369	1	0	1	0.01	0	0	0	0.00
Tarlac	1,106,339	0	0	0	0.00	0	0	0	0.00
Zambales	650,180	0	0	0	0.00	0	0	0	0.00
Angeles City	464,253	0	0	0	0.00	0	0	0	0.00
Balanga City	107,277	0	0	0	0.00	0	0	0	0.00
Cabanatuan City	337,037	0	0	0	0.00	5	0	5	1.48
City of San Fernando	345,857	0	0	0	0.00	0	0	0	0.00
Gapan City	123,009	0	0	0	0.00	0	0	0	0.00
Mabalacat City	282,858	0	0	0	0.00	0	0	0	0.00
Malolos City	283,977	0	0	0	0.00	0	0	0	0.00
Meycauayan City	235,549	0	0	0	0.00	0	0	0	0.00
Olongapo City	256,443	0	0	0	0.00	0	0	0	0.00
Palayan City	45,768	0	0	0	0.00	0	0	0	0.00
San Jose City	155,831	3	0	3	0.19	3	0	3	1.93
San Jose del Monte City	646,756	0	0	0	0.00	0	0	0	0.00
Science City of Munoz	90,867	0	0	0	0.00	0	0	0	0.00
Tarlac City	370,199	0	0	0	0.00	1	0	1	0.27
**Region 4A**	**16,361,107**	**16**	**4**	**20**	**0.01**	**17**	**7**	**24**	**0.15**
Batangas	2,144,028	3	0	3	0.01	6	0	6	0.28
Cavite	1,604,676	0	0	0	0.00	1	0	1	0.06
Laguna	1,111,798	0	0	0	0.00	2	1	3	0.27
Quezon	1,890,167	10	3	13	0.07	3	1	4	0.21
Rizal	2,386,862	3	0	3	0.01	1	0	1	0.04
Antipolo City	879,159	0	0	0	0.00	0	0	0	0.00
Bacoor City	703,029	0	0	0	0.00	0	0	0	0.00
Batangas City	380,515	0	0	0	0.00	0	0	0	0.00
Biñan City	372,804	0	0	0	0.00	2	0	2	0.54

Table 1.E.6 - Leprosy
Prevalence and Detection Rate
Philippines, 2021

Area	Population	Leprosy Cases undergoing Treatment				Newly Detected Case			
		Male	Female	Total	Prevalence Rate	Male	Female	Total	Case Detection Rate
Cabuyao City	345,623	0	0	0	0.00	0	0	0	0.00
Calamba City	508,770	0	0	0	0.00	0	2	2	0.39
Cavite City	120,342	*	*	0	0.00	0	0	0	0.00
Dasmariñas City	771,409	0	0	0	0.00	0	0	0	0.00
General Trias City	367,906	0	0	0	0.00	0	0	0	0.00
Imus City	472,646	0	0	0	0.00	0	0	0	0.00
Lipa City	383,404	0	0	0	0.00	0	0	0	0.00
Lucena City	286,460	0	1	1	0.03	2	3	5	1.75
San Pablo City	297,847	0	0	0	0.00	0	0	0	0.00
San Pedro City	364,721	0	0	0	0.00	0	0	0	0.00
Santa Rosa City	396,019	0	0	0	0.00	0	0	0	0.00
Tagaytay City	83,320	0	0	0	0.00	0	0	0	0.00
Tanauan City	199,978	0	0	0	0.00	0	0	0	0.00
Tayabas City	107,355	0	0	0	0.00	0	0	0	0.00
Trece Martires City	182,269	0	0	0	0.00	0	0	0	0.00
**Region 4B**	**3,212,211**	**3**	**0**	**3**	**0.01**	**4**	**2**	**6**	**0.19**
Marinduque	242,420	0	0	0	0.00	0	0	0	0.00
Mindoro Occidental	519,990	1	0	1	0.02	1	1	2	0.38
Mindoro Oriental	911,307	0	0	0	0.00	0	0	0	0.00
Palawan	950,209	2	0	2	0.02	3	1	4	0.42
Romblon	302,917	0	0	0	0.00	0	0	0	0.00
Puerto Princesa City	285,368	0	0	0	0.00	0	0	0	0.00
**Region 5**	**6,192,924**	**10**	**6**	**16**	**0.03**	**6**	**2**	**8**	**0.13**
Albay	1,199,362	2	1	3	0.03	0	0	0	0.00
Camarines Norte	629,402	3	0	3	0.05	2	0	2	0.32
Camarines Sur	1,750,680	*	*	0	0.00	0	0	0	0.00
Catanduanes	275,043	0	0	0	0.00	0	0	0	0.00
Masbate	944,945	2	3	5	0.05	3	1	4	0.42
Sorsogon	855,000	3	2	5	0.06	1	1	2	0.23
Iriga City	118,952	0	0	0	0.00	0	0	0	0.00
Legaspi City	210,918	0	0	0	0.00	0	0	0	0.00
Naga City	208,622	0	0	0	0.00	0	0	0	0.00
**Region 6**	**7,970,551**	**31**	**11**	**42**	**0.05**	**18**	**4**	**22**	**0.28**
Aklan	610,948	0	0	0	0.00	0	0	0	0.00
Antique	617,788	0	0	0	0.00	3	0	3	0.49
Capiz	811,948	3	1	4	0.05	0	1	1	0.12
Guimaras	187,011	0	0	0	0.00	0	0	0	0.00
Iloilo	2,069,653	1	0	1	0.00	1	0	1	0.05
Negros Occidental	2,607,671	9	3	12	0.05	11	2	13	0.50
Bacolod City	586,715	18	7	25	0.43	3	1	4	0.68
Iloilo City	478,817	0	0	0	0.00	0	0	0	0.00

Table 1.E.6 - Leprosy
Prevalence and Detection Rate
Philippines, 2021

Area	Population	Leprosy Cases undergoing Treatment				Newly Detected Case			
		Male	Female	Total	Prevalence Rate	Male	Female	Total	Case Detection Rate
**Region 7**	**8,054,772**	**10**	**4**	**14**	**0.02**	**9**	**4**	**13**	**0.16**
Bohol	1,374,101	0	0	0	0.00	0	0	0	0.00
Cebu	3,276,440	0	0	0	0.00	0	0	0	0.00
Negros Oriental	1,415,196	0	0	0	0.00	3	0	3	0.21
Siquijor	101,227	5	0	5	0.49	0	0	0	0.00
Cebu City	1,028,542	0	0	0	0.00	0	0	0	0.00
Lapu-Lapu City	454,972	0	0	0	0.00	6	4	10	2.20
Mandaue City	404,294	5	4	9	0.22	0	0	0	0.00
**Region 8**	**4,803,767**	**67**	**10**	**77**	**0.16**	**133**	**31**	**164**	**3.41**
Biliran	180,717	0	0	0	0.00	5	0	5	2.77
Eastern Samar	511,290	4	0	4	0.08	24	1	25	4.89
Northern Leyte	1,656,424	11	0	11	0.07	38	12	50	3.02
Northern Samar	675,196	26	8	34	0.50	20	4	24	3.55
Southern Leyte	358,038	13	2	15	0.42	2	0	2	0.56
Western Samar	634,040	0	0	0	0.00	15	11	26	4.10
Calbayog City	195,379	0	0	0	0.00	14	0	14	7.17
Maasin City	91,120	9	0	9	0.99	2	0	2	2.19
Ormoc City	235,937	2	0	2	0.08	3	0	3	1.27
Tacloban City	265,626	2	0	2	0.08	10	3	13	4.89
**Region 9**	**3,809,540**	**0**	**0**	**0**	**0.00**	**0**	**0**	**0**	**0.00**
Zamboanga del Norte	831,480	0	0	0	0.00	0	0	0	0.00
Zamboanga del Sur	840,050	0	0	0	0.00	0	0	0	0.00
Zamboanga Sibugay	687,546	0	0	0	0.00	0	0	0	0.00
Dapitan City	85,853	0	0	0	0.00	0	0	0	0.00
Dipolog City	136,208	0	0	0	0.00	0	0	0	0.00
Isabela City	130,380	0	0	0	0.00	0	0	0	0.00
Pagadian City	206,034	0	0	0	0.00	0	0	0	0.00
Zamboanga City	891,989	0	0	0	0.00	0	0	0	0.00
**Region 10**	**5,074,563**	**43**	**21**	**64**	**0.13**	**63**	**17**	**80**	**1.58**
Bukidnon	1,130,220	0	0	0	0.00	22	4	26	2.30
Camiguin	93,118	18	10	28	3.01	2	0	2	2.15
Lanao del Norte	735,928	0	0	0	0.00	0	2	2	0.27
Misamis Occidental	342,462	5	0	5	0.15	2	1	3	0.88
Misamis Oriental	782,065	1	0	1	0.01	16	2	18	2.30
Cagayan de Oro City	740,740	0	0	0	0.00	5	4	9	1.22
El Salvador City	55,016	11	7	18	3.27	0	0	0	0.00
Gingoog City	136,593	0	0	0	0.00	0	0	0	0.00
Iligan City	372,772	0	0	0	0.00	8	2	10	2.68
Malaybalay City	188,395	6	4	10	0.53	2	0	2	1.06
Oroquieta City	74,210	2	0	2	0.27	0	0	0	0.00
Ozamis City	148,747	0	0	0	0.00	1	0	1	0.67
Tangub City	66,085	0	0	0	0.00	0	0	0	0.00
Valencia City	208,212	0	0	0	0.00	5	2	7	3.36

Table 1.E.6 - Leprosy
Prevalence and Detection Rate
Philippines, 2021

Area	Population	Leprosy Cases undergoing Treatment				Newly Detected Case			
		Male	Female	Total	Prevalence Rate	Male	Female	Total	Case Detection Rate
**Region 11**	**5,371,254**	**36**	**0**	**36**	**0.07**	**20**	**6**	**26**	**0.48**
Davao de Oro	780,742	0	0	0	0.00	0	0	0	0.00
Davao del Norte	1,063,436	10	0	10	0.09	3	0	3	0.28
Davao del Sur	607,848	14	0	14	0.23	6	2	8	1.32
Davao Oriental	719,362	6	0	6	0.08	1	2	3	0.42
Davao Occidental	342,874	6	0	6	0.17	8	1	9	2.62
Davao City	1,856,992	0	0	0	0.00	2	1	3	0.16
**Region 12**	**4,985,878**	**14**	**6**	**20**	**0.04**	**38**	**29**	**67**	**1.34**
North Cotabato	1,553,081	0	0	0	0.00	14	13	27	1.74
Sarangani	590,746	11	4	15	0.25	9	8	17	2.88
South Cotabato	1,011,240	2	2	4	0.04	2	0	2	0.20
Sultan Kudarat	862,787	1	0	1	0.01	3	3	6	0.70
Cotabato City	311,264	0	0	0	0.00	0	0	0	0.00
Gen. Santos City	656,760	0	0	0	0.00	10	5	15	2.28
**BARMM**	**4,270,193**	**3**	**0**	**3**	**0.01**	**2**	**1**	**3**	**0.07**
Basilan	310,153	0	0	0	0.00	0	0	0	0.00
Lanao del Sur	942,654	0	0	0	0.00	0	0	0	0.00
Maguindanao	1,355,941	0	0	0	0.00	1	0	1	0.07
Sulu	932,940	3	0	3	0.03	0	0	0	0.00
Tawi-Tawi	417,702	0	0	0	0.00	0	0	0	0.00
Lamitan City	85,335	0	0	0	0.00	1	1	2	2.34
Marawi City	225,468	0	0	0	0.00	0	0	0	0.00
**CARAGA**	**2,782,218**	**2**	**2**	**4**	**0.01**	**10**	**2**	**12**	**0.43**
Agusan del Norte	382,946	0	0	0	0.00	2	1	3	0.78
Agusan del Sur	741,035	0	1	1	0.01	1	0	1	0.13
Surigao del Norte	366,639	2	1	3	0.08	2	0	2	0.55
Surigao del Sur	525,627	0	0	0	0.00	0	0	0	0.00
Province of Dinagat	131,274	0	0	0	0.00	0	0	0	0.00
Bislig City	99,839	0	0	0	0.00	0	0	0	0.00
Butuan City	364,103	0	0	0	0.00	0	0	0	0.00
Surigao City	170,755	0	0	0	0.00	5	1	6	3.51

Legend:
0 = No Case
** = No Report*

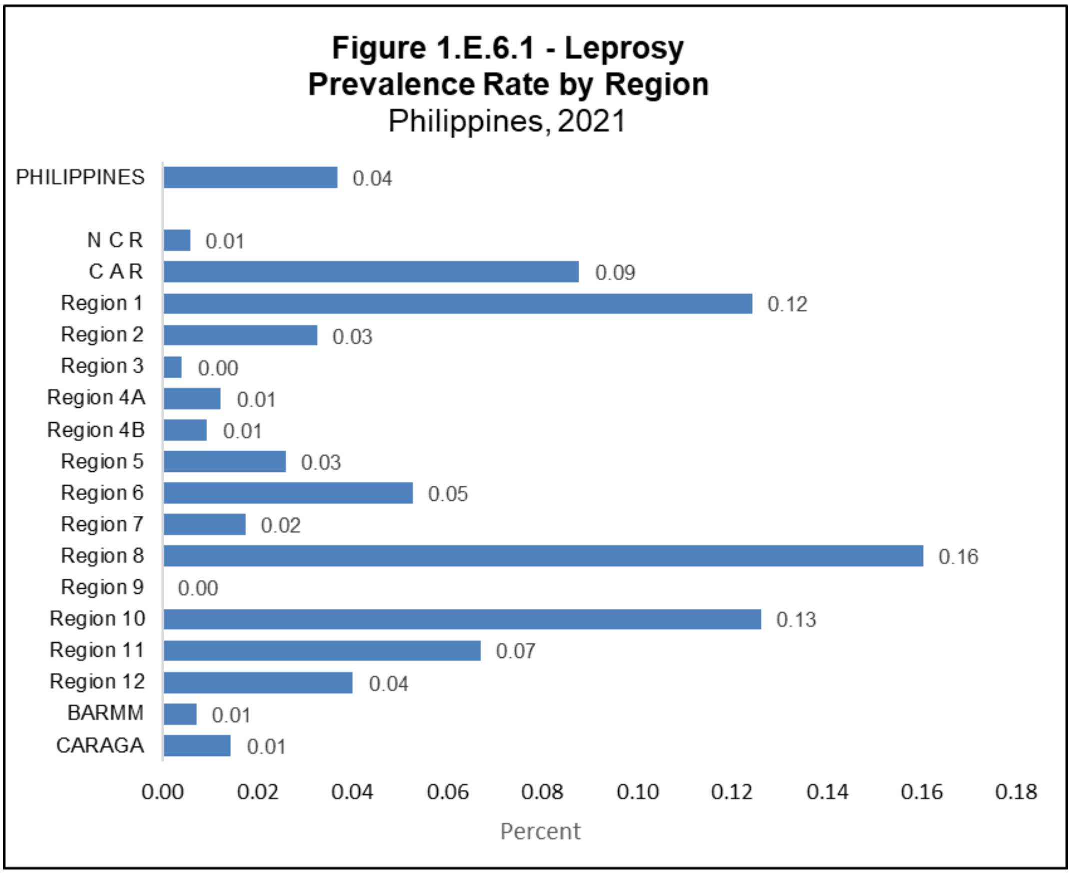
Figure 1.E.6.1 - Leprosy
Prevalence Rate by Region
Philippines, 2021
PHILIPPINES 0.04
N C R 0.01
C A R 0.09
Region 1 0.12
Region 2 0.03
Region 3 0.00
Region 4A 0.01
Region 4B 0.01
Region 5 0.03
Region 6 0.05
Region 7 0.02
Region 8 0.16
Region 9 0.00
Region 10 0.13
Region 11 0.07
Region 12 0.04
BARMM 0.01
CARAGA 0.01
0.00 0.02 0.04 0.06 0.08 0.10 0.12 0.14 0.16 0.18
Percent

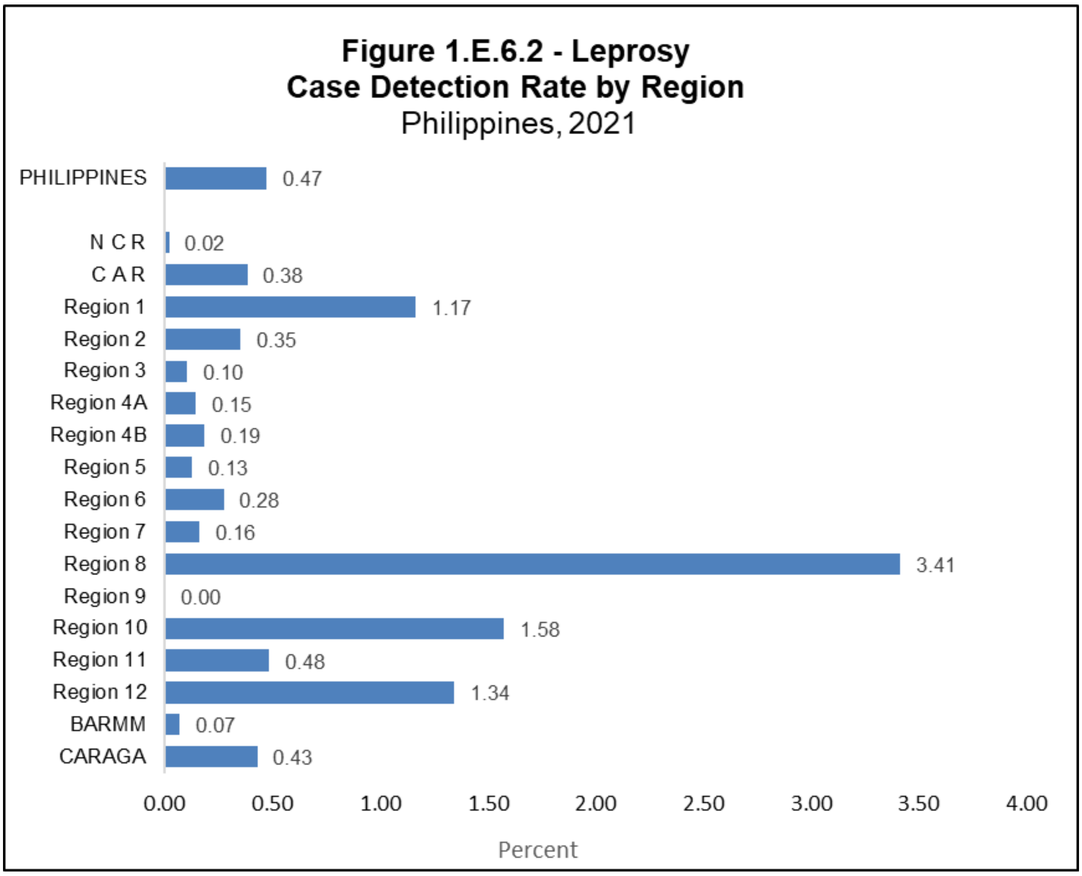
Figure 1.E.6.2 - Leprosy
Case Detection Rate by Region
Philippines, 2021
PHILIPPINES 0.47
N C R 0.02
C A R 0.38
Region 1 1.17
Region 2 0.35
Region 3 0.10
Region 4A 0.15
Region 4B 0.19
Region 5 0.13
Region 6 0.28
Region 7 0.16
Region 8 3.41
Region 9 0.00
Region 10 1.58
Region 11 0.48
Region 12 1.34
BARMM 0.07
CARAGA 0.43
0.00 0.50 1.00 1.50 2.00 2.50 3.00 3.50 4.00
Percent

Figure 1.E.6.3 - Leprosy
Case Detection Rate by Year
Philippines, 2019 - 2021

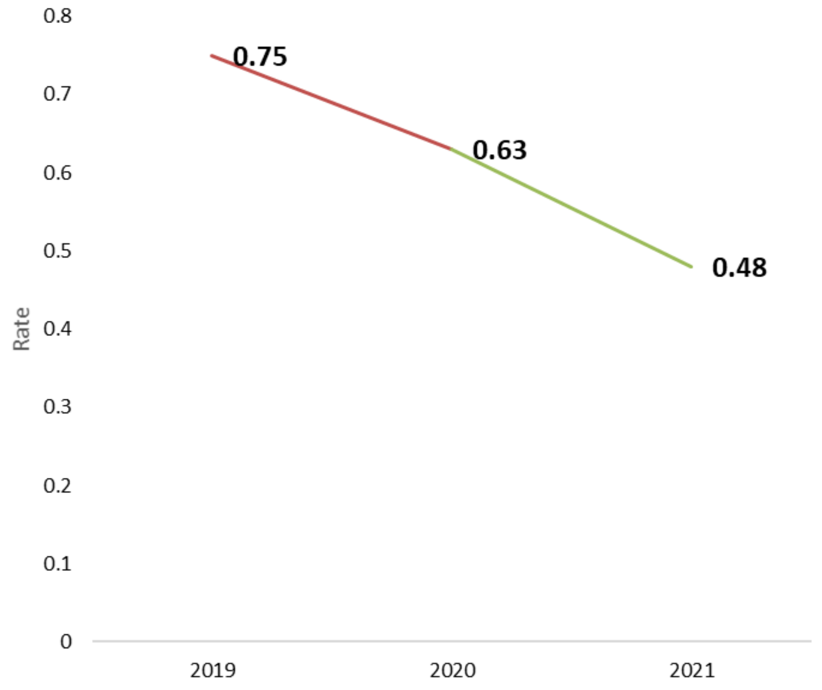

Figure 1.E.6.4 - Leprosy
Prevalence Rate by Year
Philippines, 2019 - 2021

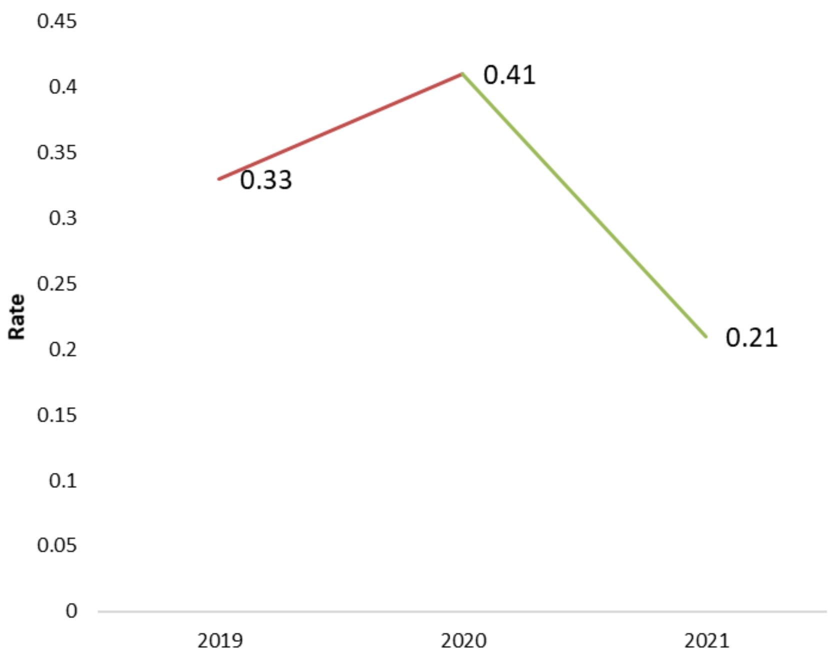

Table 1.E.7 - Rabies

Number and Proportion of Deaths due to Rabies
Philippines, 2021

Area	Animal Bites			No. of Deaths due to Rabies			Rate
	Male	Female	Total	Male	Female	Total	
**PHILIPPINES**	**346,965**	**360,380**	**707,345**	**161**	**69**	**230**	**0.03**
**N C R**	**78,903**	**87,403**	**166,306**	**6**	**1**	**7**	**0.00**
Malabon City	2,911	3,303	6,214	0	0	0	0.00
Navotas City	1,688	1,938	3,626	0	0	0	0.00
Valenzuela City	2,083	2,285	4,368	0	0	0	0.00
Caloocan City	5,458	5,566	11,024	2	0	2	0.02
Marikina City	4,301	4,436	8,737	0	0	0	0.00
Pasig City	10,524	12,424	22,948	0	0	0	0.00
Pateros	883	978	1,861	0	0	0	0.00
Taguig City	9,349	9,522	18,871	0	0	0	0.00
Quezon City	19,047	21,191	40,238	3	0	3	0.01
Makati City	1,721	2,379	4,100	0	0	0	0.00
Mandaluyong City	2,667	3,305	5,972	0	0	0	0.00
San Juan City	1,317	1,535	2,852	0	0	0	0.00
Manila City	4,034	4,446	8,480	0	1	1	0.01
Las Piñas City	2,320	2,378	4,698	0	0	0	0.00
Muntinlupa City	6,722	7,394	14,116	1	0	1	0.01
Parañaque City	1,745	1,961	3,706	0	0	0	0.00
Pasay City	2,133	2,362	4,495	0	0	0	0.00
**C A R**	**16,600**	**15,121**	**31,721**	**0**	**1**	**1**	**0.00**
Abra	1,718	1,551	3,269	0	0	0	0.00
Apayao	1,328	1,293	2,621	0	1	1	0.04
Benguet	2,385	2,034	4,419	0	0	0	0.00
Ifugao	1,869	1,575	3,444	0	0	0	0.00
Kalinga	1,422	1,256	2,678	0	0	0	0.00
Mt. Province	1,284	1,131	2,415	0	0	0	0.00
Baguio City	6,594	6,281	12,875	0	0	0	0.00
**Region 1**	**14,250**	**15,471**	**29,721**	**8**	**1**	**9**	**0.03**
Ilocos Norte	1,223	1,294	2,517	0	0	0	0.00
Ilocos Sur	2,249	2,307	4,556	1	0	1	0.02
La Union	1,349	1,441	2,790	0	0	0	0.00
Pangasinan	3,324	3,614	6,938	6	1	7	0.10
Alaminos City	211	233	444	0	0	0	0.00
Candon City	63	87	150	0	0	0	0.00
Dagupan City	2,769	2,845	5,614	0	0	0	0.00
Laoag City	516	678	1,194	0	0	0	0.00
San Carlos City	1,522	1,658	3,180	0	0	0	0.00
San Fernando City	39	59	98	1	0	1	1.02
Urdaneta City	0	0	0	0	0	0	0.00
Vigan City	985	1,255	2,240	0	0	0	0.00

Table 1.E.7 - Rabies

Number and Proportion of Deaths due to Rabies
Philippines, 2021

Area	Animal Bites			No. of Deaths due to Rabies			Rate
	Male	Female	Total	Male	Female	Total	
**Region 2**	**22,809**	**22,695**	**45,504**	**10**	**4**	**14**	**0.03**
Batanes	1	1	2	0	0	0	0.00
Cagayan	2,707	2,716	5,423	0	0	0	0.00
Isabela	12,544	12,217	24,761	9	4	13	0.05
Nueva Vizcaya	4,283	4,766	9,049	1	0	1	0.01
Quirino	36	43	79	0	0	0	0.00
Cauayan City	739	525	1,264	0	0	0	0.00
Ilagan City	938	886	1,824	0	0	0	0.00
Santiago City	1,561	1,541	3,102	0	0	0	0.00
Tuguegarao City	0	0	0	0	0	0	0.00
**Region 3**	**27,633**	**30,089**	**57,722**	**13**	**5**	**18**	**0.03**
Aurora	304	328	632	1	0	1	0.16
Bataan	2,227	2,291	4,518	2	0	2	0.04
Bulacan	1,956	1,987	3,943	0	0	0	0.00
Nueva Ecija	6,687	7,464	14,151	6	4	10	0.07
Pampanga	403	416	819	0	0	0	0.00
Tarlac	0	0	0	0	0	0	0.00
Zambales	2,669	2,770	5,439	2	1	3	0.06
Angeles City	1,138	1,169	2,307	0	0	0	0.00
Balanga City	985	1,134	2,119	0	0	0	0.00
Cabanatuan City	2,006	2,140	4,146	0	0	0	0.00
City of San Fernando	528	536	1,064	0	0	0	0.00
Gapan City	58	37	95	0	0	0	0.00
Mabalacat City	0	0	0	0	0	0	0.00
Malolos City	0	0	0	0	0	0	0.00
Meycauayan City	382	501	883	0	0	0	0.00
Olongapo City	900	991	1,891	0	0	0	0.00
Palayan City	30	21	51	0	0	0	0.00
San Jose City	1,857	1,928	3,785	0	0	0	0.00
San Jose del Monte City	1,571	1,667	3,238	1	0	1	0.03
Science City of Munoz	906	1,025	1,931	1	0	1	0.05
Tarlac City	3,026	3,684	6,710	0	0	0	0.00
**Region 4A**	**34,687**	**36,777**	**71,464**	**20**	**14**	**34**	**0.05**
Batangas	2,552	2,682	5,234	0	3	3	0.06
Cavite	3,908	4,365	8,273	0	0	0	0.00
Laguna	4,528	5,039	9,567	7	1	8	0.08
Quezon	2,746	2,858	5,604	4	4	8	0.14
Rizal	8,344	8,523	16,867	7	5	12	0.07
Antipolo City	0	0	0	0	0	0	0.00
Bacoor City	729	807	1,536	0	0	0	0.00
Batangas City	37	28	65	2	1	3	4.62
Biñan City	1,337	1,408	2,745	0	0	0	0.00

Table 1.E.7 - Rabies

Number and Proportion of Deaths due to Rabies
Philippines, 2021

Area	Animal Bites			No. of Deaths due to Rabies			Rate
	Male	Female	Total	Male	Female	Total	
Cabuyao City	0	0	0	0	0	0	0.00
Calamba City	2,924	3,265	6,189	0	0	0	0.00
Cavite City	0	0	0	0	0	0	0.00
Dasmariñas City	21	17	38	0	0	0	0.00
General Trias City	51	58	109	0	0	0	0.00
Imus City	193	229	422	0	0	0	0.00
Lipa City	138	141	279	0	0	0	0.00
Lucena City	2,396	2,408	4,804	0	0	0	0.00
San Pablo City	464	421	885	0	0	0	0.00
San Pedro City	0	0	0	0	0	0	0.00
Santa Rosa City	1,927	2,162	4,089	0	0	0	0.00
Tagaytay City	0	0	0	0	0	0	0.00
Tanauan City	1,945	2,031	3,976	0	0	0	0.00
Tayabas City	0	0	0	0	0	0	0.00
Trece Martires City	447	335	782	0	0	0	0.00
**Region 4B**	**6,243**	**6,181**	**12,424**	**3**	**4**	**7**	**0.06**
Marinduque	525	645	1,170	0	0	0	0.00
Mindoro Occidental	3,568	3,558	7,126	0	0	0	0.00
Mindoro Oriental	558	487	1,045	0	1	1	0.10
Palawan	1,498	1,387	2,885	3	3	6	0.21
Romblon	94	104	198	0	0	0	0.00
Puerto Princesa City	0	0	0	0	0	0	0.00
**Region 5**	**8,987**	**9,583**	**18,570**	**23**	**7**	**30**	**0.16**
Albay	1,738	1,791	3,529	3	0	3	0.09
Camarines Norte	535	546	1,081	2	1	3	0.28
Camarines Sur	309	345	654	7	3	10	1.53
Catanduanes	1,028	1,107	2,135	0	0	0	0.00
Masbate	133	120	253	5	0	5	1.98
Sorsogon	3,114	3,358	6,472	4	3	7	0.11
Iriga City	613	662	1,275	0	0	0	0.00
Legaspi City	75	54	129	1	0	1	0.78
Naga City	1,442	1,600	3,042	1	0	1	0.03
**Region 6**	**58,362**	**60,962**	**119,324**	**24**	**3**	**27**	**0.02**
Aklan	4,453	4,408	8,861	4	1	5	0.06
Antique	4,046	4,137	8,183	0	0	0	0.00
Capiz	6,998	7,709	14,707	5	0	5	0.03
Guimaras	804	841	1,645	0	0	0	0.00
Iloilo	19,314	20,869	40,183	10	2	12	0.03
Negros Occidental	11,031	10,628	21,659	4	0	4	0.02
Bacolod City	4,674	4,945	9,619	0	0	0	0.00
Bago City	7,042	7,425	14,467	1	0	1	0.01

Table 1.E.7 - Rabies

Number and Proportion of Deaths due to Rabies
Philippines, 2021

Area	Animal Bites			No. of Deaths due to Rabies			Rate
	Male	Female	Total	Male	Female	Total	
**Region 7**	**4,108**	**4,700**	**8,808**	**1**	**0**	**1**	**0.01**
Bohol	0	0	0	0	0	0	0.00
Cebu	0	0	0	0	0	0	0.00
Negros Oriental	2,165	2,117	4,282	1	0	1	0.02
Siquijor	0	0	0	0	0	0	0.00
Cebu City	687	669	1,356	0	0	0	0.00
Lapu-Lapu City	1,256	1,914	3,170	0	0	0	0.00
Mandaue City	0	0	0	0	0	0	0.00
**Region 8**	**18,614**	**17,411**	**36,025**	**4**	**0**	**4**	**0.01**
Biliran	530	484	1,014	0	0	0	0.00
Eastern Samar	2,288	2,107	4,395	2	0	2	0.05
Northern Leyte	2,657	2,459	5,116	0	0	0	0.00
Northern Samar	4,591	4,585	9,176	0	0	0	0.00
Southern Leyte	1,636	1,539	3,175	0	0	0	0.00
Western Samar	1,452	1,277	2,729	0	0	0	0.00
Calbayog City	1,194	1,112	2,306	0	0	0	0.00
Maasin City	863	861	1,724	0	0	0	0.00
Ormoc City	1,721	1,580	3,301	2	0	2	0.06
Tacloban City	1,682	1,407	3,089	0	0	0	0.00
**Region 9**	**3,219**	**3,343**	**6,562**	**11**	**9**	**20**	**0.30**
							0.00
Zamboanga del Norte	0	0	0	0	0	0	0.00
Zamboanga del Sur	7	8	15	0	0	0	0.00
Zamboanga Sibugay	3212	3335	6,547	11	9	20	0.31
Dapitan City	0	0	0	0	0	0	0.00
Dipolog City	0	0	0	0	0	0	0.00
Isabela City	0	0	0	0	0	0	0.00
Pagadian City	0	0	0	0	0	0	0.00
Zamboanga City	0	0	0	0	0	0	0.00
**Region 10**	**19,951**	**18,961**	**38,912**	**8**	**3**	**11**	**0.03**
Bukidnon	2,427	2,333	4,760	7	2	9	0.19
Camiguin	400	404	804	0	0	0	0.00
Lanao del Norte	1,103	946	2,049	1	0	1	0.05
Misamis Occidental	78	82	160	0	0	0	0.00
Misamis Oriental	1,102	1,071	2,173	0	0	0	0.00
Cagayan de Oro City	5,618	5,550	11,168	0	1	1	0.01
El Salvador City	445	361	806	0	0	0	0.00
Gingoog City	1,764	1,523	3,287	0	0	0	0.00
Iligan City	2,211	2,114	4,325	0	0	0	0.00
Malaybalay City	1,733	1,651	3,384	0	0	0	0.00
Oroquieta City	916	896	1,812	0	0	0	0.00

Table 1.E.7 - Rabies

Number and Proportion of Deaths due to Rabies
Philippines, 2021

Area	Animal Bites			No. of Deaths due to Rabies			Rate
	Male	Female	Total	Male	Female	Total	
Ozamis City	439	336	775	0	0	0	0.00
Tangub City	51	51	102	0	0	0	0.00
Valencia City	1,664	1,643	3,307	0	0	0	0.00
**Region 11**	**11,598**	**11,362**	**22,960**	**13**	**10**	**23**	**0.10**
Davao de Oro	1,061	946	2,007	1	1	2	0.10
Davao del Norte	3,755	3,635	7,390	1	1	2	0.03
Davao del Sur	719	683	1,402	3	1	4	0.29
Davao Oriental	1,446	1,466	2,912	1	2	3	0.10
Davao Occidental	240	259	499	4	3	7	1.40
Davao City	4,377	4,373	8,750	3	2	5	0.06
**Region 12**	**13,906**	**13,496**	**27,402**	**13**	**7**	**20**	**0.07**
North Cotabato	2,220	2,364	4,584	3	1	4	0.09
Sarangani	545	474	1,019	4	0	4	0.39
South Cotabato	4,227	4,074	8,301	2	2	4	0.05
Sultan Kudarat	5,888	5,569	11,457	0	0	0	0.00
Cotabato City	0	0	0	0	0	0	0.00
Gen. Santos City	1,026	1,015	2,041	4	4	8	0.39
**BARMM**	**538**	**414**	**952**	**1**	**0**	**1**	**0.11**
Basilan	0	0	0	0	0	0	0.00
Lanao del Sur	160	150	310	0	0	0	0.00
Maguindanao	360	242	602	0	0	0	0.00
Sulu	0	0	0	0	0	0	0.00
Tawi-Tawi	0	0	0	0	0	0	0.00
Lamitan City	0	0	0	0	0	0	0.00
Marawi City	18	22	40	1	0	1	2.50
**CARAGA**	**6,557**	**6,411**	**12,968**	**3**	**0**	**3**	**0.02**
Agusan del Norte	239	229	468	2	0	2	0.43
Agusan del Sur	130	139	269	0	0	0	0.00
Surigao del Norte	1,754	1,808	3,562	0	0	0	0.00
Surigao del Sur	2,210	2,127	4,337	0	0	0	0.00
Province of Dinagat	215	209	424	0	0	0	0.00
Bislig City	497	490	987	0	0	0	0.00
Butuan City	217	174	391	1	0	1	0.26
Surigao City	1,295	1,235	2,530	0	0	0	0.00

Legend:
0 = No Case
** = No Report*

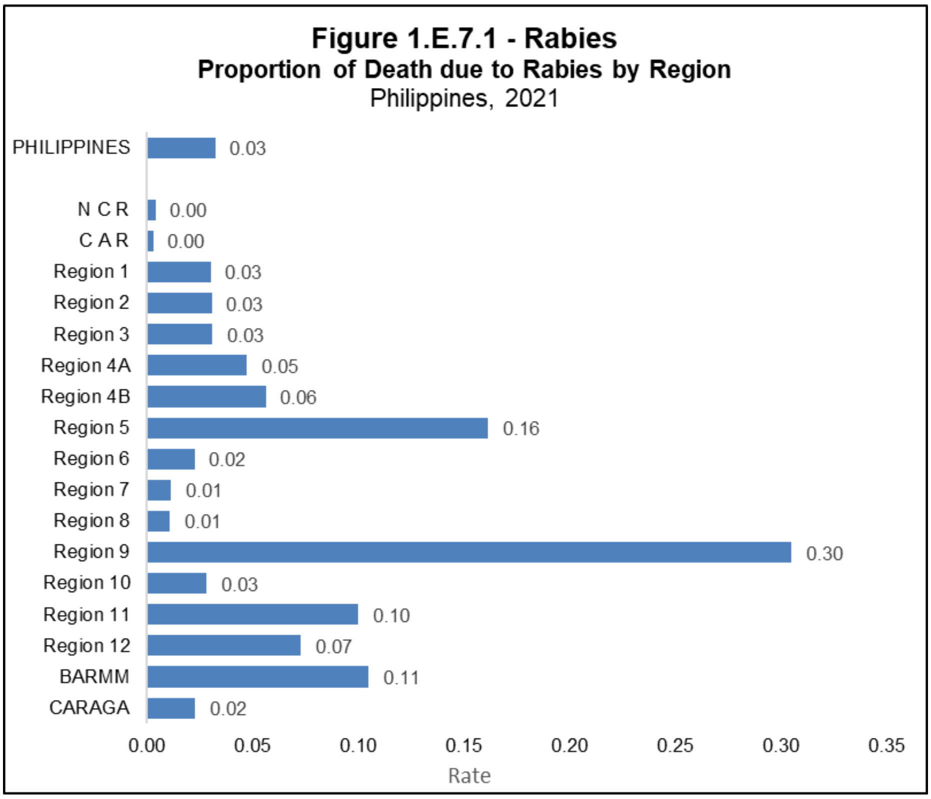
Figure 1.E.7.1 - Rabies
Proportion of Death due to Rabies by Region
Philippines, 2021
PHILIPPINES 0.03
N C R 0.00
C A R 0.00
Region 1 0.03
Region 2 0.03
Region 3 0.03
Region 4A 0.05
Region 4B 0.06
Region 5 0.16
Region 6 0.02
Region 7 0.01
Region 8 0.01
Region 9 0.30
Region 10 0.03
Region 11 0.10
Region 12 0.07
BARMM 0.11
CARAGA 0.02
0.00 0.05 0.10 0.15 0.20 0.25 0.30 0.35
Rate

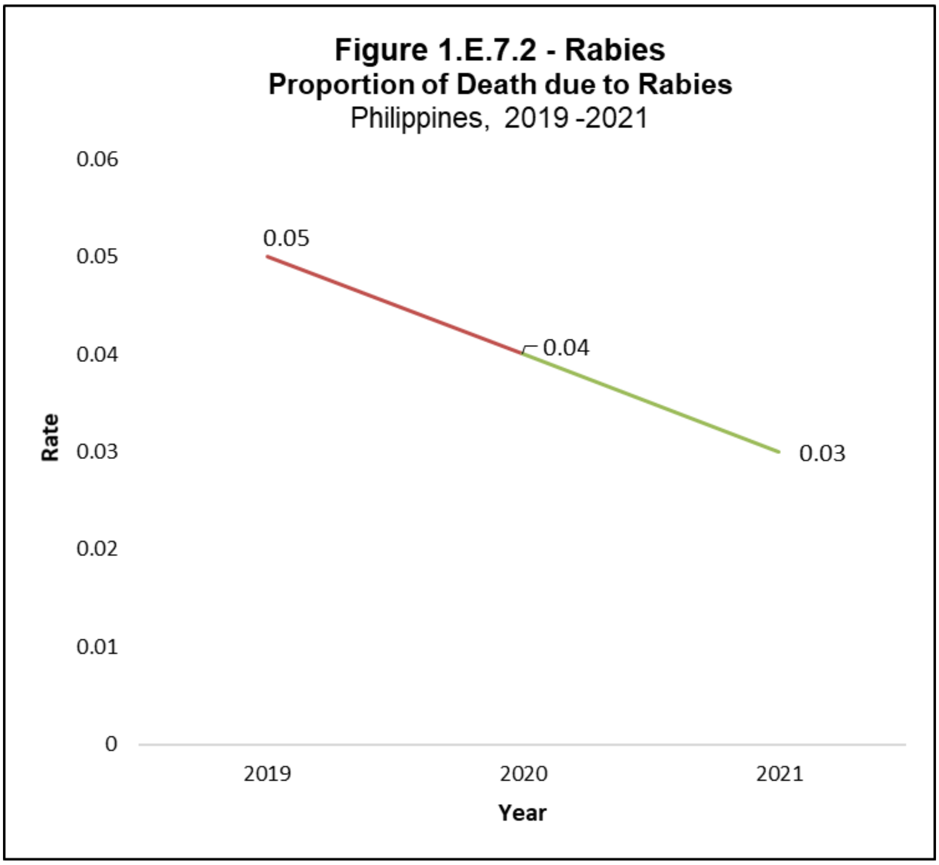
Figure 1.E.7.2 - Rabies
Proportion of Death due to Rabies
Philippines, 2019 -2021
Rate
0.06
0.05
0.04
0.03
0.02
0.01
0
0.05
0.04
0.03
2019
2020
2021
Year

Table 1.E.8.1 - Tuberculosis
Case Notification Rate (All forms)
Philippines, 2021

Area	Total Population	Notified TB cases all forms			CNR (All Forms)
		Male	Female	Total	
**PHILIPPINES**	**110,198,654**	**212,508**	**109,055**	**321,563**	**291.80**
**N C R**	**13,966,223**	**36,935**	**21,047**	**57,982**	**415.16**
Malabon City	396,434	860	505	1,365	344.32
Navotas City	270,559	1,048	589	1,637	605.04
Valenzuela City	672,888	2,248	1,214	3,462	514.50
Caloocan City	1,717,928	4,072	2,250	6,322	368.00
Marikina City	488,857	1,625	870	2,495	510.37
Pasig City	819,171	1,914	1,169	3,083	376.36
Pateros	69,239	76	23	99	142.98
Taguig City	872,980	2,577	1,448	4,025	461.06
Quezon City	3,184,407	7,484	4,323	11,807	370.78
Makati City	631,867	1,483	1,131	2,614	413.69
Mandaluyong City	418,942	1,386	562	1,948	464.98
San Juan City	132,512	524	279	803	605.98
Manila City	1,930,696	6,811	4,354	11,165	578.29
Las Piñas City	638,694	1,069	576	1,645	257.56
Muntinlupa City	547,173	1,371	473	1,844	337.00
Parañaque City	722,126	1,142	574	1,716	237.63
Pasay City	451,750	1,245	707	1,952	432.10
**C A R**	**1,822,719**	**1,661**	**743**	**2,404**	**131.89**
Abra	248,468	300	136	436	175.48
Apayao	124,728	179	111	290	232.51
Benguet	483,934	217	68	285	58.89
Ifugao	212,063	175	78	253	119.30
Kalinga	221,563	336	147	483	218.00
Mt. Province	157,413	106	39	145	92.11
Baguio City	374,550	348	164	512	136.70
**Region 1**	**5,313,223**	**7,087**	**3,524**	**10,611**	**199.71**
Ilocos Norte	501,276	341	183	524	104.53
Ilocos Sur	600,674	403	160	563	93.73
La Union	705,988	788	445	1,233	174.65
Pangasinan	2,522,027	3,620	1,833	5,453	216.21
Alaminos City	95,291	140	65	205	215.13
Candon City	63,312	0	1	1	1.58
Dagupan City	181,929	491	217	708	389.16
Laoag City	115,582	62	33	95	82.19
San Carlos City	200,310	415	203	618	308.52
San Fernando City	129,350	383	159	542	419.02
Urdaneta City	141,215	379	192	571	404.35
Vigan City	56,269	65	33	98	174.16

Table 1.E.8.1 - Tuberculosis
Case Notification Rate (All forms)
Philippines, 2021

Area	Total Population	Notified TB cases all forms			CNR (All Forms)
		Male	Female	Total	
**Region 2**	**3,693,664**	**4,469**	**2,271**	**6,740**	**182.47**
Batanes	18,013	21	16	37	205.41
Cagayan	1,121,561	1,145	552	1,697	151.31
Isabela	1,264,006	1,506	716	2,222	175.79
Nueva Vizcaya	488,238	448	241	689	141.12
Quirino	199,473	227	146	373	186.99
Cauayan City	138,317	138	68	206	148.93
Ilagan City	155,452	310	208	518	333.22
Santiago City	143,982	464	226	690	479.23
Tuguegarao City	164,622	210	98	308	187.10
**Region 3**	**12,513,847**	**26,230**	**13,764**	**39,994**	**319.60**
Aurora	229,841	515	244	759	330.23
Bataan	742,197	1,673	841	2,514	338.72
Bulacan	2,542,502	5,291	2,775	8,066	317.25
Nueva Ecija	1,646,738	1,929	956	2,885	175.19
Pampanga	1,850,369	3,024	1,483	4,507	243.57
Tarlac	1,106,339	1,821	911	2,732	246.94
Zambales	650,180	2,138	1,249	3,387	520.93
Angeles City	464,253	950	537	1,487	320.30
Balanga City	107,277	419	181	600	559.30
Cabanatuan City	337,037	947	515	1,462	433.78
City of San Fernando	345,857	911	435	1,346	389.18
Gapan City	123,009	279	161	440	357.70
Mabalacat City	282,858	594	347	941	332.68
Malolos City	283,977	773	363	1,136	400.03
Meycauayan City	235,549	548	308	856	363.41
Olongapo City	256,443	761	425	1,186	462.48
Palayan City	45,768	59	23	82	179.16
San Jose City	155,831	316	189	505	324.07
San Jose del Monte City	646,756	1,869	1,121	2,990	462.31
Science City of Munoz	90,867	193	123	316	347.76
Tarlac City	370,199	1,220	577	1,797	485.41
**Region 4A**	**16,361,107**	**39,783**	**22,127**	**61,910**	**378.40**
Batangas	2,144,028	4,777	2,451	7,228	337.12
Cavite	1,604,676	3,565	2,109	5,674	353.59
Laguna	1,111,798	3,147	1,754	4,901	440.82
Quezon	1,890,167	3,782	2,131	5,913	312.83
Rizal	2,386,862	5,803	3,267	9,070	380.00
Antipolo City	879,159	1,927	1,112	3,039	345.67
Bacoor City	703,029	1,036	590	1,626	231.28
Batangas City	380,515	1,296	656	1,952	512.99
Biñan City	372,804	1,366	732	2,098	562.76
Cabuyao City	345,623	925	505	1,430	413.75

Table 1.E.8.1 - Tuberculosis
Case Notification Rate (All forms)
Philippines, 2021

Area	Total Population	Notified TB cases all forms			CNR (All Forms)
		Male	Female	Total	
Calamba City	508,770	1,758	863	2,621	515.16
Cavite City	120,342	250	134	384	319.09
Dasmariñas City	771,409	1,691	929	2,620	339.64
General Trias City	367,906	865	495	1,360	369.66
Imus City	472,646	1,076	667	1,743	368.77
Lipa City	383,404	1,129	601	1,730	451.22
Lucena City	286,460	890	587	1,477	515.60
San Pablo City	297,847	861	454	1,315	441.50
San Pedro City	364,721	1,061	570	1,631	447.19
Santa Rosa City	396,019	962	516	1,478	373.21
Tagaytay City	83,320	130	67	197	236.44
Tanauan City	199,978	537	385	922	461.05
Tayabas City	107,355	248	133	381	354.90
Trece Martires City	182,269	701	419	1,120	614.48
**Region 4B**	**3,212,211**	**6,281**	**3,312**	**9,593**	**298.64**
Marinduque	242,420	562	256	818	337.43
Mindoro Occidental	519,990	1,225	629	1,854	356.55
Mindoro Oriental	911,307	2,107	1,170	3,277	359.59
Palawan	950,209	1,235	692	1,927	202.80
Romblon	302,917	447	201	648	213.92
				0	
Puerto Princesa City	285,368	705	364	1,069	374.60
**Region 5**	**6,192,924**	**13,466**	**6,497**	**19,963**	**322.35**
Albay	1,199,362	2,368	1,096	3,464	288.82
Camarines Norte	629,402	1,428	499	1,927	306.16
Camarines Sur	1,750,680	3,740	1,908	5,648	322.62
Catanduanes	275,043	465	252	717	260.69
Masbate	944,945	1,623	797	2,420	256.10
Sorsogon	855,000	1,553	759	2,312	270.41
				0	
Iriga City	118,952	285	135	420	353.08
Legaspi City	210,918	972	549	1,521	721.13
Naga City	208,622	1,032	502	1,534	735.30
**Region 6**	**7,970,551**	**20,117**	**8,979**	**29,096**	**365.04**
Aklan	610,948	1,139	513	1,652	270.40
Antique	617,788	1,219	653	1,872	303.02
Capiz	811,948	1,633	749	2,382	293.37
Guimaras	187,011	457	201	658	351.85
Iloilo	2,069,653	3,972	1,625	5,597	270.43
Negros Occidental	2,607,671	7,266	3,201	10,467	401.39
Bacolod City	586,715	2,508	1,148	3,656	623.13
Iloilo City	478,817	1,923	889	2,812	587.28

Table 1.E.8.1 - Tuberculosis
Case Notification Rate (All forms)
Philippines, 2021

Area	Total Population	Notified TB cases all forms			CNR (All Forms)
		Male	Female	Total	
**Region 7**	**8,054,772**	**12,301**	**5,671**	**17,972**	**223.12**
Bohol	1,374,101	1,442	658	2,100	152.83
Cebu	3,276,440	4,568	2,008	6,576	200.71
Negros Oriental	1,415,196	2,253	980	3,233	228.45
Siquijor	101,227	147	55	202	199.55
Cebu City	1,028,542	2,291	1,136	3,427	333.19
Lapu-Lapu City	454,972	890	441	1,331	292.55
Mandaue City	404,294	710	393	1,103	272.82
**Region 8**	**4,803,767**	**6,709**	**3,470**	**10,179**	**211.90**
Biliran	180,717	332	160	492	272.25
Eastern Samar	511,290	507	274	781	152.75
Northern Leyte	1,656,424	2,220	972	3,192	192.70
Northern Samar	675,196	882	547	1,429	211.64
Southern Leyte	358,038	388	170	558	155.85
Western Samar	634,040	803	389	1,192	188.00
Calbayog City	195,379	286	168	454	232.37
Maasin City	91,120	116	54	170	186.57
Ormoc City	235,937	315	203	518	219.55
Tacloban City	265,626	860	533	1,393	524.42
**Region 9**	**3,809,540**	**5,659**	**2,499**	**8,158**	**214.15**
Zamboanga del Norte	831,480	836	322	1,158	139.27
Zamboanga del Sur	840,050	1,066	464	1,530	182.13
Zamboanga Sibugay	687,546	744	347	1,091	158.68
Dapitan City	85,853	142	47	189	220.14
Dipolog City	136,208	260	93	353	259.16
Isabela City	130,380	58	31	89	68.26
Pagadian City	206,034	396	167	563	273.26
Zamboanga City	891,989	2,157	1,028	3,185	357.07
**Region 10**	**5,074,563**	**6,735**	**3,217**	**9,952**	**196.12**
Bukidnon	1,130,220	662	256	918	81.22
Camiguin	93,118	90	49	139	149.27
Lanao del Norte	735,928	830	439	1,269	172.44
Misamis Occidental	342,462	322	112	434	126.73
Misamis Oriental	782,065	899	378	1,277	163.29
Cagayan de Oro City	740,740	1,573	827	2,400	324.00
El Salvador City	55,016	90	33	123	223.57
Gingoog City	136,593	199	94	293	214.51
Iligan City	372,772	1,249	668	1,917	514.26
Malaybalay City	188,395	187	69	256	135.88
Oroquieta City	74,210	111	43	154	207.52

Table 1.E.8.1 - Tuberculosis
Case Notification Rate (All forms)
Philippines, 2021

Area	Total Population	Notified TB cases all forms			CNR (All Forms)
		Male	Female	Total	
Ozamis City	148,747	250	140	390	262.19
Tangub City	66,085	46	13	59	89.28
Valencia City	208,212	227	96	323	155.13
**Region 11**	**5,371,254**	**8,079**	**3,514**	**11,593**	**215.83**
Davao de Oro	780,742	808	285	1,093	140.00
Davao del Norte	1,063,436	2,006	781	2,787	262.08
Davao del Sur	607,848	795	312	1,107	182.12
Davao Oriental	719,362	306	131	437	60.75
Davao Occidental	342,874	731	299	1,030	300.40
Davao City	1,856,992	3,433	1,706	5,139	276.74
**Region 12**	**4,985,878**	**9,369**	**4,295**	**13,664**	**274.05**
North Cotabato	1,553,081	2,285	1,015	3,300	212.48
Sarangani	590,746	830	376	1,206	204.15
South Cotabato	1,011,240	2,085	928	3,013	297.95
Sultan Kudarat	862,787	1,493	666	2,159	250.24
Cotabato City	311,264	889	504	1,393	447.53
Gen. Santos City	656,760	1,787	806	2,593	394.82
**BARMM**	**4,270,193**	**2,227**	**1,495**	**3,722**	**87.16**
Basilan	310,153	311	234	545	175.72
Lanao del Sur	942,654	610	458	1,068	113.30
Maguindanao	1,355,941	690	375	1,065	78.54
Sulu	932,940	90	71	161	17.26
Tawi-Tawi	417,702	189	124	313	74.93
Lamitan City	85,335	133	73	206	241.40
Marawi City	225,468	204	160	364	161.44
**CARAGA**	**2,782,218**	**5,400**	**2,630**	**8,030**	**288.62**
Agusan del Norte	382,946	522	208	730	190.63
Agusan del Sur	741,035	1,170	481	1,651	222.80
Surigao del Norte	366,639	705	401	1,106	301.66
Surigao del Sur	525,627	1,035	501	1,536	292.22
Province of Dinagat	131,274	144	60	204	155.40
Bislig City	99,839	368	263	631	632.02
Butuan City	364,103	893	415	1,308	359.24
Surigao City	170,755	563	301	864	505.99

Legend:
0 = No Case
** = No Report*

Table 1.E.8.2 - Tuberculosis
Case Notification Rate (DRTB)
Philippines, 2021

Area	Registered Bacteriologically Confirmed Drug Resistant			Estimated MDR among New & Retreatment			CNR (DRTB)
	Male	Female	Total	Male	Female	Total	
**PHILIPPINES**	**4,026**	**1,697**	**5,723**	**0**	**0**	**12,125**	**47.2**
**N C R**	**912**	**433**	**1,345**	**0**	**0**	**2,318**	**58.0**
Malabon City	33	14	47			75	62.3
Navotas City	32	6	38			68	55.7
Valenzuela City	41	20	61			157	38.8
Caloocan City	85	41	126			256	49.1
Marikina City	36	15	51			102	50.1
Pasig City	39	25	64			120	53.2
Pateros			0			4	0.0
Taguig City	86	26	112			162	68.9
Quezon City	160	103	263			426	61.8
Makati City	33	20	53			91	58.0
Mandaluyong City	35	14	49			80	61.1
San Juan City	13	15	28			23	123.5
Manila City	184	84	268			469	57.2
Las Piñas City	25	17	42			54	78.4
Muntinlupa City	64	10	74			90	82.3
Parañaque City	29	11	40			65	61.4
Pasay City	17	12	29			76	38.3
**C A R**	**36**	**9**	**45**	**0**	**0**	**65**	**68.7**
Abra	7	0	7			11	61.3
Apayao	7	3	10			7	145.3
Benguet	1	0	1			7	13.7
Ifugao	4	1	5			7	67.4
Kalinga	8	3	11			13	84.1
Mt. Province	0	0	0			4	0.0
Baguio City	9	2	11			15	73.2
**Region 1**	**177**	**75**	**252**	**0**	**0**	**324**	**77.7**
Ilocos Norte	12	5	17			20	84.9
Ilocos Sur	7	0	7			19	37.4
La Union	5	8	13			35	37.5
Pangasinan	20	13	33			155	21.3
Alaminos City	18	0	18			5	347.0
Candon City	0	0	0			0	0.0
Dagupan City	32	13	45			26	172.0
Laoag City	0	0	0			2	0.0
San Carlos City	33	13	46			21	216.6
San Fernando City	19	7	26			20	129.1
Urdaneta City	25	16	41			17	235.9
Vigan City	6	0	6			4	161.1

Table 1.E.8.2 - Tuberculosis
Case Notification Rate (DRTB)
Philippines, 2021

Area	Registered Bacteriologically Confirmed Drug Resistant			Estimated MDR among New & Retreatment			CNR (DRTB)
	Male	Female	Total	Male	Female	Total	
**Region 2**	**81**	**19**	**100**	**0**	**0**	**270**	**37.0**
Batanes	0	0	0			2	0.0
Cagayan	9	2	11			59	18.7
Isabela	0	0	0			125	0.0
Nueva Vizcaya	4	5	9			23	38.4
Quirino	5	2	7			9	75.4
Cauayan City	0	0	0			8	0.0
Ilagan City	0	0	0			15	0.0
Santiago City	30	4	34			17	198.0
Tuguegarao City	33	6	39			12	332.9
**Region 3**	**508**	**230**	**738**	**0**	**0**	**1,472**	**50.1**
Aurora	6	5	11			30	36.7
Bataan	15	2	17			108	15.8
Bulacan	89	45	134			280	47.9
Nueva Ecija	24	14	38			98	38.9
Pampanga	45	15	60			157	38.1
Tarlac	27	17	44			104	42.3
Zambales	35	17	52			139	37.4
Angeles City	34	11	45			57	79.2
Balanga City	25	8	33			24	134.9
Cabanatuan City	20	15	35			43	81.6
City of San Fernando	40	25	65			44	148.2
Gapan City	0	0	0			18	0.0
Mabalacat City	11	8	19			38	49.9
Malolos City	52	10	62			43	145.5
Meycauayan City	0	0	0			39	0.0
Olongapo City	12	9	21			88	24.0
Palayan City	0	0	0			1	0.0
San Jose City	15	5	20			17	120.1
San Jose del Monte City	38	14	52			76	68.5
Science City of Munoz	0	0	0			7	0.0
Tarlac City	20	10	30			63	48.0
**Region 4A**	**698**	**297**	**995**	**0**	**0**	**2,199**	**45.2**
Batangas	41	11	52			251	20.7
Cavite	12	4	16			180	8.9
Laguna	51	19	70			177	39.5
Quezon	60	27	87			229	38.0
Rizal	124	47	171			353	48.5
Antipolo City	54	26	80			122	65.5
Bacoor City	16	9	25			61	41.3
Batangas City	31	13	44			58	75.6
Biñan City	0	0	0			89	0.0
Cabuyao City	0	0	0			38	0.0

Table 1.E.8.2 - Tuberculosis
Case Notification Rate (DRTB)
Philippines, 2021

Area	Registered Bacteriologically Confirmed Drug Resistant			Estimated MDR among New & Retreatment			CNR (DRTB)
	Male	Female	Total	Male	Female	Total	
Calamba City	61	19	80			100	80.2
Cavite City	0	0	0			17	0.0
Dasmariñas City	35	22	57			88	64.9
General Trias City	0	0	0			47	0.0
Imus City	19	11	30			43	69.3
Lipa City	38	11	49			49	99.2
Lucena City	30	17	47			74	63.2
San Pablo City	0	0	0			37	0.0
San Pedro City	0	0	0			47	0.0
Santa Rosa City	70	30	100			56	179.2
Tagaytay City	7	4	11			8	143.5
Tanauan City	0	0	0			21	0.0
Tayabas City	0	0	0			19	0.0
Trece Martires City	49	27	76			35	216.7
**Region 4B**	**103**	**42**	**145**	**0**	**0**	**432**	**33.5**
Marinduque	11	4	15			40	37.7
Mindoro Occidental	25	8	33			77	42.9
Mindoro Oriental	36	14	50			152	33.0
Palawan	15	8	23			99	23.3
Romblon	8	4	12			24	49.1
Puerto Princesa City	8	4	12			41	29.2
**Region 5**	**292**	**114**	**406**	**0**	**0**	**814**	**49.9**
Albay	32	9	41			153	26.8
Camarines Norte	53	18	71			73	97.8
Camarines Sur	13	5	18			244	7.4
Catanduanes	12	8	20			31	64.6
Masbate	6	3	9			92	9.8
Sorsogon	57	18	75			87	86.6
Iriga City	0	0	0			14	0.0
Legaspi City	38	24	62			65	95.7
Naga City	81	29	110			56	196.6
**Region 6**	**336**	**127**	**463**	**0**	**0**	**1,302**	**35.6**
Aklan	27	10	37			59	62.5
Antique	21	9	30			82	36.5
Capiz	27	10	37			76	48.9
Guimaras			0			19	0.0
Iloilo	33	17	50			216	23.2
Negros Occidental	125	42	167			539	31.0
Bacolod City	46	23	69			192	36.0
Iloilo City	57	16	73			120	61.1

Table 1.E.8.2 - Tuberculosis
Case Notification Rate (DRTB)
Philippines, 2021

Area	Registered Bacteriologically Confirmed Drug Resistant			Estimated MDR among New & Retreatment			CNR (DRTB)
	Male	Female	Total	Male	Female	Total	
**Region 7**	**214**	**64**	**278**	**0**	**0**	**663**	**41.9**
Bohol	30	10	40			71	56.6
Cebu	0	0	0			208	0.0
Negros Oriental	44	2	46			129	35.7
Siquijor	0	0	0			9	0.0
Cebu City	87	27	114			140	81.2
Lapu-Lapu City	0	0	0			61	0.0
Mandaue City	53	25	78			45	174.4
**Region 8**	**131**	**59**	**190**	**0**	**0**	**365**	**52.0**
Biliran	0	0	0			16	0.0
Eastern Samar	11	10	21			29	72.3
Northern Leyte	74	24	98			120	81.8
Northern Samar	17	8	25			49	50.9
Southern Leyte	0	0	0			18	0.0
Western Samar	0	0	0			41	0.0
Calbayog City	13	11	24			17	141.3
Maasin City	16	6	22			8	274.5
Ormoc City	0	0	0			22	0.0
Tacloban City	0	0	0			46	0.0
**Region 9**	**92**	**26**	**118**	**0**	**0**	**274**	**43.0**
Zamboanga del Norte	0	0	0			37	0.0
Zamboanga del Sur	14	10	24			54	44.7
Zamboanga Sibugay	15	8	23			37	61.8
Dapitan City	24	2	26			8	331.0
Dipolog City	0	0	0			10	0.0
Isabela City	2	1	3			4	73.9
Pagadian City	0	0	0			13	0.0
Zamboanga City	37	5	42			112	37.6
**Region 10**	**95**	**38**	**133**	**0**	**0**	**325**	**40.9**
Bukidnon	0	0	0			28	0.0
Camiguin	0	1	1			4	24.2
Lanao del Norte	16	8	24			44	54.7
Misamis Occidental	0	0	0			13	0.0
Misamis Oriental	1	1	2			45	4.5
Cagayan de Oro City	29	9	38			65	58.8
El Salvador City	0	0	0			5	0.0
Gingoog City	6	2	8			13	62.5
Iligan City	22	13	35			64	54.4
Malaybalay City	**5**	0	5			10	50.6
Oroquieta City	8	2	10			6	162.0
Ozamis City	7	2	9			17	52.6
Tangub City	1	0	1			2	50.4
Valencia City	0	0	0			9	0.0

Table 1.E.8.2 - Tuberculosis
Case Notification Rate (DRTB)
Philippines, 2021

Area	Registered Bacteriologically Confirmed Drug Resistant			Estimated MDR among New & Retreatment			CNR (DRTB)
	Male	Female	Total	Male	Female	Total	
**Region 11**	**124**	**40**	**164**	**0**	**0**	**358**	**45.8**
Davao de Oro	0	0	0			27	0.0
Davao del Norte	52	10	62			95	65.2
Davao del Sur	16	2	18			28	64.9
Davao Oriental	17	3	20			38	52.2
Davao Occidental	3		3			11	27.2
Davao City	36	25	61			159	38.4
**Region 12**	**125**	**56**	**181**	**0**	**0**	**525**	**34.5**
North Cotabato	35	12	47			117	40.3
Sarangani	6	3	9			61	14.8
South Cotabato	23	11	34			94	36.2
Sultan Kudarat	15	6	21			81	26.0
Cotabato City	18	12	30			51	58.4
Gen. Santos City	28	12	40			122	32.9
**BARMM**	**31**	**29**	**60**	**0**	**0**	**155**	**38.7**
Basilan	0	0	0			26	0.0
Lanao del Sur	2	0	2			51	3.9
Maguindanao	8	9	17			36	47.4
Sulu	3	2	5			4	119.8
Tawi-Tawi	1	2	3			8	37.9
Lamitan City	3	3	6			9	67.7
Marawi City	14	13	27			21	130.8
**CARAGA**	**71**	**39**	**110**	**0**	**0**	**263**	**41.8**
Agusan del Norte	0	0	0			18	0.0
Agusan del Sur	11	5	16			52	31.0
Surigao del Norte	4	3	7			41	16.9
Surigao del Sur	8	6	14			58	24.1
Province of Dinagat	1	0	1			6	16.9
Bislig City	14	9	23			26	88.9
Butuan City	15	4	19			34	55.6
Surigao City	18	12	30			28	108.2

Legend:
0 = No Case
** = No Report*

Table 1.E.8.3 - Tuberculosis
Treatment Success Rate DSTB - All Forms
Philippines, 2021

Area	TB all forms cured and Completely Treated			Registered TB all forms Registered during Specified Period			Treatment Success Rate
	Male	Female	Total	Male	Female	Total	
**PHILIPPINES**	**137,223**	**65,989**	**203,212**	**148,287**	**76,983**	**225,270**	**90.21**
**N C R**	**17,141**	**9,185**	**26,326**	**19,422**	**10,160**	**29,582**	**88.99**
Malabon City	625	376	1,001	700	404	1,104	90.67
Navotas City	402	213	615	442	232	674	91.25
Valenzuela City	1278	701	1,979	1381	760	2,141	92.43
Caloocan City	2094	1120	3,214	2331	1216	3,547	90.61
Marikina City	814	429	1,243	949	478	1,427	87.11
Pasig City	1041	546	1,587	1247	633	1,880	84.41
Pateros	38	26	64	47	32	79	81.01
Taguig City	1540	942	2,482	1622	976	2,598	95.54
Quezon City	2921	1691	4,612	3516	1967	5,483	84.11
Makati City	694	446	1,140	770	479	1,249	91.27
Mandaluyong City	798	353	1,151	869	392	1,261	91.28
San Juan City	167	61	228	178	64	242	94.21
Manila City	2204	1102	3,306	2555	1258	3,813	86.70
Las Piñas City	574	285	859	630	303	933	92.07
Muntinlupa City	781	310	1,091	896	355	1,251	87.21
Parañaque City	606	312	918	659	322	981	93.58
Pasay City	564	272	836	630	289	919	90.97
**C A R**	**1,442**	**683**	**2,125**	**1,607**	**740**	**2,347**	**90.54**
Abra	261	110	371	274	114	388	95.62
Apayao	148	81	229	160	85	245	93.47
Benguet	151	61	212	179	69	248	85.48
Ifugao	166	91	257	186	100	286	89.86
Kalinga	291	157	448	309	162	471	95.12
Mt. Province	116	46	162	120	48	168	96.43
Baguio City	309	137	446	379	162	541	82.44
**Region 1**	**6,315**	**3,024**	**9,339**	**6,845**	**3,214**	**10,059**	**92.84**
Ilocos Norte	357	186	543	442	221	663	81.90
Ilocos Sur	587	257	844	685	290	975	86.56
La Union	668	341	1,009	717	360	1,077	93.69
Pangasinan	3,173	1,577	4,750	3,290	1,611	4,901	96.92
Alaminos City	129	65	194	134	67	201	96.52
Candon City	52	15	67	53	15	68	77.94
Dagupan City	334	147	481	392	162	554	86.82
Laoag City	81	21	102	99	26	125	81.60
San Carlos City	301	115	416	311	124	435	95.63
San Fernando City	288	159	447	334	184	518	86.29
Urdaneta City	270	104	374	289	109	398	93.97
Vigan City	75	37	112	99	45	144	77.78
**Region 2**	**4,718**	**2,346**	**7,064**	**5,233**	**2,585**	**7,818**	**90.36**
Batanes	26	13	39	31	15	46	84.78

Table 1.E.8.3 - Tuberculosis
Treatment Success Rate DSTB - All Forms
Philippines, 2021

Area	TB all forms cured and Completely Treated			Registered TB all forms Registered during Specified Period			Treatment Success Rate
	Male	Female	Total	Male	Female	Total	
Cagayan	1,451	716	2,167	1,516	745	2,261	95.84
Isabela	1,607	854	2,461	1,929	1,000	2,929	84.02
Nueva Vizcaya	495	228	723	552	265	817	88.49
Quirino	275	115	390	292	117	409	95.35
Cauayan City	100	58	158	120	76	196	80.61
Ilagan City	211	106	317	221	109	330	96.06
Santiago City	341	165	506	351	166	517	97.87
Tuguegarao City	212	91	303	221	92	313	96.81
**Region 3**	**15,112**	**7,190**	**17,658**	**16,789**	**7,954**	**19,624**	**89.98**
Aurora	353	171	524	384	176	560	93.57
Bataan	1,074	476	1,550	1,135	489	1,624	95.44
Bulacan	3,000	1,435	4,435	3,494	1,694	5,188	85.49
Nueva Ecija	1,286	624	1,910	1,664	809	2,473	77.23
Pampanga	2,186	1,025	3,211	2,366	1,116	3,482	92.22
Tarlac	1,407	674	2,081	1,435	695	2,130	97.70
Zambales	1,023	549	1,572	1,109	593	1,702	92.36
Angeles City	443	201	644	464	205	669	96.26
Balanga City	252	106	358	265	111	376	95.21
Cabanatuan City	392	179	571	410	187	597	95.64
City of San Fernando	355	177	532	363	181	544	97.79
Gapan City	180	90	270	188	91	279	96.77
Mabalacat City	335	167	502	384	190	574	87.46
Malolos City	301	135	436	388	153	541	80.59
Meycauayan City	214	127	341	309	172	481	70.89
Olongapo City	406	197	603	457	215	672	89.73
Palayan City	71	17	88	80	18	98	89.80
San Jose City	209	100	309	236	107	343	90.09
San Jose del Monte City	925	449	1,374	942	454	1,396	98.42
Science City of Munoz	86	58	144	90	58	148	97.30
Tarlac City	614	233	847	626	240	866	97.81
**Region 4A**	**19,008**	**9,503**	**28,511**	**20,397**	**10,033**	**30,430**	**93.69**
Batangas	2,006	969	2,975	2,165	1,031	3,196	93.09
Cavite	1,795	963	2,758	1,950	1,029	2,979	92.58
Laguna	1395	769	2,164	1509	803	2,312	93.60
Quezon	2237	1039	3,276	2364	1081	3,445	95.09
Rizal	3269	1747	5,016	3512	1833	5,345	93.84
Antipolo City	1165	544	1,709	1256	585	1,841	92.83
Bacoor City	504	291	795	573	330	903	88.04
Batangas City	380	185	565	429	202	631	89.54
Biñan City	580	304	884	589	309	898	98.44
Cabuyao City	352	153	505	362	156	518	97.49
Calamba City	812	299	1,111	820	302	1,122	99.02
Cavite City	208	92	300	219	97	316	94.94
Dasmariñas City	871	530	1,401	958	561	1,519	92.23
General Trias City	367	188	555	391	206	597	92.96
Imus City	504	279	783	542	293	835	93.77
Lipa City	287	137	424	296	138	434	97.70
Lucena City	461	230	691	507	243	750	92.13
San Pablo City	297	147	444	335	156	491	90.43

Table 1.E.8.3 - Tuberculosis
Treatment Success Rate DSTB - All Forms
Philippines, 2021

Area	TB all forms cured and Completely Treated			Registered TB all forms Registered during Specified Period			Treatment Success Rate
	Male	Female	Total	Male	Female	Total	
San Pedro City	388	132	520	417	140	557	93.36
Santa Rosa City	428	186	614	457	198	655	93.74
Tagaytay City	71	31	102	71	32	103	99.03
Tanauan City	185	75	260	202	81	283	91.87
Tayabas City	132	79	211	146	89	235	89.79
Trece Martires City	314	134	448	327	138	465	96.34
**Region 4B**	**4,986**	**2,740**	**7,726**	**5,540**	**2,989**	**8,529**	**90.59**
Marinduque	417	220	637	468	248	716	88.97
Mindoro Occidental	1001	503	1,504	1086	521	1,607	93.59
Mindoro Oriental	1376	758	2,134	1475	807	2,282	93.51
Palawan	1287	782	2,069	1451	870	2,321	89.14
Romblon	397	230	627	542	292	834	75.18
Puerto Princesa City	508	247	755	518	251	769	98.18
**Region 5**	**9,545**	**4,781**	**14,326**	**10,377**	**5,060**	**15,437**	**92.80**
Albay	1861	950	2,811	1951	981	2,932	95.87
Camarines Norte	1011	359	1,370	1135	382	1,517	90.31
Camarines Sur	2,963	1,582	4,545	3,202	1,668	4,870	93.33
Catanduanes	374	208	582	416	232	648	89.81
Masbate	1,271	703	1,974	1,381	747	2,128	92.76
Sorsogon	1,212	568	1,780	1,353	614	1,967	90.49
							0.00
Iriga City	198	85	283	222	98	320	88.44
Legaspi City	306	158	464	327	167	494	93.93
Naga City	349	168	517	390	171	561	92.16
**Region 6**	**14,455**	**6,593**	**21,048**	**15,359**	**6,979**	**22,338**	**94.23**
Aklan	966	400	1,366	1059	429	1,488	91.80
Antique	1120	518	1,638	1193	546	1,739	94.19
Capiz	1061	466	1,527	1142	500	1,642	93.00
Guimaras	293	145	438	305	147	452	96.90
Iloilo	3245	1465	4,710	3396	1528	4,924	95.65
Negros Occidental	5306	2464	7,770	5619	2604	8,223	94.49
Bacolod City	1370	587	1,957	1460	633	2,093	93.50
Iloilo City	1094	548	1,642	1185	592	1,777	92.40
**Region 7**	**9,397**	**4,216**	**13,613**	**10,340**	**4,577**	**14,917**	**91.26**
Bohol	1694	859	2,553	1819	905	2,724	93.72
Cebu	3293	1443	4,736	3641	1568	5,209	90.92
Negros Oriental	2042	850	2,892	2170	919	3,089	93.62
Siquijor	149	51	200	161	56	217	92.17
Cebu City	1122	503	1,625	1284	565	1,849	87.89
Lapu-Lapu City	656	327	983	757	356	1,113	88.32
Mandaue City	441	183	624	508	208	716	87.15
**Region 8**	**5,012**	**2,483**	**7,495**	**5,724**	**2,848**	**8,572**	**87.44**
Biliran	279	139	418	295	144	439	95.22

Table 1.E.8.3 - Tuberculosis

Treatment Success Rate DSTB - All Forms

Philippines, 2021

Area	TB all forms cured and Completely Treated			Registered TB all forms Registered during Specified Period			Treatment Success Rate
	Male	Female	Total	Male	Female	Total	
Eastern Samar	428	233	661	487	274	761	86.86
Northern Leyte	1,663	680	2,343	1909	785	2,694	86.97
Northern Samar	701	388	1,089	768	427	1,195	91.13
Southern Leyte	319	142	461	417	185	602	76.58
Western Samar	537	306	843	618	350	968	87.09
Calbayog City	223	123	346	252	147	399	86.72
Maasin City	104	63	167	112	67	179	93.30
Ormoc City	263	151	414	313	173	486	85.19
Tacloban City	495	258	753	553	296	849	88.69
**Region 9**	**4,533**	**1,849**	**6,382**	**5,046**	**2,060**	**7,106**	**89.81**
Zamboanga del Norte	495	258	753	553	296	849	88.69
Zamboanga del Sur	990	400	1,390	1091	441	1,532	90.73
Zamboanga Sibugay	729	295	1,024	812	329	1,141	89.75
Dapitan City	91	52	143	97	56	153	93.46
Dipolog City	174	65	239	191	70	261	91.57
Isabela City	61	48	109	105	71	176	61.93
Pagadian City	219	109	328	233	118	351	93.45
Zamboanga City	1774	622	2,396	1964	679	2,643	90.65
**Region 10**	**5,480**	**2,430**	**7,910**	**6,013**	**2,640**	**8,653**	**91.41**
Bukidnon	661	253	914	722	267	989	92.42
Camiguin	105	40	145	110	41	151	96.03
Lanao del Norte	736	411	1,147	764	428	1,192	96.22
Misamis Occidental	382	176	558	425	200	625	89.28
Misamis Oriental	871	369	1,240	917	380	1,297	95.61
			0				
Cagayan de Oro City	1,137	474	1,611	1307	561	1,868	86.24
El Salvador City	105	49	154	108	51	159	96.86
Gingoog City	141	76	217	161	81	242	89.67
Iligan City	701	284	985	750	298	1,048	93.99
Malaybalay City	117	51	168	155	65	220	76.36
Oroquieta City	81	57	138	93	58	151	91.39
Ozamis City	204	98	302	216	101	317	95.27
Tangub City	47	26	73	73	39	112	65.18
Valencia City	192	66	258	212	70	282	91.49
**Region 11**	**6,449**	**2,419**	**8,868**	**7,093**	**2,650**	**9,743**	**91.02**
Davao de Oro	895	286	1,181	960	311	1,271	92.92
Davao del Norte	1485	531	2,016	1642	566	2,208	91.30
Davao del Sur	776	258	1,034	813	269	1,082	95.56
Davao Occidental	309	139	448	328	146	474	94.51
Davao Oriental	786	279	1,065	825	290	1,115	95.52
Davao City	2198	926	3,124	2525	1068	3,593	86.95
**Region 12**	**7,437**	**3,332**	**10,769**	**8,174**	**3,630**	**11,804**	**91.23**
North Cotabato	1946	909	2,855	2178	1006	3,184	89.67
Sarangani	683	360	1,043	739	391	1,130	92.30
South Cotabato	1598	626	2,224	1702	660	2,362	94.16

Table 1.E.8.3 - Tuberculosis
Treatment Success Rate DSTB - All Forms
Philippines, 2021

Area	TB all forms cured and Completely Treated			Registered TB all forms Registered during Specified Period			Treatment Success Rate
	Male	Female	Total	Male	Female	Total	
Sultan Kudarat	1309	695	2,004	1380	724	2,104	95.25
Cotabato City	474	260	734	521	296	817	89.84
Gen. Santos City	1427	482	1,909	1654	553	2,207	86.50
**BARMM**	**1,935**	**1,207**	**3,142**	**2,392**	**1,507**	**3,899**	**80.58**
Basilan	181	130	311	185	135	320	97.19
Lanao del Sur	568	446	1,014	588	465	1,053	96.30
Maguindanao	1028	542	1,570	1073	557	1,630	96.32
Sulu	4	4	8	240	173	413	1.94
Tawi-Tawi			0	120	72	192	0.00
Lamitan City	90	39	129	93	42	135	95.56
Marawi City	64	46	110	93	63	156	70.51
**CARAGA**	**4,258**	**2,008**	**6,266**	**4,584**	**2,124**	**6,708**	**93.41**
Agusan del Norte	393	144	537	440	157	597	89.95
Agusan del Sur	1034	462	1,496	1077	475	1,552	96.39
Surigao del Norte	615	280	895	669	296	965	92.75
Surigao del Sur	924	462	1,386	992	481	1,473	94.09
Province of Dinagat	140	74	214	146	79	225	95.11
			0				
Bislig City	220	144	364	233	147	380	95.79
Butuan City	581	281	862	619	301	920	93.70
Surigao City	351	161	512	408	188	596	85.91

Legend:
0 = No Case
** = No Report*

Table 1.E.8.4 - Tuberculosis

Treatment Success Rate - MDRTB

Philippines, 2021

Area	Registered Bacteriology Confirmed Drug Resistant			Registered Bacteriologically Confirmed Drug Resistant Specified Period			Treatment Success Rate
	Male	Female	Total	Male	Female	Total	
**PHILIPPINES**	**3,789**	**1,585**	**5,374**	**5,154**	**2,186**	**7,340**	**73.22**
**N C R**	**682**	**293**	**975**	**1,019**	**447**	**1,466**	**66.51**
Malabon City	27	17	44	40	30	70	62.86
Navotas City	20	8	28	36	15	51	54.90
Valenzuela City	26	16	42	46	24	70	60.00
Caloocan City	66	31	97	93	45	138	70.29
Marikina City	28	14	42	40	22	62	67.74
Pasig City	31	13	44	39	21	60	73.33
Pateros	0	0	0	0	0	0	0.00
Taguig City	44	7	51	72	10	82	62.20
Quezon City	137	77	214	200	107	307	69.71
Makati City	22	18	40	35	24	59	67.80
Mandaluyong City	20	8	28	32	13	45	62.22
San Juan City	7	5	12	8	7	15	80.00
Manila City	131	55	186	219	90	309	60.19
Las Piñas City	17	7	24	18	13	31	77.42
Muntinlupa City	55	8	63	71	12	83	75.90
Parañaque City	27	3	30	36	7	43	69.77
Pasay City	24	6	30	34	7	41	73.17
**C A R**	**18**	**5**	**23**	**26**	**7**	**33**	**69.70**
Abra	5	0	5	6	0	6	83.33
Apayao	2	0	2	4	0	4	50.00
Benguet	0	0	0	0	0	0	0.00
Ifugao	1	1	2	1	2	3	66.67
Kalinga	2	3	5	2	3	5	100.00
Mt. Province	1	0	1	1	0	1	100.00
						0	
Baguio City	7	1	8	12	2	14	57.14
**Region 1**	**283**	**121**	**404**	**356**	**136**	**492**	**82.11**
Ilocos Norte	16	7	23	17	7	24	95.83
Ilocos Sur	12	5	17	19	5	24	70.83
La Union	18	11	29	25	13	38	76.32
Pangasinan	109	44	153	135	50	185	82.70
Alaminos City	12	2	14	15	4	19	73.68
Candon City	0	0	0	0	0	0	0.00
Dagupan City	22	12	34	29	15	44	77.27
Laoag City	0	0	0	0	0	0	0.00
San Carlos City	40	20	60	49	20	69	86.96
San Fernando City	15	9	24	20	10	30	80.00
Urdaneta City	35	10	45	42	11	53	84.91
Vigan City	4	1	5	5	1	6	83.33

Table 1.E.8.4 - Tuberculosis
Treatment Success Rate - MDRTB
Philippines, 2021

Area	Registered Bacteriology Confirmed Drug Resistant			Registered Bacteriologically Confirmed Drug Resistant Specified Period			Treatment Success Rate
	Male	Female	Total	Male	Female	Total	
**Region 2**	**114**	**44**	**158**	**136**	**55**	**191**	**82.72**
Batanes	0	0	0	0	0	0	0.00
Cagayan	35	7	42	41	10	51	82.35
Isabela	24	13	37	28	16	44	84.09
Nueva Vizcaya	4	2	6	5	3	8	75.00
Quirino	2	2	4	3	2	5	80.00
Cauayan City	0	0	0	0	0	0	0.00
Ilagan City	0	0	0	0	0	0	0.00
Santiago City	24	13	37	28	16	44	84.09
Tuguegarao City	25	7	32	31	8	39	82.05
**Region 3**	**636**	**265**	**901**	**891**	**394**	**1,285**	**70.12**
Aurora	10	4	14	12	4	16	75.00
Bataan	35	17	52	43	24	67	64.18
Bulacan	138	52	190	199	78	277	68.59
Nueva Ecija	47	23	70	76	36	112	67.86
Pampanga	86	36	122	123	55	178	69.10
Tarlac	45	20	65	55	26	81	78.00
Zambales	42	13	55	52	21	73	71.23
						0	
Angeles City	30	7	37	37	7	44	84.09
Balanga City	28	11	39	34	17	51	76.47
Cabanatuan City	19	9	28	32	15	47	59.57
City of San Fernando	37	17	54	53	27	80	67.50
Gapan City	0	0	0	0	0	0	0.00
Mabalacat City	16	11	27	21	15	36	75.00
Malolos City	48	14	62	67	29	96	64.58
Meycauayan City	0	0	0			0	0.00
Olongapo City	12	5	17	16	6	22	77.27
Palayan City	0	0	0	0	0	0	0.00
San Jose City	5	6	11	11	10	21	52.38
San Jose del Monte City	18	10	28	32	12	44	63.64
Science City of Munoz	0	0	0	0	0	0	0.00
Tarlac City	20	10	30	28	12	40	75.00
**Region 4A**	**766**	**342**	**1,108**	**1,064**	**472**	**1,536**	**72.14**
Batangas	82	36	118	116	54	170	69.41
Cavite	132	78	210	174	100	274	76.64
Laguna	121	44	165	165	56	221	74.66
Quezon	42	12	54	52	15	67	80.60
Rizal	121	57	178	180	81	261	68.20
Antipolo City	31	9	40	50	17	67	59.70
Bacoor City	16	13	29	21	16	37	78.38
Batangas City	24	11	35	36	19	55	63.64
Biñan City	0	0	0	0	0	0	0.00
Cabuyao City	0	0	0	0	0	0	0.00
Calamba City	25	4	29	34	7	41	70.73
Cavite City	0	0	0	0	0	0	0.00

Table 1.E.8.4 - Tuberculosis
Treatment Success Rate - MDRTB
Philippines, 2021

Area	Registered Bacteriology Confirmed Drug Resistant			Registered Bacteriologically Confirmed Drug Resistant Specified Period			Treatment Success Rate
	Male	Female	Total	Male	Female	Total	
Dasmariñas City	33	15	48	53	20	73	65.75
General Trias City	0	0	0	0	0	0	0.00
Imus City	22	7	29	26	10	36	80.56
Lipa City	27	9	36	39	14	53	67.92
Lucena City	0	0	42	0	0	0	0.00
San Pablo City	0	0	0	0	0	0	0.00
San Pedro City	39	12	51	55	18	73	69.86
Santa Rosa City	6	1	7	8	3	11	63.64
Tagaytay City	0	0	0	0	0	0	0.00
Tanauan City	0	0	0	0	0	0	0.00
Tayabas City	45	34	79	55	42	97	81.44
Trece Martires City	0	0	0	0	0	0	0.00
**Region 4B**	**124**	**48**	**172**	**151**	**64**	**215**	**80.00**
Marinduque	11	1	12	14	1	15	80.00
Mindoro Occidental	30	8	38	36	10	46	82.61
Mindoro Oriental	33	13	46	40	15	55	83.64
Palawan	13	14	27	16	18	34	79.41
Romblon	15	3	18	19	6	25	72.00
Puerto Princesa City	22	9	31	26	14	40	77.50
**Region 5**	**263**	**93**	**356**	**357**	**132**	**489**	**72.80**
Albay	40	14	54	62	23	85	63.53
Camarines Norte	36	7	43	43	11	54	79.63
Camarines Sur	59	25	84	81	35	116	72.41
Catanduanes	8	4	12	8	4	12	100.00
Masbate	15	4	19	22	4	26	73.08
Sorsogon	26	9	35	28	11	39	89.74
Iriga City	0	0	0	0	0	0	0.00
Legaspi City	27	9	36	44	15	59	61.02
Naga City	52	21	0	69	29	98	0.00
**Region 6**	**255**	**87**	**342**	**305**	**109**	**414**	**82.61**
Aklan	29	4	33	34	8	42	78.57
Antique	20	8	28	24	10	34	82.35
Capiz	21	6	27	21	6	27	100.00
Guimaras	0	0	0	0	0	0	0.00
Iloilo	21	10	31	30	13	43	72.09
Negros Occidental	96	36	132	110	40	150	88.00
			0			0	
Bacolod City	39	10	49	47	15	62	79.03
Iloilo City	29	13	42	39	17	56	75.00
**Region 7**	**131**	**54**	**185**	**174**	**64**	**238**	**77.73**
Bohol	23	7	30	36	7	43	69.77
Cebu	0	1	1	0	1	1	100.00
Negros Oriental	15	6	21	21	10	31	67.74
Siquijor			0			0	0.00

Table 1.E.8.4 - Tuberculosis
Treatment Success Rate - MDRTB
Philippines, 2021

Area	Registered Bacteriology Confirmed Drug Resistant			Registered Bacteriologically Confirmed Drug Resistant Specified Period			Treatment Success Rate
	Male	Female	Total	Male	Female	Total	
Cebu City	37	19	56	61	25	86	65.12
Lapu-Lapu City	0	0	0	0	0	0	0.00
Mandaue City	56	21	77	56	21	77	100.00
**Region 8**	**47**	**12**	**59**	**77**	**25**	**102**	**57.84**
Biliran	0	0	0	0	0	0	0.00
Eastern Samar	17	5	22	19	6	25	88.00
Northern Leyte			0			0	0.00
Northern Samar	8	3	11	8	5	13	84.62
Southern Leyte	6	2	8	10	3	13	61.54
Western Samar	0	0	0	15	4	19	0.00
Calbayog City	10	0	10	15	4	19	52.63
Maasin City	6	2	8	10	3	13	61.54
Ormoc City	0	0	0	0	0	0	0.00
Tacloban City	0	0	0	0	0	0	0.00
**Region 9**	**69**	**26**	**95**	**97**	**36**	**133**	**71.43**
Zamboanga del Norte	15	4	19	23	7	30	63.33
Zamboanga del Sur	7	3	10	12	4	16	62.50
Zamboanga Sibugay	7	5	12	13	8	21	57.14
						0	
Dapitan City	15	4	19	23	7	30	63.33
Dipolog City	0	0	0	0	0	0	0.00
Isabela City	3	5	8	4	5	9	88.89
Pagadian City	0	0	0	0	0	0	0.00
Zamboanga City	22	5	27	22	5	27	100.00
**Region 10**	**81**	**37**	**118**	**112**	**50**	**162**	**72.84**
Bukidnon	5	0	5	7	0	7	71.43
Camiguin	0	0	0	0	0	0	0.00
Lanao del Norte	10	8	18	15	10	25	72.00
Misamis Occidental	6	6	12	11	11	22	54.55
Misamis Oriental	5	1	6	7	1	8	75.00
			0				
Cagayan de Oro City	29	9	38	34	10	44	86.36
El Salvador City	0	0	0	0	0	0	0.00
Gingoog City	5	1	6	7	1	8	75.00
Iligan City	10	6	16	13	6	19	84.21
Malaybalay City	5		5	7		7	71.43
Oroquieta City	4	3	7	5	5	10	70.00
Ozamis City	2	3	5	6	6	12	41.67
Tangub City	0	0	0	0	0	0	0.00
Valencia City	0	0	0	0	0	0	0.00
**Region 11**	**102**	**37**	**139**	**121**	**43**	**164**	**84.76**
Davao de Oro	0	0	0	0	0	0	0.00
Davao del Norte	46	12	58	52	12	64	90.63
Davao del Sur	13	4	17	15	7	22	77.27

Table 1.E.8.4 - Tuberculosis
Treatment Success Rate - MDRTB
Philippines, 2021

Area	Registered Bacteriology Confirmed Drug Resistant			Registered Bacteriologically Confirmed Drug Resistant Specified Period			Treatment Success Rate
	Male	Female	Total	Male	Female	Total	
Davao Occidental	0	0	0	0	0	0	0.00
Davao Oriental	8	4	12	11	5	16	75.00
Davao City	35	17	52	43	19	62	83.87
**Region 12**	**94**	**52**	**146**	**116**	**63**	**179**	**81.56**
North Cotabato	21	15	36	25	19	44	81.82
Sarangani	0	0	0	0	0	0	0.00
South Cotabato	22	10	32	30	13	43	74.42
Sultan Kudarat	10	6	16	12	8	20	80.00
Cotabato City	24	11	35	28	13	41	85.37
Gen. Santos City	17	10	27	21	10	31	87.10
**BARMM**	**26**	**33**	**59**	**31**	**41**	**72**	**81.94**
Basilan	2	1	3	2	1	3	100.00
Lanao del Sur	8	12	20	9	15	24	83.33
Maguindanao	5	2	7	5	3	8	87.50
Sulu	0	2	2	3	3	6	33.33
Tawi-Tawi	1	3	4	1	3	4	100.00
Lamitan City	2	1	3	2	1	3	100.00
Marawi City	8	12	20	9	15	24	83.33
**CARAGA**	**98**	**36**	**134**	**121**	**48**	**169**	**79.29**
Agusan del Norte	0	0	0	0	0	0	0.00
Agusan del Sur	4	1	5	7	3	10	50.00
Surigao del Norte	18	6	24	21	7	28	85.71
Surigao del Sur	23	11	34	29	15	44	77.27
Province of Dinagat	1	1	2	1	1	2	100.00
Bislig City	17	5	22	21	8	29	75.86
Butuan City	19	7	26	24	8	32	81.25
Surigao City	16	5	21	18	6	24	87.50

Legend:
0 = No Case
** = No Report*

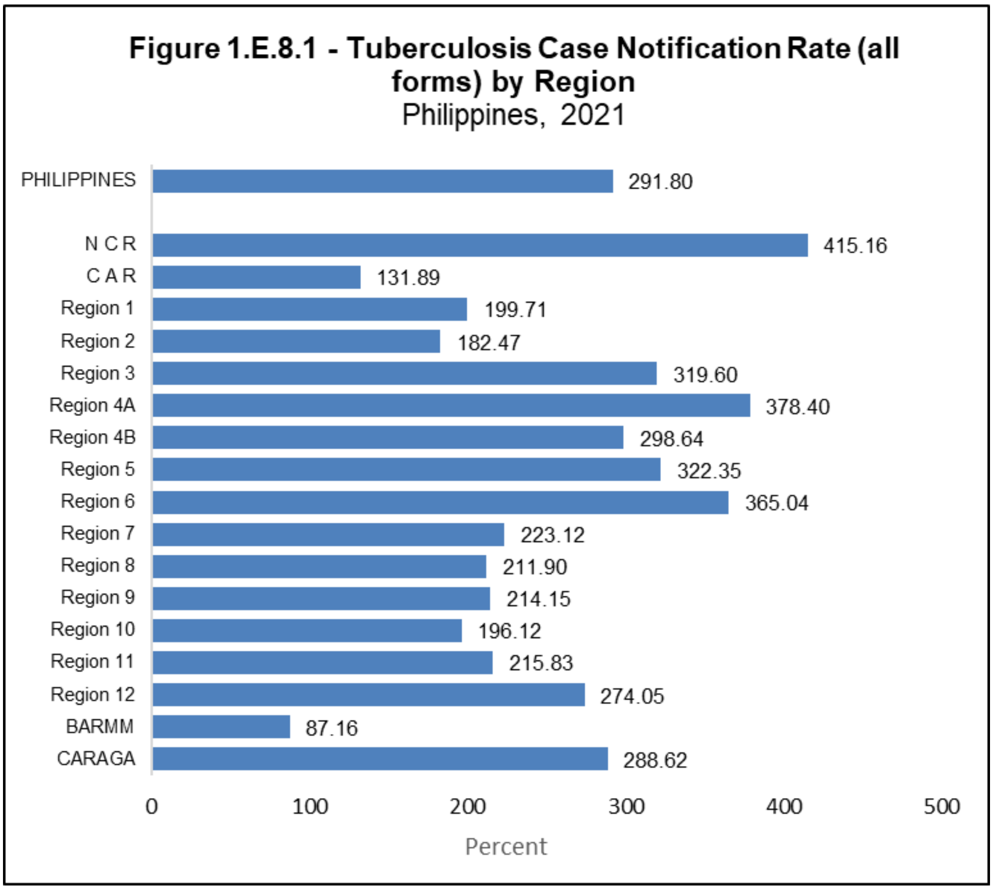

Figure 1.E.8.1 - Tuberculosis Case Notification Rate (all forms) by Region
Philippines, 2021
PHILIPPINES 291.80
N C R 415.16
C A R 131.89
Region 1 199.71
Region 2 182.47
Region 3 319.60
Region 4A 378.40
Region 4B 298.64
Region 5 322.35
Region 6 365.04
Region 7 223.12
Region 8 211.90
Region 9 214.15
Region 10 196.12
Region 11 215.83
Region 12 274.05
BARMM 87.16
CARAGA 288.62
0
100
200
300
400
500
Percent

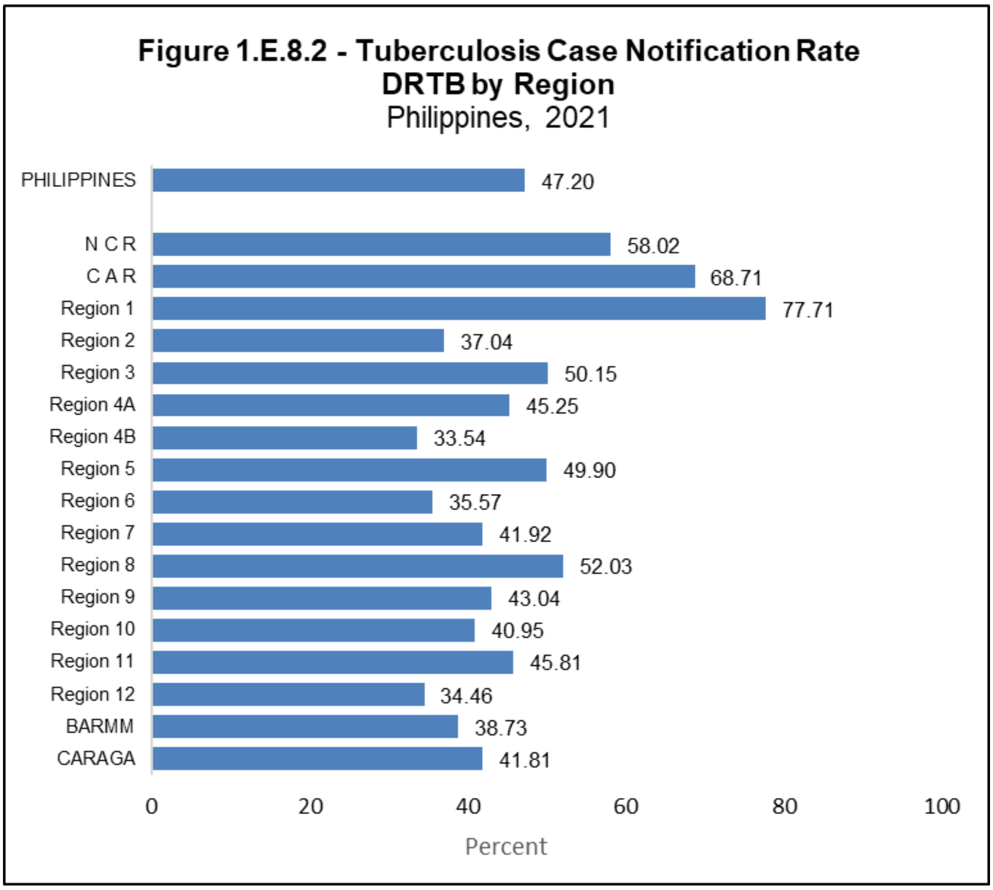

Figure 1.E.8.2 - Tuberculosis Case Notification Rate DRTB by Region
Philippines, 2021
PHILIPPINES 47.20
N C R 58.02
C A R 68.71
Region 1 77.71
Region 2 37.04
Region 3 50.15
Region 4A 45.25
Region 4B 33.54
Region 5 49.90
Region 6 35.57
Region 7 41.92
Region 8 52.03
Region 9 43.04
Region 10 40.95
Region 11 45.81
Region 12 34.46
BARMM 38.73
CARAGA 41.81
0
20
40
60
80
100
Percent

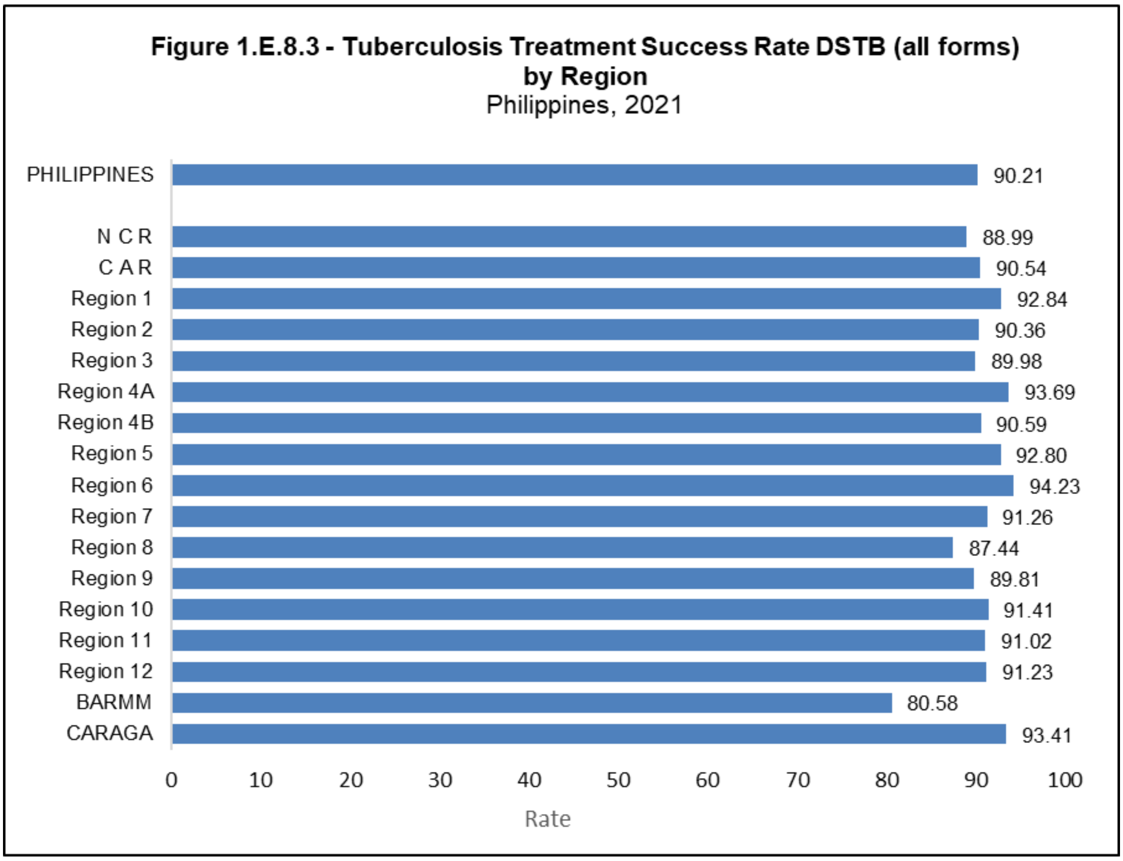
Figure 1.E.8.3 - Tuberculosis Treatment Success Rate DSTB (all forms) by Region
Philippines, 2021
PHILIPPINES
90.21
N C R
88.99
C A R
90.54
Region 1
92.84
Region 2
90.36
Region 3
89.98
Region 4A
93.69
Region 4B
90.59
Region 5
92.80
Region 6
94.23
Region 7
91.26
Region 8
87.44
Region 9
89.81
Region 10
91.41
Region 11
91.02
Region 12
91.23
BARMM
80.58
CARAGA
93.41
0
10
20
30
40
50
60
70
80
90
100
Rate

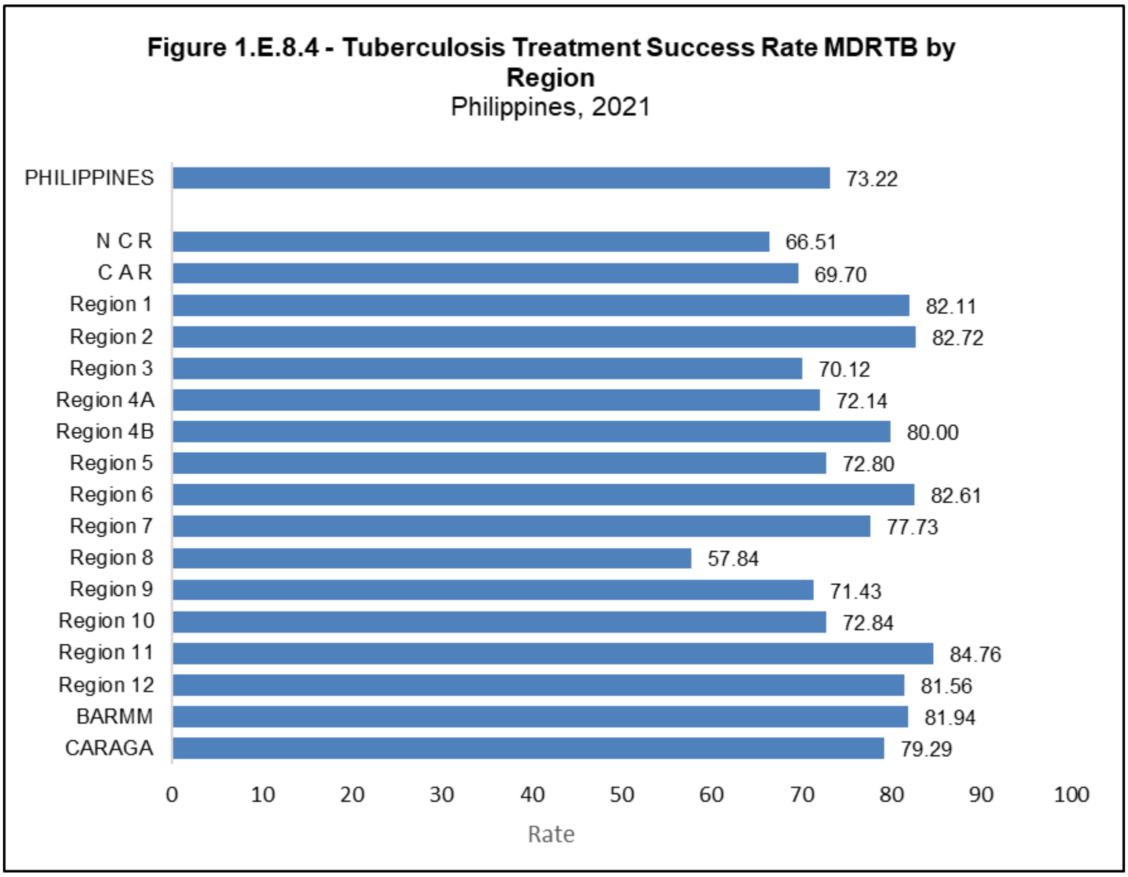
Figure 1.E.8.4 - Tuberculosis Treatment Success Rate MDRTB by Region
Philippines, 2021
PHILIPPINES
73.22
N C R
66.51
C A R
69.70
Region 1
82.11
Region 2
82.72
Region 3
70.12
Region 4A
72.14
Region 4B
80.00
Region 5
72.80
Region 6
82.61
Region 7
77.73
Region 8
57.84
Region 9
71.43
Region 10
72.84
Region 11
84.76
Region 12
81.56
BARMM
81.94
CARAGA
79.29
0
10
20
30
40
50
60
70
80
90
100
Rate

NON-COMMUNICABLE DISEASE PREVENTION AND CONTROL SERVICES

FHSIS ANNUAL REPORT 2021

1.E. Non-Communicable Disease Prevention and Control Services

A total of 4,429,060 adults 20 years old and above was risk assessed using the PhilPEN protocol. Of these, 23.23% were identified as Current Smokers, 20.40% were Binge Drinkers and 9.27% were Overweight or Obese based on the Body Mass Index of the Asia Pacific Standard.

Figure 1.E.1 Proportion of Adults 20 Years Old and Above Who Were Risk Assessed* as Current Smokers, Binge Drinkers and Overweight/Obese Philippines, 2021

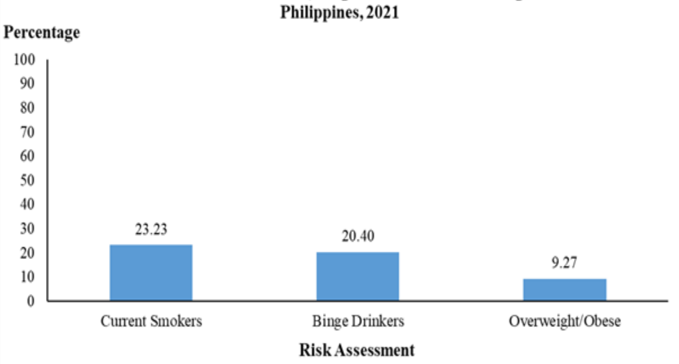

The highest percentage distribution for Current Smokers, among 20 years old and above was registered in Region 4A, Binge Drinkers in registered in Region 4B and Overweight/Obese in NCR, while the least percentage distribution for the same classifications was registered in BARMM.

Table 1.E.1 Proportion of Adults 20 Years Old and Above Who Were Risk Assessed* as Current Smokers, Binge Drinkers and Overweight/Obese by Region Philippines, 2021

Region	Current Smoker	Binge Drinker	Overweight/Obese
NCR	11.82	13.02	36.04
CAR	14.42	17.29	21.47
Region 1	20.26	20.62	12.13
Region 2	12.74	12.87	7.22
Region 3	15.21	12.34	8.26
Region 4A	60.34	57.09	11.81
Region 4B	54.81	74.49	10.23
Region 5	17.58	15.86	9.14
Region 6	24.91	20.99	8.03
Region 7	15.67	19.85	14.82
Region 8	32.97	46.14	12.08
Region 9	36.66	28.71	10.52
Region 10	37.02	31.63	3.96
Region 11	46.49	26.50	12.55
Region 12	29.90	24.61	11.05
BARMM	3.78	0.35	0.64
CARAGA	56.71	47.63	10.46

There were 74,900 women aged 20 years old and above screened for cervical cancer. This is less than 1% of the estimated number of women aged 20 years old and above. Among the regions, Region 8 had the highest proportion of women found positive or suspected for cervical cancer with 23.53%.

For women screened for breast mass, a total of 181,102 were registered (0.54%). Region 4B had the highest proportion of women with suspicious breast mass with 25.76%.

Table 1.E.2 Number of Women 20 Years Old and Above Who Were Screened for Cervical Cancer and Breast Mass with their Corresponding Results by Region Philippines, 2021

Region	No. Screened for Cervical Cancer	No. Positive or Suspected for Cervical Cancer	%	No. Screened for Breast Mass	No. With Suspicious Breast Mass	%
NCR	4,215	278	6.60	11,723	663	5.66
CAR	1,347	78	5.79	17,700	376	2.12
Region 1	663	24	3.62	5,936	129	2.17
Region 2	960	29	3.02	16,857	515	3.06
Region 3	2,242	72	3.21	5,955	258	4.33
Region 4A	2,150	190	8.84	6,164	249	4.04
Region 4B	130	7	5.38	198	52	25.76
Region 5	4,970	109	2.19	13,924	286	2.05
Region 6	548	41	7.48	21,300	413	1.94
Region 7	3,728	51	1.37	29,242	481	1.64
Region 8	95	16	23.53	450	31	7.88
Region 9	62	5	8.06	2,860	72	2.52
Region 10	404	17	4.21	2,192	216	9.85
Region 11	50,227	216	0.43	22,007	1,304	5.93
Region 12	1,603	82	5.12	15,226	774	5.08
BARMM	1,224	5	0.77	5,904	123	1.61
CARAGA	332	33	9.94	3,464	50	1.44
TOTAL	74,900	1,253	1.67	181,102	5,992	3.31

Among the adults aged 20 years old and above who were risk assessed, there were 429,925 (8.42%) newly identified Hypertensive adults while 101,259 (1.98%) were newly identified with Type 2 Diabetes Mellitus. Region 4B had the highest proportion of newly identified Hypertensive adults with 23.82%, followed by Region 8 with 23.02%. For newly identified Type 2 Diabetes Mellitus adults, Region 4B had the highest proportion with 23.82%, followed by Region 3 with 5.7%.

Figure 1.E.2 Proportion of Adults 20 Years Old and Above Newly Identified with Hypertension and Type II Diabetes Mellitus (DM) by Region Philippines, 2021

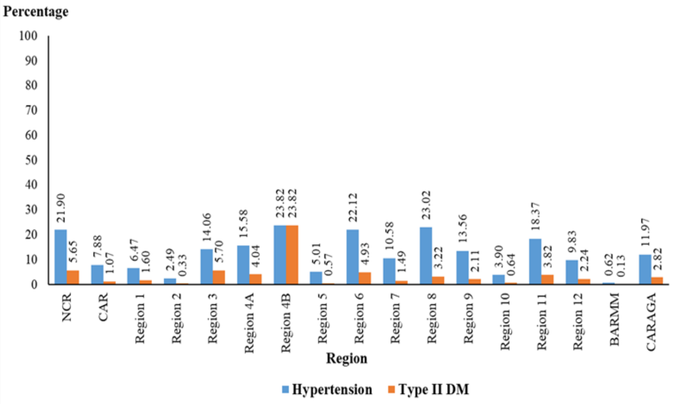

There was a total of 126,135 Senior Citizens 60 years old and above screened for Eye Problems. Among the regions that reported, Region 7 had the highest percentage (73.22%) of Senior Citizens who were diagnosed with eye problems followed by Region 4B with 69.04%.

Table 1.E.3 Number of Senior Citizens 60 Years Old and Above Who Were Screened and Diagnosed with Eye Problems by Region Philippines, 2021

Region	No. Screened for Eye Problems	No. Diagnosed with Eye Problems	%
NCR	6,848	3,421	49.96
CAR	2,920	304	10.41
Region 1	2,309	482	20.87
Region 2	8,197	758	9.25
Region 3	1,222	321	26.27
Region 4A	1,756	411	23.41
Region 4B	197	136	69.04
Region 5	4,218	1,095	25.96
Region 6	1,682	438	26.04
Region 7	422	309	73.22
Region 8	535	106	19.81
Region 9	901	215	23.86
Region 10	5,335	884	16.57
Region 11	86,238	3,735	4.33
Region 12	2,201	529	24.03
BARMM	767	120	15.65
CARAGA	387	139	35.92
TOTAL	126,135	13,403	10.63

A total of 185,776 senior citizens 60 years old and above received one dose of Pneumococcal Polysaccharide Vaccine (PPV) or 1.88% of the estimated number of senior citizens, while 751,650 (7.62%) of them received one dose of Influenza vaccine. The coverage for all regions was less than 3% for PPV and less than 13% for Influenza vaccines.

Figure 1.E.3 Proportion of Senior Citizens 60 Years Old and Above Who Received One Dose of Pneumococcal Polysacharide Vaccine (PPV) and Influenza Vaccine by Region Philippines, 2021

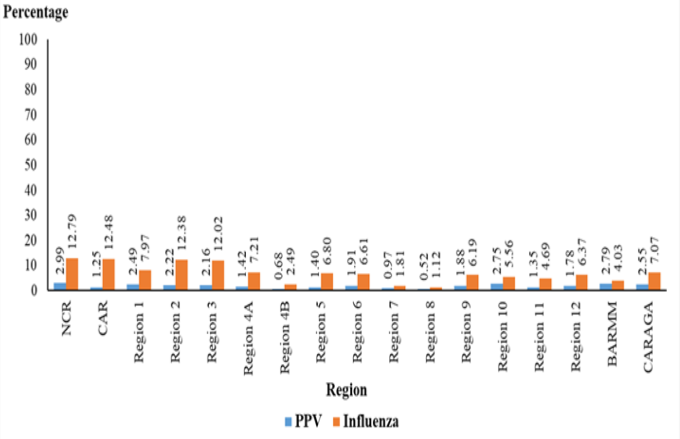

Table 1.F.1.1 - Lifestyle Related Diseases (Risk Assessment)

Number and proportion of adults aged 20 yrs old and above who were risk-assessed using the PhilPEN protocol

Philippines, 2021

Area	Eligible Pop. (20 y/o & above)	Adults who were risk-assessed			
		Male	Female	Total	%
**PHILIPPINES**	**66,807,585**	**1,946,700**	**1,929,520**	**3,876,220**	**5.80**
**N C R**	**9,105,826**	**59,886**	**90,393**	**150,279**	**1.65**
Malabon	258,468	2,324	2,481	4,805	1.86
Navotas	176,401	4,689	5,961	10,650	6.04
Valenzuela City	438,716	4,117	6,165	10,282	2.34
Caloocan City	1,120,068	4,091	10,085	14,176	1.27
Marikina City	318,731	1,263	1,478	2,741	0.86
Pasig City	534,094	7,537	13,106	20,643	3.87
Pateros	45,144	471	612	1,083	2.40
Taguig	569,171	6,983	11,778	18,761	3.30
Quezon City	2,076,201	9,394	14,559	23,953	1.15
Makati City	411,970	1,082	1,340	2,422	0.59
Mandaluyong City	273,145	5,005	5,515	10,520	3.85
San Juan	86,397	886	1,326	2,212	2.56
Manila City	1,258,791	4,740	7,902	12,642	1.00
Las Piñas City	416,423	1,473	1,994	3,467	0.83
Muntinlupa City	356,752	2,486	2,451	4,937	1.38
Parañaque City	470,819	880	1,421	2,301	0.49
Pasay City	294,535	2,465	2,219	4,684	1.59
**C A R**	**1,111,513**	**45,805**	**59,407**	**105,212**	**9.47**
Abra	154,649	10,614	11,048	21,662	14.01
Apayao	73,087	12,517	17,678	30,195	41.31
Benguet	305,877	6,186	9,178	15,364	5.02
Ifugao	121,825	2,565	3,720	6,285	5.16
Kalinga	127,380	4,966	4,650	9,616	7.55
Mt. Province	91,960	6,777	8,338	15,115	16.44
Baguio City	236,735	2,180	4,795	6,975	2.95
**Region 1**	**3,289,261**	**156,687**	**140,471**	**297,158**	**9.03**
Ilocos Norte	333,265	5,327	5,766	11,093	3.33
Ilocos Sur	393,473	25,859	25,587	51,446	13.07
La Union	458,101	9,274	10,934	20,208	4.41
Pangasinan	1,497,835	91,383	79,227	170,610	11.39
Alaminos City	56,597	13,546	5,468	19,014	33.60
Candon City	41,471	4,550	5,049	9,599	23.15
Dagupan City	108,048	153	104	257	0.24
Laoag City	76,843	471	504	975	1.27
San Carlos City	118,968	356	644	1,000	0.84
San Fernando City	83,934	302	339	641	0.76
Urdaneta City	83,867	5,059	6,540	11,599	13.83
Vigan City	36,859	407	309	716	1.94

Table 1.F.1.1 - Lifestyle Related Diseases (Risk Assessment)

Number and proportion of adults aged 20 yrs old and above who were risk-assessed using the PhilPEN protocol

Philippines, 2021

Area	Eligible Pop. (20 y/o & above)	Adults who were risk-assessed			
		Male	Female	Total	%
**Region 2**	**2,281,400**	**197,451**	**235,177**	**432,628**	**18.96**
Batanes	10,780	1,211	1,452	2,663	24.70
Cagayan	699,125	32,311	34,237	66,548	9.52
Isabela	783,323	44,131	60,220	104,351	13.32
Nueva Vizcaya	295,810	23,365	29,442	52,807	17.85
Quirino	118,461	39,176	41,350	80,526	67.98
Cauayan City	85,715	1,147	1,790	2,937	3.43
Ilagan City	96,337	12,390	21,314	33,704	34.99
Santiago City	89,233	43,289	44,938	88,227	98.87
Tuguegarao City	102,616	431	434	865	0.84
**Region 3**	**7,899,941**	**124,965**	**148,881**	**273,846**	**3.47**
Aurora	134,216	468	509	977	0.73
Bataan	467,670	7,505	11,022	18,527	3.96
Bulacan	1,614,712	17,299	16,456	33,755	2.09
Nueva Ecija	1,035,964	2,911	3,211	6,122	0.59
Pampanga	1,176,789	6,871	5,743	12,614	1.07
Tarlac	697,552	34,303	38,787	73,090	10.48
Zambales	403,626	17,836	27,722	45,558	11.29
Angeles City	295,255	825	762	1,587	0.54
Balanga City	67,592	670	792	1,462	2.16
Cabanatuan City	212,032	5,247	7,237	12,484	5.89
City of San Fernando	219,963	2,244	2,702	4,946	2.25
Gapan City	77,385	130	83	213	0.28
Mabalacat City	179,886	12,494	19,126	31,620	17.58
Malolos City	180,352	2,392	2,686	5,078	2.82
Meycauayan	149,594	493	477	970	0.65
Olongapo	159,196	499	645	1,144	0.72
Palayan City	28,792	42	47	89	0.31
San Jose City	98,035	1,221	528	1,749	1.78
San Jose del Monte City	410,752	9,187	8,024	17,211	4.19
Science City of Munoz	57,166	1,603	1,577	3,180	5.56
Tarlac City	233,412	725	745	1,470	0.63
**Region 4A**	**10,222,196**	**162,004**	**68,356**	**230,360**	**2.25**
Batangas	1,339,887	57,477	10,686	68,163	5.09
Cavite	1,020,364	7,408	9,432	16,840	1.65
Laguna	710,248	4,449	2,850	7,299	1.03
Quezon	1,111,931	25,240	9,487	34,727	3.12
Rizal	1,482,152	10,192	7,842	18,034	1.22
Antipolo City	545,920	51	52	103	0.02
Bacoor City	447,028	210	216	426	0.10
Batangas City	237,798	1,244	811	2,055	0.86
Biñan City	238,159	2,025	1,840	3,865	1.62
Cabuyao City	220,795	1,620	1,182	2,802	1.27

Table 1.F.1.1 - Lifestyle Related Diseases (Risk Assessment)

Number and proportion of adults aged 20 yrs old and above who were risk-assessed using the PhilPEN protocol

Philippines, 2021

Area	Eligible Pop. (20 y/o & above)	Adults who were risk-assessed			
		Male	Female	Total	%
Calamba City	325,016	4,253	1,532	5,785	1.78
Cavite City	76,524	1,070	926	1,996	2.61
Dasmariñas City	490,507	2,339	1,863	4,202	0.86
General Trias City	233,936	258	134	392	0.17
Imus City	300,535	2,080	1,718	3,798	1.26
Lipa City	239,602	25,923	3,422	29,345	12.25
Lucena City	168,519	57	52	109	0.06
San Pablo City	190,275	131	188	319	0.17
San Pedro City	232,996	283	280	563	0.24
Santa Rosa City	252,989	475	800	1,275	0.50
Tagaytay City	52,983	7,098	6,170	13,268	25.04
Tanauan City	124,973	6,367	5,716	12,083	9.67
Tayabas City	63,161	655	584	1,239	1.96
Trece Martires City	115,898	1,099	573	1,672	1.44
**Region 4B**	**1,795,961**	**23,280**	**9,640**	**32,920**	**1.83**
Marinduque	138,935	2,580	1,322	3,902	2.81
Mindoro Occidental	284,200	7,265	2,949	10,214	3.59
Mindoro Oriental	515,270	752	506	1,258	0.24
Palawan	528,871	12,166	4,471	16,637	3.15
Romblon	169,854	407	293	700	0.41
Puerto Princesa City	158,831	110	99	209	0.13
**Region 5**	**3,454,854**	**202,141**	**253,784**	**455,925**	**13.20**
Albay	699,855	44,303	63,996	108,299	15.47
Camarines Norte	355,257	17,766	24,677	42,443	11.95
Camarines Sur	971,745	63,346	80,255	143,601	14.78
Catanduanes	152,163	3,736	4,304	8,040	5.28
Masbate	498,451	12,706	7,810	20,516	4.12
Sorsogon	472,490	50,493	60,236	110,729	23.44
Iriga City	66,025	5,646	7,430	13,076	19.80
Legaspi City	123,074	3,072	3,803	6,875	5.59
Naga City	115,794	1,073	1,273	2,346	2.03
**Region 6**	**4,919,699**	**216,520**	**187,826**	**404,346**	**8.22**
Aklan	376,905	20,452	23,748	44,200	11.73
Antique	359,878	19,510	24,628	44,138	12.26
Capiz	514,896	15,898	22,241	38,139	7.41
Guimaras	117,842	16,214	19,468	35,682	30.28
Iloilo	1,307,795	67,439	40,546	107,985	8.26
Negros Occidental	1,583,530	67,904	44,746	112,650	7.11
Bacolod City	356,288	1,201	2,601	3,802	1.07
Iloilo City	302,565	7,902	9,848	17,750	5.87

Table 1.F.1.1 - Lifestyle Related Diseases (Risk Assessment)

Number and proportion of adults aged 20 yrs old and above who were risk-assessed using the PhilPEN protocol

Philippines, 2021

Area	Eligible Pop. (20 y/o & above)	Adults who were risk-assessed			
		Male	Female	Total	%
**Region 7**	**4,886,423**	**63,909**	**106,766**	**170,675**	**3.49**
Bohol	811,951	1,583	1,741	3,324	0.41
Cebu	1,999,896	44,158	84,996	129,154	6.46
Negros Oriental	858,211	7,829	6,653	14,482	1.69
Siquijor	64,052	2,541	3,952	6,493	10.14
Cebu City	627,819	2,247	3,503	5,750	0.92
Lapu-Lapu City	277,714	2,999	3,654	6,653	2.40
Mandaue City	246,780	2,552	2,267	4,819	1.95
**Region 8**	**2,747,517**	**92,099**	**102,701**	**194,800**	**7.09**
Biliran	103,808	5,029	5,863	10,892	10.49
Eastern Samar	289,015	27,465	26,164	53,629	18.56
Northern Leyte	969,261	14,855	18,812	33,667	3.47
Northern Samar	362,424	9,034	10,537	19,571	5.40
Southern Leyte	217,084	23,521	25,218	48,739	22.45
Western Samar	349,490	6,314	7,340	13,654	3.91
Calbayog City	107,701	527	885	1,412	1.31
Maasin City	55,249	915	1,078	1,993	3.61
Ormoc City	138,055	1,033	1,086	2,119	1.53
Tacloban City	155,430	3,406	5,718	9,124	5.87
**Region 9**	**2,206,437**	**67,999**	**57,903**	**125,902**	**5.71**
Zamboanga del Norte	483,000	16,130	15,599	31,729	6.57
Zamboanga del Sur	492,672	39,765	28,418	68,183	13.84
Zamboanga Sibugay	383,451	4,530	5,426	9,956	2.60
Dapitan City	49,873	3,086	2,250	5,336	10.70
Dipolog City	79,122	567	517	1,084	1.37
Isabela City	74,331	102	110	212	0.29
Pagadian City	120,838	247	284	531	0.44
Zamboanga City	523,150	3,572	5,299	8,871	1.70
**Region 10**	**3,019,626**	**159,116**	**183,492**	**342,608**	**11.35**
Bukidnon	664,088	37,520	12,879	50,399	7.59
Camiguin	55,717	96	81	177	0.32
Lanao del Norte	418,343	13,944	4,667	18,611	4.45
Misamis Occidental	208,554	15,474	7,937	23,411	11.23
Misamis Oriental	479,883	43,256	9,005	52,261	10.89
Cagayan de Oro City	454,528	31,741	137,499	169,240	37.23
El Salvador City	33,758	39	46	85	0.25
Gingoog City	83,808	318	219	537	0.64
Iligan City	211,898	2,151	1,481	3,632	1.71
Malaybalay City	110,696	6,890	705	7,595	6.86
Oroquieta City	45,190	0	0	0	0.00
Ozamis City	90,578	4,206	5,338	9,544	10.54

Table 1.F.1.1 - Lifestyle Related Diseases (Risk Assessment)

Number and proportion of adults aged 20 yrs old and above who were risk-assessed using the PhilPEN protocol

Philippines, 2021

Area	Eligible Pop. (20 y/o & above)	Adults who were risk-assessed			
		Male	Female	Total	%
Tangub City	40,242	311	282	593	1.47
Valencia City	122,343	3,170	3,353	6,523	5.33
**Region 11**	**3,208,131**	**188,928**	**123,427**	**312,355**	**9.74**
Davao de Oro	462,405	37,650	37,222	74,872	16.19
Davao del Norte	643,061	75,030	28,154	103,184	16.05
Davao Oriental	344,800	20,155	18,184	38,339	11.12
Davao del Sur	435,398	30,649	25,682	56,331	12.94
Davao Occidental	198,500	13,841	4,542	18,383	9.26
Davao City	1,123,967	11,603	9,643	21,246	1.89
**Region 12**	**2,897,731**	**125,432**	**126,464**	**251,896**	**8.69**
North Cotabato	889,314	39,276	40,347	79,623	8.95
Sarangani	331,565	38,227	29,808	68,035	20.52
South Cotabato	604,607	12,801	11,087	23,888	3.95
Sultan Kudarat	502,693	30,683	38,460	69,143	13.75
Cotabato City	176,890	3,236	4,973	8,209	4.64
Gen. Santos City	392,662	1,209	1,789	2,998	0.76
**BARMM**	**2,165,760**	**19,842**	**19,239**	**39,081**	**1.80**
Basilan	154,497	3,065	934	3,999	2.59
Lanao del Sur	473,310	7,422	7,982	15,404	3.25
Maguindanao	690,595	6,202	5,377	11,579	1.68
Sulu	474,517	100	8	108	0.02
Tawi-Tawi	217,133	0	0	0	0.00
Lamitan City	42,503	43	122	165	0.39
Marawi City	113,205	3,010	4,816	7,826	6.91
**CARAGA**	**1,595,309**	**40,636**	**15,593**	**56,229**	**3.52**
Agusan del Norte	224,353	4,702	3,995	8,697	3.88
Agusan del Sur	406,876	20,904	4,388	25,292	6.22
Surigao del Norte	214,410	5,858	1,423	7,281	3.40
Surigao del Sur	301,720	3,469	2,634	6,103	2.02
Province of Dinagat	77,473	891	382	1,273	1.64
Bislig City	57,308	952	134	1,086	1.90
Butuan City	213,316	3,236	1,249	4,485	2.10
Surigao City	99,853	624	1,388	2,012	2.01

[1] Based on the PhilPEN protocol

Note: Put asterisk (*) for No Report and Zero (0) for No Case

Table 1.F.1.2 - Lifestyle Related Diseases (Tobacco Use, Harmful Use of Alcohol, Unhealthy Diet)

Number and proportion of adults aged 20 yrs old and above who are current smokers, binge drinkers, and overweight or obese
Philippines, 2021

Area	Total No. of clients ≥ 20 y/o risk-assesed[1]	Current Smokers				Binge Drinkers				Overweight or obese			
		Male	Female	Total	%	Male	Female	Total	%	Male	Female	Total	%
**PHILIPPINES**	**4,429,060**	**823,562**	**205,138**	**1,028,700**	**23.23**	**713,799**	**189,563**	**903,362**	**20.40**	**185,769**	**224,597**	**410,366**	**9.27**
**N C R**	**150,279**	**12,098**	**5,671**	**17,769**	**11.82**	**12,459**	**7,112**	**19,571**	**13.02**	**21,454**	**32,701**	**54,155**	**36.04**
Malabon	4,805	243	133	376	7.83	190	90	280	5.83	660	680	1,340	27.89
Navotas	10,650	1,209	616	1,825	17.14	1,356	769	2,125	19.95	2,030	2,586	4,616	43.34
Valenzuela City	10,282	815	380	1,195	11.62	1,586	788	2,374	23.09	1,882	2,708	4,590	44.64
Caloocan City	14,176	632	411	1,043	7.36	952	1,022	1,974	13.92	1,245	3,204	4,449	31.38
Marikina City	2,741	419	121	540	19.70	336	80	416	15.18	627	804	1,431	52.21
Pasig City	20,643	942	532	1,474	7.14	967	626	1,593	7.72	1,711	3,738	5,449	26.40
Pateros	1,083	92	19	111	10.25	113	27	140	12.93	265	350	615	56.79
Taguig	18,761	1,724	876	2,600	13.86	2,469	1,842	4,311	22.98	3,928	5,555	9,483	50.55
Quezon City	23,953	983	390	1,373	5.73	1,157	471	1,628	6.80	2,440	3,951	6,391	26.68
Makati City	2,422	361	191	552	22.79	157	47	204	8.42	564	1,102	1,666	68.79
Mandaluyong City	10,520	1,745	962	2,707	25.73	897	414	1,311	12.46	2,023	2,109	4,132	39.28
San Juan	2,212	180	97	277	12.52	186	92	278	12.57	545	674	1,219	55.11
Manila City	12,642	907	464	1,371	10.84	909	380	1,289	10.20	1,333	2,150	3,483	27.55
Las Piñas City	3,467	277	117	394	11.36	263	90	353	10.18	495	645	1,140	32.88
Muntinlupa City	4,937	639	149	788	15.96	290	92	382	7.74	818	1,063	1,881	38.10
Parañaque City	2,301	216	88	304	13.21	308	113	421	18.30	249	444	693	30.12
Pasay City	4,684	714	125	839	17.91	323	169	492	10.50	639	938	1,577	33.67
**C A R**	**105,212**	**11,429**	**3,746**	**15,175**	**14.42**	**13,899**	**4,291**	**18,190**	**17.29**	**8,364**	**14,227**	**22,591**	**21.47**
Abra	21,662	4,207	1,738	5,945	27.44	4,354	1,646	6,000	27.70	2,028	3,041	5,069	23.40
Apayao	30,195	2,802	998	3,800	12.58	3,723	1,164	4,887	16.18	1,350	2,158	3,508	11.62
Benguet	15,364	1,167	228	1,395	9.08	1,398	371	1,769	11.51	2,105	3,681	5,786	37.66
Ifugao	6,285	177	48	225	3.58	275	84	359	5.71	243	414	657	10.45
Kalinga	9,616	1,693	400	2,093	21.77	2,343	522	2,865	29.79	285	398	683	7.10
Mt. Province	15,115	954	73	1,027	6.79	1,325	103	1,428	9.45	1,508	2,420	3,928	25.99
Baguio City	6,975	429	261	690	9.89	481	401	882	12.65	845	2,115	2,960	42.44
**Region 1**	**295,633**	**43,271**	**16,639**	**59,910**	**20.26**	**46,701**	**14,247**	**60,948**	**20.62**	**18,913**	**16,951**	**35,864**	**12.13**
Ilocos Norte	10,969	584	175	759	6.92	830	197	1,027	9.36	312	490	802	7.31
Ilocos Sur	49,936	7,908	3,189	11,097	22.22	6,737	1,307	8,044	16.11	1,739	2,396	4,135	8.28
La Union	20,033	1,983	559	2,542	12.69	1,842	654	2,496	12.46	1,194	1,470	2,664	13.30
Pangasinan	170,610	28,296	10,579	38,875	22.79	32,894	10,362	43,256	25.35	13,172	9,878	23,050	13.51
Alaminos City	19,014	586	227	813	4.28	488	251	739	3.89	109	98	207	1.09
Candon City	9,599	1,448	1,120	2,568	26.75	1,819	554	2,373	24.72	923	599	1,522	15.86
Dagupan City	330	139	31	170	51.52	139	39	178	53.94	6	5	11	3.33
Laoag City	975	20	4	24	2.46	9	2	11	1.13	4	4	8	0.82
San Carlos City	1,000	121	44	165	16.50	131	56	187	18.70	120	306	426	42.60
San Fernando City	795	27	5	32	4.03	20	20	40	5.03	0	0	0	0.00
Urdaneta City	11,599	2,139	702	2,841	24.49	1,788	805	2,593	22.36	1,334	1,705	3,039	26.20
Vigan City	773	20	4	24	3.10	4	0	4	0.52	0	0	0	0.00
**Region 2**	**432,628**	**43,749**	**11,386**	**55,135**	**12.74**	**45,150**	**10,544**	**55,694**	**12.87**	**14,217**	**17,014**	**31,231**	**7.22**
Batanes	2,663	163	19	182	6.83	407	139	546	20.50	289	329	618	23.21
Cagayan	66,548	9,450	1,475	10,925	16.42	9,375	928	10,303	15.48	1,512	1,641	3,153	4.74
Isabela	104,351	13,584	2,353	15,937	15.27	12,306	2,927	15,233	14.60	4,176	6,108	10,284	9.86
Nueva Vizcaya	52,807	3,331	3,082	6,413	12.14	3,942	1,167	5,109	9.67	1,766	2,421	4,187	7.93
Quirino	80,526	3,851	903	4,754	5.90	4,200	906	5,106	6.34	1,766	2,169	3,935	4.89
Cauayan City	2,937	68	13	81	2.76	62	15	77	2.62	9	10	19	0.65
Ilagan City	33,704	2,927	799	3,726	11.06	2,949	580	3,529	10.47	129	196	325	0.96
Santiago City	88,227	10,300	2,724	13,024	14.76	11,814	3,860	15,674	17.77	4,461	4,016	8,477	9.61
Tuguegarao City	865	75	18	93	10.75	95	22	117	13.53	109	124	233	26.94
**Region 3**	**273,846**	**28,502**	**13,148**	**41,650**	**15.21**	**23,389**	**10,403**	**33,792**	**12.34**	**10,151**	**12,480**	**22,631**	**8.26**
Aurora	977	68	13	81	8.29	74	40	114	11.67	28	34	62	6.35
Bataan	18,527	1,937	526	2,463	13.29	1,237	280	1,517	8.19	819	1,086	1,905	10.28
Bulacan	33,755	4,171	2,298	6,469	19.16	2,365	1,006	3,371	9.99	717	953	1,670	4.95
Nueva Ecija	6,122	303	73	376	6.14	244	30	274	4.48	101	186	287	4.69
Pampanga	12,614	755	184	939	7.44	493	103	596	4.72	530	652	1,182	9.37
Tarlac	73,090	5,659	2,556	8,215	11.24	4,931	2,327	7,258	9.93	3,219	2,430	5,649	7.73
Zambales	45,558	3,541	1,376	4,917	10.79	2,753	1,066	3,819	8.38	792	1,598	2,390	5.25
Angeles City	1,587	337	158	495	31.19	216	115	331	20.86	106	207	313	19.72
Balanga City	1,462	232	38	270	18.47	247	77	324	22.16	165	249	414	28.32

Table 1.F.1.2 - Lifestyle Related Diseases (Tobacco Use, Harmful Use of Alcohol, Unhealthy Diet)

Number and proportion of adults aged 20 yrs old and above who are current smokers, binge drinkers, and overweight or obese
Philippines, 2021

Area	Total No. of clients ≥ 20 y/o risk-assesed[1]	Current Smokers				Binge Drinkers				Overweight or obese			
		Male	Female	Total	%	Male	Female	Total	%	Male	Female	Total	%
Cabanatuan City	12,484	2,091	685	2,776	22.24	2,322	885	3,207	25.69	96	105	201	1.61
City of San Fernando	4,946	687	352	1,039	21.01	375	152	527	10.66	171	219	390	7.89
Gapan City	213	99	44	143	67.14	84	40	124	58.22	52	54	106	49.77
Mabalacat City	31,620	2,852	1,246	4,098	12.96	3,283	1,337	4,620	14.61	2,177	3,218	5,395	17.06
Malolos City	5,078	53	13	66	1.30	24	13	37	0.73	12	5	17	0.33
Meycauayan	970	213	119	332	34.23	194	119	313	32.27	90	67	157	16.19
Olongapo	1,144	128	46	174	15.21	56	26	82	7.17	17	15	32	2.80
Palayan City	89	11	11	22	24.72	2	0	2	2.25	1	0	1	1.12
San Jose City	1,749	515	221	736	42.08	684	279	963	55.06	55	36	91	5.20
San Jose del Monte City	17,211	4,007	3,039	7,046	40.94	3,165	2,348	5,513	32.03	754	905	1,659	9.64
Science City of Munoz	3,180	550	87	637	20.03	485	118	603	18.96	124	162	286	8.99
Tarlac City	1,470	293	63	356	24.22	155	42	197	13.40	125	299	424	28.84
**Region 4A**	**230,360**	**121,109**	**17,881**	**138,990**	**60.34**	**110,702**	**20,806**	**131,508**	**57.09**	**13,187**	**14,010**	**27,197**	**11.81**
Batangas	68,163	57,477	3,788	61,265	89.88	49,599	3,381	52,980	77.73	2,000	1,238	3,238	4.75
Cavite	16,840	2,354	1,445	3,799	22.56	1,517	777	2,294	13.62	2,149	2,953	5,102	30.30
Laguna	7,299	1,974	169	2,143	29.36	1,756	422	2,178	29.84	68	89	157	2.15
Quezon	34,727	25,240	4,665	29,905	86.11	22,334	9,172	31,506	90.72	3,725	3,532	7,257	20.90
Rizal	18,034	1,150	853	2,003	11.11	498	174	672	3.73	453	605	1,058	5.87
Antipolo City	103	0	0	0	0.00	0	0	0	0.00	0	0	0	0.00
Bacoor City	426	34	27	61	14.32	7	1	8	1.88	14	12	26	6.10
Batangas City	2,055	343	264	607	29.54	171	12	183	8.91	91	134	225	10.95
Biñan City	3,865	370	209	579	14.98	421	256	677	17.52	124	35	159	4.11
Cabuyao City	2,802	240	93	333	11.88	205	49	254	9.06	1,297	838	2,135	76.20
Calamba City	5,785	4,253	1,501	5,754	99.46	2,683	1,039	3,722	64.34	567	521	1,088	18.81
Cavite City	1,996	106	40	146	7.31	96	28	124	6.21	58	89	147	7.36
Dasmariñas City	4,202	2,040	1,213	3,253	77.42	1,657	711	2,368	56.35	391	344	735	17.49
General Trias City	392	206	84	290	73.98	200	65	265	67.60	18	36	54	13.78
Imus City	3,798	1,370	1,182	2,552	67.19	1,974	1,168	3,142	82.73	132	133	265	6.98
Lipa City	29,345	22,511	1,914	24,425	83.23	25,923	3,145	29,068	99.06	1,799	3,071	4,870	16.60
Lucena City	109	43	10	53	48.62	33	7	40	36.70	14	11	25	22.94
San Pablo City	319	51	38	89	27.90	24	2	26	8.15	48	57	105	32.92
San Pedro City	563	0	0	0	0.00	0	0	0	0.00	0	0	0	0.00
Santa Rosa City	1,275	104	46	150	11.76	70	12	82	6.43	26	17	43	3.37
Tagaytay City	13,268	396	134	530	3.99	569	180	749	5.65	39	34	73	0.55
Tanauan City	12,083	176	76	252	2.09	208	60	268	2.22	65	66	131	1.08
Tayabas City	1,239	3	2	5	0.40	6	31	37	2.99	93	178	271	21.87
Trece Martires City	1,672	668	128	796	47.61	751	114	865	51.73	16	17	33	1.97
**Region 4B**	**32,920**	**15,197**	**2,846**	**18,043**	**54.81**	**18,870**	**5,652**	**24,522**	**74.49**	**1,328**	**2,040**	**3,368**	**10.23**
Marinduque	3,902	660	156	816	20.91	1,201	395	1,596	40.90	94	223	317	8.12
Mindoro Occidental	10,214	4,771	1,524	6,295	61.63	7,001	2,730	9,731	95.27	58	94	152	1.49
Mindoro Oriental	1,258	290	37	327	25.99	290	14	304	24.17	4	3	7	0.56
Palawan	16,637	9,151	1,002	10,153	61.03	10,157	2,414	12,571	75.56	1,098	1,637	2,735	16.44
Romblon	700	244	81	325	46.43	188	84	272	38.86	74	83	157	22.43
Puerto Princesa City	209	81	46	127	60.77	33	15	48	22.97	0	0	0	0.00
**Region 5**	**455,925**	**67,799**	**12,364**	**80,163**	**17.58**	**54,674**	**17,649**	**72,323**	**15.86**	**16,378**	**25,271**	**41,649**	**9.14**
Albay	108,299	7,126	1,324	8,450	7.80	5,901	1,501	7,402	6.83	1,466	1,213	2,679	2.47
Camarines Norte	42,443	7,086	1,379	8,465	19.94	3,616	1,249	4,865	11.46	2,761	4,270	7,031	16.57
Camarines Sur	143,601	20,011	3,006	23,017	16.03	16,797	6,159	22,956	15.99	4,892	9,115	14,007	9.75
Catanduanes	8,040	1,654	213	1,867	23.22	1,773	561	2,334	29.03	333	777	1,110	13.81
Masbate	20,516	11,870	3,005	14,875	72.50	8,064	3,262	11,326	55.21	876	1,085	1,961	9.56
Sorsogon	110,729	18,263	3,109	21,372	19.30	16,989	4,620	21,609	19.52	4,758	7,036	11,794	10.65
Iriga City	13,076	780	90	870	6.65	807	111	918	7.02	952	1,275	2,227	17.03
Legaspi City	6,875	634	123	757	11.01	393	73	466	6.78	216	307	523	7.61
Naga City	2,346	375	115	490	20.89	334	113	447	19.05	124	193	317	13.51
**Region 6**	**404,346**	**84,904**	**15,814**	**100,718**	**24.91**	**70,553**	**14,327**	**84,880**	**20.99**	**15,247**	**17,218**	**32,465**	**8.03**
Aklan	44,200	2,710	542	3,252	7.36	4,286	835	5,121	11.59	1,722	813	2,535	5.74
Antique	44,138	5,061	1,167	6,228	14.11	4,161	841	5,002	11.33	580	536	1,116	2.53
Capiz	38,139	1,648	162	1,810	4.75	2,293	475	2,768	7.26	869	1,081	1,950	5.11
Guimaras	35,682	4,116	260	4,376	12.26	4,750	370	5,120	14.35	1,481	1,699	3,180	8.91
Iloilo	107,985	34,373	4,618	38,991	36.11	21,925	4,071	25,996	24.07	4,681	3,750	8,431	7.81
Negros Occidental	112,650	35,179	8,318	43,497	38.61	31,552	7,107	38,659	34.32	5,178	8,043	13,221	11.74
Bacolod City	3,802	489	262	751	19.75	432	237	669	17.60	234	674	908	23.88
Iloilo City	17,750	1,328	485	1,813	10.21	1,154	391	1,545	8.70	502	622	1,124	6.33

Table 1.F.1.2 - Lifestyle Related Diseases (Tobacco Use, Harmful Use of Alcohol, Unhealthy Diet)

Number and proportion of adults aged 20 yrs old and above who are current smokers, binge drinkers, and overweight or obese
Philippines, 2021

Area	Total No. of clients ≥ 20 y/o risk-assesed[1]	Current Smokers				Binge Drinkers				Overweight or obese			
		Male	Female	Total	%	Male	Female	Total	%	Male	Female	Total	%
**Region 7**	**168,470**	**21,218**	**5,181**	**26,399**	**15.67**	**25,357**	**8,080**	**33,437**	**19.85**	**9,888**	**15,083**	**24,971**	**14.82**
Bohol	3,324	429	82	511	15.37	426	57	483	14.53	179	311	490	14.74
Cebu	129,084	13,790	3,175	16,965	13.14	18,621	6,440	25,061	19.41	7,996	11,744	19,740	15.29
Negros Oriental	14,482	4,240	660	4,900	33.84	3,903	591	4,494	31.03	848	1,900	2,748	18.98
Siquijor	6,493	907	496	1,403	21.61	486	213	699	10.77	279	471	750	11.55
Cebu City	5,750	806	310	1,116	19.41	843	409	1,252	21.77	336	421	757	13.17
Lapu-Lapu City	4,518	692	363	1,055	23.35	663	253	916	20.27	198	195	393	8.70
Mandaue City	4,819	354	95	449	9.32	415	117	532	11.04	52	41	93	1.93
**Region 8**	**146,231**	**39,386**	**8,825**	**48,211**	**32.97**	**45,608**	**21,859**	**67,467**	**46.14**	**9,640**	**8,031**	**17,671**	**12.08**
Biliran	3,311	703	143	846	25.55	460	129	589	17.79	88	105	193	5.83
Eastern Samar	39,365	14,493	2,565	17,058	43.33	23,718	12,855	36,573	92.91	652	842	1,494	3.80
Northern Leyte	7,696	1,450	1,337	2,787	36.21	117	144	261	3.39	28	32	60	0.78
Northern Samar	19,578	9,799	1,258	11,057	56.48	7,534	2,605	10,139	51.79	1,910	1,333	3,243	16.56
Southern Leyte	48,739	7,785	2,149	9,934	20.38	8,797	3,588	12,385	25.41	4,154	4,623	8,777	18.01
Western Samar	13,654	2,942	716	3,658	26.79	2,280	1,055	3,335	24.43	2,113	404	2,517	18.43
Calbayog City	652	95	54	149	22.85	143	116	259	39.72	223	60	283	43.40
Maasin City	1,993	885	104	989	49.62	992	114	1,106	55.49	175	294	469	23.53
Ormoc City	2,119	173	38	211	9.96	286	252	538	25.39	55	63	118	5.57
Tacloban City	9,124	1,061	461	1,522	16.68	1,281	1,001	2,282	25.01	242	275	517	5.67
**Region 9**	**109,789**	**31,650**	**8,602**	**40,252**	**36.66**	**24,853**	**6,666**	**31,519**	**28.71**	**5,033**	**6,520**	**11,553**	**10.52**
Zamboanga del Norte	31,729	7,513	2,977	10,490	33.06	5,188	2,201	7,389	23.29	1,388	1,842	3,230	10.18
Zamboanga del Sur	53,990	20,906	4,684	25,590	47.40	17,255	3,835	21,090	39.06	2,521	3,328	5,849	10.83
Zamboanga Sibugay	9,956	1,222	290	1,512	15.19	922	175	1,097	11.02	180	255	435	4.37
Dapitan City	4,371	33	3	36	0.82	2	2	4	0.09	10	7	17	0.39
Dipolog City	1,084	291	79	370	34.13	212	49	261	24.08	87	80	167	15.41
Isabela City	212	21	5	26	12.26	15	2	17	8.02	8	13	21	9.91
Pagadian City	531	56	12	68	12.81	62	17	79	14.88	84	107	191	35.97
Zamboanga City	7,916	1,608	552	2,160	27.29	1,197	385	1,582	19.98	755	888	1,643	20.76
**Region 10**	**342,608**	**108,655**	**18,184**	**126,839**	**37.02**	**89,223**	**19,161**	**108,384**	**31.63**	**7,049**	**6,507**	**13,556**	**3.96**
Bukidnon	50,399	38,748	4,137	42,885	85.09	33,021	4,598	37,619	74.64	1,736	1,544	3,280	6.51
Camiguin	177	0	0	0	0.00	0	0	0	0.00	20	17	37	20.90
Lanao del Norte	18,611	13,713	2,565	16,278	87.46	6,033	1,898	7,931	42.61	766	639	1,405	7.55
Misamis Occidental	23,411	6,983	2,416	9,399	40.15	2,655	387	3,042	12.99	548	665	1,213	5.18
Misamis Oriental	52,261	30,531	4,513	35,044	67.06	31,030	7,251	38,281	73.25	909	1,172	2,081	3.98
Cagayan de Oro City	169,240	11,086	3,344	14,430	8.53	10,476	3,920	14,396	8.51	1,347	899	2,246	1.33
El Salvador City	85	0	0	0	0.00	0	0	0	0.00	0	0	0	0.00
Gingoog City	537	128	69	197	36.69	154	98	252	46.93	108	81	189	35.20
Iligan City	3,632	1,303	421	1,724	47.47	920	221	1,141	31.42	314	374	688	18.94
Malaybalay City	7,595	4,630	374	5,004	65.89	3,232	462	3,694	48.64	190	248	438	5.77
Oroquieta City	0	0	0	0	0.00	0	0	0	0.00	0	0	0	0.00
Ozamis City	9,544	607	144	751	7.87	509	127	636	6.66	129	187	316	3.31
Tangub City	593	56	50	106	17.88	46	54	100	16.86	128	112	240	40.47
Valencia City	6,523	870	151	1,021	15.65	1,147	145	1,292	19.81	854	569	1,423	21.82
**Region 11**	**283,427**	**90,069**	**41,683**	**131,752**	**46.49**	**63,009**	**12,113**	**75,122**	**26.50**	**19,400**	**16,168**	**35,568**	**12.55**
Davao de Oro	57,938	17,586	10,915	28,501	49.19	9,159	1,242	10,401	17.95	2,659	3,377	6,036	10.42
Davao del Norte	83,758	26,888	14,810	41,698	49.78	25,368	4,353	29,721	35.48	9,109	6,048	15,157	18.10
Davao Oriental	45,771	25,774	9,813	35,587	77.75	12,674	2,317	14,991	32.75	1,317	1,435	2,752	6.01
Davao del Sur	56,331	6,007	3,389	9,396	16.68	6,192	1,636	7,828	13.90	2,611	991	3,602	6.39
Davao Occidental	18,383	2,087	960	3,047	16.58	4,044	955	4,999	27.19	53	52	105	0.57
Davao City	21,246	11,727	1,796	13,523	63.65	5,572	1,610	7,182	33.80	3,651	4,265	7,916	37.26
**Region 12**	**230,479**	**59,653**	**9,270**	**68,923**	**29.90**	**47,305**	**9,426**	**56,731**	**24.61**	**11,023**	**14,441**	**25,464**	**11.05**
North Cotabato	56,843	23,071	3,531	26,602	46.80	17,275	2,933	20,208	35.55	3,155	4,172	7,327	12.89
Sarangani	68,038	22,460	3,155	25,615	37.65	18,784	4,009	22,793	33.50	3,188	3,808	6,996	10.28
South Cotabato	25,248	5,664	893	6,557	25.97	3,862	845	4,707	18.64	905	1,402	2,307	9.14
Sultan Kudarat	69,143	7,824	1,610	9,434	13.64	7,098	1,529	8,627	12.48	3,310	4,302	7,612	11.01
Cotabato City	8,209	504	48	552	6.72	232	97	329	4.01	430	693	1,123	13.68
Gen. Santos City	2,998	130	33	163	5.44	54	13	67	2.23	35	64	99	3.30

Table 1.F.1.2 - Lifestyle Related Diseases (Tobacco Use, Harmful Use of Alcohol, Unhealthy Diet)

Number and proportion of adults aged 20 yrs old and above who are current smokers, binge drinkers, and overweight or obese
Philippines, 2021

Area	Total No. of clients ≥ 20 y/o risk-assesed[1]	Current Smokers				Binge Drinkers				Overweight or obese			
		Male	Female	Total	%	Male	Female	Total	%	Male	Female	Total	%
**BARMM**	**710,678**	**19,097**	**7,786**	**26,883**	**3.78**	**2,073**	**420**	**2,493**	**0.35**	**1,844**	**2,709**	**4,553**	**0.64**
Basilan	0	3,288	1,066	4,354	0.00	228	47	275	0.00	21	50	71	0.00
Lanao del Sur	15,504	7,572	2,993	10,565	68.14	644	87	731	4.71	489	676	1,165	7.51
Maguindanao	690,595	6,789	2,130	8,919	1.29	1,077	166	1,243	0.18	757	1,095	1,852	0.27
Sulu	0	0	0	0	0.00	0	0	0	0.00	0	0	0	0.00
Tawi-Tawi	0	0	0	0	0.00	0	0	0	0.00	0	0	0	0.00
Lamitan City	0	393	153	546	0.00	44	31	75	0.00	9	8	17	0.00
Marawi City	4,579	1,055	1,444	2,499	54.58	80	89	169	3.69	568	880	1,448	31.62
**CARAGA**	**56,229**	**25,776**	**6,112**	**31,888**	**56.71**	**19,974**	**6,807**	**26,781**	**47.63**	**2,653**	**3,226**	**5,879**	**10.46**
Agusan del Norte	8,697	3,499	1,977	5,476	62.96	2,278	1,747	4,025	46.28	1,392	1,841	3,233	37.17
Agusan del Sur	25,292	12,192	1,620	13,812	54.61	11,282	2,712	13,994	55.33	187	168	355	1.40
Surigao del Norte	7,281	3,857	464	4,321	59.35	1,927	568	2,495	34.27	189	202	391	5.37
Surigao del Sur	6,103	1,713	615	2,328	38.15	1,395	572	1,967	32.23	282	304	586	9.60
Province of Dinagat	1,273	797	164	961	75.49	808	271	1,079	84.76	114	146	260	20.42
Bislig City	1,086	818	98	916	84.35	162	26	188	17.31	63	58	121	11.14
Butuan City	4,485	2,703	622	3,325	74.14	1,905	345	2,250	50.17	332	391	723	16.12
Surigao City	2,012	197	552	749	37.23	217	566	783	38.92	94	116	210	10.44

[1] Based on the PhilPEN protocol
Note: Put asterisk (*) for No Report and Zero (0) for No Case

Table 1.F.2 - Cancer Prevention and Control (Cervical and Breast Cancer Screening)

Number and proportion of women aged 20 yrs old and above screened for Cervical and Breast Cancer
Philippines, 2021

Area	Eligible Pop. (20 y/o and above female)	Women screened for Cervical Cancer		Women found positive or suspected for Cervical Cancer		Women screened for breast mass		Women with suspicious breast mass	
		Total	%	Total	%	Total	%	Total	%
**PHILIPPINES**	**33,455,399**	**74,900**	**0.22**	**1,253**	**1.67**	**181,102**	**0.54**	**5,992**	**3.31**
**N C R**	**4,695,065**	**4,215**	**0.09**	**278**	**6.60**	**11,723**	**0.25**	**663**	**5.66**
Malabon	133,268	0	0.00	0	0.00	0	0.00	0	0.00
Navotas	90,954	108	0.12	0	0.00	2	0.00	2	100.00
Valenzuela City	226,206	0	0.00	0	0.00	23	0.01	12	52.17
Caloocan City	577,521	758	0.13	5	0.66	2,107	0.36	0	0.00
Marikina City	164,342	0	0.00	0	0.00	0	0.00	0	0.00
Pasig City	275,385	19	0.01	16	84.21	36	0.01	24	66.67
Pateros	23,276	2	0.01	0	0.00	185	0.79	0	0.00
Taguig	293,471	1,917	0.65	138	7.20	2,931	1.00	365	12.45
Quezon City	1,070,514	111	0.01	3	2.70	39	0.00	0	0.00
Makati City	212,417	332	0.16	44	13.25	2,223	1.05	107	4.81
Mandaluyong City	140,837	208	0.15	0	0.00	323	0.23	3	0.93
San Juan	44,547	2	0.00	0	0.00	3	0.01	1	33.33
Manila City	649,047	691	0.11	64	9.26	3,491	0.54	130	3.72
Las Piñas City	214,713	19	0.01	5	26.32	248	0.12	4	1.61
Muntinlupa City	183,945	40	0.02	3	7.50	48	0.03	15	31.25
Parañaque City	242,760	0	0.00	0	0.00	0	0.00	0	0.00
Pasay City	151,862	8	0.01	0	0.00	64	0.04	0	0.00
**C A R**	**547,905**	**1,347**	**0.25**	**78**	**5.79**	**17,700**	**3.23**	**376**	**2.12**
Abra	76,222	1	0.00	0	0.00	383	0.50	27	7.05
Apayao	35,354	11	0.03	0	0.00	3,581	10.13	17	0.47
Benguet	152,647	510	0.33	35	6.86	6,775	4.44	79	1.17
Ifugao	58,962	9	0.02	0	0.00	137	0.23	22	16.06
Kalinga	61,907	0	0.00	0	0.00	475	0.77	20	4.21
Mt. Province	44,670	21	0.05	0	0.00	942	2.11	33	3.50
Baguio City	118,143	795	0.67	43	5.41	5,407	4.58	178	3.29
**Region 1**	**1,654,296**	**663**	**0.04**	**24**	**3.62**	**5,936**	**0.36**	**129**	**2.17**
Ilocos Norte	168,384	0	0.00	0	0.00	30	0.02	7	23.33
Ilocos Sur	198,541	117	0.06	11	9.40	1,793	0.90	31	1.73
La Union	230,414	42	0.02	1	2.38	2,953	1.28	13	0.44
Pangasinan	751,912	240	0.03	11	4.58	457	0.06	69	15.10
Alaminos City	28,412	0	0.00	0	0.00	70	0.25	0	0.00
Candon City	20,927	205	0.98	1	0.49	185	0.88	3	1.62
Dagupan City	54,241	0	0.00	0	0.00	286	0.53	6	2.10
Laoag City	38,826	0	0.00	0	0.00	0	0.00	0	0.00
San Carlos City	59,723	0	0.00	0	0.00	103	0.17	0	0.00
San Fernando City	42,217	0	0.00	0	0.00	0	0.00	0	0.00
Urdaneta City	42,099	0	0.00	0	0.00	0	0.00	0	0.00
Vigan City	18,600	59	0.32	0	0.00	59	0.32	0	0.00
**Region 2**	**1,131,127**	**960**	**0.08**	**29**	**3.02**	**16,857**	**1.49**	**515**	**3.06**
Batanes	5,359	3	0.06	3	100.00	10	0.19	10	100.00
Cagayan	348,462	305	0.09	4	1.31	2,784	0.80	26	0.93
Isabela	387,826	102	0.03	10	9.80	100	0.03	23	23.00

Table 1.F.2 - Cancer Prevention and Control (Cervical and Breast Cancer Screening)
Number and proportion of women aged 20 yrs old and above screened for Cervical and Breast Cancer
Philippines, 2021

Area	Eligible Pop. (20 y/o and above female)	Women screened for Cervical Cancer		Women found positive or suspected for Cervical Cancer		Women screened for breast mass		Women with suspicious breast mass	
		Total	%	Total	%	Total	%	Total	%
Nueva Vizcaya	145,934	515	0.35	8	1.55	3,407	2.33	29	0.85
Quirino	58,087	4	0.01	4	100.00	994	1.71	5	0.50
Cauayan City	42,438	4	0.01	0	0.00	105	0.25	0	0.00
Ilagan City	47,697	0	0.00	0	0.00	604	1.27	3	0.50
Santiago City	44,178	27	0.06	0	0.00	8,853	20.04	419	4.73
Tuguegarao City	51,146	0	0.00	0	0.00	0	0.00	0	0.00
**Region 3**	**3,955,879**	**2,242**	**0.06**	**72**	**3.21**	**5,955**	**0.15**	**258**	**4.33**
Aurora	65,675	0	0.00	0	0.00	233	0.35	8	3.43
Bataan	234,928	11	0.00	0	0.00	2,254	0.96	47	2.09
Bulacan	814,727	373	0.05	14	3.75	234	0.03	67	28.63
Nueva Ecija	516,702	0	0.00	0	0.00	8	0.00	8	100.00
Pampanga	590,488	0	0.00	0	0.00	116	0.02	6	5.17
Tarlac	345,548	61	0.02	11	18.03	240	0.07	23	9.58
Zambales	200,514	0	0.00	4	0.00	916	0.46	12	1.31
Angeles City	148,153	146	0.10	4	2.74	807	0.54	30	3.72
Balanga City	33,951	322	0.95	38	11.80	824	2.43	21	2.55
Cabanatuan City	105,753	0	0.00	0	0.00	0	0.00	0	0.00
City of San Fernando	110,374	2	0.00	0	0.00	2	0.00	2	100.00
Gapan City	38,597	0	0.00	0	0.00	78	0.20	0	0.00
Mabalacat City	90,260	7	0.01	0	0.00	0	0.00	0	0.00
Malolos City	90,998	0	0.00	0	0.00	0	0.00	0	0.00
Meycauayan	75,479	0	0.00	0	0.00	3	0.00	3	100.00
Olongapo	79,086	33	0.04	0	0.00	59	0.07	2	3.39
Palayan City	14,360	0	0.00	0	0.00	0	0.00	0	0.00
San Jose City	48,898	0	0.00	0	0.00	0	0.00	0	0.00
San Jose del Monte City	207,251	1,280	0.62	1	0.08	170	0.08	29	17.06
Science City of Munoz	28,511	7	0.02	0	0.00	6	0.02	0	0.00
Tarlac City	115,626	0	0.00	0	0.00	5	0.00	0	0.00
**Region 4A**	**5,195,392**	**2,150**	**0.04**	**190**	**8.84**	**6,164**	**0.12**	**249**	**4.04**
Batangas	677,675	8	0.00	4	50.00	307	0.05	6	1.95
Cavite	524,047	429	0.08	5	1.17	465	0.09	5	1.08
Laguna	363,655	75	0.02	6	8.00	96	0.03	7	7.29
Quezon	551,555	632	0.11	119	18.83	2,890	0.52	125	4.33
Rizal	752,160	182	0.02	20	10.99	992	0.13	37	3.73
Antipolo City	277,047	0	0.00	0	0.00	0	0.00	0	0.00
Bacoor City	229,584	3	0.00	0	0.00	10	0.00	8	80.00
Batangas City	120,273	0	0.00	0	0.00	202	0.17	4	1.98
Biñan City	121,938	141	0.12	2	1.42	53	0.04	4	7.55
Cabuyao City	113,048	3	0.00	1	33.33	14	0.01	1	7.14
Calamba City	166,408	15	0.01	5	33.33	54	0.03	11	20.37
Cavite City	39,302	0	0.00	0	0.00	0	0.00	0	0.00
Dasmariñas City	251,916	374	0.15	7	1.87	366	0.15	8	2.19
General Trias City	120,146	0	0.00	1	0.00	1	0.00	1	100.00
Imus City	154,349	0	0.00	0	0.00	0	0.00	0	0.00
Lipa City	121,179	0	0.00	0	0.00	33	0.03	2	6.06
Lucena City	83,589	0	0.00	0	0.00	61	0.07	22	36.07
San Pablo City	97,421	0	0.00	0	0.00	0	0.00	0	0.00
San Pedro City	119,294	0	0.00	0	0.00	0	0.00	0	0.00
Santa Rosa City	129,531	96	0.07	4	4.17	459	0.35	1	0.22
Tagaytay City	27,212	25	0.09	0	0.00	1	0.00	1	100.00
Tanauan City	63,209	0	0.00	11	0.00	5	0.01	3	60.00

Table 1.F.2 - Cancer Prevention and Control (Cervical and Breast Cancer Screening)

Number and proportion of women aged 20 yrs old and above screened for Cervical and Breast Cancer
Philippines, 2021

Area	Eligible Pop. (20 y/o and above female)	Women screened for Cervical Cancer		Women found positive or suspected for Cervical Cancer		Women screened for breast mass		Women with suspicious breast mass	
		Total	%	Total	%	Total	%	Total	%
Tayabas City	31,330	167	0.53	4	2.40	155	0.49	3	1.94
Trece Martires City	59,524	0	0.00	1	0.00	0	0.00	0	0.00
**Region 4B**	**881,264**	**130**	**0.01**	**7**	**5.38**	**198**	**0.02**	**52**	**26.26**
Marinduque	69,747	0	0.00	0	0.00	2	0.00	2	100.00
Mindoro Occidental	137,748	10	0.01	0	0.00	7	0.01	7	100.00
Mindoro Oriental	256,386	1	0.00	0	0.00	15	0.01	4	26.67
Palawan	255,846	9	0.00	7	77.78	152	0.06	37	24.34
Romblon	84,699	110	0.13	0	0.00	22	0.03	2	9.09
Puerto Princesa City	76,838	0	0.00	0	0.00	0	0.00	0	0.00
**Region 5**	**1,712,762**	**4,970**	**0.29**	**109**	**2.19**	**13,924**	**0.81**	**286**	**2.05**
Albay	350,068	164	0.05	7	4.27	9,545	2.73	94	0.98
Camarines Norte	174,763	1	0.00	1	100.00	63	0.04	1	1.59
Camarines Sur	481,534	312	0.06	3	0.96	1,676	0.35	64	3.82
Catanduanes	74,860	52	0.07	1	1.92	58	0.08	5	8.62
Masbate	245,682	3,833	1.56	1	0.03	398	0.16	11	2.76
Sorsogon	234,197	375	0.16	95	25.33	518	0.22	79	15.25
Iriga City	32,717	9	0.03	0	0.00	711	2.17	15	2.11
Legaspi City	61,560	2	0.00	1	50.00	39	0.06	12	30.77
Naga City	57,381	222	0.39	0	0.00	916	1.60	5	0.55
**Region 6**	**2,445,667**	**548**	**0.02**	**41**	**7.48**	**21,300**	**0.87**	**413**	**1.94**
Aklan	187,098	0	0.00	0	0.00	91	0.05	9	9.89
Antique	176,447	31	0.02	0	0.00	3,252	1.84	41	1.26
Capiz	256,589	23	0.01	0	0.00	1,803	0.70	9	0.50
Guimaras	57,598	0	0.00	0	0.00	2,551	4.43	37	1.45
Iloilo	655,569	100	0.02	21	21.00	4,511	0.69	75	1.66
Negros Occidental	784,244	393	0.05	19	4.83	3,841	0.49	144	3.75
Bacolod City	176,450	1	0.00	1	100.00	76	0.04	31	40.79
Iloilo City	151,672	0	0.00	0	0.00	5,175	3.41	67	1.29
**Region 7**	**2,437,732**	**3,728**	**0.15**	**51**	**1.37**	**29,242**	**1.20**	**481**	**1.64**
Bohol	402,769	0	0.00	0	0.00	51	0.01	16	31.37
Cebu	1,002,404	3,507	0.35	24	0.68	23,169	2.31	301	1.30
Negros Oriental	423,152	0	0.00	0	0.00	607	0.14	63	10.38
Siquijor	31,841	0	0.00	0	0.00	75	0.24	17	22.67
Cebu City	314,676	202	0.06	27	13.37	4,203	1.34	80	1.90
Lapu-Lapu City	139,198	0	0.00	0	0.00	1,137	0.82	4	0.35
Mandaue City	123,692	19	0.02	0	0.00	0	0.00	0	0.00
**Region 8**	**1,341,232**	**95**	**0.01**	**16**	**16.84**	**450**	**0.03**	**31**	**6.89**
Biliran	51,230	29	0.06	12	41.38	340	0.66	7	2.06
Eastern Samar	139,585	0	0.00	0	0.00	64	0.05	3	4.69
Northern Leyte	473,537	3	0.00	0	0.00	3	0.00	2	66.67
Northern Samar	177,699	26	0.01	4	15.38	32	0.02	12	37.50
Southern Leyte	107,291	0	0.00	0	0.00	3	0.00	1	33.33
Western Samar	169,094	37	0.02	0	0.00	0	0.00	0	0.00

Table 1.F.2 - Cancer Prevention and Control (Cervical and Breast Cancer Screening)

Number and proportion of women aged 20 yrs old and above screened for Cervical and Breast Cancer
Philippines, 2021

Area	Eligible Pop. (20 y/o and above female)	Women screened for Cervical Cancer		Women found positive or suspected for Cervical Cancer		Women screened for breast mass		Women with suspicious breast mass	
		Total	%	Total	%	Total	%	Total	%
Calbayog City	52,109	0	0.00	0	0.00	4	0.01	2	50.00
Maasin City	27,307	0	0.00	0	0.00	4	0.01	4	100.00
Ormoc City	67,447	0	0.00	0	0.00	0	0.00	0	0.00
Tacloban City	75,933	0	0.00	0	0.00	0	0.00	0	0.00
**Region 9**	**1,090,436**	**62**	**0.01**	**5**	**8.06**	**2,860**	**0.26**	**72**	**2.52**
Zamboanga del Norte	236,156	25	0.01	2	8.00	692	0.29	21	3.03
Zamboanga del Sur	245,311	11	0.00	1	9.09	279	0.11	24	8.60
Zamboanga Sibugay	187,072	0	0.00	0	0.00	522	0.28	8	1.53
Dapitan City	24,386	0	0.00	0	0.00	0	0.00	0	0.00
Dipolog City	38,684	0	0.00	0	0.00	21	0.05	2	9.52
Isabela City	38,172	0	0.00	0	0.00	4	0.01	4	100.00
Pagadian City	60,166	0	0.00	0	0.00	0	0.00	0	0.00
Zamboanga City	260,489	26	0.01	2	7.69	1,342	0.52	13	0.97
**Region 10**	**1,483,373**	**404**	**0.03**	**17**	**4.21**	**2,192**	**0.15**	**216**	**9.85**
Bukidnon	317,249	201	0.06	3	1.49	627	0.20	132	21.05
Camiguin	27,472	0	0.00	0	0.00	2	0.01	1	50.00
Lanao del Norte	209,572	6	0.00	6	100.00	663	0.32	1	0.15
Misamis Occidental	103,366	17	0.02	0	0.00	10	0.01	1	10.00
Misamis Oriental	237,661	118	0.05	0	0.00	354	0.15	28	7.91
Cagayan de Oro City	225,105	51	0.02	4	7.84	312	0.14	10	3.21
El Salvador City	16,719	0	0.00	0	0.00	90	0.54	0	0.00
Gingoog City	41,505	3	0.01	0	0.00	10	0.02	8	80.00
Iligan City	106,152	3	0.00	0	0.00	18	0.02	2	11.11
Malaybalay City	52,883	0	0.00	0	0.00	6	0.01	1	16.67
Oroquieta City	22,402	0	0.00	0	0.00	0	0.00	3	0.00
Ozamis City	44,895	5	0.01	4	80.00	98	0.22	27	27.55
Tangub City	19,945	0	0.00	0	0.00	2	0.01	2	100.00
Valencia City	58,447	0	0.00	0	0.00	0	0.00	0	0.00
**Region 11**	**1,571,790**	**50,227**	**3.20**	**216**	**0.43**	**22,007**	**1.40**	**1,304**	**5.93**
Davao de Oro	220,081	21,896	9.95	21	0.10	6,078	2.76	209	3.44
Davao del Norte	314,030	7,792	2.48	122	1.57	3,210	1.02	164	5.11
Davao Oriental	166,208	8,223	4.95	9	0.11	3,222	1.94	187	5.80
Davao del Sur	216,201	5,779	2.67	4	0.07	1,162	0.54	92	7.92
Davao Occidental	97,138	4,289	4.42	25	0.58	569	0.59	11	1.93
Davao City	558,132	2,248	0.40	35	1.56	7,766	1.39	641	8.25
**Region 12**	**1,428,635**	**1,603**	**0.11**	**82**	**5.12**	**15,226**	**1.07**	**774**	**5.08**
North Cotabato	436,294	323	0.07	55	17.03	1,354	0.31	80	5.91
Sarangani	159,736	556	0.35	0	0.00	0	0.00	640	0.00
South Cotabato	299,439	140	0.05	0	0.00	2,641	0.88	25	0.95
Sultan Kudarat	246,647	369	0.15	24	6.50	7,179	2.91	18	0.25
Cotabato City	92,050	33	0.04	1	3.03	202	0.22	3	1.49
Gen. Santos City	194,469	182	0.09	2	1.10	3,850	1.98	8	0.21

Table 1.F.2 - Cancer Prevention and Control (Cervical and Breast Cancer Screening)

Number and proportion of women aged 20 yrs old and above screened for Cervical and Breast Cancer
Philippines, 2021

Area	Eligible Pop. (20 y/o and above female)	Women screened for Cervical Cancer		Women found positive or suspected for Cervical Cancer		Women screened for breast mass		Women with suspicious breast mass	
		Total	%	Total	%	Total	%	Total	%
**BARMM**	**1,105,658**	**1,224**	**0.11**	**5**	**0.41**	**5,904**	**0.53**	**123**	**2.08**
Basilan	78,843	0	0.00	0	0.00	6	0.01	0	0.00
Lanao del Sur	246,150	147	0.06	0	0.00	5,818	2.36	79	1.36
Maguindanao	345,163	1,020	0.30	5	0.49	30	0.01	10	33.33
Sulu	245,394	0	0.00	0	0.00	0	0.00	0	0.00
Tawi-Tawi	109,545	0	0.00	0	0.00	0	0.00	0	0.00
Lamitan City	21,691	27	0.12	0	0.00	0	0.00	0	0.00
Marawi City	58,872	30	0.05	0	0.00	50	0.08	34	68.00
**CARAGA**	**777,186**	**332**	**0.04**	**33**	**9.94**	**3,464**	**0.45**	**50**	**1.44**
Agusan del Norte	109,871	125	0.11	1	0.80	2,358	2.15	10	0.42
Agusan del Sur	195,222	44	0.02	11	25.00	36	0.02	6	16.67
Surigao del Norte	105,755	39	0.04	0	0.00	37	0.03	4	10.81
Surigao del Sur	146,414	26	0.02	7	26.92	177	0.12	21	11.86
Province of Dinagat	38,402	23	0.06	0	0.00	26	0.07	0	0.00
Bislig City	27,808	27	0.10	9	33.33	26	0.09	7	26.92
Butuan City	104,461	2	0.00	2	100.00	167	0.16	0	0.00
Surigao City	49,253	46	0.09	3	6.52	637	1.29	2	0.31

Note: Put asterisk (*) for No Report and Zero (0) for No Case

Table 1.F.3 - Cardiovascular Disease and Diabetes Mellitus Prevention and Control

Number and proportion of newly-identified 20 yrs old and above adults with Hypertension and Type 2 Diabetes Mellitus
Philippines, 2021

Area	Total No. of clients ≥ 20 y/o risk-assesed[1]	Newly identified Hypertensive Adults				Newly identified with Type 2 Diabetes Mellitus			
		Male	Female	Total	%	Male	Female	Total	%
**PHILIPPINES**	**5,107,512**	**197,108**	**232,817**	**429,925**	**8.42**	**47,234**	**54,025**	**101,259**	**1.98**
**N C R**	**150,279**	**14,331**	**18,586**	**32,917**	**21.90**	**3,512**	**4,980**	**8,492**	**5.65**
Malabon	4,805	501	522	1,023	21.29	122	156	278	5.79
Navotas	10,650	678	790	1,468	13.78	187	292	479	4.50
Valenzuela City	10,282	726	958	1,684	16.38	243	291	534	5.19
Caloocan City	14,176	466	688	1,154	8.14	81	111	192	1.35
Marikina City	2,741	429	486	915	33.38	78	94	172	6.28
Pasig City	20,643	1,503	2,383	3,886	18.82	470	787	1,257	6.09
Pateros	1,083	156	153	309	28.53	38	37	75	6.93
Taguig	18,761	2,152	2,480	4,632	24.69	656	1,067	1,723	9.18
Quezon City	23,953	2,962	5,117	8,079	33.73	350	770	1,120	4.68
Makati City	2,422	302	381	683	28.20	170	208	378	15.61
Mandaluyong City	10,520	1,262	1,197	2,459	23.37	314	297	611	5.81
San Juan	2,212	86	61	147	6.65	6	12	18	0.81
Manila City	12,642	1,283	1,516	2,799	22.14	382	378	760	6.01
Las Piñas City	3,467	912	1,055	1,967	56.73	215	252	467	13.47
Muntinlupa City	4,937	356	256	612	12.40	64	73	137	2.77
Parañaque City	2,301	71	96	167	7.26	27	25	52	2.26
Pasay City	4,684	486	447	933	19.92	109	130	239	5.10
**C A R**	**105,212**	**3,830**	**4,460**	**8,290**	**7.88**	**484**	**642**	**1,126**	**1.07**
Abra	21,662	970	1,000	1,970	9.09	131	102	233	1.08
Apayao	30,195	269	287	556	1.84	51	67	118	0.39
Benguet	15,364	1,406	1,926	3,332	21.69	174	266	440	2.86
Ifugao	6,285	145	164	309	4.92	10	12	22	0.35
Kalinga	9,616	350	323	673	7.00	59	81	140	1.46
Mt. Province	15,115	390	271	661	4.37	31	69	100	0.66
Baguio City	6,975	300	489	789	11.31	28	45	73	1.05
**Region 1**	**295,633**	**8,975**	**10,154**	**19,129**	**6.47**	**2,056**	**2,683**	**4,739**	**1.60**
Ilocos Norte	10,969	683	833	1,516	13.82	98	127	225	2.05
Ilocos Sur	49,936	934	1,006	1,940	3.88	181	300	481	0.96
La Union	20,033	951	957	1,908	9.52	122	141	263	1.31
Pangasinan	170,610	5,352	6,189	11,541	6.76	1,496	1,879	3,375	1.98
Alaminos City	19,014	238	263	501	2.63	31	38	69	0.36
Candon City	9,599	173	147	320	3.33	40	57	97	1.01
Dagupan City	330	82	80	162	49.09	13	9	22	6.67
Laoag City	975	110	102	212	21.74	5	5	10	1.03
San Carlos City	1,000	17	55	72	7.20	1	1	2	0.20
San Fernando City	795	258	259	517	65.03	44	80	124	15.60
Urdaneta City	11,599	129	204	333	2.87	9	17	26	0.22
Vigan City	773	48	59	107	13.84	16	29	45	5.82
**Region 2**	**432,628**	**5,274**	**5,477**	**10,751**	**2.49**	**678**	**755**	**1,433**	**0.33**
Batanes	2,663	35	20	55	2.07	4	8	12	0.45
Cagayan	66,548	764	528	1,292	1.94	98	90	188	0.28
Isabela	104,351	1,953	2,269	4,222	4.05	155	189	344	0.33
Nueva Vizcaya	52,807	770	800	1,570	2.97	127	148	275	0.52

Table 1.F.3 - Cardiovascular Disease and Diabetes Mellitus Prevention and Control
Number and proportion of newly-identified 20 yrs old and above adults with Hypertension and Type 2 Diabetes Mellitus
Philippines, 2021

Area	Total No. of clients ≥ 20 y/o risk-assesed[1]	Newly identified Hypertensive Adults				Newly identified with Type 2 Diabetes Mellitus			
		Male	Female	Total	%	Male	Female	Total	%
Quirino	80,526	1,139	1,159	2,298	2.85	196	185	381	0.47
Cauayan City	2,937	191	227	418	14.23	24	25	49	1.67
Ilagan City	33,704	139	194	333	0.99	27	44	71	0.21
Santiago City	88,227	230	245	475	0.54	45	60	105	0.12
Tuguegarao City	865	53	35	88	10.17	2	6	8	0.92
**Region 3**	**273,846**	**18,398**	**20,105**	**38,503**	**14.06**	**9,470**	**6,149**	**15,619**	**5.70**
Aurora	977	197	350	547	55.99	51	66	117	11.98
Bataan	18,527	805	998	1,803	9.73	164	235	399	2.15
Bulacan	33,755	2,191	2,166	4,357	12.91	645	722	1,367	4.05
Nueva Ecija	6,122	2,286	2,590	4,876	79.65	2,731	219	2,950	48.19
Pampanga	12,614	907	1,119	2,026	16.06	1,150	269	1,419	11.25
Tarlac	73,090	7,216	7,334	14,550	19.91	3,528	3,012	6,540	8.95
Zambales	45,558	985	1,250	2,235	4.91	144	198	342	0.75
Angeles City	1,587	353	376	729	45.94	71	92	163	10.27
Balanga City	1,462	282	391	673	46.03	60	102	162	11.08
Cabanatuan City	12,484	748	846	1,594	12.77	135	170	305	2.44
City of San Fernando	4,946	272	308	580	11.73	105	114	219	4.43
Gapan City	213	91	68	159	74.65	34	29	63	29.58
Mabalacat City	31,620	336	372	708	2.24	103	133	236	0.75
Malolos City	5,078	185	249	434	8.55	50	96	146	2.88
Meycauayan	970	237	254	491	50.62	74	83	157	16.19
Olongapo	1,144	258	268	526	45.98	26	24	50	4.37
Palayan City	89	28	31	59	66.29	3	9	12	13.48
San Jose City	1,749	24	27	51	2.92	7	17	24	1.37
San Jose del Monte City	17,211	436	472	908	5.28	155	204	359	2.09
Science City of Munoz	3,180	116	158	274	8.62	57	92	149	4.69
Tarlac City	1,470	445	478	923	62.79	177	263	440	29.93
**Region 4A**	**230,360**	**16,209**	**19,671**	**35,880**	**15.58**	**4,377**	**4,939**	**9,316**	**4.04**
Batangas	68,163	2,681	3,395	6,076	8.91	779	941	1,720	2.52
Cavite	16,840	1,529	2,383	3,912	23.23	628	728	1,356	8.05
Laguna	7,299	1,586	1,707	3,293	45.12	336	375	711	9.74
Quezon	34,727	1,600	2,369	3,969	11.43	242	373	615	1.77
Rizal	18,034	3,883	4,176	8,059	44.69	1,496	1,305	2,801	15.53
Antipolo City	103	0	0	0	0.00	0	0	0	0.00
Bacoor City	426	116	158	274	64.32	39	61	100	23.47
Batangas City	2,055	163	201	364	17.71	36	39	75	3.65
Biñan City	3,865	452	418	870	22.51	128	143	271	7.01
Cabuyao City	2,802	1,242	1,396	2,638	94.15	64	58	122	4.35
Calamba City	5,785	576	589	1,165	20.14	181	184	365	6.31
Cavite City	1,996	199	203	402	20.14	40	50	90	4.51
Dasmariñas City	4,202	656	682	1,338	31.84	132	164	296	7.04
General Trias City	392	49	38	87	22.19	9	15	24	6.12
Imus City	3,798	398	521	919	24.20	83	239	322	8.48
Lipa City	29,345	29	25	54	0.18	4	16	20	0.07
Lucena City	109	89	118	207	189.91	6	19	25	22.94
San Pablo City	319	114	205	319	100.00	28	50	78	24.45
San Pedro City	563	0	0	0	0.00	0	0	0	0.00
Santa Rosa City	1,275	240	376	616	48.31	48	62	110	8.63
Tagaytay City	13,268	130	107	237	1.79	27	35	62	0.47
Tanauan City	12,083	13	3	16	0.13	1	0	1	0.01
Tayabas City	1,239	436	578	1,014	81.84	64	73	137	11.06
Trece Martires City	1,672	28	23	51	3.05	6	9	15	0.90

Table 1.F.3 - Cardiovascular Disease and Diabetes Mellitus Prevention and Control

Number and proportion of newly-identified 20 yrs old and above adults with Hypertension and Type 2 Diabetes Mellitus
Philippines, 2021

Area	Total No. of clients ≥ 20 y/o risk-assesed[1]	Newly identified Hypertensive Adults				Newly identified with Type 2 Diabetes Mellitus			
		Male	Female	Total	%	Male	Female	Total	%
**Region 4B**	**32,920**	**4,255**	**3,588**	**7,843**	**23.82**	**4,255**	**3,588**	**7,843**	**23.82**
Marinduque	3,902	174	200	374	9.58	174	200	374	9.58
Mindoro Occidental	10,214	2,479	1,678	4,157	40.70	2,479	1,678	4,157	40.70
Mindoro Oriental	1,258	411	462	873	69.40	411	462	873	69.40
Palawan	16,637	875	944	1,819	10.93	875	944	1,819	10.93
Romblon	700	227	211	438	62.57	227	211	438	62.57
Puerto Princesa City	209	89	93	182	87.08	89	93	182	87.08
**Region 5**	**455,925**	**9,684**	**13,176**	**22,860**	**5.01**	**1,064**	**1,527**	**2,591**	**0.57**
Albay	108,299	2,253	3,688	5,941	5.49	207	297	504	0.47
Camarines Norte	42,443	1,010	1,186	2,196	5.17	113	157	270	0.64
Camarines Sur	143,601	2,165	2,850	5,015	3.49	194	351	545	0.38
Catanduanes	8,040	338	407	745	9.27	29	36	65	0.81
Masbate	20,516	948	1,291	2,239	10.91	119	193	312	1.52
Sorsogon	110,729	1,882	2,299	4,181	3.78	284	357	641	0.58
Iriga City	13,076	314	491	805	6.16	34	42	76	0.58
Legaspi City	6,875	456	599	1,055	15.35	47	51	98	1.43
Naga City	2,346	318	365	683	29.11	37	43	80	3.41
**Region 6**	**404,346**	**40,748**	**48,711**	**89,459**	**22.12**	**8,496**	**11,450**	**19,946**	**4.93**
Aklan	44,200	5,902	7,433	13,335	30.17	761	1,033	1,794	4.06
Antique	44,138	8,277	11,025	19,302	43.73	1,543	2,143	3,686	8.35
Capiz	38,139	8,263	12,175	20,438	53.59	2,176	3,495	5,671	14.87
Guimaras	35,682	2,731	2,872	5,603	15.70	358	626	984	2.76
Iloilo	107,985	11,570	10,394	21,964	20.34	2,627	2,739	5,366	4.97
Negros Occidental	112,650	2,287	2,704	4,991	4.43	499	667	1,166	1.04
Bacolod City	3,802	321	477	798	20.99	56	104	160	4.21
Iloilo City	17,750	1,397	1,631	3,028	17.06	476	643	1,119	6.30
**Region 7**	**168,470**	**8,019**	**9,805**	**17,824**	**10.58**	**1,028**	**1,475**	**2,503**	**1.49**
Bohol	3,324	350	415	765	23.01	16	23	39	1.17
Cebu	129,084	4,769	5,425	10,194	7.90	551	777	1,328	1.03
Negros Oriental	14,482	731	1,302	2,033	14.04	109	218	327	2.26
Siquijor	6,493	182	248	430	6.62	38	50	88	1.36
Cebu City	5,750	917	1,182	2,099	36.50	178	232	410	7.13
Lapu-Lapu City	4,518	847	1,040	1,887	41.77	127	165	292	6.46
Mandaue City	4,819	223	193	416	8.63	9	10	19	0.39
**Region 8**	**132,217**	**13,529**	**16,908**	**30,437**	**23.02**	**1,754**	**2,501**	**4,255**	**3.22**
Biliran	3,316	64	76	140	4.22	37	38	75	2.26
Eastern Samar	19,292	509	656	1,165	6.04	107	114	221	1.15
Northern Leyte	14,083	5,710	8,373	14,083	100.00	1,113	1,668	2,781	19.75
Northern Samar	15,258	442	570	1,012	6.63	73	138	211	1.38
Southern Leyte	48,262	803	856	1,659	3.44	143	197	340	0.70
Western Samar	19,817	4,925	5,236	10,161	51.27	41	40	81	0.41
Calbayog City	652	119	160	279	42.79	32	108	140	21.47
Maasin City	1,993	110	97	207	10.39	26	43	69	3.46
Ormoc City	2,119	632	612	1,244	58.71	133	114	247	11.66

Table 1.F.3 - Cardiovascular Disease and Diabetes Mellitus Prevention and Control

Number and proportion of newly-identified 20 yrs old and above adults with Hypertension and Type 2 Diabetes Mellitus
Philippines, 2021

Area	Total No. of clients ≥ 20 y/o risk-assesed[1]	Newly identified Hypertensive Adults				Newly identified with Type 2 Diabetes Mellitus			
		Male	Female	Total	%	Male	Female	Total	%
Tacloban City	7,425	215	272	487	6.56	49	41	90	1.21
**Region 9**	**112,869**	**7,106**	**8,194**	**15,300**	**13.56**	**987**	**1,394**	**2,381**	**2.11**
Zamboanga del Norte	28,067	1,837	2,236	4,073	14.51	304	393	697	2.48
Zamboanga del Sur	59,824	1,483	1,758	3,241	5.42	222	379	601	1.00
Zamboanga Sibugay	10,593	918	1,091	2,009	18.97	100	176	276	2.61
Dapitan City	5,336	1,411	1,419	2,830	53.04	43	31	74	1.39
Dipolog City	995	330	344	674	67.74	51	41	92	9.25
Isabela City	196	60	75	135	68.88	16	24	40	20.41
Pagadian City	201	167	175	342	170.15	50	71	121	60.20
Zamboanga City	7,657	900	1,096	1,996	26.07	201	279	480	6.27
**Region 10**	**342,640**	**6,996**	**6,368**	**13,364**	**3.90**	**986**	**1,215**	**2,201**	**0.64**
Bukidnon	50,399	1,791	1,268	3,059	6.07	105	145	250	0.50
Camiguin	177	96	81	177	100.00	11	34	45	25.42
Lanao del Norte	18,611	805	887	1,692	9.09	95	121	216	1.16
Misamis Occidental	23,411	687	554	1,241	5.30	103	102	205	0.88
Misamis Oriental	52,261	796	527	1,323	2.53	199	117	316	0.60
Cagayan de Oro City	169,240	361	344	705	0.42	49	54	103	0.06
El Salvador City	85	37	47	84	98.82	4	2	6	7.06
Gingoog City	537	99	81	180	33.52	43	127	170	31.66
Iligan City	3,632	1,174	1,231	2,405	66.22	202	253	455	12.53
Malaybalay City	7,595	63	73	136	1.79	6	10	16	0.21
Oroquieta City	32	0	32	32	100.00	0	0	0	0.00
Ozamis City	9,544	743	808	1,551	16.25	119	180	299	3.13
Tangub City	593	102	90	192	32.38	24	34	58	9.78
Valencia City	6,523	242	345	587	9.00	26	36	62	0.95
**Region 11**	**256,162**	**21,610**	**25,437**	**47,047**	**18.37**	**4,460**	**5,327**	**9,787**	**3.82**
Davao de Oro	46,858	3,735	2,779	6,514	13.90	407	542	949	2.03
Davao del Norte	78,086	5,775	6,283	12,058	15.44	1,804	1,769	3,573	4.58
Davao Oriental	48,409	3,738	4,599	8,337	17.22	283	517	800	1.65
Davao del Sur	50,050	4,481	7,118	11,599	23.17	910	1,164	2,074	4.14
Davao Occidental	12,829	466	625	1,091	8.50	86	108	194	1.51
Davao City	19,930	3,415	4,033	7,448	37.37	970	1,227	2,197	11.02
**Region 12**	**253,256**	**11,159**	**13,735**	**24,894**	**9.83**	**2,206**	**3,468**	**5,674**	**2.24**
North Cotabato	79,623	2,308	2,531	4,839	6.08	341	462	803	1.01
Sarangani	68,035	2,202	2,764	4,966	7.30	397	530	927	1.36
South Cotabato	25,248	1,819	1,985	3,804	15.07	342	365	707	2.80
Sultan Kudarat	69,143	4,110	5,217	9,327	13.49	997	1,659	2,656	3.84
Cotabato City	8,209	362	528	890	10.84	60	100	160	1.95
Gen. Santos City	2,998	358	710	1,068	35.62	69	352	421	14.04

Table 1.F.3 - Cardiovascular Disease and Diabetes Mellitus Prevention and Control

Number and proportion of newly-identified 20 yrs old and above adults with Hypertension and Type 2 Diabetes Mellitus
Philippines, 2021

Area	Total No. of clients ≥ 20 y/o risk-assesed[1]	Newly identified Hypertensive Adults				Newly identified with Type 2 Diabetes Mellitus			
		Male	Female	Total	%	Male	Female	Total	%
**BARMM**	**1,404,520**	**3,783**	**4,911**	**8,694**	**0.62**	**806**	**961**	**1,767**	**0.13**
Basilan	0	283	413	696	0.00	25	39	64	0.00
Lanao del Sur	15,504	1,814	2,270	4,084	26.34	427	562	989	6.38
Maguindanao	1,381,190	1,387	1,710	3,097	0.22	285	288	573	0.04
Sulu	0	0	0	0	0.00	0	0	0	0.00
Tawi-Tawi	0	0	0	0	0.00	0	0	0	0.00
Lamitan City	0	21	16	37	0.00	5	2	7	0.00
Marawi City	7,826	278	502	780	9.97	64	70	134	1.71
**CARAGA**	**56,229**	**3,202**	**3,531**	**6,733**	**11.97**	**615**	**971**	**1,586**	**2.82**
Agusan del Norte	8,697	360	388	748	8.60	79	130	209	2.40
Agusan del Sur	25,292	416	431	847	3.35	65	80	145	0.57
Surigao del Norte	7,281	675	696	1,371	18.83	138	186	324	4.45
Surigao del Sur	6,103	855	1,175	2,030	33.26	172	357	529	8.67
Province of Dinagat	1,273	137	132	269	21.13	40	67	107	8.41
Bislig City	1,086	88	67	155	14.27	29	33	62	5.71
Butuan City	4,485	463	464	927	20.67	37	67	104	2.32
Surigao City	2,012	208	178	386	19.18	55	51	106	5.27

1Total adults 20 yrs old and above who were risk-assessed using the PhilPEN protocol
Note: Put asterisk (*) for No Report and Zero (0) for No Case

Table 1.F.4 - Blindness Prevention Program

Number and proportion of senior citizens 60 yrs old and above screened visual acuity and diagnosed with eye diseases

Philippines, 2021

Area	Eligible Pop.(60 y/o and above)	Screened for Visual Acuity				Diagnosed with eye diseases			
		Male	Female	Total	%	Male	Female	Total	%
**PHILIPPINES**	**9,863,061**	**58,657**	**67,478**	**126,135**	**1.28**	**5,763**	**7,640**	**13,403**	**10.63**
**N C R**	**1,283,374**	**2,774**	**4,074**	**6,848**	**0.53**	**1,275**	**2,146**	**3,421**	**49.96**
Malabon	36,430	15	16	31	0.09	5	2	7	22.58
Navotas	24,865	19	24	43	0.17	2	4	6	13.95
Valenzuela City	61,826	7	19	26	0.04	7	19	26	100.00
Caloocan City	157,866	320	375	695	0.44	45	47	92	13.24
Marikina City	44,923	0	1	1	0.00	0	1	1	100.00
Pasig City	75,277	1,474	2,201	3,675	4.88	410	897	1,307	35.56
Pateros	6,362	0	0	0	0.00	0	0	0	0.00
Taguig	80,220	7	32	39	0.05	7	7	14	35.90
Quezon City	292,619	611	1,021	1,632	0.56	611	1,021	1,632	100.00
Makati City	58,062	84	143	227	0.39	49	59	108	47.58
Mandaluyong City	38,498	1	1	2	0.01	1	1	2	100.00
San Juan	12,176	0	0	0	0.00	0	0	0	0.00
Manila City	177,414	128	168	296	0.17	30	21	51	17.23
Las Piñas City	58,691	45	59	104	0.18	45	59	104	100.00
Muntinlupa City	50,281	61	8	69	0.14	61	8	69	100.00
Parañaque City	66,358	2	0	2	0.00	2	0	2	100.00
Pasay City	41,506	0	6	6	0.01	0	0	0	0.00
**C A R**	**155,330**	**1,221**	**1,699**	**2,920**	**1.88**	**136**	**168**	**304**	**10.41**
Abra	26,995	72	91	163	0.60	3	1	4	2.45
Apayao	10,310	321	409	730	7.08	20	29	49	6.71
Benguet	39,401	115	229	344	0.87	24	58	82	23.84
Ifugao	16,163	40	51	91	0.56	19	24	43	47.25
Kalinga	17,855	35	60	95	0.53	9	19	28	29.47
Mt. Province	14,115	373	626	999	7.08	15	37	52	5.21
Baguio City	30,491	265	233	498	1.63	46	0	46	9.24
**Region 1**	**576,290**	**981**	**1,328**	**2,309**	**0.40**	**216**	**266**	**482**	**20.87**
Ilocos Norte	63,187	5	4	9	0.01	2	0	2	22.22
Ilocos Sur	74,192	336	567	903	1.22	38	61	99	10.96
La Union	81,852	67	134	201	0.25	13	13	26	12.94
Pangasinan	251,107	304	335	639	0.25	121	159	280	43.82
Alaminos City	9,490	63	51	114	1.20	18	11	29	25.44
Candon City	7,820	206	237	443	5.66	23	21	44	9.93
Dagupan City	18,116	0	0	0	0.00	0	0	0	0.00
Laoag City	14,576	0	0	0	0.00	0	0	0	0.00
San Carlos City	19,945	0	0	0	0.00	1	1	2	0.00
San Fernando City	14,997	0	0	0	0.00	0	0	0	0.00
Urdaneta City	14,062	0	0	0	0.00	0	0	0	0.00
Vigan City	6,946	0	0	0	0.00	0	0	0	0.00
**Region 2**	**366,093**	**3,516**	**4,681**	**8,197**	**2.24**	**334**	**424**	**758**	**9.25**
Batanes	2,050	0	0	0	0.00	0	0	0	0.00
Cagayan	118,251	437	463	900	0.76	31	45	76	8.44
Isabela	122,393	350	573	923	0.75	59	115	174	18.85

Table 1.F.4 - Blindness Prevention Program

Number and proportion of senior citizens 60 yrs old and above screened visual acuity and diagnosed with eye diseases

Philippines, 2021

Area	Eligible Pop.(60 y/o and above)	Screened for Visual Acuity				Diagnosed with eye diseases			
		Male	Female	Total	%	Male	Female	Total	%
Nueva Vizcaya	46,314	90	144	234	0.51	19	19	38	16.24
Quirino	17,353	105	191	296	1.71	45	24	69	23.31
Cauayan City	13,393	0	0	0	0.00	0	0	0	0.00
Ilagan City	15,047	385	199	584	3.88	16	18	34	5.82
Santiago City	13,938	2,149	3,111	5,260	37.74	164	203	367	6.98
Tuguegarao City	17,354	0	0	0	0.00	0	0	0	0.00
**Region 3**	**1,196,922**	**607**	**615**	**1,222**	**0.10**	**151**	**170**	**321**	**26.27**
Aurora	19,355	0	0	0	0.00	0	0	0	0.00
Bataan	70,333	58	60	118	0.17	20	22	42	35.59
Bulacan	237,039	98	112	210	0.09	19	28	47	22.38
Nueva Ecija	163,833	102	50	152	0.09	25	22	47	30.92
Pampanga	175,014	51	56	107	0.06	13	12	25	23.36
Tarlac	110,293	174	201	375	0.34	54	61	115	30.67
Zambales	62,379	2	2	4	0.01	2	2	4	100.00
Angeles City	43,920	0	0	0	0.00	0	0	0	0.00
Balanga City	10,166	0	0	0	0.00	0	0	0	0.00
Cabanatuan City	33,532	0	0	0	0.00	0	0	0	0.00
City of San Fernando	32,715	10	15	25	0.08	5	7	12	48.00
Gapan City	12,240	0	0	0	0.00	0	0	0	0.00
Mabalacat City	26,760	4	6	10	0.04	3	6	9	90.00
Malolos City	26,480	0	0	0	0.00	0	0	0	0.00
Meycauayan	21,963	1	1	2	0.01	0	0	0	0.00
Olongapo	24,605	21	20	41	0.17	0	0	0	0.00
Palayan City	4,555	0	0	0	0.00	0	0	0	0.00
San Jose City	15,502	0	0	0	0.00	0	0	0	0.00
San Jose del Monte City	60,300	78	80	158	0.26	4	2	6	3.80
Science City of Munoz	9,036	8	12	20	0.22	6	6	12	60.00
Tarlac City	36,902	0	0	0	0.00	0	2	2	0.00
**Region 4A**	**1,425,123**	**751**	**1,005**	**1,756**	**0.12**	**176**	**235**	**411**	**23.41**
Batangas	201,100	79	119	198	0.10	25	35	60	30.30
Cavite	136,403	48	97	145	0.11	0	2	2	1.38
Laguna	96,210	60	61	121	0.13	10	11	21	17.36
Quezon	169,590	105	194	299	0.18	22	74	96	32.11
Rizal	196,387	20	23	43	0.02	1	1	2	4.65
Antipolo City	72,336	0	0	0	0.00	0	0	0	0.00
Bacoor City	59,758	46	56	102	0.17	2	2	4	3.92
Batangas City	35,685	0	0	0	0.00	0	0	0	0.00
Biñan City	32,260	104	104	208	0.64	33	31	64	30.77
Cabuyao City	29,911	4	3	7	0.02	1	1	2	28.57
Calamba City	44,024	214	256	470	1.07	37	33	70	14.89
Cavite City	10,222	0	0	0	0.00	1	0	1	0.00
Dasmariñas City	65,573	13	9	22	0.03	10	9	19	86.36
General Trias City	31,271	0	0	0	0.00	0	0	0	0.00
Imus City	40,176	0	0	0	0.00	0	0	0	0.00
Lipa City	35,958	27	29	56	0.16	24	30	54	96.43
Lucena City	25,705	3	2	5	0.02	3	1	4	80.00
San Pablo City	25,770	26	50	76	0.29	0	0	0	0.00
San Pedro City	31,559	0	0	0	0.00	0	0	0	0.00
Santa Rosa City	34,270	0	0	0	0.00	0	0	0	0.00
Tagaytay City	7,076	2	2	4	0.06	6	5	11	275.00
Tanauan City	18,753	0	0	0	0.00	1	0	1	0.00

Table 1.F.4 - Blindness Prevention Program

Number and proportion of senior citizens 60 yrs old and above screened visual acuity and diagnosed with eye diseases

Philippines, 2021

Area	Eligible Pop.(60 y/o and above)	Screened for Visual Acuity				Diagnosed with eye diseases			
		Male	Female	Total	%	Male	Female	Total	%
Tayabas City	9,632	0	0	0	0.00	0	0	0	0.00
Trece Martires City	15,494	0	0	0	0.00	0	0	0	0.00
**Region 4B**	**268,267**	**96**	**101**	**197**	**0.07**	**61**	**75**	**136**	**69.04**
Marinduque	26,815	0	0	0	0.00	0	0	0	0.00
Mindoro Occidental	40,501	17	0	17	0.04	0	0	0	0.00
Mindoro Oriental	78,522	2	6	8	0.01	1	2	3	37.50
Palawan	70,564	75	89	164	0.23	58	69	127	77.44
Romblon	30,678	2	6	8	0.03	2	4	6	75.00
		0	0			0	0		
Puerto Princesa City	21,187	0	0	0	0.00	0	0	0	0.00
**Region 5**	**536,548**	**1,685**	**2,533**	**4,218**	**0.79**	**409**	**686**	**1,095**	**25.96**
Albay	114,224	460	615	1,075	0.94	79	125	204	18.98
Camarines Norte	53,524	66	129	195	0.36	8	25	33	16.92
Camarines Sur	147,958	455	741	1,196	0.81	82	168	250	20.90
Catanduanes	24,626	10	6	16	0.06	6	1	7	43.75
Masbate	74,206	114	151	265	0.36	2	14	16	6.04
Sorsogon	74,241	277	470	747	1.01	75	119	194	25.97
Iriga City	10,050	186	270	456	4.54	118	174	292	64.04
Legaspi City	20,090	114	151	265	1.32	39	60	99	37.36
Naga City	17,629	3	0	3	0.02	0	0	0	0.00
**Region 6**	**843,227**	**653**	**1,029**	**1,682**	**0.20**	**174**	**264**	**438**	**26.04**
Aklan	63,617	117	134	251	0.39	14	9	23	9.16
Antique	61,386	96	156	252	0.41	45	83	128	50.79
Capiz	90,290	28	38	66	0.07	1	2	3	4.55
Guimaras	20,021	23	33	56	0.28	0	0	0	0.00
Iloilo	232,571	68	95	163	0.07	37	46	83	50.92
Negros Occidental	262,479	300	537	837	0.32	68	106	174	20.79
Bacolod City	59,054	8	12	20	0.03	6	8	14	70.00
Iloilo City	53,809	13	24	37	0.07	3	10	13	35.14
**Region 7**	**771,468**	**191**	**231**	**422**	**0.05**	**139**	**170**	**309**	**73.22**
Bohol	148,855	0	0	0	0.00	0	0	0	0.00
Cebu	294,062	103	132	235	0.08	103	132	235	100.00
Negros Oriental	146,058	61	57	118	0.08	34	33	67	56.78
Siquijor	13,077	8	9	17	0.13	1	1	2	11.76
Cebu City	92,303	12	27	39	0.04	1	3	4	10.26
Lapu-Lapu City	40,831	7	6	13	0.03	0	1	1	7.69
Mandaue City	36,282	0	0	0	0.00	0	0	0	0.00
**Region 8**	**447,308**	**233**	**302**	**535**	**0.12**	**60**	**46**	**106**	**19.81**
Biliran	17,454	3	1	4	0.02	2	0	2	50.00
Eastern Samar	48,109	118	117	235	0.49	11	14	25	10.64
Northern Leyte	160,069	0	0	0	0.00	0	0	0	0.00
Northern Samar	52,294	45	29	74	0.14	44	28	72	97.30
Southern Leyte	40,971	58	147	205	0.50	3	4	7	3.41
Western Samar	53,138	4	2	6	0.01	0	0	0	0.00

Table 1.F.4 - Blindness Prevention Program

Number and proportion of senior citizens 60 yrs old and above screened visual acuity and diagnosed with eye diseases

Philippines, 2021

Area	Eligible Pop.(60 y/o and above)	Screened for Visual Acuity				Diagnosed with eye diseases			
		Male	Female	Total	%	Male	Female	Total	%
Calbayog City	16,375	5	6	11	0.07	0	0	0	0.00
Maasin City	10,434	0	0	0	0.00	0	0	0	0.00
Ormoc City	22,800	0	0	0	0.00	0	0	0	0.00
Tacloban City	25,664	0	0	0	0.00	0	0	0	0.00
**Region 9**	**311,996**	**380**	**521**	**901**	**0.29**	**89**	**126**	**215**	**23.86**
Zamboanga del Norte	74,069	182	241	423	0.57	50	58	108	25.53
Zamboanga del Sur	68,881	78	104	182	0.26	32	58	90	49.45
Zamboanga Sibugay	49,678	41	40	81	0.16	0	1	1	1.23
Dapitan City	7,648	0	0	0	0.00	0	0	0	0.00
Dipolog City	12,134	65	109	174	1.43	0	1	1	0.57
Isabela City	9,583	4	5	9	0.09	4	4	8	88.89
Pagadian City	16,889	0	0	0	0.00	0	0	0	0.00
Zamboanga City	73,114	10	22	32	0.04	3	4	7	21.88
**Region 10**	**434,177**	**2,232**	**3,103**	**5,335**	**1.23**	**386**	**498**	**884**	**16.57**
Bukidnon	87,168	213	297	510	0.59	117	134	251	49.22
Camiguin	10,332	0	0	0	0.00	0	0	0	0.00
Lanao del Norte	54,320	767	878	1,645	3.03	39	60	99	6.02
Misamis Occidental	36,852	37	55	92	0.25	37	55	92	100.00
Misamis Oriental	71,302	47	84	131	0.18	21	31	52	39.69
Cagayan de Oro City	67,526	31	40	71	0.11	16	19	35	49.30
El Salvador City	5,016	0	0	0	0.00	0	0	0	0.00
Gingoog City	12,455	0	0	0	0.00	0	0	0	0.00
Iligan City	27,512	25	34	59	0.21	10	6	16	27.12
Malaybalay City	14,531	91	88	179	1.23	10	10	20	11.17
Oroquieta City	7,983	0	0	0	0.00	0	0	0	0.00
Ozamis City	16,007	990	1,597	2,587	16.16	130	178	308	11.91
Tangub City	7,113	15	11	26	0.37	6	4	10	38.46
Valencia City	16,060	16	19	35	0.22	0	1	1	2.86
**Region 11**	**458,573**	**41,914**	**44,324**	**86,238**	**18.81**	**1,829**	**1,906**	**3,735**	**4.33**
Davao de Oro	62,728	4,325	4,352	8,677	13.83	377	227	604	6.96
Davao del Norte	92,328	16,526	18,309	34,835	37.73	375	459	834	2.39
Davao Oriental	52,133	11,024	10,692	21,716	41.65	138	223	361	1.66
Davao del Sur	62,437	4,779	5,174	9,953	15.94	253	289	542	5.45
Davao Occidental	27,794	3,703	4,156	7,859	28.28	456	444	900	11.45
Davao City	161,153	1,557	1,641	3,198	1.98	230	264	494	15.45
**Region 12**	**365,094**	**960**	**1,241**	**2,201**	**0.60**	**217**	**312**	**529**	**24.03**
North Cotabato	116,481	102	159	261	0.22	84	142	226	86.59
Sarangani	39,460	453	555	1,008	2.55	62	65	127	12.60
South Cotabato	78,396	233	281	514	0.66	5	1	6	1.17
Sultan Kudarat	60,895	85	127	212	0.35	65	99	164	77.36
Cotabato City	18,952	56	88	144	0.76	1	5	6	4.17
Gen. Santos City	50,910	31	31	62	0.12	0	0	0	0.00

Table 1.F.4 - Blindness Prevention Program

Number and proportion of senior citizens 60 yrs old and above screened visual acuity and diagnosed with eye diseases
Philippines, 2021

Area	Eligible Pop.(60 y/o and above)	Screened for Visual Acuity				Diagnosed with eye diseases			
		Male	Female	Total	%	Male	Female	Total	%
**BARMM**	**186,087**	**309**	**458**	**767**	**0.41**	**57**	**63**	**120**	**15.65**
Basilan	14,423	13	14	27	0.19	0	0	0	0.00
Lanao del Sur	37,784	5	9	14	0.04	6	9	15	107.14
Maguindanao	58,962	247	369	616	1.04	49	48	97	15.75
Sulu	42,187	0	0	0	0.00	0	0	0	0.00
Tawi-Tawi	19,727	0	0	0	0.00	0	0	0	0.00
Lamitan City	3,973	0	0	0	0.00	0	0	0	0.00
Marawi City	9,031	44	66	110	1.22	2	6	8	7.27
**CARAGA**	**237,184**	**154**	**233**	**387**	**0.16**	**54**	**85**	**139**	**35.92**
Agusan del Norte	33,726	4	16	20	0.06	6	9	15	75.00
Agusan del Sur	51,807	20	33	53	0.10	12	12	24	45.28
Surigao del Norte	34,096	27	60	87	0.26	2	17	19	21.84
Surigao del Sur	47,096	43	44	87	0.18	8	10	18	20.69
Province of Dinagat	13,570	37	45	82	0.60	8	9	17	20.73
Bislig City	8,948	12	13	25	0.28	11	12	23	92.00
Butuan City	32,059	9	20	29	0.09	5	14	19	65.52
Surigao City	15,882	2	2	4	0.03	2	2	4	100.00

Note: Put asterisk (*) for No Report and Zero (0) for No Case

Table 1.F.5 - Immunization for Senior Citizen

Number and proportion of senior citizen 60 yrs old and above who received one (1) dose of Pneumococcal Polysaccharide Vaccine (PPV) and one (1) dose of Influenza Vaccine

Philippines, 2021

Area	Eligible Pop. (60 y/o & above)	Received one (1) dose of PPV				Received one (1) dose of Influenza Vaccine			
		Male	Female	Total	%	Male	Female	Total	%
**PHILIPPINES**	**9,863,061**	**78,189**	**107,587**	**185,776**	**1.88**	**307,654**	**443,996**	**751,650**	**7.62**
**N C R**	**1,283,374**	**16,256**	**22,123**	**38,379**	**2.99**	**67,968**	**96,146**	**164,114**	**12.79**
Malabon	36,430	222	312	534	1.47	1,563	1,926	3,489	9.58
Navotas	24,865	208	282	490	1.97	2,072	3,083	5,155	20.73
Valenzuela City	61,826	1,471	1,916	3,387	5.48	3,355	4,888	8,243	13.33
Caloocan City	157,866	888	1,259	2,147	1.36	7,740	12,028	19,768	12.52
Marikina City	44,923	714	982	1,696	3.78	3,000	4,763	7,763	17.28
Pasig City	75,277	1,375	2,100	3,475	4.62	7,367	11,061	18,428	24.48
Pateros	6,362	107	162	269	4.23	758	1,008	1,766	27.76
Taguig	80,220	519	855	1,374	1.71	3,197	4,854	8,051	10.04
Quezon City	292,619	4,762	6,971	11,733	4.01	21,004	29,470	50,474	17.25
Makati City	58,062	1	0	1	0.00	1	4	5	0.01
Mandaluyong City	38,498	1,184	1,088	2,272	5.90	2,291	2,726	5,017	13.03
San Juan	12,176	83	120	203	1.67	829	1,058	1,887	15.50
Manila City	177,414	2,914	3,418	6,332	3.57	10,476	12,488	22,964	12.94
Las Piñas City	58,691	1,407	1,869	3,276	5.58	3,214	4,848	8,062	13.74
Muntinlupa City	50,281	339	654	993	1.97	388	788	1,176	2.34
Parañaque City	66,358	2	0	2	0.00	2	0	2	0.00
Pasay City	41,506	60	135	195	0.47	711	1,153	1,864	4.49
**C A R**	**155,330**	**813**	**1,126**	**1,939**	**1.25**	**7,291**	**12,100**	**19,391**	**12.48**
Abra	26,995	67	43	110	0.41	1,208	2,052	3,260	12.08
Apayao	10,310	85	114	199	1.93	548	923	1,471	14.27
Benguet	39,401	92	154	246	0.62	963	1,862	2,825	7.17
Ifugao	16,163	41	77	118	0.73	435	676	1,111	6.87
Kalinga	17,855	37	57	94	0.53	647	1,010	1,657	9.28
Mt. Province	14,115	112	187	299	2.12	326	771	1,097	7.77
Baguio City	30,491	379	494	873	2.86	3,164	4,806	7,970	26.14
**Region 1**	**576,290**	**5,680**	**8,646**	**14,326**	**2.49**	**18,042**	**27,891**	**45,933**	**7.97**
Ilocos Norte	63,187	898	1,329	2,227	3.52	1,871	3,007	4,878	7.72
Ilocos Sur	74,192	447	834	1,281	1.73	2,124	2,841	4,965	6.69
La Union	81,852	919	1,407	2,326	2.84	2,928	5,151	8,079	9.87
Pangasinan	251,107	825	1,313	2,138	0.85	7,313	11,431	18,744	7.46
Alaminos City	9,490	48	54	102	1.07	778	1,003	1,781	18.77
Candon City	7,820	0	0	0	0.00	0	0	0	0.00
Dagupan City	18,116	435	740	1,175	6.49	0	0	0	0.00
Laoag City	14,576	111	168	279	1.91	606	875	1,481	10.16
San Carlos City	19,945	32	82	114	0.57	198	511	709	3.55
San Fernando City	14,997	0	0	0	0.00	0	0	0	0.00
Urdaneta City	14,062	1,965	2,719	4,684	33.31	2,224	3,072	5,296	37.66
Vigan City	6,946	0	0	0	0.00	0	0	0	0.00
**Region 2**	**366,093**	**3,561**	**4,559**	**8,120**	**2.22**	**17,740**	**27,576**	**45,316**	**12.38**
Batanes	2,050	24	35	59	2.88	239	369	608	29.66
Cagayan	118,251	452	581	1,033	0.87	1,874	2,788	4,662	3.94
Isabela	122,393	1,019	1,567	2,586	2.11	6,095	10,510	16,605	13.57
Nueva Vizcaya	46,314	383	552	935	2.02	1,736	2,607	4,343	9.38
Quirino	17,353	236	294	530	3.05	665	811	1,476	8.51

Table 1.F.5 - Immunization for Senior Citizen

Number and proportion of senior citizen 60 yrs old and above who received one (1) dose of Pneumococcal Polysaccharide Vaccine (PPV) and one (1) dose of Influenza Vaccine

Philippines, 2021

Area	Eligible Pop. (60 y/o & above)	Received one (1) dose of PPV				Received one (1) dose of Influenza Vaccine			
		Male	Female	Total	%	Male	Female	Total	%
Cauayan City	13,393	415	321	736	5.50	1,833	1,796	3,629	27.10
Ilagan City	15,047	186	194	380	2.53	1,111	1,720	2,831	18.81
Santiago City	13,938	473	633	1,106	7.94	2,328	3,725	6,053	43.43
Tuguegarao City	17,354	373	382	755	4.35	1,859	3,250	5,109	29.44
**Region 3**	**1,196,922**	**11,442**	**14,356**	**25,798**	**2.16**	**62,604**	**81,212**	**143,816**	**12.02**
Aurora	19,355	313	586	899	4.64	1,738	2,595	4,333	22.39
Bataan	70,333	1,312	1,900	3,212	4.57	6,500	10,225	16,725	23.78
Bulacan	237,039	990	1,332	2,322	0.98	9,176	10,245	19,421	8.19
Nueva Ecija	163,833	251	171	422	0.26	546	1,037	1,583	0.97
Pampanga	175,014	1,038	1,022	2,060	1.18	7,993	10,128	18,121	10.35
Tarlac	110,293	441	507	948	0.86	4,829	6,058	10,887	9.87
Zambales	62,379	754	1,176	1,930	3.09	1,167	1,941	3,108	4.98
Angeles City	43,920	1,380	1,607	2,987	6.80	4,952	6,651	11,603	26.42
Balanga City	10,166	192	267	459	4.52	2,464	3,732	6,196	60.95
Cabanatuan City	33,532	883	939	1,822	5.43	3,165	4,385	7,550	22.52
City of San Fernando	32,715	188	297	485	1.48	1,307	2,226	3,533	10.80
Gapan City	12,240	110	155	265	2.17	383	451	834	6.81
Mabalacat City	26,760	0	0	0	0.00	583	617	1,200	4.48
Malolos City	26,480	244	289	533	2.01	4,045	4,519	8,564	32.34
Meycauayan	21,963	386	514	900	4.10	2,655	3,065	5,720	26.04
Olongapo	24,605	307	355	662	2.69	862	819	1,681	6.83
Palayan City	4,555	57	79	136	2.99	395	627	1,022	22.44
San Jose City	15,502	0	0	0	0.00	1,144	1,224	2,368	15.28
San Jose del Monte City	60,300	1,628	2,261	3,889	6.45	4,352	6,341	10,693	17.73
Science City of Munoz	9,036	254	305	559	6.19	1,410	1,349	2,759	30.53
Tarlac City	36,902	714	594	1,308	3.54	2,938	2,977	5,915	16.03
**Region 4A**	**1,425,123**	**8,365**	**11,834**	**20,199**	**1.42**	**42,098**	**60,616**	**102,714**	**7.21**
Batangas	201,100	1,177	1,787	2,964	1.47	7,026	10,319	17,345	8.63
Cavite	136,403	948	1,025	1,973	1.45	1,966	3,304	5,270	3.86
Laguna	96,210	786	1,136	1,922	2.00	2,683	4,469	7,152	7.43
Quezon	169,590	385	733	1,118	0.66	1,125	1,984	3,109	1.83
Rizal	196,387	642	835	1,477	0.75	4,300	6,681	10,981	5.59
Antipolo City	72,336	0	0	0	0.00	0	0	0	0.00
Bacoor City	59,758	531	685	1,216	2.03	2,338	3,591	5,929	9.92
Batangas City	35,685	121	175	296	0.83	947	1,529	2,476	6.94
Biñan City	32,260	252	355	607	1.88	1,865	1,836	3,701	11.47
Cabuyao City	29,911	184	218	402	1.34	3,317	3,713	7,030	23.50
Calamba City	44,024	623	947	1,570	3.57	2,708	4,471	7,179	16.31
Cavite City	10,222	12	20	32	0.31	24	26	50	0.49
Dasmariñas City	65,573	390	507	897	1.37	2,492	3,567	6,059	9.24
General Trias City	31,271	83	175	258	0.83	549	867	1,416	4.53
Imus City	40,176	93	118	211	0.53	2,360	3,234	5,594	13.92
Lipa City	35,958	363	455	818	2.27	488	758	1,246	3.47
Lucena City	25,705	59	89	148	0.58	122	180	302	1.17
San Pablo City	25,770	365	671	1,036	4.02	1,334	2,525	3,859	14.97
San Pedro City	31,559	0	0	0	0.00	0	0	0	0.00
Santa Rosa City	34,270	859	928	1,787	5.21	3,202	3,586	6,788	19.81
Tagaytay City	7,076	3	2	5	0.07	24	17	41	0.58
Tanauan City	18,753	258	502	760	4.05	1,832	2,646	4,478	23.88
Tayabas City	9,632	135	362	497	5.16	1,256	1,055	2,311	23.99
Trece Martires City	15,494	96	109	205	1.32	140	258	398	2.57

Table 1.F.5 - Immunization for Senior Citizen

Number and proportion of senior citizen 60 yrs old and above who received one (1) dose of Pneumococcal Polysaccharide Vaccine (PPV) and one (1) dose of Influenza Vaccine

Philippines, 2021

Area	Eligible Pop. (60 y/o & above)	Received one (1) dose of PPV				Received one (1) dose of Influenza Vaccine			
		Male	Female	Total	%	Male	Female	Total	%
**Region 4B**	**268,267**	**737**	**1,100**	**1,837**	**0.68**	**2,785**	**3,897**	**6,682**	**2.49**
Marinduque	26,815	219	342	561	2.09	922	1,300	2,222	8.29
Mindoro Occidental	40,501	42	43	85	0.21	198	320	518	1.28
Mindoro Oriental	78,522	96	170	266	0.34	700	1,159	1,859	2.37
Palawan	70,564	267	307	574	0.81	850	884	1,734	2.46
Romblon	30,678	113	238	351	1.14	115	234	349	1.14
Puerto Princesa City	21,187	0	0	0	0.00	0	0	0	0.00
**Region 5**	**536,548**	**2,951**	**4,571**	**7,522**	**1.40**	**12,984**	**23,499**	**36,483**	**6.80**
Albay	114,224	578	904	1,482	1.30	2,912	5,555	8,467	7.41
Camarines Norte	53,524	480	826	1,306	2.44	1,841	3,228	5,069	9.47
Camarines Sur	147,958	196	328	524	0.35	3,308	6,652	9,960	6.73
Catanduanes	24,626	204	442	646	2.62	361	523	884	3.59
Masbate	74,206	399	577	976	1.32	981	1,413	2,394	3.23
Sorsogon	74,241	250	416	666	0.90	1,389	2,653	4,042	5.44
Iriga City	10,050	160	242	402	4.00	380	542	922	9.17
Legaspi City	20,090	299	291	590	2.94	567	992	1,559	7.76
Naga City	17,629	385	545	930	5.28	1,245	1,941	3,186	18.07
**Region 6**	**843,227**	**6,459**	**9,660**	**16,119**	**1.91**	**22,409**	**33,334**	**55,743**	**6.61**
Aklan	63,617	159	356	515	0.81	1,511	2,446	3,957	6.22
Antique	61,386	1,338	2,204	3,542	5.77	1,687	2,764	4,451	7.25
Capiz	90,290	172	258	430	0.48	1,495	1,552	3,047	3.37
Guimaras	20,021	88	116	204	1.02	186	341	527	2.63
Iloilo	232,571	1,757	1,886	3,643	1.57	5,455	7,726	13,181	5.67
Negros Occidental	262,479	1,570	2,577	4,147	1.58	8,771	12,763	21,534	8.20
Bacolod City	59,054	111	288	399	0.68	2,051	3,505	5,556	9.41
Iloilo City	53,809	1,264	1,975	3,239	6.02	1,253	2,237	3,490	6.49
**Region 7**	**771,468**	**3,132**	**4,356**	**7,488**	**0.97**	**4,833**	**9,113**	**13,946**	**1.81**
Bohol	148,855	1,490	1,954	3,444	2.31	932	2,239	3,171	2.13
Cebu	294,062	499	711	1,210	0.41	671	1,963	2,634	0.90
Negros Oriental	146,058	340	511	851	0.58	1,427	2,235	3,662	2.51
Siquijor	13,077	19	73	92	0.70	50	63	113	0.86
Cebu City	92,303	229	301	530	0.57	311	535	846	0.92
Lapu-Lapu City	40,831	242	369	611	1.50	849	1,288	2,137	5.23
Mandaue City	36,282	313	437	750	2.07	593	790	1,383	3.81
**Region 8**	**447,308**	**865**	**1,439**	**2,304**	**0.52**	**1,732**	**3,277**	**5,009**	**1.12**
Biliran	17,454	58	119	177	1.01	151	290	441	2.53
Eastern Samar	48,109	28	61	89	0.18	71	309	380	0.79
Northern Leyte	160,069	0	0	0	0.00	0	0	0	0.00
Northern Samar	52,294	134	210	344	0.66	385	628	1,013	1.94
Southern Leyte	40,971	107	175	282	0.69	539	1,005	1,544	3.77
Western Samar	53,138	40	58	98	0.18	148	236	384	0.72
Calbayog City	16,375	116	215	331	2.02	0	0	0	0.00
Maasin City	10,434	42	146	188	1.80	66	195	261	2.50
Ormoc City	22,800	253	349	602	2.64	372	614	986	4.32
Tacloban City	25,664	87	106	193	0.75	0	0	0	0.00

Table 1.F.5 - Immunization for Senior Citizen

Number and proportion of senior citizen 60 yrs old and above who received one (1) dose of Pneumococcal Polysaccharide Vaccine (PPV) and one (1) dose of Influenza Vaccine

Philippines, 2021

Area	Eligible Pop. (60 y/o & above)	Received one (1) dose of PPV				Received one (1) dose of Influenza Vaccine			
		Male	Female	Total	%	Male	Female	Total	%
**Region 9**	**311,996**	**2,403**	**3,464**	**5,867**	**1.88**	**8,023**	**11,298**	**19,321**	**6.19**
Zamboanga del Norte	74,069	640	935	1,575	2.13	2,661	3,239	5,900	7.97
Zamboanga del Sur	68,881	233	328	561	0.81	410	563	973	1.41
Zamboanga Sibugay	49,678	122	204	326	0.66	727	1,165	1,892	3.81
Dapitan City	7,648	20	30	50	0.65	568	932	1,500	19.61
Dipolog City	12,134	258	299	557	4.59	787	932	1,719	14.17
Isabela City	9,583	175	231	406	4.24	412	434	846	8.83
Pagadian City	16,889	0	0	0	0.00	0	0	0	0.00
Zamboanga City	73,114	955	1,437	2,392	3.27	2,458	4,033	6,491	8.88
**Region 10**	**434,177**	**5,300**	**6,639**	**11,939**	**2.75**	**10,470**	**13,657**	**24,127**	**5.56**
Bukidnon	87,168	1,090	1,294	2,384	2.73	1,546	1,624	3,170	3.64
Camiguin	10,332	51	64	115	1.11	356	393	749	7.25
Lanao del Norte	54,320	1,224	1,322	2,546	4.69	1,615	2,137	3,752	6.91
Misamis Occidental	36,852	219	218	437	1.19	364	445	809	2.20
Misamis Oriental	71,302	700	1,085	1,785	2.50	583	889	1,472	2.06
Cagayan de Oro City	67,526	777	927	1,704	2.52	1,474	1,667	3,141	4.65
El Salvador City	5,016	76	76	152	3.03	100	213	313	6.24
Gingoog City	12,455	0	0	0	0.00	0	0	0	0.00
Iligan City	27,512	555	791	1,346	4.89	2,267	3,220	5,487	19.94
Malaybalay City	14,531	195	235	430	2.96	696	877	1,573	10.83
Oroquieta City	7,983	102	169	271	3.39	552	808	1,360	17.04
Ozamis City	16,007	300	423	723	4.52	809	1,231	2,040	12.74
Tangub City	7,113	11	35	46	0.65	97	148	245	3.44
Valencia City	16,060	0	0	0	0.00	11	5	16	0.10
**Region 11**	**458,573**	**2,703**	**3,494**	**6,197**	**1.35**	**9,365**	**12,165**	**21,530**	**4.69**
Davao de Oro	62,728	896	1,273	2,169	3.46	2,374	3,057	5,431	8.66
Davao del Norte	92,328	315	378	693	0.75	1,192	1,600	2,792	3.02
Davao Oriental	52,133	457	524	981	1.88	1,372	1,583	2,955	5.67
Davao del Sur	62,437	230	265	495	0.79	876	1,105	1,981	3.17
Davao Occidental	27,794	18	12	30	0.11	27	44	71	0.26
Davao City	161,153	787	1,042	1,829	1.13	3,524	4,776	8,300	5.15
**Region 12**	**365,094**	**2,781**	**3,735**	**6,516**	**1.78**	**9,435**	**13,830**	**23,265**	**6.37**
North Cotabato	116,481	550	720	1,270	1.09	1,942	2,961	4,903	4.21
Sarangani	39,460	869	1,152	2,021	5.12	1,970	2,701	4,671	11.84
South Cotabato	78,396	666	1,108	1,774	2.26	3,772	5,681	9,453	12.06
Sultan Kudarat	60,895	619	654	1,273	2.09	1,228	1,522	2,750	4.52
Cotabato City	18,952	21	13	34	0.18	278	460	738	3.89
Gen. Santos City	50,910	56	88	144	0.28	245	505	750	1.47
**BARMM**	**186,087**	**2,214**	**2,970**	**5,184**	**2.79**	**3,190**	**4,303**	**7,493**	**4.03**
Basilan	14,423	0	0	0	0.00	0	0	0	0.00
Lanao del Sur	37,784	158	236	394	1.04	239	480	719	1.90
Maguindanao	58,962	1,832	2,382	4,214	7.15	2,699	3,451	6,150	10.43
Sulu	42,187	0	0	0	0.00	0	0	0	0.00
Tawi-Tawi	19,727	0	0	0	0.00	0	0	0	0.00
Lamitan City	3,973	0	0	0	0.00	0	0	0	0.00
Marawi City	9,031	224	352	576	6.38	252	372	624	6.91

Table 1.F.5 - Immunization for Senior Citizen

Number and proportion of senior citizen 60 yrs old and above who received one (1) dose of Pneumococcal Polysaccharide Vaccine (PPV) and one (1) dose of Influenza Vaccine

Philippines, 2021

Area	Eligible Pop. (60 y/o & above)	Received one (1) dose of PPV				Received one (1) dose of Influenza Vaccine			
		Male	Female	Total	%	Male	Female	Total	%
**CARAGA**	**237,184**	**2,527**	**3,515**	**6,042**	**2.55**	**6,685**	**10,082**	**16,767**	**7.07**
Agusan del Norte	33,726	51	81	132	0.39	450	753	1,203	3.57
Agusan del Sur	51,807	186	207	393	0.76	389	826	1,215	2.35
Surigao del Norte	34,096	175	267	442	1.30	1,209	1,878	3,087	9.05
Surigao del Sur	47,096	125	244	369	0.78	1,903	2,583	4,486	9.53
Province of Dinagat	13,570	39	99	138	1.02	262	366	628	4.63
Bislig City	8,948	95	98	193	2.16	66	172	238	2.66
Butuan City	32,059	630	914	1,544	4.82	1,308	1,800	3,108	9.69
Surigao City	15,882	1,226	1,605	2,831	17.83	1,098	1,704	2,802	17.64

Note: Put asterisk (*) for No Report and Zero (0) for No Case

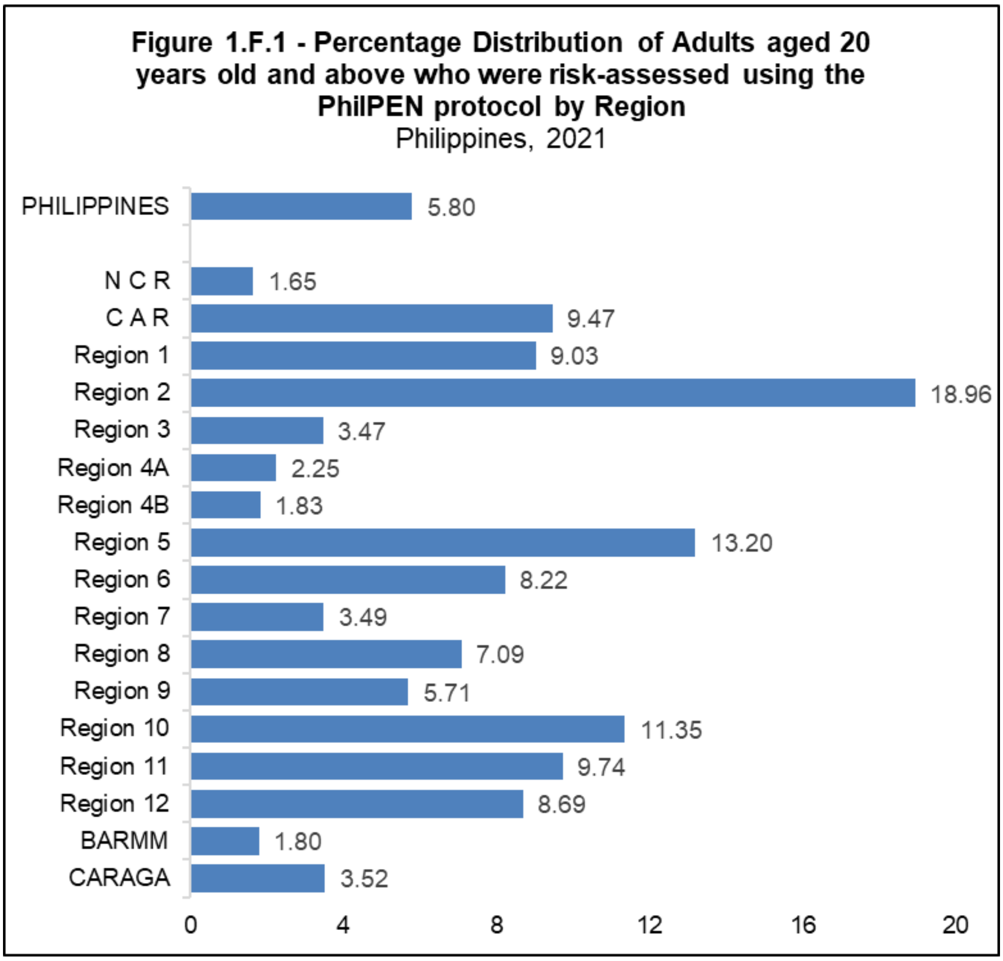
Figure 1.F.1 - Percentage Distribution of Adults aged 20 years old and above who were risk-assessed using the PhilPEN protocol by Region
Philippines, 2021
PHILIPPINES
5.80
N C R
1.65
C A R
9.47
Region 1
9.03
Region 2
18.96
Region 3
3.47
Region 4A
2.25
Region 4B
1.83
Region 5
13.20
Region 6
8.22
Region 7
3.49
Region 8
7.09
Region 9
5.71
Region 10
11.35
Region 11
9.74
Region 12
8.69
BARMM
1.80
CARAGA
3.52
0
4
8
12
16
20

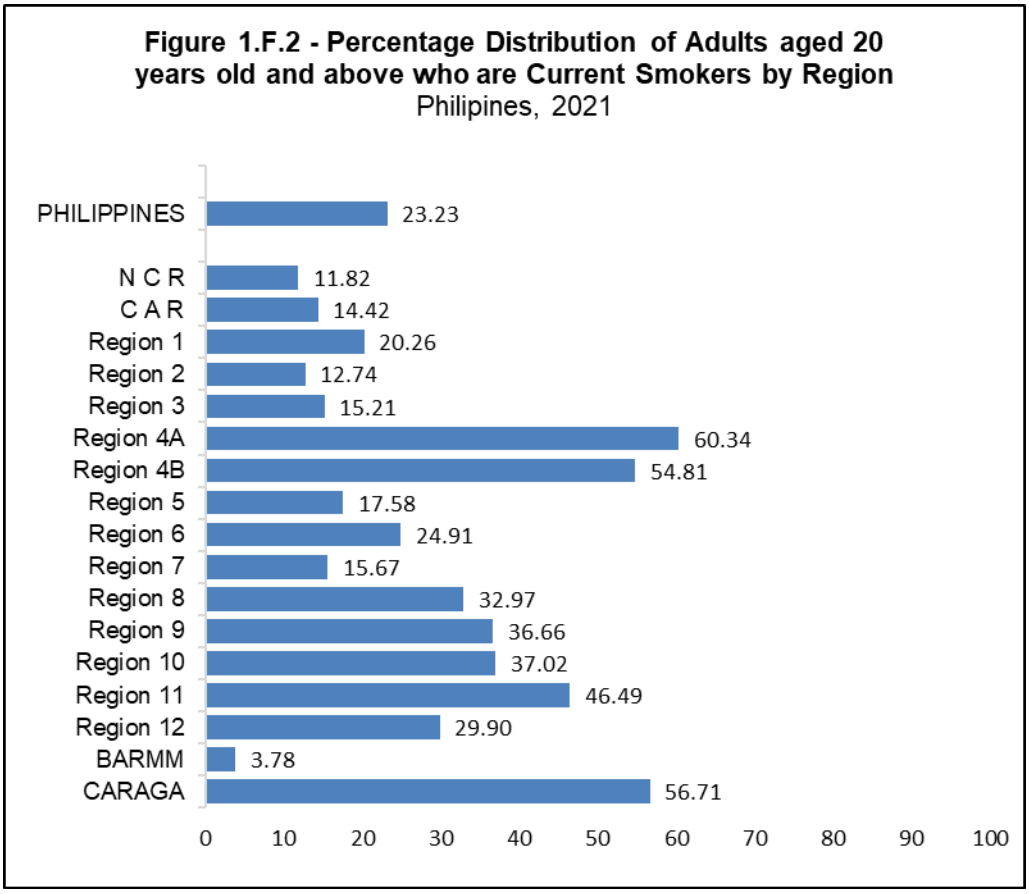
Figure 1.F.2 - Percentage Distribution of Adults aged 20 years old and above who are Current Smokers by Region
Philipines, 2021
PHILIPPINES
23.23
N C R
11.82
C A R
14.42
Region 1
20.26
Region 2
12.74
Region 3
15.21
Region 4A
60.34
Region 4B
54.81
Region 5
17.58
Region 6
24.91
Region 7
15.67
Region 8
32.97
Region 9
36.66
Region 10
37.02
Region 11
46.49
Region 12
29.90
BARMM
3.78
CARAGA
56.71
0
10
20
30
40
50
60
70
80
90
100

Figure 1.F.3 - Percentage Distribution of Adults aged 20 years old and above who are Binge Drinkers by Region
Philippines, 2021

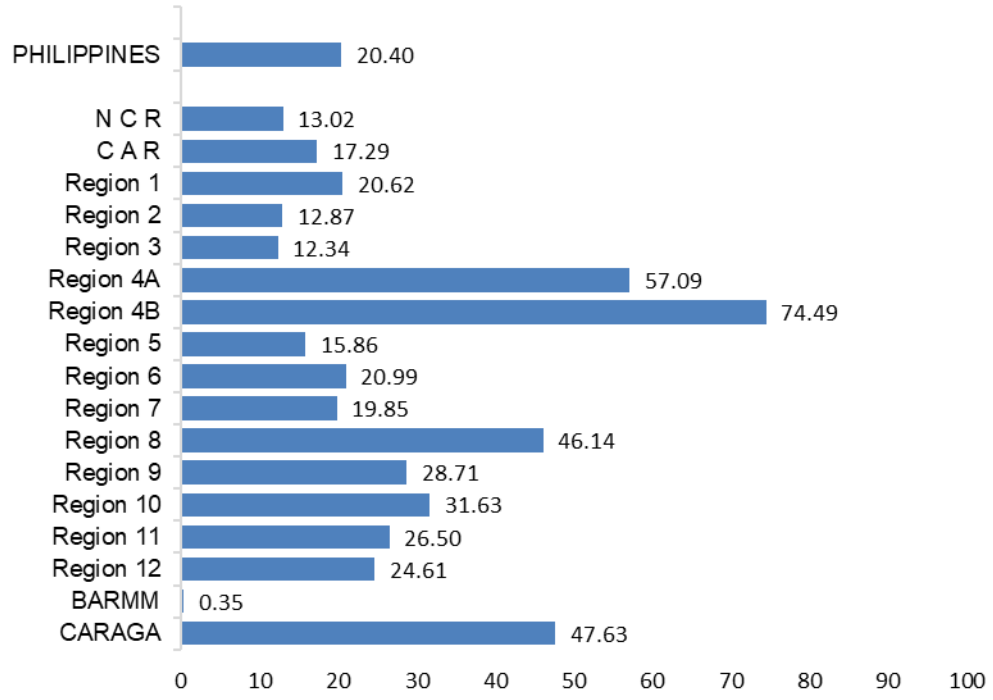

Figure 1.F.4 - Percentage Distribution of Adults aged 20 years old and above who are Overweight or Obese by Region
Philippines, 2021

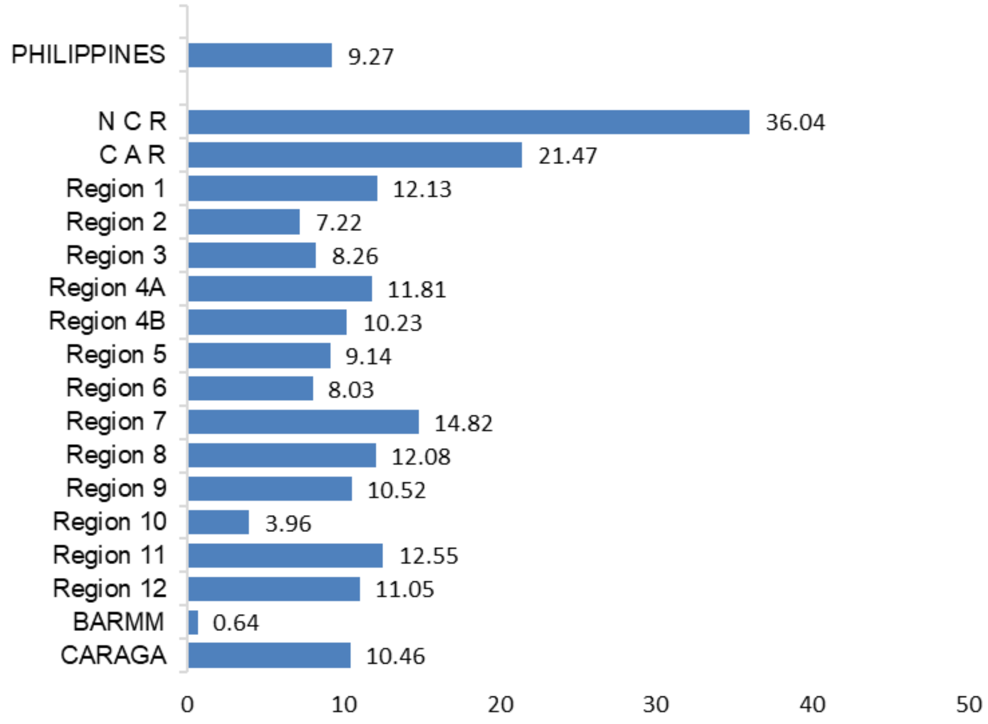

Figure 1.F.5 - Percentage Distribution of adults aged 20 years old and above who are Current Smokers, Binge Drinkers, and Overweight or Obese
Philippines, 2021

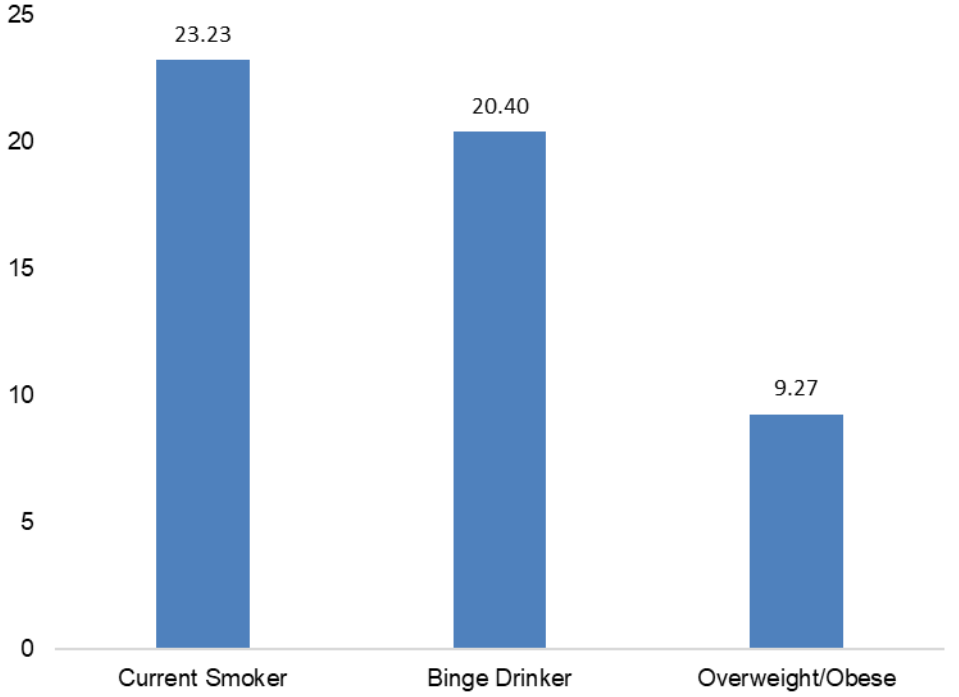

Figure 1.F.6 - Percentage Distribution of Women aged 20 years old and above screened for Breast Mass by Region
Philippines, 2021

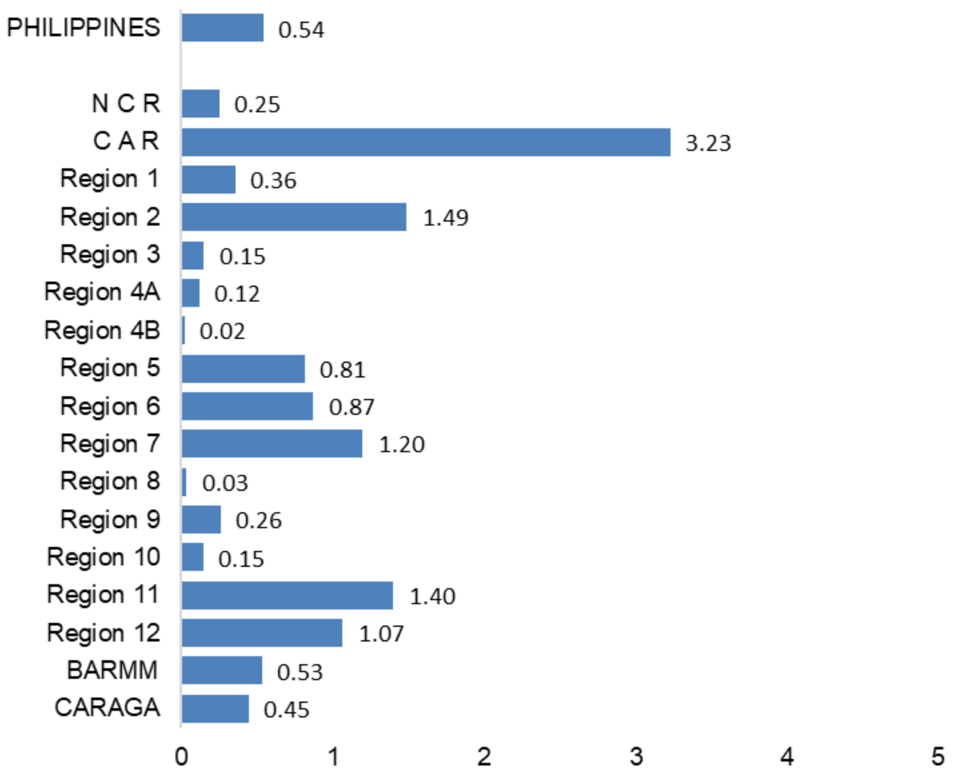

Figure 1.F.7 - Percentage Distribution of Women aged 20 years old and above with Suspicious Breast Mass by Region
Philippines, 2021

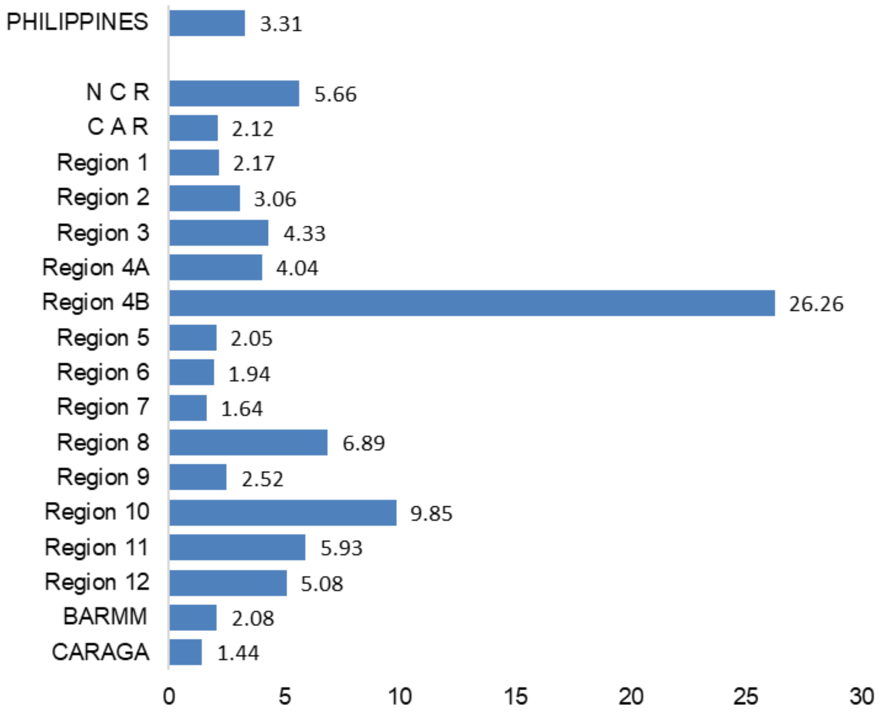

Figure 1.F.8 - Percentage Distribution of Women aged 20 years old and above screened for cervical cancer, found positive or suspected for cervical cancer, screened for breast mass, and women with suspicious breast mass
Philippines, 2021

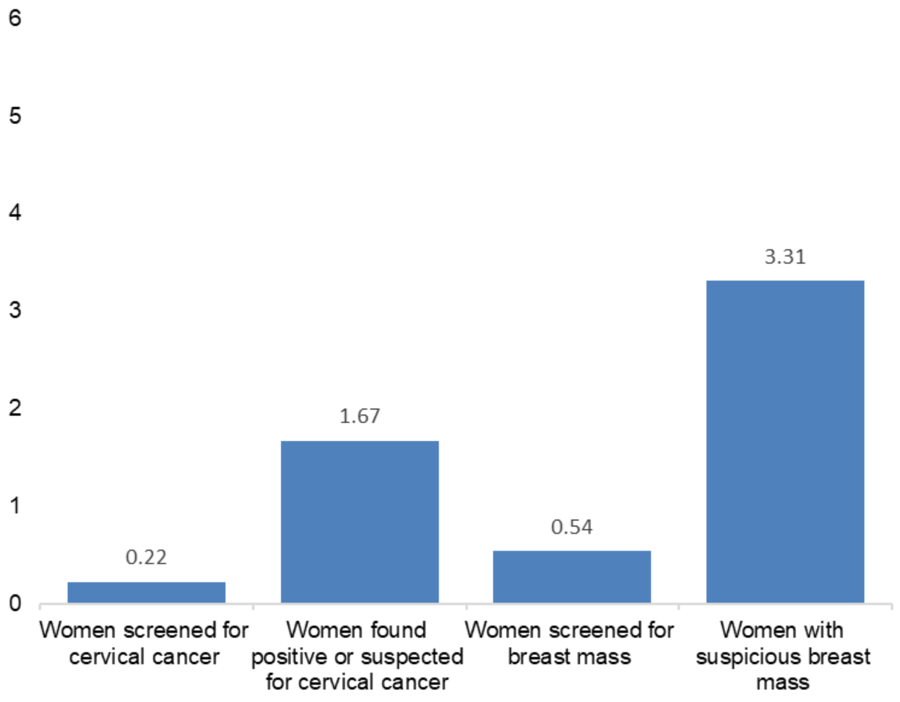

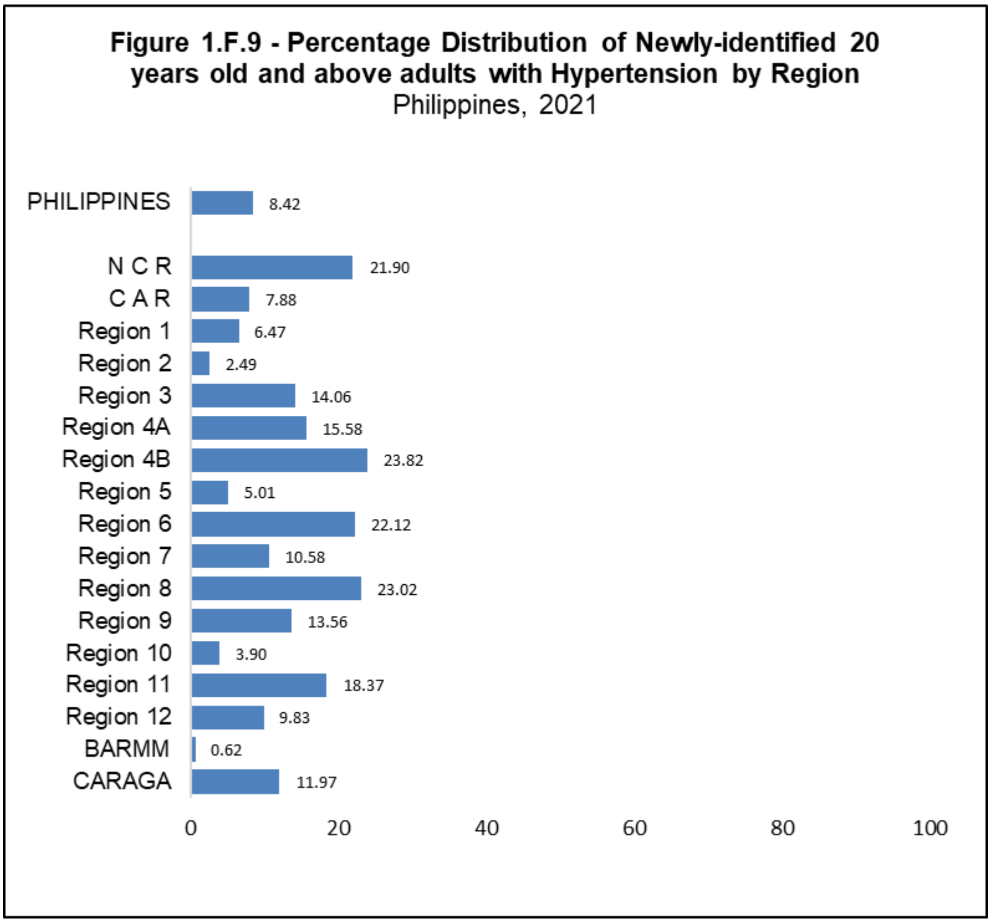
Figure 1.F.9 - Percentage Distribution of Newly-identified 20 years old and above adults with Hypertension by Region
Philippines, 2021
PHILIPPINES 8.42
N C R 21.90
C A R 7.88
Region 1 6.47
Region 2 2.49
Region 3 14.06
Region 4A 15.58
Region 4B 23.82
Region 5 5.01
Region 6 22.12
Region 7 10.58
Region 8 23.02
Region 9 13.56
Region 10 3.90
Region 11 18.37
Region 12 9.83
BARMM 0.62
CARAGA 11.97
0
20
40
60
80
100

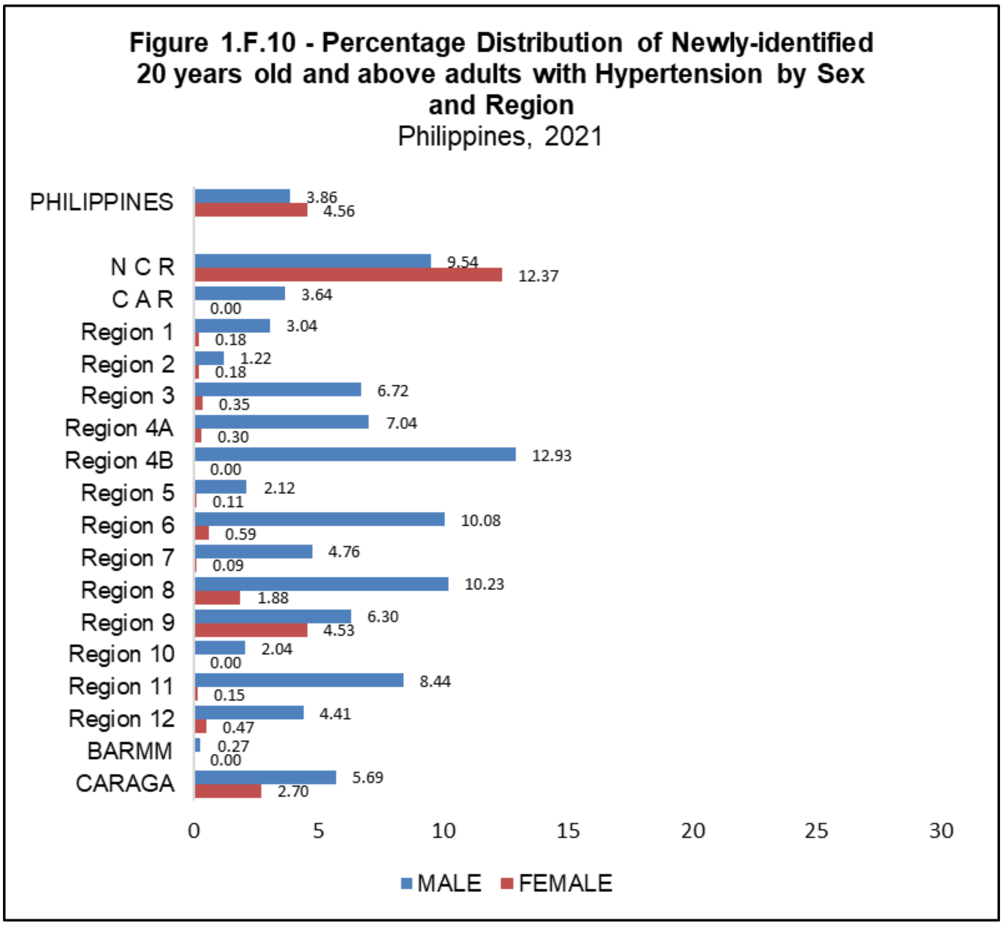
Figure 1.F.10 - Percentage Distribution of Newly-identified 20 years old and above adults with Hypertension by Sex and Region
Philippines, 2021
PHILIPPINES 3.86 4.56
N C R 9.54 12.37
C A R 3.64 0.00
Region 1 3.04 0.18
Region 2 1.22 0.18
Region 3 6.72 0.35
Region 4A 7.04 0.30
Region 4B 12.93 0.00
Region 5 2.12 0.11
Region 6 10.08 0.59
Region 7 4.76 0.09
Region 8 10.23 1.88
Region 9 6.30 4.53
Region 10 2.04 0.00
Region 11 8.44 0.15
Region 12 4.41 0.47
BARMM 0.27 0.00
CARAGA 5.69 2.70
0
5
10
15
20
25
30
MALE
FEMALE

Figure 1.F.11 - Percentage Distribution of Newly-identified 20 years old and above adults with Diabetes Mellitus II by Region
Philippines, 2021

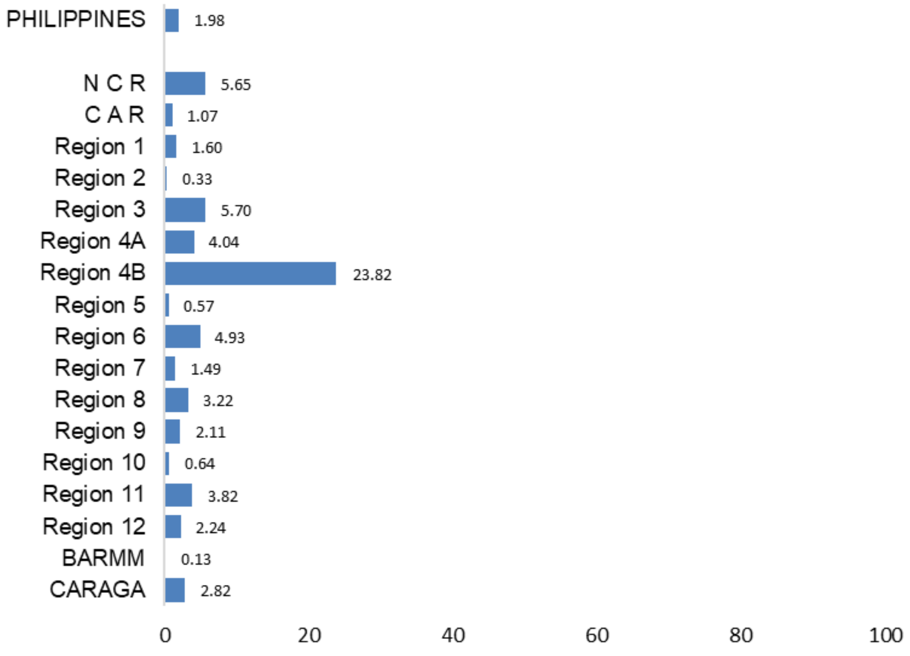

Figure 1.F.12 - Percentage Distribution of Newly-identified 20 years old and above adults with Diabetes Mellitus II by Sex and Region
Philippines, 2021

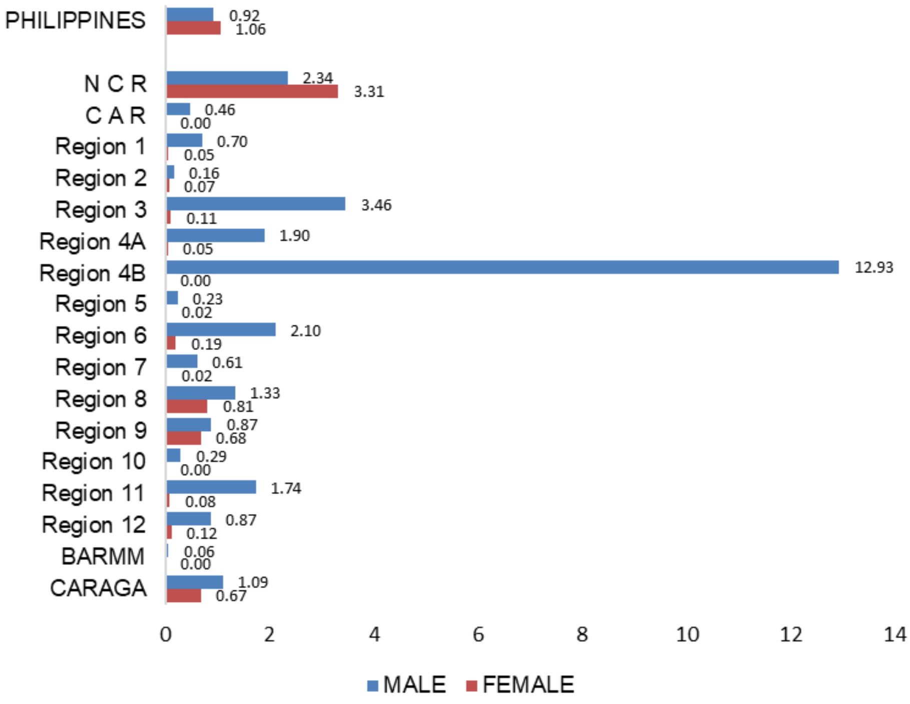

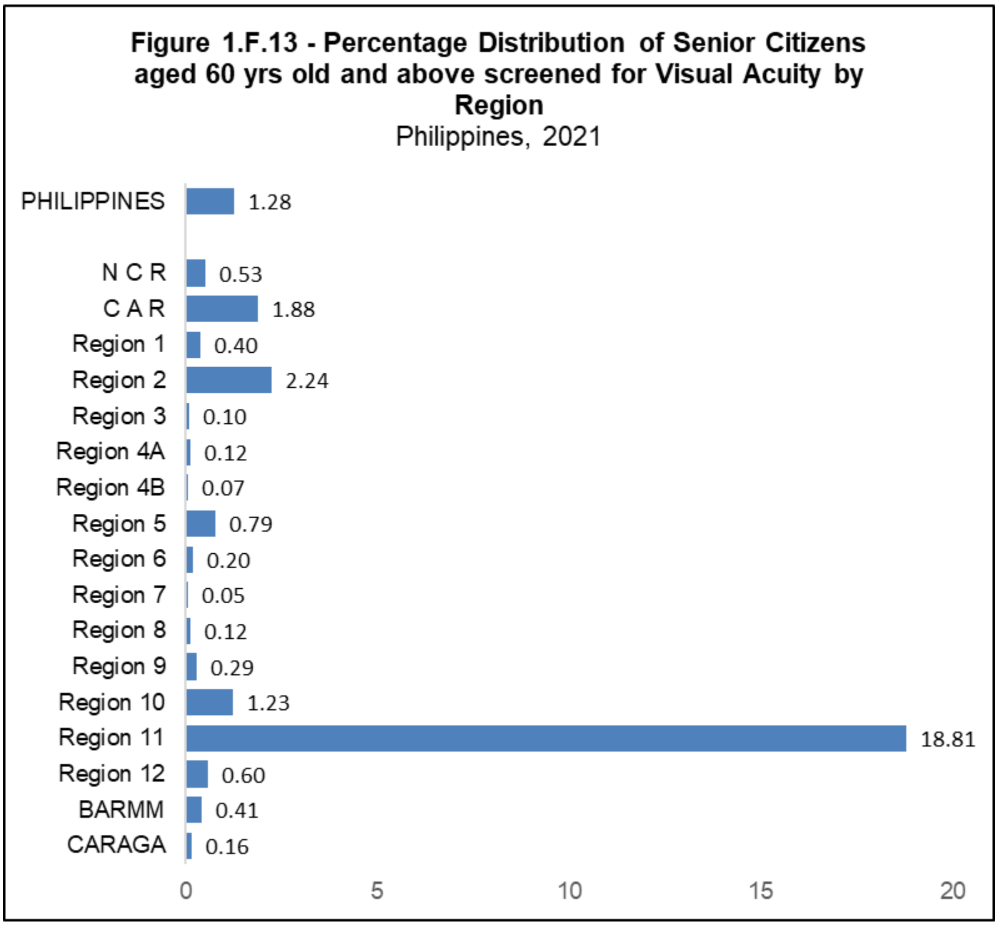
Figure 1.F.13 - Percentage Distribution of Senior Citizens aged 60 yrs old and above screened for Visual Acuity by Region
Philippines, 2021
PHILIPPINES
1.28
N C R
0.53
C A R
1.88
Region 1
0.40
Region 2
2.24
Region 3
0.10
Region 4A
0.12
Region 4B
0.07
Region 5
0.79
Region 6
0.20
Region 7
0.05
Region 8
0.12
Region 9
0.29
Region 10
1.23
Region 11
18.81
Region 12
0.60
BARMM
0.41
CARAGA
0.16
0
5
10
15
20

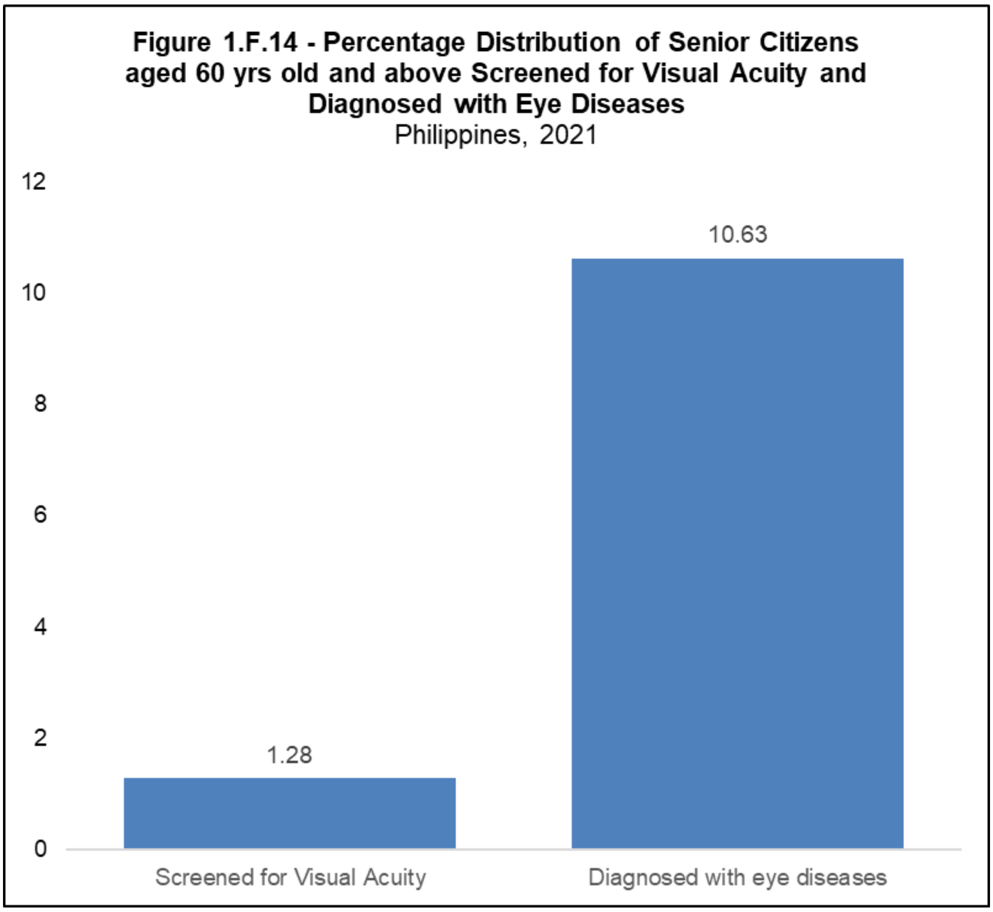
Figure 1.F.14 - Percentage Distribution of Senior Citizens aged 60 yrs old and above Screened for Visual Acuity and Diagnosed with Eye Diseases
Philippines, 2021
12
10
8
6
4
2
0
1.28
10.63
Screened for Visual Acuity
Diagnosed with eye diseases

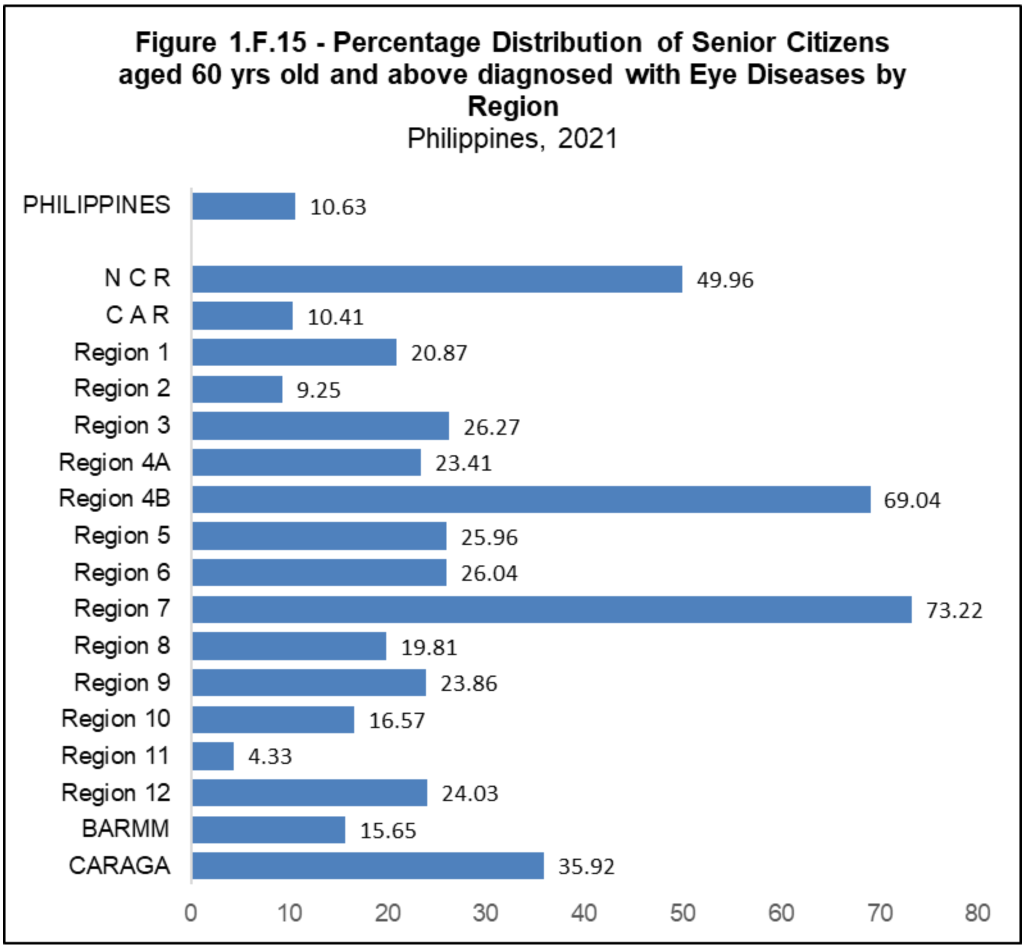
Figure 1.F.15 - Percentage Distribution of Senior Citizens aged 60 yrs old and above diagnosed with Eye Diseases by Region
Philippines, 2021
PHILIPPINES 10.63
N C R 49.96
C A R 10.41
Region 1 20.87
Region 2 9.25
Region 3 26.27
Region 4A 23.41
Region 4B 69.04
Region 5 25.96
Region 6 26.04
Region 7 73.22
Region 8 19.81
Region 9 23.86
Region 10 16.57
Region 11 4.33
Region 12 24.03
BARMM 15.65
CARAGA 35.92
0 10 20 30 40 50 60 70 80

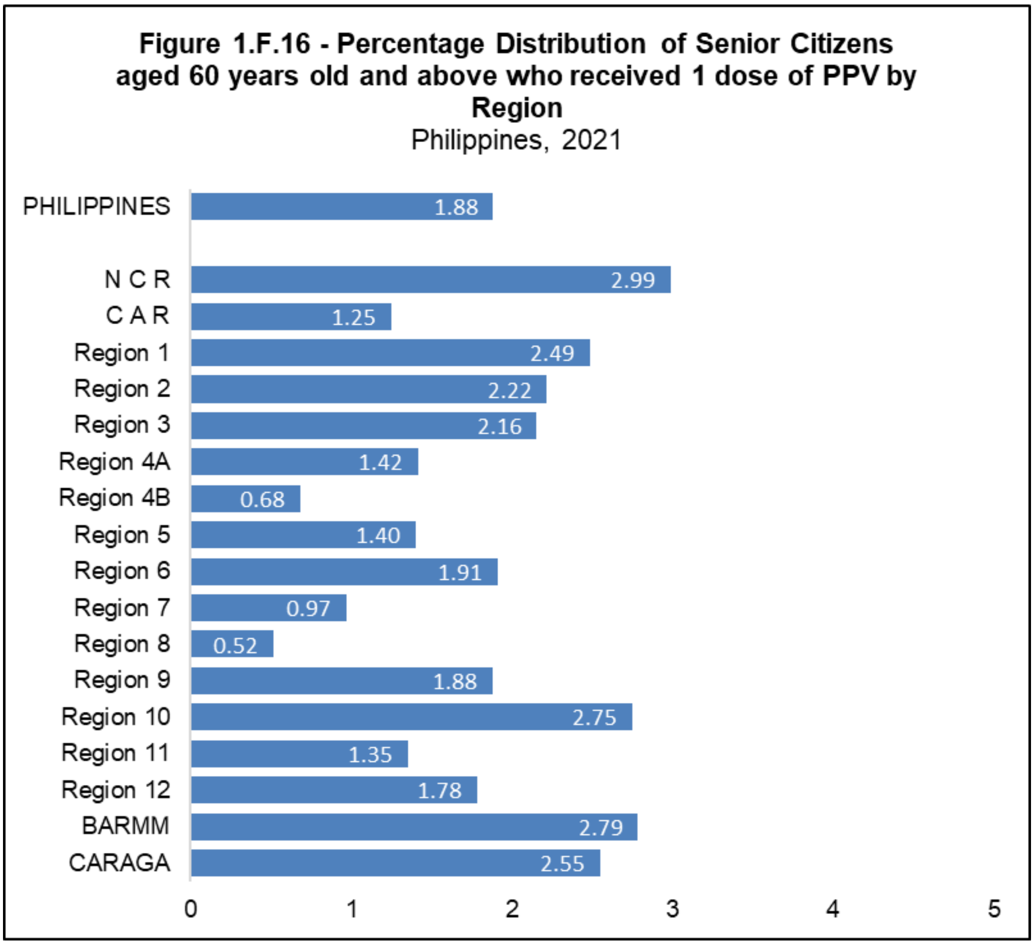
Figure 1.F.16 - Percentage Distribution of Senior Citizens aged 60 years old and above who received 1 dose of PPV by Region
Philippines, 2021
PHILIPPINES 1.88
N C R 2.99
C A R 1.25
Region 1 2.49
Region 2 2.22
Region 3 2.16
Region 4A 1.42
Region 4B 0.68
Region 5 1.40
Region 6 1.91
Region 7 0.97
Region 8 0.52
Region 9 1.88
Region 10 2.75
Region 11 1.35
Region 12 1.78
BARMM 2.79
CARAGA 2.55
0 1 2 3 4 5

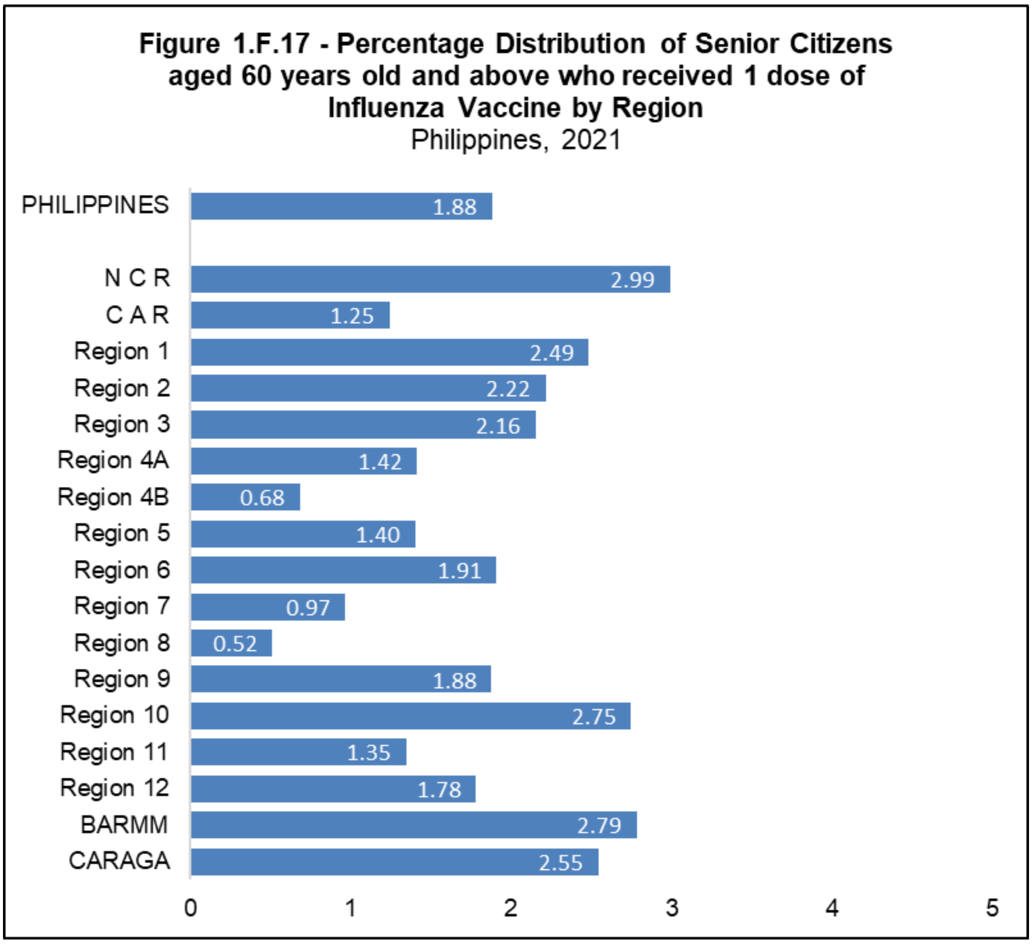

Figure 1.F.17 - Percentage Distribution of Senior Citizens aged 60 years old and above who received 1 dose of Influenza Vaccine by Region
Philippines, 2021
PHILIPPINES
1.88
N C R
2.99
C A R
1.25
Region 1
2.49
Region 2
2.22
Region 3
2.16
Region 4A
1.42
Region 4B
0.68
Region 5
1.40
Region 6
1.91
Region 7
0.97
Region 8
0.52
Region 9
1.88
Region 10
2.75
Region 11
1.35
Region 12
1.78
BARMM
2.79
CARAGA
2.55
0
1
2
3
4
5

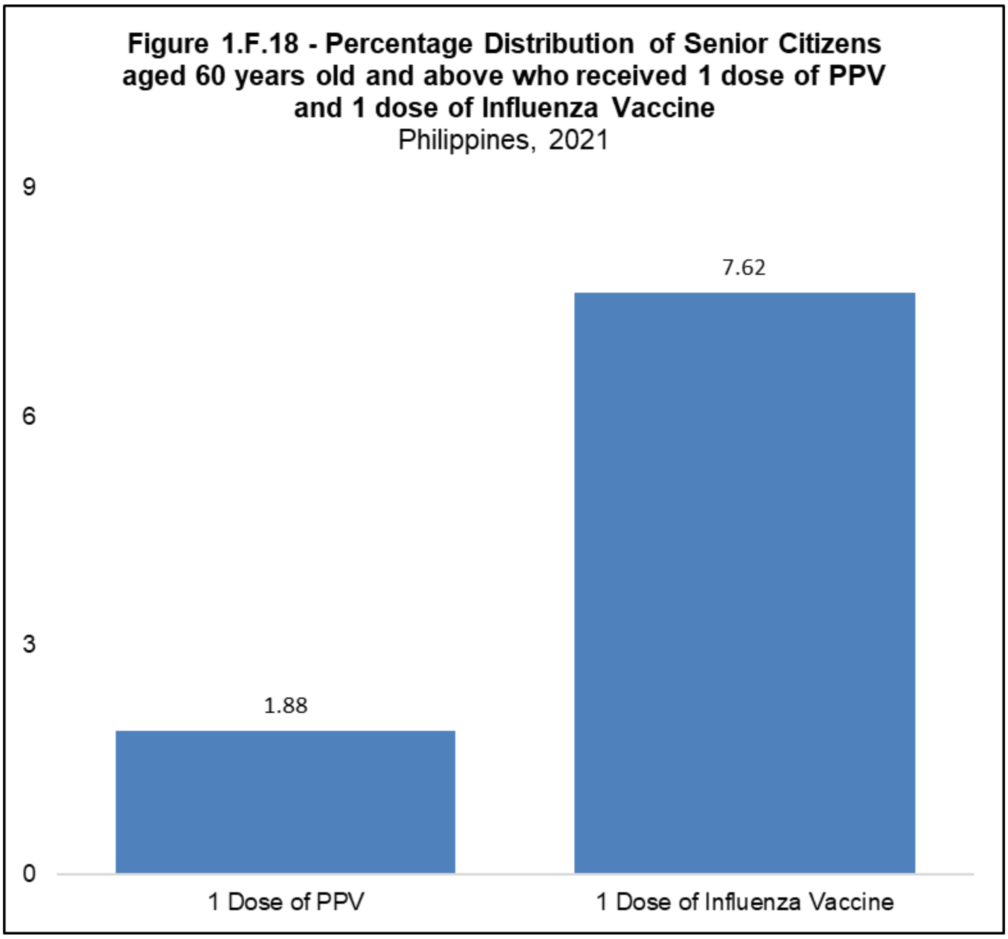

Figure 1.F.18 - Percentage Distribution of Senior Citizens aged 60 years old and above who received 1 dose of PPV and 1 dose of Influenza Vaccine
Philippines, 2021
9
6
3
0
7.62
1.88
1 Dose of PPV
1 Dose of Influenza Vaccine

ENVIRONMENTAL HEALTH AND SANITATION SERVICES

FHSIS ANNUAL REPORT 2021

1.G Environmental Health and Sanitation Services

The percentage of households that have access to basic safe water supply (drinking from an improved drinking-water source) was 84.17% for 2021. This was a continuous increase since 2019. For households with basic sanitation facilities, despite the steep decrease observed in 2019, an increase was seen in the following years (2020 with 68.03% and 2021 with 75.00%).

Figure 1.G.1 Proportion of Households with Access to Basic Safe Water Supply and Basic Sanitation Facility Philippines, 2017 - 2021

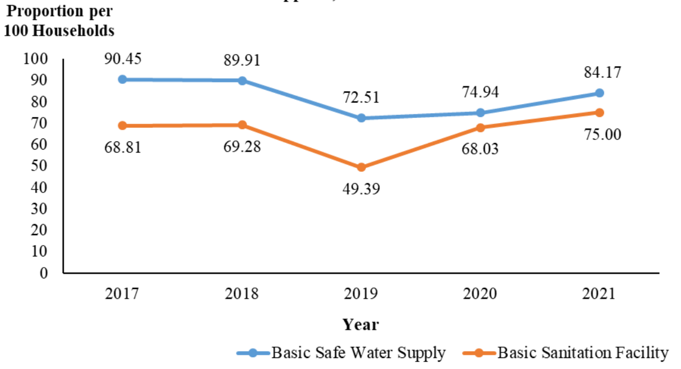

Improved drinking-water sources can be disaggregated into three levels. In 2021, for the Philippines, majority (61.20%) were registered having a Level III water source. Among regions, 12 (71%) had Level III water source as the leading source in their area. The NCR registered highest (96.34%) for Level III water source. For 5 (29%) regions however, Level I was noted to be the leading water source in their area.

Figure 1.G.2 Proportion of Households with Access to Basic Safe Water Supply by Source Type and Region Philippines, 2021

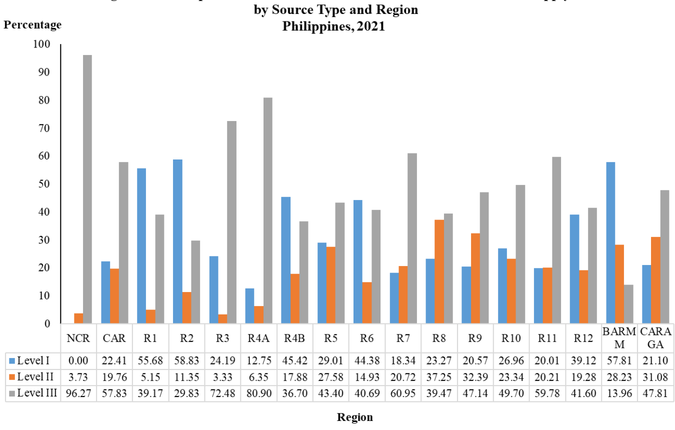

	NCR	CAR	R1	R2	R3	R4A	R4B	R5	R6	R7	R8	R9	R10	R11	R12	BARMM	CARAGA
Level I	0.00	22.41	55.68	58.83	24.19	12.75	45.42	29.01	44.38	18.34	23.27	20.57	26.96	20.01	39.12	57.81	21.10
Level II	3.73	19.76	5.15	11.35	3.33	6.35	17.88	27.58	14.93	20.72	37.25	32.39	23.34	20.21	19.28	28.23	31.08
Level III	96.27	57.83	39.17	29.83	72.48	80.90	36.70	43.40	40.69	60.95	39.47	47.14	49.70	59.78	41.60	13.96	47.81

For the proportion of households using safely managed drinking water services and sanitation services, a continuous increase in the trend was noted since 2019.

Figure 1.G.3 Proportion of Households Using Safely Managed Drinking Water Services and Sanitation Services Philippines, 2019 - 2021

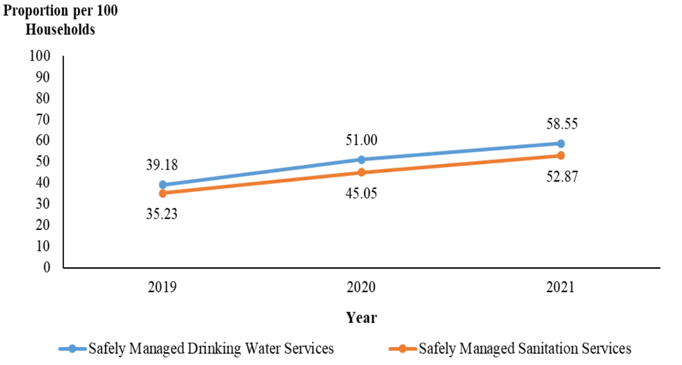

In 2021, the proportion of industrial establishments with sanitary permits reached 59.40%. For Region 2, all (100%) existing industrial establishments in the area were issued with sanitary permits. It was also observed that 9 (52%) regions achieved at least 80% coverage.

Figure 1.G.4 Proportion of Industrial Establishments with Sanitary Permits by Region Philippines, 2021

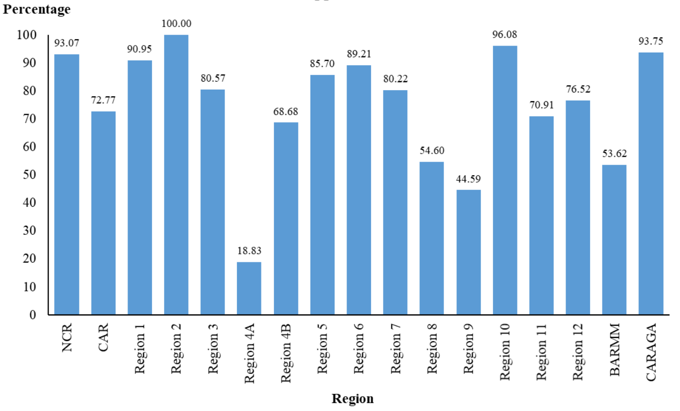

The Philippines registered 24.04% of barangay certified as Zero Open Defecation (ZOD) area. Compared to other regions, Region 1 had a significantly high coverage with 75.30% followed by Region 6 with 51.91%. The regions with the lowest percentage proportion were Region 4B with 2.88%, Region 3 with 6.21% and Region 9 with 8.51%.

Figure 1.G.5 Proportion of Barangay Declared Zero Open Defecation Area by Region Philippines, 2021

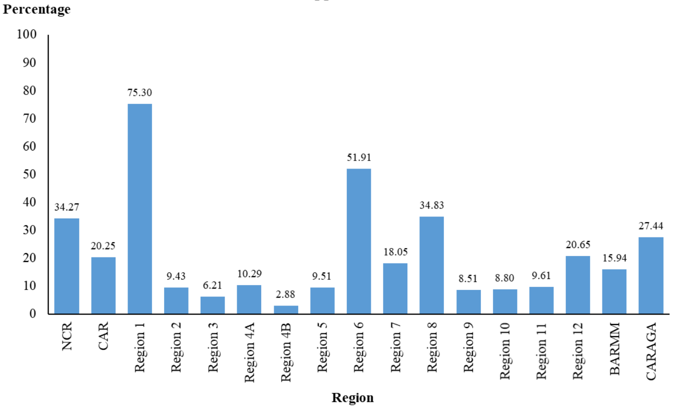

Table 1.G.1- ENVIRONMENTAL HEALTH AND SANITATION SERVICES

Proportion and Total Number of Households (HHs) with access to basic safe water supply and manage drinking water
Philippines, 2021

Area	Projected No. of HHs	HHs with Access to Basic Safe Water Supply								HHs using Safely Managed Drinking Water Services	
		Level 1		Level 2		Level 3		Total	%		
		No.	%	No.	%	No.	%			No.	%
**PHILIPPINES**	**25,216,517**	**5,203,514**	**24.52**	**3,030,942**	**14.28**	**12,990,845**	**61.20**	**21,225,301**	**84.17**	**15,032,441**	**59.61**
**NCR**	**3,379,343**	**0**	**0.00**	**117,365**	**3.73**	**3,029,655**	**96.27**	**3,147,020**	**93.13**	**2,985,685**	**88.35**
Malabon	93,797	0	0.00	0	0.00	93,797	100.00	93,797	100.00	93,797	100.00
Navotas	66,132	0	0.00	0	0.00	65,272	100.00	65,272	98.70	65,272	98.70
Valenzuela City	166,278	0	0.00	0	0.00	166,278	100.00	166,278	100.00	166,278	100.00
Caloocan City	399,732	0	0.00	0	0.00	399,732	100.00	399,732	100.00	339,772	85.00
Marikina City	106,984	0	0.00	0	0.00	106,978	100.00	106,978	99.99	74,984	70.09
Pasig City	196,479	0	0.00	0	0.00	196,479	100.00	196,479	100.00	196,479	100.00
Pateros	15,433	0	0.00	0	0.00	15,433	100.00	15,433	100.00	14,120	91.49
Taguig City	218,248	0	0.00	0	0.00	172,946	100.00	172,946	79.24	172,946	79.24
Quezon City	740,559	0	0.00	0	0.00	689,497	100.00	689,497	93.10	689,511	93.11
Makati City	168,040	0	0.00	2,420	1.44	165,610	98.56	168,030	99.99	168,038	100.00
Mandaluyong City	111,267	0	0.00	675	0.61	110,592	99.39	111,267	100.00	110,592	99.39
San Juan City	31,425	0	0.00	0	0.00	31,425	100.00	31,425	100.00	31,425	100.00
Manila City	476,545	0	0.00	95,093	22.73	323,225	77.27	418,318	87.78	418,318	87.78
Las Piñas City	154,249	0	0.00	1,488	1.40	104,959	98.60	106,447	69.01	106,447	69.01
Muntinlupa City	138,889	0	0.00	0	0.00	137,320	100.00	137,320	98.87	137,320	98.87
Parañaque City	177,422	0	0.00	17,689	9.97	159,733	90.03	177,422	100.00	177,422	100.00
Pasay City	117,864	0	0.00	0	0.00	90,379	100.00	90,379	76.68	22,964	19.48
**CAR**	**421,691**	**89,652**	**22.41**	**79,041**	**19.76**	**231,355**	**57.83**	**400,048**	**94.87**	**258,920**	**61.40**
Abra	54,884	10,241	23.52	12,578	28.88	20,730	47.60	43,549	79.35	19,560	35.64
Apayao	26,865	15,354	61.83	5,500	22.15	3,977	16.02	24,831	92.43	946	3.52
Benguet	116,296	20,256	19.95	12,158	11.97	69,135	68.08	101,549	87.32	67,652	58.17
Ifugao	45,312	16,689	36.54	22,187	48.58	6,798	14.88	45,674	100.80	42,610	94.04
Kalinga	44,012	15,579	32.13	15,152	31.25	17,754	36.62	48,485	110.16	24,603	55.90
Mt. Province	35,832	5,652	15.08	4,884	13.03	26,946	71.89	37,482	104.60	13,975	39.00
Baguio City	98,490	5,881	5.97	6,582	6.68	86,015	87.34	98,478	99.99	89,574	90.95
**Region 1**	**1,220,692**	**642,177**	**55.68**	**59,349**	**5.15**	**451,770**	**39.17**	**1,153,296**	**94.48**	**621,650**	**50.93**
Ilocos Norte	118,905	42,791	47.73	12,021	13.41	34,833	38.86	89,645	75.39	50,279	42.29
Ilocos Sur	137,682	66,015	56.96	10,751	9.28	39,137	33.77	115,903	84.18	52,523	38.15
La Union	162,189	109,622	68.37	18,382	11.47	32,322	20.16	160,326	98.85	41,779	25.76
Pangasinan	577,046	332,676	58.89	17,996	3.19	214,218	37.92	564,890	97.89	318,642	55.22
Alaminos City	22,970	0	0.00	0	0.00	22,583	100.00	22,583	98.32	20,668	89.98
Candon City	14,479	12,098	82.89	0	0.00	2,498	17.11	14,596	100.81	3,617	24.98
Dagupan City	40,810	1,350	3.14	0	0.00	41,659	96.86	43,009	105.39	41,659	102.08
Laoag City	26,469	3,185	13.77	0	0.00	19,946	86.23	23,131	87.39	23,131	87.39
San Carlos City	44,740	33,719	75.37	93	0.21	10,928	24.43	44,740	100.00	39,100	87.39
San Fernando City	30,415	18,884	62.60	106	0.35	11,174	37.04	30,164	99.17	4,064	13.36
Urdaneta City	32,400	21,442	67.69	0	0.00	10,237	32.31	31,679	97.77	13,558	41.85
Vigan City	12,587	395	3.13	0	0.00	12,235	96.87	12,630	100.34	12,630	100.34
**Region 2**	**910,994**	**326,837**	**58.83**	**63,037**	**11.35**	**165,724**	**29.83**	**555,598**	**60.99**	**479,806**	**52.67**
Batanes	5,015	0	0.00	0	0.00	5,007	100.00	5,007	99.84	0	0.00
Cagayan	256,379	65,791	55.85	25,225	21.41	26,779	22.73	117,795	45.95	158,839	61.95
Isabela	296,310	109,885	67.59	8,059	4.96	44,639	27.46	162,583	54.87	175,681	59.29
Nueva Vizcaya	168,501	59,770	61.10	14,199	14.51	23,854	24.38	97,823	58.05	25,613	15.20
Quirino	45,979	28,069	68.21	5,849	14.21	7,234	17.58	41,152	89.50	6,130	13.33

Table 1.G.1- ENVIRONMENTAL HEALTH AND SANITATION SERVICES

Proportion and Total Number of Households (HHs) with access to basic safe water supply and manage drinking water
Philippines, 2021

Area	Projected No. of HHs	HHs with Access to Basic Safe Water Supply								HHs using Safely Managed Drinking Water Services	
		Level 1		Level 2		Level 3		Total	%		
		No.	%	No.	%	No.	%			No.	%
Cauayan City	32,820	18,281	59.89	415	1.36	11,829	38.75	30,525	93.01	30,123	91.78
Ilagan City	37,107	24,409	65.78	9,290	25.04	3,408	9.18	37,107	100.00	34,732	93.60
Santiago City	32,662	18,100	55.94	0	0.00	14,258	44.06	32,358	99.07	19,972	61.15
Tuguegarao City	36,221	2,532	8.10	0	0.00	28,716	91.90	31,248	86.27	28,716	79.28
**Region 3**	**2,869,852**	**627,363**	**24.19**	**86,344**	**3.33**	**1,880,098**	**72.48**	**2,593,805**	**90.38**	**2,161,323**	**75.31**
Aurora	53,151	23,508	50.13	12,601	26.87	10,785	23.00	46,894	88.23	43,654	82.13
Bataan	170,621	29,254	18.77	8,008	5.14	118,589	76.09	155,851	91.34	131,841	77.27
Bulacan	591,197	79,959	14.90	5,814	1.08	450,736	84.01	536,509	90.75	401,144	67.85
Nueva Ecija	382,195	130,008	43.32	24,200	8.06	145,926	48.62	300,134	78.53	231,212	60.50
Pampanga	399,962	51,296	16.16	9,441	2.97	256,677	80.87	317,414	79.36	304,366	76.10
Tarlac	256,271	109,994	47.75	4,646	2.02	115,690	50.23	230,330	89.88	177,521	69.27
Zambales	156,149	106,976	56.73	12,391	6.57	69,198	36.70	188,565	120.76	120,575	77.22
Angeles City	109,251	2,598	2.42	4,708	4.39	99,972	93.19	107,278	98.19	99,972	91.51
Balanga City	24,218	1,368	7.98	309	1.80	15,460	90.21	17,137	70.76	16,656	68.78
Cabanatuan City	76,501	23,011	32.02	0	0.00	48,848	67.98	71,859	93.93	48,848	63.85
City of San Fernando	77,618	80	0.11	2,094	2.93	69,196	96.95	71,370	91.95	71,370	91.95
Gapan City	27,786	17,551	63.40	0	0.00	10,130	36.60	27,681	99.62	27,680	99.62
Mabalacat City	63,928	1,213	2.05	0	0.00	58,030	97.95	59,243	92.67	58,510	91.52
Malolos City	65,803	4,491	7.07	0	0.00	59,027	92.93	63,518	96.53	63,518	96.53
Meycauayan	59,239	628	1.08	0	0.00	57,270	98.92	57,898	97.74	57,253	96.65
Olongapo	62,481	0	0.00	0	0.00	63,310	100.00	63,310	101.33	63,310	101.33
Palayan City	10,556	884	10.36	0	0.00	7,647	89.64	8,531	80.82	7,647	72.44
San Jose City	36,704	11,813	36.24	0	0.00	20,787	63.76	32,600	88.82	32,150	87.59
San Jose del Monte Cit	142,734	0	0.00	1,276	0.89	141,458	99.11	142,734	100.00	142,734	100.00
Science City of Munoz	21,467	1,216	6.09	856	4.29	17,893	89.62	19,965	93.00	17,893	83.35
Tarlac City	82,020	31,515	42.03	0	0.00	43,469	57.97	74,984	91.42	43,469	53.00
**Region 4A**	**3,868,351**	**374,365**	**12.75**	**186,343**	**6.35**	**2,374,348**	**80.90**	**2,935,056**	**75.87**	**2,581,319**	**66.73**
Batangas	490,939	63,811	29.86	19,674	9.21	130,218	60.93	213,703	43.53	227,337	46.31
Cavite	379,650	25,994	9.12	4,540	1.59	254,585	89.29	285,119	75.10	229,766	60.52
Laguna	267,297	51,268	20.83	19,978	8.12	174,873	71.05	246,119	92.08	137,198	51.33
Quezon	433,749	129,883	41.09	70,568	22.32	115,649	36.59	316,100	72.88	234,626	54.09
Rizal	547,200	18,255	6.38	24,804	8.67	243,193	84.96	286,252	52.31	259,601	47.44
Antipolo City	193,506	10,790	5.91	26,618	14.57	145,274	79.52	182,682	94.41	177,916	91.94
Bacoor City	174,970	2,671	2.13	0	0.00	122,483	97.87	125,154	71.53	125,256	71.59
Batangas City	90,577	711	0.83	343	0.40	84,461	98.77	85,515	94.41	86,164	95.13
Biñan City	97,365	3,027	3.18	16	0.02	92,124	96.80	95,167	97.74	94,106	96.65
Cabuyao City	91,540	33,996	40.10	0	0.00	50,780	59.90	84,776	92.61	82,426	90.04
Calamba City	139,174	13,410	11.44	3,137	2.68	100,724	85.89	117,271	84.26	139,172	100.00
Cavite City	30,521	*	0.00	*	0.00	*	0.00	0	0.00	*	0.00
Dasmariñas City	177,028	298	0.20	0	0.00	148,073	99.80	148,371	83.81	135,962	76.80
General Trias City	94,336	0	0.00	393	0.42	93,943	99.58	94,336	100.00	94,336	100.00
Imus City	114,297	515	0.39	0	0.00	133,079	99.61	133,594	116.88	92,982	81.35
Lipa City	85,434	0	0.00	0	0.00	81,750	100.00	81,750	95.69	70,995	83.10
Lucena City	64,680	609	0.95	10,891	17.02	52,488	82.03	63,988	98.93	52,488	81.15
San Pablo City	70,048	2,065	3.22	0	0.00	62,165	96.78	64,230	91.69	41,180	58.79
San Pedro City	81,892	2,755	3.74	0	0.00	70,929	96.26	73,684	89.98	73,686	89.98
Santa Rosa City	113,549	11,501	10.66	0	0.00	96,374	89.34	107,875	95.00	96,374	84.87
Tagaytay City	19,263	140	0.73	0	0.00	19,123	99.27	19,263	100.00	19,152	99.42
Tanauan City	44,204	476	1.11	0	0.00	42,499	98.89	42,975	97.22	48,594	109.93
Tayabas City	25,675	2,190	8.53	5,381	20.96	18,104	70.51	25,675	100.00	20,545	80.02
Trece Martires City	41,457	0	0.00	0	0.00	41,457	100.00	41,457	100.00	41,457	100.00
**Region 4B**	**744,408**	**250,378**	**45.42**	**98,591**	**17.88**	**202,308**	**36.70**	**551,277**	**74.06**	**288,313**	**38.73**
Marinduque	56,464	14,197	25.73	14,269	25.86	26,710	48.41	55,176	97.72	35,482	62.84

Table 1.G.1- ENVIRONMENTAL HEALTH AND SANITATION SERVICES

Proportion and Total Number of Households (HHs) with access to basic safe water supply and manage drinking water
Philippines, 2021

Area	Projected No. of HHs	HHs with Access to Basic Safe Water Supply								HHs using Safely Managed Drinking Water Services	
		Level 1		Level 2		Level 3		Total	%		
		No.	%	No.	%	No.	%			No.	%
Mindoro Occidental	120,149	67,747	62.75	12,379	11.47	27,840	25.79	107,966	89.86	96,008	79.91
Mindoro Oriental	206,567	114,033	53.82	40,276	19.01	57,577	27.17	211,886	102.57	28,341	13.72
Palawan	219,189	30,055	51.73	10,425	17.94	17,621	30.33	58,101	26.51	28,722	13.10
Romblon	71,033	18,207	33.74	14,667	27.18	21,086	39.08	53,960	75.96	47,142	66.37
Puerto Princesa City	71,006	6,139	9.56	6,575	10.24	51,474	80.19	64,188	90.40	52,618	74.10
**Region 5**	**1,303,222**	**307,027**	**29.01**	**291,871**	**27.58**	**459,313**	**43.40**	**1,058,211**	**81.20**	**695,713**	**53.38**
Albay	252,871	77,197	31.66	65,840	27.00	100,783	41.34	243,820	96.42	243,820	96.42
Camarines Norte	140,632	27,101	20.30	46,955	35.17	59,452	44.53	133,508	94.93	131,089	93.21
Camarines Sur	358,907	63,709	33.86	52,040	27.66	72,412	38.48	188,161	52.43	62,518	17.42
Catanduanes	57,176	11,771	22.06	13,386	25.09	28,199	52.85	53,356	93.32	23,718	41.48
Masbate	199,577	66,775	47.73	44,644	31.91	28,490	20.36	139,909	70.10	75,036	37.60
Sorsogon	180,509	44,516	23.85	56,558	30.30	85,566	45.85	186,640	103.40	80,387	44.53
Iriga City	23,359	2,467	11.42	3,879	17.96	15,249	70.61	21,595	92.45	15,249	65.28
Legaspi City	44,944	13,051	28.28	7,507	16.27	25,595	55.46	46,153	102.69	18,827	41.89
Naga City	45,247	440	0.98	1,062	2.36	43,567	96.67	45,069	99.61	45,069	99.61
**Region 6**	**1,810,835**	**757,140**	**44.38**	**254,634**	**14.93**	**694,083**	**40.69**	**1,705,857**	**94.20**	**871,602**	**48.13**
Aklan	142,649	51,766	39.64	24,204	18.53	54,631	41.83	130,601	91.55	55,574	38.96
Antique	138,024	48,493	34.14	50,376	35.47	43,172	30.39	142,041	102.91	72,658	52.64
Capiz	192,326	101,686	54.08	19,878	10.57	66,472	35.35	188,036	97.77	78,079	40.60
Guimaras	43,533	25,235	51.43	3,362	6.85	20,466	41.71	49,063	112.70	16,206	37.23
Iloilo	466,874	270,412	61.00	55,852	12.60	117,032	26.40	443,296	94.95	166,340	35.63
Negros Occidental	589,468	220,523	41.23	97,079	18.15	217,247	40.62	534,849	90.73	320,999	54.46
Bacolod City	135,511	10,071	8.19	3,883	3.16	108,996	88.65	122,950	90.73	95,603	70.55
Iloilo City	102,450	28,954	30.47	0	0.00	66,067	69.53	95,021	92.75	66,143	64.56
**Region 7**	**1,860,669**	**280,290**	**18.34**	**316,720**	**20.72**	**931,674**	**60.95**	**1,528,684**	**82.16**	**714,704**	**38.41**
Bohol	302,905	51,900	22.58	28,480	12.39	149,499	65.03	229,879	75.89	209,064	69.02
Cebu	748,994	112,191	15.87	175,910	24.89	418,627	59.23	706,728	94.36	176,935	23.62
Negros Oriental	325,455	49,861	21.78	55,869	24.40	123,225	53.82	228,955	70.35	187,800	57.70
Siquijor	24,135	823	3.42	4,853	20.19	18,365	76.39	24,041	99.61	24,041	99.61
Cebu City	241,450	14,005	10.52	37,959	28.52	81,155	60.96	133,119	55.13	5,876	2.43
Lapu-Lapu City	111,486	45,152	40.70	10,479	9.45	55,297	49.85	110,928	99.50	110,928	99.50
Mandaue City	106,244	6,358	6.69	3,170	3.34	85,506	89.97	95,034	89.45	60	0.06
**Region 8**	**1,071,642**	**192,930**	**23.27**	**308,807**	**37.25**	**327,230**	**39.47**	**828,967**	**77.35**	**608,024**	**56.74**
Biliran	40,772	9,128	16.82	39,011	71.87	6,142	11.32	54,281	133.13	30,912	75.82
Eastern Samar	116,802	8,533	18.97	14,856	33.03	21,585	47.99	44,974	38.50	42,092	36.04
Northern Leyte	392,077	77,004	23.35	117,875	35.75	134,879	40.90	329,758	84.11	221,523	56.50
Northern Samar	135,532	56,392	55.68	32,202	31.80	12,678	12.52	101,272	74.72	98,273	72.51
Southern Leyte	79,220	6,940	9.11	17,260	22.64	52,021	68.25	76,221	96.21	59,556	75.18
Western Samar	135,083	16,376	28.97	18,767	33.20	21,379	37.82	56,522	41.84	3,079	2.28
Calbayog City	40,978	6,311	17.46	14,549	40.26	15,276	42.27	36,136	88.18	29,825	72.78
Maasin City	19,972	2,880	14.10	4,581	22.43	12,960	63.46	20,421	102.25	19,972	100.00
Ormoc City	55,381	5,038	9.15	18,523	33.64	31,500	57.21	55,061	99.42	54,640	98.66
Tacloban City	55,825	4,328	7.97	31,183	57.41	18,810	34.63	54,321	97.31	48,152	86.26
**Region 9**	**842,036**	**137,268**	**20.57**	**215,475**	**32.29**	**314,569**	**47.14**	**667,312**	**79.25**	**433,837**	**51.52**
Zamboanga del Norte	184,496	41,105	38.99	38,893	36.89	25,429	24.12	105,427	57.14	80,747	43.77
Zamboanga del Sur	181,894	33,059	18.28	83,496	46.18	64,264	35.54	180,819	99.41	107,180	58.92

Table 1.G.1- ENVIRONMENTAL HEALTH AND SANITATION SERVICES

Proportion and Total Number of Households (HHs) with access to basic safe water supply and manage drinking water
Philippines, 2021

Area	Projected No. of HHs	HHs with Access to Basic Safe Water Supply								HHs using Safely Managed Drinking Water Services	
		Level 1		Level 2		Level 3		Total	%		
		No.	%	No.	%	No.	%			No.	%
Zamboanga Sibugay	153,129	24,460	27.79	31,764	36.09	31,785	36.12	88,009	57.47	54,064	35.31
Dapitan City	18,963	2,063	12.40	4,347	26.13	10,224	61.46	16,634	87.72	16,634	87.72
Dipolog City	31,525	9,015	28.67	1,296	4.12	21,130	67.21	31,441	99.73	31,441	99.73
Isabela City	26,649	3,362	12.66	11,496	43.30	11,694	44.04	26,552	99.64	14,678	55.08
Pagadian City	43,122	1,945	7.21	3,824	14.18	21,202	78.61	26,971	62.55	20,653	47.89
Zamboanga City	202,258	22,259	11.63	40,359	21.08	128,841	67.29	191,459	94.66	108,440	53.61
**Region 10**	**1,135,195**	**298,615**	**26.96**	**258,511**	**23.34**	**550,582**	**49.70**	**1,107,708**	**97.58**	**483,776**	**42.62**
Bukidnon	244,466	146,680	60.00	48,893	20.00	48,893	20.00	244,466	100.00	945	0.39
Camiguin	21,180	114	0.54	2,035	9.64	18,956	89.82	21,105	99.65	13,516	63.81
Lanao del Norte	153,079	56,503	40.54	35,087	25.18	47,779	34.28	139,369	91.04	28,230	18.44
Misamis Occidental	78846	21,309	27.94	24,850	32.59	30,102	39.47	76,261	96.72	46,272	58.69
Misamis Oriental	176,853	33,699	18.94	48,641	27.33	95,609	53.73	177,949	100.62	86,026	48.64
Cagayan de Oro City	180,310	3,237	1.97	16,251	9.87	145,121	88.16	164,609	91.29	96,844	53.71
El Salvador City	13,110	143	1.09	1,534	11.70	11,433	87.21	13,110	100.00	12,761	97.34
Gingoog City	29,156	3,086	10.21	11,288	37.35	15,852	52.44	30,226	103.67	27,450	94.15
Iligan City	84,210	2,115	2.68	32,030	40.61	44,723	56.71	78,868	93.66	64,677	76.80
Malaybalay City	42,685	6,827	15.63	14,454	33.10	22,389	51.27	43,670	102.31	33,223	77.83
Oroquieta City	17,521	1,046	5.61	4,165	22.36	13,418	72.03	18,629	106.32	11,908	67.96
Ozamis City	32,051	9,722	30.50	3,194	10.02	18,964	59.49	31,880	99.47	16,886	52.68
Tangub City	13,722	3,882	25.75	7,425	49.25	3,770	25.00	15,077	109.87	15,077	109.87
Valencia City	48,006	10,252	19.53	8,664	16.51	33,573	63.96	52,489	109.34	29,961	62.41
**Region 11**	**1,301,197**	**234,491**	**20.01**	**236,894**	**20.21**	**700,757**	**59.78**	**1,172,142**	**90.08**	**950,699**	**73.06**
Davao de Oro	180,169	69,364	37.76	56,356	30.68	57,961	31.56	183,681	101.95	121,590	67.49
Davao del Norte	246,313	54,288	28.81	22,988	12.20	111,140	58.99	188,416	76.49	175,583	71.28
Davao Oriental	140,304	50,226	35.32	43,800	30.80	48,162	33.87	142,188	101.34	35,207	25.09
Davao del Sur	181,121	40,752	25.23	37,161	23.00	83,635	51.77	161,548	89.19	122,331	67.54
Davao Occidental	84,075	16,771	27.61	37,765	62.17	6,208	10.22	60,744	72.25	59,965	71.32
Davao City	469,215	3,090	0.71	38,824	8.91	393,651	90.38	435,565	92.83	436,023	92.93
**Region 12**	**1,156,886**	**317,653**	**39.12**	**156,569**	**19.28**	**337,860**	**41.60**	**812,082**	**70.20**	**419,771**	**36.28**
North Cotabato	362,360	106,390	40.96	62,080	23.90	91,281	35.14	259,751	71.68	184,667	50.96
Sarangani	135,144	49,882	46.72	35,619	33.36	21,272	19.92	106,773	79.01	75,393	55.79
South Cotabato	241,416	30,318	26.62	21,237	18.65	62,318	54.73	113,873	47.17	105,125	43.55
Sultan Kudarat	196,635	103,692	73.91	23,440	16.71	13,162	9.38	140,294	71.35	25,784	13.11
Cotabato City	61,440	3,044	8.88	5,195	15.16	26,035	75.96	34,274	55.78	28,802	46.88
Gen. Santos City	159,891	24,327	15.48	8,998	5.73	123,792	78.79	157,117	98.27	*	0.00
**BARMM**	**701,960**	**243,395**	**57.81**	**118,854**	**28.23**	**58,751**	**13.96**	**421,000**	**59.97**	**158,285**	**22.55**
Basilan	51,158	23,849	54.12	14,548	33.01	5,668	12.86	44,065	86.14	9,503	18.58
Lanao del Sur	145,994	4,804	20.50	11,824	50.45	6,807	29.05	23,435	16.05	2,389	1.64
Maguindanao	223,608	99,249	55.46	56,888	31.79	22,805	12.74	178,942	80.02	69,173	30.93
Sulu	157,584	62,999	0.00	13,989	0.00	6,784	0.00	*	0.00	*	0.00
Tawi-Tawi	72,380	43,518	80.69	8,563	15.88	1,853	3.44	53,934	74.52	45,604	63.01
Lamitan City	17,214	3,130	28.93	3,060	28.28	4,629	42.79	10,819	62.85	5,411	31.43
Marawi City	34,022	5,846	22.46	9,982	38.34	10,205	39.20	26,033	76.52	26,205	77.02

Table 1.G.1- ENVIRONMENTAL HEALTH AND SANITATION SERVICES

Proportion and Total Number of Households (HHs) with access to basic safe water supply and manage drinking water
Philippines, 2021

Area	Projected No. of HHs	HHs with Access to Basic Safe Water Supply								HHs using Safely Managed Drinking Water Services	
		Level 1		Level 2		Level 3		Total	%		
		No.	%	No.	%	No.	%			No.	%
**CARAGA**	**617,544**	**123,933**	**21.10**	**182,537**	**31.08**	**280,768**	**47.81**	**587,238**	**95.09**	**319,014**	**51.66**
Agusan del Norte	84,078	13,791	18.75	20,601	28.01	39,163	53.24	73,555	87.48	54,015	64.24
Agusan del Sur	162,957	58,220	39.07	42,497	28.52	48,284	32.41	149,001	91.44	48,887	30.00
Surigao del Norte	81,219	10,851	12.12	36,173	40.39	42,536	47.49	89,560	110.27	60,645	74.67
Surigao del Sur	114,459	19,509	17.51	34,641	31.09	57,259	51.40	111,409	97.34	57,259	50.03
Province of Dinagat	29,539	3,748	13.30	10,880	38.61	13,549	48.09	28,177	95.39	18,231	61.72
Bislig City	22,646	878	4.38	3,818	19.05	15,343	76.57	20,039	88.49	15,343	67.75
Butuan City	82,739	15,565	19.59	21,467	27.02	42,420	53.39	79,452	96.03	42,420	51.27
Surigao City	39,907	1,371	3.80	12,460	34.57	22,214	61.63	36,045	90.32	22,214	55.66

Legend:
0 = No Case
** = No Report*

Table 1.G.2 - ENVIRONMENTAL HEALTH AND SANITATION SERVICES

Proportion and Total Number of Households (HHs) with access to basic sanitation facility
Philippines, 2021

Area	Projected No. of HHs	HH with access to Basic Sanitation Facility								HHs using Safely Managed Sanitation Service	
		Pour/Flush Toilet									
		Total	%	Septic Tank		Community sewer/sewerage system		Ventillated improved Pit Latrine (VIP)			
				No.	%	No.	%	No.	%	No.	%
**PHILIPPINES**	**25,216,517**	**18,911,890**	**75.00**	**17,353,729**	**91.76**	**737,298**	**3.90**	**820,863**	**4.34**	**13,333,172**	**52.87**
**NCR**	**3,379,343**	**2,976,639**	**88.08**	**2,634,445**	**88.50**	**342,194**	**11.50**	**0**	**0.00**	**1,876,564**	**55.53**
Malabon	93,797	88,169	94.00	75,037	85.11	13,132	14.89	0	0.00	84,417	90.00
Navotas	66,132	60,775	91.90	60,775	100.00	0	0.00	0	0.00	60,775	91.90
Valenzuela City	166,278	166,196	99.95	166,196	100.00	0	0.00	0	0.00	166,196	99.95
Caloocan City	399,732	339,772	85.00	339,772	100.00	0	0.00	0	0.00	339,772	85.00
Marikina City	106,984	77,588	72.52	76,605	98.73	983	1.27	0	0.00	0	0.00
Pasig City	196,479	178,706	90.95	178,706	100.00	0	0.00	0	0.00	178,808	91.01
Pateros	15,433	15,406	99.83	15,406	100.00	0	0.00	0	0.00	14,282	92.54
Taguig	218,248	216,032	98.98	216,032	100.00	0	0.00	0	0.00	216,032	98.98
Quezon City	740,559	686,100	92.65	686,100	100.00	0	0.00	0	0.00	31,895	4.31
Makati City	168,040	154,525	91.96	141,160	91.35	13,365	8.65	0	0.00	138,020	82.14
Mandaluyong City	111,267	111,267	100.00	104,755	94.15	6,512	5.85	0	0.00	110,592	99.39
San Juan	31,425	31,425	100.00	31,425	100.00	0	0.00	0	0.00	31,425	100.00
Manila City	476,545	343,036	71.98	50,197	14.63	292,839	85.37	0	0.00		0.00
Las Piñas City	154,249	100,860	65.39	100,860	100.00	0	0.00	0	0.00	106,013	68.73
Muntinlupa City	138,889	138,681	99.85	138,681	100.00	0	0.00	0	0.00	137,333	98.88
Parañaque City	177,422	177,422	100.00	162,059	91.34	15,363	8.66	0	0.00	170,325	96.00
Pasay City	117,864	90,679	76.94	90,679	100.00	0	0.00	0	0.00	90,679	76.94
**CAR**	**421,691**	**383,291**	**90.89**	**339,423**	**88.55**	**15,042**	**3.92**	**28,826**	**7.52**	**341,159**	**80.90**
Abra	54,884	43,083	78.50	40,810	94.72	250	0.58	2,023	4.70	31,670	57.70
Apayao	26,865	23,350	86.92	18,768	80.38	0	0.00	4,582	19.62	23,350	86.92
Benguet	116,296	104,028	89.45	97,024	93.27	0	0.00	7,004	6.73	92,604	79.63
Ifugao	45,312	42,700	94.24	36,014	84.34	0	0.00	6,686	15.66	29,696	65.54
Kalinga	44,012	38,123	86.62	31,824	83.48	0	0.00	6,299	16.52	33,608	76.36
Mt. Province	35,832	34,539	96.39	32,132	93.03	175	0.51	2,232	6.46	34,135	95.26
Baguio City	98,490	97,468	98.96	82,851	85.00	14,617	15.00	0	0.00	96,096	97.57
**Region 1**	**1,220,692**	**1,146,311**	**93.91**	**1,127,001**	**98.32**	**6,156**	**0.54**	**13,154**	**1.15**	**454,249**	**37.21**
Ilocos Norte	118,905	112,690	94.77	112,577	99.90	0	0.00	113	0.10	28,789	24.21
Ilocos Sur	137,682	110,117	79.98	109,952	99.85	0	0.00	165	0.15	107,997	78.44
La Union	162,189	159,068	98.08	148,329	93.25	1,066	0.67	9,673	6.08	10,739	6.62
Pangasinan	577,046	551,854	95.63	549,250	99.53	0	0.00	2,604	0.47	161,054	27.91
Alaminos City	22,970	20,668	89.98	20,668	100.00	0	0.00	0	0.00	10,137	44.13
Candon City	14,479	14,090	97.31	14,090	100.00	0	0.00	0	0.00	14,090	97.31
Dagupan City	40,810	41,096	100.70	41,096	100.00	0	0.00	0	0.00	39,398	96.54
Laoag City	26,469	23,110	87.31	23,110	100.00	0	0.00	0	0.00	23,110	87.31
San Carlos City	44,740	43,200	96.56	43,200	100.00	0	0.00	0	0.00	28,100	62.81
San Fernando City	30,415	26,603	87.47	20,914	78.62	5,090	19.13	599	2.25	5,689	18.70
Urdaneta City	32,400	31,411	96.95	31,411	100.00	0	0.00	0	0.00	12,742	39.33
Vigan City	12,587	12,404	98.55	12,404	100.00	0	0.00	0	0.00	12,404	98.55
**Region 2**	**910,994**	**435,294**	**47.78**	**413,617**	**95.02**	**4,688**	**1.08**	**16,989**	**3.90**	**345,194**	**37.89**
Batanes	5,015	5,007	99.84	5,007	100.00	0	0.00	0	0.00	5,007	99.84

Table 1.G.2 - ENVIRONMENTAL HEALTH AND SANITATION SERVICES

Proportion and Total Number of Households (HHs) with access to basic sanitation facility
Philippines, 2021

Area	Projected No. of HHs	HH with access to Basic Sanitation Facility									HHs using Safely Managed Sanitation Service	
		Pour/Flush Toilet										
		Total	%	Septic Tank		Community sewer/sewerage system		Ventillated improved Pit Latrine (VIP)				
				No.	%	No.	%	No.	%		No.	%
Cagayan	256,379	52,563	20.50	45,381	86.34	568	1.08	6,614	12.58		75,770	29.55
Isabela	296,310	146,729	49.52	141,571	96.48	3,219	2.19	1,939	1.32		66,215	22.35
Nueva Vizcaya	168,501	77,789	46.17	77,033	99.03	0	0.00	756	0.97		43,609	25.88
Quirino	45,979	45,952	99.94	38,103	82.92	901	1.96	6,948	15.12		34,261	74.51
Cauayan City	32,820	30,946	94.29	30,946	100.00	0	0.00	0	0.00		28,175	85.85
Ilagan City	37,107	18,386	49.55	17,654	96.02	0	0.00	732	3.98		34,977	94.26
Santiago City	32,662	28,464	87.15	28,464	100.00	0	0.00	0	0.00		28,464	87.15
Tuguegarao City	36,221	29,458	81.33	29,458	100.00	0	0.00	0	0.00		28,716	79.28
**Region 3**	**2,869,852**	**2,339,155**	**81.51**	**2,295,275**	**98.12**	**18,165**	**0.78**	**25,715**	**1.10**		**2,052,046**	**71.50**
Aurora	53,151	45,845	86.25	40,463	88.26	358	0.78	5,024	10.96		42,711	80.36
Bataan	170,621	163,810	96.01	163,810	100.00	0	0.00	0	0.00		152,278	89.25
Bulacan	591,197	314,528	53.20	314,234	99.91	86	0.03	208	0.07		163,708	27.69
Nueva Ecija	382,195	283,370	74.14	274,654	96.92	0	0.00	8,716	3.08		253,107	66.22
Pampanga	399,962	330,198	82.56	325,196	98.49	3,617	1.10	1,385	0.42		268,955	67.25
Tarlac	256,271	234,910	91.66	234,374	99.77	0	0.00	536	0.23		229,721	89.64
Zambales	156,149	171,998	110.15	153,058	88.99	14,104	8.20	4,836	2.81		137,731	88.20
Angeles City	109,251	106,827	97.78	106,827	100.00	0	0.00	0	0.00		106,827	97.78
Balanga City	24,218	16,350	67.51	16,350	100.00	0	0.00	0	0.00		16,322	67.40
Cabanatuan City	76,501	57,486	75.14	56,223	97.80	0	0.00	1,263	2.20		70,135	91.68
City of San Fernando	77,618	65,326	84.16	63,226	96.79	0	0.00	2,100	3.21		63,810	82.21
Gapan City	27,786	26,663	95.96	26,663	100.00	0	0.00	0	0.00		26,682	96.03
Mabalacat City	63,928	57,832	90.46	56,496	97.69	0	0.00	1,336	2.31		57,542	90.01
Malolos City	65,803	62,040	94.28	62,040	100.00	0	0.00	0	0.00		62,040	94.28
Meycauayan	59,239	58,013	97.93	58,013	100.00	0	0.00	0	0.00		58,013	97.93
Olongapo	62,481	64,004	102.44	63,744	99.59	0	0.00	260	0.41		63,310	101.33
Palayan City	10,556	9,521	90.20	9,521	100.00	0	0.00	0	0.00		9,064	85.87
San Jose City	36,704	32,220	87.78	32,220	100.00	0	0.00	0	0.00		32,220	87.78
San Jose del Monte City	142,734	142,734	100.00	142,734	100.00	0	0.00	0	0.00		142,734	100.00
Science City of Munoz	21,467	20,987	97.76	20,936	99.76	0	0.00	51	0.24		20,936	97.53
Tarlac City	82,020	74,493	90.82	74,493	100.00	0	0.00	0	0.00		74,200	90.47
**Region 4A**	**3,868,351**	**2,813,702**	**72.74**	**2,634,400**	**93.63**	**80,568**	**2.86**	**98,734**	**3.51**		**2,306,308**	**59.62**
Batangas	490,939	175,615	35.77	170,005	96.81	890	0.51	4,720	2.69		141,288	28.78
Cavite	379,650	278,774	73.43	272,901	97.89	5,873	2.11	0	0.00		241,426	63.59
Laguna	267,297	238,644	89.28	236,650	99.16	0	0.00	1,994	0.84		71,274	26.66
Quezon	433,749	311,029	71.71	300,938	96.76	4,256	1.37	5,835	1.88		190,648	43.95
Rizal	547,200	246,158	44.99	178,147	72.37	66,959	27.20	1,052	0.43		244,268	44.64
Antipolo City	193,506	177,916	91.94	177,916	100.00	0	0.00	0	0.00		177,916	91.94
Bacoor City	174,970	124,688	71.26	124,688	100.00	0	0.00	0	0.00		124,688	71.26
Batangas City	90,577	86,164	95.13	85,574	99.32	0	0.00	590	0.68		85,937	94.88
Biñan City	97,365	94,879	97.45	94,870	99.99	0	0.00	9	0.01		94,166	96.71
Cabuyao City	91,540	86,782	94.80	86,782	100.00	0	0.00	0	0.00		87,610	95.71
Calamba City	139,174	115,684	83.12	115,684	100.00	0	0.00	0	0.00		139,173	100.00
Cavite City	30,521	0	0.00	*	0.00	*	0.00	*	0.00		*	0.00
Dasmariñas City	177,028	148,386	83.82	148,386	100.00	0	0.00	0	0.00		135,842	76.73
General Trias City	94,336	96,011	101.78	93,726	97.62	2,285	2.38	0	0.00		88,734	94.06
Imus City	114,297	133,166	116.51	133,166	100.00	0	0.00	0	0.00		131,260	114.84
Lipa City	85,434	80,050	93.70	0	0.00	0	0.00	80,050	100.00		79,051	92.53
Lucena City	64,680	49,198	76.06	49,198	100.00	0	0.00	0	0.00		49,198	76.06
San Pablo City	70,048	63,971	91.32	63,666	99.52	305	0.48	0	0.00		62,675	89.47

Table 1.G.2 - ENVIRONMENTAL HEALTH AND SANITATION SERVICES

Proportion and Total Number of Households (HHs) with access to basic sanitation facility
Philippines, 2021

Area	Projected No. of HHs	HH with access to Basic Sanitation Facility								HHs using Safely Managed Sanitation Service	
		Pour/Flush Toilet									
		Total	%	Septic Tank		Community sewer/sewerage system		Ventillated improved Pit Latrine (VIP)			
				No.	%	No.	%	No.	%	No.	%
San Pedro City	81,892	73,135	89.31	73,135	100.00	0	0.00	0	0.00	0	0.00
Santa Rosa City	113,549	106,904	94.15	106,904	100.00	0	0.00	0	0.00	57,698	50.81
Tagaytay City	19,263	18,953	98.39	18,953	100.00	0	0.00	0	0.00	19,174	99.54
Tanauan City	44,204	42,526	96.20	42,526	100.00	0	0.00	0	0.00	42,825	96.88
Tayabas City	25,675	23,612	91.96	19,128	81.01	0	0.00	4,484	18.99	0	0.00
Trece Martires City	41,457	41,457	100.00	41,457	100.00	0	0.00	0	0.00	41,457	100.00
**Region 4B**	**744,408**	**546,095**	**73.36**	**532,519**	**97.51**	**1,298**	**0.24**	**12,278**	**2.25**	**248,902**	**33.44**
Marinduque	56,464	52,453	92.90	52,453	100.00	0	0.00	0	0.00	52,453	92.90
Mindoro Occidental	120,149	101,819	84.74	100,188	98.40	0	0.00	1,631	1.60	61,864	51.49
Mindoro Oriental	206,567	183,011	88.60	182,749	99.86	245	0.13	17	0.01	0	0.00
Palawan	219,189	80,194	36.59	74,049	92.34	1,053	1.31	5,092	6.35	33,842	15.44
Romblon	71,033	69,424	97.73	64,803	93.34	0	0.00	4,621	6.66	44,988	63.33
Puerto Princesa City	71,006	59,194	83.36	58,277	98.45	0	0.00	917	1.55	55,755	78.52
**Region 5**	**1,303,222**	**933,565**	**71.64**	**809,219**	**86.68**	**86,028**	**9.21**	**38,318**	**4.10**	**634,323**	**48.67**
Albay	252,871	219,003	86.61	124596	56.89	80,751	36.87	13,656	6.24	124696	49.31
Camarines Norte	140,632	127,112	90.39	124011	97.56	1,760	1.38	1,341	1.05	103177	73.37
Camarines Sur	358,907	151,398	42.18	143555	94.82	2,830	1.87	5,013	3.31	58113	16.19
Catanduanes	57,176	50,572	88.45	50464	99.79	17	0.03	91	0.18	44017	76.99
Masbate	199,577	117,145	58.70	108,702	92.79	148	0.13	8,295	7.08	78,105	39.14
Sorsogon	180,509	164,174	90.95	163,555	99.62	522	0.32	97	0.06	121,651	67.39
Iriga City	23,359	20,198	86.47	10,373	51.36	0	0.00	9,825	48.64	20,601	88.19
Legaspi City	44,944	38,894	86.54	38894	100.00	0	0.00	0	0.00	38894	86.54
Naga City	45,247	45,069	99.61	45069	100.00	0	0.00	0	0.00	45069	99.61
**Region 6**	**1,810,835**	**1,568,125**	**86.60**	**1,381,550**	**88.10**	**83,539**	**5.33**	**103,036**	**6.57**	**961,914**	**53.12**
Aklan	142,649	131,049	91.87	52,510	40.07	59,421	45.34	19,118	14.59	12,458	8.73
Antique	138,024	117,561	85.17	115,509	98.25	1	0.00	2,051	1.74	100,426	72.76
Capiz	192,326	178,934	93.04	144,017	80.49	0	0.00	34,917	19.51	159,490	82.93
Guimaras	43,533	47,490	109.09	46,446	97.80	0	0.00	1,044	2.20	42,986	98.74
Iloilo	466,874	442,879	94.86	396,252	89.47	22,421	5.06	24,206	5.47	160,118	34.30
Negros Occidental	589,468	441,470	74.89	422,211	95.64	1,696	0.38	17,563	3.98	296,771	50.35
Bacolod City	135,511	120,511	88.93	116,910	97.01	0	0.00	3,601	2.99	102,010	75.28
Iloilo City	102,450	88,231	86.12	87,695	99.39	0	0.00	536	0.61	87,655	85.56
**Region 7**	**1,860,669**	**894,743**	**48.09**	**842,051**	**94.11**	**21,795**	**2.44**	**30,897**	**3.45**	**715,811**	**38.47**
Bohol	302,905	222,513	73.46	222,490	99.99	5	0.00	18	0.01	85,354	28.18
Cebu	748,994	31,515	4.21	25,335	80.39	5,913	18.76	267	0.85	173,030	23.10
Negros Oriental	325,455	228,307	70.15	181,818	79.64	15,877	6.95	30,612	13.41	112,492	34.56
Siquijor	24,135	23,343	96.72	23,343	100.00	0	0.00	0	0.00	22,943	95.06
Cebu City	241,450	200,192	82.91	200,192	100.00	0	0.00	0	0.00	133,119	55.13
Lapu-Lapu City	111,486	96,546	86.60	96,546	100.00	0	0.00	0	0.00	96,546	86.60
Mandaue City	106,244	92,327	86.90	92,327	100.00	0	0.00	0	0.00	92,327	86.90
**Region 8**	**1,071,642**	**785,934**	**73.34**	**610,253**	**77.65**	**49,200**	**6.26**	**126,481**	**16.09**	**442,761**	**41.32**
Biliran	40,772	114,508	280.85	114,508	100.00	0	0.00	0	0.00	34,123	83.69

Table 1.G.2 - ENVIRONMENTAL HEALTH AND SANITATION SERVICES

Proportion and Total Number of Households (HHs) with access to basic sanitation facility
Philippines, 2021

Area	Projected No. of HHs	HH with access to Basic Sanitation Facility									HHs using Safely Managed Sanitation Service	
		Pour/Flush Toilet										
		Total	%	Septic Tank		Community sewer/sewerage system		Ventillated improved Pit Latrine (VIP)				
				No.	%	No.	%	No.	%		No.	%
Eastern Samar	116,802	38,246	32.74	38,118	99.67	128	0.33	0	0.00		42,204	36.13
Northern Leyte	392,077	324,513	82.77	279,151	86.02	18,971	5.85	26,391	8.13		96,682	24.66
Northern Samar	135,532	86,185	63.59	73,166	84.89	2	0.00	13,017	15.10		38,397	28.33
Southern Leyte	79,220	75,442	95.23	-	0.00	0	0.00	75,442	100.00		75,442	95.23
Western Samar	135,083	20,792	15.39	15,563	74.85	5,229	25.15	0	0.00		23,601	17.47
Calbayog City	40,978	6,000	14.64	6,000	100.00	0	0.00	0	0.00		14,531	35.46
Maasin City	19,972	18,819	94.23	18,819	100.00	0	0.00	0	0.00		18,819	94.23
Ormoc City	55,381	49,101	88.66	42,829	87.23	0	0.00	6,272	12.77		52,015	93.92
Tacloban City	55,825	52,328	93.74	22,099	42.23	24,870	47.53	5,359	10.24		46,947	84.10
**Region 9**	**842,036**	**584,178**	**69.38**	**465,502**	**79.68**	**18,307**	**3.13**	**100,369**	**17.18**		**601,667**	**71.45**
Zamboanga del Norte	184,496	137,080	74.30	117,434	85.67	8,484	6.19	11,162	8.14		142,854	77.43
Zamboanga del Sur	181,894	144,901	79.66	82,762	57.12	3,550	2.45	58,589	40.43		119,449	65.67
Zamboanga Sibugay	153,129	52,631	34.37	33,419	63.50	6,273	11.92	12,939	24.58		93,862	61.30
Dapitan City	18,963	9,933	52.38	9,555	96.19	0	0.00	378	3.81		7,392	38.98
Dipolog City	31,525	31,525	100.00	31,372	99.51	0	0.00	153	0.49		31,372	99.51
Isabela City	26,649	11,114	41.71	4,347	39.11	0	0.00	6,767	60.89		14,678	55.08
Pagadian City	43,122	25,364	58.82	15,072	59.42	0	0.00	10,292	40.58		20,430	47.38
Zamboanga City	202,258	171,630	84.86	171,541	99.95	0	0.00	89	0.05		171,630	84.86
**Region 10**	**1,135,195**	**798,903**	**70.38**	**704,338**	**88.16**	**18**	**0.00**	**94,547**	**11.83**		**188,774**	**16.63**
Bukidnon	244,466	119,788	49.00	73,340	61.22	0	0.00	46,448	38.78		945	0.39
Camiguin	21,180	19,555	92.33	19,555	100.00	0	0.00	0	0.00		13,032	61.53
Lanao del Norte	153,079	51,970	33.95	41,620	80.08	0	0.00	10,350	19.92		28,956	18.92
Misamis Occidental	78846	71,803	91.07	62,569	87.14	0	0.00	9,234	12.86		61,500	78.00
Misamis Oriental	176,853	160,862	90.96	151,123	93.95	18	0.01	9,721	6.04		29,148	16.48
Cagayan de Oro City	180,310	159,980	88.72	158,945	99.35	0	0.00	1,035	0.65		3,470	1.92
El Salvador City	13,110	12,723	97.05	12,571	98.81	0	0.00	152	1.19		12,948	98.76
Gingoog City	29,156	29,026	99.55	28,526	98.28	0	0.00	500	1.72		1,693	5.81
Iligan City	84,210	59,929	71.17	59,494	99.27	0	0.00	435	0.73		1,036	1.23
Malaybalay City	42,685	36,052	84.46	35,632	98.84	0	0.00	420	1.16		7,479	17.52
Oroquieta City	17,521	17,876	102.03	12,870	72.00	0	0.00	5,006	28.00		7,704	43.97
Ozamis City	32,051	28,256	88.16	25,685	90.90	0	0.00	2,571	9.10		9,386	29.28
Tangub City	13,722	18,695	136.24	11,457	61.28	0	0.00	7,238	38.72		11,457	83.49
Valencia City	48,006	12,388	25.81	10,951	88.40	0	0.00	1,437	11.60		20	0.04
**Region 11**	**1,301,197**	**1,153,288**	**88.63**	**1,121,439**	**97.24**	**513**	**0.04**	**31,336**	**2.72**		**1,126,662**	**86.59**
Davao de Oro	180,169	174,747	96.99	163,261	93.43	0	0.00	11,486	6.57		162,877	90.40
Davao del Norte	246,313	234,648	95.26	224,969	95.88	513	0.22	9,166	3.91		233,605	94.84
Davao Oriental	140,304	134,167	95.63	133,612	99.59	0	0.00	555	0.41		133,735	95.32
Davao del Sur	181,121	125,561	69.32	117,904	93.90	0	0.00	7,657	6.10		132,296	73.04
Davao Occidental	84,075	49,653	59.06	47,181	95.02	0	0.00	2,472	4.98		29,637	35.25
Davao City	469,215	434,512	92.60	434,512	100.00	*	0.00	*	0.00		434,512	92.60
**Region 12**	**1,156,886**	**753,615**	**65.14**	**703,033**	**93.29**	**3,710**	**0.49**	**46,872**	**6.22**		**523,202**	**45.23**
North Cotabato	362,360	221,551	61.14	194,199	87.65	0	0.00	27,352	12.35		151,400	41.78
Sarangani	135,144	82,120	60.76	79,795	97.17	0	0.00	2,325	2.83		68,736	50.86
South Cotabato	241,416	100,852	41.78	95,758	94.95	0	0.00	5,094	5.05		60,496	25.06

Table 1.G.2 - ENVIRONMENTAL HEALTH AND SANITATION SERVICES

Proportion and Total Number of Households (HHs) with access to basic sanitation facility
Philippines, 2021

Area	Projected No. of HHs	HH with access to Basic Sanitation Facility								HHs using Safely Managed Sanitation Service	
		Pour/Flush Toilet									
		Total	%	Septic Tank		Community sewer/sewerage system		Ventillated improved Pit Latrine (VIP)			
				No.	%	No.	%	No.	%	No.	%
Sultan Kudarat	196,635	166,945	84.90	157,134	94.12	0	0.00	9,811	5.88	79,617	40.49
Cotabato City	61,440	25,426	41.38	25,426	100.00	0	0.00	0	0.00	23,051	37.52
Gen. Santos City	159,891	156,721	98.02	150,721	96.17	3,710	2.37	2,290	1.46	139,902	87.50
**BARMM**	**701,960**	**246,717**	**35.15**	**202,168**	**81.94**	**4,505**	**1.83**	**40,044**	**16.23**	**141,205**	**20.12**
Basilan	51,158	16,718	32.68	14,264	85.32	2158	12.91	296	1.77	7,207	14.09
Lanao del Sur	145,994	22,094	15.13	12,354	55.92	0	0.00	9740	44.08	4,971	3.40
Maguindanao	223,608	132,190	59.12	108,590	82.15	2337	1.77	21263	16.09	82,212	36.77
Sulu	157,584	33,353	21.17	30,720	92.11	10	0.03	2623	7.86	319	0.20
Tawi-Tawi	72,380	11,902	16.44	9,936	83.48	0	0.00	1966	16.52	11,031	15.24
Lamitan City	17,214	7,072	41.08	3,836	54.24	0	0.00	3236	45.76	5,635	32.73
Marawi City	34,022	23,388	68.74	22,468	96.07	0	0.00	920	3.93	29,830	87.68
**CARAGA**	**617,544**	**552,335**	**89.44**	**537,496**	**97.31**	**1,572**	**0.28**	**13,267**	**2.40**	**372,431**	**60.31**
Agusan del Norte	84,078	74,609	88.74	71,963	96.45	1,570	2.10	1,076	1.44	55,850	66.43
Agusan del Sur	162,957	149,848	91.96	143,322	95.64	0	0.00	6,526	4.36	149,848	91.96
Surigao del Norte	81,219	74,926	92.25	73,482	98.07	2	0.00	1,442	1.92	68,066	83.81
Surigao del Sur	114,459	104,499	91.30	100,800	96.46	0	0.00	3,699	3.54	0	0.00
Province of Dinagat	29,539	22,768	77.08	22,768	100.00	0	0.00	0	0.00	0	0.00
Bislig City	22,646	18,188	80.31	17,664	97.12	0	0.00	524	2.88	0	0.00
Butuan City	82,739	74,132	89.60	74,132	100.00	0	0.00	0	0.00	65,302	78.93
Surigao City	39,907	33,365	83.61	33,365	100.00	0	0.00	0	0.00	33,365	83.61

Legend:
0 = No Case
** = No Report*

Table 1.G.3 - ENVIRONMENTAL HEALTH AND SANITATION SERVICES

Number and Proportion of Total Industrial Establishment, Industrial Establishment with Sanitary Permit, Total Number of Barangays and Number of Barangays certified as ZOD*

Philippines, 2021

Area	Total No. of Industrial Establishment	Industrial Establishment with Sanitary Permit		Total No. of Barangays	No. of Barangays Certified as ZOD areas	
		No.	%		No.	%
**PHILIPPINES**	**406,800**	**241,622**	**59.40**	**42,067**	**10,112**	**24.04**
**NCR**	**2,539**	**2,363**	**93.07**	**1,710**	**586**	**34.27**
Malabon	370	370	100.00	21	0	0.00
Navotas	142	142	100.00	18	0	0.00
Valenzuela City	563	415	73.71	33	33	100.00
Caloocan City	549	549	100.00	188	110	58.51
Marikina City	377	377	100.00	16	16	100.00
Pasig City	125	97	77.60	30	30	100.00
Pateros	15	15	100.00	10	6	60.00
Taguig	*	*	0.00	28	28	100.00
Quezon City	*	*	0.00	142	137	96.48
Makati City	*	*	0.00	33	21	63.64
Mandaluyong City	8	8	100.00	27	27	100.00
San Juan	*	*	0.00	21	0	0.00
Manila City	254	254	100.00	897	160	17.84
Las Piñas City	42	42	100.00	20	0	0.00
Muntinlupa City	24	24	100.00	9	9	100.00
Parañaque City	62	62	100.00	16	0	0.00
Pasay City	8	8	100.00	201	9	4.48
**CAR**	**7,562**	**5,503**	**72.77**	**1,180**	**239**	**20.25**
Abra	681	681	100.00	303	18	5.94
Apayao	1,262	1,103	87.40	134	4	2.99
Benguet	3,073	2,376	77.32	134	0	0.00
Ifugao	227	167	73.57	179	26	14.53
Kalinga	144	112	77.78	157	55	35.03
Mt. Province	802	690	86.03	144	52	36.11
Baguio City	1,373	374	27.24	129	84	65.12
**Region 1**	**25,190**	**22,911**	**90.95**	**3,267**	**2,460**	**75.30**
Ilocos Norte	3,233	2,526	78.13	479	353	73.70
Ilocos Sur	3,994	2,517	63.02	687	376	54.73
La Union	2,299	2,299	100.00	517	514	99.42
Pangasinan	9,965	9,943	99.78	1,174	907	77.26
		-				
Alaminos City	64	64	100.00	39	39	100.00
Candon City	436	433	99.31	42	32	76.19
Dagupan City	103	103	100.00	31	12	38.71
Laoag City	3,405	3,405	100.00	80	36	45.00
San Carlos City	108	108	100.00	86	79	91.86

Table 1.G.3 - ENVIRONMENTAL HEALTH AND SANITATION SERVICES

Number and Proportion of Total Industrial Establishment, Industrial Establishment with Sanitary Permit, Total Number of Barangays and Number of Barangays certified as ZOD*
Philippines, 2021

Area	Total No. of Industrial Establishment	Industrial Establishment with Sanitary Permit		Total No. of Barangays	No. of Barangays Certified as ZOD areas	
		No.	%		No.	%
San Fernando City	468	468	100.00	59	59	100.00
Urdaneta City	188	118	62.77	34	22	64.71
Vigan City	927	927	100.00	39	31	79.49
**Region 2**	**2,963**	**2,963**	**100.00**	**2,311**	**218**	**9.43**
Batanes	0	0	0.00	29	0	0.00
Cagayan	304	304	100.00	771	17	2.20
Isabela	2,564	2,564	100.00	862	70	8.12
Nueva Vizcaya	63	63	100.00	275	15	5.45
Quirino	28	28	100.00	132	25	18.94
Cauayan City	0	0	0.00	65	0	0.00
Ilagan City	4	4	100.00	91	91	100.00
Santiago City	0	0	0.00	37	0	0.00
Tuguegarao City	0	0	0.00	49	0	0.00
**Region 3**	**55,666**	**44,852**	**80.57**	**3,139**	**195**	**6.21**
Aurora	3,077	3,077	100.00	151	8	5.30
Bataan	190	190	100.00	212	0	0.00
Bulacan	16,131	10,255	63.57	433	0	0.00
Nueva Ecija	1,683	1,474	87.58	680	78	11.47
Pampanga	19,881	18,362	92.36	443	0	0.00
Tarlac	741	679	91.63	435	6	1.38
Zambales	1,487	1,365	91.80	230	1	0.43
Angeles City	102	102	100.00	33	0	0.00
Balanga City	77	77	100.00	25	2	8.00
Cabanatuan City	128	13	10.16	89	89	100.00
City of San Fernando	151	151	100.00	35	0	0.00
Gapan City	27	27	100.00	23	0	0.00
Mabalacat City	216	204	94.44	27	0	0.00
Malolos City	3,282	481	14.66	51	0	0.00
Meycauayan	132	124	93.94	26	0	0.00
Olongapo	1,556	1,556	100.00	17	0	0.00
Palayan City	1	1	100.00	19	0	0.00
San Jose City	450	449	99.78	38	0	0.00
San Jose del Monte City	6,125	6,125	100.00	59	0	0.00
Science City of Munoz	109	20	18.35	37	0	0.00
Tarlac City	120	120	100.00	76	11	14.47
**Region 4A**	**137,608**	**25,914**	**18.83**	**4,023**	**414**	**10.29**
Batangas	103,055	1,900	1.84	853	120	14.07
Cavite	189	189	100.00	420	0	0.00
Laguna	1,524	990	64.96	460	25	5.43
Quezon	1,797	1,078	59.99	1,144	210	18.36
Rizal	6,659	6,447	96.82	172	22	12.79

Table 1.G.3 - ENVIRONMENTAL HEALTH AND SANITATION SERVICES

Number and Proportion of Total Industrial Establishment, Industrial Establishment with Sanitary Permit, Total Number of Barangays and Number of Barangays certified as ZOD*

Philippines, 2021

Area	Total No. of Industrial Establishment	Industrial Establishment with Sanitary Permit		Total No. of Barangays	No. of Barangays Certified as ZOD areas	
		No.	%		No.	%
Antipolo City	*	*	0.00	16	0	0.00
Bacoor City	2	2	100.00	73	0	0.00
Batangas City	44	44	100.00	105	0	0.00
Biñan City	4,443	202	4.55	24	0	0.00
Cabuyao City	4,269	4,269	100.00	18	0	0.00
Calamba City	8,888	8,888	100.00	54	0	0.00
Cavite City	*	*	0.00	84	*	0.00
Dasmariñas City	1,521	389	25.58	75	0	0.00
General Trias City	*	*	0.00	33	0	0.00
Imus City	65	65	100.00	97	0	0.00
Lipa City	4,749	1,118	23.54	72	0	0.00
Lucena City	9	9	100.00	33	28	84.85
San Pablo City	120	120	100.00	84	0	0.00
San Pedro City	93	26	27.96	27	0	0.00
Santa Rosa City	30	27	90.00	18	0	0.00
Tagaytay City	*	*	0.00	34	0	0.00
Tanauan City	31	31	100.00	48	0	0.00
Tayabas City	87	87	100.00	66	9	13.64
Trece Martires City	33	33	100.00	13	0	0.00
**Region 4B**	**8,312**	**5,709**	**68.68**	**1,459**	**42**	**2.88**
Marinduque	365	365	100.00	218	0	0.00
Mindoro Occidental	3,459	1,540	44.52	163	2	1.23
Mindoro Oriental	680	680	100.00	426	9	2.11
Palawan	3,491	2,812	80.55	367	26	7.08
Romblon	306	301	98.37	219	5	2.28
Puerto Princesa City	11	11	100.00	66	0	0.00
**Region 5**	**5,126**	**4,393**	**85.70**	**3,471**	**330**	**9.51**
Albay	585	585	100.00	650	110	16.92
Camarines Norte	71	71	100.00	282	0	0.00
Camarines Sur	0	619	0.00	1,000	45	4.50
Catanduanes	322	297	92.24	315	0	0.00
Masbate	514	0	0.00	550	59	10.73
Sorsogon	2,620	2,045	78.05	541	99	18.30
Iriga City	923	685	74.21	36	0	0.00
Legaspi City	73	73	100.00	70	17	24.29
Naga City	18	18	100.00	27	0	0.00
**Region 6**	**15,788**	**14,085**	**89.21**	**4,051**	**2,103**	**51.91**
Aklan	807	197	24.41	327	102	31.19
Antique	730	620	84.93	590	246	41.69
Capiz	1,676	1,485	88.60	473	389	82.24

Table 1.G.3 - ENVIRONMENTAL HEALTH AND SANITATION SERVICES

Number and Proportion of Total Industrial Establishment, Industrial Establishment with Sanitary Permit, Total Number of Barangays and Number of Barangays certified as ZOD*

Philippines, 2021

Area	Total No. of Industrial Establishment	Industrial Establishment with Sanitary Permit		Total No. of Barangays	No. of Barangays Certified as ZOD areas	
		No.	%		No.	%
Guimaras	526	507	96.39	98	53	54.08
Iloilo	7,704	7,274	94.42	1,721	1,260	73.21
Negros Occidental	2,216	2,032	91.70	601	51	8.49
Bacolod City	1,799	1,772	98.50	61	2	3.28
Iloilo City	330	198	60.00	180	0	0.00
**Region 7**	**94,814**	**76,061**	**80.22**	**3,003**	**542**	**18.05**
Bohol	74,352	56,605	76.13	1,109	0	0.00
Cebu	5,532	4,546	82.18	1,051	18	1.71
Negros Oriental	1,401	1,386	98.93	557	410	73.61
Siquijor	3,014	3,014	100.00	134	102	76.12
Cebu City	9,899	9,899	100.00	80	0	0.00
Lapu-Lapu City	616	611	99.19	30	12	40.00
Mandaue City	*	*	0.00	42	0	0.00
**Region 8**	**15,648**	**8,544**	**54.60**	**4,390**	**1,529**	**34.83**
Biliran	4,280	606	14.16	132	132	100.00
Eastern Samar	400	276	69.00	597	76	12.73
Northern Leyte	2,450	2,000	81.63	1,393	625	44.87
Northern Samar	1,388	981	70.68	569	137	24.08
Southern Leyte	1,081	1,001	92.60	430	367	85.35
Western Samar	3,165	1,101	34.79	794	104	13.10
Calbayog City	246	98	39.84	157.00	2.00	1.27
Maasin City	1,875	1,872	99.84	70.00	39.00	55.71
Ormoc City	244	90	36.89	110.00	27.00	24.55
Tacloban City	519	519	100.00	138.00	20.00	14.49
**Region 9**	**3,848**	**1,716**	**44.59**	**1,904**	**162**	**8.51**
Zamboanga del Norte	*	*	0.00	620	126	20.32
Zamboanga del Sur	817	740	90.58	627	17	2.71
Zamboanga Sibugay	1,265	382	30.20	389	5	1.29
Dapitan City	39	13	33.33	50	0	0.00
Dipolog City	1,717	573	33.37	21	14	66.67
Isabela City	*	*	0.00	45	0	0.00
Pagadian City	*	*	0.00	54	0	0.00
Zamboanga City	10	8	80.00	98	0	0.00
**Region 10**	**12,549**	**12,057**	**96.08**	**2,022**	**178**	**8.80**
Bukidnon	596	574	96.31	387	12	3.10
Camiguin	79	79	100.00	58	1	1.72
Lanao del Norte	654	600	91.74	462	7	1.52

Table 1.G.3 - ENVIRONMENTAL HEALTH AND SANITATION SERVICES

Number and Proportion of Total Industrial Establishment, Industrial Establishment with Sanitary Permit, Total Number of Barangays and Number of Barangays certified as ZOD*

Philippines, 2021

Area	Total No. of Industrial Establishment	Industrial Establishment with Sanitary Permit		Total No. of Barangays	No. of Barangays Certified as ZOD areas	
		No.	%		No.	%
Misamis Occidental	109	109	100.00	337	34	10.09
Misamis Oriental	2,252	1,904	84.55	330	28	8.48
					0	
Cagayan de Oro City	6,069	6,069	100.00	80	17	21.25
El Salvador City	1,583	1,583	100.00	15	0	0.00
Gingoog City	438	438	100.00	79	64	81.01
Iligan City	12	6	50.00	44	10	22.73
Malaybalay City	462	462	100.00	46	0	0.00
Oroquieta City	14	14	100.00	47	3	6.38
Ozamis City	185	185	100.00	51	0	0.00
Tangub City	4	4	100.00	55	0	0.00
Valencia City	92	30	32.61	31	2	6.45
**Region 11**	**3,651**	**2,589**	**70.91**	**1,134**	**109**	**9.61**
Davao de Oro	843	513	60.85	210	20	9.52
Davao del Norte	699	524	74.96	223	17	7.62
Davao Oriental	846	588	69.50	183	64	34.97
Davao del Sur	204	204	100.00	232	8	3.45
Davao Occidental	844	548	64.93	105	0	0.00
Davao City	215	212	98.60	181	0	0.00
**Region 12**	**9,381**	**7,178**	**76.52**	**1,201**	**248**	**20.65**
North Cotabato	1,796	1,544	85.97	543	154	28.36
Sarangani	118	118	100.00	141	86	60.99
South Cotabato	3,445	3,445	100.00	199	2	1.01
Sultan Kudarat	1,143	891	77.95	255	6	2.35
Cotabato City	*	*	0.00	37	0	0.00
Gen. Santos City	2,879	1,180	40.99	26	0	0.00
**BARMM**	**2,458**	**1,318**	**53.62**	**2,490**	**397**	**15.94**
Basilan	*	*	0.00	165	0	0.00
Lanao del Sur	14	14	100.00	1,063	31	2.92
Maguindanao	0	0	0.00	508	366	72.05
Sulu	*	*	0.00	410	0	0.00
Tawi-Tawi	2,444	1,304	53.36	203	0	0.00
Lamitan City	*	*	0.00	45	0	0.00
Marawi City	*	*	0.00	96	0	0.00

Table 1.G.3 - ENVIRONMENTAL HEALTH AND SANITATION SERVICES

Number and Proportion of Total Industrial Establishment, Industrial Establishment with Sanitary Permit, Total Number of Barangays and Number of Barangays certified as ZOD*

Philippines, 2021

Area	Total No. of Industrial Establishment	Industrial Establishment with Sanitary Permit		Total No. of Barangays	No. of Barangays Certified as ZOD areas	
		No.	%		No.	%
**CARAGA**	**3,697**	**3,466**	**93.75**	**1,312**	**360**	**27.44**
Agusan del Norte	213	184	86.38	168	23	13.69
Agusan del Sur	276	263	95.29	314	30	9.55
Surigao del Norte	128	127	99.22	281	92	32.74
Surigao del Sur	2,640	2,452	92.88	285	173	60.70
Province of Dinagat	341	341	100.00	100	17	17.00
Bislig City	58	58	100.00	24	1	4.17
Butuan City	32	32	100.00	86	16	18.60
Surigao City	9	9	100.00	54	8	14.81

Legend: *ZOD - Zero Open Defecation*

0 = No Case

** = No Report*

Figure 1.G.1 - Percentage of Household with access to basic safe water supply (Level 1) by Region
Philippines, 2021

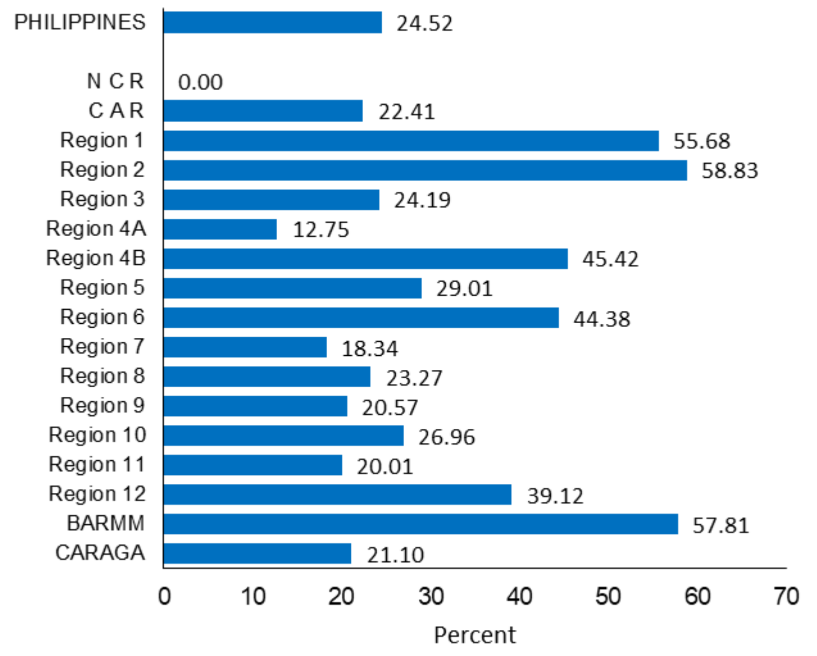

Figure 1.G.2 - Percentage of Household with access to basic safe water supply (Level 2) by Region
Philippines, 2021

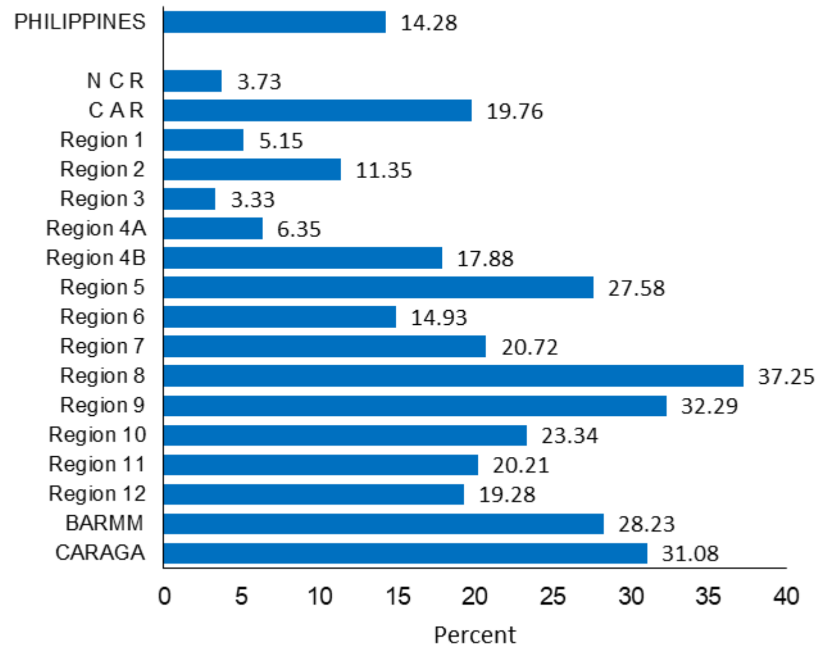

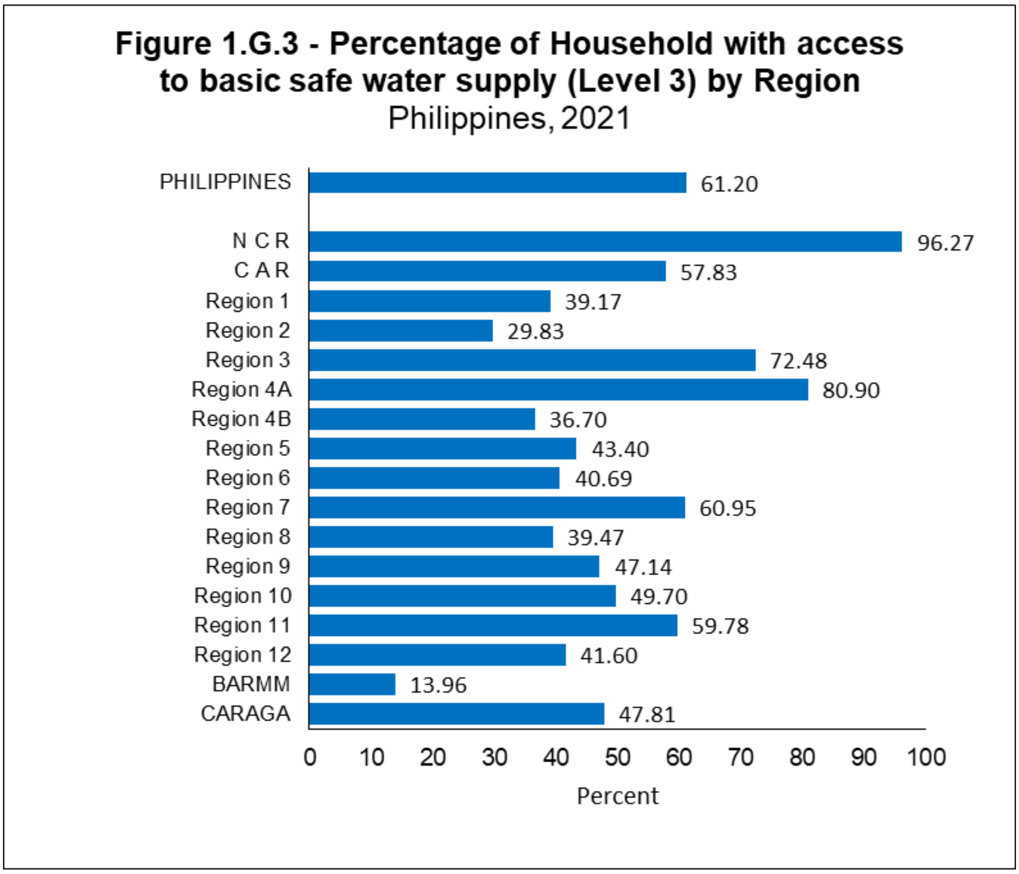
Figure 1.G.3 - Percentage of Household with access to basic safe water supply (Level 3) by Region
Philippines, 2021
PHILIPPINES 61.20
N C R 96.27
C A R 57.83
Region 1 39.17
Region 2 29.83
Region 3 72.48
Region 4A 80.90
Region 4B 36.70
Region 5 43.40
Region 6 40.69
Region 7 60.95
Region 8 39.47
Region 9 47.14
Region 10 49.70
Region 11 59.78
Region 12 41.60
BARMM 13.96
CARAGA 47.81
0 10 20 30 40 50 60 70 80 90 100
Percent

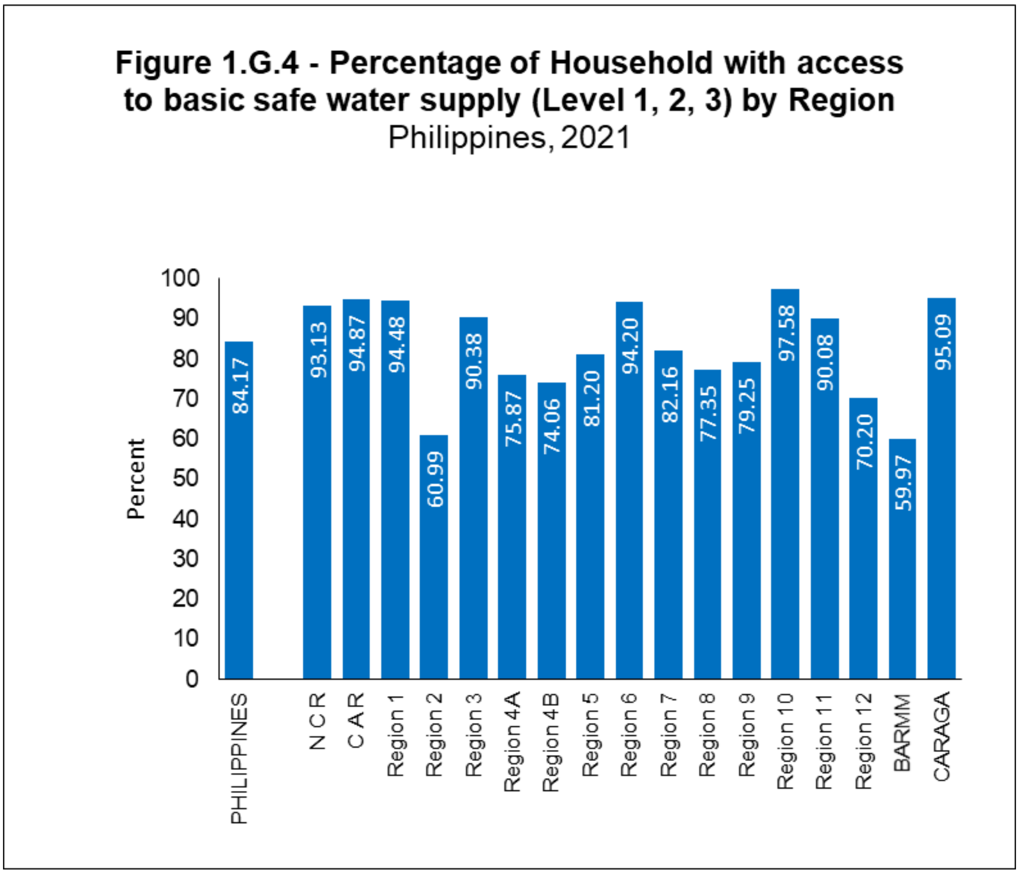
Figure 1.G.4 - Percentage of Household with access to basic safe water supply (Level 1, 2, 3) by Region
Philippines, 2021
Percent
100 90 80 70 60 50 40 30 20 10 0
PHILIPPINES 84.17
N C R 93.13
C A R 94.87
Region 1 94.48
Region 2 60.99
Region 3 90.38
Region 4A 75.87
Region 4B 74.06
Region 5 81.20
Region 6 94.20
Region 7 82.16
Region 8 77.35
Region 9 79.25
Region 10 97.58
Region 11 90.08
Region 12 70.20
BARMM 59.97
CARAGA 95.09

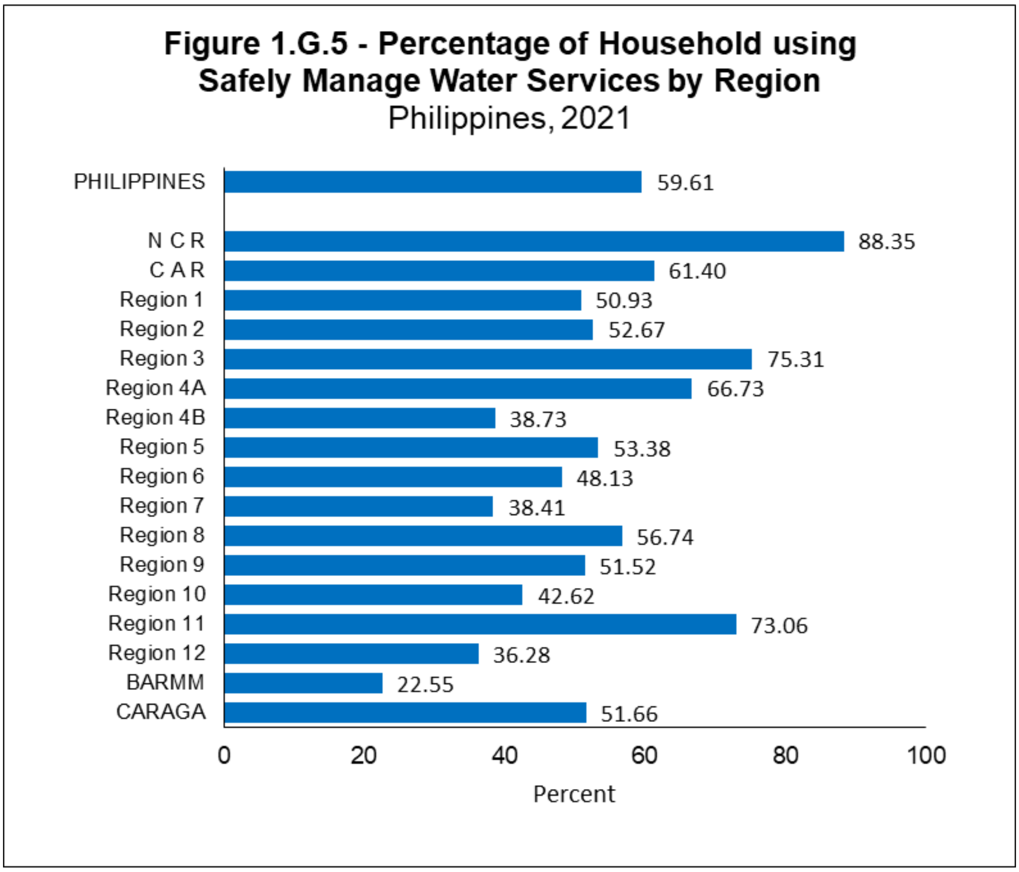
Figure 1.G.5 - Percentage of Household using Safely Manage Water Services by Region
Philippines, 2021
PHILIPPINES 59.61
N C R 88.35
C A R 61.40
Region 1 50.93
Region 2 52.67
Region 3 75.31
Region 4A 66.73
Region 4B 38.73
Region 5 53.38
Region 6 48.13
Region 7 38.41
Region 8 56.74
Region 9 51.52
Region 10 42.62
Region 11 73.06
Region 12 36.28
BARMM 22.55
CARAGA 51.66
0
20
40
60
80
100
Percent

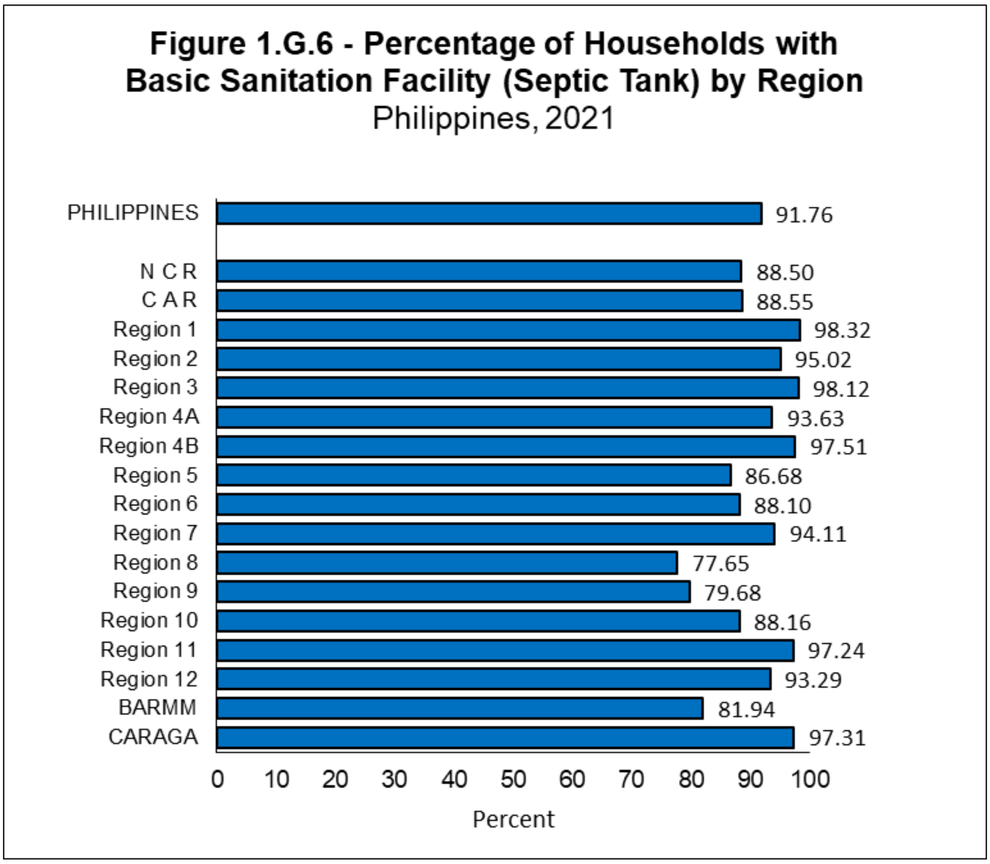
Figure 1.G.6 - Percentage of Households with Basic Sanitation Facility (Septic Tank) by Region
Philippines, 2021
PHILIPPINES 91.76
N C R 88.50
C A R 88.55
Region 1 98.32
Region 2 95.02
Region 3 98.12
Region 4A 93.63
Region 4B 97.51
Region 5 86.68
Region 6 88.10
Region 7 94.11
Region 8 77.65
Region 9 79.68
Region 10 88.16
Region 11 97.24
Region 12 93.29
BARMM 81.94
CARAGA 97.31
0
10
20
30
40
50
60
70
80
90
100
Percent

Figure 1.G.7 - Percentage of Households with Basic Sanitation Facility (Community Sewerage System) by Region
Philippines, 2021

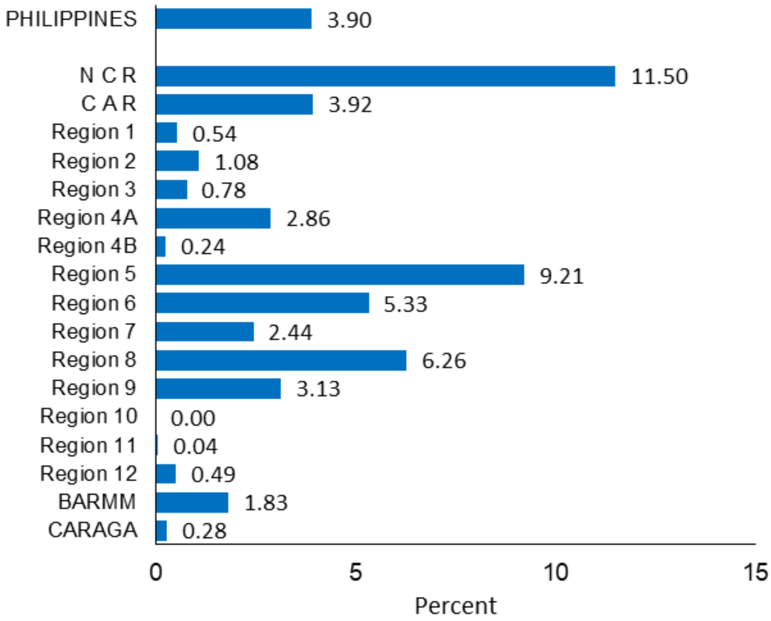

Figure 1.G.8 - Percentage of Households with Basic Sanitation Facility (Ventillated Improve Pit Latrine) by Region
Philippines, 2021

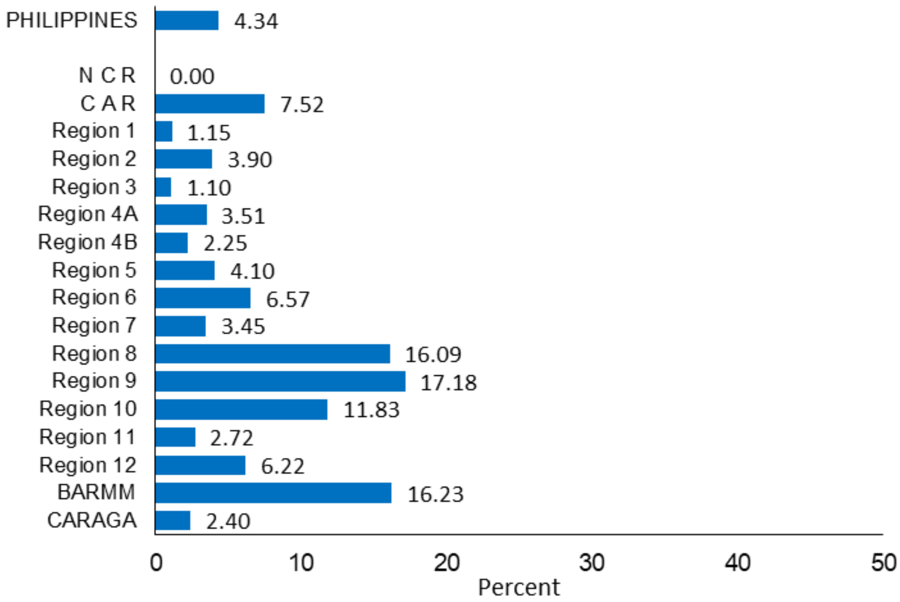

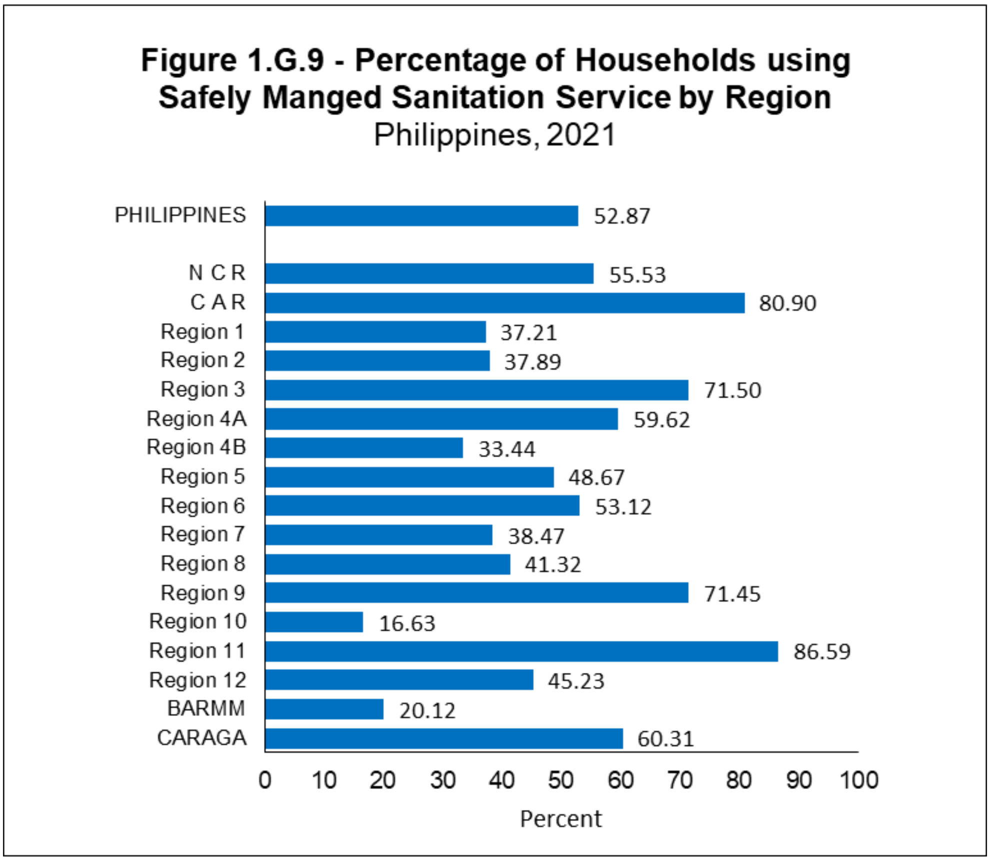
Figure 1.G.9 - Percentage of Households using Safely Manged Sanitation Service by Region
Philippines, 2021
PHILIPPINES
N C R
C A R
Region 1
Region 2
Region 3
Region 4A
Region 4B
Region 5
Region 6
Region 7
Region 8
Region 9
Region 10
Region 11
Region 12
BARMM
CARAGA
52.87
55.53
80.90
37.21
37.89
71.50
59.62
33.44
48.67
53.12
38.47
41.32
71.45
16.63
86.59
45.23
20.12
60.31
0 10 20 30 40 50 60 70 80 90 100
Percent

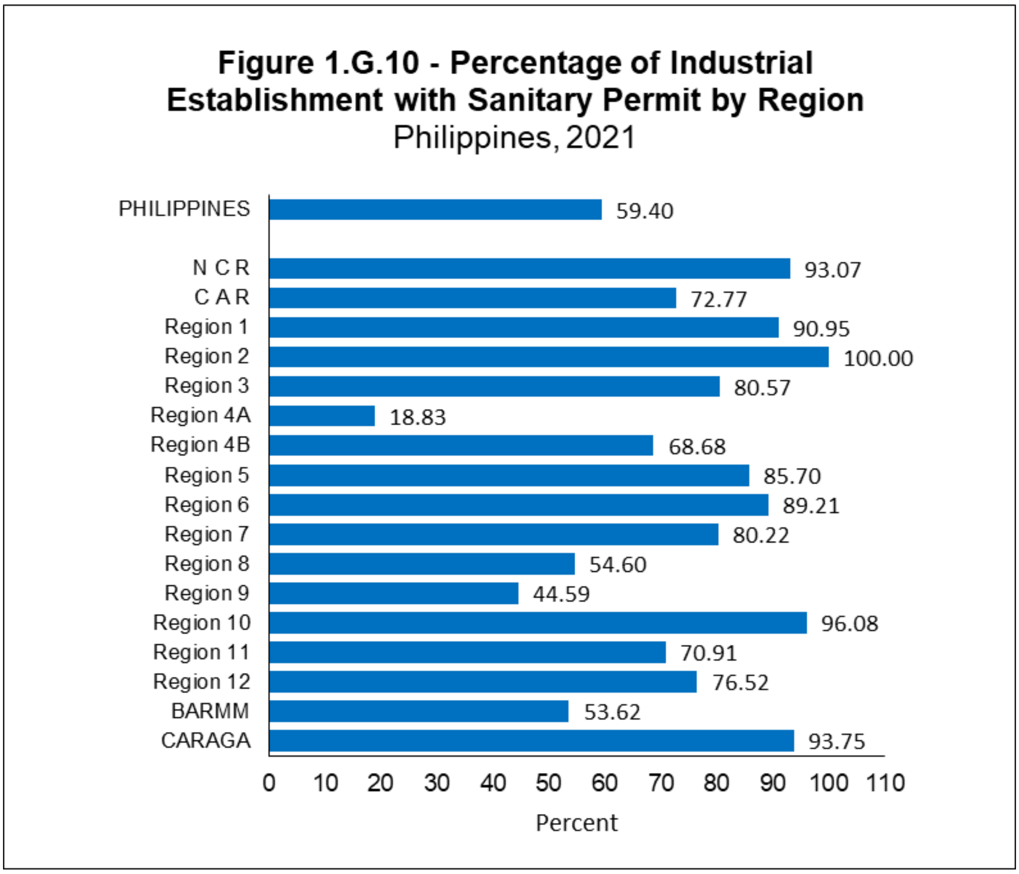
Figure 1.G.10 - Percentage of Industrial Establishment with Sanitary Permit by Region
Philippines, 2021
PHILIPPINES
N C R
C A R
Region 1
Region 2
Region 3
Region 4A
Region 4B
Region 5
Region 6
Region 7
Region 8
Region 9
Region 10
Region 11
Region 12
BARMM
CARAGA
59.40
93.07
72.77
90.95
100.00
80.57
18.83
68.68
85.70
89.21
80.22
54.60
44.59
96.08
70.91
76.52
53.62
93.75
0 10 20 30 40 50 60 70 80 90 100 110
Percent

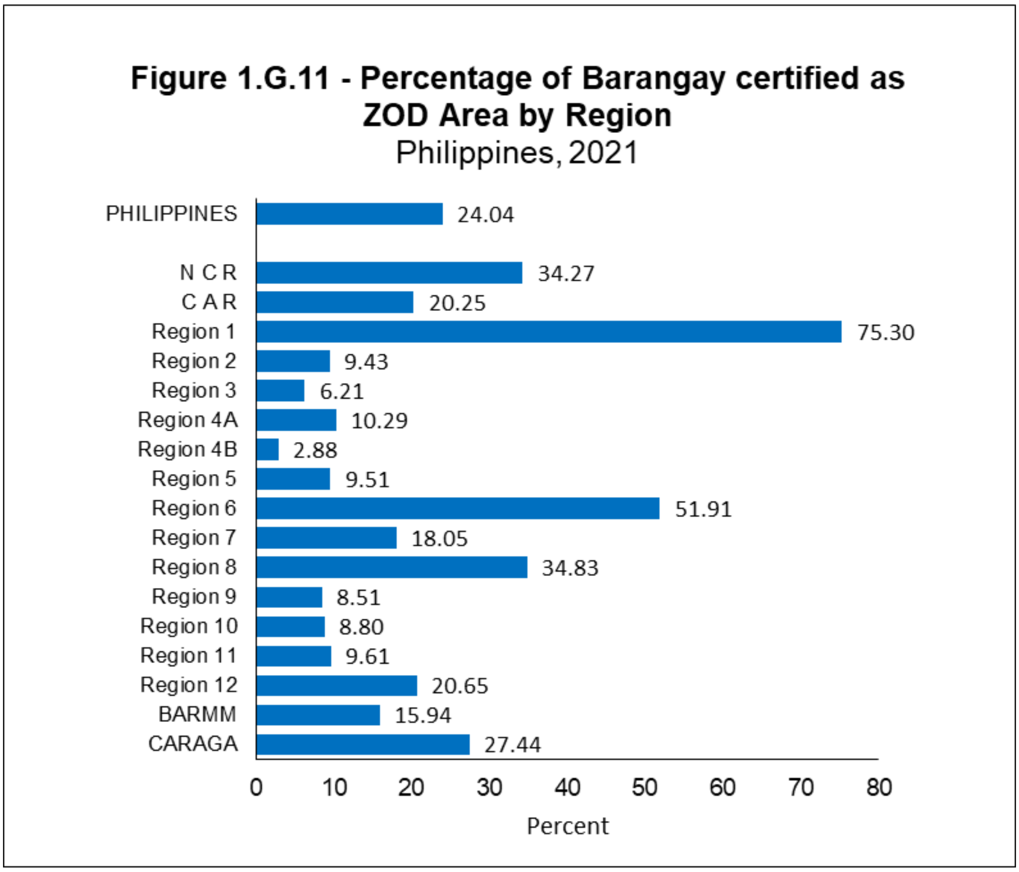
Figure 1.G.11 - Percentage of Barangay certified as ZOD Area by Region
Philippines, 2021
PHILIPPINES
24.04
N C R
34.27
C A R
20.25
Region 1
75.30
Region 2
9.43
Region 3
6.21
Region 4A
10.29
Region 4B
2.88
Region 5
9.51
Region 6
51.91
Region 7
18.05
Region 8
34.83
Region 9
8.51
Region 10
8.80
Region 11
9.61
Region 12
20.65
BARMM
15.94
CARAGA
27.44
0
10
20
30
40
50
60
70
80
Percent

FIELD HEALTH SERVICE INFORMATION SYSTEM

CHAPTER 2

Annual Report 2021

MORBIDITY, MORTALITY AND NATALITY

FHSIS ANNUAL REPORT 2021

2.A Morbidity

In 2021, the leading cause of Morbidity was Acute Respiratory Infection at 543.19 per 100,000 population followed by Hypertension (341.23) and Animal Bites (244.11).

Figure 2.A.1 Top 10 Leading Causes of Morbidity Philippines, 2021

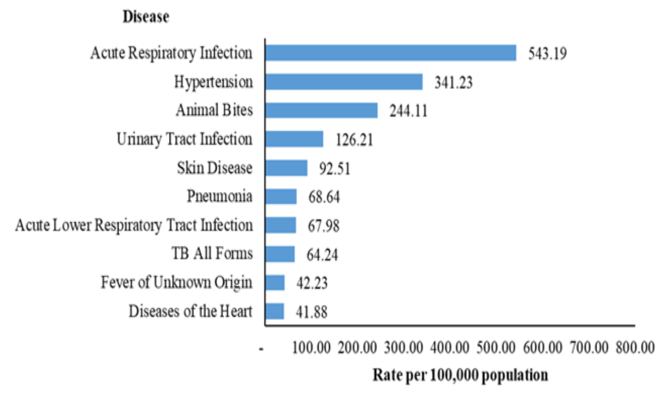

2.B Mortality

In 2021, a total of 534,368 deaths were recorded in the FHSIS, with a Mortality Rate of 4.85%. An increasing trend was observed from 2019 to 2021.

Figure 2.B.1 Mortality Rate Philippines, 2017 - 2021

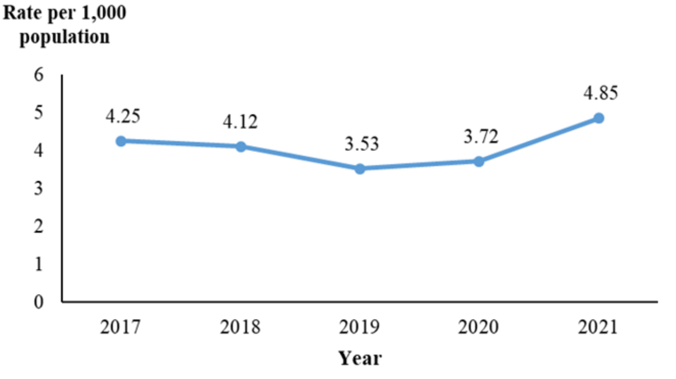

For the proportion of deaths in the country, a higher percentage was observed in Males (55.28%) than Females (44.72%). Region 12 had the highest percentage of death among males at 58.72% while Region 7 had the highest percentage of death among females at 46.69%.

Figure 2.B.2 Proportion of Deaths by Sex and Region Philippines, 2021

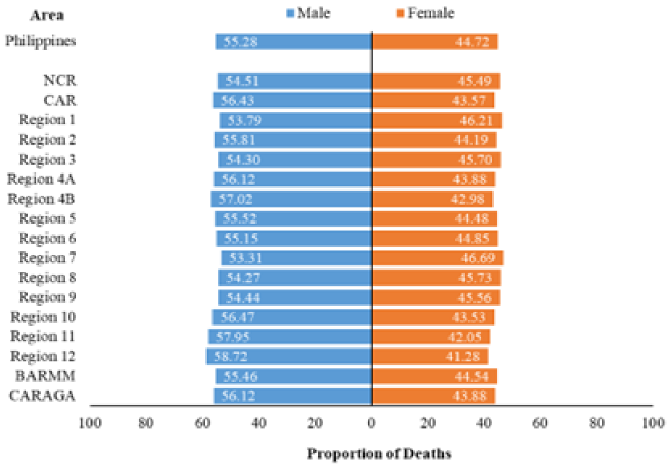

The Maternal Mortality Ratio (MMR) for 2021 was 84.86. It was the highest recorded MMR for the past 5 years (2017 – 2021).

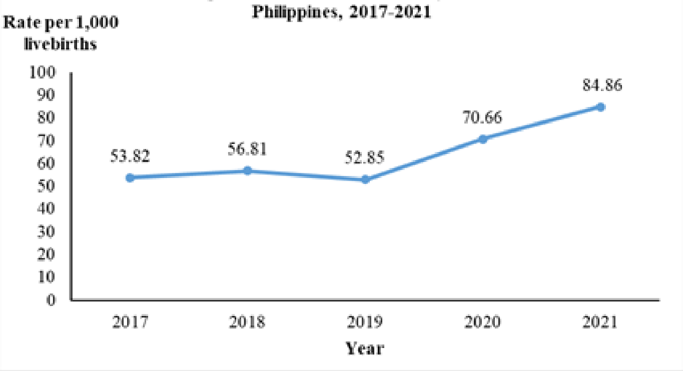

Figure 2.B.3 Maternal Mortality Ratio Philippines, 2017-2021

The region with the highest MMR was Region 9 (150.38 per 100,000 live births) followed by NCR (150.32 per 100,000 live births) and Region 5 (144.02 per 100,000 live births).

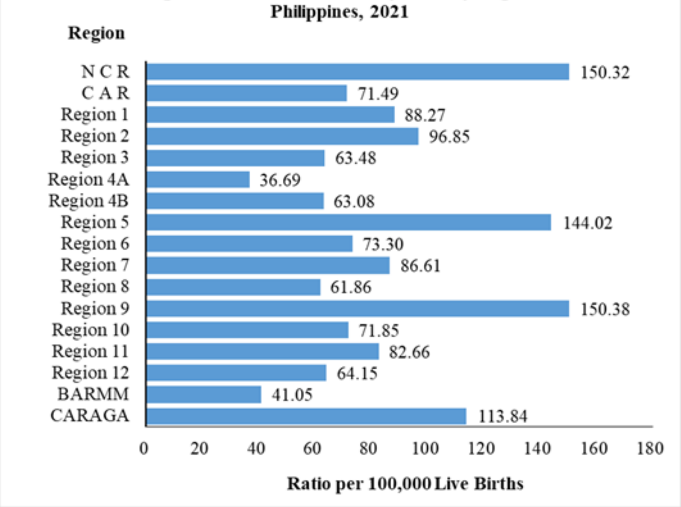

Figure 2.B.4 Maternal Motality Ratio by Region Philippines, 2021

A total of 16,273 Under-Five deaths were recorded in the death registration logbook of public health facilities. This corresponds to 13.04 Under-Five Mortality Rate (U5MR) in 2021, being the highest for the past 5 years (2017 – 2021).

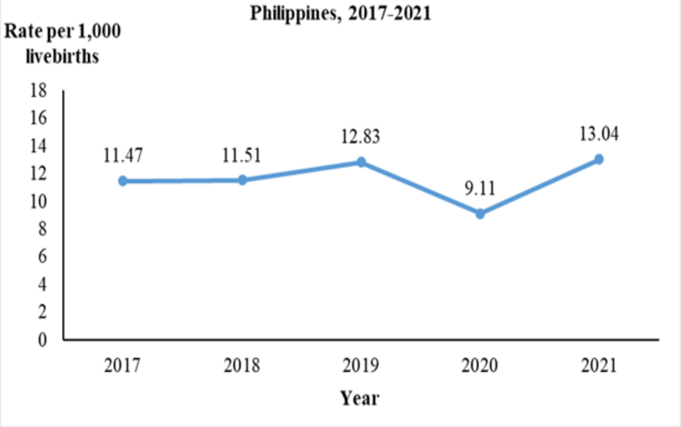

Figure 2.B.5 Under-Five Mortality Rate Philippines, 2017-2021

Among all regions, the National Capital Region has recorded the highest U5MR with 27.81% followed by Region 5 (25.52%) and Region 1 (23.90%).

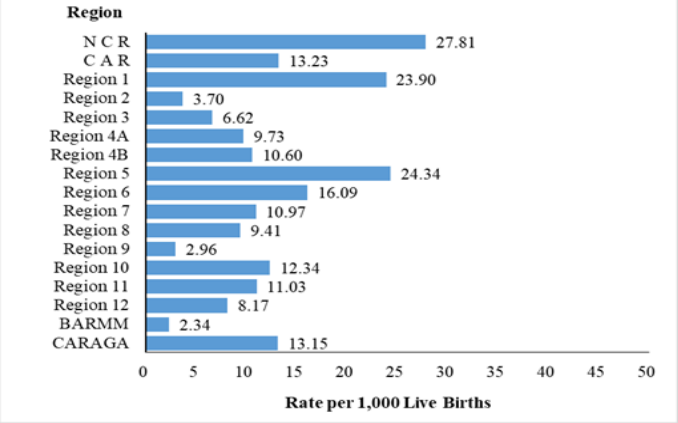

Figure 2.B.6 Under-Five Mortality Rate by Region Philippines, 2021

The Infant Mortality Rate (IMR) decreased from 10.69 per 1,000 live births in 2020 to 8.77 per 1,000 live births this year.

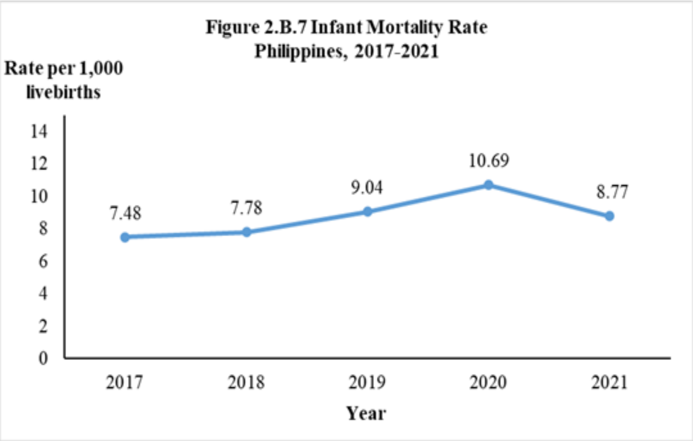

Figure 2.B.7 Infant Mortality Rate Philippines, 2017-2021

The Region with the highest registered IMR in 2021 was NCR with 21.64 per 1,000 live births followed by Region 5 (18.01%) and Region 1 (16.69%).

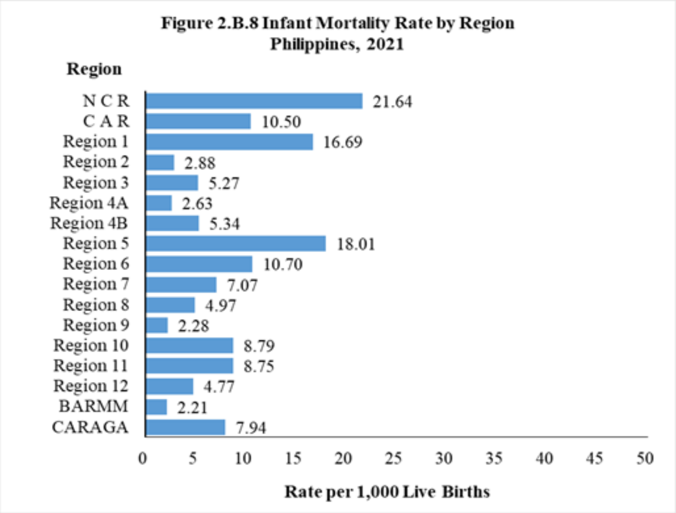

Figure 2.B.8 Infant Mortality Rate by Region Philippines, 2021

An increase in the Neonatal Mortality Rate (NMR) was observed in 2021 (5.59%) compared to the previous year (4.47%).

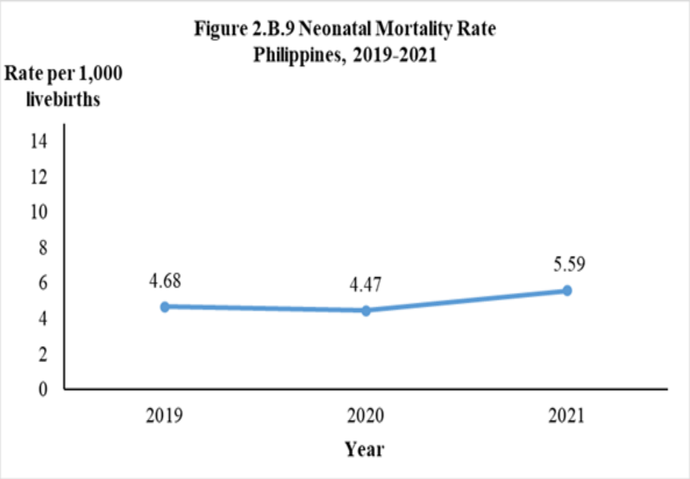

Figure 2.B.9 Neonatal Mortality Rate Philippines, 2019-2021

The Region with the highest registered NMR in 2021 was NCR with 11.34 per 1,000 live births followed by CAR (8.94%) and Region 5 (7.83%).

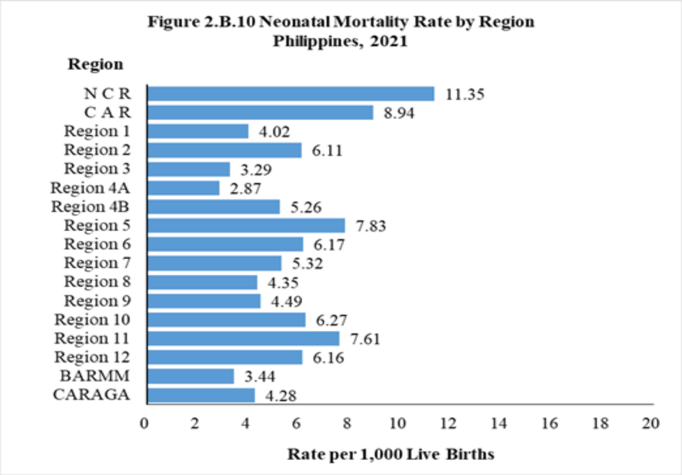

Figure 2.B.10 Neonatal Mortality Rate by Region Philippines, 2021

The Perinatal Mortality Rate (PMR) comprises fetal deaths and early neonatal deaths (0-6 days of life). In the Philippines, the PMR is gradually increasing from 5.01% in 2017 to 9.47% in 2021.

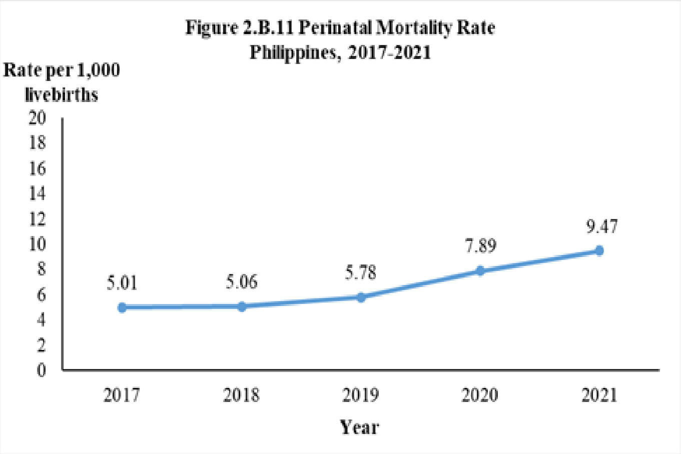

Among all regions, the National Capital Region has recorded the highest PMR with 19.87% followed by CAR (13.94%) and Region 1 (12.17%).

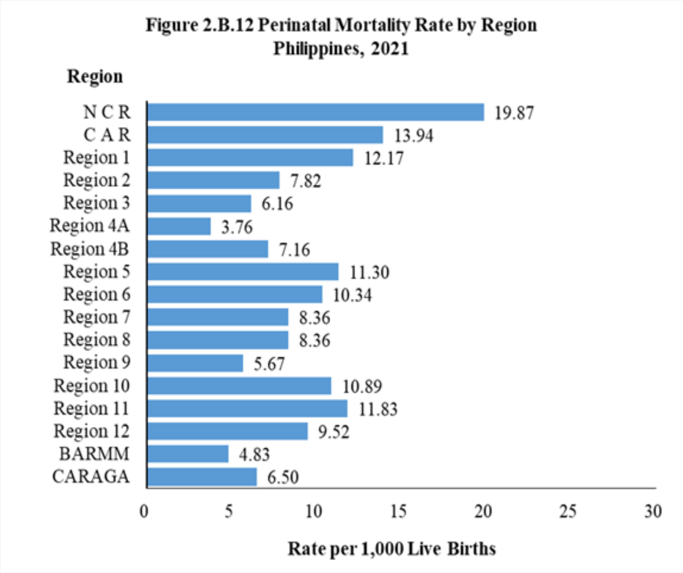

2.C Natality

In 2021 a total of 1,246,351 live births were recorded which can be translated into 11.31 or 11 births per 1,000 live births. A steady decline was noted from 2017 to 2021.

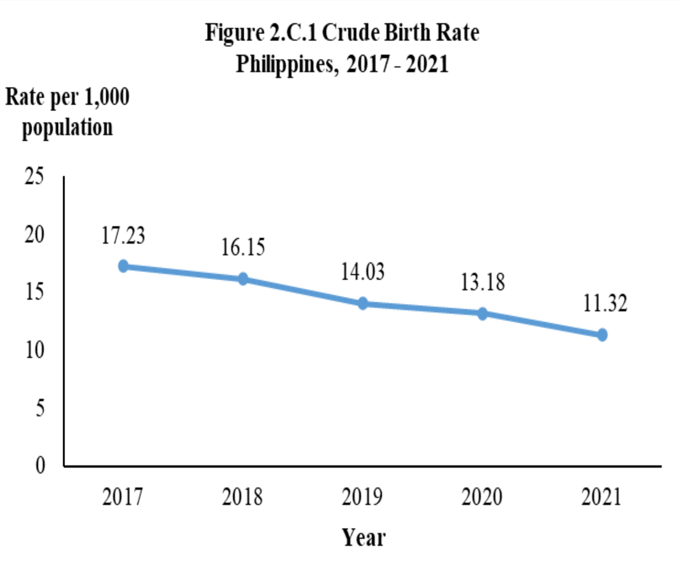

For the proportion of live births in the country, a higher percentage was observed in Males (51.22%) than Females (48.78%). Region 4A had the highest percentage of live births among males at 57.36% while Region 8 had the highest percentage of live births among females at 52.92%.

Figure 2.C.2 Proportion of Livebirths by Sex and Region Philippines, 2021

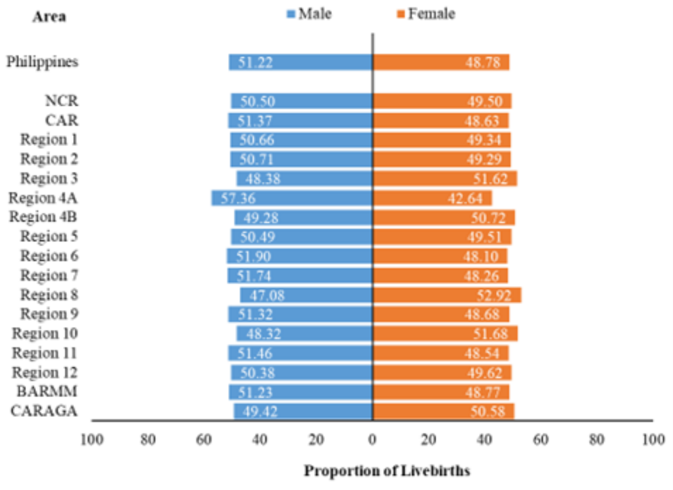

In 2021, 113,403 live births were born of adolescent pregnancies which is equivalent to an Adolescent Birth Rate (ABR) of 22.39%. A decrease in ABR was observed in 2021 compared to the previous year (24.08%).

Figure 2.C.3 Adolescent Birth Rate Philippines, 2019 - 2021

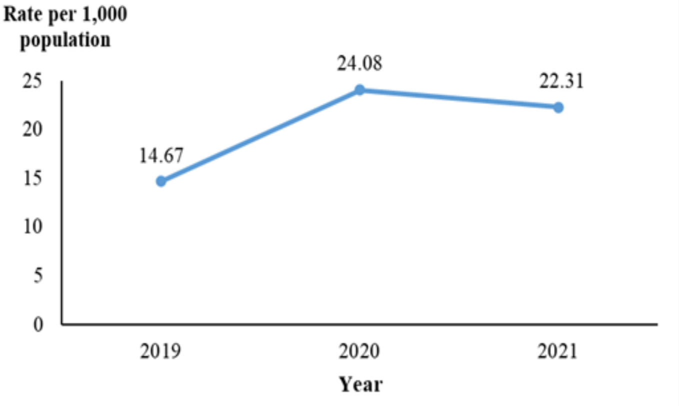

Table 2.A.1 - Ten Leading Causes of Morbidity

Philippines, 2021

	Disease	Number of Case	Rate per 100,000 population
1	Acute Respiratory Infection	598,591	543.19
2	Hypertension	376,033	341.23
3	Animal Bites	269,010	244.11
4	Urinary Tract Infection	139,081	126.21
5	Skin Disease	101,948	92.51
6	Pneumonia	75,645	68.64
7	Acute Lower Respiratory Tract Infection	74,918	67.98
8	TB All Forms	70,791	64.24
9	Fever of Unknown Origin	46,534	42.23
10	Diseases of the Heart	46,153	41.88

2021 FHSIS

Table 2.A.2 Ten Leading Causes of Morbidity by Gender

Table 2.A.2.1 - Male
Philippines, 2021

	Disease	Number of case	Rate per 100,000 population
1	Acute Respiratory Infection	279,364	502.08
2	Hypertension	159,822	287.24
3	Animal Bites	133,671	240.24
4	Skin Diseases	47,938	86.16
5	TB All Forms	45,725	82.18
6	Urinary Tract Infection	43,366	77.94
7	Pneumonia	38,102	68.48
8	Acute Lower Respiratoty Tract Infection	35,711	64.18
9	Fever of Unknown Origin	22,980	41.30
10	Acute Watery Diarrhea	20,975	37.70

2021 FHSIS

Table 2.A.2.2 - Female
Philippines, 2021

	Disease	Number of case	Rate per 100,000 population
1	Acute Respiratory Infection	319,227	585.12
2	Hypertension	216,211	396.30
3	Animal Bites	135,339	248.07
4	Urinary Tract Infection	95,734	175.47
5	Skin Diseases	54,010	99.00
6	Acute Lower Respiratoty Tract Infection	39,207	71.86
7	Pneumonia	37,543	68.81
8	Diseases of the Heart	26,851	49.22
9	TB All Forms	25,066	45.94
10	Fever of Unknown Origin	23,554	43.17

2021 FHSIS

Table 2.A.3 Ten Leading Causes of Morbidity by Region

Philippines, 2021

Table 2.A.3.1 - National Capital Region (NCR)

	Disease	Number of Case	Rate per 100,000 population
1	Hypertension	69,453	497.29
2	Acute Respiratory Infection	63,615	455.49
3	Animal Bites	18,561	132.90
4	Urinary Tract Infection	17,502	125.32
5	Skin Diseases	12,258	87.77
6	Acute Lower Respiratory Tract Infection	9,352	66.96
7	TB All Forms	7,390	52.91
8	Diseases of the Heart	4,547	32.56
9	Bronchitis	3,296	23.60
10	Acute Watery Diarrhea	3,026	21.67

Table 2.A.3.2 - Cordillera Administrative Region (CAR)

	Disease	Number of Case	Rate per 100,000 population
1	Acute Respiratory Infection	75,436	4,138.65
2	Hypertension	22,548	1,237.05
3	Urinary Tract Infection	16,061	881.16
4	Skin Diseases	10,457	573.70
5	Fever of Unknown Origin	8,323	456.63
6	Animal Bites	6,327	347.12
7	Acute Watery Diarrhea	5,887	322.98
8	Pneumonia	5,134	281.67
9	Influenza Like-Illness	3,033	166.40
10	Acute Lower Respiratory Tract Infection	2,022	110.93

Table 2.A.3.3 - Region 1

	Disease	Number of Case	Rate per 100,000 population
1	Acute Respiratory Infection	47,081	886.10
2	Hypertension	25,820	485.96
3	Acute Lower Respiratory Tract Infection	16,082	302.68
4	Animal Bites	10,921	205.54
5	Urinary Tract Infection	10,443	196.55
6	Skin Diseases	8,063	151.75
7	TB All Forms	2,477	46.62
8	Pneumonia	1,472	27.70
9	Acute watery diarrhea	1,402	26.39
10	Bronchitis	1,389	26.14

Table 2.A.3 Ten Leading Causes of Morbidity by Region

Philippines, 2021

Table 2.A.3.4 - Region 2

	Disease	Number of Case	Rate per 100,000 population
1	Acute Respiratory Infection	65,441	1,771.71
2	Hypertension	26,697	722.78
3	Animal Bites	15,965	432.23
4	Skin Diseases	12,251	331.68
5	Urinary Tract Infection	11,227	303.95
6	Acute Watery Diarrhea	4,586	124.16
7	Pneumonia	4,280	115.87
8	TB All Forms	2,921	79.08
9	Influenza	2,552	69.09
10	Acute Lower Respiratory Tract Infection	2,340	63.35

Table 2.A.3.5 - Region 3

	Disease	Number of Case	Rate per 100,000 population
1	Acute Respiratory Infection	29,892	238.87
2	TB All Forms	8,142	65.06
3	Animal Bites	3,680	29.41
4	Hypertension	3,604	28.80
5	Urinary Tract Infection	2,493	19.92
6	Skin Disease	2,356	18.83
7	Acute Lower Respiratory Tract Infection	1,174	9.38
8	Acute Watery Diarrhea	658	5.26
9	Acute Hemorrhagic Fever	462	3.69
10	Influenza Like-Illness	384	3.07

Table 2.A.3.6 - Region 4A

	Disease	Number of Case	Rate per 100,000 population
1	Acute Respiratory Infection	45,597	278.69
2	Diseases of the Heart	29,375	179.54
3	Hypertension	21,102	128.98
4	Acute Bloody Diarrhea	15,548	95.03
5	Acute Febrille Illness	15,155	92.63
6	Acute Lower Respiratory Tract Infection	14,397	88.00
7	Pneumonia	13,443	82.16
8	Urinary Tract Infection	9,572	58.50
9	Animal Bites	8,529	52.13
10	Skin Diseases	7,399	45.22

Table 2.A.3 Ten Leading Causes of Morbidity by Region

Philippines, 2021

Table 2.A.3.7 - Region 4B

	Disease	Number of Case	Rate per 100,000 population
1	Acute Respiratory Infection	29,621	922.14
2	Hypertension	8,539	265.83
3	Animal Bites	6,605	205.62
4	Urinary Tract Infection	4,794	149.24
5	Influenza	4,159	129.47
6	Influenza Like-Illness	3,075	95.73
7	Acute Lower Respiratory Tract Infection	2,751	85.64
8	Chronic Obstructive Pulmonary Infection	2,214	68.92
9	Skin Diseases	2,162	67.31
10	TB All Forms	1,796	55.91

Table 2.A.3.8 - Region 5

	Disease	Number of Case	Rate per 100,000 population
1	Acute Respiratory Infection	106,063	1,712.65
2	Hypertension	68,594	1,107.62
3	TB All Forms	22,345	360.82
4	Fever Of Unknown Origin	20,086	324.34
5	Animal Bites	14,005	226.15
6	Urinary Tract Infection	13,505	218.07
7	Skin Diseases	7,715	124.58
8	Bronchitis	5,034	81.29
9	Acute Febrille Illness	4,783	77.23
10	Influenza Like-Illness	4,353	70.29

Table 2.A.3.9 - Region 6

	Disease	Number of Case	Rate per 100,000 population
1	Animal Bites	119,342	1497.06
2	Hypertension	28,756	360.78
3	Acute Respiratory Infection	22,726	285.12
4	Urinary Tract Infection	17,089	214.40
5	TB All Forms	12,420	155.82
6	Pneumonia	11,855	148.74
7	Skin Diseases	8,923	111.95
8	Bronchitis	3,304	41.45
9	Influenza	2,881	36.15
10	Fever Of Unknown Origin	2,340	29.36

Table 2.A.3 Ten Leading Causes of Morbidity by Region

Philippines, 2021

Table 2.A.3.10 - Region 7

	Disease	Number of Case	Rate per 100,000 population
1	Animal Bites	8,957	111.20
2	Hypertension	4,563	56.65
3	Pneumonia	3,465	43.02
4	Acute Lower Respiratory Tract Infection	3,202	39.75
5	Acute Respiratory Infection	2,707	33.61
6	Urinary Tract Infection	1,293	16.05
7	Skin Diseases	1,191	14.79
8	Bronchitis	1,149	14.26
9	TB All Forms	547	6.79
10	Fever of Unknown Origin	300	3.72

Table 2.A.3.11 - Region 8

	Disease	Number of Case	Rate per 100,000 population
1	Acute Respiratory Infection	20,792	432.83
2	Hypertension	17,498	364.26
3	Urinary Tract Infection	7,123	148.28
4	Pneumonia	7,005	145.82
5	Animal Bites	6,582	137.02
6	Skin Diseases	5,852	121.82
7	Acute Febrille Illness	4,231	88.08
8	Bronchitis	2,814	58.58
9	Acute Watery Diarrhea	2,311	48.11
10	TB All Forms	1,905	39.66

Table 2.A.3.12 - Region 9

	Disease	Number of Case	Rate per 100,000 population
1	Acute Respiratory Infection	29,026	761.93
2	Animal Bites	10,214	268.12
3	Hypertension	8,997	236.17
4	Urinary Tract Infection	4,565	119.83
5	Skin Diseases	4,030	105.79
6	Pneumonia	2,172	57.01
7	Fever of Unknown Origin	976	25.62
8	Influenza Like-Illness	750	19.69
9	Influenza	606	15.91
10	Acute Bloody Diarrhea	305	8.01

Table 2.A.3 Ten Leading Causes of Morbidity by Region

Philippines, 2021

Table 2.A.3.13 - Region 10

	Disease	Number of Case	Rate per 100,000 population
1	Hypertension	24,672	486.19
2	Acute Respiratory Infection	17,074	336.46
3	Animal Bites	15,947	314.25
4	Skin Diseases	8,751	172.45
5	Urinary Tract Infection	7,272	143.30
6	Influenza Like-Illness	5,469	107.77
7	Pneumonia	3,589	70.73
8	Acute Lower Respiratory Tract Infection	3,053	60.16
9	Acute Febrille Illness	2,983	58.78
10	Acute Watery Diarrhea	2,451	48.30

Table 2.A.3.14 - Region 11

	Disease	Number of Case	Rate per 100,000 population
1	Hypertension	18,786	349.75
2	Acute Respiratory Infection	10,473	194.98
3	Urinary Tract Infection	5,144	95.77
4	Acute Lower Respiratory Tract Infection	3,564	66.35
5	Skin Diseases	3,509	65.33
6	Pneumonia	3,333	62.05
7	Animal Bites	2,953	54.98
8	Acute Hemorrhagic Fever	2,087	38.85
9	Acute Watery Diarrhea	2,000	37.24
10	Diseases of the Heart	1,969	36.66

Table 2.A.3.15 - Region 12

	Disease	Number of Case	Rate per 100,000 population
1	Acute Respiratory Infection	13,718	275.14
2	Animal Bites	12,612	252.95
3	Hypertension	10,134	203.25
4	Pneumonia	6,850	137.39
5	Acute Lower Respiratory Tract Infection	5,856	117.45
6	Urinary Tract Infection	5,553	111.37
7	Fever of Unknown Origin	5,343	107.16
8	Influenza	4,105	82.33
9	Skin Diseases	2,933	58.83
10	Acute Watery Diarrhea	2,785	55.86

Table 2.A.3 Ten Leading Causes of Morbidity by Region

Philippines, 2021

Table 2.A.3.16 - Bangsamoro Autonomous Region in Muslim Mindanao (BARMM)

	Disease	Number of Case	Rate per 100,000 population
1	Acute Watery Diarrhea	7,739	197.84
2	Pneumonia	4,296	140.74
3	Hypertension	4126	100.60
4	TB All Forms	1,265	29.62
5	Acute Lower Respiratory Tract Infection	1,152	26.98
6	Skin Diseases	1,116	26.13
7	Urinary Tract Infection	797	18.66
8	Acute Respiratory Infection	732	17.14
9	Bronchitis	632	14.80
10	Fever of Unknown Origin	447	10.47

Table 2.A.3.17 - Caraga

	Disease	Number of Case	Rate per 100,000 population
1	Acute Respiratory Infection	18,597	668.42
2	Hypertension	12,144	436.49
3	Animal Bites	6,977	250.77
4	Urinary Tract Infection	5,445	195.71
5	Acute Lower Respiratory Tract Infection	3,858	138.67
6	Influenza	3,037	109.16
7	Skin Diseases	2,982	107.18
8	Influenza Like-Illness	2,425	87.16
9	Pneumonia	1,903	68.40
10	TB All Forms	1,276	45.86

Table 2.A.4.1 - Acute Bloody Diarrhea
By Age group, Sex, Province and City
Philippines, 2021

Region/Province/City	0 - 6 days		7 - 28 days		29 days - 11 mos.		1 - 4 yrs.		5 - 9 yrs.		10 -14 yrs.		15 - 19 yrs.		20 - 24 yrs.		25 - 29 yrs.		30 - 34 yrs.	
	Male	Female	Male	Female	Male	Female	Male	Female	Male	Female	Male	Female	Male	Female	Male	Female	Male	Female	Male	Female
**PHILIPPINES**	**181**	**133**	**188**	**208**	**1,561**	**1,403**	**3,085**	**2,945**	**1,533**	**1,440**	**646**	**493**	**321**	**295**	**228**	**280**	**184**	**244**	**153**	**268**
**NCR**	**0**	**0**	**0**	**0**	**0**	**0**	**6**	**7**	**3**	**4**	**0**	**0**	**0**	**0**	**1**	**1**	**1**	**0**	**0**	**0**
Malabon City	0	0	0	0	0	0	0	0	0	0	0	0	0	0	0	0	0	0	0	0
Navotas City	0	0	0	0	0	0	0	0	0	0	0	0	0	0	0	0	0	0	0	0
Valenzuela City	0	0	0	0	0	0	1	0	1	1	0	0	0	0	0	0	0	0	0	0
Caloocan City	0	0	0	0	0	0	0	0	0	0	0	0	0	0	0	0	0	0	0	0
Marikina City	0	0	0	0	0	0	0	0	0	0	0	0	0	0	0	0	0	0	0	0
Pasig City	0	0	0	0	0	0	0	0	0	0	0	0	0	0	0	0	0	0	0	0
Pateros	0	0	0	0	0	0	0	0	0	0	0	0	0	0	0	0	0	0	0	0
Taguig City	0	0	0	0	0	0	0	0	0	0	0	0	0	0	0	0	0	0	0	0
Quezon City	0	0	0	0	0	0	0	0	0	0	0	0	0	0	0	0	0	0	0	0
Makati City	0	0	0	0	0	0	0	0	0	0	0	0	0	0	0	0	0	0	0	0
Mandaluyong City	0	0	0	0	0	0	0	1	0	1	0	0	0	0	0	0	0	0	0	0
San Juan City	0	0	0	0	0	0	0	0	0	0	0	0	0	0	0	0	0	0	0	0
Manila City	0	0	0	0	0	0	0	0	0	0	0	0	0	0	0	0	0	0	0	0
Las Piñas City	0	0	0	0	0	0	0	0	0	0	0	0	0	0	0	0	0	0	0	0
Muntinlupa City	0	0	0	0	0	0	0	0	0	0	0	0	0	0	0	0	0	0	0	0
Parañaque City	0	0	0	0	0	0	0	0	0	0	0	0	0	0	0	0	0	0	0	0
Pasay City	0	0	0	0	0	0	5	6	2	2	0	0	0	0	1	1	1	0	0	0
**CAR**	**0**	**0**	**1**	**0**	**6**	**7**	**39**	**34**	**22**	**12**	**6**	**5**	**5**	**6**	**4**	**5**	**5**	**6**	**2**	**14**
Abra	0	0	0	0	0	0	0	0	0	0	0	0	0	0	0	0	0	0	0	0
Apayao	0	0	0	0	0	0	4	6	2	3	1	2	0	2	0	2	1	1	0	0
Benguet	0	0	0	0	1	1	1	2	1	1	0	1	0	0	0	0	0	0	0	0
Ifugao	0	0	1	0	5	5	22	15	8	5	3	2	4	3	2	2	2	2	0	3
Kalinga	0	0	0	0	0	1	8	9	11	3	0	0	0	1	1	1	2	0	1	11
Mt. Province	0	0	0	0	0	0	4	2	0	0	2	0	1	0	1	0	0	3	1	0
Baguio City	0	0	0	0	0	0	0	0	0	0	0	0	0	0	0	0	0	0	0	0
**Region 1**	**1**	**0**	**0**	**0**	**1**	**2**	**8**	**6**	**3**	**0**	**0**	**2**	**1**	**1**	**2**	**0**	**0**	**1**	**1**	**0**
Ilocos Note	0	0	0	0	0	0	0	0	0	0	0	0	0	0	0	0	0	1	0	0
Ilocos Sur	0	0	0	0	1	0	4	3	0	0	0	0	1	1	0	0	0	0	0	0
La Union	0	0	0	0	0	0	0	0	0	0	0	0	0	0	0	0	0	0	0	0
Pangasinan	1	0	0	0	0	0	4	3	3	0	0	2	0	0	0	0	0	0	1	0
Alaminos City	0	0	0	0	0	0	0	0	0	0	0	0	0	0	0	0	0	0	0	0
Candon City	0	0	0	0	0	0	0	0	0	0	0	0	0	0	0	0	0	0	0	0
Dagupan City	0	0	0	0	0	0	0	0	0	0	0	0	0	0	0	0	0	0	0	0
Laoag City	0	0	0	0	0	0	0	0	0	0	0	0	0	0	0	0	0	0	0	0
San Carlos City	0	0	0	0	0	0	0	0	0	0	0	0	0	0	0	0	0	0	0	0
San Fernando City	0	0	0	0	0	0	0	0	0	0	0	0	0	0	0	0	0	0	0	0
Urdaneta City	0	0	0	0	0	0	0	0	0	0	0	0	0	0	0	0	0	0	0	0
Vigan City	0	0	0	0	0	2	0	0	0	0	0	0	0	0	2	0	0	0	0	0
**Region 2**	**0**	**0**	**1**	**0**	**4**	**3**	**21**	**19**	**14**	**5**	**1**	**0**	**4**	**1**	**3**	**4**	**1**	**2**	**3**	**2**
Batanes	0	0	0	0	0	0	0	0	0	0	0	0	0	0	0	0	0	0	0	0
Cagayan	0	0	0	0	0	0	4	2	0	0	0	0	2	0	0	2	0	0	0	0
Isabela	0	0	0	0	3	3	6	8	3	2	1	0	0	0	0	0	0	1	1	0
Nueva Vizcaya	0	0	1	0	1	0	6	3	1	0	0	0	0	0	0	0	0	0	0	0
Quirino	0	0	0	0	0	0	0	0	0	0	0	0	0	0	0	0	0	0	0	0
Cauayan City	0	0	0	0	0	0	5	6	10	3	0	0	2	1	3	2	1	1	2	2
Ilagan City	0	0	0	0	0	0	0	0	0	0	0	0	0	0	0	0	0	0	0	0
Santiago City	0	0	0	0	0	0	0	0	0	0	0	0	0	0	0	0	0	0	0	0
Tuguegarao City	0	0	0	0	0	0	0	0	0	0	0	0	0	0	0	0	0	0	0	0
**Region 3**	**0**	**0**	**0**	**0**	**0**	**0**	**0**	**2**	**0**	**0**	**0**	**1**	**0**	**0**	**0**	**0**	**0**	**0**	**0**	**0**
Aurora	0	0	0	0	0	0	0	0	0	0	0	0	0	0	0	0	0	0	0	0
Bataan	0	0	0	0	0	0	0	2	0	0	0	1	0	0	0	0	0	0	0	0
Bulacan	0	0	0	0	0	0	0	0	0	0	0	0	0	0	0	0	0	0	0	0
Nueva Ecija	0	0	0	0	0	0	0	0	0	0	0	0	0	0	0	0	0	0	0	0
Pampanga	0	0	0	0	0	0	0	0	0	0	0	0	0	0	0	0	0	0	0	0
Tarlac	0	0	0	0	0	0	0	0	0	0	0	0	0	0	0	0	0	0	0	0
Zambales	0	0	0	0	0	0	0	0	0	0	0	0	0	0	0	0	0	0	0	0
Angeles City	0	0	0	0	0	0	0	0	0	0	0	0	0	0	0	0	0	0	0	0
Balanga City	0	0	0	0	0	0	0	0	0	0	0	0	0	0	0	0	0	0	0	0
Cabanatuan City	0	0	0	0	0	0	0	0	0	0	0	0	0	0	0	0	0	0	0	0
City of San Fernando	0	0	0	0	0	0	0	0	0	0	0	0	0	0	0	0	0	0	0	0
Gapan City	0	0	0	0	0	0	0	0	0	0	0	0	0	0	0	0	0	0	0	0
Mabalacat City	0	0	0	0	0	0	0	0	0	0	0	0	0	0	0	0	0	0	0	0
Malolos City	0	0	0	0	0	0	0	0	0	0	0	0	0	0	0	0	0	0	0	0
Meycauayan City	0	0	0	0	0	0	0	0	0	0	0	0	0	0	0	0	0	0	0	0
Olongapo City	0	0	0	0	0	0	0	0	0	0	0	0	0	0	0	0	0	0	0	0
Palayan City	0	0	0	0	0	0	0	0	0	0	0	0	0	0	0	0	0	0	0	0
San Jose City	0	0	0	0	0	0	0	0	0	0	0	0	0	0	0	0	0	0	0	0
San Jose del Monte City	0	0	0	0	0	0	0	0	0	0	0	0	0	0	0	0	0	0	0	0
Science City of Munoz	0	0	0	0	0	0	0	0	0	0	0	0	0	0	0	0	0	0	0	0
Tarlac City	0	0	0	0	0	0	0	0	0	0	0	0	0	0	0	0	0	0	0	0
**Region 4A**	**157**	**115**	**182**	**205**	**1,477**	**1,328**	**2,698**	**2,626**	**1,371**	**1,323**	**610**	**425**	**250**	**249**	**182**	**230**	**128**	**186**	**127**	**188**
Batangas	3	3	6	15	20	29	119	119	91	93	46	24	19	17	29	39	8	16	18	12
Cavite	6	3	6	15	53	40	148	141	59	60	39	17	10	7	15	27	10	8	5	10
Laguna	67	13	15	24	51	55	79	86	38	39	15	18	4	27	8	12	2	14	3	9
Quezon	76	90	139	131	438	502	1,444	1,398	692	738	347	216	123	104	61	83	70	81	52	71
Rizal	0	0	12	7	730	491	384	404	204	183	40	51	39	39	29	11	16	30	22	45
Antipolo City	0	0	0	0	0	0	0	0	0	0	0	0	0	0	0	0	0	0	0	0
Bacoor City	0	0	0	0	3	3	9	8	1	0	0	0	0	0	0	0	0	0	0	0
Batangas City	0	0	0	0	0	0	1	1	0	0	0	0	0	0	0	0	1	0	0	0
Biñan City	0	0	2	1	24	22	85	85	47	36	21	15	16	15	14	10	7	11	6	10
Cabuyao City	0	0	0	0	0	0	0	0	0	0	0	0	0	0	0	0	0	0	0	0
Calamba City	0	0	0	0	11	8	21	22	2	3	2	0	0	0	2	0	0	0	0	0
Cavite City	0	0	0	0	0	0	0	0	0	0	0	0	0	0	0	0	0	0	0	0
Dasmariñas City	0	0	0	0	0	0	1	0	0	0	0	0	0	0	0	0	0	0	0	0
General Trias City	0	0	0	0	13	11	20	23	7	5	2	0	0	0	1	1	0	0	2	0
Imus City	0	0	0	0	6	7	9	10	7	4	3	2	1	0	0	2	0	0	1	0
Lipa City	0	0	0	0	0	0	0	0	0	0	0	0	0	0	0	0	0	0	0	0
Lucena City	1	0	2	0	31	27	50	50	19	17	3	12	2	4	0	2	2	1	3	4
San Pablo City	0	6	0	12	11	13	65	43	42	20	12	6	6	0	6	18	6	4	3	9
San Pedro City	0	0	0	0	0	0	0	0	0	0	0	0	0	0	0	0	0	0	0	0
Santa Rosa City	0	0	0	0	7	6	20	11	20	13	10	3	5	3	6	4	0	8	8	4
Tagaytay City	0	0	0	0	13	21	23	28	13	4	6	3	0	2	2	0	3	2	4	2
Tanauan City	4	0	0	0	66	78	198	171	110	101	61	54	25	31	9	18	3	10	0	12
Tayabas City	0	0	0	0	0	15	20	26	19	7	3	4	0	0	0	3	0	1	0	0
Trece Martires City	0	0	0	0	0	0	2	0	0	0	0	0	0	0	0	0	0	0	0	0

Table 2.A.4.1 - Acute Bloody Diarrhea
By Age group, Sex, Province and City
Philippines, 2021

Region/Province/City	0 - 6 days		7 - 28 days		29 days - 11 mos.		1 - 4 yrs.		5 - 9 yrs.		10 -14 yrs.		15 - 19 yrs.		20 - 24 yrs.		25 - 29 yrs.		30 - 34 yrs.	
	Male	Female	Male	Female	Male	Female	Male	Female	Male	Female	Male	Female	Male	Female	Male	Female	Male	Female	Male	Female
**Region 4B**	**0**	**0**	**0**	**0**	**1**	**0**	**0**	**0**	**0**	**1**	**0**	**0**	**0**	**1**	**0**	**1**	**0**	**0**	**0**	**1**
Marinduque	0	0	0	0	0	0	0	0	0	0	0	0	0	0	0	0	0	0	0	0
Mindoro Occidental	0	0	0	0	0	0	0	0	0	0	0	0	0	0	0	0	0	0	0	0
Mindoro Oriental	0	0	0	0	0	0	0	0	0	0	0	0	0	0	0	0	0	0	0	0
Palawan	0	0	0	0	1	0	0	0	0	1	0	0	0	1	0	1	0	0	0	1
Romblon	0	0	0	0	0	0	0	0	0	0	0	0	0	0	0	0	0	0	0	0
Puerto Princesa City	0	0	0	0	0	0	0	0	0	0	0	0	0	0	0	0	0	0	0	0
**Region 5**	**0**	**0**	**0**	**1**	**10**	**9**	**36**	**56**	**13**	**15**	**6**	**7**	**7**	**4**	**3**	**5**	**8**	**6**	**1**	**6**
Albay	0	0	0	0	0	0	1	5	3	3	0	1	0	0	0	0	0	1	1	1
Camarines Norte	0	0	0	0	4	5	22	39	4	10	3	6	3	2	0	2	3	3	0	1
Camarines Sur	0	0	0	0	3	2	4	3	1	1	2	0	2	1	0	1	1	1	0	0
Catanduanes	0	0	0	0	0	0	3	0	0	0	0	0	0	0	1	0	0	0	0	1
Masbate	0	0	0	0	0	0	0	0	0	0	0	0	0	0	0	0	0	0	0	0
Sorsogon	0	0	0	1	3	2	6	9	5	1	1	0	2	1	2	2	4	1	0	3
Iriga City	0	0	0	0	0	0	0	0	0	0	0	0	0	0	0	0	0	0	0	0
Legaspi City	0	0	0	0	0	0	0	0	0	0	0	0	0	0	0	0	0	0	0	0
Naga City	0	0	0	0	0	0	0	0	0	0	0	0	0	0	0	0	0	0	0	0
**Region 6**	**0**	**0**	**0**	**0**	**1**	**2**	**20**	**5**	**6**	**6**	**4**	**2**	**4**	**1**	**2**	**5**	**8**	**1**	**2**	**0**
Aklan	0	0	0	0	0	0	0	0	0	0	0	0	0	0	0	0	0	0	0	0
Antique	0	0	0	0	0	0	1	1	0	0	0	0	0	0	0	0	0	0	0	0
Capiz	0	0	0	0	0	0	0	0	0	0	0	0	0	0	0	0	0	0	0	0
Guimaras	0	0	0	0	0	0	0	0	0	0	0	0	0	0	0	0	0	0	0	0
Iloilo	0	0	0	0	1	0	12	3	4	1	0	0	2	0	0	3	3	0	2	0
Negros Occidental	0	0	0	0	0	2	4	1	2	1	2	2	1	1	2	2	5	1	0	0
Bacolod City	0	0	0	0	0	0	0	0	0	0	0	0	0	0	0	0	0	0	0	0
Iloilo City	0	0	0	0	0	0	3	0	0	4	2	0	1	0	0	0	0	0	0	0
**Region 7**	**0**	**0**	**0**	**0**	**0**	**0**	**1**	**0**	**0**	**0**	**0**	**0**	**0**	**0**	**0**	**0**	**0**	**0**	**0**	**0**
Bohol	0	0	0	0	0	0	0	0	0	0	0	0	0	0	0	0	0	0	0	0
Cebu	0	0	0	0	0	0	0	0	0	0	0	0	0	0	0	0	0	0	0	0
Negros Oriental	0	0	0	0	0	0	0	0	0	0	0	0	0	0	0	0	0	0	0	0
Siquijor	0	0	0	0	0	0	0	0	0	0	0	0	0	0	0	0	0	0	0	0
Cebu City	0	0	0	0	0	0	1	0	0	0	0	0	0	0	0	0	0	0	0	0
Lapu-Lapu City	0	0	0	0	0	0	0	0	0	0	0	0	0	0	0	0	0	0	0	0
Mandaue City	0	0	0	0	0	0	0	0	0	0	0	0	0	0	0	0	0	0	0	0
**Region 8**	**0**	**0**	**1**	**0**	**6**	**5**	**23**	**16**	**10**	**20**	**3**	**4**	**2**	**6**	**8**	**2**	**10**	**9**	**12**	**22**
Biliran	0	0	0	0	0	2	5	2	1	0	0	0	0	0	0	0	0	1	1	0
Eastern Samar	0	0	0	0	3	0	2	2	1	0	0	0	0	0	0	0	0	0	0	0
Northern Leyte	0	0	0	0	1	1	4	0	0	1	1	0	0	0	0	0	0	0	1	0
Northern Samar	0	0	0	0	0	0	3	2	1	2	0	0	0	0	0	0	0	0	1	1
Southern Leyte	0	0	0	0	0	0	0	0	0	1	0	0	0	0	0	0	0	0	0	2
Western Samar	0	0	0	0	0	0	1	7	2	12	2	4	1	3	6	1	4	6	9	16
Calbayog City	0	0	0	0	0	0	0	0	0	0	0	0	0	0	0	0	0	0	0	0
Maasin City	0	0	1	0	2	1	6	3	5	4	0	0	1	2	2	0	6	2	0	3
Ormoc City	0	0	0	0	0	1	2	0	0	0	0	0	0	1	0	1	0	0	0	0
Tacloban City	0	0	0	0	0	0	0	0	0	0	0	0	0	0	0	0	0	0	0	0
**Region 9**	**0**	**0**	**1**	**1**	**14**	**15**	**45**	**40**	**18**	**16**	**7**	**10**	**4**	**6**	**4**	**11**	**11**	**10**	**2**	**11**
Zamboanga del Norte	0	0	0	0	1	1	1	1	0	0	1	0	0	0	0	0	1	1	0	1
Zamboanga del Sur	0	0	0	0	2	3	8	1	2	3	2	1	1	0	2	2	5	2	1	1
Zamboanga Sibugay	0	0	1	1	7	3	24	16	7	10	3	3	0	2	1	3	2	2	1	1
Dapitan City	0	0	0	0	0	0	0	1	0	0	0	0	0	0	0	0	0	0	0	0
Dipolog City	0	0	0	0	0	3	1	3	1	0	0	1	0	0	1	0	0	0	0	1
Isabela City	0	0	0	0	3	4	8	16	7	3	1	4	3	3	0	6	3	4	0	7
Pagadian City	0	0	0	0	0	1	2	0	0	0	0	0	0	1	0	0	0	0	0	0
Zamboanga City	0	0	0	0	1	0	1	2	1	0	0	1	0	0	0	0	0	1	0	0
**Region 10**	**0**	**0**	**1**	**1**	**9**	**15**	**40**	**30**	**9**	**21**	**8**	**19**	**5**	**3**	**5**	**4**	**7**	**11**	**2**	**15**
Bukidnon	0	0	0	0	0	0	3	0	0	1	0	0	1	1	0	0	0	0	0	2
Camiguin	0	0	0	0	0	0	0	0	0	0	0	0	0	0	0	0	0	0	0	0
Lanao del Norte	0	0	0	1	0	1	4	2	0	1	0	0	1	0	0	1	2	2	0	3
Misamis Occidental	0	0	0	0	0	0	0	0	0	0	0	3	0	1	0	0	0	4	0	0
Misamis Oriental	0	0	0	0	0	2	4	2	0	0	0	0	0	0	0	0	0	0	0	0
Cagayan de Oro City	0	0	0	0	0	1	2	0	1	0	0	0	1	0	0	0	0	0	1	0
El Salvador City	0	0	0	0	0	0	0	0	0	0	0	0	0	0	0	0	0	0	0	0
Gingoog City	0	0	0	0	0	0	0	8	8	15	7	15	1	0	1	2	0	2	0	7
Iligan City	0	0	1	0	9	11	25	18	0	4	1	1	1	1	4	1	5	3	1	3
Malaybalay City	0	0	0	0	0	0	2	0	0	0	0	0	0	0	0	0	0	0	0	0
Oroquieta City	0	0	0	0	0	0	0	0	0	0	0	0	0	0	0	0	0	0	0	0
Ozamis City	0	0	0	0	0	0	0	0	0	0	0	0	0	0	0	0	0	0	0	0
Tangub City	0	0	0	0	0	0	0	0	0	0	0	0	0	0	0	0	0	0	0	0
Valencia City	0	0	0	0	0	0	0	0	0	0	0	0	0	0	0	0	0	0	0	0
**Region 11**	**23**	**18**	**1**	**0**	**25**	**7**	**120**	**75**	**53**	**5**	**1**	**14**	**39**	**17**	**11**	**7**	**3**	**5**	**1**	**4**
Davao de Oro	0	0	0	0	0	0	2	0	0	0	0	0	0	0	0	0	0	0	0	0
Davao del Norte	0	0	0	0	0	0	1	1	0	0	0	0	0	0	0	0	0	0	0	0
Davao del Sur	0	0	0	0	6	2	11	7	6	3	1	0	0	3	4	1	2	1	0	3
Davao Oriental	0	0	0	0	0	0	0	0	0	0	0	0	0	0	0	0	0	0	0	0
Davao Occidental	0	0	0	0	0	0	0	0	0	0	0	0	0	1	0	0	0	0	0	0
Davao City	23	18	1	0	19	5	106	67	47	2	0	14	39	13	7	6	1	4	1	1
**Region 12**	**0**	**0**	**0**	**0**	**7**	**4**	**11**	**16**	**3**	**4**	**0**	**2**	**0**	**0**	**0**	**4**	**1**	**2**	**0**	**0**
North Cotabato	0	0	0	0	0	0	0	0	0	1	0	0	0	0	0	0	0	0	0	0
Sarangani	0	0	0	0	4	4	4	8	2	1	0	0	0	0	0	4	1	2	0	0
South Cotabato	0	0	0	0	3	0	5	7	0	1	0	0	0	0	0	0	0	0	0	0
Sultan Kudarat	0	0	0	0	0	0	0	0	0	0	0	0	0	0	0	0	0	0	0	0
Cotabato City	0	0	0	0	0	0	0	0	0	0	0	0	0	0	0	0	0	0	0	0
Gen. Santos City	0	0	0	0	0	0	2	1	1	1	0	2	0	0	0	0	0	0	0	0

Table 2.A.4.1 - Acute Bloody Diarrhea

By Age group, Sex, Province and City
Philippines, 2021

Region/Province/City	0 - 6 days		7 - 28 days		29 days - 11 mos.		1 - 4 yrs.		5 - 9 yrs.		10 -14 yrs.		15 - 19 yrs.		20 - 24 yrs.		25 - 29 yrs.		30 - 34 yrs.	
	Male	Female	Male	Female	Male	Female	Male	Female	Male	Female	Male	Female	Male	Female	Male	Female	Male	Female	Male	Female
**BARMM**	**0**	**0**	**0**	**0**	**0**	**0**	**0**	**0**	**0**	**0**	**0**	**0**	**0**	**0**	**0**	**0**	**0**	**0**	**0**	**0**
Basilan	0	0	0	0	0	0	0	0	0	0	0	0	0	0	0	0	0	0	0	0
Lanao del Sur	0	0	0	0	0	0	0	0	0	0	0	0	0	0	0	0	0	0	0	0
Maguindanao	0	0	0	0	0	0	0	0	0	0	0	0	0	0	0	0	0	0	0	0
Sulu	0	0	0	0	0	0	0	0	0	0	0	0	0	0	0	0	0	0	0	0
Tawi-Tawi	0	0	0	0	0	0	0	0	0	0	0	0	0	0	0	0	0	0	0	0
Lamitan City	0	0	0	0	0	0	0	0	0	0	0	0	0	0	0	0	0	0	0	0
Marawi City	0	0	0	0	0	0	0	0	0	0	0	0	0	0	0	0	0	0	0	0
**CARAGA**	**0**	**0**	**0**	**0**	**0**	**6**	**17**	**13**	**8**	**8**	**0**	**2**	**0**	**0**	**3**	**1**	**1**	**5**	**0**	**5**
Agusan del Norte	0	0	0	0	0	0	0	0	0	0	0	0	0	0	0	0	0	0	0	0
Agusan del Sur	0	0	0	0	0	0	0	0	0	0	0	0	0	0	0	0	0	0	0	0
Surigao del Norte	0	0	0	0	0	0	2	0	0	4	0	0	0	0	1	0	1	0	0	1
Surigao del Sur	0	0	0	0	0	4	9	7	5	4	0	1	0	0	2	0	0	4	0	3
Province of Dinagat	0	0	0	0	0	0	0	0	0	0	0	0	0	0	0	0	0	0	0	0
Bislig City	0	0	0	0	0	2	3	5	3	0	0	1	0	0	0	0	0	1	0	1
Butuan City	0	0	0	0	0	0	3	1	0	0	0	0	0	0	0	1	0	0	0	0
Surigao City	0	0	0	0	0	0	0	0	0	0	0	0	0	0	0	0	0	0	0	0

Legend:
0 = No Data
* = No Report

Table 2.A.4.1 - Acute Bloody Diarrhea

By Age group, Sex, Province and City
Philippines, 2021

Region/Province/City	35 -39 yrs.		40 - 44 yrs.		45 -49 yrs.		50 -54 yrs.		55 - 59 yrs.		60 - 64. yrs.		65 - 69 yrs.		70 yrs above		Total		Total Both Sexes	
	Male	Female	Male	Female	Male	Female	Male	Female	Male	Female	Male	Female	Male	Female	Male	Female	Male	Female	Number	Rate
**PHILIPPINES**	**125**	**206**	**149**	**185**	**77**	**244**	**120**	**177**	**79**	**198**	**122**	**149**	**97**	**132**	**105**	**128**	**8,954**	**9,128**	**18,082**	**16.41**
**NCR**	**1**	**1**	**0**	**0**	**0**	**0**	**0**	**0**	**0**	**0**	**0**	**1**	**0**	**0**	**0**	**0**	**12**	**14**	**26**	**0.19**
Malabon City	0	0	0	0	0	0	0	0	0	0	0	0	0	0	0	0	0	0	0	0.00
Navotas City	0	0	0	0	0	0	0	0	0	0	0	0	0	0	0	0	0	0	0	0.00
Valenzuela City	0	0	0	0	0	0	0	0	0	0	0	0	0	0	0	0	2	1	3	0.45
Caloocan City	0	0	0	0	0	0	0	0	0	0	0	0	0	0	0	0	0	0	0	0.00
Marikina City	0	0	0	0	0	0	0	0	0	0	0	0	0	0	0	0	0	0	0	0.00
Pasig City	0	0	0	0	0	0	0	0	0	0	0	0	0	0	0	0	0	0	0	0.00
Pateros	0	0	0	0	0	0	0	0	0	0	0	0	0	0	0	0	0	0	0	0.00
Taguig City	0	0	0	0	0	0	0	0	0	0	0	0	0	0	0	0	0	0	0	0.00
Quezon City	0	0	0	0	0	0	0	0	0	0	0	0	0	0	0	0	0	0	0	0.00
Makati City	0	0	0	0	0	0	0	0	0	0	0	0	0	0	0	0	0	0	0	0.00
Mandaluyong City	0	0	0	0	0	0	0	0	0	0	0	0	0	0	0	0	0	2	2	0.48
San Juan City	0	0	0	0	0	0	0	0	0	0	0	0	0	0	0	0	0	0	0	0.00
Manila City	0	0	0	0	0	0	0	0	0	0	0	0	0	0	0	0	0	0	0	0.00
Las Piñas City	0	0	0	0	0	0	0	0	0	0	0	0	0	0	0	0	0	0	0	0.00
Muntinlupa City	0	0	0	0	0	0	0	0	0	0	0	0	0	0	0	0	0	0	0	0.00
Parañaque City	0	0	0	0	0	0	0	0	0	0	0	0	0	0	0	0	0	0	0	0.00
Pasay City	1	1	0	0	0	0	0	0	0	0	0	1	0	0	0	0	10	11	21	4.65
**CAR**	**4**	**5**	**5**	**2**	**5**	**6**	**8**	**12**	**3**	**7**	**4**	**6**	**3**	**10**	**7**	**16**	**129**	**153**	**282**	**15.47**
Abra	0	0	0	0	0	0	0	0	0	0	0	0	0	0	0	0	0	0	0	0.00
Apayao	2	0	3	0	0	1	3	6	1	0	0	0	0	1	0	3	17	27	44	35.28
Benguet	0	0	0	0	0	0	0	0	0	1	2	0	0	0	1	0	6	6	12	2.48
Ifugao	1	5	1	1	3	1	2	0	0	1	1	1	0	4	3	11	58	61	119	56.12
Kalinga	1	0	1	0	2	2	2	3	1	3	1	4	1	1	1	0	33	39	72	32.50
Mt. Province	0	0	0	1	0	2	1	3	0	1	0	1	1	4	2	2	13	19	32	20.33
Baguio City	0	0	0	0	0	0	0	0	1	1	0	0	1	0	0	0	2	1	3	0.80
**Region 1**	**1**	**0**	**0**	**2**	**0**	**0**	**1**	**1**	**2**	**0**	**0**	**0**	**1**	**2**	**1**	**0**	**23**	**17**	**40**	**0.75**
Ilocos Note	0	0	0	0	0	0	0	0	0	0	0	0	1	0	0	0	1	1	2	0.40
Ilocos Sur	0	0	0	1	0	0	1	1	2	0	0	0	0	2	1	0	10	8	18	3.00
La Union	0	0	0	0	0	0	0	0	0	0	0	0	0	0	0	0	0	0	0	0.00
Pangasinan	1	0	0	1	0	0	0	0	0	0	0	0	0	0	0	0	10	6	16	0.63
Alaminos City	0	0	0	0	0	0	0	0	0	0	0	0	0	0	0	0	0	0	0	0.00
Candon City	0	0	0	0	0	0	0	0	0	0	0	0	0	0	0	0	0	0	0	0.00
Dagupan City	0	0	0	0	0	0	0	0	0	0	0	0	0	0	0	0	0	0	0	0.00
Laoag City	0	0	0	0	0	0	0	0	0	0	0	0	0	0	0	0	0	0	0	0.00
San Carlos City	0	0	0	0	0	0	0	0	0	0	0	0	0	0	0	0	0	0	0	0.00
San Fernando City	0	0	0	0	0	0	0	0	0	0	0	0	0	0	0	0	0	0	0	0.00
Urdaneta City	0	0	0	0	0	0	0	0	0	0	0	0	0	0	0	0	0	0	0	0.00
Vigan City	0	0	0	0	0	0	0	0	0	0	0	0	0	0	0	0	2	2	4	7.11
**Region 2**	**2**	**1**	**1**	**3**	**1**	**1**	**2**	**3**	**1**	**0**	**0**	**0**	**3**	**2**	**1**	**5**	**63**	**51**	**114**	**3.09**
Batanes	0	0	0	0	0	0	0	0	0	0	0	0	0	0	0	0	0	0	0	0.00
Cagayan	0	0	0	0	0	0	2	2	0	0	0	0	2	0	0	4	10	10	20	1.78
Isabela	1	0	0	0	0	0	0	0	1	0	0	0	0	1	0	0	16	15	31	2.45
Nueva Vizcaya	0	0	0	0	1	0	0	0	0	0	0	0	1	1	1	1	12	5	17	3.48
Quirino	0	0	0	0	0	0	0	0	0	0	0	0	0	0	0	0	0	0	0	0.00
Cauayan City	1	1	1	3	0	1	0	1	0	0	0	0	0	0	0	0	25	21	46	33.26
Ilagan City	0	0	0	0	0	0	0	0	0	0	0	0	0	0	0	0	0	0	0	0.00
Santiago City	0	0	0	0	0	0	0	0	0	0	0	0	0	0	0	0	0	0	0	0.00
Tuguegarao City	0	0	0	0	0	0	0	0	0	0	0	0	0	0	0	0	0	0	0	0.00
**Region 3**	**0**	**0**	**0**	**0**	**0**	**0**	**0**	**0**	**0**	**0**	**0**	**0**	**0**	**0**	**0**	**0**	**0**	**3**	**3**	**0.02**
Aurora	0	0	0	0	0	0	0	0	0	0	0	0	0	0	0	0	0	0	0	0.00
Bataan	0	0	0	0	0	0	0	0	0	0	0	0	0	0	0	0	0	3	3	0.40
Bulacan	0	0	0	0	0	0	0	0	0	0	0	0	0	0	0	0	0	0	0	0.00
Nueva Ecija	0	0	0	0	0	0	0	0	0	0	0	0	0	0	0	0	0	0	0	0.00
Pampanga	0	0	0	0	0	0	0	0	0	0	0	0	0	0	0	0	0	0	0	0.00
Tarlac	0	0	0	0	0	0	0	0	0	0	0	0	0	0	0	0	0	0	0	0.00
Zambales	0	0	0	0	0	0	0	0	0	0	0	0	0	0	0	0	0	0	0	0.00
Angeles City	0	0	0	0	0	0	0	0	0	0	0	0	0	0	0	0	0	0	0	0.00
Balanga City	0	0	0	0	0	0	0	0	0	0	0	0	0	0	0	0	0	0	0	0.00
Cabanatuan City	0	0	0	0	0	0	0	0	0	0	0	0	0	0	0	0	0	0	0	0.00
City of San Fernando	0	0	0	0	0	0	0	0	0	0	0	0	0	0	0	0	0	0	0	0.00
Gapan City	0	0	0	0	0	0	0	0	0	0	0	0	0	0	0	0	0	0	0	0.00
Mabalacat City	0	0	0	0	0	0	0	0	0	0	0	0	0	0	0	0	0	0	0	0.00
Malolos City	0	0	0	0	0	0	0	0	0	0	0	0	0	0	0	0	0	0	0	0.00
Meycauayan City	0	0	0	0	0	0	0	0	0	0	0	0	0	0	0	0	0	0	0	0.00
Olongapo City	0	0	0	0	0	0	0	0	0	0	0	0	0	0	0	0	0	0	0	0.00
Palayan City	0	0	0	0	0	0	0	0	0	0	0	0	0	0	0	0	0	0	0	0.00
San Jose City	0	0	0	0	0	0	0	0	0	0	0	0	0	0	0	0	0	0	0	0.00
San Jose del Monte City	0	0	0	0	0	0	0	0	0	0	0	0	0	0	0	0	0	0	0	0.00
Science City of Munoz	0	0	0	0	0	0	0	0	0	0	0	0	0	0	0	0	0	0	0	0.00
Tarlac City	0	0	0	0	0	0	0	0	0	0	0	0	0	0	0	0	0	0	0	0.00
**Region 4A**	**93**	**154**	**123**	**145**	**51**	**143**	**84**	**119**	**45**	**119**	**85**	**83**	**63**	**66**	**61**	**57**	**7,787**	**7,761**	**15,548**	**95.03**
Batangas	19	14	22	14	10	10	20	5	11	10	10	7	3	19	13	4	467	450	917	42.77
Cavite	7	8	2	7	3	3	9	4	0	0	6	3	3	3	6	3	387	359	746	46.49
Laguna	1	17	3	4	2	2	0	1	2	2	2	10	1	2	2	4	295	339	634	57.02
Quezon	31	56	29	81	20	59	18	66	14	57	29	26	16	15	16	17	3,615	3,791	7,406	391.82
Rizal	9	25	39	16	2	32	17	21	5	27	16	6	14	7	2	16	1,580	1,411	2,991	125.31
Antipolo City	0	0	0	0	0	0	0	0	0	0	0	0	0	0	0	0	0	0	0	0.00
Bacoor City	1	0	0	0	0	0	0	0	0	0	0	0	0	0	0	0	14	11	25	3.56
Batangas City	0	0	0	0	0	0	0	0	0	0	0	0	1	0	0	0	3	1	4	1.05
Biñan City	3	3	4	3	4	2	0	0	2	0	0	3	3	2	0	0	238	218	456	122.32
Cabuyao City	0	0	0	0	0	0	0	0	0	0	0	0	0	0	0	0	0	0	0	0.00
Calamba City	0	0	1	0	0	0	0	3	0	0	1	0	1	0	0	0	41	36	77	15.13
Cavite City	0	0	0	0	0	0	0	0	0	0	0	0	0	0	0	0	0	0	0	0.00
Dasmariñas City	0	0	0	0	0	0	0	0	0	0	0	0	0	0	0	0	1	0	1	0.13
General Trias City	0	1	0	0	0	0	0	2	0	0	0	1	0	2	0	0	45	46	91	24.73
Imus City	1	0	2	0	0	0	0	1	0	0	0	0	0	0	0	0	30	26	56	11.85
Lipa City	0	0	0	0	0	0	0	0	0	0	0	0	0	0	0	0	0	0	0	0.00
Lucena City	2	2	2	0	0	2	1	0	3	0	0	0	0	0	0	0	121	121	242	84.48
San Pablo City	3	6	3	0	3	15	6	0	0	0	0	0	0	0	6	0	172	152	324	108.78
San Pedro City	0	0	0	0	0	0	0	0	0	0	0	0	0	0	0	0	0	0	0	0.00
Santa Rosa City	2	5	1	4	3	3	4	7	7	11	10	11	11	8	6	6	120	107	227	57.32
Tagaytay City	5	2	3	4	0	6	6	0	0	0	5	5	4	2	0	0	87	81	168	201.63
Tanauan City	9	15	12	12	4	9	3	9	1	12	6	11	6	6	10	7	527	556	1,083	541.56
Tayabas City	0	0	0	0	0	0	0	0	0	0	0	0	0	0	0	0	42	56	98	91.29
Trece Martires City	0	0	0	0	0	0	0	0	0	0	0	0	0	0	0	0	2	0	2	1.10

Table 2.A.4.1 - Acute Bloody Diarrhea
By Age group, Sex, Province and City
Philippines, 2021

Region/Province/City	35 -39 yrs.		40 - 44 yrs.		45 -49 yrs.		50 -54 yrs.		55 - 59 yrs.		60 - 64. yrs.		65 - 69 yrs.		70 yrs above		Total		Total Both Sexes	
	Male	Female	Male	Female	Male	Female	Male	Female	Male	Female	Male	Female	Male	Female	Male	Female	Male	Female	Number	Rate
**Region 4B**	**0**	**1**	**0**	**0**	**0**	**0**	**0**	**0**	**0**	**0**	**1**	**0**	**0**	**0**	**0**	**0**	**2**	**5**	**7**	**0.22**
Marinduque	0	0	0	0	0	0	0	0	0	0	0	0	0	0	0	0	0	0	0	0.00
Mindoro Occidental	0	0	0	0	0	0	0	0	0	0	0	0	0	0	0	0	0	0	0	0.00
Mindoro Oriental	0	0	0	0	0	0	0	0	0	0	0	0	0	0	0	0	0	0	0	0.00
Palawan	0	1	0	0	0	0	0	0	0	0	1	0	0	0	0	0	2	5	7	0.74
Romblon	0	0	0	0	0	0	0	0	0	0	0	0	0	0	0	0	0	0	0	0.00
Puerto Princesa City	0	0	0	0	0	0	0	0	0	0	0	0	0	0	0	0	0	0	0	0.00
**Region 5**	**0**	**5**	**1**	**2**	**1**	**3**	**1**	**6**	**2**	**6**	**0**	**4**	**1**	**7**	**1**	**4**	**91**	**146**	**237**	**3.83**
Albay	0	2	0	1	0	0	0	0	0	1	0	0	0	0	0	0	5	15	20	1.67
Camarines Norte	0	1	0	0	0	0	0	1	1	1	0	0	0	2	1	0	41	73	114	18.11
Camarines Sur	0	0	0	1	0	2	0	5	1	3	0	4	0	5	0	0	14	29	43	2.46
Catanduanes	0	0	0	0	0	0	0	0	0	0	0	0	0	0	0	0	4	1	5	1.82
Masbate	0	0	0	0	0	0	0	0	0	0	0	0	0	0	0	0	0	0	0	0.00
Sorsogon	0	2	1	0	1	1	1	0	0	1	0	0	1	0	0	4	27	28	55	6.43
Iriga City	0	0	0	0	0	0	0	0	0	0	0	0	0	0	0	0	0	0	0	0.00
Legaspi City	0	0	0	0	0	0	0	0	0	0	0	0	0	0	0	0	0	0	0	0.00
Naga City	0	0	0	0	0	0	0	0	0	0	0	0	0	0	0	0	0	0	0	0.00
**Region 6**	**2**	**4**	**0**	**0**	**2**	**1**	**2**	**3**	**4**	**0**	**2**	**1**	**0**	**2**	**0**	**4**	**59**	**37**	**96**	**1.20**
Aklan	0	0	0	0	0	0	0	0	0	0	0	0	0	0	0	0	0	0	0	0.00
Antique	0	0	0	0	0	0	0	0	0	0	0	0	0	0	0	0	1	1	2	0.32
Capiz	0	0	0	0	0	0	0	0	0	0	0	0	0	0	0	0	0	0	0	0.00
Guimaras	0	0	0	0	0	0	0	0	0	0	0	0	0	0	0	0	0	0	0	0.00
Iloilo	0	0	0	0	0	0	0	1	1	0	0	1	0	1	0	1	21	8	30	1.45
Negros Occidental	1	4	0	0	2	1	2	2	2	0	1	0	0	0	0	2	24	18	42	1.61
Bacolod City	0	0	0	0	0	0	0	0	0	0	0	0	0	0	0	0	0	0	0	0.00
Iloilo City	1	0	0	0	0	0	0	0	1	0	1	0	0	1	0	1	5	3	8	1.67
**Region 7**	**0**	**0**	**0**	**0**	**0**	**0**	**0**	**0**	**0**	**1**	**0**	**0**	**0**	**0**	**0**	**0**	**1**	**1**	**2**	**0.02**
Bohol	0	0	0	0	0	0	0	0	0	0	0	0	0	0	0	0	0	0	0	0.00
Cebu	0	0	0	0	0	0	0	0	0	0	0	0	0	0	0	0	0	0	0	0.00
Negros Oriental	0	0	0	0	0	0	0	0	0	0	0	0	0	0	0	0	0	0	0	0.00
Siquijor	0	0	0	0	0	0	0	0	0	0	0	0	0	0	0	0	0	0	0	0.00
Cebu City	0	0	0	0	0	0	0	0	0	1	0	0	0	0	0	0	1	1	2	0.19
Lapu-Lapu City	0	0	0	0	0	0	0	0	0	0	0	0	0	0	0	0	0	0	0	0.00
Mandaue City	0	0	0	0	0	0	0	0	0	0	0	0	0	0	0	0	0	0	0	0.00
**Region 8**	**11**	**21**	**8**	**13**	**6**	**21**	**7**	**13**	**12**	**48**	**11**	**33**	**14**	**28**	**13**	**21**	**157**	**282**	**439**	**9.14**
Biliran	0	0	1	0	0	0	0	0	0	0	0	1	0	0	0	0	8	6	14	7.75
Eastern Samar	0	0	0	0	0	1	0	2	0	0	0	0	0	0	0	0	6	5	11	2.15
Northern Leyte	2	1	0	0	1	0	0	1	0	0	0	0	0	0	0	0	10	4	14	0.85
Northern Samar	0	0	0	0	1	0	0	0	0	0	0	1	0	0	0	0	6	6	12	1.78
Southern Leyte	0	0	0	0	0	0	0	0	0	0	0	0	0	0	0	0	0	3	3	0.84
Western Samar	7	19	1	12	4	18	5	8	11	46	9	29	14	27	13	20	89	228	317	50.00
Calbayog City	0	0	0	0	0	0	0	0	0	0	0	0	0	0	0	0	0	0	0	0.00
Maasin City	2	1	5	1	0	2	2	2	1	2	1	2	0	1	0	0	34	26	60	65.85
Ormoc City	0	0	1	0	0	0	0	0	0	0	1	0	0	0	0	1	4	4	8	3.39
Tacloban City	0	0	0	0	0	0	0	0	0	0	0	0	0	0	0	0	0	0	0	0.00
**Region 9**	**5**	**1**	**6**	**4**	**5**	**5**	**5**	**13**	**4**	**5**	**8**	**3**	**2**	**2**	**3**	**8**	**144**	**161**	**305**	**8.01**
Zamboanga del Norte	0	0	0	0	0	0	0	1	0	0	0	0	0	1	0	3	4	9	13	1.56
Zamboanga del Sur	4	0	2	2	0	1	1	3	3	1	3	1	2	0	0	2	38	23	61	7.26
Zamboanga Sibugay	1	1	2	2	0	0	3	3	1	1	4	0	0	0	0	0	57	48	105	15.27
Dapitan City	0	0	0	0	0	0	0	0	0	0	0	0	0	0	0	0	0	1	1	1.16
Dipolog City	0	0	0	0	1	1	1	0	0	0	0	0	0	0	0	2	5	11	16	11.75
Isabela City	0	0	1	0	4	2	0	5	0	3	0	2	0	1	1	1	31	61	92	70.56
Pagadian City	0	0	1	0	0	1	0	1	0	0	1	0	0	0	1	0	5	4	9	4.37
Zamboanga City	0	0	0	0	0	0	0	0	0	0	0	0	0	0	1	0	4	4	8	0.90
**Region 10**	**0**	**7**	**1**	**6**	**3**	**9**	**4**	**4**	**6**	**10**	**3**	**7**	**8**	**6**	**7**	**7**	**118**	**175**	**293**	**5.77**
Bukidnon	0	0	0	1	0	2	0	0	1	2	0	0	0	1	0	0	5	10	15	1.33
Camiguin	0	0	0	0	0	0	0	0	0	0	0	0	0	0	0	0	0	0	0	0.00
Lanao del Norte	0	1	0	1	0	1	0	2	0	2	1	1	0	1	1	2	9	22	31	4.21
Misamis Occidental	0	0	0	0	0	0	0	0	0	0	0	0	5	0	0	0	5	8	13	3.80
Misamis Oriental	0	0	0	0	0	0	0	0	0	0	0	0	0	0	0	0	4	4	8	1.02
Cagayan de Oro City	0	0	0	0	1	0	1	0	0	0	0	1	0	0	3	1	10	3	13	1.76
El Salvador City	0	0	0	0	0	0	0	0	0	0	0	0	0	0	0	0	0	0	0	0.00
Gingoog City	0	6	0	4	1	6	0	2	2	4	2	4	2	3	0	1	24	79	103	75.41
Iligan City	0	0	1	0	1	0	3	0	3	2	0	1	1	1	3	3	59	49	108	28.97
Malaybalay City	0	0	0	0	0	0	0	0	0	0	0	0	0	0	0	0	2	0	2	1.06
Oroquieta City	0	0	0	0	0	0	0	0	0	0	0	0	0	0	0	0	0	0	0	0.00
Ozamis City	0	0	0	0	0	0	0	0	0	0	0	0	0	0	0	0	0	0	0	0.00
Tangub City	0	0	0	0	0	0	0	0	0	0	0	0	0	0	0	0	0	0	0	0.00
Valencia City	0	0	0	0	0	0	0	0	0	0	0	0	0	0	0	0	0	0	0	0.00
**Region 11**	**6**	**4**	**3**	**2**	**1**	**49**	**2**	**1**	**0**	**2**	**6**	**8**	**0**	**1**	**3**	**5**	**298**	**224**	**522**	**9.72**
Davao de Oro	0	0	0	0	0	0	0	0	0	0	0	0	0	0	1	0	3	0	3	0.38
Davao del Norte	0	0	0	1	0	0	0	0	0	0	0	0	0	0	0	0	1	2	3	0.28
Davao del Sur	4	3	3	1	1	0	0	1	0	2	3	1	0	0	1	1	42	29	71	11.68
Davao Oriental	0	0	0	0	0	0	0	0	0	0	0	0	0	0	0	0	0	0	0	0.00
Davao Occidental	1	0	0	0	0	0	0	0	0	0	0	0	0	0	0	0	1	1	2	0.58
Davao City	1	1	0	0	0	49	2	0	0	0	3	7	0	1	1	4	251	192	443	23.86
**Region 12**	**0**	**0**	**1**	**2**	**2**	**2**	**0**	**0**	**0**	**0**	**1**	**2**	**2**	**4**	**0**	**0**	**28**	**42**	**70**	**1.40**
North Cotabato	0	0	0	0	0	0	0	0	0	0	0	0	0	0	0	0	0	1	1	0.06
Sarangani	0	0	0	2	2	2	0	0	0	0	0	2	2	4	0	0	15	29	44	7.45
South Cotabato	0	0	1	0	0	0	0	0	0	0	1	0	0	0	0	0	10	8	18	1.78
Sultan Kudarat	0	0	0	0	0	0	0	0	0	0	0	0	0	0	0	0	0	0	0	0.00
Cotabato City	0	0	0	0	0	0	0	0	0	0	0	0	0	0	0	0	0	0	0	0.00
Gen. Santos City	0	0	0	0	0	0	0	0	0	0	0	0	0	0	0	0	3	4	7	1.07

Table 2.A.4.1 - Acute Bloody Diarrhea

By Age group, Sex, Province and City
Philippines, 2021

Region/Province/City	35 -39 yrs.		40 - 44 yrs.		45 -49 yrs.		50 -54 yrs.		55 - 59 yrs.		60 - 64. yrs.		65 - 69 yrs.		70 yrs above		Total		Total Both Sexes	
	Male	Female	Male	Female	Male	Female	Male	Female	Male	Female	Male	Female	Male	Female	Male	Female	Male	Female	Number	Rate
**BARMM**	**0**	**0**	**0**	**0**	**0**	**0**	**0**	**0**	**0**	**0**	**0**	**0**	**0**	**0**	**0**	**0**	**0**	**0**	**0**	**0.00**
Basilan	0	0	0	0	0	0	0	0	0	0	0	0	0	0	0	0	0	0	0	0.00
Lanao del Sur	0	0	0	0	0	0	0	0	0	0	0	0	0	0	0	0	0	0	0	0.00
Maguindanao	0	0	0	0	0	0	0	0	0	0	0	0	0	0	0	0	0	0	0	0.00
Sulu	0	0	0	0	0	0	0	0	0	0	0	0	0	0	0	0	0	0	0	0.00
Tawi-Tawi	0	0	0	0	0	0	0	0	0	0	0	0	0	0	0	0	0	0	0	0.00
Lamitan City	0	0	0	0	0	0	0	0	0	0	0	0	0	0	0	0	0	0	0	0.00
Marawi City	0	0	0	0	0	0	0	0	0	0	0	0	0	0	0	0	0	0	0	0.00
**CARAGA**	**0**	**2**	**0**	**4**	**0**	**4**	**4**	**2**	**0**	**0**	**1**	**1**	**0**	**2**	**8**	**1**	**42**	**56**	**98**	**3.52**
Agusan del Norte	0	0	0	0	0	0	0	0	0	0	0	0	0	0	0	0	0	0	0	0.00
Agusan del Sur	0	0	0	0	0	0	0	0	0	0	0	0	0	0	0	0	0	0	0	0.00
Surigao del Norte	0	0	0	0	0	0	0	0	0	0	0	0	0	0	0	0	4	5	9	2.45
Surigao del Sur	0	2	0	2	0	3	3	1	0	0	1	1	0	2	5	1	25	35	60	11.41
Province of Dinagat	0	0	0	0	0	0	0	0	0	0	0	0	0	0	0	0	0	0	0	0.00
Bislig City	0	0	0	2	0	1	1	1	0	0	0	0	0	0	3	0	10	14	24	24.04
Butuan City	0	0	0	0	0	0	0	0	0	0	0	0	0	0	0	0	3	2	5	1.37
Surigao City	0	0	0	0	0	0	0	0	0	0	0	0	0	0	0	0	0	0	0	0.00

Legend:
0 = No Data
* = No Report

Table 2.A.4.2 - Acute Febrile Illness

By Age group, Sex, Province and City

Philippines, 2021

Region/Province/City	0 - 6 days		7 - 28 days		29 days - 11 mos.		1 - 4 yrs.		5 - 9 yrs.		10 -14 yrs.		15 - 19 yrs.		20 - 24 yrs.		25 - 29 yrs.		30 - 34 yrs.	
	Male	Female	Male	Female	Male	Female	Male	Female	Male	Female	Male	Female	Male	Female	Male	Female	Male	Female	Male	Female
**PHILIPPINES**	**164**	**128**	**206**	**261**	**2,180**	**2,058**	**4,628**	**4,453**	**2,511**	**2,443**	**1,094**	**895**	**594**	**596**	**374**	**524**	**351**	**557**	**350**	**460**
**NCR**	**0**	**0**	**0**	**0**	**2**	**4**	**12**	**9**	**9**	**10**	**4**	**4**	**2**	**5**	**6**	**9**	**5**	**2**	**1**	**4**
Malabon City	0	0	0	0	0	0	0	0	0	0	0	0	0	0	0	0	0	0	0	0
Navotas City	0	0	0	0	0	0	0	0	0	0	0	0	0	0	0	0	0	0	0	0
Valenzuela City	0	0	0	0	0	0	1	2	1	1	0	0	0	0	0	0	1	0	0	0
Caloocan City	0	0	0	0	0	0	0	0	0	0	0	0	0	0	0	0	0	0	0	0
Marikina City	0	0	0	0	0	0	0	0	0	0	0	0	0	0	0	0	0	0	0	0
Pasig City	0	0	0	0	0	0	0	0	0	0	0	0	0	0	0	0	0	0	0	0
Pateros	0	0	0	0	0	0	0	0	0	0	0	0	0	0	0	0	0	0	0	0
Taguig City	0	0	0	0	0	0	0	0	3	2	1	1	2	3	4	6	3	2	1	3
Quezon City	0	0	0	0	0	0	0	0	0	0	0	0	0	0	0	0	0	0	0	0
Makati City	0	0	0	0	0	0	0	0	0	0	0	0	0	0	0	0	0	0	0	0
Mandaluyong City	0	0	0	0	0	0	0	0	0	0	0	0	0	0	0	0	0	0	0	0
San Juan City	0	0	0	0	0	0	0	0	0	0	0	0	0	0	0	0	0	0	0	0
Manila City	0	0	0	0	0	0	0	0	0	0	0	0	0	0	0	0	0	0	0	0
Las Piñas City	0	0	0	0	2	4	11	7	5	7	3	3	0	2	2	3	1	0	0	1
Muntinlupa City	0	0	0	0	0	0	0	0	0	0	0	0	0	0	0	0	0	0	0	0
Parañaque City	0	0	0	0	0	0	0	0	0	0	0	0	0	0	0	0	0	0	0	0
Pasay City	0	0	0	0	0	0	0	0	0	0	0	0	0	0	0	0	0	0	0	0
**CAR**	**0**	**0**	**0**	**0**	**9**	**17**	**47**	**44**	**27**	**16**	**8**	**5**	**5**	**6**	**1**	**0**	**2**	**0**	**1**	**1**
Abra	0	0	0	0	0	0	0	0	0	0	0	0	0	0	0	0	0	0	0	0
Apayao	0	0	0	0	0	0	0	0	0	0	0	0	0	0	0	0	0	0	0	0
Benguet	0	0	0	0	0	0	0	0	0	0	0	0	0	0	0	0	0	0	0	0
Ifugao	0	0	0	0	0	0	0	0	0	0	0	0	0	0	0	0	0	0	0	0
Kalinga	0	0	0	0	0	0	0	0	0	0	0	0	0	0	0	0	0	0	0	0
Mt. Province	0	0	0	0	9	17	47	44	27	16	8	5	5	6	1	0	2	0	1	1
Baguio City	0	0	0	0	0	0	0	0	0	0	0	0	0	0	0	0	0	0	0	0
**Region 1**	**1**	**1**	**0**	**0**	**10**	**12**	**46**	**19**	**36**	**37**	**19**	**17**	**5**	**10**	**1**	**6**	**1**	**2**	**0**	**3**
Ilocos Note	0	0	0	0	0	0	0	0	0	0	0	0	0	0	0	0	0	0	0	0
Ilocos Sur	1	1	0	0	10	12	46	19	36	37	19	17	5	10	1	6	1	2	0	3
La Union	0	0	0	0	0	0	0	0	0	0	0	0	0	0	0	0	0	0	0	0
Pangasinan	0	0	0	0	0	0	0	0	0	0	0	0	0	0	0	0	0	0	0	0
Alaminos City	0	0	0	0	0	0	0	0	0	0	0	0	0	0	0	0	0	0	0	0
Candon City	0	0	0	0	0	0	0	0	0	0	0	0	0	0	0	0	0	0	0	0
Dagupan City	0	0	0	0	0	0	0	0	0	0	0	0	0	0	0	0	0	0	0	0
Laoag City	0	0	0	0	0	0	0	0	0	0	0	0	0	0	0	0	0	0	0	0
San Carlos City	0	0	0	0	0	0	0	0	0	0	0	0	0	0	0	0	0	0	0	0
San Fernando City	0	0	0	0	0	0	0	0	0	0	0	0	0	0	0	0	0	0	0	0
Urdaneta City	0	0	0	0	0	0	0	0	0	0	0	0	0	0	0	0	0	0	0	0
Vigan City	0	0	0	0	0	0	0	0	0	0	0	0	0	0	0	0	0	0	0	0
**Region 2**	**1**	**0**	**0**	**0**	**9**	**12**	**44**	**28**	**20**	**22**	**5**	**11**	**3**	**2**	**3**	**0**	**0**	**2**	**1**	**3**
Batanes	0	0	0	0	0	0	0	0	0	0	0	0	0	0	0	0	0	0	0	0
Cagayan	0	0	0	0	0	0	2	0	0	0	0	0	2	0	0	0	0	0	0	0
Isabela	1	0	0	0	3	4	8	7	5	4	1	2	1	1	2	0	0	0	1	1
Nueva Vizcaya	0	0	0	0	6	8	34	21	14	15	3	9	0	1	1	0	0	2	0	2
Quirino	0	0	0	0	0	0	0	0	1	3	1	0	0	0	0	0	0	0	0	0
Cauayan City	0	0	0	0	0	0	0	0	0	0	0	0	0	0	0	0	0	0	0	0
Ilagan City	0	0	0	0	0	0	0	0	0	0	0	0	0	0	0	0	0	0	0	0
Santiago City	0	0	0	0	0	0	0	0	0	0	0	0	0	0	0	0	0	0	0	0
Tuguegarao City	0	0	0	0	0	0	0	0	0	0	0	0	0	0	0	0	0	0	0	0
**Region 3**	**0**	**0**	**0**	**0**	**0**	**0**	**5**	**3**	**2**	**7**	**0**	**0**	**0**	**0**	**0**	**0**	**0**	**0**	**0**	**0**
Aurora	0	0	0	0	0	0	5	3	2	7	0	0	0	0	0	0	0	0	0	0
Bataan	0	0	0	0	0	0	0	0	0	0	0	0	0	0	0	0	0	0	0	0
Bulacan	0	0	0	0	0	0	0	0	0	0	0	0	0	0	0	0	0	0	0	0
Nueva Ecija	0	0	0	0	0	0	0	0	0	0	0	0	0	0	0	0	0	0	0	0
Pampanga	0	0	0	0	0	0	0	0	0	0	0	0	0	0	0	0	0	0	0	0
Tarlac	0	0	0	0	0	0	0	0	0	0	0	0	0	0	0	0	0	0	0	0
Zambales	0	0	0	0	0	0	0	0	0	0	0	0	0	0	0	0	0	0	0	0
Angeles City	0	0	0	0	0	0	0	0	0	0	0	0	0	0	0	0	0	0	0	0
Balanga City	0	0	0	0	0	0	0	0	0	0	0	0	0	0	0	0	0	0	0	0
Cabanatuan City	0	0	0	0	0	0	0	0	0	0	0	0	0	0	0	0	0	0	0	0
City of San Fernando	0	0	0	0	0	0	0	0	0	0	0	0	0	0	0	0	0	0	0	0
Gapan City	0	0	0	0	0	0	0	0	0	0	0	0	0	0	0	0	0	0	0	0
Mabalacat City	0	0	0	0	0	0	0	0	0	0	0	0	0	0	0	0	0	0	0	0
Malolos City	0	0	0	0	0	0	0	0	0	0	0	0	0	0	0	0	0	0	0	0
Meycauayan City	0	0	0	0	0	0	0	0	0	0	0	0	0	0	0	0	0	0	0	0
Olongapo City	0	0	0	0	0	0	0	0	0	0	0	0	0	0	0	0	0	0	0	0
Palayan City	0	0	0	0	0	0	0	0	0	0	0	0	0	0	0	0	0	0	0	0
San Jose City	0	0	0	0	0	0	0	0	0	0	0	0	0	0	0	0	0	0	0	0
San Jose del Monte City	0	0	0	0	0	0	0	0	0	0	0	0	0	0	0	0	0	0	0	0
Science City of Munoz	0	0	0	0	0	0	0	0	0	0	0	0	0	0	0	0	0	0	0	0
Tarlac City	0	0	0	0	0	0	0	0	0	0	0	0	0	0	0	0	0	0	0	0
**Region 4A**	**159**	**123**	**181**	**224**	**1,499**	**1,364**	**2,585**	**2,555**	**1,330**	**1,310**	**574**	**426**	**241**	**247**	**157**	**209**	**130**	**171**	**114**	**188**
Batangas	3	3	6	15	20	29	113	117	90	93	46	24	18	16	28	38	8	16	18	12
Cavite	5	9	6	17	42	42	110	115	39	37	16	12	9	12	9	16	13	7	3	10
Laguna	67	13	15	24	47	48	60	78	34	33	14	18	2	27	6	7	2	13	3	9
Quezon	76	92	140	134	461	519	1,495	1,461	723	783	360	234	134	112	66	92	76	87	57	78
Rizal	0	0	12	7	762	535	395	417	206	188	41	51	41	42	29	10	17	30	22	48
Antipolo City	0	0	0	0	0	0	0	0	0	0	0	0	0	0	0	0	0	0	0	0
Bacoor City	0	0	0	0	0	0	0	0	0	0	0	0	0	0	0	0	0	0	0	0
Batangas City	0	0	0	0	0	0	0	0	0	0	0	0	0	0	0	0	0	0	0	0
Biñan City	0	0	0	15	13	15	9	1	1	4	0	1	1	0	0	0	1	0	0	0
Cabuyao City	0	0	0	0	0	0	0	0	0	0	0	0	0	0	0	0	0	0	0	0
Calamba City	0	0	0	0	0	0	0	0	0	0	0	0	0	0	0	0	0	0	0	0
Cavite City	0	0	0	0	0	0	0	0	0	0	0	0	0	0	0	0	0	0	0	0
Dasmariñas City	0	0	0	0	0	0	0	0	0	0	0	0	0	0	0	0	0	0	0	0
General Trias City	0	0	0	0	13	11	20	23	7	5	2	0	0	0	1	1	0	0	2	0
Imus City	0	0	0	0	6	7	9	10	7	4	3	2	1	0	0	2	0	0	1	0
Lipa City	0	0	0	0	0	0	0	0	0	0	0	0	0	0	0	0	0	0	0	0
Lucena City	1	0	2	0	31	18	43	47	15	16	4	10	2	4	0	2	1	1	1	4
San Pablo City	0	6	0	12	11	13	65	43	42	20	12	6	6	0	6	18	6	4	3	9
San Pedro City	0	0	0	0	0	0	0	0	0	0	0	0	0	0	0	0	0	0	0	0
Santa Rosa City	0	0	0	0	6	3	9	3	9	6	3	3	0	0	0	0	0	0	0	3
Tagaytay City	0	0	0	0	13	21	23	28	13	4	6	3	0	2	2	0	3	2	4	2
Tanauan City	7	0	0	0	74	88	214	189	125	110	64	56	27	32	10	20	3	10	0	13
Tayabas City	0	0	0	0	0	15	18	23	19	7	3	6	0	0	0	3	0	1	0	0
Trece Martires City	0	0	0	0	0	0	2	0	0	0	0	0	0	0	0	0	0	0	0	0
**Region 4B**	**1**	**0**	**0**	**0**	**6**	**7**	**21**	**24**	**16**	**18**	**2**	**3**	**2**	**4**	**3**	**6**	**0**	**7**	**1**	**5**
Marinduque	0	0	0	0	0	0	0	0	0	0	0	0	0	0	0	0	0	0	0	0

Table 2.A.4.2 - Acute Febrile Illness
By Age group, Sex, Province and City
Philippines, 2021

Region/Province/City	0 - 6 days		7 - 28 days		29 days - 11 mos.		1 - 4 yrs.		5 - 9 yrs.		10 -14 yrs.		15 - 19 yrs.		20 - 24 yrs.		25 - 29 yrs.		30 - 34 yrs.	
	Male	Female	Male	Female	Male	Female	Male	Female	Male	Female	Male	Female	Male	Female	Male	Female	Male	Female	Male	Female
Mindoro Occidental	0	0	0	0	0	0	0	0	0	0	0	0	0	0	0	0	0	0	0	0
Mindoro Oriental	0	0	0	0	0	0	0	0	0	0	0	0	0	0	0	0	0	0	0	0
Palawan	1	0	0	0	6	5	19	22	15	18	2	3	2	4	3	6	0	6	1	4
Romblon	0	0	0	0	0	2	2	2	1	0	0	0	0	0	0	0	0	1	0	1
Puerto Princesa City	0	0	0	0	0	0	0	0	0	0	0	0	0	0	0	0	0	0	0	0
**Region 5**	**0**	**2**	**6**	**9**	**214**	**196**	**837**	**808**	**463**	**441**	**202**	**169**	**104**	**120**	**72**	**98**	**55**	**113**	**65**	**62**
Albay	0	0	0	0	17	15	82	84	67	40	30	19	14	19	2	10	2	5	5	3
Camarines Norte	0	0	0	1	20	13	37	36	28	25	14	15	9	10	5	9	3	2	5	3
Camarines Sur	0	1	3	2	76	94	365	390	196	209	70	67	27	24	21	34	16	36	22	18
Catanduanes	0	0	0	0	4	2	8	8	7	7	0	1	0	2	0	1	0	1	0	0
Masbate	0	0	0	0	0	0	0	0	0	0	0	0	0	0	0	0	0	0	0	0
Sorsogon	0	0	0	0	14	12	52	33	18	16	4	5	2	2	2	1	3	6	0	2
Iriga City	0	0	0	0	0	0	0	0	0	0	0	0	0	0	0	0	0	0	0	0
Legaspi City	0	1	3	6	83	60	293	257	147	144	84	62	52	62	42	43	31	63	33	36
Naga City	0	0	0	0	0	0	0	0	0	0	0	0	0	1	0	0	0	0	0	0
**Region 6**	**0**	**0**	**7**	**0**	**9**	**15**	**43**	**38**	**15**	**8**	**1**	**2**	**1**	**0**	**2**	**1**	**0**	**0**	**1**	**0**
Aklan	0	0	0	0	0	1	2	1	1	1	0	1	0	0	0	0	0	0	0	0
Antique	0	0	0	0	0	0	0	0	0	0	0	0	0	0	0	0	0	0	0	0
Capiz	0	0	0	0	0	0	0	0	0	0	0	0	0	0	0	0	0	0	0	0
Guimaras	0	0	0	0	0	0	0	0	0	0	0	0	0	0	0	0	0	0	0	0
Iloilo	0	0	0	0	0	0	0	0	0	0	0	0	0	0	0	0	0	0	0	0
Negros Occidental	0	0	7	0	9	14	41	37	14	7	1	1	1	0	2	1	0	0	1	0
Bacolod City	0	0	0	0	0	0	0	0	0	0	0	0	0	0	0	0	0	0	0	0
Iloilo City	0	0	0	0	0	0	0	0	0	0	0	0	0	0	0	0	0	0	0	0
**Region 7**	**0**	**0**	**0**	**0**	**0**	**0**	**0**	**0**	**0**	**0**	**0**	**0**	**0**	**0**	**0**	**0**	**0**	**0**	**0**	**0**
Bohol	0	0	0	0	0	0	0	0	0	0	0	0	0	0	0	0	0	0	0	0
Cebu	0	0	0	0	0	0	0	0	0	0	0	0	0	0	0	0	0	0	0	0
Negros Oriental	0	0	0	0	0	0	0	0	0	0	0	0	0	0	0	0	0	0	0	0
Siquijor	0	0	0	0	0	0	0	0	0	0	0	0	0	0	0	0	0	0	0	0
Cebu City	0	0	0	0	0	0	0	0	0	0	0	0	0	0	0	0	0	0	0	0
Lapu-Lapu City	0	0	0	0	0	0	0	0	0	0	0	0	0	0	0	0	0	0	0	0
Mandaue City	0	0	0	0	0	0	0	0	0	0	0	0	0	0	0	0	0	0	0	0
**Region 8**	**1**	**0**	**4**	**12**	**201**	**250**	**510**	**481**	**361**	**312**	**146**	**120**	**82**	**111**	**80**	**76**	**93**	**96**	**95**	**94**
Biliran	0	0	0	0	1	0	3	2	0	2	1	0	0	0	0	0	0	0	0	0
Eastern Samar	0	0	0	0	5	7	8	15	6	0	1	0	0	0	0	0	0	0	0	0
Northern Leyte	0	0	0	3	11	17	60	61	36	29	10	13	5	5	3	6	7	18	5	10
Northern Samar	0	0	0	4	67	77	152	135	91	71	31	23	10	9	6	14	7	15	8	22
Southern Leyte	1	0	2	1	5	16	38	30	22	29	9	9	4	4	5	3	2	4	1	6
Western Samar	0	0	0	3	20	29	73	85	66	57	30	23	16	18	10	15	13	11	14	11
Calbayog City	0	0	0	0	0	0	0	0	0	0	1	0	0	0	0	0	0	0	0	0
Maasin City	0	0	2	1	23	35	116	92	84	78	30	17	29	43	23	14	32	25	26	29
Ormoc City	0	0	0	0	69	69	60	61	56	46	33	35	18	32	33	24	32	23	41	16
Tacloban City	0	0	0	0	0	0	0	0	0	0	0	0	0	0	0	0	0	0	0	0
**Region 9**	**0**	**0**	**0**	**0**	**0**	**0**	**0**	**0**	**0**	**0**	**0**	**0**	**0**	**0**	**0**	**0**	**0**	**0**	**0**	**0**
Zamboanga del Norte	0	0	0	0	0	0	0	0	0	0	0	0	0	0	0	0	0	0	0	0
Zamboanga del Sur	0	0	0	0	0	0	0	0	0	0	0	0	0	0	0	0	0	0	0	0
Zamboanga Sibugay	0	0	0	0	0	0	0	0	0	0	0	0	0	0	0	0	0	0	0	0
Dapitan City	0	0	0	0	0	0	0	0	0	0	0	0	0	0	0	0	0	0	0	0
Dipolog City	0	0	0	0	0	0	0	0	0	0	0	0	0	0	0	0	0	0	0	0
Isabela City	0	0	0	0	0	0	0	0	0	0	0	0	0	0	0	0	0	0	0	0
Pagadian City	0	0	0	0	0	0	0	0	0	0	0	0	0	0	0	0	0	0	0	0
Zamboanga City	0	0	0	0	0	0	0	0	0	0	0	0	0	0	0	0	0	0	0	0
**Region 10**	**0**	**1**	**5**	**5**	**153**	**104**	**392**	**354**	**187**	**211**	**110**	**115**	**130**	**83**	**38**	**101**	**52**	**142**	**59**	**77**
Bukidnon	0	0	2	2	33	19	109	71	42	45	16	4	3	6	0	16	7	5	7	20
Camiguin	0	0	0	0	0	0	0	0	0	0	0	0	0	0	0	0	0	0	0	0
Lanao del Norte	0	0	2	2	90	65	212	224	83	114	65	78	103	59	21	57	36	127	43	50
Misamis Occidental	0	0	0	0	2	2	3	8	2	6	3	4	0	0	1	1	3	1	0	1
Misamis Oriental	0	0	1	1	2	3	19	17	12	14	7	9	2	3	3	3	2	1	2	1
Cagayan de Oro City	0	0	0	0	1	0	3	3	2	0	1	0	0	0	0	0	0	0	1	0
El Salvador City	0	0	0	0	0	0	0	0	0	0	0	0	0	0	0	0	0	0	0	0
Gingoog City	0	0	0	0	3	2	6	2	7	5	0	1	2	0	0	1	0	3	1	0
Iligan City	0	1	0	0	13	7	14	14	21	15	11	11	8	5	4	4	0	2	1	0
Malaybalay City	0	0	0	0	0	0	0	0	0	0	0	0	0	0	0	0	0	0	0	0
Oroquieta City	0	0	0	0	0	0	0	0	0	0	0	0	0	0	0	0	0	0	0	0
Ozamis City	0	0	0	0	6	4	10	7	10	5	4	8	12	9	9	16	3	3	3	1
Tangub City	0	0	0	0	0	0	0	0	0	0	0	0	0	0	0	0	0	0	0	0
Valencia City	0	0	0	0	3	2	16	8	8	7	3	0	0	1	0	3	1	0	1	4
**Region 11**	**1**	**1**	**3**	**11**	**66**	**76**	**76**	**76**	**34**	**42**	**20**	**14**	**9**	**1**	**3**	**9**	**6**	**14**	**5**	**16**
Davao de Oro	0	0	0	0	0	4	5	3	3	1	0	0	2	0	0	0	0	0	0	0
Davao del Norte	0	0	0	0	2	1	10	9	0	3	0	6	0	0	0	8	1	7	0	5
Davao del Sur	0	0	0	0	12	12	2	13	5	10	4	0	1	0	3	0	0	0	0	5
Davao Oriental	0	0	0	4	36	46	48	38	17	23	6	4	3	0	0	0	0	4	1	0
Davao Occidental	1	1	3	7	15	12	11	12	9	5	10	4	2	1	0	1	3	3	2	3
Davao City	0	0	0	0	1	1	0	1	0	0	0	0	1	0	0	0	2	0	2	3
**Region 12**	**0**	**0**	**0**	**0**	**2**	**1**	**10**	**14**	**11**	**9**	**3**	**9**	**10**	**7**	**8**	**9**	**7**	**8**	**7**	**7**
North Cotabato	0	0	0	0	2	0	7	11	11	9	3	9	10	7	8	9	7	8	7	7
Sarangani	0	0	0	0	0	0	0	0	0	0	0	0	0	0	0	0	0	0	0	0
South Cotabato	0	0	0	0	0	0	0	0	0	0	0	0	0	0	0	0	0	0	0	0
Sultan Kudarat	0	0	0	0	0	0	0	0	0	0	0	0	0	0	0	0	0	0	0	0
Cotabato City	0	0	0	0	0	1	3	3	0	0	0	0	0	0	0	0	0	0	0	0
Gen. Santos City	0	0	0	0	0	0	0	0	0	0	0	0	0	0	0	0	0	0	0	0
**BARMM**	**0**	**0**	**0**	**0**	**0**	**0**	**0**	**0**	**0**	**0**	**0**	**0**	**0**	**0**	**0**	**0**	**0**	**0**	**0**	**0**
Basilan	0	0	0	0	0	0	0	0	0	0	0	0	0	0	0	0	0	0	0	0
Lanao del Sur	0	0	0	0	0	0	0	0	0	0	0	0	0	0	0	0	0	0	0	0
Maguindanao	0	0	0	0	0	0	0	0	0	0	0	0	0	0	0	0	0	0	0	0
Sulu	0	0	0	0	0	0	0	0	0	0	0	0	0	0	0	0	0	0	0	0
Tawi-Tawi	0	0	0	0	0	0	0	0	0	0	0	0	0	0	0	0	0	0	0	0
Lamitan City	0	0	0	0	0	0	0	0	0	0	0	0	0	0	0	0	0	0	0	0
Marawi City	0	0	0	0	0	0	0	0	0	0	0	0	0	0	0	0	0	0	0	0

Table 2.A.4.2 - Acute Febrile Illness
By Age group, Sex, Province and City
Philippines, 2021

Region/Province/City	0 - 6 days		7 - 28 days		29 days - 11 mos.		1 - 4 yrs.		5 - 9 yrs.		10 -14 yrs.		15 - 19 yrs.		20 - 24 yrs.		25 - 29 yrs.		30 - 34 yrs.	
	Male	Female	Male	Female	Male	Female	Male	Female	Male	Female	Male	Female	Male	Female	Male	Female	Male	Female	Male	Female
**CARAGA**	**0**	**0**	**0**	**0**	**0**	**0**	**0**	**0**	**0**	**0**	**0**	**0**	**0**	**0**	**0**	**0**	**0**	**0**	**0**	**0**
Agusan del Norte	0	0	0	0	0	0	0	0	0	0	0	0	0	0	0	0	0	0	0	0
Agusan del Sur	0	0	0	0	0	0	0	0	0	0	0	0	0	0	0	0	0	0	0	0
Surigao del Norte	0	0	0	0	0	0	0	0	0	0	0	0	0	0	0	0	0	0	0	0
Surigao del Sur	0	0	0	0	0	0	0	0	0	0	0	0	0	0	0	0	0	0	0	0
Province of Dinagat	0	0	0	0	0	0	0	0	0	0	0	0	0	0	0	0	0	0	0	0
Bislig City	0	0	0	0	0	0	0	0	0	0	0	0	0	0	0	0	0	0	0	0
Butuan City	0	0	0	0	0	0	0	0	0	0	0	0	0	0	0	0	0	0	0	0
Surigao City	0	0	0	0	0	0	0	0	0	0	0	0	0	0	0	0	0	0	0	0

Legend:
0 = No Data
* = No Report

Table 2.A.4.2 - Acute Febrile Illness
By Age group, Sex, Province and City
Philippines, 2021

Region/Province/City	35 -39 yrs.		40 - 44 yrs.		45 -49 yrs.		50 -54 yrs.		55 - 59 yrs.		60 - 64. yrs.		65 - 69 yrs.		70 yrs above		Total		Total Both Sexes	
	Male	Female	Male	Female	Male	Female	Male	Female	Male	Female	Male	Female	Male	Female	Male	Female	Male	Female	Number	Rate
**PHILIPPINES**	**289**	**398**	**306**	**438**	**185**	**349**	**296**	**337**	**158**	**274**	**200**	**254**	**144**	**186**	**137**	**189**	**14,167**	**14,800**	**28,967**	**26.29**
**NCR**	**4**	**3**	**1**	**1**	**2**	**2**	**7**	**2**	**0**	**0**	**2**	**3**	**1**	**1**	**0**	**0**	**58**	**59**	**117**	**0.84**
Malabon City	0	0	0	0	0	0	0	0	0	0	0	0	0	0	0	0	0	0	0	0.00
Navotas City	0	0	0	0	0	0	0	0	0	0	0	0	0	0	0	0	0	0	0	0.00
Valenzuela City	0	0	1	0	1	0	0	0	0	0	0	0	0	0	0	0	5	3	8	1.19
Caloocan City	0	0	0	0	0	0	0	0	0	0	0	0	0	0	0	0	0	0	0	0.00
Marikina City	0	0	0	0	0	0	0	0	0	0	0	0	0	0	0	0	0	0	0	0.00
Pasig City	0	0	0	0	0	0	0	0	0	0	0	0	0	0	0	0	0	0	0	0.00
Pateros	0	0	0	0	0	0	0	0	0	0	0	0	0	0	0	0	0	0	0	0.00
Taguig City	4	3	0	0	1	2	3	2	0	0	2	3	1	1	0	0	25	28	53	6.07
Quezon City	0	0	0	0	0	0	0	0	0	0	0	0	0	0	0	0	0	0	0	0.00
Makati City	0	0	0	0	0	0	0	0	0	0	0	0	0	0	0	0	0	0	0	0.00
Mandaluyong City	0	0	0	0	0	0	0	0	0	0	0	0	0	0	0	0	0	0	0	0.00
[truncated: 1,149,584 more chars]
